# Supplementary material for: Applying univariate vs. multivariate statistics to investigate therapeutic efficacy in (pre)clinical trials: A Monte Carlo simulation study on the example of a controlled preclinical neurotrauma trial
Source: PLoS One. 2020 Mar 26;15(3):e0230798. doi: 10.1371/journal.pone.0230798 (PMC7098614; doi:10.1371/journal.pone.0230798)
Supplement: S1 Appendix — Figs 1–4 show comparisons of type I error rate and empirical power of the four different multivariate statistics used to evaluate the significance of MANOVA tests. (PDF) [file pone.0230798.s001.pdf]

Applying univariate vs. multivariate statistics to investigate therapeutic efficacy in (pre)clinical trials: A Monte Carlo simulation study on the example of a controlled preclinical neurotrauma trial.

Hristo Todorov, Emily Searle-White, Susanne Gerber

Table S1: An overview of outcome measures from a traumatic brain injury rat model included in the simulation study. The mean and covariance vectors were obtained by averaging values from a non-parametric bootstrap with 10,000 samples drawn from the original animal study using the control group.

Nr.	Variable	Mean	Variance
1	20 point neuroscore day 1	15.862	2.612
2	20 point neuroscore day 7	18.446	1.304
3	20 point neuroscore day 14	19.196	1.337
4	Limb placing score day 1	8.78	4.62
5	Limb placing score day 7	11.057	2.945
6	Limb placing score day 14	12.504	1.917
7	Lesion volume day 1 [mm^3]	89.554	1169.808
8	Lesion volume day 7 [mm^3]	43.541	72.754
9	Lesion volume day 14 [mm^3]	24.852	147.991
10	Edema volume day 1 [mm^3]	46.448	696.146
11	Edema volume day 7 [mm^3]	16.926	136.323
12	Edema volume day 14 [mm^3]	14.295	103.103
13	T2 lesion day 1 [ms]	59.447	8.83
14	T2 lesion day 7 [ms]	54.58	5.888
15	T2 lesion day 14 [ms]	61.292	129.32
16	T2 lesion contralateral cortex day 1 [ms]	53.544	1.191
17	T2 lesion contralateral cortex day 7 [ms]	52.859	0.838
18	T2 lesion contralateral cortex day 14 [ms]	54.21	0.422

Table S2 Correlation matrix used for drawing samples from multivariate distributions. The matrix was calculated from the covariance matrix obtained from the non-parametric bootstrap procedure Positive correlations are colored red and negative correlations are colored blue. The numbering of columns and rows corresponds to the variables in Table S1.

	**1**	**2**	**3**	**4**	**5**	**6**	**7**	**8**	**9**	**10**	**11**	**12**	**13**	**14**	**15**	**16**	**17**	**18**
**1**	1	0.509	0.32	0.658	0.566	0.407	-0.318	-0.257	-0.194	-0.341	-0.208	0.106	-0.057	-0.44	-0.372	0.159	-0.202	-0.29
**2**	0.509	1	0.829	0.596	0.74	0.513	-0.42	-0.111	-0.511	-0.562	-0.142	0.481	-0.107	0.066	0.059	0.345	0.022	0.123
**3**	0.32	0.829	1	0.375	0.681	0.669	-0.709	-0.26	-0.558	-0.77	0.071	0.511	-0.3	0.032	-0.099	0.449	0.102	0.226
**4**	0.658	0.596	0.375	1	0.712	0.449	-0.262	-0.273	-0.17	-0.242	-0.191	0.105	0.034	-0.108	-0.065	0.034	0.086	-0.033
**5**	0.566	0.74	0.681	0.712	1	0.785	-0.644	-0.261	-0.538	-0.619	-0.02	0.39	-0.102	-0.03	-0.104	0.211	-0.011	-0.102
**6**	0.407	0.513	0.669	0.449	0.785	1	-0.649	-0.336	-0.326	-0.557	0.116	0.307	-0.055	0.052	-0.084	0.235	-0.009	0.067
**7**	-0.318	-0.42	-0.709	-0.262	-0.644	-0.649	1	0.354	0.656	0.907	-0.144	-0.45	0.566	0.27	0.415	0.044	0.144	0.04
**8**	-0.257	-0.111	-0.26	-0.273	-0.261	-0.336	0.354	1	0.378	0.239	0.11	-0.074	0.069	0.312	0.476	-0.002	0.038	-0.06
**9**	-0.194	-0.511	-0.558	-0.17	-0.538	-0.326	0.656	0.378	1	0.678	0.013	-0.297	0.248	0.357	0.519	0.063	0.244	-0.014
**10**	-0.341	-0.562	-0.77	-0.242	-0.619	-0.557	0.907	0.239	0.678	1	0.095	-0.418	0.63	0.319	0.412	-0.056	0.134	0.071
**11**	-0.208	-0.142	0.071	-0.191	-0.02	0.116	-0.144	0.11	0.013	0.095	1	0.321	0.13	0.537	0.177	0.017	0.227	0.002
**12**	0.106	0.481	0.511	0.105	0.39	0.307	-0.45	-0.074	-0.297	-0.418	0.321	1	-0.344	0.28	0.236	0.142	0.114	-0.134
**13**	-0.057	-0.107	-0.3	0.034	-0.102	-0.055	0.566	0.069	0.248	0.63	0.13	-0.344	1	0.282	0.315	0.153	-0.091	0.178
**14**	-0.44	0.066	0.032	-0.108	-0.03	0.052	0.27	0.312	0.357	0.319	0.537	0.28	0.282	1	0.81	0.066	0.476	0.097
**15**	-0.372	0.059	-0.099	-0.065	-0.104	-0.084	0.415	0.476	0.519	0.412	0.177	0.236	0.315	0.81	1	0.125	0.127	0.25
**16**	0.159	0.345	0.449	0.034	0.211	0.235	0.044	-0.002	0.063	-0.056	0.017	0.142	0.153	0.066	0.125	1	0.138	0.296
**17**	-0.202	0.022	0.102	0.086	-0.011	-0.009	0.144	0.038	0.244	0.134	0.227	0.114	-0.091	0.476	0.127	0.138	1	-0.245
**18**	-0.29	0.123	0.226	-0.033	-0.102	0.067	0.04	-0.06	-0.014	0.071	0.002	-0.134	0.178	0.097	0.25	0.296	-0.245	1

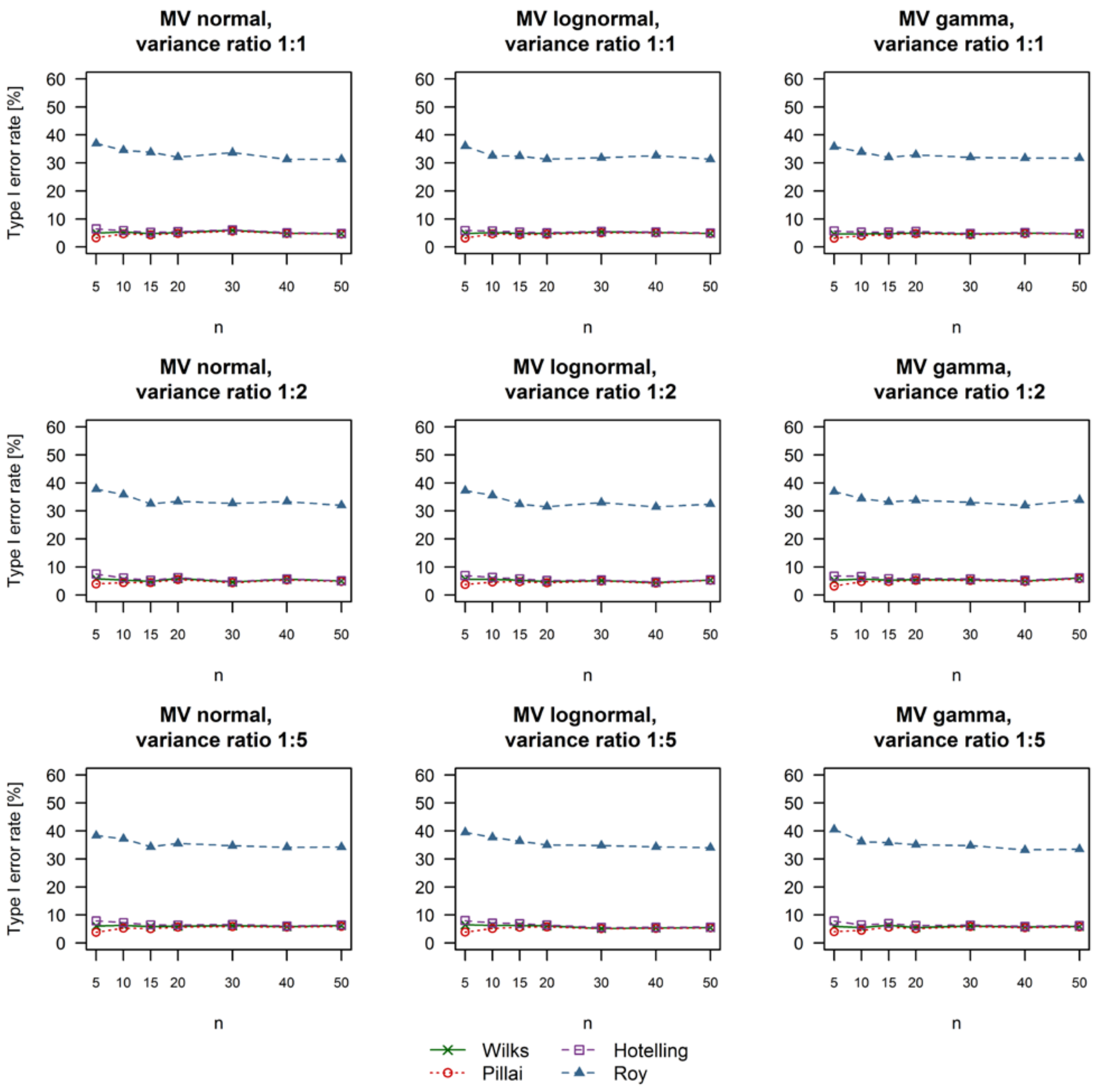

Fig S. Type I error rate of different multivariate statistics used to evaluate the significance of MANOVA tests. The multivariate distribution from which variables were sampled and the variance ratio between control and treatment groups are shown in the title of each plot.

MANOVA: Multivariate analysis of variance; Wilks: Wilks' lambda; Pillai: Pillai's trace; Hotelling: Lawley-Hotelling trace; Roy: Roy's largest root; MV: Multivariate.

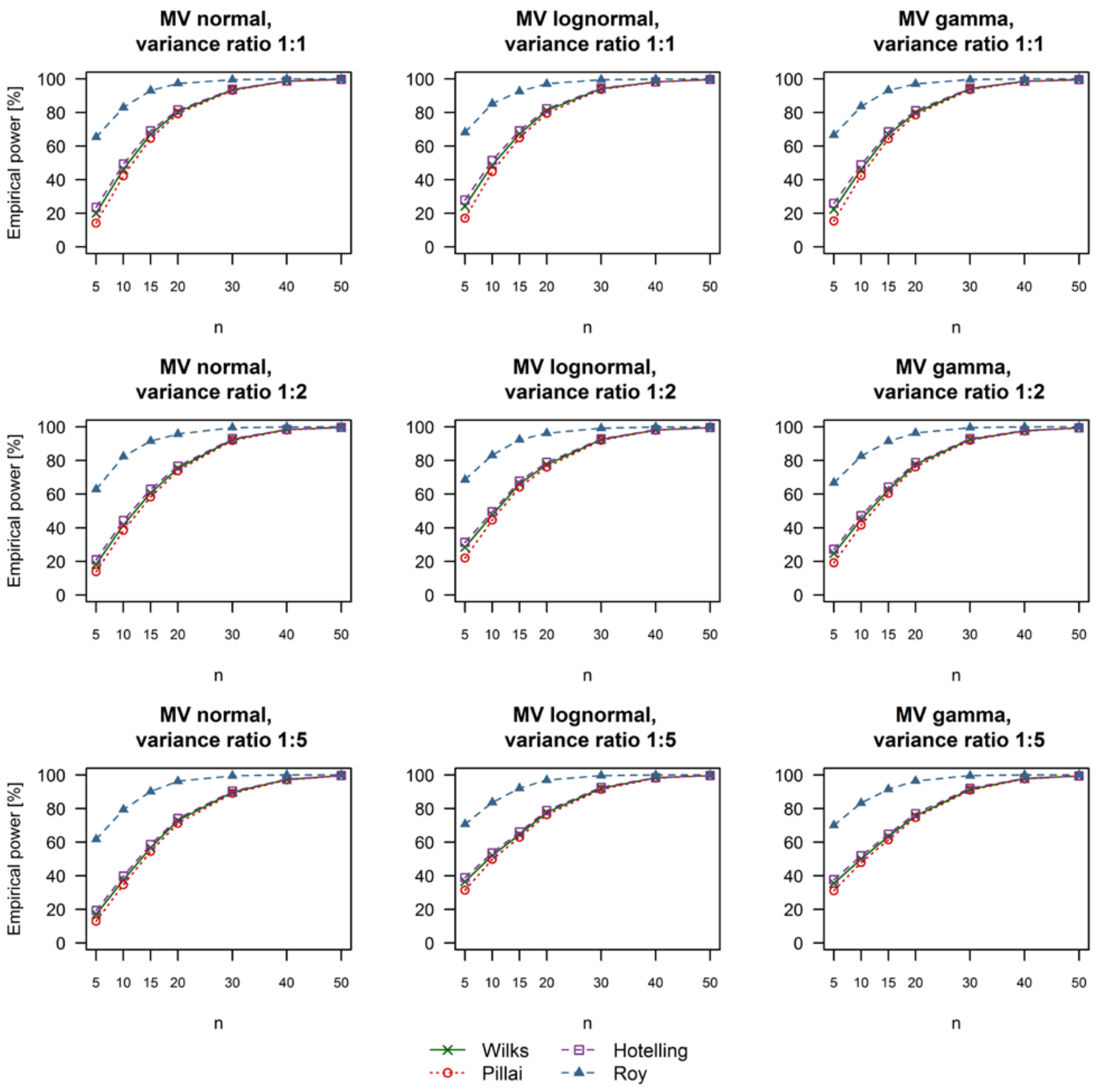

Fig S2. Empirical power of different multivariate statistics used to evaluate the significance of MANOVA with simulated large treatment effects (Cohen's d equal to 0.8). The multivariate distribution from which variables were sampled and the variance ratio between control and treatment groups are shown in the title of each plot.

MANOVA: Multivariate analysis of variance; Wilks: Wilks' lambda; Pillai: Pillai's trace; Hotelling: Lawley-Hotelling trace; Roy: Roy's largest root; MV: Multivariate.

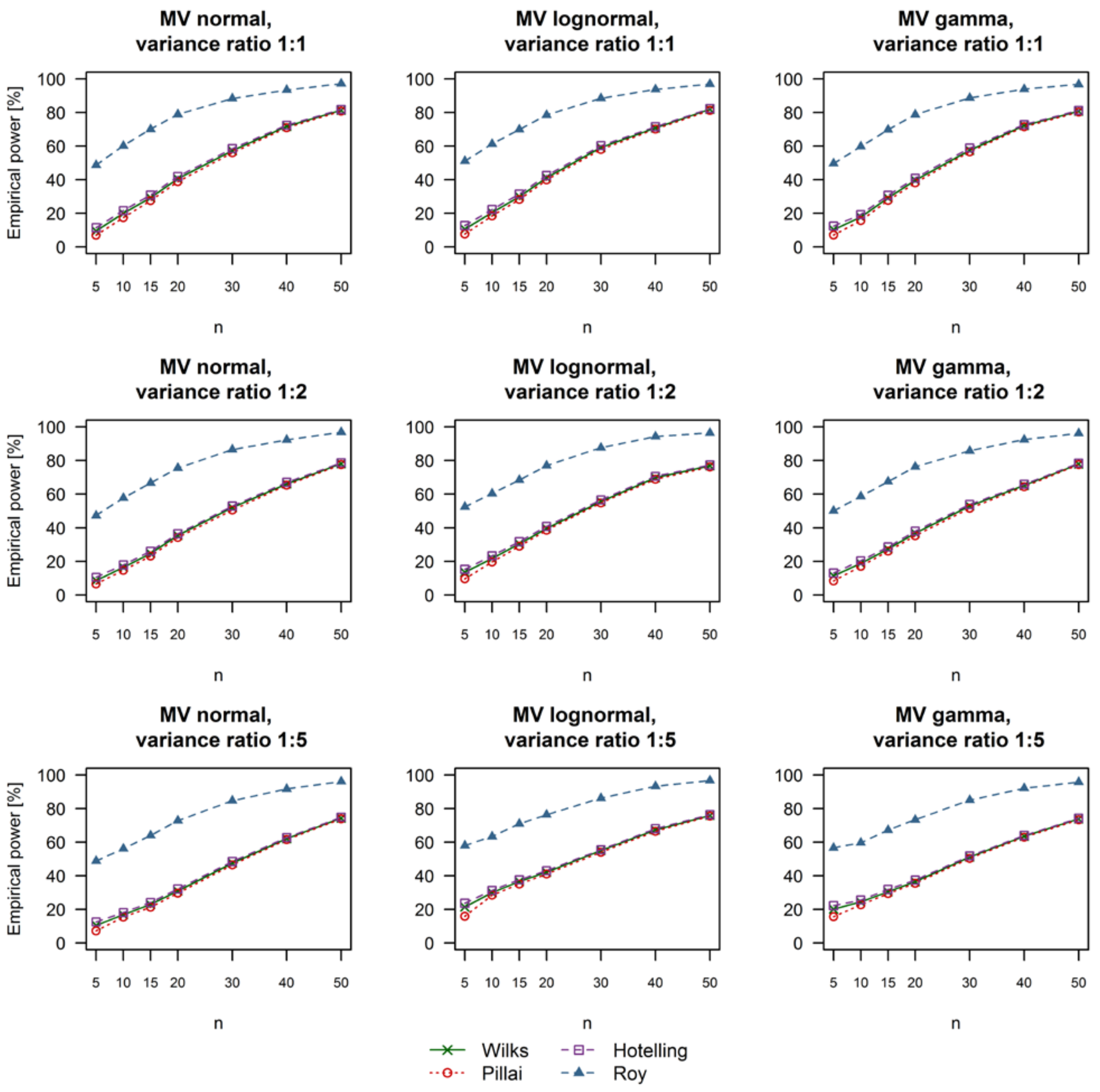

Fig S3. Empirical power of different multivariate statistics used to evaluate the significance of MANOVA with simulated moderate treatment effects (Cohen's d equal to 0.5). The multivariate distribution from which variables were sampled and the variance ratio between control and treatment groups are shown in the title of each plot.

MANOVA: Multivariate analysis of variance; Wilks: Wilks' lambda; Pillai: Pillai's trace; Hotelling: Lawley-Hotelling trace; Roy: Roy's largest root; MV: Multivariate.

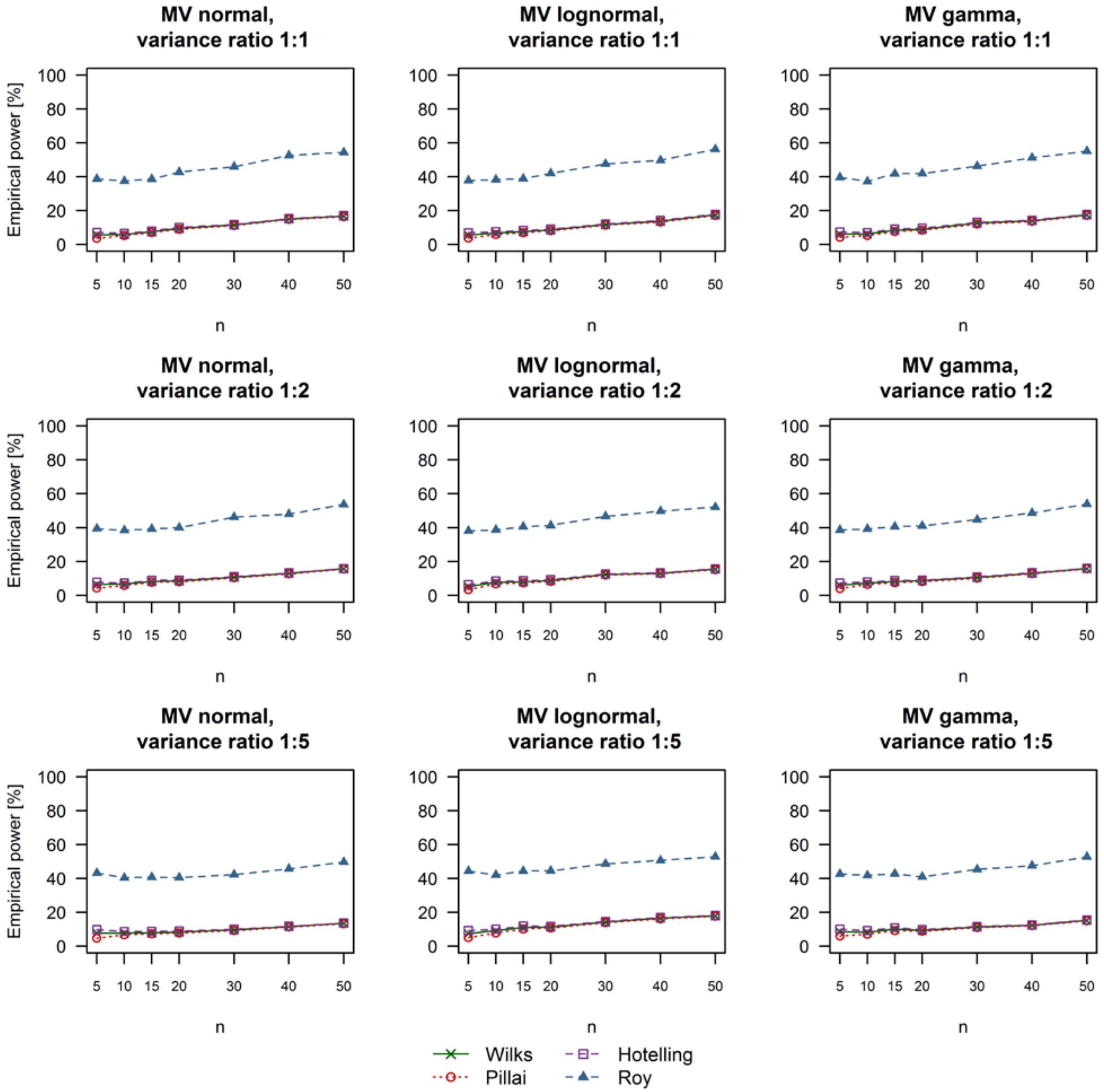

Fig S4. Empirical power of different multivariate statistics used to evaluate the significance of MANOVA with simulated small treatment effects (Cohen's d equal to 0.2). The multivariate distribution from which variables were sampled and the variance ratio between control and treatment groups are shown in the title of each plot.

MANOVA: Multivariate analysis of variance; Wilks: Wilks' lambda; Pillai: Pillai's trace; Hotelling: Lawley-Hotelling trace; Roy: Roy's largest root; MV: Multivariate.
