# Supplementary material for: Applying univariate vs. multivariate statistics to investigate therapeutic efficacy in (pre)clinical trials: A Monte Carlo simulation study on the example of a controlled preclinical neurotrauma trial
Source: PLoS One. 2020 Mar 26;15(3):e0230798. doi: 10.1371/journal.pone.0230798 (PMC7098614; doi:10.1371/journal.pone.0230798)
Supplement: S2 Appendix — The file contains the results from 1000 simulated data sets without treatment effects, 5 measurements per group with data obtained from a multivariate normal distribution with equal variance in all groups. LDA, PCA, RDA, or PLS-DA were considered to falsely capture a non-existing treatment effect pattern if the 95% confidence ellipse around the control group did not overlap with the confidence ellipses of treatment groups (dose1 to dose3). (PDF) [file pone.0230798.s002.pdf]

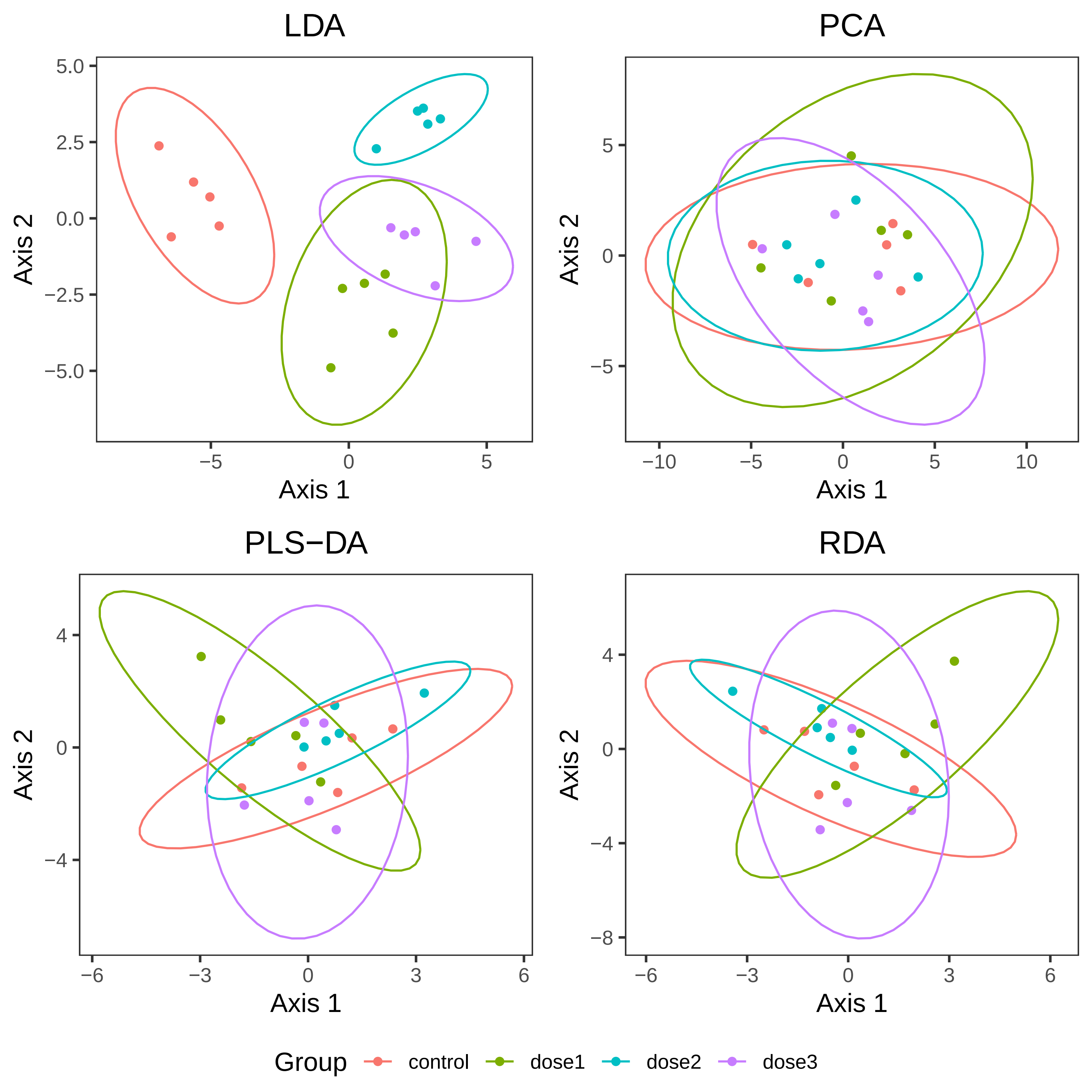

LDA
PCA
PLS−DA
RDA
Axis 1
Axis 2
5.0
2.5
0.0
−2.5
−5.0
−5
0
5
−10
10
−6
−3
3
6
4
−4
−8
Group
control
dose1
dose2
dose3

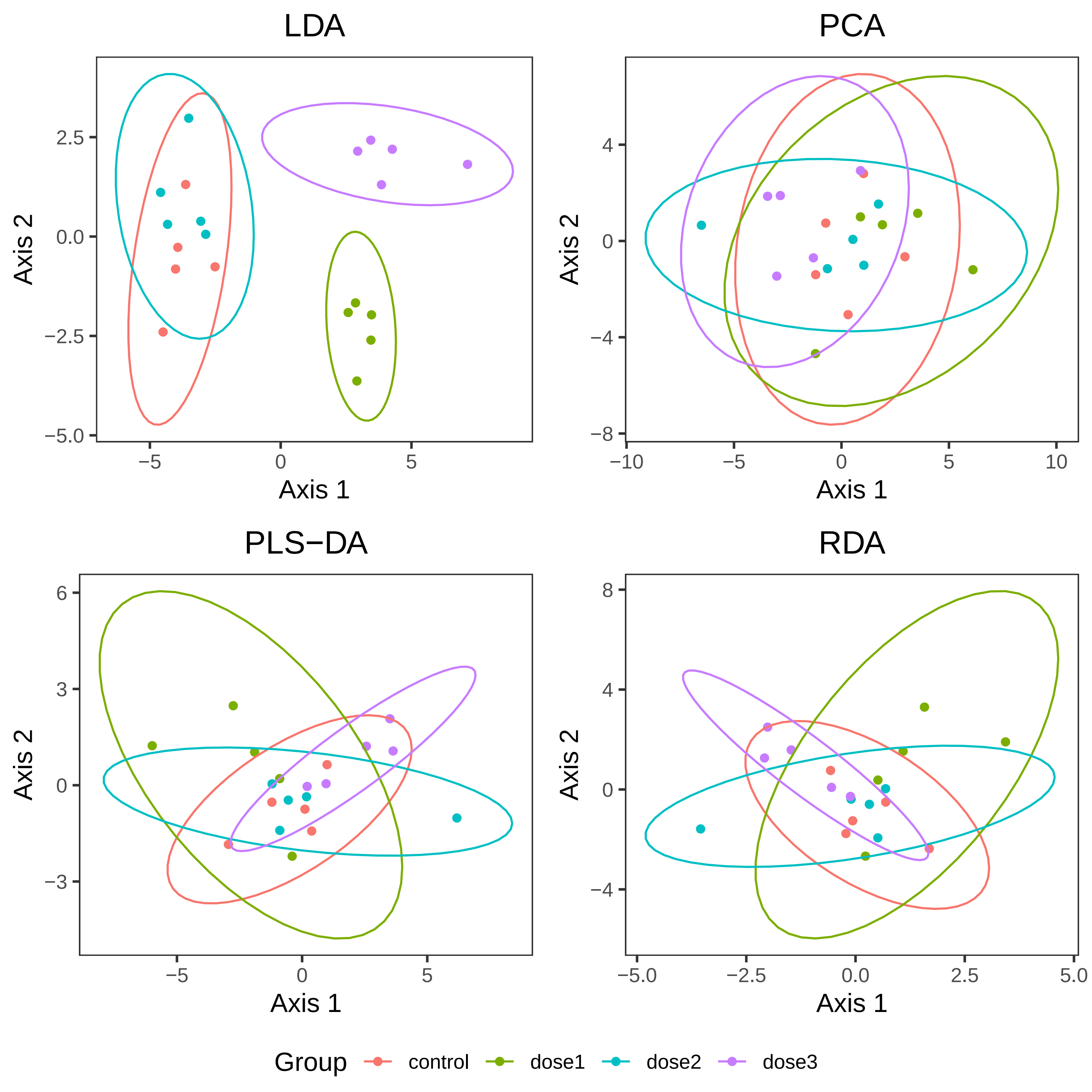
LDA
PCA
PLS−DA
RDA
Axis 1
Axis 2
Group
control
dose1
dose2
dose3

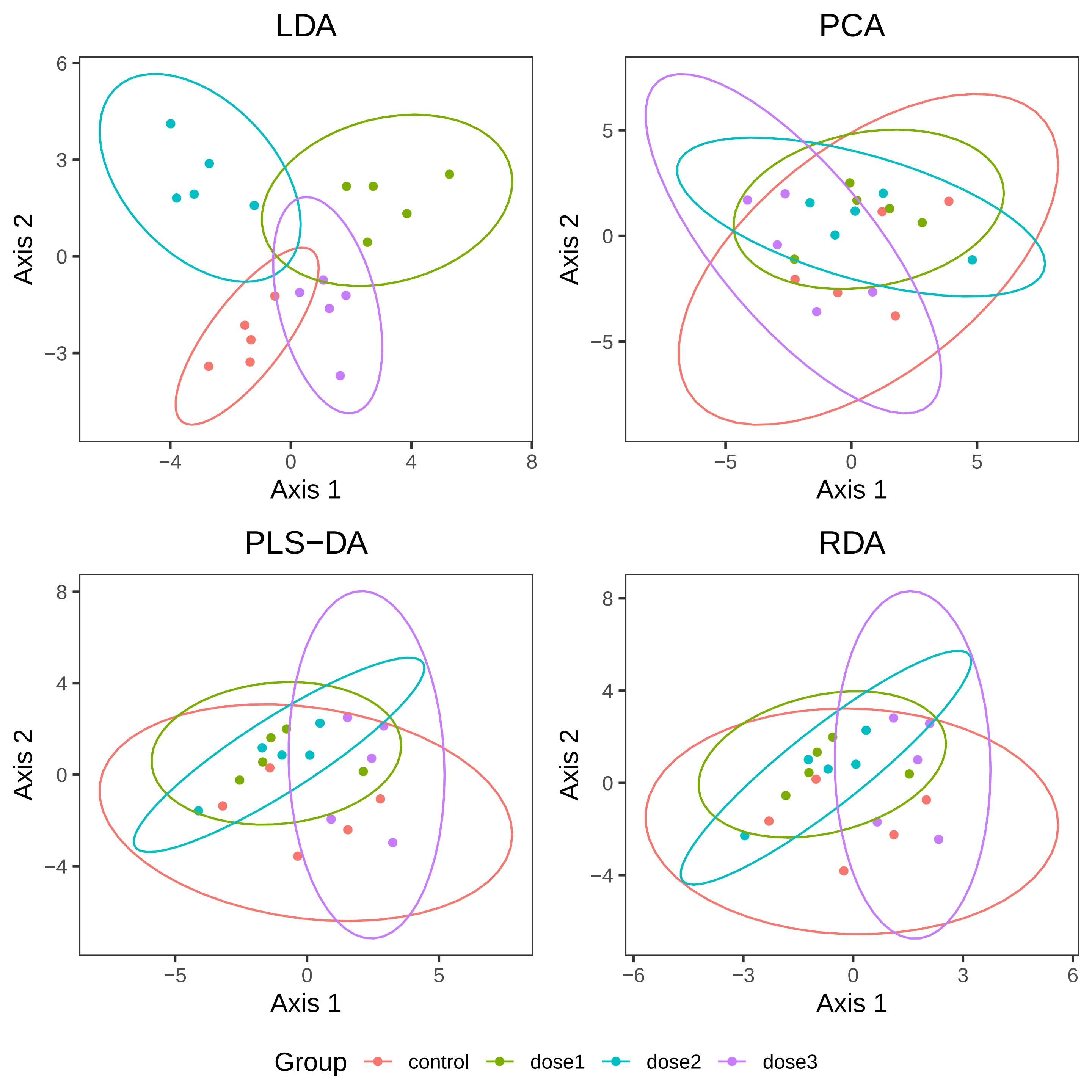

LDA
PCA
PLS−DA
RDA
Axis 1
Axis 2
Group
control
dose1
dose2
dose3

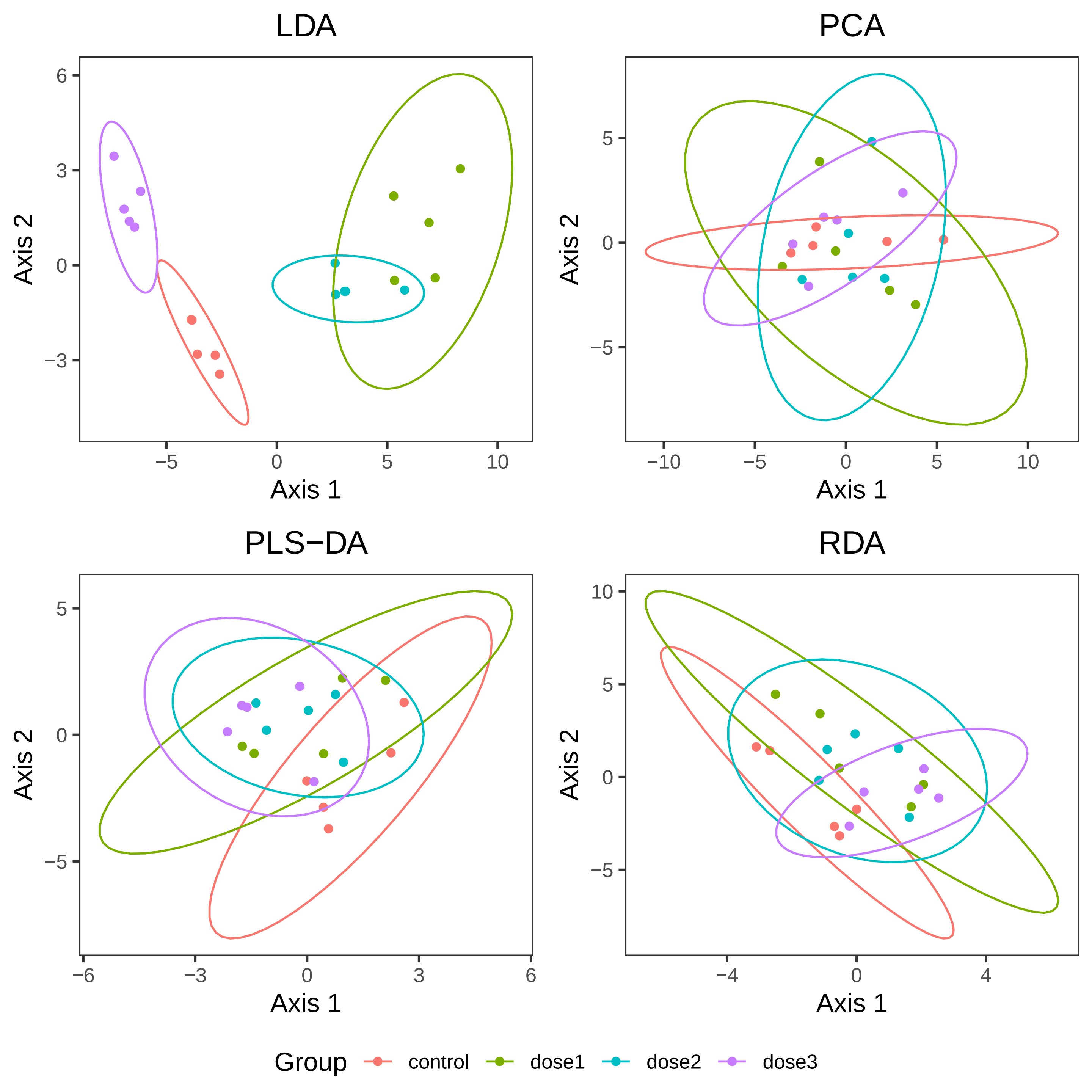

LDA
PCA
PLS−DA
RDA
Axis 1
Axis 2
6
3
0
−3
−5
5
10
−10
−6
−4
4
Group
control
dose1
dose2
dose3

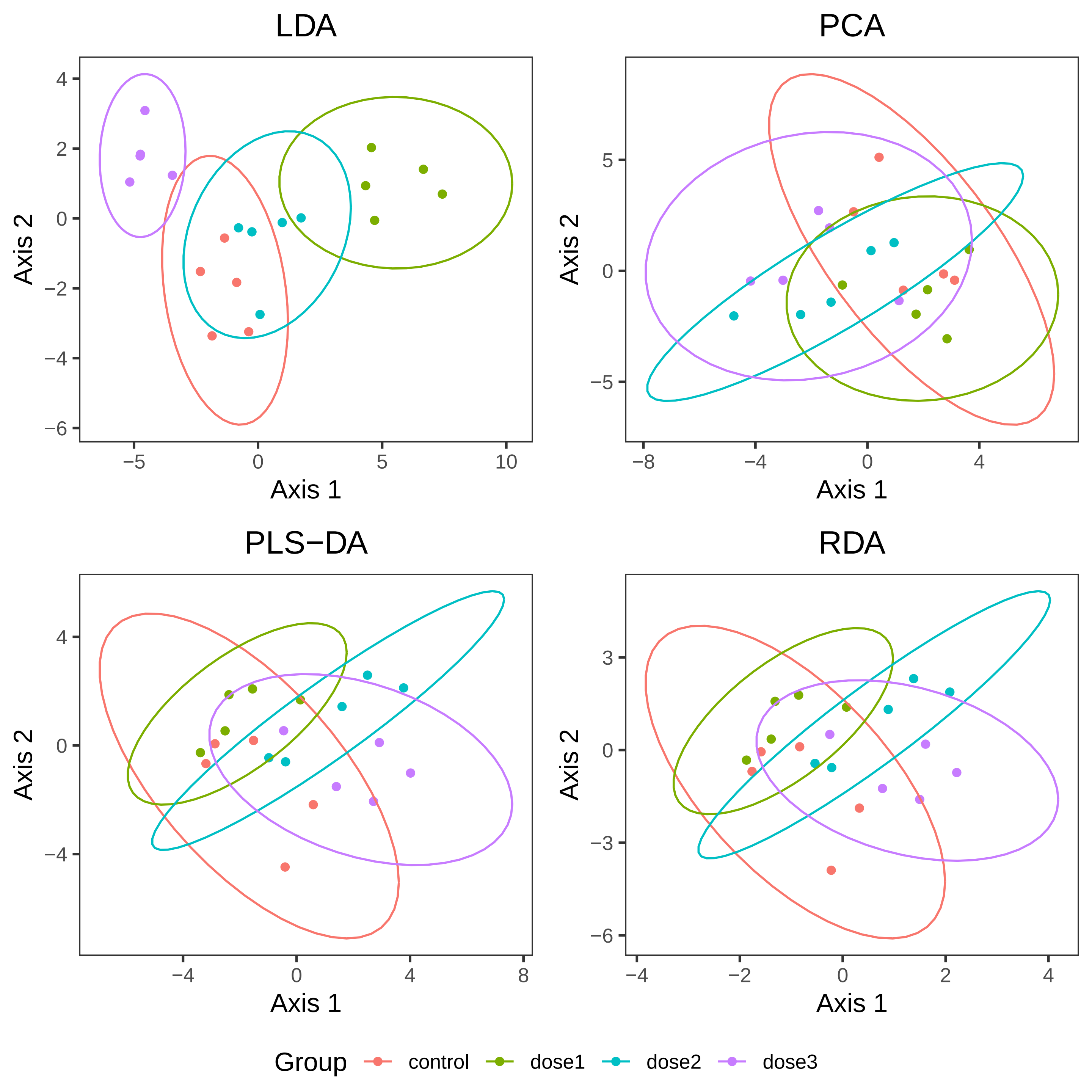
LDA
PCA
PLS−DA
RDA
Axis 1
Axis 2
Group
control
dose1
dose2
dose3

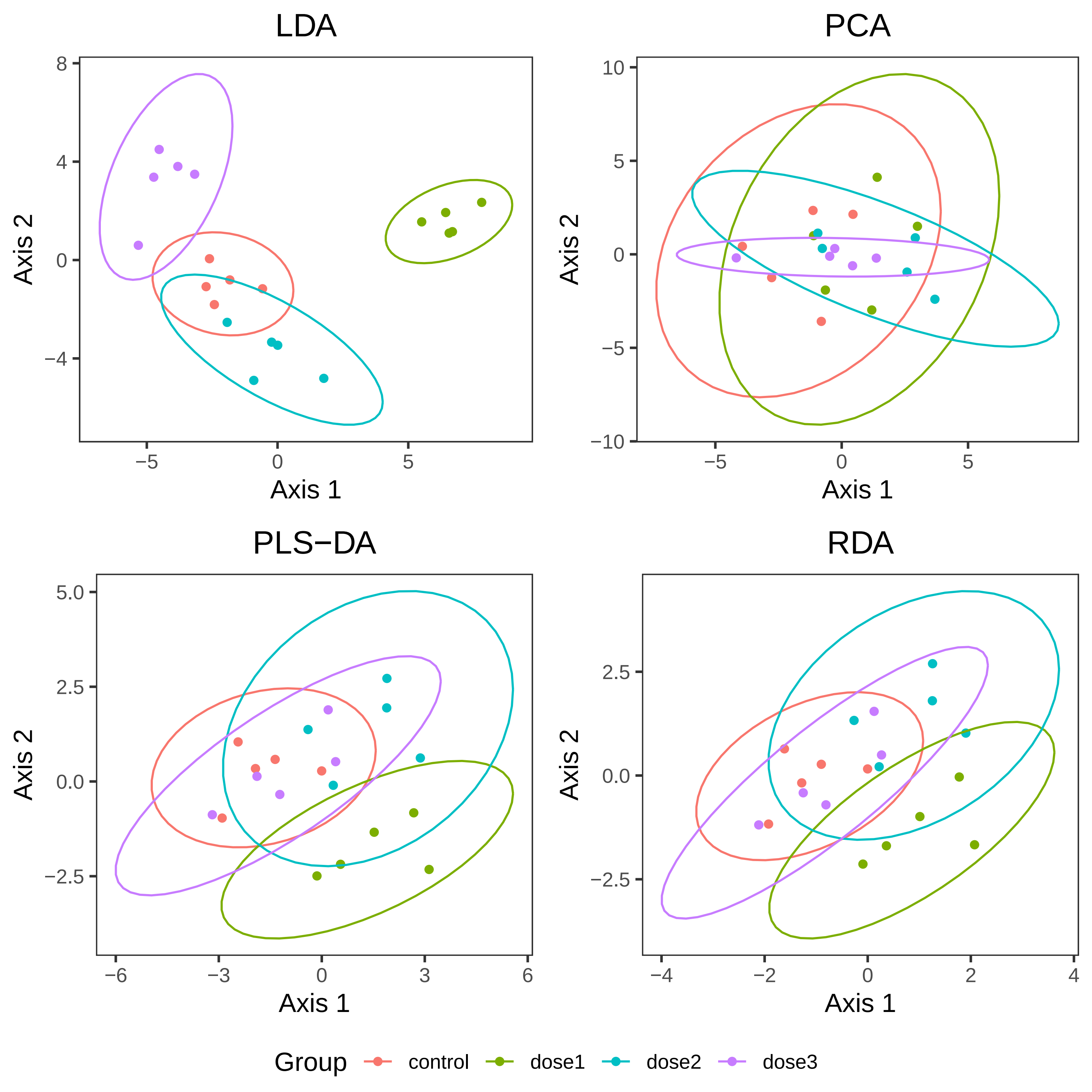
LDA
PCA
PLS−DA
RDA
Axis 1
Axis 2
Group
control
dose1
dose2
dose3

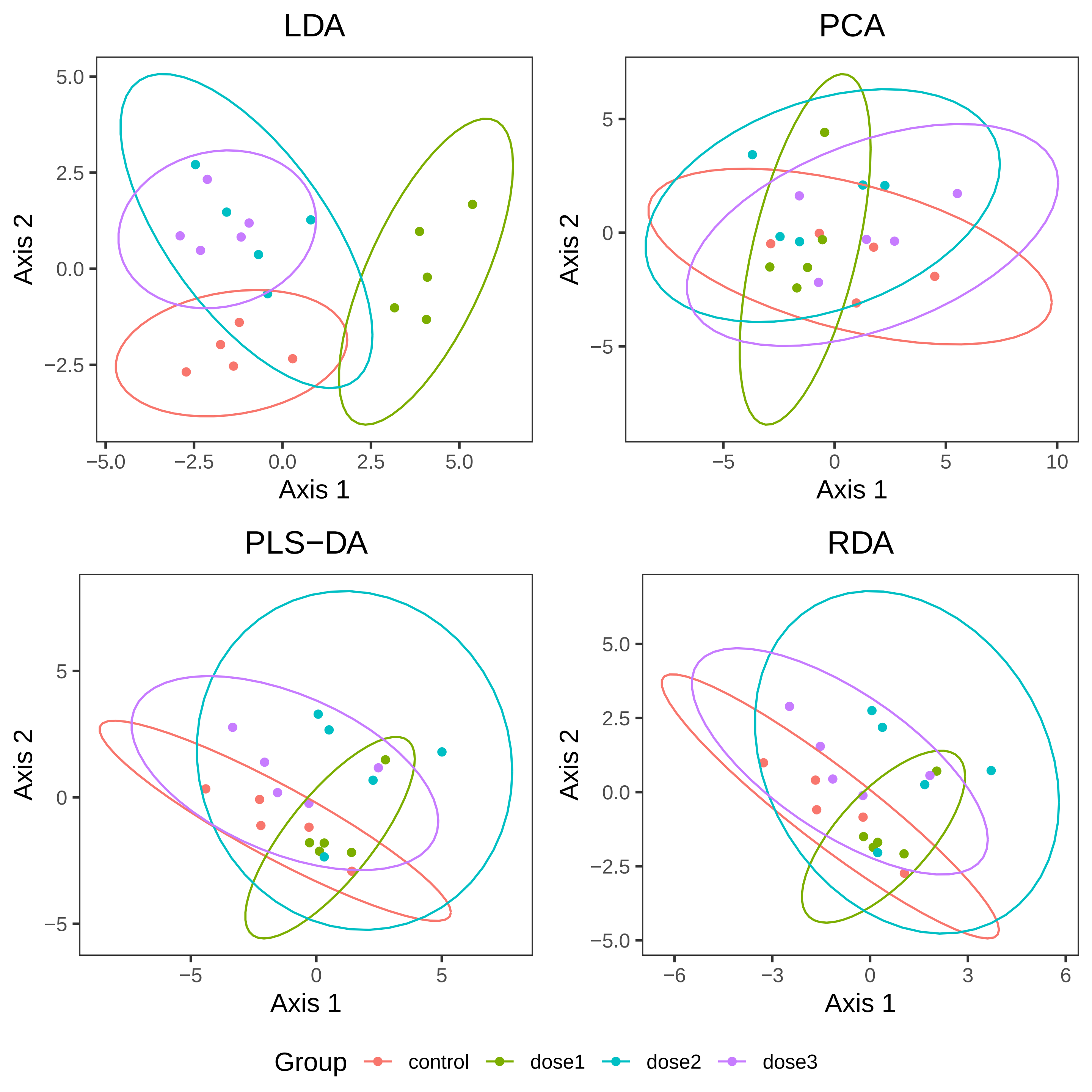
LDA
PCA
PLS−DA
RDA
Axis 1
Axis 2
Group
control
dose1
dose2
dose3

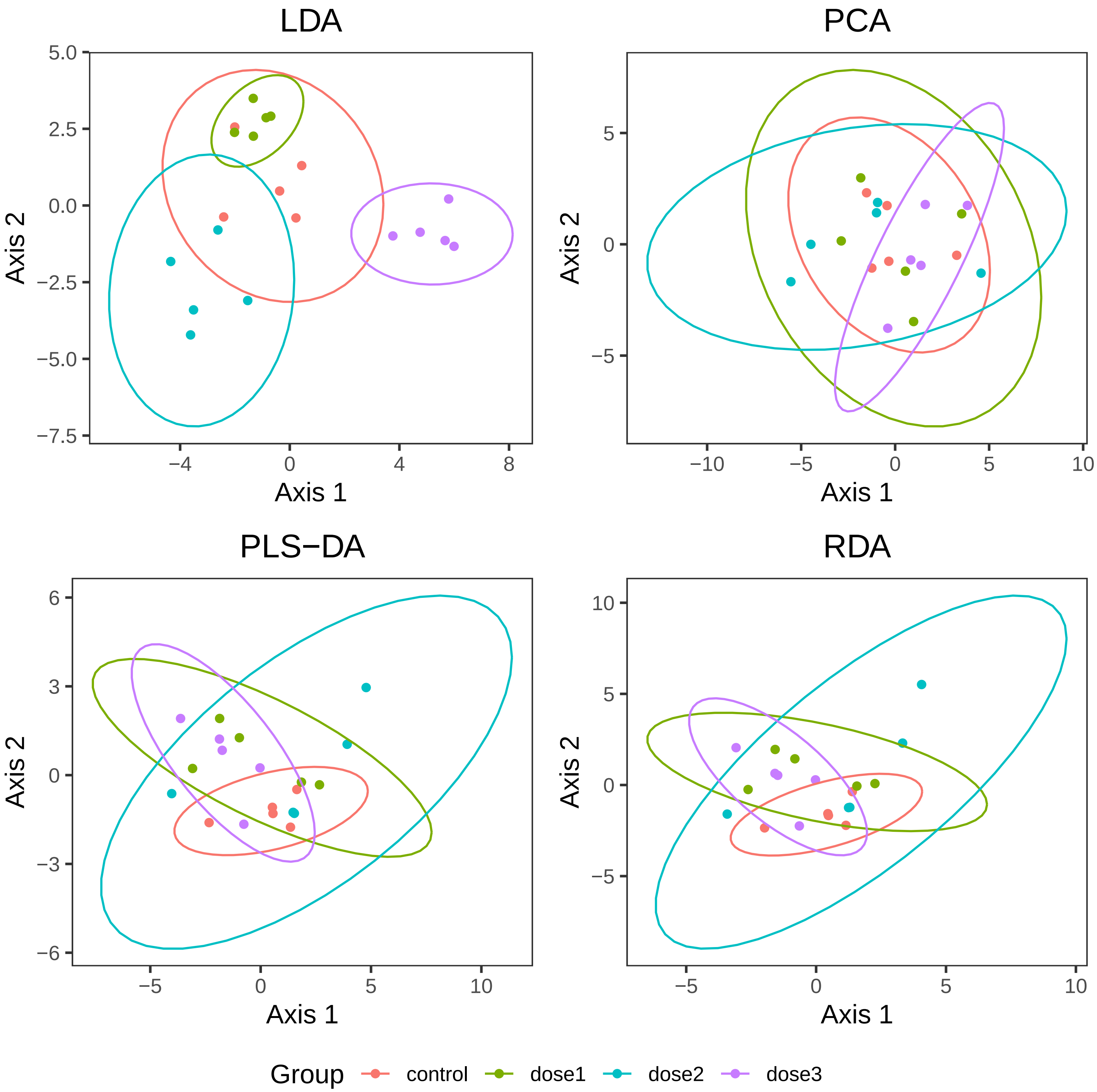
LDA
PCA
PLS−DA
RDA
Axis 1
Axis 2
5.0
2.5
0.0
−2.5
−5.0
−7.5
−4
0
4
8
5
0
−5
−10
10
6
3
−3
−6
Group
control
dose1
dose2
dose3

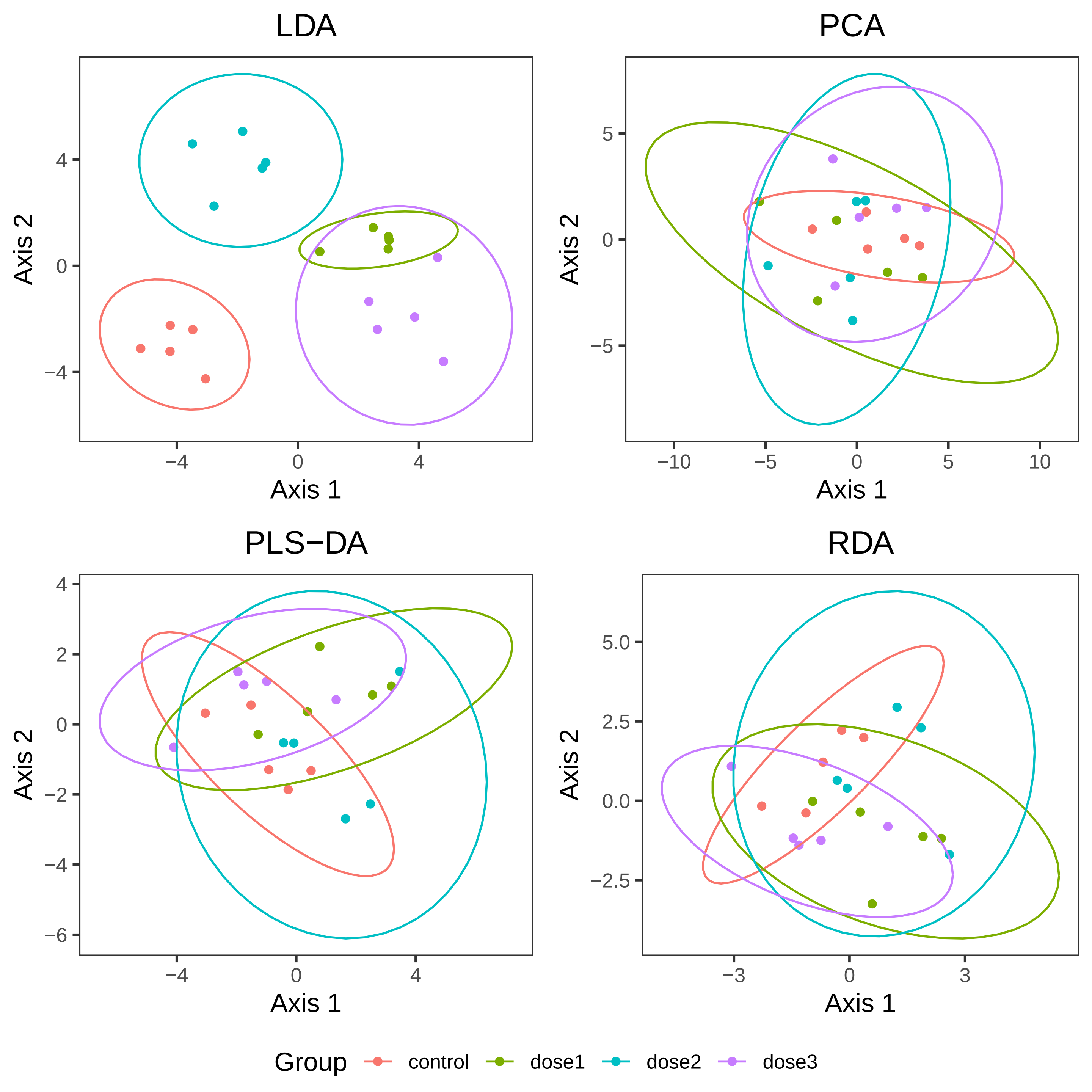
LDA
PCA
PLS−DA
RDA
Axis 1
Axis 2
Group
control
dose1
dose2
dose3

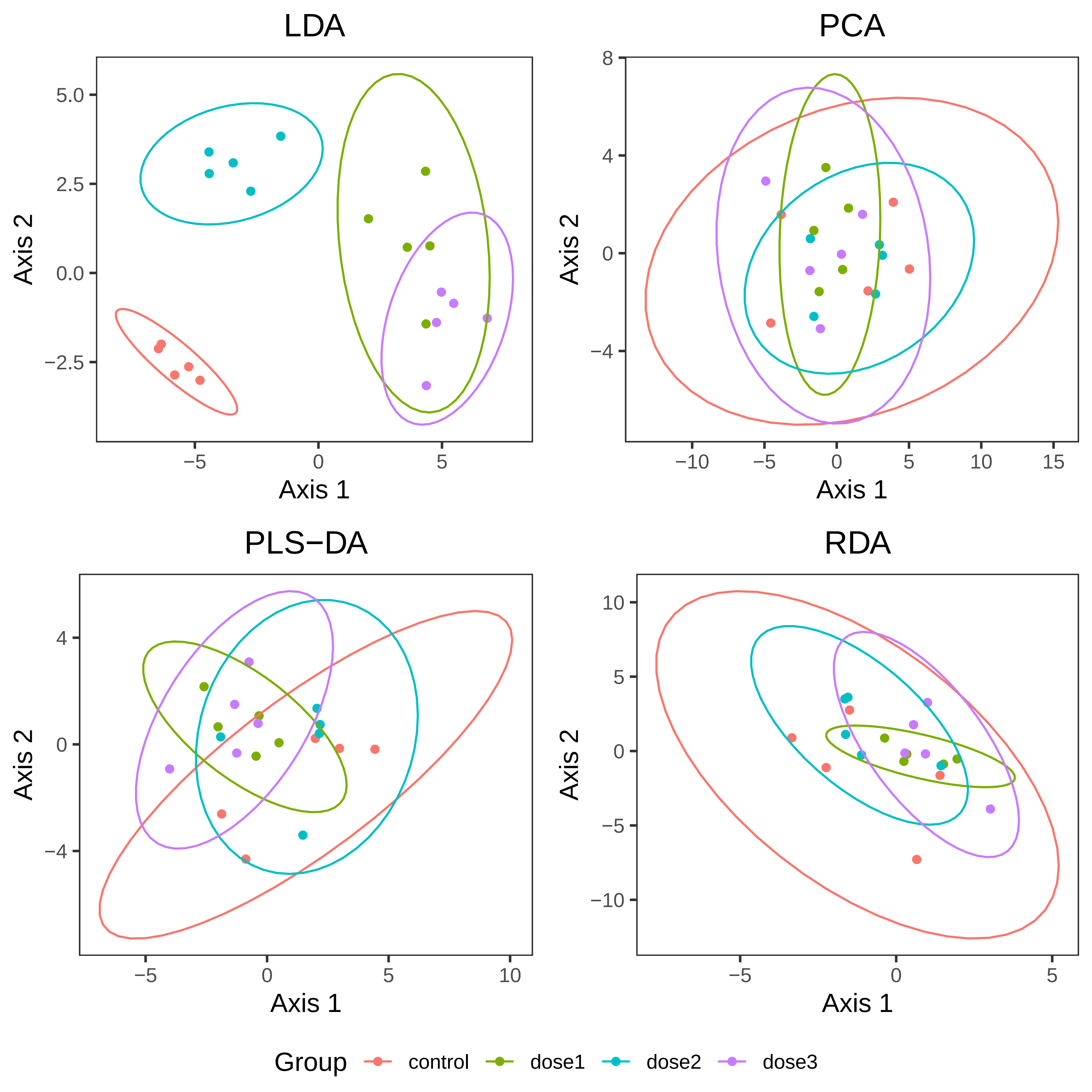
LDA
PCA
PLS−DA
RDA
Axis 1
Axis 2
5.0
2.5
0.0
−2.5
−5
0
5
8
4
0
−4
−10
−5
0
5
10
15
4
0
−4
−5
0
5
10
10
5
0
−5
−10
−5
0
5
Group
control
dose1
dose2
dose3

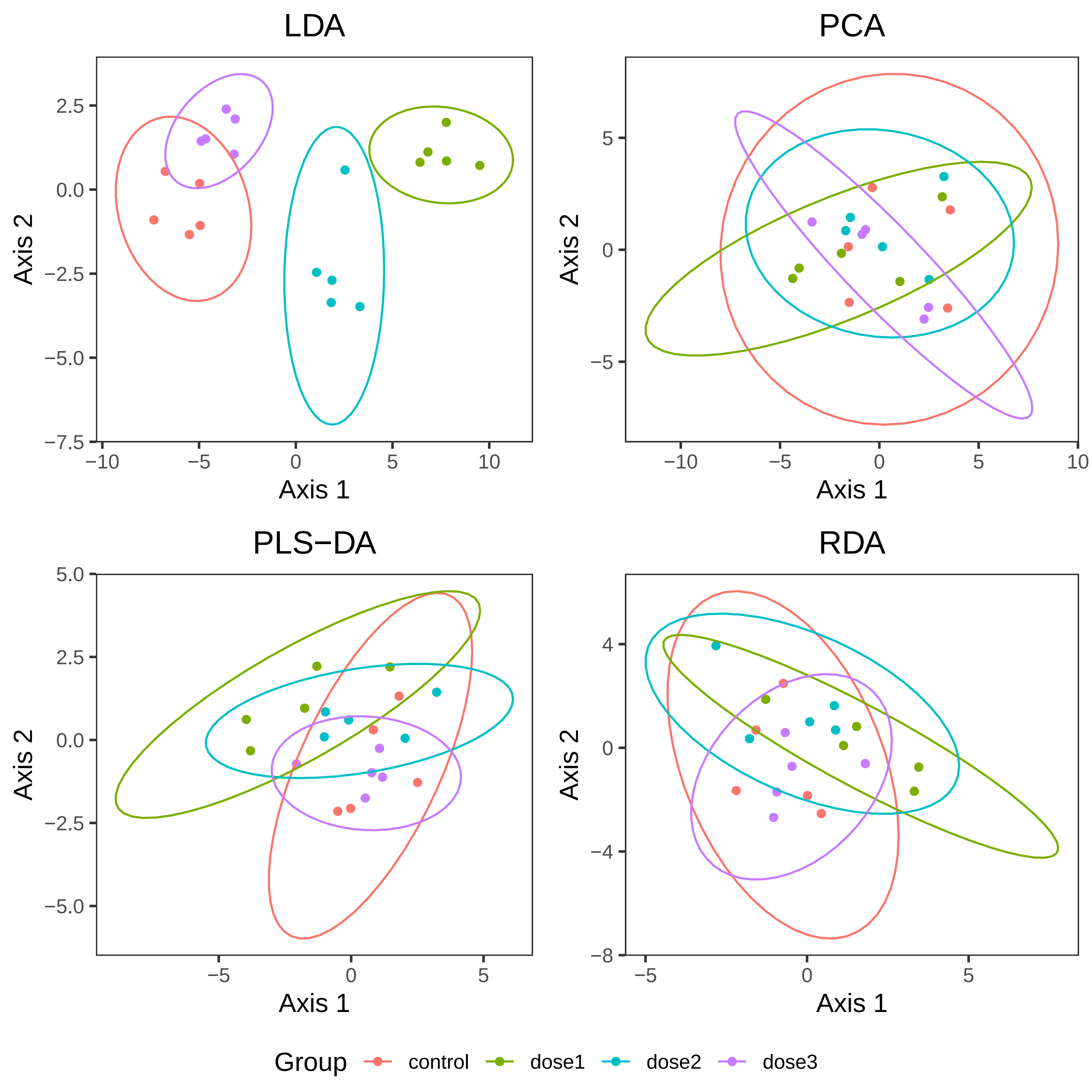
LDA
PCA
PLS−DA
RDA
Axis 1
Axis 2
Group
control
dose1
dose2
dose3

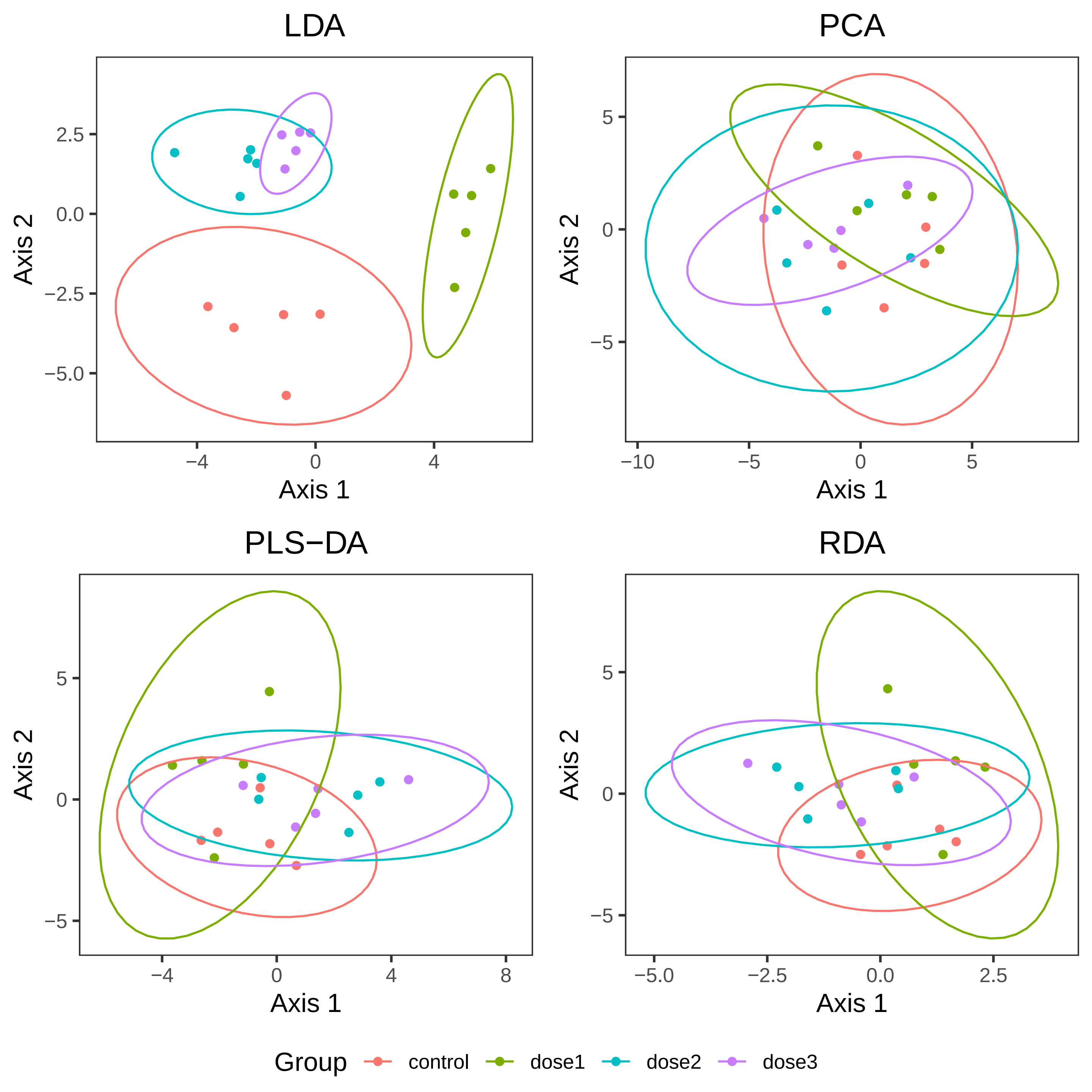
LDA
PCA
PLS−DA
RDA
Axis 1
Axis 2
2.5
0.0
−2.5
−5.0
−4
0
4
5
−5
−10
8
−5.0
Group
control
dose1
dose2
dose3

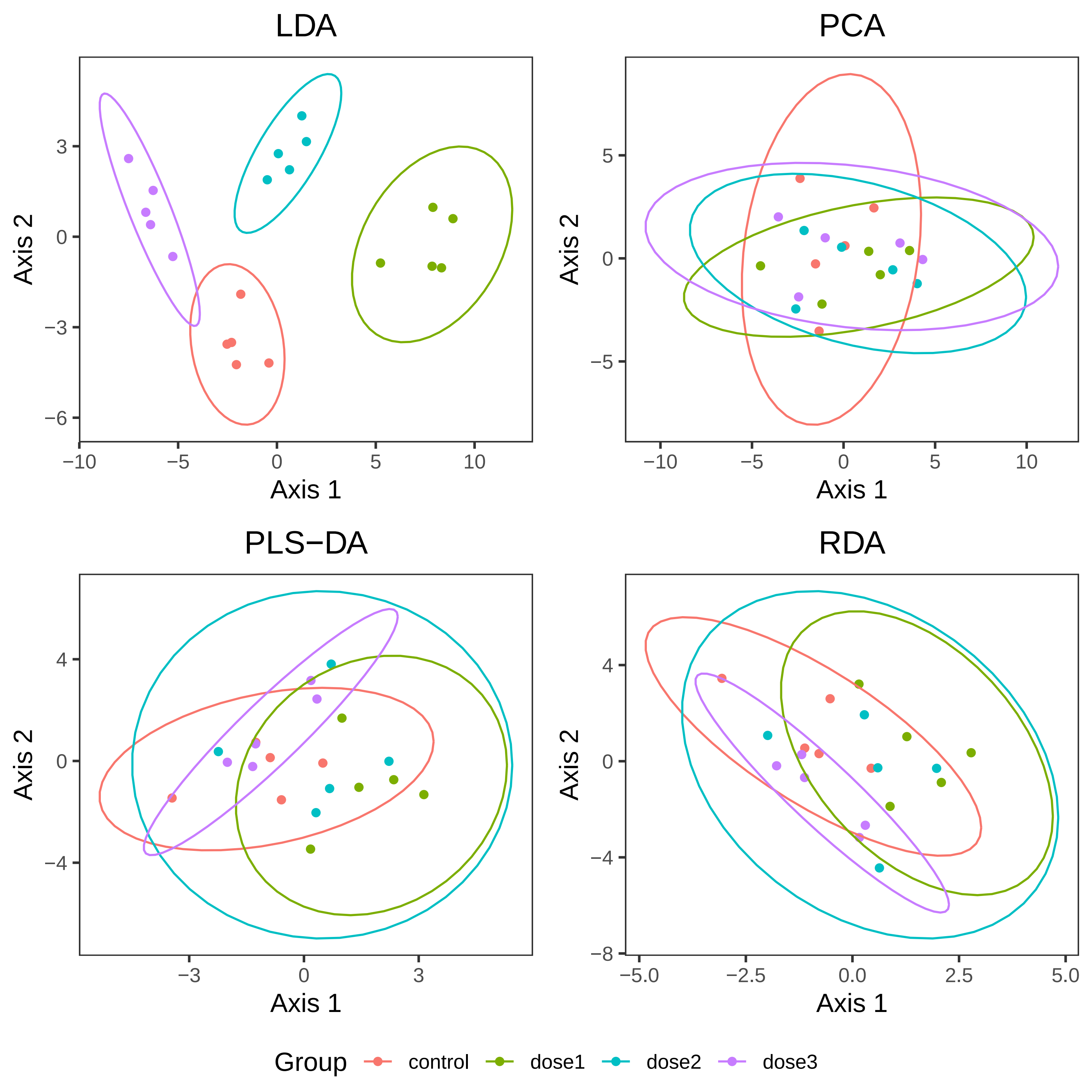
LDA
PCA
PLS−DA
RDA
Axis 1
Axis 2
Group
control
dose1
dose2
dose3

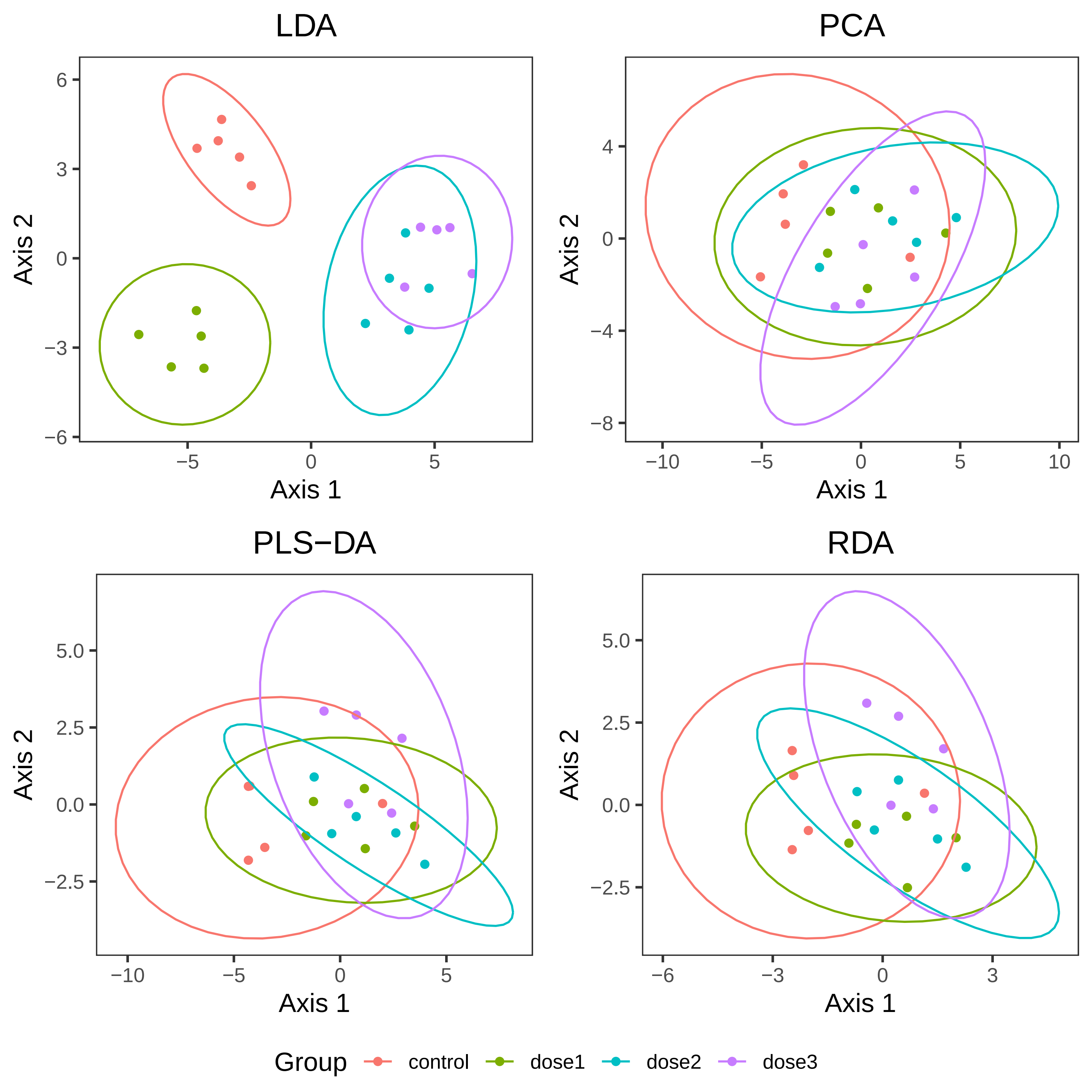
LDA
PCA
PLS−DA
RDA
Axis 1
Axis 2
Group
control
dose1
dose2
dose3

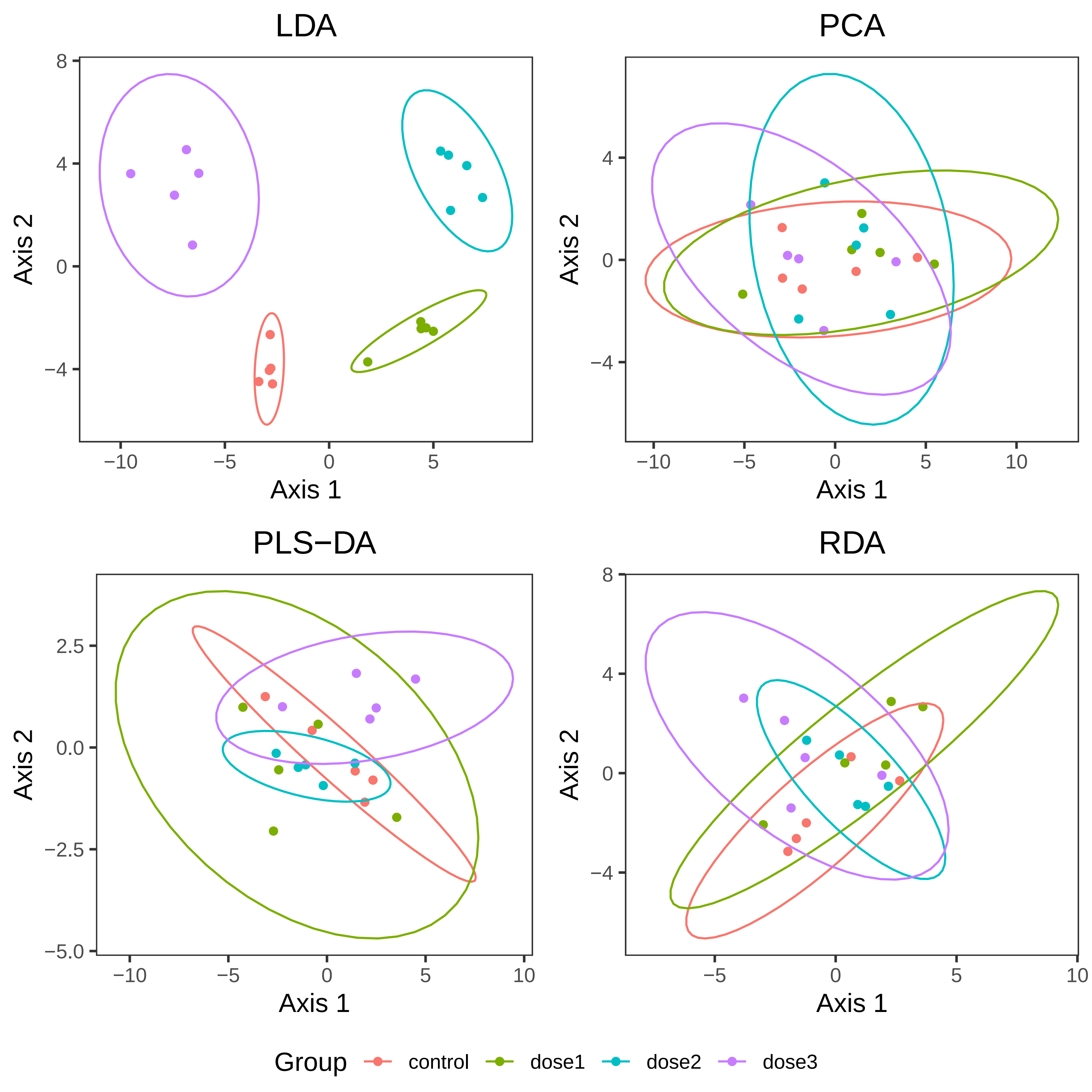
LDA
PCA
PLS−DA
RDA
Axis 1
Axis 2
Group
control
dose1
dose2
dose3

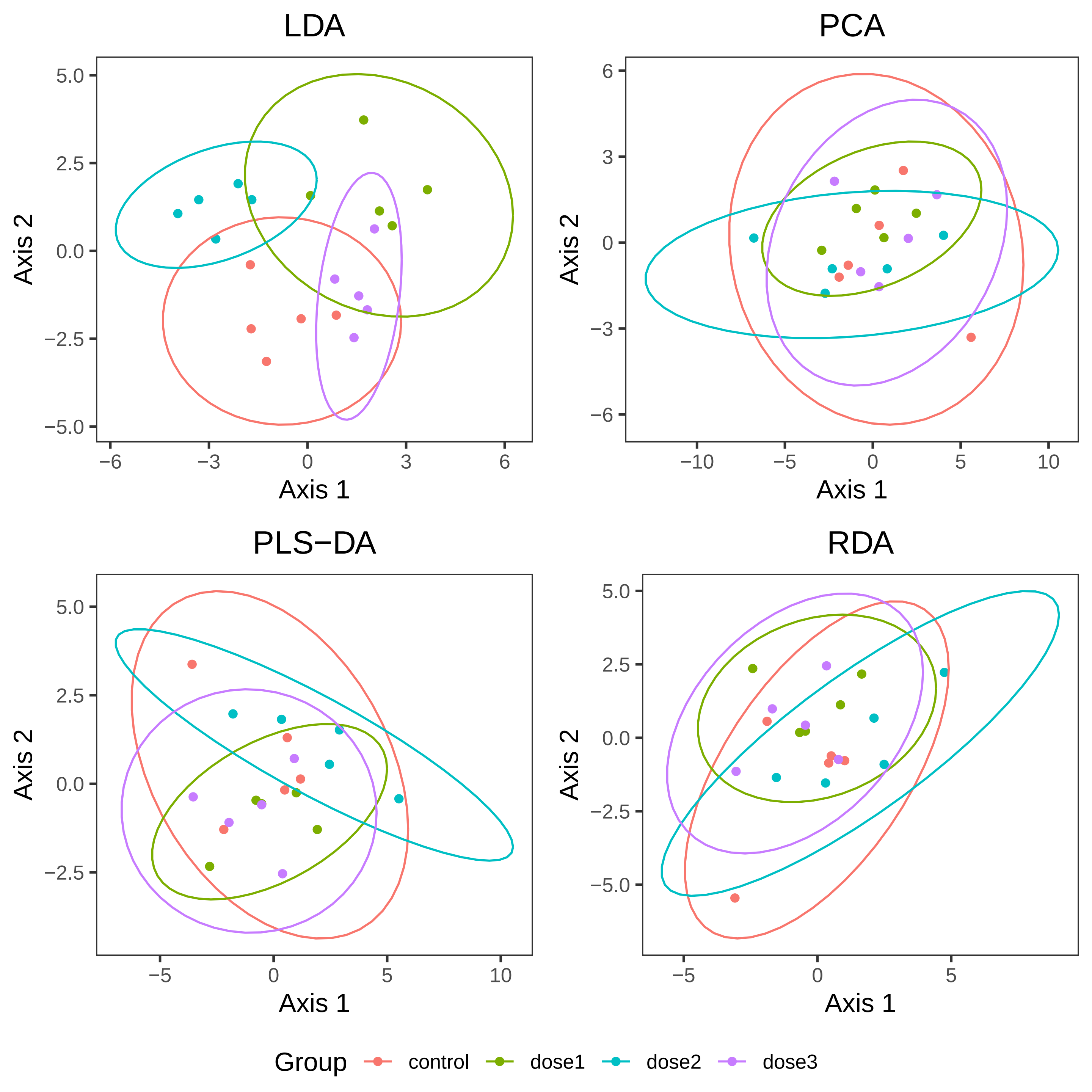

LDA
PCA
PLS−DA
RDA
Axis 1
Axis 2
5.0
2.5
0.0
−2.5
−5.0
−6
−3
0
3
6
−10
−5
5
10
Group
control
dose1
dose2
dose3

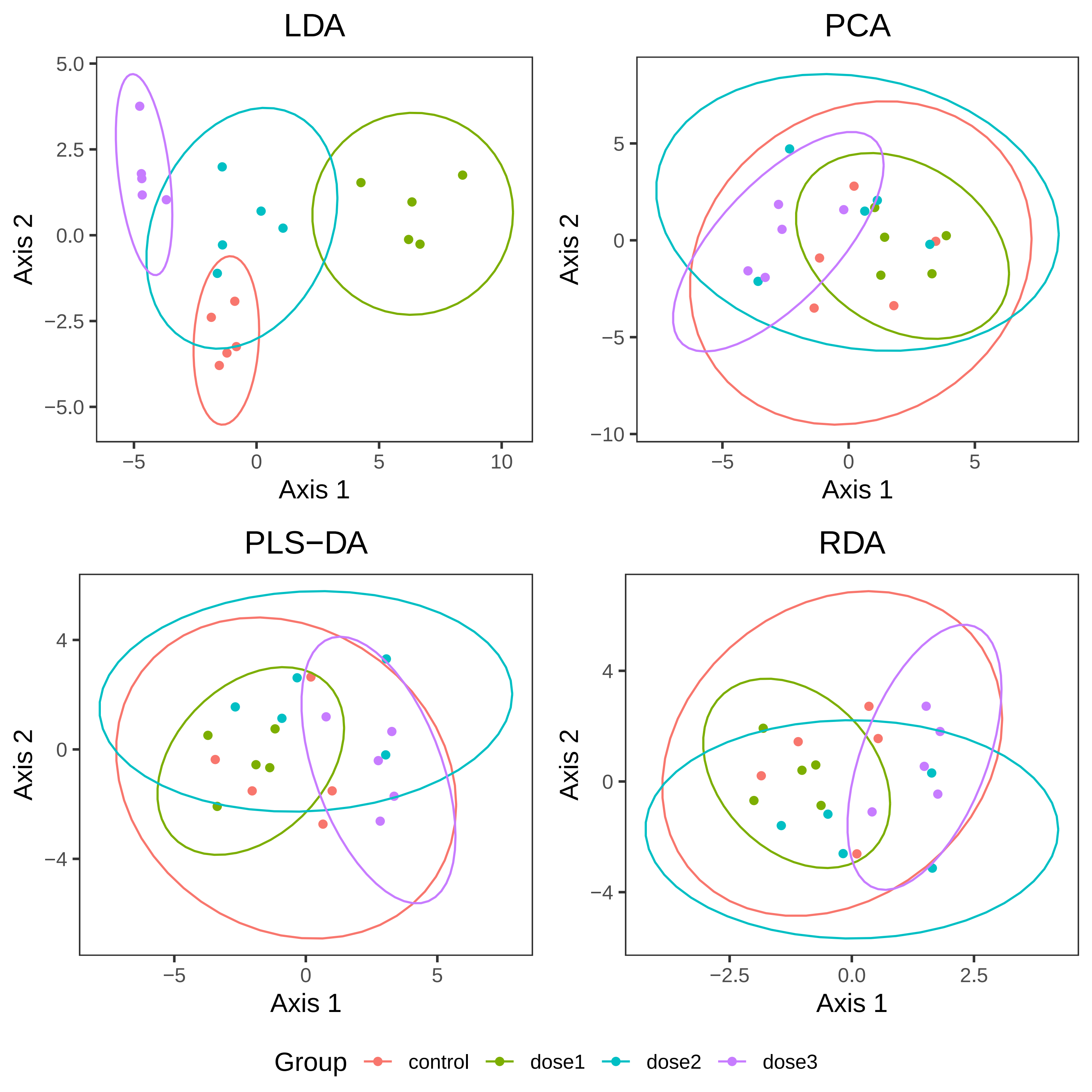

LDA
PCA
PLS−DA
RDA
Axis 1
Axis 2
Group
control
dose1
dose2
dose3

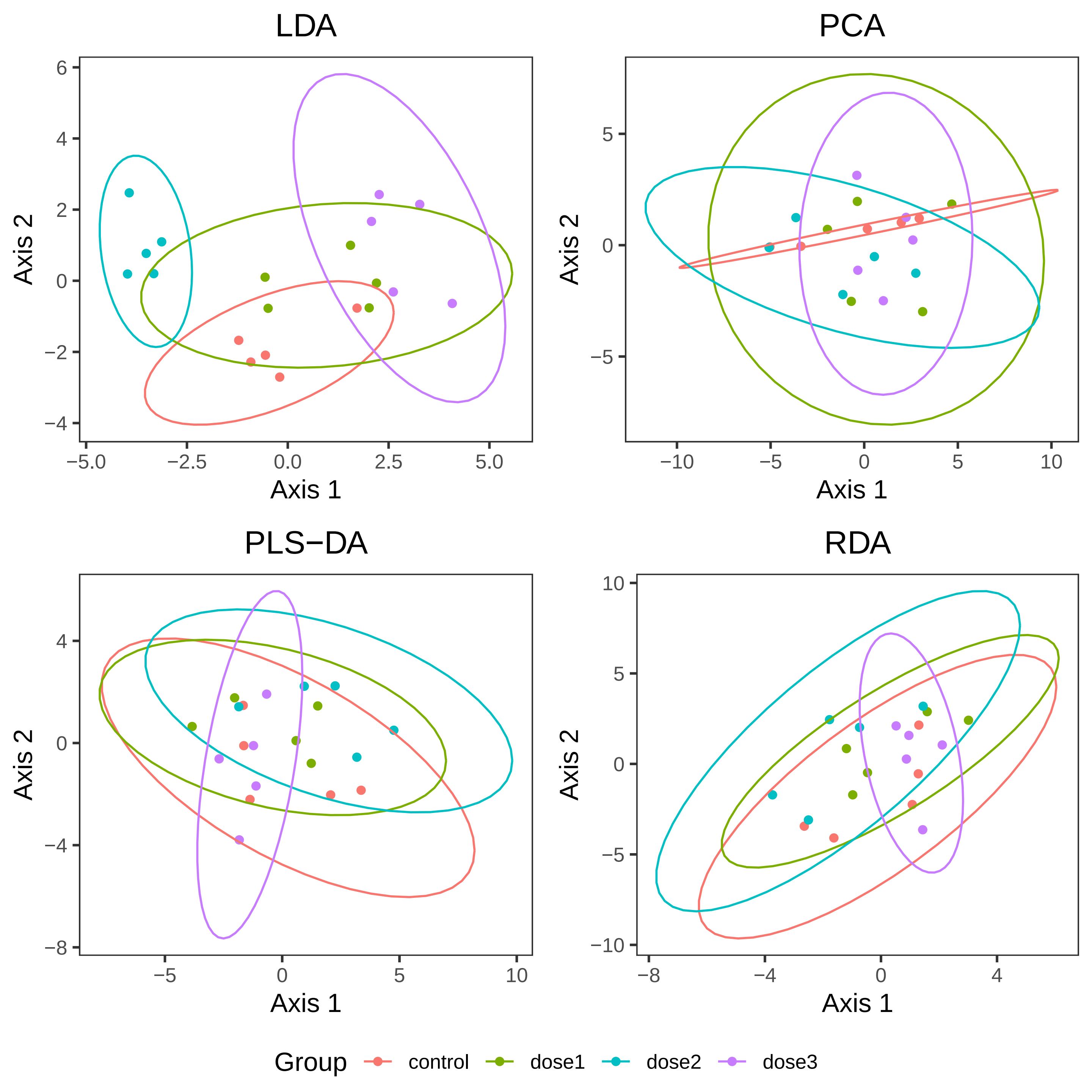
LDA
PCA
PLS−DA
RDA
Axis 1
Axis 2
Group
control
dose1
dose2
dose3

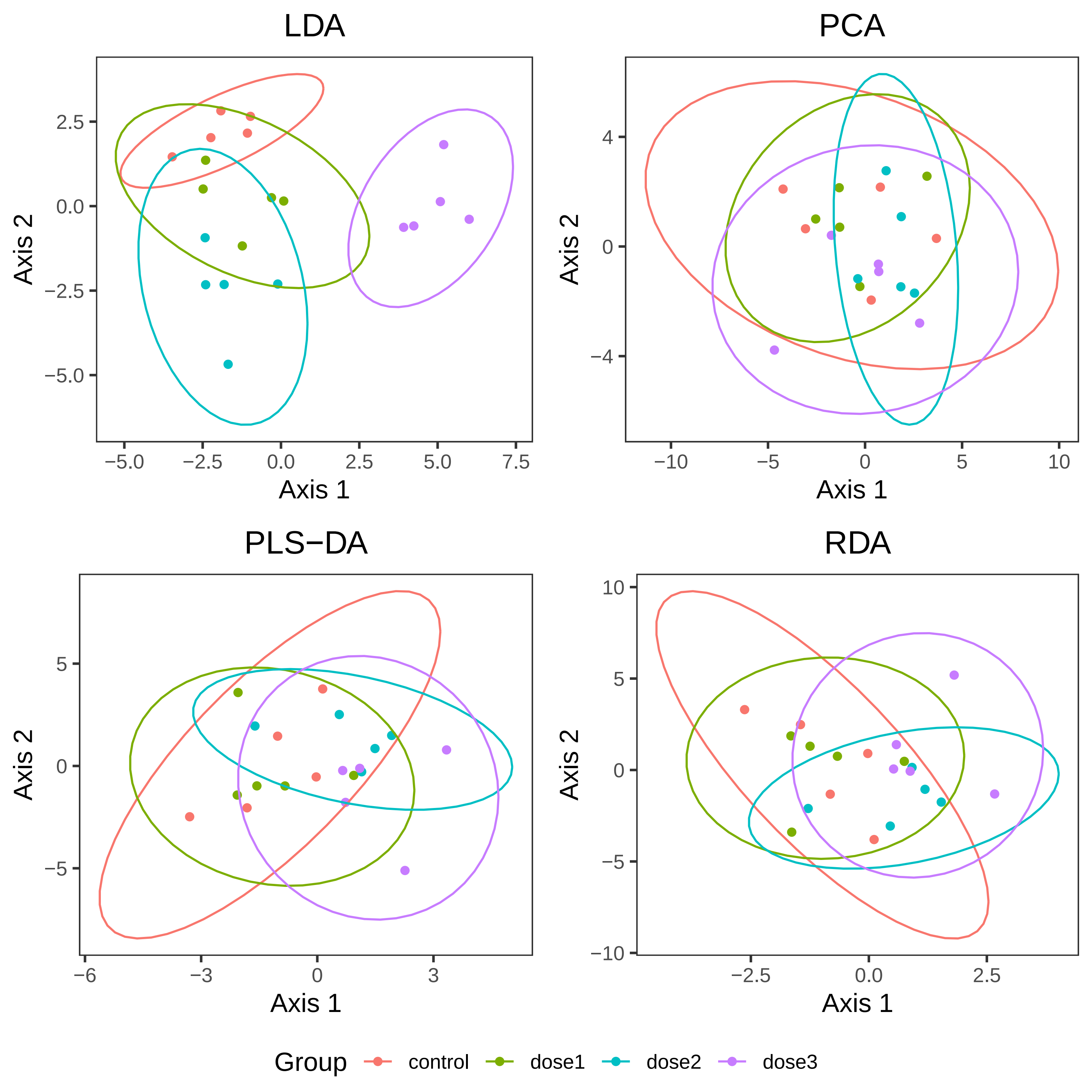
LDA
PCA
PLS−DA
RDA
Axis 1
Axis 2
Group
control
dose1
dose2
dose3

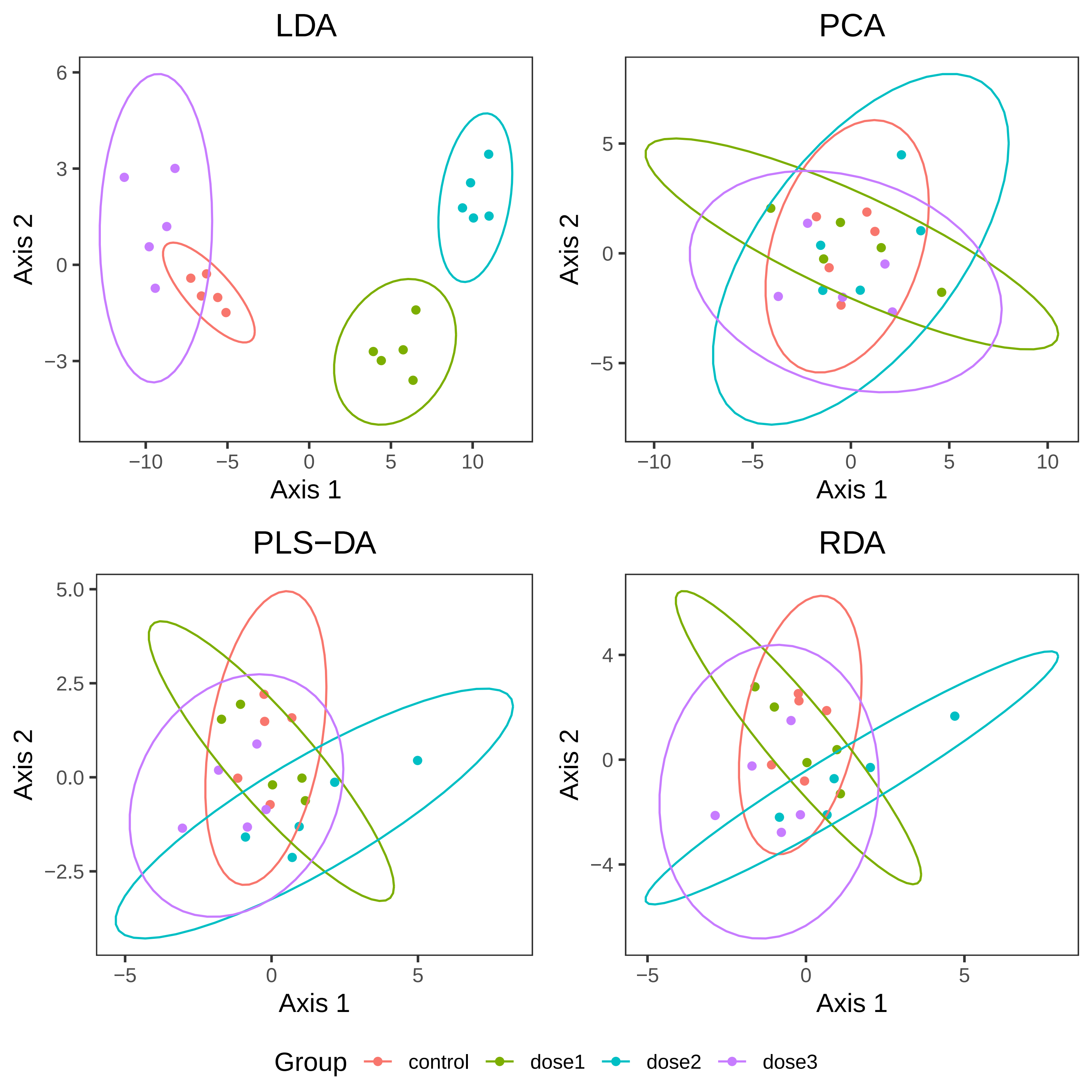
LDA
PCA
PLS−DA
RDA
Axis 1
Axis 2
6
3
0
−3
−10
−5
0
5
10
5.0
2.5
0.0
−2.5
4
−4
Group
control
dose1
dose2
dose3

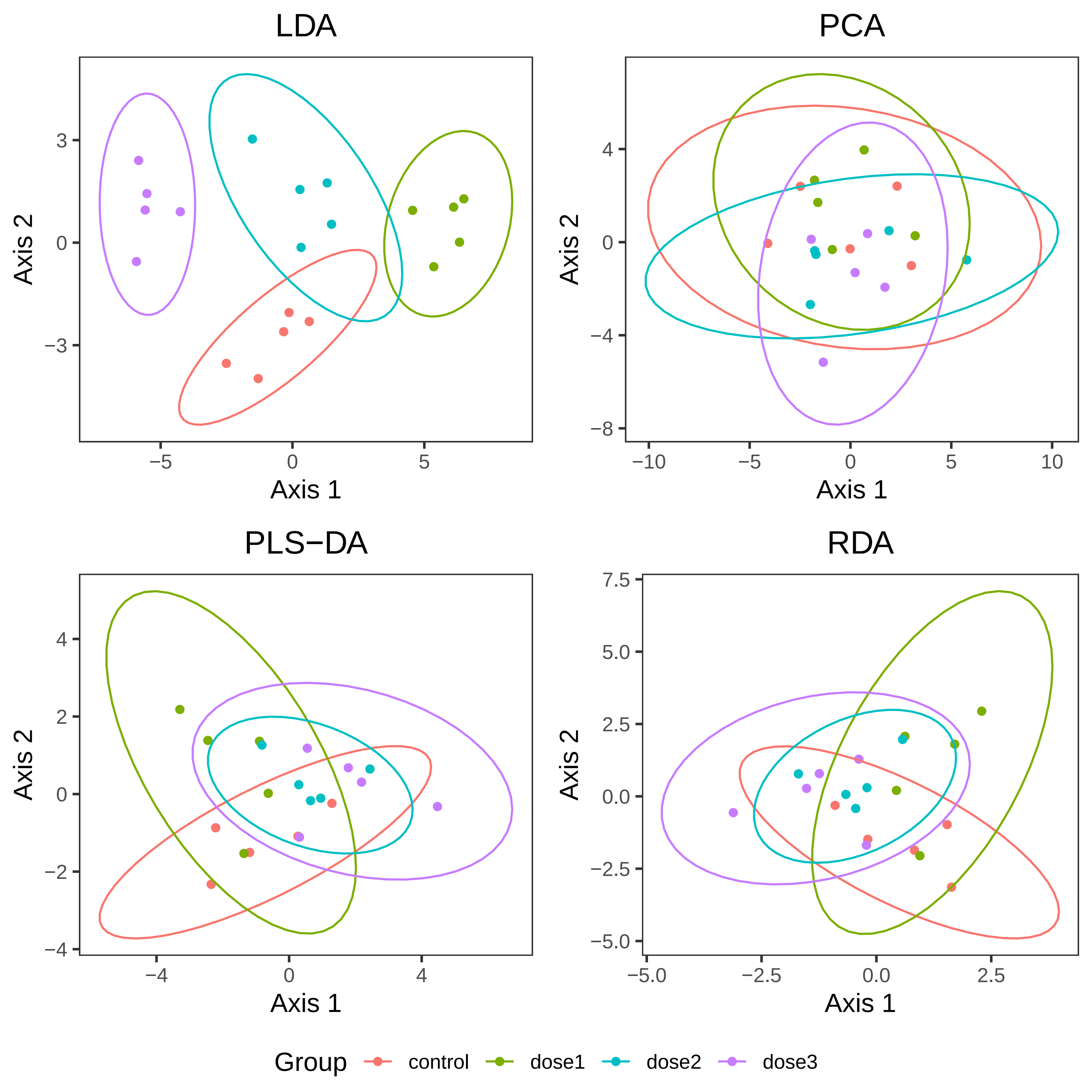
LDA
PCA
PLS−DA
RDA
Axis 1
Axis 2
−5
0
5
10
−10
3
−3
4
−4
−8
2
−2
7.5
5.0
2.5
0.0
−2.5
−5.0
Group
control
dose1
dose2
dose3

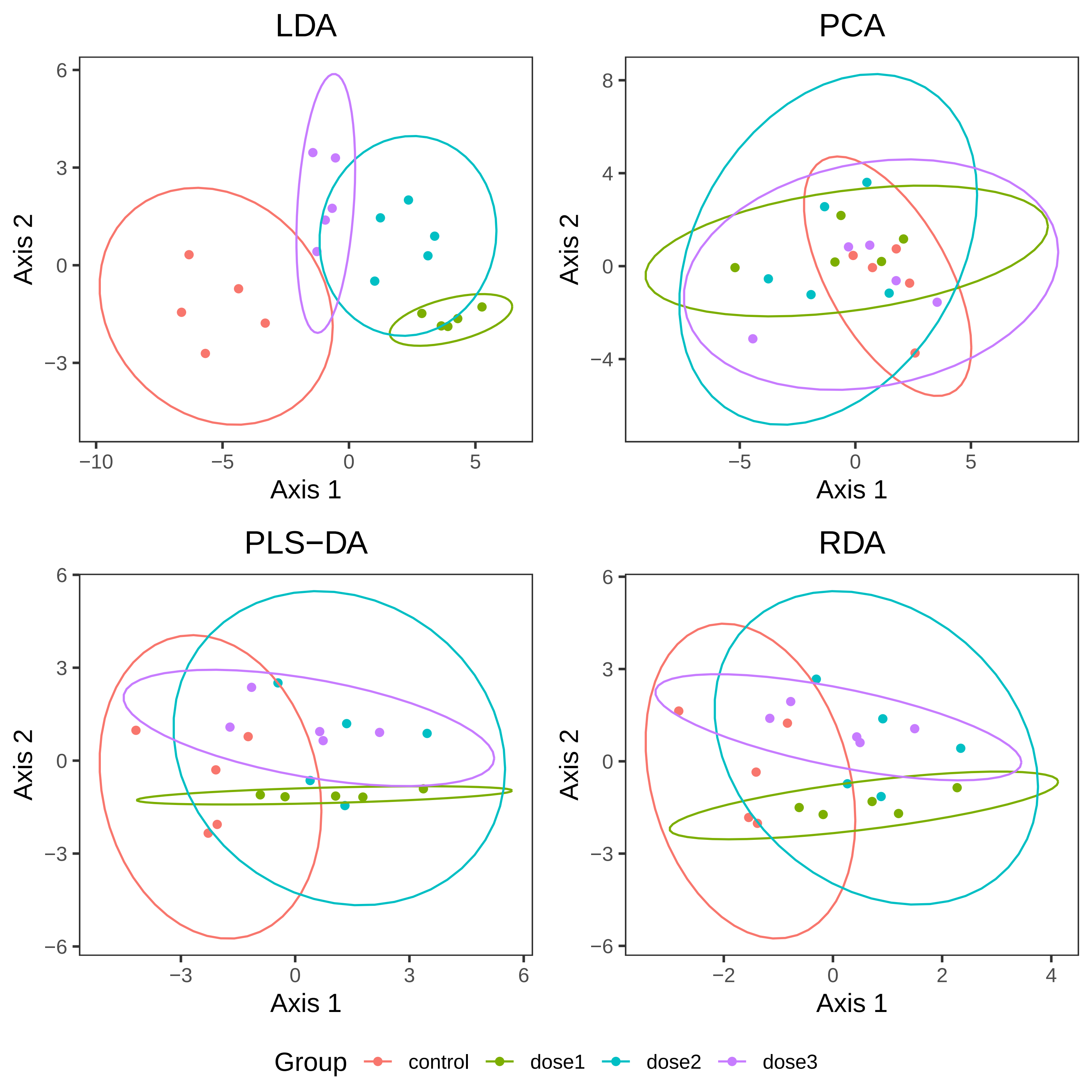

LDA
PCA
PLS−DA
RDA
Axis 1
Axis 2
Group
control
dose1
dose2
dose3

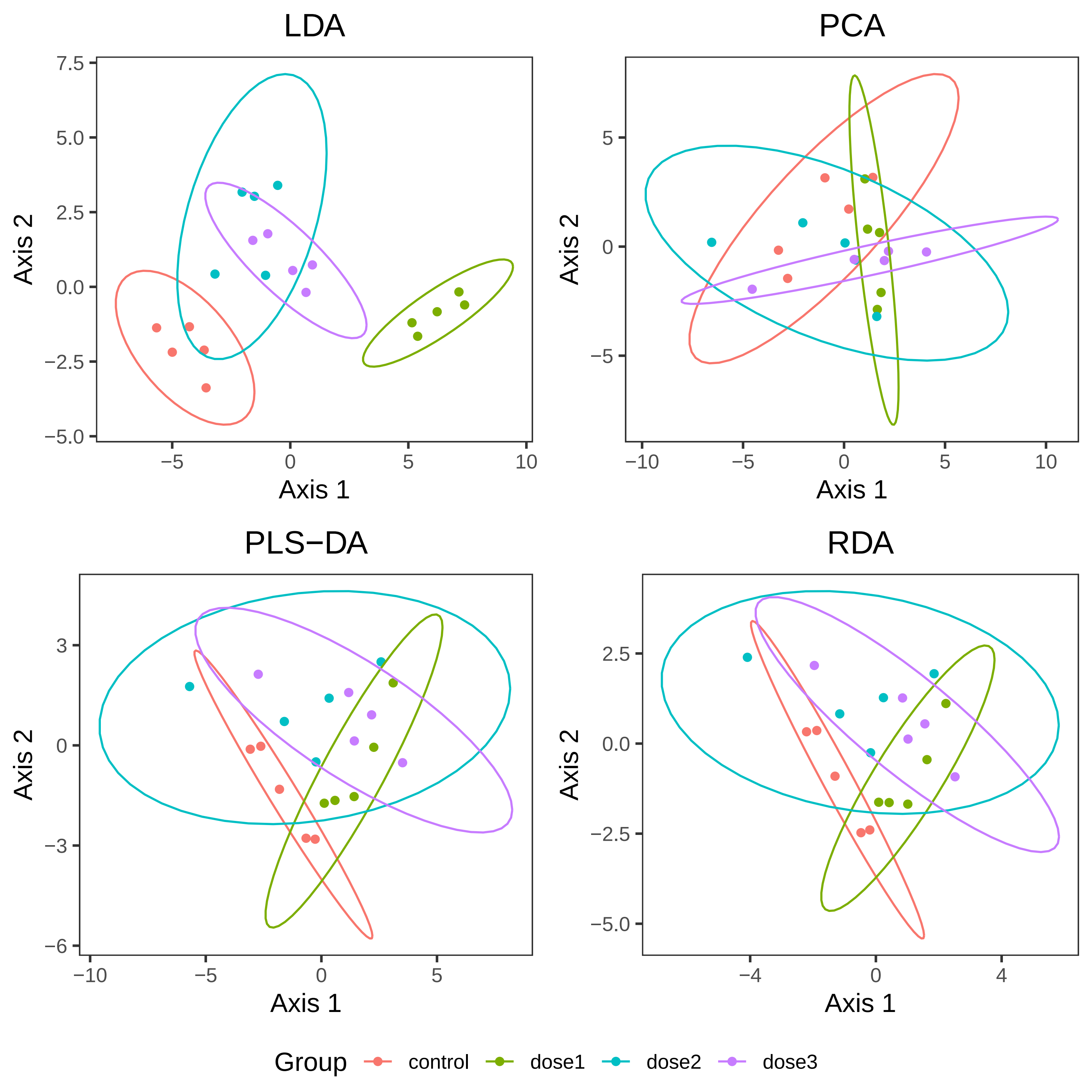
LDA
PCA
PLS−DA
RDA
Axis 1
Axis 2
7.5
5.0
2.5
0.0
−2.5
−5.0
−5
0
5
10
−10
3
−3
−6
−4
4
Group
control
dose1
dose2
dose3

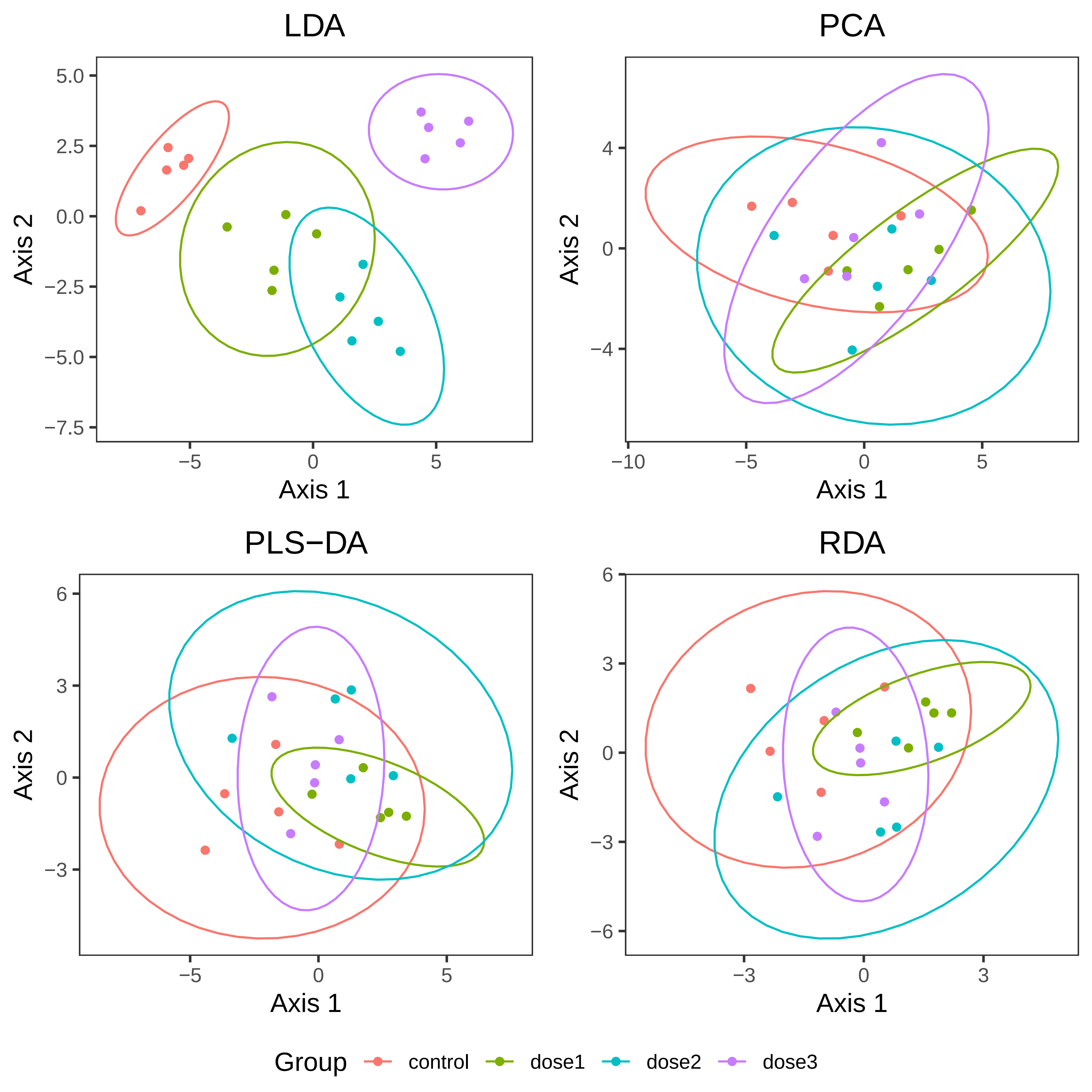

LDA
PCA
PLS−DA
RDA
Axis 1
Axis 2
Group
control
dose1
dose2
dose3

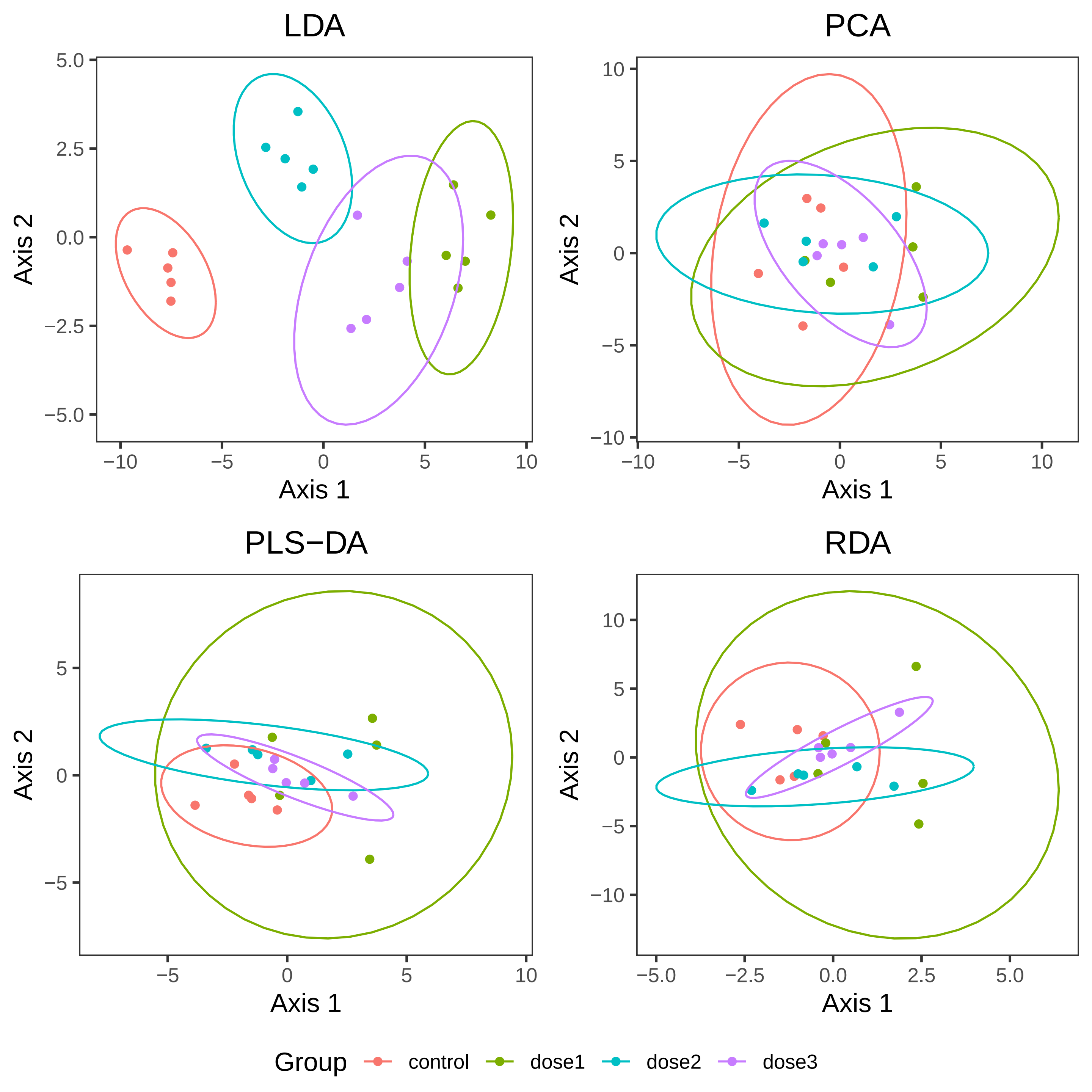
LDA
PCA
PLS−DA
RDA
Axis 1
Axis 2
Group
control
dose1
dose2
dose3

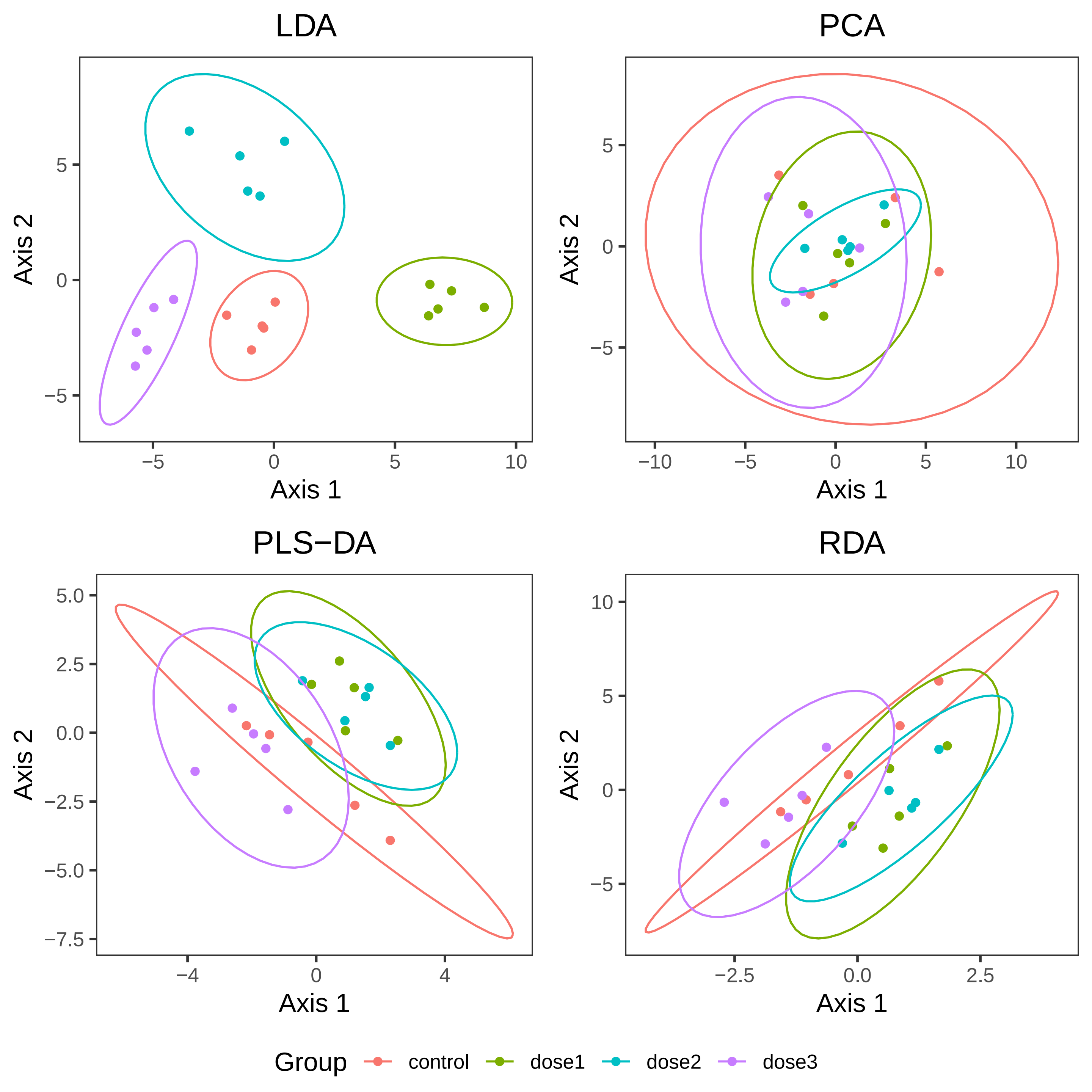

LDA
PCA
PLS−DA
RDA
Axis 1
Axis 2
Group
control
dose1
dose2
dose3

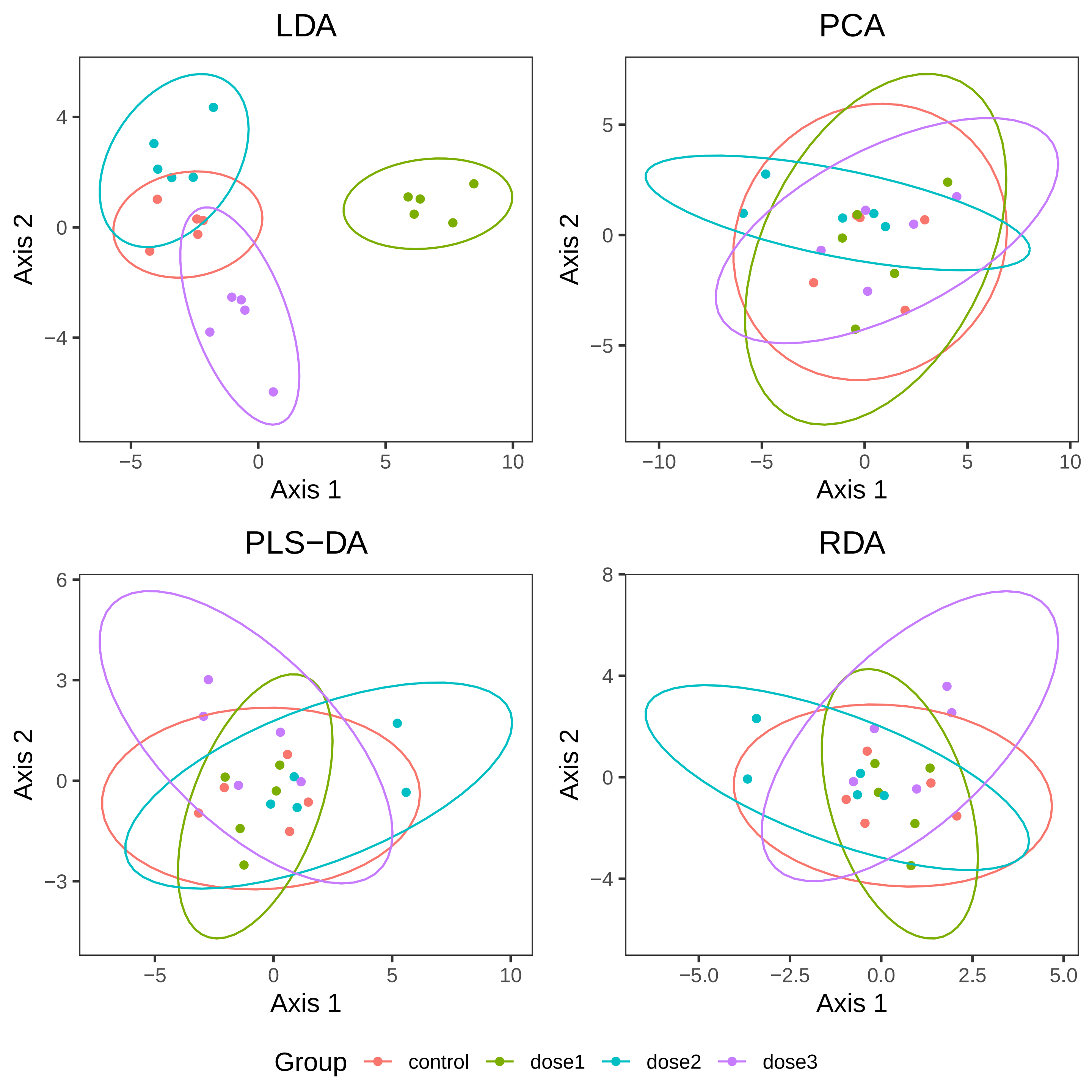

LDA
PCA
PLS−DA
RDA
Axis 1
Axis 2
Group
control
dose1
dose2
dose3

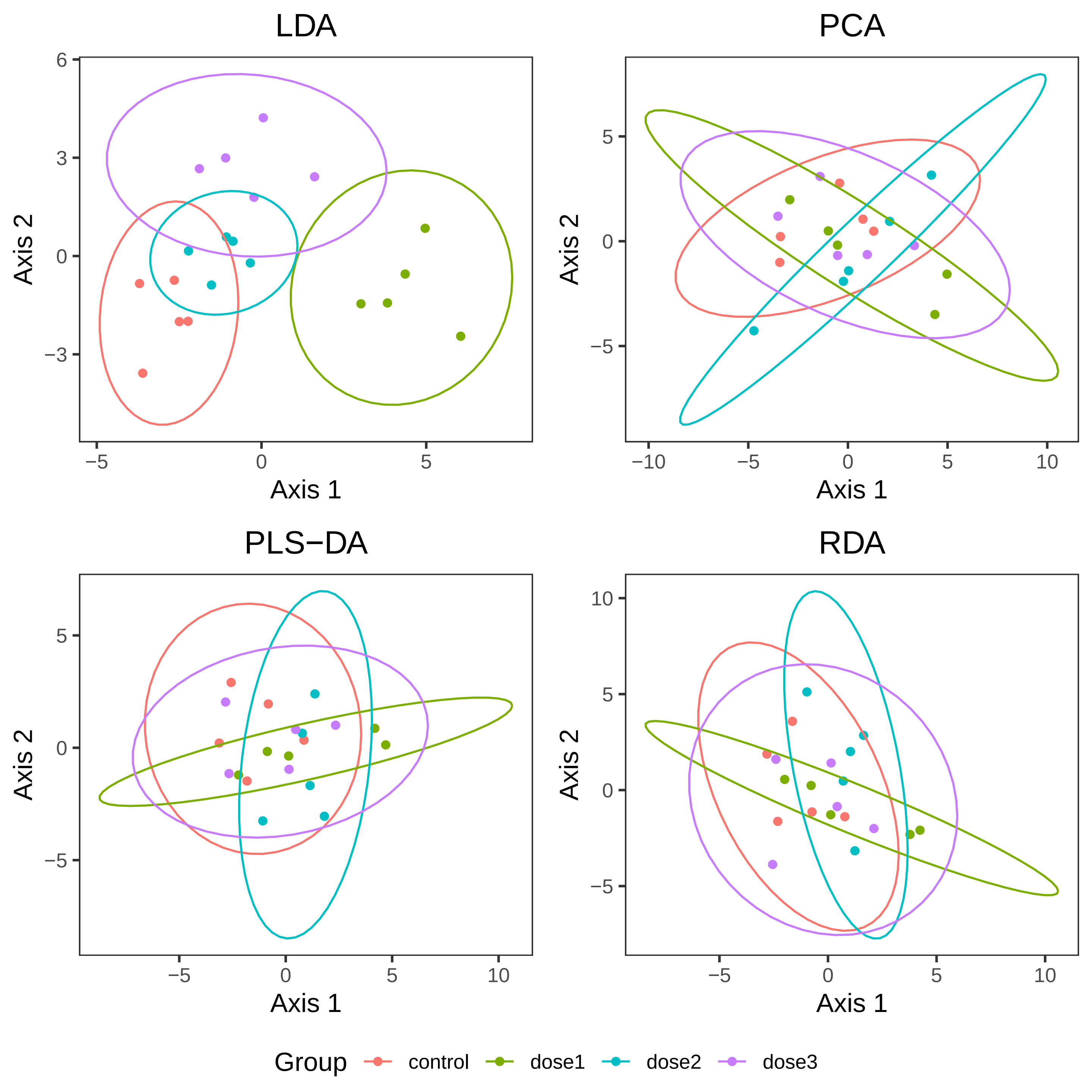
LDA
PCA
PLS−DA
RDA
Axis 1
Axis 2
Group
control
dose1
dose2
dose3

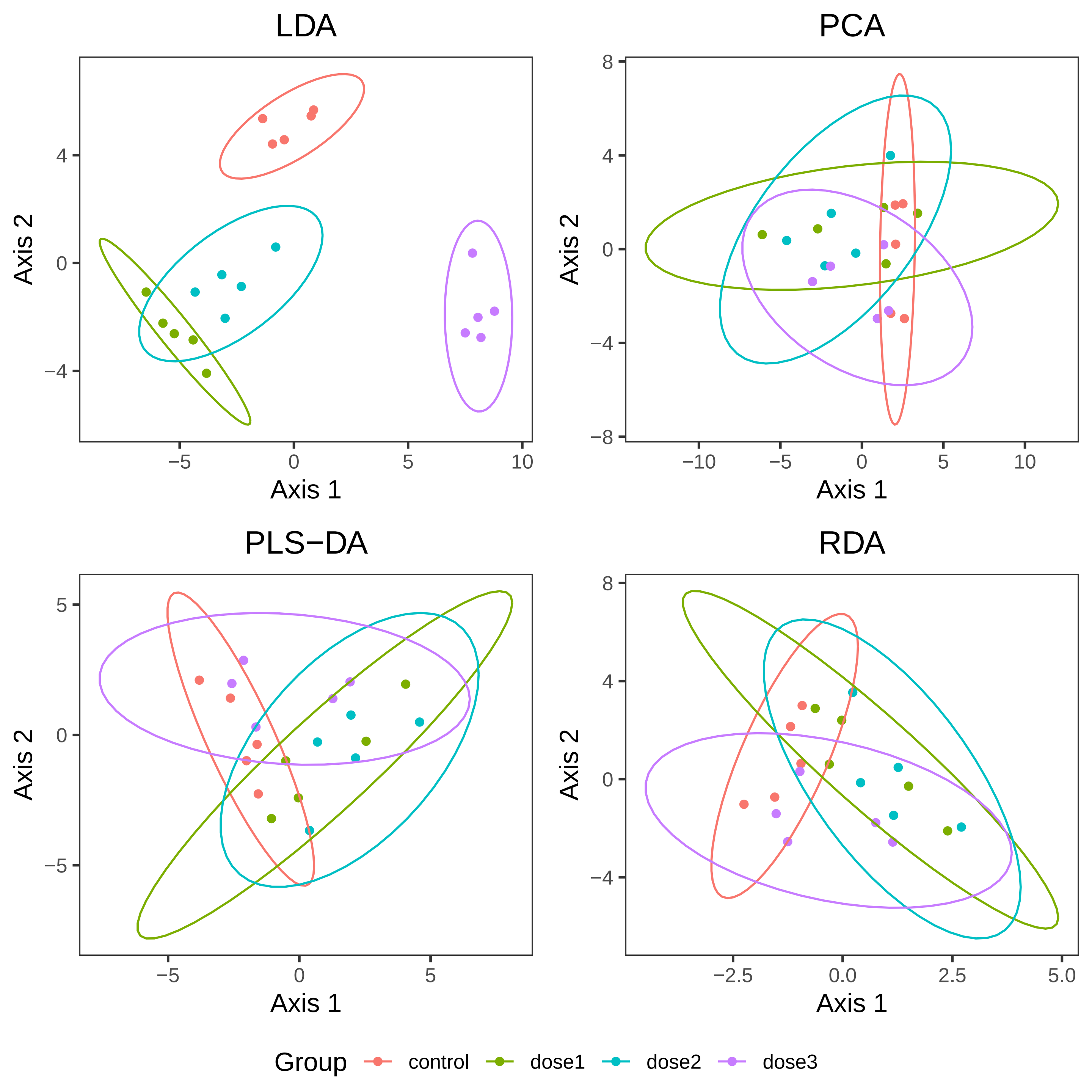

LDA
PCA
PLS−DA
RDA
Axis 1
Axis 2
Group
control
dose1
dose2
dose3

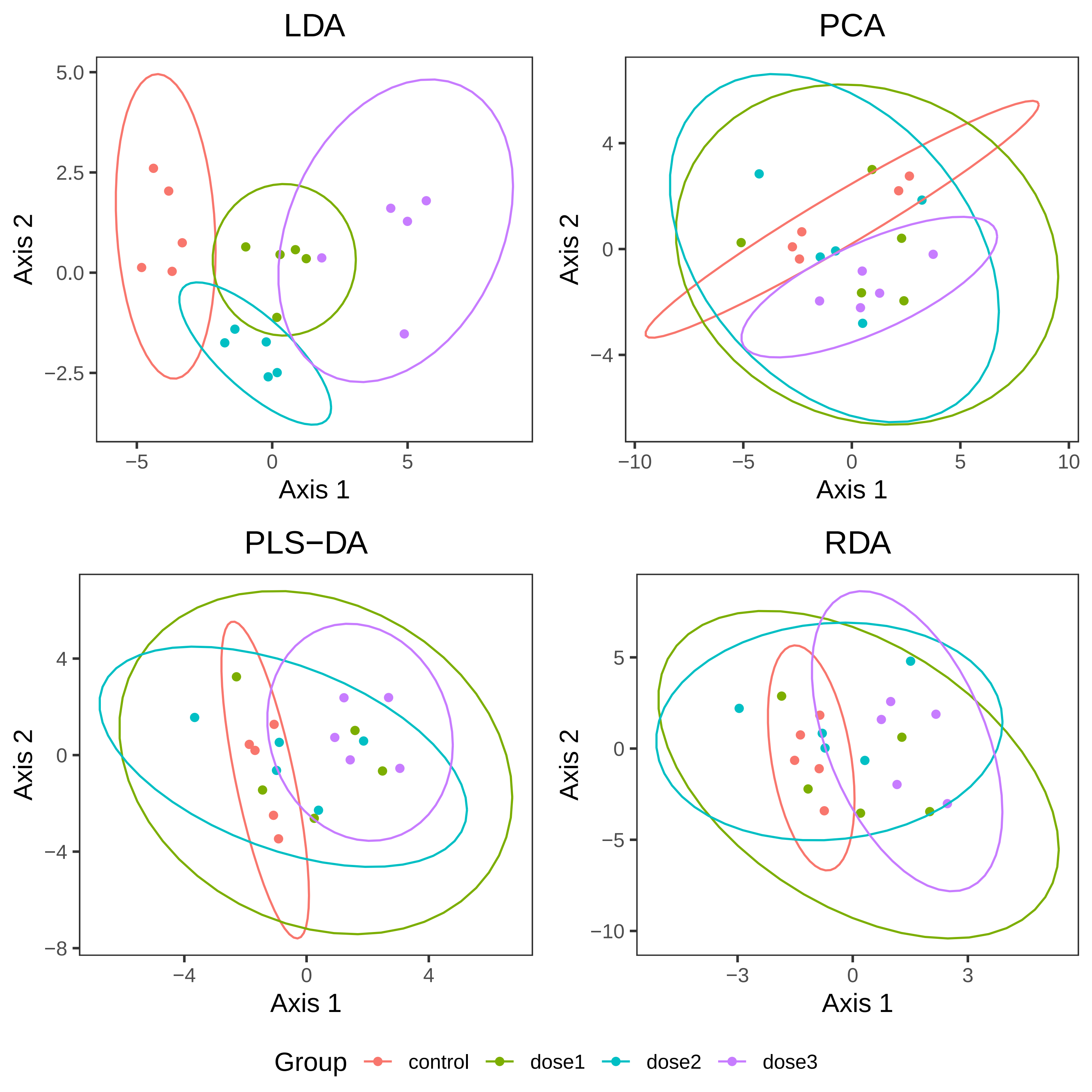

LDA
PCA
PLS−DA
RDA
Axis 1
Axis 2
Group
control
dose1
dose2
dose3

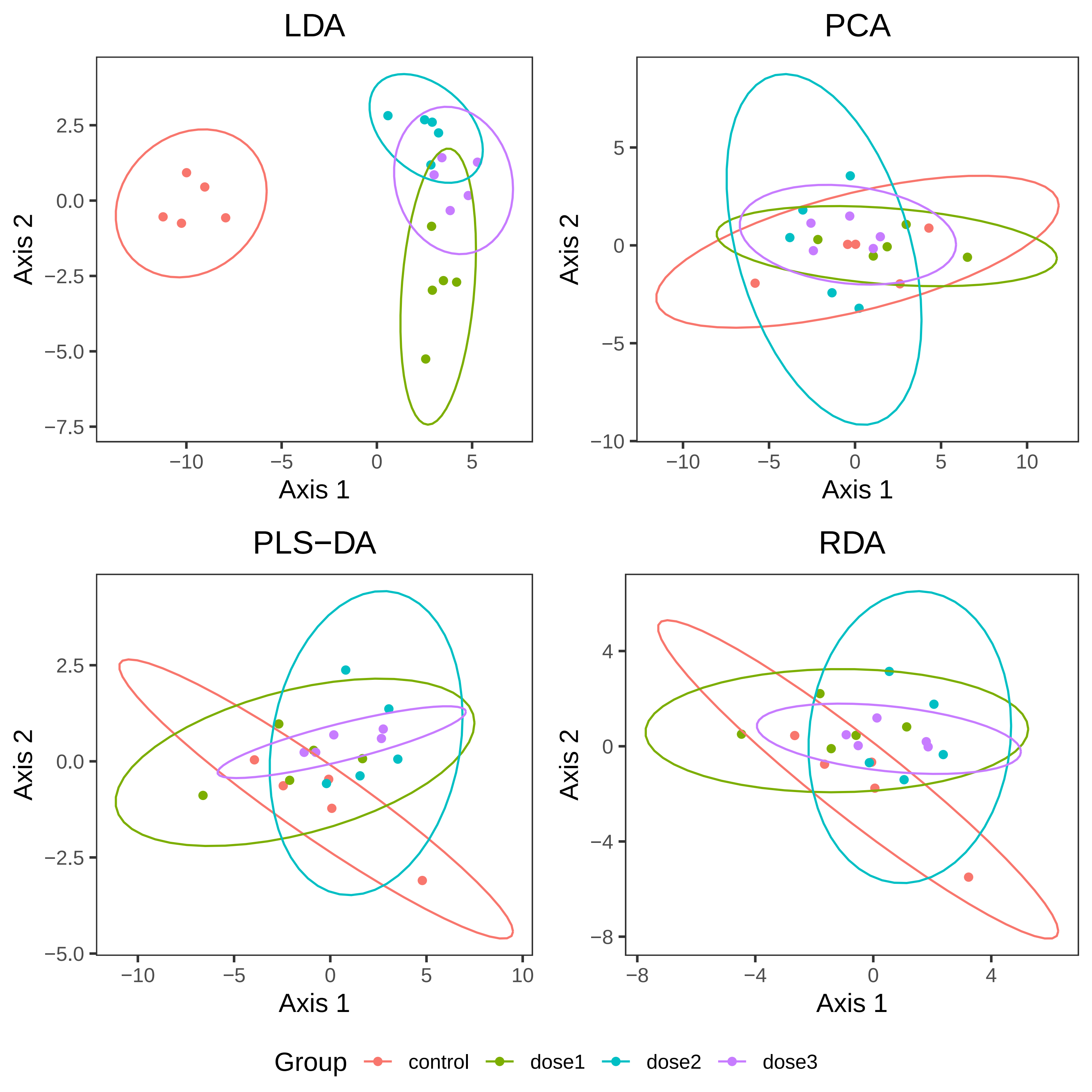
LDA
PCA
PLS−DA
RDA
Axis 1
Axis 2
2.5
0.0
−2.5
−5.0
−7.5
−10
−5
0
5
10
−8
−4
4
Group
control
dose1
dose2
dose3

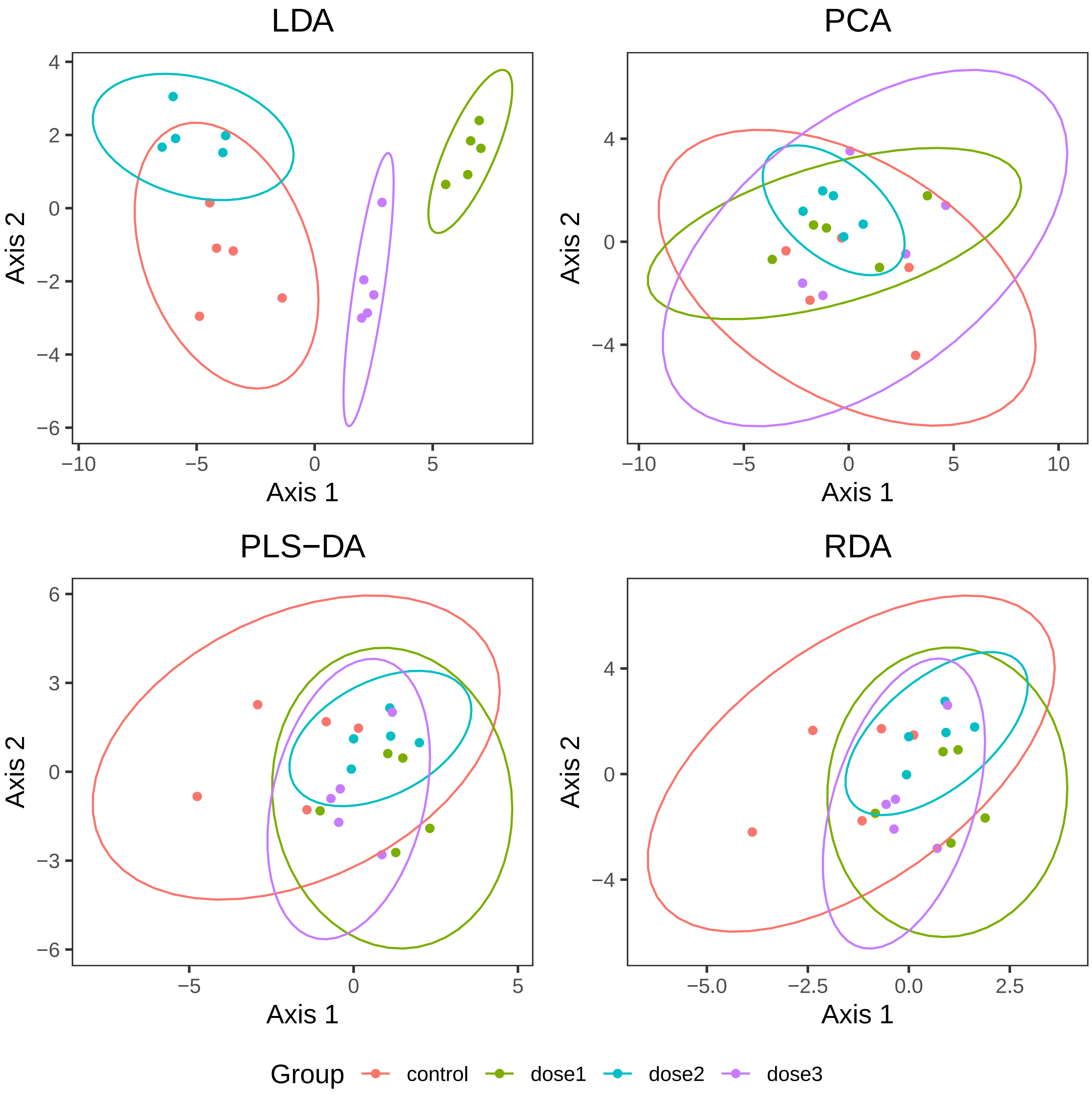

LDA
PCA
PLS−DA
RDA
Axis 1
Axis 2
Group
control
dose1
dose2
dose3

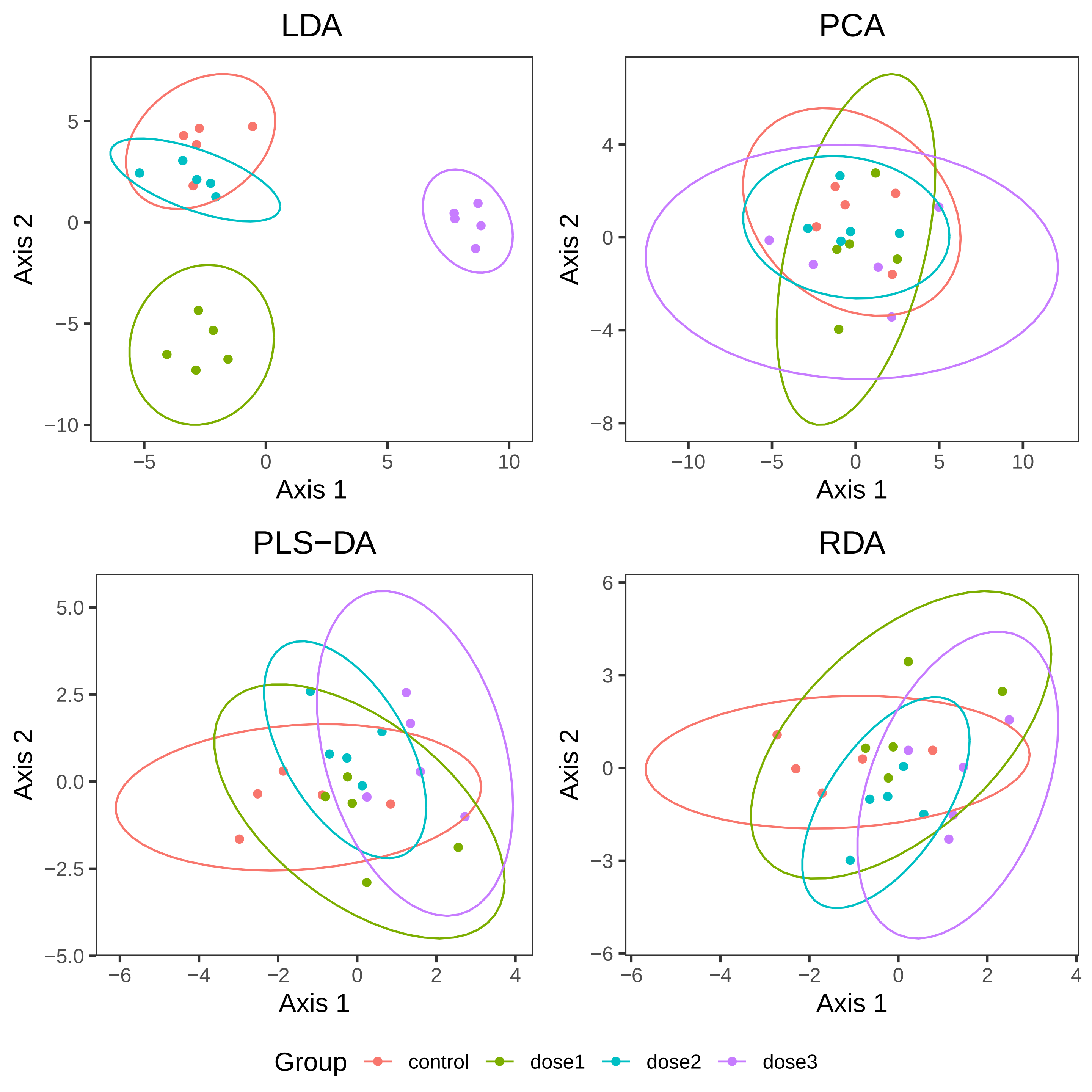
LDA
PCA
PLS−DA
RDA
Axis 1
Axis 2
Group
control
dose1
dose2
dose3

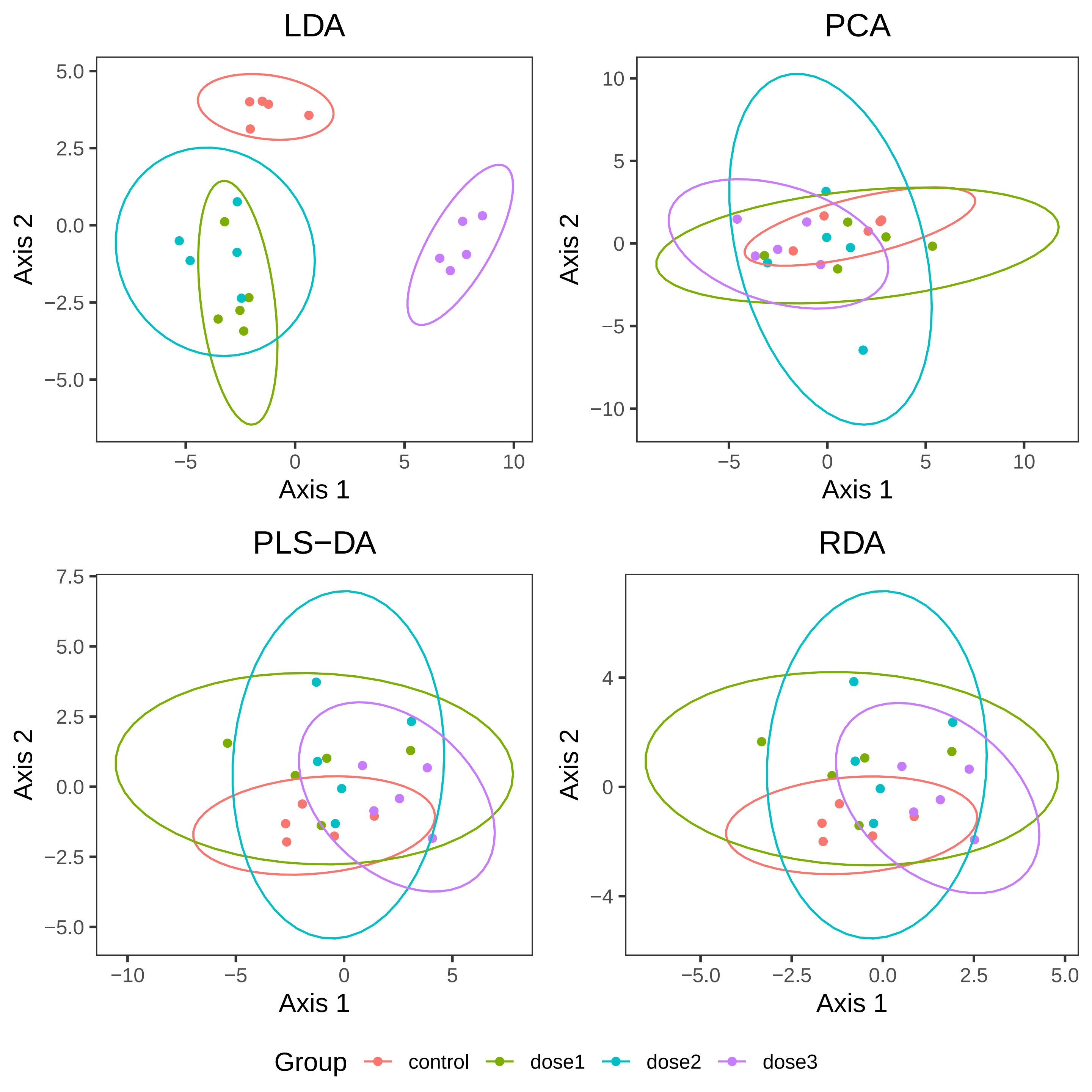
LDA
PCA
PLS−DA
RDA
Axis 1
Axis 2
Group
control
dose1
dose2
dose3

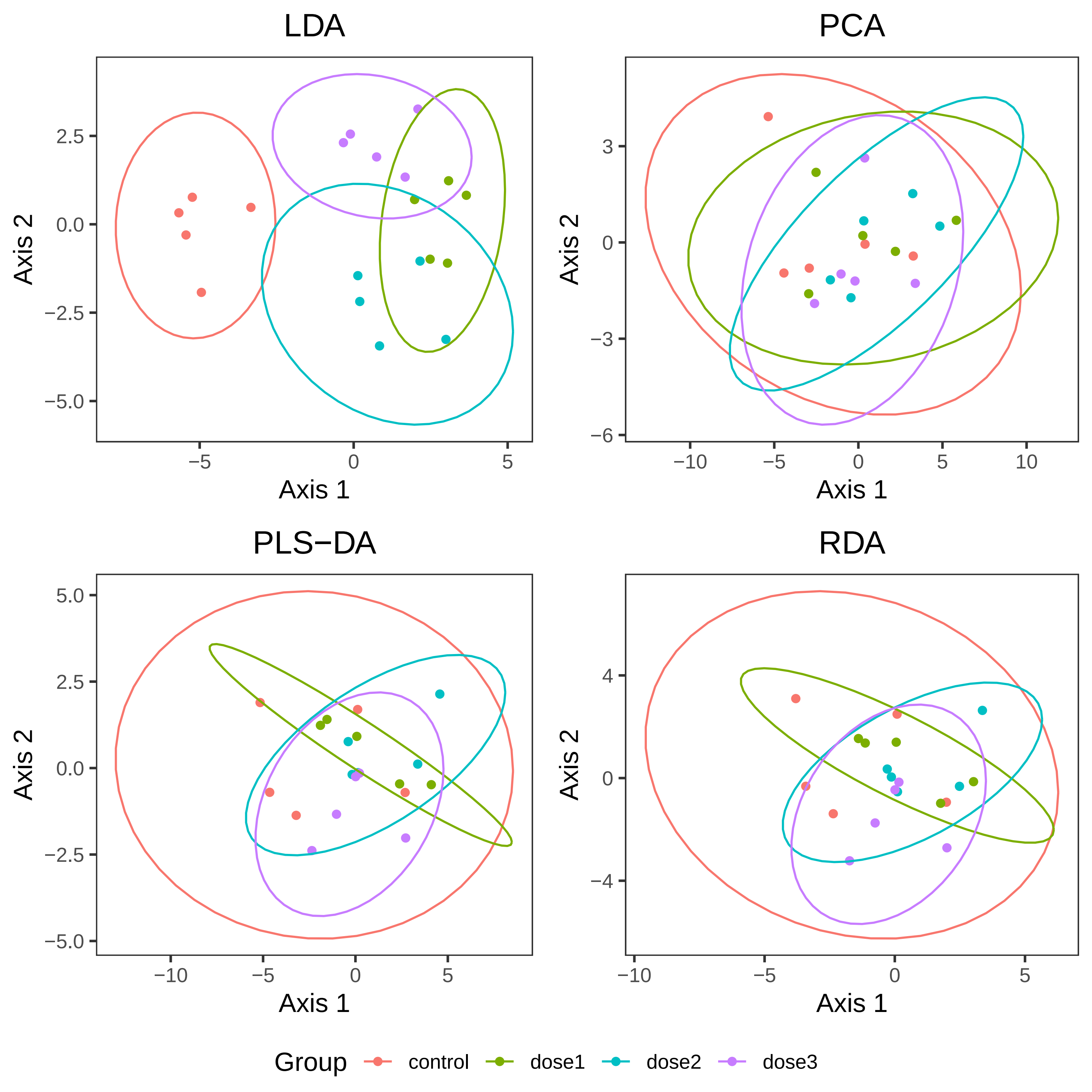

LDA
PCA
PLS−DA
RDA
Axis 1
Axis 2
Group
control
dose1
dose2
dose3

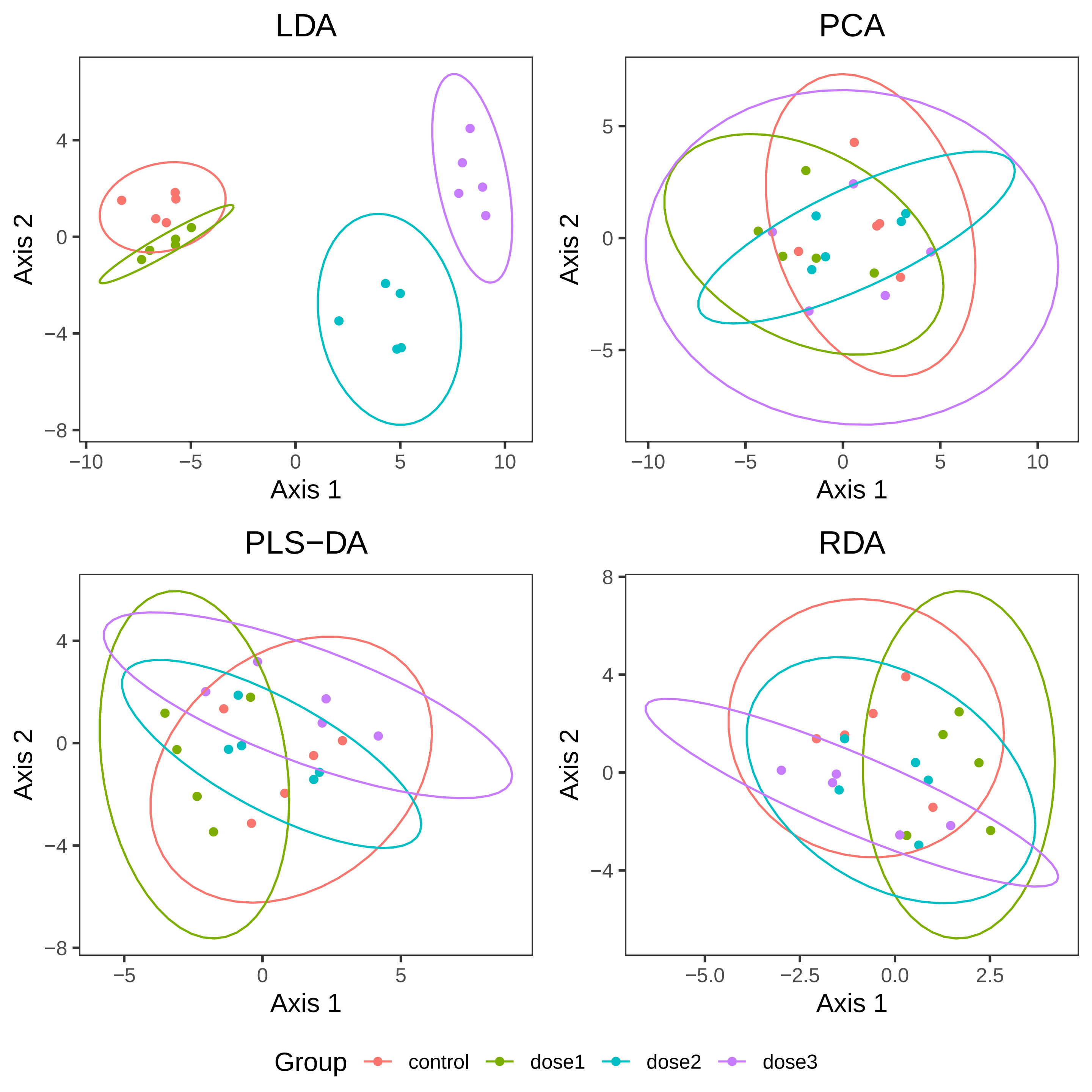

LDA
PCA
PLS−DA
RDA
Axis 1
Axis 2
Group
control
dose1
dose2
dose3

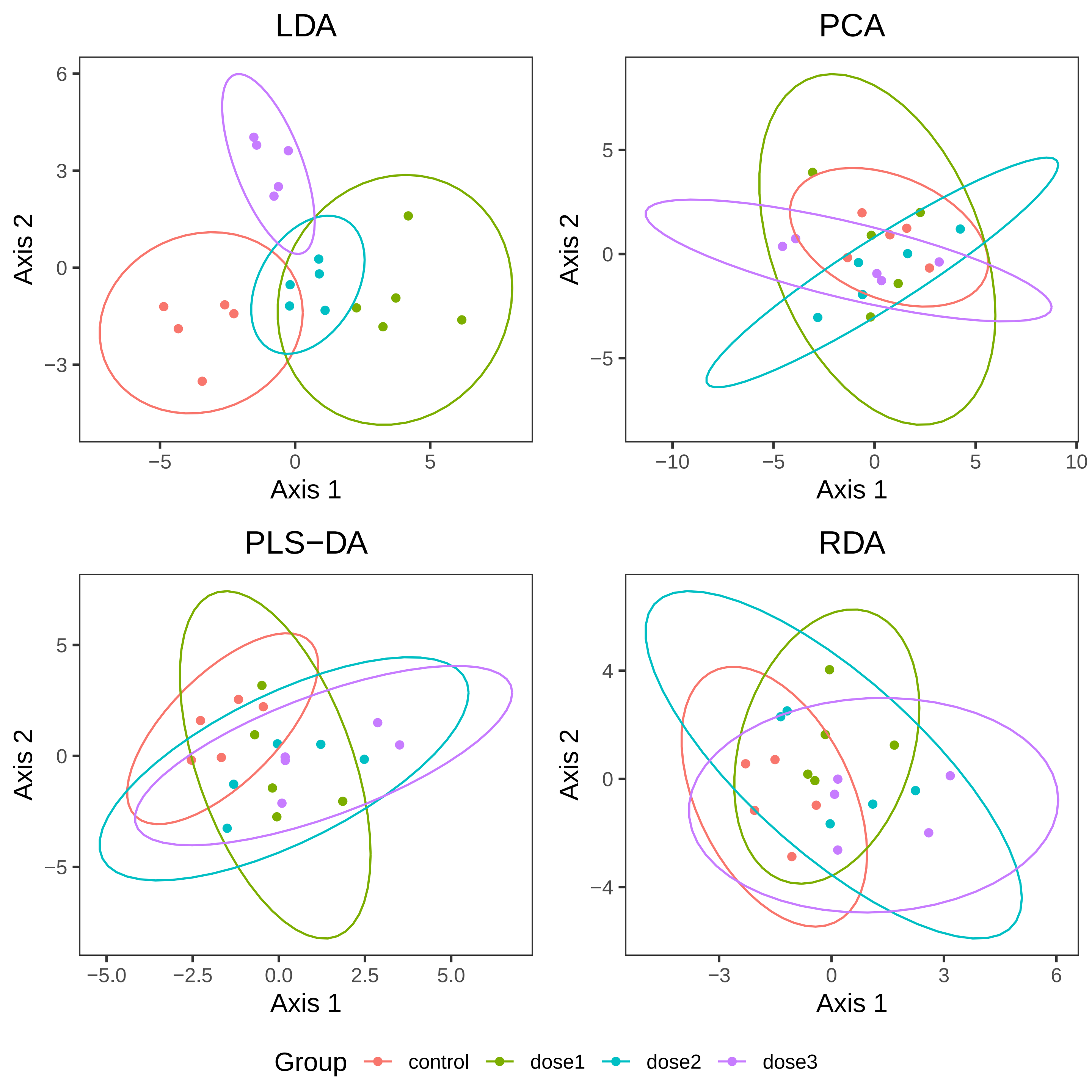
LDA
PCA
PLS−DA
RDA
Axis 1
Axis 2
Group
control
dose1
dose2
dose3

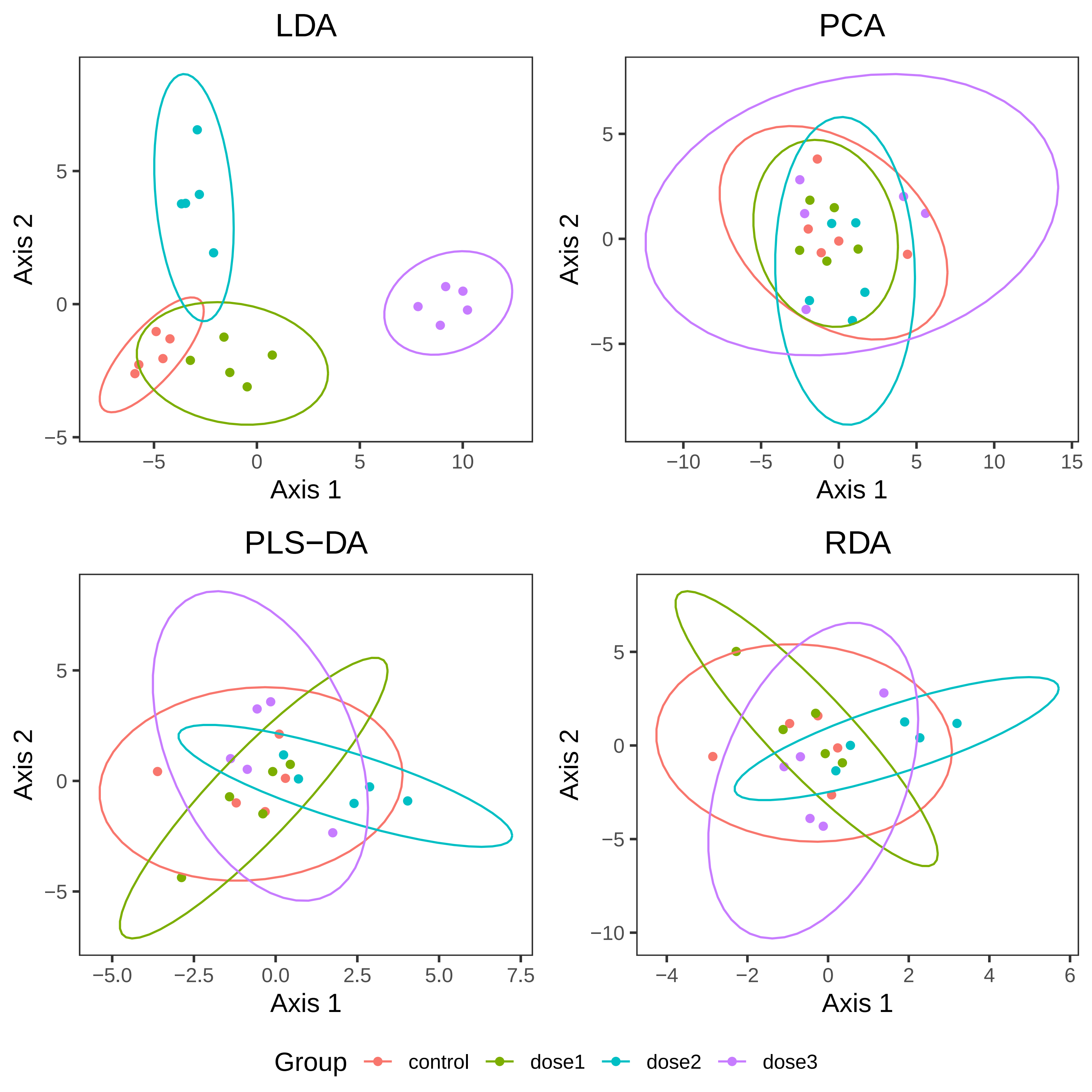
LDA
PCA
PLS−DA
RDA
Axis 1
Axis 2
Group
control
dose1
dose2
dose3

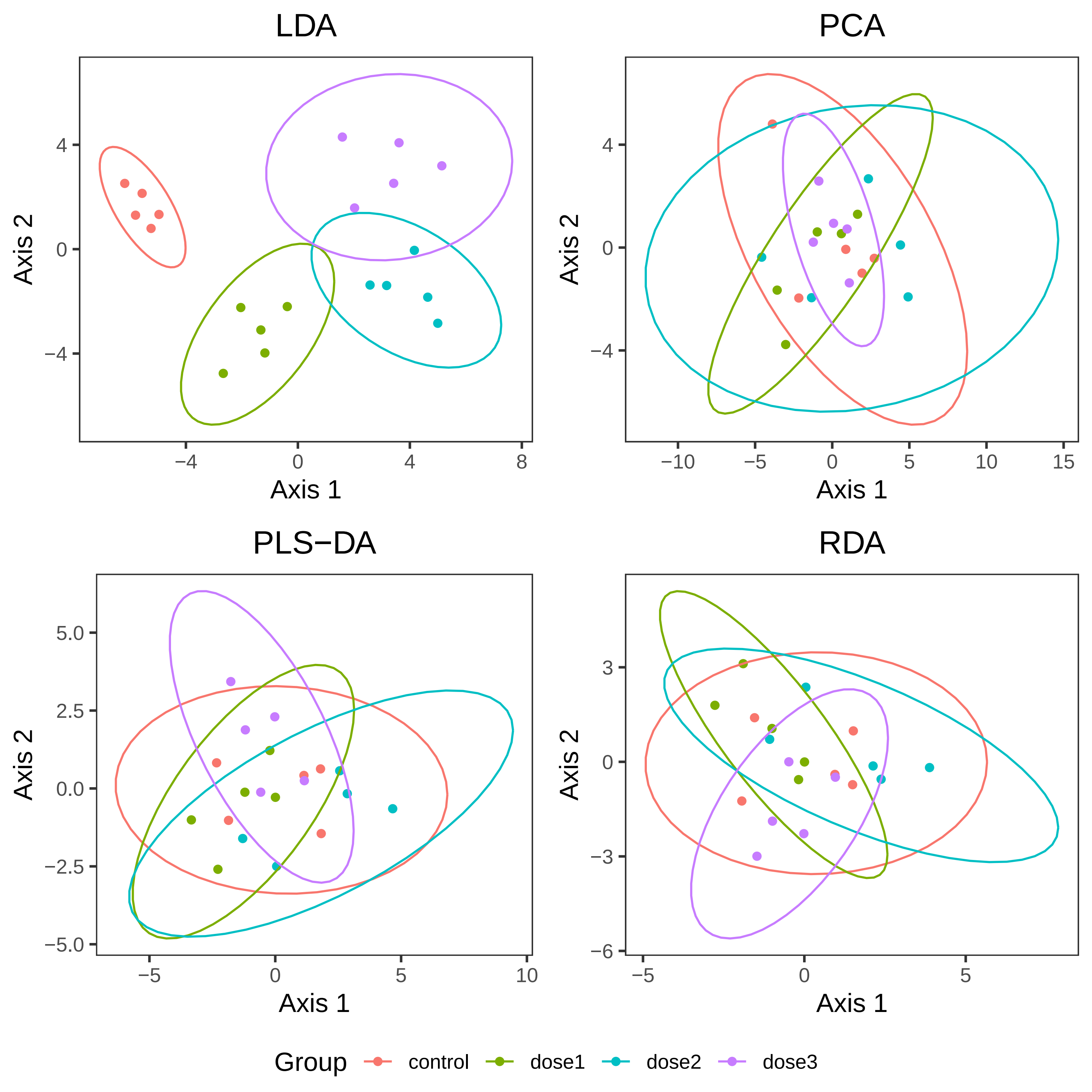

LDA
PCA
PLS−DA
RDA
Axis 1
Axis 2
Group
control
dose1
dose2
dose3

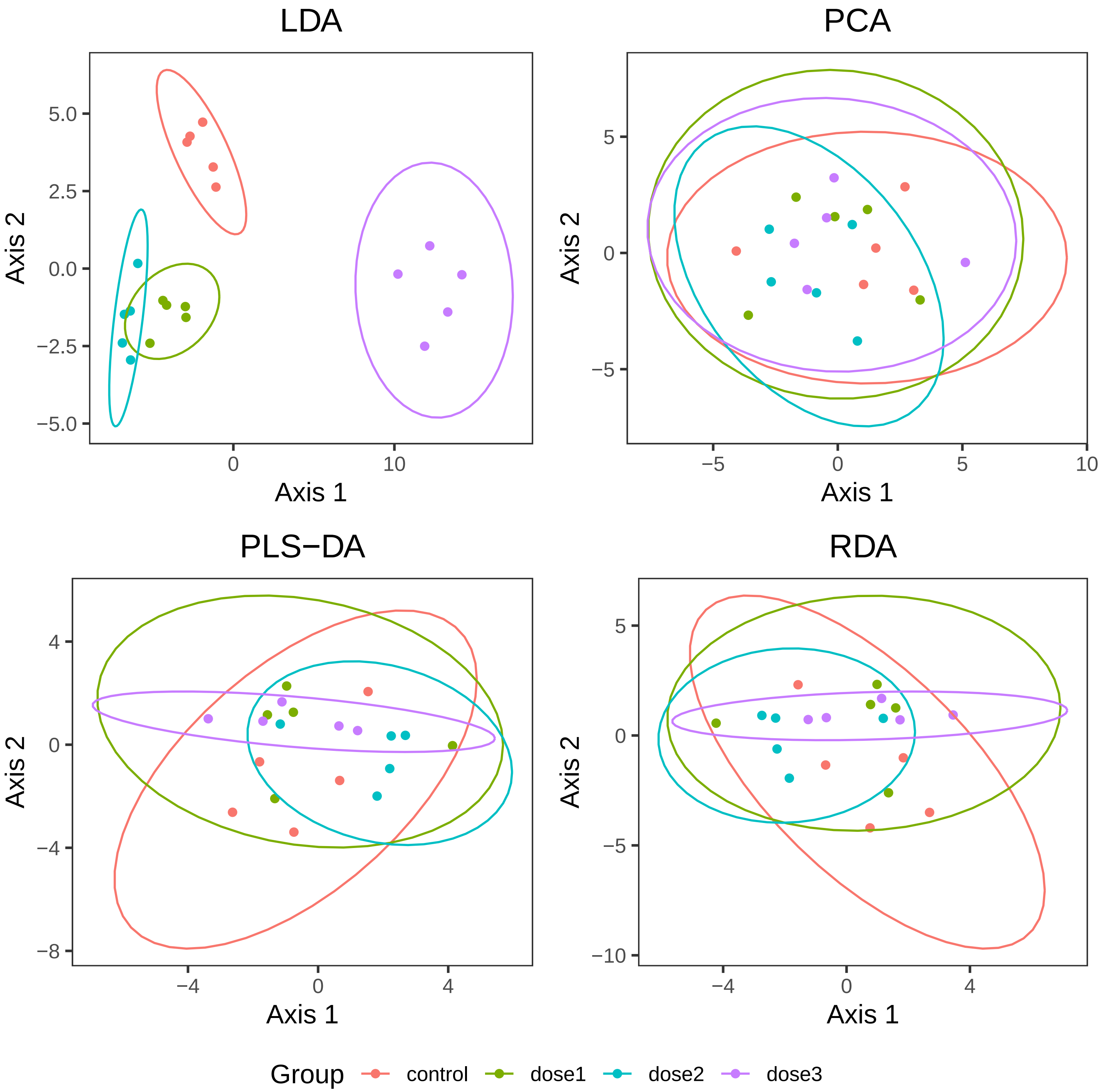

LDA
PCA
PLS−DA
RDA
Axis 1
Axis 2
5.0
2.5
0.0
−2.5
−5.0
0
10
5
0
−5
−5
0
5
10
4
0
−4
−8
−4
0
4
5
0
−5
−10
−4
0
4
Group
control
dose1
dose2
dose3

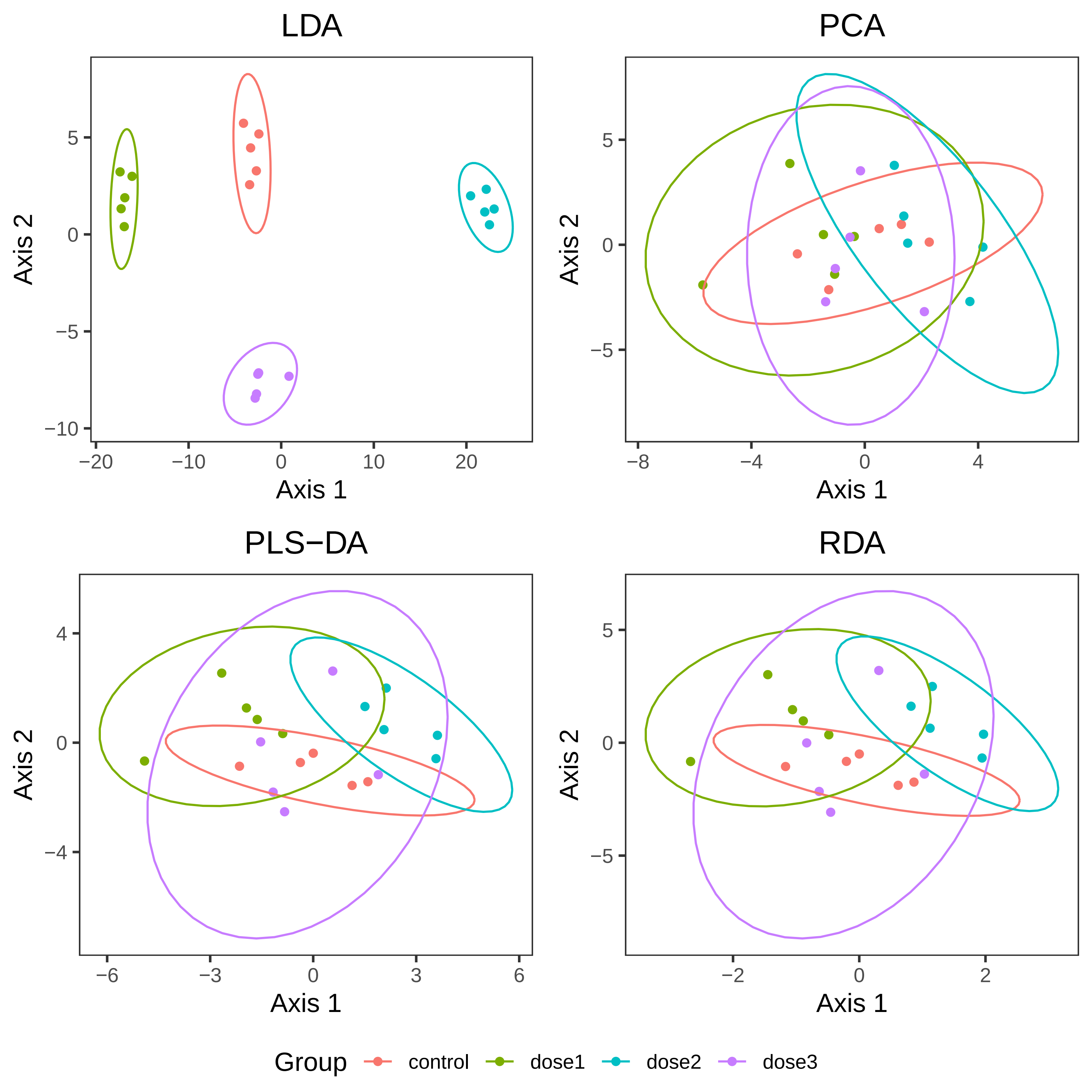
LDA
PCA
PLS−DA
RDA
Axis 1
Axis 2
5
0
−5
−10
−20
−10
0
10
20
−8
−4
0
4
4
0
−4
−6
−3
0
3
6
−2
2
Group
control
dose1
dose2
dose3

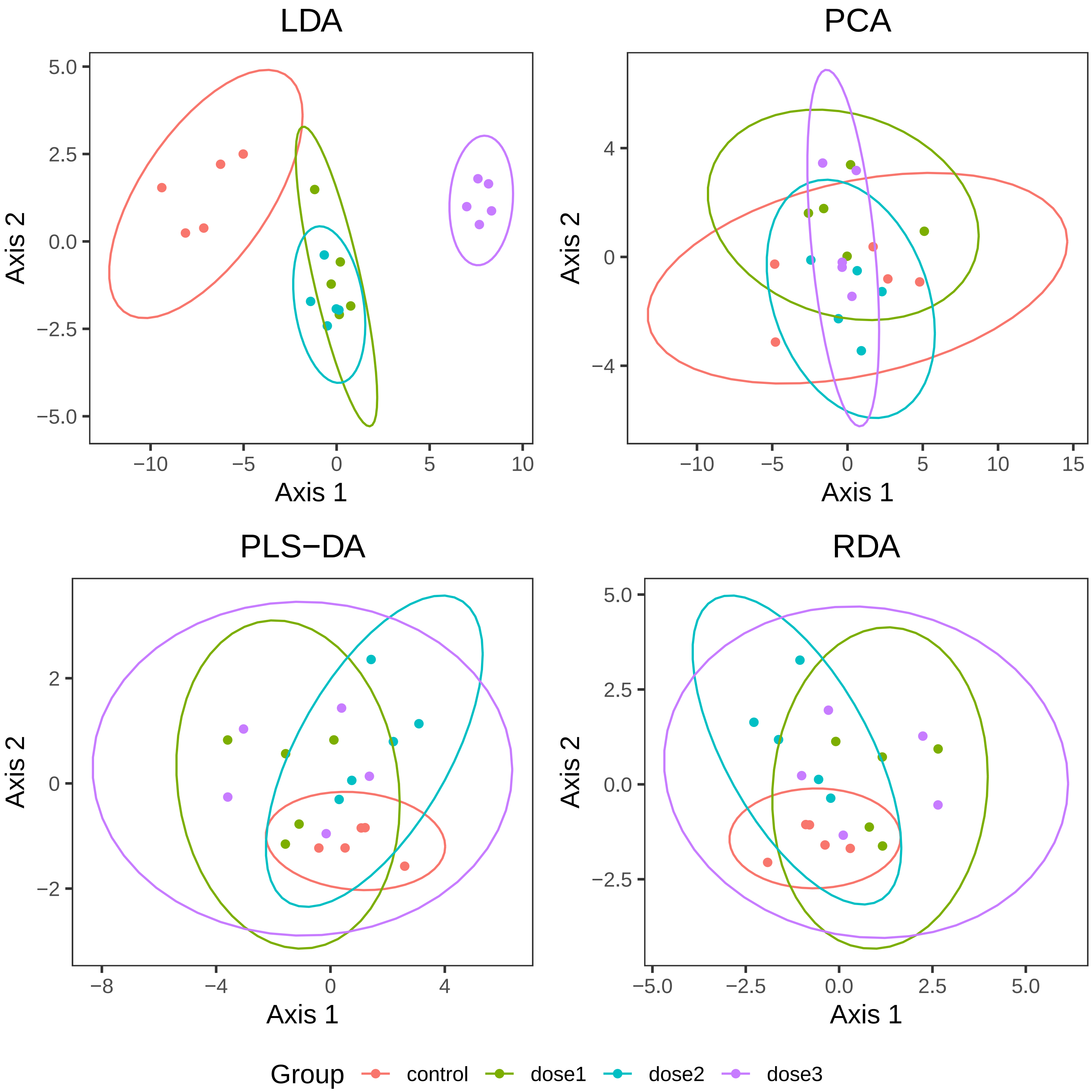
LDA
PCA
PLS−DA
RDA
Axis 1
Axis 2
Group
control
dose1
dose2
dose3
5.0
2.5
0.0
−2.5
−5.0
−10
−5
0
5
10
15
4
−4
−8
2
−2

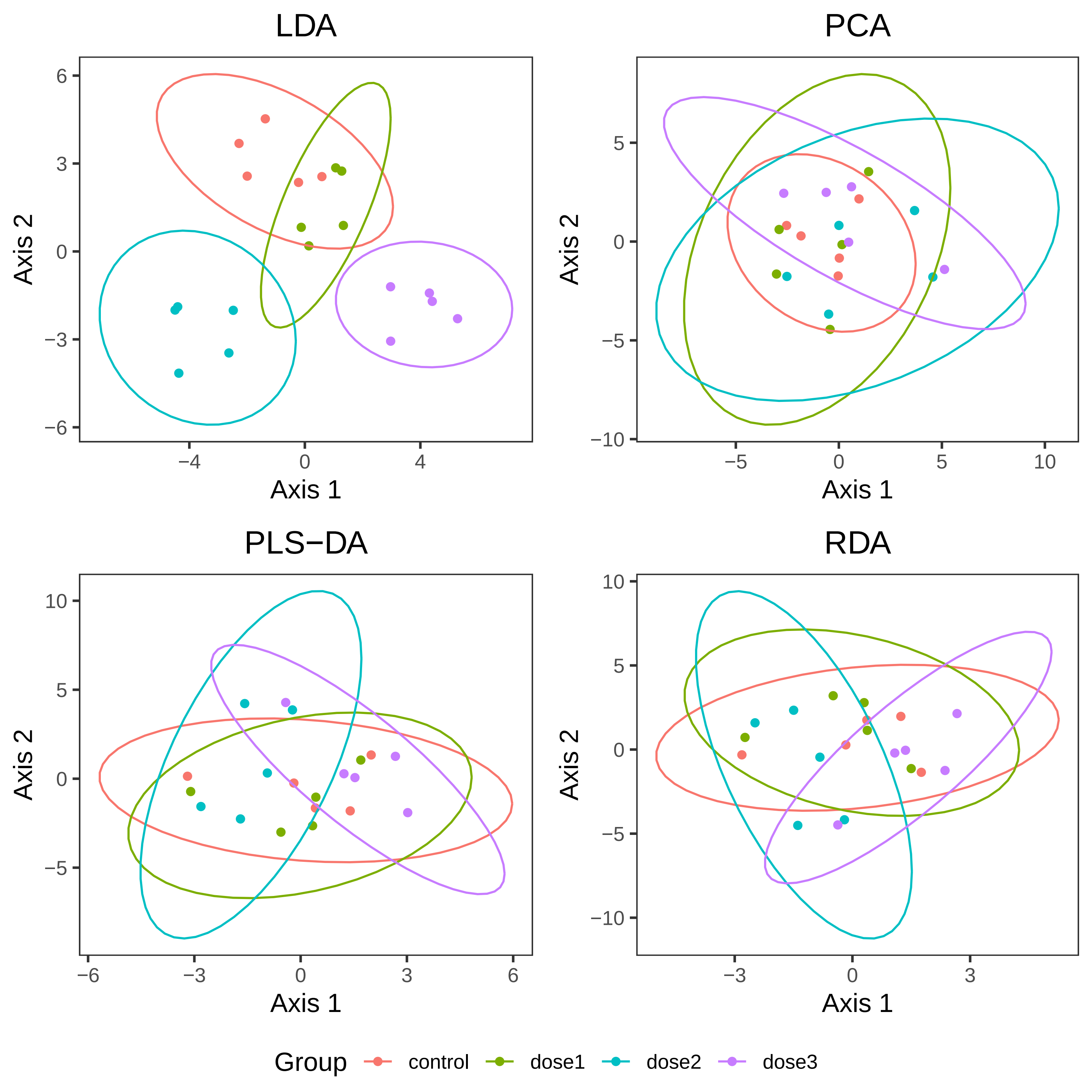

LDA
PCA
PLS−DA
RDA
Axis 1
Axis 2
Group
control
dose1
dose2
dose3

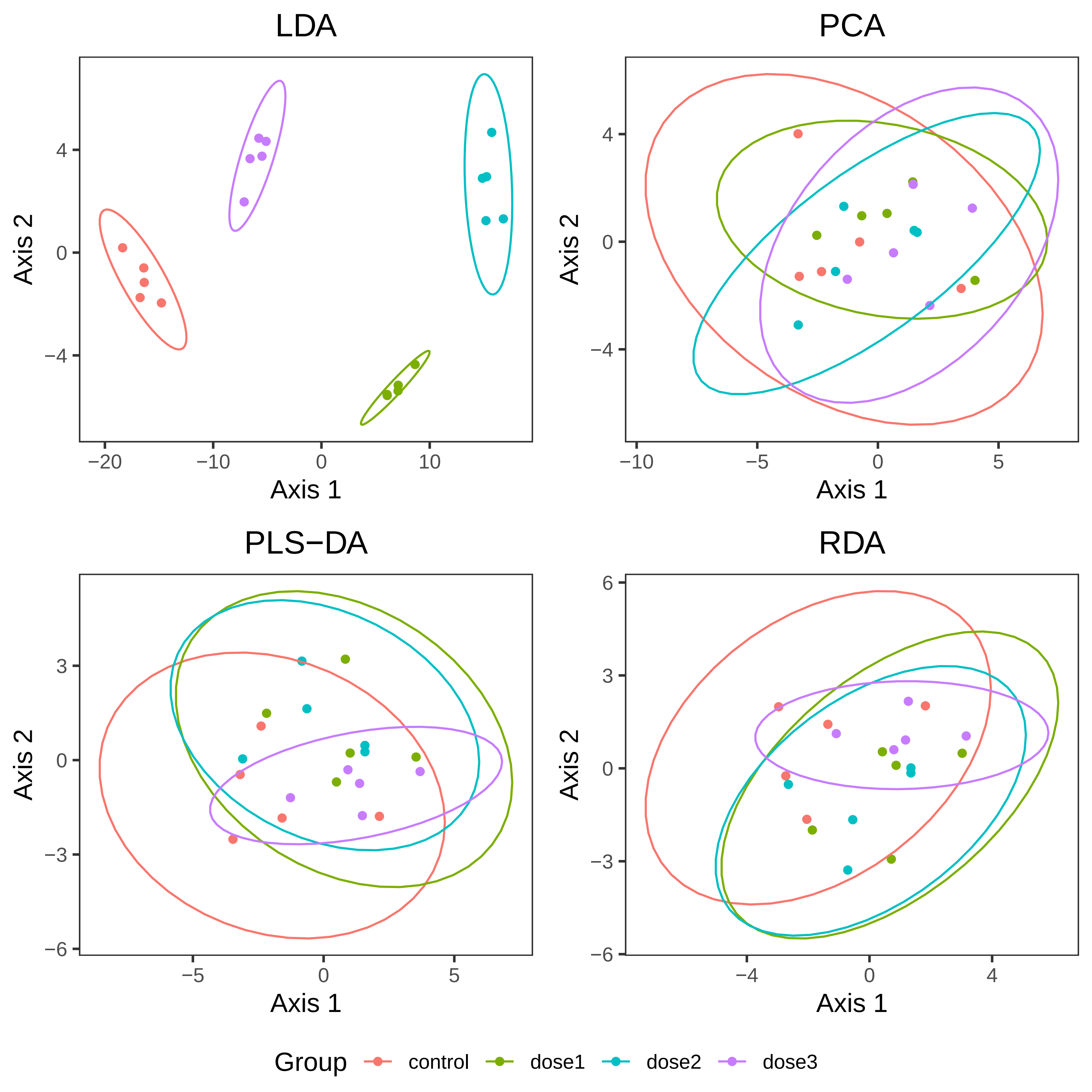

LDA
PCA
PLS−DA
RDA
Axis 1
Axis 2
Group
control
dose1
dose2
dose3

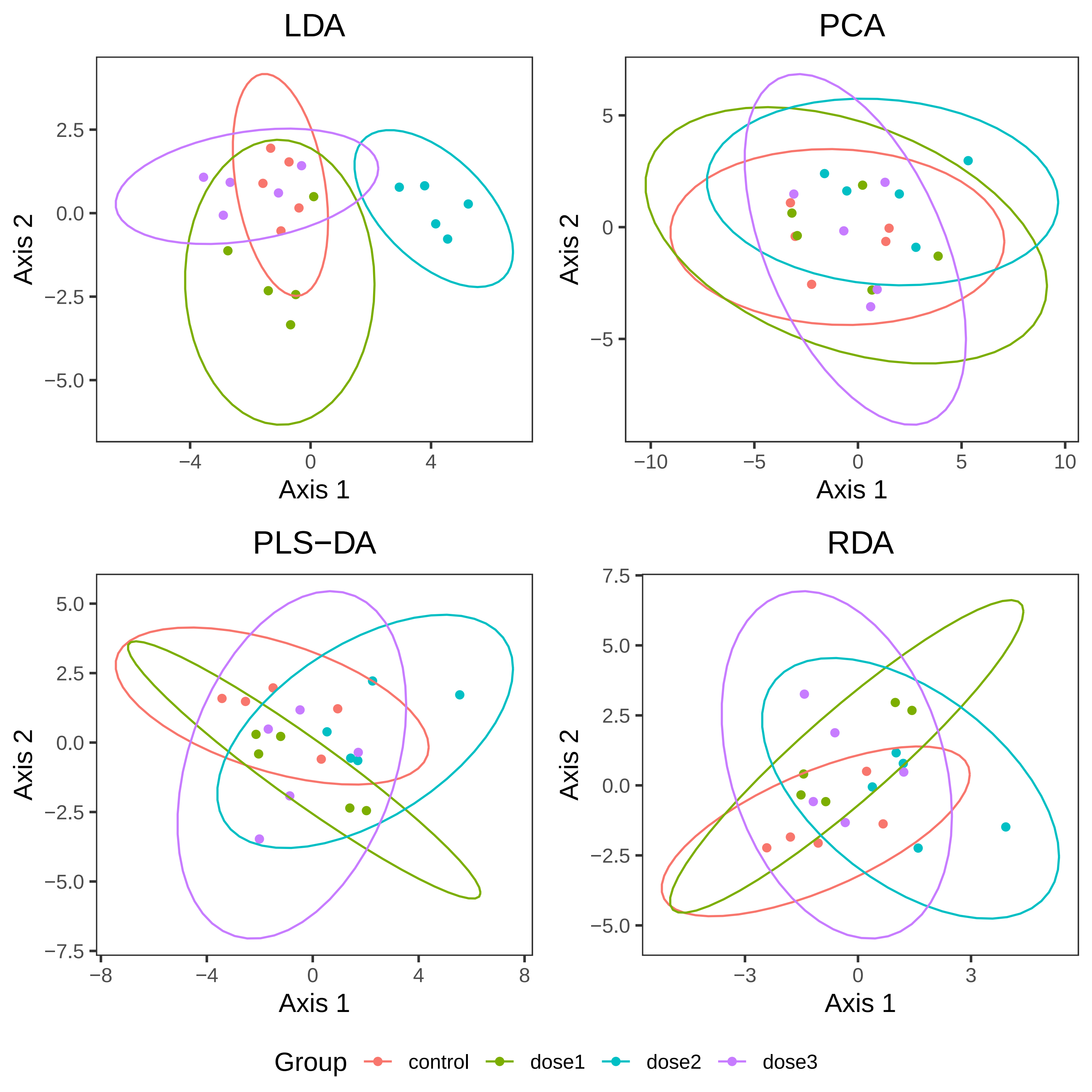

LDA
PCA
PLS−DA
RDA
Axis 1
Axis 2
Group
control
dose1
dose2
dose3

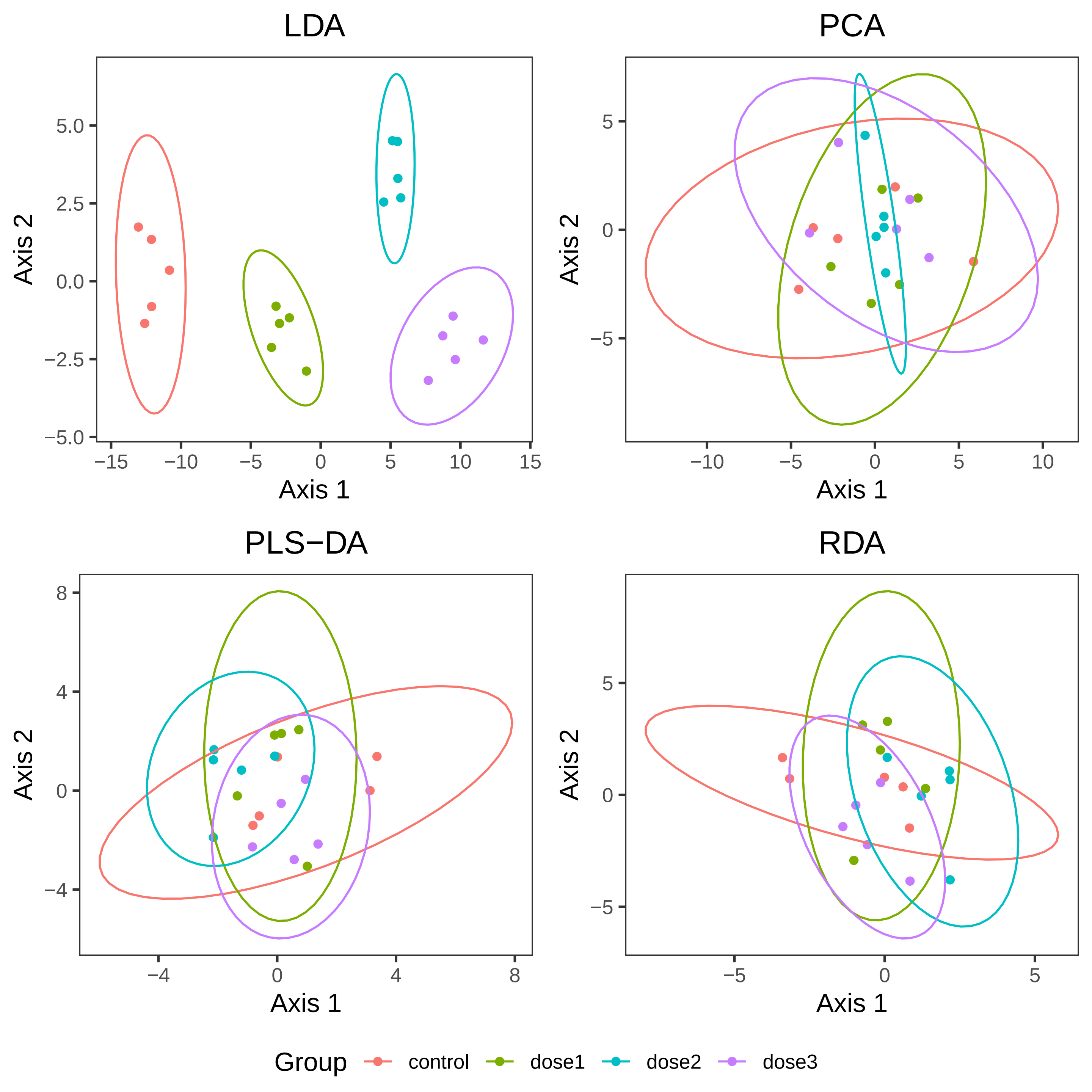
LDA
PCA
PLS−DA
RDA
Axis 1
Axis 2
5.0
2.5
0.0
−2.5
−5.0
−15
−10
−5
0
5
10
15
−4
4
8
Group
control
dose1
dose2
dose3

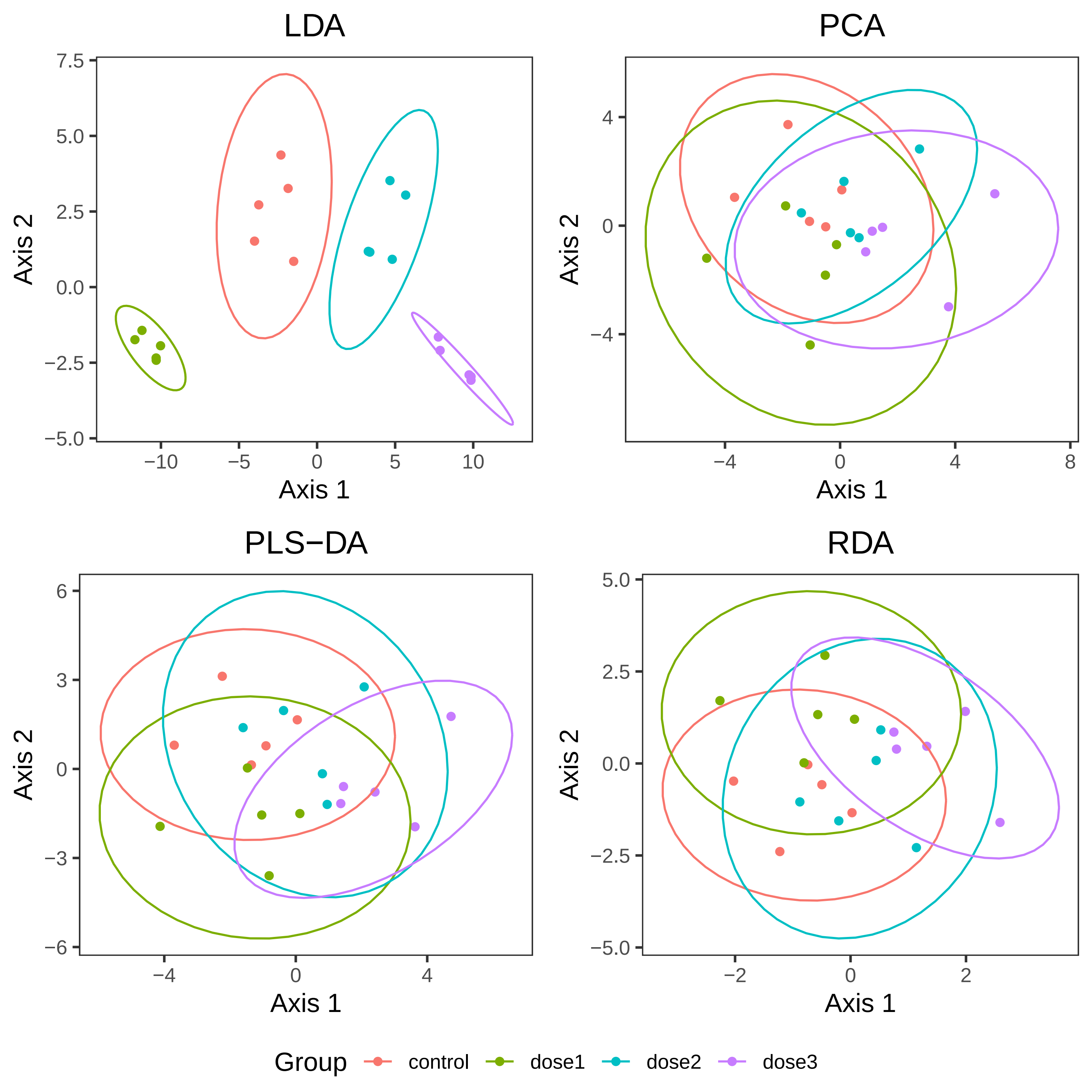

LDA
PCA
PLS−DA
RDA
Axis 1
Axis 2
Group
control
dose1
dose2
dose3

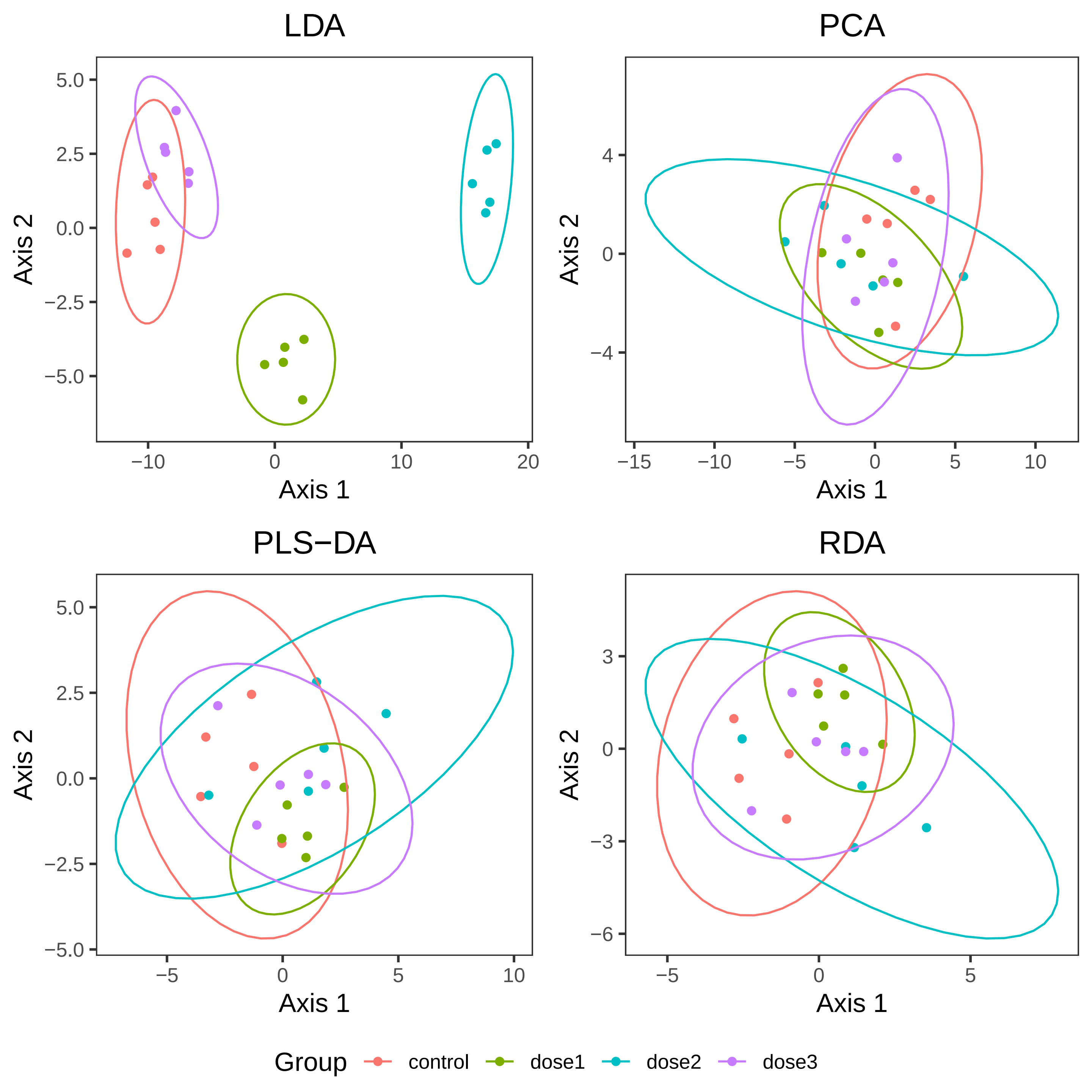
LDA
PCA
PLS−DA
RDA
Axis 1
Axis 2
5.0
2.5
0.0
−2.5
−5.0
−10
0
10
20
4
0
−4
−15
−10
−5
5
3
−3
−6
Group
control
dose1
dose2
dose3

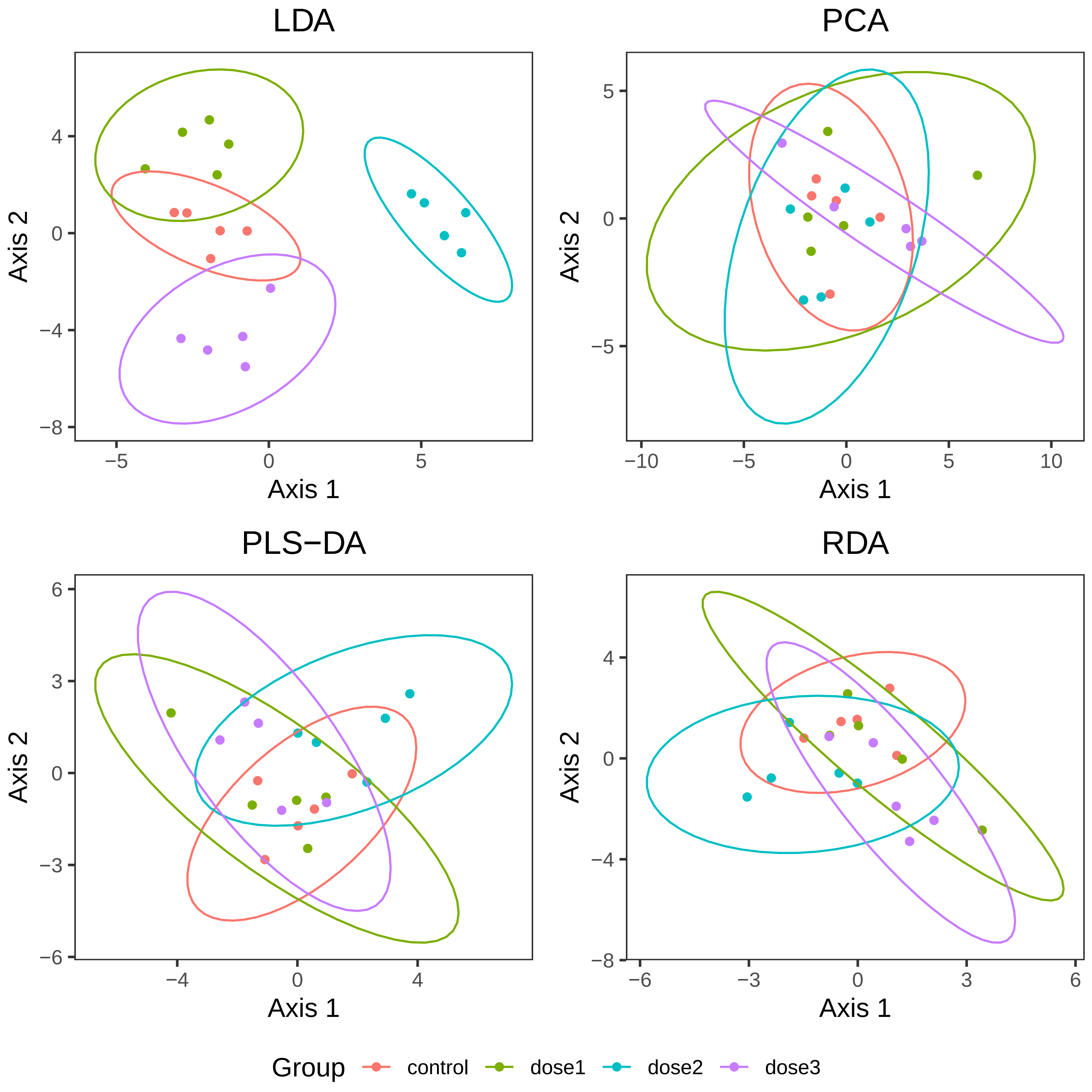

LDA
PCA
PLS−DA
RDA
Axis 1
Axis 2
4
0
−4
−8
−5
0
5
−10
−5
0
5
10
6
3
0
−3
−6
−4
0
4
−6
−3
0
3
6
Group
control
dose1
dose2
dose3

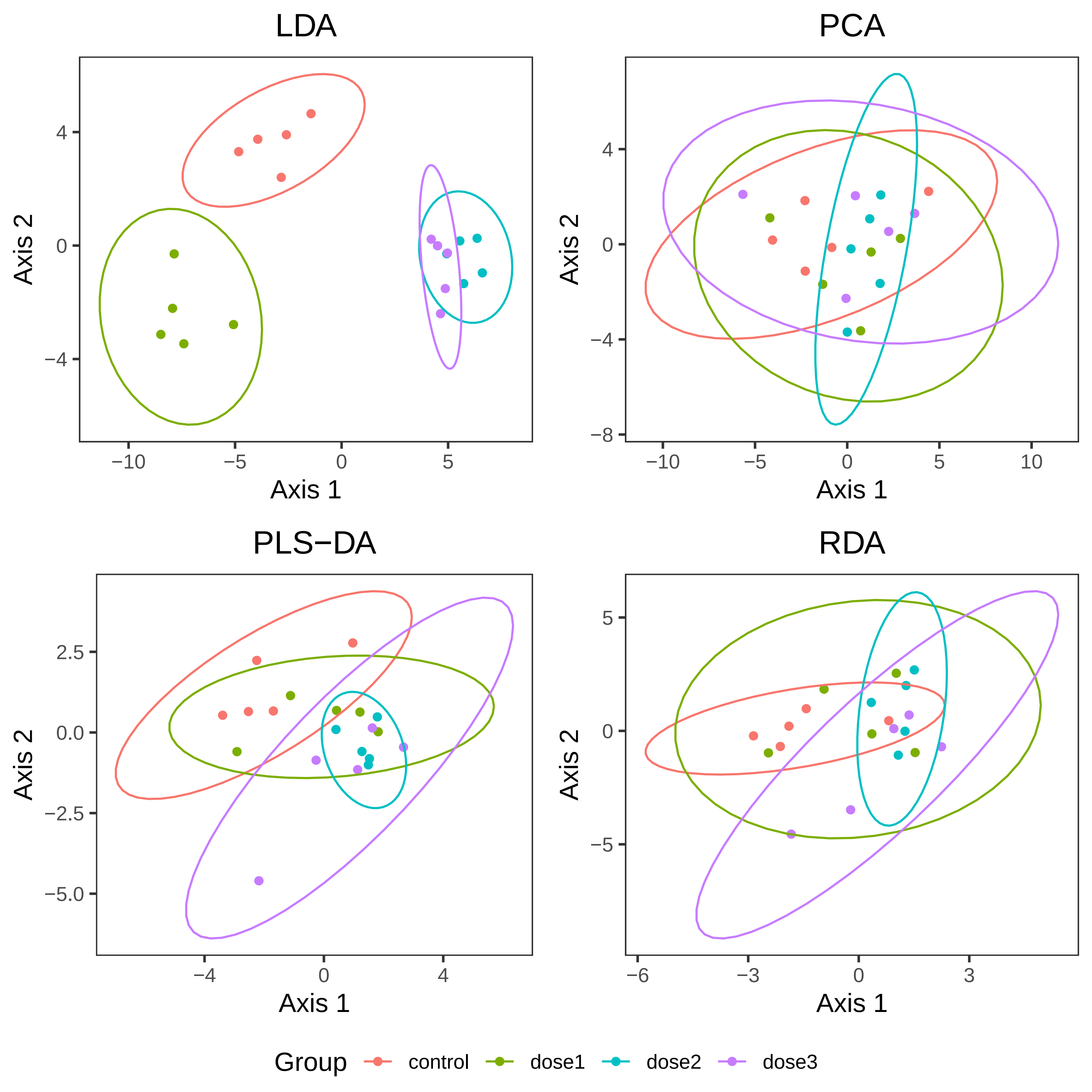

LDA
PCA
PLS−DA
RDA
Axis 1
Axis 2
Group
control
dose1
dose2
dose3

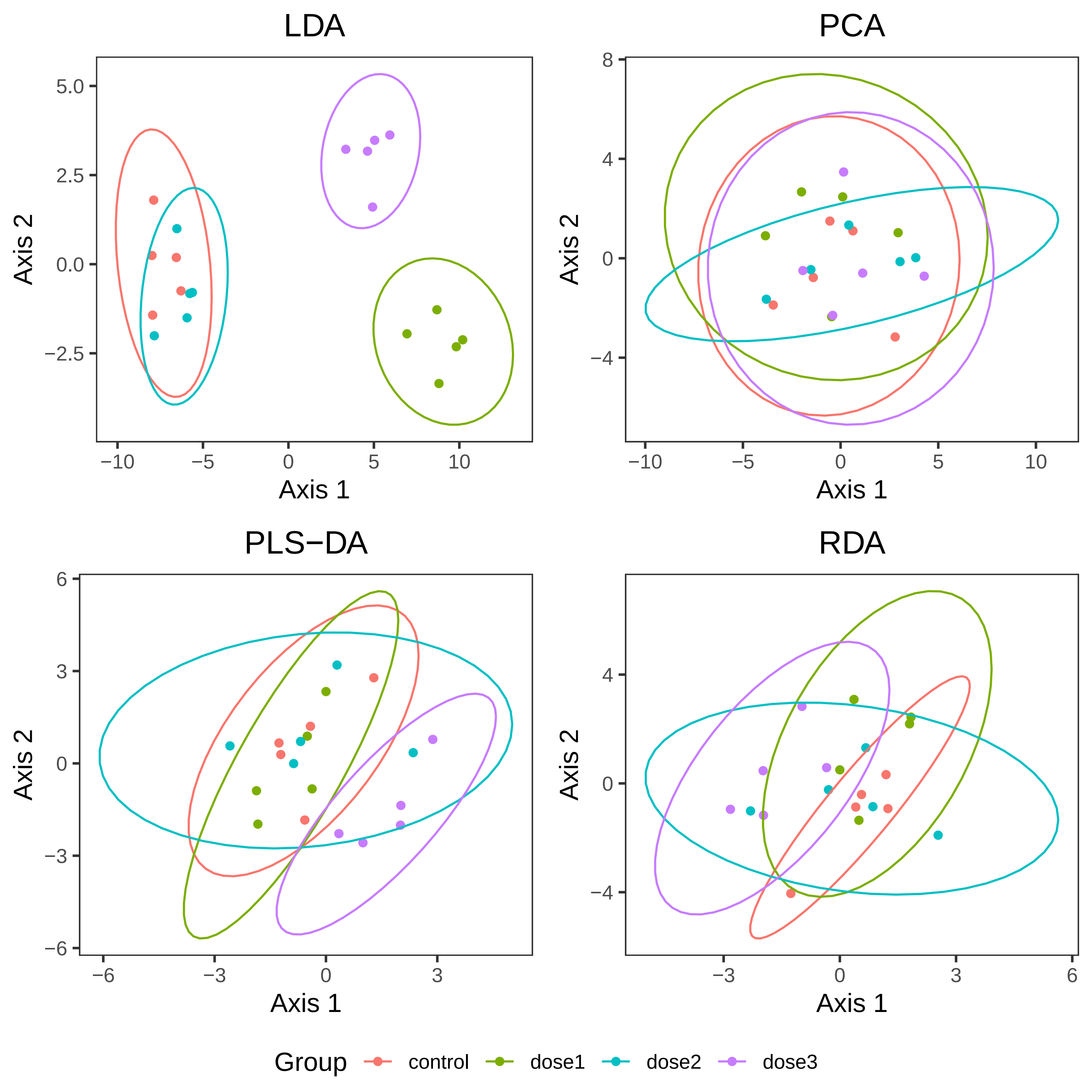

LDA
PCA
PLS−DA
RDA
Axis 1
Axis 2
Group
control
dose1
dose2
dose3

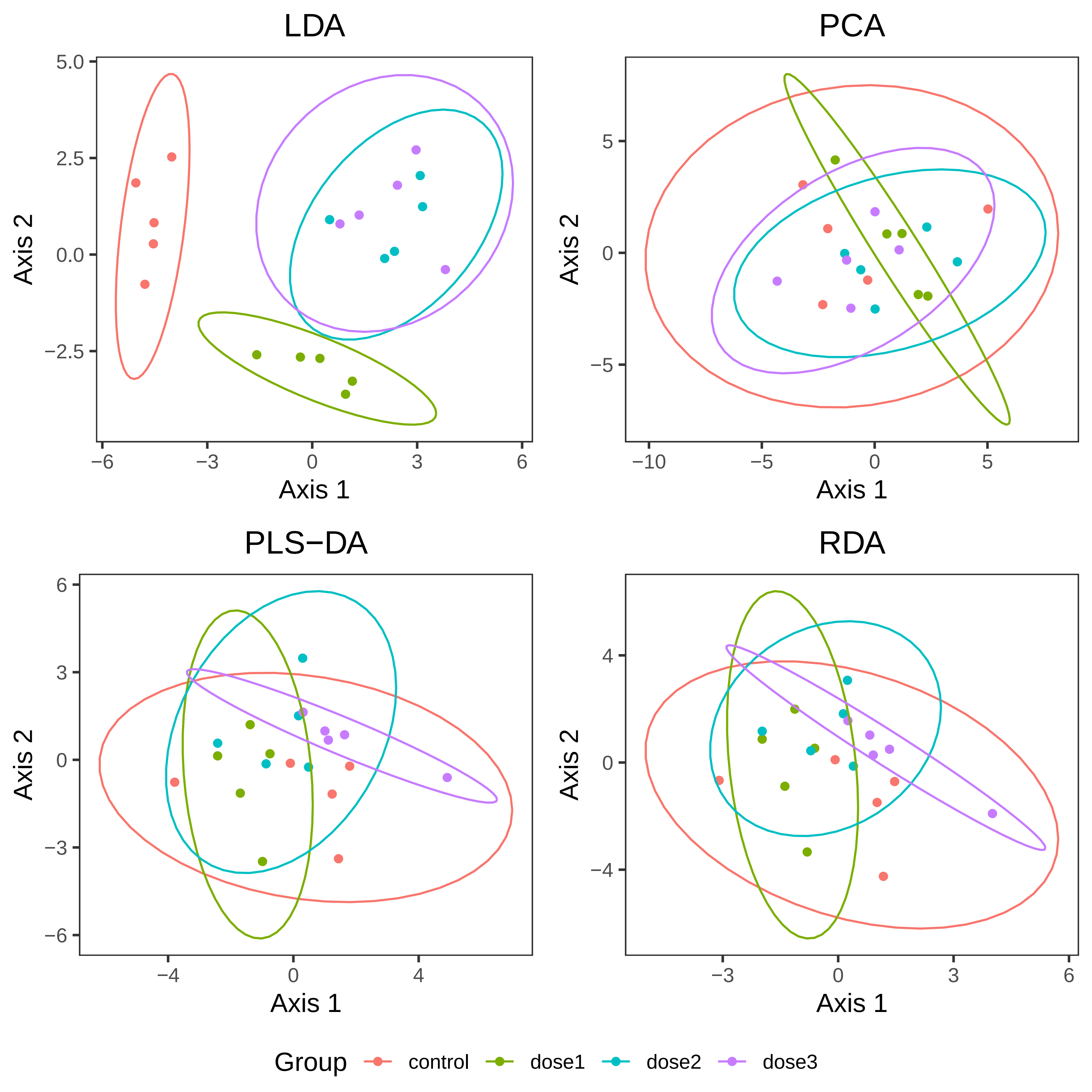

LDA
PCA
PLS−DA
RDA
Axis 1
Axis 2
5.0
2.5
0.0
−2.5
−6
−3
0
3
6
5
0
−5
−10
−5
0
5
6
3
0
−3
−6
−4
0
4
4
0
−4
−3
0
3
6
Group
control
dose1
dose2
dose3

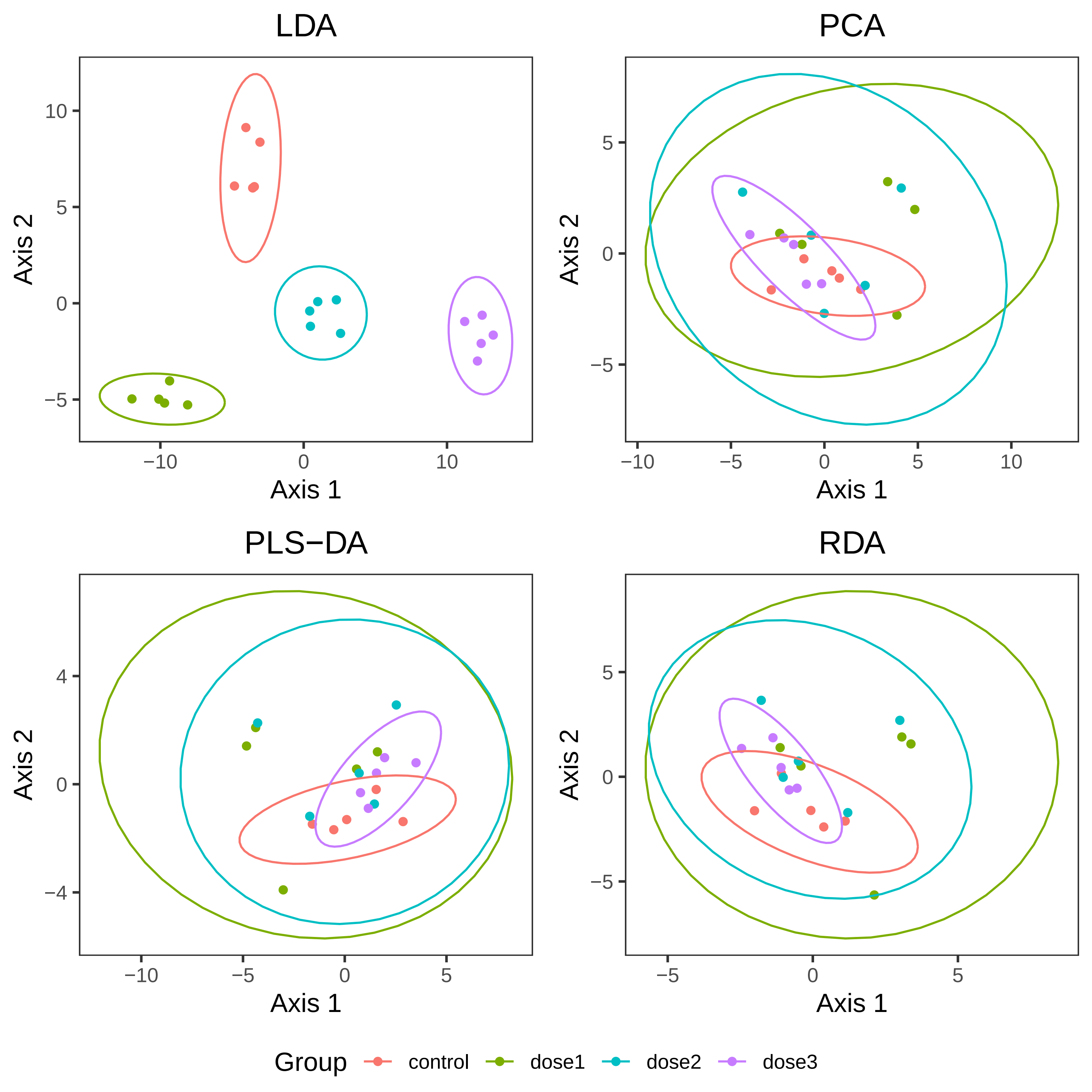

LDA
PCA
PLS−DA
RDA
Axis 1
Axis 2
Group
control
dose1
dose2
dose3

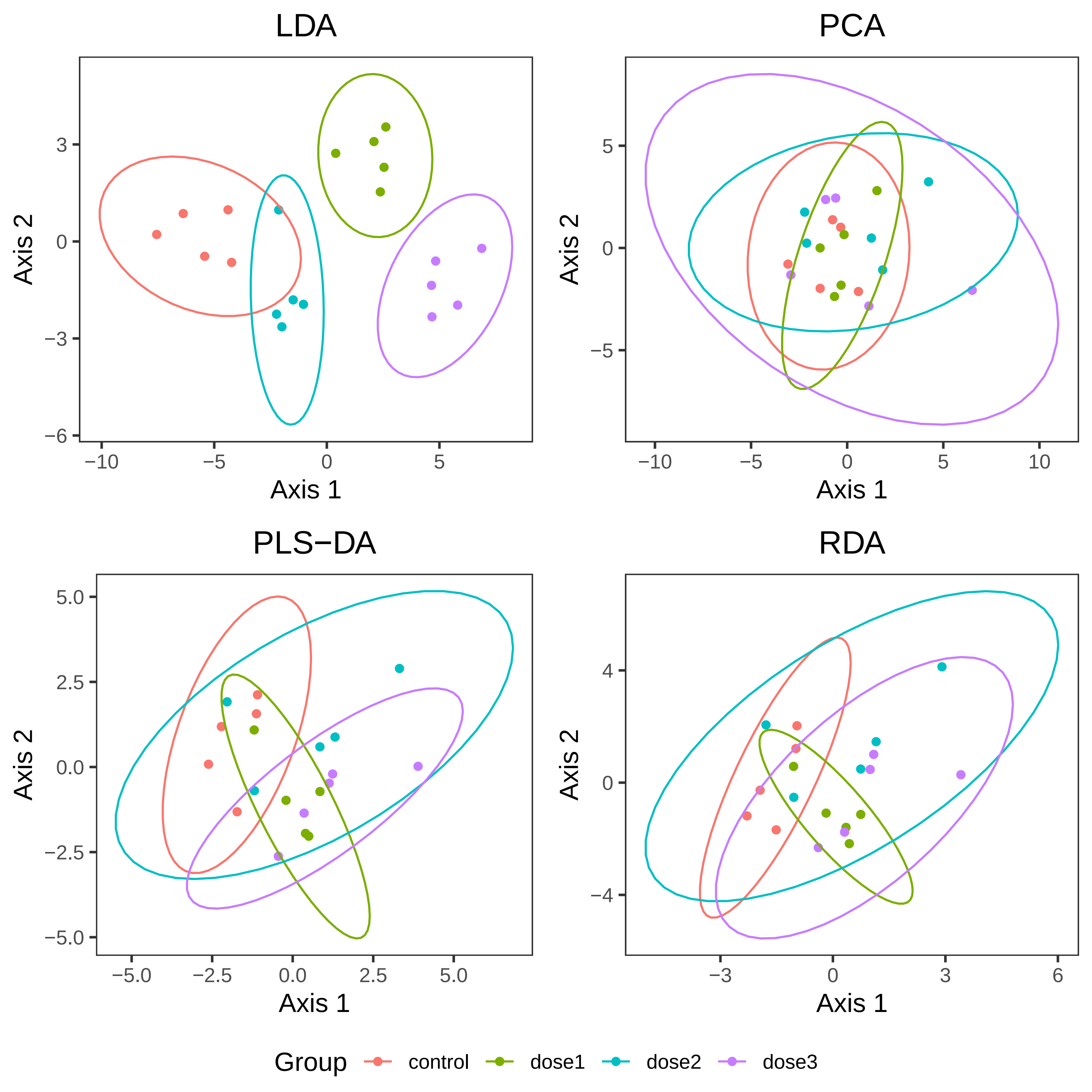

LDA
PCA
PLS−DA
RDA
Axis 1
Axis 2
Group
control
dose1
dose2
dose3

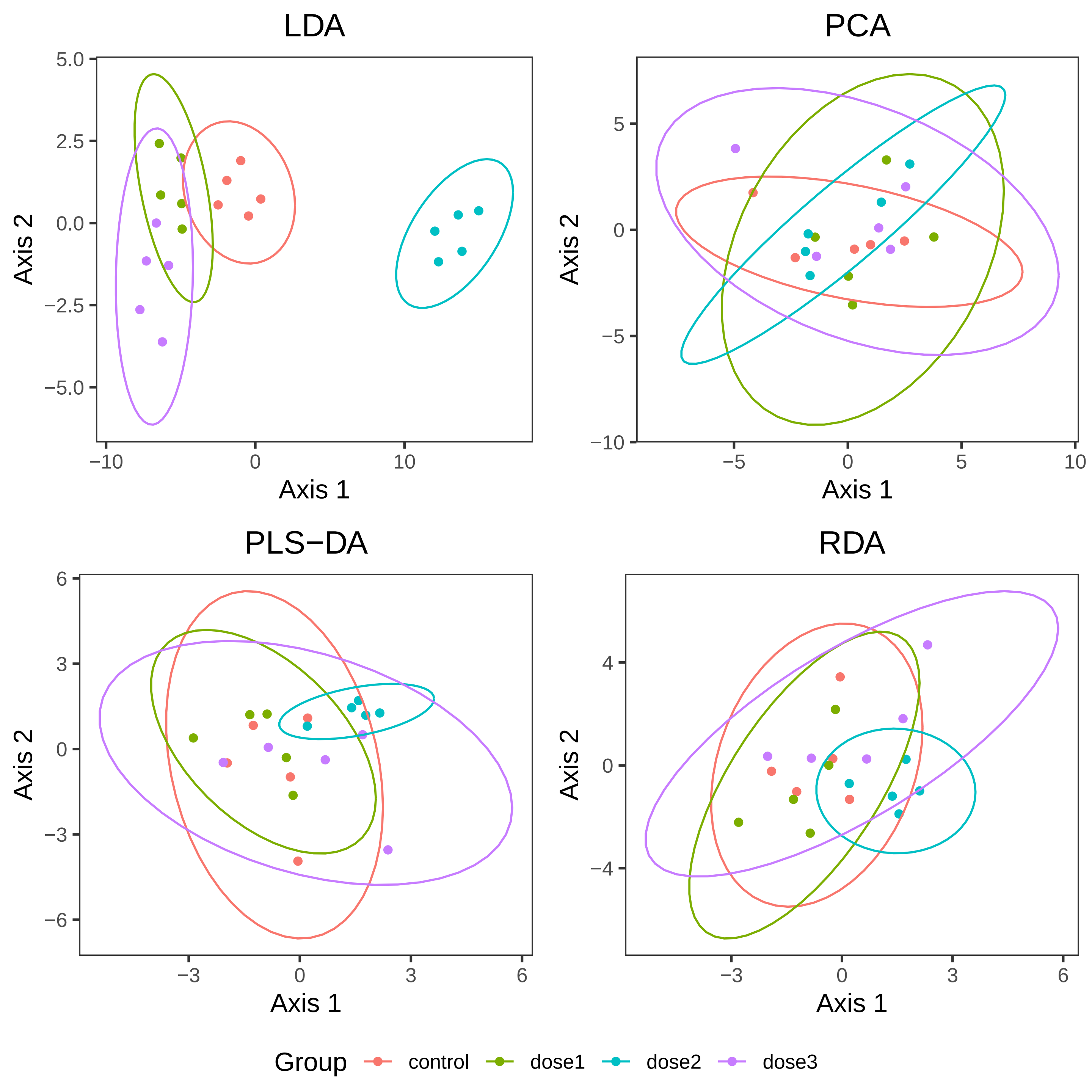

LDA
PCA
PLS−DA
RDA
Axis 1
Axis 2
5.0
2.5
0.0
−2.5
−5.0
−10
0
10
5
0
−5
−10
−5
0
5
10
6
3
0
−3
−6
−3
0
3
6
4
0
−4
−3
0
3
6
Group
control
dose1
dose2
dose3

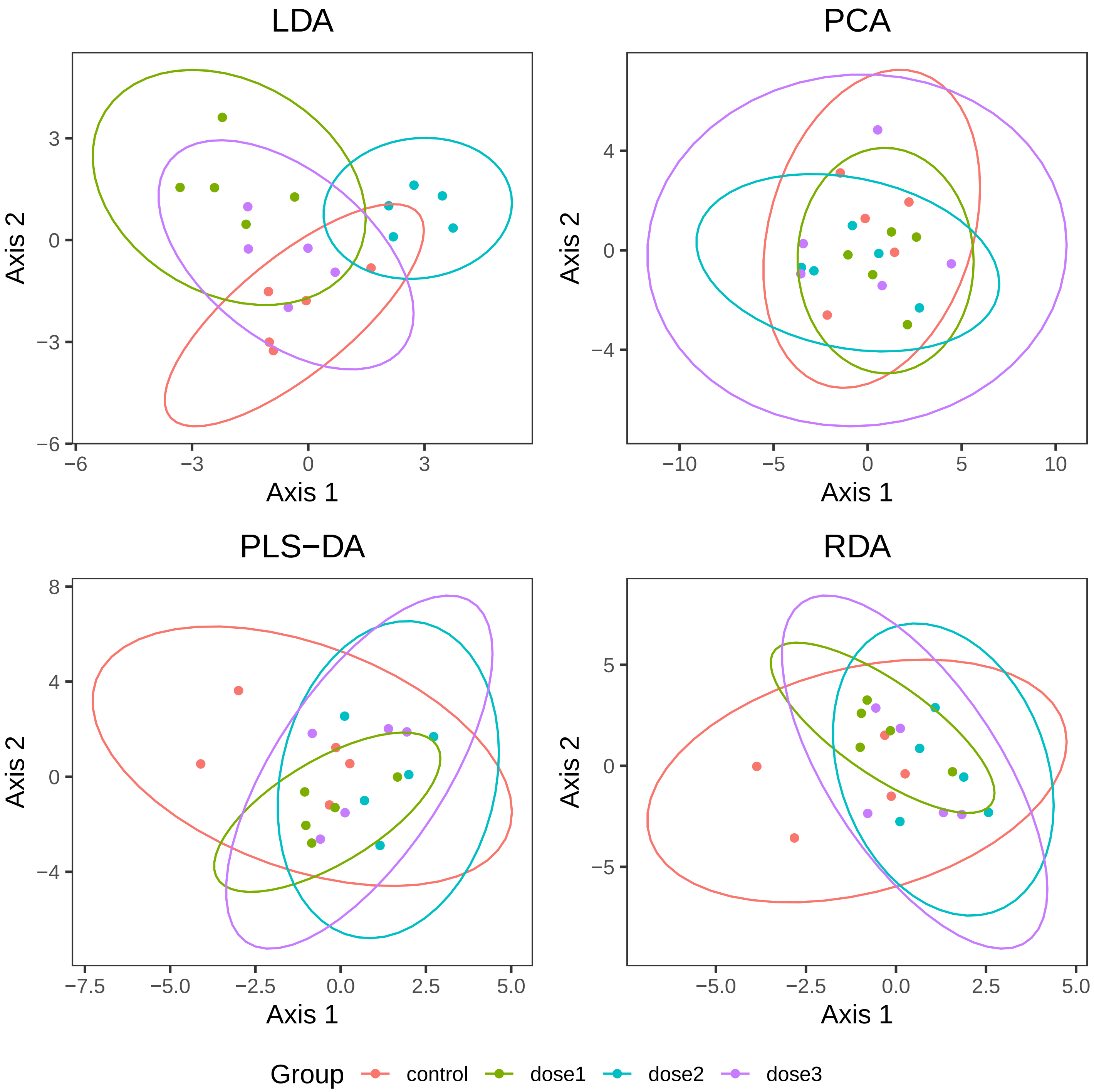

LDA
PCA
PLS−DA
RDA
Axis 1
Axis 2
Group
control
dose1
dose2
dose3

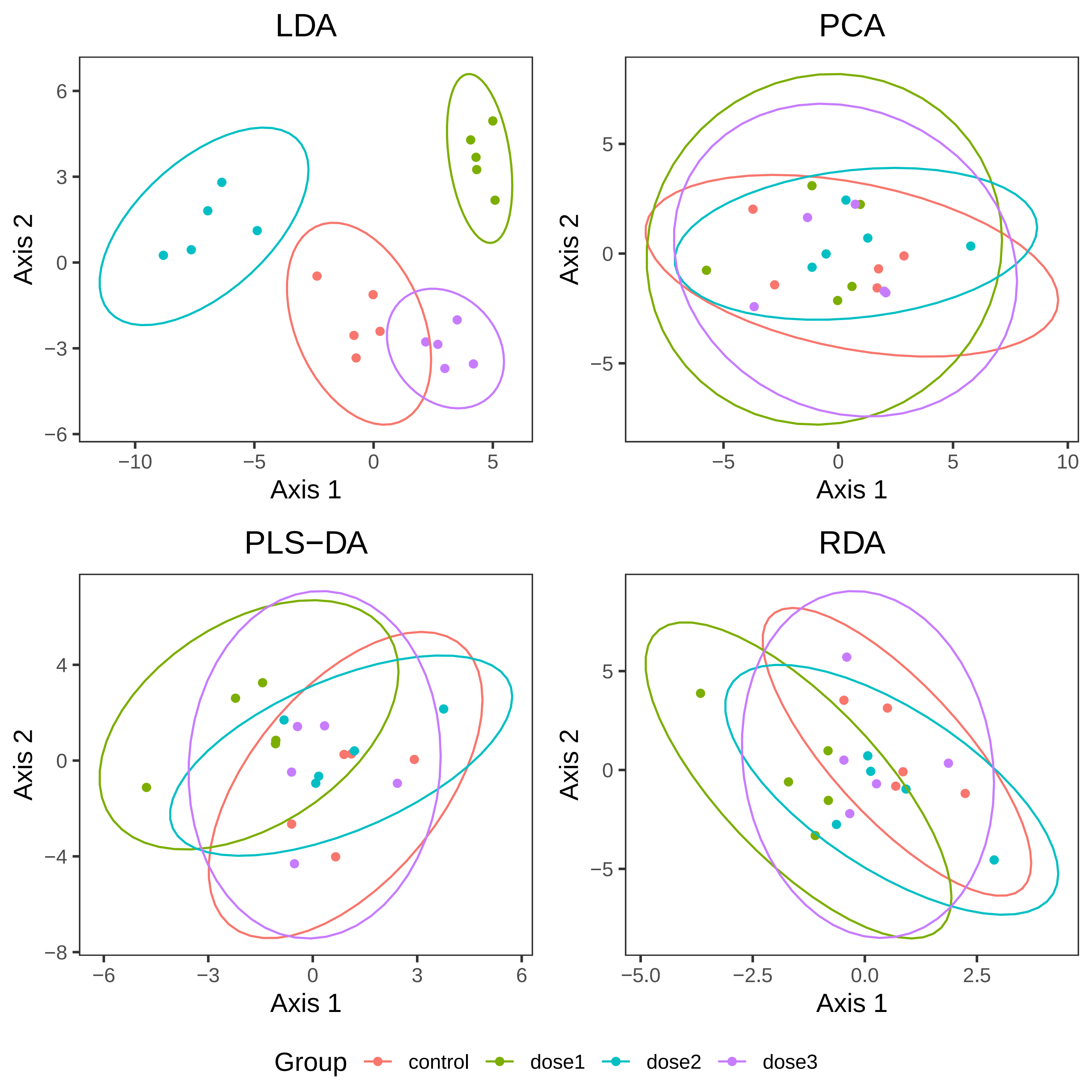

LDA
PCA
PLS−DA
RDA
Axis 1
Axis 2
Group
control
dose1
dose2
dose3

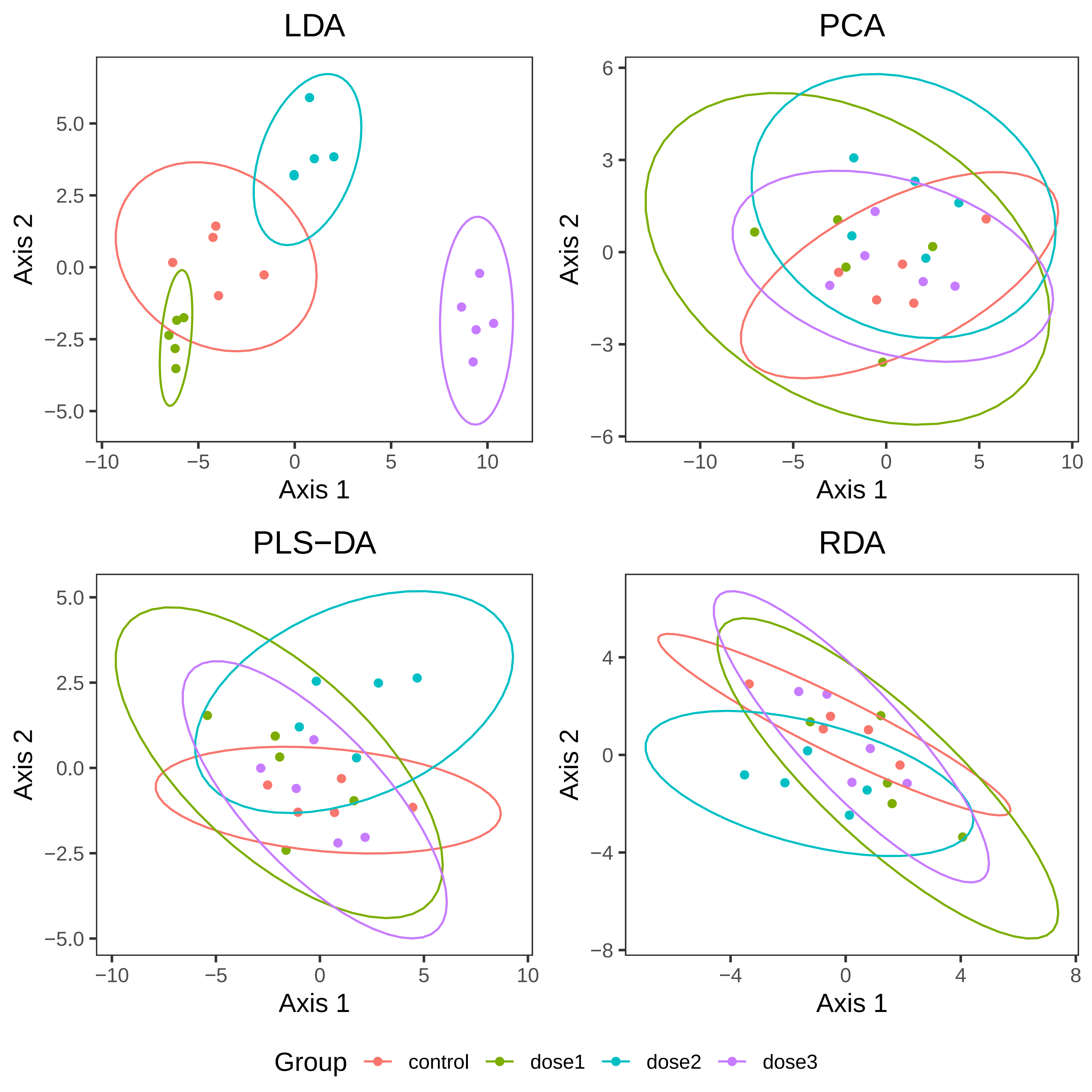

LDA
PCA
PLS−DA
RDA
Axis 1
Axis 2
Group
control
dose1
dose2
dose3

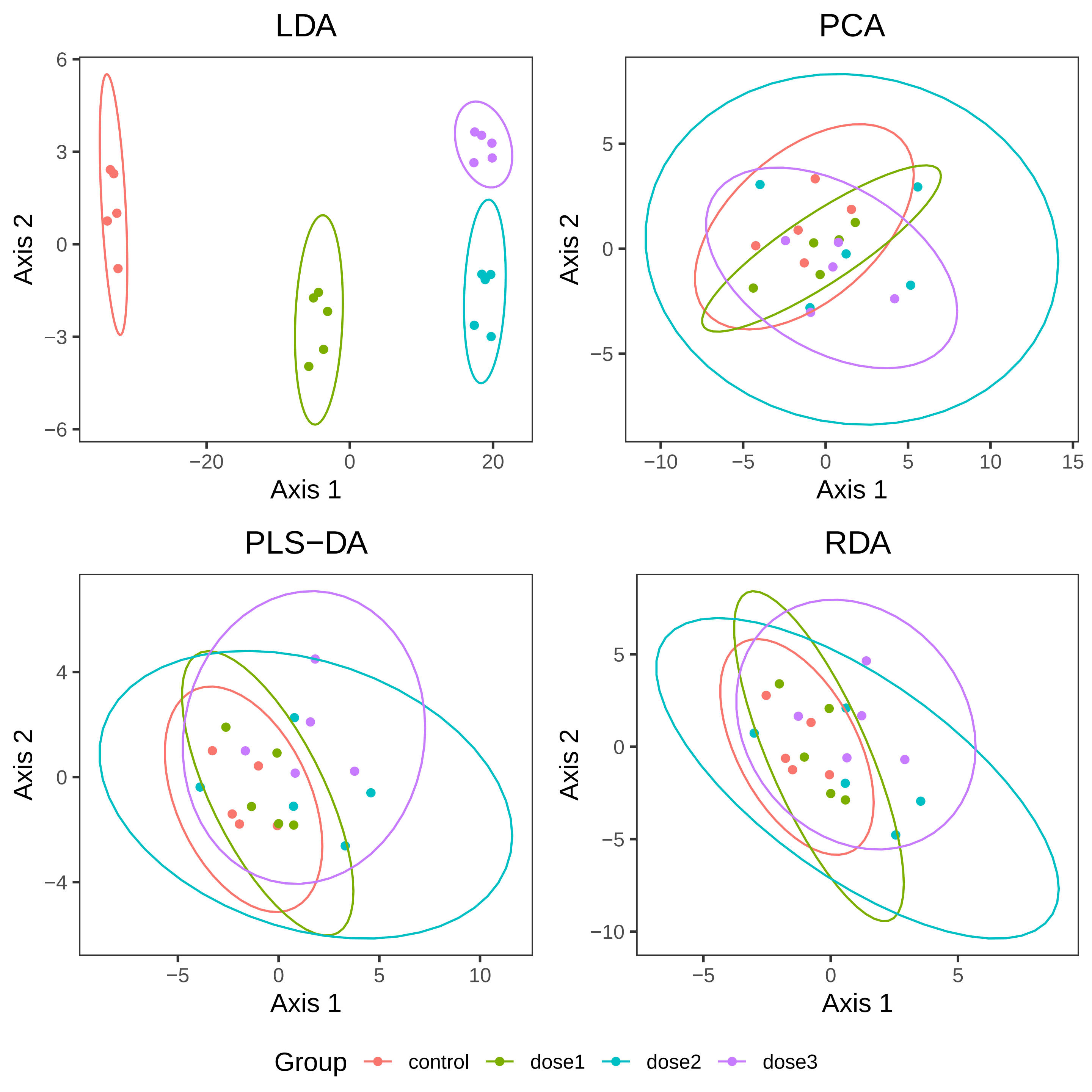

LDA
PCA
PLS−DA
RDA
Axis 1
Axis 2
6
3
0
−3
−6
−20
0
20
5
0
−5
−10
−5
0
5
10
15
4
0
−4
−5
0
5
10
5
0
−5
−10
−5
0
5
Group
control
dose1
dose2
dose3

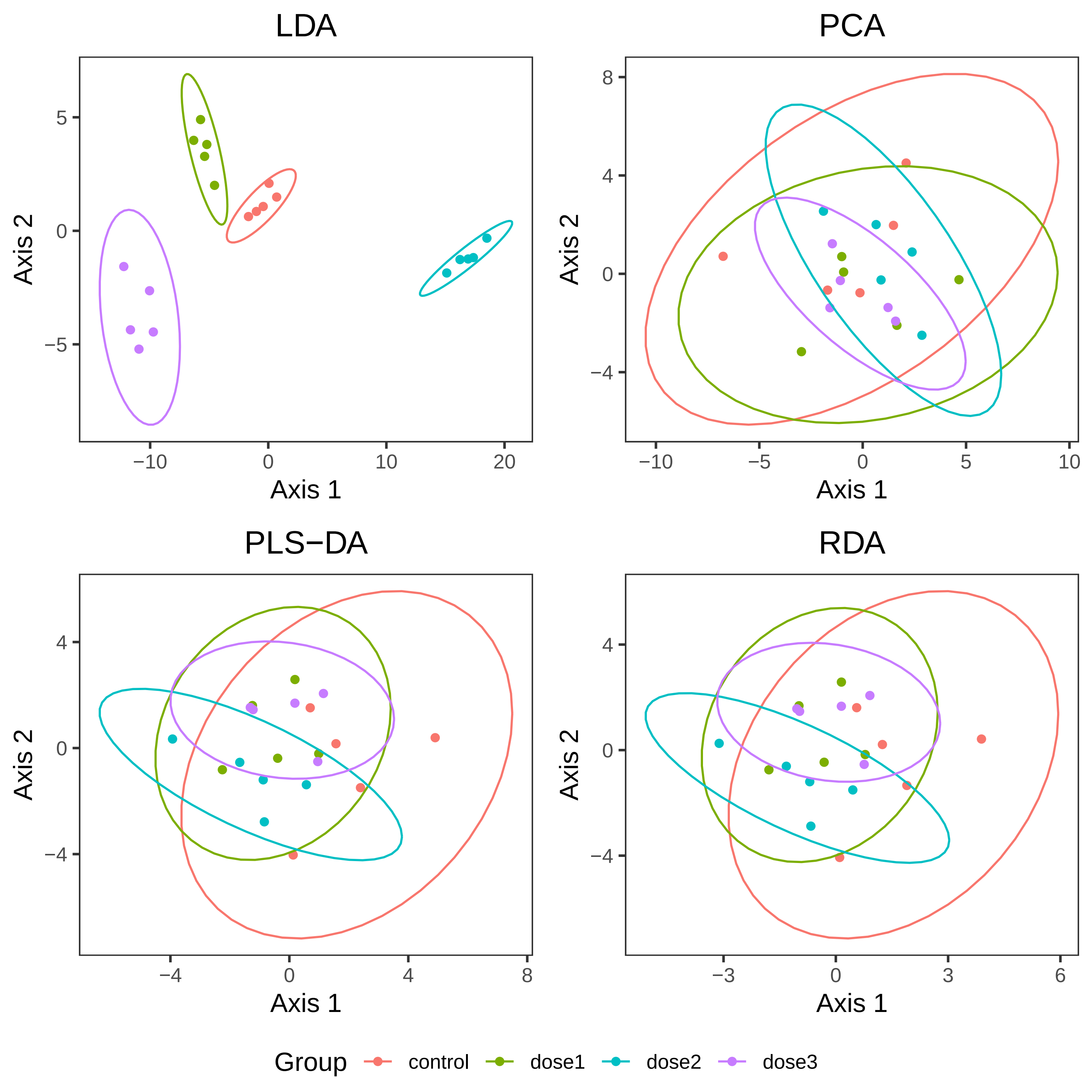
LDA
PCA
PLS−DA
RDA
Axis 1
Axis 2
Group
control
dose1
dose2
dose3

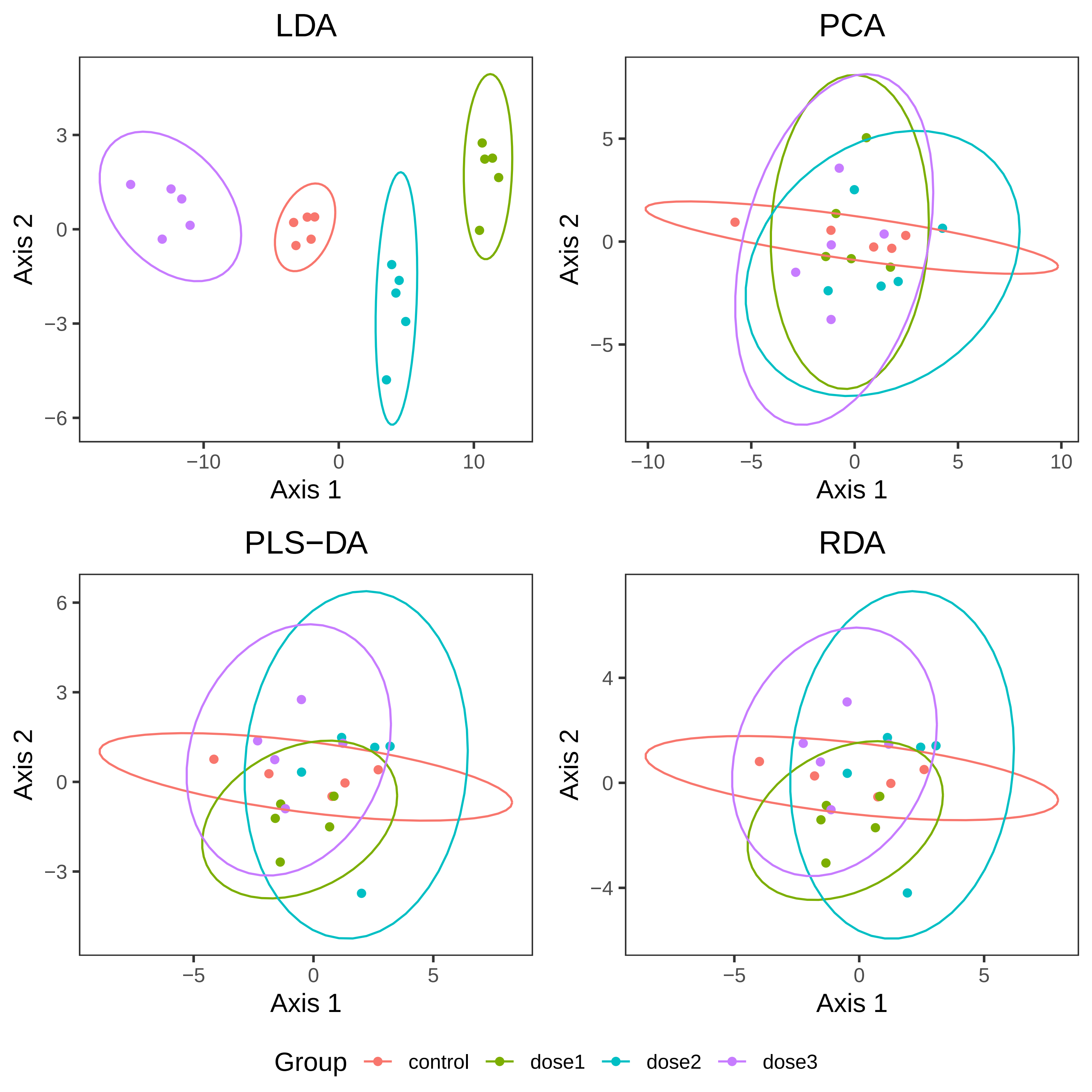

LDA
PCA
PLS−DA
RDA
Axis 1
Axis 2
3
0
−3
−6
−10
10
5
−5
6
4
−4
Group
control
dose1
dose2
dose3

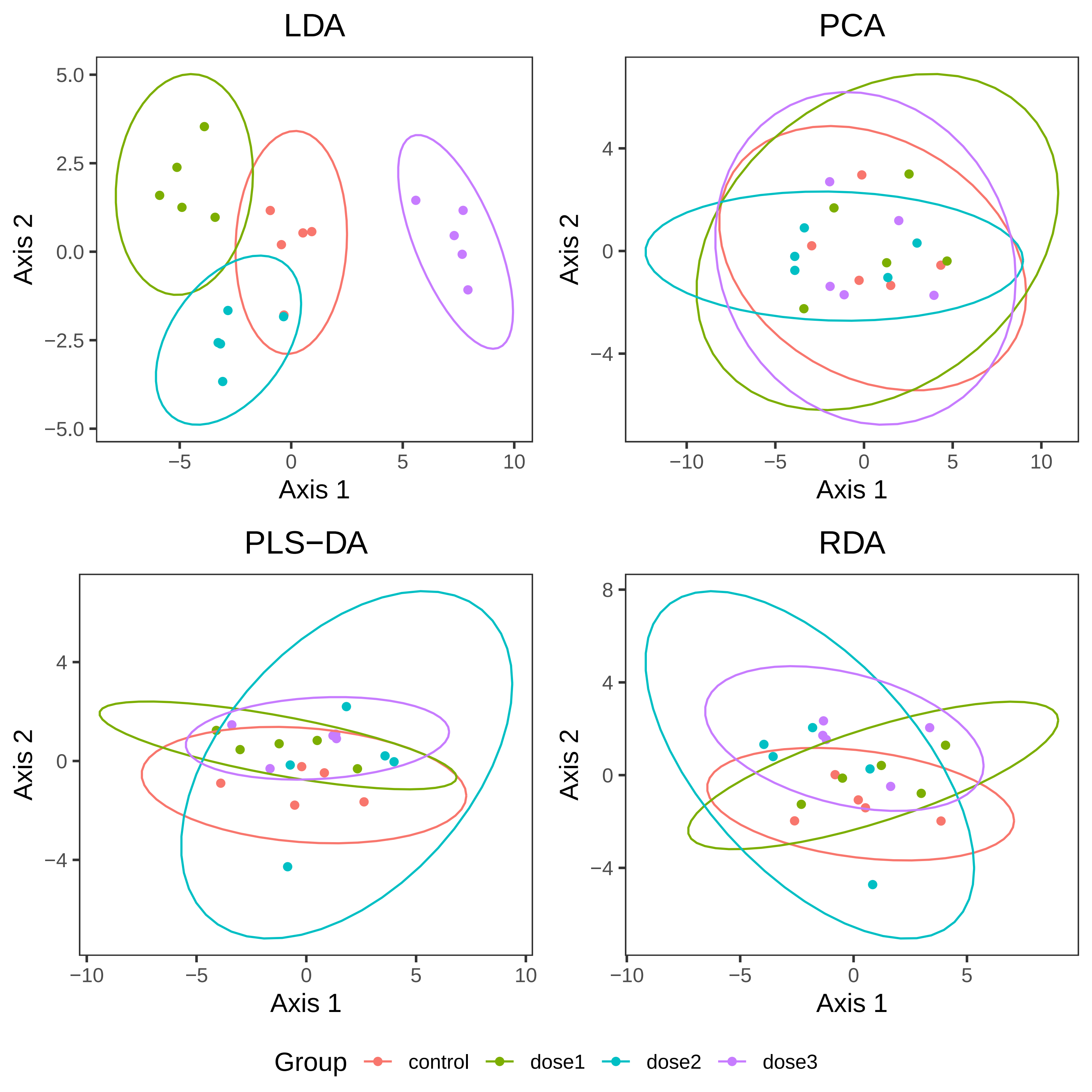
LDA
PCA
PLS−DA
RDA
Axis 1
Axis 2
Group
control
dose1
dose2
dose3

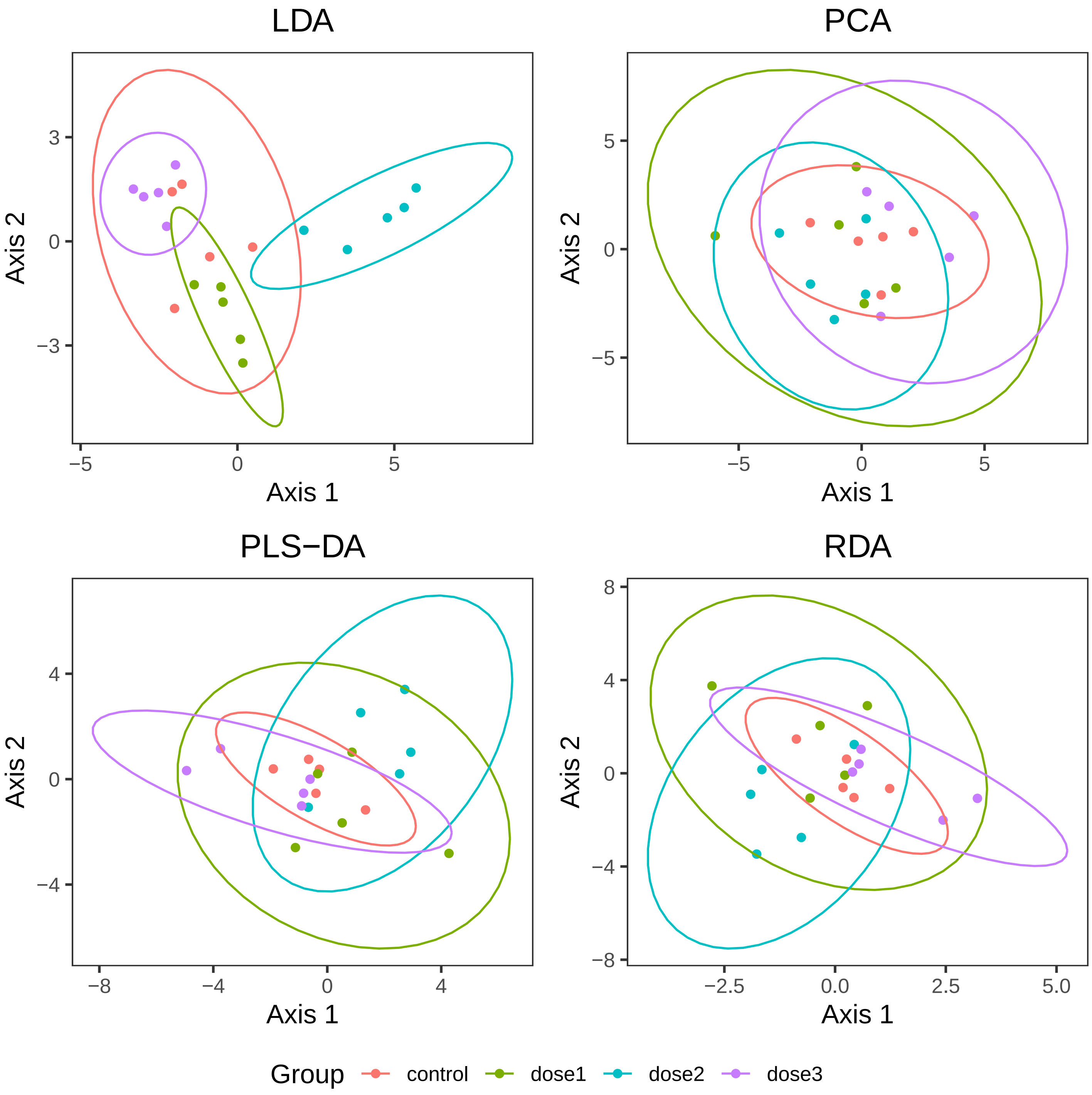
LDA
PCA
PLS−DA
RDA
Axis 1
Axis 2
Group
control
dose1
dose2
dose3

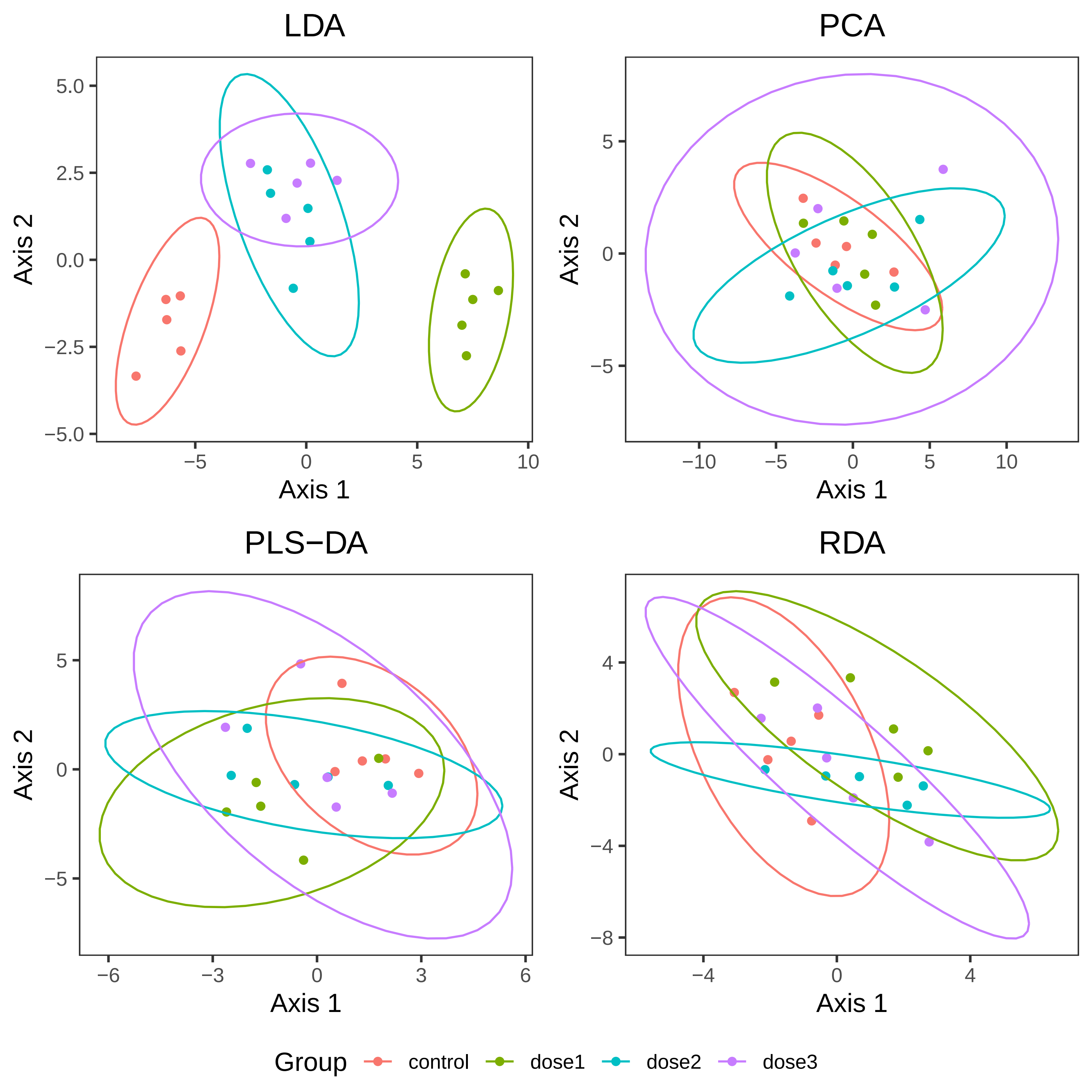

LDA
PCA
PLS−DA
RDA
Axis 1
Axis 2
Group
control
dose1
dose2
dose3

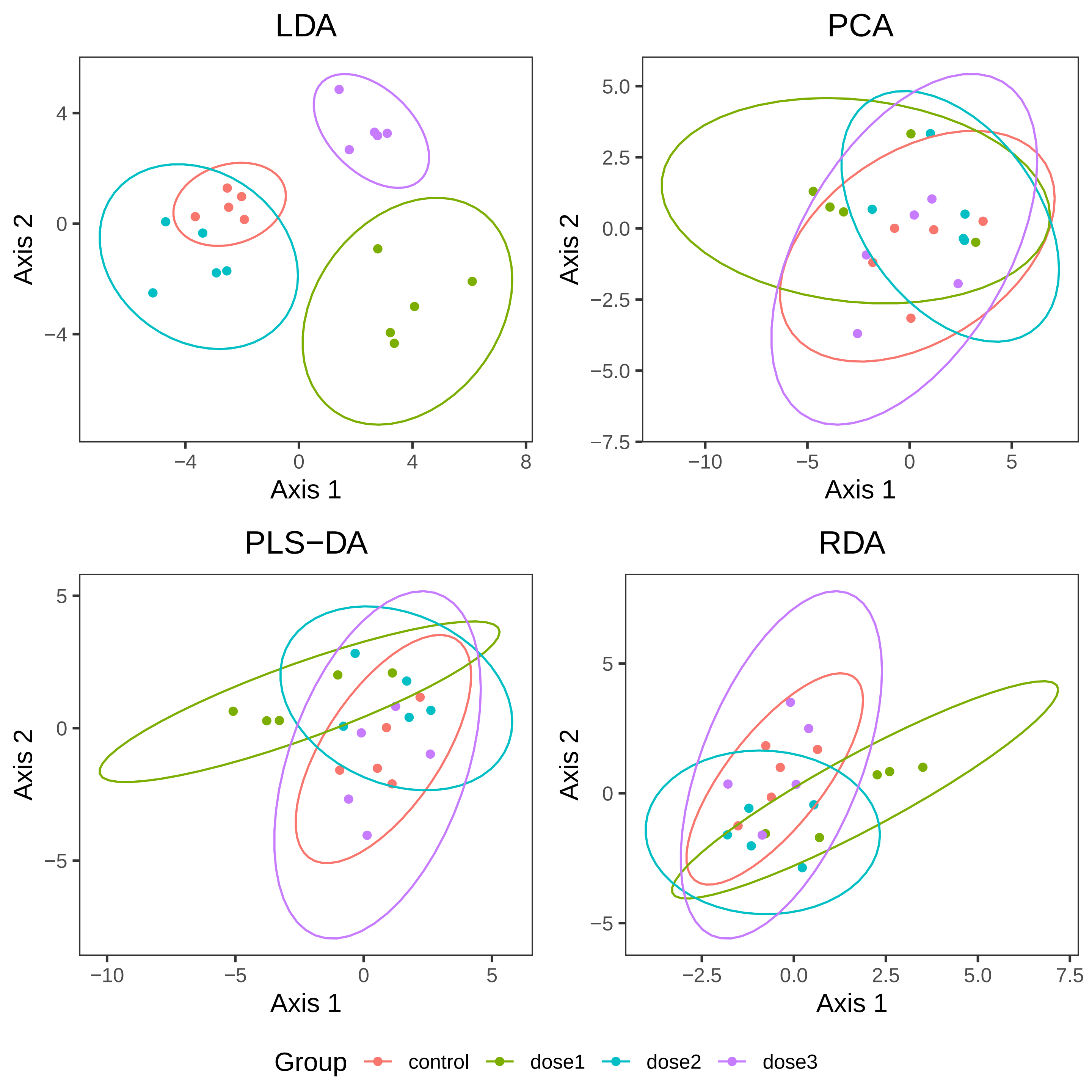
LDA
PCA
PLS−DA
RDA
Axis 1
Axis 2
Group
control
dose1
dose2
dose3

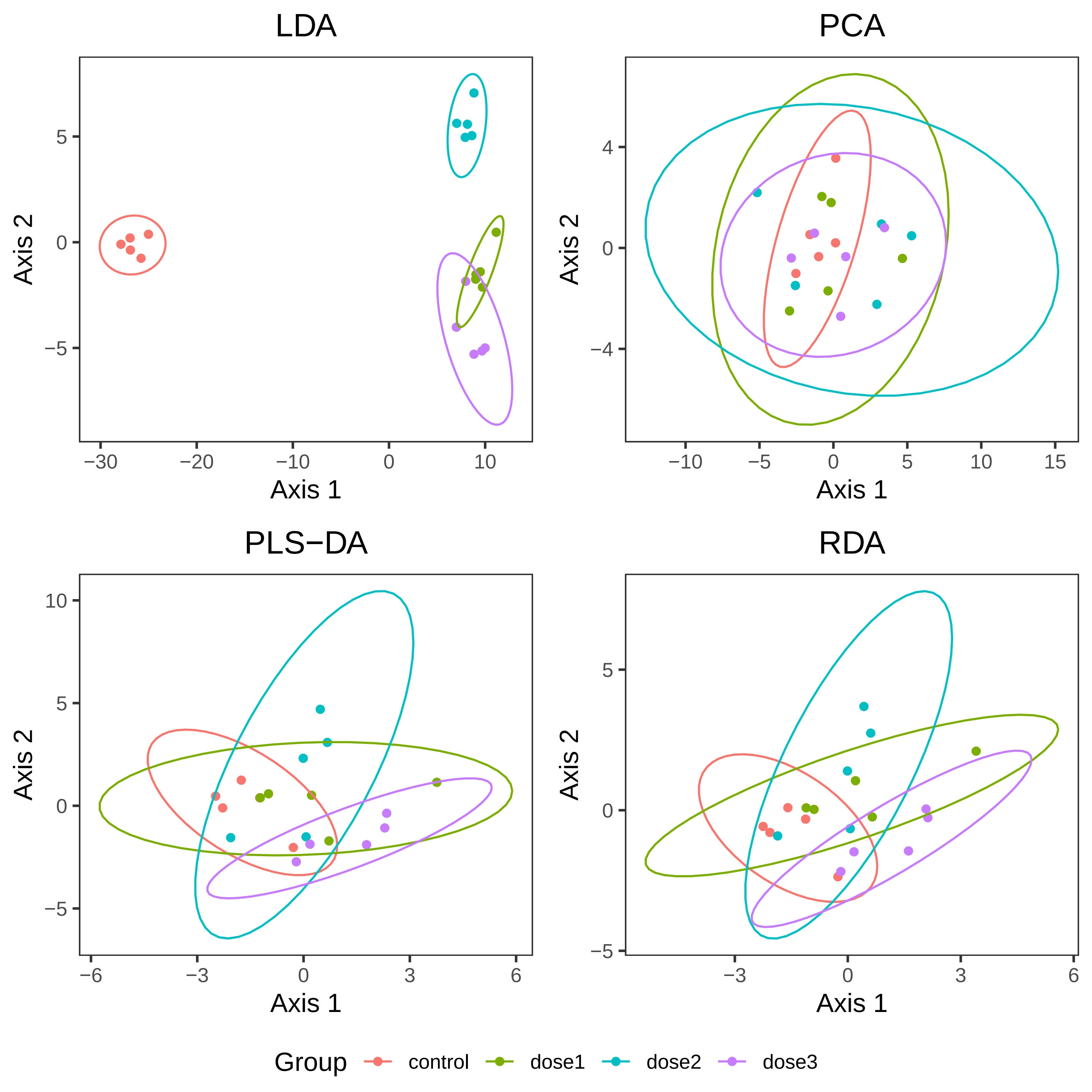
LDA
PCA
PLS−DA
RDA
Axis 1
Axis 2
−30
−20
−10
0
10
5
0
−5
−10
−5
0
5
10
15
4
0
−4
−6
−3
0
3
6
10
5
0
−5
−3
0
3
6
5
0
−5
Group
control
dose1
dose2
dose3

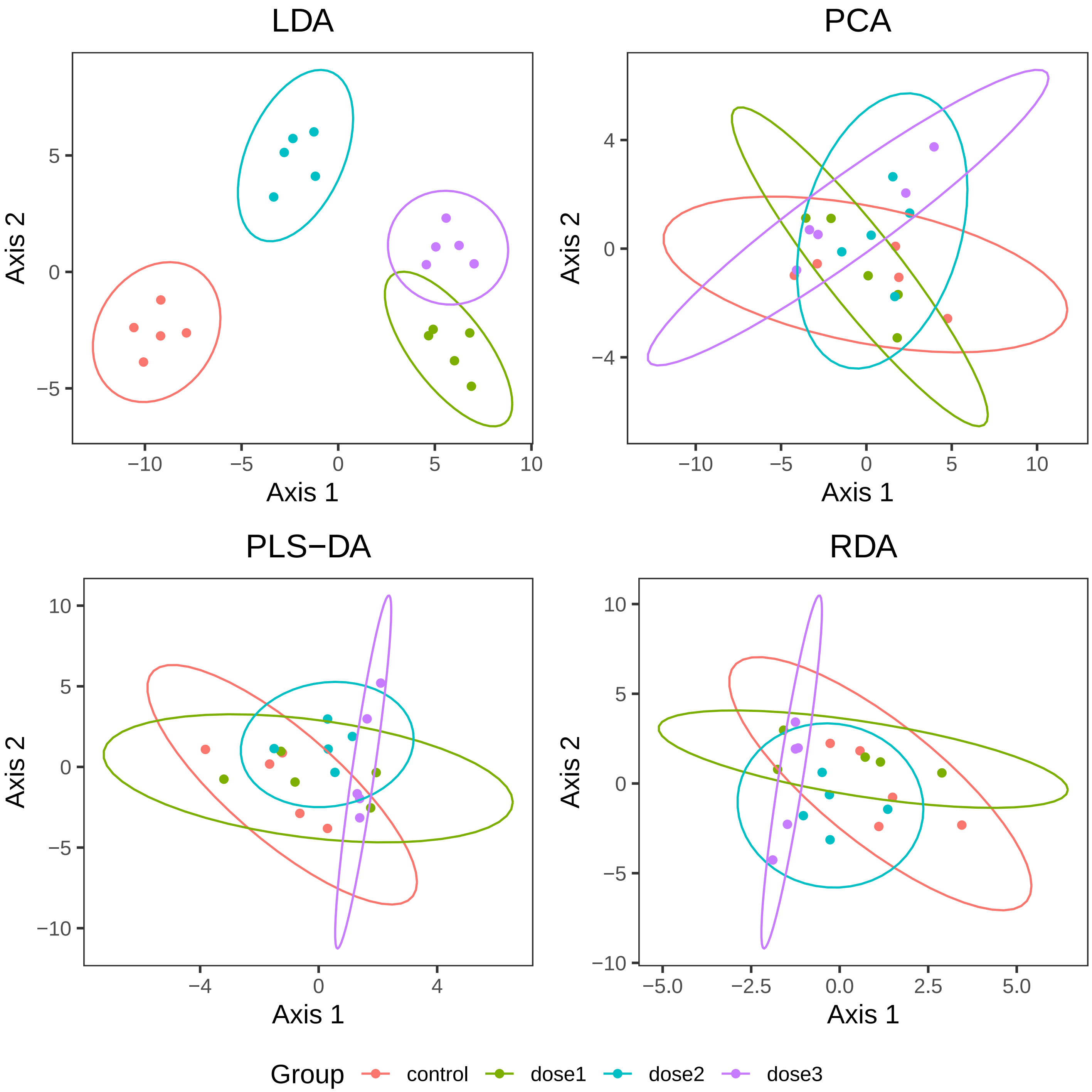

LDA
PCA
PLS−DA
RDA
Axis 1
Axis 2
Group
control
dose1
dose2
dose3

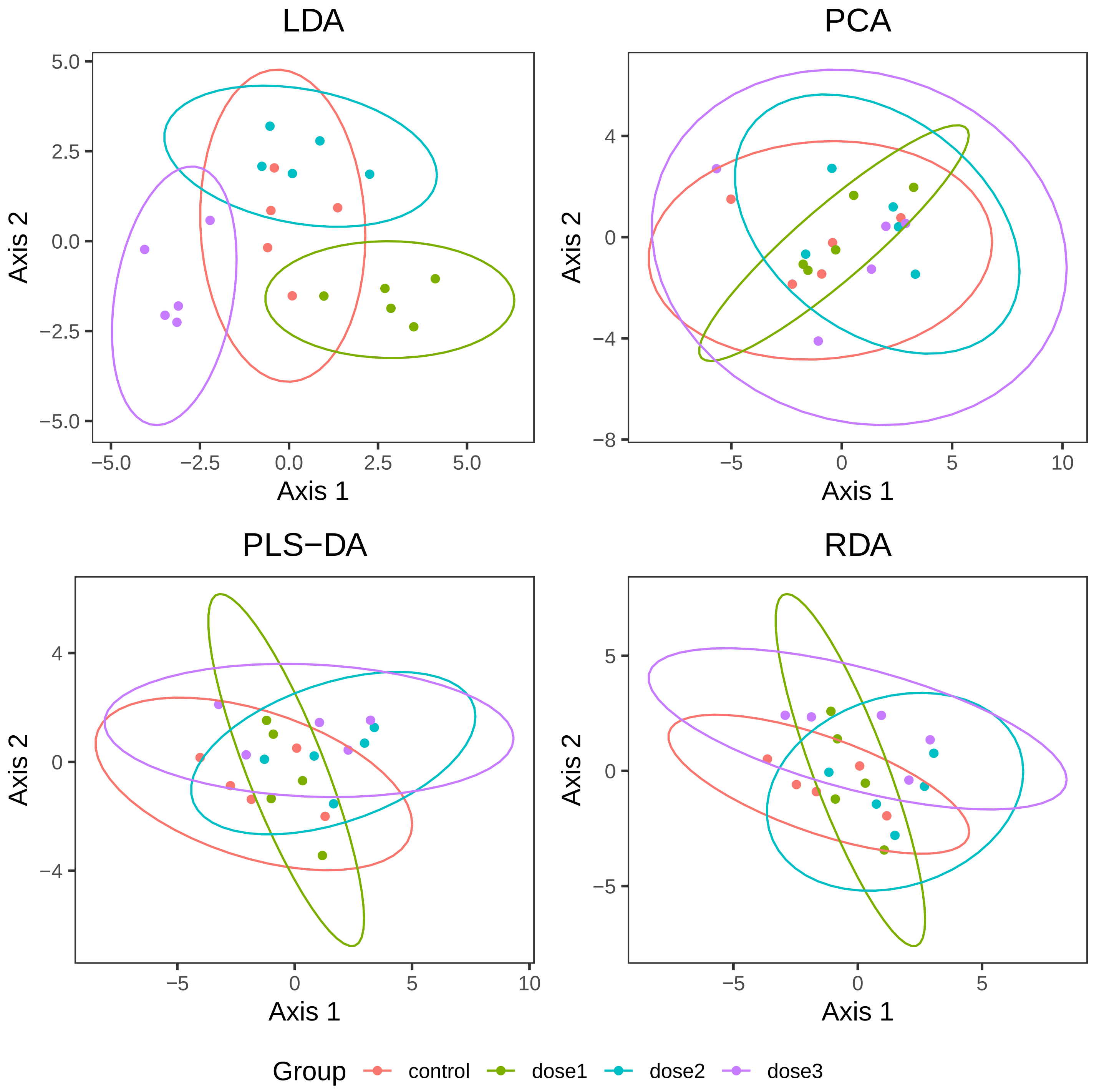
LDA
PCA
PLS−DA
RDA
Axis 1
Axis 2
5.0
2.5
0.0
−2.5
−5.0
4
0
−4
−8
5
−5
10
Group
control
dose1
dose2
dose3

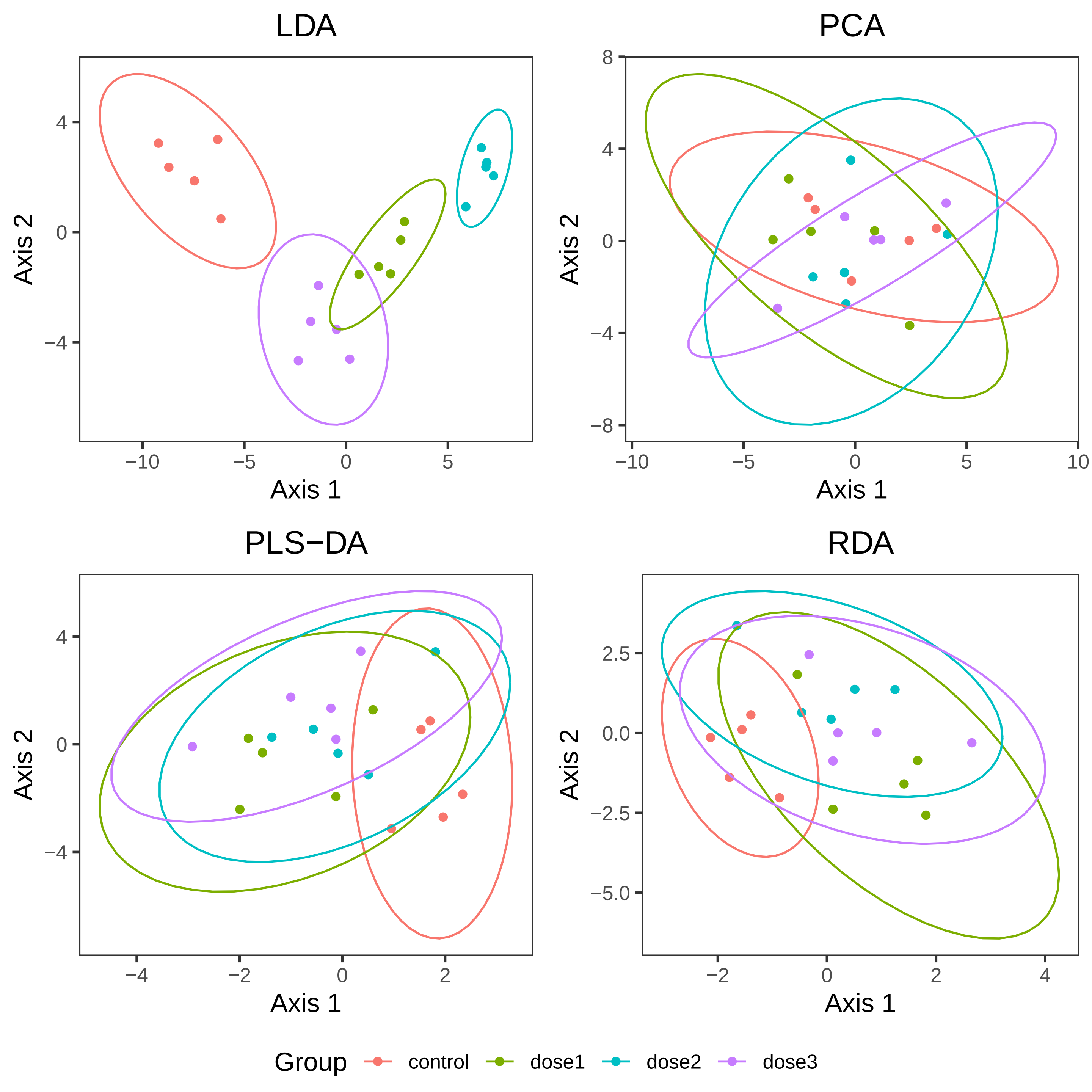
LDA
PCA
PLS−DA
RDA
Axis 1
Axis 2
Group
control
dose1
dose2
dose3

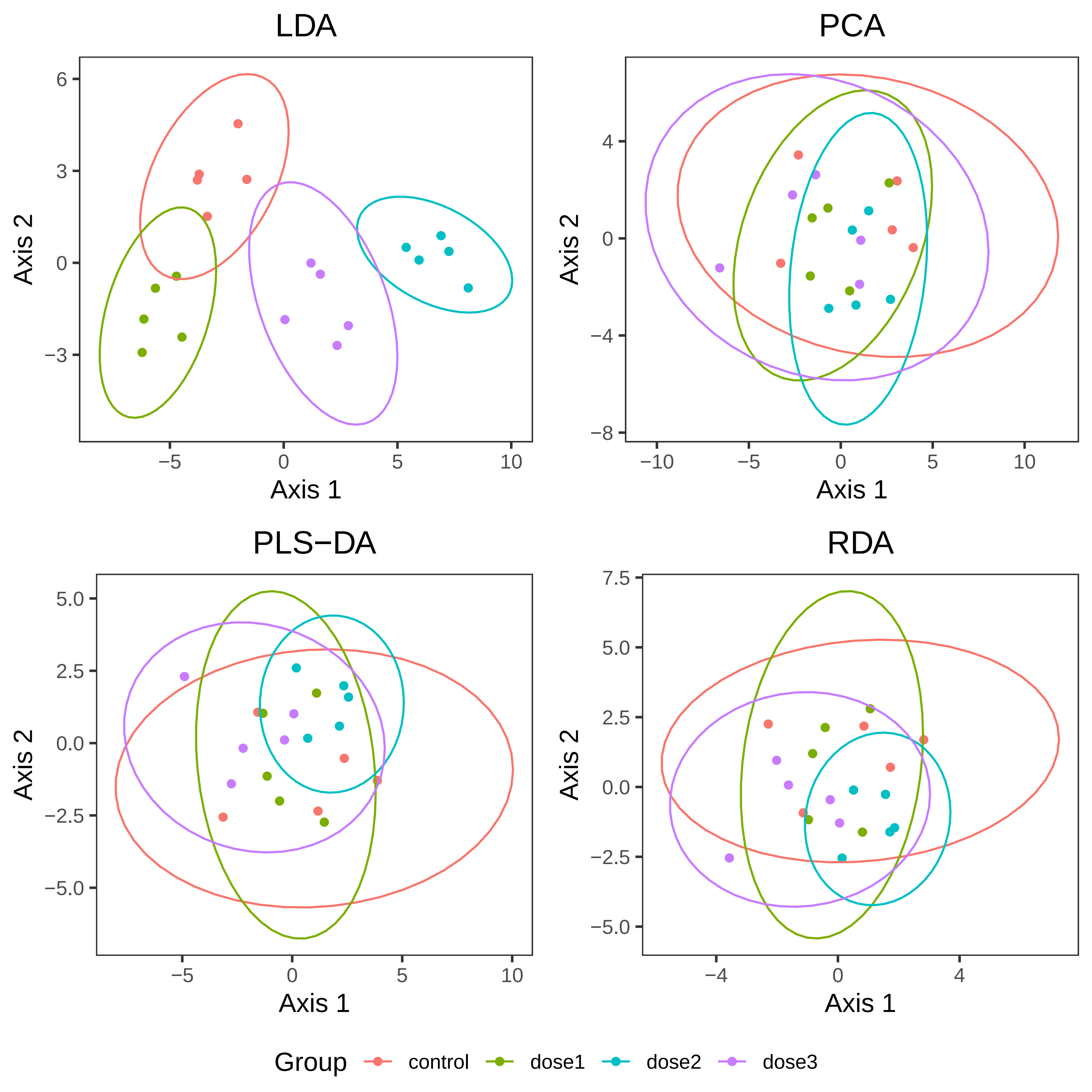
LDA
PCA
PLS−DA
RDA
Axis 1
Axis 2
Group
control
dose1
dose2
dose3

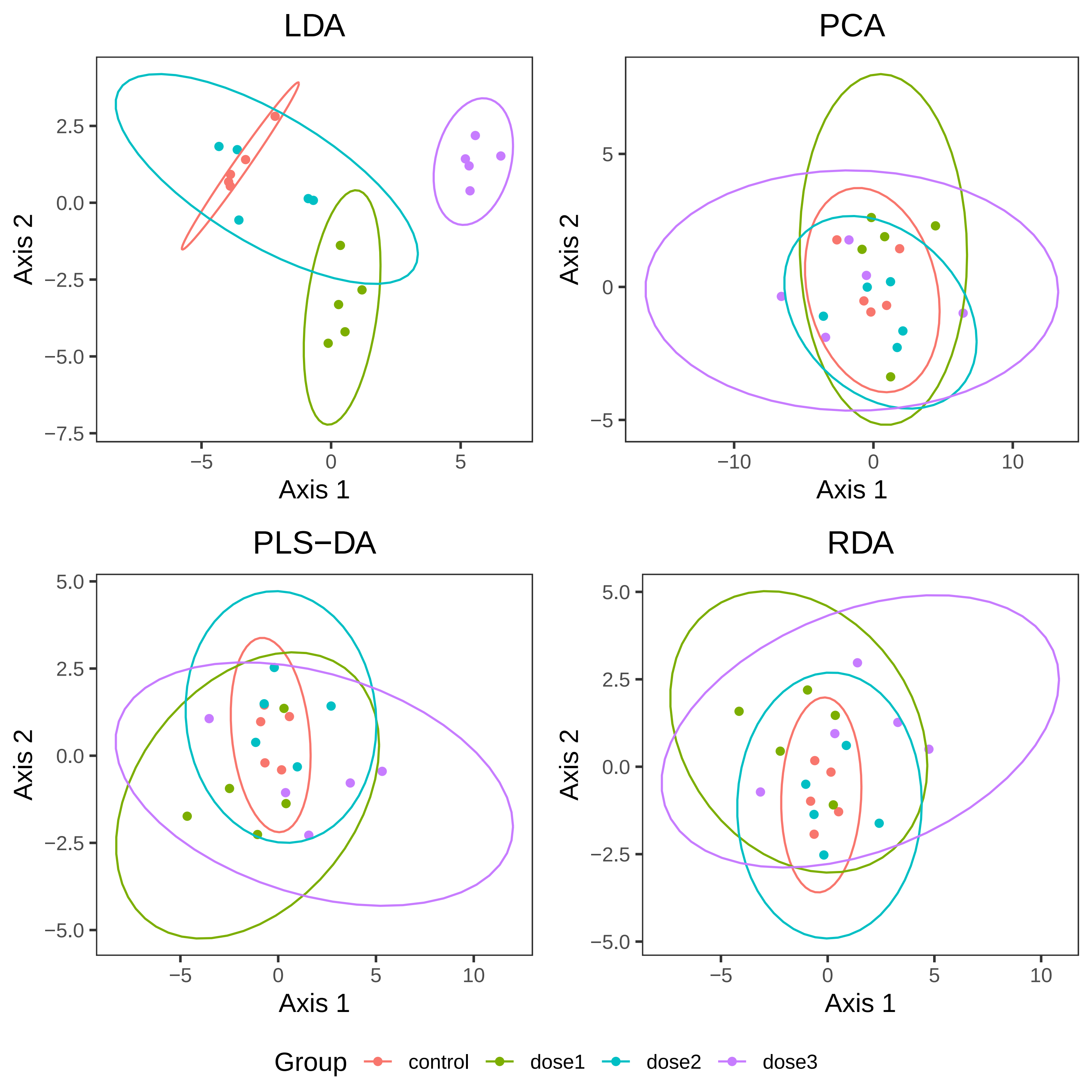

LDA
PCA
PLS−DA
RDA
Axis 1
Axis 2
Group
control
dose1
dose2
dose3

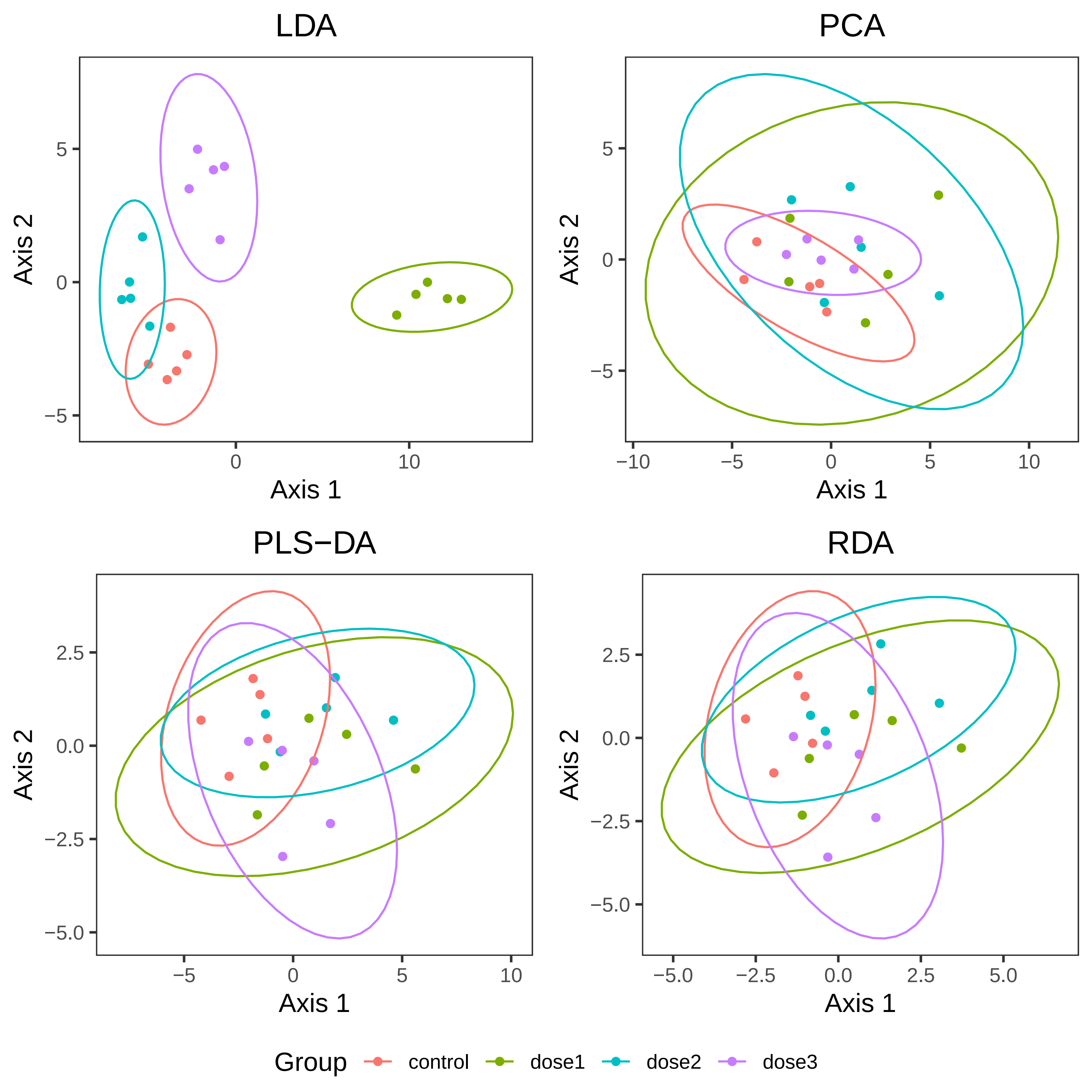
LDA
PCA
PLS−DA
RDA
Axis 1
Axis 2
Group
control
dose1
dose2
dose3

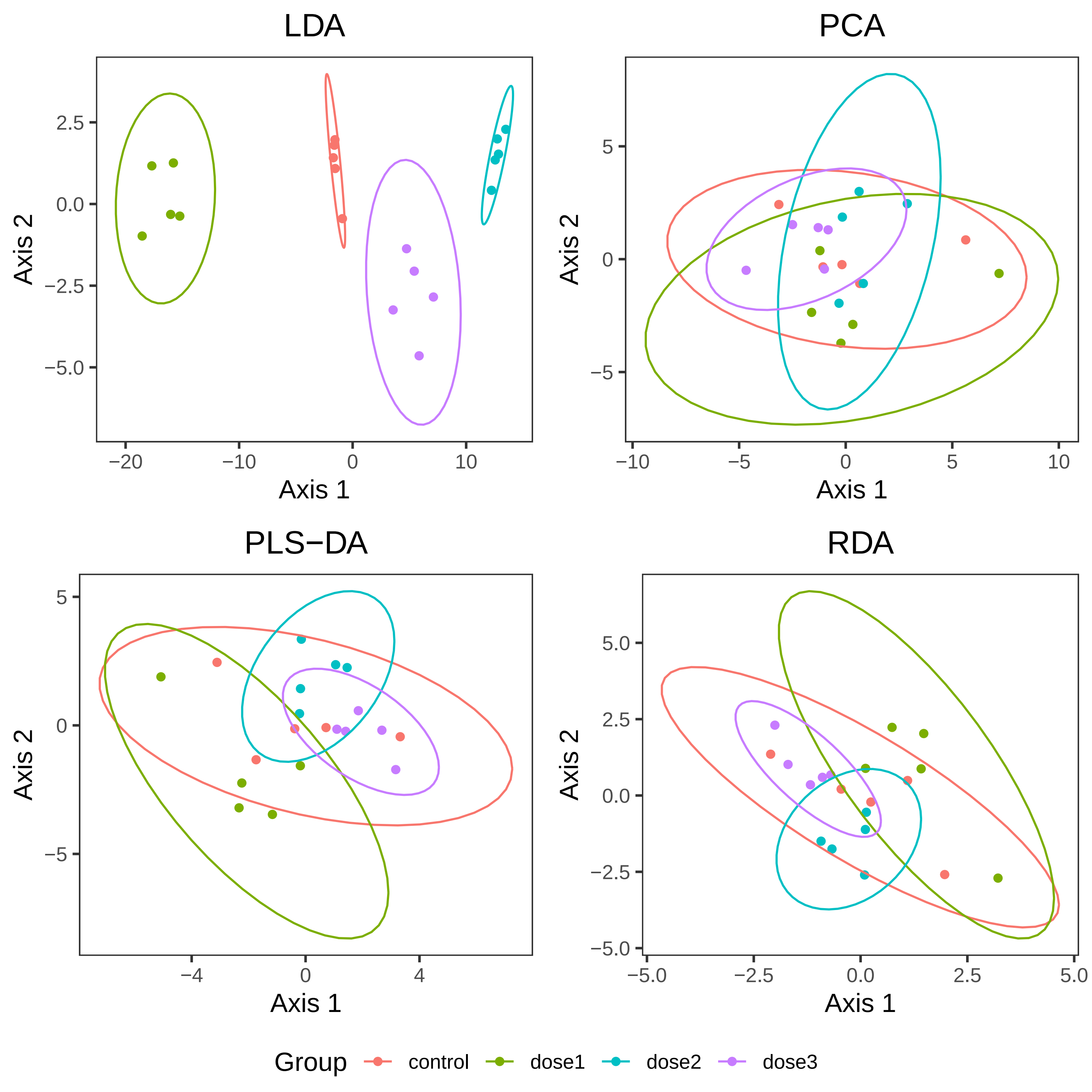
LDA
PCA
PLS−DA
RDA
Axis 1
Axis 2
Group
control
dose1
dose2
dose3

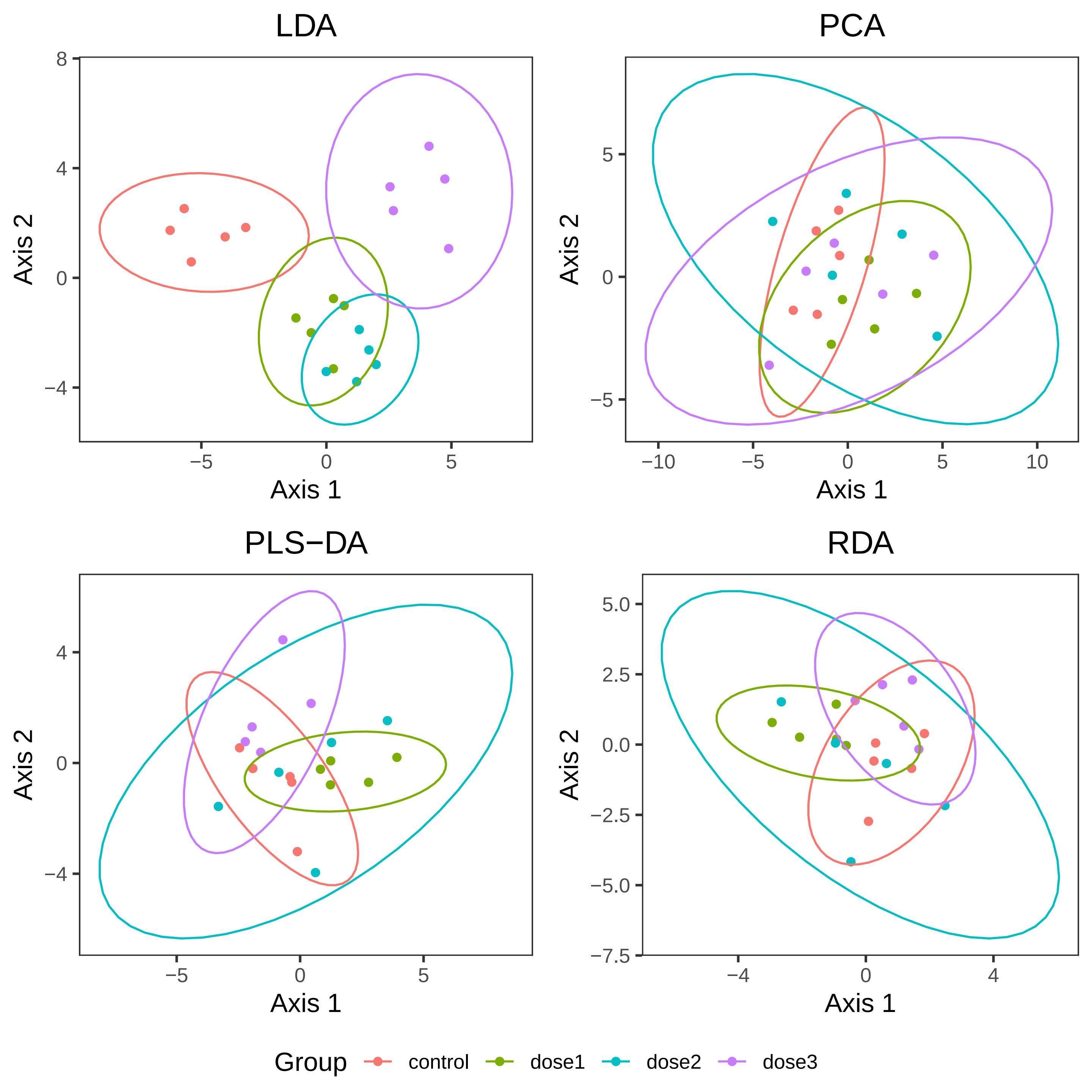

LDA
PCA
PLS−DA
RDA
Axis 1
Axis 2
8
4
0
−4
−5
0
5
10
−10
5.0
2.5
0.0
−2.5
−5.0
−7.5
Group
control
dose1
dose2
dose3

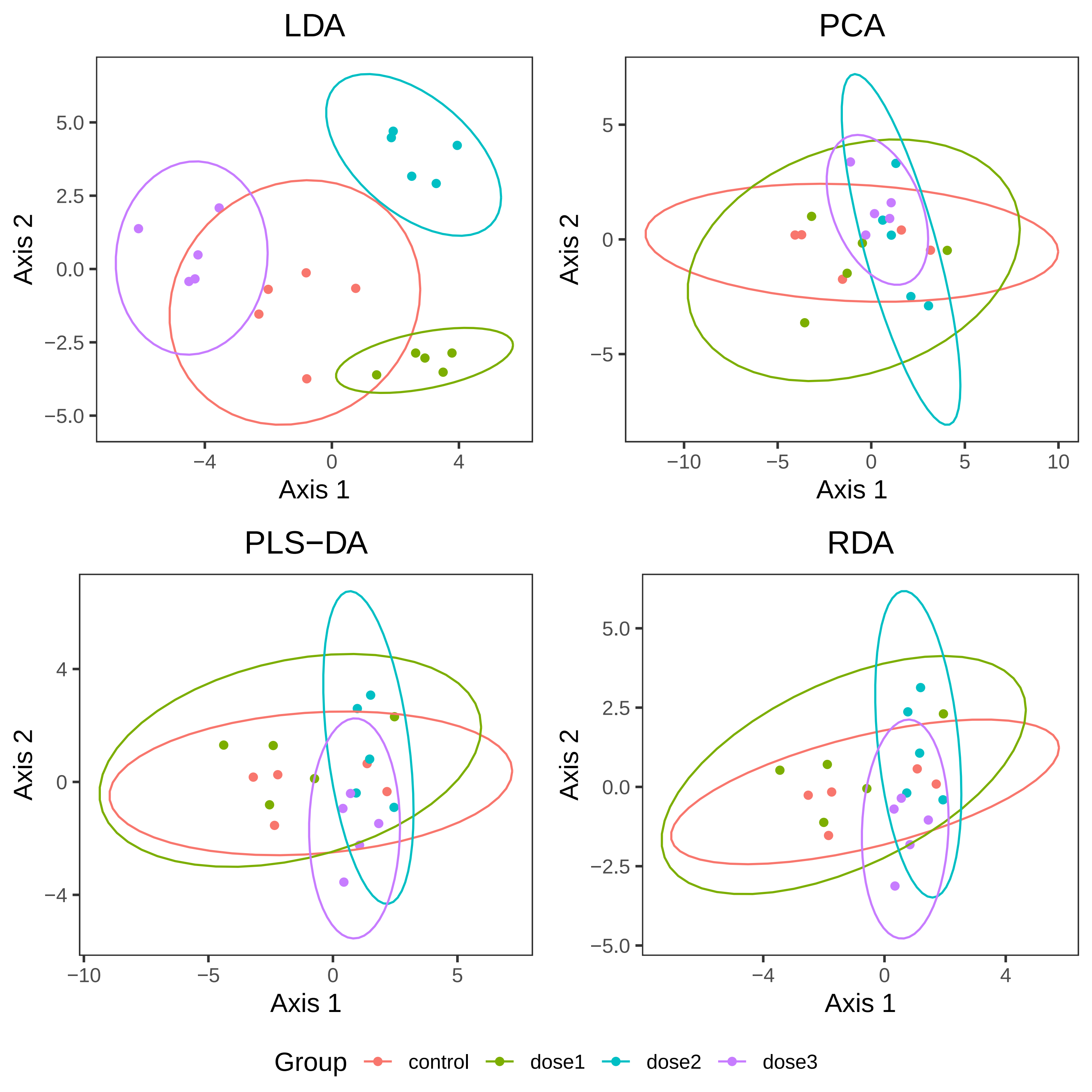
LDA
PCA
PLS−DA
RDA
Axis 1
Axis 2
5.0
2.5
0.0
−2.5
−5.0
−4
0
4
5
0
−5
−10
10
−4
Group
control
dose1
dose2
dose3

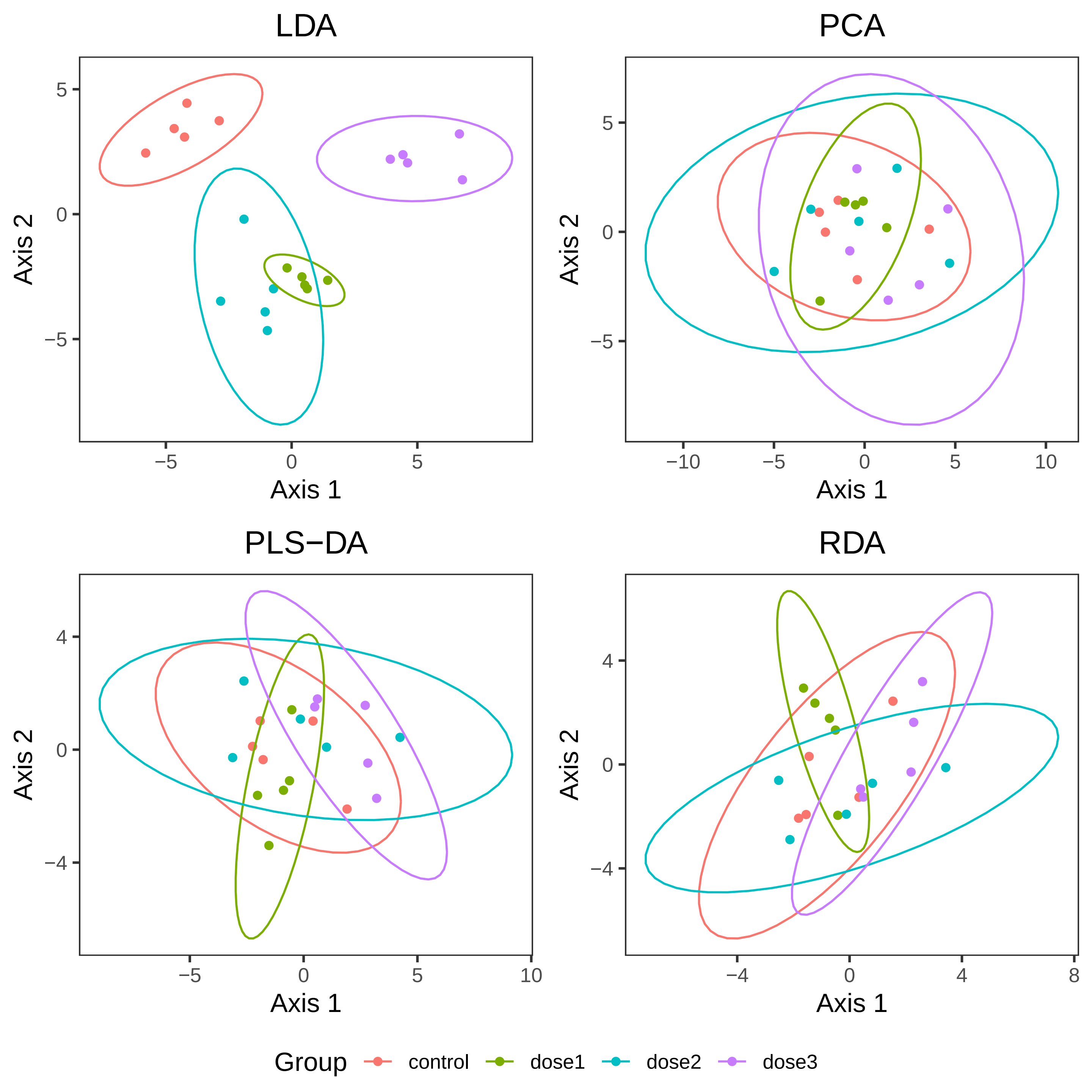
LDA
PCA
PLS−DA
RDA
Axis 1
Axis 2
5
0
−5
−10
10
4
−4
8
Group
control
dose1
dose2
dose3

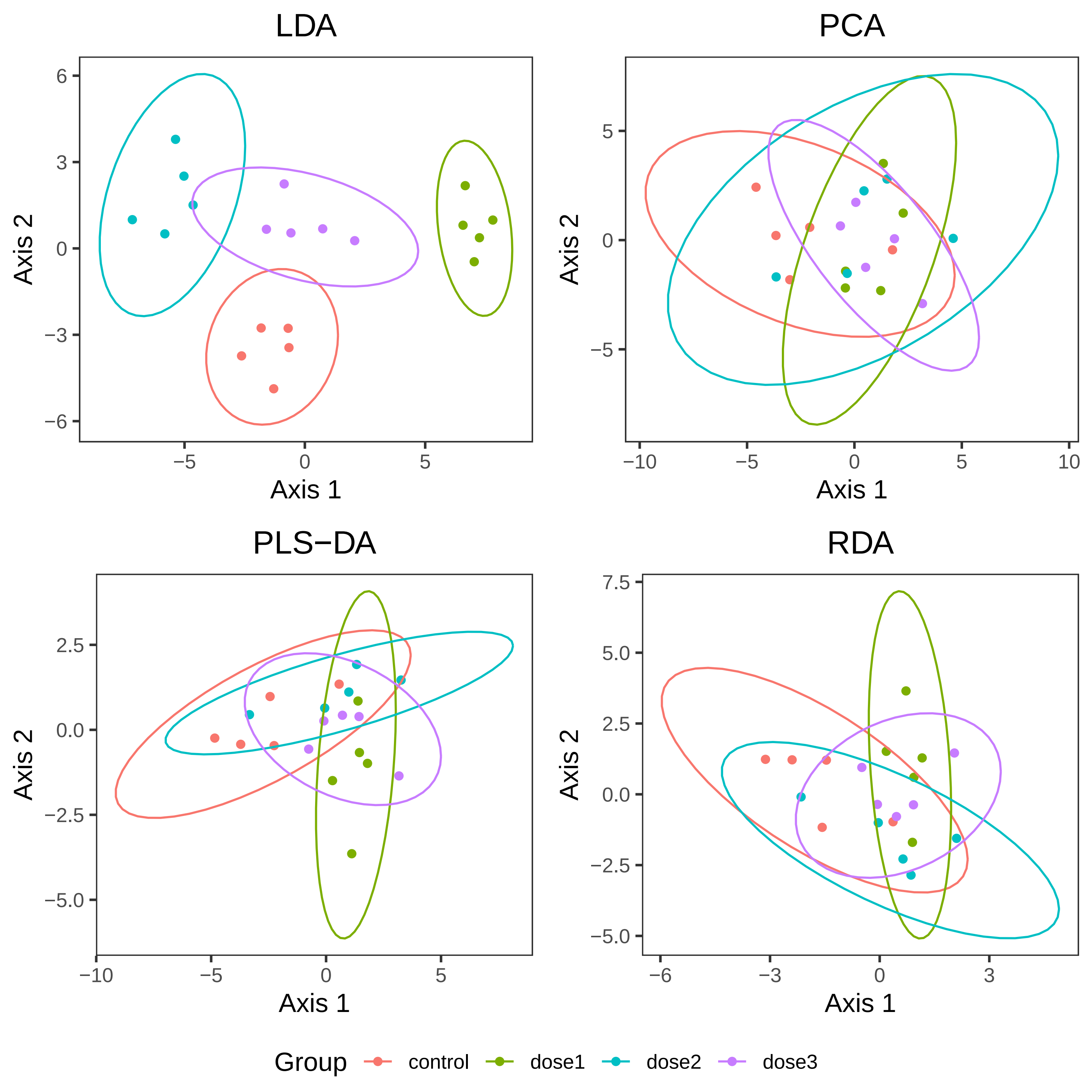
LDA
PCA
PLS−DA
RDA
Axis 1
Axis 2
6
3
0
−3
−6
−5
5
−10
10
2.5
0.0
−2.5
−5.0
7.5
5.0
−6
Group
control
dose1
dose2
dose3

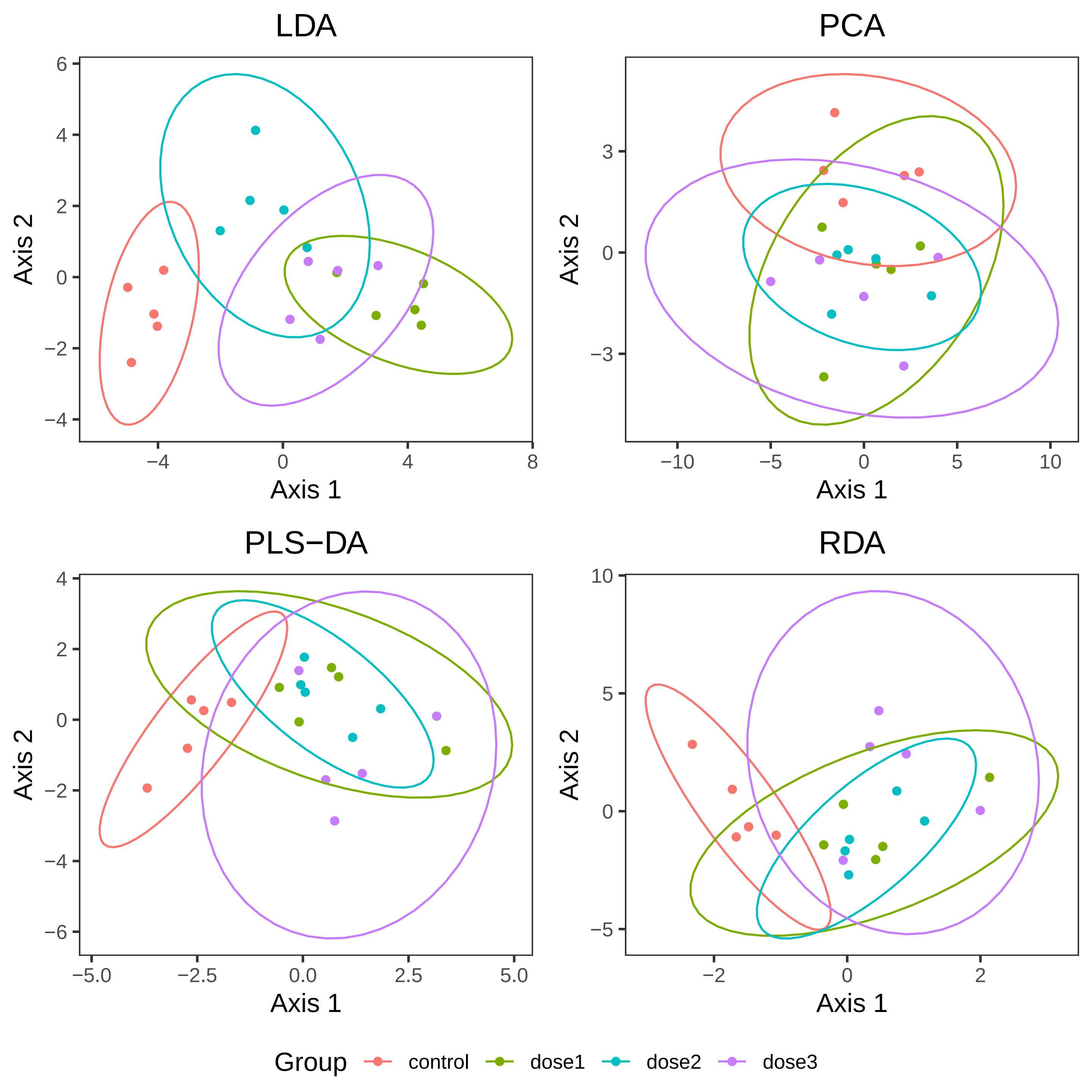

LDA
PCA
PLS−DA
RDA
Axis 1
Axis 2
6
4
2
0
−2
−4
8
3
−3
−10
−5
5
10
−6
−2.5
−5.0
0.0
2.5
5.0
Group
control
dose1
dose2
dose3

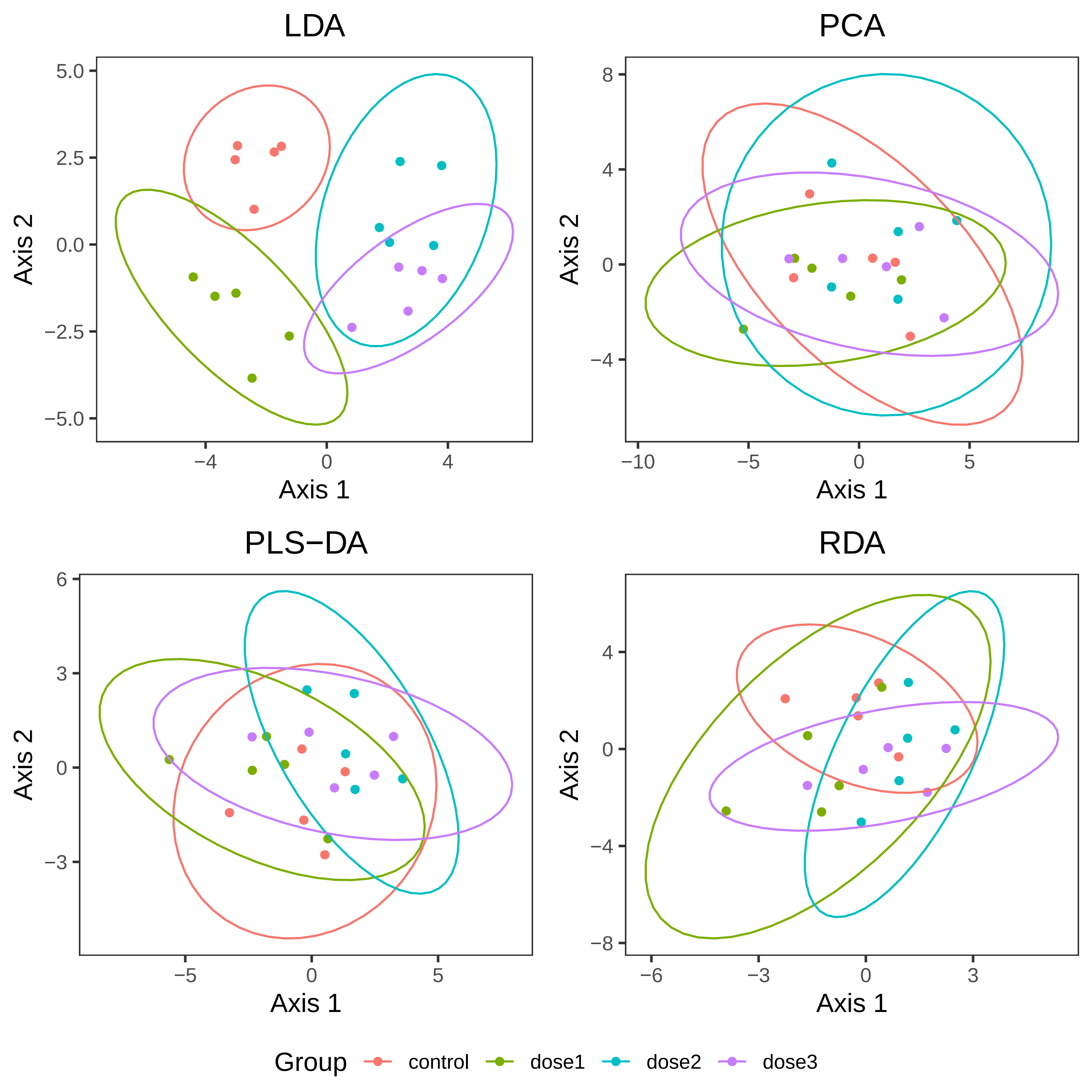
LDA
PCA
PLS−DA
RDA
Axis 1
Axis 2
5.0
2.5
0.0
−2.5
−5.0
−4
0
4
8
−10
−5
5
6
3
−3
−6
−8
Group
control
dose1
dose2
dose3

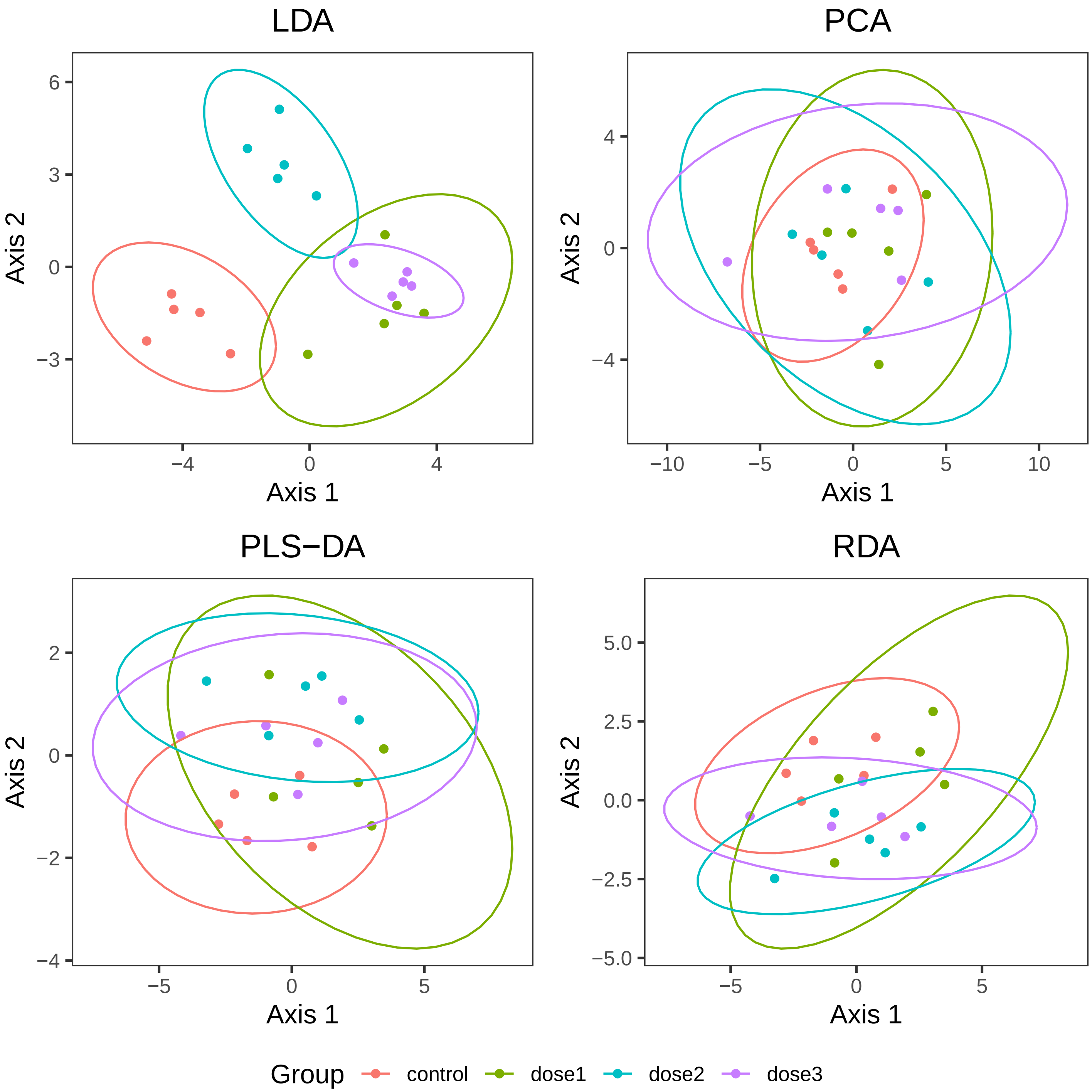

LDA
PCA
PLS−DA
RDA
Axis 1
Axis 2
6
3
0
−3
−4
4
−10
−5
5
10
2
−2
−4
5.0
2.5
0.0
−2.5
−5.0
Group
control
dose1
dose2
dose3

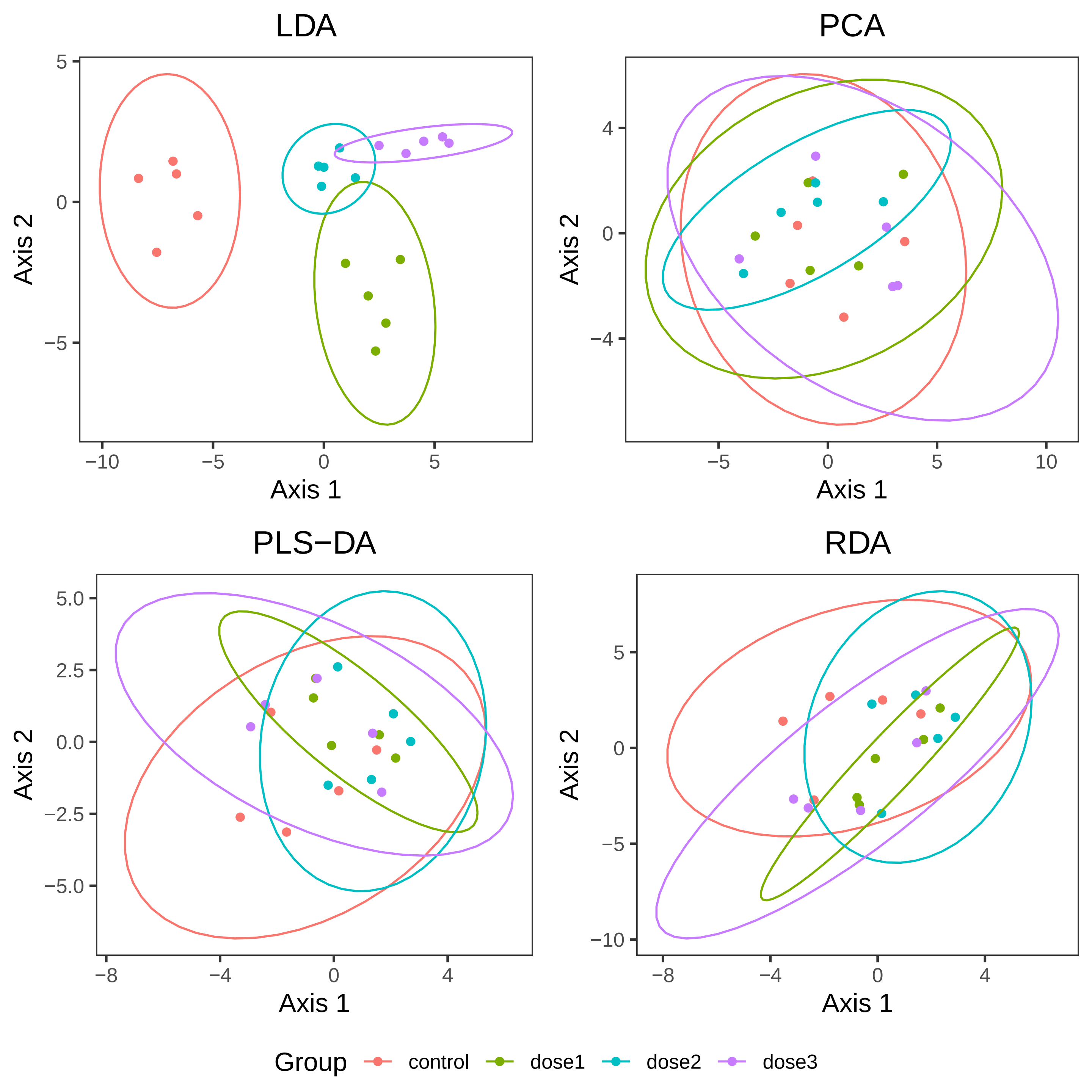

LDA
PCA
PLS−DA
RDA
Axis 1
Axis 2
Group
control
dose1
dose2
dose3

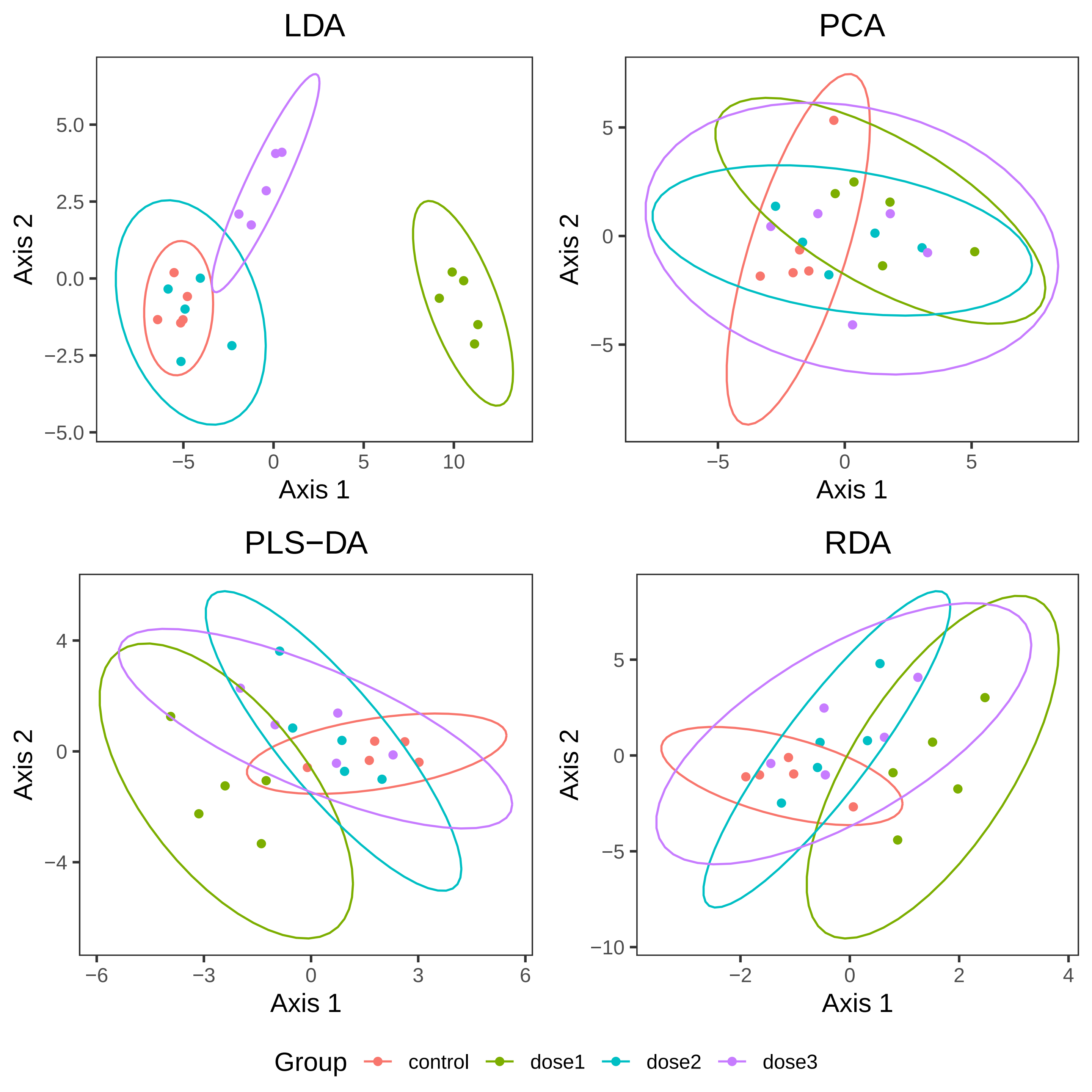

LDA
PCA
PLS−DA
RDA
Axis 1
Axis 2
Group
control
dose1
dose2
dose3

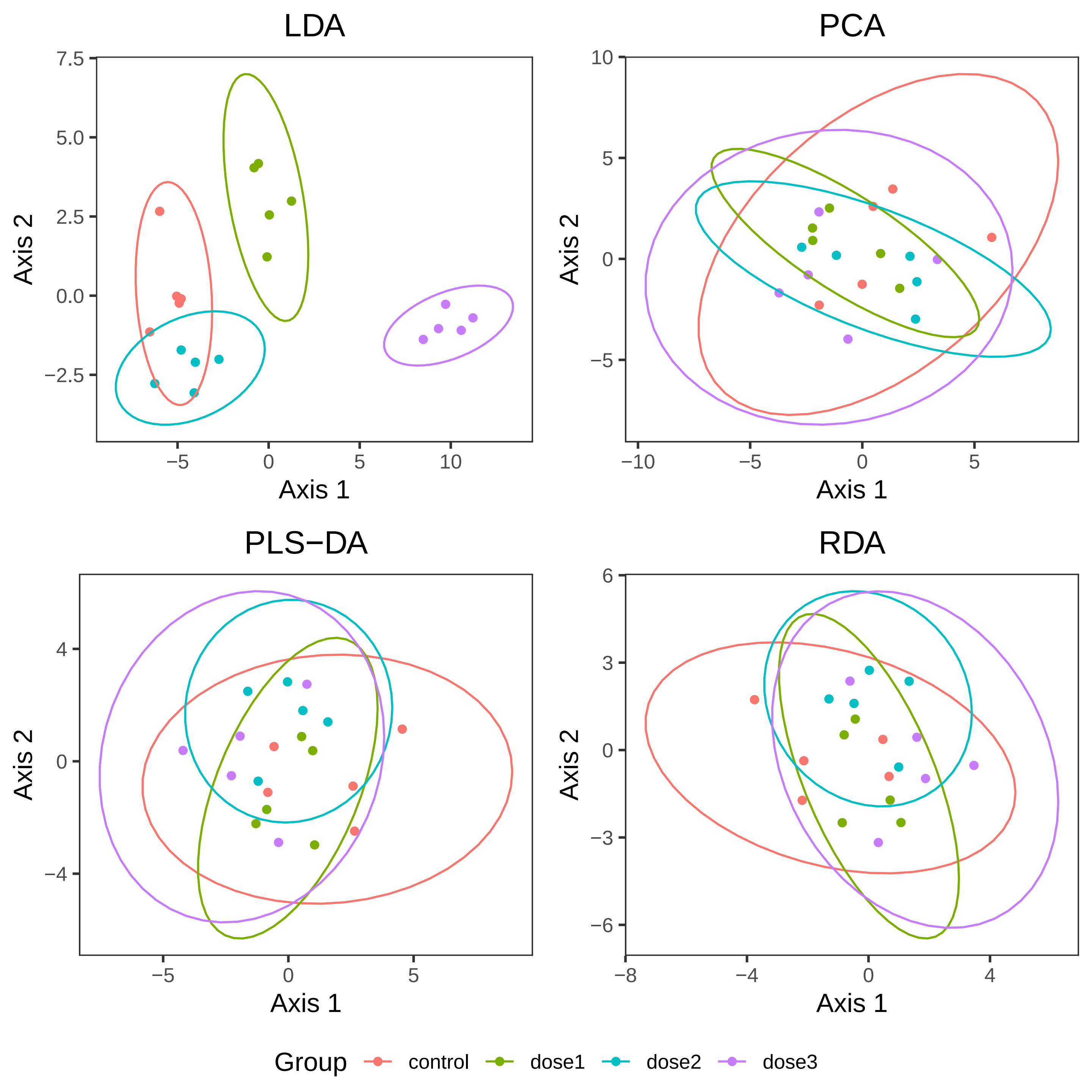
LDA
PCA
PLS−DA
RDA
Axis 1
Axis 2
7.5
5.0
2.5
0.0
−2.5
−5
0
5
10
−10
−4
4
−8
6
3
−3
−6
Group
control
dose1
dose2
dose3

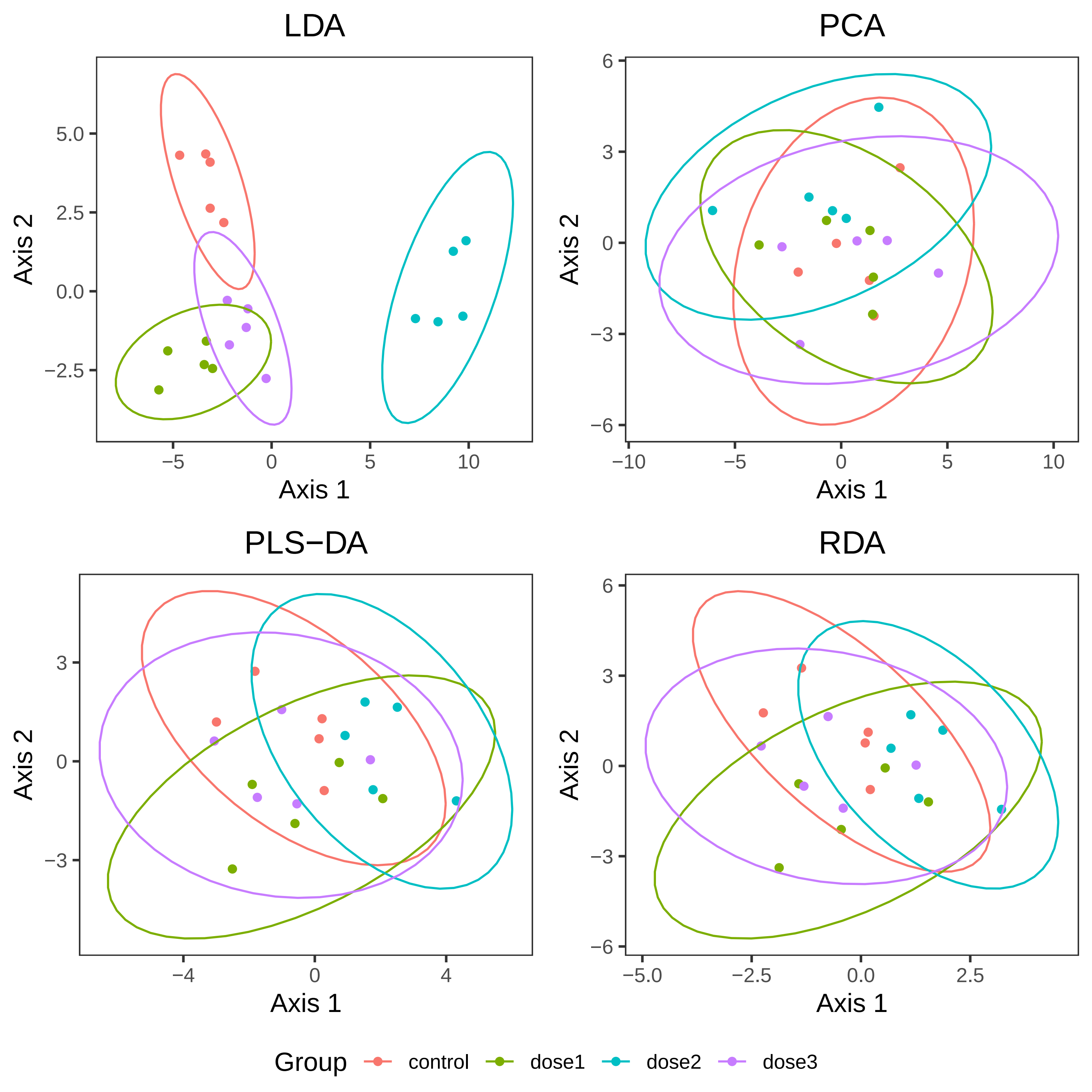
LDA
PCA
PLS−DA
RDA
Axis 1
Axis 2
5.0
2.5
0.0
−2.5
−5
0
5
10
6
3
0
−3
−6
−10
−5
0
5
10
3
0
−3
−4
0
4
6
3
0
−3
−6
−5.0
−2.5
0.0
2.5
Group
control
dose1
dose2
dose3

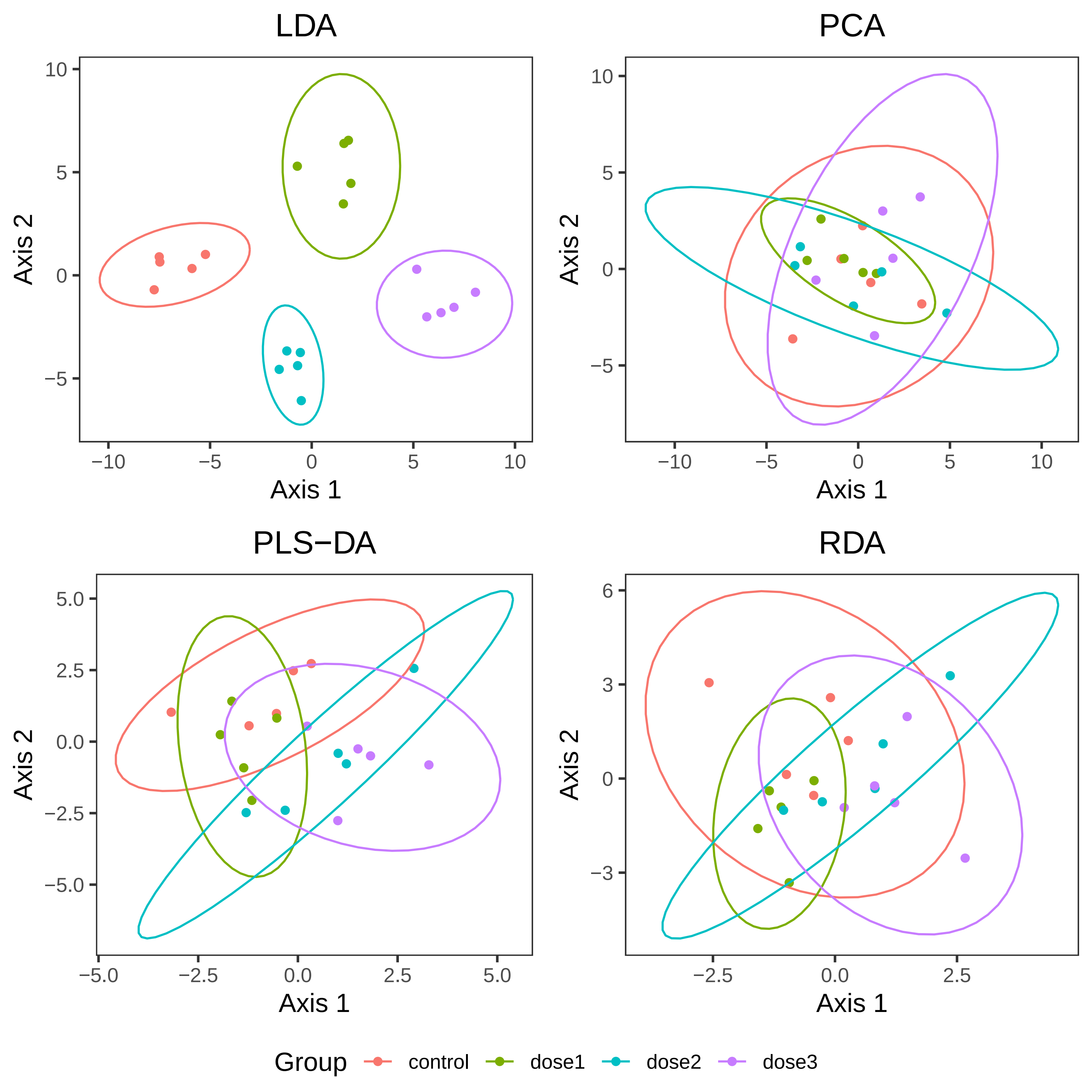
LDA
PCA
PLS−DA
RDA
Axis 1
Axis 2
Group
control
dose1
dose2
dose3

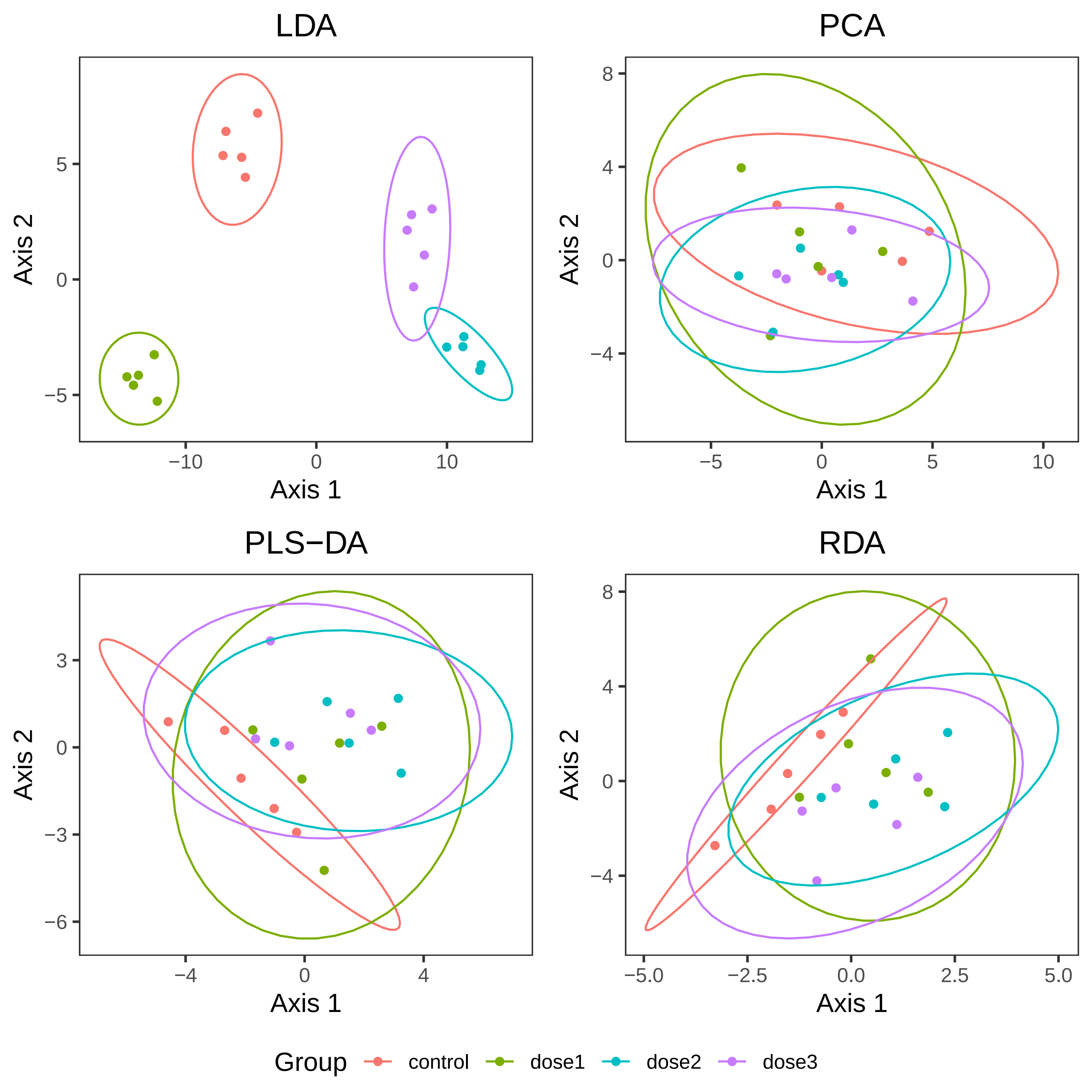

LDA
PCA
PLS−DA
RDA
Axis 1
Axis 2
Group
control
dose1
dose2
dose3

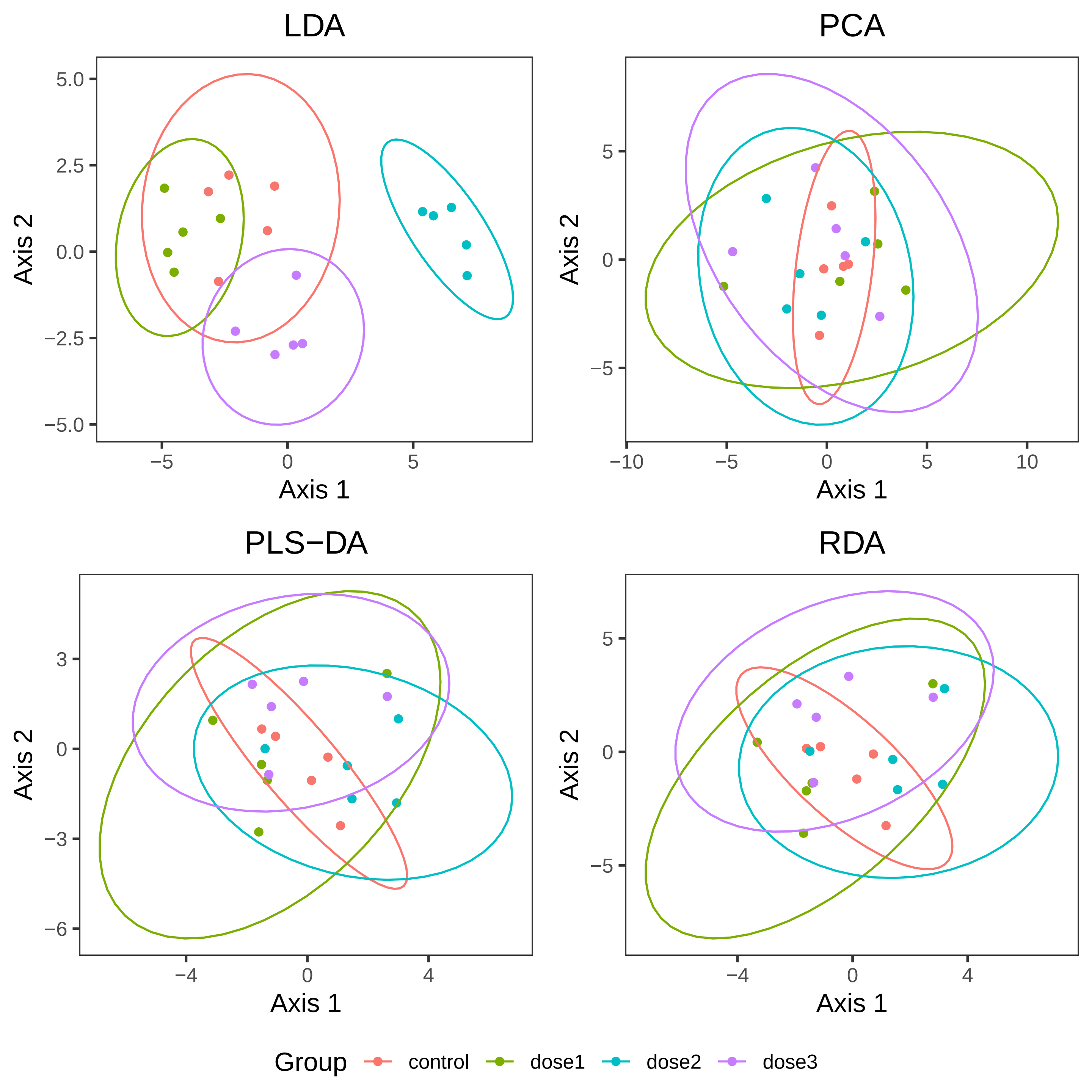
LDA
PCA
PLS−DA
RDA
Axis 1
Axis 2
5.0
2.5
0.0
−2.5
−5.0
−5
0
5
−10
10
3
−3
−6
−4
4
Group
control
dose1
dose2
dose3

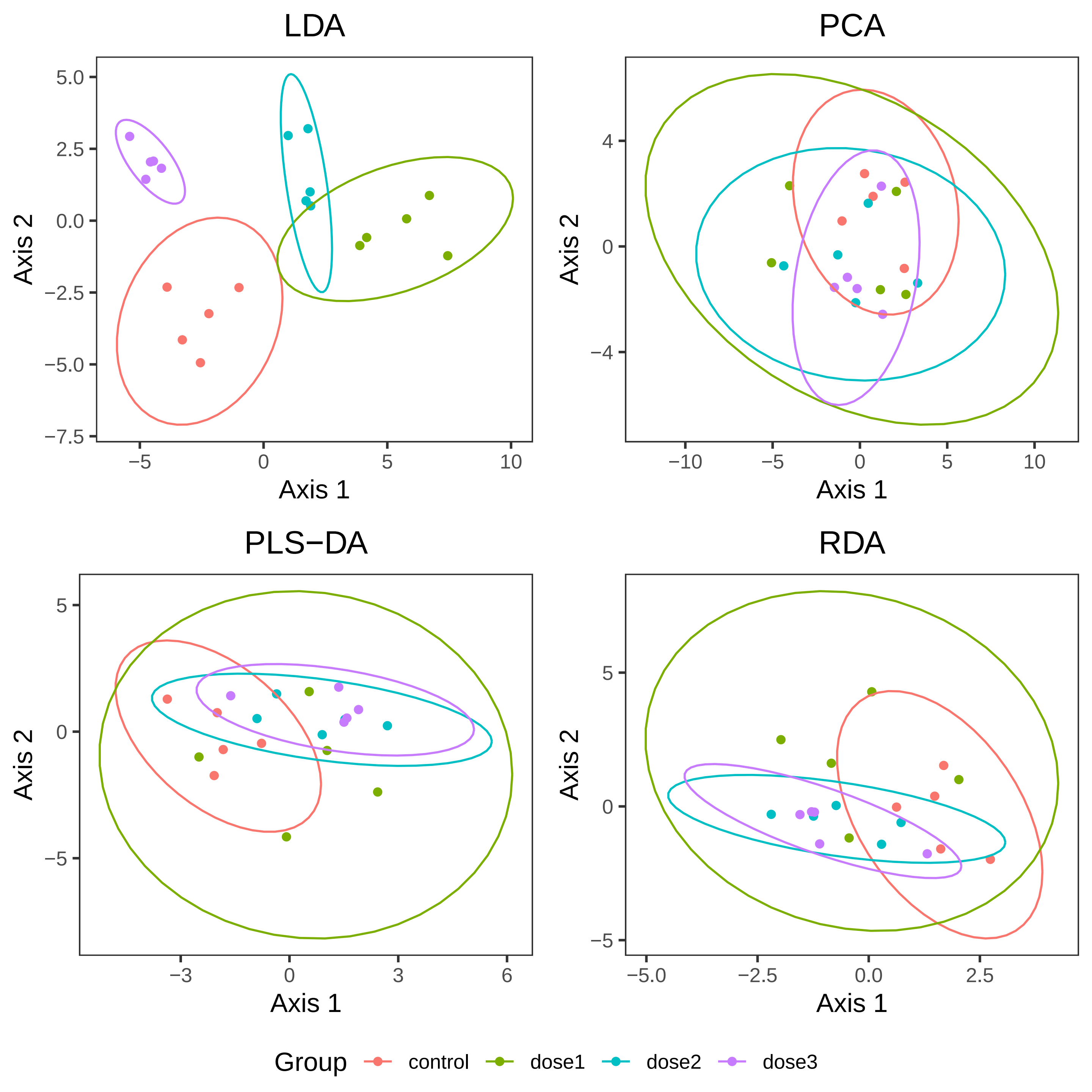
LDA
PCA
PLS−DA
RDA
Axis 1
Axis 2
5.0
2.5
0.0
−2.5
−5.0
−7.5
−5
0
5
10
4
0
−4
−10
−3
3
6
−5.0
−2.5
0.0
2.5
Group
control
dose1
dose2
dose3

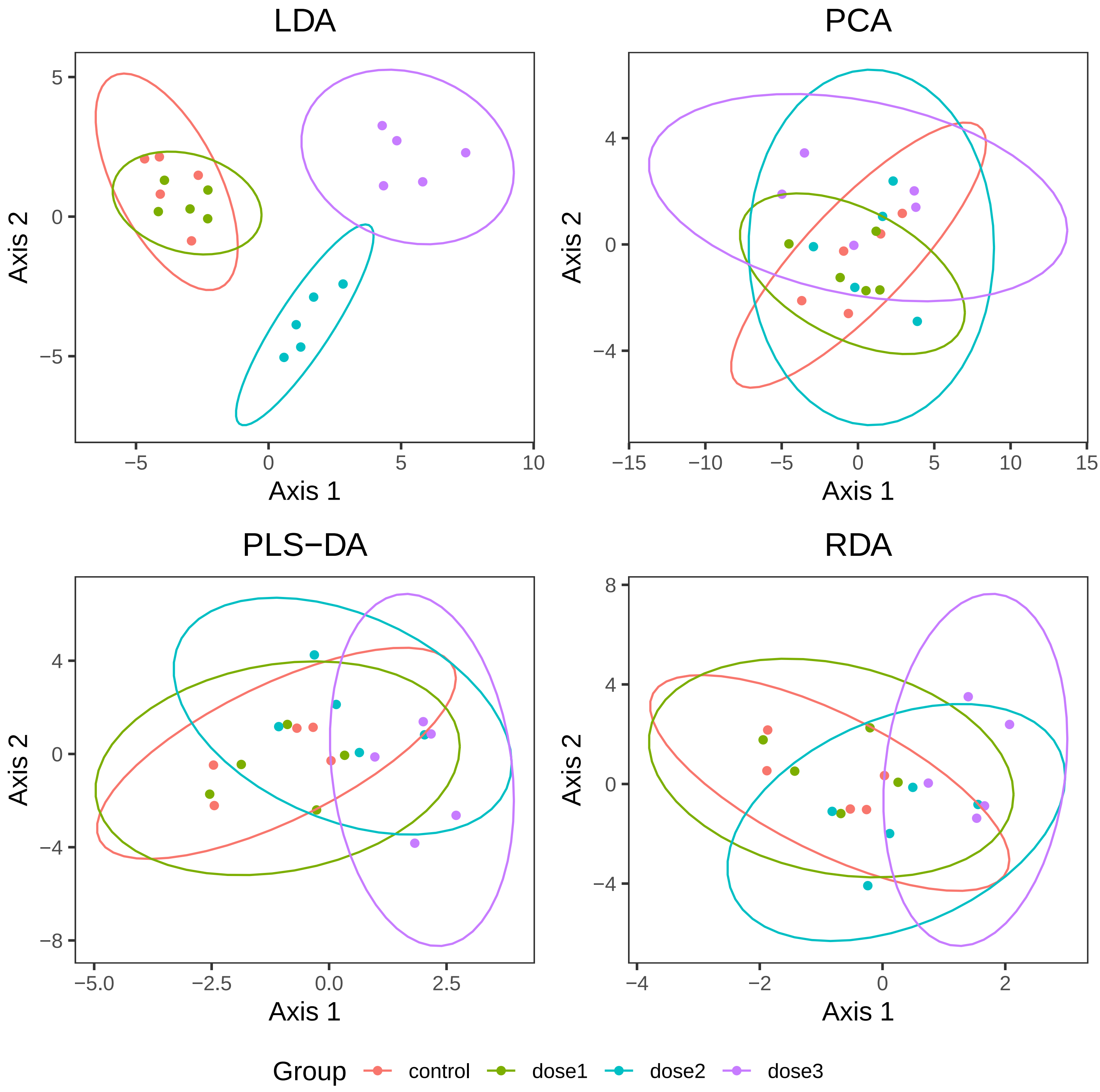
LDA
PCA
PLS−DA
RDA
Axis 1
Axis 2
Group
control
dose1
dose2
dose3

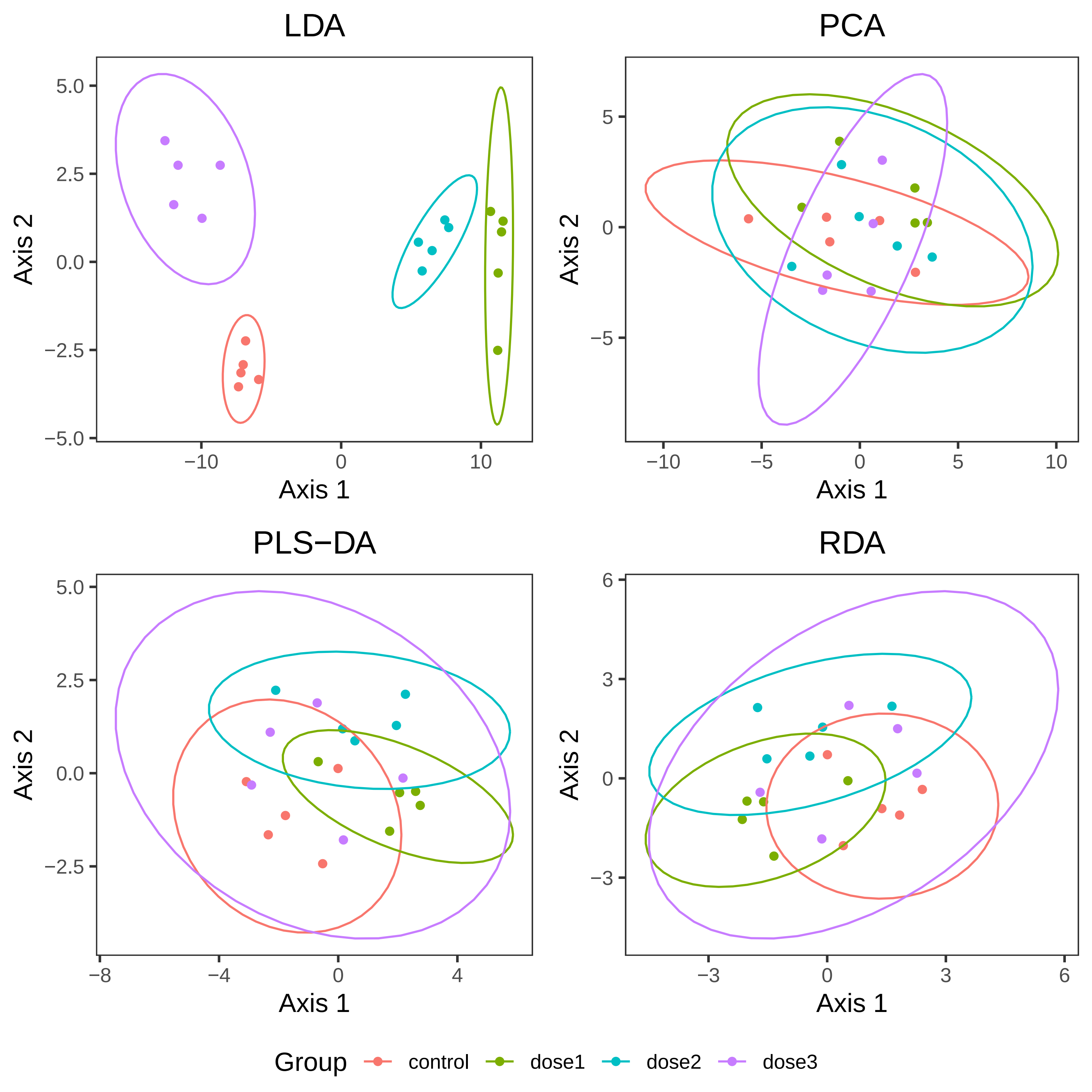
LDA
PCA
PLS−DA
RDA
Axis 1
Axis 2
5.0
2.5
0.0
−2.5
−5.0
−10
0
10
5
−5
−8
−4
4
6
3
−3
Group
control
dose1
dose2
dose3

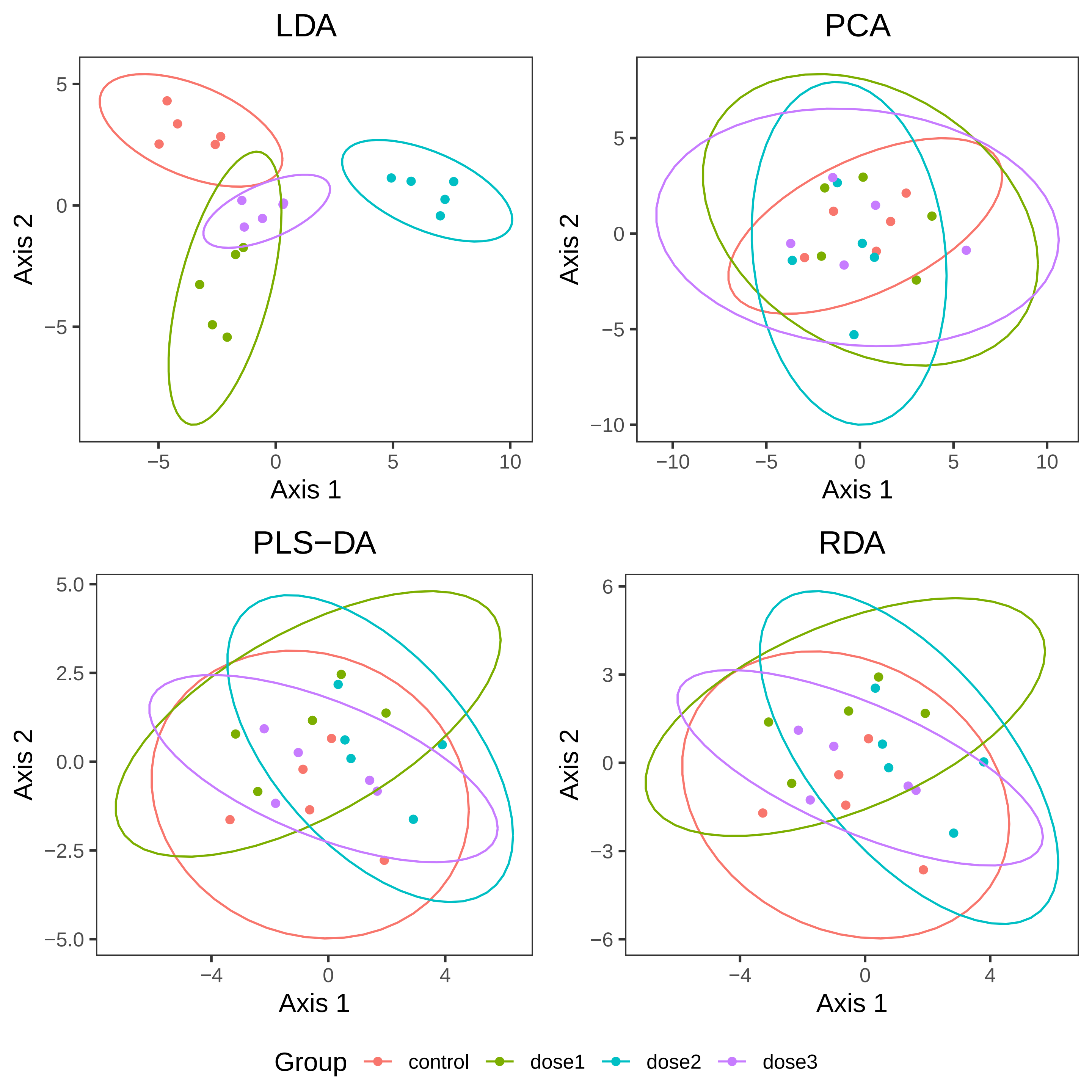

LDA
PCA
PLS−DA
RDA
Axis 1
Axis 2
Group
control
dose1
dose2
dose3

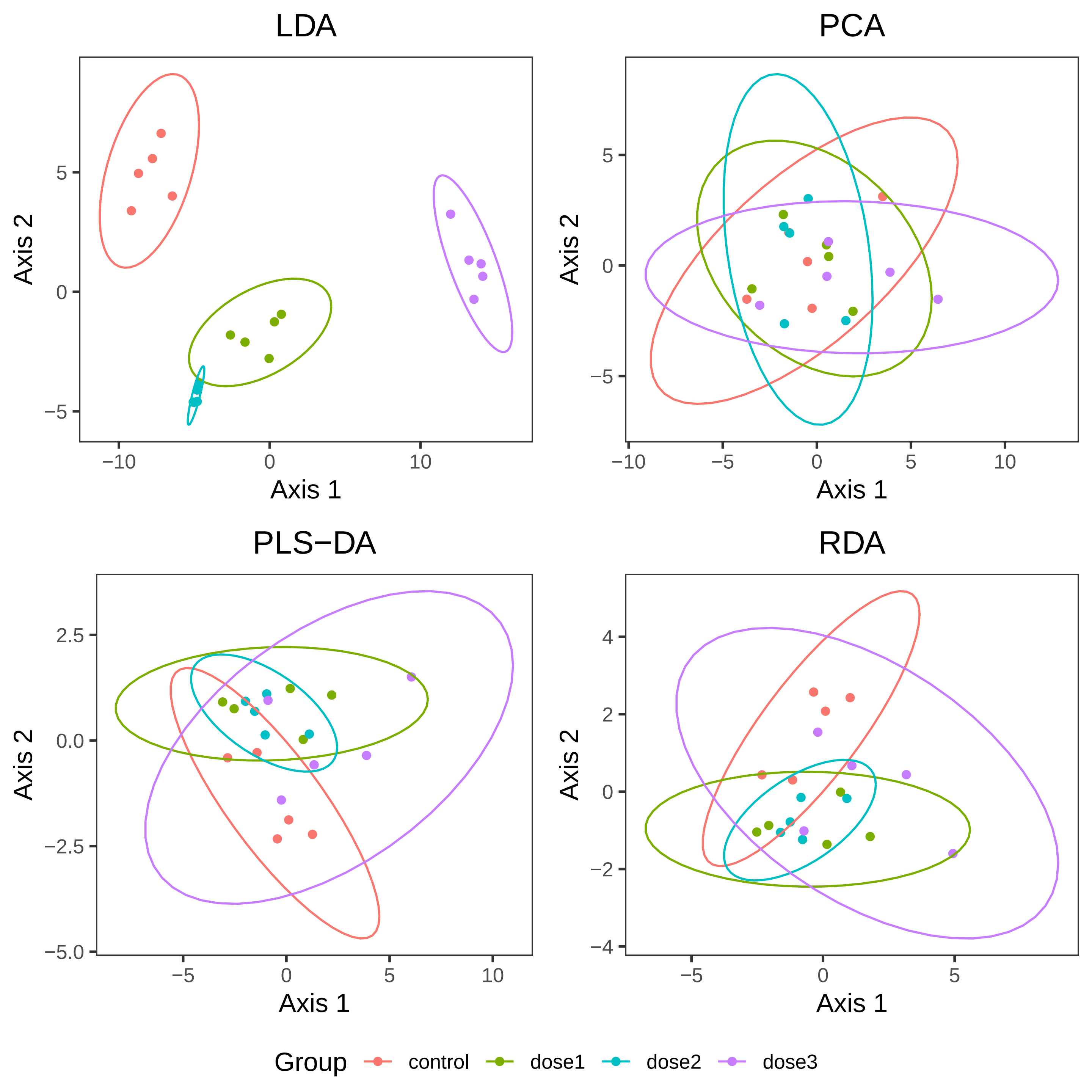

LDA
PCA
PLS−DA
RDA
Axis 1
Axis 2
−10
0
10
5
−5
2.5
0.0
−2.5
−5.0
4
2
−2
−4
Group
control
dose1
dose2
dose3

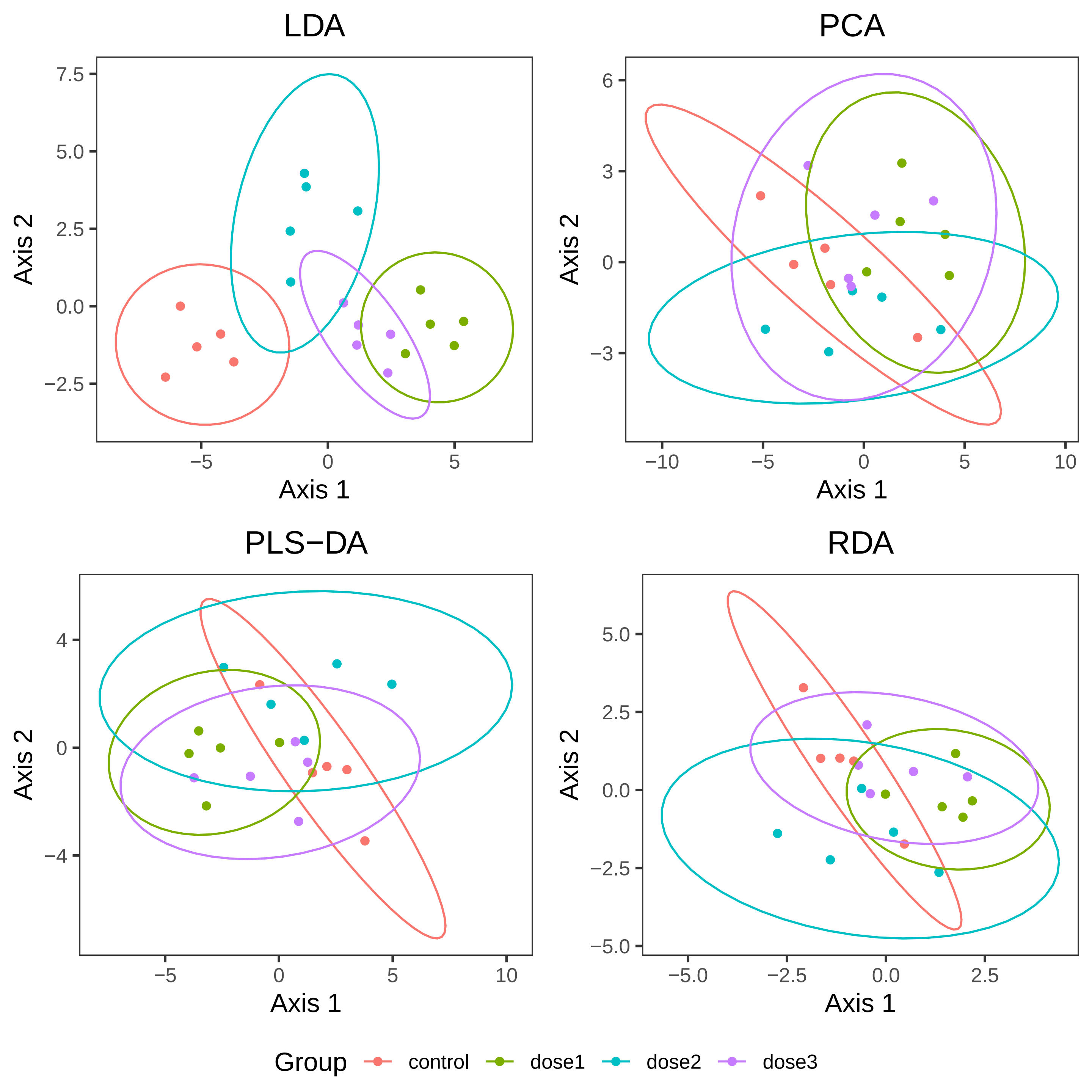

LDA
PCA
PLS−DA
RDA
Axis 1
Axis 2
Group
control
dose1
dose2
dose3

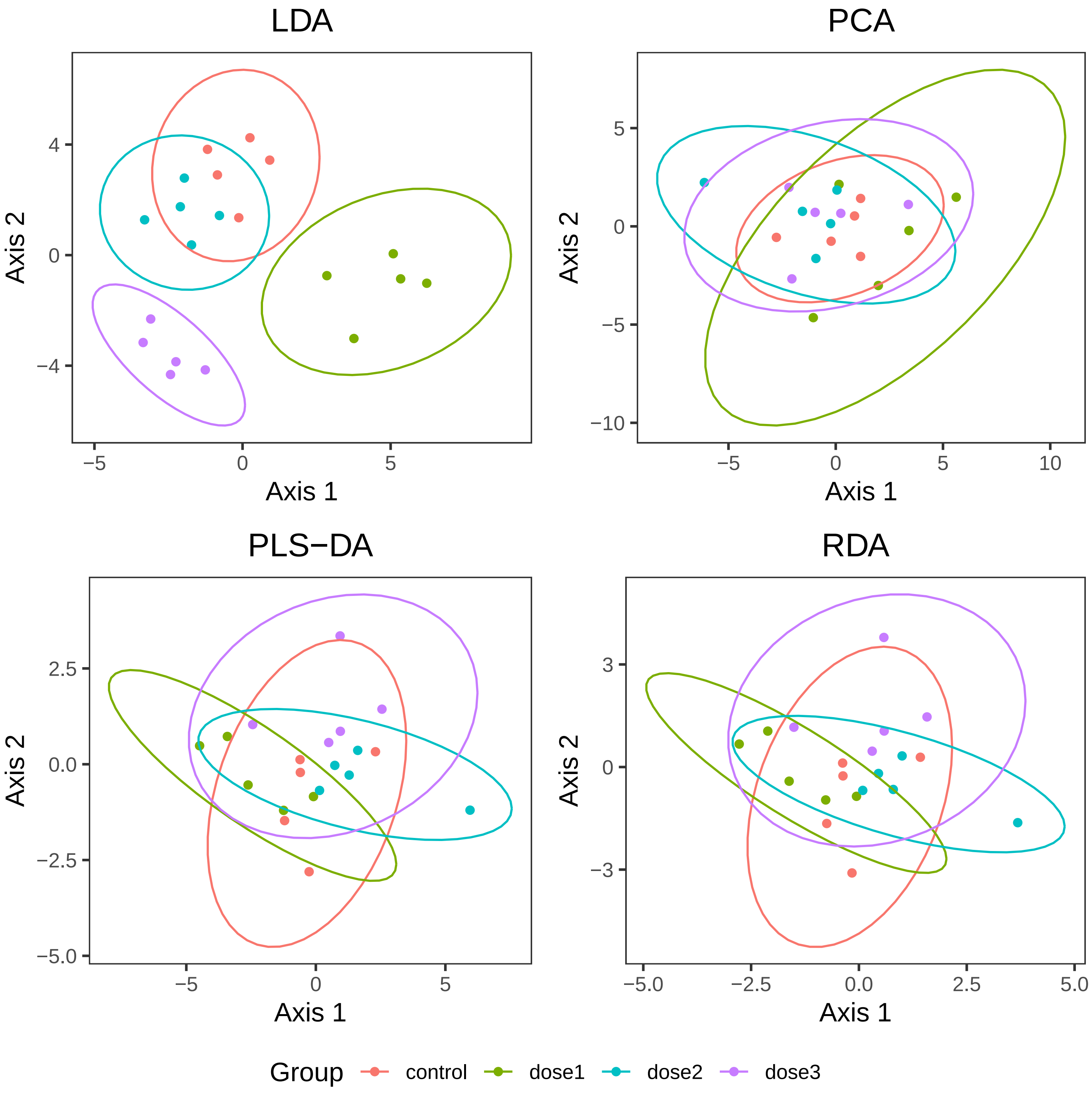
LDA
PCA
PLS−DA
RDA
Axis 1
Axis 2
Group
control
dose1
dose2
dose3

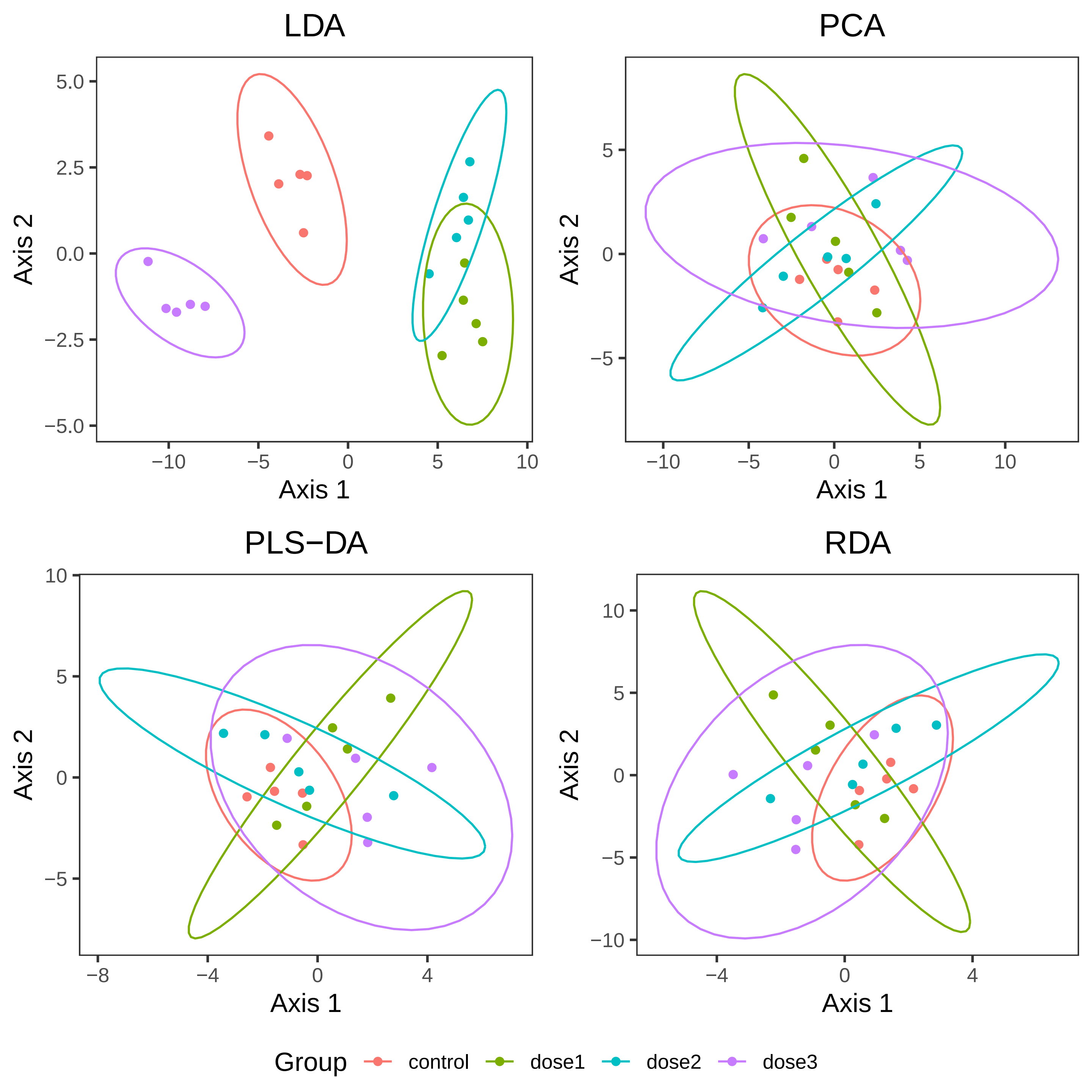

LDA
PCA
PLS−DA
RDA
Axis 1
Axis 2
5.0
2.5
0.0
−2.5
−5.0
−10
−5
0
5
10
−8
−4
4
−10
Group
control
dose1
dose2
dose3

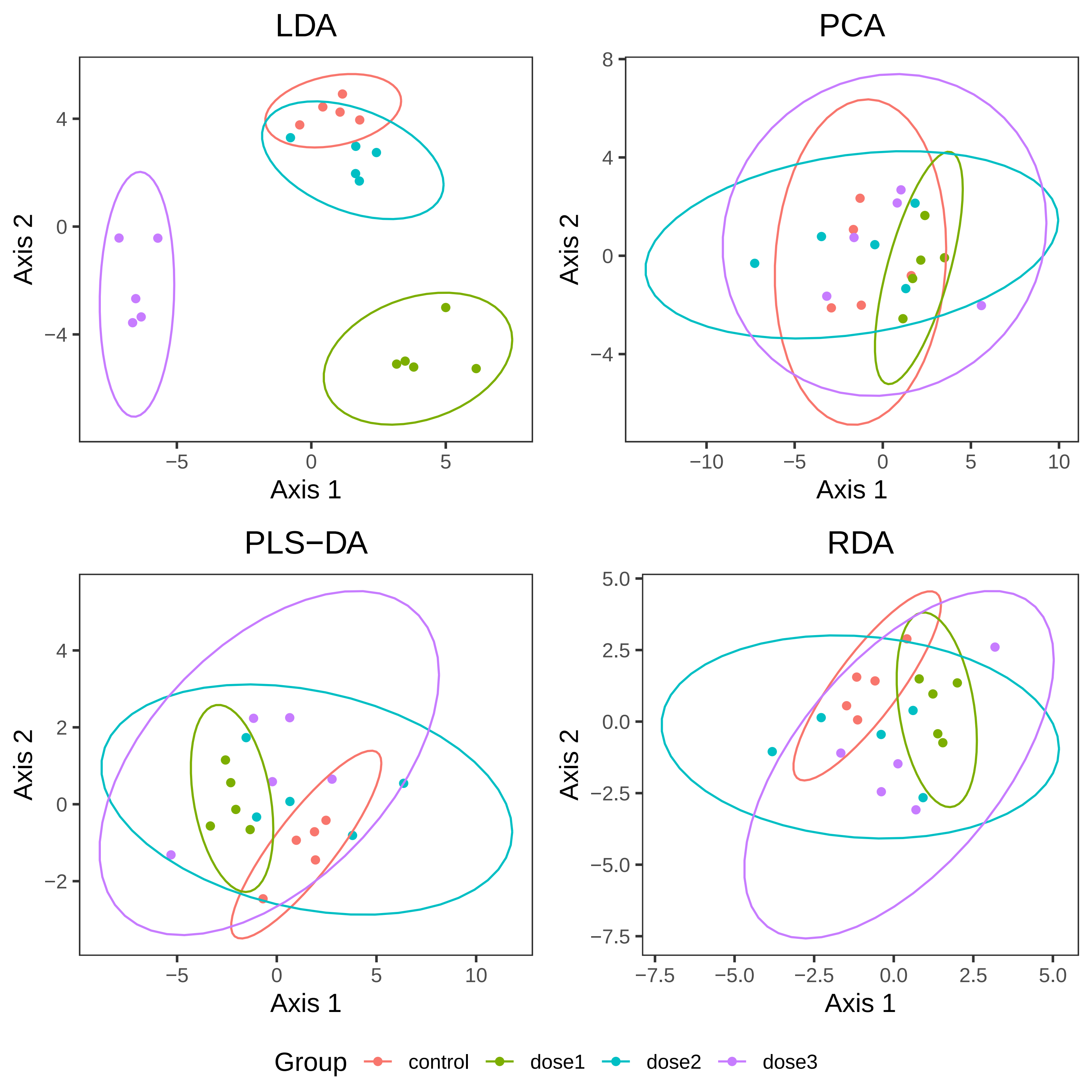

LDA
PCA
PLS−DA
RDA
Axis 1
Axis 2
Group
control
dose1
dose2
dose3

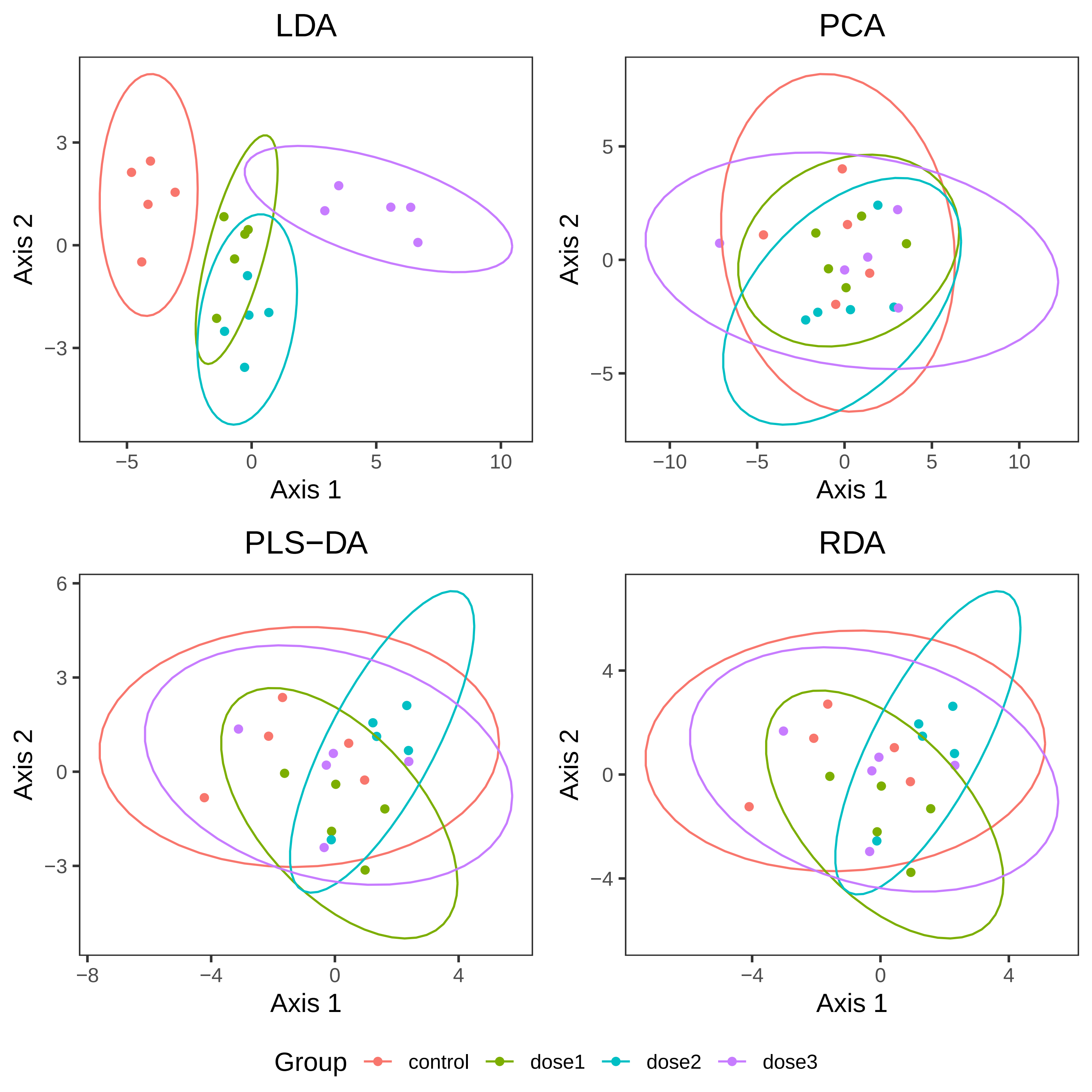
LDA
PCA
PLS−DA
RDA
Axis 1
Axis 2
3
0
−3
−5
0
5
10
5
0
−5
−10
−5
0
5
10
6
3
0
−3
−8
−4
0
4
4
0
−4
−4
0
4
Group
control
dose1
dose2
dose3

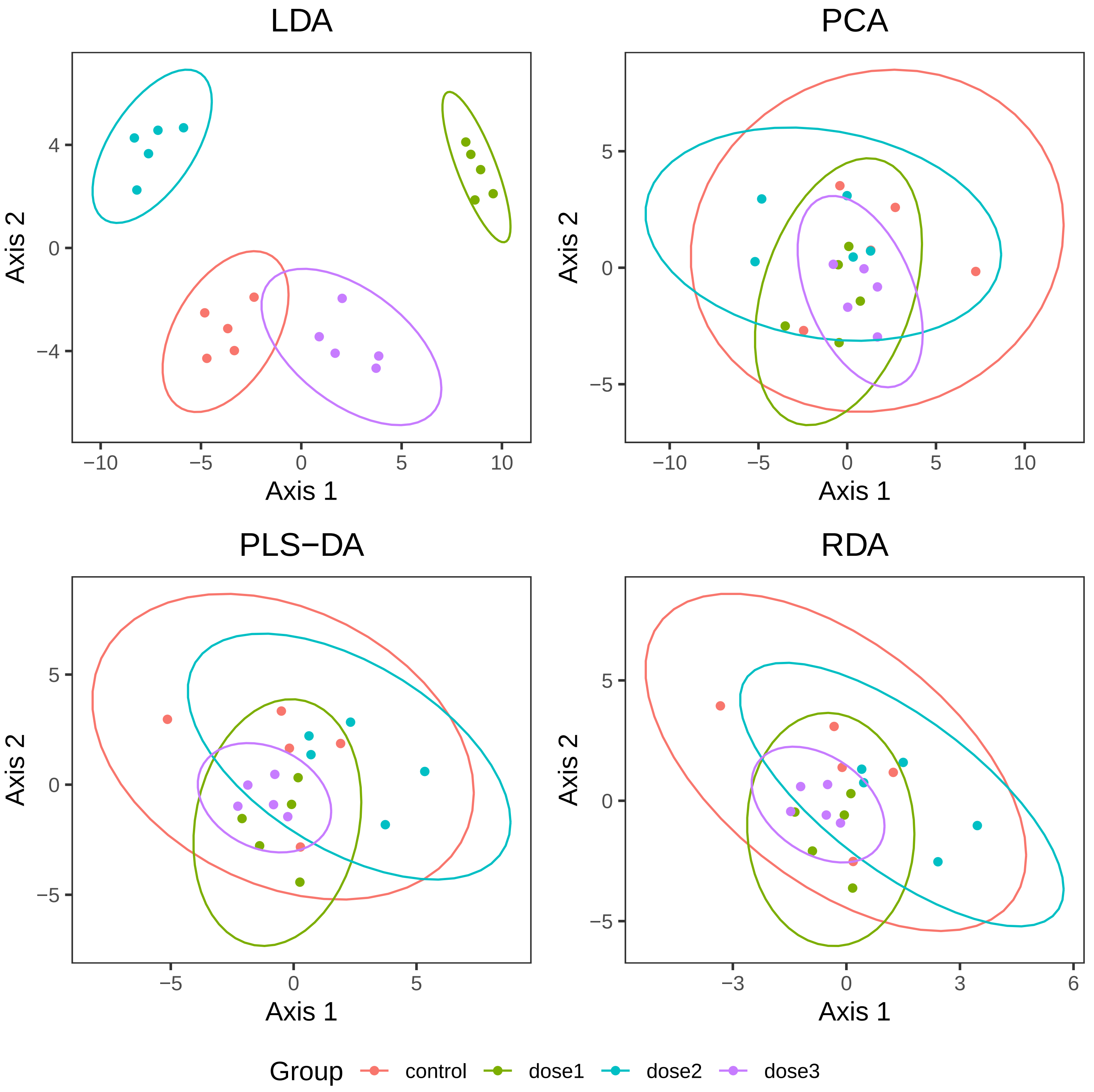
LDA
PCA
PLS−DA
RDA
Axis 1
Axis 2
Group
control
dose1
dose2
dose3

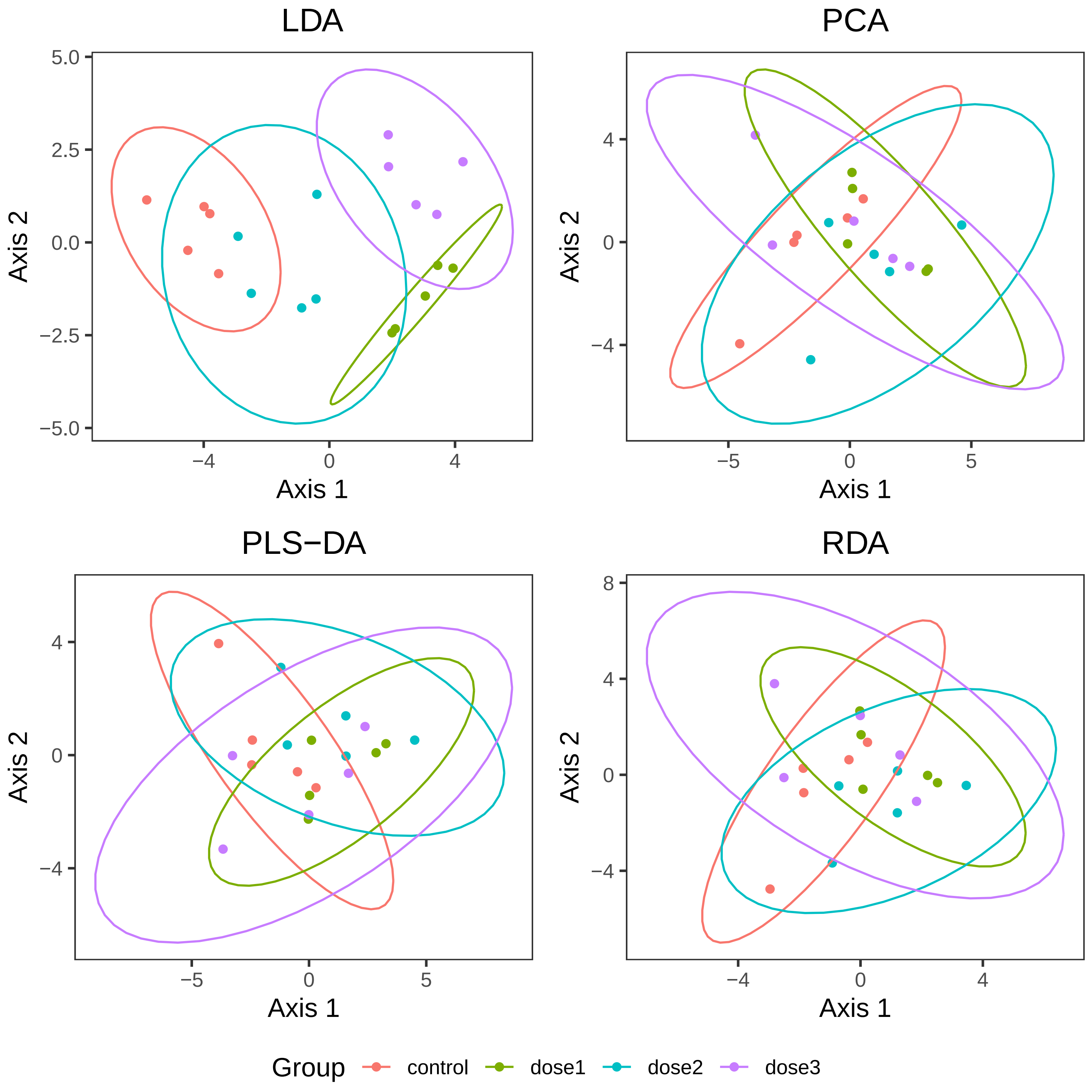

LDA
PCA
PLS−DA
RDA
Axis 1
Axis 2
Group
control
dose1
dose2
dose3

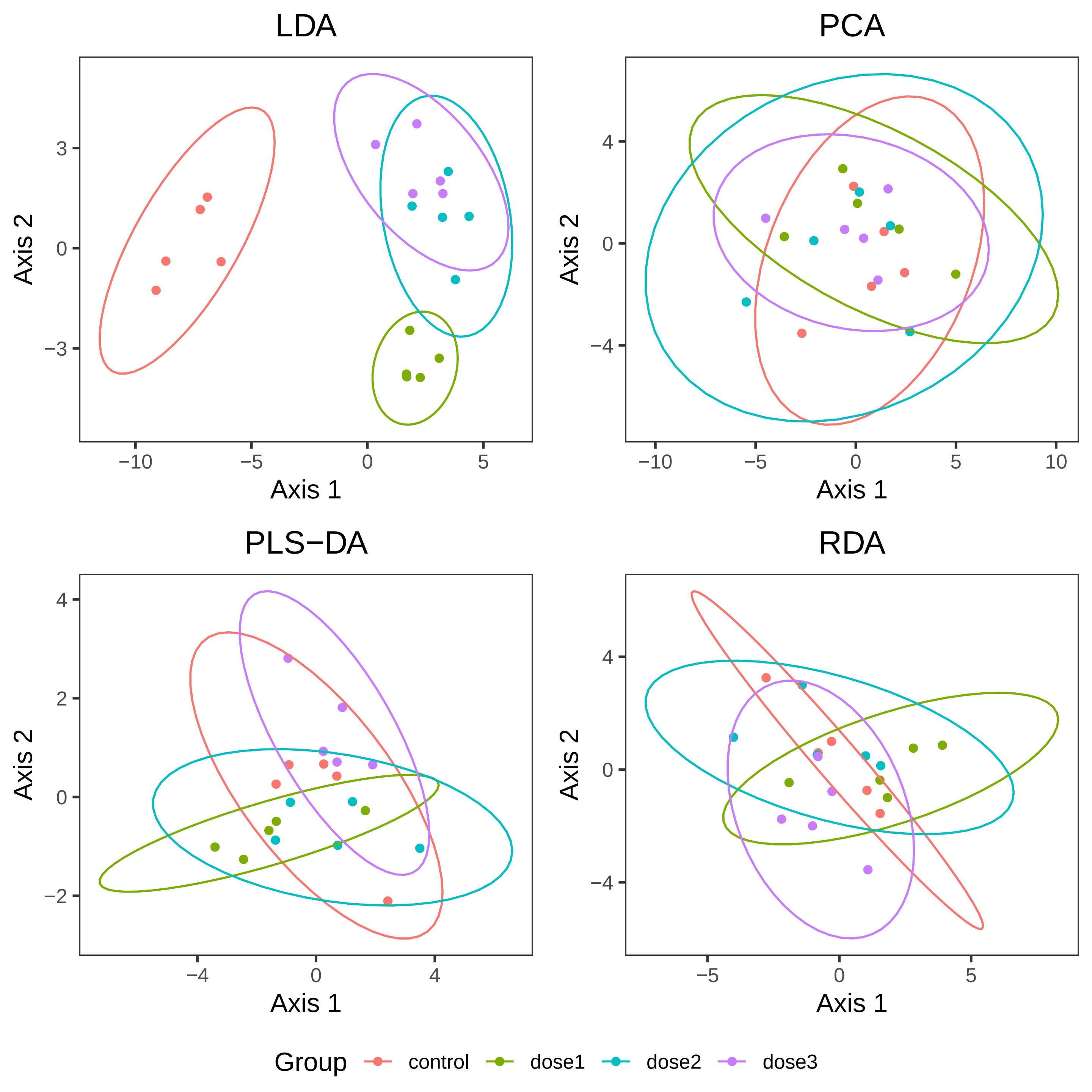
LDA
PCA
PLS−DA
RDA
Axis 1
Axis 2
Group
control
dose1
dose2
dose3

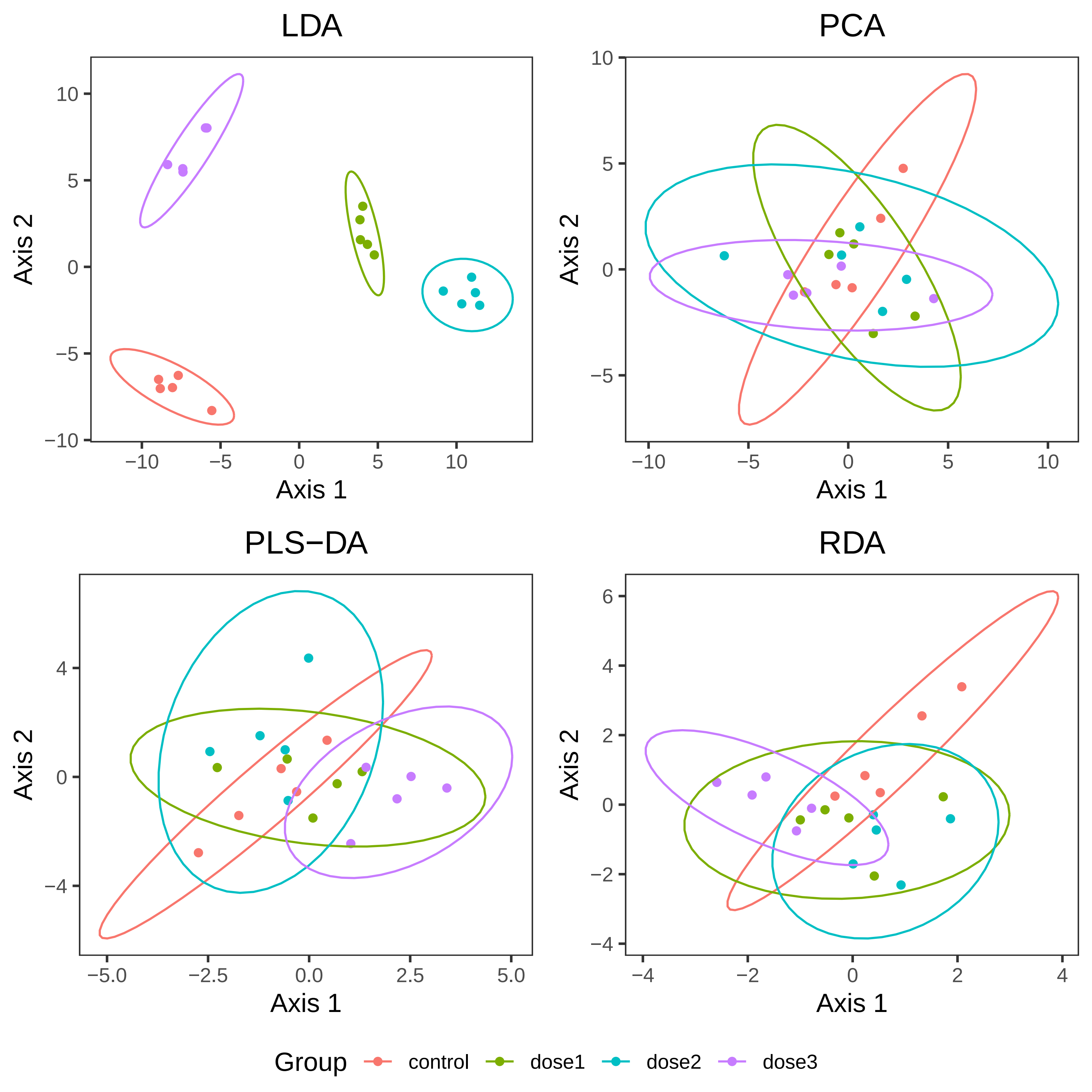

LDA
PCA
PLS−DA
RDA
Axis 1
Axis 2
Group
control
dose1
dose2
dose3

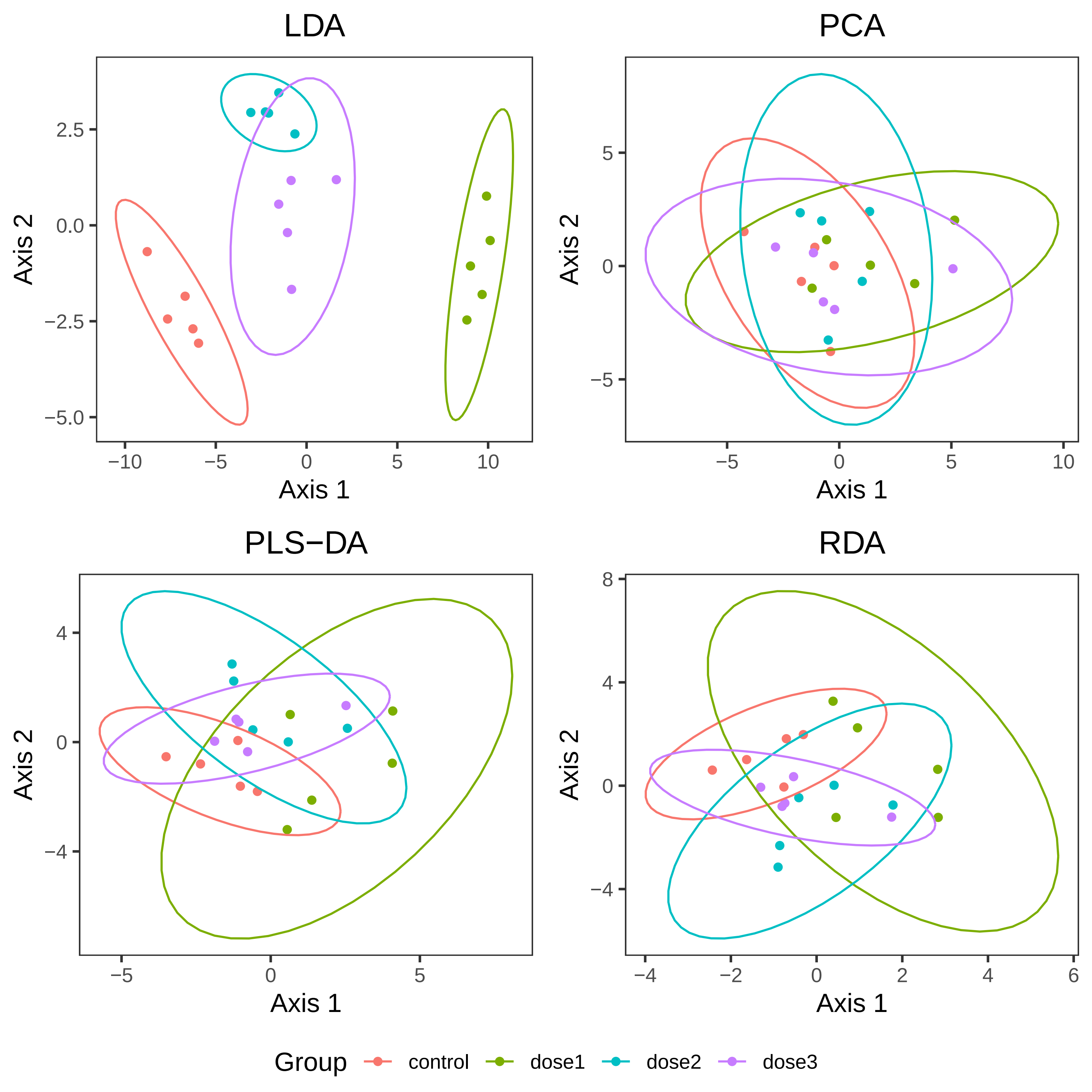

LDA
PCA
PLS−DA
RDA
Axis 1
Axis 2
Group
control
dose1
dose2
dose3

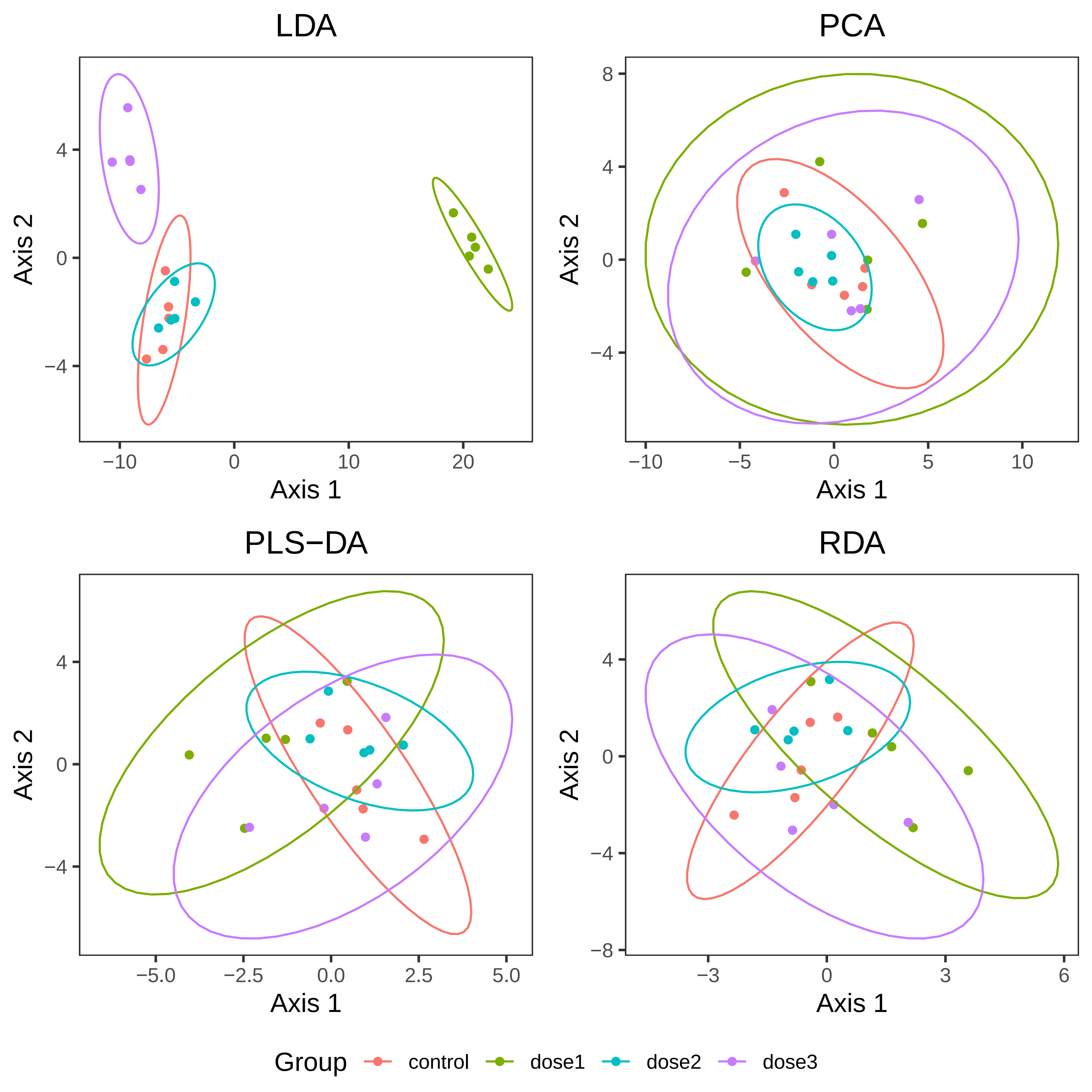
LDA
PCA
PLS−DA
RDA
Axis 1
Axis 2
Group
control
dose1
dose2
dose3

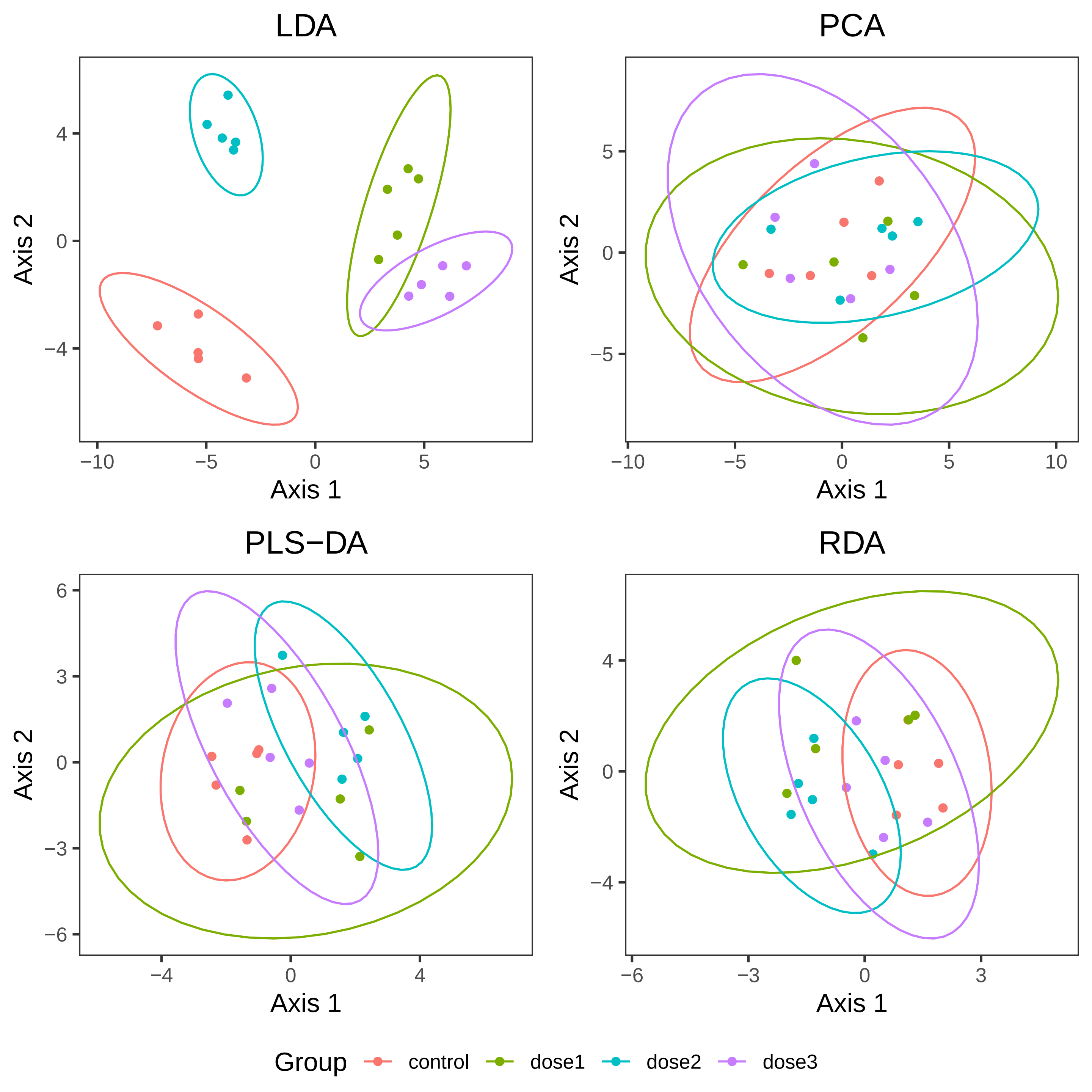
LDA
PCA
PLS−DA
RDA
Axis 1
Axis 2
Group
control
dose1
dose2
dose3

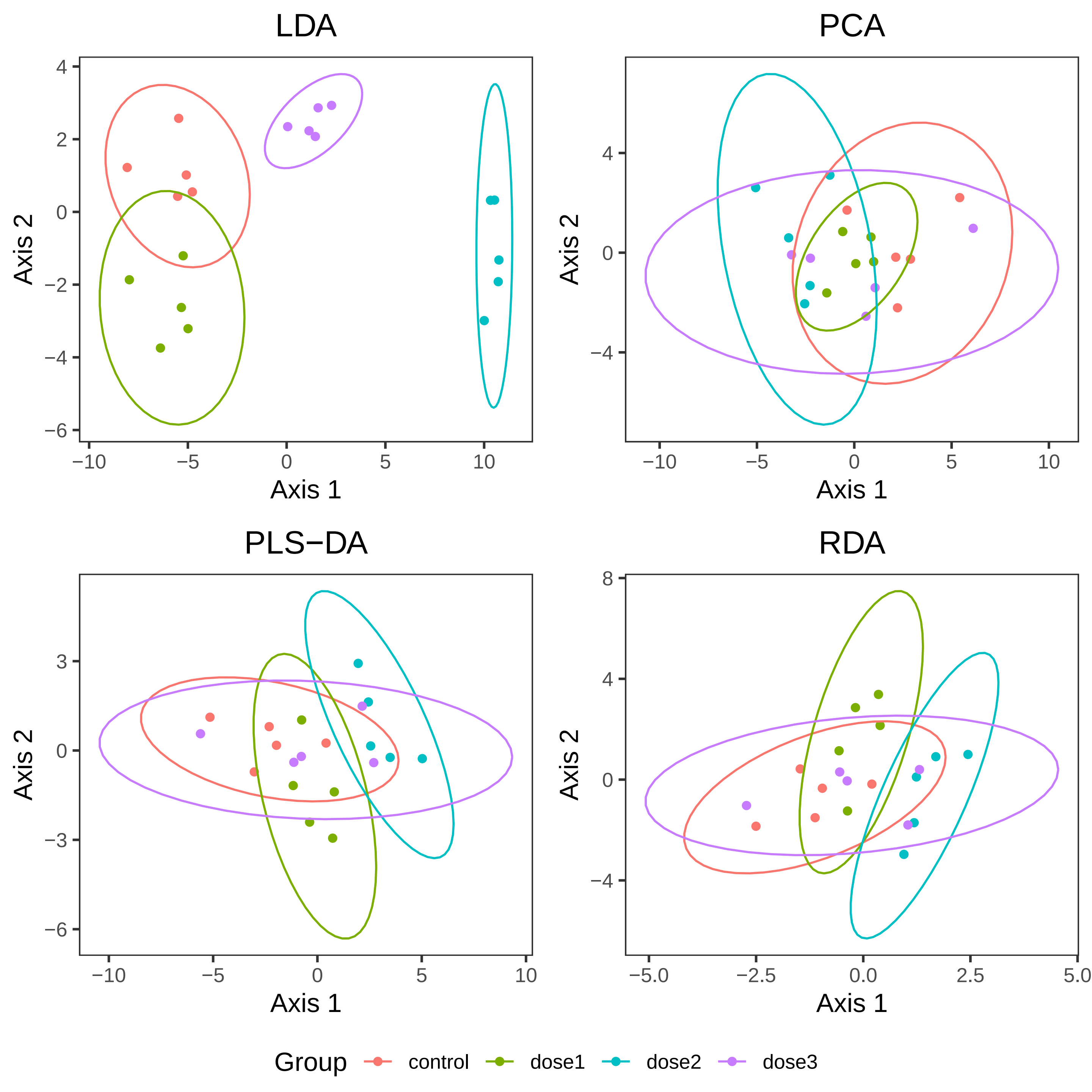

LDA
PCA
PLS−DA
RDA
Axis 1
Axis 2
Group
control
dose1
dose2
dose3

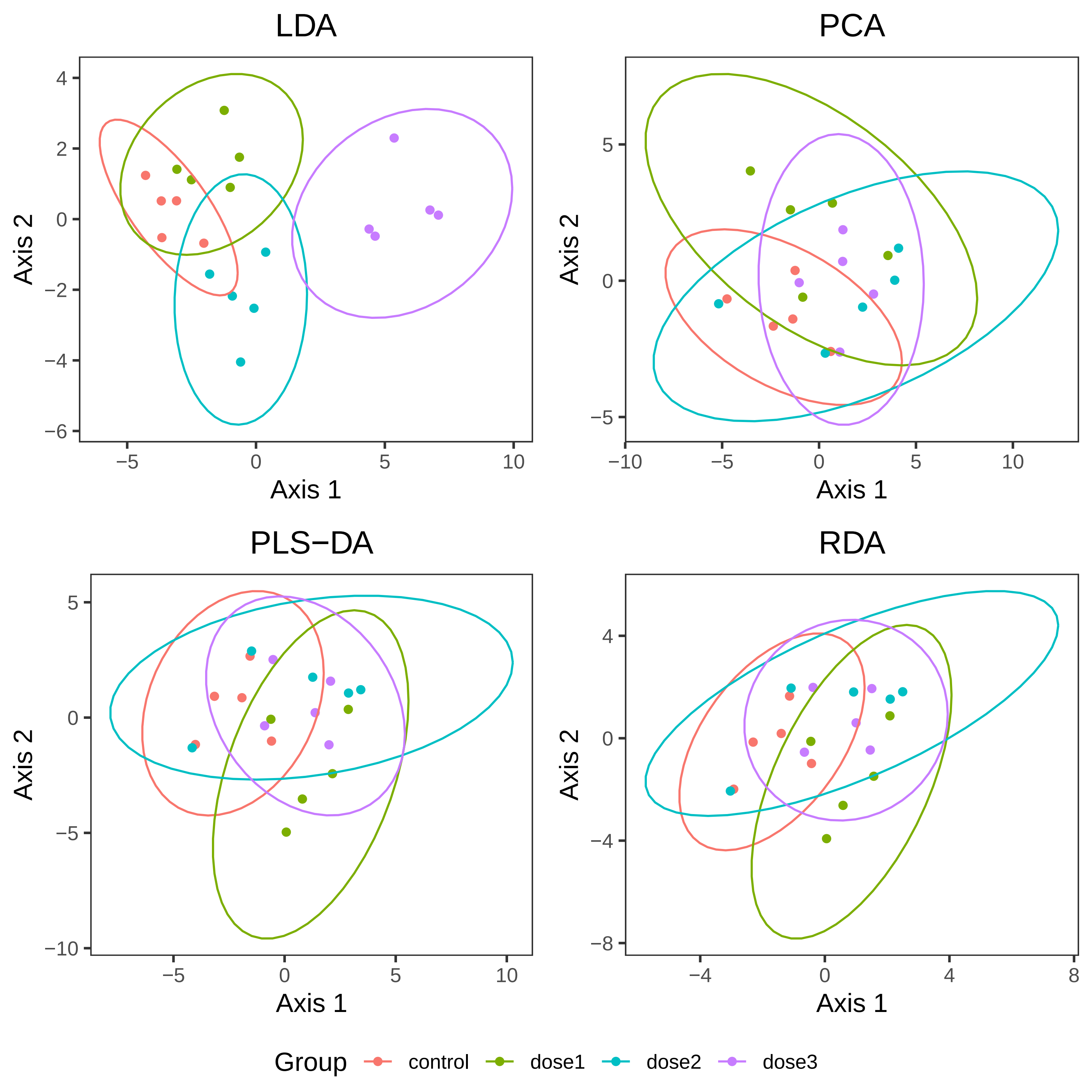

LDA
PCA
PLS−DA
RDA
Axis 1
Axis 2
Group
control
dose1
dose2
dose3

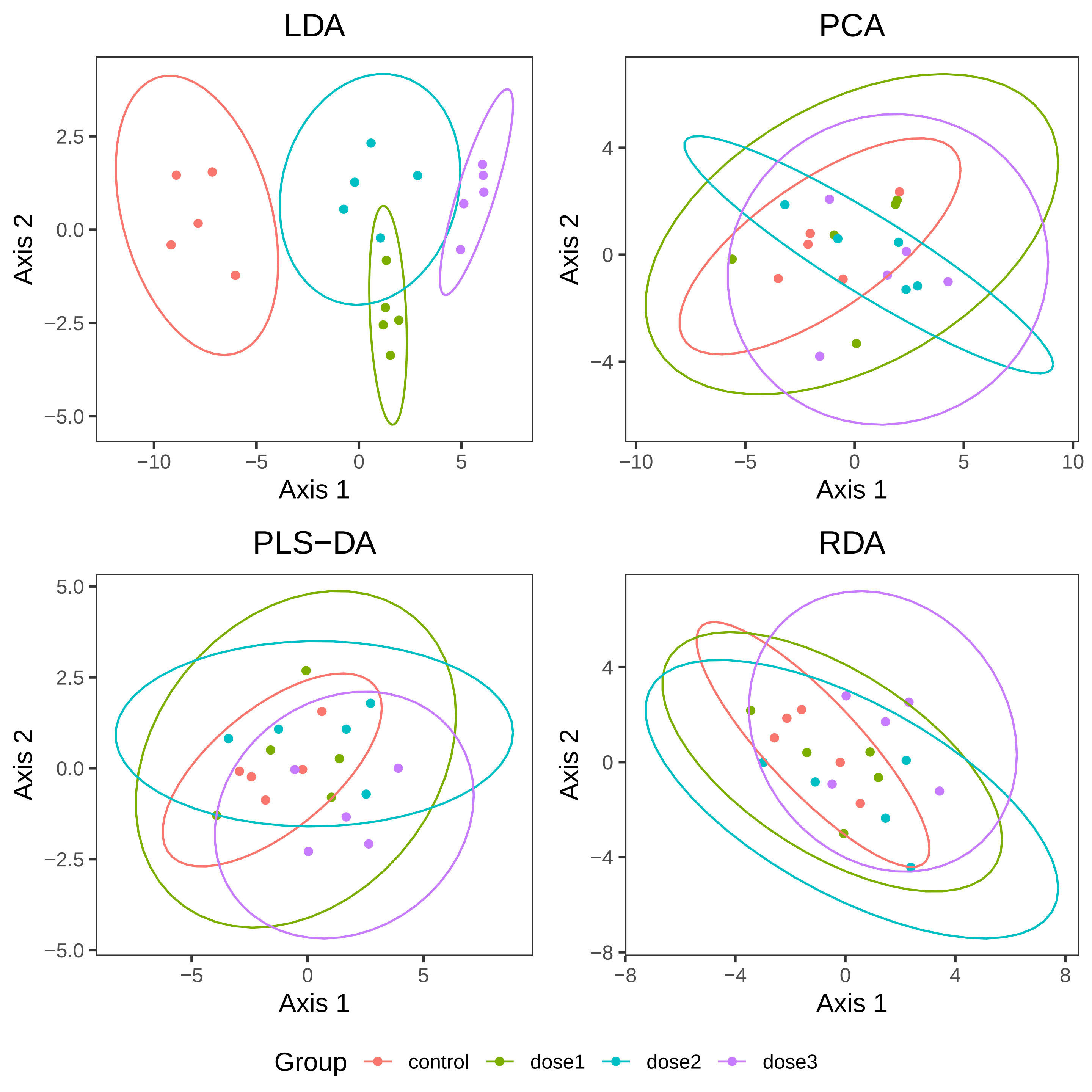
LDA
PCA
PLS−DA
RDA
Axis 1
Axis 2
2.5
0.0
−2.5
−5.0
−10
−5
0
5
10
4
−4
5.0
−8
8
Group
control
dose1
dose2
dose3

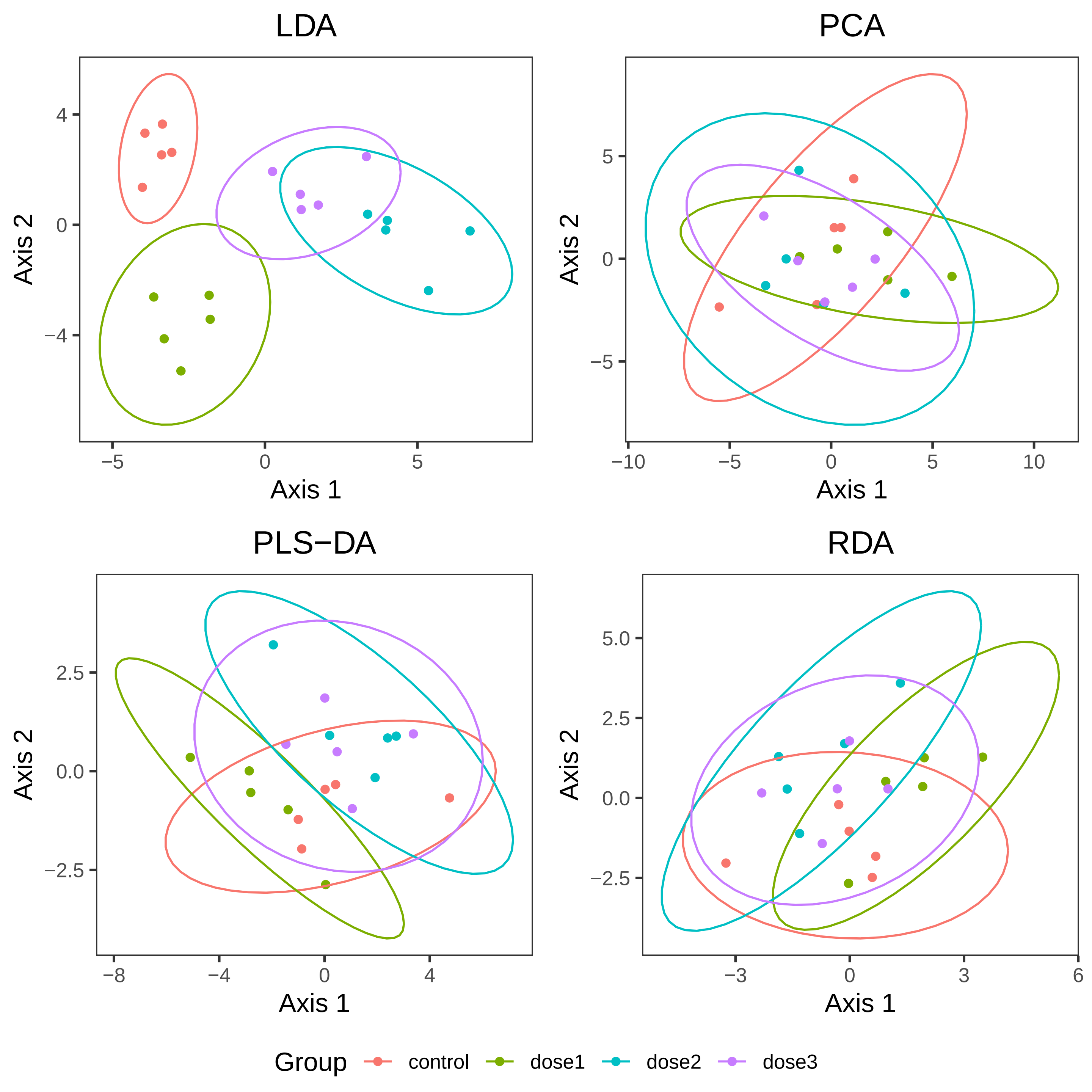
LDA
PCA
PLS−DA
RDA
Axis 1
Axis 2
Group
control
dose1
dose2
dose3

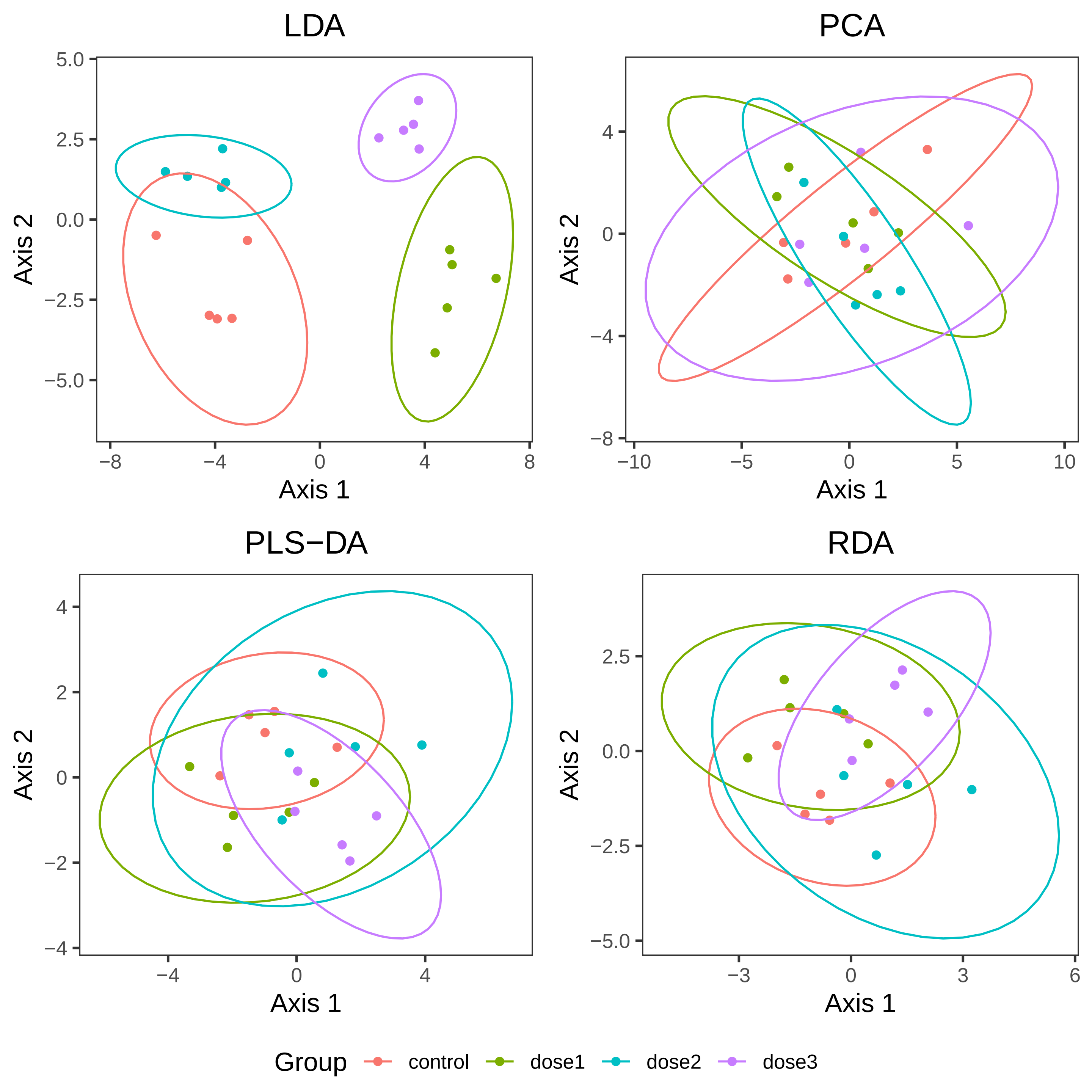
LDA
PCA
PLS−DA
RDA
Axis 1
Axis 2
5.0
2.5
0.0
−2.5
−5.0
−8
−4
0
4
8
−10
−5
5
10
Group
control
dose1
dose2
dose3

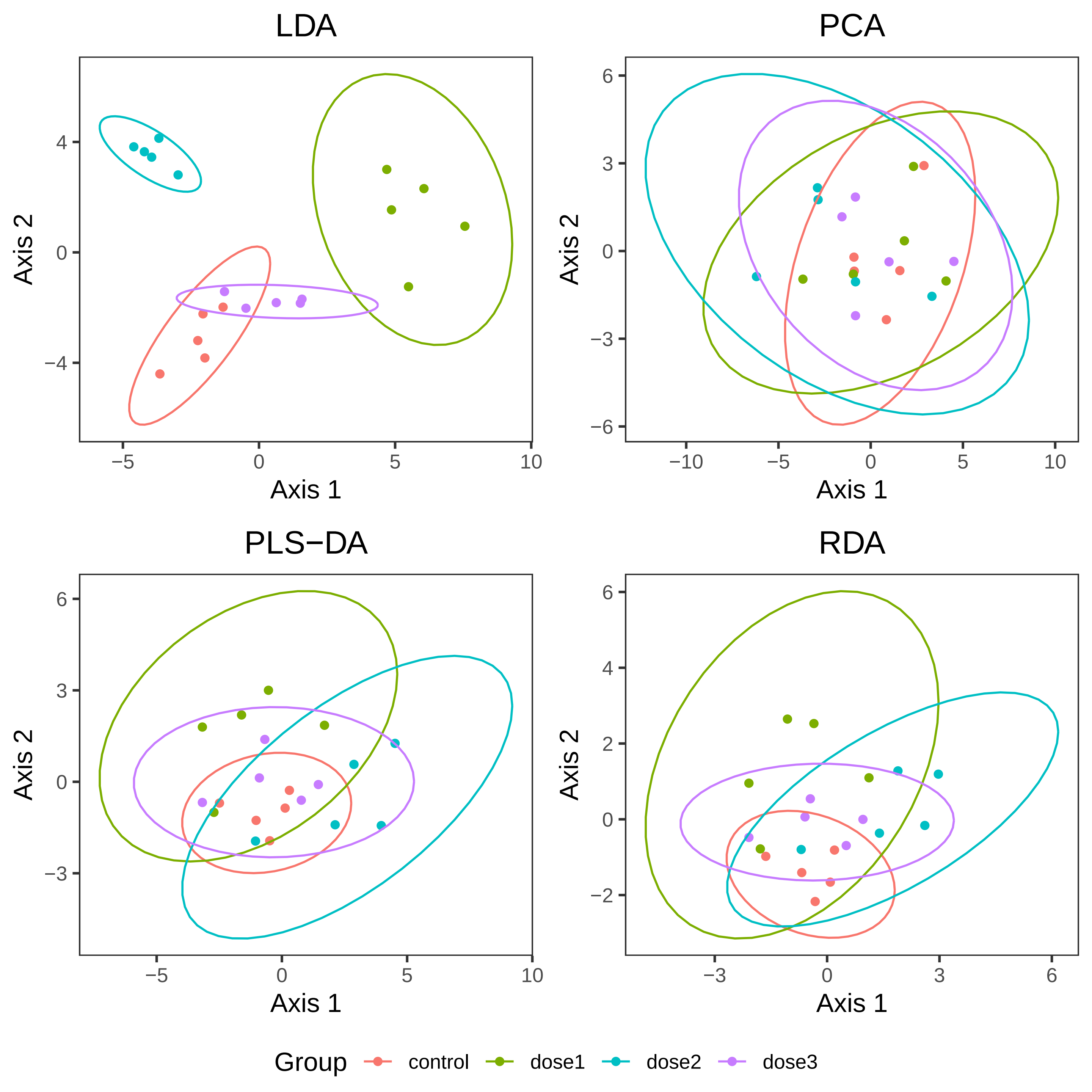
LDA
PCA
PLS−DA
RDA
Axis 1
Axis 2
Group
control
dose1
dose2
dose3

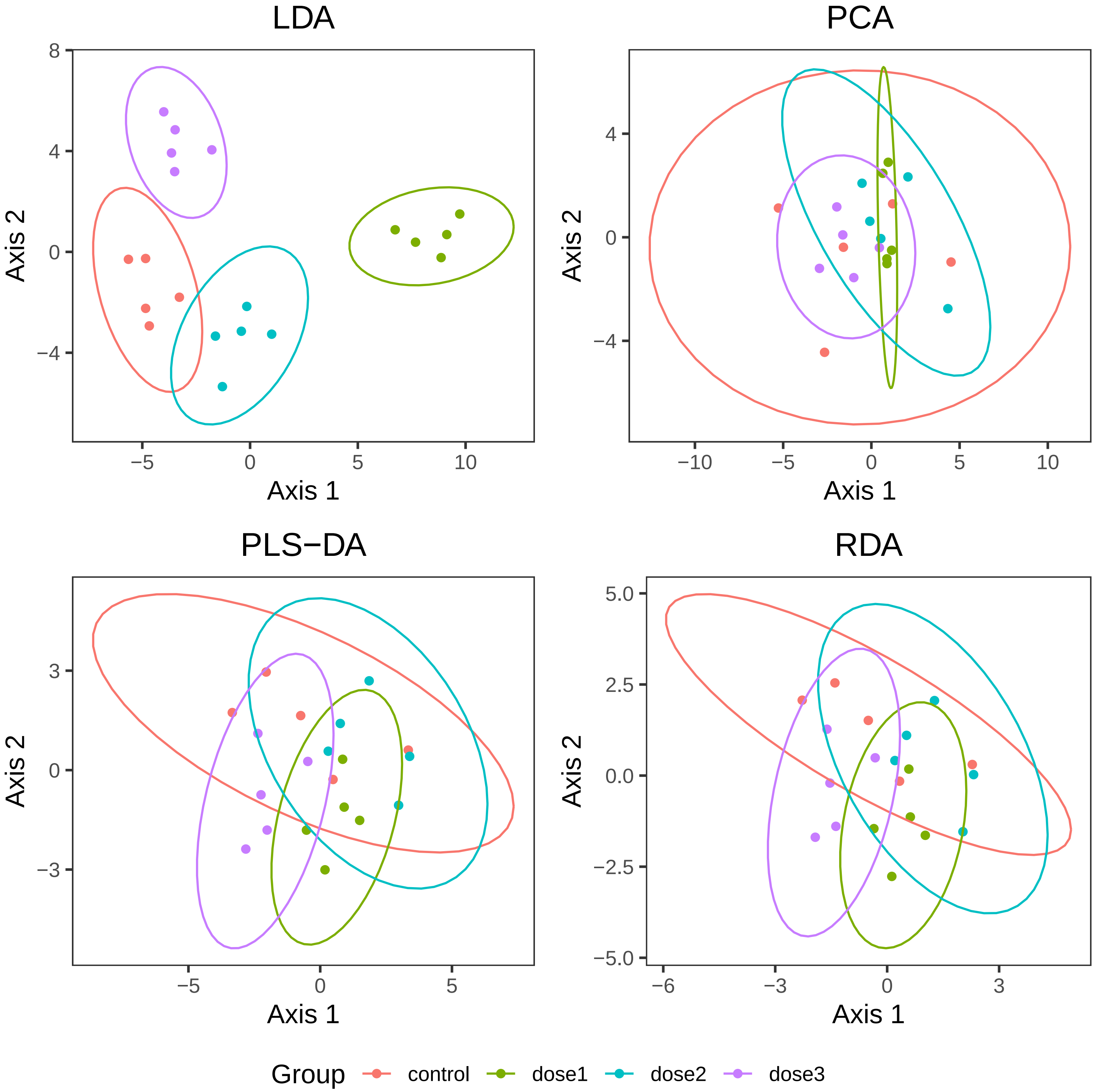

LDA
PCA
PLS−DA
RDA
Axis 1
Axis 2
8
4
0
−4
−5
0
5
10
−10
−2.5
2.5
5.0
0.0
−5.0
3
−3
−6
Group
control
dose1
dose2
dose3

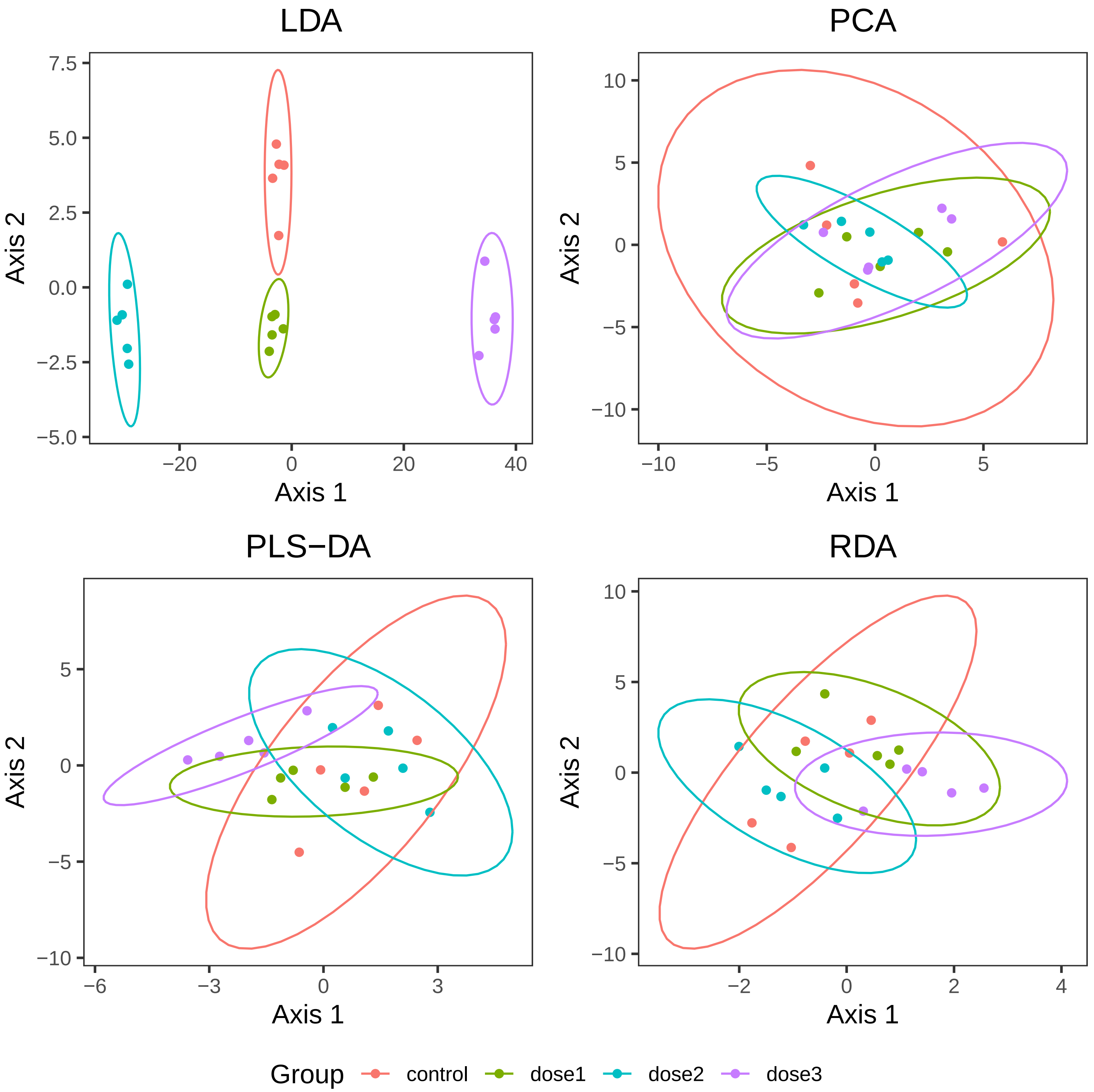
LDA
PCA
PLS−DA
RDA
Axis 1
Axis 2
Group
control
dose1
dose2
dose3

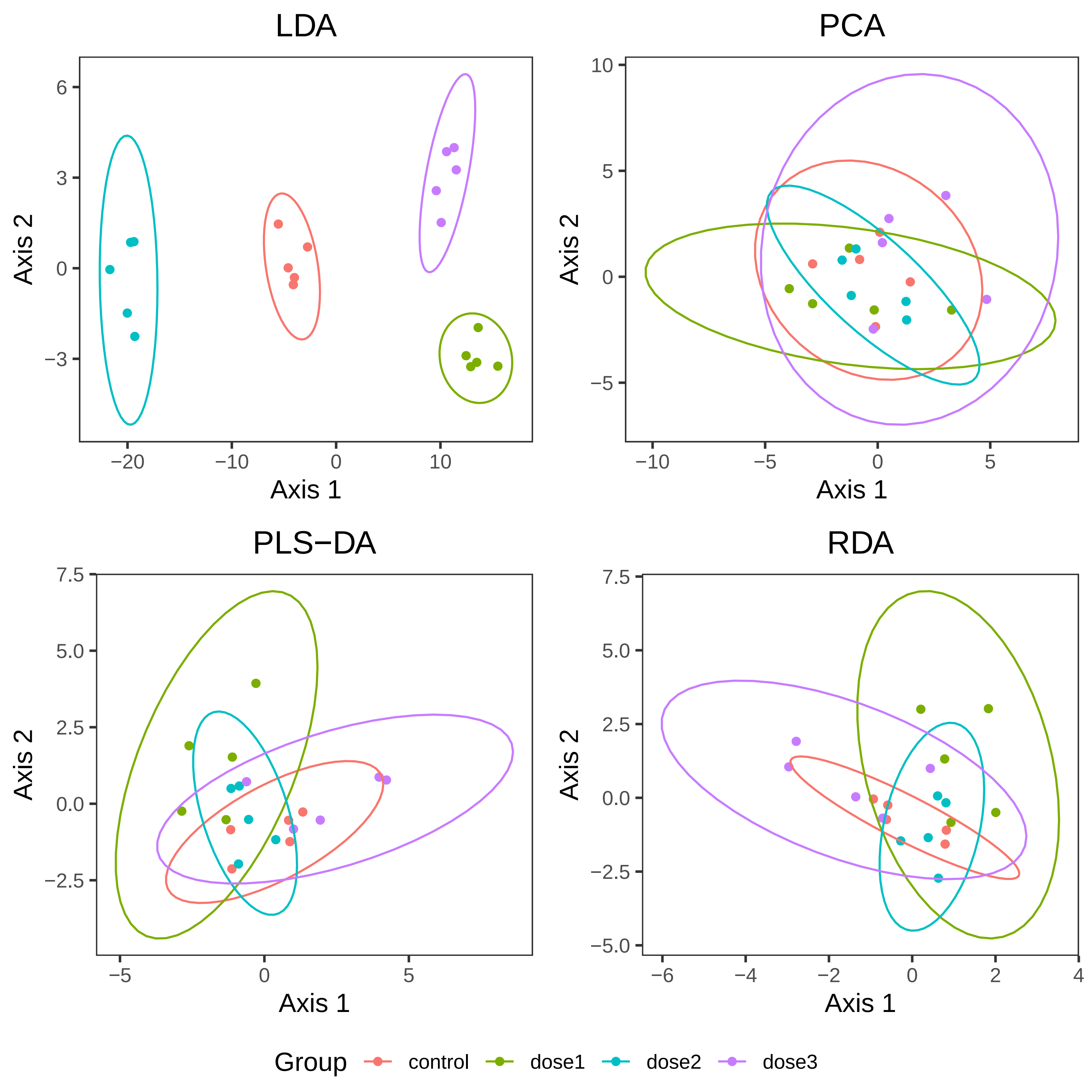
LDA
PCA
PLS−DA
RDA
Axis 1
Axis 2
Group
control
dose1
dose2
dose3

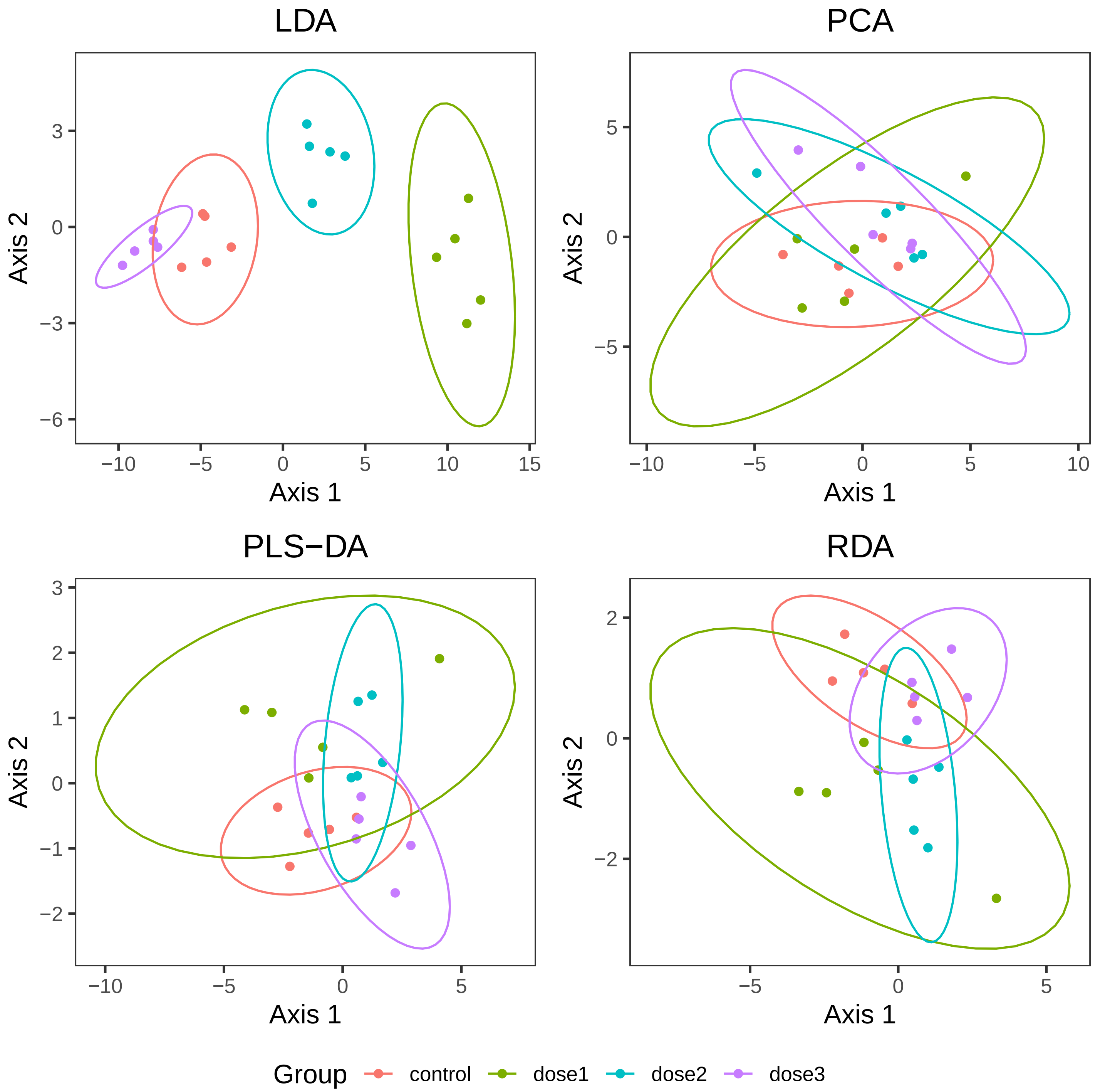
LDA
PCA
PLS−DA
RDA
Axis 1
Axis 2
Group
control
dose1
dose2
dose3

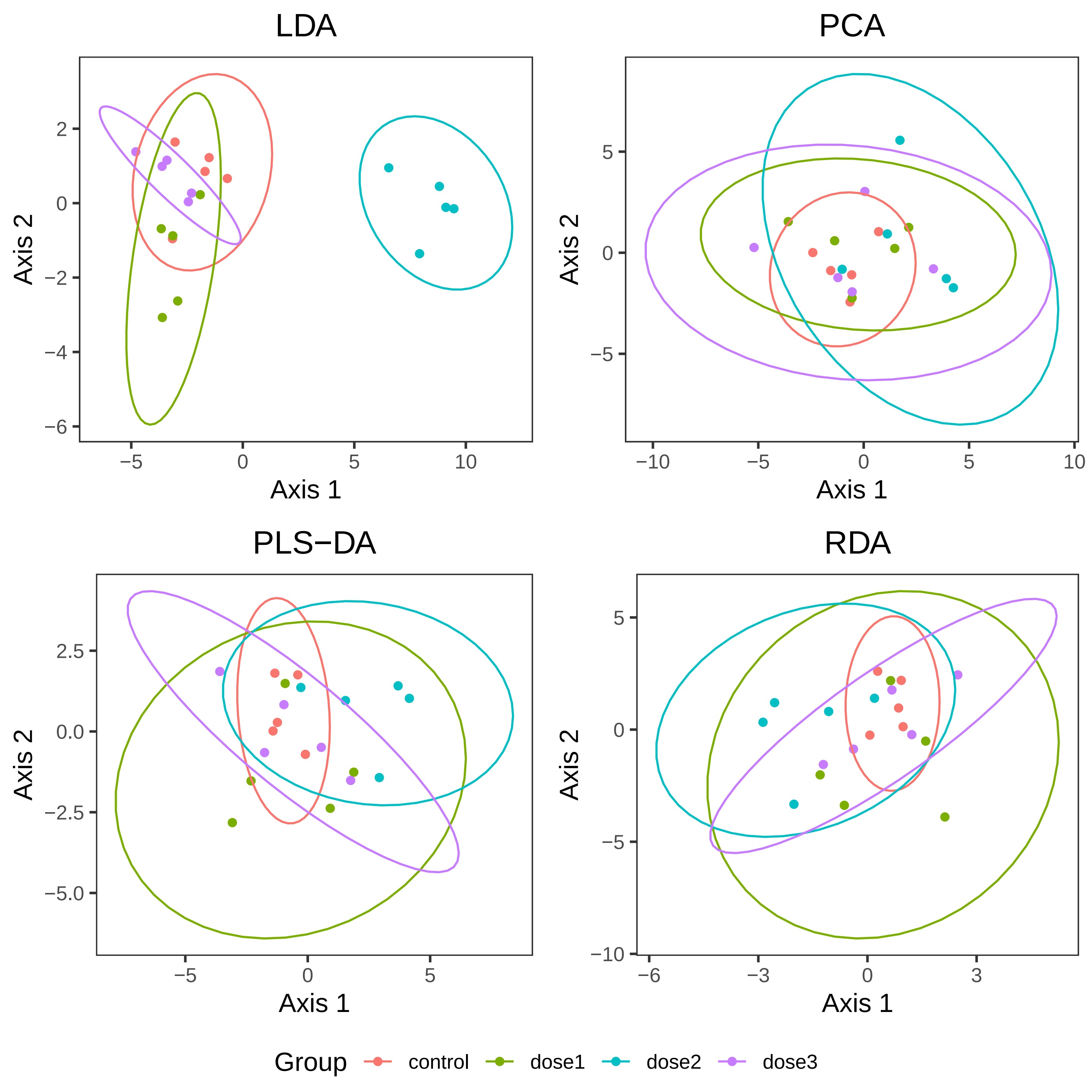

LDA
Axis 1
Axis 2
−5
0
5
10
2
0
−2
−4
−6
PCA
Axis 1
Axis 2
−10
−5
0
5
10
5
0
−5
PLS−DA
Axis 1
Axis 2
−5
0
5
2.5
0.0
−2.5
−5.0
RDA
Axis 1
Axis 2
−6
−3
0
3
5
0
−5
−10
Group
control
dose1
dose2
dose3

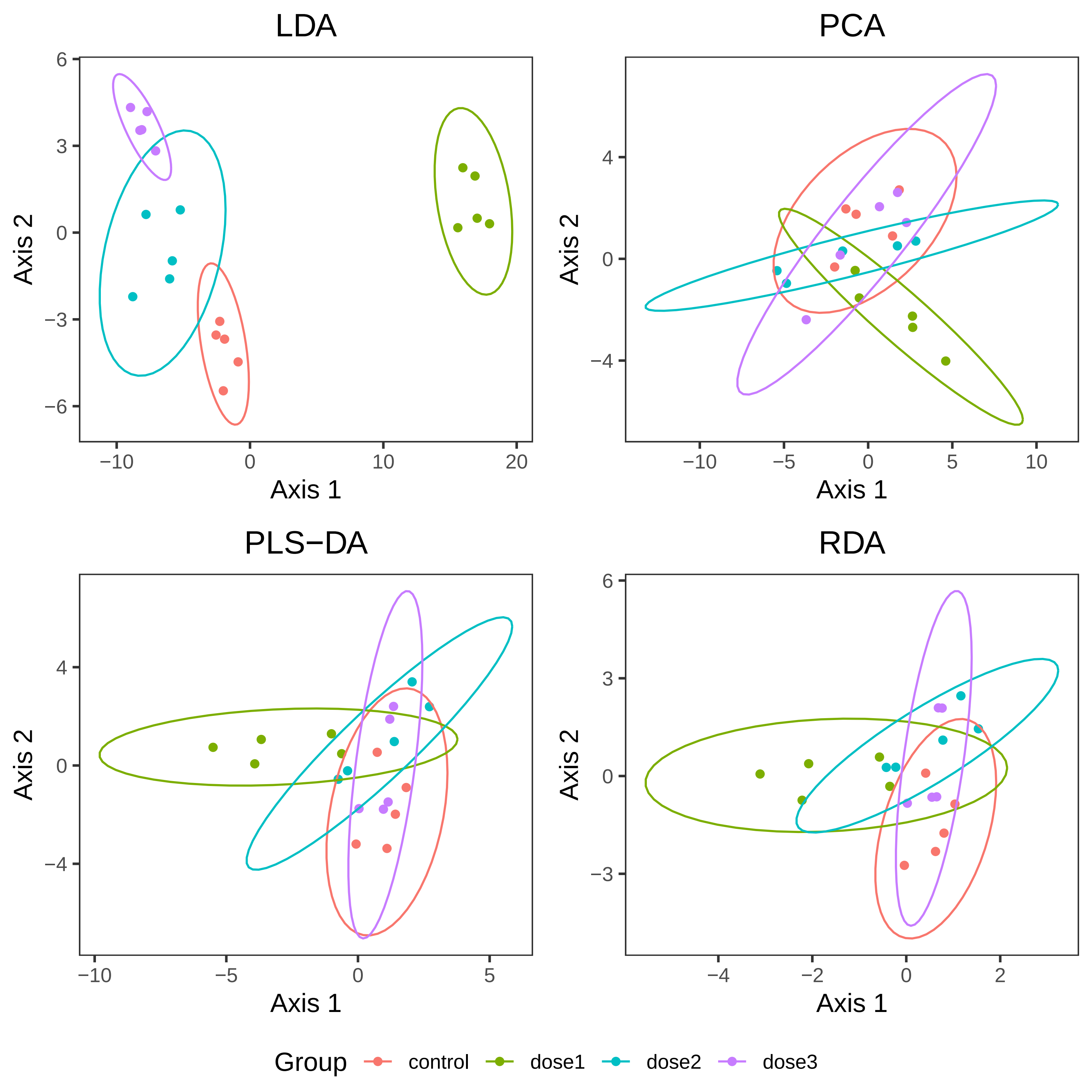
LDA
PCA
PLS−DA
RDA
Axis 1
Axis 2
Group
control
dose1
dose2
dose3

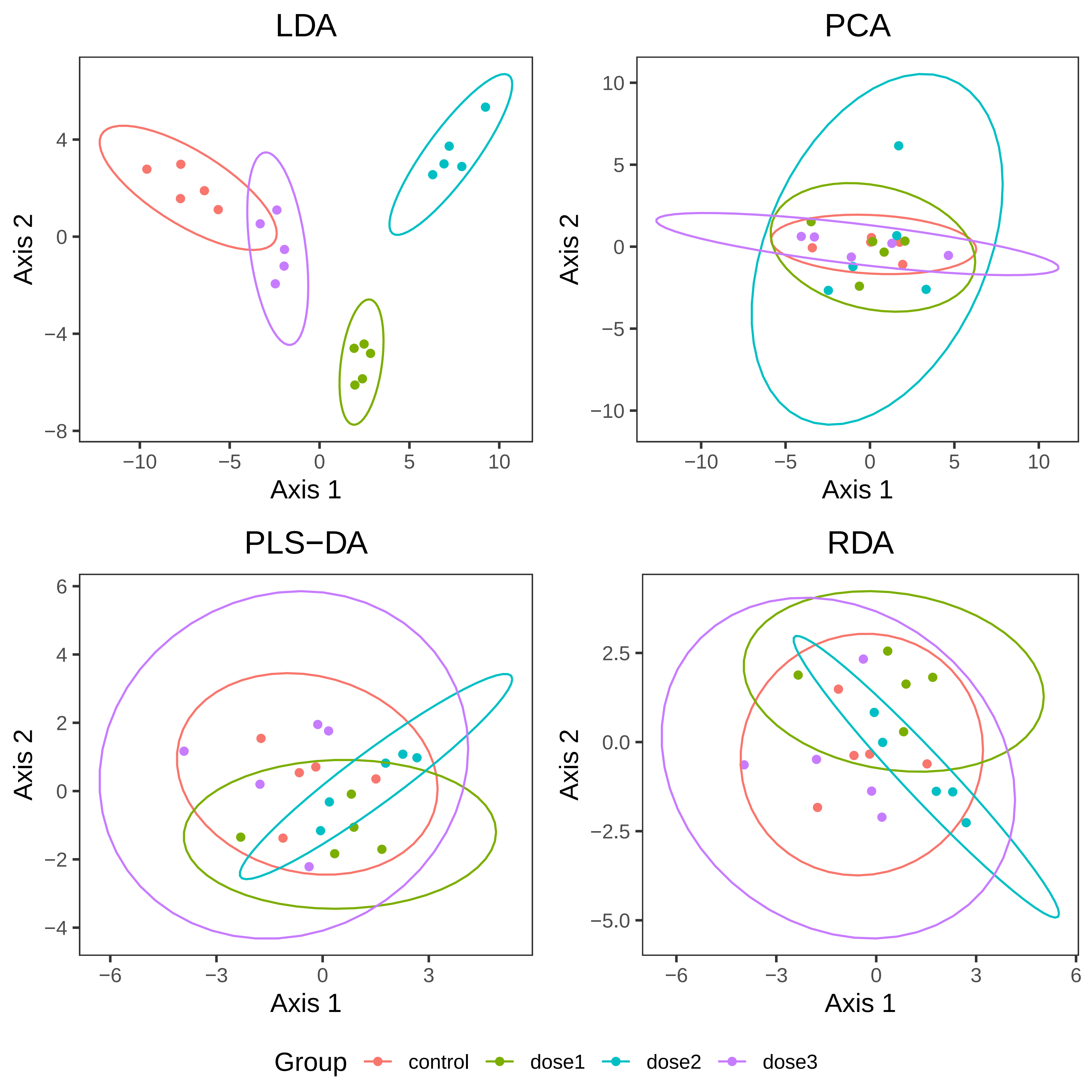
LDA
PCA
PLS−DA
RDA
Axis 1
Axis 2
Group
control
dose1
dose2
dose3

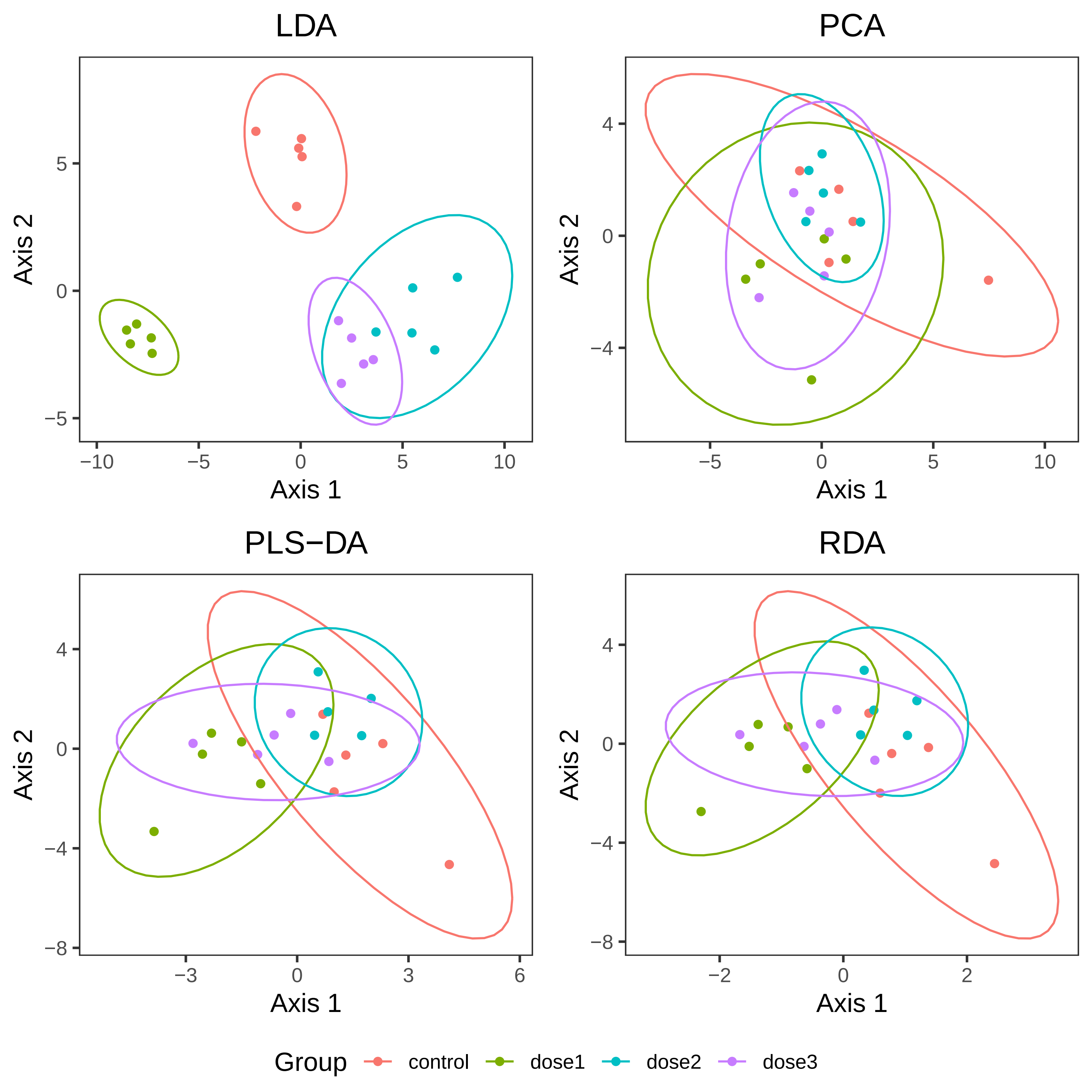

LDA
PCA
PLS−DA
RDA
Axis 1
Axis 2
−10
−5
0
5
10
−2
2
3
−3
6
4
−4
−8
Group
control
dose1
dose2
dose3

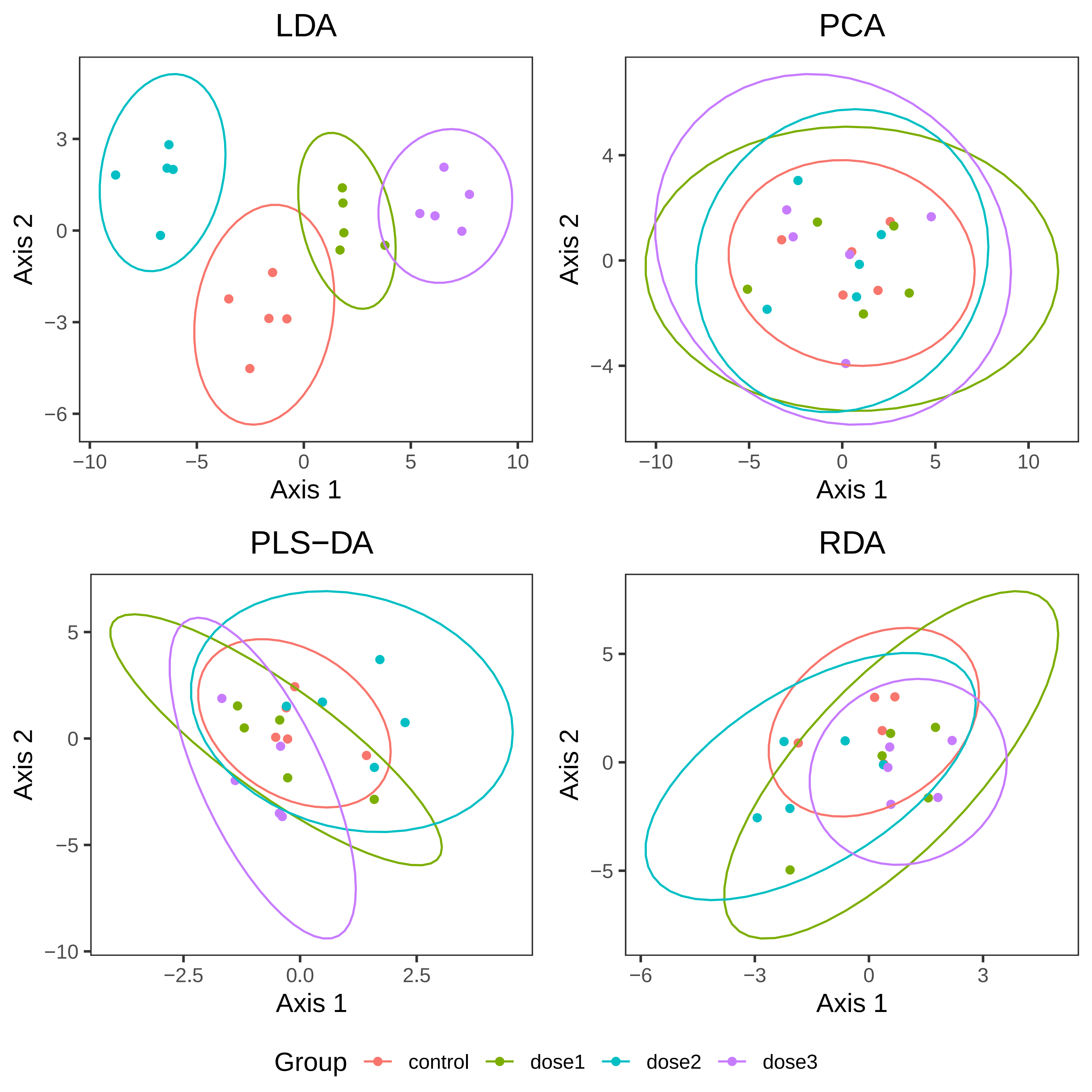
LDA
PCA
PLS−DA
RDA
Axis 1
Axis 2
Group
control
dose1
dose2
dose3

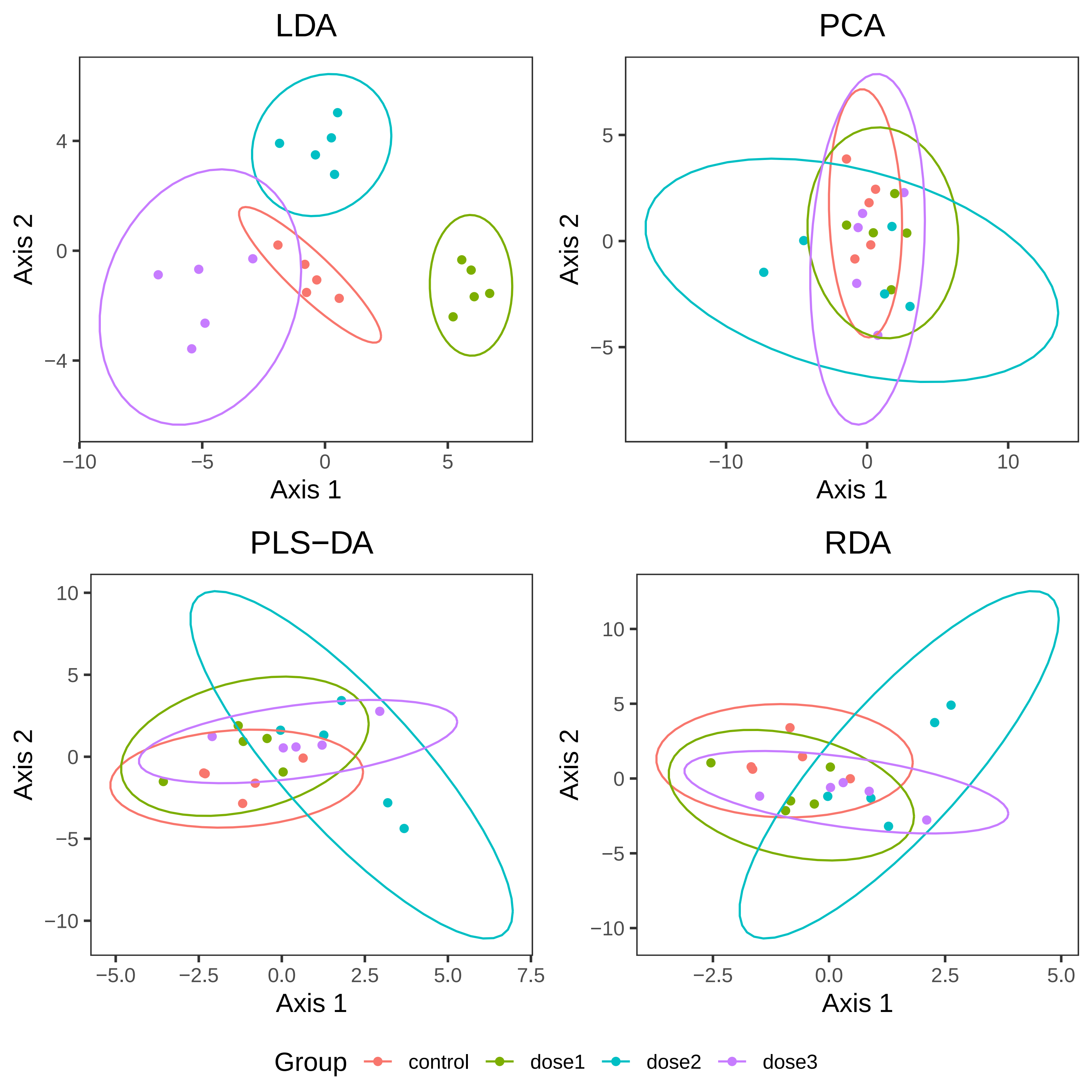

LDA
PCA
PLS−DA
RDA
Axis 1
Axis 2
Group
control
dose1
dose2
dose3

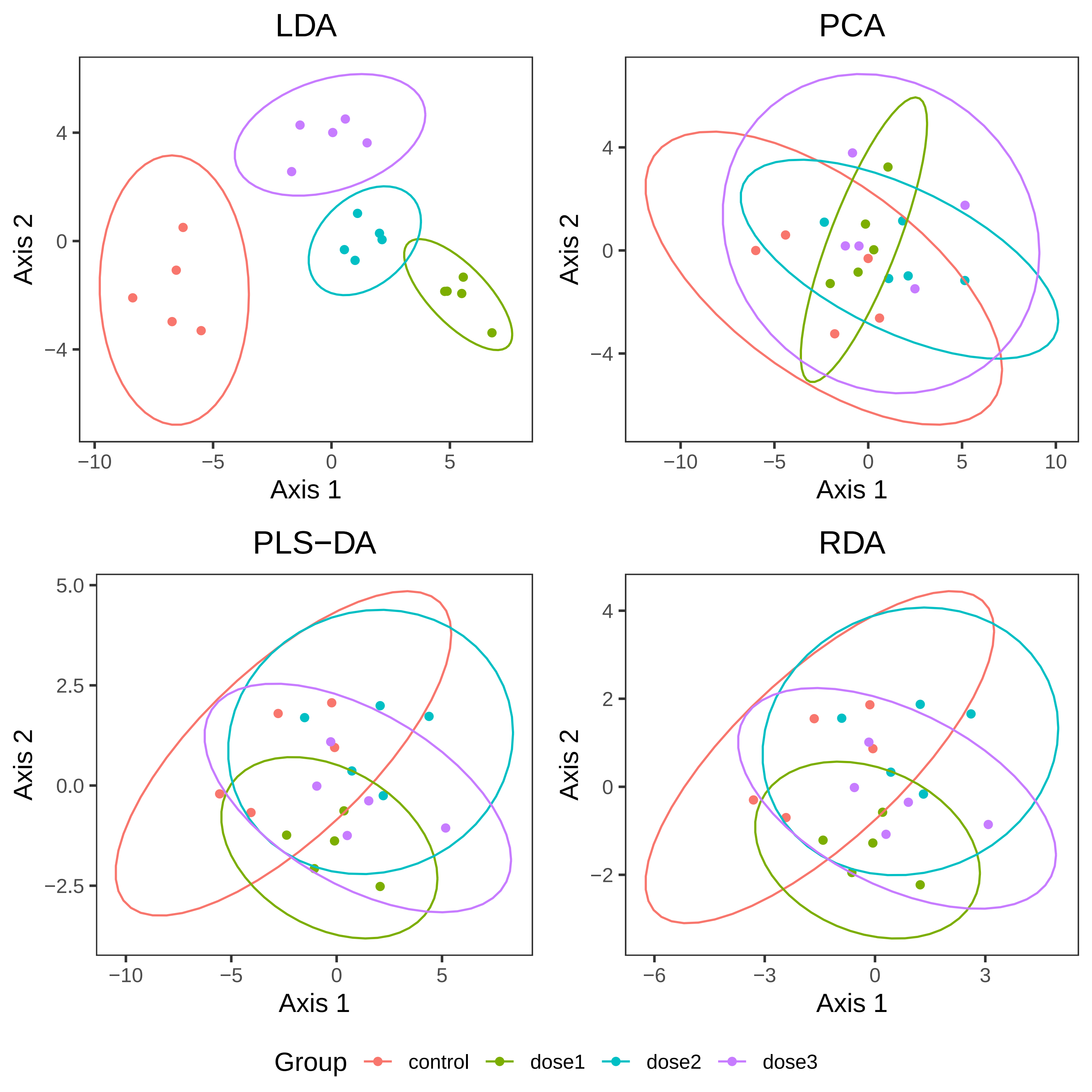

LDA
PCA
PLS−DA
RDA
Axis 1
Axis 2
Group
control
dose1
dose2
dose3

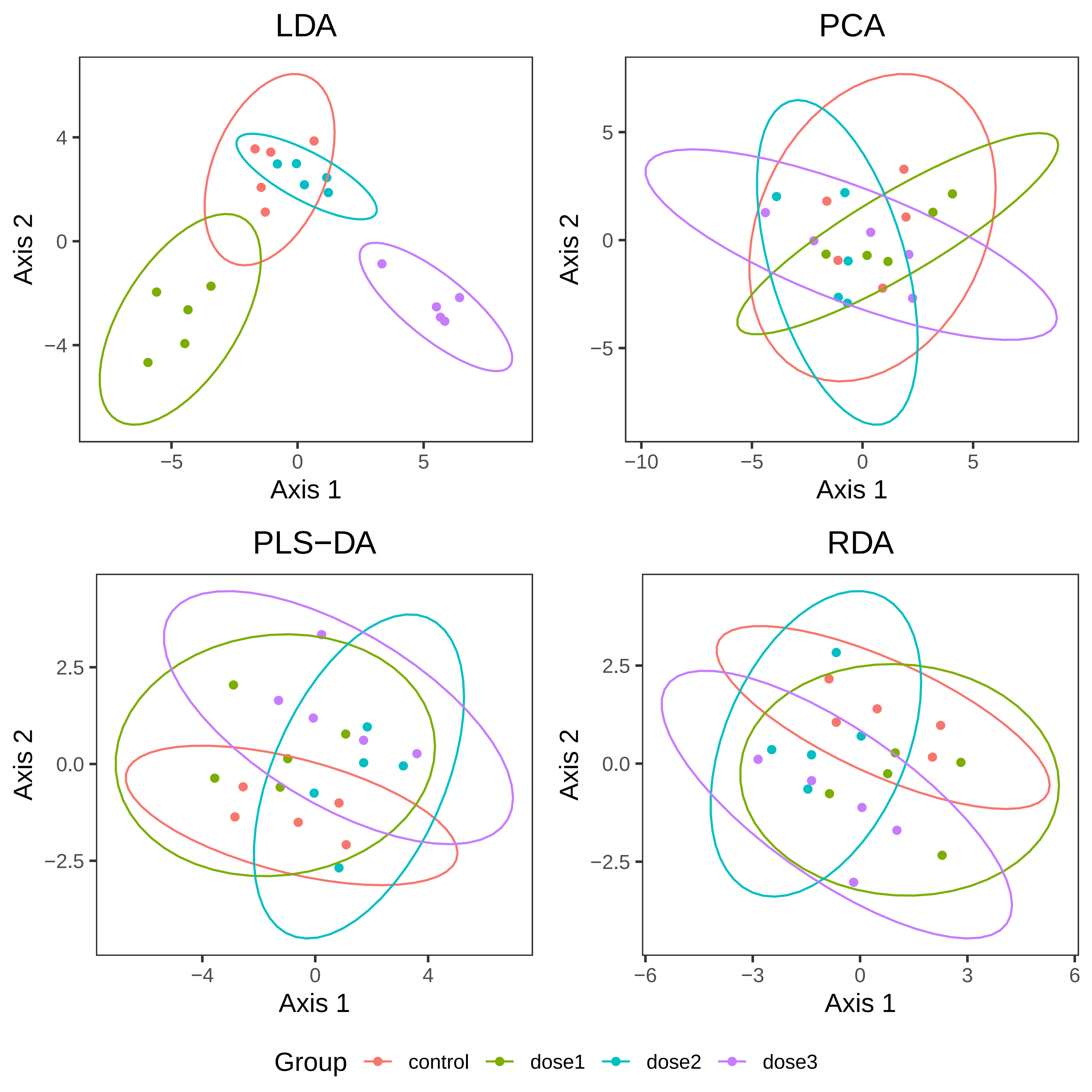

LDA
PCA
PLS−DA
RDA
Axis 1
Axis 2
Group
control
dose1
dose2
dose3

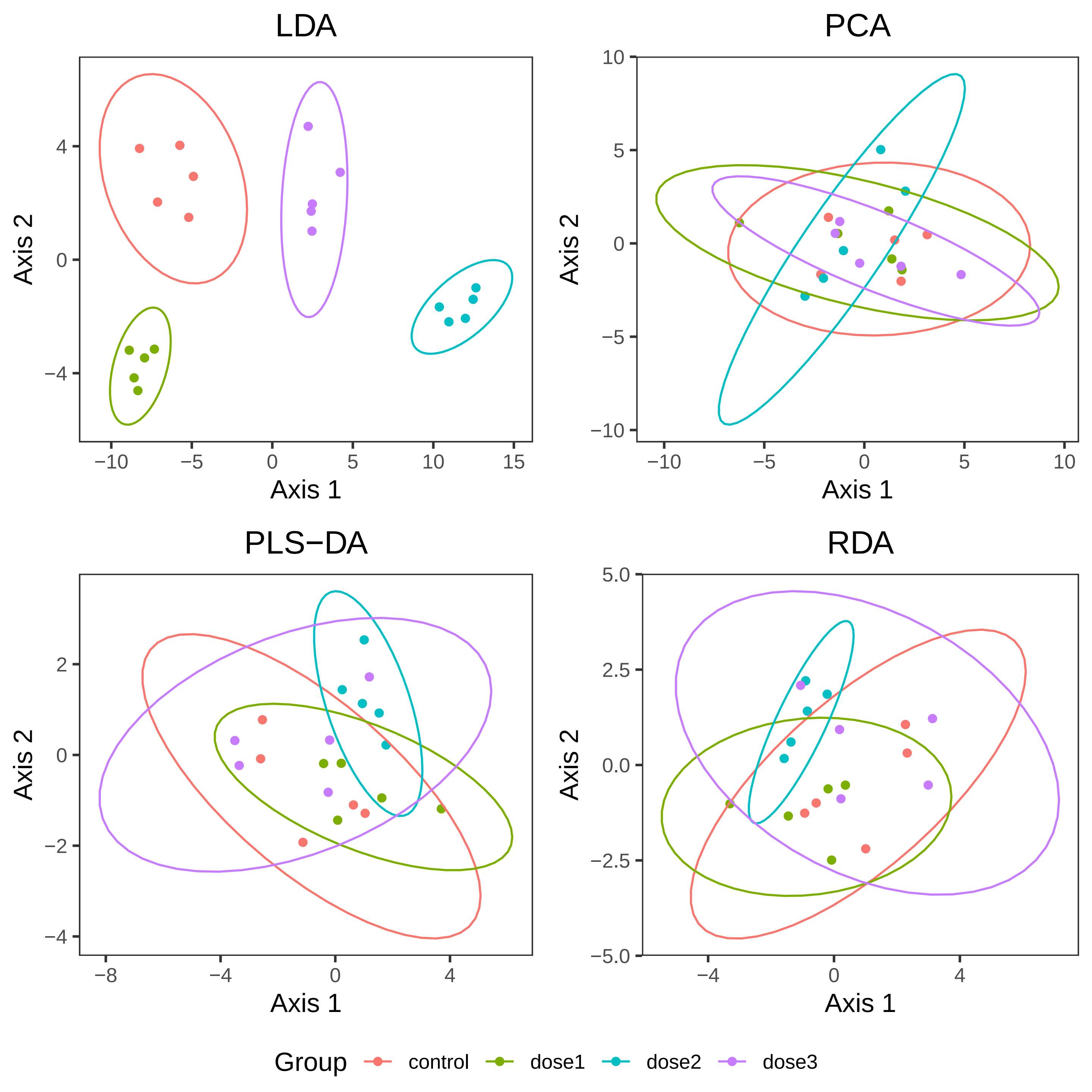

LDA
PCA
PLS−DA
RDA
Axis 1
Axis 2
−10
−5
0
5
10
15
4
−4
−8
2
−2
2.5
5.0
0.0
−2.5
−5.0
Group
control
dose1
dose2
dose3

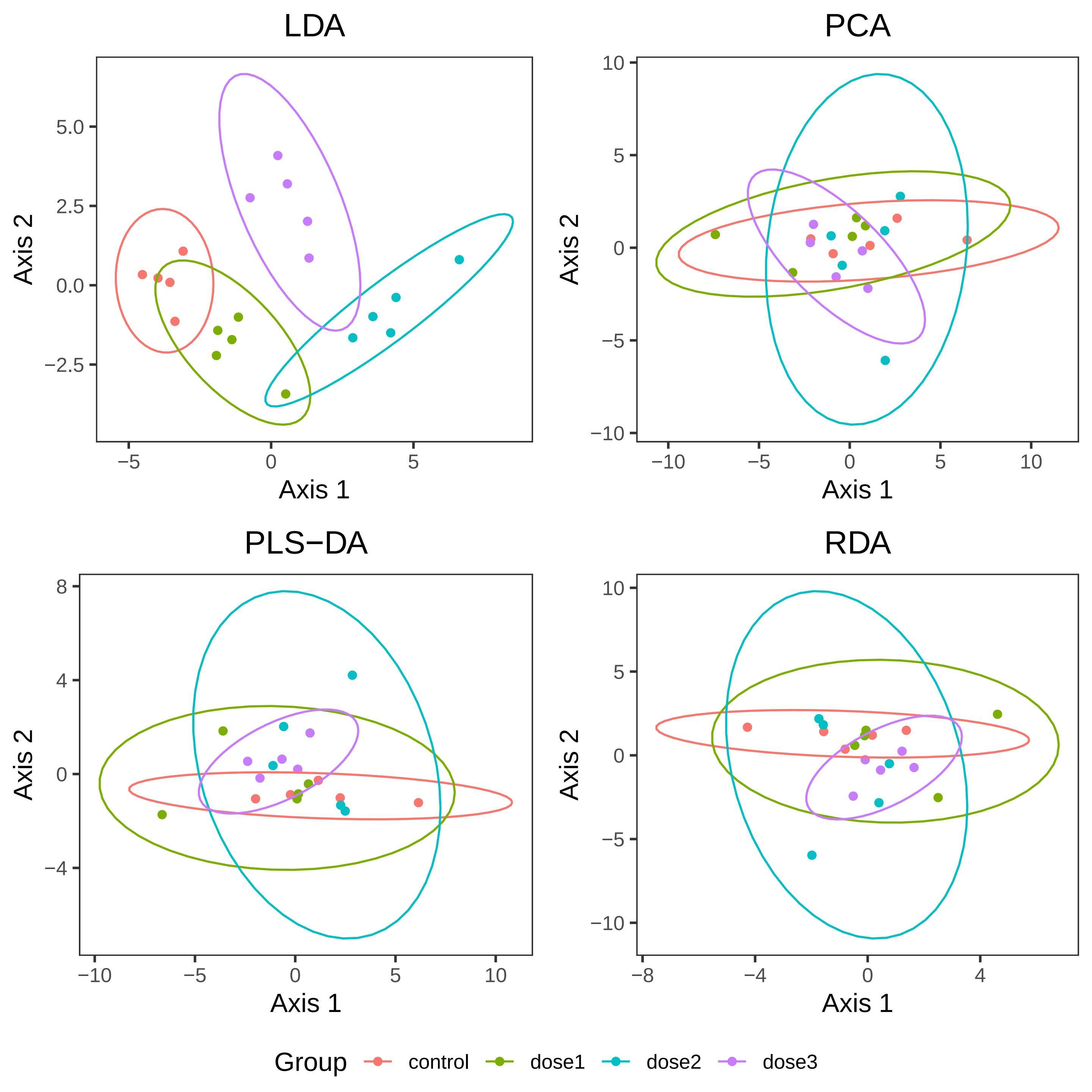
LDA
PCA
PLS−DA
RDA
Axis 1
Axis 2
Group
control
dose1
dose2
dose3

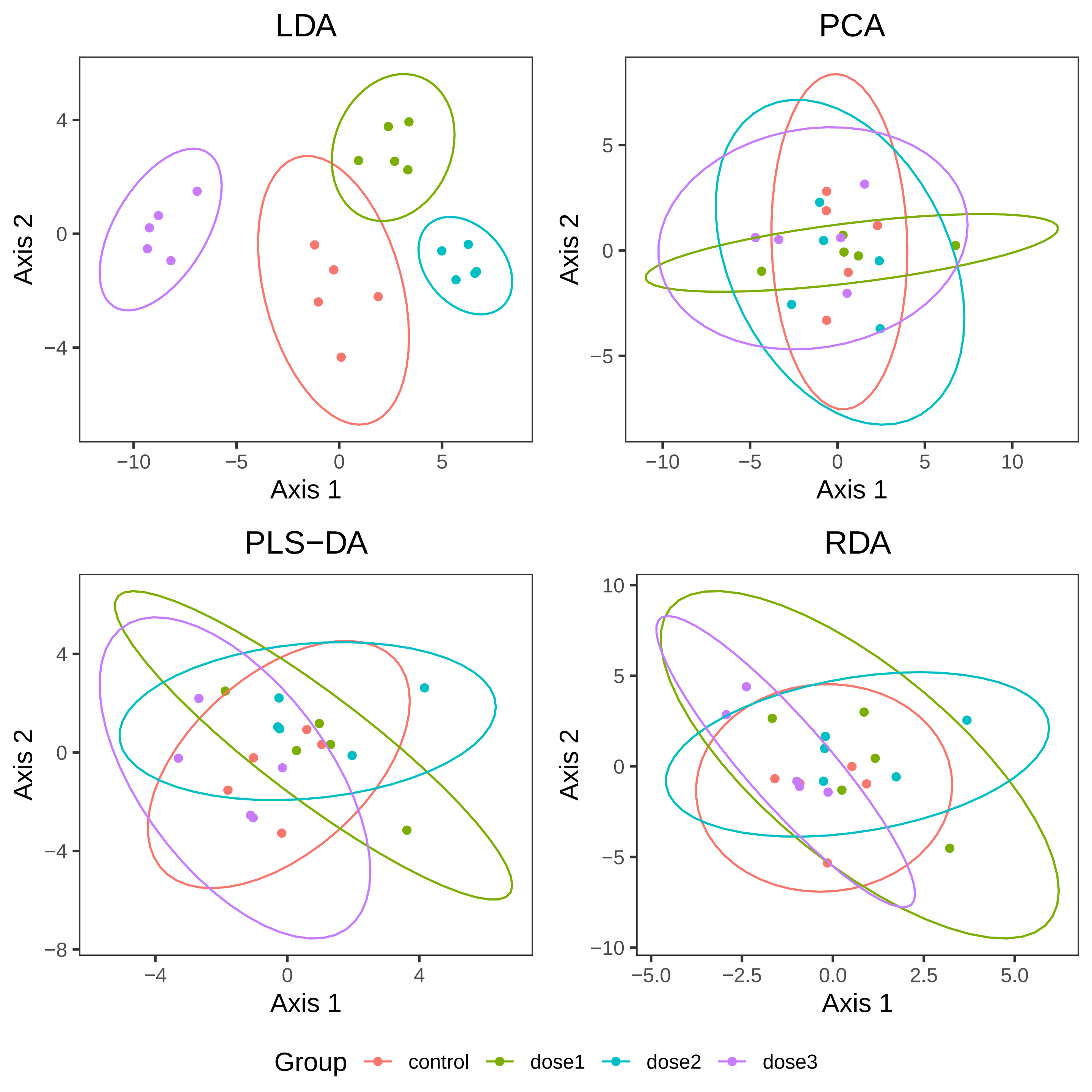
LDA
PCA
PLS−DA
RDA
Axis 1
Axis 2
Group
control
dose1
dose2
dose3

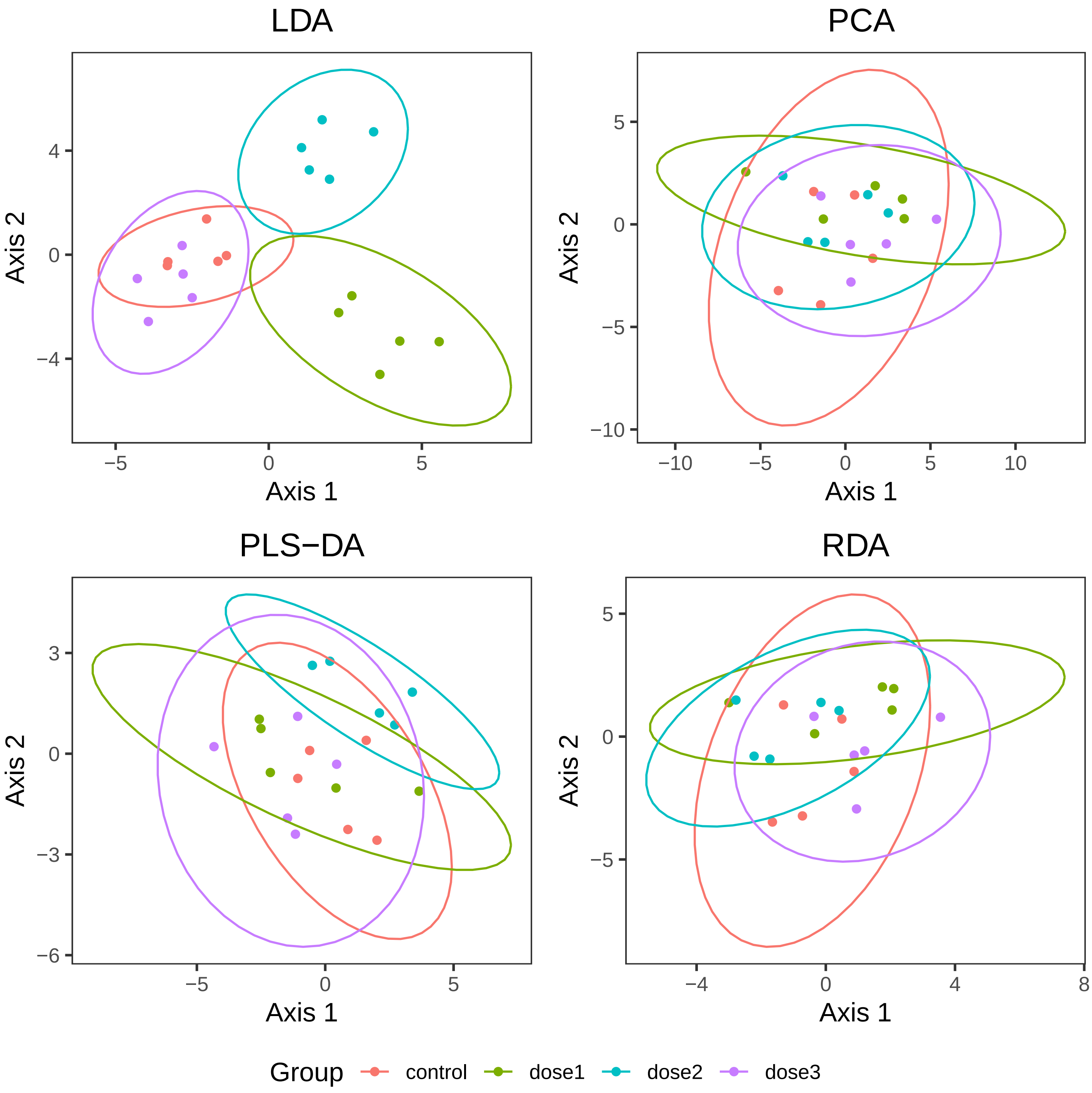
LDA
PCA
PLS−DA
RDA
Axis 1
Axis 2
Group
control
dose1
dose2
dose3

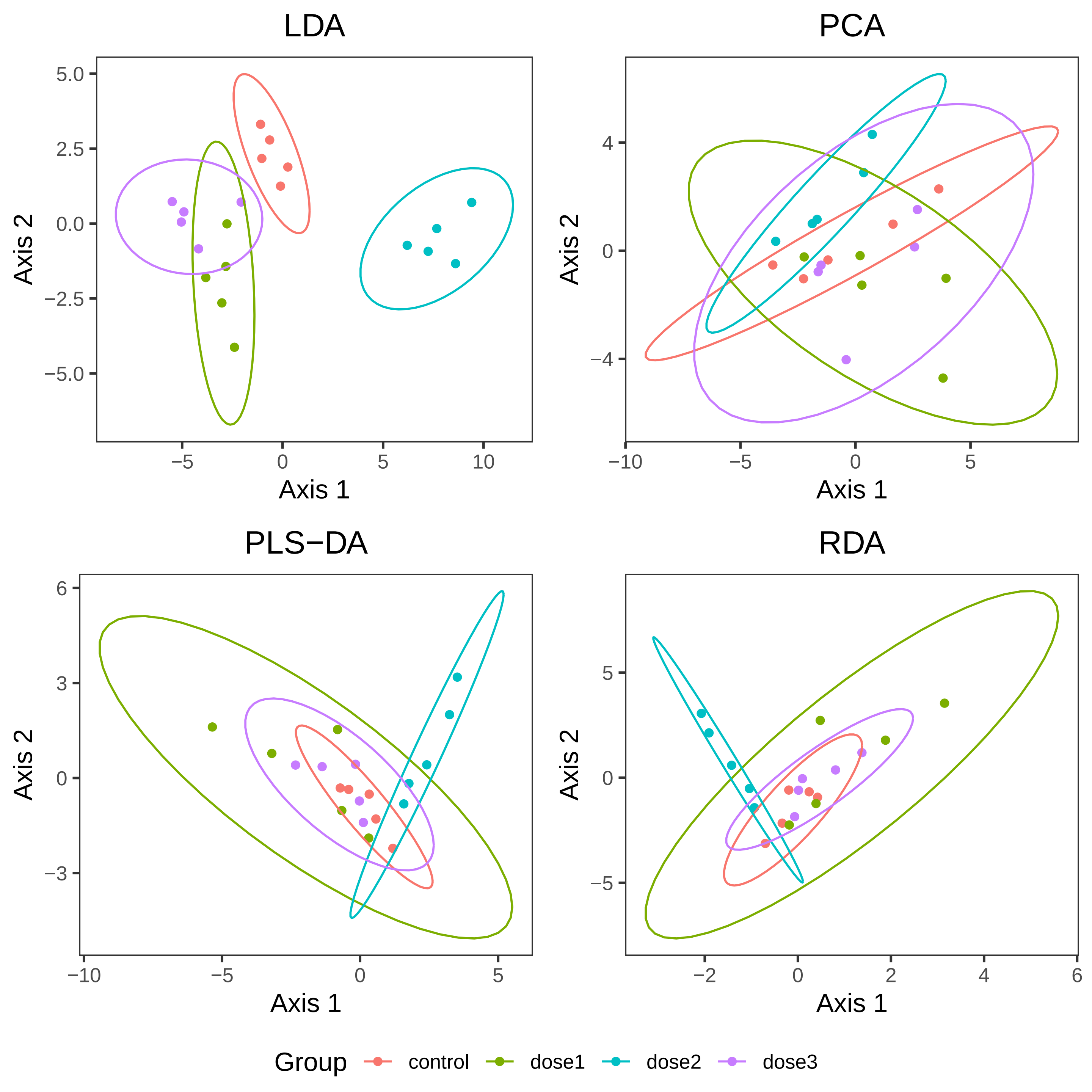
LDA
PCA
PLS−DA
RDA
Axis 1
Axis 2
Group
control
dose1
dose2
dose3

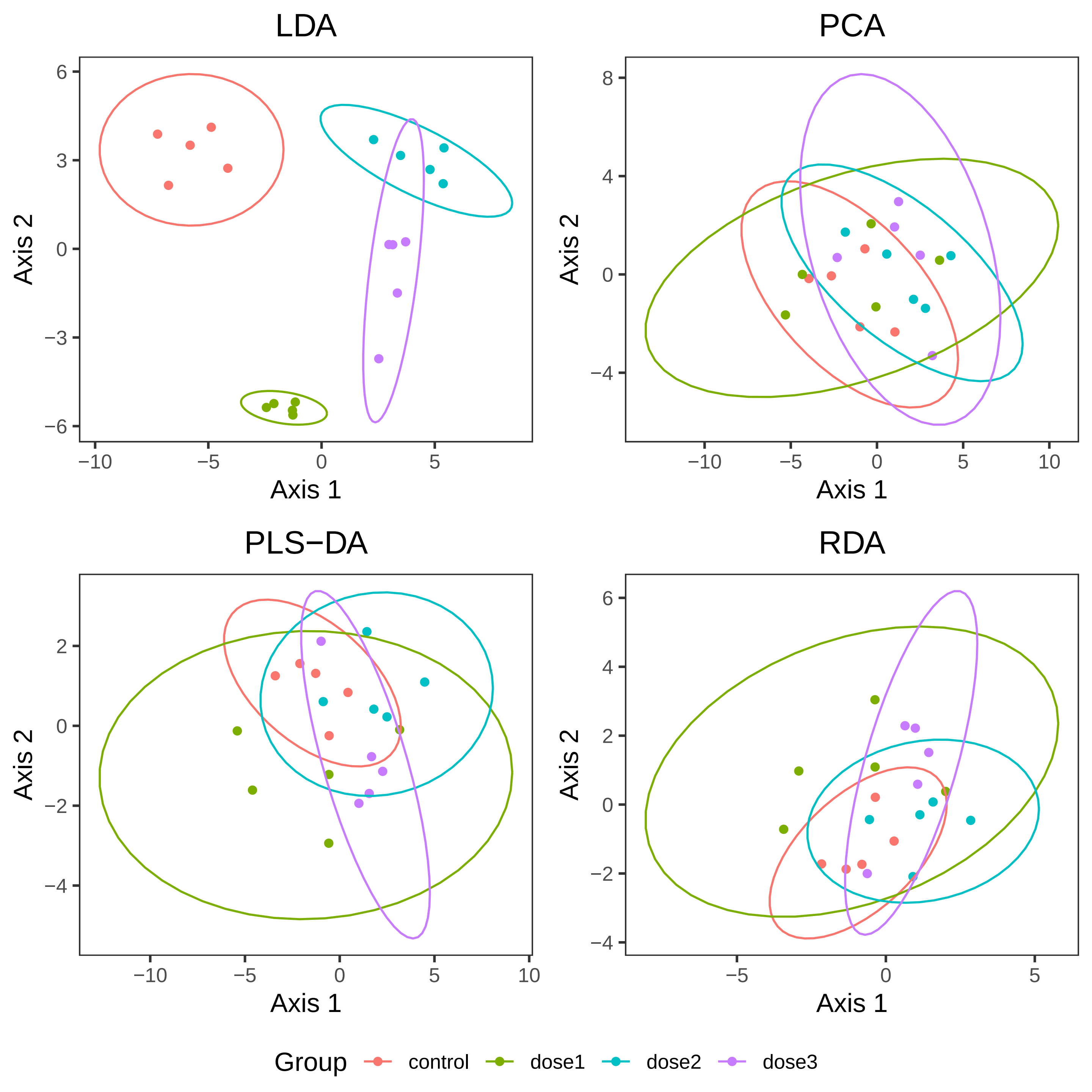

LDA
PCA
PLS−DA
RDA
Axis 1
Axis 2
Group
control
dose1
dose2
dose3

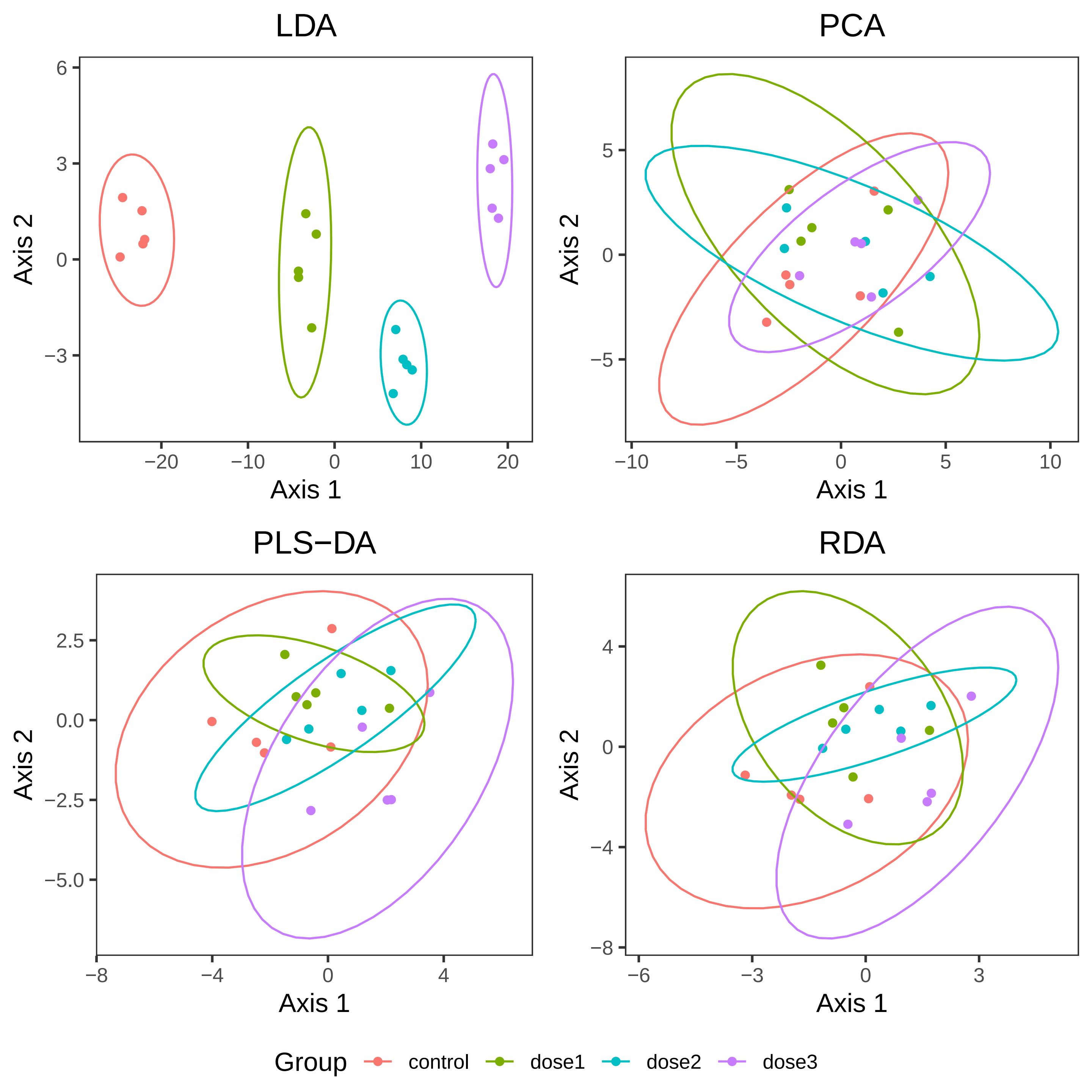
LDA
PCA
PLS−DA
RDA
Axis 1
Axis 2
Group
control
dose1
dose2
dose3

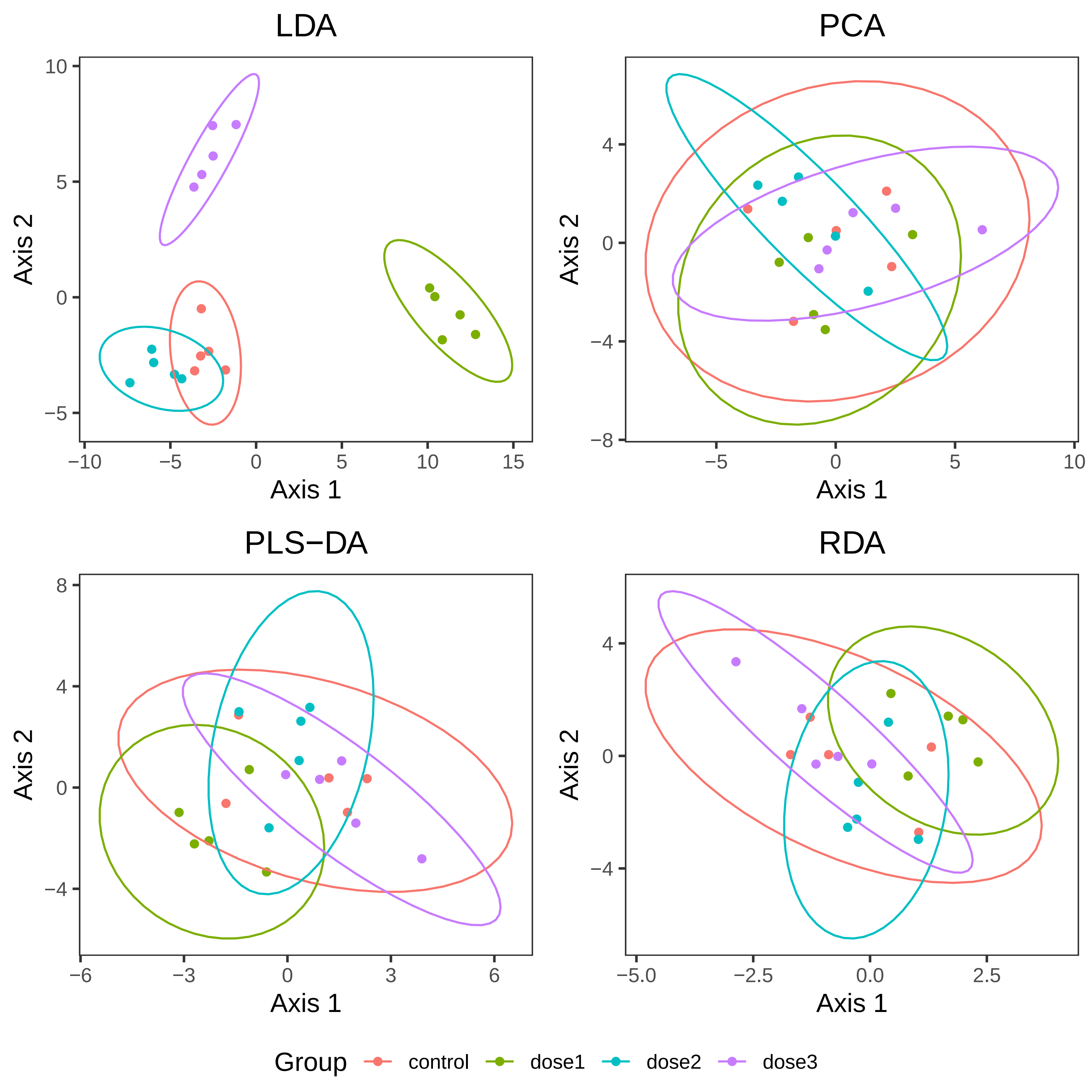

LDA
PCA
PLS−DA
RDA
Axis 1
Axis 2
Group
control
dose1
dose2
dose3

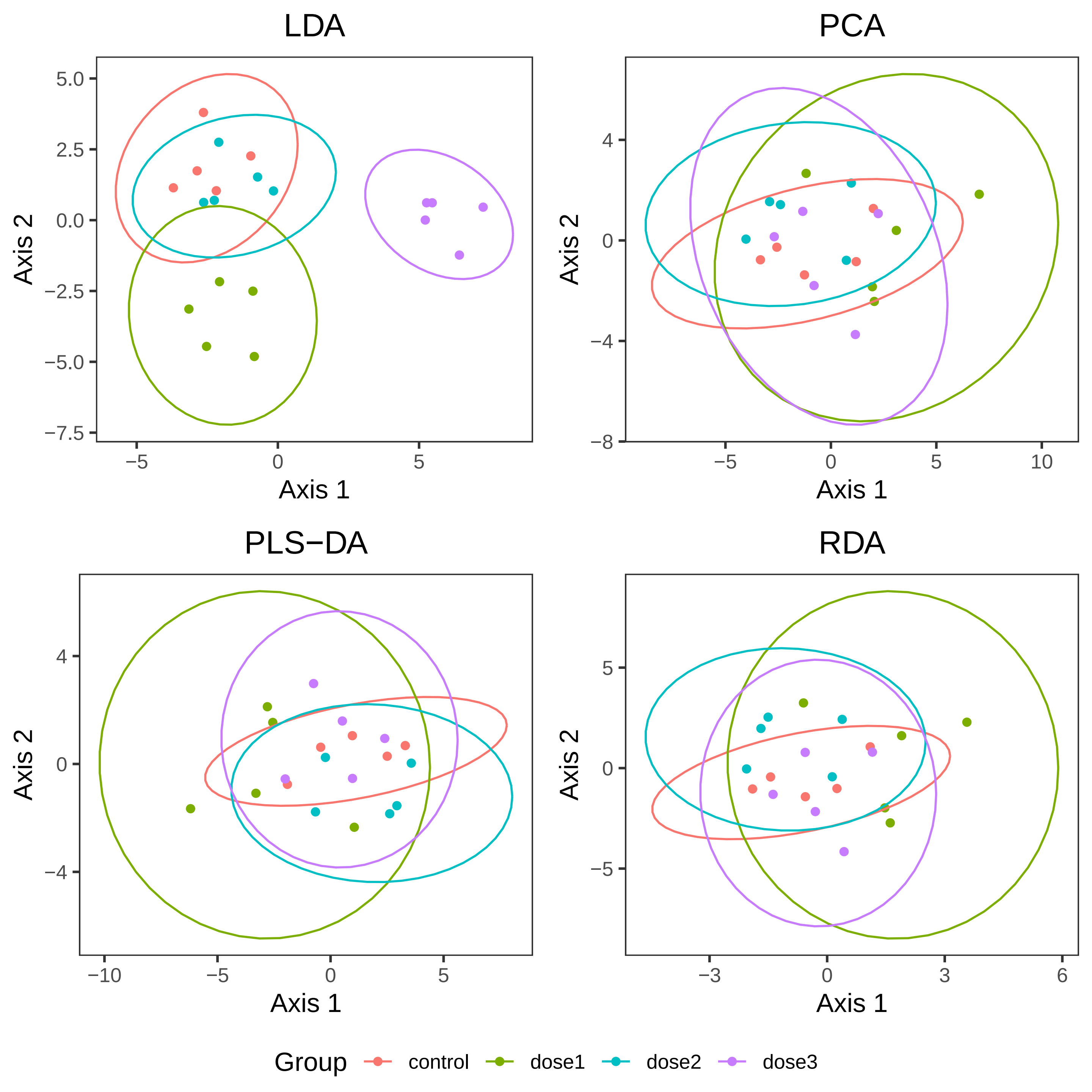

LDA
PCA
PLS−DA
RDA
Axis 1
Axis 2
Group
control
dose1
dose2
dose3

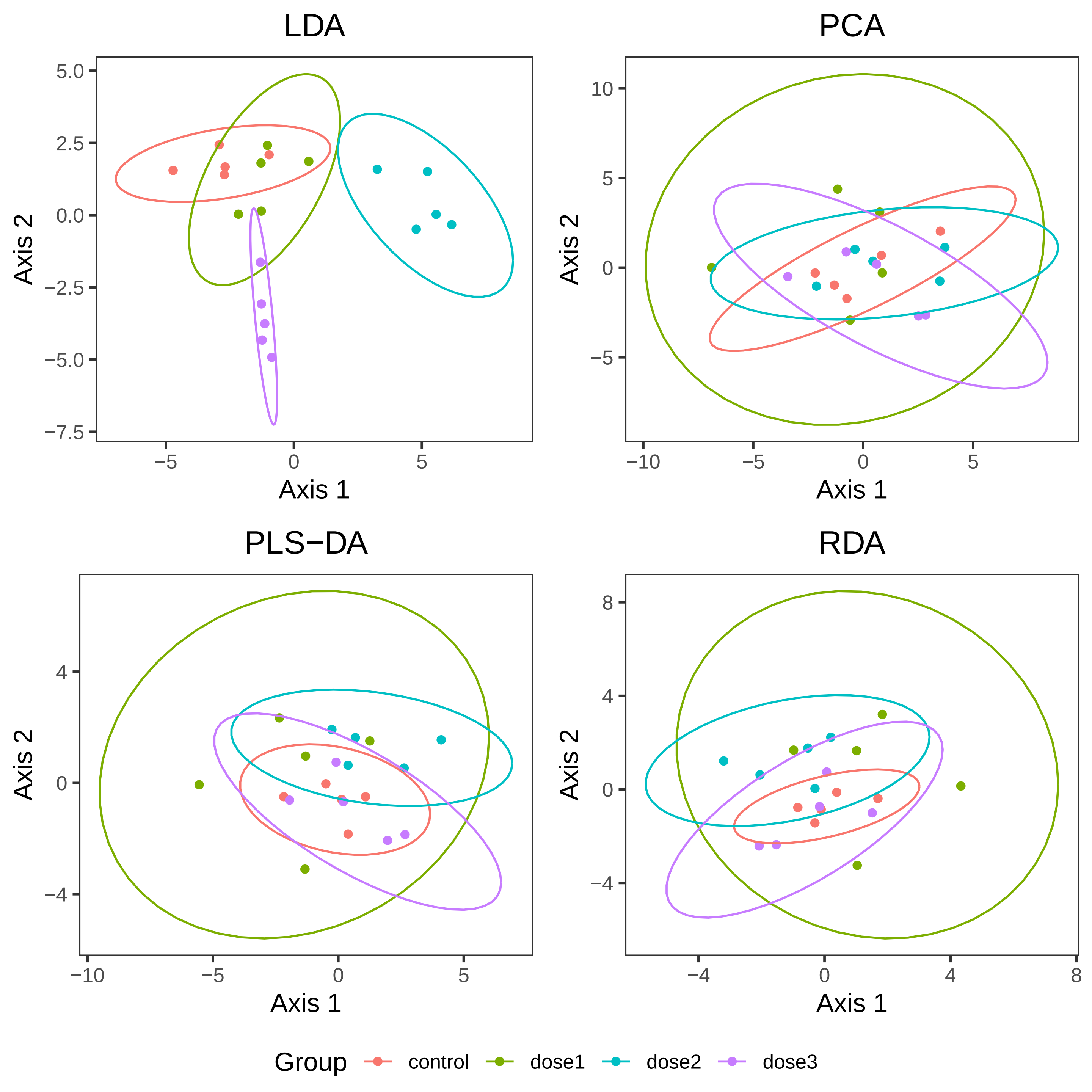

LDA
PCA
PLS−DA
RDA
Axis 1
Axis 2
5.0
2.5
0.0
−2.5
−5.0
−7.5
−5
0
5
10
−10
4
−4
8
Group
control
dose1
dose2
dose3

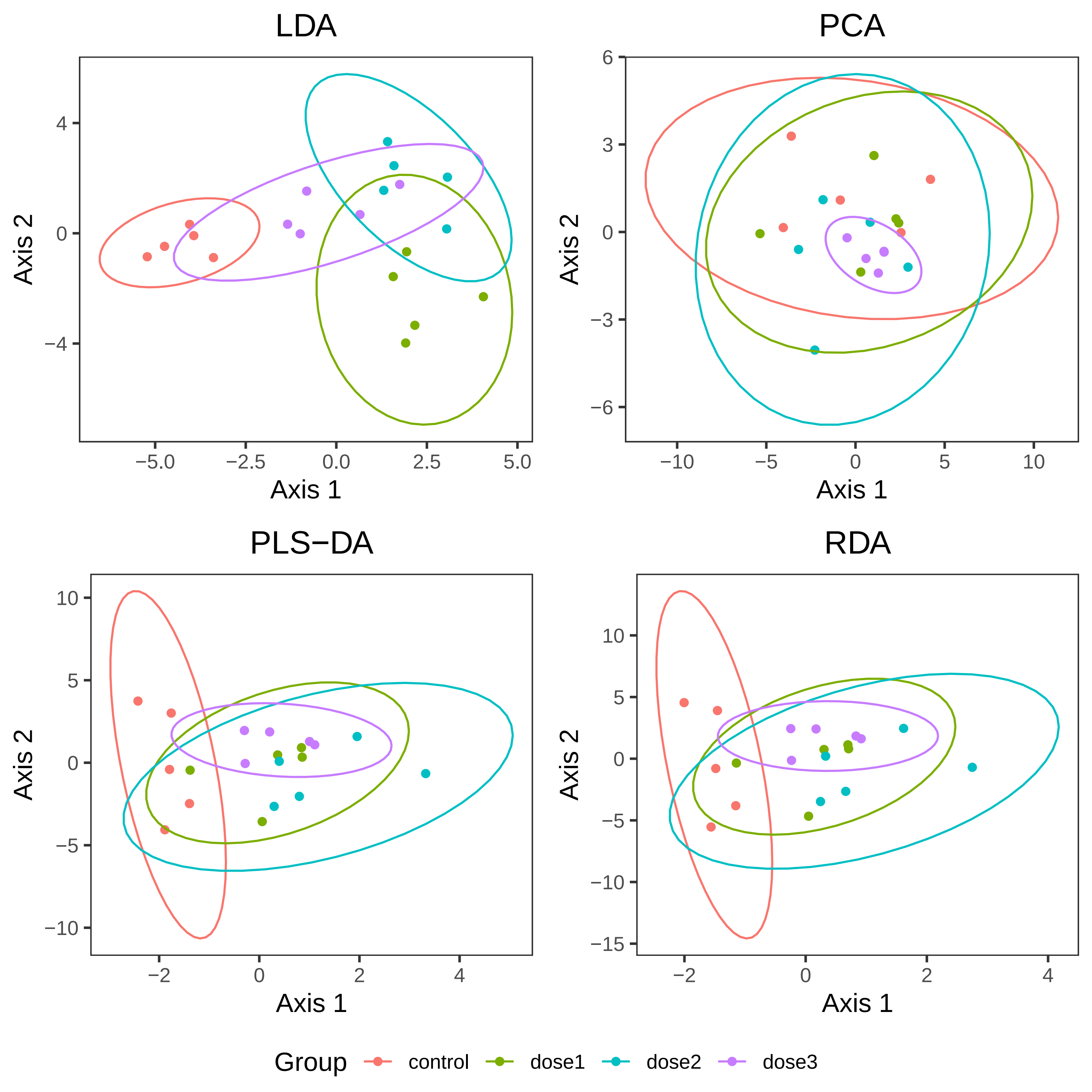
LDA
PCA
PLS−DA
RDA
Axis 1
Axis 2
Group
control
dose1
dose2
dose3

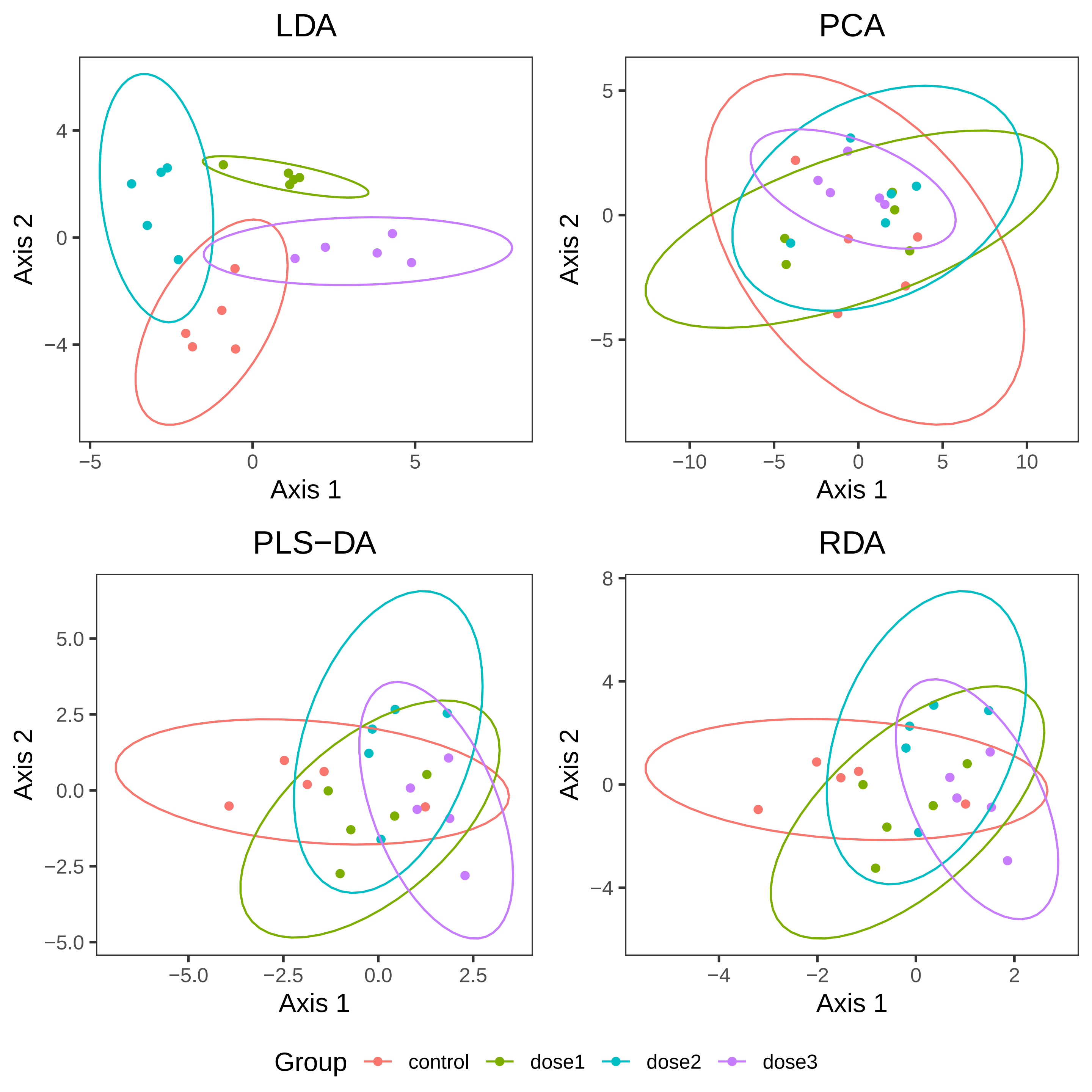

LDA
PCA
PLS−DA
RDA
Axis 1
Axis 2
4
0
−4
−5
0
5
5
0
−5
−10
−5
0
5
10
5.0
2.5
0.0
−2.5
−5.0
−5.0
−2.5
0.0
2.5
8
4
0
−4
−4
−2
0
2
Group
control
dose1
dose2
dose3

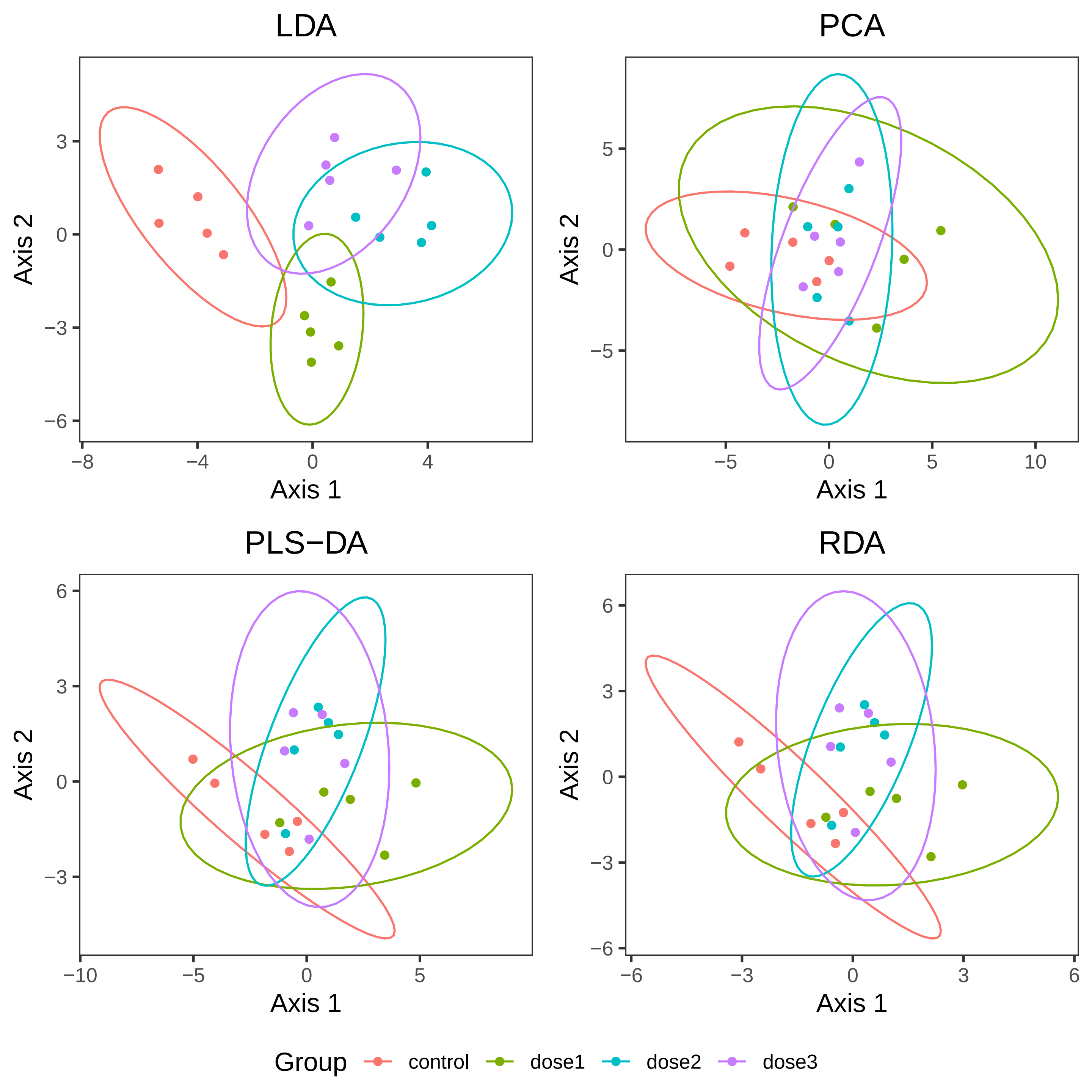

LDA
PCA
PLS−DA
RDA
Axis 1
Axis 2
Group
control
dose1
dose2
dose3

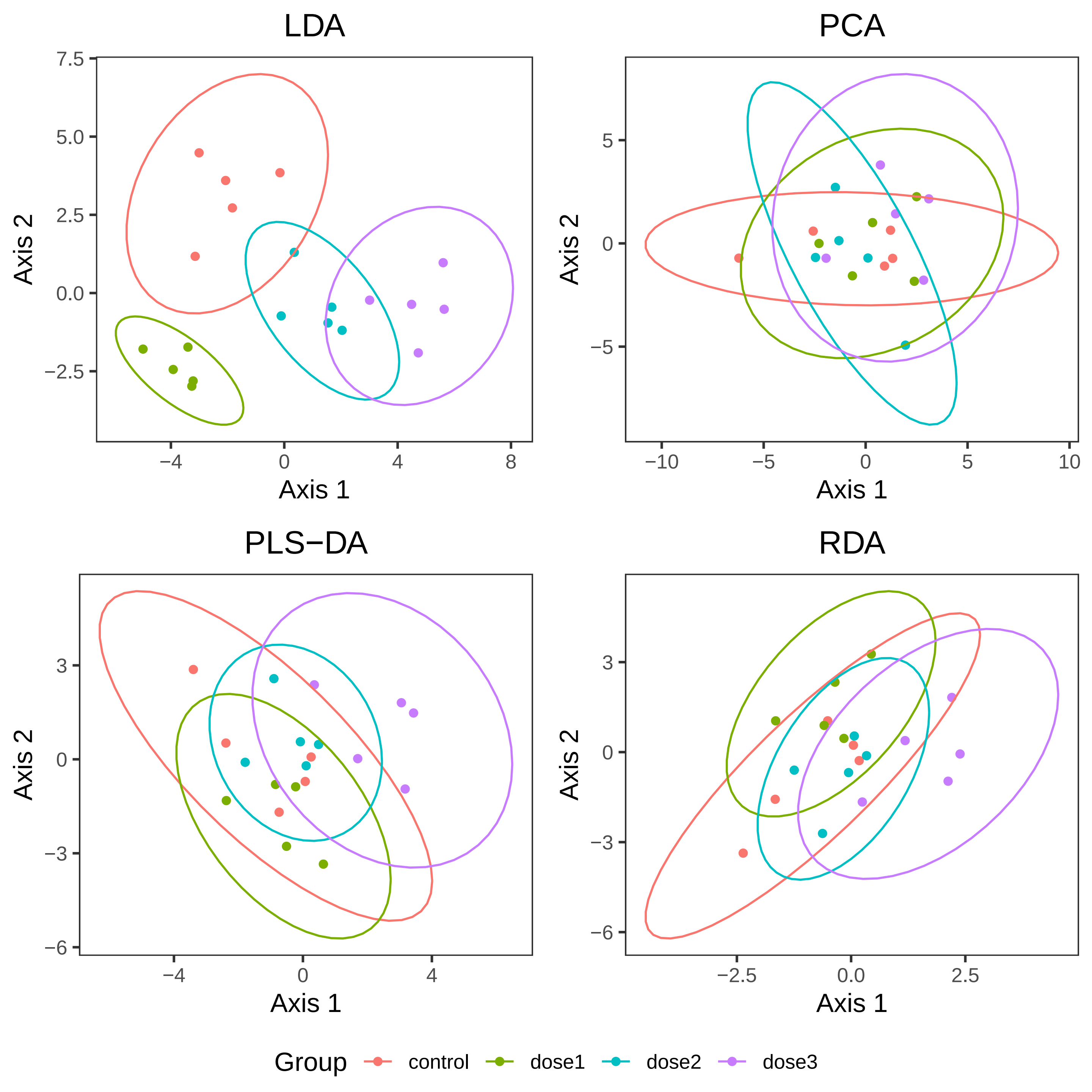

LDA
PCA
PLS−DA
RDA
Axis 1
Axis 2
7.5
5.0
2.5
0.0
−2.5
−4
0
4
8
5
0
−5
−10
10
3
−3
−6
−2.5
Group
control
dose1
dose2
dose3

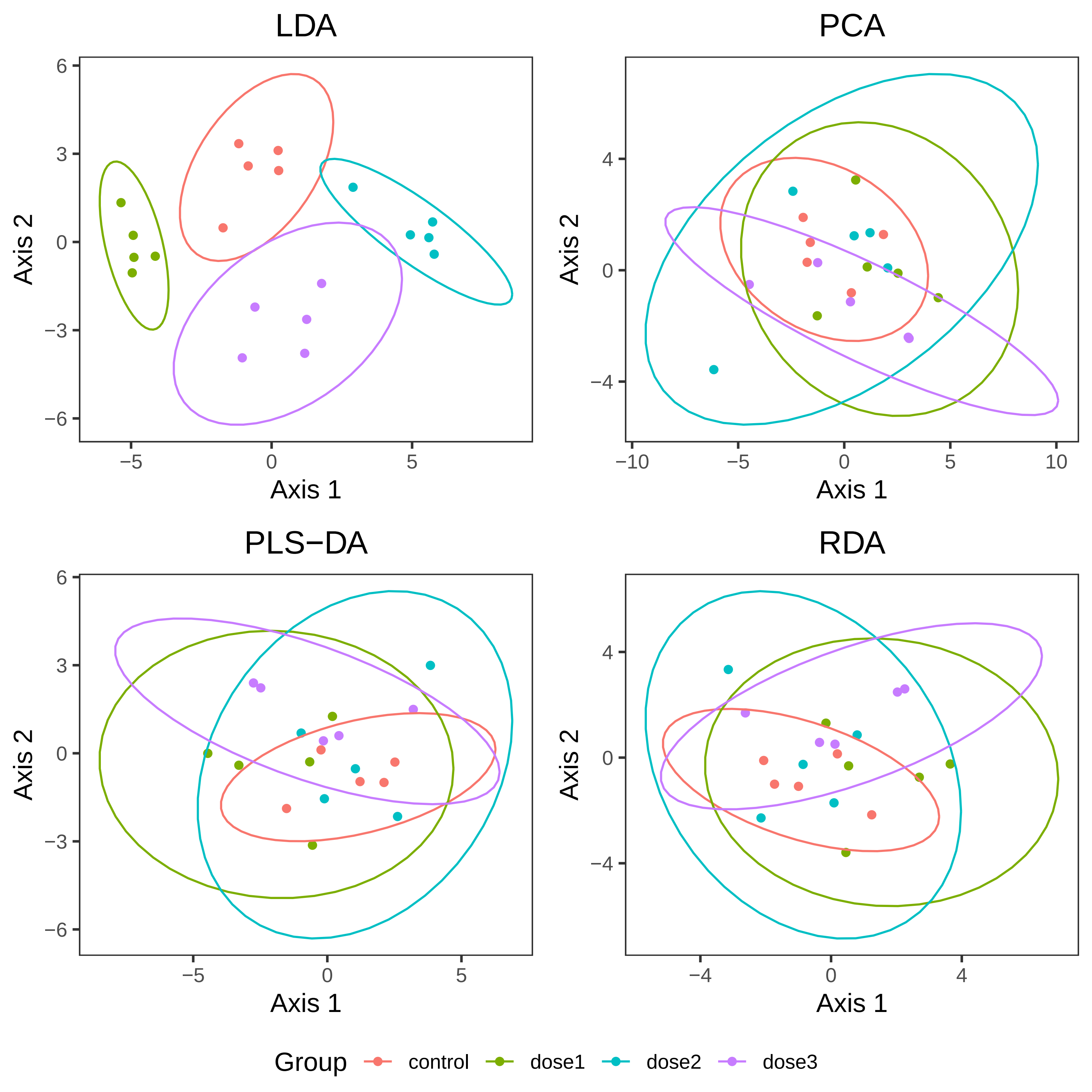
LDA
PCA
PLS−DA
RDA
Axis 1
Axis 2
Group
control
dose1
dose2
dose3

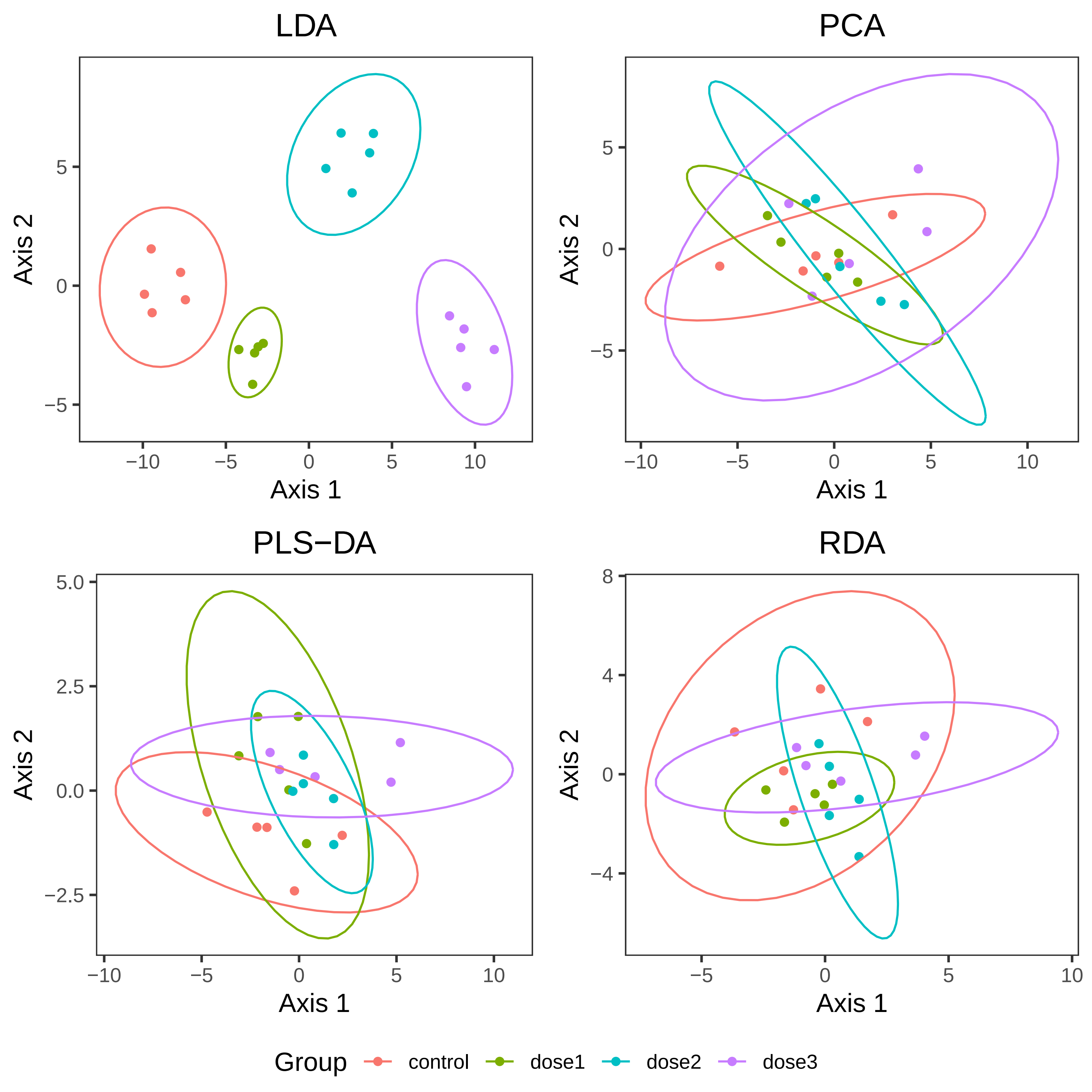

LDA
PCA
PLS−DA
RDA
Axis 1
Axis 2
−10
−5
0
5
10
2.5
5.0
0.0
−2.5
8
4
−4
Group
control
dose1
dose2
dose3

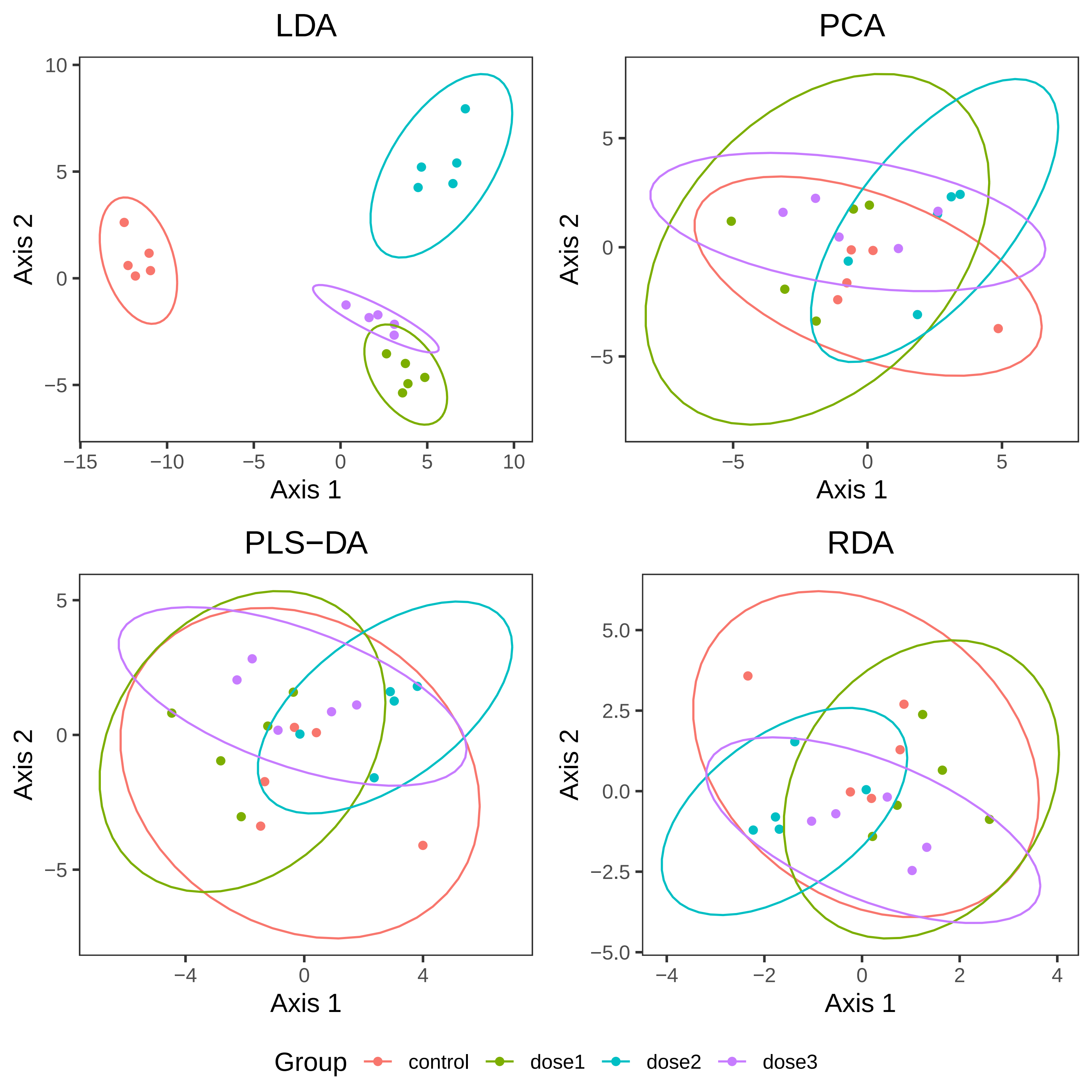

LDA
PCA
PLS−DA
RDA
Axis 1
Axis 2
Group
control
dose1
dose2
dose3

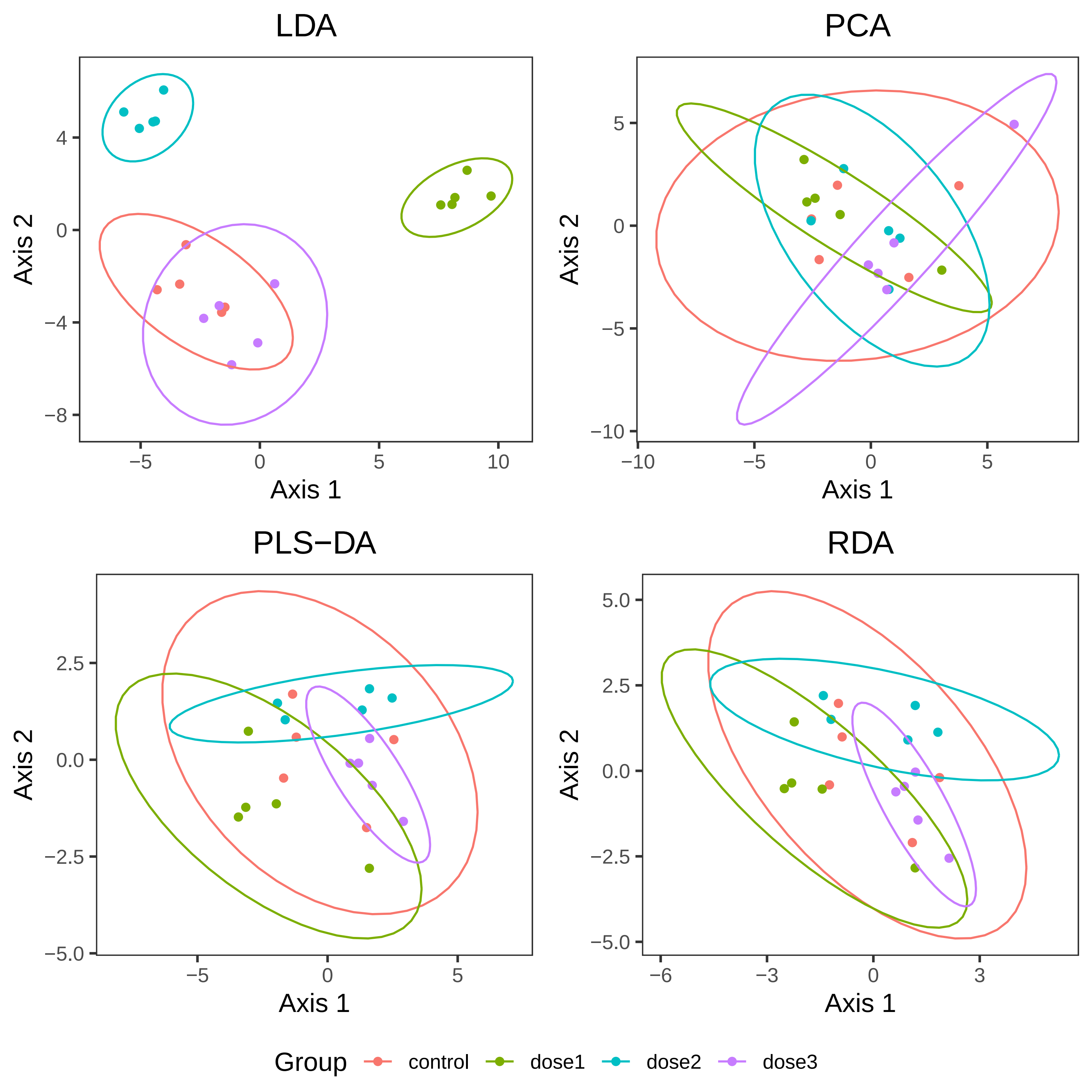

LDA
PCA
PLS−DA
RDA
Axis 1
Axis 2
4
0
−4
−8
−5
0
5
10
5
0
−5
−10
2.5
0.0
−2.5
−5.0
5.0
−6
−3
3
Group
control
dose1
dose2
dose3

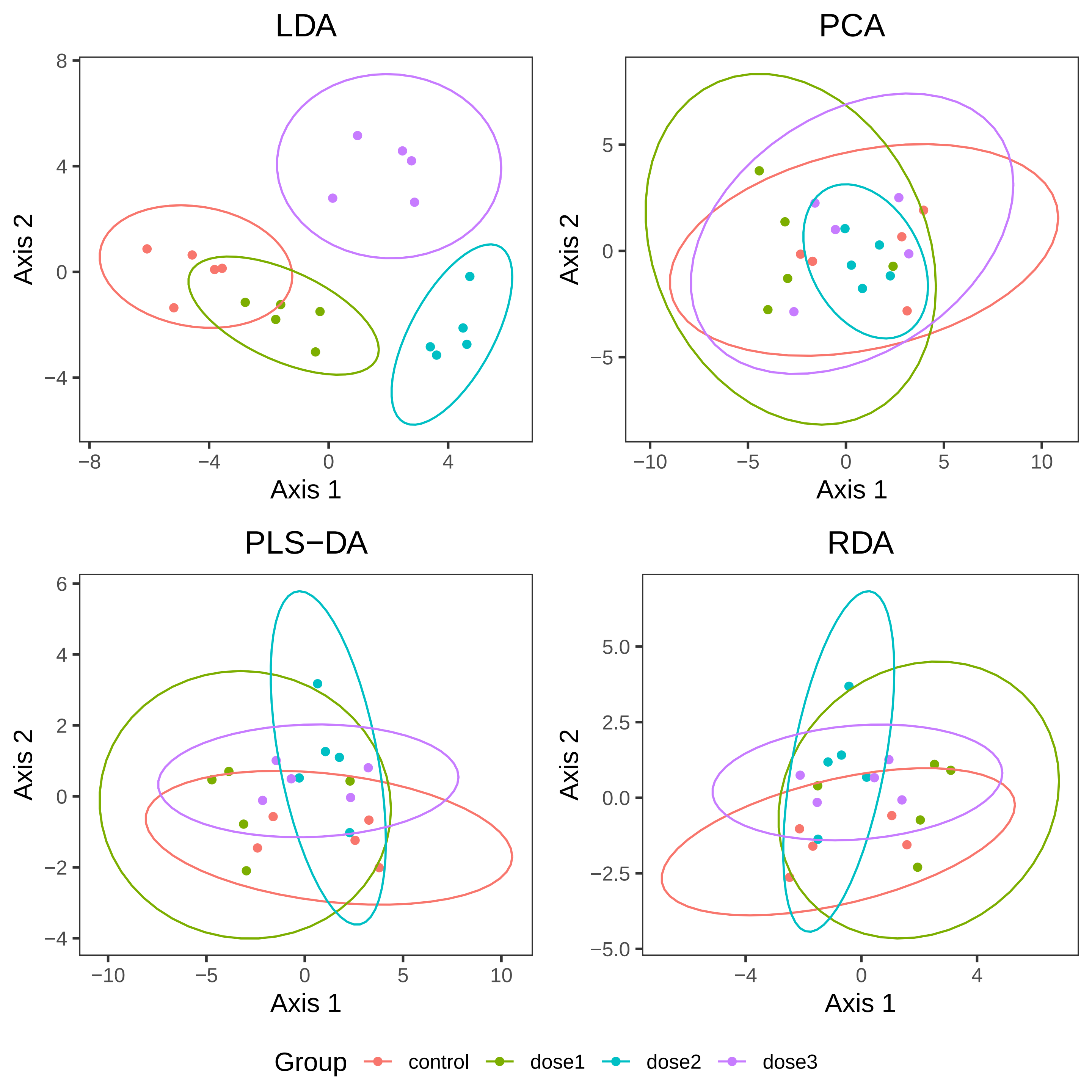
LDA
PCA
PLS−DA
RDA
Axis 1
Axis 2
Group
control
dose1
dose2
dose3

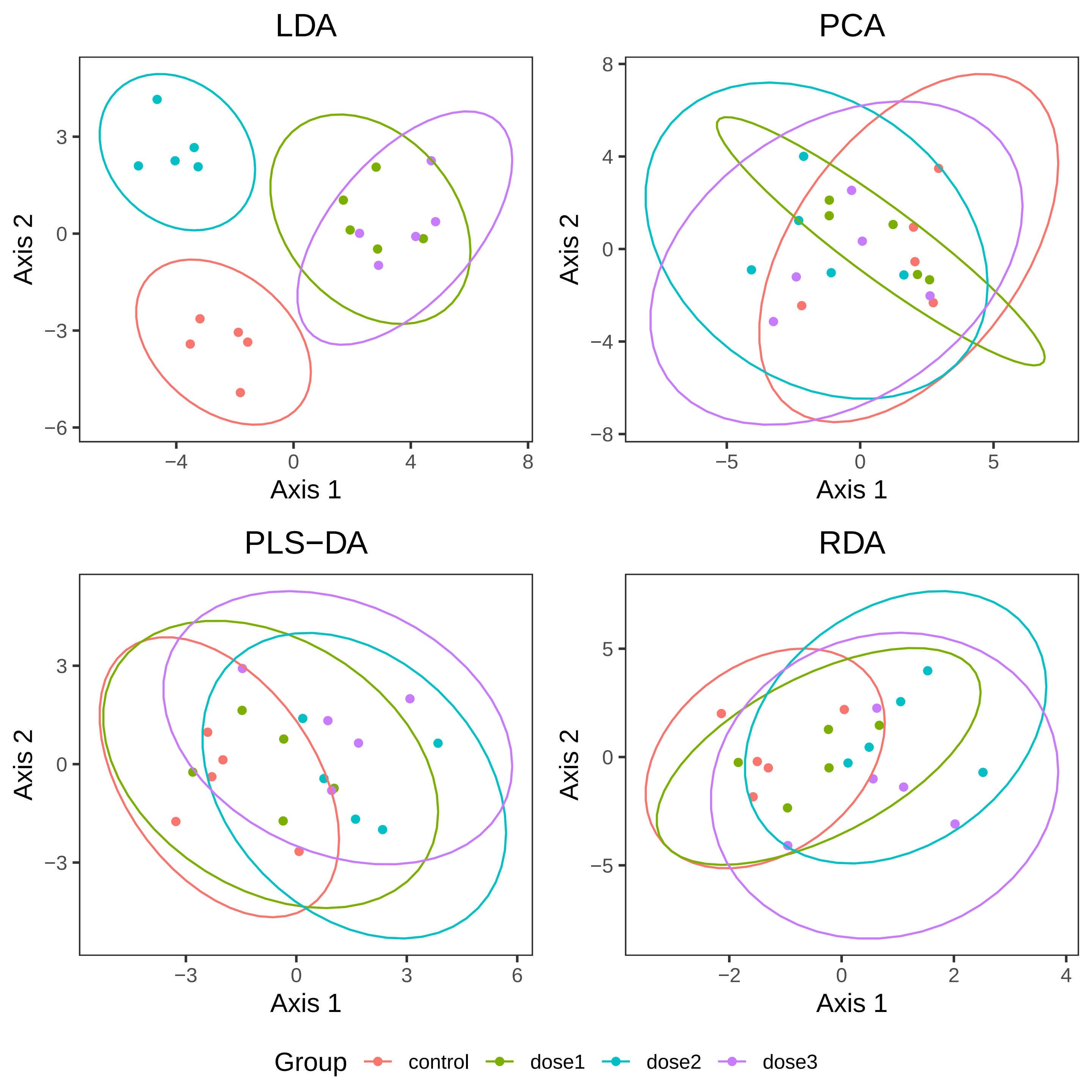
LDA
PCA
PLS−DA
RDA
Axis 1
Axis 2
Group
control
dose1
dose2
dose3

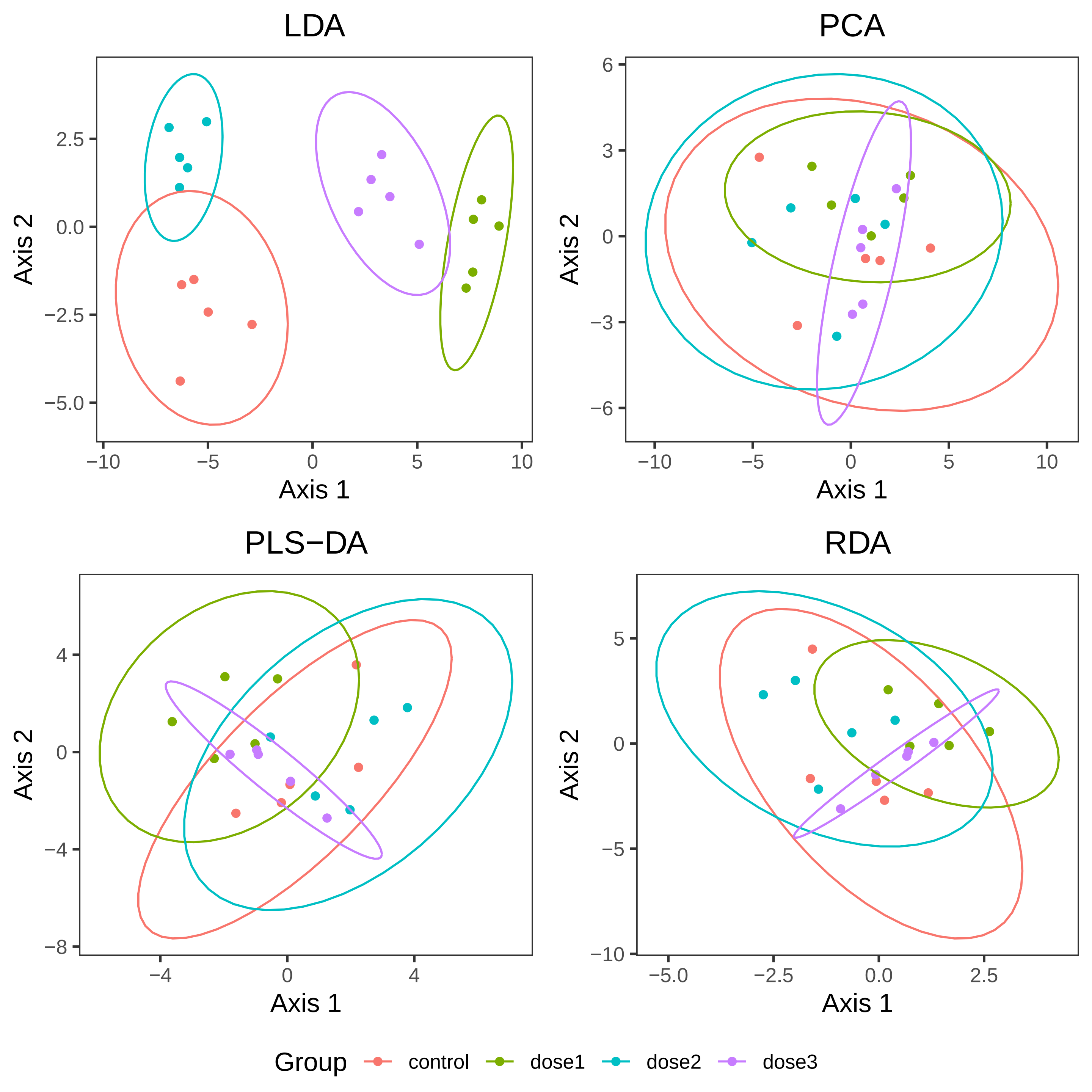

LDA
PCA
PLS−DA
RDA
Axis 1
Axis 2
Group
control
dose1
dose2
dose3

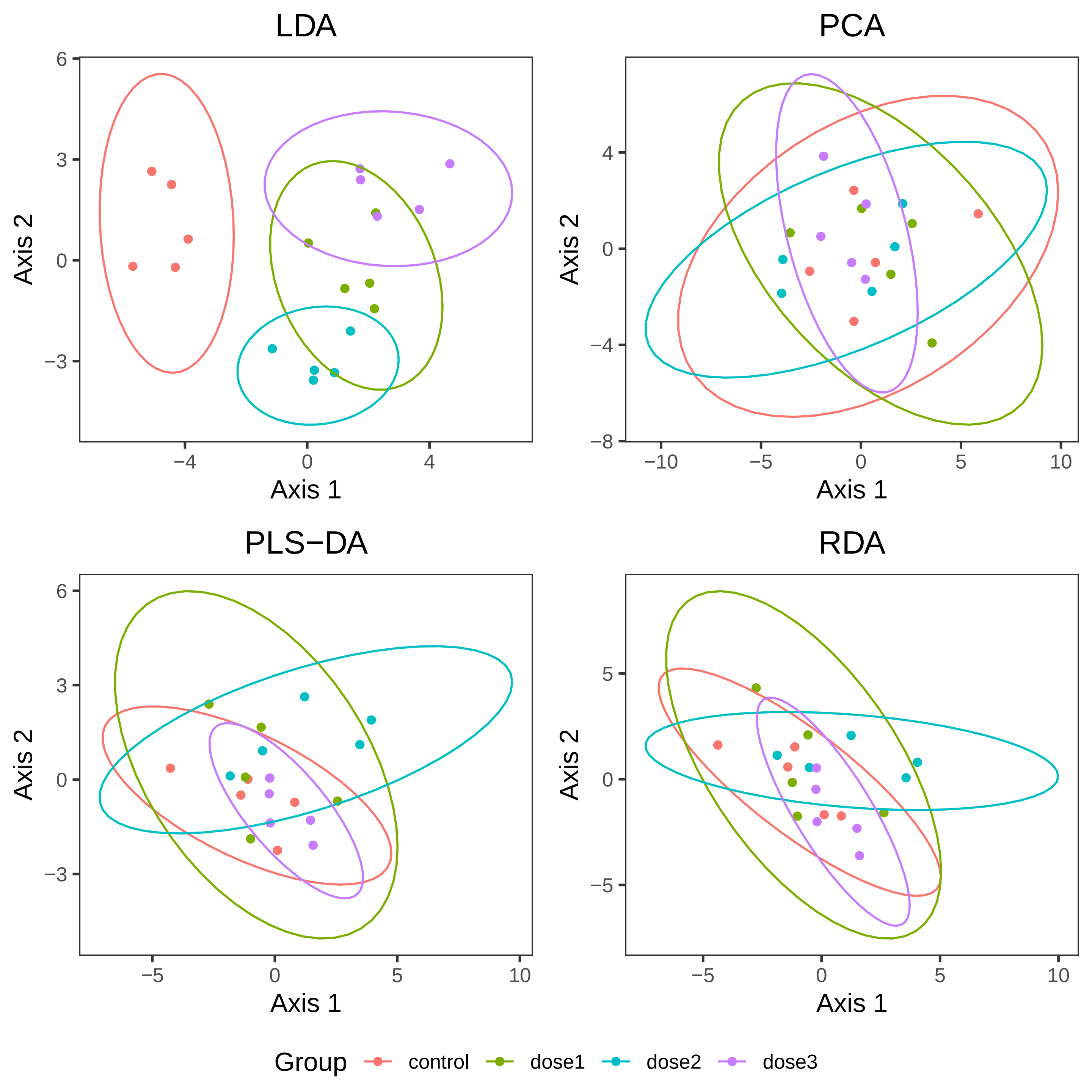
LDA
PCA
PLS−DA
RDA
Axis 1
Axis 2
Group
control
dose1
dose2
dose3

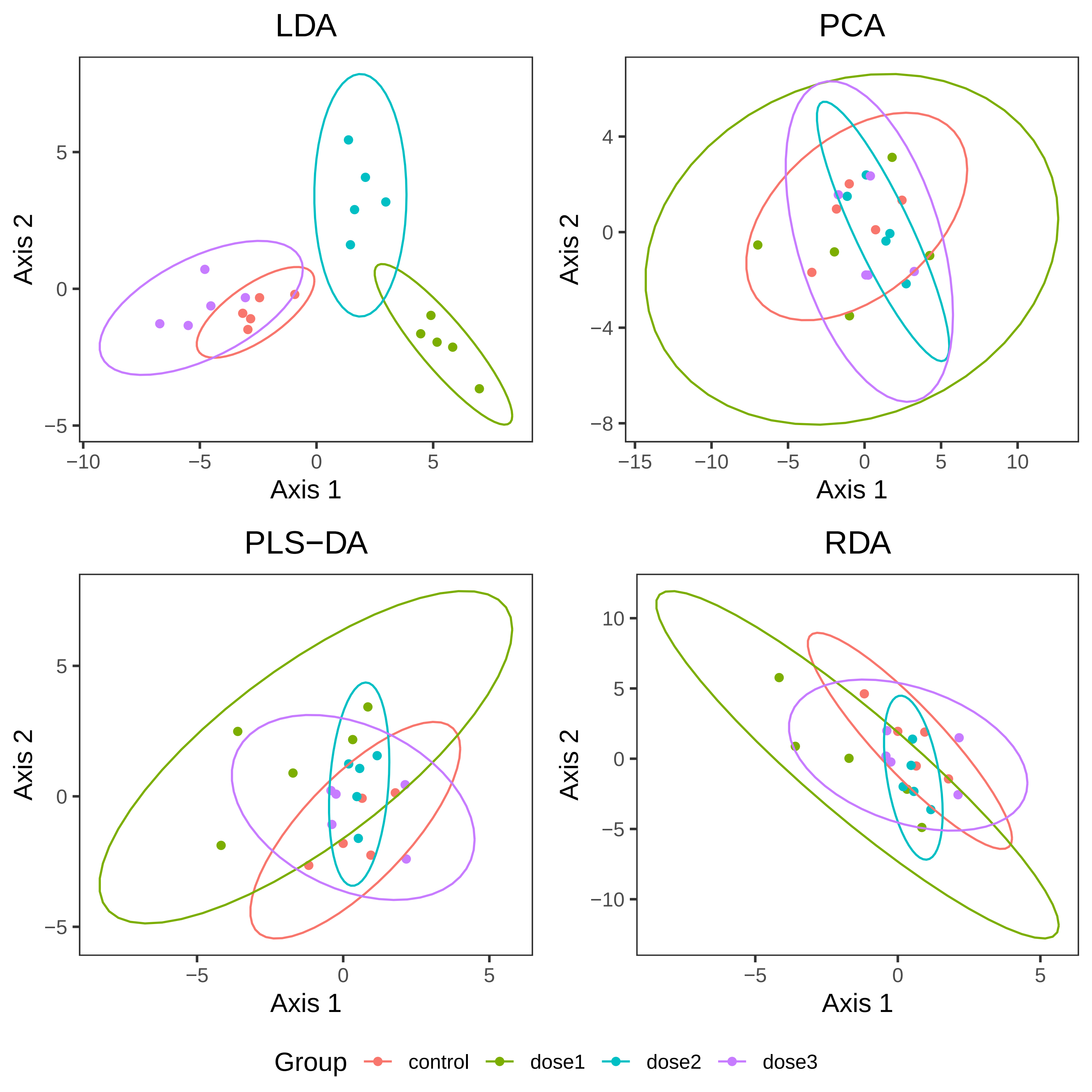
LDA
PCA
PLS−DA
RDA
Axis 1
Axis 2
Group
control
dose1
dose2
dose3

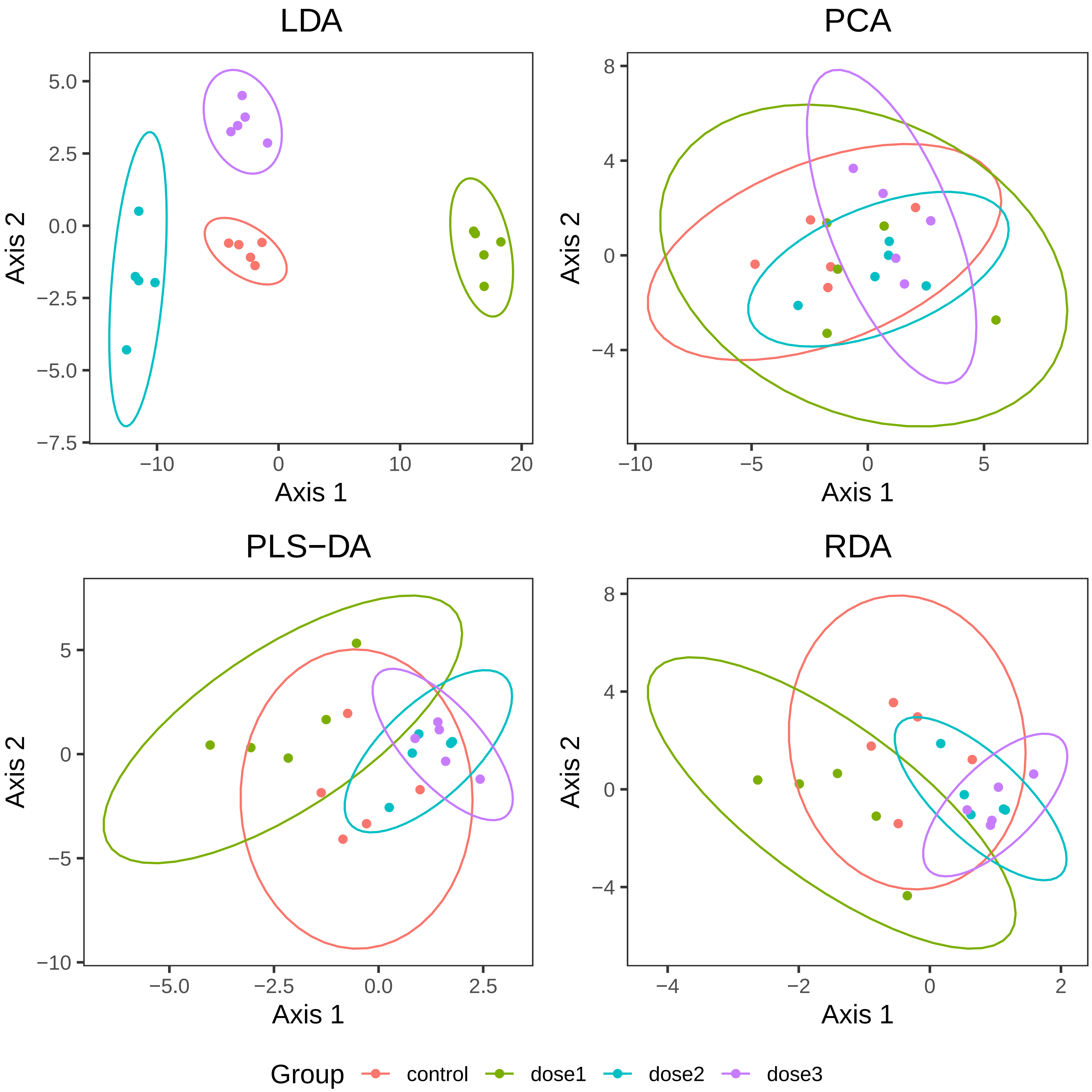

LDA
PCA
PLS−DA
RDA
Axis 1
Axis 2
5.0
2.5
0.0
−2.5
−5.0
−7.5
−10
0
10
20
8
4
0
−4
−10
−5
0
5
5
0
−5
−10
−5.0
−2.5
0.0
2.5
8
4
0
−4
−4
−2
0
2
Group
control
dose1
dose2
dose3

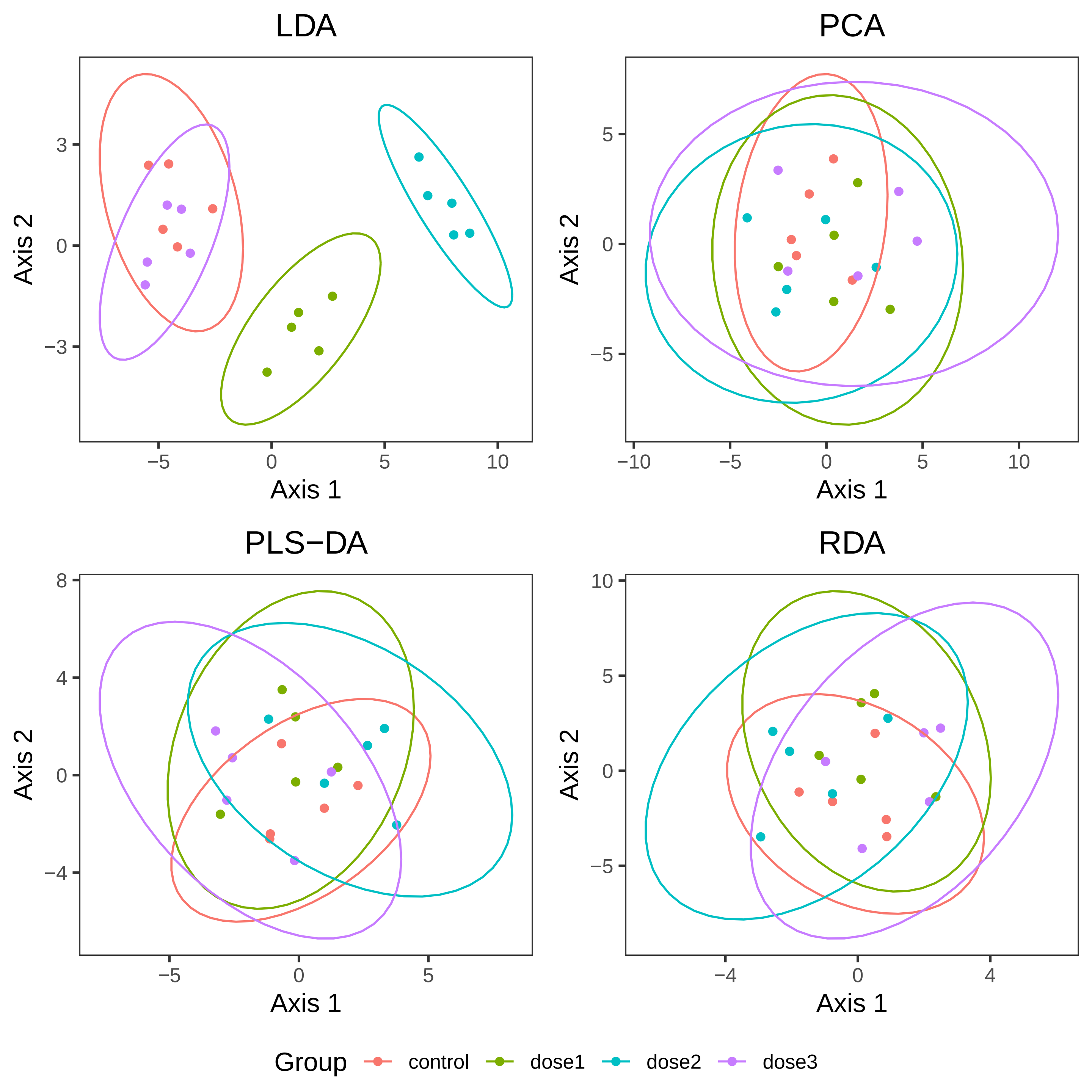

LDA
PCA
PLS−DA
RDA
Axis 1
Axis 2
Group
control
dose1
dose2
dose3

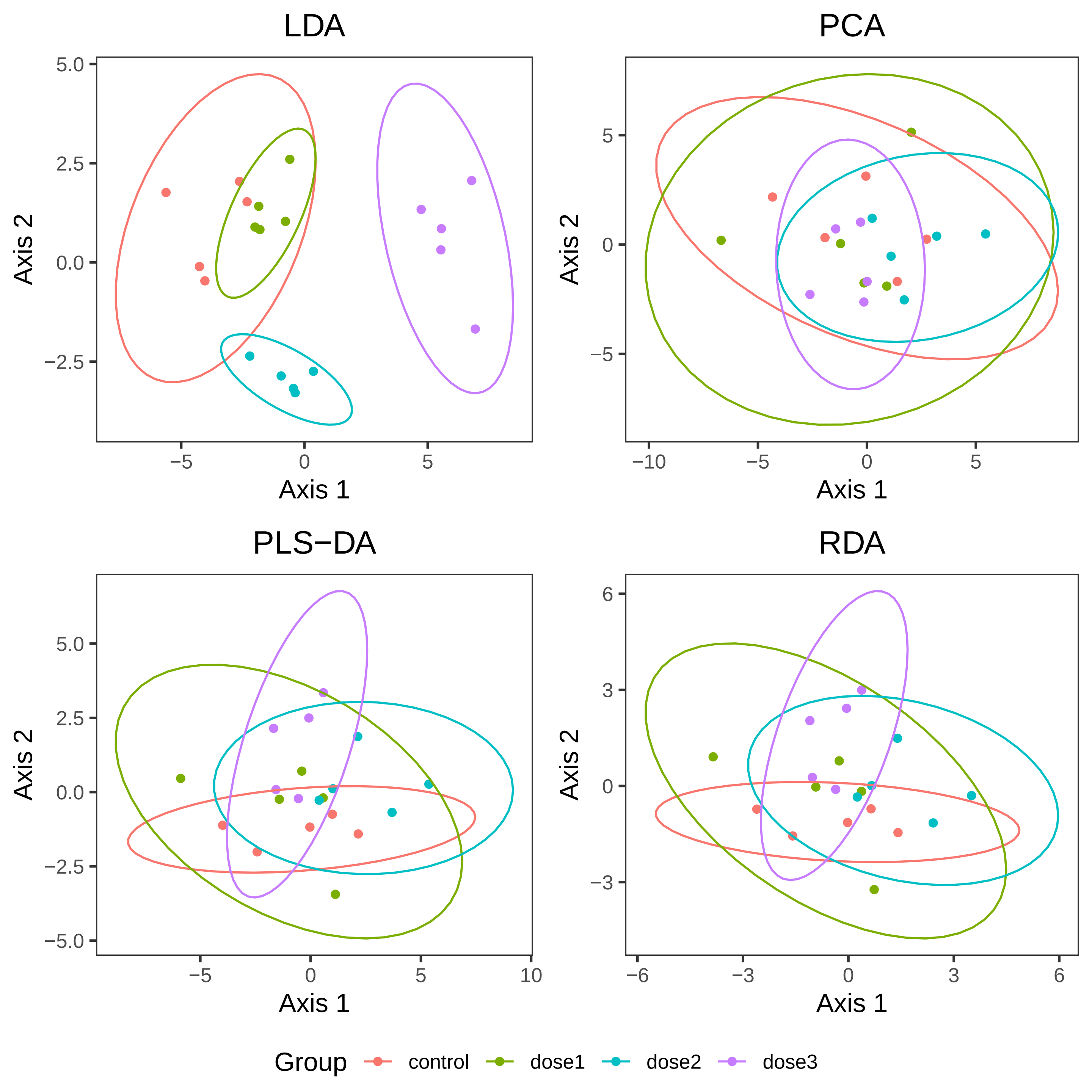
LDA
PCA
PLS−DA
RDA
Axis 1
Axis 2
5.0
2.5
0.0
−2.5
−5.0
−10
−5
0
5
10
−6
−3
3
6
Group
control
dose1
dose2
dose3

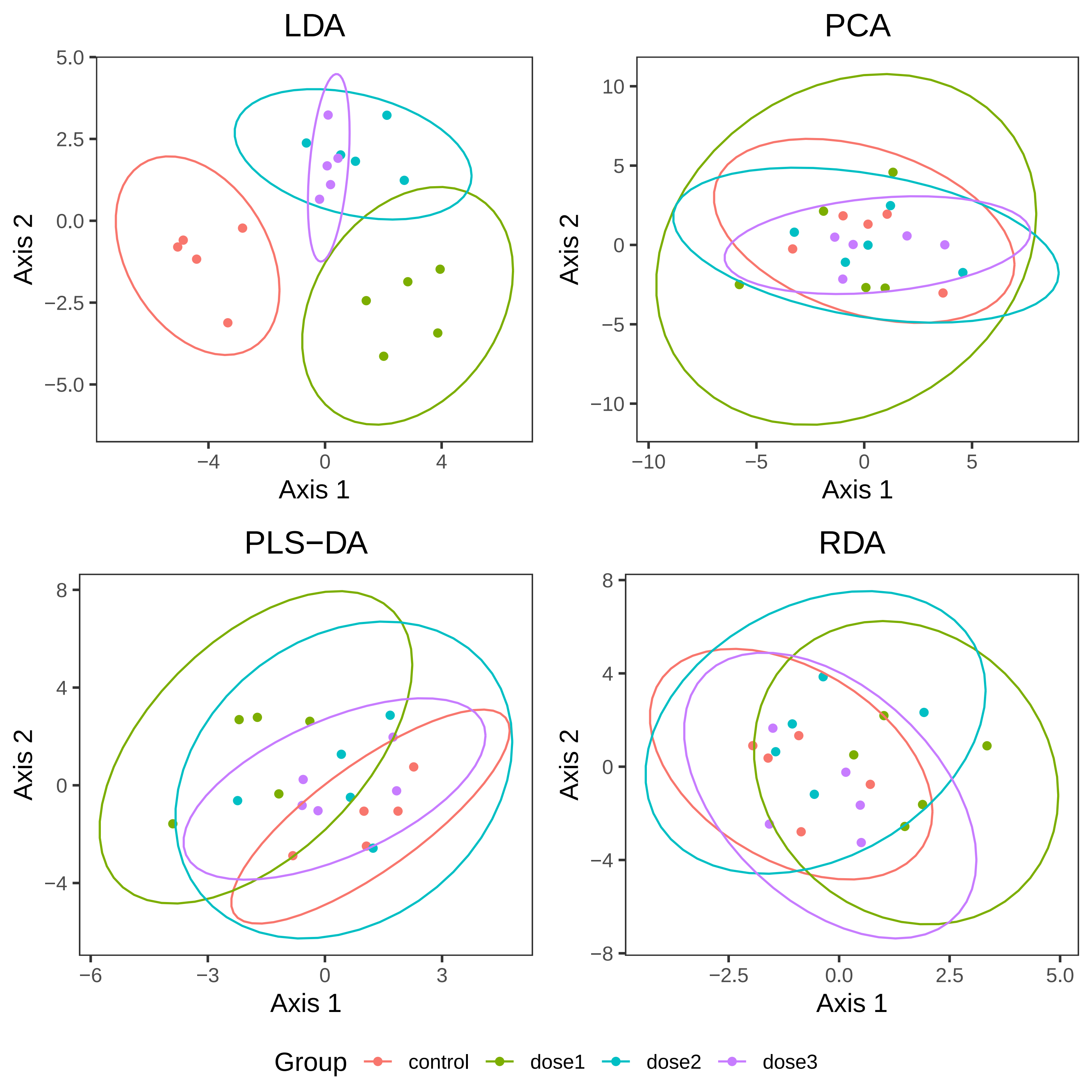
LDA
PCA
PLS−DA
RDA
Axis 1
Axis 2
Group
control
dose1
dose2
dose3

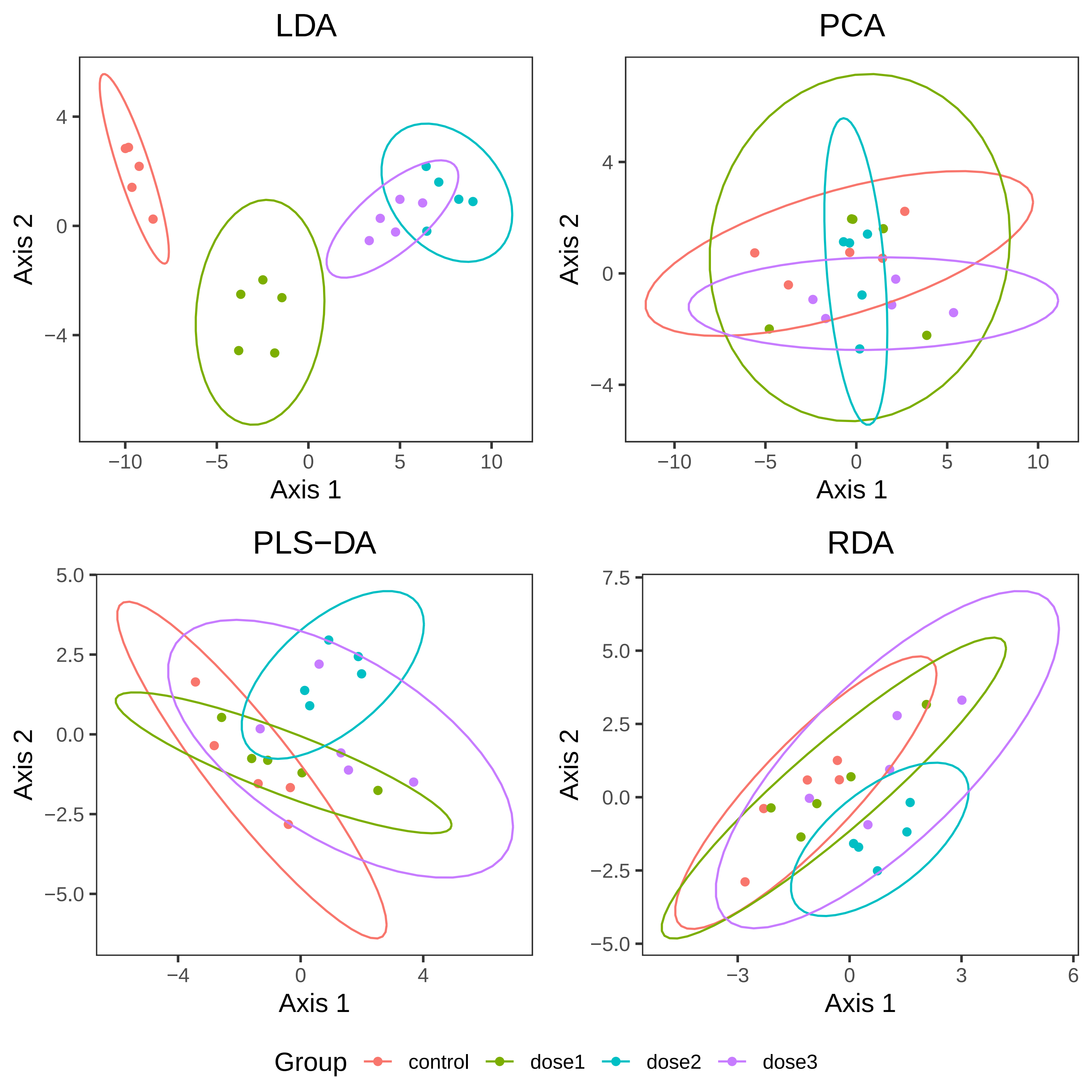
LDA
PCA
PLS−DA
RDA
Axis 1
Axis 2
−10
−5
0
5
10
4
0
−4
5.0
2.5
0.0
−2.5
−5.0
7.5
−3
3
6
Group
control
dose1
dose2
dose3

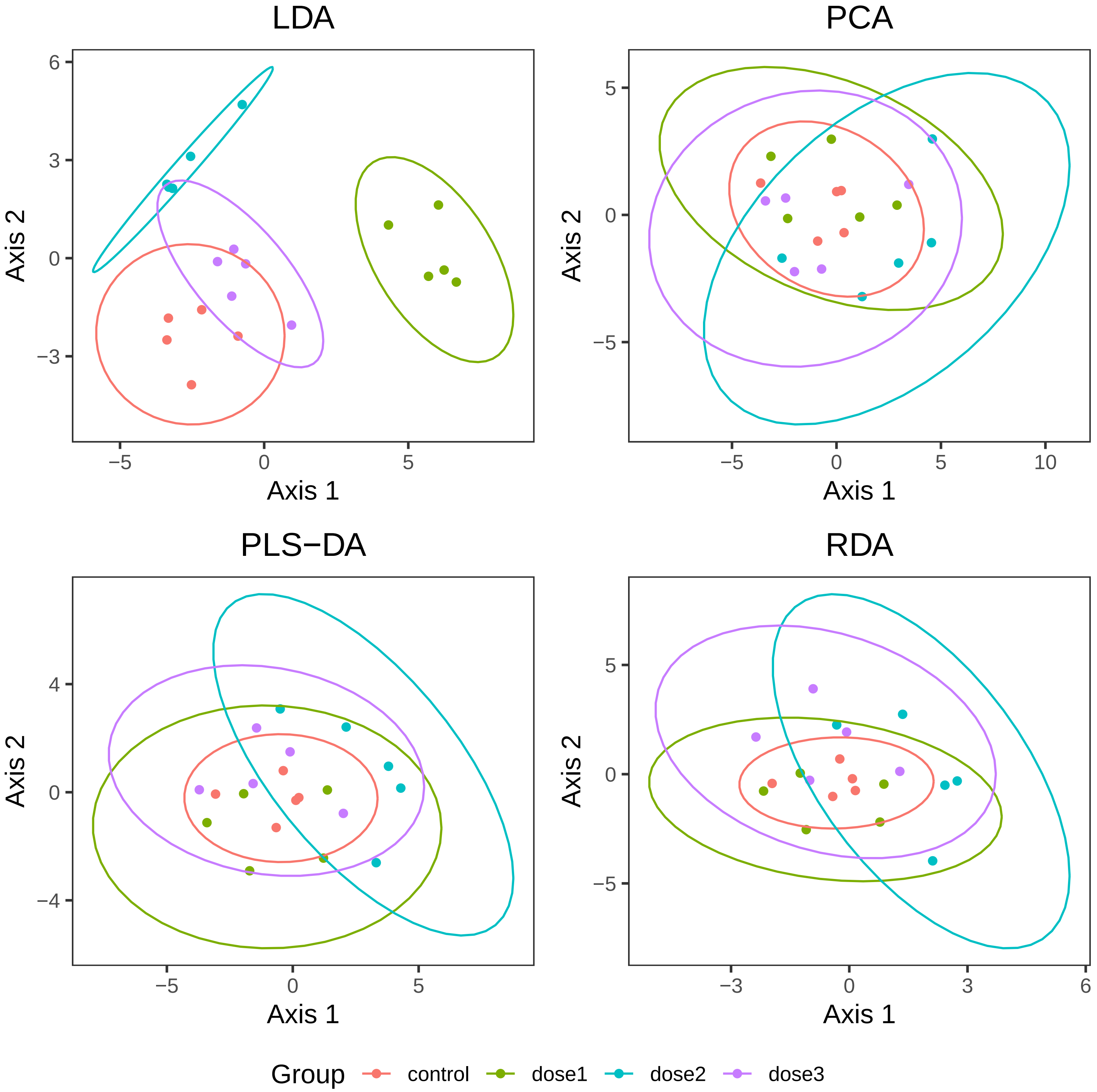

LDA
PCA
PLS−DA
RDA
Axis 1
Axis 2
6
3
0
−3
−5
5
10
4
−4
Group
control
dose1
dose2
dose3

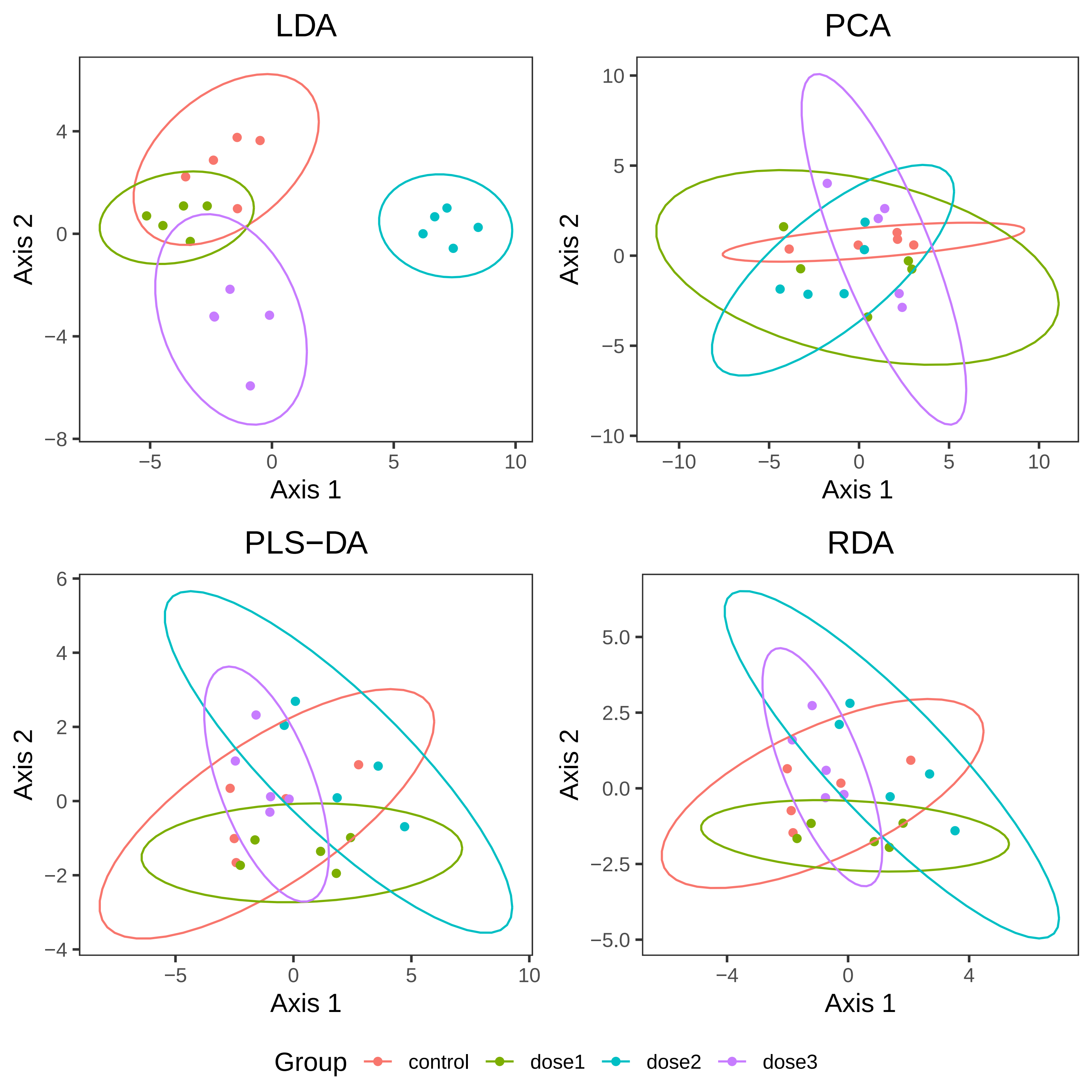

LDA
PCA
PLS−DA
RDA
Axis 1
Axis 2
Group
control
dose1
dose2
dose3

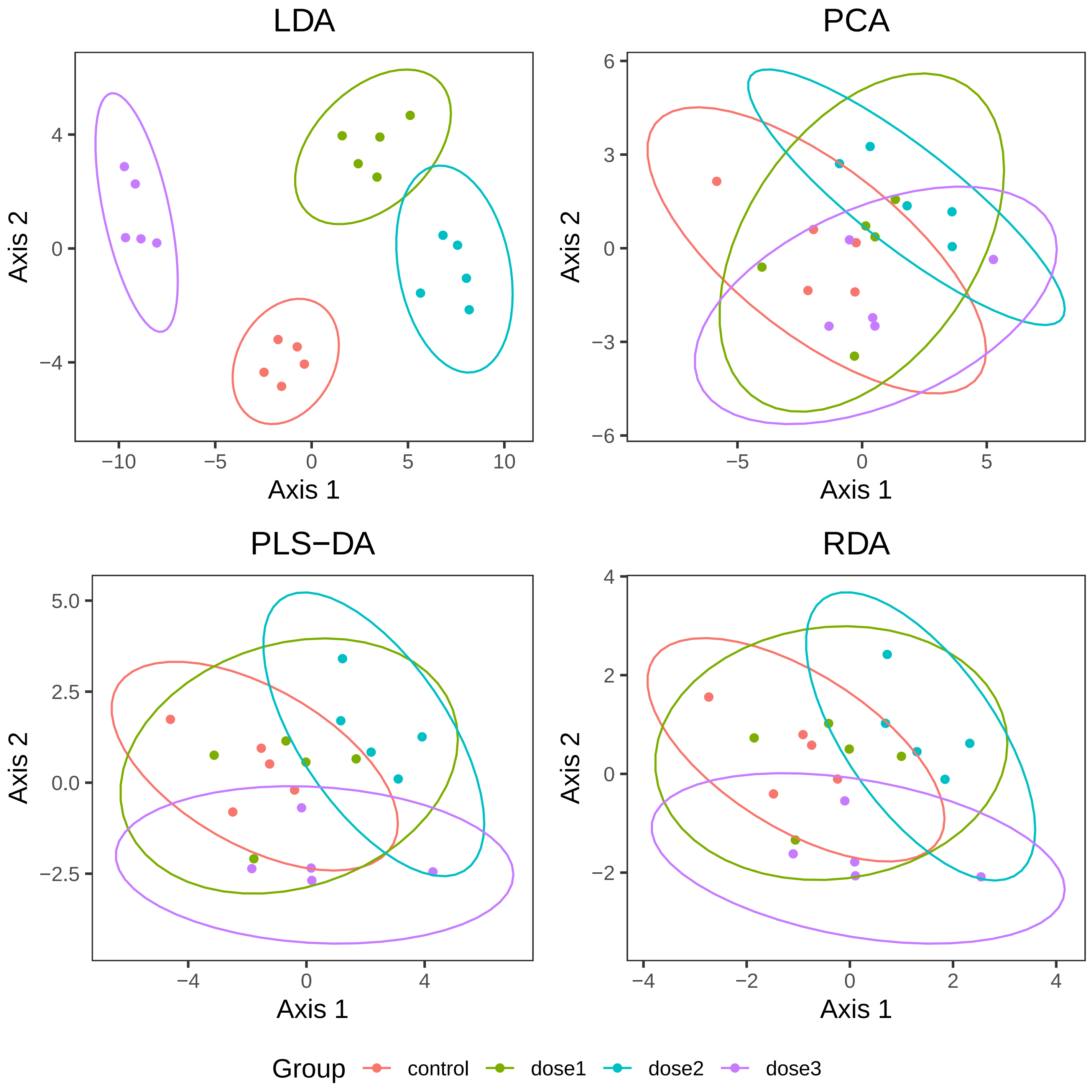

LDA
PCA
PLS−DA
RDA
Axis 1
Axis 2
−10
−5
0
5
10
4
0
−4
6
3
−3
−6
5.0
2.5
0.0
−2.5
2
−2
Group
control
dose1
dose2
dose3

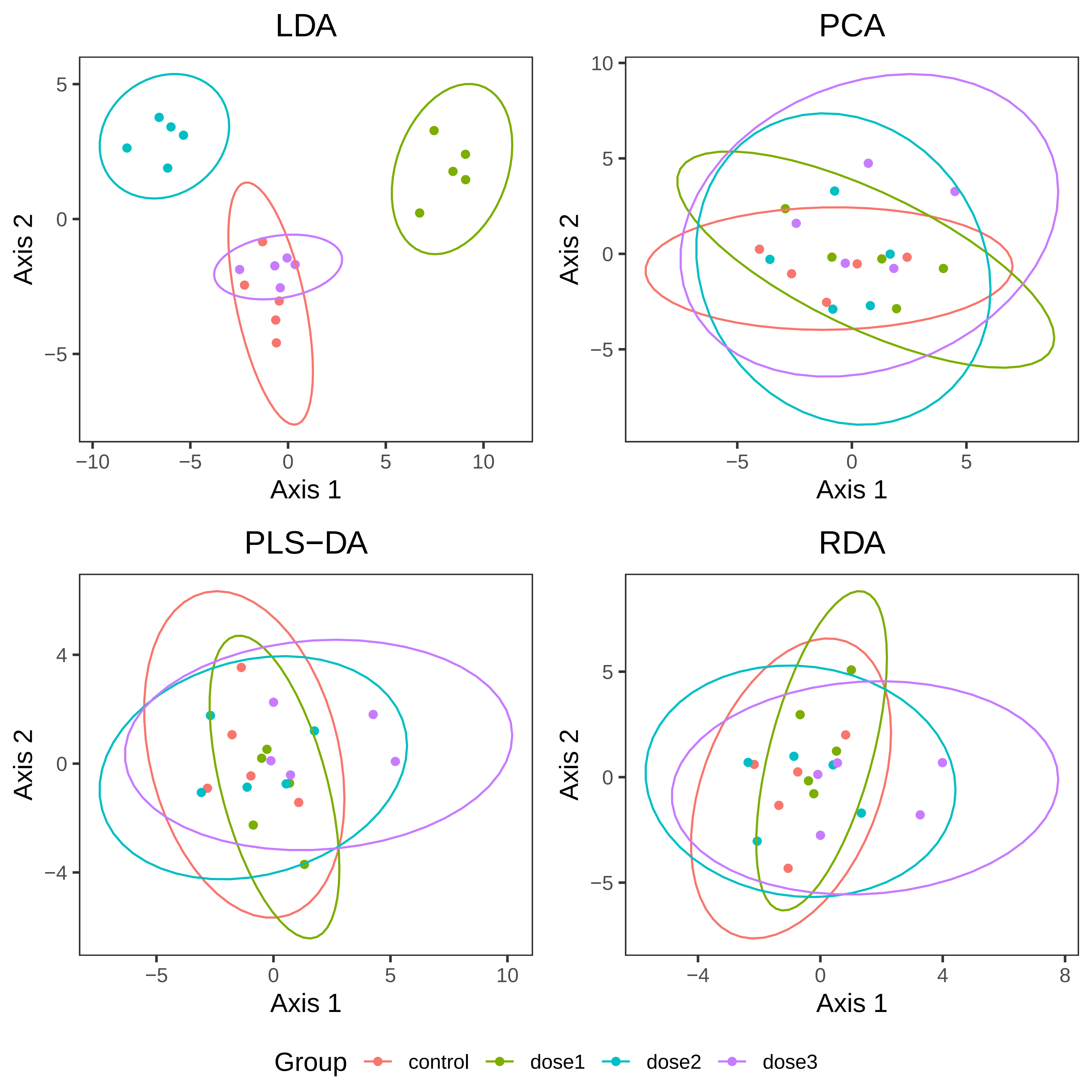

LDA
PCA
PLS−DA
RDA
Axis 1
Axis 2
Group
control
dose1
dose2
dose3

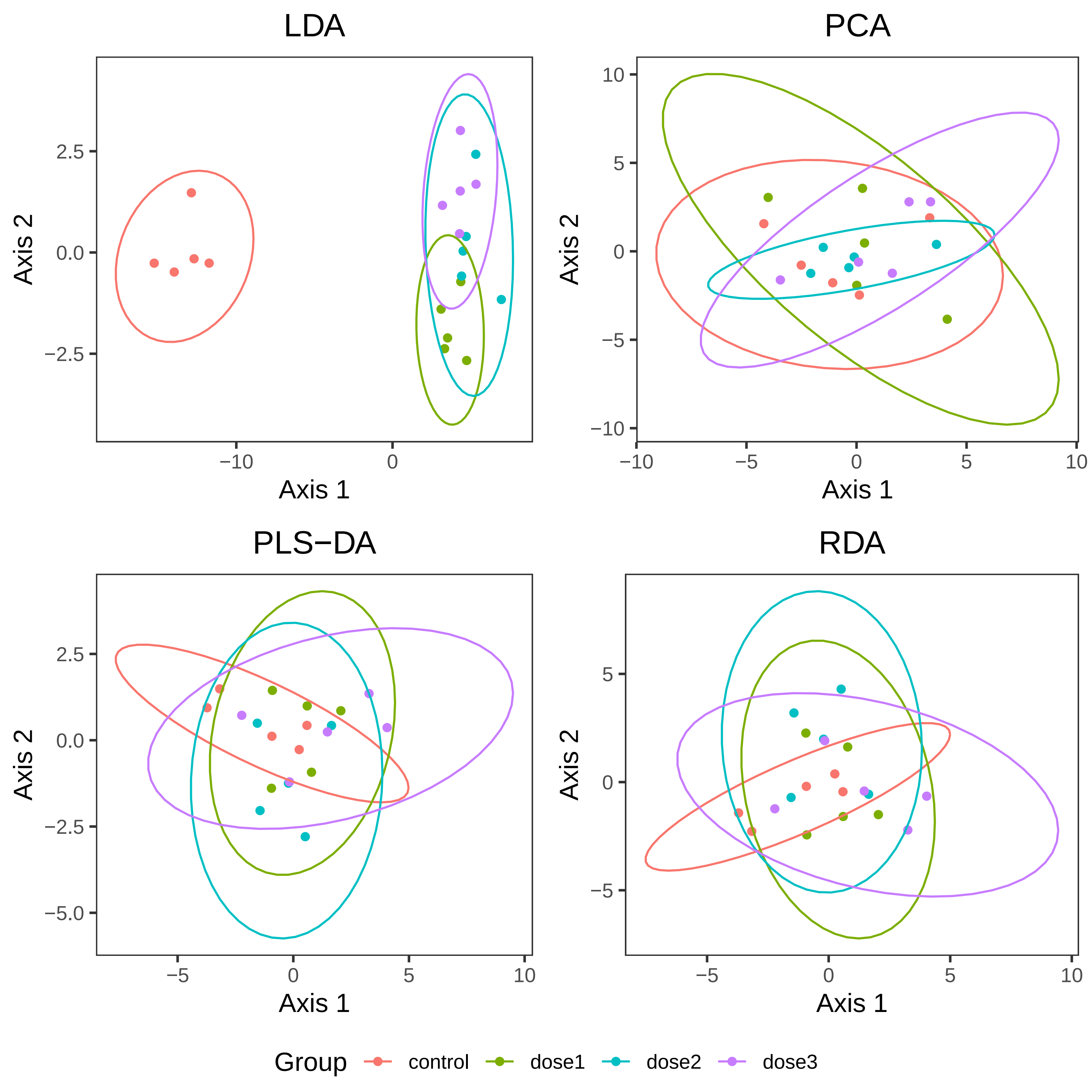
LDA
PCA
PLS−DA
RDA
Axis 1
Axis 2
−10
0
−2.5
0.0
2.5
−5
5
10
−5.0
Group
control
dose1
dose2
dose3

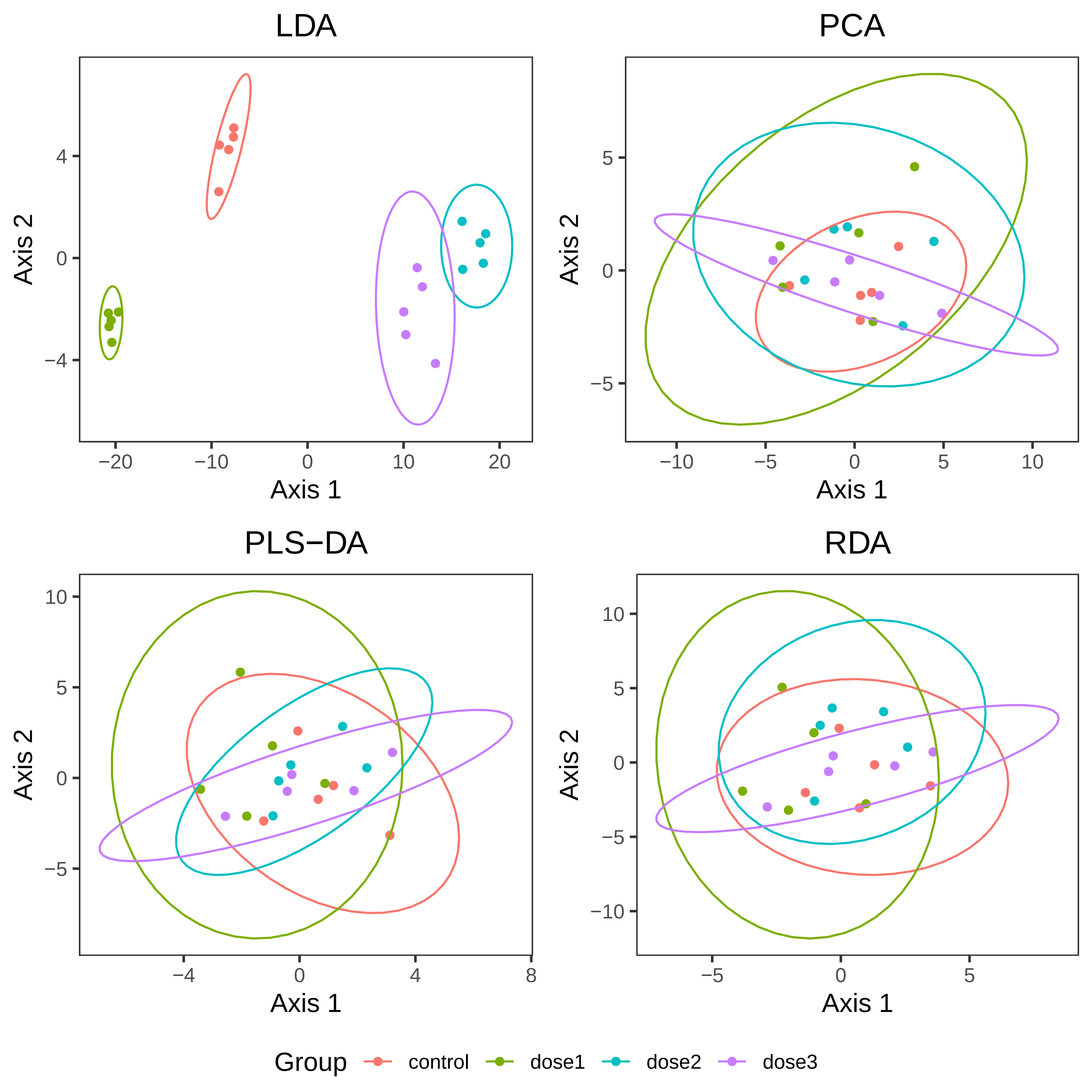
LDA
PCA
PLS−DA
RDA
Axis 1
Axis 2
Group
control
dose1
dose2
dose3

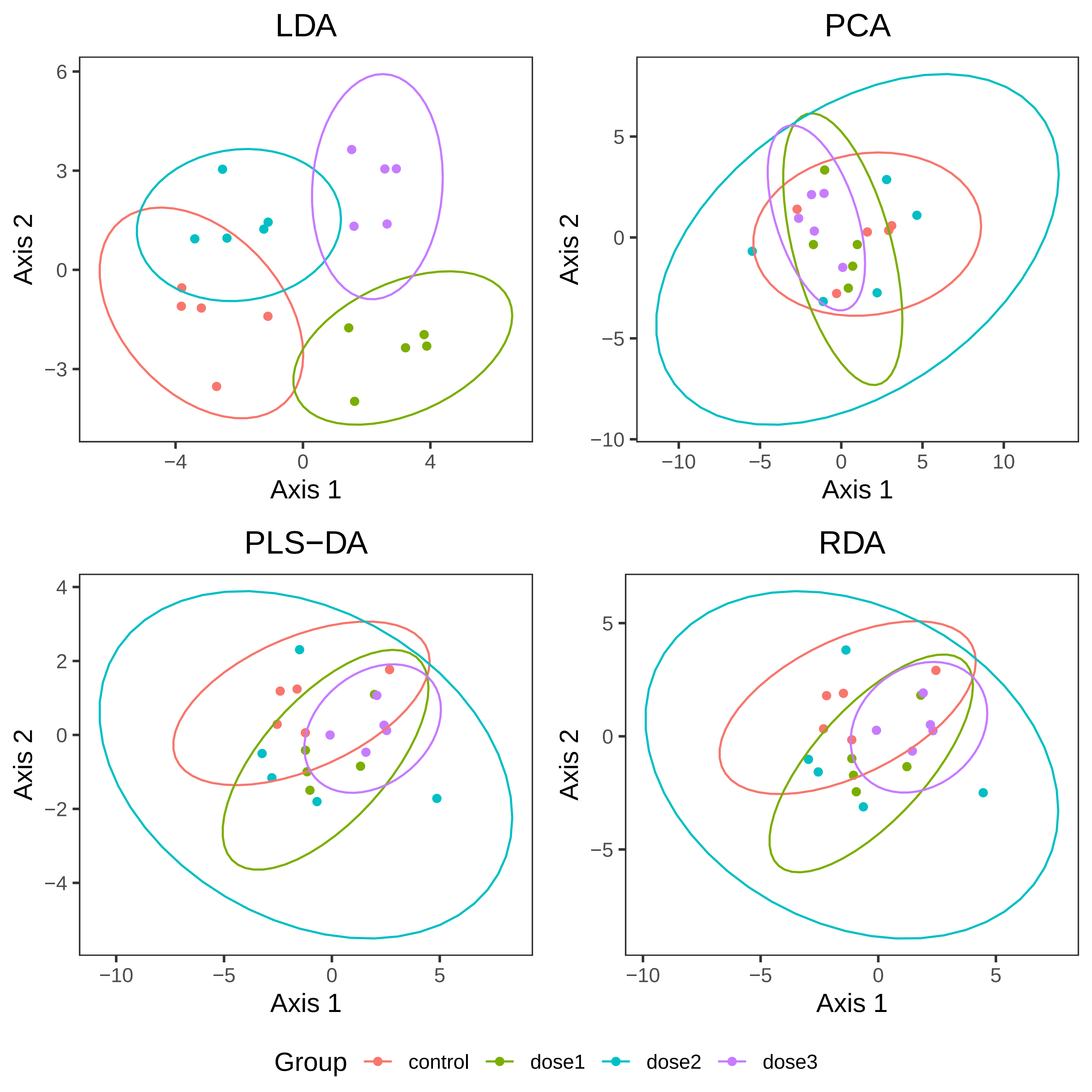
LDA
PCA
PLS−DA
RDA
Axis 1
Axis 2
Group
control
dose1
dose2
dose3

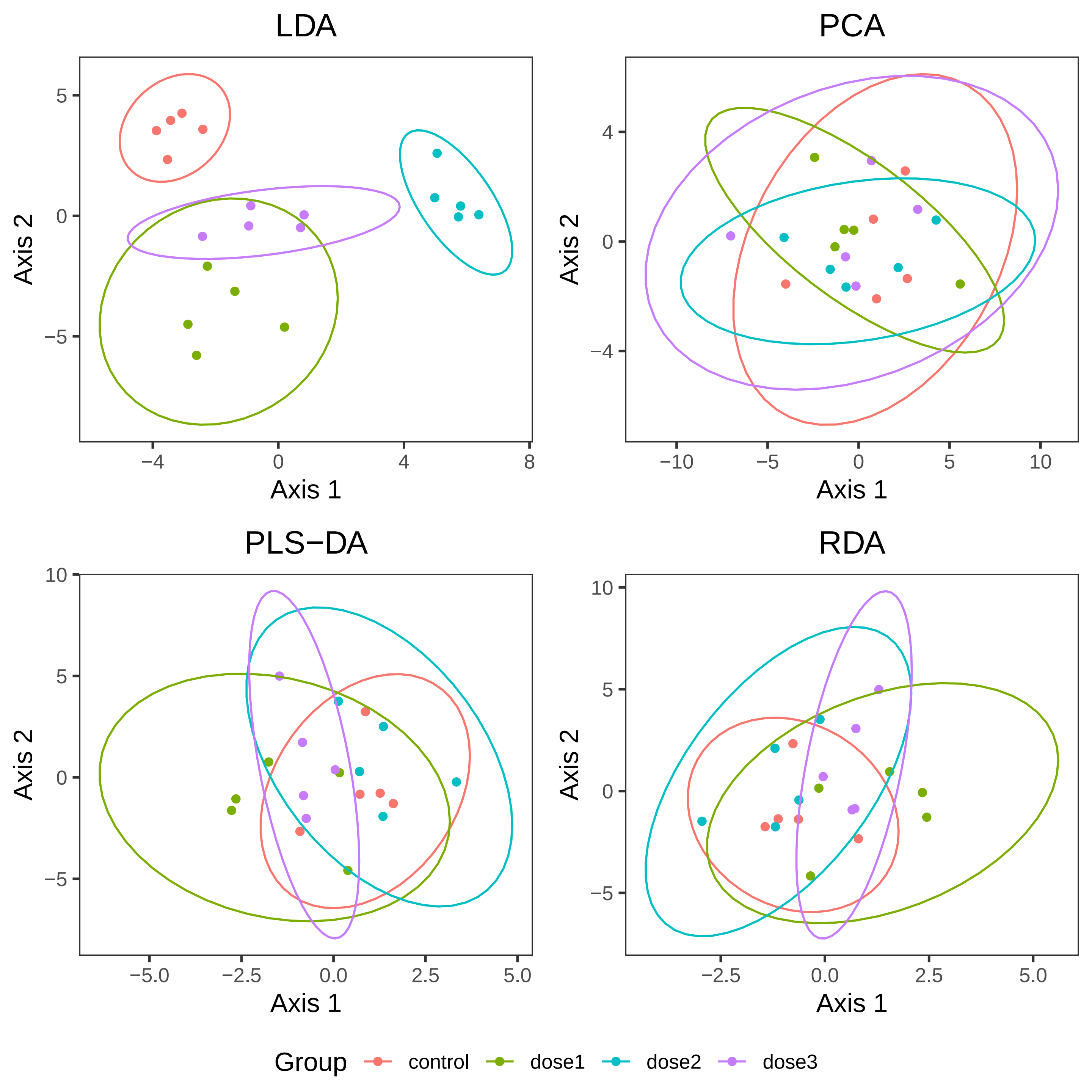

LDA
PCA
PLS−DA
RDA
Axis 1
Axis 2
Group
control
dose1
dose2
dose3

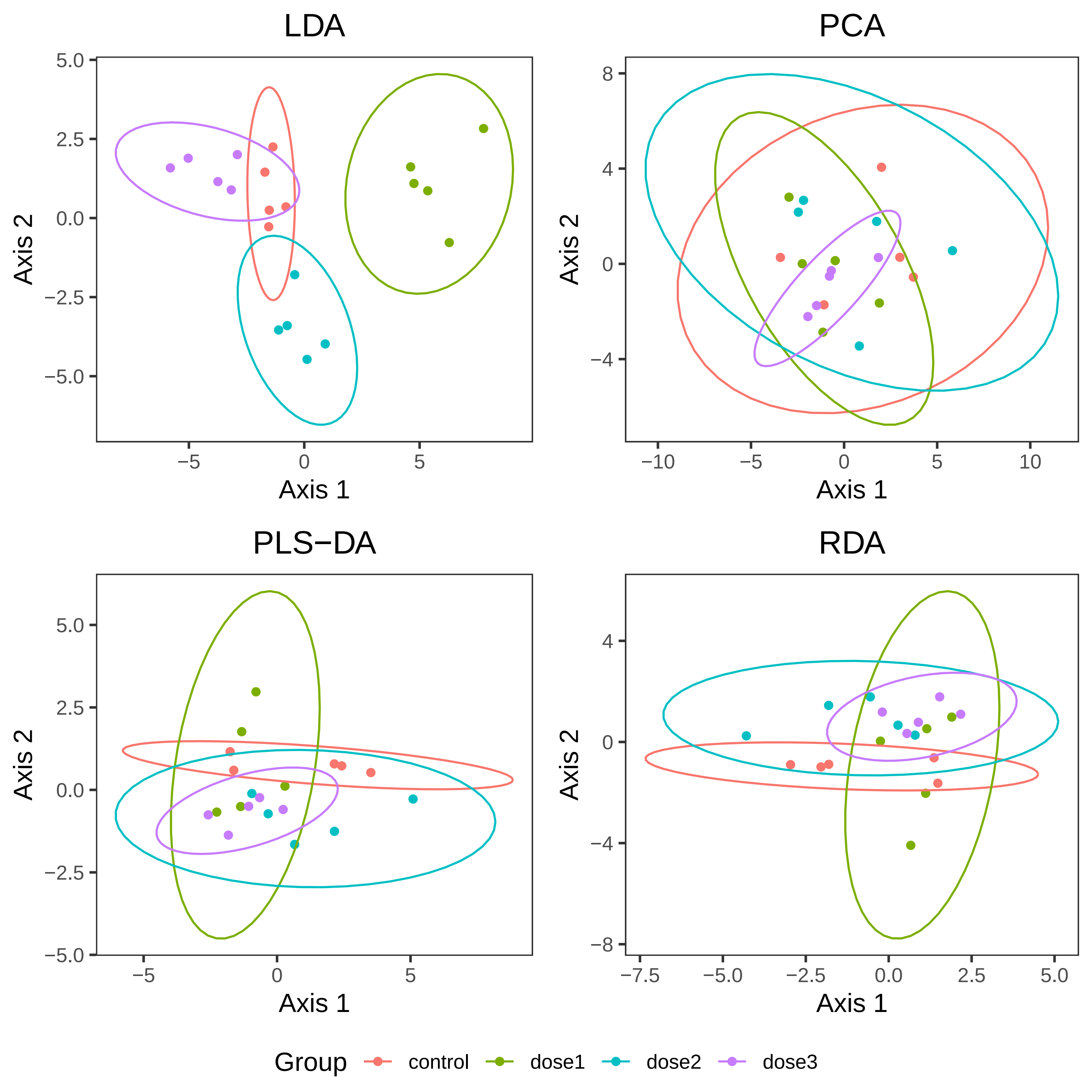

LDA
PCA
PLS−DA
RDA
Axis 1
Axis 2
5.0
2.5
0.0
−2.5
−5.0
−5
0
5
8
4
−4
−10
10
−8
−7.5
Group
control
dose1
dose2
dose3

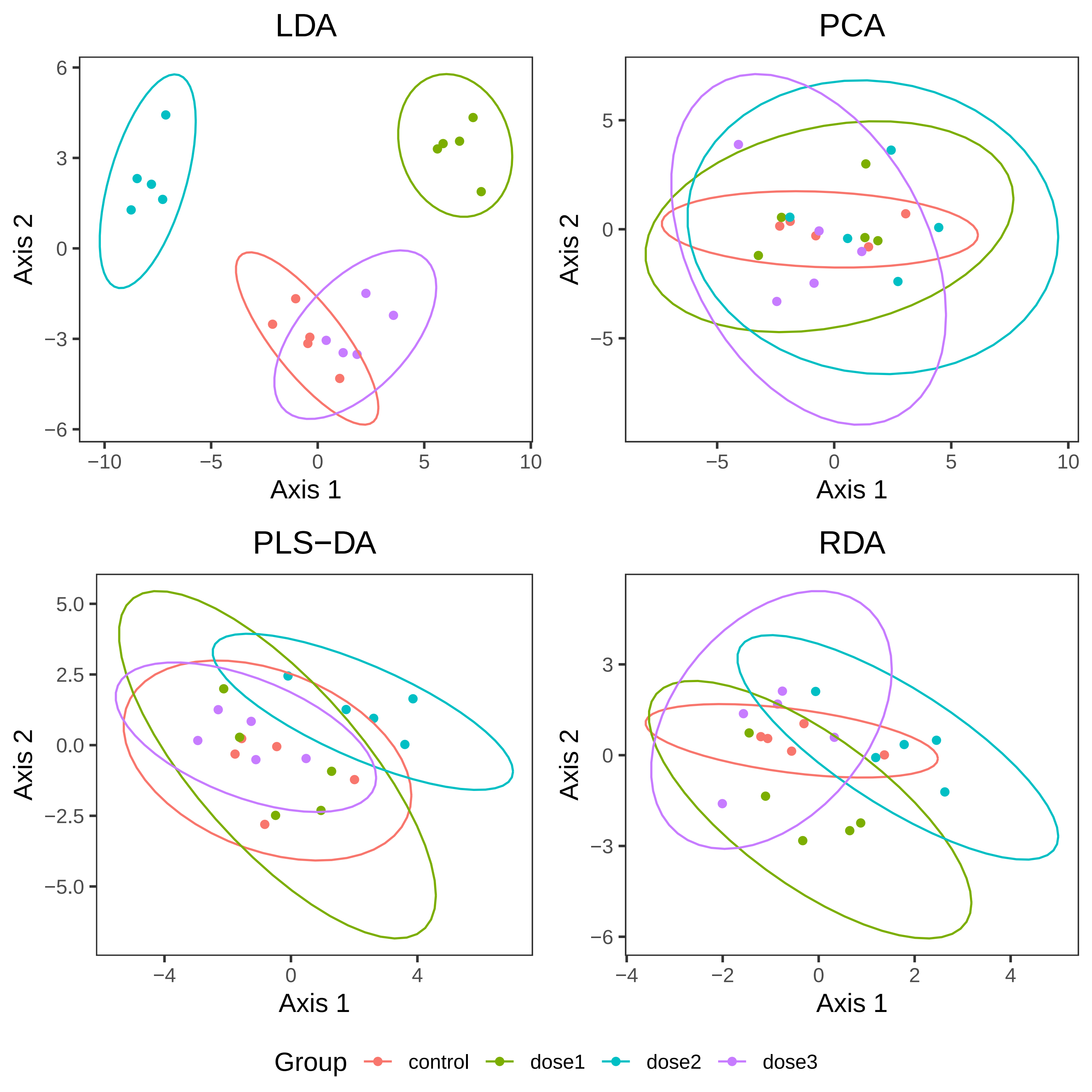

LDA
PCA
PLS−DA
RDA
Axis 1
Axis 2
Group
control
dose1
dose2
dose3

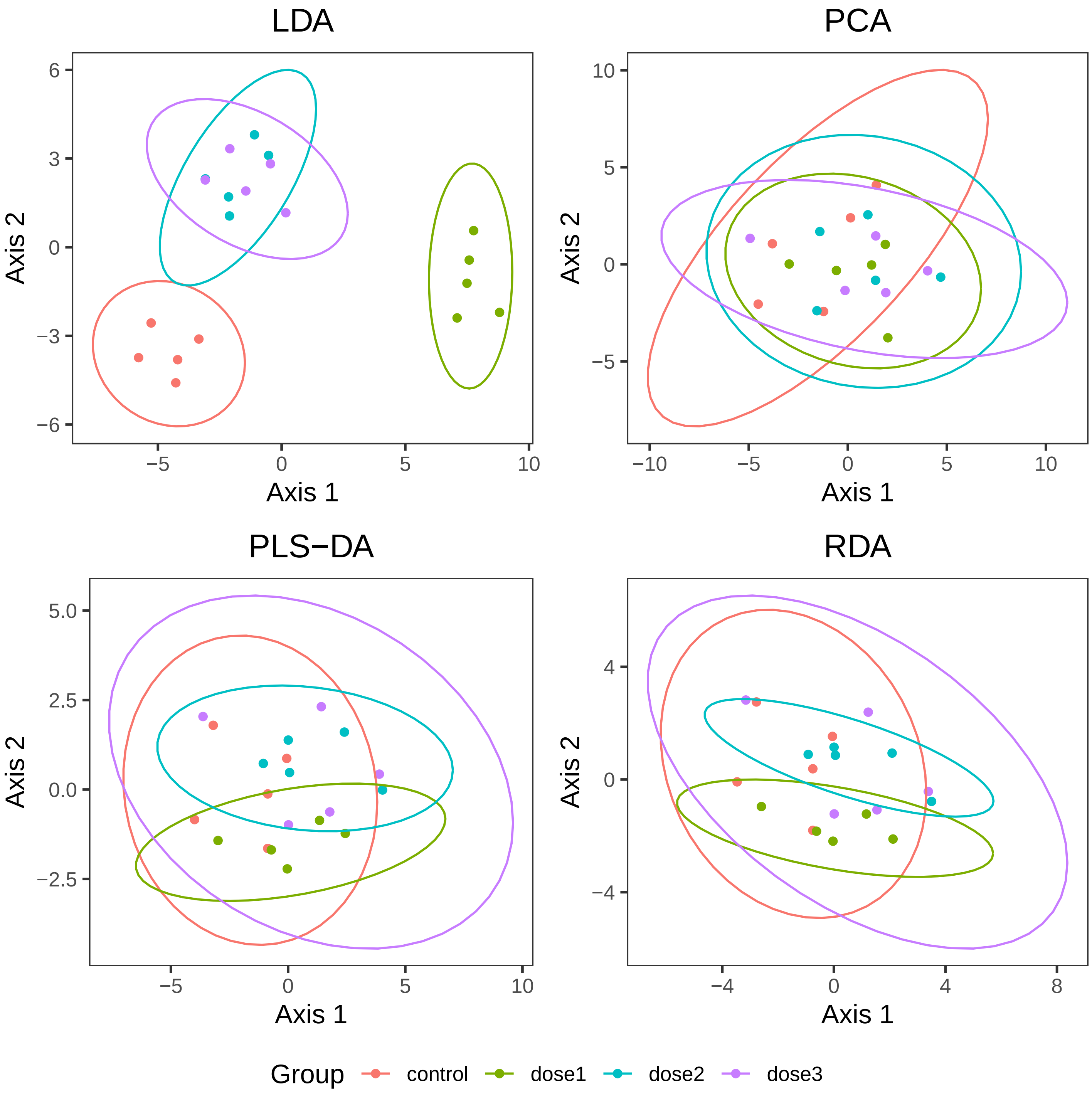

LDA
PCA
PLS−DA
RDA
Axis 1
Axis 2
Group
control
dose1
dose2
dose3

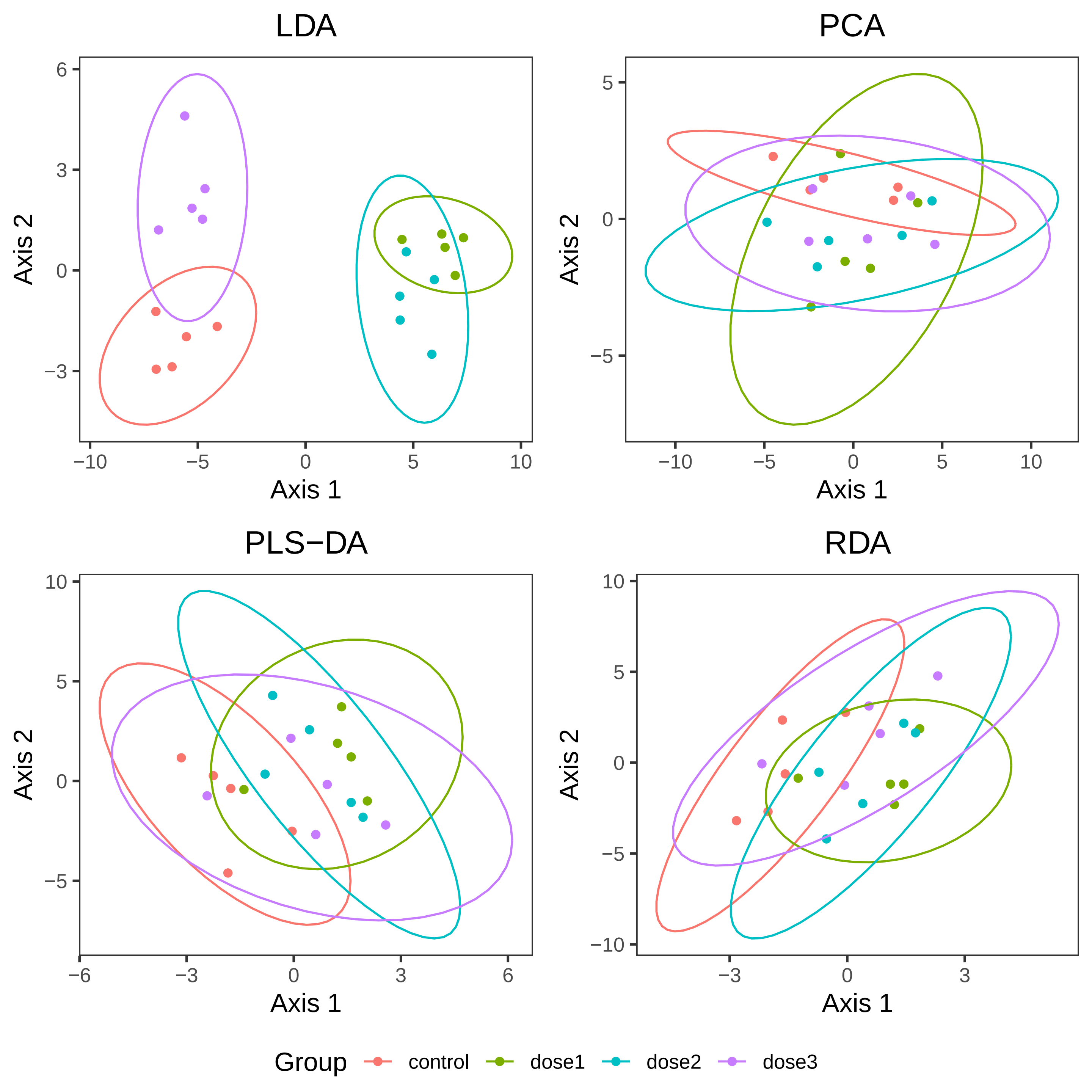
LDA
PCA
PLS−DA
RDA
Axis 1
Axis 2
Group
control
dose1
dose2
dose3

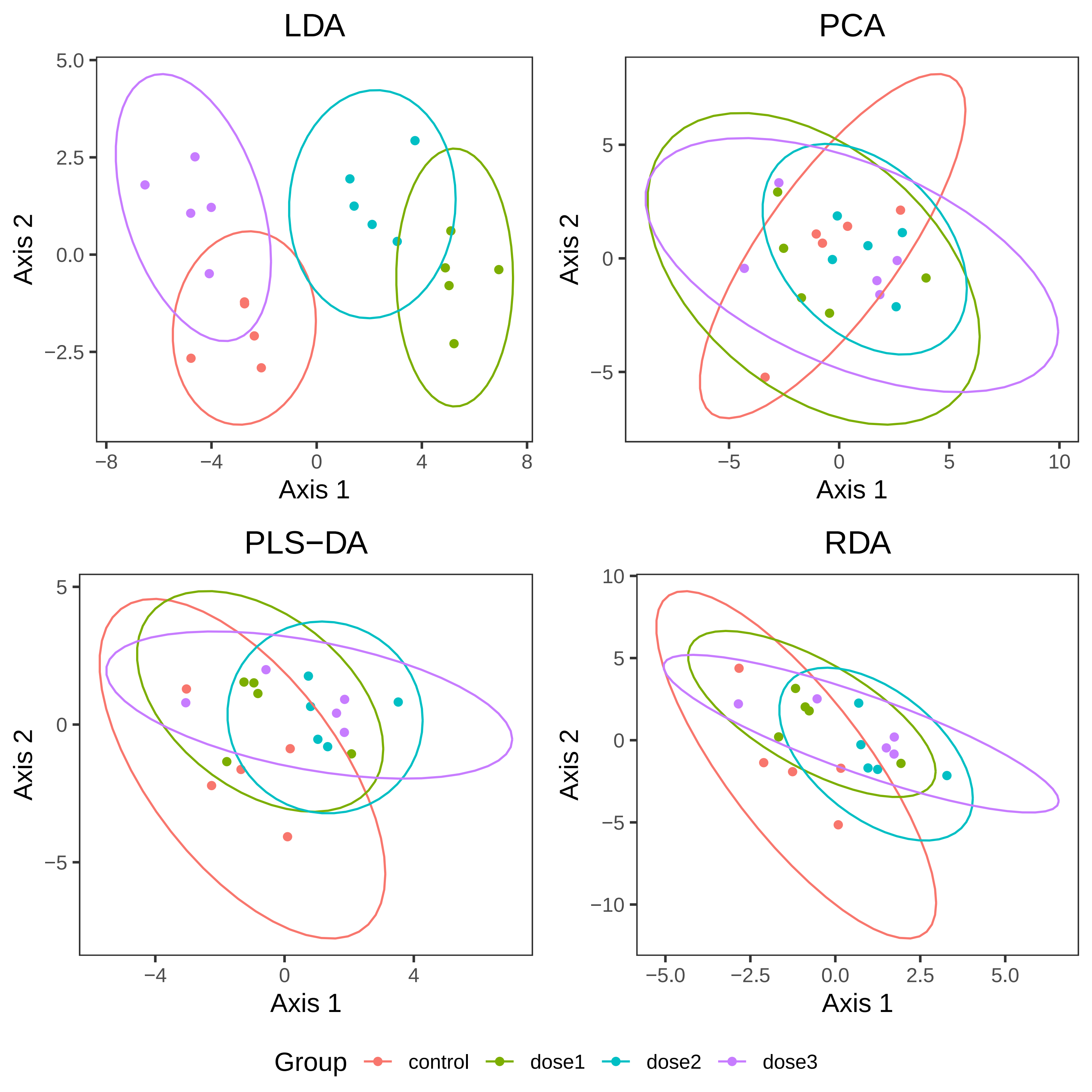
LDA
PCA
PLS−DA
RDA
Axis 1
Axis 2
Group
control
dose1
dose2
dose3

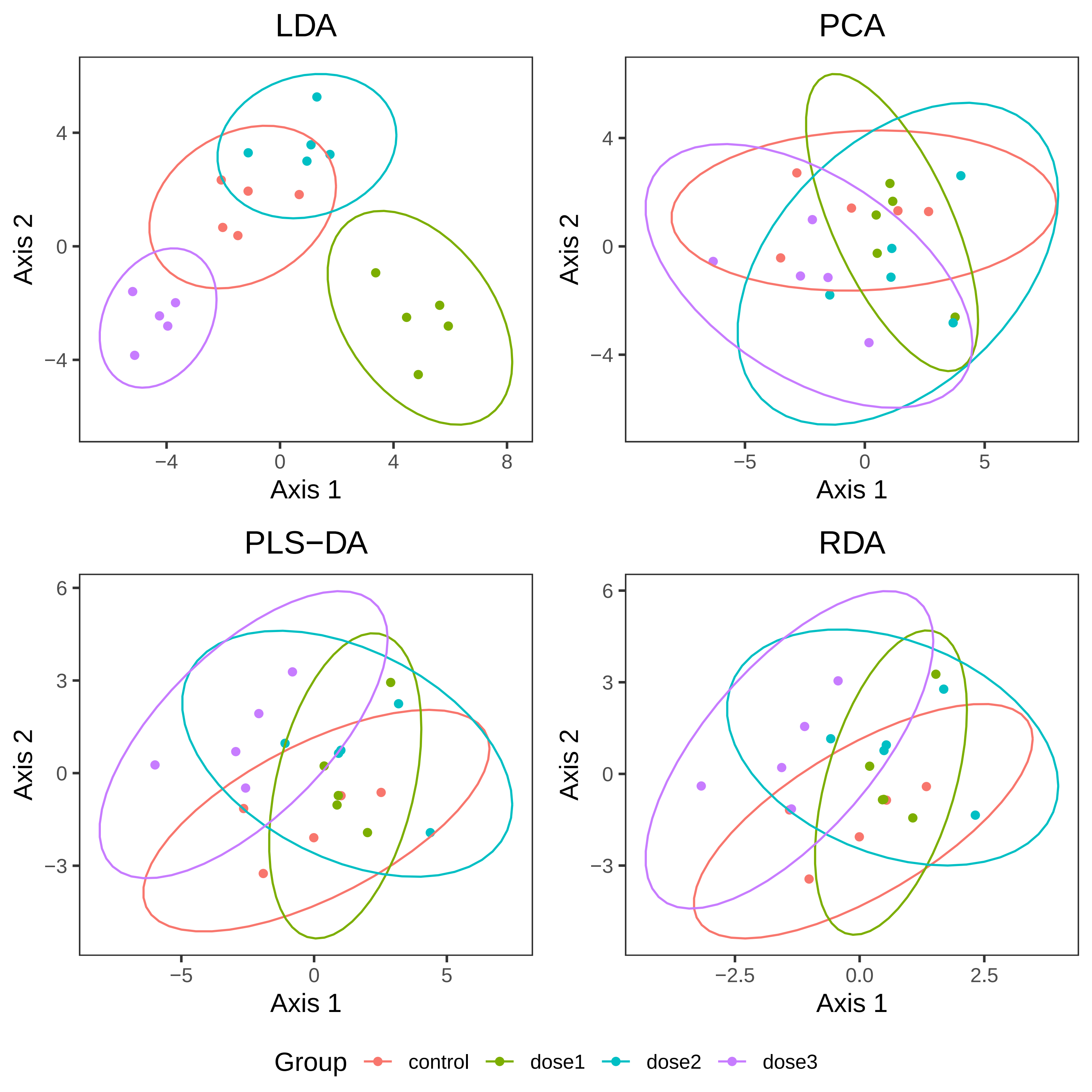

LDA
PCA
PLS−DA
RDA
Axis 1
Axis 2
4
0
−4
8
−5
5
6
3
−3
−2.5
0.0
2.5
Group
control
dose1
dose2
dose3

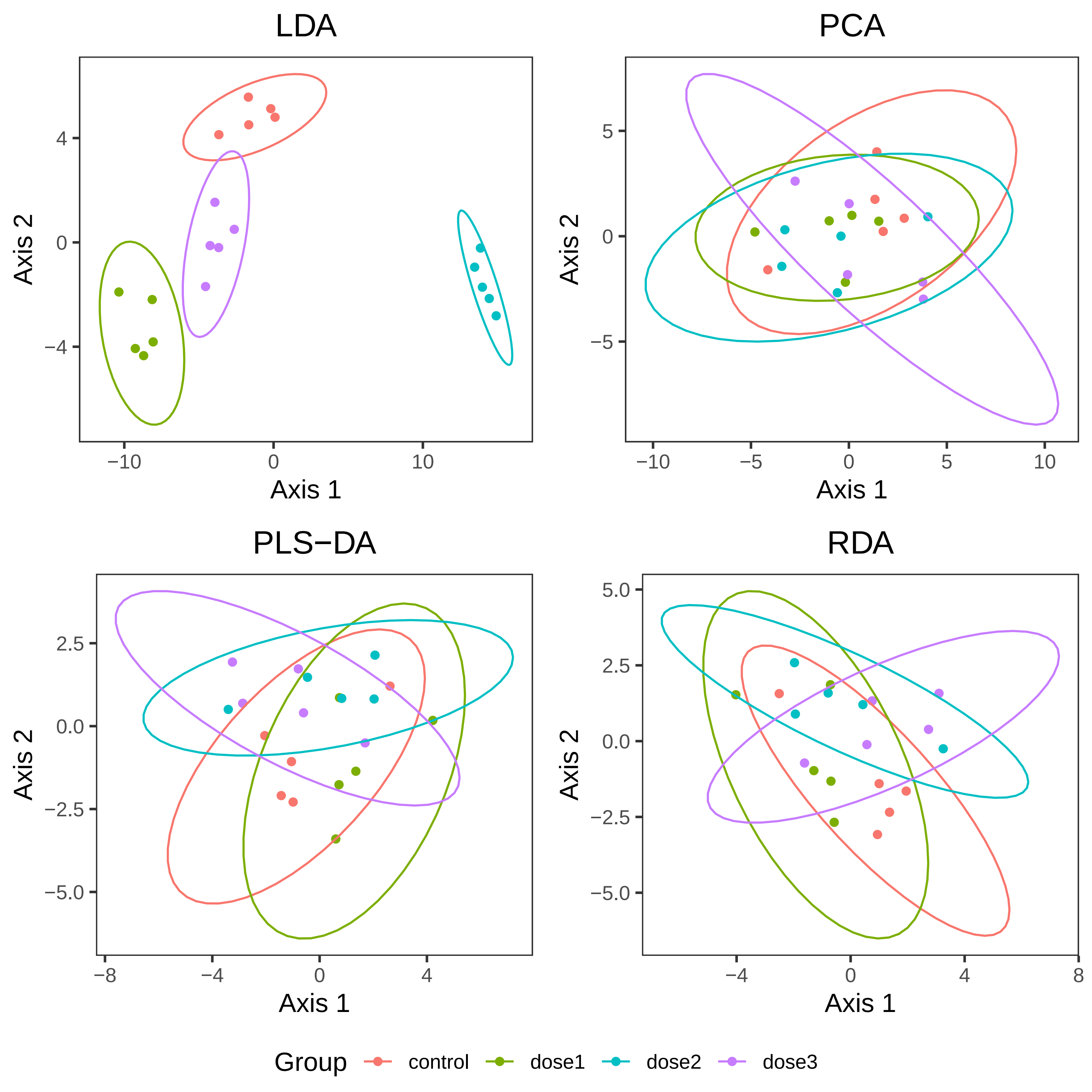

LDA
PCA
PLS−DA
RDA
Axis 1
Axis 2
Group
control
dose1
dose2
dose3

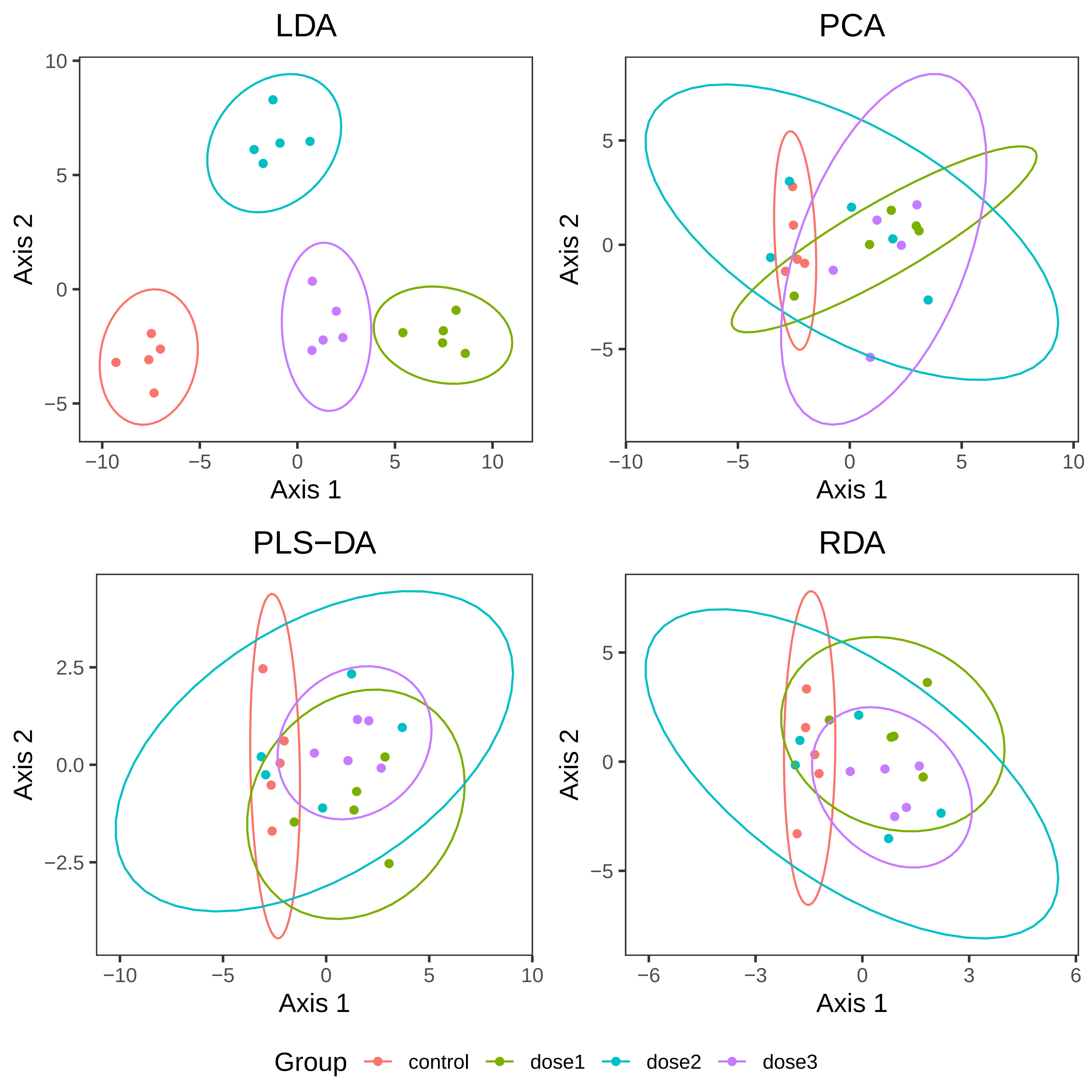

LDA
PCA
PLS−DA
RDA
Axis 1
Axis 2
−10
−5
0
5
10
2.5
0.0
−2.5
−6
−3
3
6
Group
control
dose1
dose2
dose3

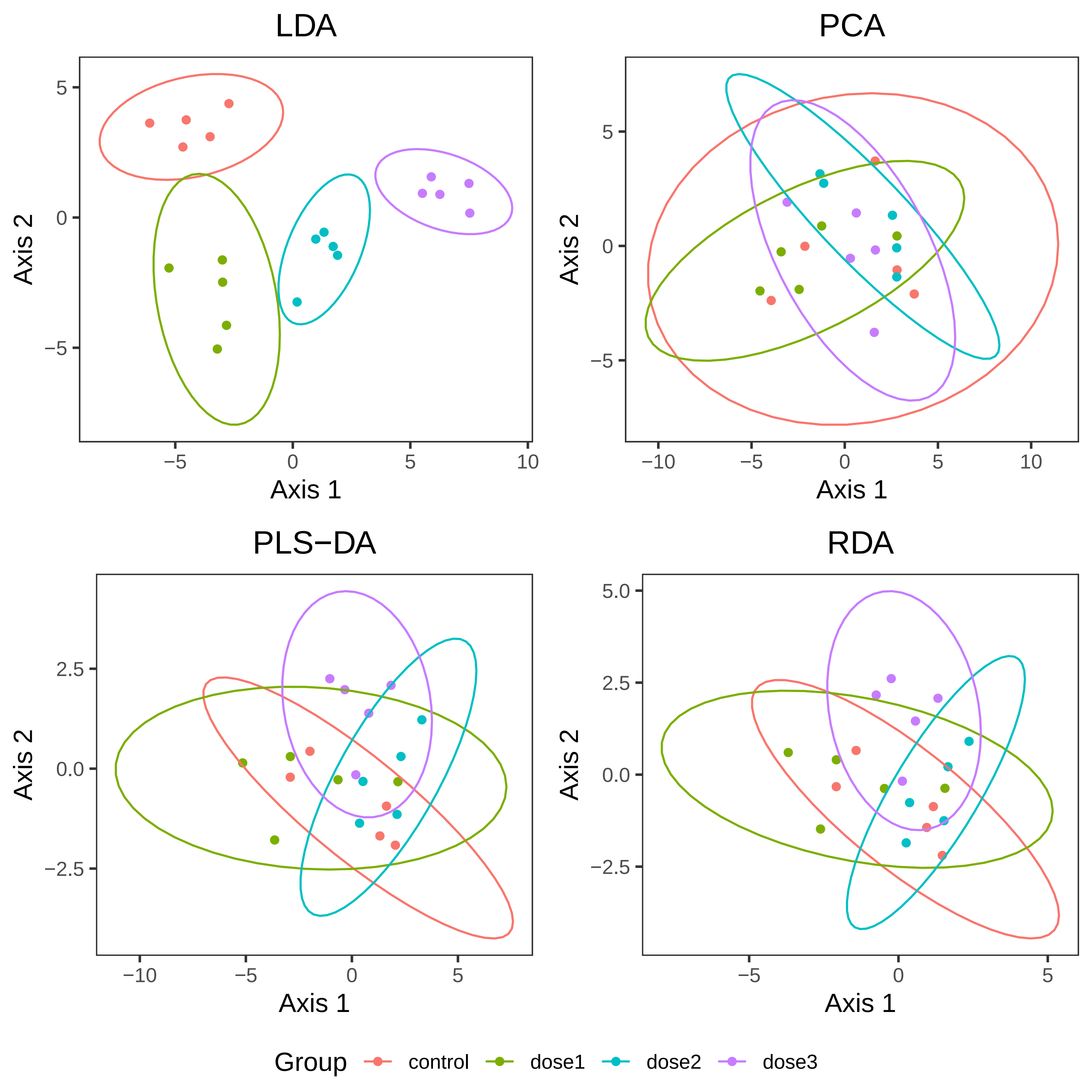
LDA
PCA
PLS−DA
RDA
Axis 1
Axis 2
5
0
−5
10
−10
2.5
0.0
−2.5
5.0
Group
control
dose1
dose2
dose3

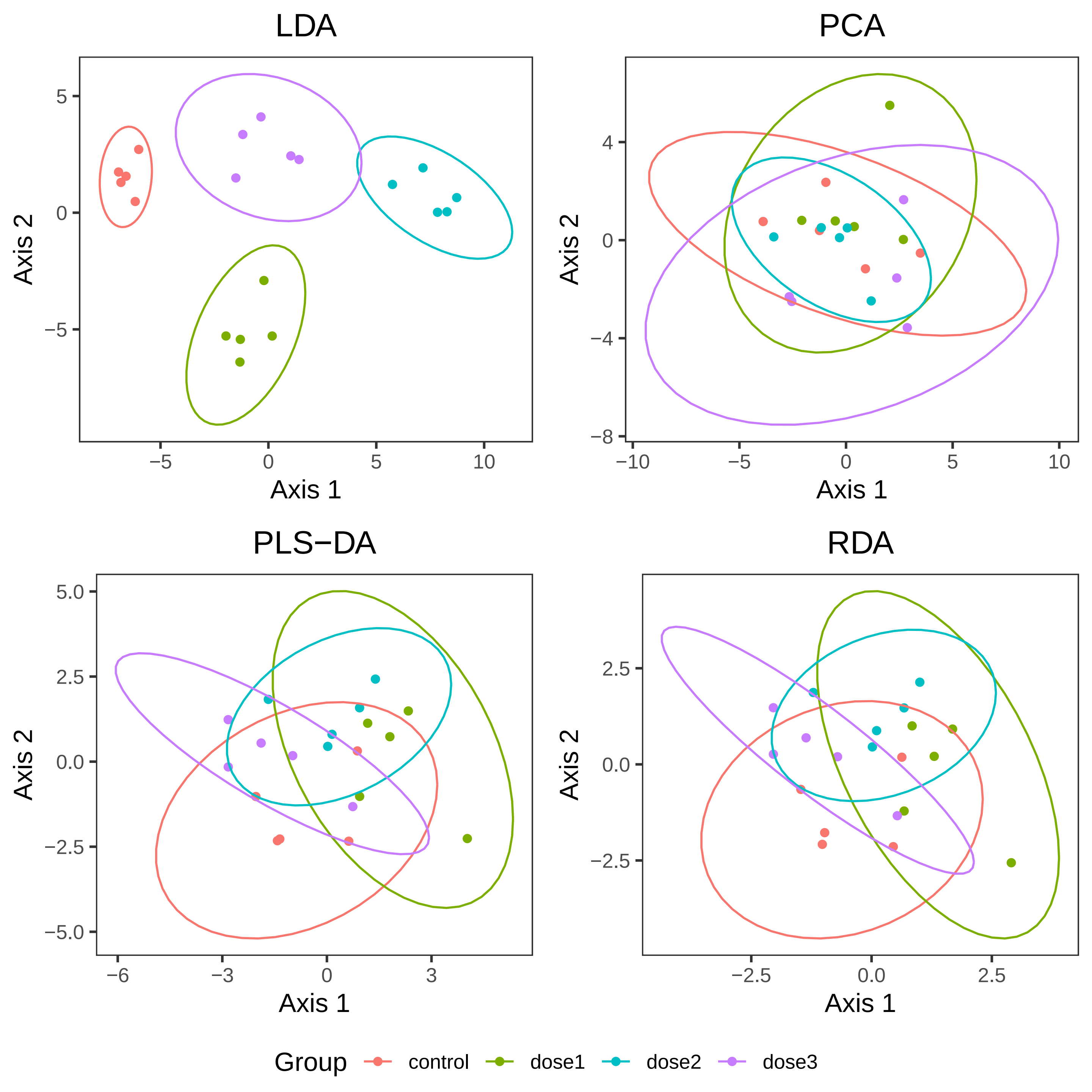
LDA
PCA
PLS−DA
RDA
Axis 1
Axis 2
5
0
−5
−5
0
5
10
4
0
−4
−8
−10
−5
0
5
10
5.0
2.5
0.0
−2.5
−5.0
−6
−3
0
3
2.5
0.0
−2.5
−2.5
0.0
2.5
Group
control
dose1
dose2
dose3

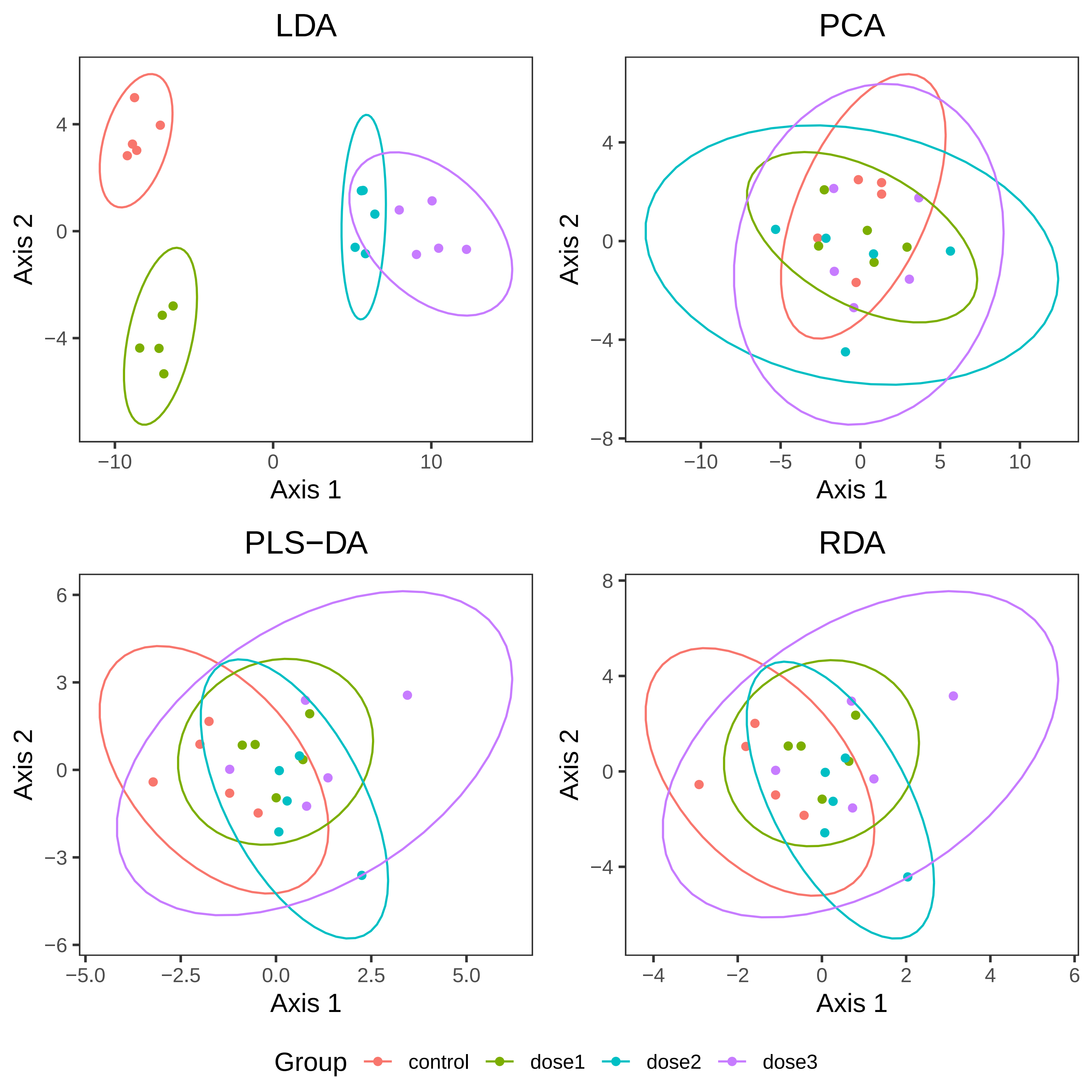

LDA
PCA
PLS−DA
RDA
Axis 1
Axis 2
Group
control
dose1
dose2
dose3

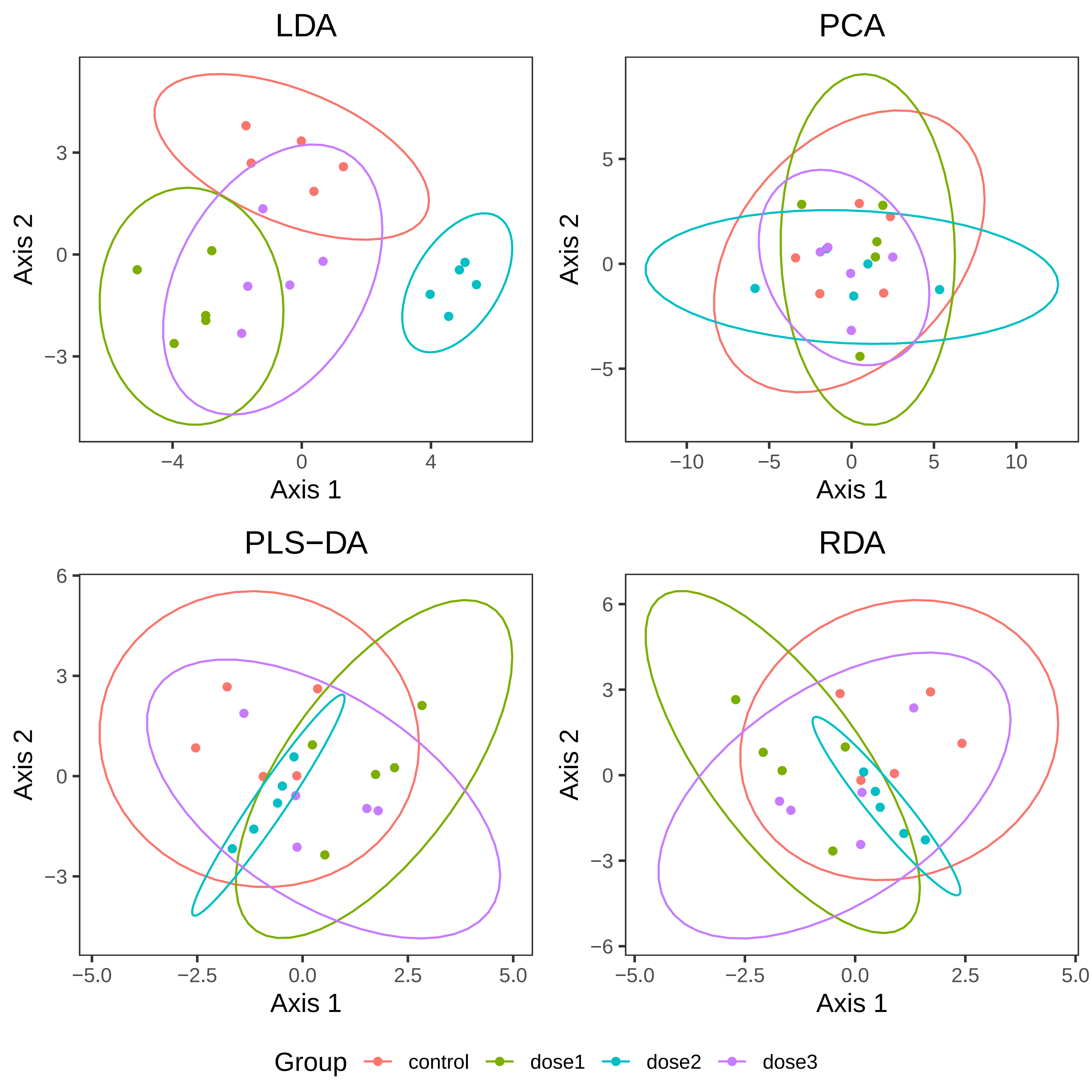
LDA
PCA
PLS−DA
RDA
Axis 1
Axis 2
Group
control
dose1
dose2
dose3

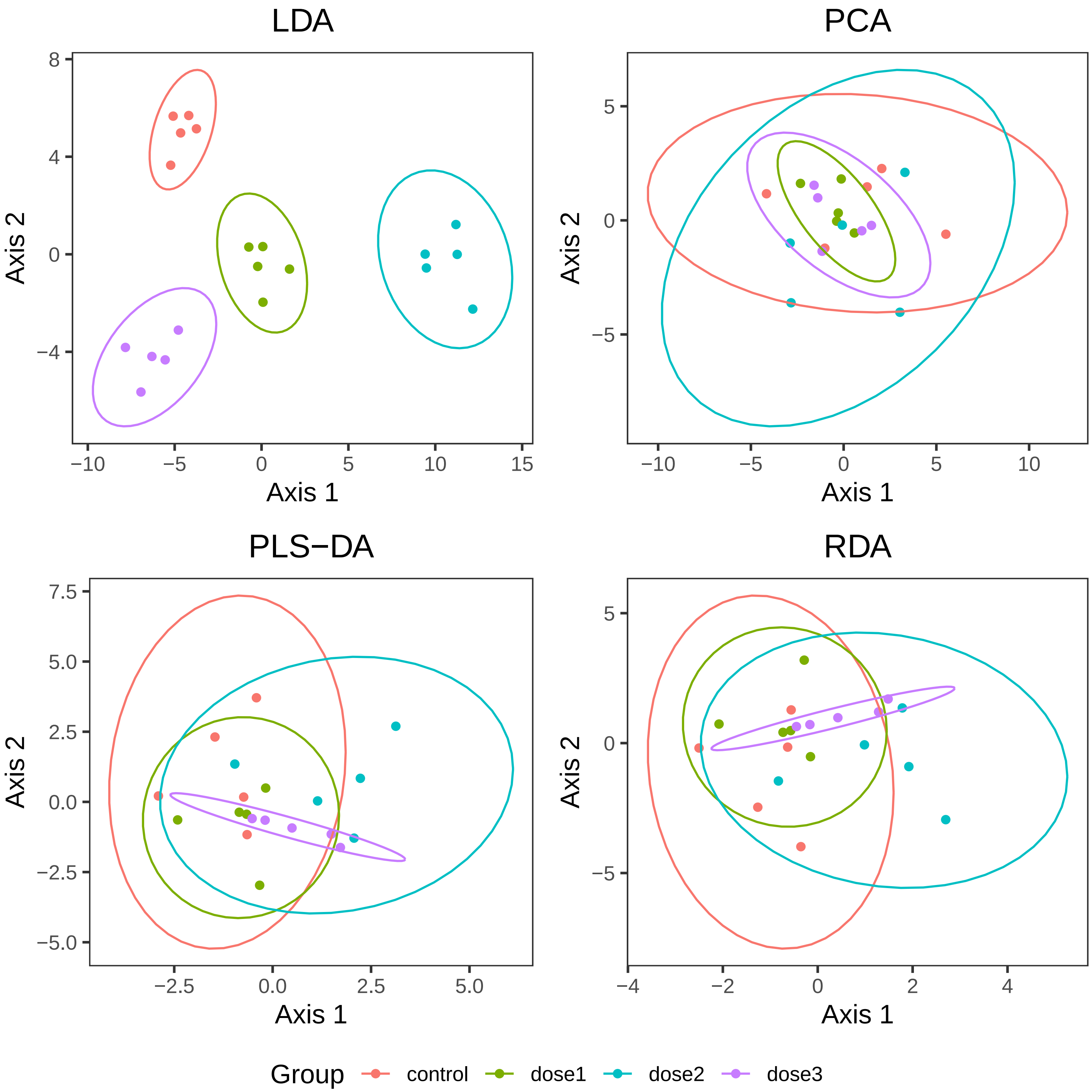
LDA
PCA
PLS−DA
RDA
Axis 1
Axis 2
Group
control
dose1
dose2
dose3

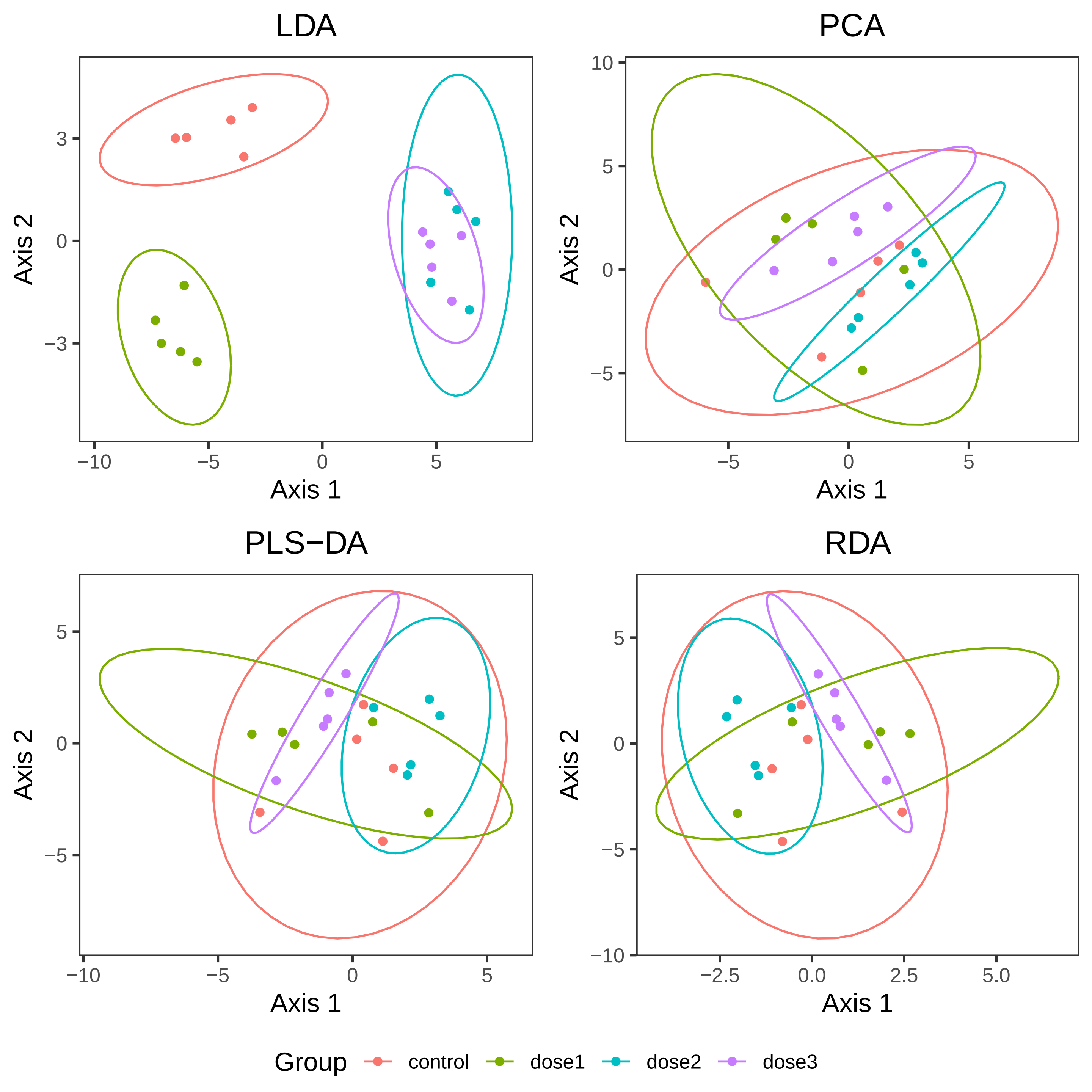

LDA
PCA
PLS−DA
RDA
Axis 1
Axis 2
3
0
−3
−10
−5
0
5
10
5
0
−5
−10
−2.5
0.0
2.5
5.0
Group
control
dose1
dose2
dose3

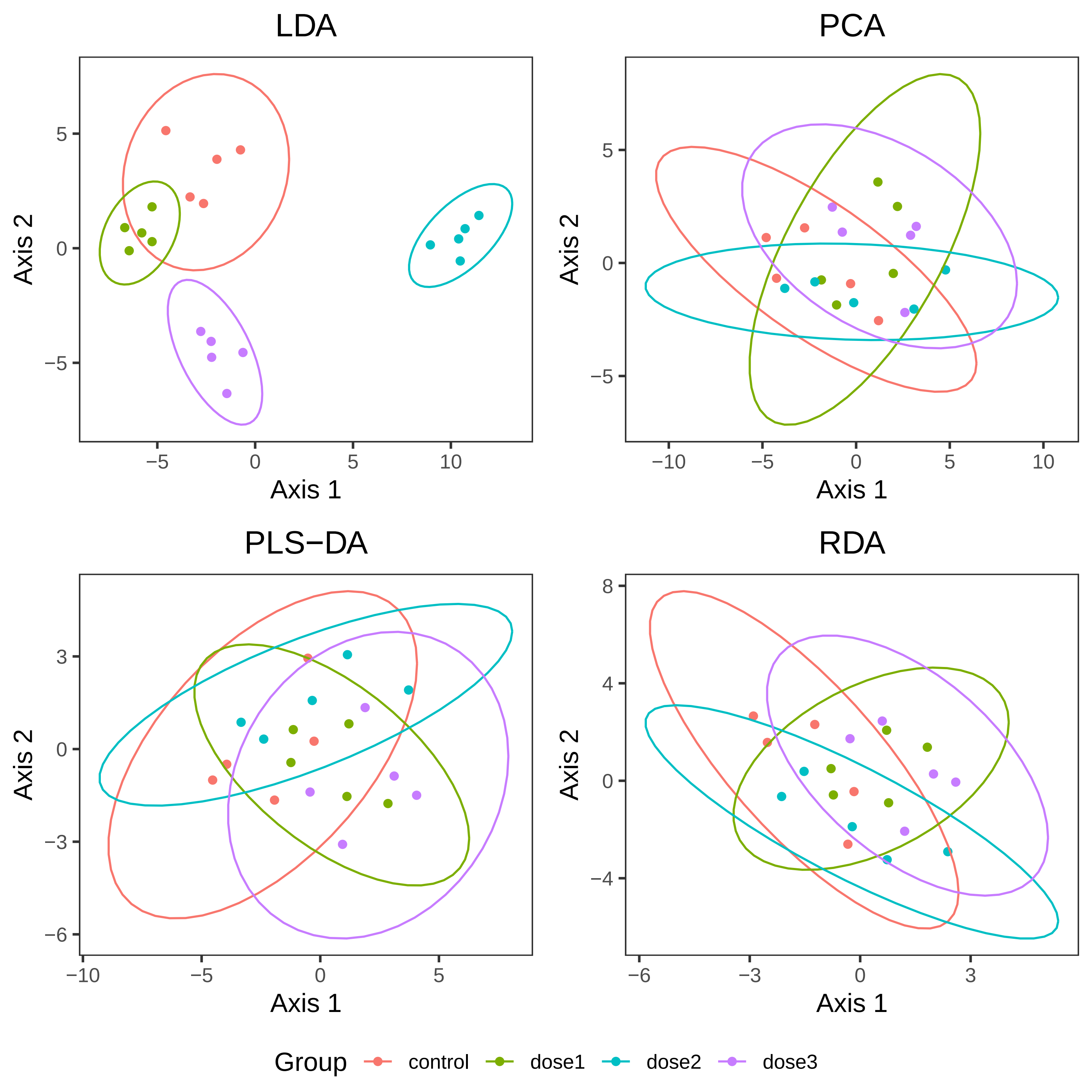
LDA
PCA
PLS−DA
RDA
Axis 1
Axis 2
Group
control
dose1
dose2
dose3

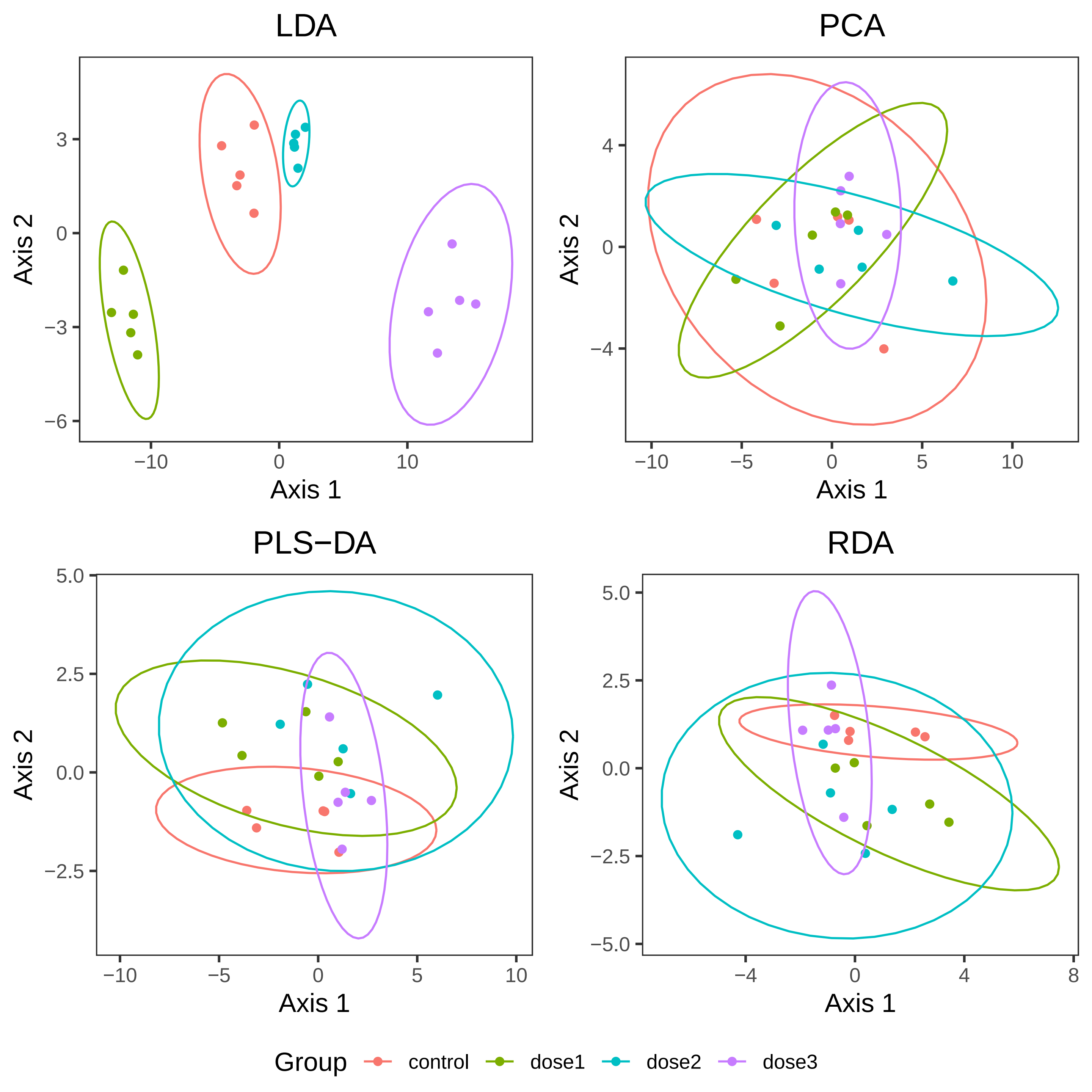
LDA
PCA
PLS−DA
RDA
Axis 1
Axis 2
Group
control
dose1
dose2
dose3

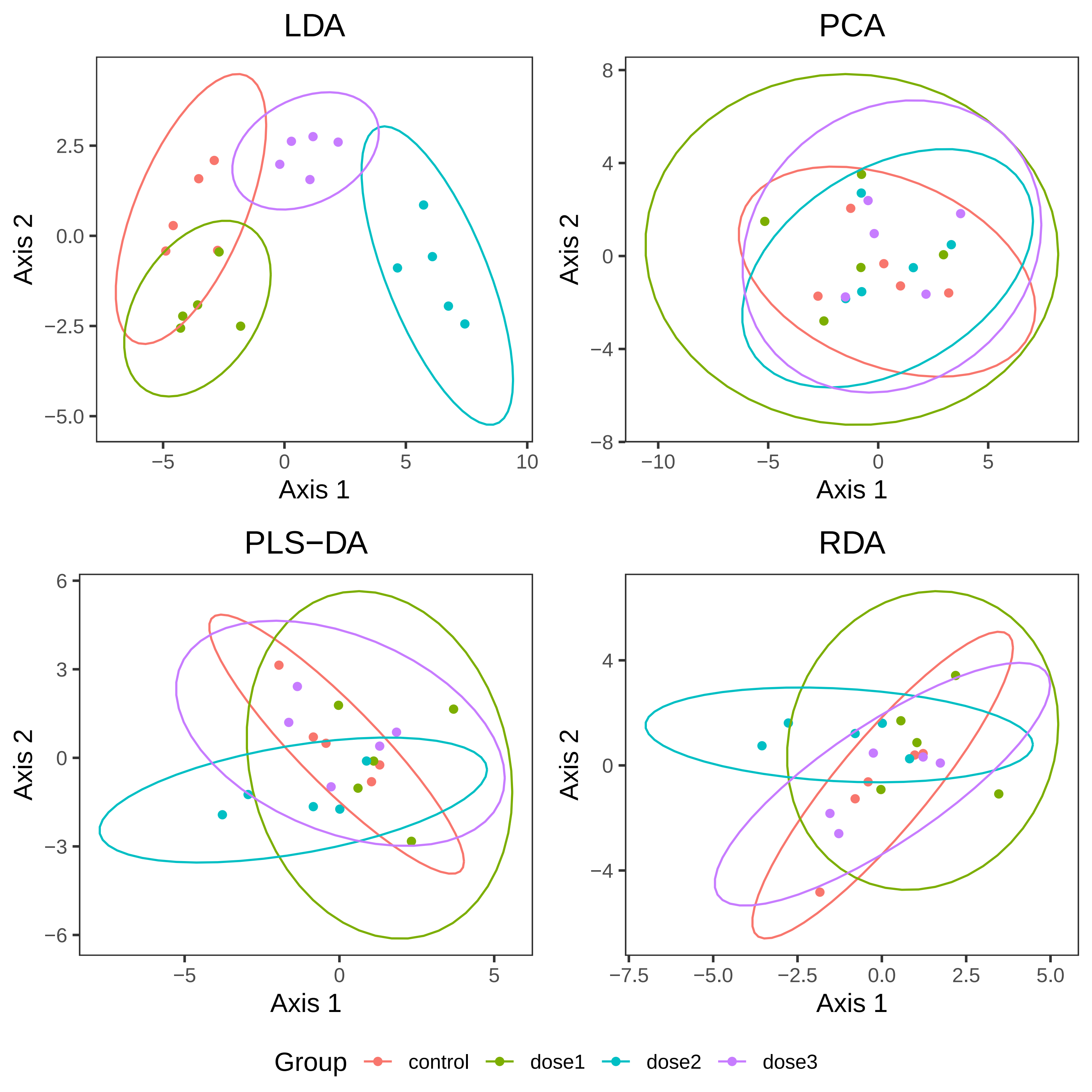
LDA
PCA
PLS−DA
RDA
Axis 1
Axis 2
Group
control
dose1
dose2
dose3

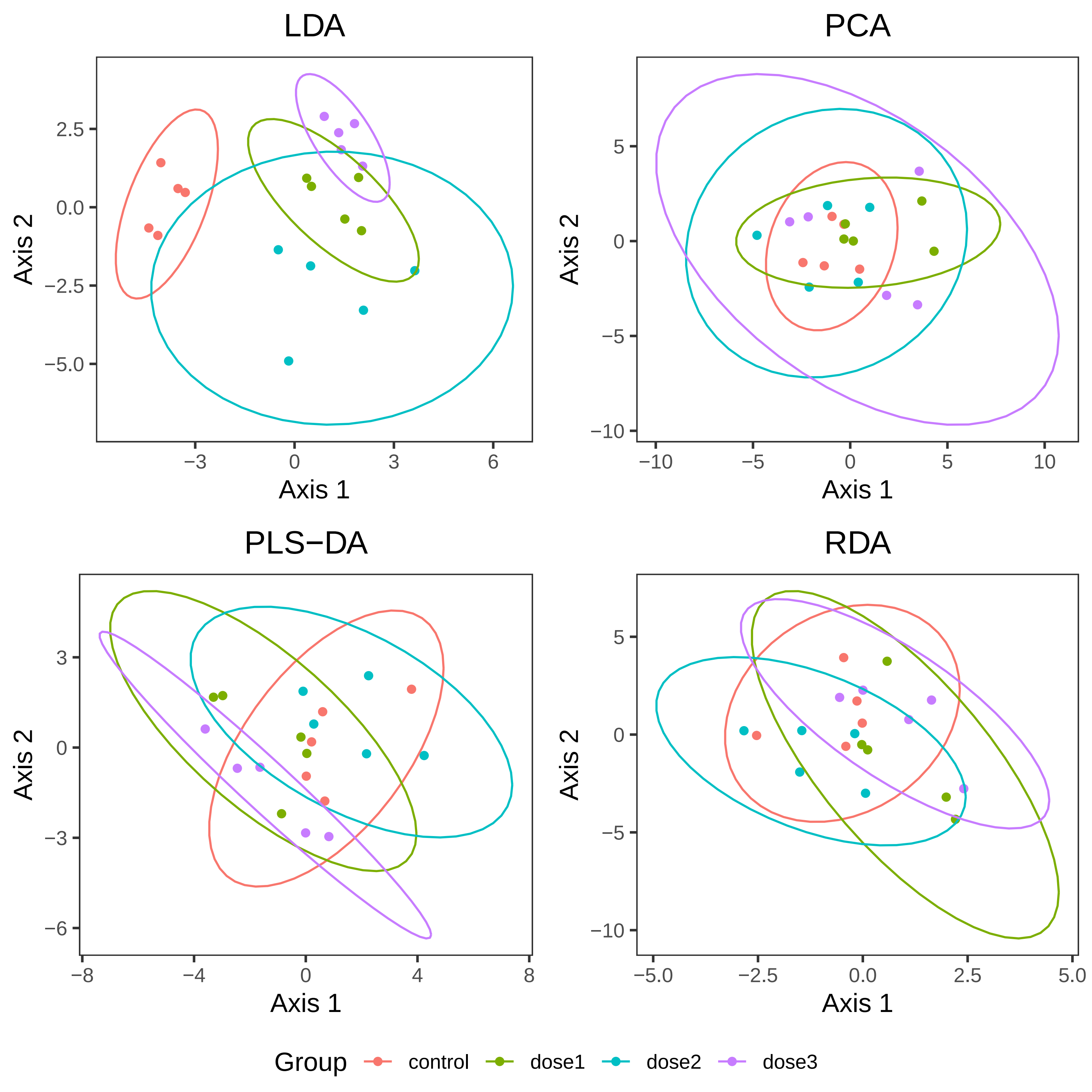
LDA
PCA
PLS−DA
RDA
Axis 1
Axis 2
2.5
0.0
−2.5
−5.0
−3
0
3
6
5
0
−5
−10
−10
−5
0
5
10
3
0
−3
−6
−8
−4
0
4
8
5
0
−5
−10
−5.0
−2.5
0.0
2.5
5.0
Group
control
dose1
dose2
dose3

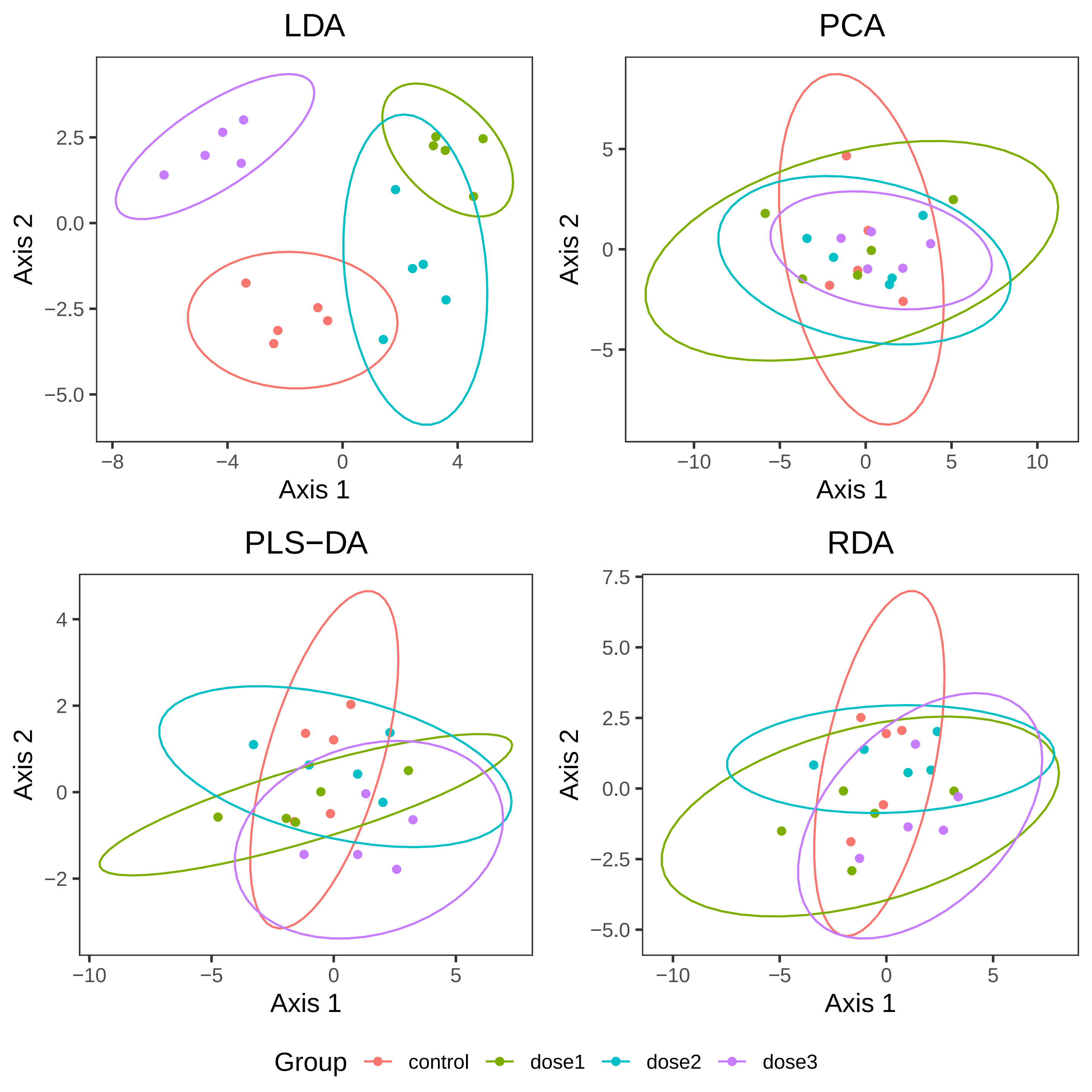

LDA
PCA
PLS−DA
RDA
Axis 1
Axis 2
Group
control
dose1
dose2
dose3

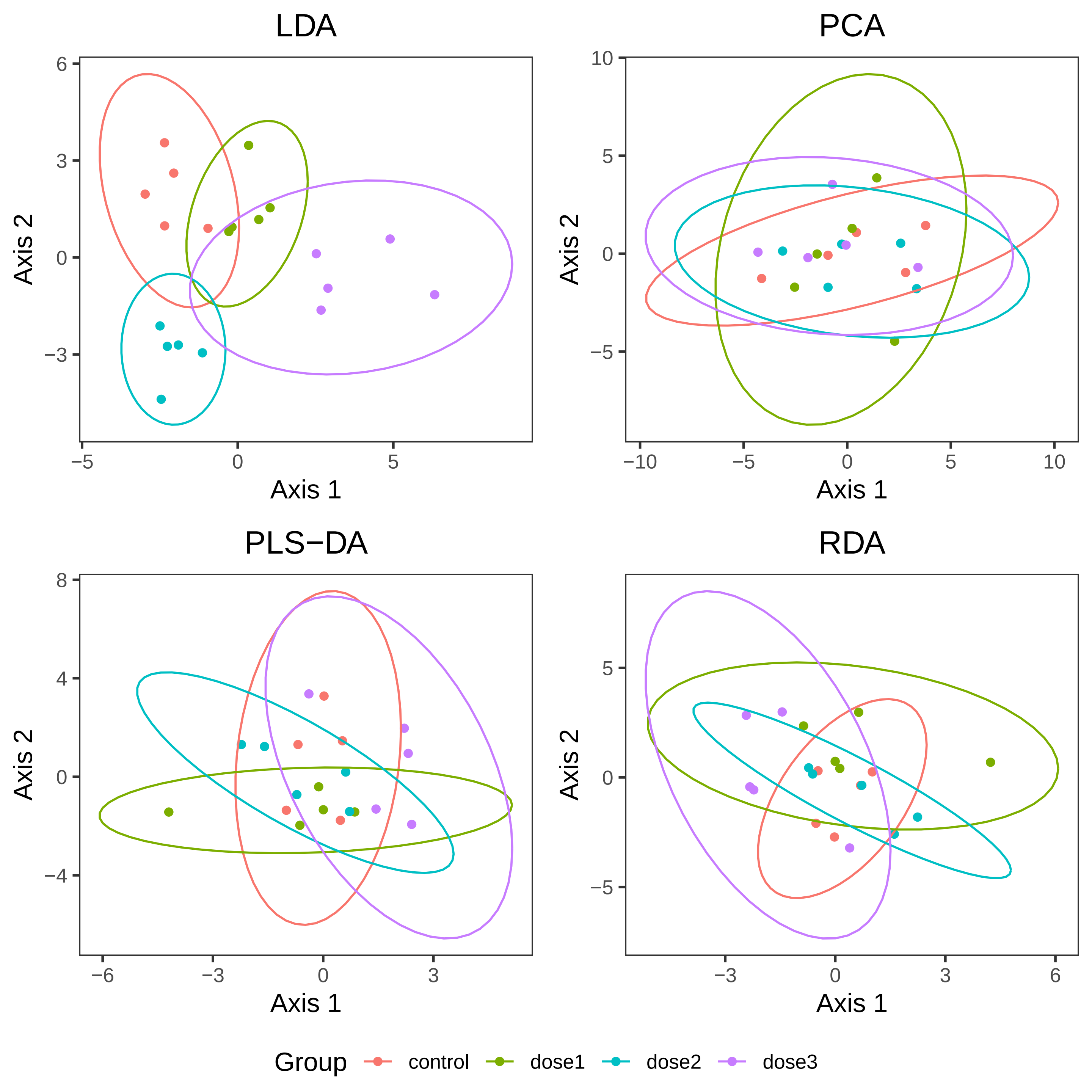

LDA
PCA
PLS−DA
RDA
Axis 1
Axis 2
Group
control
dose1
dose2
dose3

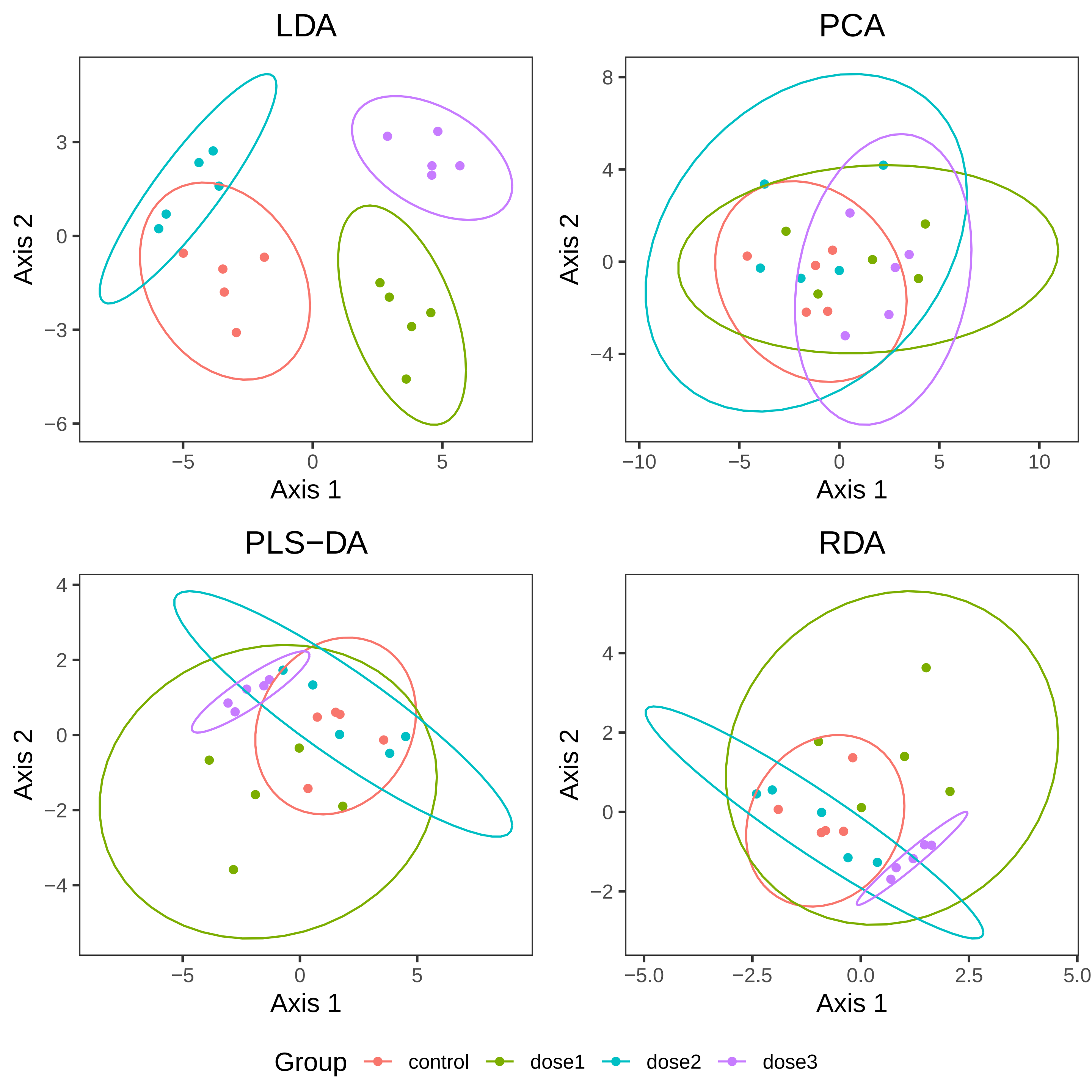

LDA
PCA
PLS−DA
RDA
Axis 1
Axis 2
Group
control
dose1
dose2
dose3

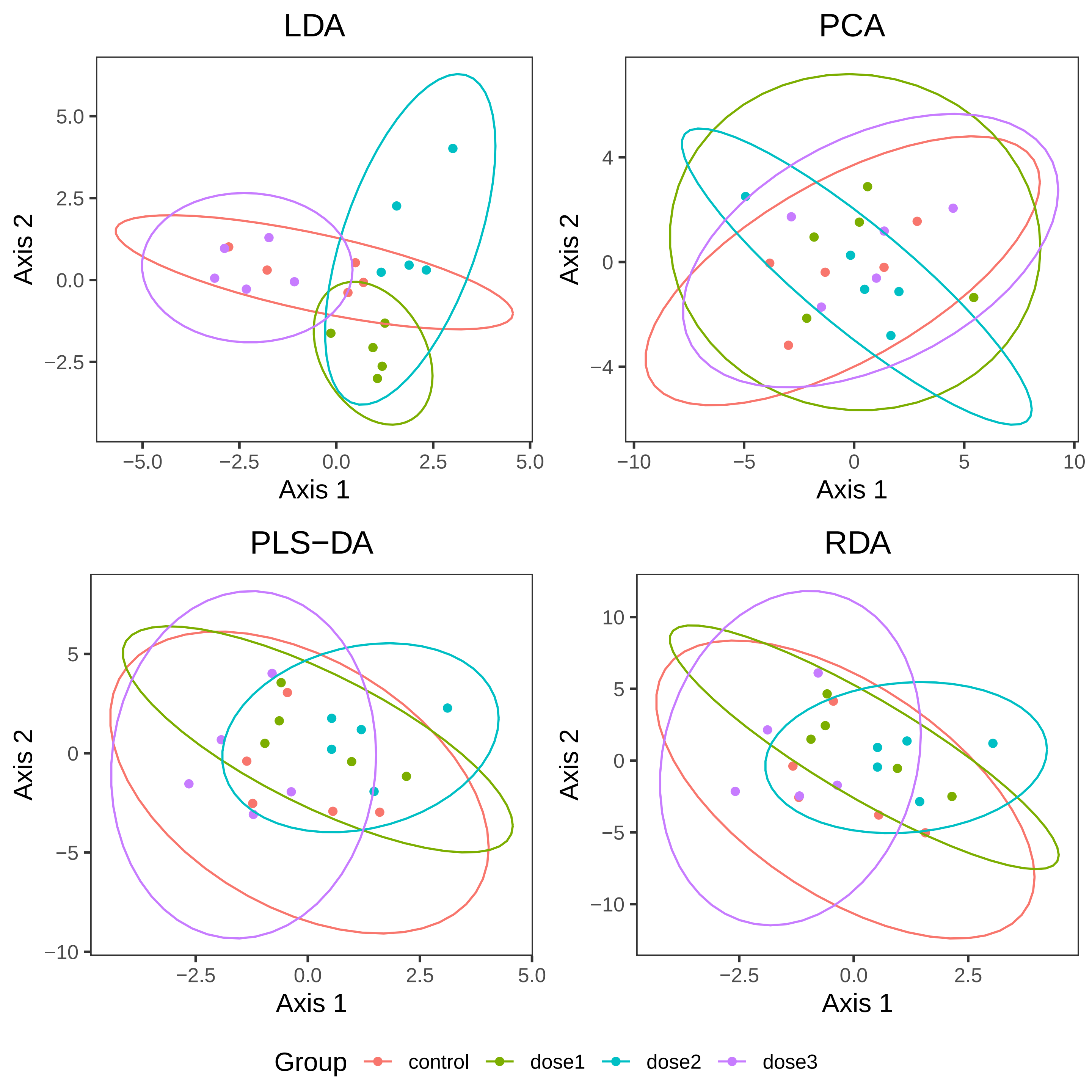

LDA
PCA
PLS−DA
RDA
Axis 1
Axis 2
5.0
2.5
0.0
−2.5
−5.0
4
0
−4
−10
−5
5
10
Group
control
dose1
dose2
dose3

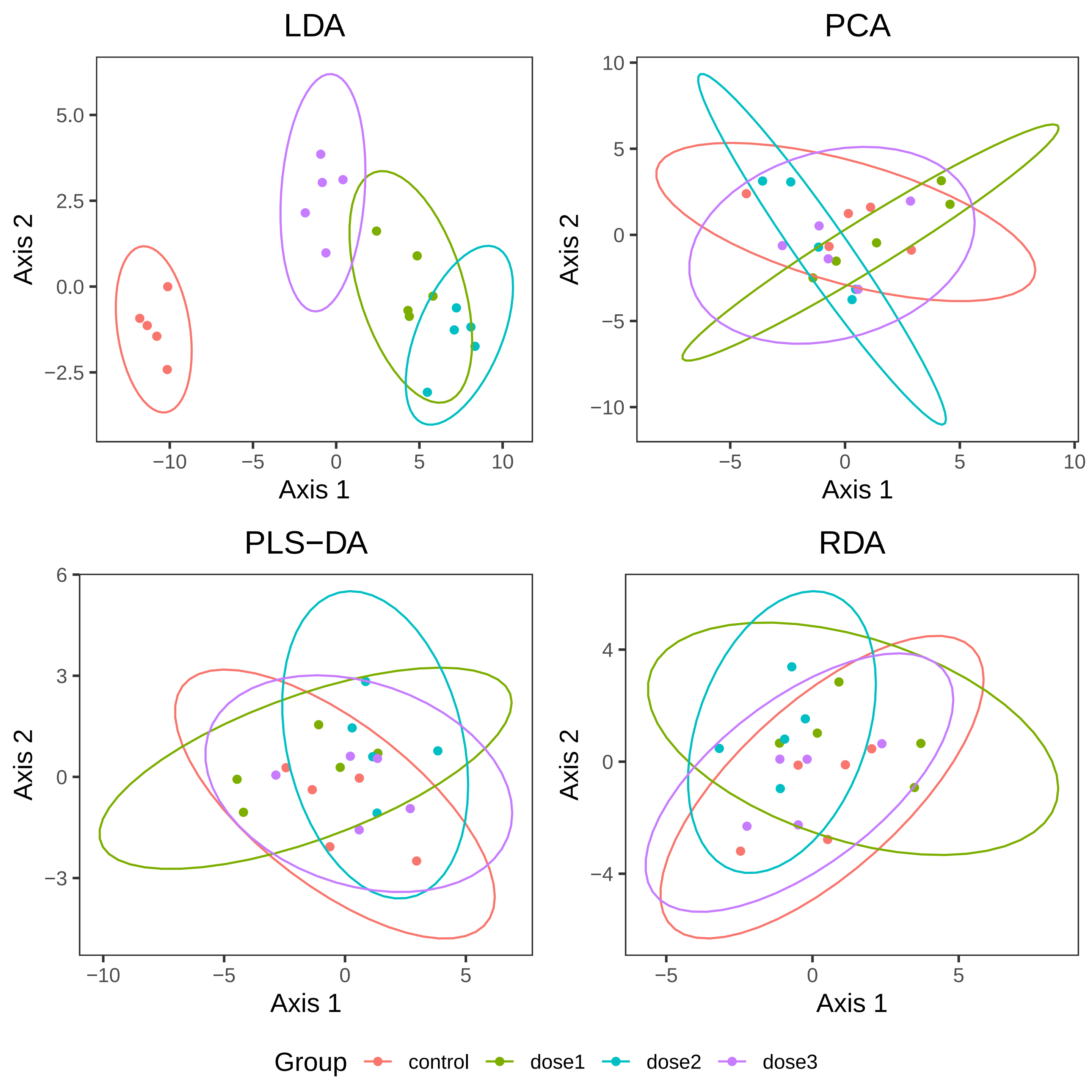

LDA
PCA
PLS−DA
RDA
Axis 1
Axis 2
5.0
2.5
0.0
−2.5
−10
−5
0
5
10
−5
−10
6
3
−3
4
−4
Group
control
dose1
dose2
dose3

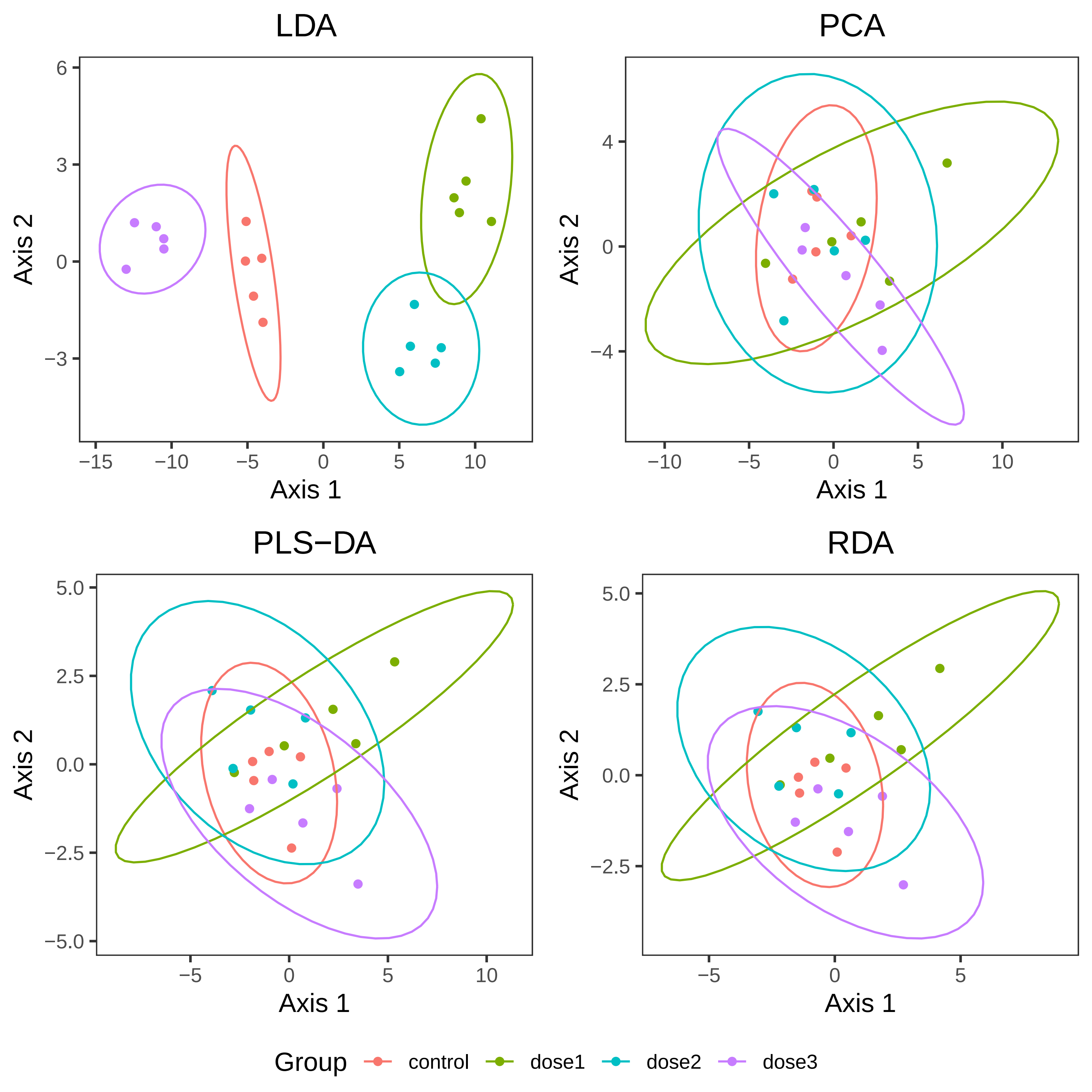
LDA
PCA
PLS−DA
RDA
Axis 1
Axis 2
6
3
0
−3
−15
−10
−5
0
5
10
4
−4
5.0
2.5
0.0
−2.5
−5.0
Group
control
dose1
dose2
dose3

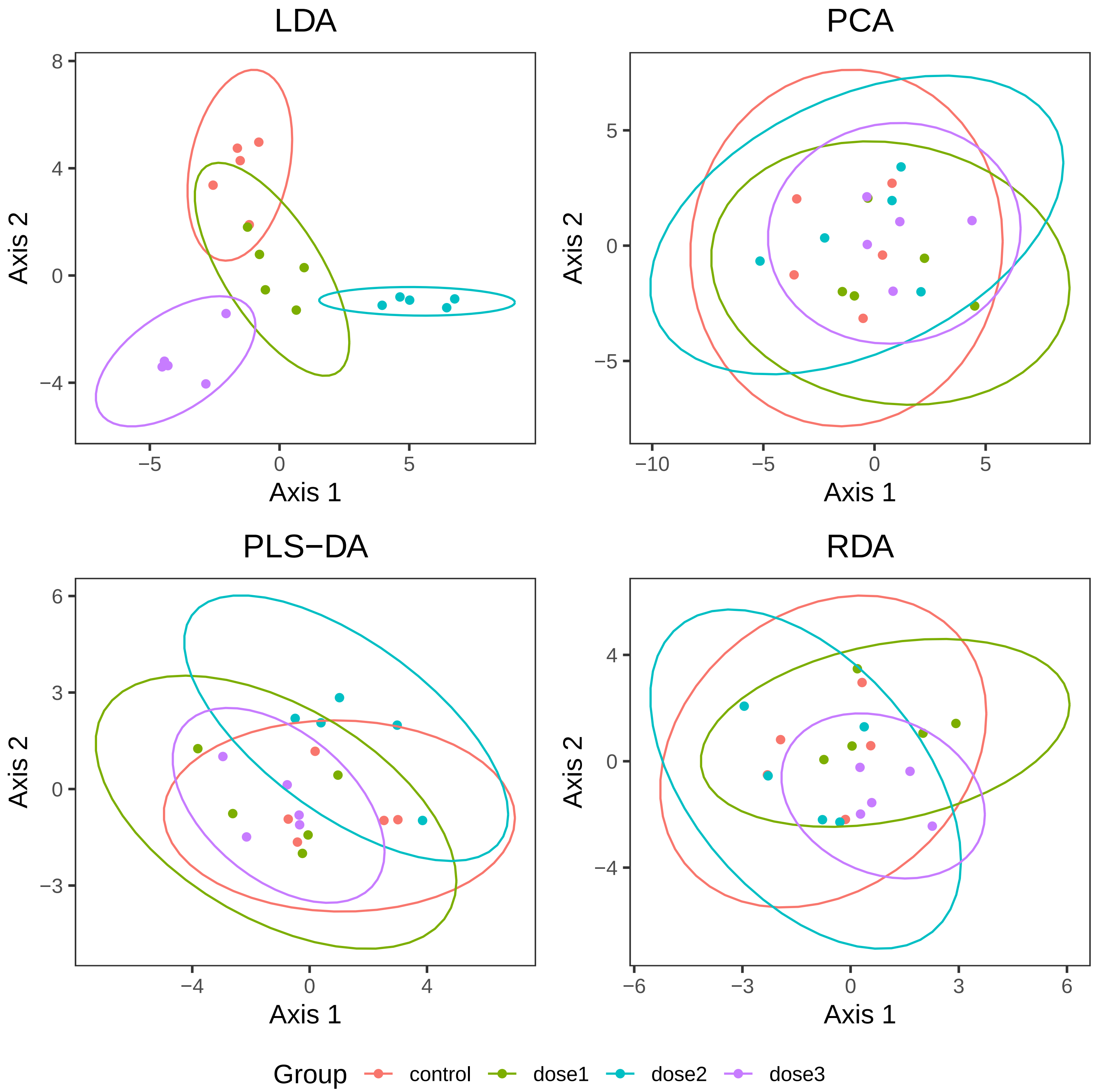
LDA
PCA
PLS−DA
RDA
Axis 1
Axis 2
8
4
0
−4
−5
5
−10
−5
6
3
−3
−4
−6
−3
Group
control
dose1
dose2
dose3

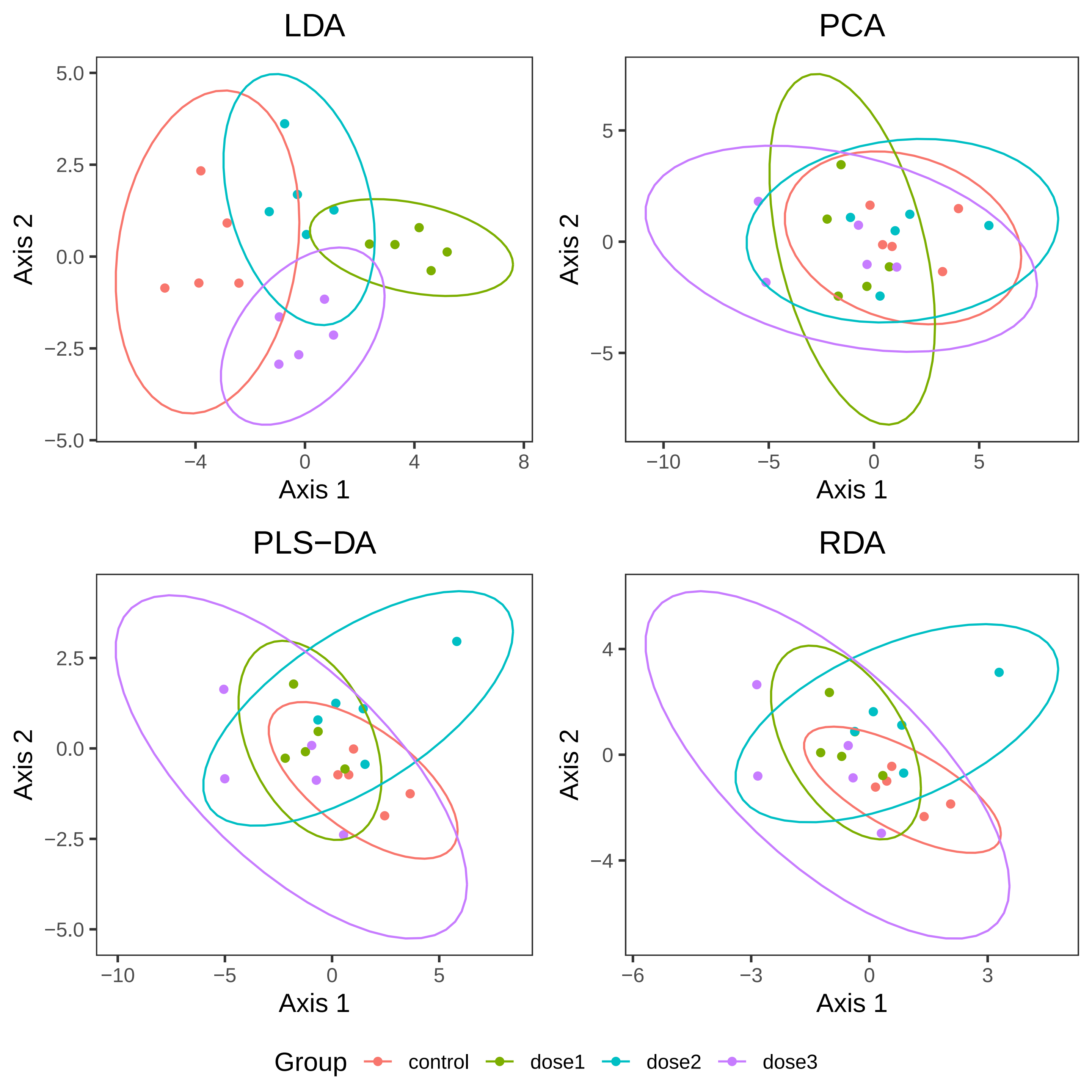
LDA
PCA
PLS−DA
RDA
Axis 1
Axis 2
5.0
2.5
0.0
−2.5
−5.0
−4
0
4
8
5
0
−5
−10
−5
0
5
2.5
0.0
−2.5
−5.0
−10
−5
0
5
4
0
−4
−6
−3
0
3
Group
control
dose1
dose2
dose3

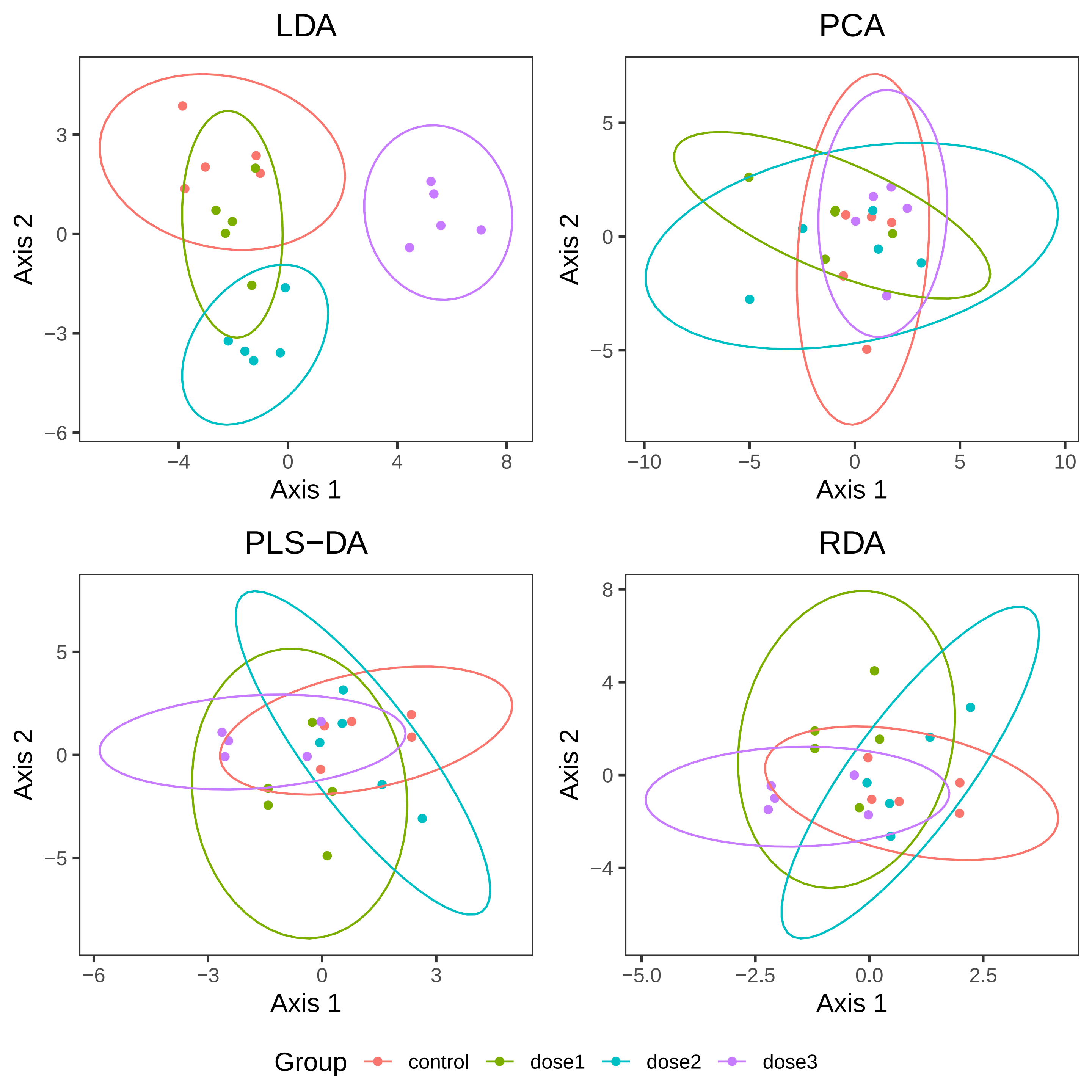

LDA
PCA
PLS−DA
RDA
Axis 1
Axis 2
Group
control
dose1
dose2
dose3

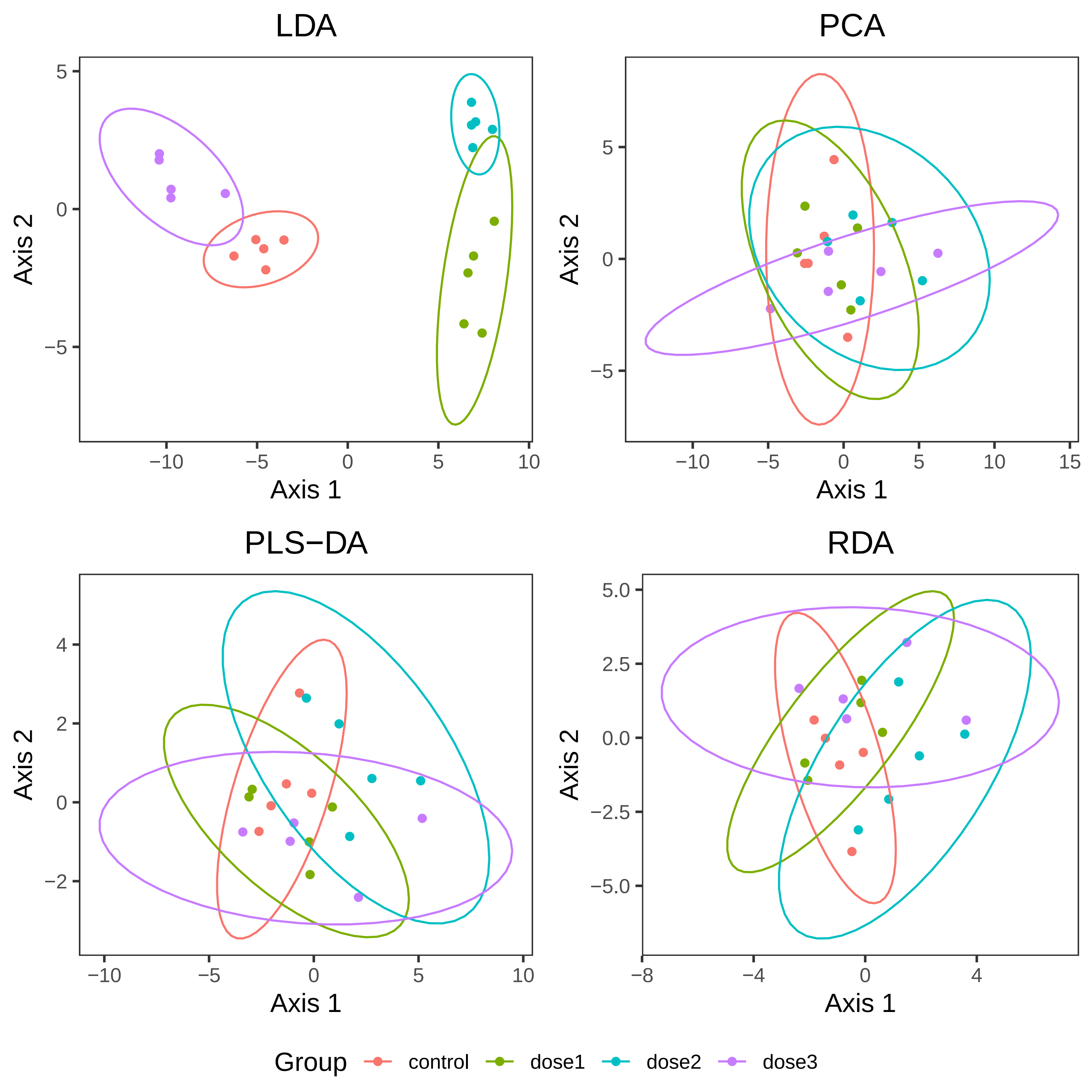

LDA
PCA
PLS−DA
RDA
Axis 1
Axis 2
Group
control
dose1
dose2
dose3

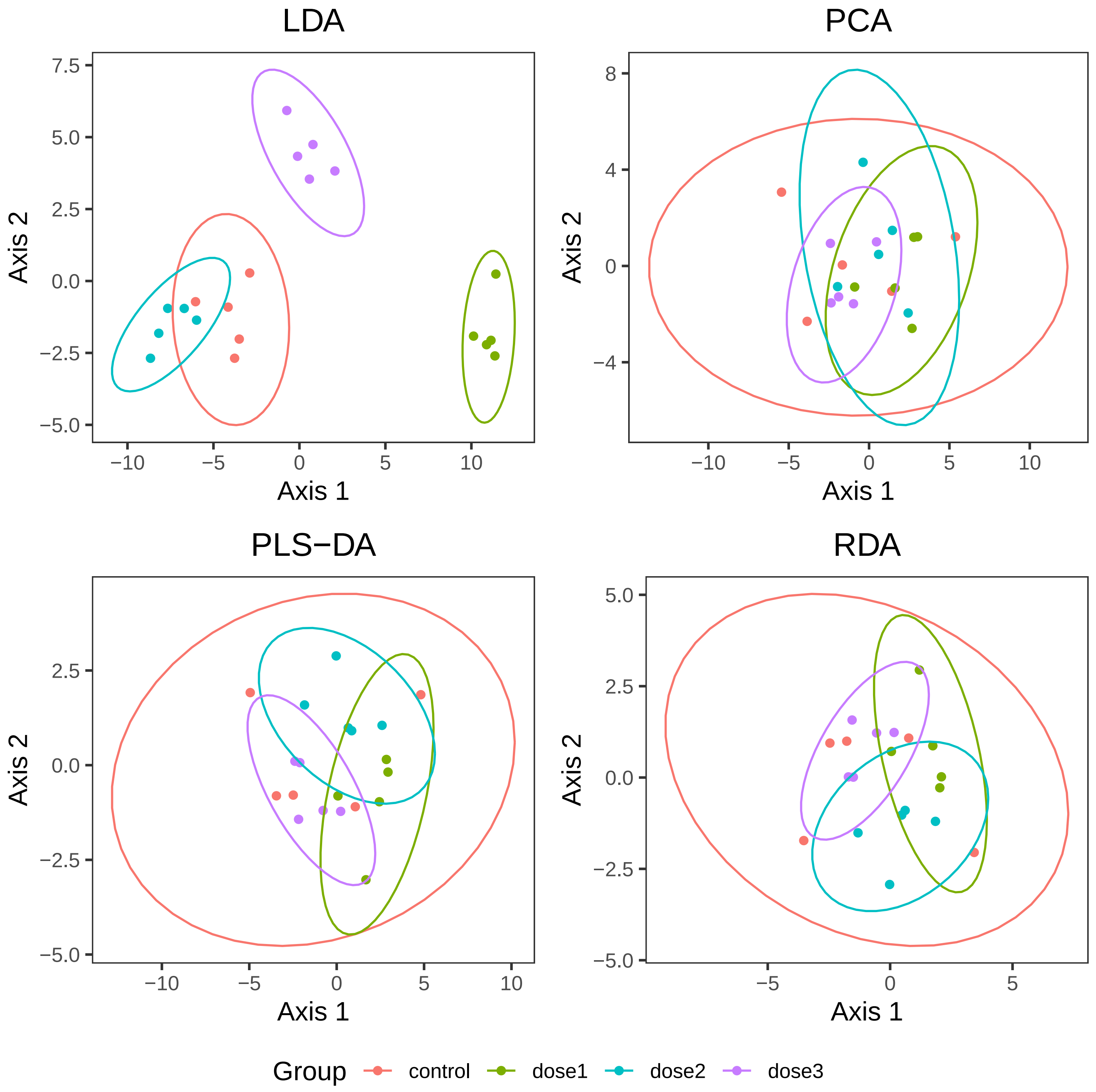
LDA
PCA
PLS−DA
RDA
Axis 1
Axis 2
Group
control
dose1
dose2
dose3

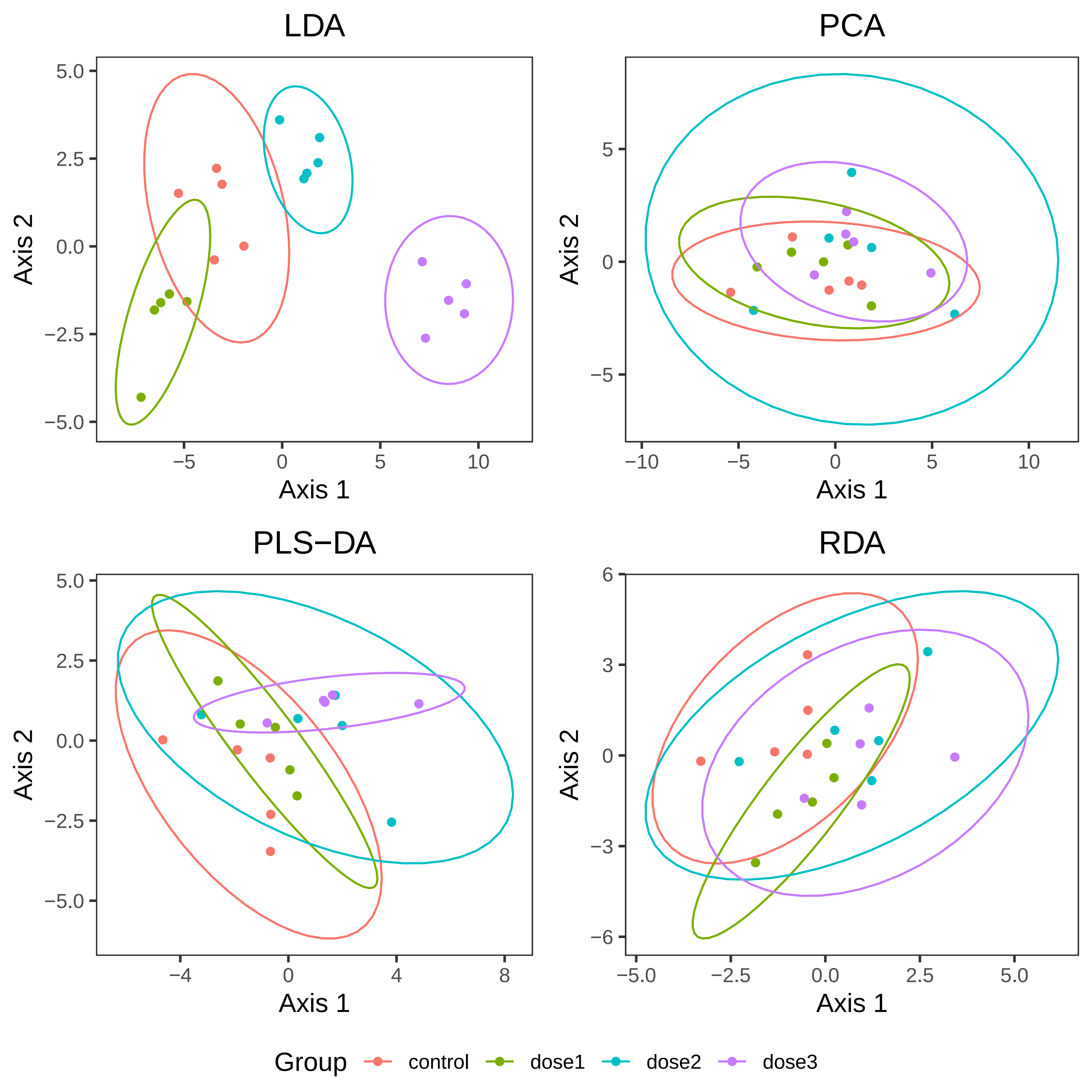

LDA
PCA
PLS−DA
RDA
Axis 1
Axis 2
Group
control
dose1
dose2
dose3

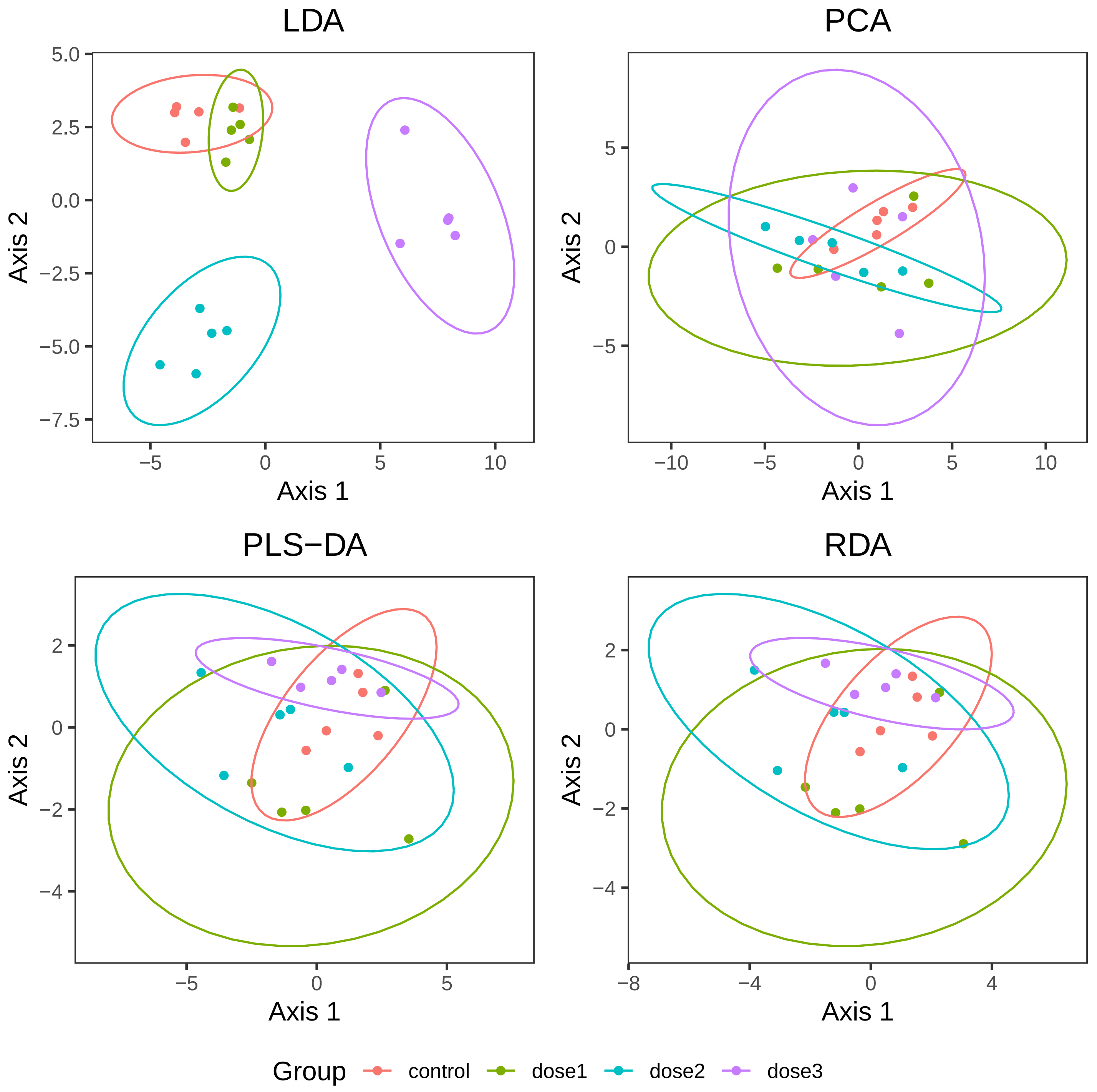
LDA
5.0
2.5
0.0
−2.5
−5.0
−7.5
−5
0
5
10
Axis 1
Axis 2
PCA
5
0
−5
−10
−5
0
5
10
Axis 1
Axis 2
PLS−DA
2
0
−2
−4
−5
0
5
Axis 1
Axis 2
RDA
2
0
−2
−4
−8
−4
0
4
Axis 1
Axis 2
Group
control
dose1
dose2
dose3

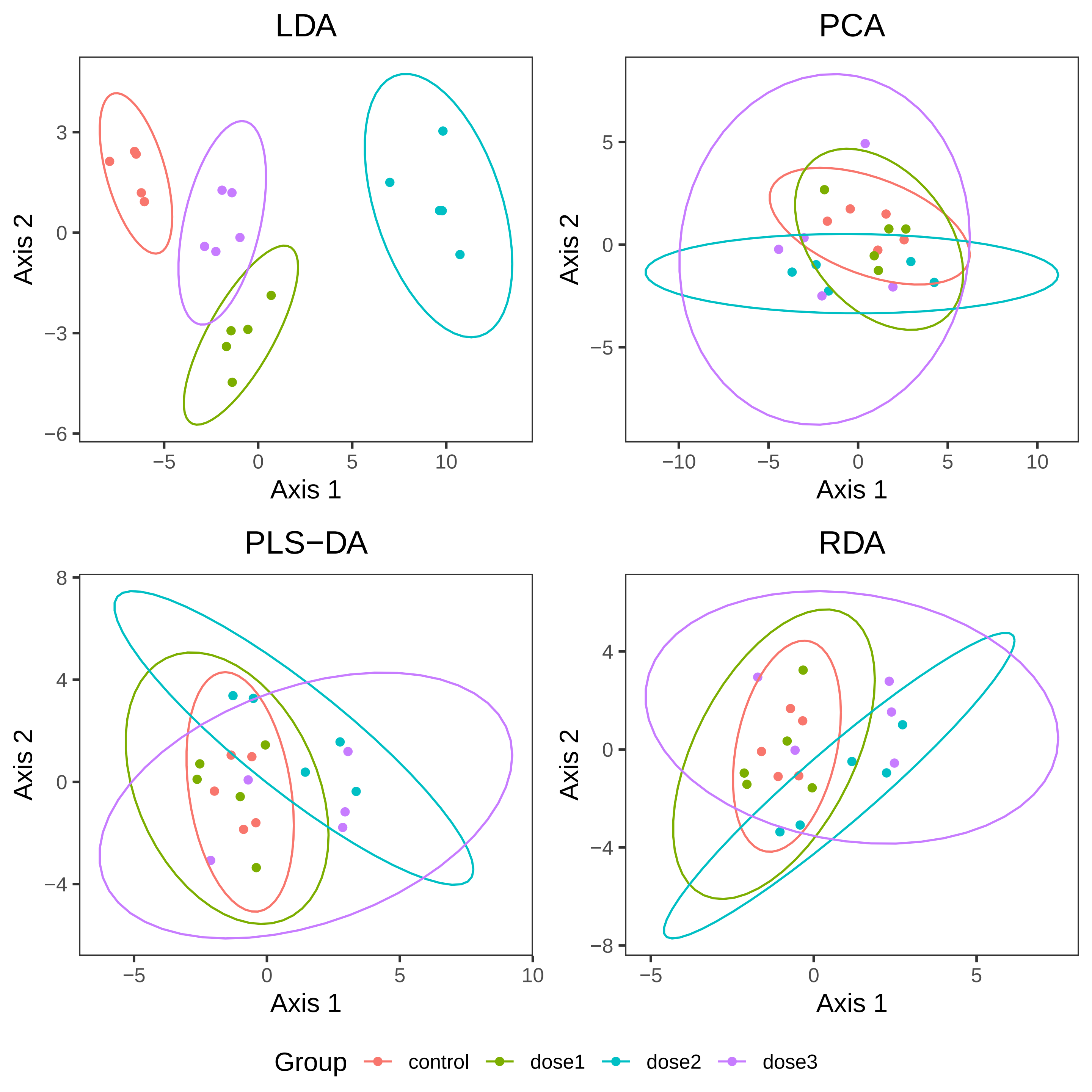

LDA
PCA
PLS−DA
RDA
Axis 1
Axis 2
3
0
−3
−6
−5
0
5
10
−10
−5
0
5
10
8
4
0
−4
−8
Group
control
dose1
dose2
dose3

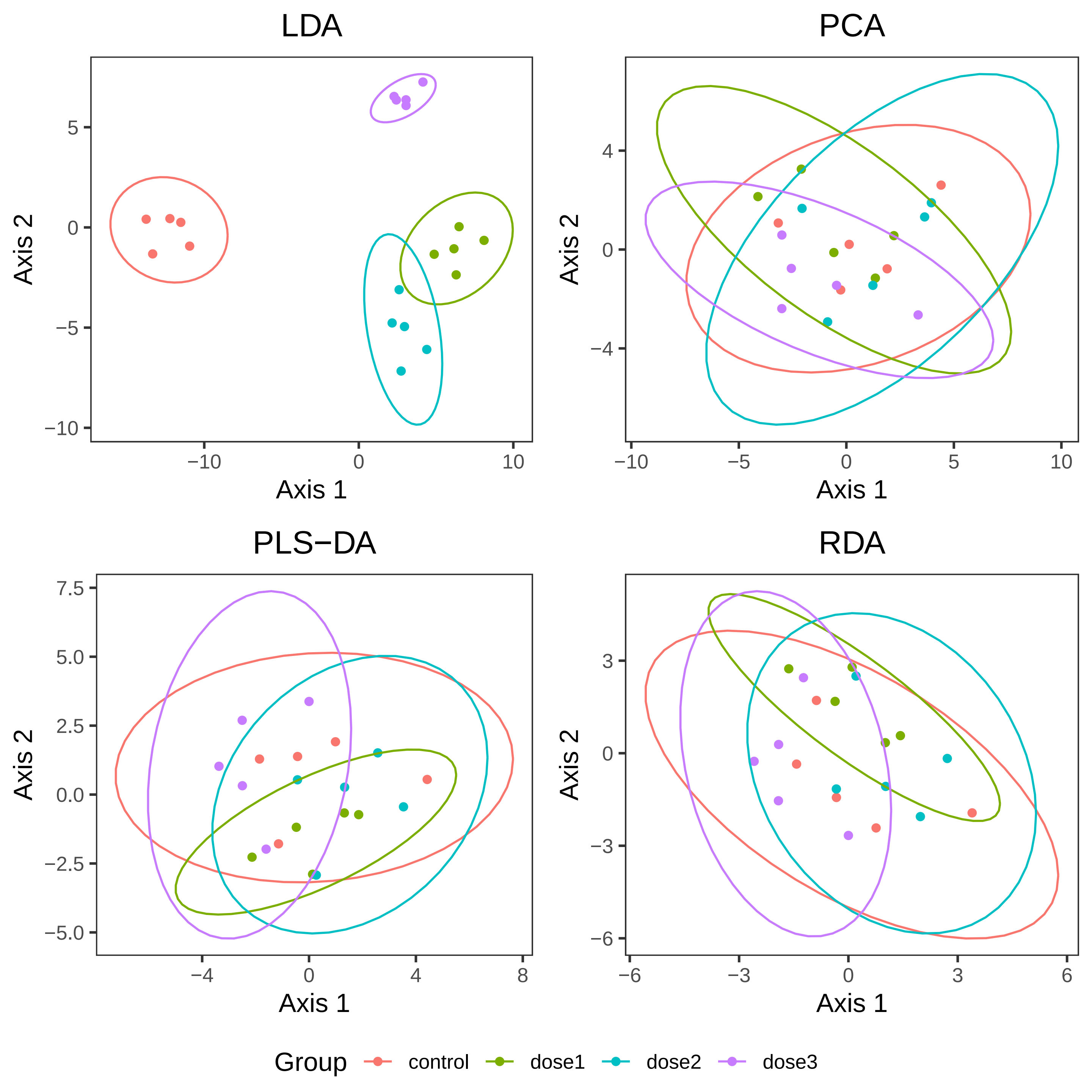

LDA
PCA
PLS−DA
RDA
Axis 1
Axis 2
5
0
−5
−10
−10
0
10
4
0
−4
−10
−5
0
5
10
7.5
5.0
2.5
0.0
−2.5
−5.0
−4
0
4
8
3
0
−3
−6
−6
−3
0
3
6
Group
control
dose1
dose2
dose3

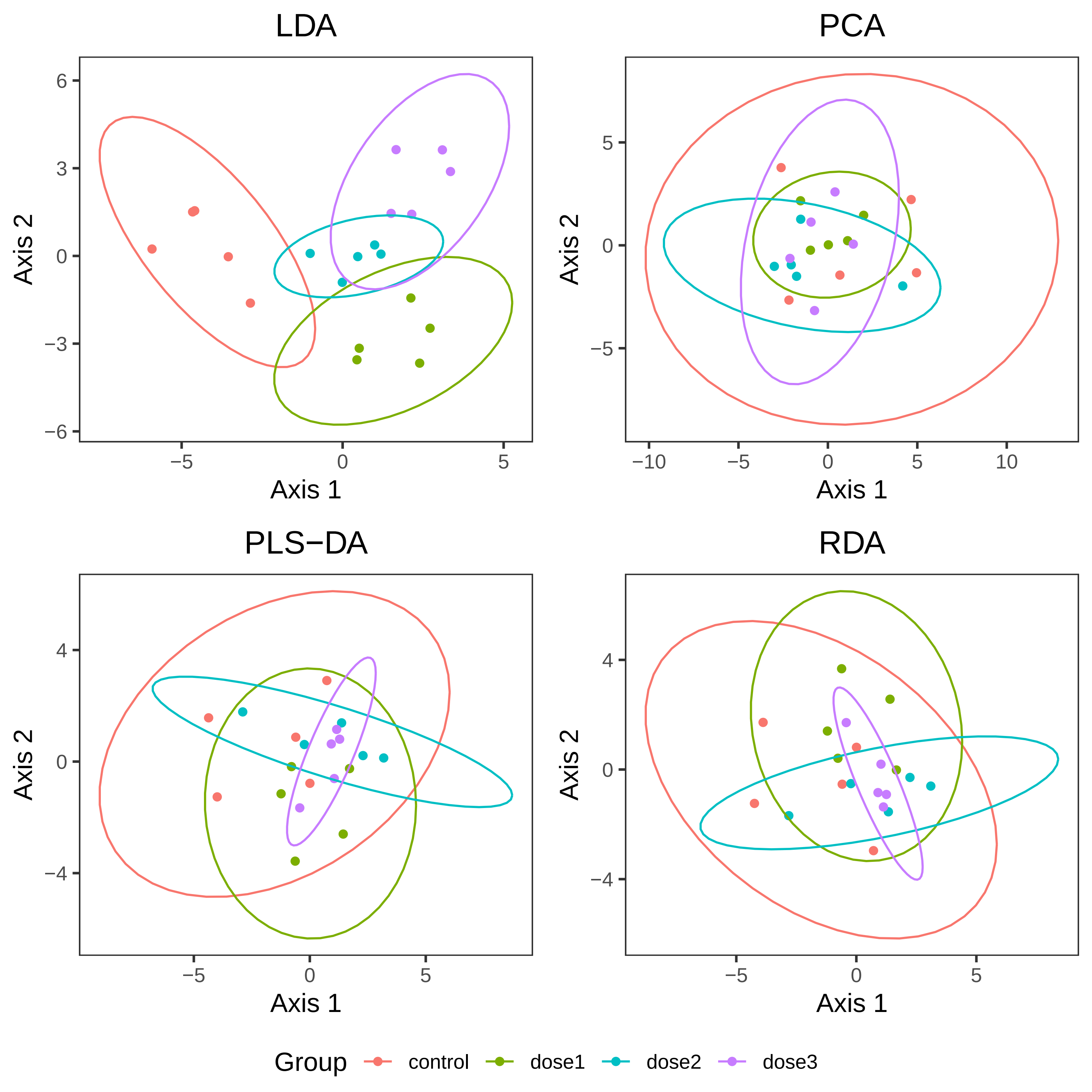

LDA
PCA
PLS−DA
RDA
Axis 1
Axis 2
6
3
0
−3
−6
−5
5
−10
10
4
−4
Group
control
dose1
dose2
dose3

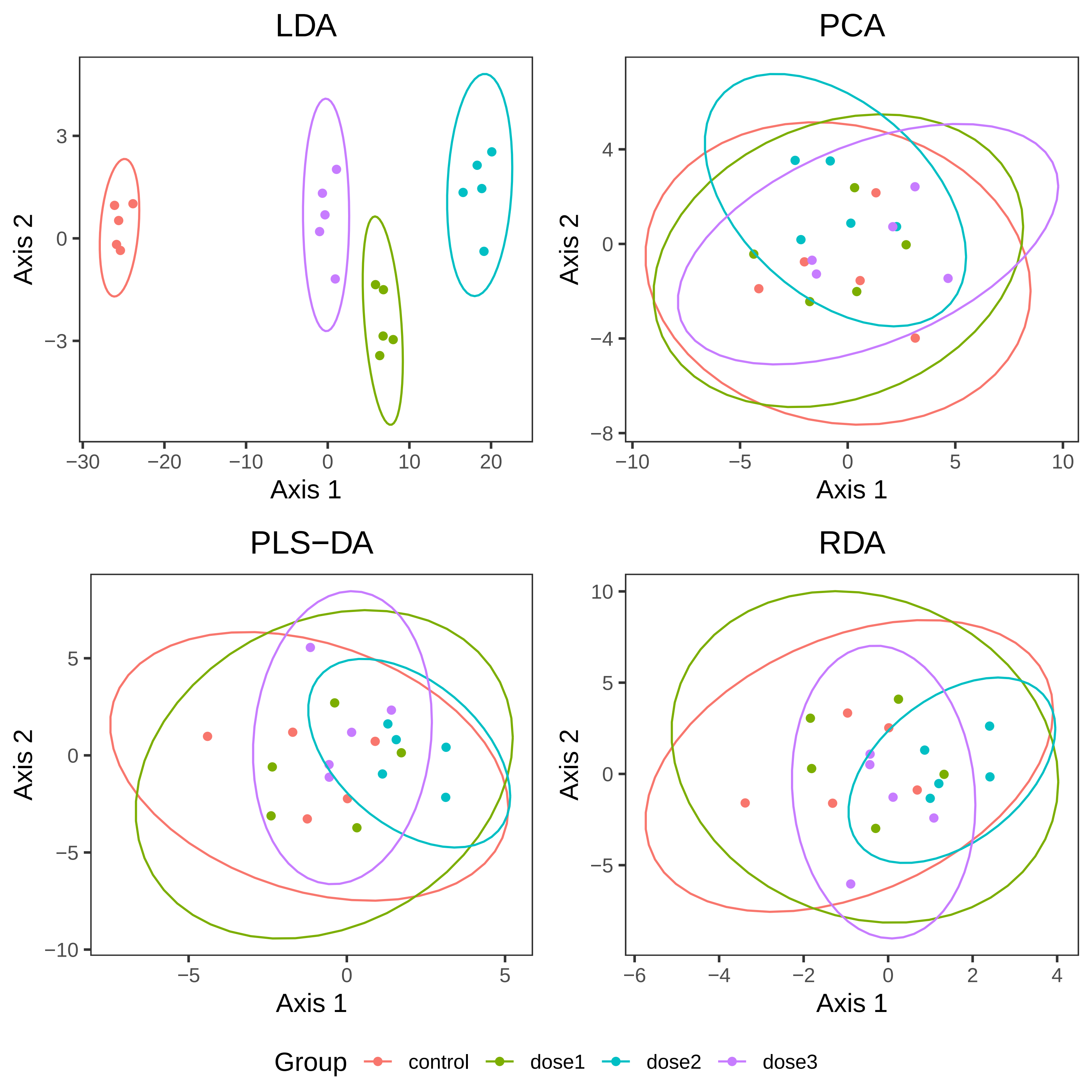
LDA
PCA
PLS−DA
RDA
Axis 1
Axis 2
Group
control
dose1
dose2
dose3

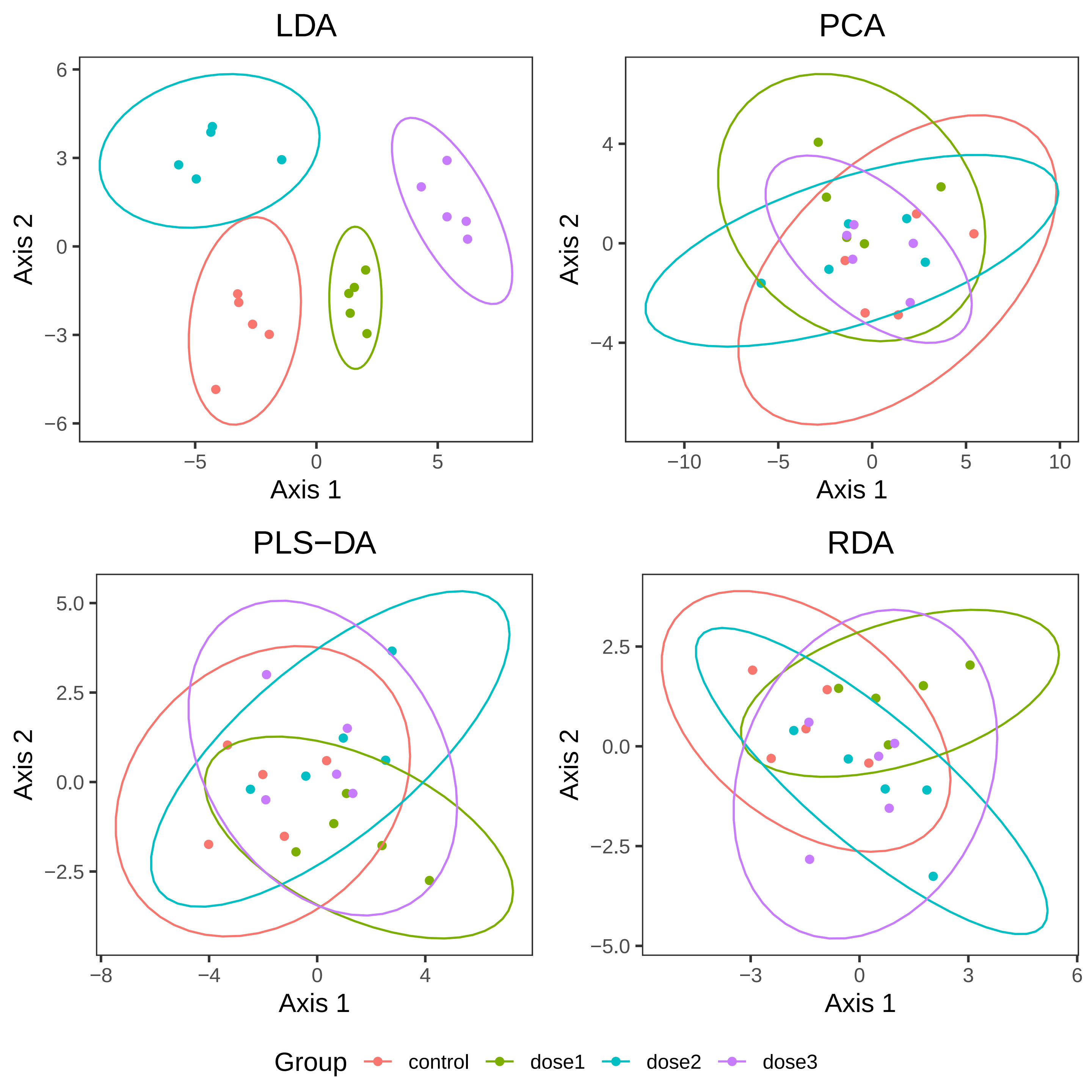

LDA
PCA
PLS−DA
RDA
Axis 1
Axis 2
Group
control
dose1
dose2
dose3

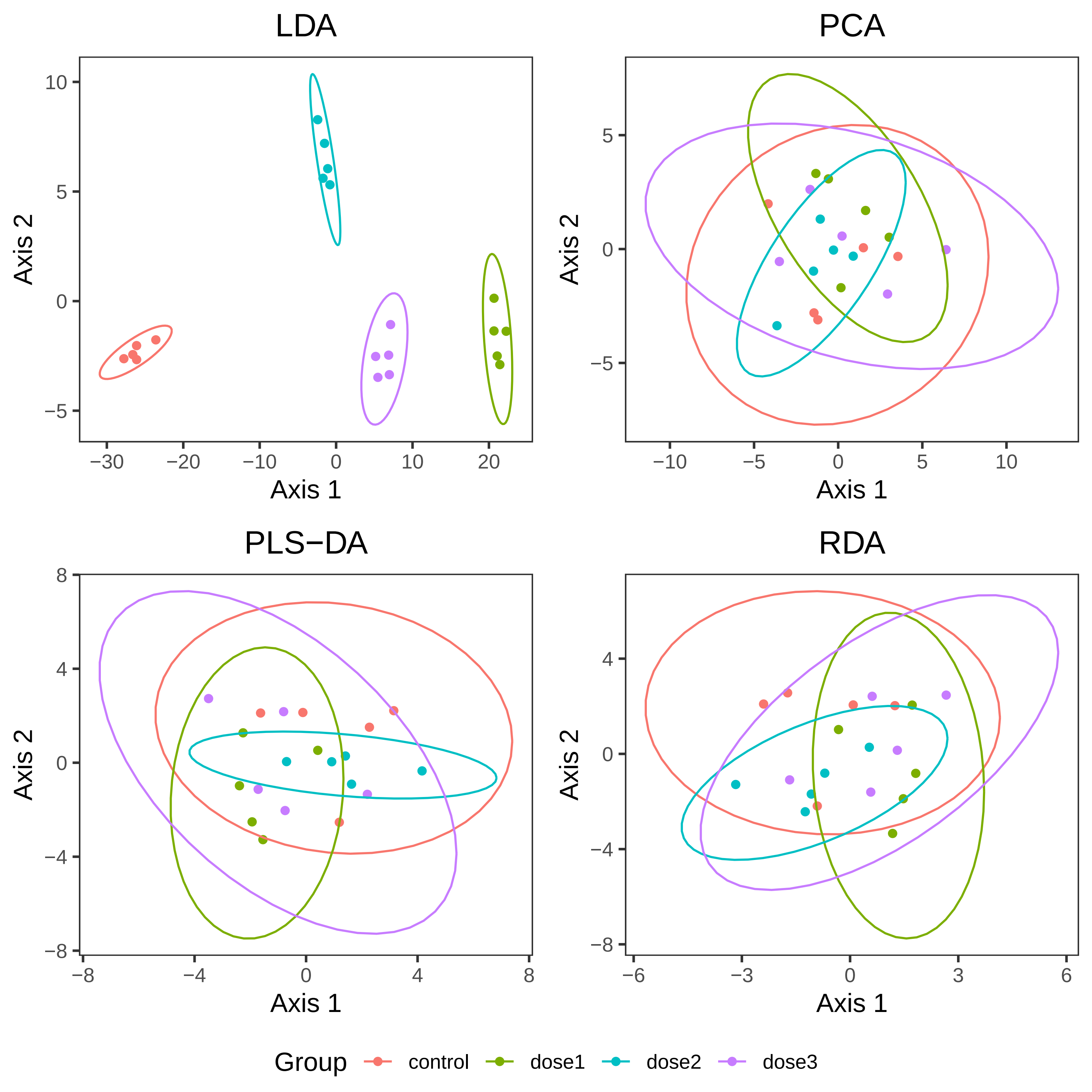
LDA
PCA
PLS−DA
RDA
Axis 1
Axis 2
Group
control
dose1
dose2
dose3

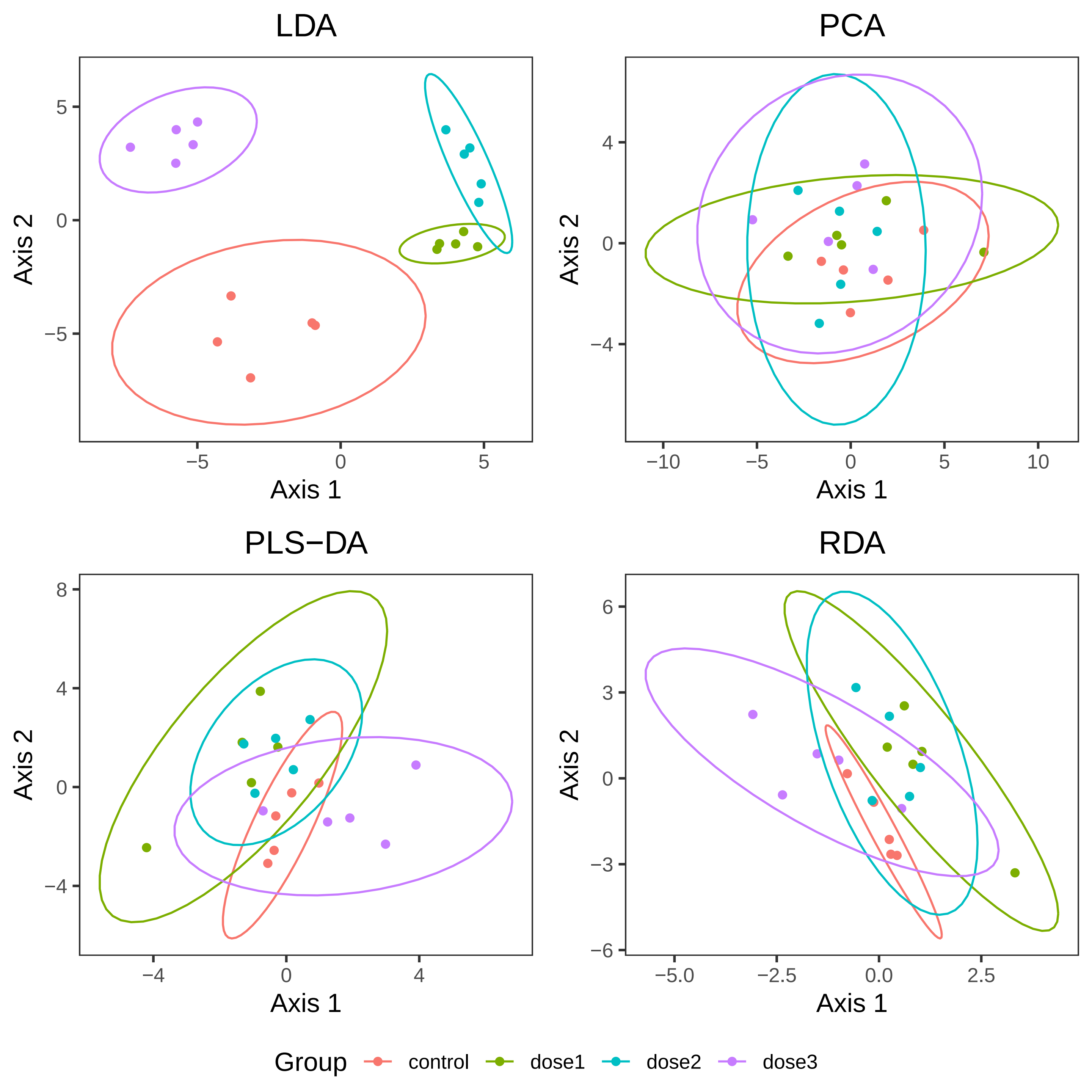

LDA
PCA
PLS−DA
RDA
Axis 1
Axis 2
Group
control
dose1
dose2
dose3

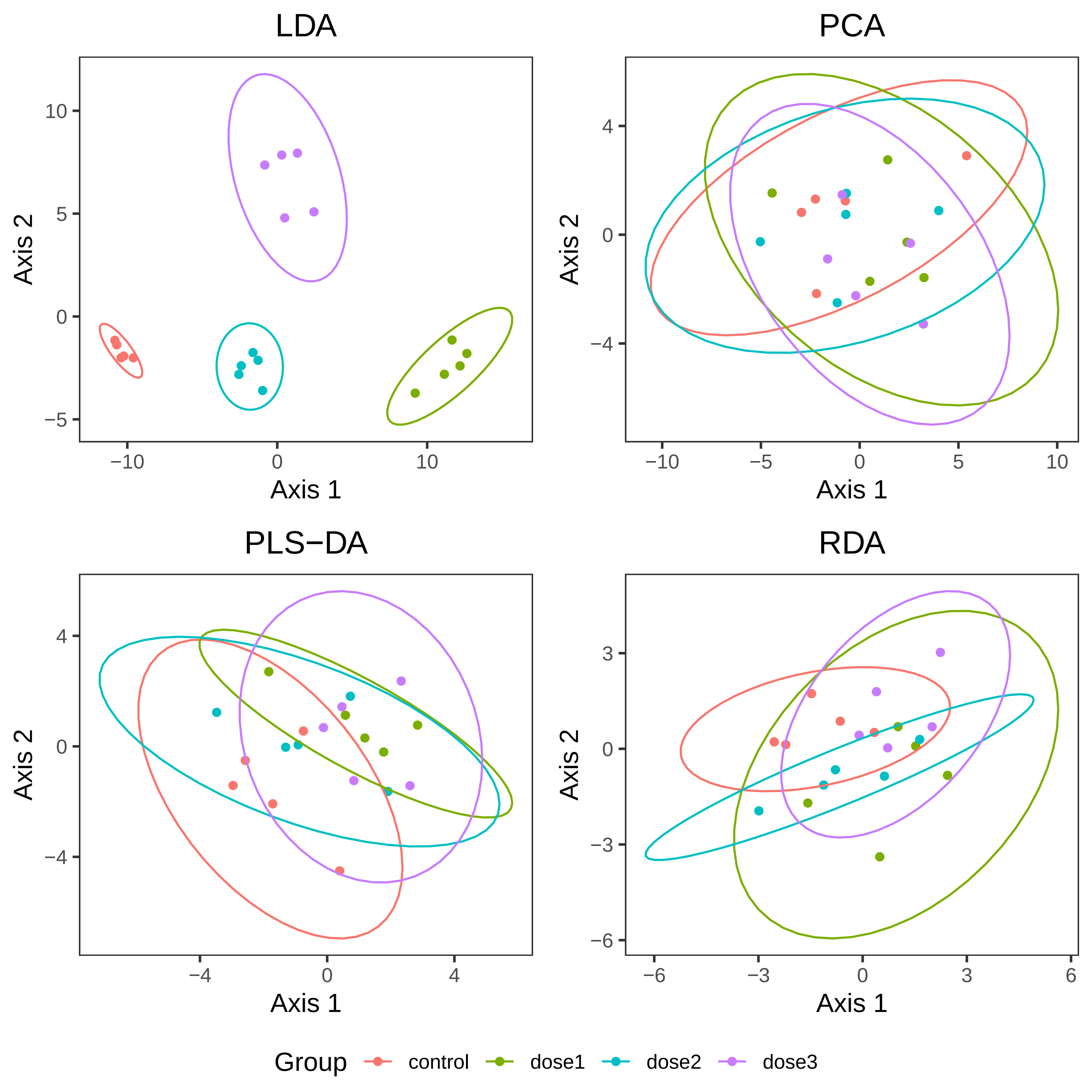
LDA
PCA
PLS−DA
RDA
Axis 1
Axis 2
Group
control
dose1
dose2
dose3

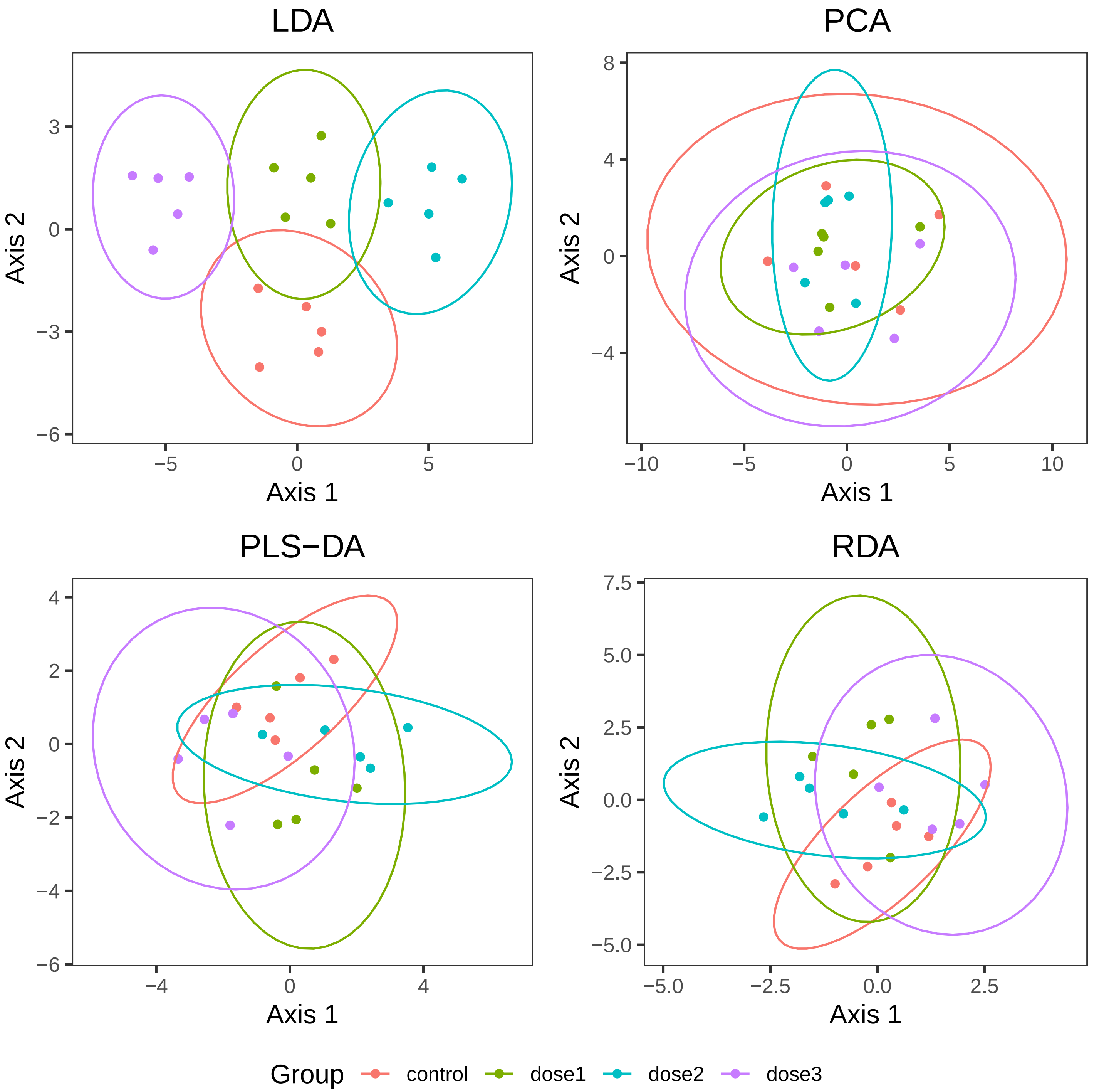
LDA
PCA
PLS−DA
RDA
Axis 1
Axis 2
Group
control
dose1
dose2
dose3

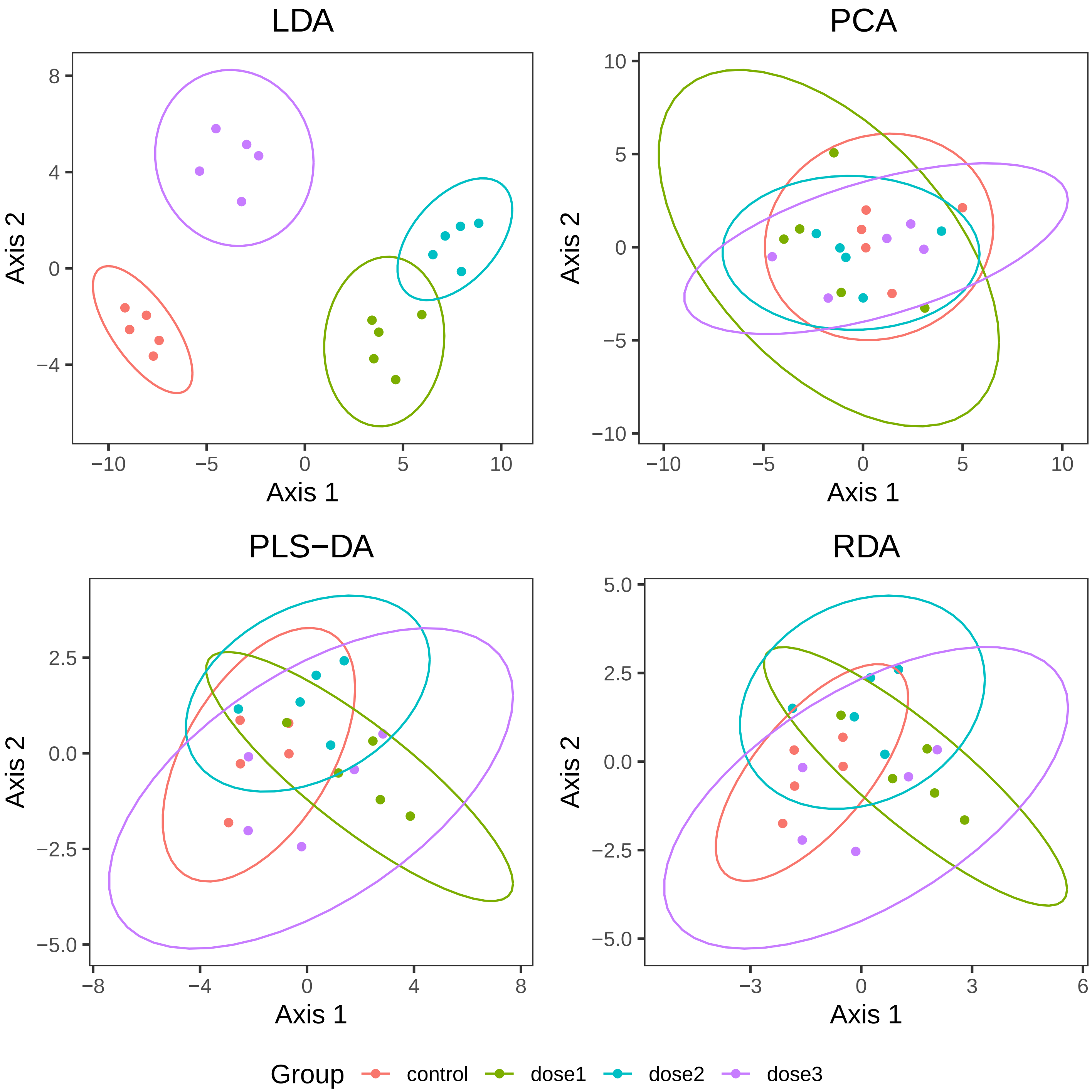
LDA
PCA
PLS−DA
RDA
Axis 1
Axis 2
Group
control
dose1
dose2
dose3

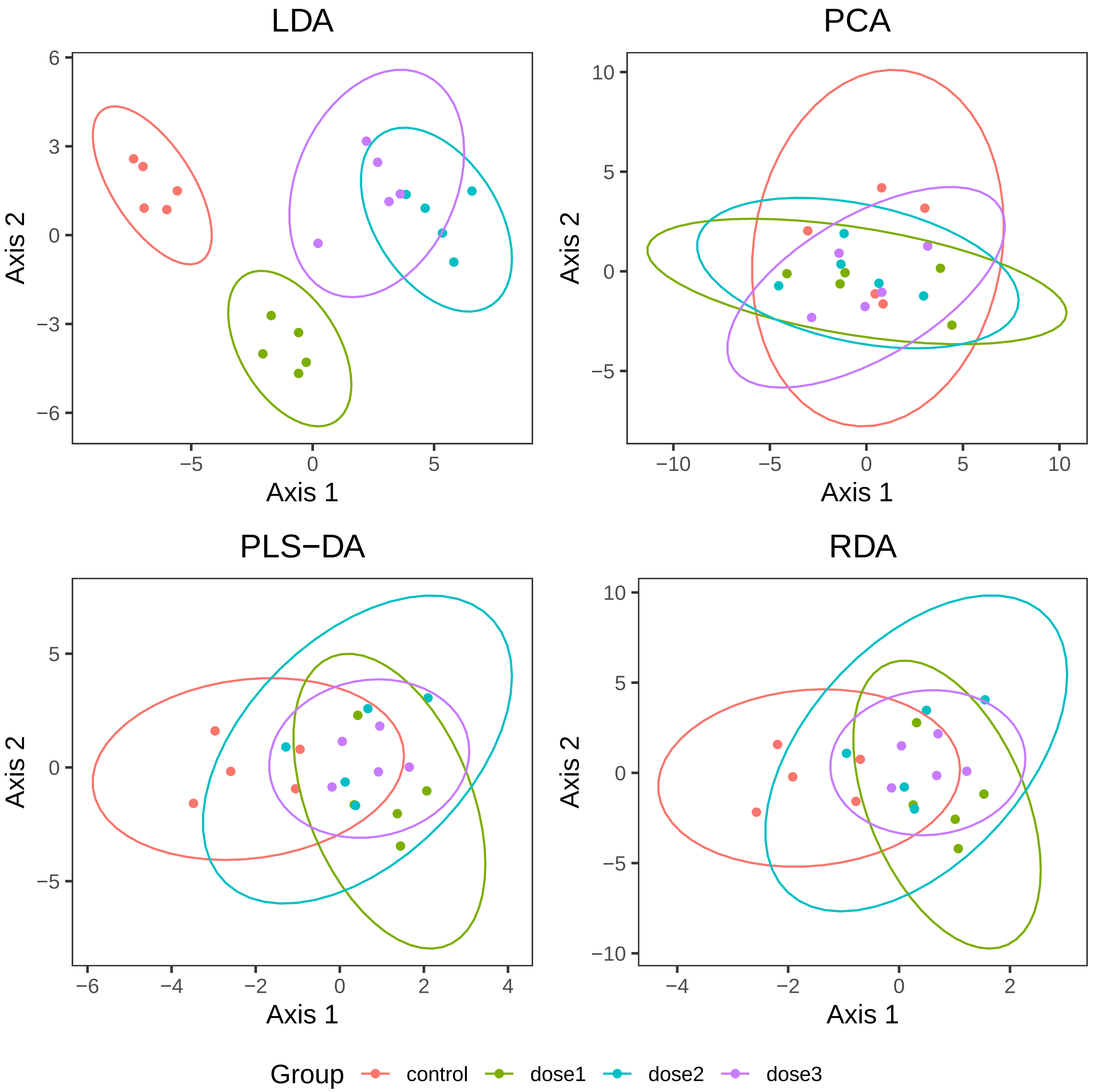

LDA
PCA
PLS−DA
RDA
Axis 1
Axis 2
Group
control
dose1
dose2
dose3

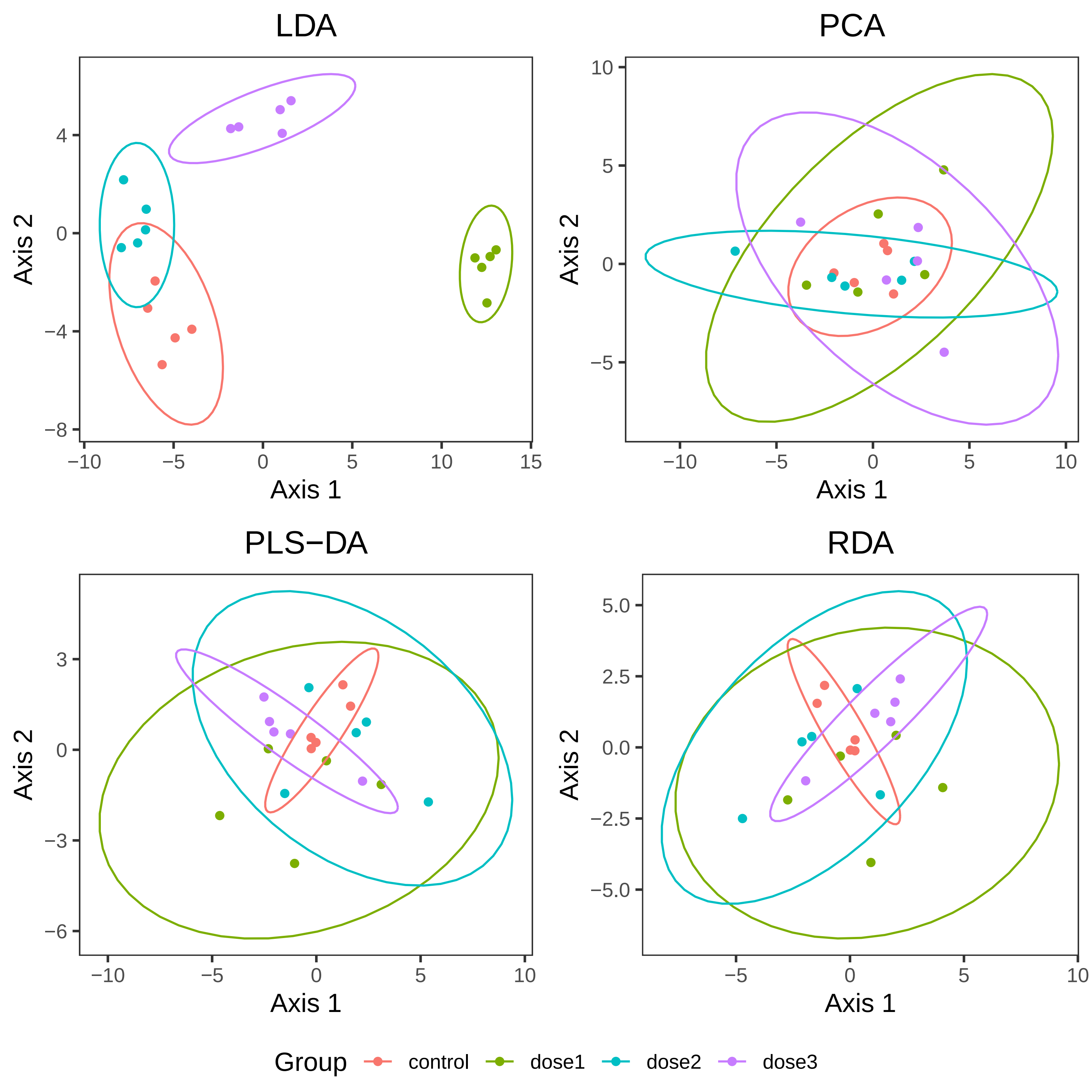
LDA
PCA
PLS−DA
RDA
Axis 1
Axis 2
Group
control
dose1
dose2
dose3

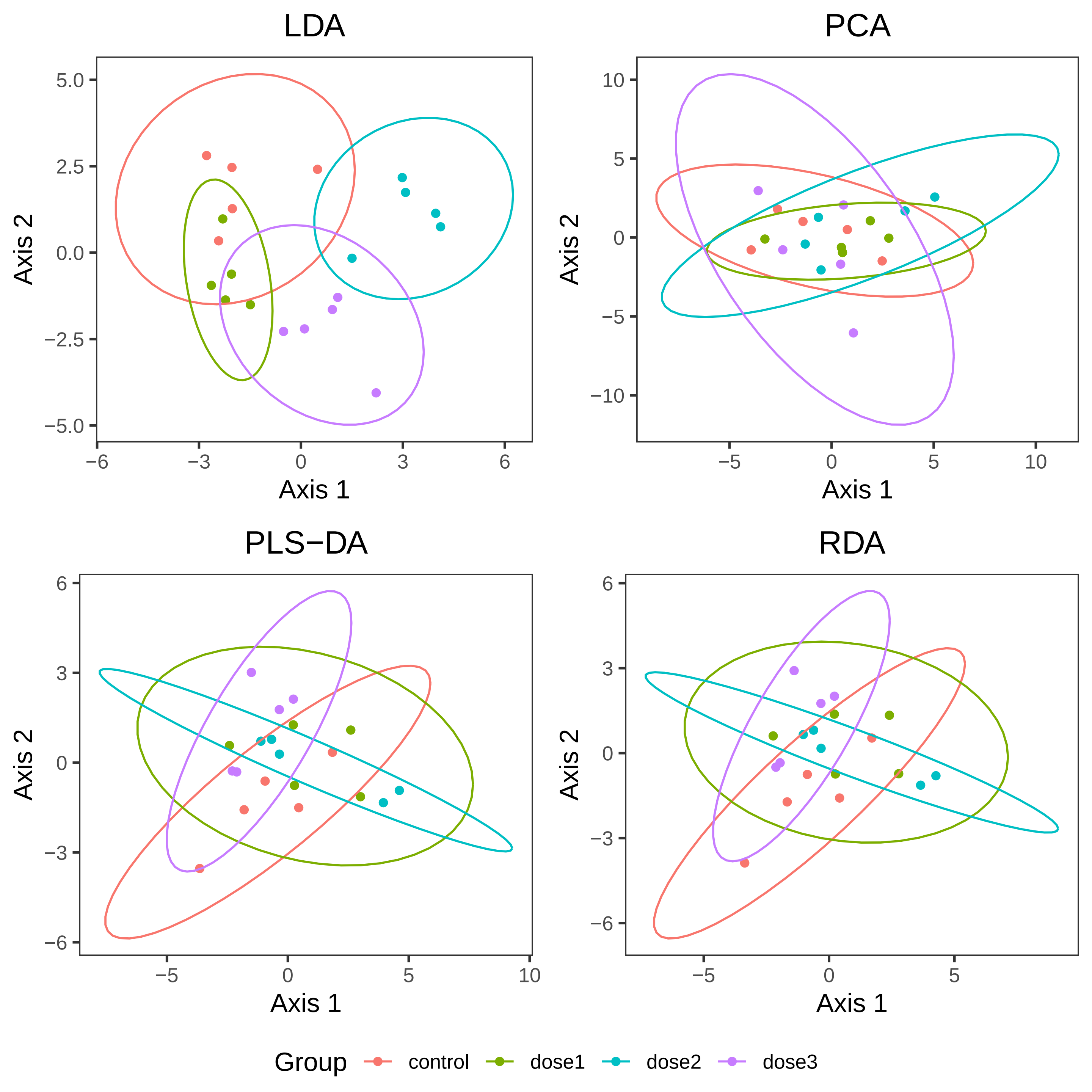

LDA
PCA
PLS−DA
RDA
Axis 1
Axis 2
5.0
2.5
0.0
−2.5
−5.0
−6
−3
0
3
6
10
5
−5
−10
Group
control
dose1
dose2
dose3

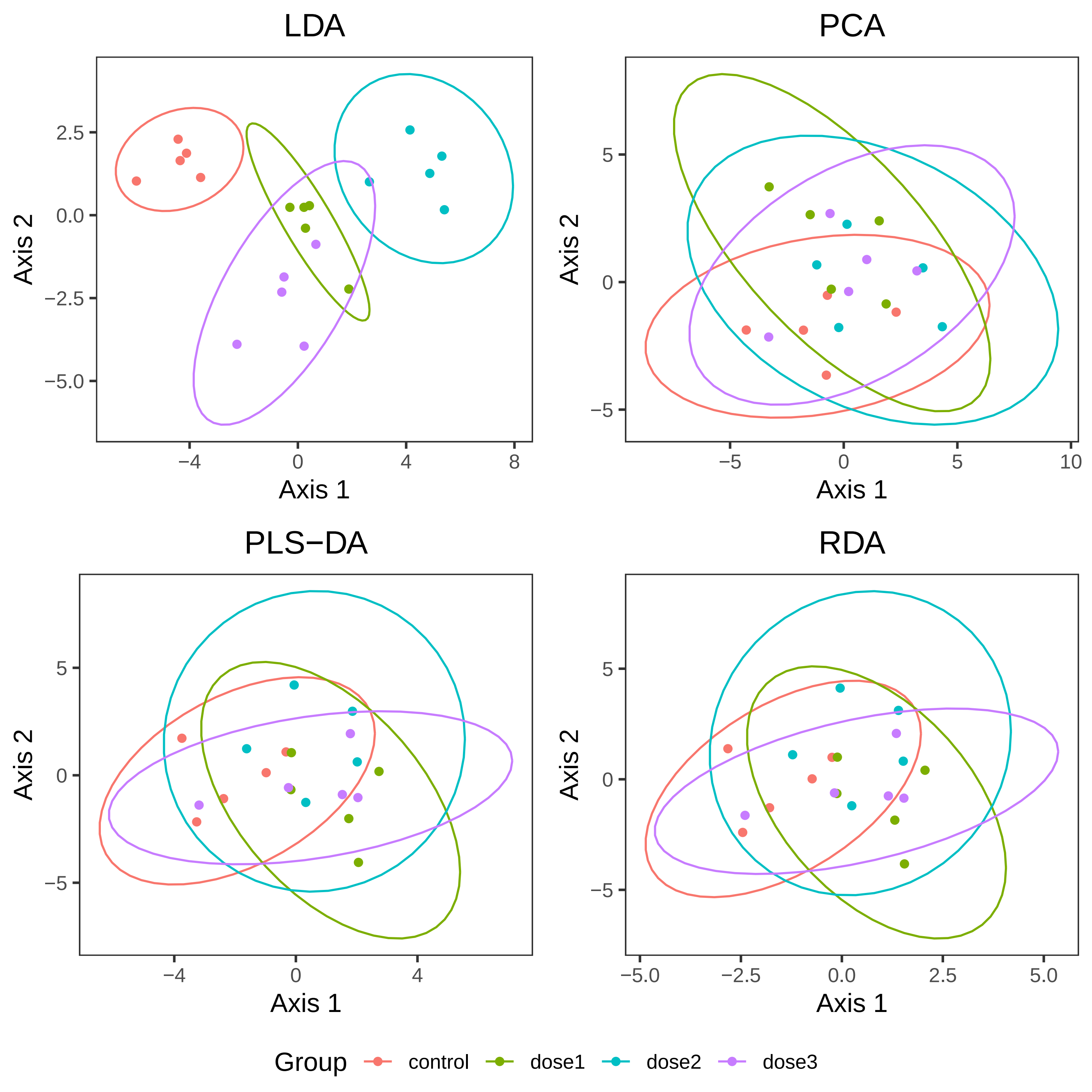
LDA
PCA
PLS−DA
RDA
Axis 1
Axis 2
2.5
0.0
−2.5
−5.0
−4
0
4
8
5
0
−5
10
−5.0
−2.5
0.0
2.5
5.0
Group
control
dose1
dose2
dose3

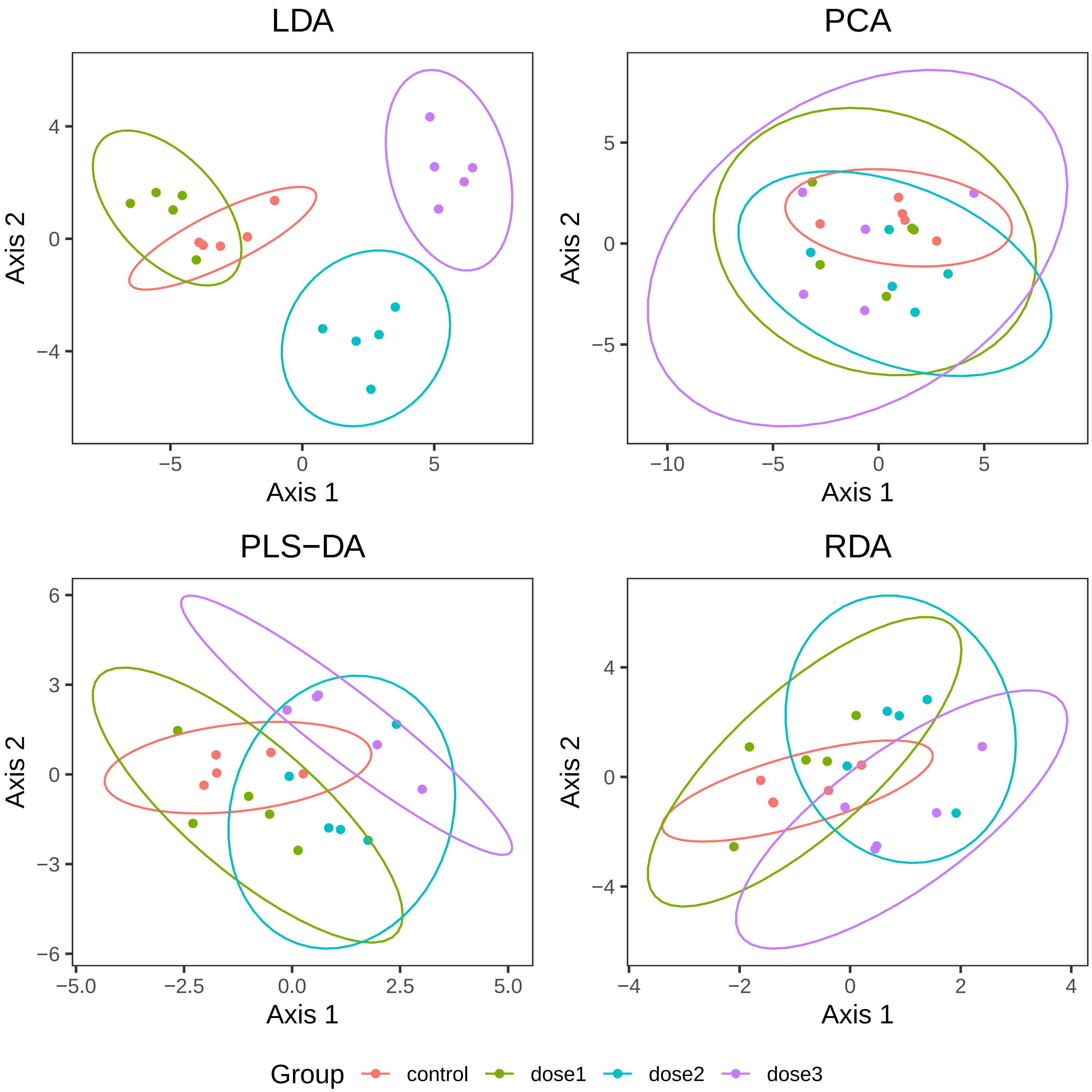

LDA
PCA
PLS−DA
RDA
Axis 1
Axis 2
Group
control
dose1
dose2
dose3

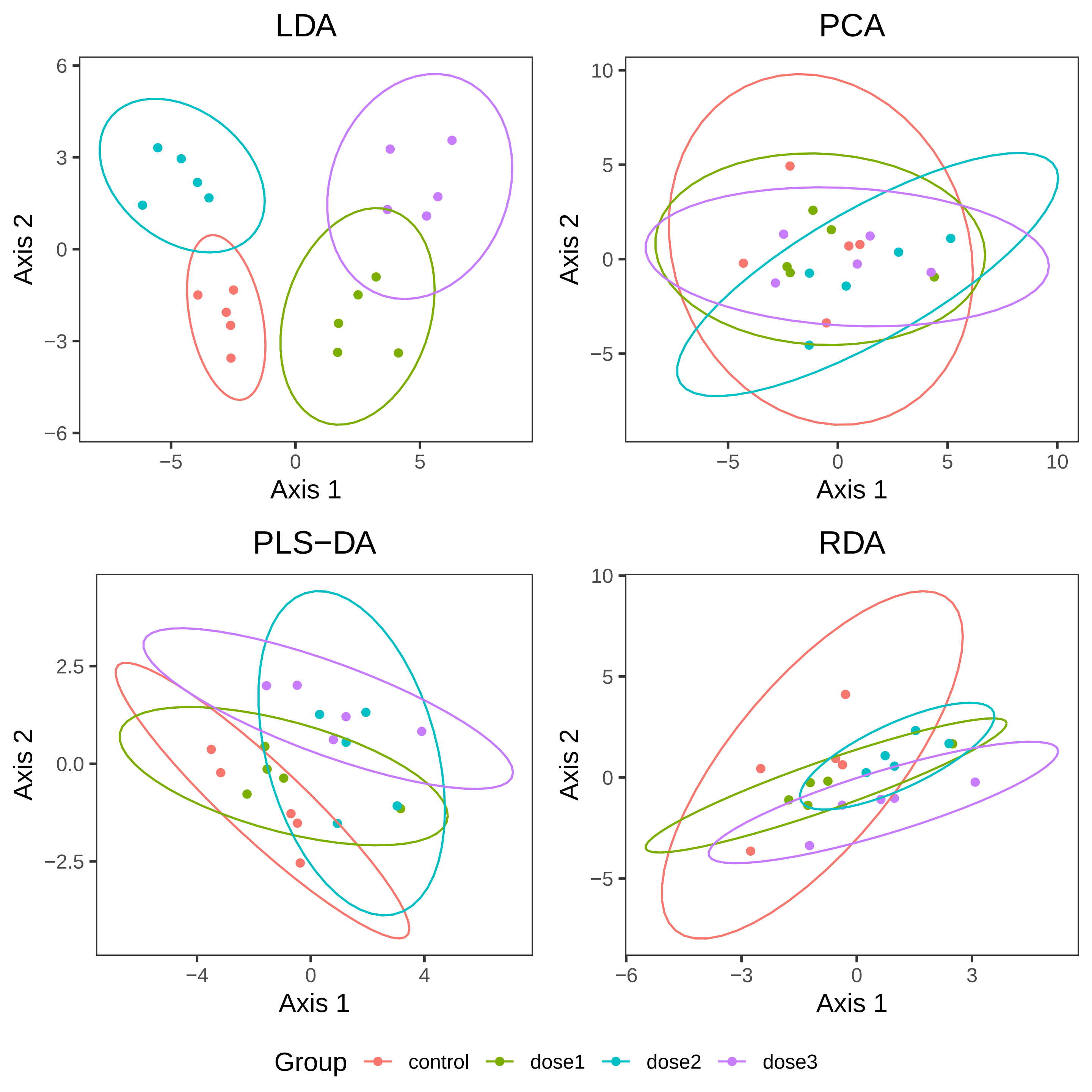

LDA
PCA
PLS−DA
RDA
Axis 1
Axis 2
6
3
0
−3
−6
−5
0
5
10
−4
4
2.5
0.0
−2.5
−6
Group
control
dose1
dose2
dose3

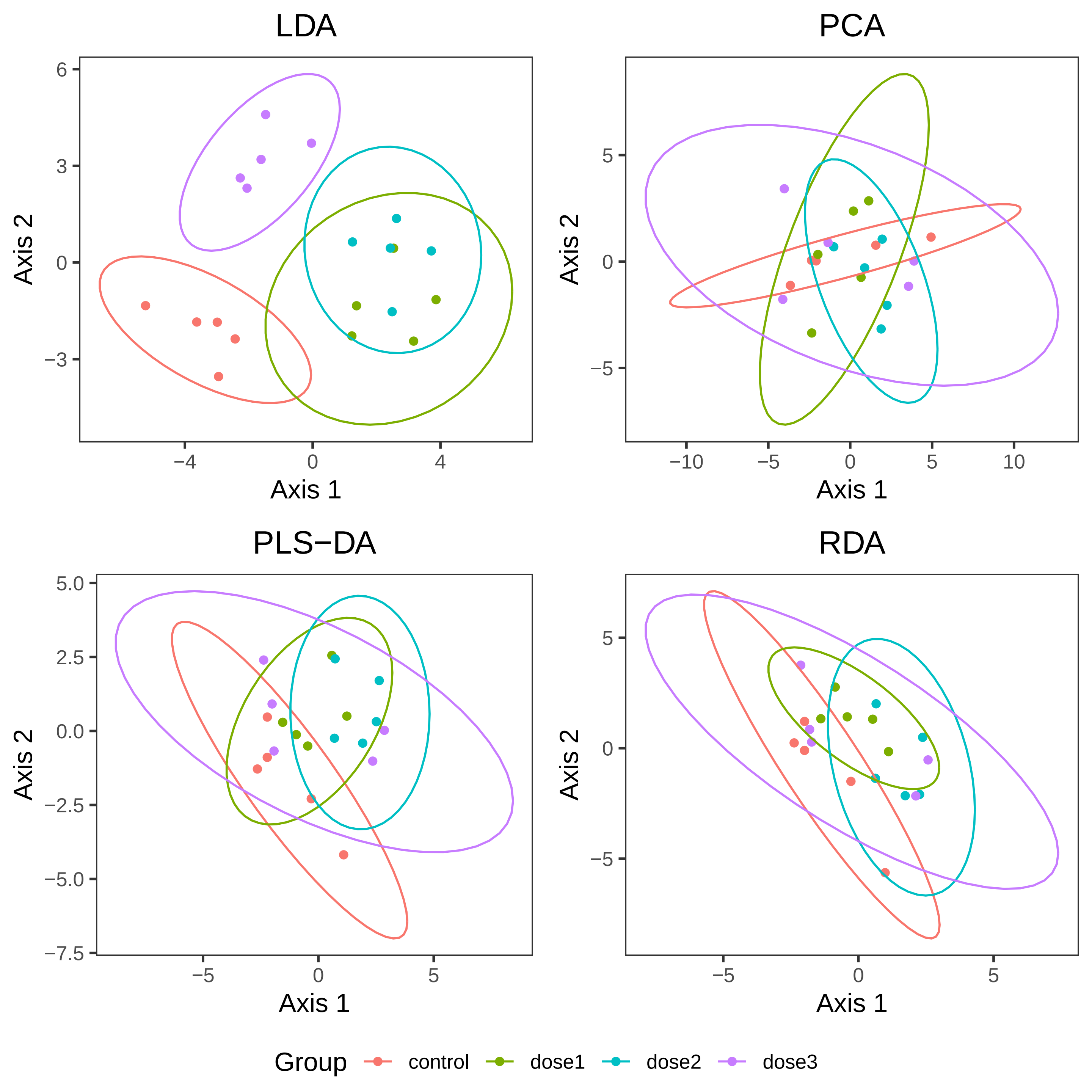
LDA
PCA
PLS−DA
RDA
Axis 1
Axis 2
Group
control
dose1
dose2
dose3

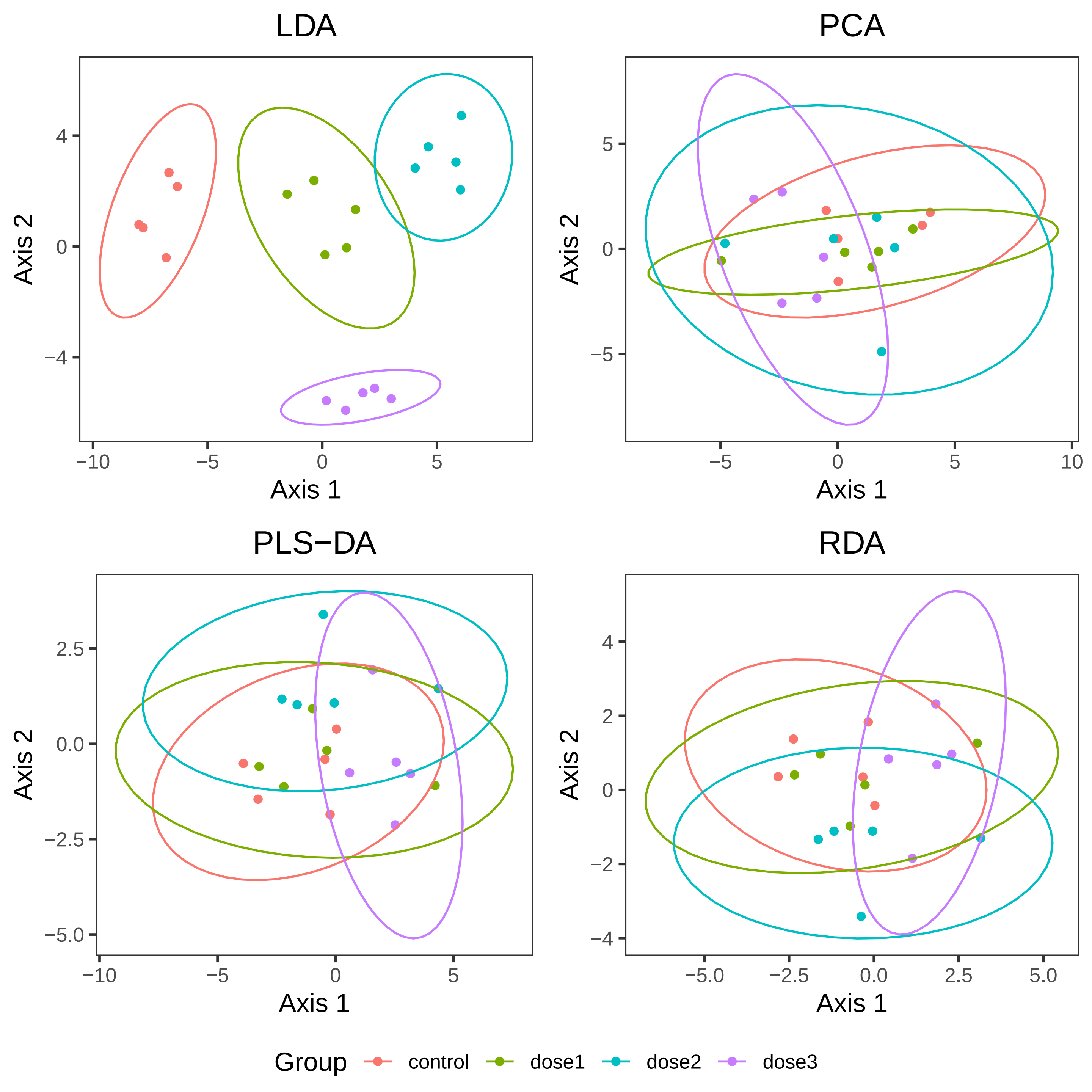
LDA
PCA
PLS−DA
RDA
Axis 1
Axis 2
4
0
−4
−10
−5
0
5
10
−5
2.5
0.0
−2.5
−5.0
−5.0
−2.5
0.0
2.5
5.0
2
−2
Group
control
dose1
dose2
dose3

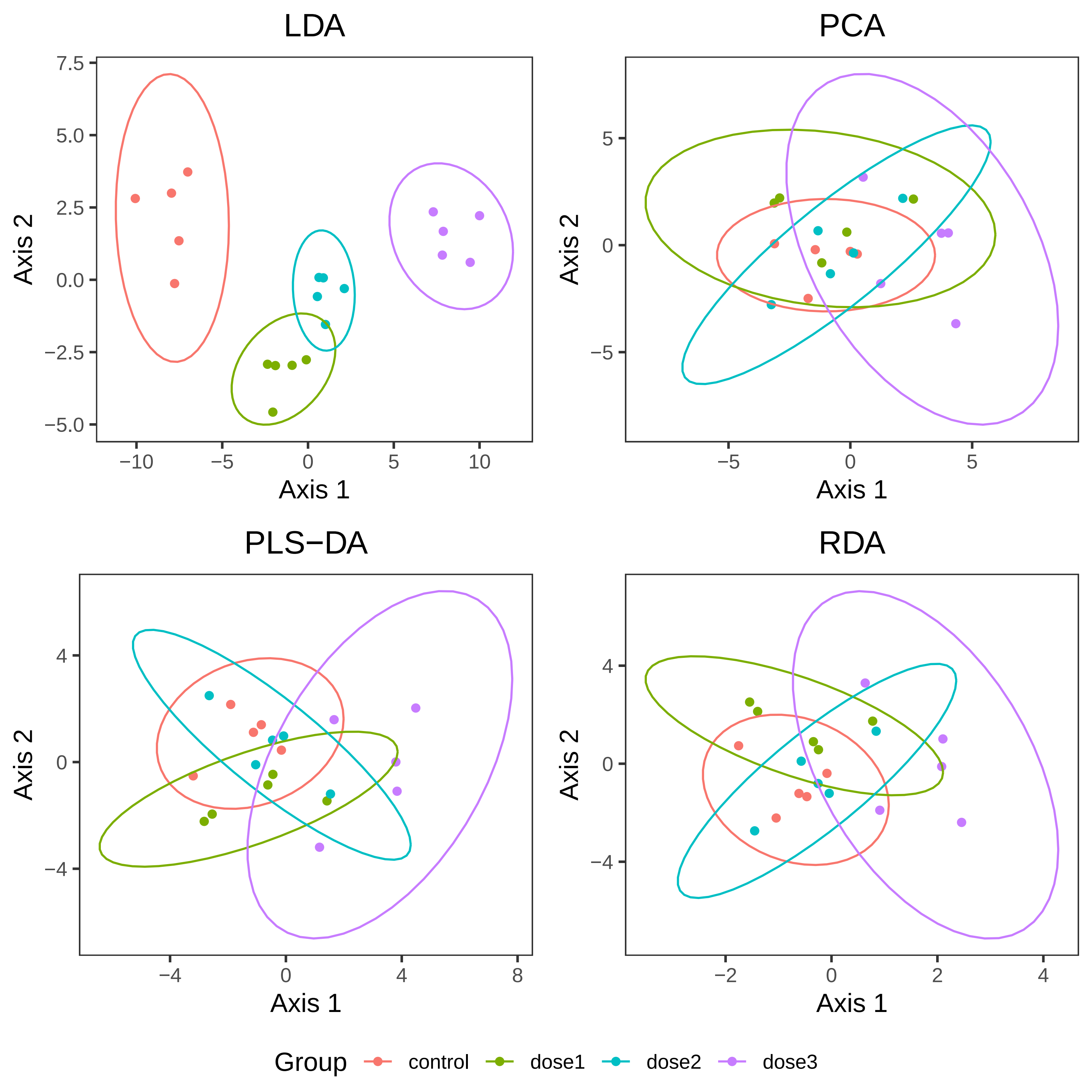

LDA
PCA
PLS−DA
RDA
Axis 1
Axis 2
Group
control
dose1
dose2
dose3

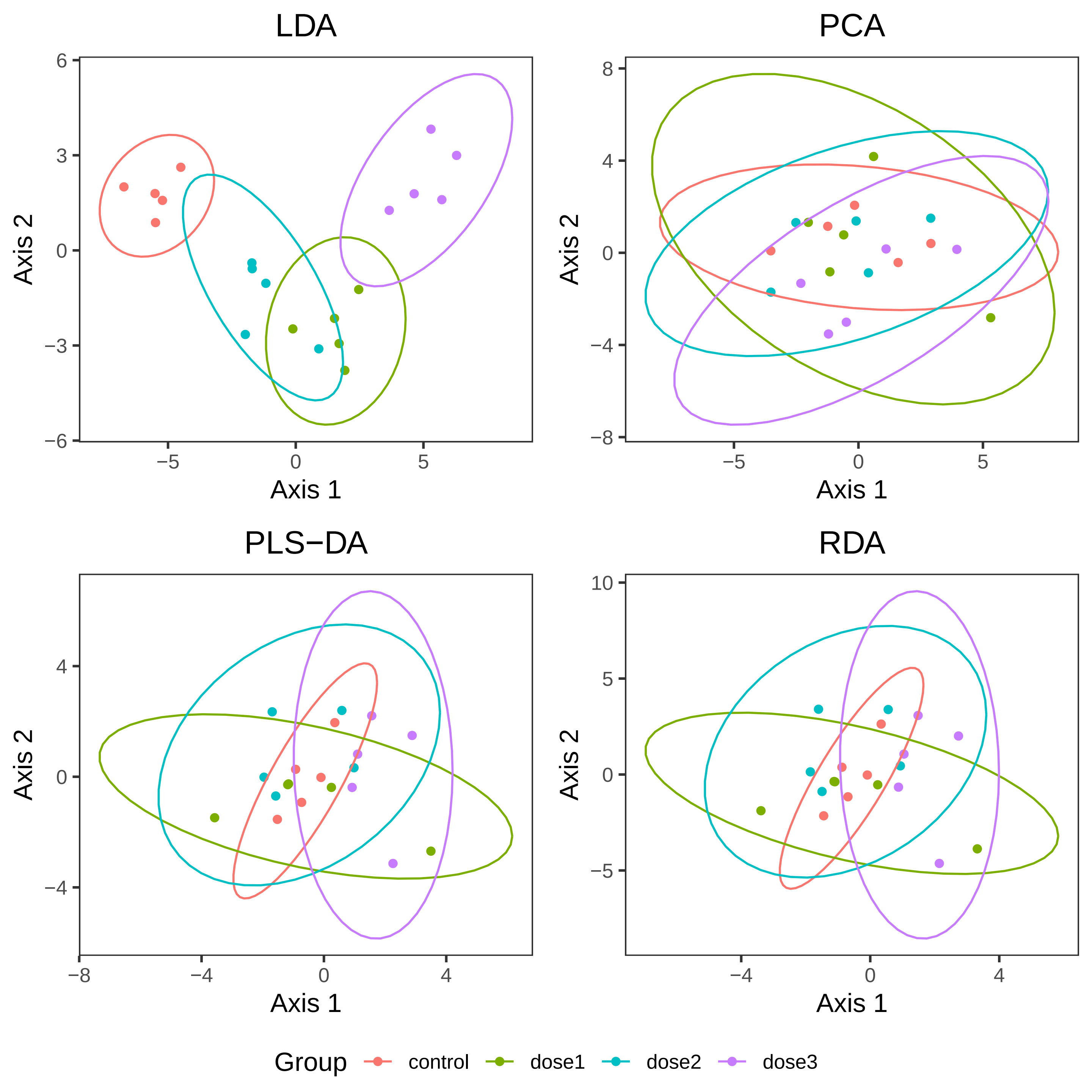
LDA
PCA
PLS−DA
RDA
Axis 1
Axis 2
6
3
0
−3
−6
−5
0
5
8
4
0
−4
−8
4
0
−4
−8
−4
0
4
10
5
0
−5
−4
0
4
Group
control
dose1
dose2
dose3

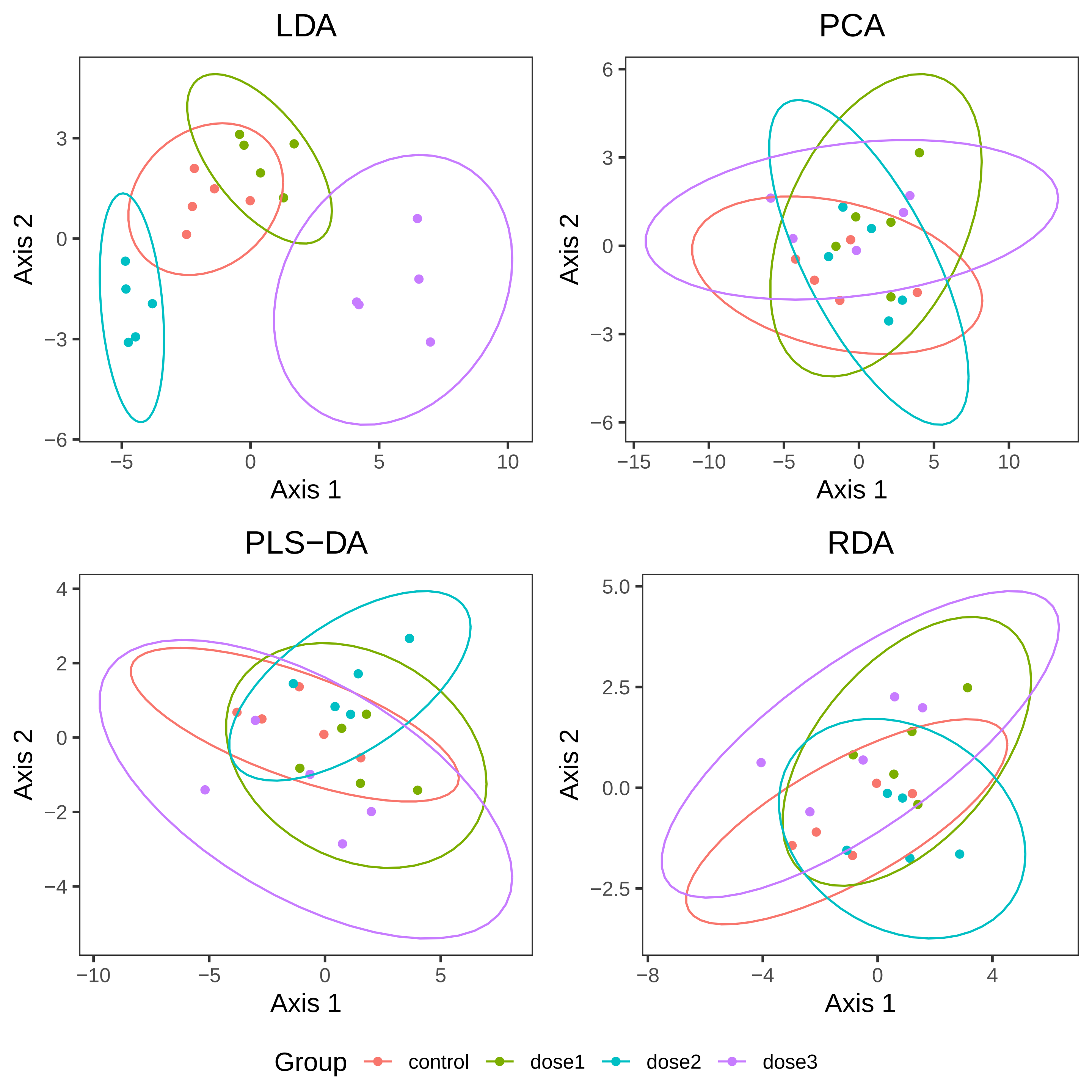
LDA
PCA
PLS−DA
RDA
Axis 1
Axis 2
Group
control
dose1
dose2
dose3

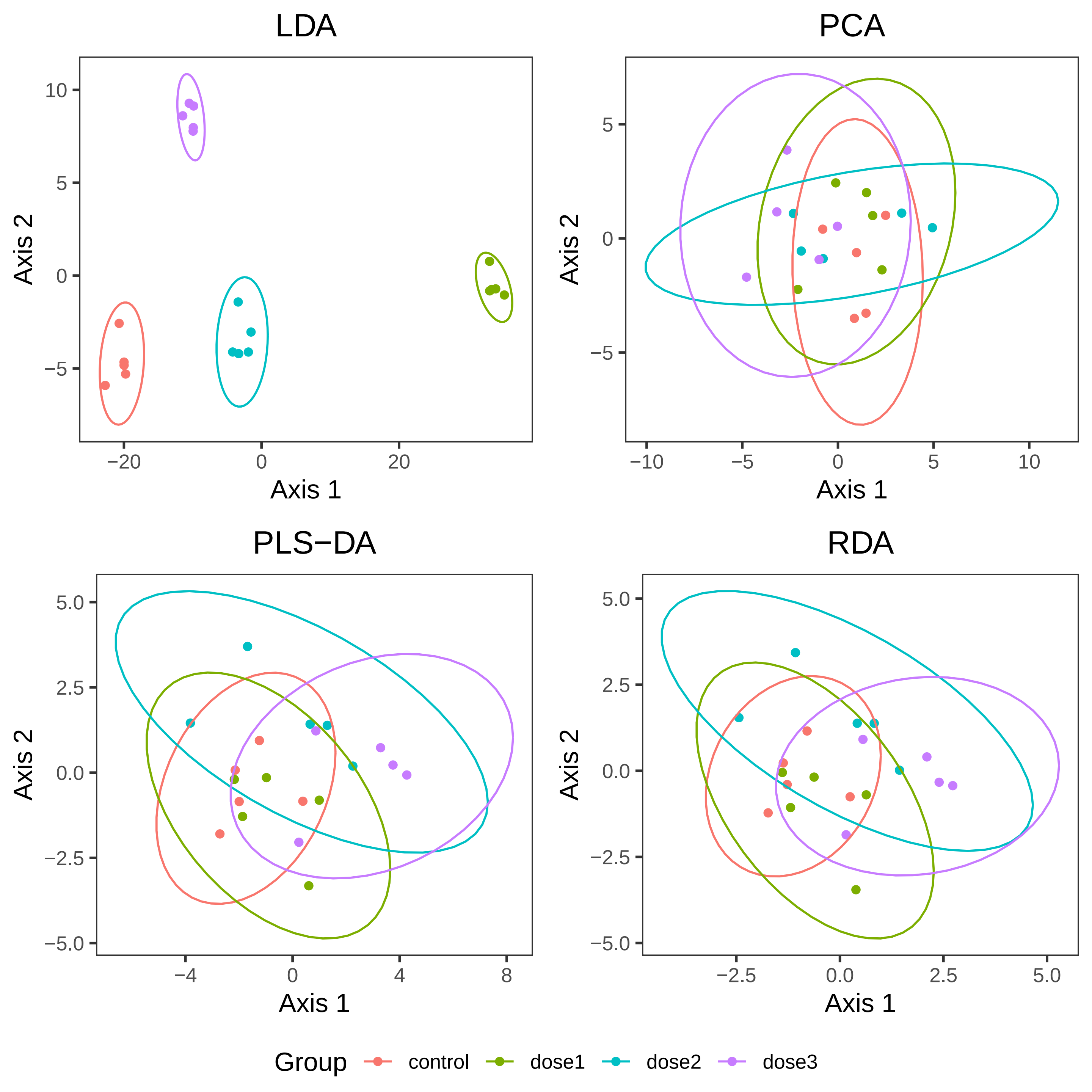
LDA
PCA
PLS−DA
RDA
Axis 1
Axis 2
Group
control
dose1
dose2
dose3

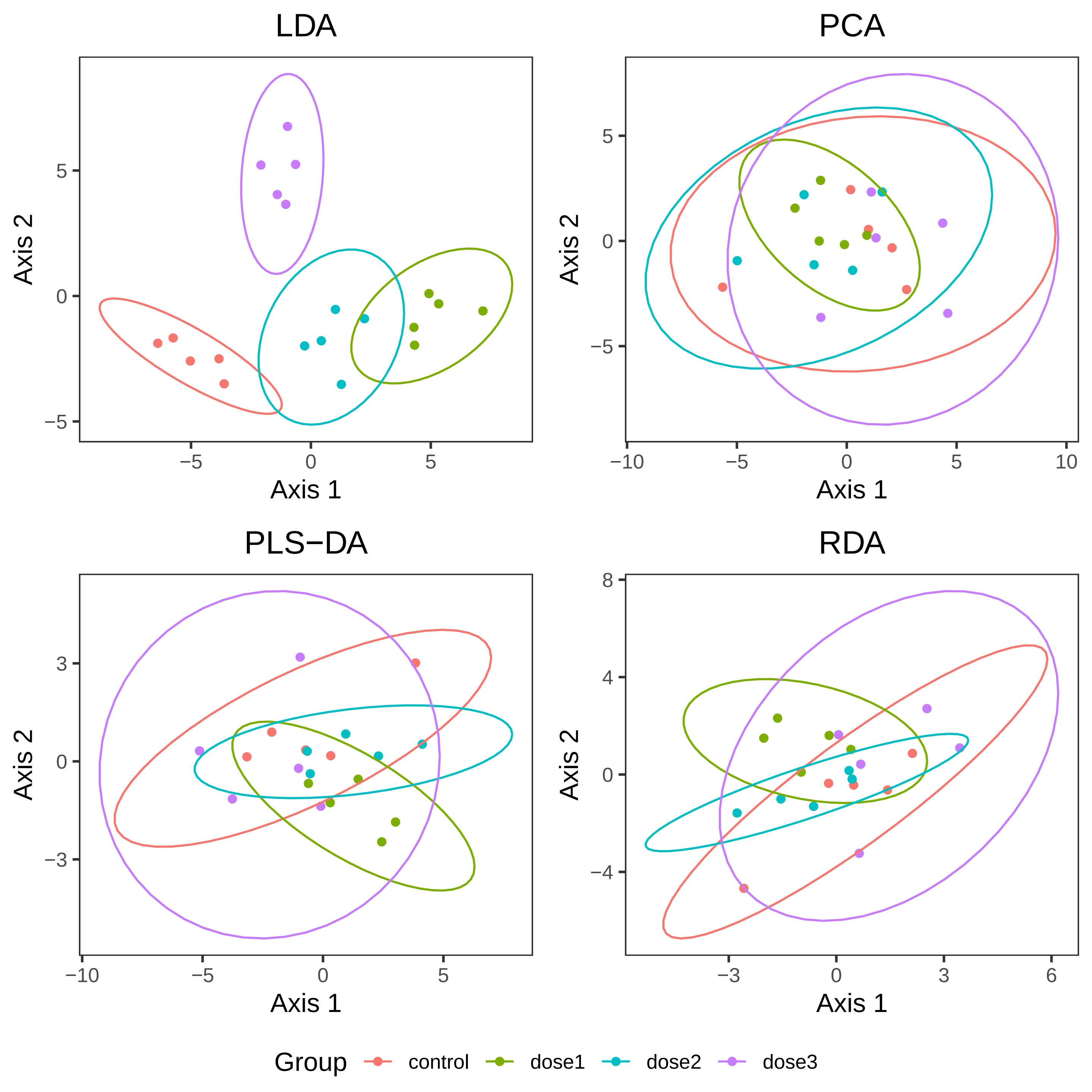

LDA
PCA
PLS−DA
RDA
Axis 1
Axis 2
Group
control
dose1
dose2
dose3

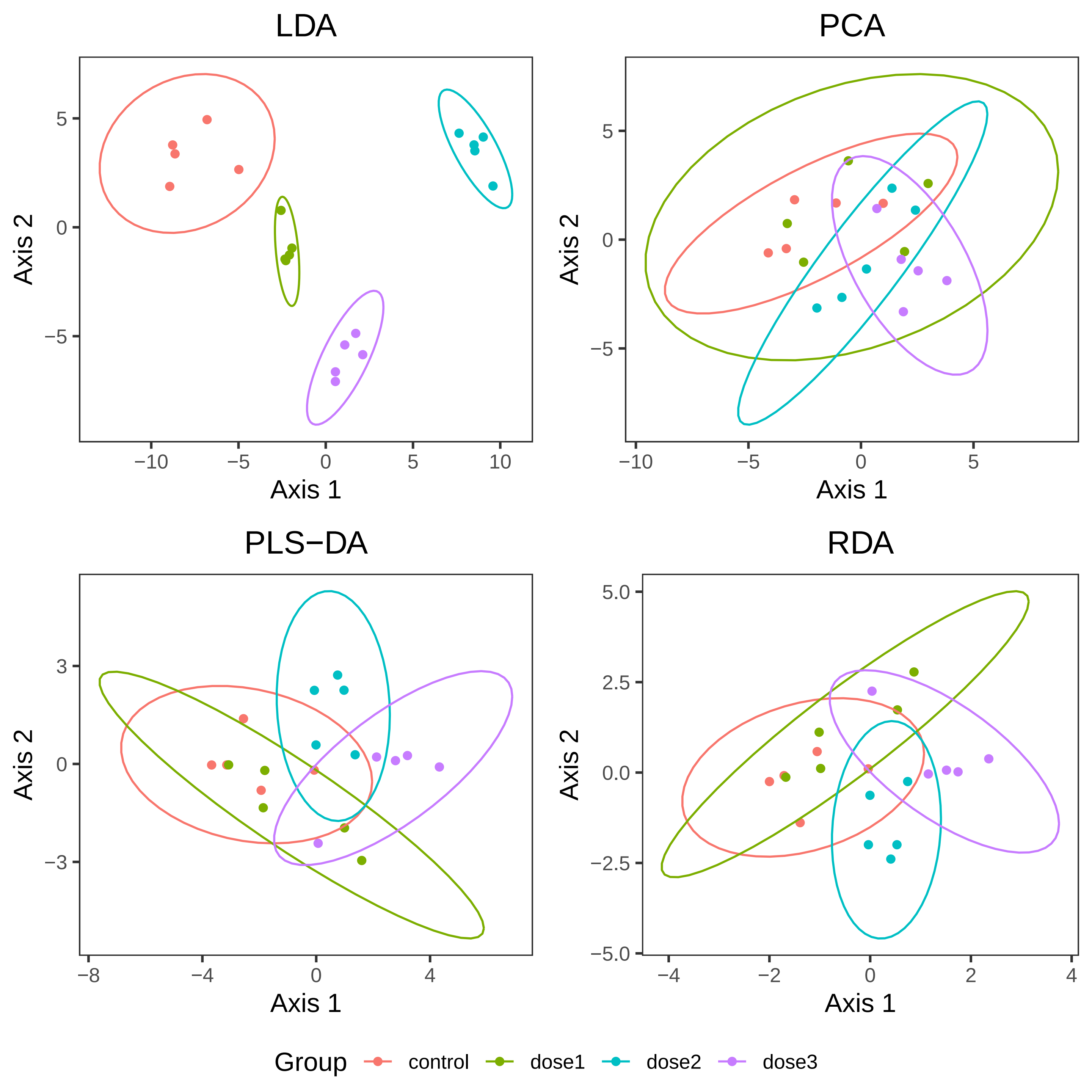
LDA
PCA
PLS−DA
RDA
Axis 1
Axis 2
5
0
−5
−10
10
3
−3
−8
−4
4
5.0
2.5
0.0
−2.5
−5.0
−2
2
Group
control
dose1
dose2
dose3

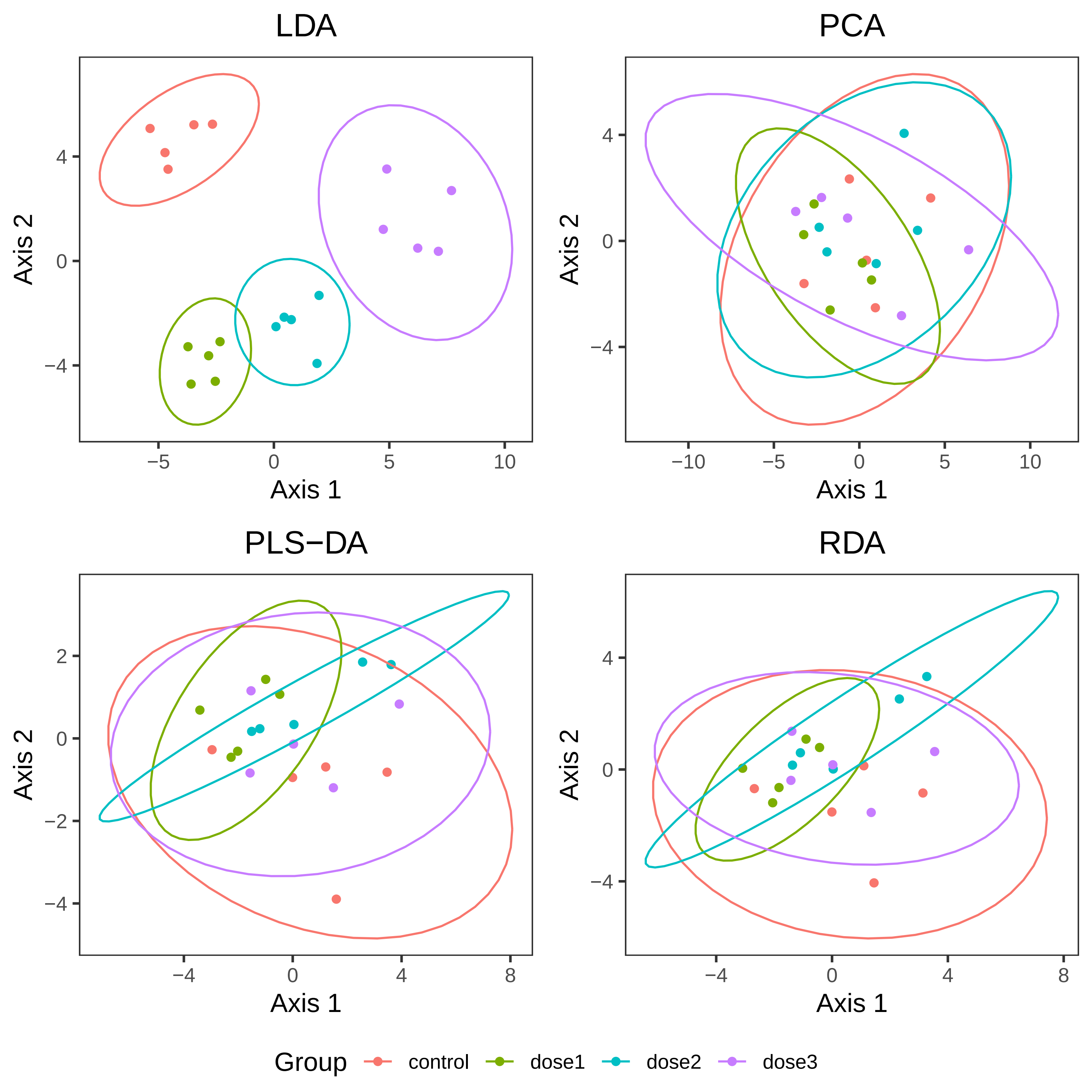

LDA
PCA
PLS−DA
RDA
Axis 1
Axis 2
−5
0
5
10
4
0
−4
−10
−8
8
2
−2
Group
control
dose1
dose2
dose3

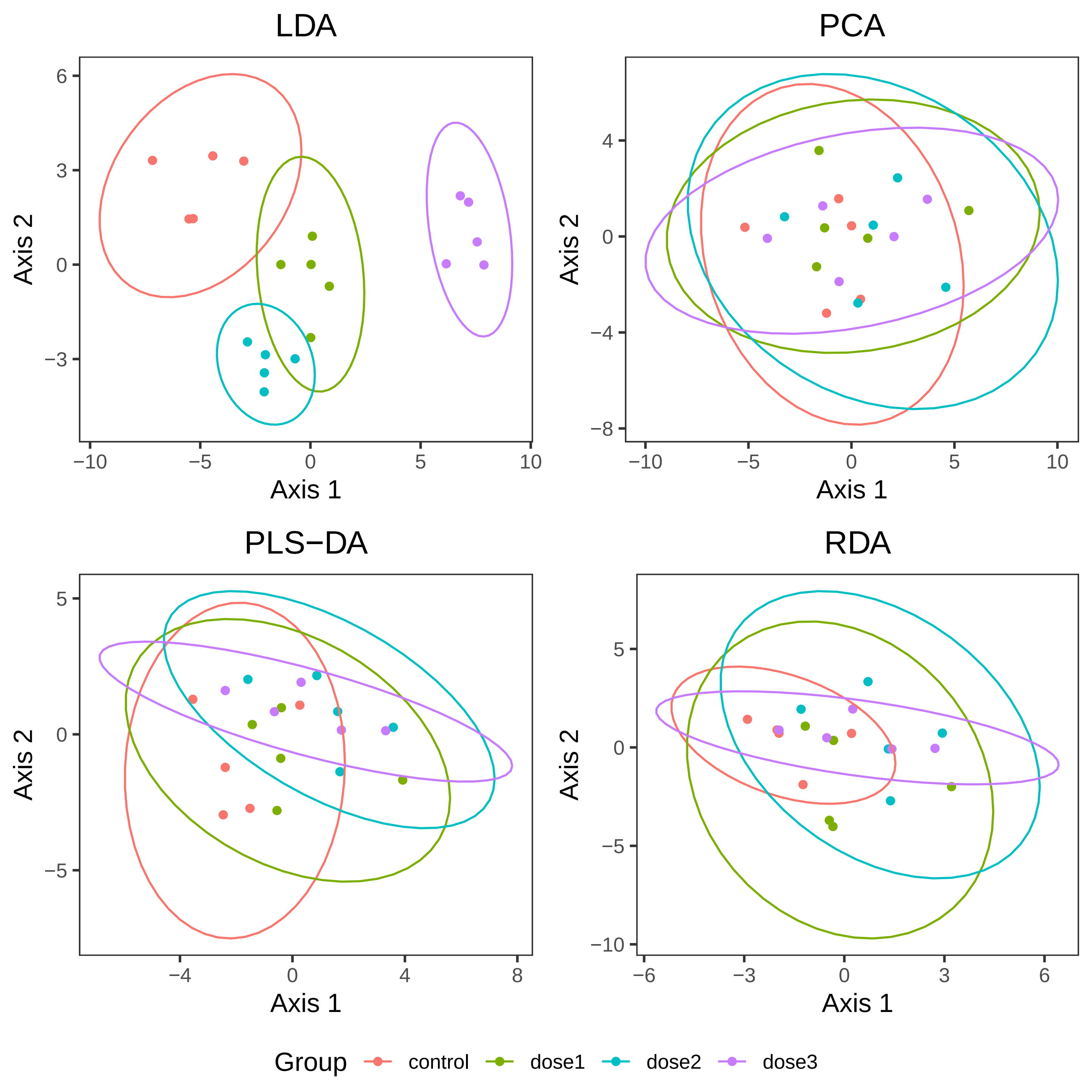

LDA
PCA
PLS−DA
RDA
Axis 1
Axis 2
Group
control
dose1
dose2
dose3

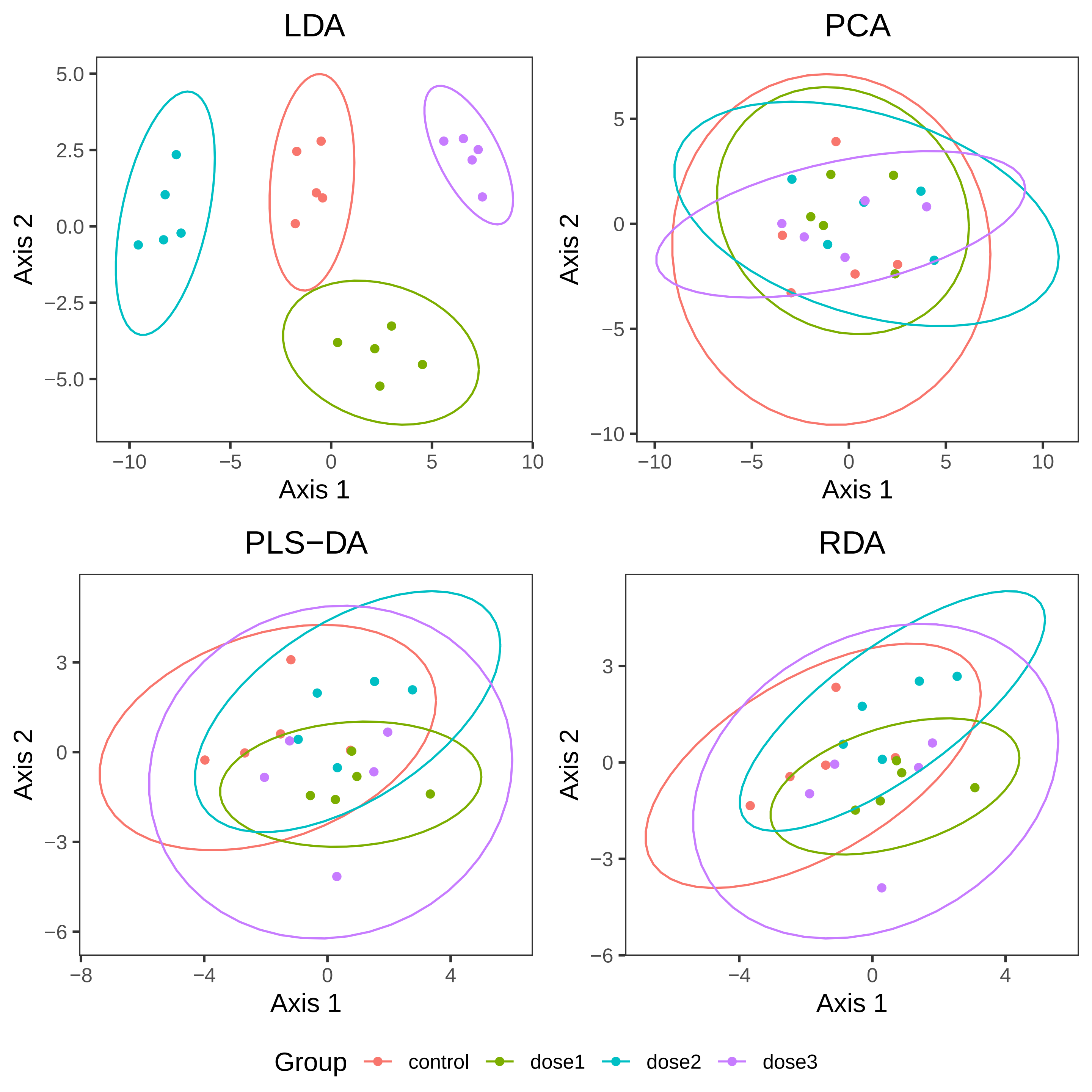
LDA
PCA
PLS−DA
RDA
Axis 1
Axis 2
5.0
2.5
0.0
−2.5
−5.0
−10
−5
0
5
10
−8
−4
4
3
−3
−6
Group
control
dose1
dose2
dose3

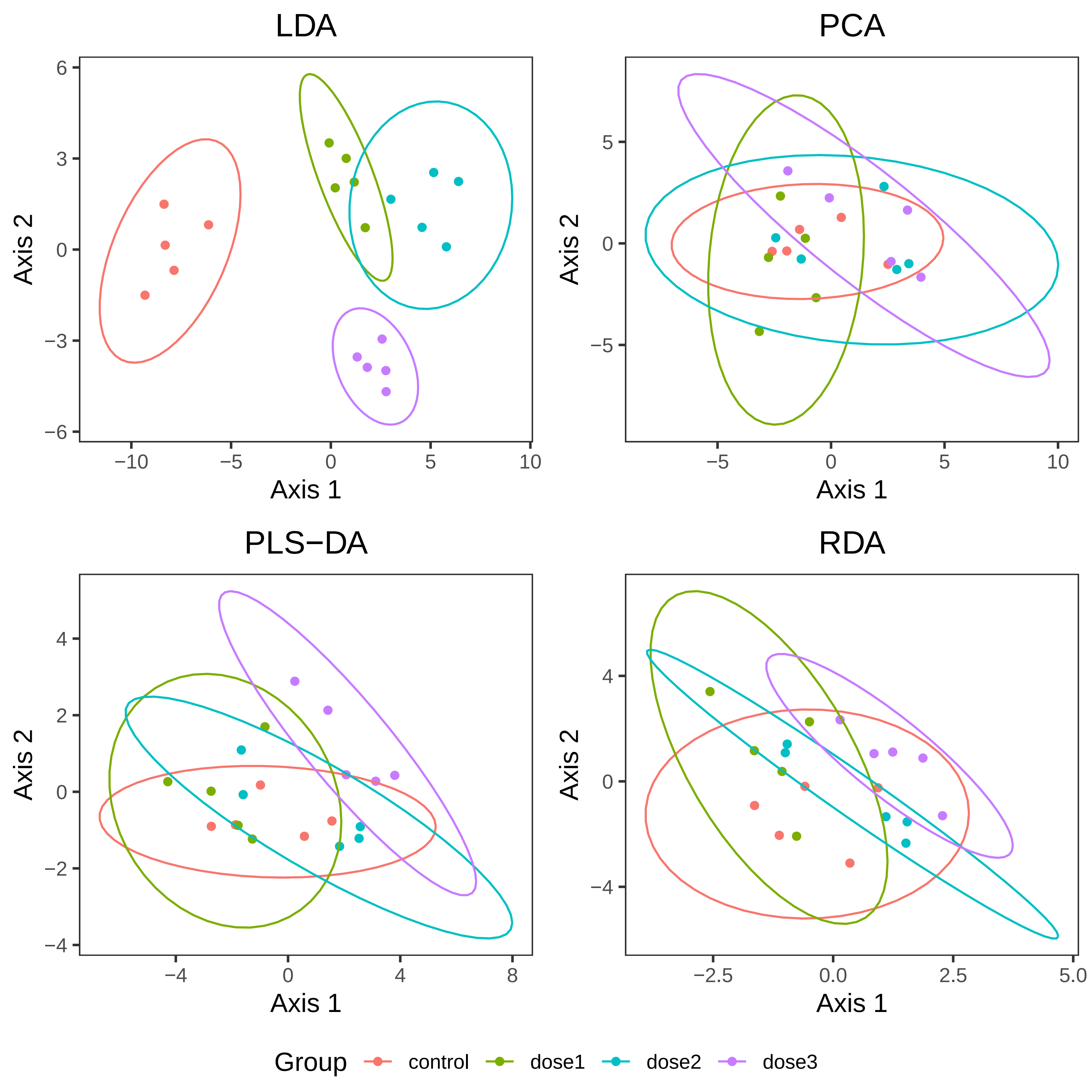

LDA
PCA
PLS−DA
RDA
Axis 1
Axis 2
Group
control
dose1
dose2
dose3

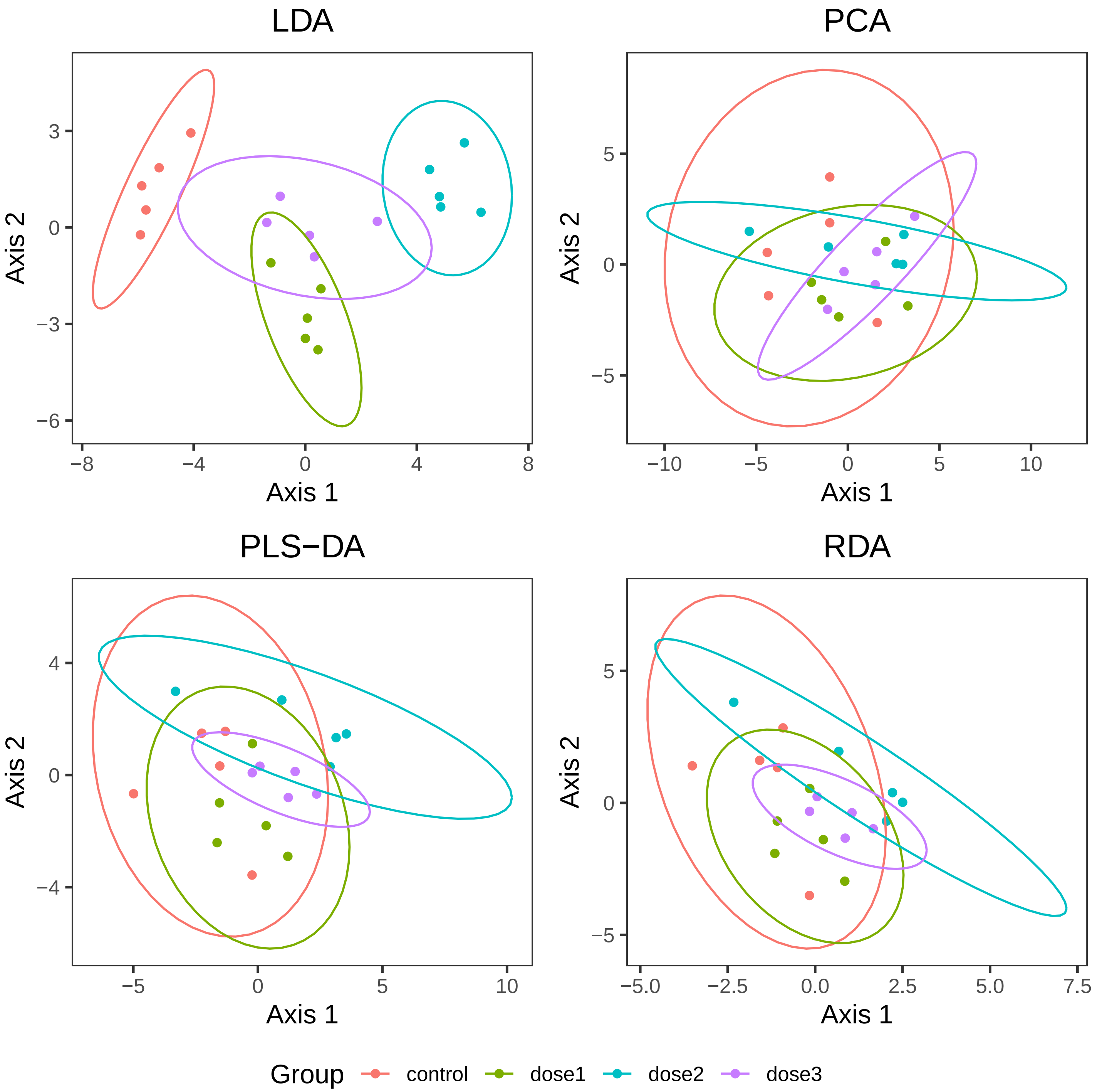
LDA
PCA
PLS−DA
RDA
Axis 1
Axis 2
Group
control
dose1
dose2
dose3

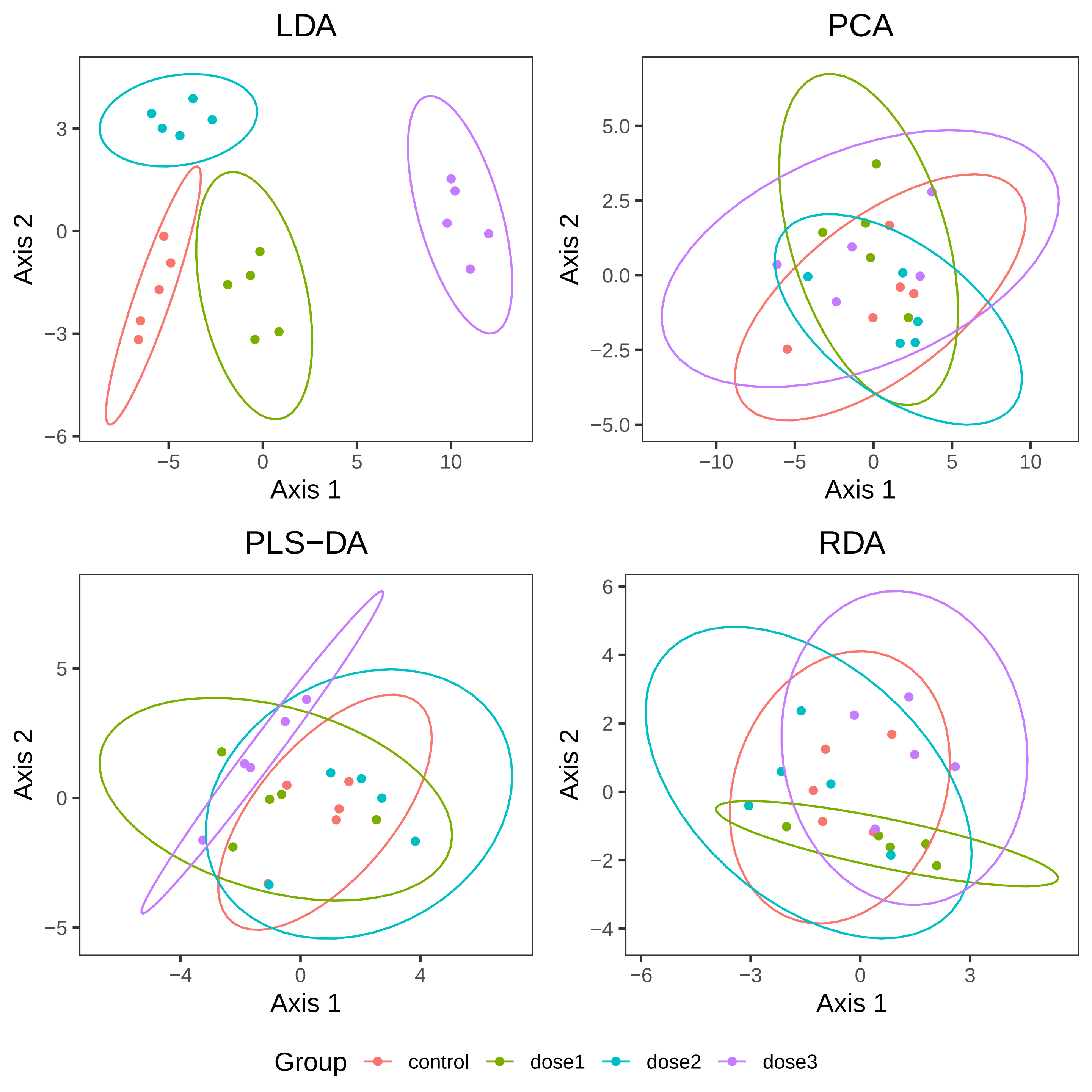

LDA
PCA
PLS−DA
RDA
Axis 1
Axis 2
Group
control
dose1
dose2
dose3

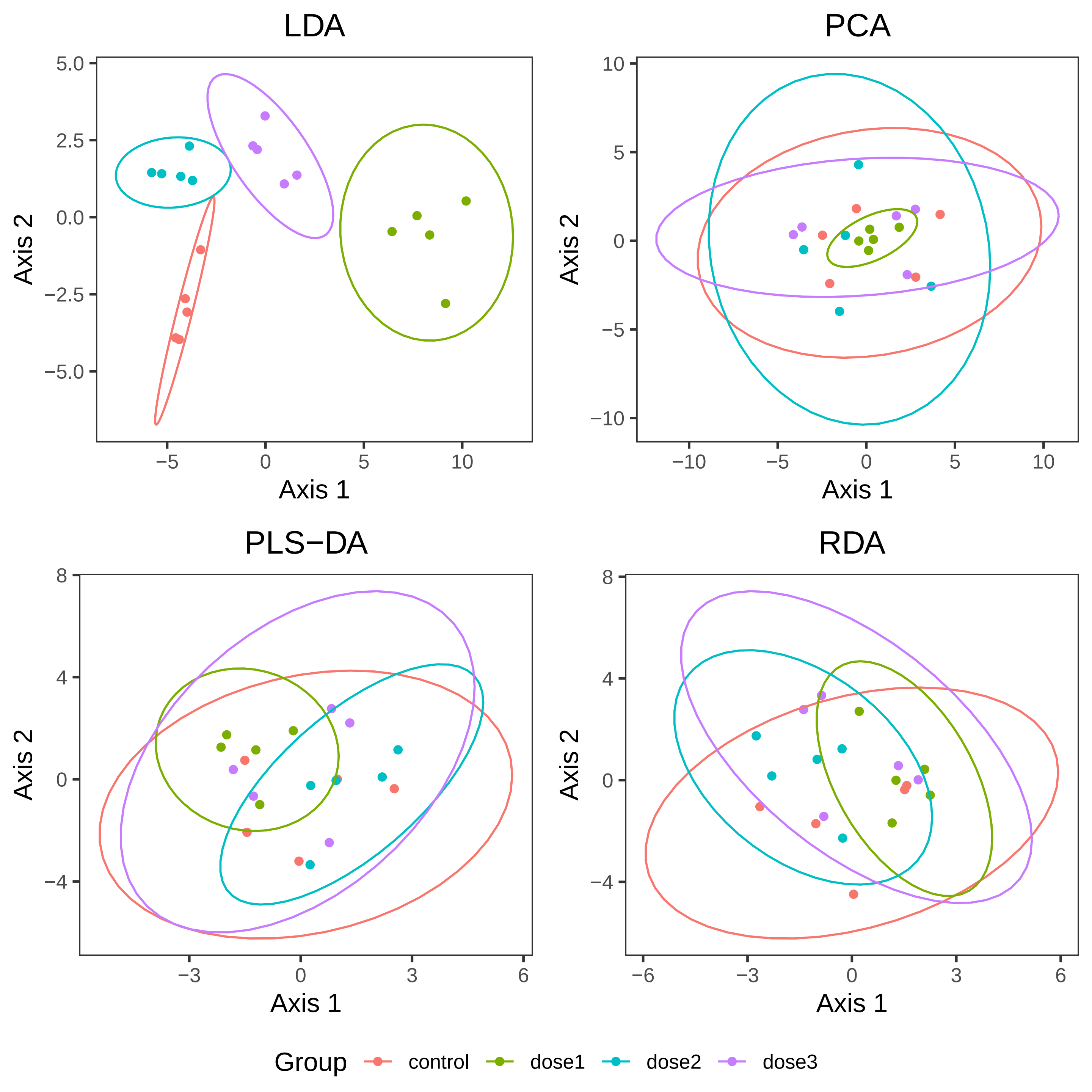
LDA
PCA
PLS−DA
RDA
Axis 1
Axis 2
Group
control
dose1
dose2
dose3

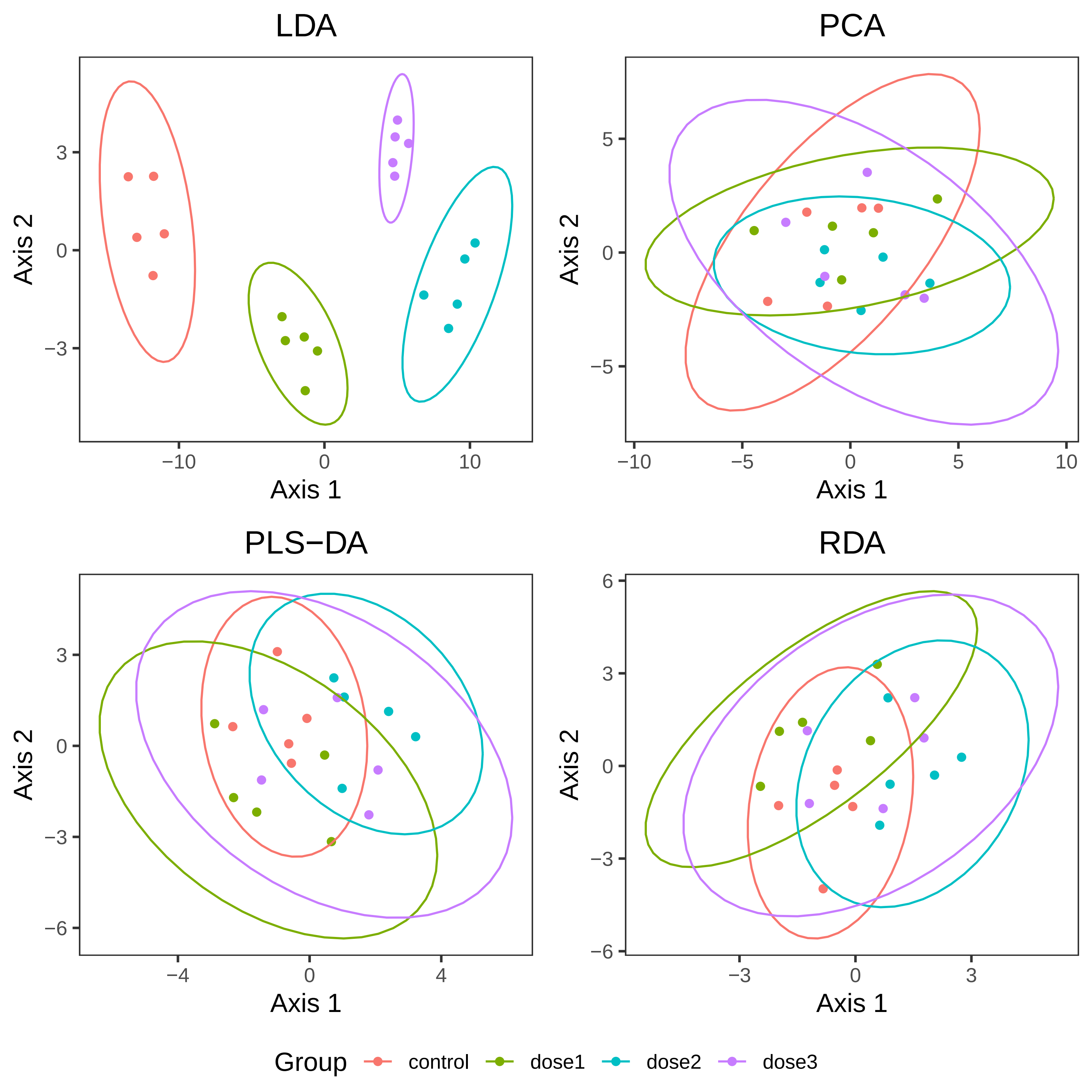
LDA
PCA
PLS−DA
RDA
Axis 1
Axis 2
Group
control
dose1
dose2
dose3

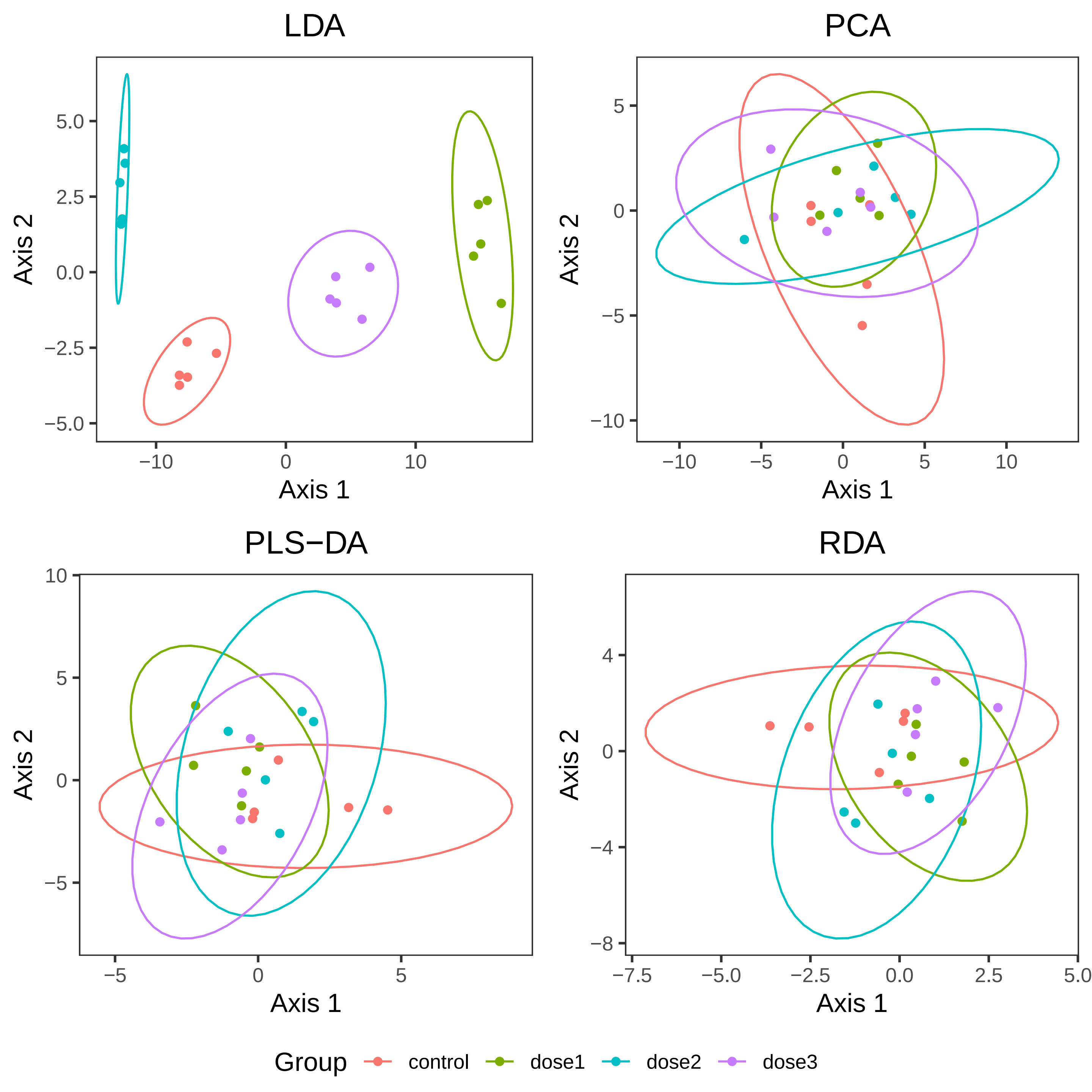
LDA
PCA
PLS−DA
RDA
Axis 1
Axis 2
Group
control
dose1
dose2
dose3

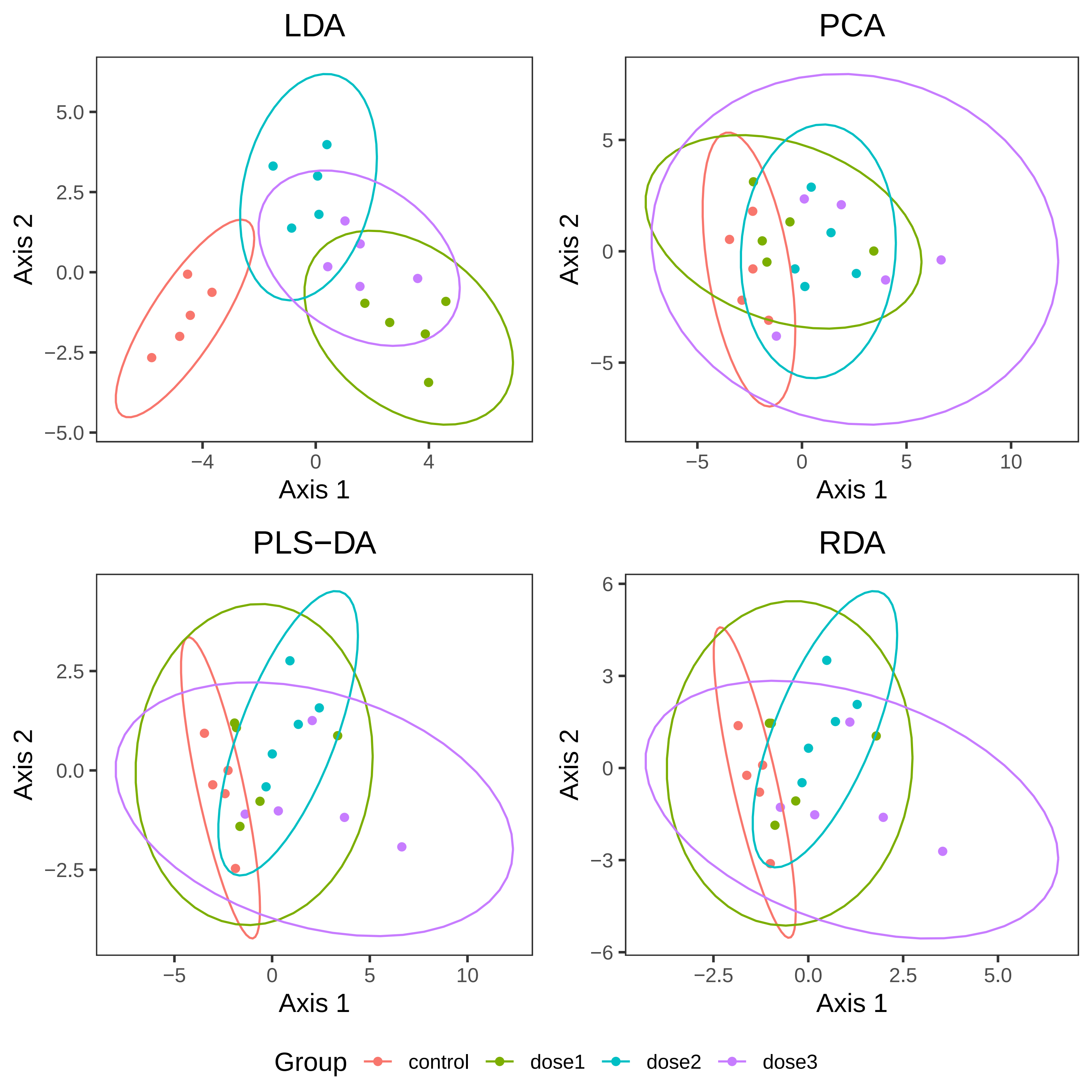
LDA
PCA
PLS−DA
RDA
Axis 1
Axis 2
Group
control
dose1
dose2
dose3

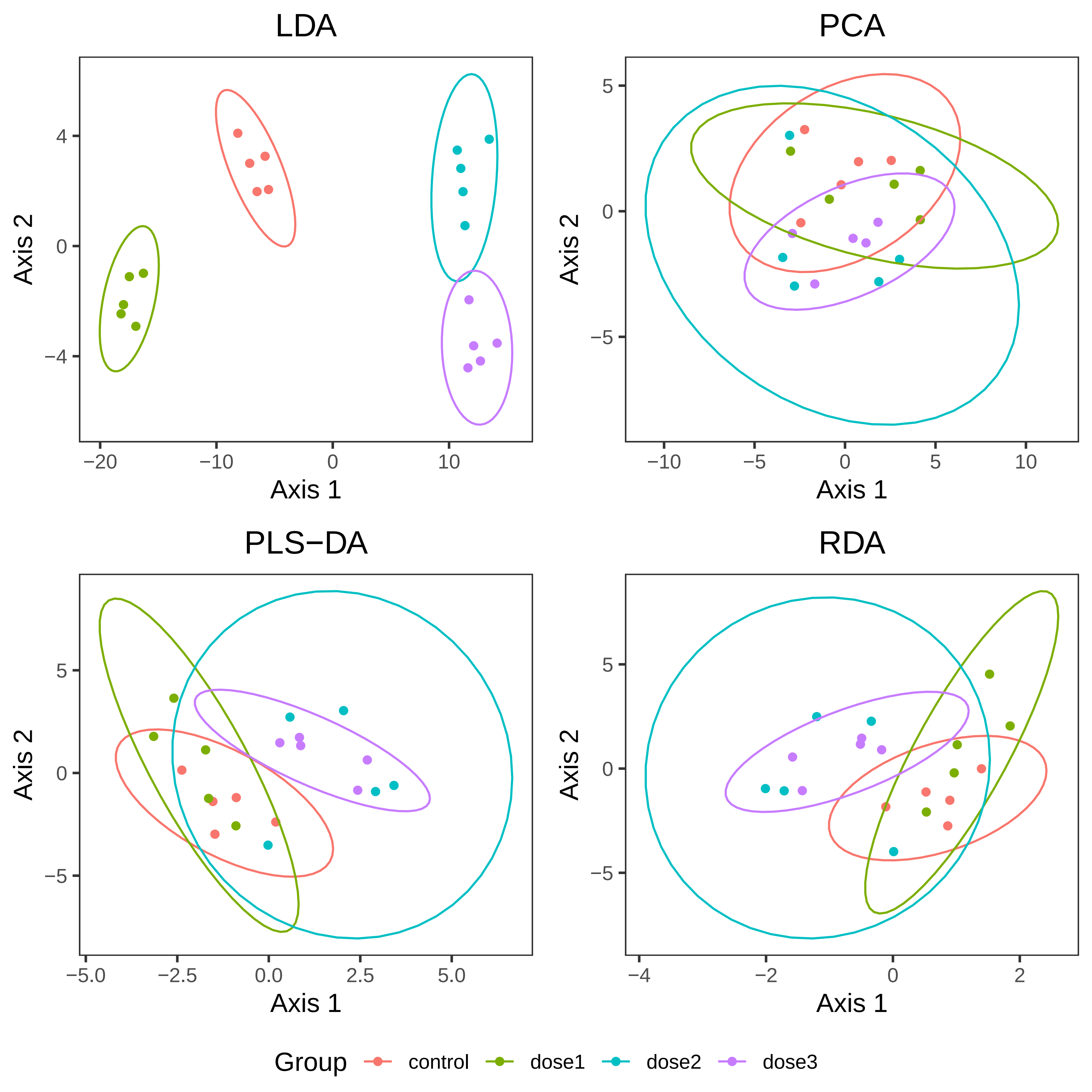

LDA
PCA
PLS−DA
RDA
Axis 1
Axis 2
Group
control
dose1
dose2
dose3

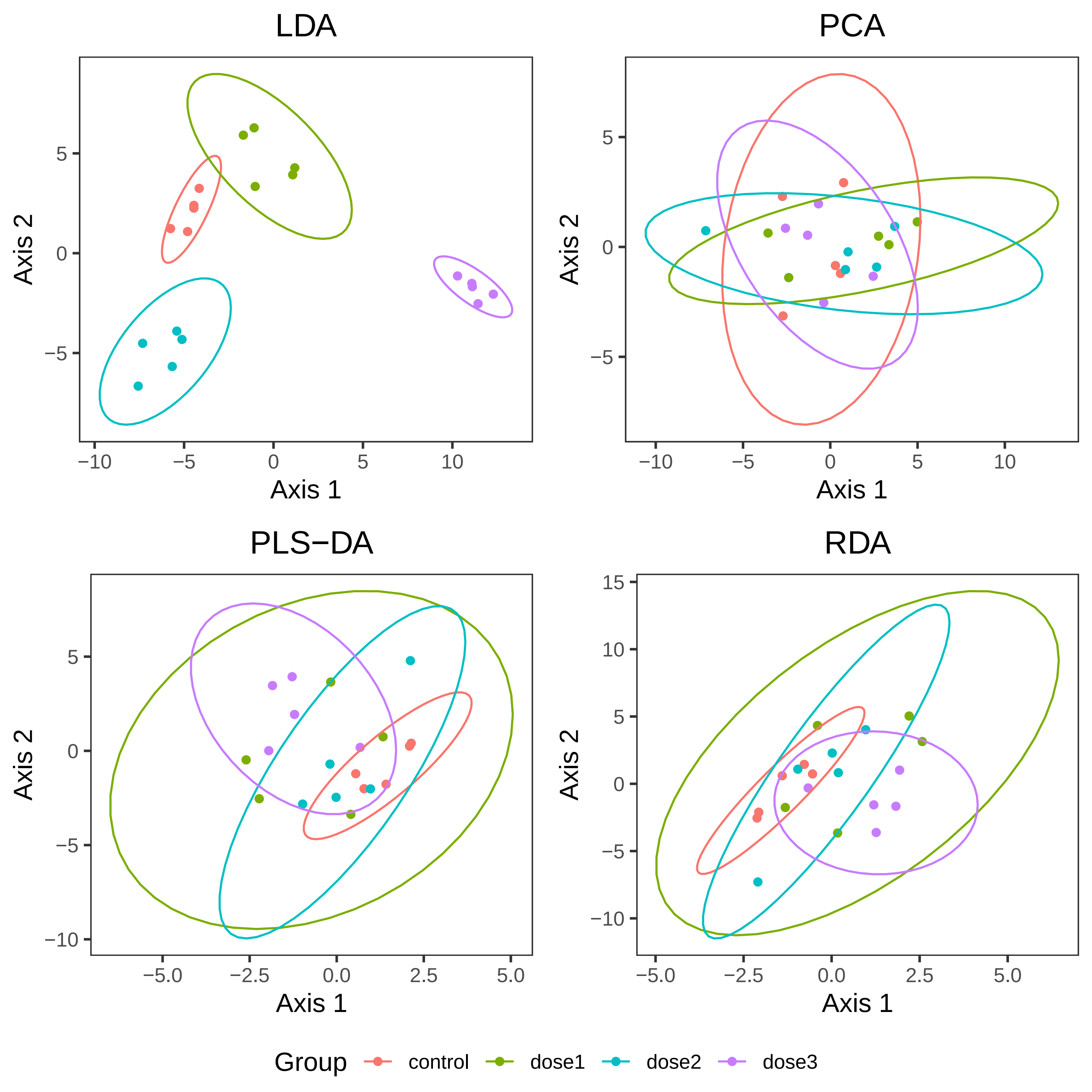

LDA
PCA
PLS−DA
RDA
Axis 1
Axis 2
Group
control
dose1
dose2
dose3

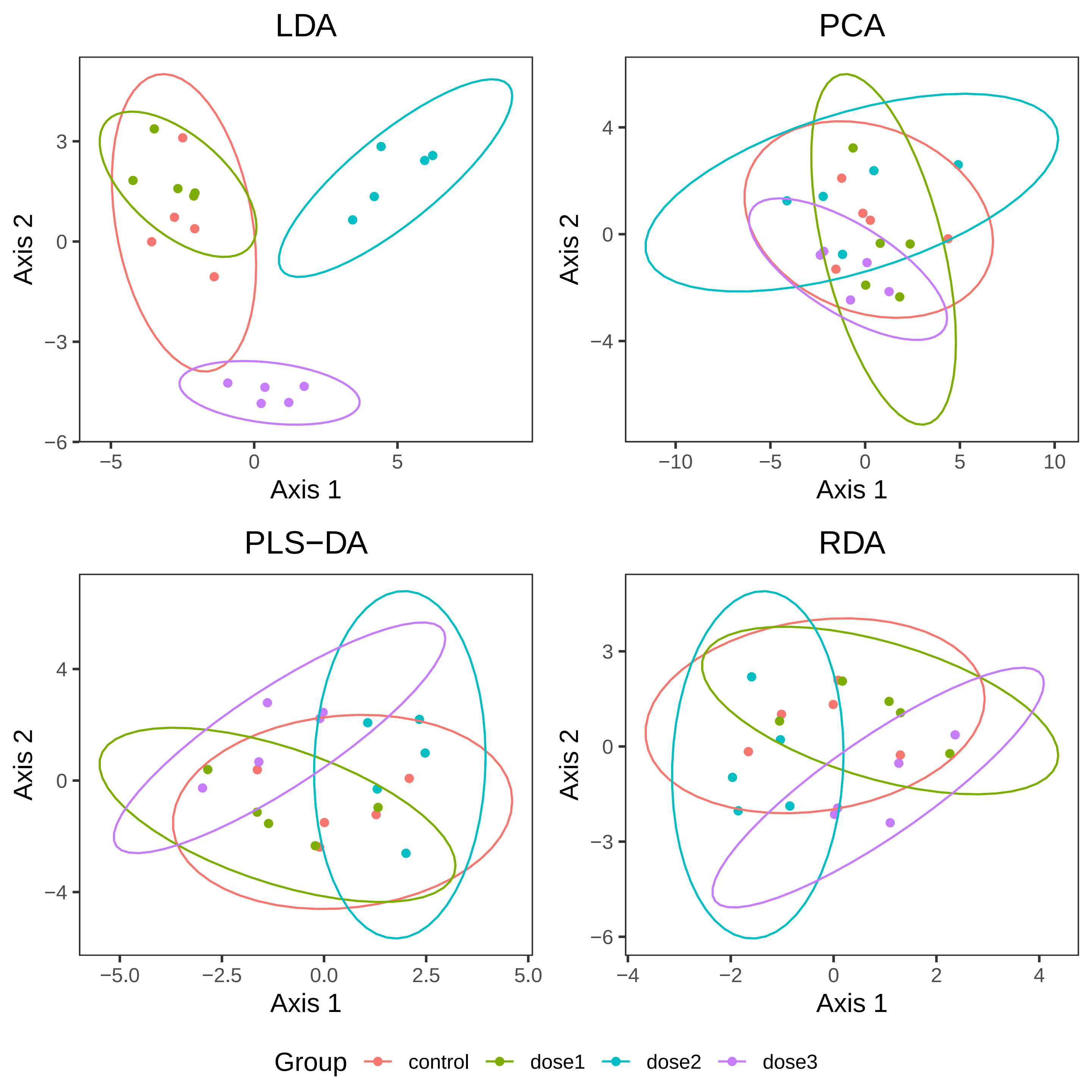
LDA
PCA
PLS−DA
RDA
Axis 1
Axis 2
Group
control
dose1
dose2
dose3

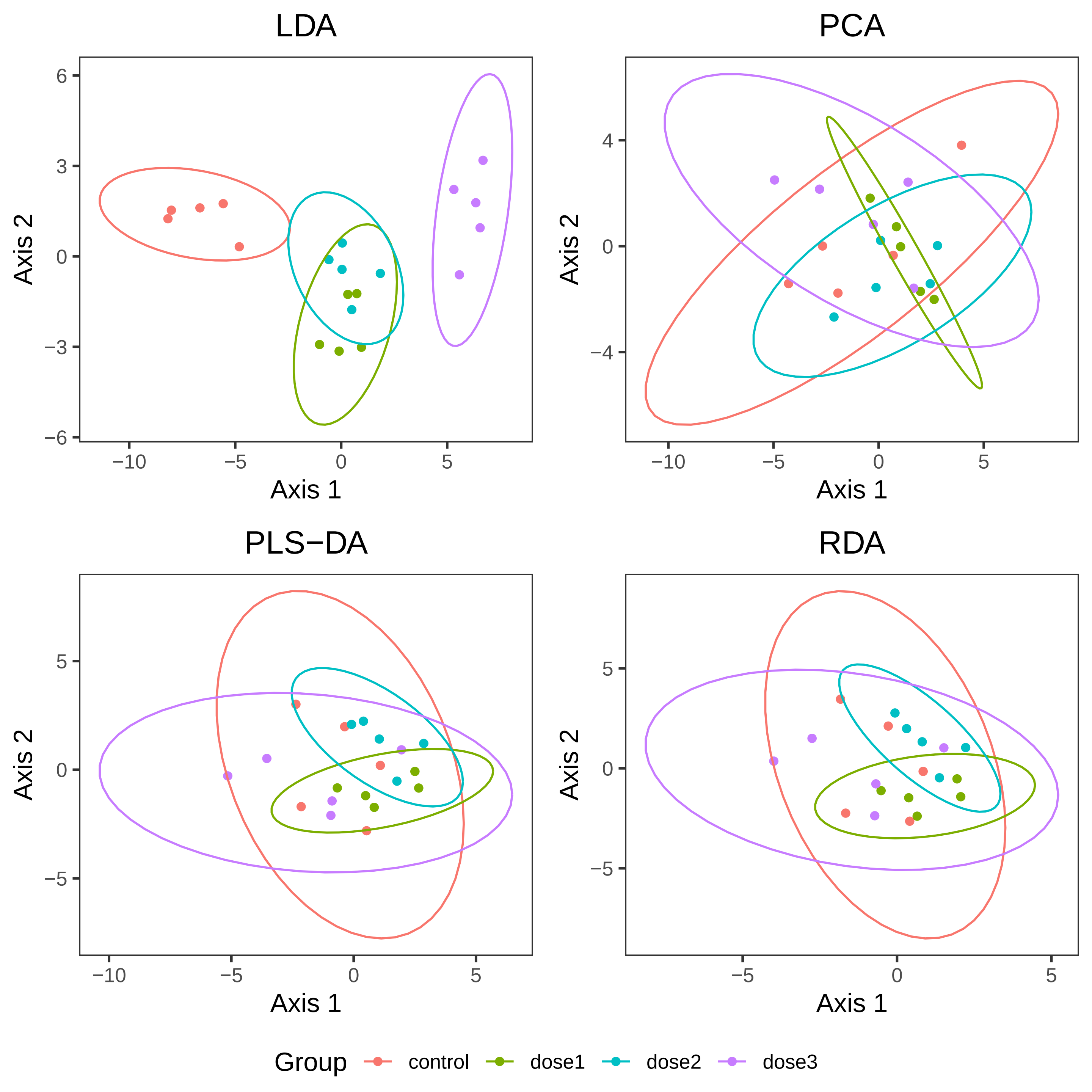
LDA
PCA
PLS−DA
RDA
Axis 1
Axis 2
Group
control
dose1
dose2
dose3

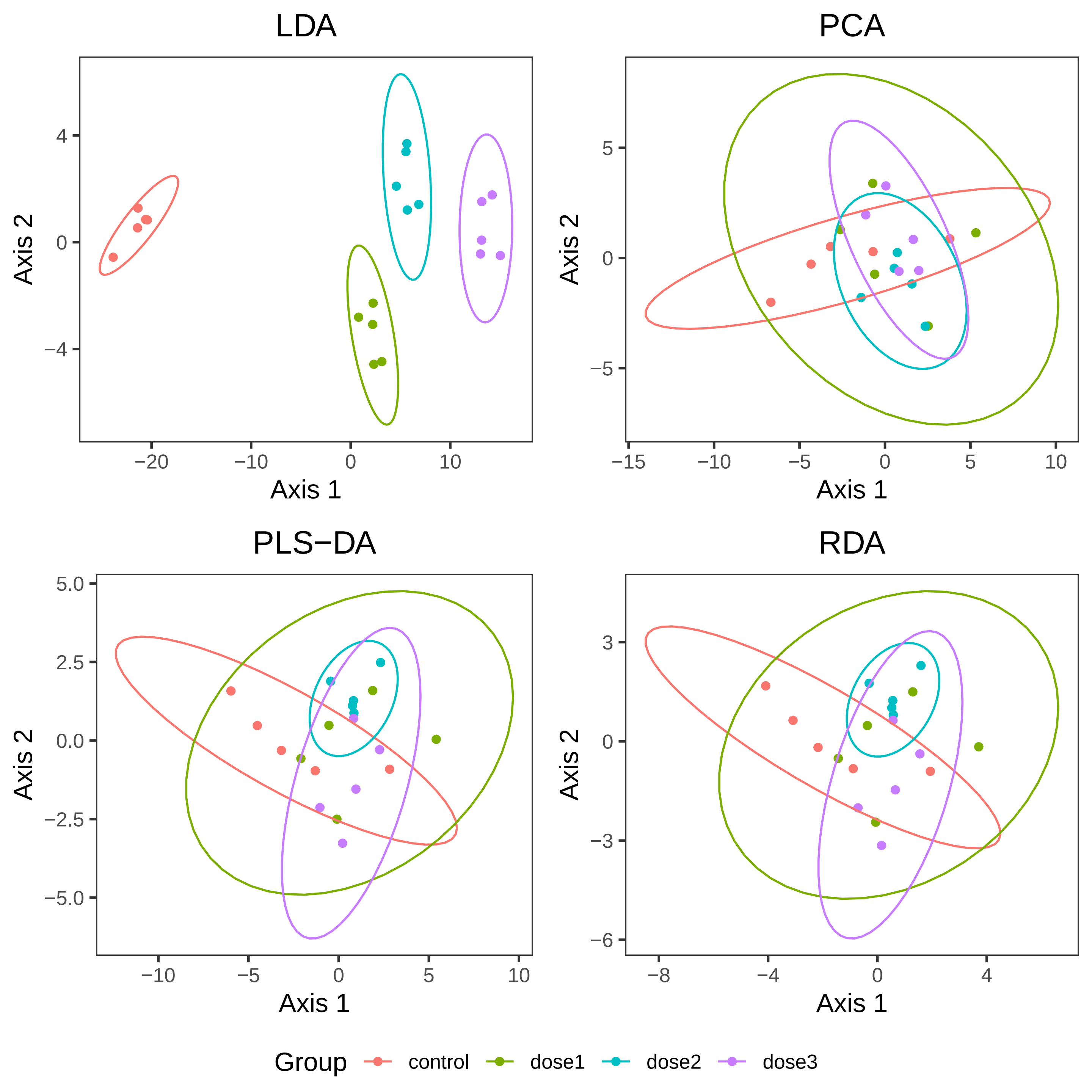
LDA
PCA
PLS−DA
RDA
Axis 1
Axis 2
4
0
−4
−20
−10
0
10
5
−5
−15
5.0
2.5
0.0
−2.5
−5.0
3
−3
−6
−8
Group
control
dose1
dose2
dose3

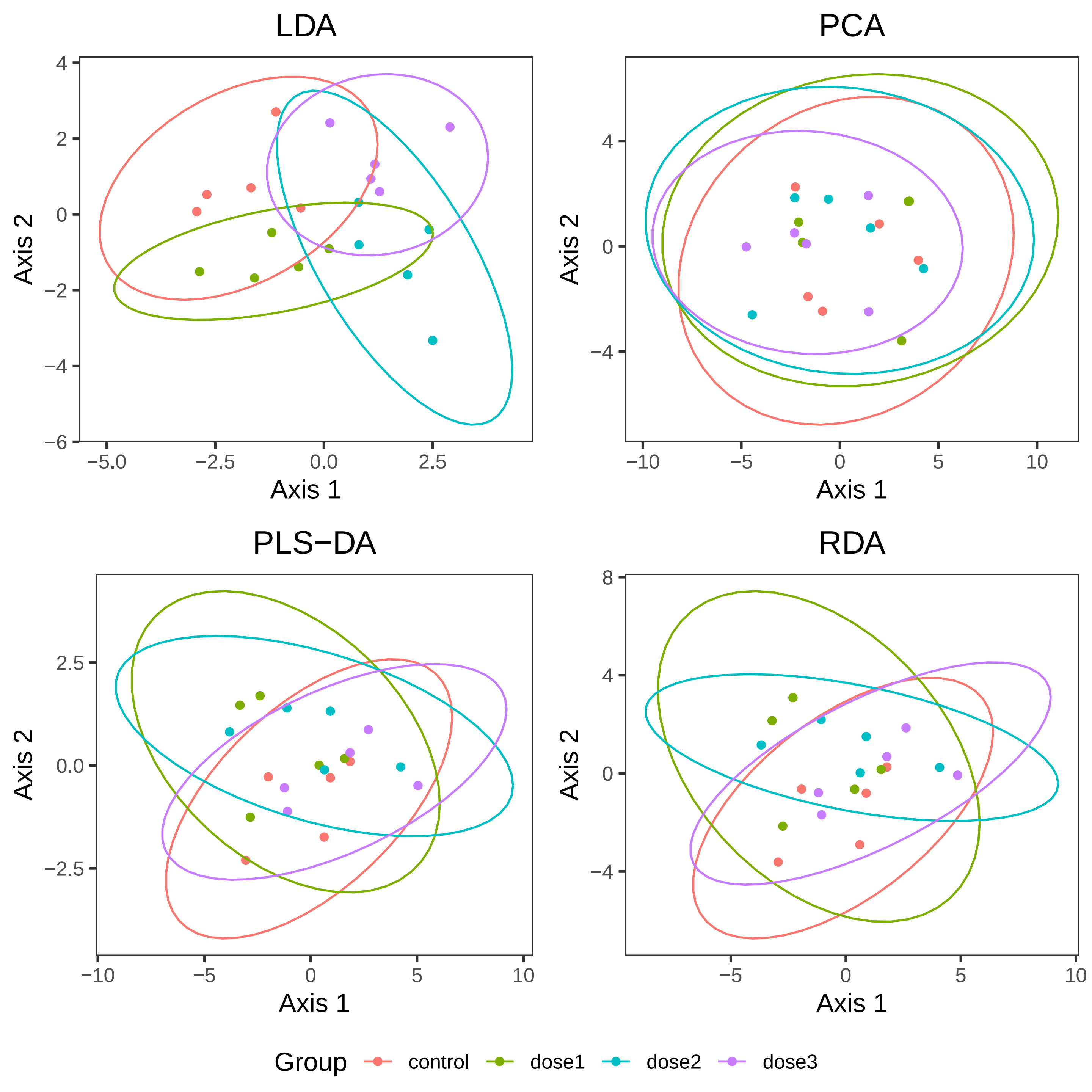
LDA
PCA
PLS−DA
RDA
Axis 1
Axis 2
Group
control
dose1
dose2
dose3

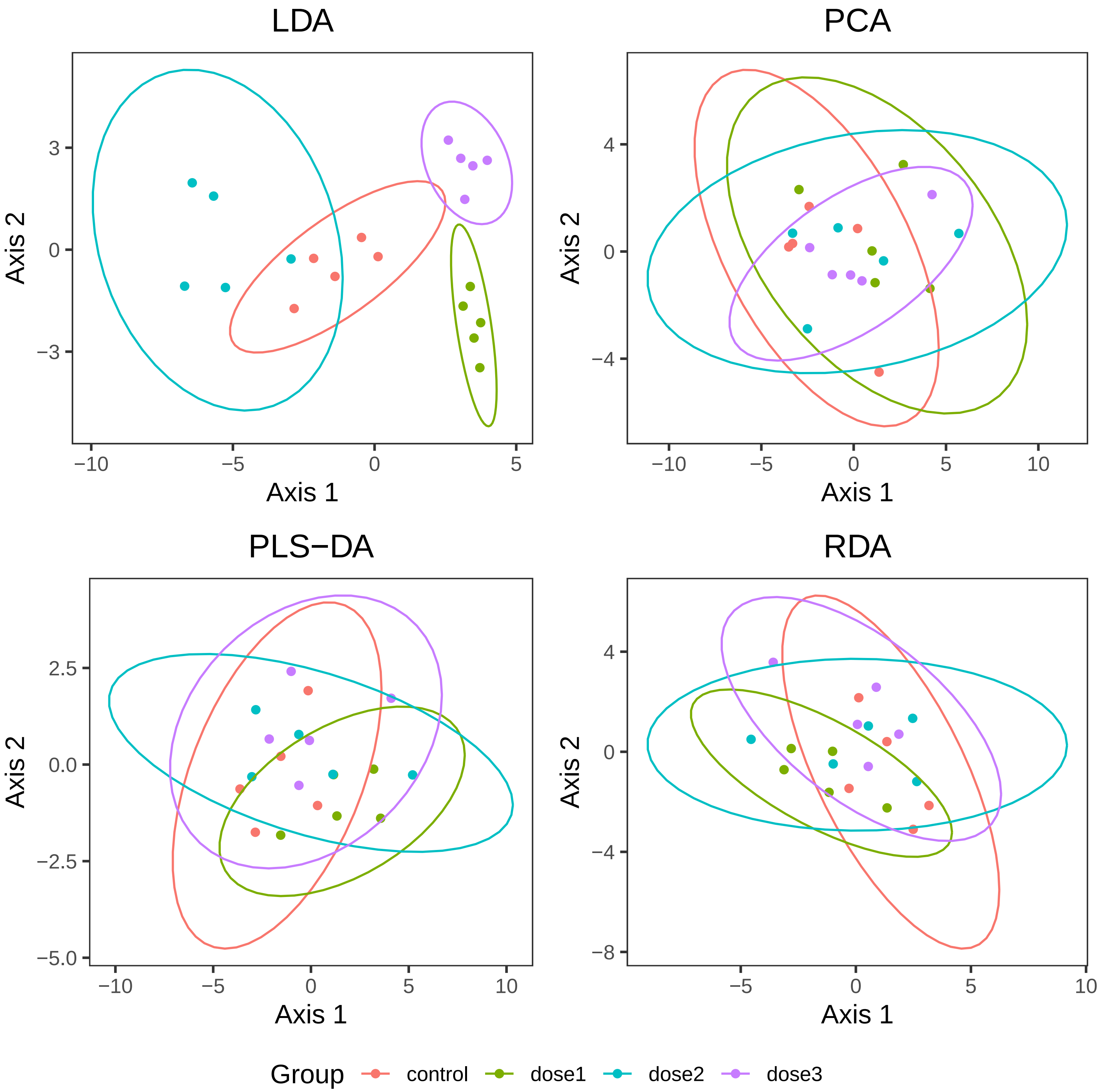

LDA
PCA
PLS−DA
RDA
Axis 1
Axis 2
Group
control
dose1
dose2
dose3

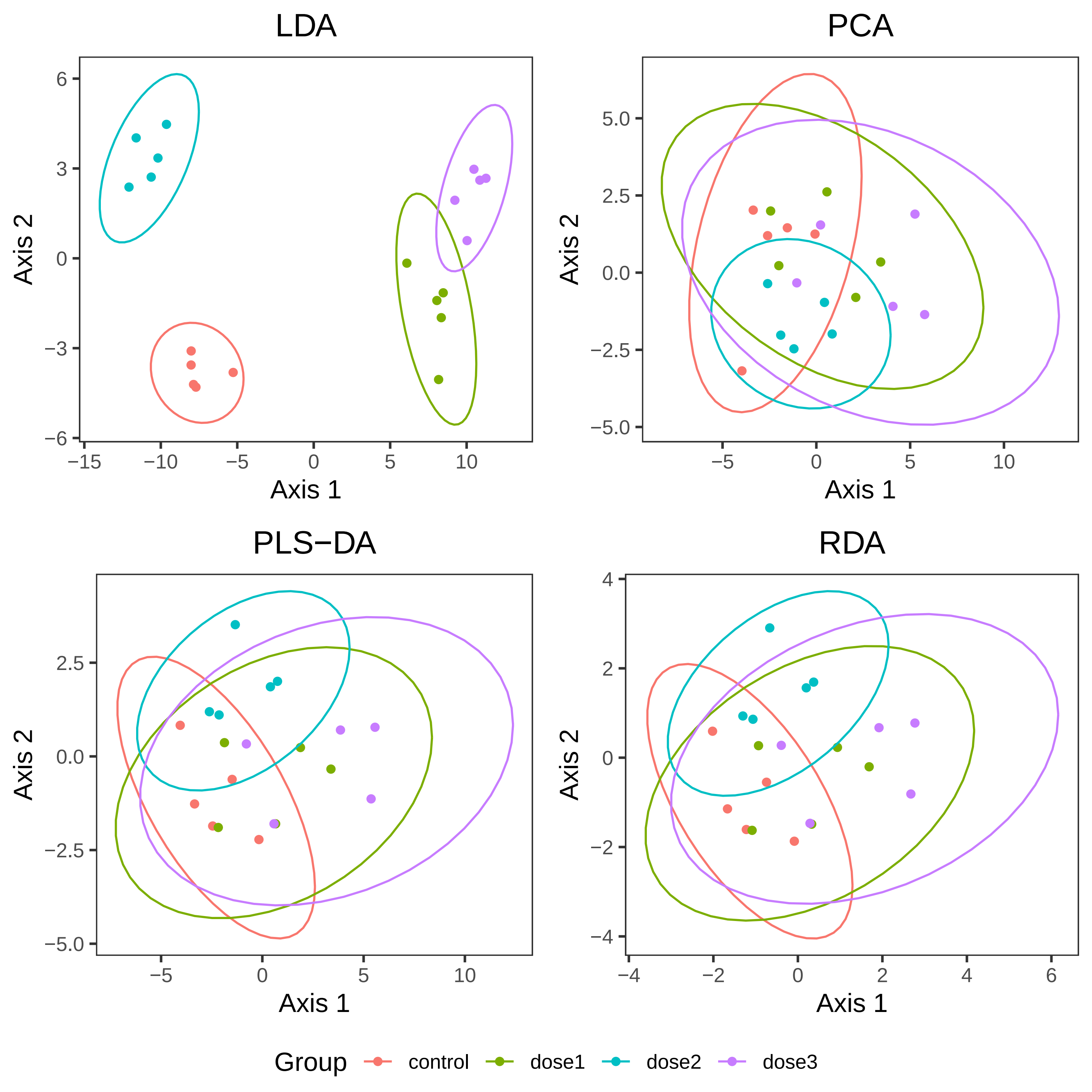

LDA
PCA
PLS−DA
RDA
Axis 1
Axis 2
6
3
0
−3
−6
−15
−10
−5
0
5
10
5.0
2.5
0.0
−2.5
−5.0
2.5
0.0
−2.5
−5.0
4
2
0
−2
−4
−4
−2
0
2
4
6
Group
control
dose1
dose2
dose3

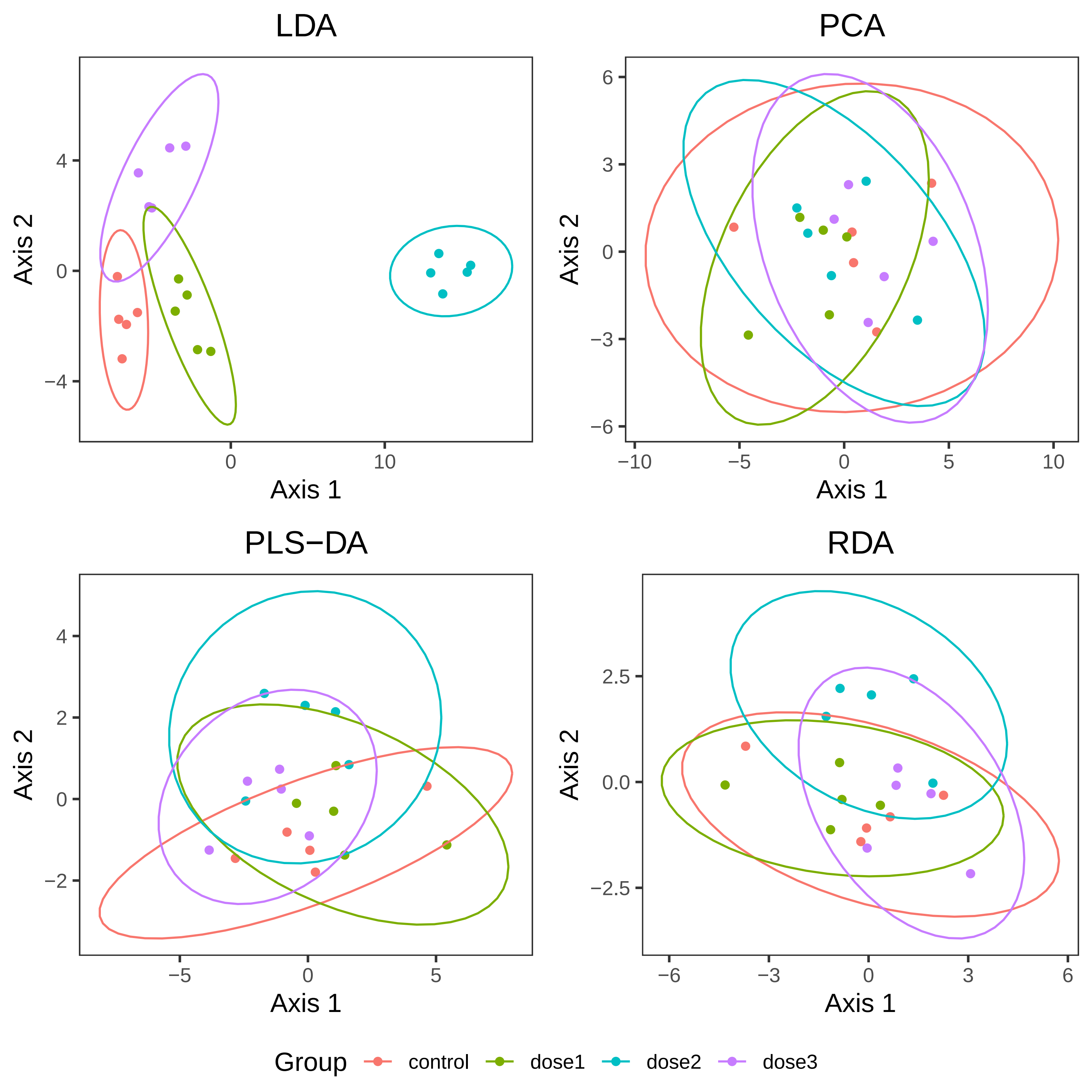

LDA
PCA
PLS−DA
RDA
Axis 1
Axis 2
Group
control
dose1
dose2
dose3

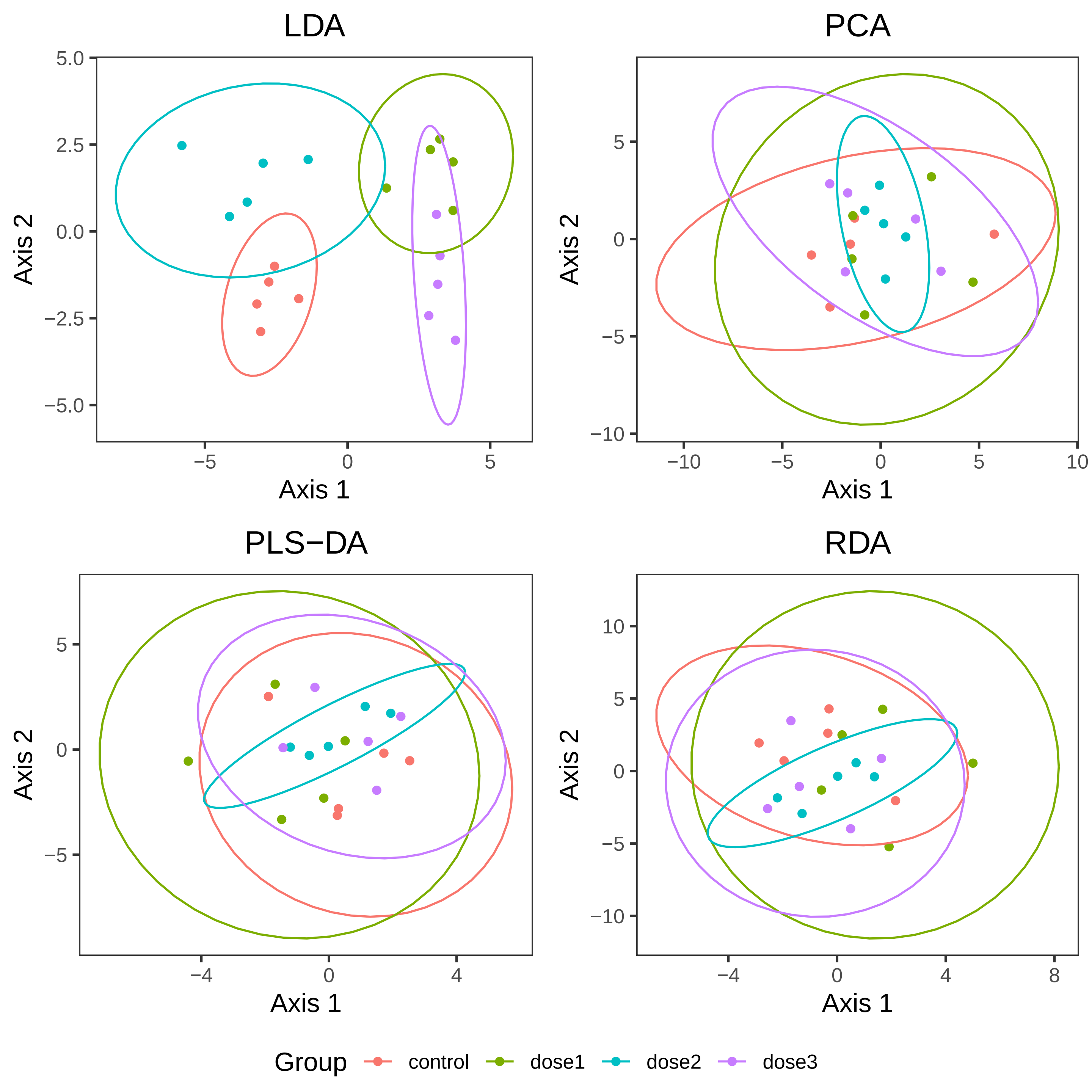
LDA
PCA
PLS−DA
RDA
Axis 1
Axis 2
5.0
2.5
0.0
−2.5
−5.0
−5
0
5
10
−10
−4
4
8
Group
control
dose1
dose2
dose3

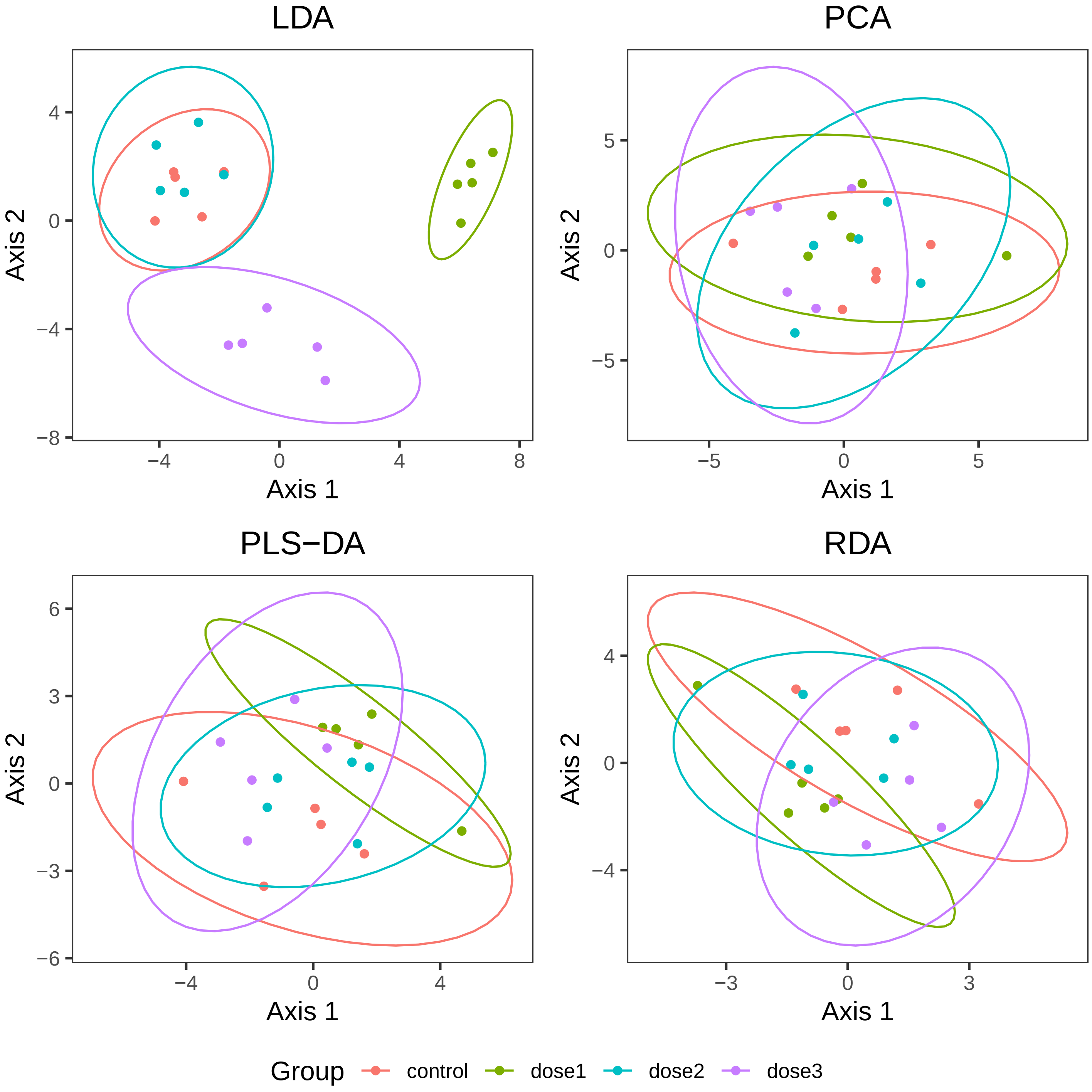

LDA
PCA
PLS−DA
RDA
Axis 1
Axis 2
4
0
−4
−8
8
5
−5
6
3
−3
−6
Group
control
dose1
dose2
dose3

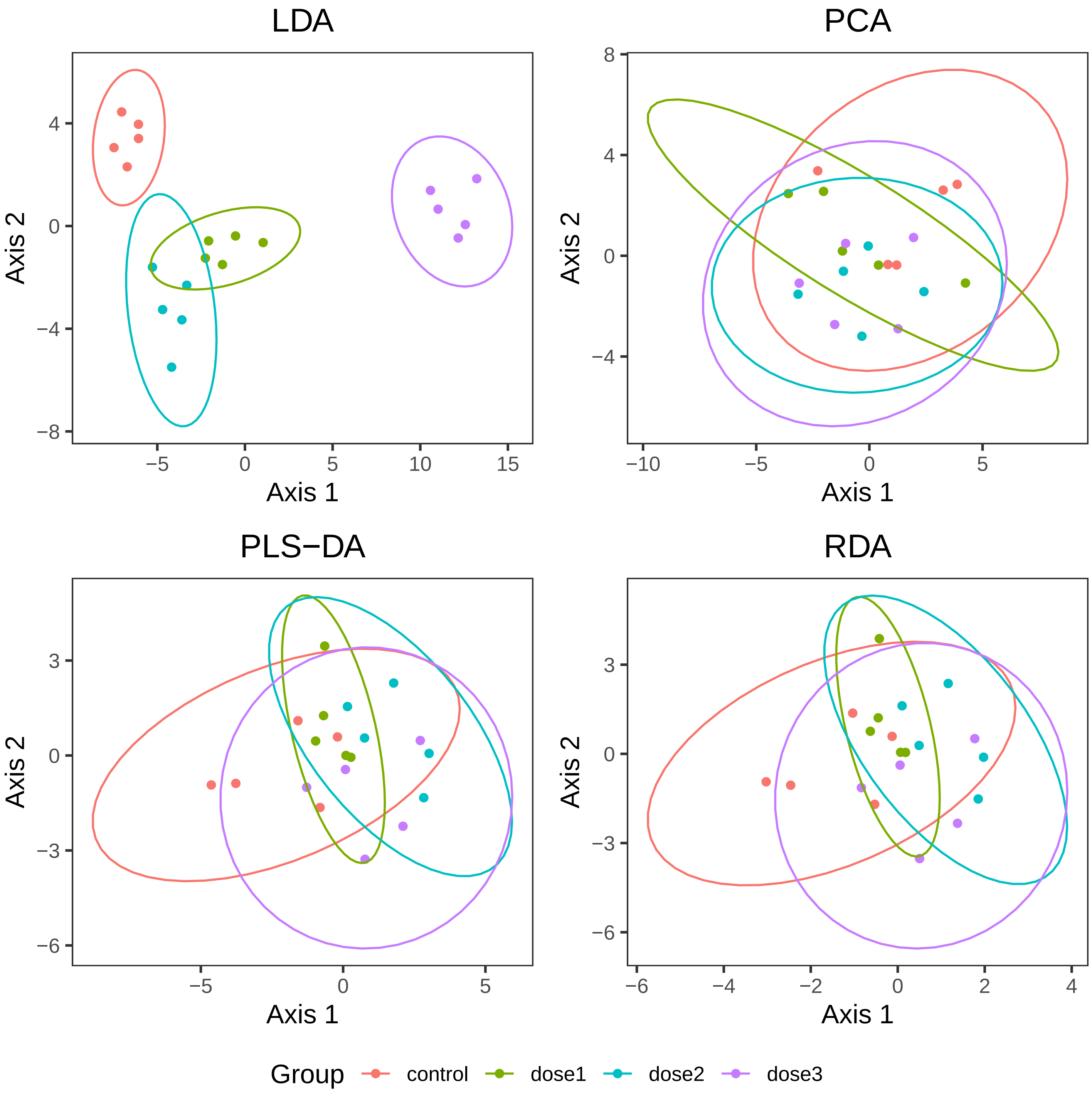
LDA
PCA
PLS−DA
RDA
Axis 1
Axis 2
Group
control
dose1
dose2
dose3

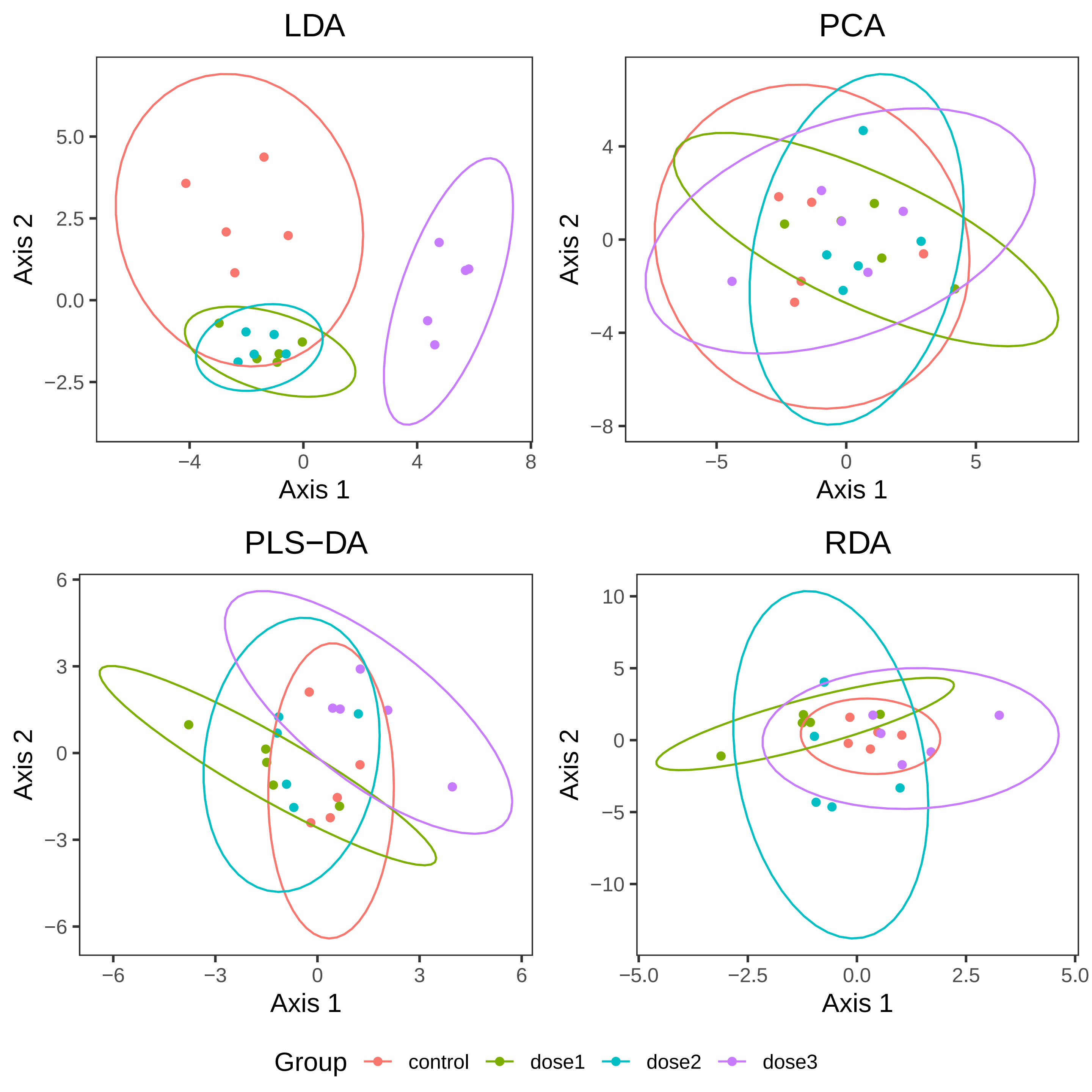
LDA
PCA
PLS−DA
RDA
Axis 1
Axis 2
Group
control
dose1
dose2
dose3

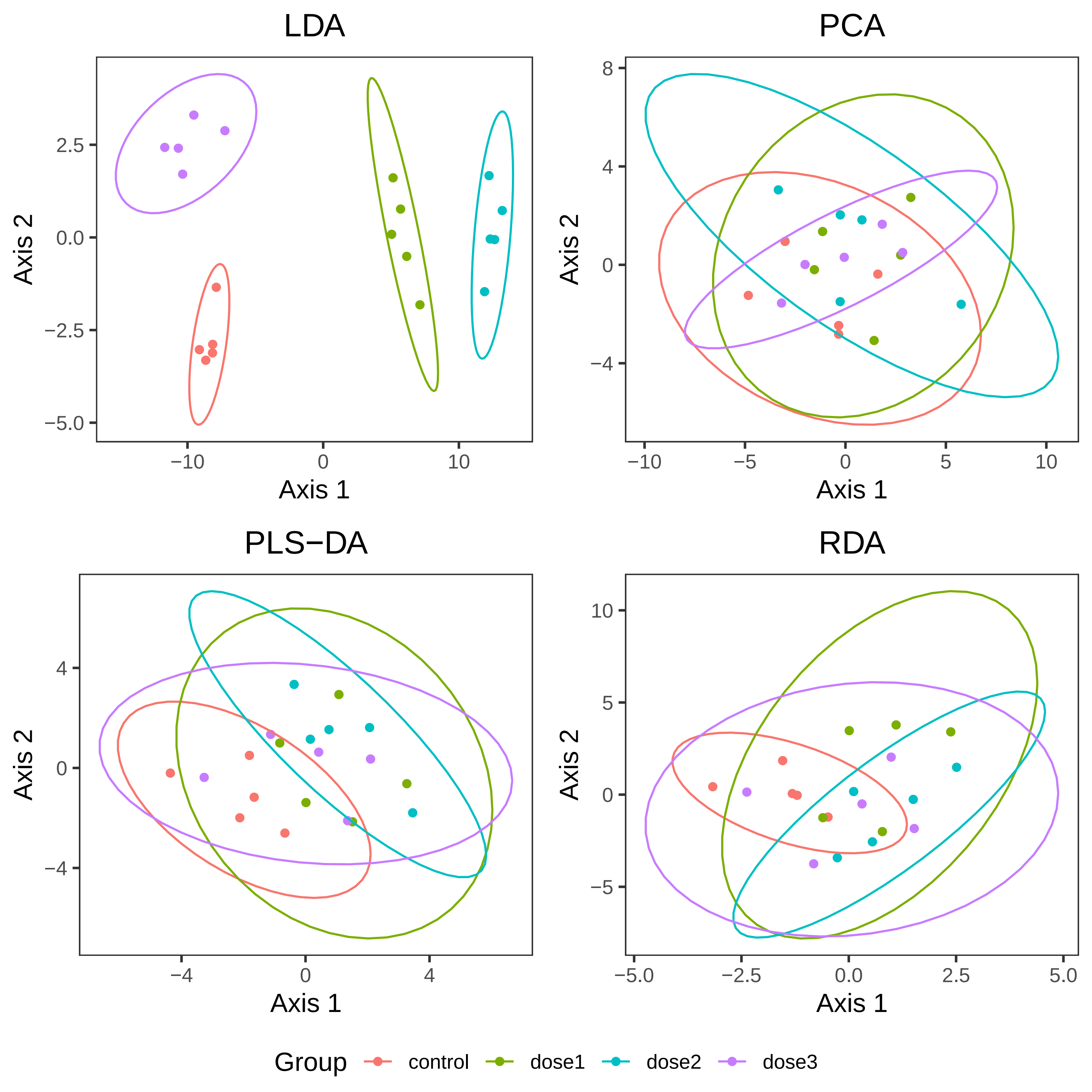

LDA
PCA
PLS−DA
RDA
Axis 1
Axis 2
−10
0
10
2.5
0.0
−2.5
−5.0
−5
5
8
4
−4
−5.0
5.0
Group
control
dose1
dose2
dose3

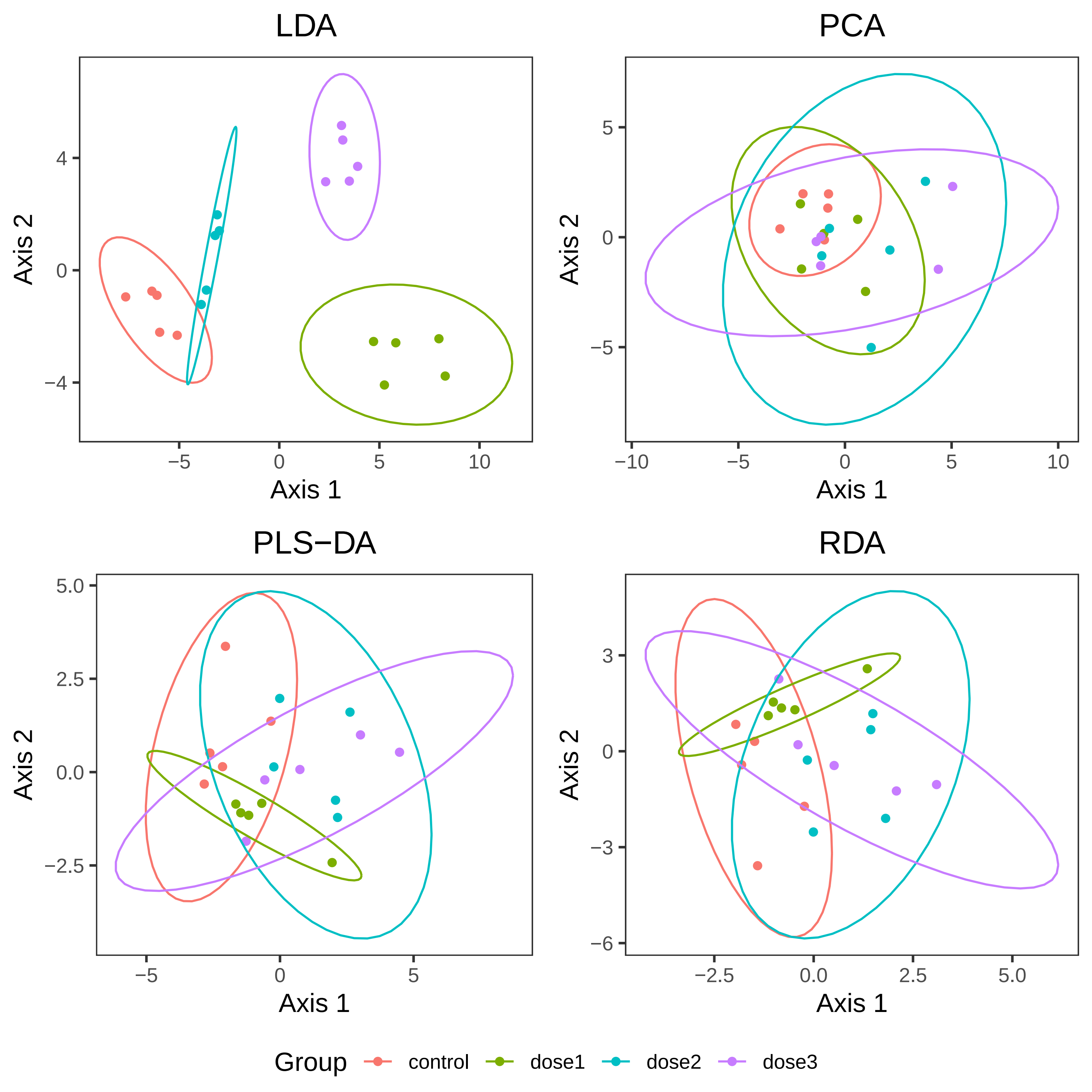

LDA
PCA
PLS−DA
RDA
Axis 1
Axis 2
Group
control
dose1
dose2
dose3

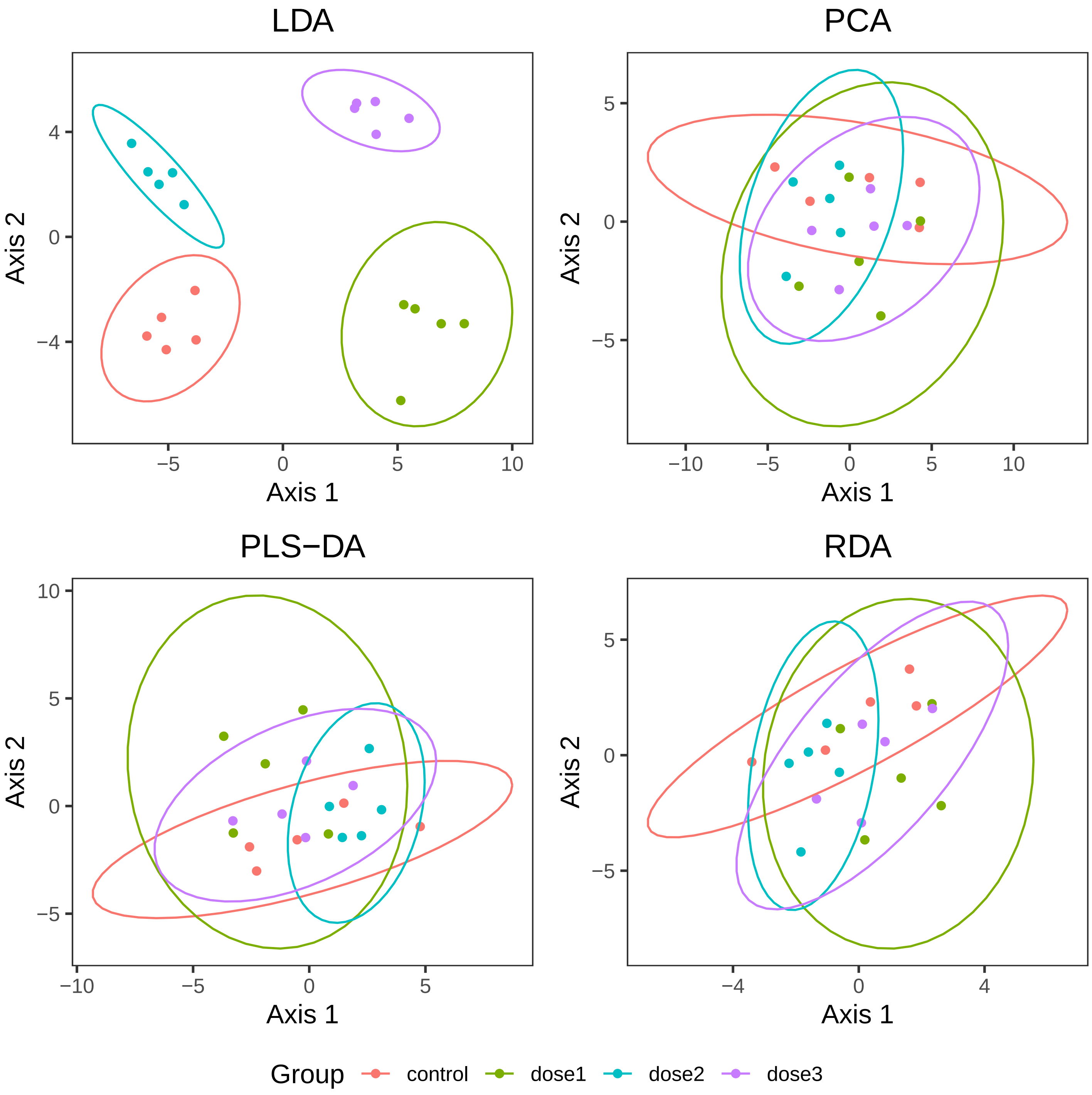

LDA
PCA
PLS−DA
RDA
Axis 1
Axis 2
Group
control
dose1
dose2
dose3

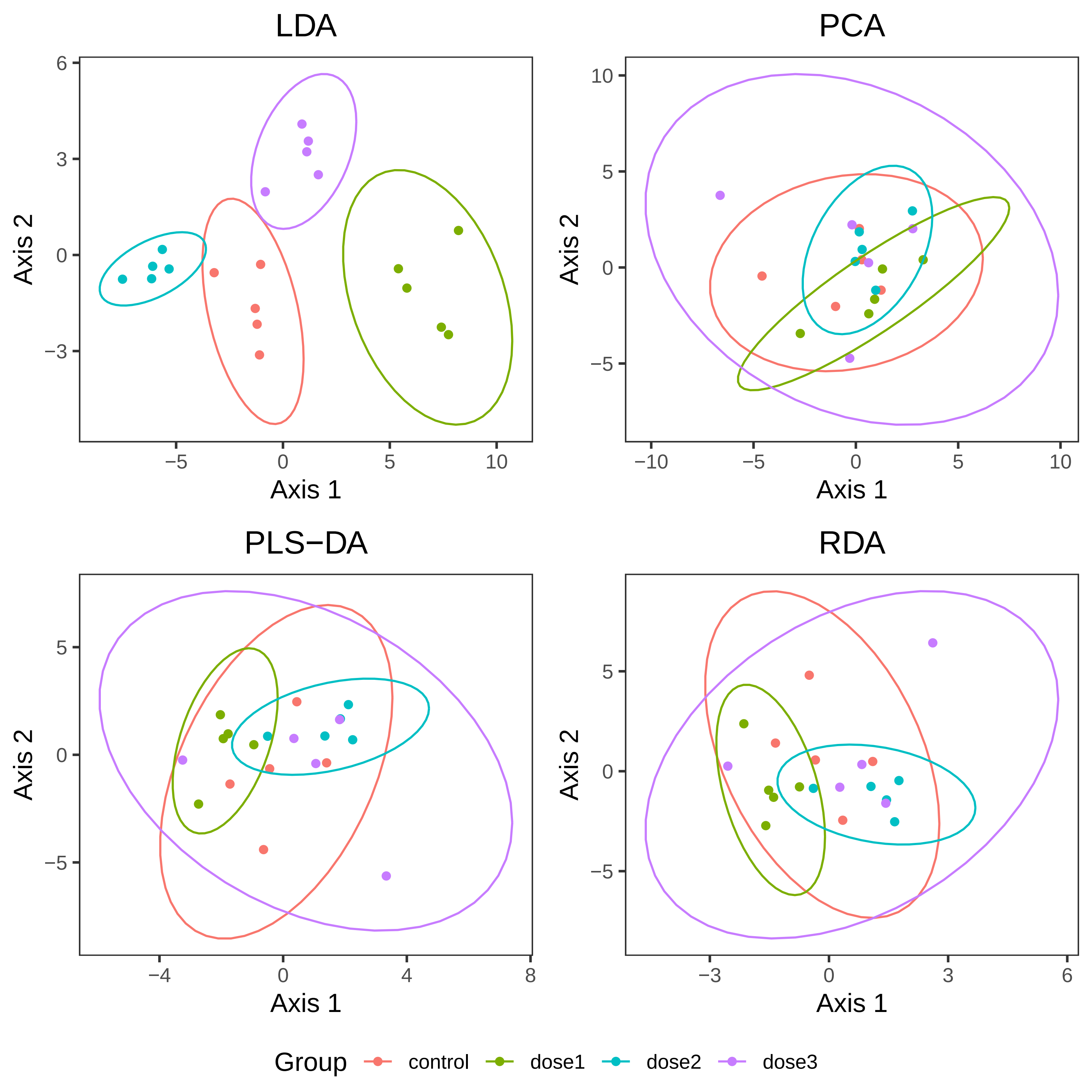
LDA
PCA
PLS−DA
RDA
Axis 1
Axis 2
Group
control
dose1
dose2
dose3

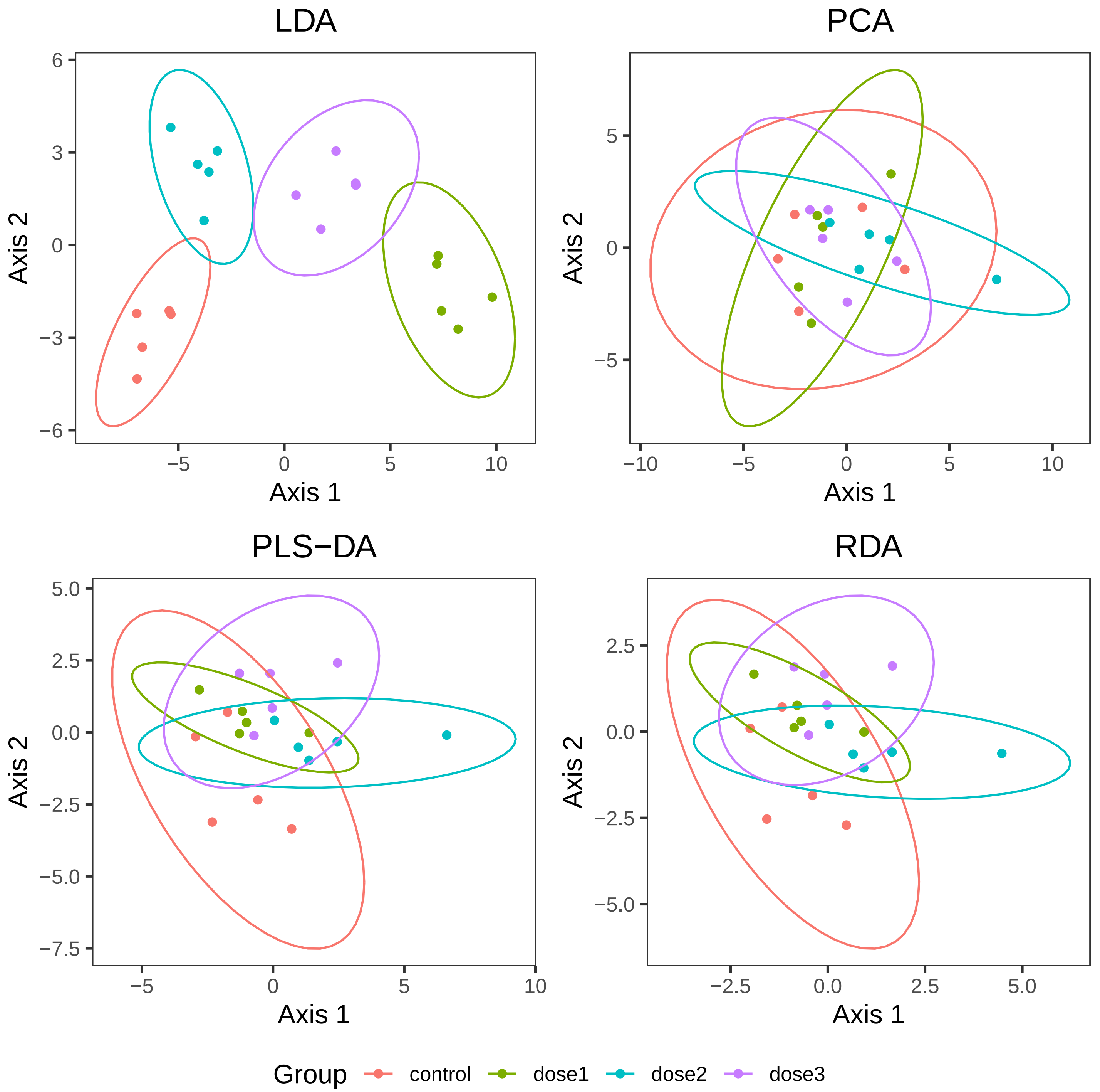
LDA
PCA
PLS−DA
RDA
Axis 1
Axis 2
6
3
0
−3
−6
−5
0
5
10
−10
2.5
5.0
−2.5
−5.0
−7.5
0.0
Group
control
dose1
dose2
dose3

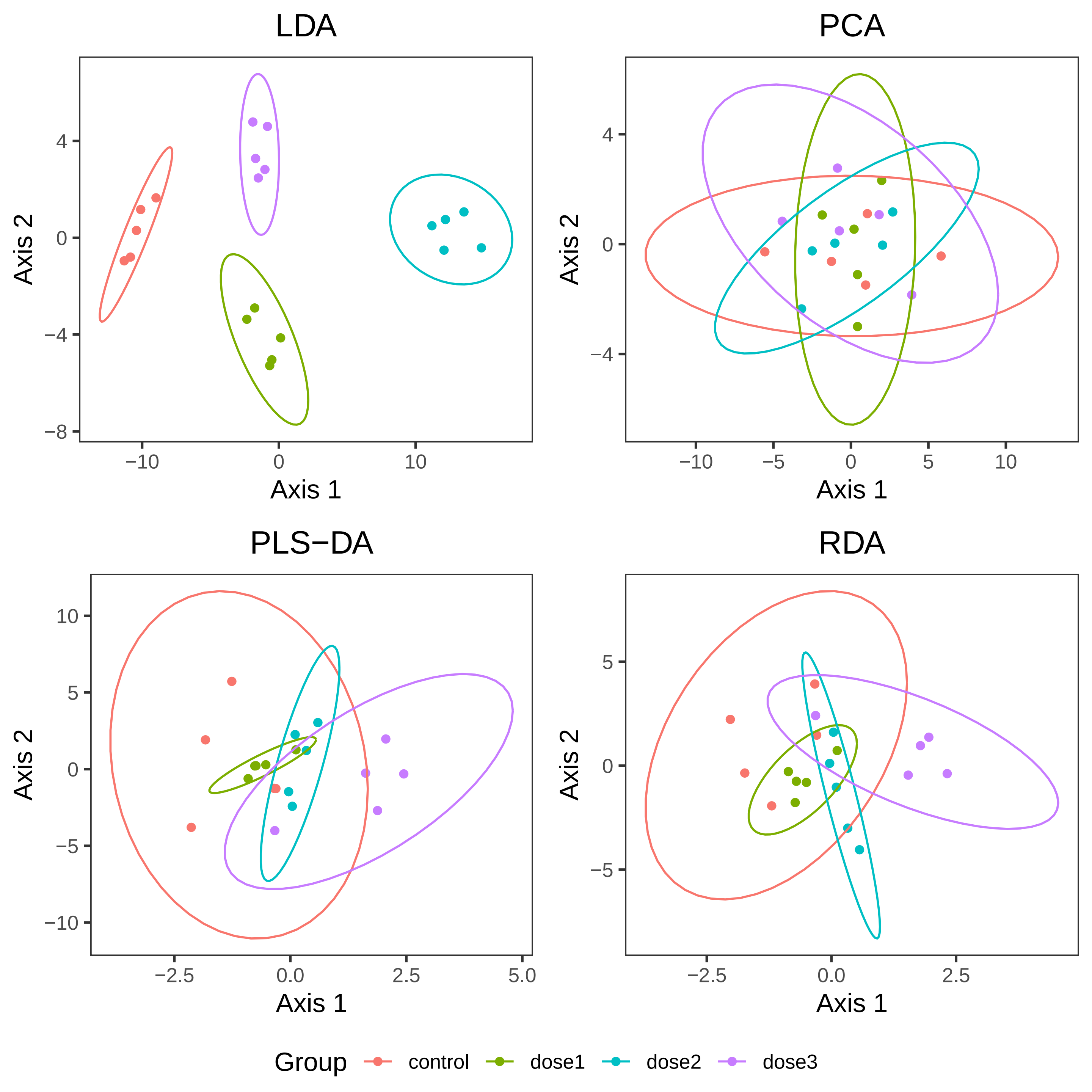

LDA
PCA
PLS−DA
RDA
Axis 1
Axis 2
Group
control
dose1
dose2
dose3

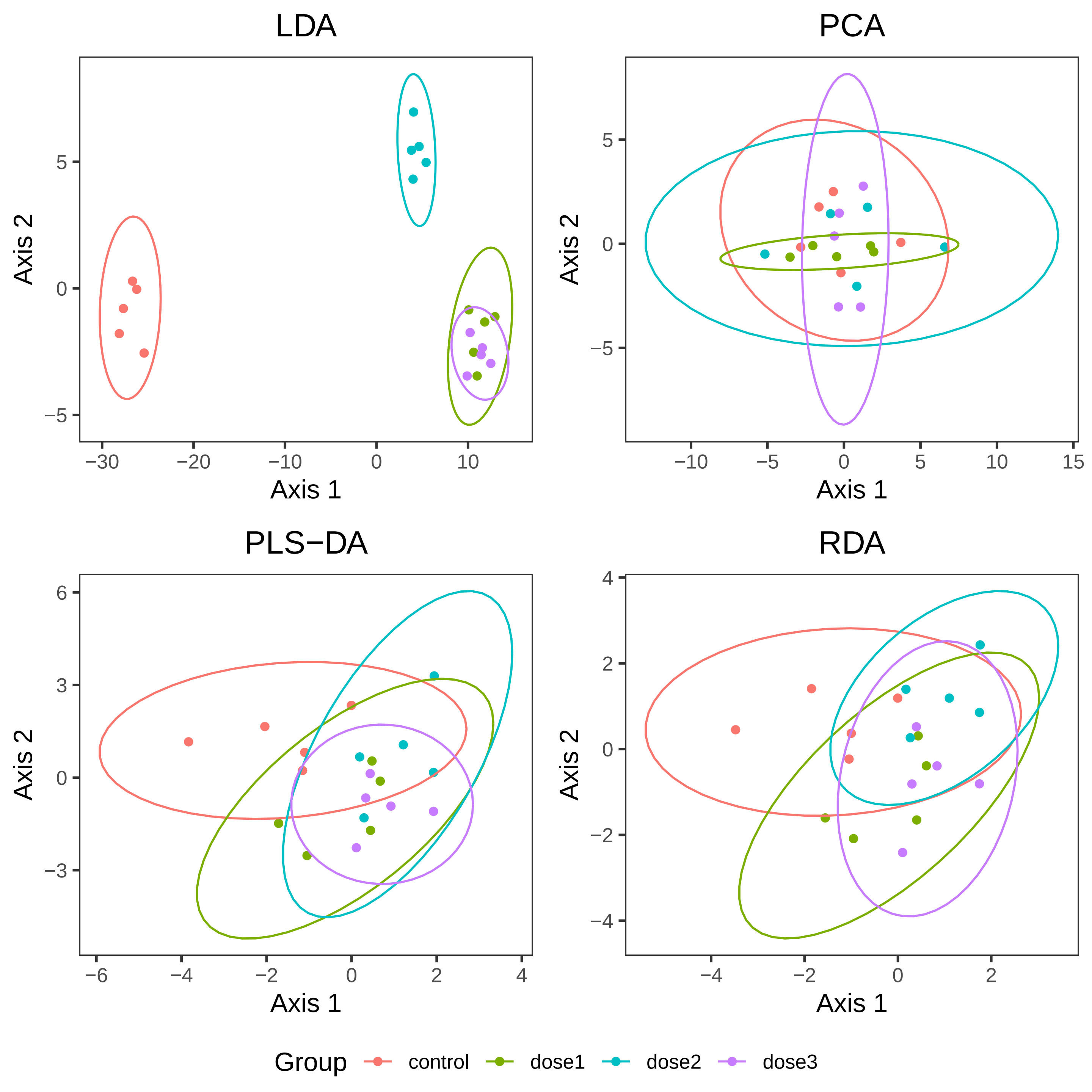
LDA
PCA
PLS−DA
RDA
Axis 1
Axis 2
Group
control
dose1
dose2
dose3

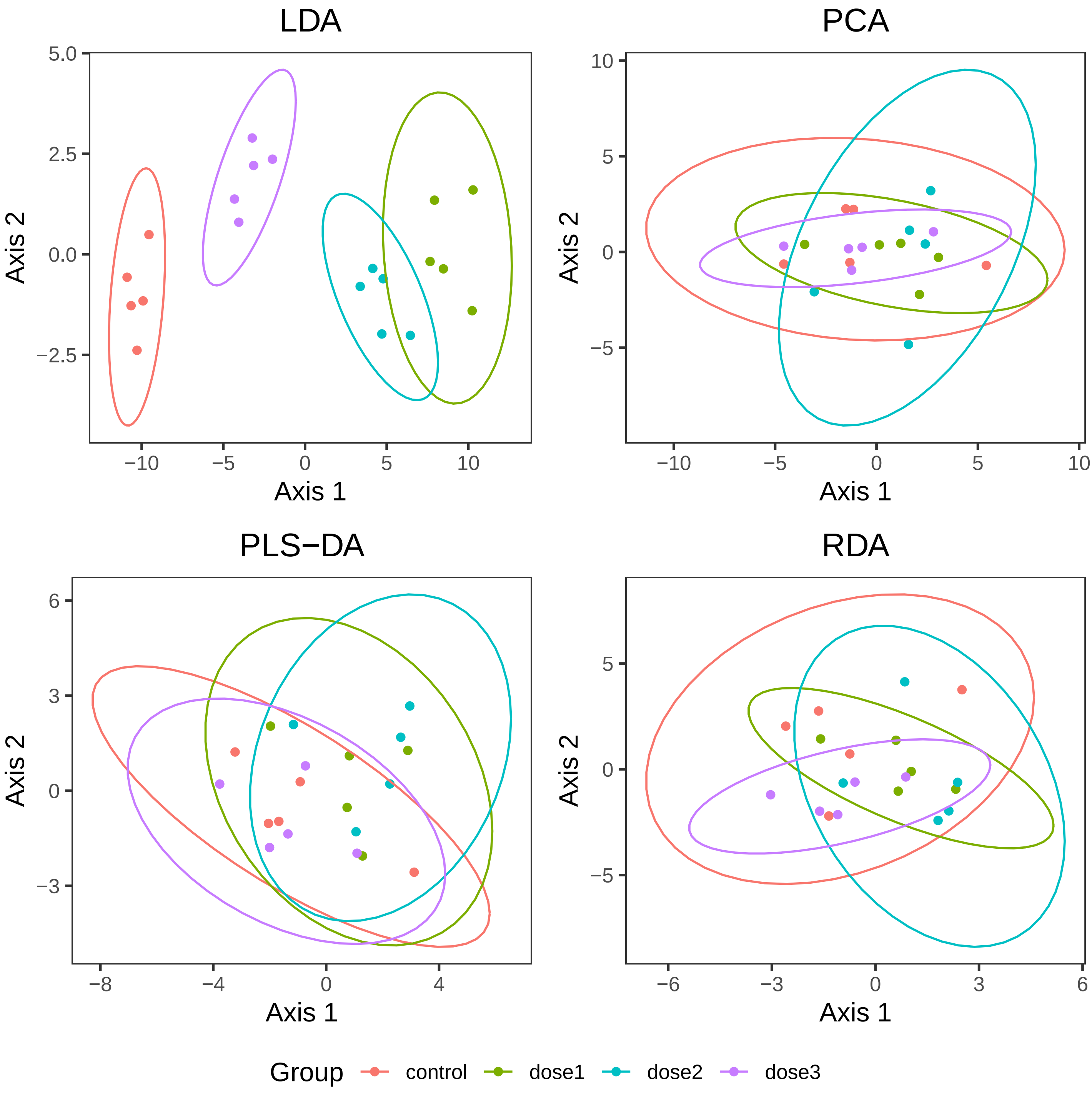
LDA
PCA
PLS−DA
RDA
Axis 1
Axis 2
5.0
2.5
0.0
−2.5
−10
−5
0
5
10
−5
0
5
10
−8
−4
0
4
6
3
0
−3
−6
−3
0
3
6
5
0
−5
Group
control
dose1
dose2
dose3

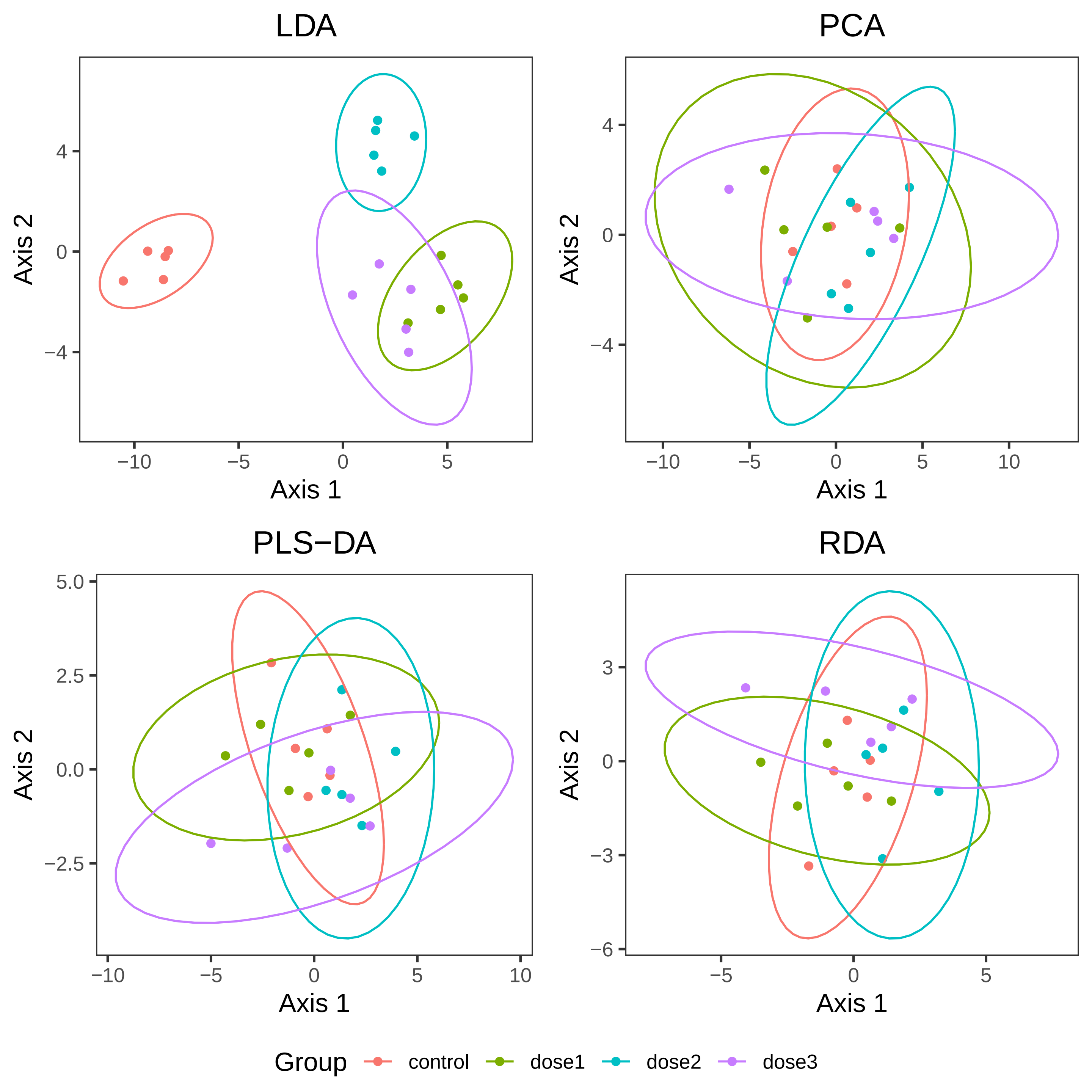
LDA
PCA
PLS−DA
RDA
Axis 1
Axis 2
Group
control
dose1
dose2
dose3

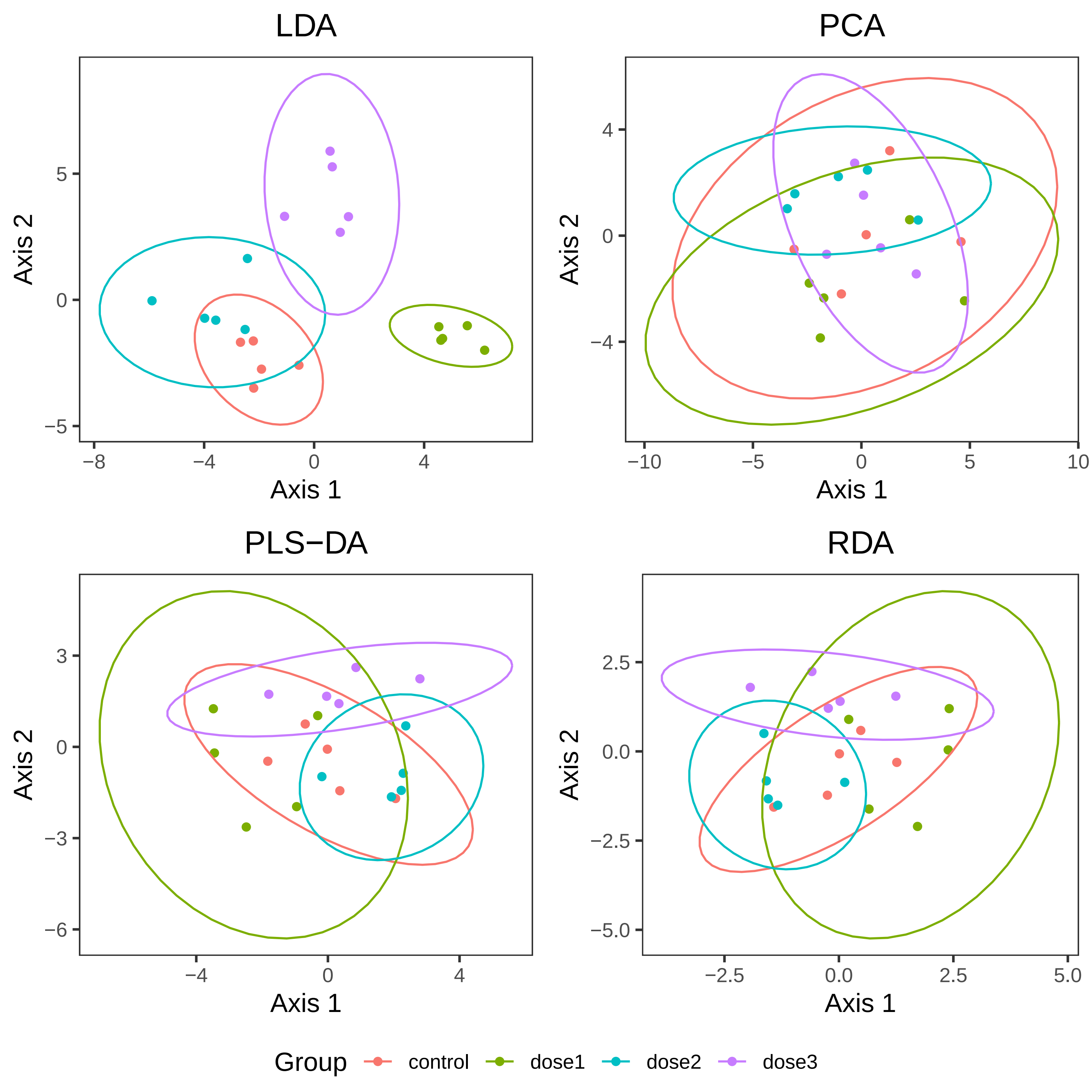
LDA
PCA
PLS−DA
RDA
Axis 1
Axis 2
Group
control
dose1
dose2
dose3

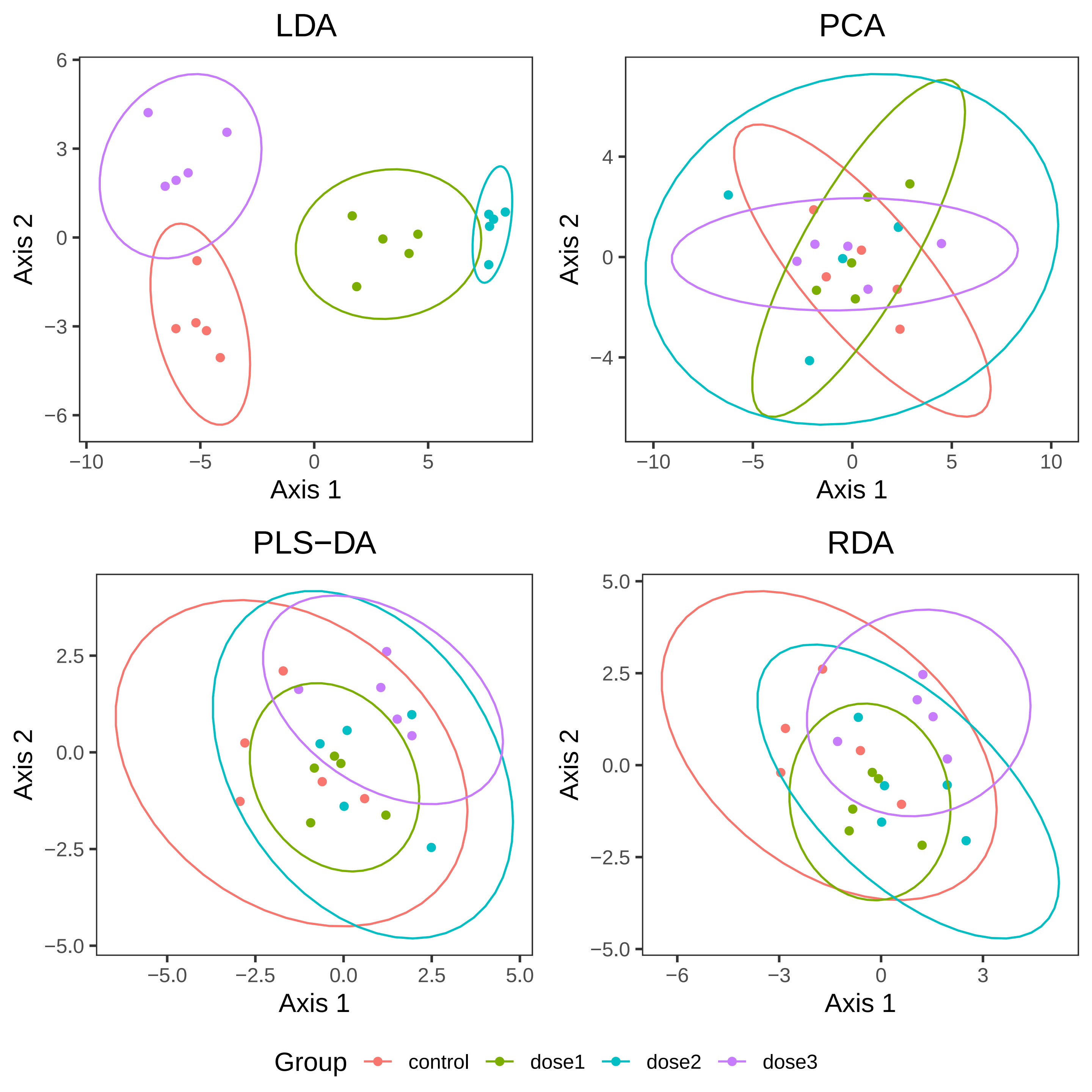
LDA
PCA
PLS−DA
RDA
Axis 1
Axis 2
Group
control
dose1
dose2
dose3

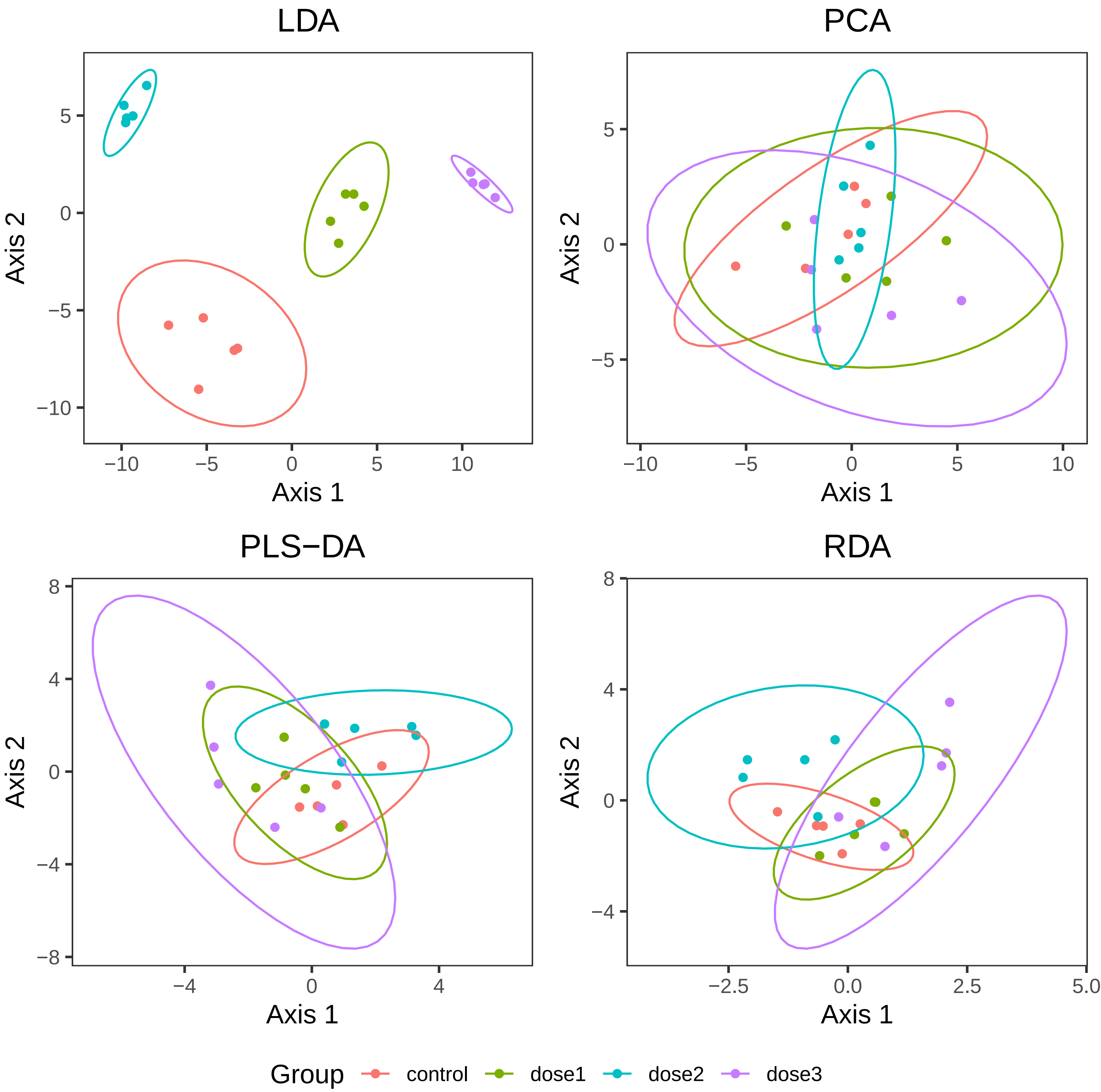

LDA
PCA
PLS−DA
RDA
Axis 1
Axis 2
Group
control
dose1
dose2
dose3

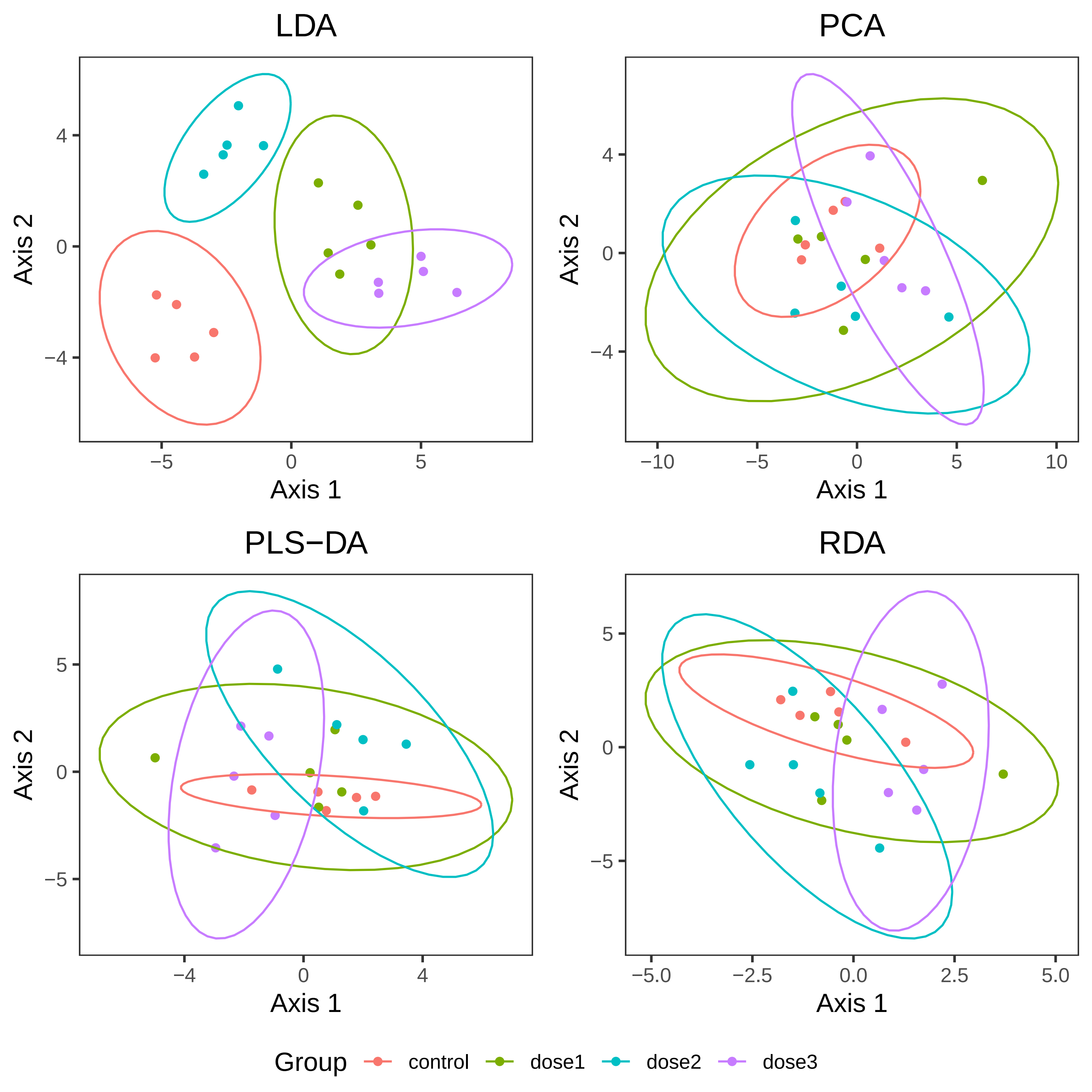

LDA
PCA
PLS−DA
RDA
Axis 1
Axis 2
−5
0
5
4
−4
−10
10
−2.5
2.5
−5.0
0.0
5.0
Group
control
dose1
dose2
dose3

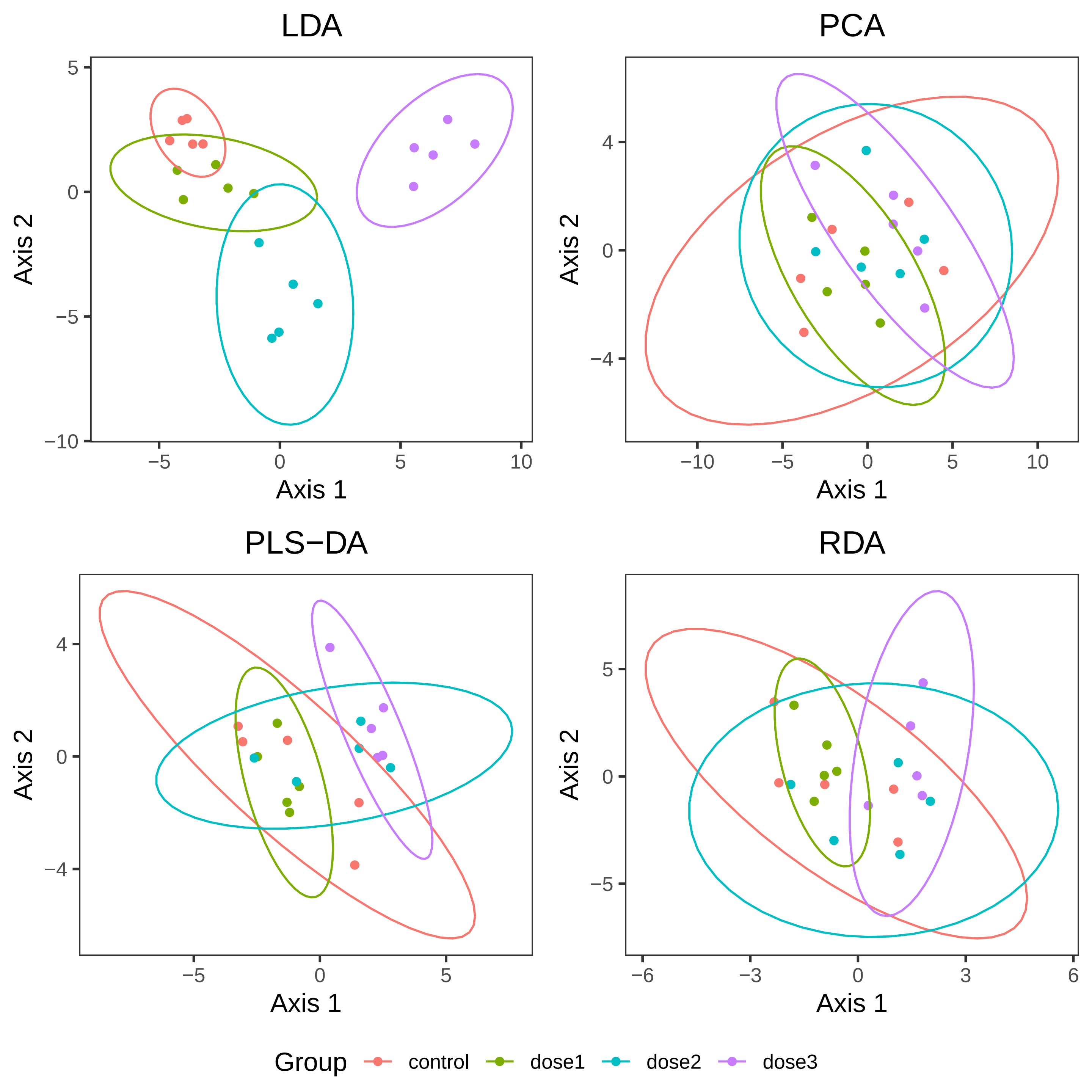

LDA
PCA
PLS−DA
RDA
Axis 1
Axis 2
Group
control
dose1
dose2
dose3

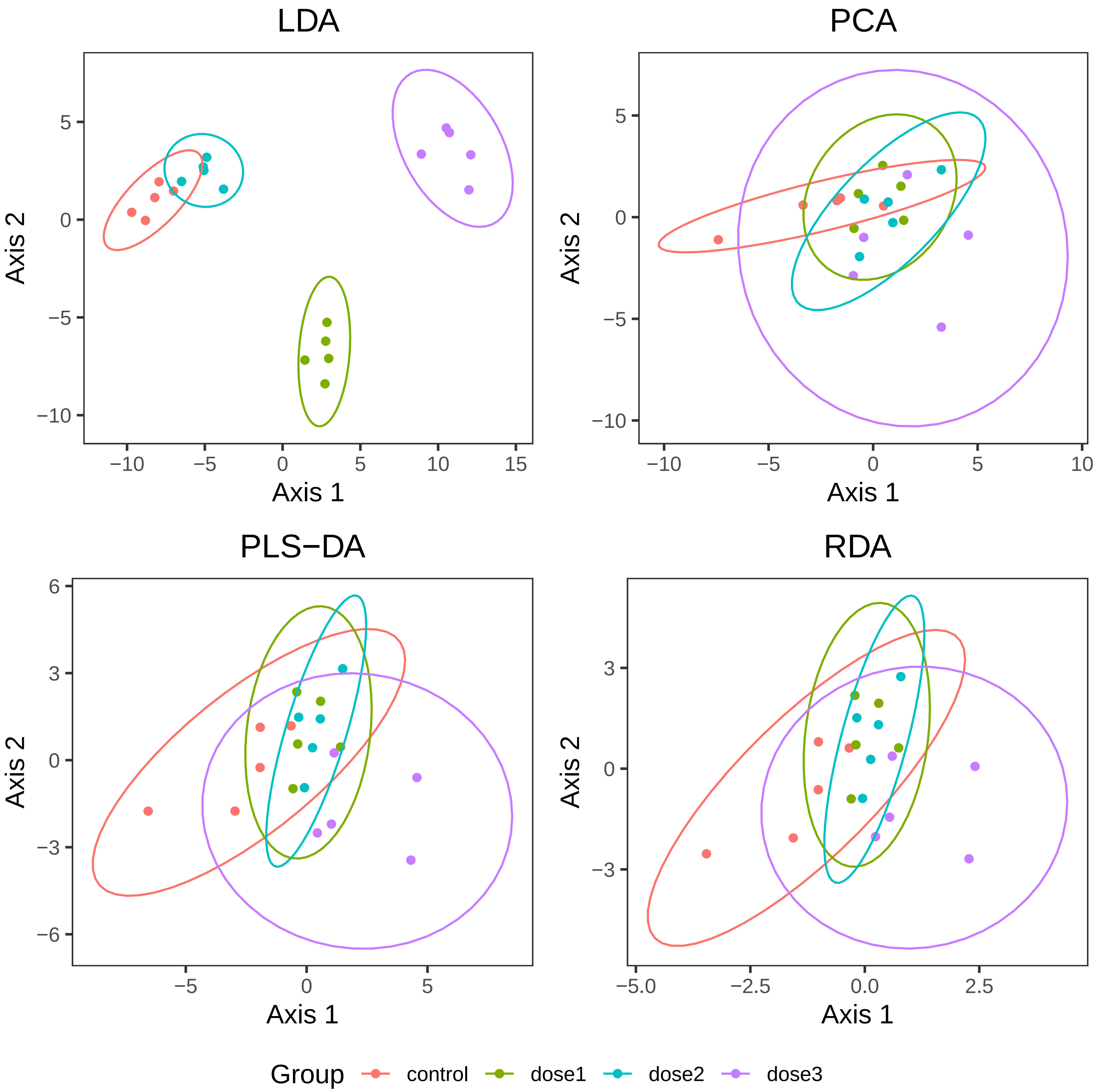
LDA
PCA
PLS−DA
RDA
Axis 1
Axis 2
−10
−5
0
5
10
15
−6
−3
3
6
−5.0
−2.5
0.0
2.5
Group
control
dose1
dose2
dose3

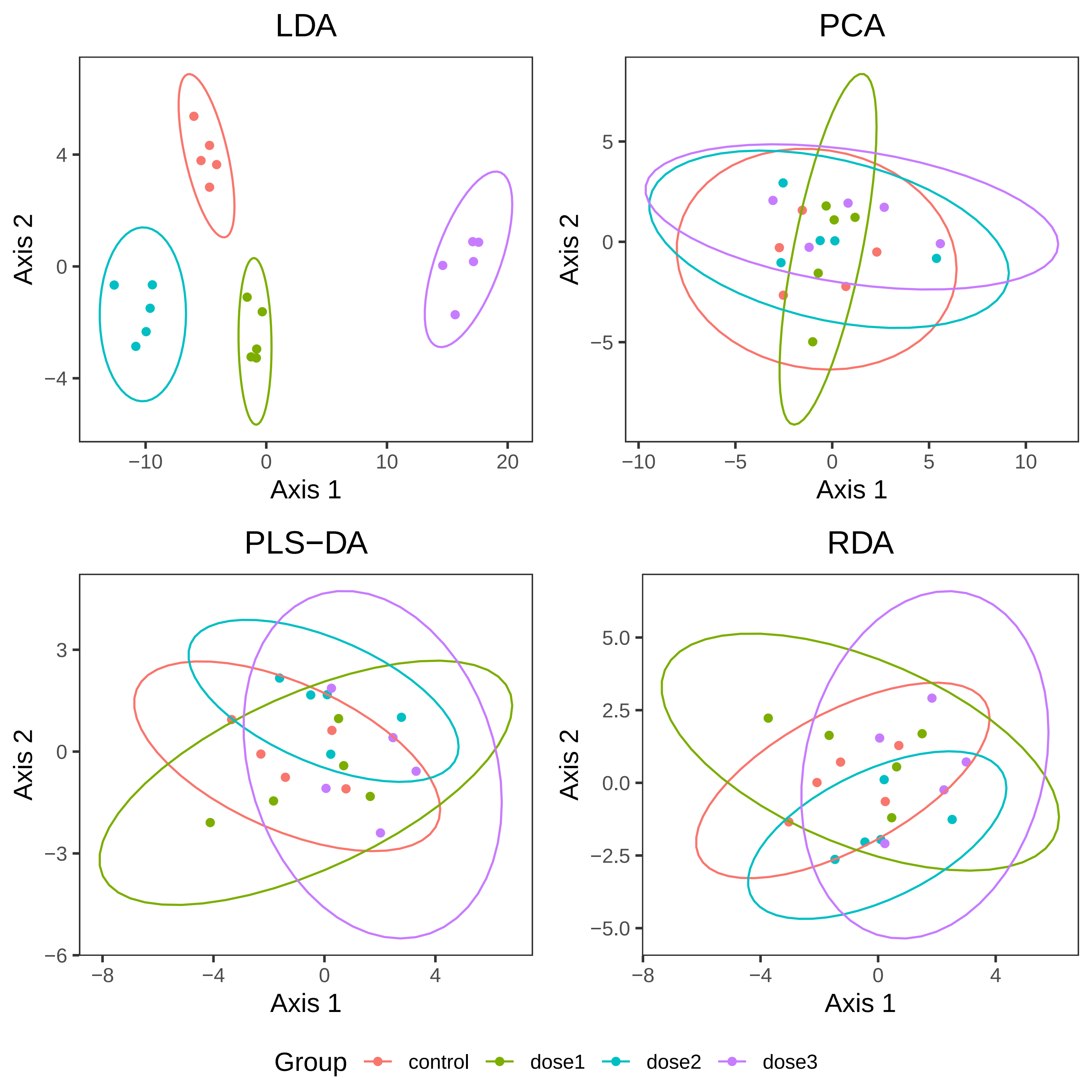
LDA
PCA
PLS−DA
RDA
Axis 1
Axis 2
Group
control
dose1
dose2
dose3

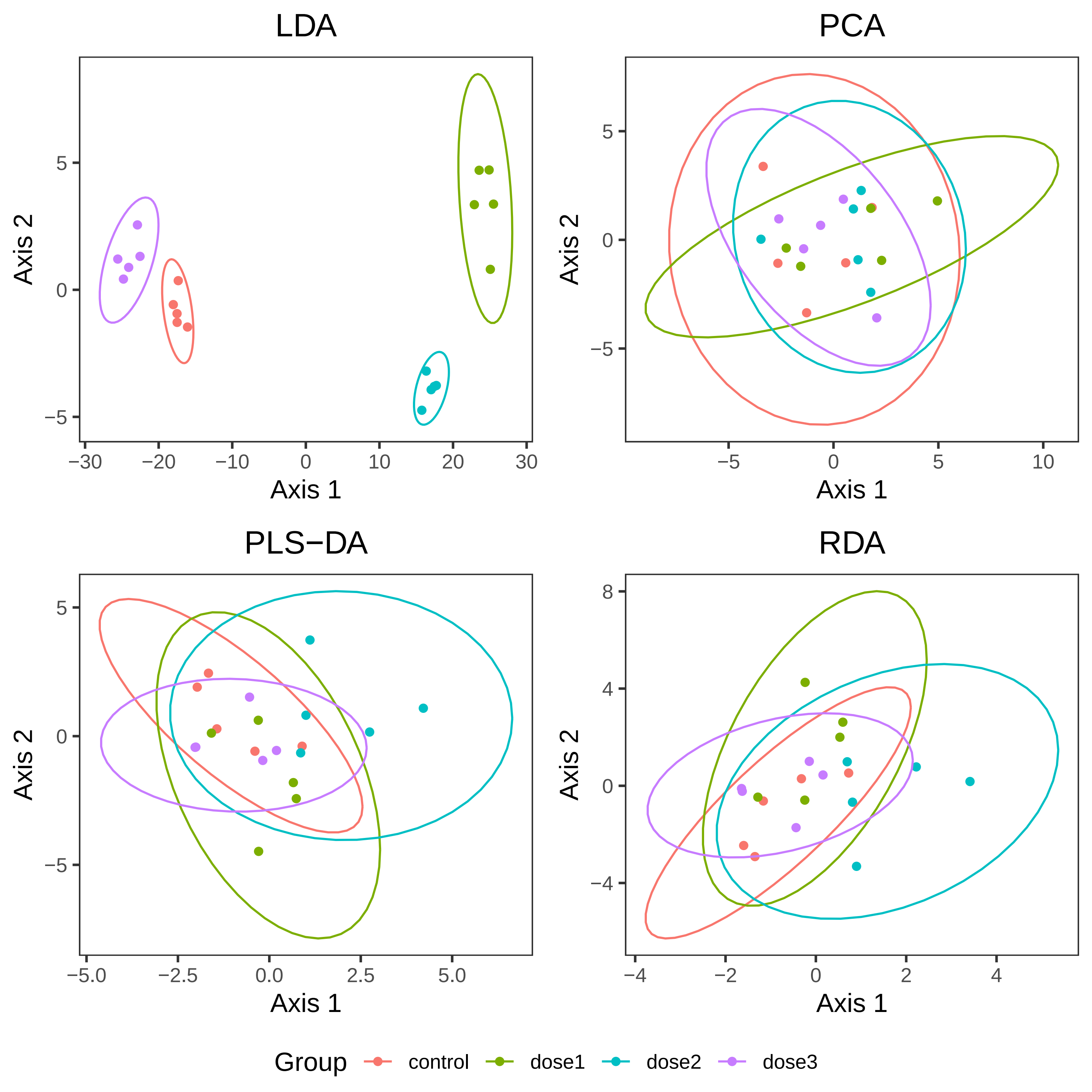

LDA
PCA
PLS−DA
RDA
Axis 1
Axis 2
Group
control
dose1
dose2
dose3

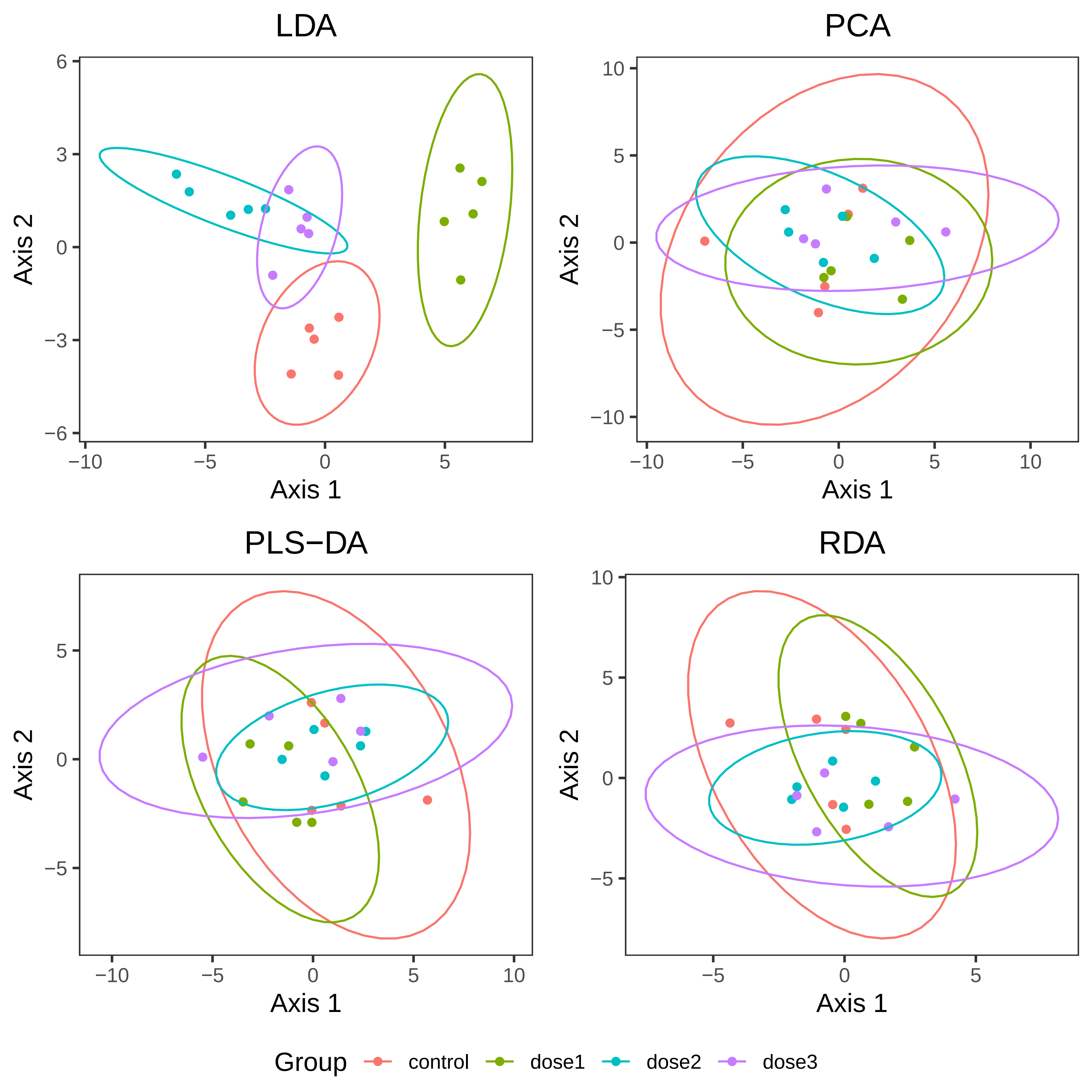

LDA
PCA
PLS−DA
RDA
Axis 1
Axis 2
Group
control
dose1
dose2
dose3

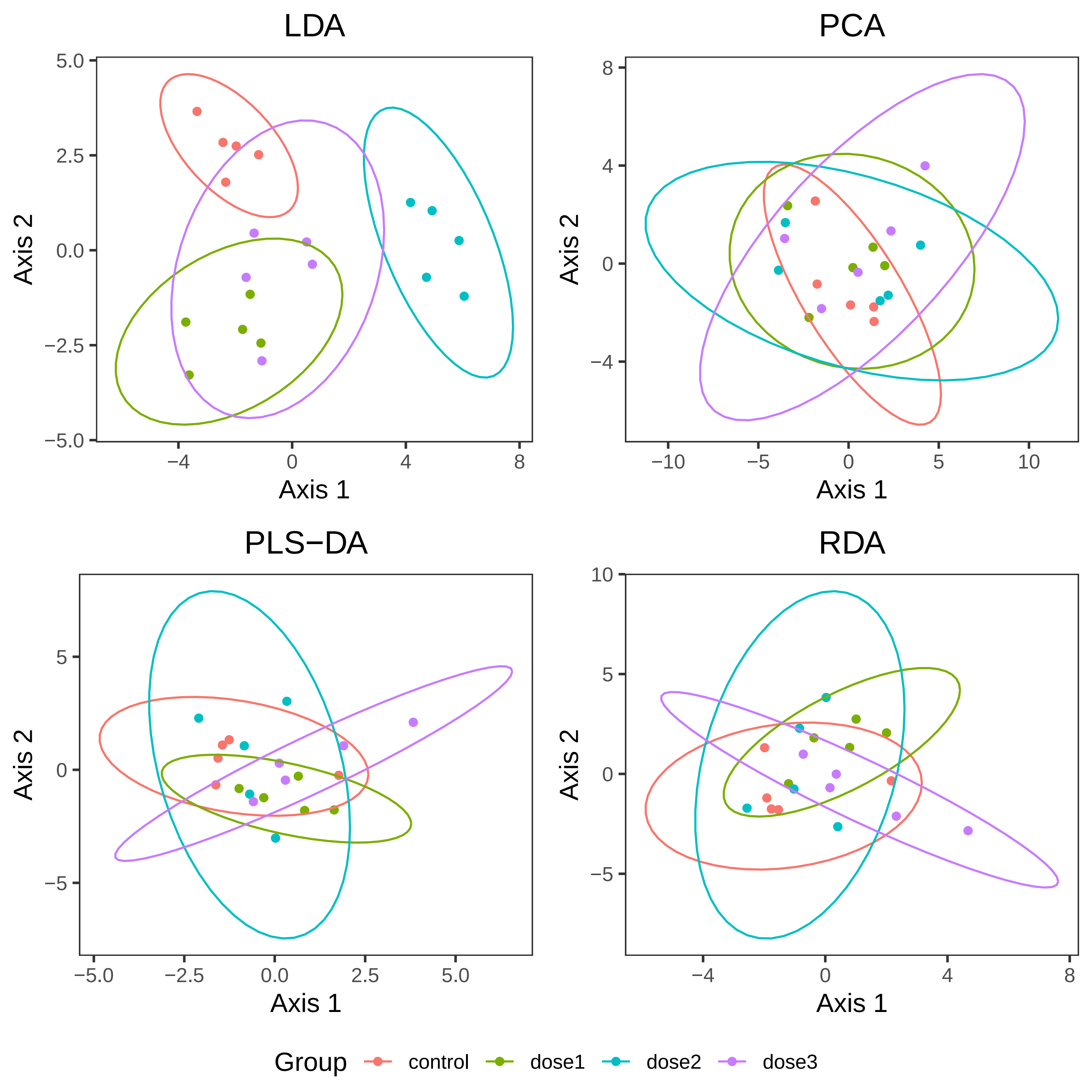

LDA
PCA
PLS−DA
RDA
Axis 1
Axis 2
5.0
2.5
0.0
−2.5
−5.0
−4
0
4
8
−10
−5
5
10
−5.0
−2.5
0.0
2.5
5.0
Group
control
dose1
dose2
dose3

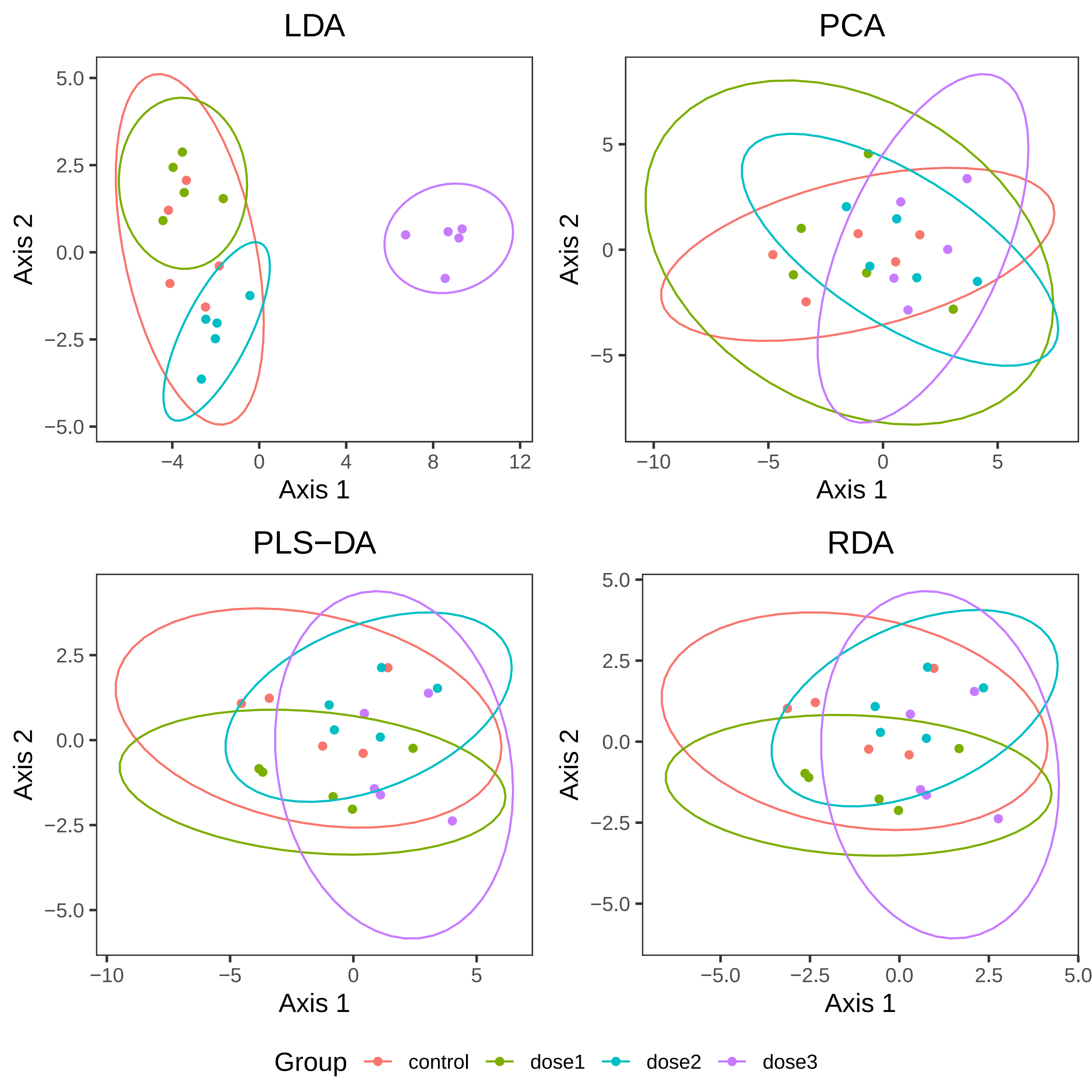

LDA
PCA
PLS−DA
RDA
Axis 1
Axis 2
5.0
2.5
0.0
−2.5
−5.0
−4
0
4
8
12
5
0
−5
−10
−5
0
5
−10
−5
0
5
−5.0
−2.5
0.0
2.5
5.0
Group
control
dose1
dose2
dose3

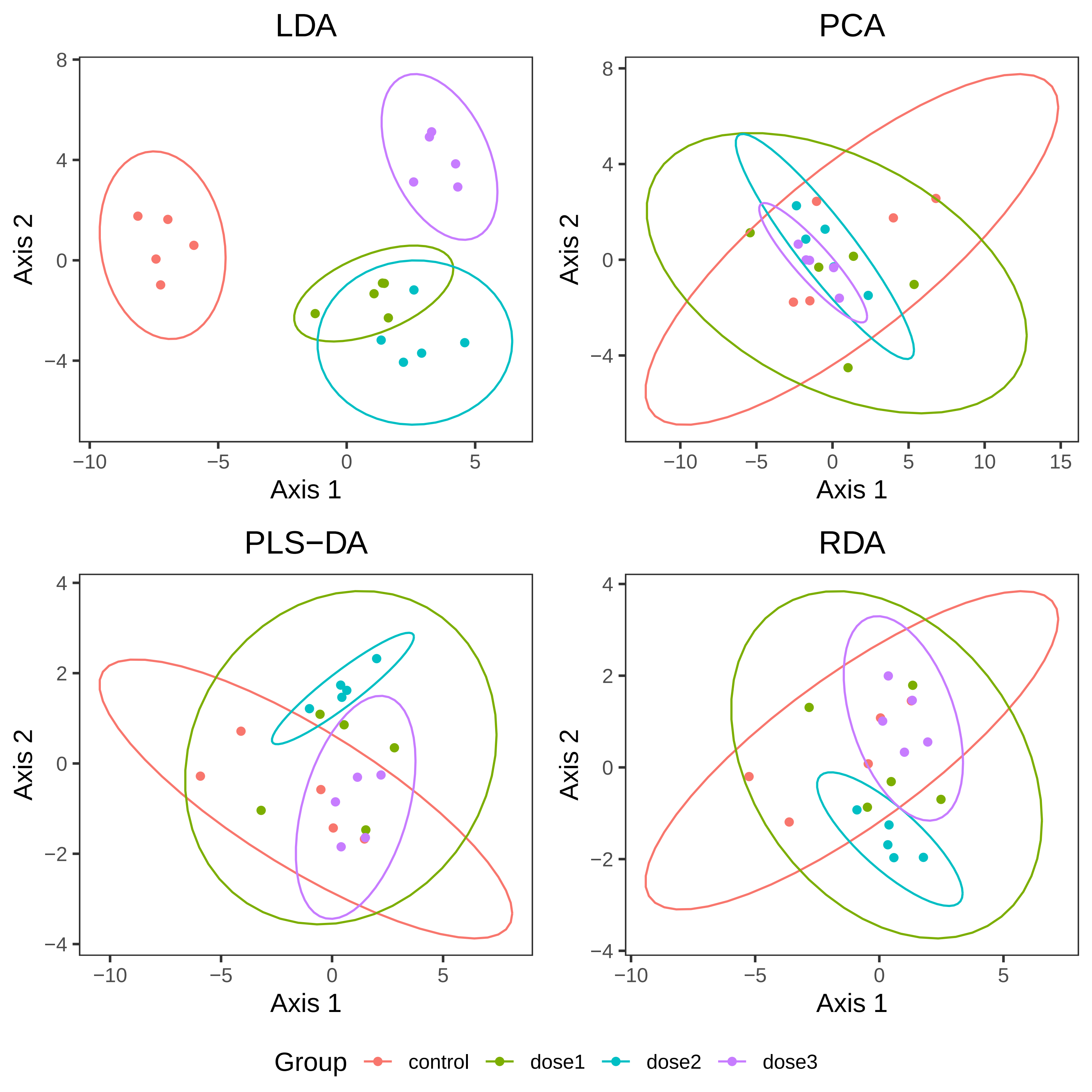
LDA
PCA
PLS−DA
RDA
Axis 1
Axis 2
Group
control
dose1
dose2
dose3
−10
−5
0
5
10
15
8
4
0
−4
2
−2

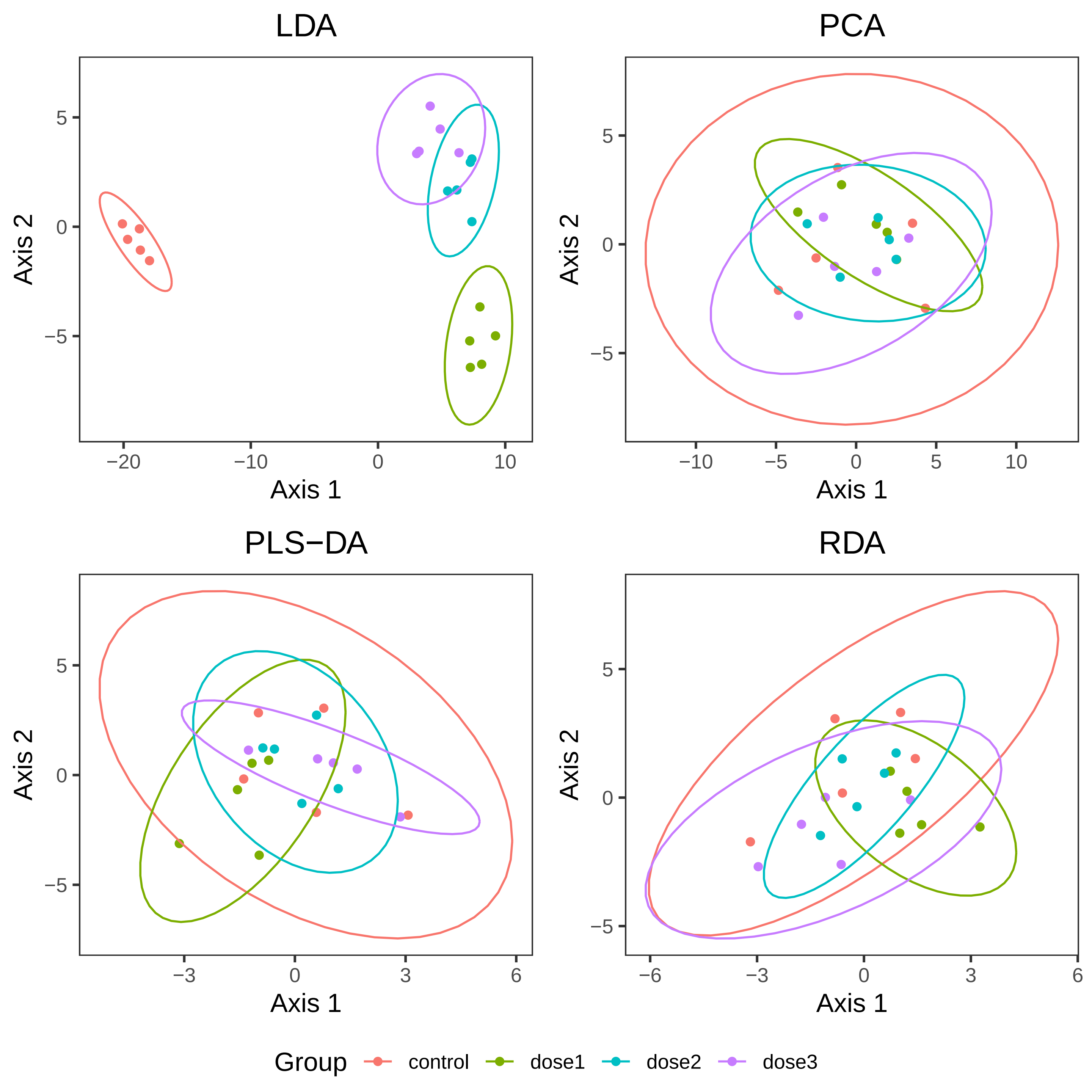

LDA
PCA
PLS−DA
RDA
Axis 1
Axis 2
Group
control
dose1
dose2
dose3

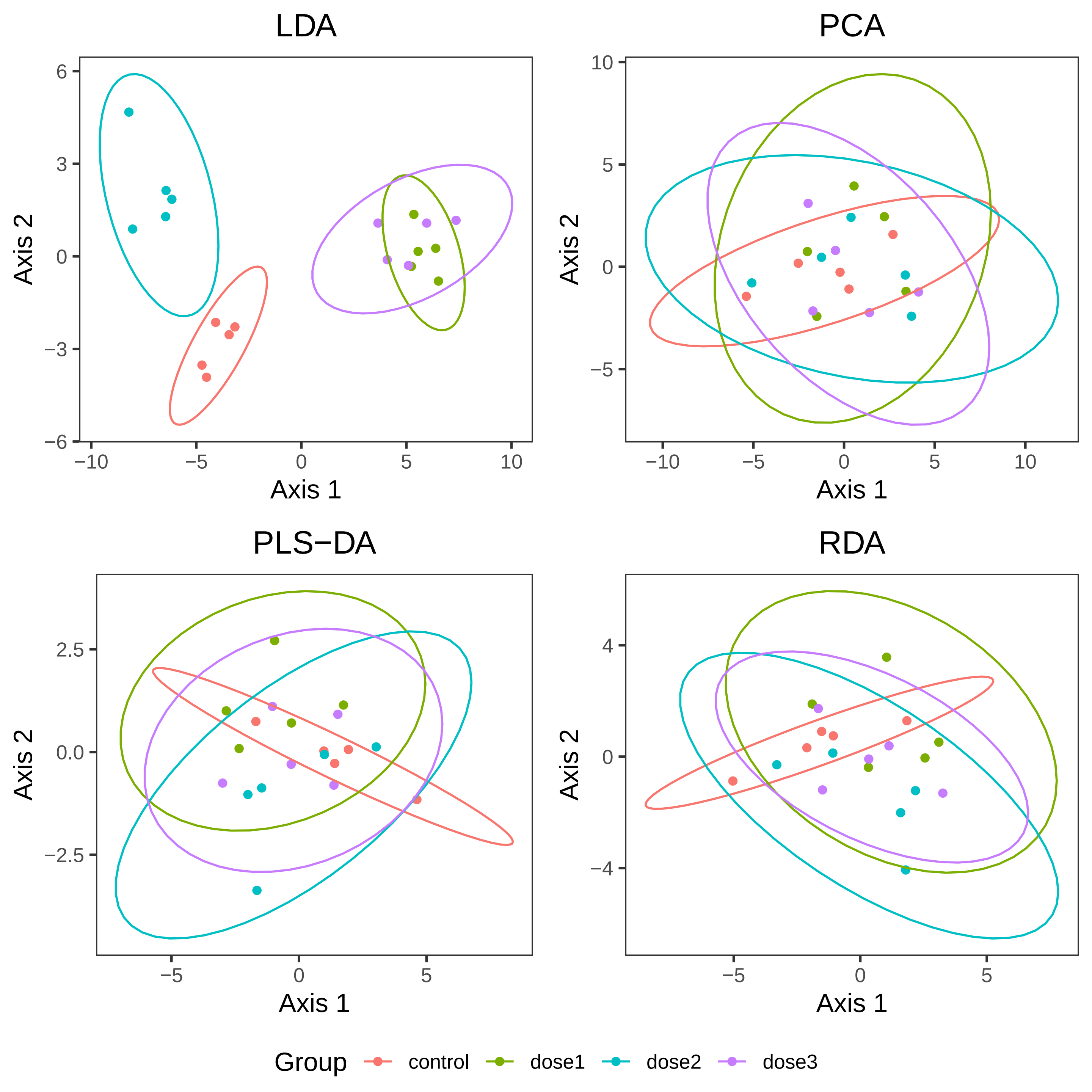

LDA
PCA
PLS−DA
RDA
Axis 1
Axis 2
Group
control
dose1
dose2
dose3

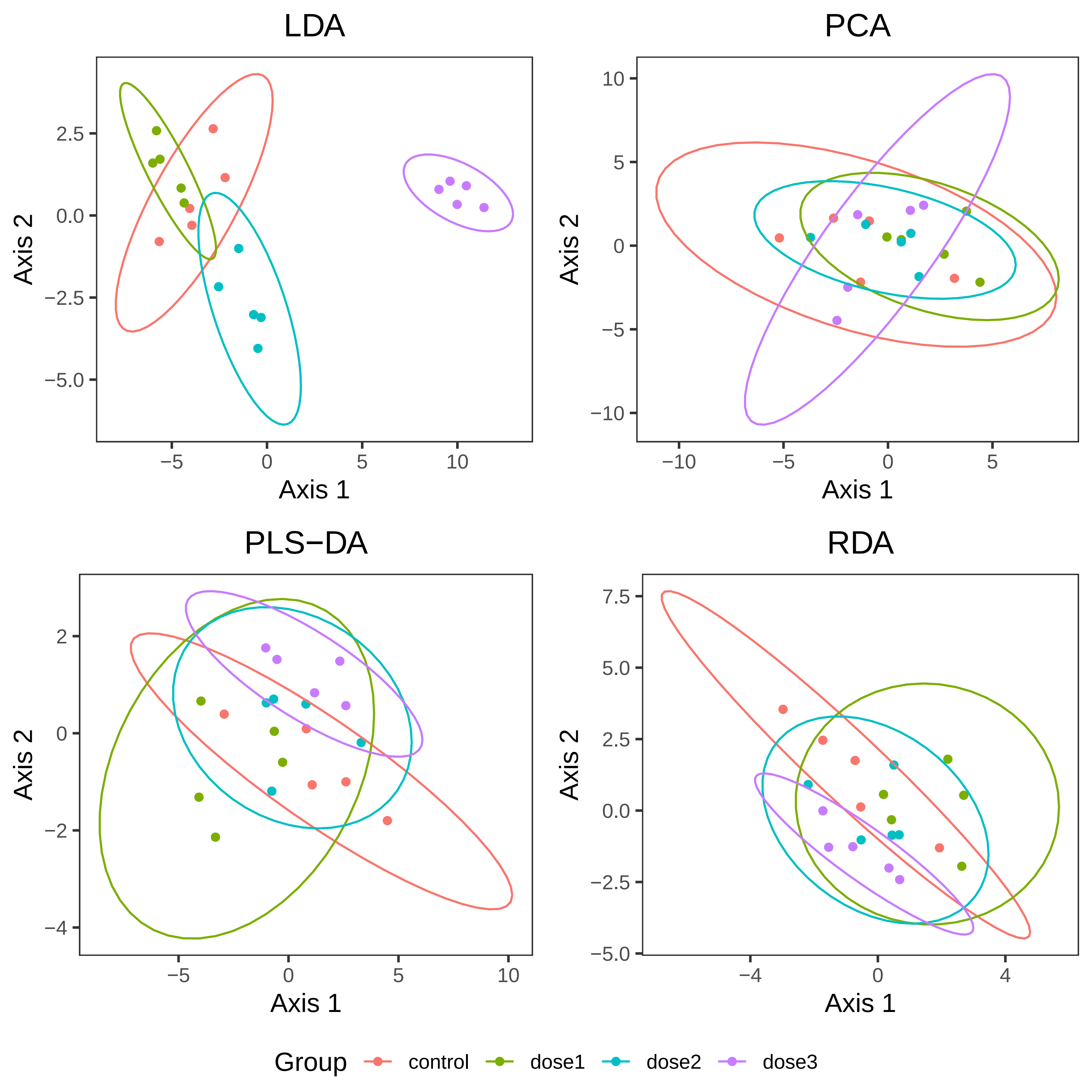

LDA
PCA
PLS−DA
RDA
Axis 1
Axis 2
Group
control
dose1
dose2
dose3

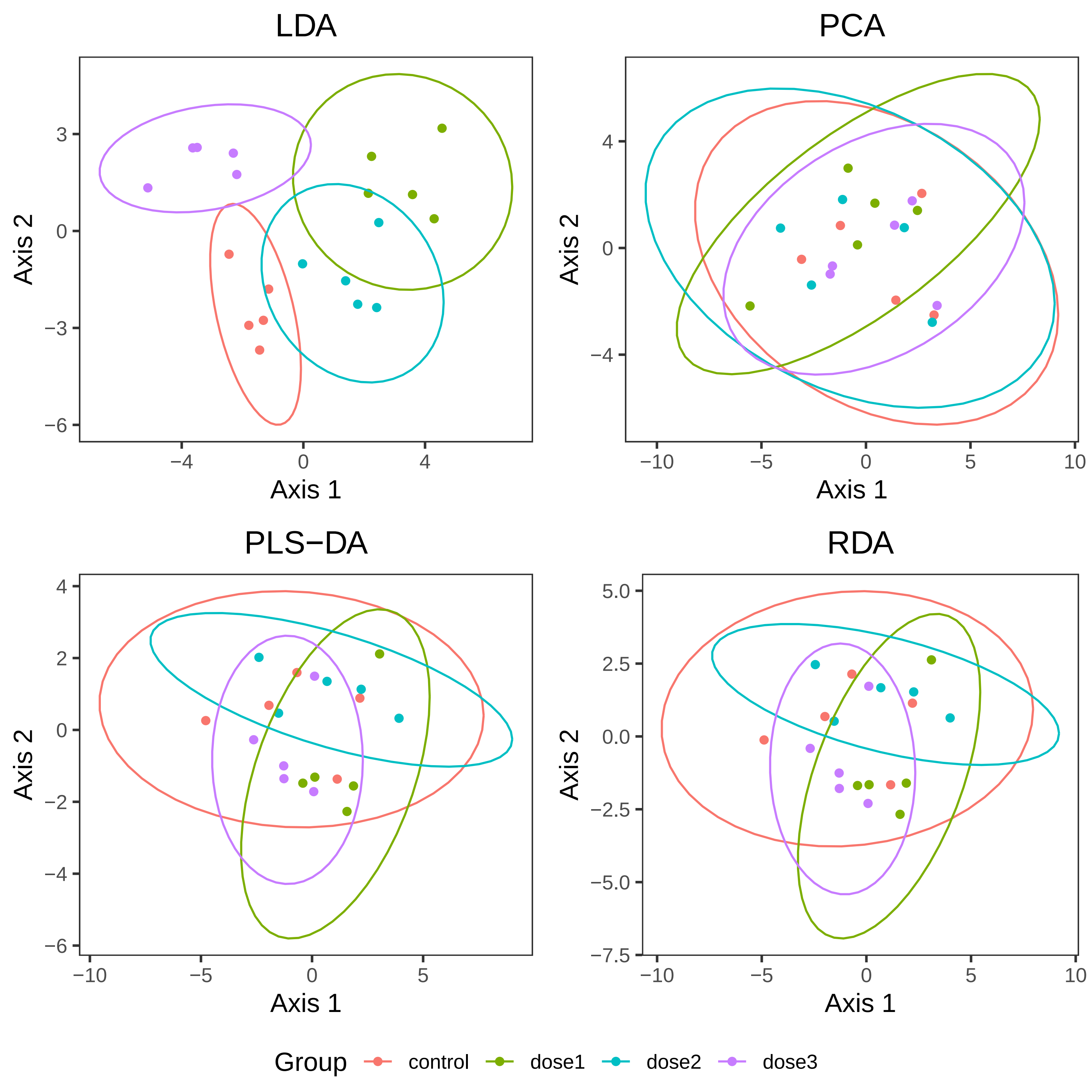

LDA
PCA
PLS−DA
RDA
Axis 1
Axis 2
3
0
−3
−6
−4
0
4
−10
−5
5
10
2
−2
−4
−6
5.0
2.5
0.0
−2.5
−5.0
−7.5
Group
control
dose1
dose2
dose3

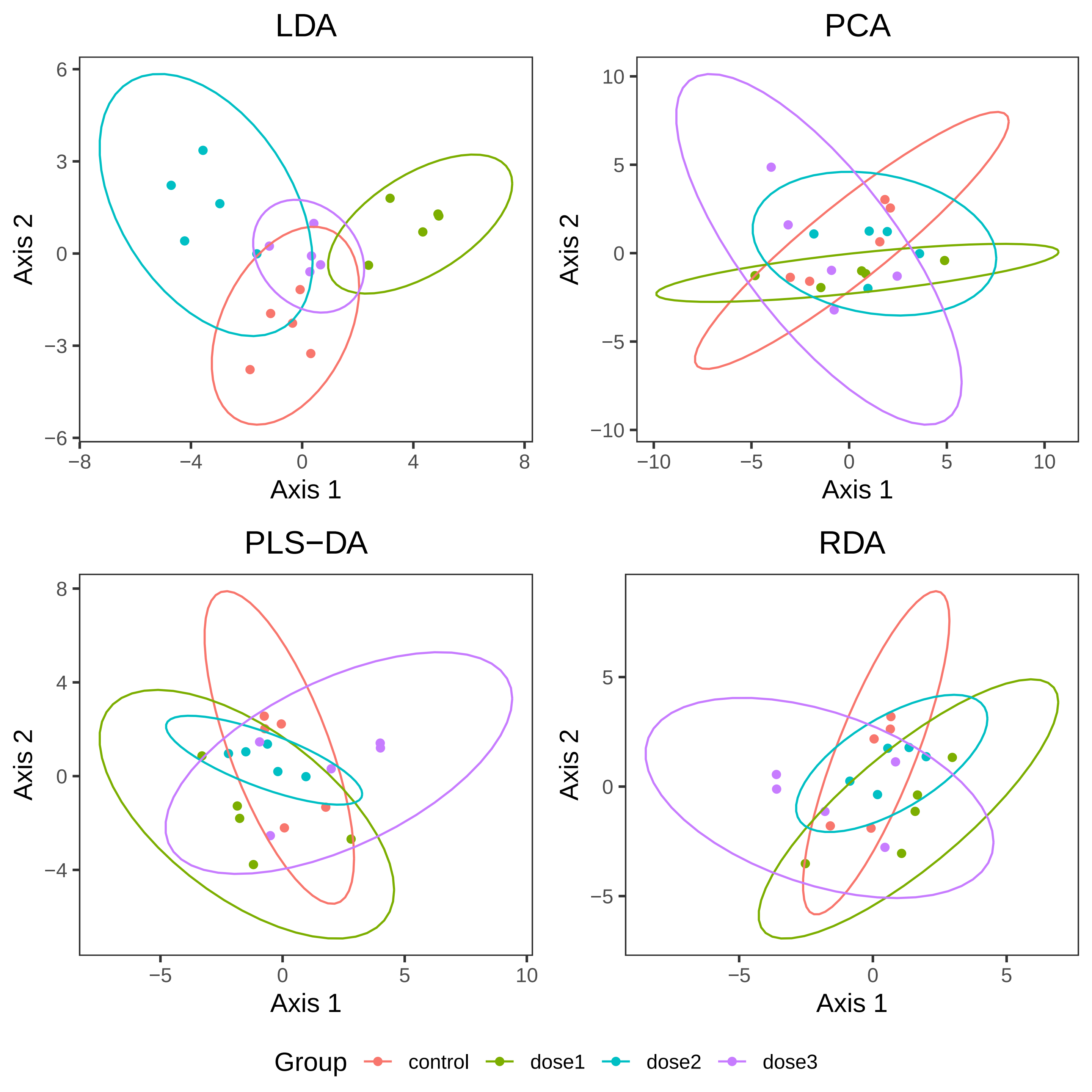

LDA
PCA
PLS−DA
RDA
Axis 1
Axis 2
6
3
0
−3
−6
−8
−4
0
4
8
10
5
0
−5
−10
−10
−5
0
5
10
8
4
0
−4
−5
0
5
10
5
0
−5
−5
0
5
Group
control
dose1
dose2
dose3

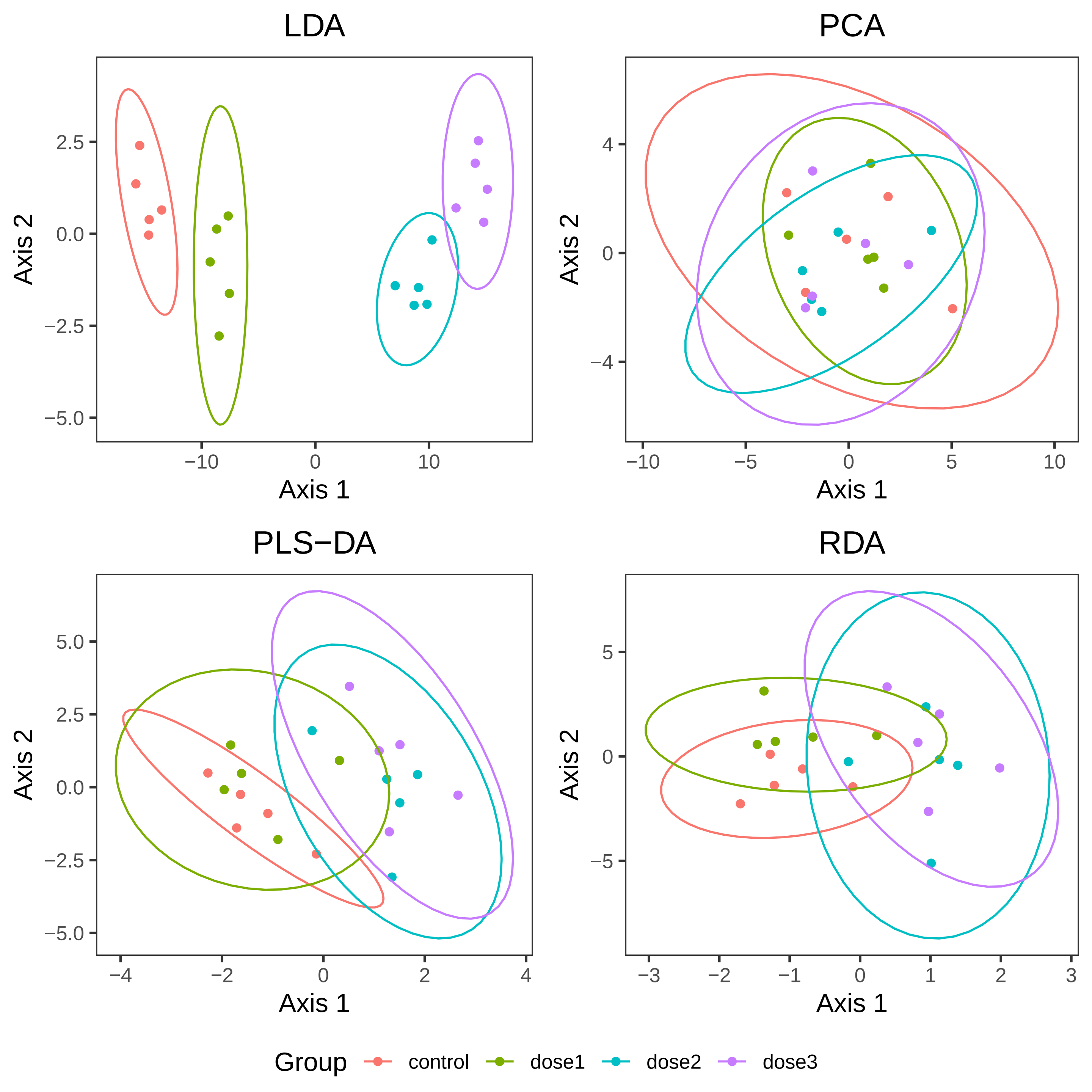

LDA
PCA
PLS−DA
RDA
Axis 1
Axis 2
Group
control
dose1
dose2
dose3

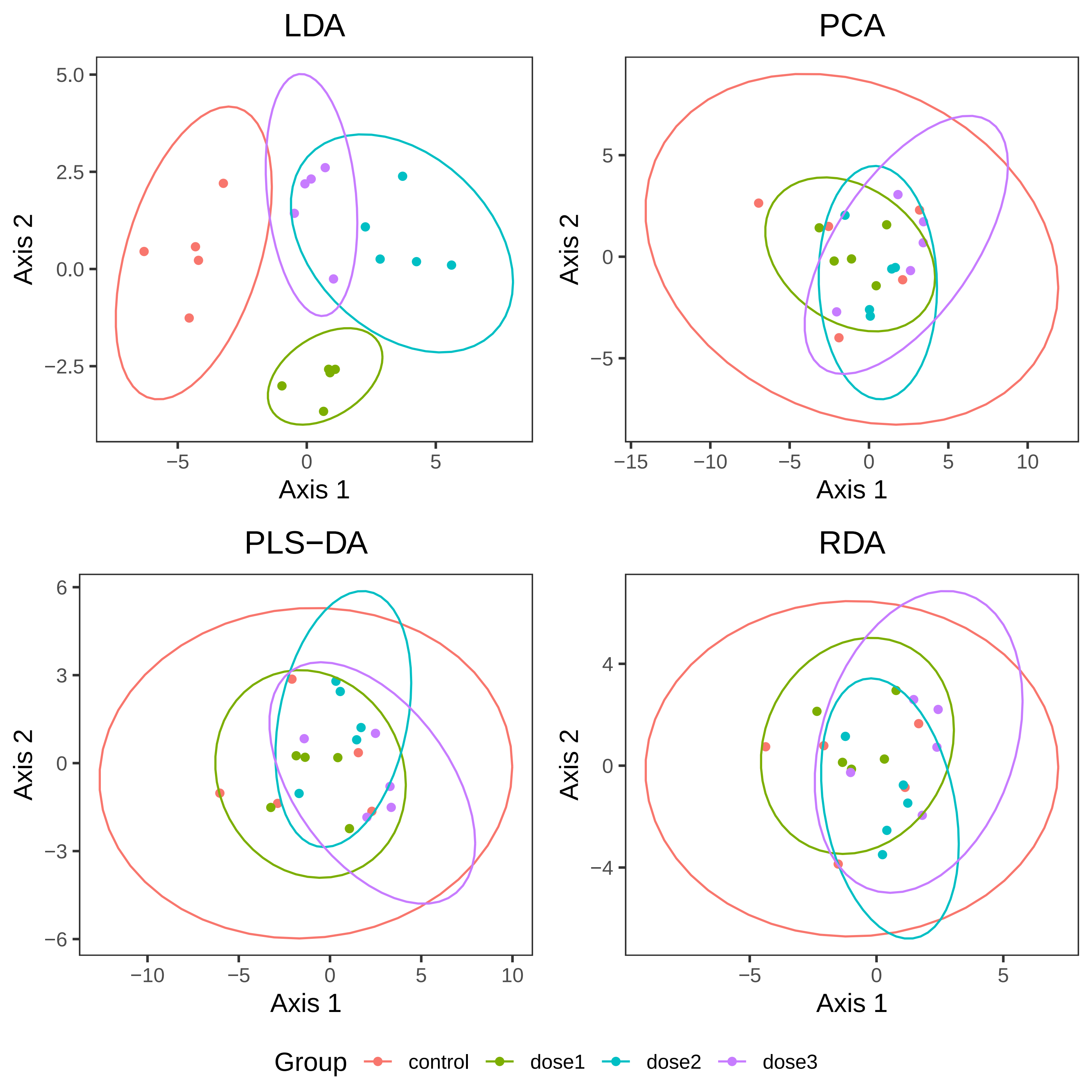

LDA
PCA
PLS−DA
RDA
Axis 1
Axis 2
Group
control
dose1
dose2
dose3

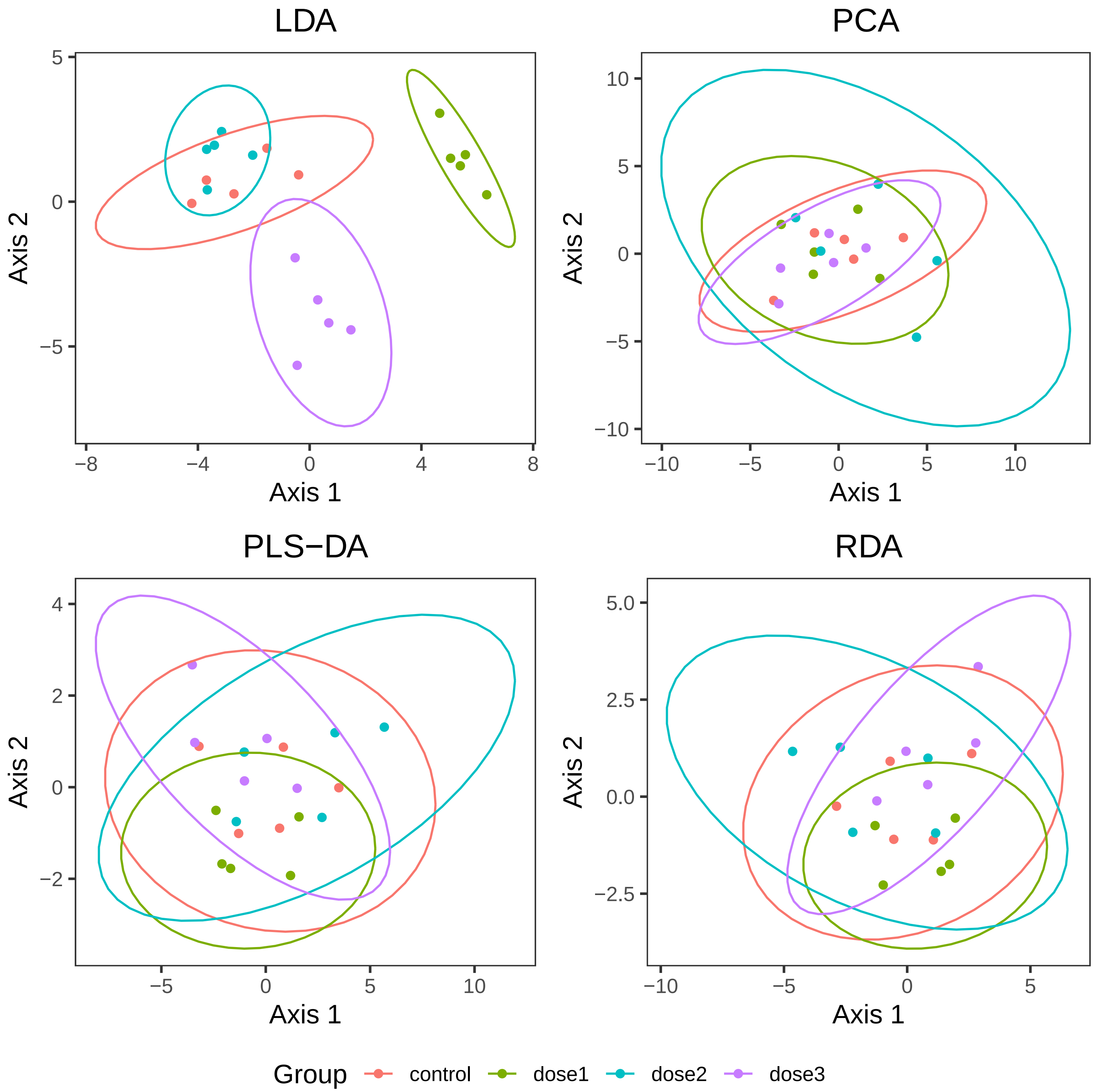

LDA
PCA
PLS−DA
RDA
Axis 1
Axis 2
Group
control
dose1
dose2
dose3

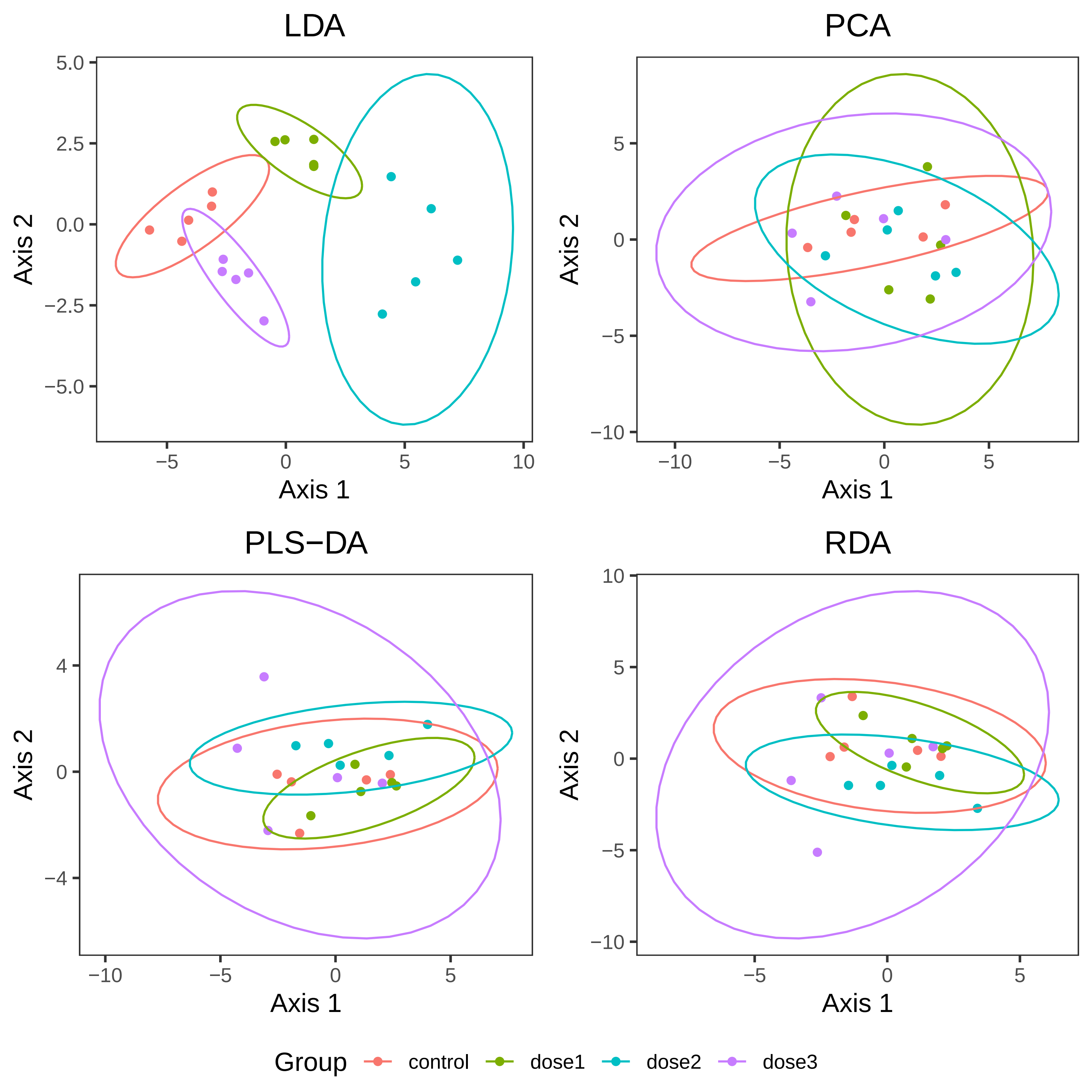
LDA
PCA
PLS−DA
RDA
Axis 1
Axis 2
Group
control
dose1
dose2
dose3

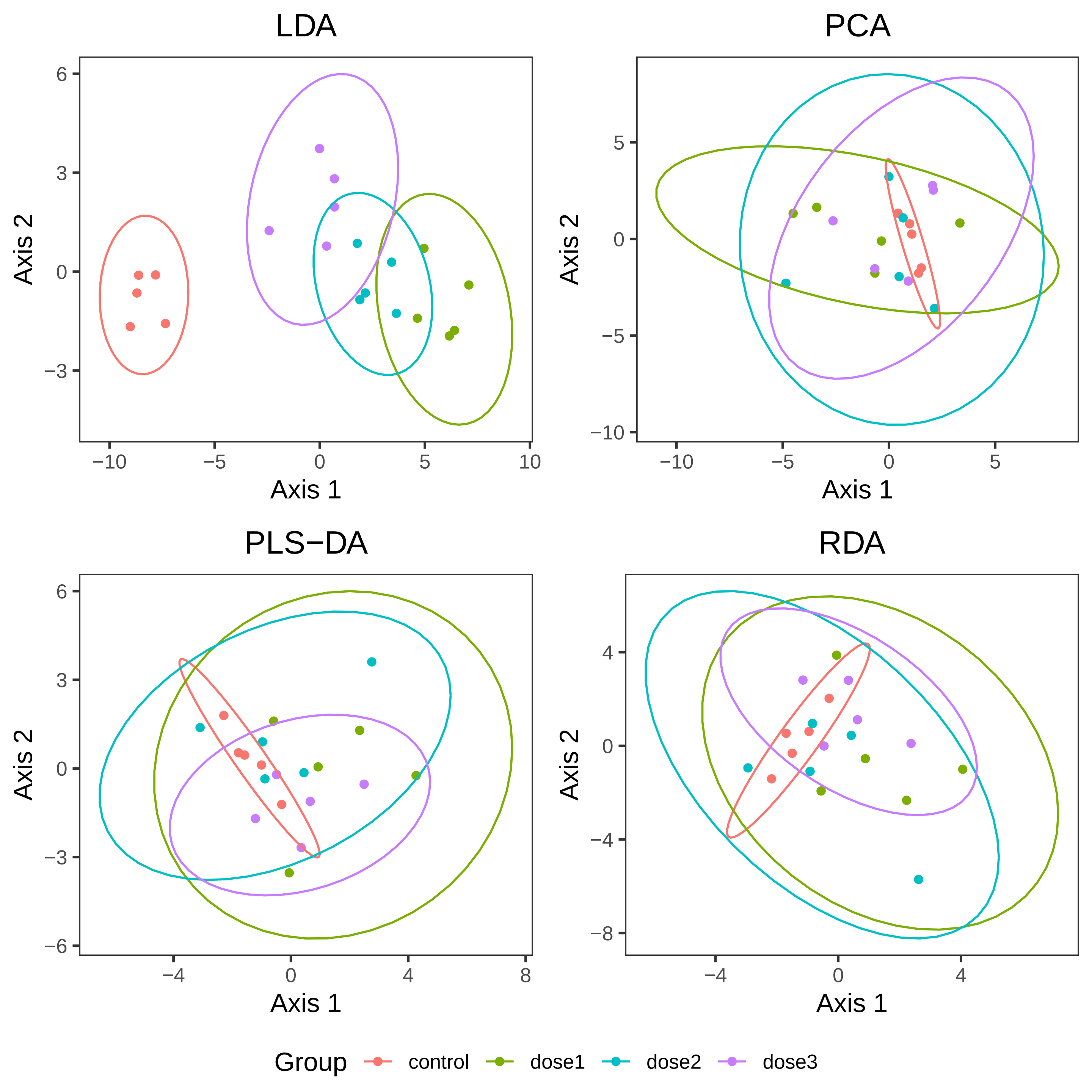
LDA
PCA
PLS−DA
RDA
Axis 1
Axis 2
Group
control
dose1
dose2
dose3

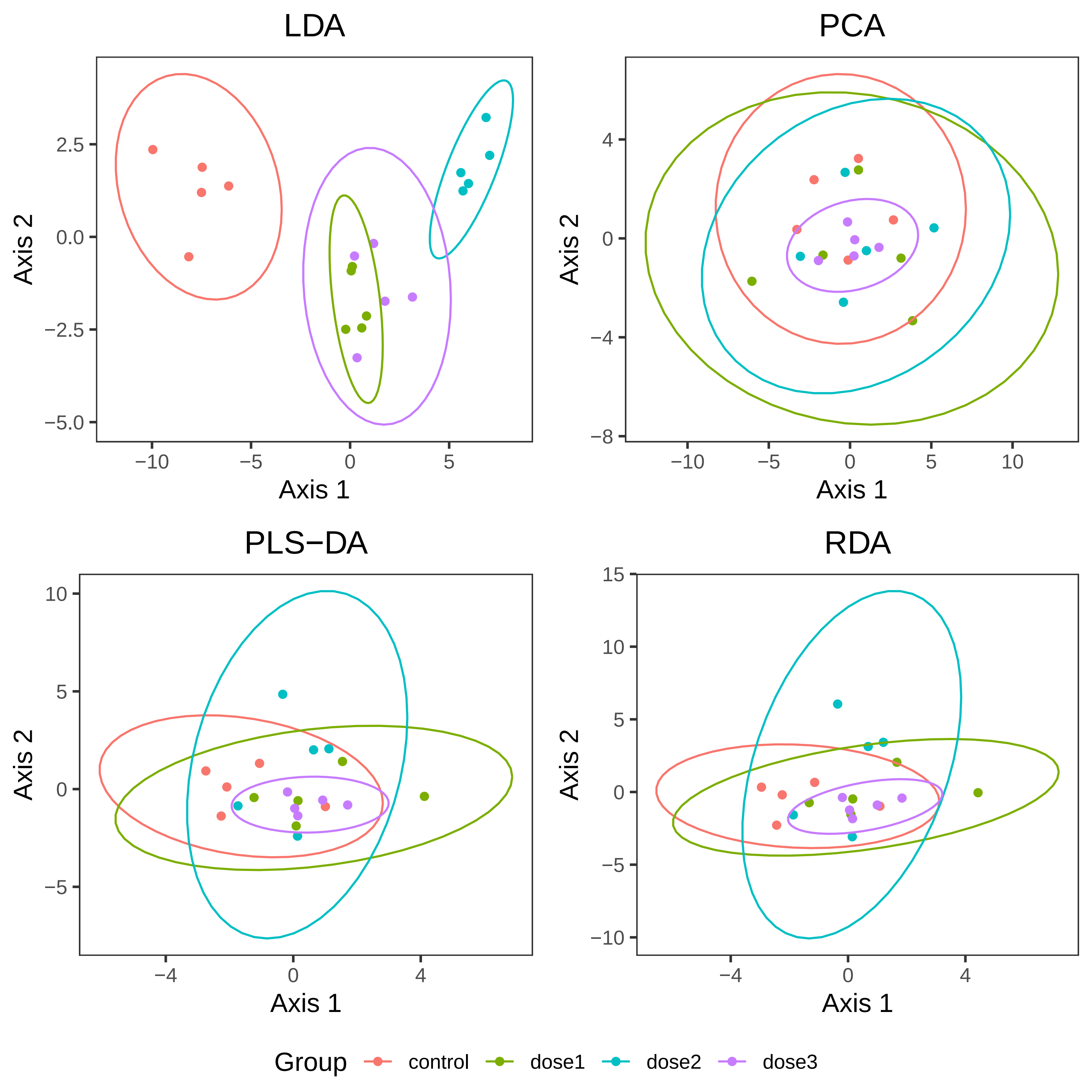

LDA
PCA
PLS−DA
RDA
Axis 1
Axis 2
2.5
0.0
−2.5
−5.0
−10
−5
0
5
10
4
−4
−8
15
Group
control
dose1
dose2
dose3

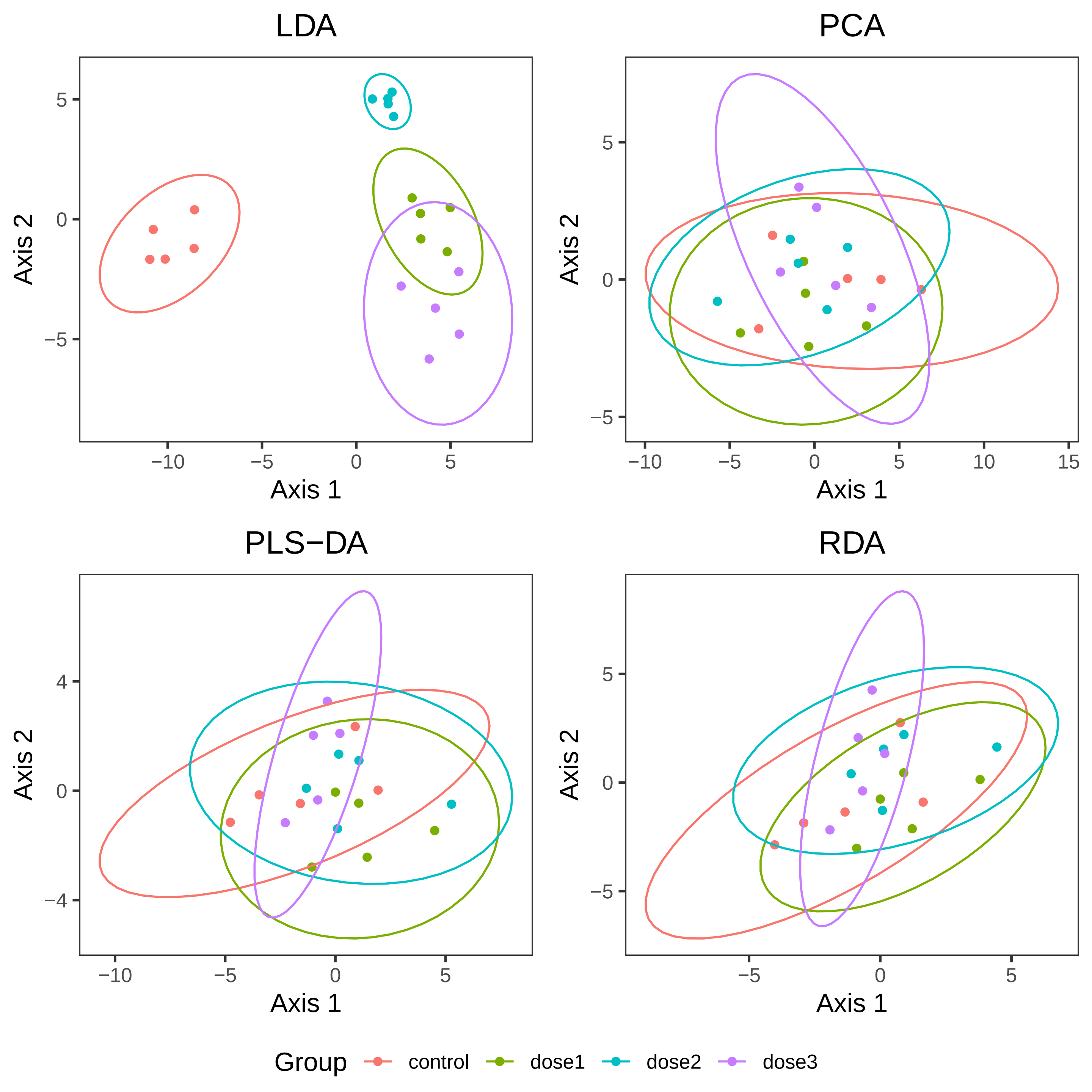

LDA
PCA
PLS−DA
RDA
Axis 1
Axis 2
Group
control
dose1
dose2
dose3

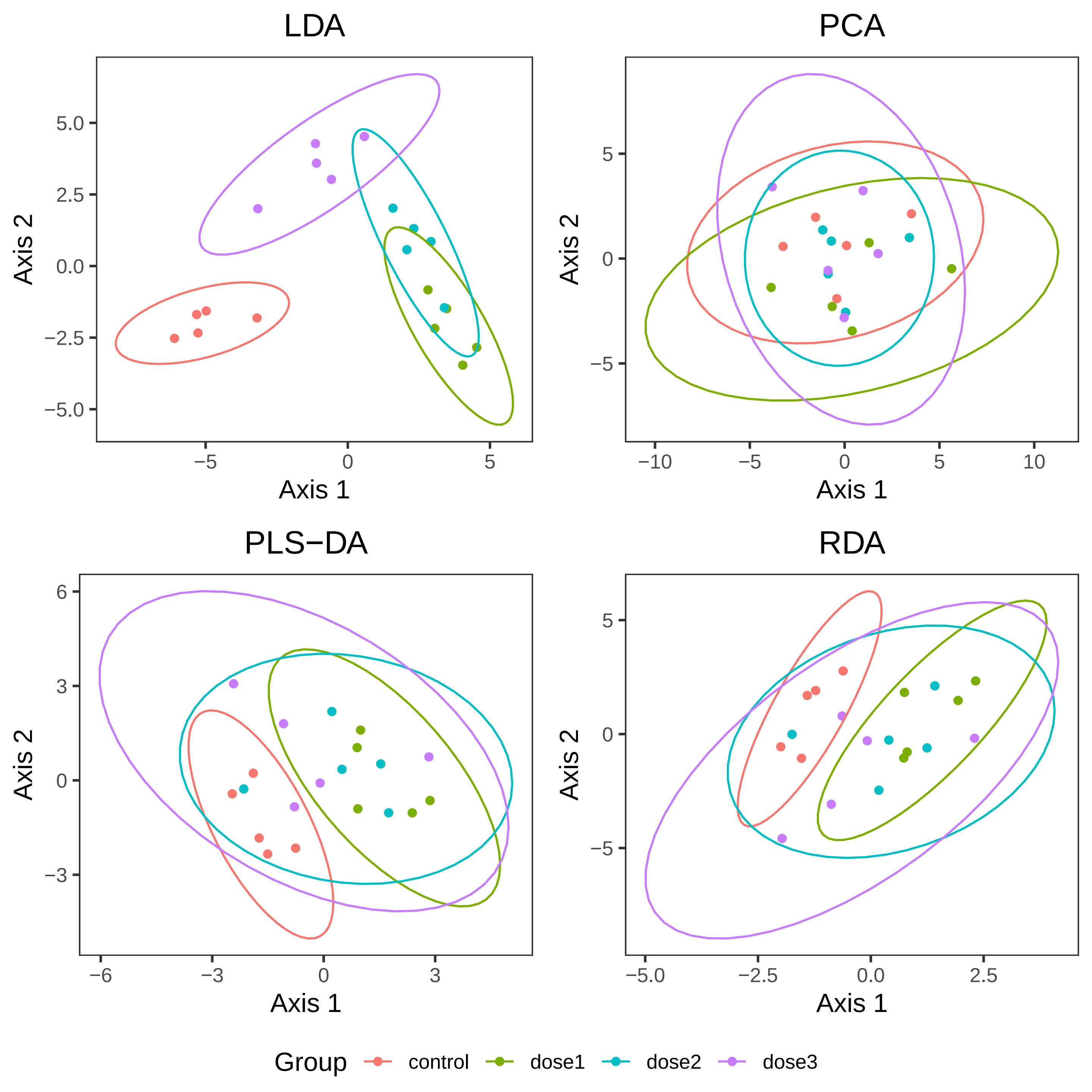
LDA
PCA
PLS−DA
RDA
Axis 1
Axis 2
5.0
2.5
0.0
−2.5
−5.0
−5
0
5
−10
10
6
3
−3
−6
−5.0
−2.5
0.0
2.5
Group
control
dose1
dose2
dose3

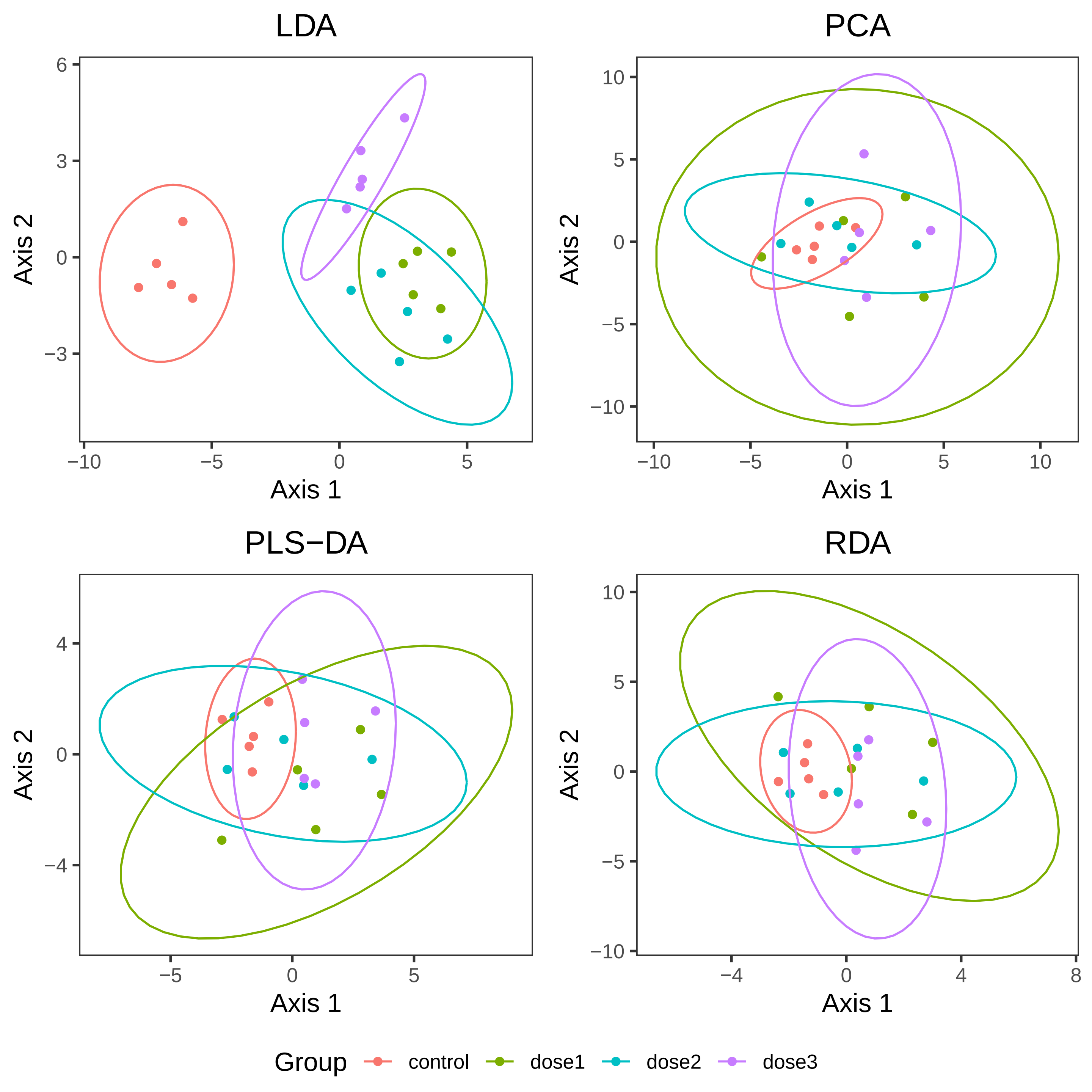
LDA
PCA
PLS−DA
RDA
Axis 1
Axis 2
Group
control
dose1
dose2
dose3

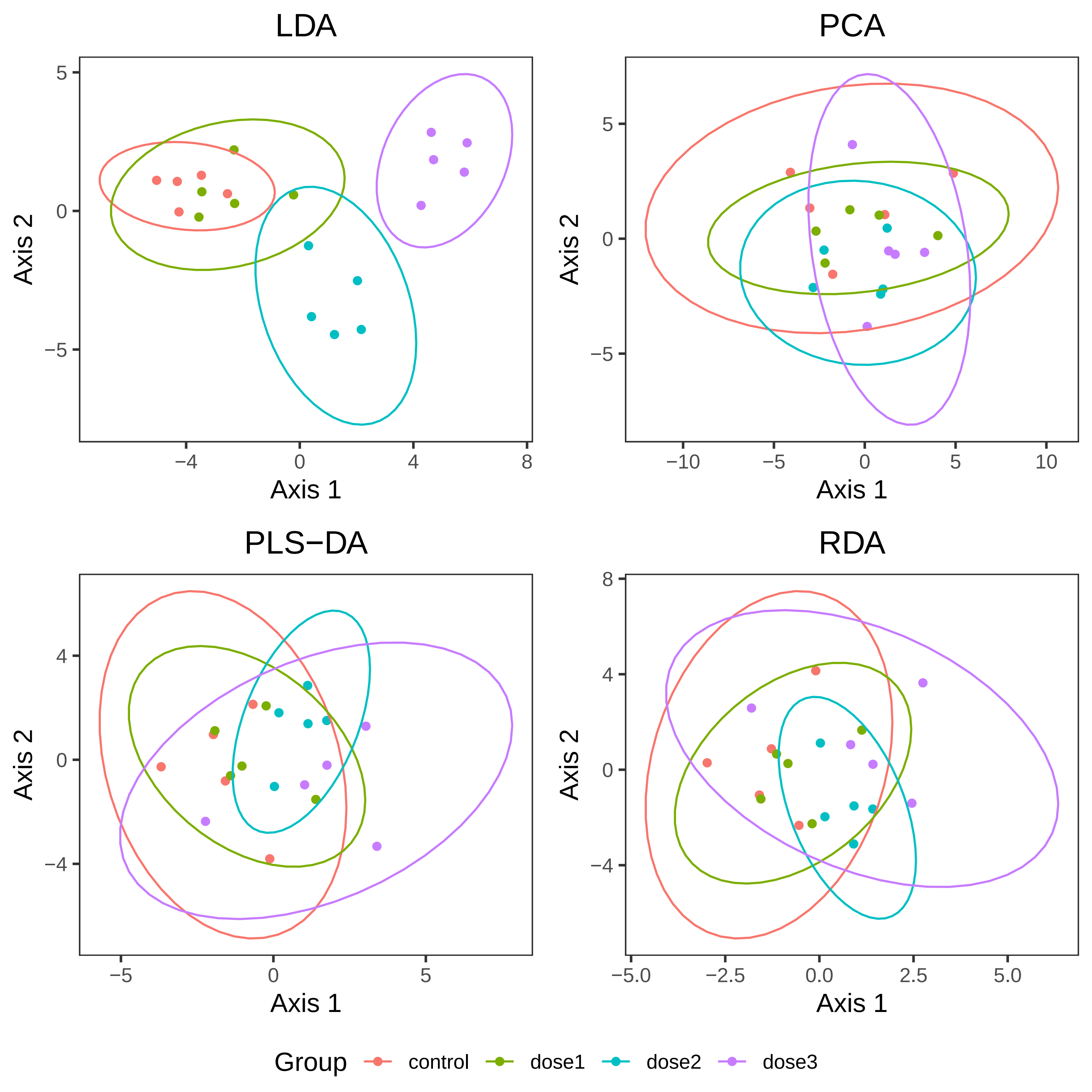
LDA
PCA
PLS−DA
RDA
Axis 1
Axis 2
Group
control
dose1
dose2
dose3

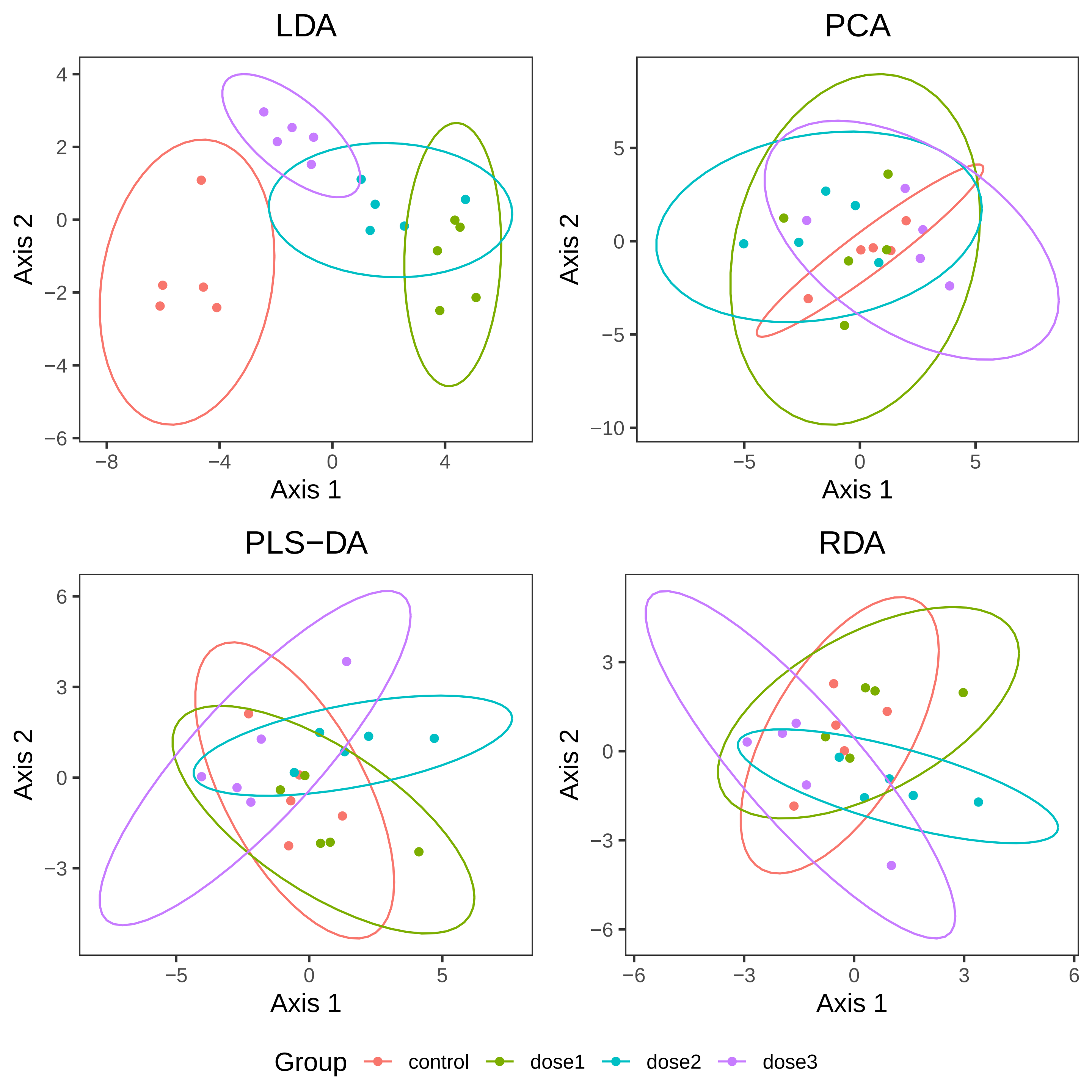
LDA
PCA
PLS−DA
RDA
Axis 1
Axis 2
Group
control
dose1
dose2
dose3

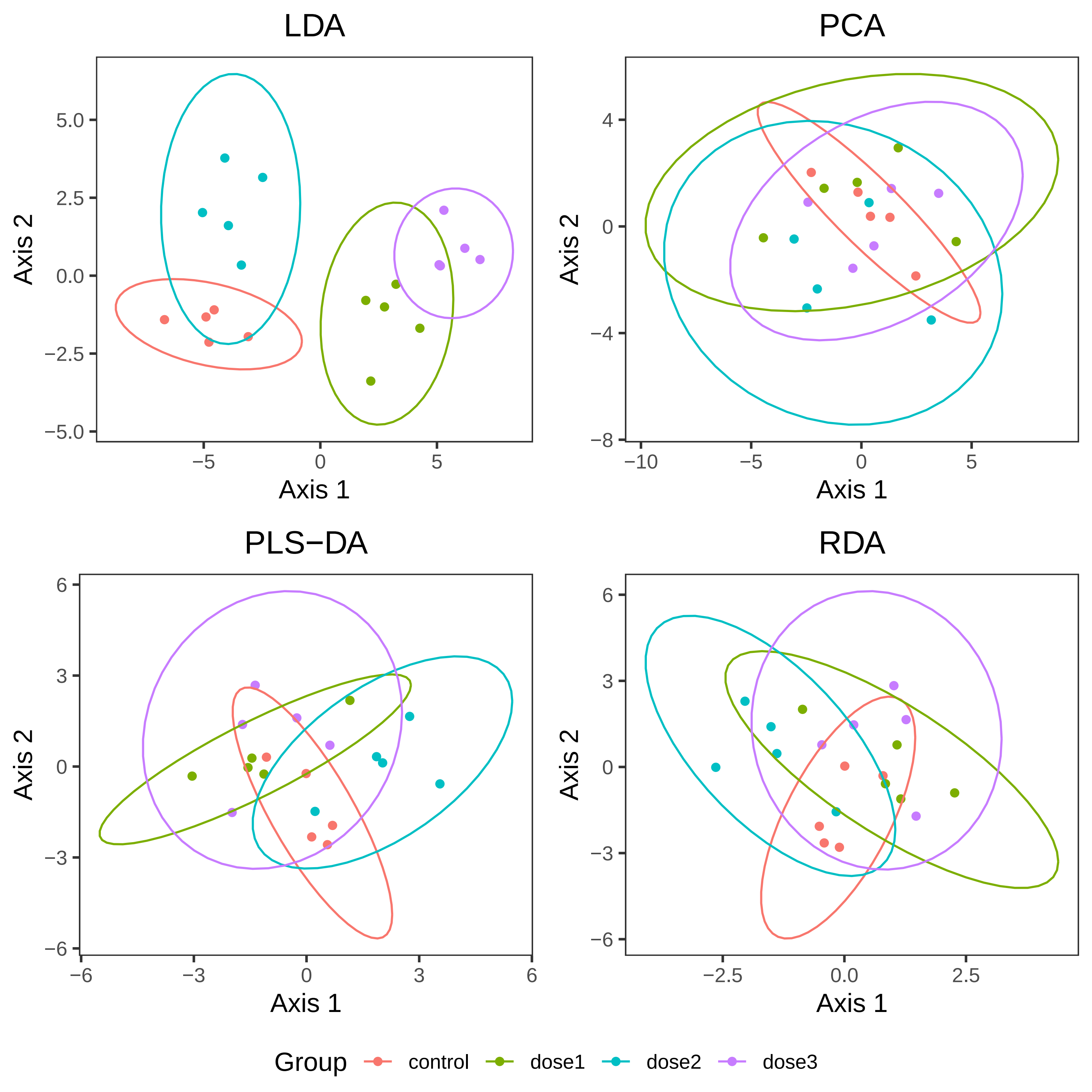

LDA
PCA
PLS−DA
RDA
Axis 1
Axis 2
5.0
2.5
0.0
−2.5
−5.0
−5
0
5
4
0
−4
−8
−10
6
3
−3
−6
−2.5
Group
control
dose1
dose2
dose3

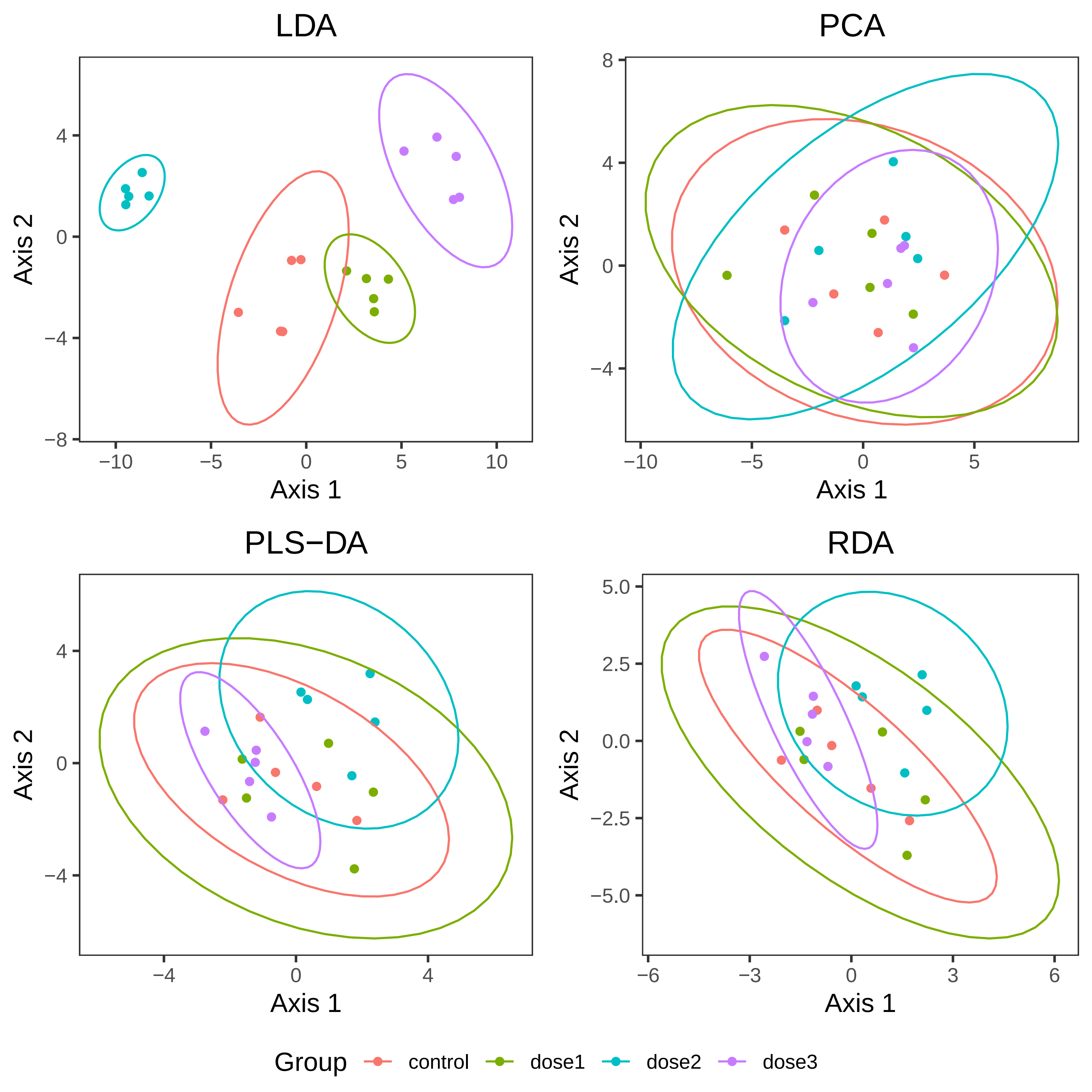
LDA
PCA
PLS−DA
RDA
Axis 1
Axis 2
−10
−5
0
5
10
4
0
−4
−8
8
−6
−3
3
6
5.0
2.5
0.0
−2.5
−5.0
Group
control
dose1
dose2
dose3

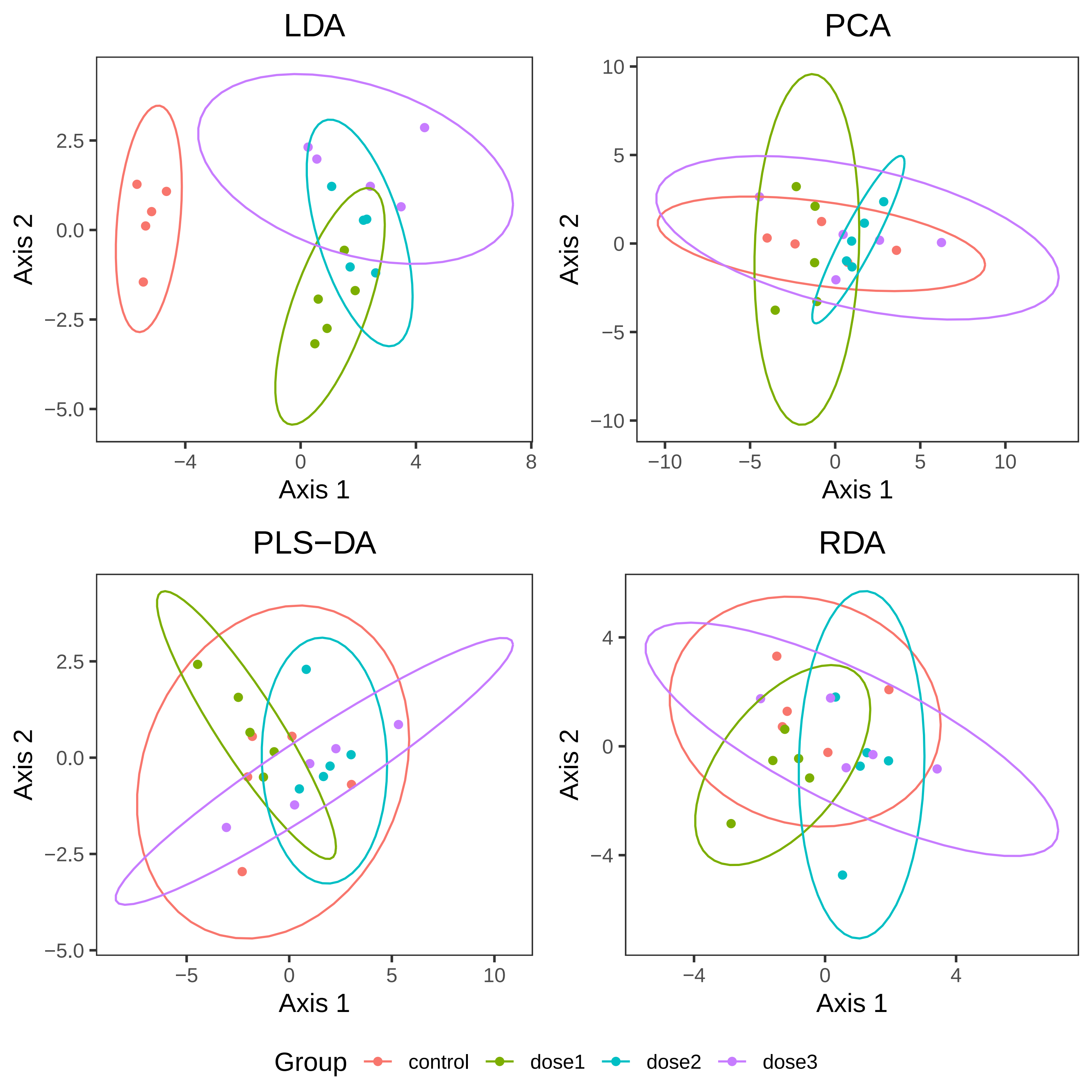

LDA
PCA
PLS−DA
RDA
Axis 1
Axis 2
2.5
0.0
−2.5
−5.0
−4
0
4
8
10
5
0
−5
−10
−10
−5
0
5
10
2.5
0.0
−2.5
−5.0
−5
0
5
10
4
0
−4
−4
0
4
Group
control
dose1
dose2
dose3

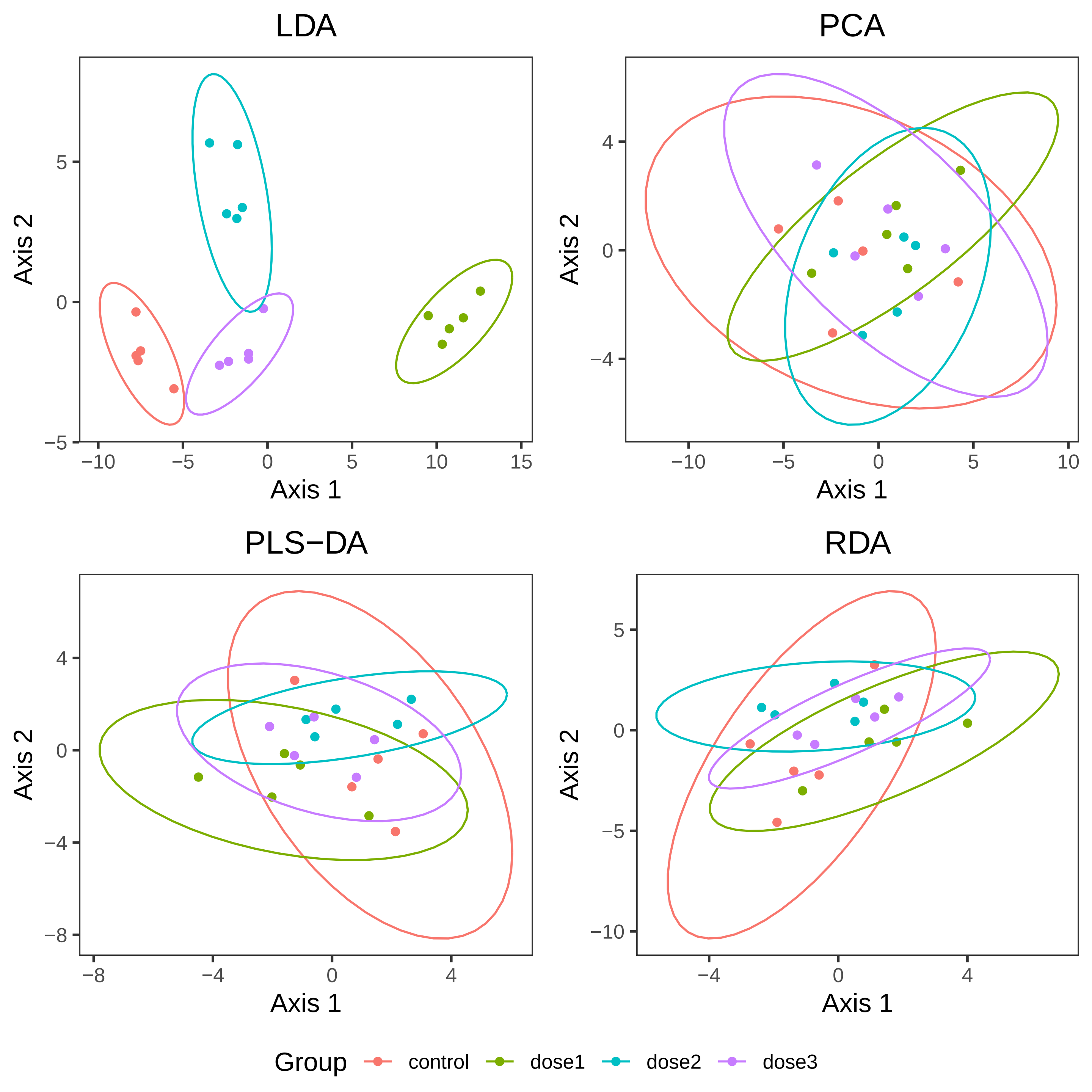
LDA
PCA
PLS−DA
RDA
Axis 1
Axis 2
Group
control
dose1
dose2
dose3

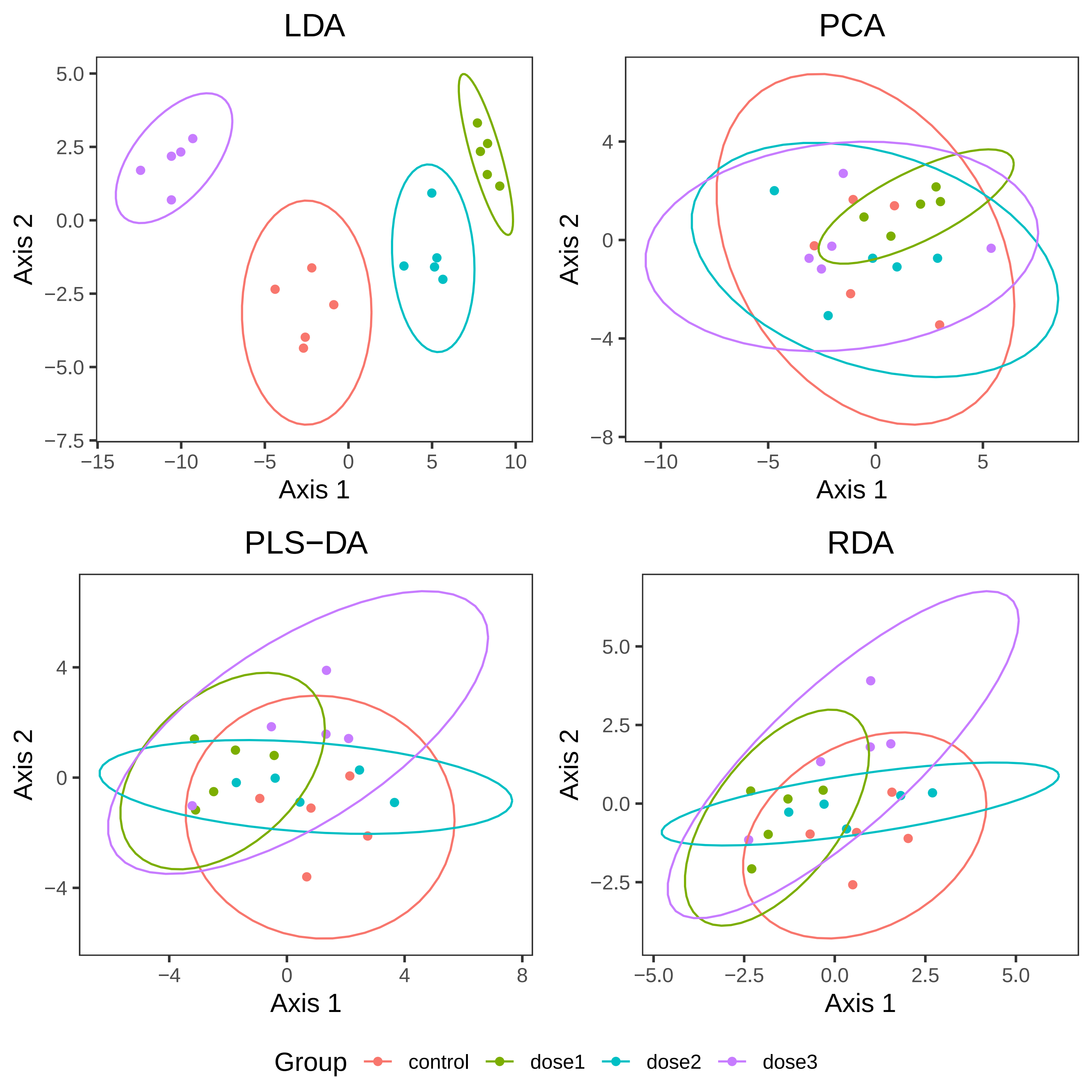

LDA
PCA
PLS−DA
RDA
Axis 1
Axis 2
Group
control
dose1
dose2
dose3

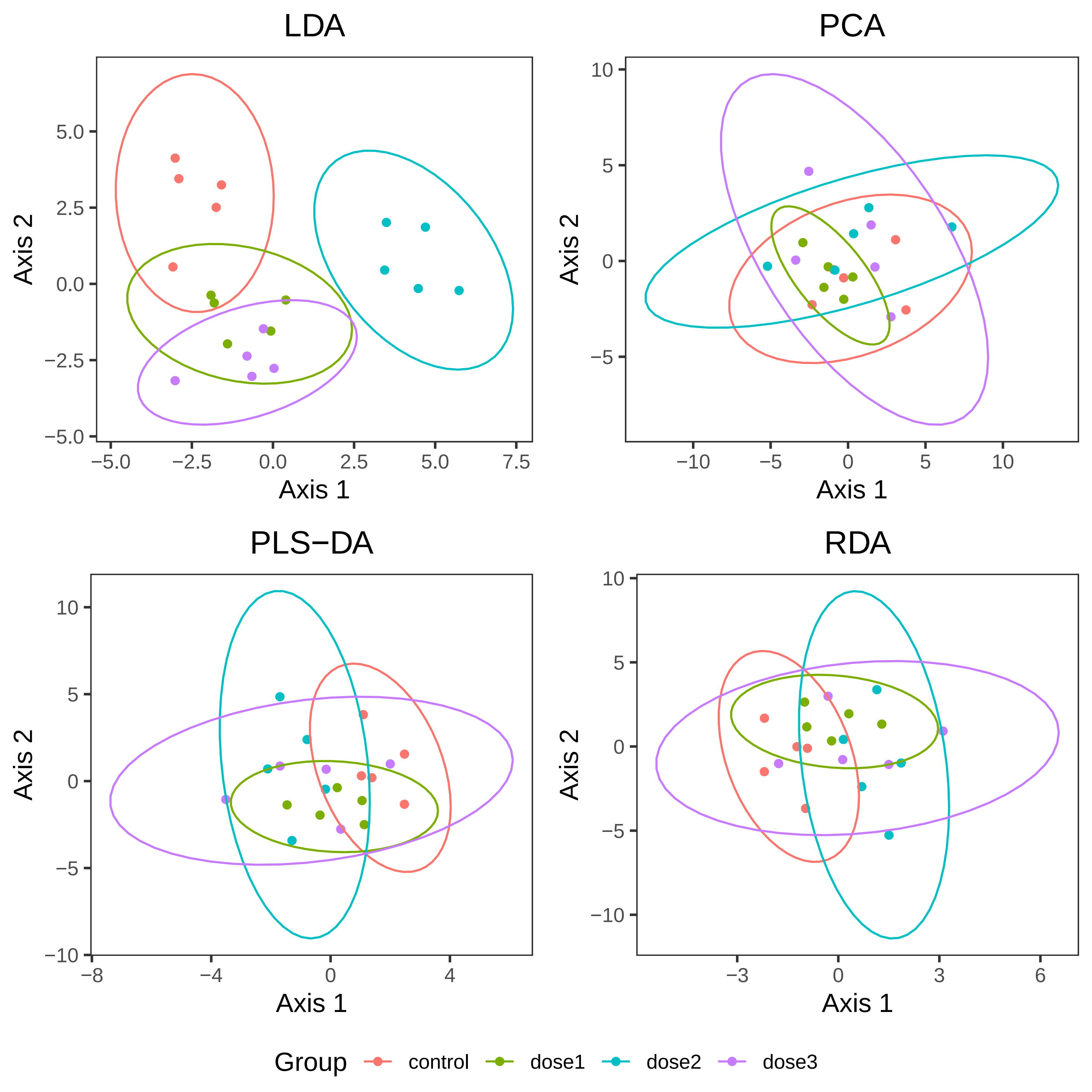
LDA
PCA
PLS−DA
RDA
Axis 1
Axis 2
Group
control
dose1
dose2
dose3

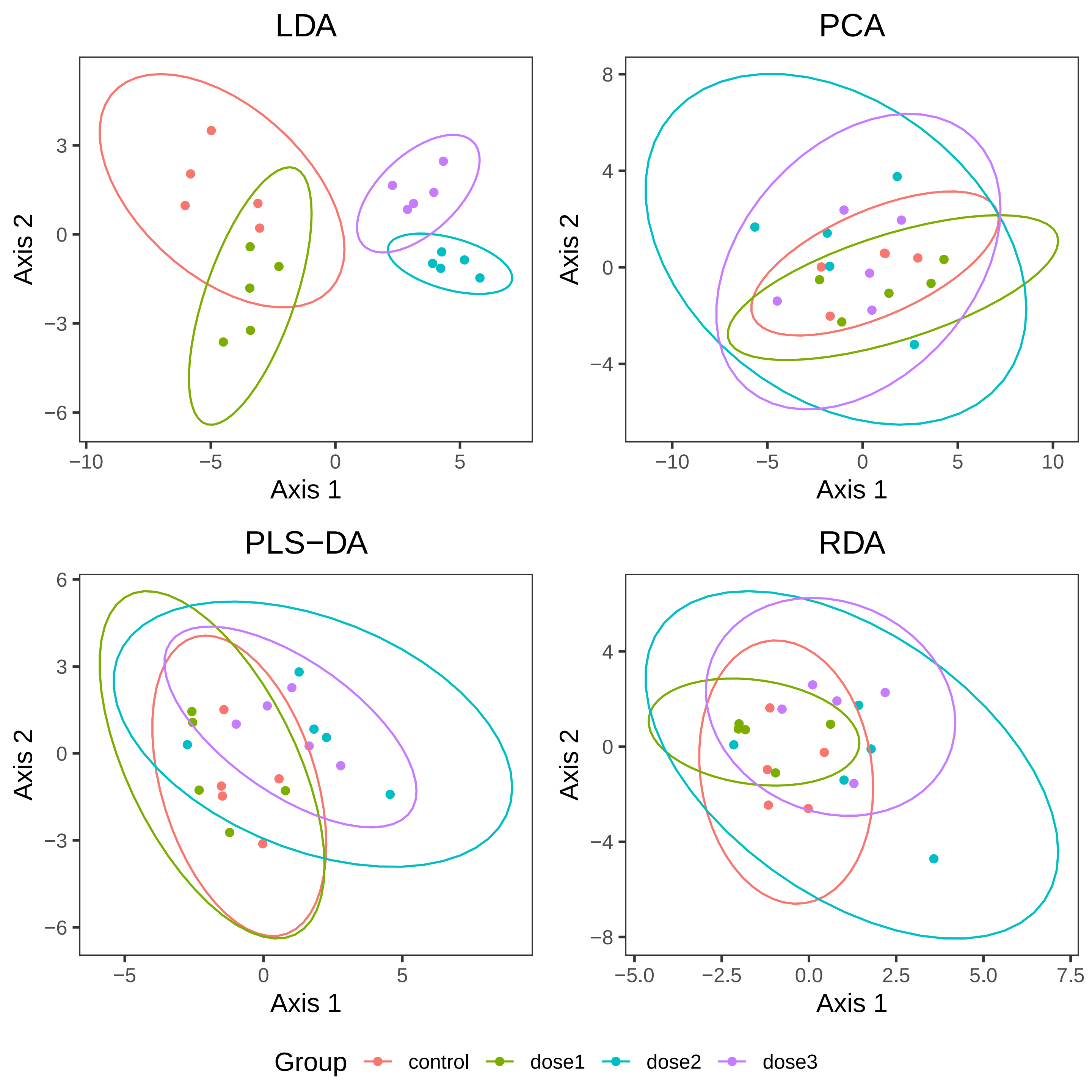

LDA
PCA
PLS−DA
RDA
Axis 1
Axis 2
Group
control
dose1
dose2
dose3

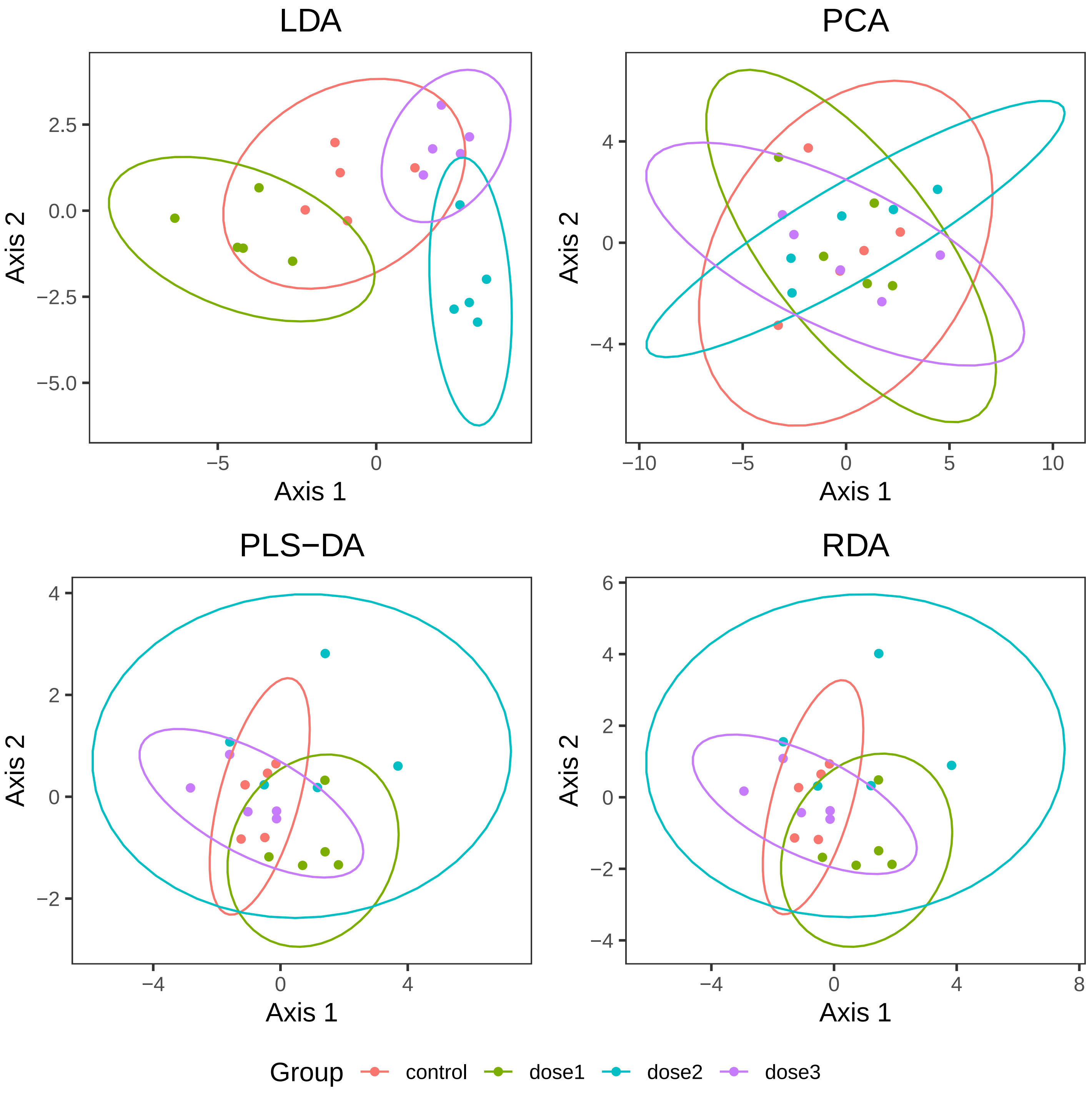
LDA
PCA
PLS−DA
RDA
Axis 1
Axis 2
Group
control
dose1
dose2
dose3

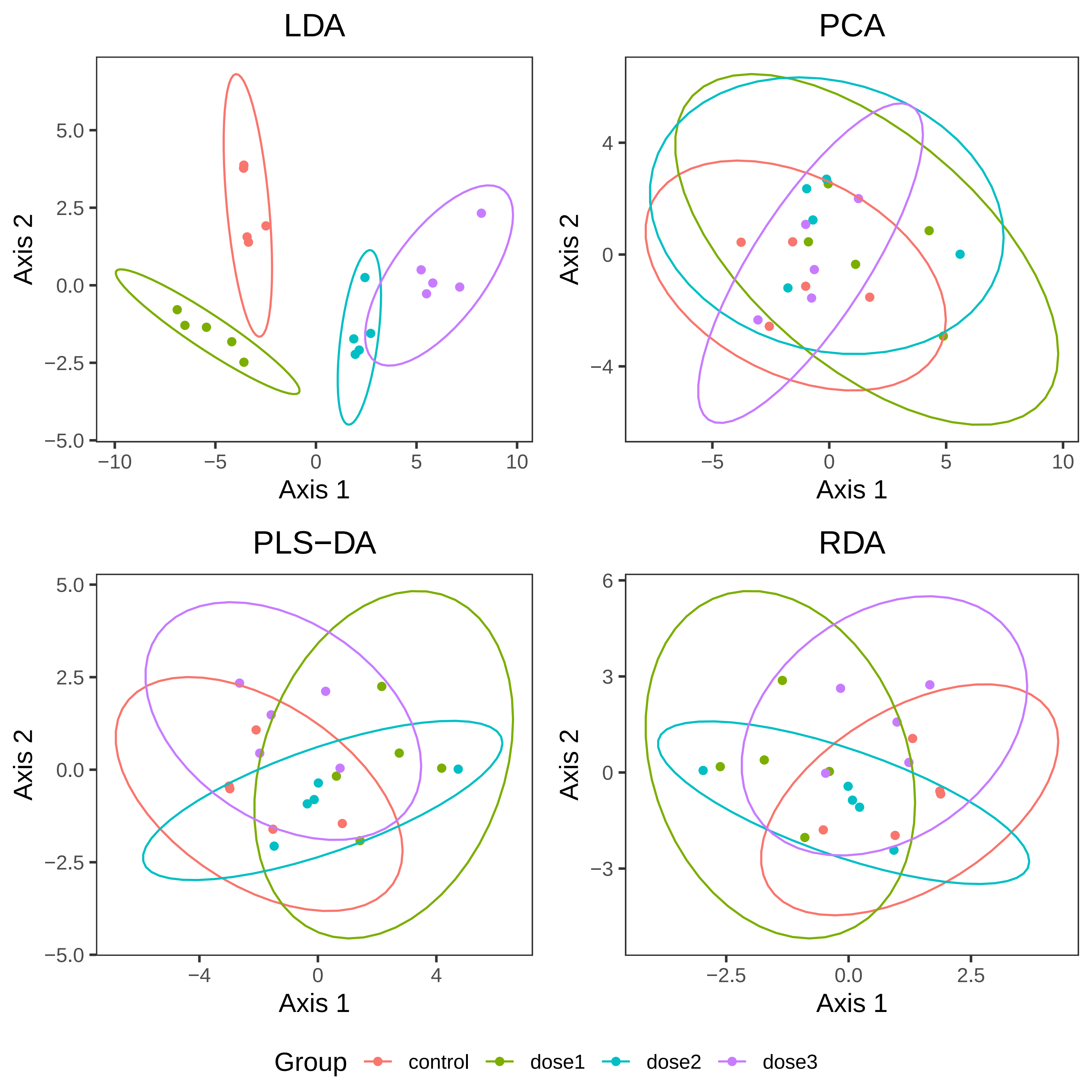

LDA
PCA
PLS−DA
RDA
Axis 1
Axis 2
5.0
2.5
0.0
−2.5
−5.0
−10
−5
0
5
10
4
−4
6
3
−3
−2.5
2.5
Group
control
dose1
dose2
dose3

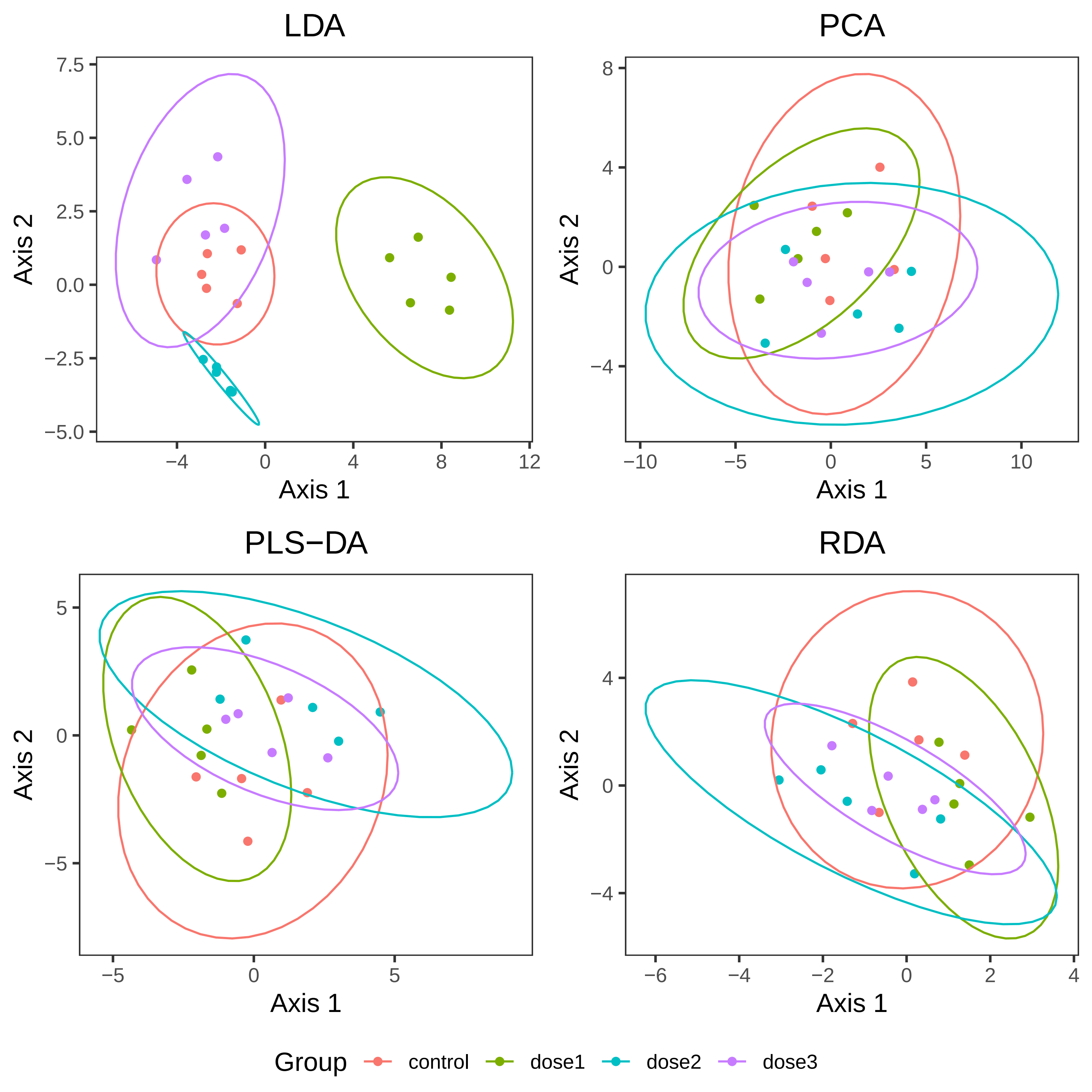
LDA
PCA
PLS−DA
RDA
Axis 1
Axis 2
7.5
5.0
2.5
0.0
−2.5
−5.0
−4
0
4
8
12
−10
−5
5
10
Group
control
dose1
dose2
dose3

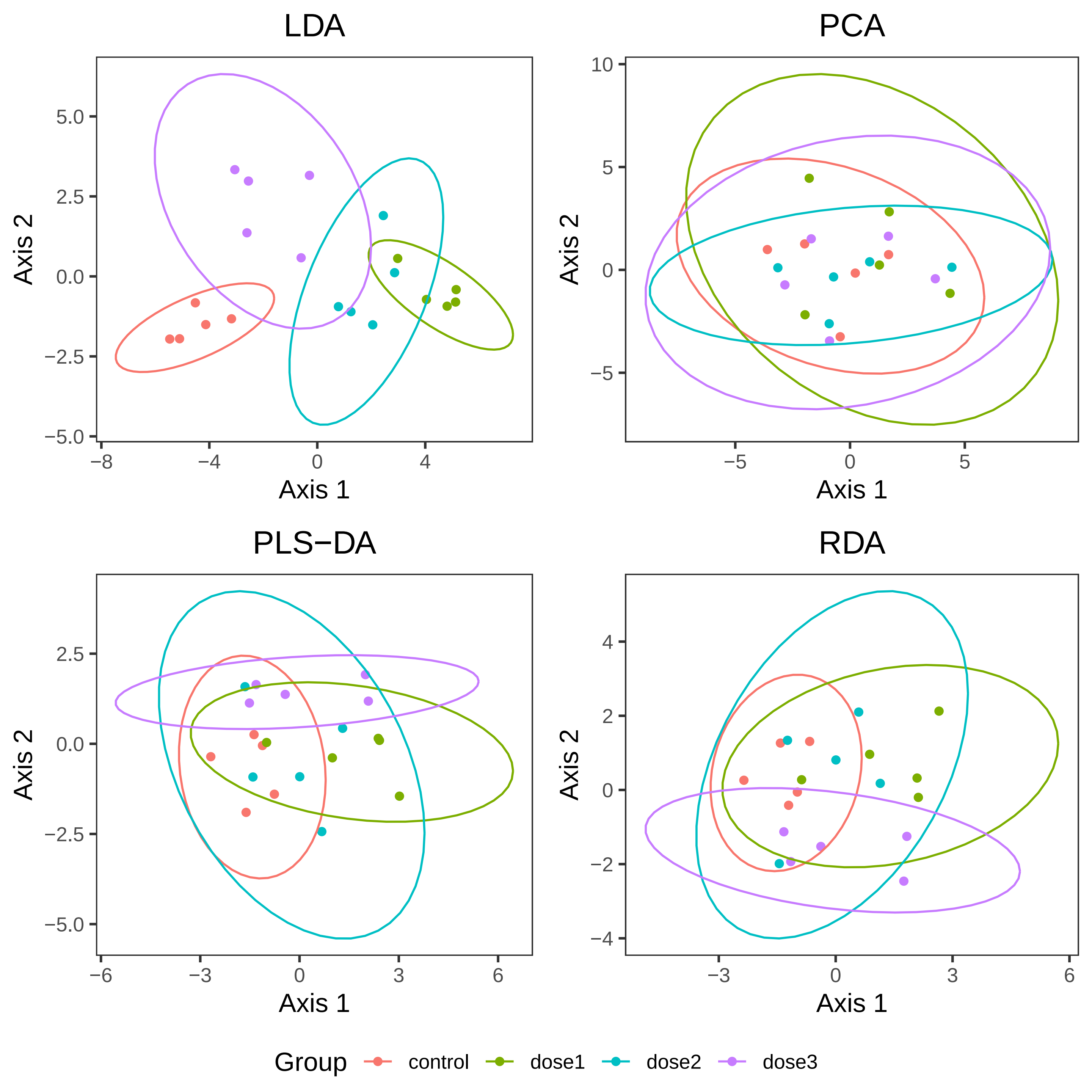
LDA
PCA
PLS−DA
RDA
Axis 1
Axis 2
Group
control
dose1
dose2
dose3

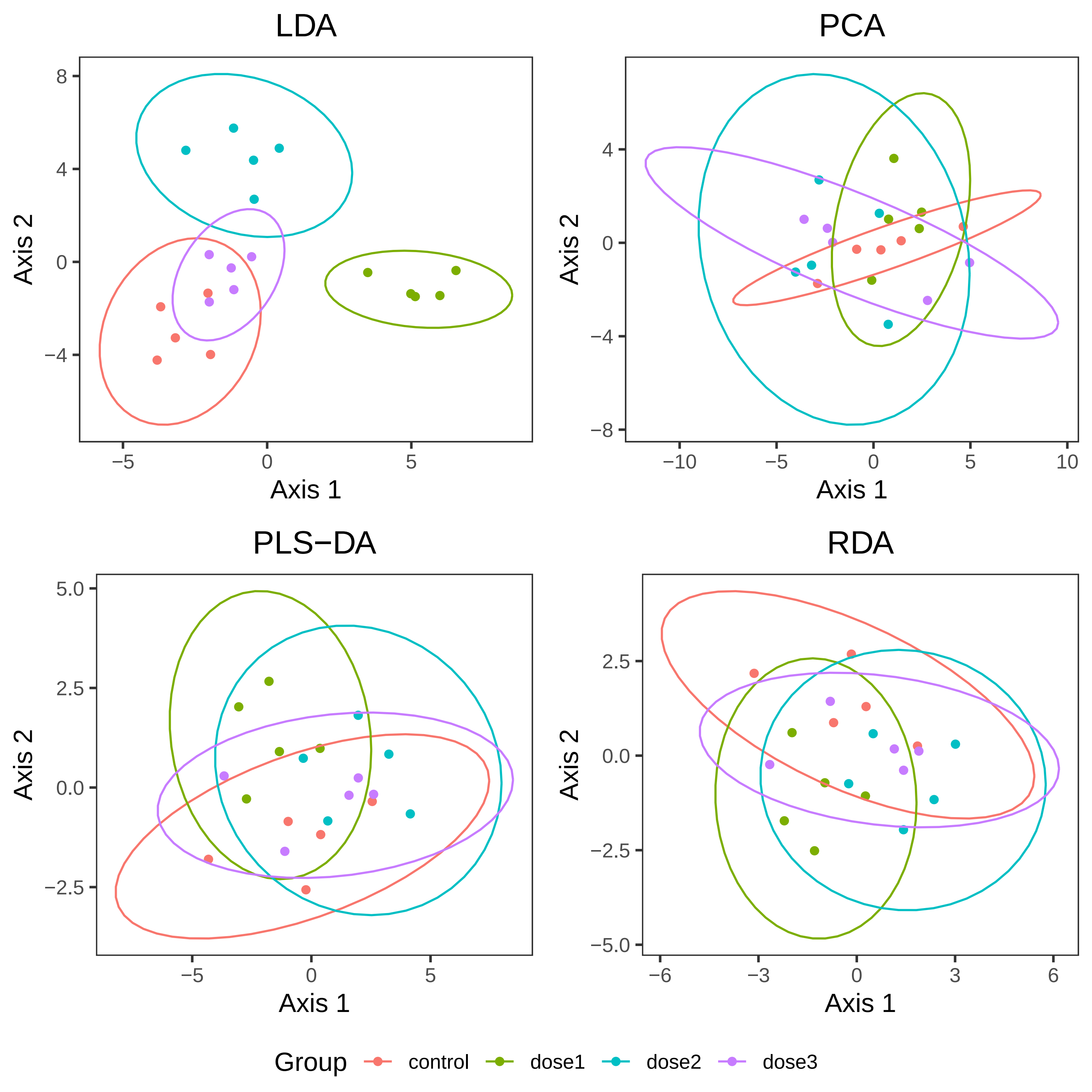

LDA
PCA
PLS−DA
RDA
Axis 1
Axis 2
Group
control
dose1
dose2
dose3

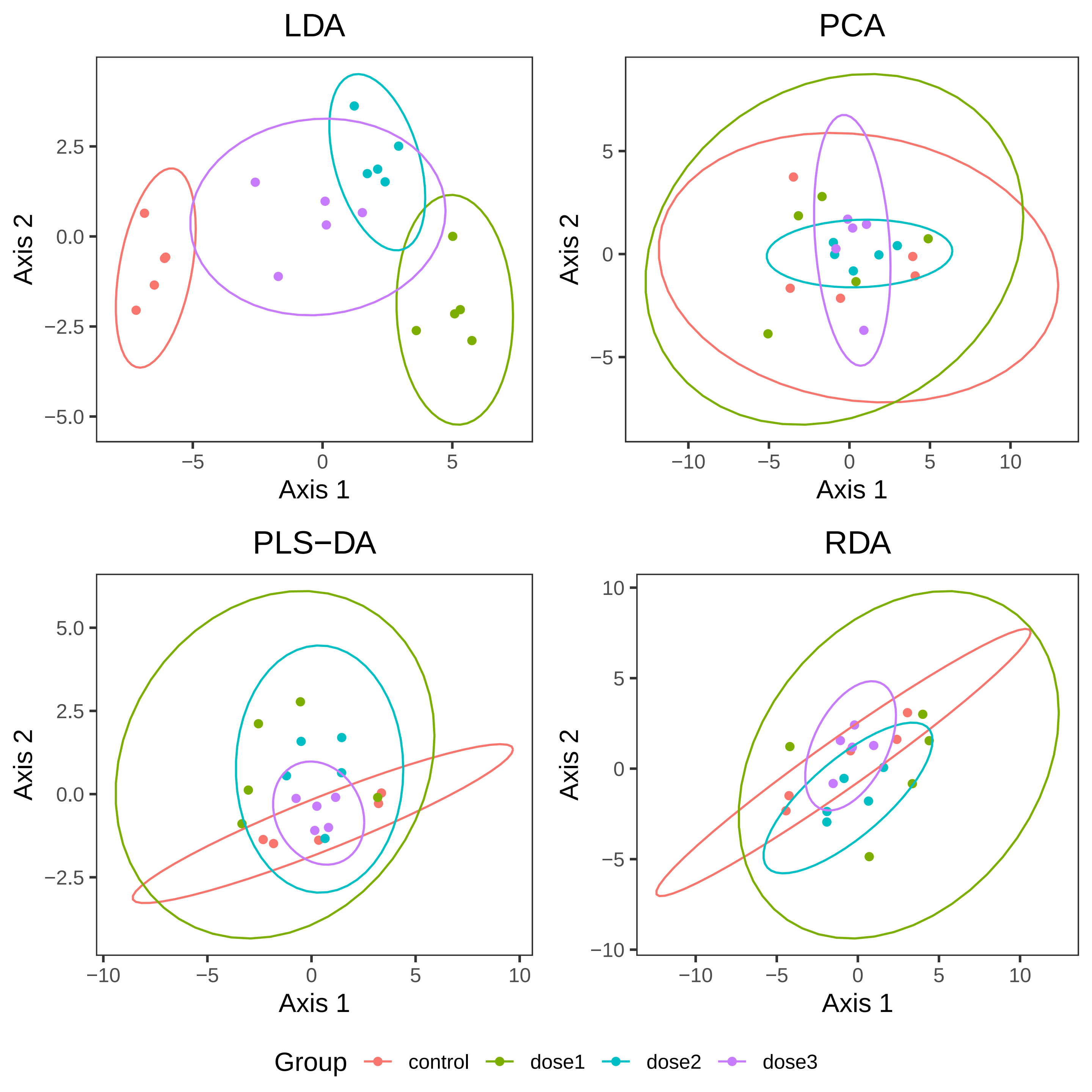
LDA
PCA
PLS−DA
RDA
Axis 1
Axis 2
Group
control
dose1
dose2
dose3

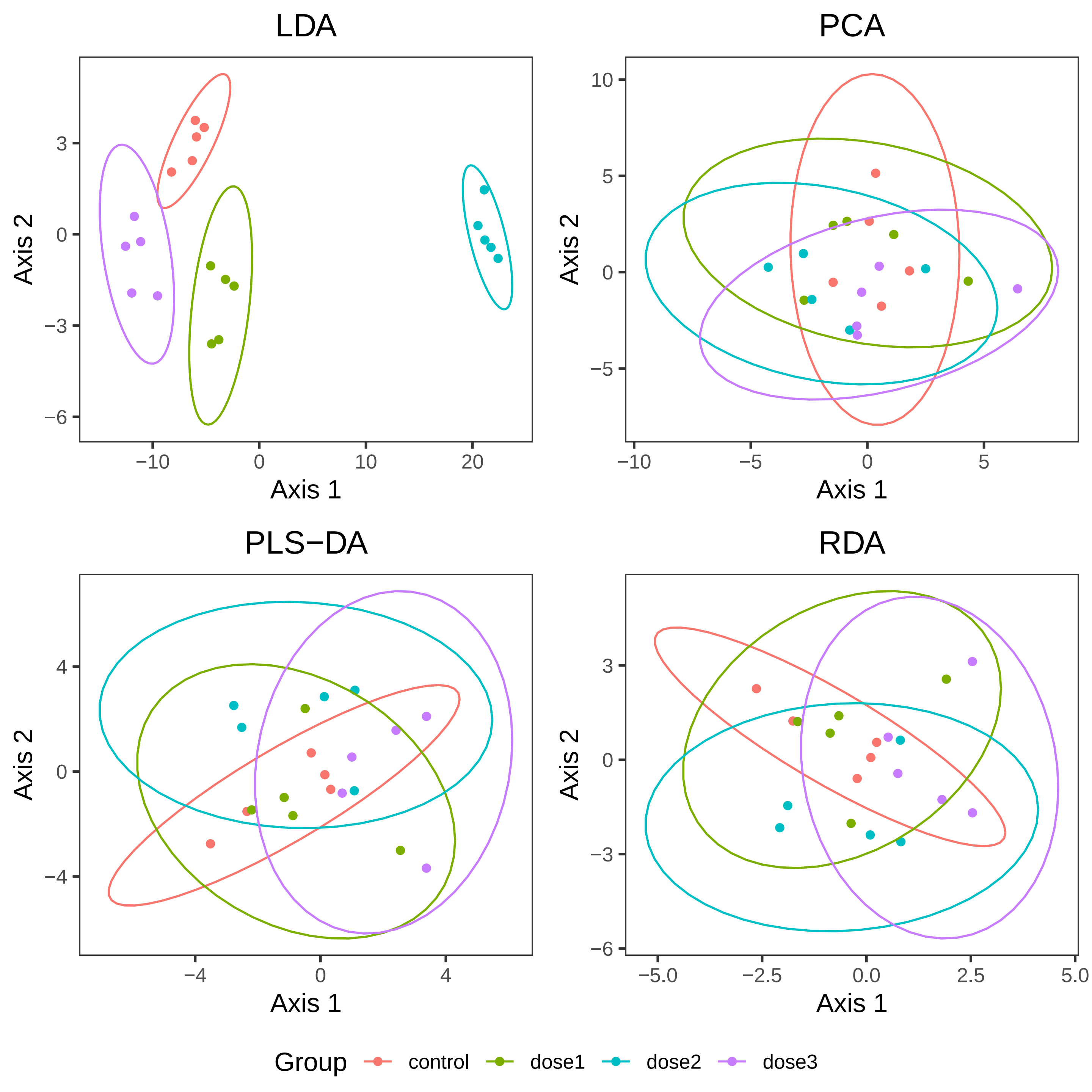
LDA
PCA
PLS−DA
RDA
Axis 1
Axis 2
Group
control
dose1
dose2
dose3

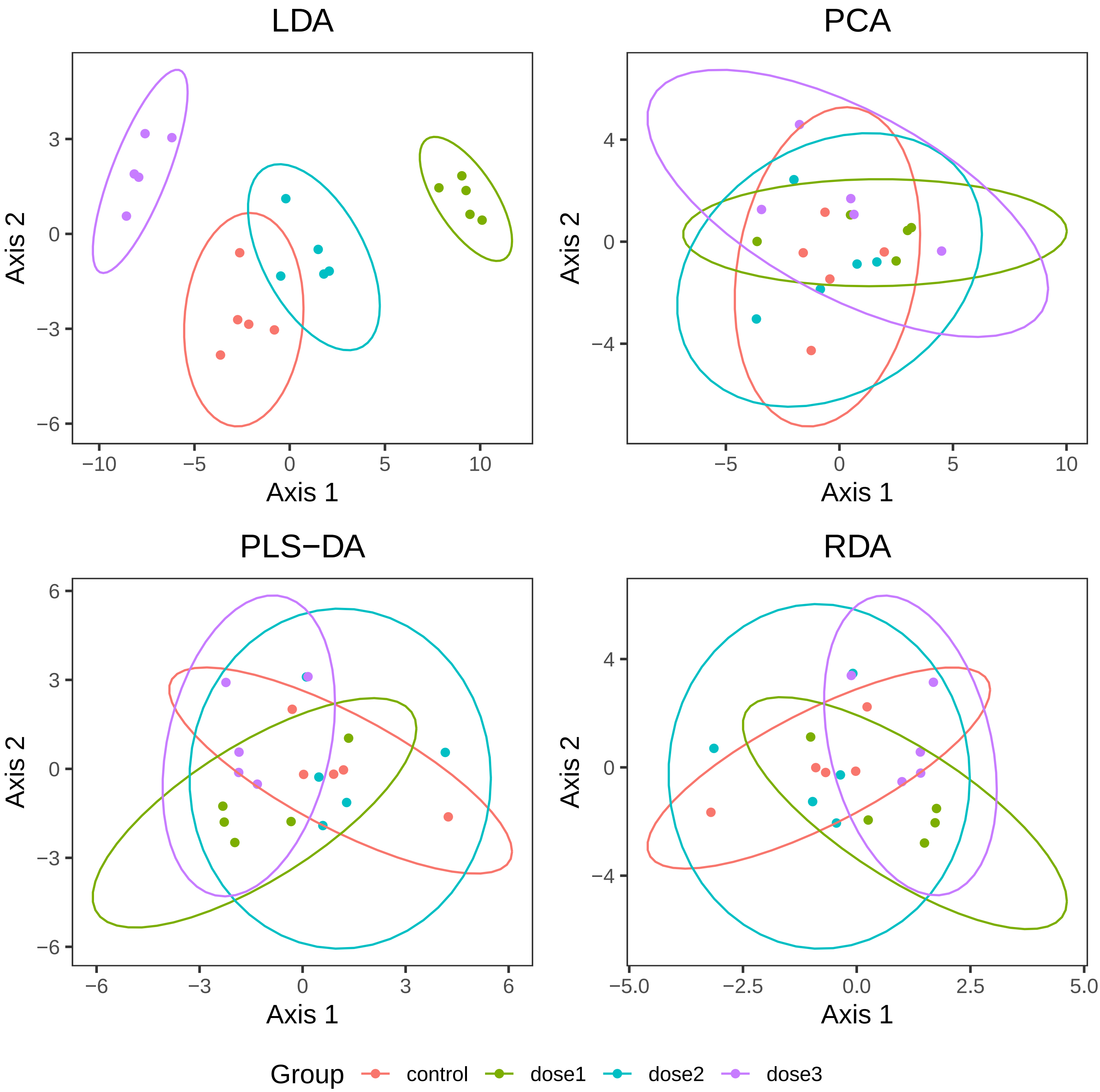
LDA
PCA
PLS−DA
RDA
Axis 1
Axis 2
3
0
−3
−6
−10
−5
0
5
10
4
−4
6
−5.0
−2.5
0.0
2.5
5.0
Group
control
dose1
dose2
dose3

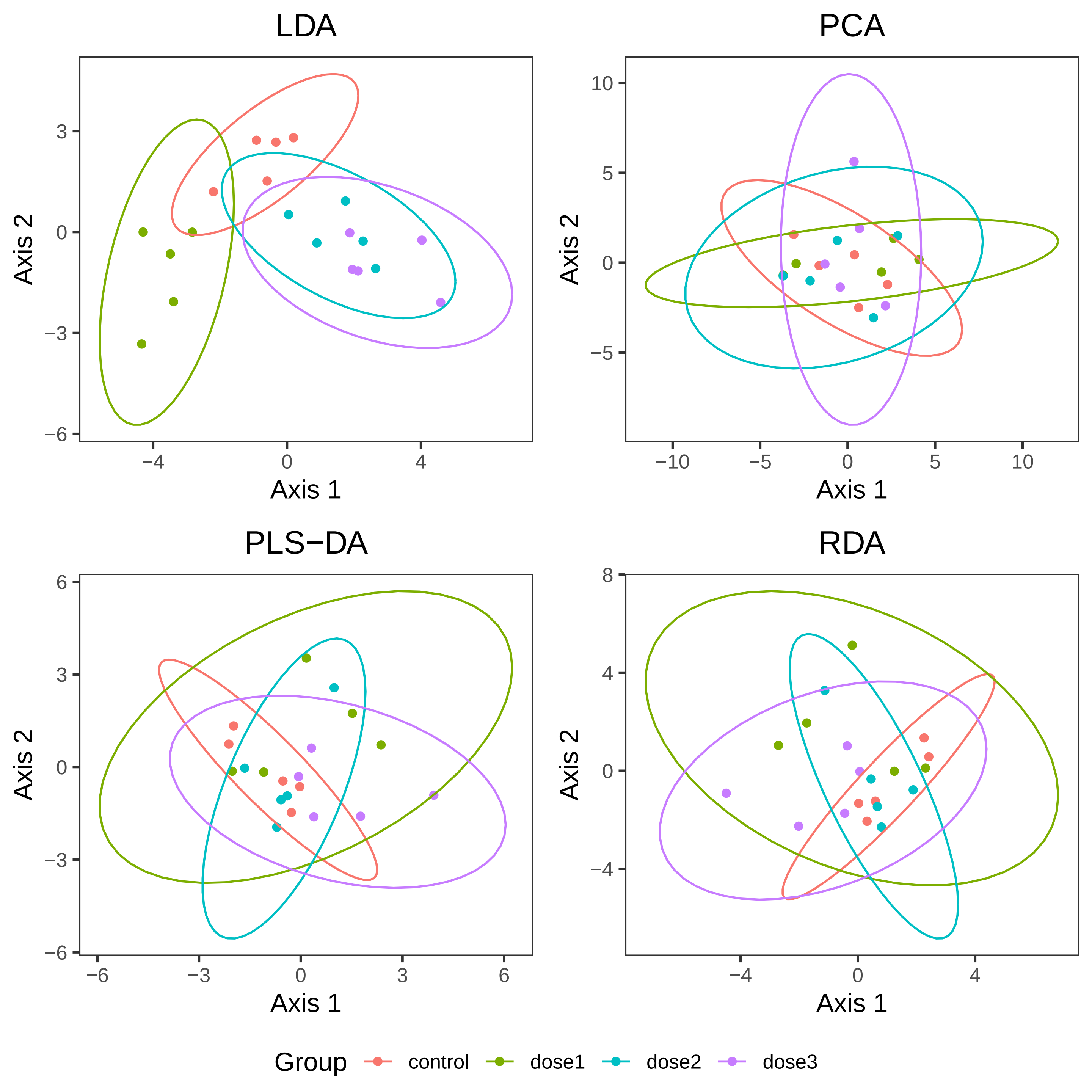

LDA
PCA
PLS−DA
RDA
Axis 1
Axis 2
3
0
−3
−6
−4
0
4
10
5
0
−5
−10
−5
0
5
10
6
3
0
−3
−6
−6
−3
0
3
6
8
4
0
−4
−4
0
4
Group
control
dose1
dose2
dose3

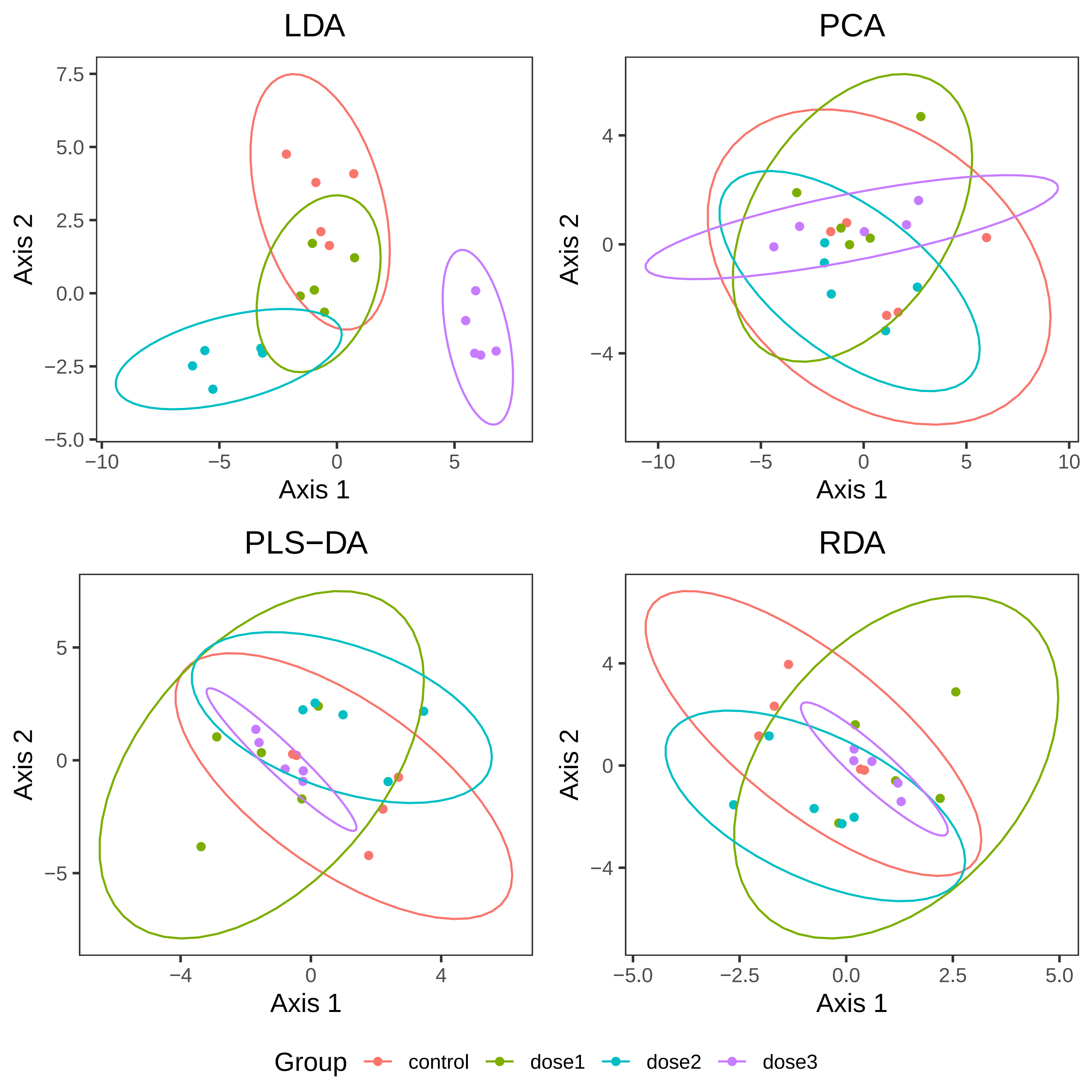

LDA
PCA
PLS−DA
RDA
Axis 1
Axis 2
Group
control
dose1
dose2
dose3

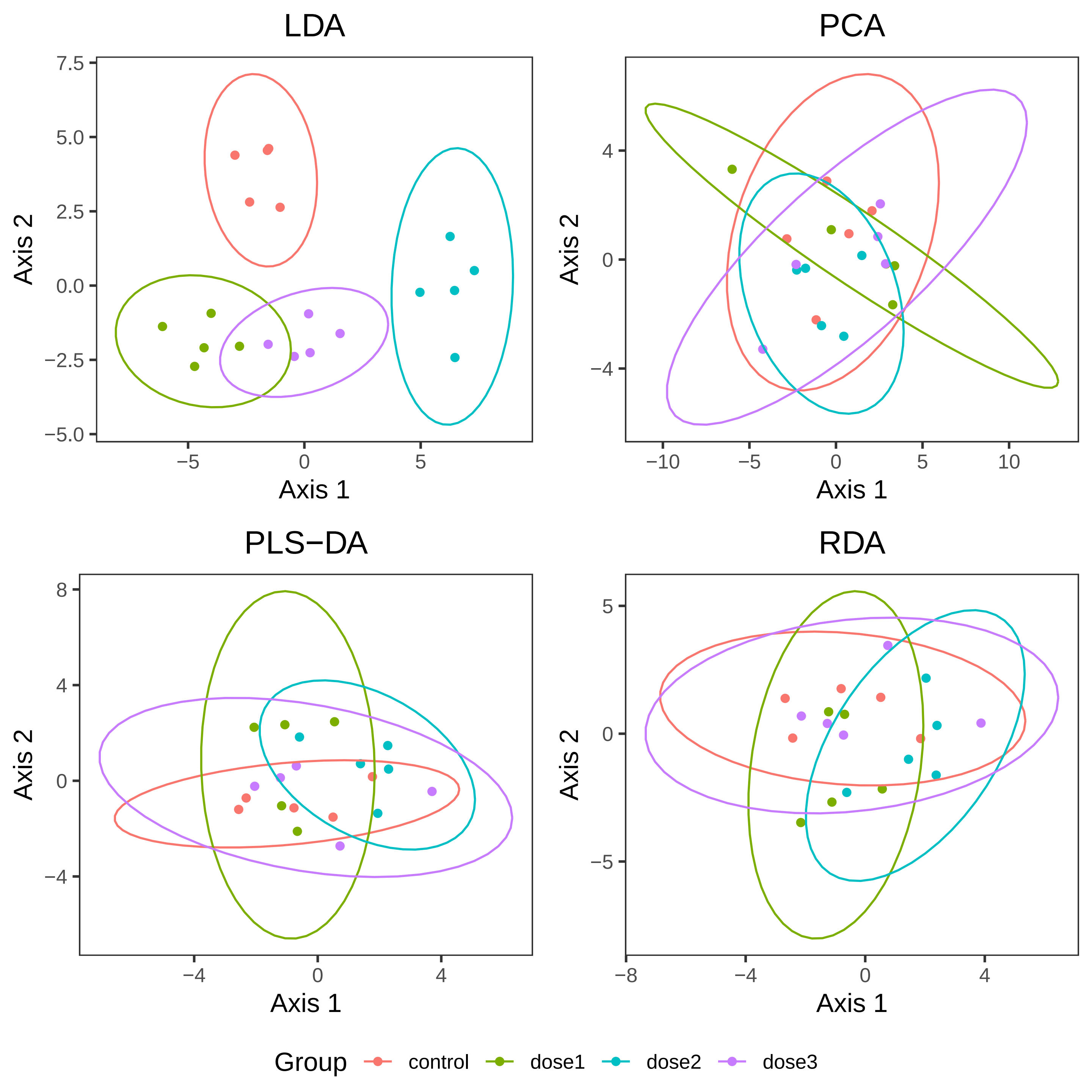

LDA
PCA
PLS−DA
RDA
Axis 1
Axis 2
7.5
5.0
2.5
0.0
−2.5
−5.0
−5
0
5
4
0
−4
−10
−5
0
5
10
8
4
0
−4
−4
0
4
5
0
−5
−8
−4
0
4
Group
control
dose1
dose2
dose3

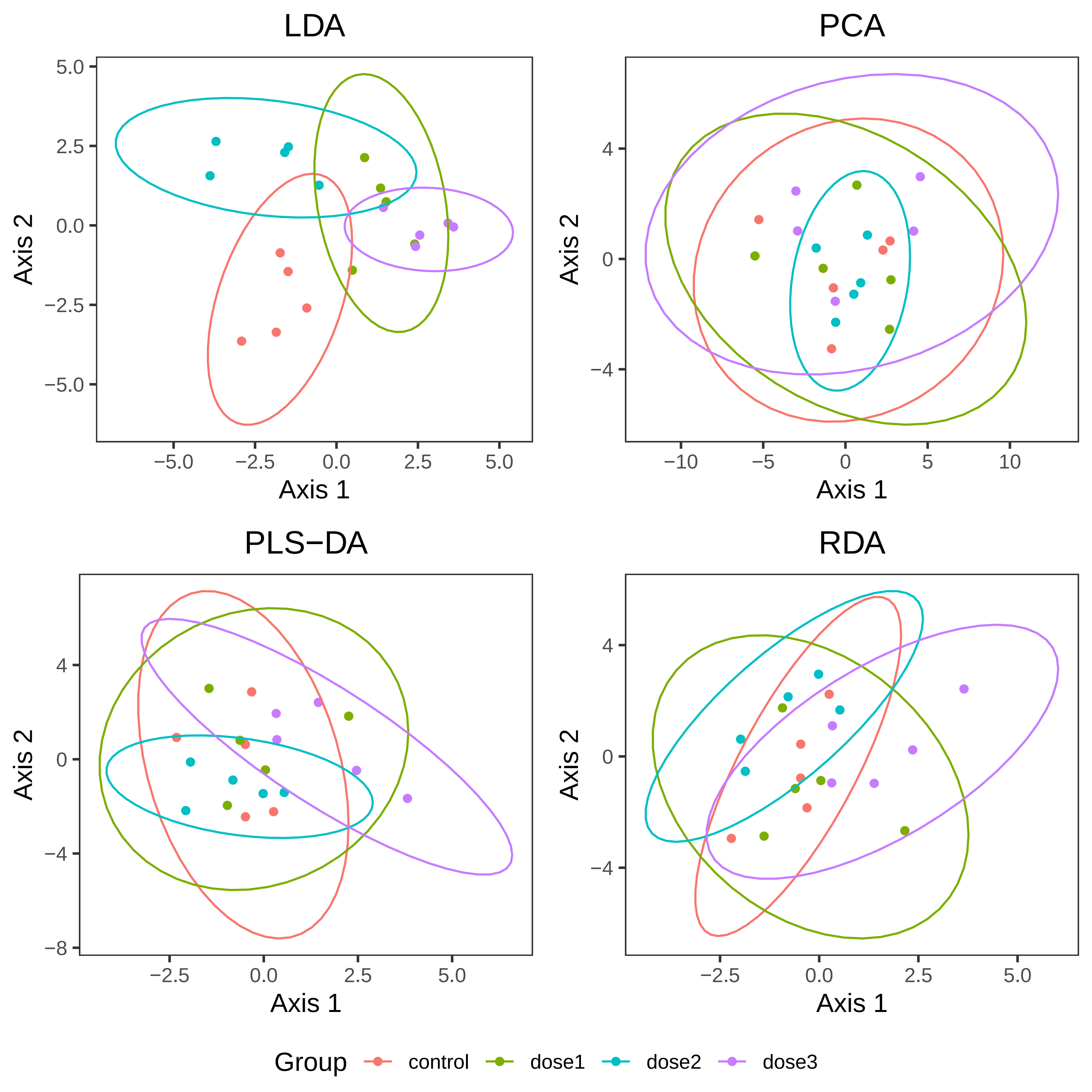

LDA
PCA
PLS−DA
RDA
Axis 1
Axis 2
5.0
2.5
0.0
−2.5
−5.0
4
0
−4
−8
−10
−5
5
10
Group
control
dose1
dose2
dose3

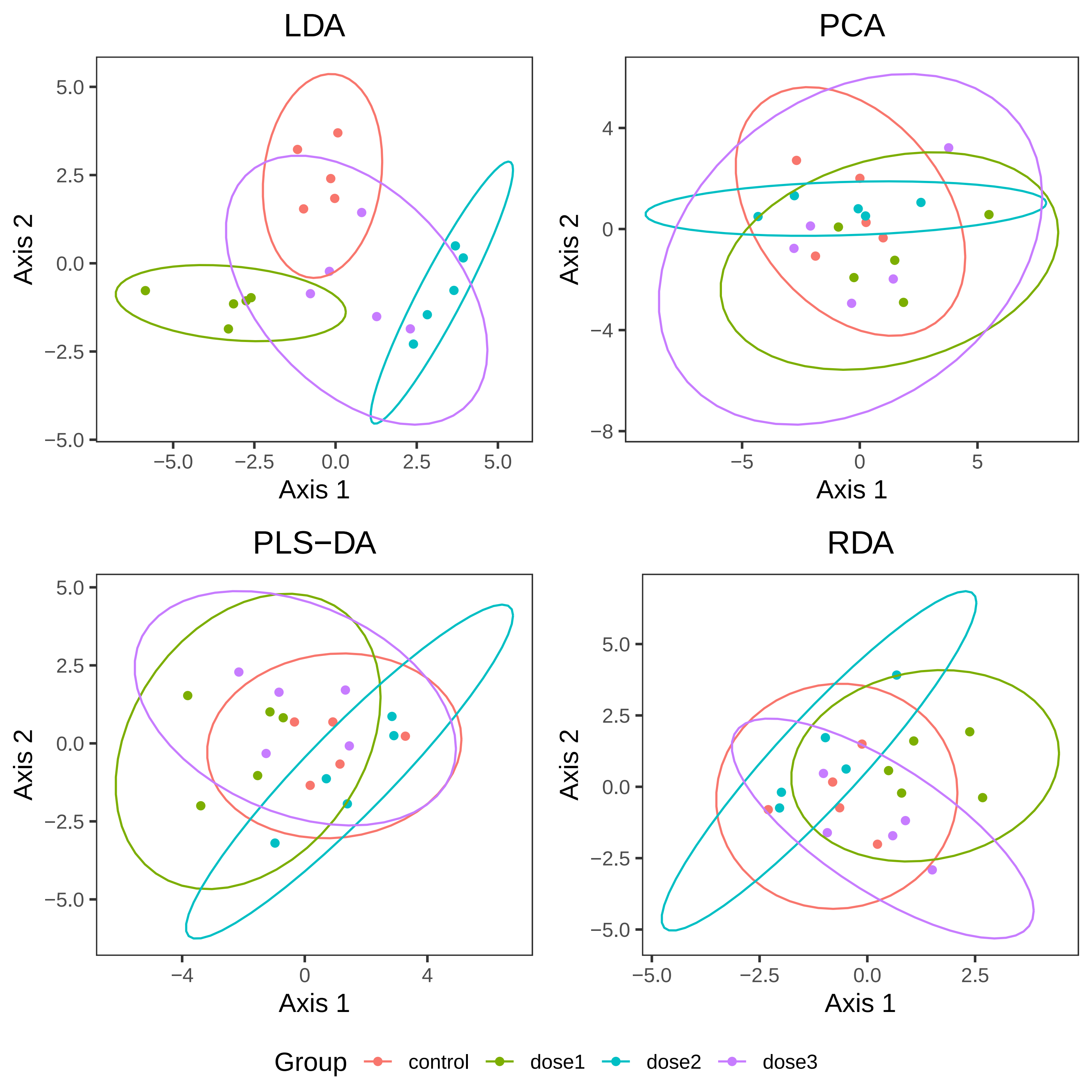

LDA
PCA
PLS−DA
RDA
Axis 1
Axis 2
−5.0
−2.5
0.0
2.5
5.0
−8
−4
0
4
−5
5
Group
control
dose1
dose2
dose3

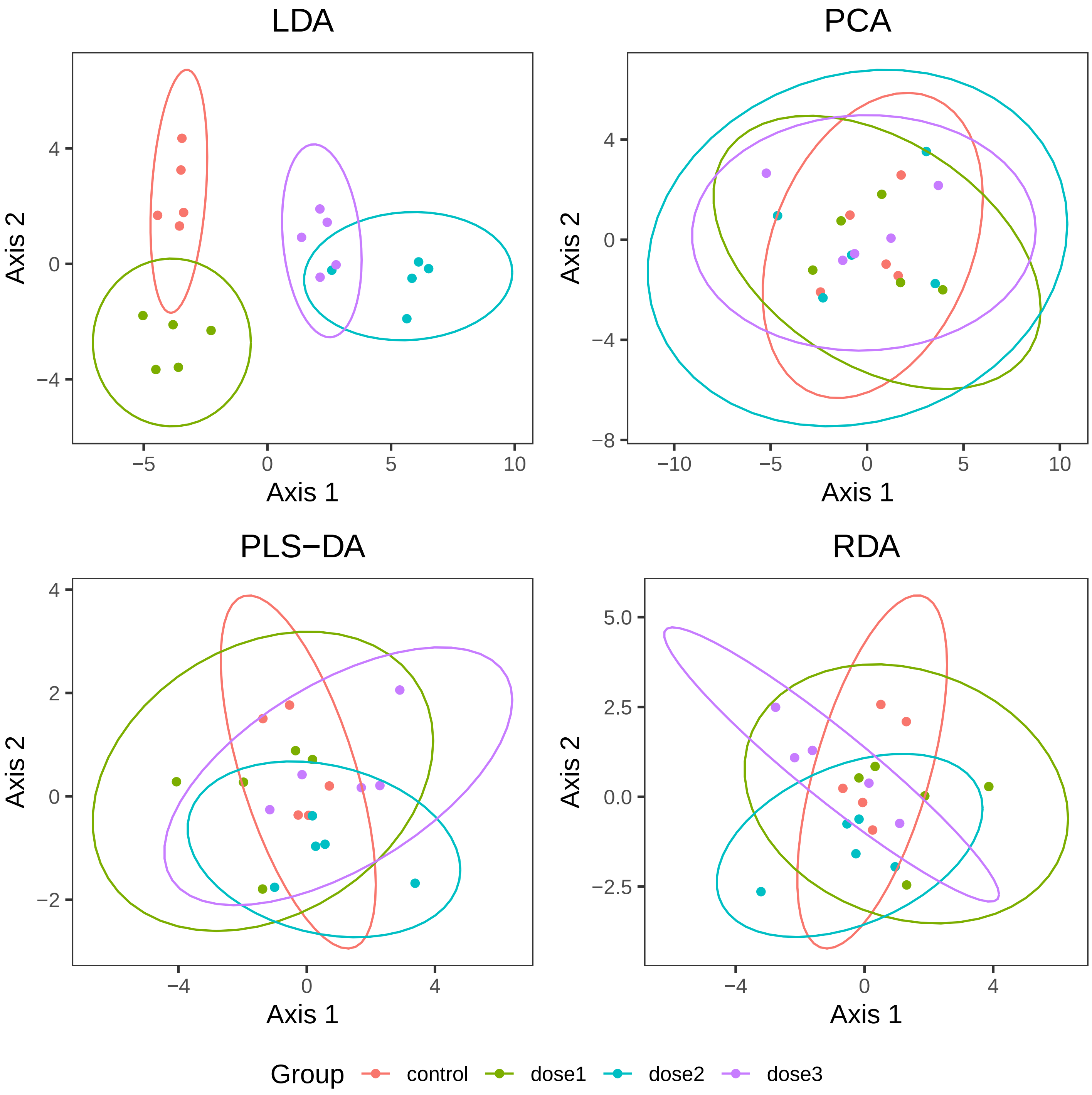

LDA
PCA
PLS−DA
RDA
Axis 1
Axis 2
Group
control
dose1
dose2
dose3

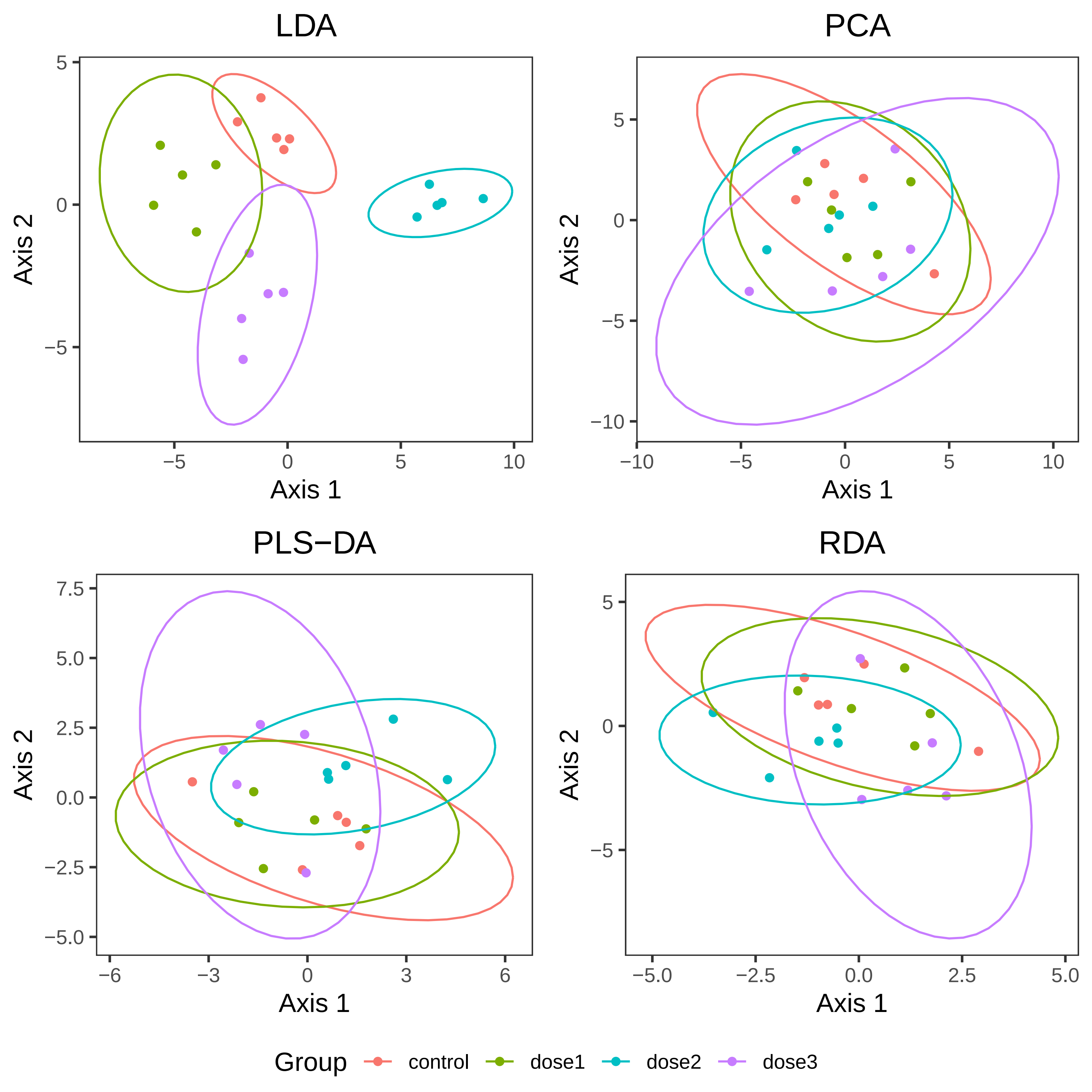
LDA
PCA
PLS−DA
RDA
Axis 1
Axis 2
Group
control
dose1
dose2
dose3

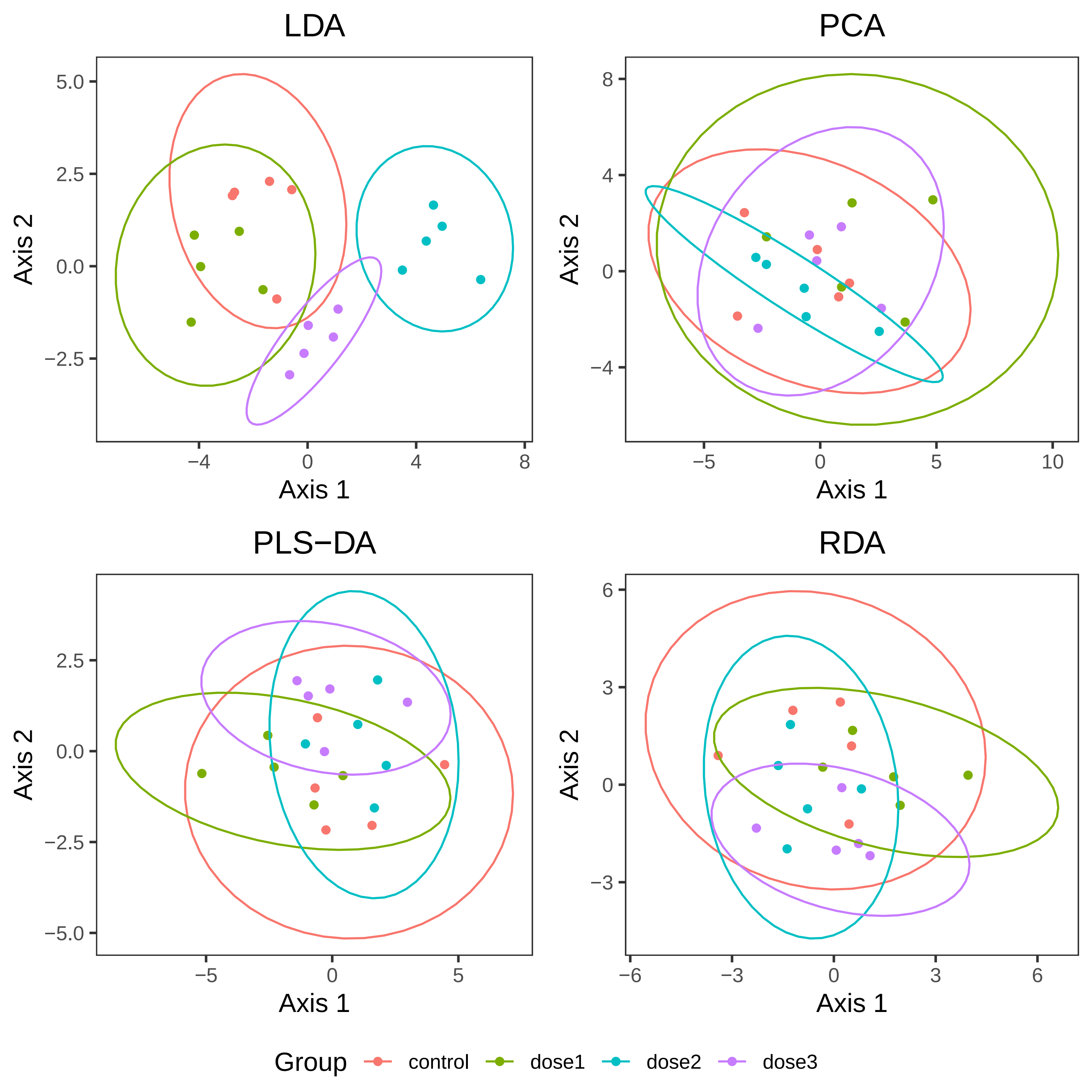

LDA
PCA
PLS−DA
RDA
Axis 1
Axis 2
5.0
2.5
0.0
−2.5
−4
0
4
8
−4
−5
5
10
−5.0
−6
−3
3
6
Group
control
dose1
dose2
dose3

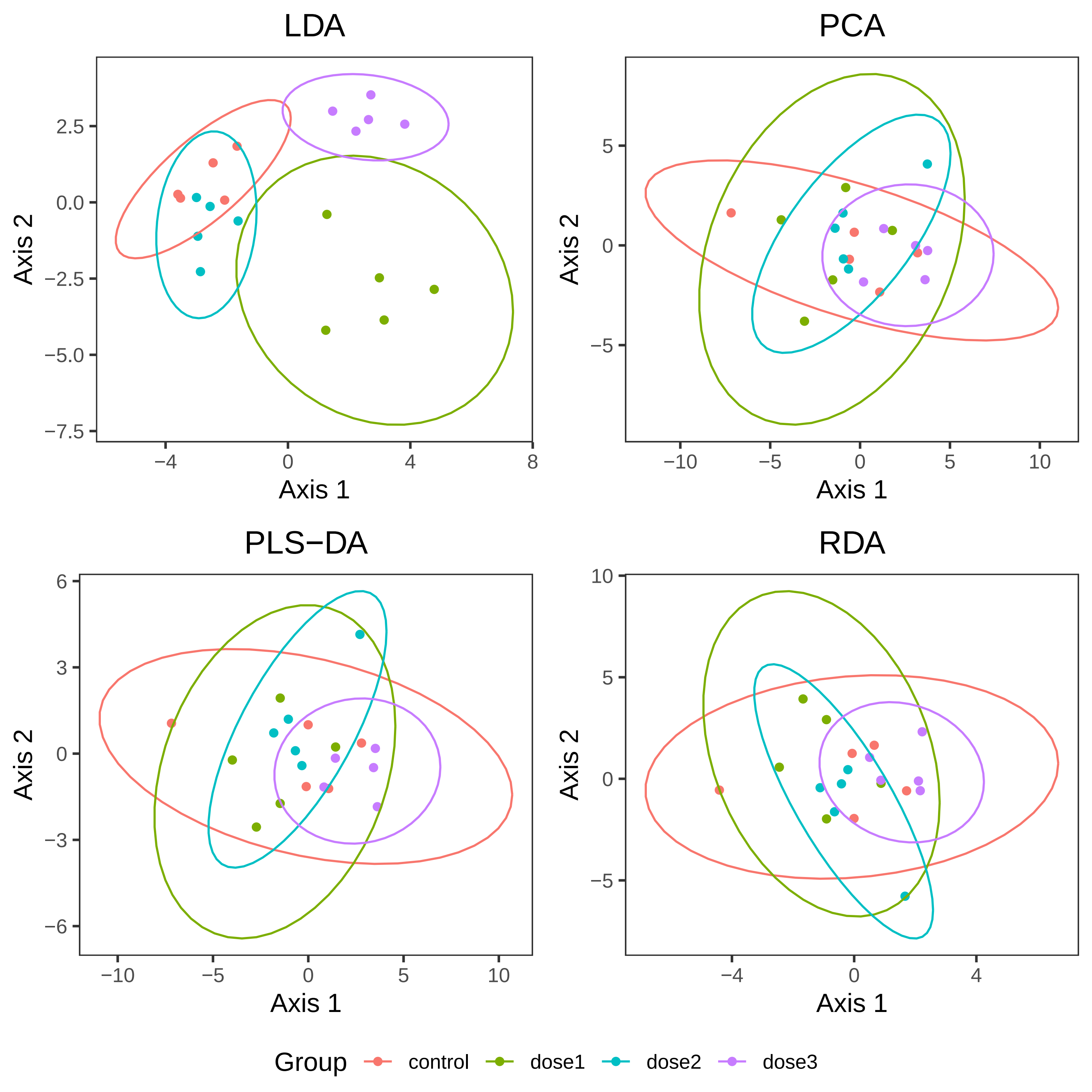

LDA
PCA
PLS−DA
RDA
Axis 1
Axis 2
2.5
0.0
−2.5
−5.0
−7.5
−4
0
4
8
5
0
−5
−10
−5
0
5
10
6
3
0
−3
−6
−10
−5
0
5
10
10
5
0
−5
−4
0
4
Group
control
dose1
dose2
dose3

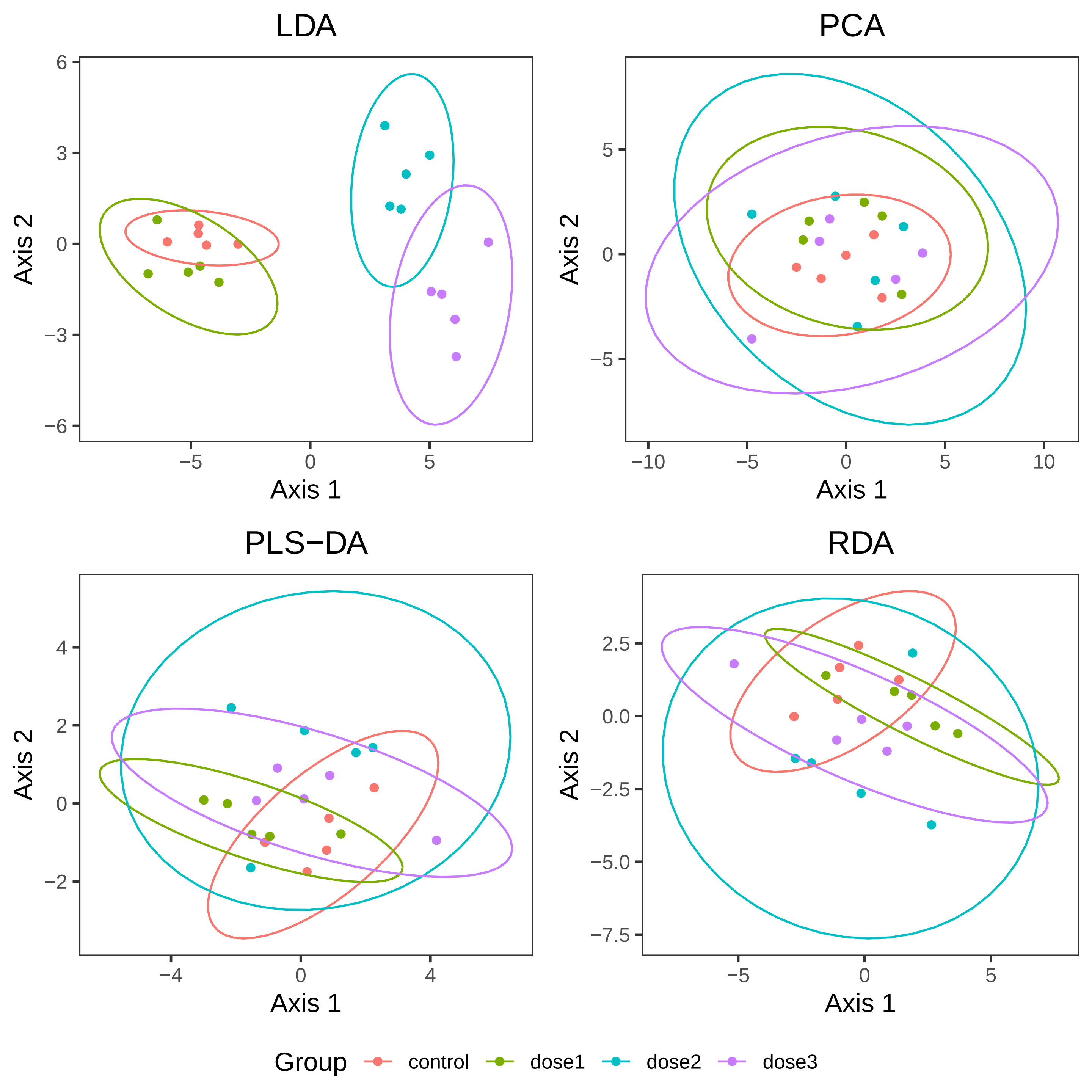

LDA
PCA
PLS−DA
RDA
Axis 1
Axis 2
6
3
0
−3
−6
−5
0
5
−10
10
4
2
−2
−4
2.5
0.0
−2.5
−5.0
−7.5
Group
control
dose1
dose2
dose3

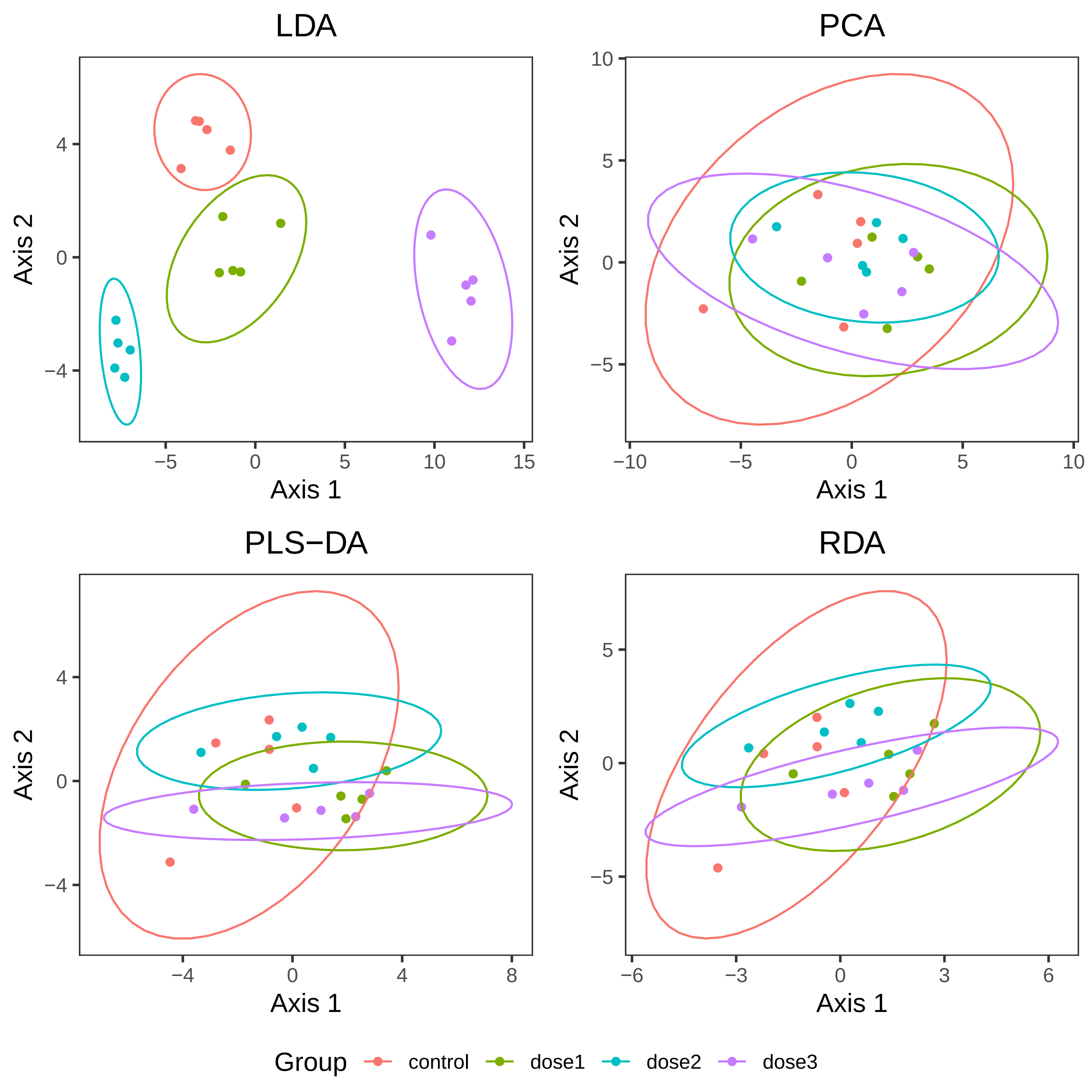

LDA
PCA
PLS−DA
RDA
Axis 1
Axis 2
Group
control
dose1
dose2
dose3

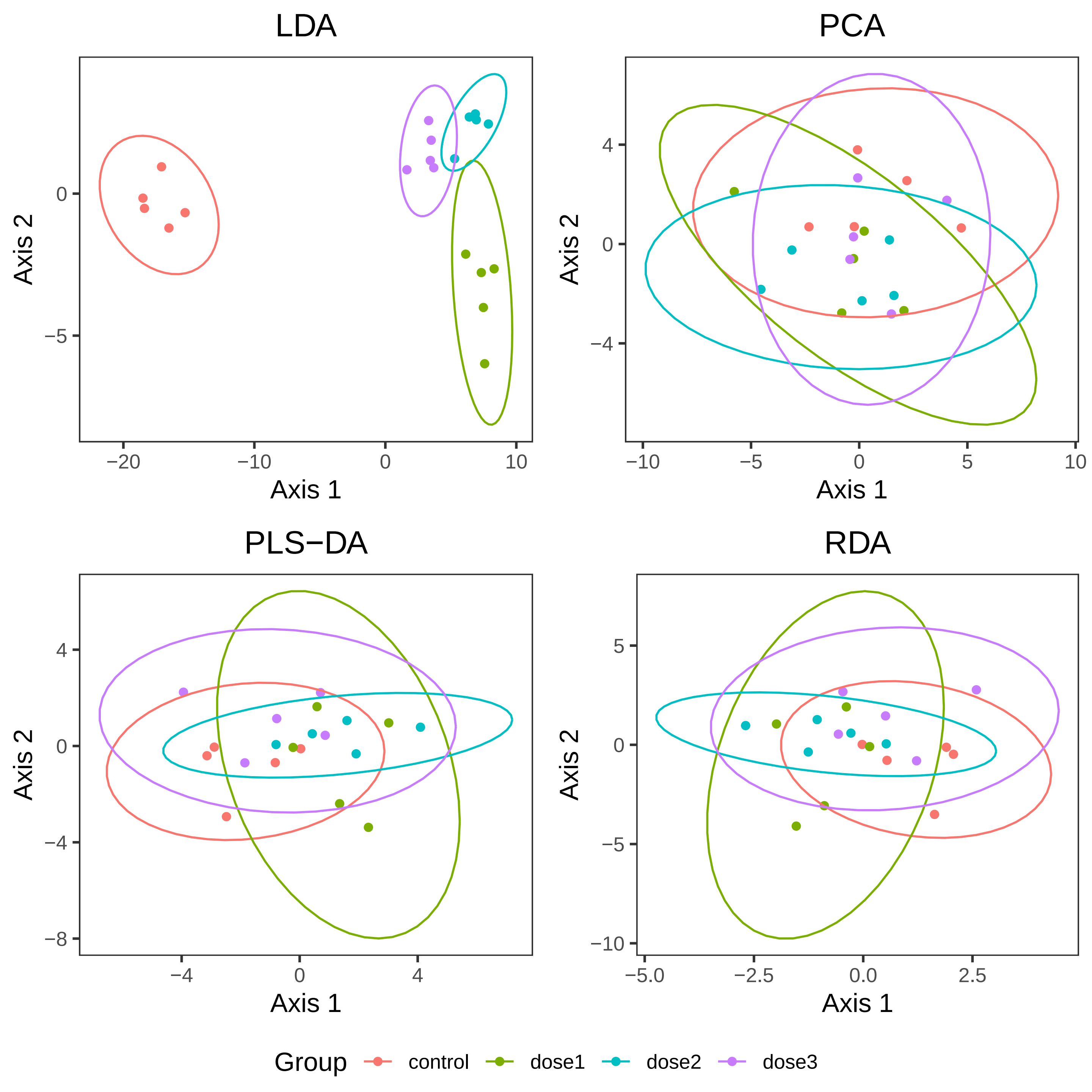
LDA
PCA
PLS−DA
RDA
Axis 1
Axis 2
−20
−10
0
10
−5
−10
5
−4
4
−8
−5.0
−2.5
0.0
2.5
Group
control
dose1
dose2
dose3

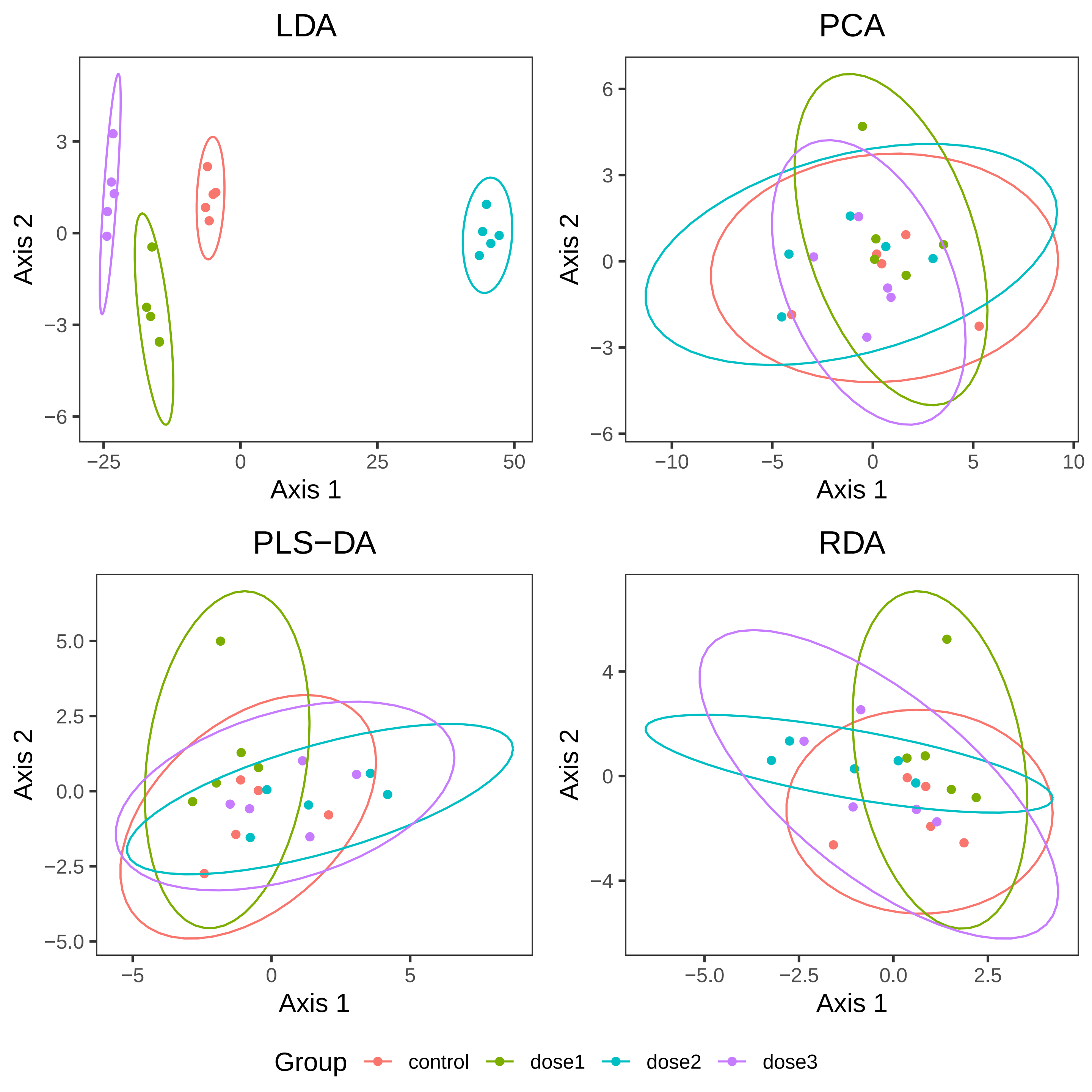
LDA
PCA
PLS−DA
RDA
Axis 1
Axis 2
Group
control
dose1
dose2
dose3

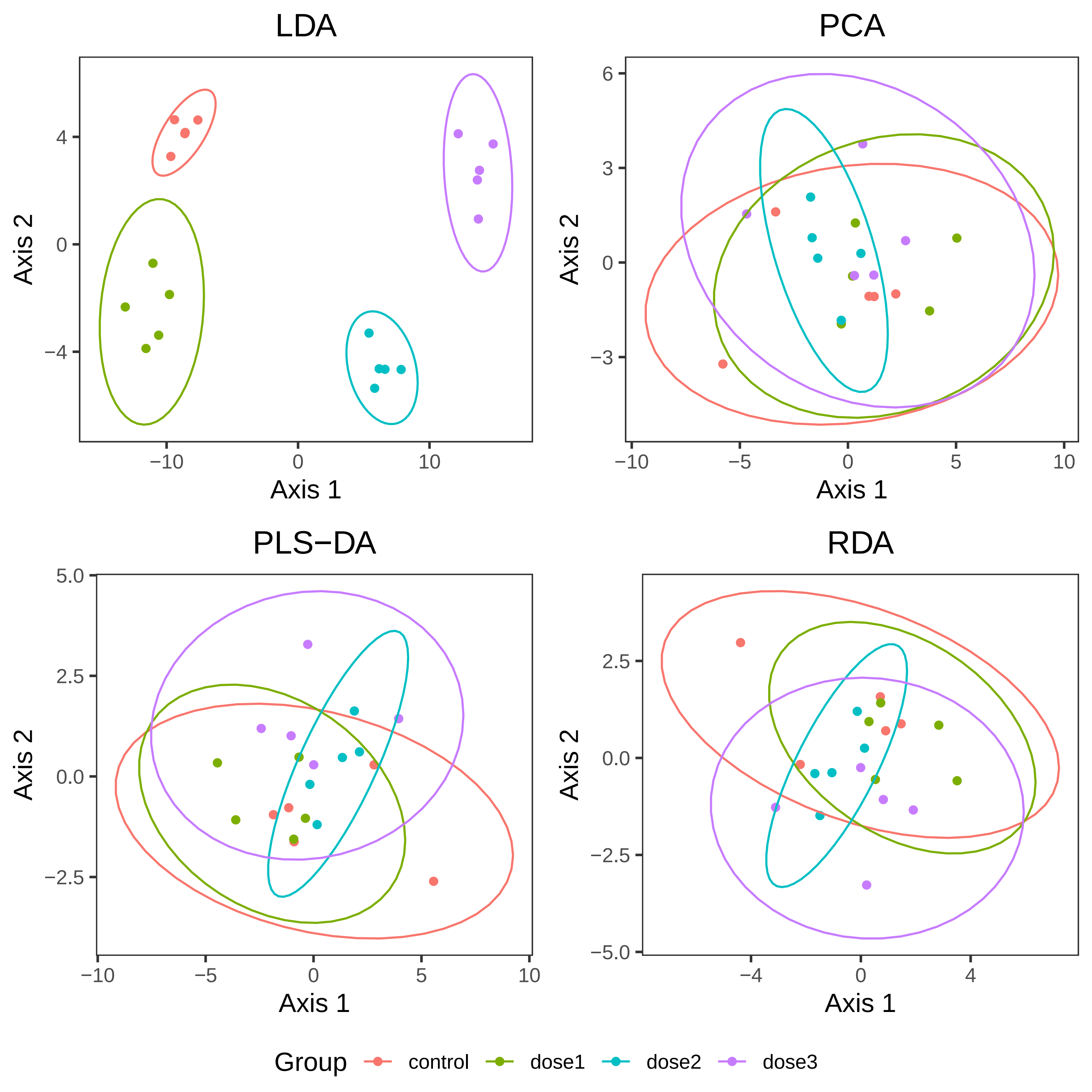

LDA
PCA
PLS−DA
RDA
Axis 1
Axis 2
4
0
−4
−10
0
10
6
3
0
−3
−10
−5
0
5
10
5.0
2.5
0.0
−2.5
−10
−5
0
5
10
2.5
0.0
−2.5
−5.0
−4
0
4
Group
control
dose1
dose2
dose3

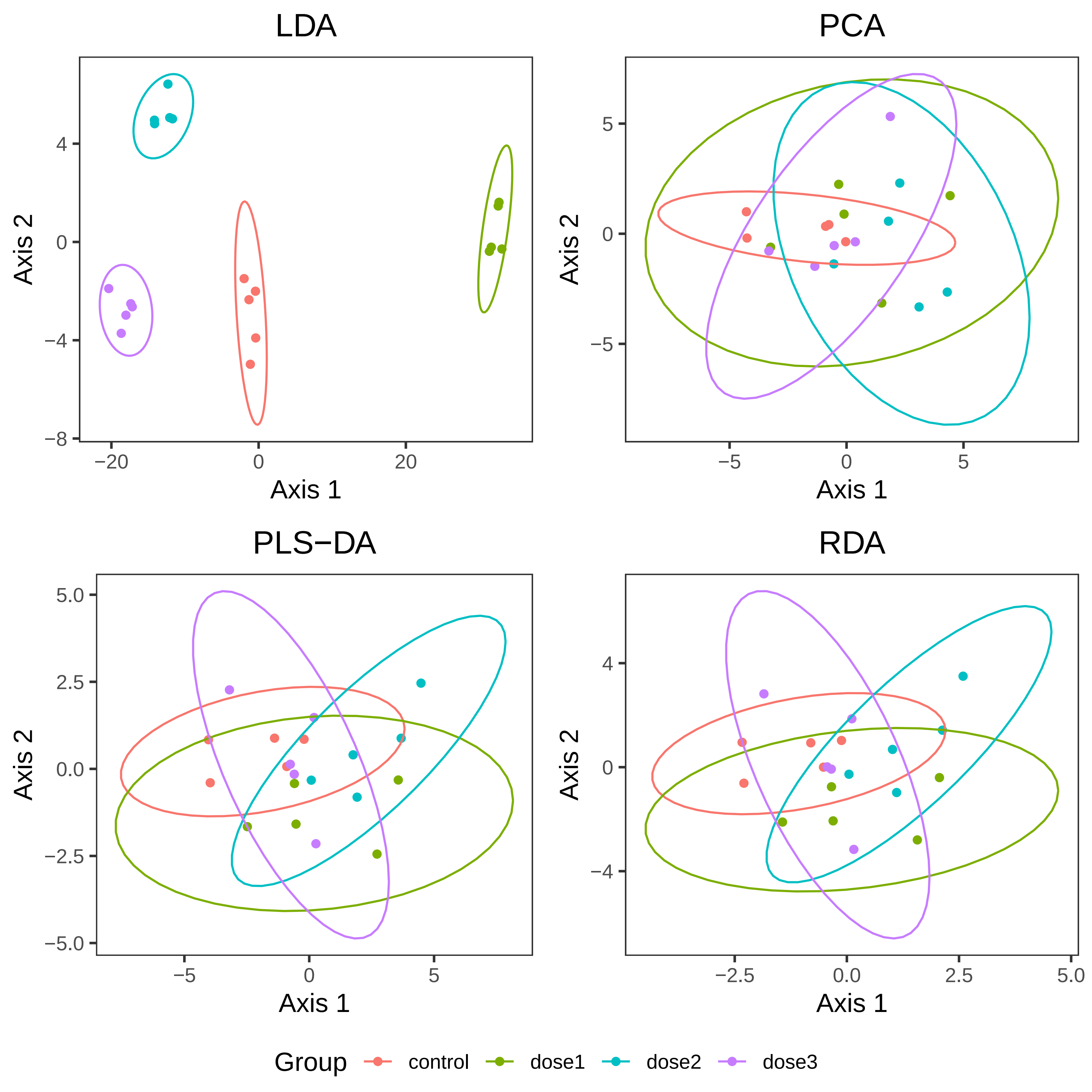

LDA
PCA
PLS−DA
RDA
Axis 1
Axis 2
Group
control
dose1
dose2
dose3

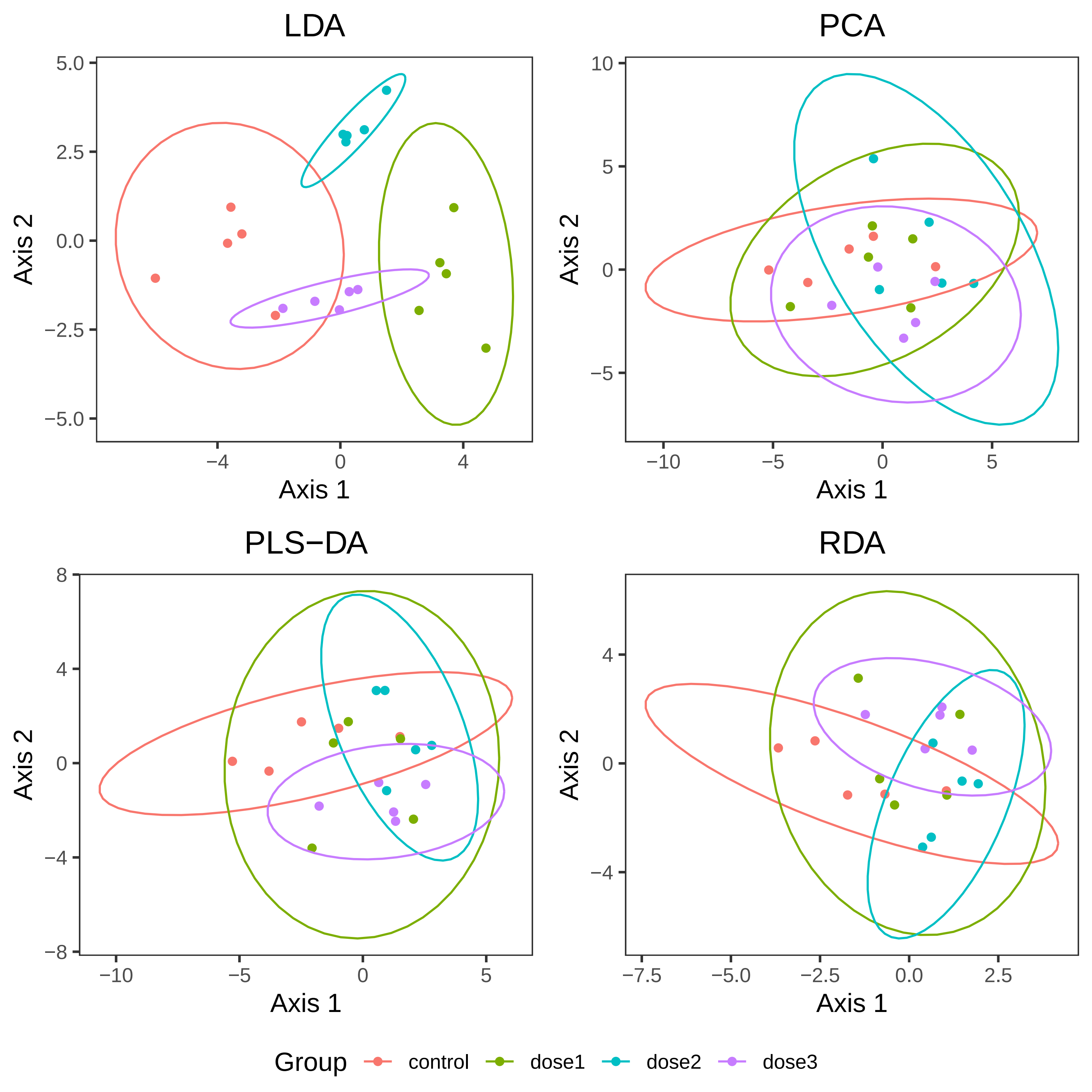
LDA
PCA
PLS−DA
RDA
Axis 1
Axis 2
Group
control
dose1
dose2
dose3

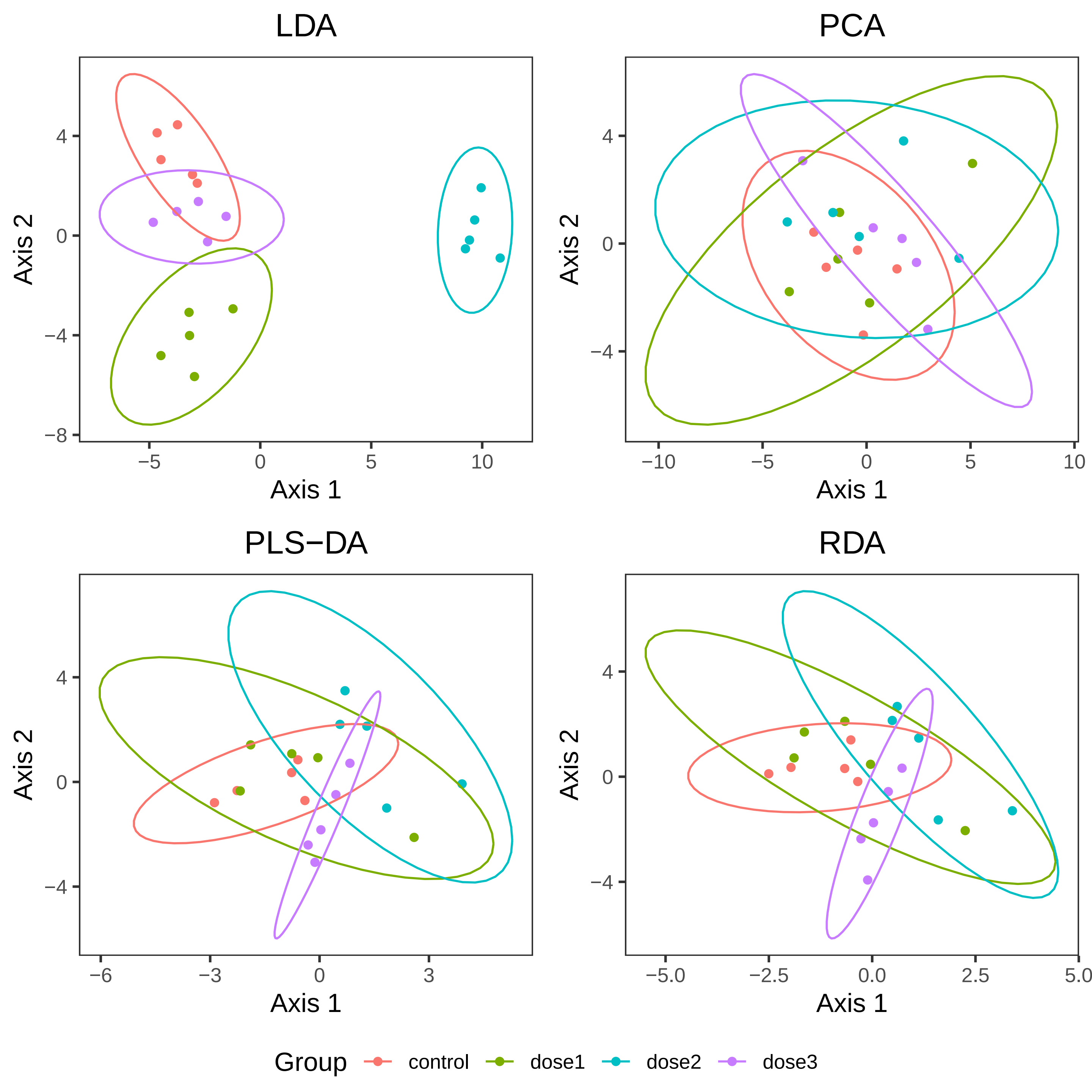
LDA
PCA
PLS−DA
RDA
Axis 1
Axis 2
Group
control
dose1
dose2
dose3

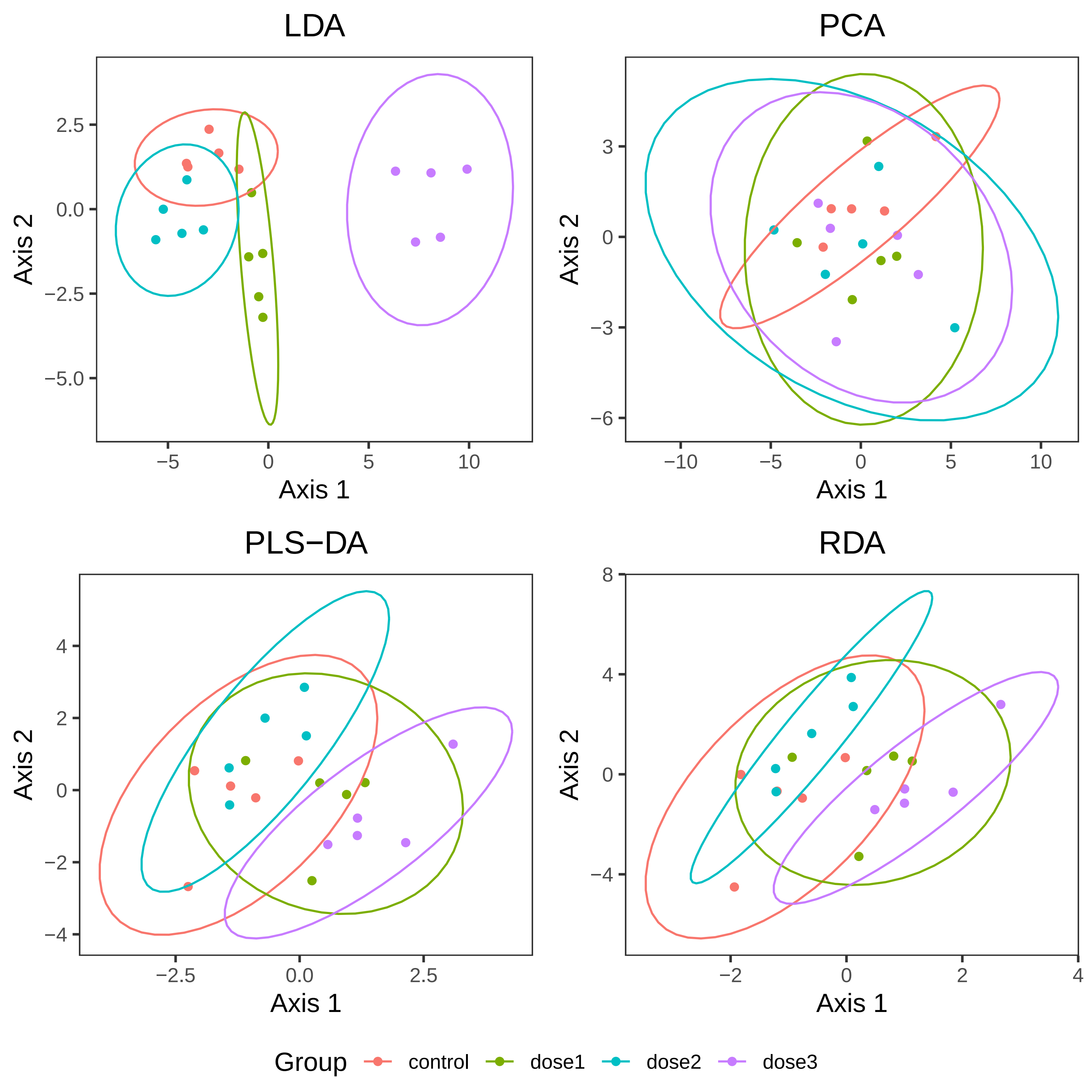
LDA
PCA
PLS−DA
RDA
Axis 1
Axis 2
Group
control
dose1
dose2
dose3

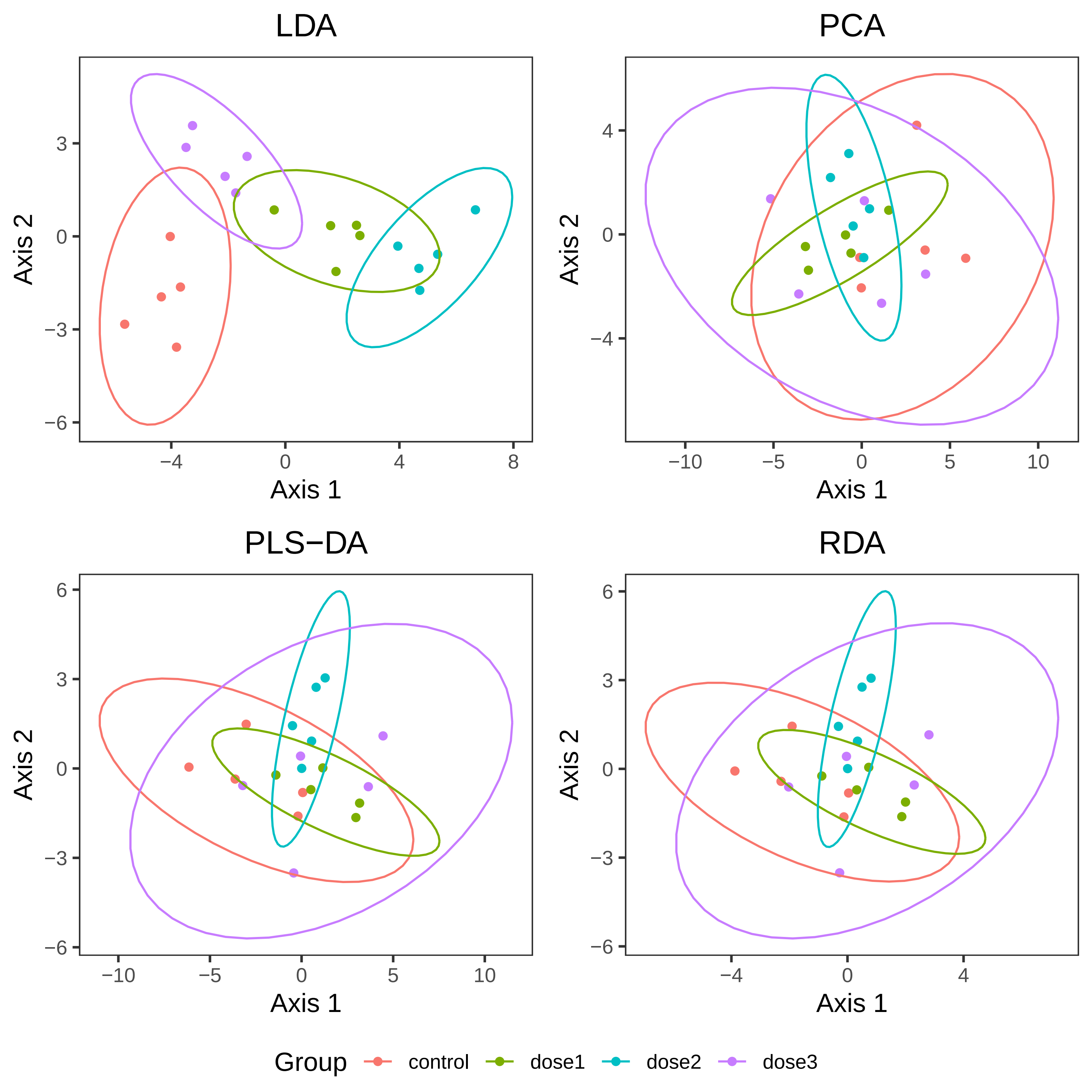
LDA
PCA
PLS−DA
RDA
Axis 1
Axis 2
Group
control
dose1
dose2
dose3

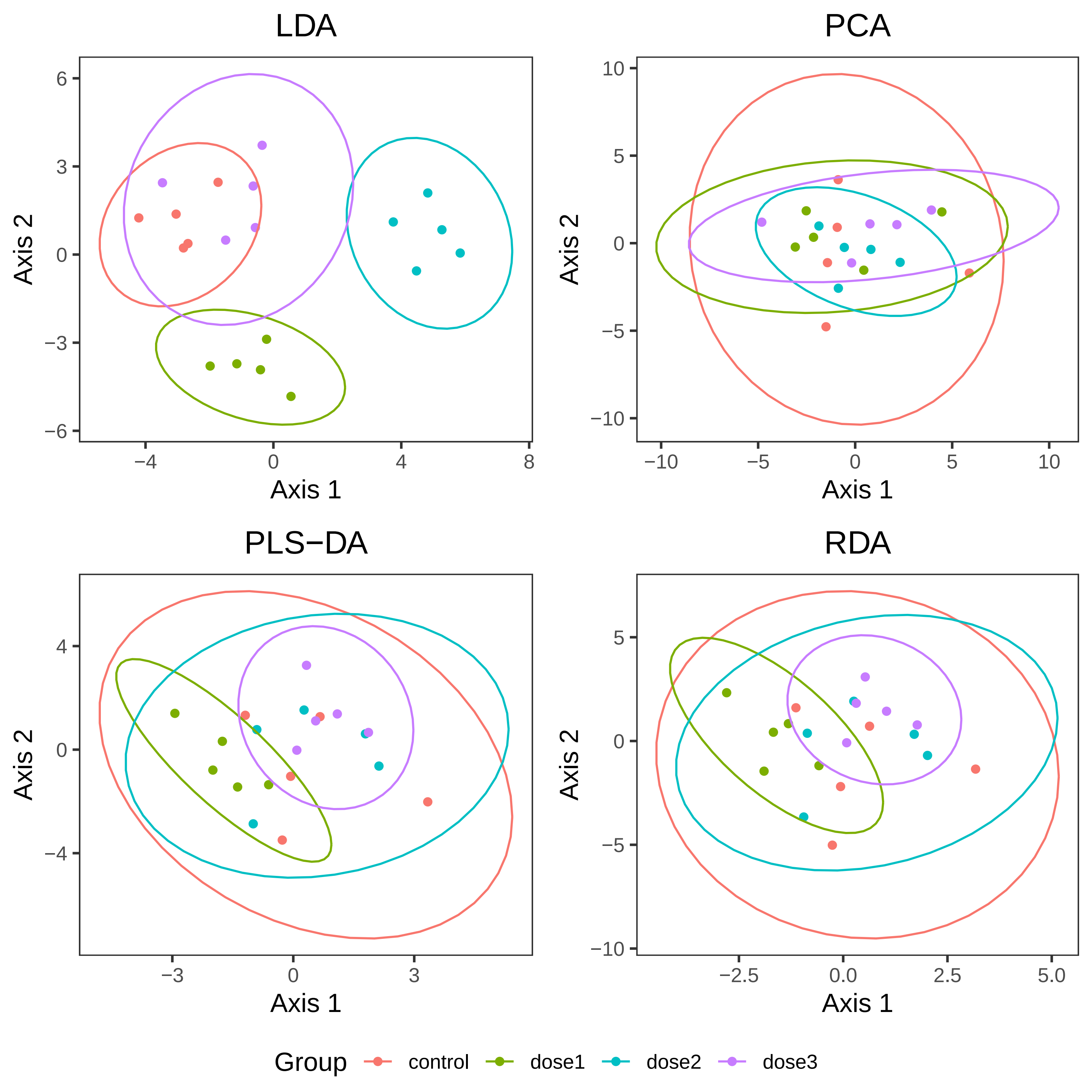
LDA
PCA
PLS−DA
RDA
Axis 1
Axis 2
6
3
0
−3
−6
−4
0
4
8
10
5
0
−5
−10
−10
−5
0
5
10
4
0
−4
−3
0
3
5
0
−5
−10
−2.5
0.0
2.5
5.0
Group
control
dose1
dose2
dose3

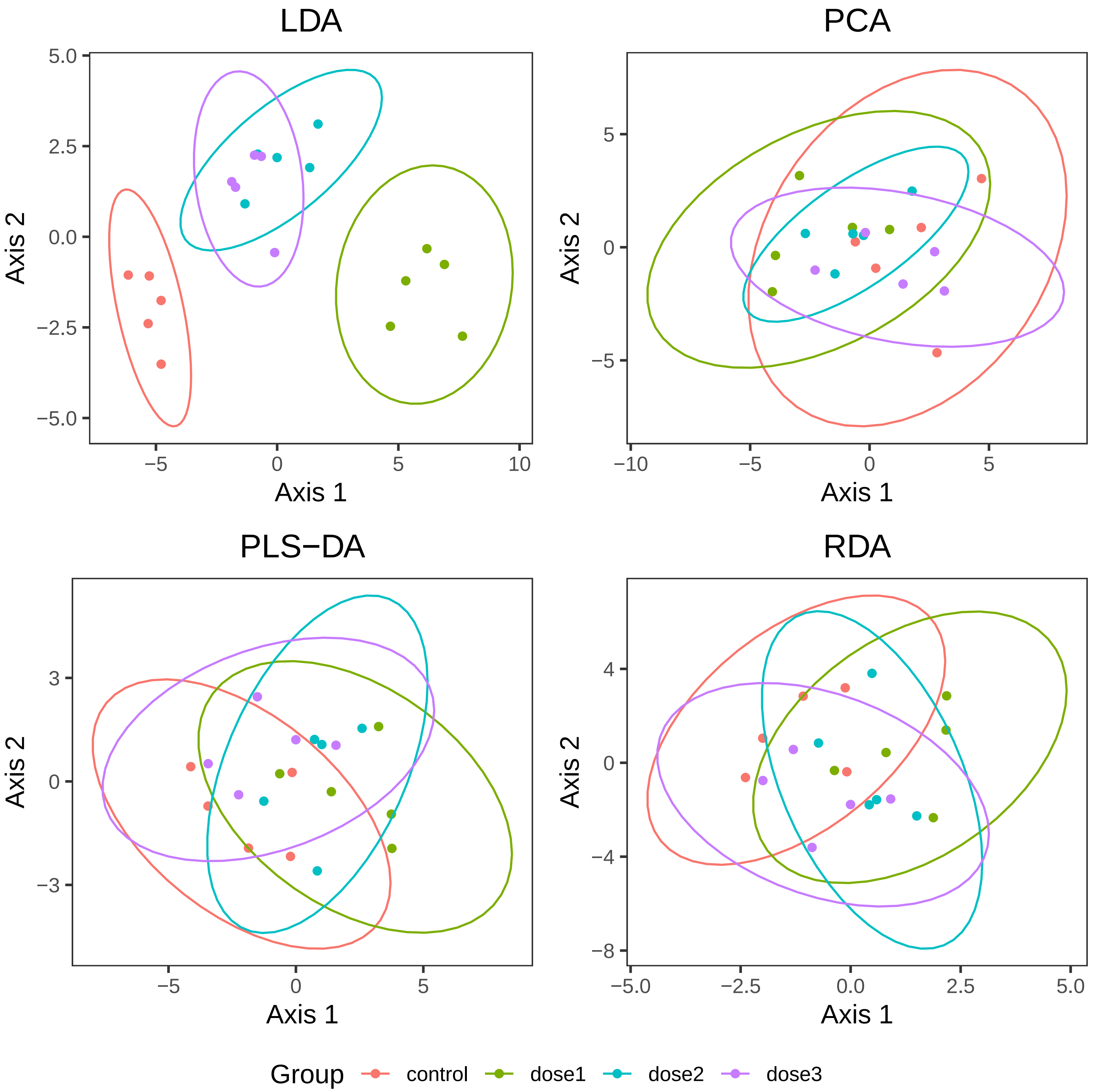

LDA
PCA
PLS−DA
RDA
Axis 1
Axis 2
5.0
2.5
0.0
−2.5
−5.0
−5
0
5
10
−10
−3
3
−8
−4
4
Group
control
dose1
dose2
dose3

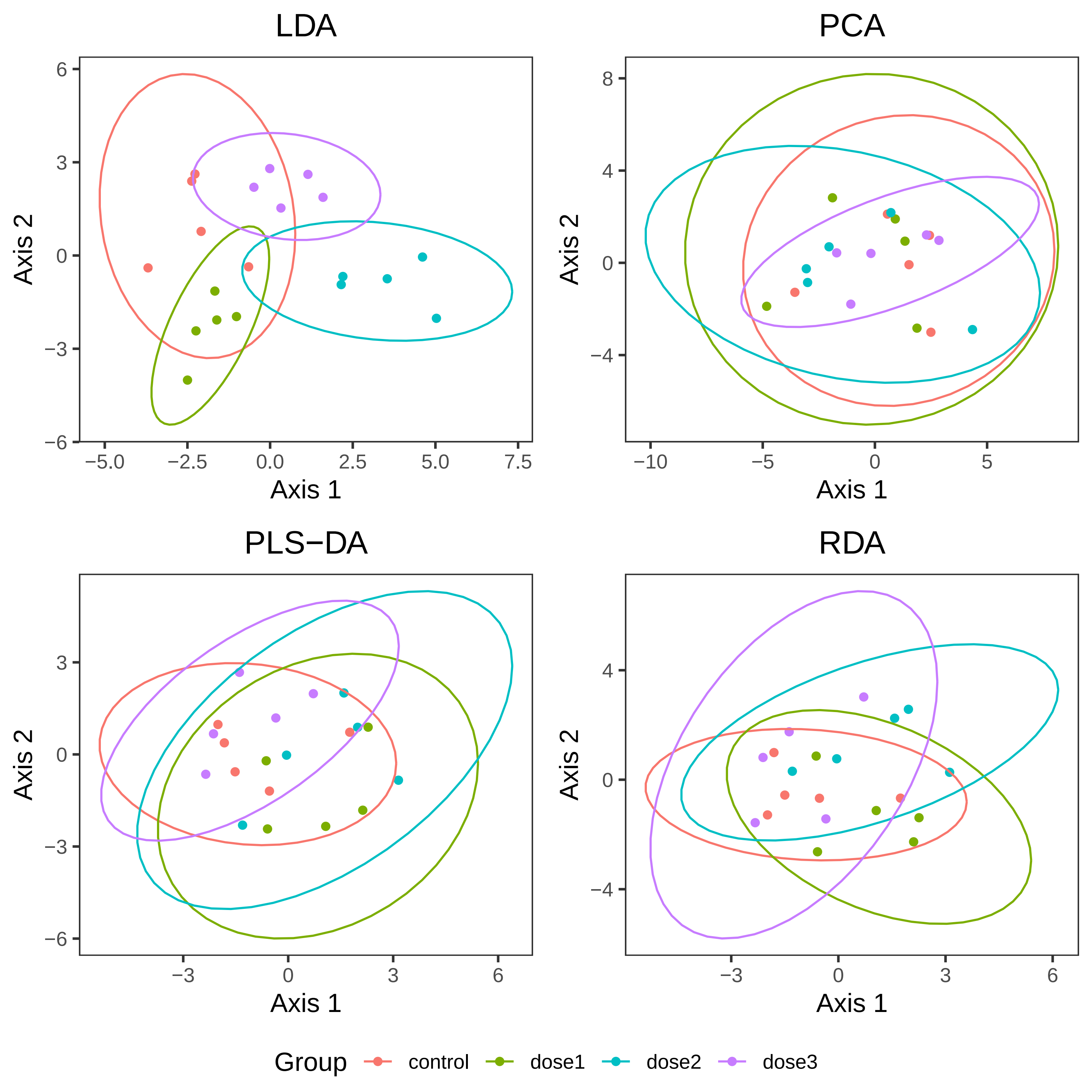

LDA
PCA
PLS−DA
RDA
Axis 1
Axis 2
6
3
0
−3
−6
−5.0
−2.5
0.0
2.5
5.0
7.5
8
4
0
−4
−10
−5
0
5
−3
0
3
6
4
0
−4
Group
control
dose1
dose2
dose3

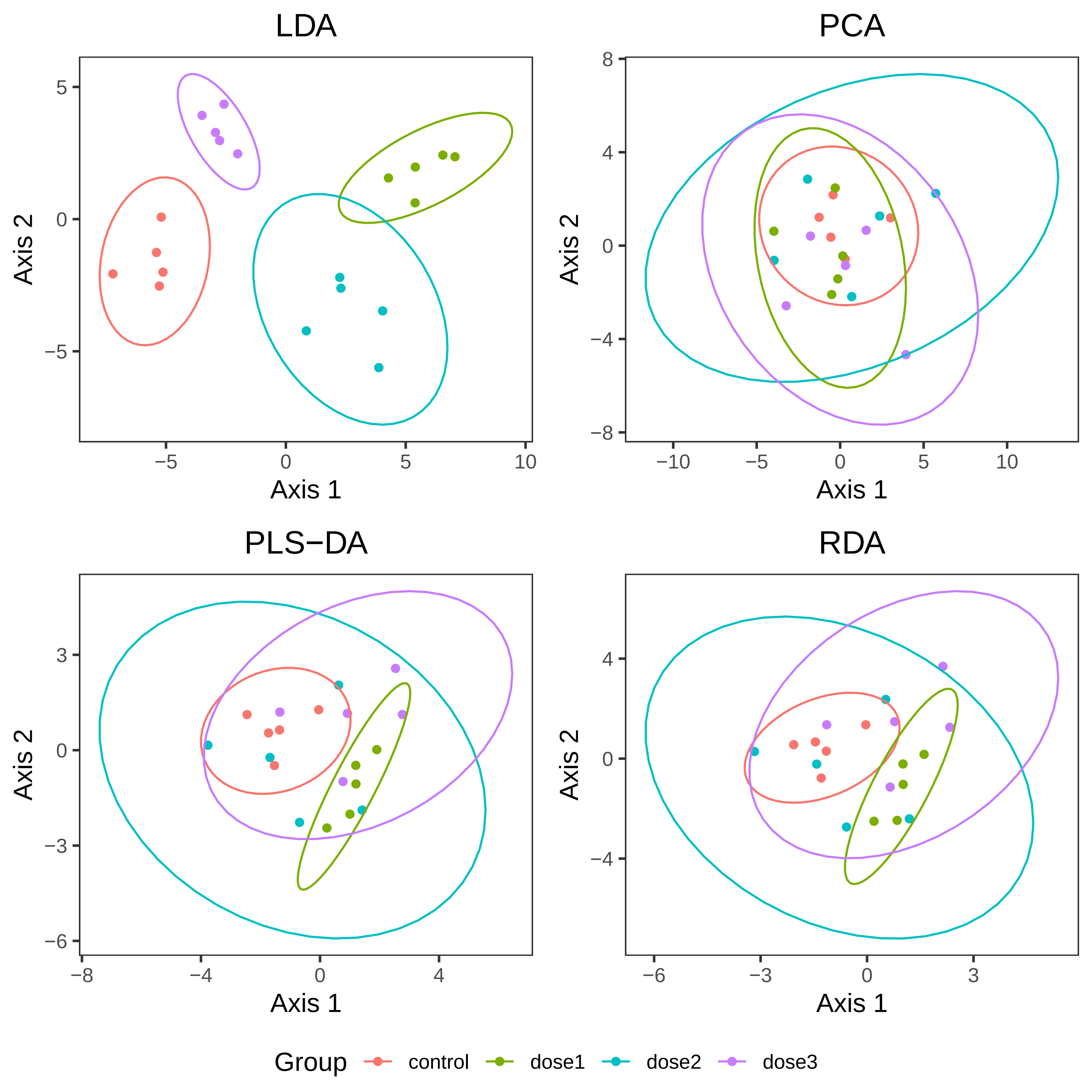

LDA
PCA
PLS−DA
RDA
Axis 1
Axis 2
Group
control
dose1
dose2
dose3

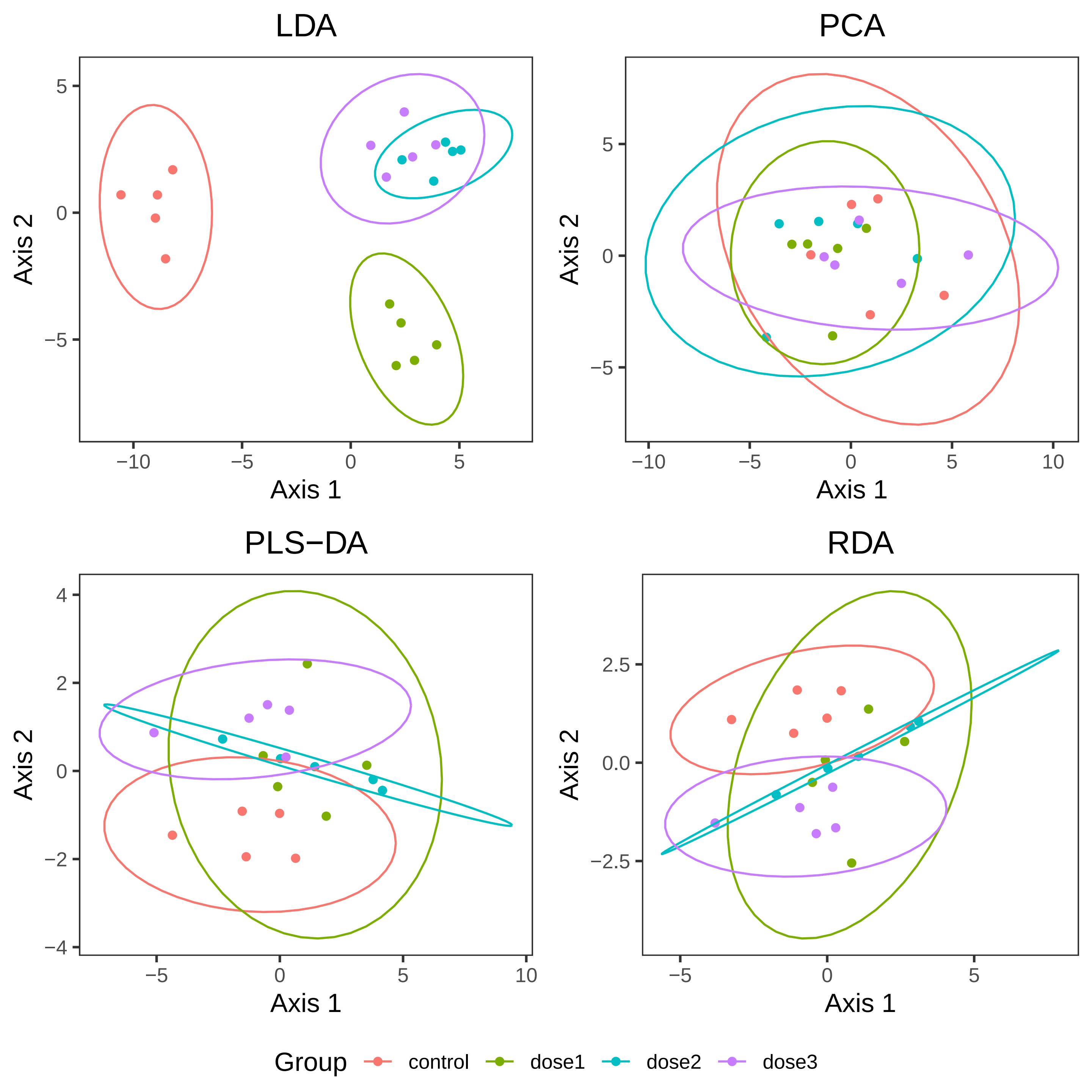
LDA
PCA
PLS−DA
RDA
Axis 1
Axis 2
Group
control
dose1
dose2
dose3

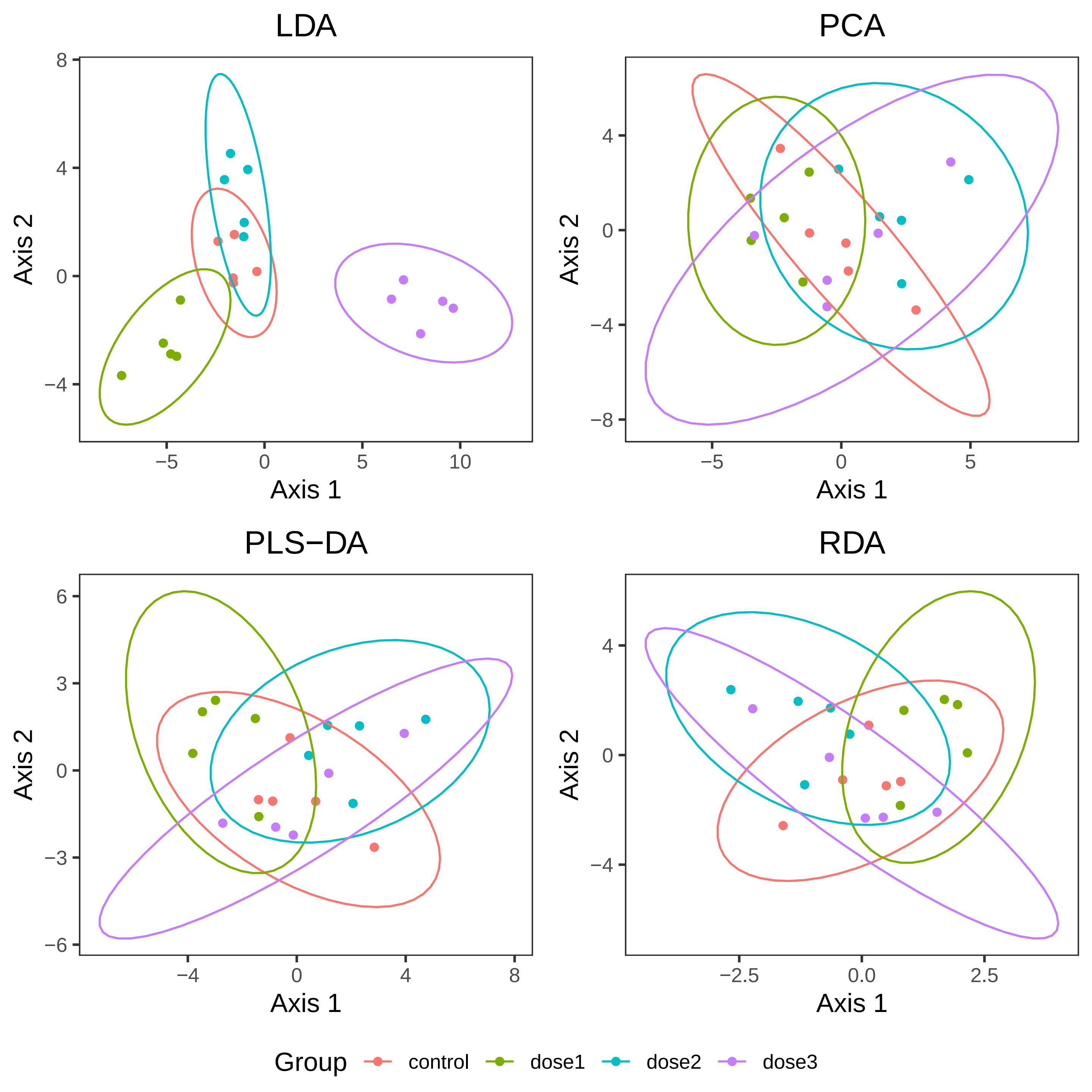

LDA
PCA
PLS−DA
RDA
Axis 1
Axis 2
8
4
0
−4
−5
0
5
10
−8
−6
−3
3
6
−2.5
0.0
2.5
Group
control
dose1
dose2
dose3

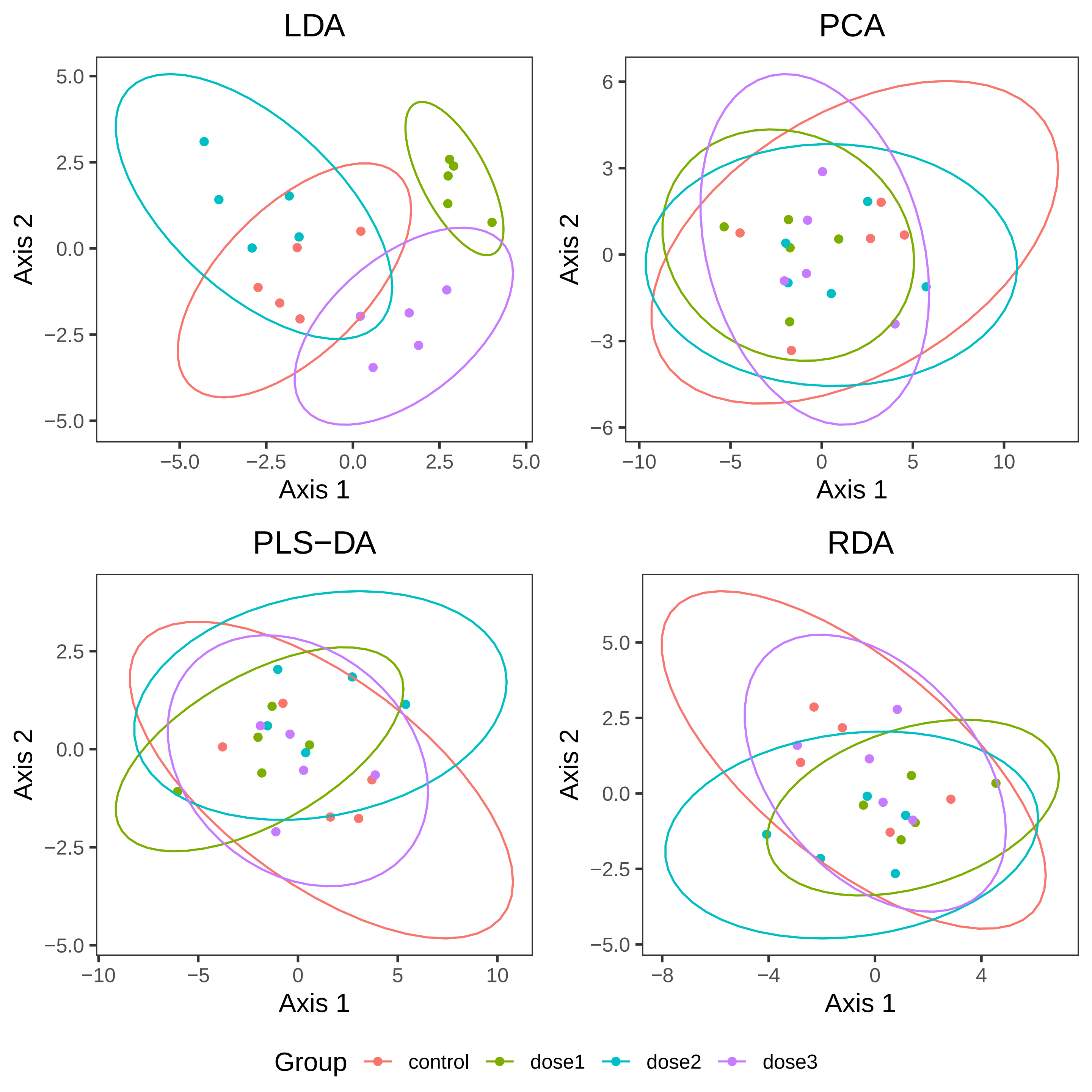

LDA
PCA
PLS−DA
RDA
Axis 1
Axis 2
Group
control
dose1
dose2
dose3

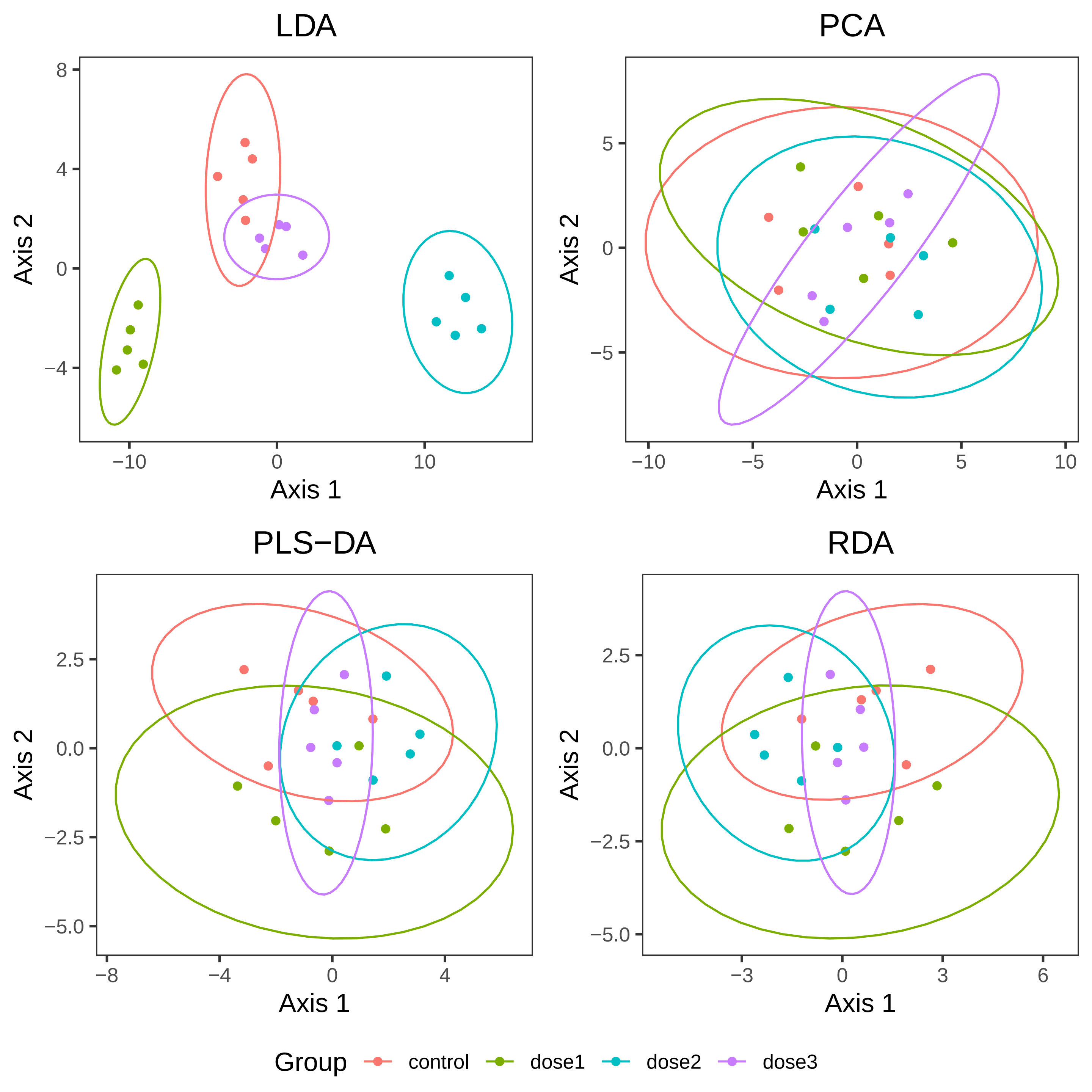
LDA
PCA
PLS−DA
RDA
Axis 1
Axis 2
Group
control
dose1
dose2
dose3

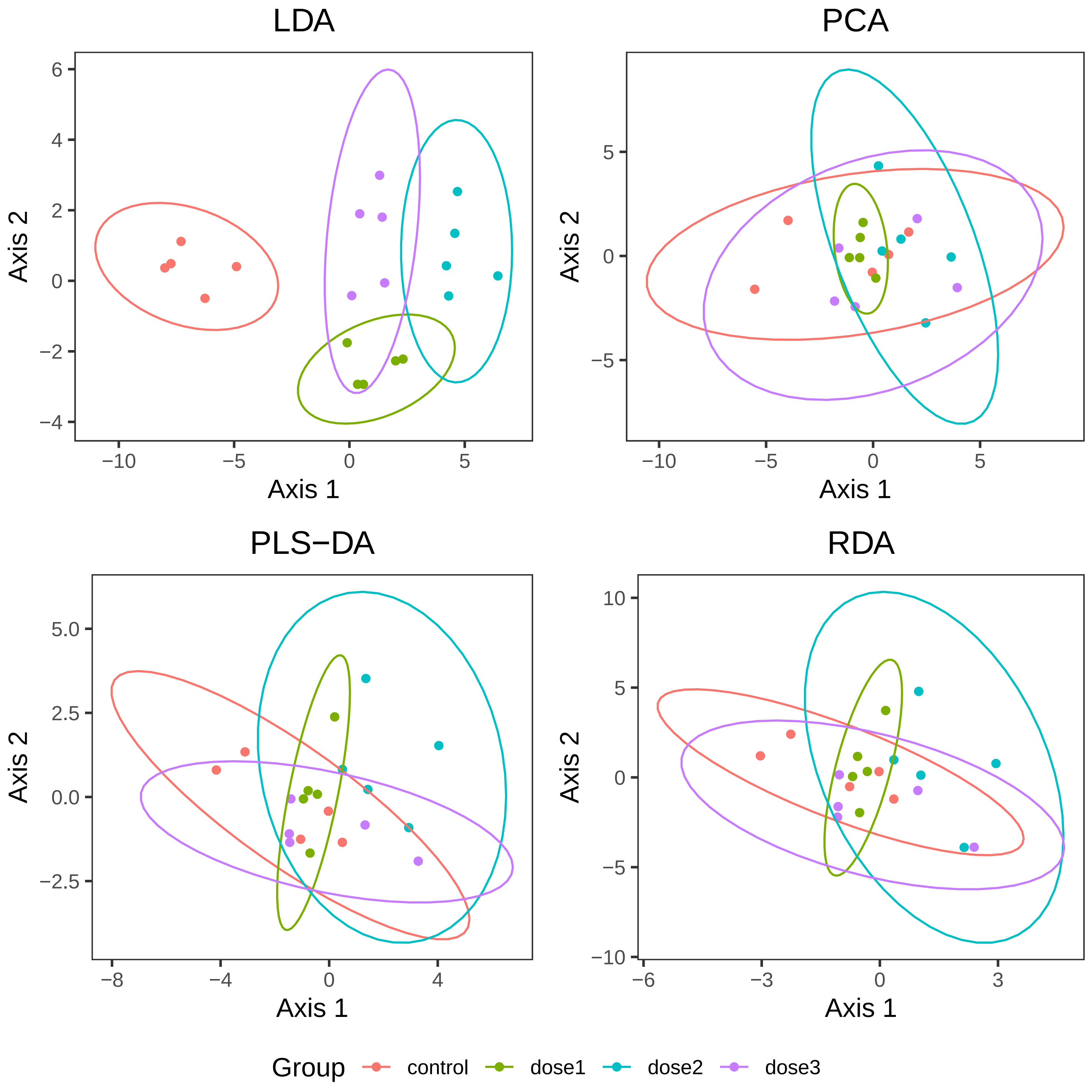
LDA
PCA
PLS−DA
RDA
Axis 1
Axis 2
Group
control
dose1
dose2
dose3

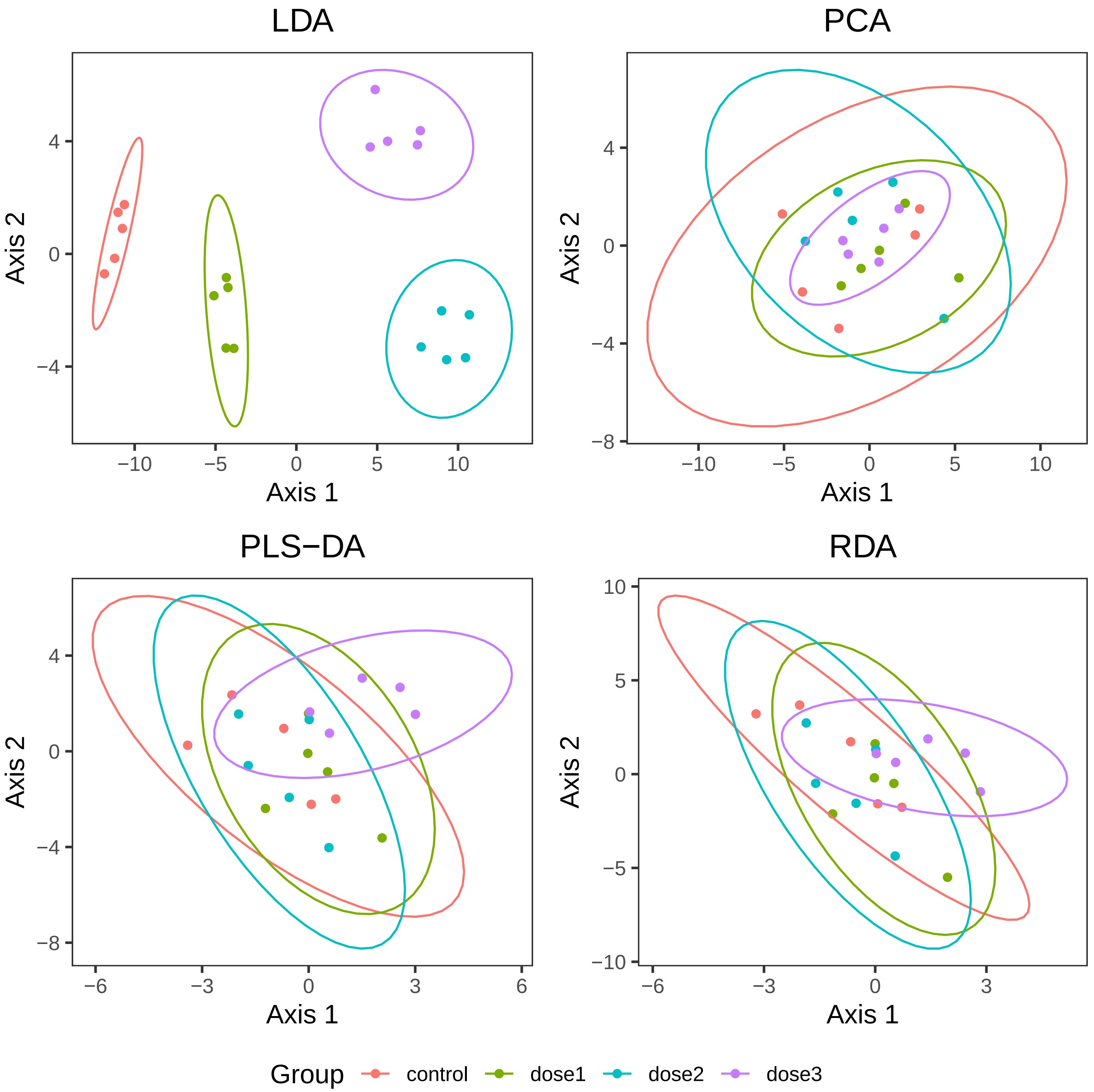
LDA
PCA
PLS−DA
RDA
Axis 1
Axis 2
Group
control
dose1
dose2
dose3

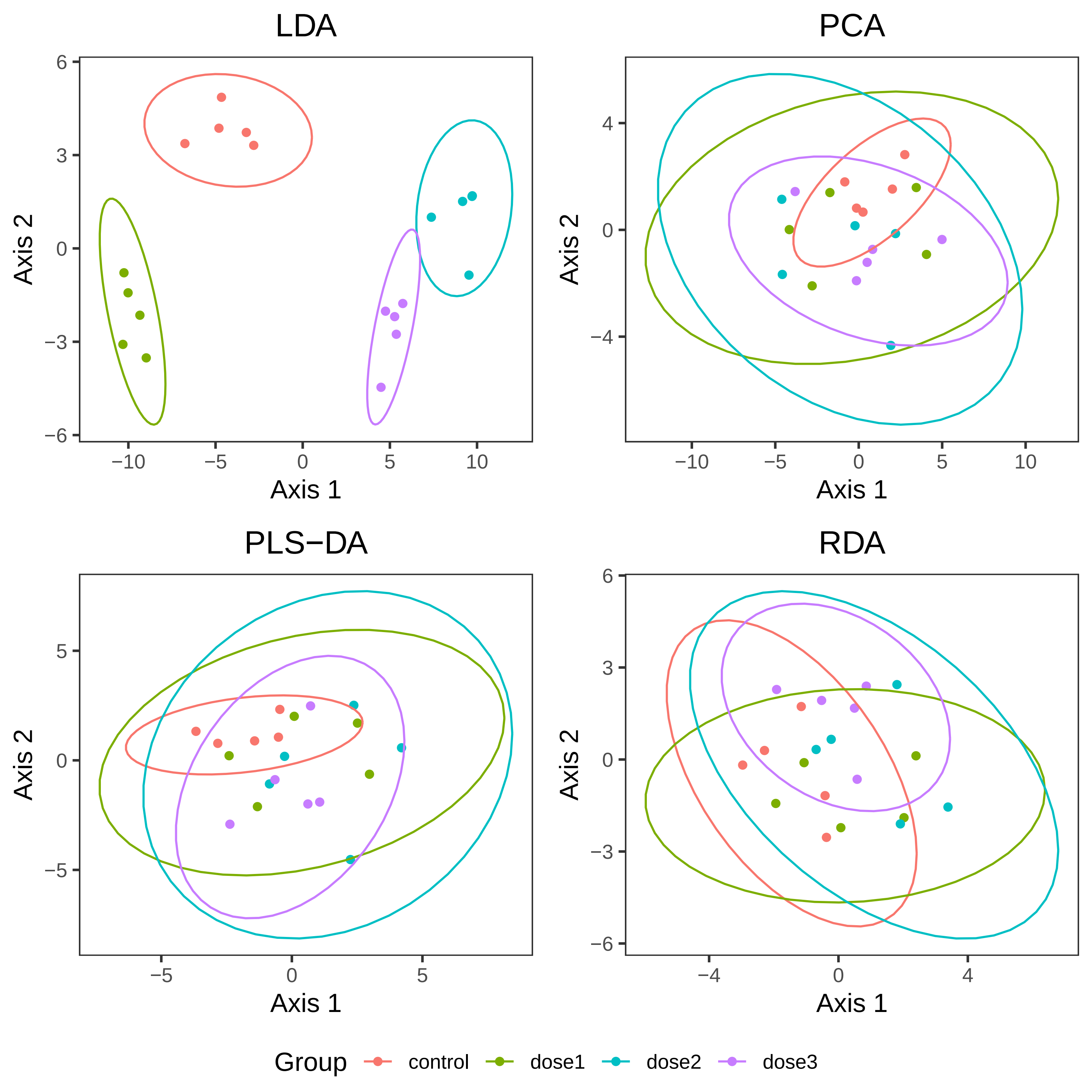

LDA
PCA
PLS−DA
RDA
Axis 1
Axis 2
Group
control
dose1
dose2
dose3

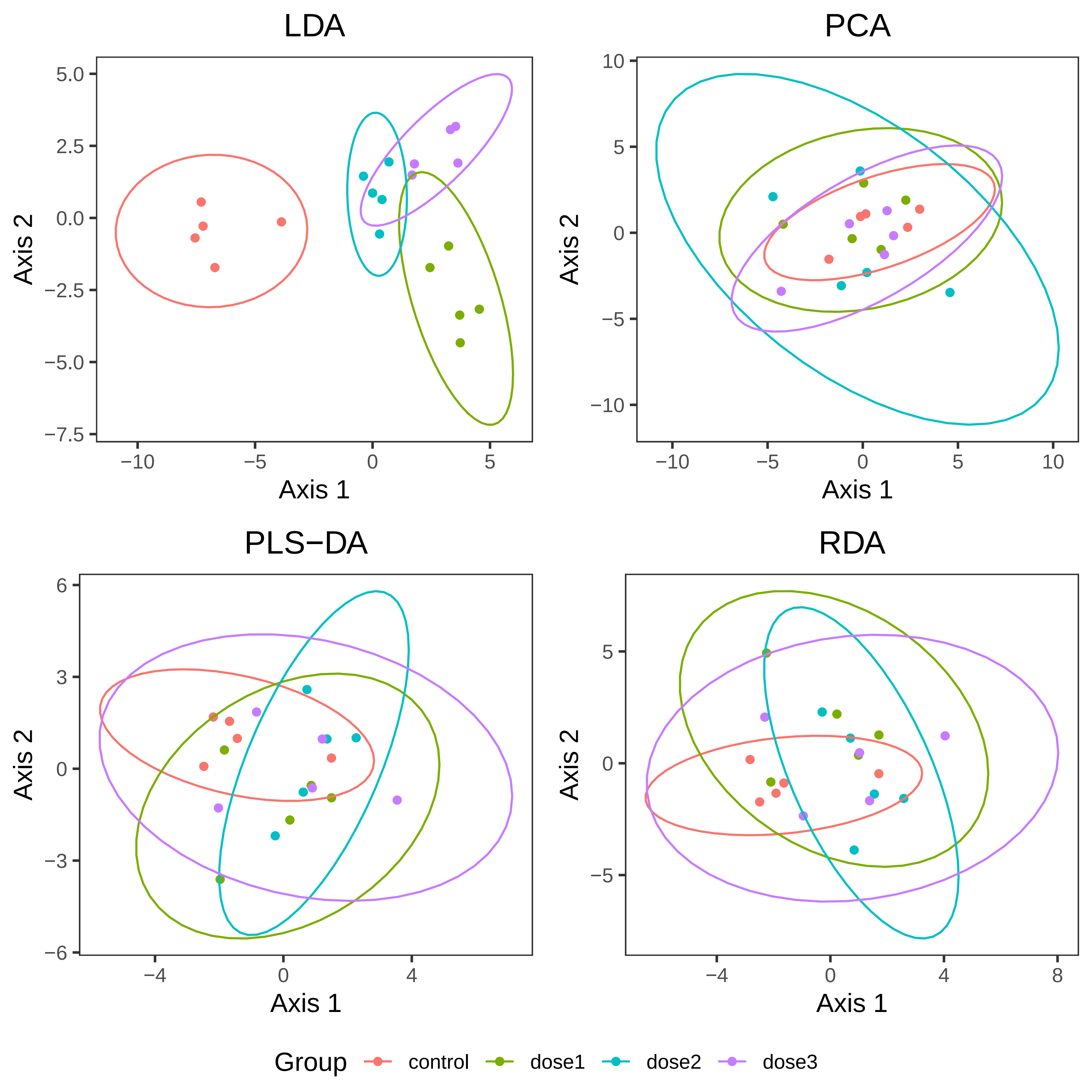
LDA
PCA
PLS−DA
RDA
Axis 1
Axis 2
Group
control
dose1
dose2
dose3

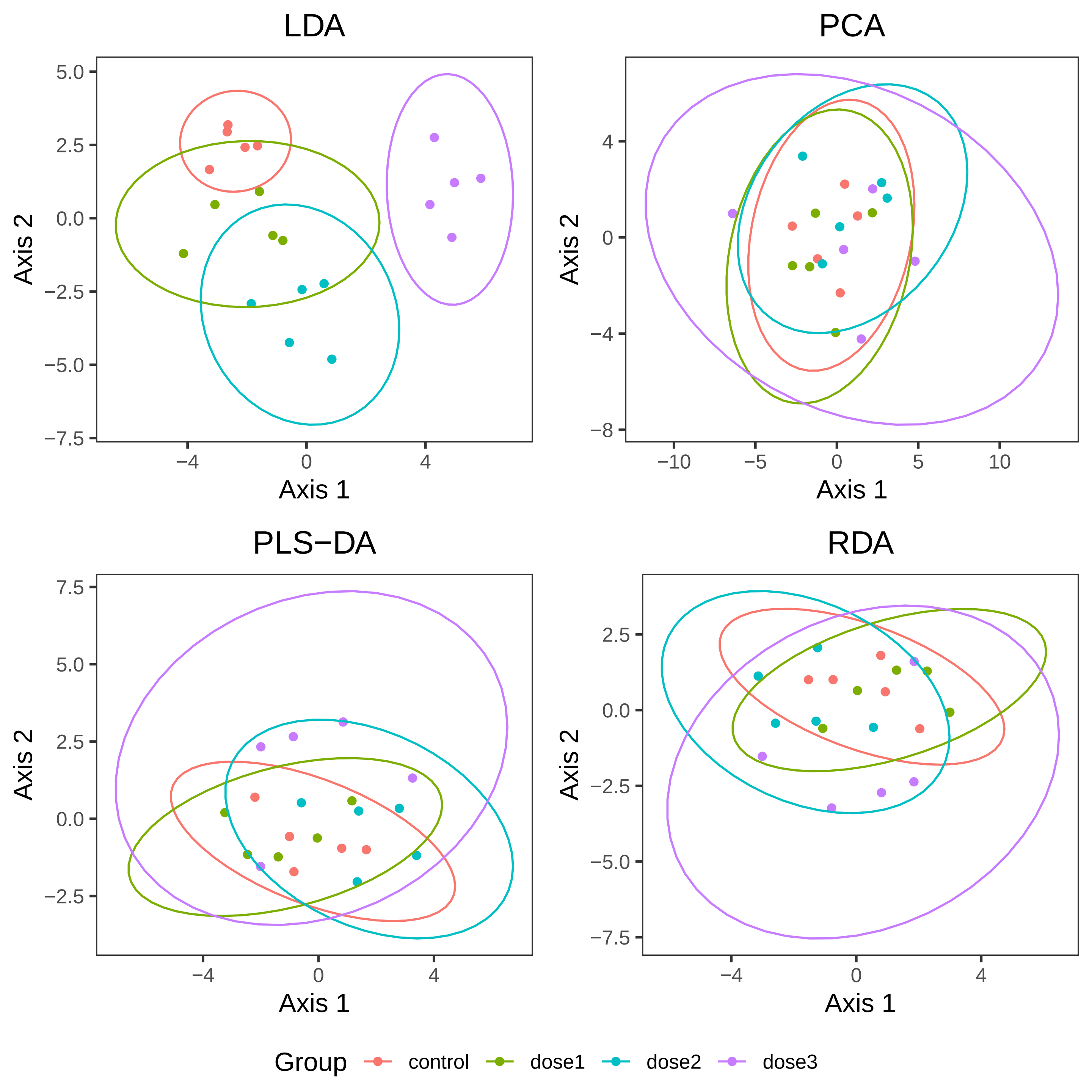

LDA
PCA
PLS−DA
RDA
Axis 1
Axis 2
5.0
2.5
0.0
−2.5
−5.0
−7.5
−4
0
4
−10
−5
5
10
−8
7.5
Group
control
dose1
dose2
dose3

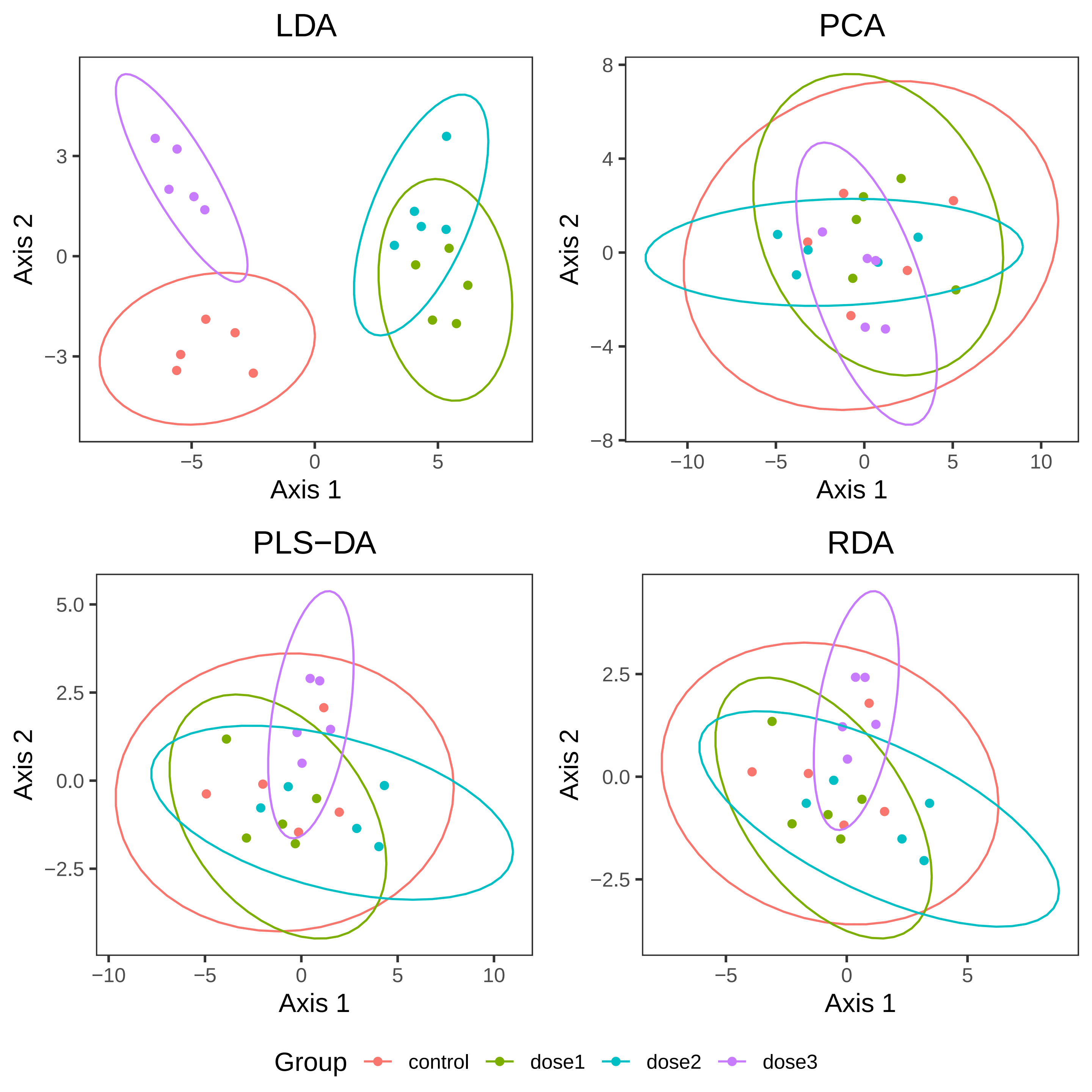
LDA
PCA
PLS−DA
RDA
Axis 1
Axis 2
Group
control
dose1
dose2
dose3

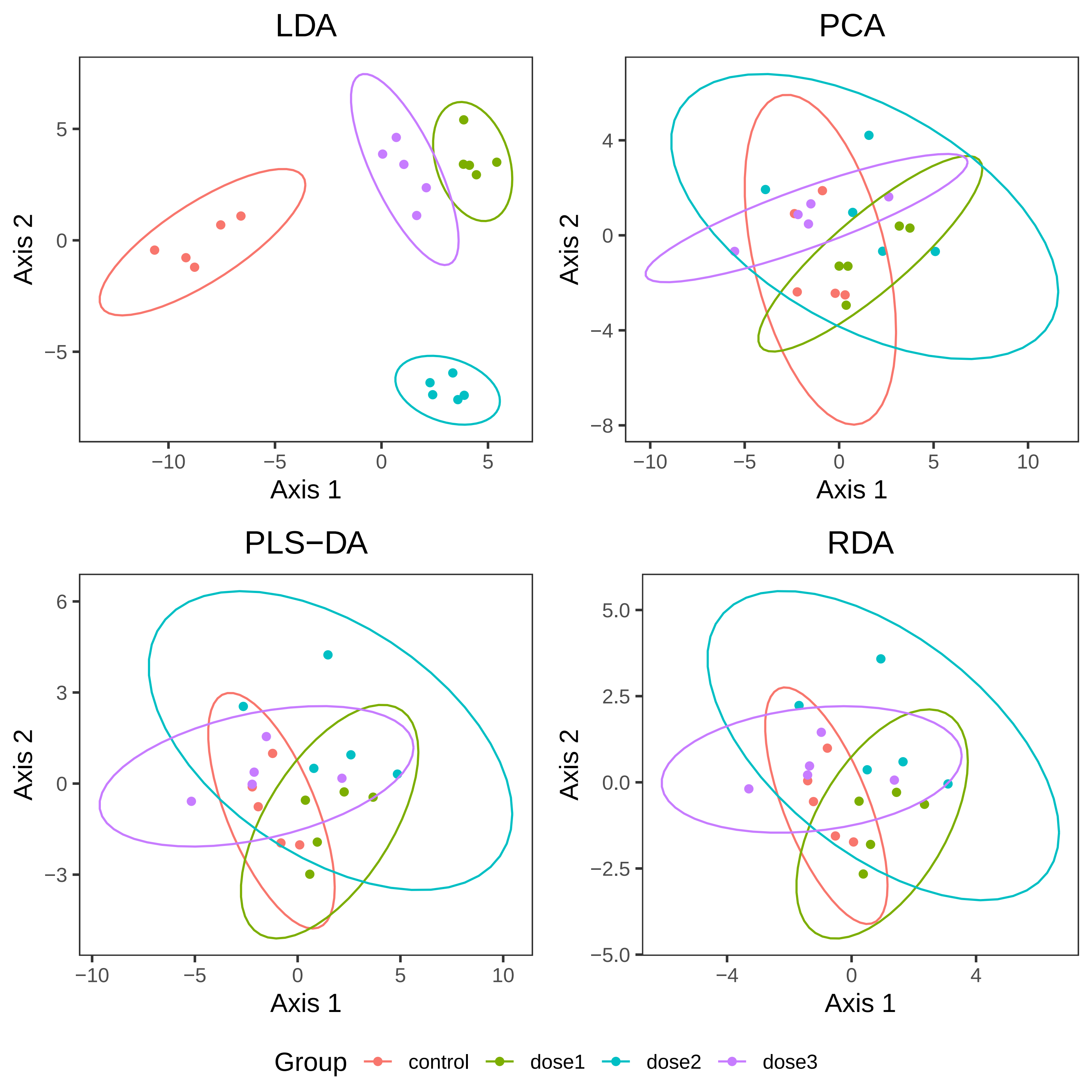
LDA
PCA
PLS−DA
RDA
Axis 1
Axis 2
Group
control
dose1
dose2
dose3

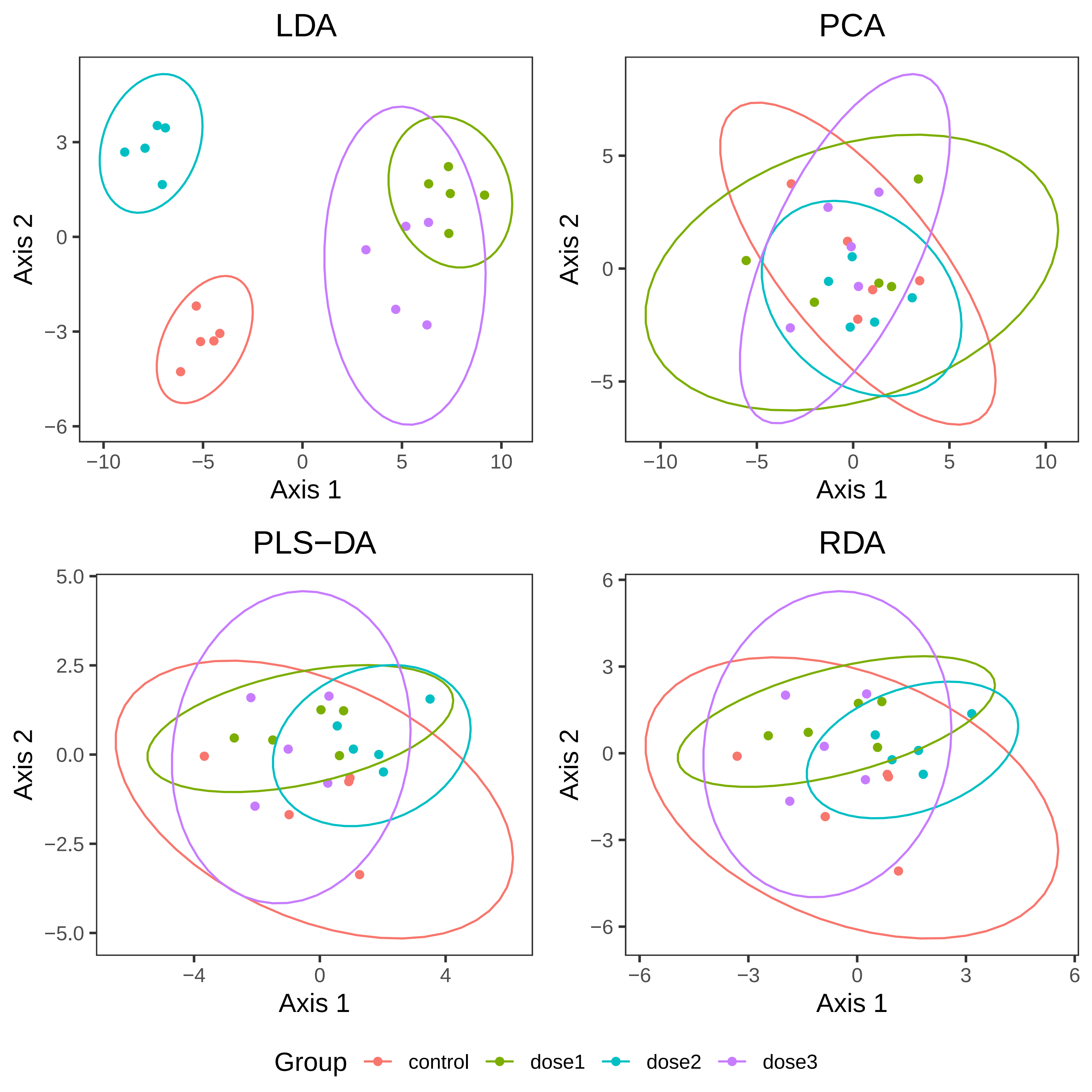

LDA
PCA
PLS−DA
RDA
Axis 1
Axis 2
−10
−5
0
5
10
3
−3
−6
−4
4
5.0
2.5
0.0
−2.5
−5.0
6
Group
control
dose1
dose2
dose3

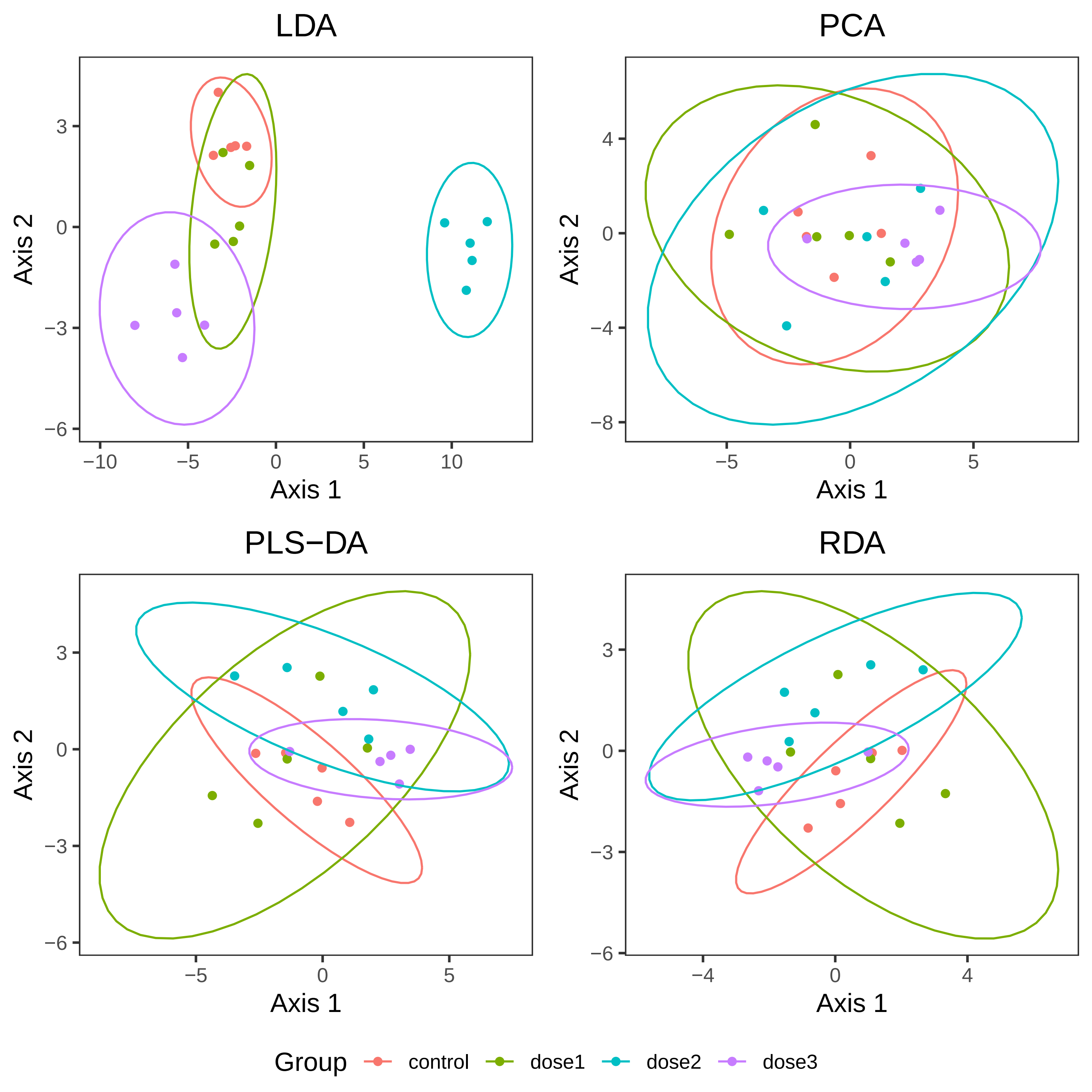
LDA
PCA
PLS−DA
RDA
Axis 1
Axis 2
Group
control
dose1
dose2
dose3

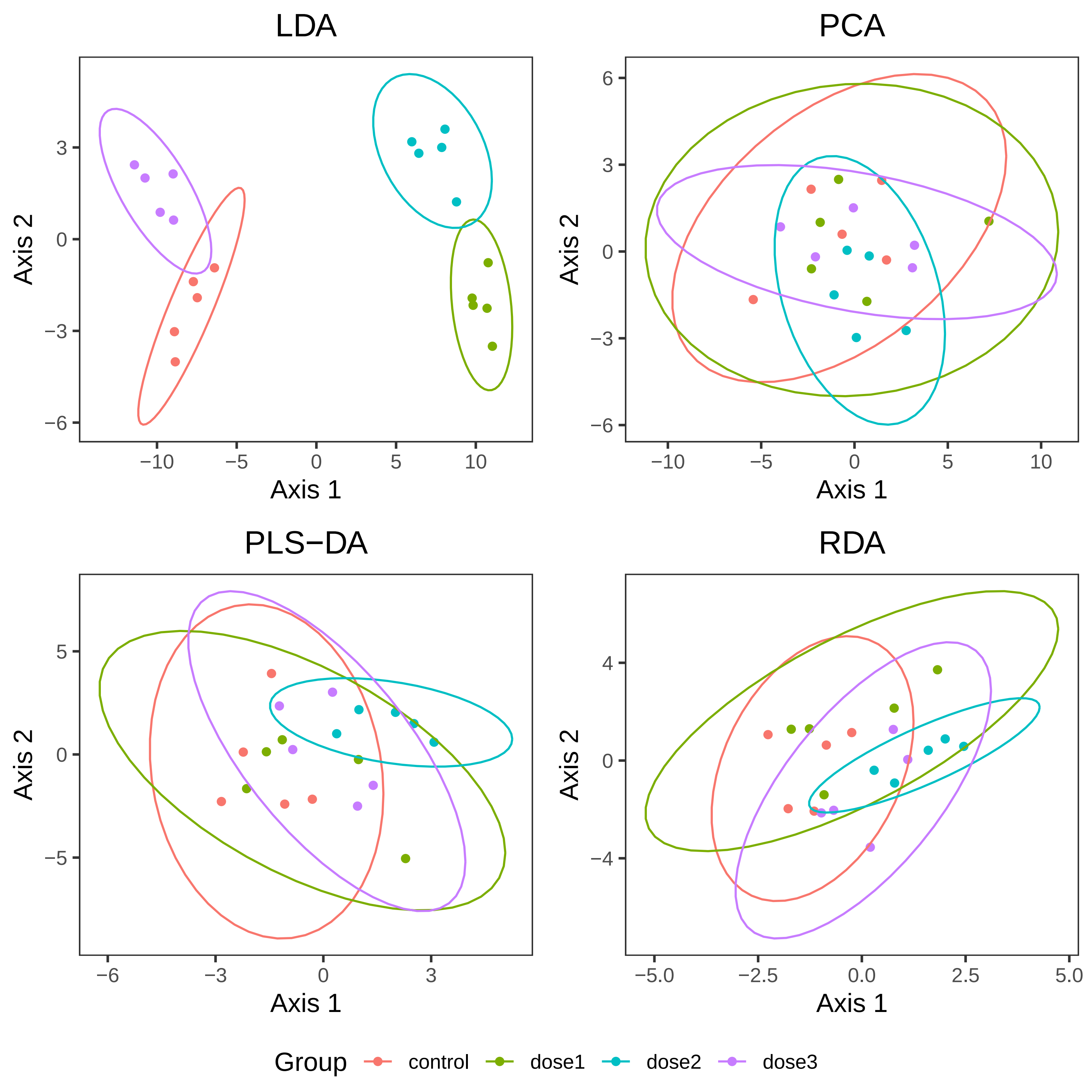

LDA
PCA
PLS−DA
RDA
Axis 1
Axis 2
−10
−5
0
5
10
3
0
−3
−6
6
−6
−3
5
−5
−5.0
−2.5
0.0
2.5
5.0
4
−4
Group
control
dose1
dose2
dose3

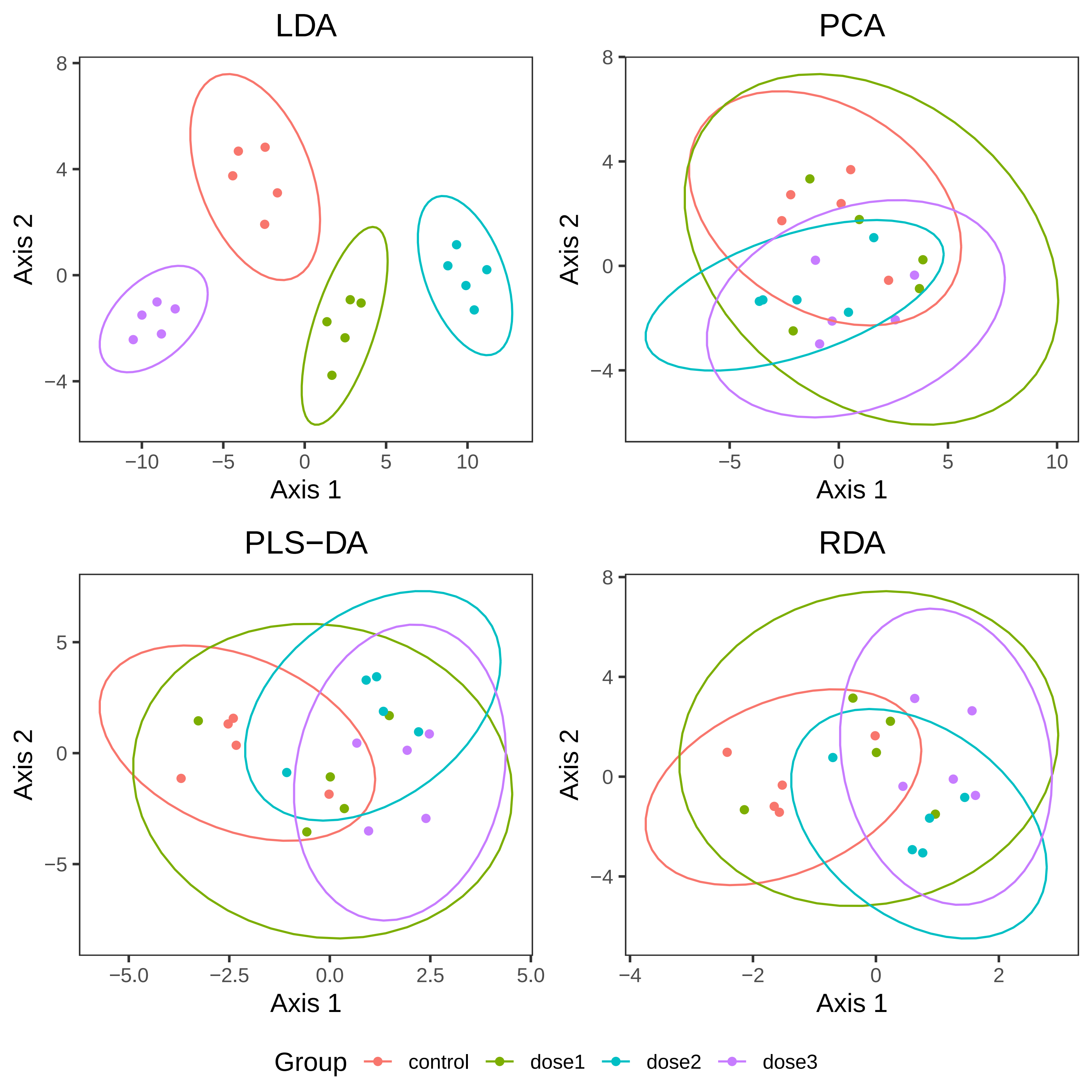

LDA
PCA
PLS−DA
RDA
Axis 1
Axis 2
Group
control
dose1
dose2
dose3

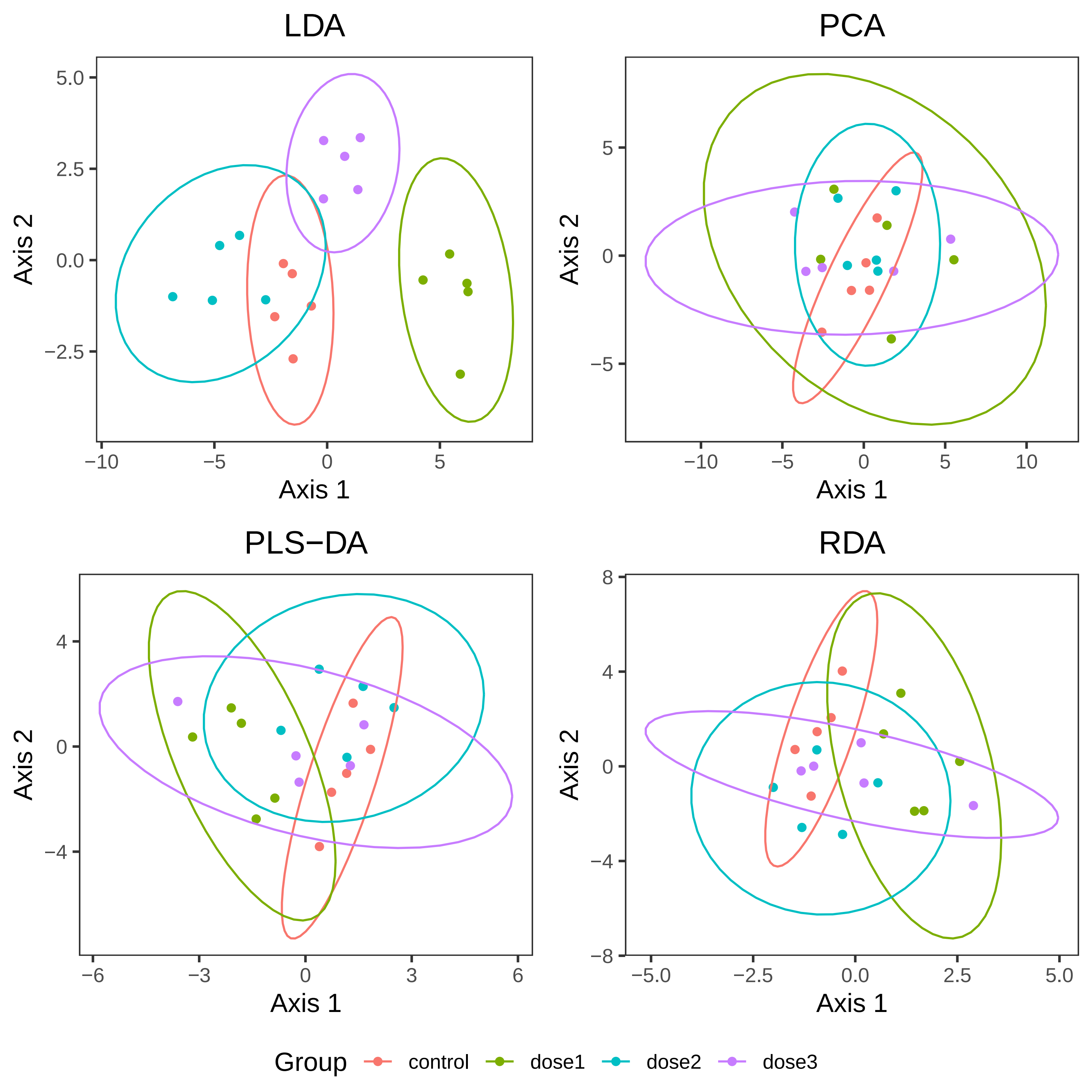

LDA
PCA
PLS−DA
RDA
Axis 1
Axis 2
5.0
2.5
0.0
−2.5
−10
−5
0
5
10
−6
−3
3
6
4
−4
8
−8
−5.0
−2.5
Group
control
dose1
dose2
dose3

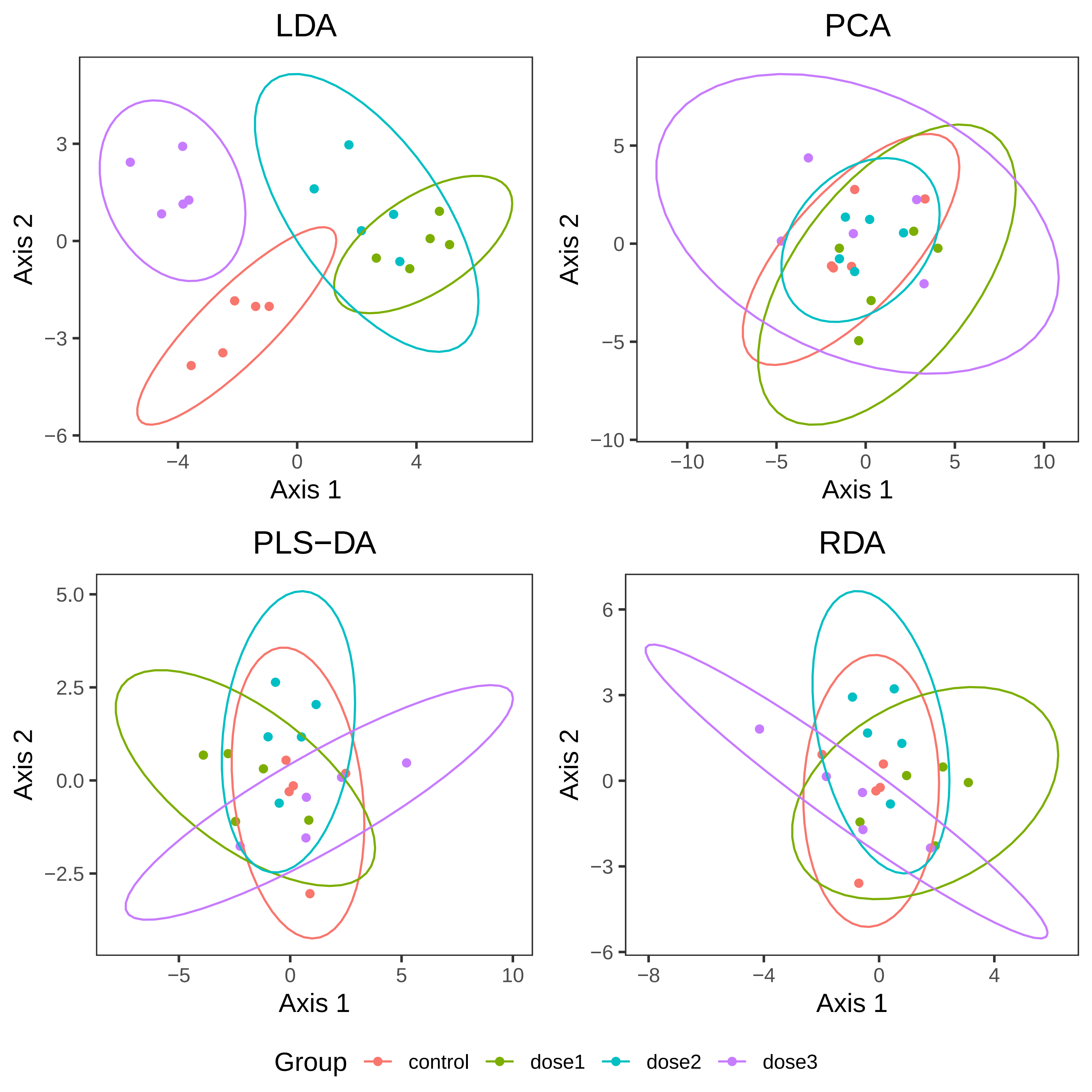
LDA
PCA
PLS−DA
RDA
Axis 1
Axis 2
Group
control
dose1
dose2
dose3

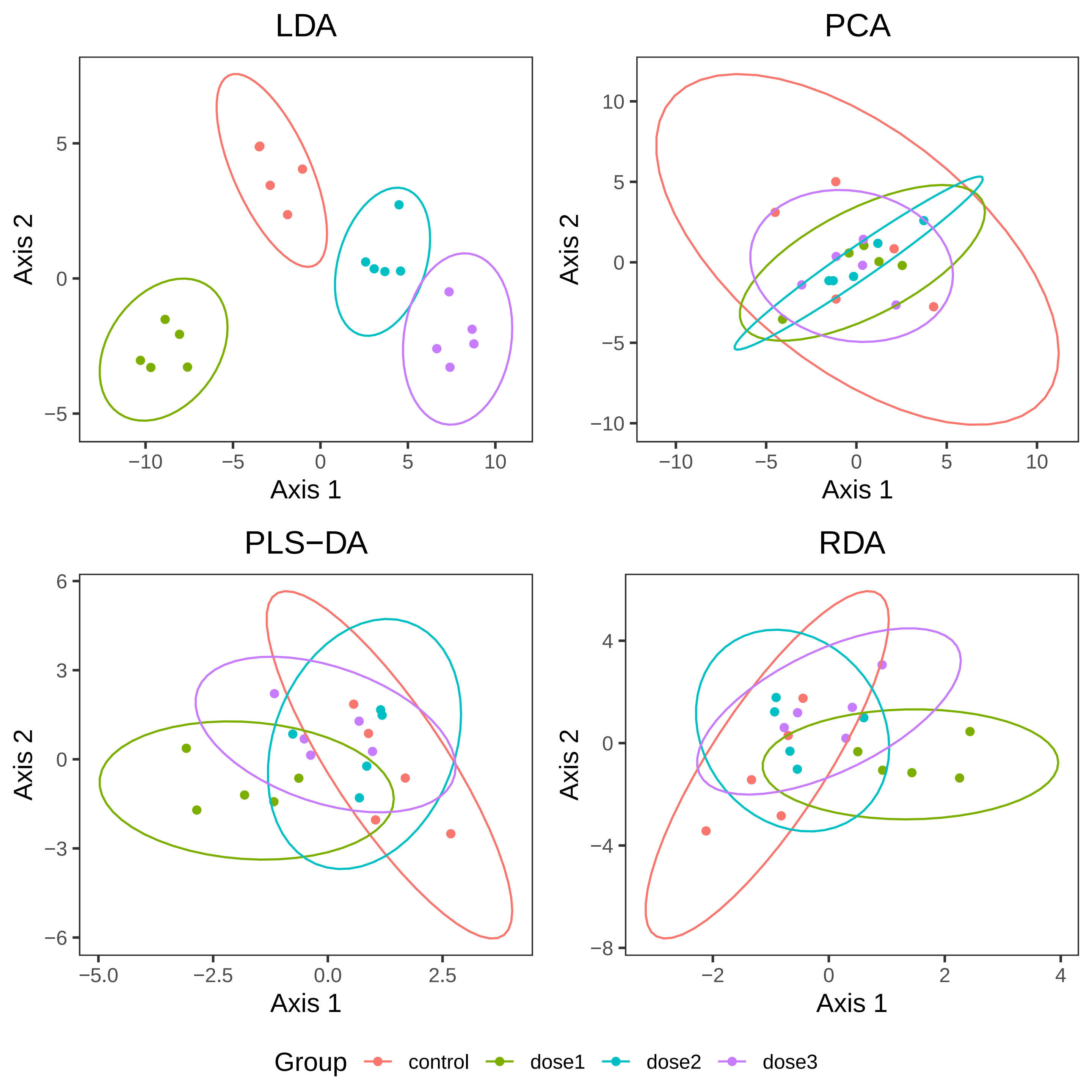

LDA
PCA
PLS−DA
RDA
Axis 1
Axis 2
Group
control
dose1
dose2
dose3

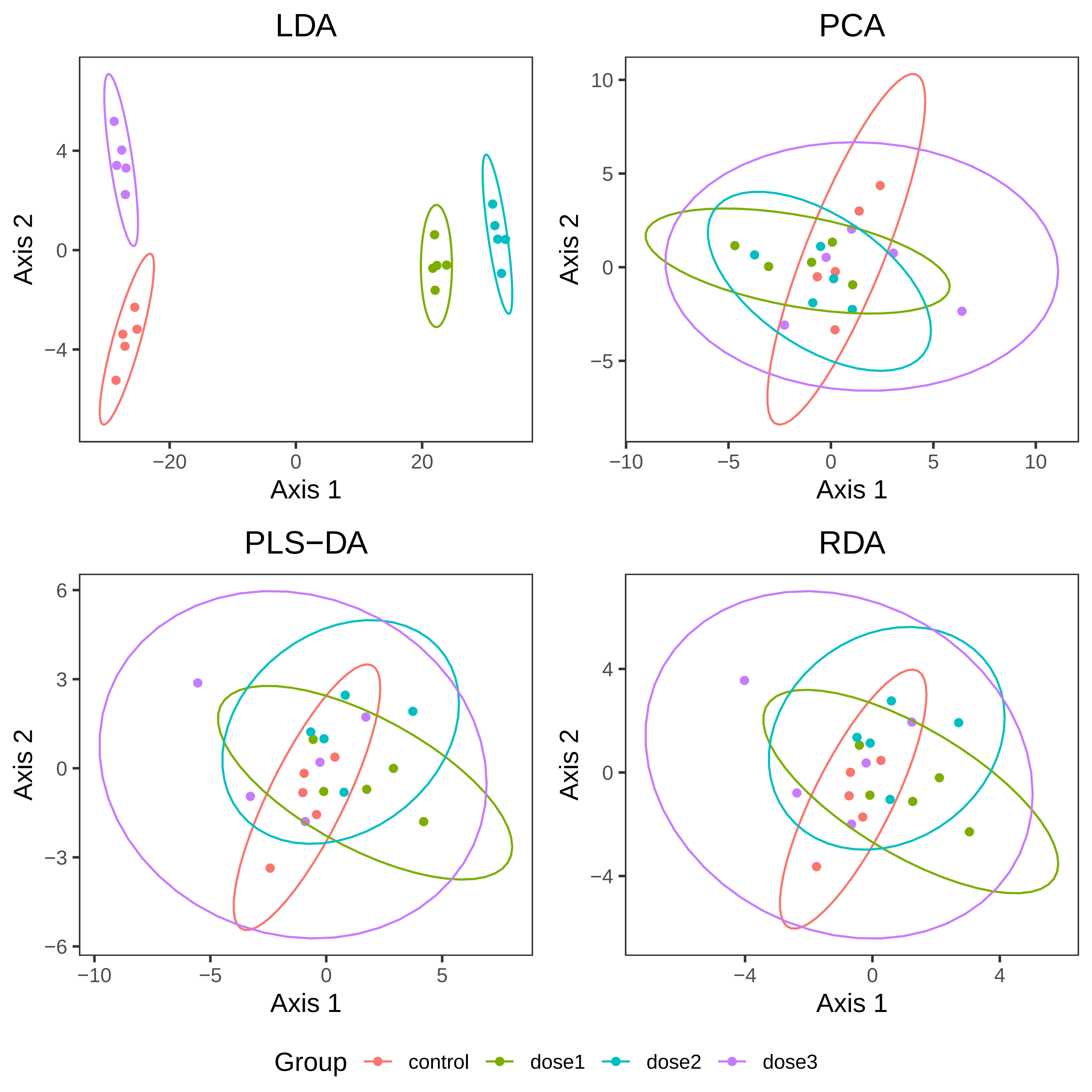
LDA
PCA
PLS−DA
RDA
Axis 1
Axis 2
Group
control
dose1
dose2
dose3

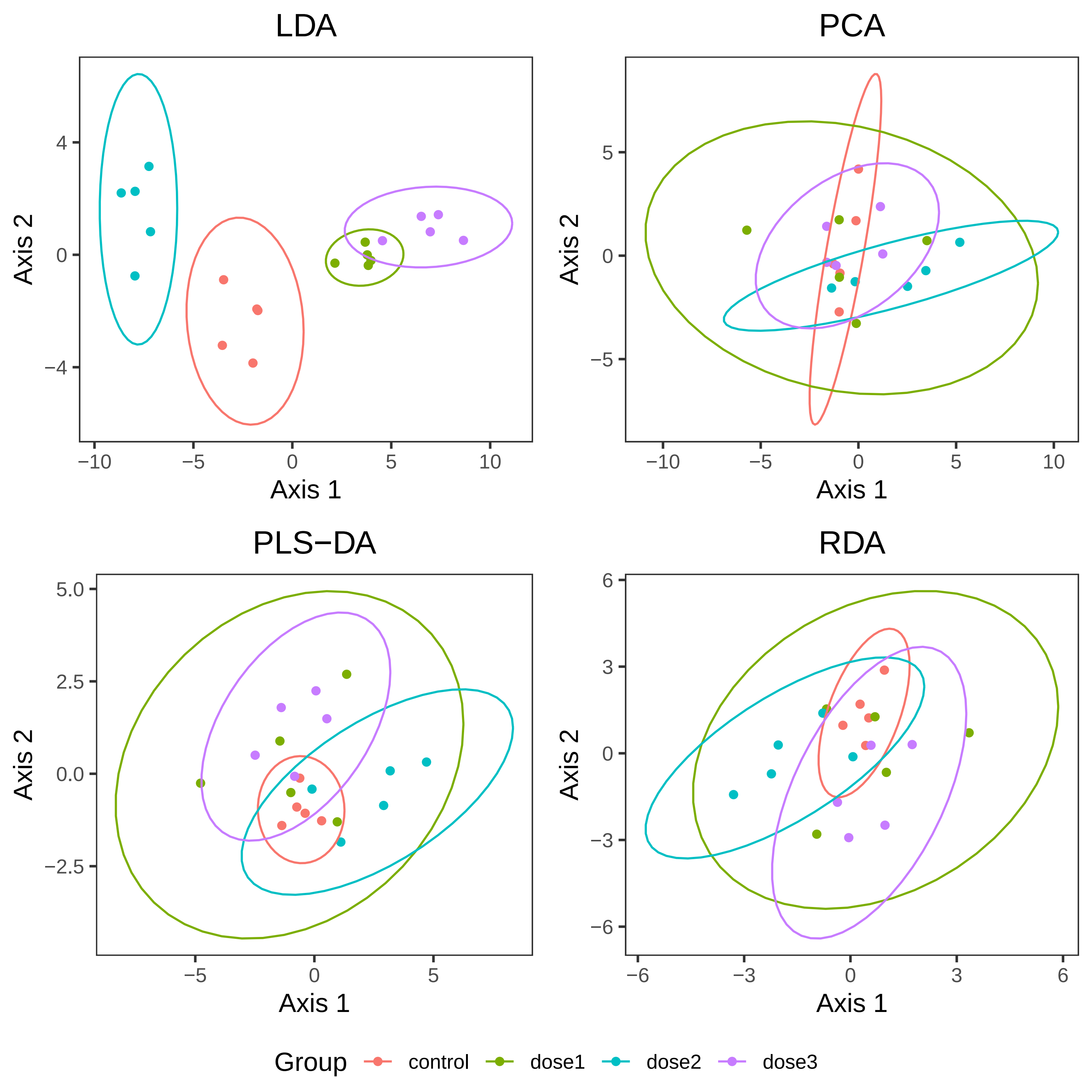

LDA
PCA
PLS−DA
RDA
Axis 1
Axis 2
4
0
−4
−10
−5
5
10
−5
2.5
5.0
0.0
−2.5
6
3
−3
−6
Group
control
dose1
dose2
dose3

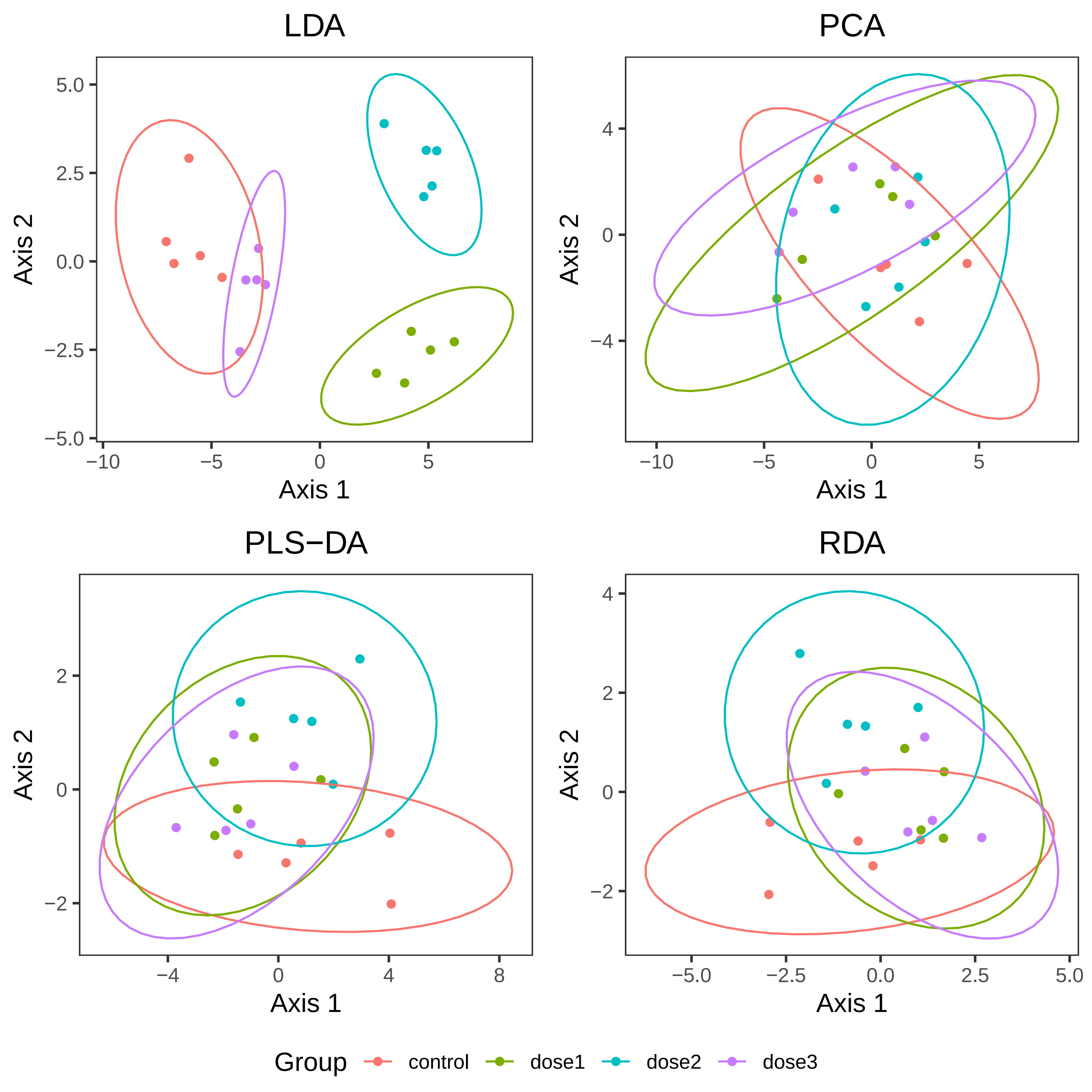
LDA
PCA
PLS−DA
RDA
Axis 1
Axis 2
5.0
2.5
0.0
−2.5
−5.0
−10
−5
0
5
4
−4
2
−2
8
−5.0
−2.5
0.0
2.5
5.0
Group
control
dose1
dose2
dose3

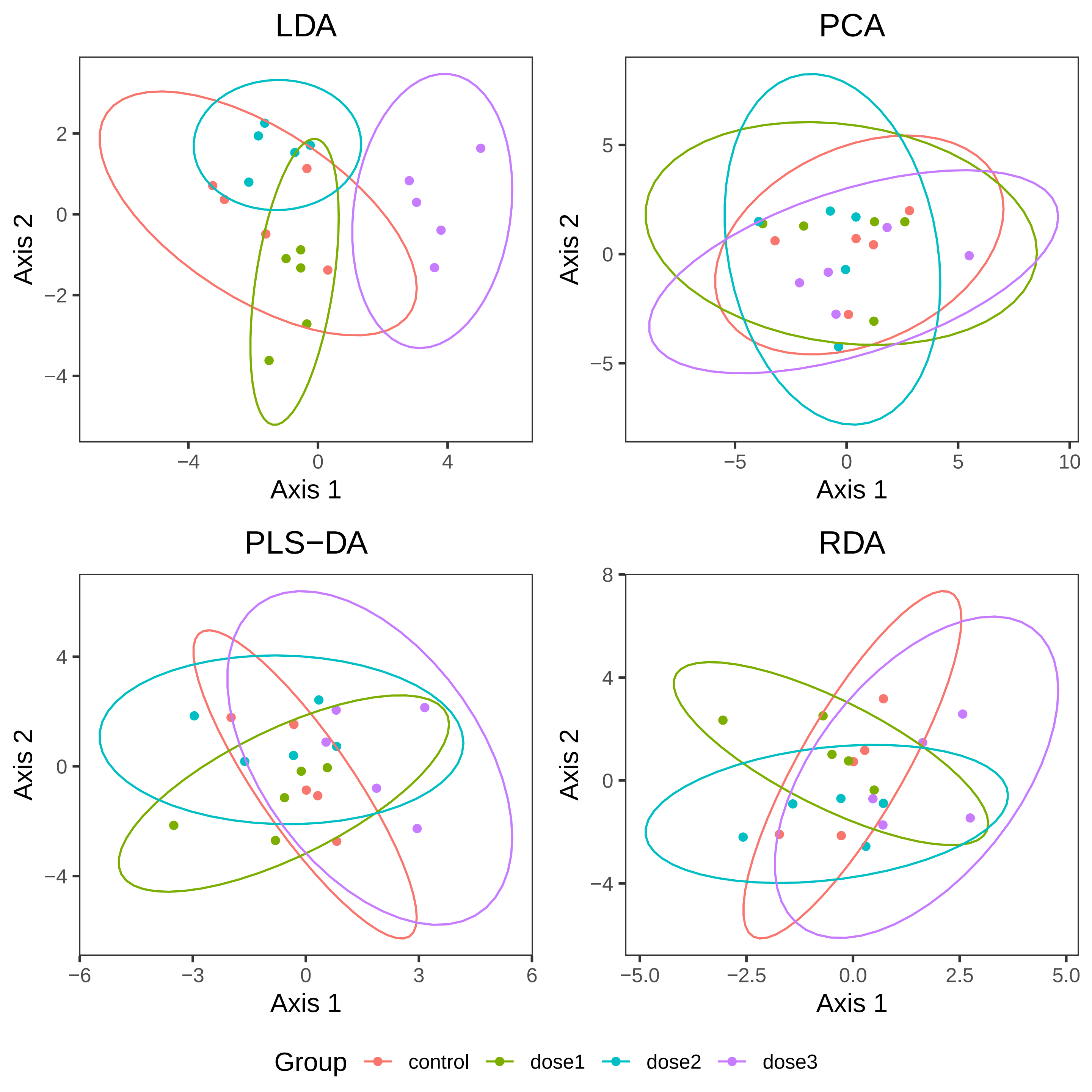

LDA
PCA
PLS−DA
RDA
Axis 1
Axis 2
Group
control
dose1
dose2
dose3

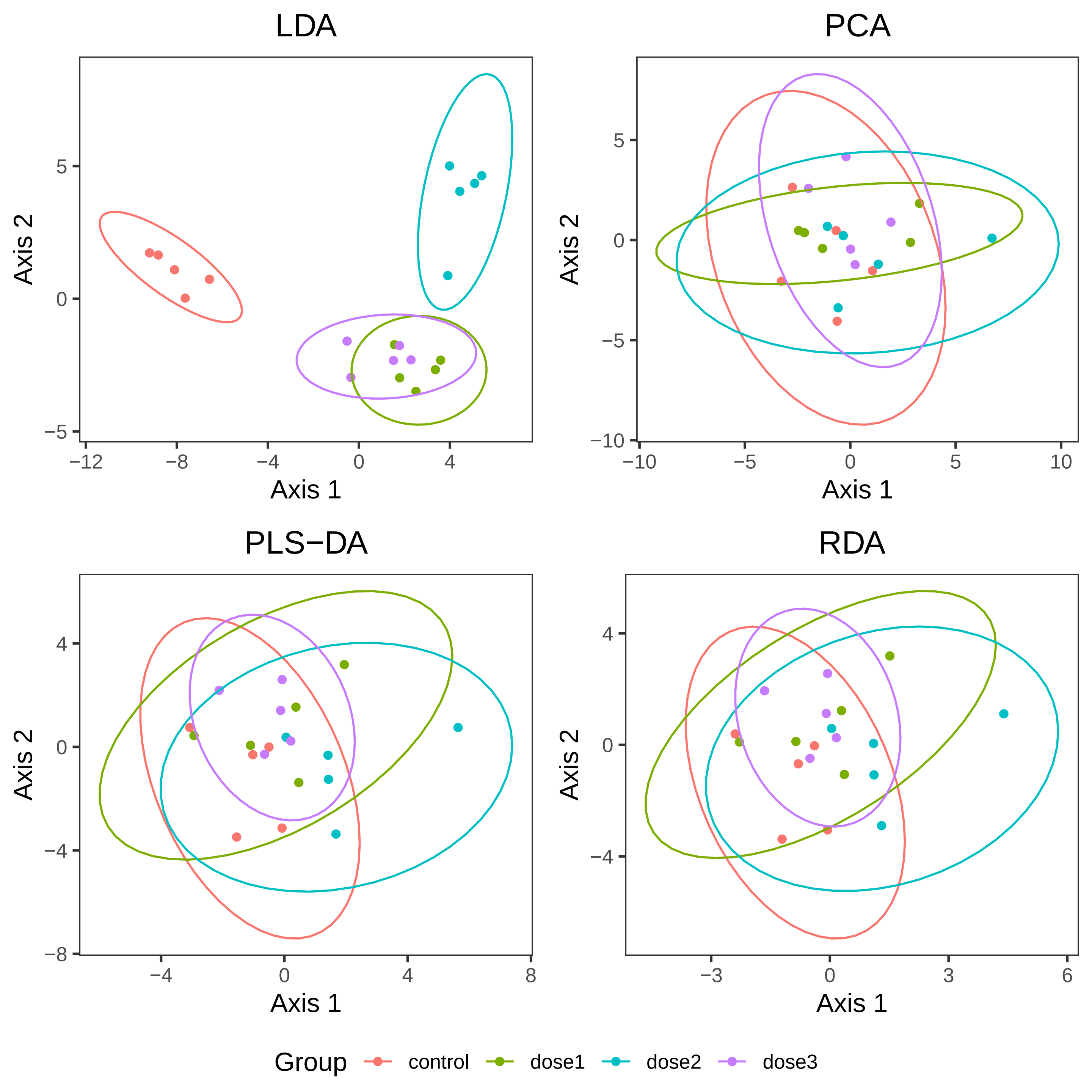

LDA
PCA
PLS−DA
RDA
Axis 1
Axis 2
Group
control
dose1
dose2
dose3

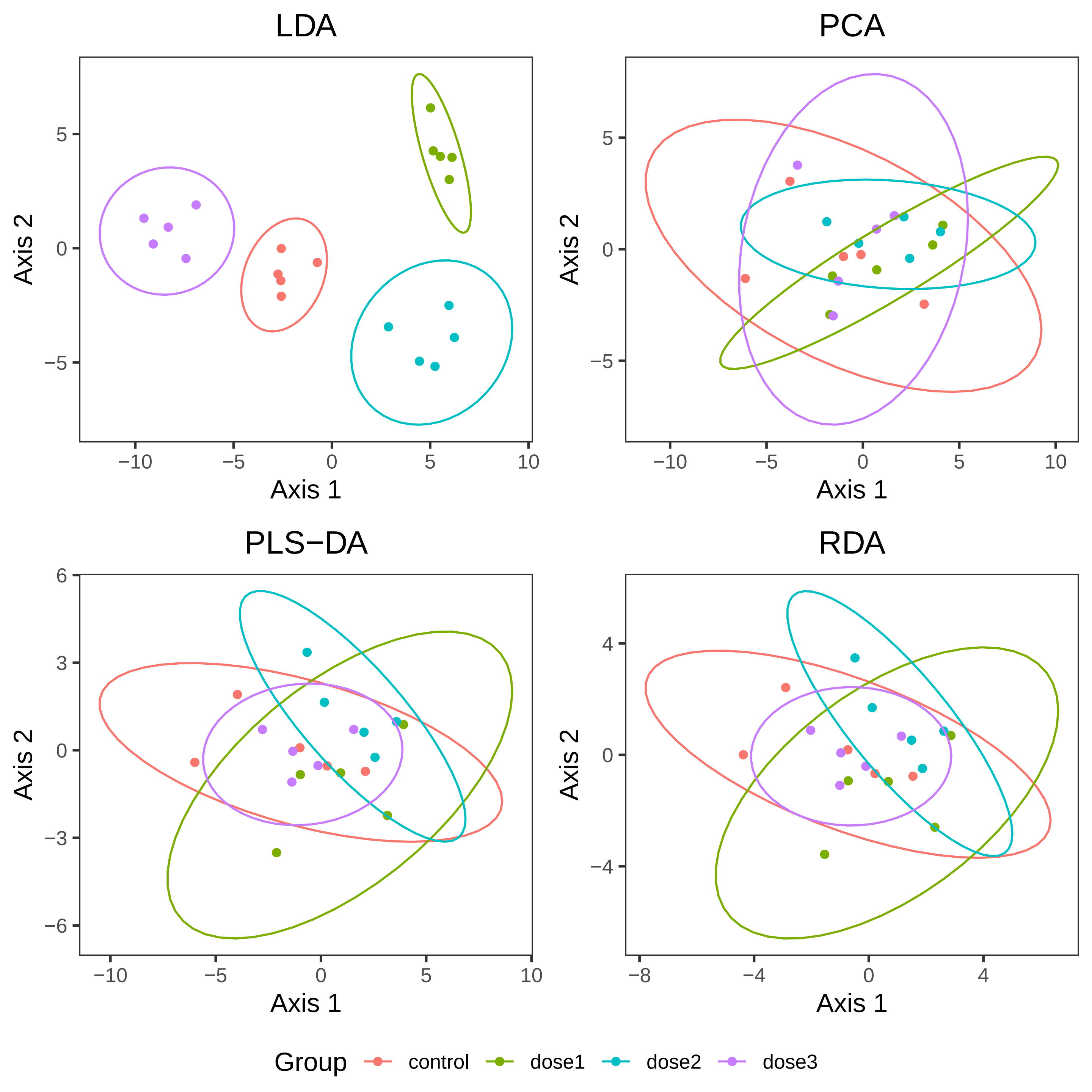

LDA
PCA
PLS−DA
RDA
Axis 1
Axis 2
Group
control
dose1
dose2
dose3

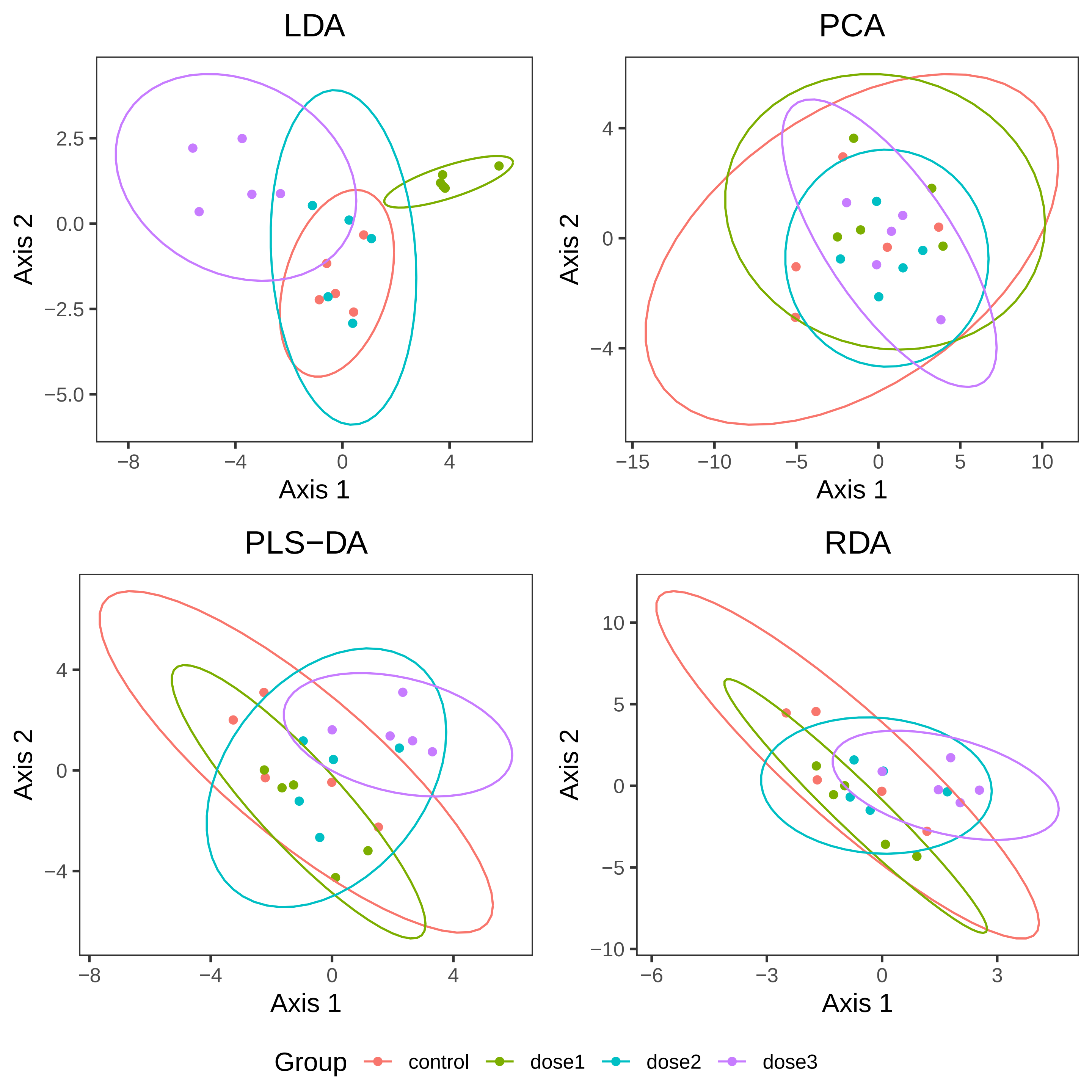
LDA
PCA
PLS−DA
RDA
Axis 1
Axis 2
2.5
0.0
−2.5
−5.0
−8
−4
0
4
−15
−10
−5
5
10
−10
−6
−3
3
Group
control
dose1
dose2
dose3

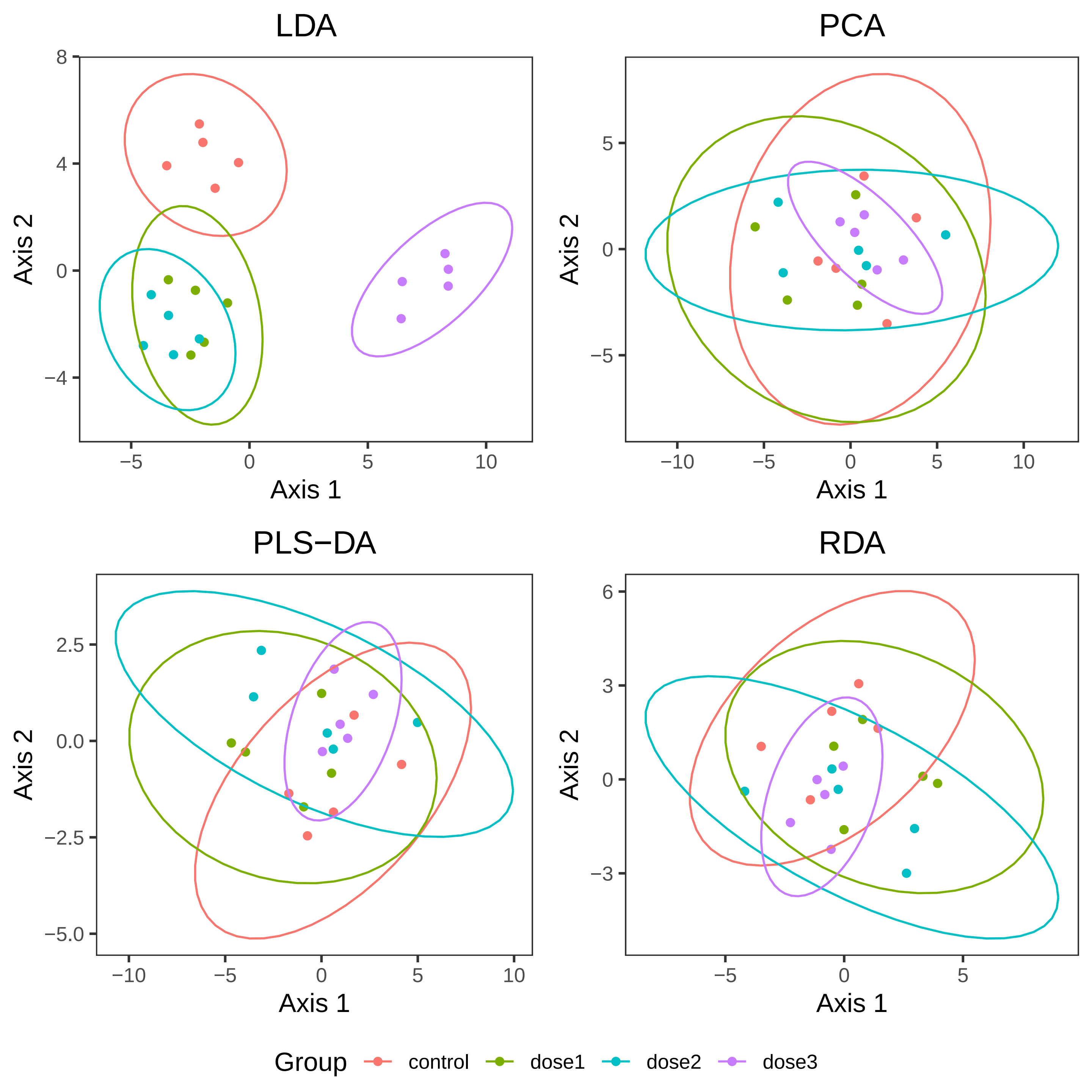

LDA
PCA
PLS−DA
RDA
Axis 1
Axis 2
Group
control
dose1
dose2
dose3

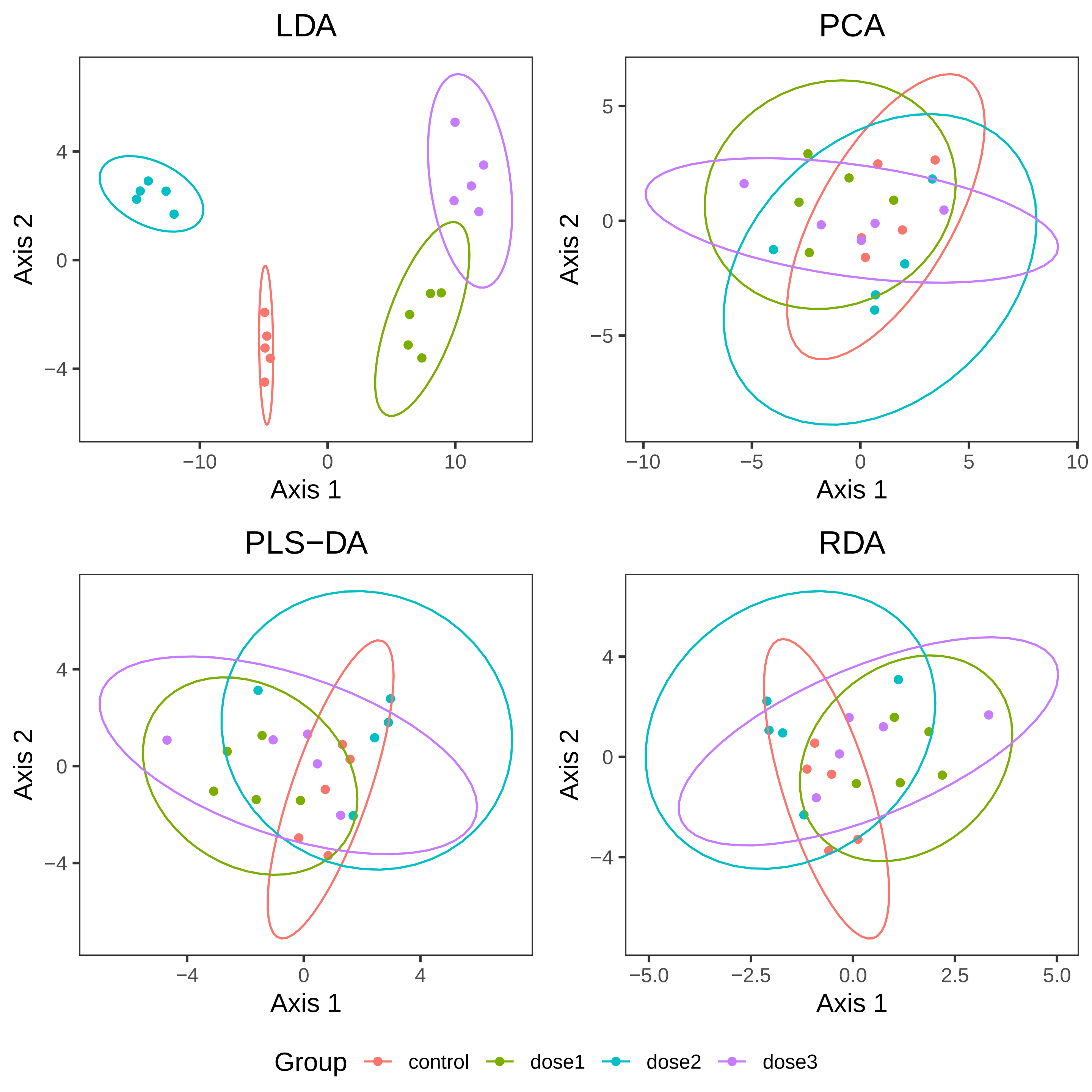

LDA
PCA
PLS−DA
RDA
Axis 1
Axis 2
−10
0
10
4
−4
−5
5
−5.0
−2.5
0.0
2.5
5.0
Group
control
dose1
dose2
dose3

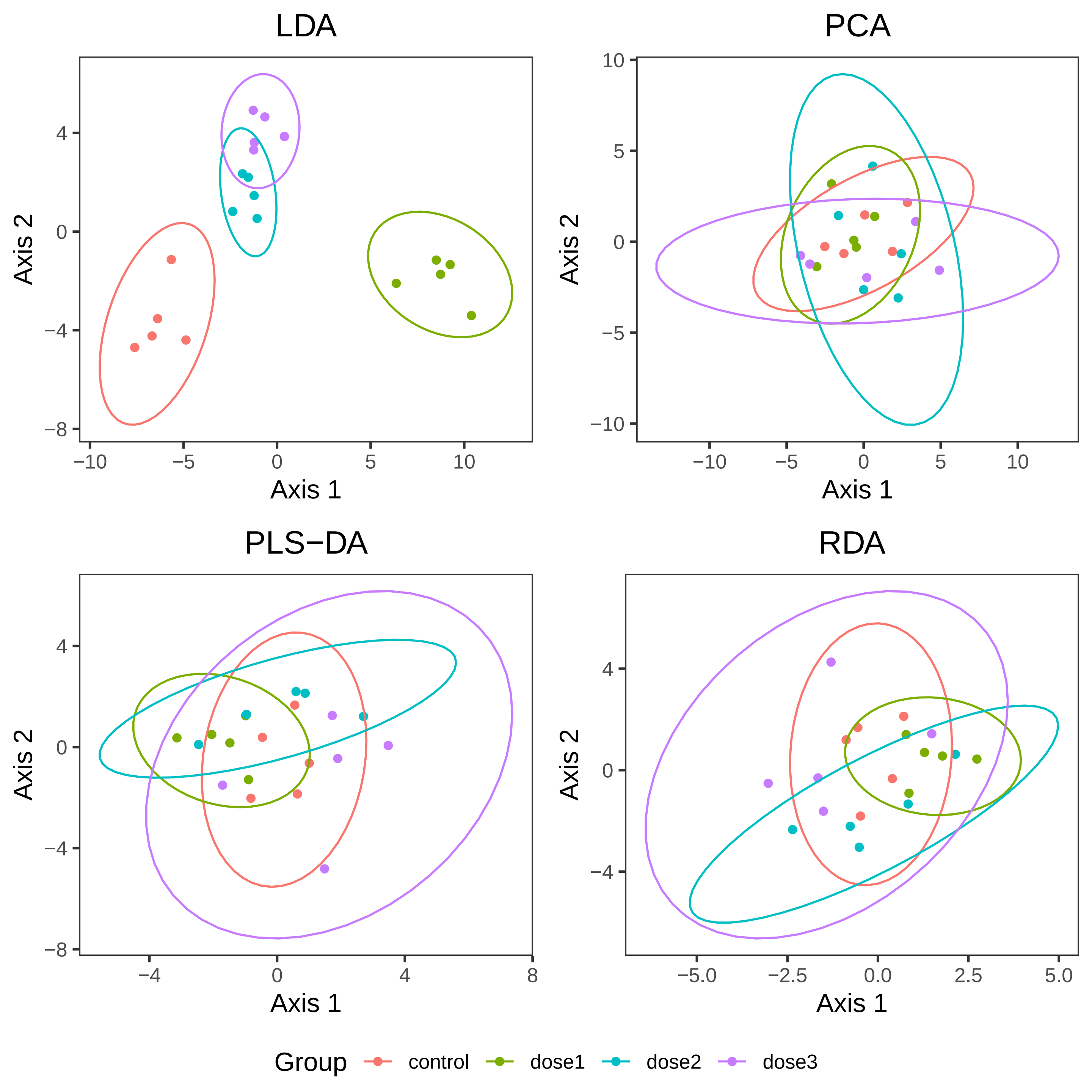
LDA
PCA
PLS−DA
RDA
Axis 1
Axis 2
Group
control
dose1
dose2
dose3

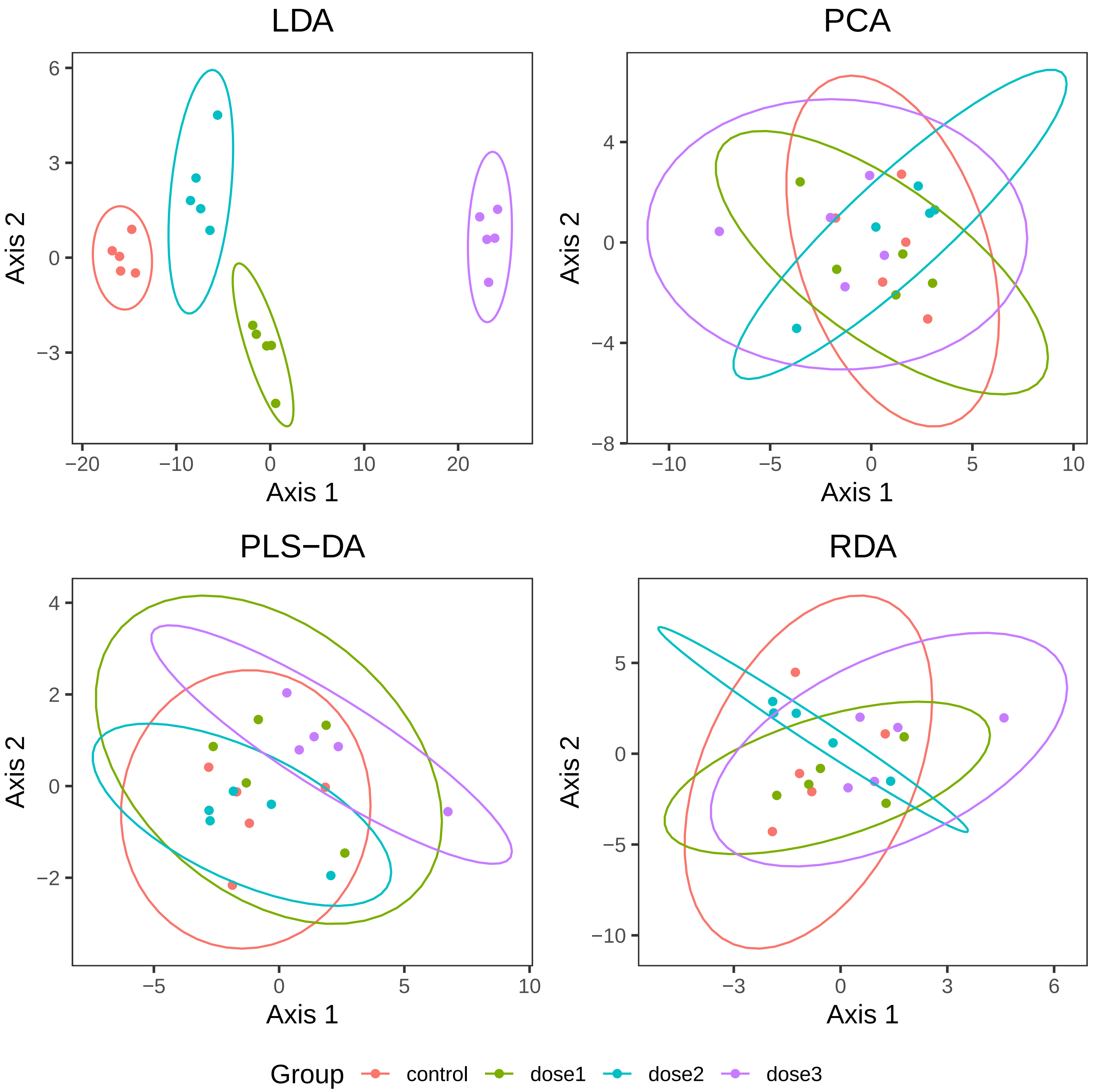
LDA
PCA
PLS−DA
RDA
Axis 1
Axis 2
Group
control
dose1
dose2
dose3

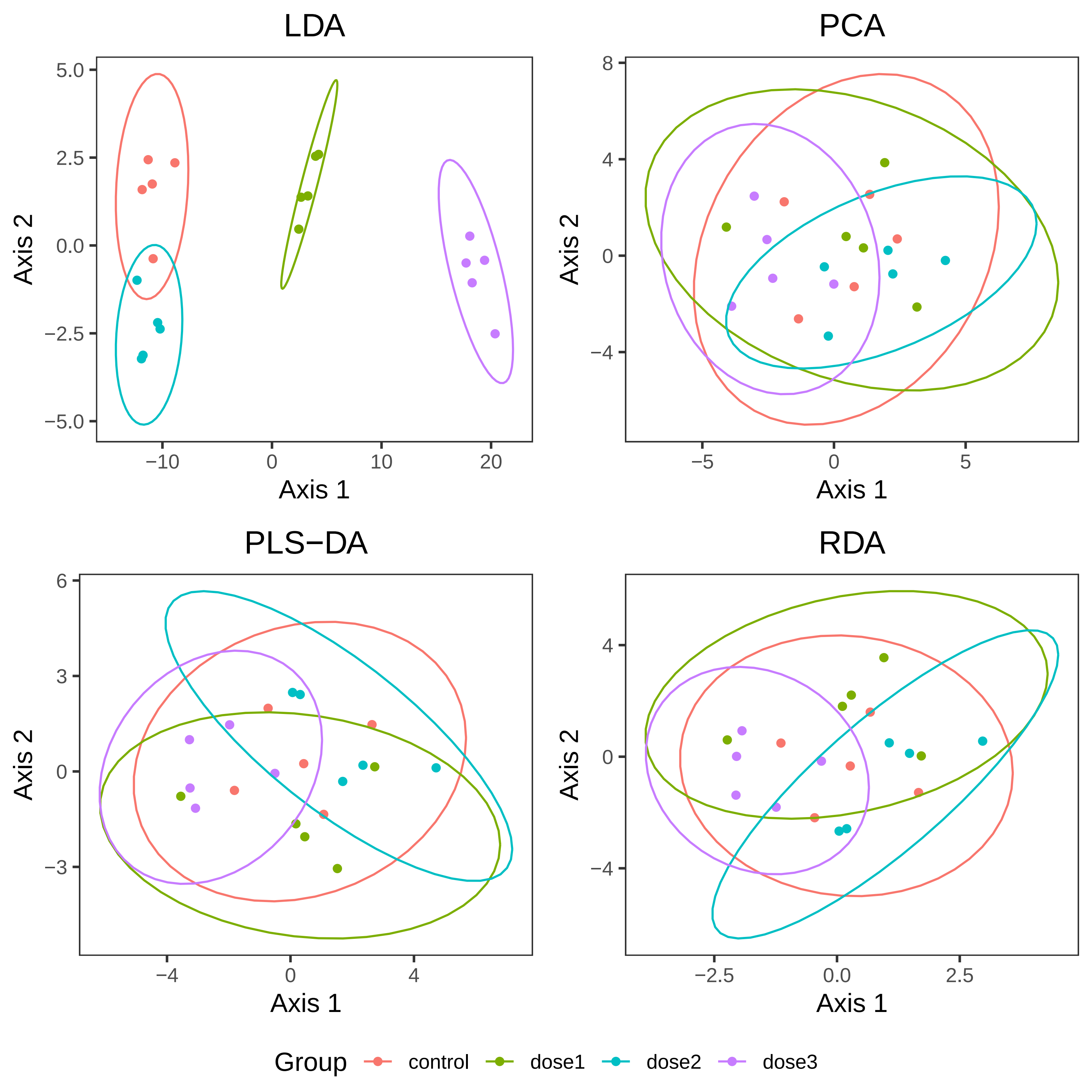

LDA
PCA
PLS−DA
RDA
Axis 1
Axis 2
5.0
2.5
0.0
−2.5
−5.0
−10
0
10
20
8
4
0
−4
−5
5
6
3
−3
−4
4
−2.5
2.5
Group
control
dose1
dose2
dose3

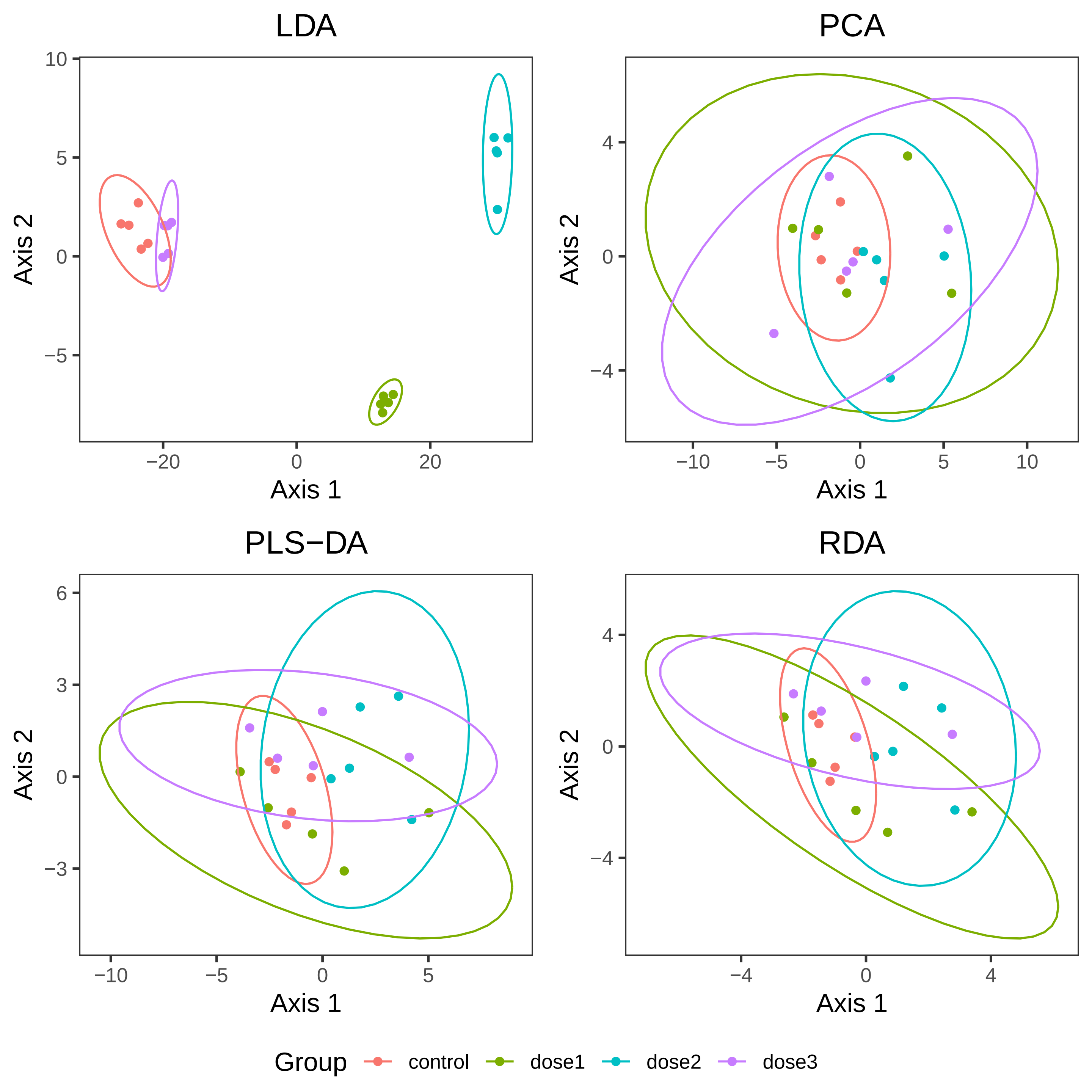

LDA
PCA
PLS−DA
RDA
Axis 1
Axis 2
Group
control
dose1
dose2
dose3

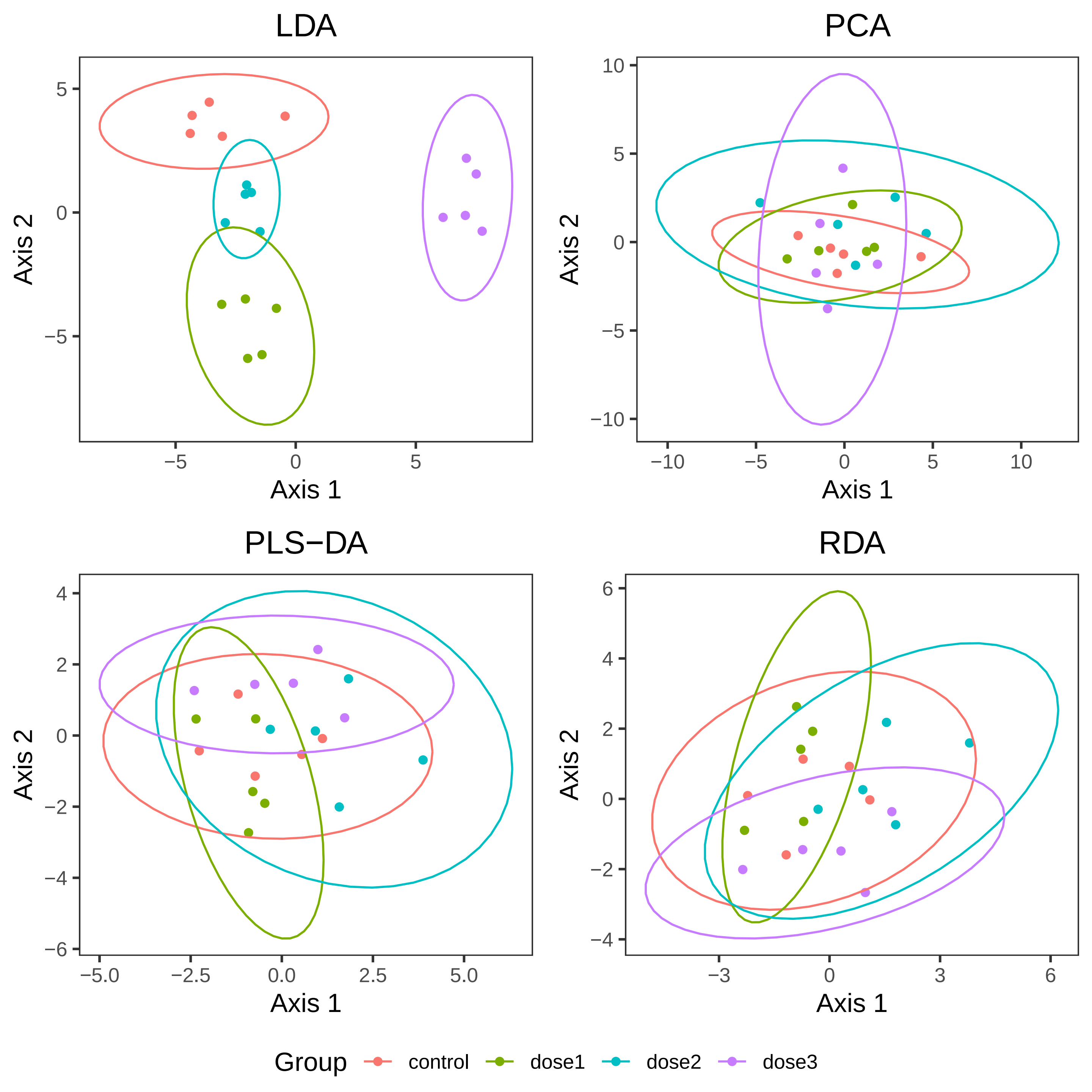

LDA
PCA
PLS−DA
RDA
Axis 1
Axis 2
5
0
−5
10
−10
2.5
−2.5
5.0
−5.0
0.0
4
2
−2
−4
−6
6
3
−3
Group
control
dose1
dose2
dose3

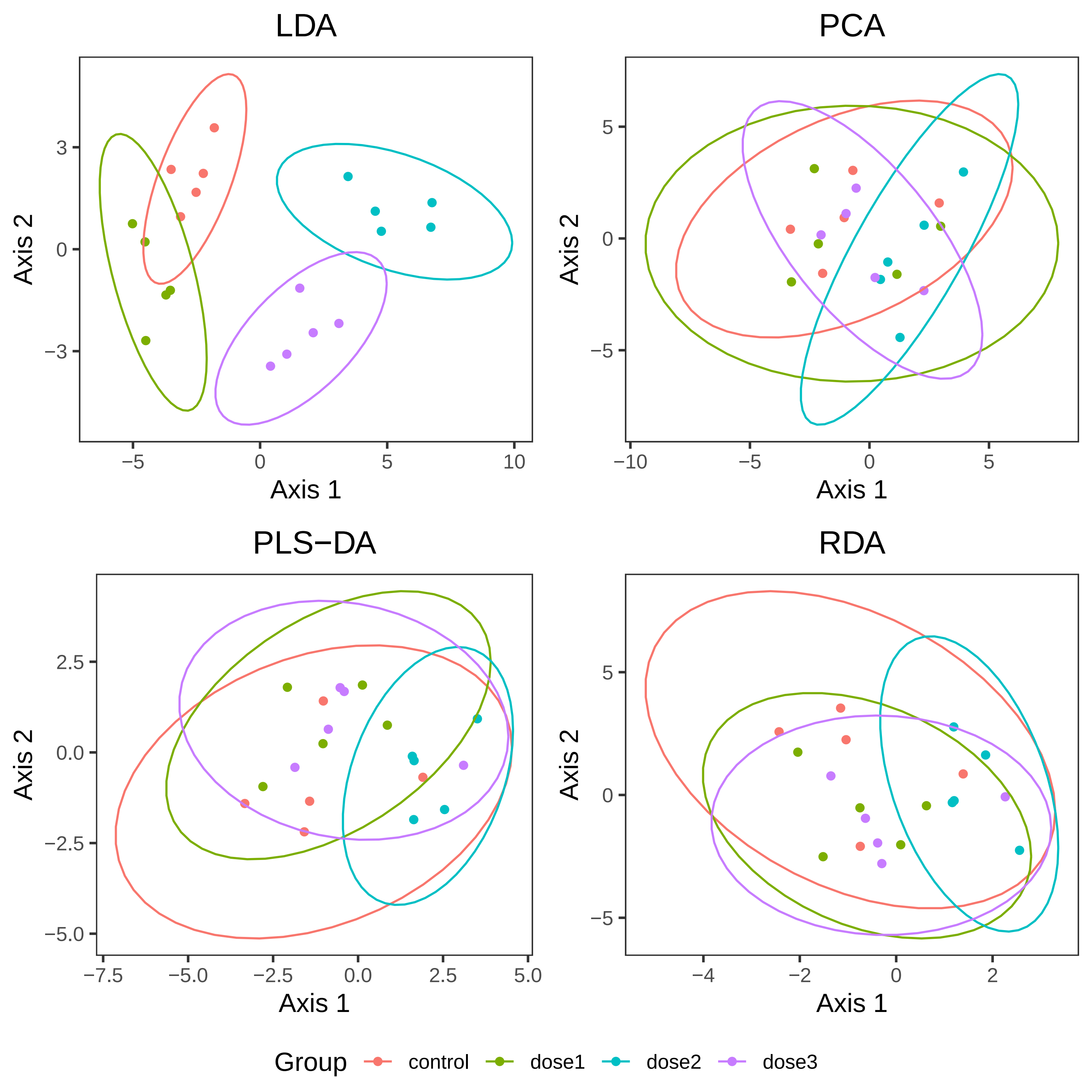

LDA
PCA
PLS−DA
RDA
Axis 1
Axis 2
3
0
−3
−5
0
5
10
5
0
−5
−10
−5
0
5
2.5
0.0
−2.5
−5.0
−7.5
−5.0
−2.5
0.0
2.5
5.0
5
0
−5
−4
−2
0
2
Group
control
dose1
dose2
dose3

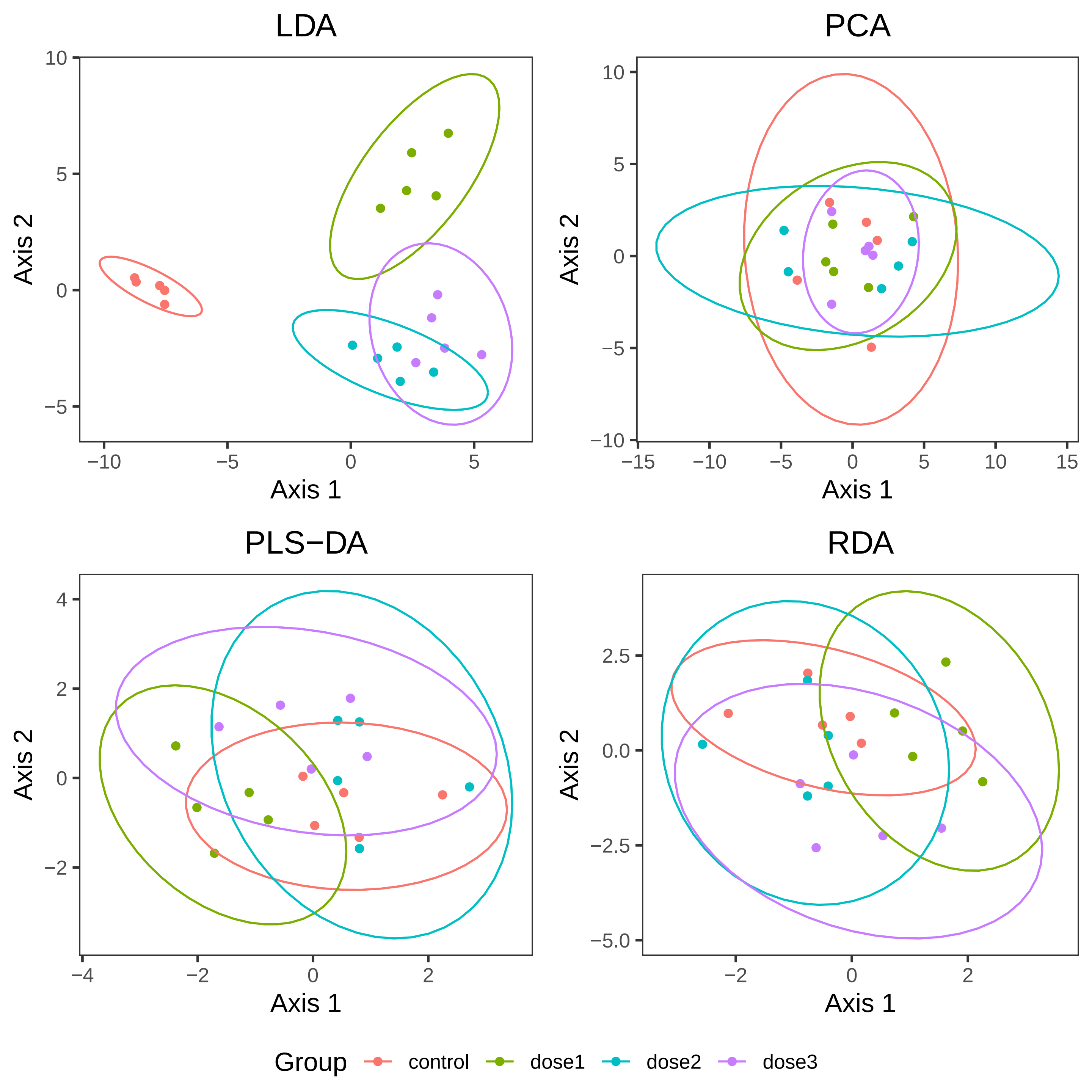

LDA
PCA
PLS−DA
RDA
Axis 1
Axis 2
Group
control
dose1
dose2
dose3

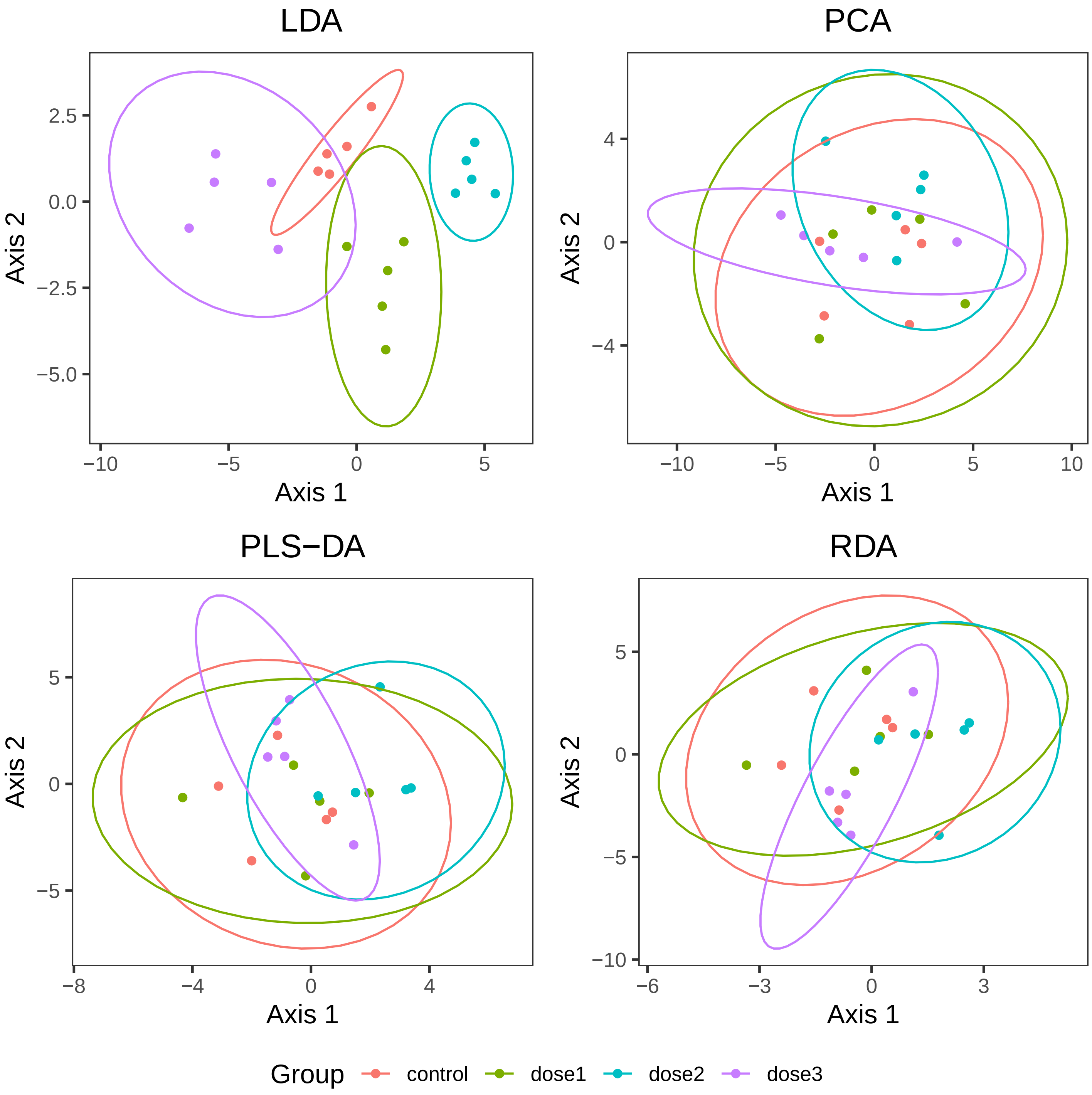

LDA
PCA
PLS−DA
RDA
Axis 1
Axis 2
Group
control
dose1
dose2
dose3

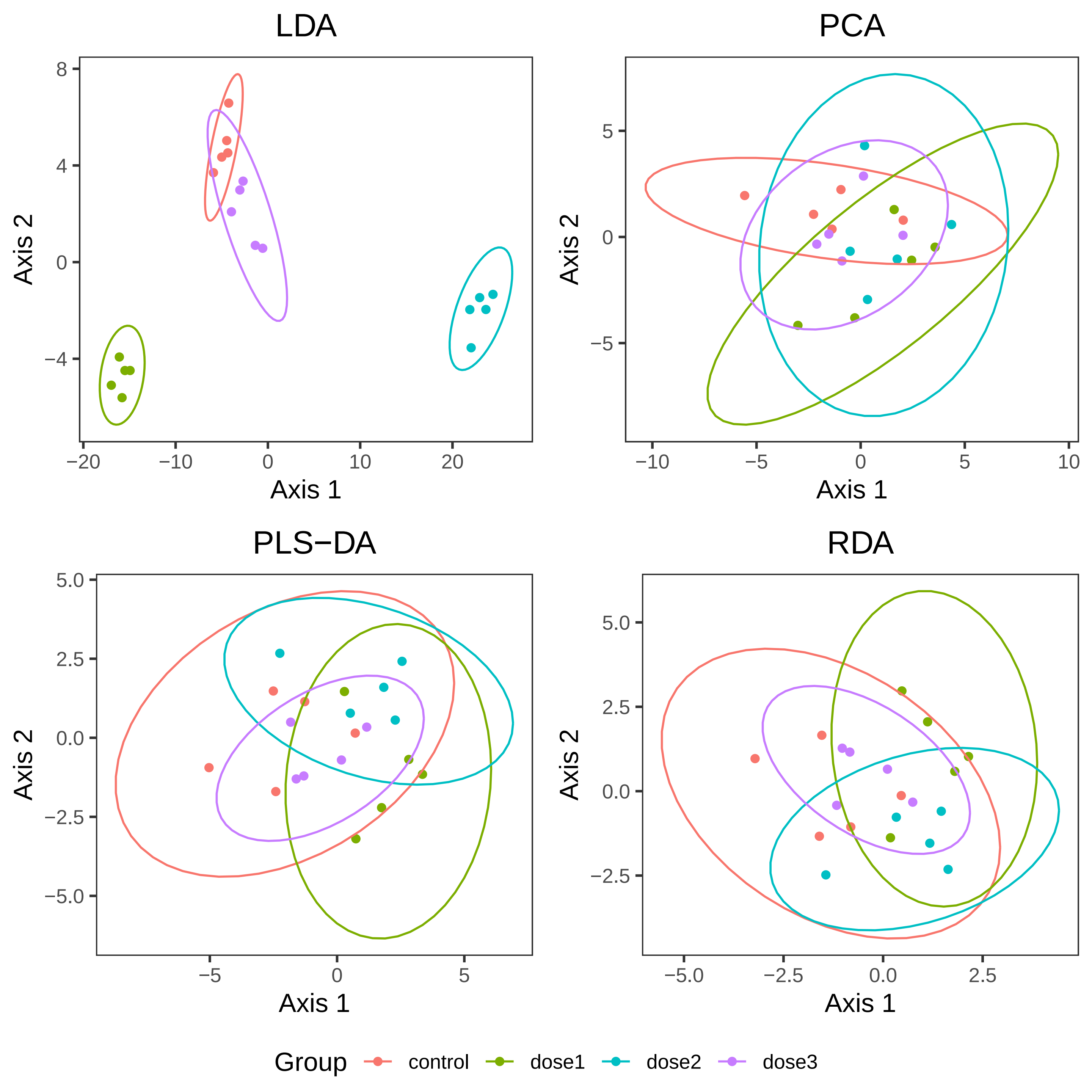

LDA
PCA
PLS−DA
RDA
Axis 1
Axis 2
Group
control
dose1
dose2
dose3

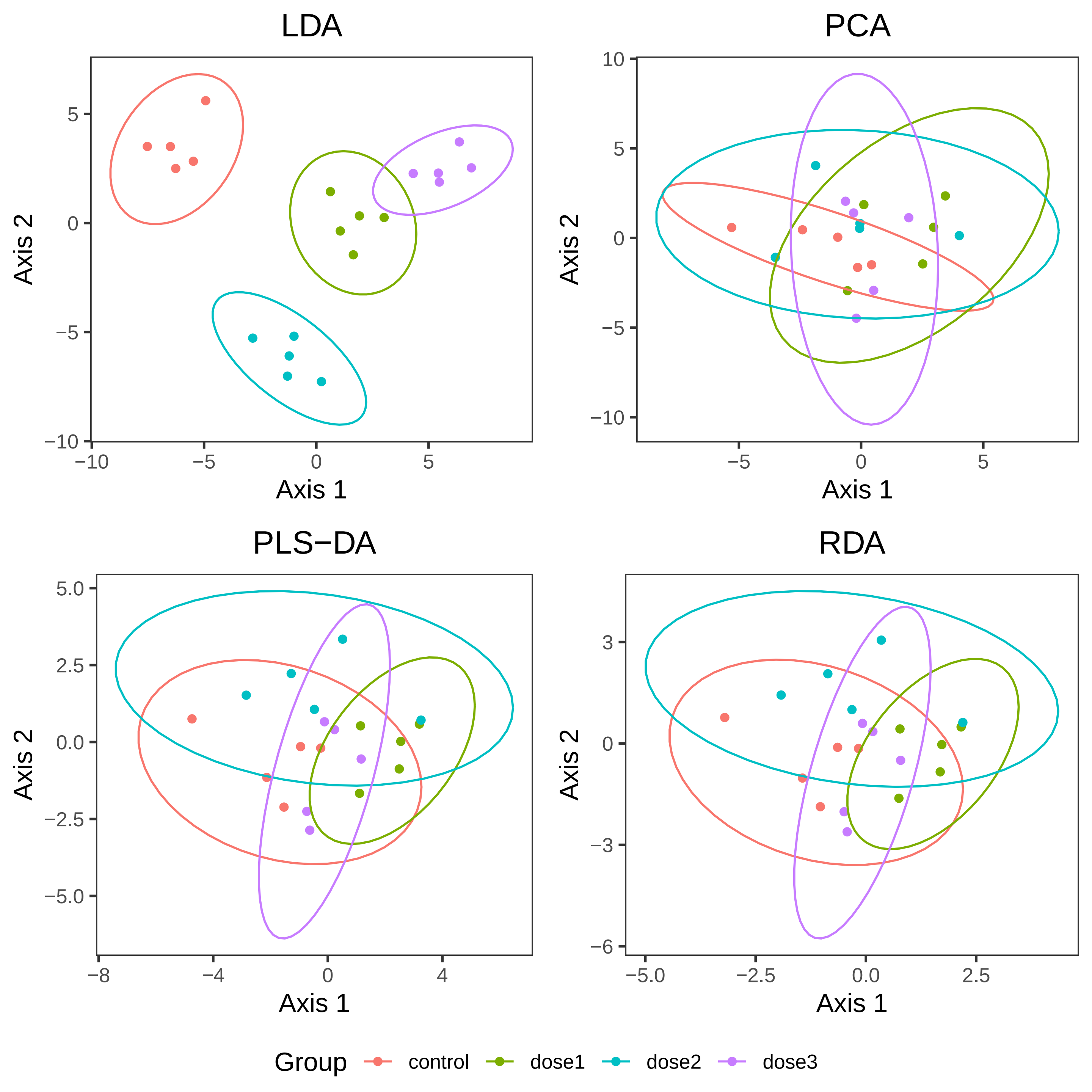
LDA
PCA
PLS−DA
RDA
Axis 1
Axis 2
Group
control
dose1
dose2
dose3

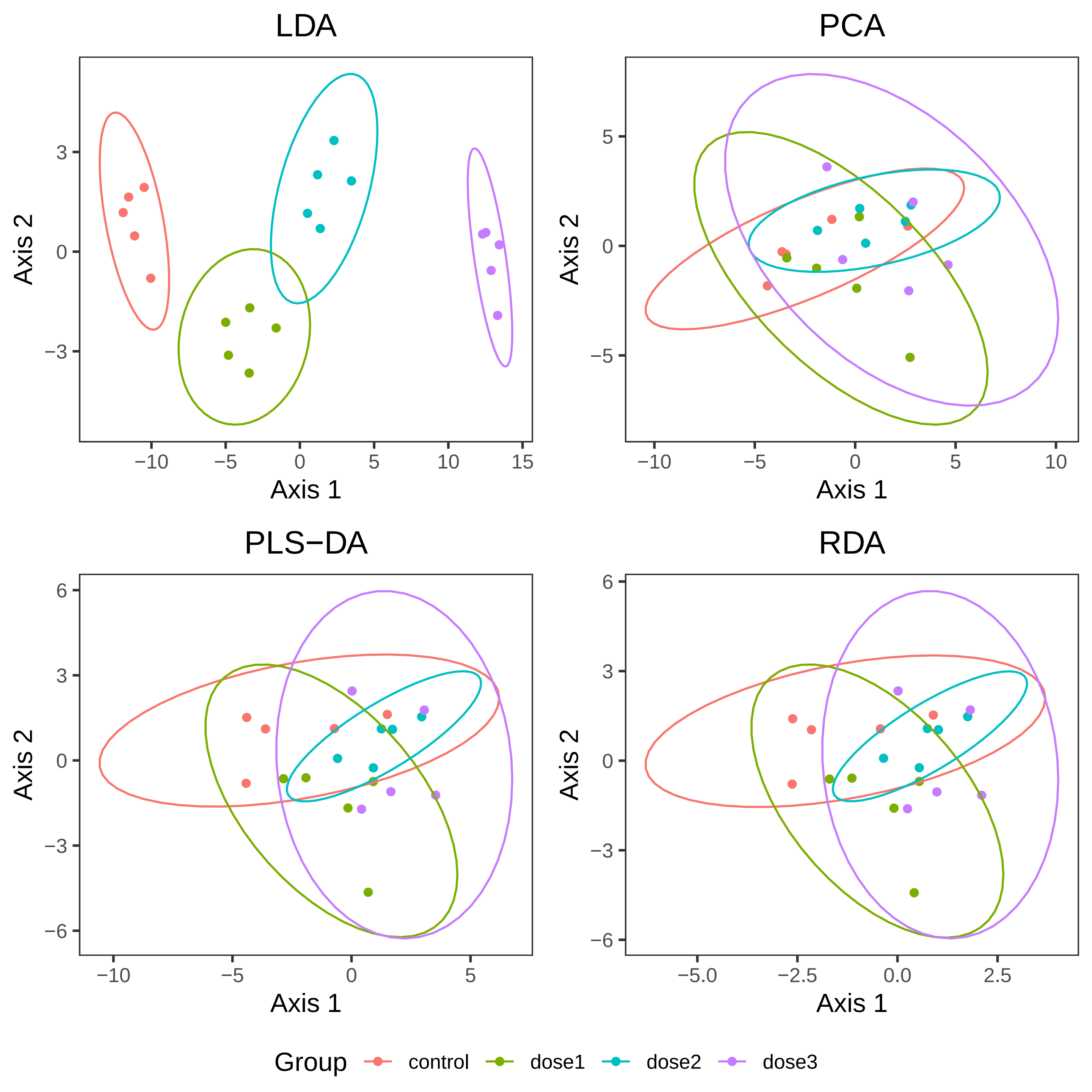
LDA
PCA
PLS−DA
RDA
Axis 1
Axis 2
Group
control
dose1
dose2
dose3

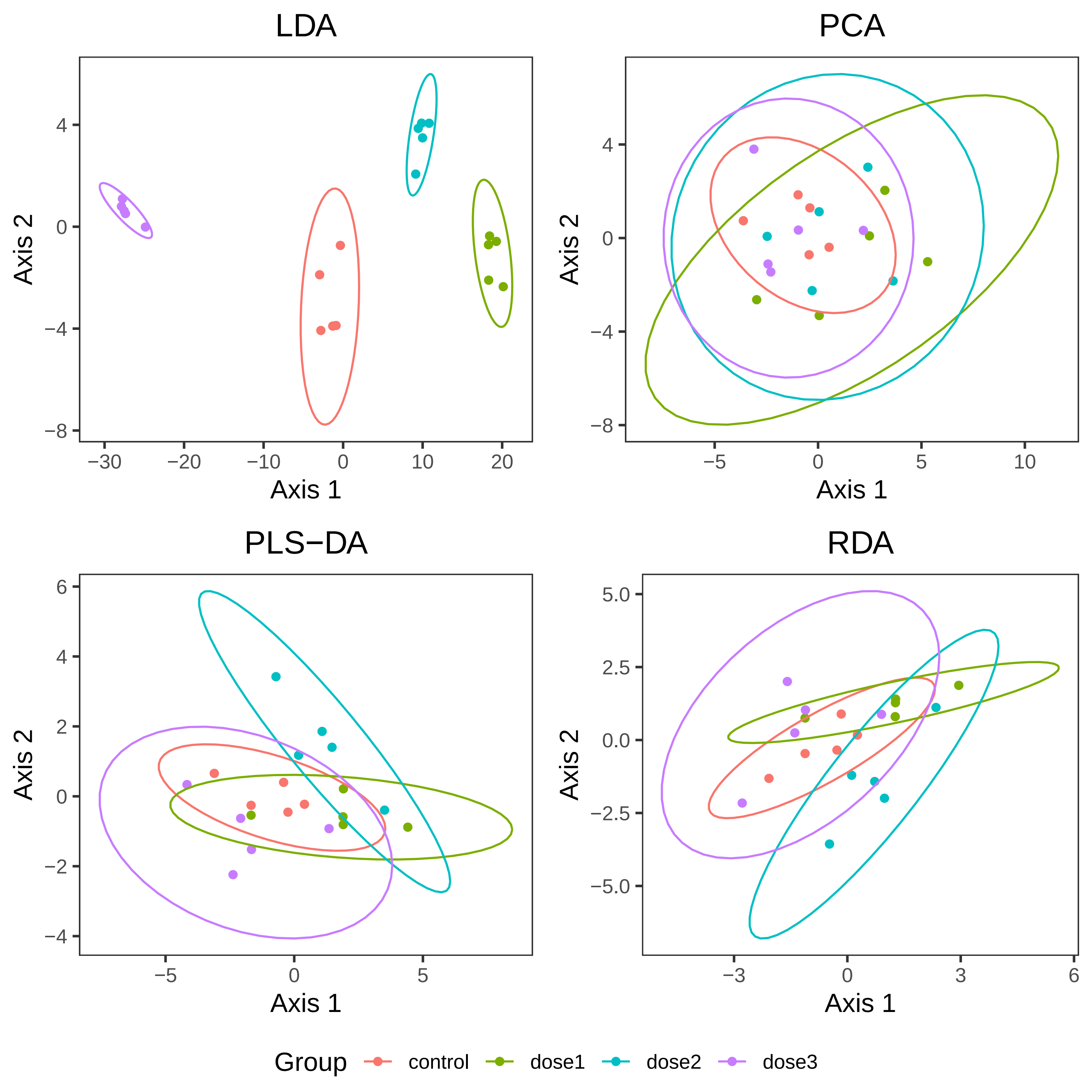
LDA
PCA
PLS−DA
RDA
Axis 1
Axis 2
Group
control
dose1
dose2
dose3

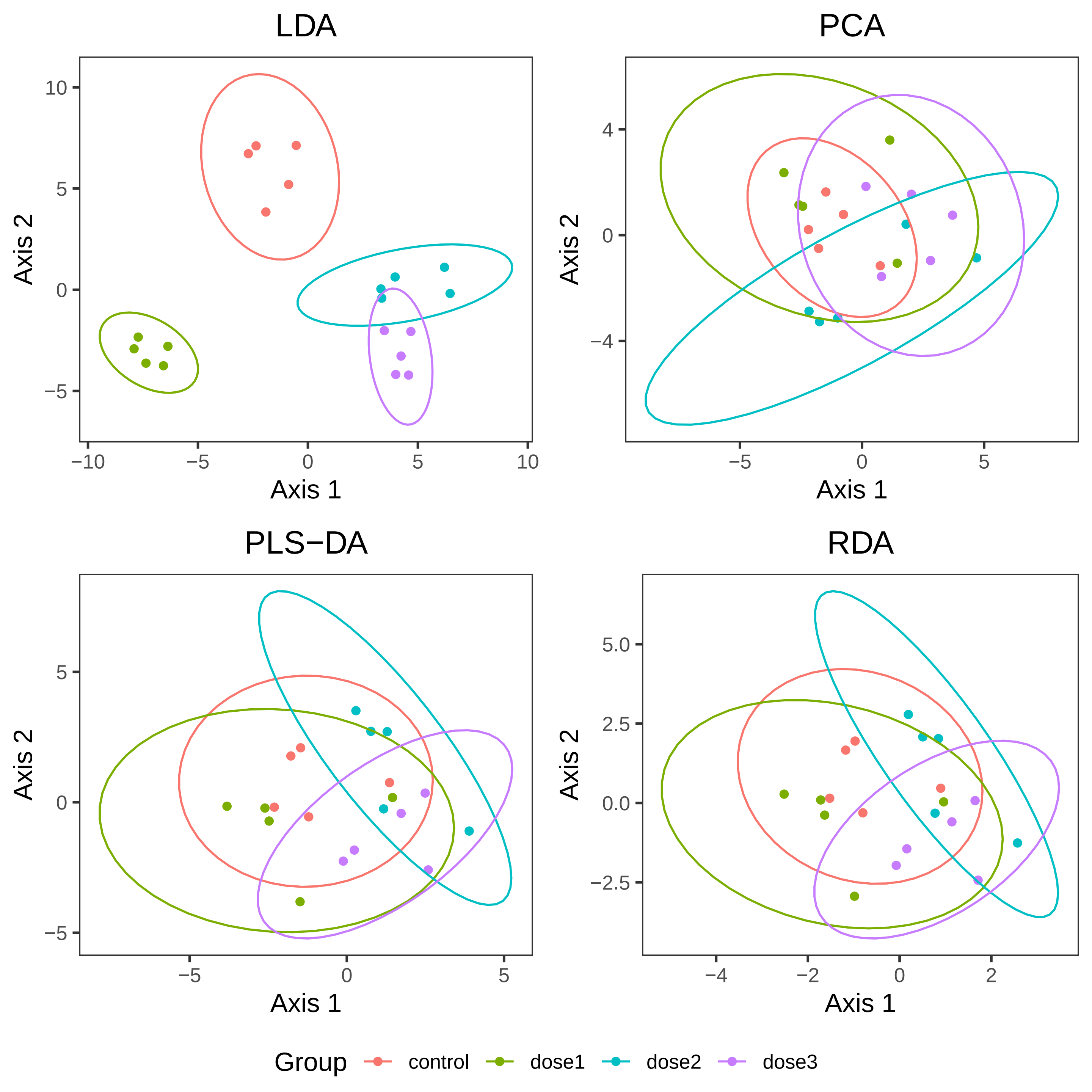
LDA
PCA
PLS−DA
RDA
Axis 1
Axis 2
Group
control
dose1
dose2
dose3

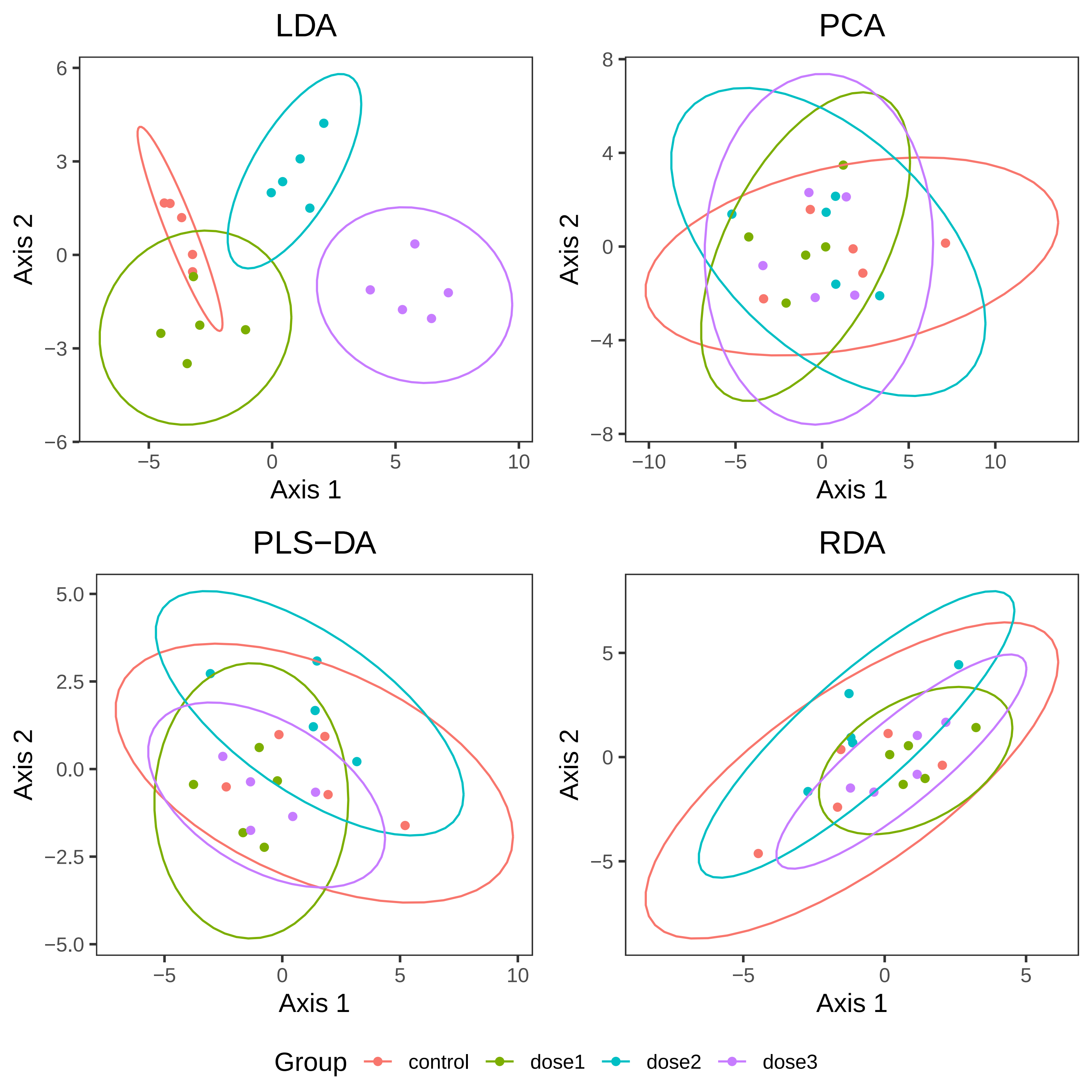

LDA
PCA
PLS−DA
RDA
Axis 1
Axis 2
6
3
0
−3
−6
−5
0
5
10
8
4
−4
−8
−10
5.0
2.5
0.0
−2.5
−5.0
Group
control
dose1
dose2
dose3

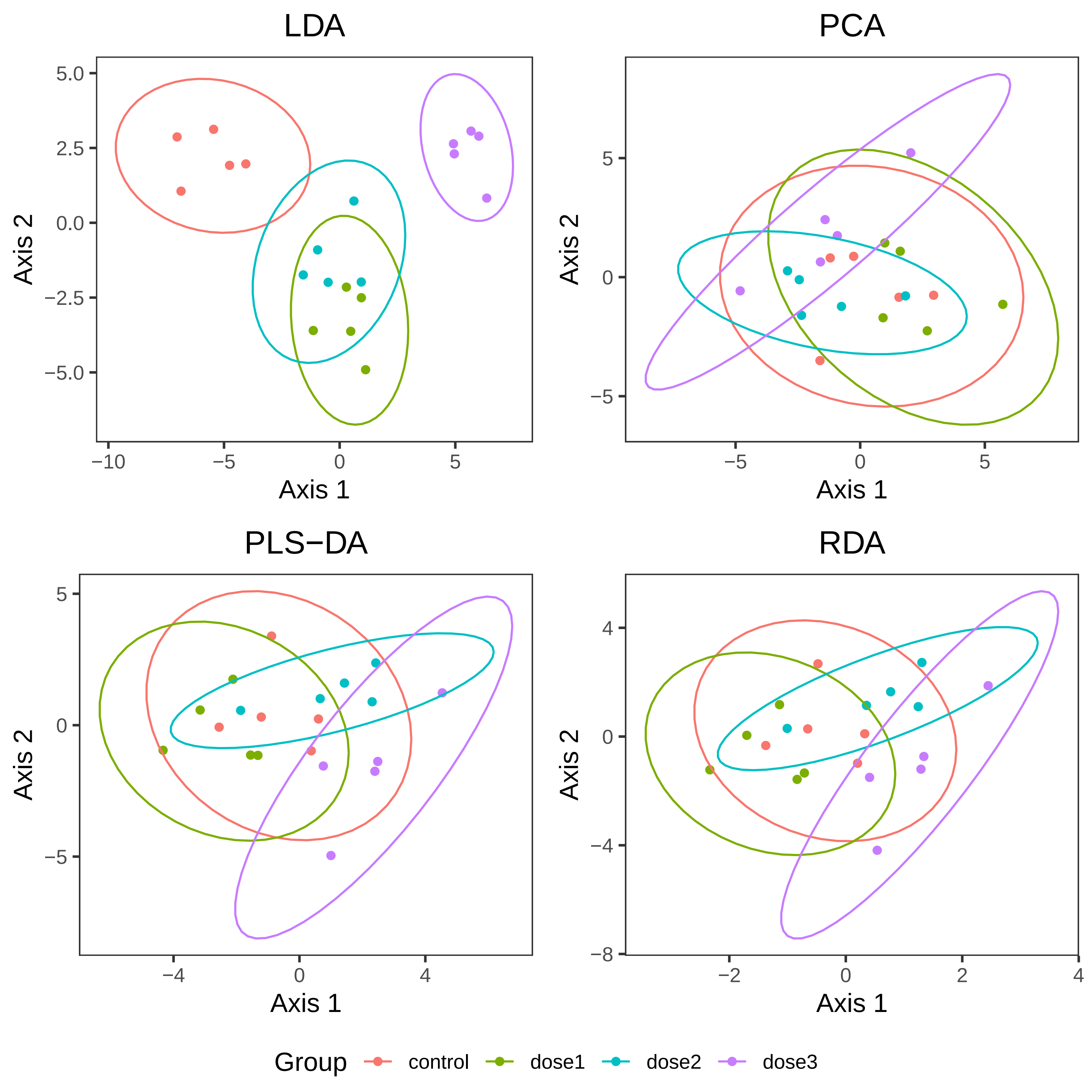

LDA
PCA
PLS−DA
RDA
Axis 1
Axis 2
Group
control
dose1
dose2
dose3

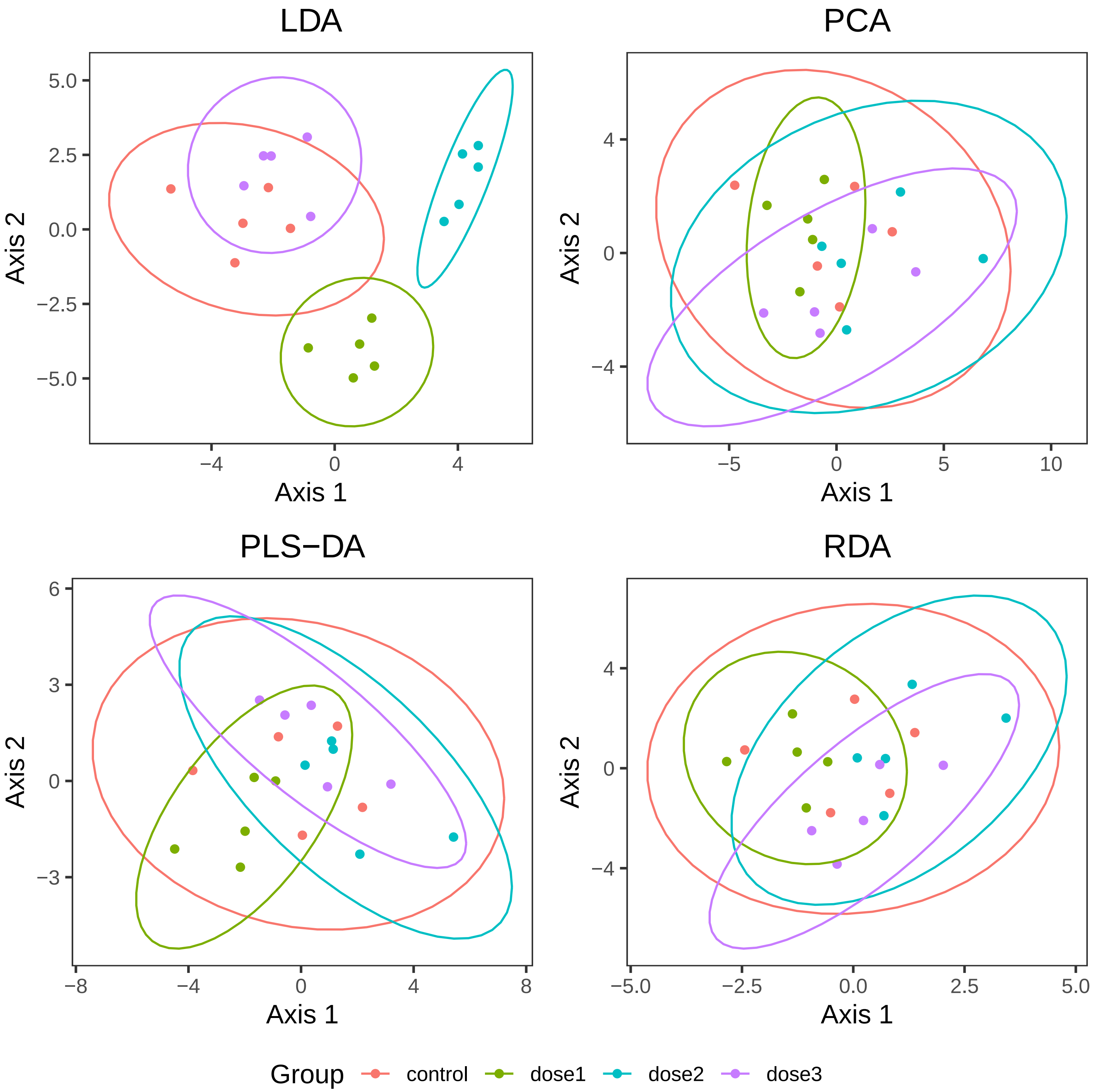
LDA
PCA
PLS−DA
RDA
Axis 1
Axis 2
5.0
2.5
0.0
−2.5
−5.0
−4
0
4
−5
5
10
6
3
−3
−8
8
−2.5
−5.0
Group
control
dose1
dose2
dose3

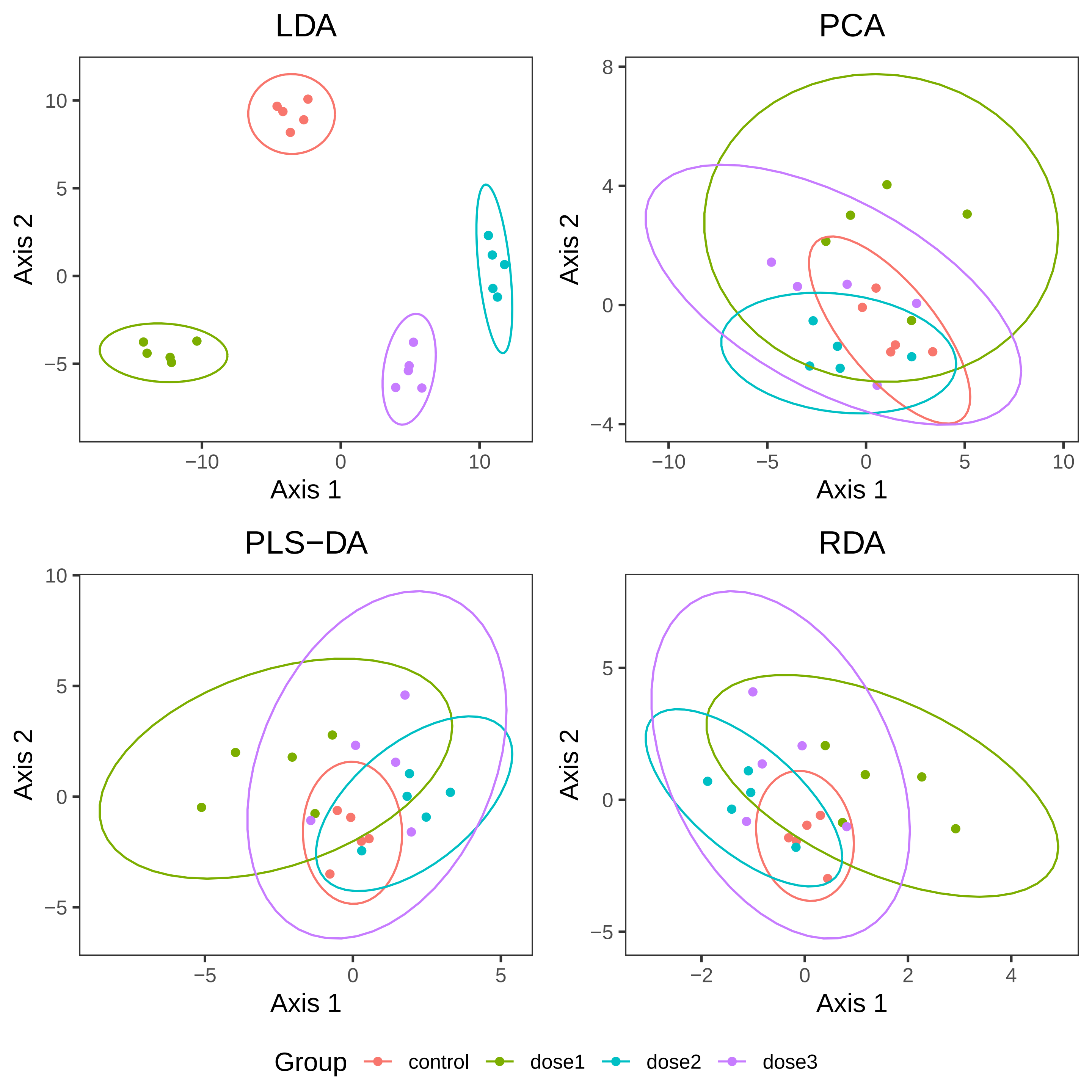

LDA
PCA
PLS−DA
RDA
Axis 1
Axis 2
Group
control
dose1
dose2
dose3

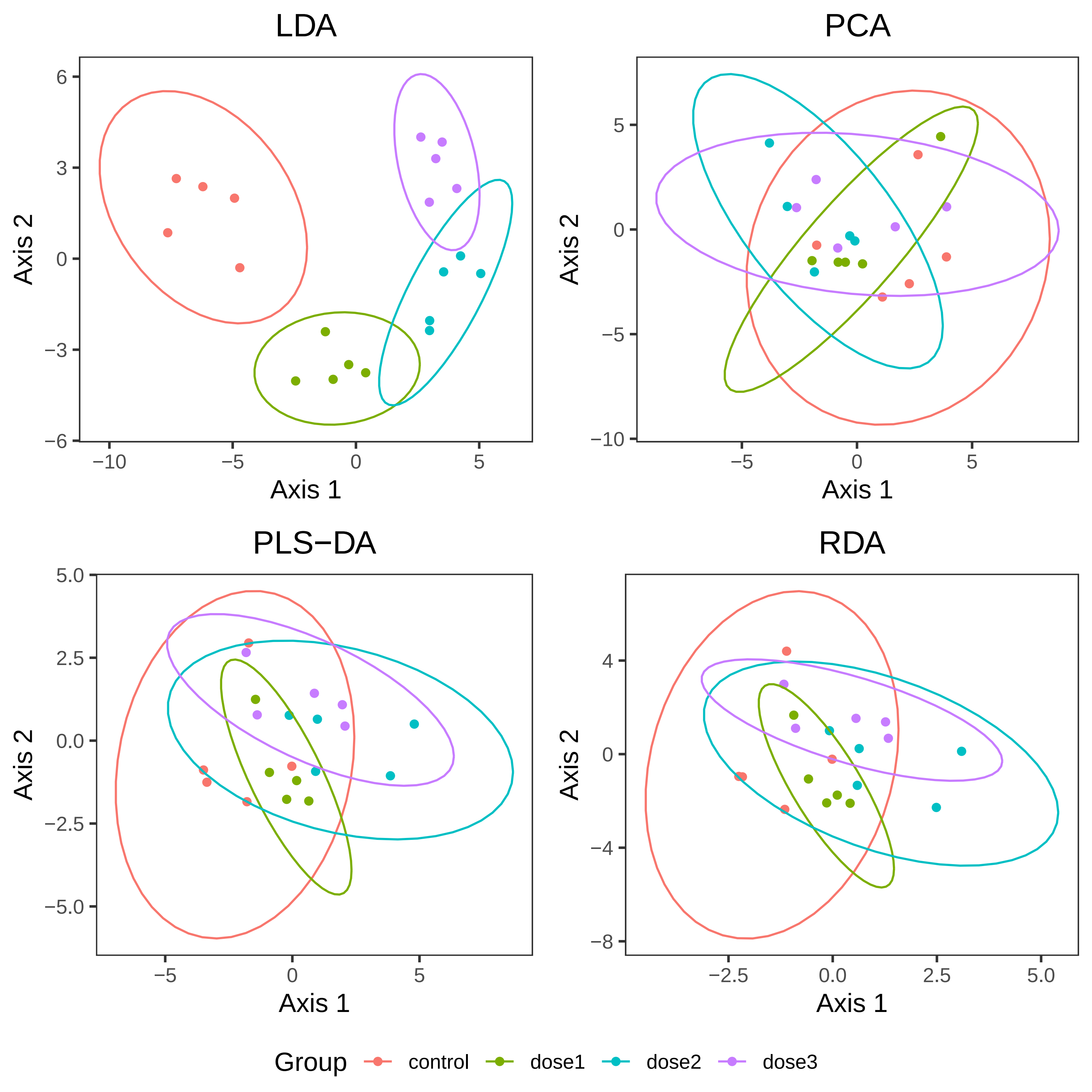
LDA
PCA
PLS−DA
RDA
Axis 1
Axis 2
Group
control
dose1
dose2
dose3

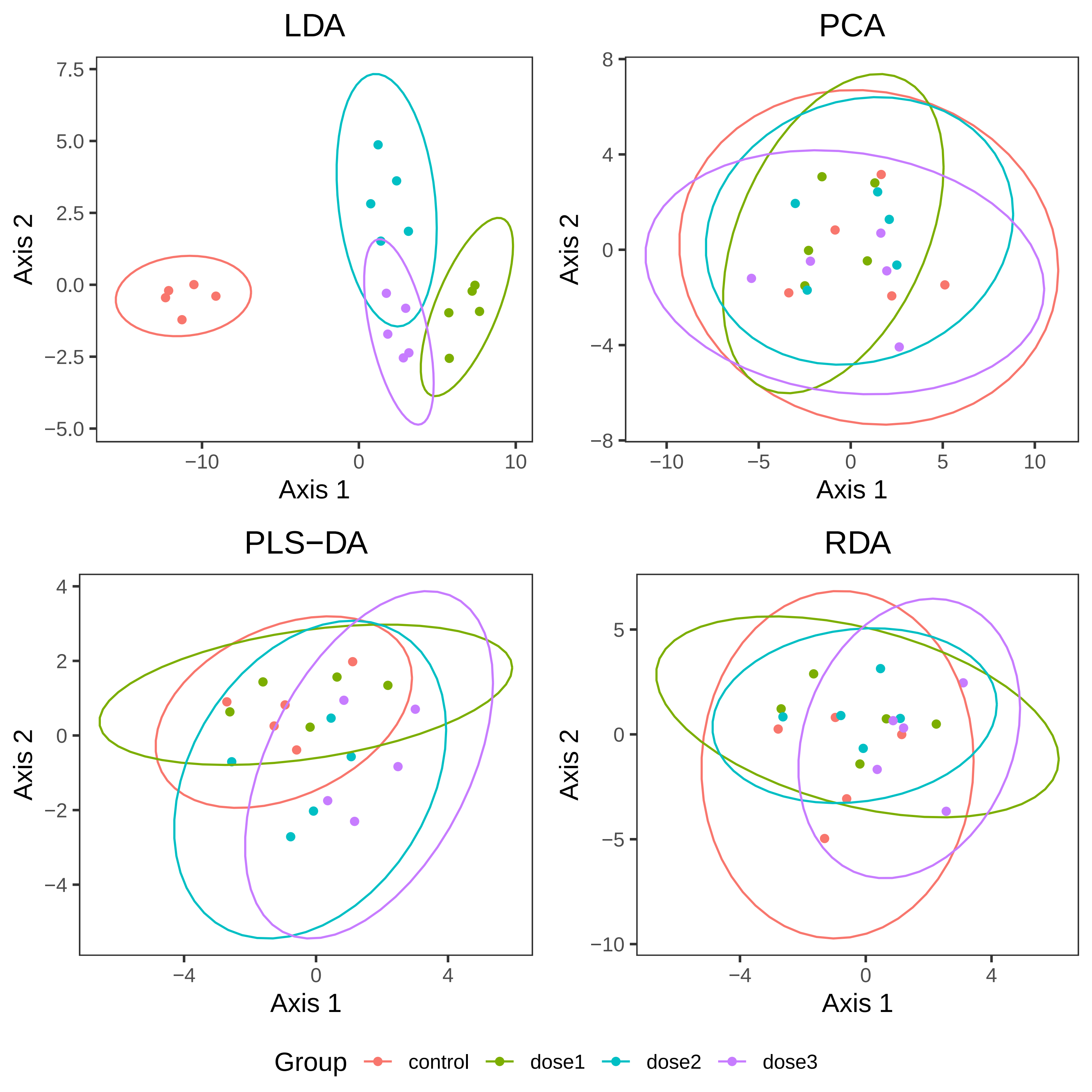

LDA
PCA
PLS−DA
RDA
Axis 1
Axis 2
7.5
5.0
2.5
0.0
−2.5
−5.0
−10
0
10
8
4
0
−4
−8
−5
5
2
−2
−6
−10
Group
control
dose1
dose2
dose3

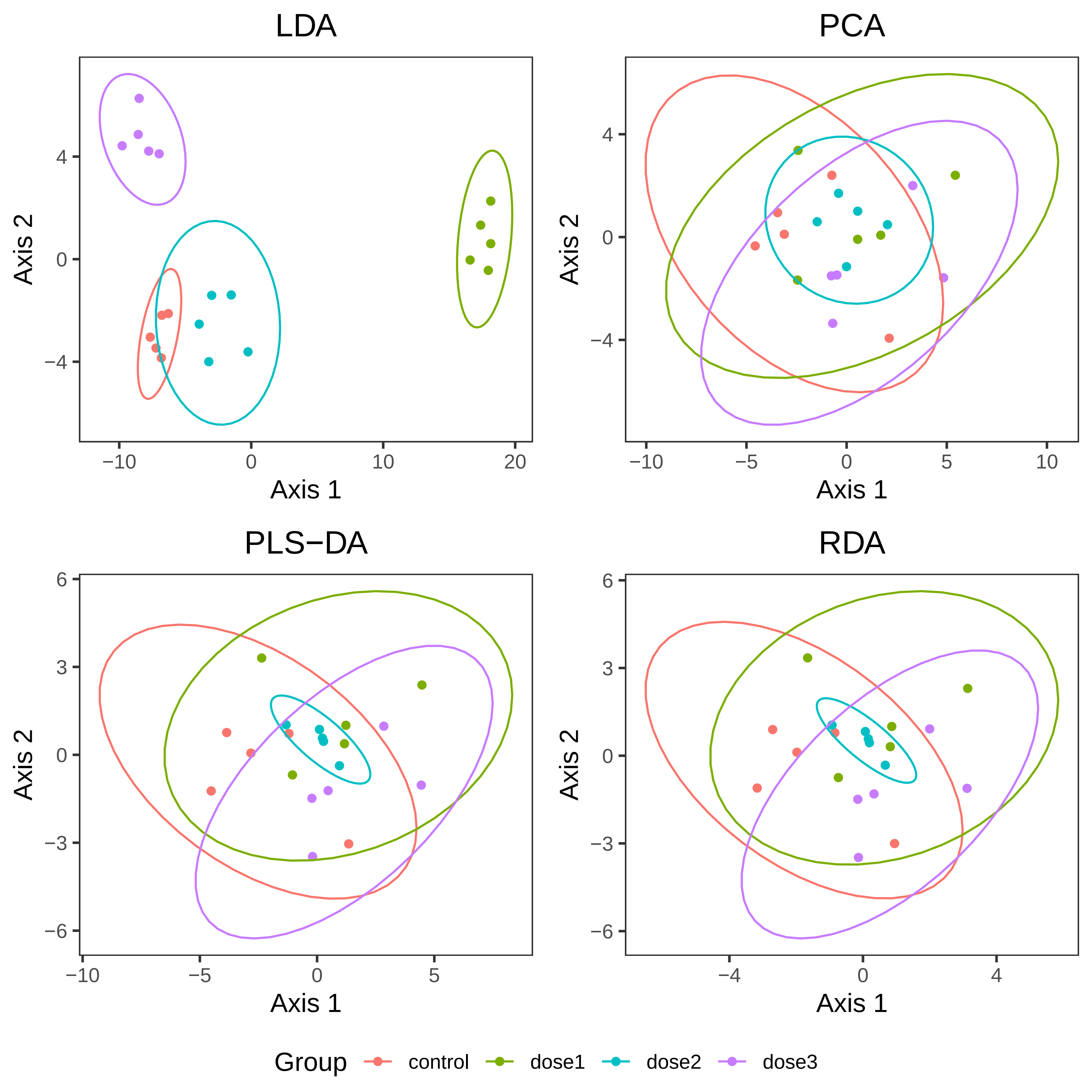

LDA
PCA
PLS−DA
RDA
Axis 1
Axis 2
Group
control
dose1
dose2
dose3

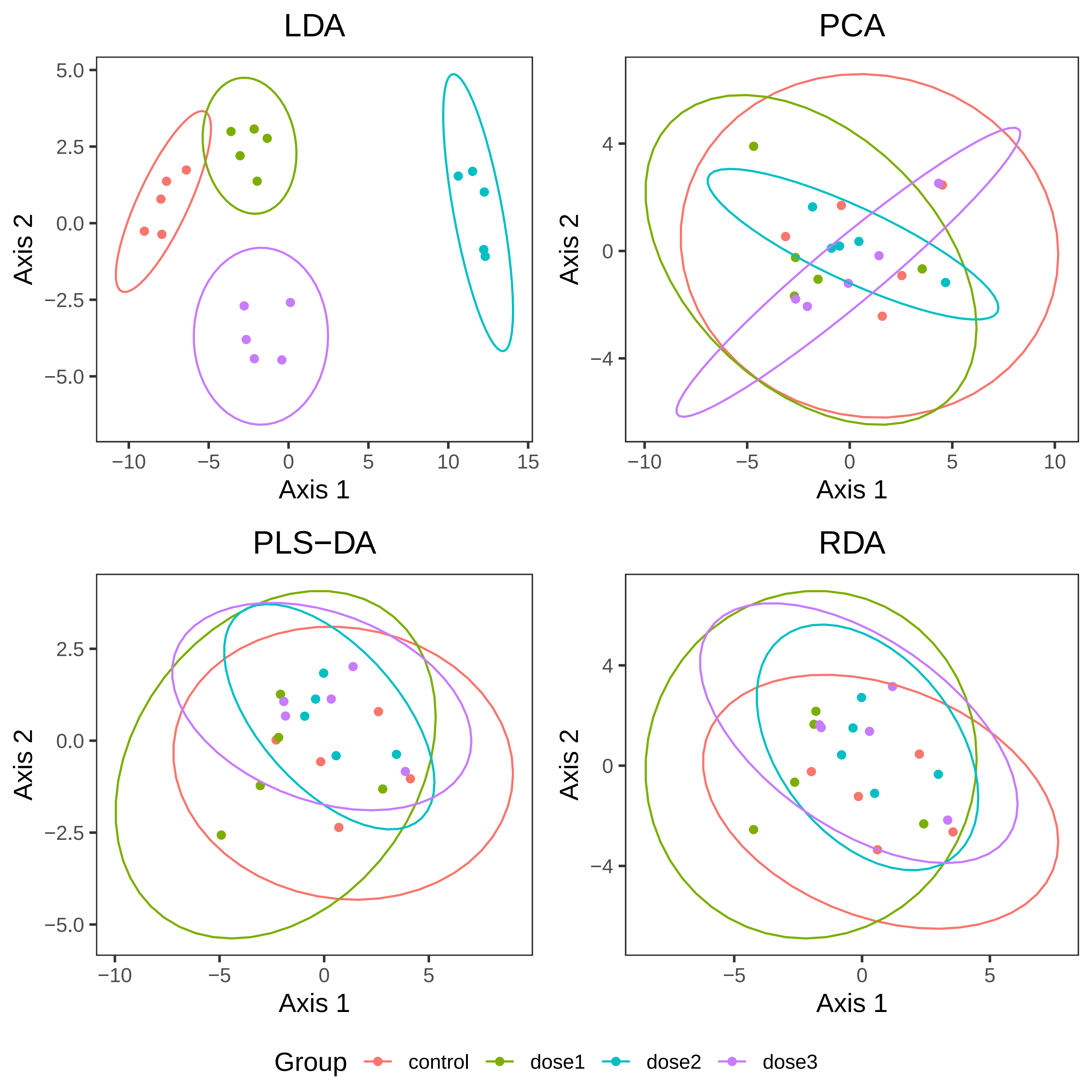

LDA
PCA
PLS−DA
RDA
Axis 1
Axis 2
Group
control
dose1
dose2
dose3

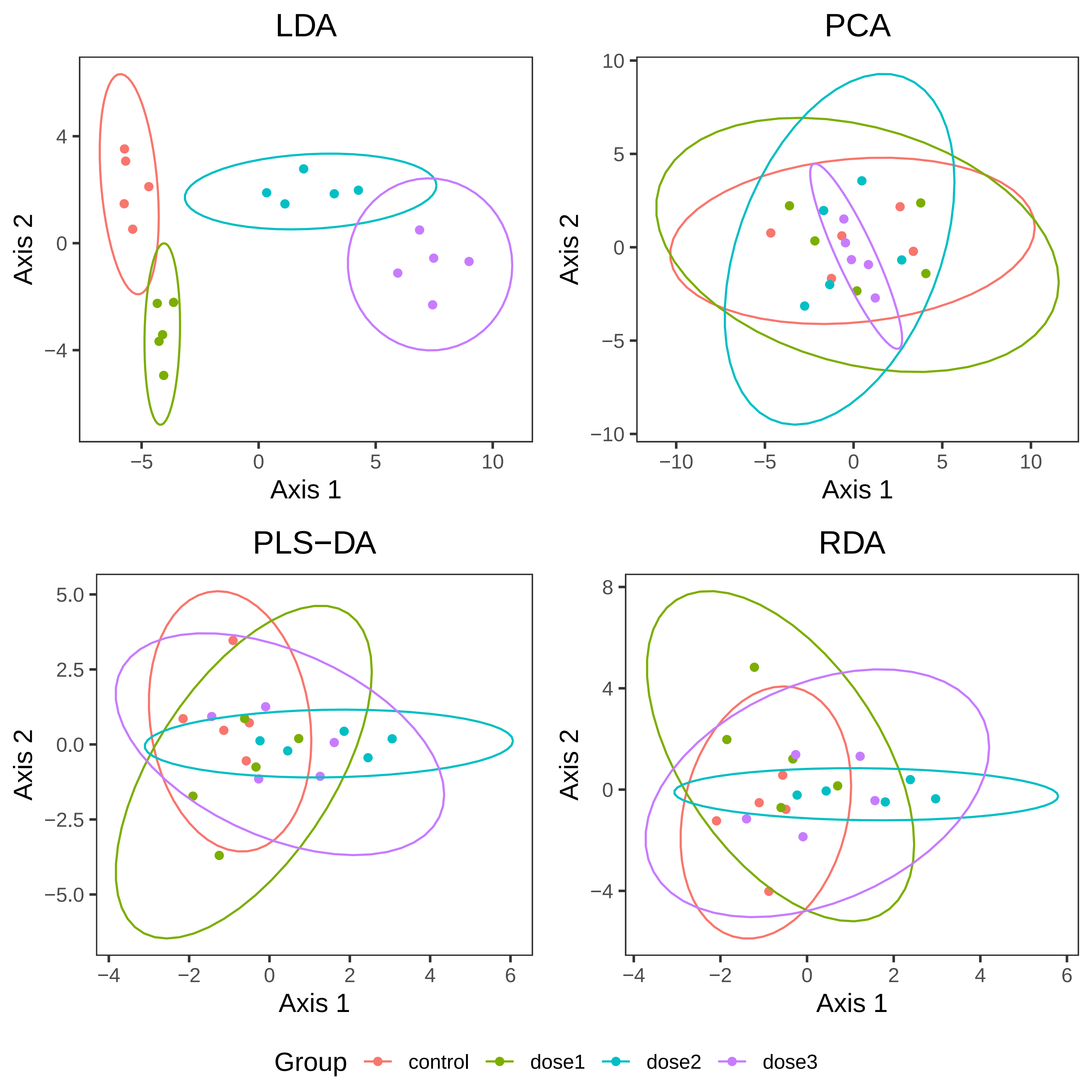

LDA
PCA
PLS−DA
RDA
Axis 1
Axis 2
Group
control
dose1
dose2
dose3

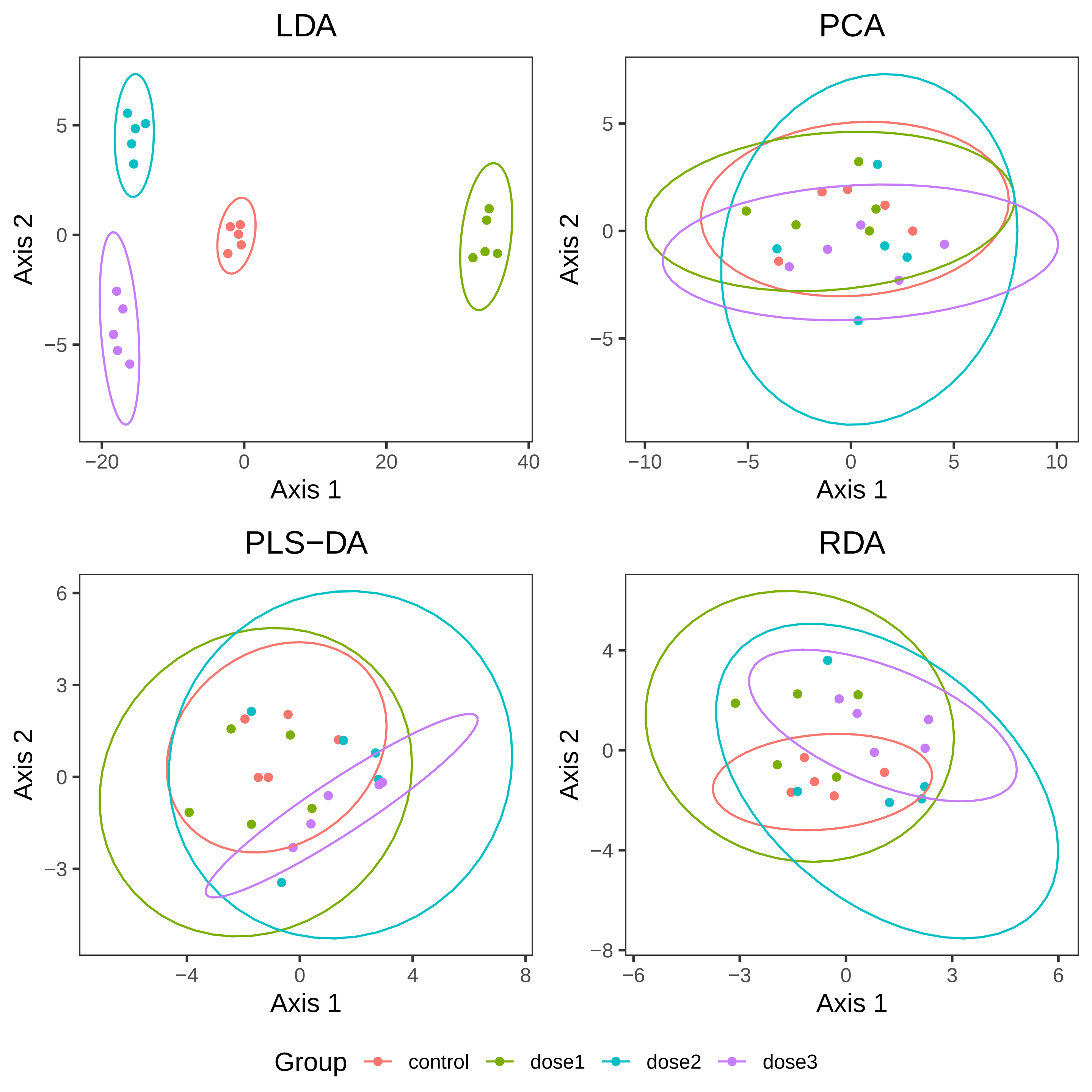

LDA
PCA
PLS−DA
RDA
Axis 1
Axis 2
5
0
−5
−20
0
20
40
−10
−5
0
5
10
6
3
0
−3
−4
0
4
8
4
0
−4
−8
−6
−3
0
3
6
Group
control
dose1
dose2
dose3

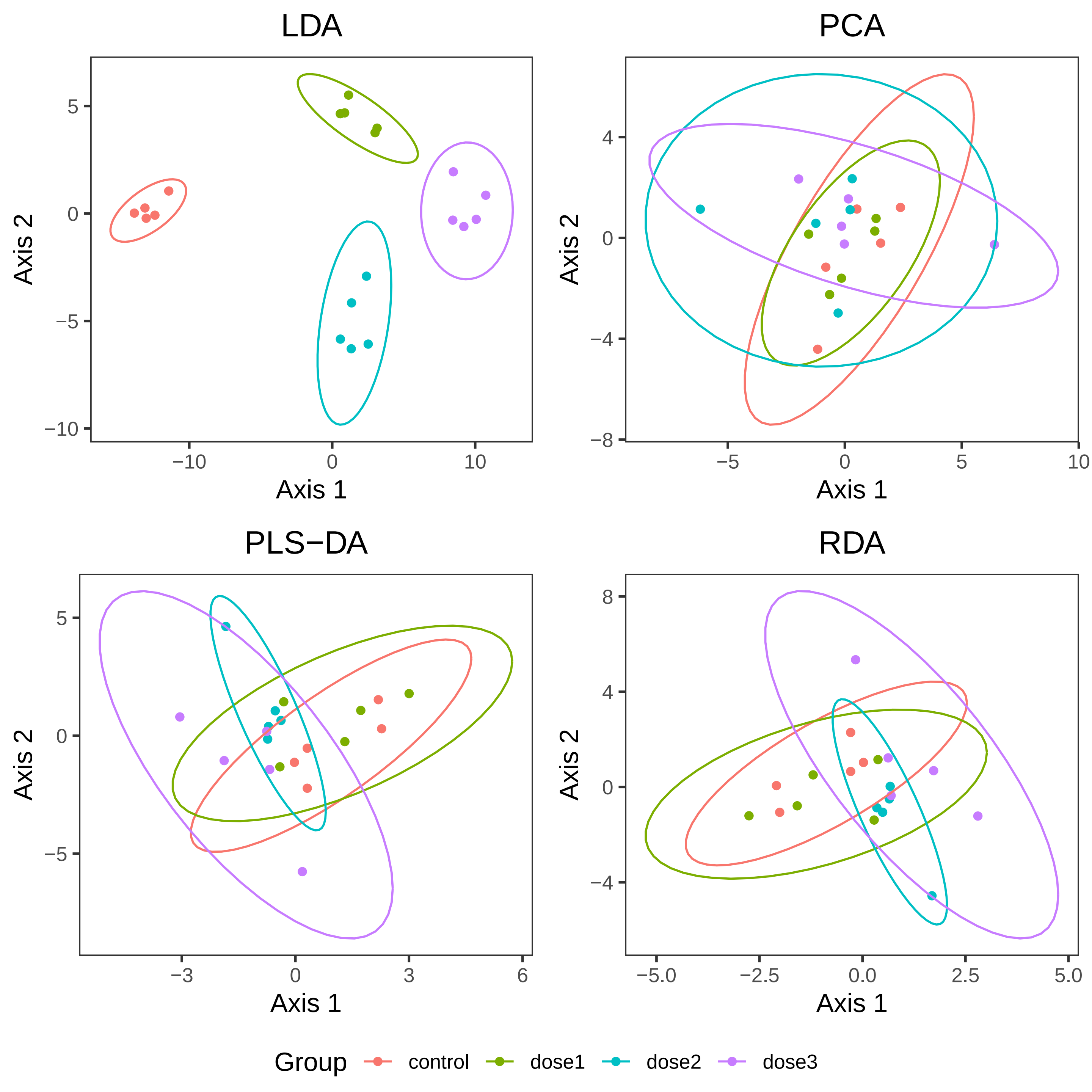

LDA
PCA
PLS−DA
RDA
Axis 1
Axis 2
5
0
−5
−10
10
4
−4
−8
−3
3
6
8
−5.0
−2.5
0.0
2.5
5.0
Group
control
dose1
dose2
dose3

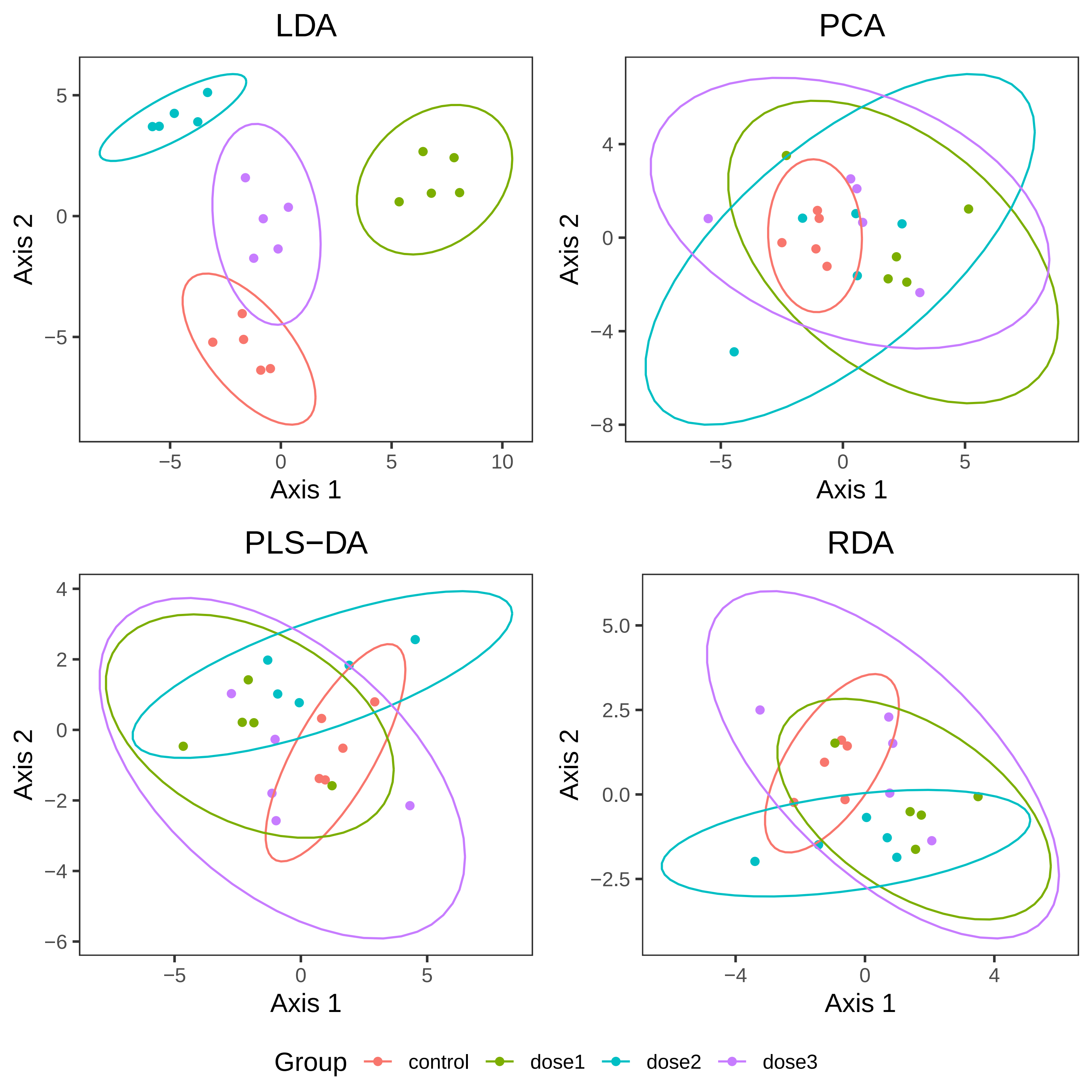

LDA
PCA
PLS−DA
RDA
Axis 1
Axis 2
5
0
−5
10
4
−4
−8
2
−2
−6
5.0
2.5
0.0
−2.5
Group
control
dose1
dose2
dose3

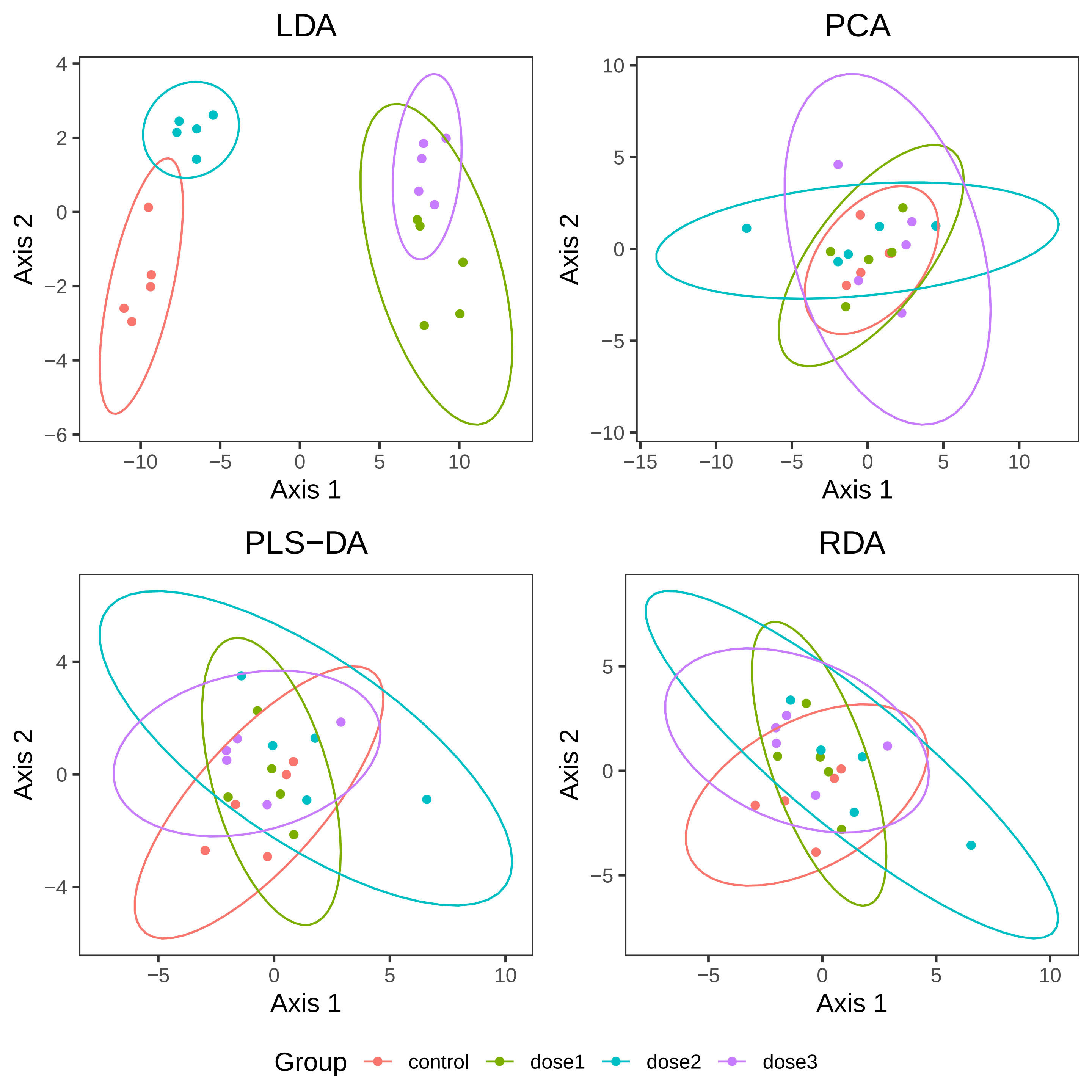
LDA
PCA
PLS−DA
RDA
Axis 1
Axis 2
−10
−5
0
5
10
4
2
−2
−4
−6
−15
−10
Group
control
dose1
dose2
dose3

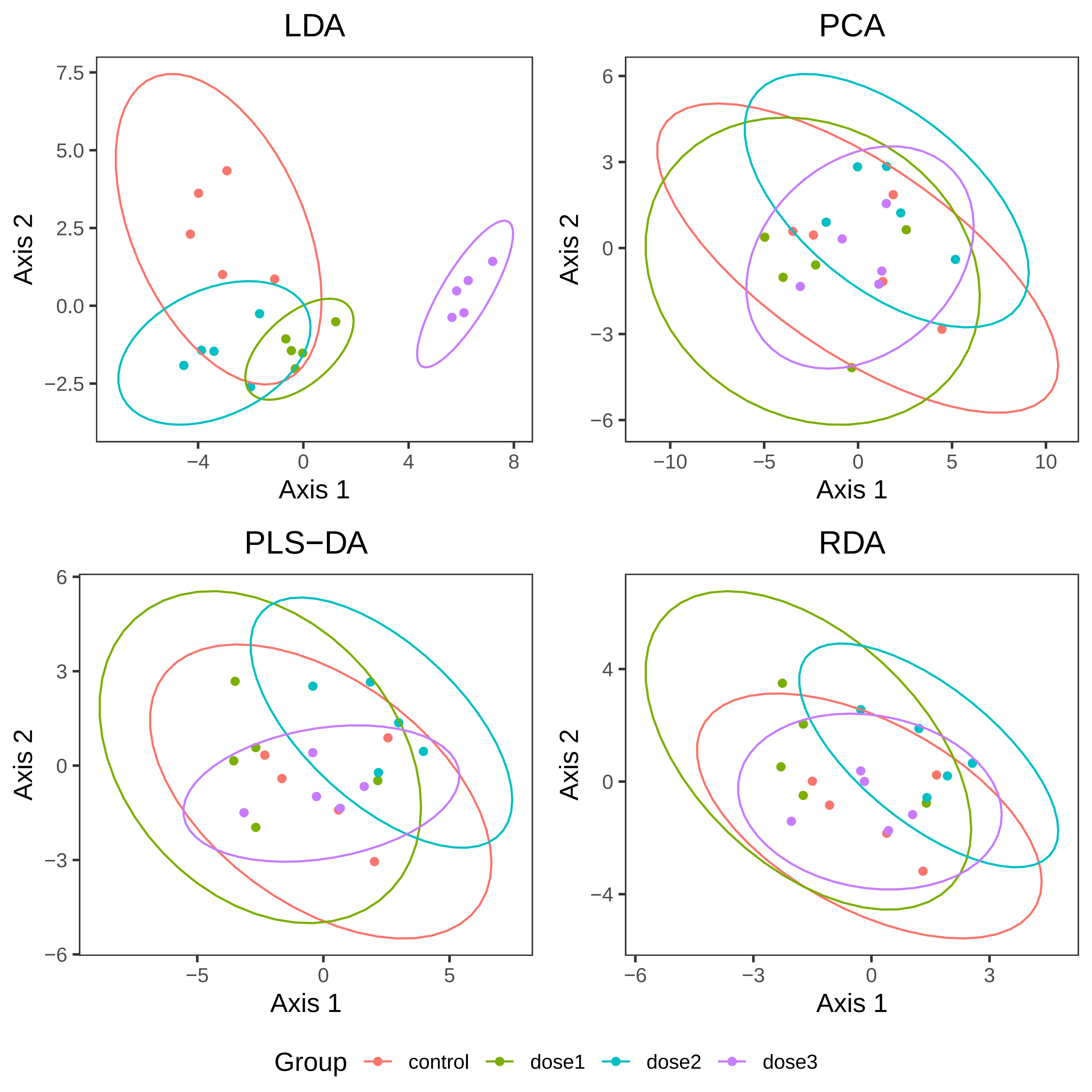

LDA
PCA
PLS−DA
RDA
Axis 1
Axis 2
Group
control
dose1
dose2
dose3

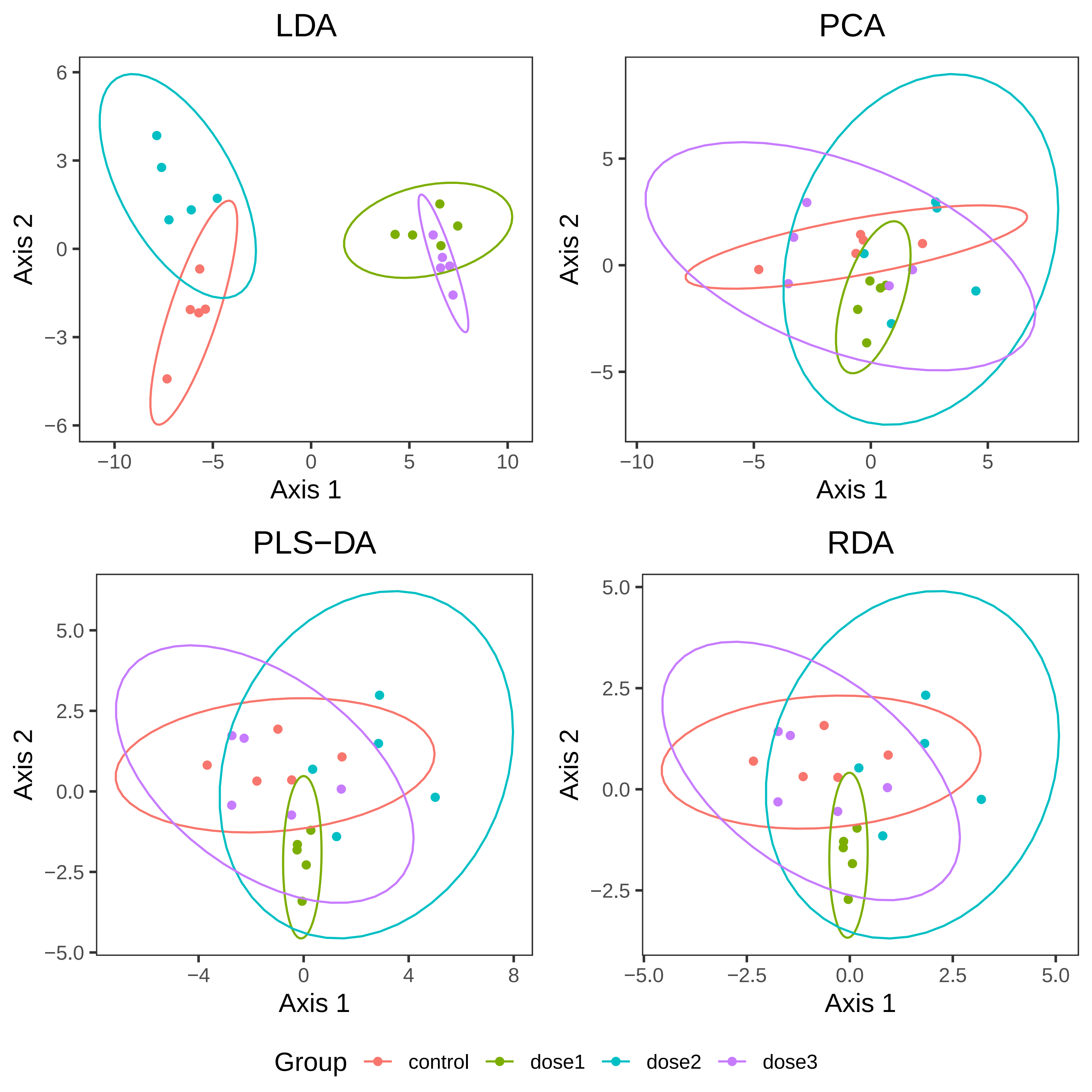

LDA
PCA
PLS−DA
RDA
Axis 1
Axis 2
Group
control
dose1
dose2
dose3

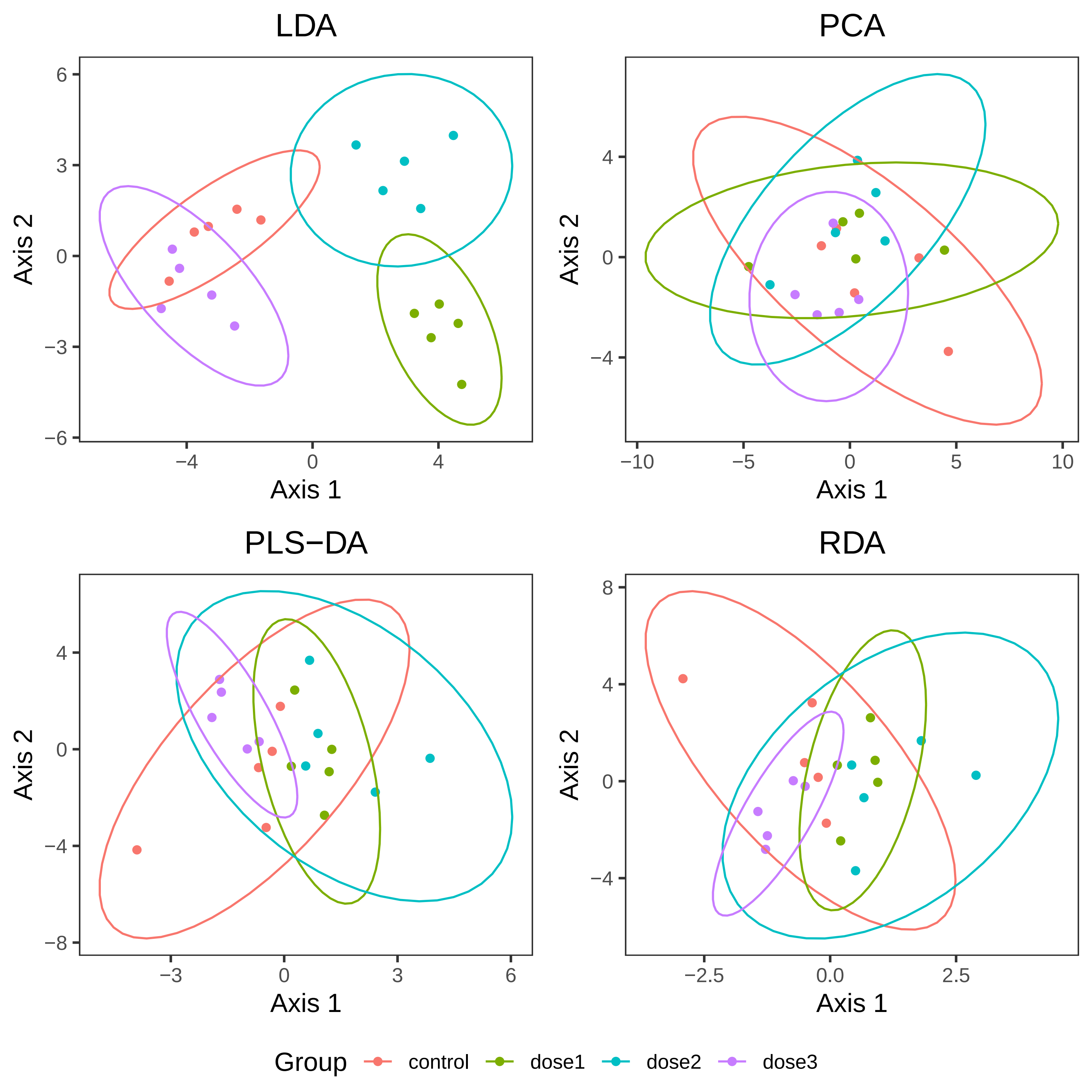
LDA
PCA
PLS−DA
RDA
Axis 1
Axis 2
Group
control
dose1
dose2
dose3

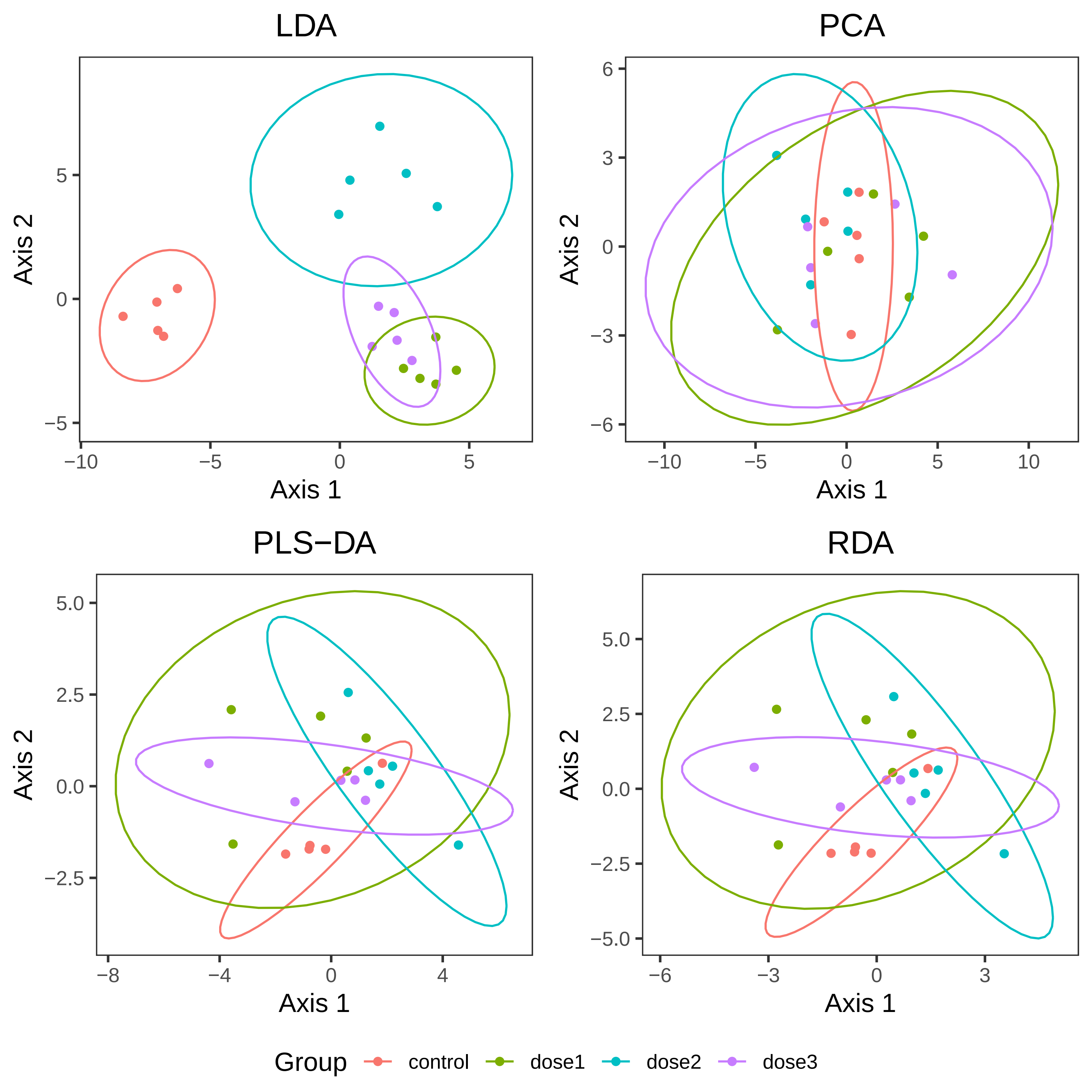

LDA
PCA
PLS−DA
RDA
Axis 1
Axis 2
Group
control
dose1
dose2
dose3

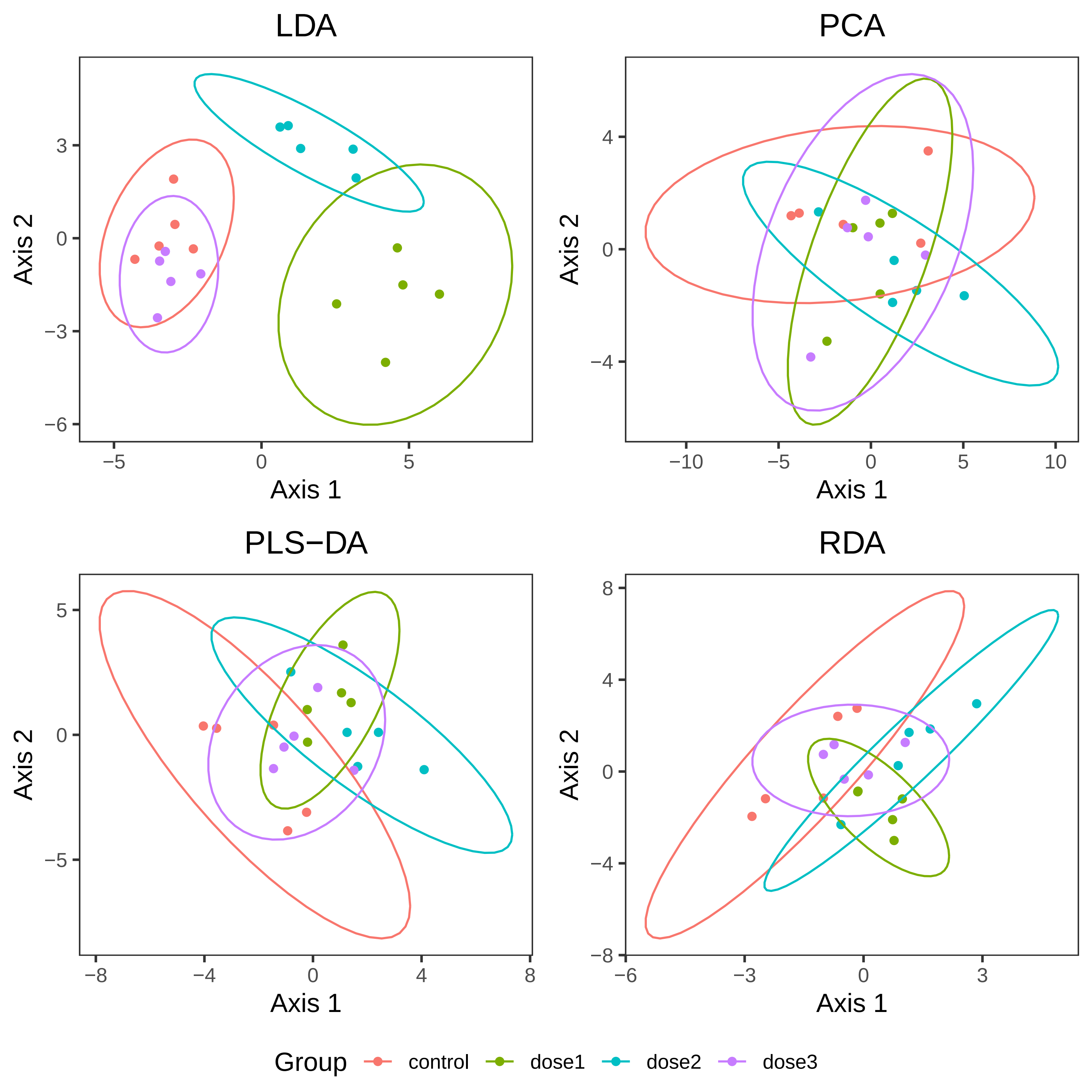

LDA
PCA
PLS−DA
RDA
Axis 1
Axis 2
Group
control
dose1
dose2
dose3

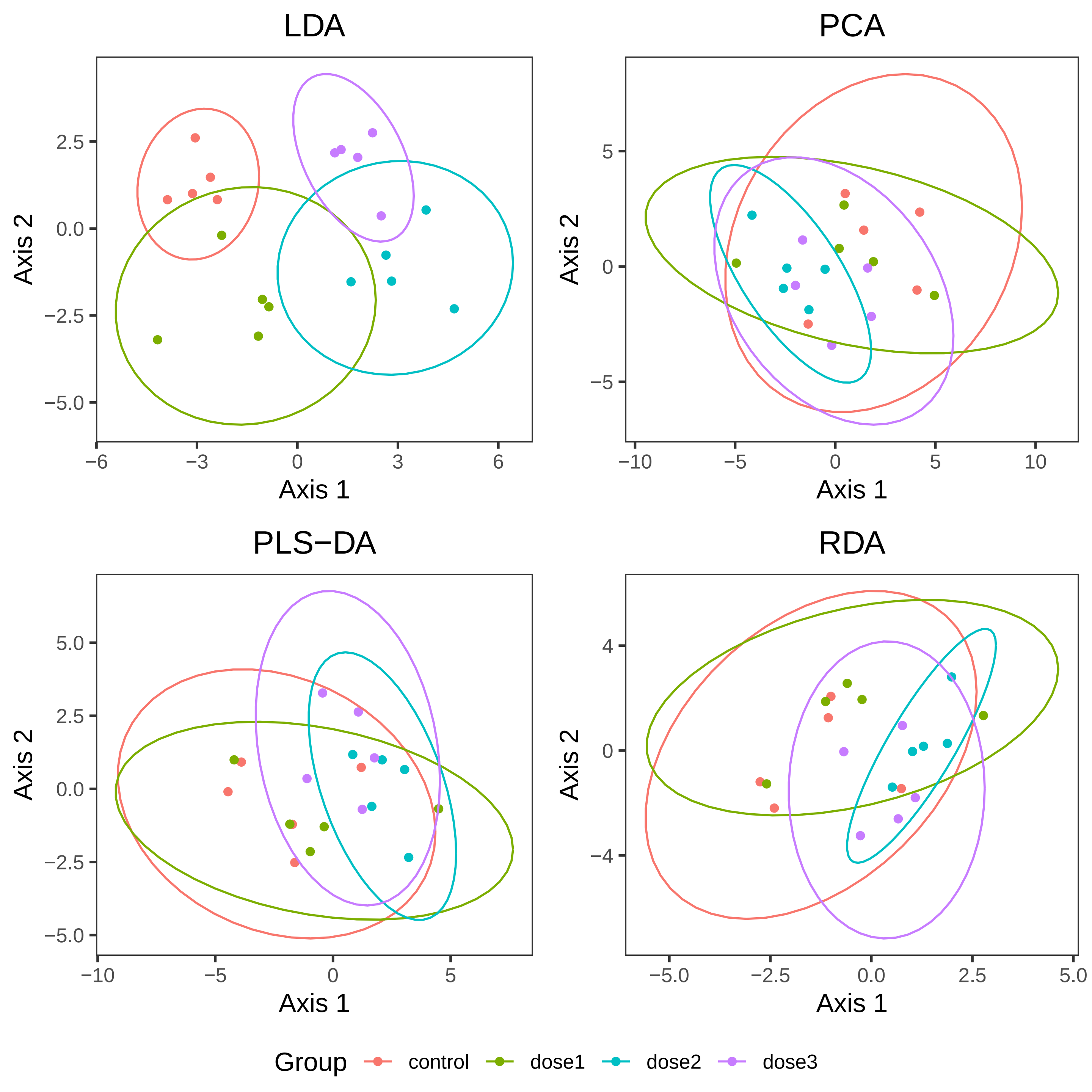

LDA
PCA
PLS−DA
RDA
Axis 1
Axis 2
Group
control
dose1
dose2
dose3

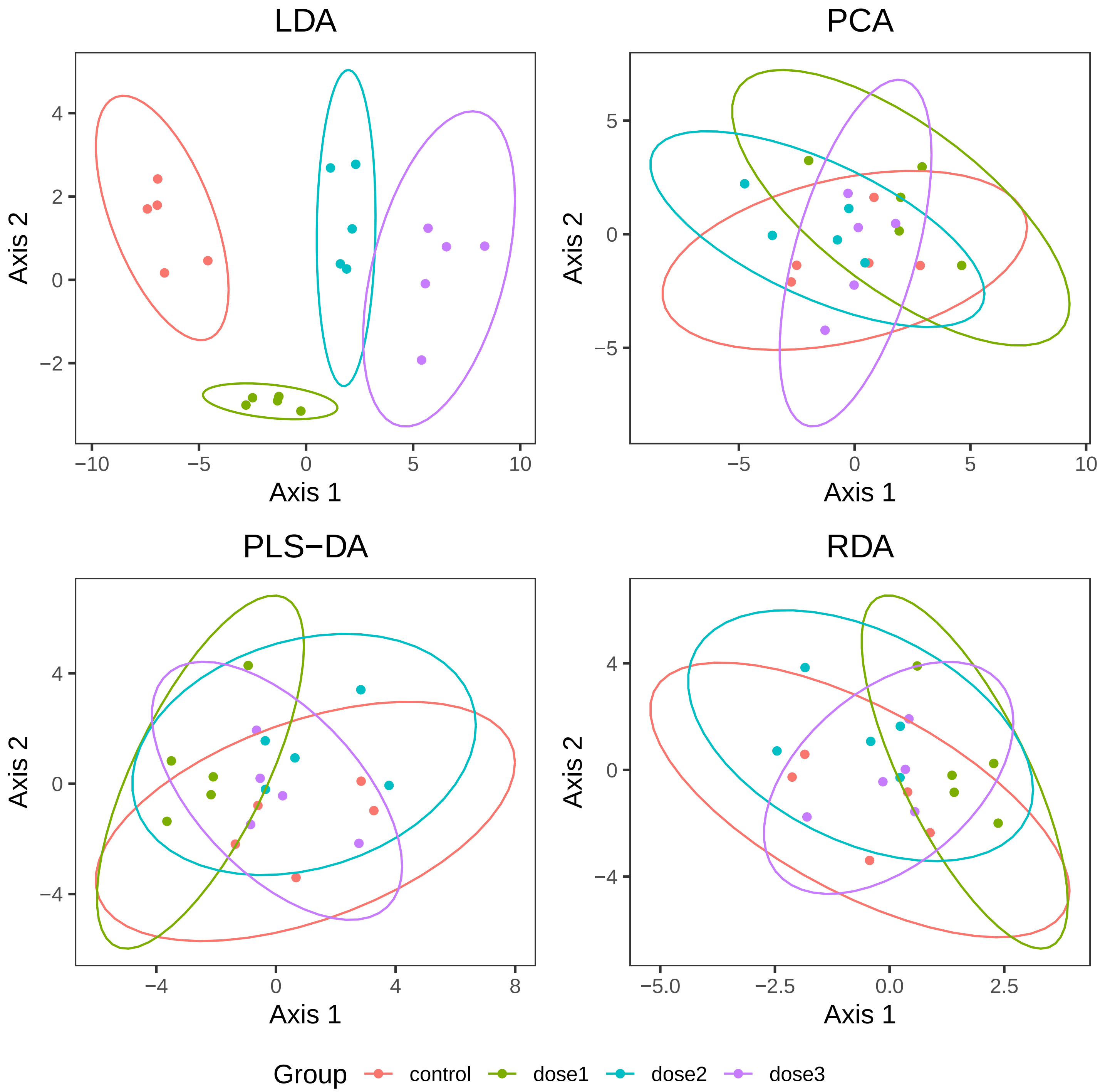

LDA
PCA
PLS−DA
RDA
Axis 1
Axis 2
Group
control
dose1
dose2
dose3

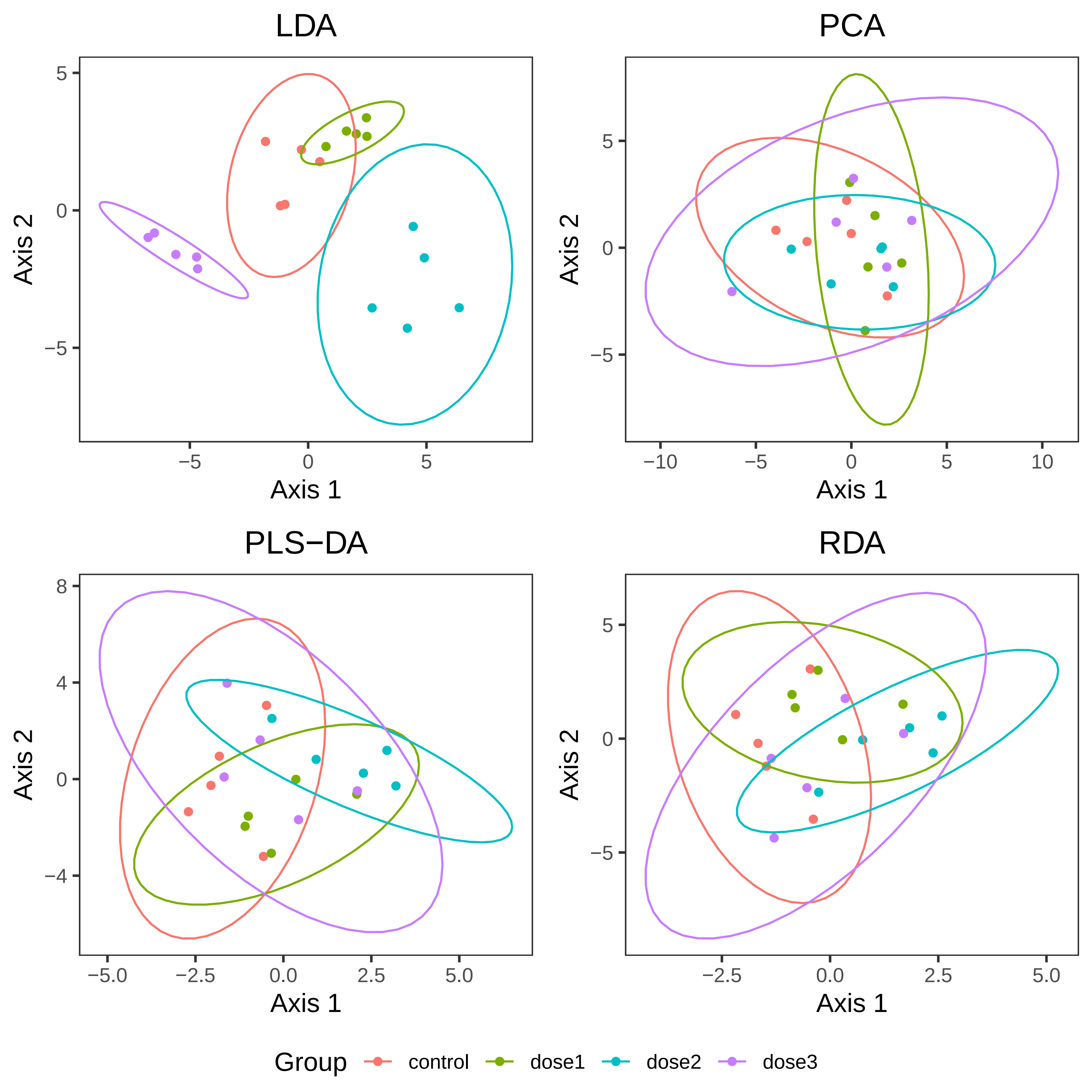
LDA
PCA
PLS−DA
RDA
Axis 1
Axis 2
Group
control
dose1
dose2
dose3

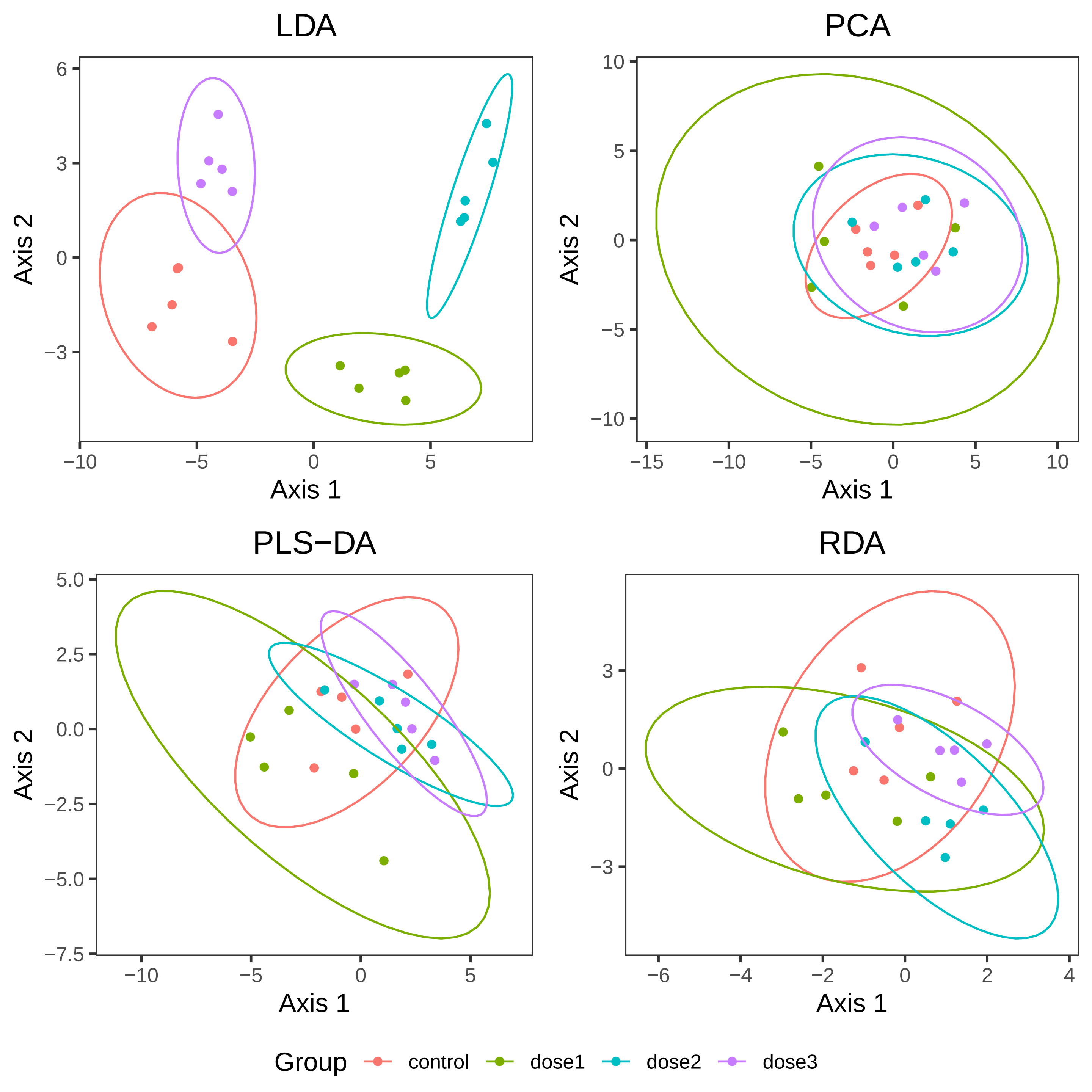

LDA
PCA
PLS−DA
RDA
Axis 1
Axis 2
Group
control
dose1
dose2
dose3

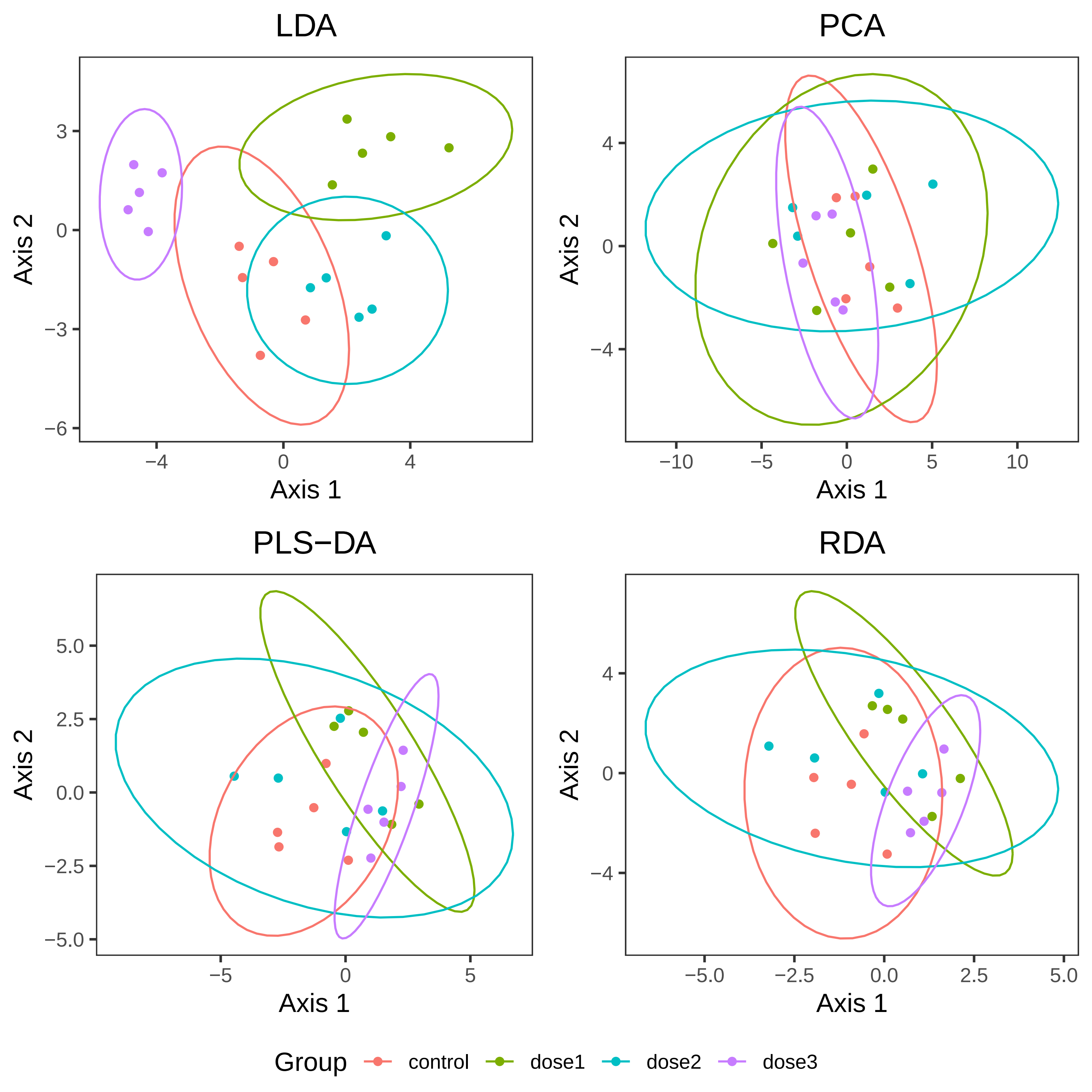
LDA
PCA
PLS−DA
RDA
Axis 1
Axis 2
3
0
−3
−6
−4
0
4
−10
−5
5
10
5.0
2.5
0.0
−2.5
−5.0
Group
control
dose1
dose2
dose3

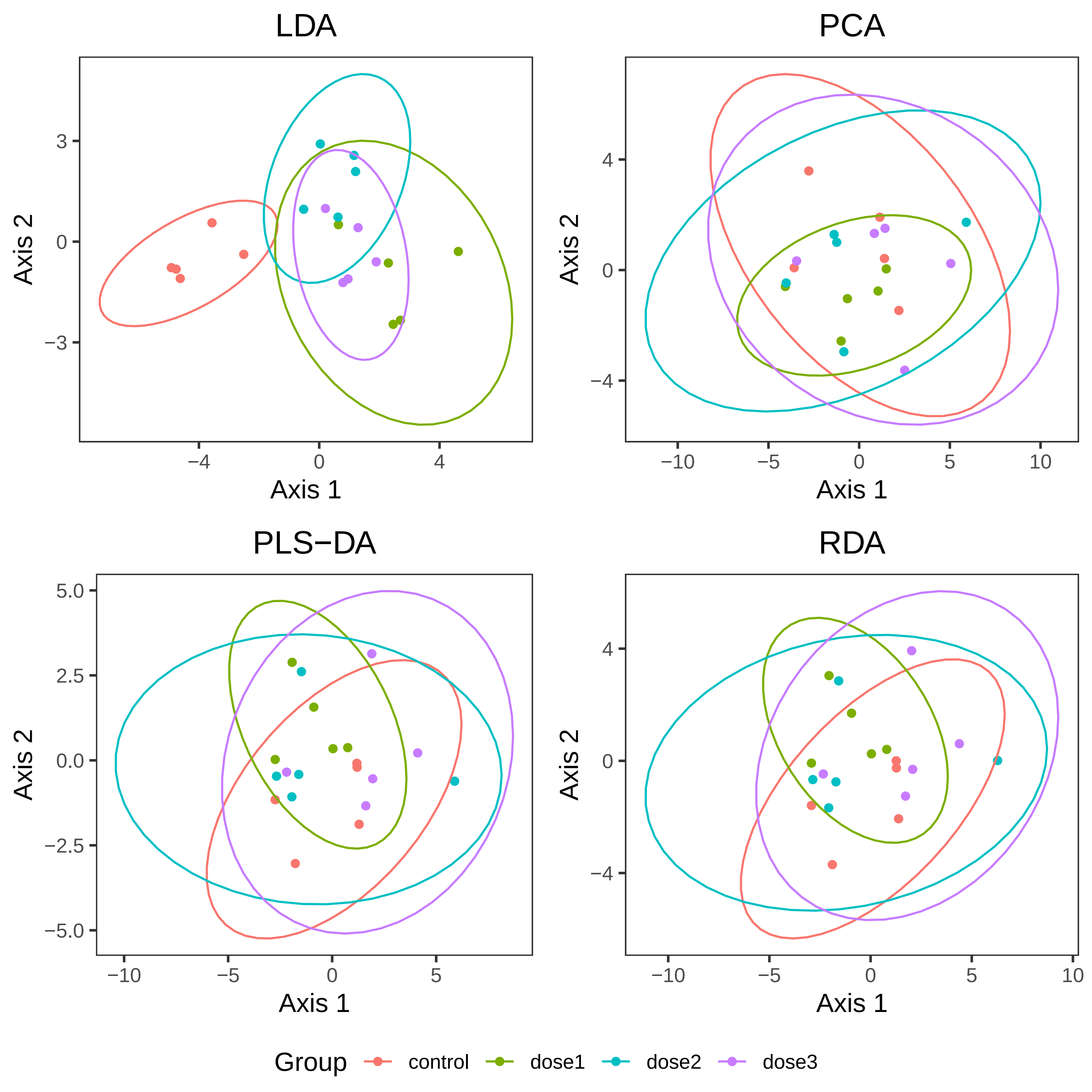

LDA
PCA
PLS−DA
RDA
Axis 1
Axis 2
3
0
−3
−4
0
4
−10
−5
5
10
5.0
2.5
0.0
−2.5
−5.0
Group
control
dose1
dose2
dose3

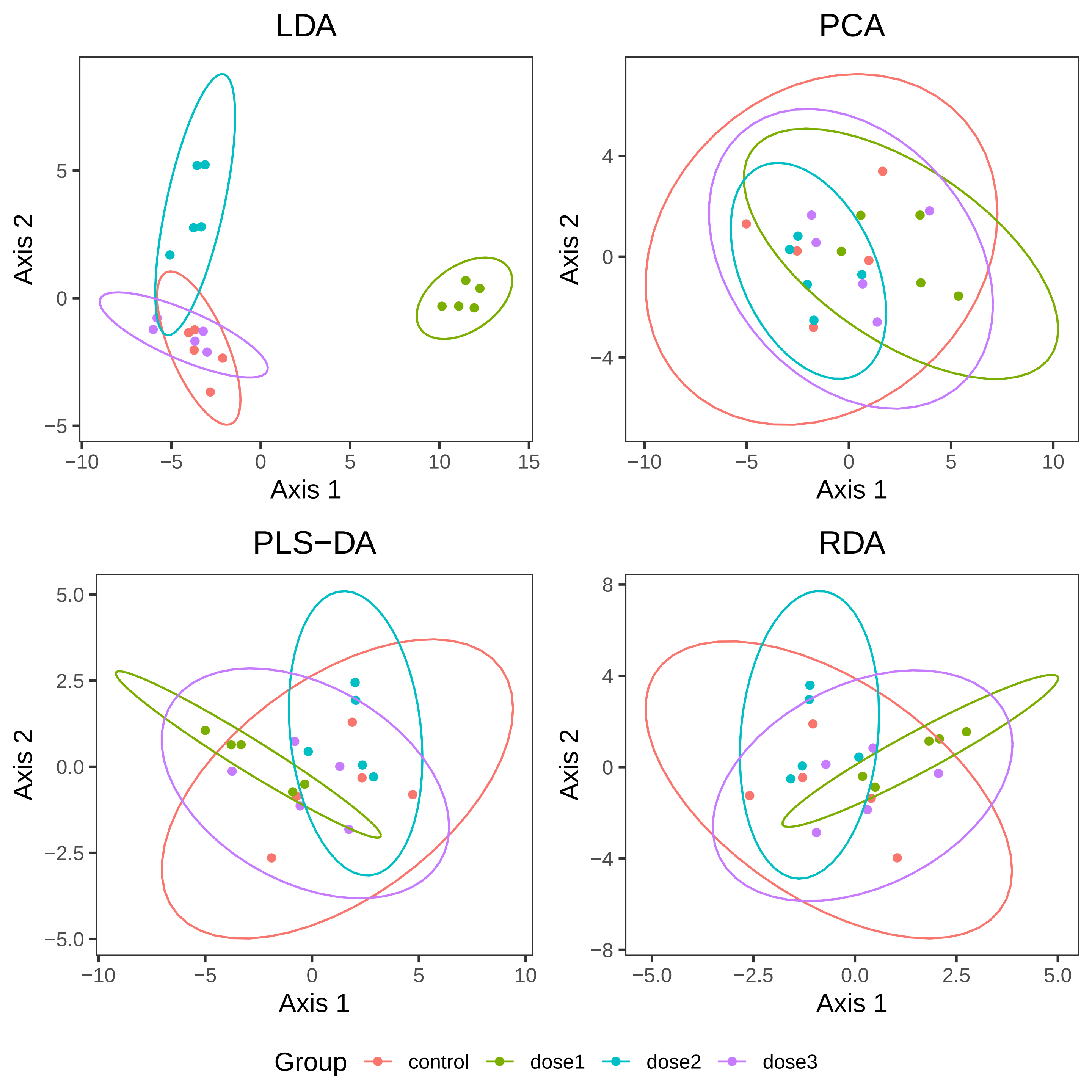

LDA
PCA
PLS−DA
RDA
Axis 1
Axis 2
Group
control
dose1
dose2
dose3

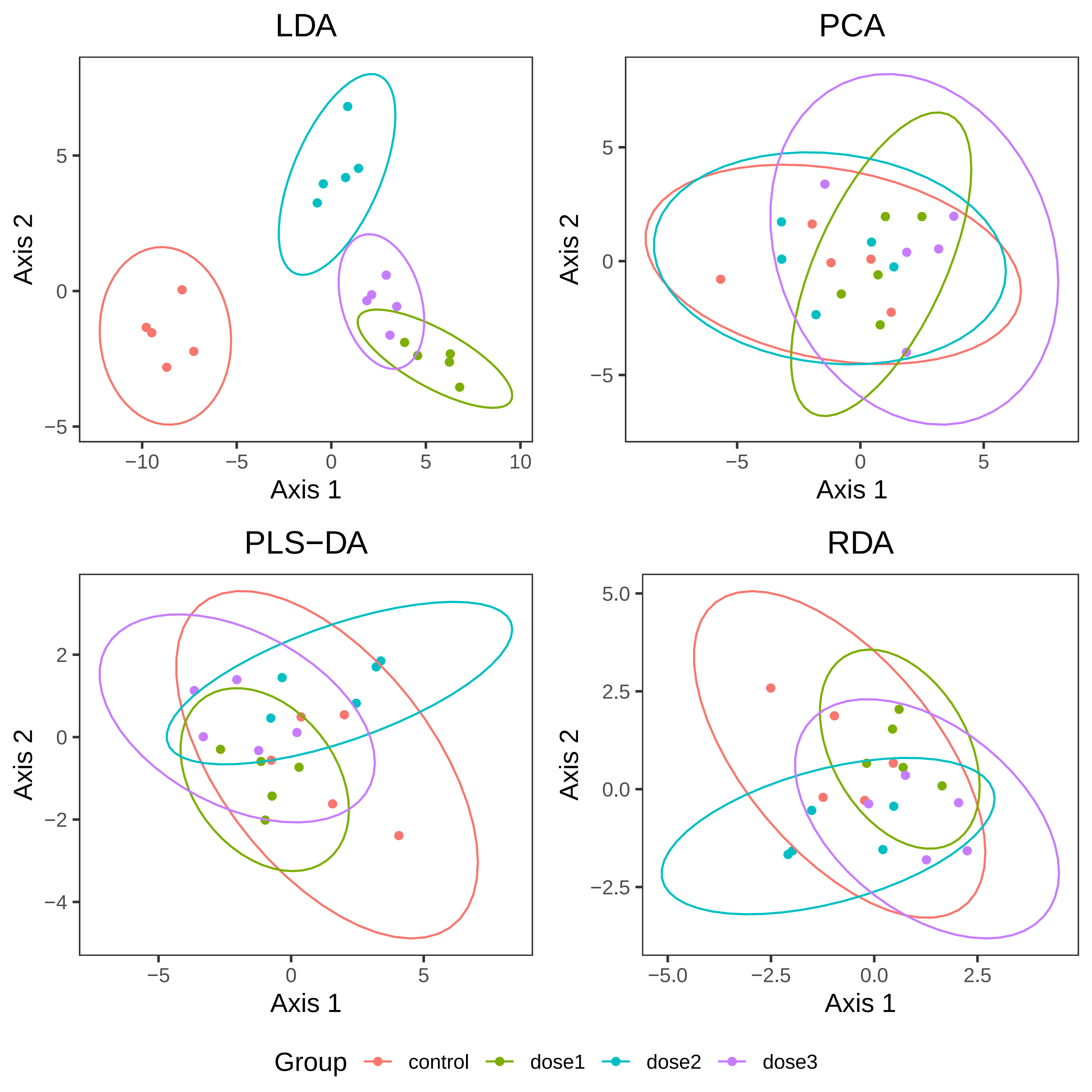
LDA
PCA
PLS−DA
RDA
Axis 1
Axis 2
Group
control
dose1
dose2
dose3

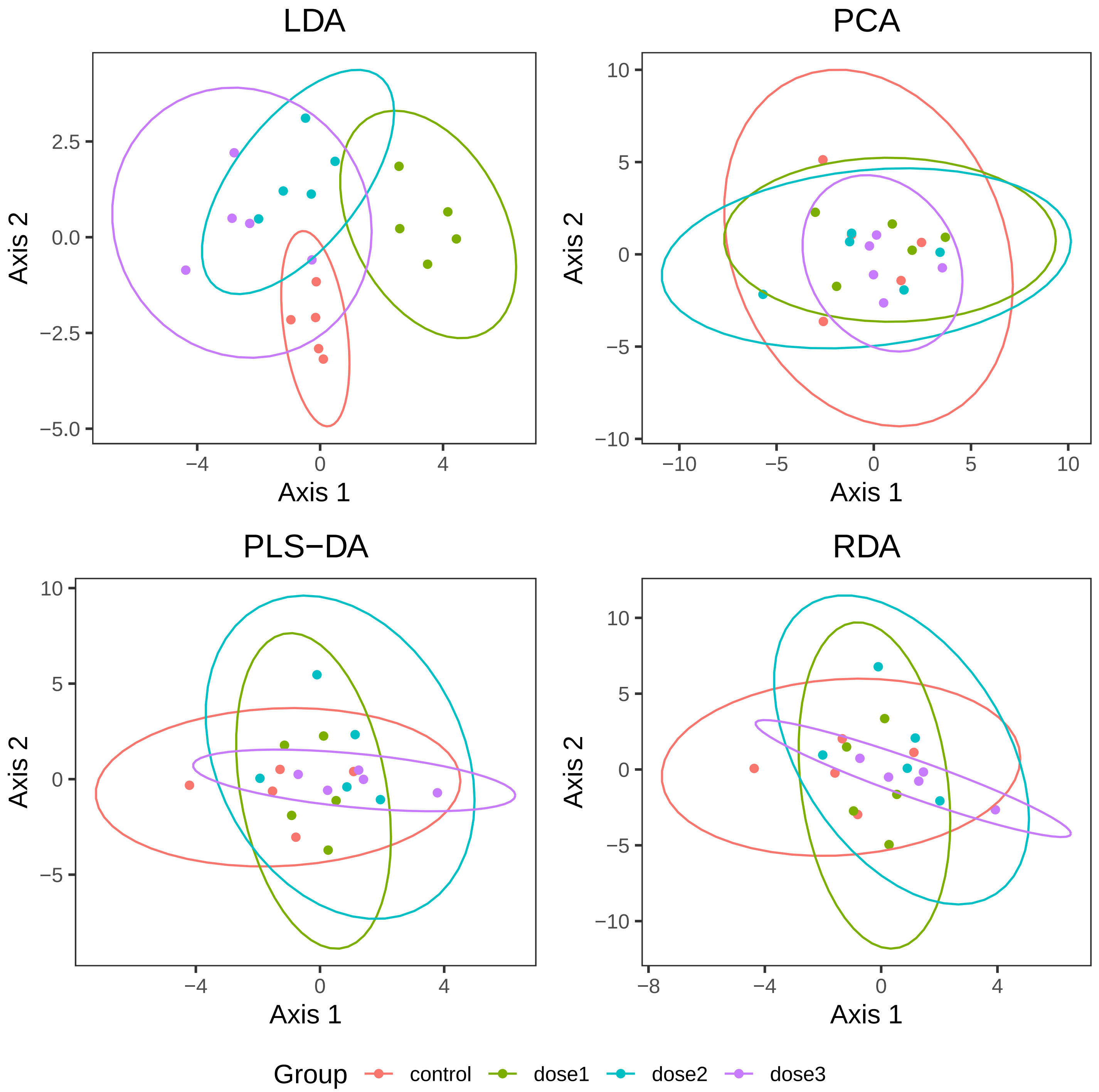
LDA
PCA
PLS−DA
RDA
Axis 1
Axis 2
2.5
0.0
−2.5
−5.0
−4
0
4
10
5
0
−5
−10
−10
−5
0
5
10
−8
Group
control
dose1
dose2
dose3

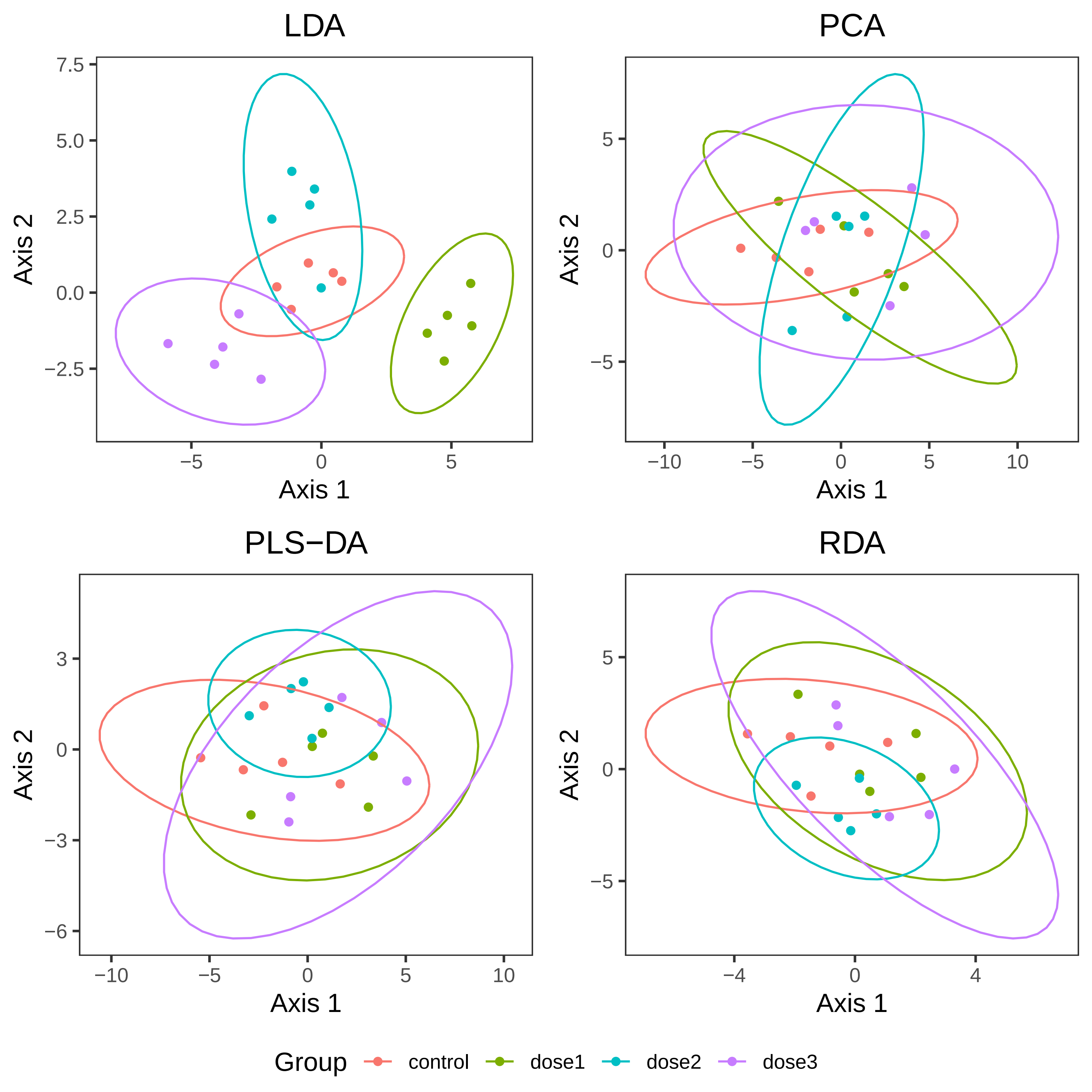

LDA
PCA
PLS−DA
RDA
Axis 1
Axis 2
7.5
5.0
2.5
0.0
−2.5
−5
0
5
−10
10
3
−3
−6
−4
4
Group
control
dose1
dose2
dose3

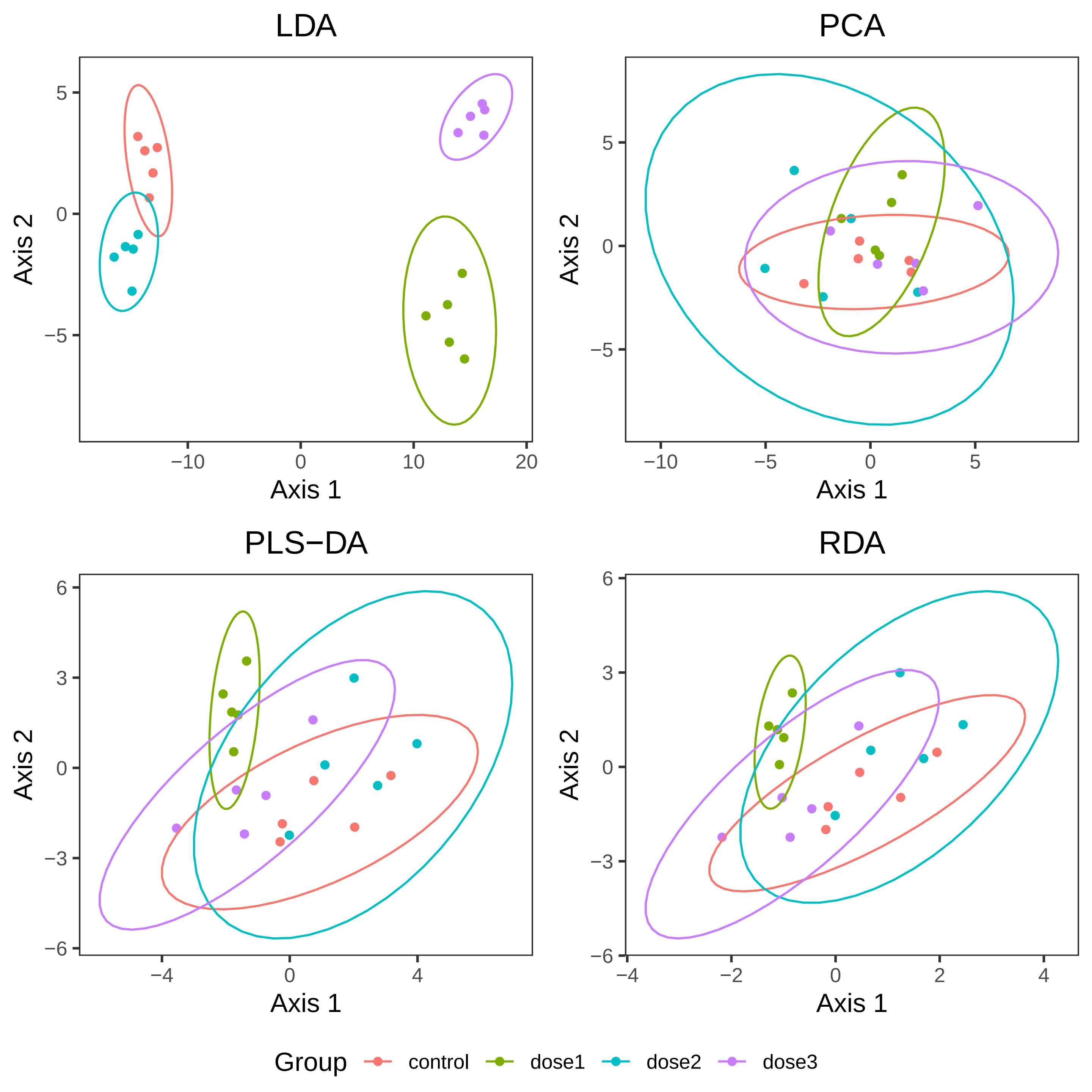

LDA
PCA
PLS−DA
RDA
Axis 1
Axis 2
Group
control
dose1
dose2
dose3

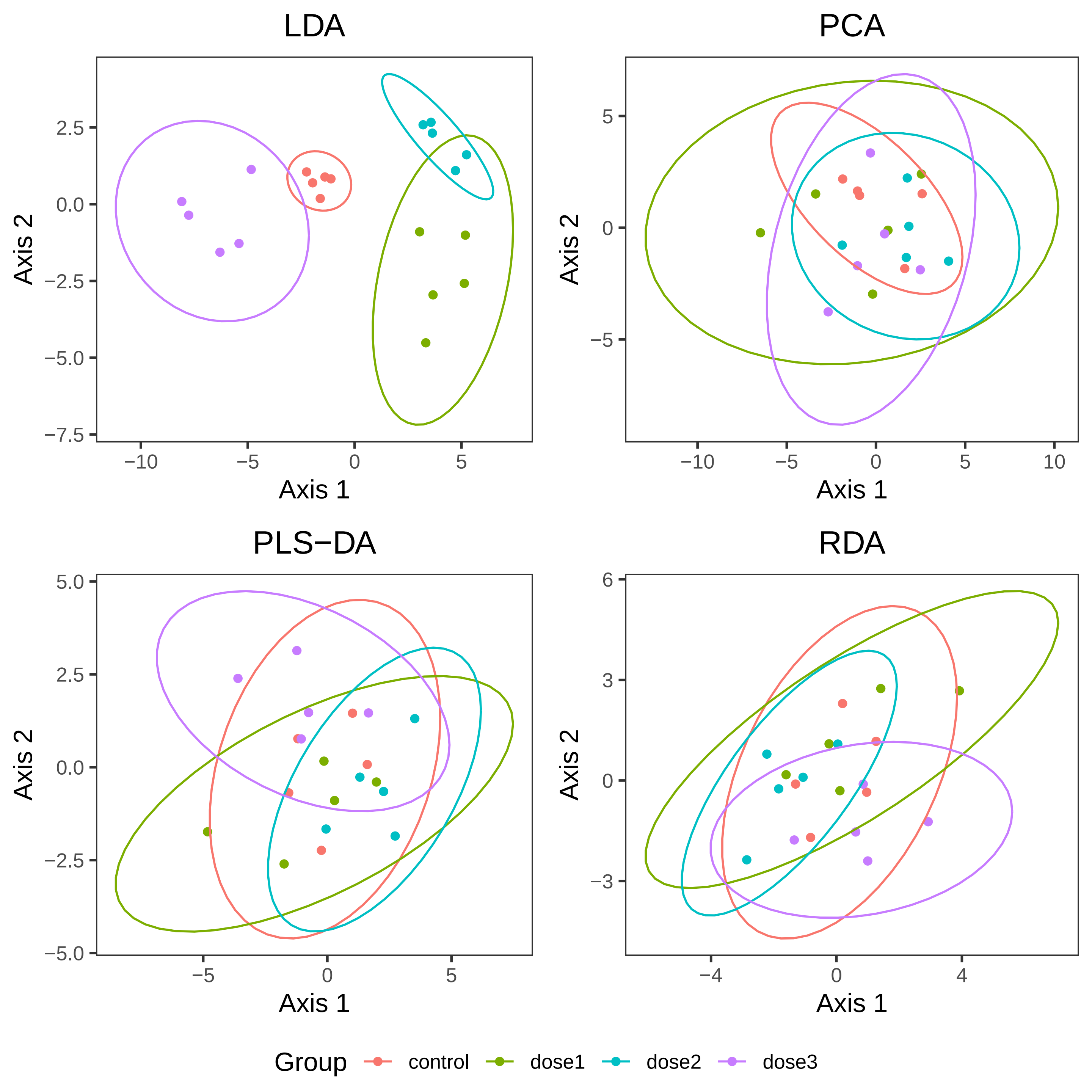

LDA
PCA
PLS−DA
RDA
Axis 1
Axis 2
Group
control
dose1
dose2
dose3

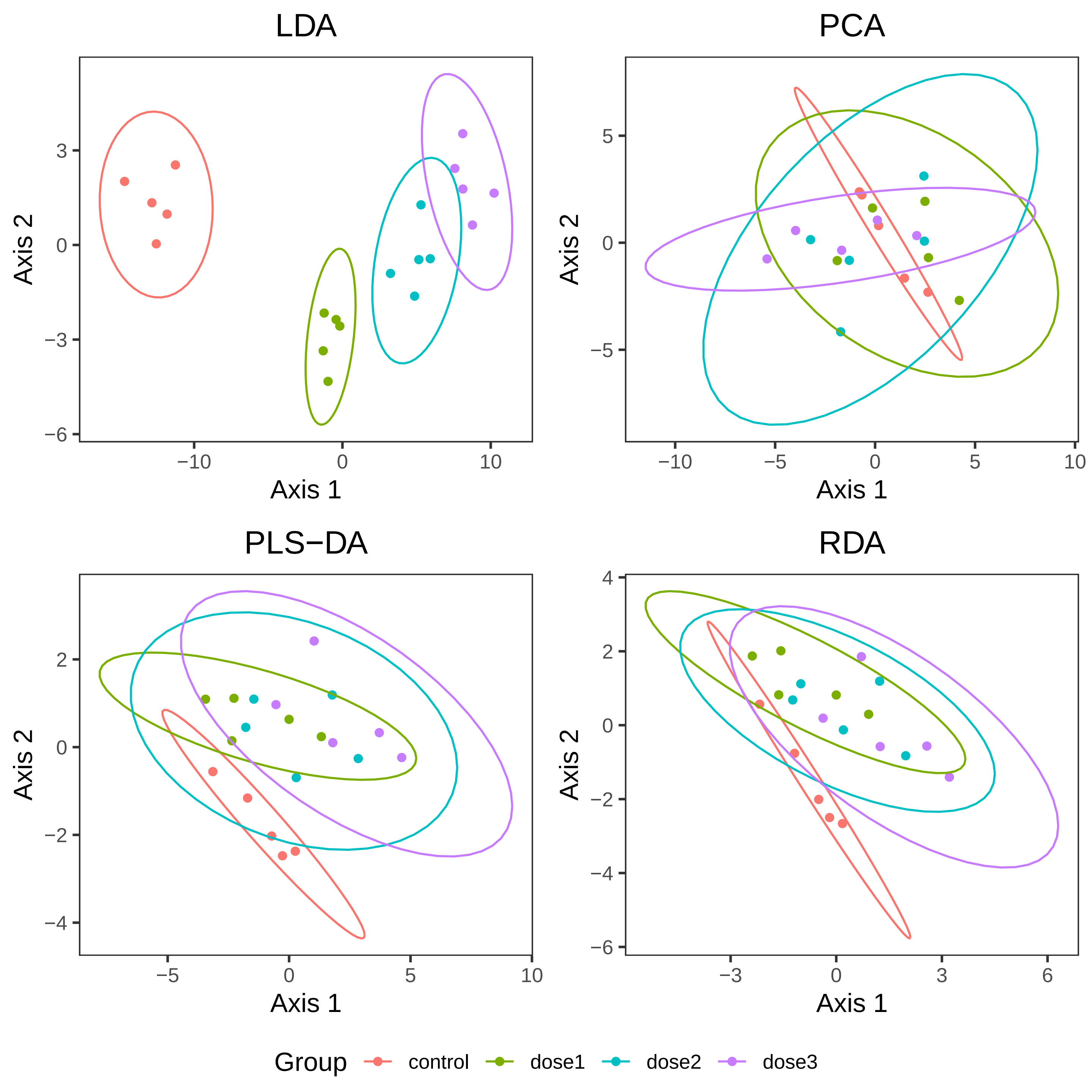

LDA
PCA
PLS−DA
RDA
Axis 1
Axis 2
3
0
−3
−6
−10
10
5
−5
2
−2
−4
4
6
Group
control
dose1
dose2
dose3

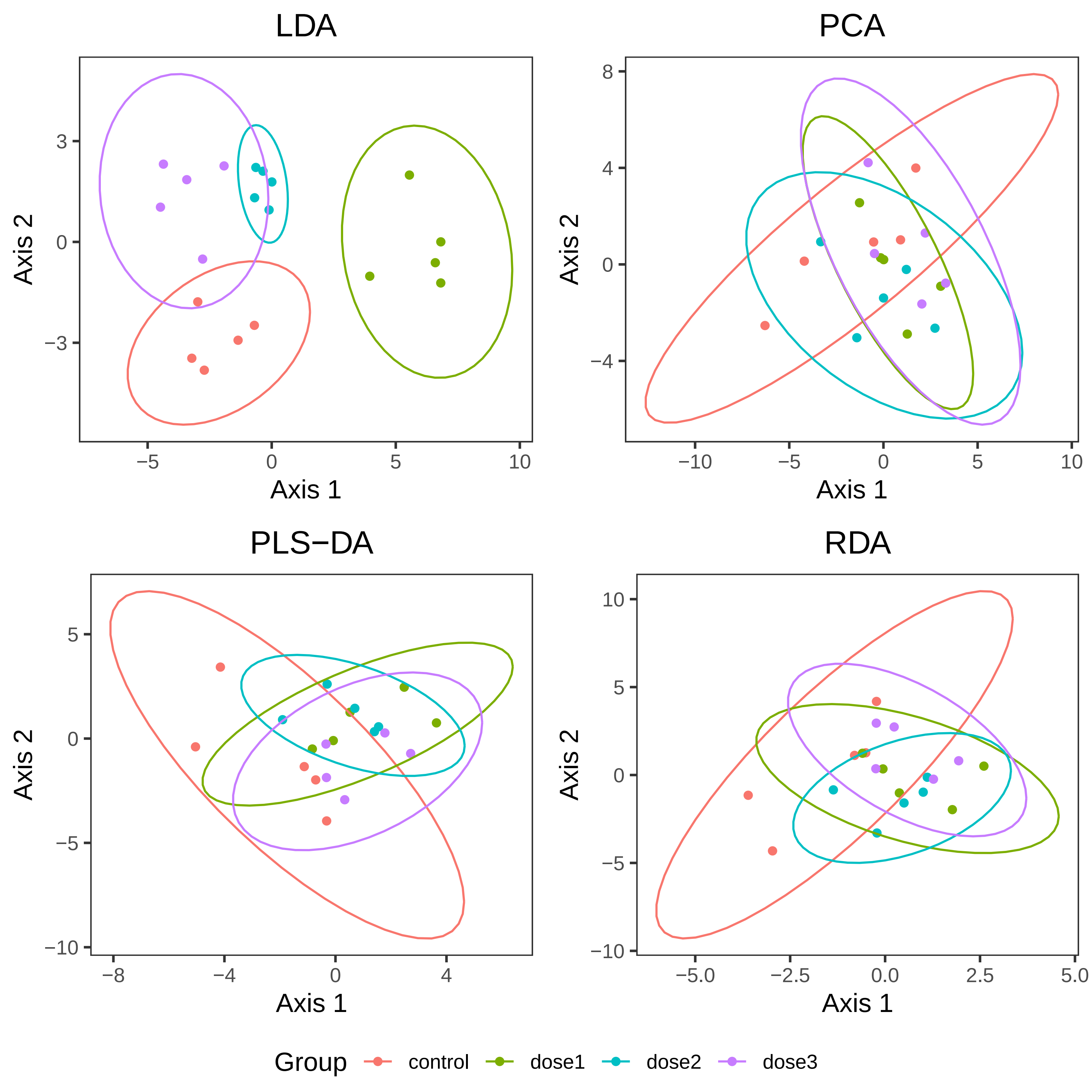
LDA
PCA
PLS−DA
RDA
Axis 1
Axis 2
3
0
−3
−5
5
10
8
4
−4
−10
−8
10
5.0
2.5
0.0
−2.5
−5.0
Group
control
dose1
dose2
dose3

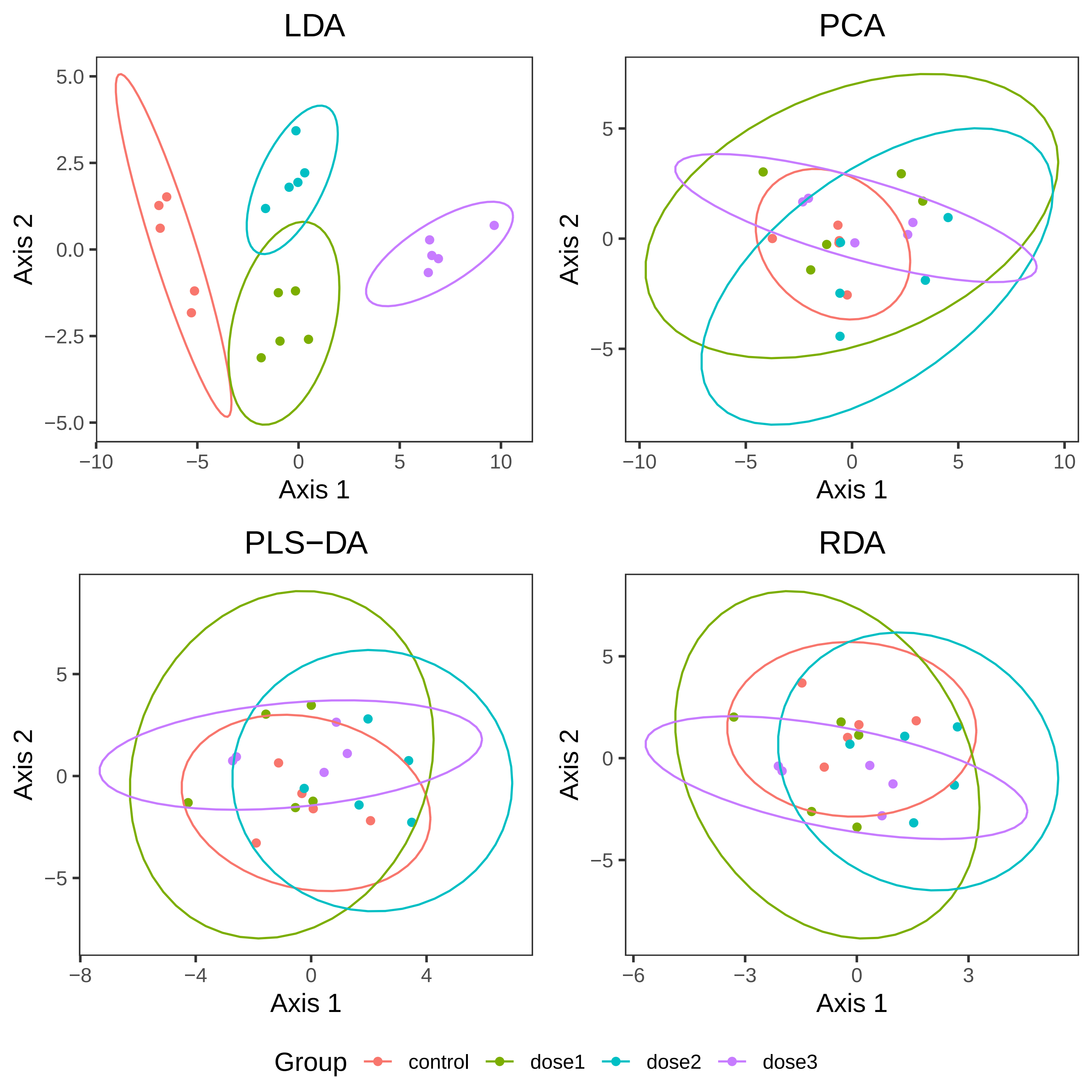
LDA
PCA
PLS−DA
RDA
Axis 1
Axis 2
5.0
2.5
0.0
−2.5
−5.0
−10
−5
0
5
10
−8
−4
4
−6
−3
3
Group
control
dose1
dose2
dose3

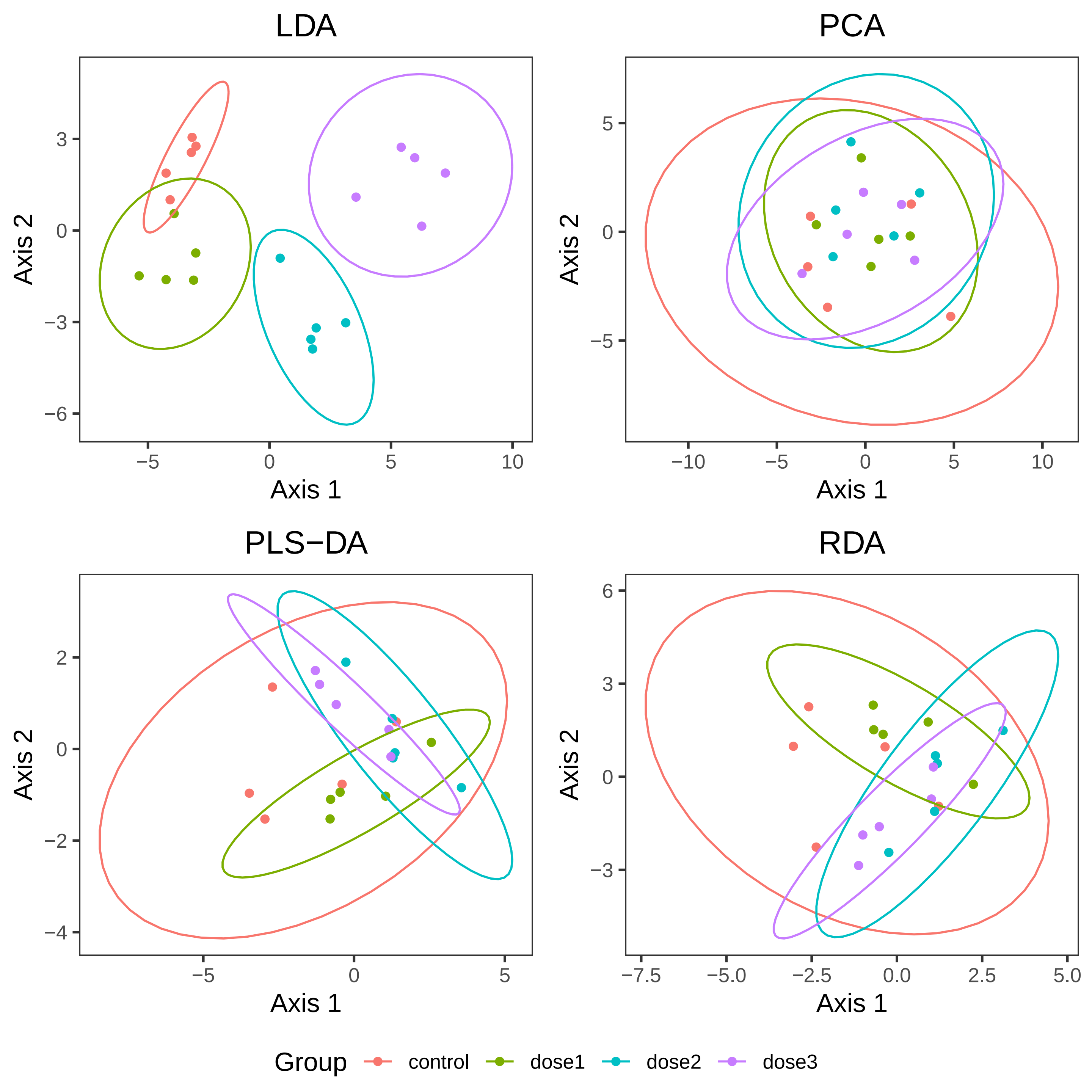

LDA
PCA
PLS−DA
RDA
Axis 1
Axis 2
Group
control
dose1
dose2
dose3

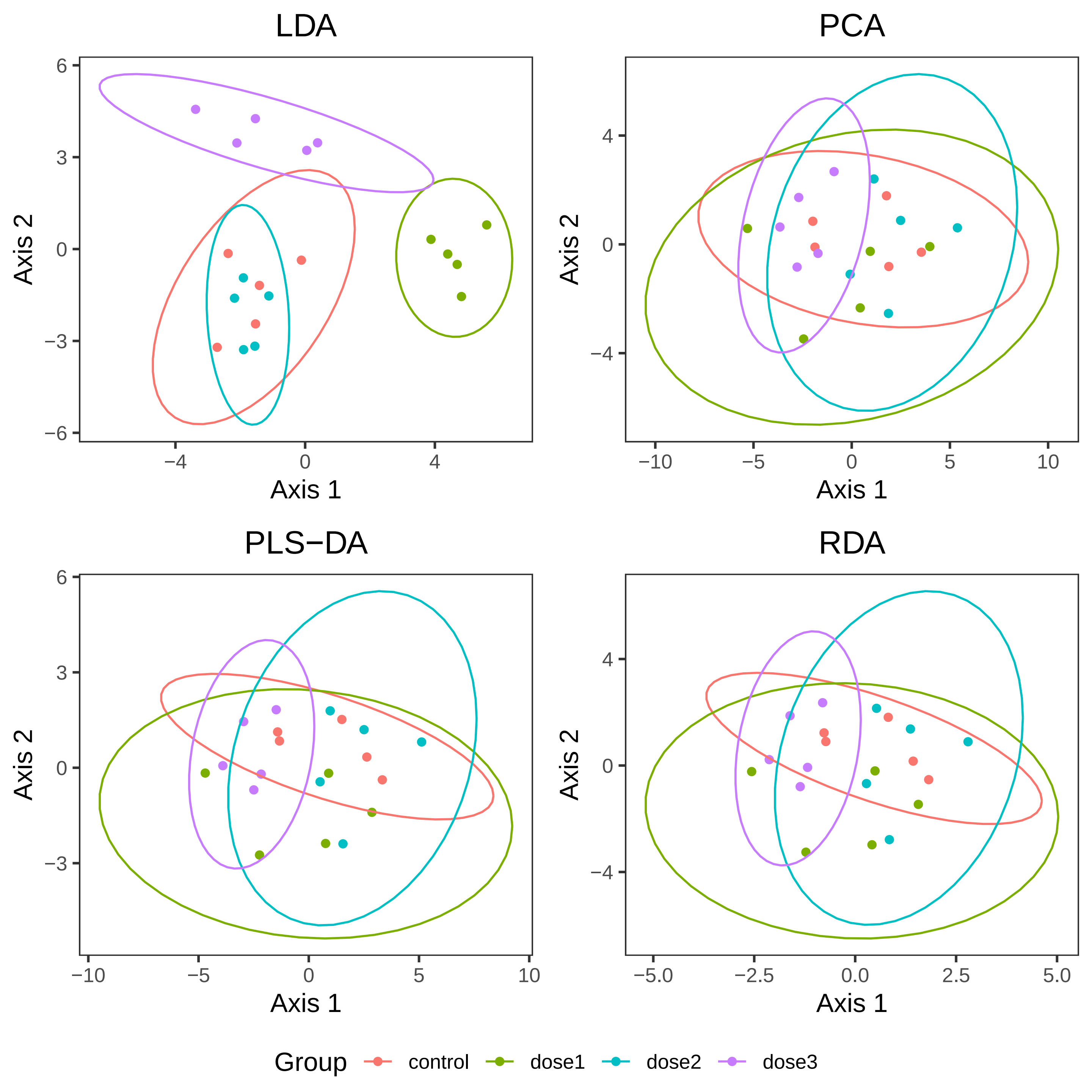
LDA
PCA
PLS−DA
RDA
Axis 1
Axis 2
Group
control
dose1
dose2
dose3

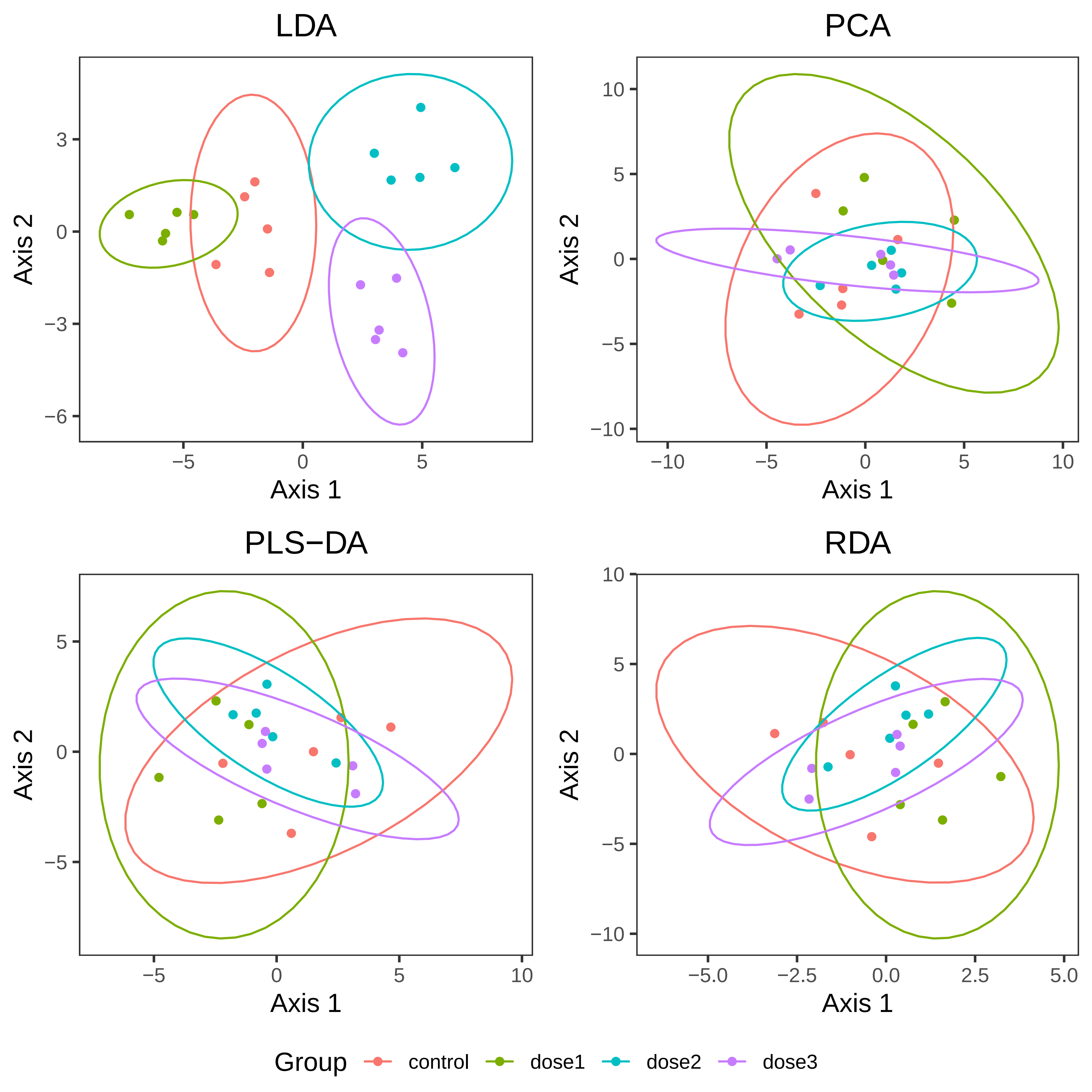

LDA
PCA
PLS−DA
RDA
Axis 1
Axis 2
Group
control
dose1
dose2
dose3

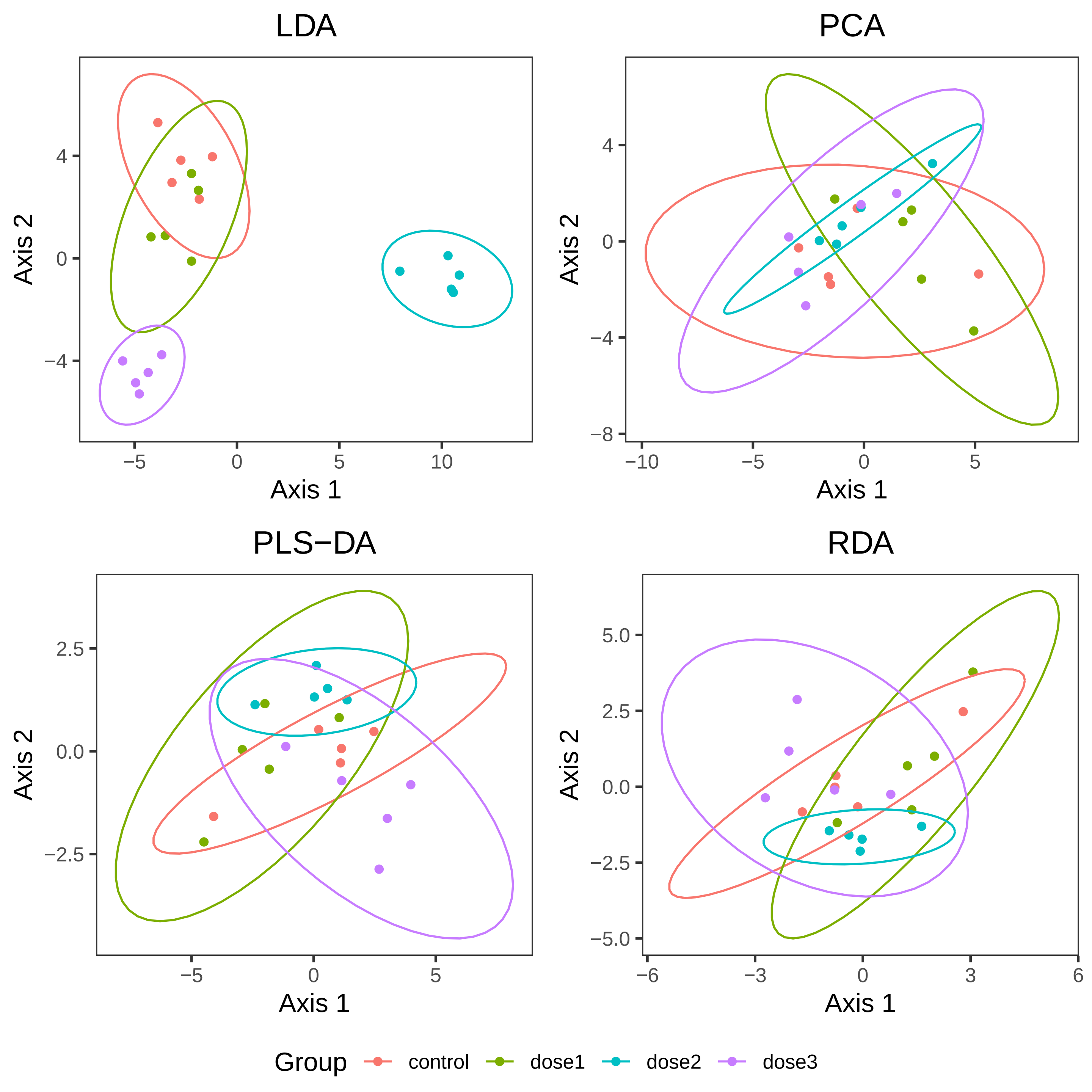
LDA
PCA
PLS−DA
RDA
Axis 1
Axis 2
Group
control
dose1
dose2
dose3

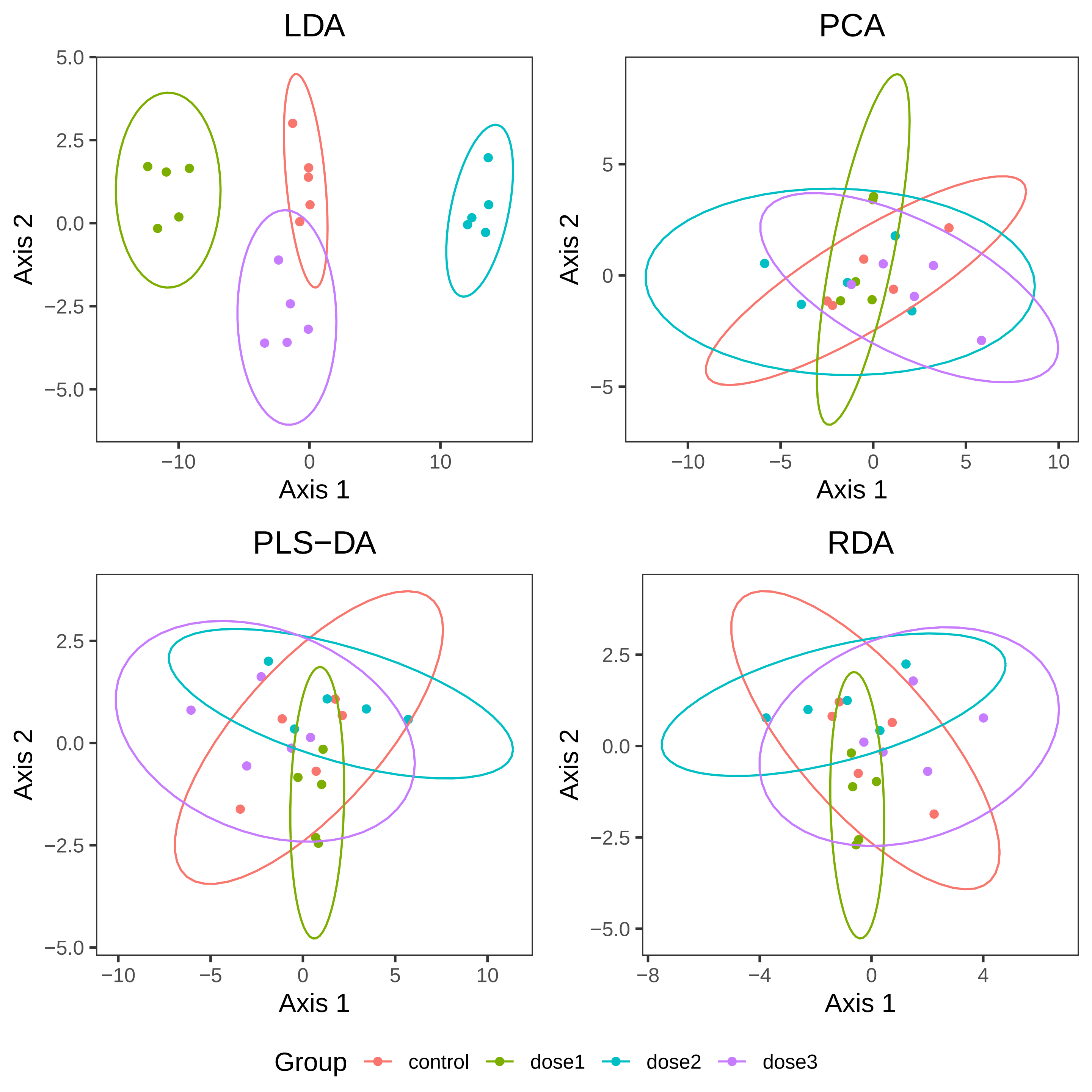

LDA
PCA
PLS−DA
RDA
Axis 1
Axis 2
5.0
2.5
0.0
−2.5
−5.0
−10
0
10
5
−5
−8
−4
4
Group
control
dose1
dose2
dose3

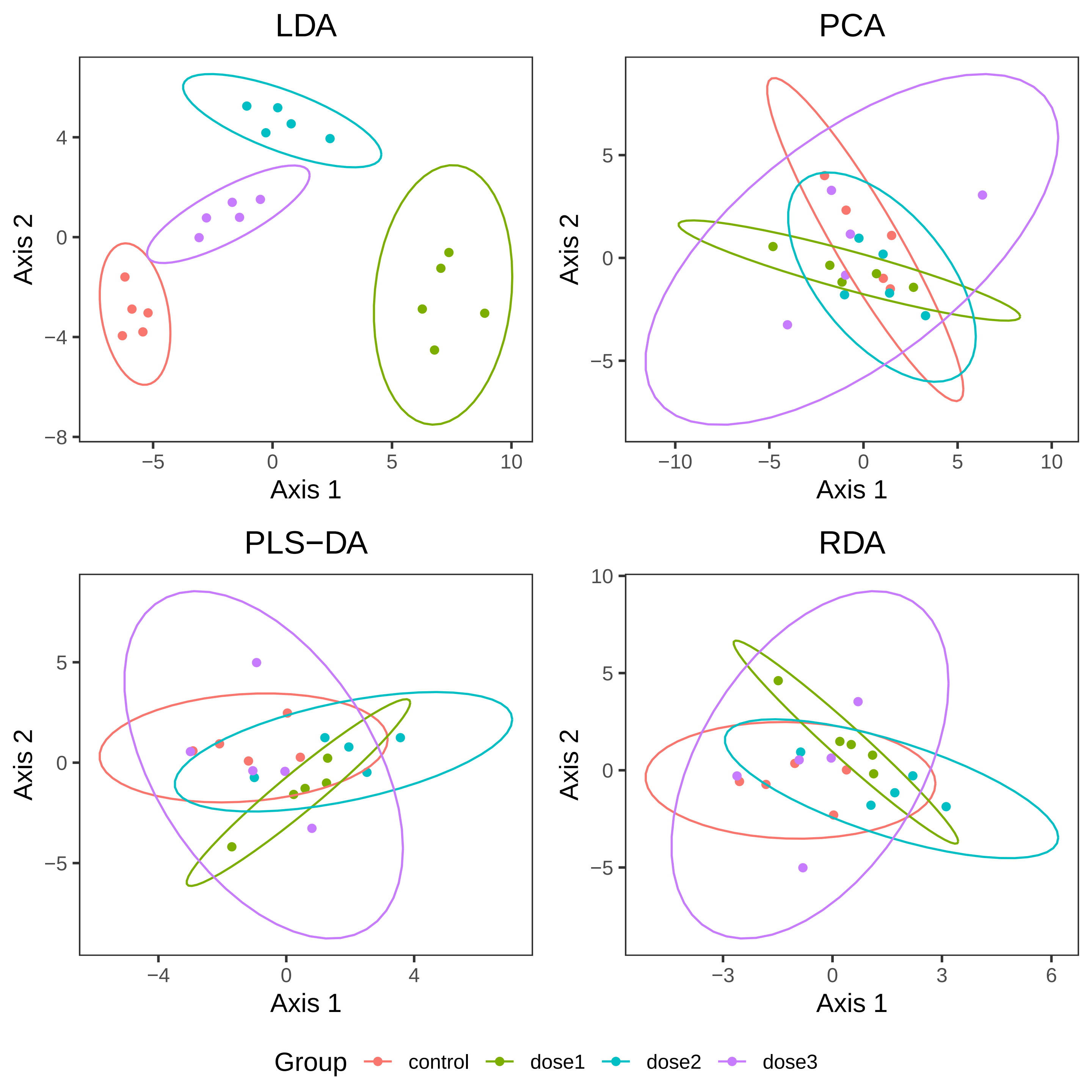
LDA
PCA
PLS−DA
RDA
Axis 1
Axis 2
4
0
−4
−8
−5
0
5
10
−10
−5
0
5
10
5
0
−5
−4
0
4
10
5
0
−5
−3
0
3
6
Group
control
dose1
dose2
dose3

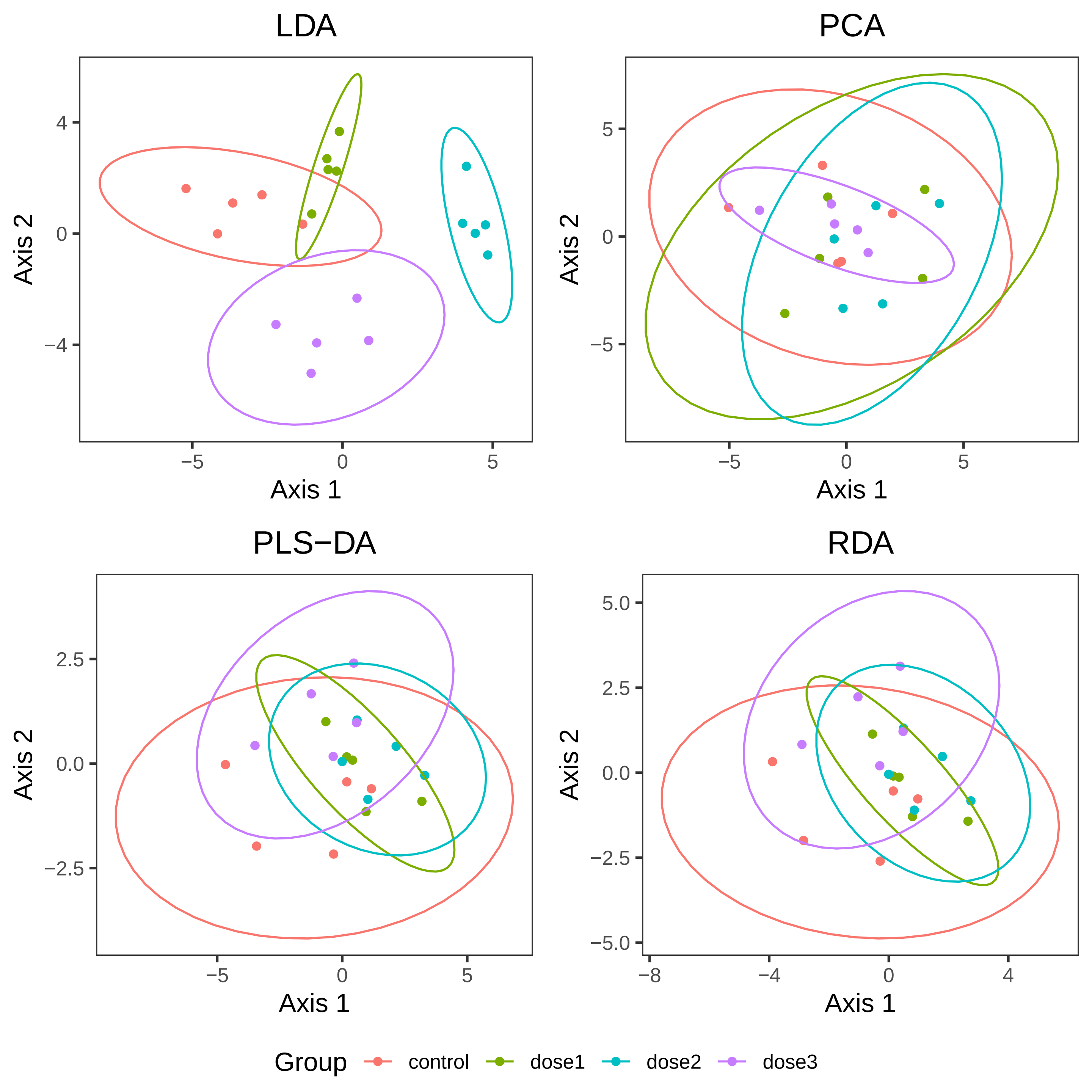

LDA
PCA
PLS−DA
RDA
Axis 1
Axis 2
−5
0
5
4
−4
2.5
0.0
−2.5
5.0
−5.0
−8
Group
control
dose1
dose2
dose3

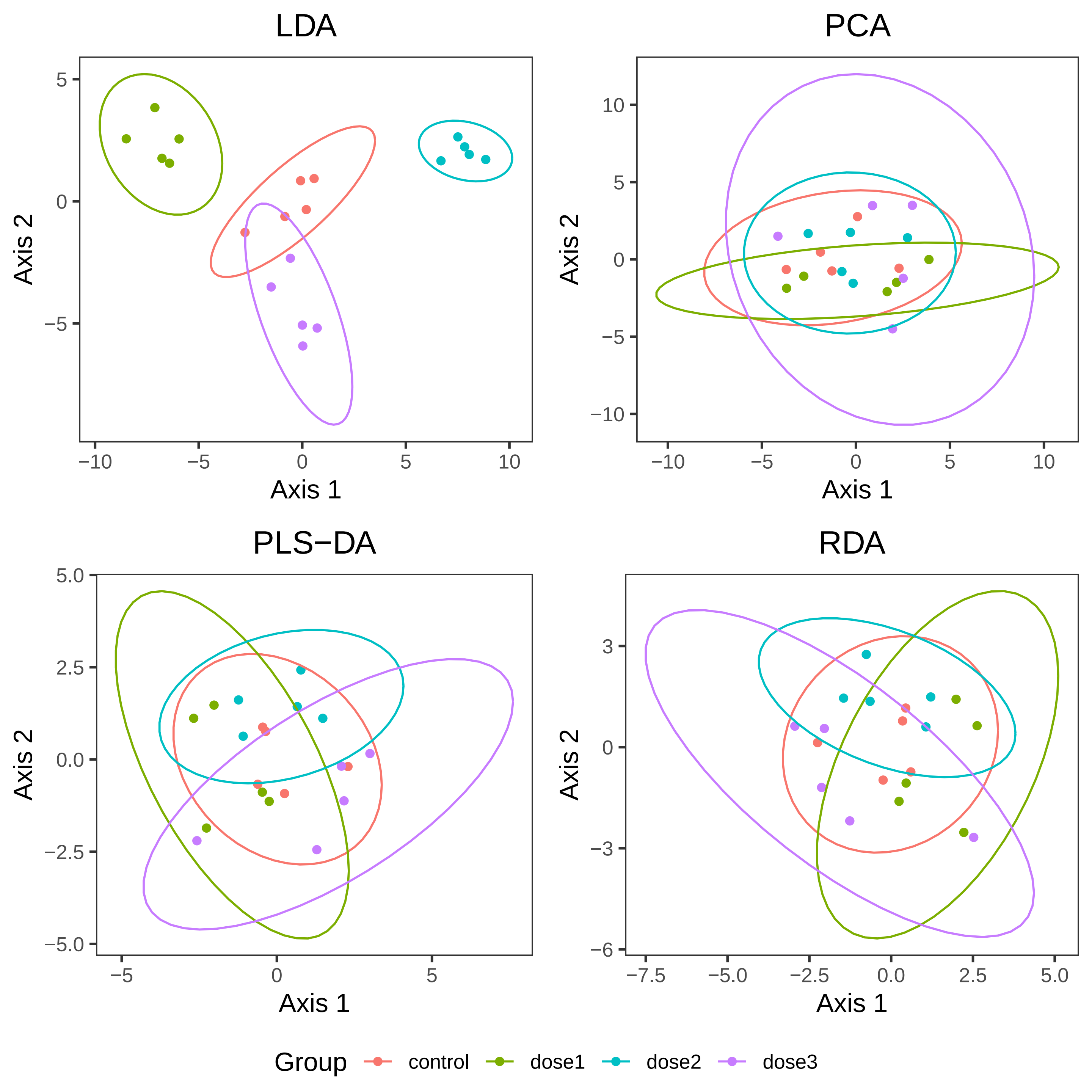
LDA
PCA
PLS−DA
RDA
Axis 1
Axis 2
Group
control
dose1
dose2
dose3

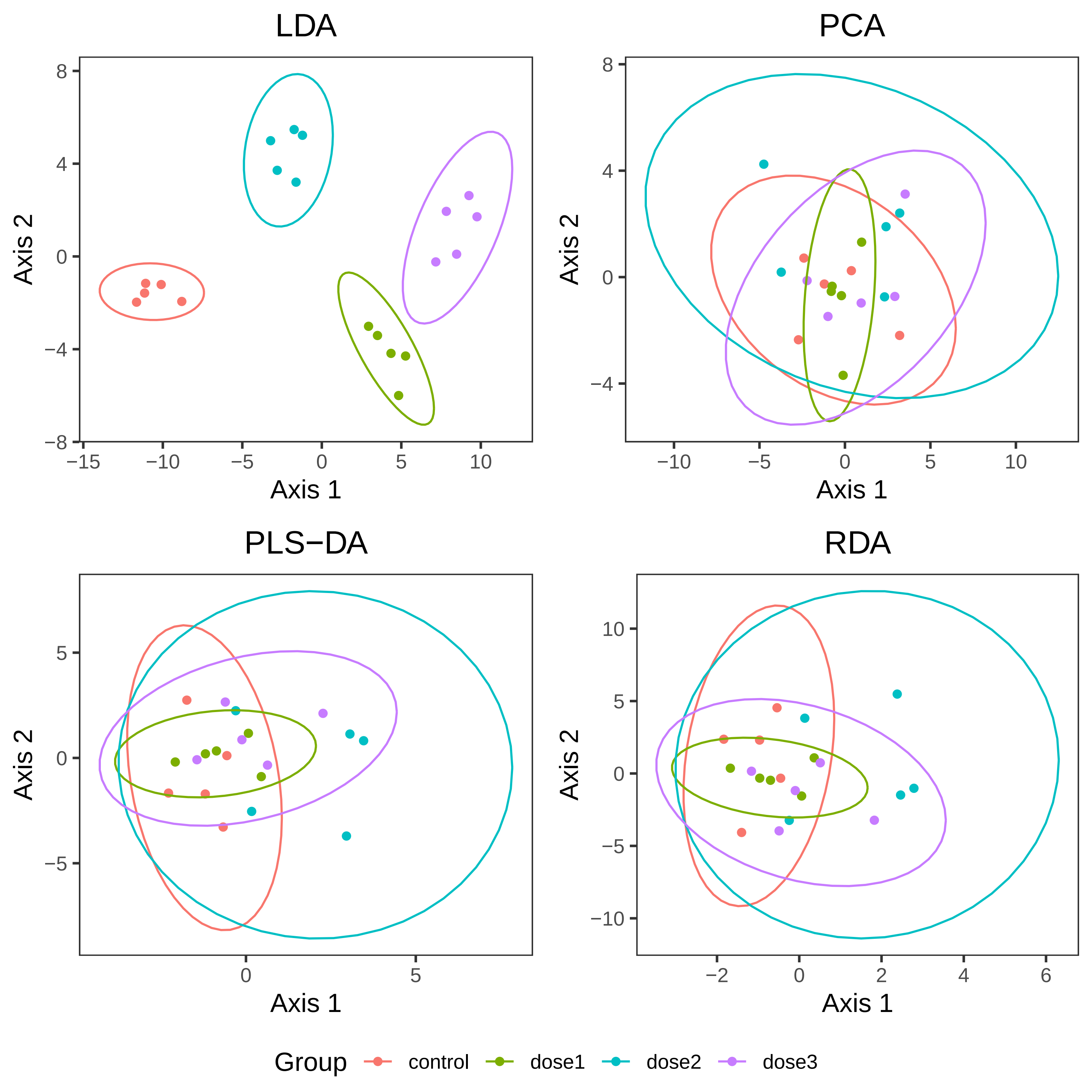
LDA
PCA
PLS−DA
RDA
Axis 1
Axis 2
8
4
0
−4
−8
−15
−10
−5
0
5
10
−4
5
−5
10
−2
2
4
6
Group
control
dose1
dose2
dose3

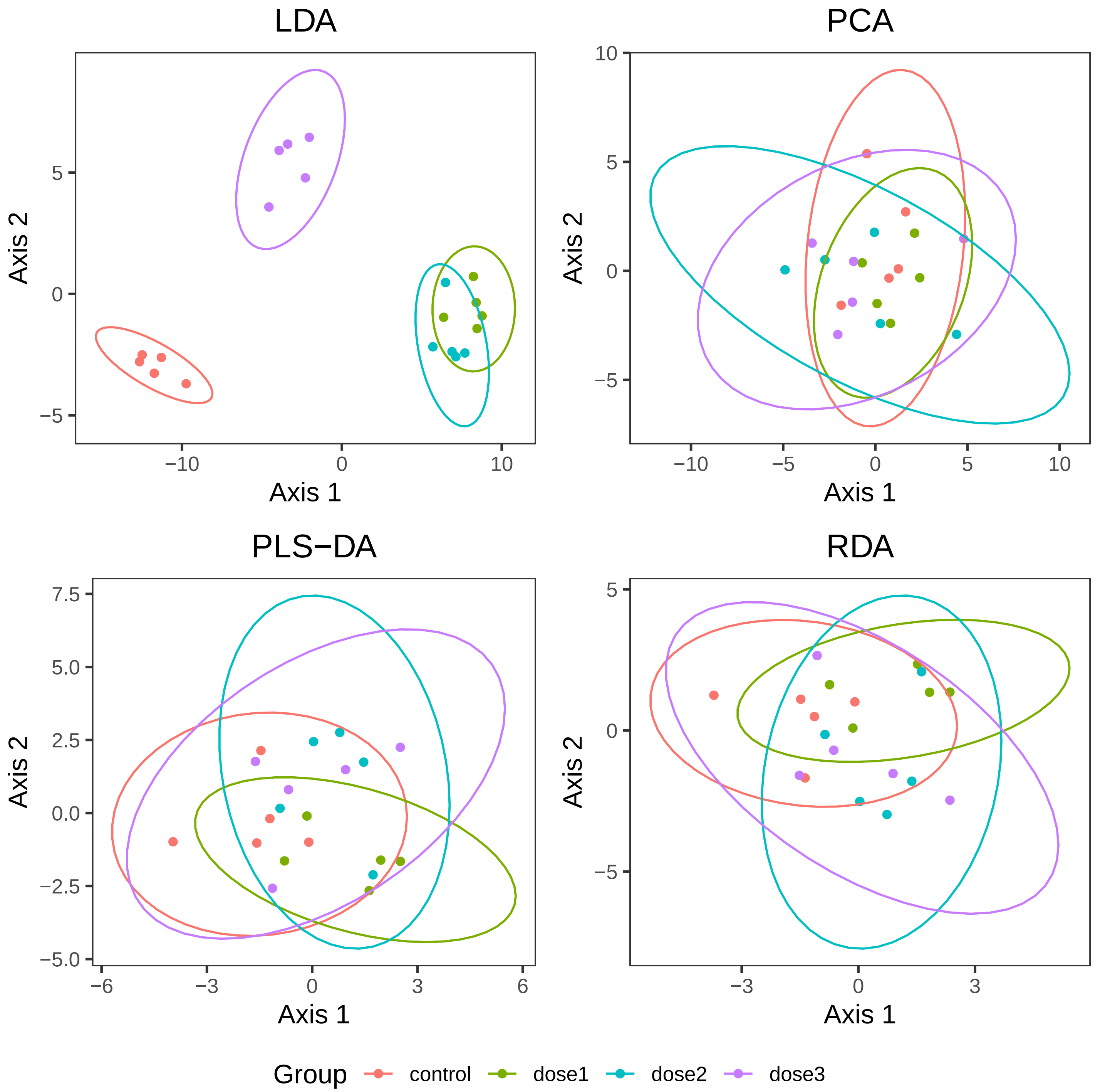

LDA
PCA
PLS−DA
RDA
Axis 1
Axis 2
Group
control
dose1
dose2
dose3

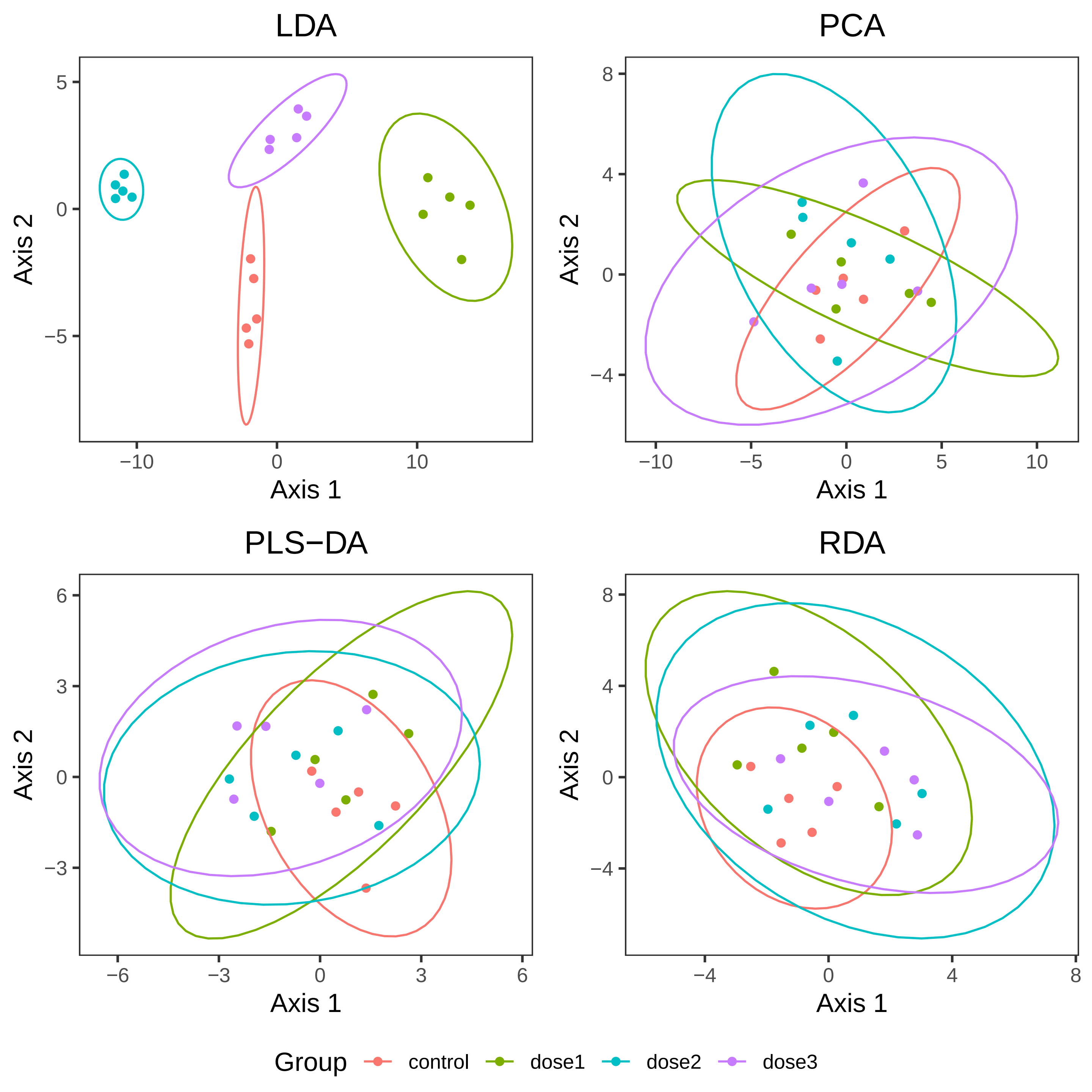

LDA
PCA
PLS−DA
RDA
Axis 1
Axis 2
Group
control
dose1
dose2
dose3

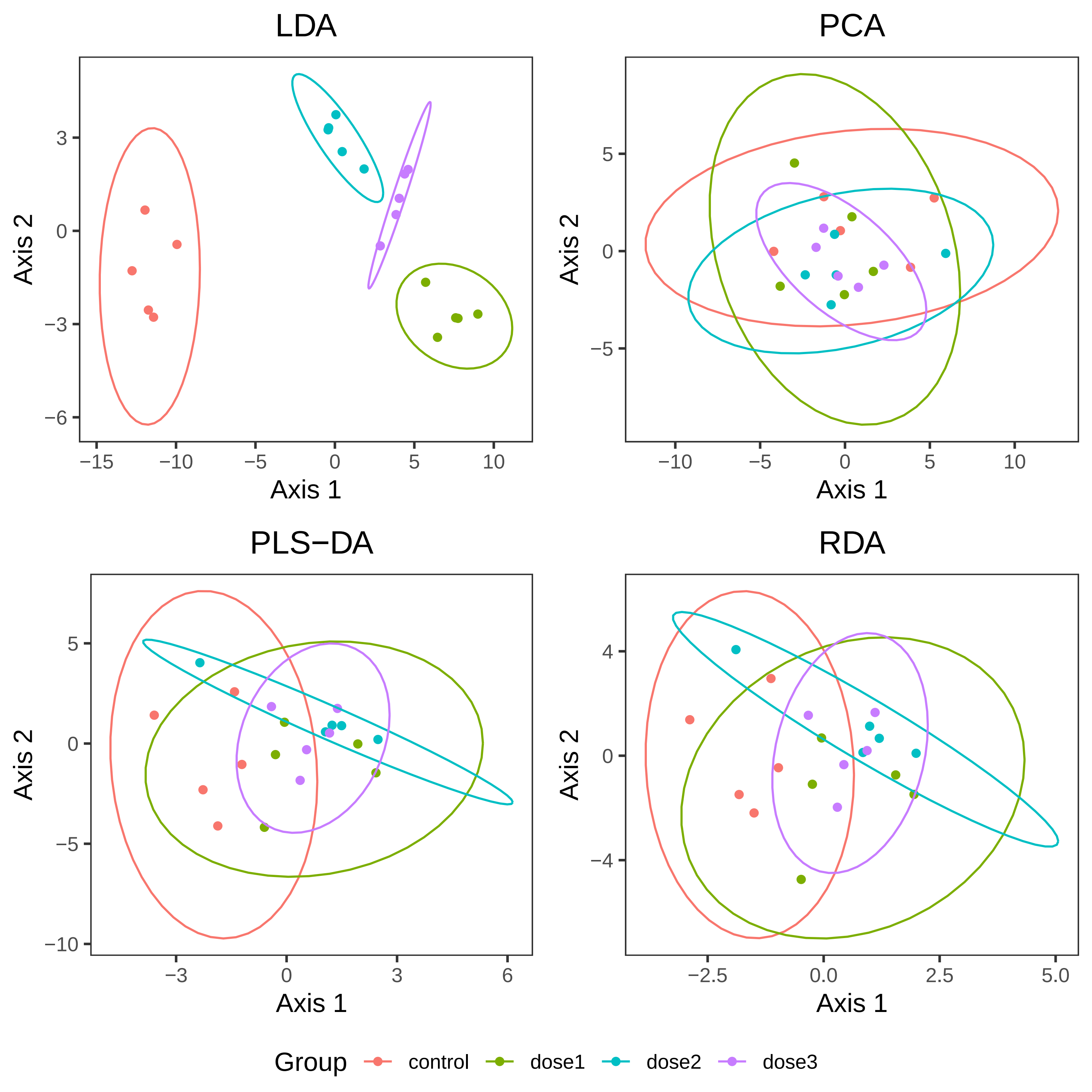
LDA
PCA
PLS−DA
RDA
Axis 1
Axis 2
Group
control
dose1
dose2
dose3

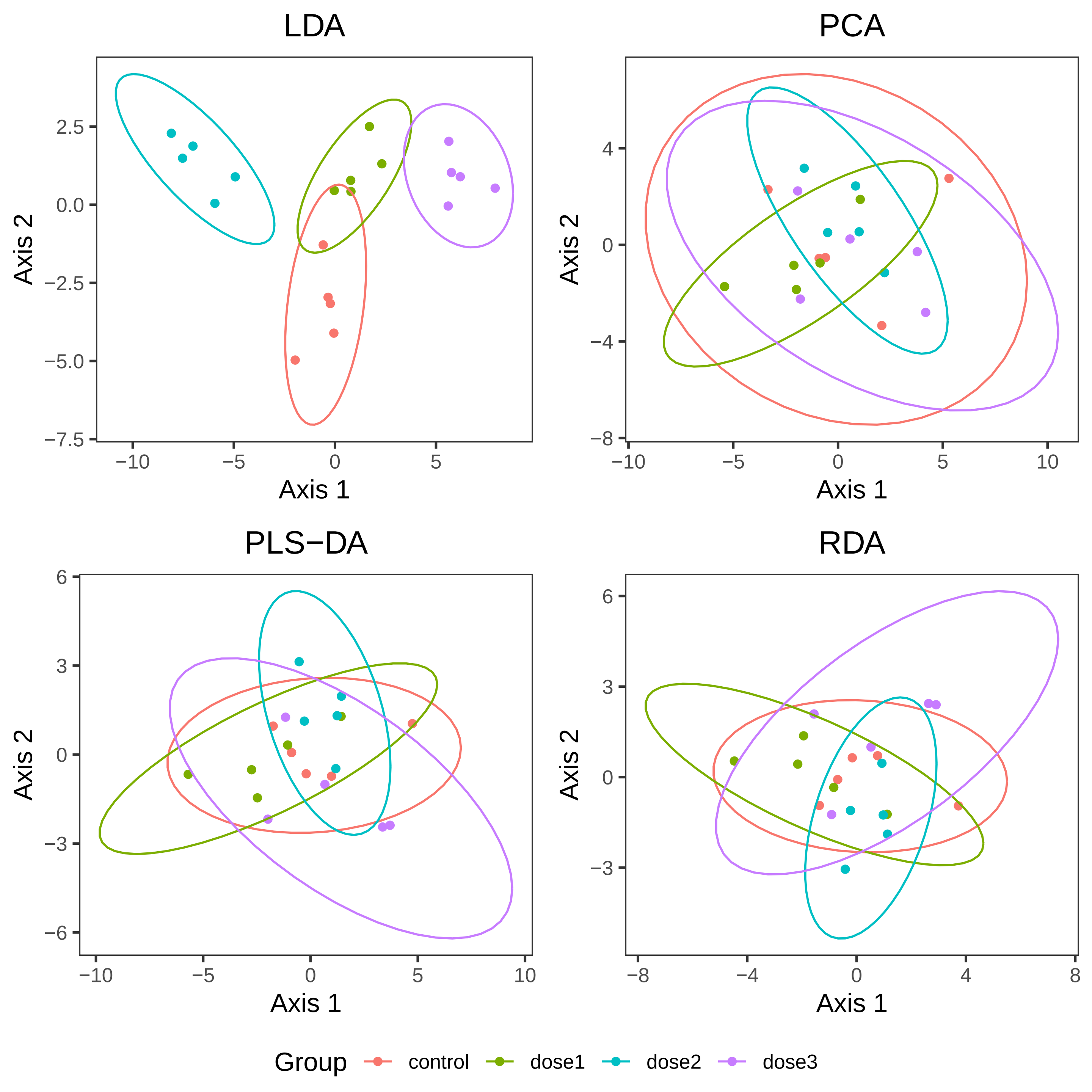

LDA
PCA
PLS−DA
RDA
Axis 1
Axis 2
2.5
0.0
−2.5
−5.0
−7.5
−10
−5
0
5
4
−4
−8
10
6
3
−3
−6
8
Group
control
dose1
dose2
dose3

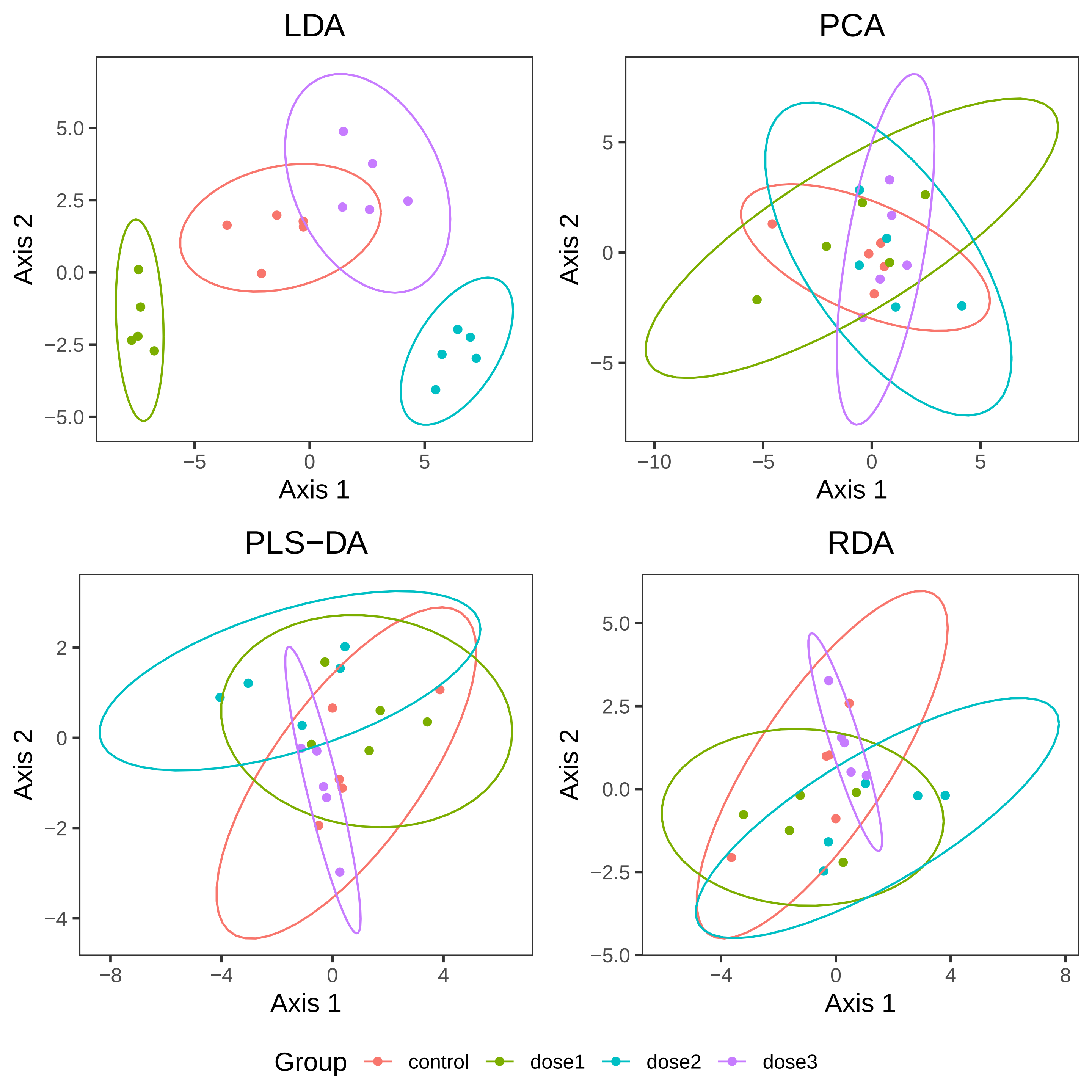
LDA
PCA
PLS−DA
RDA
Axis 1
Axis 2
Group
control
dose1
dose2
dose3

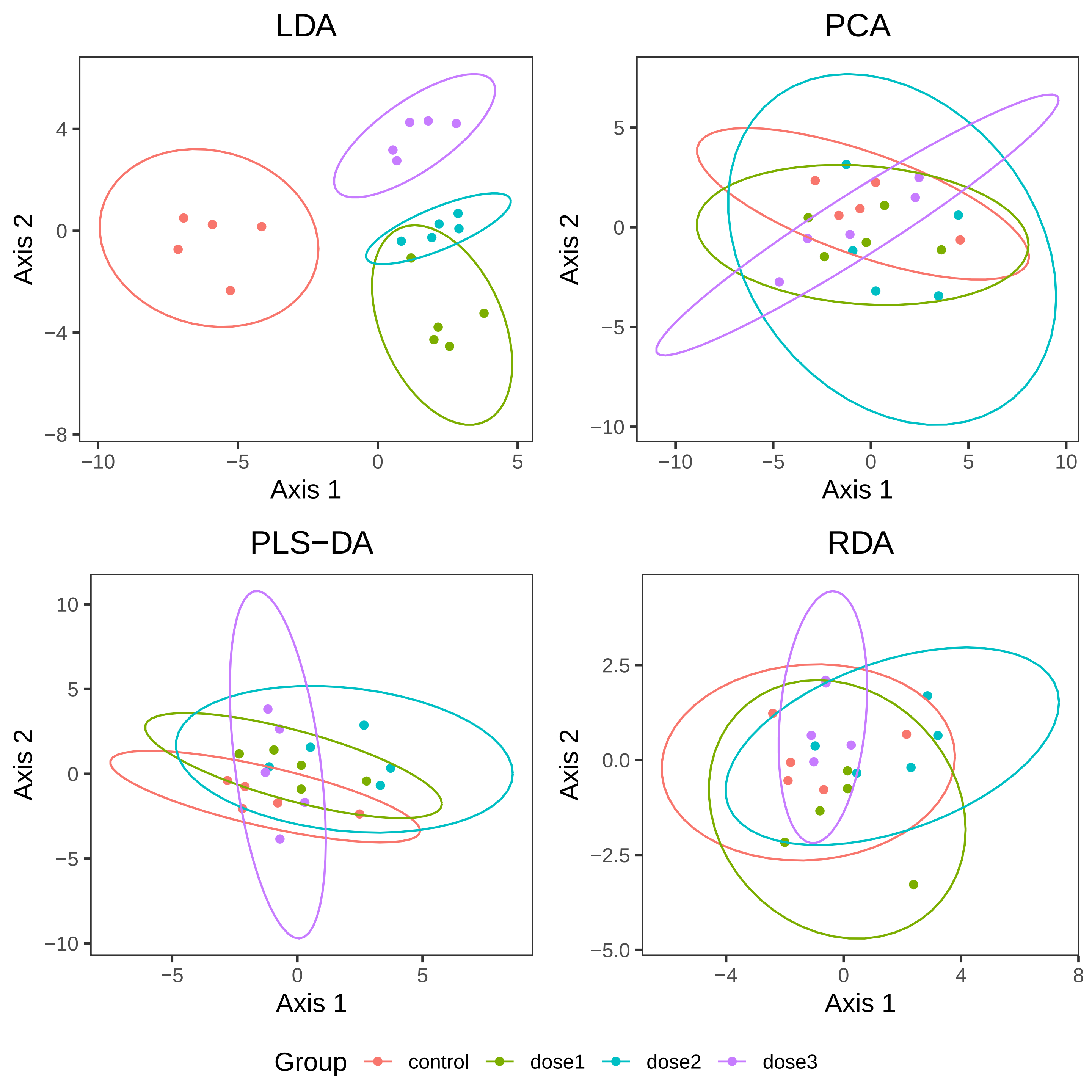

LDA
PCA
PLS−DA
RDA
Axis 1
Axis 2
Group
control
dose1
dose2
dose3

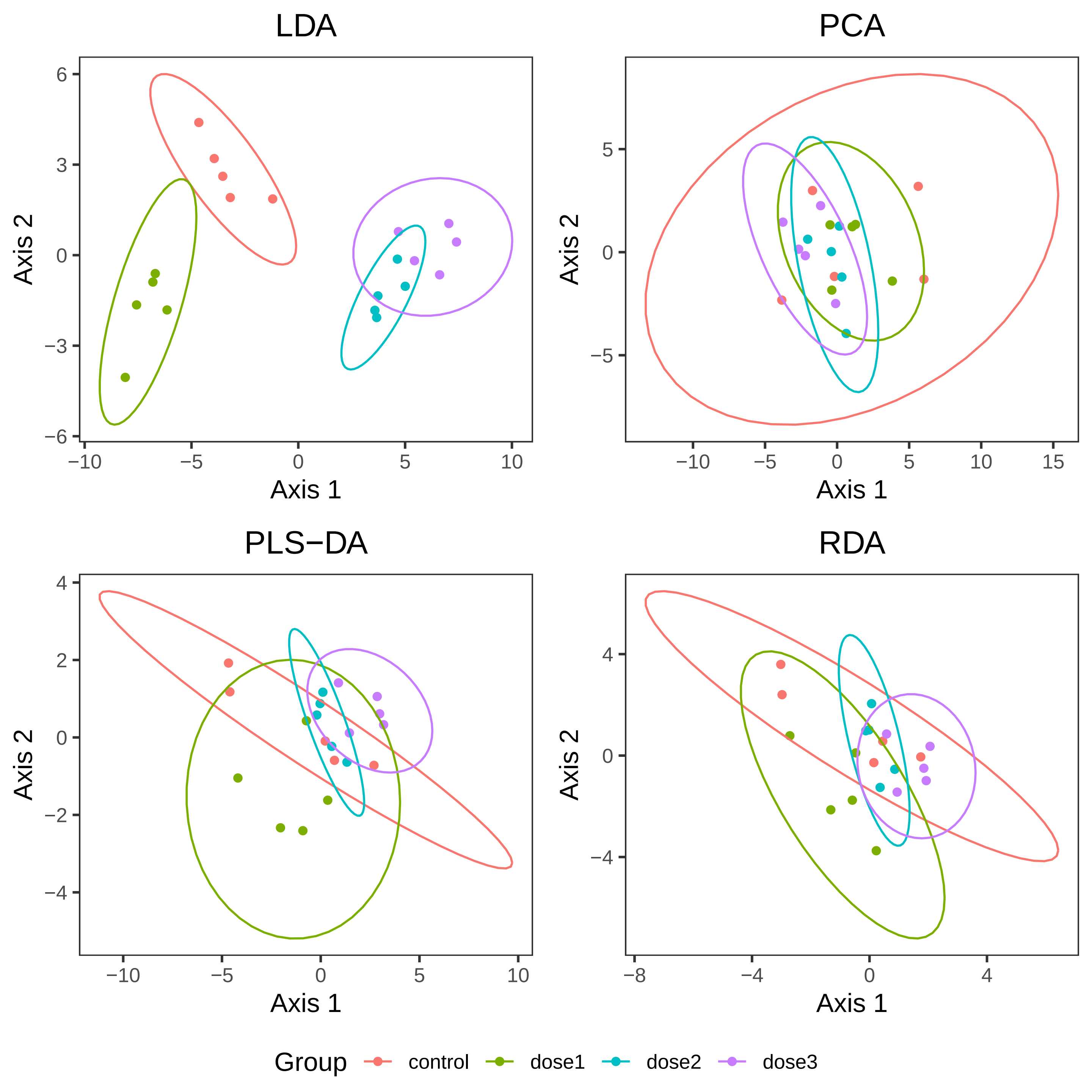

LDA
PCA
PLS−DA
RDA
Axis 1
Axis 2
Group
control
dose1
dose2
dose3

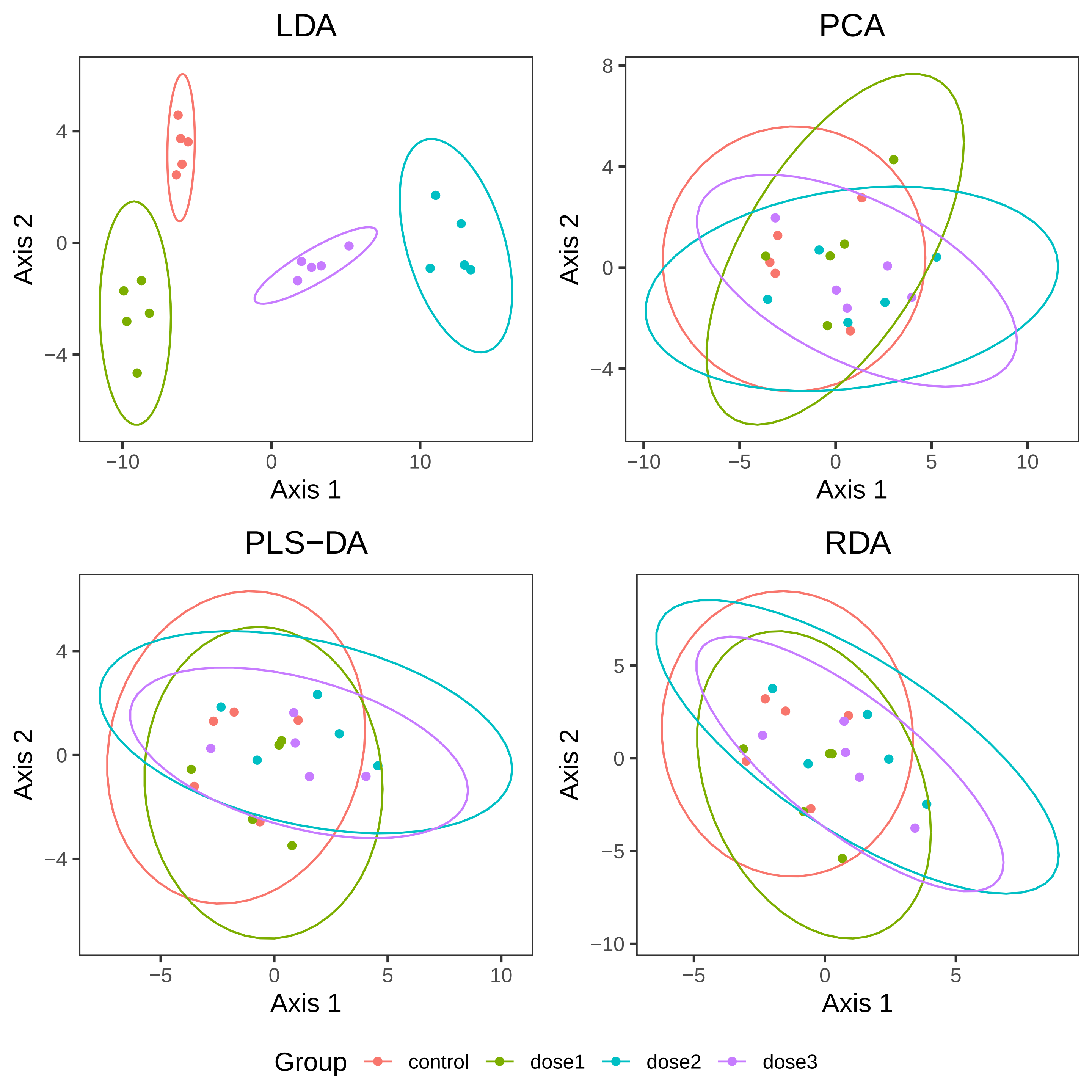

LDA
PCA
PLS−DA
RDA
Axis 1
Axis 2
−10
0
10
−5
5
8
4
−4
−10
Group
control
dose1
dose2
dose3

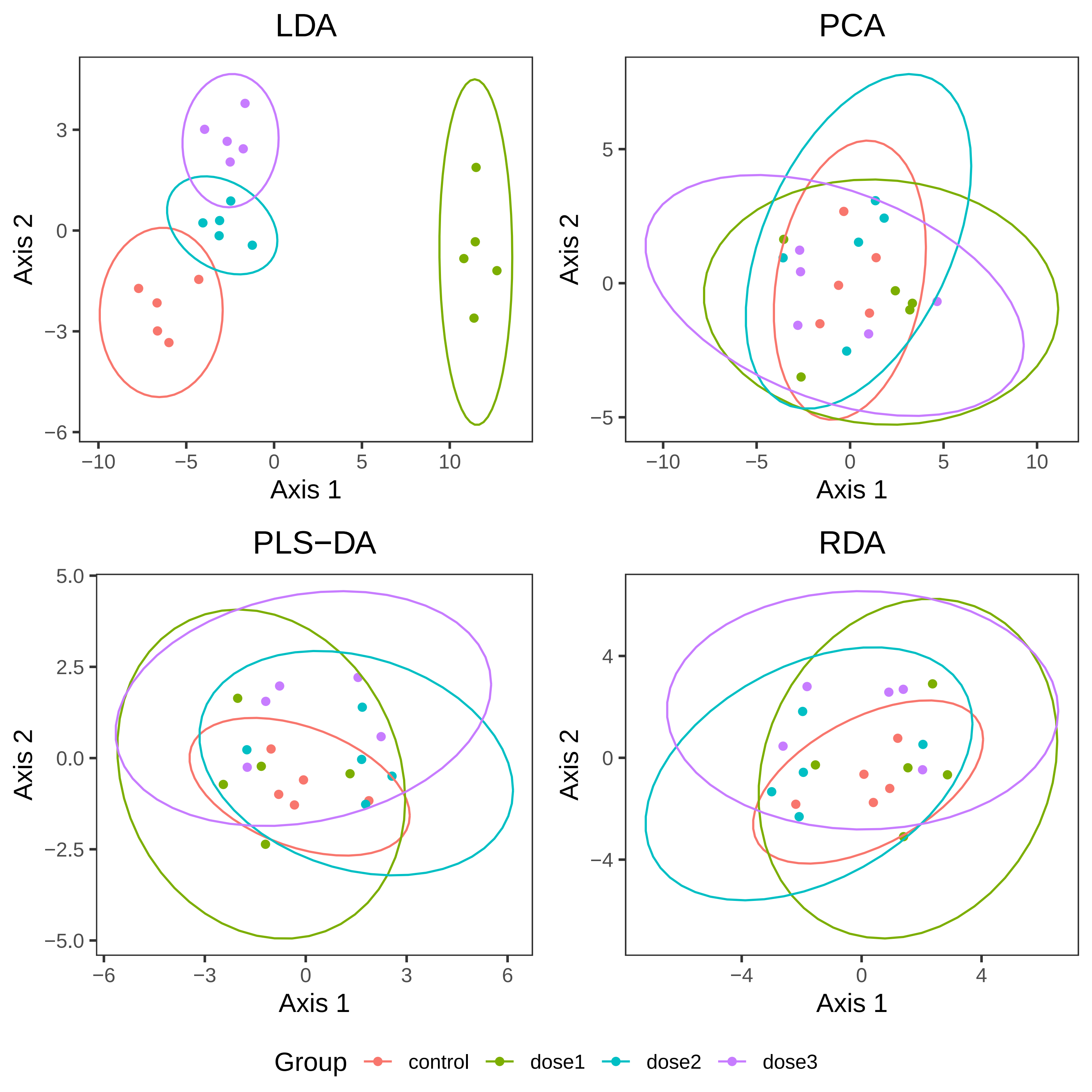

LDA
PCA
PLS−DA
RDA
Axis 1
Axis 2
3
0
−3
−6
−10
−5
0
5
10
5.0
2.5
0.0
−2.5
−5.0
−6
6
4
−4
Group
control
dose1
dose2
dose3

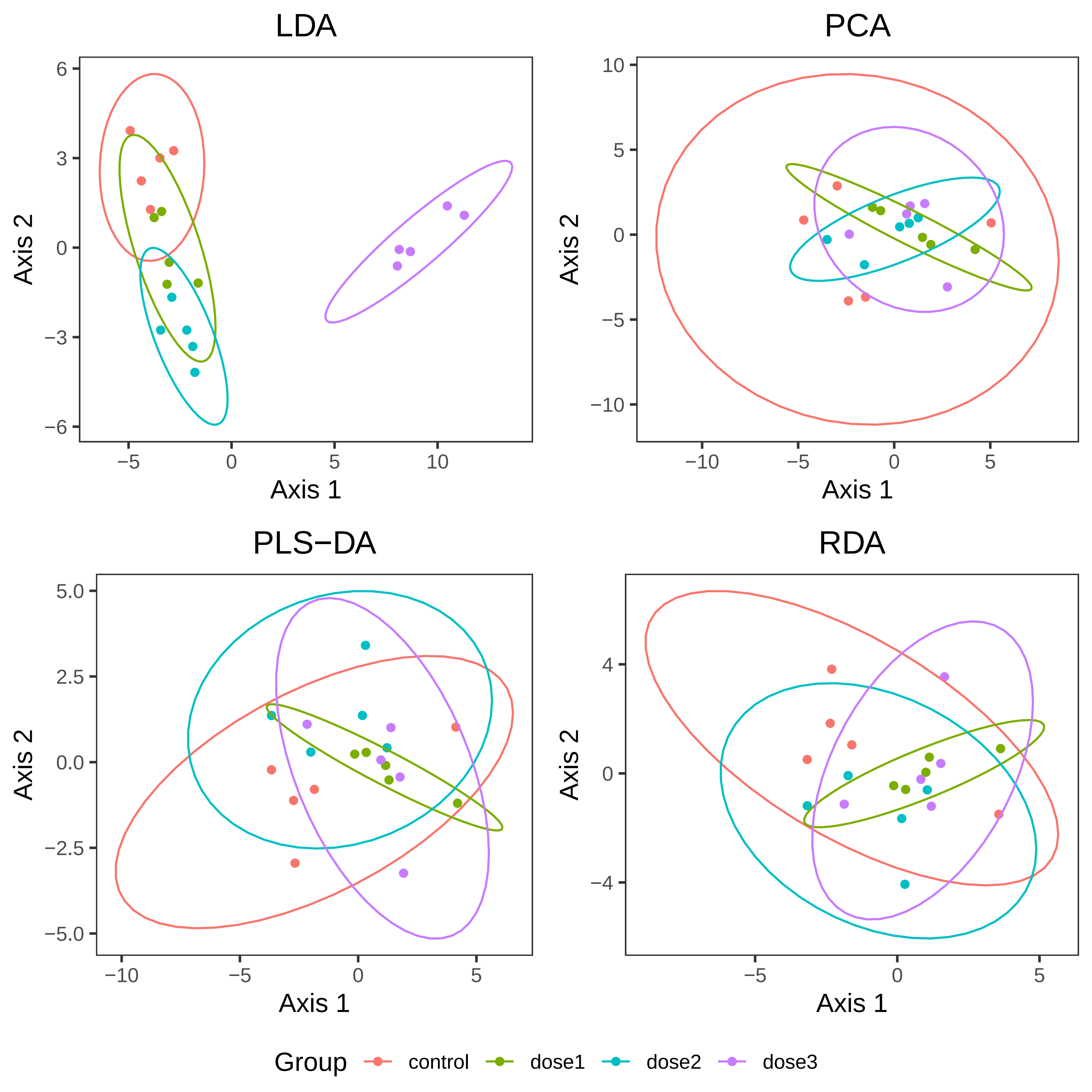
LDA
PCA
PLS−DA
RDA
Axis 1
Axis 2
Group
control
dose1
dose2
dose3

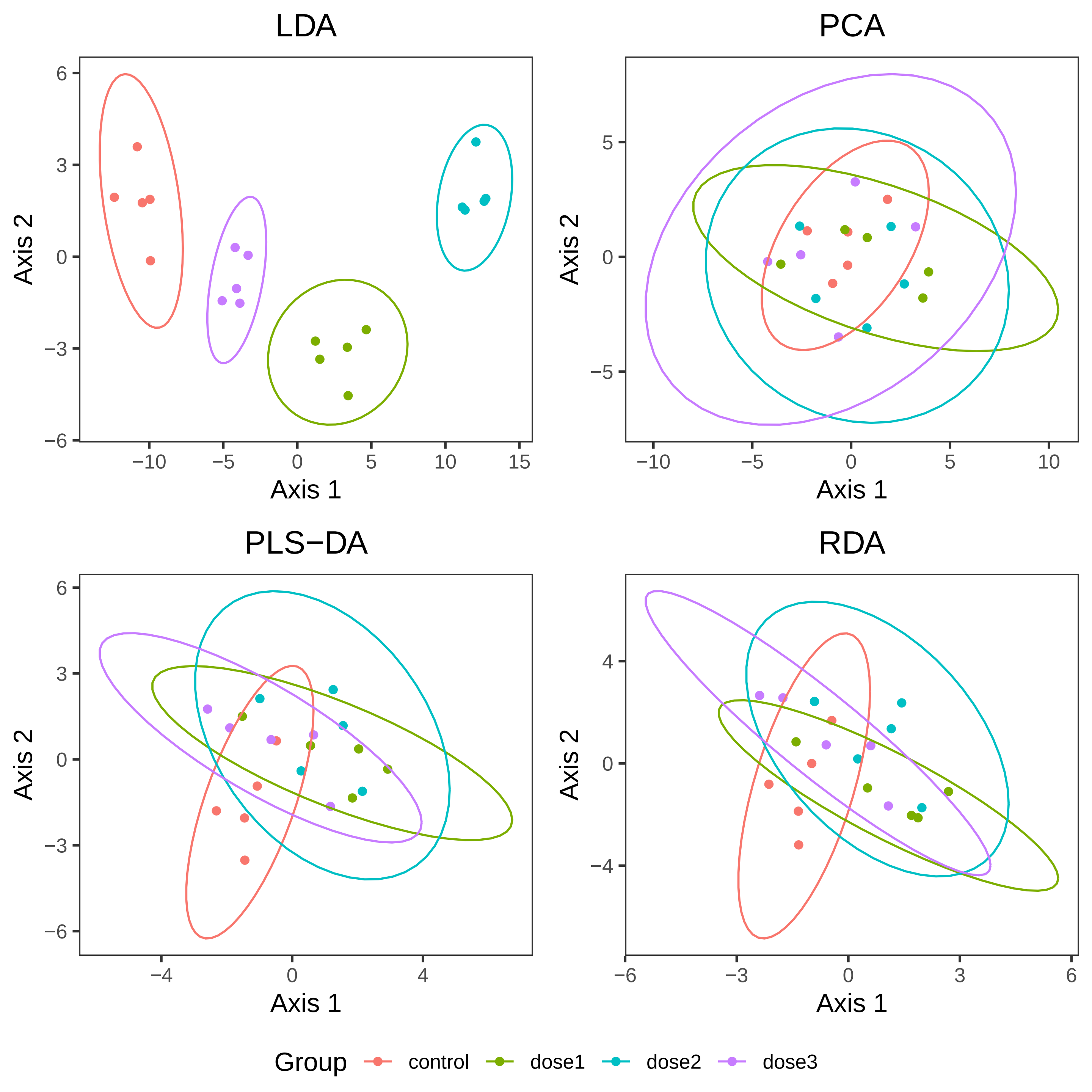

LDA
PCA
PLS−DA
RDA
Axis 1
Axis 2
6
3
0
−3
−6
−10
−5
0
5
10
15
5
−5
−4
4
−6
−3
Group
control
dose1
dose2
dose3

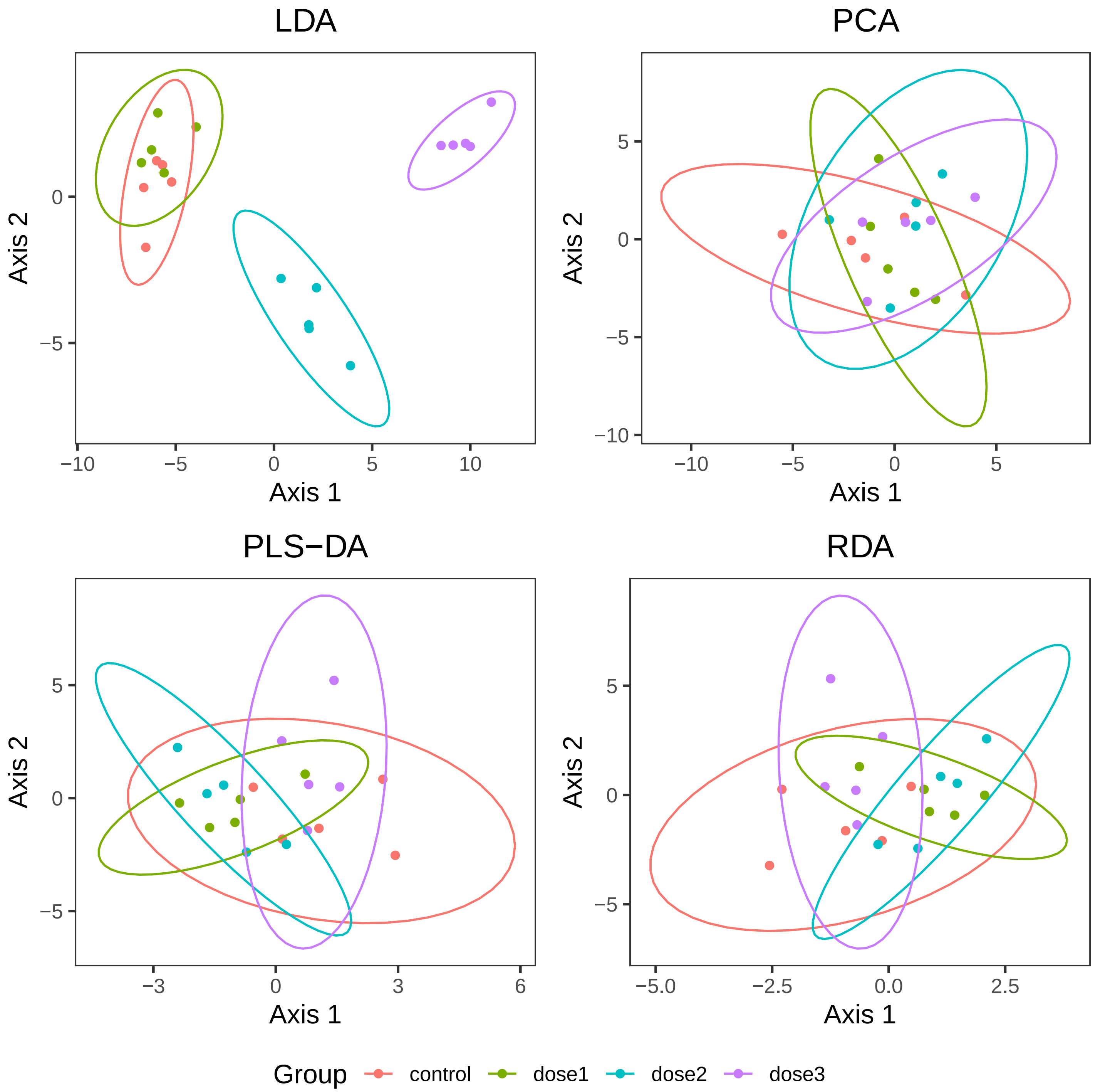

LDA
PCA
PLS−DA
RDA
Axis 1
Axis 2
Group
control
dose1
dose2
dose3

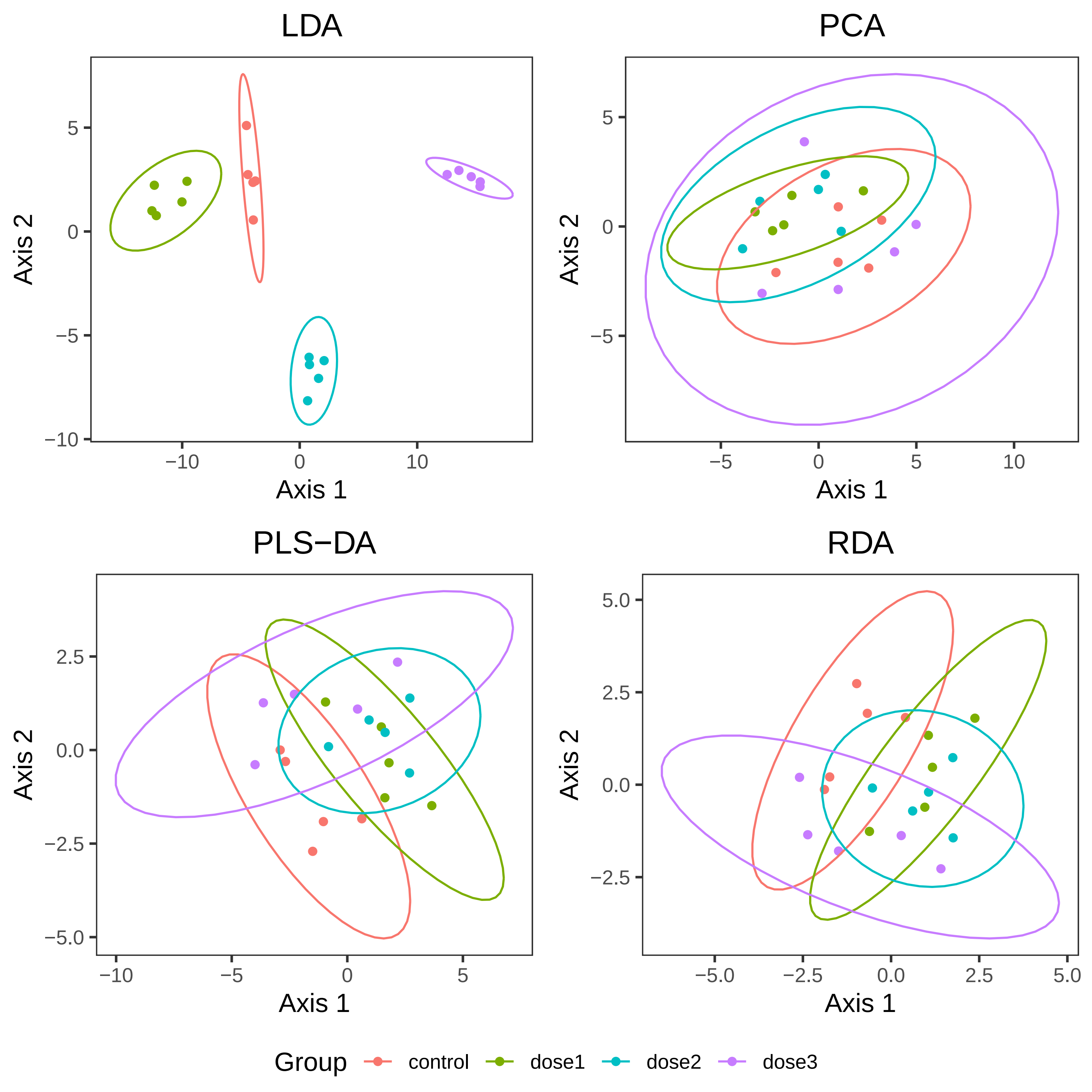

LDA
PCA
PLS−DA
RDA
Axis 1
Axis 2
Group
control
dose1
dose2
dose3

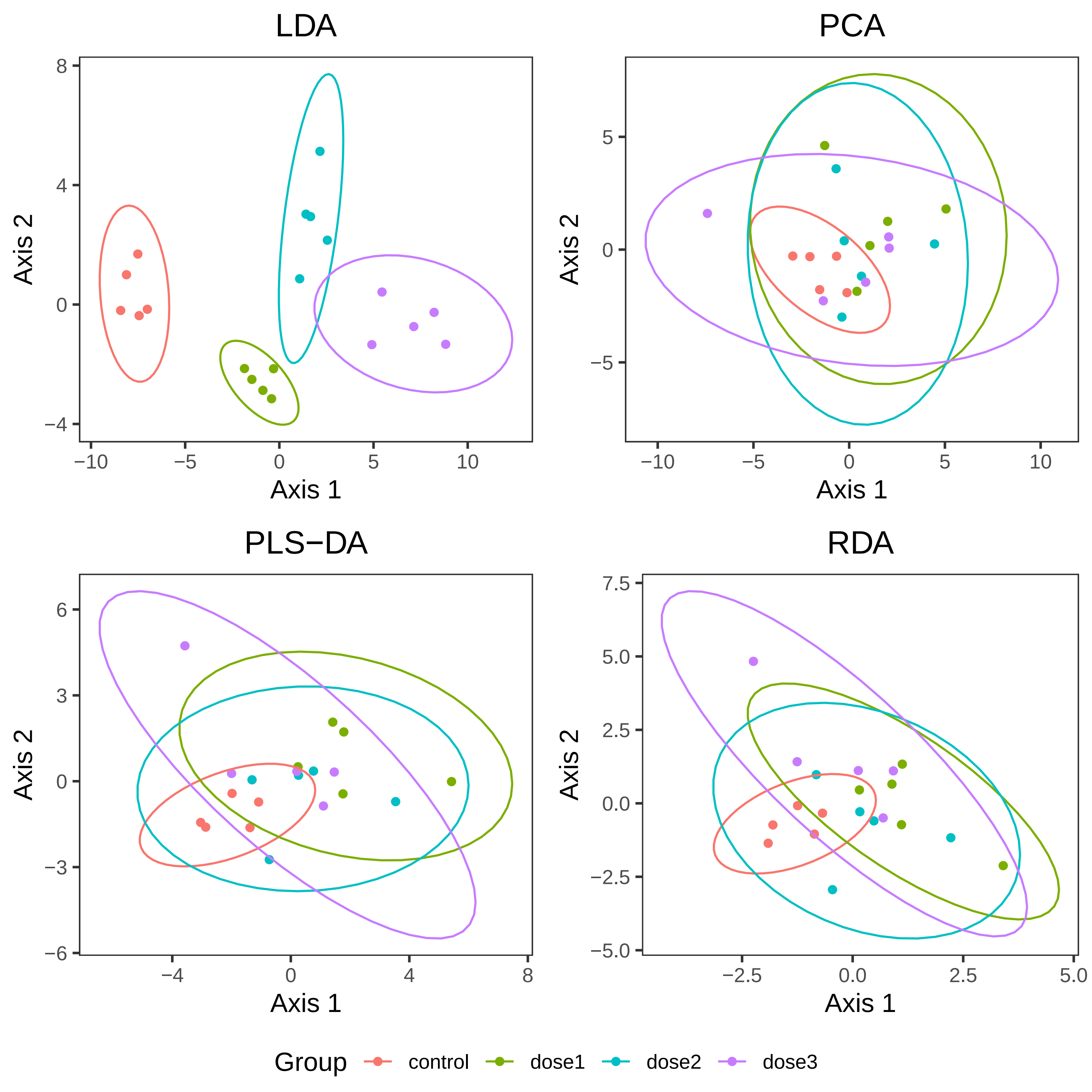

LDA
PCA
PLS−DA
RDA
Axis 1
Axis 2
−10
−5
0
5
10
8
4
−4
−2.5
0.0
2.5
5.0
7.5
−5.0
−6
−3
3
6
−4
8
Group
control
dose1
dose2
dose3

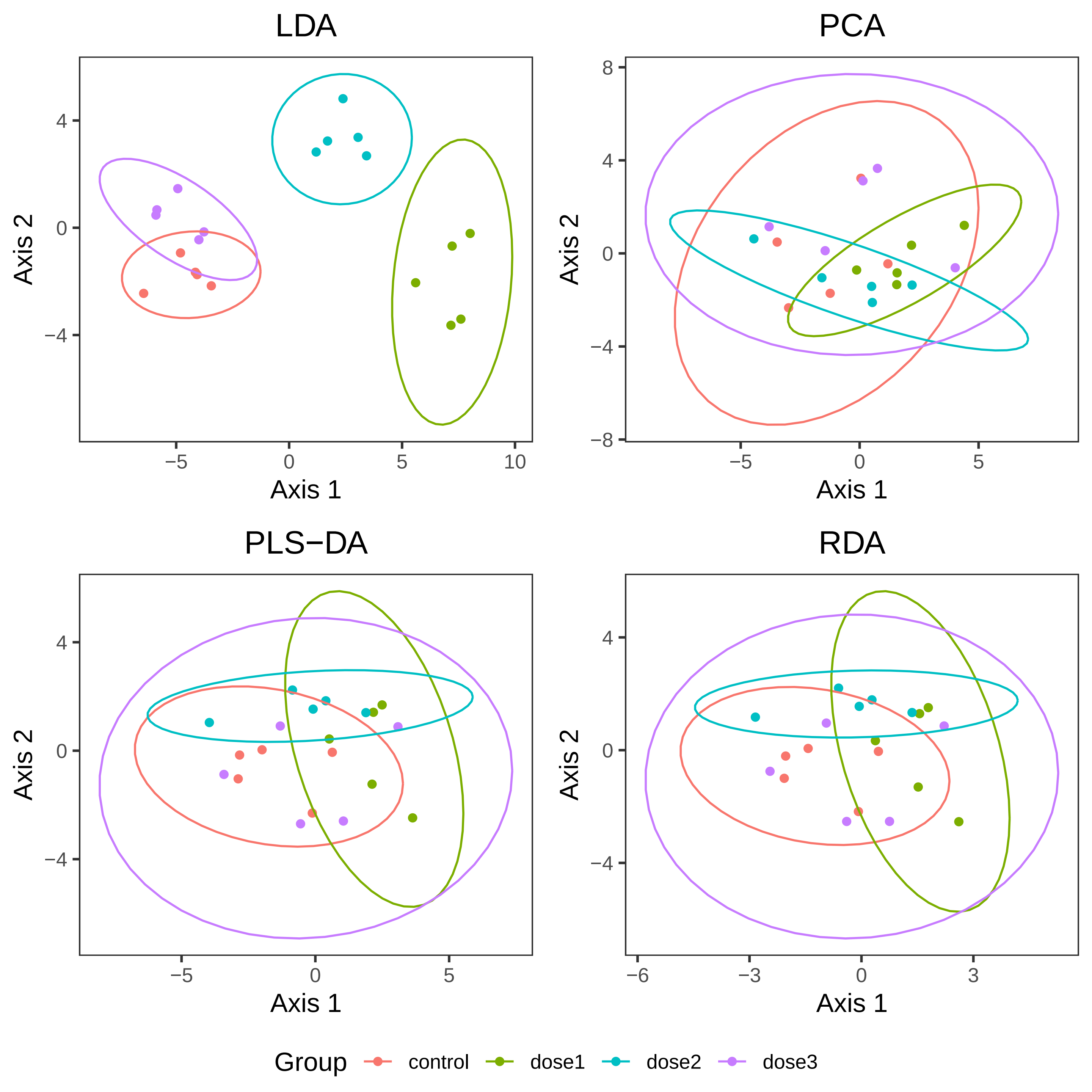

LDA
PCA
PLS−DA
RDA
Axis 1
Axis 2
Group
control
dose1
dose2
dose3

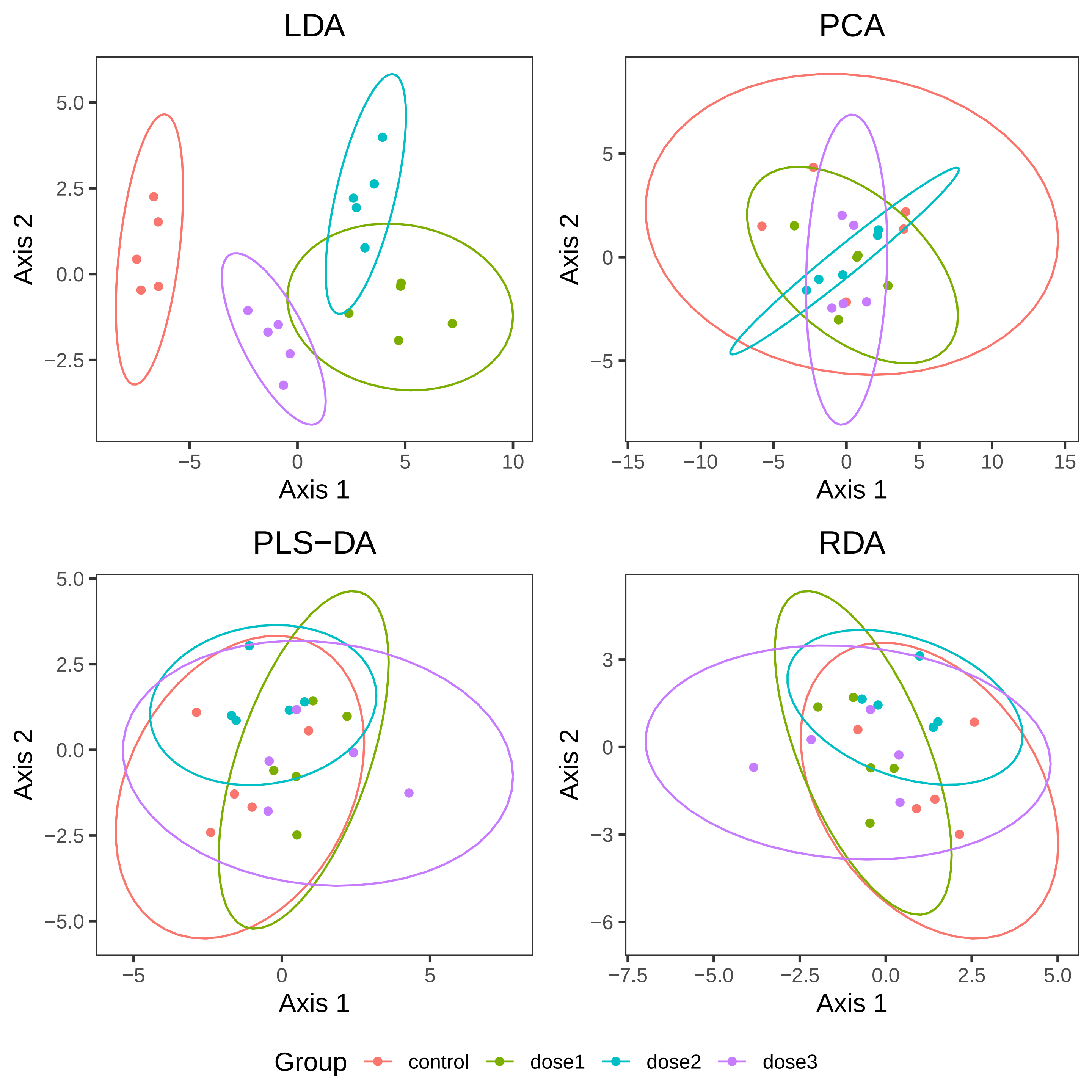
LDA
PCA
PLS−DA
RDA
Axis 1
Axis 2
Group
control
dose1
dose2
dose3

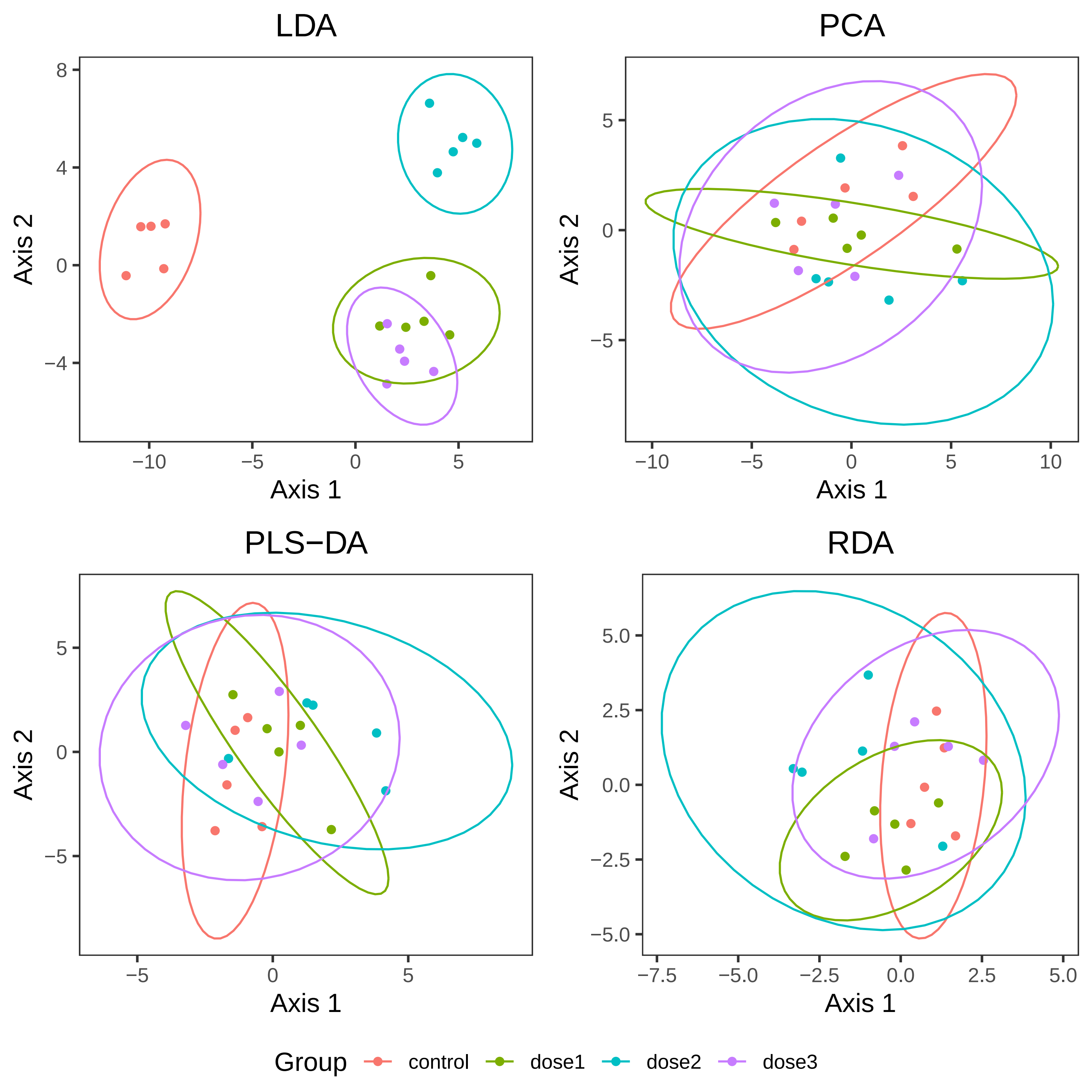
LDA
PCA
PLS−DA
RDA
Axis 1
Axis 2
Group
control
dose1
dose2
dose3

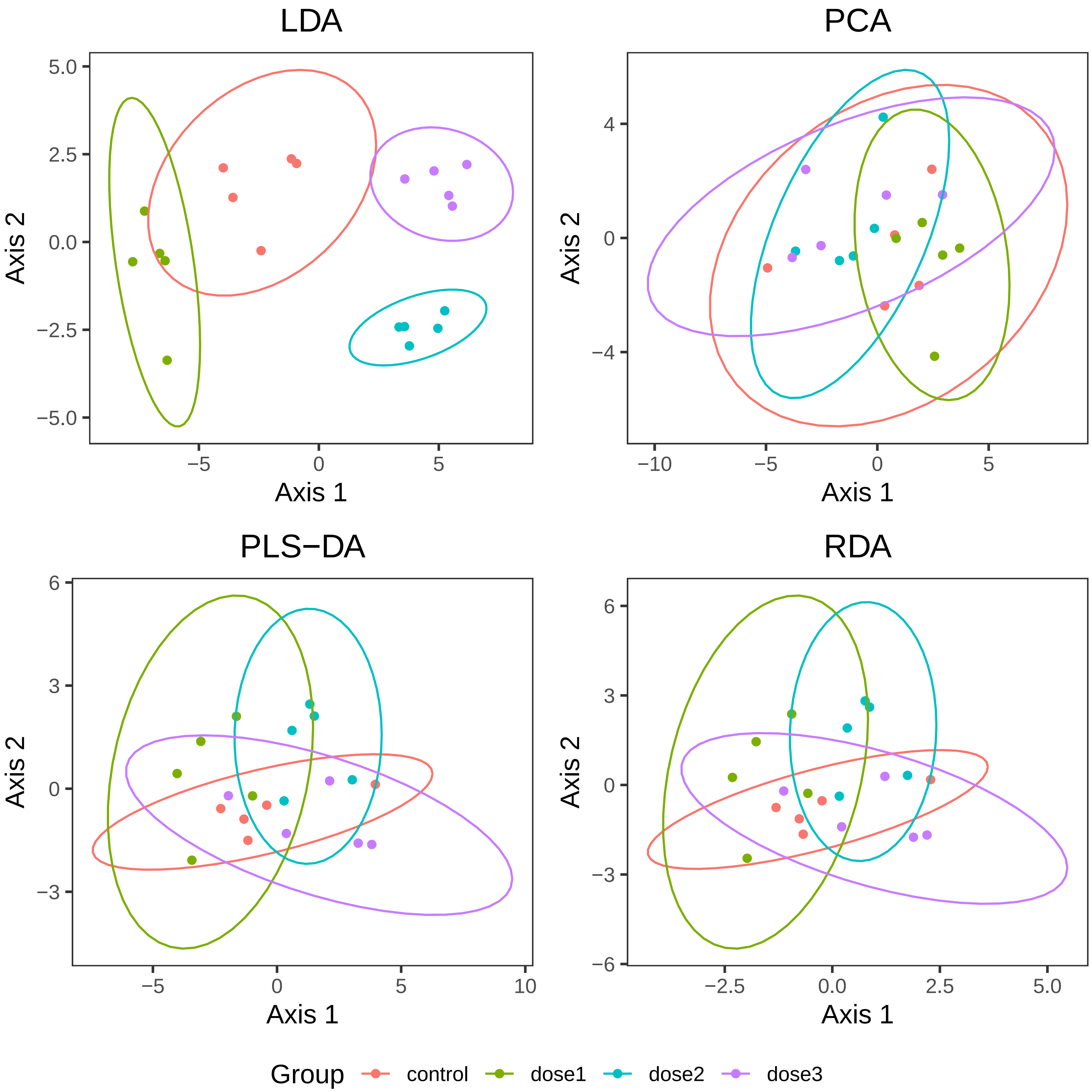
LDA
PCA
PLS−DA
RDA
Axis 1
Axis 2
5.0
2.5
0.0
−2.5
−5.0
−5
0
5
4
0
−4
−10
6
3
−3
10
−6
−2.5
0.0
2.5
5.0
Group
control
dose1
dose2
dose3

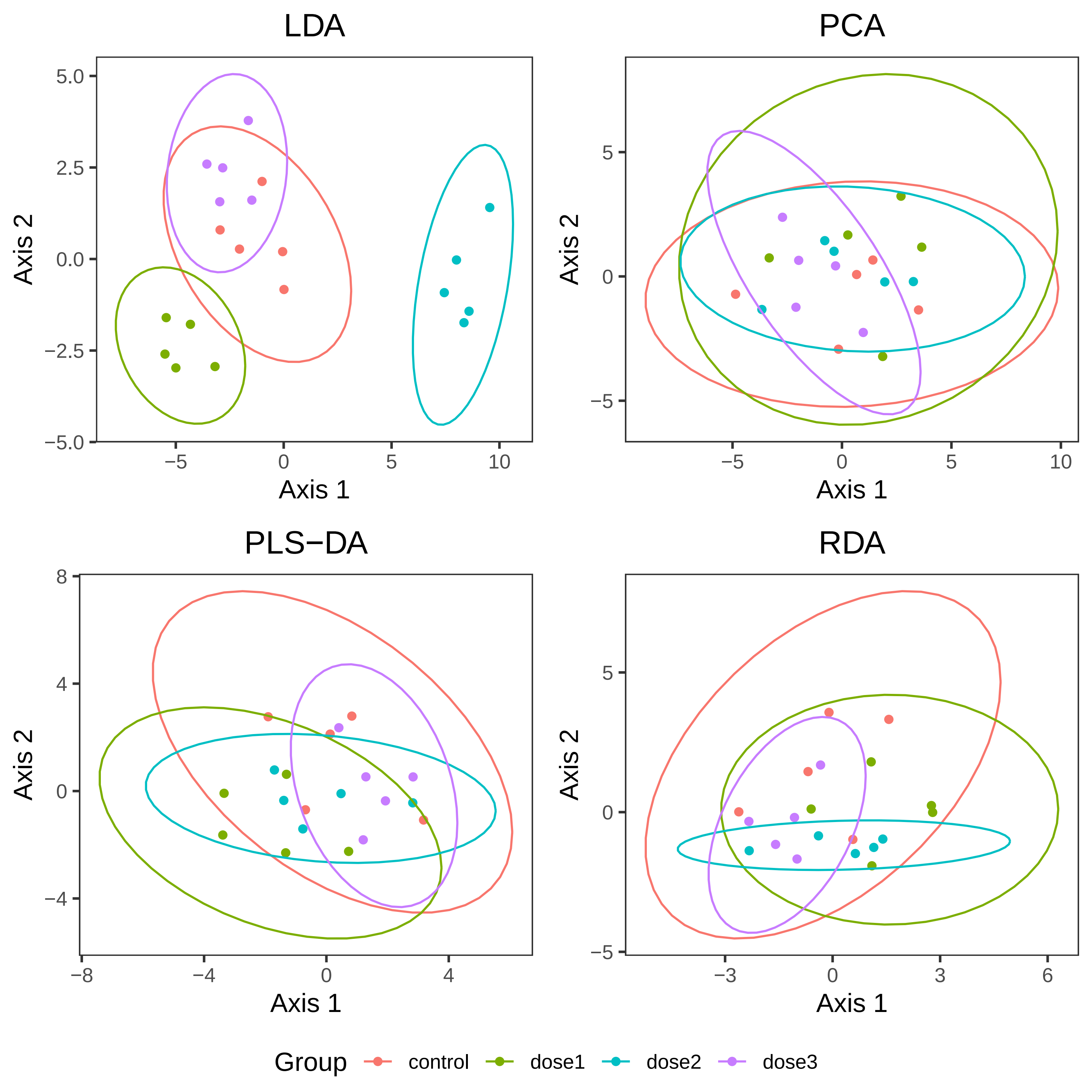

LDA
PCA
PLS−DA
RDA
Axis 1
Axis 2
Group
control
dose1
dose2
dose3

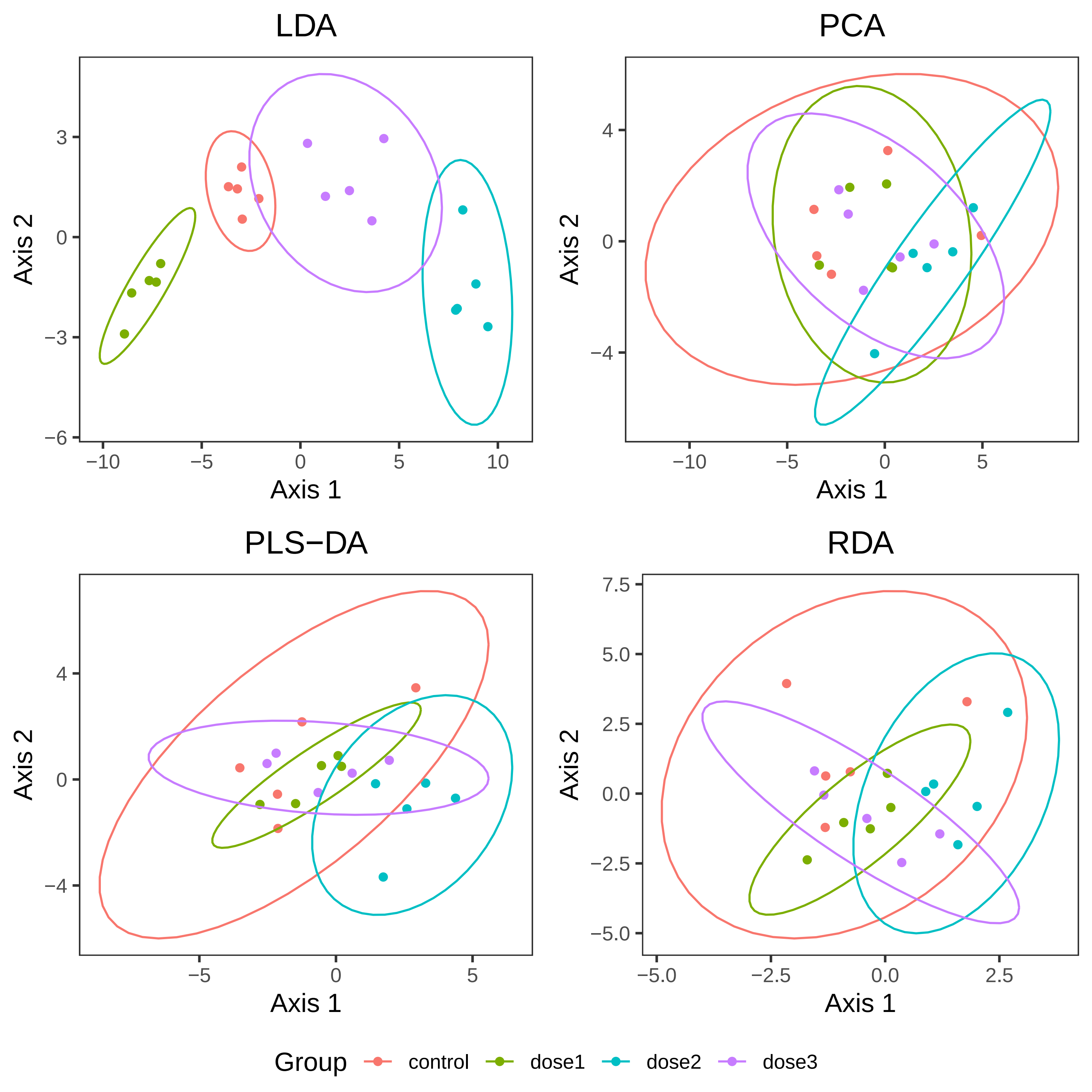
LDA
PCA
PLS−DA
RDA
Axis 1
Axis 2
Group
control
dose1
dose2
dose3

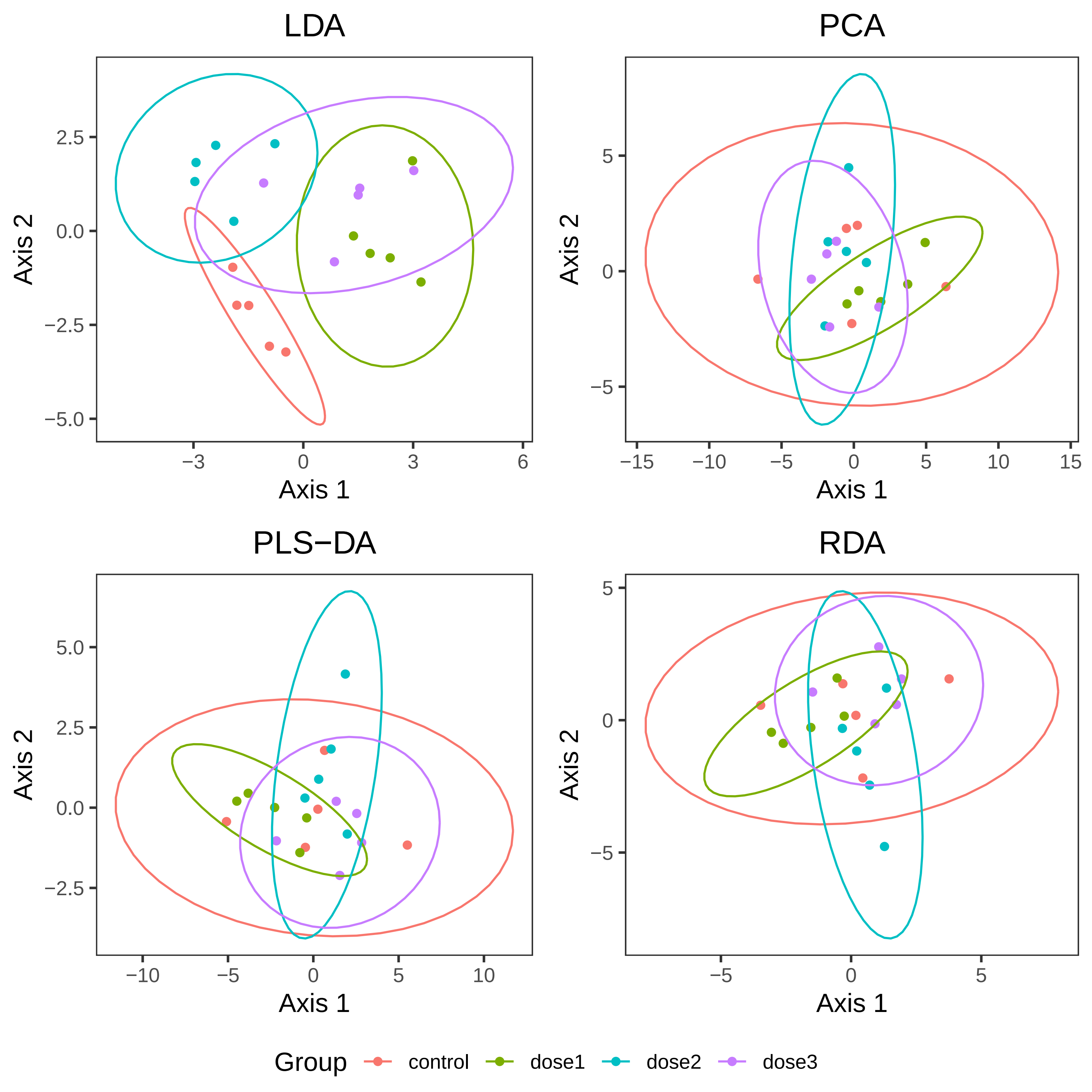

LDA
PCA
PLS−DA
RDA
Axis 1
Axis 2
2.5
0.0
−2.5
−5.0
−3
0
3
6
5
0
−5
−15
−10
−5
0
5
10
15
5.0
2.5
0.0
−2.5
−10
−5
0
5
10
5
0
−5
−5
0
5
Group
control
dose1
dose2
dose3

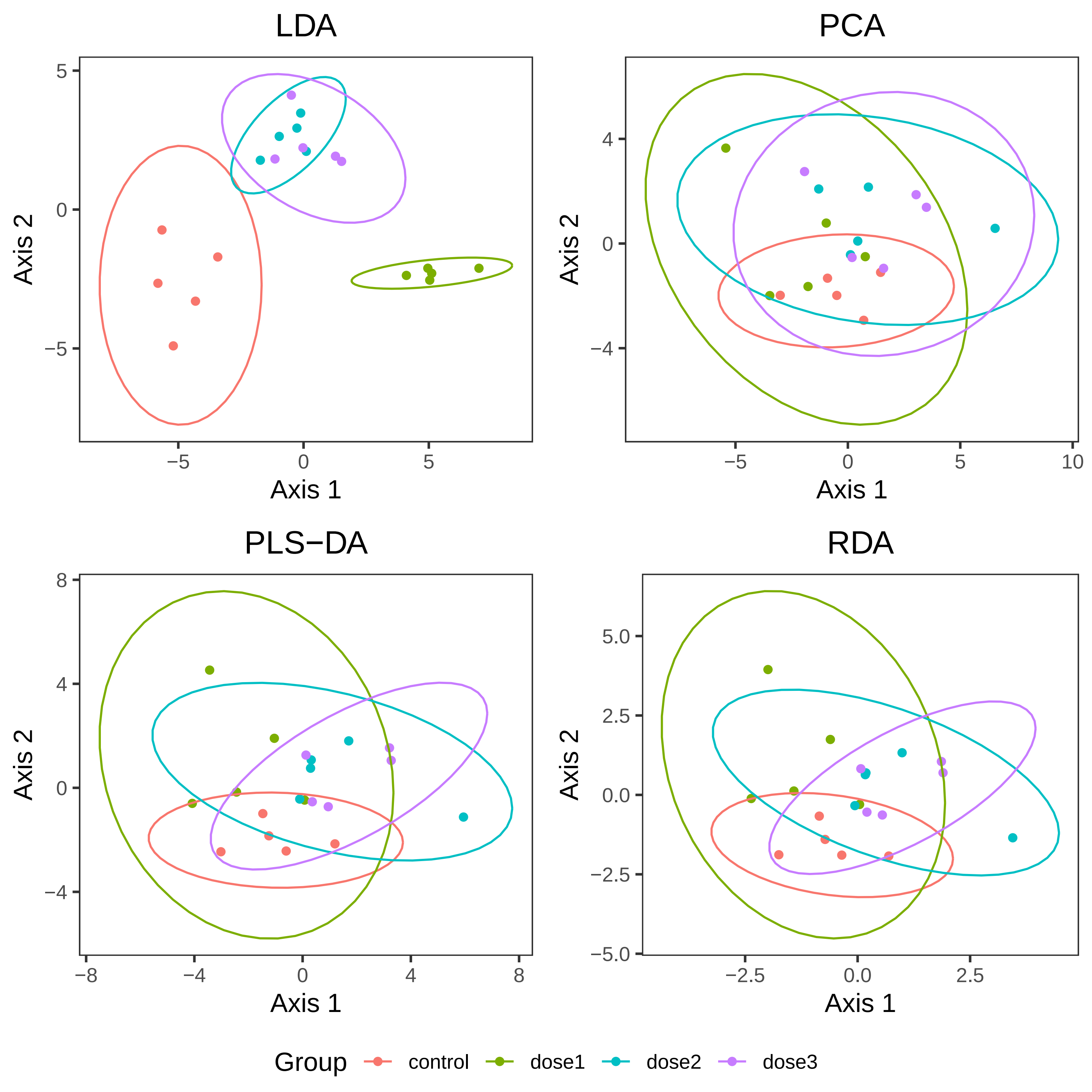

LDA
PCA
PLS−DA
RDA
Axis 1
Axis 2
5
0
−5
10
4
−4
8
−8
5.0
2.5
0.0
−2.5
−5.0
Group
control
dose1
dose2
dose3

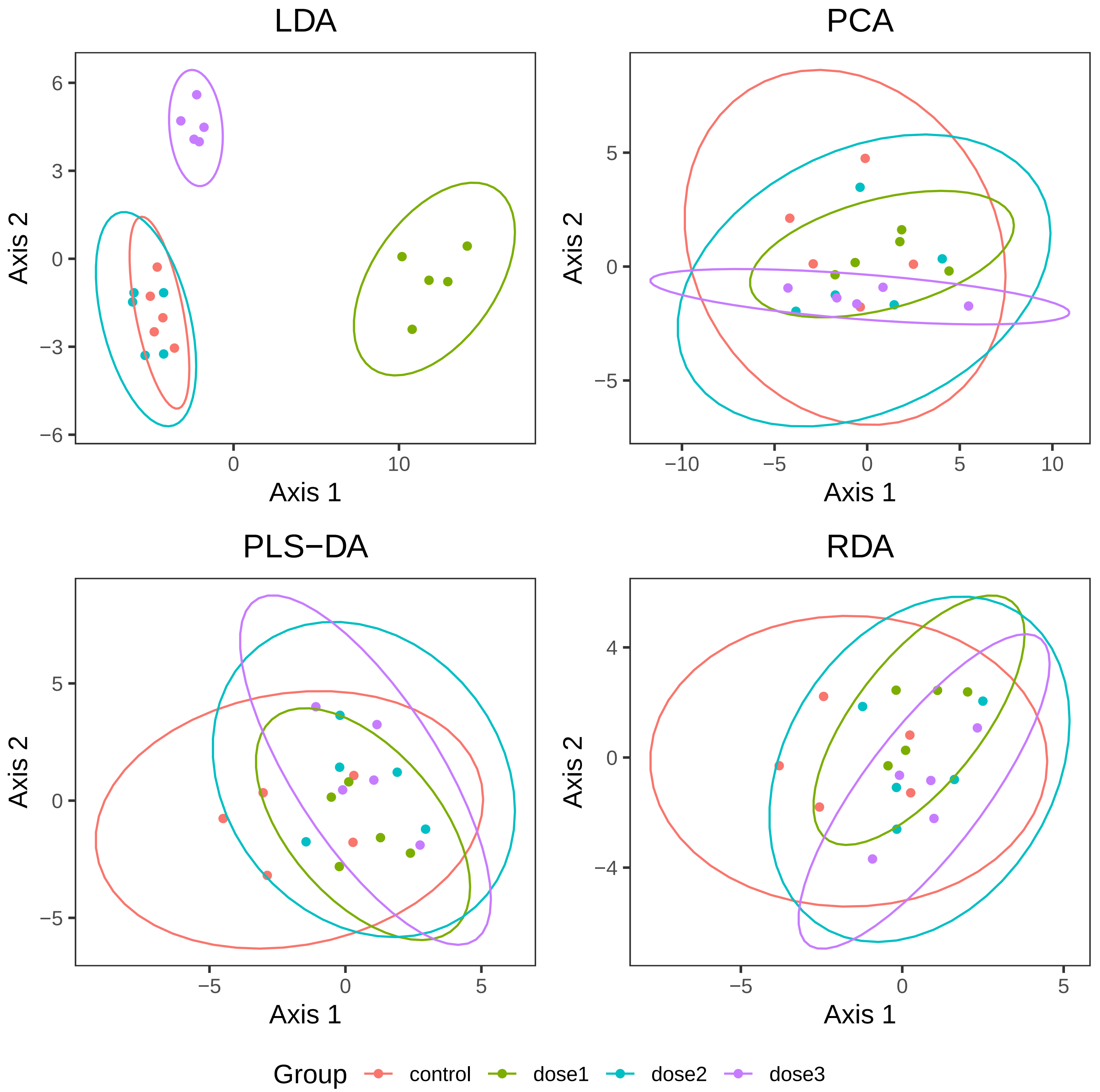

LDA
PCA
PLS−DA
RDA
Axis 1
Axis 2
6
3
0
−3
−6
0
10
5
−5
−10
4
−4
Group
control
dose1
dose2
dose3

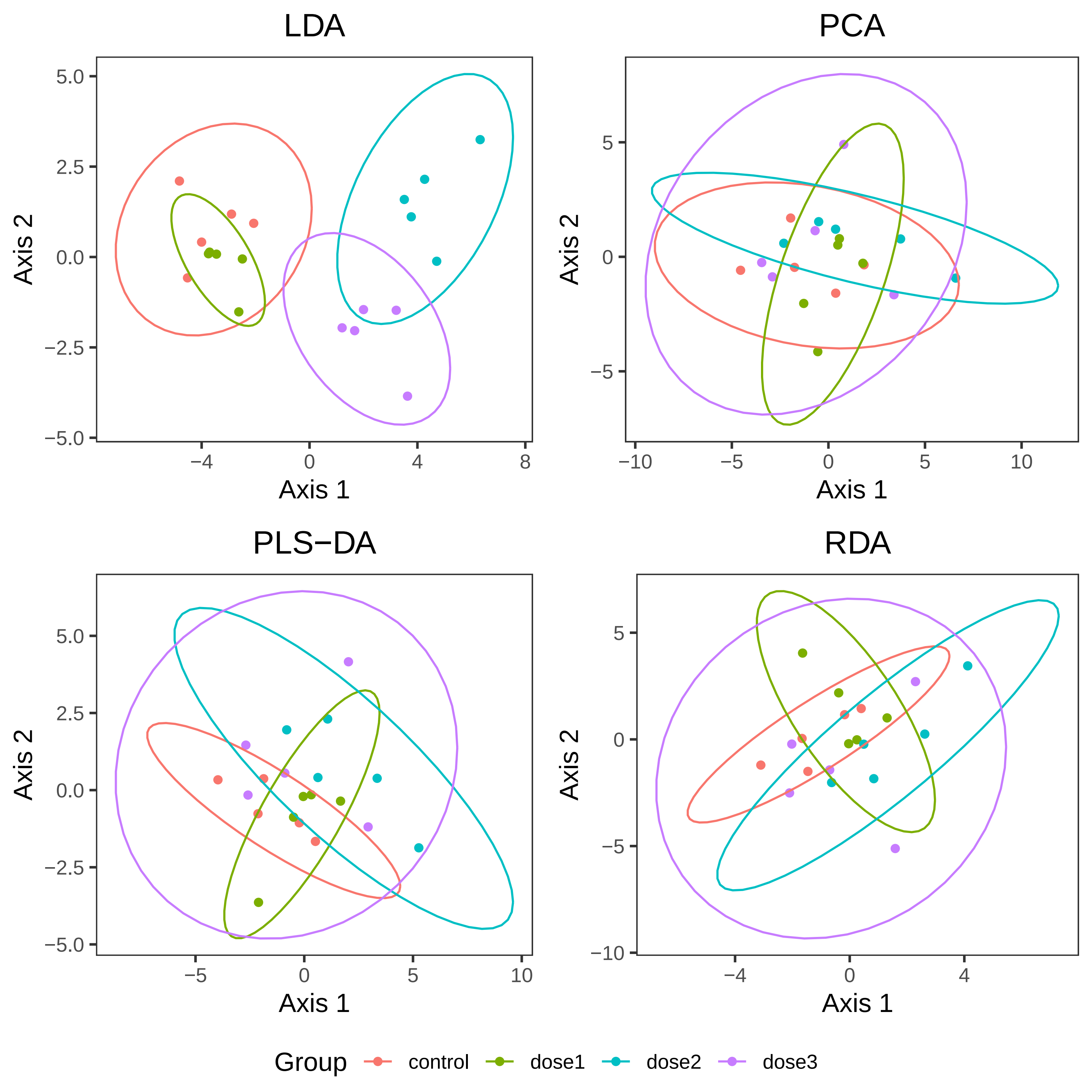
LDA
PCA
PLS−DA
RDA
Axis 1
Axis 2
Group
control
dose1
dose2
dose3

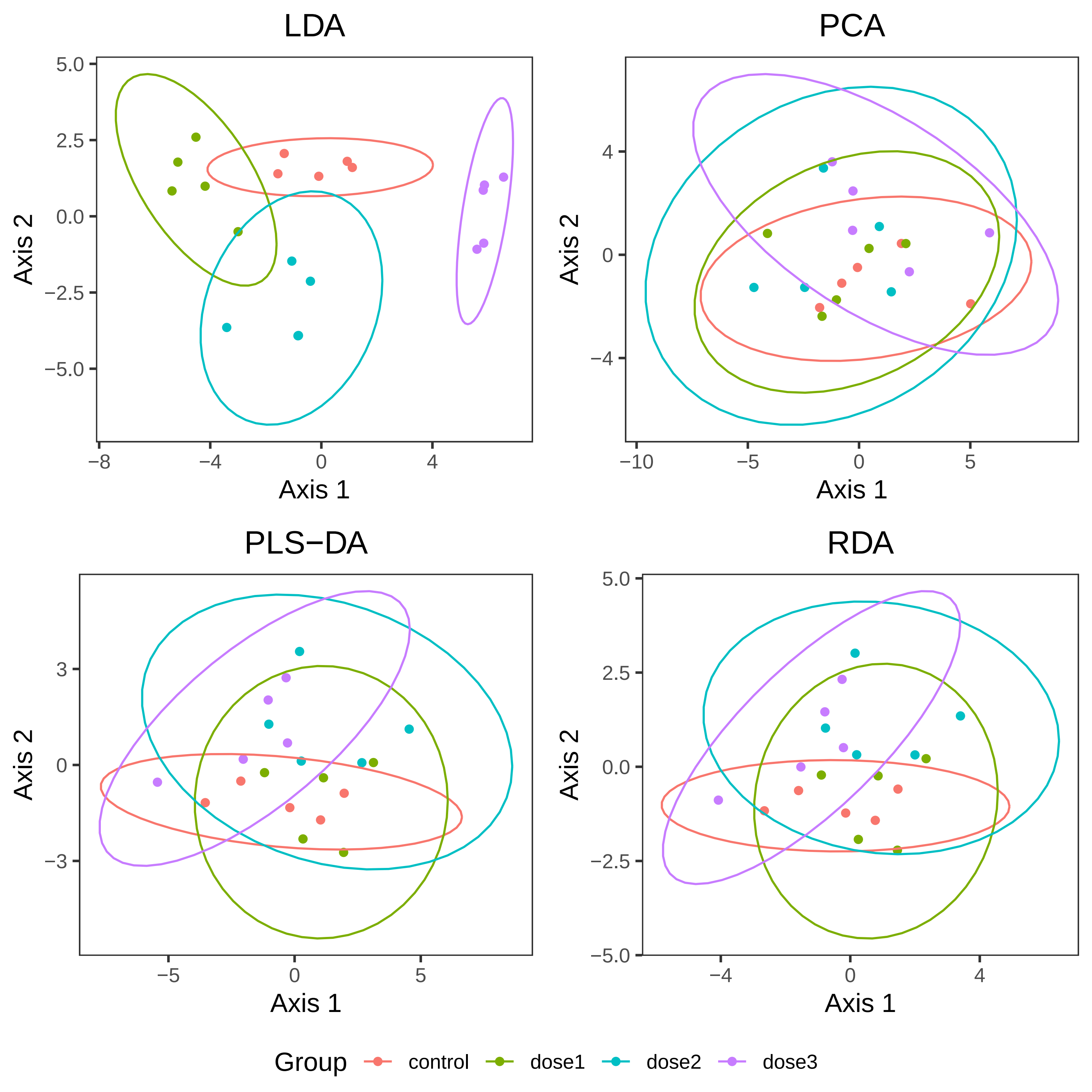

LDA
PCA
PLS−DA
RDA
Axis 1
Axis 2
5.0
2.5
0.0
−2.5
−5.0
−8
−4
0
4
−10
−5
5
3
−3
Group
control
dose1
dose2
dose3

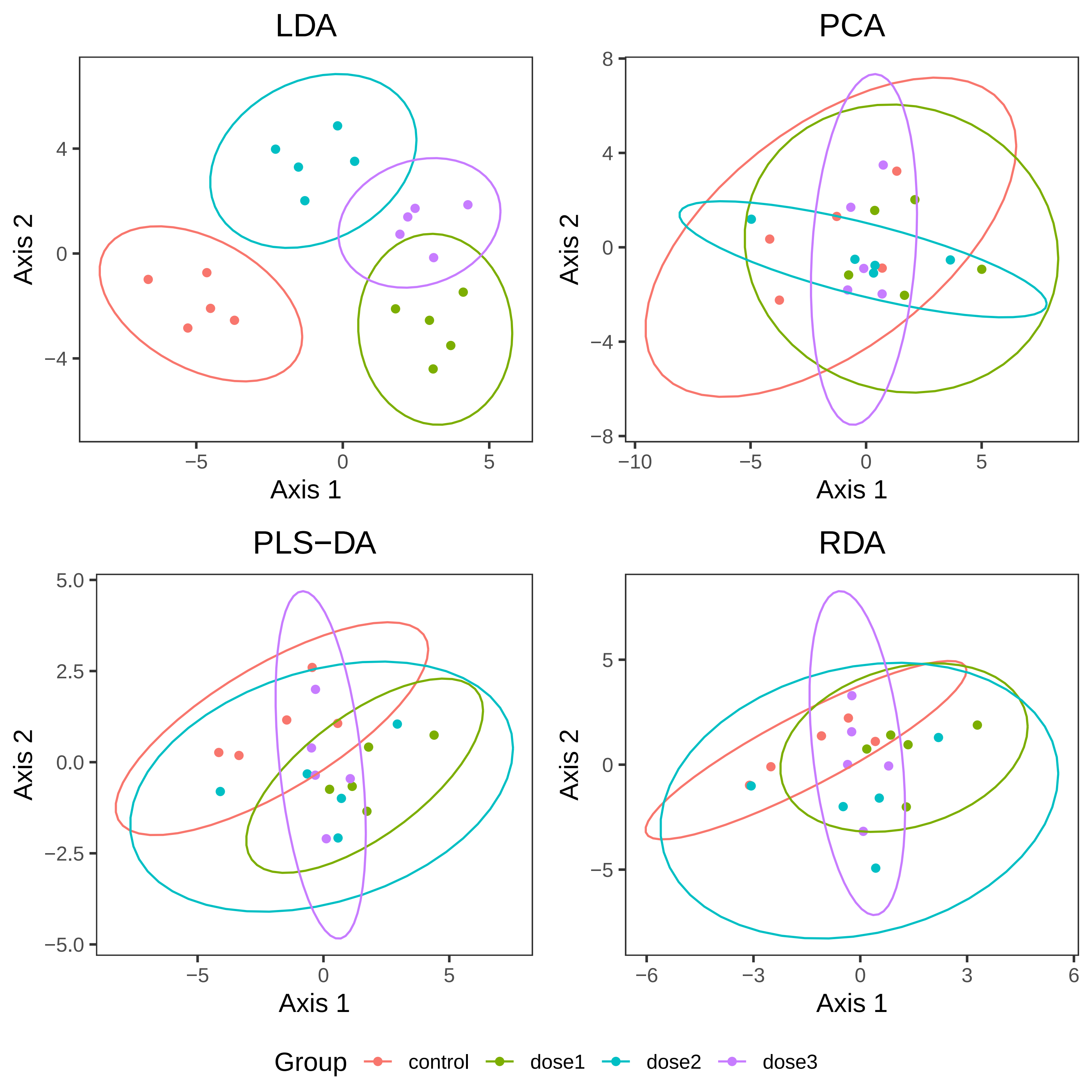

LDA
PCA
PLS−DA
RDA
Axis 1
Axis 2
Group
control
dose1
dose2
dose3

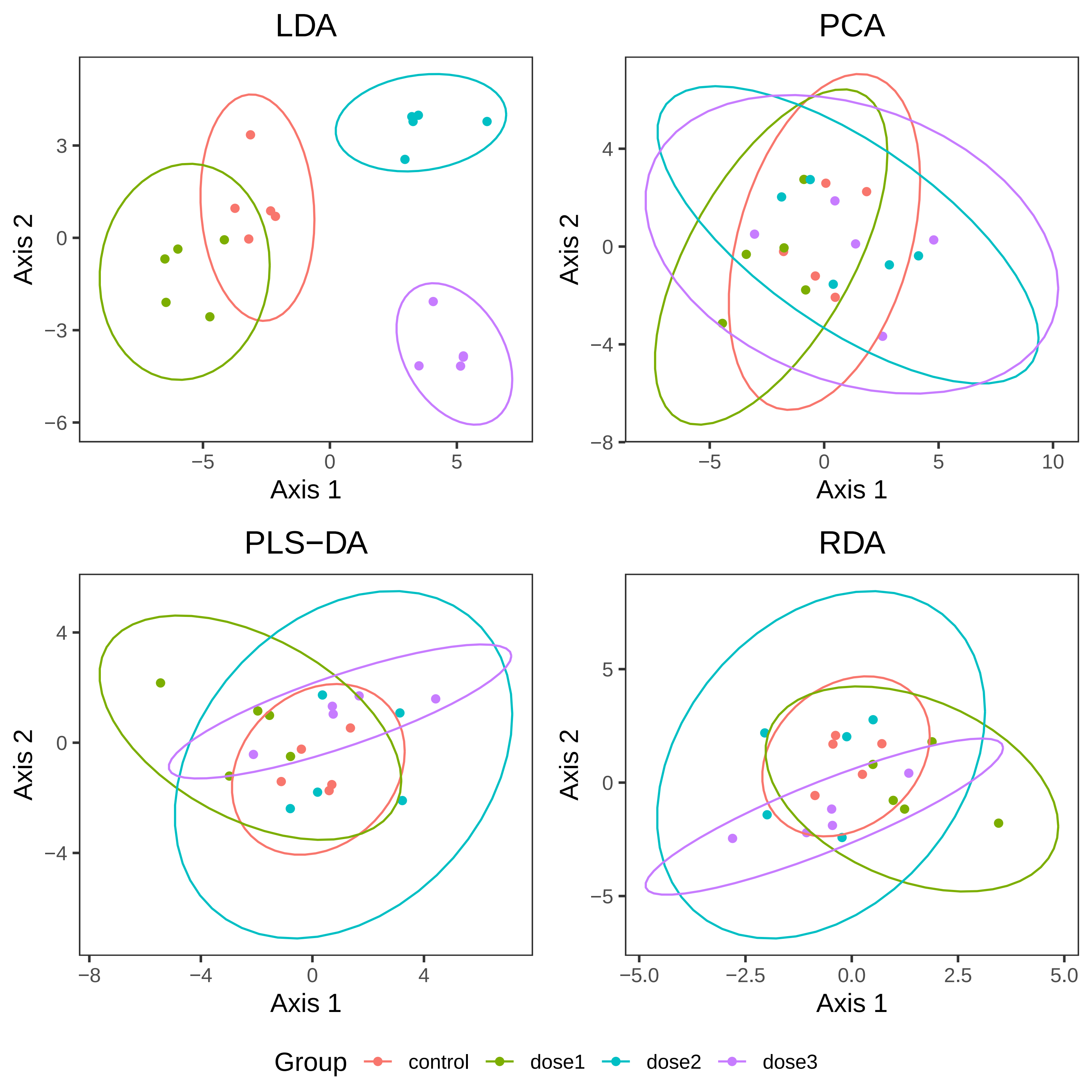

LDA
PCA
PLS−DA
RDA
Axis 1
Axis 2
Group
control
dose1
dose2
dose3

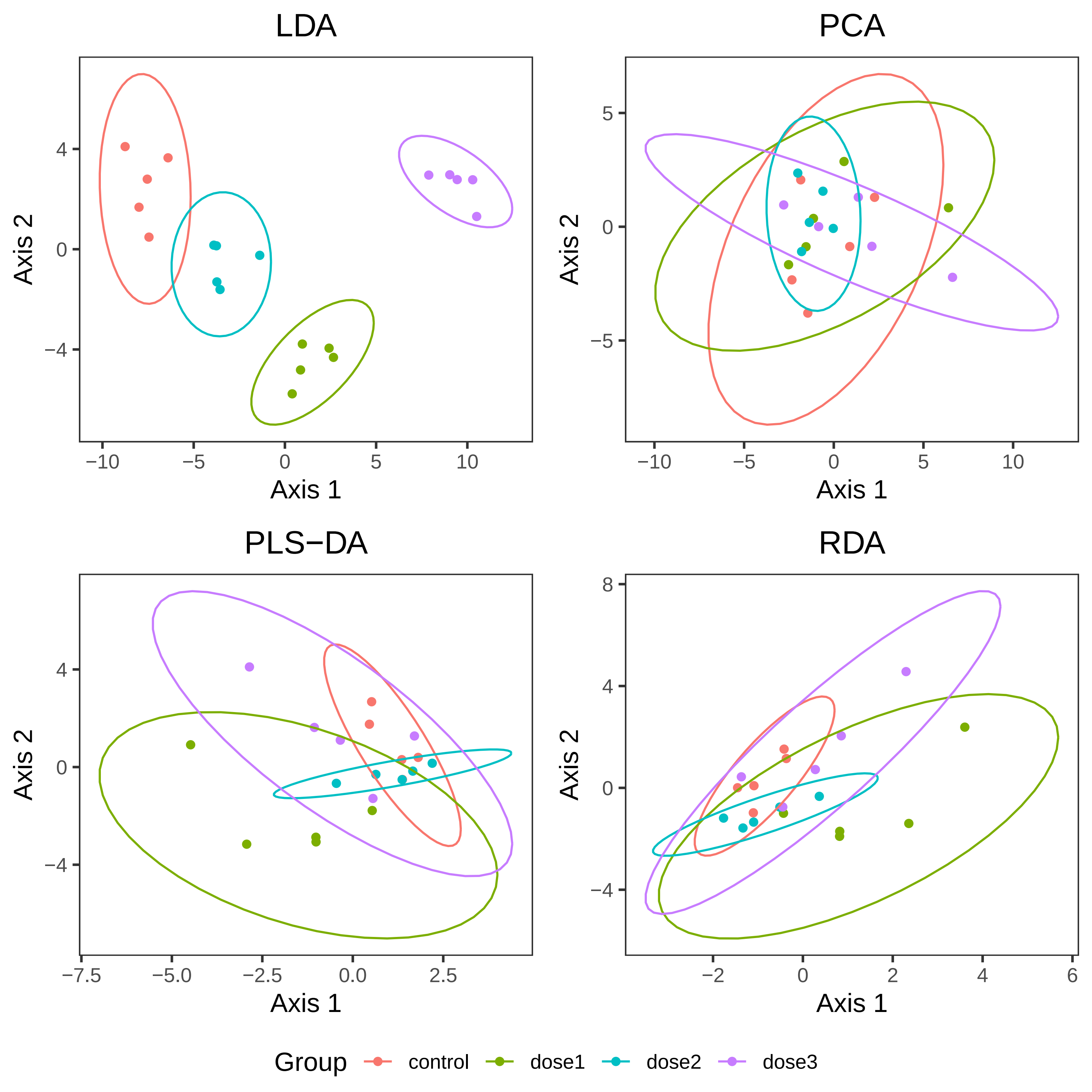

LDA
PCA
PLS−DA
RDA
Axis 1
Axis 2
4
0
−4
5
−5
8
−10
10
−7.5
−5.0
−2.5
0.0
2.5
−2
2
6
Group
control
dose1
dose2
dose3

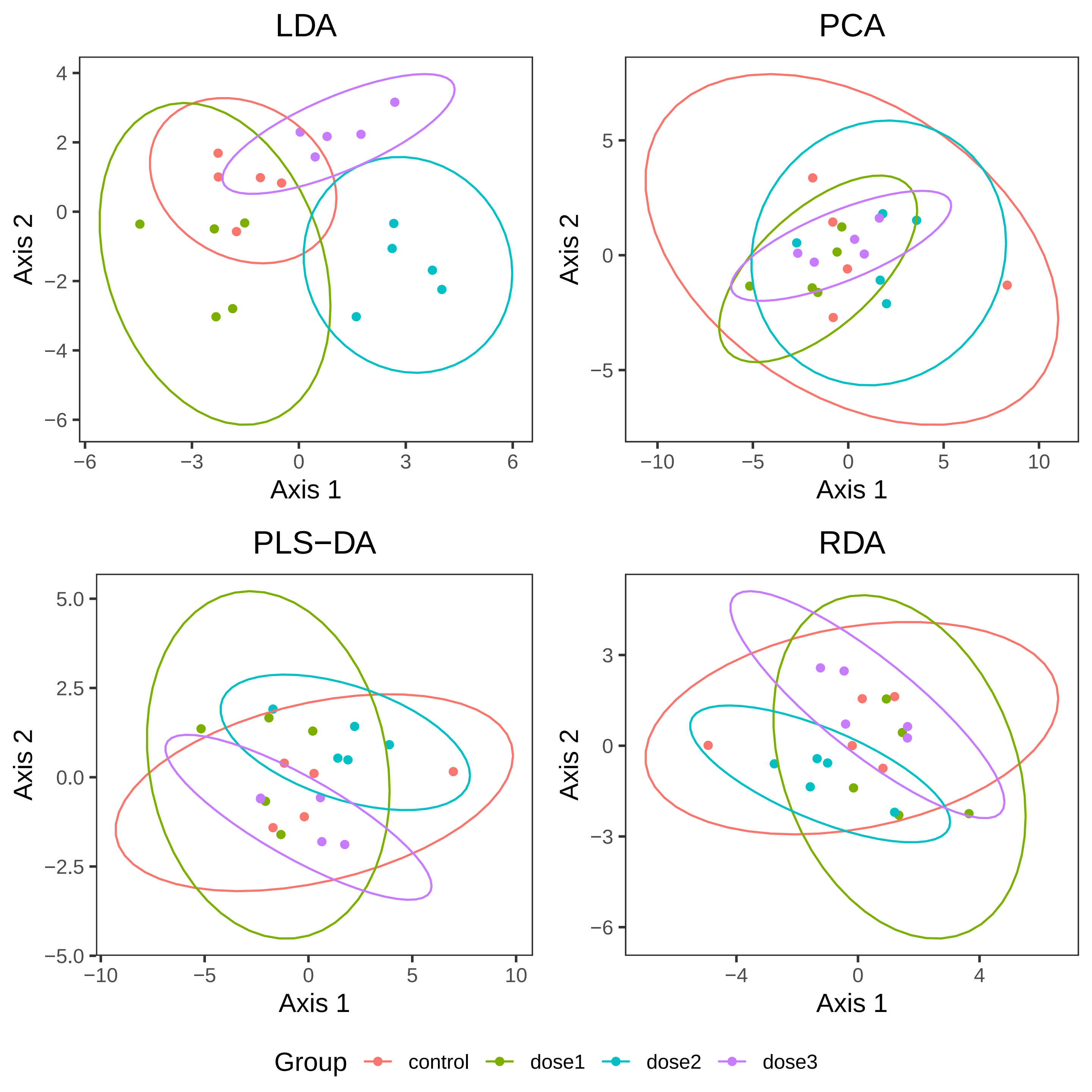

LDA
PCA
PLS−DA
RDA
Axis 1
Axis 2
Group
control
dose1
dose2
dose3

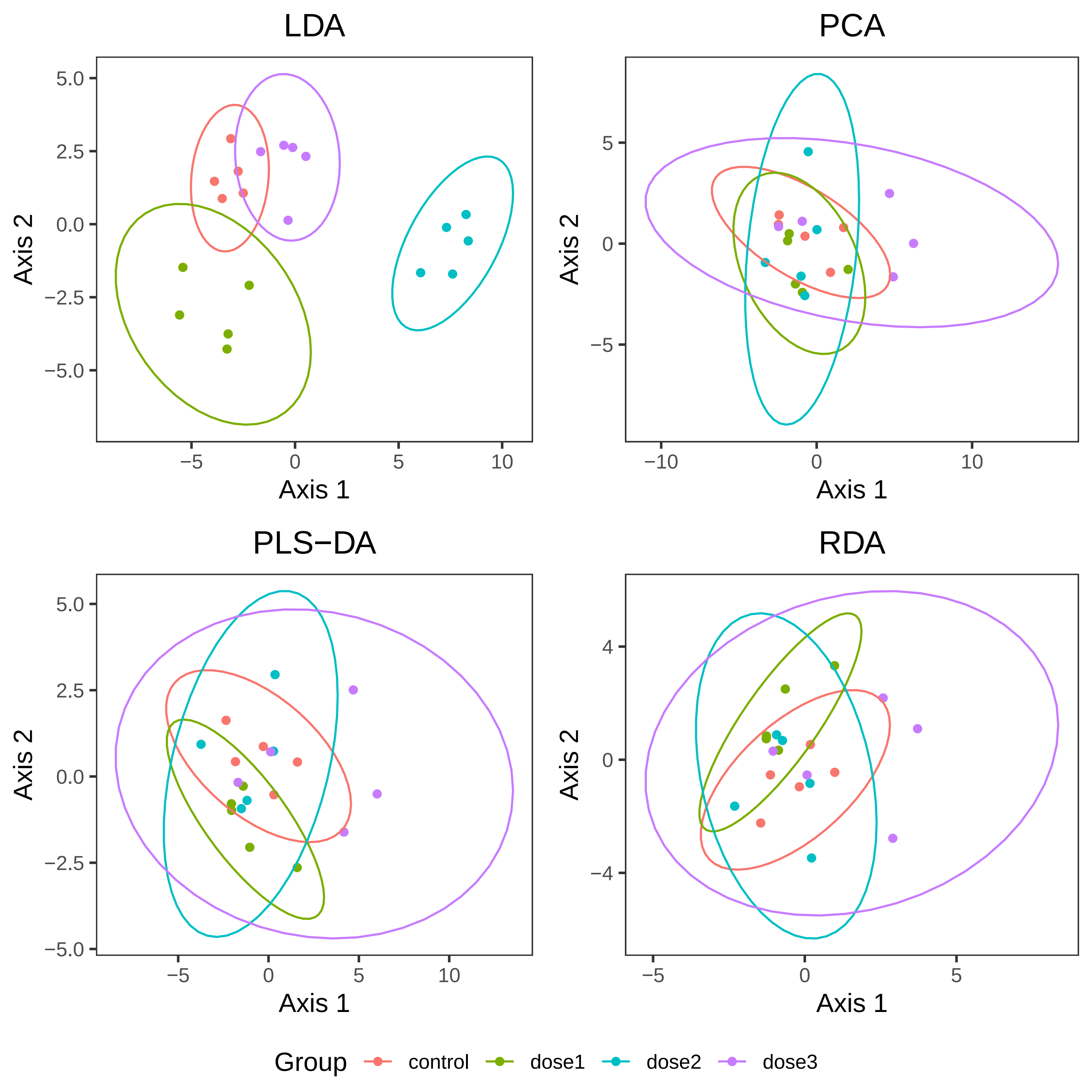
LDA
PCA
PLS−DA
RDA
Axis 1
Axis 2
Group
control
dose1
dose2
dose3

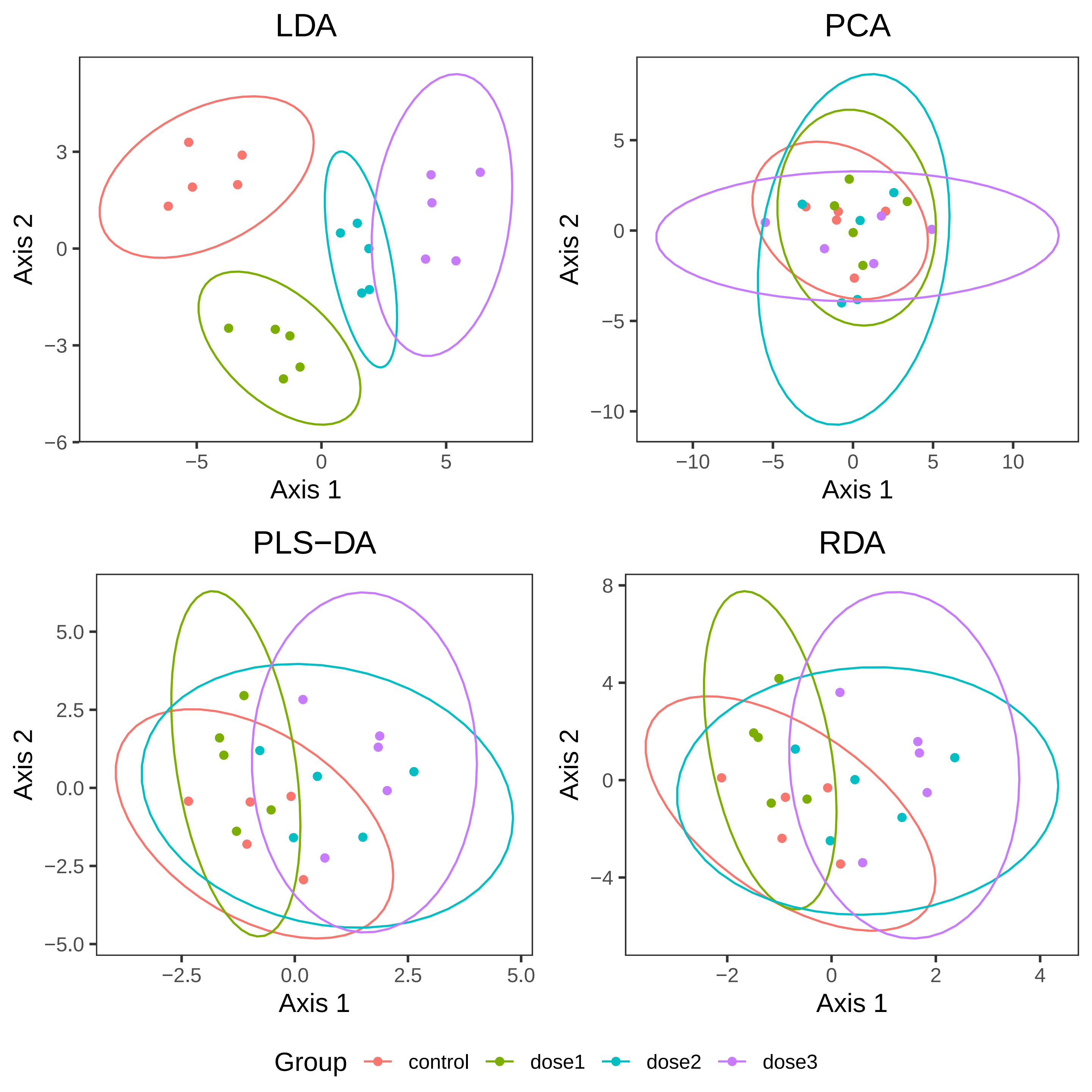

LDA
PCA
PLS−DA
RDA
Axis 1
Axis 2
Group
control
dose1
dose2
dose3

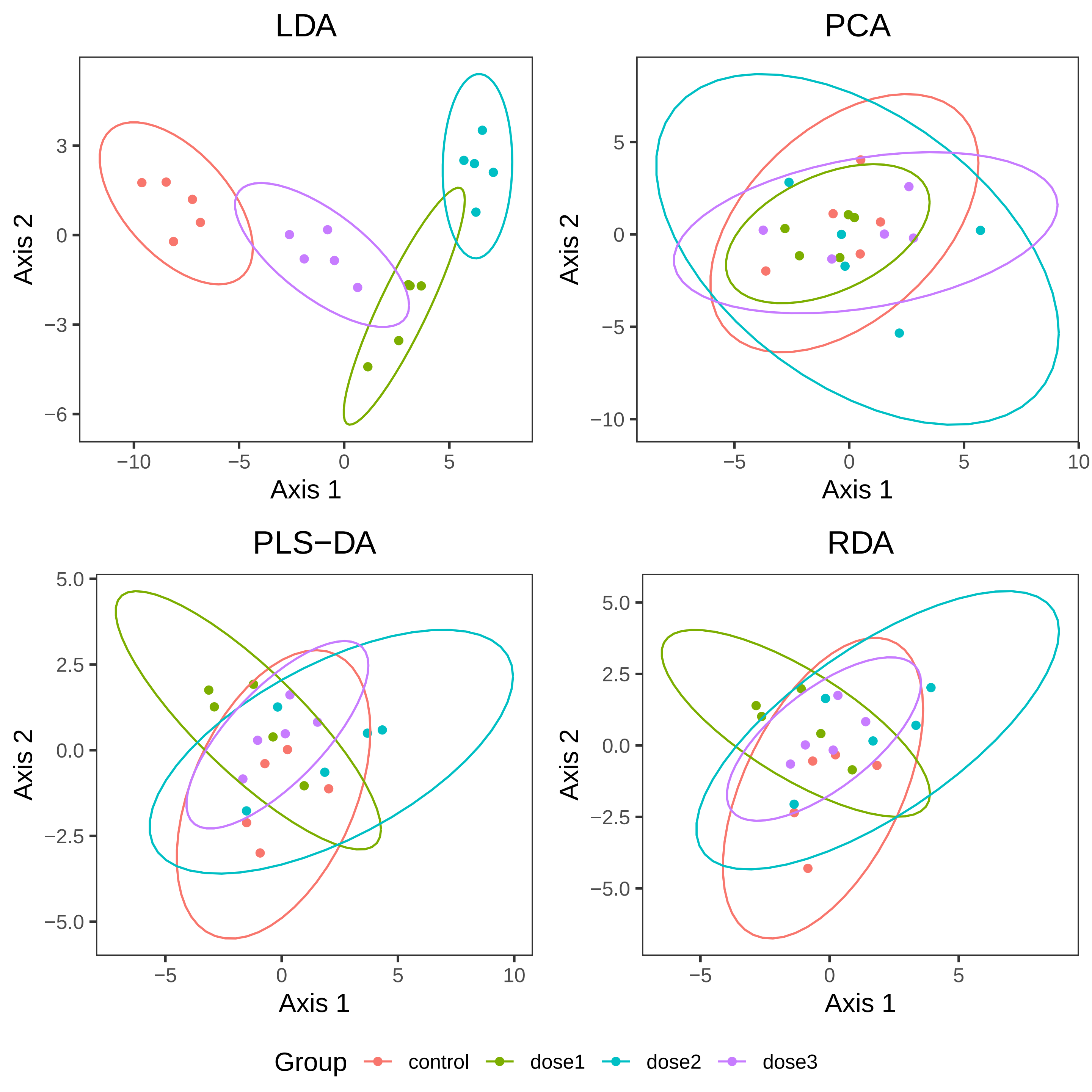
LDA
PCA
PLS−DA
RDA
Axis 1
Axis 2
3
0
−3
−6
−10
−5
0
5
10
−10
2.5
5.0
0.0
−2.5
−5.0
Group
control
dose1
dose2
dose3

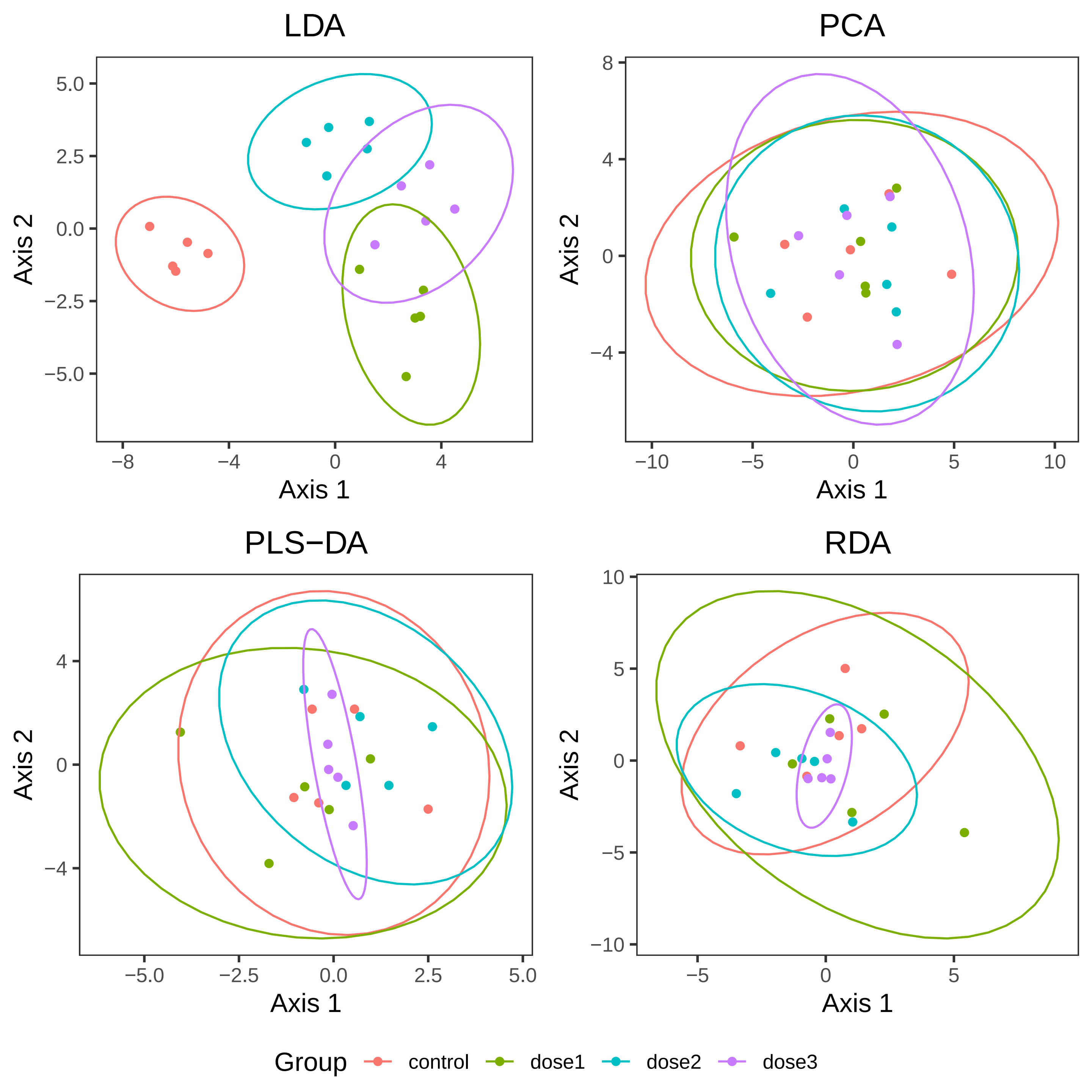
LDA
PCA
PLS−DA
RDA
Axis 1
Axis 2
Group
control
dose1
dose2
dose3

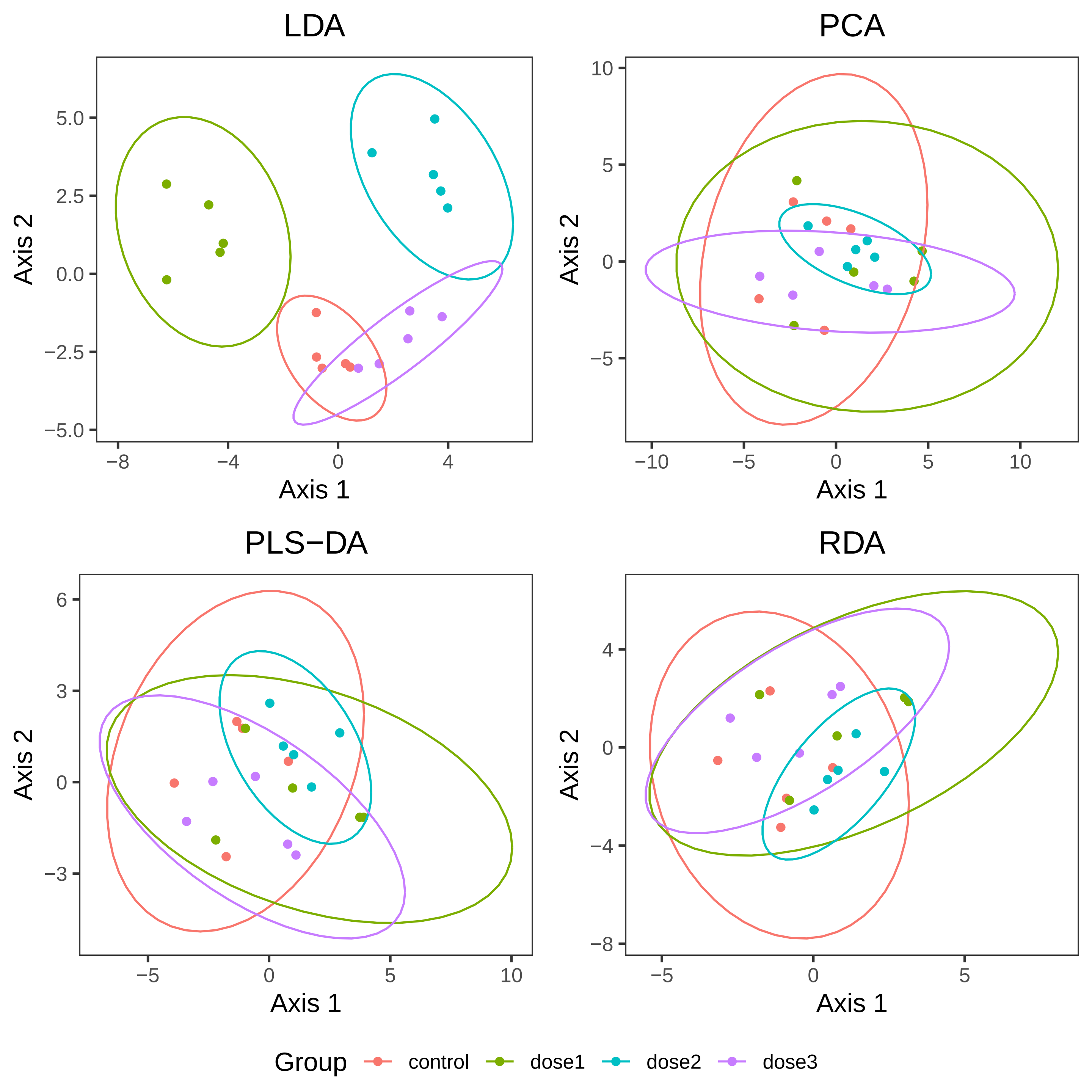

LDA
PCA
PLS−DA
RDA
Axis 1
Axis 2
Group
control
dose1
dose2
dose3

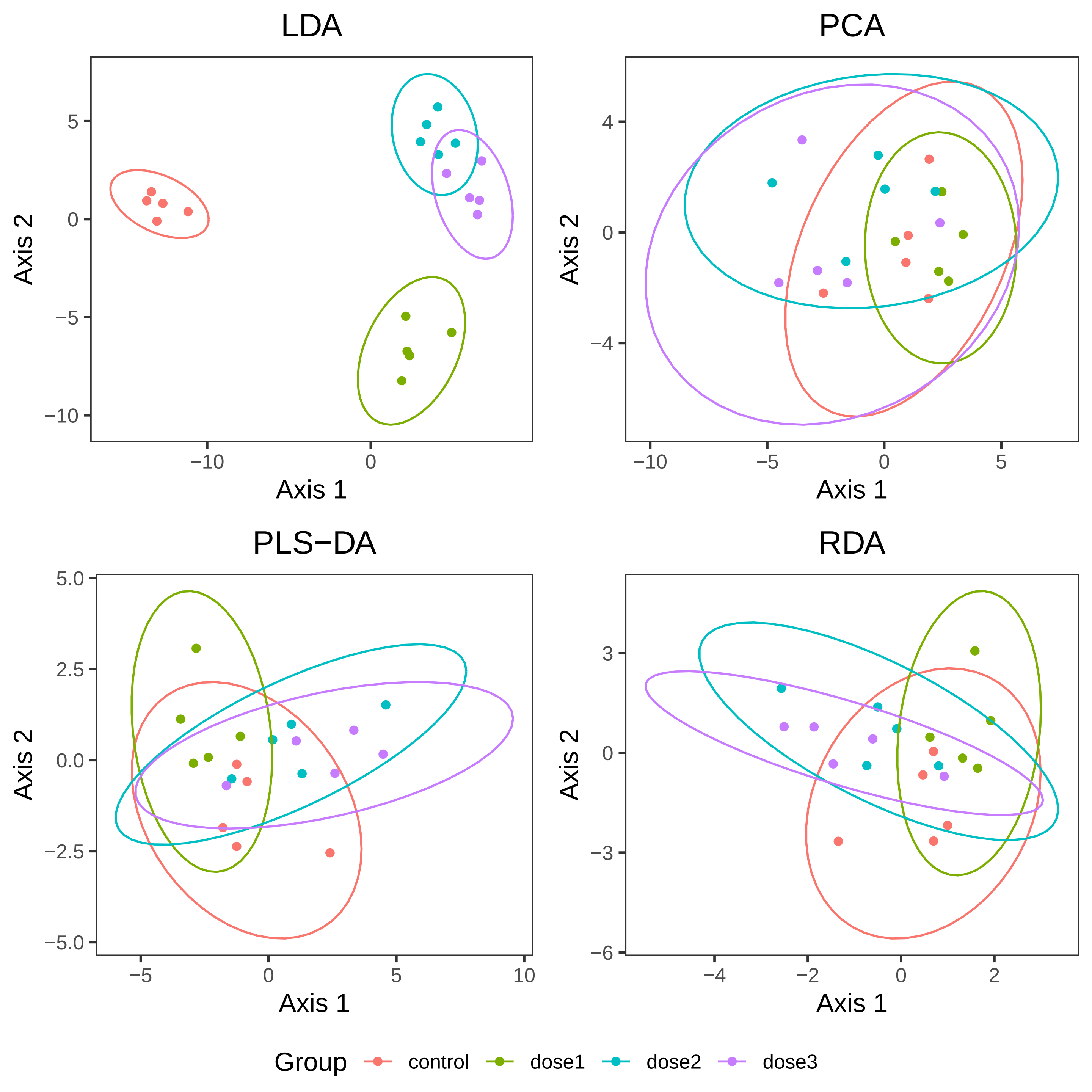

LDA
PCA
PLS−DA
RDA
Axis 1
Axis 2
5
0
−5
−10
−10
0
4
0
−4
−10
−5
0
5
5.0
2.5
0.0
−2.5
−5.0
−5
0
5
10
3
0
−3
−6
−4
−2
0
2
Group
control
dose1
dose2
dose3

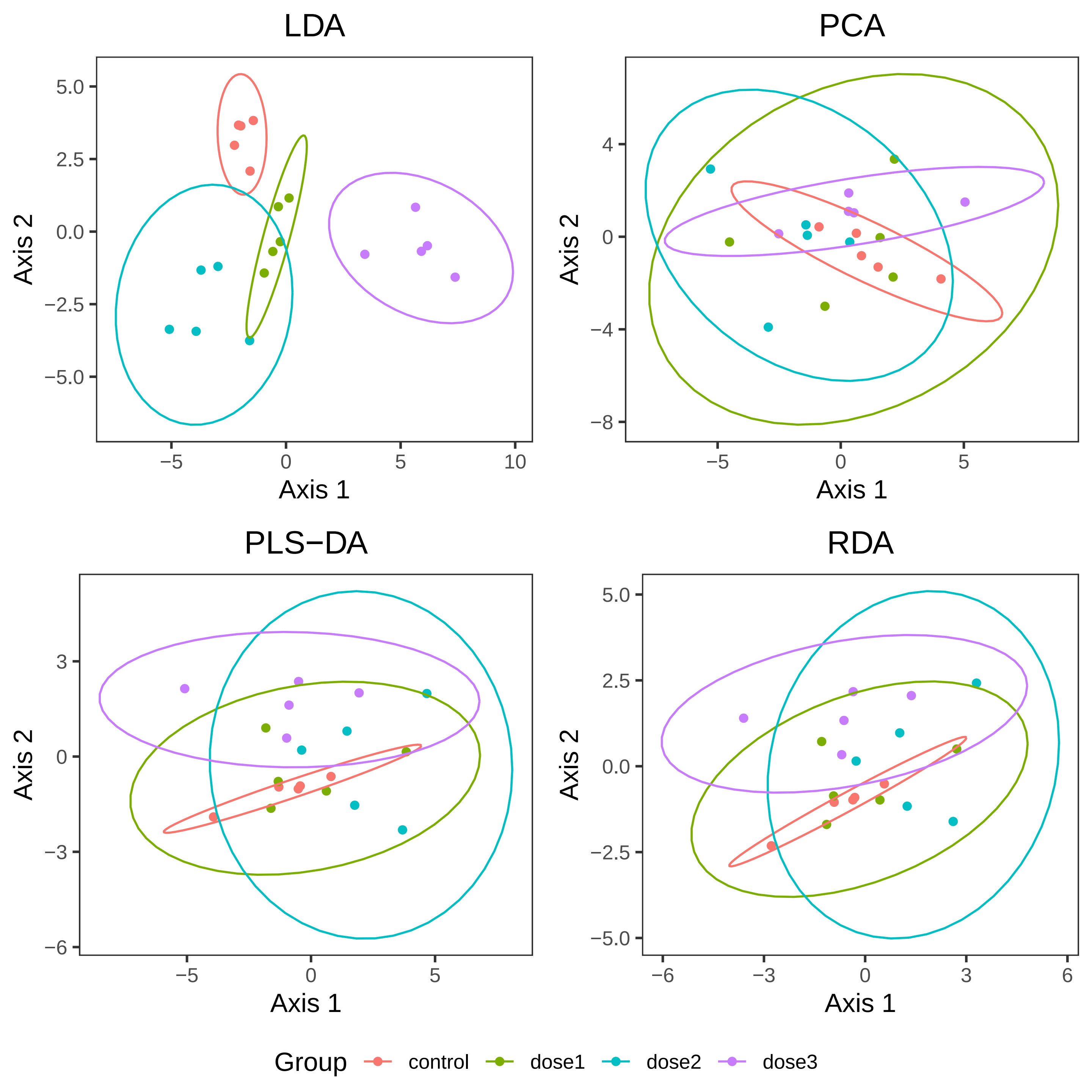

LDA
PCA
PLS−DA
RDA
Axis 1
Axis 2
5.0
2.5
0.0
−2.5
−5.0
−5
0
5
10
4
0
−4
−8
3
−3
−6
6
Group
control
dose1
dose2
dose3

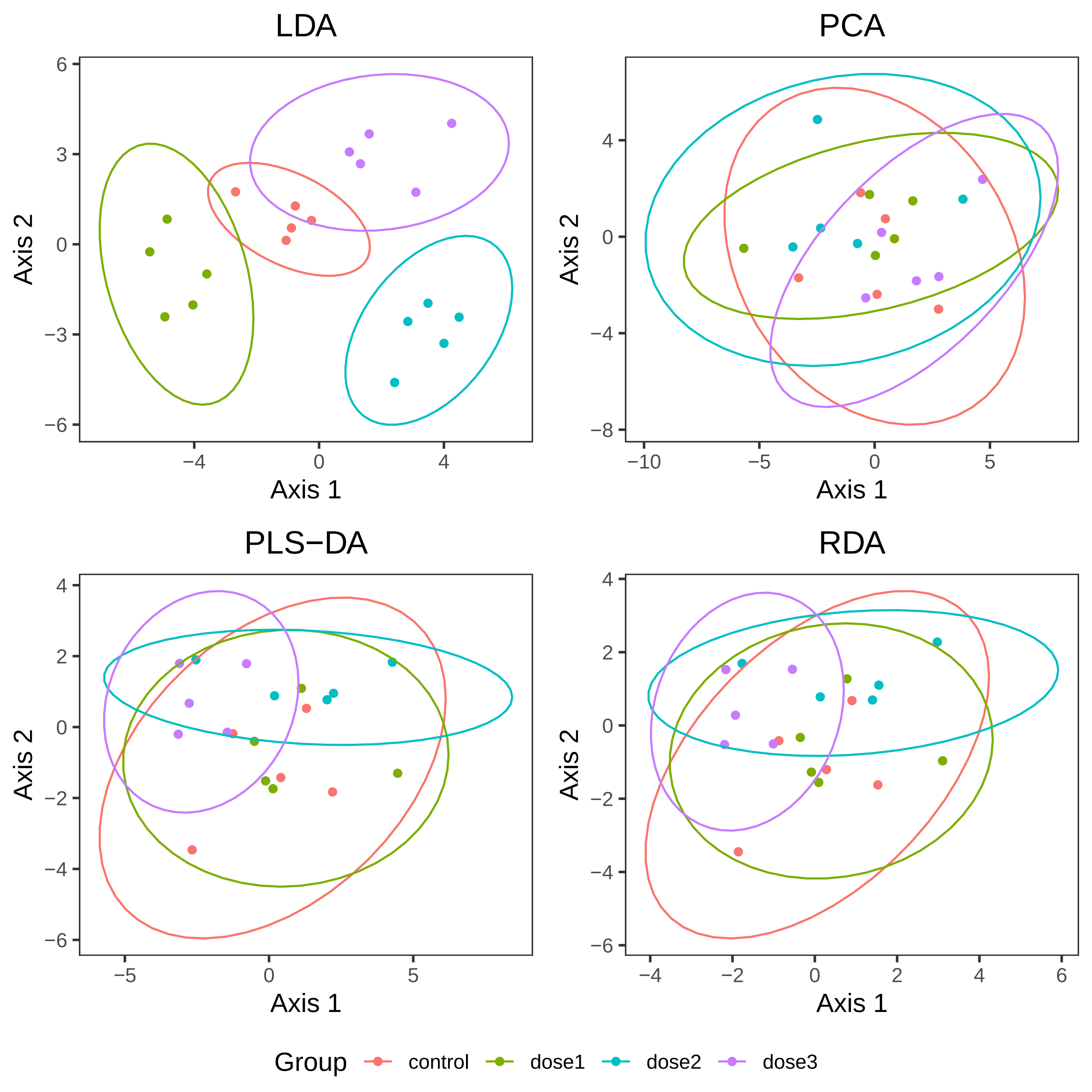
LDA
PCA
PLS−DA
RDA
Axis 1
Axis 2
6
3
0
−3
−6
−4
4
−8
−10
−5
5
2
−2
Group
control
dose1
dose2
dose3

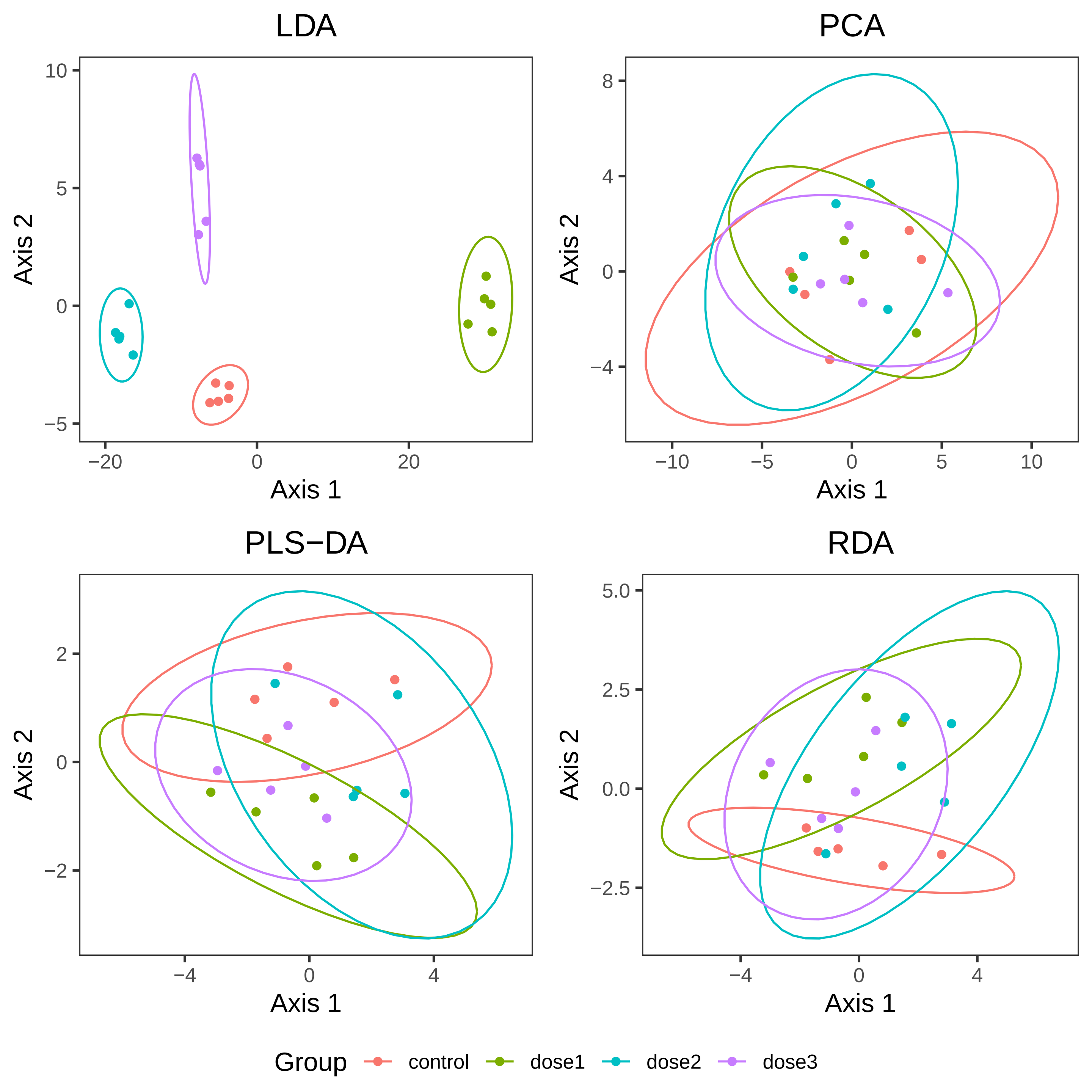

LDA
PCA
PLS−DA
RDA
Axis 1
Axis 2
Group
control
dose1
dose2
dose3

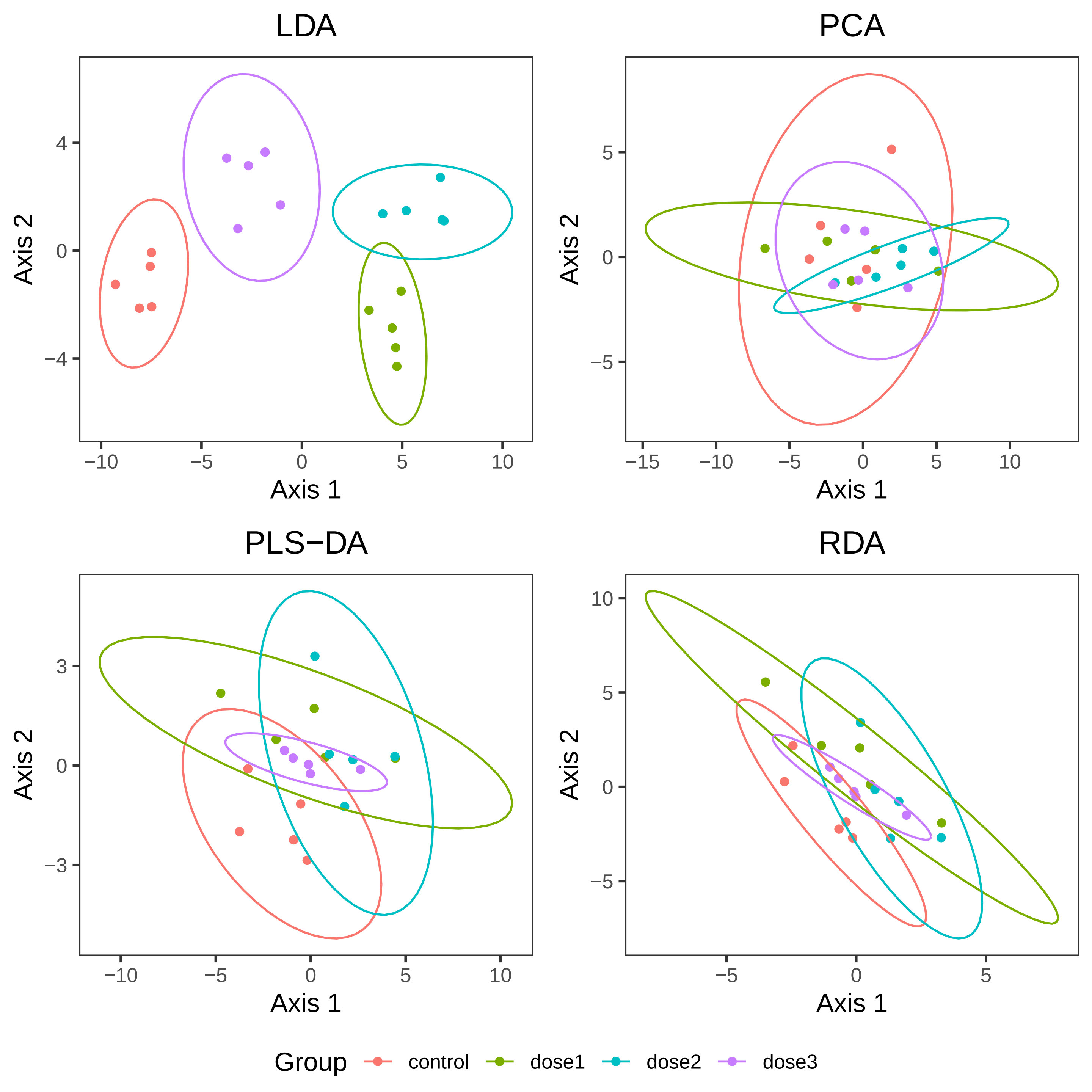

LDA
PCA
PLS−DA
RDA
Axis 1
Axis 2
Group
control
dose1
dose2
dose3

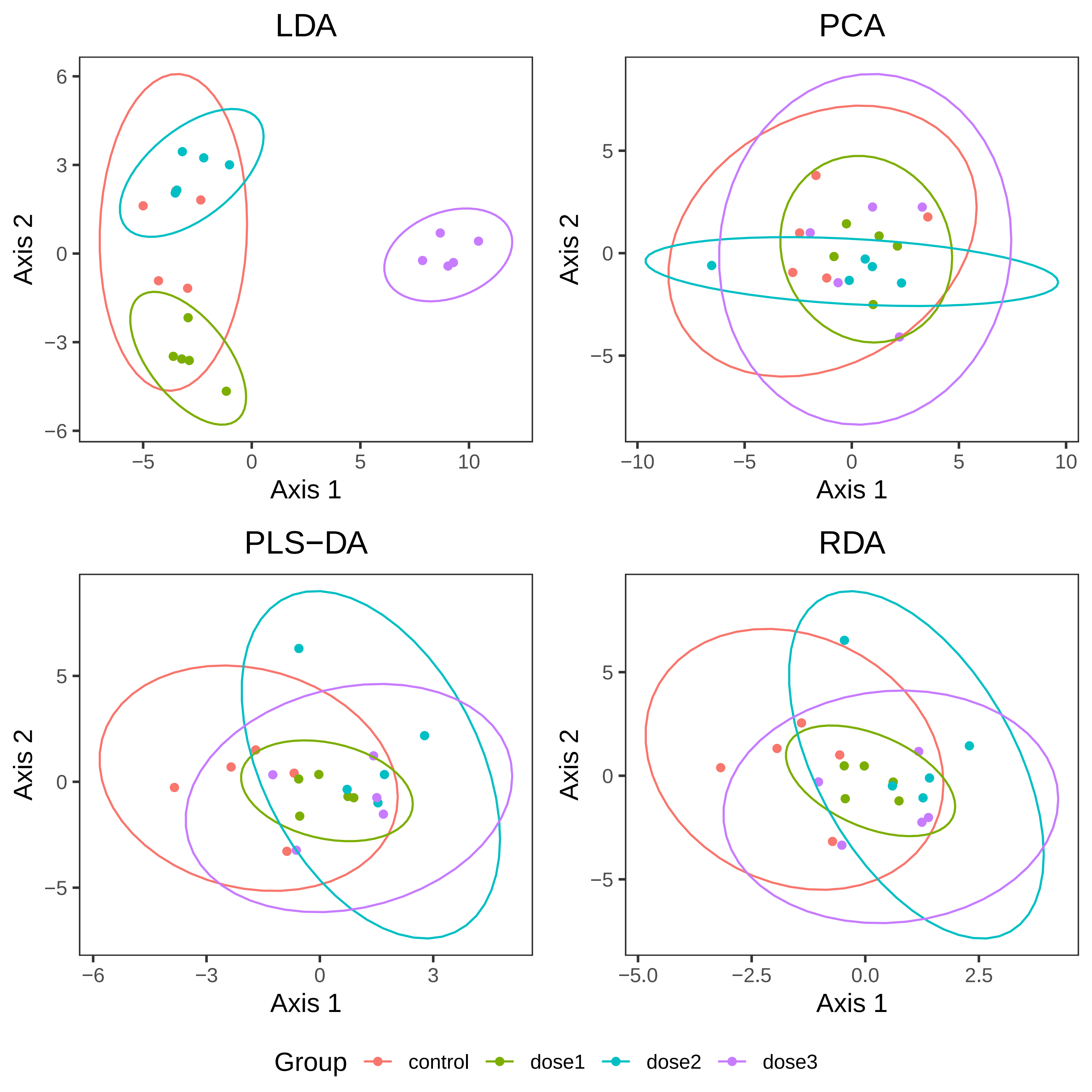

LDA
PCA
PLS−DA
RDA
Axis 1
Axis 2
Group
control
dose1
dose2
dose3

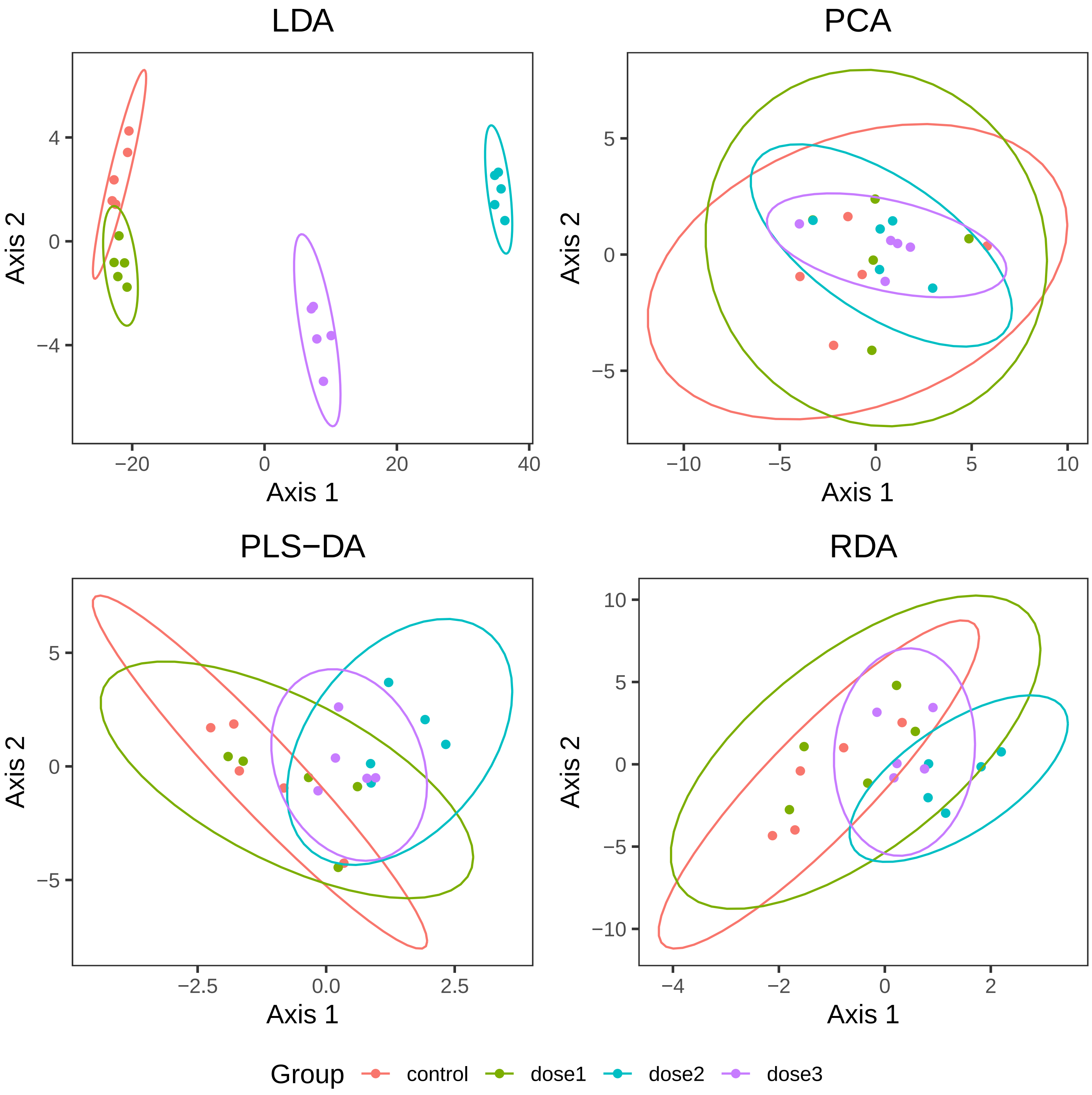
LDA
PCA
PLS−DA
RDA
Axis 1
Axis 2
Group
control
dose1
dose2
dose3

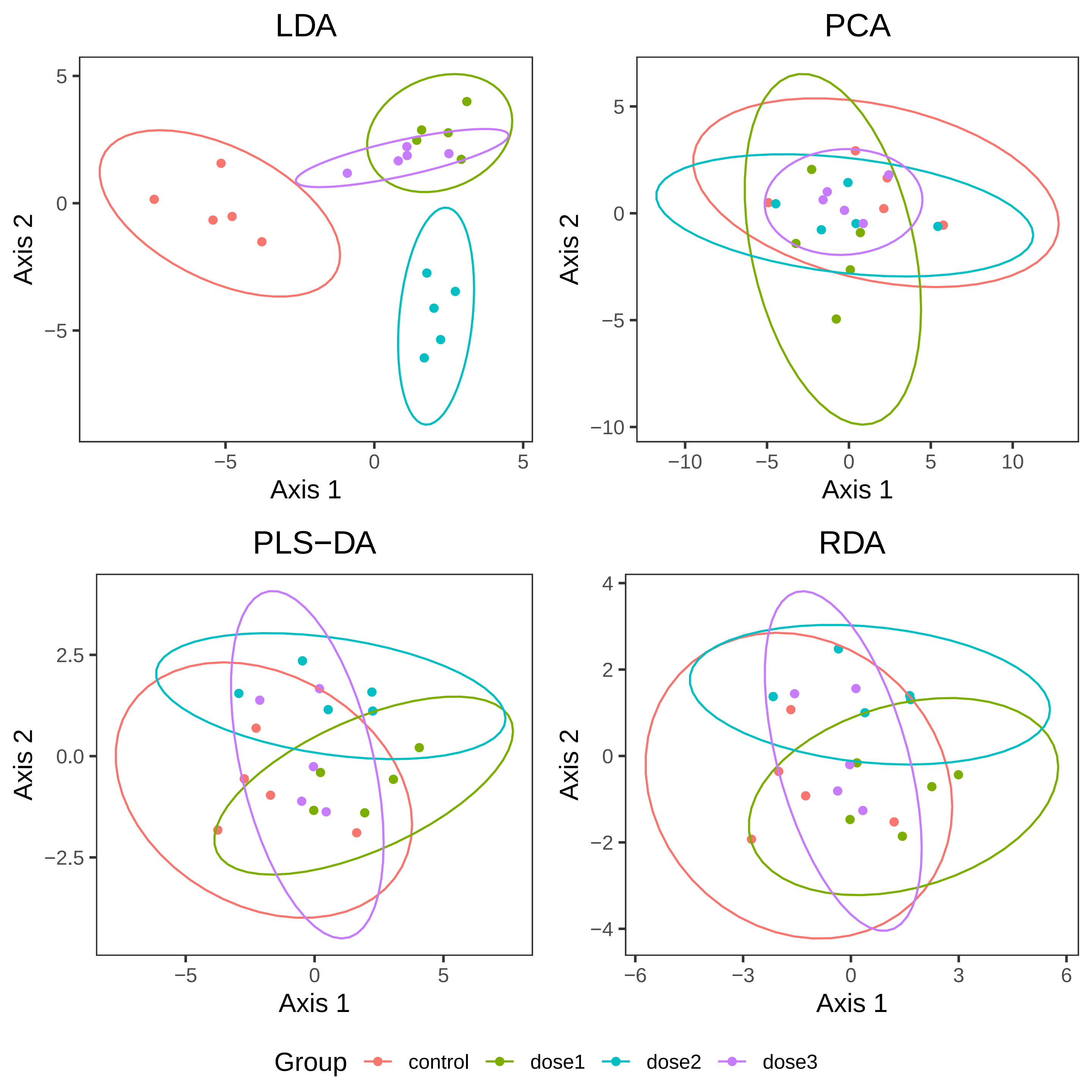
LDA
PCA
PLS−DA
RDA
Axis 1
Axis 2
5
0
−5
−10
10
2.5
0.0
−2.5
4
2
−2
−4
−6
−3
3
6
Group
control
dose1
dose2
dose3

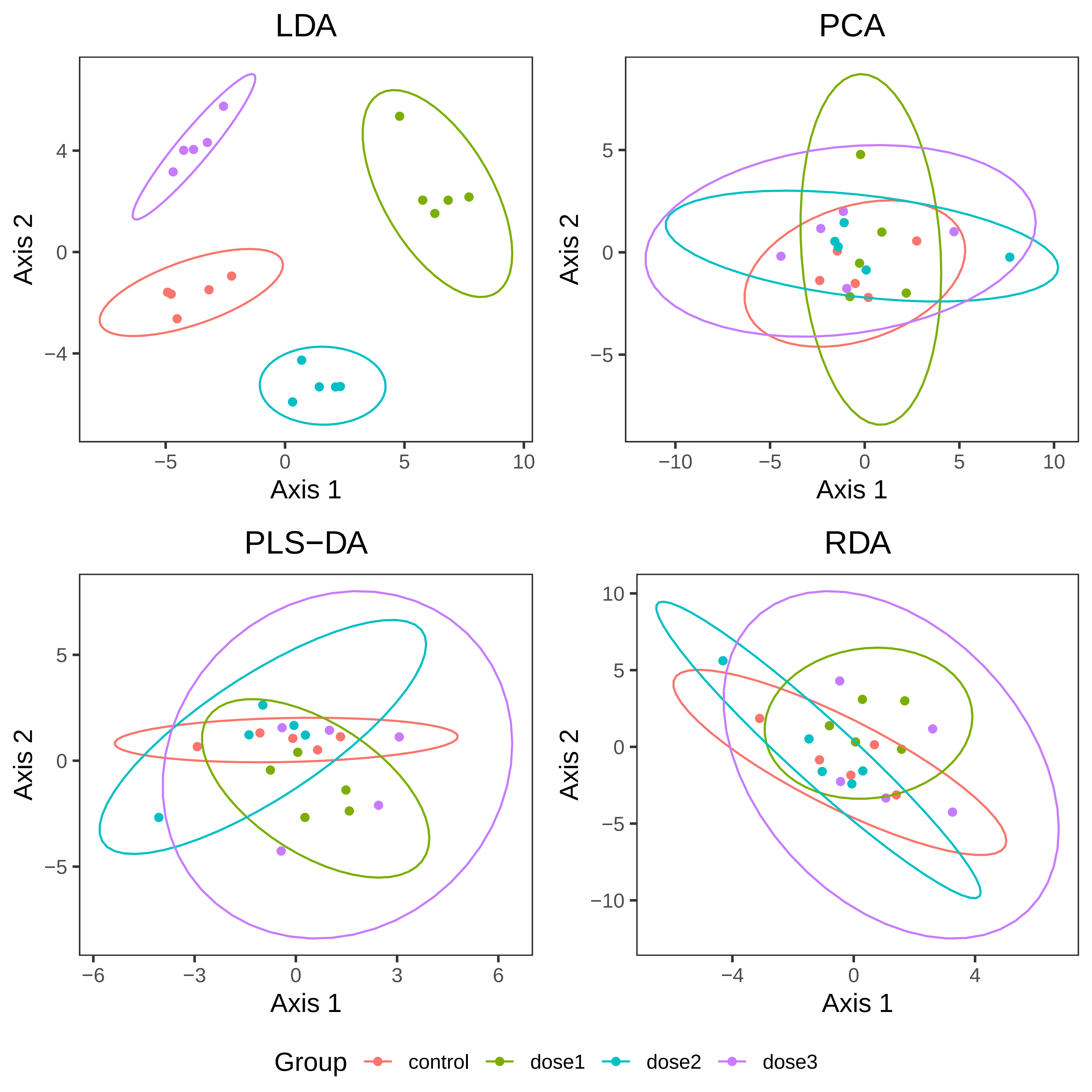
LDA
PCA
PLS−DA
RDA
Axis 1
Axis 2
Group
control
dose1
dose2
dose3

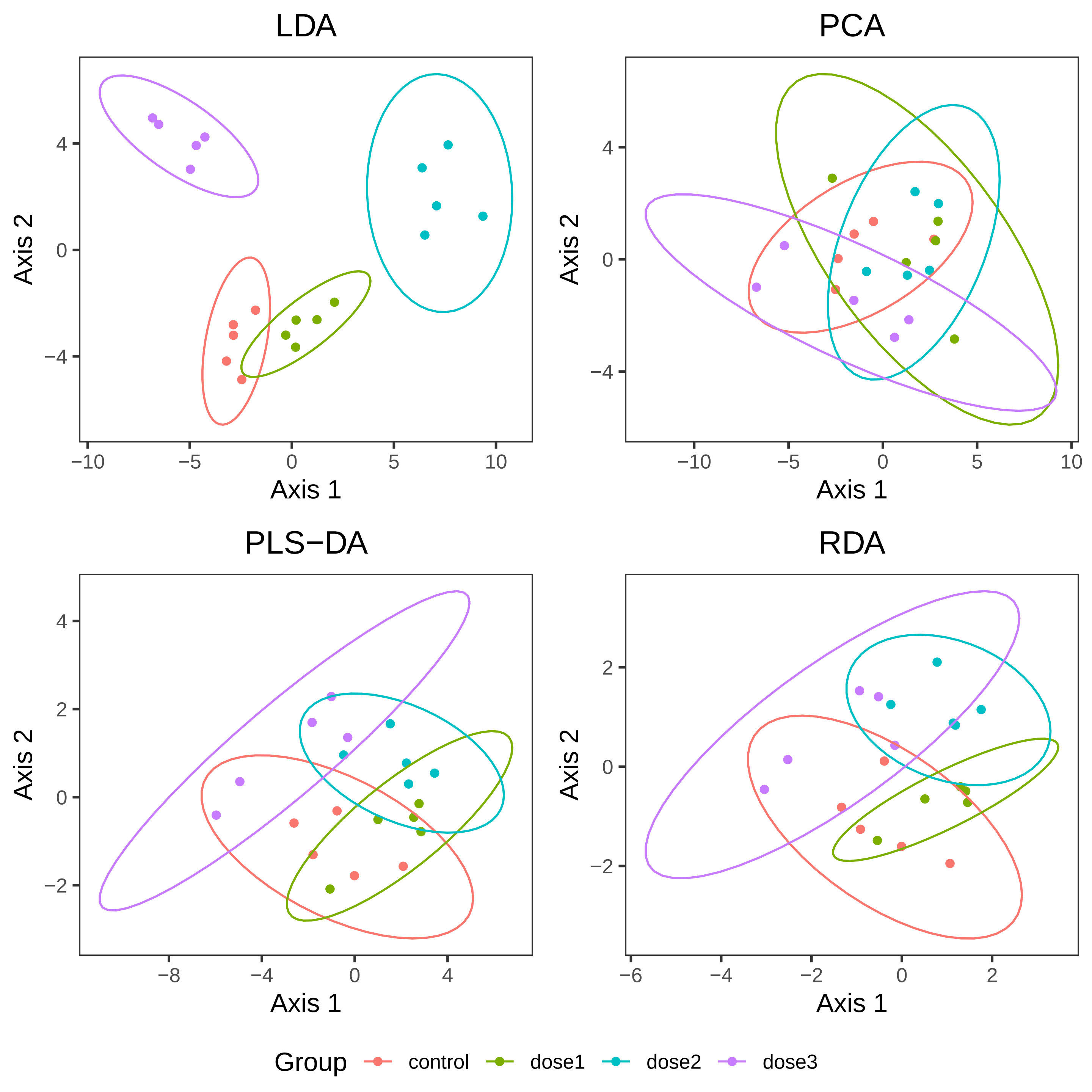
LDA
PCA
PLS−DA
RDA
Axis 1
Axis 2
Group
control
dose1
dose2
dose3

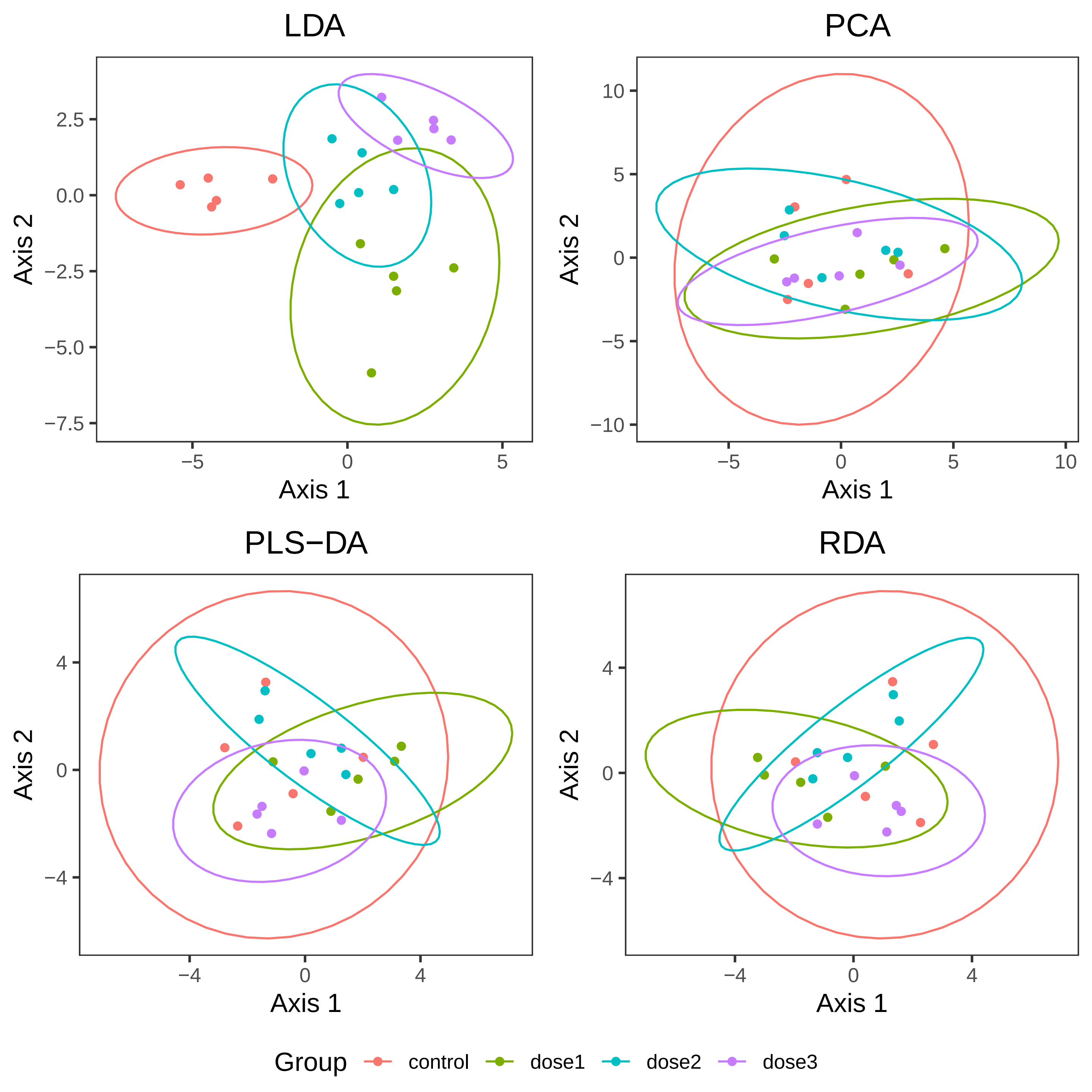
LDA
PCA
PLS−DA
RDA
Axis 1
Axis 2
2.5
0.0
−2.5
−5.0
−7.5
−5
0
5
10
−10
4
−4
Group
control
dose1
dose2
dose3

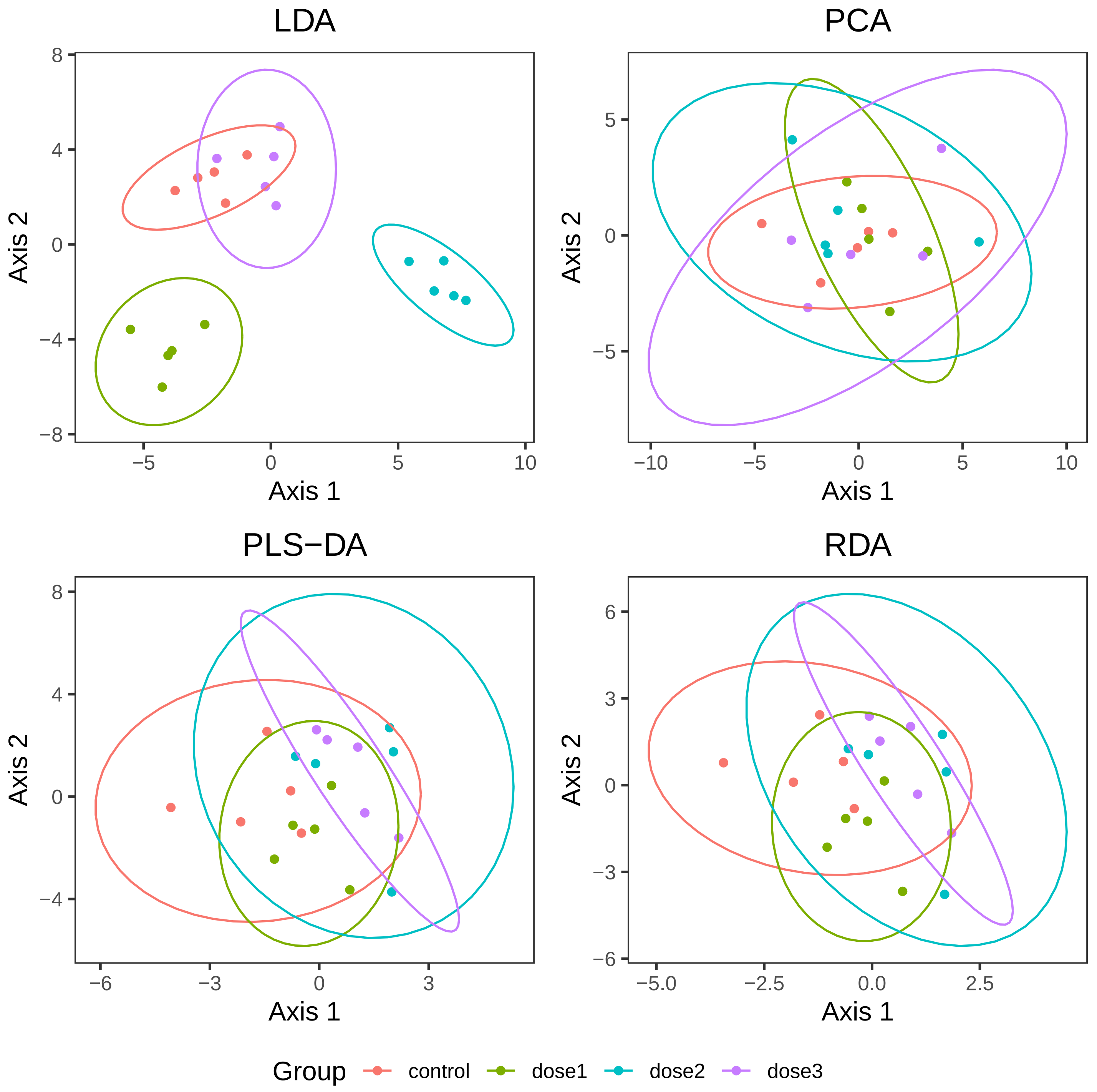
LDA
PCA
PLS−DA
RDA
Axis 1
Axis 2
Group
control
dose1
dose2
dose3

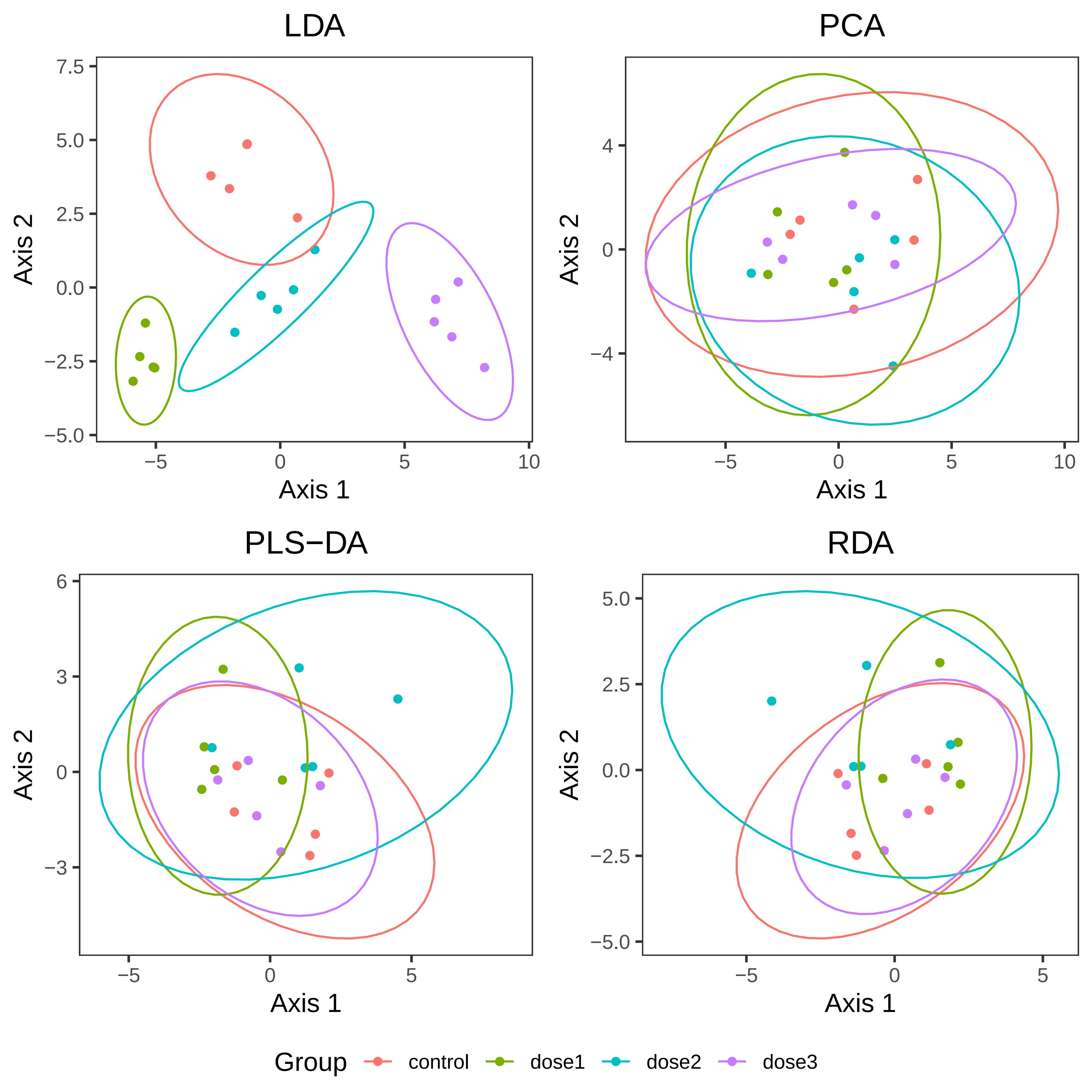

LDA
PCA
PLS−DA
RDA
Axis 1
Axis 2
Group
control
dose1
dose2
dose3

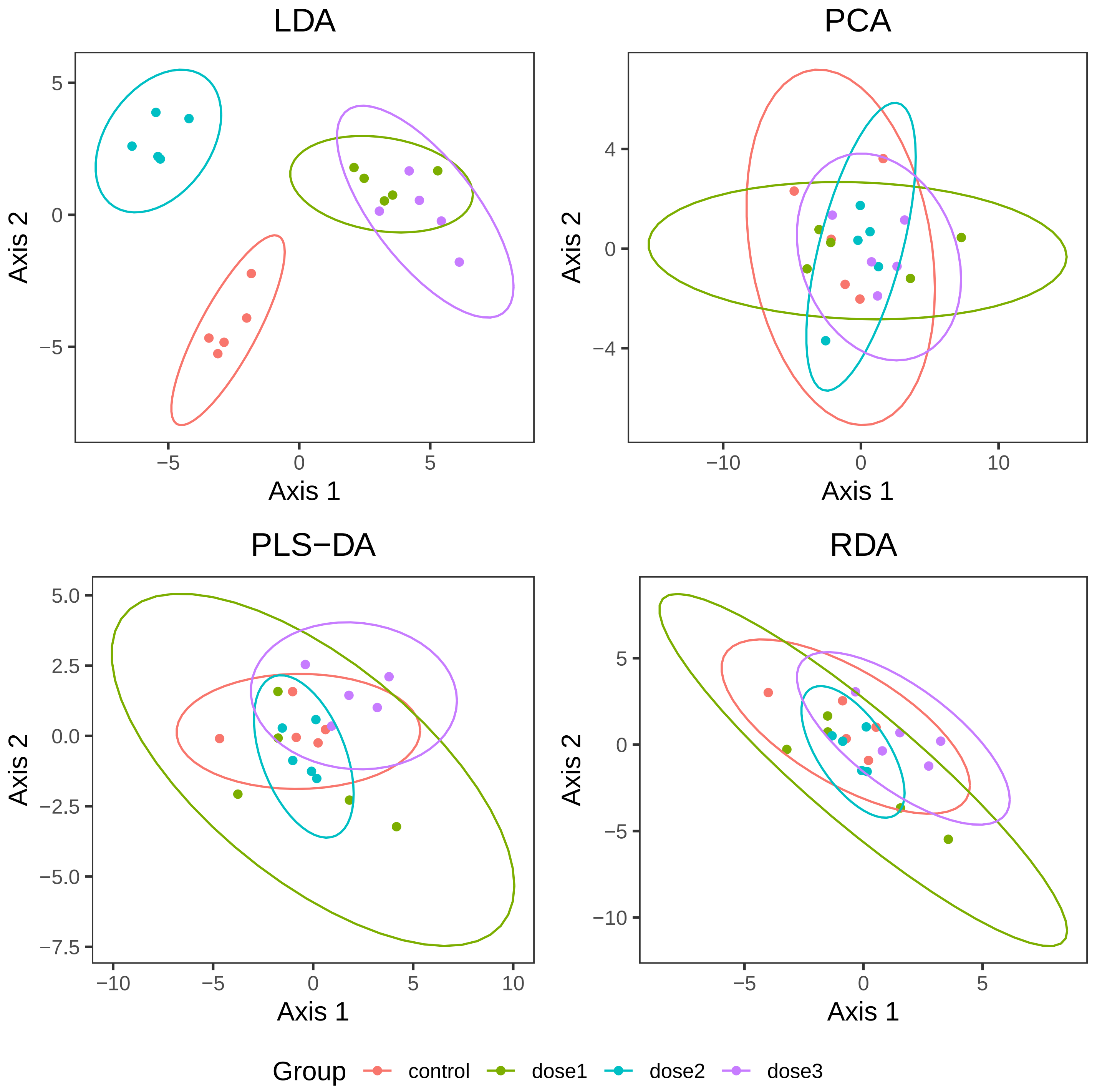
LDA
PCA
PLS−DA
RDA
Axis 1
Axis 2
Group
control
dose1
dose2
dose3

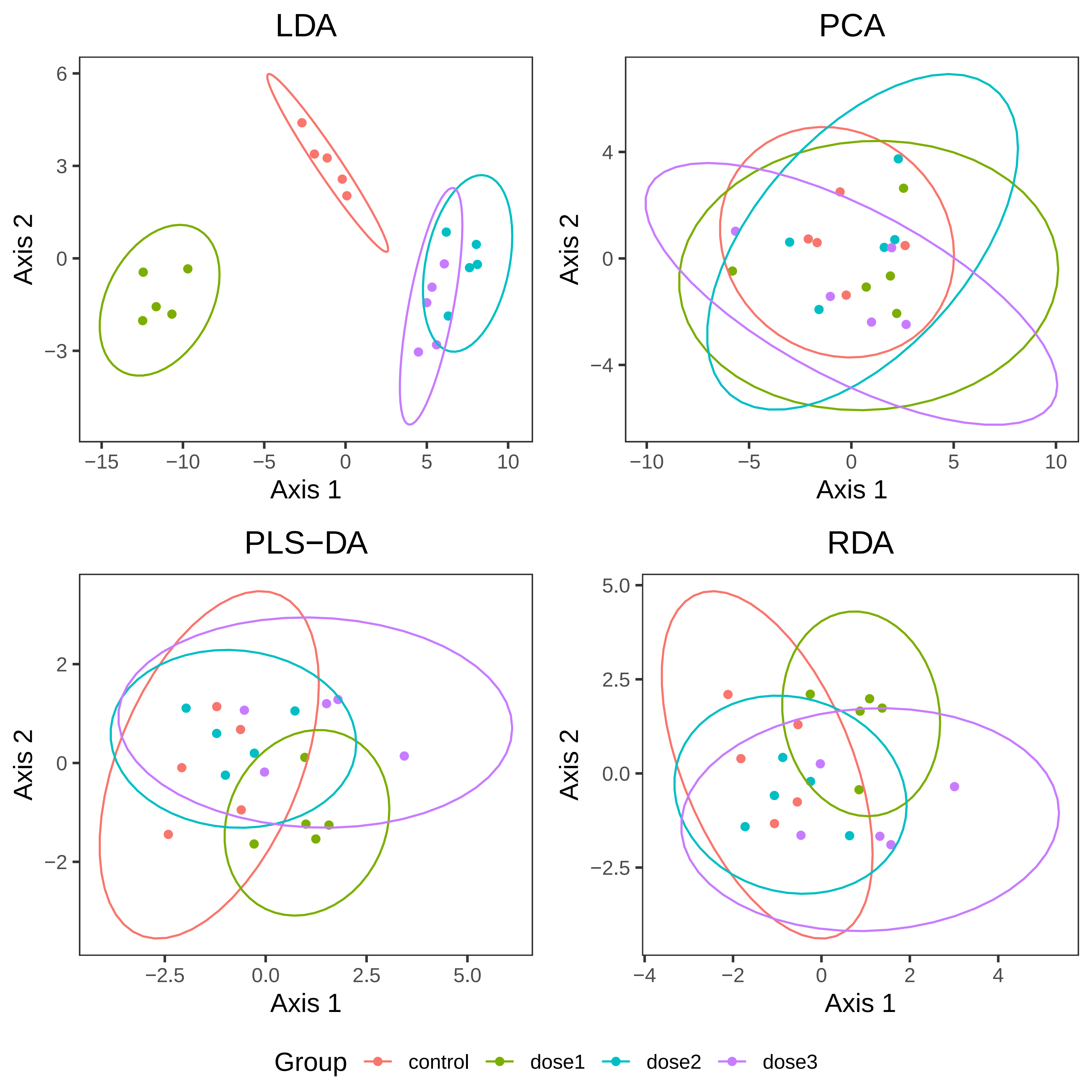
LDA
PCA
PLS−DA
RDA
Axis 1
Axis 2
6
3
0
−3
−15
−10
−5
0
5
10
4
0
−4
2
0
−2
−2.5
0.0
2.5
5.0
−2.5
0.0
2.5
5.0
−4
−2
0
2
4
Group
control
dose1
dose2
dose3

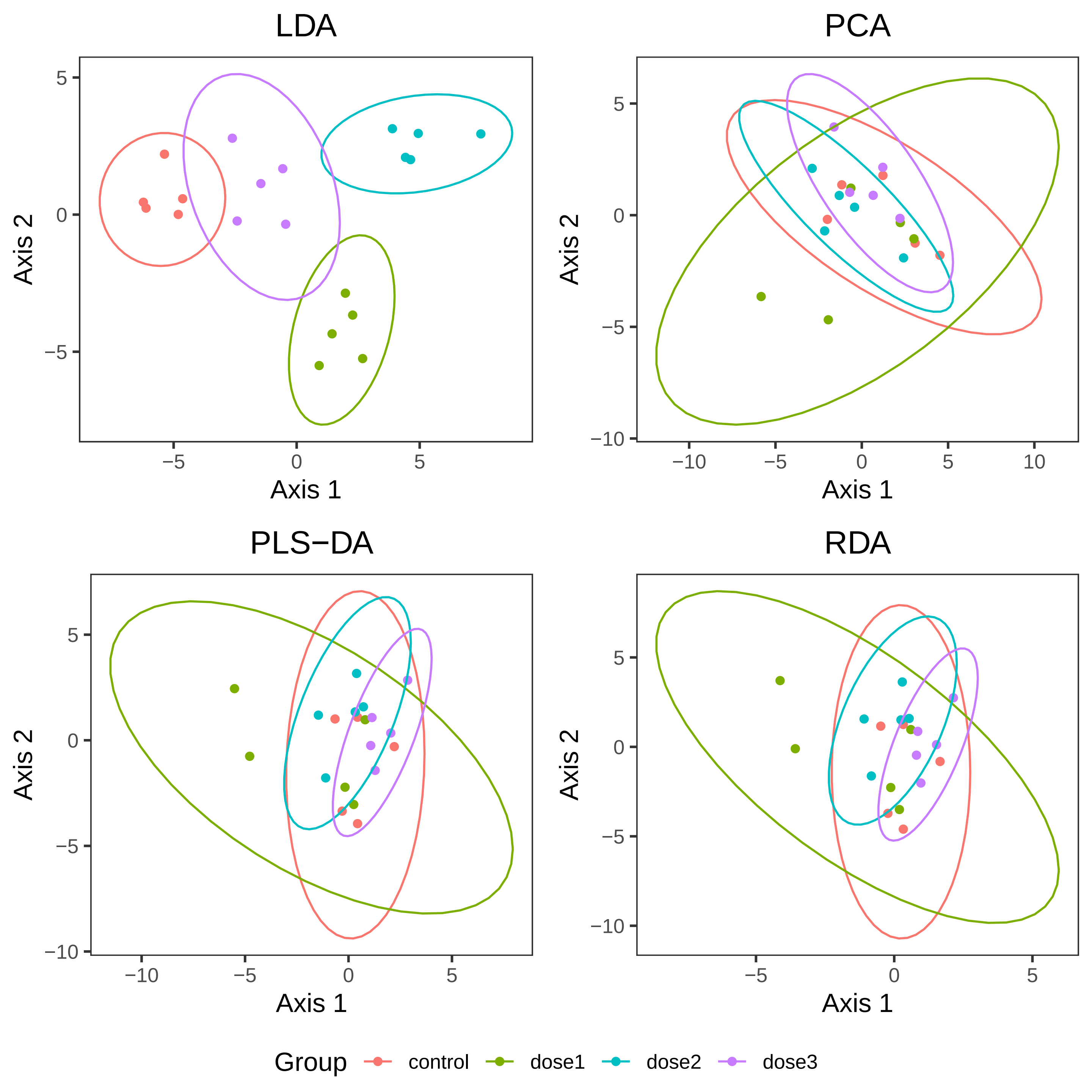

LDA
PCA
PLS−DA
RDA
Axis 1
Axis 2
Group
control
dose1
dose2
dose3

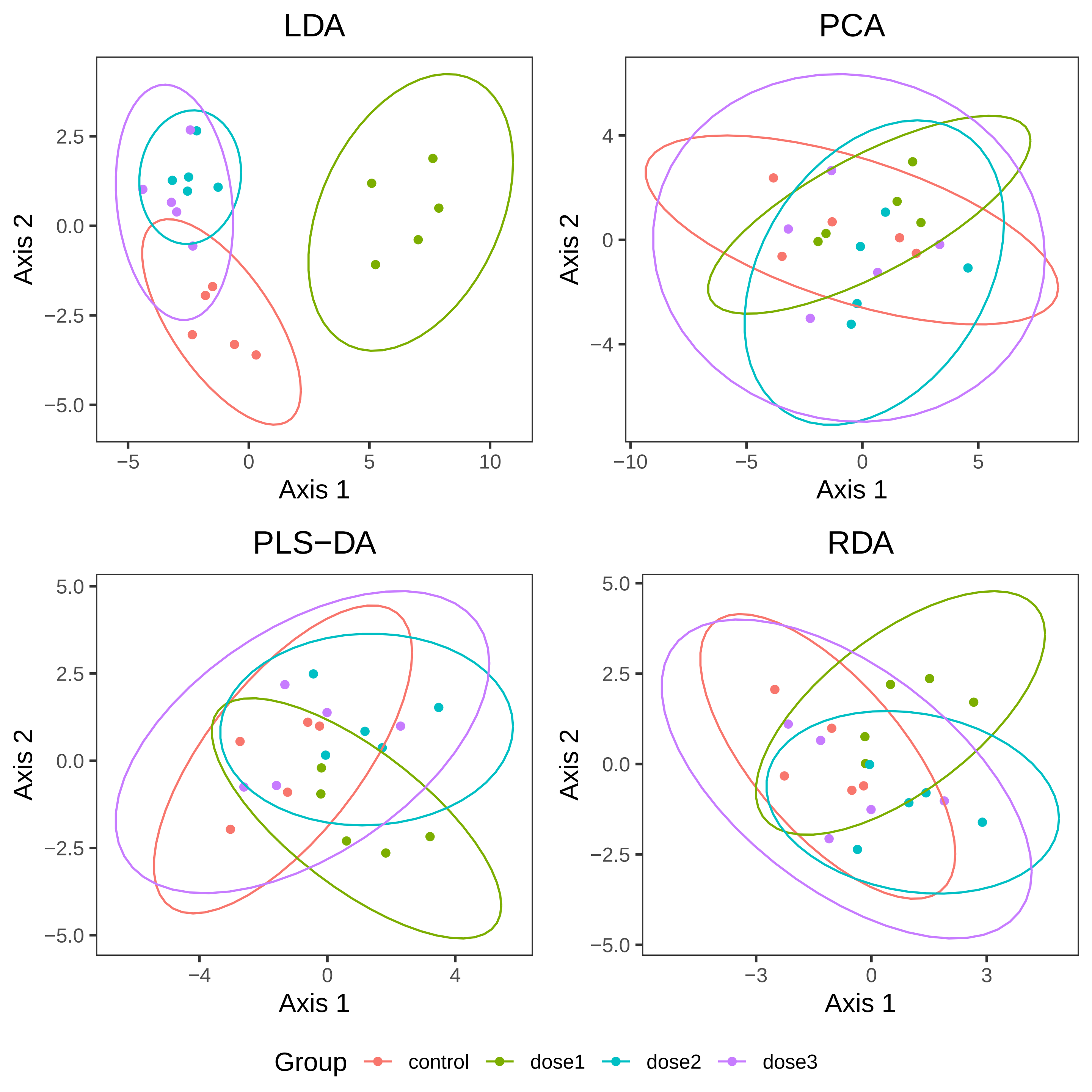
LDA
PCA
PLS−DA
RDA
Axis 1
Axis 2
2.5
0.0
−2.5
−5.0
−5
0
5
10
4
0
−4
−10
5.0
−4
3
−3
Group
control
dose1
dose2
dose3

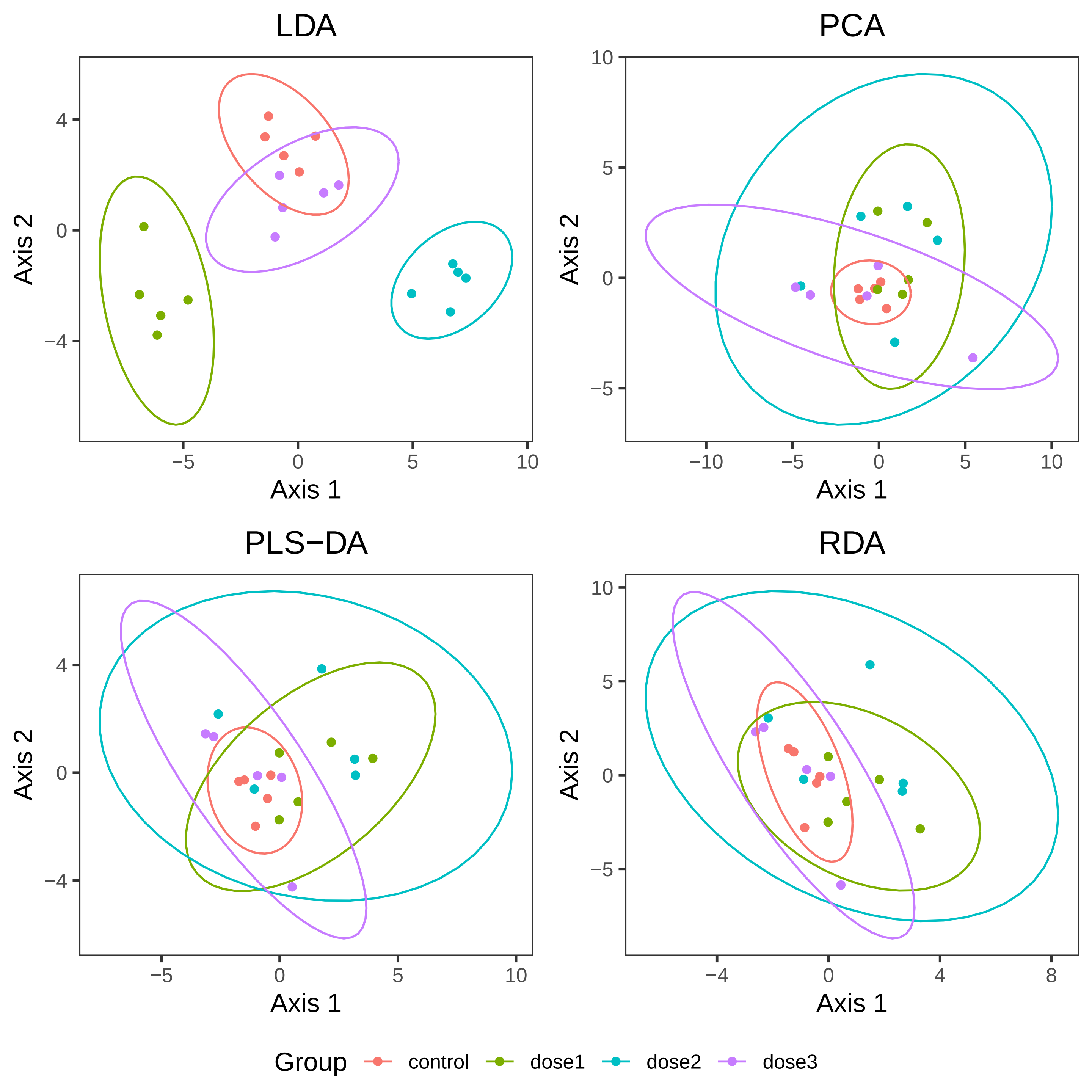
LDA
PCA
PLS−DA
RDA
Axis 1
Axis 2
Group
control
dose1
dose2
dose3

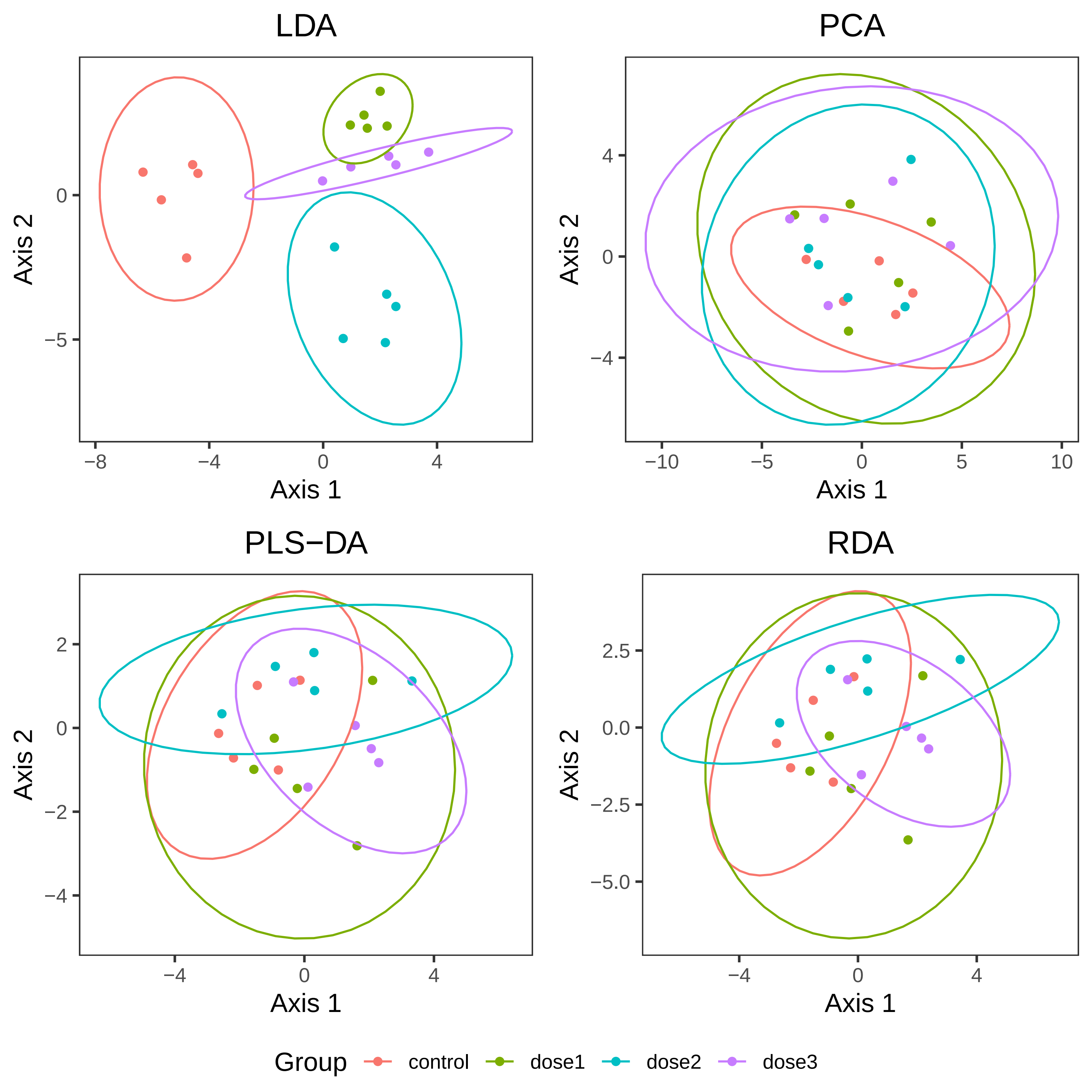

LDA
PCA
PLS−DA
RDA
Axis 1
Axis 2
Group
control
dose1
dose2
dose3

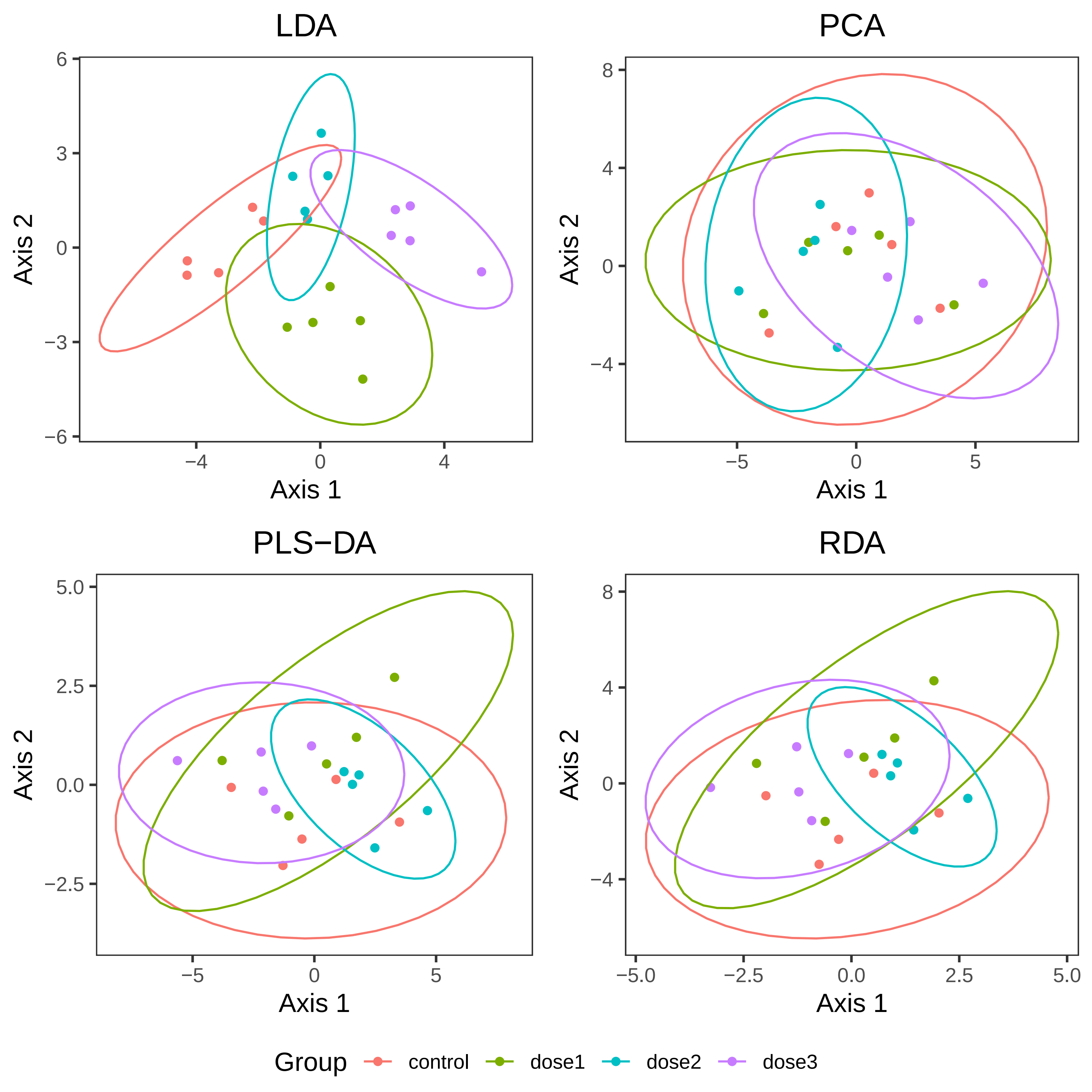

LDA
PCA
PLS−DA
RDA
Axis 1
Axis 2
Group
control
dose1
dose2
dose3

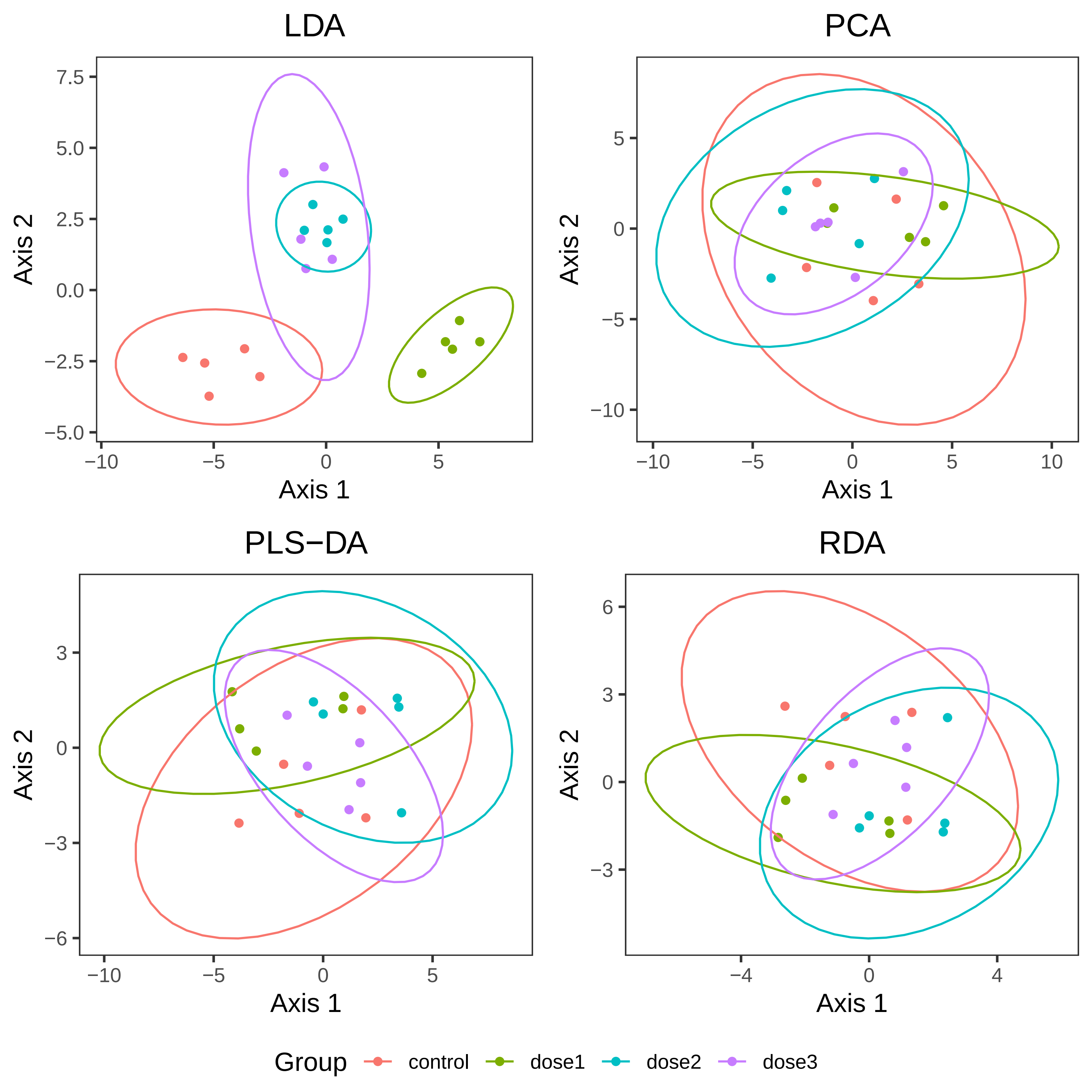
LDA
PCA
PLS−DA
RDA
Axis 1
Axis 2
Group
control
dose1
dose2
dose3

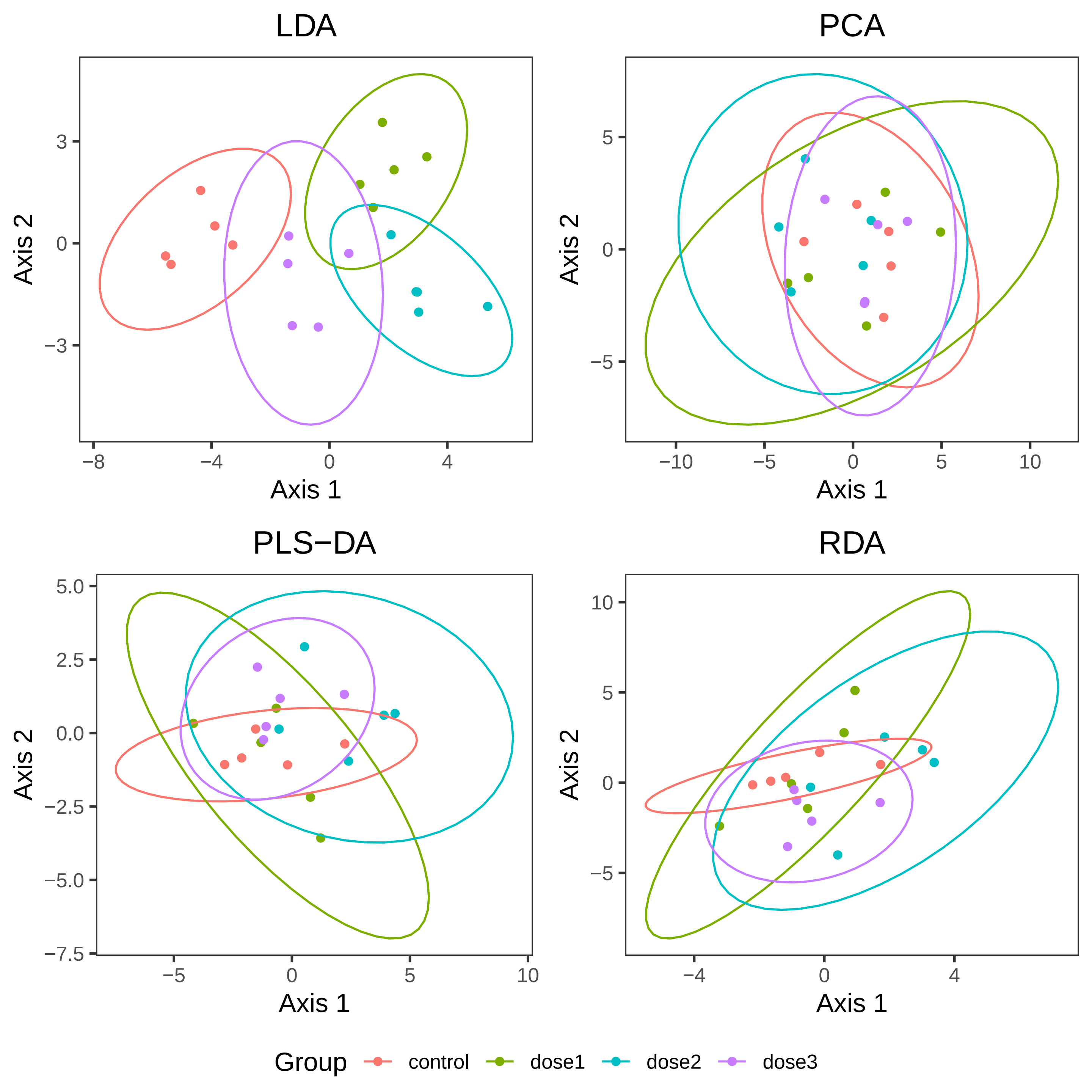

LDA
PCA
PLS−DA
RDA
Axis 1
Axis 2
−8
−4
0
4
3
−3
−10
−5
5
10
5.0
2.5
0.0
−2.5
−5.0
−7.5
Group
control
dose1
dose2
dose3

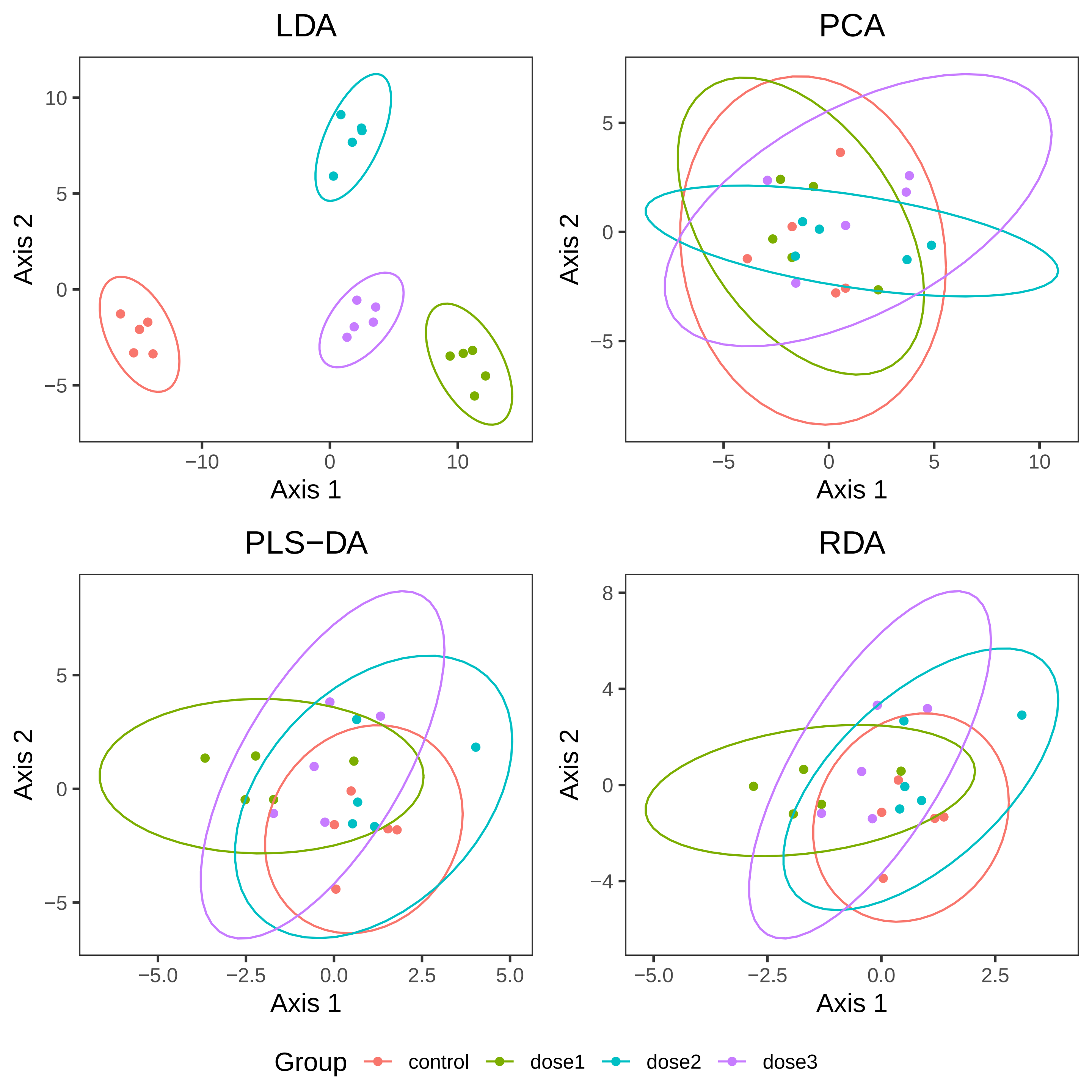
LDA
PCA
PLS−DA
RDA
Axis 1
Axis 2
Group
control
dose1
dose2
dose3

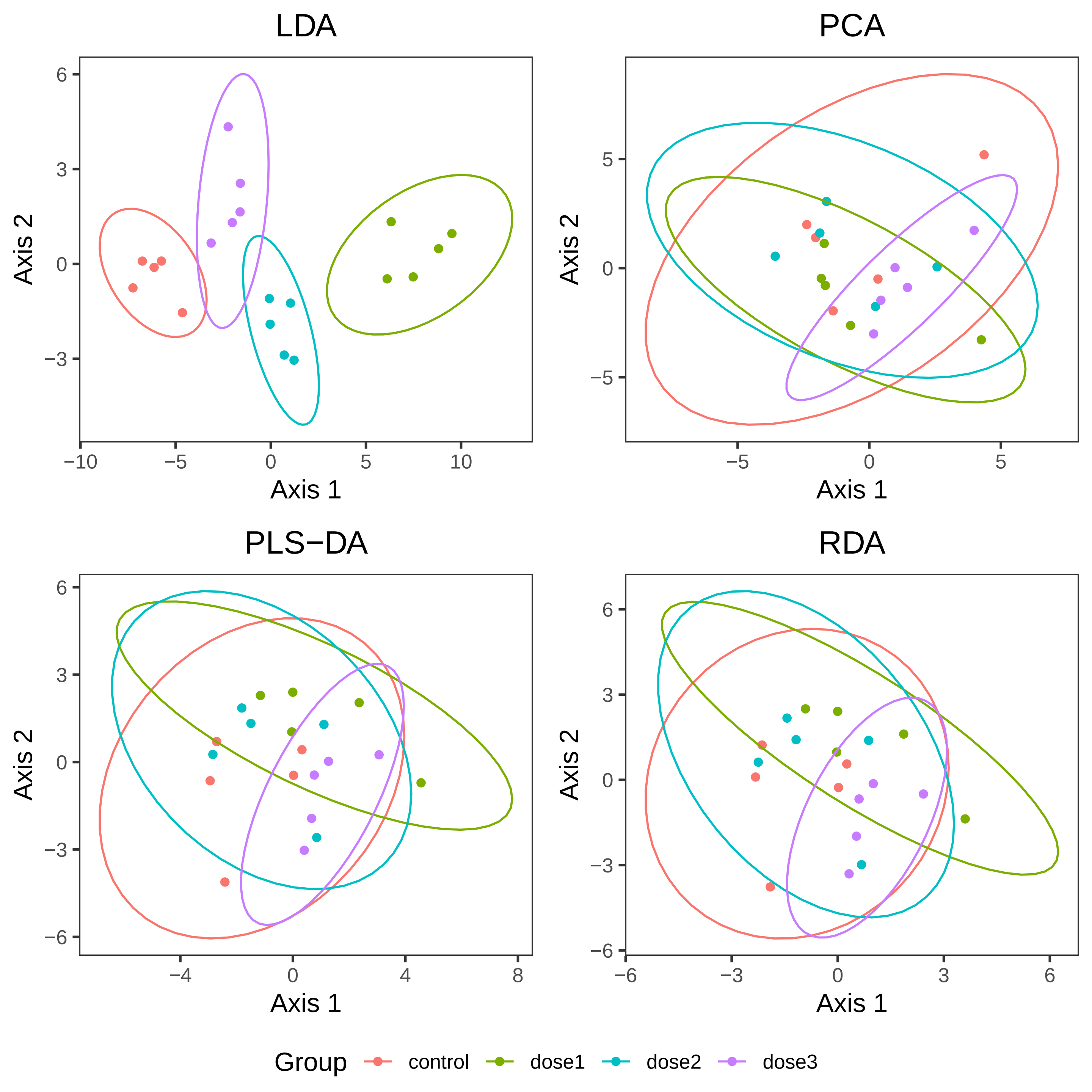
LDA
PCA
PLS−DA
RDA
Axis 1
Axis 2
Group
control
dose1
dose2
dose3

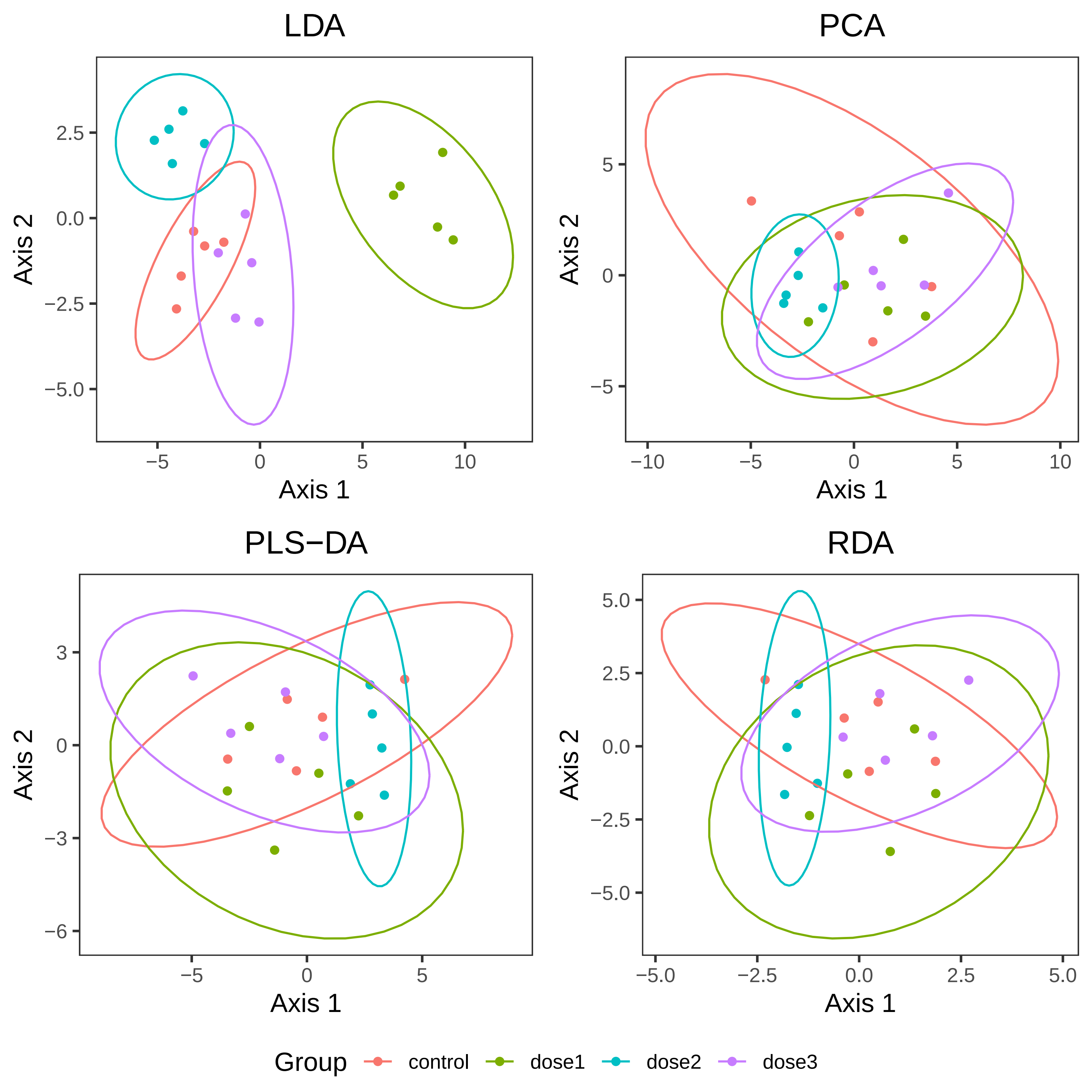
LDA
PCA
PLS−DA
RDA
Axis 1
Axis 2
2.5
0.0
−2.5
−5.0
−5
0
5
10
−10
3
−6
−3
5.0
Group
control
dose1
dose2
dose3

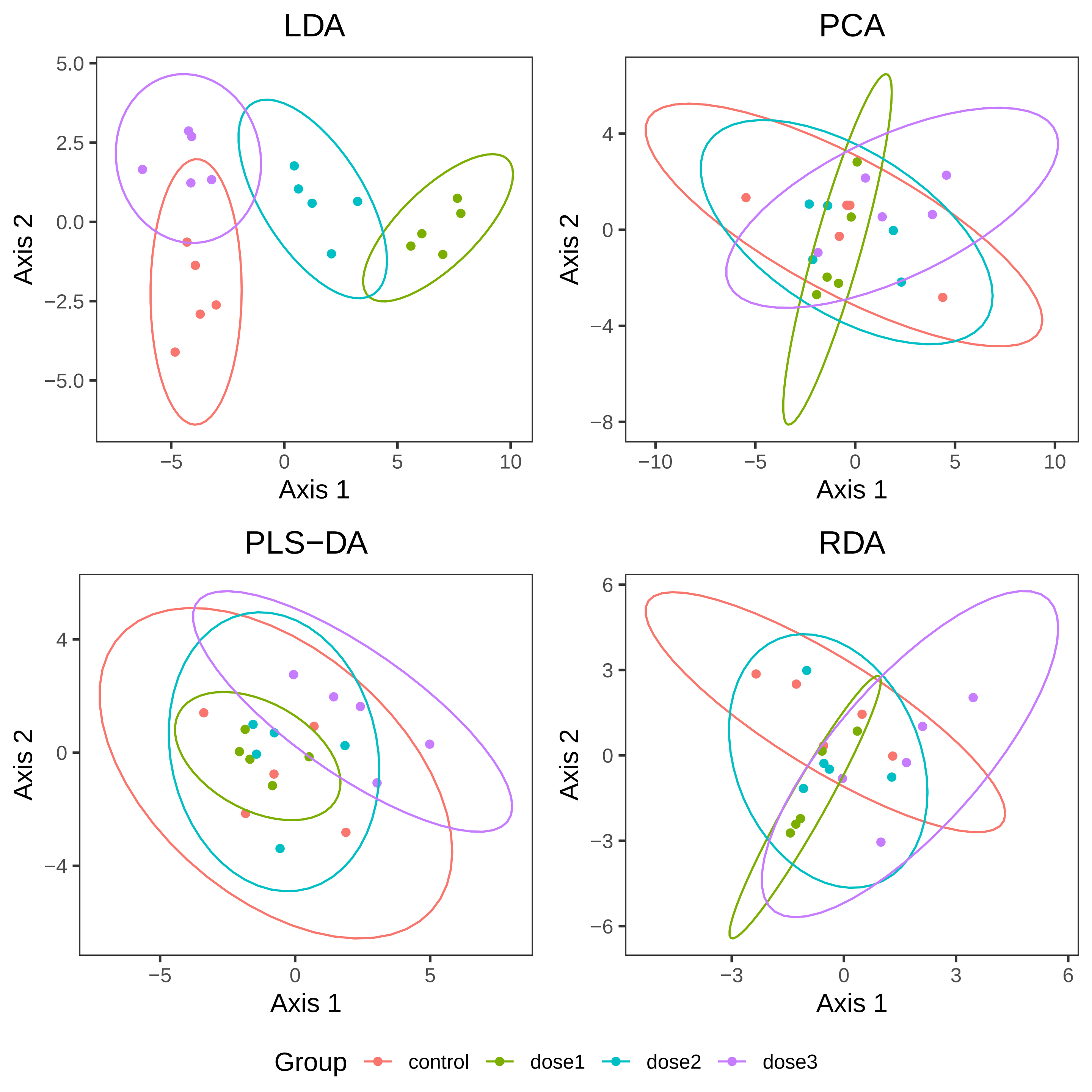

LDA
PCA
PLS−DA
RDA
Axis 1
Axis 2
Group
control
dose1
dose2
dose3

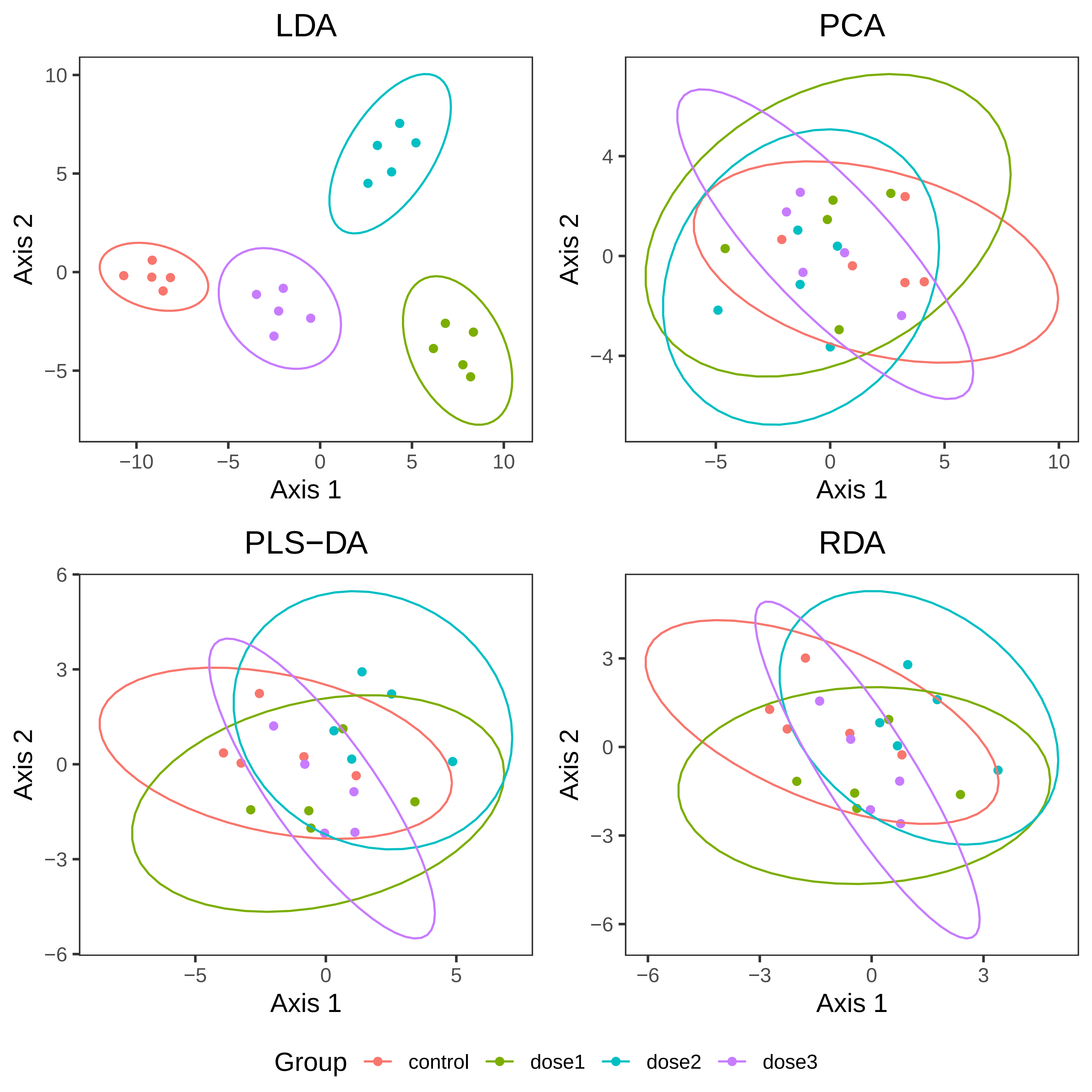
LDA
PCA
PLS−DA
RDA
Axis 1
Axis 2
Group
control
dose1
dose2
dose3

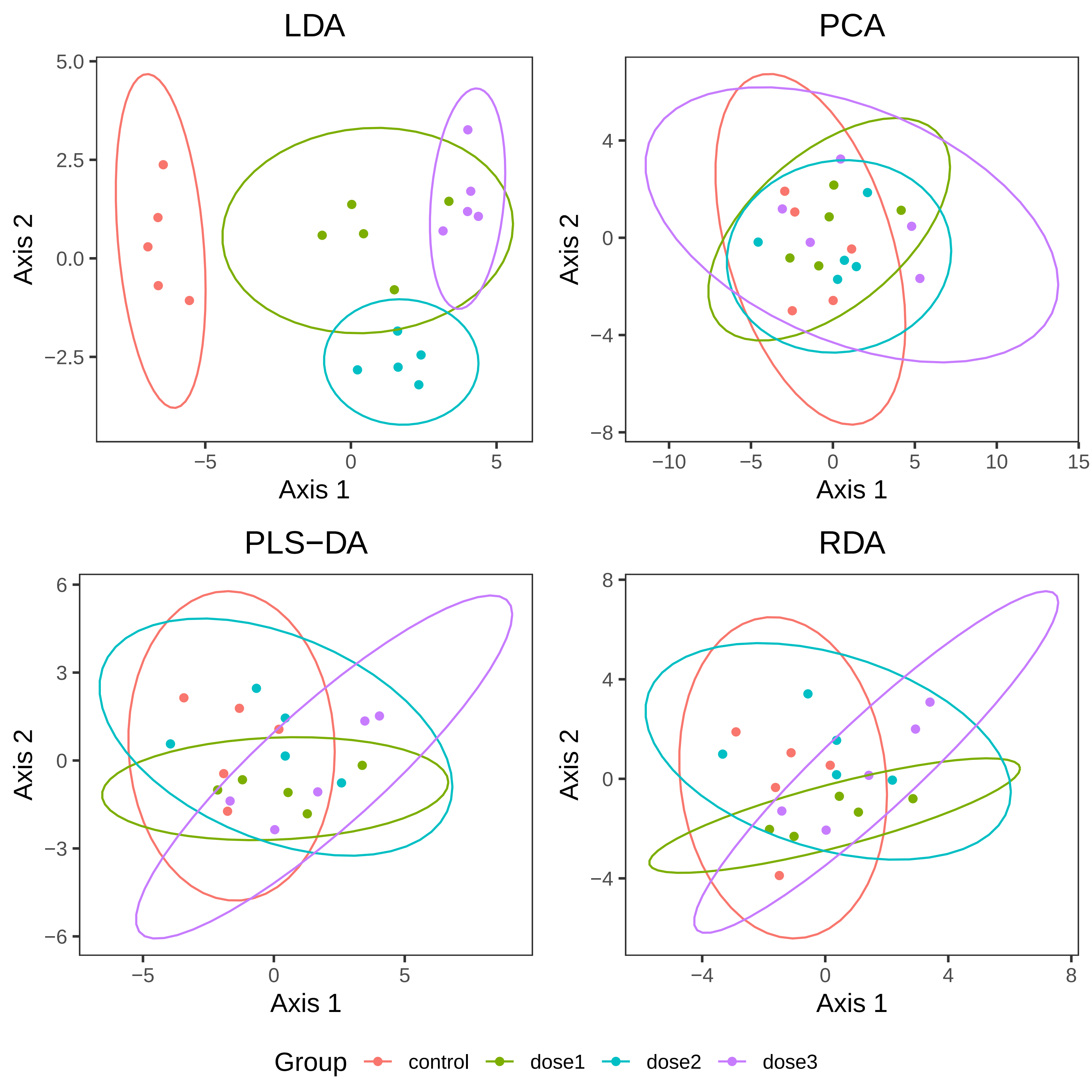
LDA
PCA
PLS−DA
RDA
Axis 1
Axis 2
Group
control
dose1
dose2
dose3

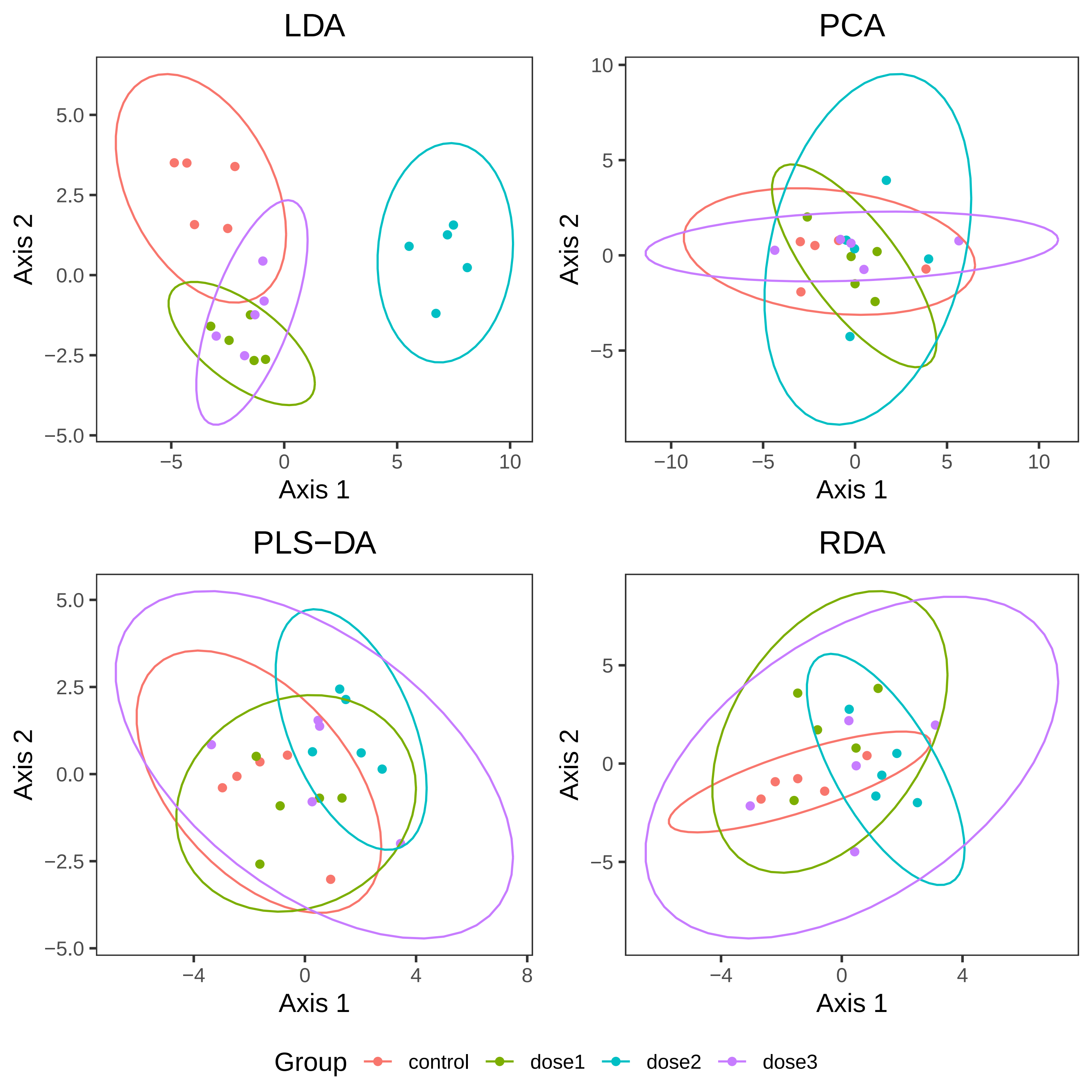
LDA
PCA
PLS−DA
RDA
Axis 1
Axis 2
Group
control
dose1
dose2
dose3

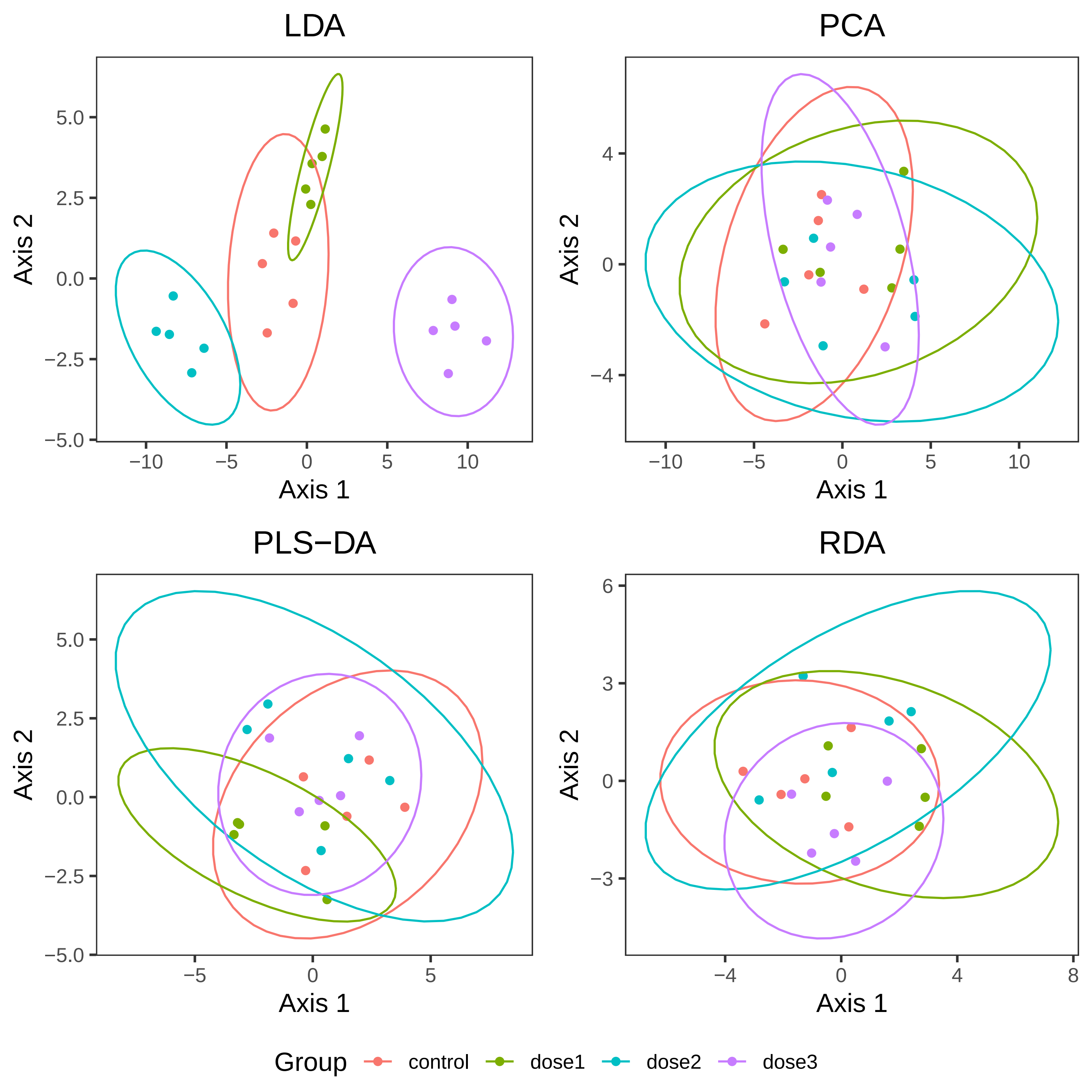

LDA
PCA
PLS−DA
RDA
Axis 1
Axis 2
Group
control
dose1
dose2
dose3

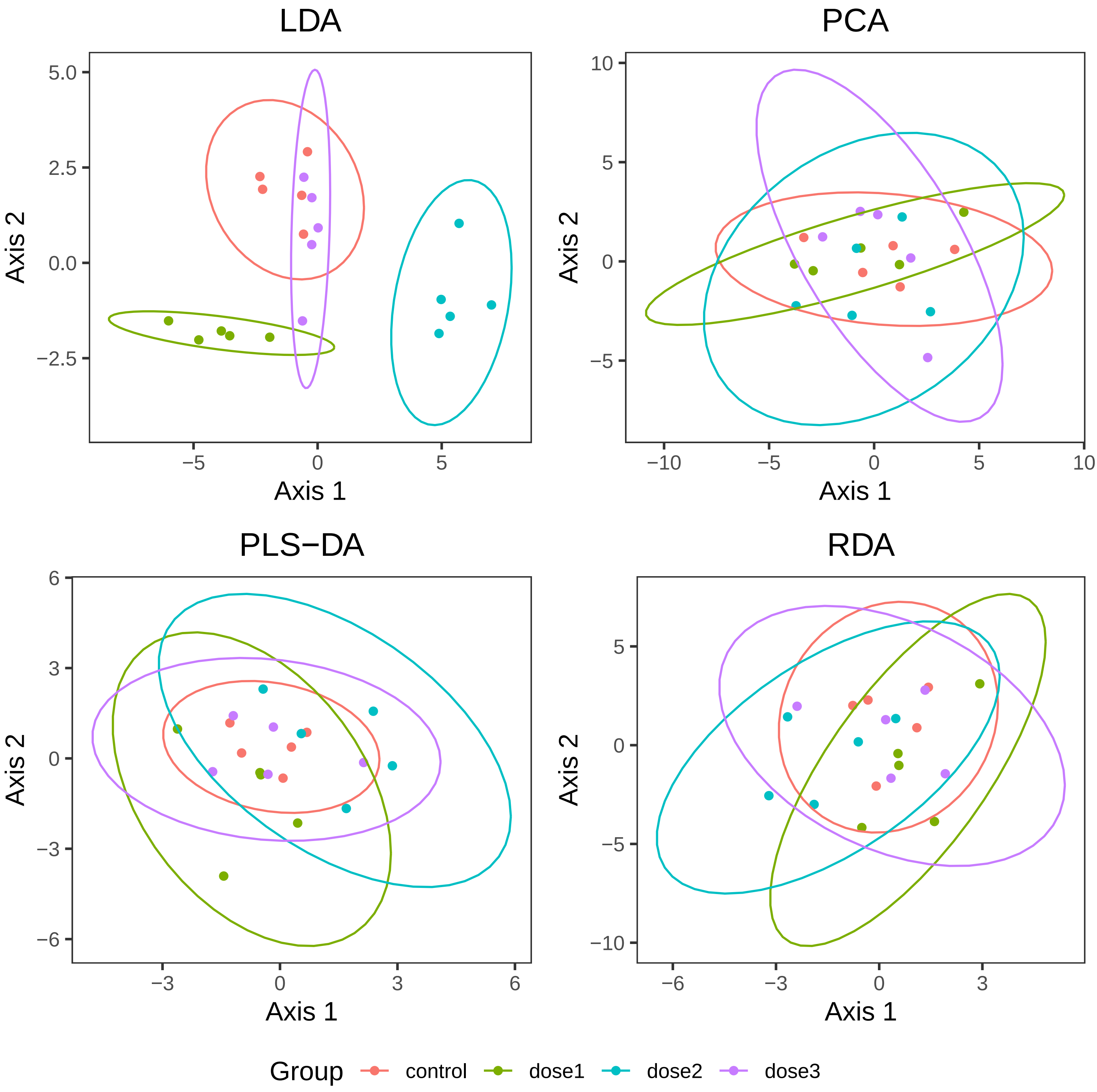

LDA
PCA
PLS−DA
RDA
Axis 1
Axis 2
5.0
2.5
0.0
−2.5
−5
0
5
10
−10
−6
−3
3
6
Group
control
dose1
dose2
dose3

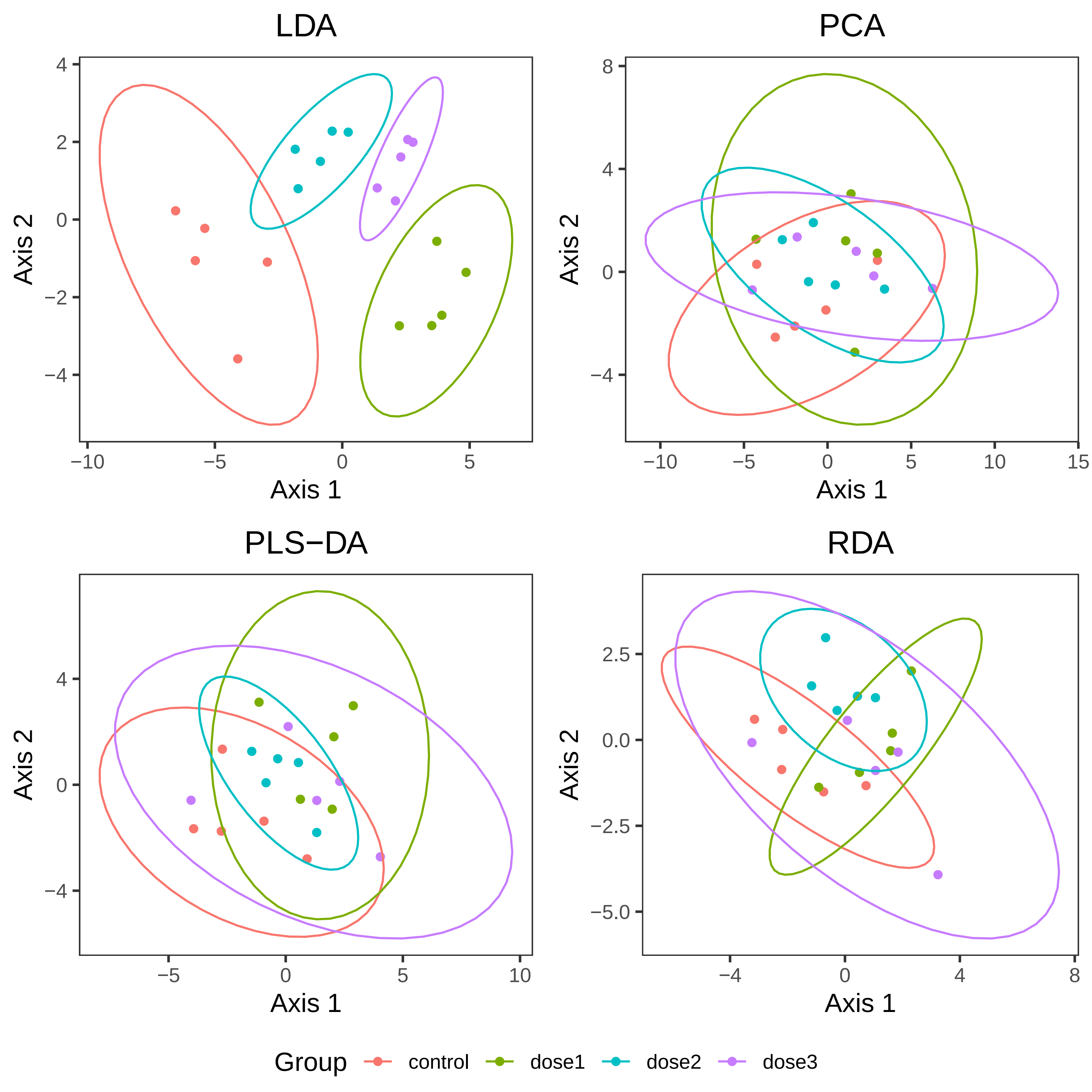

LDA
PCA
PLS−DA
RDA
Axis 1
Axis 2
Group
control
dose1
dose2
dose3

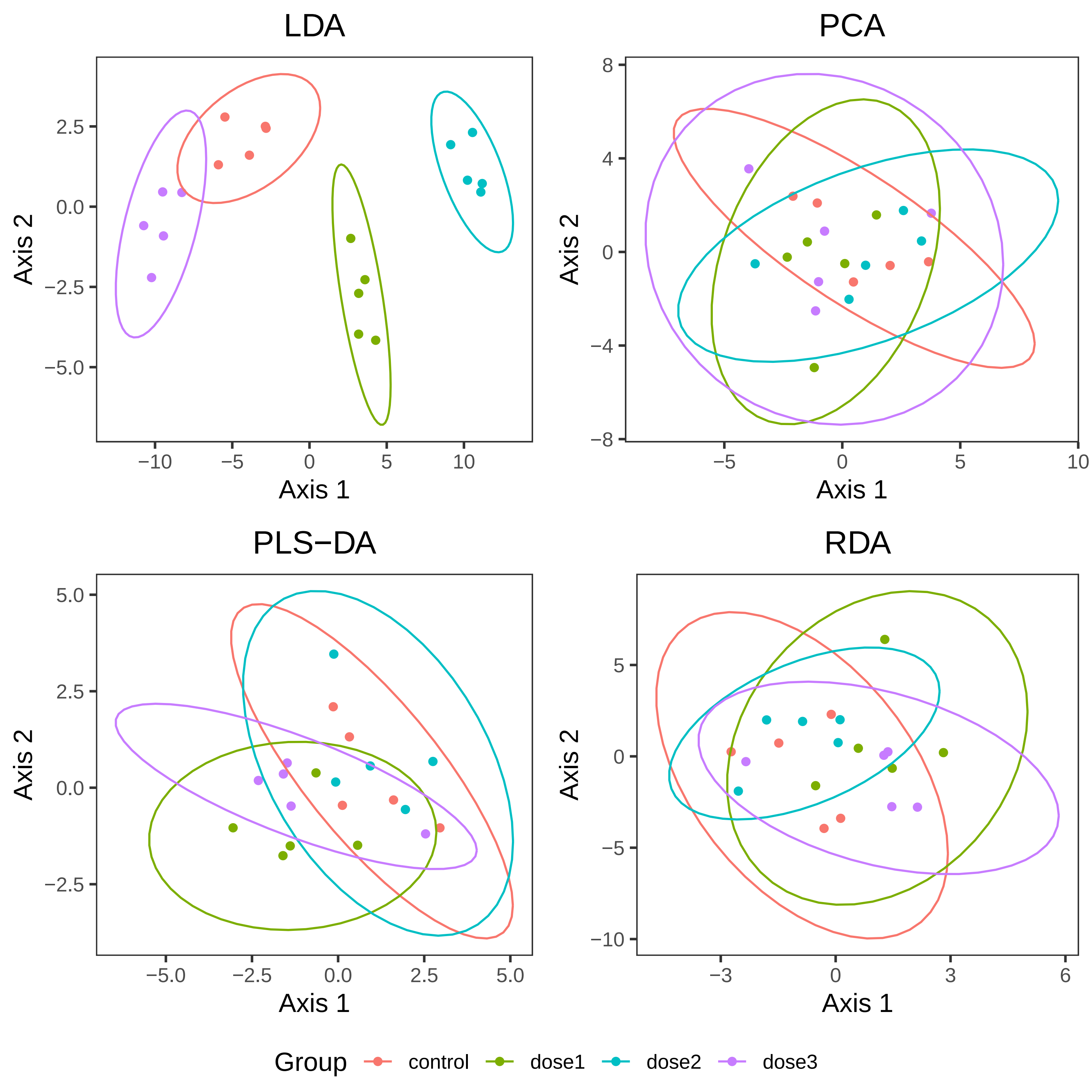

LDA
PCA
PLS−DA
RDA
Axis 1
Axis 2
Group
control
dose1
dose2
dose3

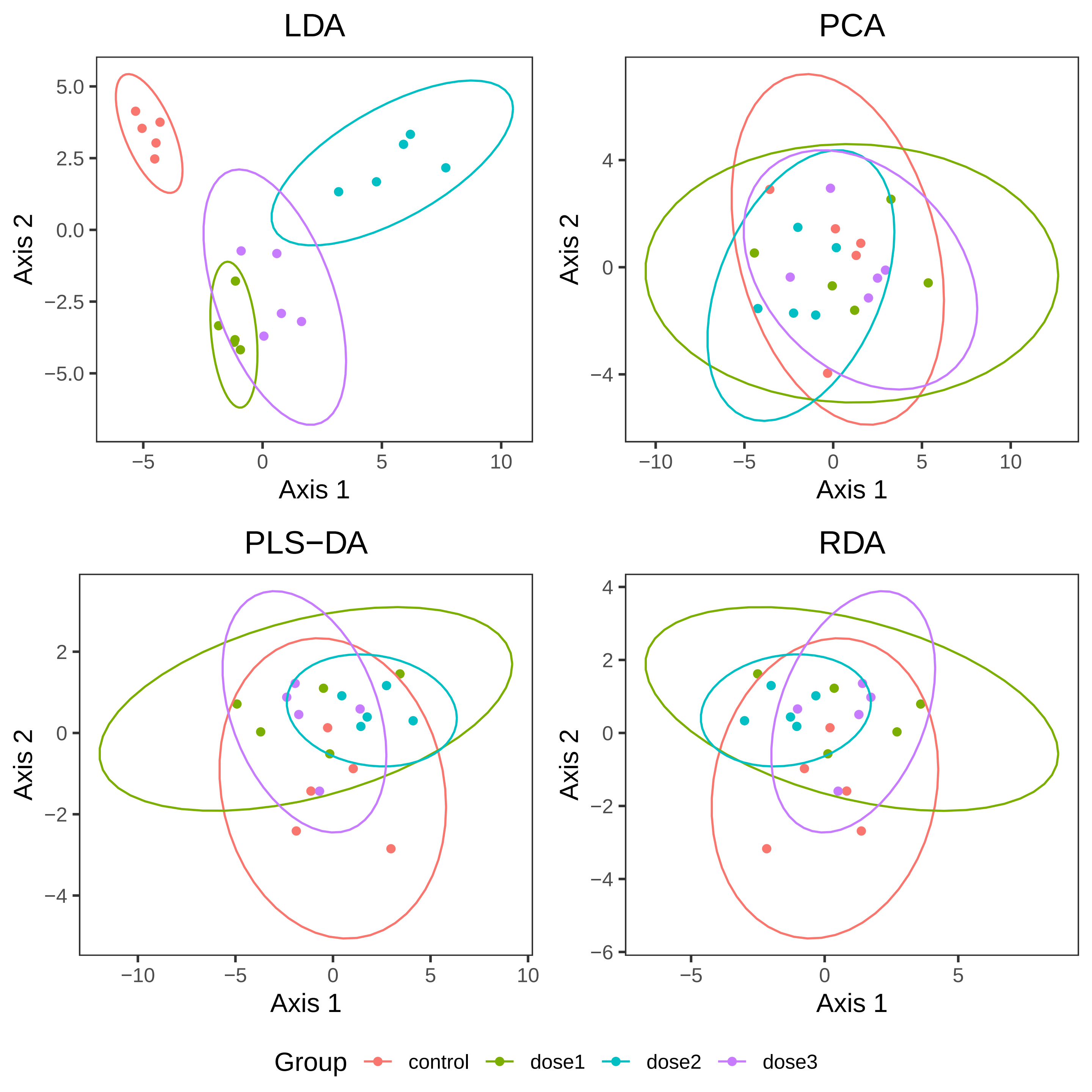

LDA
PCA
PLS−DA
RDA
Axis 1
Axis 2
Group
control
dose1
dose2
dose3

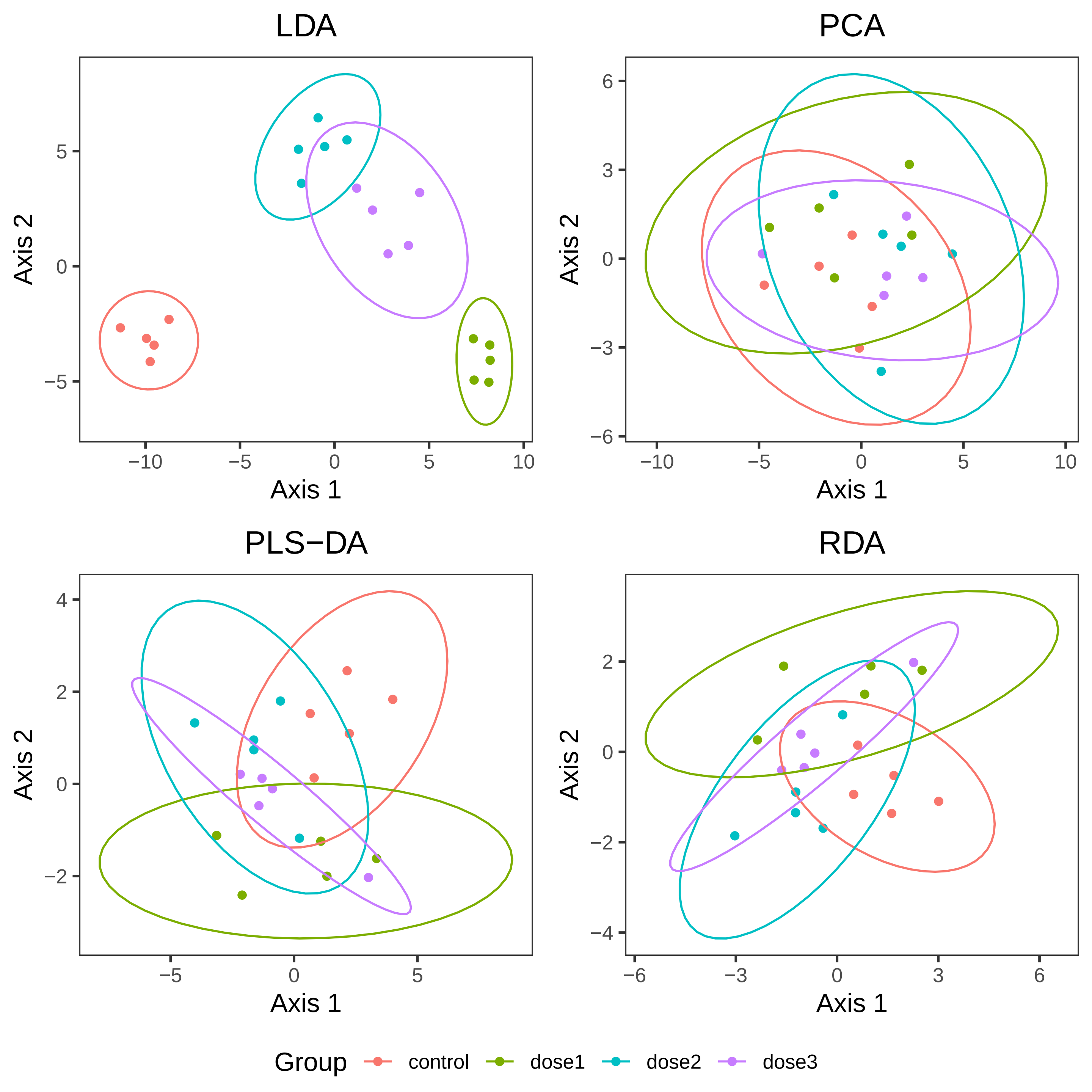

LDA
PCA
PLS−DA
RDA
Axis 1
Axis 2
Group
control
dose1
dose2
dose3

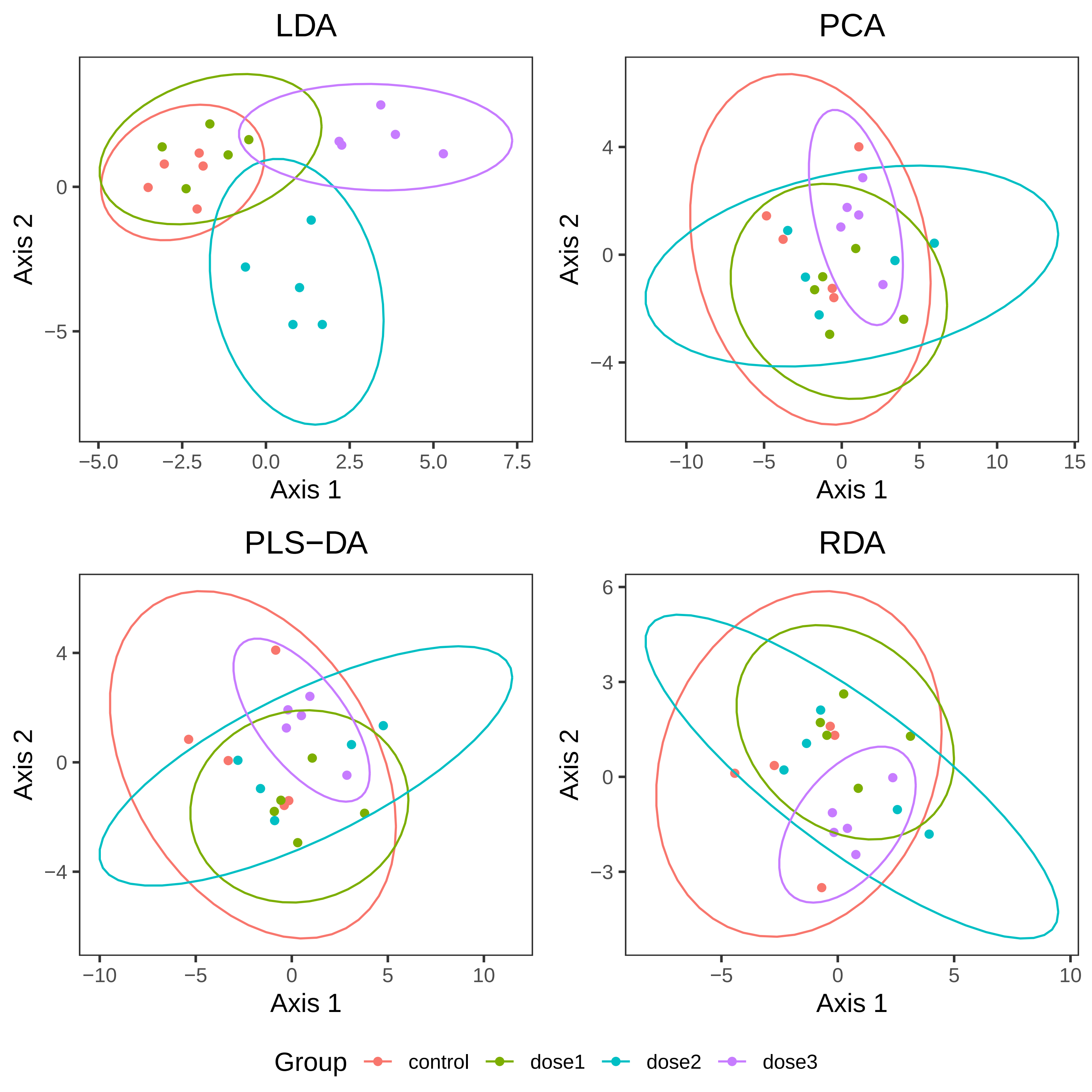

LDA
PCA
PLS−DA
RDA
Axis 1
Axis 2
−5.0
−2.5
0.0
2.5
5.0
7.5
0
−5
−10
−5
0
5
10
15
4
0
−4
−10
−5
0
5
10
4
0
−4
−5
0
5
10
6
3
0
−3
Group
control
dose1
dose2
dose3

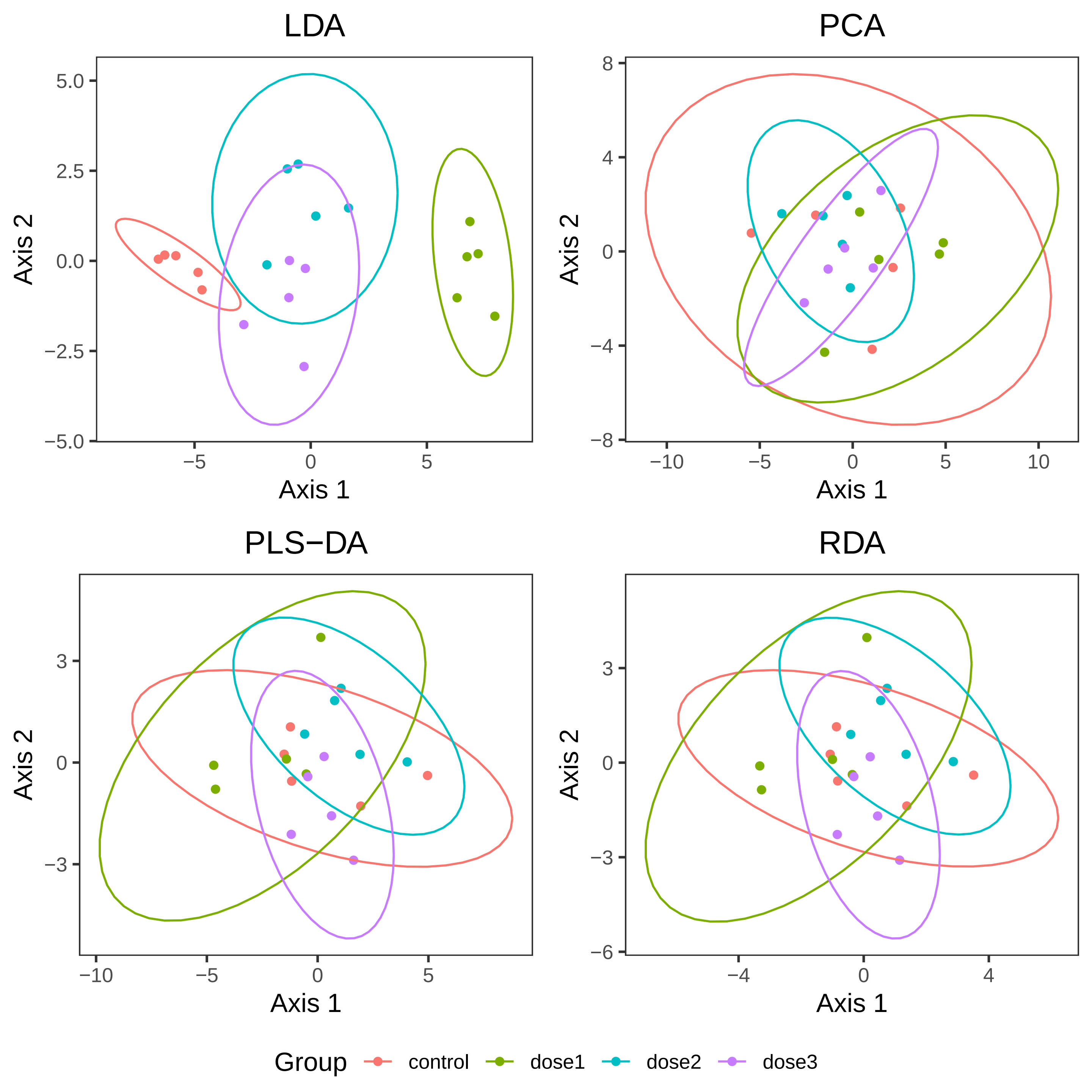

LDA
PCA
PLS−DA
RDA
Axis 1
Axis 2
5.0
2.5
0.0
−2.5
−5.0
−5
0
5
8
4
0
−4
−8
−10
−5
0
5
10
3
0
−3
−10
−5
0
5
3
0
−3
−6
−4
0
4
Group
control
dose1
dose2
dose3

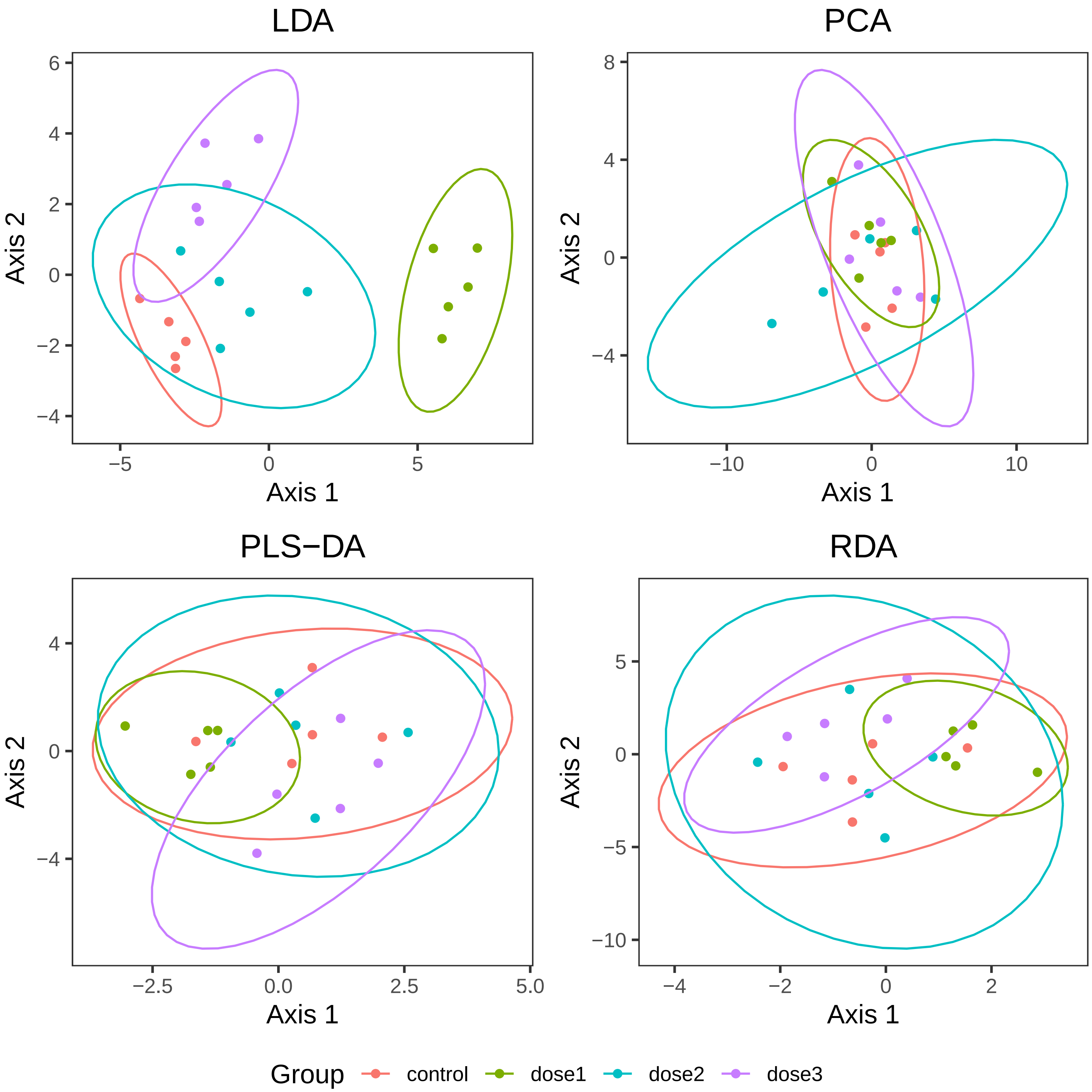
LDA
PCA
PLS−DA
RDA
Axis 1
Axis 2
Group
control
dose1
dose2
dose3

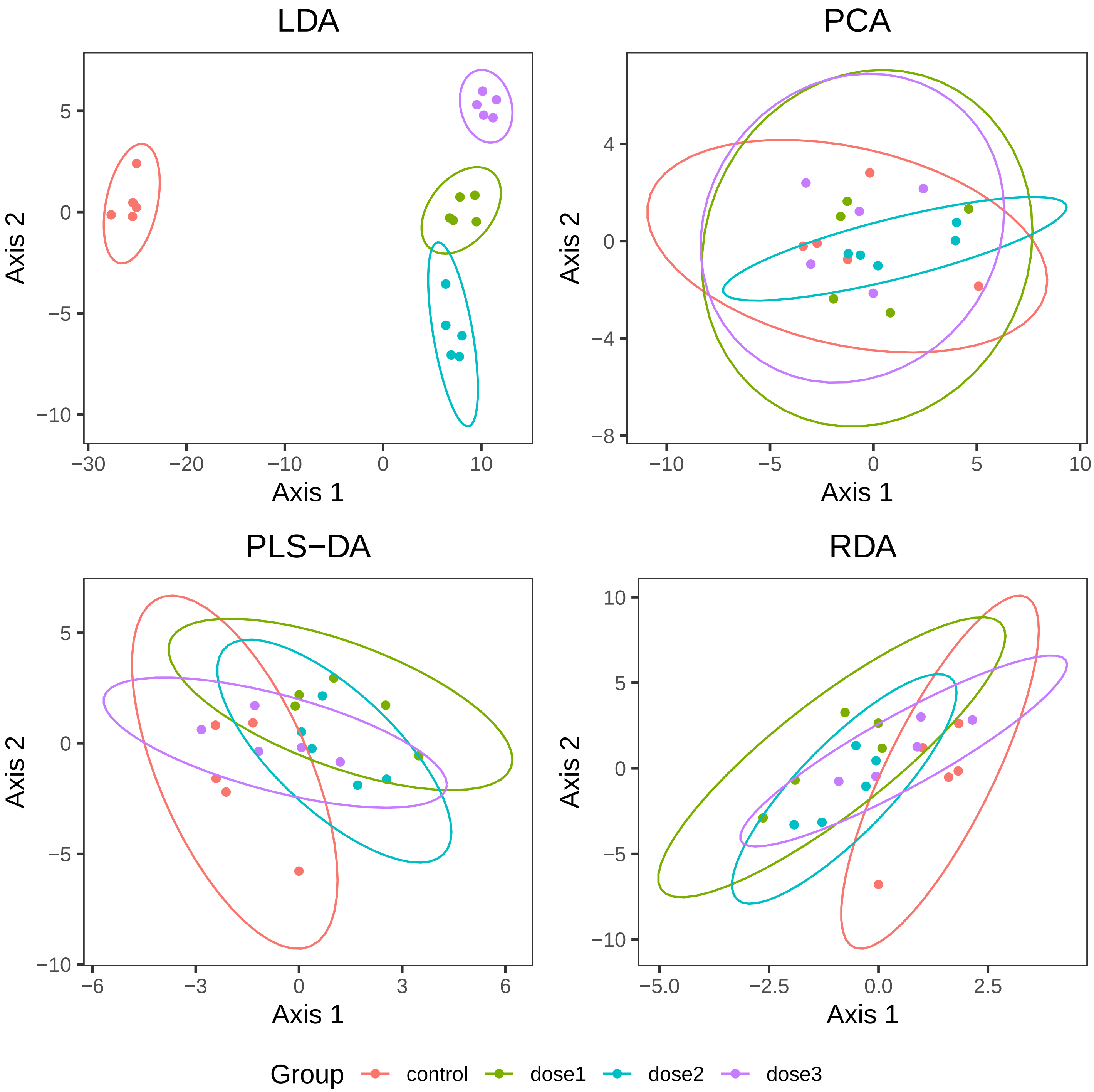

LDA
PCA
PLS−DA
RDA
Axis 1
Axis 2
5
0
−5
−10
−30
−20
−10
0
10
4
0
−4
−8
−10
−5
0
5
10
5
0
−5
−10
−6
−3
0
3
6
10
5
0
−5
−10
−5.0
−2.5
0.0
2.5
Group
control
dose1
dose2
dose3

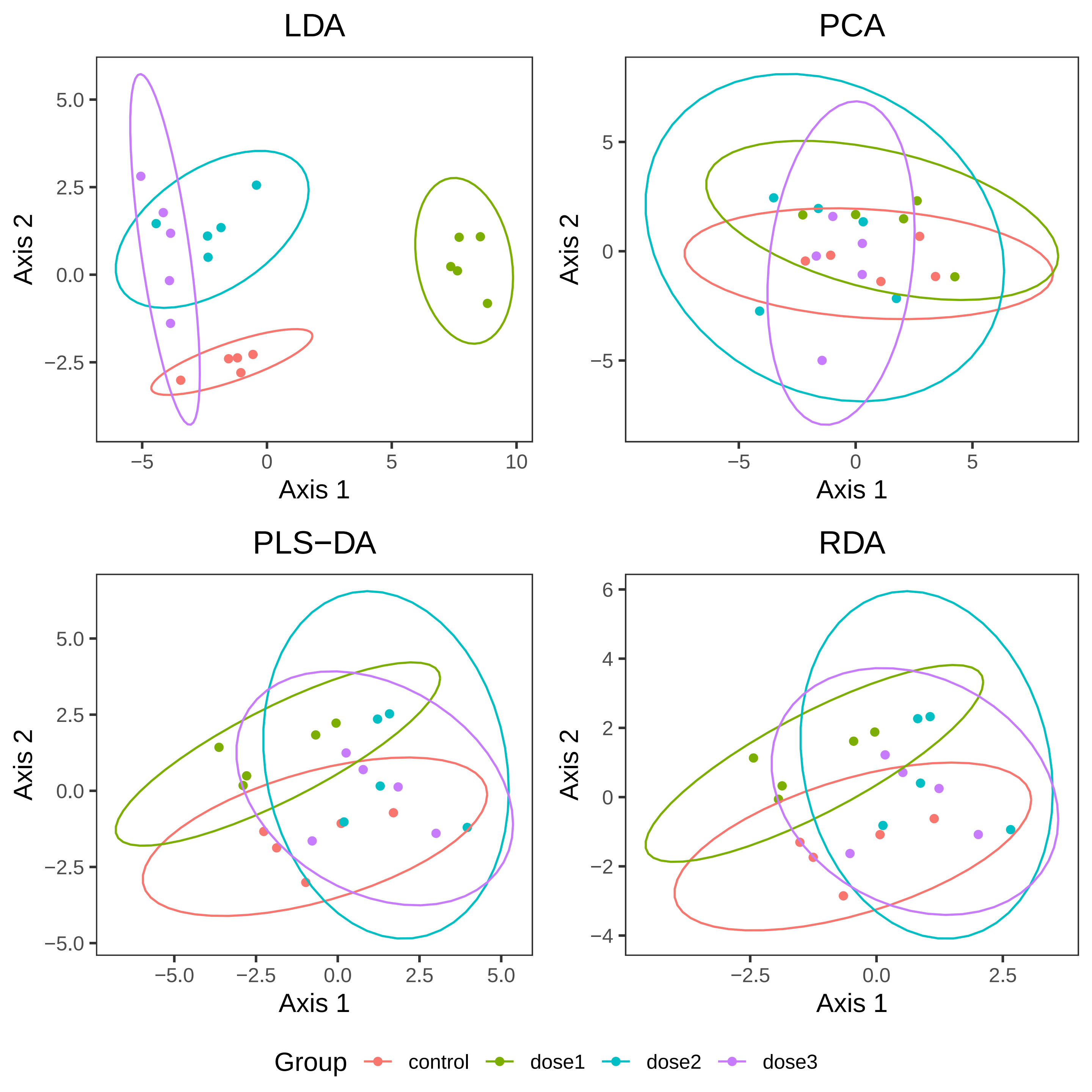

LDA
PCA
PLS−DA
RDA
Axis 1
Axis 2
5.0
2.5
0.0
−2.5
−5.0
−5
0
5
10
6
4
2
−2
−4
Group
control
dose1
dose2
dose3

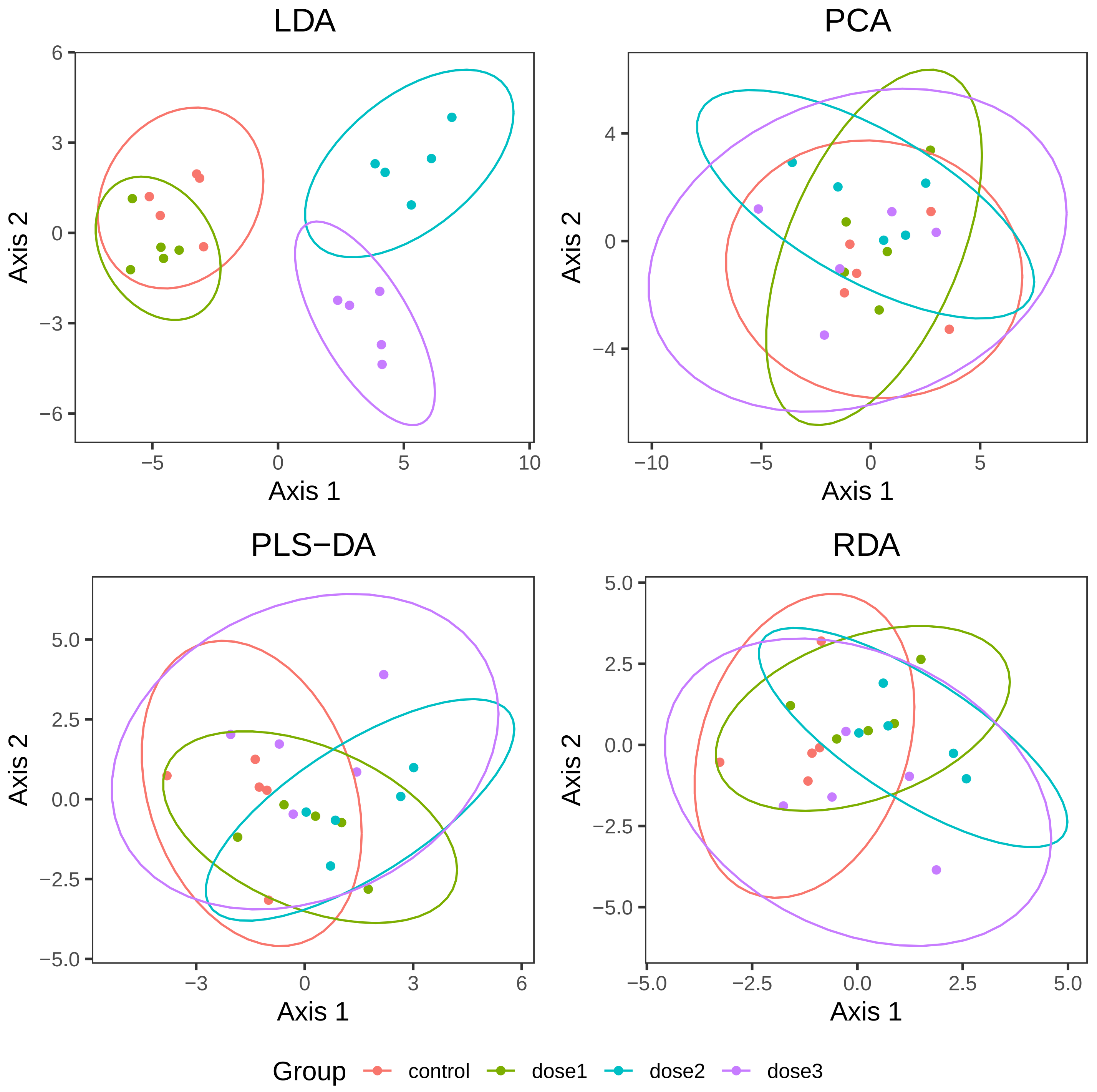

LDA
PCA
PLS−DA
RDA
Axis 1
Axis 2
6
3
0
−3
−6
−5
0
5
10
4
0
−4
−10
−5
0
5
5.0
2.5
0.0
−2.5
−5.0
−3
0
3
6
5.0
2.5
0.0
−2.5
−5.0
−5.0
−2.5
0.0
2.5
5.0
Group
control
dose1
dose2
dose3

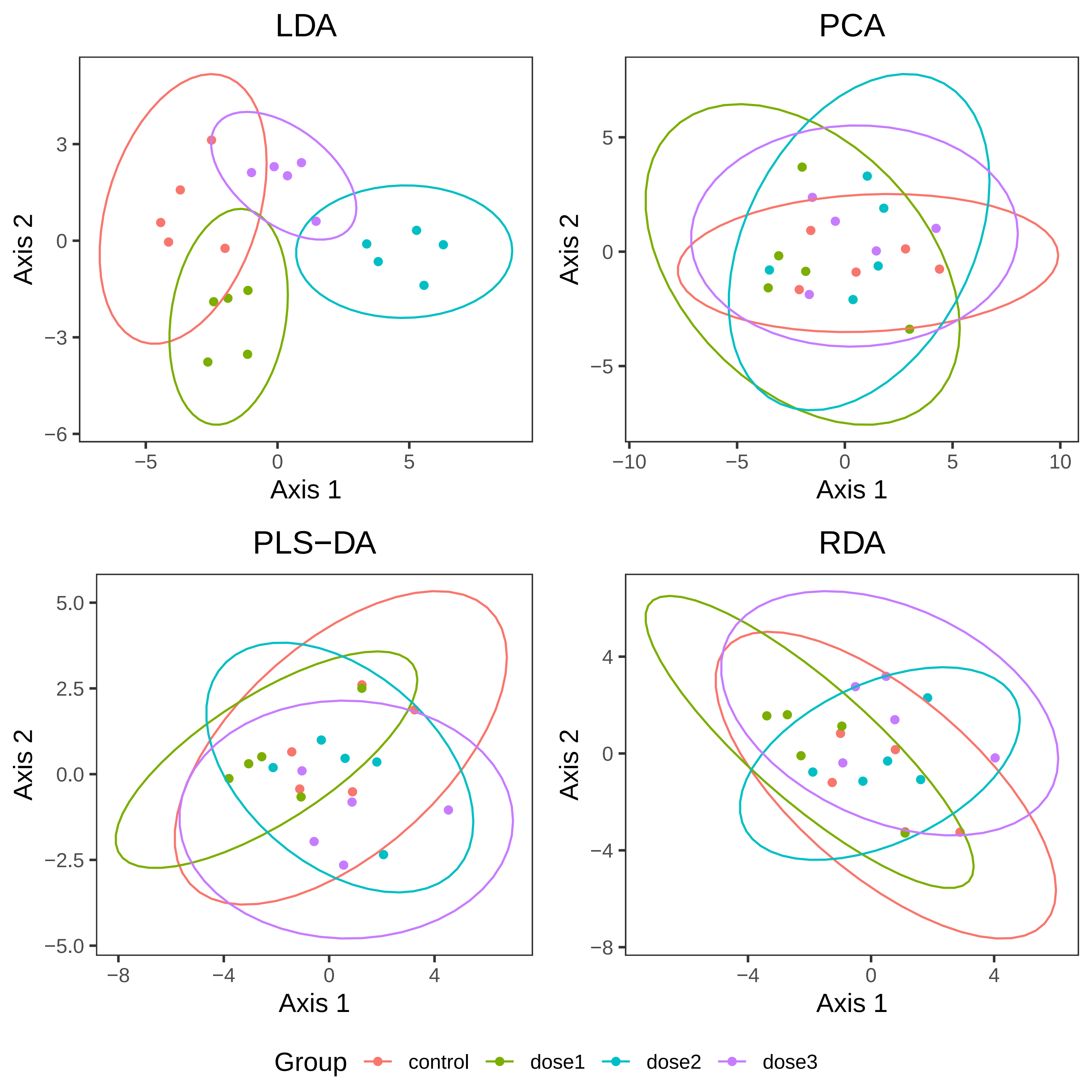

LDA
PCA
PLS−DA
RDA
Axis 1
Axis 2
3
0
−3
−6
−5
0
5
5
0
−5
−10
−5
0
5
10
5.0
2.5
0.0
−2.5
−5.0
−8
−4
0
4
4
0
−4
−8
−4
0
4
Group
control
dose1
dose2
dose3

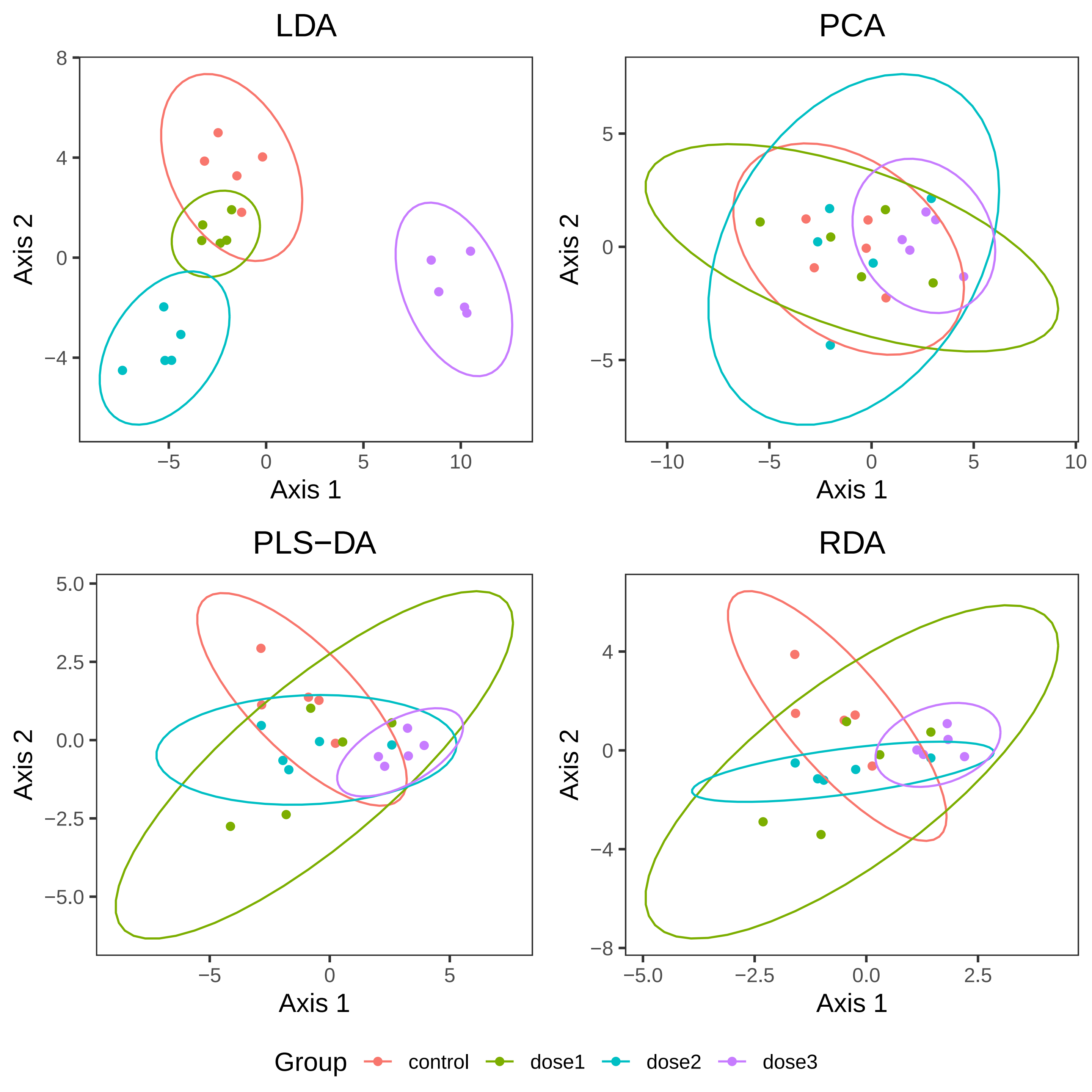

LDA
PCA
PLS−DA
RDA
Axis 1
Axis 2
Group
control
dose1
dose2
dose3

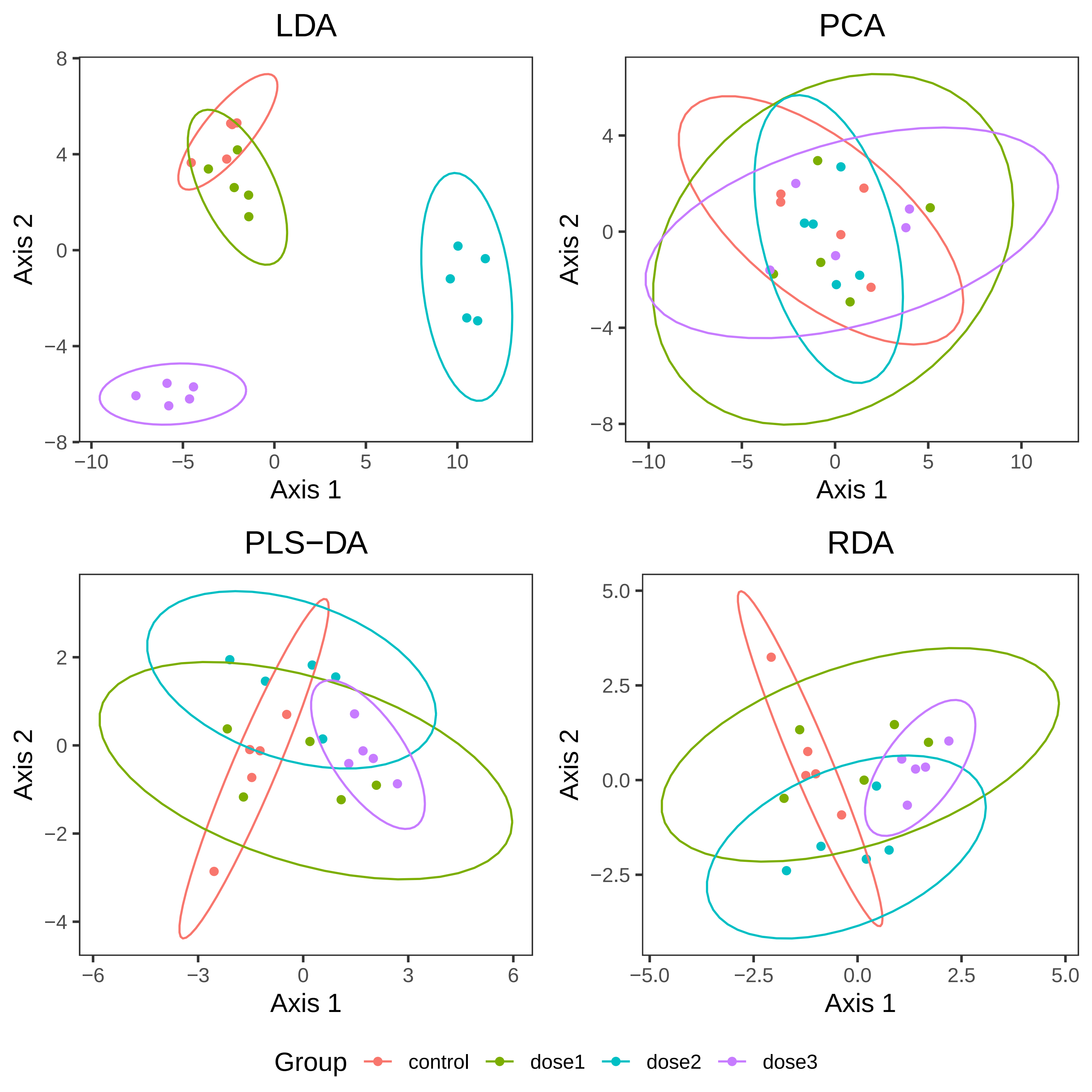

LDA
PCA
PLS−DA
RDA
Axis 1
Axis 2
Group
control
dose1
dose2
dose3

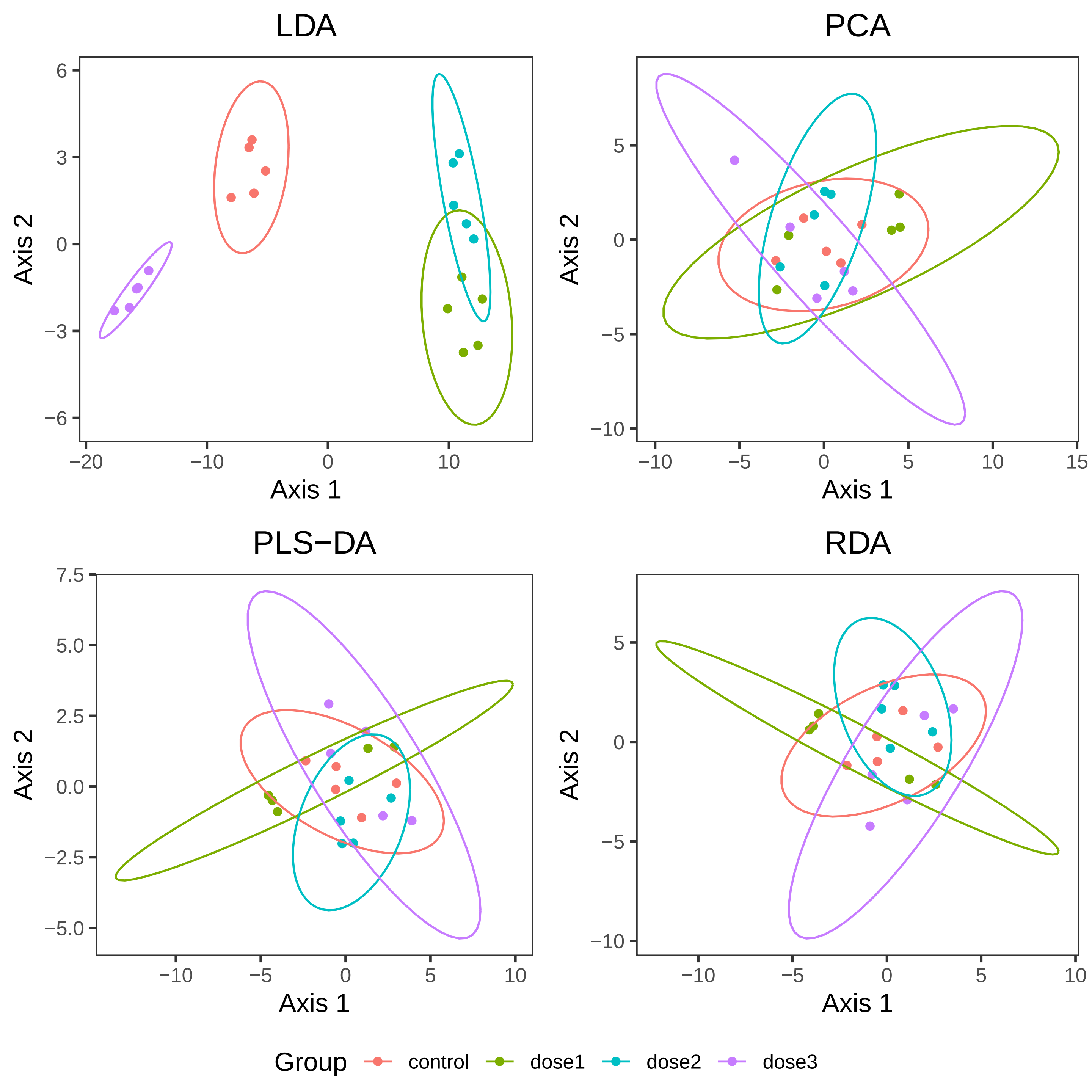

LDA
PCA
PLS−DA
RDA
Axis 1
Axis 2
Group
control
dose1
dose2
dose3

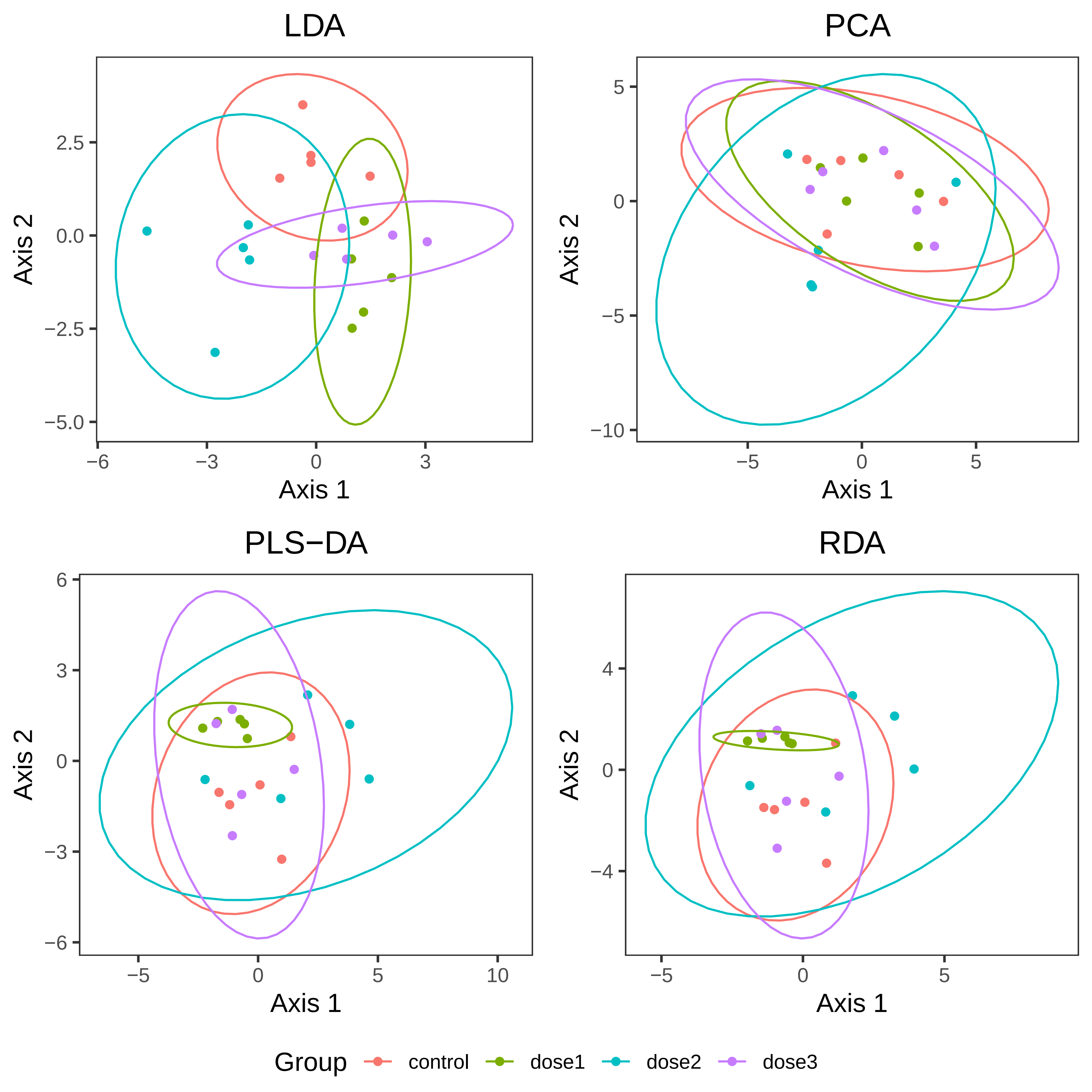
LDA
PCA
PLS−DA
RDA
Axis 1
Axis 2
Group
control
dose1
dose2
dose3

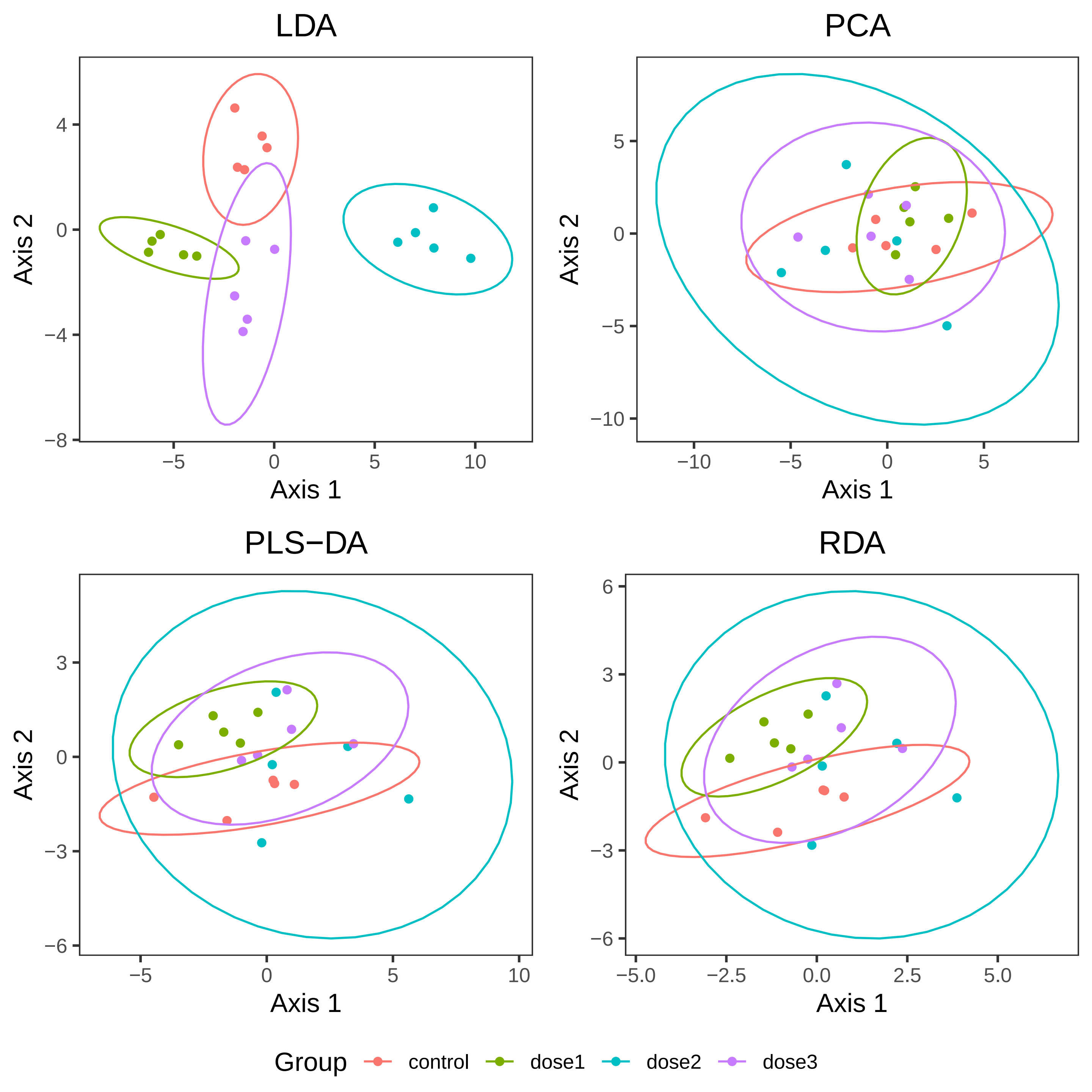
LDA
PCA
PLS−DA
RDA
Axis 1
Axis 2
Group
control
dose1
dose2
dose3

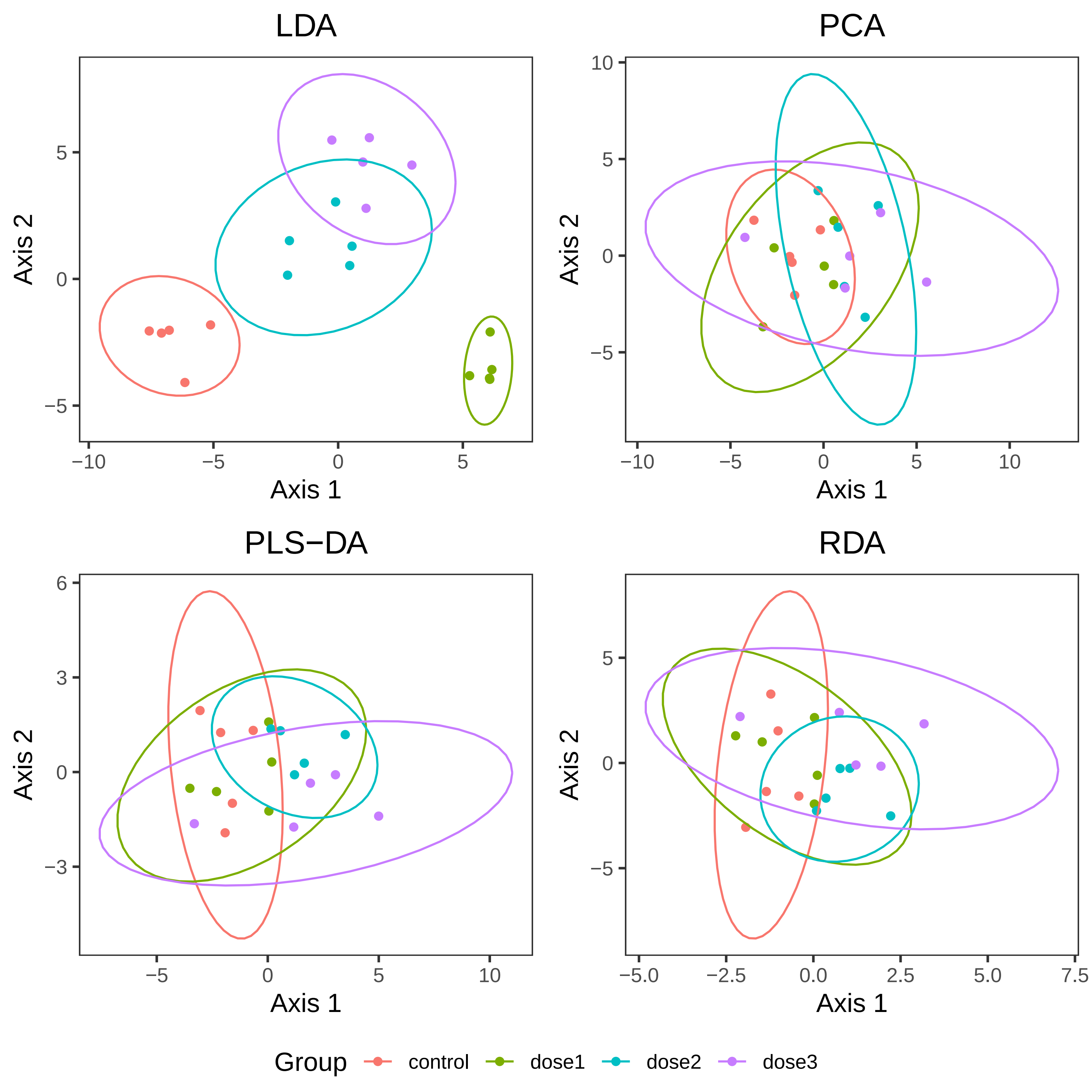
LDA
PCA
PLS−DA
RDA
Axis 1
Axis 2
Group
control
dose1
dose2
dose3

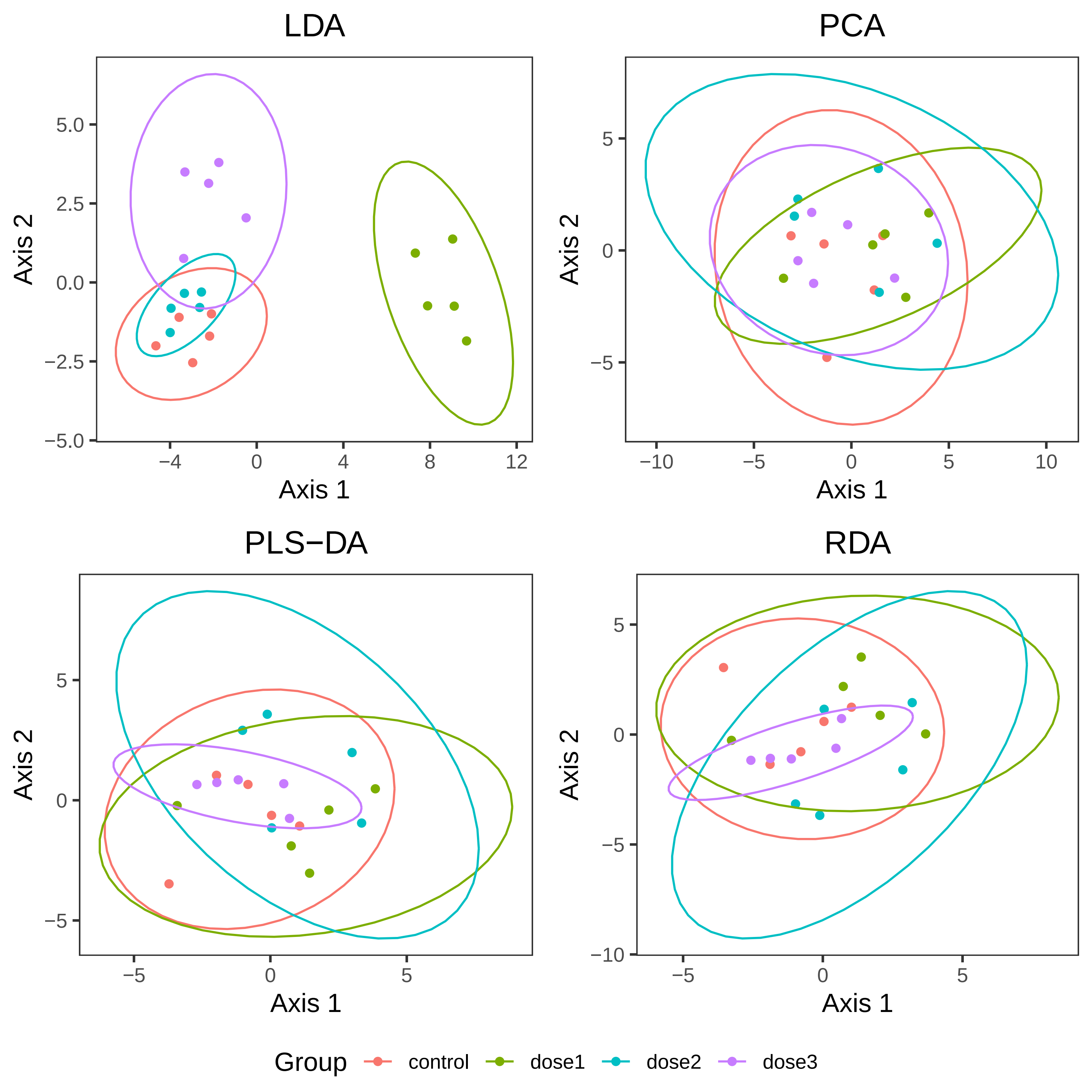
LDA
PCA
PLS−DA
RDA
Axis 1
Axis 2
Group
control
dose1
dose2
dose3

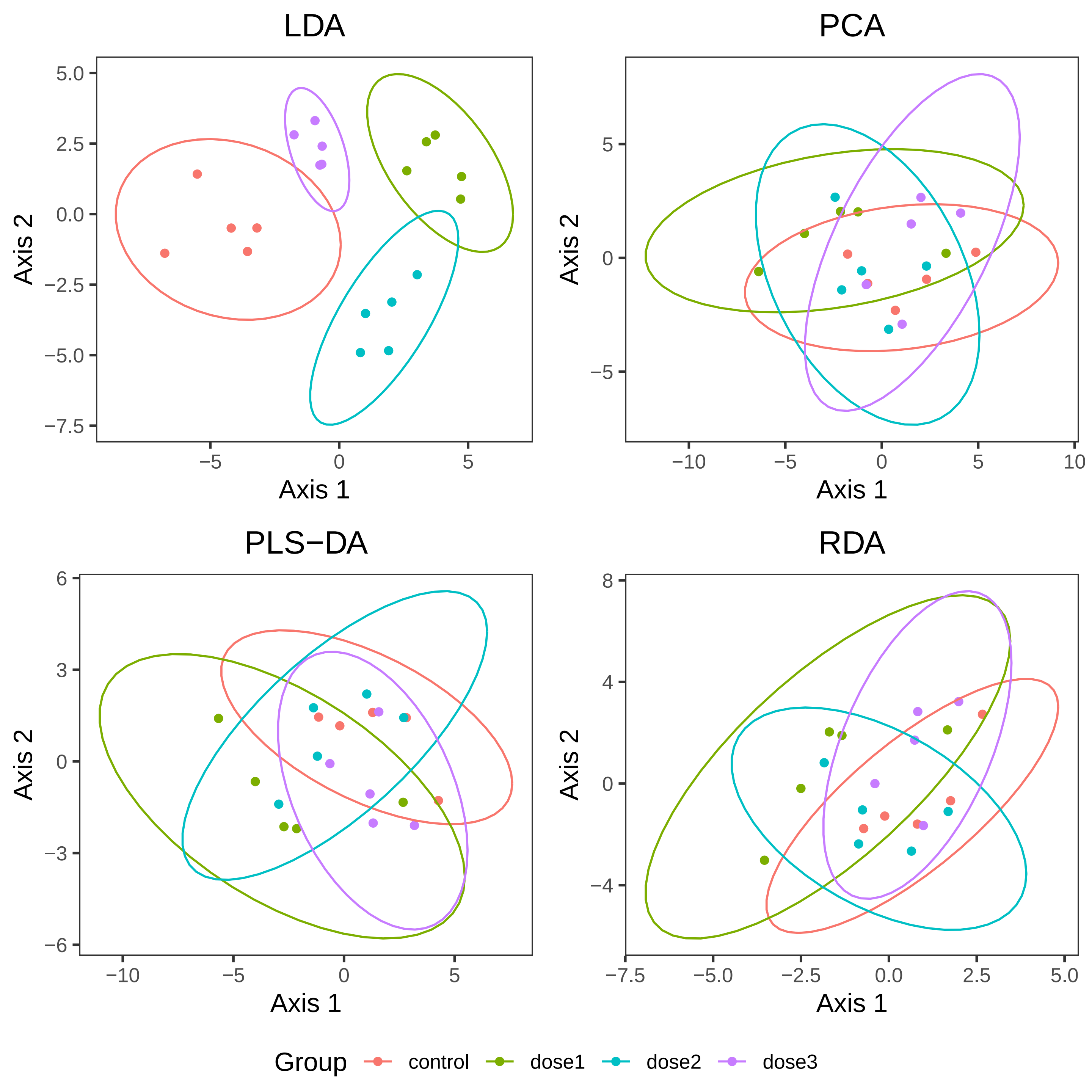
LDA
PCA
PLS−DA
RDA
Axis 1
Axis 2
Group
control
dose1
dose2
dose3

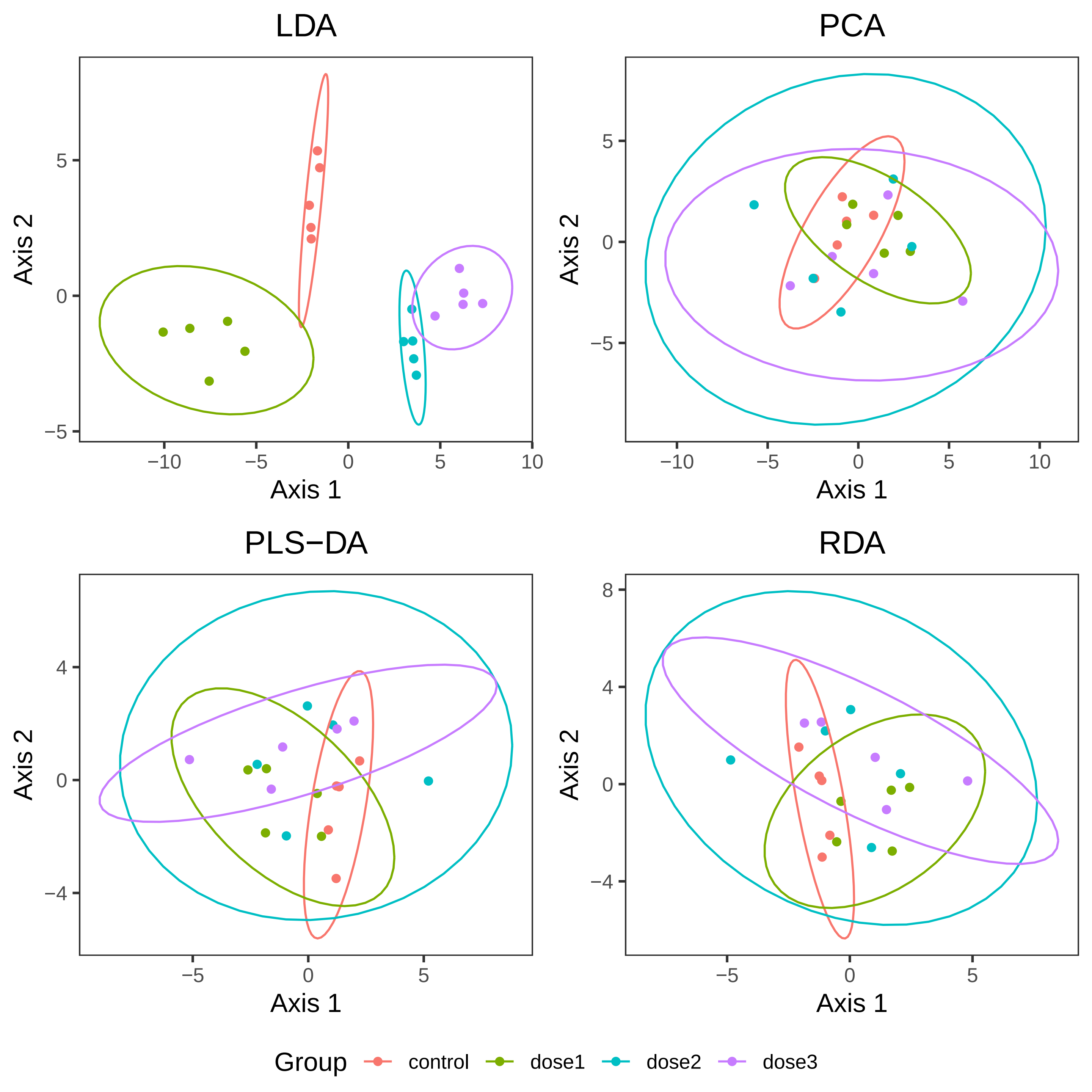

LDA
PCA
PLS−DA
RDA
Axis 1
Axis 2
−10
−5
0
5
10
−4
4
8
Group
control
dose1
dose2
dose3

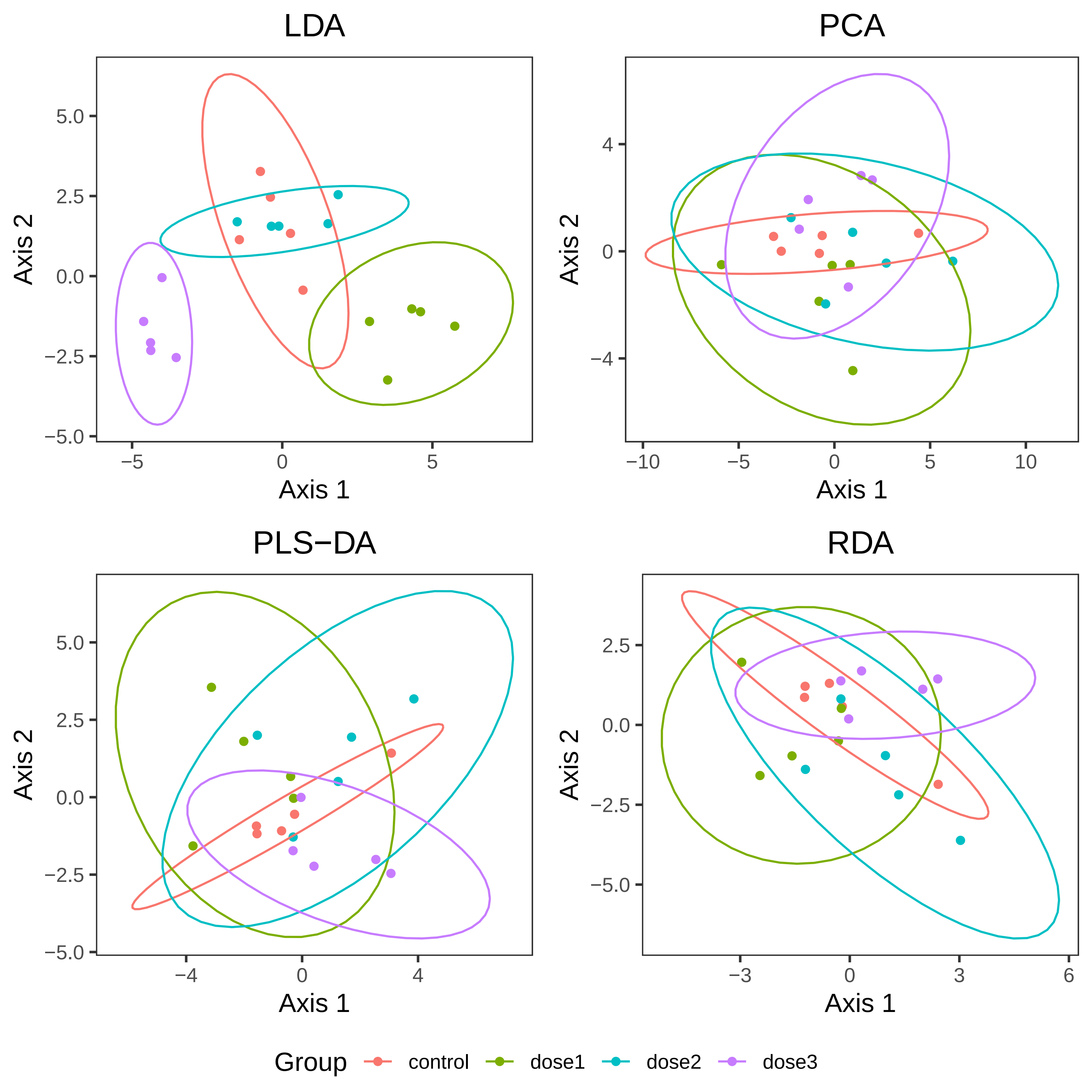
LDA
PCA
PLS−DA
RDA
Axis 1
Axis 2
Group
control
dose1
dose2
dose3

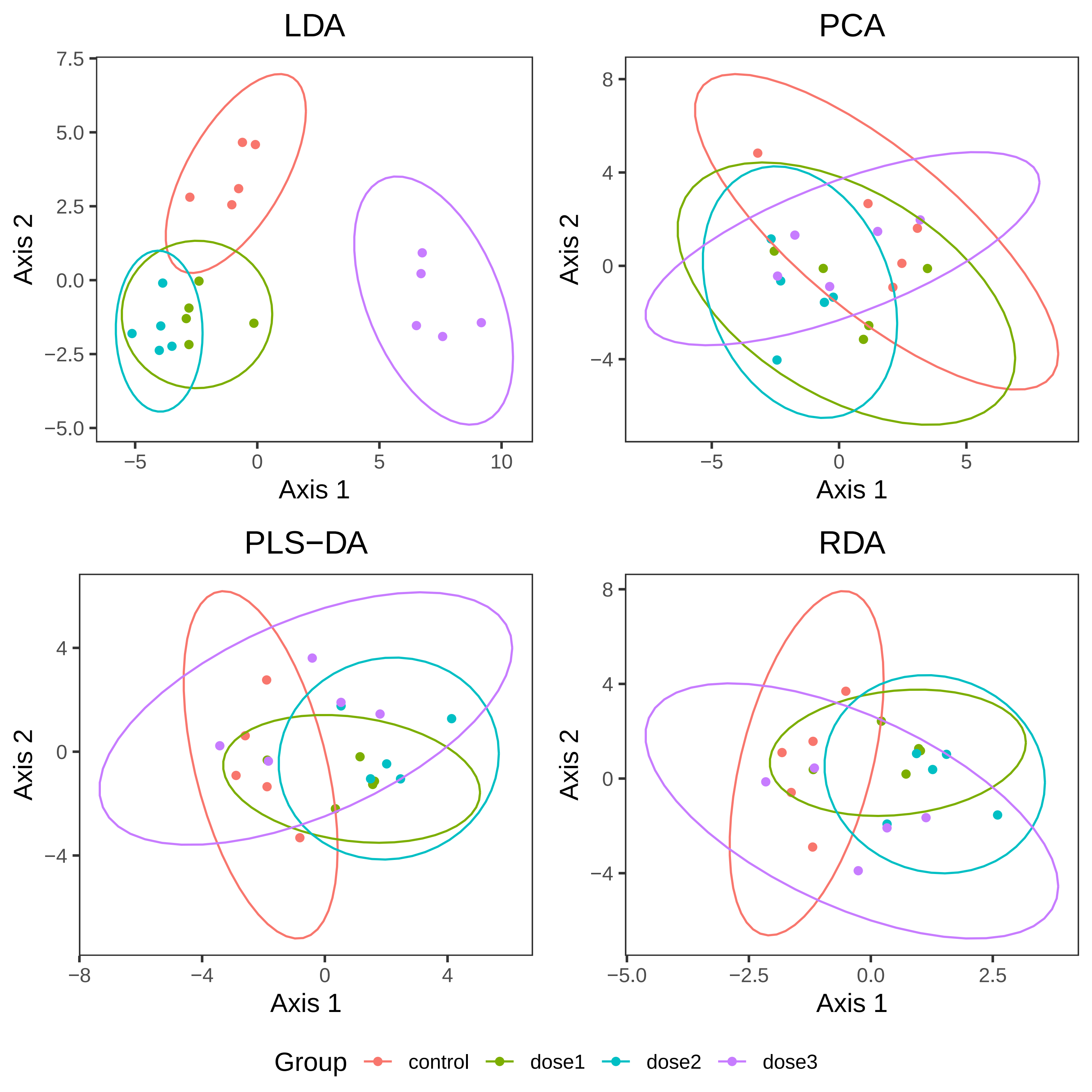
LDA
PCA
PLS−DA
RDA
Axis 1
Axis 2
7.5
5.0
2.5
0.0
−2.5
−5.0
−5
0
5
10
8
4
0
−4
−8
2.5
Group
control
dose1
dose2
dose3

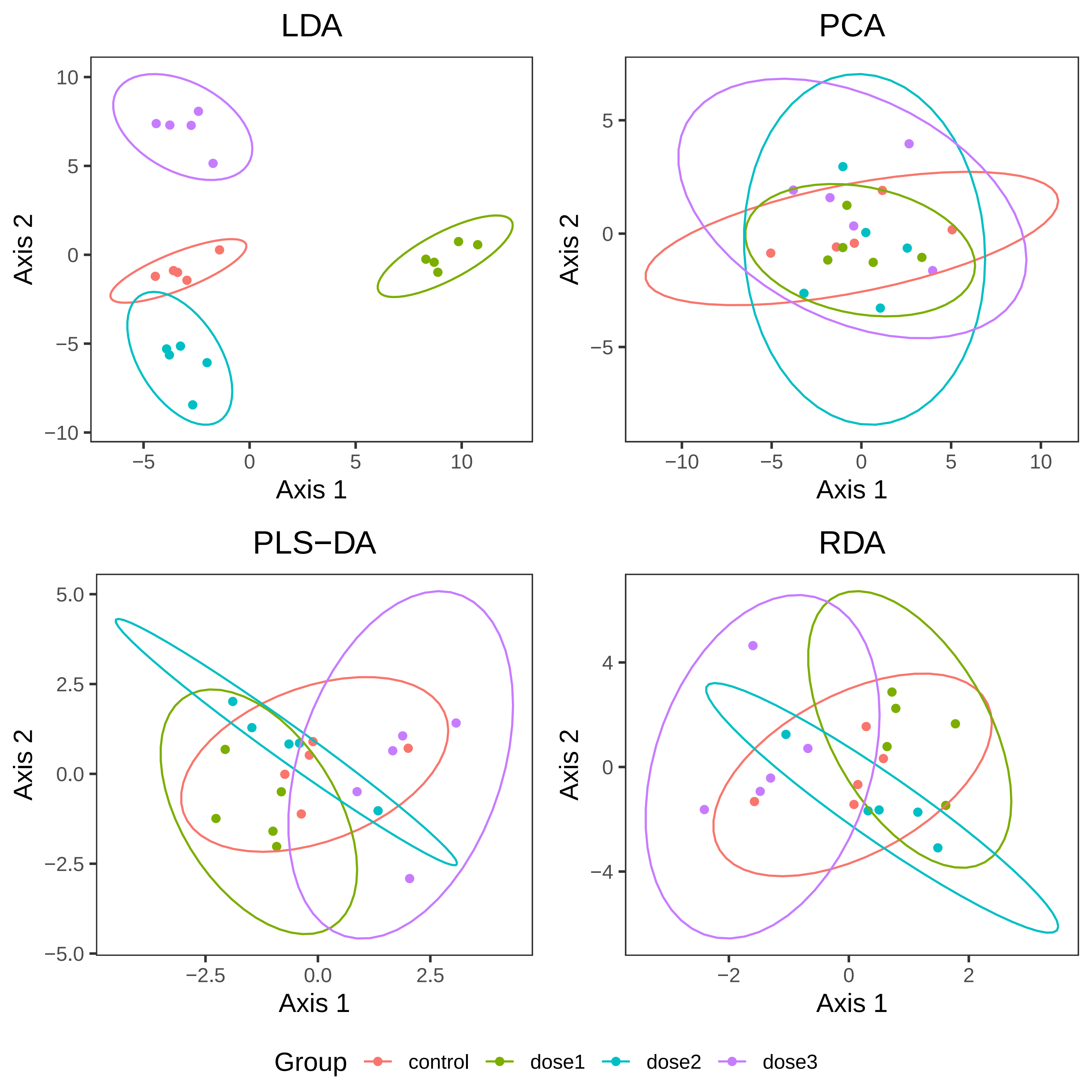

LDA
PCA
PLS−DA
RDA
Axis 1
Axis 2
Group
control
dose1
dose2
dose3

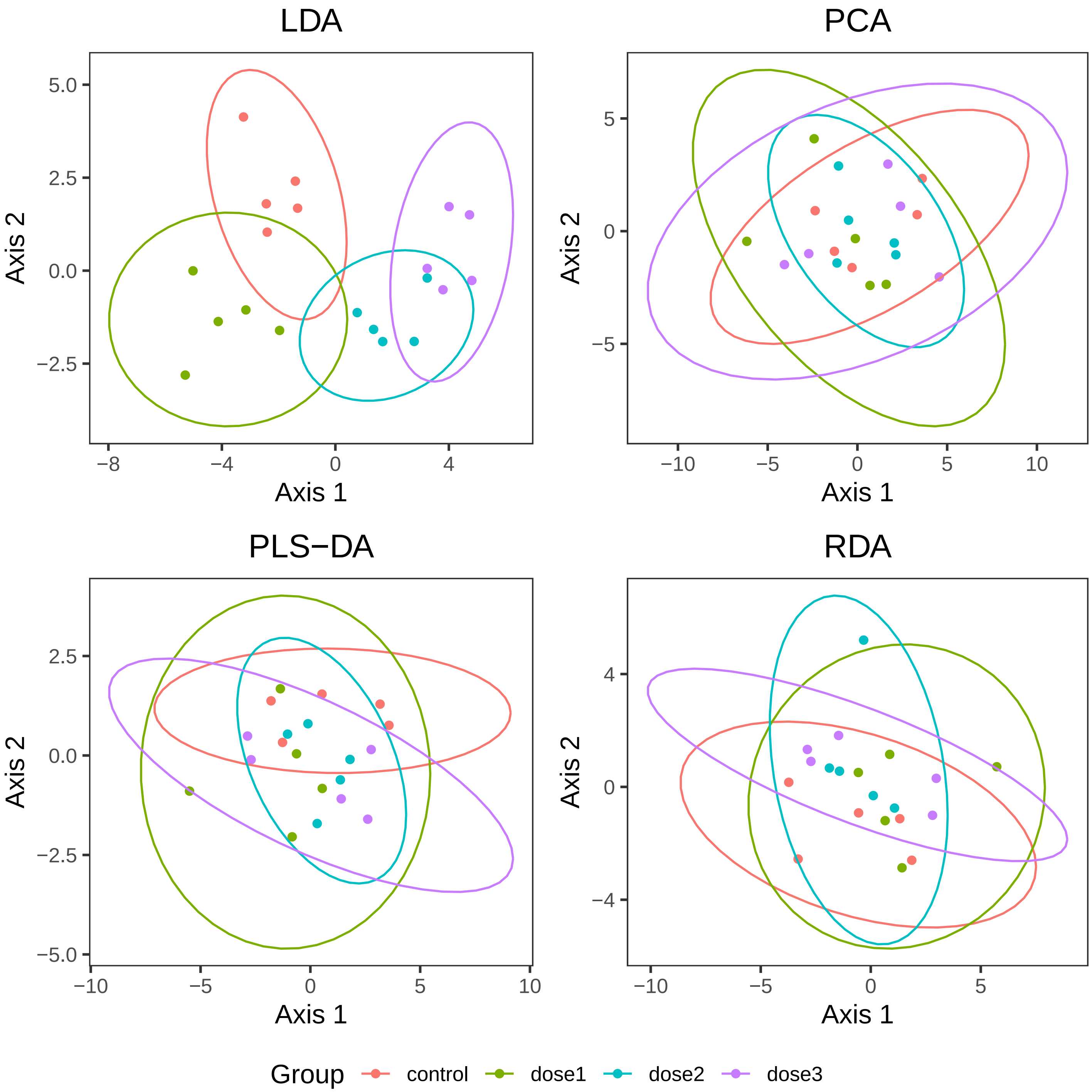

LDA
PCA
PLS−DA
RDA
Axis 1
Axis 2
5.0
2.5
0.0
−2.5
−8
−4
0
4
5
0
−5
−10
−5
0
5
10
2.5
0.0
−2.5
−5.0
−10
−5
0
5
10
4
0
−4
−10
−5
0
5
Group
control
dose1
dose2
dose3

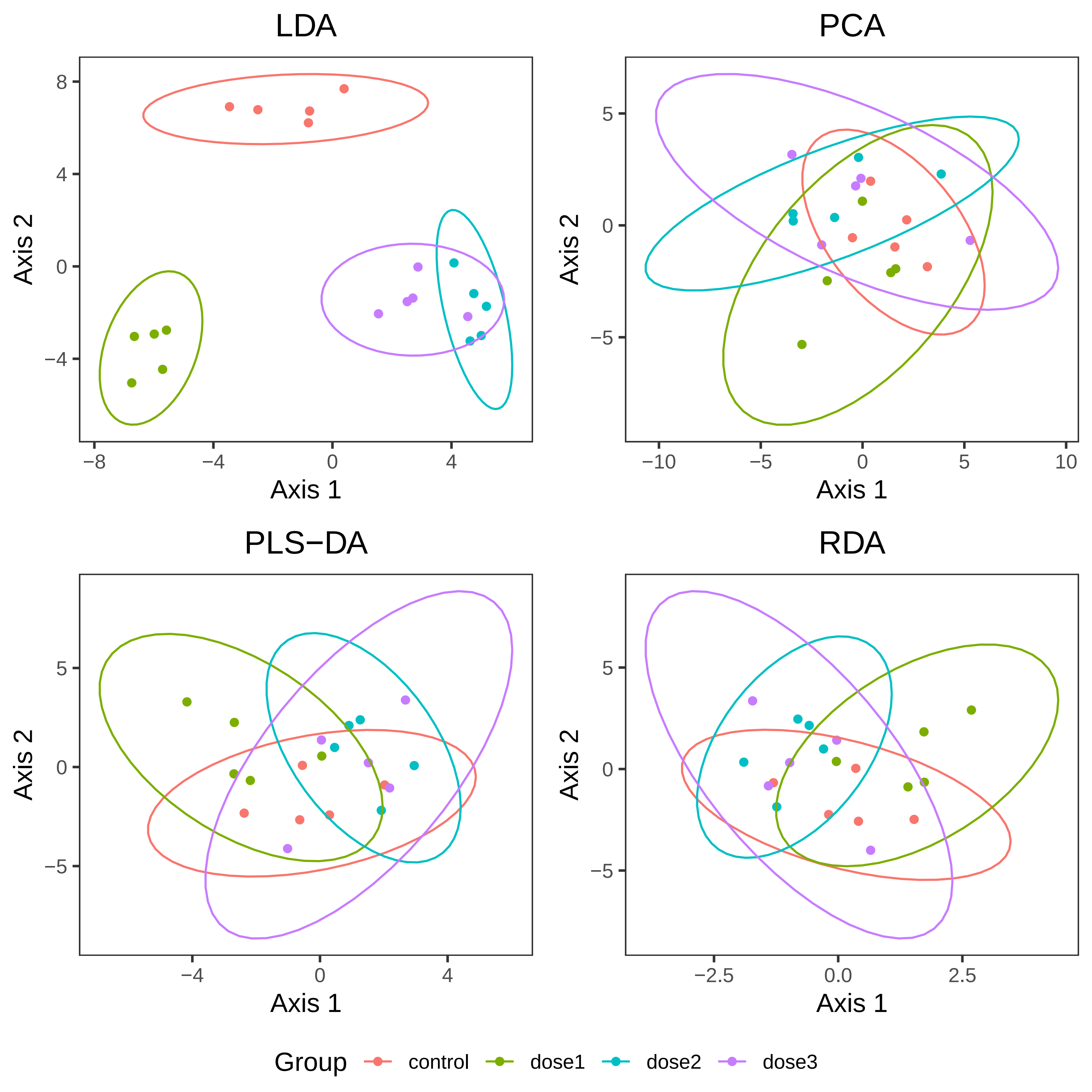
LDA
PCA
PLS−DA
RDA
Axis 1
Axis 2
8
4
0
−4
−8
5
−5
10
−10
2.5
0.0
−2.5
Group
control
dose1
dose2
dose3

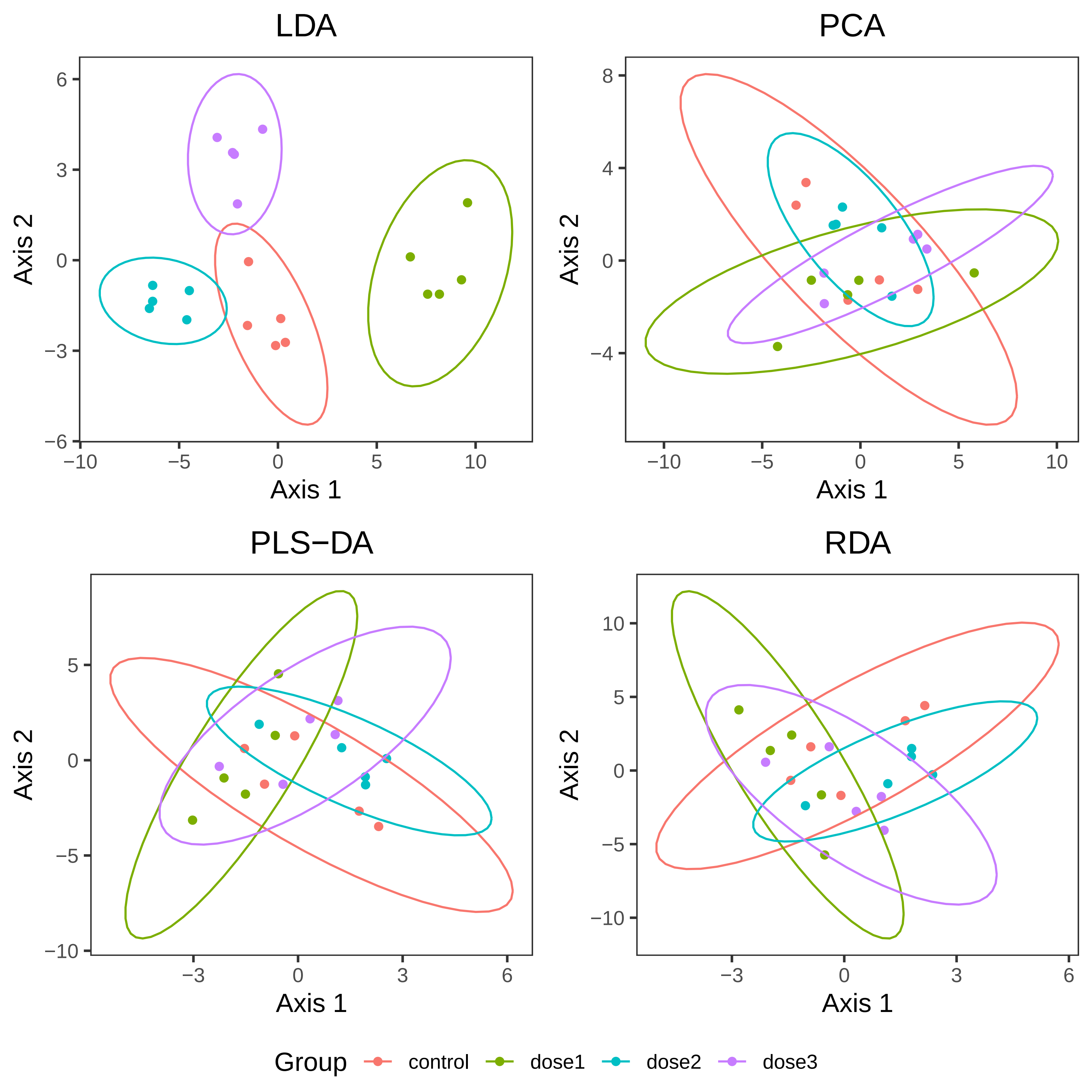

LDA
PCA
PLS−DA
RDA
Axis 1
Axis 2
Group
control
dose1
dose2
dose3

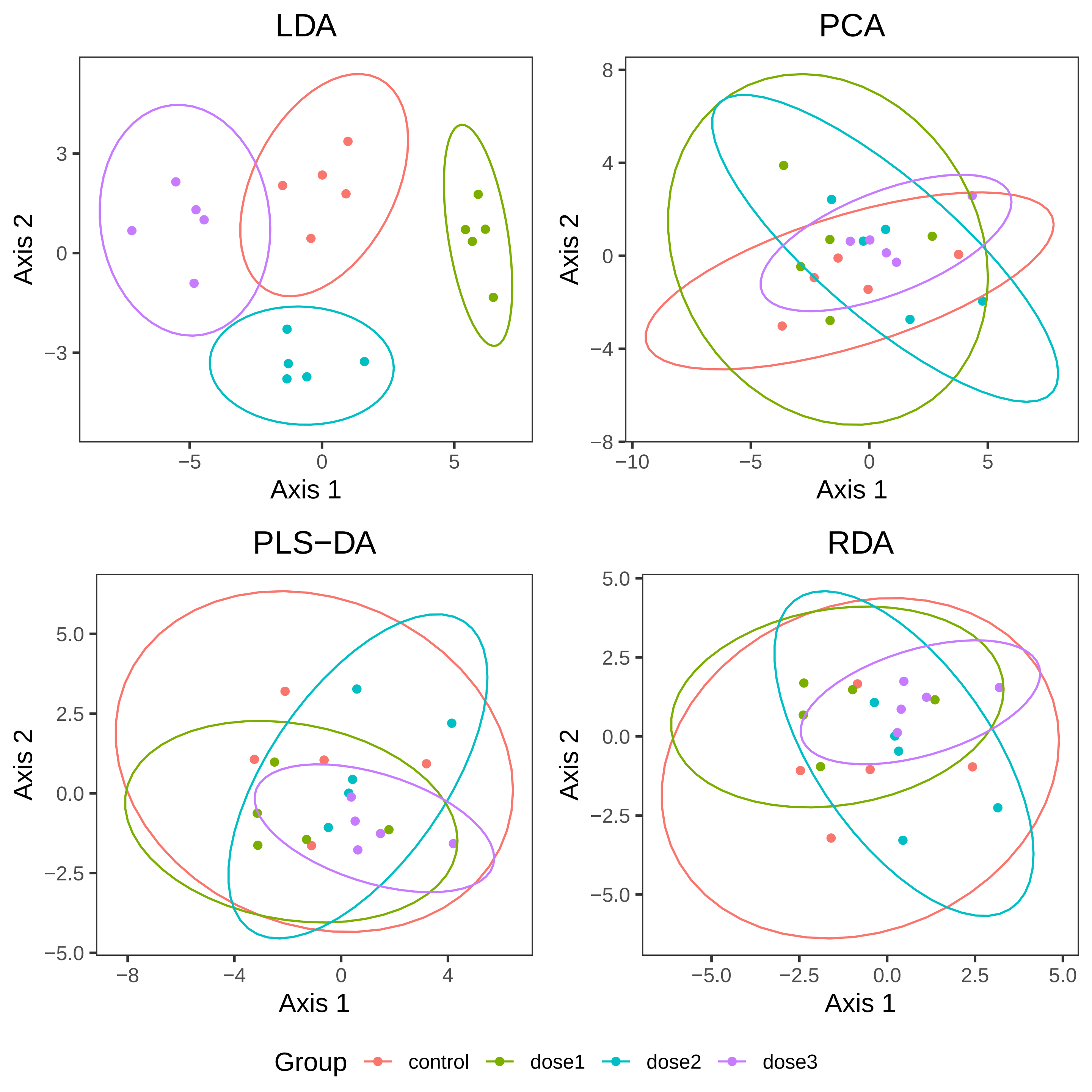

LDA
PCA
PLS−DA
RDA
Axis 1
Axis 2
Group
control
dose1
dose2
dose3

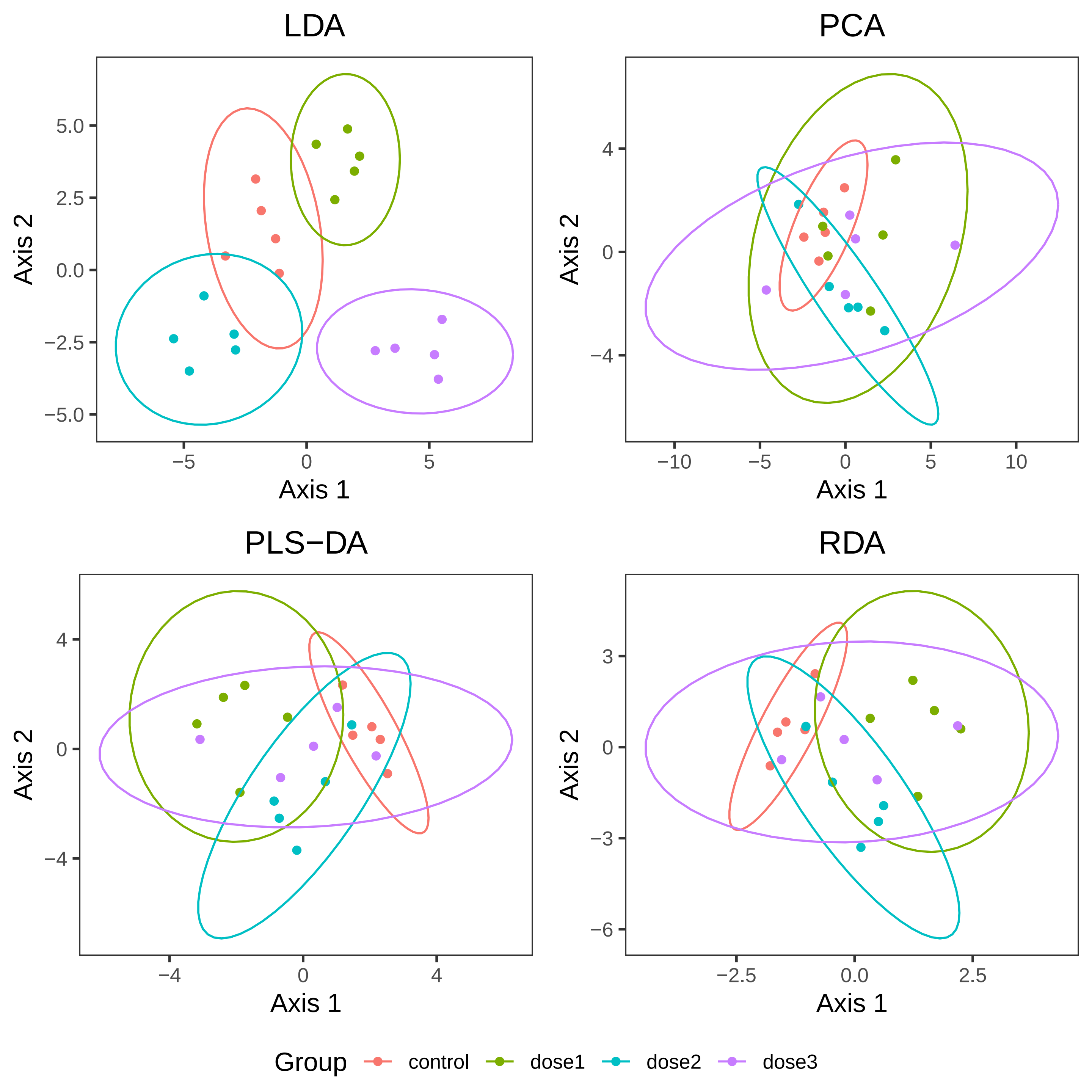

LDA
PCA
PLS−DA
RDA
Axis 1
Axis 2
5.0
2.5
0.0
−2.5
−5.0
−5
0
5
4
0
−4
−10
10
3
−3
−6
−2.5
0.0
2.5
Group
control
dose1
dose2
dose3

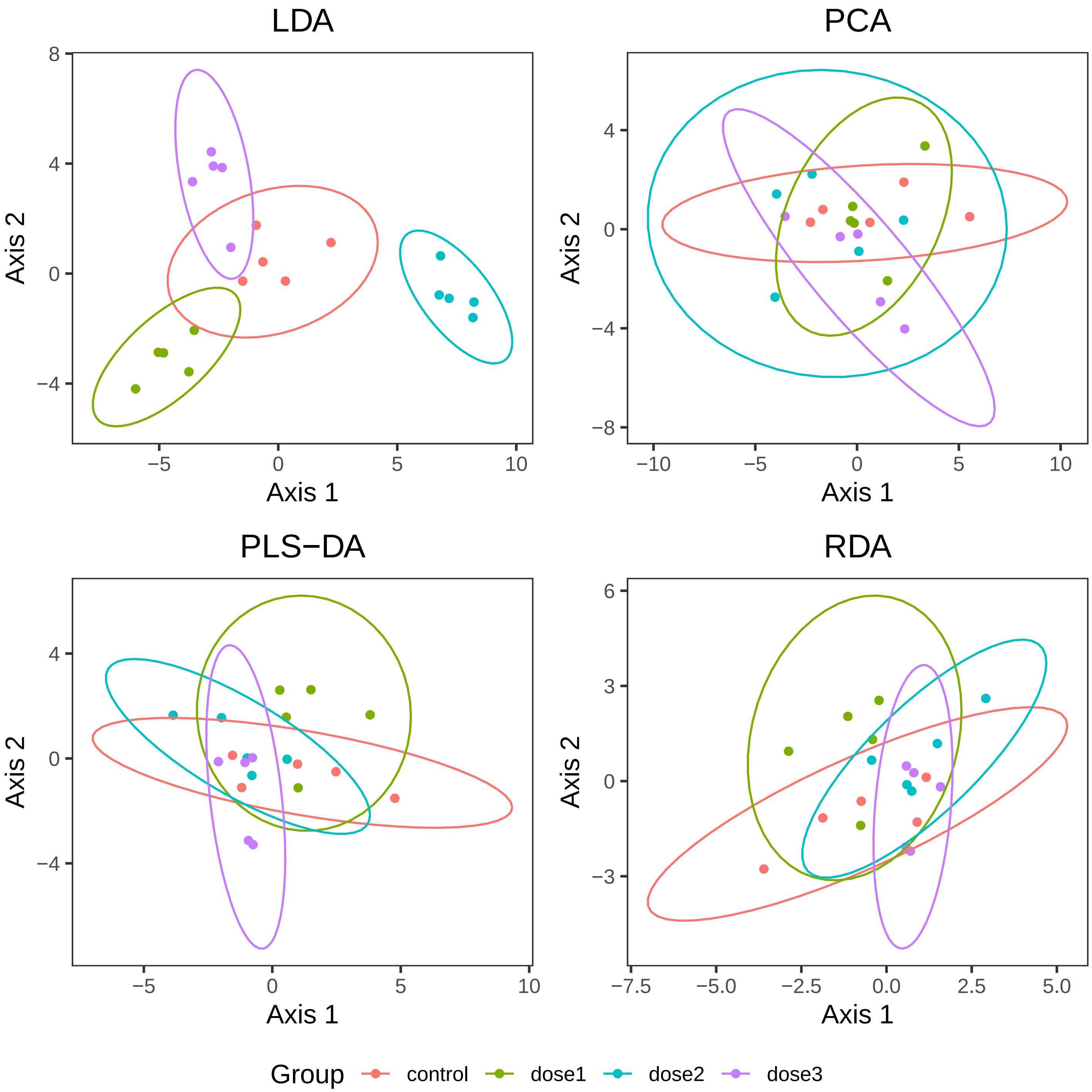

LDA
PCA
PLS−DA
RDA
Axis 1
Axis 2
Group
control
dose1
dose2
dose3

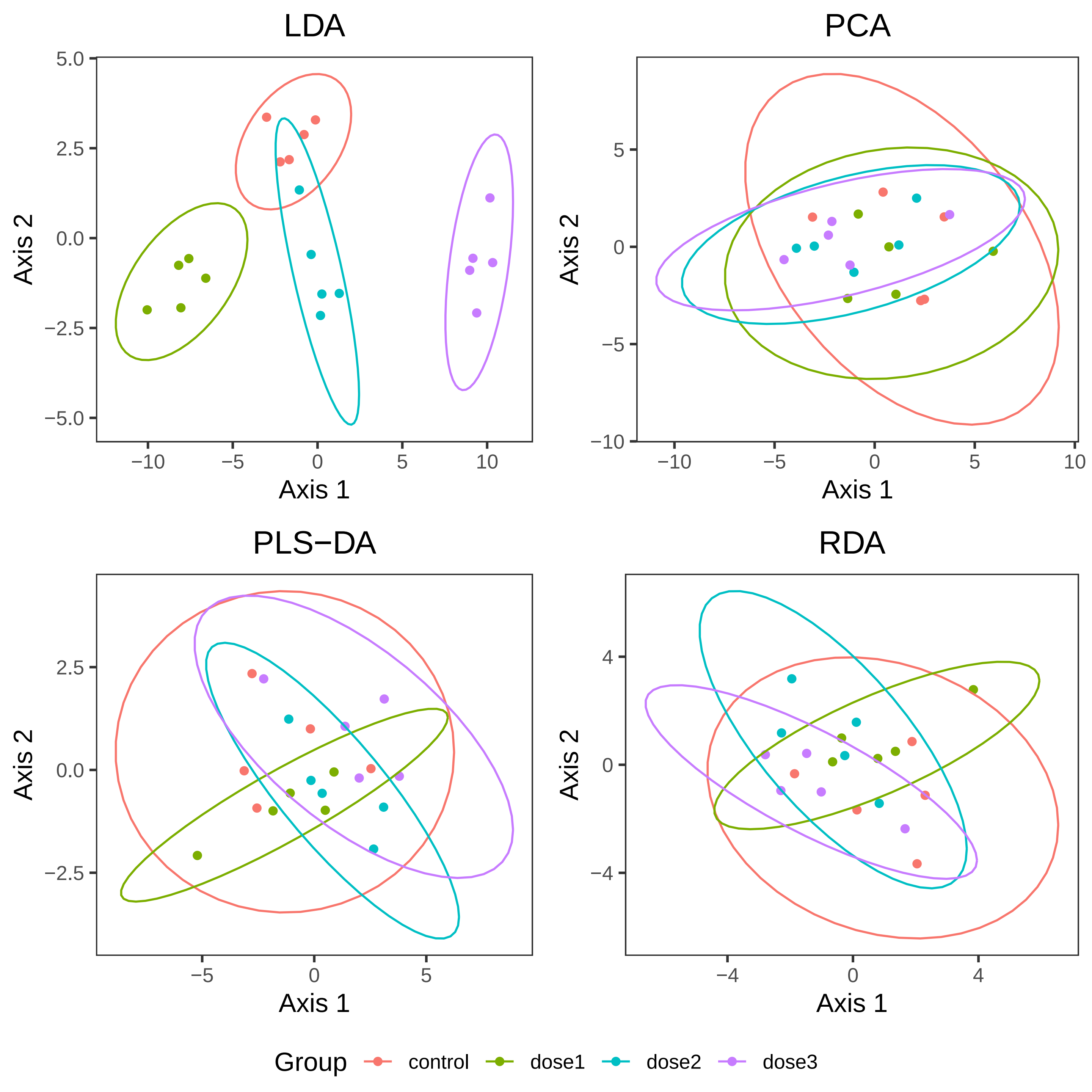

LDA
PCA
PLS−DA
RDA
Axis 1
Axis 2
Group
control
dose1
dose2
dose3

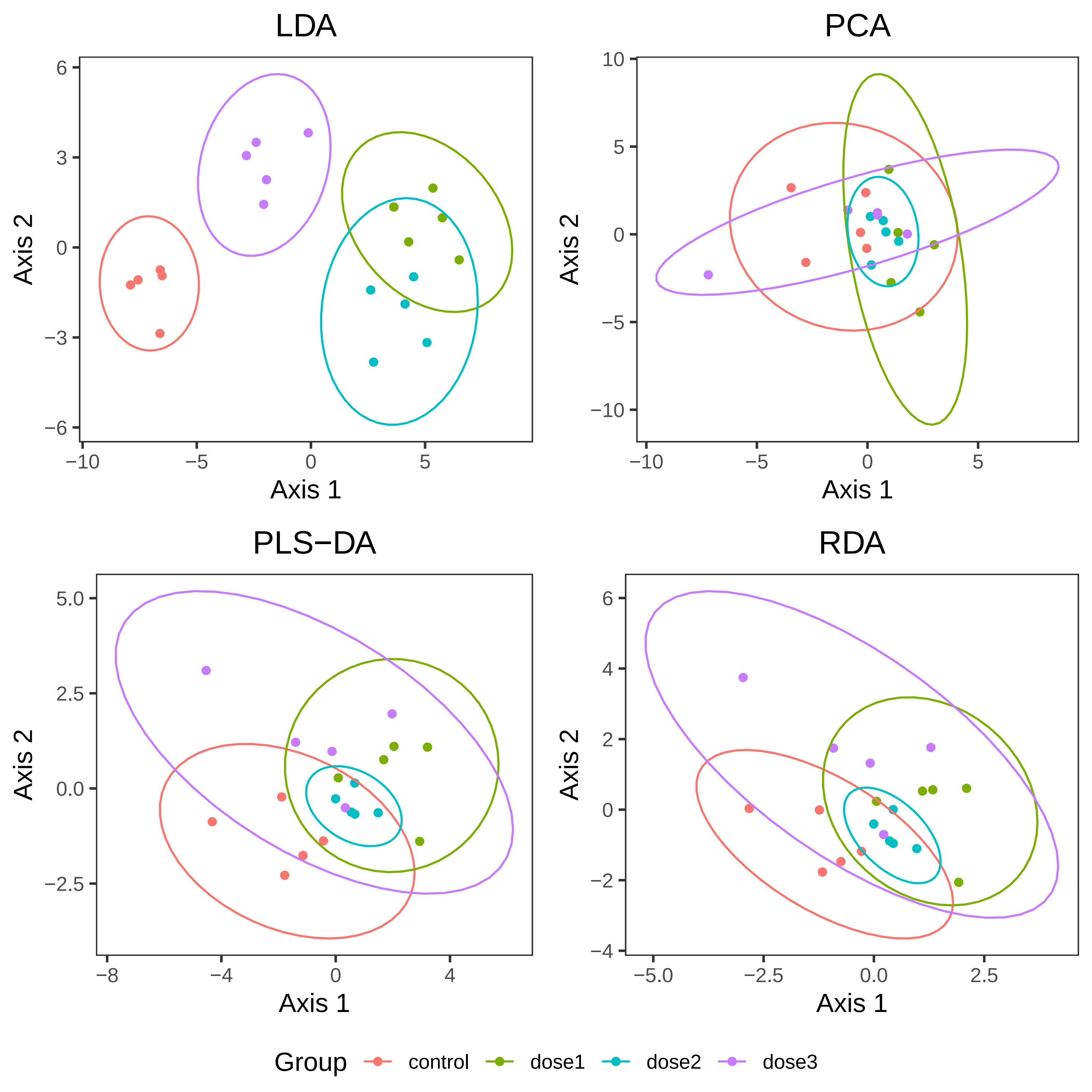
LDA
PCA
PLS−DA
RDA
Axis 1
Axis 2
6
3
0
−3
−6
−10
−5
0
5
10
5
0
−5
−10
5.0
2.5
0.0
−2.5
−8
−4
0
4
6
4
2
0
−2
−4
−5.0
−2.5
0.0
2.5
Group
control
dose1
dose2
dose3

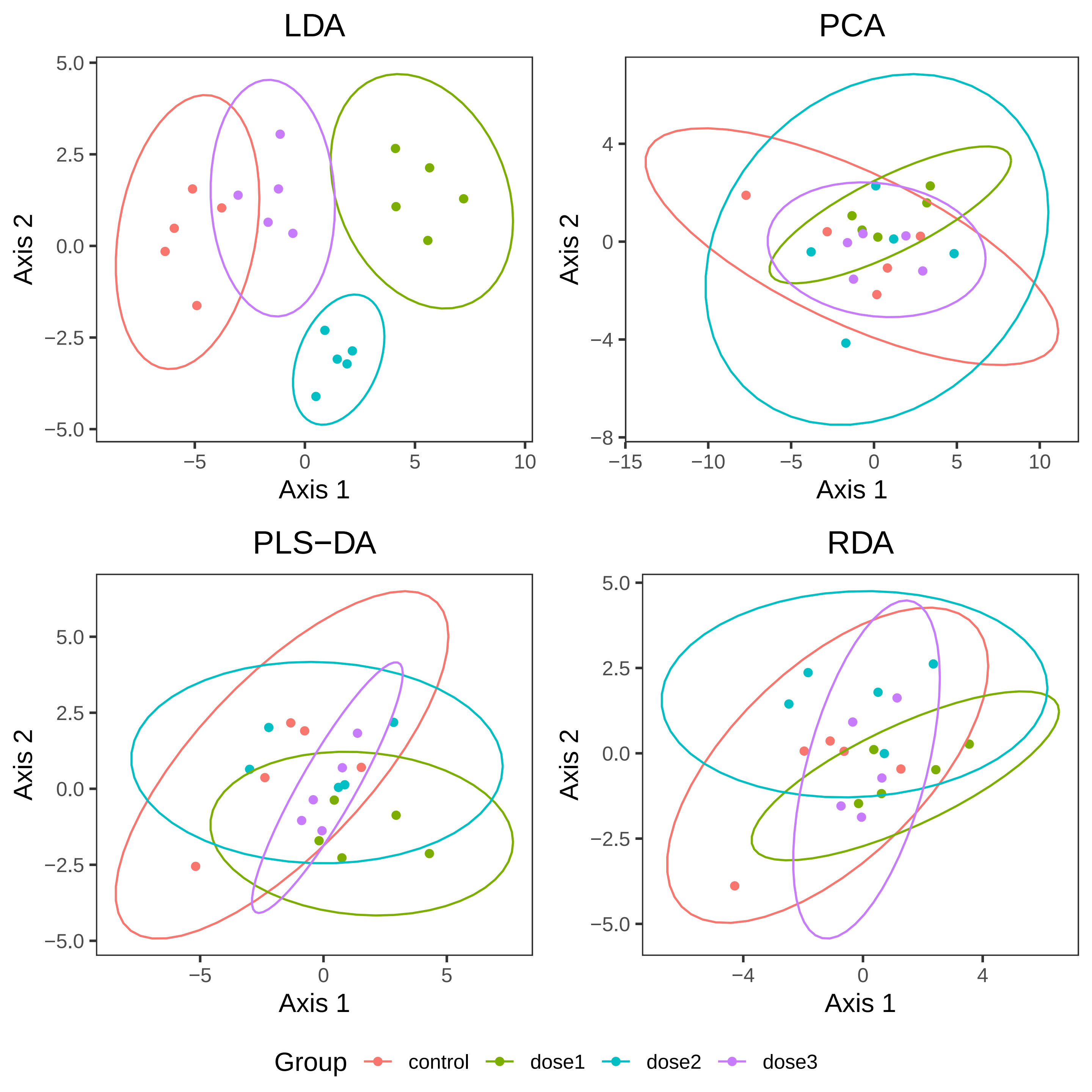
LDA
PCA
PLS−DA
RDA
Axis 1
Axis 2
5.0
2.5
0.0
−2.5
−5.0
−5
0
5
10
4
0
−4
−8
−15
−10
Group
control
dose1
dose2
dose3

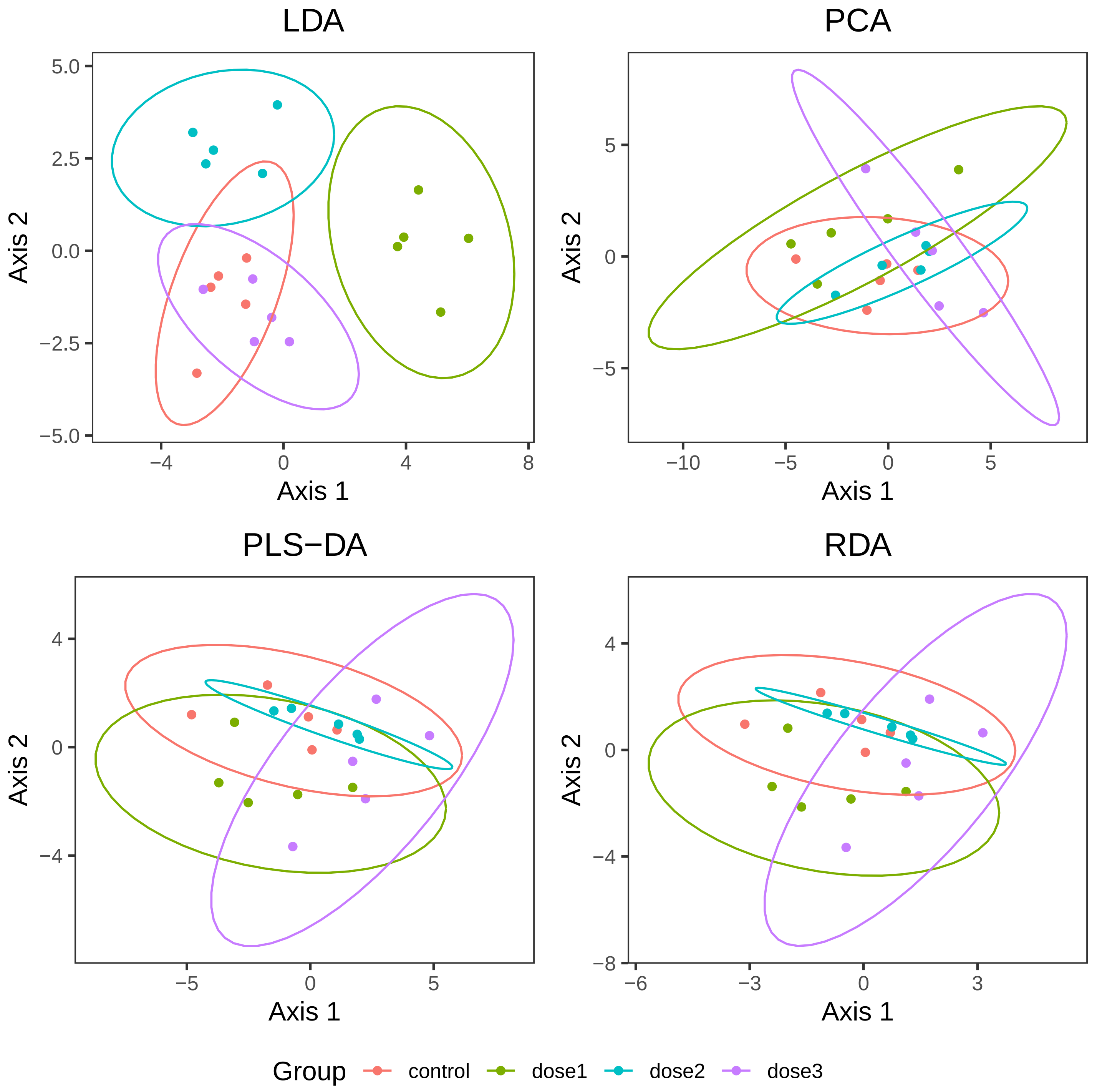

LDA
PCA
PLS−DA
RDA
Axis 1
Axis 2
5.0
2.5
0.0
−2.5
−5.0
−4
0
4
8
5
−5
−10
−4
−8
−6
−3
3
Group
control
dose1
dose2
dose3

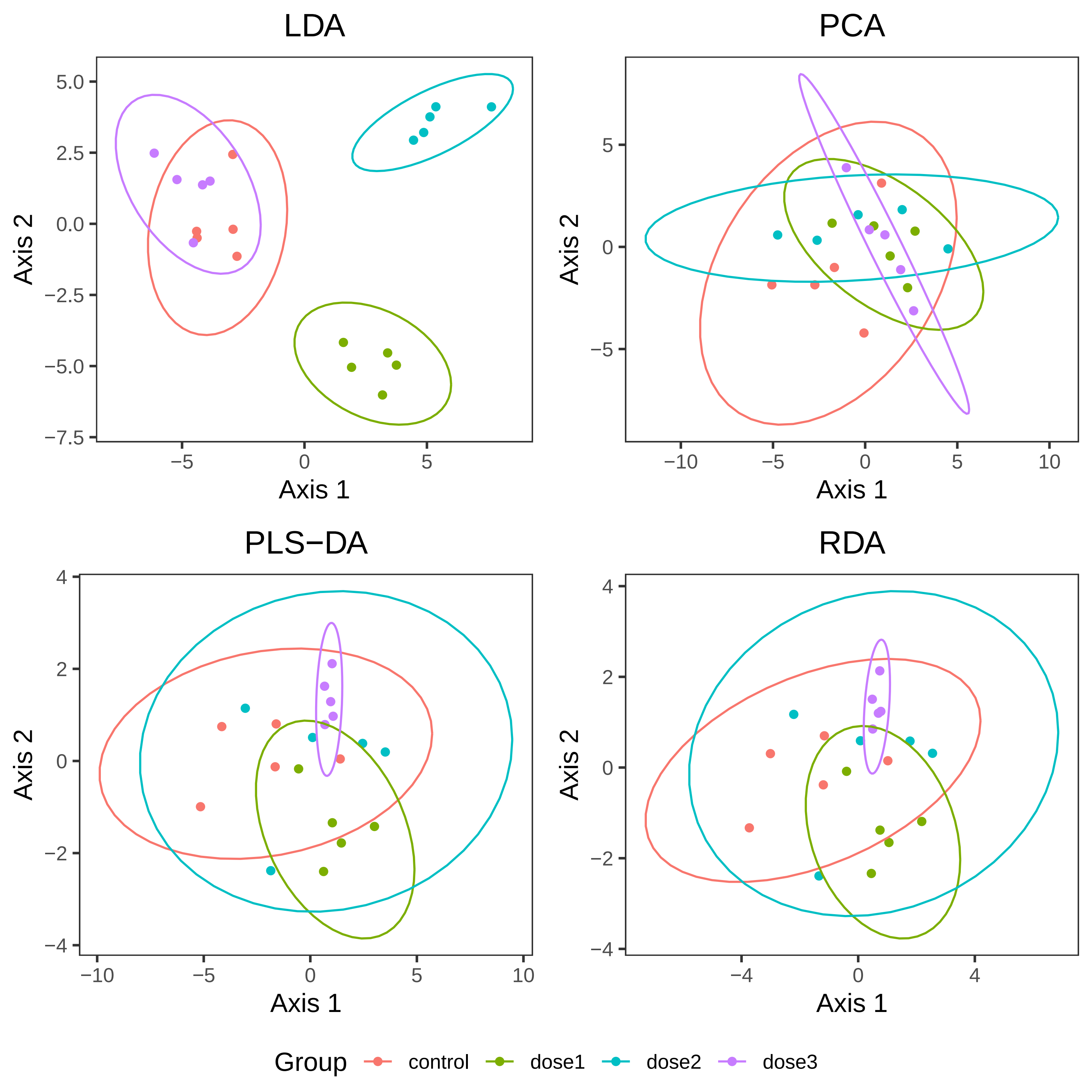

LDA
PCA
PLS−DA
RDA
Axis 1
Axis 2
Group
control
dose1
dose2
dose3

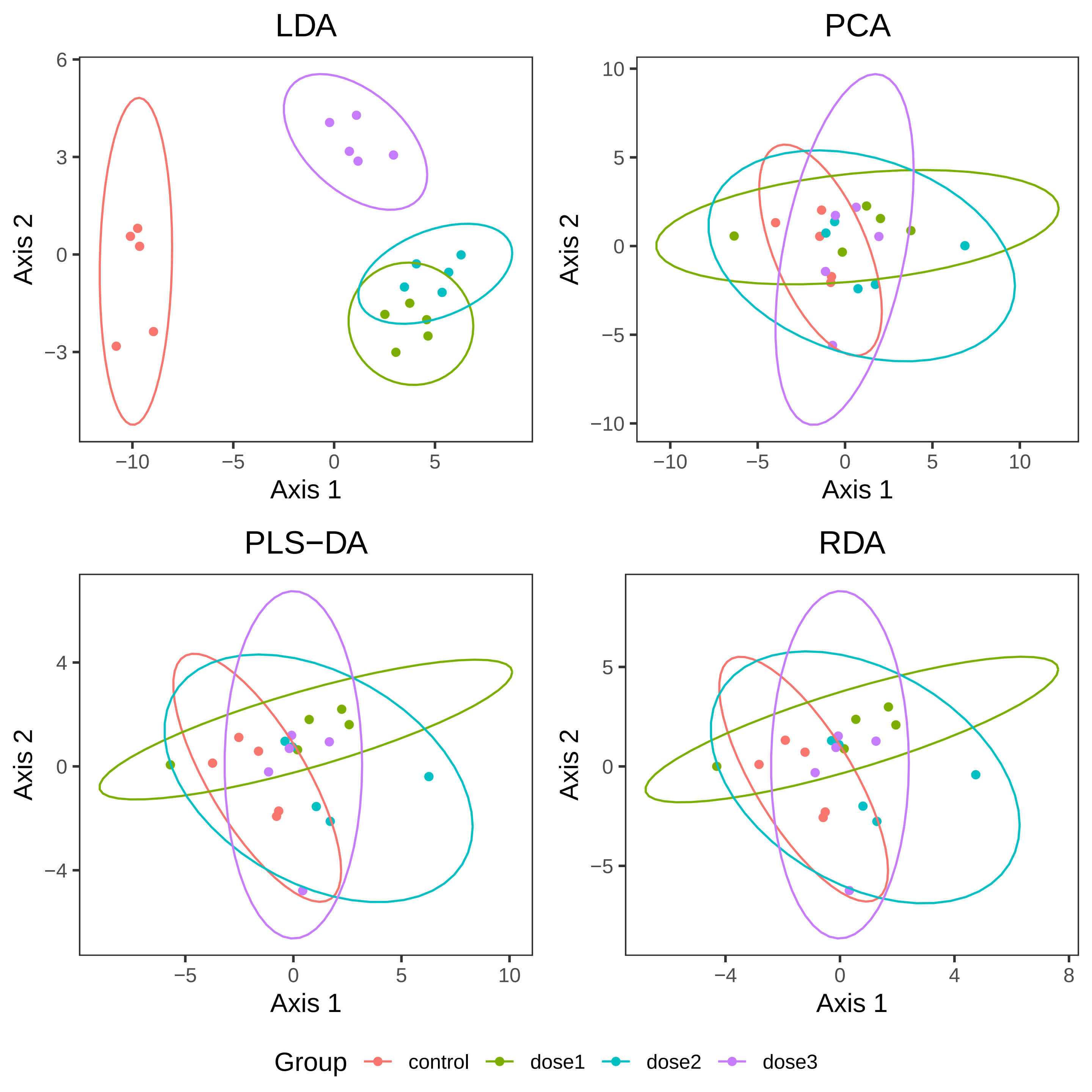
LDA
PCA
PLS−DA
RDA
Axis 1
Axis 2
Group
control
dose1
dose2
dose3

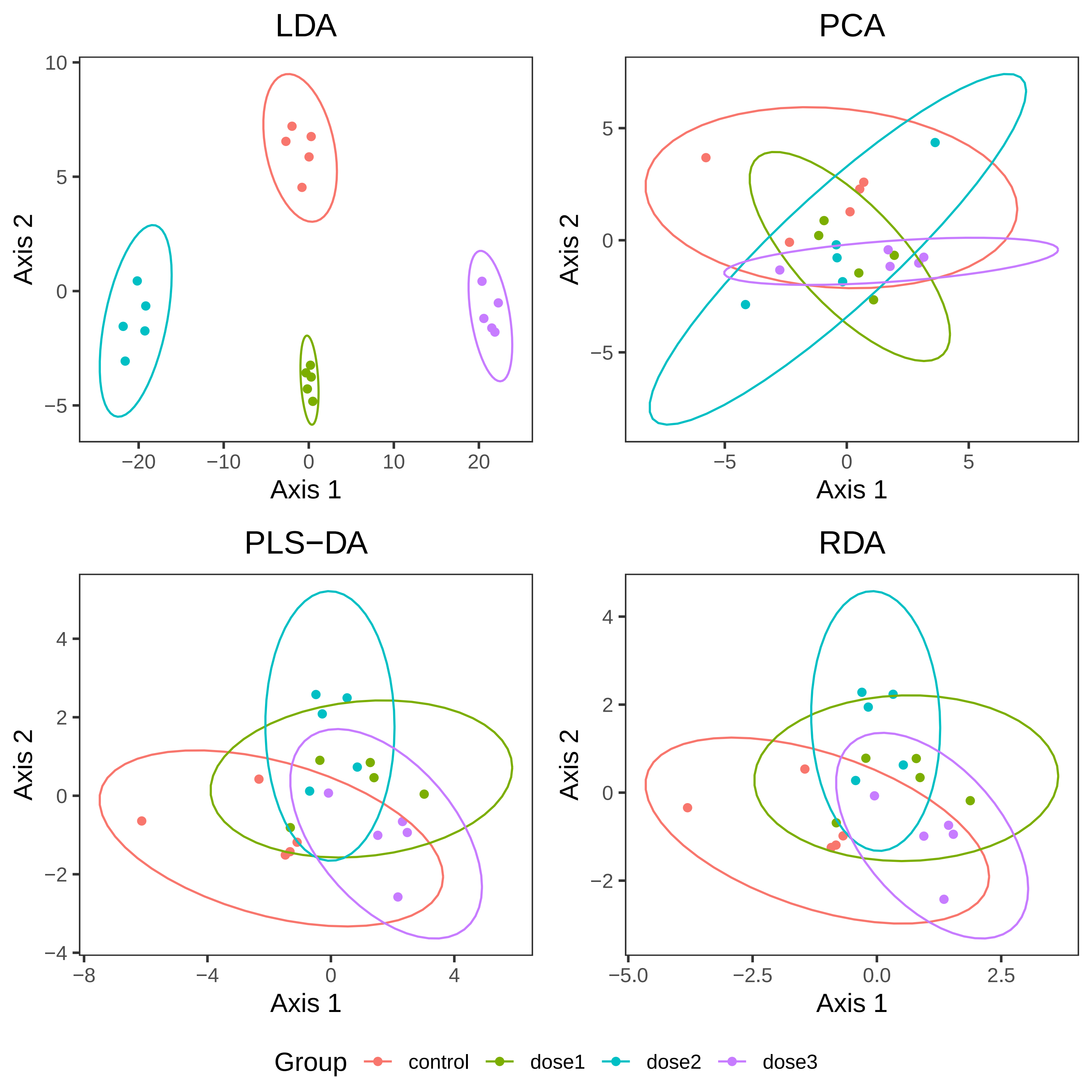
LDA
PCA
PLS−DA
RDA
Axis 1
Axis 2
Group
control
dose1
dose2
dose3

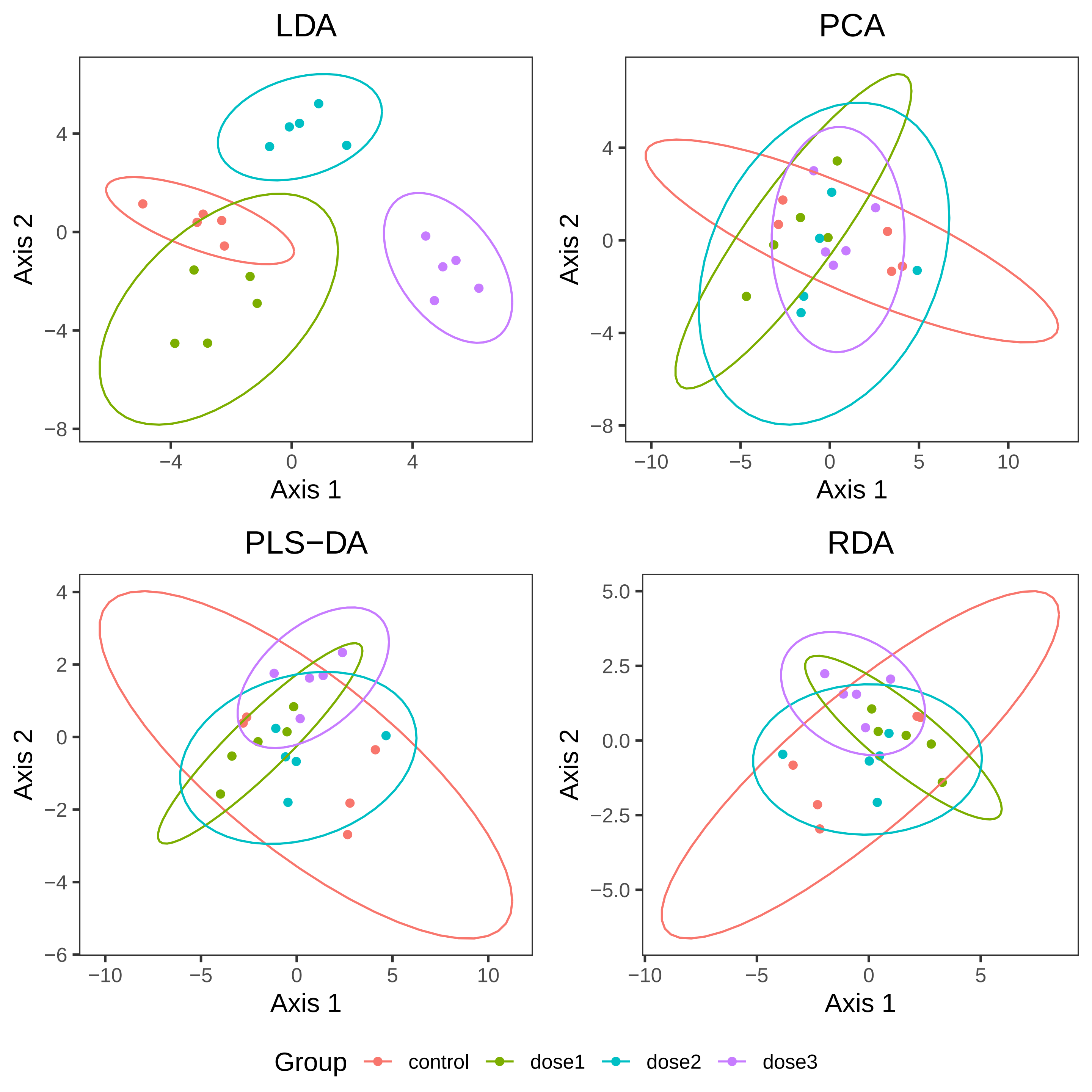
LDA
PCA
PLS−DA
RDA
Axis 1
Axis 2
Group
control
dose1
dose2
dose3

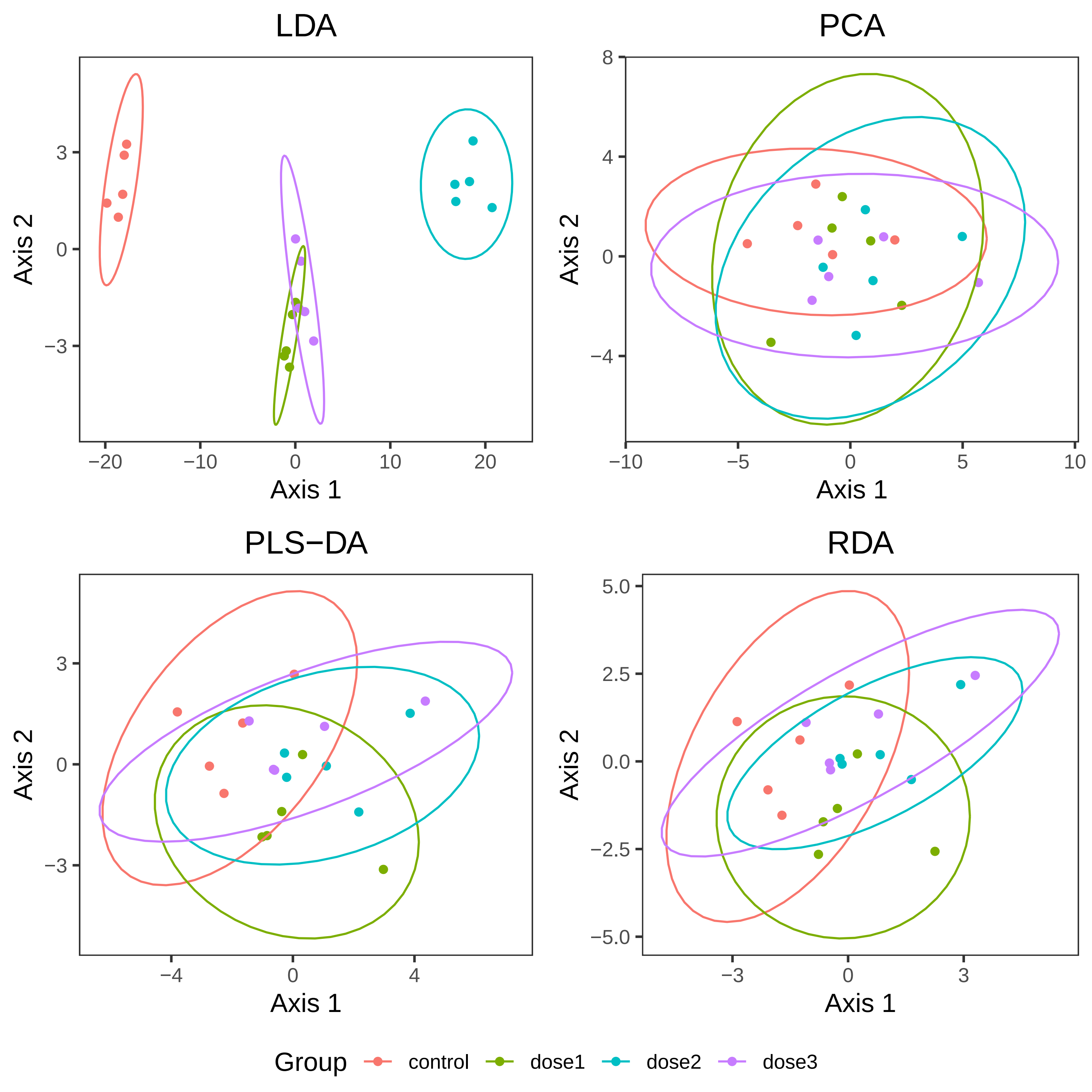
LDA
PCA
PLS−DA
RDA
Axis 1
Axis 2
Group
control
dose1
dose2
dose3

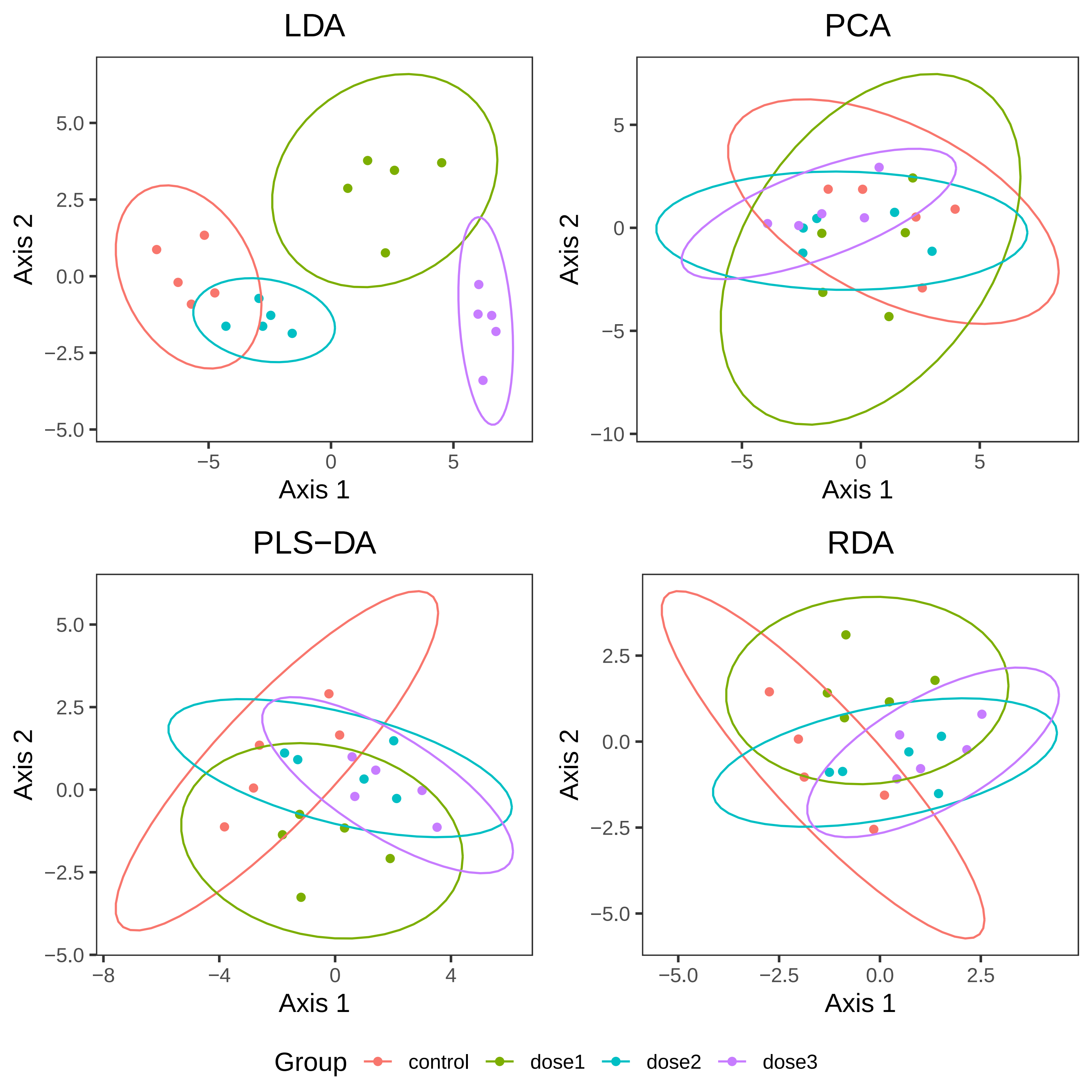

LDA
PCA
PLS−DA
RDA
Axis 1
Axis 2
Group
control
dose1
dose2
dose3

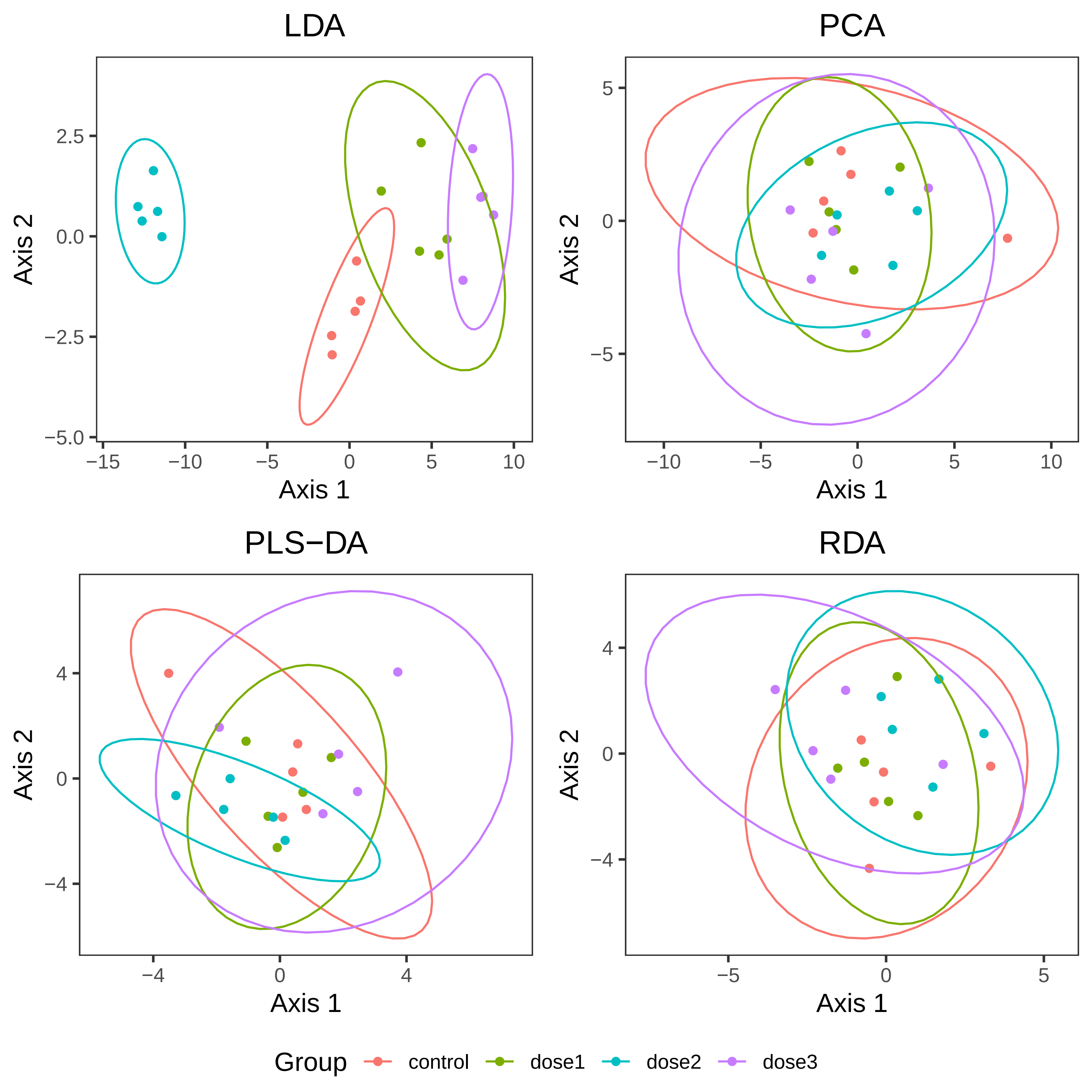
LDA
PCA
PLS−DA
RDA
Axis 1
Axis 2
2.5
0.0
−2.5
−5.0
−15
−10
−5
0
5
10
−4
4
Group
control
dose1
dose2
dose3

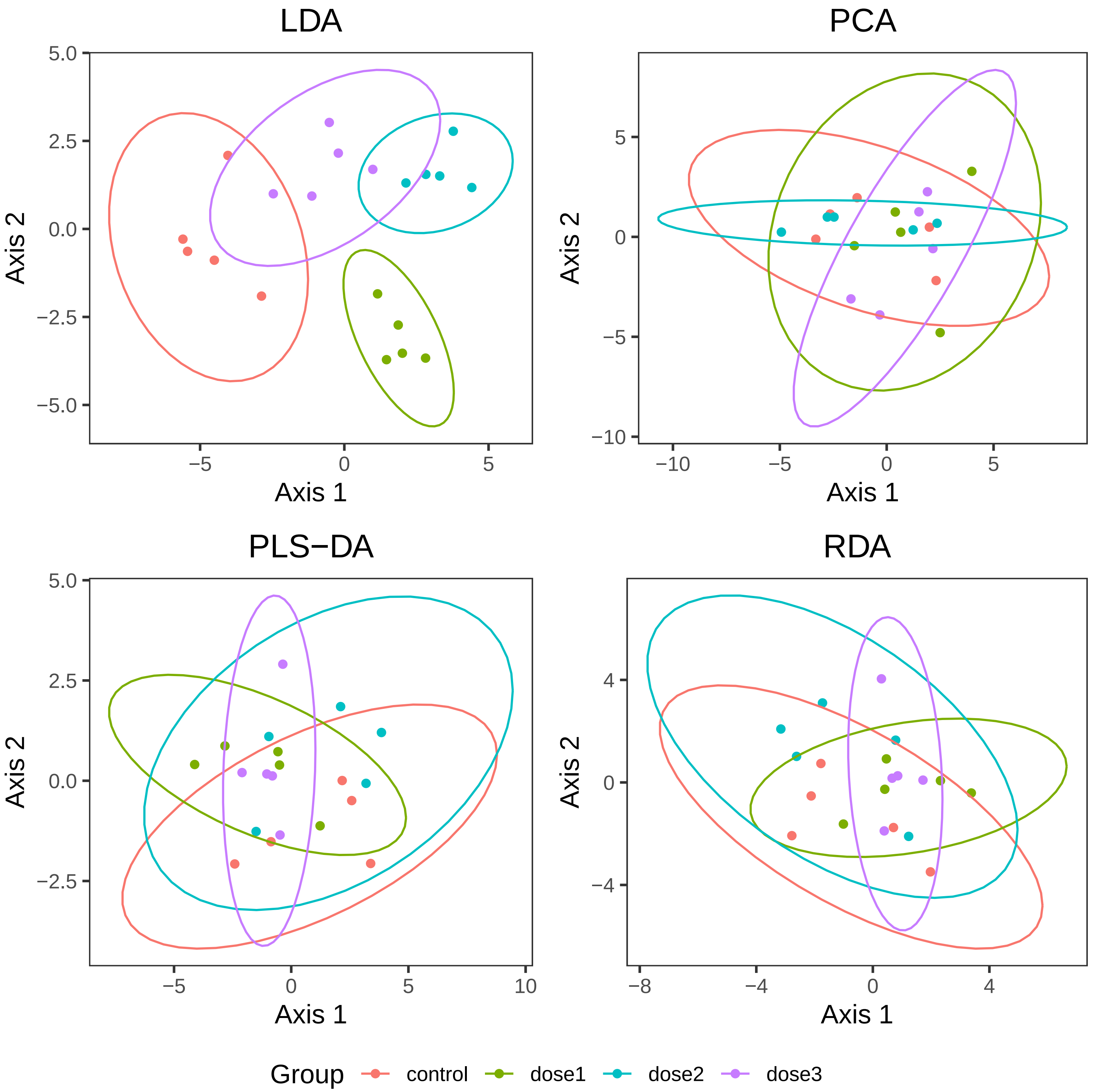
LDA
PCA
PLS−DA
RDA
Axis 1
Axis 2
Group
control
dose1
dose2
dose3

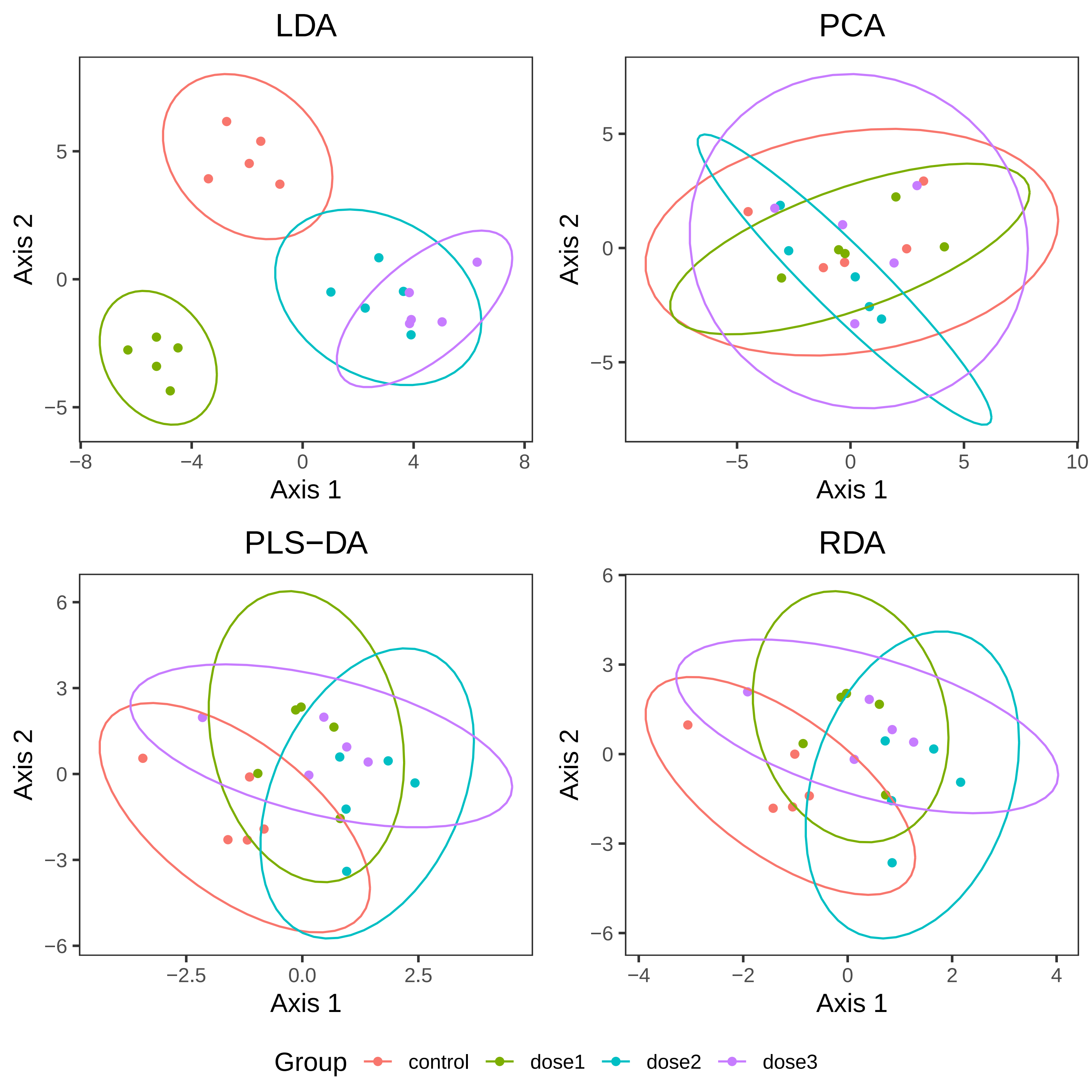

LDA
PCA
PLS−DA
RDA
Axis 1
Axis 2
Group
control
dose1
dose2
dose3

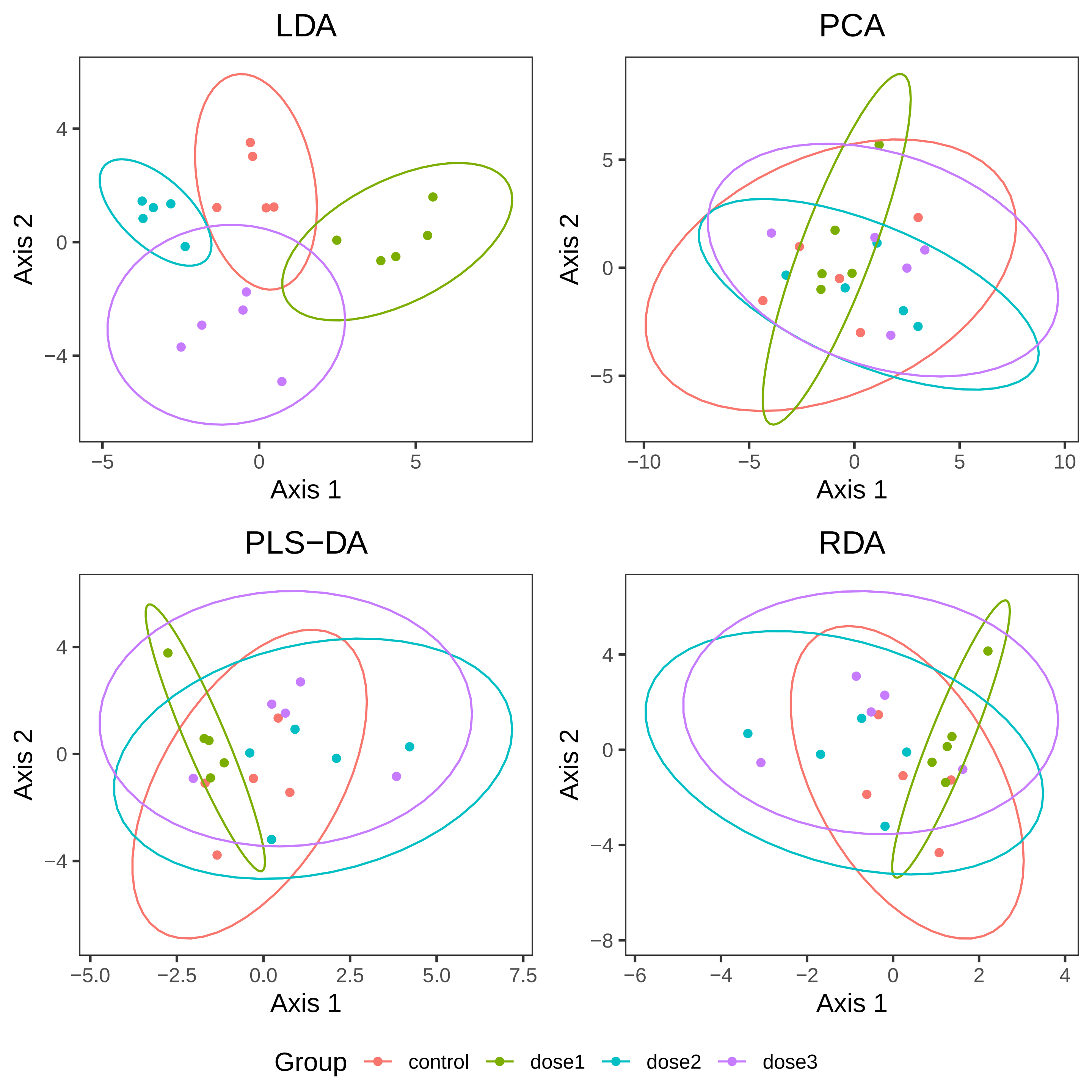

LDA
PCA
PLS−DA
RDA
Axis 1
Axis 2
Group
control
dose1
dose2
dose3

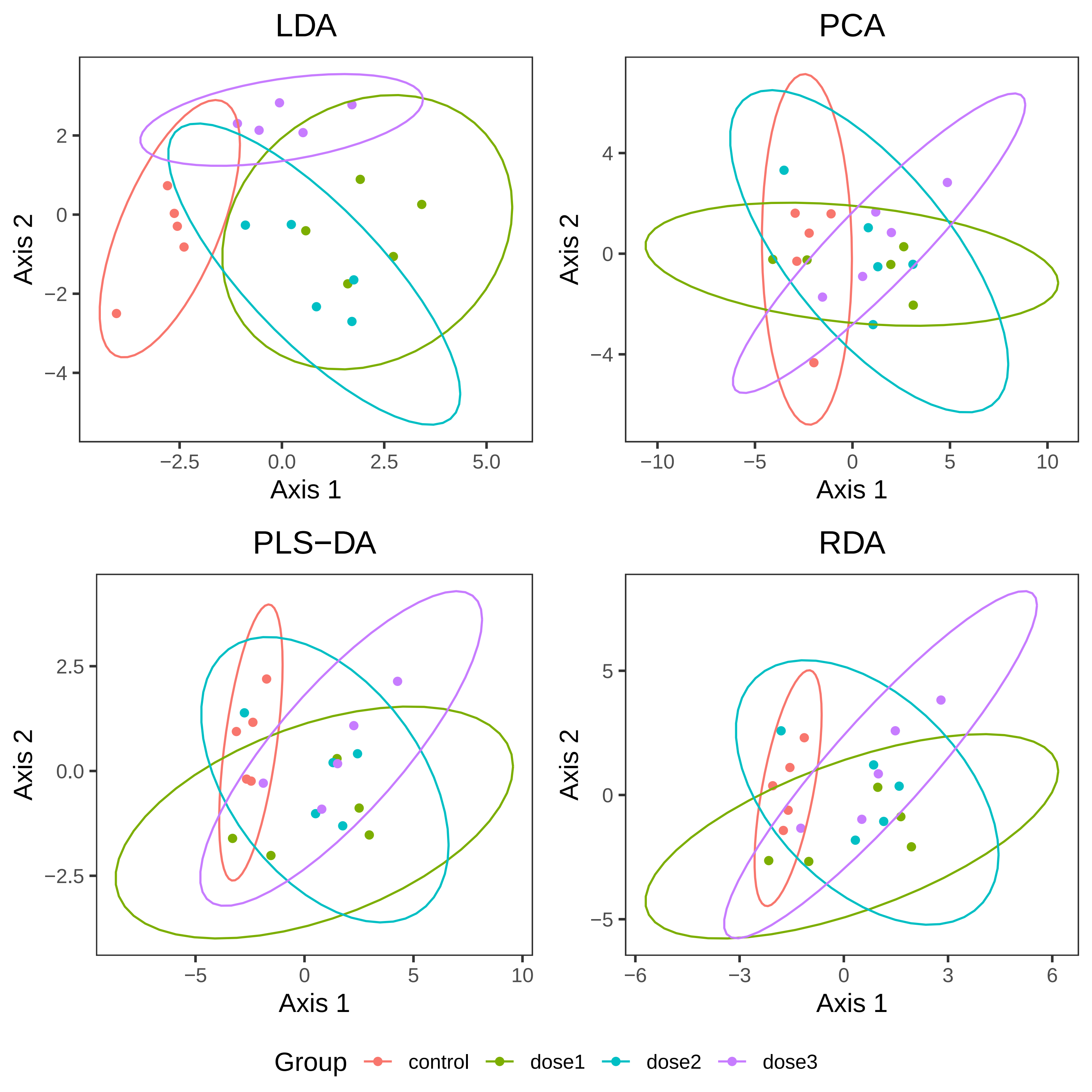

LDA
PCA
PLS−DA
RDA
Axis 1
Axis 2
Group
control
dose1
dose2
dose3

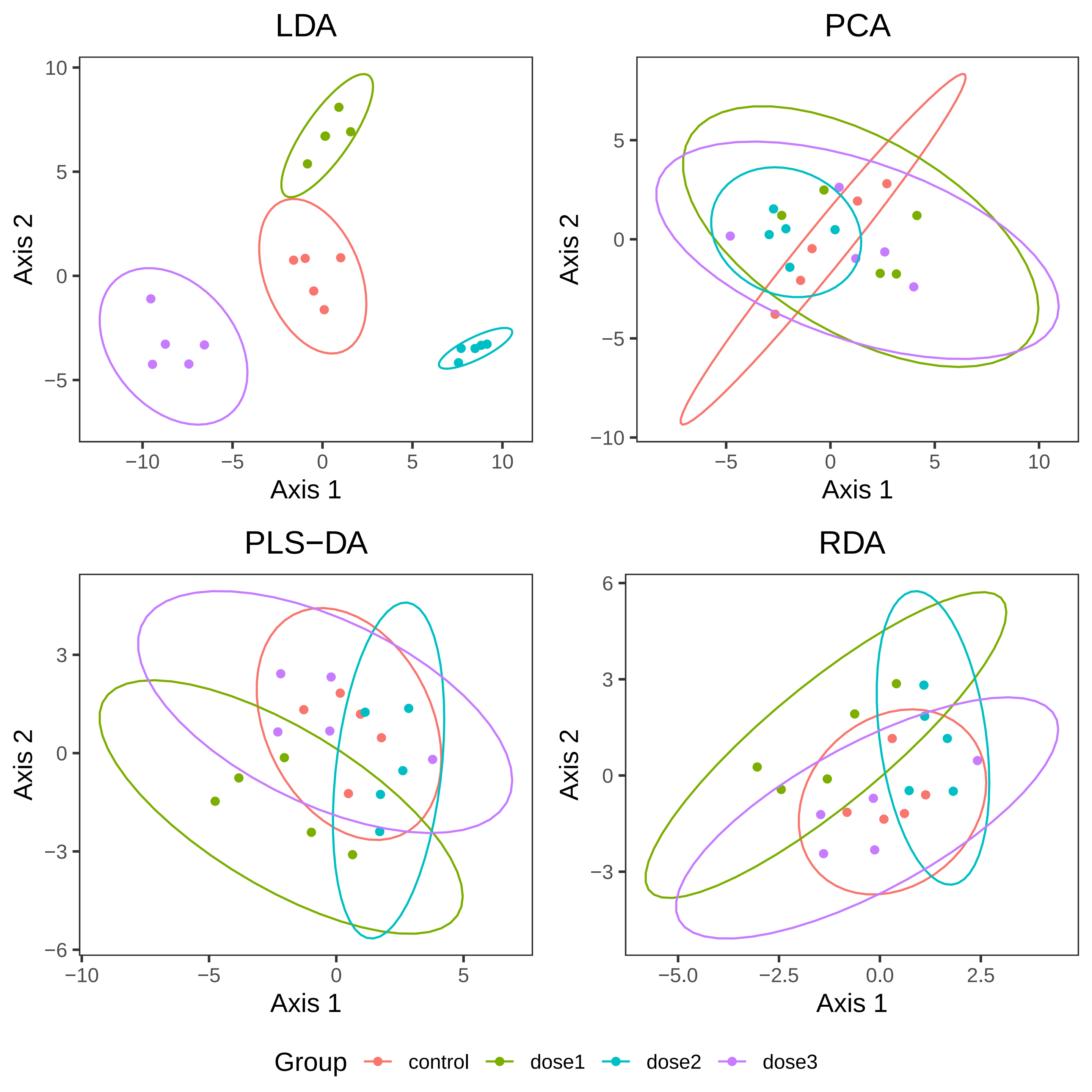
LDA
PCA
PLS−DA
RDA
Axis 1
Axis 2
Group
control
dose1
dose2
dose3

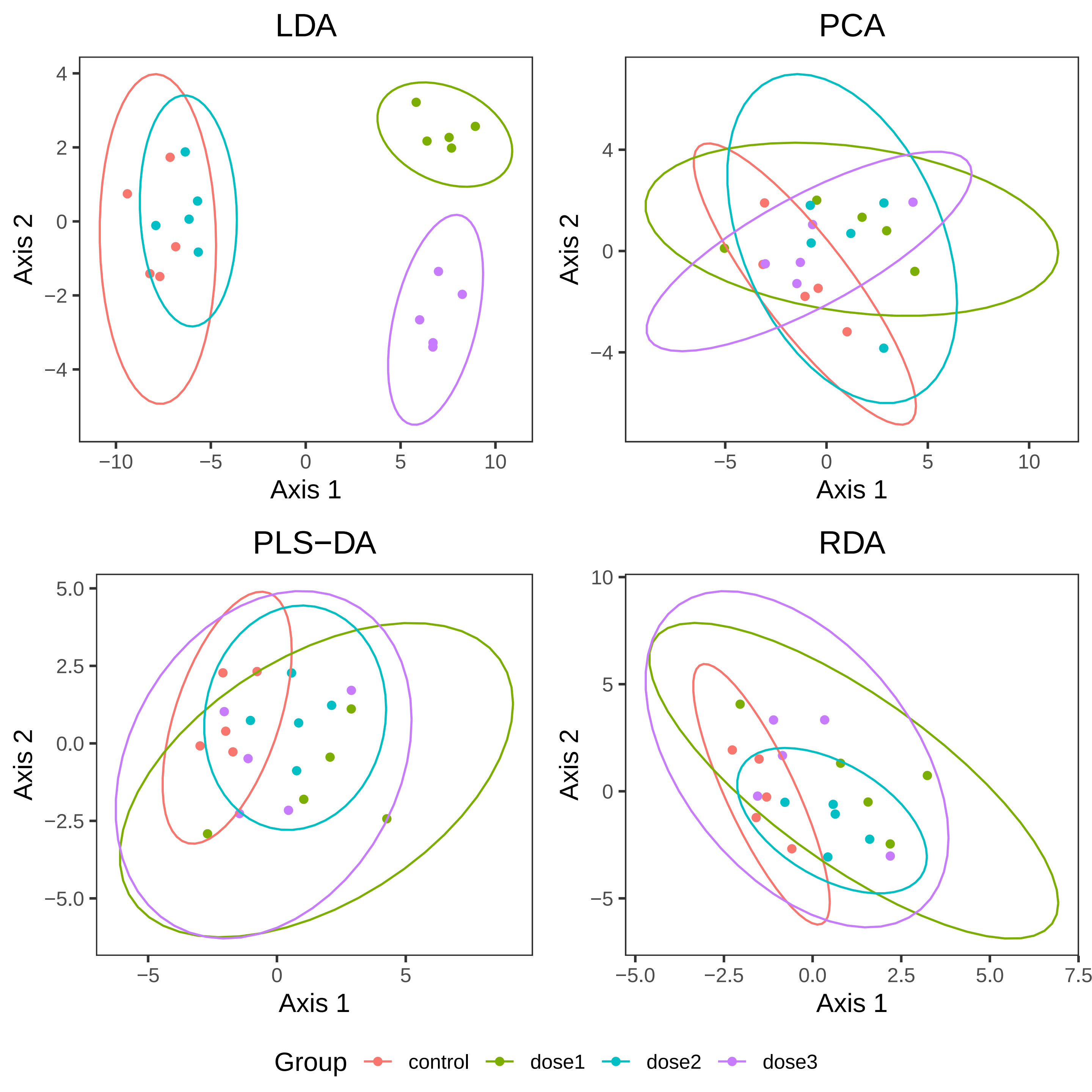

LDA
PCA
PLS−DA
RDA
Axis 1
Axis 2
−10
−5
0
5
10
4
2
0
−2
−4
5.0
2.5
0.0
−2.5
−5.0
7.5
Group
control
dose1
dose2
dose3

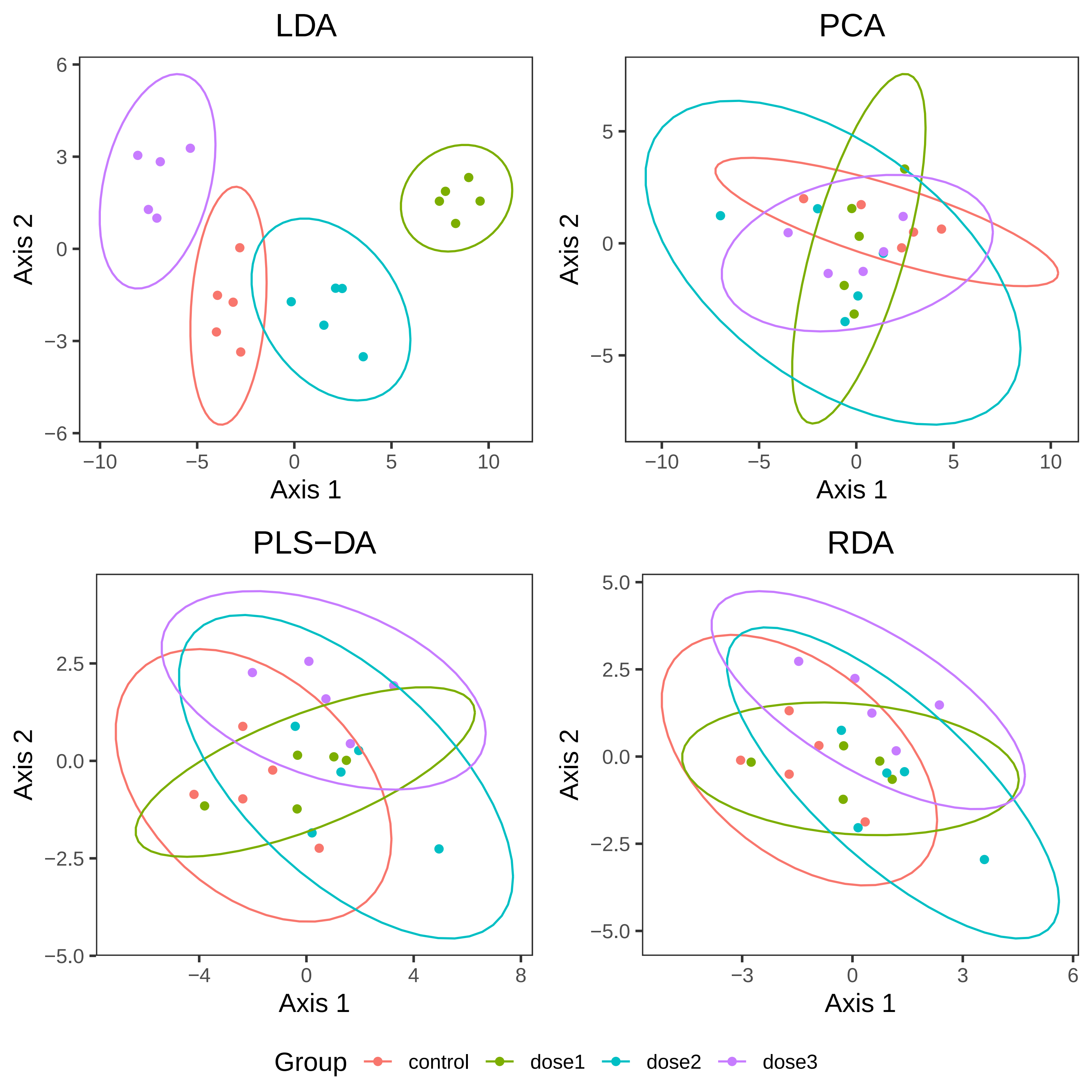
LDA
PCA
PLS−DA
RDA
Axis 1
Axis 2
Group
control
dose1
dose2
dose3

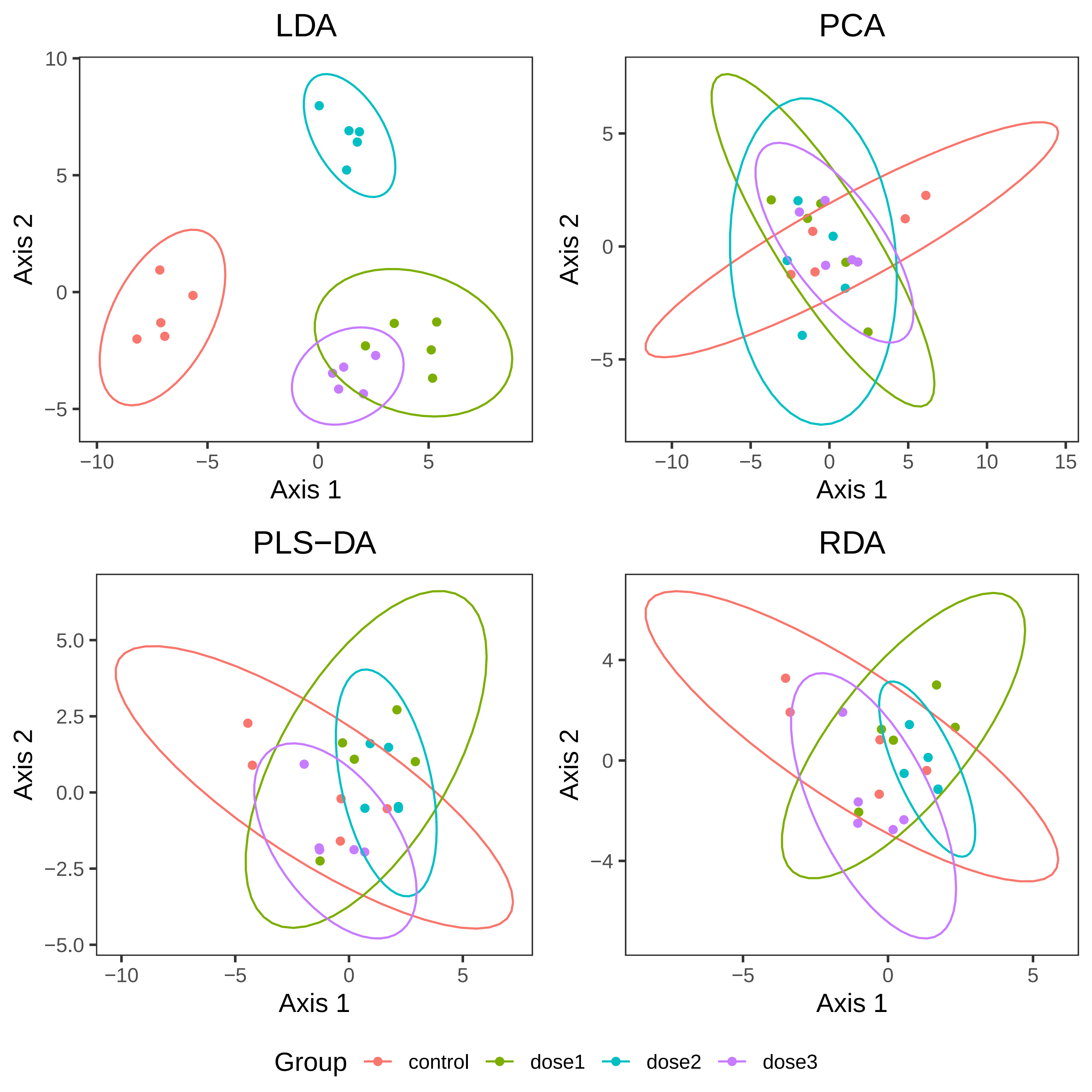

LDA
PCA
PLS−DA
RDA
Axis 1
Axis 2
Group
control
dose1
dose2
dose3

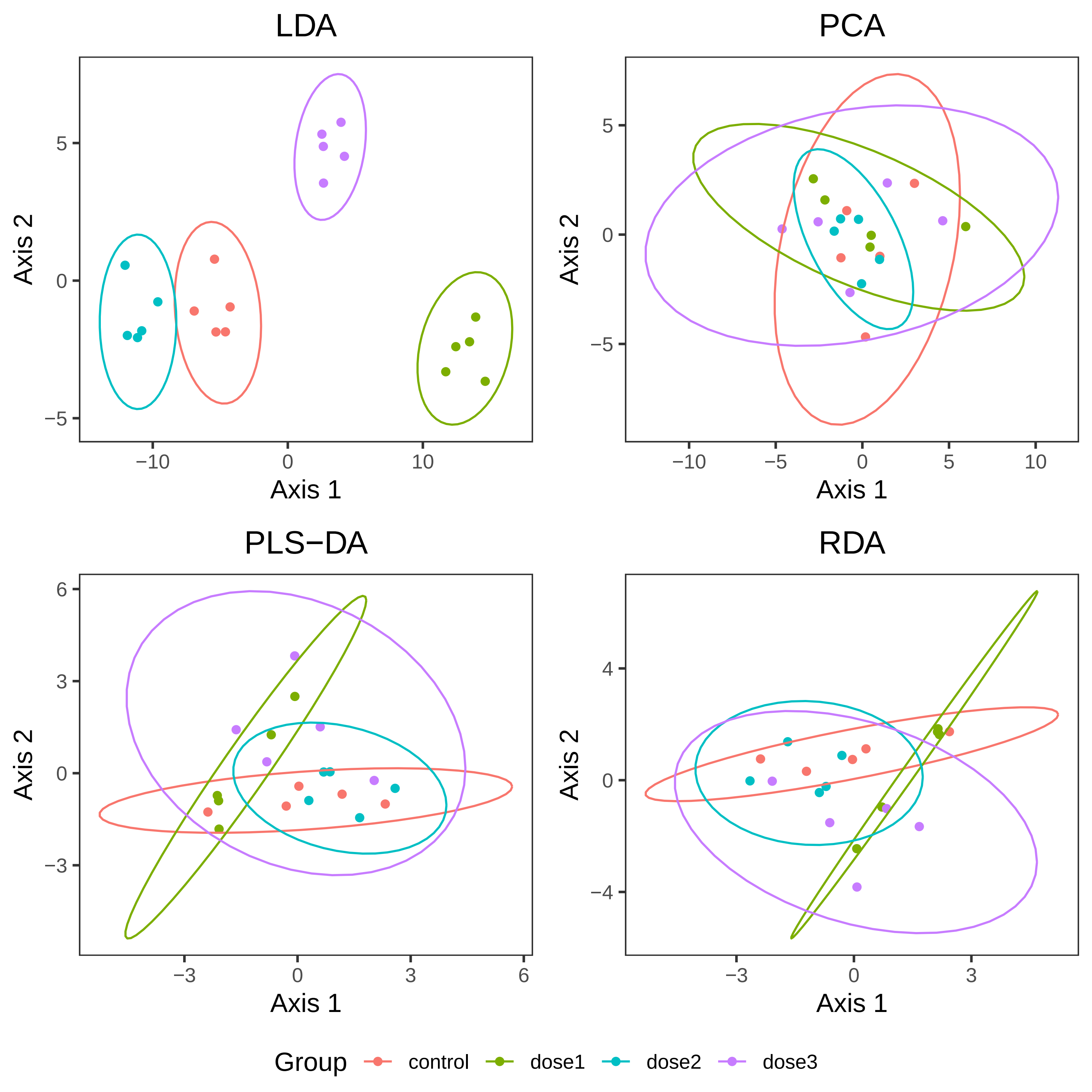

LDA
PCA
PLS−DA
RDA
Axis 1
Axis 2
Group
control
dose1
dose2
dose3

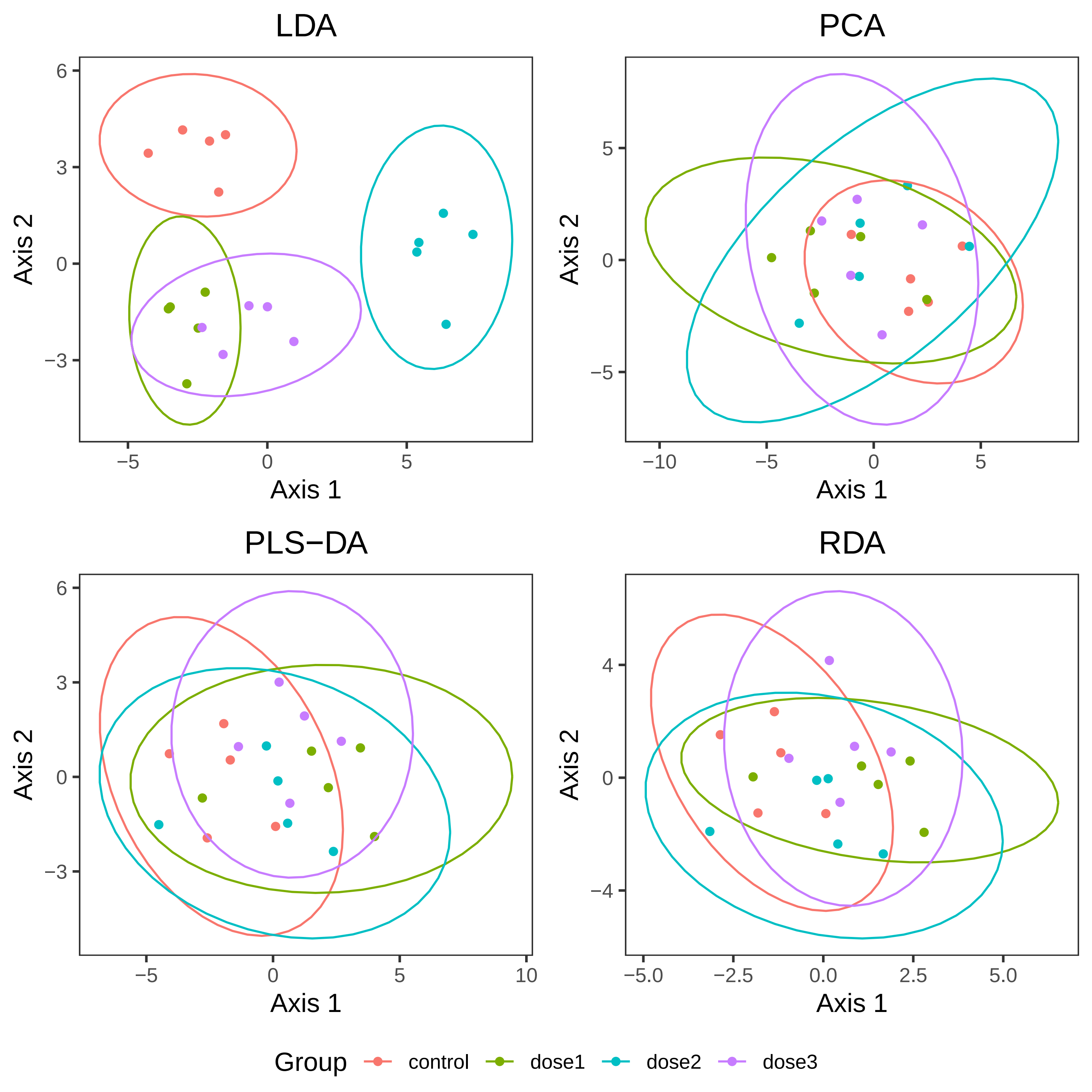
LDA
PCA
PLS−DA
RDA
Axis 1
Axis 2
Group
control
dose1
dose2
dose3

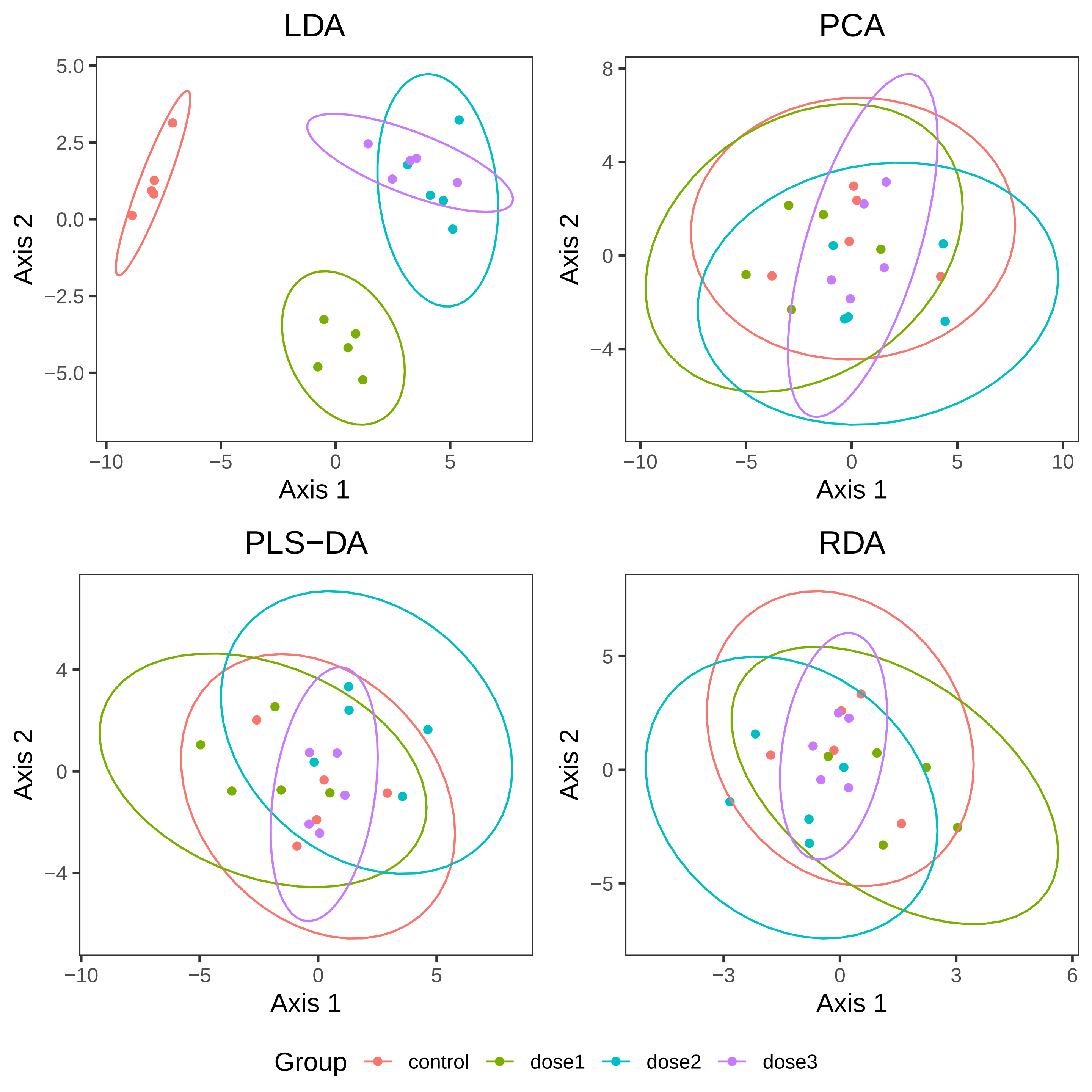
LDA
PCA
PLS−DA
RDA
Axis 1
Axis 2
5.0
2.5
0.0
−2.5
−5.0
−10
−5
0
5
10
8
4
−4
−3
3
6
Group
control
dose1
dose2
dose3

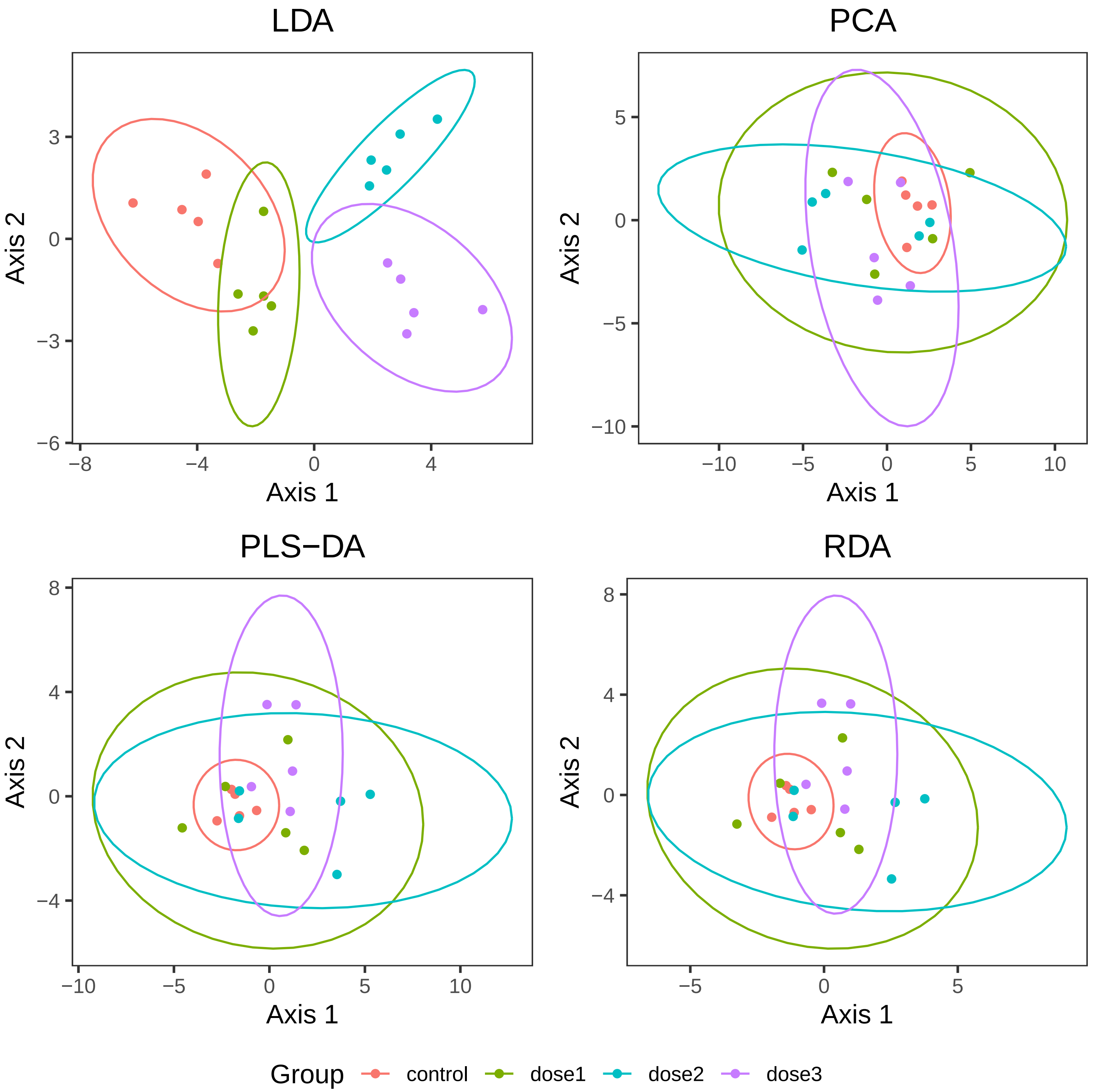

LDA
PCA
PLS−DA
RDA
Axis 1
Axis 2
Group
control
dose1
dose2
dose3

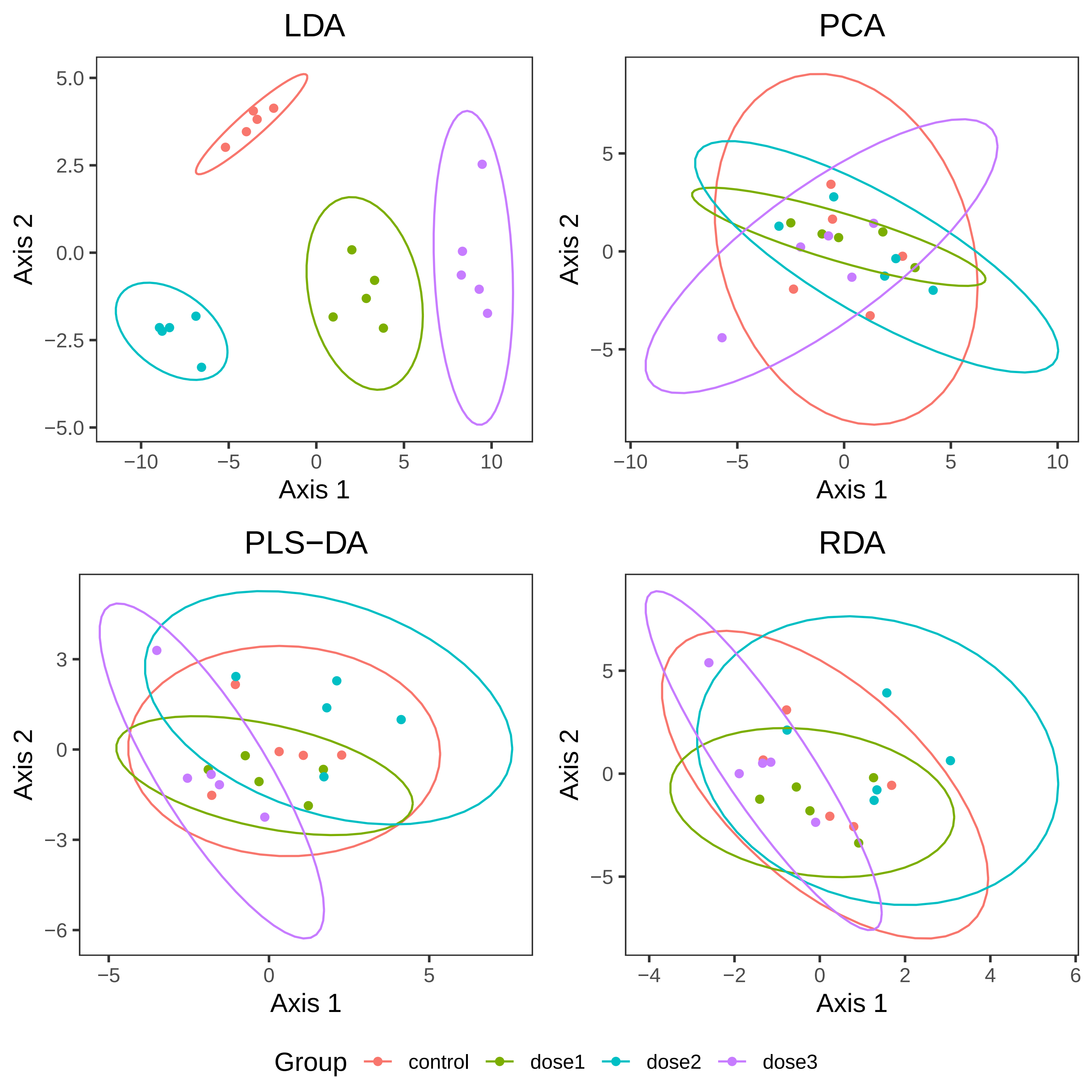
LDA
PCA
PLS−DA
RDA
Axis 1
Axis 2
5.0
2.5
0.0
−2.5
−5.0
−10
−5
0
5
10
−6
−3
3
−4
−2
2
4
6
Group
control
dose1
dose2
dose3

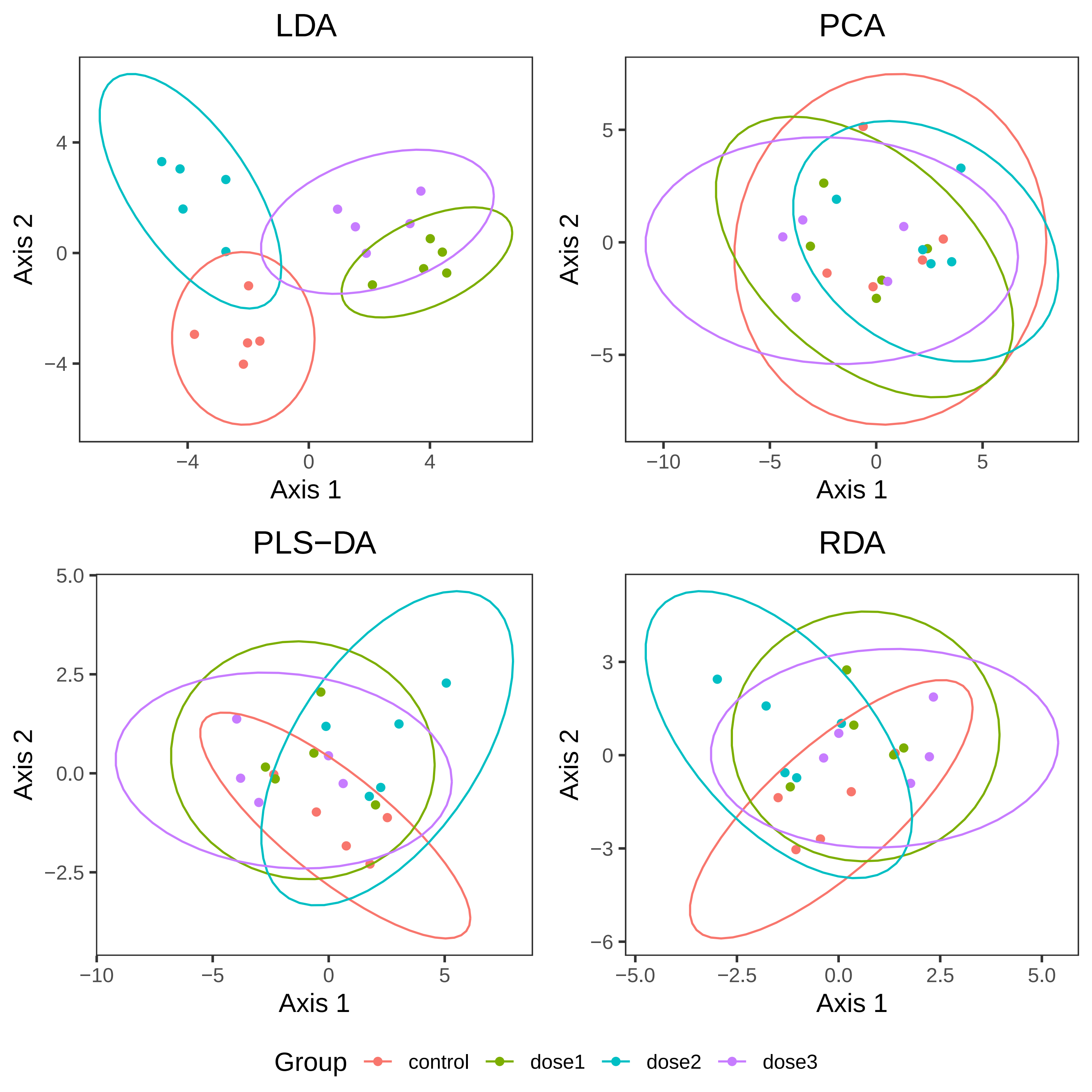

LDA
PCA
PLS−DA
RDA
Axis 1
Axis 2
4
0
−4
−10
−5
5
5.0
2.5
0.0
−2.5
−5.0
3
−3
−6
Group
control
dose1
dose2
dose3

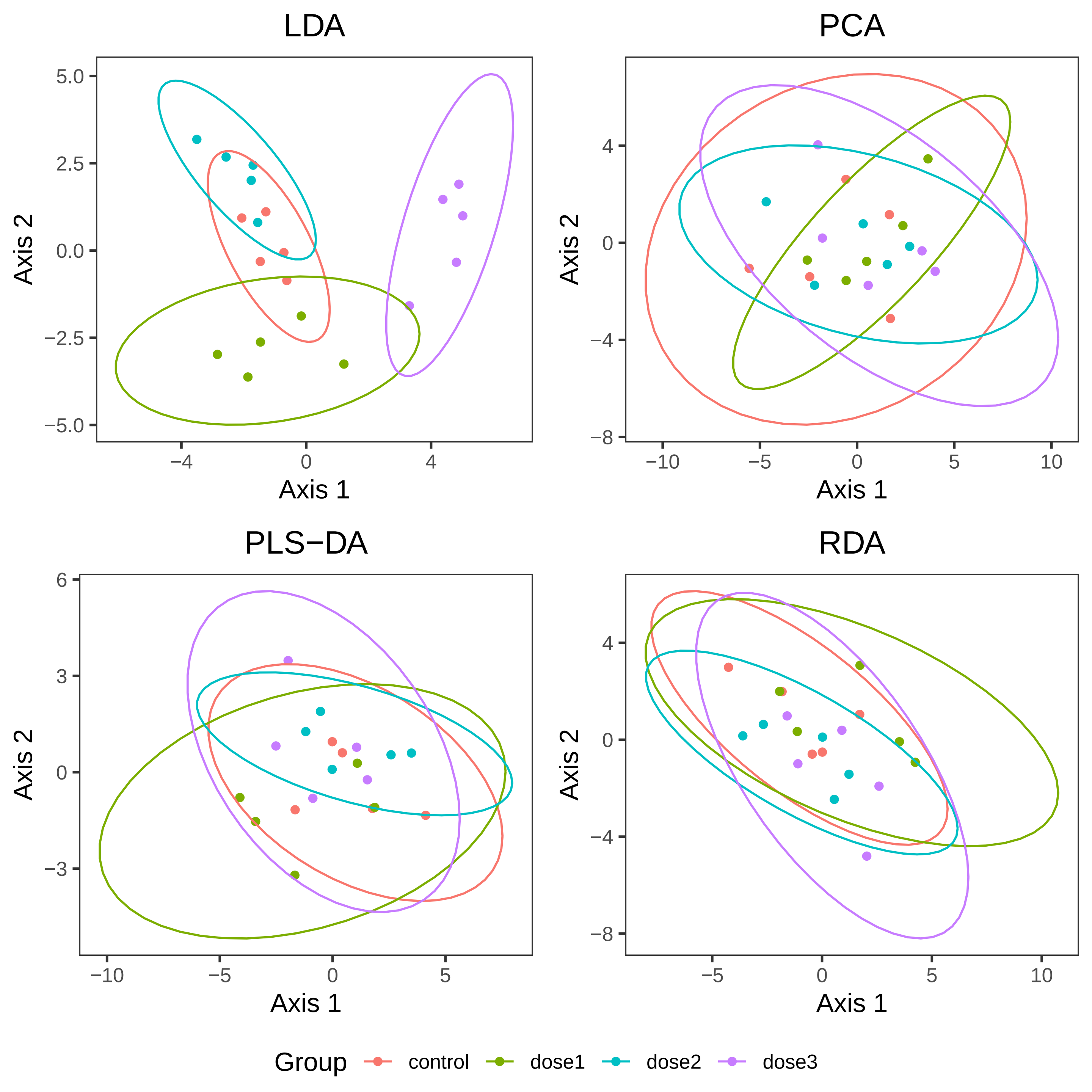
LDA
PCA
PLS−DA
RDA
Axis 1
Axis 2
Group
control
dose1
dose2
dose3

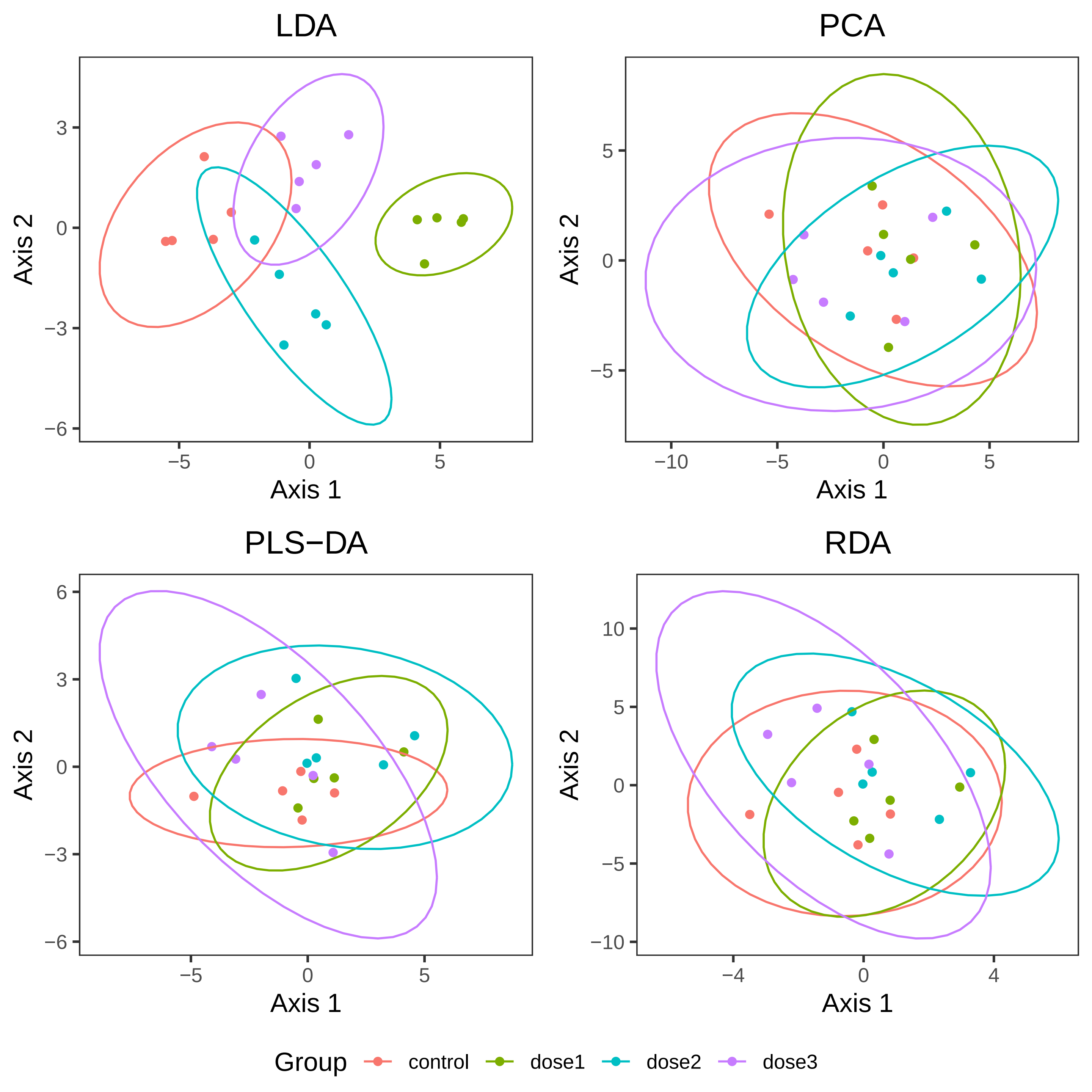
LDA
PCA
PLS−DA
RDA
Axis 1
Axis 2
Group
control
dose1
dose2
dose3

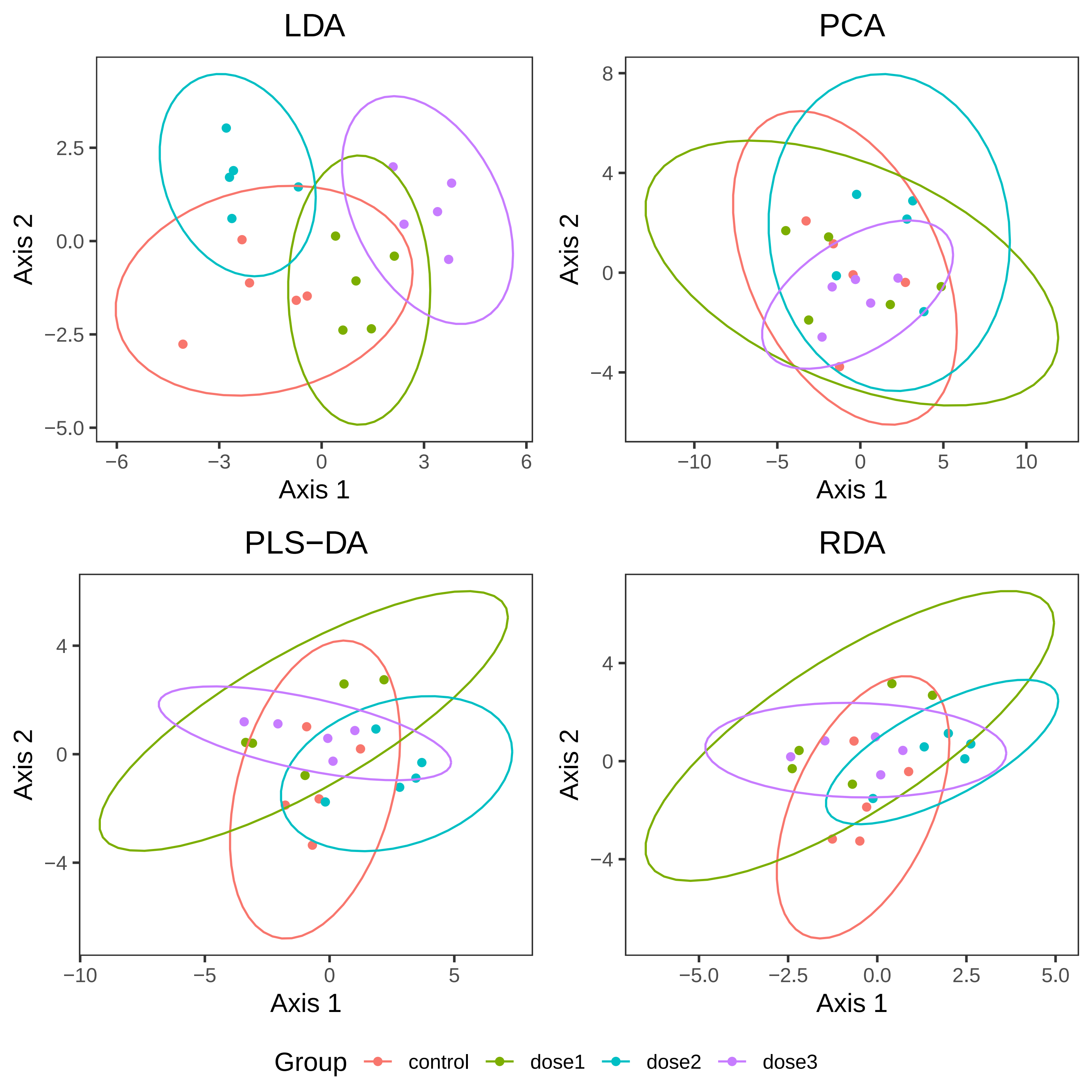

LDA
PCA
PLS−DA
RDA
Axis 1
Axis 2
2.5
0.0
−2.5
−5.0
−6
−3
0
3
6
8
4
0
−4
−10
−5
0
5
10
4
0
−4
−10
−5
0
5
4
0
−4
−5.0
−2.5
0.0
2.5
5.0
Group
control
dose1
dose2
dose3

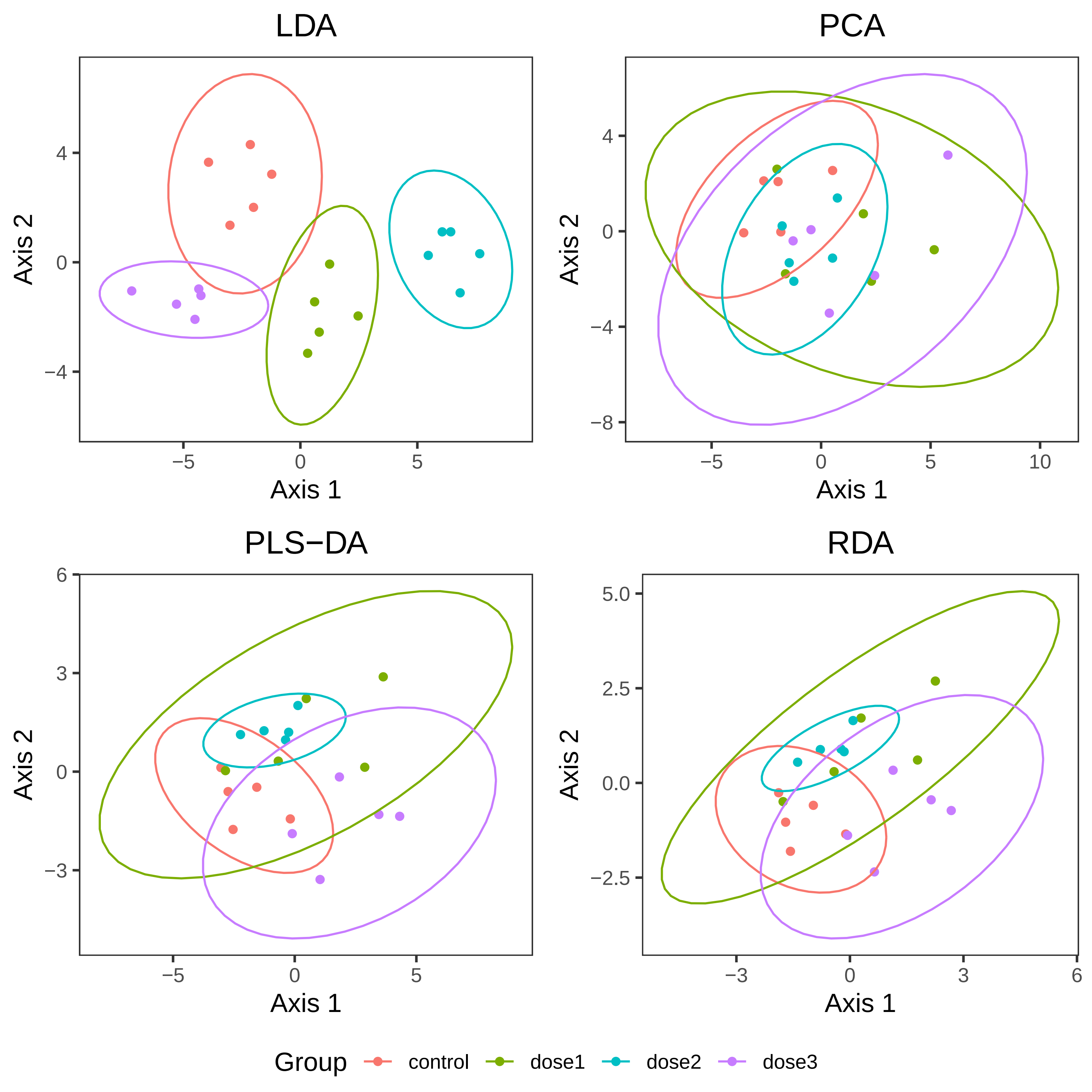

LDA
PCA
PLS−DA
RDA
Axis 1
Axis 2
Group
control
dose1
dose2
dose3

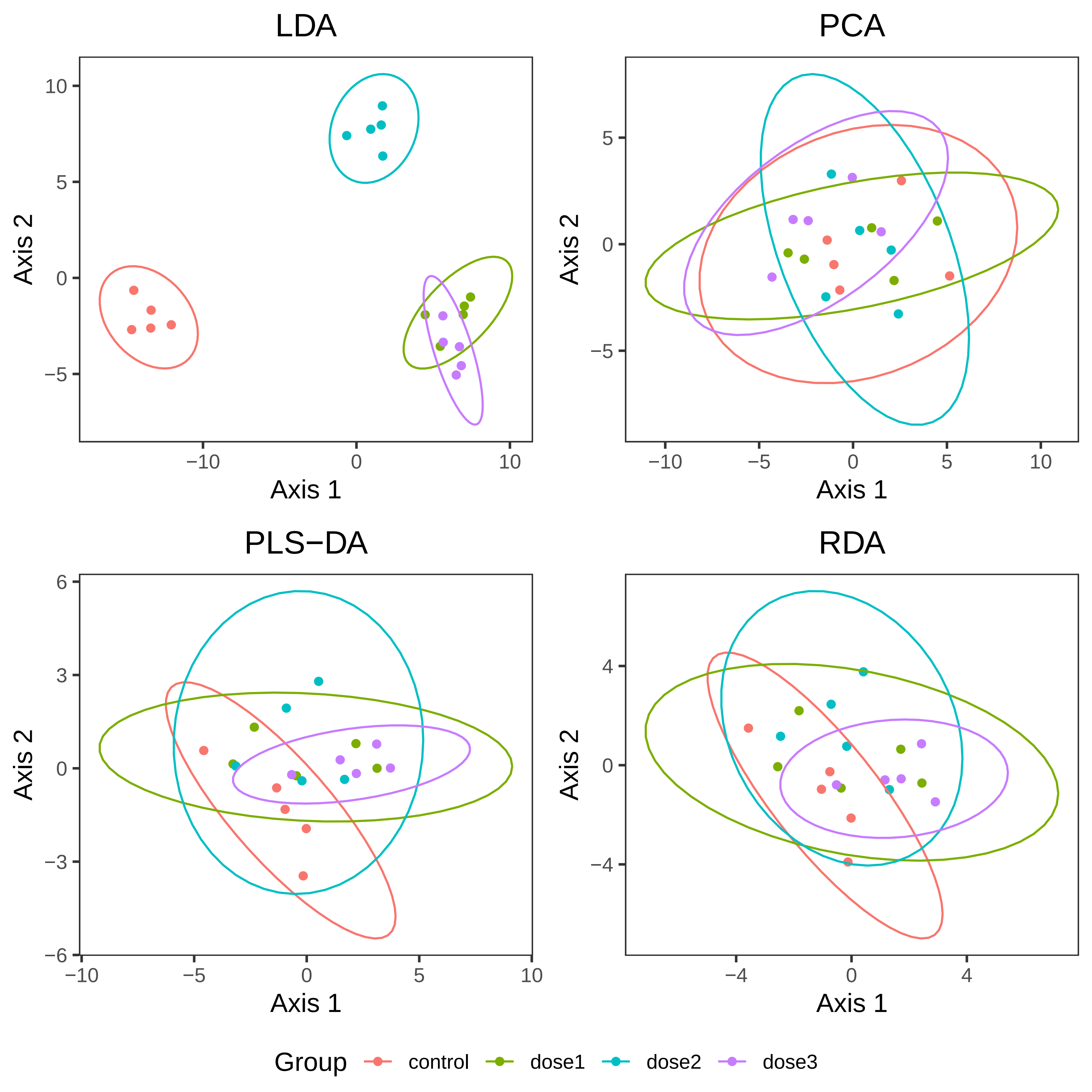

LDA
PCA
PLS−DA
RDA
Axis 1
Axis 2
10
5
0
−5
−10
−10
−5
5
6
3
−3
−6
4
−4
Group
control
dose1
dose2
dose3

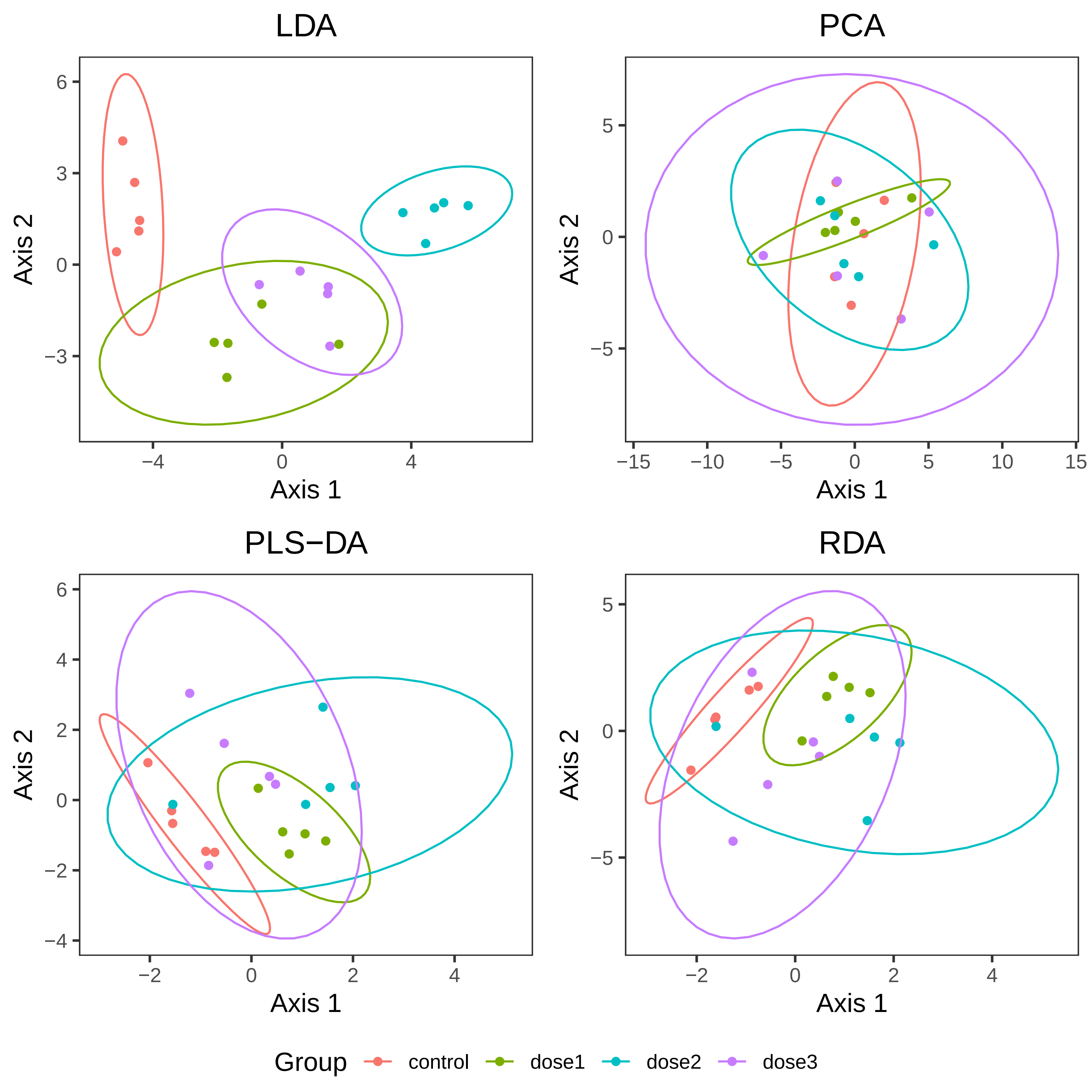

LDA
PCA
PLS−DA
RDA
Axis 1
Axis 2
Group
control
dose1
dose2
dose3

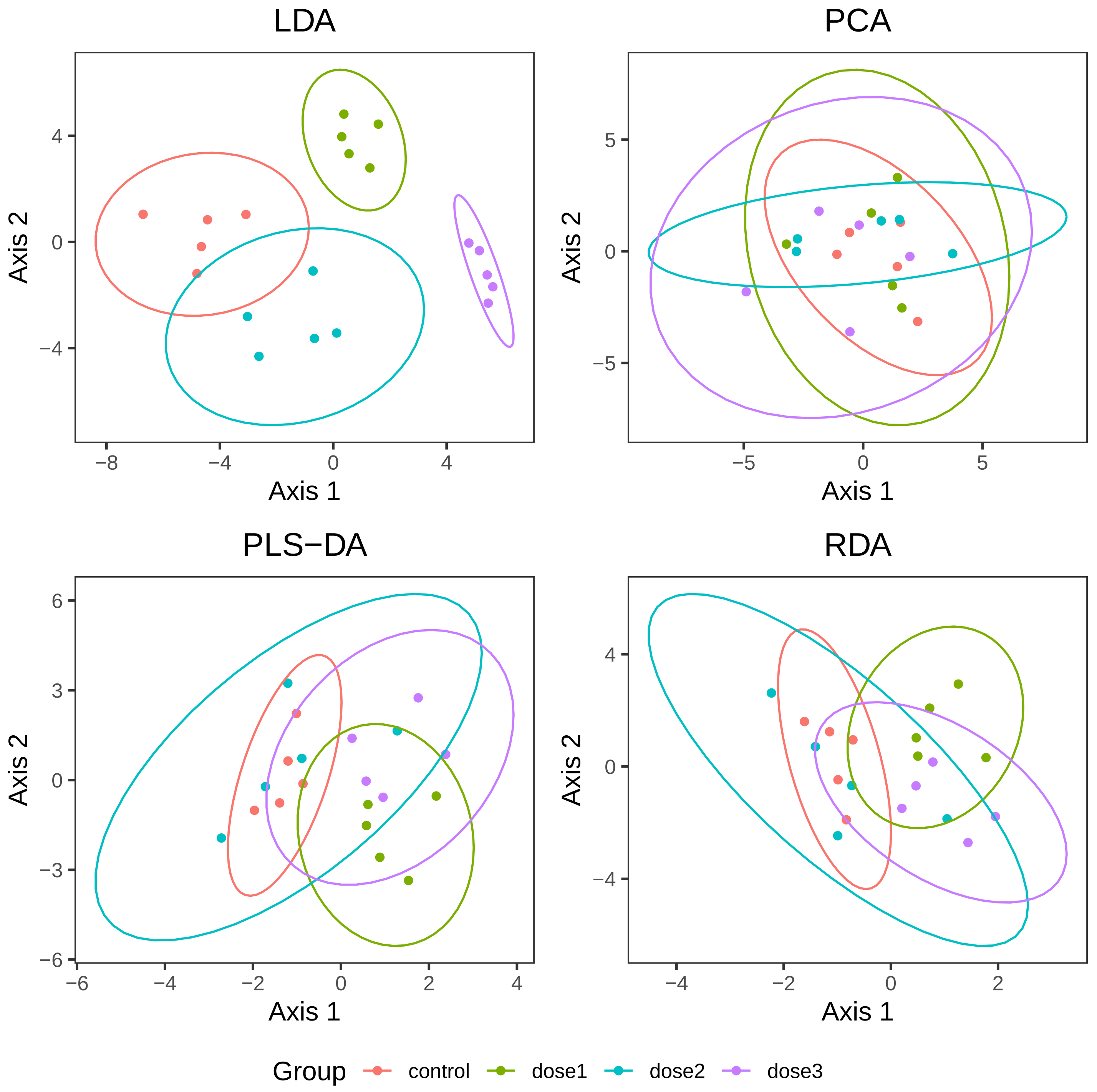

LDA
PCA
PLS−DA
RDA
Axis 1
Axis 2
−8
−4
0
4
−5
5
6
3
−3
−6
−6
−2
2
Group
control
dose1
dose2
dose3

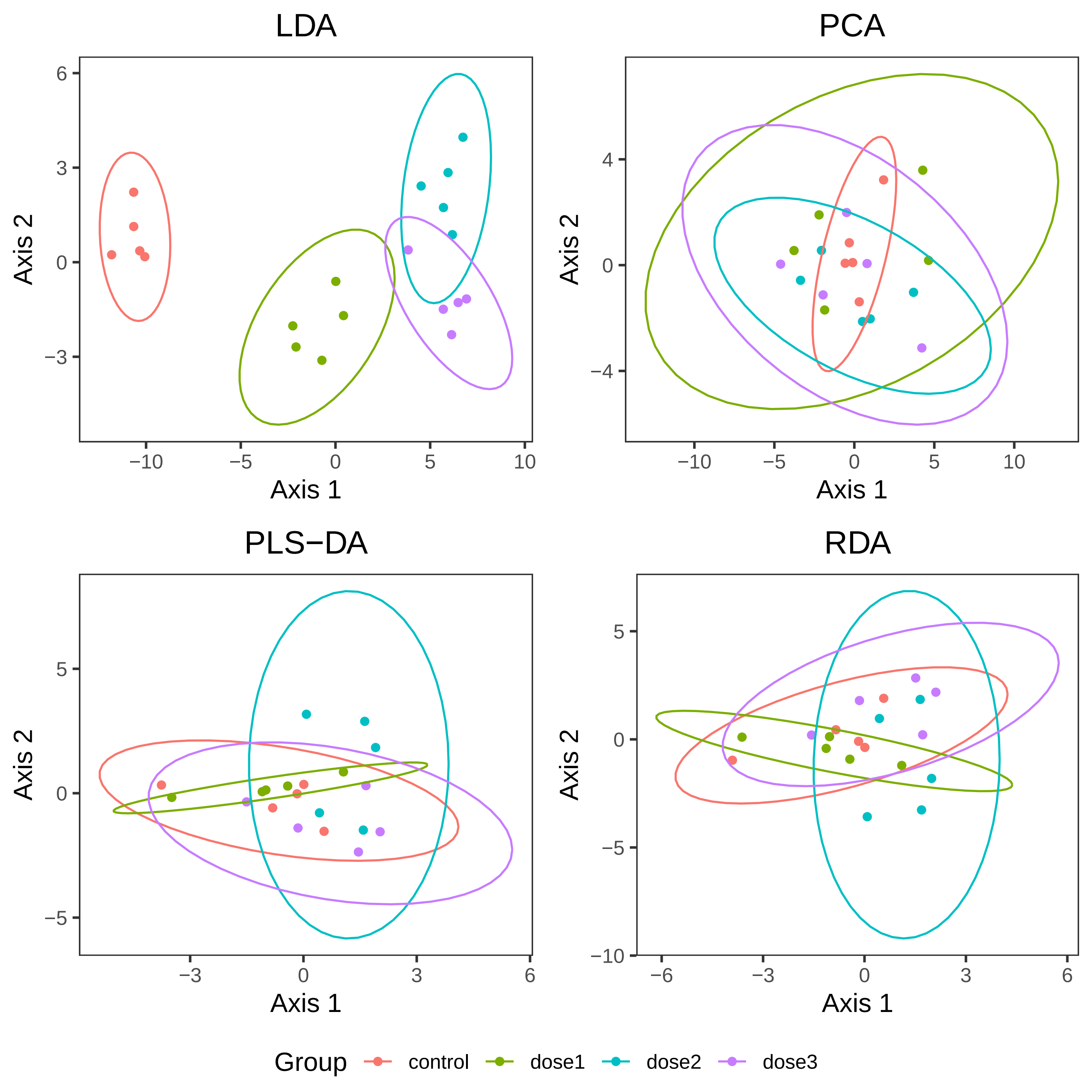
LDA
PCA
PLS−DA
RDA
Axis 1
Axis 2
Group
control
dose1
dose2
dose3

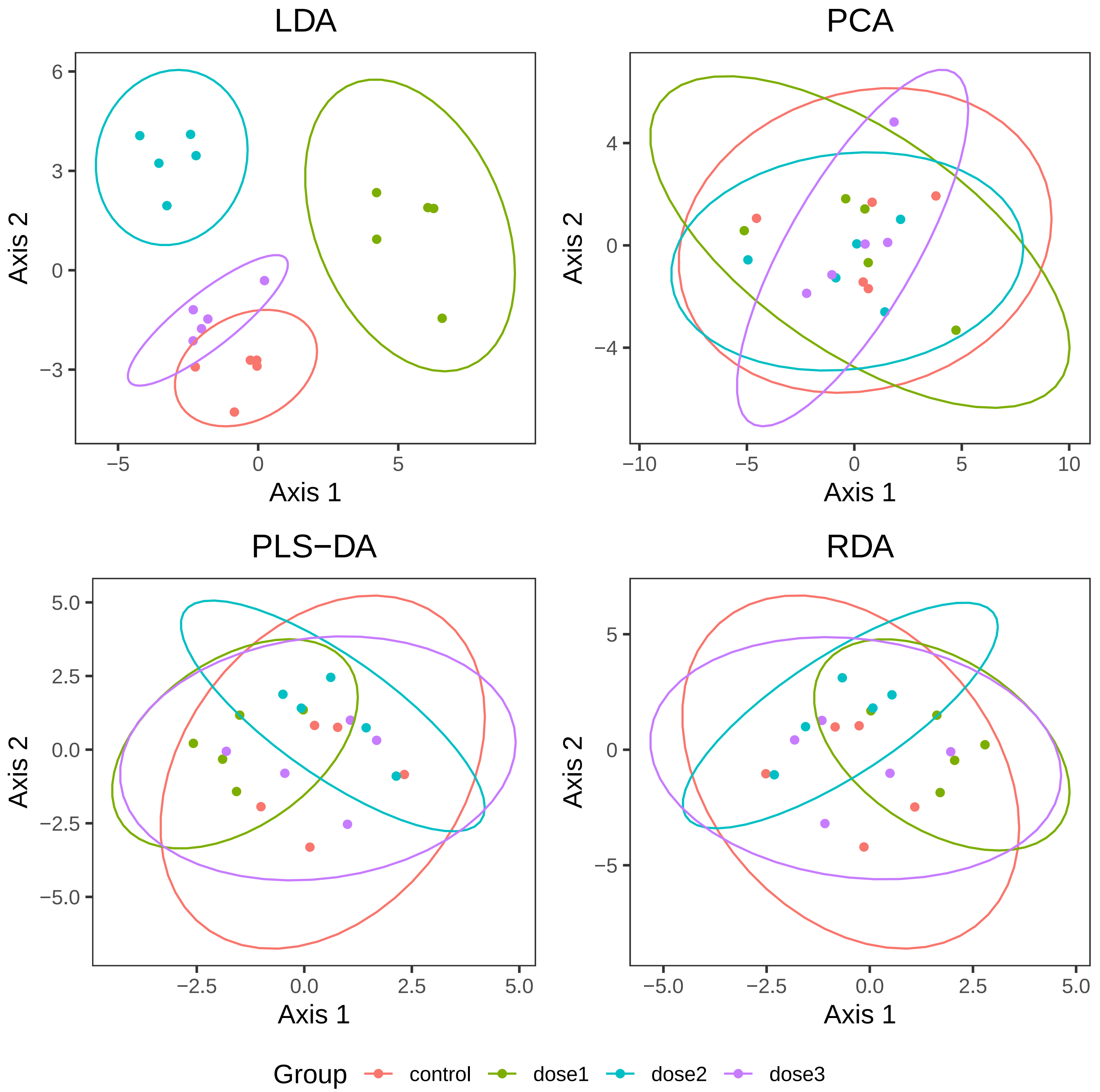

LDA
PCA
PLS−DA
RDA
Axis 1
Axis 2
6
3
0
−3
−5
0
5
4
0
−4
−10
−5
0
5
10
5.0
2.5
0.0
−2.5
−5.0
2.5
5.0
5
0
−5
Group
control
dose1
dose2
dose3

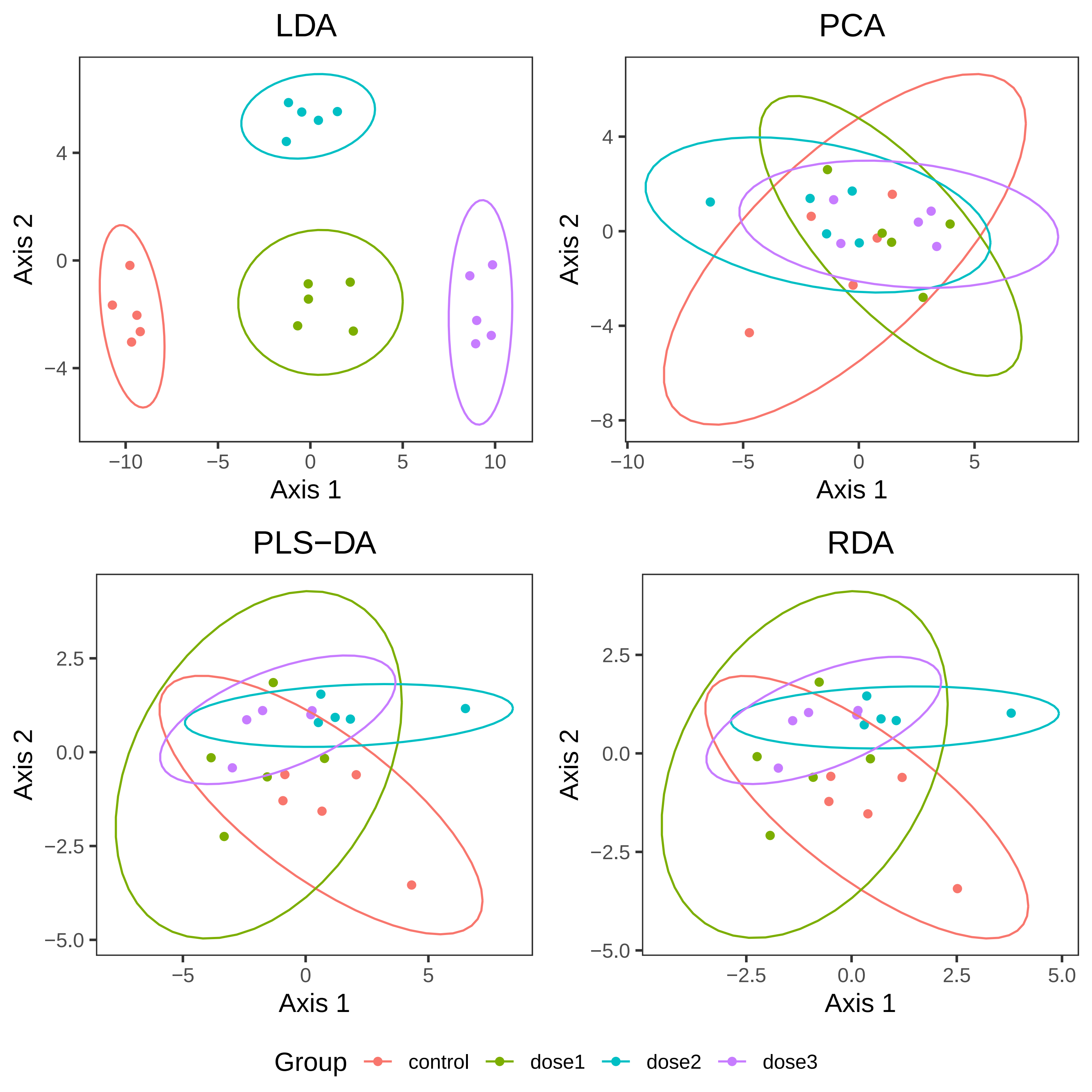
LDA
PCA
PLS−DA
RDA
Axis 1
Axis 2
Group
control
dose1
dose2
dose3

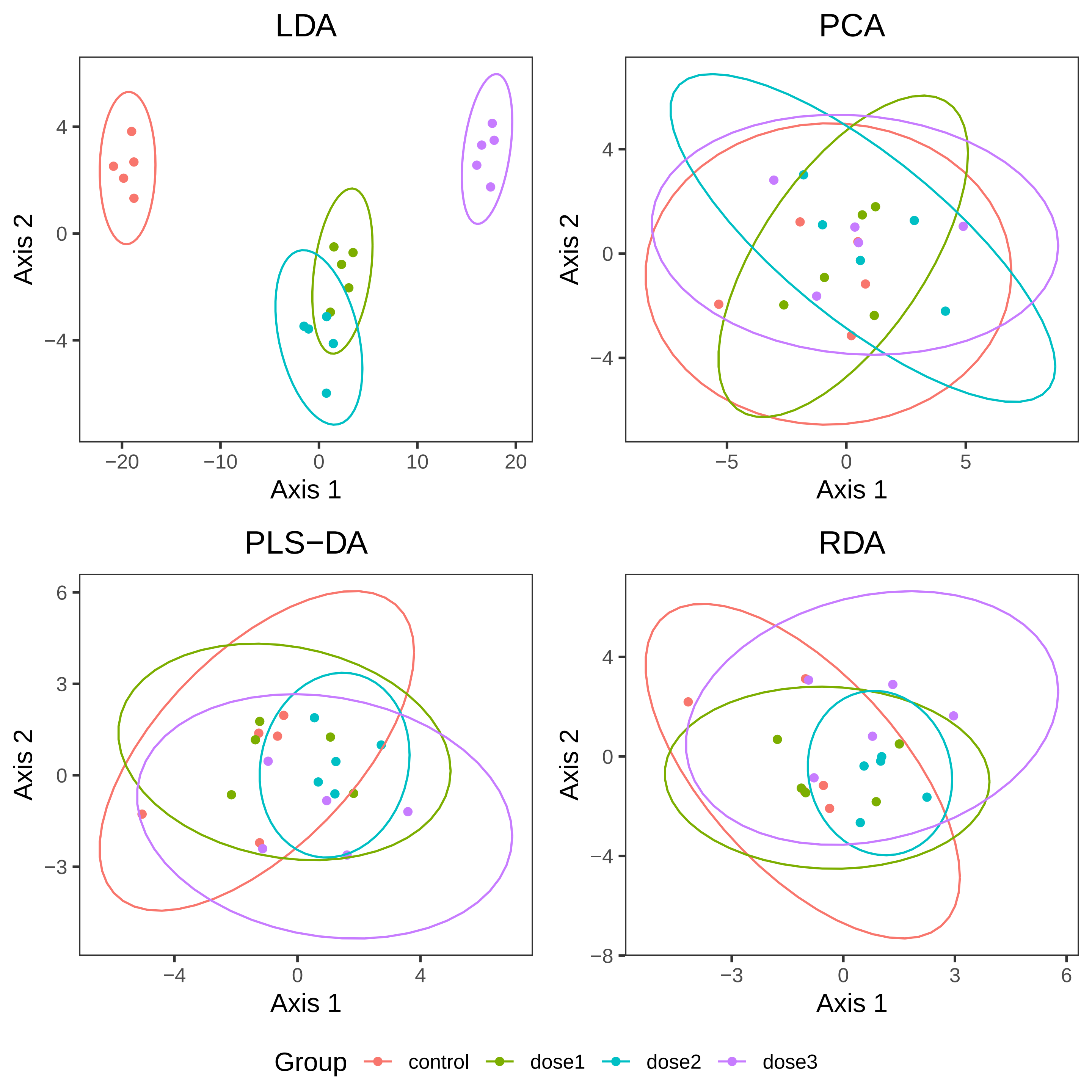

LDA
PCA
PLS−DA
RDA
Axis 1
Axis 2
−20
−10
0
10
20
4
0
−4
−5
5
6
3
−3
−8
Group
control
dose1
dose2
dose3

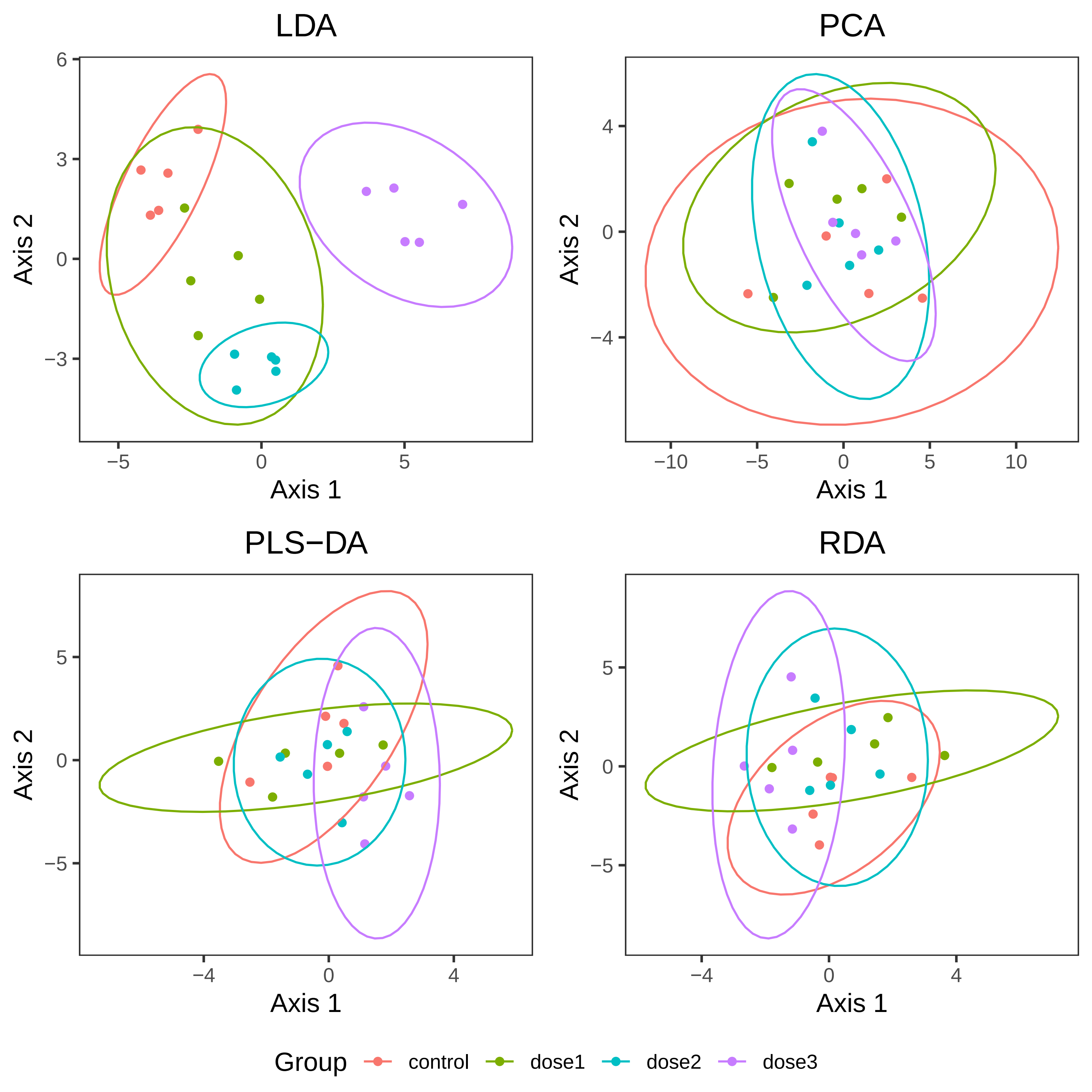

LDA
PCA
PLS−DA
RDA
Axis 1
Axis 2
Group
control
dose1
dose2
dose3

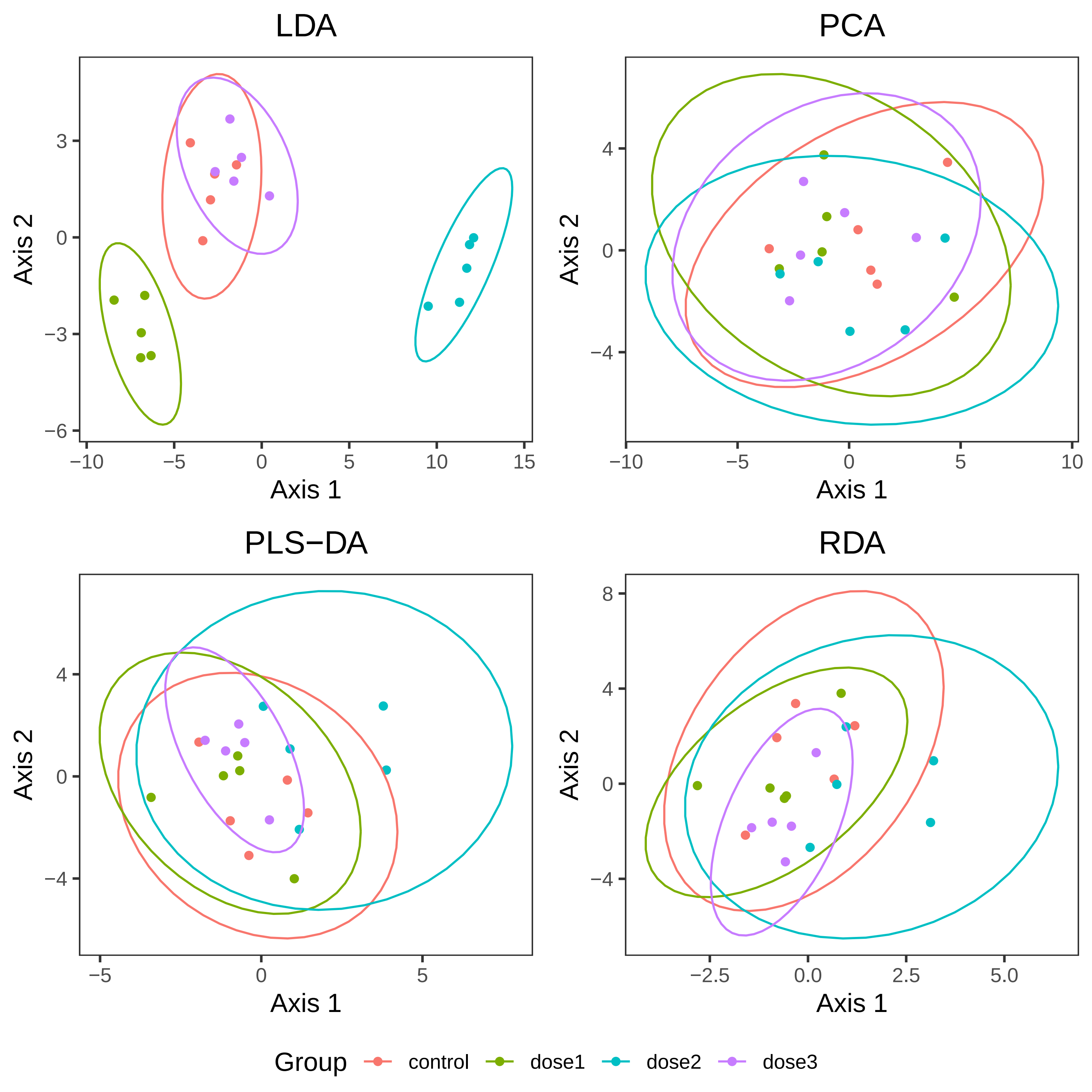
LDA
PCA
PLS−DA
RDA
Axis 1
Axis 2
−10
−5
0
5
10
15
3
0
−3
−6
4
−4
8
−2.5
0.0
2.5
5.0
Group
control
dose1
dose2
dose3

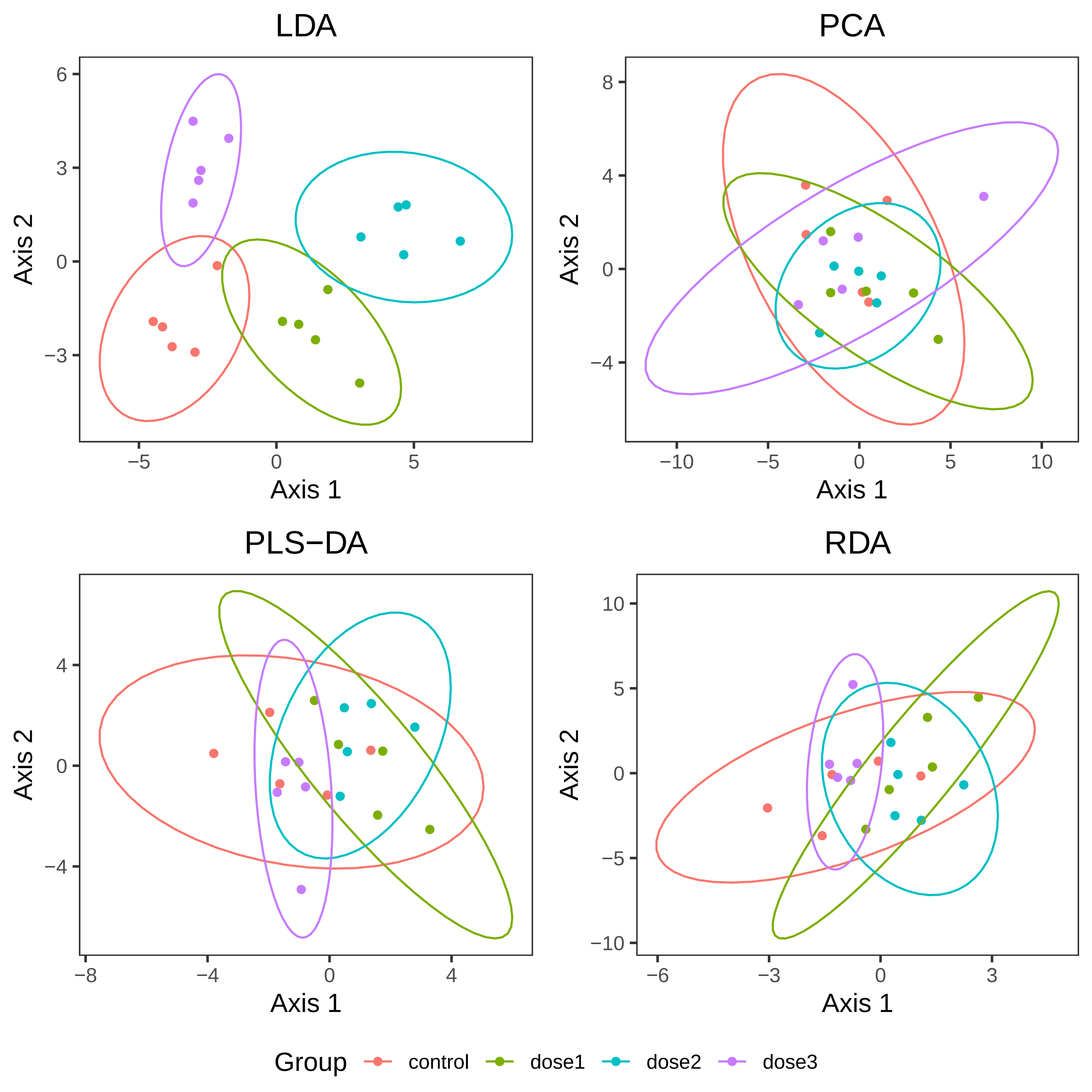

LDA
PCA
PLS−DA
RDA
Axis 1
Axis 2
6
3
0
−3
−5
0
5
8
4
0
−4
−10
−5
0
5
10
4
0
−4
−8
−4
0
4
10
5
0
−5
−10
−6
−3
0
3
Group
control
dose1
dose2
dose3

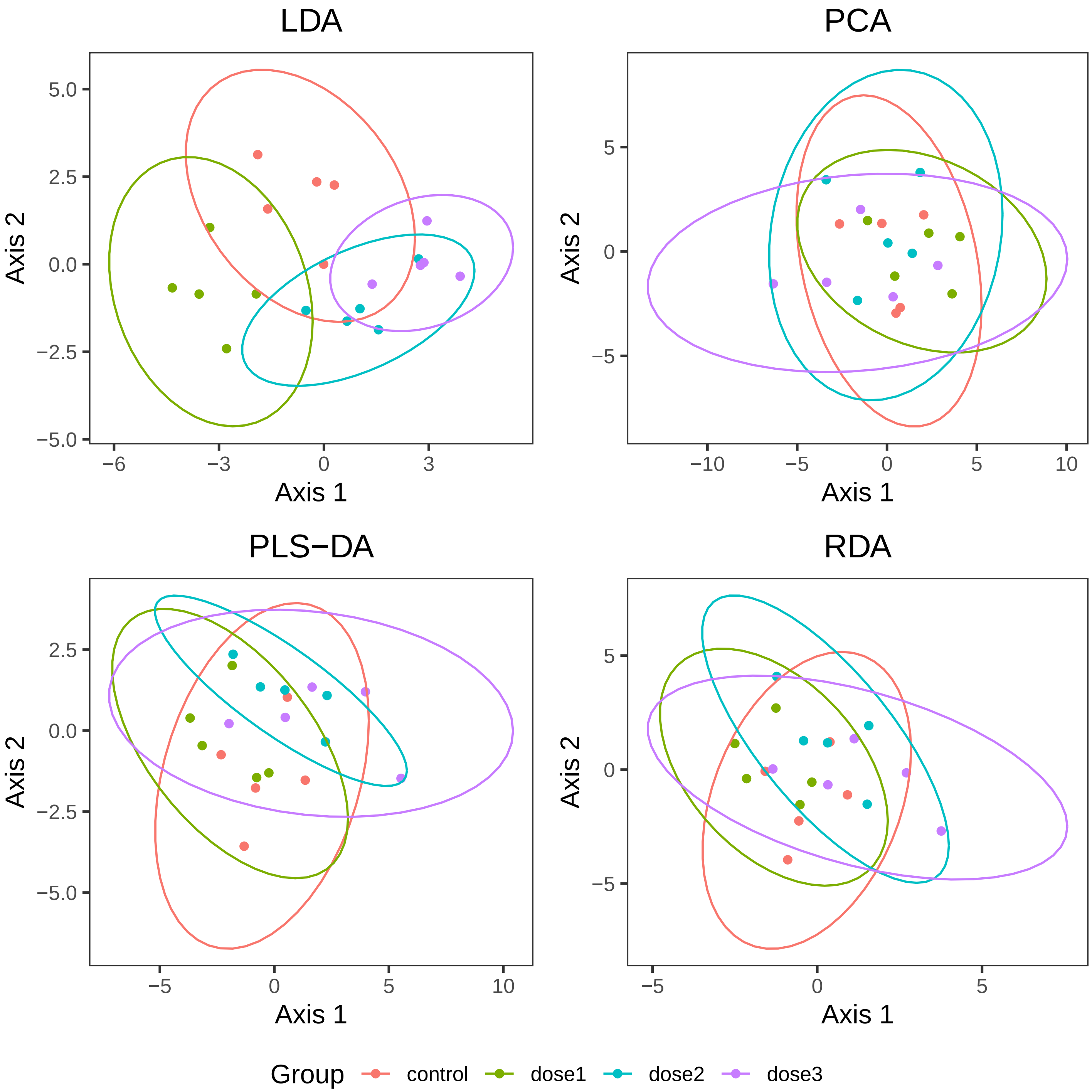
LDA
PCA
PLS−DA
RDA
Axis 1
Axis 2
5.0
2.5
0.0
−2.5
−5.0
−6
−3
0
3
5
−5
−10
10
Group
control
dose1
dose2
dose3

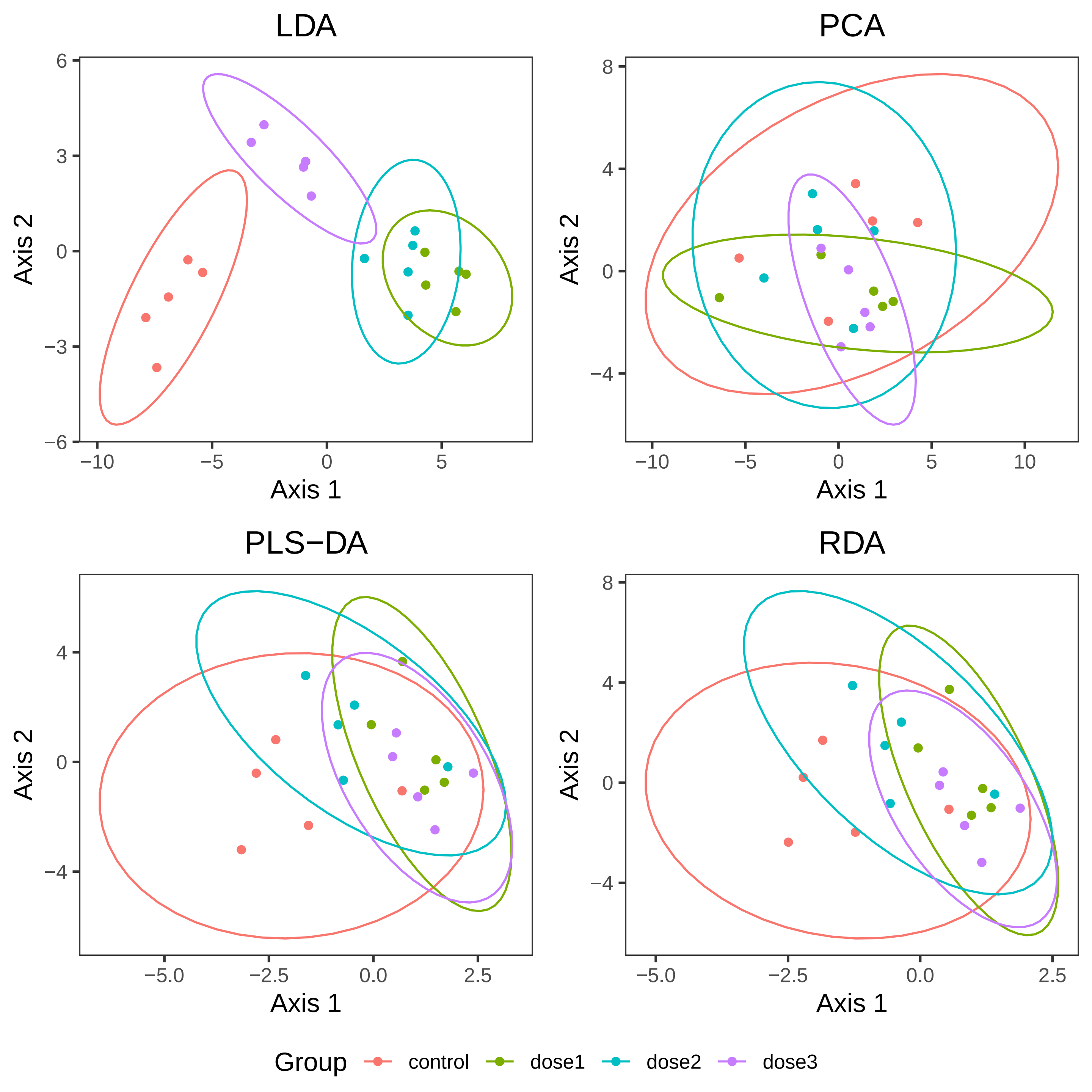
LDA
PCA
PLS−DA
RDA
Axis 1
Axis 2
−10
−5
0
5
10
6
3
−3
−6
8
4
−4
−5.0
−2.5
0.0
2.5
Group
control
dose1
dose2
dose3

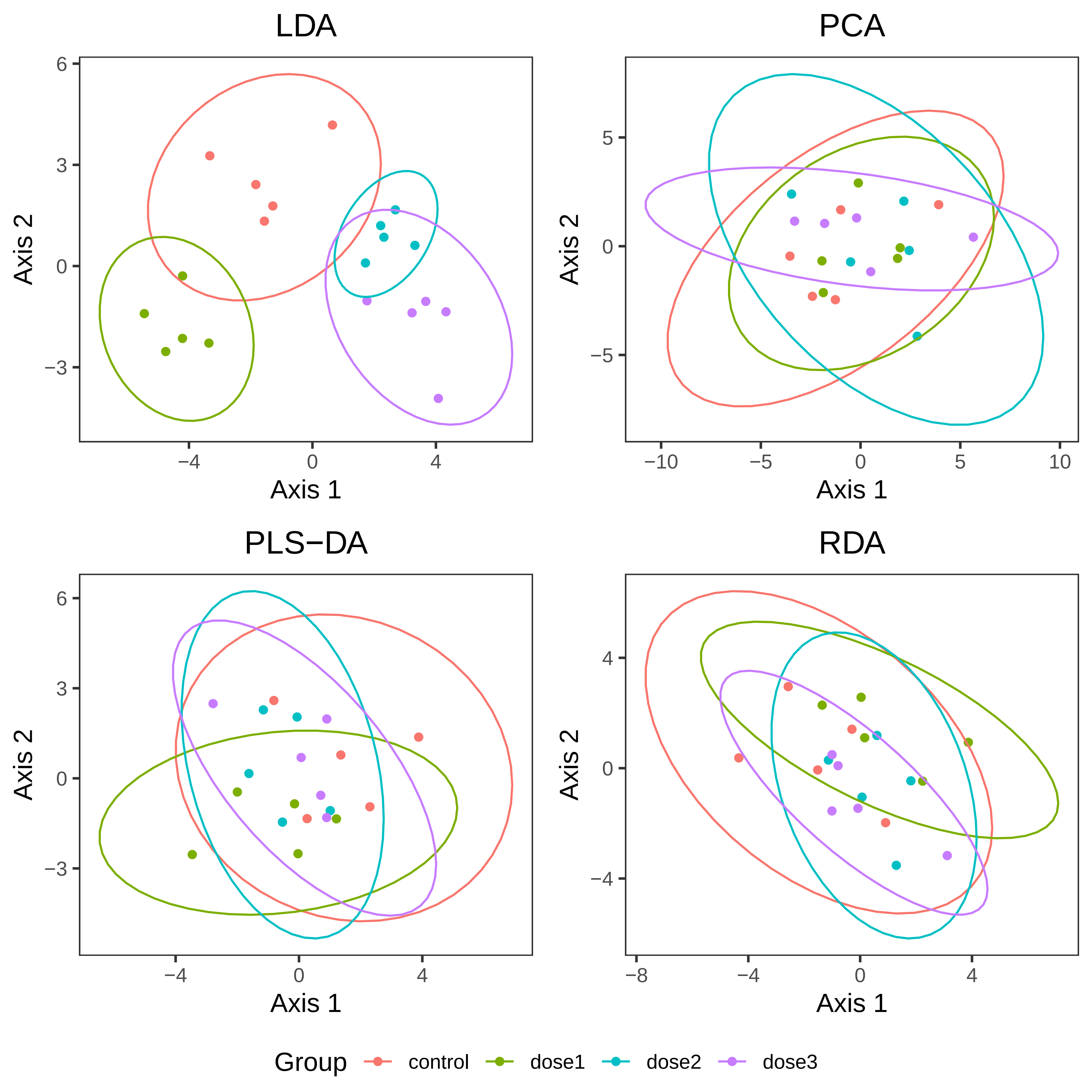
LDA
PCA
PLS−DA
RDA
Axis 1
Axis 2
−4
0
4
6
3
−3
−10
−5
5
10
−8
Group
control
dose1
dose2
dose3

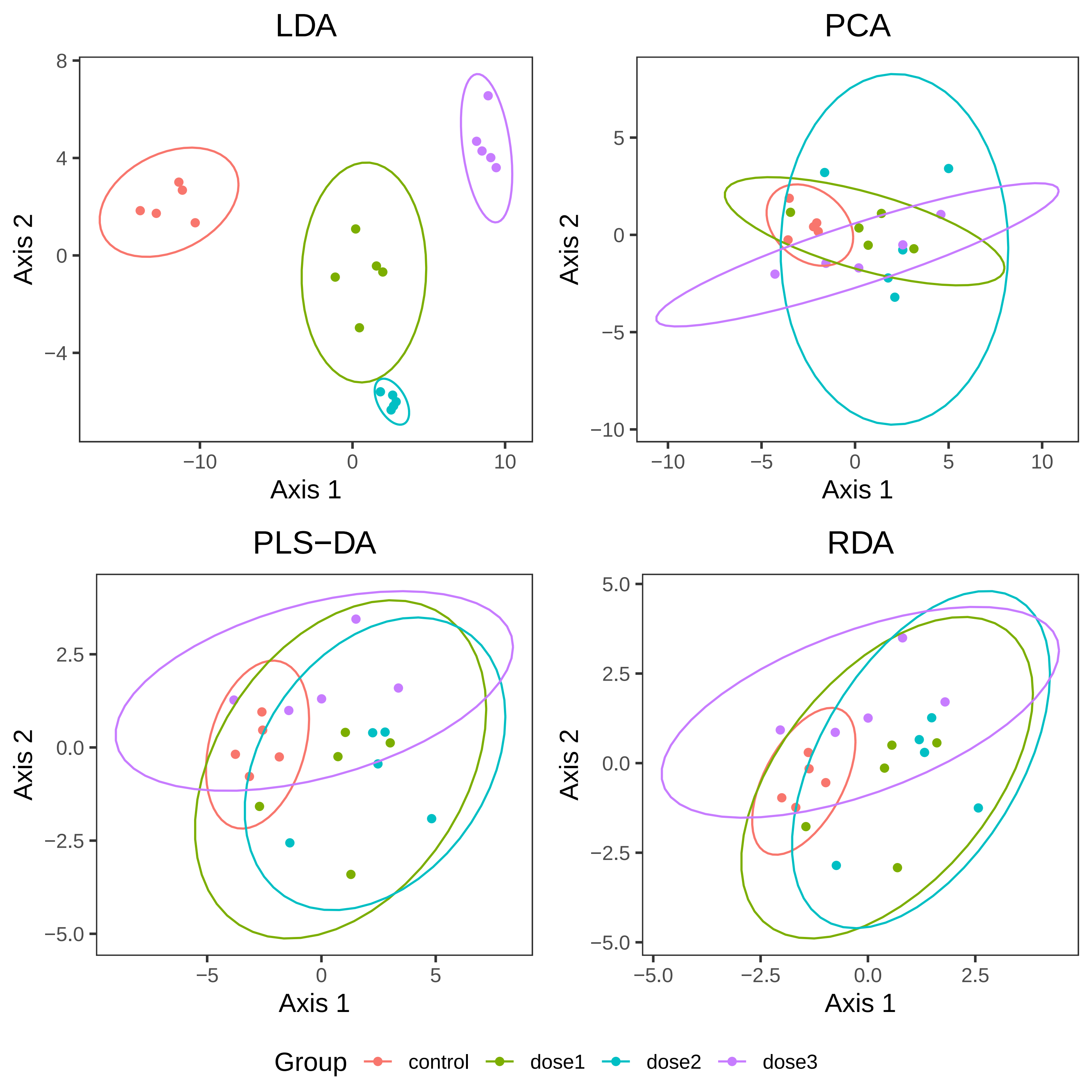

LDA
PCA
PLS−DA
RDA
Axis 1
Axis 2
Group
control
dose1
dose2
dose3

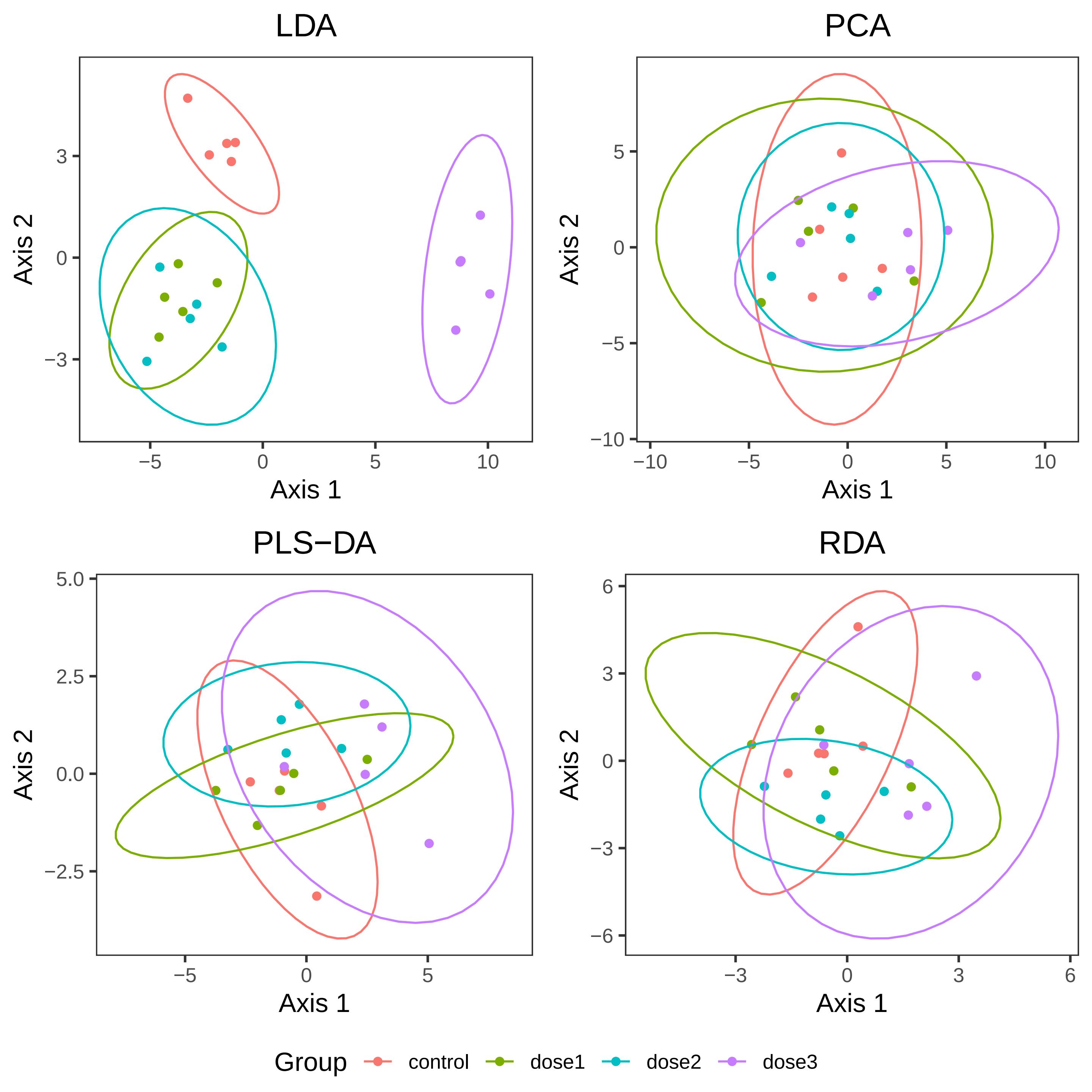
LDA
PCA
PLS−DA
RDA
Axis 1
Axis 2
Group
control
dose1
dose2
dose3

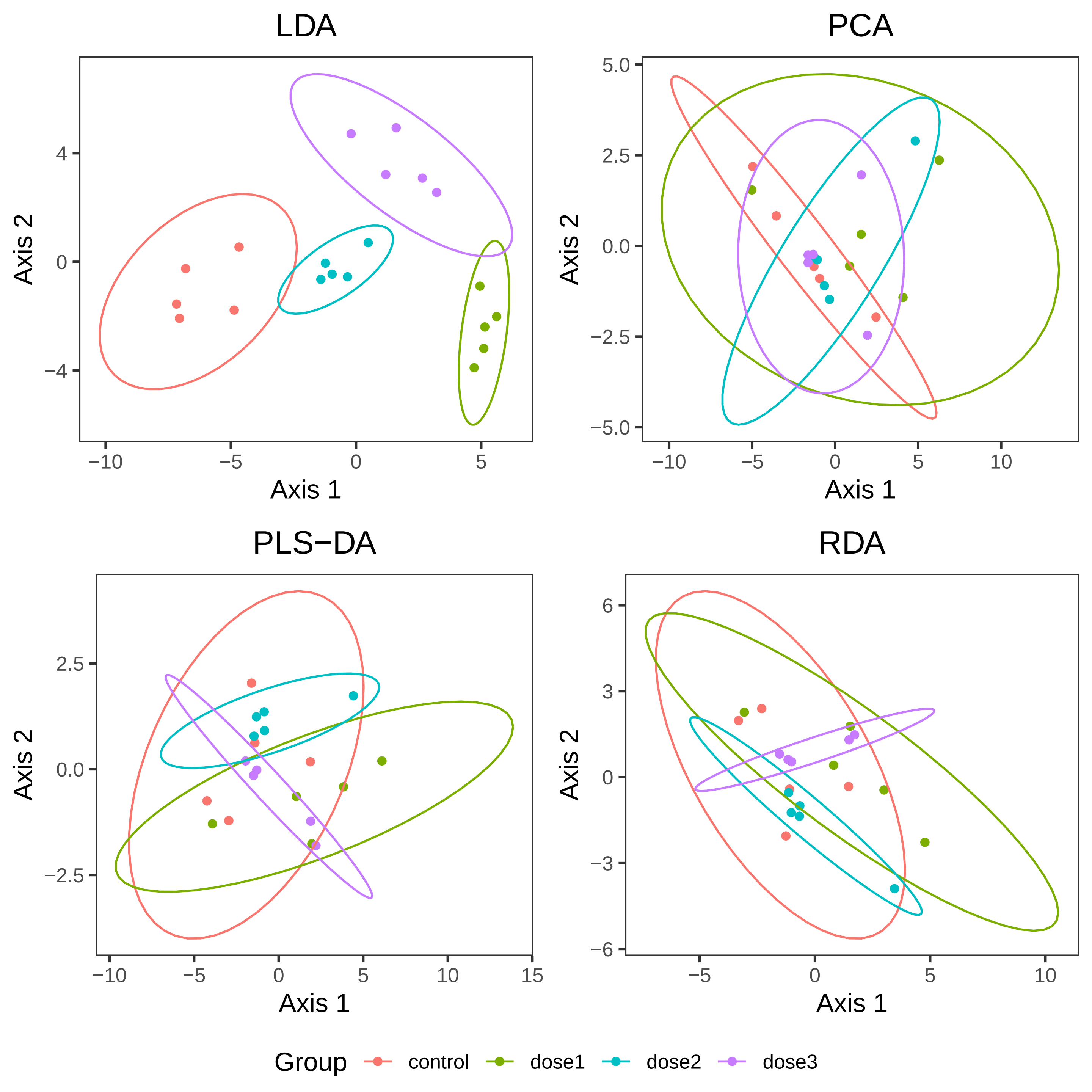

LDA
PCA
PLS−DA
RDA
Axis 1
Axis 2
Group
control
dose1
dose2
dose3

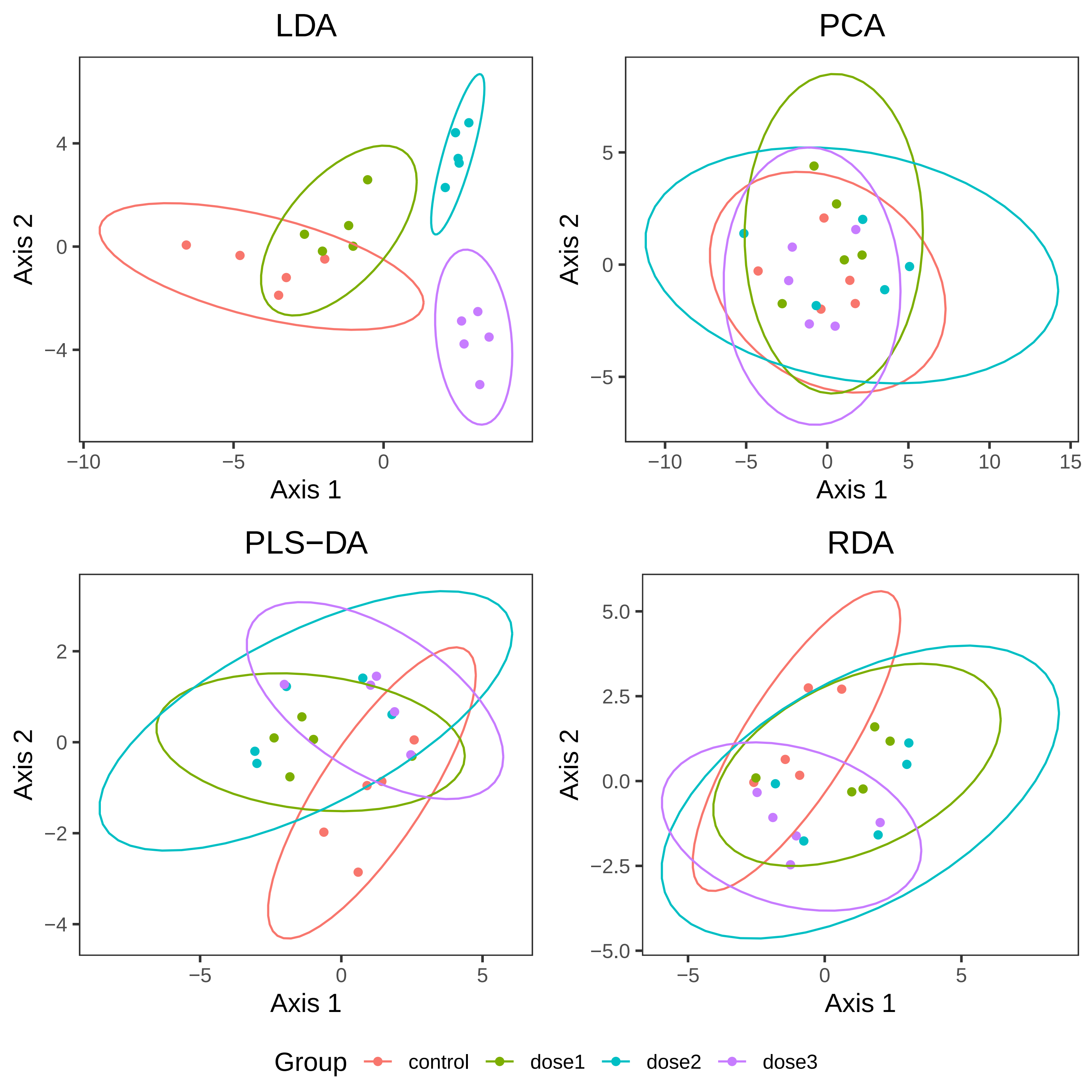
LDA
PCA
PLS−DA
RDA
Axis 1
Axis 2
Group
control
dose1
dose2
dose3

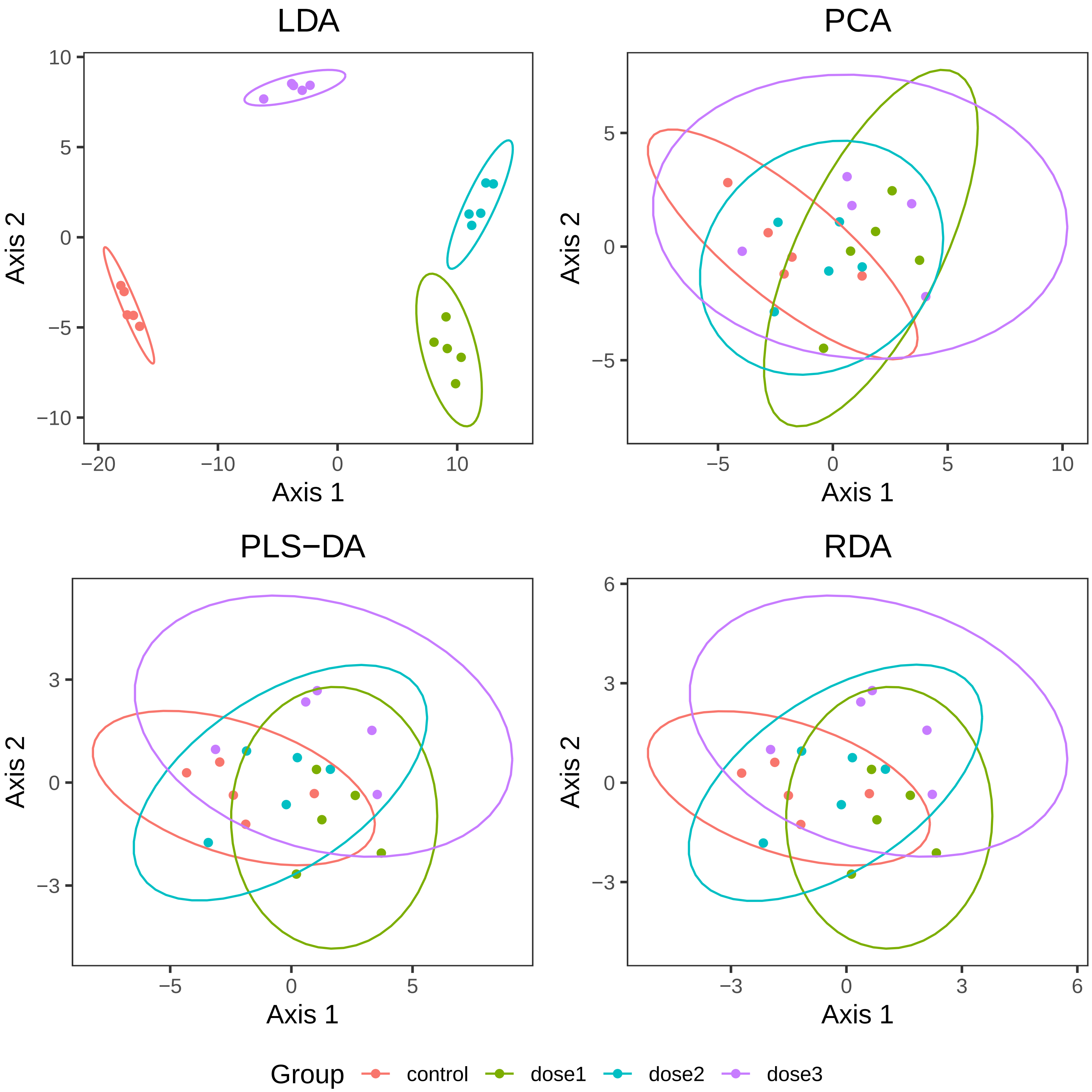

LDA
PCA
PLS−DA
RDA
Axis 1
Axis 2
Group
control
dose1
dose2
dose3

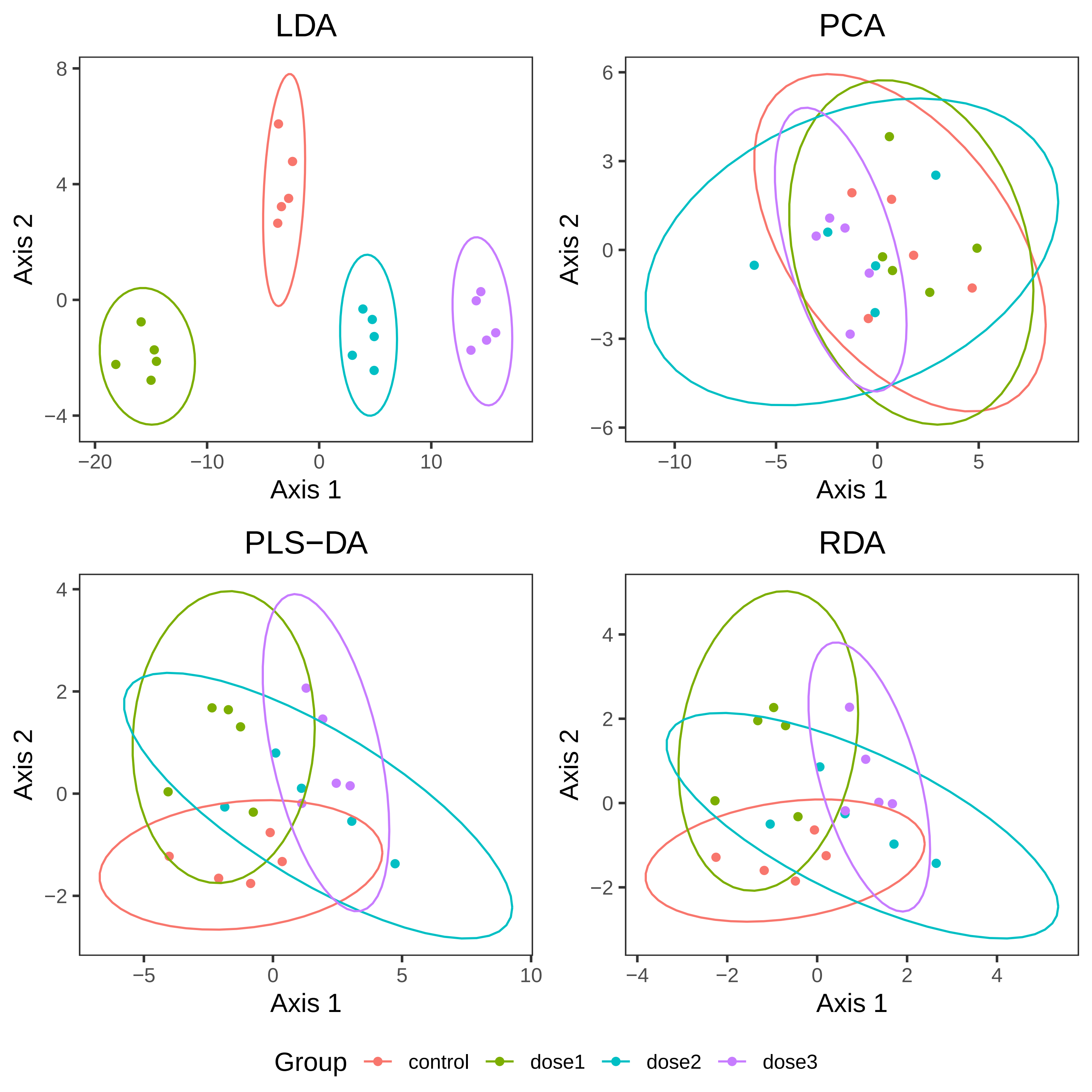
LDA
PCA
PLS−DA
RDA
Axis 1
Axis 2
8
4
0
−4
−20
−10
0
10
6
3
0
−3
−6
−10
−5
0
5
4
2
0
−2
−5
0
5
10
4
2
0
−2
−4
−2
0
2
4
Group
control
dose1
dose2
dose3

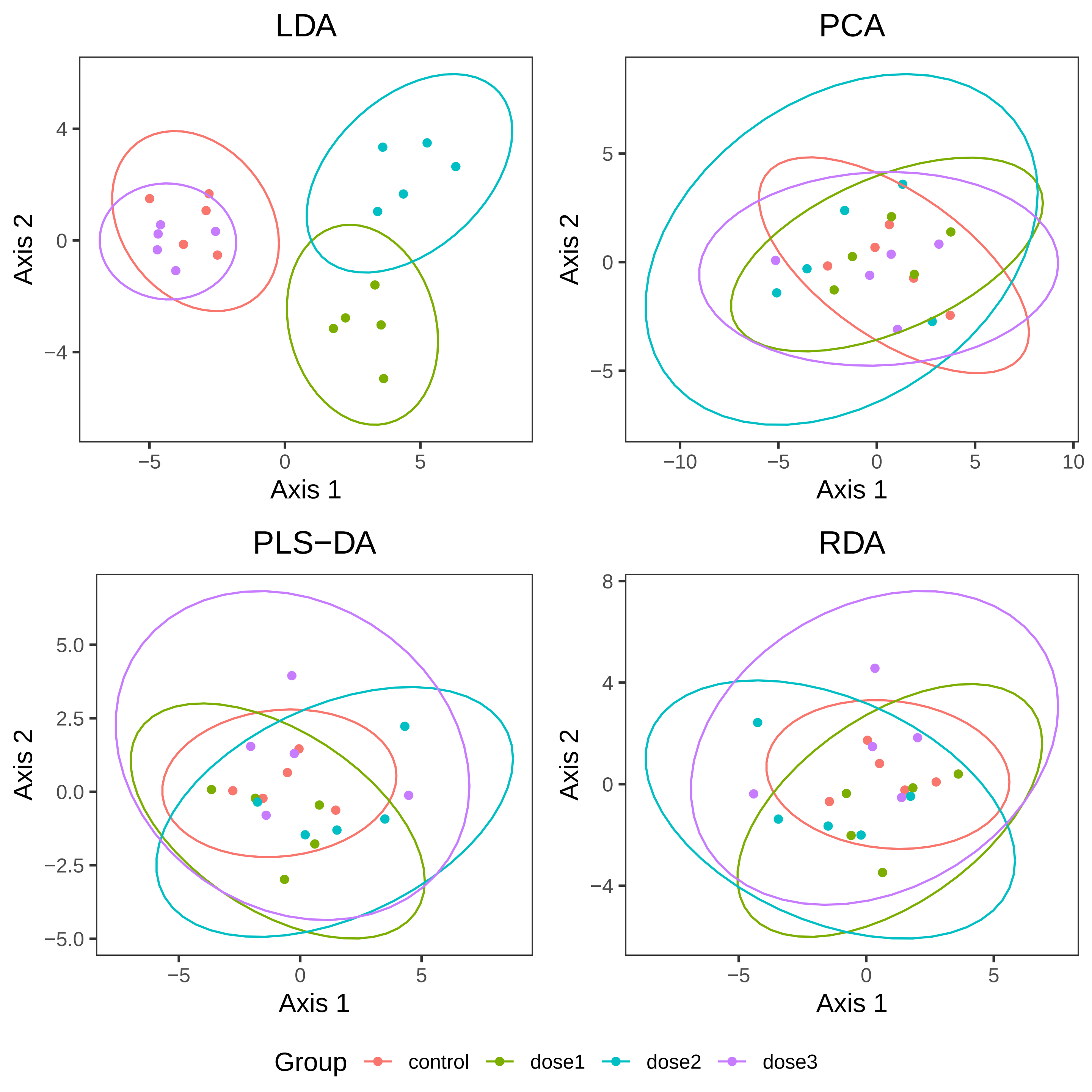
LDA
PCA
PLS−DA
RDA
Axis 1
Axis 2
Group
control
dose1
dose2
dose3

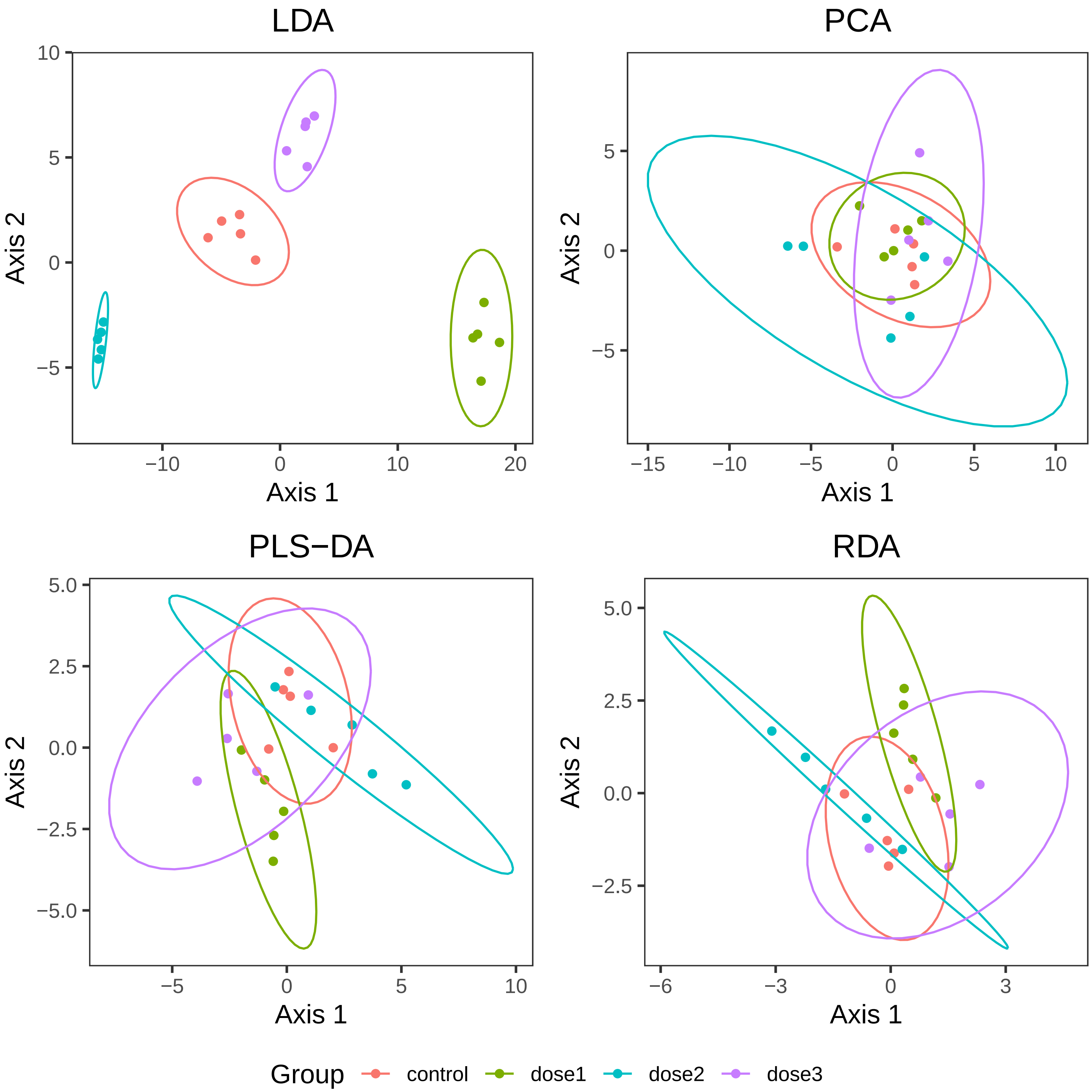
LDA
PCA
PLS−DA
RDA
Axis 1
Axis 2
Group
control
dose1
dose2
dose3

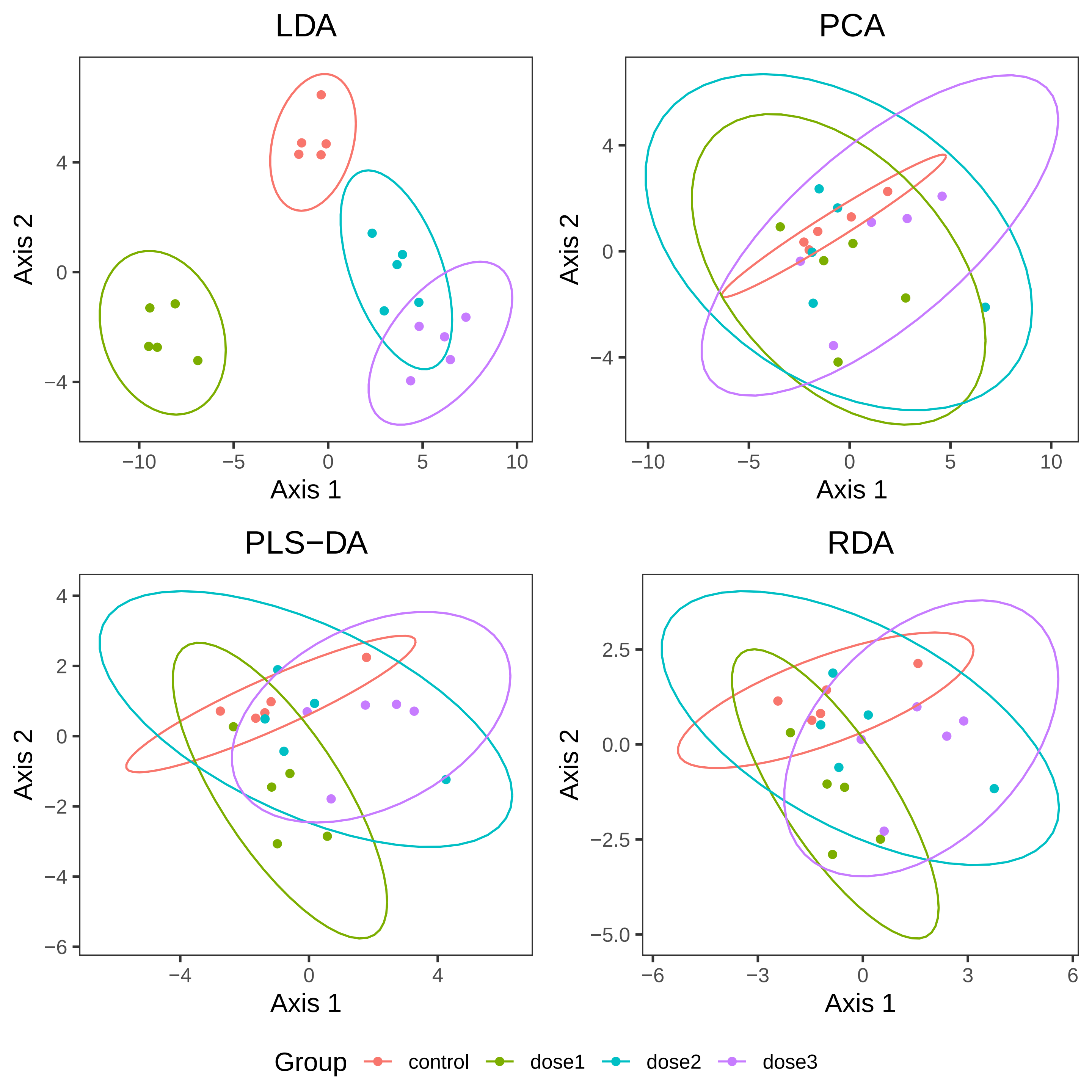

LDA
PCA
PLS−DA
RDA
Axis 1
Axis 2
Group
control
dose1
dose2
dose3

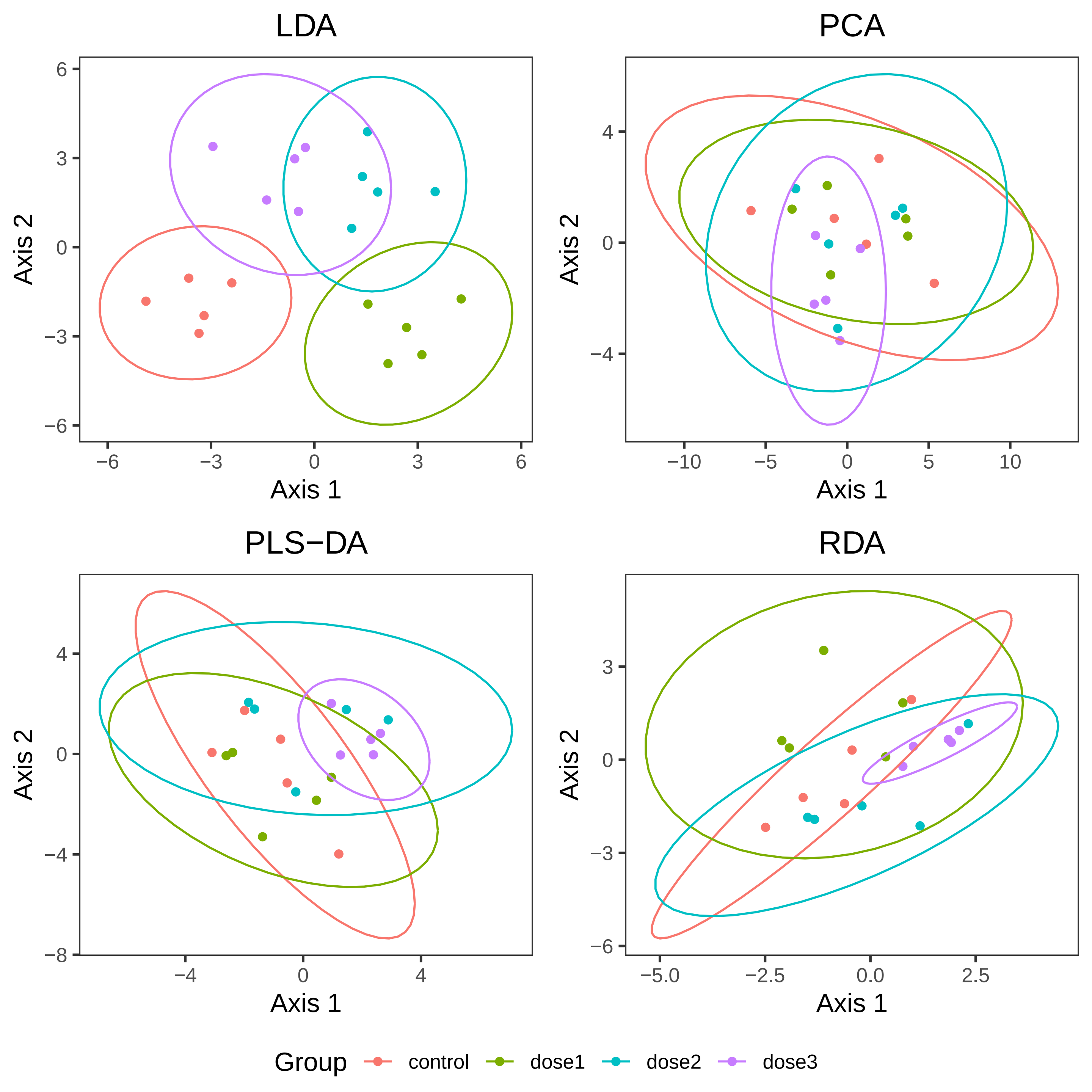

LDA
PCA
PLS−DA
RDA
Axis 1
Axis 2
Group
control
dose1
dose2
dose3

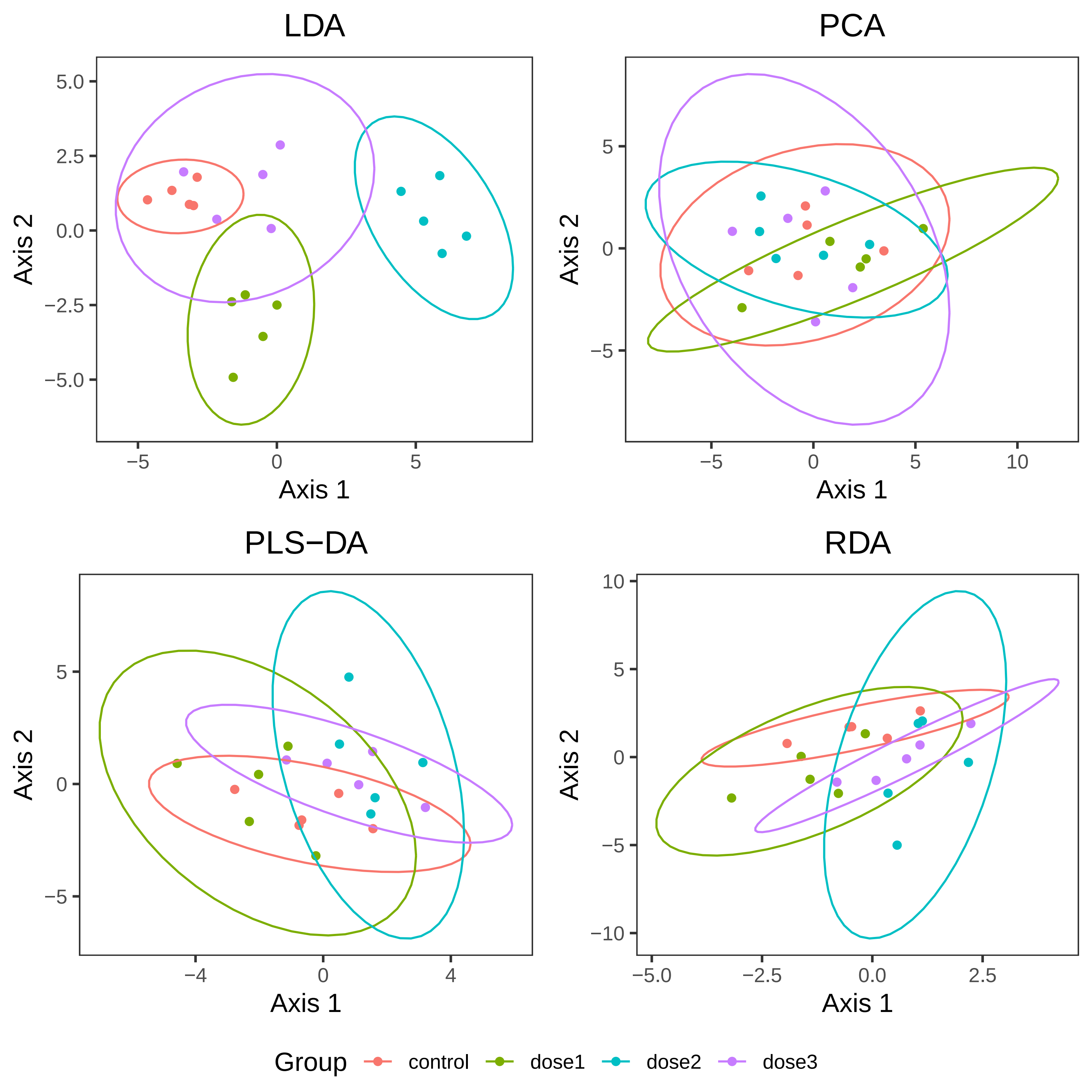

LDA
PCA
PLS−DA
RDA
Axis 1
Axis 2
5.0
2.5
0.0
−2.5
−5.0
−5
0
5
10
−4
4
−10
Group
control
dose1
dose2
dose3

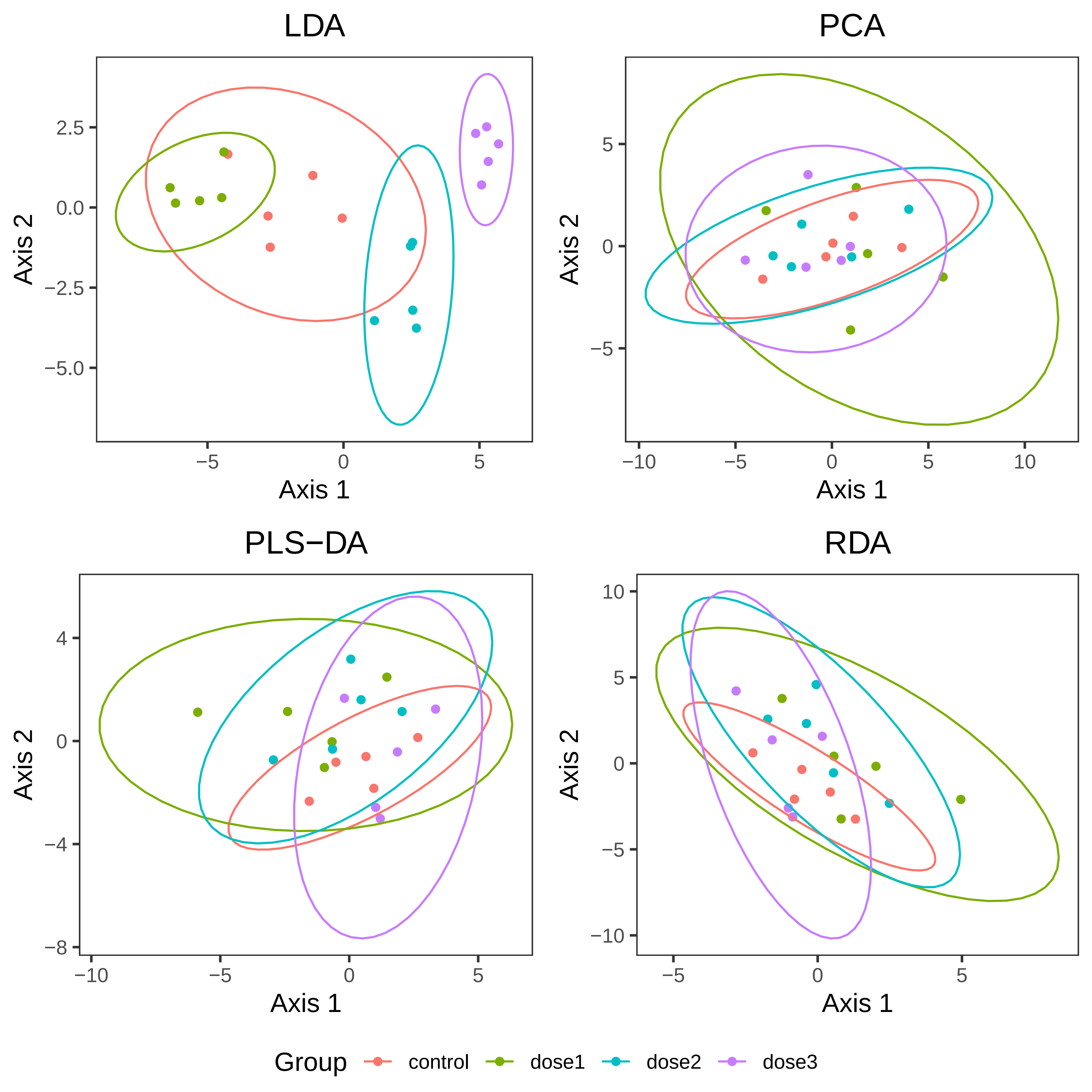
LDA
PCA
PLS−DA
RDA
Axis 1
Axis 2
Group
control
dose1
dose2
dose3

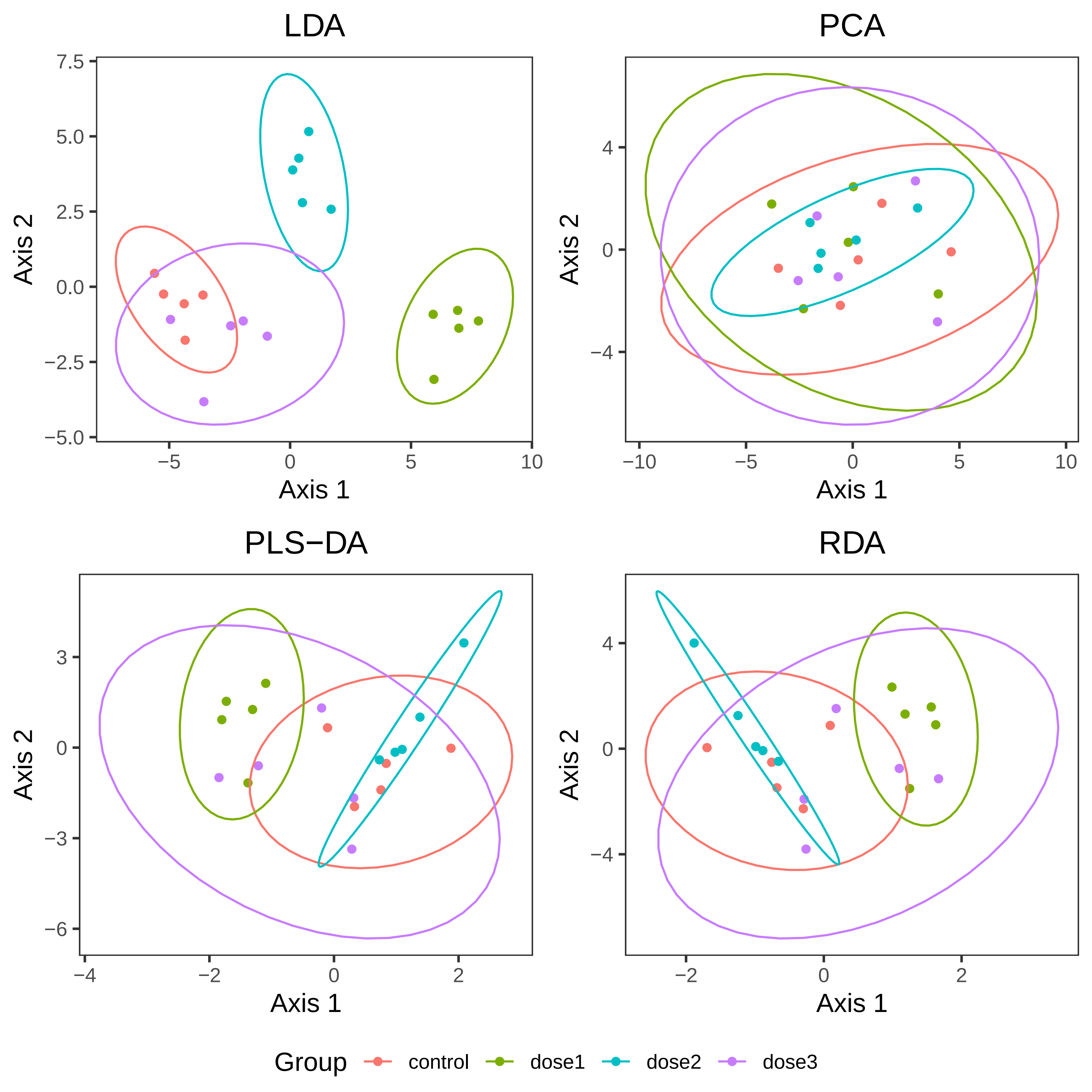

LDA
PCA
PLS−DA
RDA
Axis 1
Axis 2
7.5
5.0
2.5
0.0
−2.5
−5.0
−5
0
5
10
4
0
−4
−10
3
−6
−3
−2
2
Group
control
dose1
dose2
dose3

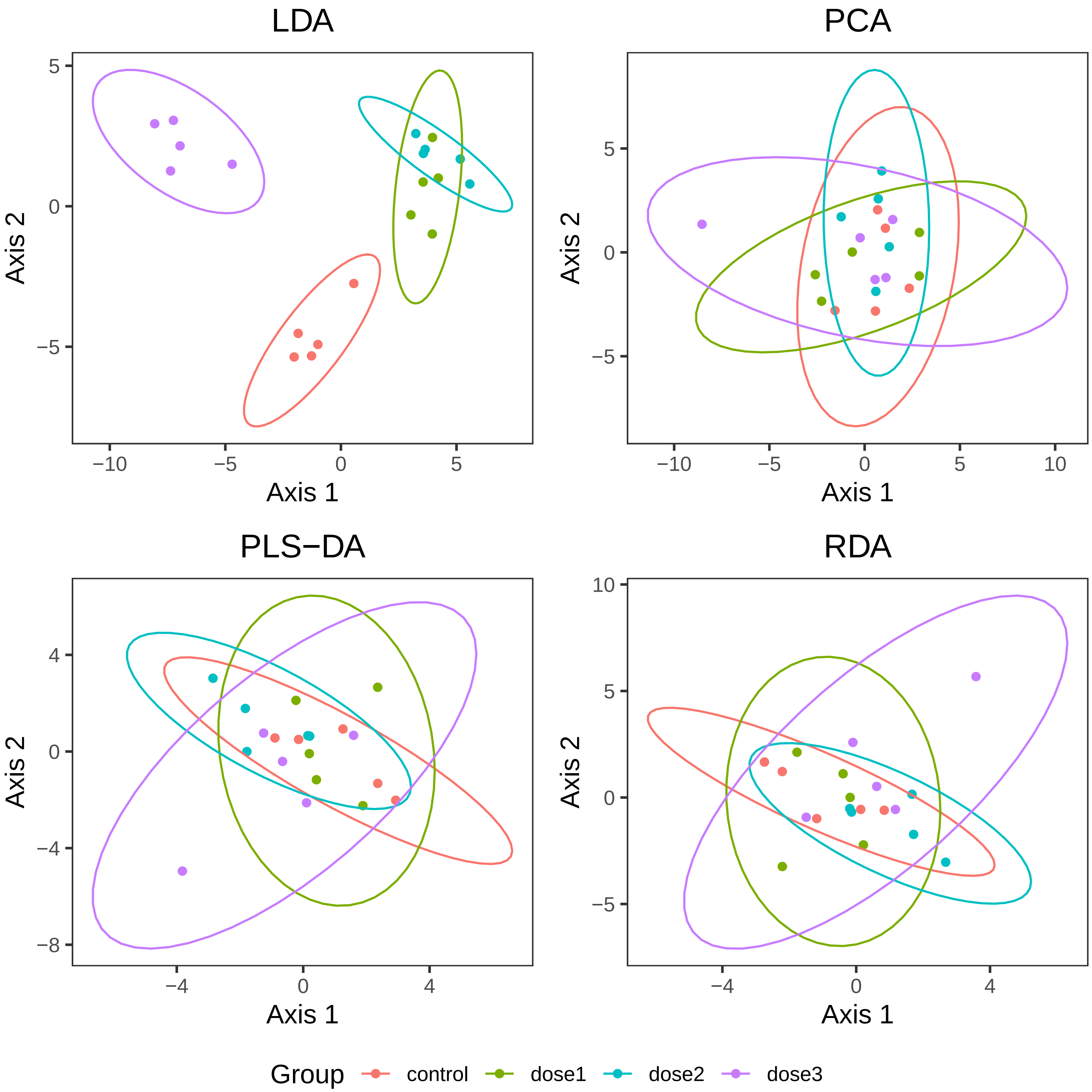
LDA
PCA
PLS−DA
RDA
Axis 1
Axis 2
Group
control
dose1
dose2
dose3

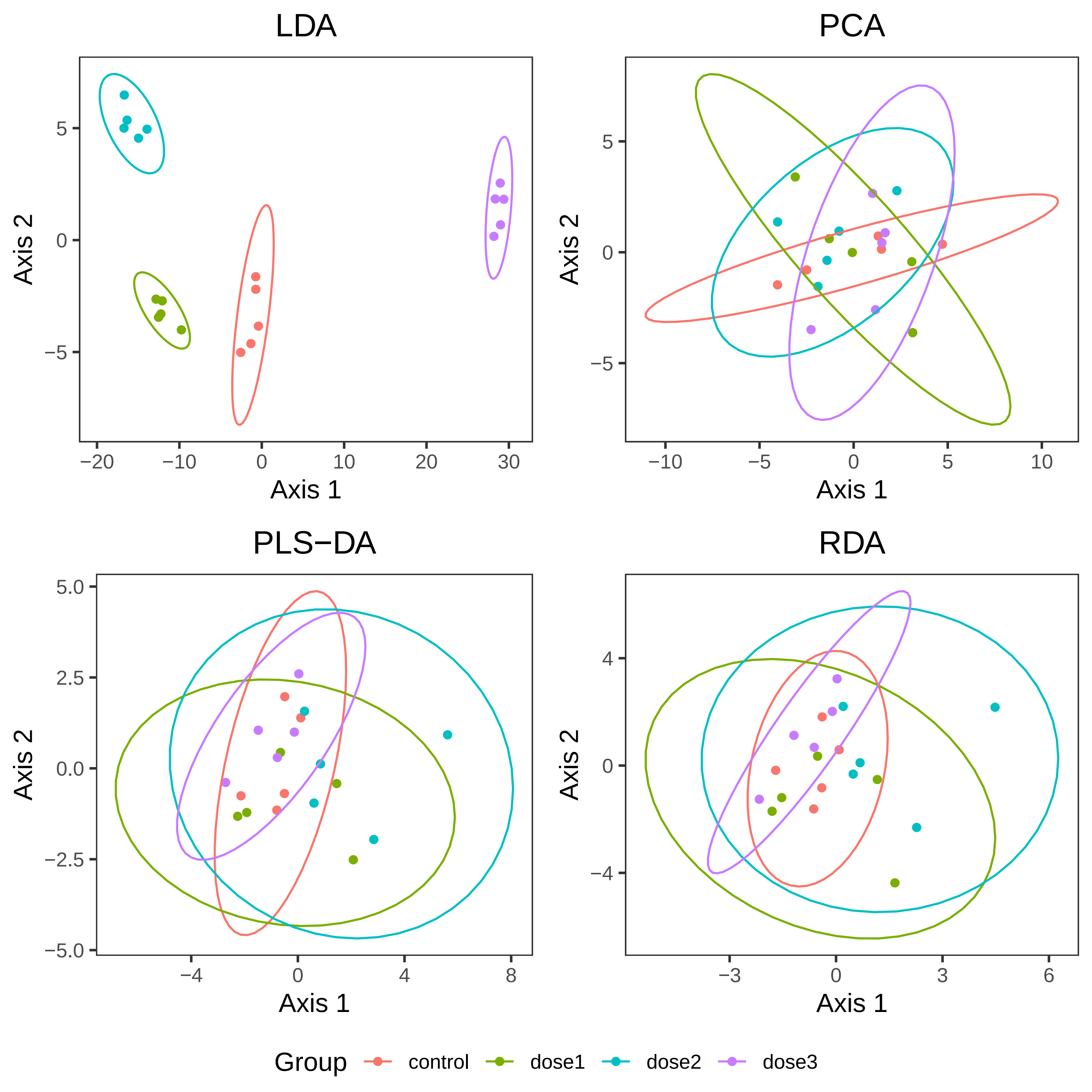
LDA
PCA
PLS−DA
RDA
Axis 1
Axis 2
5
0
−5
−20
−10
0
10
20
30
−10
−5
0
5
10
5.0
2.5
0.0
−2.5
−5.0
−4
0
4
8
4
0
−4
−3
0
3
6
Group
control
dose1
dose2
dose3

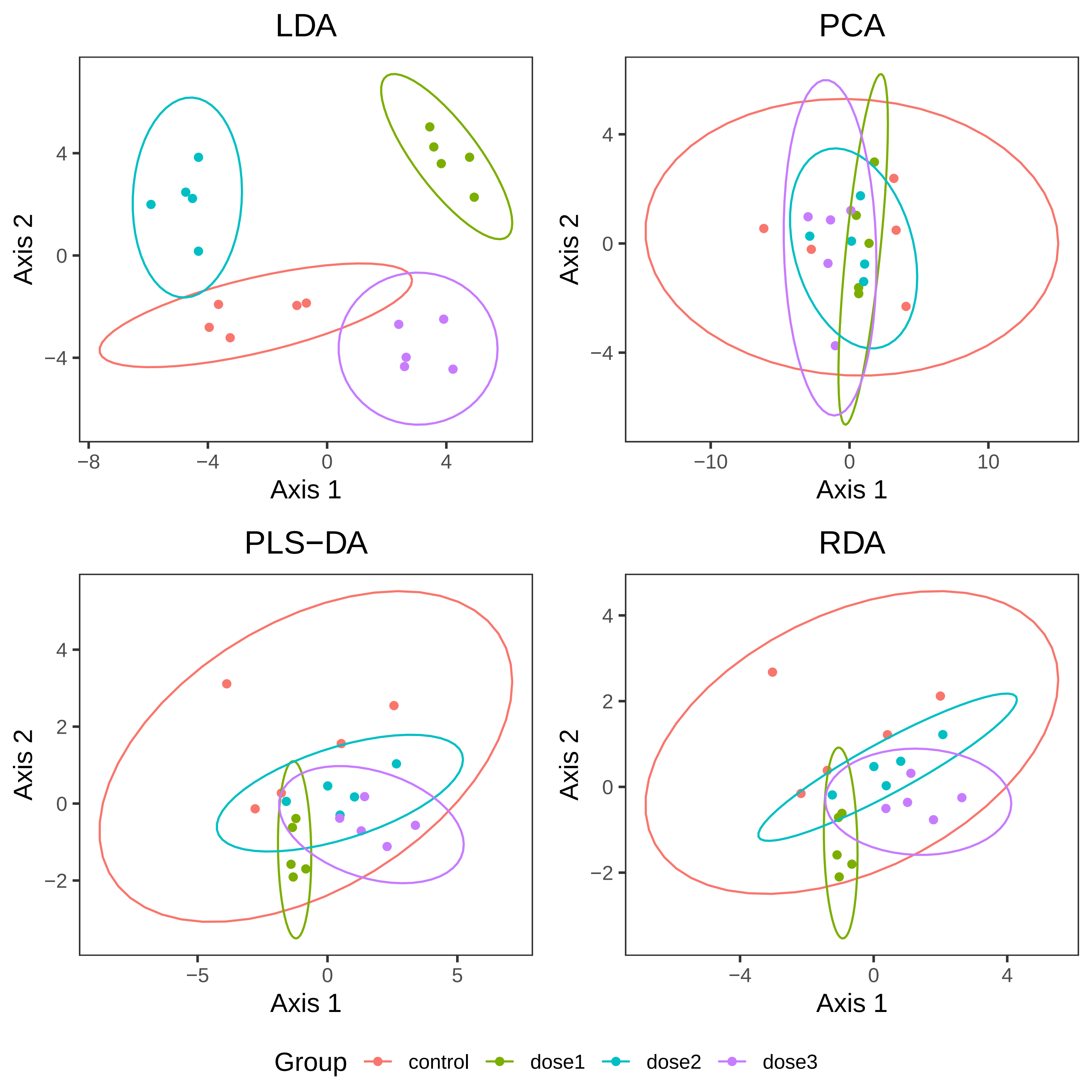
LDA
PCA
PLS−DA
RDA
Axis 1
Axis 2
−8
−4
0
4
−10
10
−5
5
2
−2
Group
control
dose1
dose2
dose3

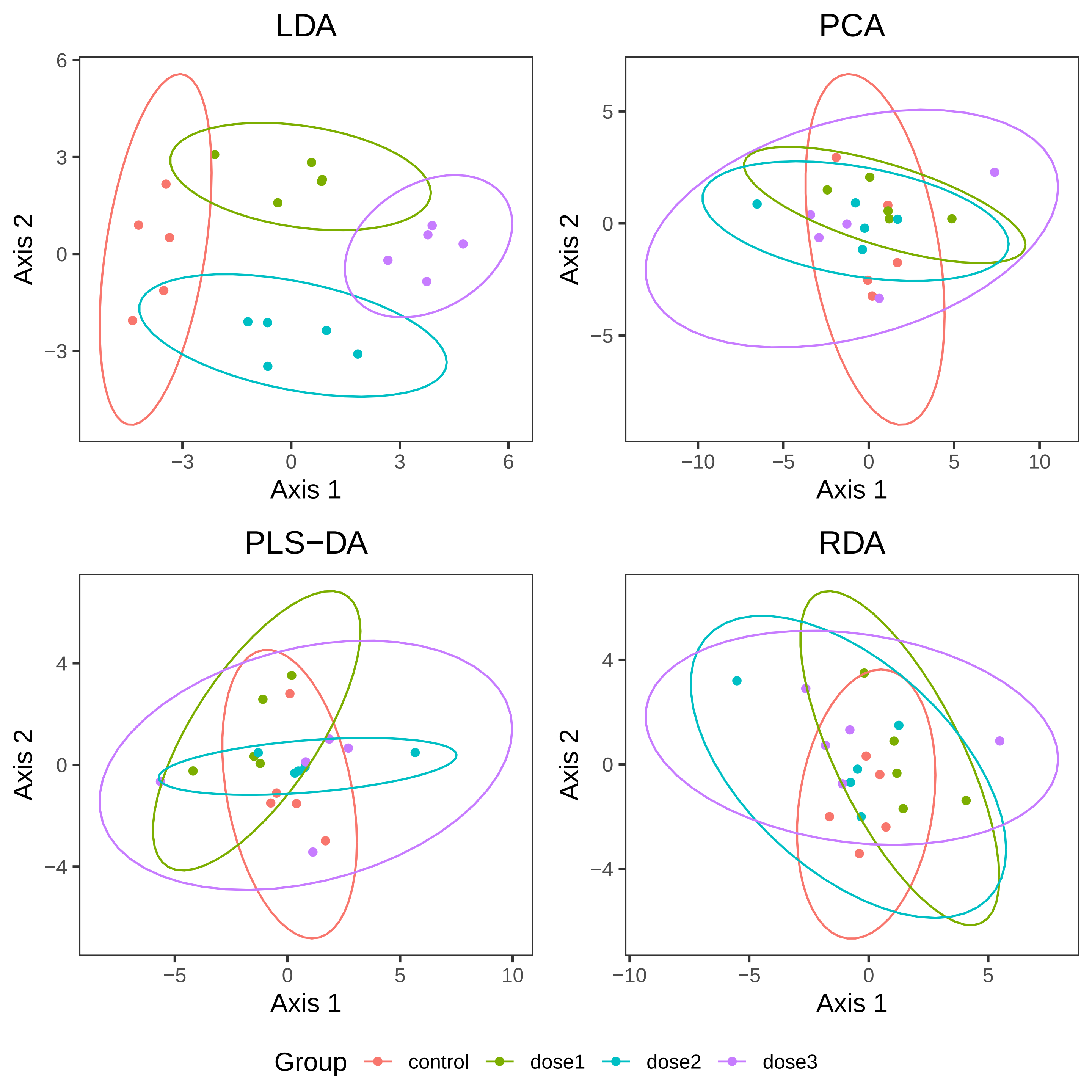

LDA
PCA
PLS−DA
RDA
Axis 1
Axis 2
6
3
0
−3
−10
−5
5
10
4
−4
Group
control
dose1
dose2
dose3

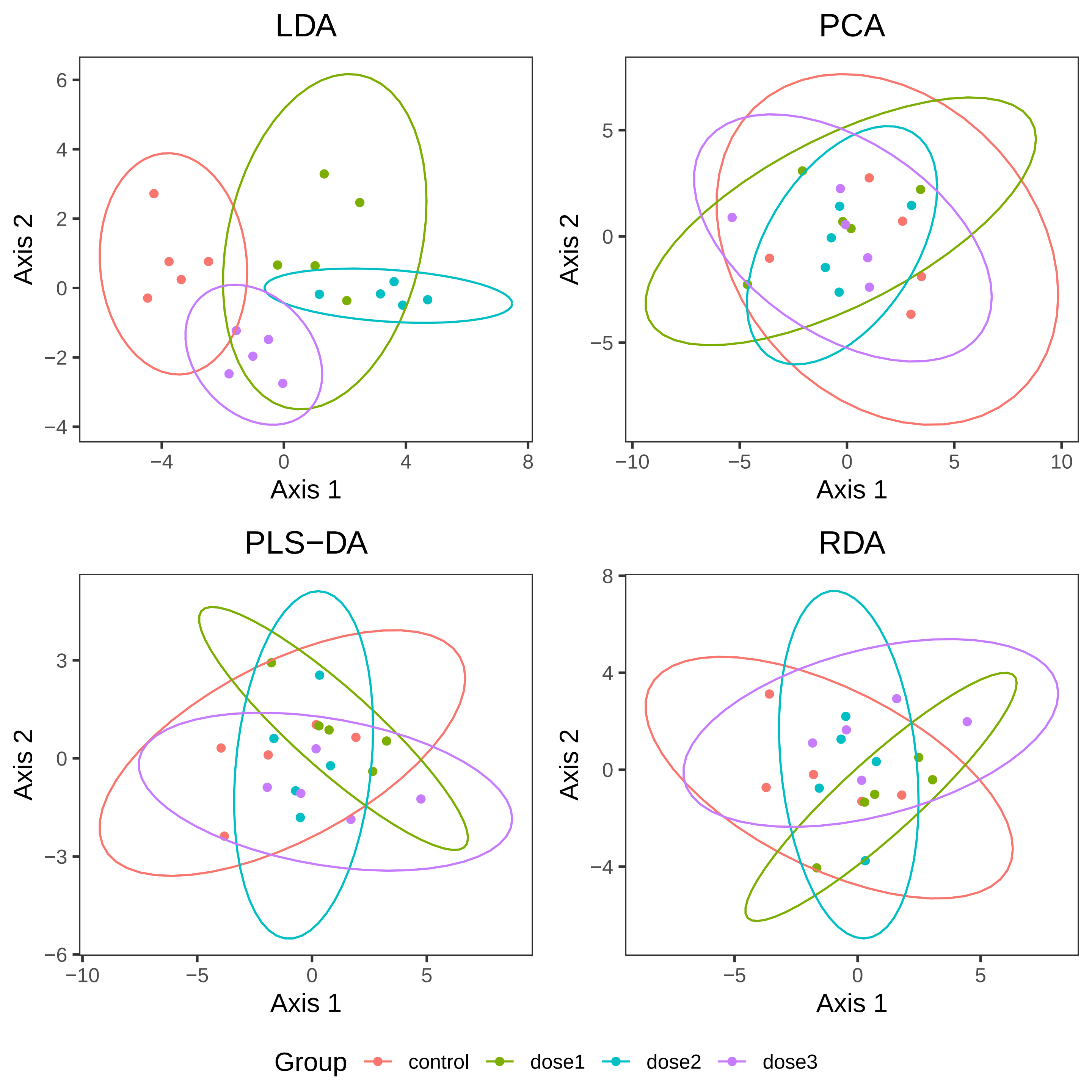

LDA
PCA
PLS−DA
RDA
Axis 1
Axis 2
Group
control
dose1
dose2
dose3

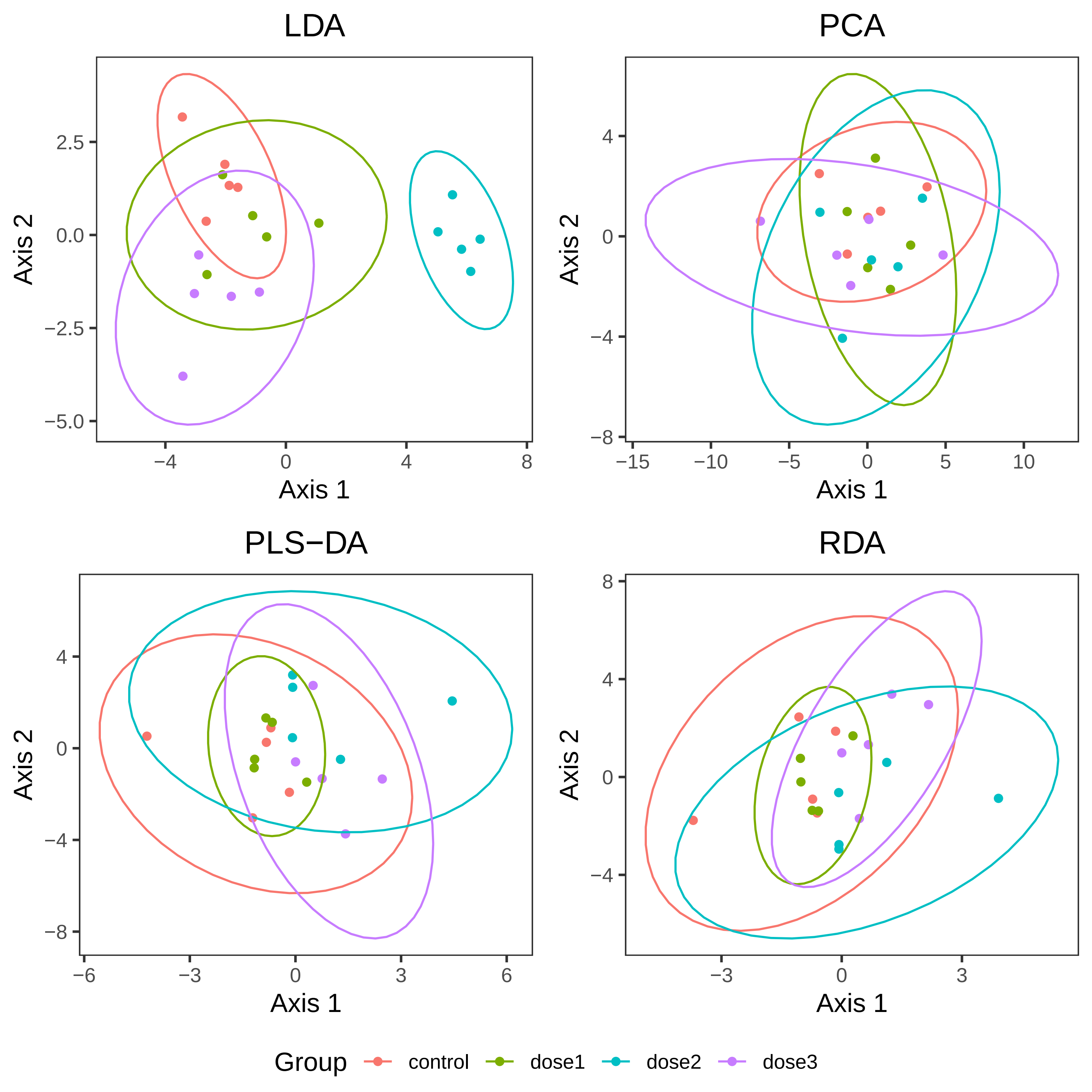

LDA
PCA
PLS−DA
RDA
Axis 1
Axis 2
Group
control
dose1
dose2
dose3

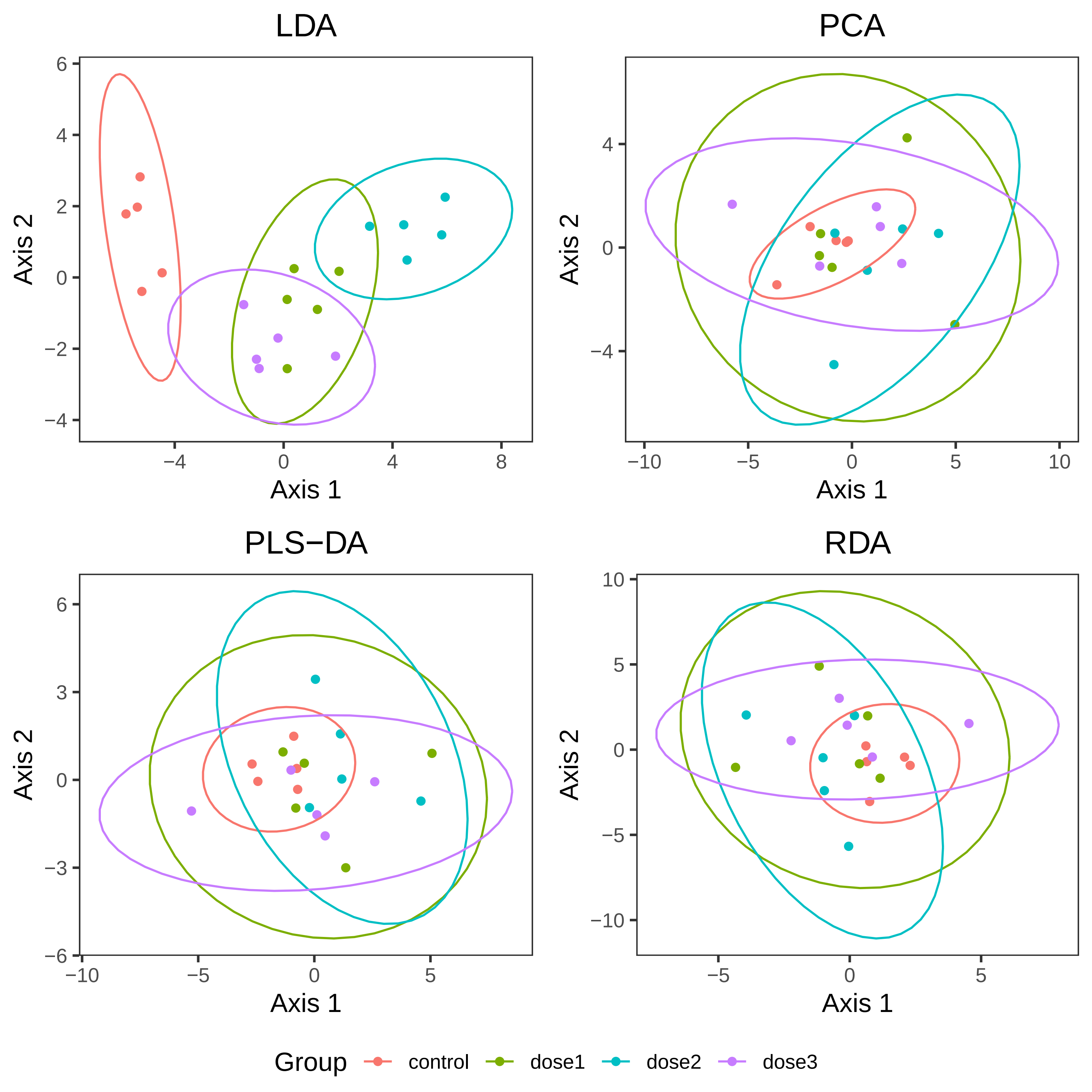
LDA
PCA
PLS−DA
RDA
Axis 1
Axis 2
Group
control
dose1
dose2
dose3

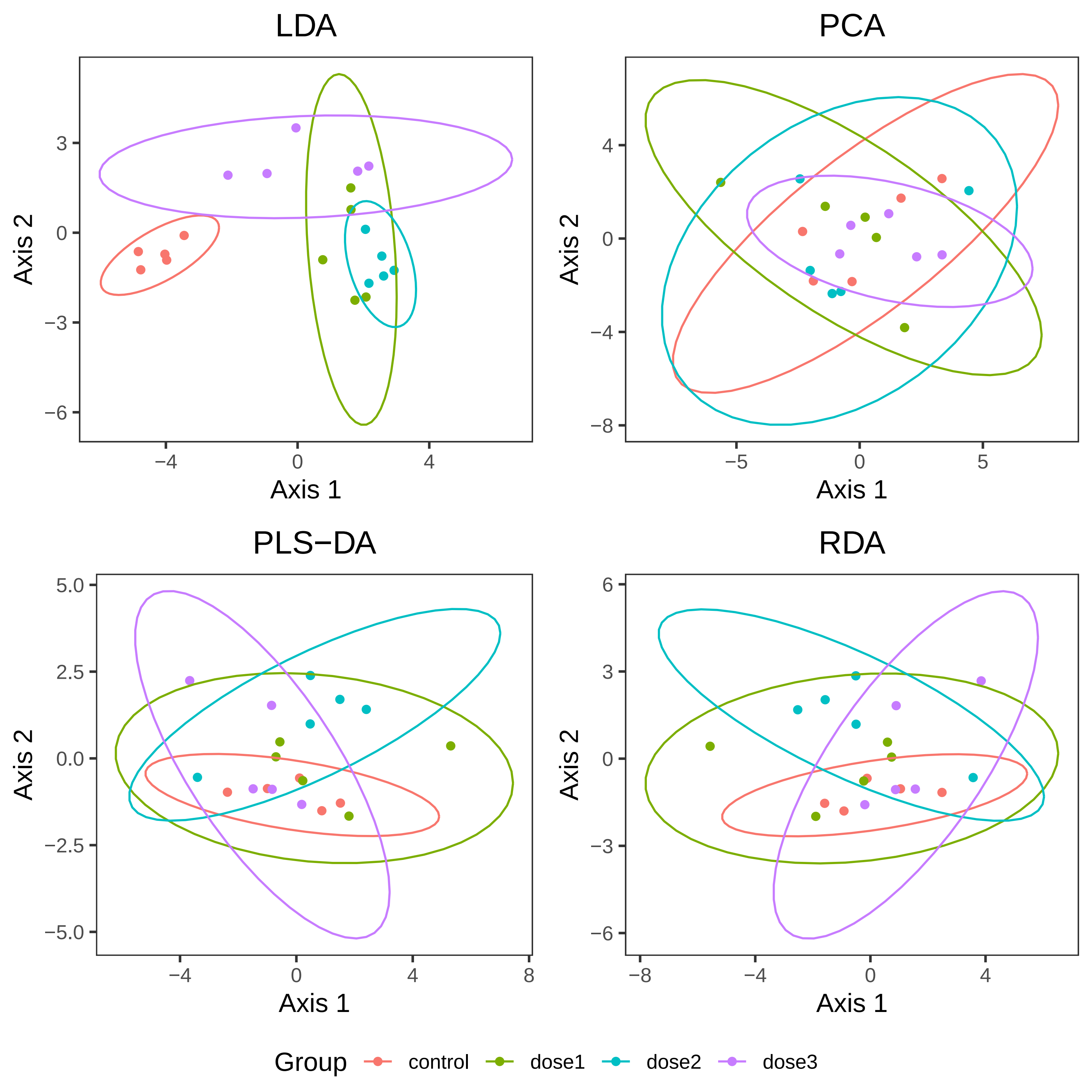

LDA
PCA
PLS−DA
RDA
Axis 1
Axis 2
3
0
−3
−6
−4
0
4
−5
5
−8
5.0
2.5
0.0
−2.5
−5.0
8
6
Group
control
dose1
dose2
dose3

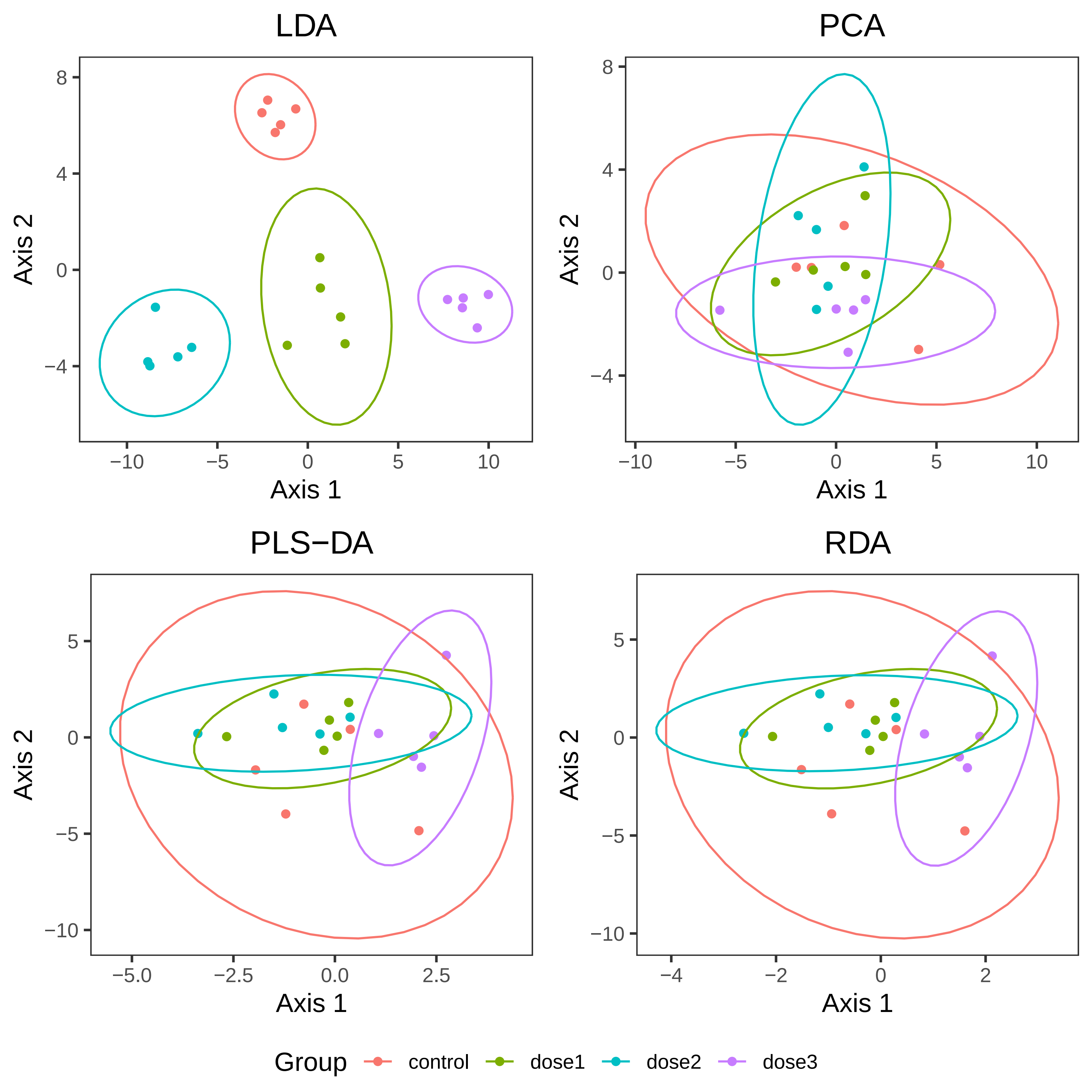
LDA
PCA
PLS−DA
RDA
Axis 1
Axis 2
Group
control
dose1
dose2
dose3

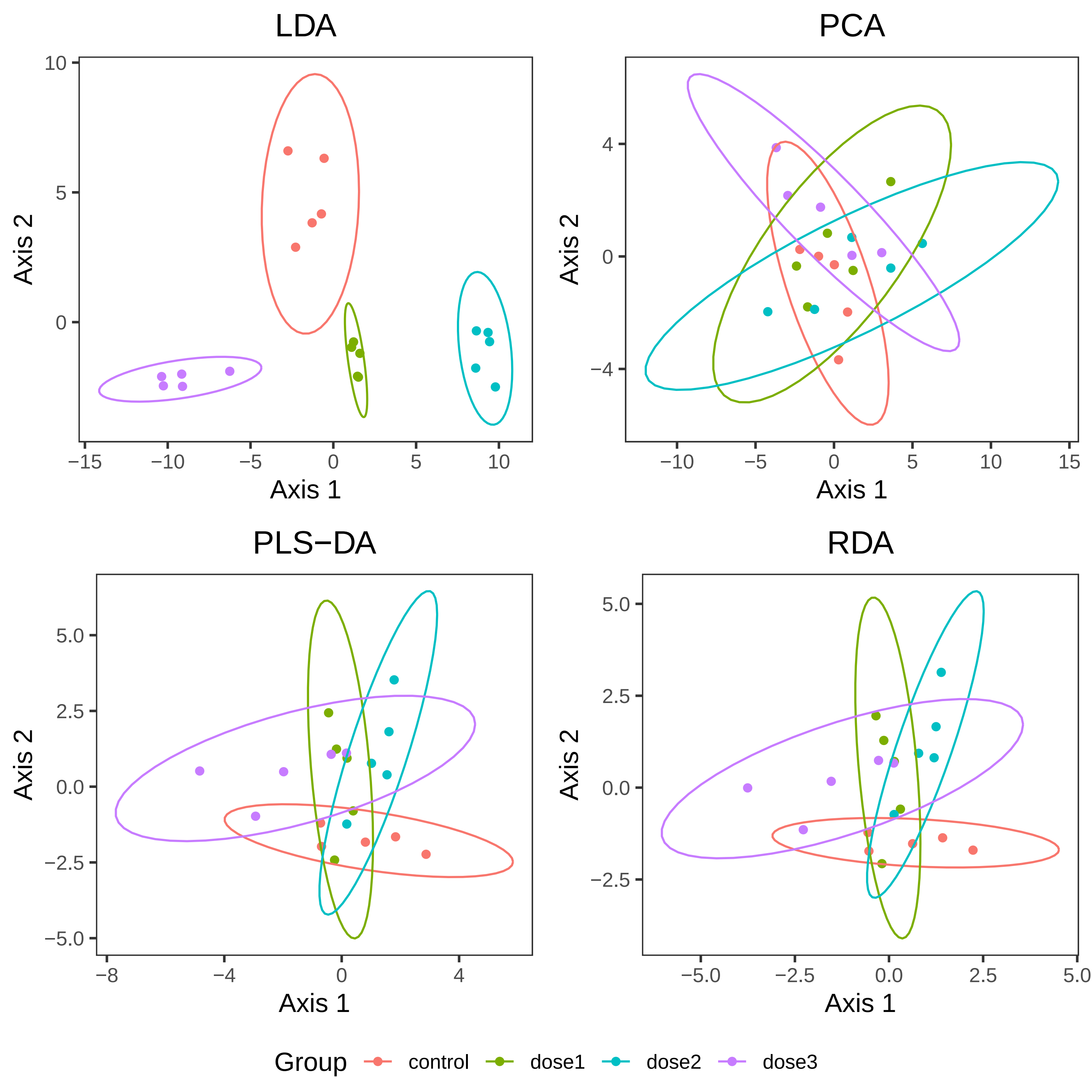

LDA
PCA
PLS−DA
RDA
Axis 1
Axis 2
Group
control
dose1
dose2
dose3

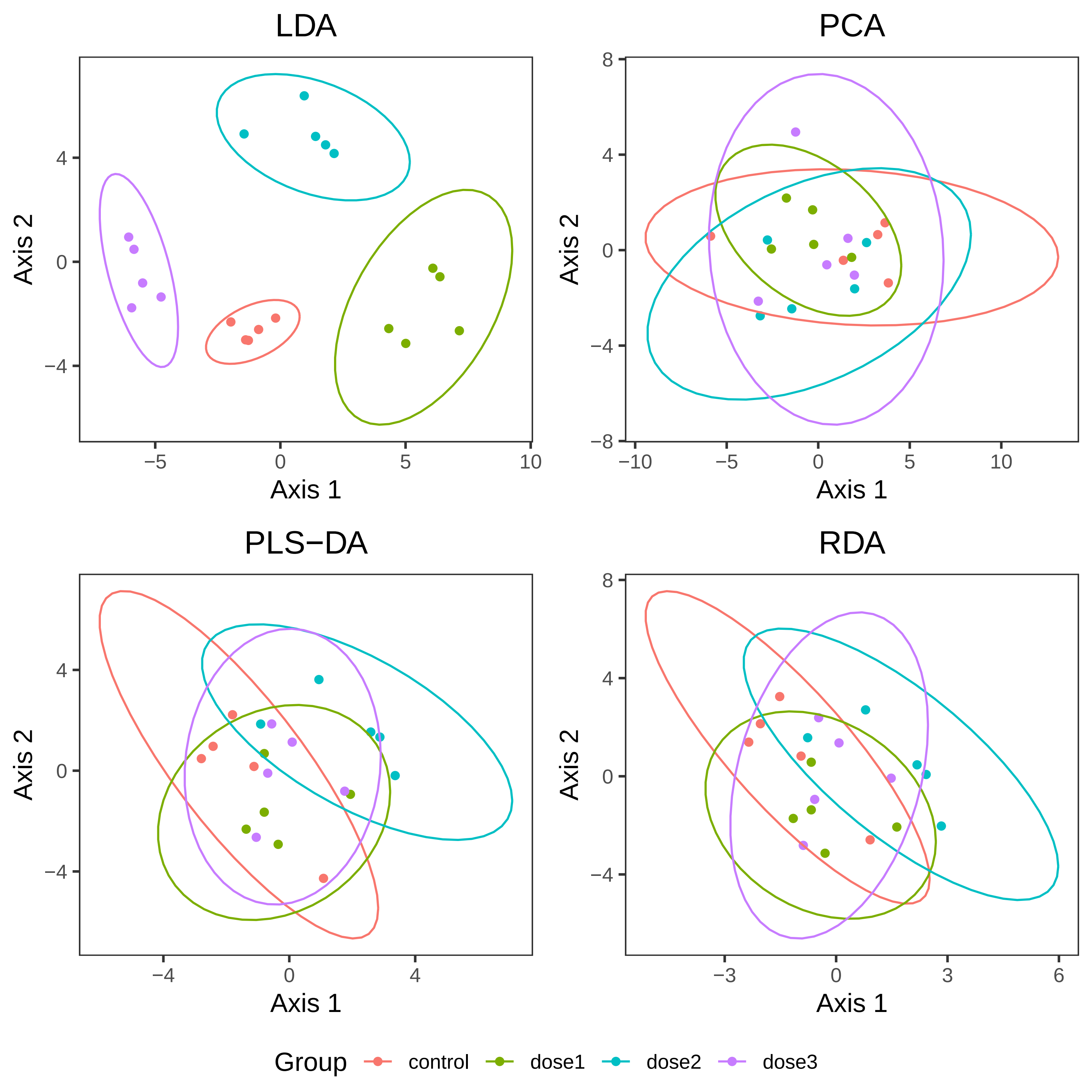
LDA
PCA
PLS−DA
RDA
Axis 1
Axis 2
Group
control
dose1
dose2
dose3

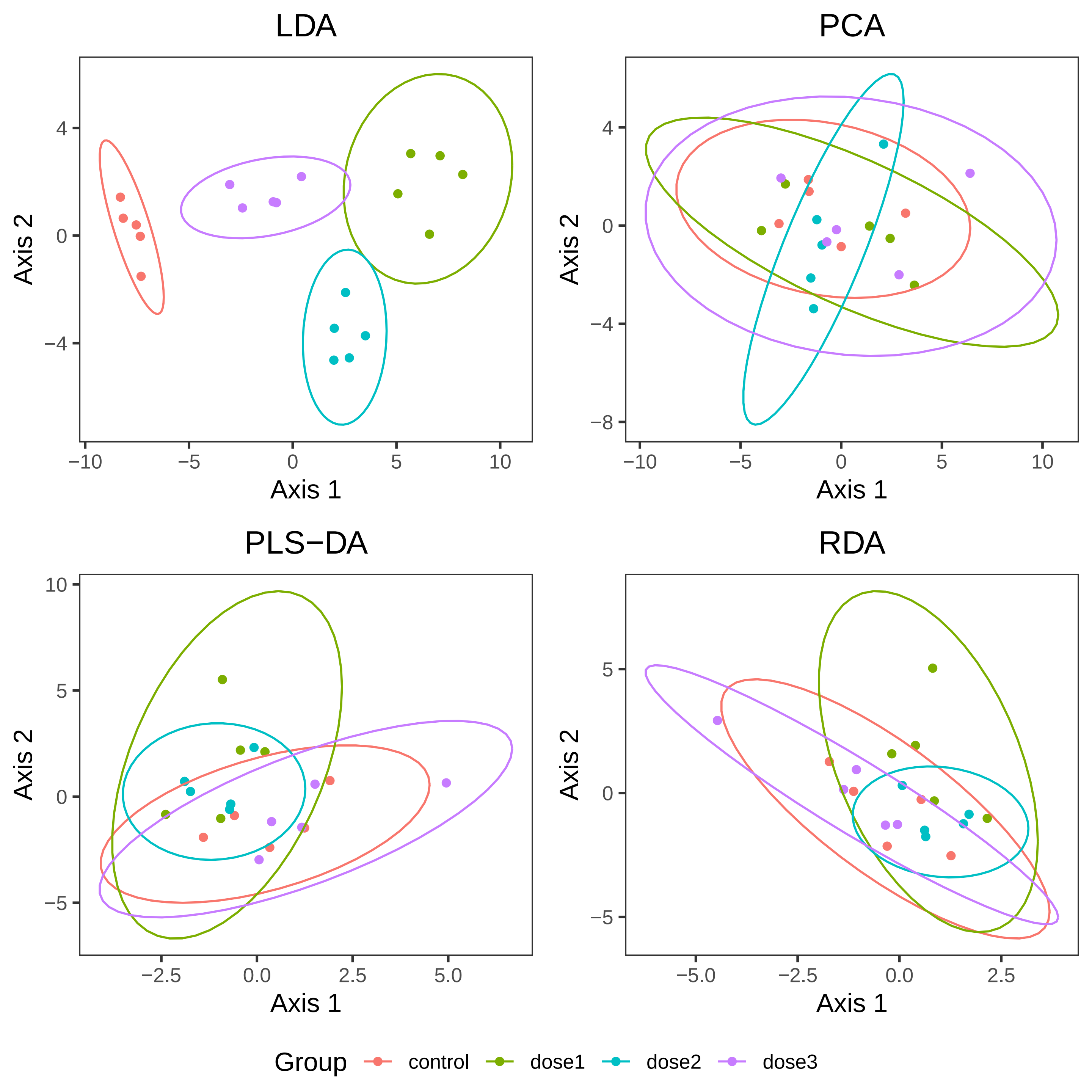

LDA
PCA
PLS−DA
RDA
Axis 1
Axis 2
4
0
−4
−8
5
10
−10
−5
−2.5
0.0
2.5
5.0
Group
control
dose1
dose2
dose3

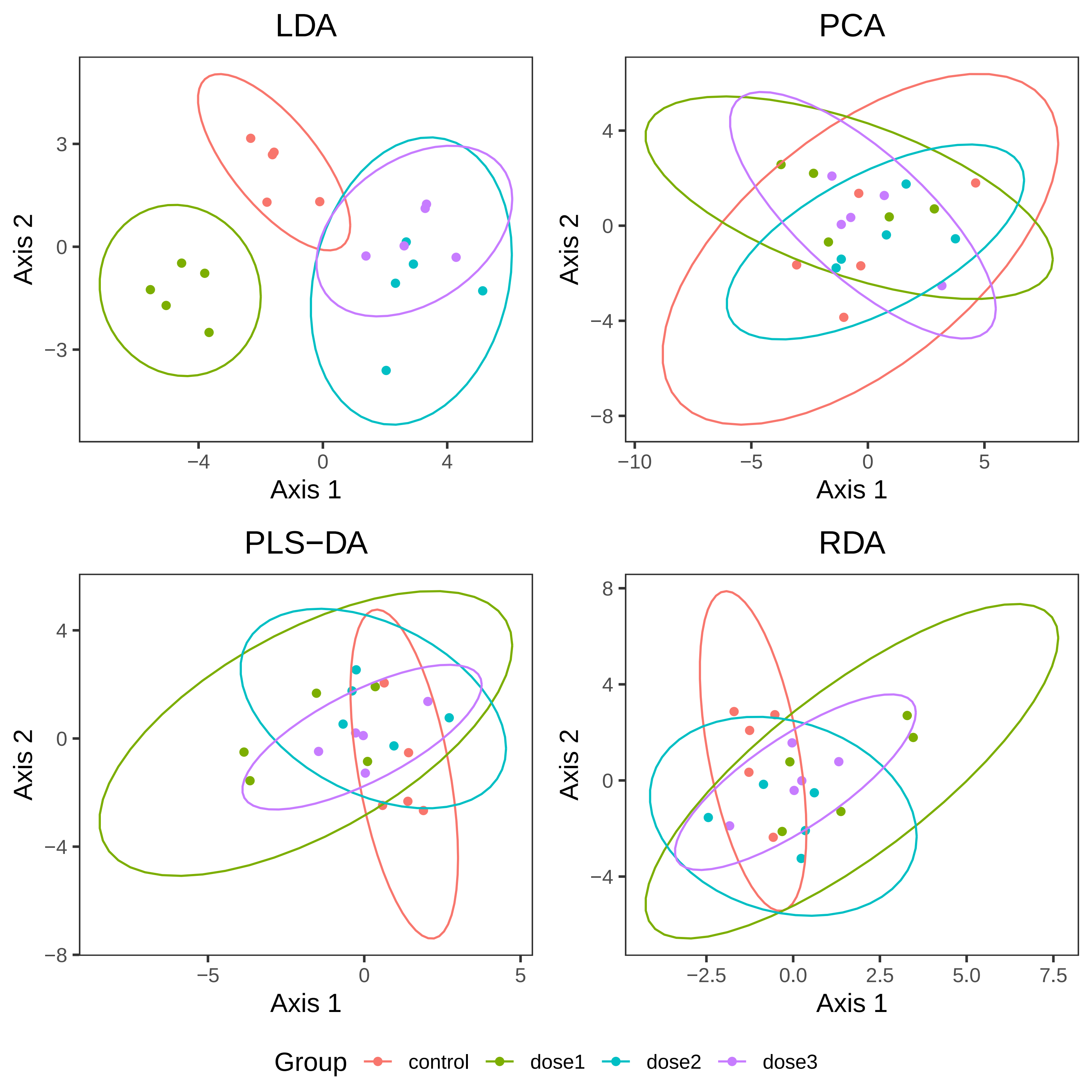
LDA
PCA
PLS−DA
RDA
Axis 1
Axis 2
3
0
−3
−4
0
4
4
0
−4
−8
−10
−5
0
5
4
0
−4
−8
−5
0
5
8
4
0
−4
−2.5
0.0
2.5
5.0
7.5
Group
control
dose1
dose2
dose3

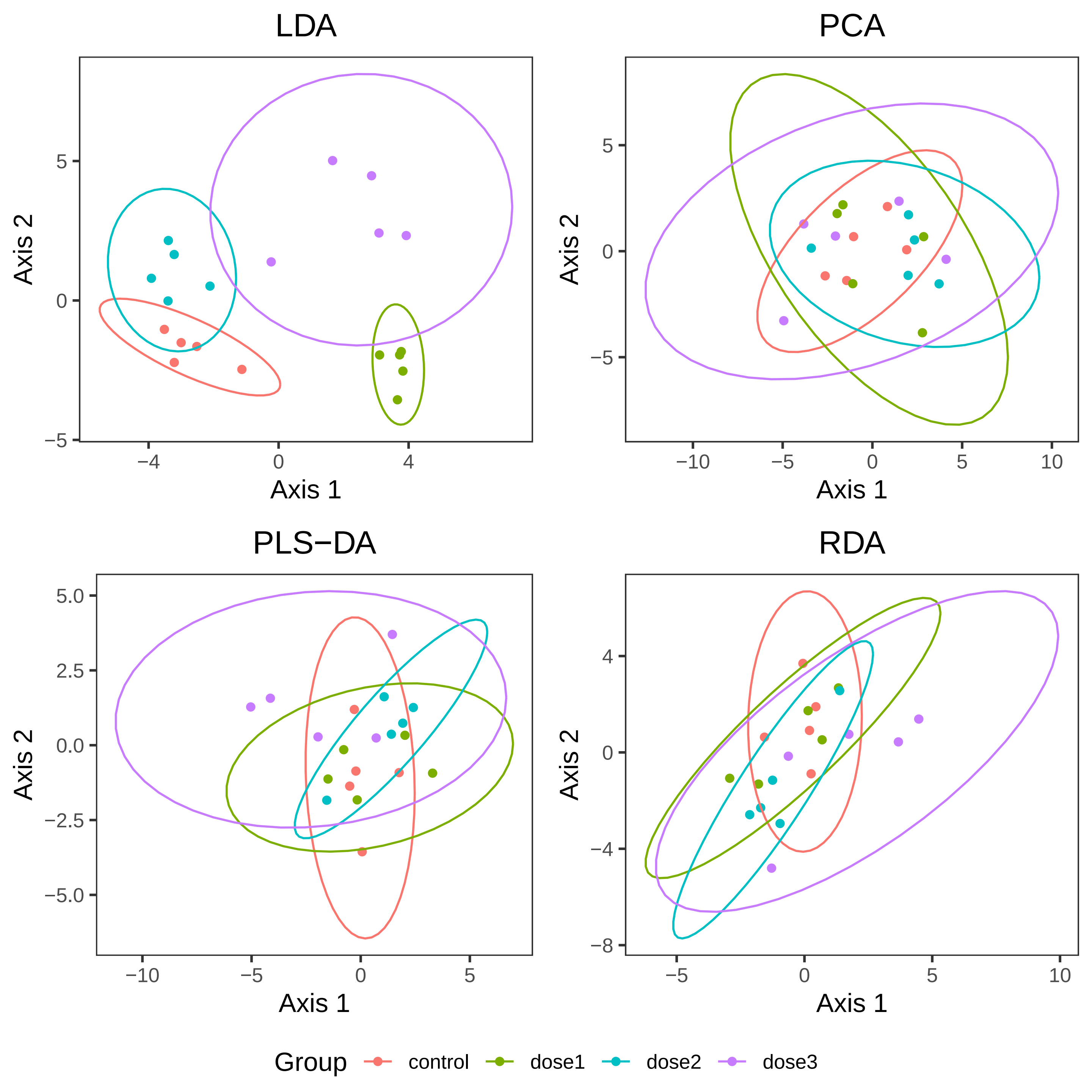

LDA
PCA
PLS−DA
RDA
Axis 1
Axis 2
Group
control
dose1
dose2
dose3

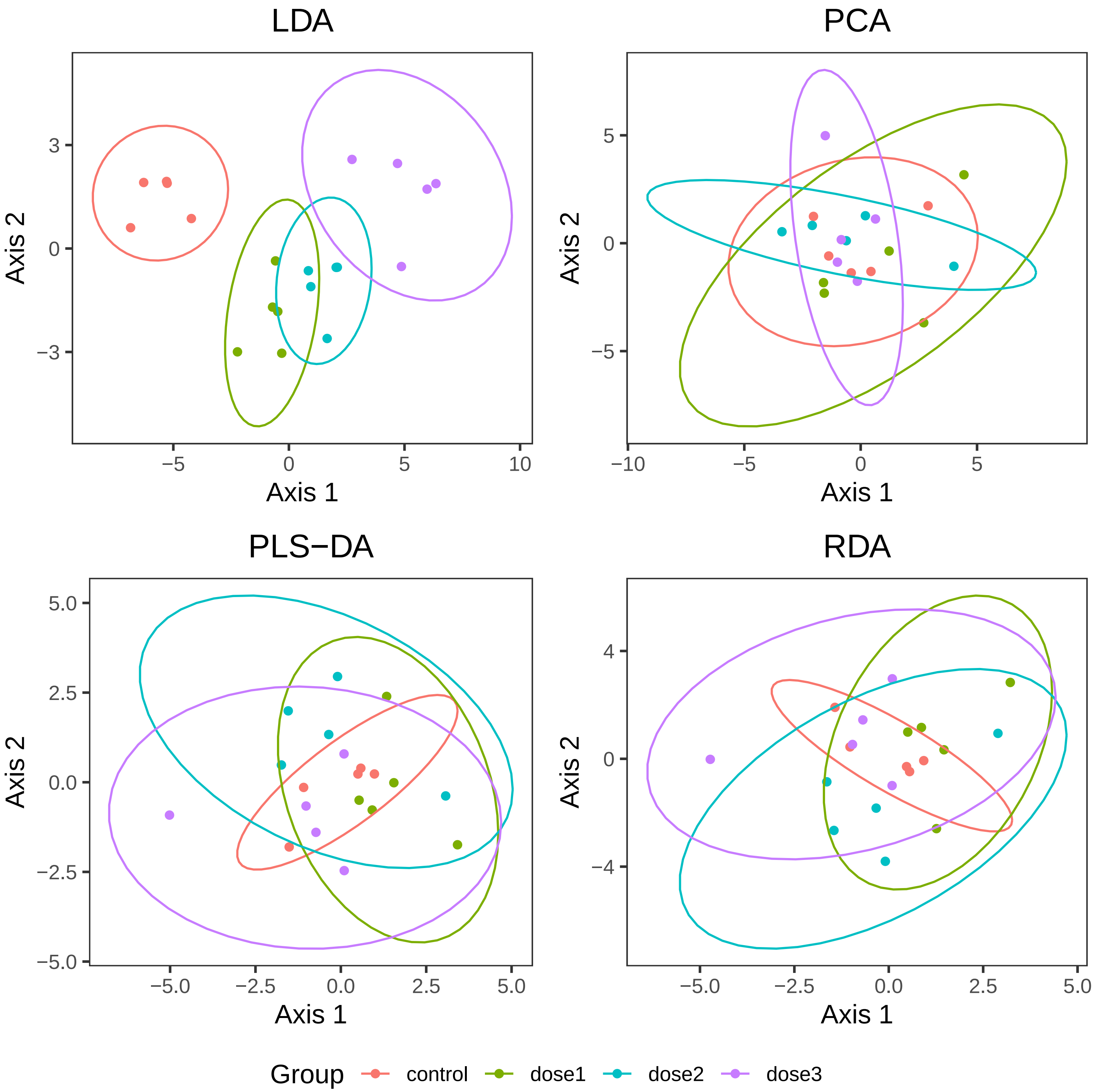

LDA
PCA
PLS−DA
RDA
Axis 1
Axis 2
Group
control
dose1
dose2
dose3

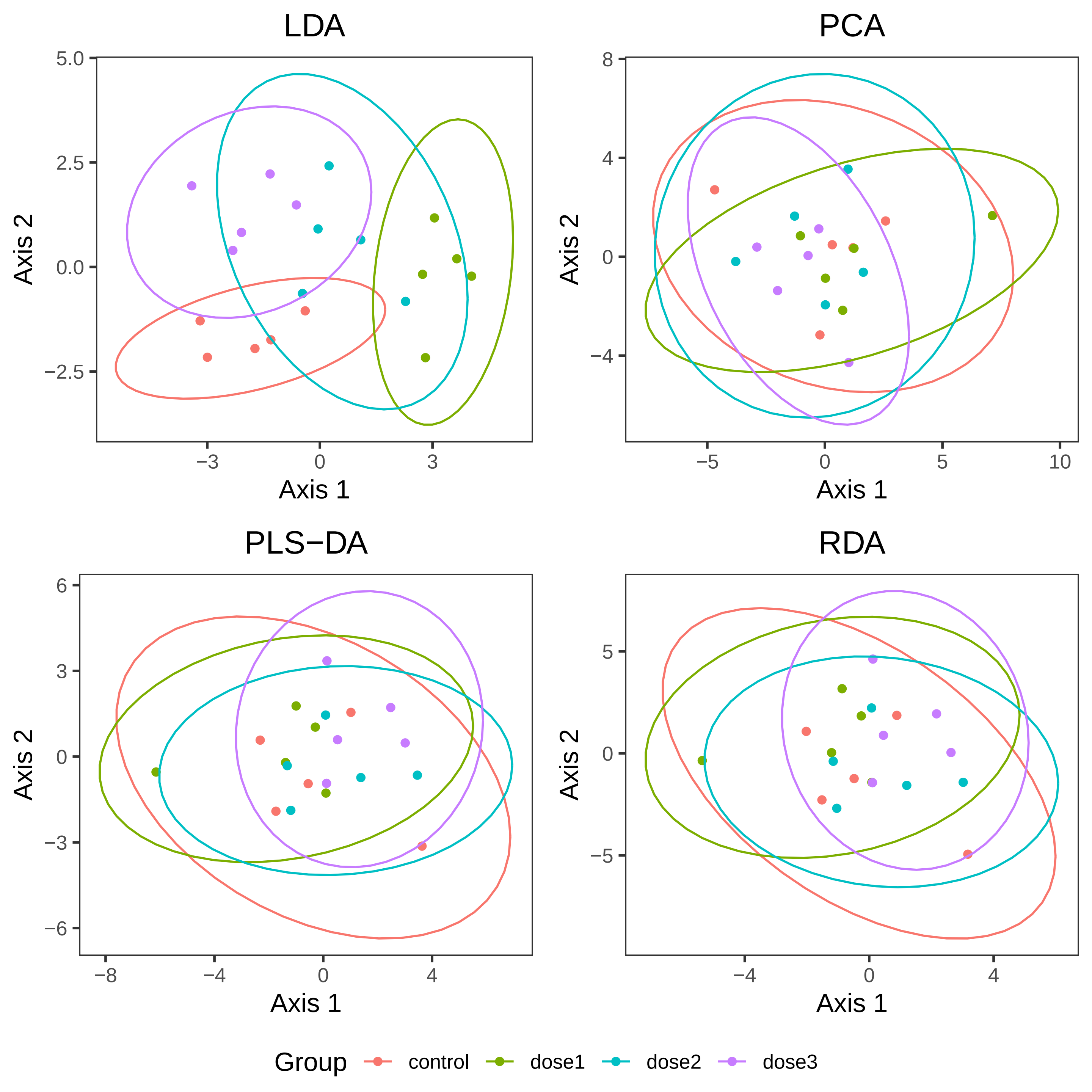

LDA
PCA
PLS−DA
RDA
Axis 1
Axis 2
5.0
2.5
0.0
−2.5
−3
0
3
8
4
0
−4
−5
0
5
10
6
3
0
−3
−6
−8
−4
0
4
5
0
−5
−4
0
4
Group
control
dose1
dose2
dose3

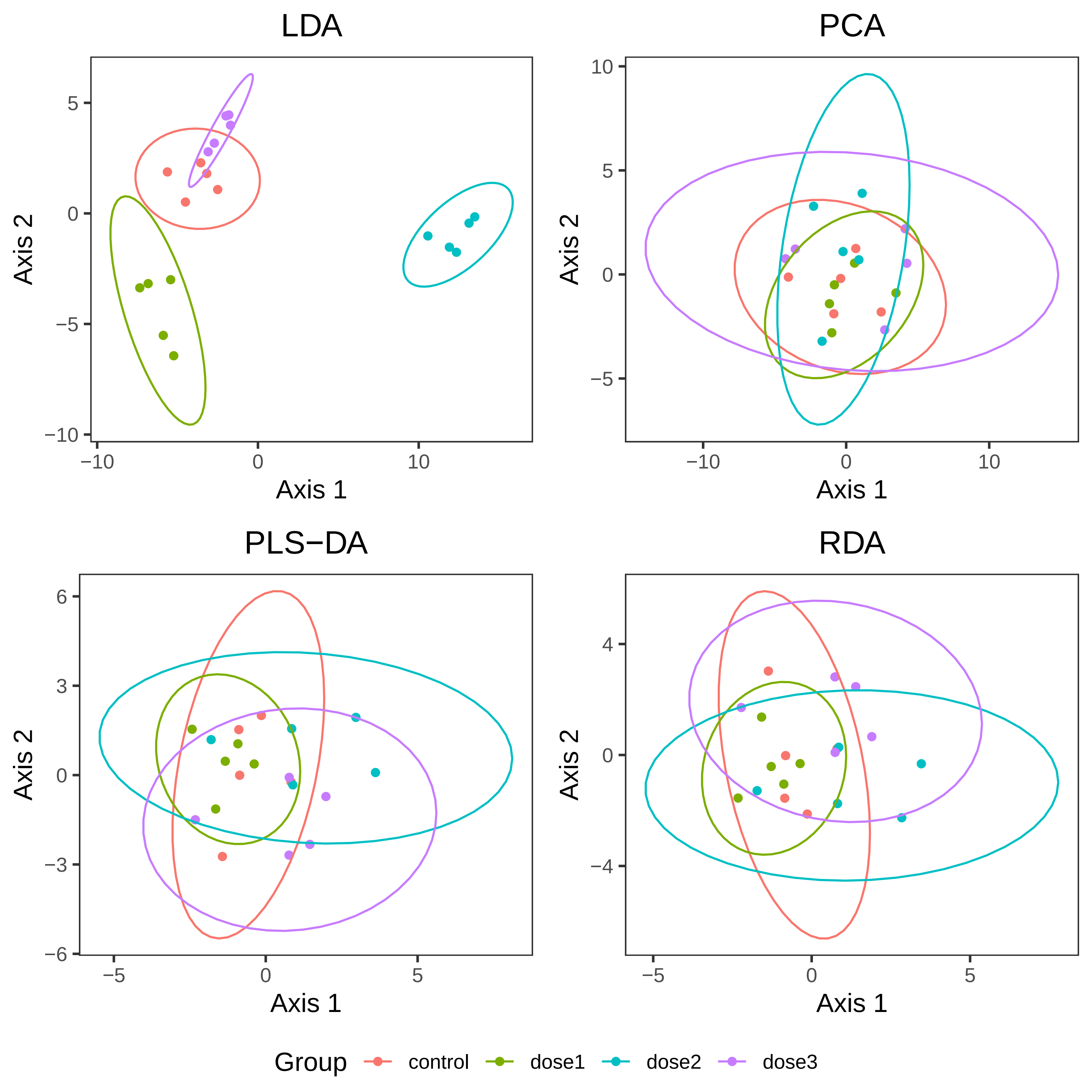
LDA
PCA
PLS−DA
RDA
Axis 1
Axis 2
Group
control
dose1
dose2
dose3

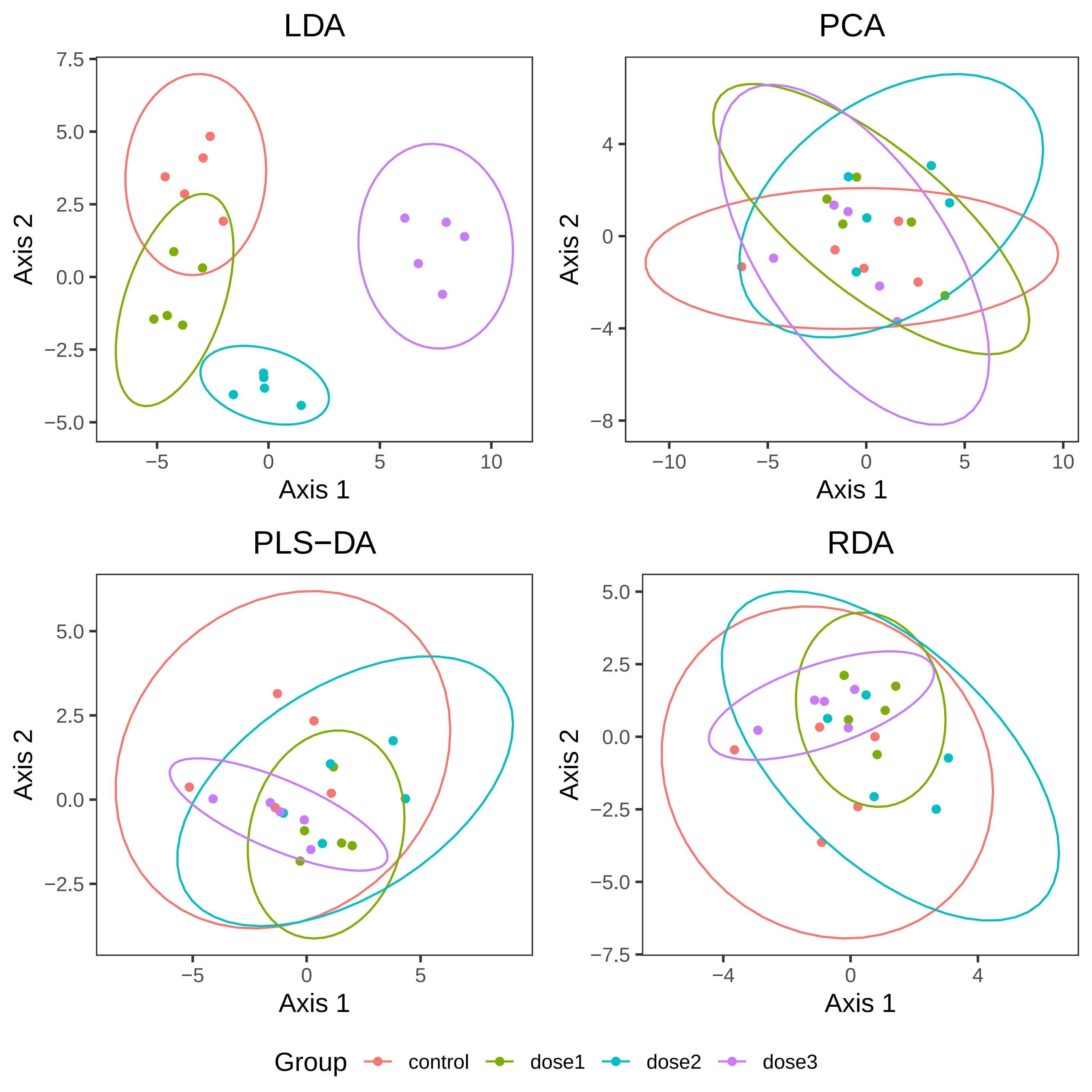
LDA
PCA
PLS−DA
RDA
Axis 1
Axis 2
Group
control
dose1
dose2
dose3

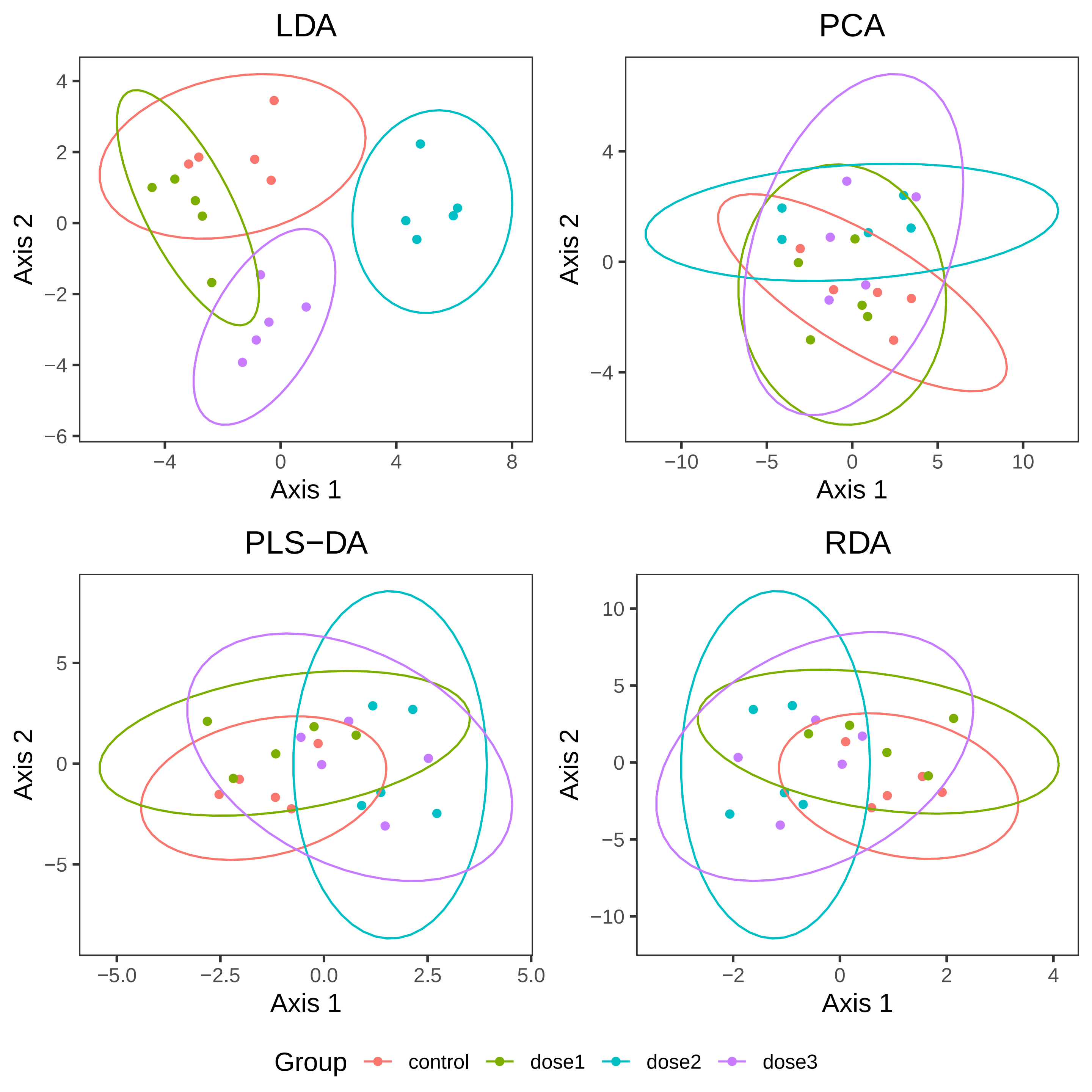

LDA
PCA
PLS−DA
RDA
Axis 1
Axis 2
Group
control
dose1
dose2
dose3

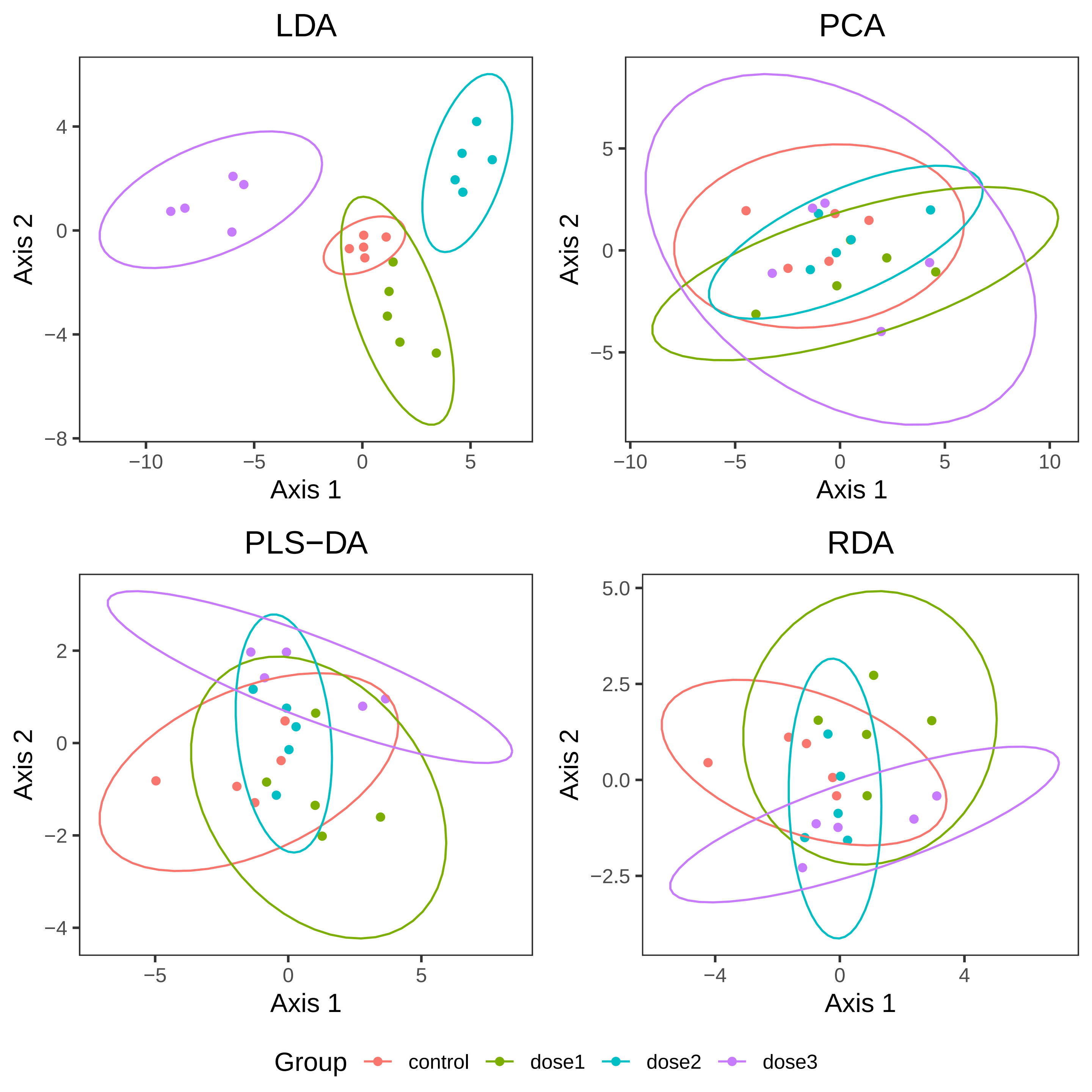

LDA
PCA
PLS−DA
RDA
Axis 1
Axis 2
Group
control
dose1
dose2
dose3

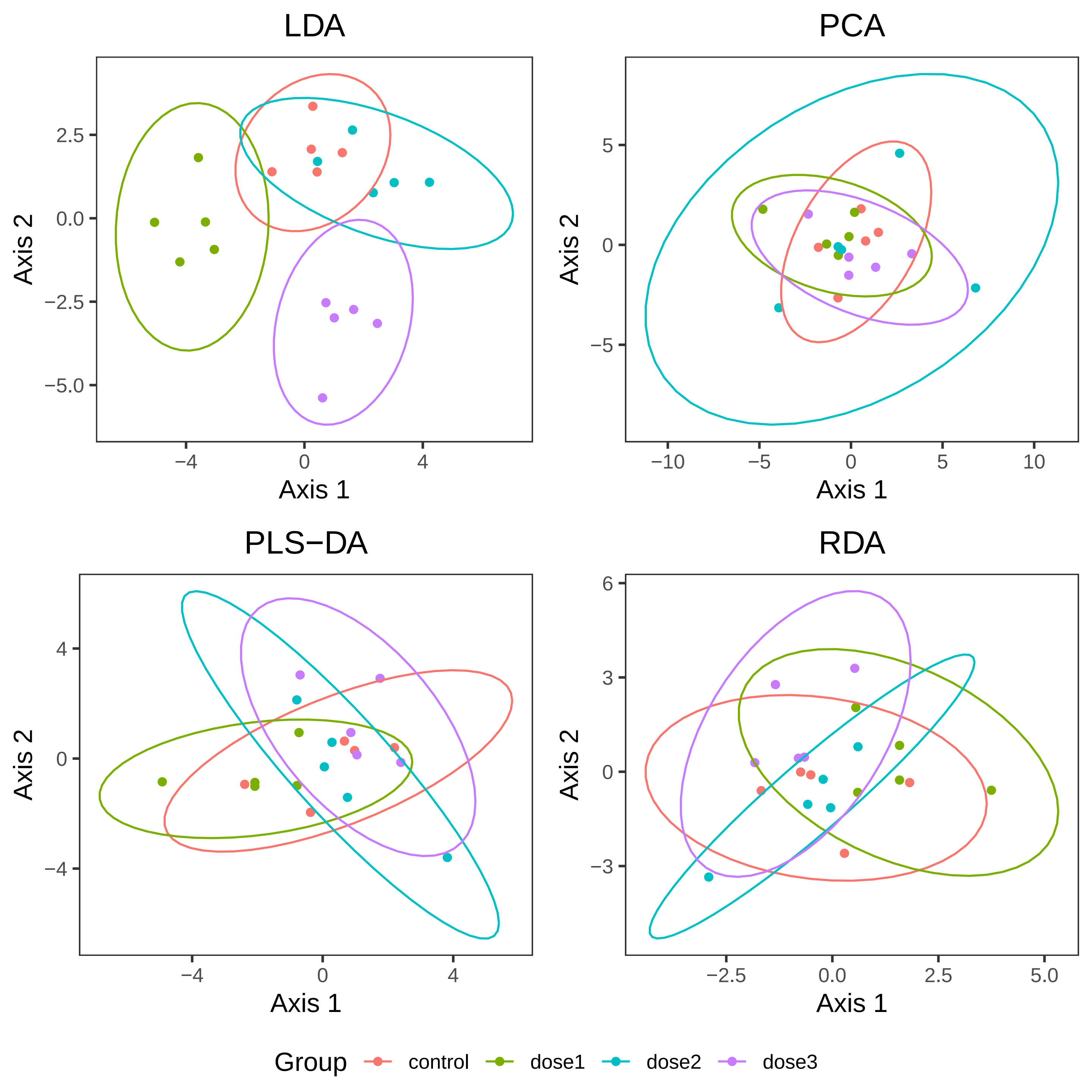
LDA
PCA
PLS−DA
RDA
Axis 1
Axis 2
2.5
0.0
−2.5
−5.0
−4
0
4
5
0
−5
−10
−5
0
5
10
4
0
−4
−4
0
4
6
3
0
−3
−2.5
0.0
2.5
5.0
Group
control
dose1
dose2
dose3

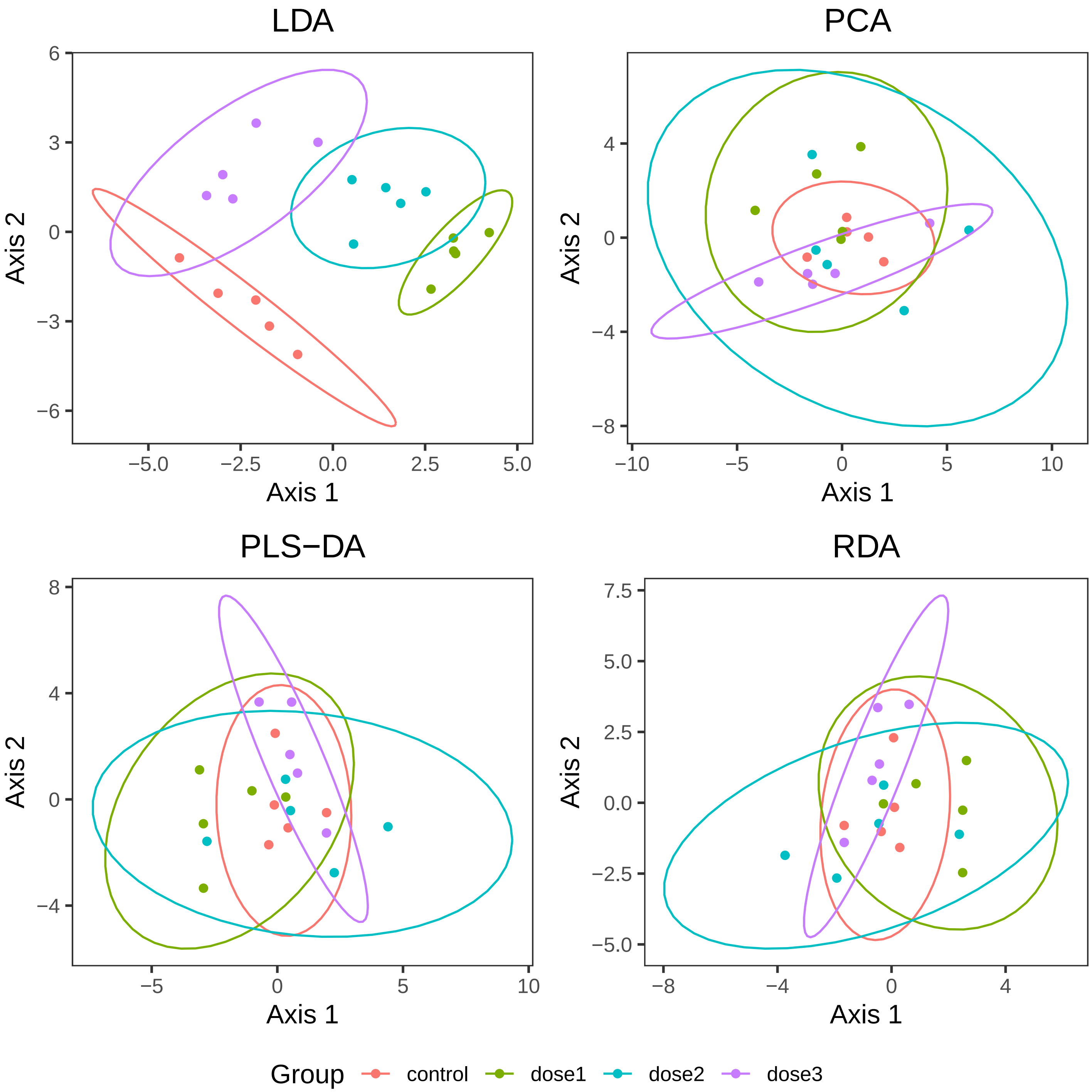
LDA
PCA
PLS−DA
RDA
Axis 1
Axis 2
Group
control
dose1
dose2
dose3

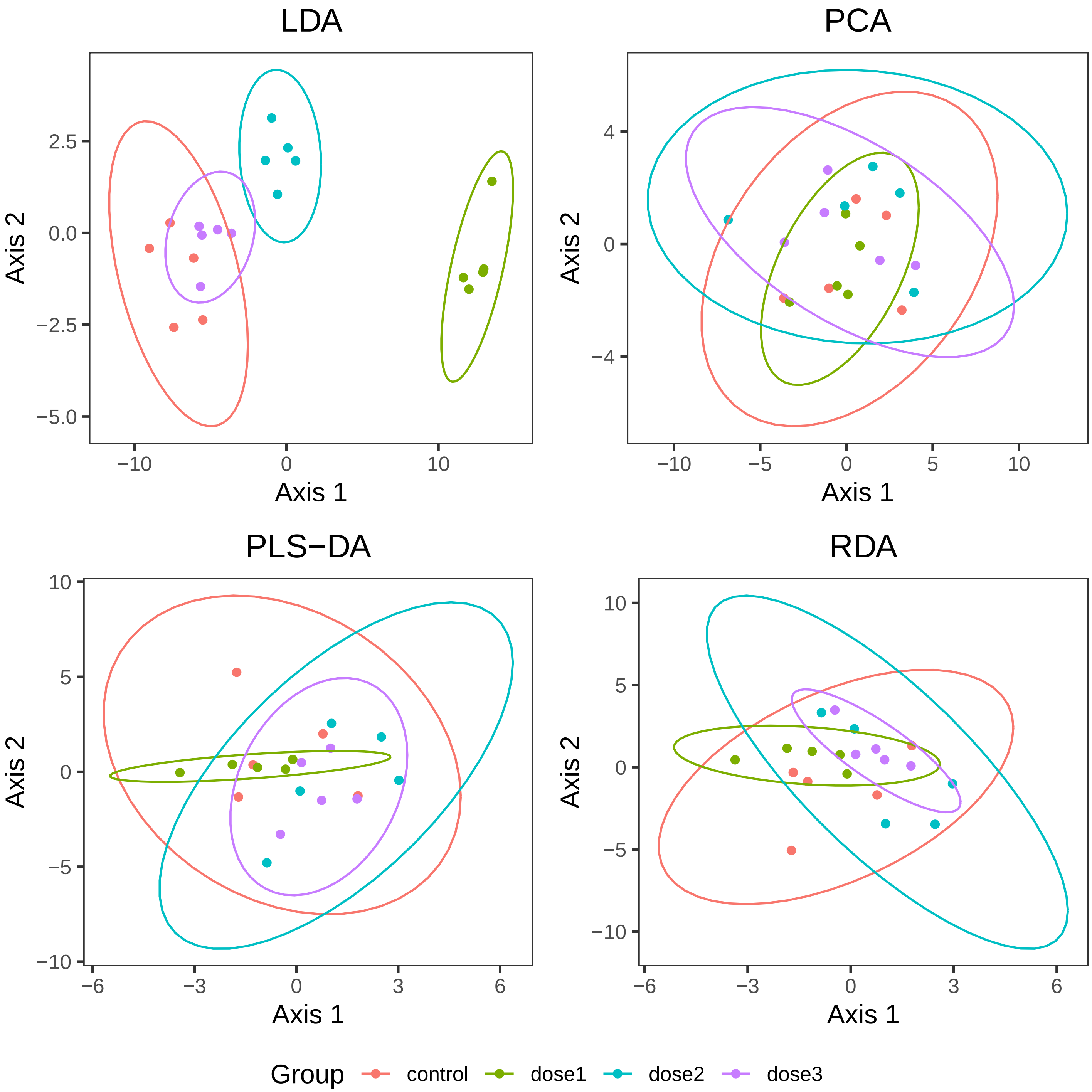
LDA
PCA
PLS−DA
RDA
Axis 1
Axis 2
2.5
0.0
−2.5
−5.0
−10
0
10
4
0
−4
−10
−5
0
5
10
10
5
0
−5
−10
−6
−3
0
3
6
Group
control
dose1
dose2
dose3

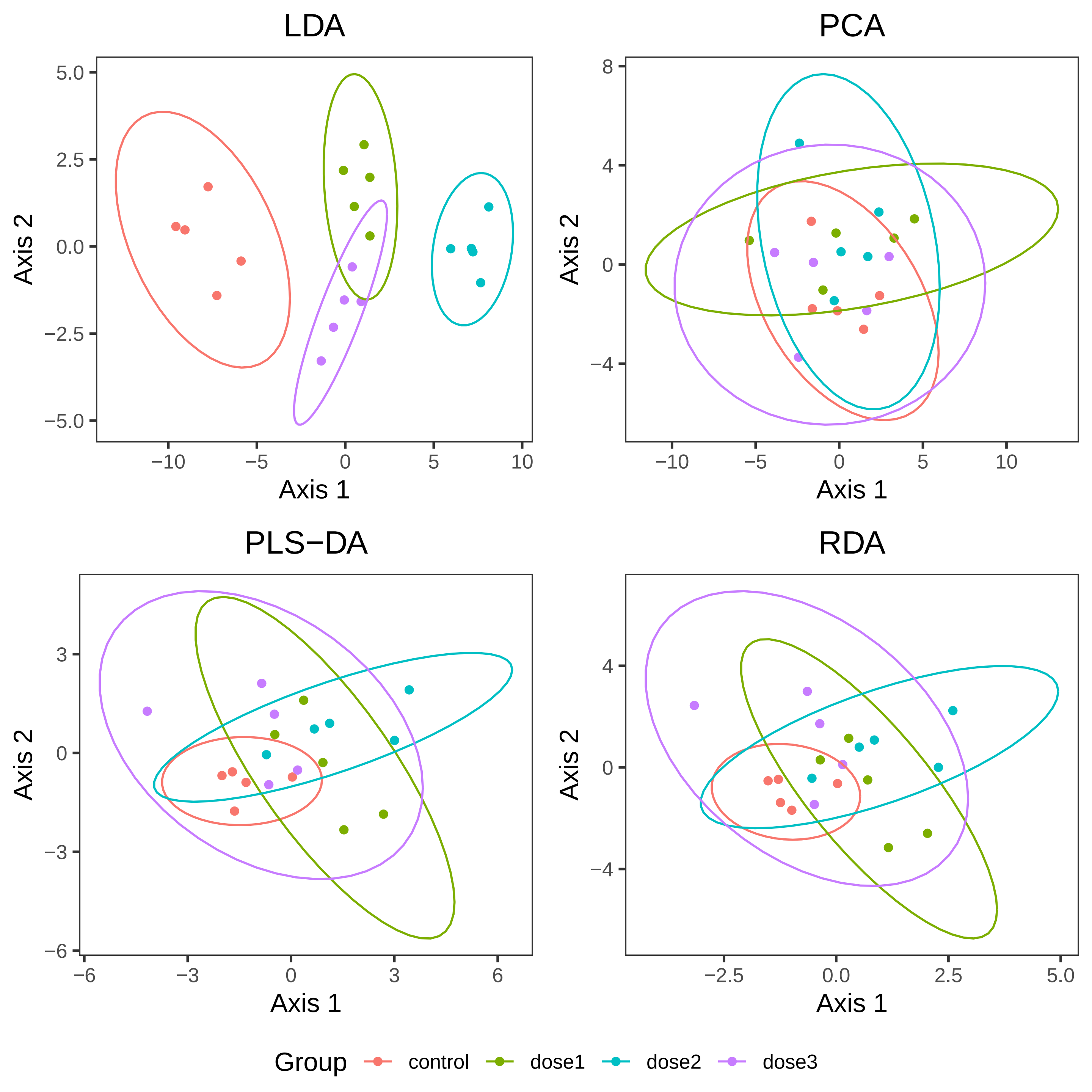

LDA
PCA
PLS−DA
RDA
Axis 1
Axis 2
Group
control
dose1
dose2
dose3

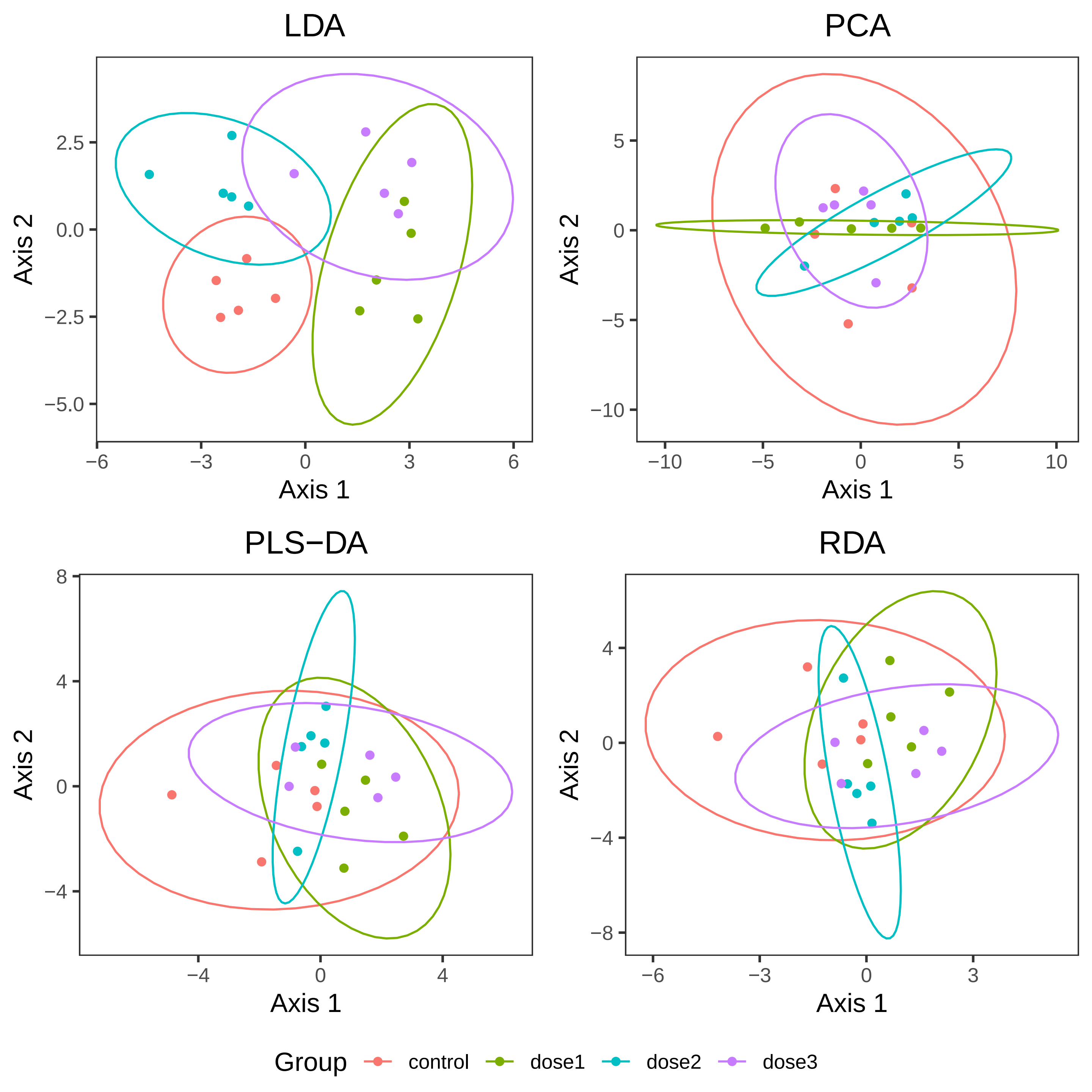
LDA
PCA
PLS−DA
RDA
Axis 1
Axis 2
Group
control
dose1
dose2
dose3

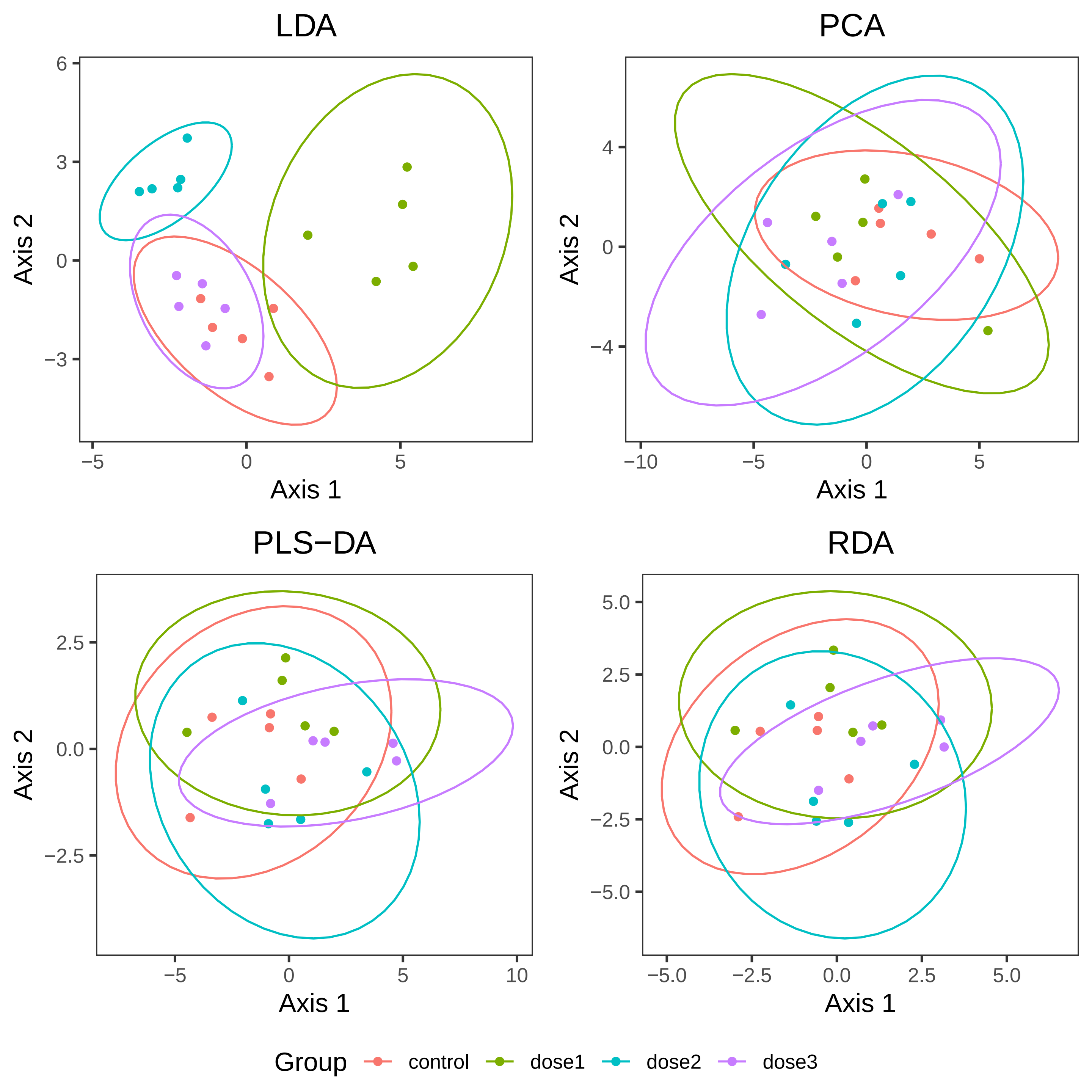

LDA
PCA
PLS−DA
RDA
Axis 1
Axis 2
Group
control
dose1
dose2
dose3

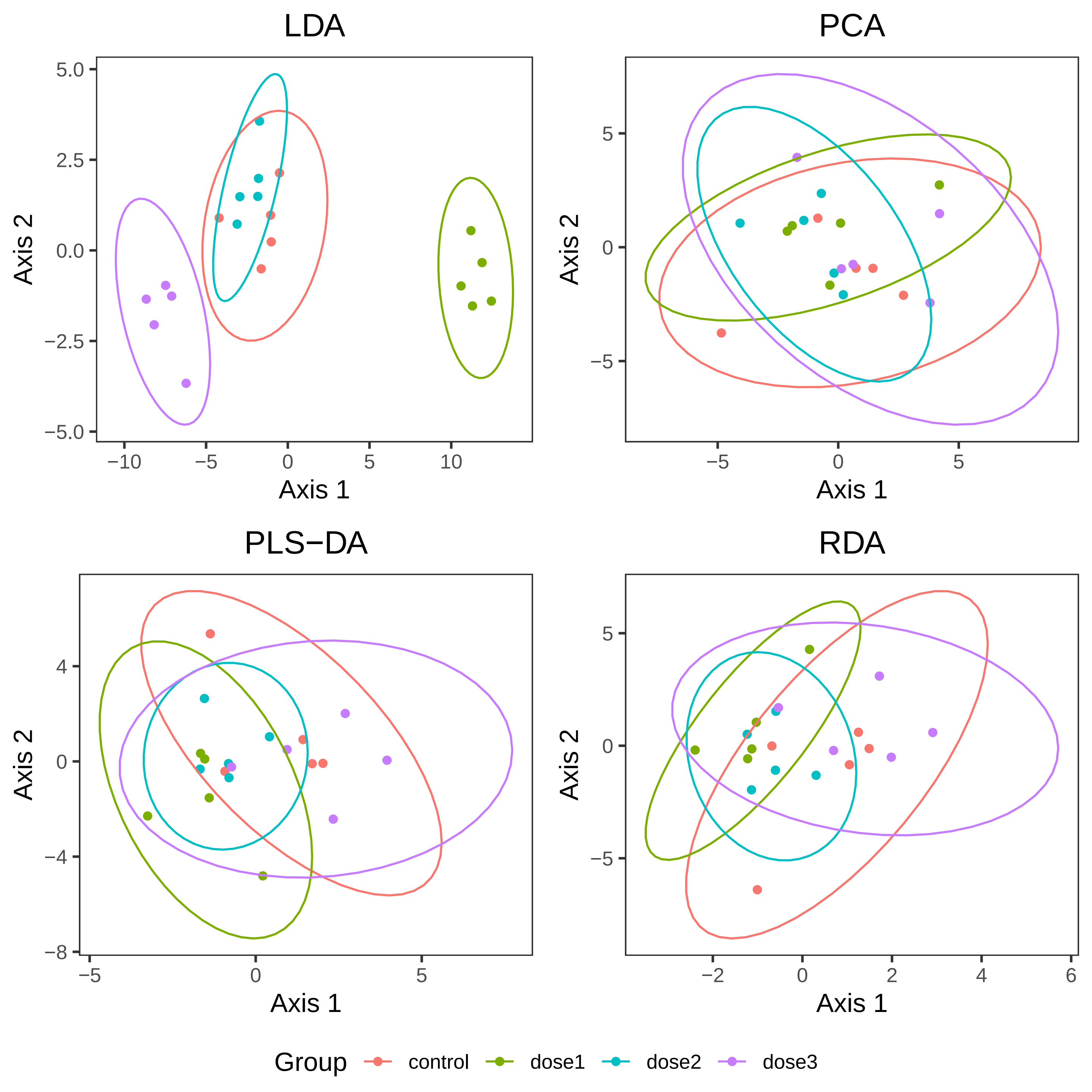

LDA
PCA
PLS−DA
RDA
Axis 1
Axis 2
5.0
2.5
0.0
−2.5
−5.0
−10
−5
0
5
10
4
−4
−8
−2
2
6
Group
control
dose1
dose2
dose3

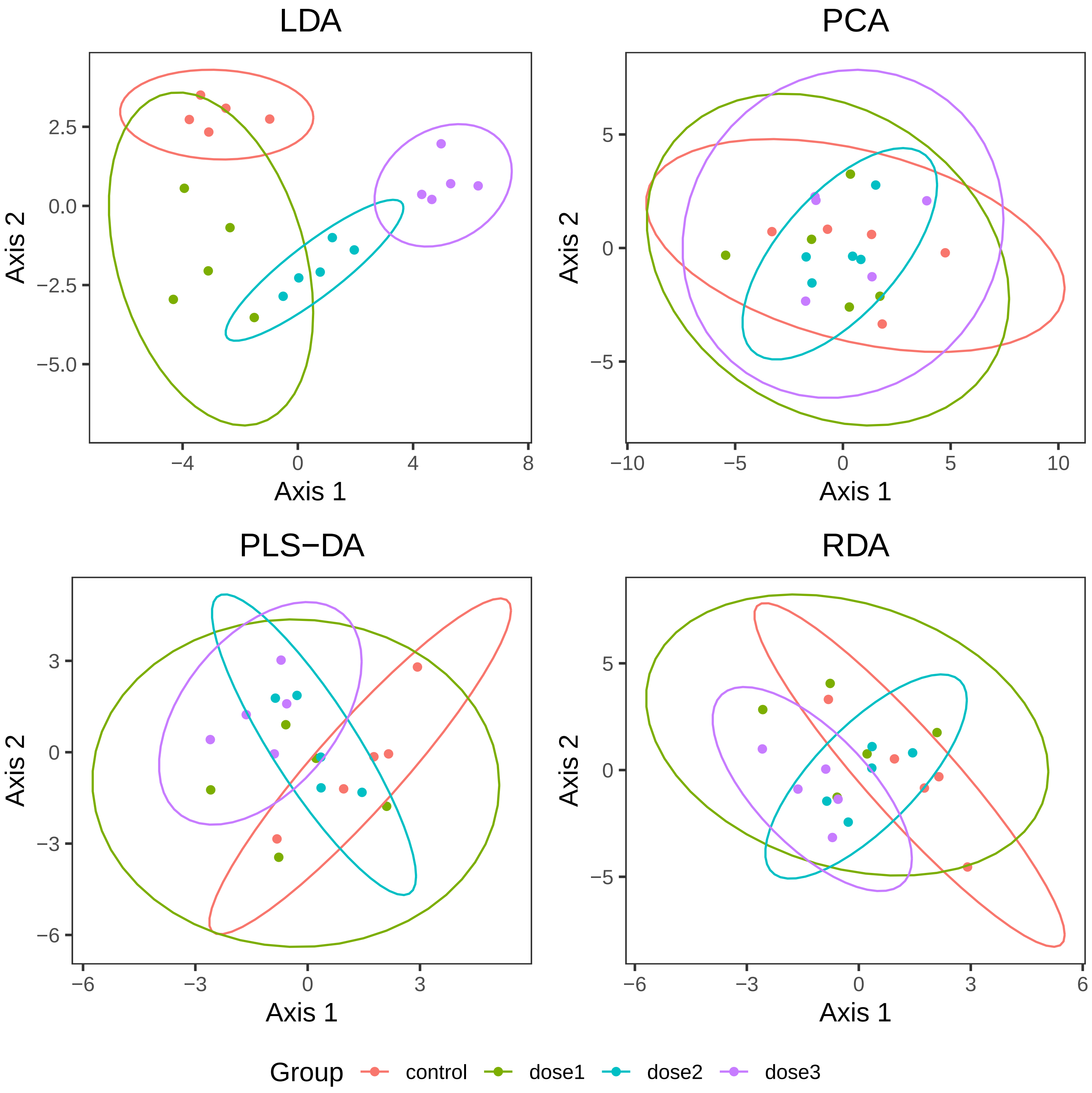
LDA
PCA
PLS−DA
RDA
Axis 1
Axis 2
Group
control
dose1
dose2
dose3

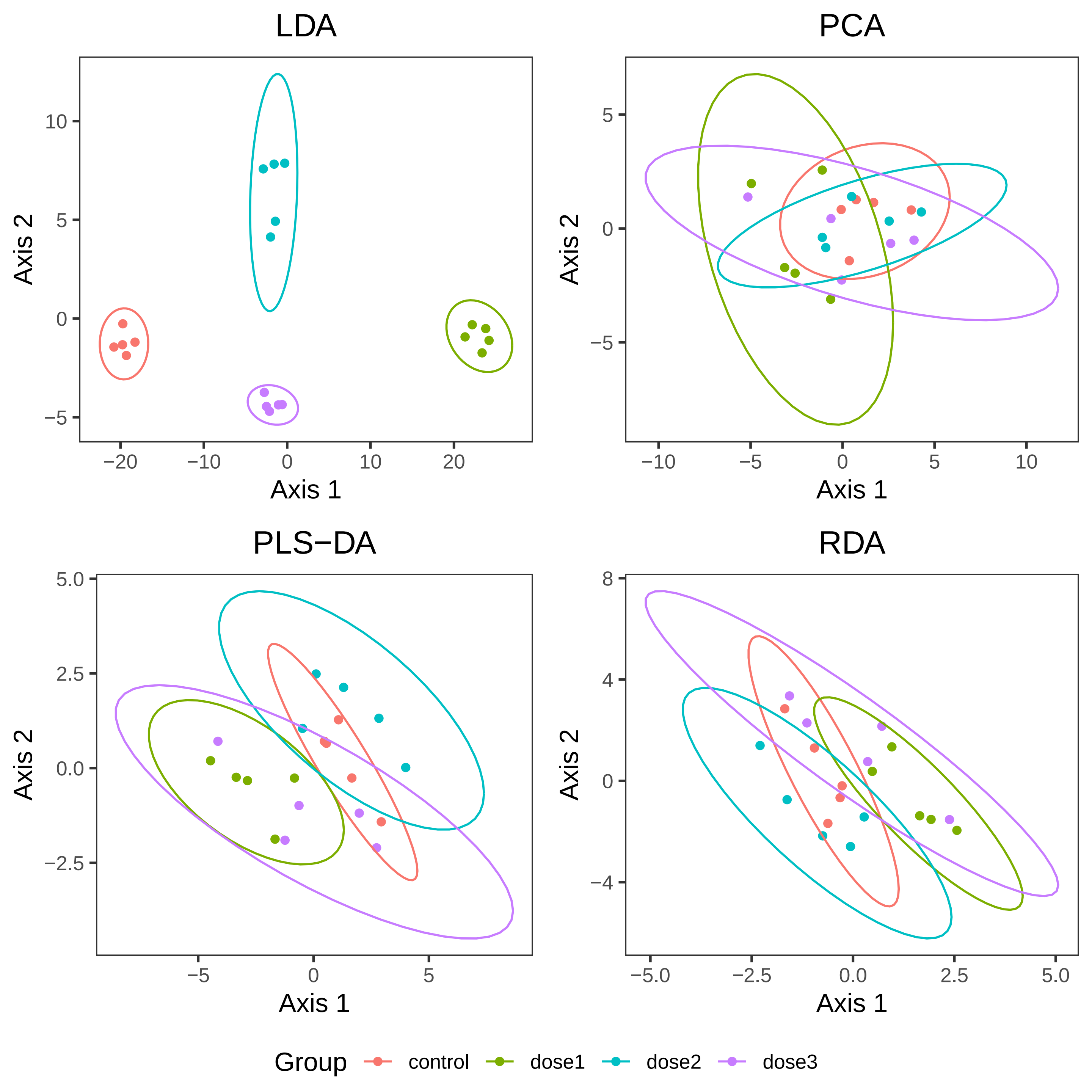

LDA
PCA
PLS−DA
RDA
Axis 1
Axis 2
Group
control
dose1
dose2
dose3

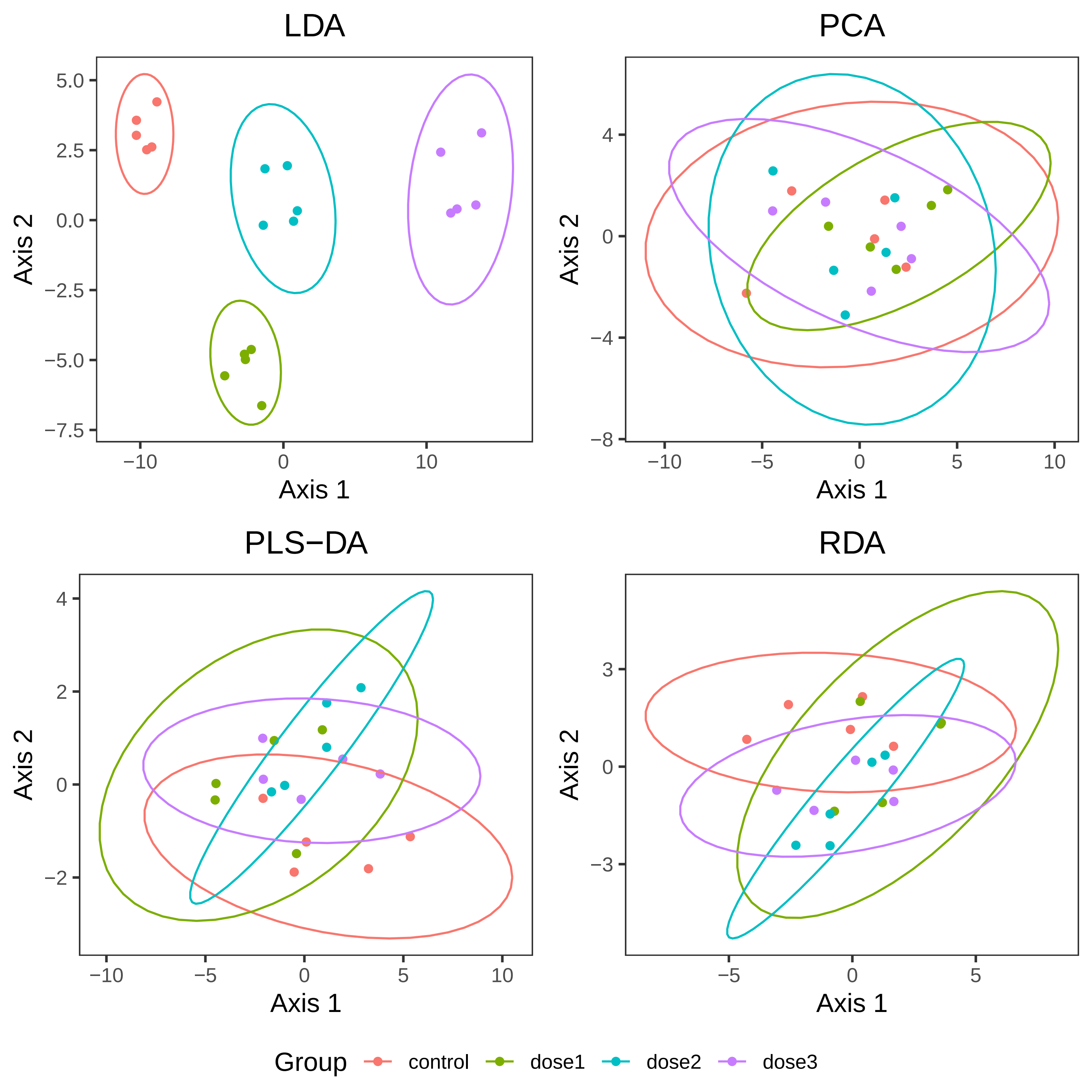

LDA
PCA
PLS−DA
RDA
Axis 1
Axis 2
5.0
2.5
0.0
−2.5
−5.0
−7.5
−10
0
10
4
0
−4
−8
−5
5
2
−2
3
−3
Group
control
dose1
dose2
dose3

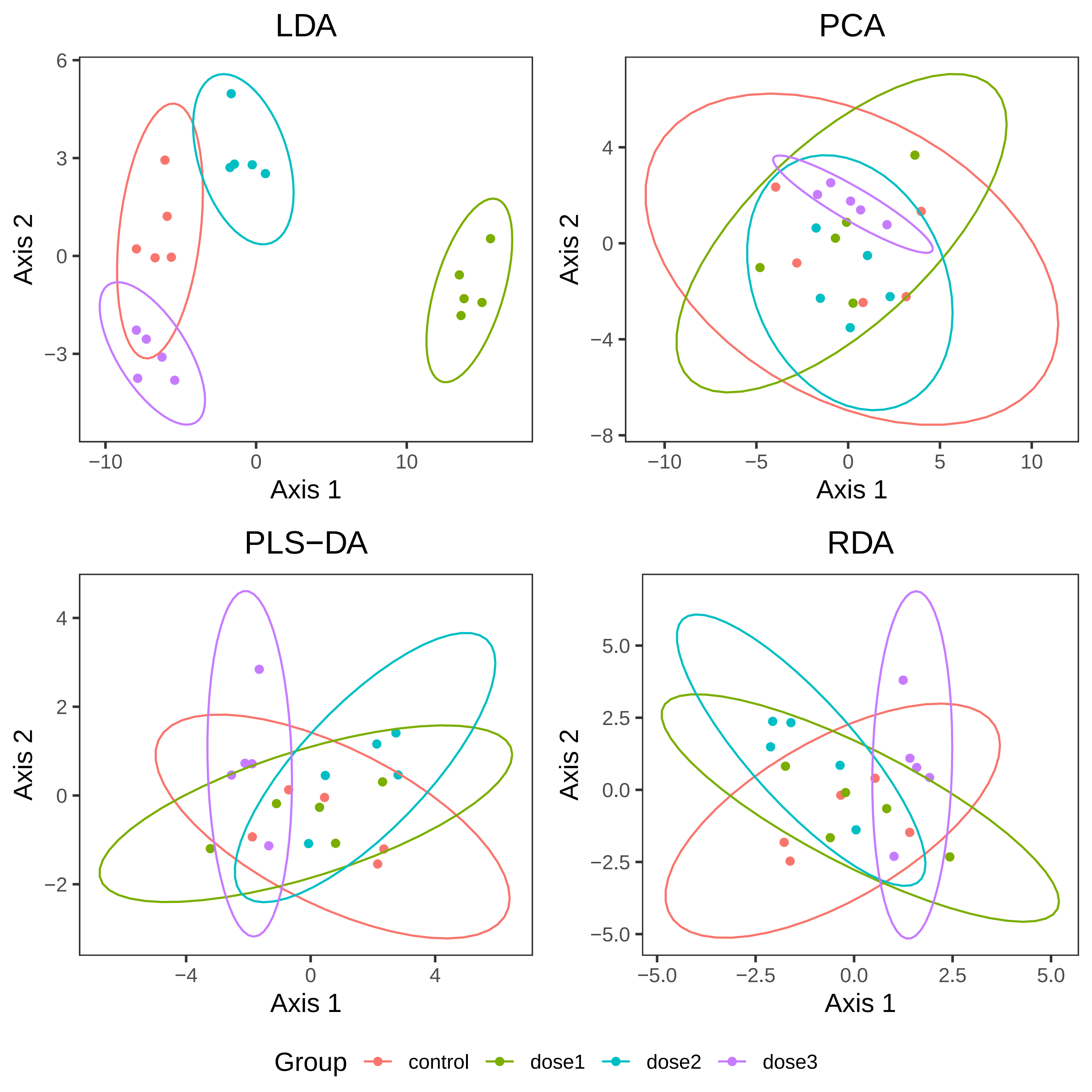

LDA
PCA
PLS−DA
RDA
Axis 1
Axis 2
6
3
0
−3
−10
0
10
4
0
−4
−8
−5
5
2
−2
−4
−5.0
−2.5
0.0
2.5
5.0
Group
control
dose1
dose2
dose3

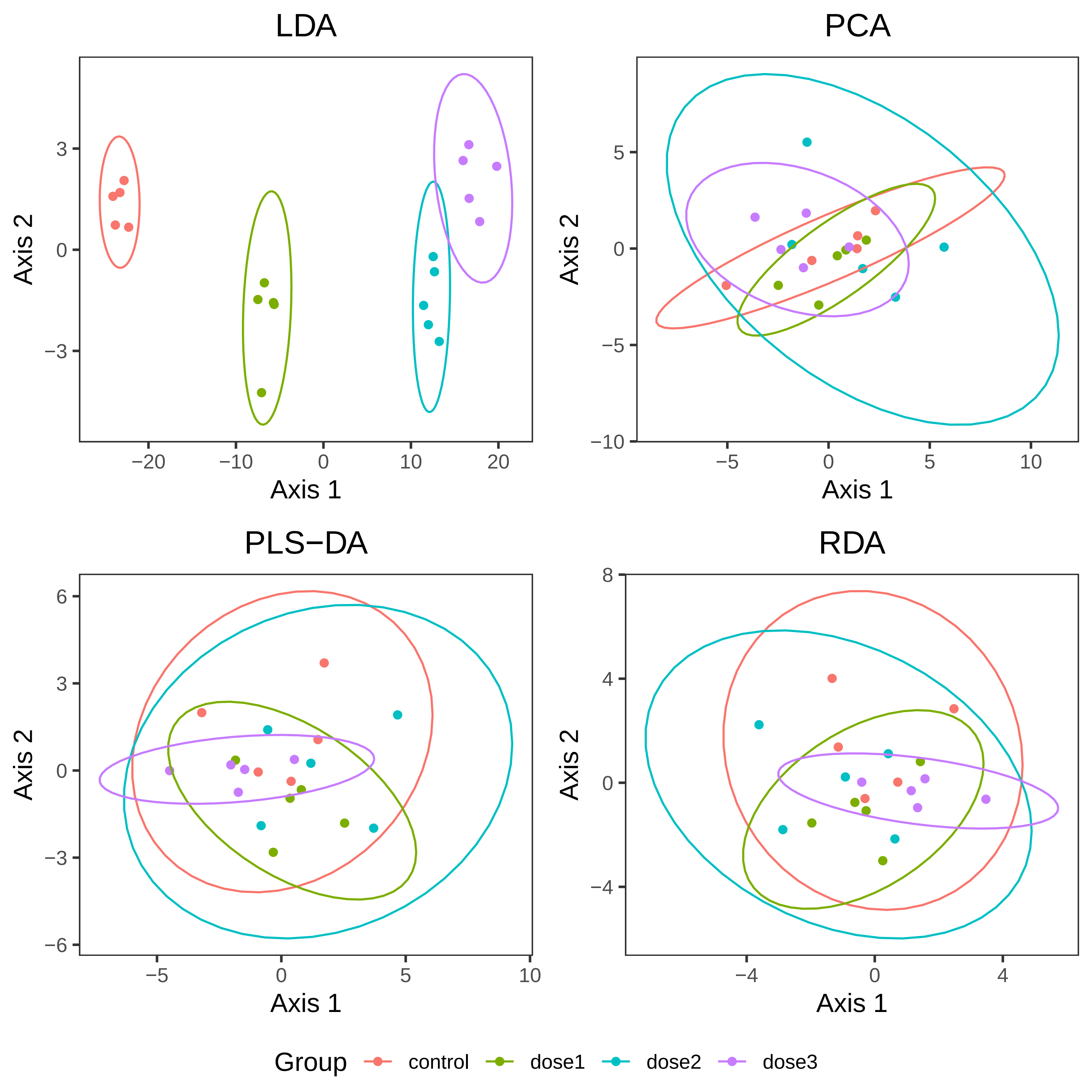
LDA
PCA
PLS−DA
RDA
Axis 1
Axis 2
Group
control
dose1
dose2
dose3

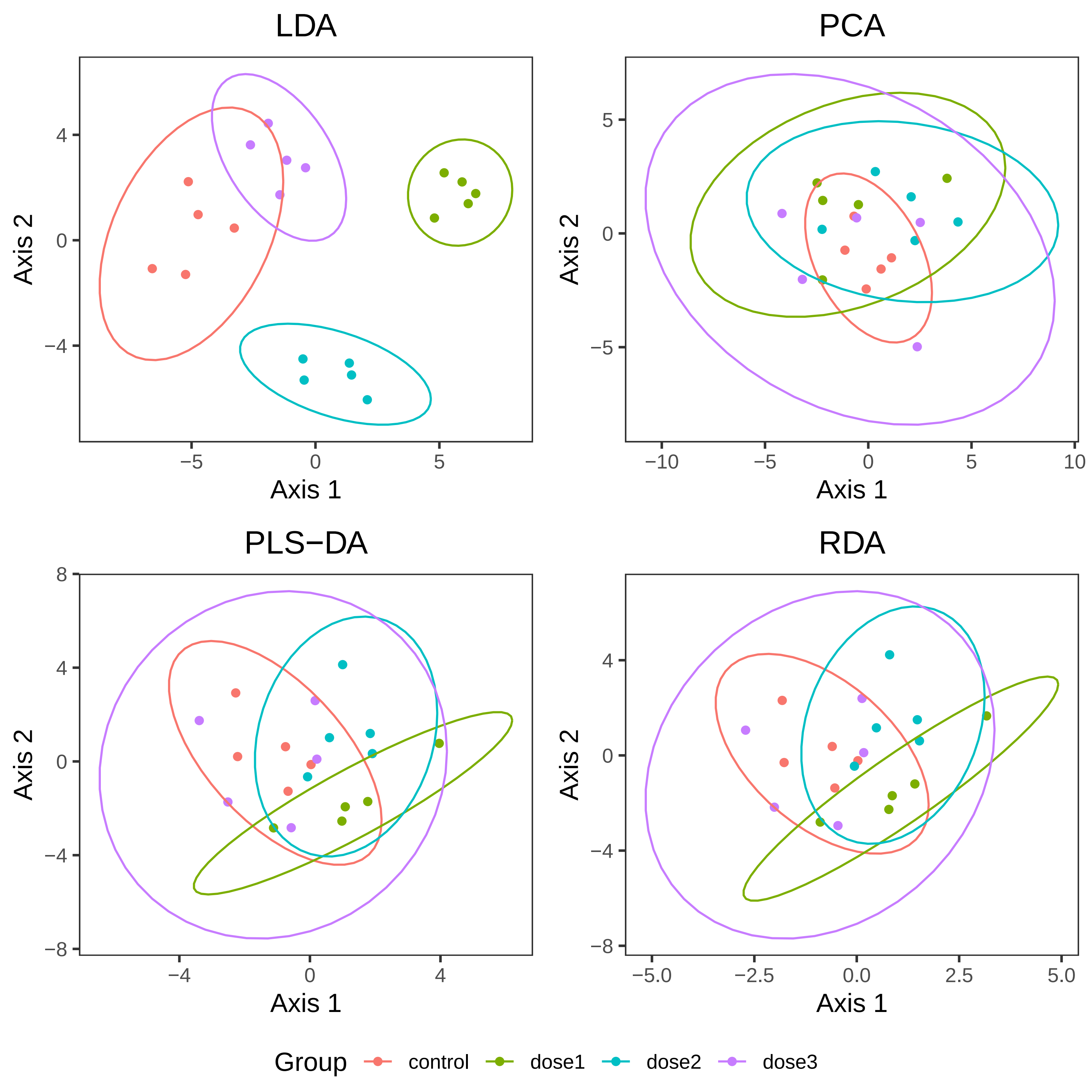
LDA
PCA
PLS−DA
RDA
Axis 1
Axis 2
Group
control
dose1
dose2
dose3

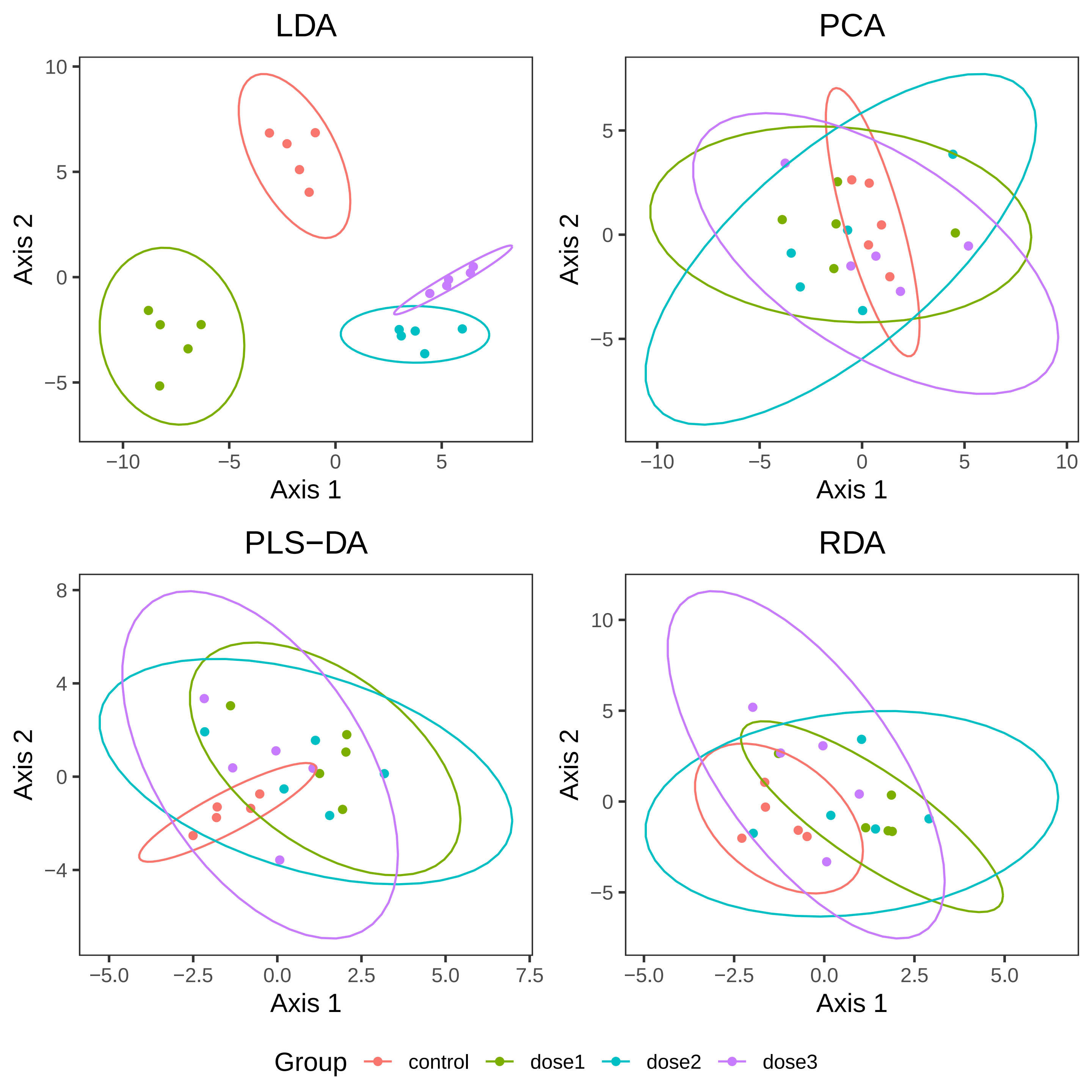

LDA
PCA
PLS−DA
RDA
Axis 1
Axis 2
Group
control
dose1
dose2
dose3

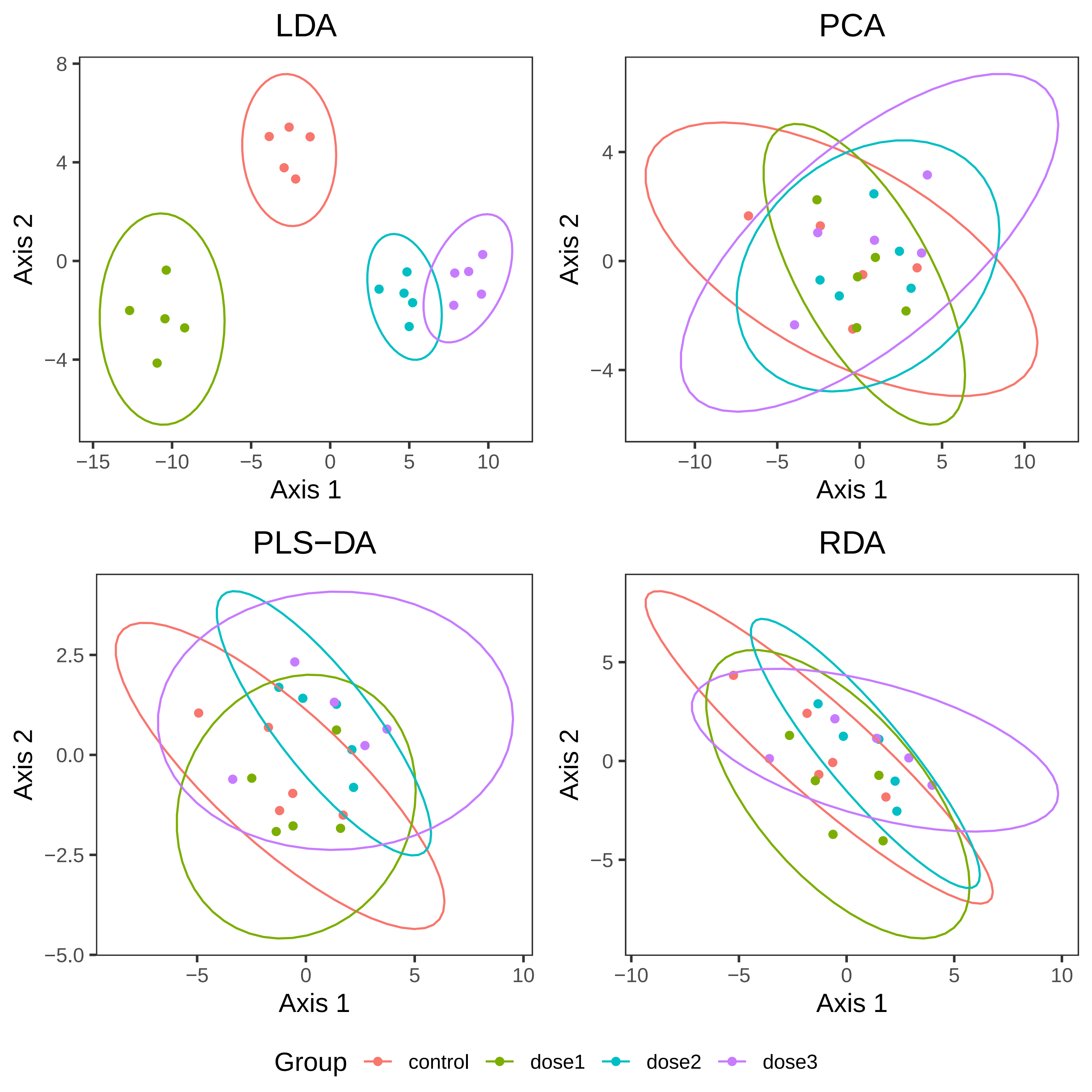

LDA
PCA
PLS−DA
RDA
Axis 1
Axis 2
8
4
0
−4
−15
−10
−5
0
5
10
2.5
0.0
−2.5
−5.0
Group
control
dose1
dose2
dose3

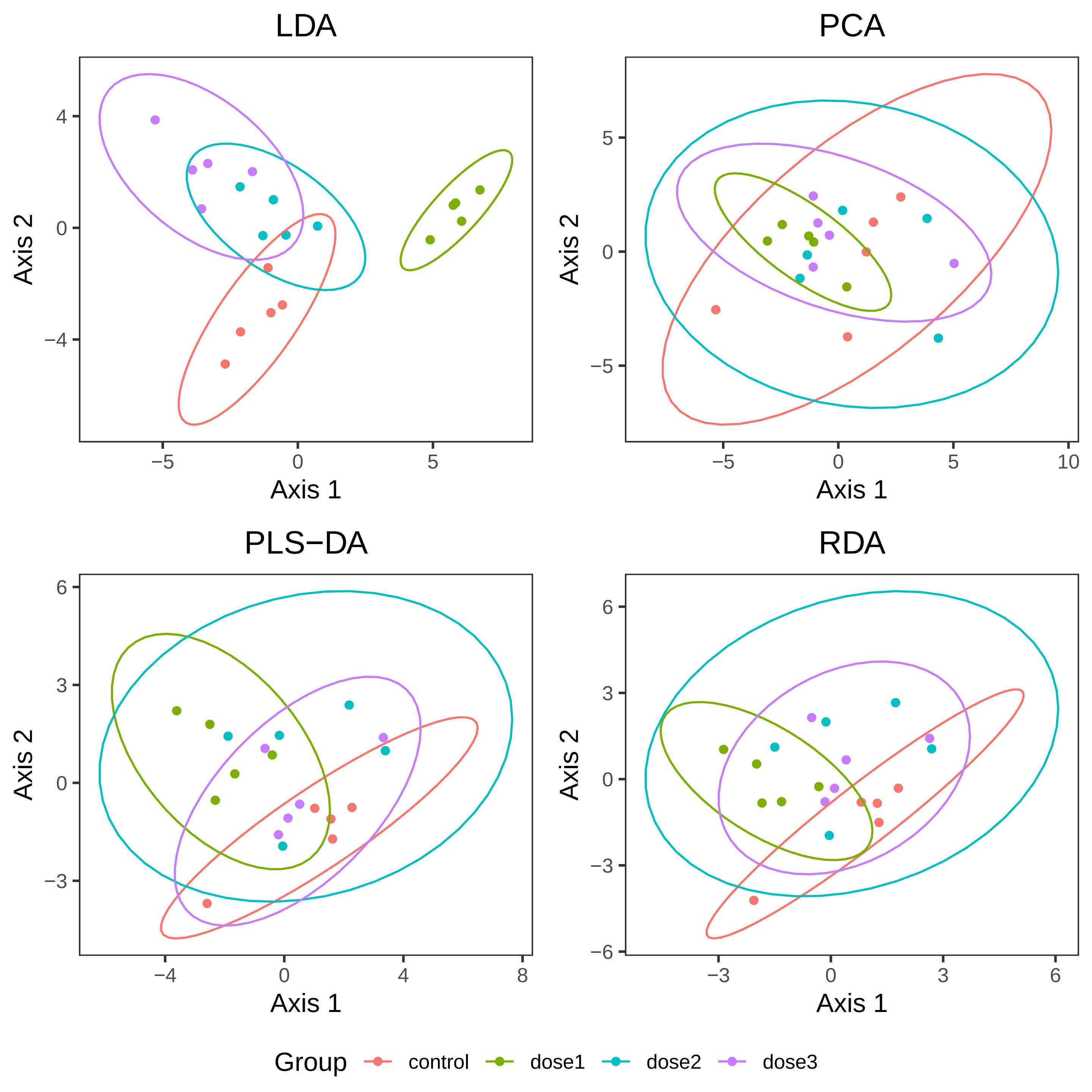

LDA
PCA
PLS−DA
RDA
Axis 1
Axis 2
Group
control
dose1
dose2
dose3

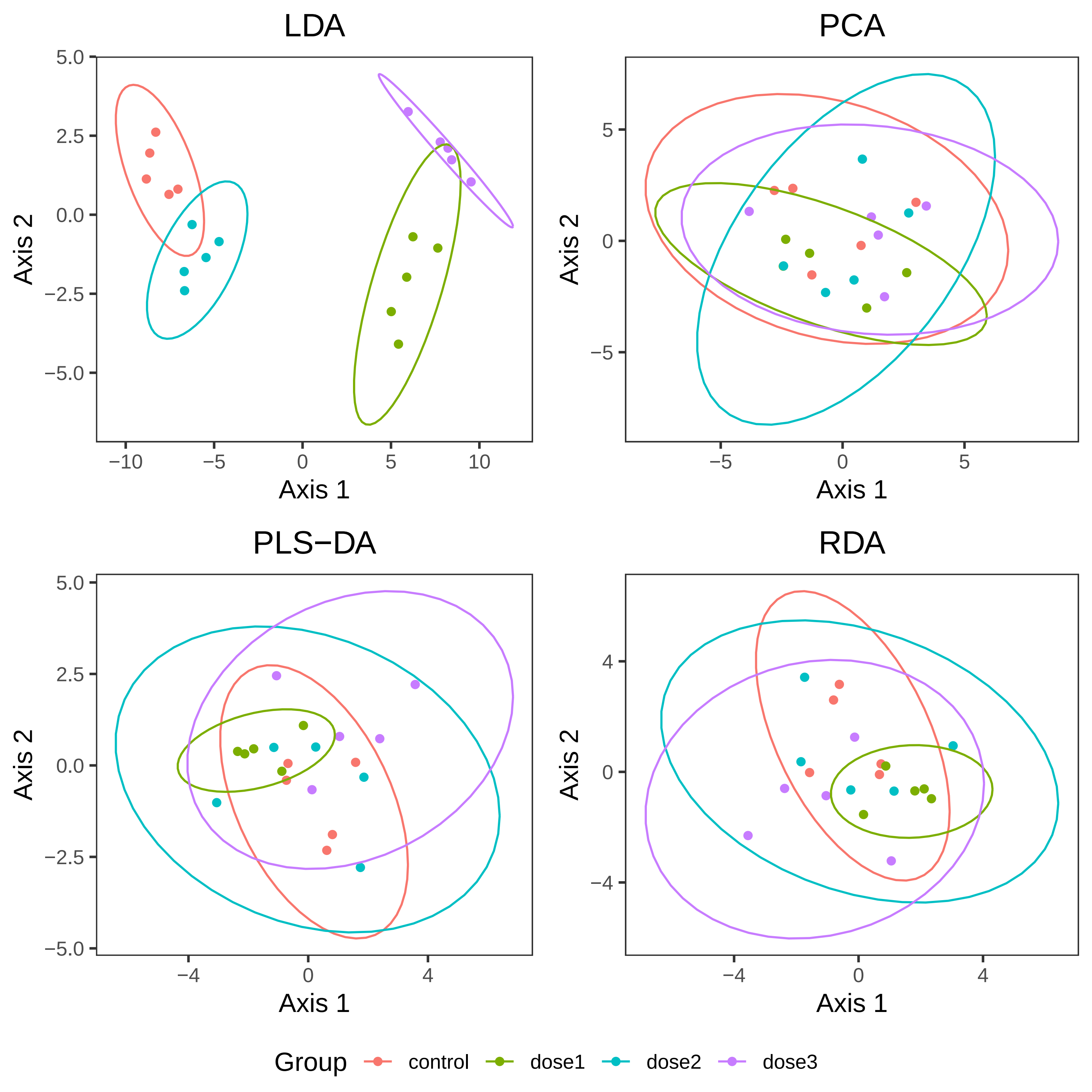
LDA
PCA
PLS−DA
RDA
Axis 1
Axis 2
5.0
2.5
0.0
−2.5
−5.0
−10
−5
0
5
10
−4
4
Group
control
dose1
dose2
dose3

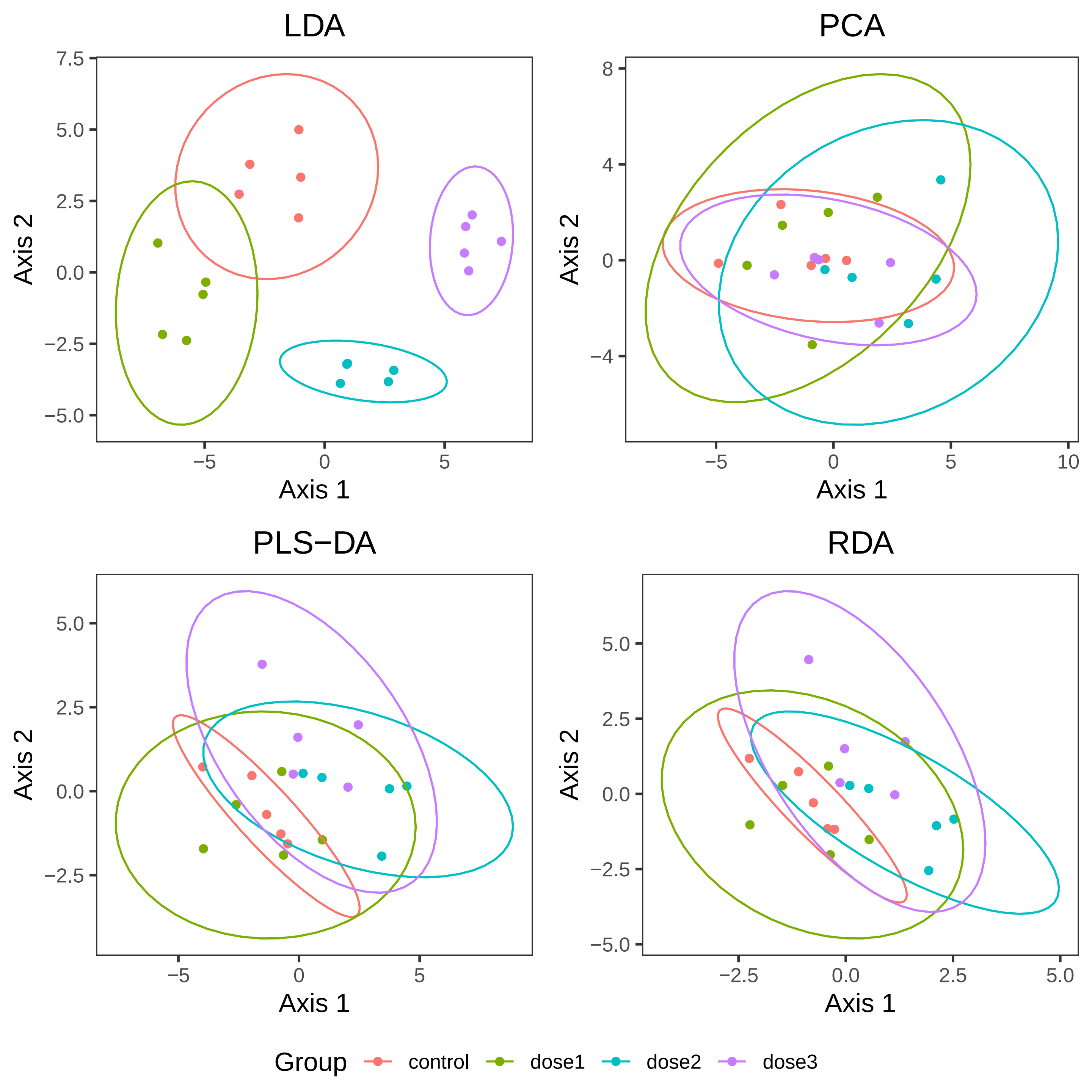
LDA
PCA
PLS−DA
RDA
Axis 1
Axis 2
7.5
5.0
2.5
0.0
−2.5
−5.0
−5
0
5
8
4
−4
10
−2.5
0.0
2.5
5.0
Group
control
dose1
dose2
dose3

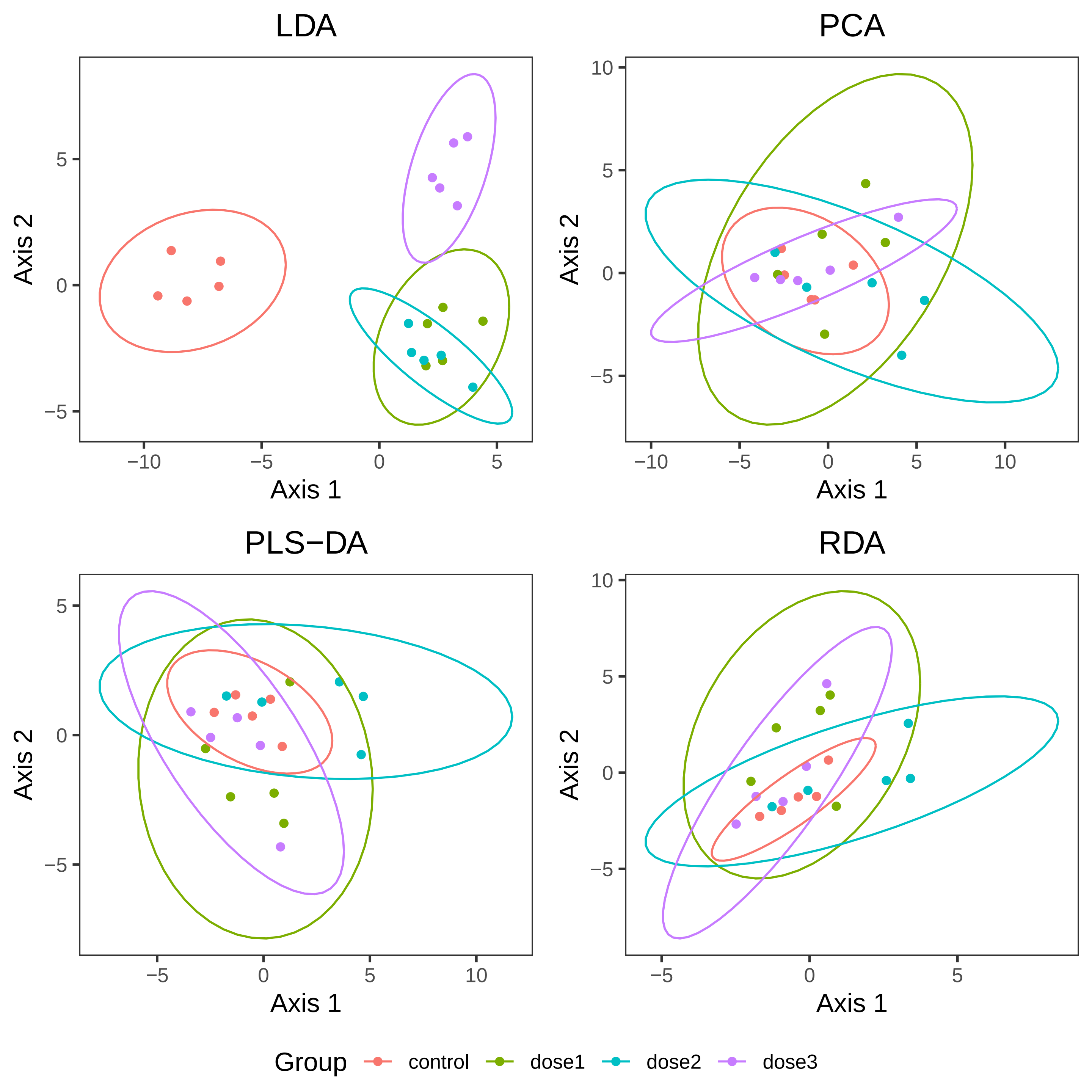
LDA
PCA
PLS−DA
RDA
Axis 1
Axis 2
Group
control
dose1
dose2
dose3

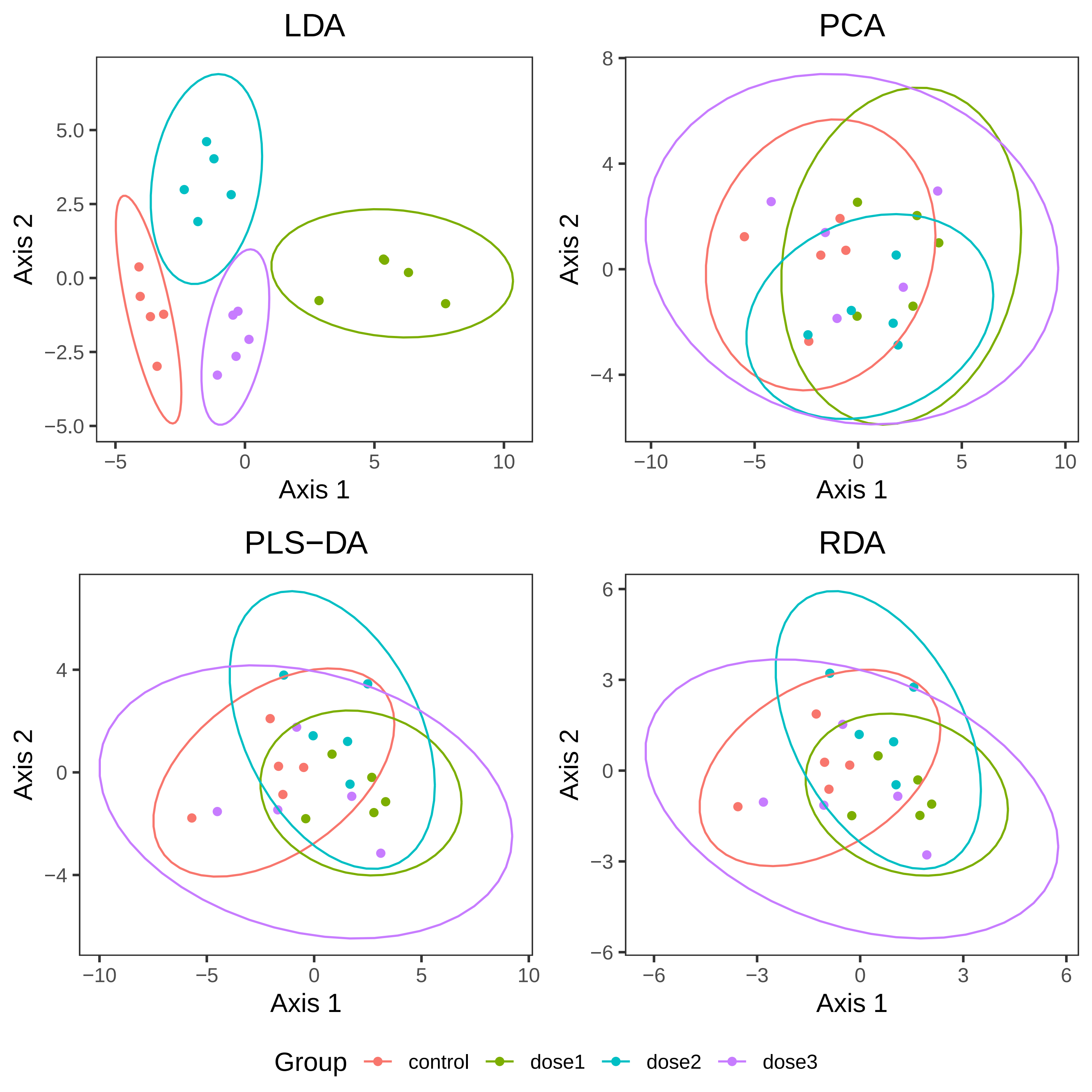
LDA
PCA
PLS−DA
RDA
Axis 1
Axis 2
5.0
2.5
0.0
−2.5
−5.0
−5
0
5
10
8
4
0
−4
−10
6
3
−3
−6
Group
control
dose1
dose2
dose3

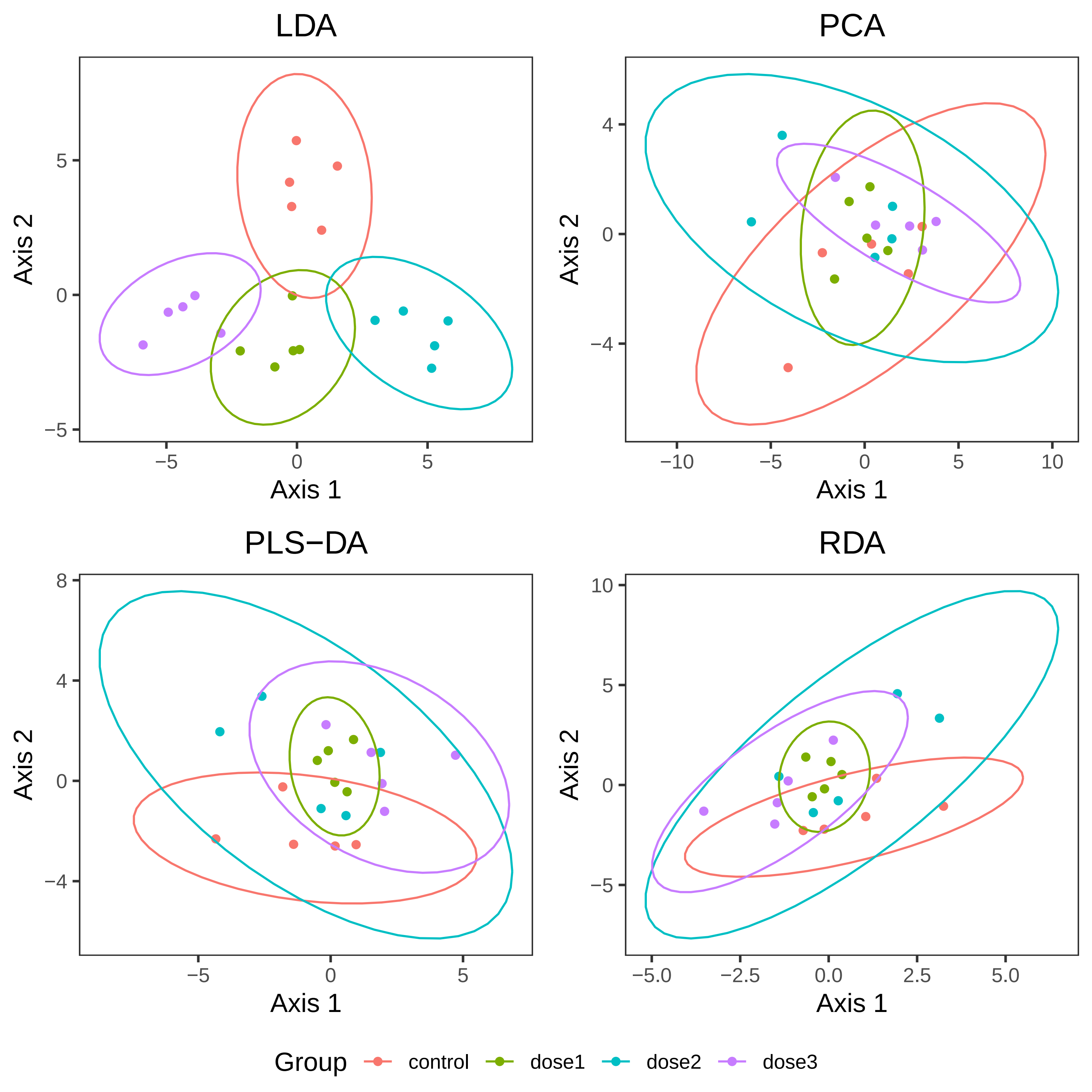

LDA
PCA
PLS−DA
RDA
Axis 1
Axis 2
Group
control
dose1
dose2
dose3

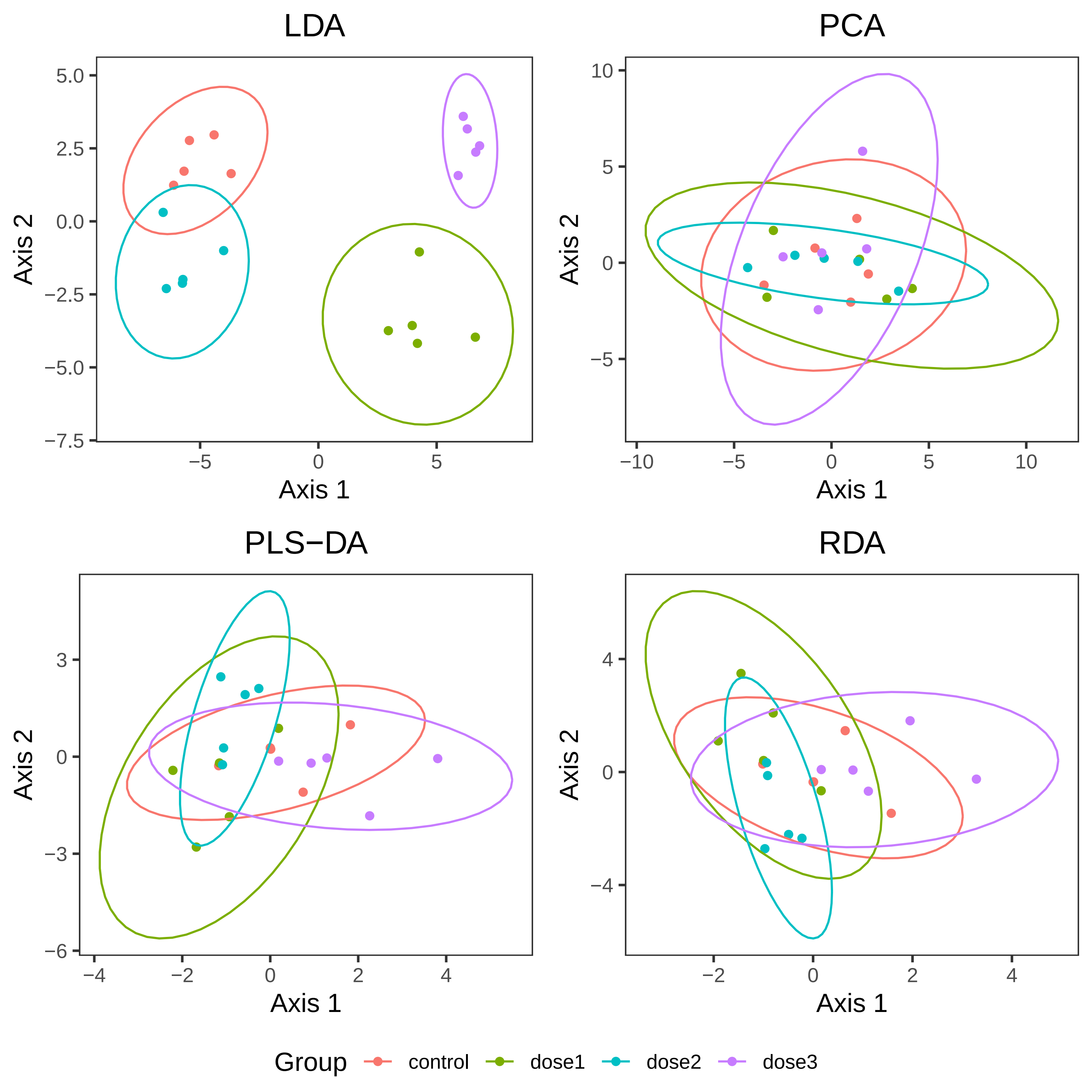

LDA
PCA
PLS−DA
RDA
Axis 1
Axis 2
Group
control
dose1
dose2
dose3

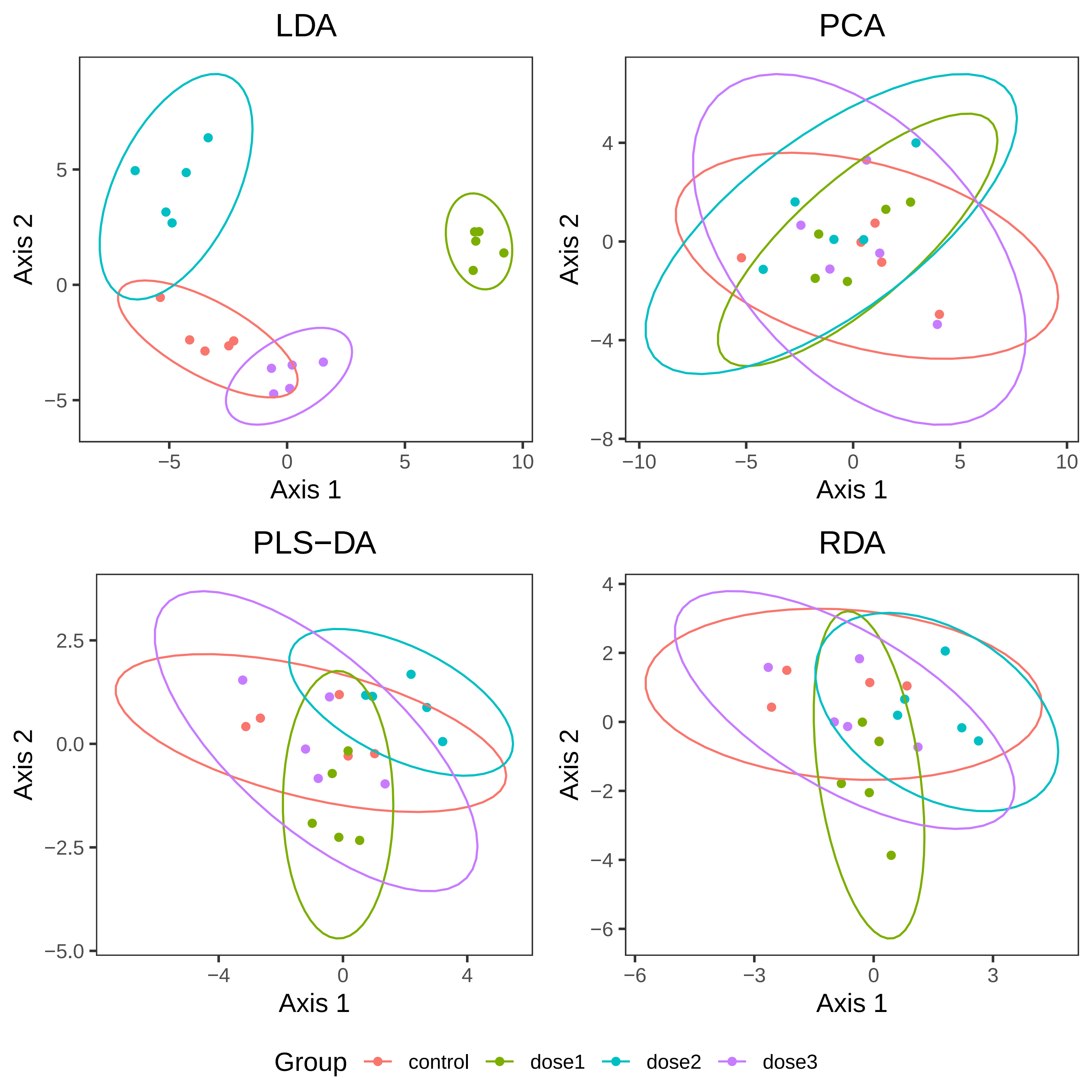

LDA
PCA
PLS−DA
RDA
Axis 1
Axis 2
Group
control
dose1
dose2
dose3

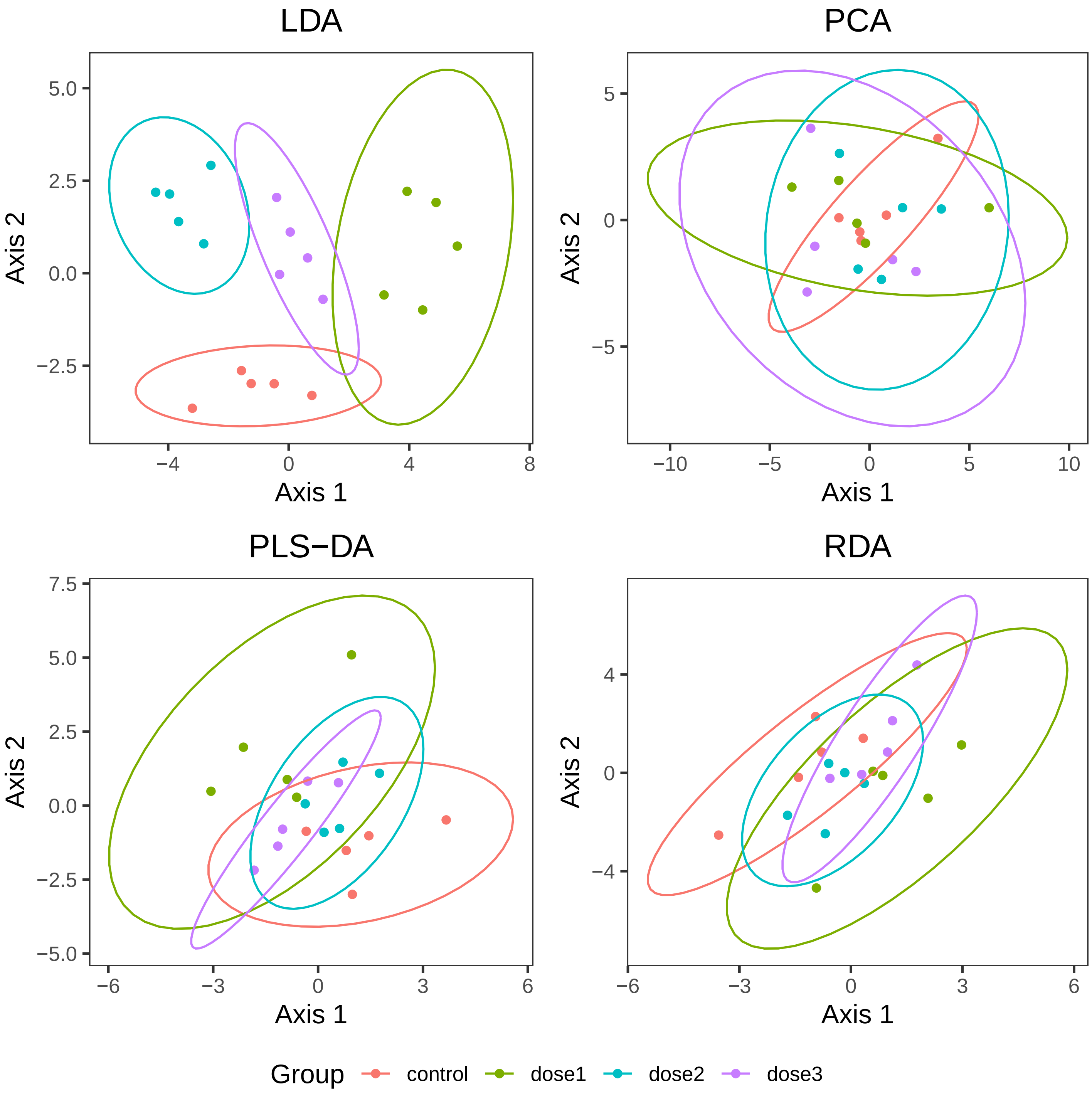
LDA
PCA
PLS−DA
RDA
Axis 1
Axis 2
5.0
2.5
0.0
−2.5
−4
0
4
8
5
0
−5
−10
−5
0
5
10
7.5
5.0
2.5
0.0
−2.5
−5.0
−6
−3
0
3
6
4
0
−4
−6
−3
0
3
6
Group
control
dose1
dose2
dose3

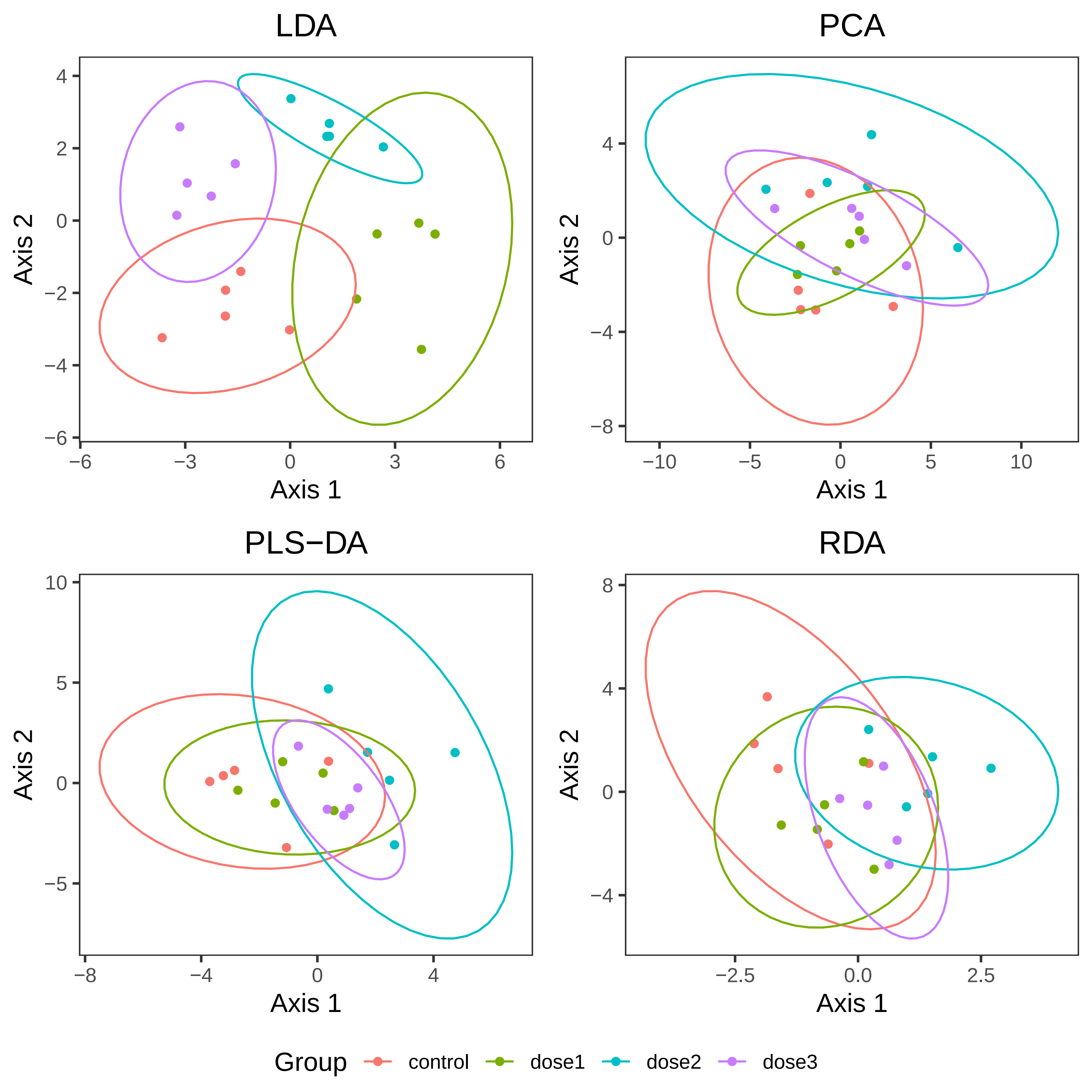

LDA
PCA
PLS−DA
RDA
Axis 1
Axis 2
Group
control
dose1
dose2
dose3

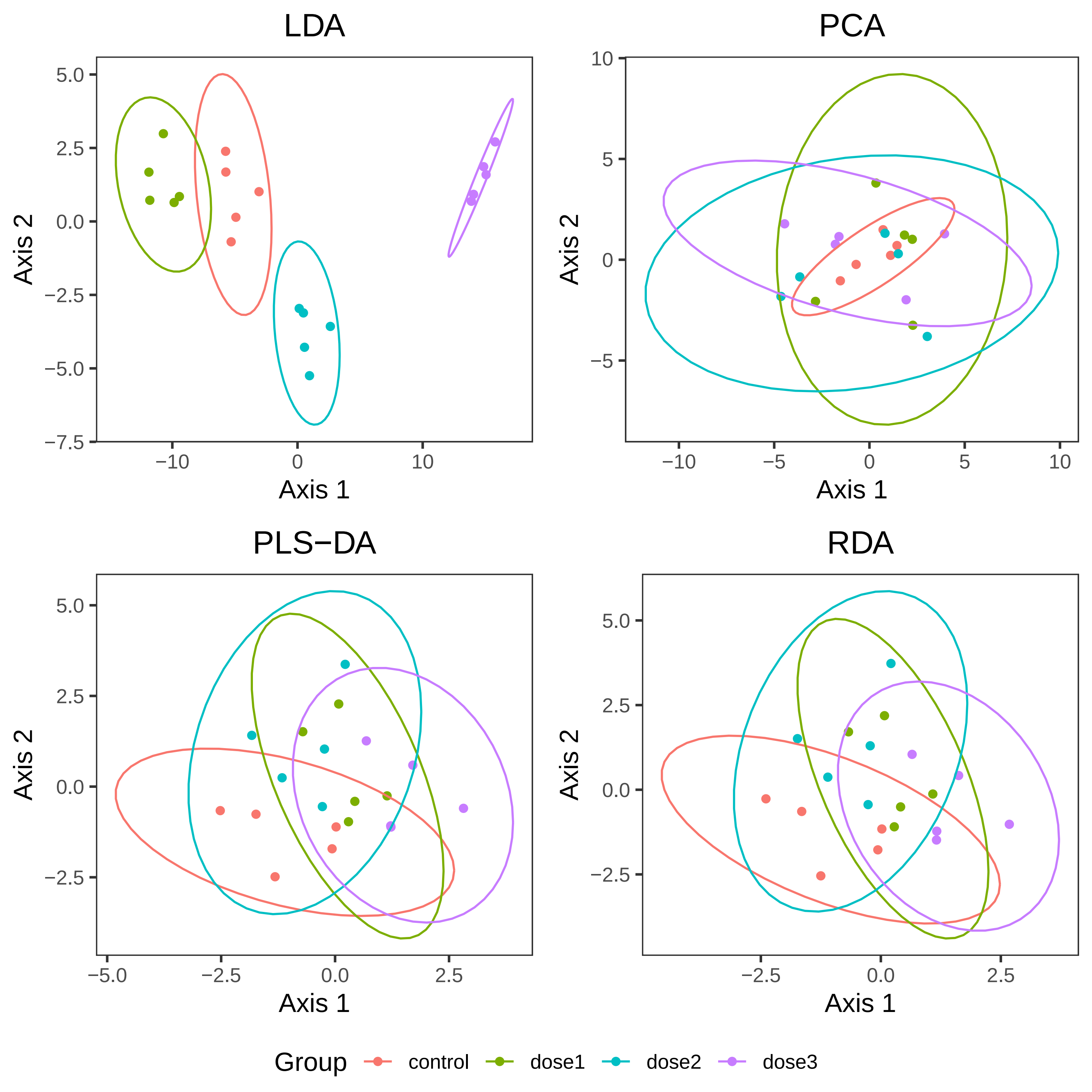

LDA
PCA
PLS−DA
RDA
Axis 1
Axis 2
5.0
2.5
0.0
−2.5
−5.0
−7.5
−10
0
10
−5
5
Group
control
dose1
dose2
dose3

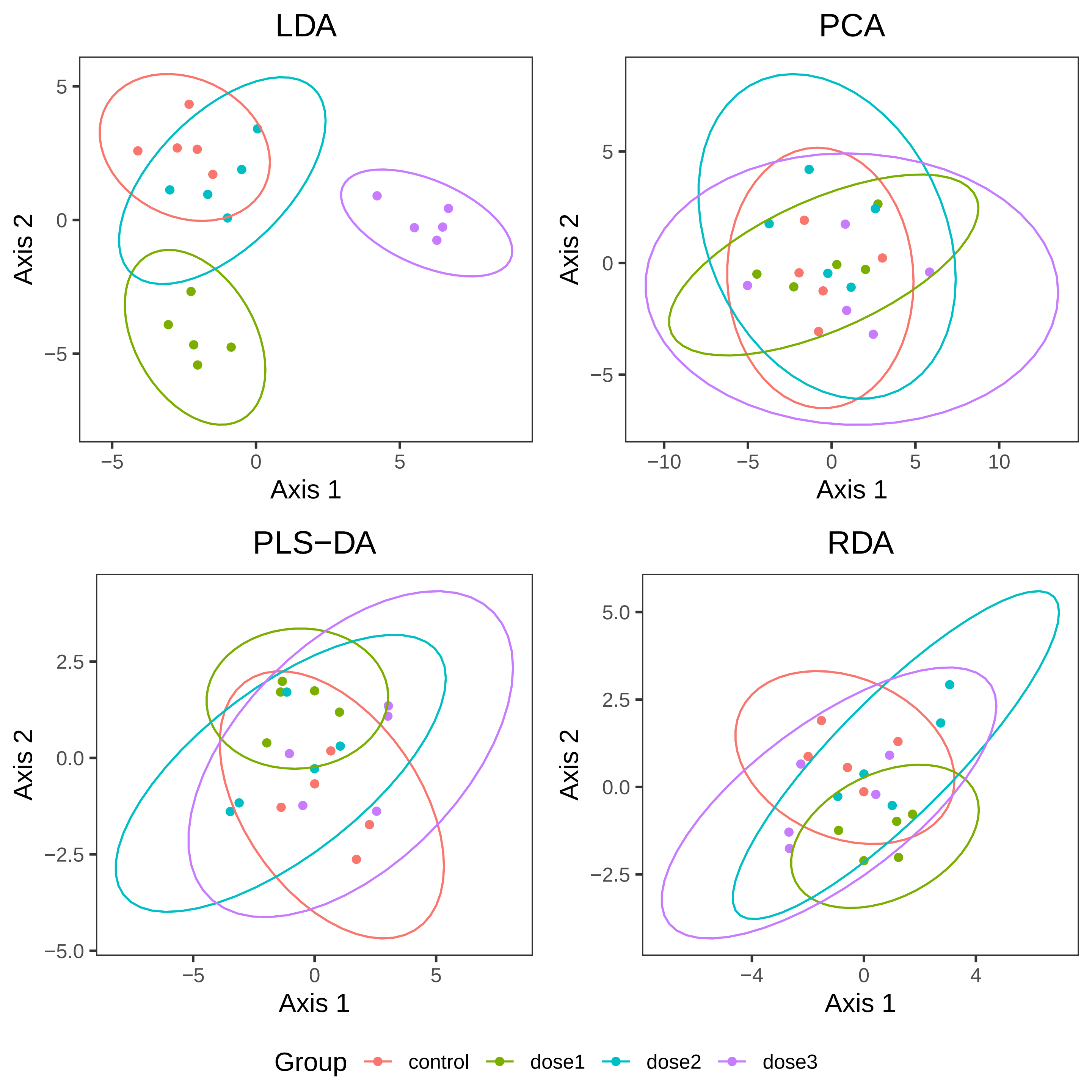
LDA
PCA
PLS−DA
RDA
Axis 1
Axis 2
5
0
−5
10
−10
2.5
0.0
−2.5
−5.0
5.0
4
−4
Group
control
dose1
dose2
dose3

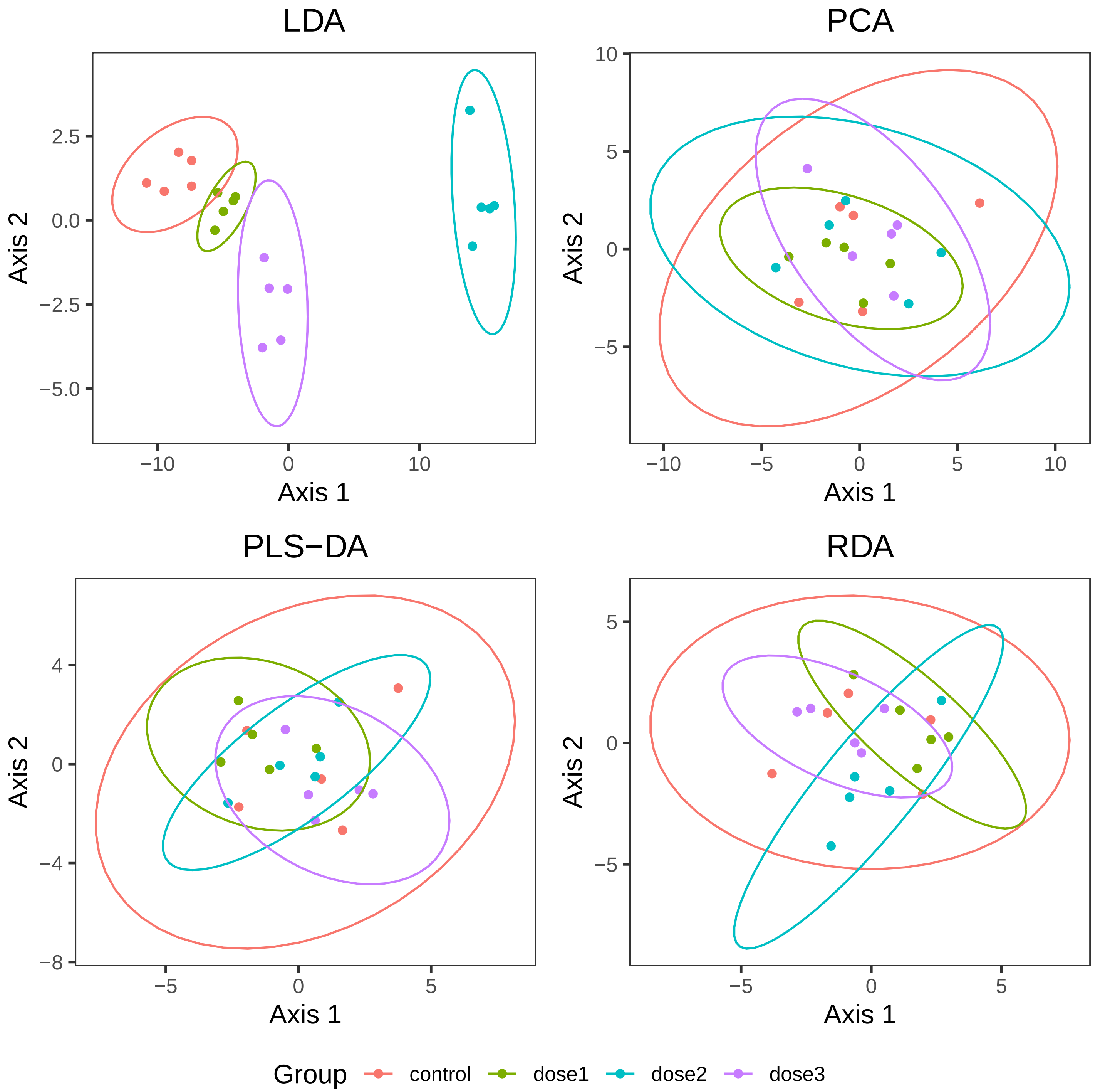
LDA
PCA
PLS−DA
RDA
Axis 1
Axis 2
Group
control
dose1
dose2
dose3

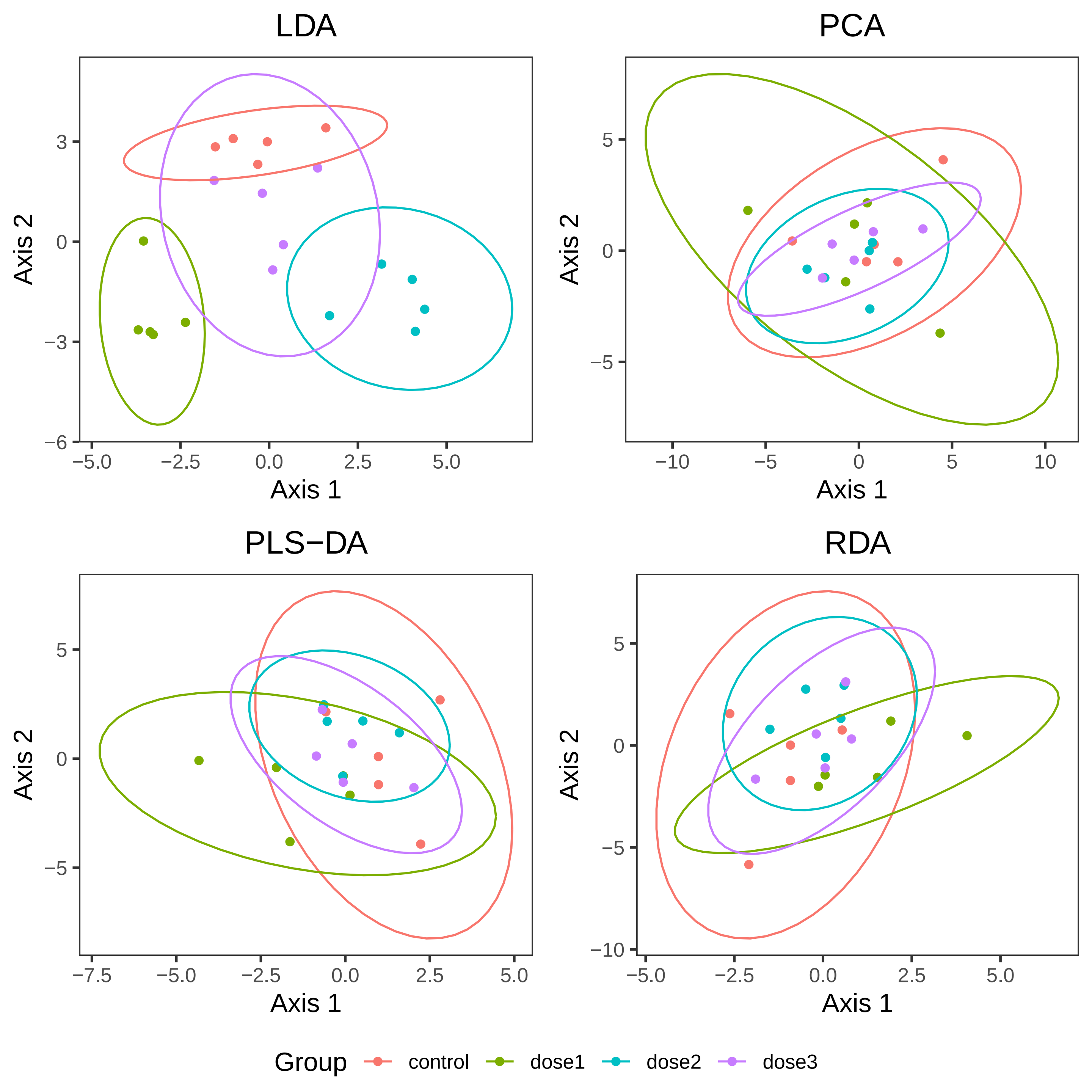
LDA
PCA
PLS−DA
RDA
Axis 1
Axis 2
3
0
−3
−6
−5.0
−2.5
0.0
2.5
5.0
5
−5
−10
10
−7.5
Group
control
dose1
dose2
dose3

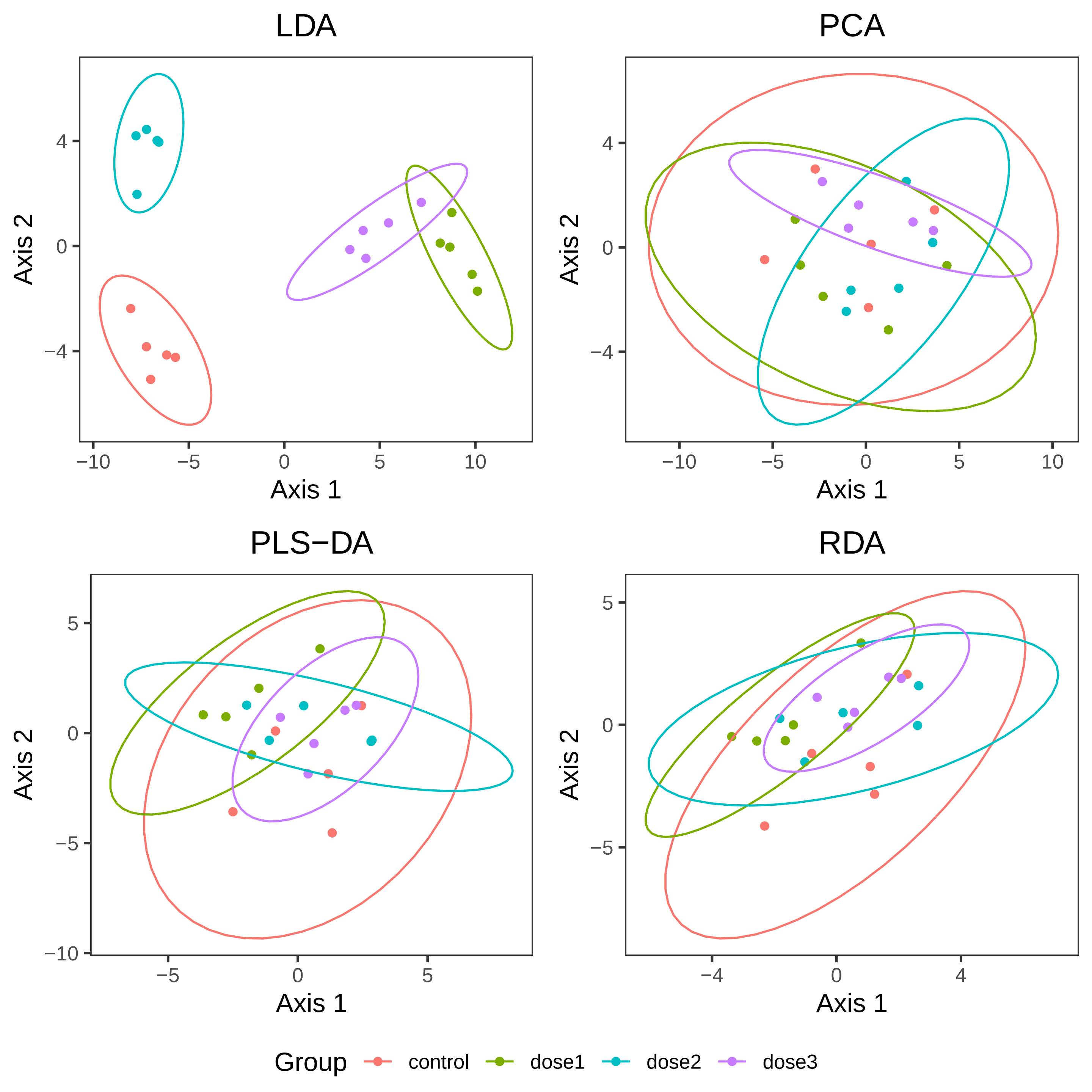

LDA
PCA
PLS−DA
RDA
Axis 1
Axis 2
Group
control
dose1
dose2
dose3

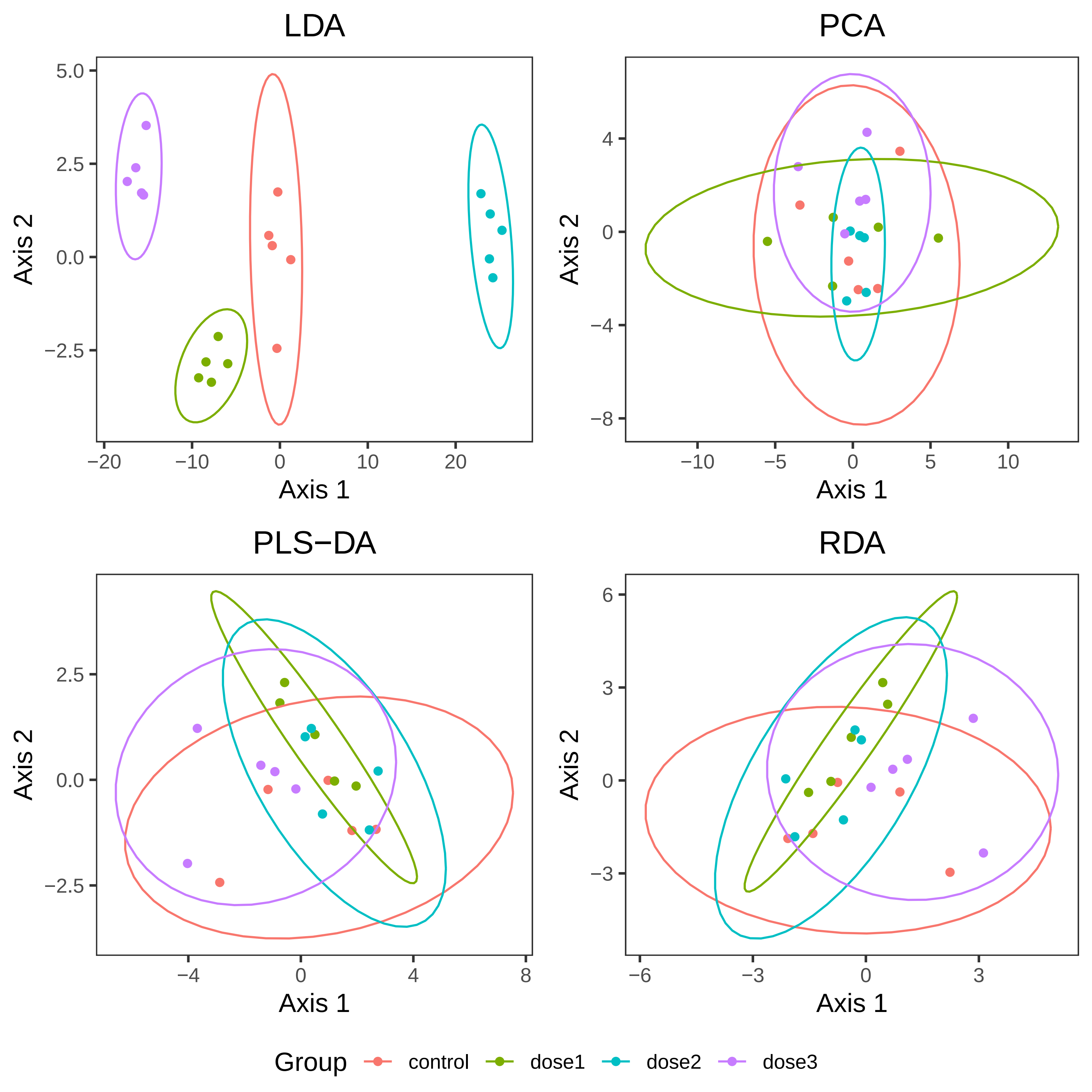
LDA
PCA
PLS−DA
RDA
Axis 1
Axis 2
Group
control
dose1
dose2
dose3

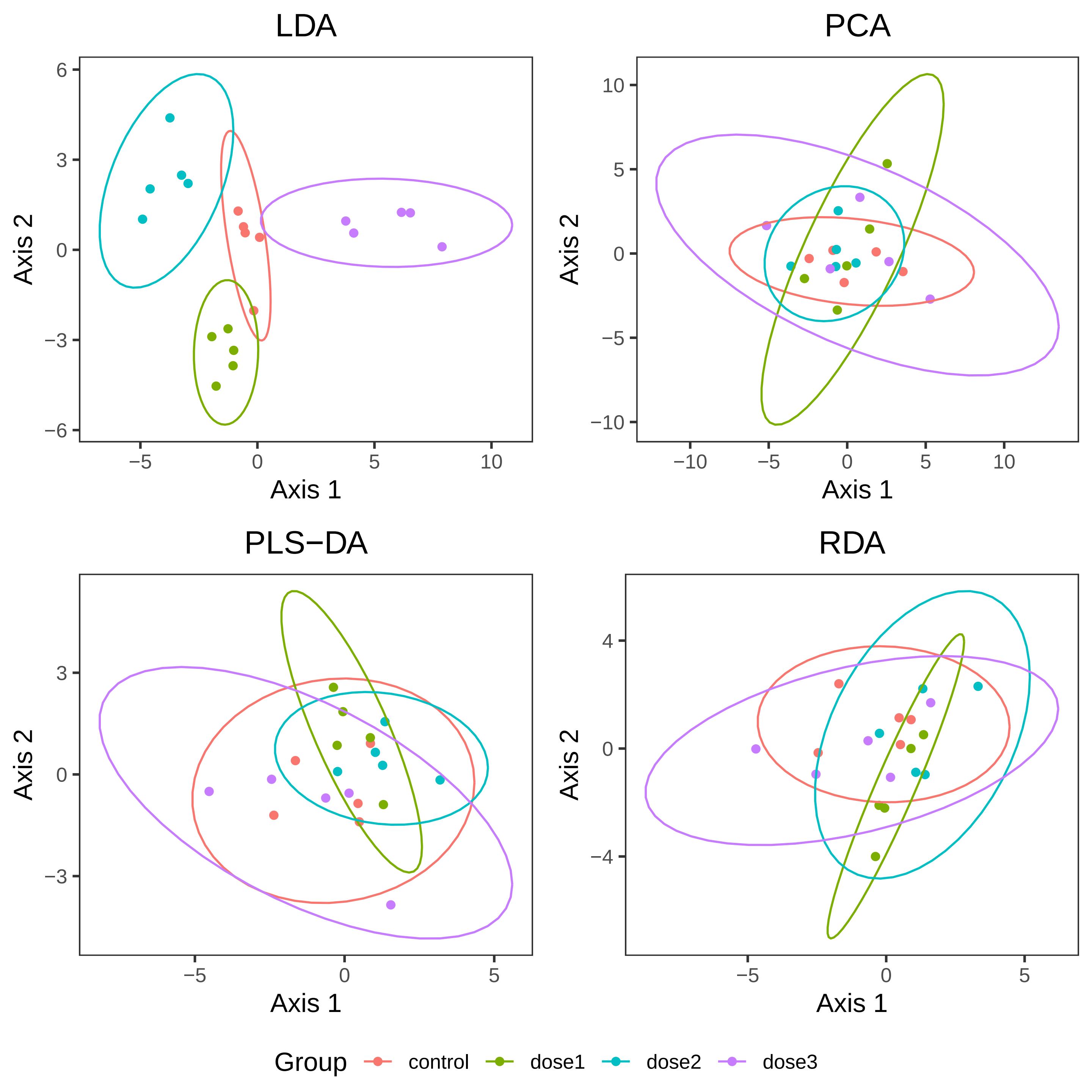
LDA
PCA
PLS−DA
RDA
Axis 1
Axis 2
Group
control
dose1
dose2
dose3

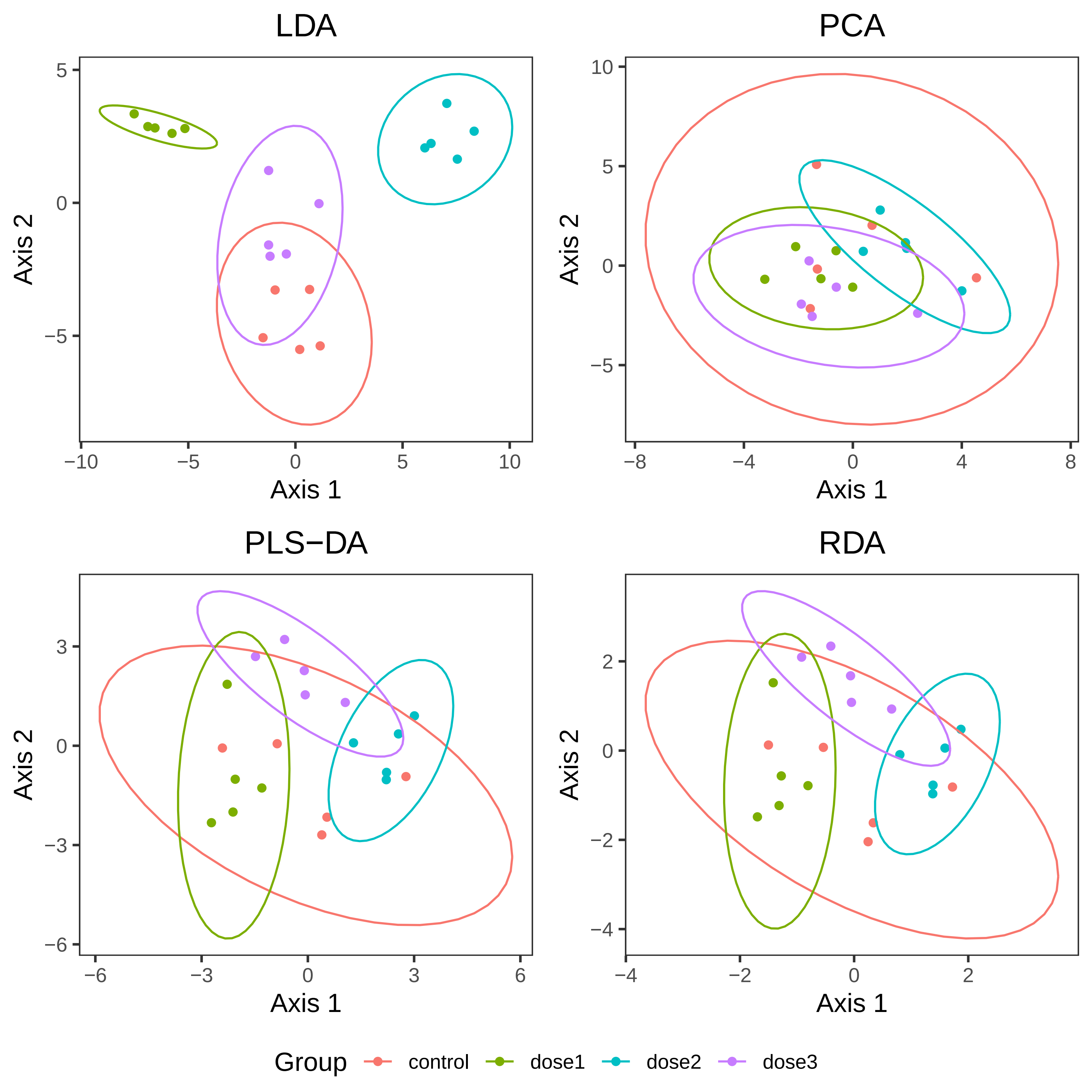

LDA
PCA
PLS−DA
RDA
Axis 1
Axis 2
Group
control
dose1
dose2
dose3

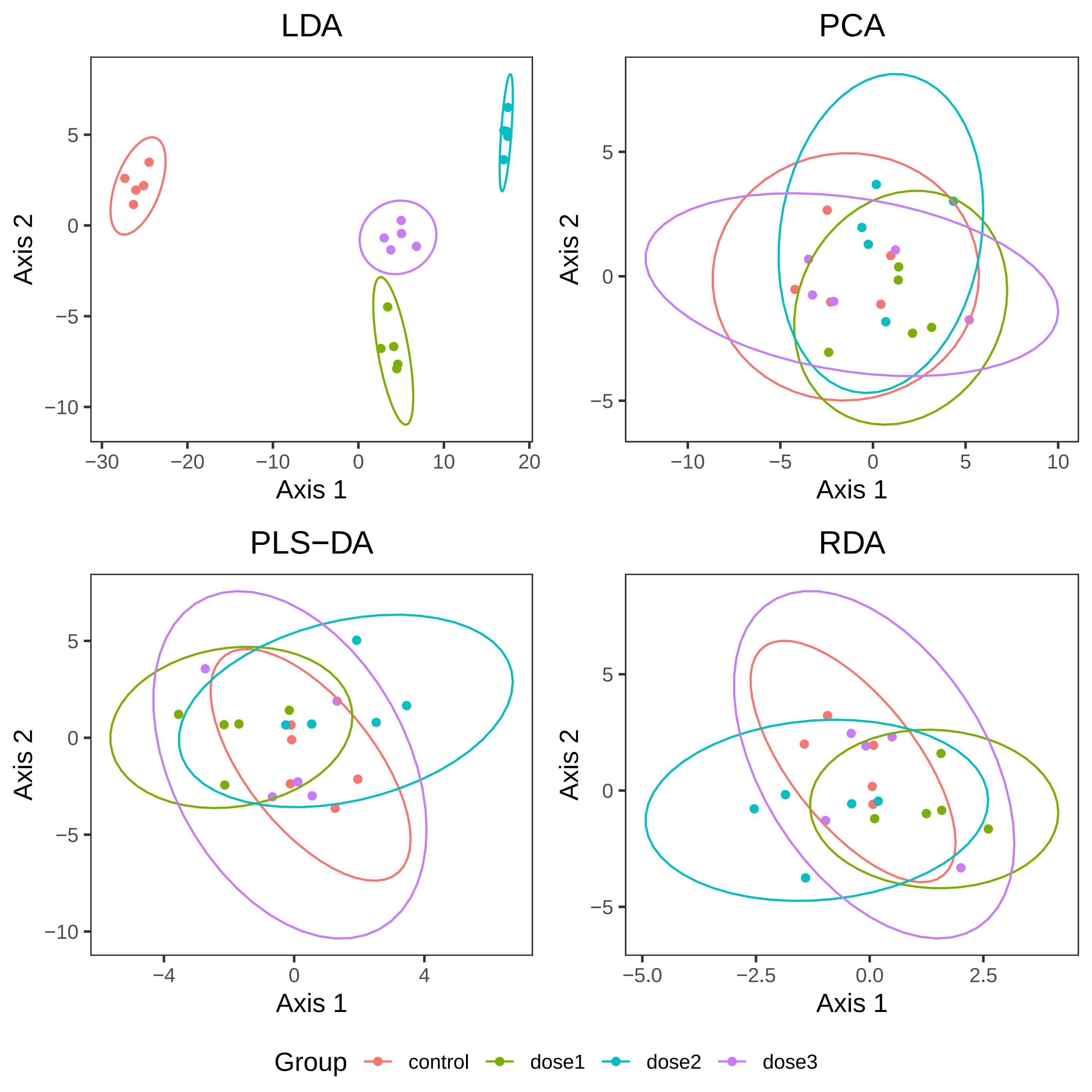

LDA
PCA
PLS−DA
RDA
Axis 1
Axis 2
Group
control
dose1
dose2
dose3

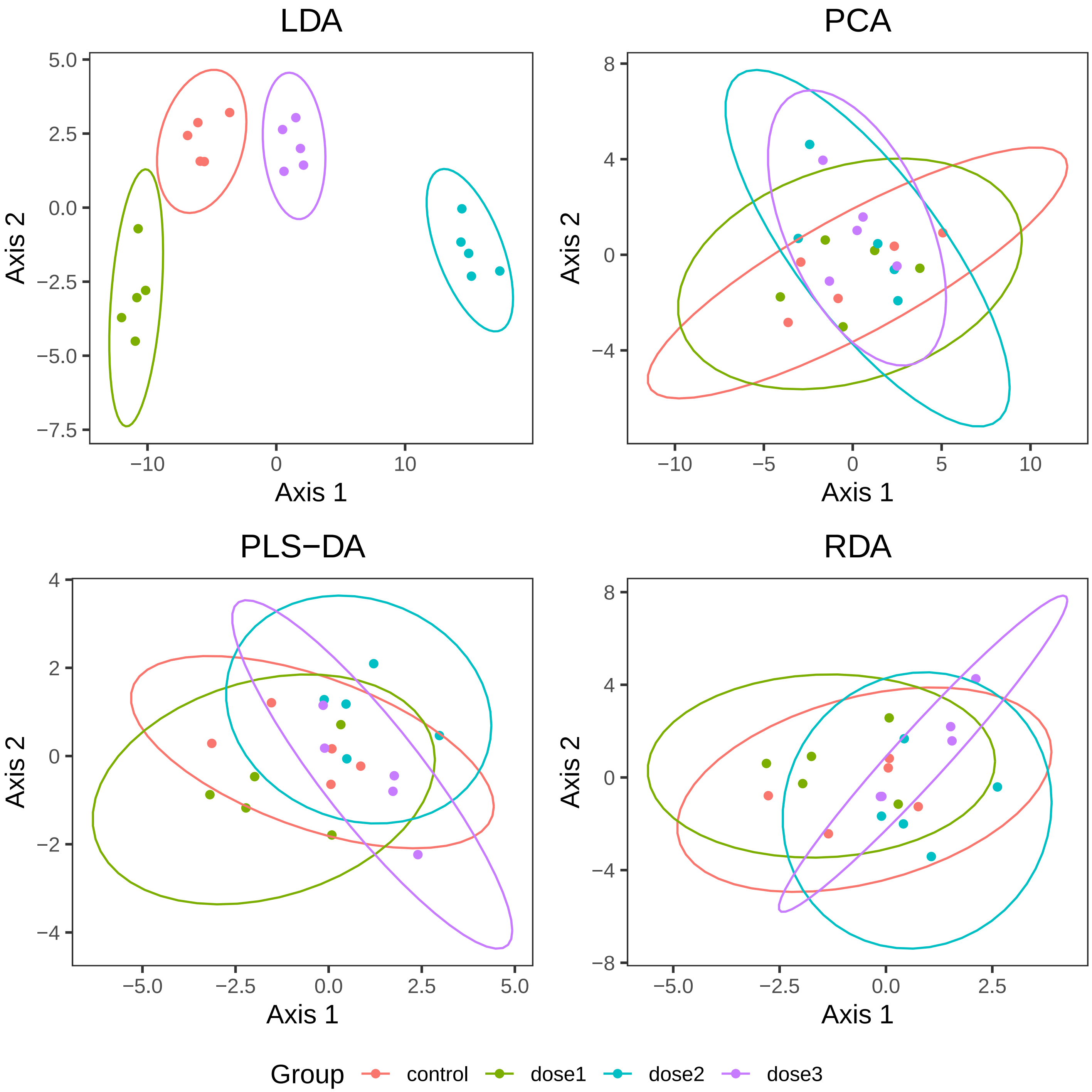

LDA
PCA
PLS−DA
RDA
Axis 1
Axis 2
Group
control
dose1
dose2
dose3

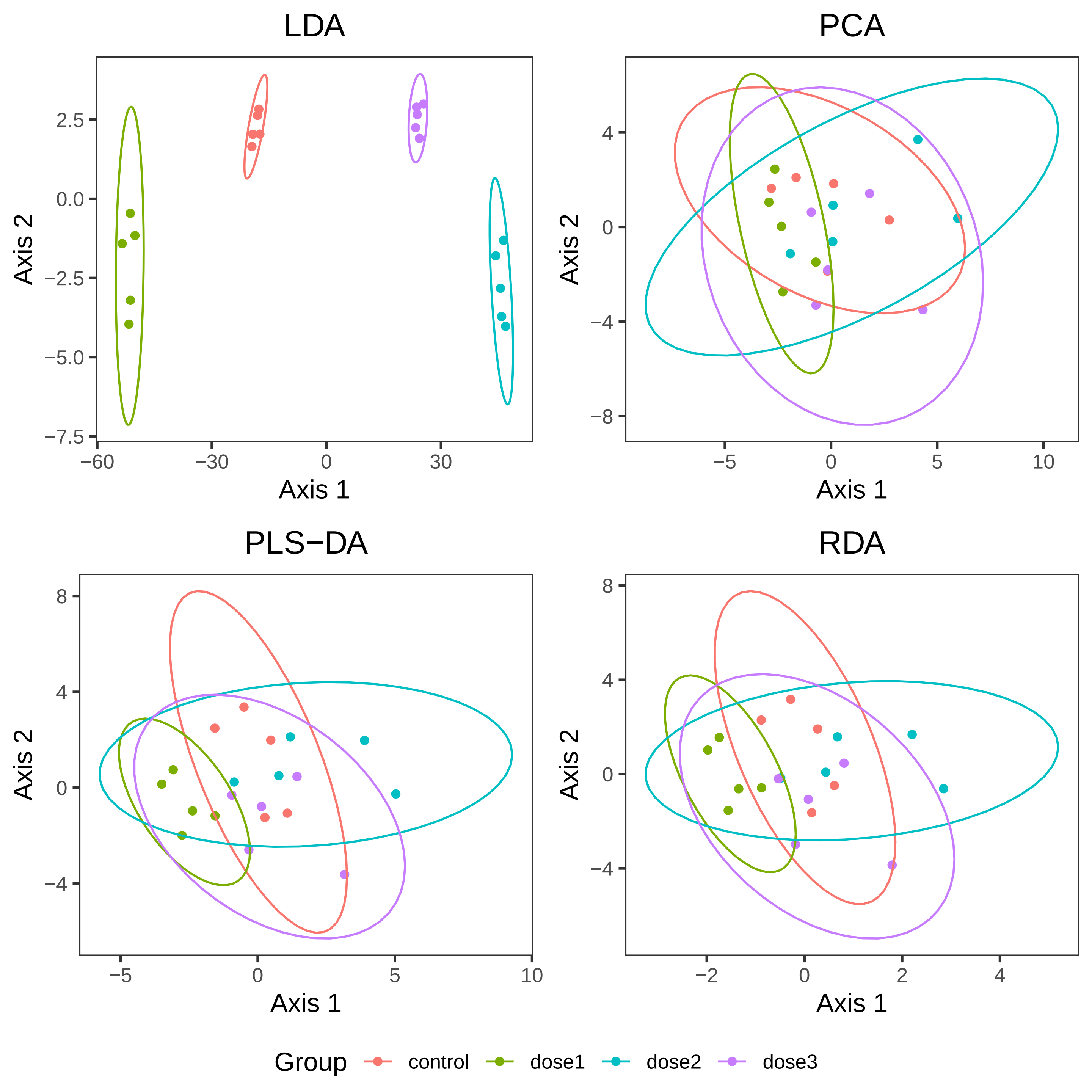

LDA
PCA
PLS−DA
RDA
Axis 1
Axis 2
2.5
0.0
−2.5
−5.0
−7.5
−60
−30
0
30
4
0
−4
−8
−5
0
5
10
8
4
0
−4
−5
0
5
10
8
4
0
−4
−2
0
2
4
Group
control
dose1
dose2
dose3

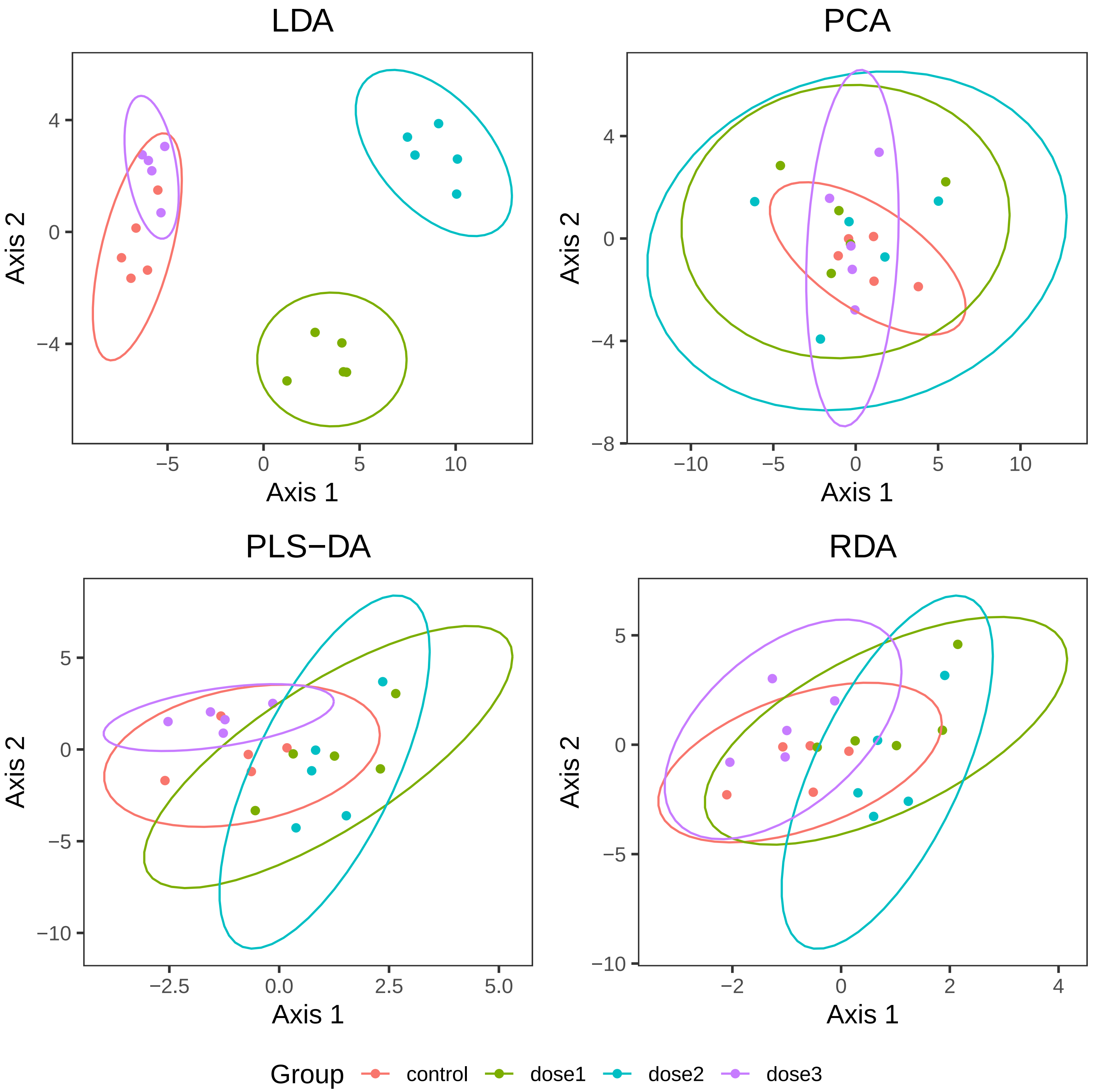

LDA
PCA
PLS−DA
RDA
Axis 1
Axis 2
Group
control
dose1
dose2
dose3

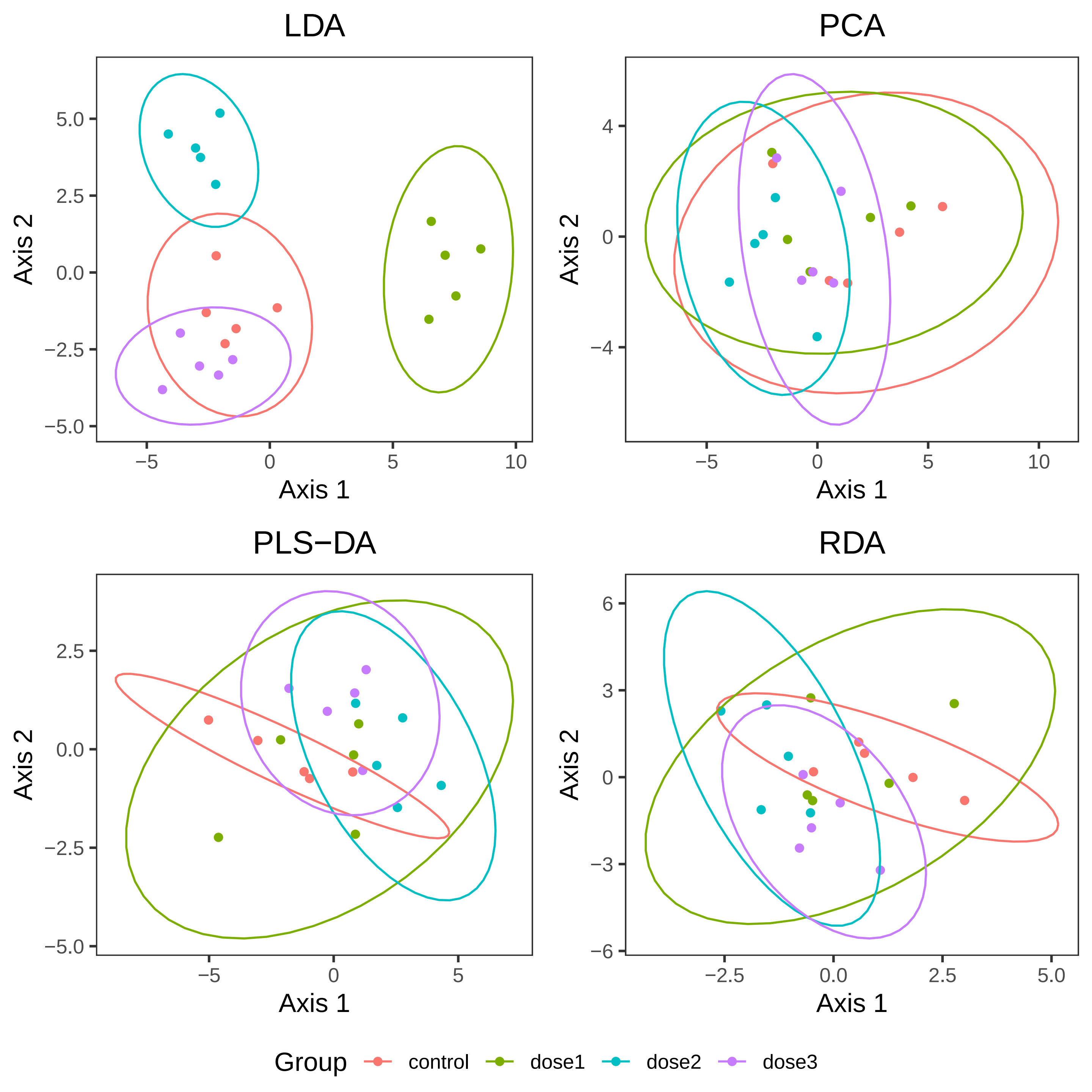
LDA
PCA
PLS−DA
RDA
Axis 1
Axis 2
Group
control
dose1
dose2
dose3

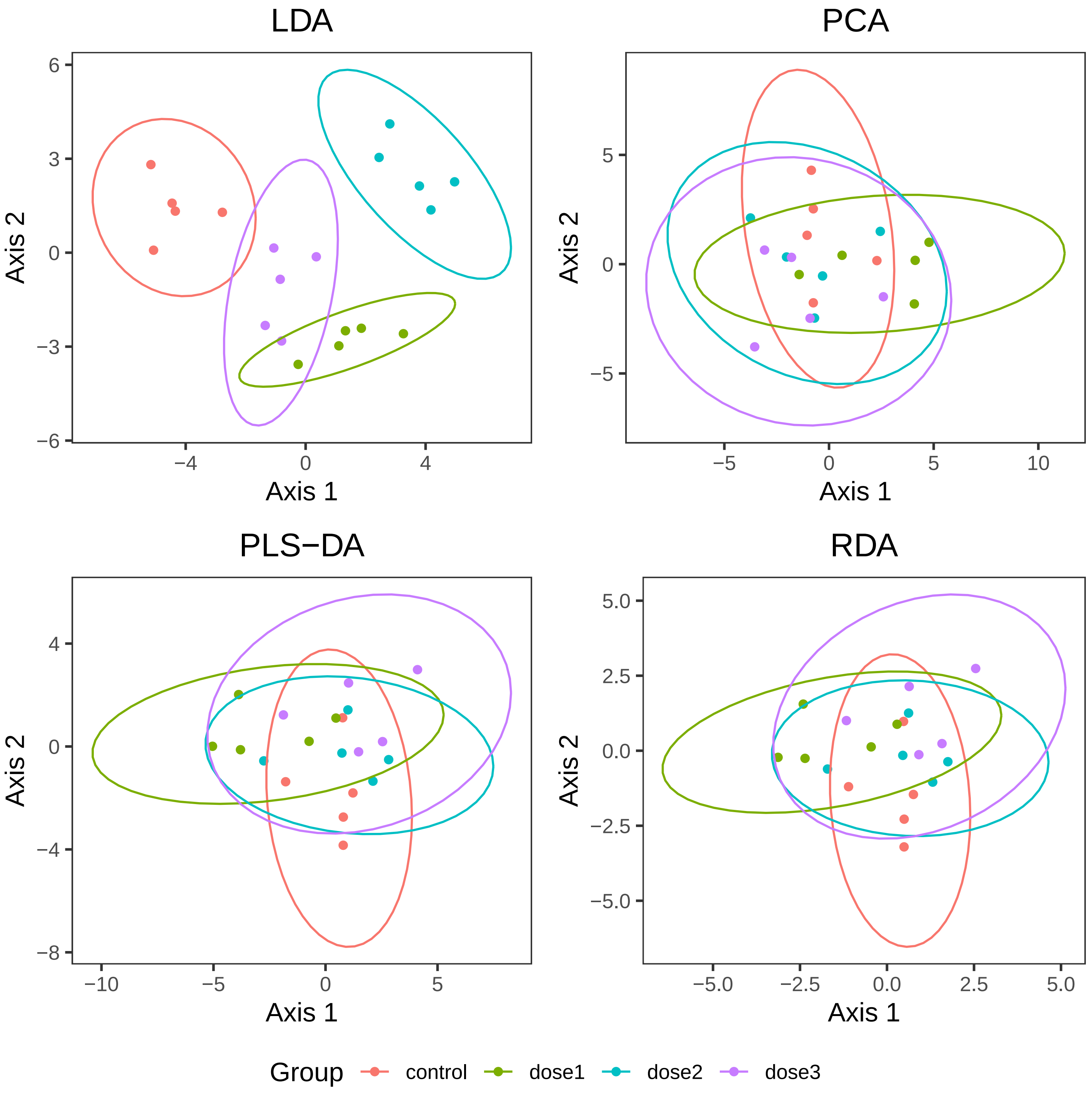
LDA
PCA
PLS−DA
RDA
Axis 1
Axis 2
6
3
0
−3
−6
−4
0
4
5
0
−5
10
−10
−8
−4
4
5.0
2.5
0.0
−2.5
−5.0
Group
control
dose1
dose2
dose3

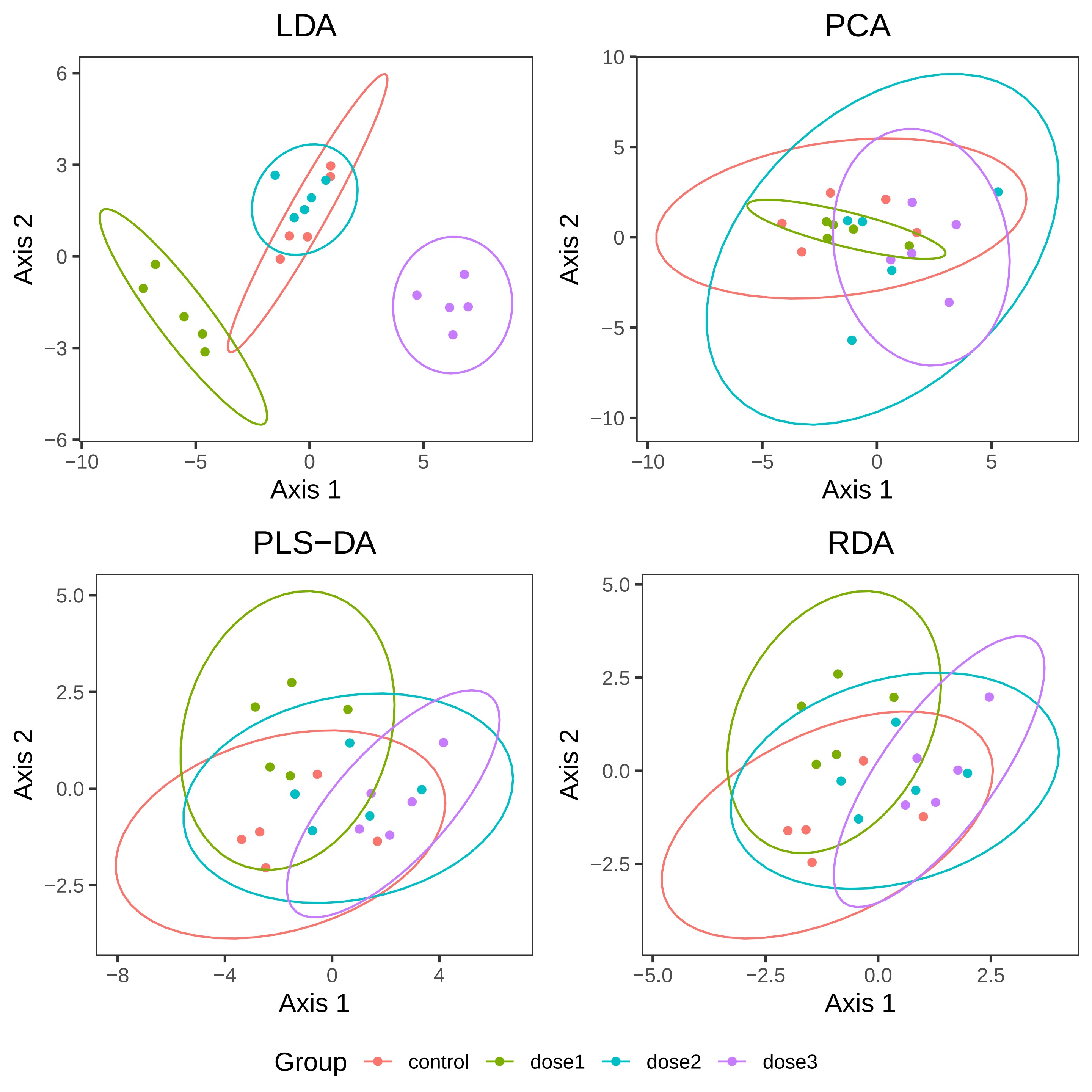

LDA
PCA
PLS−DA
RDA
Axis 1
Axis 2
Group
control
dose1
dose2
dose3

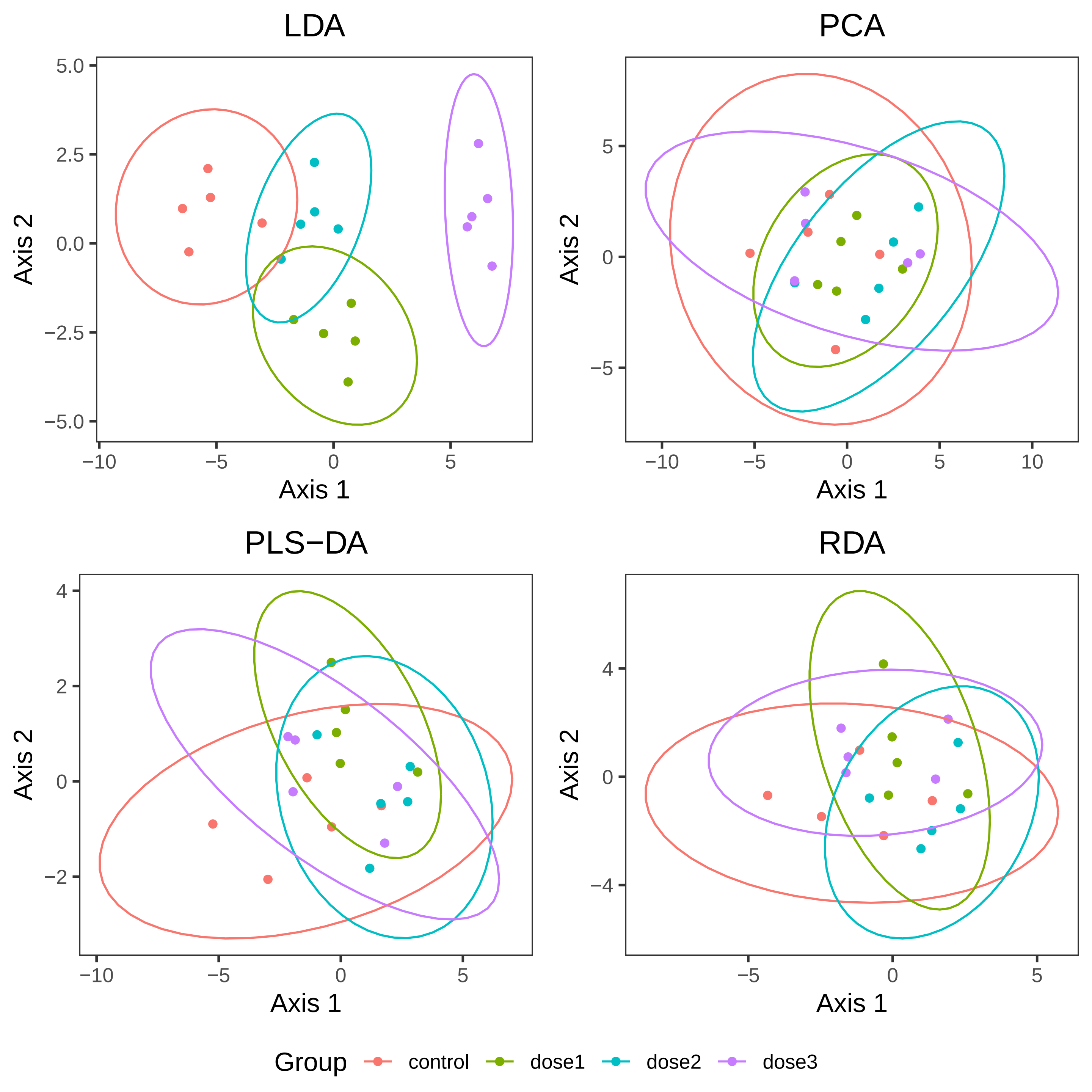

LDA
PCA
PLS−DA
RDA
Axis 1
Axis 2
Group
control
dose1
dose2
dose3

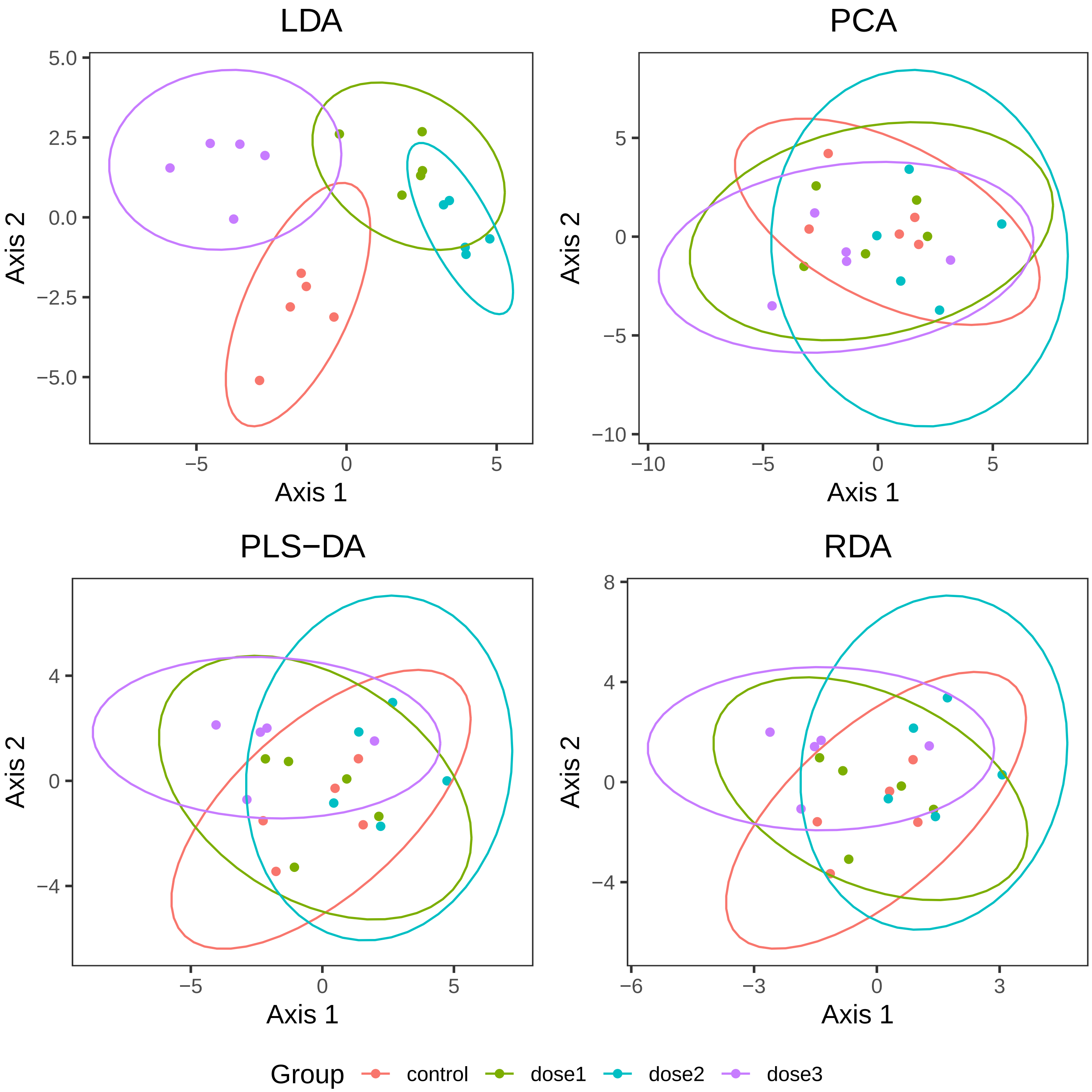

LDA
PCA
PLS−DA
RDA
Axis 1
Axis 2
5.0
2.5
0.0
−2.5
−5.0
−5
0
5
−10
Group
control
dose1
dose2
dose3

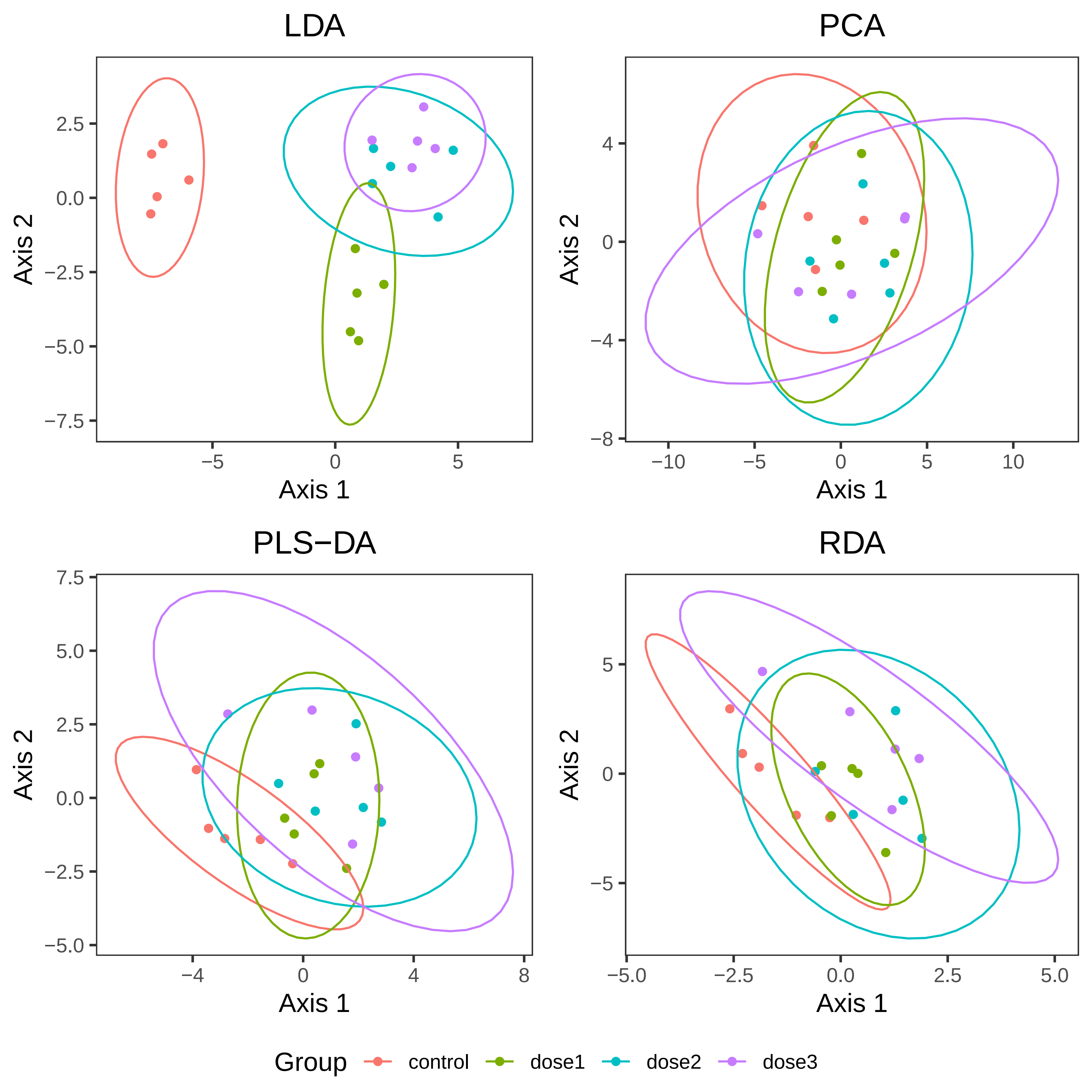

LDA
PCA
PLS−DA
RDA
Axis 1
Axis 2
Group
control
dose1
dose2
dose3

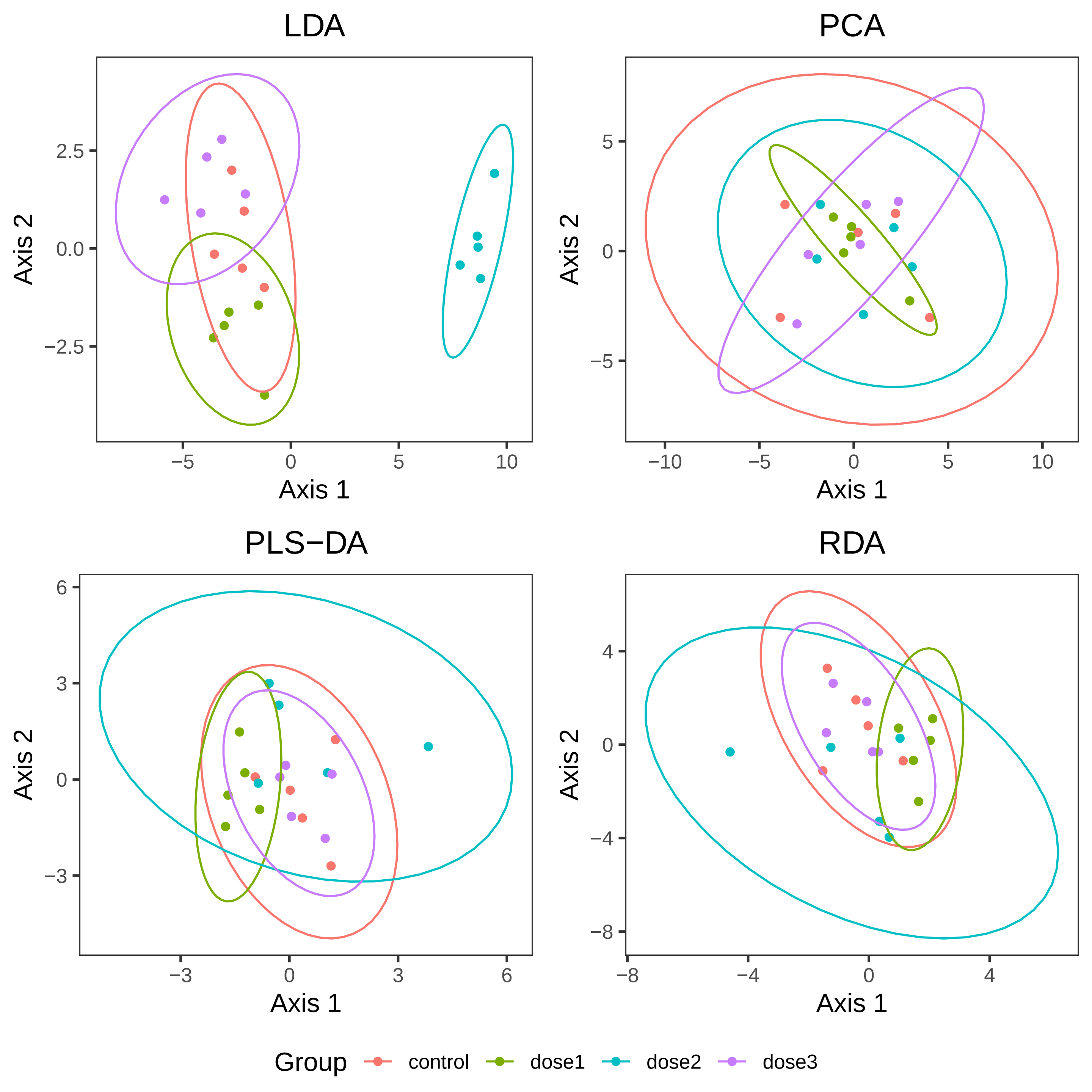
LDA
PCA
PLS−DA
RDA
Axis 1
Axis 2
Group
control
dose1
dose2
dose3

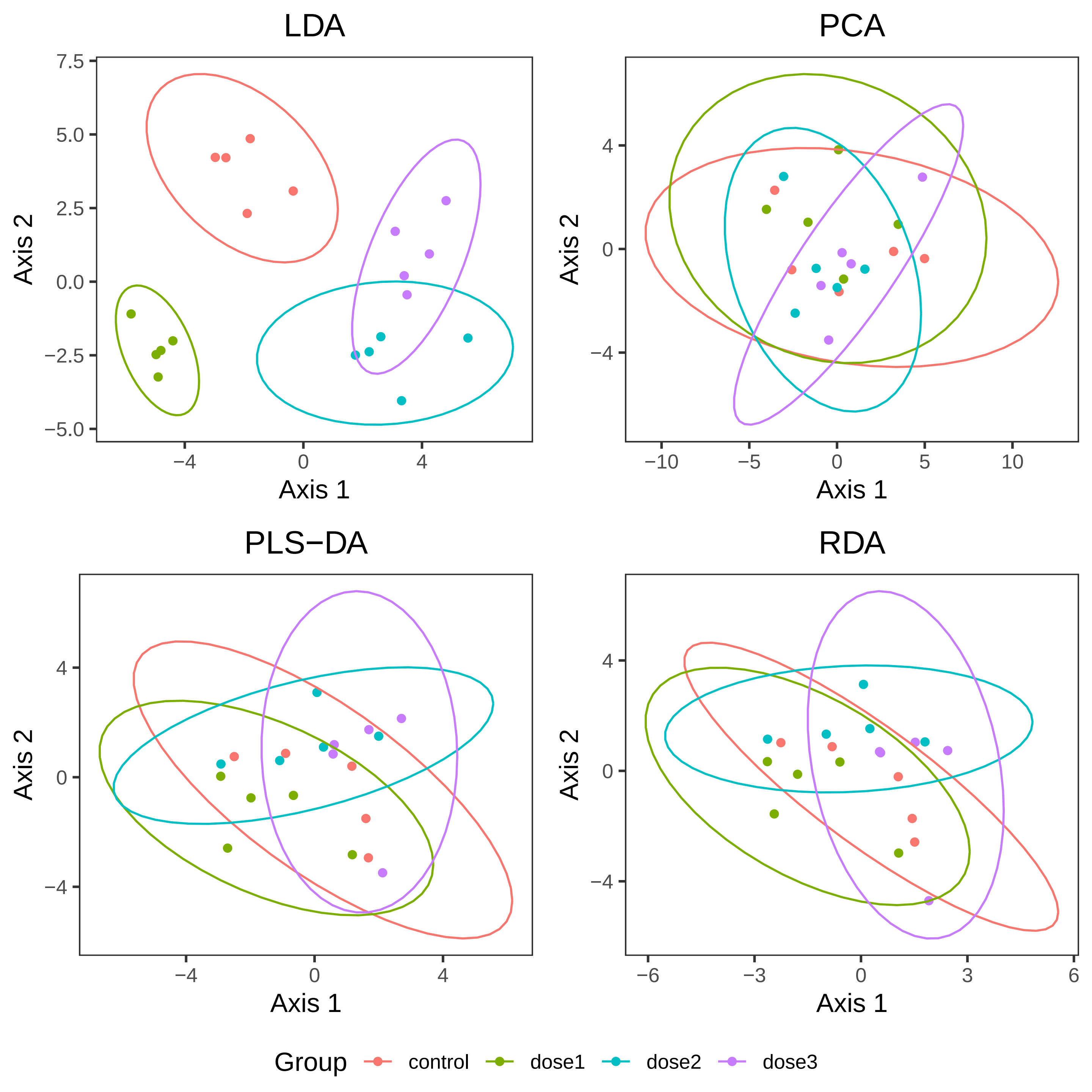

LDA
PCA
PLS−DA
RDA
Axis 1
Axis 2
7.5
5.0
2.5
0.0
−2.5
−5.0
−4
0
4
−10
−5
5
10
−6
−3
3
6
Group
control
dose1
dose2
dose3

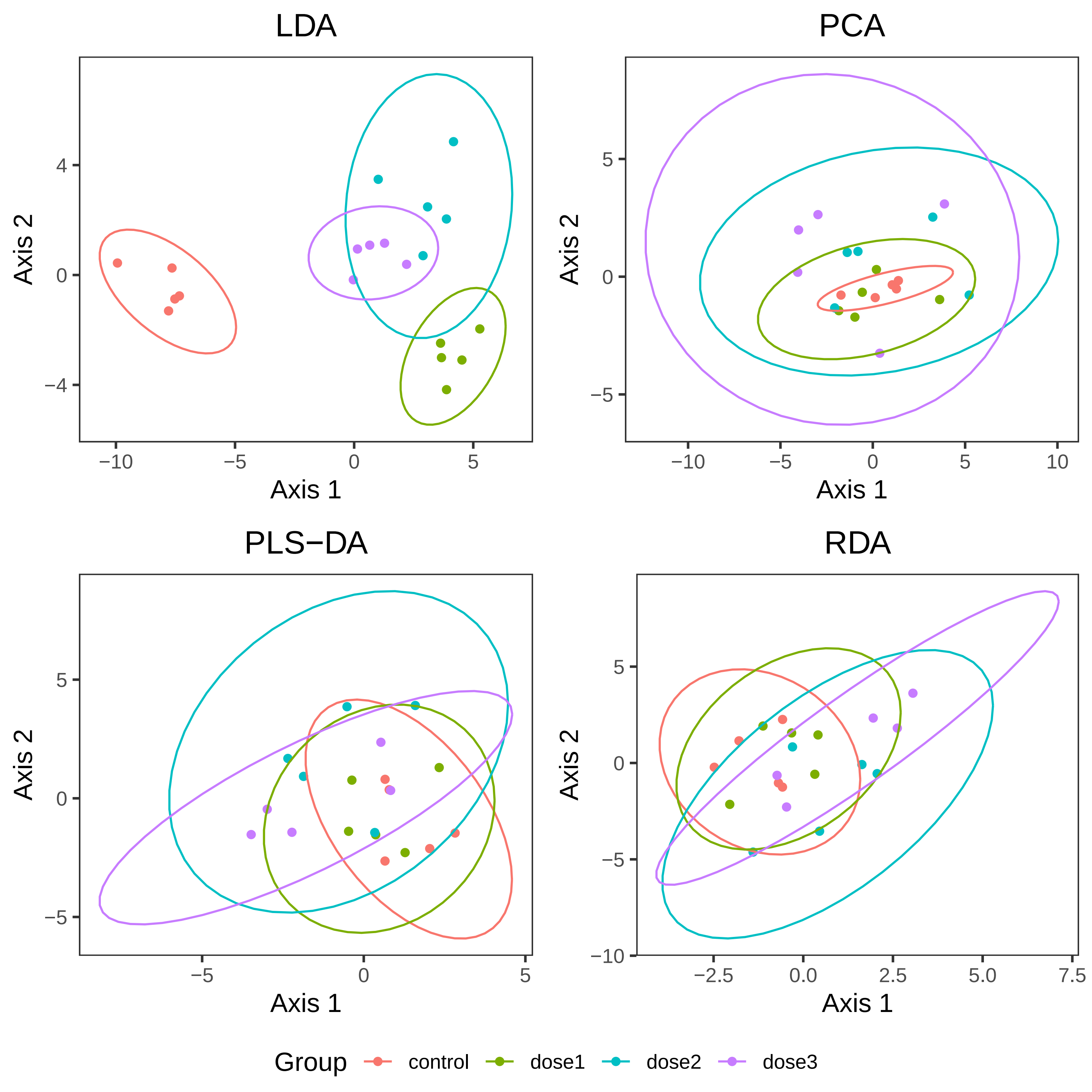
LDA
PCA
PLS−DA
RDA
Axis 1
Axis 2
Group
control
dose1
dose2
dose3

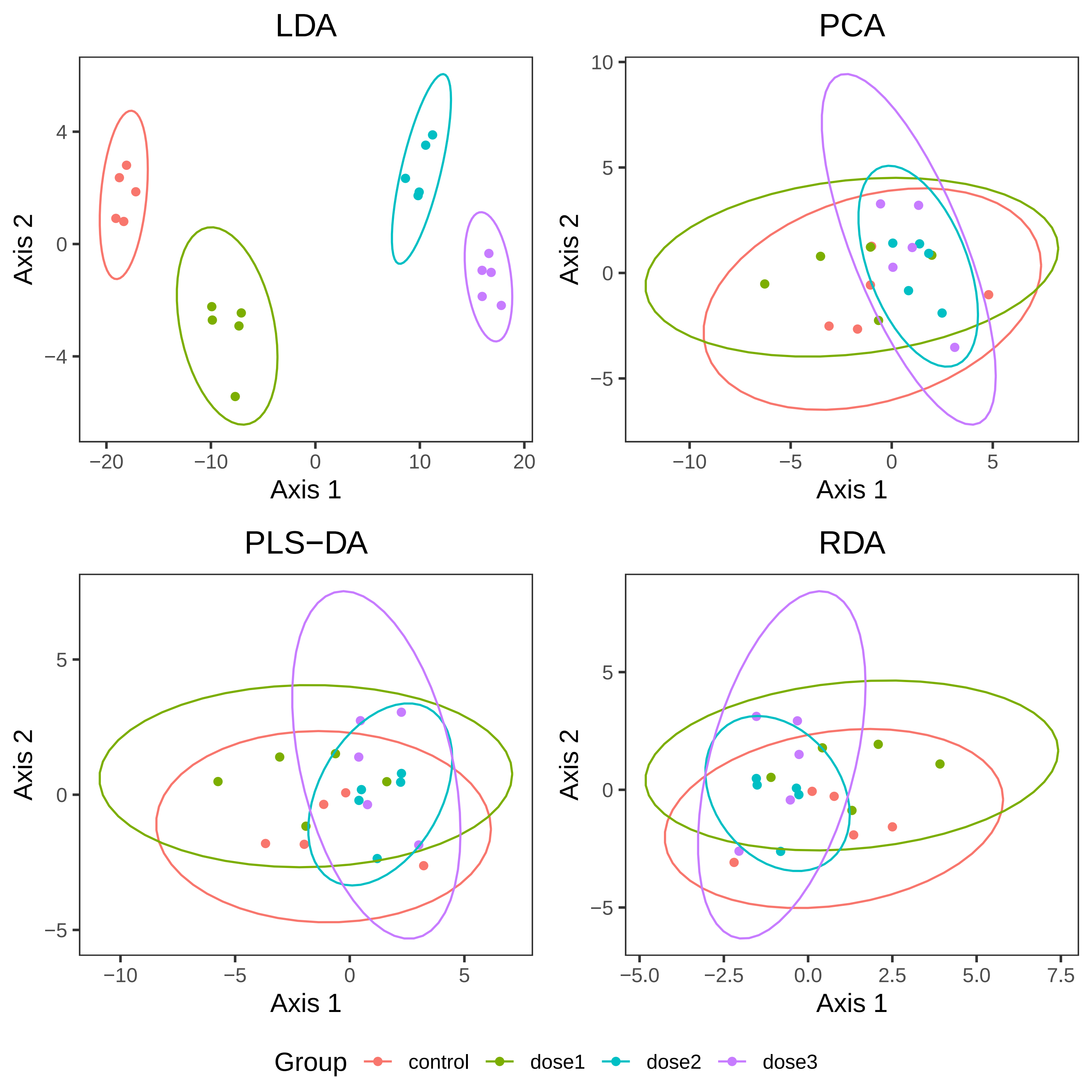

LDA
PCA
PLS−DA
RDA
Axis 1
Axis 2
Group
control
dose1
dose2
dose3

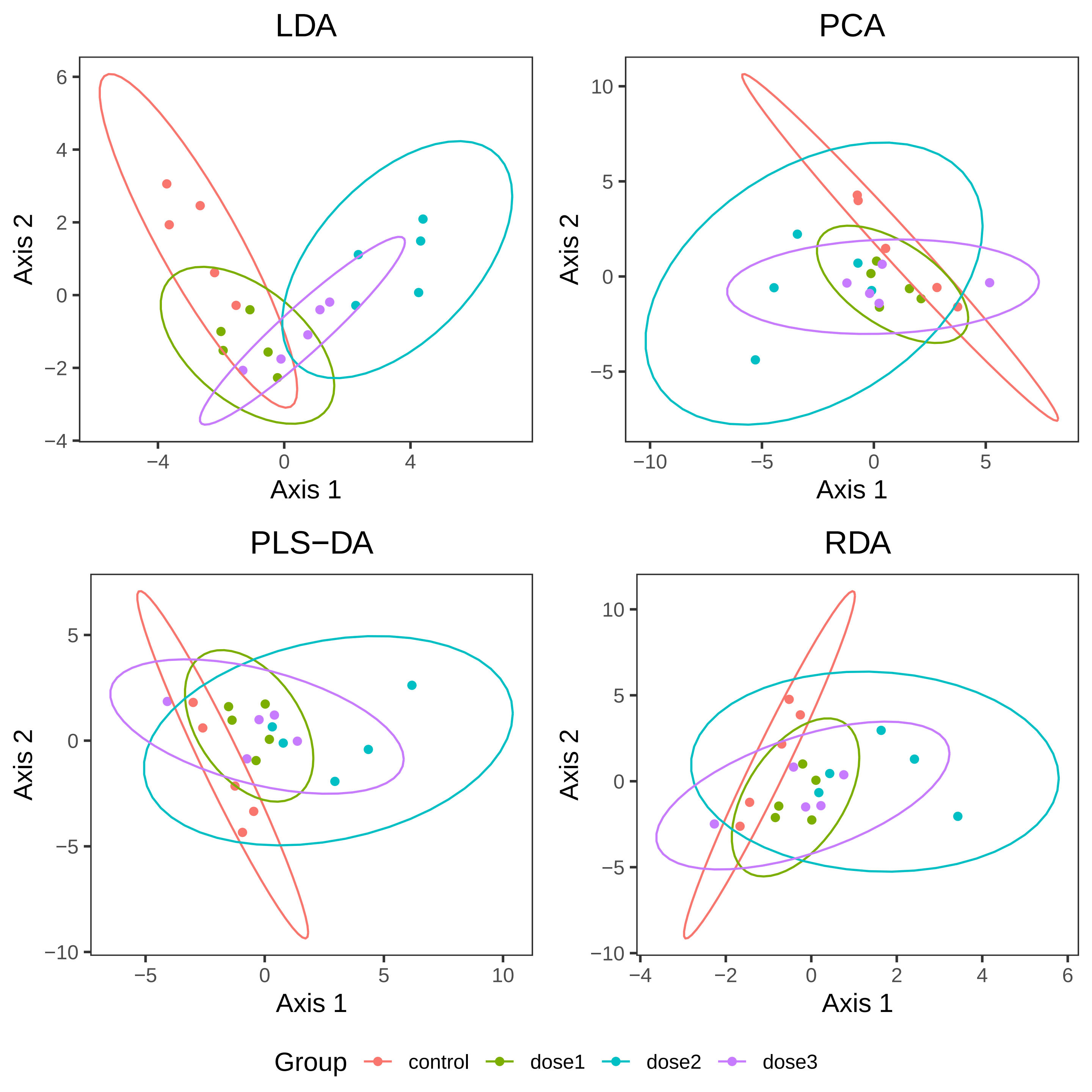
LDA
PCA
PLS−DA
RDA
Axis 1
Axis 2
Group
control
dose1
dose2
dose3

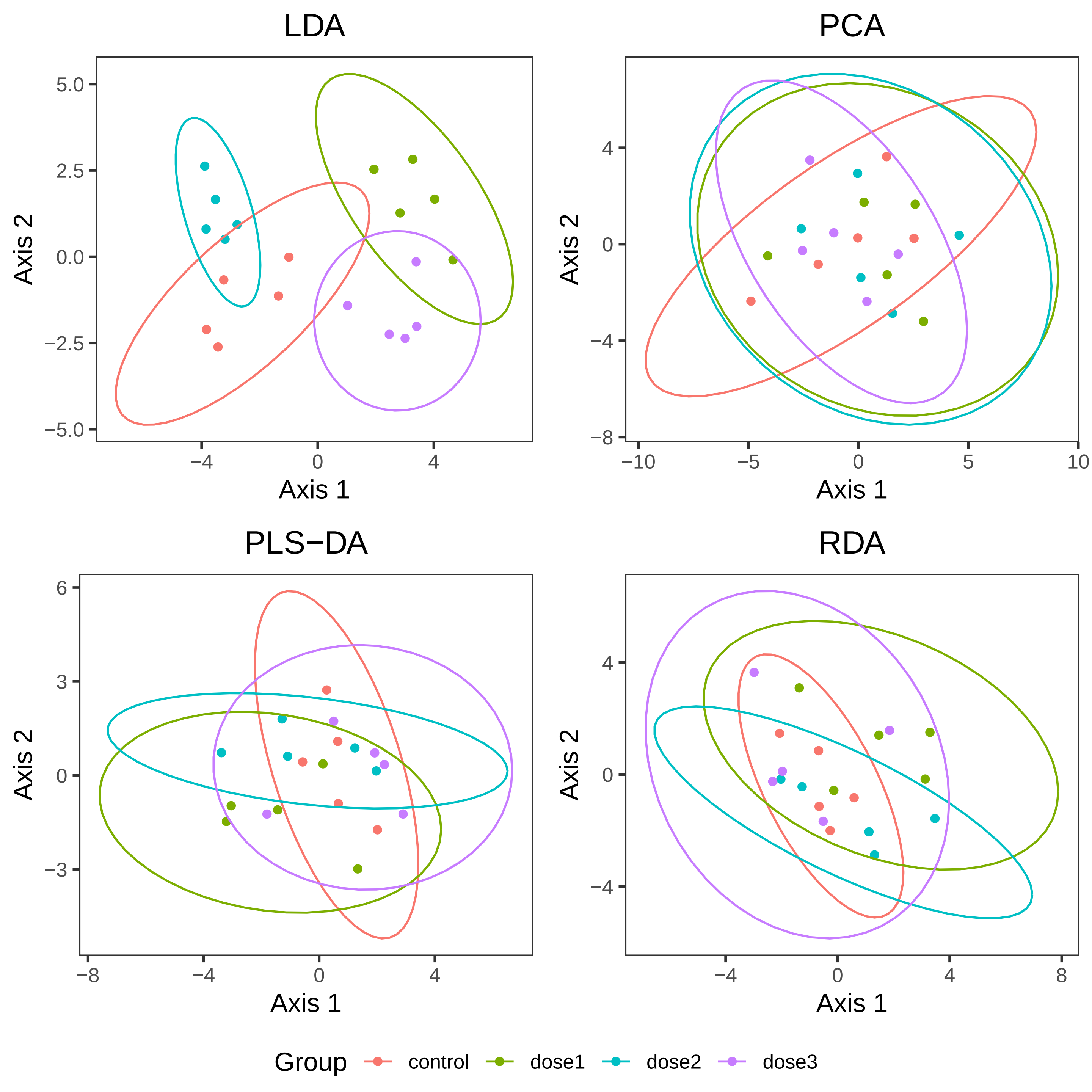

LDA
PCA
PLS−DA
RDA
Axis 1
Axis 2
5.0
2.5
0.0
−2.5
−5.0
−4
0
4
−10
−5
5
10
−8
−8
−3
3
6
8
Group
control
dose1
dose2
dose3

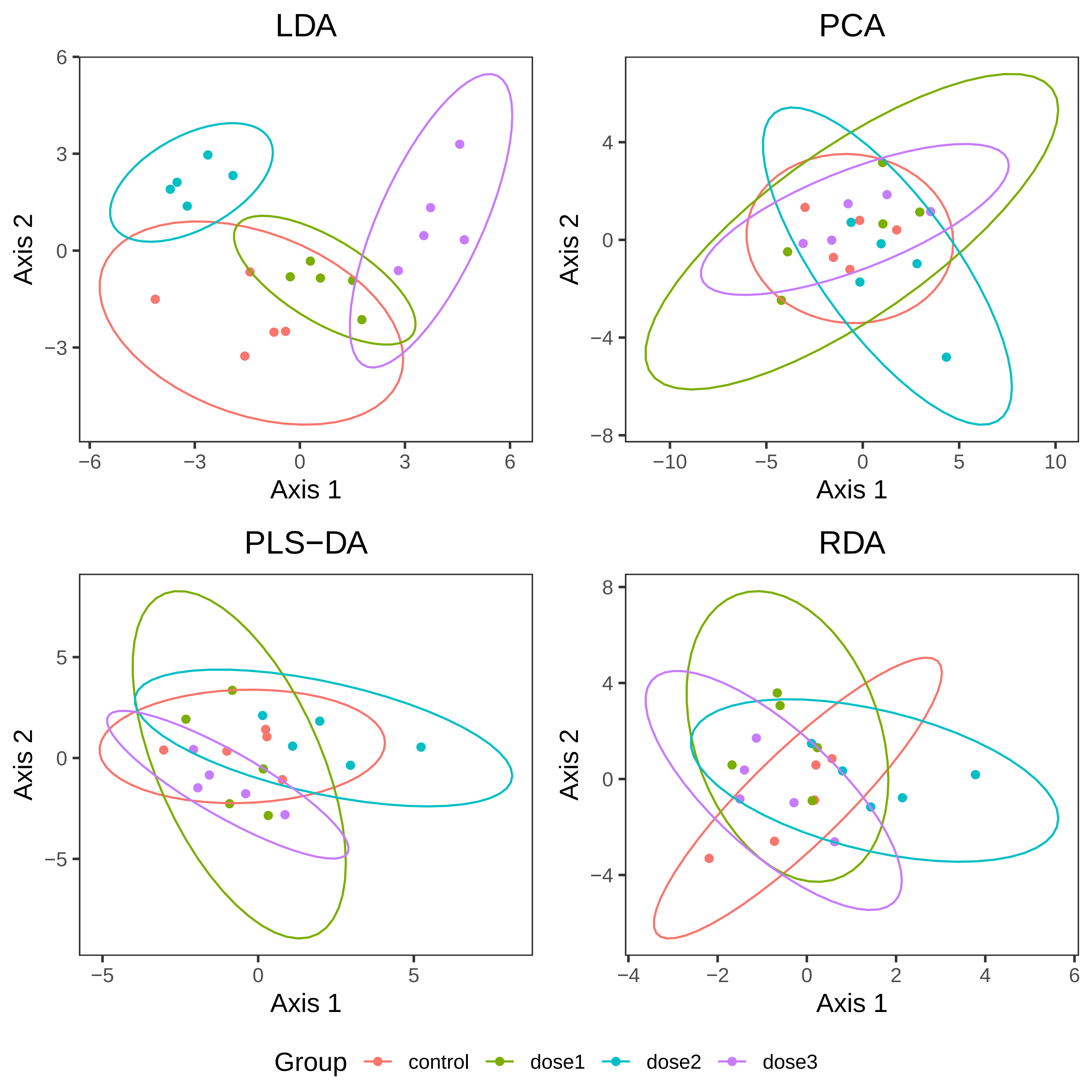

LDA
PCA
PLS−DA
RDA
Axis 1
Axis 2
Group
control
dose1
dose2
dose3

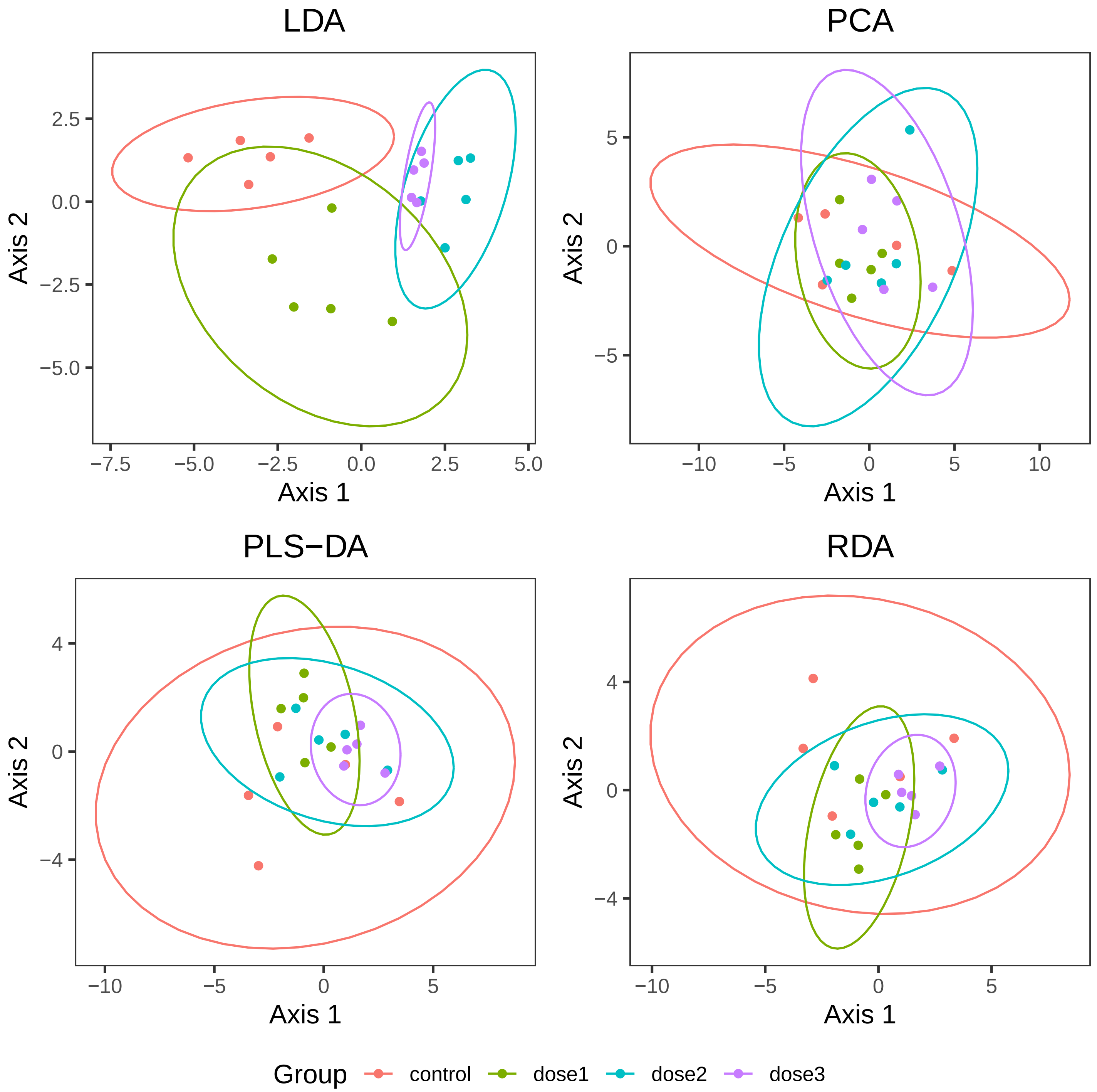

LDA
PCA
PLS−DA
RDA
Axis 1
Axis 2
2.5
0.0
−2.5
−5.0
−7.5
5.0
5
0
−5
−10
10
4
−4
Group
control
dose1
dose2
dose3

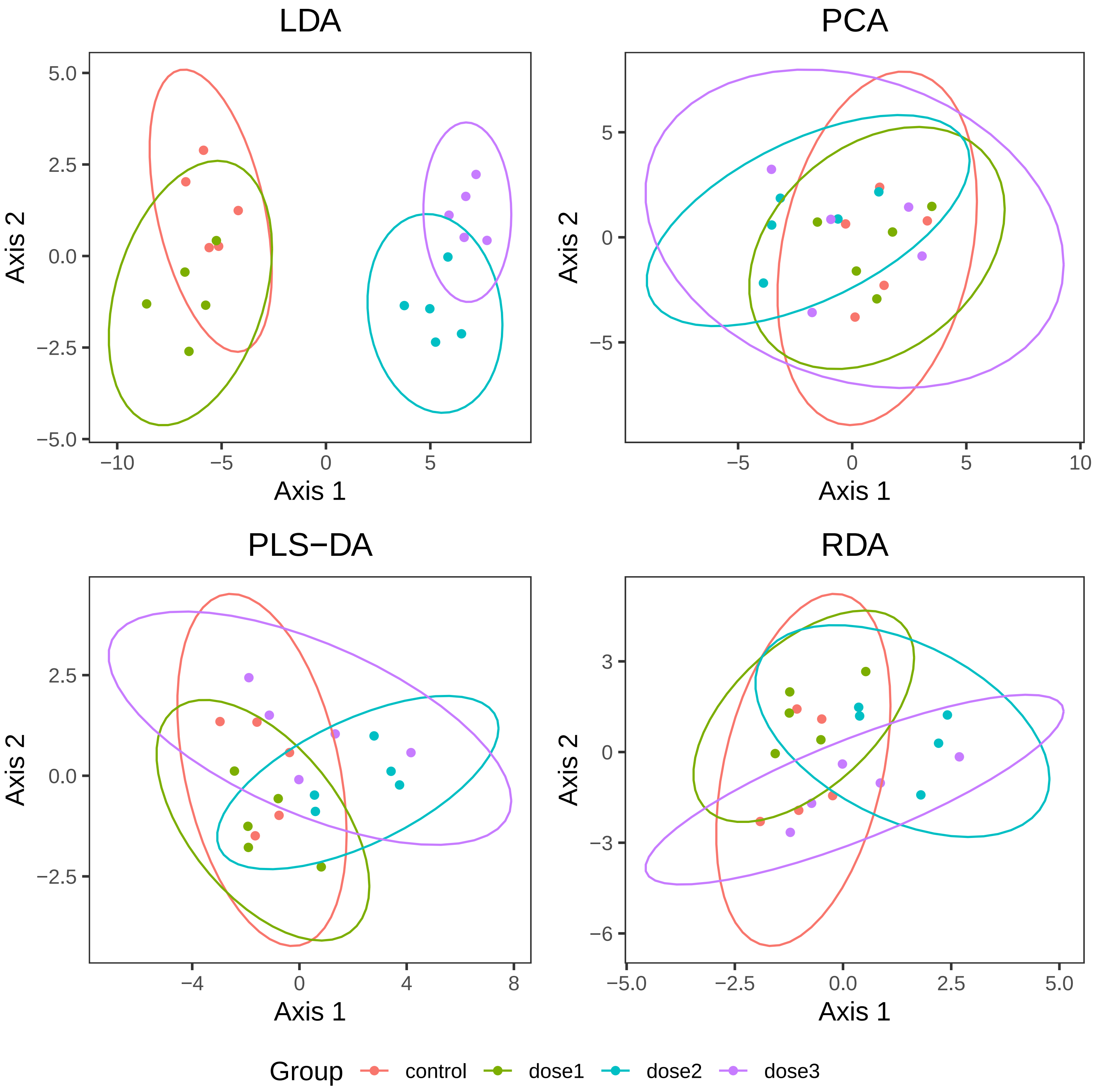

LDA
PCA
PLS−DA
RDA
Axis 1
Axis 2
5.0
2.5
0.0
−2.5
−5.0
−10
−5
0
5
10
−4
4
8
3
−3
−6
Group
control
dose1
dose2
dose3

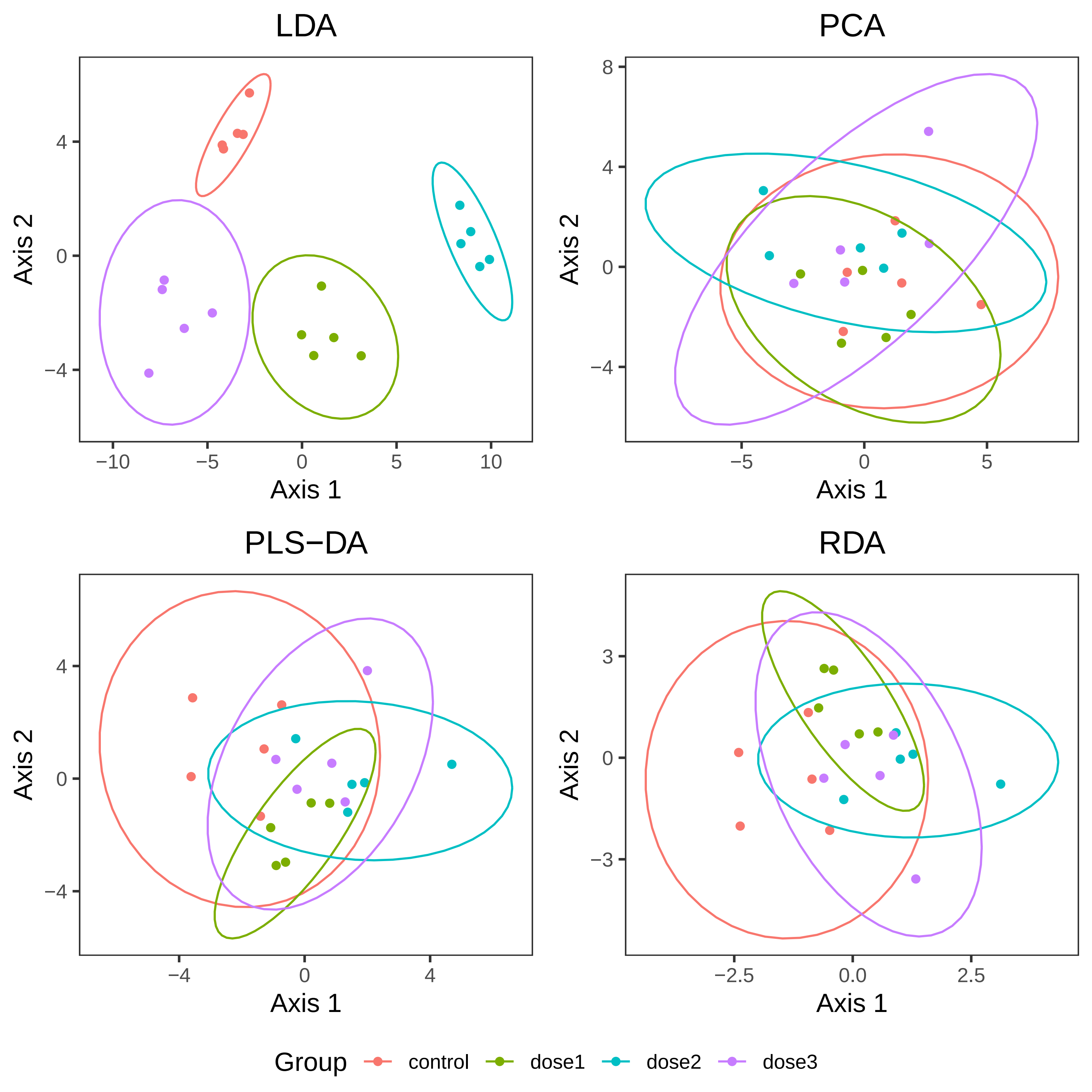
LDA
PCA
PLS−DA
RDA
Axis 1
Axis 2
−10
−5
0
5
10
4
0
−4
8
−2.5
0.0
2.5
3
−3
Group
control
dose1
dose2
dose3

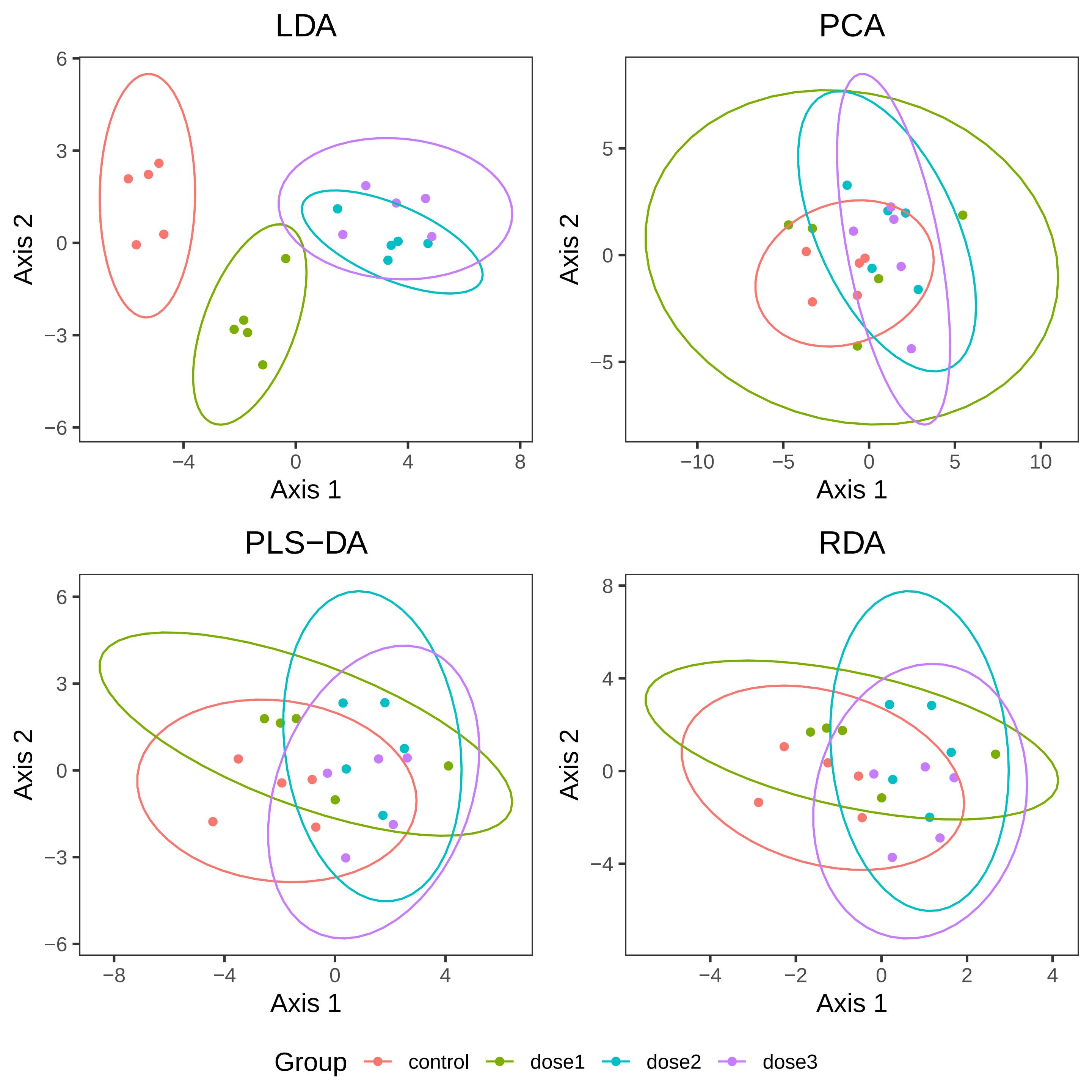

LDA
PCA
PLS−DA
RDA
Axis 1
Axis 2
6
3
0
−3
−6
−4
4
8
5
−5
−10
10
−8
2
−2
Group
control
dose1
dose2
dose3

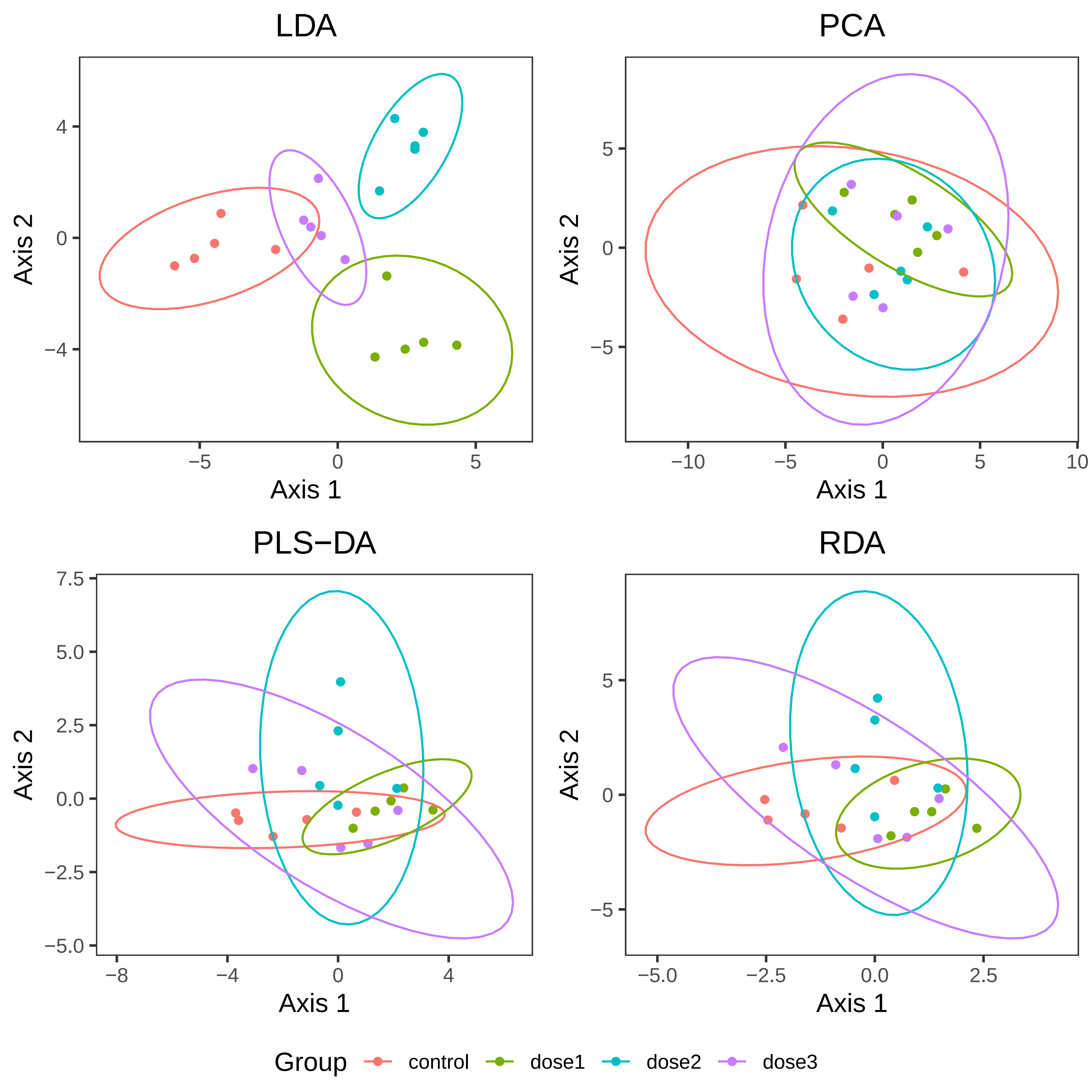
LDA
PCA
PLS−DA
RDA
Axis 1
Axis 2
4
0
−4
−5
0
5
5
0
−5
−10
−5
0
5
10
7.5
5.0
2.5
0.0
−2.5
−5.0
−8
−4
0
4
5
0
−5
−5.0
−2.5
0.0
2.5
Group
control
dose1
dose2
dose3

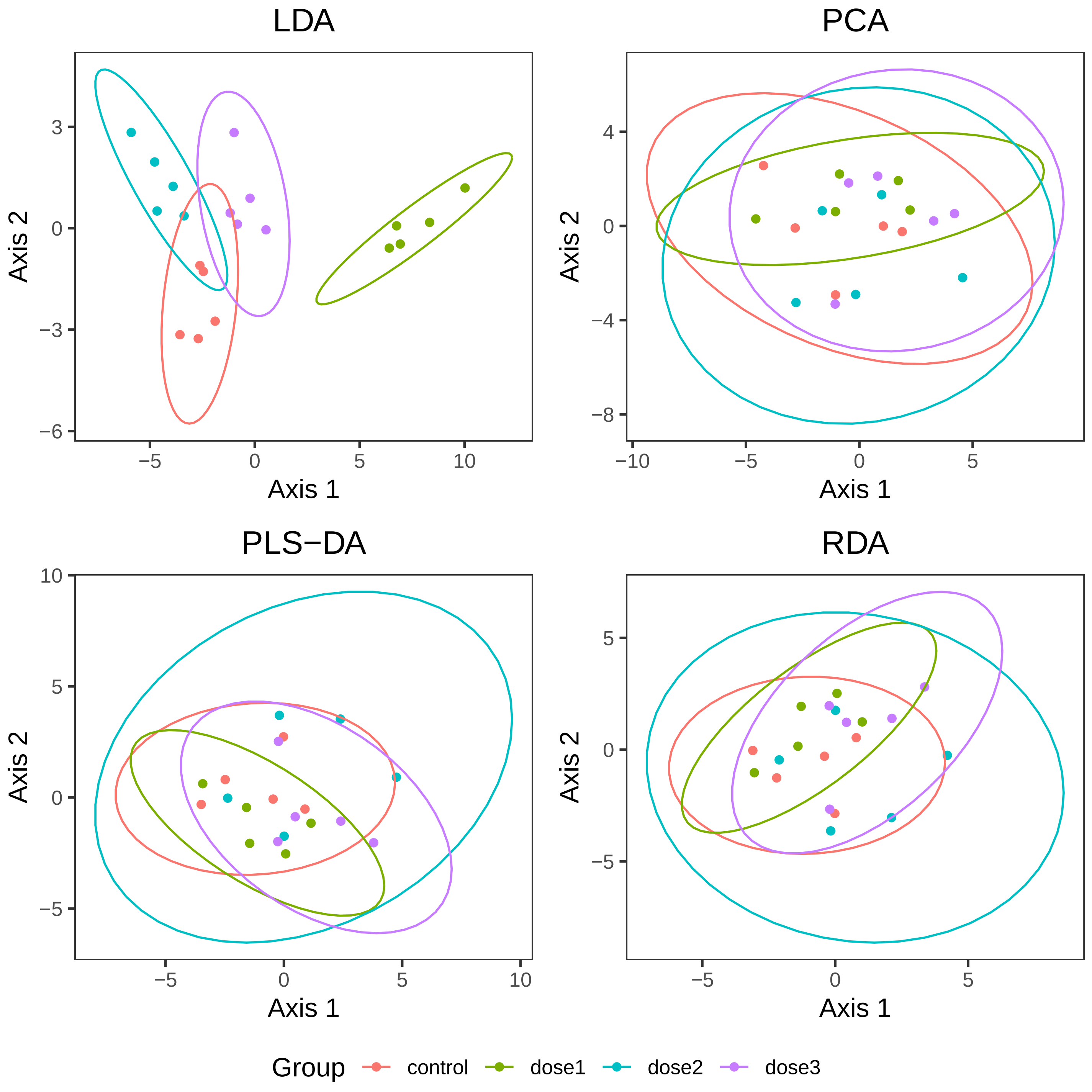
LDA
PCA
PLS−DA
RDA
Axis 1
Axis 2
Group
control
dose1
dose2
dose3

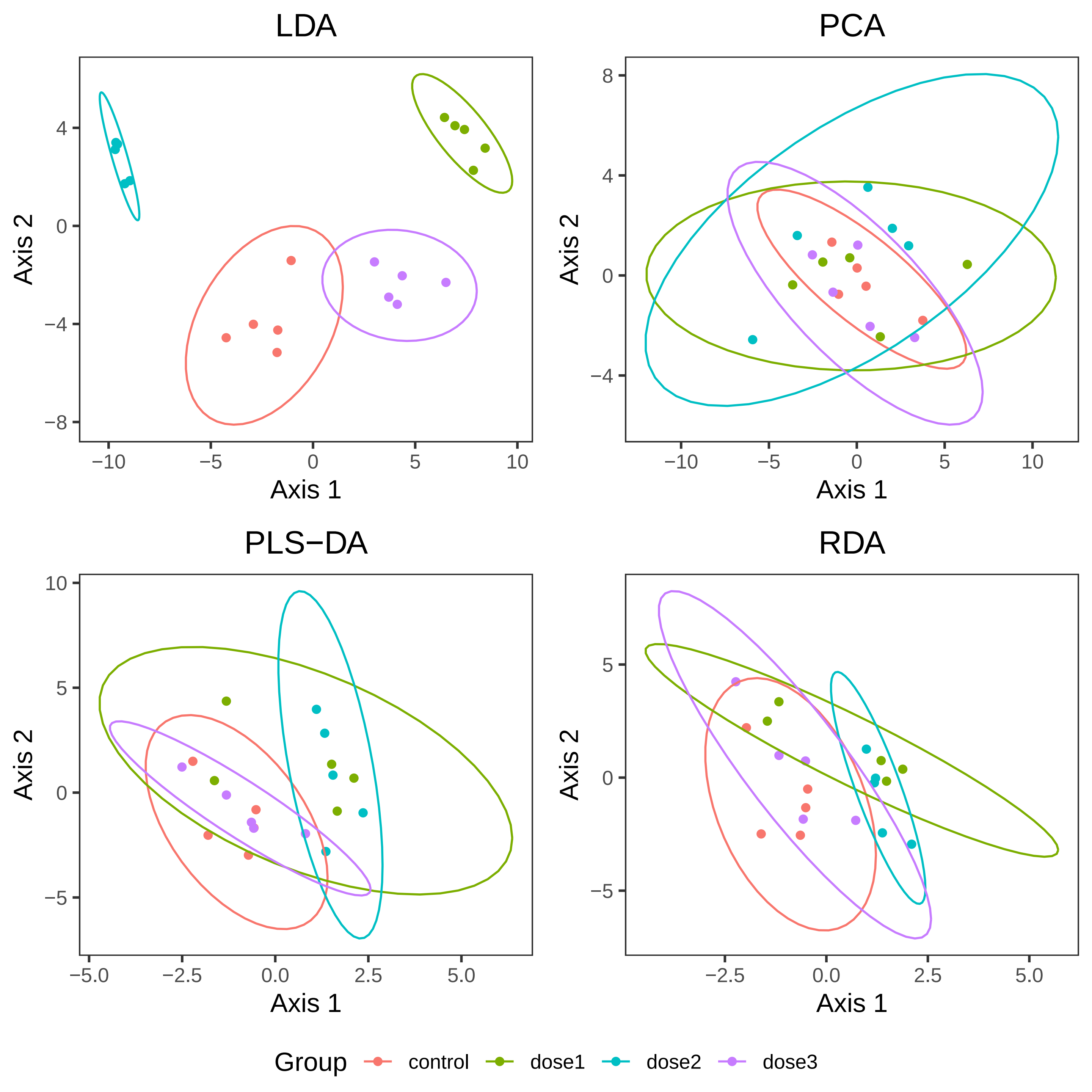

LDA
PCA
PLS−DA
RDA
Axis 1
Axis 2
Group
control
dose1
dose2
dose3

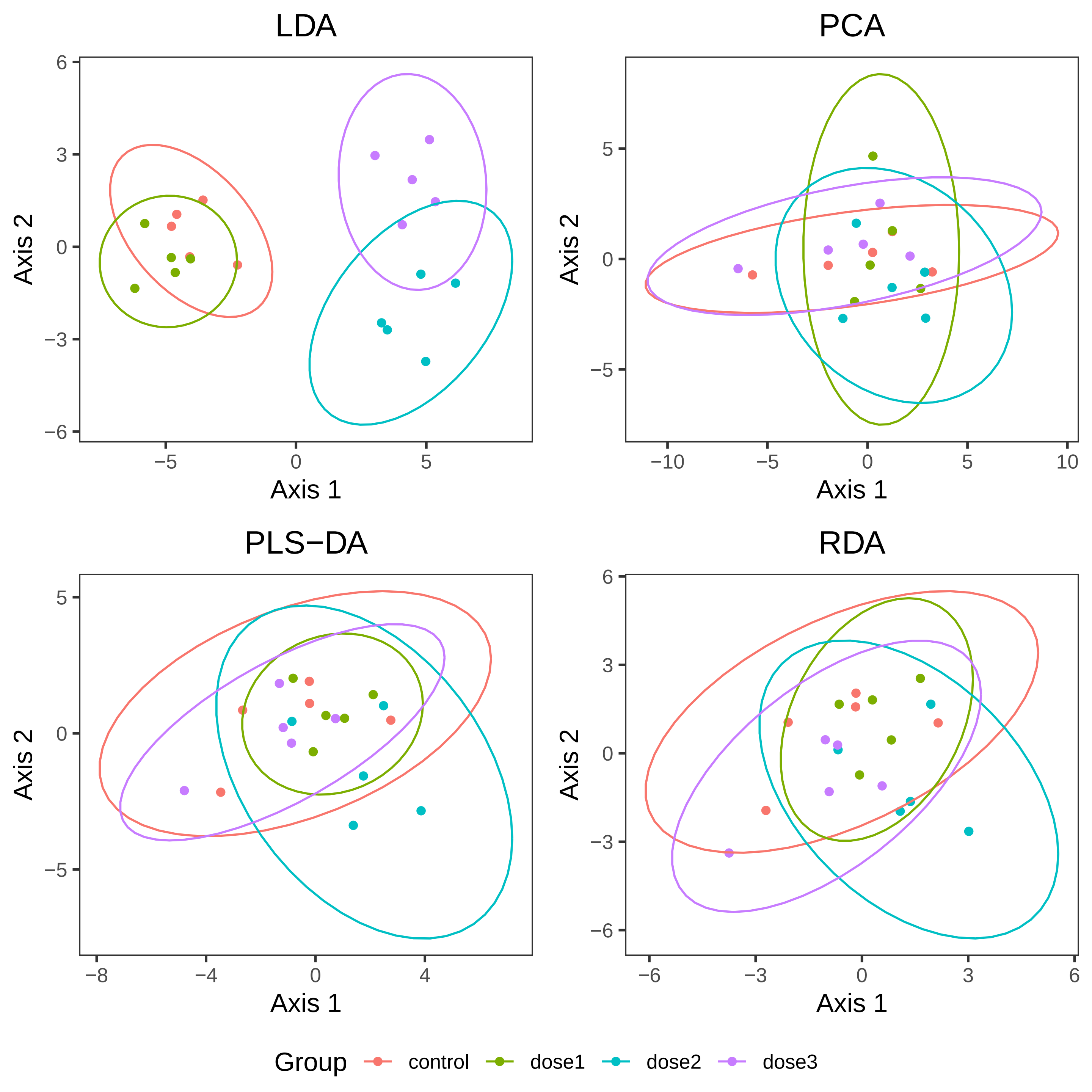
LDA
PCA
PLS−DA
RDA
Axis 1
Axis 2
Group
control
dose1
dose2
dose3

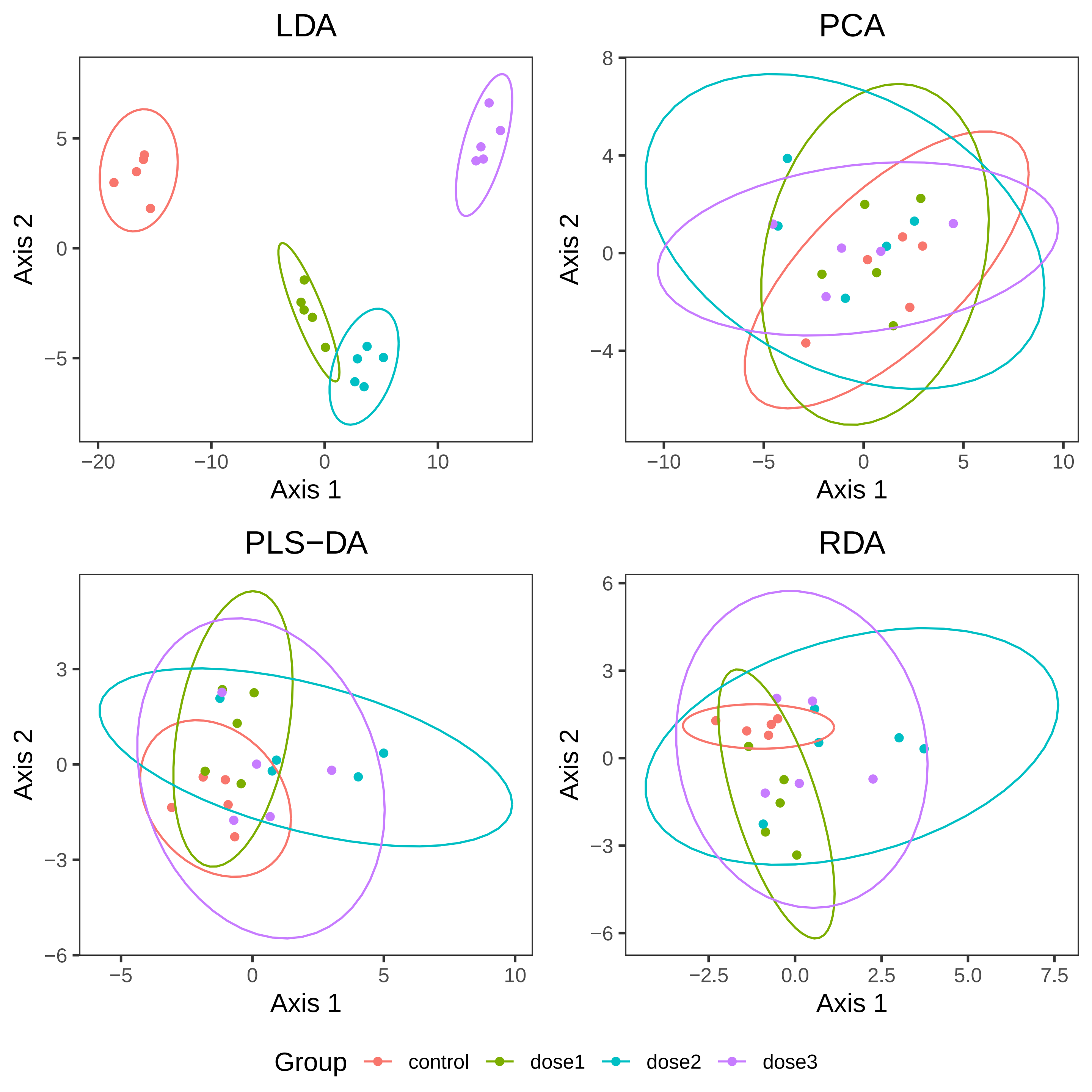

LDA
PCA
PLS−DA
RDA
Axis 1
Axis 2
Group
control
dose1
dose2
dose3

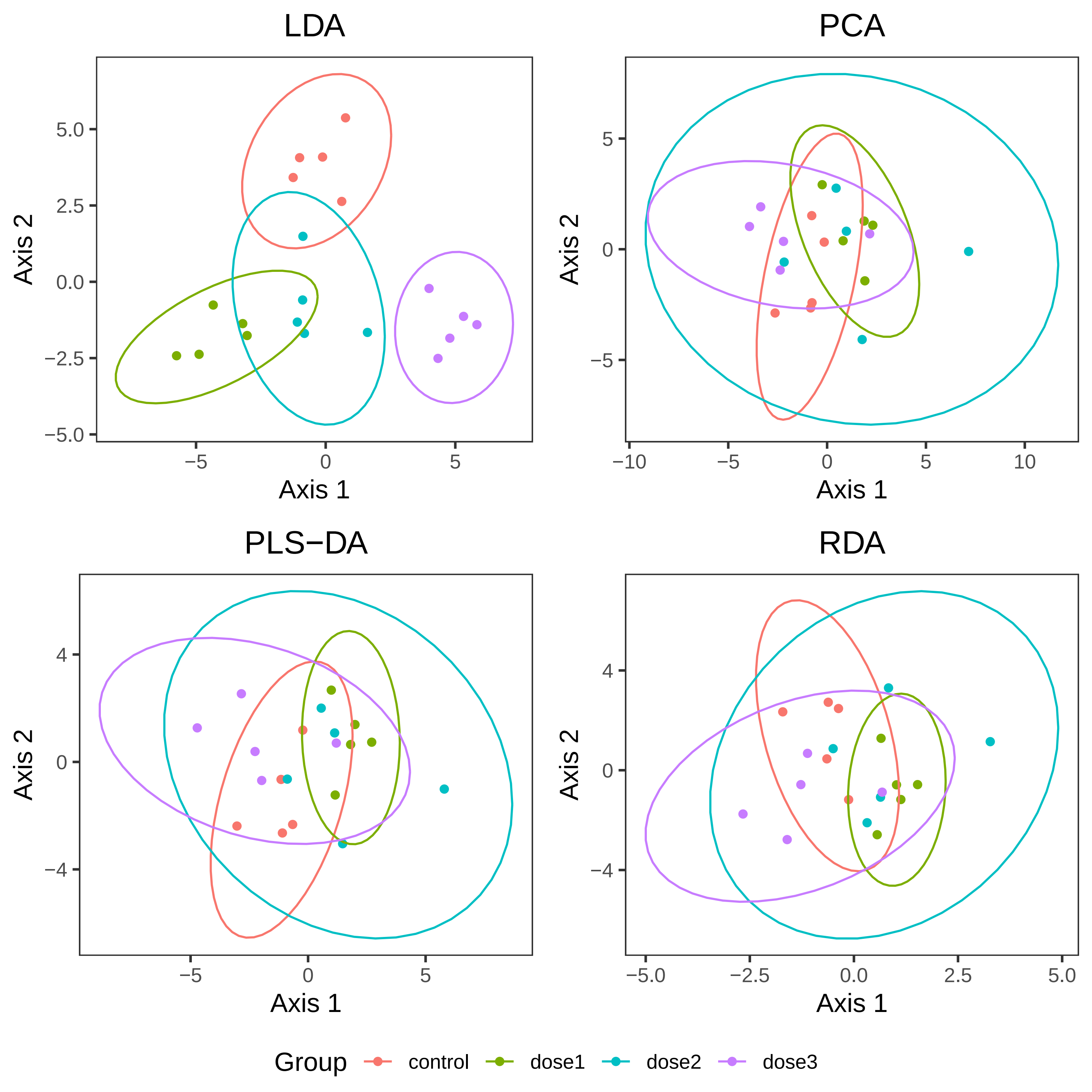
LDA
PCA
PLS−DA
RDA
Axis 1
Axis 2
5.0
2.5
0.0
−2.5
−5.0
−5
0
5
10
−10
4
−4
Group
control
dose1
dose2
dose3

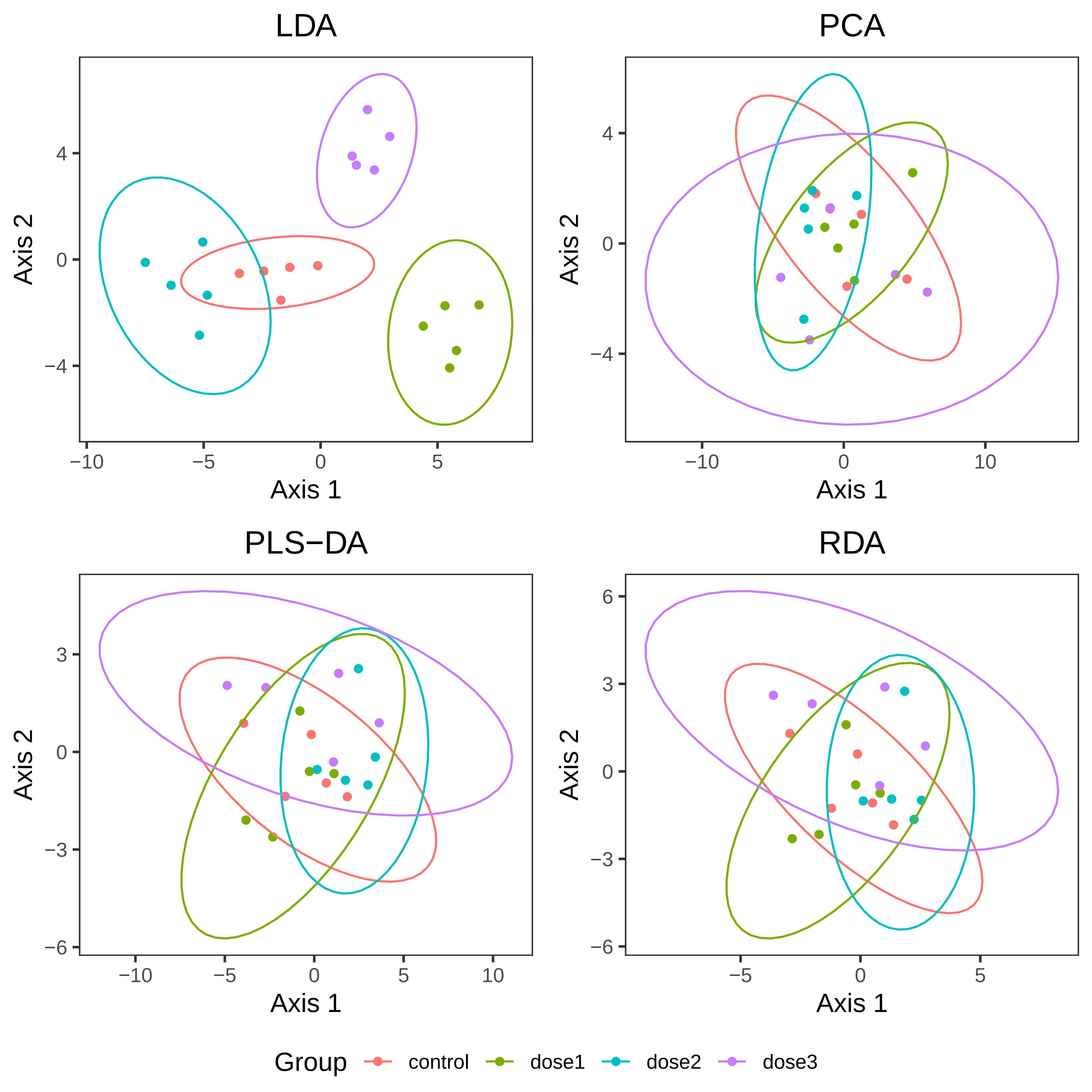

LDA
PCA
PLS−DA
RDA
Axis 1
Axis 2
−10
−5
0
5
10
4
−4
3
−3
6
−6
Group
control
dose1
dose2
dose3

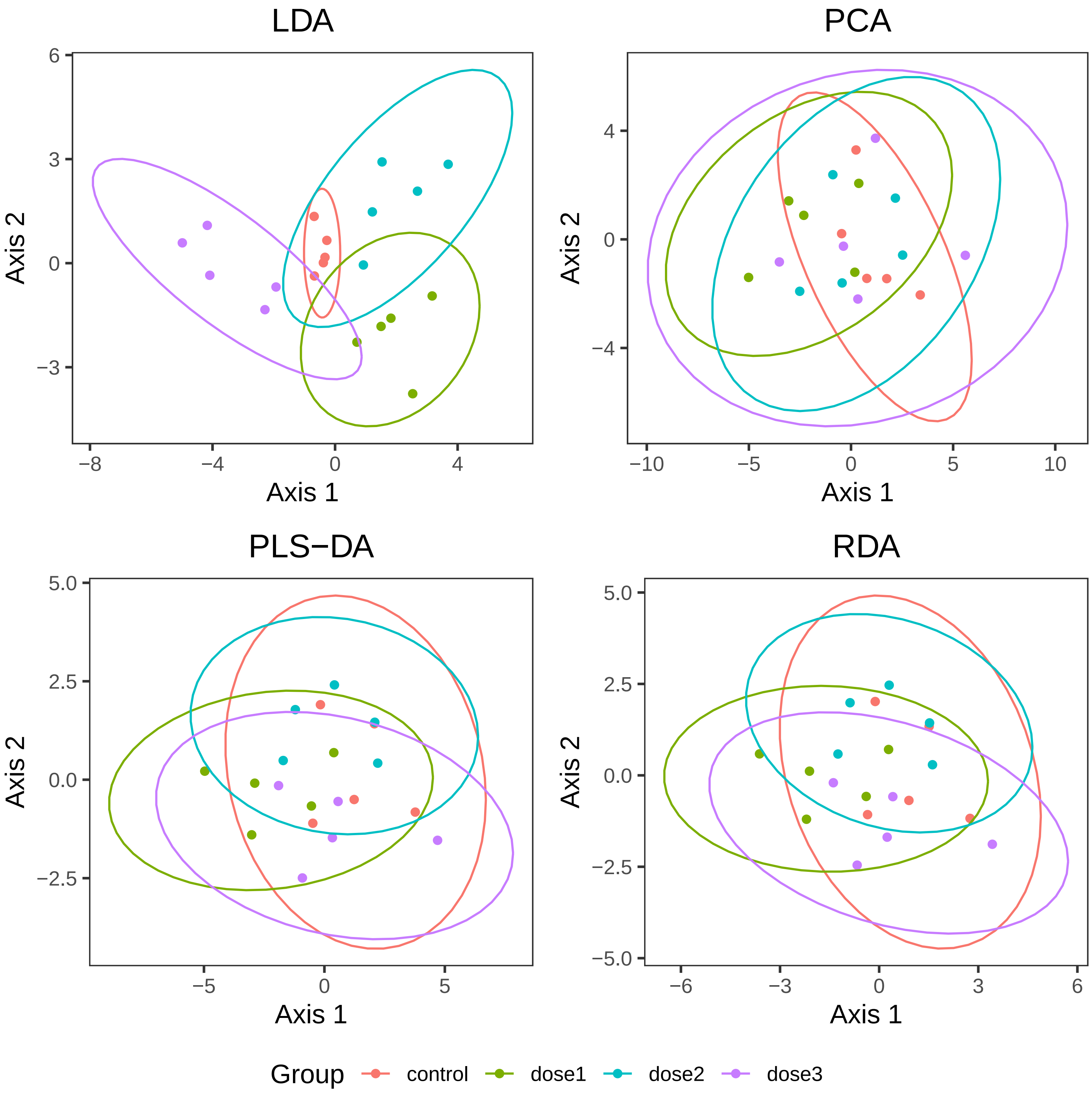
LDA
PCA
PLS−DA
RDA
Axis 1
Axis 2
Group
control
dose1
dose2
dose3

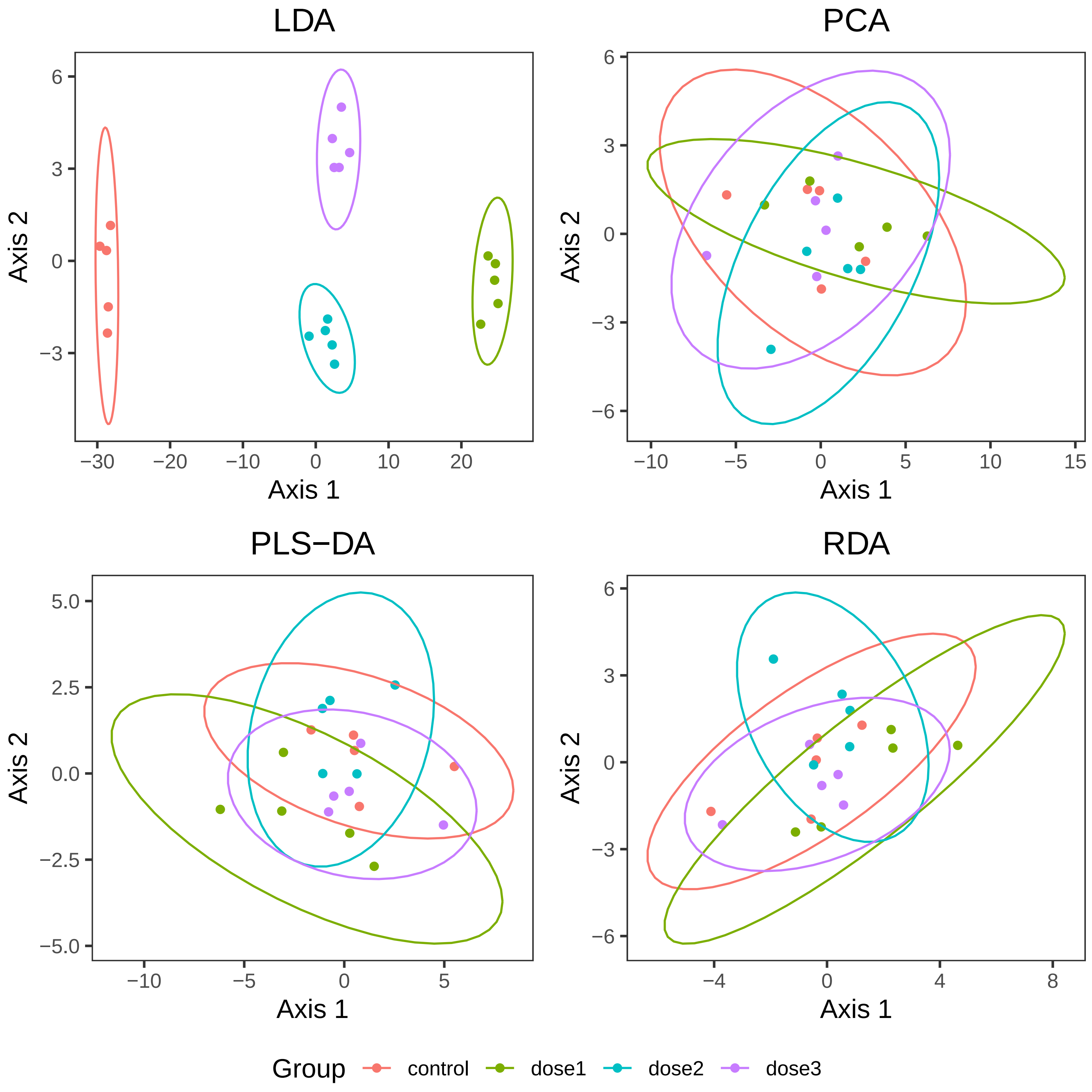

LDA
PCA
PLS−DA
RDA
Axis 1
Axis 2
Group
control
dose1
dose2
dose3

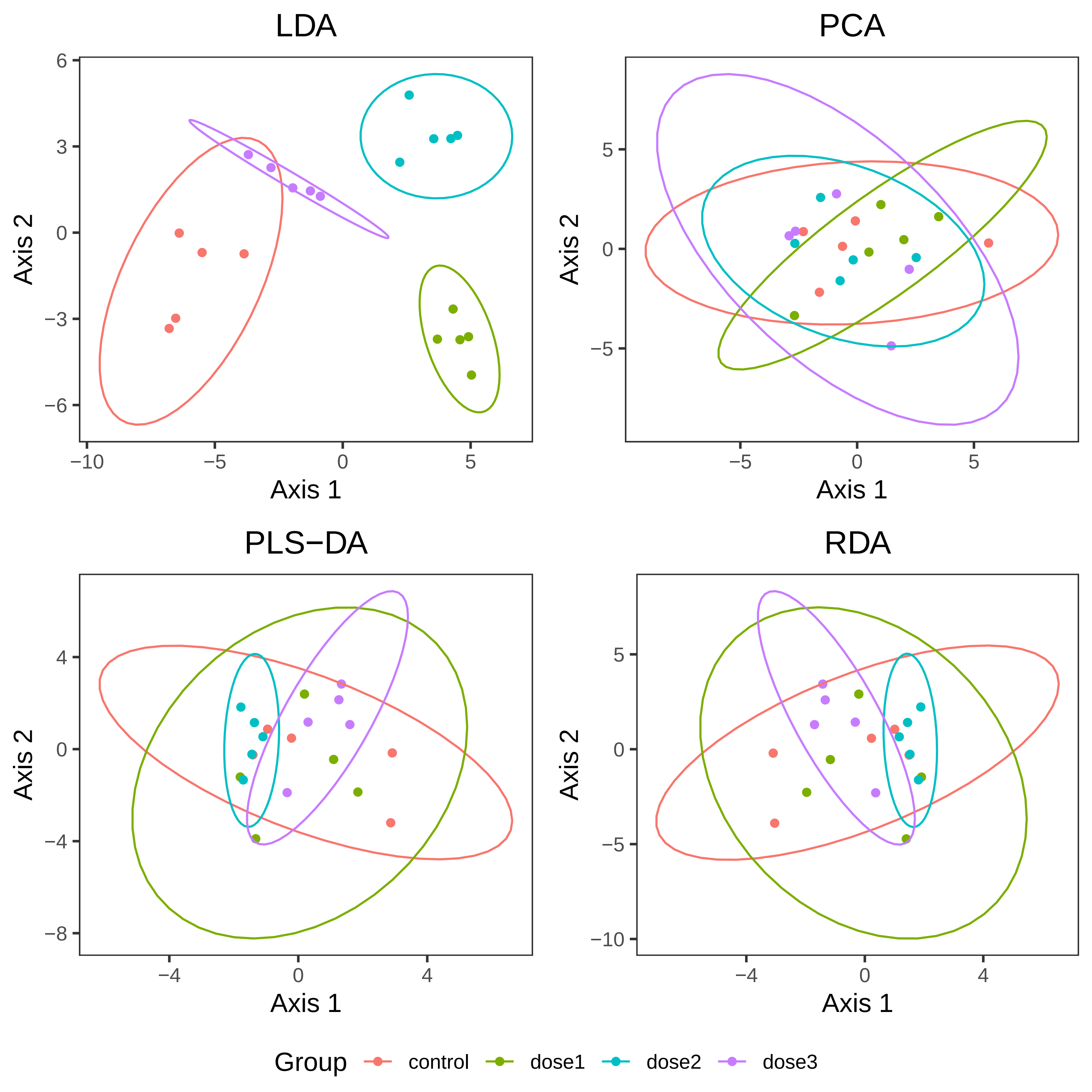

LDA
PCA
PLS−DA
RDA
Axis 1
Axis 2
6
3
0
−3
−6
−10
−5
0
5
−4
4
−8
−10
Group
control
dose1
dose2
dose3

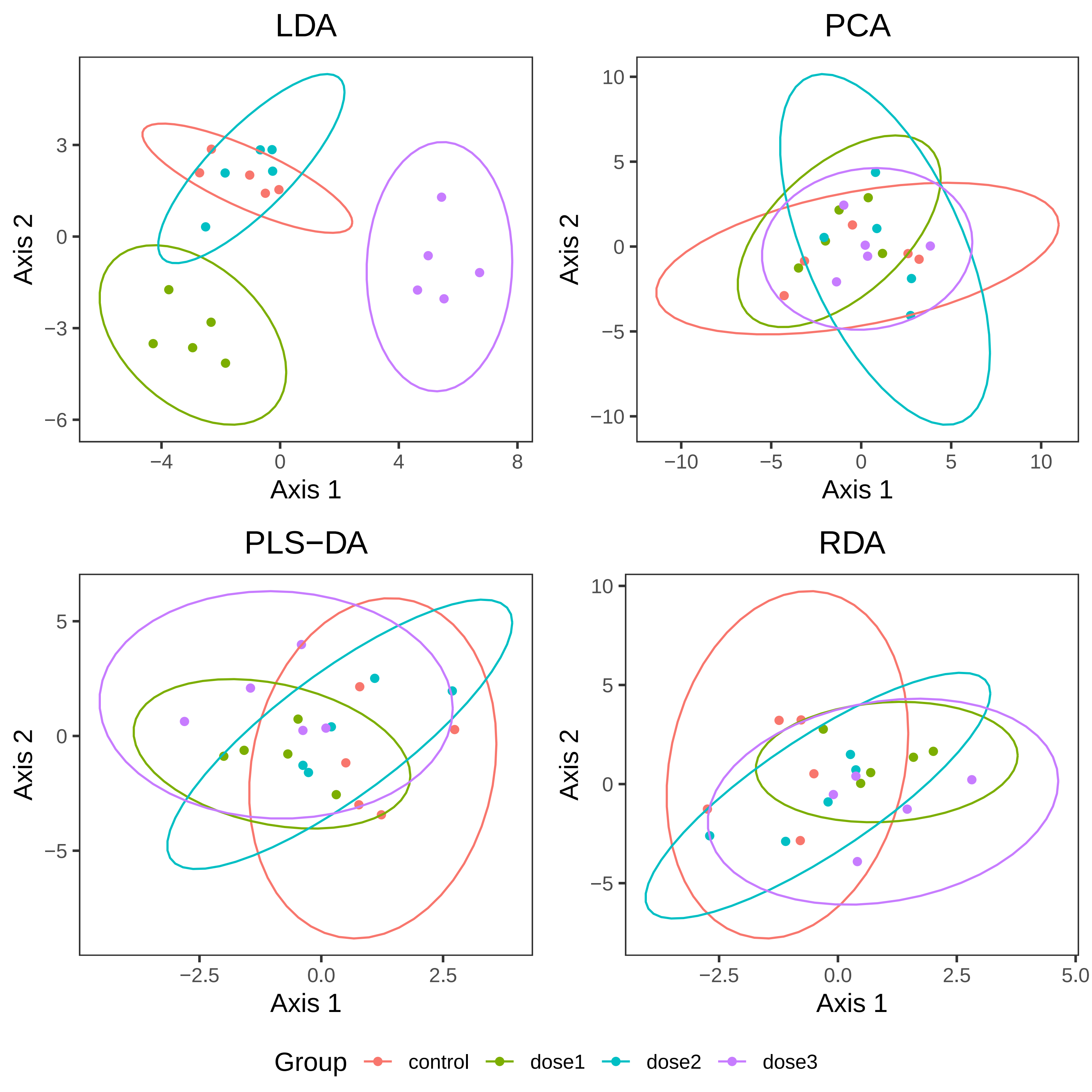
LDA
PCA
PLS−DA
RDA
Axis 1
Axis 2
Group
control
dose1
dose2
dose3

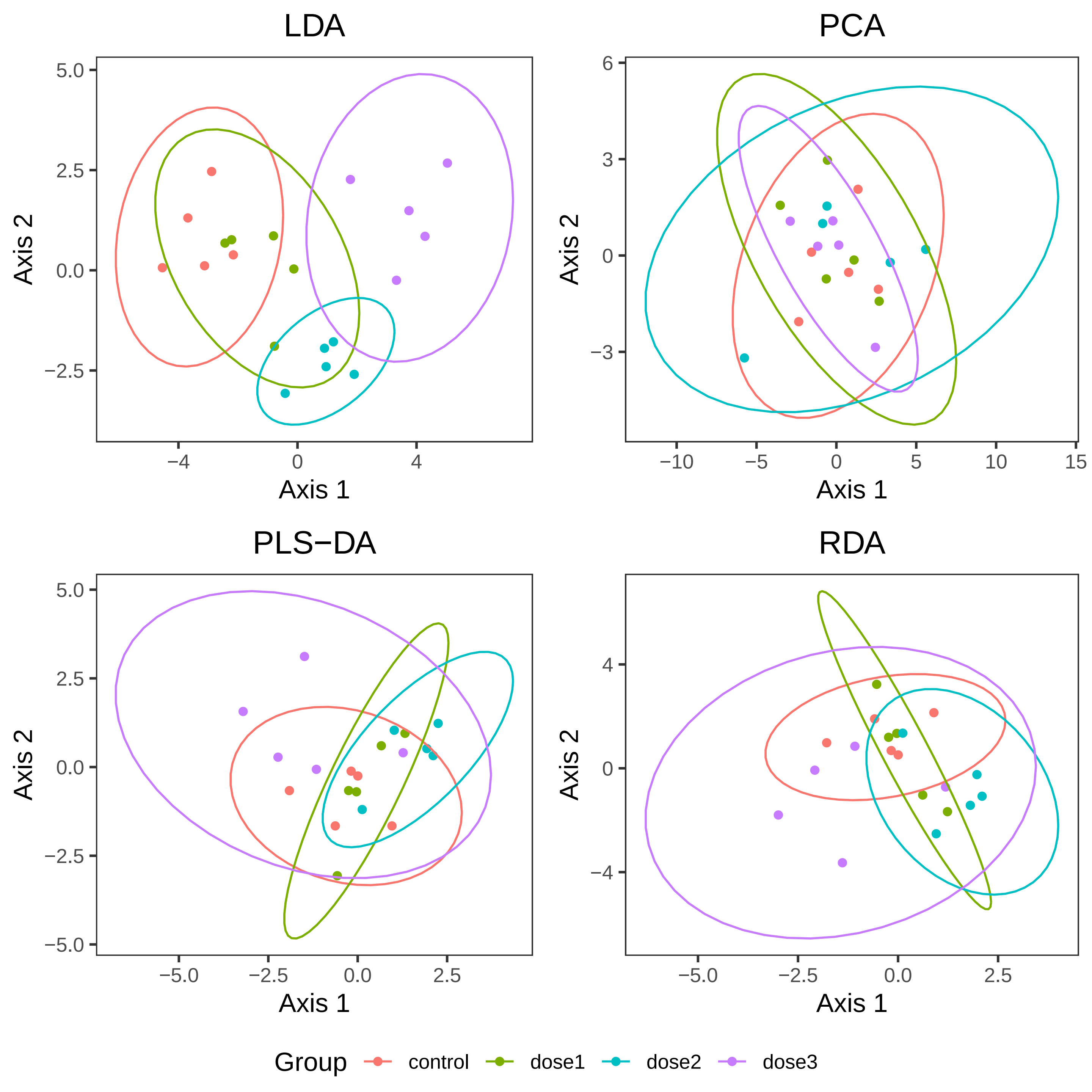
LDA
PCA
PLS−DA
RDA
Axis 1
Axis 2
5.0
2.5
0.0
−2.5
−5.0
−4
0
4
6
3
−3
−10
−5
5
10
15
2.5
−2.5
Group
control
dose1
dose2
dose3

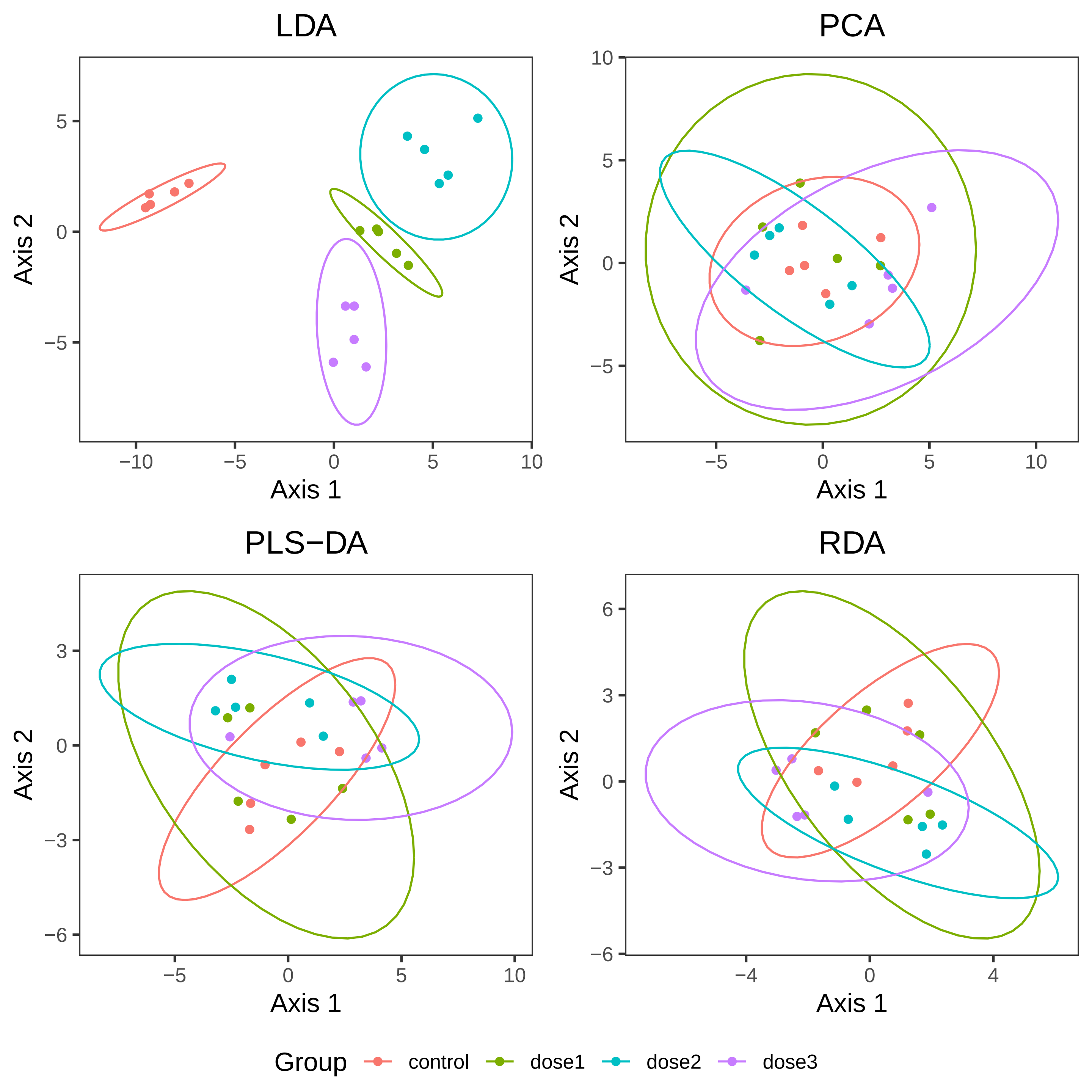
LDA
PCA
PLS−DA
RDA
Axis 1
Axis 2
Group
control
dose1
dose2
dose3

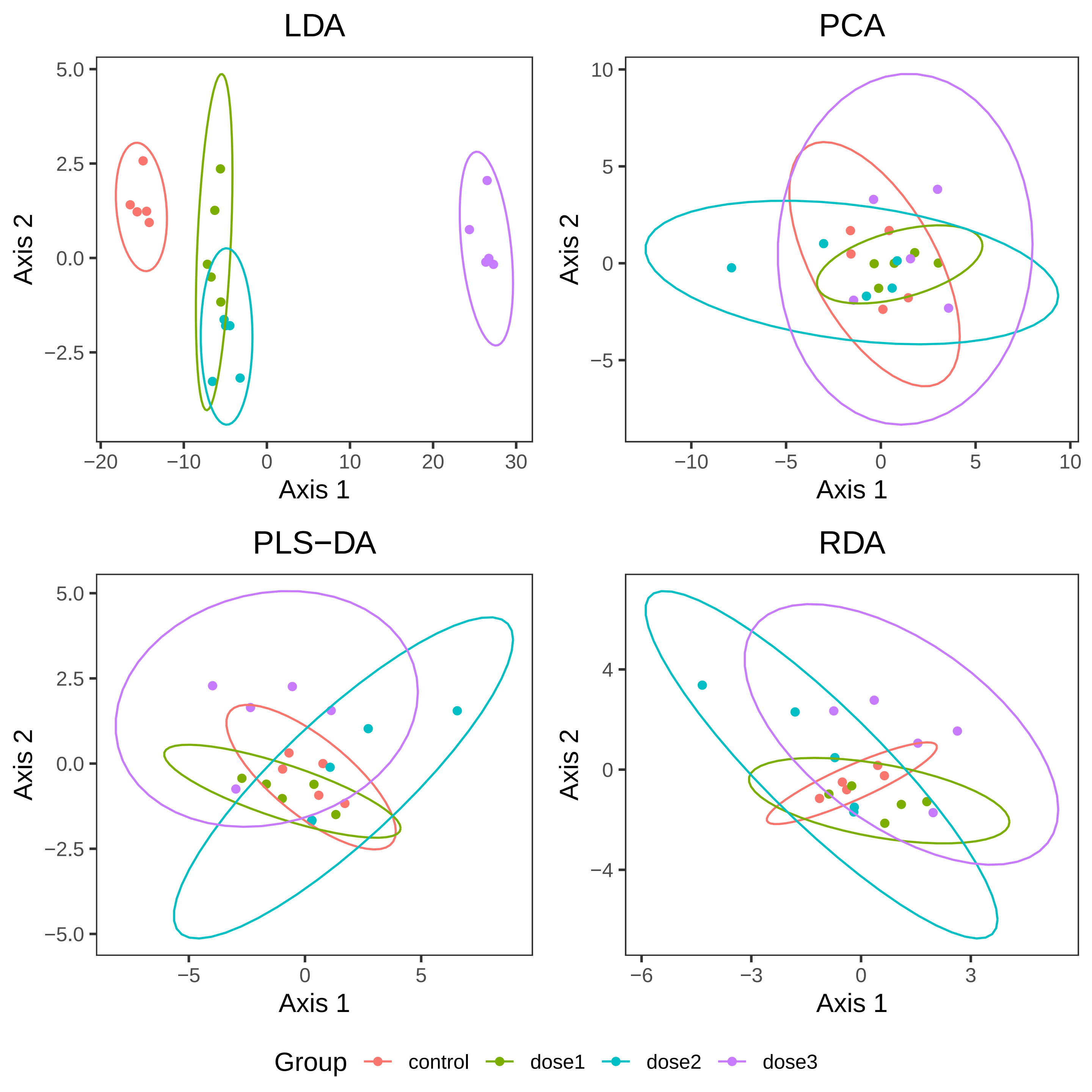

LDA
PCA
PLS−DA
RDA
Axis 1
Axis 2
Group
control
dose1
dose2
dose3

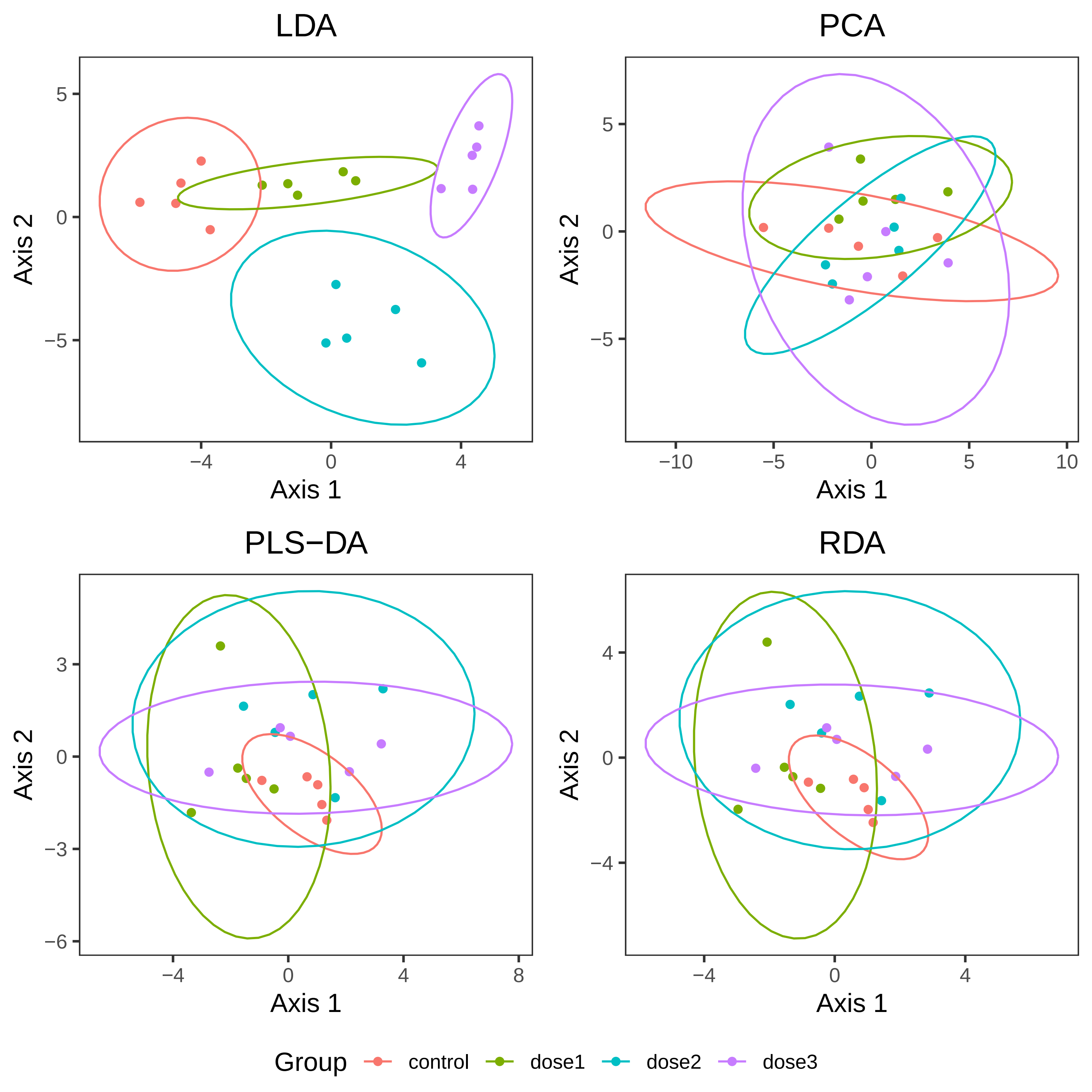

LDA
PCA
PLS−DA
RDA
Axis 1
Axis 2
Group
control
dose1
dose2
dose3

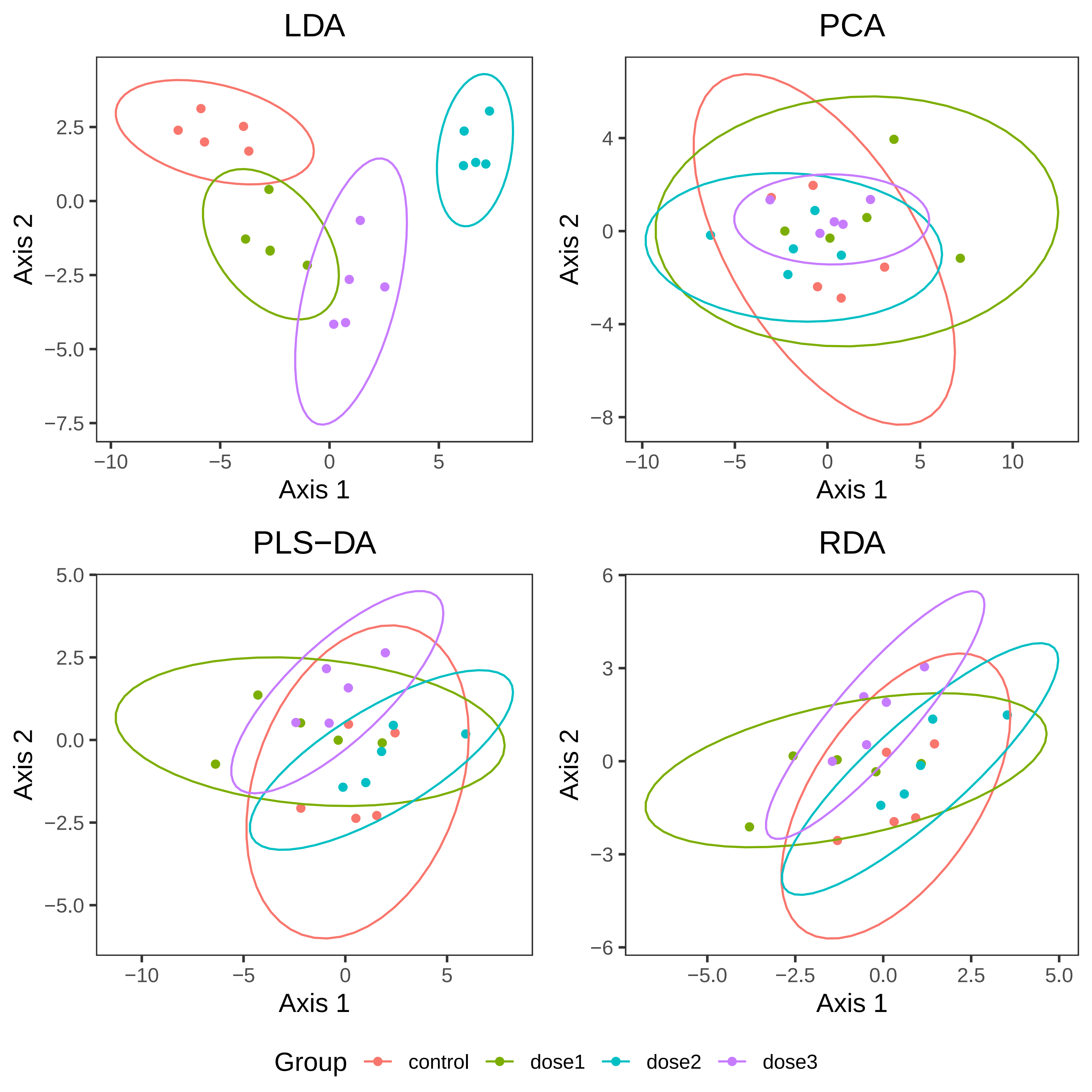
LDA
PCA
PLS−DA
RDA
Axis 1
Axis 2
Group
control
dose1
dose2
dose3

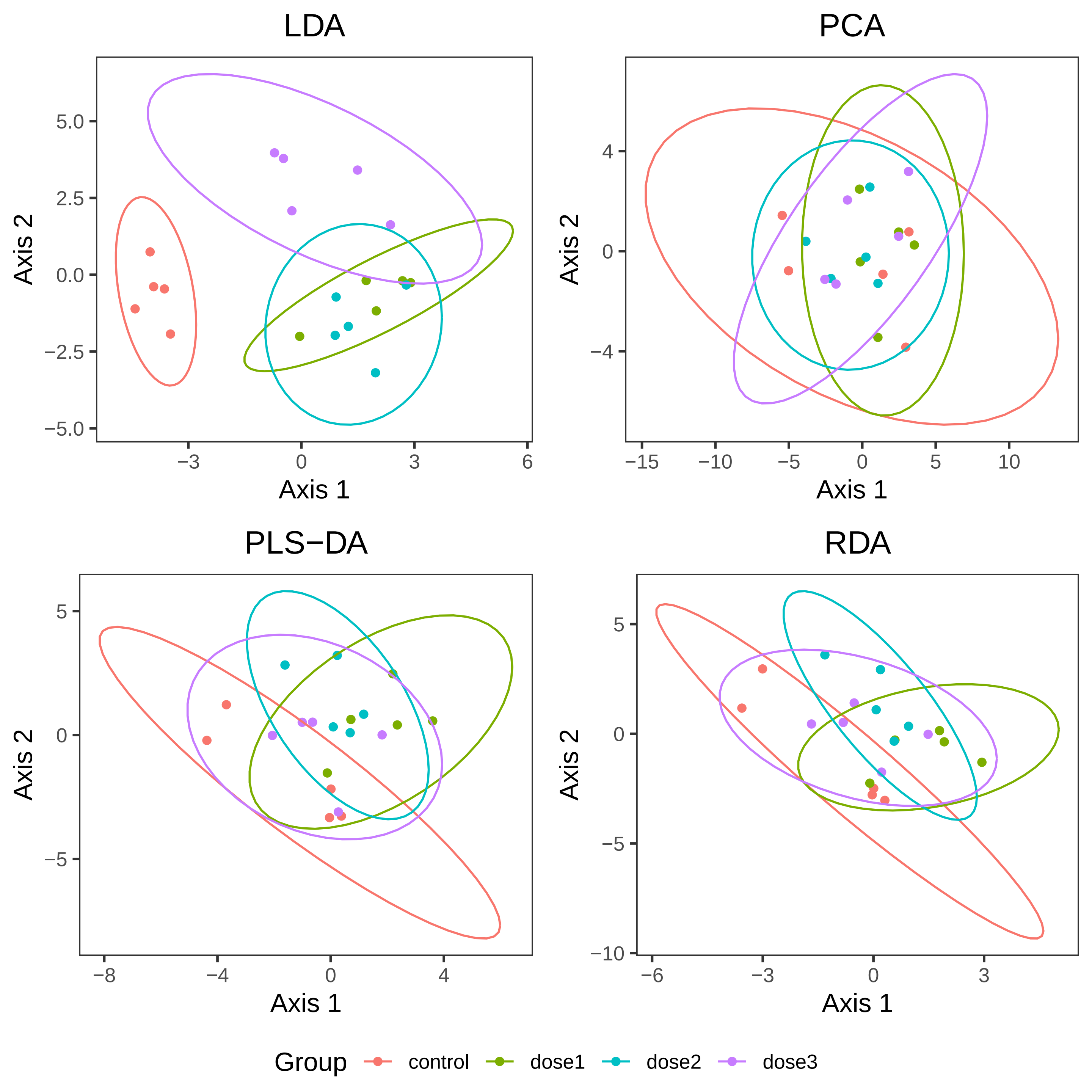

LDA
PCA
PLS−DA
RDA
Axis 1
Axis 2
Group
control
dose1
dose2
dose3

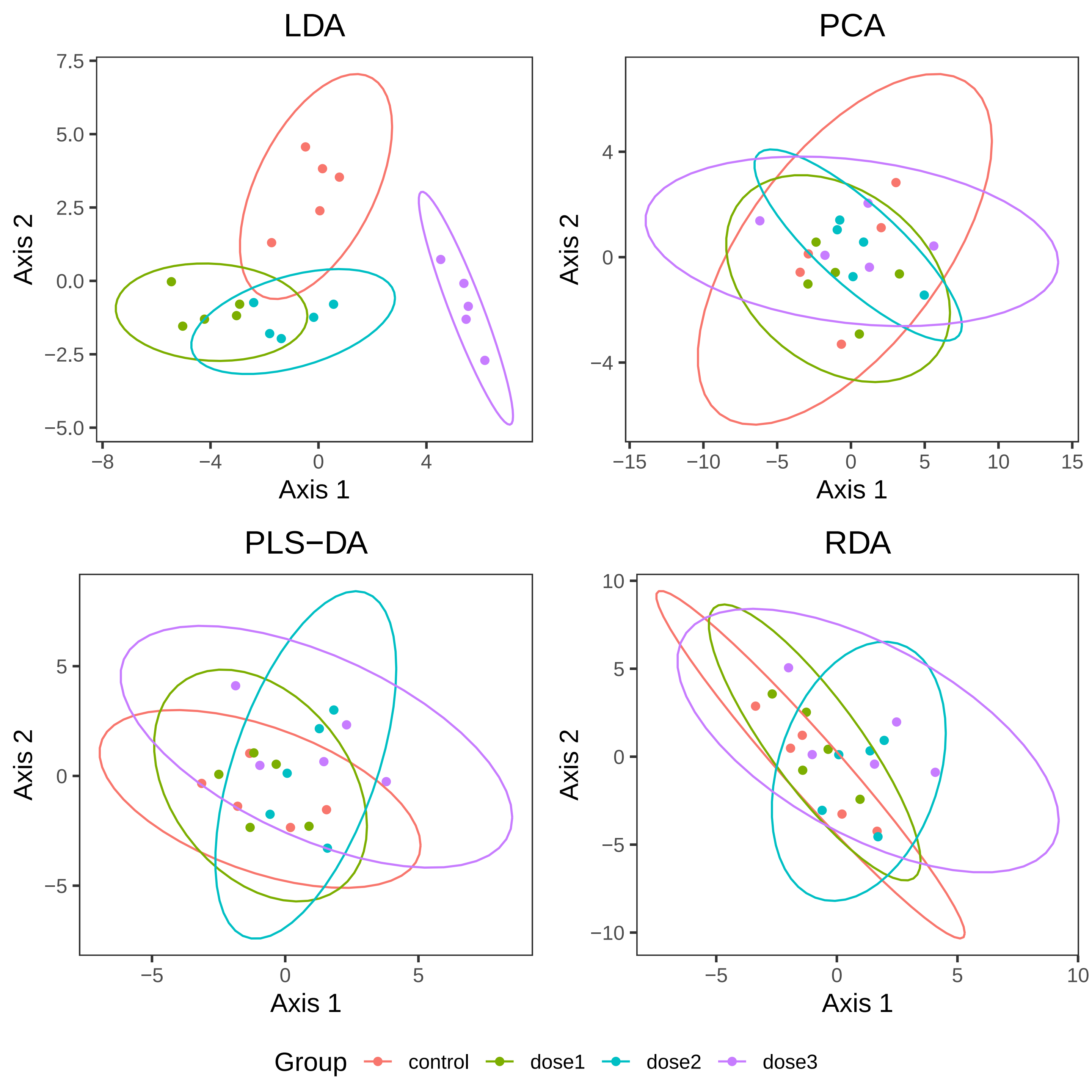

LDA
PCA
PLS−DA
RDA
Axis 1
Axis 2
7.5
5.0
2.5
0.0
−2.5
−5.0
−8
−4
0
4
−15
−10
−5
0
5
10
15
−10
Group
control
dose1
dose2
dose3

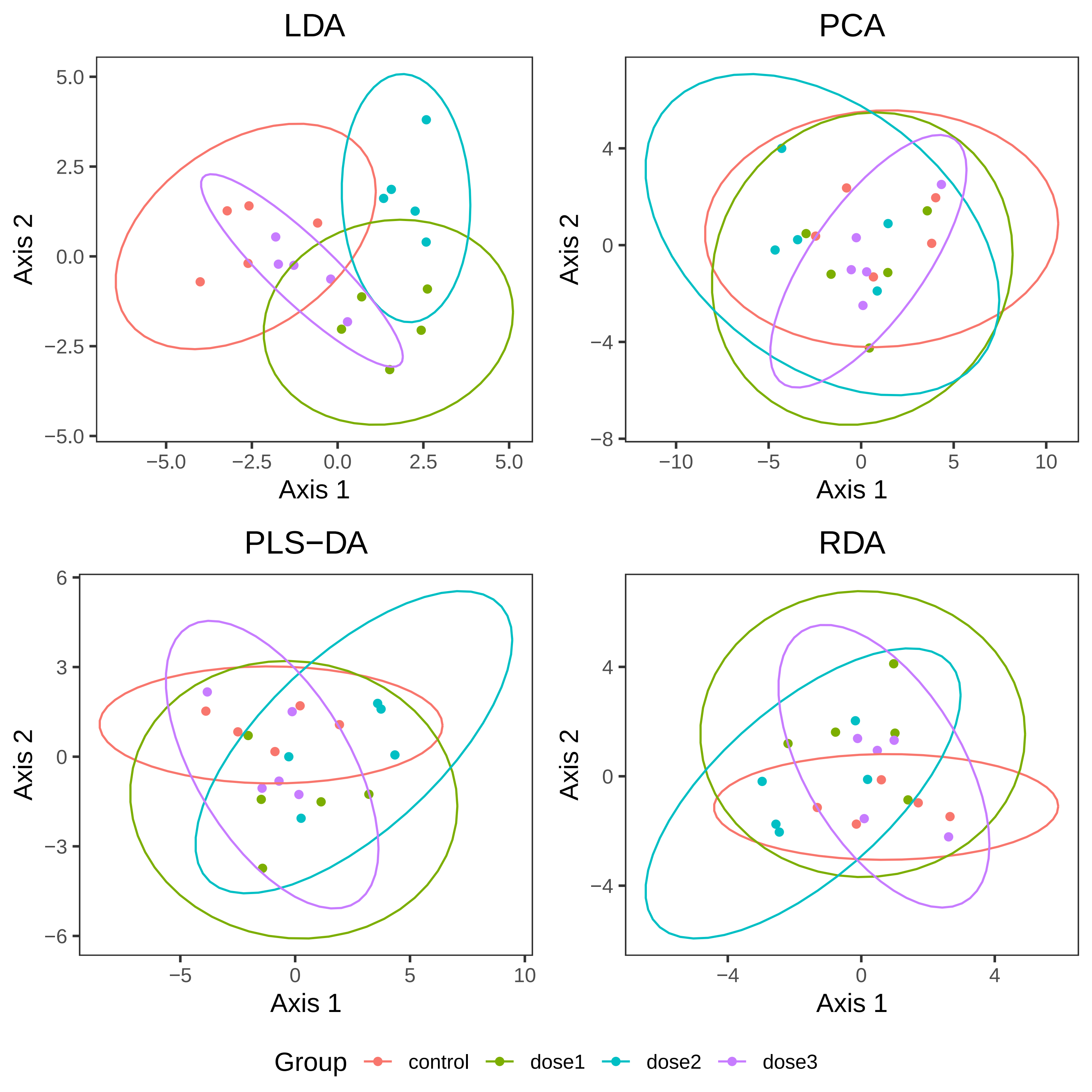

LDA
PCA
PLS−DA
RDA
Axis 1
Axis 2
5.0
2.5
0.0
−2.5
−5.0
4
0
−4
−8
−10
−5
5
10
6
3
−3
−6
Group
control
dose1
dose2
dose3

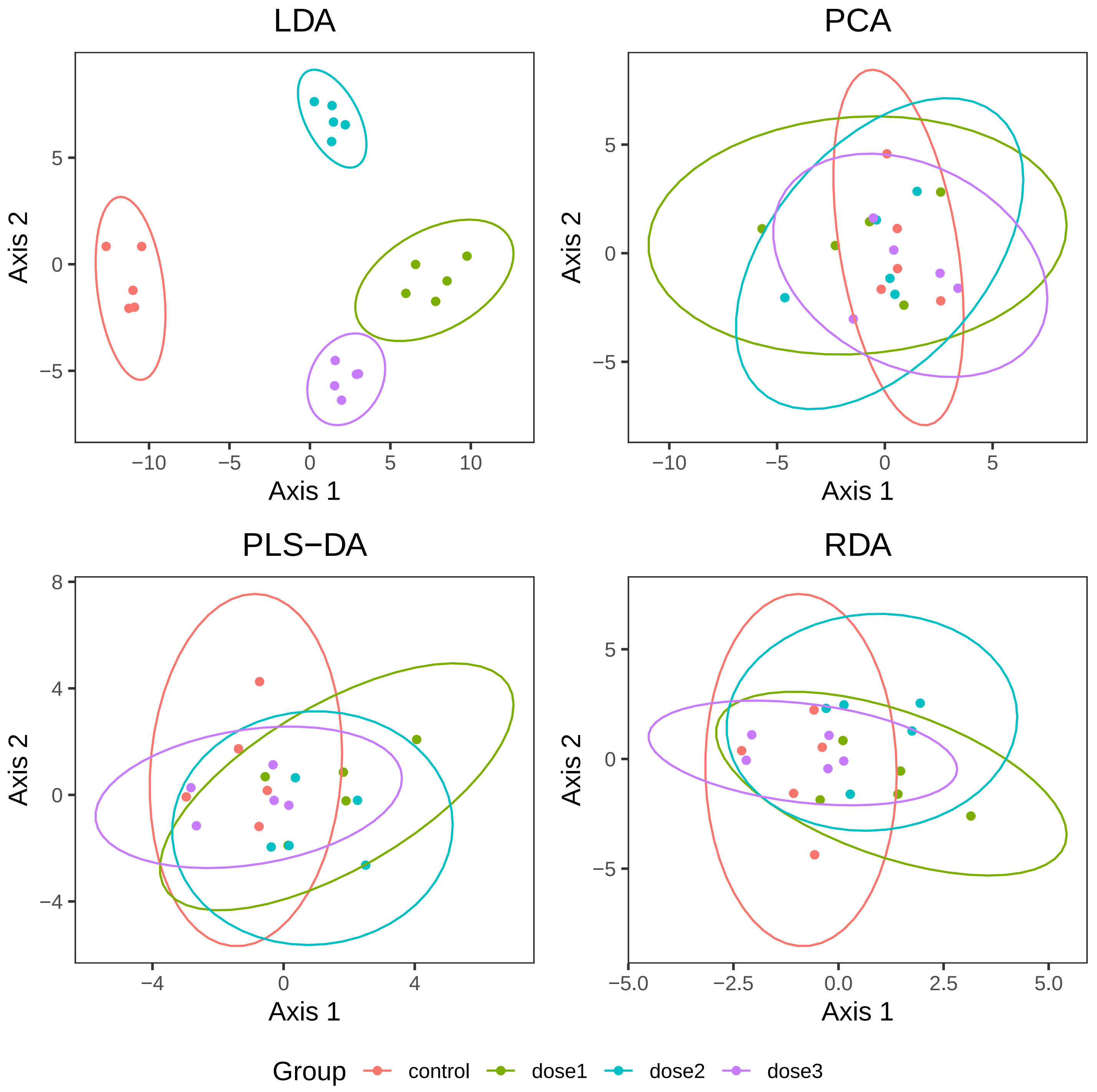

LDA
PCA
PLS−DA
RDA
Axis 1
Axis 2
Group
control
dose1
dose2
dose3

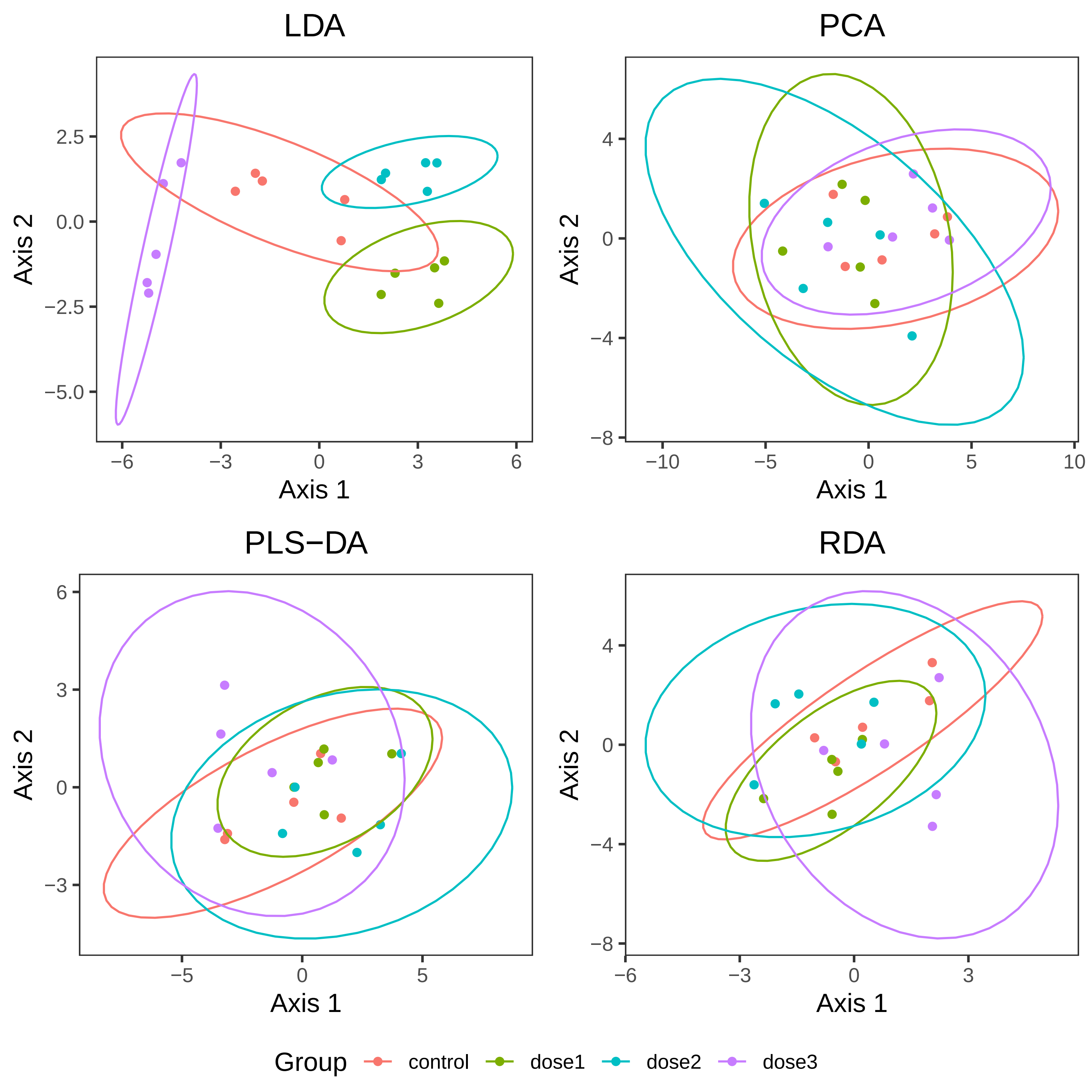

LDA
PCA
PLS−DA
RDA
Axis 1
Axis 2
Group
control
dose1
dose2
dose3

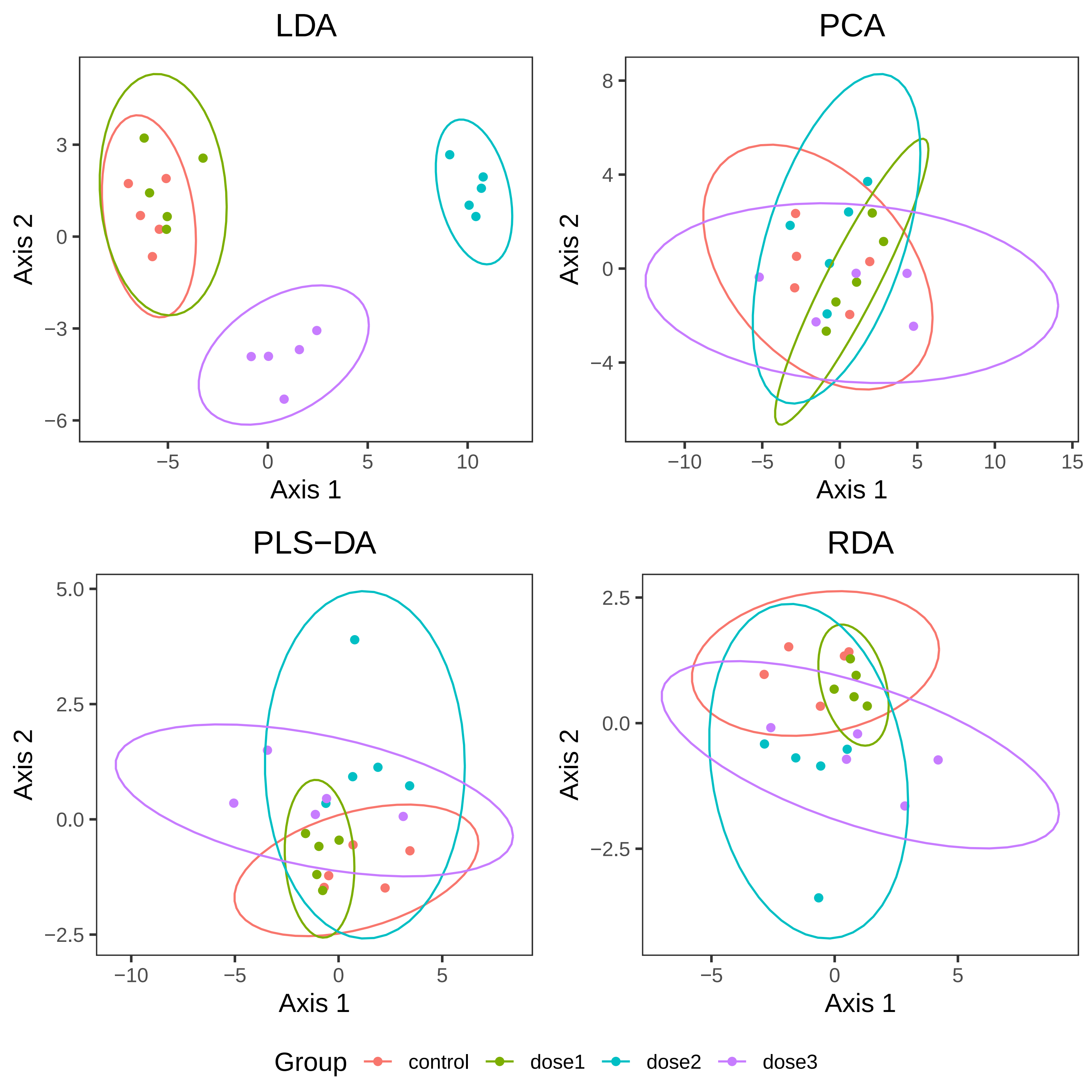
LDA
PCA
PLS−DA
RDA
Axis 1
Axis 2
3
0
−3
−6
−5
0
5
10
8
4
0
−4
−10
−5
0
5
10
15
5.0
2.5
0.0
−2.5
−10
−5
0
5
2.5
0.0
−2.5
−5
0
5
Group
control
dose1
dose2
dose3

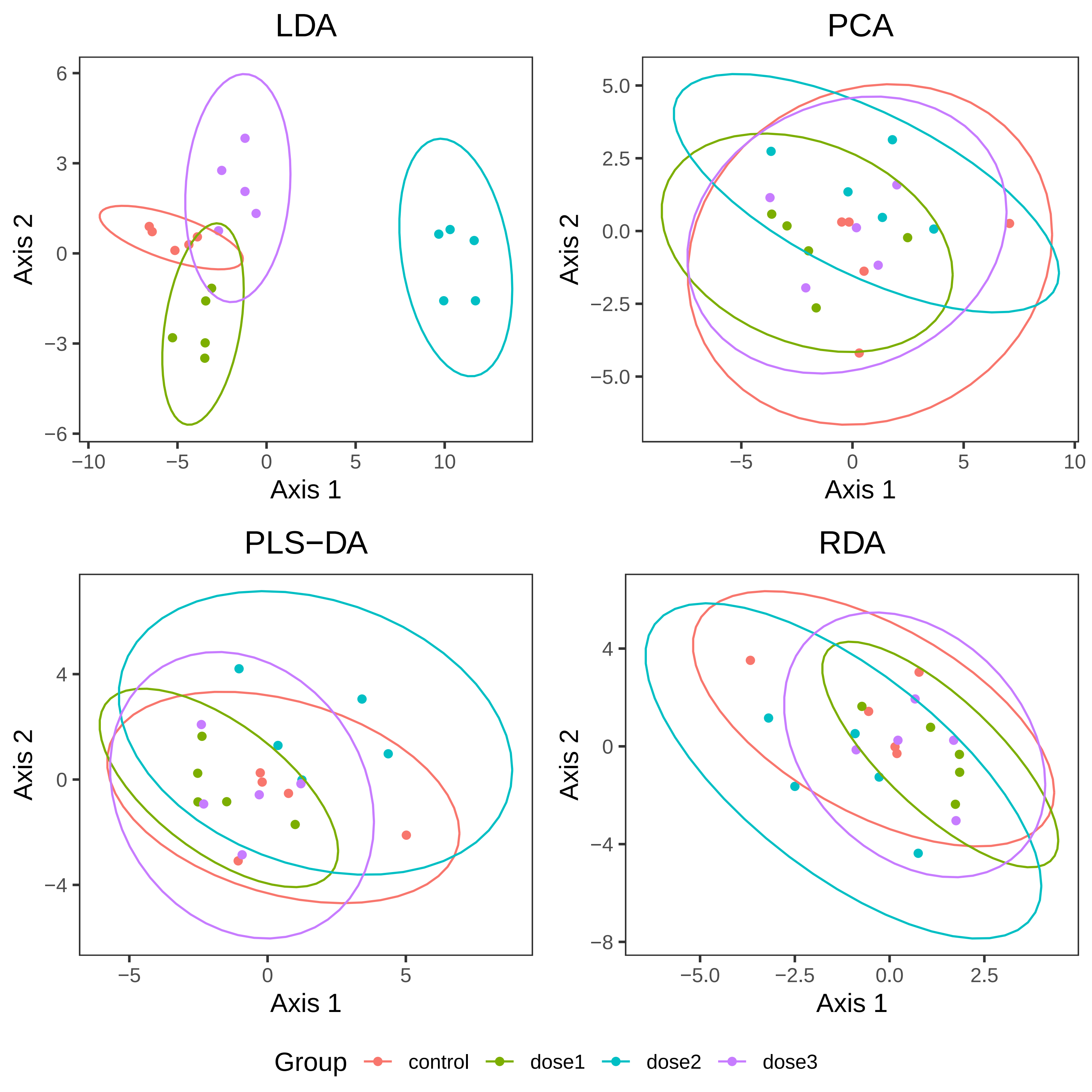
LDA
PCA
PLS−DA
RDA
Axis 1
Axis 2
Group
control
dose1
dose2
dose3

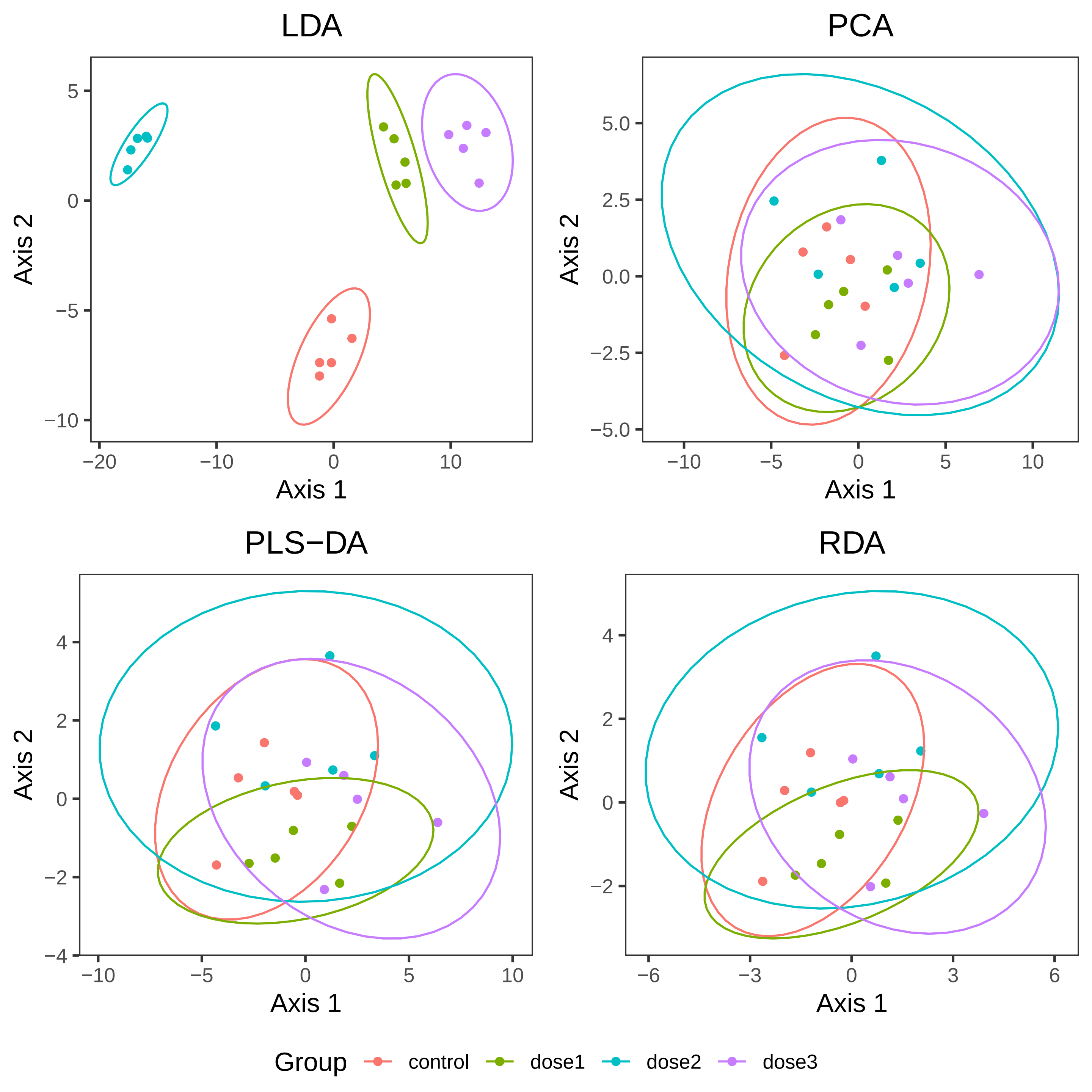

LDA
PCA
PLS−DA
RDA
Axis 1
Axis 2
Group
control
dose1
dose2
dose3

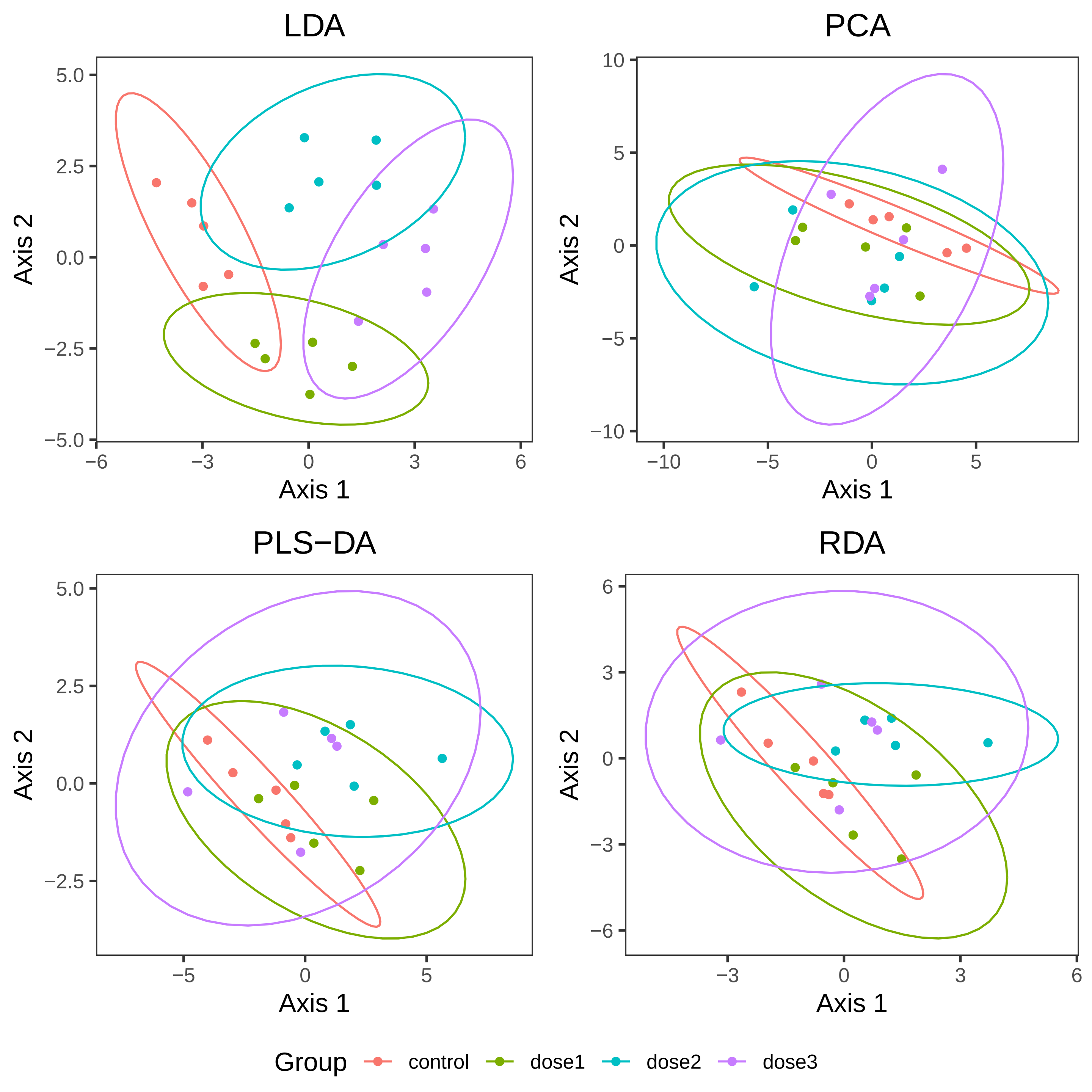

LDA
PCA
PLS−DA
RDA
Axis 1
Axis 2
Group
control
dose1
dose2
dose3

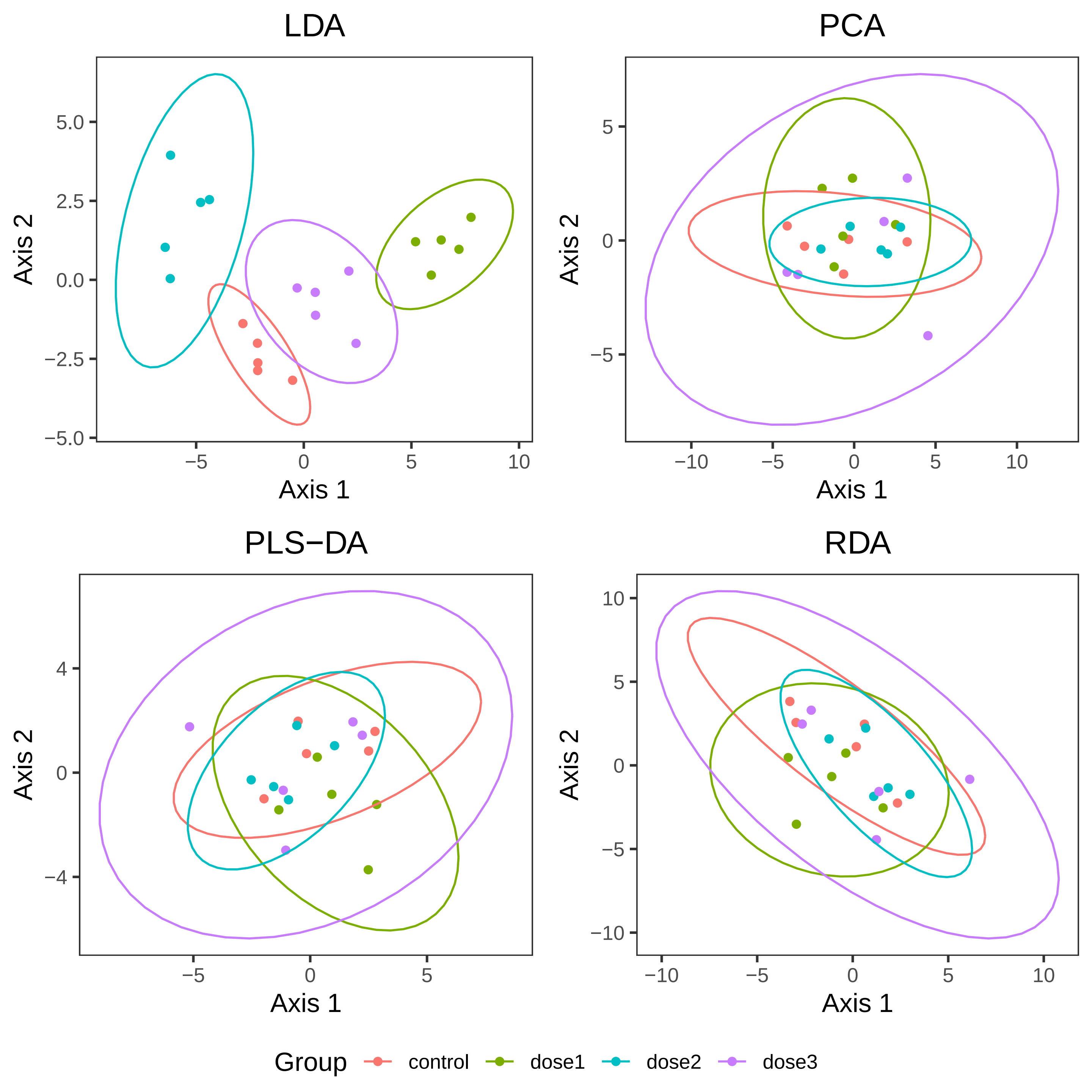
LDA
PCA
PLS−DA
RDA
Axis 1
Axis 2
5.0
2.5
0.0
−2.5
−5.0
−5
0
5
10
−10
4
−4
Group
control
dose1
dose2
dose3

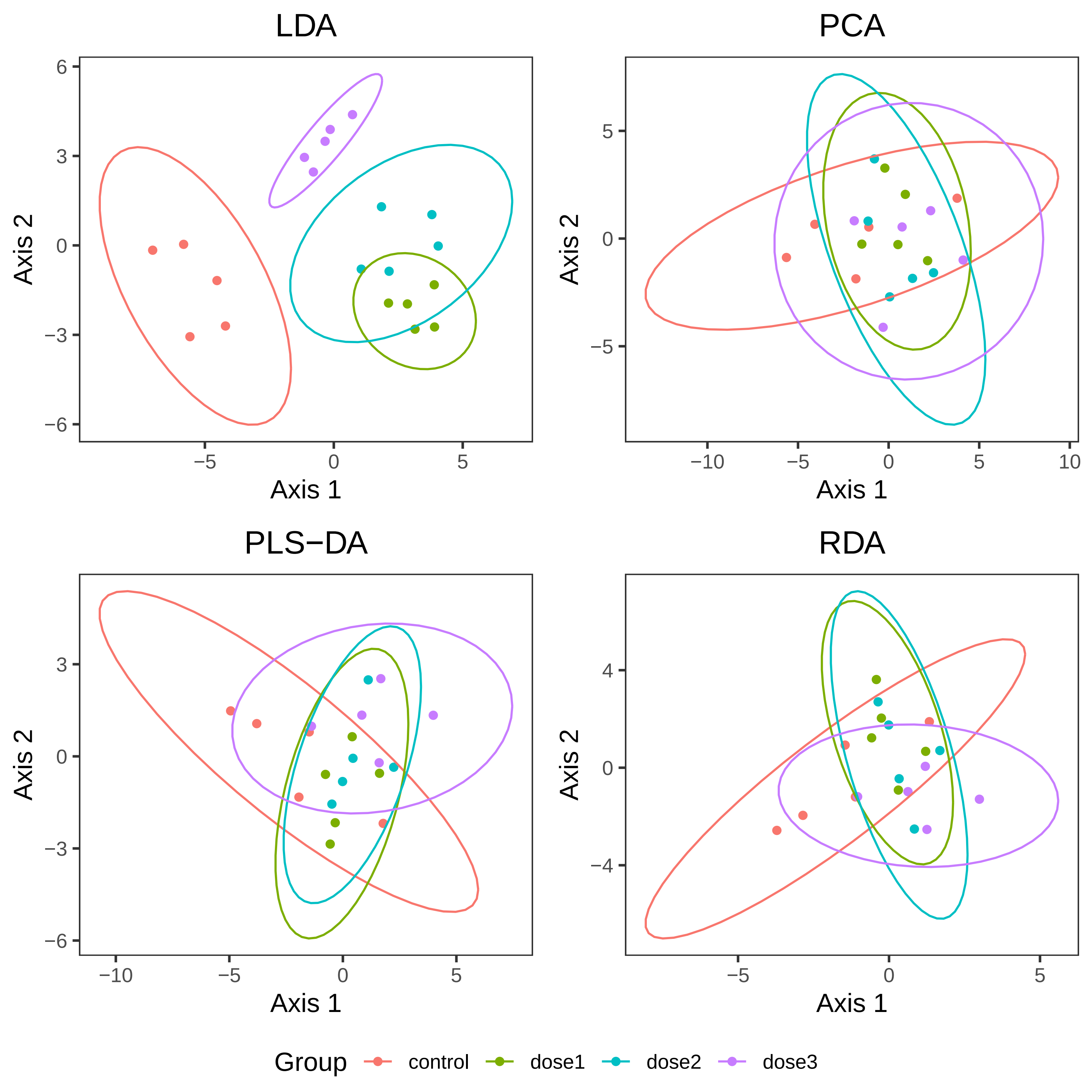
LDA
PCA
PLS−DA
RDA
Axis 1
Axis 2
Group
control
dose1
dose2
dose3

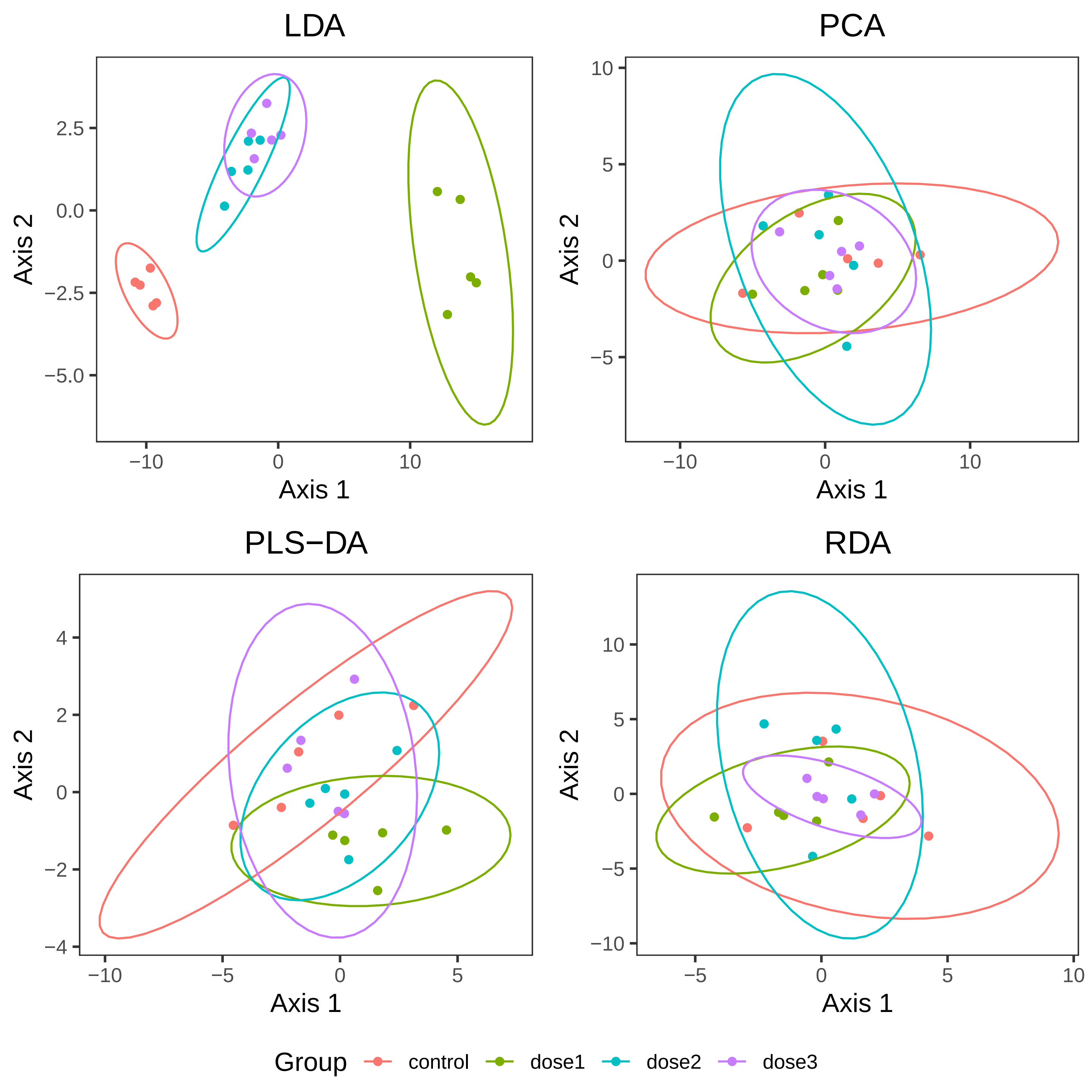

LDA
PCA
PLS−DA
RDA
Axis 1
Axis 2
Group
control
dose1
dose2
dose3

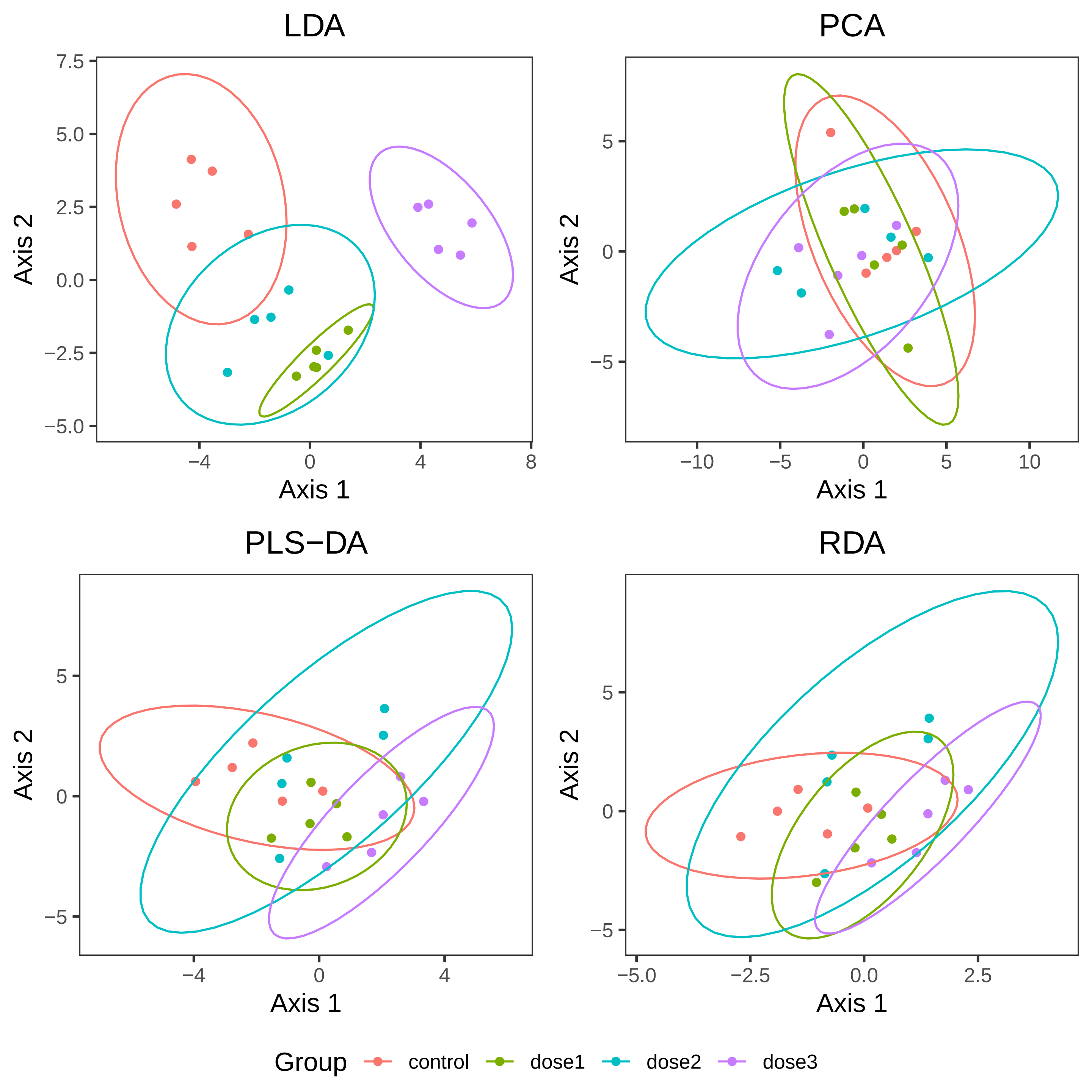

LDA
PCA
PLS−DA
RDA
Axis 1
Axis 2
7.5
5.0
2.5
0.0
−2.5
−5.0
−4
0
4
8
5
0
−5
−10
10
−5.0
2.5
Group
control
dose1
dose2
dose3

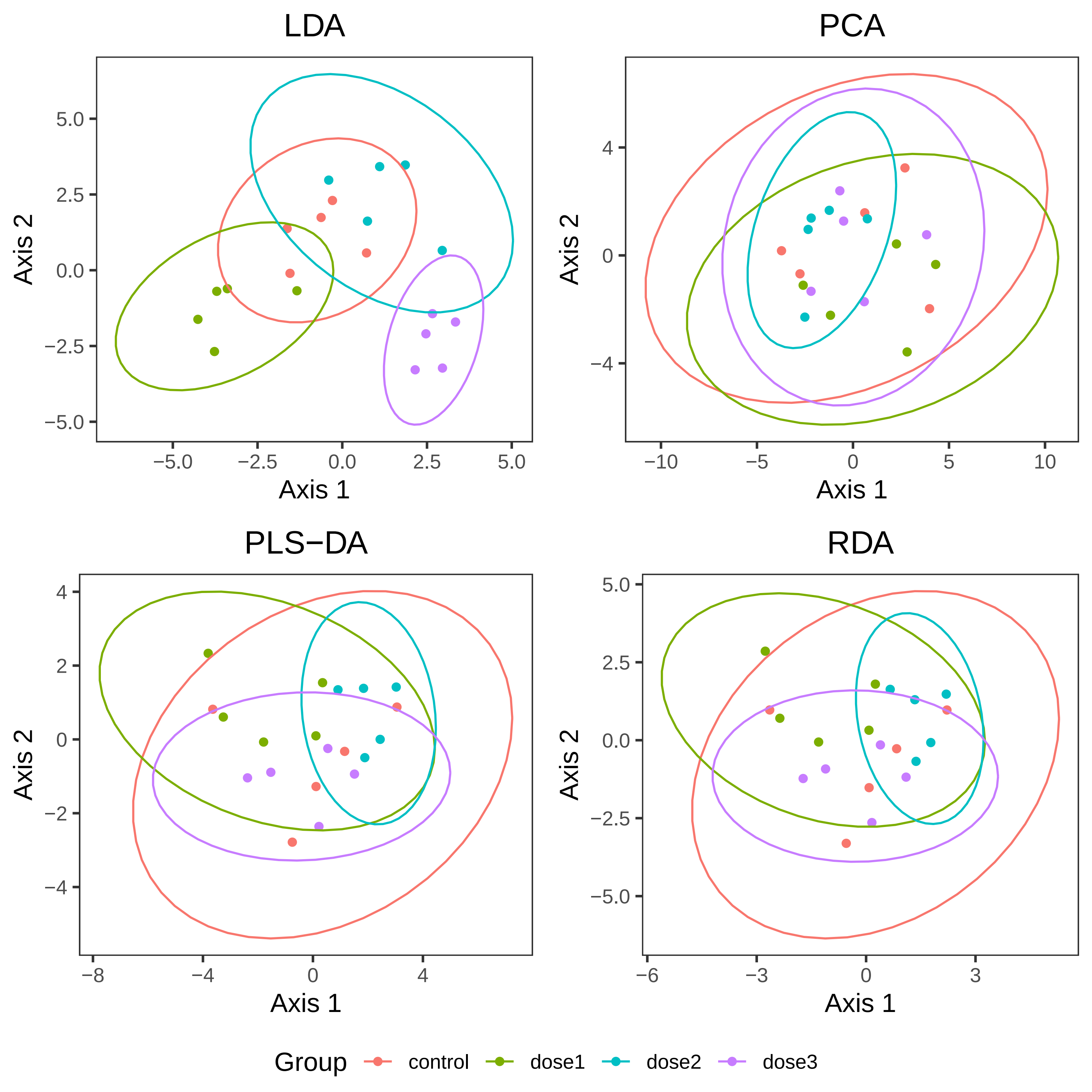

LDA
PCA
PLS−DA
RDA
Axis 1
Axis 2
−5.0
−2.5
0.0
2.5
5.0
−10
−5
0
5
10
4
−4
−8
2
−2
−6
−3
3
Group
control
dose1
dose2
dose3

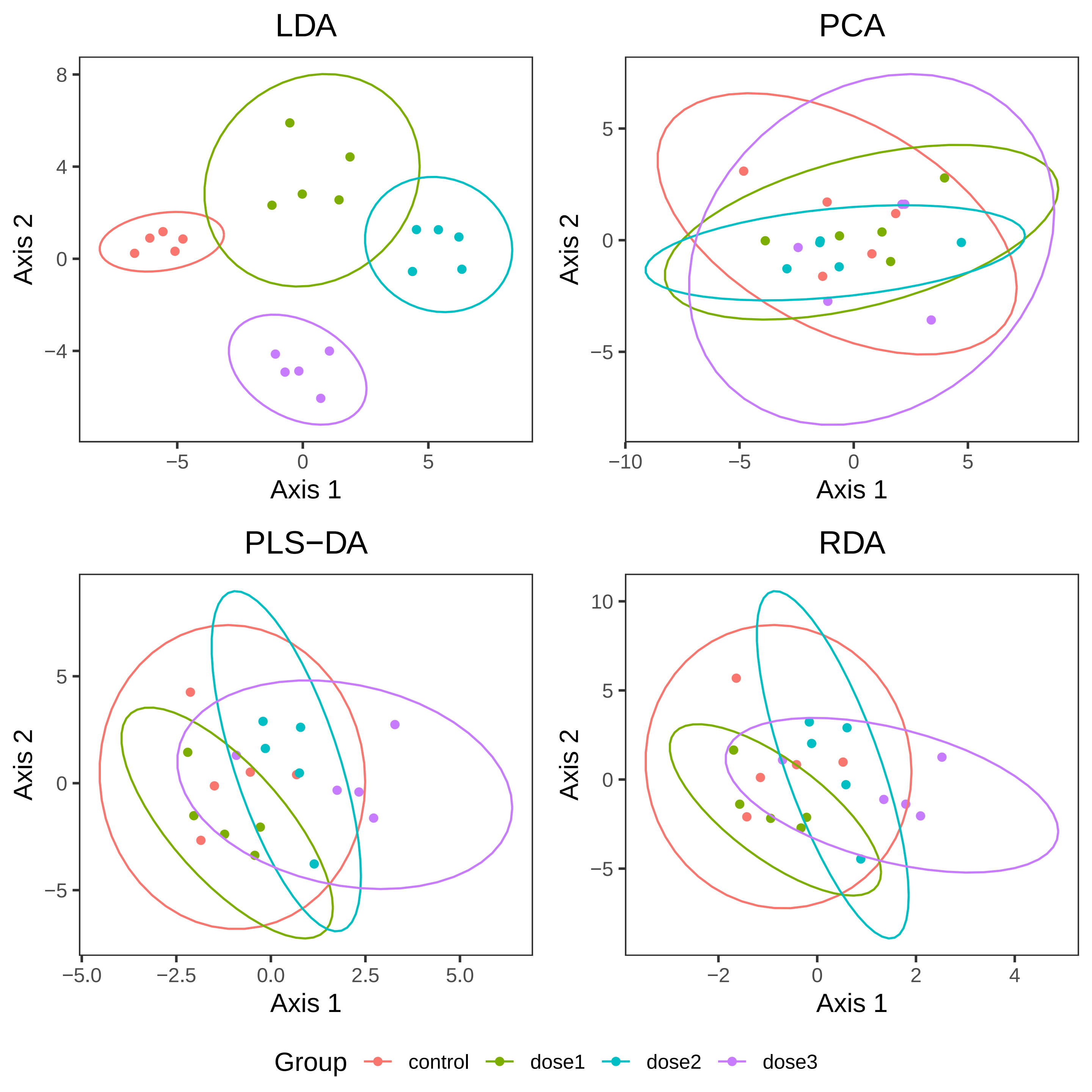

LDA
PCA
PLS−DA
RDA
Axis 1
Axis 2
8
4
0
−4
−5
5
−10
10
−5.0
−2.5
0.0
2.5
5.0
−2
2
4
Group
control
dose1
dose2
dose3

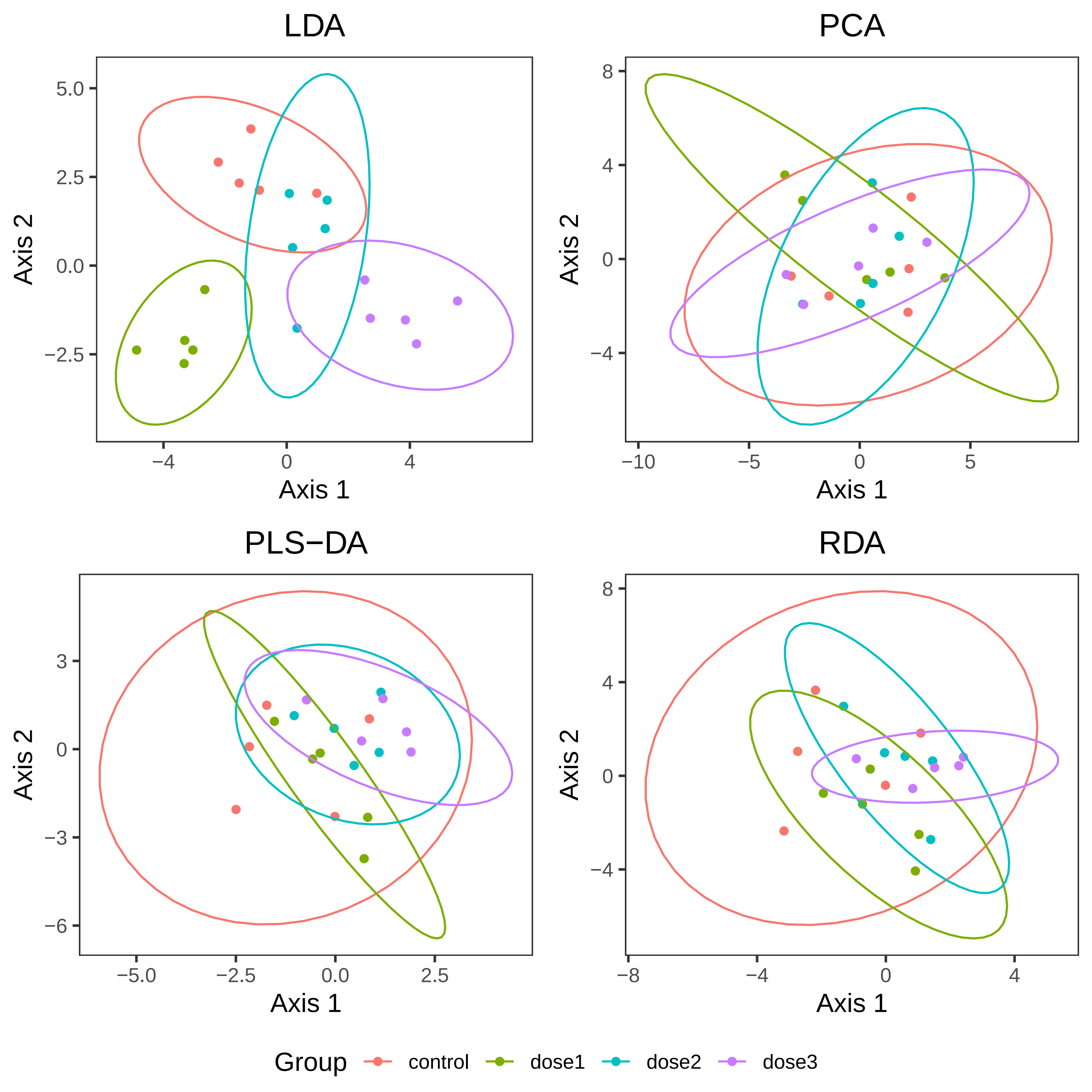
LDA
PCA
PLS−DA
RDA
Axis 1
Axis 2
5.0
2.5
0.0
−2.5
−4
0
4
8
−10
−5
5
3
−3
−6
−5.0
−2.5
−8
Group
control
dose1
dose2
dose3

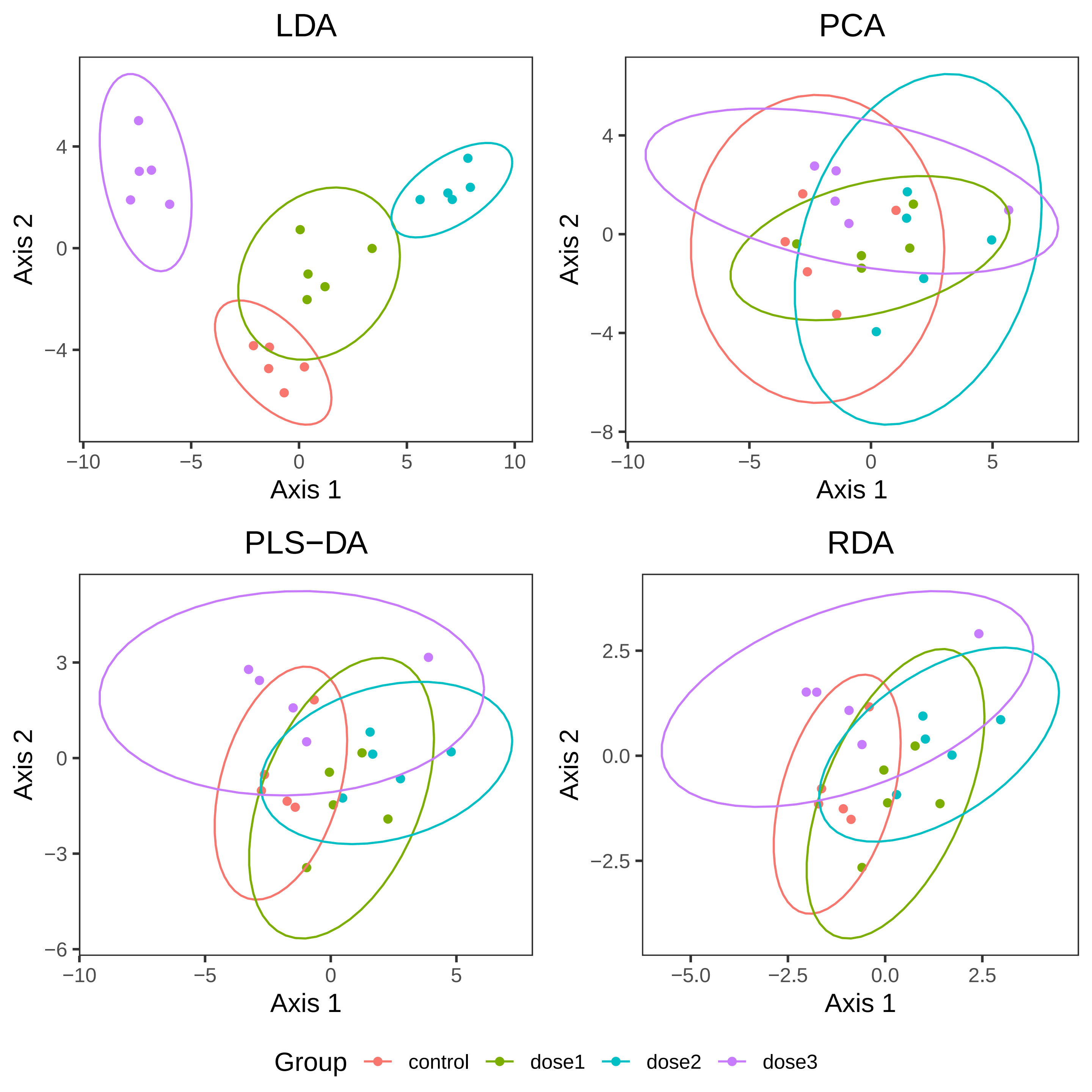

LDA
PCA
PLS−DA
RDA
Axis 1
Axis 2
Group
control
dose1
dose2
dose3

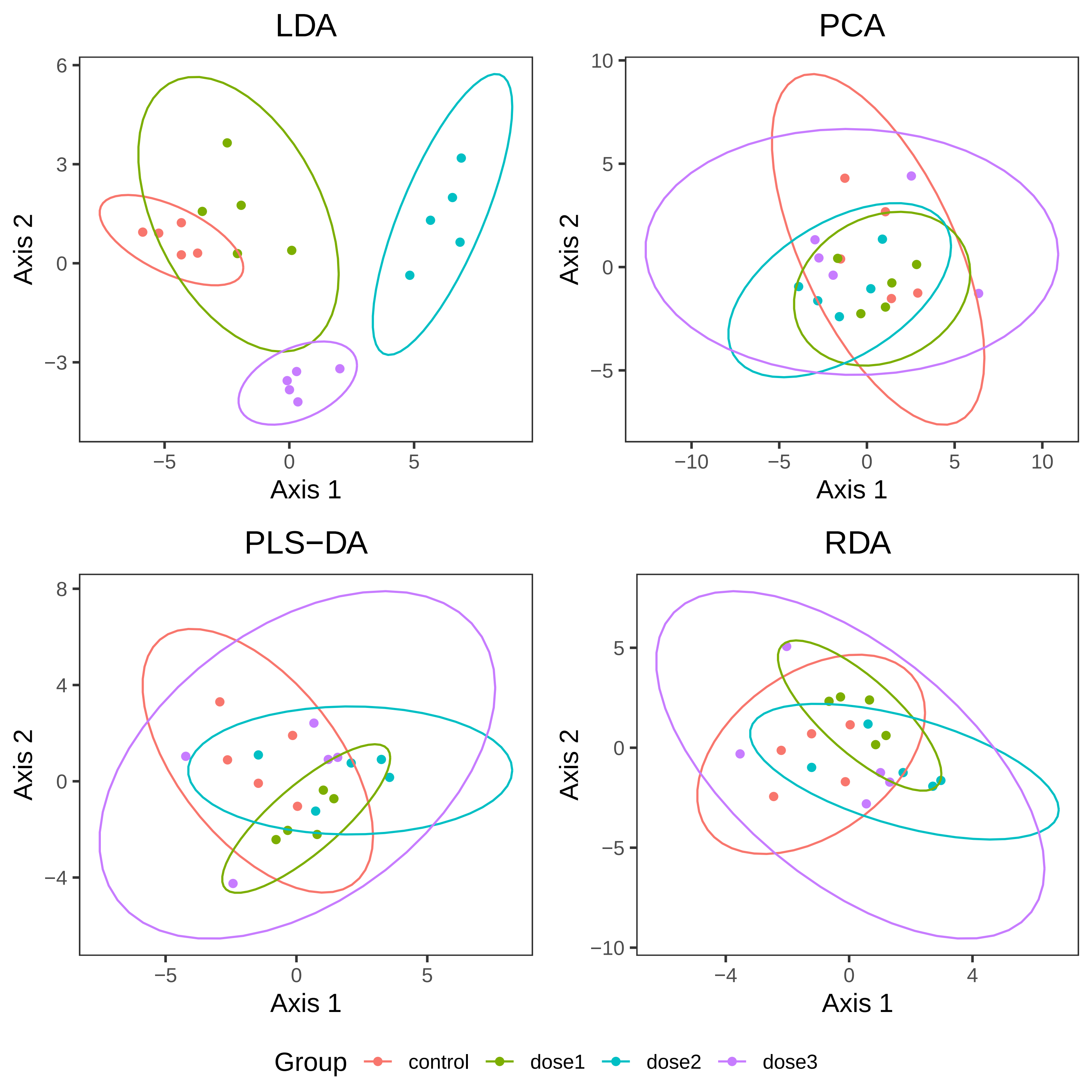

LDA
PCA
PLS−DA
RDA
Axis 1
Axis 2
Group
control
dose1
dose2
dose3

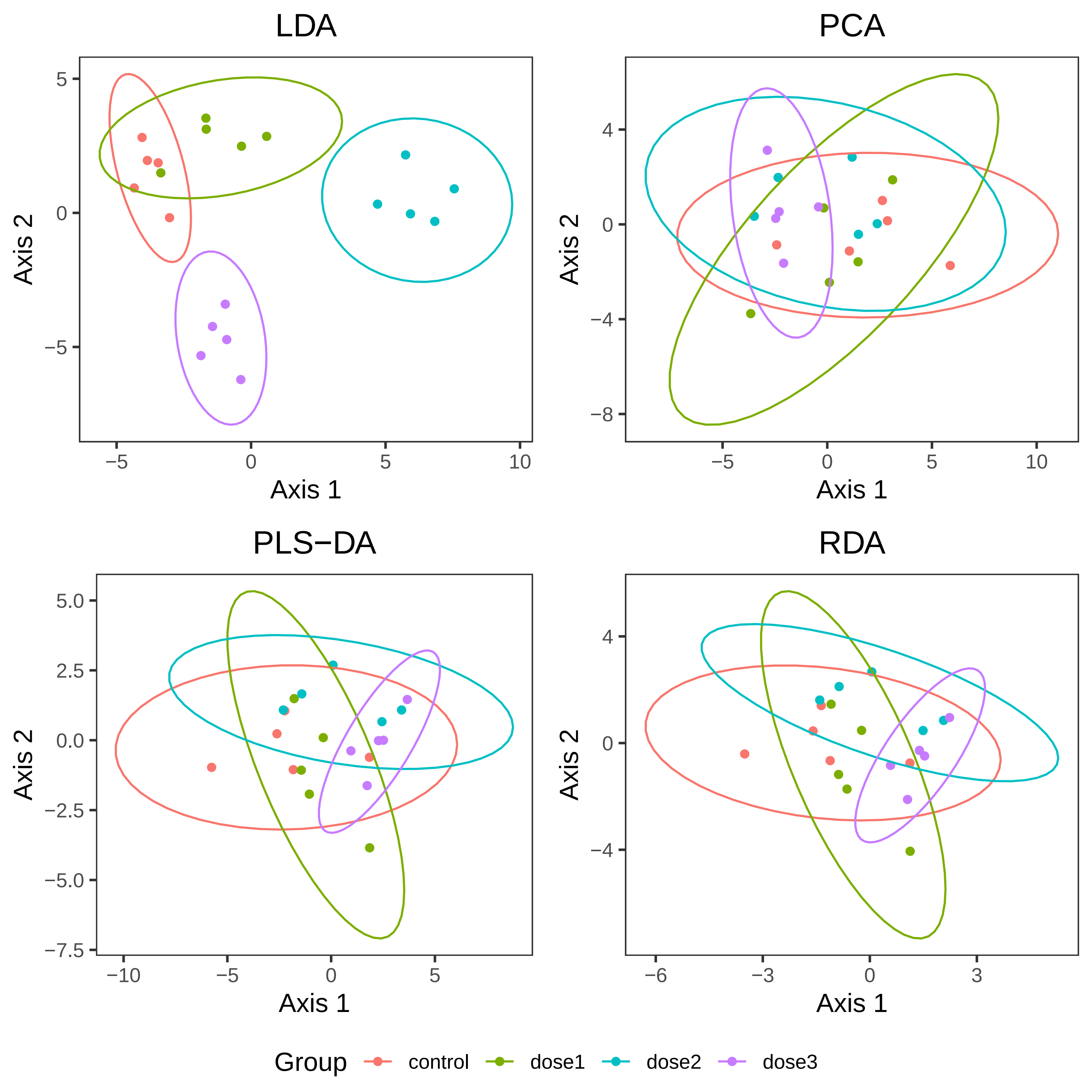

LDA
PCA
PLS−DA
RDA
Axis 1
Axis 2
Group
control
dose1
dose2
dose3

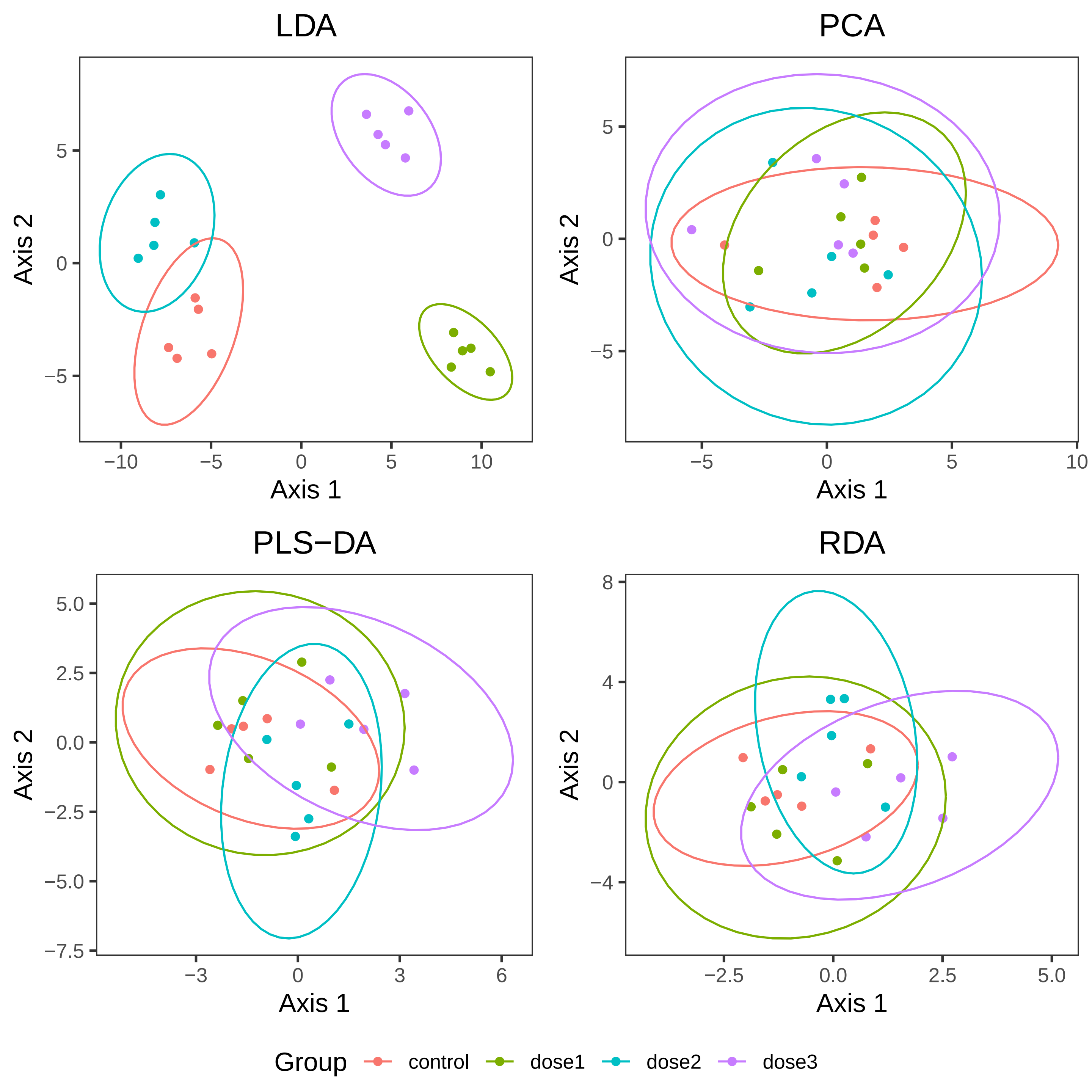

LDA
PCA
PLS−DA
RDA
Axis 1
Axis 2
Group
control
dose1
dose2
dose3

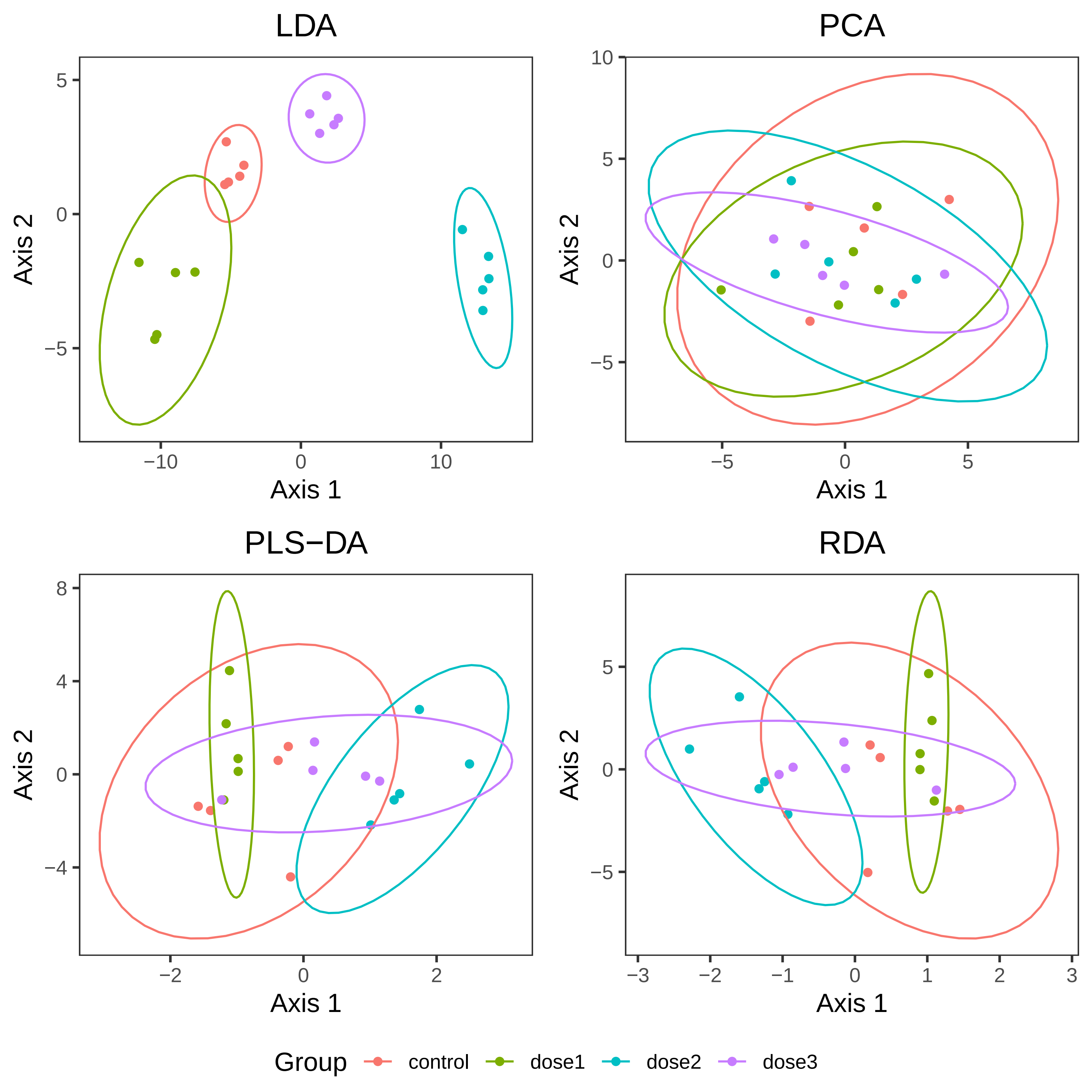

LDA
PCA
PLS−DA
RDA
Axis 1
Axis 2
Group
control
dose1
dose2
dose3

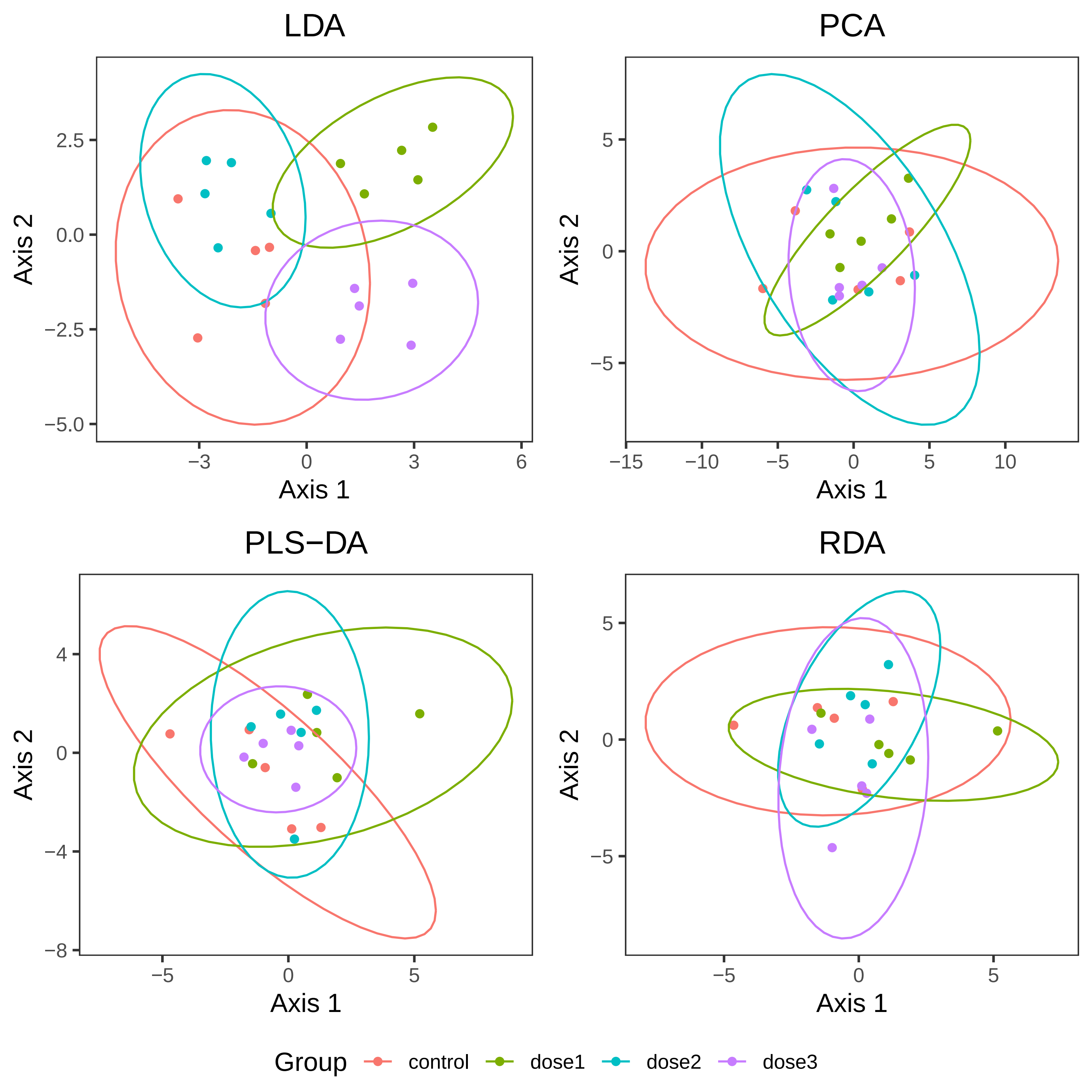
LDA
PCA
PLS−DA
RDA
Axis 1
Axis 2
2.5
0.0
−2.5
−5.0
−3
0
3
6
5
0
−5
−15
−10
−5
0
5
10
4
0
−4
−8
−5
0
5
Group
control
dose1
dose2
dose3

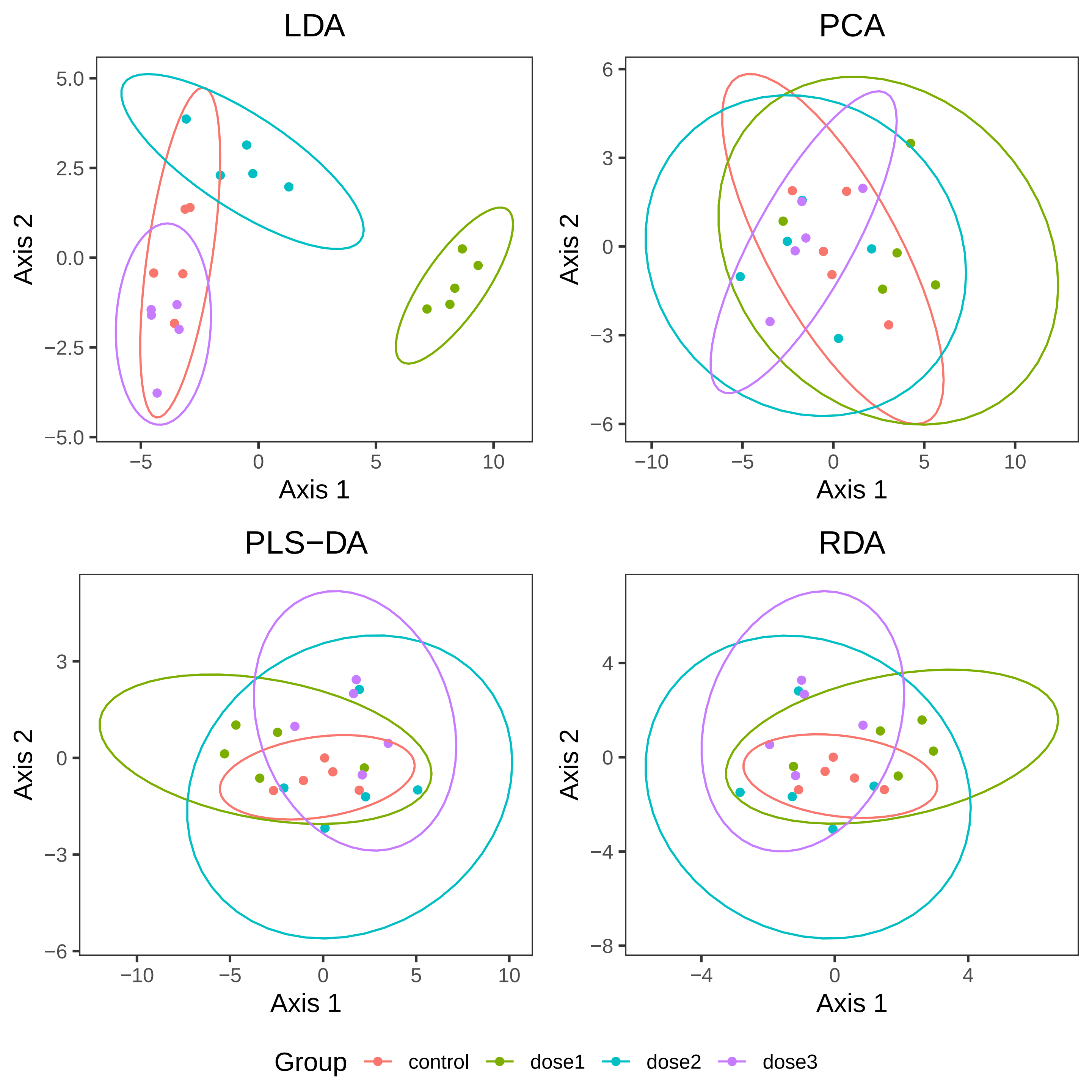

LDA
PCA
PLS−DA
RDA
Axis 1
Axis 2
5.0
2.5
0.0
−2.5
−5.0
−5
0
5
10
6
3
0
−3
−6
−10
4
−4
−8
Group
control
dose1
dose2
dose3

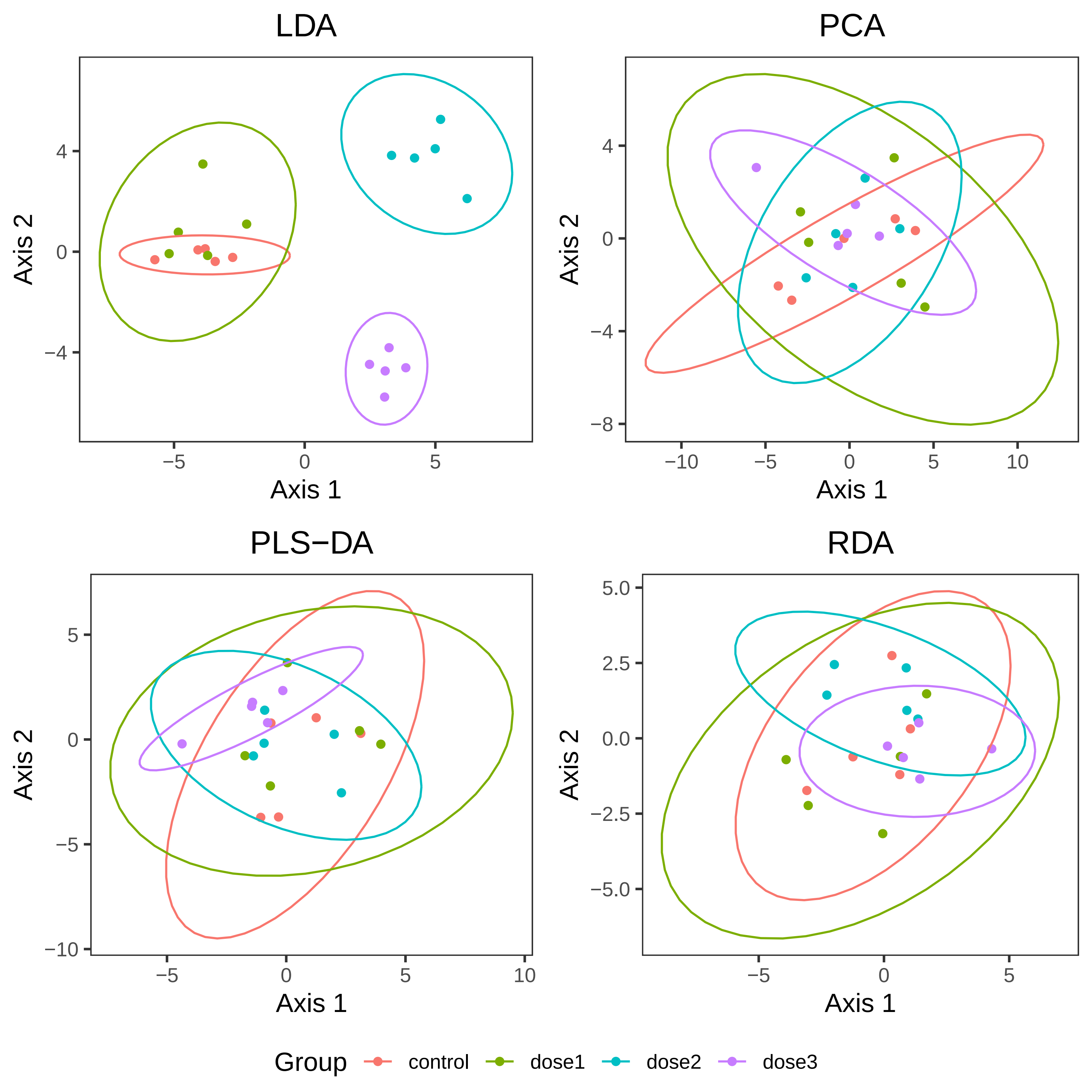
LDA
PCA
PLS−DA
RDA
Axis 1
Axis 2
Group
control
dose1
dose2
dose3

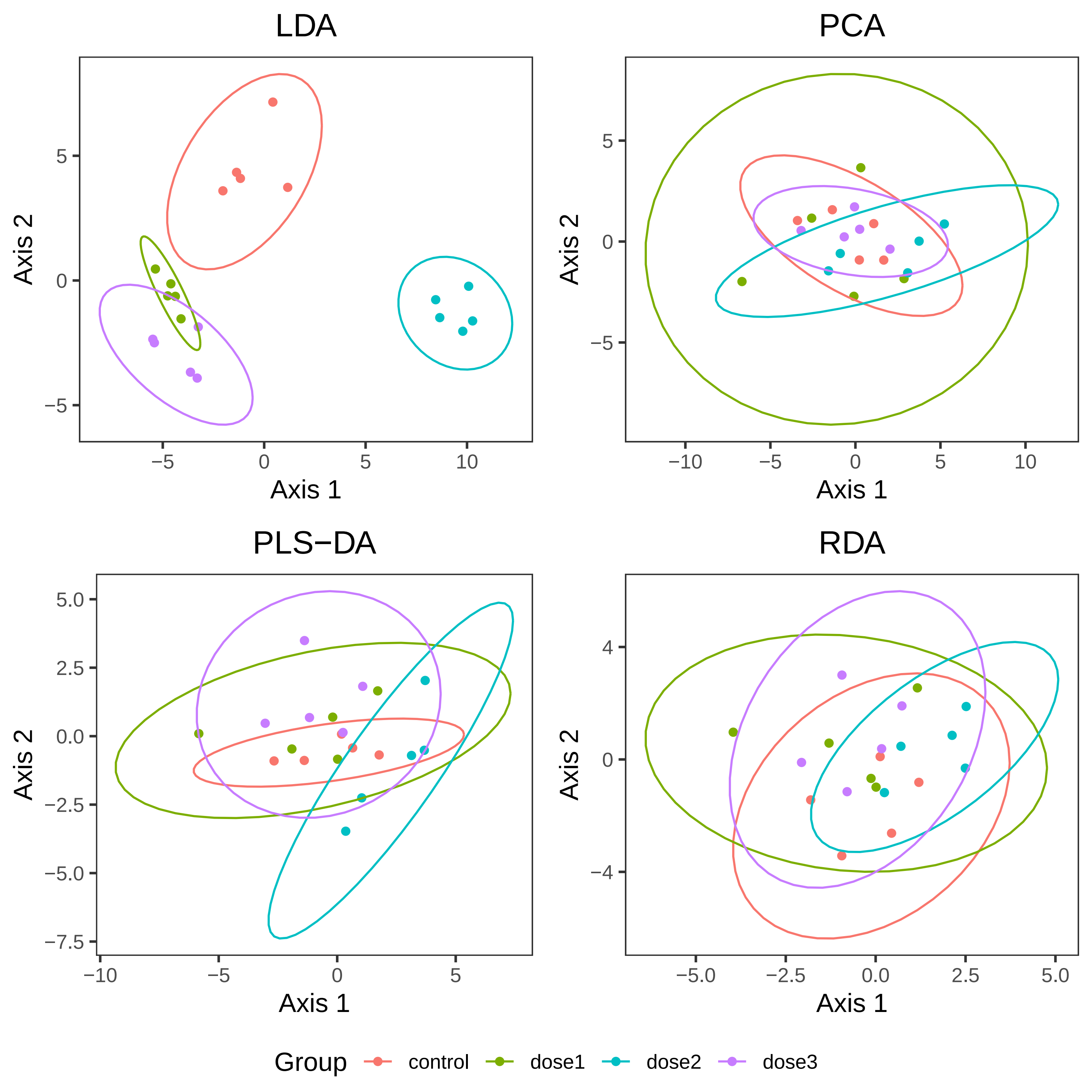
LDA
PCA
PLS−DA
RDA
Axis 1
Axis 2
Group
control
dose1
dose2
dose3

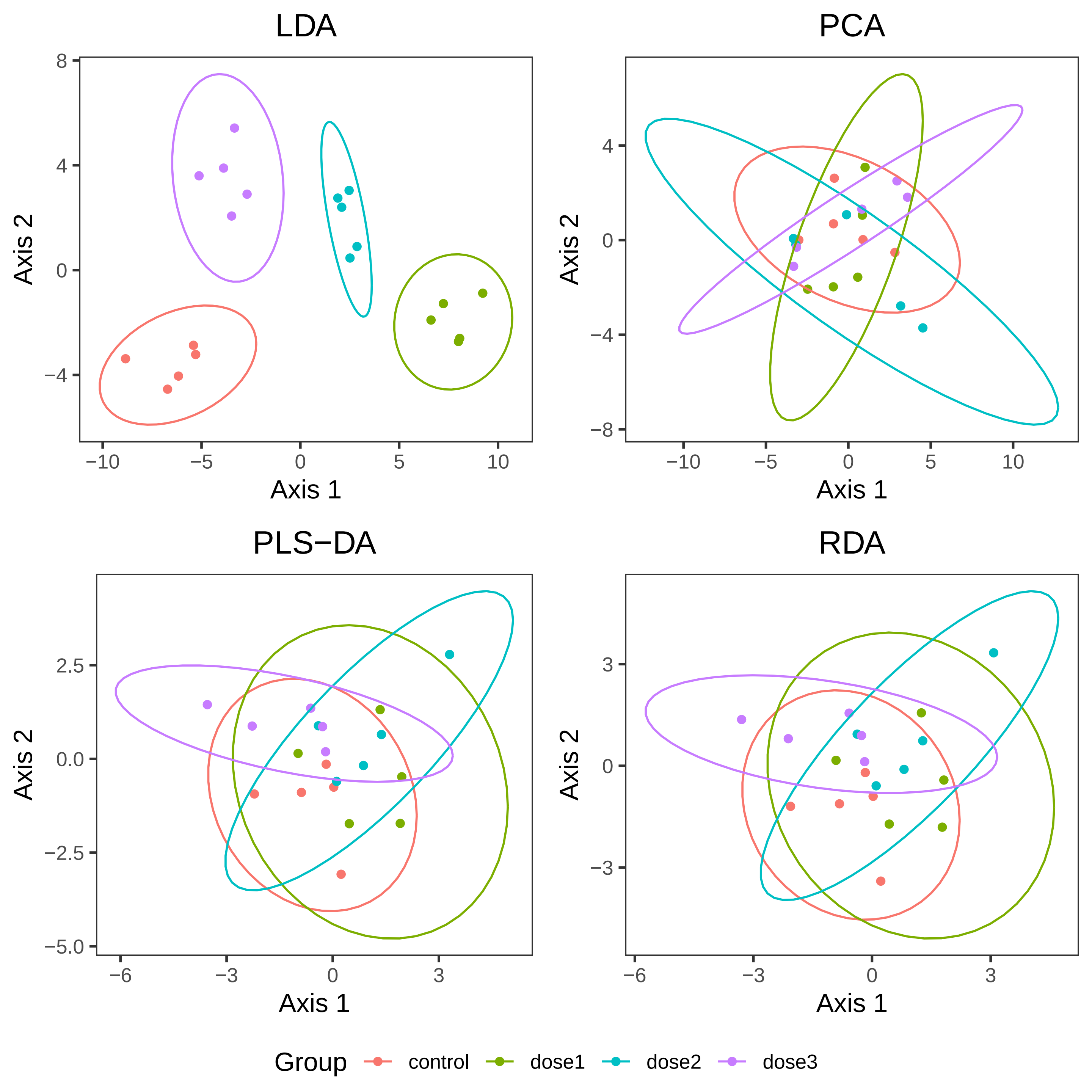

LDA
PCA
PLS−DA
RDA
Axis 1
Axis 2
Group
control
dose1
dose2
dose3

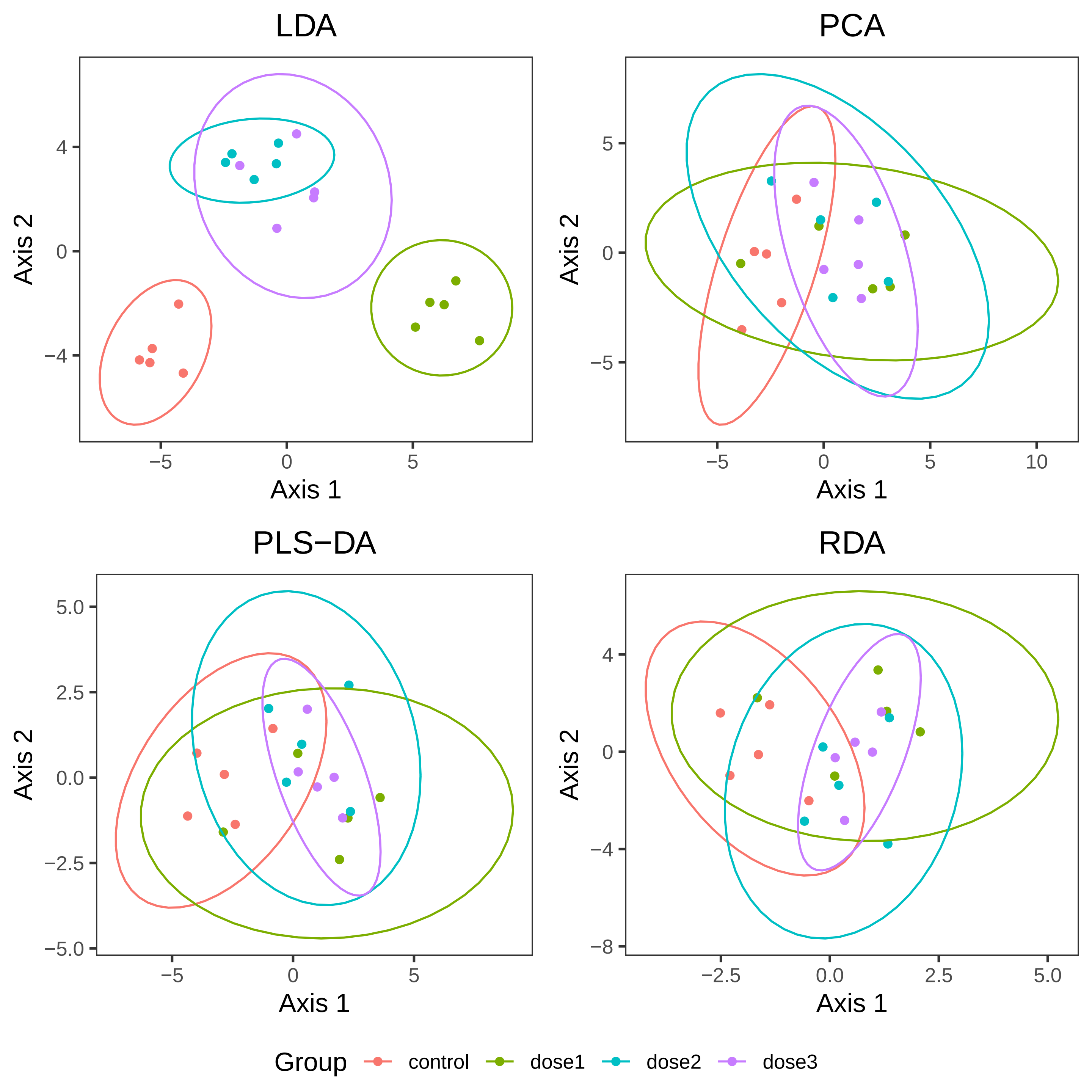
LDA
PCA
PLS−DA
RDA
Axis 1
Axis 2
Group
control
dose1
dose2
dose3

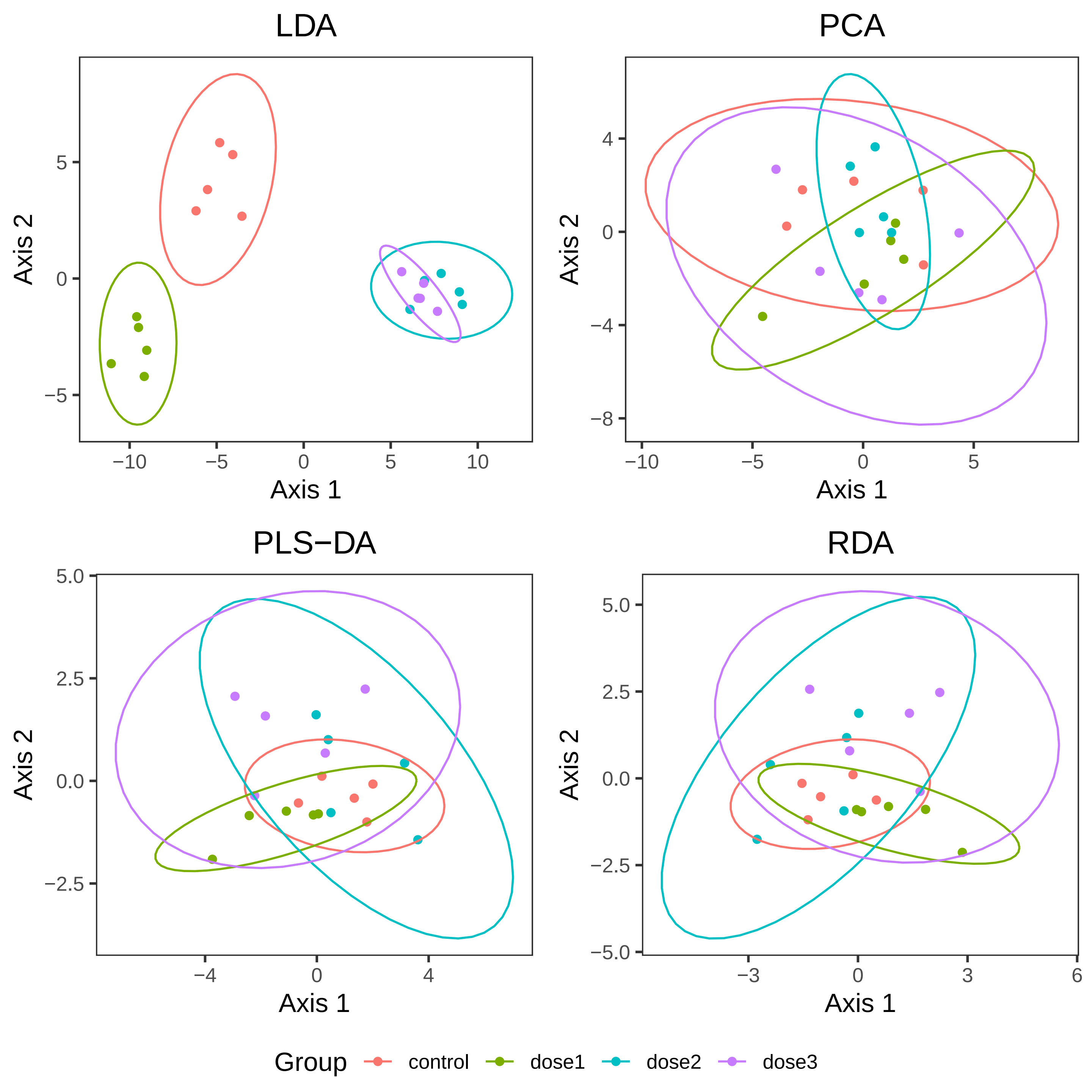

LDA
PCA
PLS−DA
RDA
Axis 1
Axis 2
−10
−5
0
5
10
−8
−4
4
5.0
2.5
0.0
−2.5
−5.0
−3
3
6
Group
control
dose1
dose2
dose3

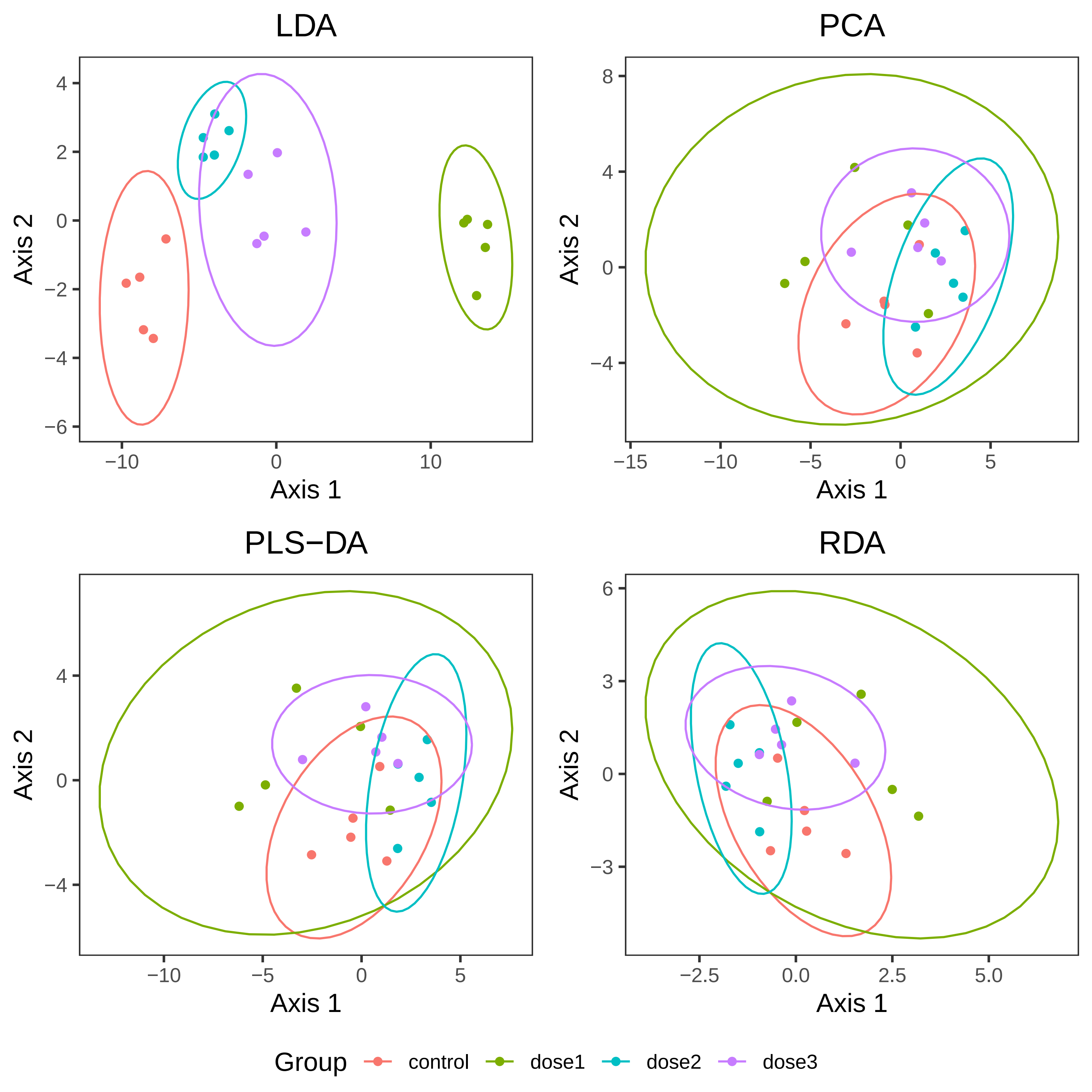

LDA
PCA
PLS−DA
RDA
Axis 1
Axis 2
Group
control
dose1
dose2
dose3

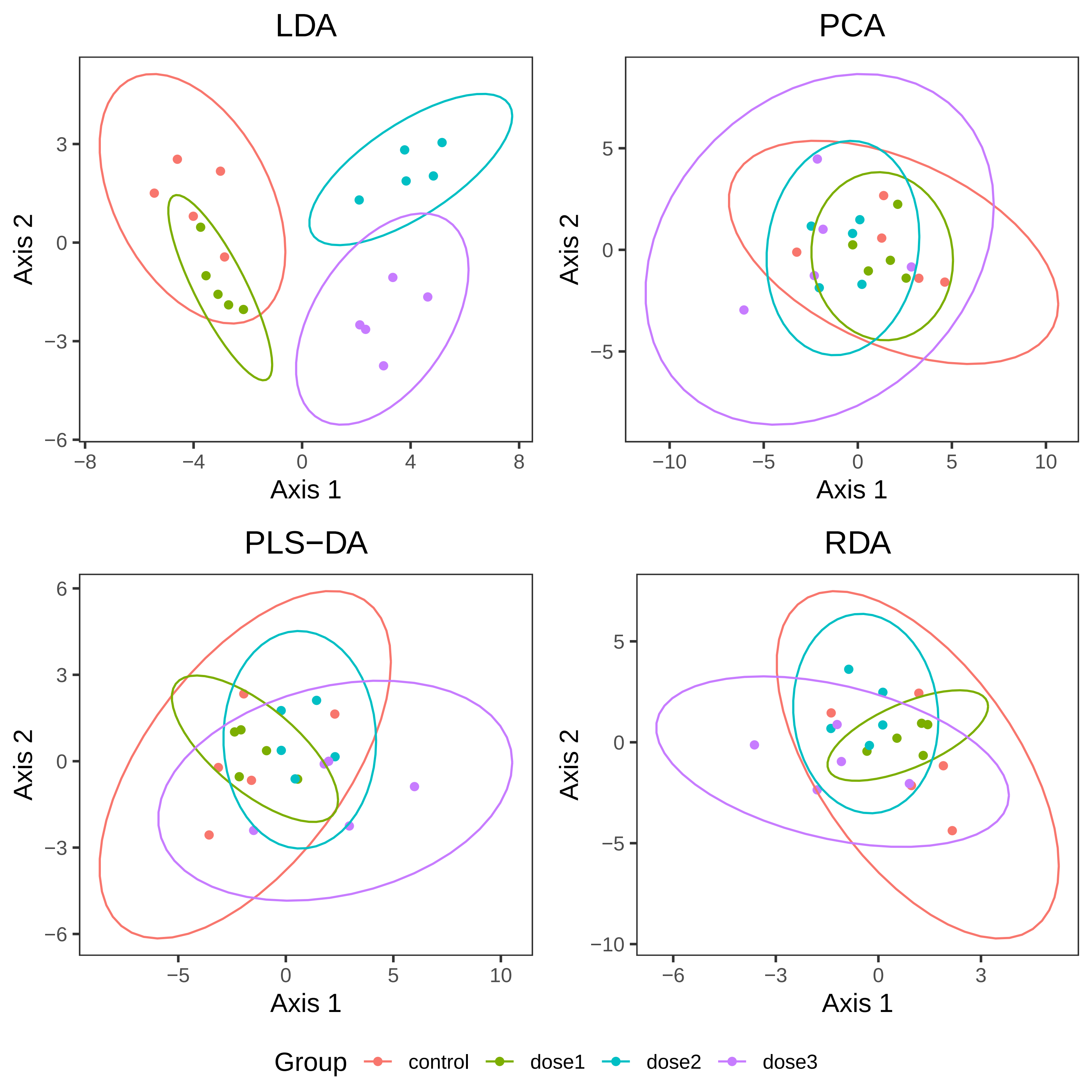

LDA
PCA
PLS−DA
RDA
Axis 1
Axis 2
3
0
−3
−6
−8
−4
0
4
8
5
0
−5
−10
−5
0
5
10
6
3
0
−3
−6
−5
0
5
10
5
0
−5
−10
−6
−3
0
3
Group
control
dose1
dose2
dose3

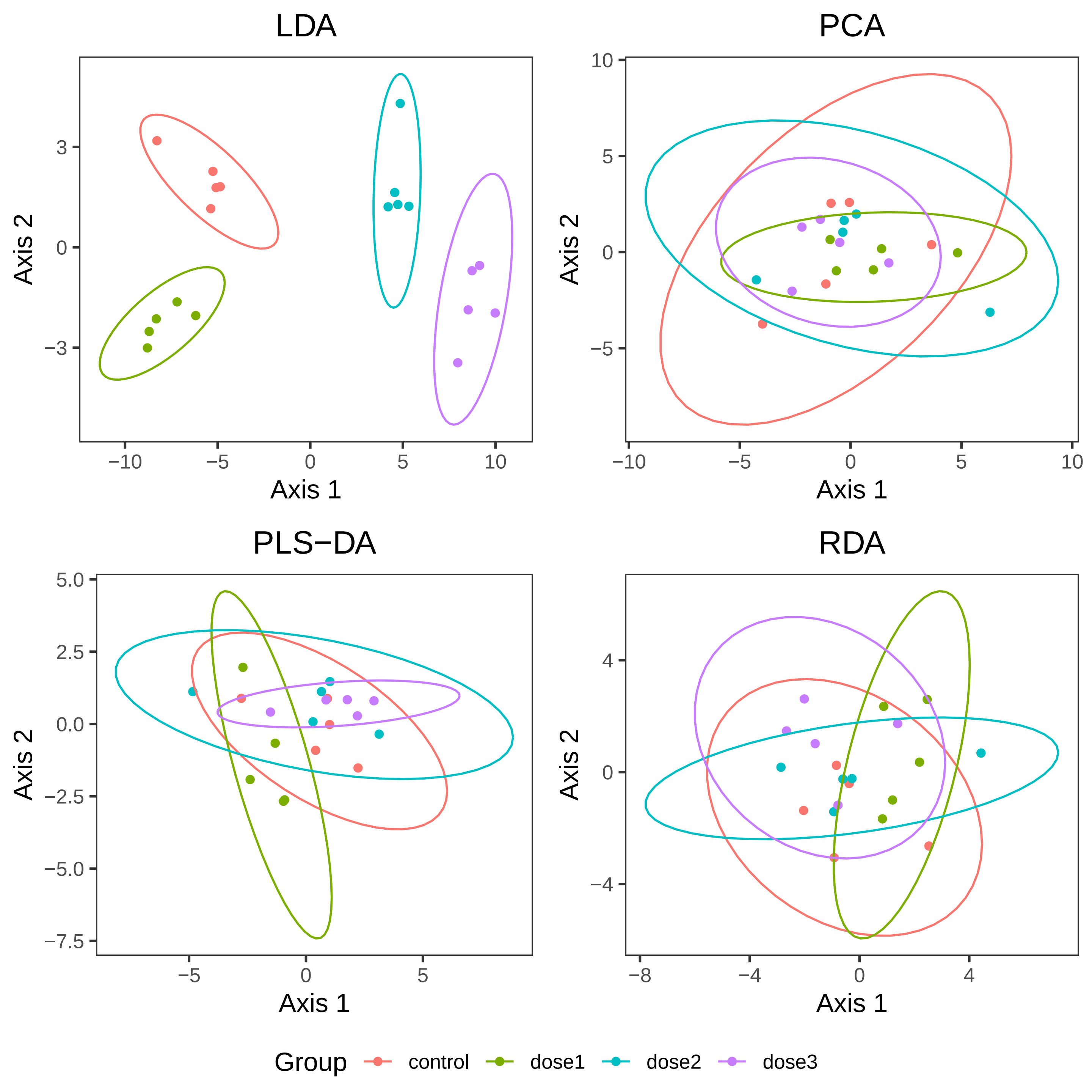

LDA
PCA
PLS−DA
RDA
Axis 1
Axis 2
Group
control
dose1
dose2
dose3

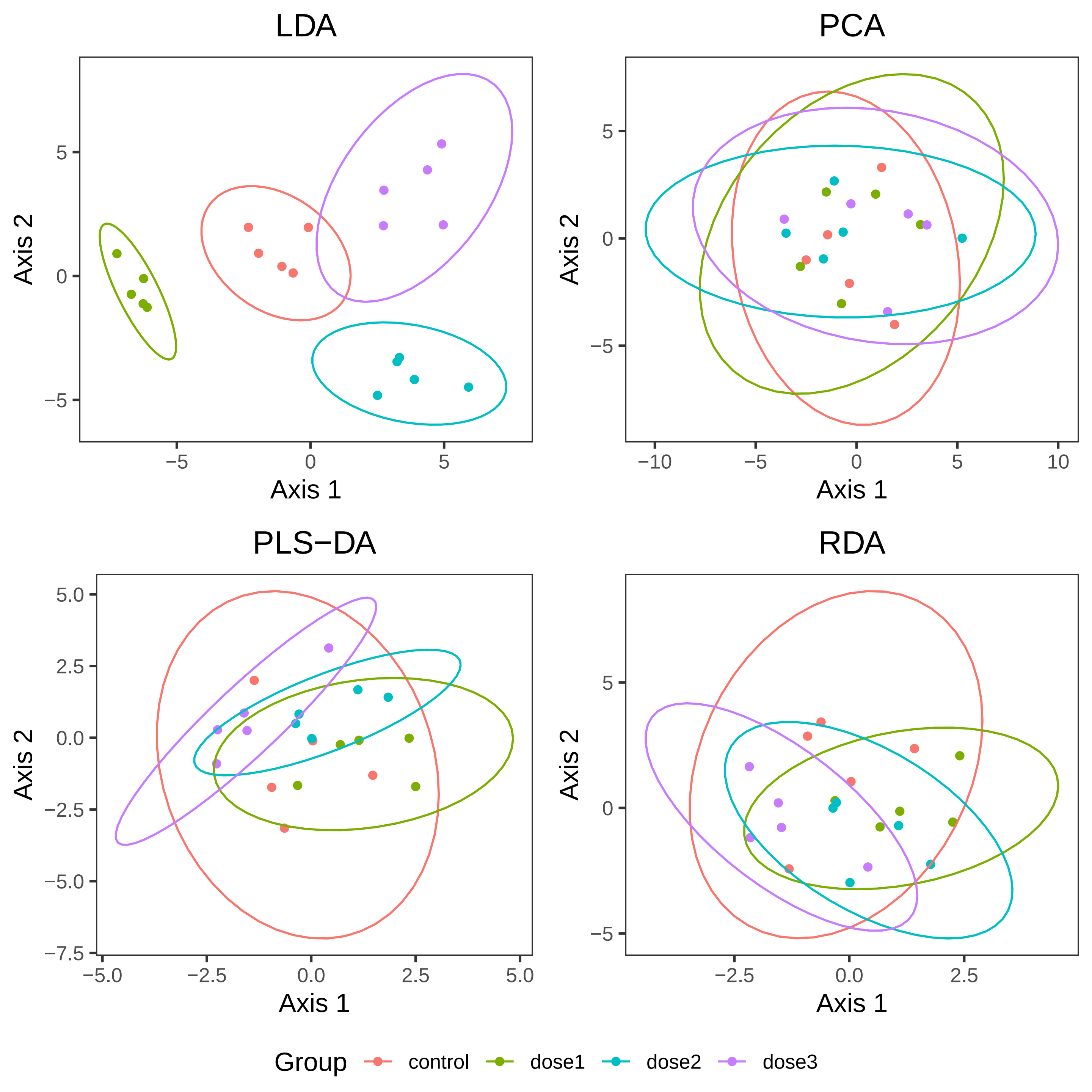

LDA
PCA
PLS−DA
RDA
Axis 1
Axis 2
−5
0
5
−10
10
5.0
2.5
0.0
−2.5
−5.0
−7.5
Group
control
dose1
dose2
dose3

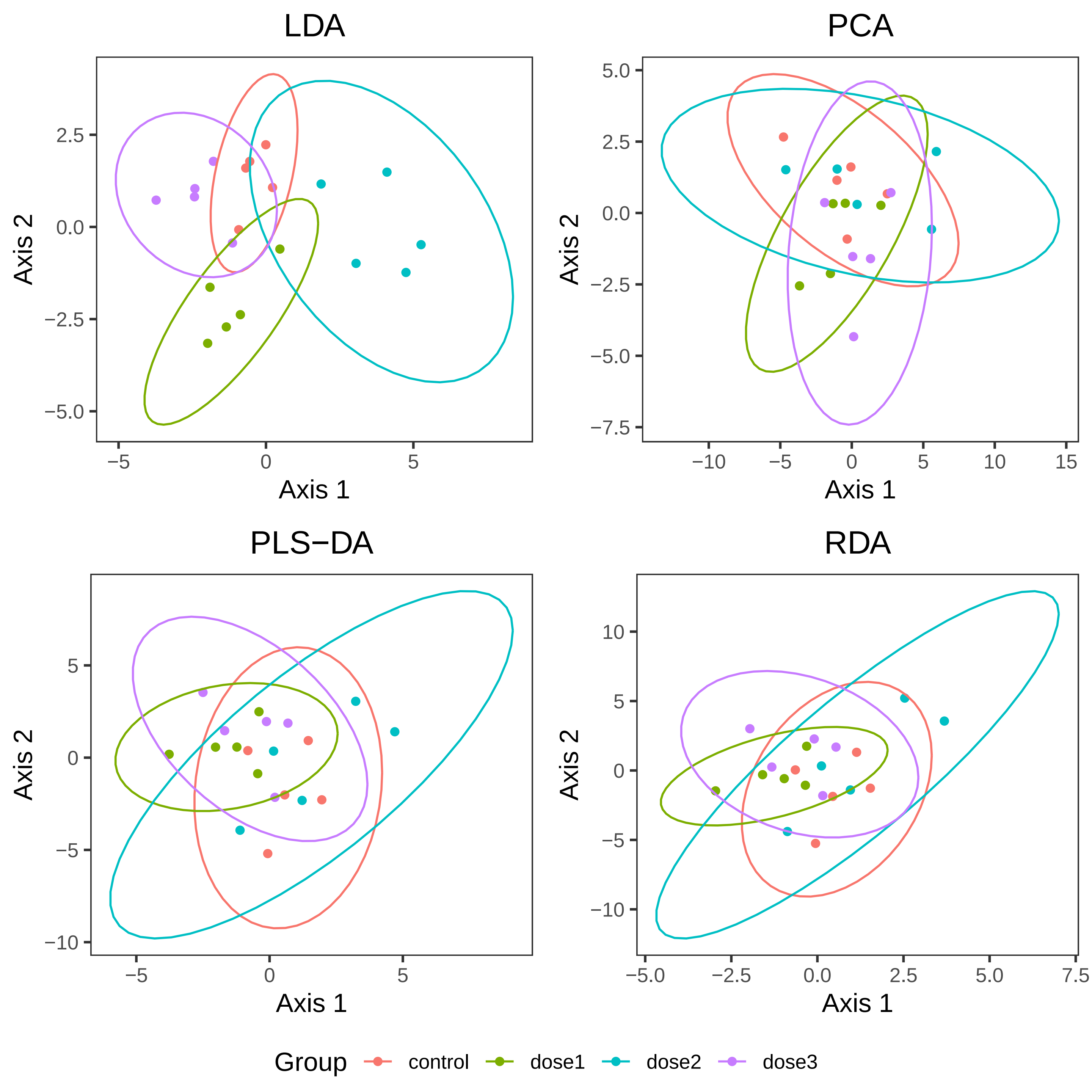

LDA
PCA
PLS−DA
RDA
Axis 1
Axis 2
2.5
0.0
−2.5
−5.0
−5
0
5
5.0
−7.5
−10
10
15
−10
7.5
Group
control
dose1
dose2
dose3

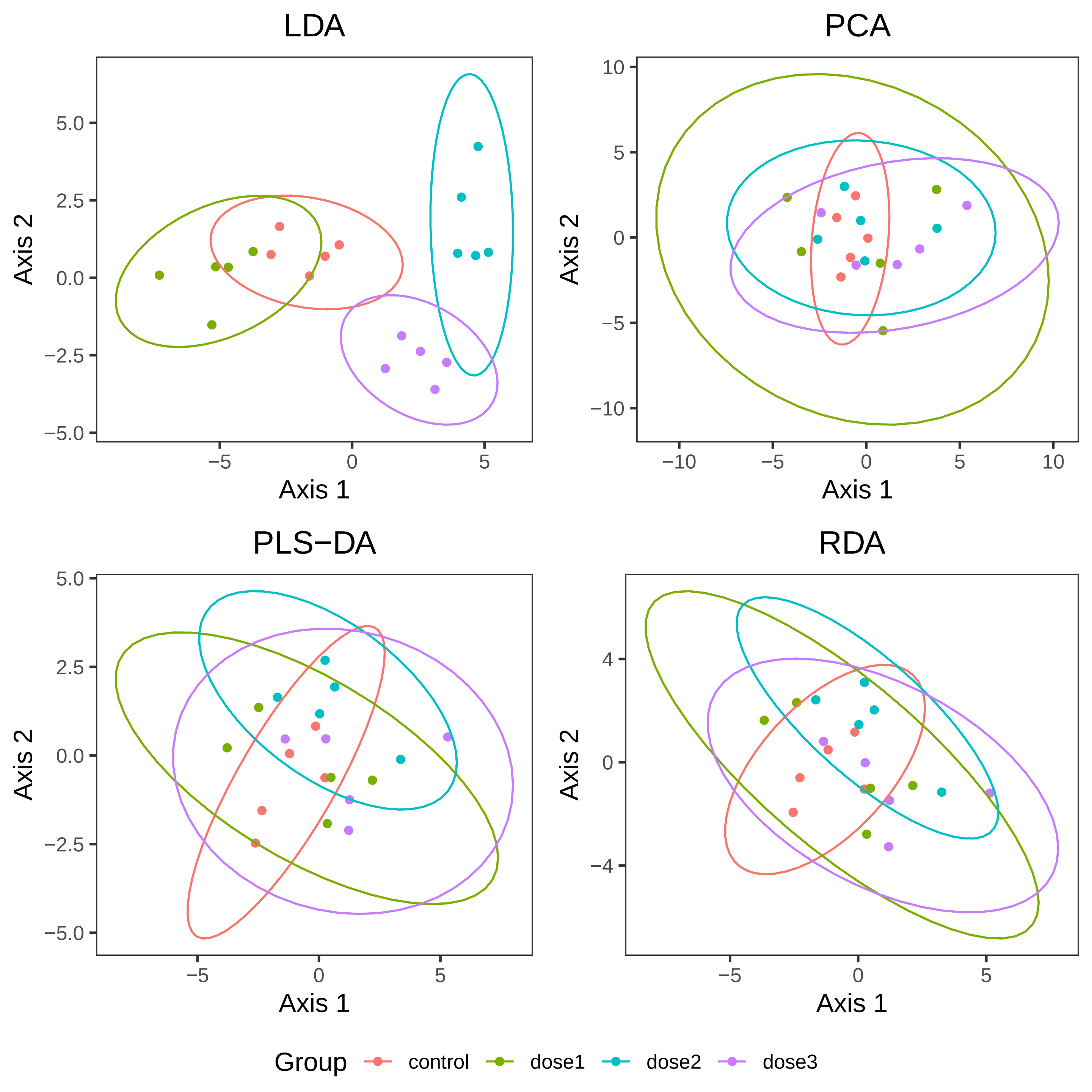

LDA
PCA
PLS−DA
RDA
Axis 1
Axis 2
5.0
2.5
0.0
−2.5
−5.0
−5
0
5
10
−10
4
−4
Group
control
dose1
dose2
dose3

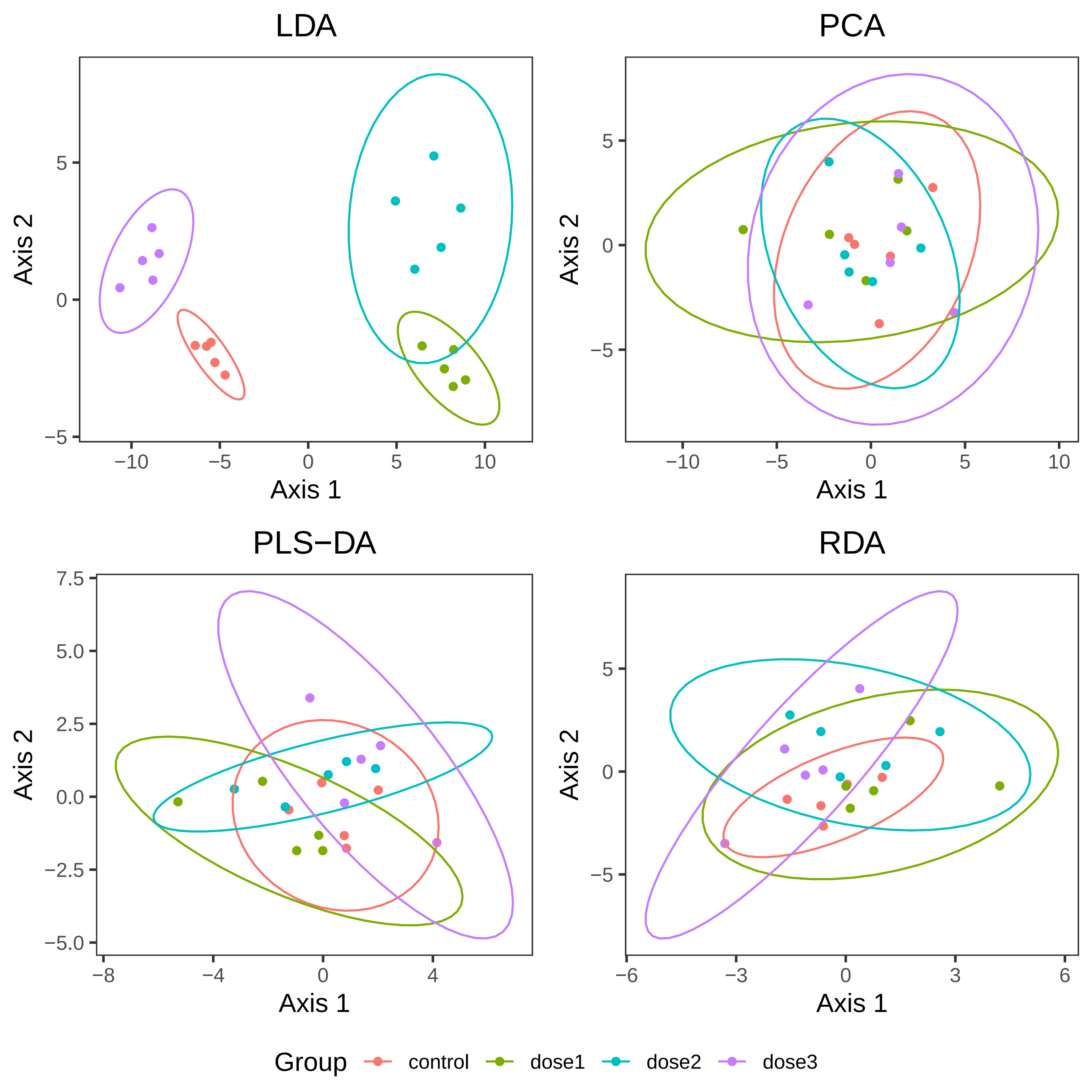

LDA
PCA
PLS−DA
RDA
Axis 1
Axis 2
−10
−5
0
5
10
7.5
5.0
2.5
0.0
−2.5
−5.0
−8
−4
4
−6
−3
3
6
Group
control
dose1
dose2
dose3

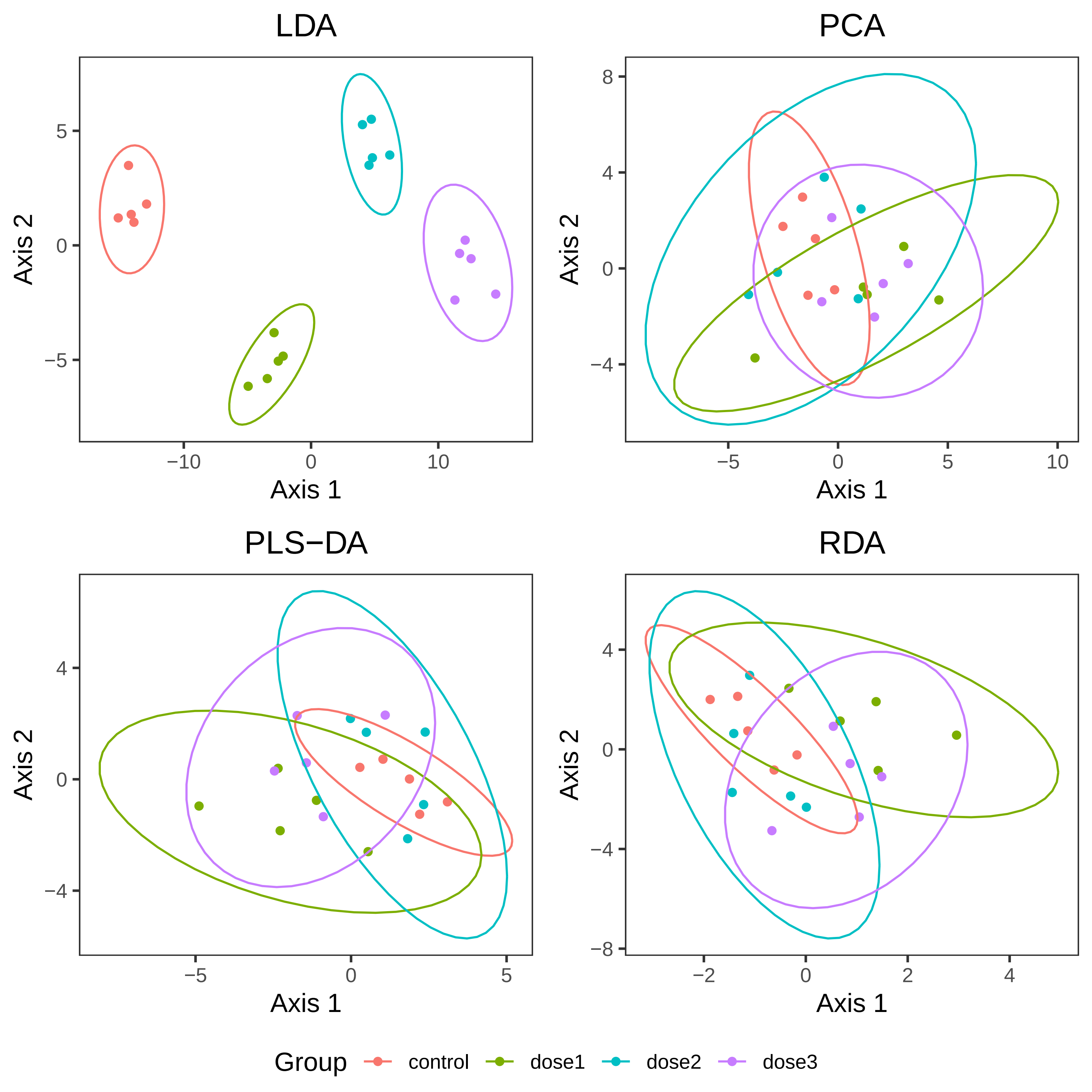

LDA
PCA
PLS−DA
RDA
Axis 1
Axis 2
−10
0
10
5
−5
8
4
−4
−8
2
−2
Group
control
dose1
dose2
dose3

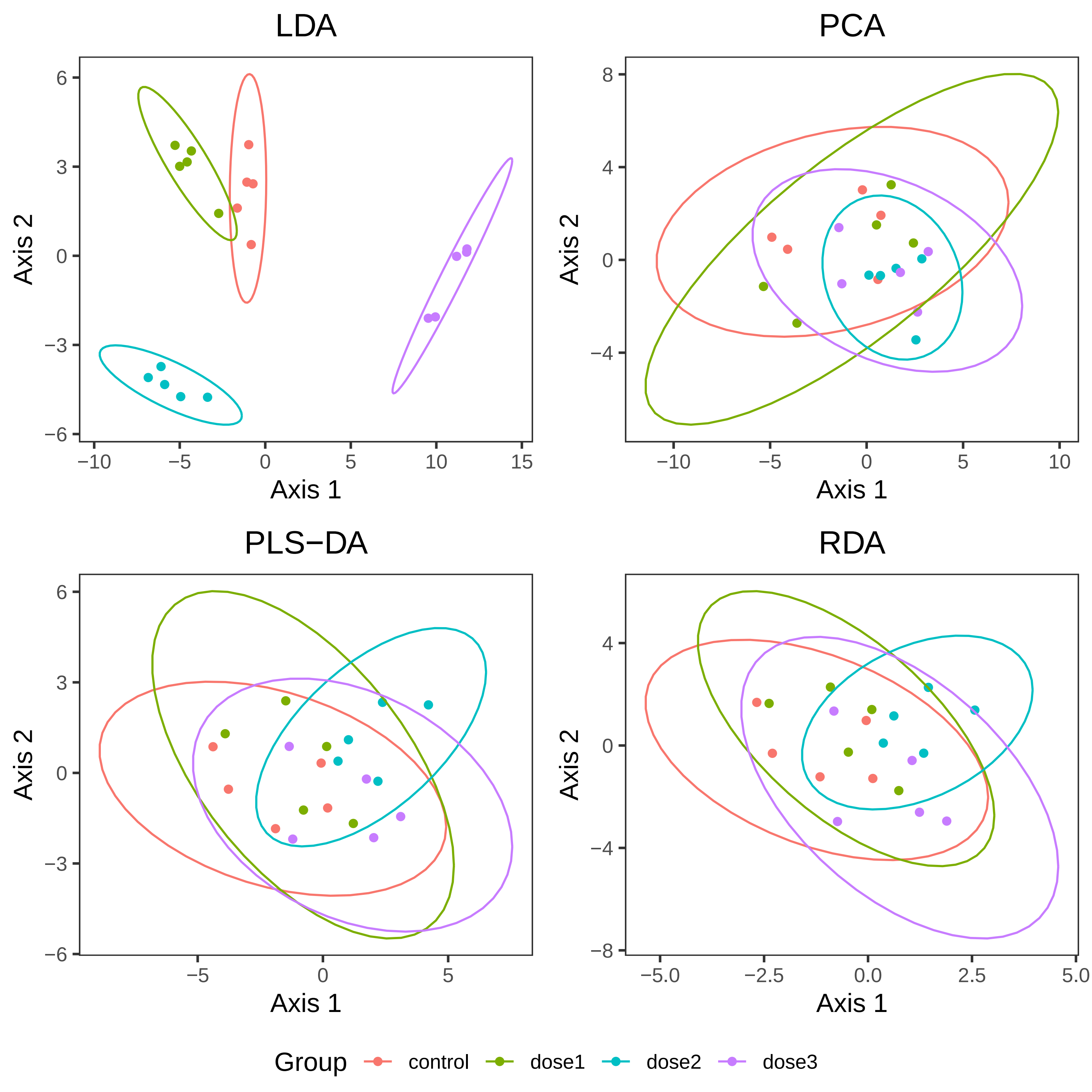
LDA
PCA
PLS−DA
RDA
Axis 1
Axis 2
Group
control
dose1
dose2
dose3

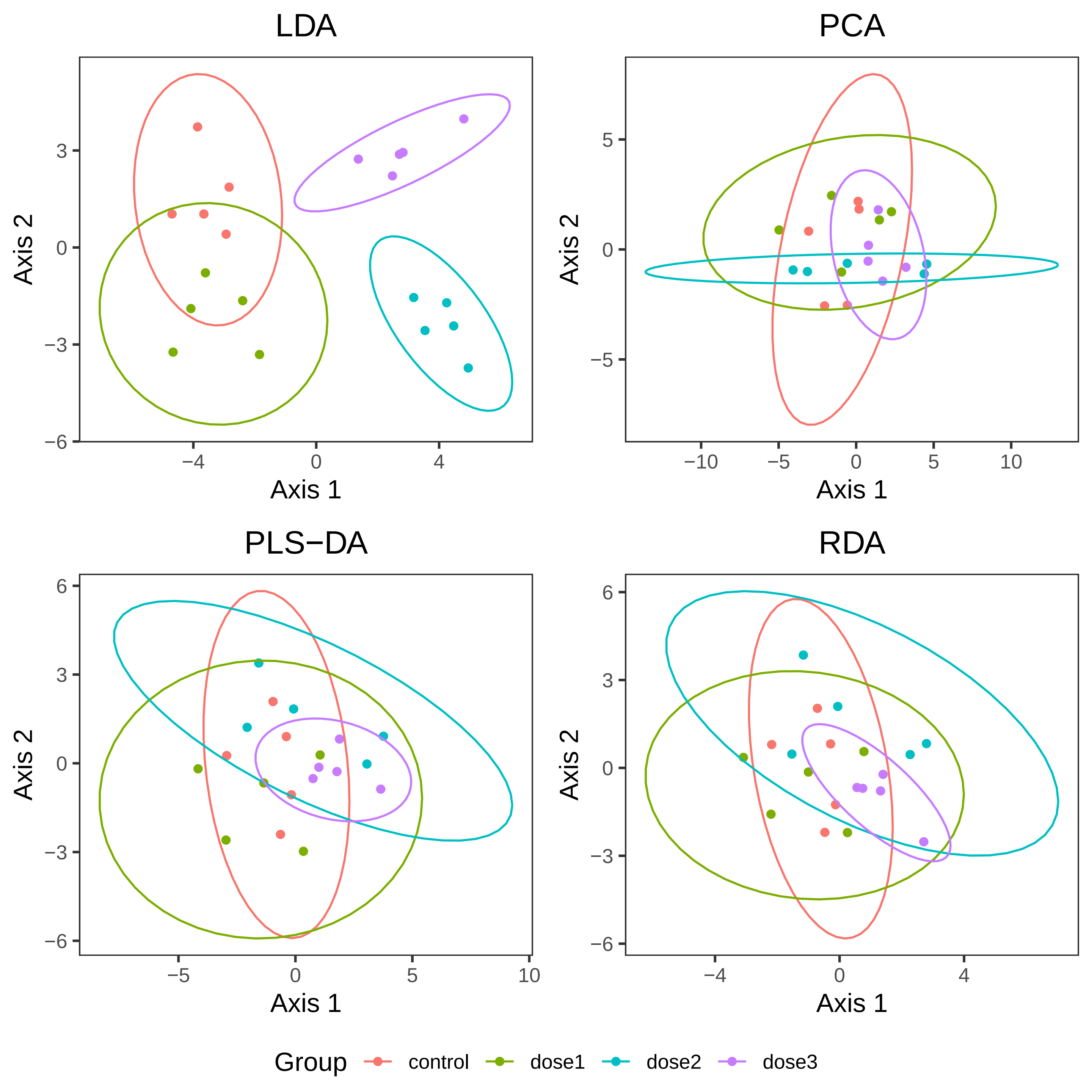

LDA
PCA
PLS−DA
RDA
Axis 1
Axis 2
3
0
−3
−6
−4
0
4
5
−5
−10
10
6
Group
control
dose1
dose2
dose3

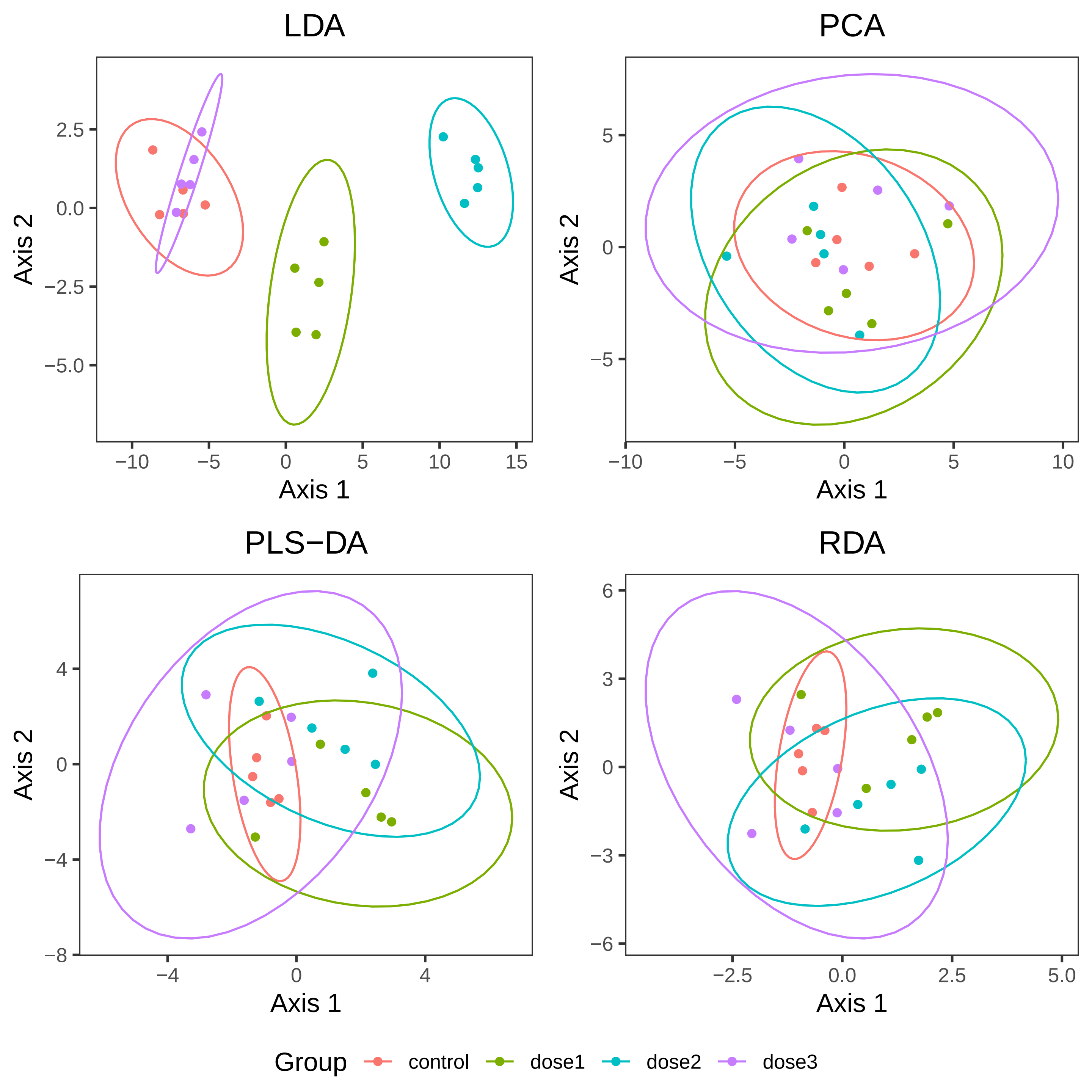
LDA
PCA
PLS−DA
RDA
Axis 1
Axis 2
Group
control
dose1
dose2
dose3

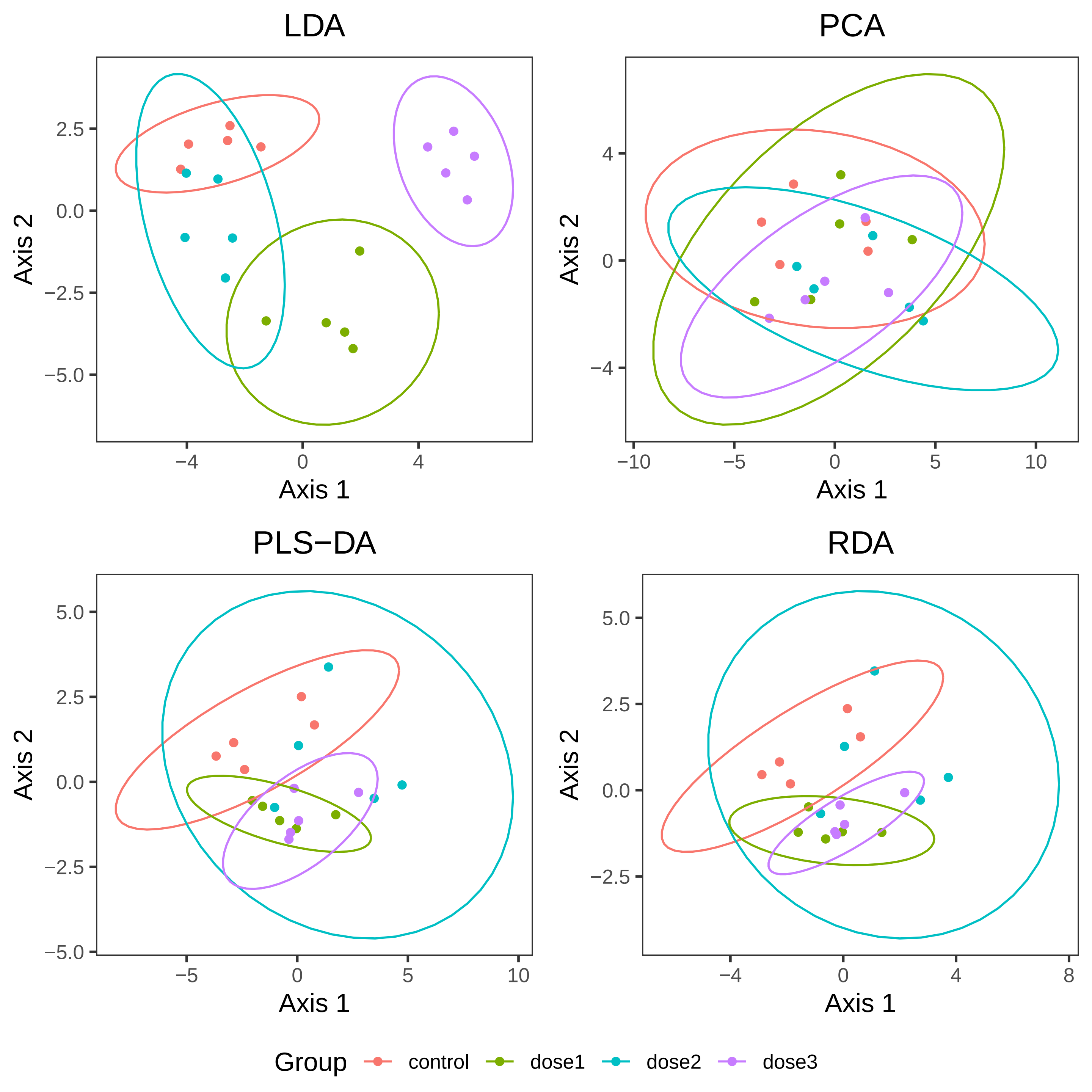

LDA
PCA
PLS−DA
RDA
Axis 1
Axis 2
Group
control
dose1
dose2
dose3

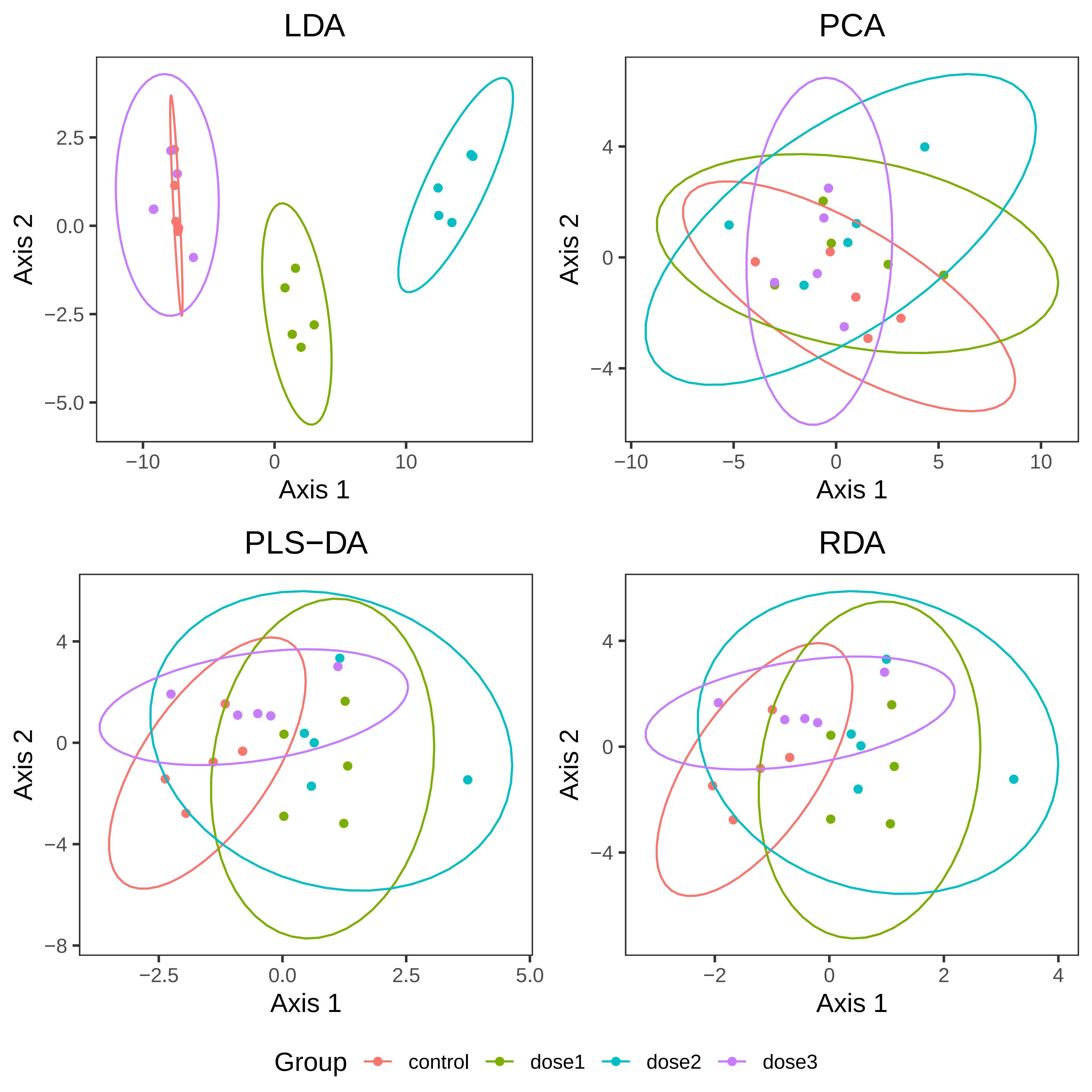
LDA
PCA
PLS−DA
RDA
Axis 1
Axis 2
2.5
0.0
−2.5
−5.0
−10
0
10
4
0
−4
−5
5
−8
5.0
−2
2
Group
control
dose1
dose2
dose3

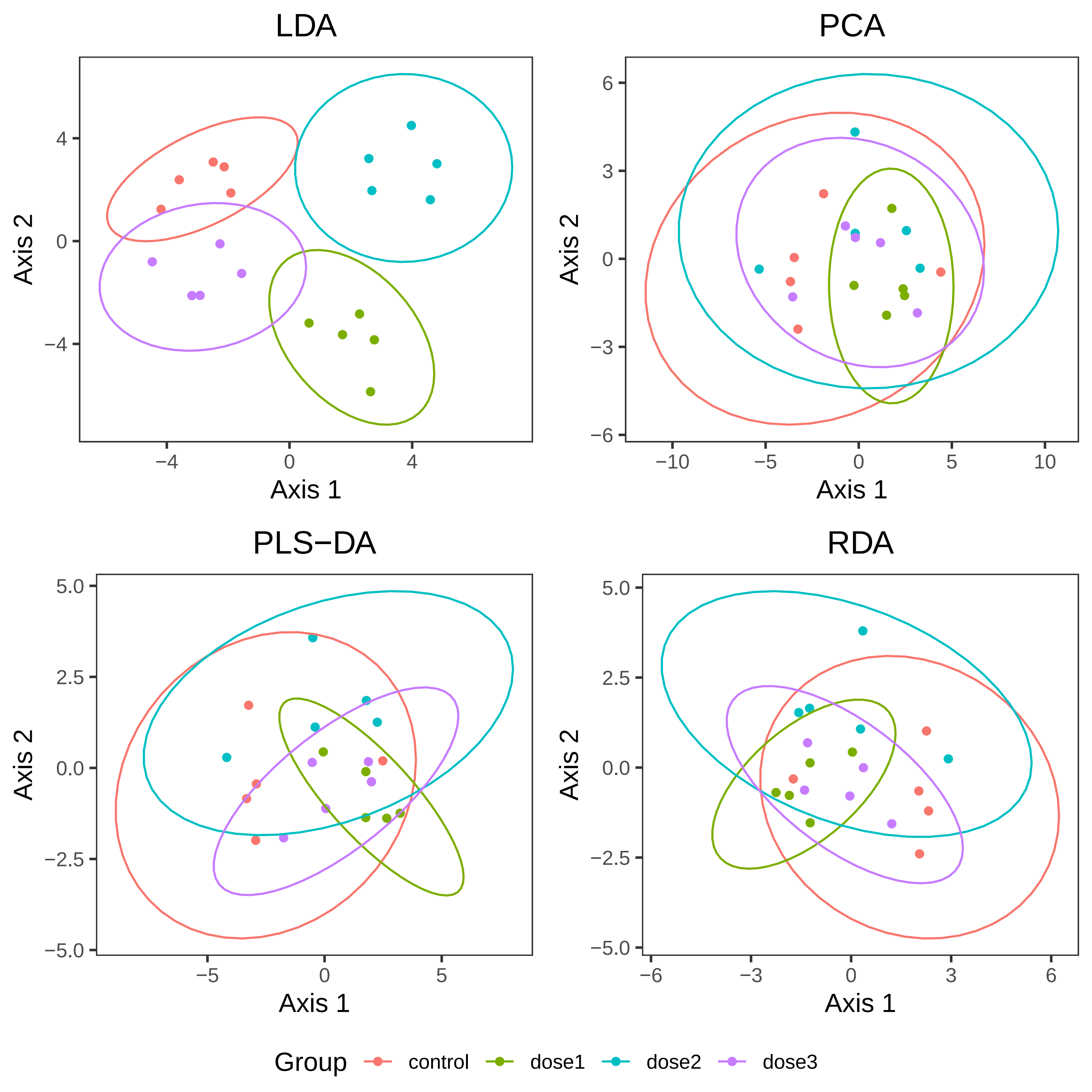
LDA
PCA
PLS−DA
RDA
Axis 1
Axis 2
Group
control
dose1
dose2
dose3

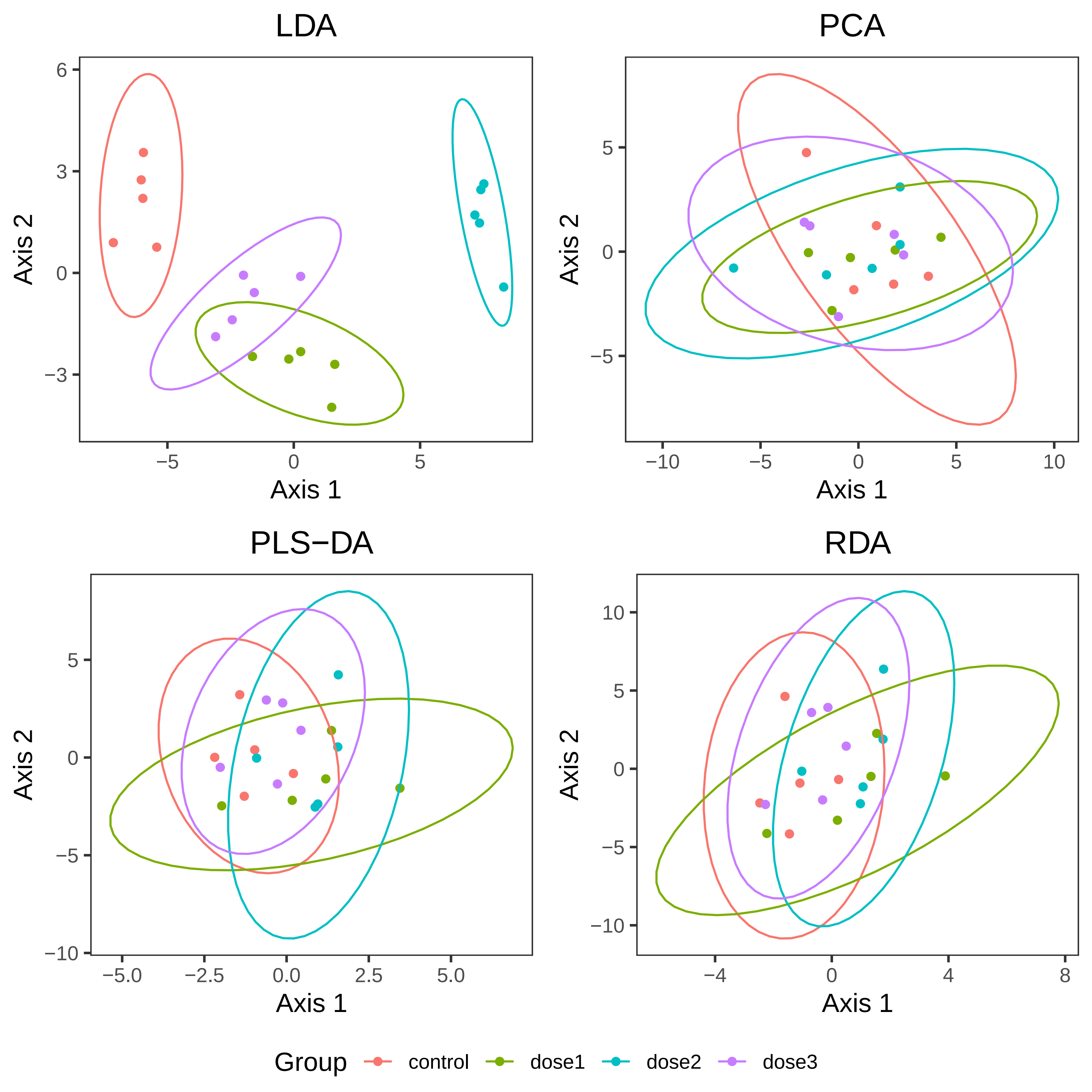

LDA
PCA
PLS−DA
RDA
Axis 1
Axis 2
Group
control
dose1
dose2
dose3

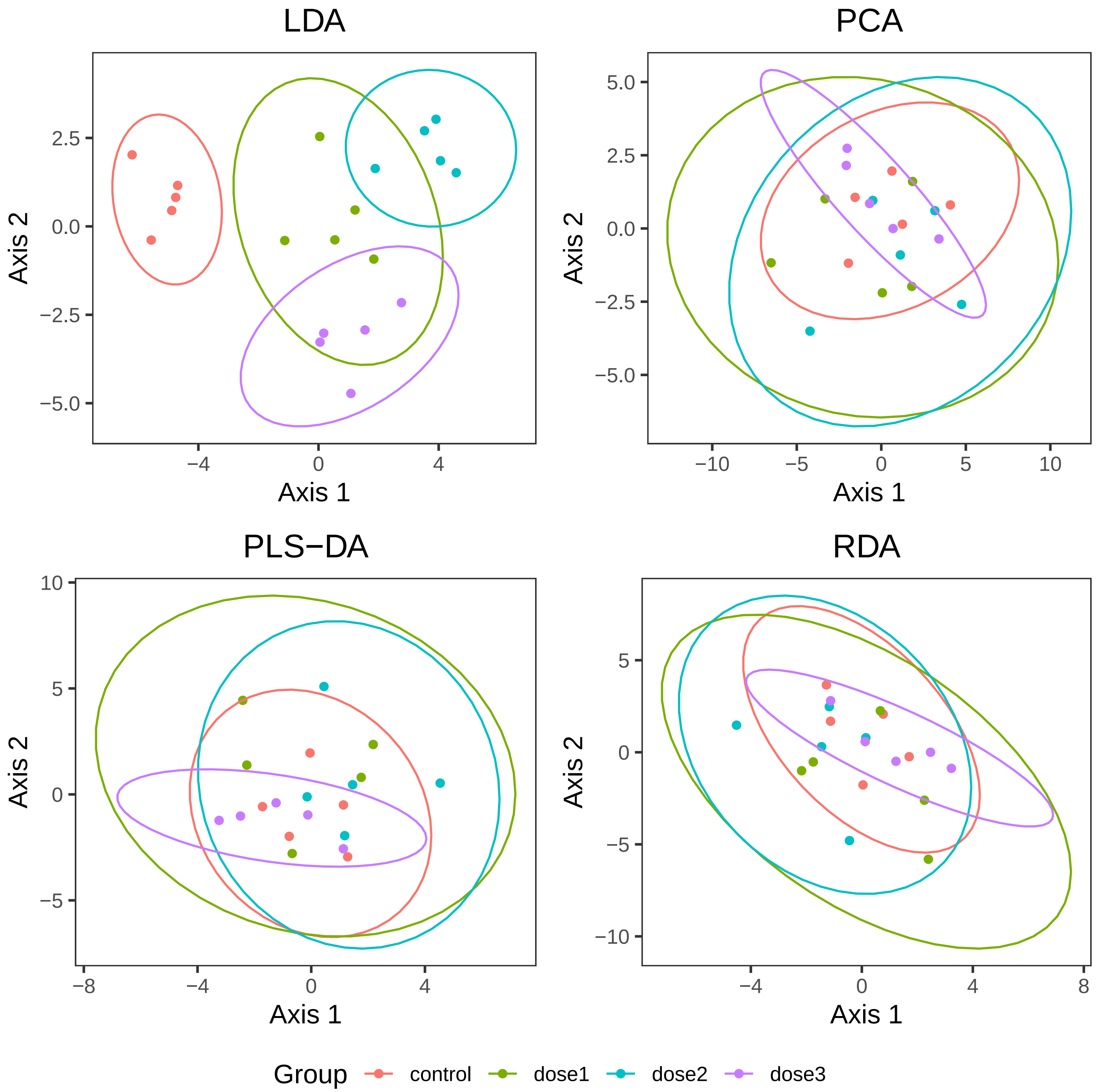
LDA
PCA
PLS−DA
RDA
Axis 1
Axis 2
−4
0
4
2.5
0.0
−2.5
−5.0
−10
−5
0
5
10
5.0
2.5
0.0
−2.5
−5.0
−8
−4
0
4
10
5
0
−5
−4
0
4
8
5
0
−5
−10
Group
control
dose1
dose2
dose3

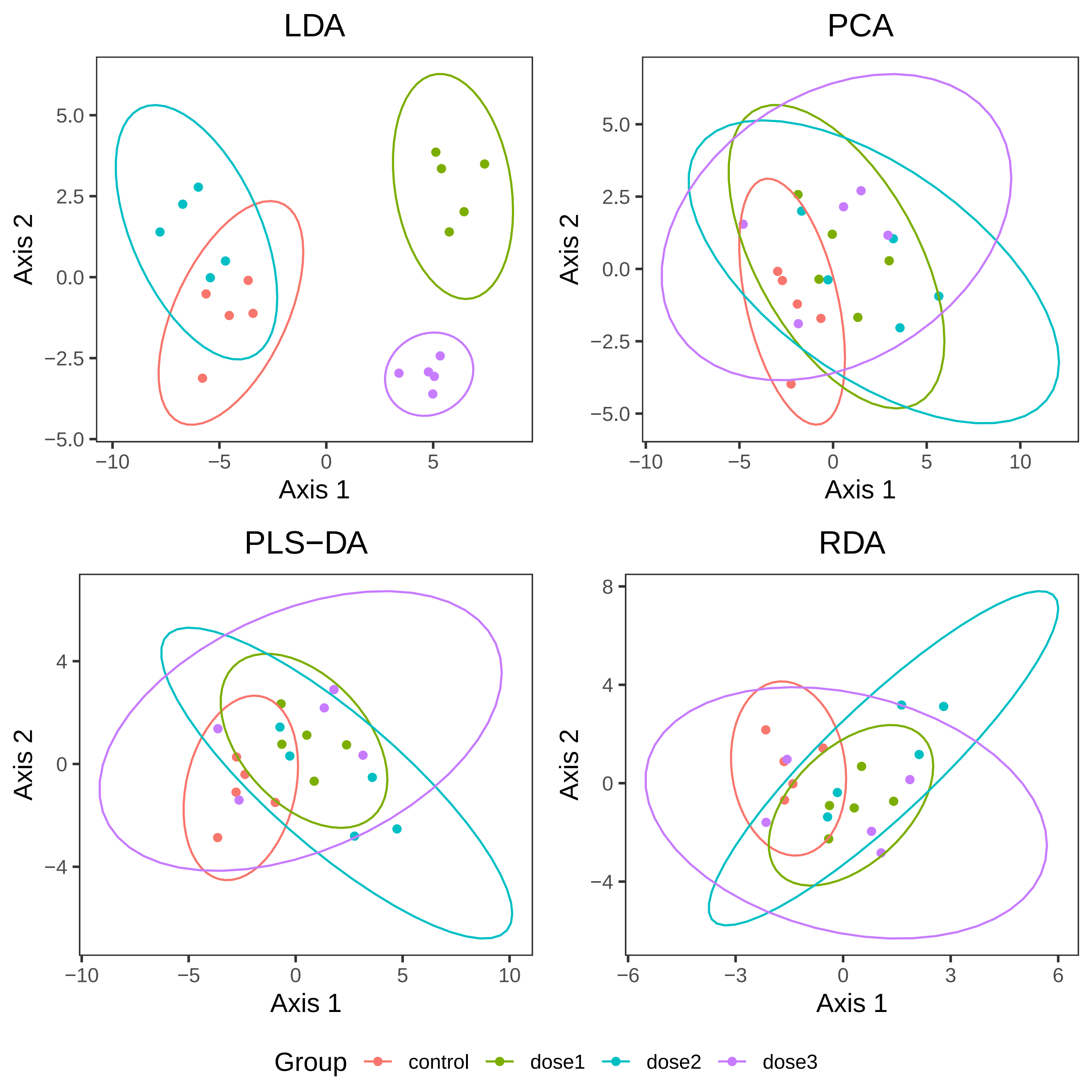
LDA
PCA
PLS−DA
RDA
Axis 1
Axis 2
5.0
2.5
0.0
−2.5
−5.0
−10
−5
0
5
10
4
−4
8
−6
−3
3
6
Group
control
dose1
dose2
dose3

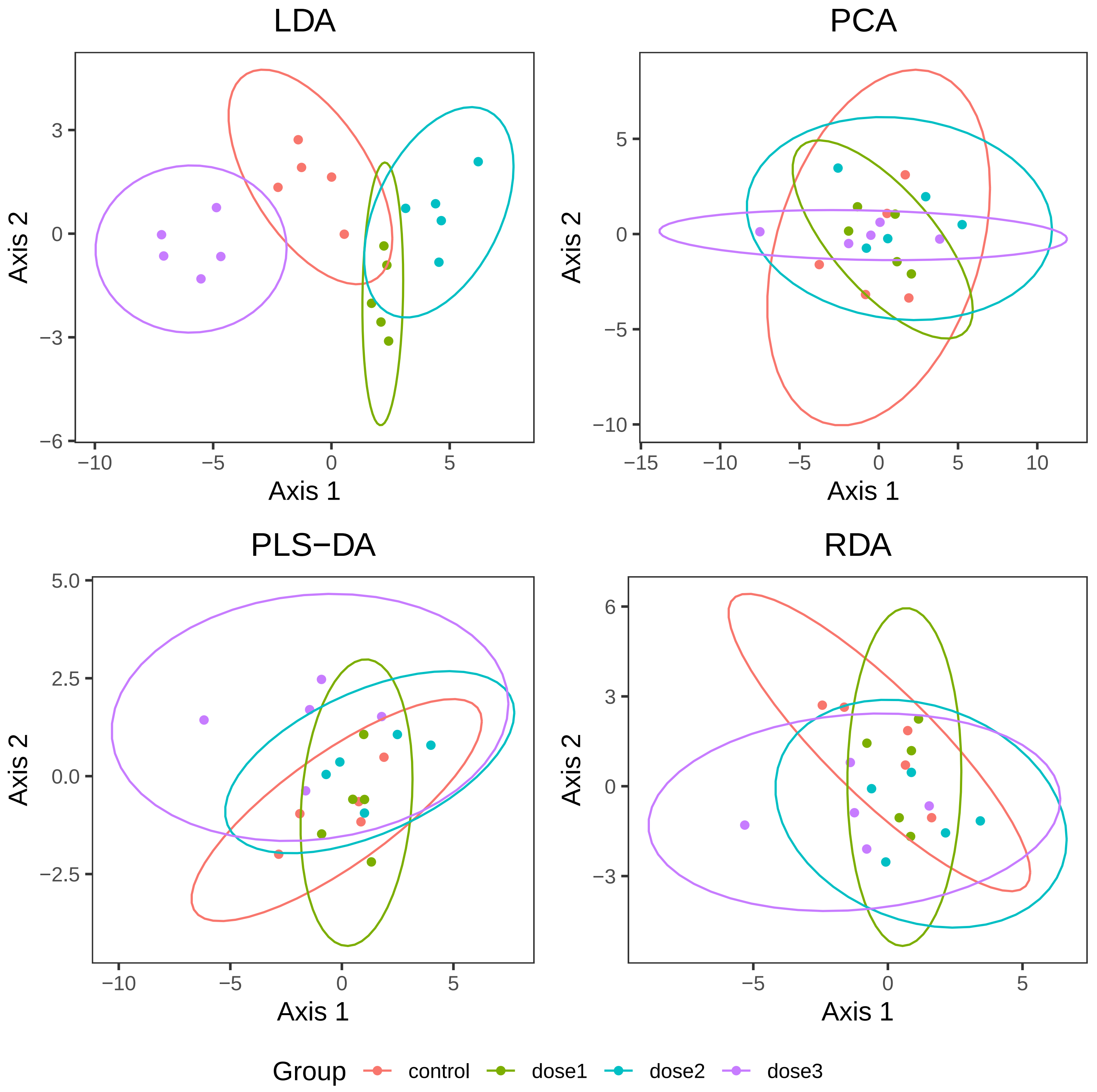
LDA
PCA
PLS−DA
RDA
Axis 1
Axis 2
3
0
−3
−6
−10
−5
0
5
10
−15
2.5
5.0
0.0
−2.5
6
Group
control
dose1
dose2
dose3

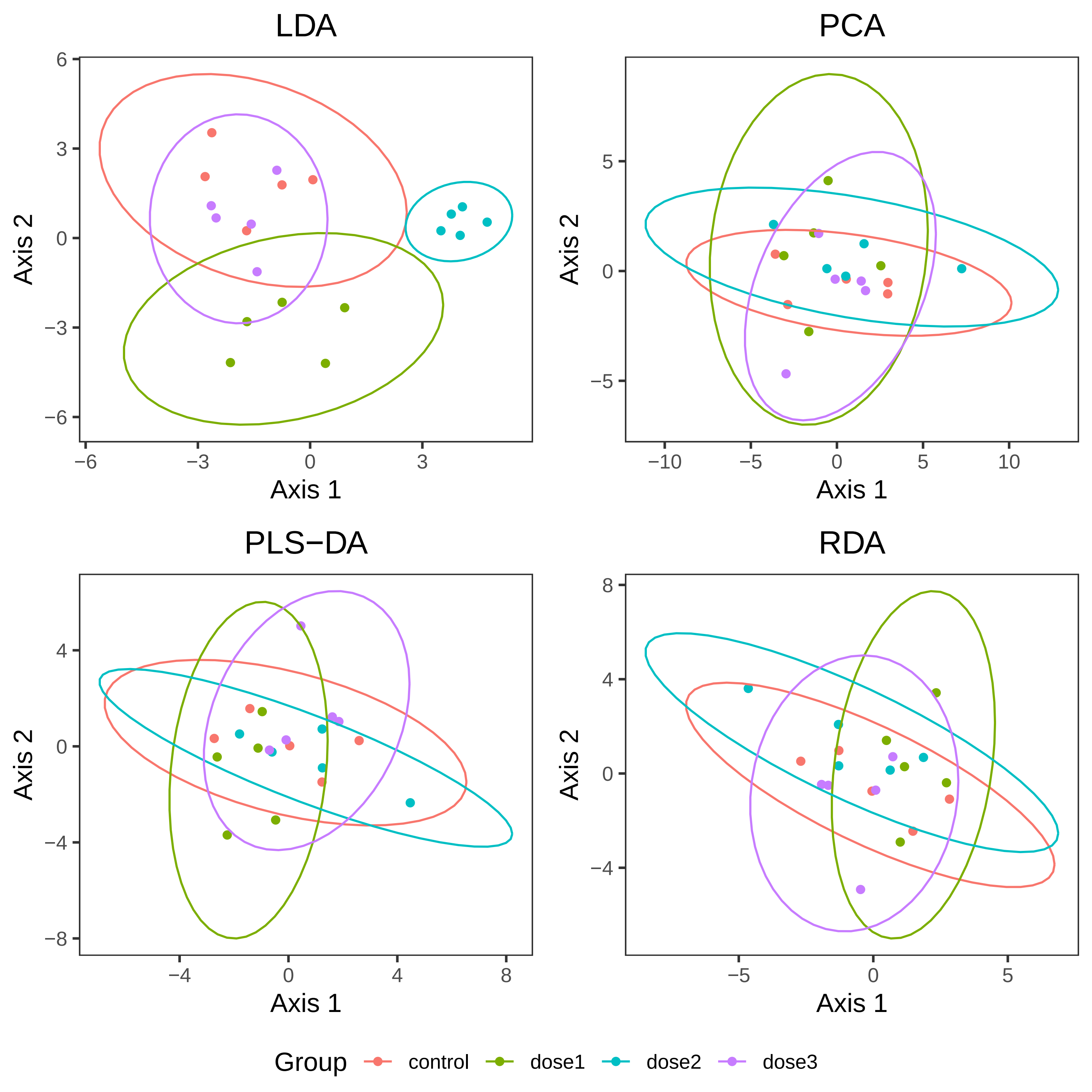

LDA
PCA
PLS−DA
RDA
Axis 1
Axis 2
6
3
0
−3
−6
5
−5
−10
10
4
−4
−8
8
Group
control
dose1
dose2
dose3

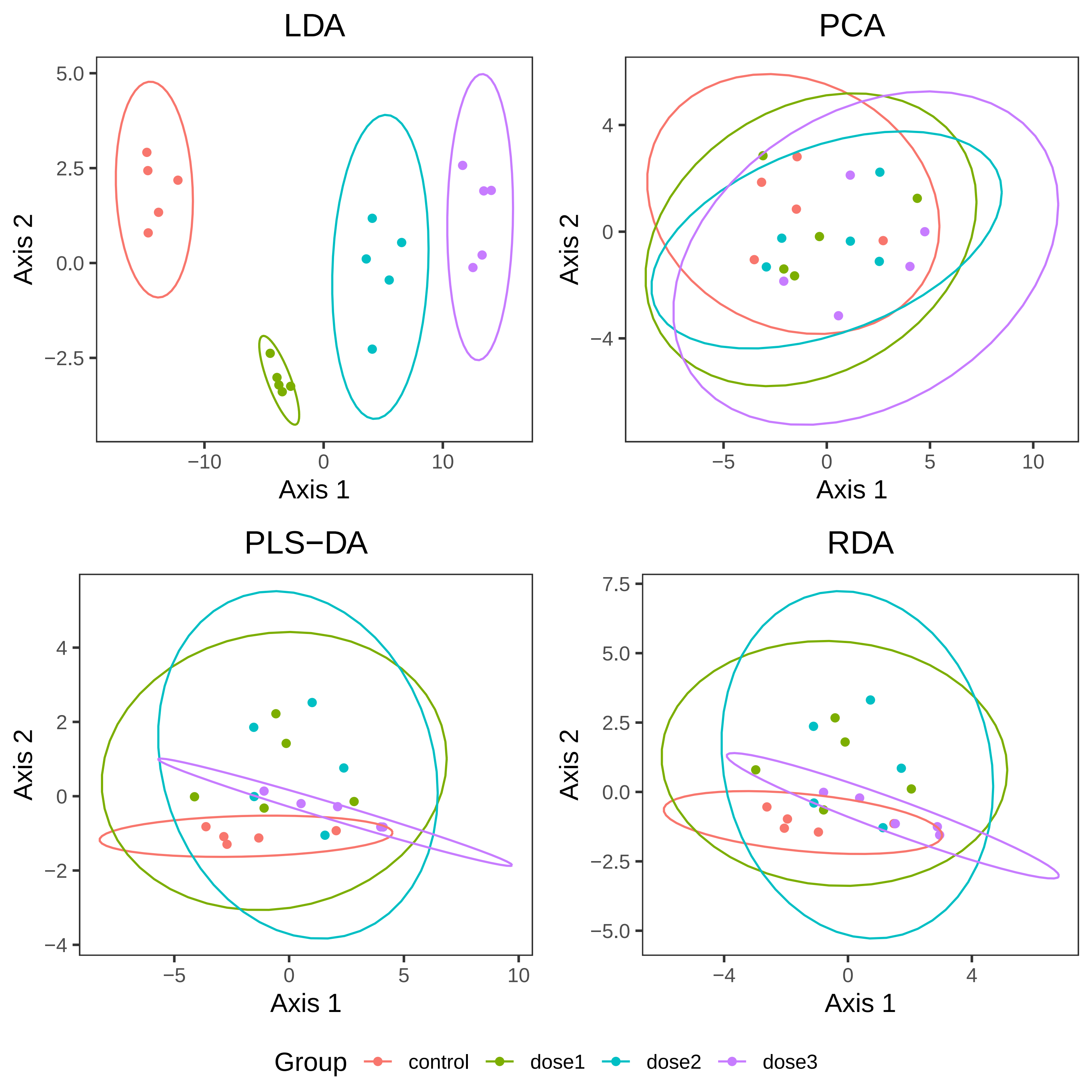

LDA
PCA
PLS−DA
RDA
Axis 1
Axis 2
5.0
2.5
0.0
−2.5
−10
0
10
4
0
−4
−5
5
10
2
−2
7.5
−5.0
Group
control
dose1
dose2
dose3

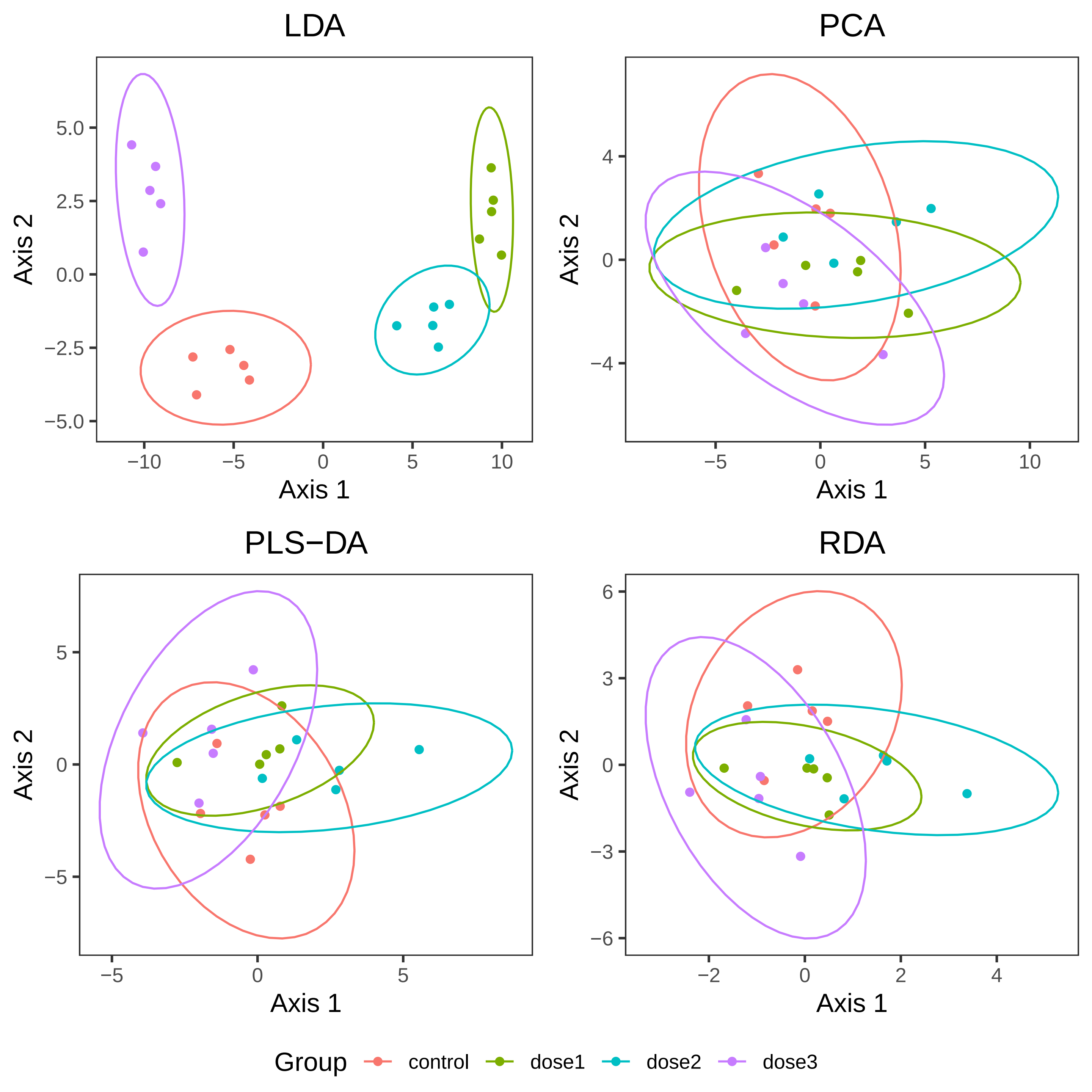

LDA
PCA
PLS−DA
RDA
Axis 1
Axis 2
Group
control
dose1
dose2
dose3

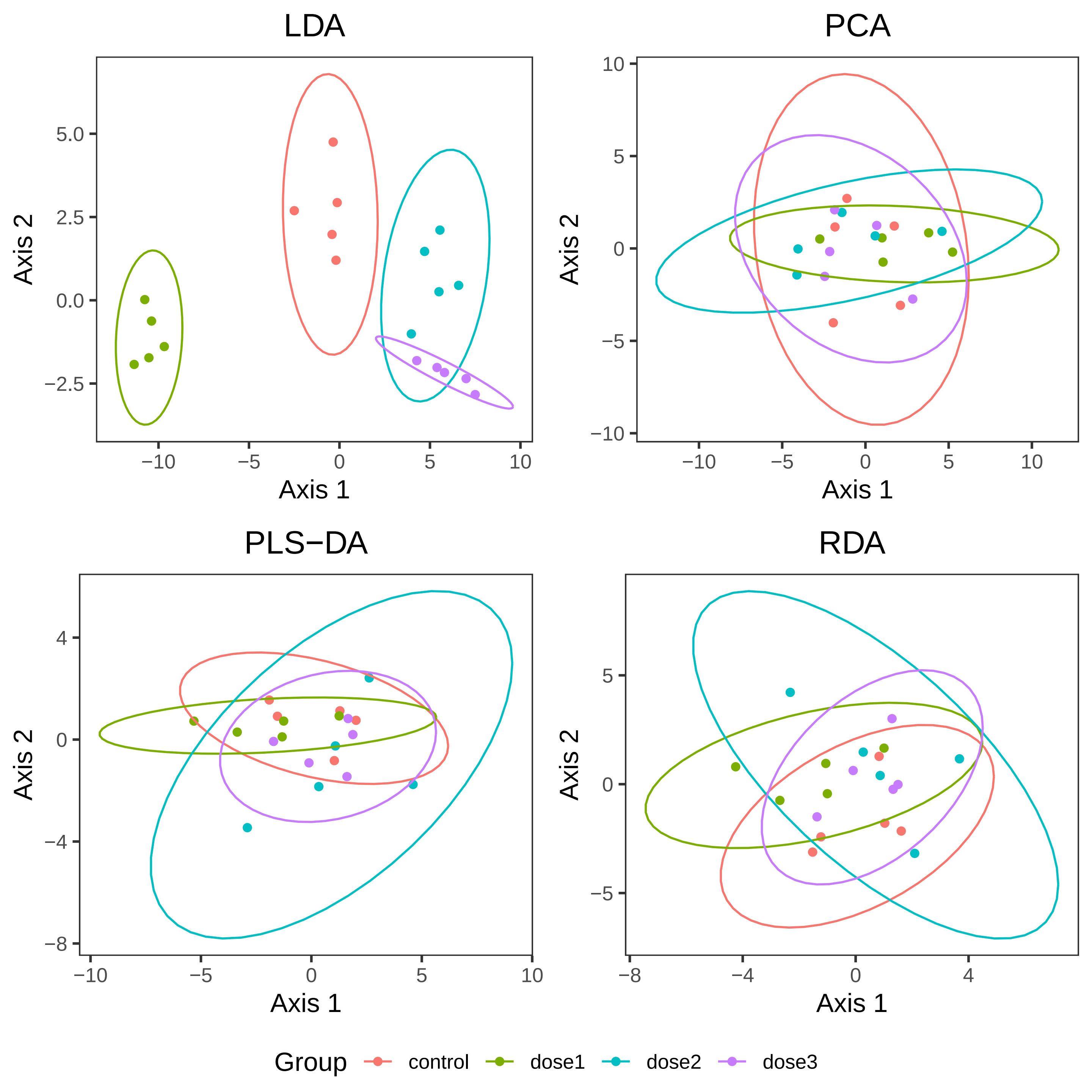

LDA
PCA
PLS−DA
RDA
Axis 1
Axis 2
Group
control
dose1
dose2
dose3

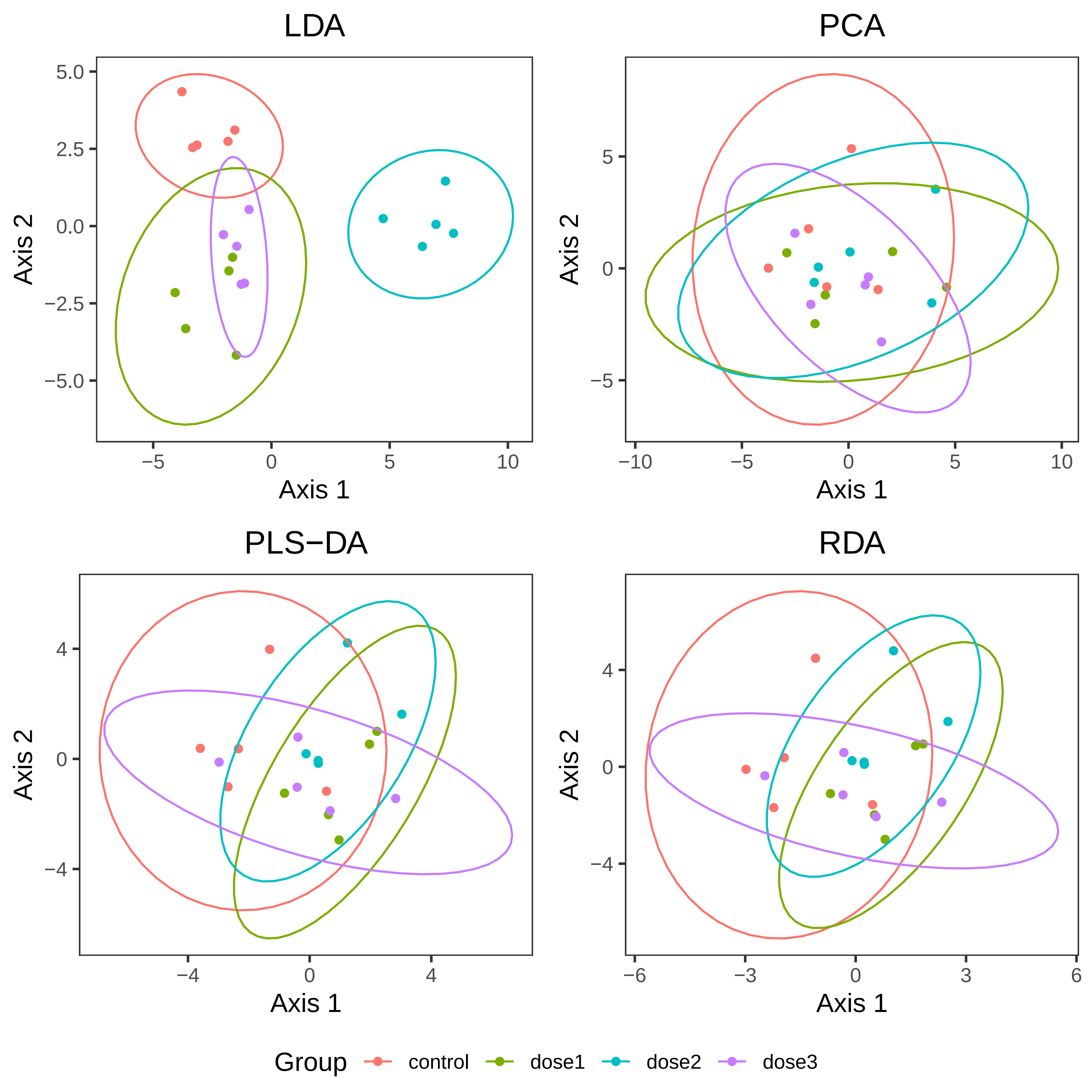
LDA
PCA
PLS−DA
RDA
Axis 1
Axis 2
Group
control
dose1
dose2
dose3

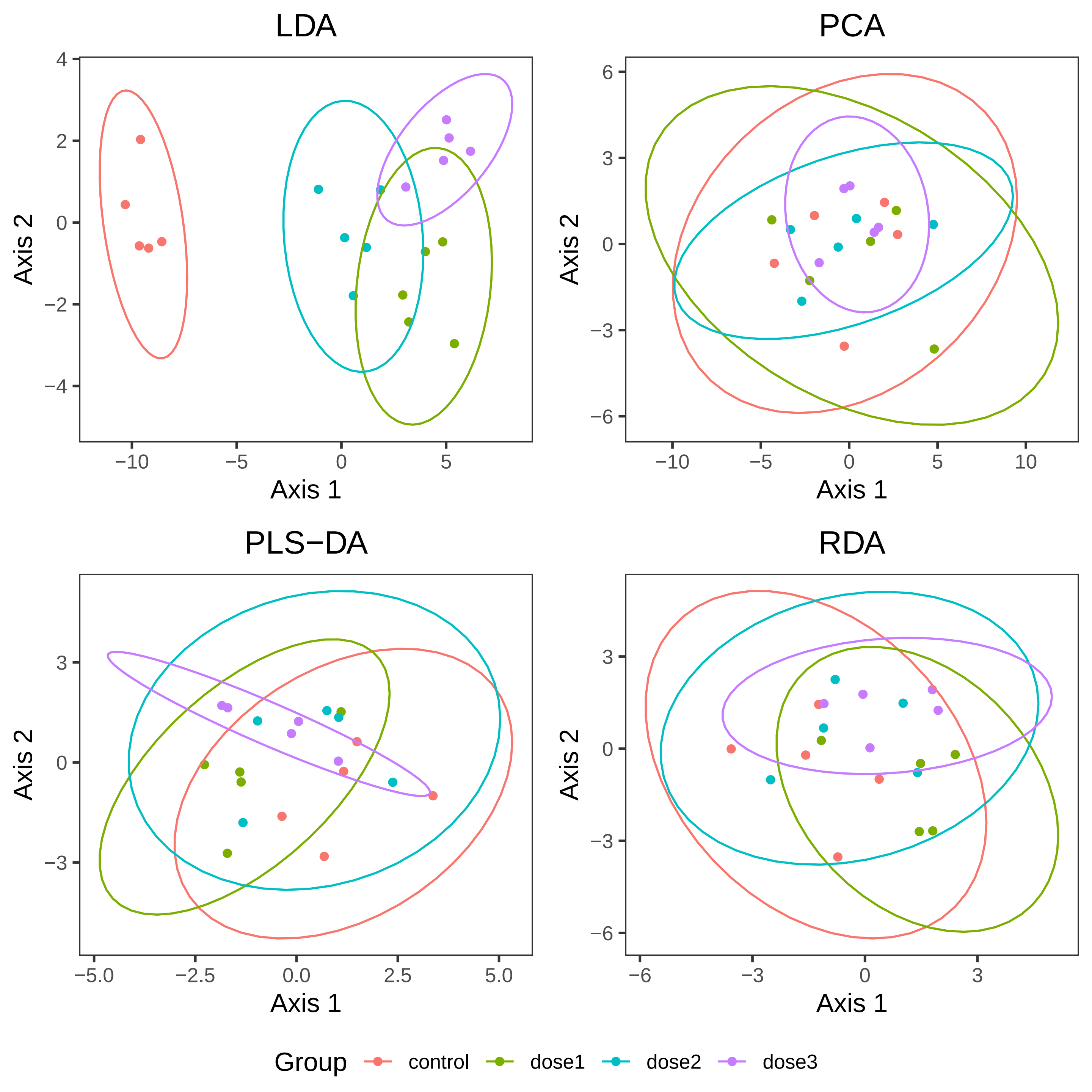

LDA
PCA
PLS−DA
RDA
Axis 1
Axis 2
Group
control
dose1
dose2
dose3

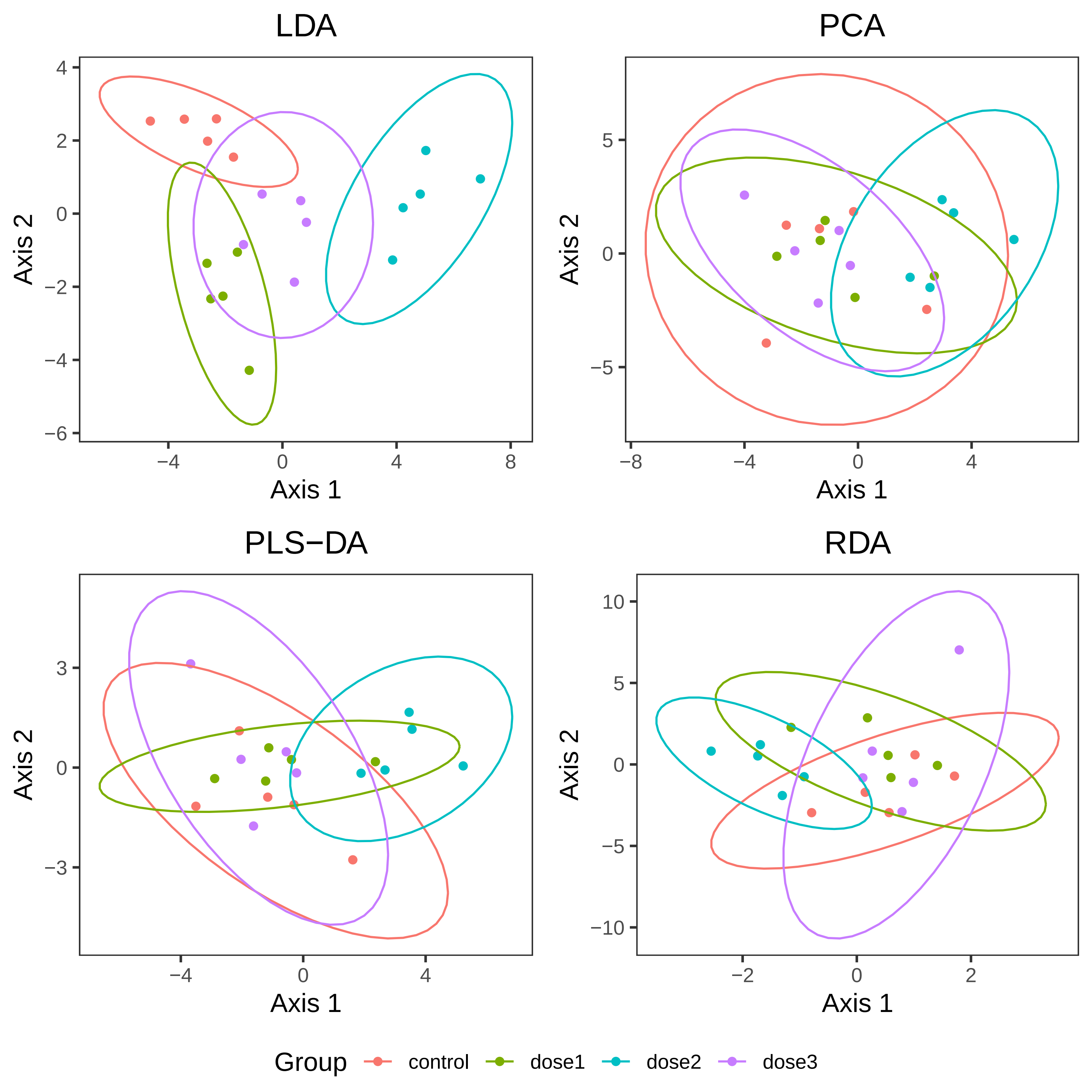

LDA
PCA
PLS−DA
RDA
Axis 1
Axis 2
4
2
0
−2
−4
−6
8
5
−5
−8
3
−3
10
−10
Group
control
dose1
dose2
dose3

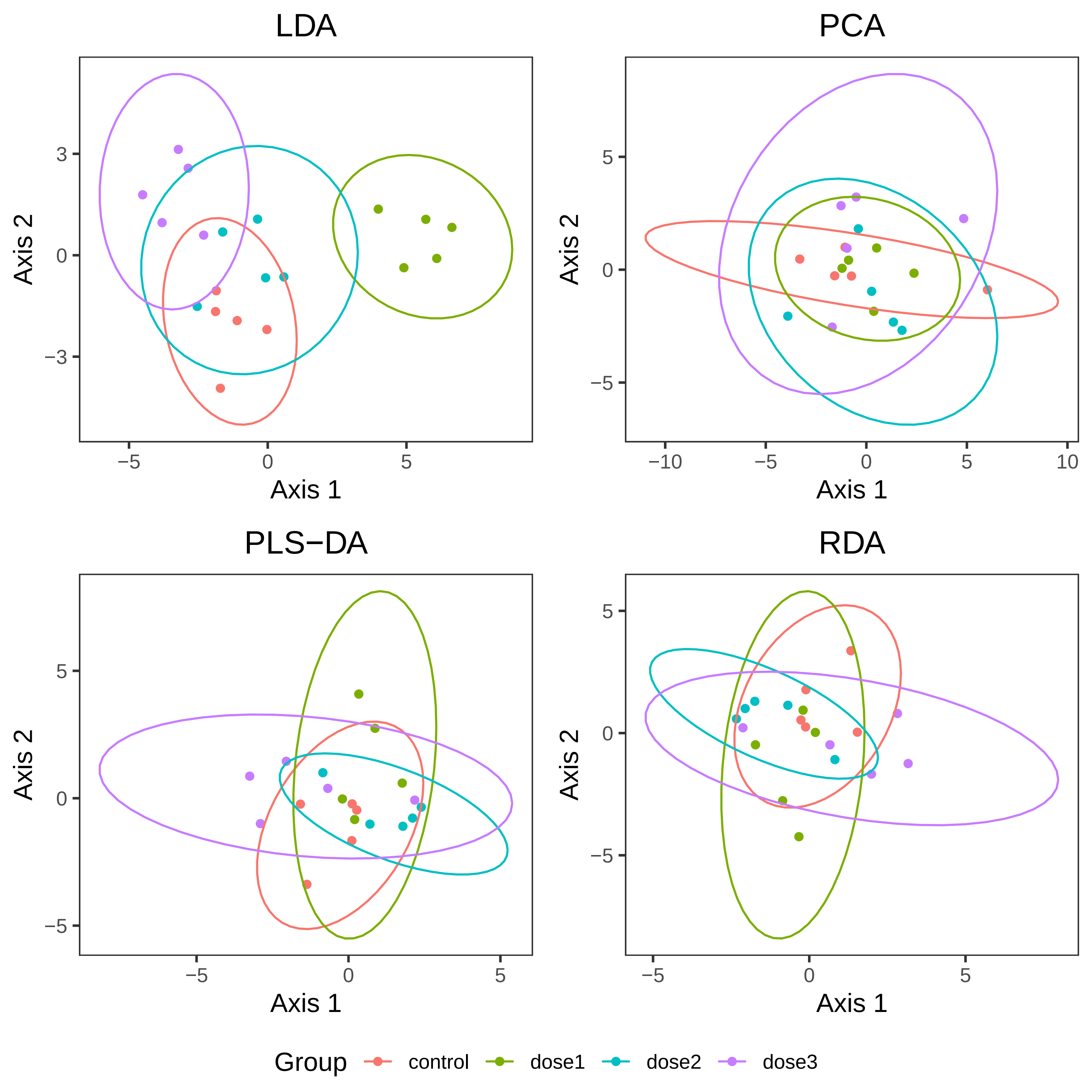
LDA
PCA
PLS−DA
RDA
Axis 1
Axis 2
Group
control
dose1
dose2
dose3

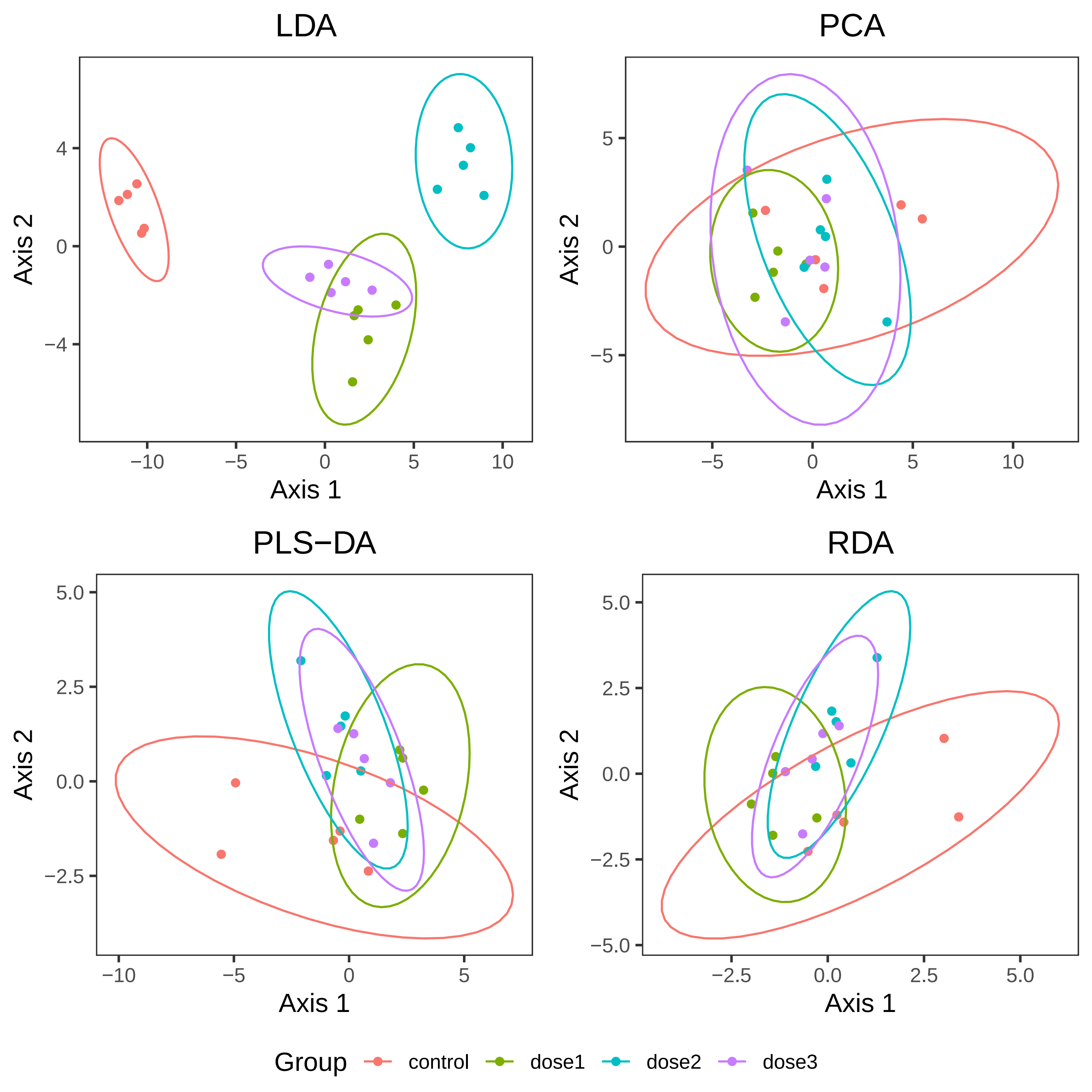

LDA
PCA
PLS−DA
RDA
Axis 1
Axis 2
Group
control
dose1
dose2
dose3

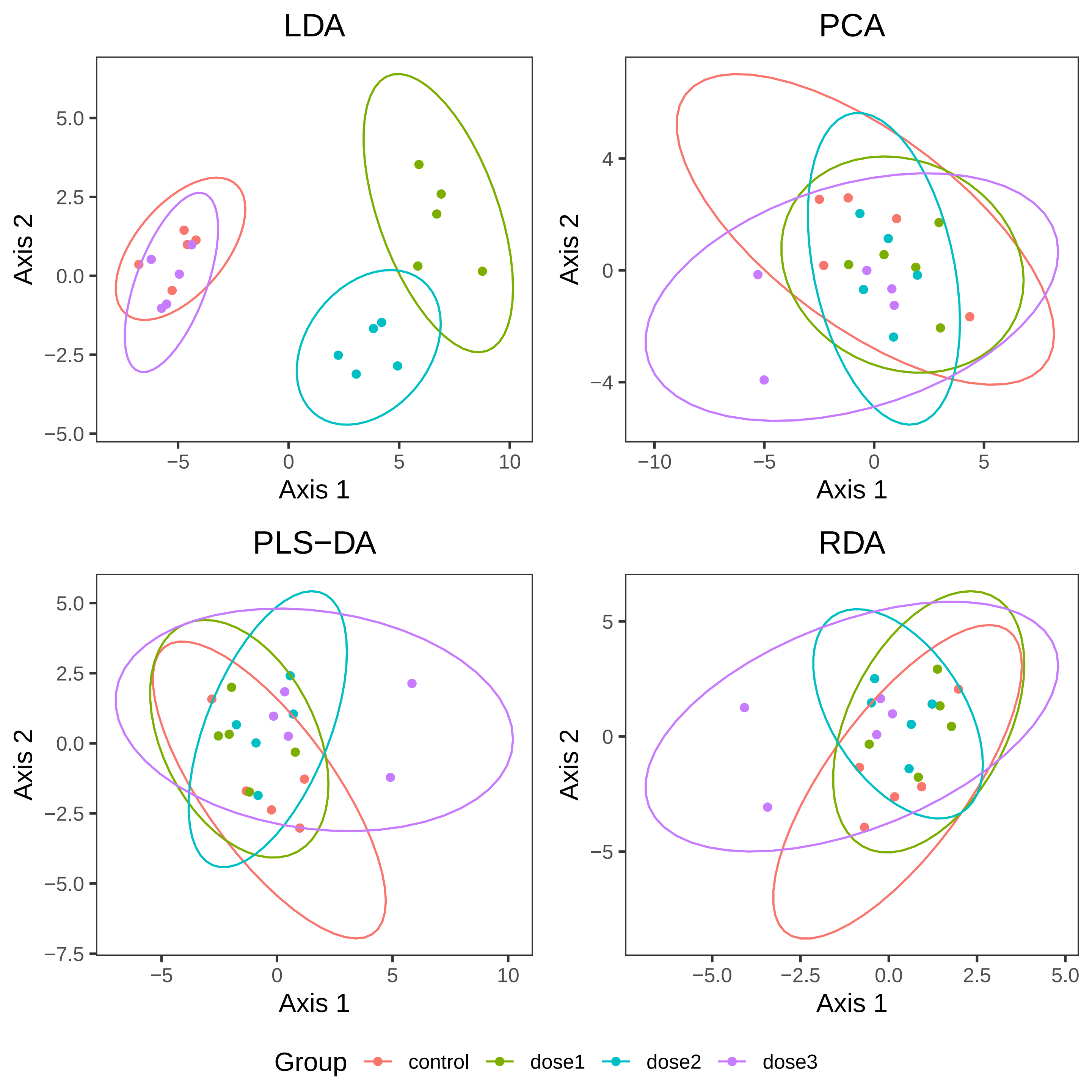
LDA
PCA
PLS−DA
RDA
Axis 1
Axis 2
5.0
2.5
0.0
−2.5
−5.0
−7.5
−10
−5
0
5
10
4
−4
Group
control
dose1
dose2
dose3

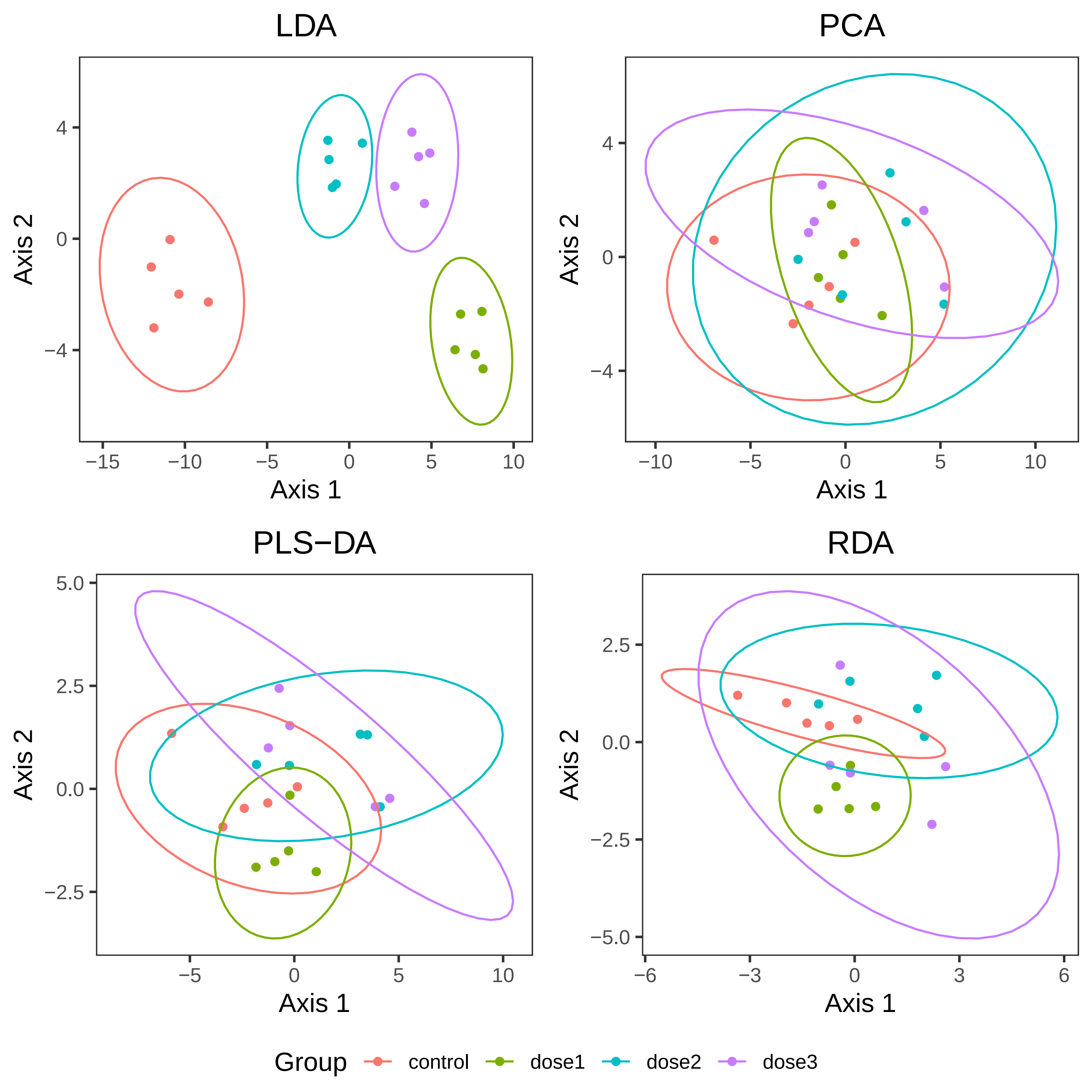

LDA
PCA
PLS−DA
RDA
Axis 1
Axis 2
4
0
−4
−15
−10
−5
0
5
10
−4
0
4
−10
−5
0
5
10
5.0
2.5
0.0
−2.5
−5
0
5
10
2.5
0.0
−2.5
−5.0
−6
−3
0
3
6
Group
control
dose1
dose2
dose3

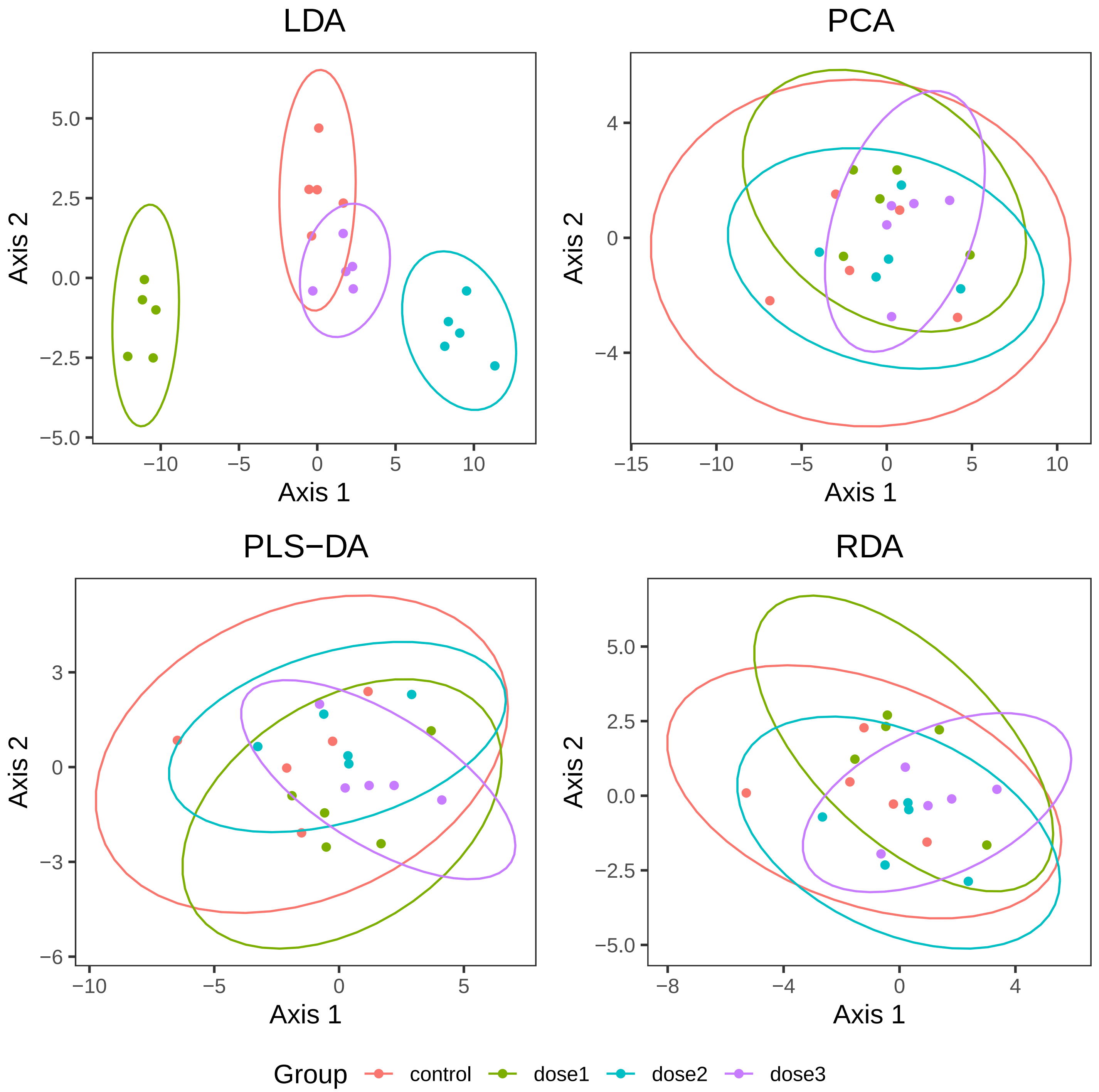

LDA
PCA
PLS−DA
RDA
Axis 1
Axis 2
Group
control
dose1
dose2
dose3

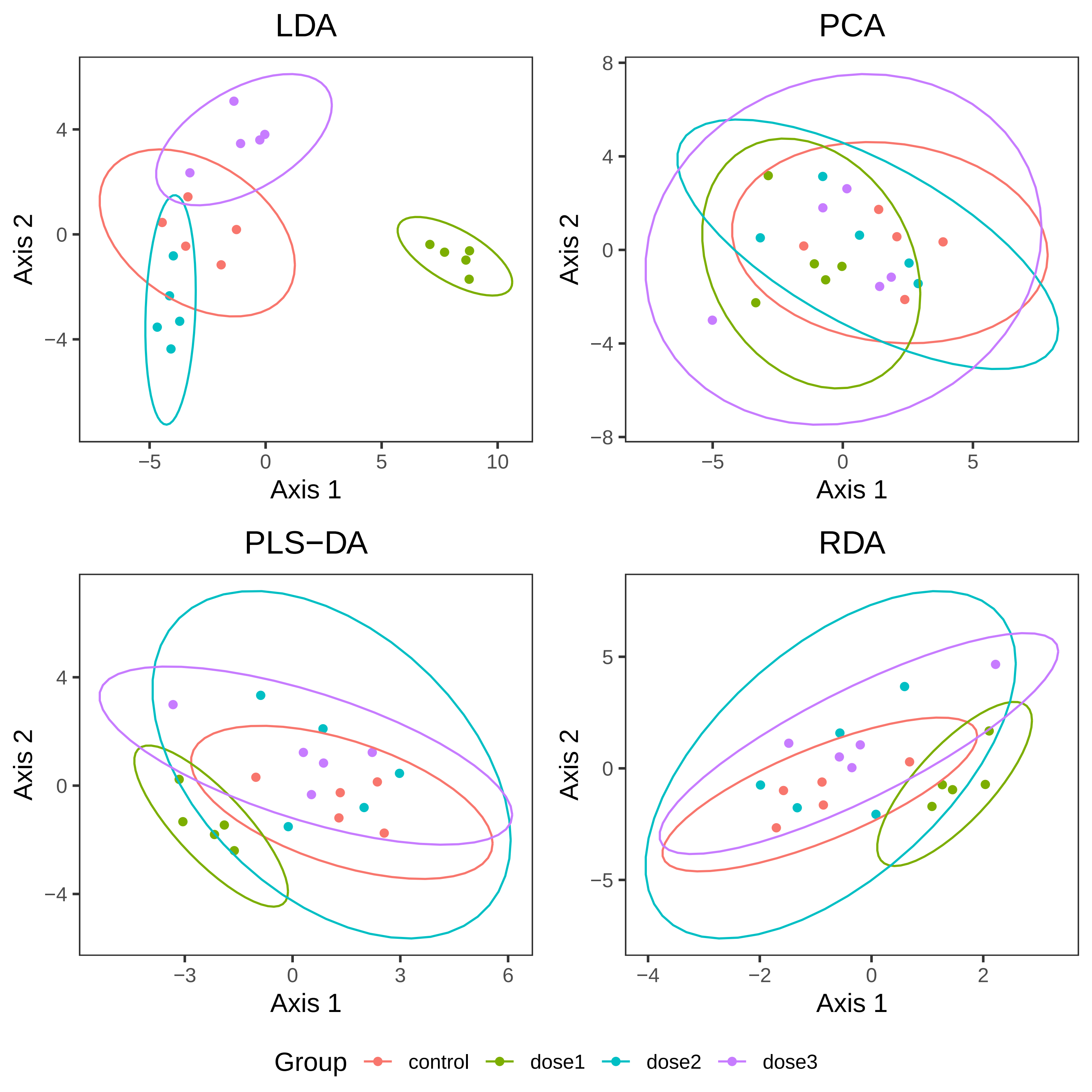
LDA
PCA
PLS−DA
RDA
Axis 1
Axis 2
Group
control
dose1
dose2
dose3

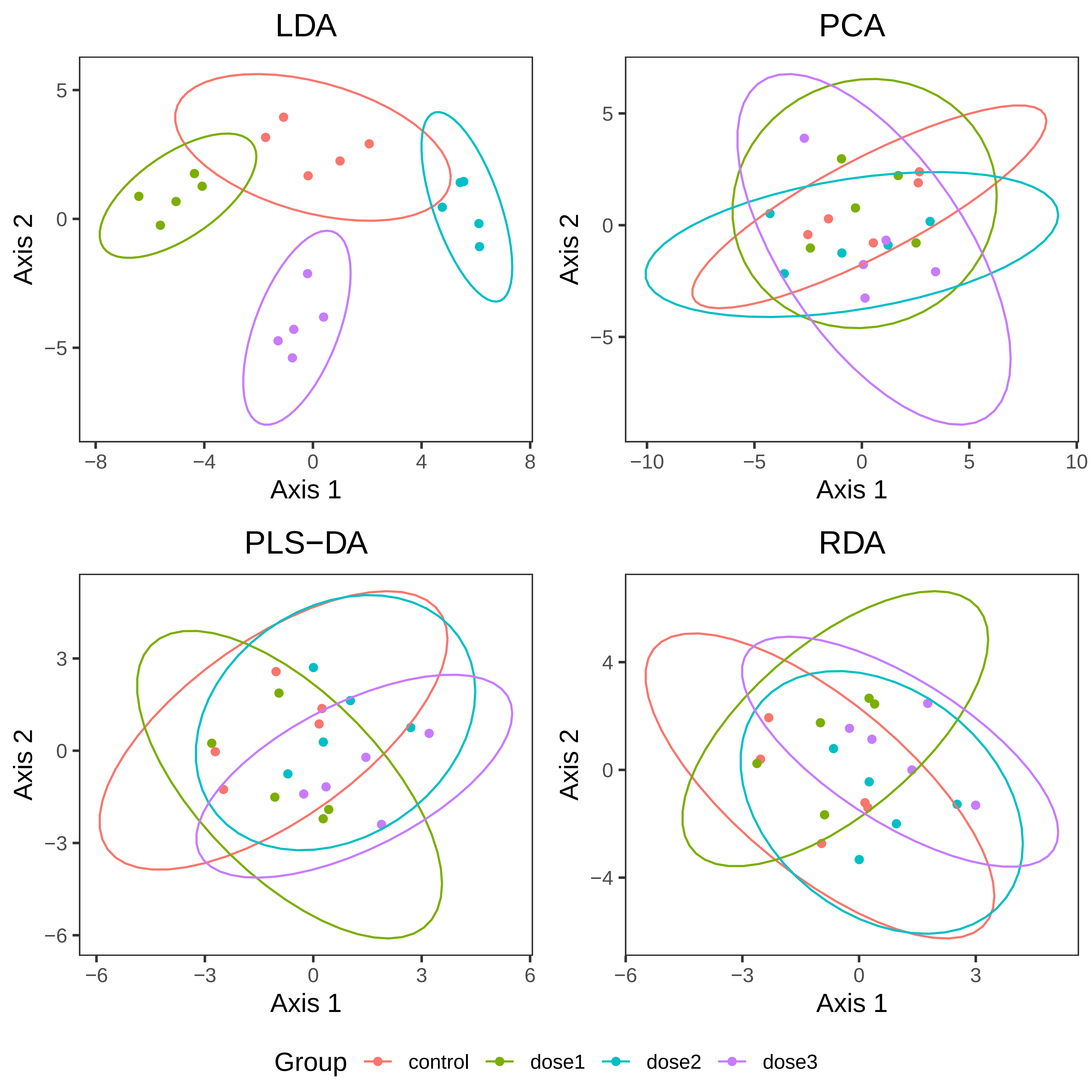

LDA
PCA
PLS−DA
RDA
Axis 1
Axis 2
Group
control
dose1
dose2
dose3

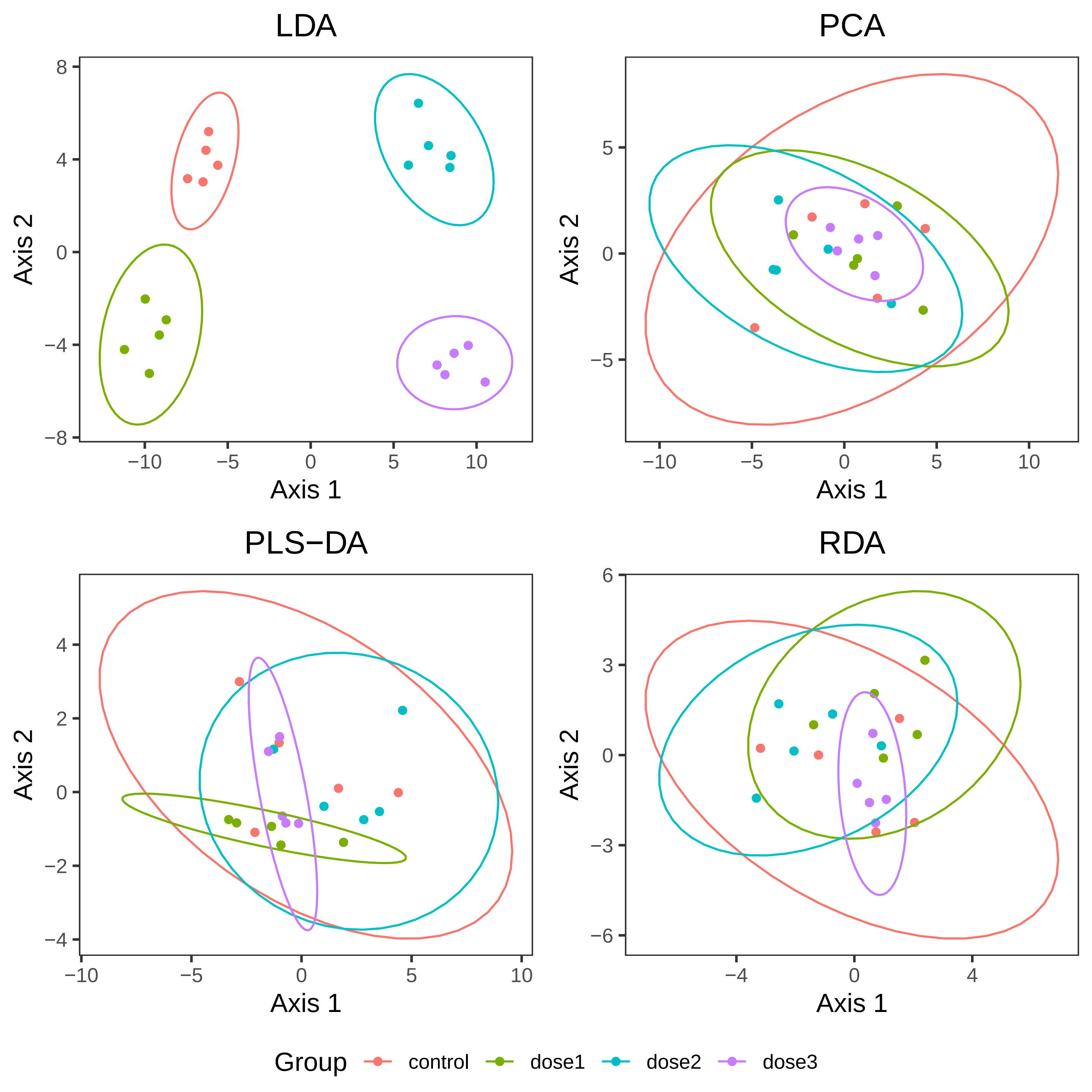

LDA
PCA
PLS−DA
RDA
Axis 1
Axis 2
8
4
0
−4
−8
−10
−5
0
5
10
6
3
−3
−6
2
−2
Group
control
dose1
dose2
dose3

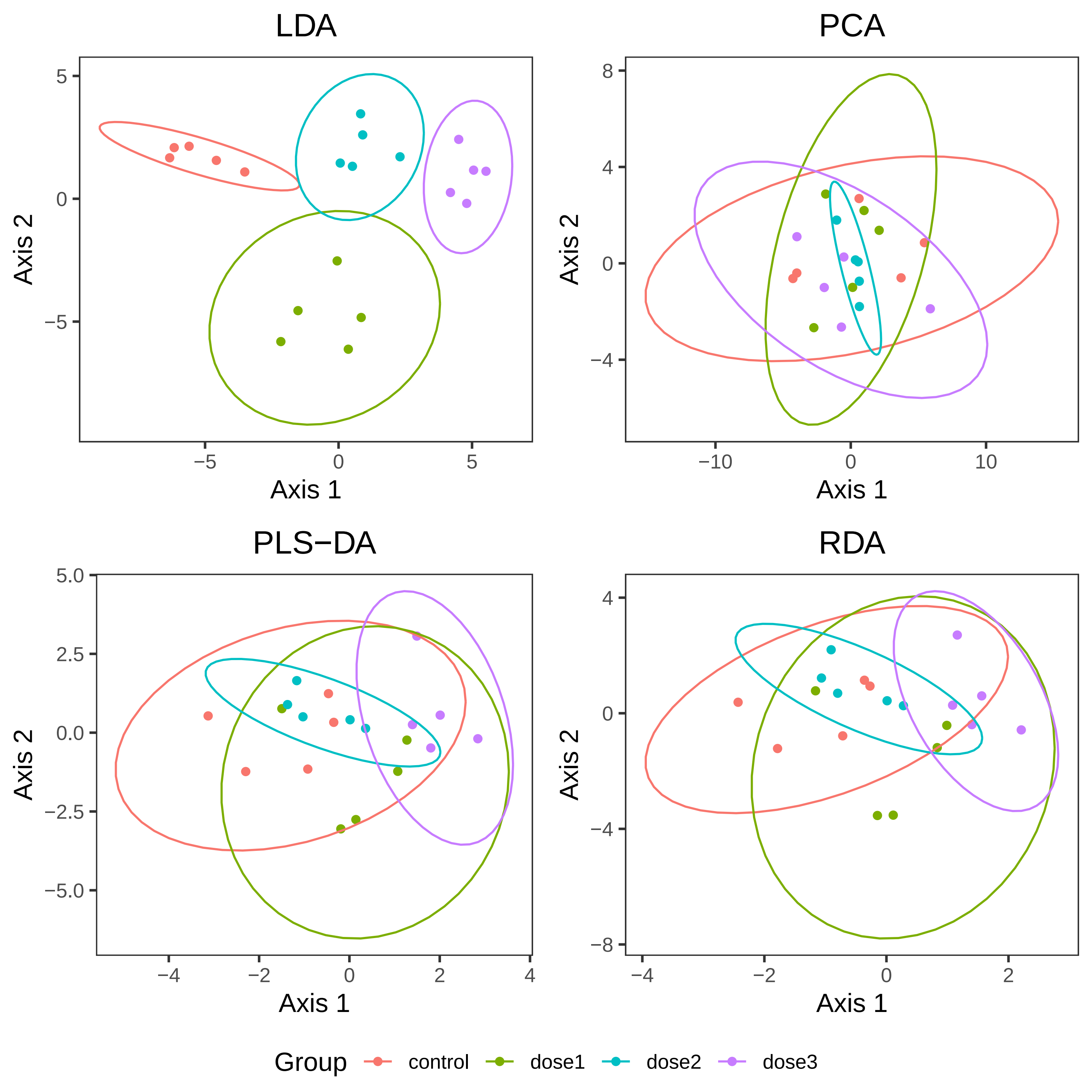

LDA
PCA
PLS−DA
RDA
Axis 1
Axis 2
−5
0
5
8
4
−4
−10
10
5.0
2.5
0.0
−2.5
−5.0
−4
−2
2
4
−8
Group
control
dose1
dose2
dose3

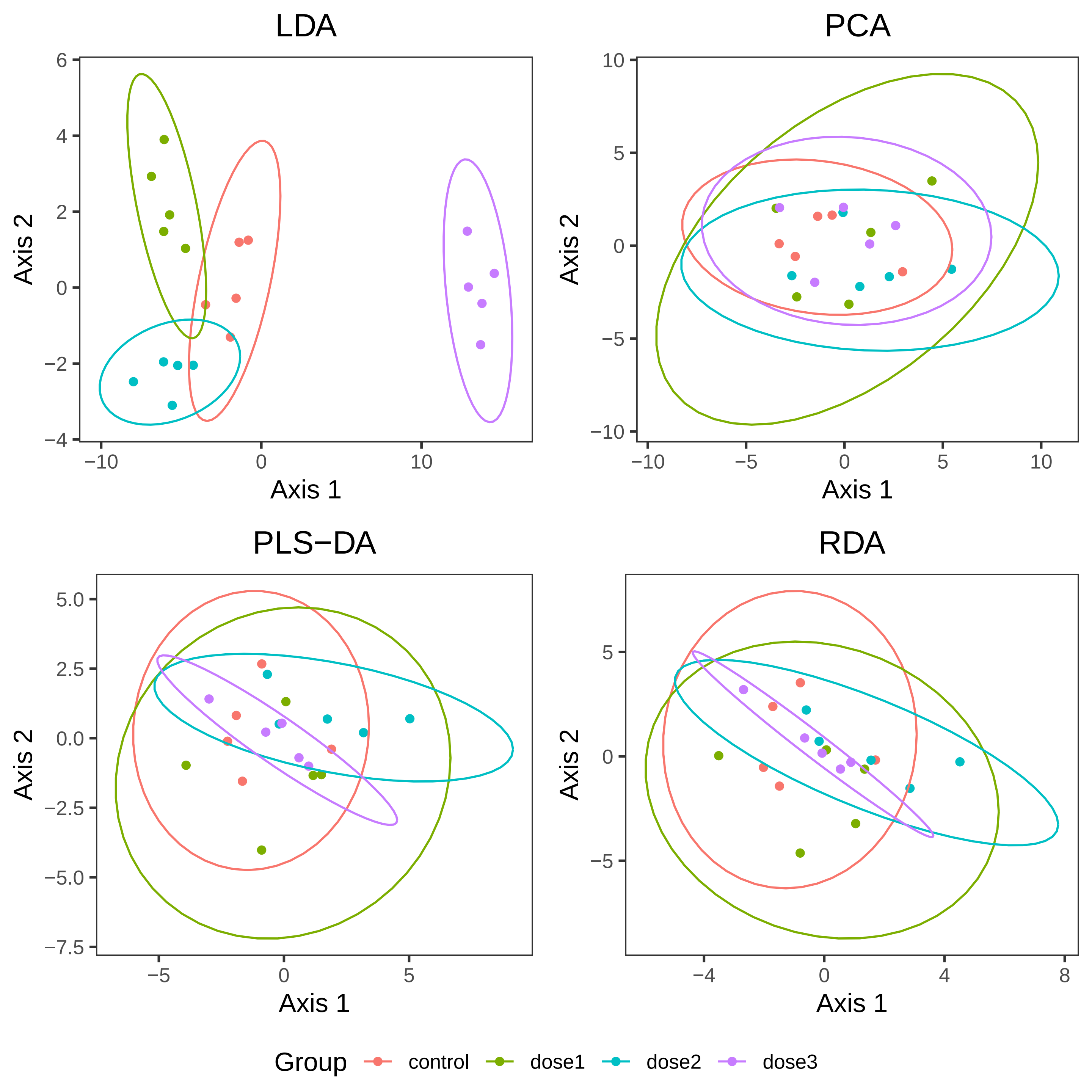

LDA
PCA
PLS−DA
RDA
Axis 1
Axis 2
Group
control
dose1
dose2
dose3

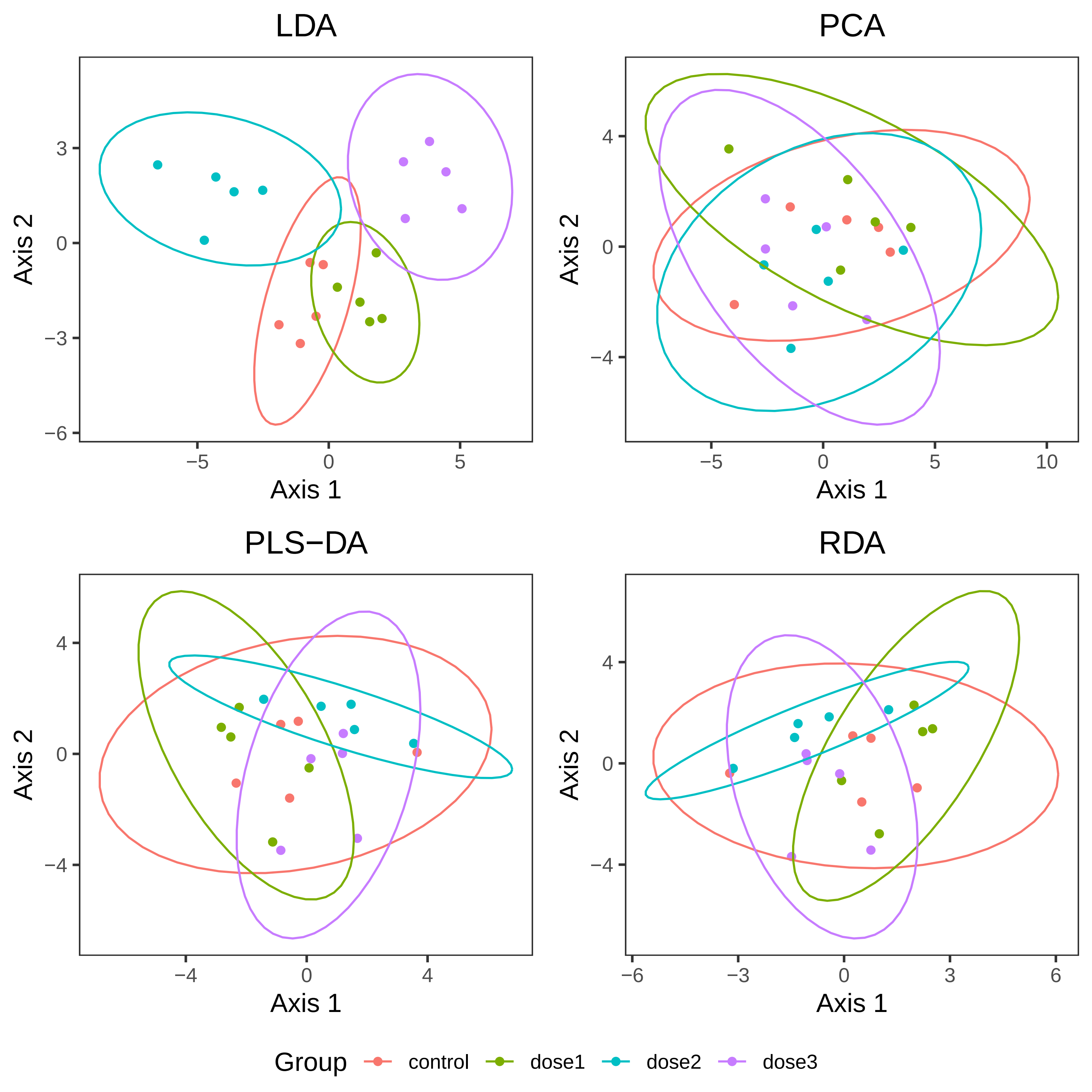
LDA
PCA
PLS−DA
RDA
Axis 1
Axis 2
Group
control
dose1
dose2
dose3

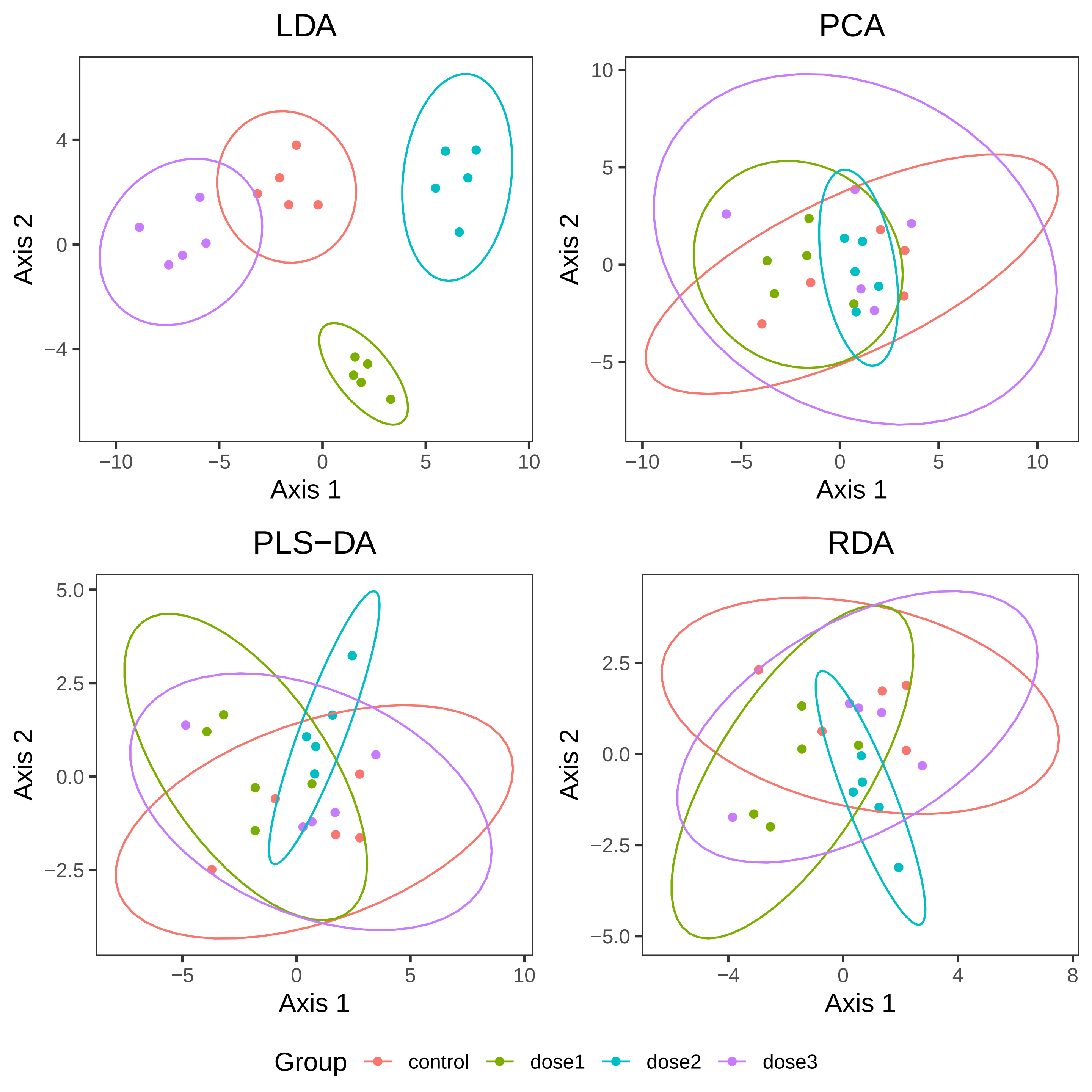

LDA
PCA
PLS−DA
RDA
Axis 1
Axis 2
Group
control
dose1
dose2
dose3

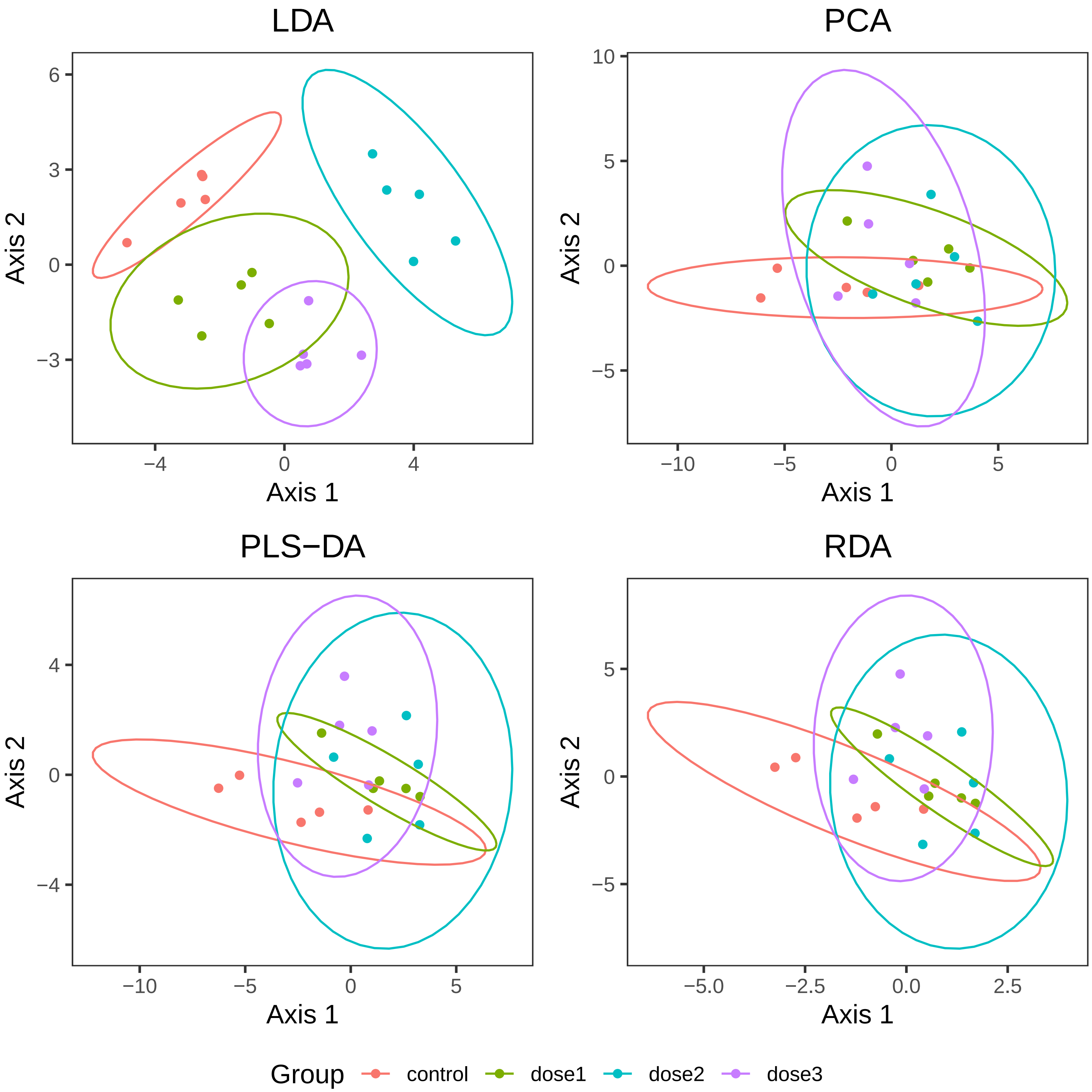

LDA
PCA
PLS−DA
RDA
Axis 1
Axis 2
Group
control
dose1
dose2
dose3

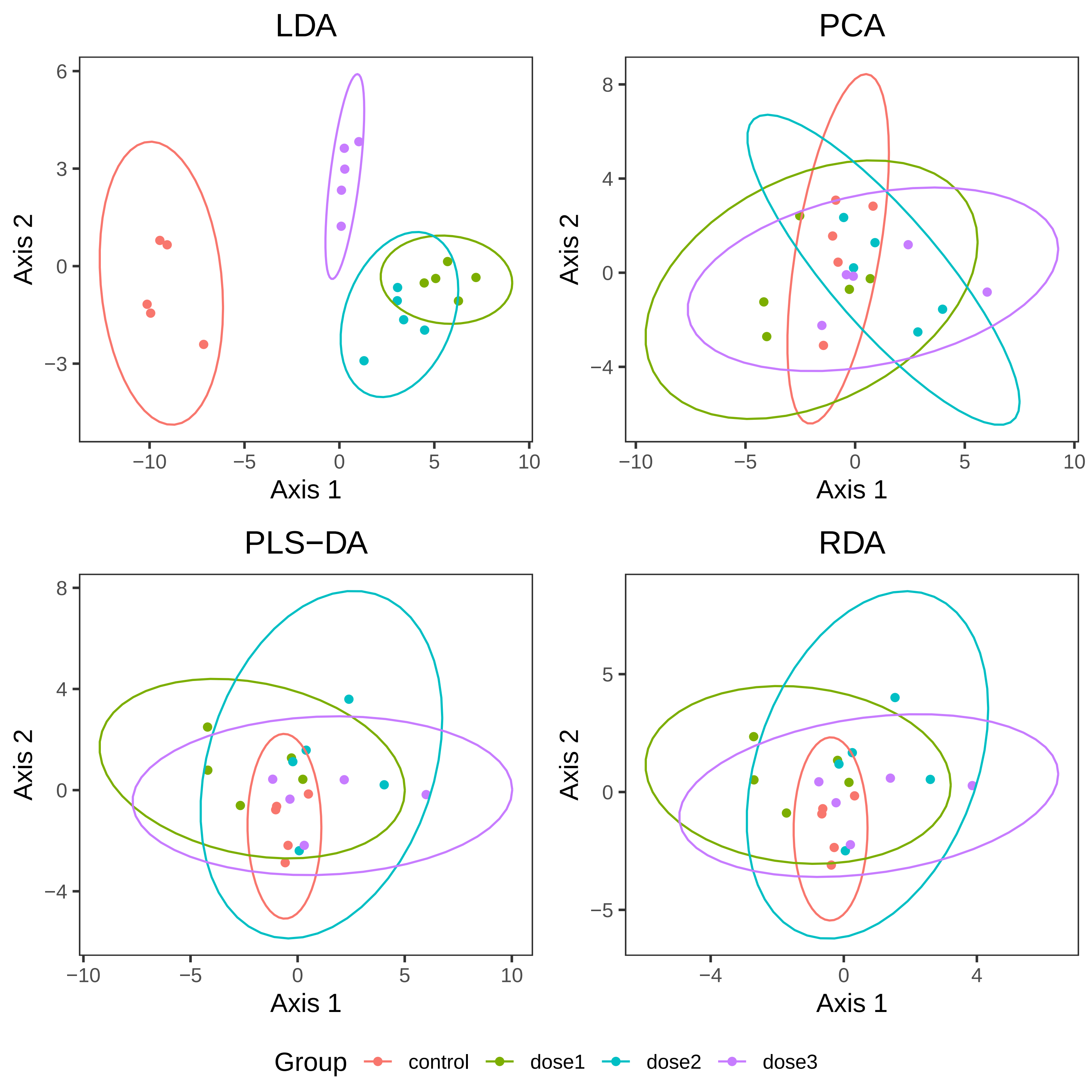

LDA
PCA
PLS−DA
RDA
Axis 1
Axis 2
Group
control
dose1
dose2
dose3

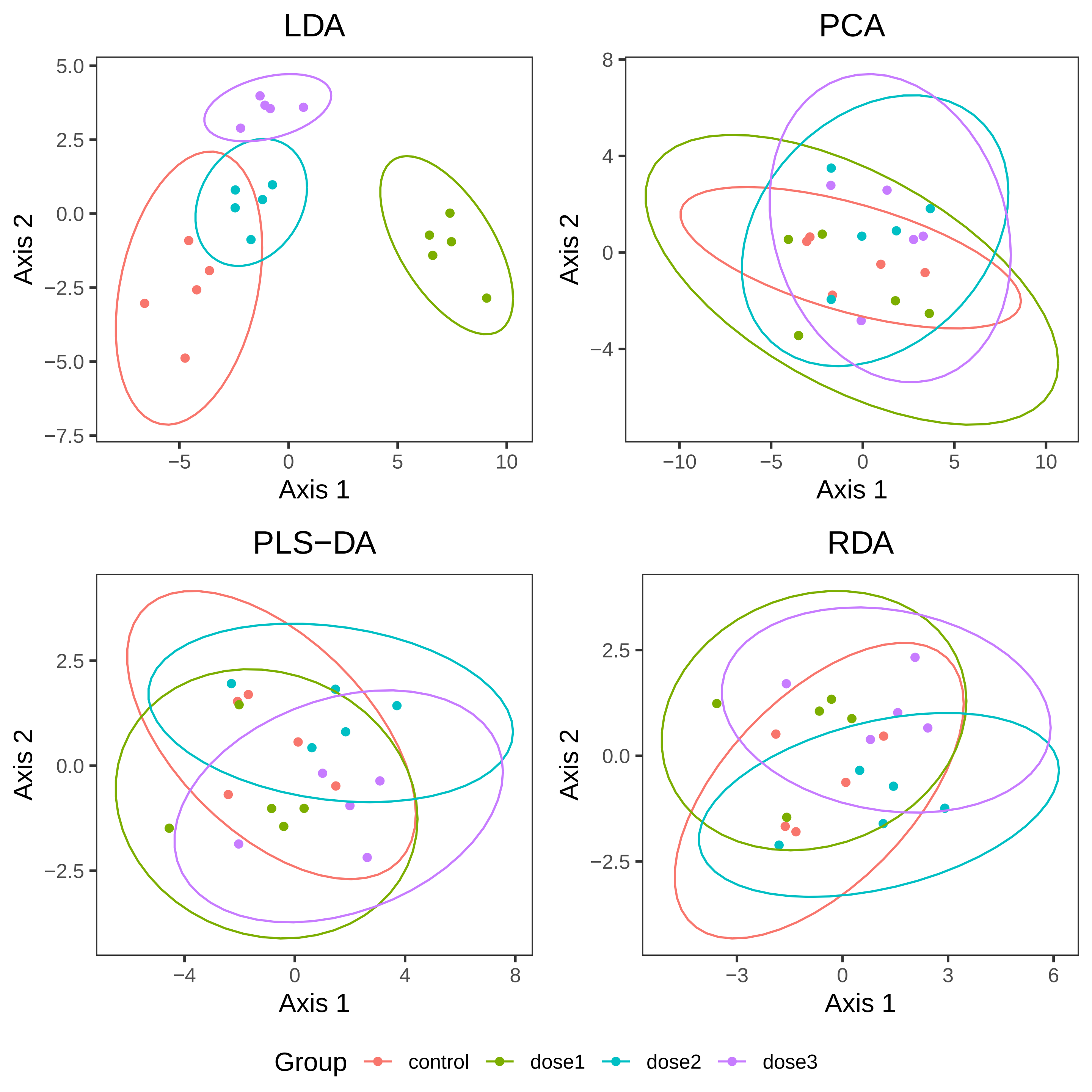
LDA
PCA
PLS−DA
RDA
Axis 1
Axis 2
Group
control
dose1
dose2
dose3

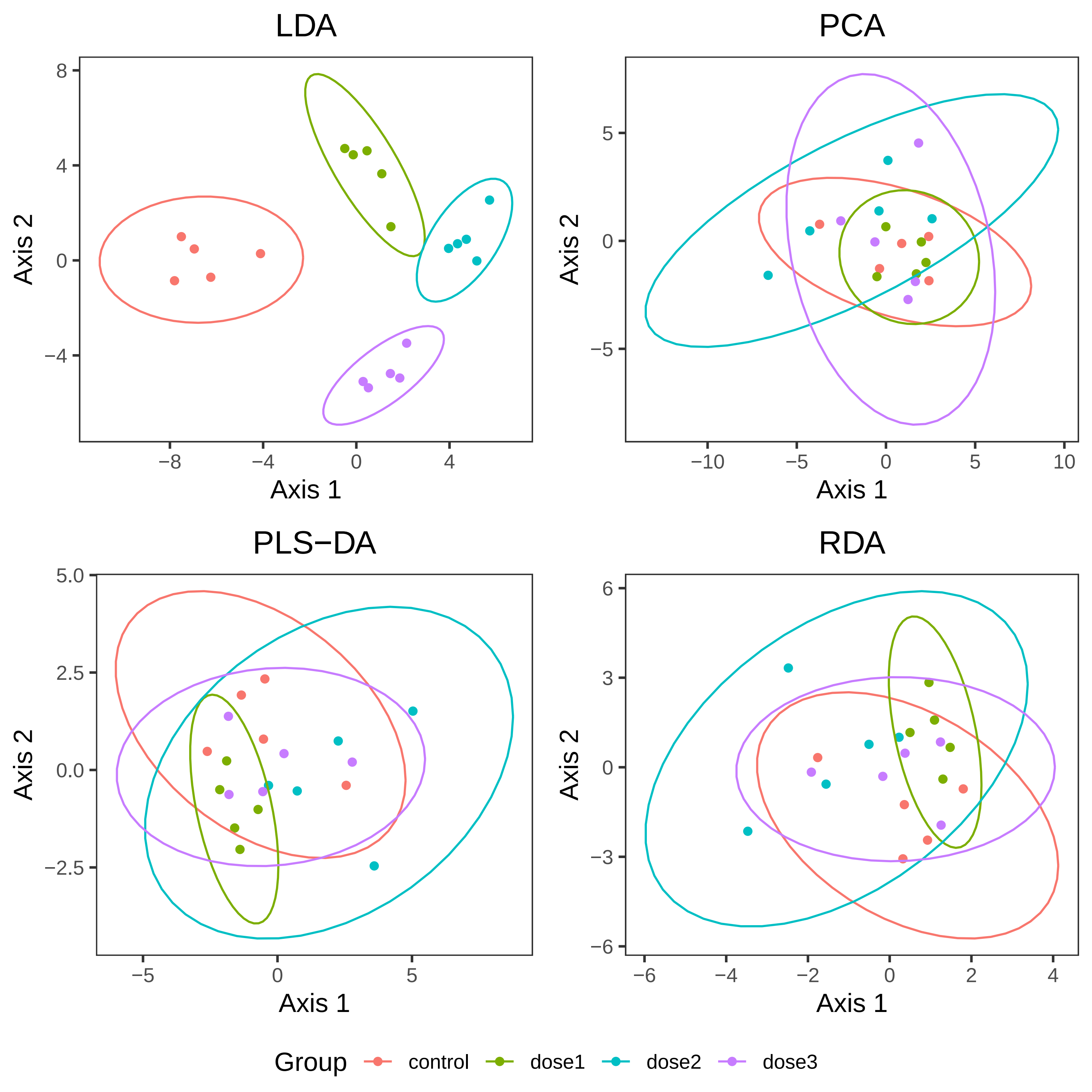
LDA
PCA
PLS−DA
RDA
Axis 1
Axis 2
8
4
0
−4
−8
5
−5
−10
10
5.0
2.5
0.0
−2.5
6
3
−3
−6
−2
2
Group
control
dose1
dose2
dose3

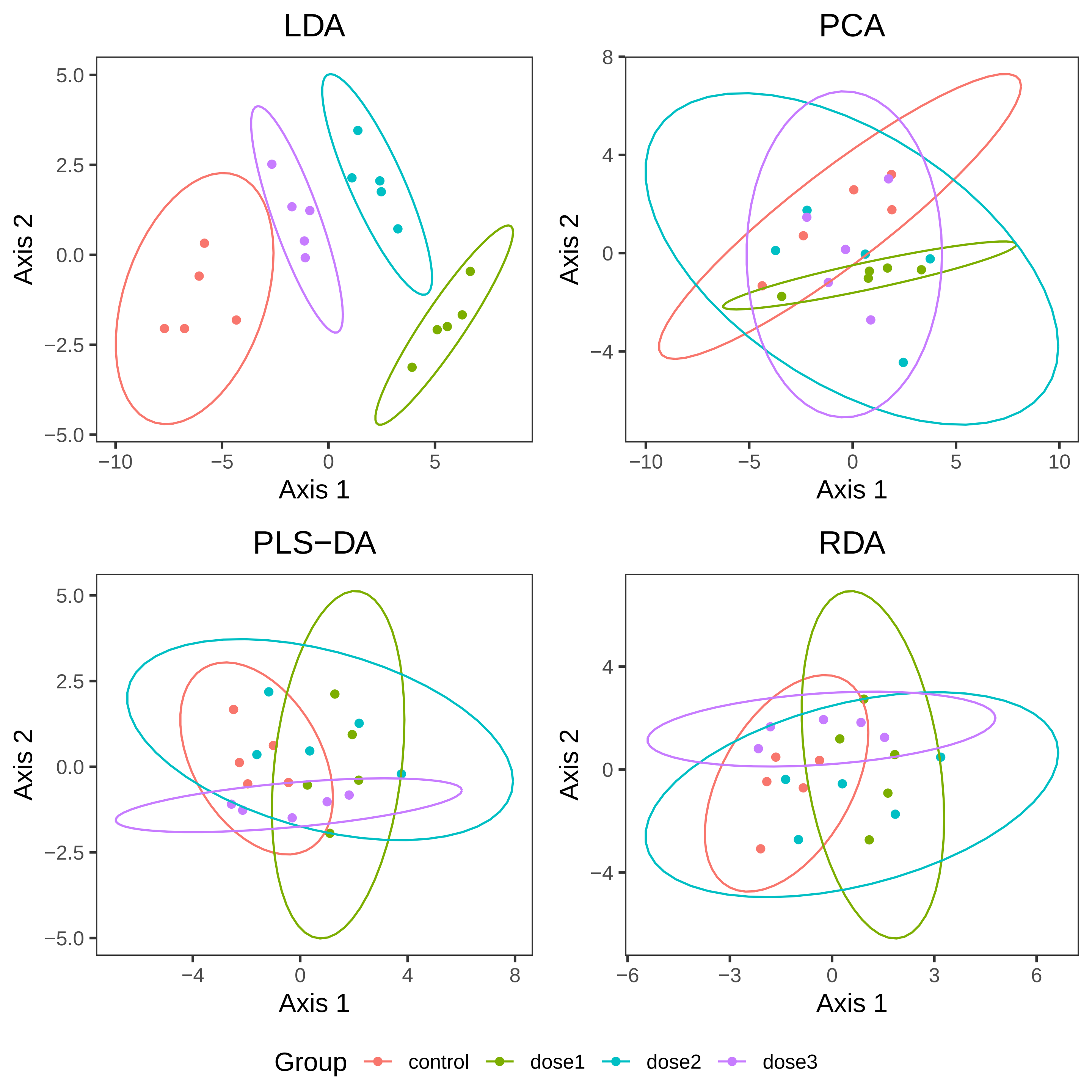
LDA
PCA
PLS−DA
RDA
Axis 1
Axis 2
Group
control
dose1
dose2
dose3

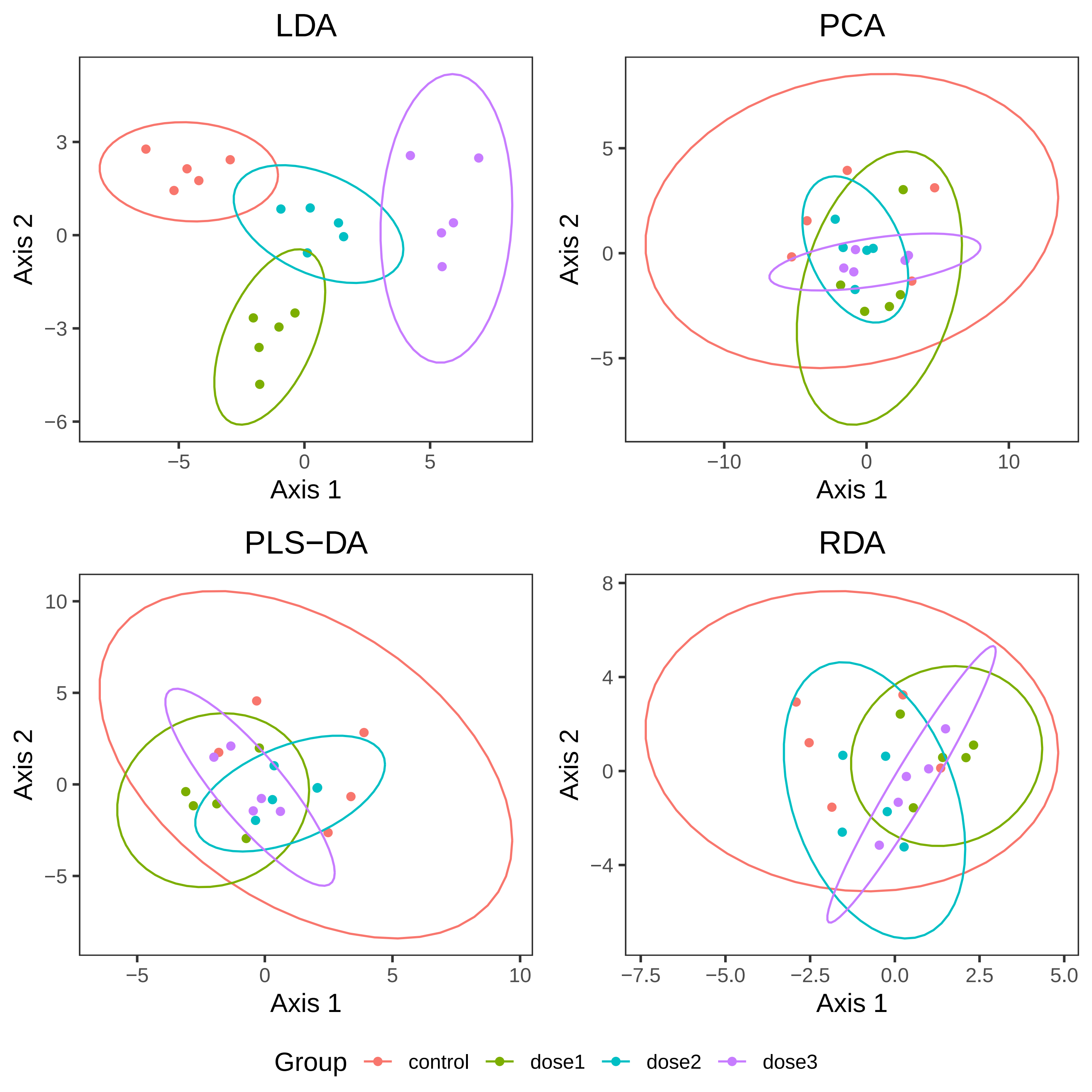

LDA
PCA
PLS−DA
RDA
Axis 1
Axis 2
Group
control
dose1
dose2
dose3

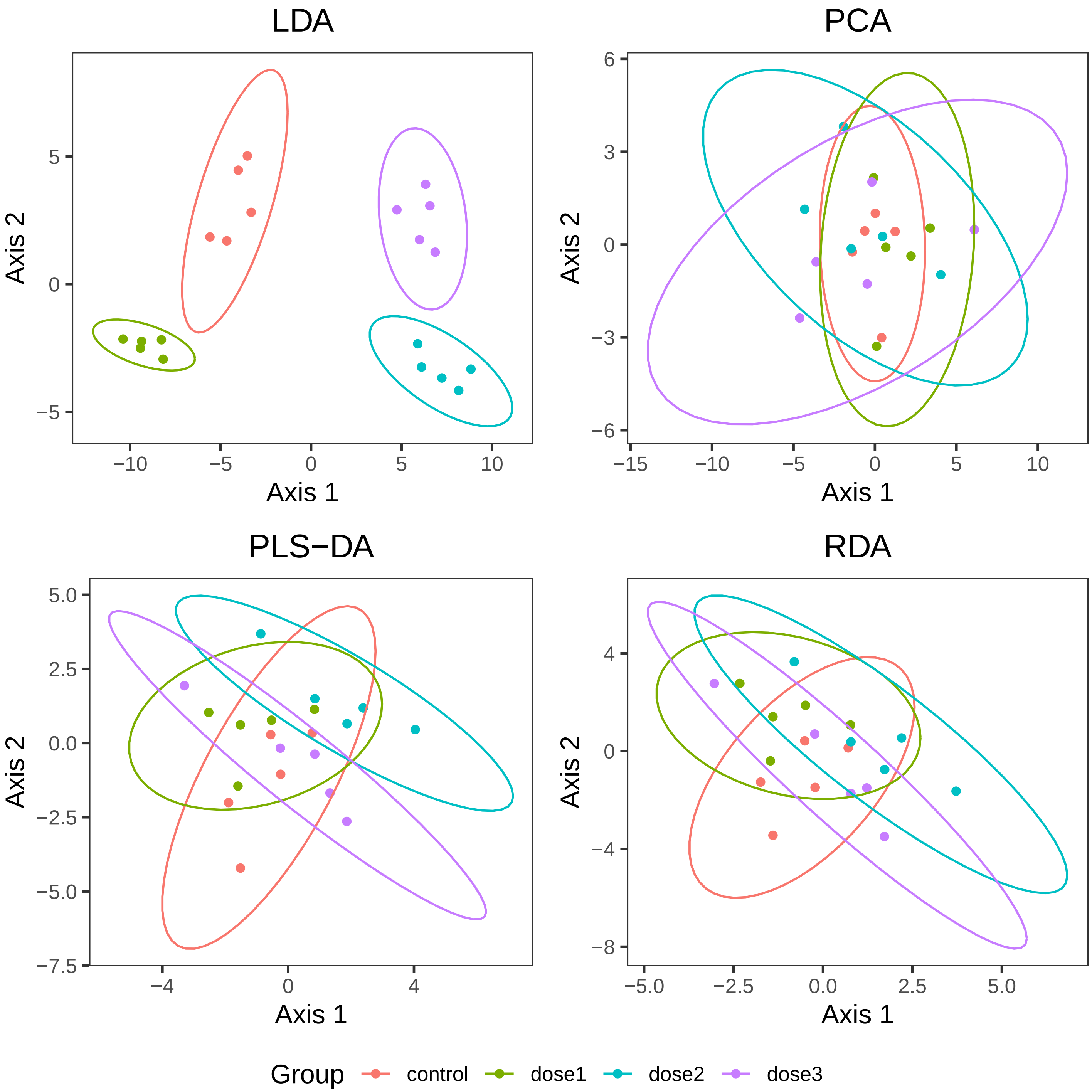
LDA
PCA
PLS−DA
RDA
Axis 1
Axis 2
Group
control
dose1
dose2
dose3

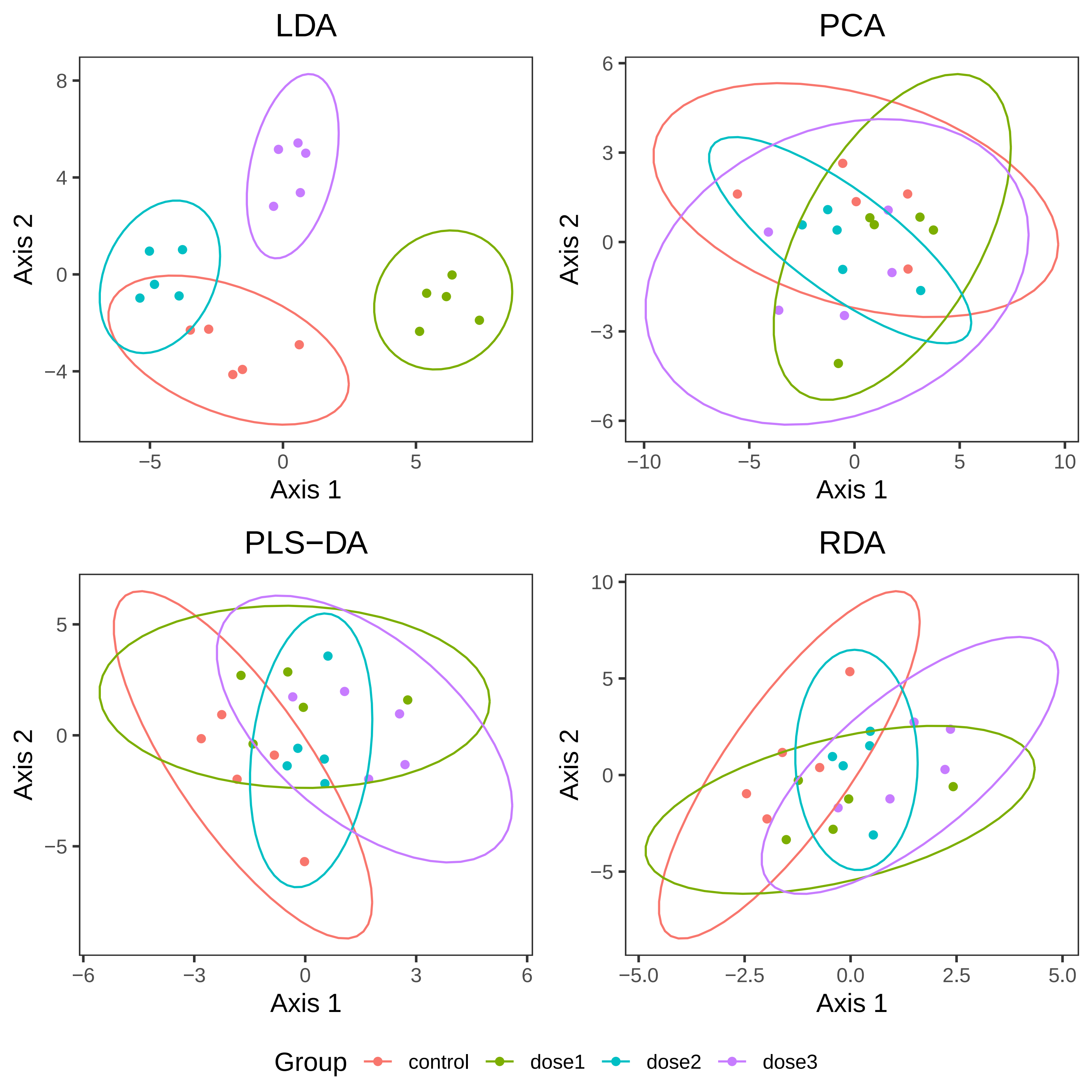

LDA
PCA
PLS−DA
RDA
Axis 1
Axis 2
Group
control
dose1
dose2
dose3

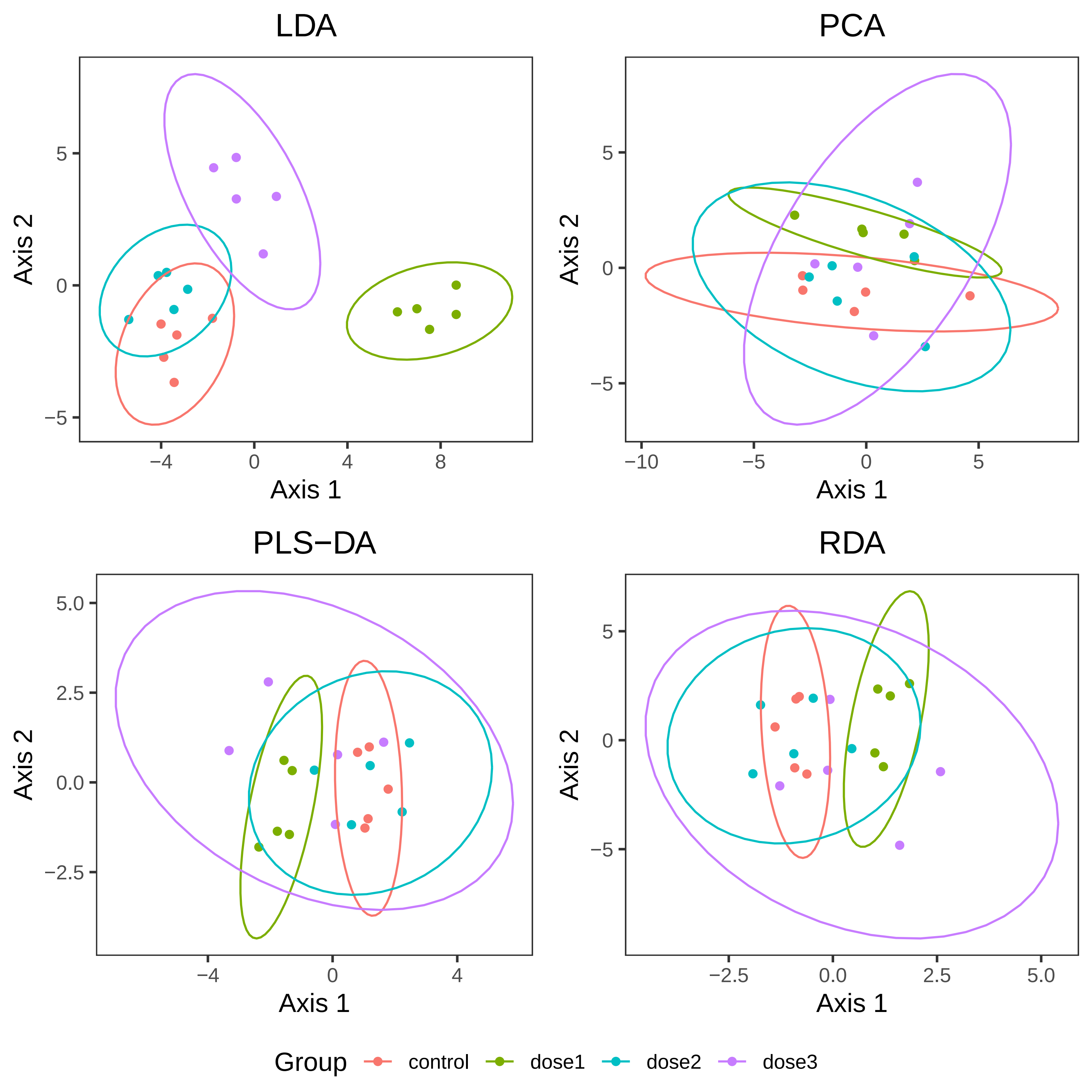

LDA
PCA
PLS−DA
RDA
Axis 1
Axis 2
−4
0
4
8
5
0
−5
−10
−5
0
5
5.0
2.5
0.0
−2.5
−4
0
4
−2.5
0.0
2.5
5.0
Group
control
dose1
dose2
dose3

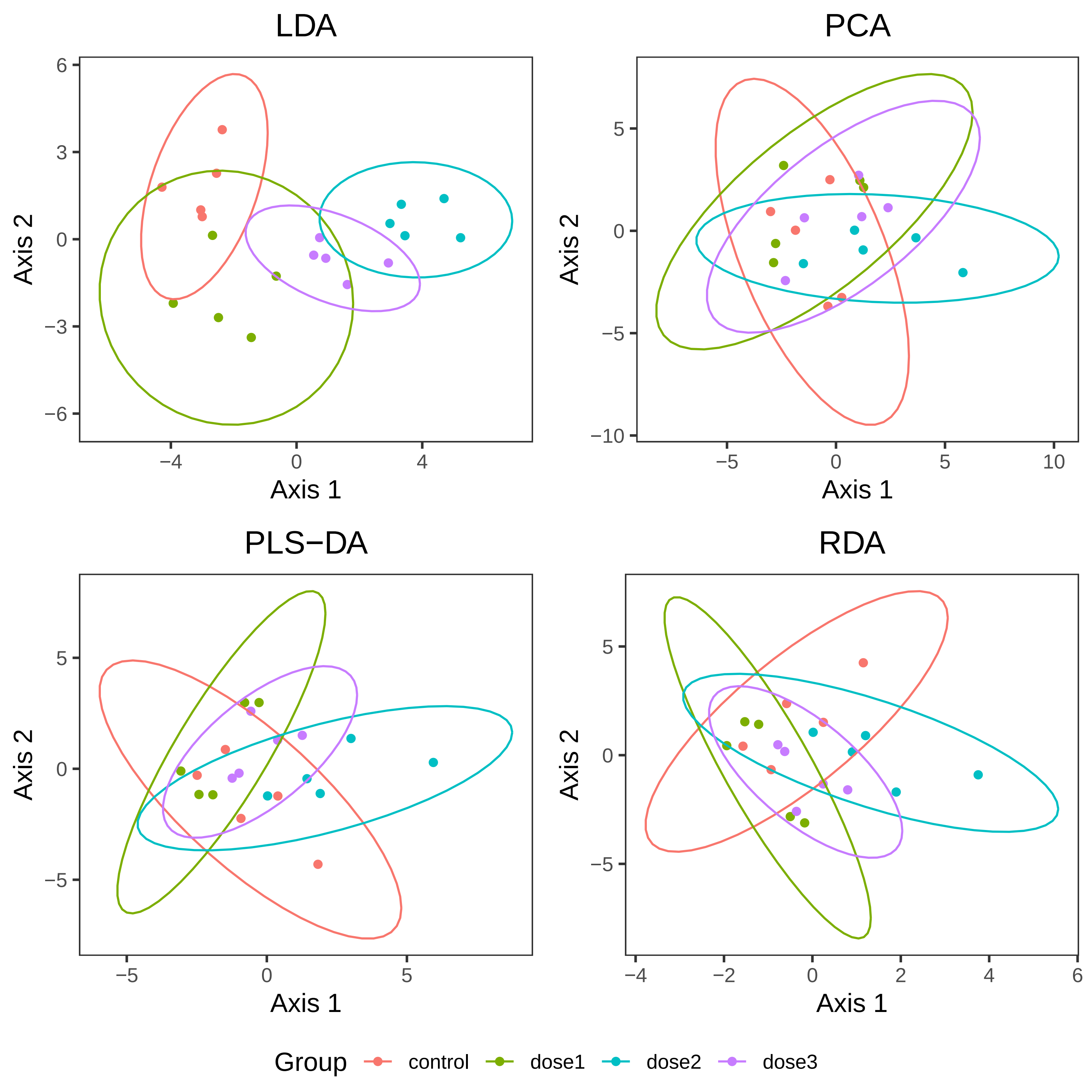

LDA
PCA
PLS−DA
RDA
Axis 1
Axis 2
Group
control
dose1
dose2
dose3

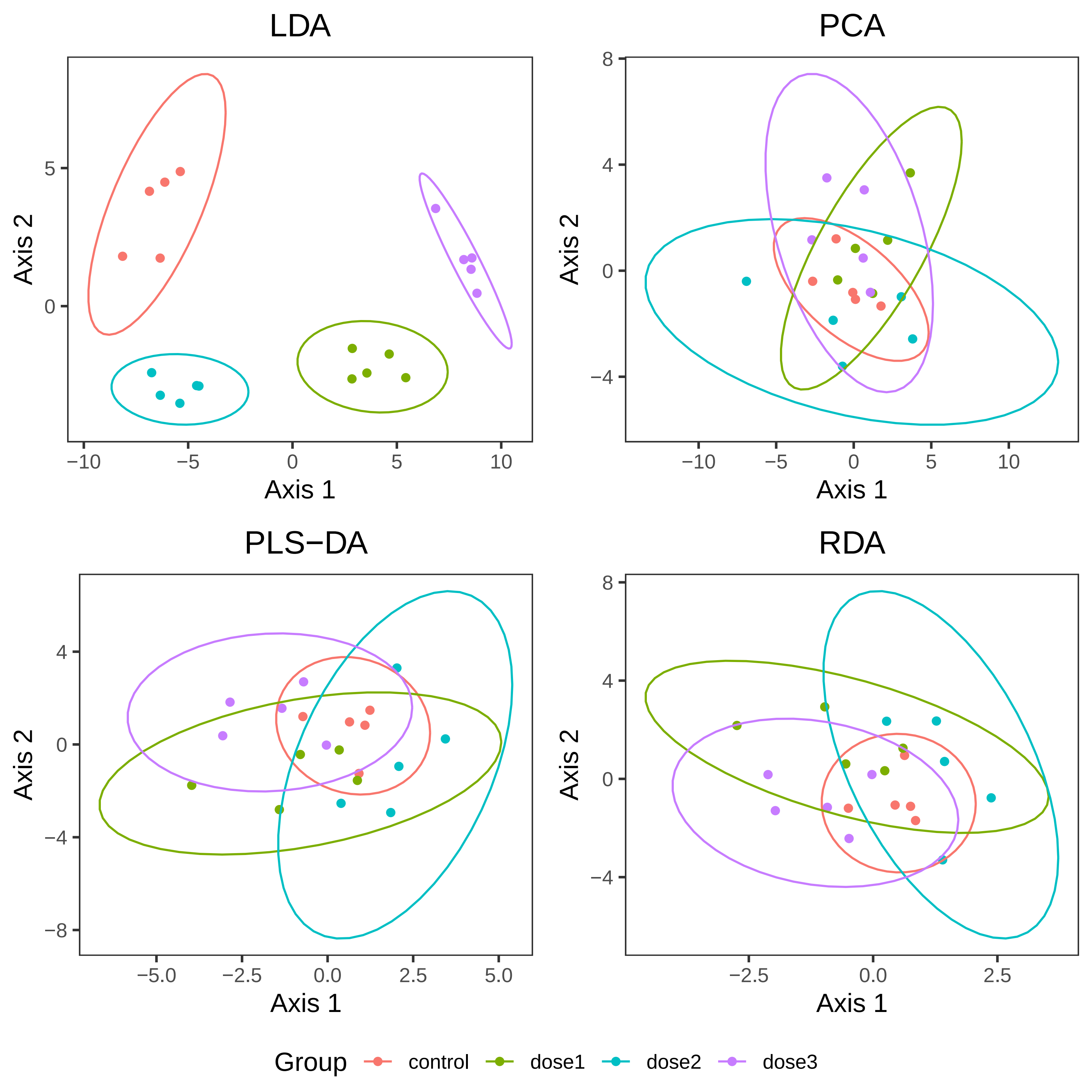

LDA
PCA
PLS−DA
RDA
Axis 1
Axis 2
Group
control
dose1
dose2
dose3

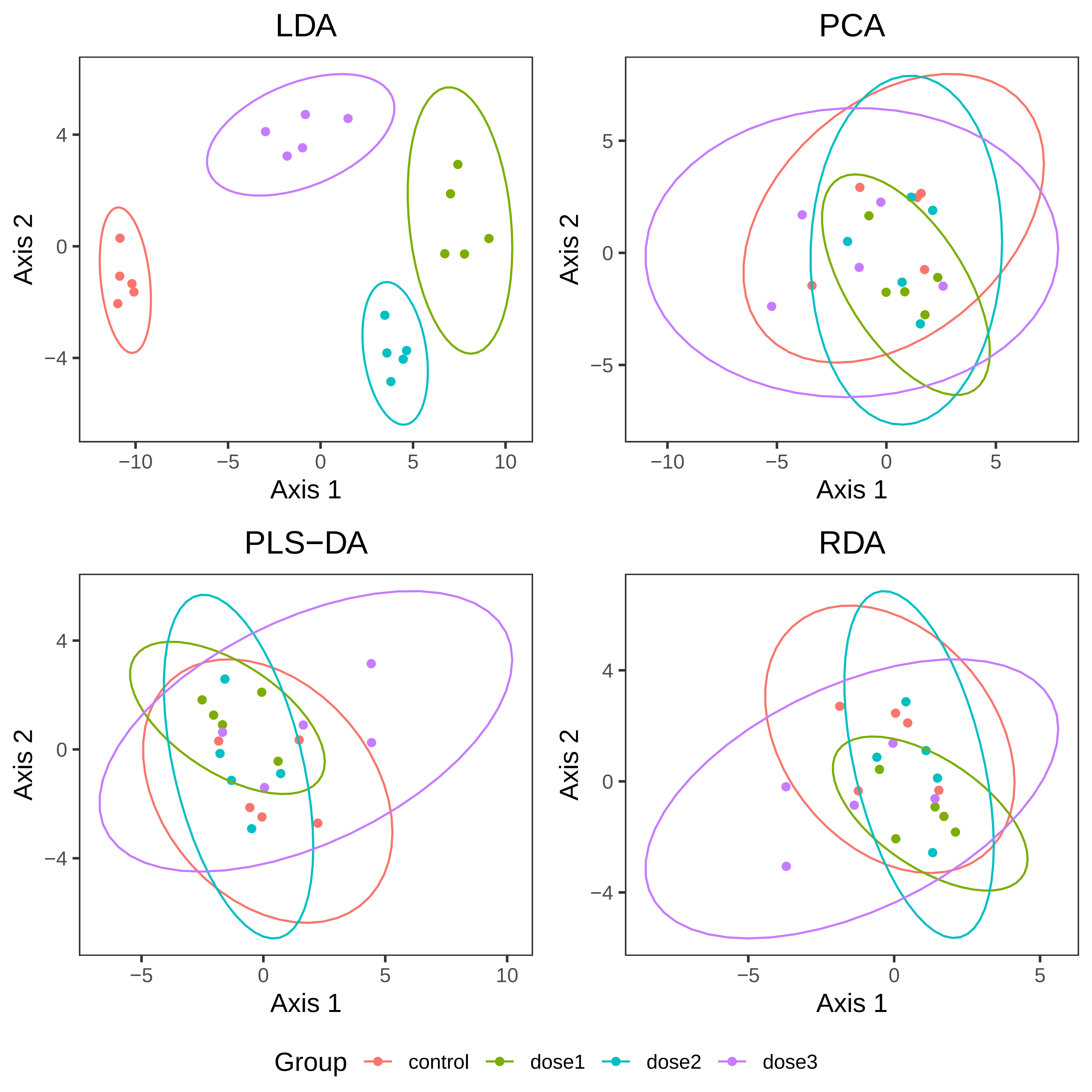
LDA
PCA
PLS−DA
RDA
Axis 1
Axis 2
Group
control
dose1
dose2
dose3

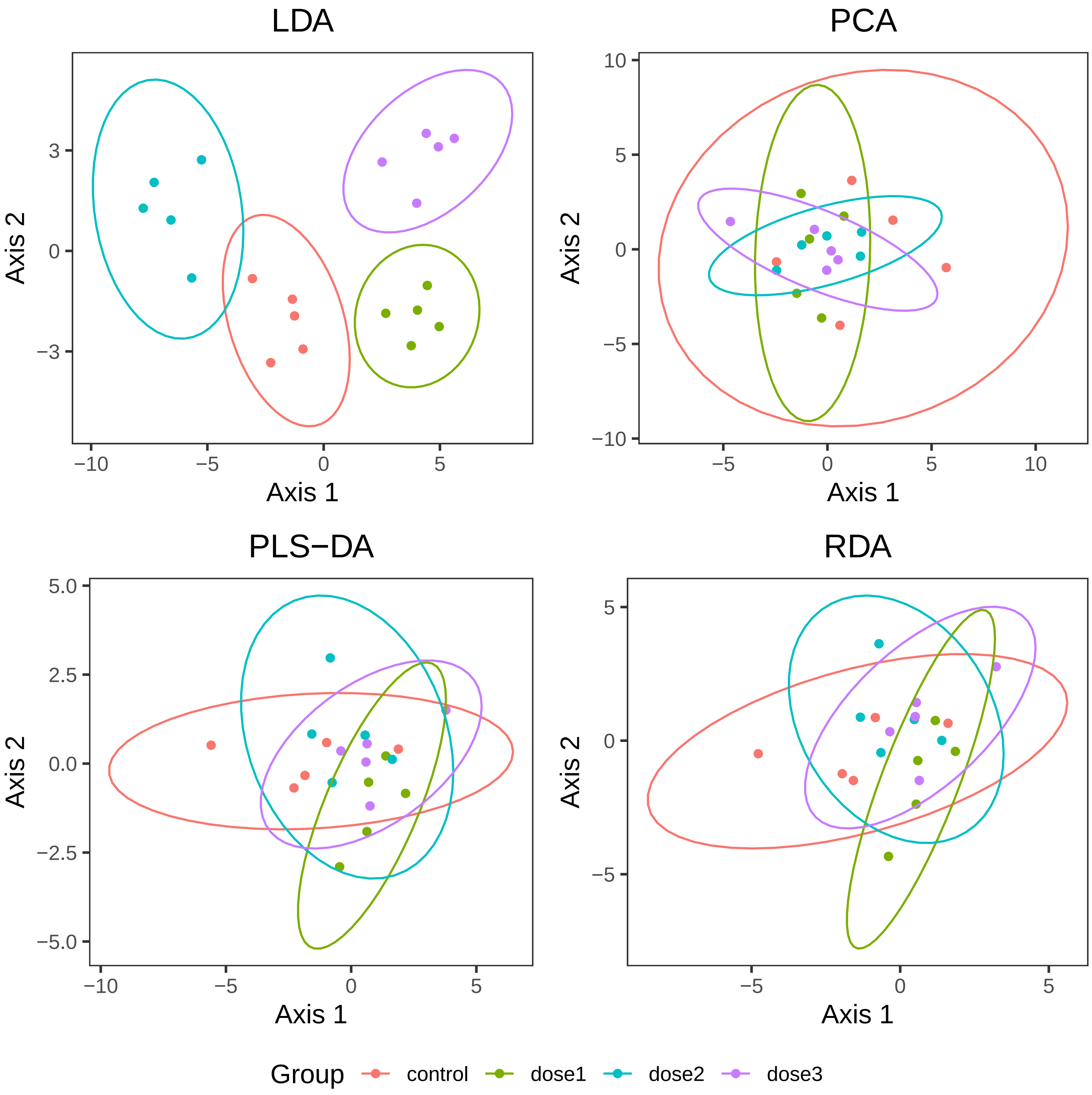

LDA
PCA
PLS−DA
RDA
Axis 1
Axis 2
Group
control
dose1
dose2
dose3

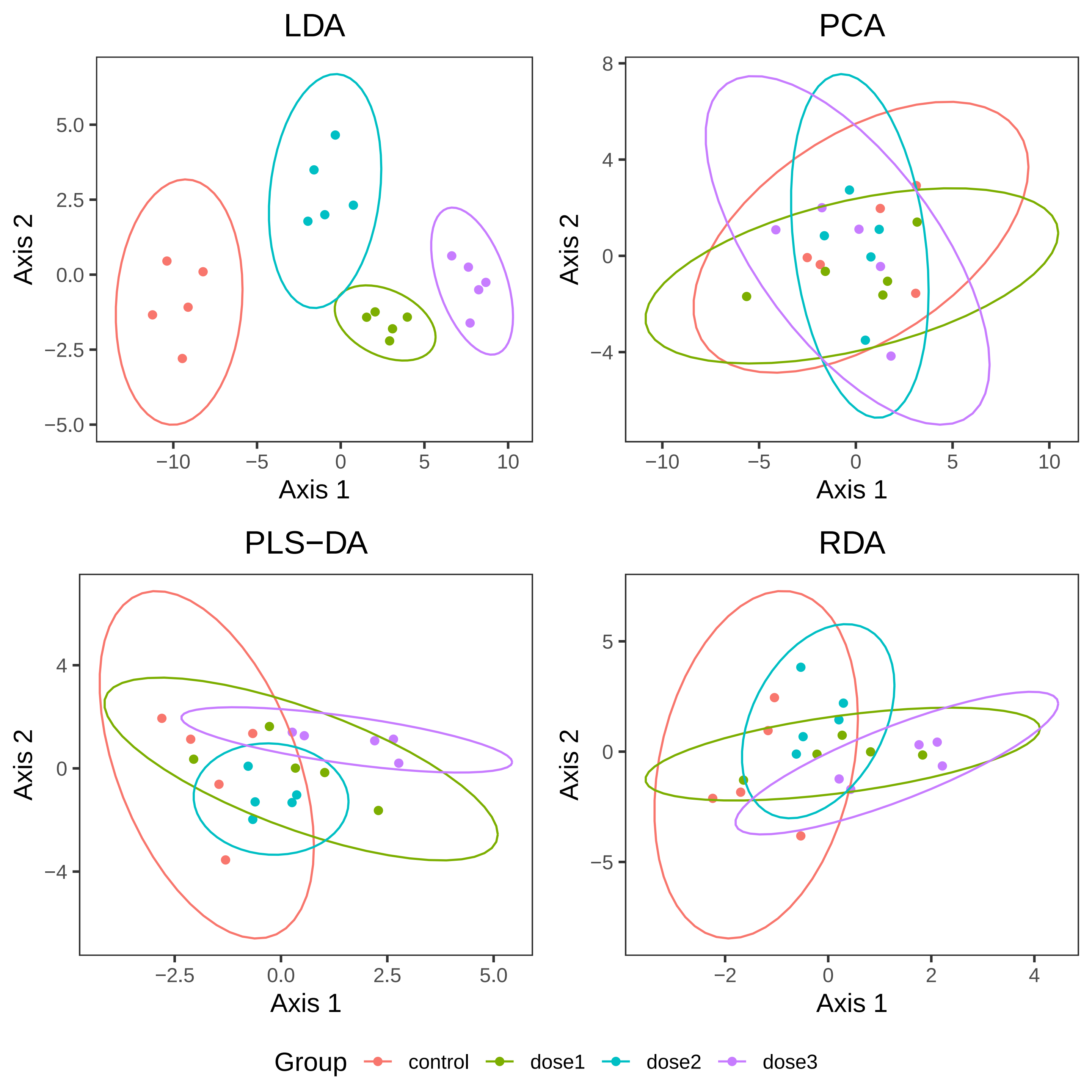
LDA
PCA
PLS−DA
RDA
Axis 1
Axis 2
5.0
2.5
0.0
−2.5
−5.0
−10
−5
0
5
10
8
4
0
−4
−2
2
Group
control
dose1
dose2
dose3

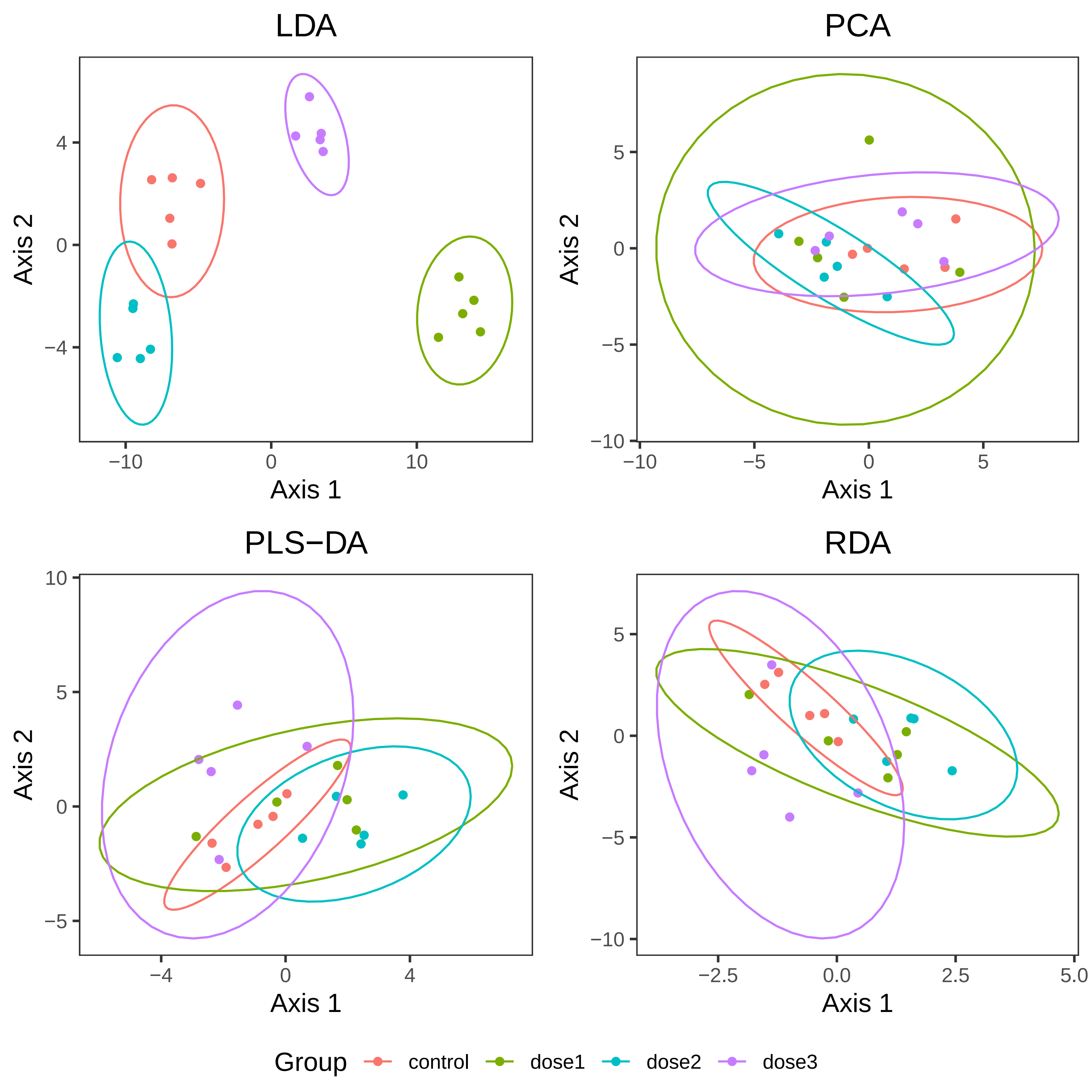

LDA
PCA
PLS−DA
RDA
Axis 1
Axis 2
Group
control
dose1
dose2
dose3

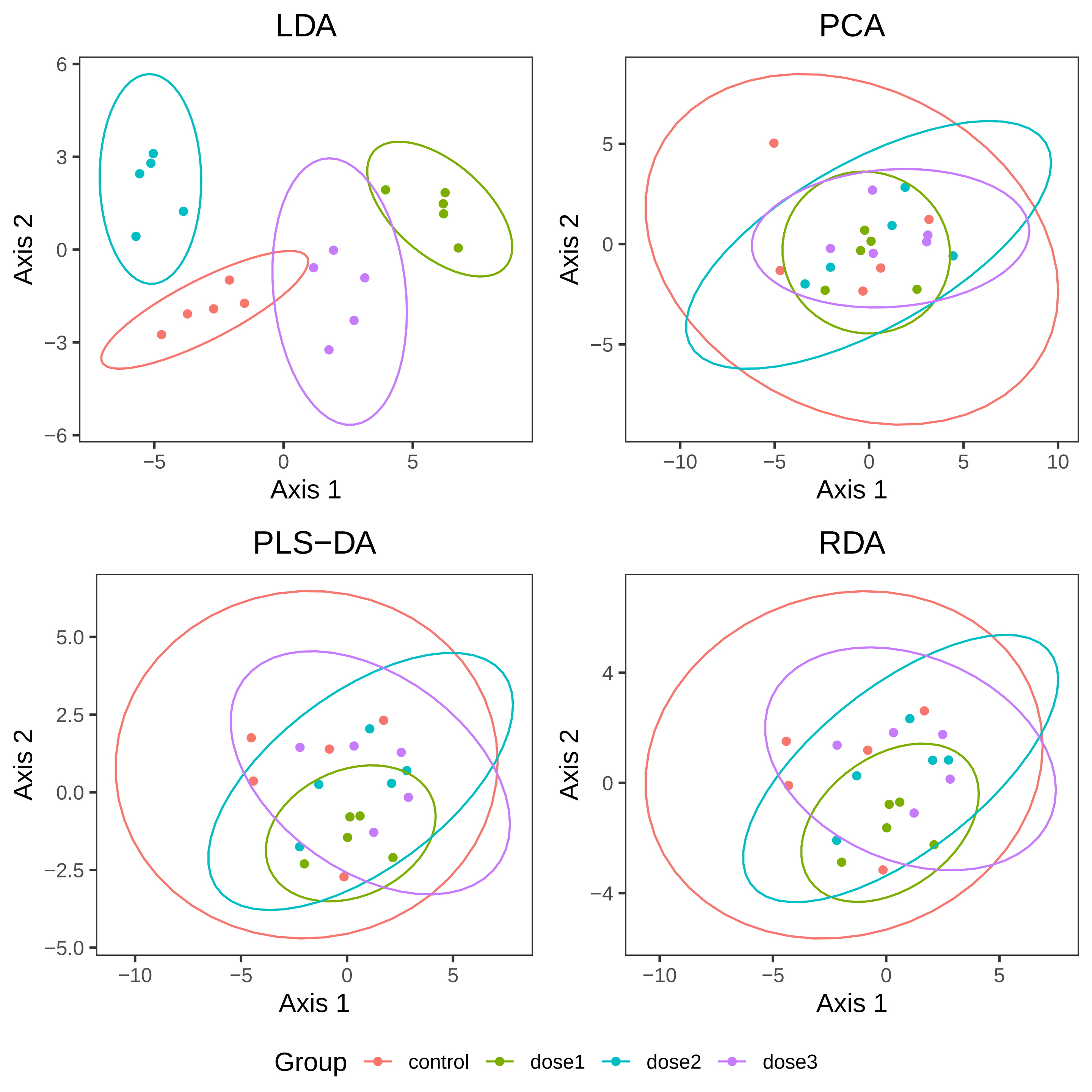

LDA
PCA
PLS−DA
RDA
Axis 1
Axis 2
Group
control
dose1
dose2
dose3

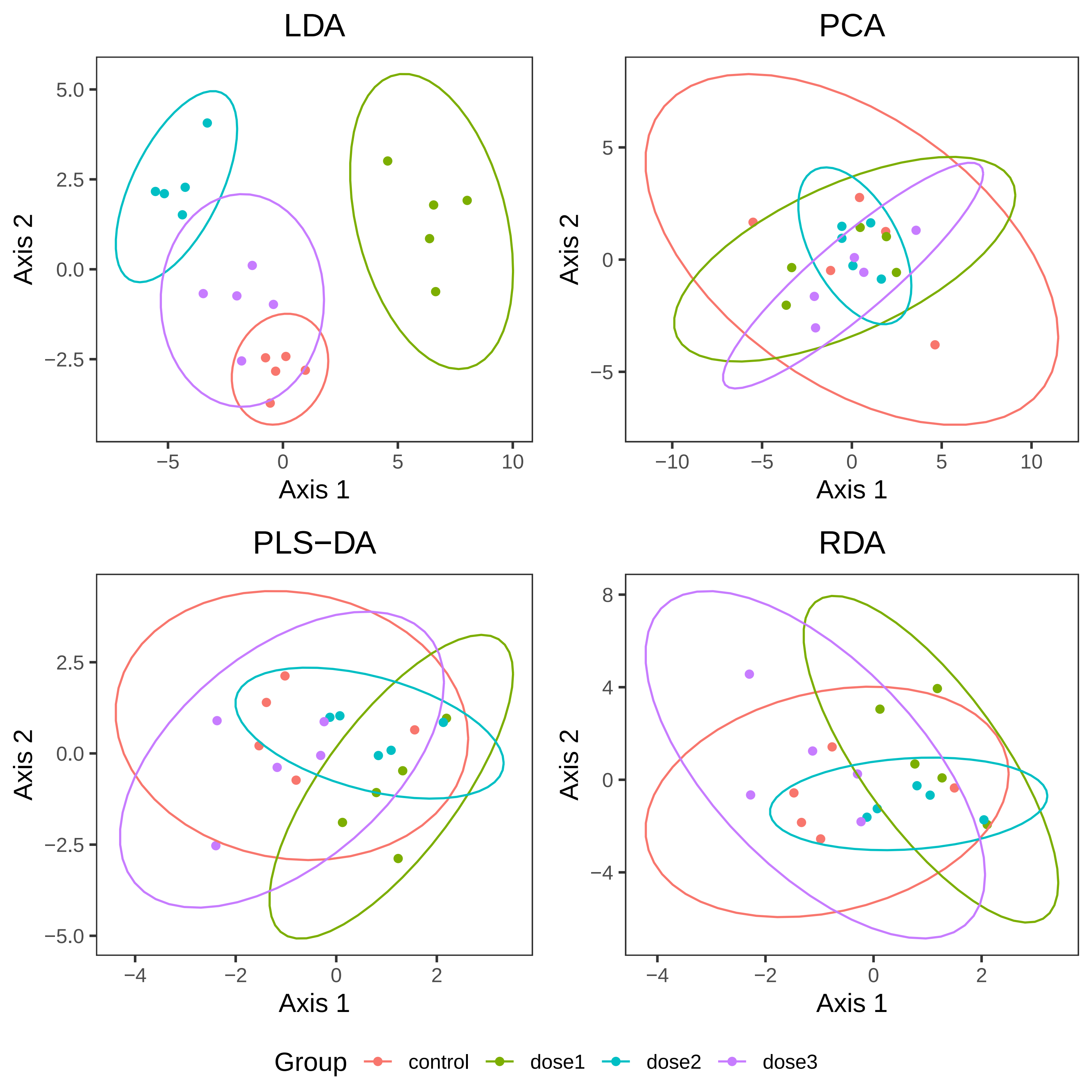

LDA
PCA
PLS−DA
RDA
Axis 1
Axis 2
5.0
2.5
0.0
−2.5
−5
0
5
10
−10
−4
−2
2
4
8
−5.0
Group
control
dose1
dose2
dose3

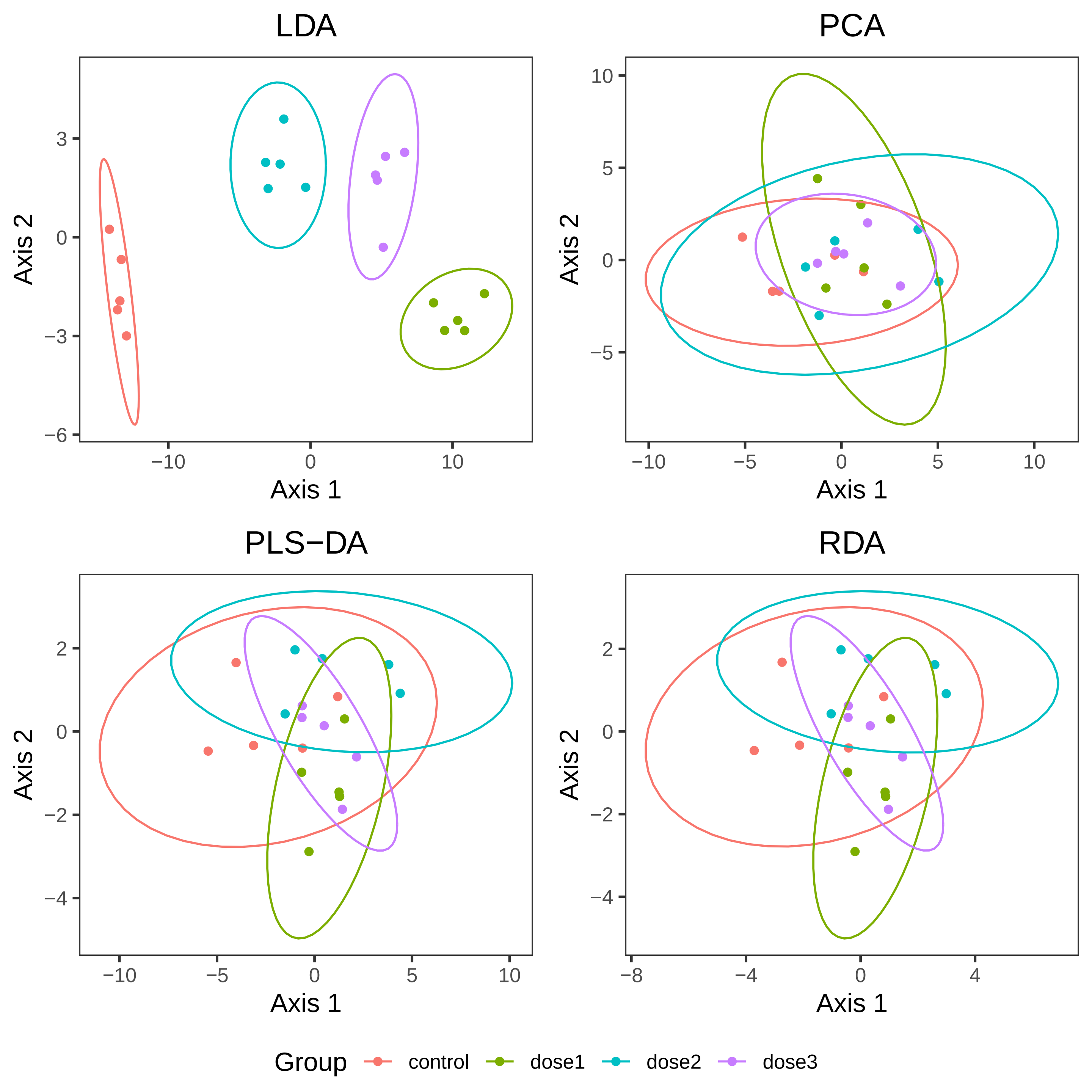
LDA
PCA
PLS−DA
RDA
Axis 1
Axis 2
−10
−5
0
5
10
3
−3
−6
−8
−4
4
2
−2
Group
control
dose1
dose2
dose3

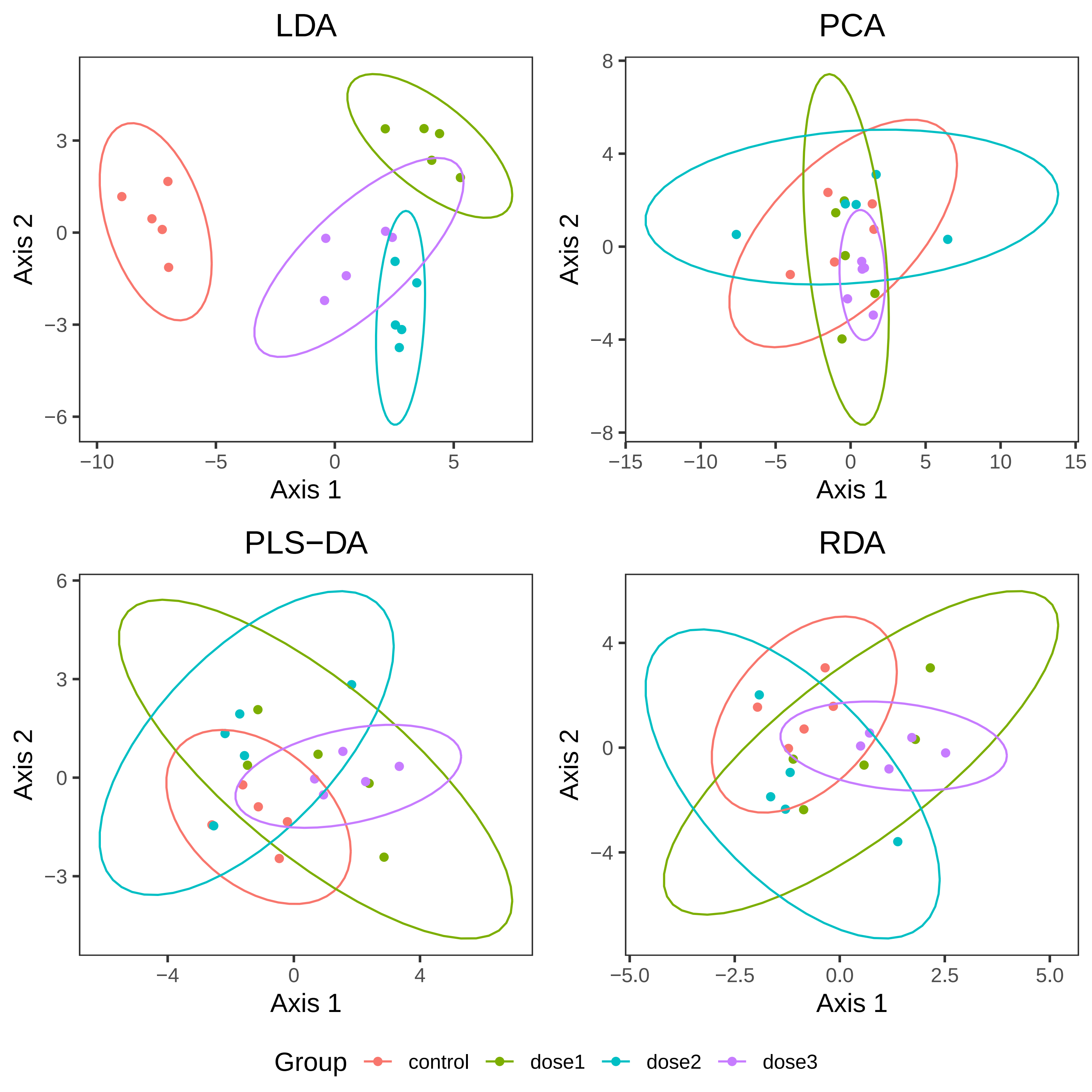

LDA
PCA
PLS−DA
RDA
Axis 1
Axis 2
Group
control
dose1
dose2
dose3

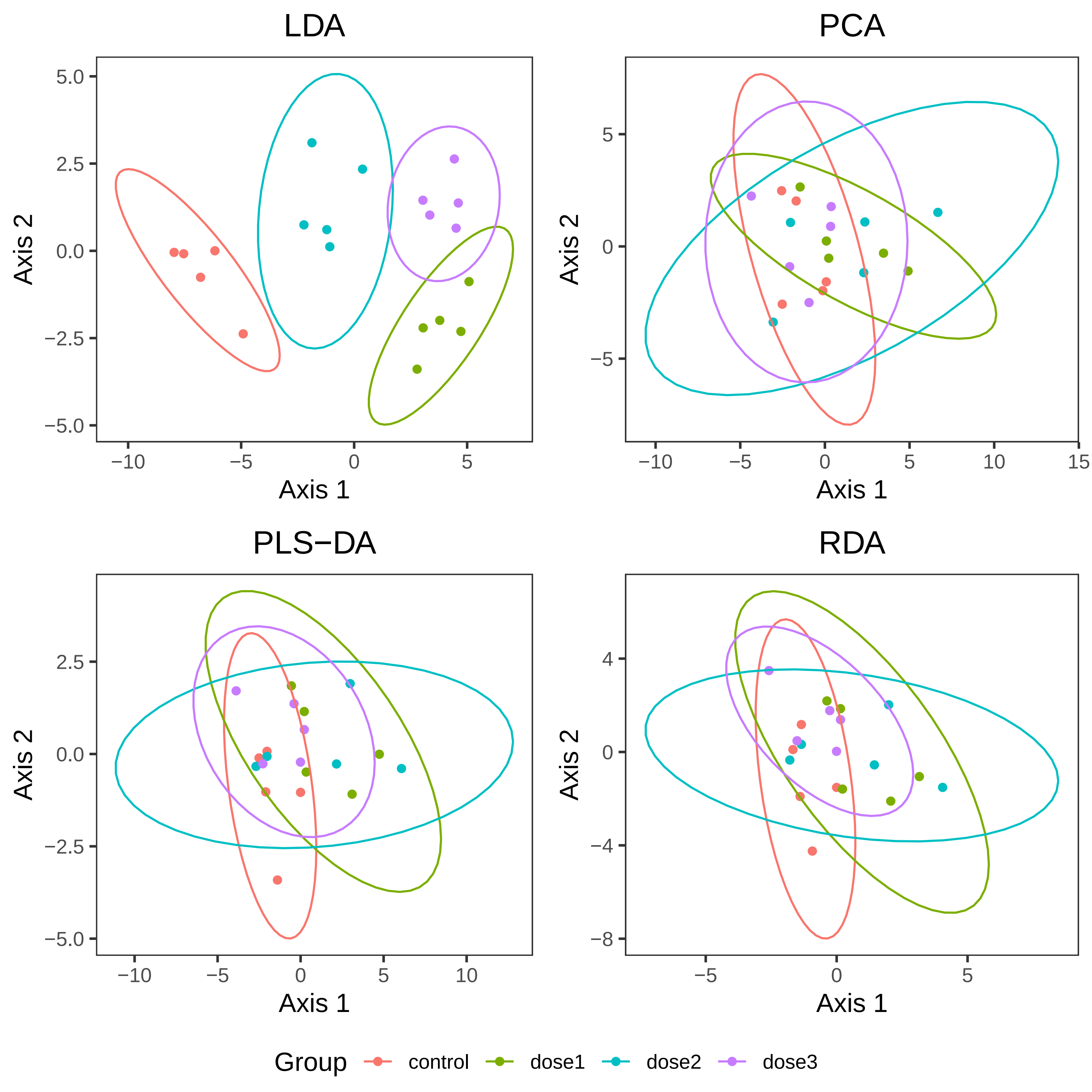

LDA
PCA
PLS−DA
RDA
Axis 1
Axis 2
5.0
2.5
0.0
−2.5
−5.0
−10
−5
0
5
10
15
4
−4
−8
Group
control
dose1
dose2
dose3

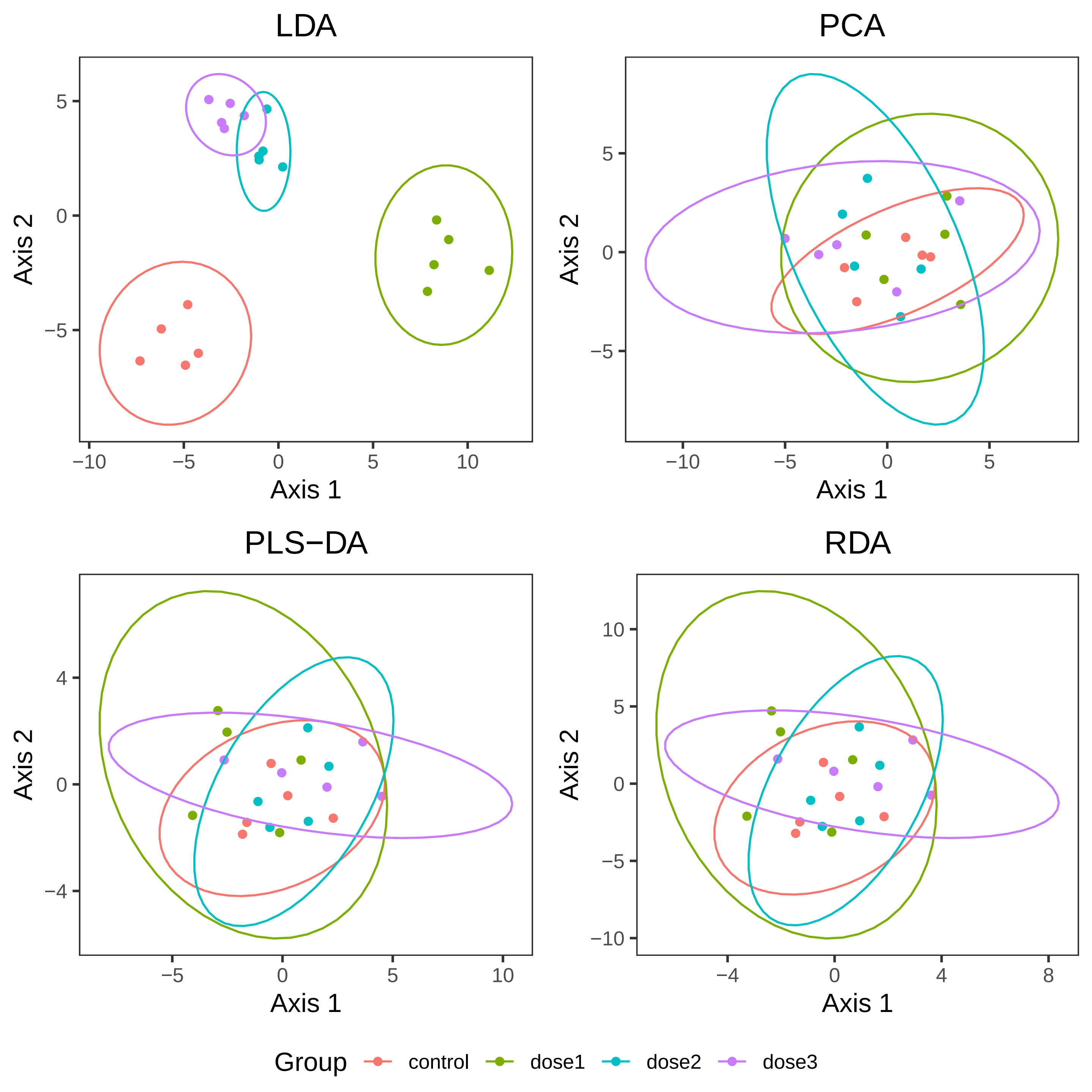

LDA
PCA
PLS−DA
RDA
Axis 1
Axis 2
Group
control
dose1
dose2
dose3

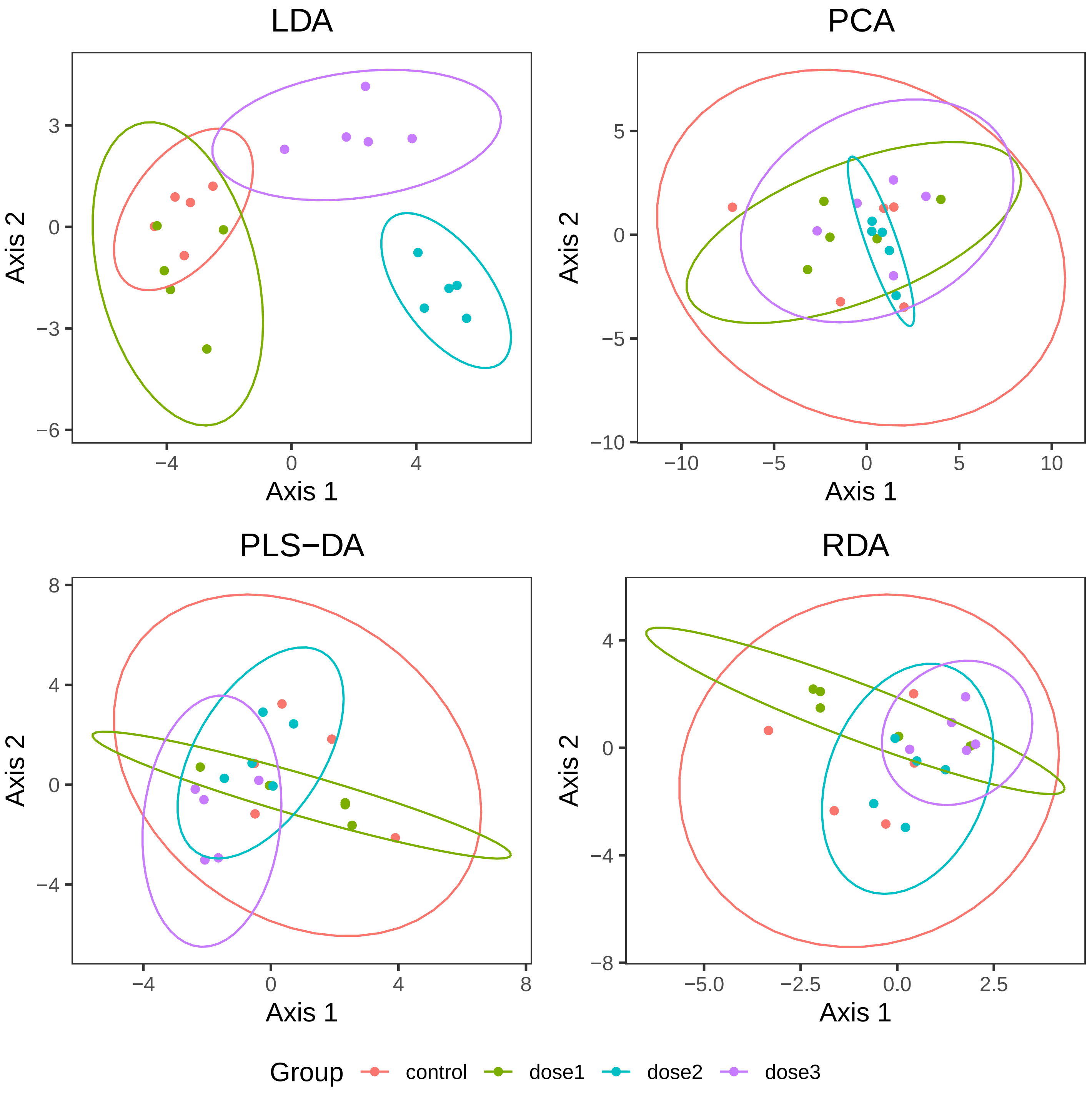
LDA
PCA
PLS−DA
RDA
Axis 1
Axis 2
Group
control
dose1
dose2
dose3

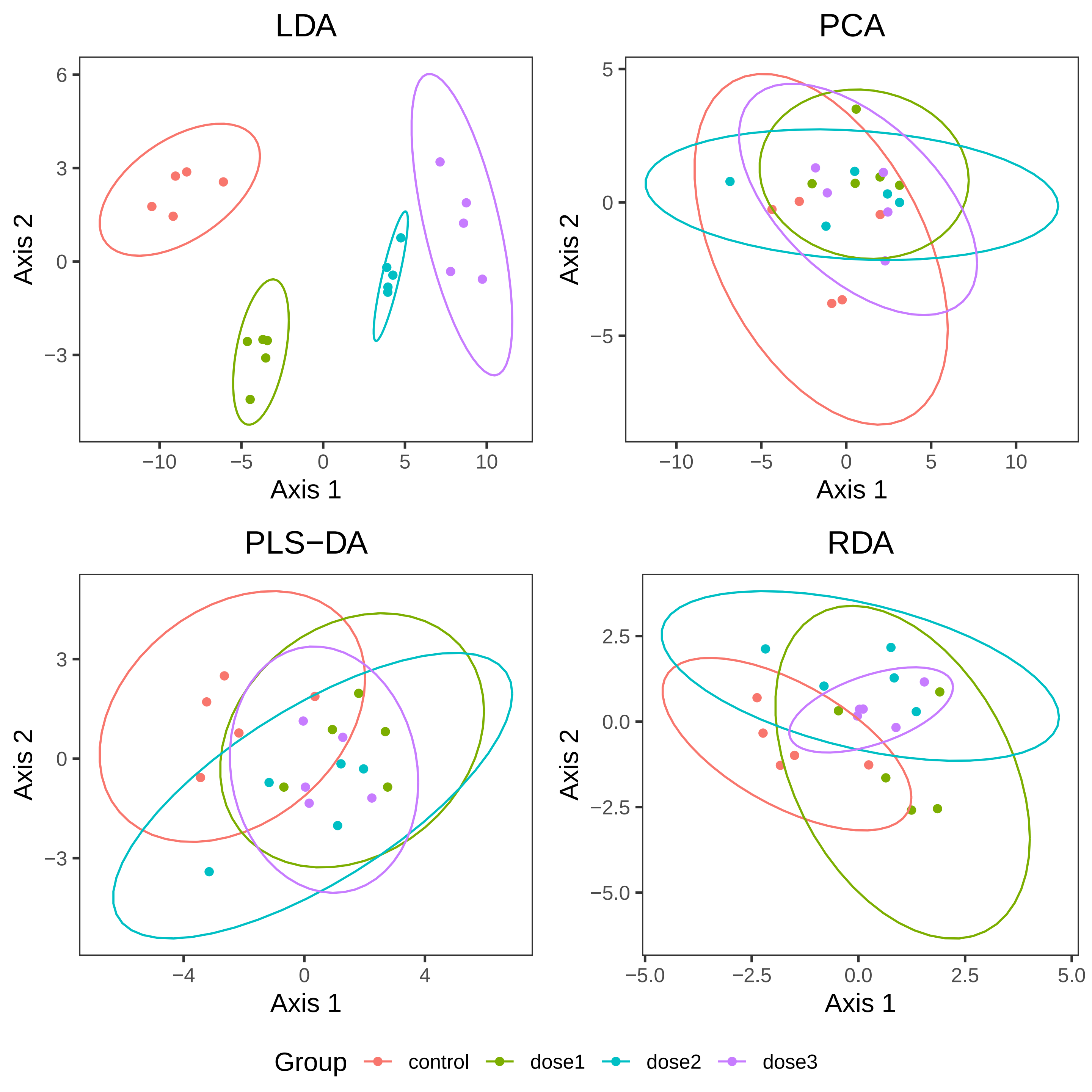

LDA
PCA
PLS−DA
RDA
Axis 1
Axis 2
Group
control
dose1
dose2
dose3

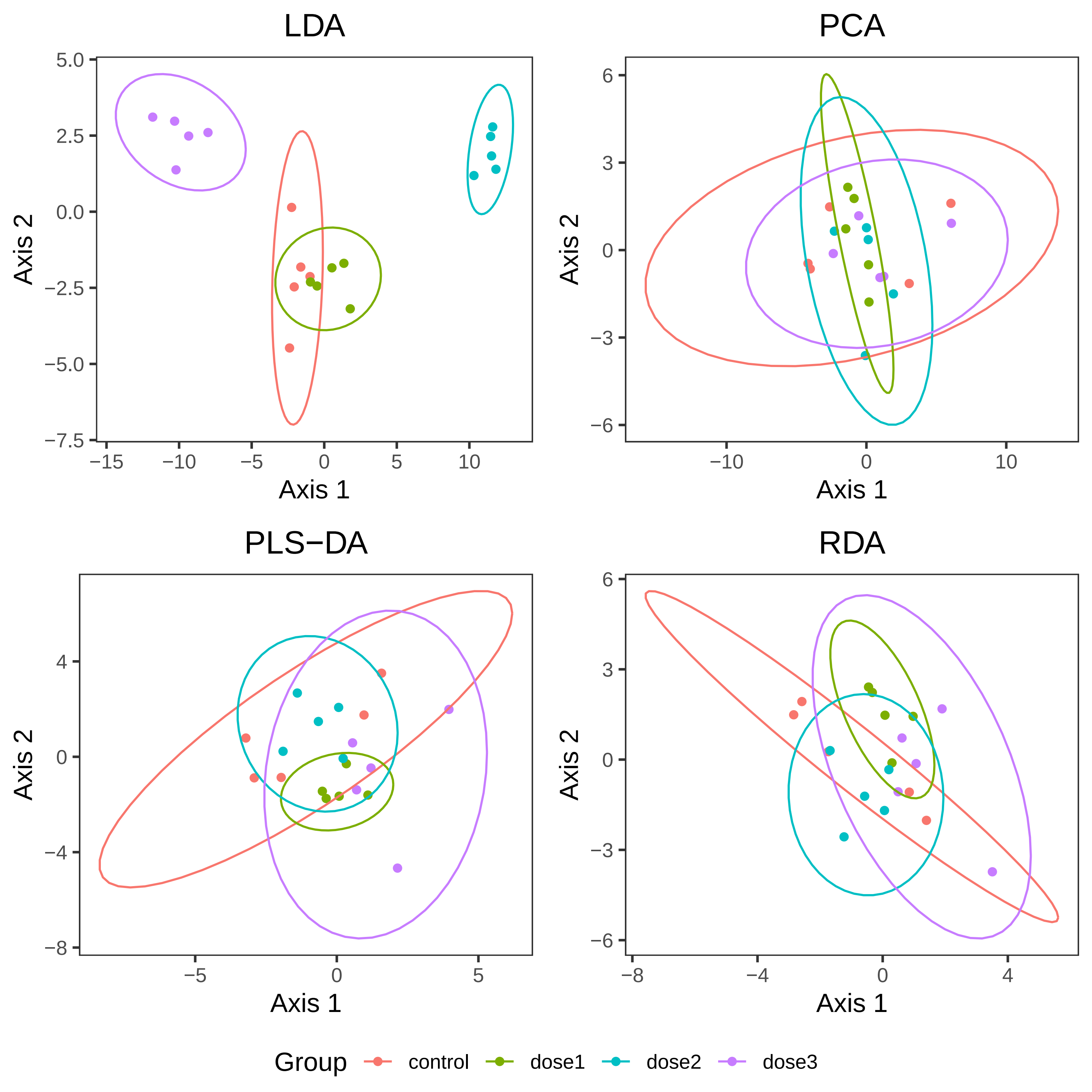

LDA
PCA
PLS−DA
RDA
Axis 1
Axis 2
5.0
2.5
0.0
−2.5
−5.0
−7.5
−15
−10
−5
0
5
10
6
3
0
−3
−6
4
0
−4
−8
−8
−4
Group
control
dose1
dose2
dose3

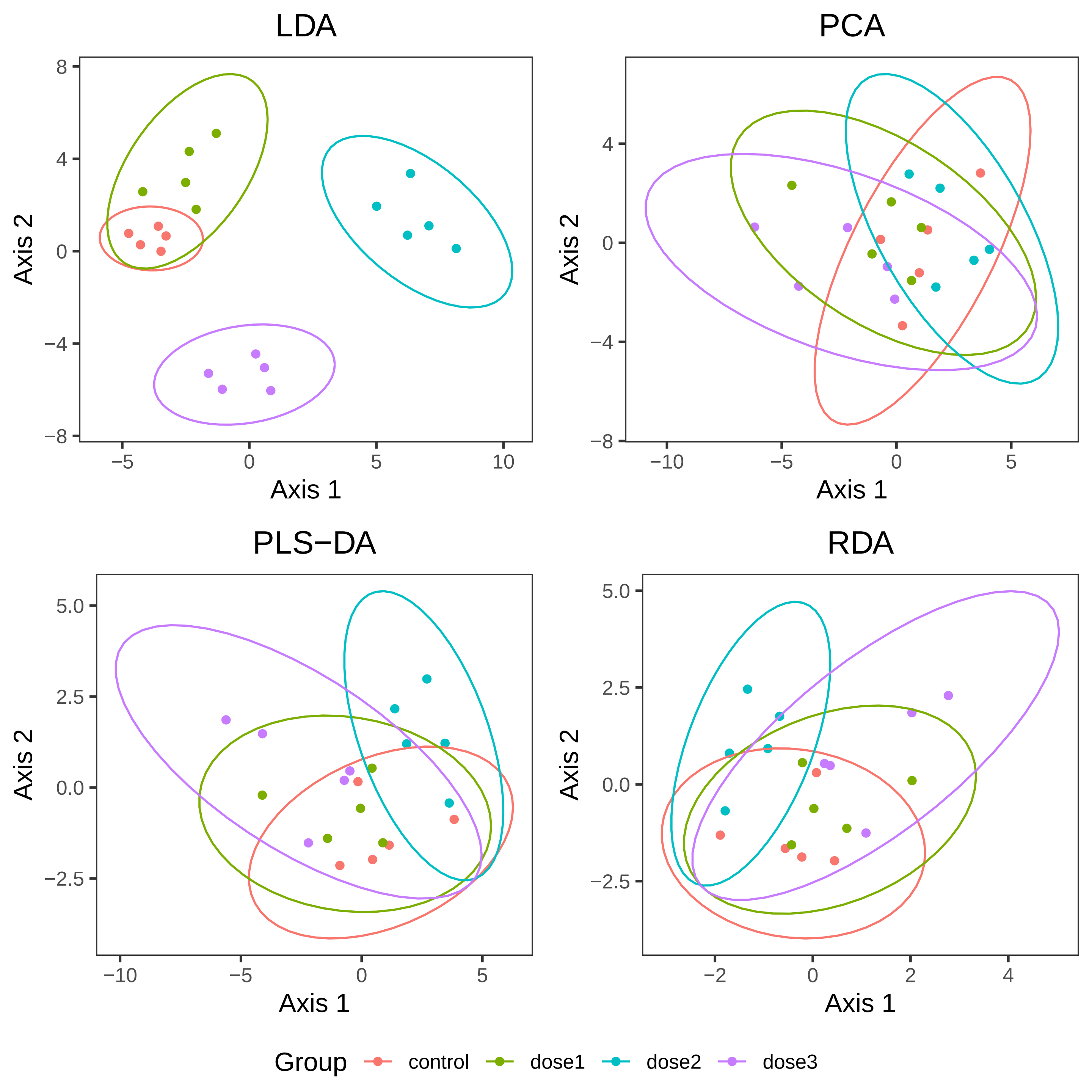

LDA
PCA
PLS−DA
RDA
Axis 1
Axis 2
8
4
0
−4
−8
−5
0
5
10
−10
5.0
2.5
0.0
−2.5
−2
2
Group
control
dose1
dose2
dose3

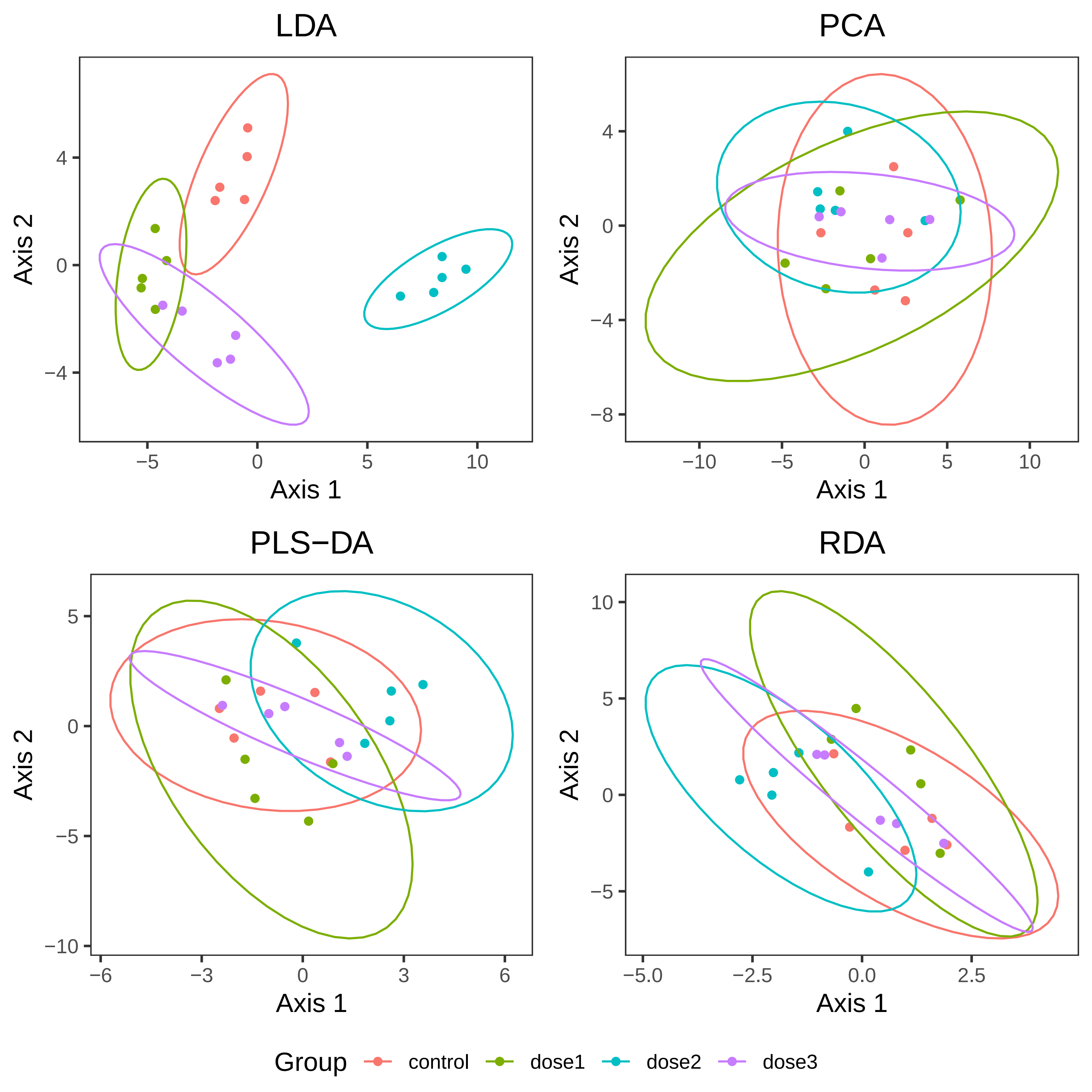

LDA
PCA
PLS−DA
RDA
Axis 1
Axis 2
Group
control
dose1
dose2
dose3

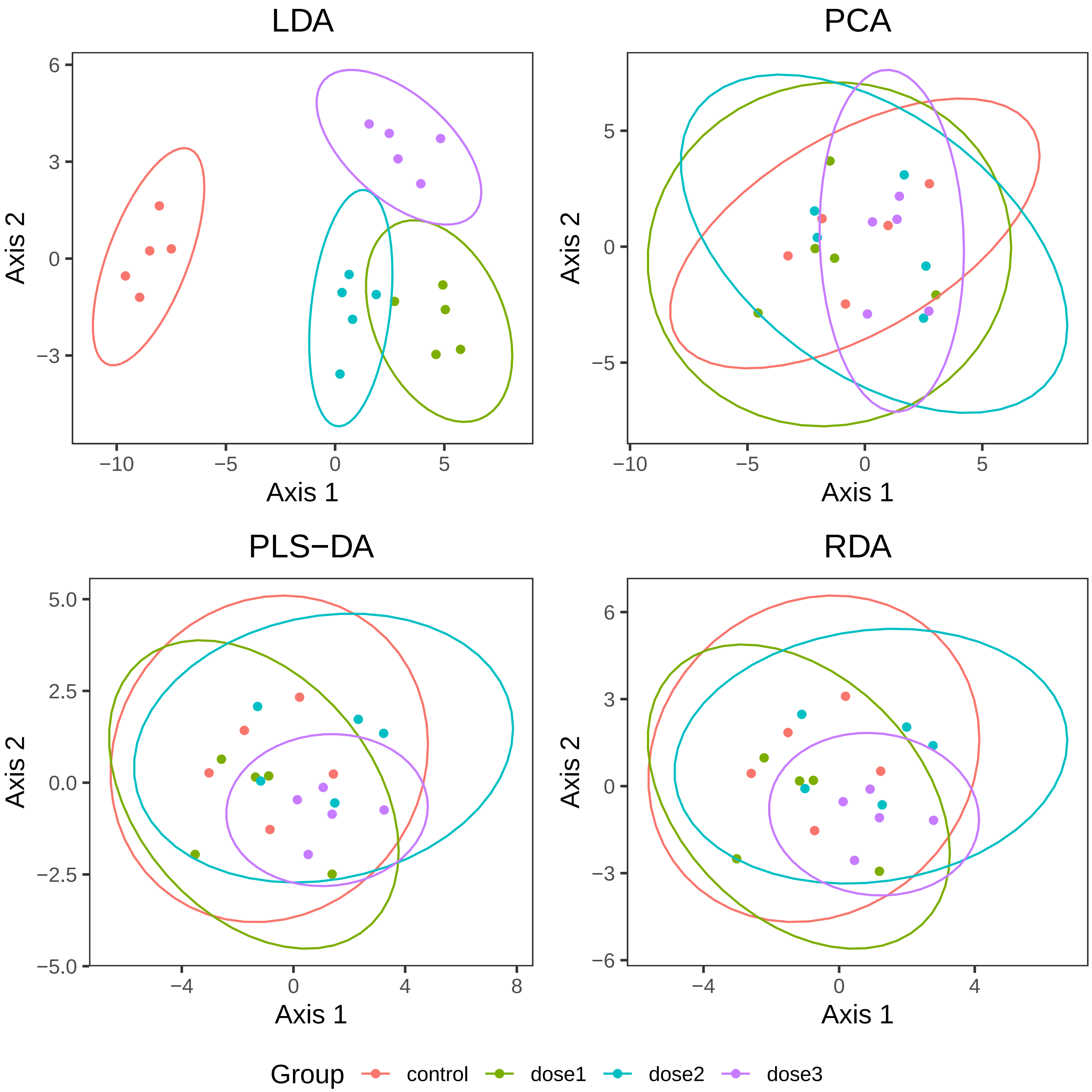
LDA
PCA
PLS−DA
RDA
Axis 1
Axis 2
Group
control
dose1
dose2
dose3

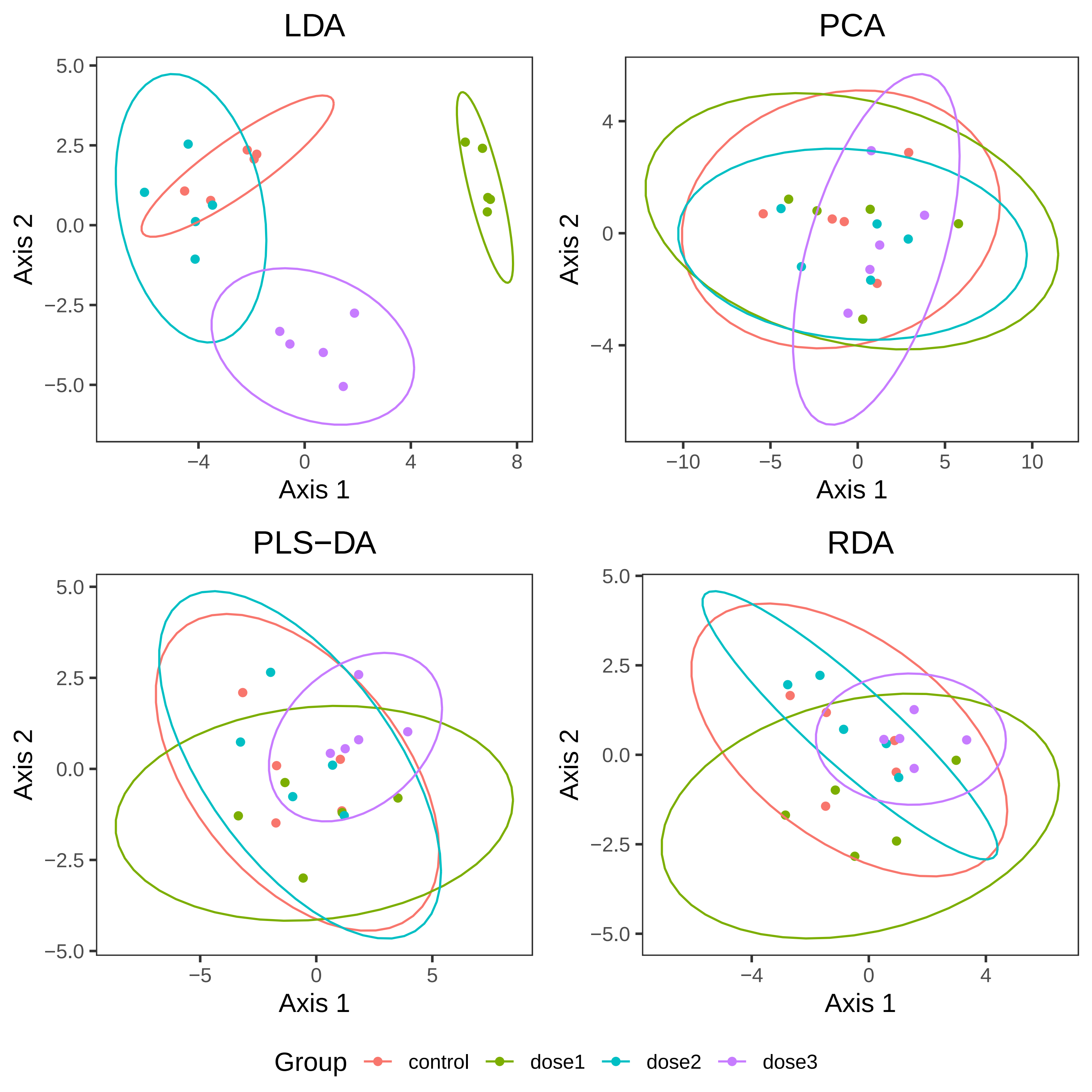

LDA
PCA
PLS−DA
RDA
Axis 1
Axis 2
5.0
2.5
0.0
−2.5
−5.0
−4
0
4
8
−10
−5
5
10
Group
control
dose1
dose2
dose3

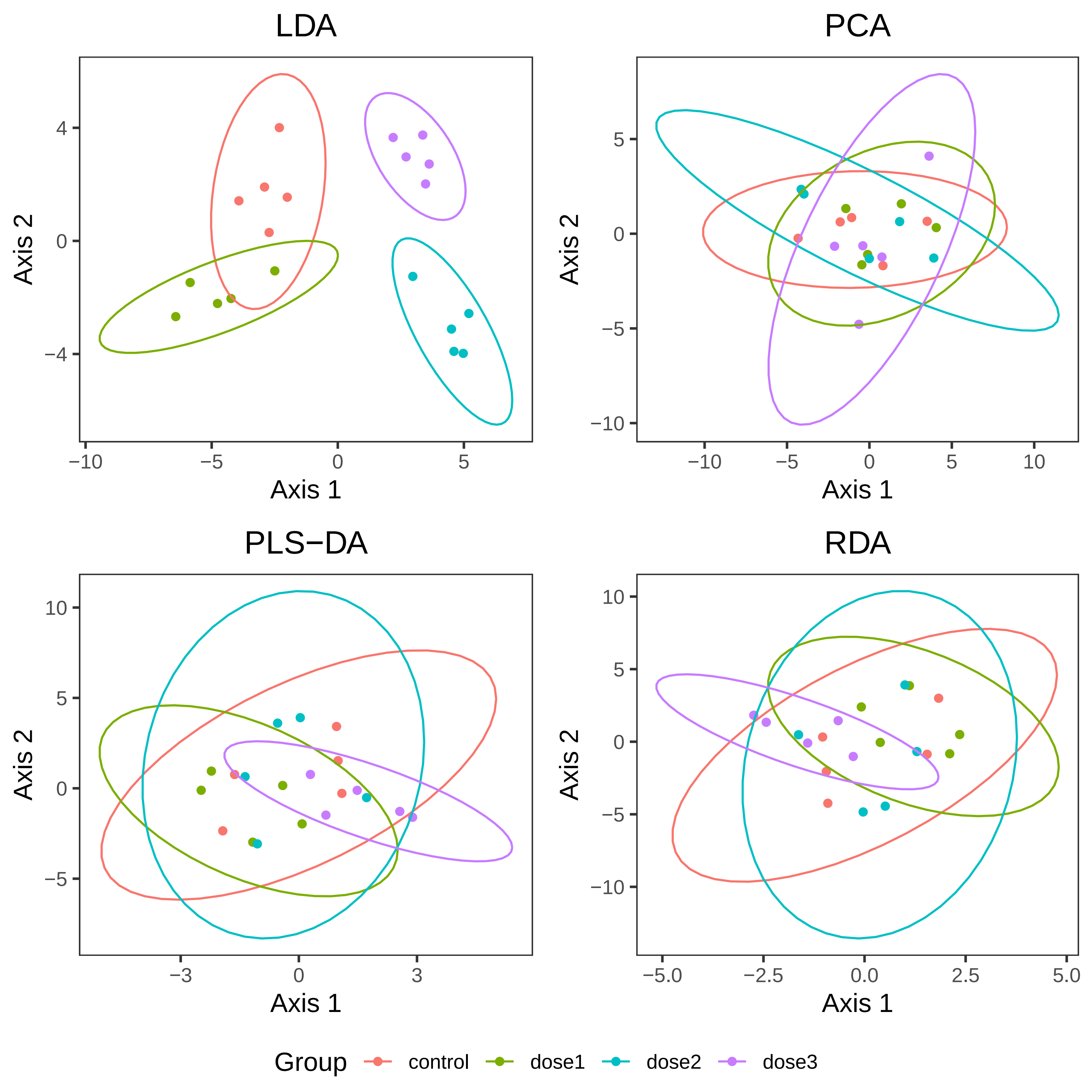
LDA
PCA
PLS−DA
RDA
Axis 1
Axis 2
4
0
−4
−10
−5
0
5
10
−3
3
−5.0
−2.5
0.0
2.5
5.0
Group
control
dose1
dose2
dose3

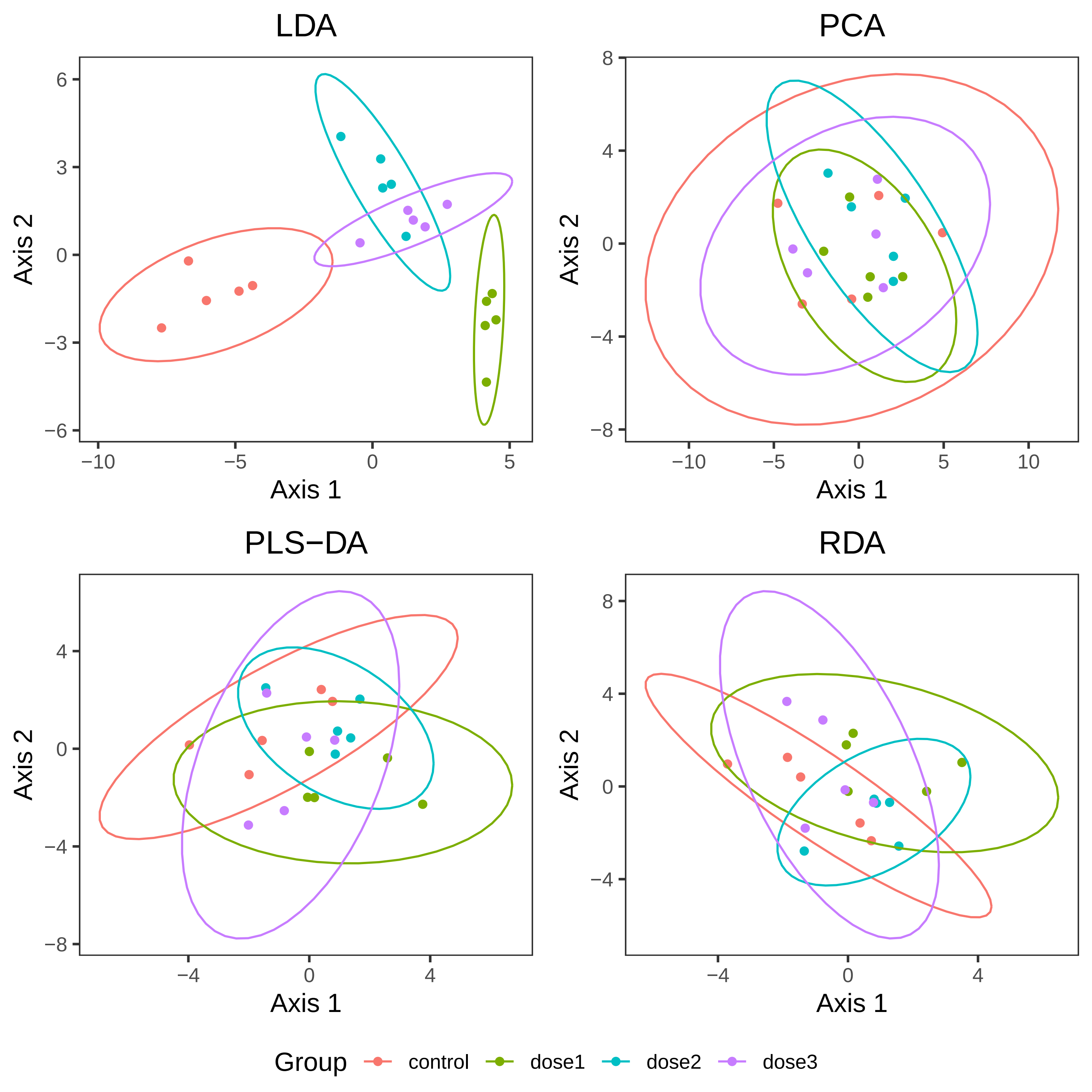

LDA
PCA
PLS−DA
RDA
Axis 1
Axis 2
−10
−5
0
5
10
6
3
−3
−6
8
4
−4
−8
Group
control
dose1
dose2
dose3

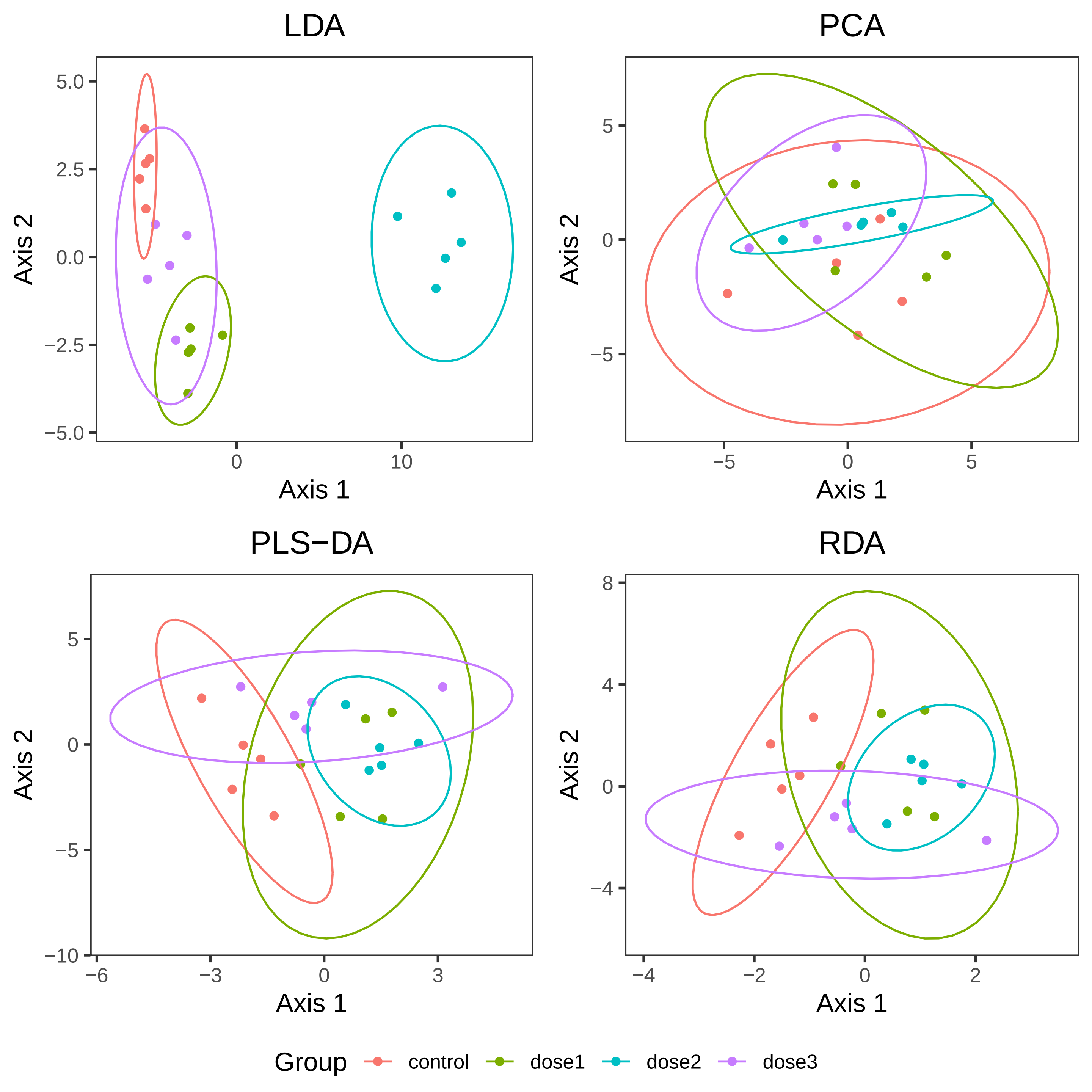
LDA
PCA
PLS−DA
RDA
Axis 1
Axis 2
5.0
2.5
0.0
−2.5
−5.0
0
10
5
0
−5
−5
0
5
5
0
−5
−10
−6
−3
0
3
8
4
0
−4
−4
−2
0
2
Group
control
dose1
dose2
dose3

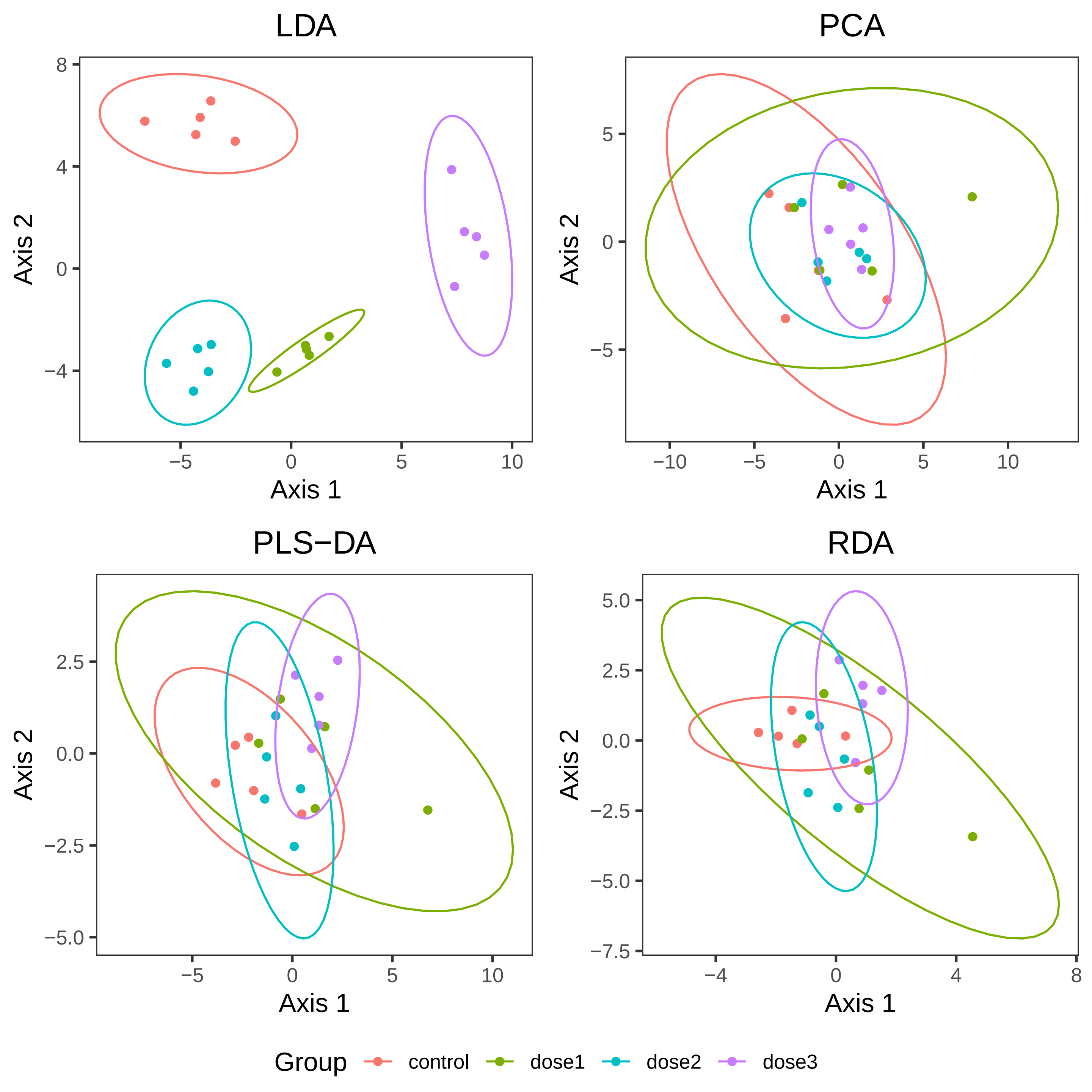
LDA
PCA
PLS−DA
RDA
Axis 1
Axis 2
Group
control
dose1
dose2
dose3

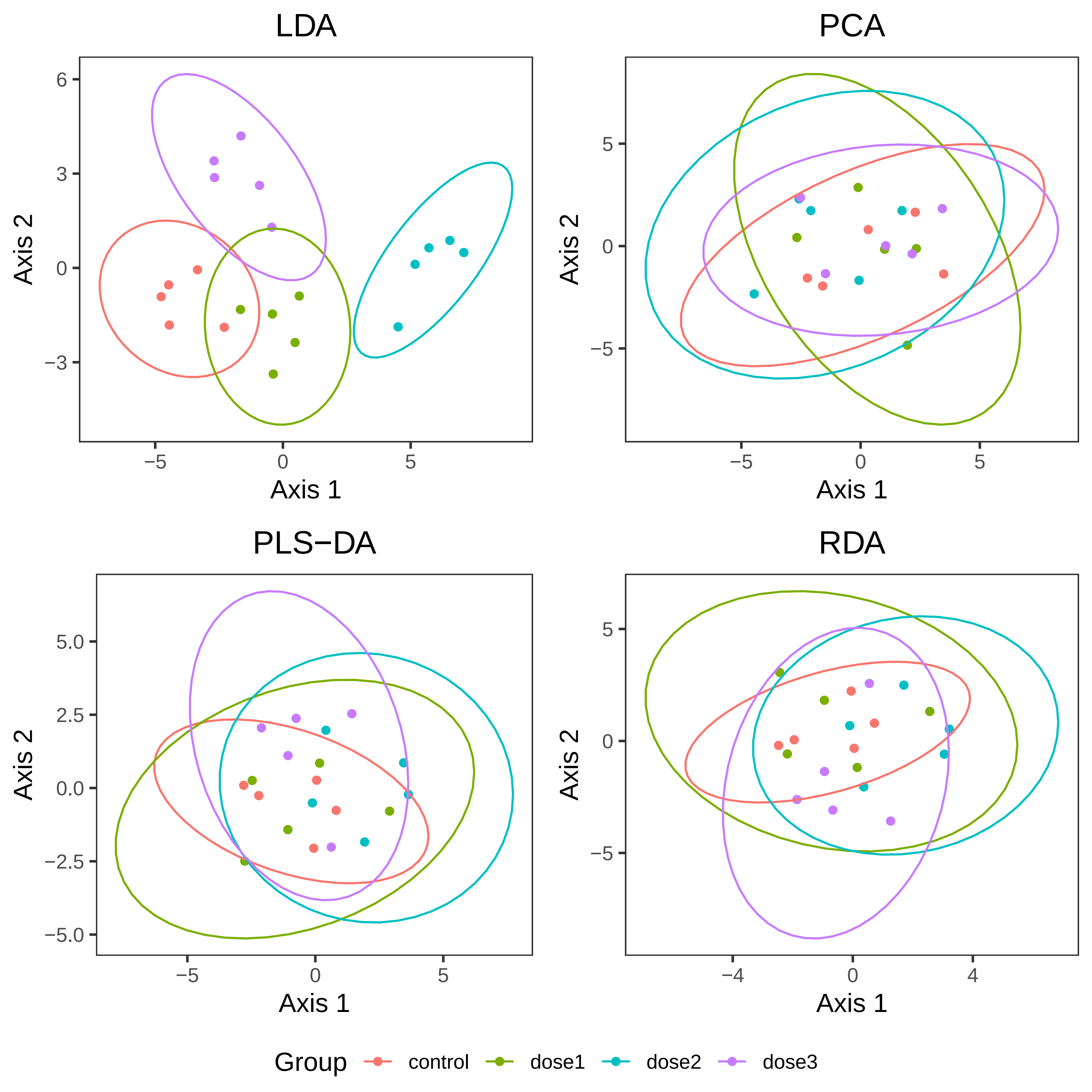
LDA
PCA
PLS−DA
RDA
Axis 1
Axis 2
6
3
0
−3
−5
0
5
−5
0
5
−5
0
5
5.0
2.5
0.0
−2.5
−5.0
−5
0
5
−4
0
4
Group
control
dose1
dose2
dose3

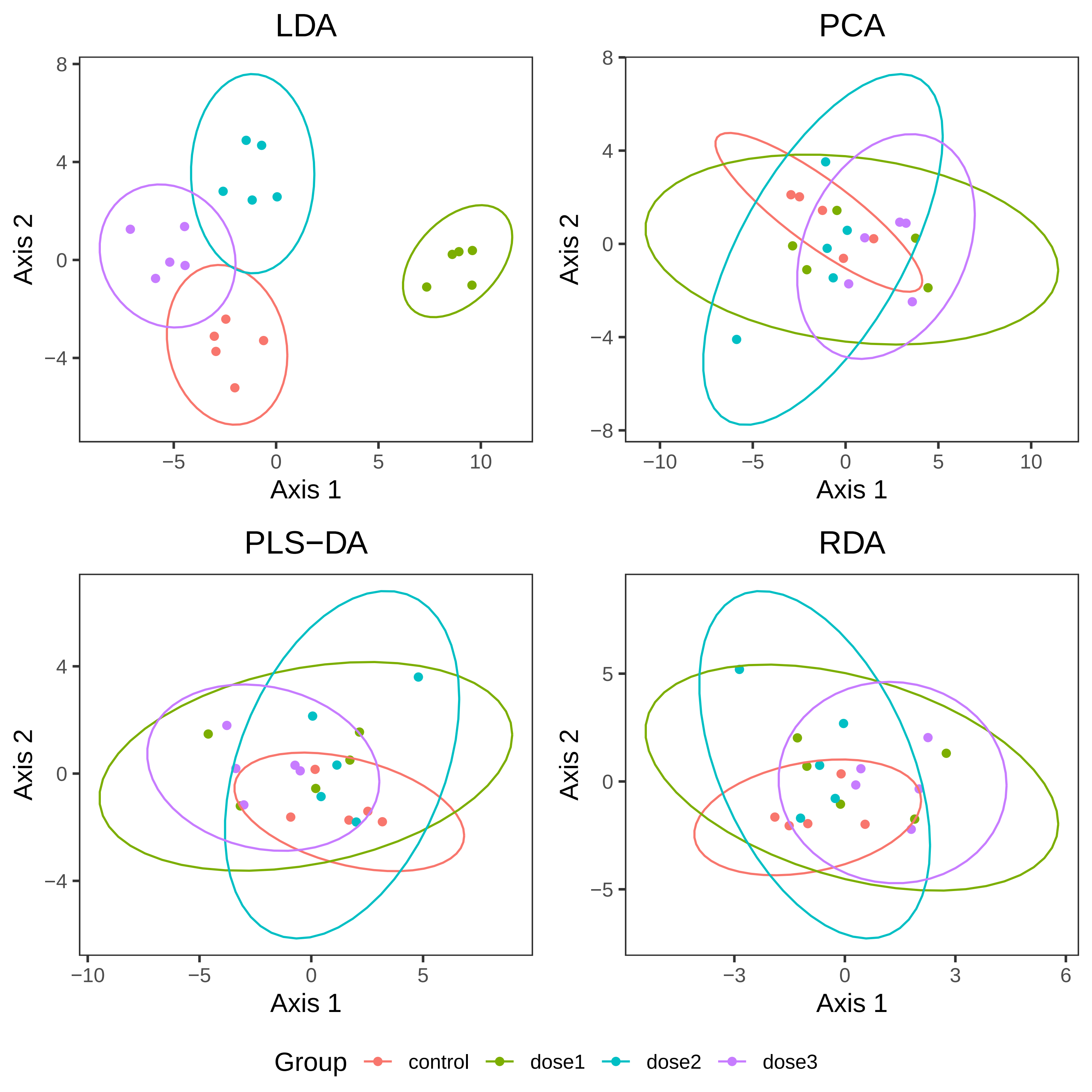
LDA
PCA
PLS−DA
RDA
Axis 1
Axis 2
8
4
0
−4
−8
−10
−5
0
5
10
−3
3
6
Group
control
dose1
dose2
dose3

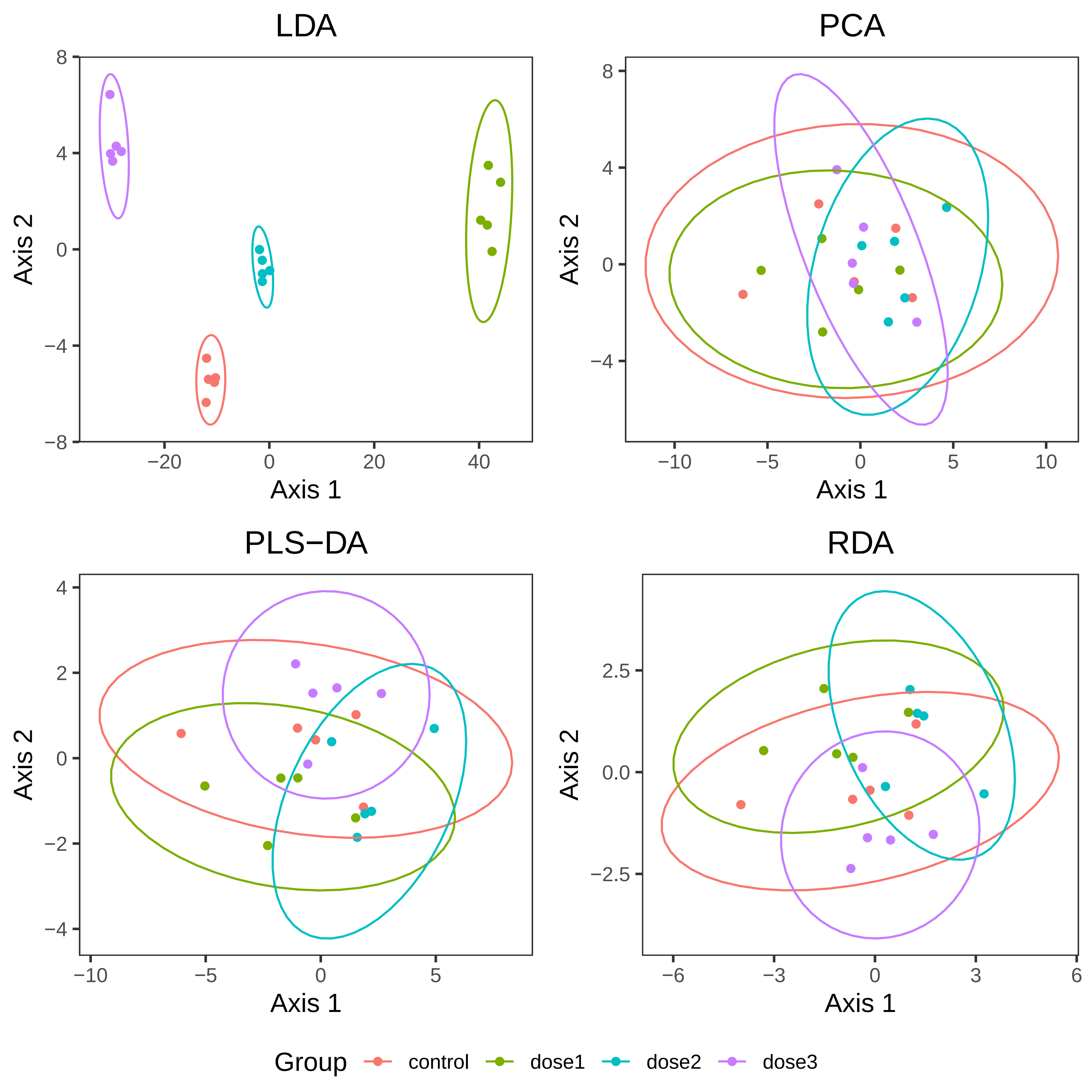
LDA
PCA
PLS−DA
RDA
Axis 1
Axis 2
Group
control
dose1
dose2
dose3

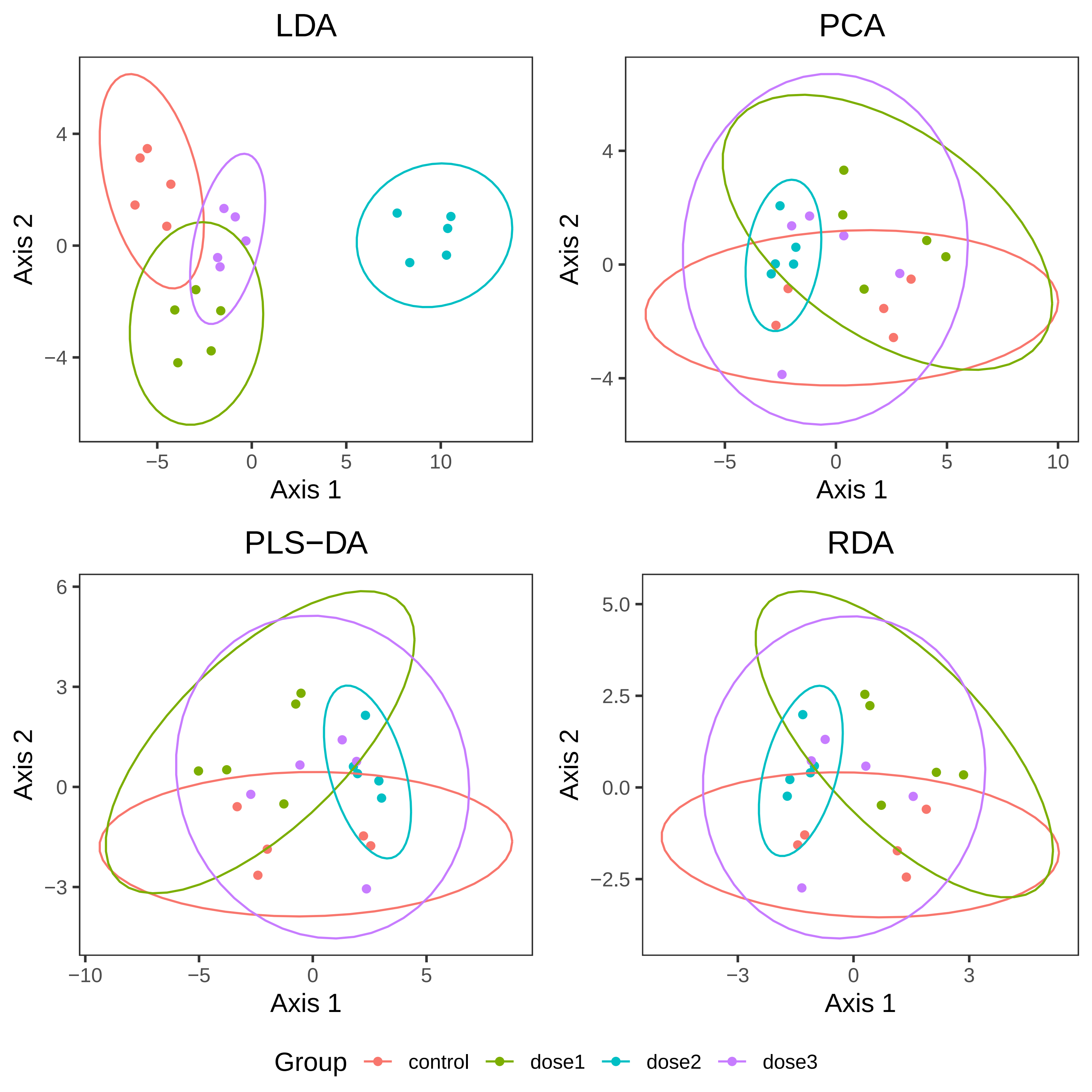

LDA
PCA
PLS−DA
RDA
Axis 1
Axis 2
4
0
−4
−5
0
5
10
6
3
−3
−10
5.0
2.5
0.0
−2.5
Group
control
dose1
dose2
dose3

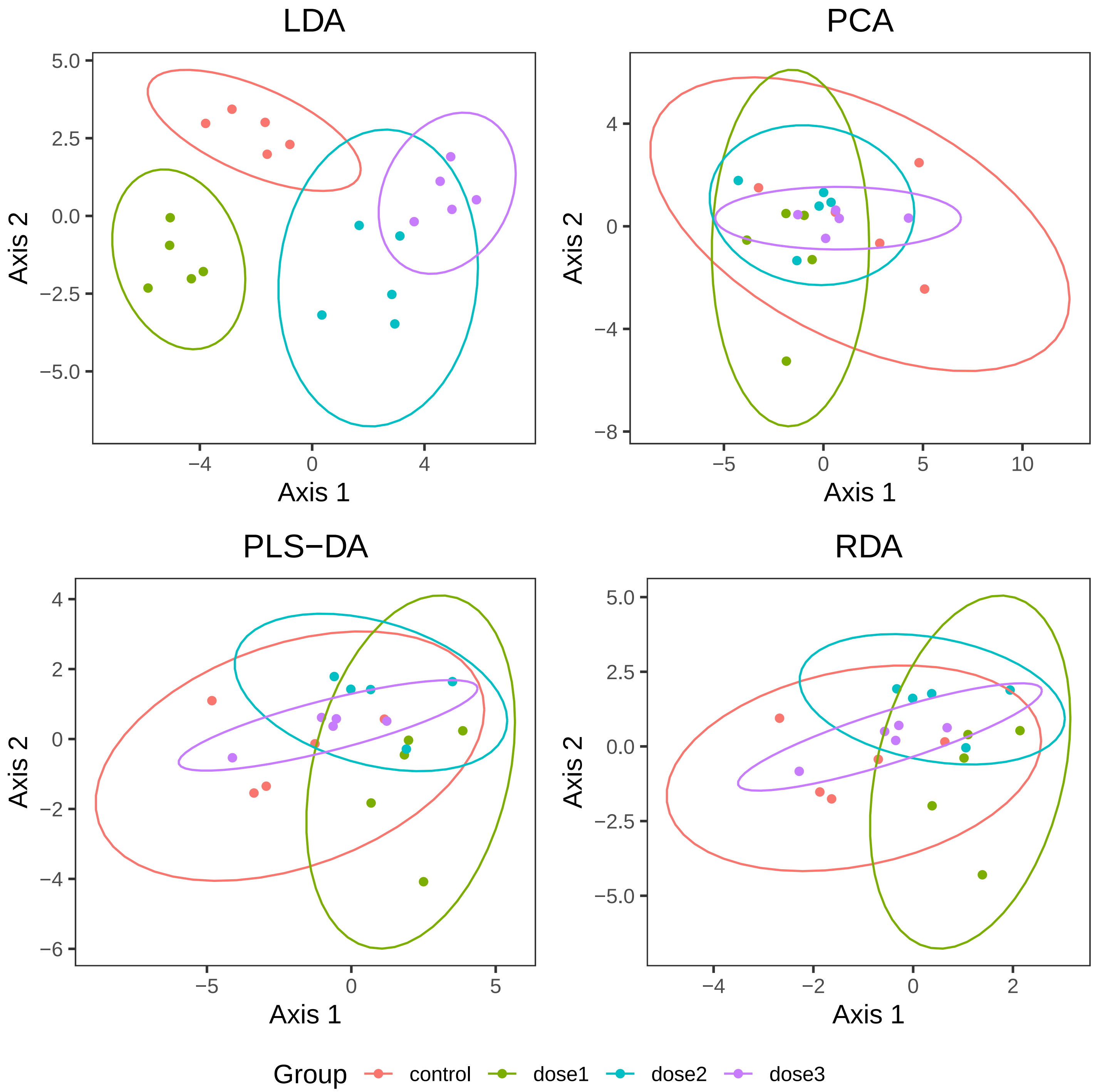
LDA
PCA
PLS−DA
RDA
Axis 1
Axis 2
Group
control
dose1
dose2
dose3

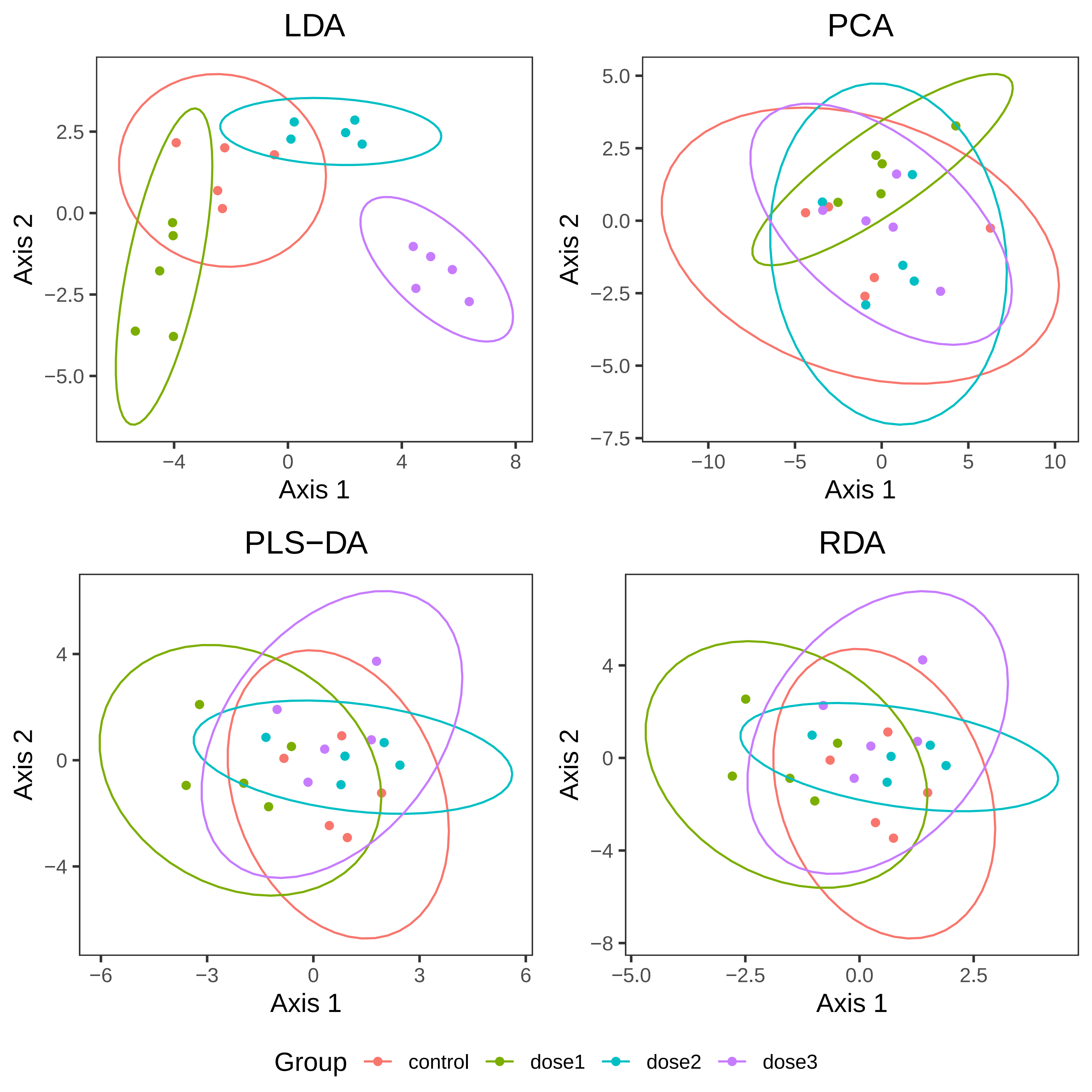

LDA
PCA
PLS−DA
RDA
Axis 1
Axis 2
Group
control
dose1
dose2
dose3

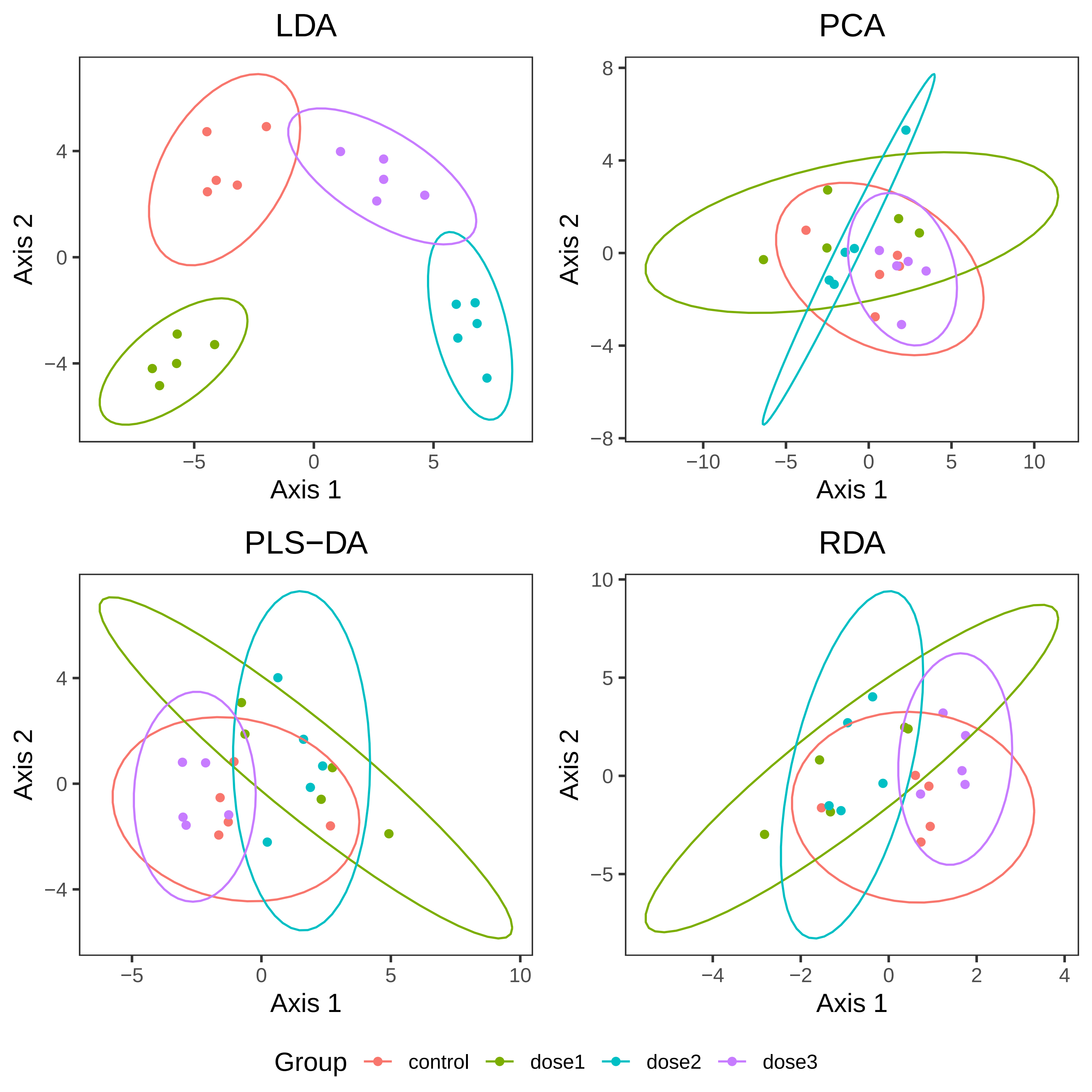

LDA
PCA
PLS−DA
RDA
Axis 1
Axis 2
Group
control
dose1
dose2
dose3

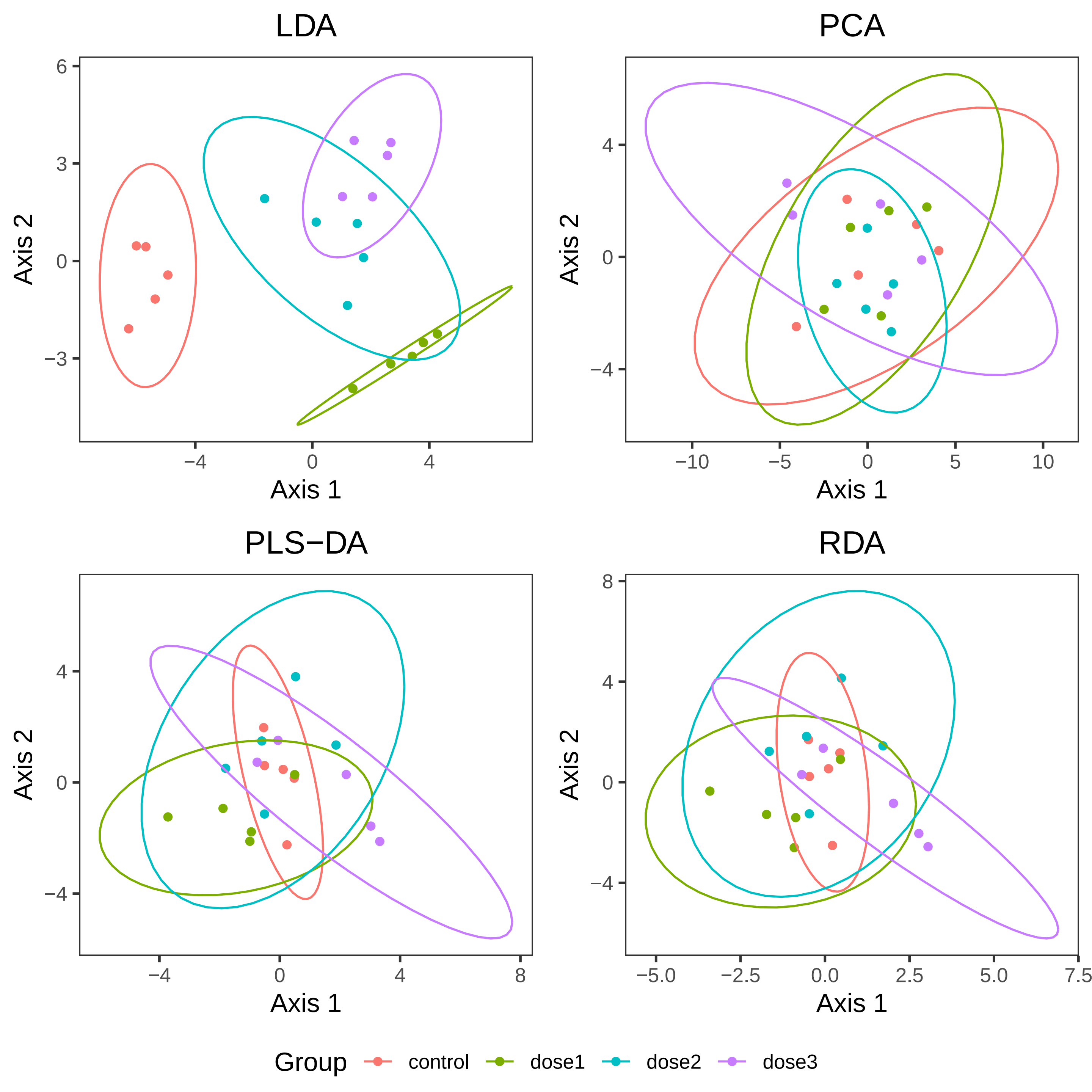

LDA
PCA
PLS−DA
RDA
Axis 1
Axis 2
6
3
0
−3
−4
0
4
−10
−5
5
10
−4
8
−5.0
−2.5
0.0
2.5
5.0
7.5
Group
control
dose1
dose2
dose3

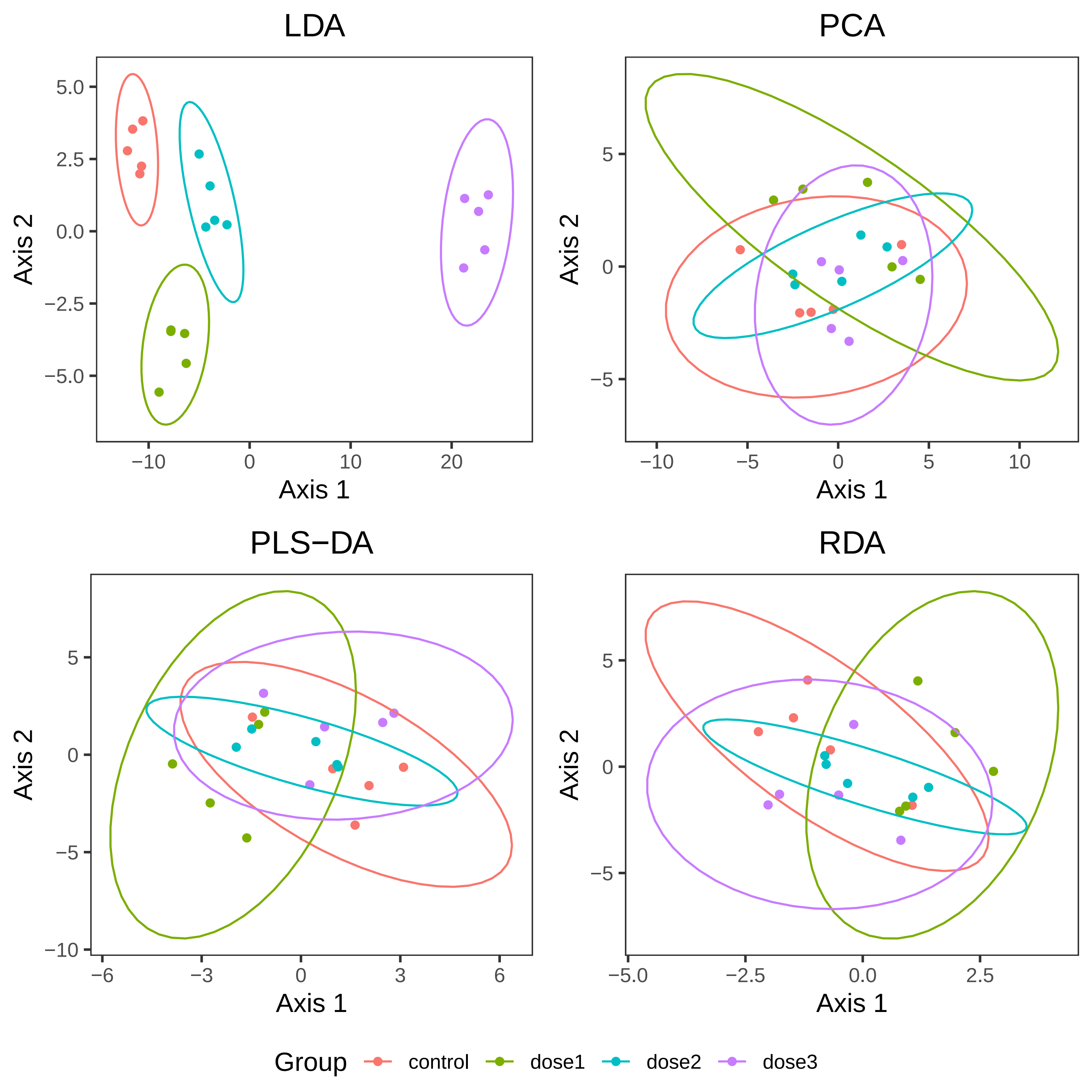
LDA
PCA
PLS−DA
RDA
Axis 1
Axis 2
Group
control
dose1
dose2
dose3

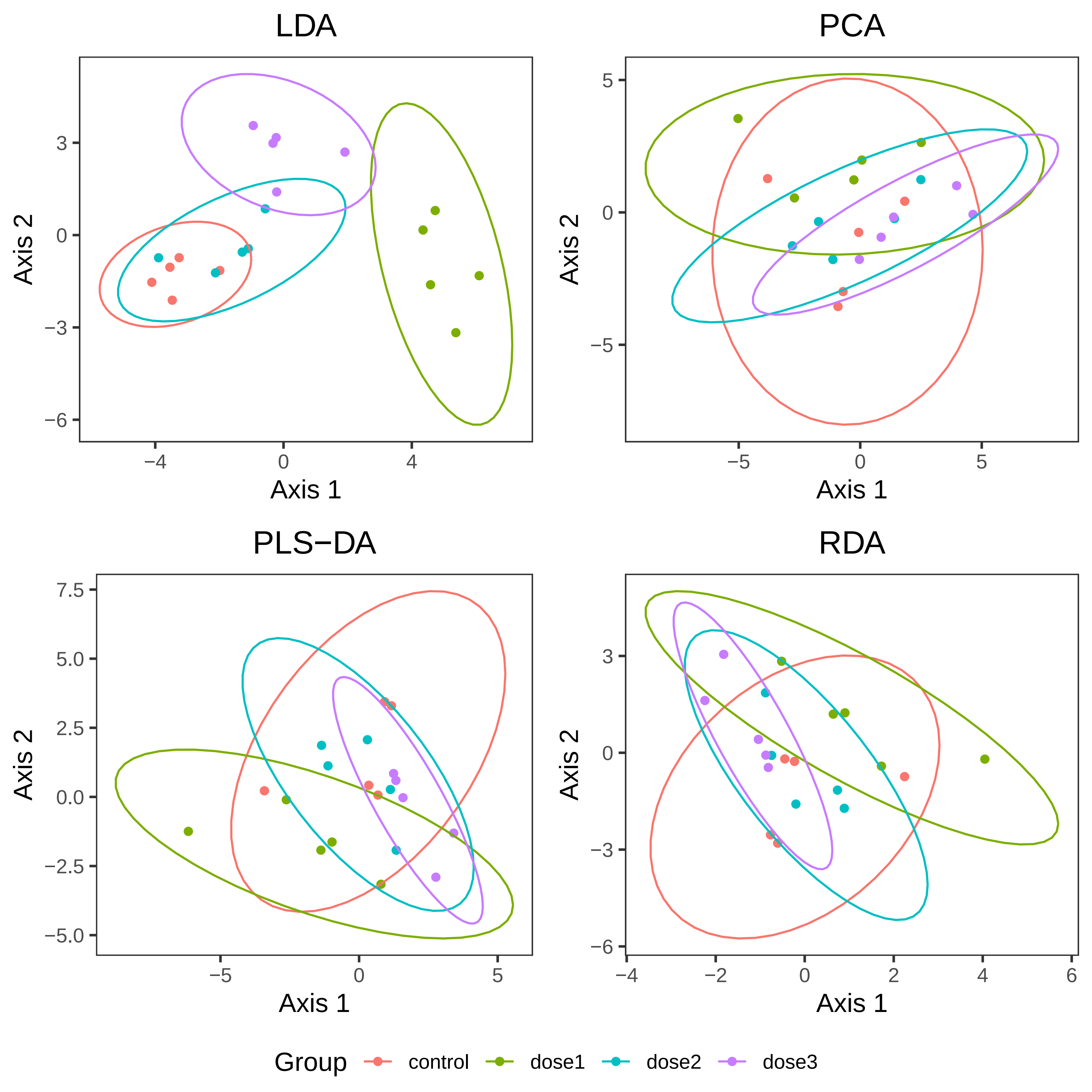

LDA
PCA
PLS−DA
RDA
Axis 1
Axis 2
Group
control
dose1
dose2
dose3

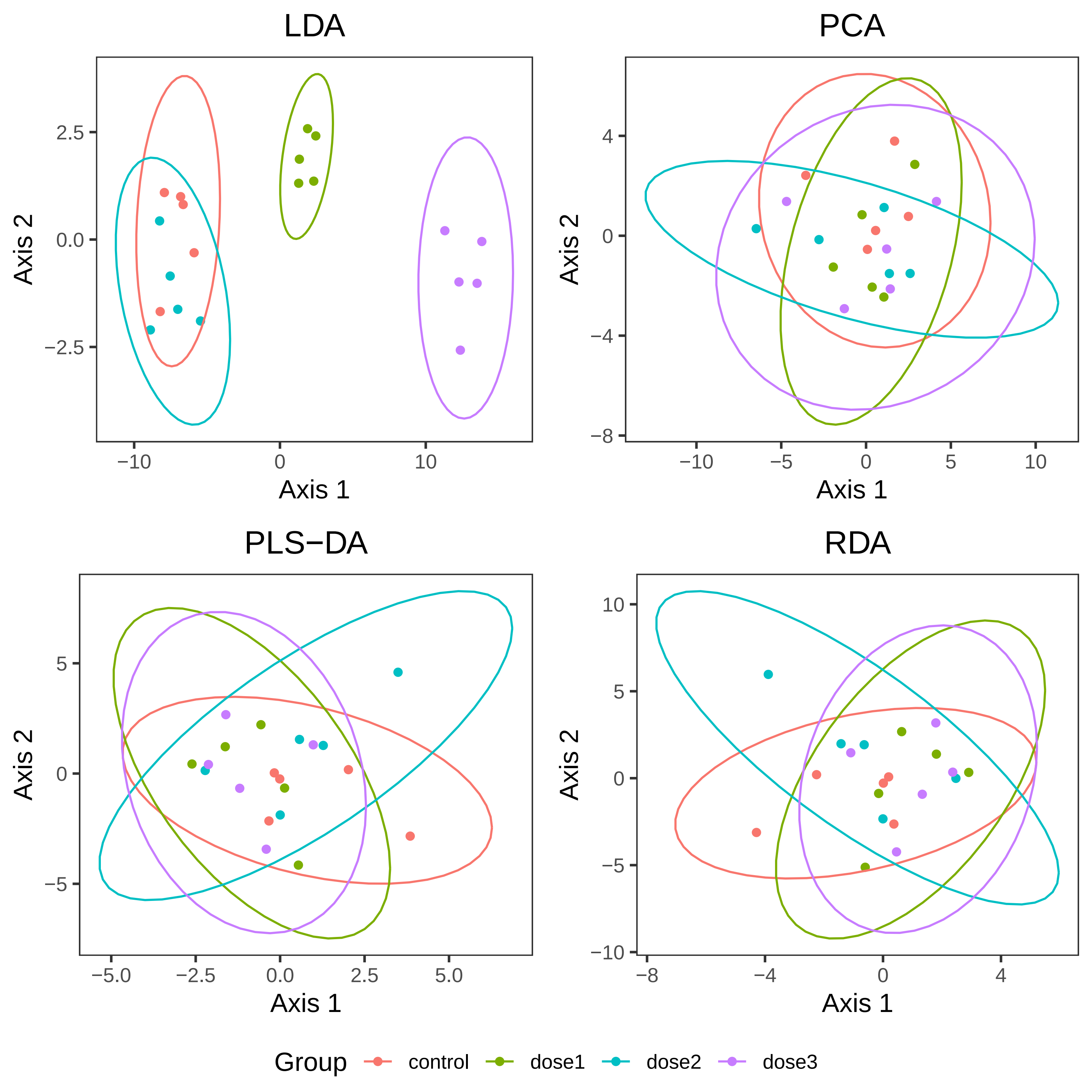

LDA
PCA
PLS−DA
RDA
Axis 1
Axis 2
2.5
0.0
−2.5
−10
0
10
4
0
−4
−8
−5
5
−5.0
−2.5
0.0
2.5
5.0
10
−10
−8
−4
Group
control
dose1
dose2
dose3

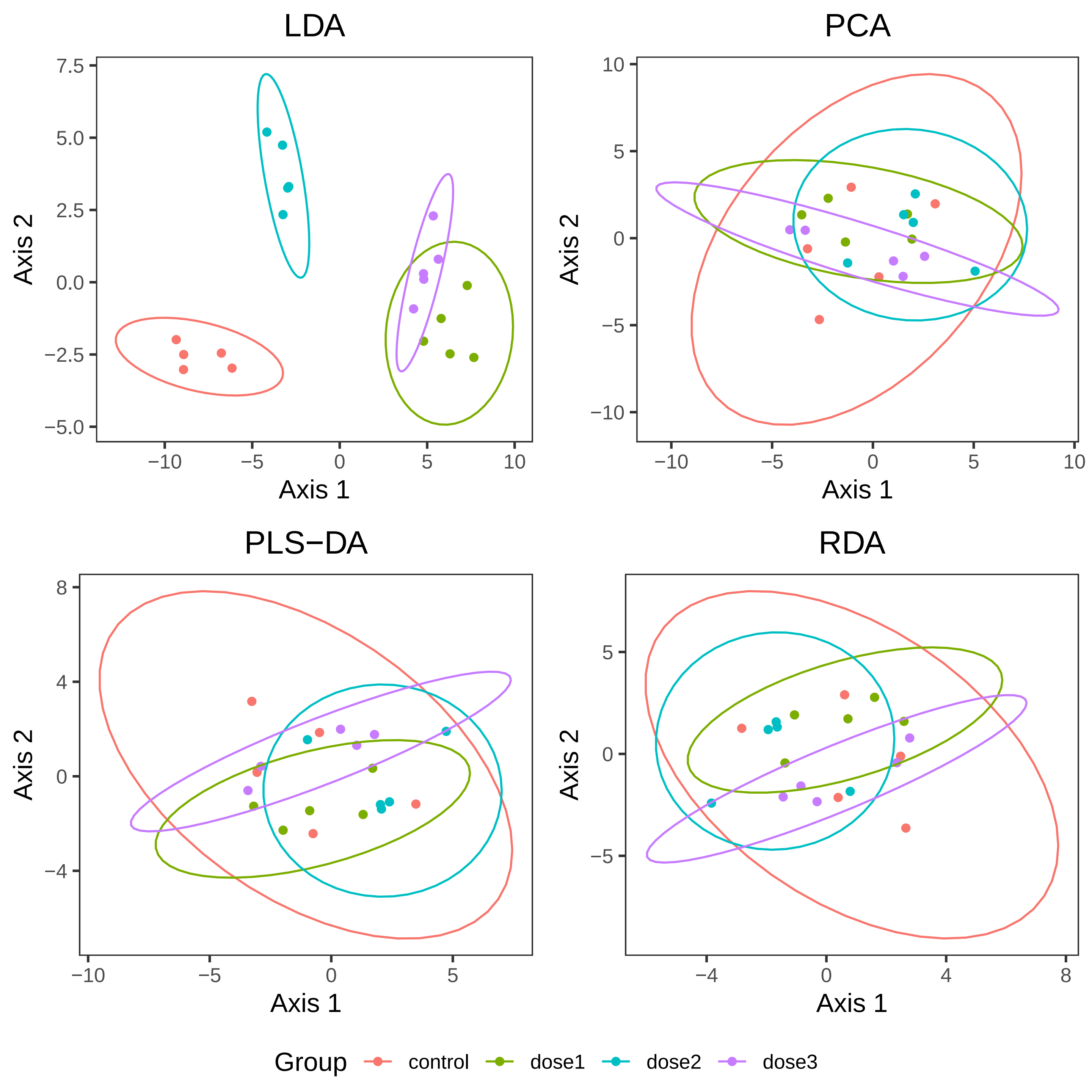

LDA
PCA
PLS−DA
RDA
Axis 1
Axis 2
Group
control
dose1
dose2
dose3

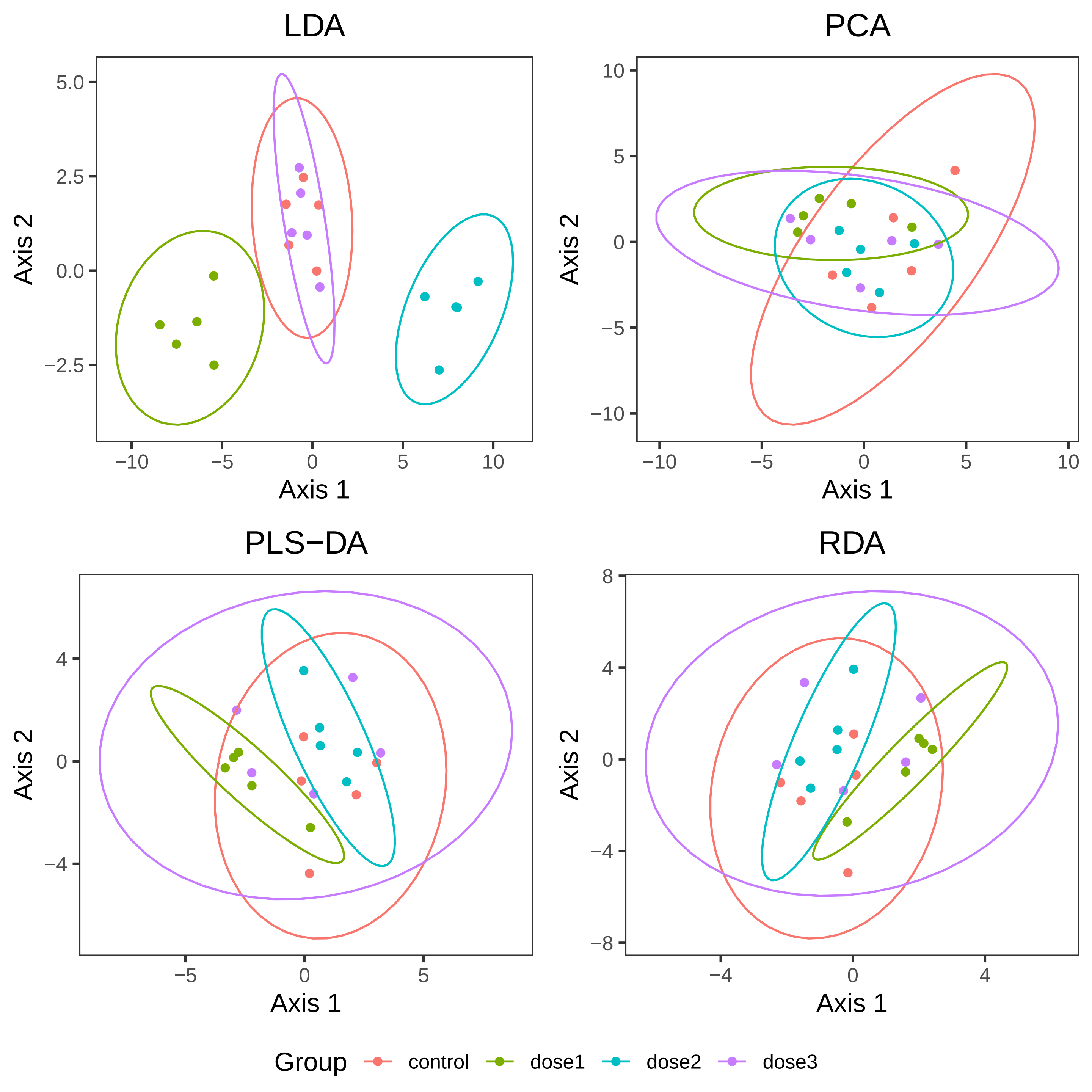
LDA
PCA
PLS−DA
RDA
Axis 1
Axis 2
Group
control
dose1
dose2
dose3

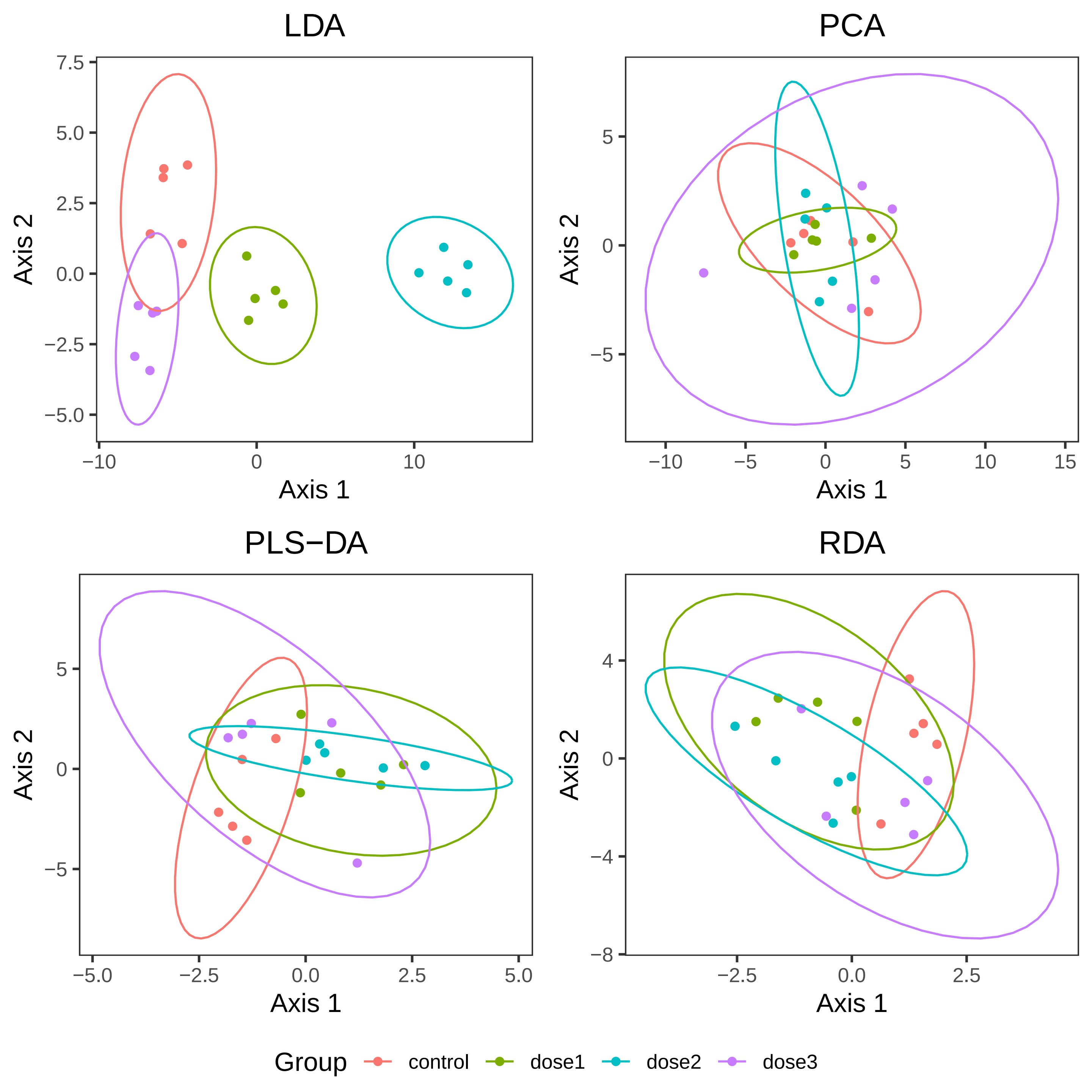

LDA
PCA
PLS−DA
RDA
Axis 1
Axis 2
7.5
5.0
2.5
0.0
−2.5
−5.0
−10
0
10
5
−5
−10
15
−5.0
2.5
4
−4
−8
Group
control
dose1
dose2
dose3

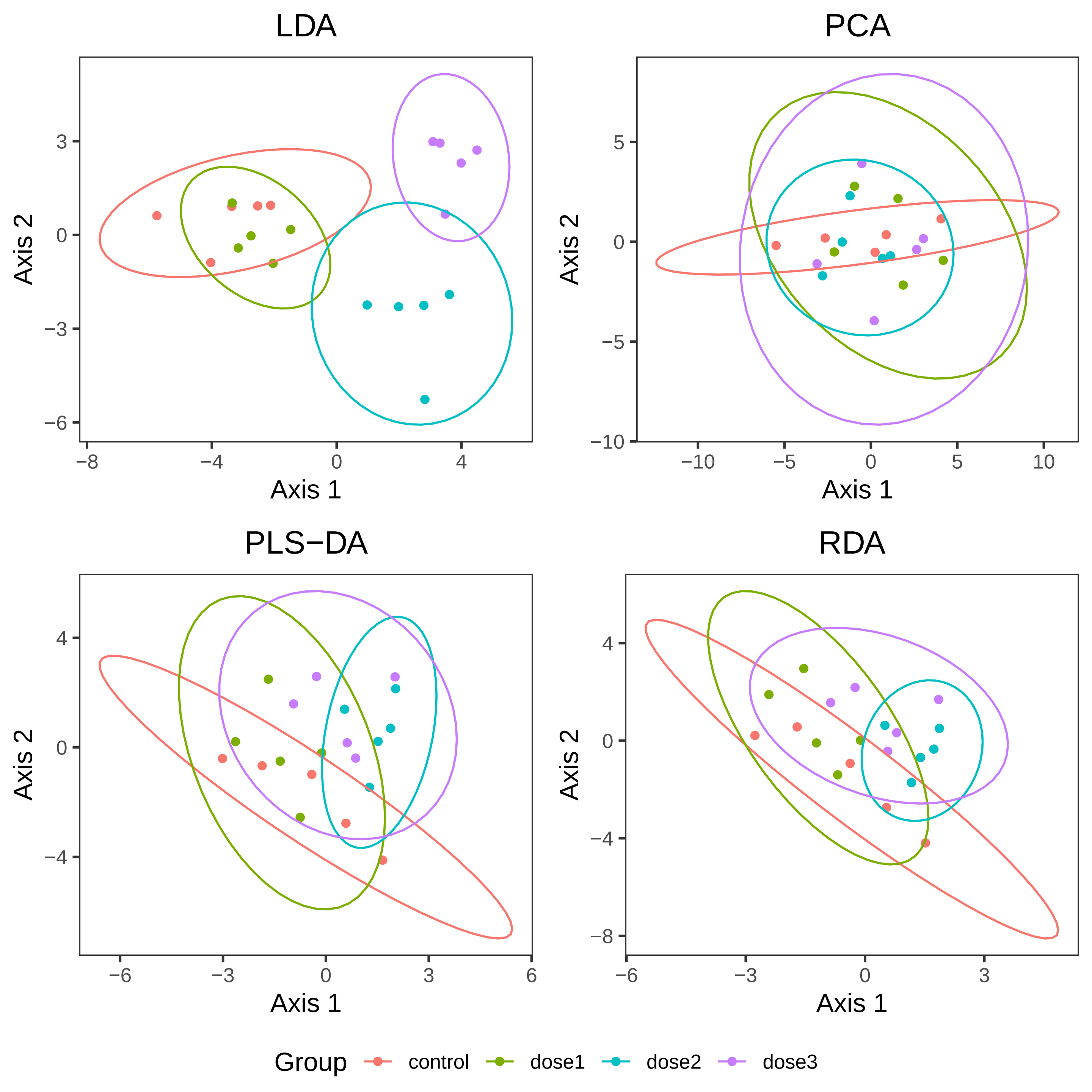

LDA
PCA
PLS−DA
RDA
Axis 1
Axis 2
3
0
−3
−6
−8
−4
0
4
5
0
−5
−10
−10
−5
0
5
10
4
0
−4
−6
−3
0
3
6
4
0
−4
−8
−6
−3
0
3
Group
control
dose1
dose2
dose3

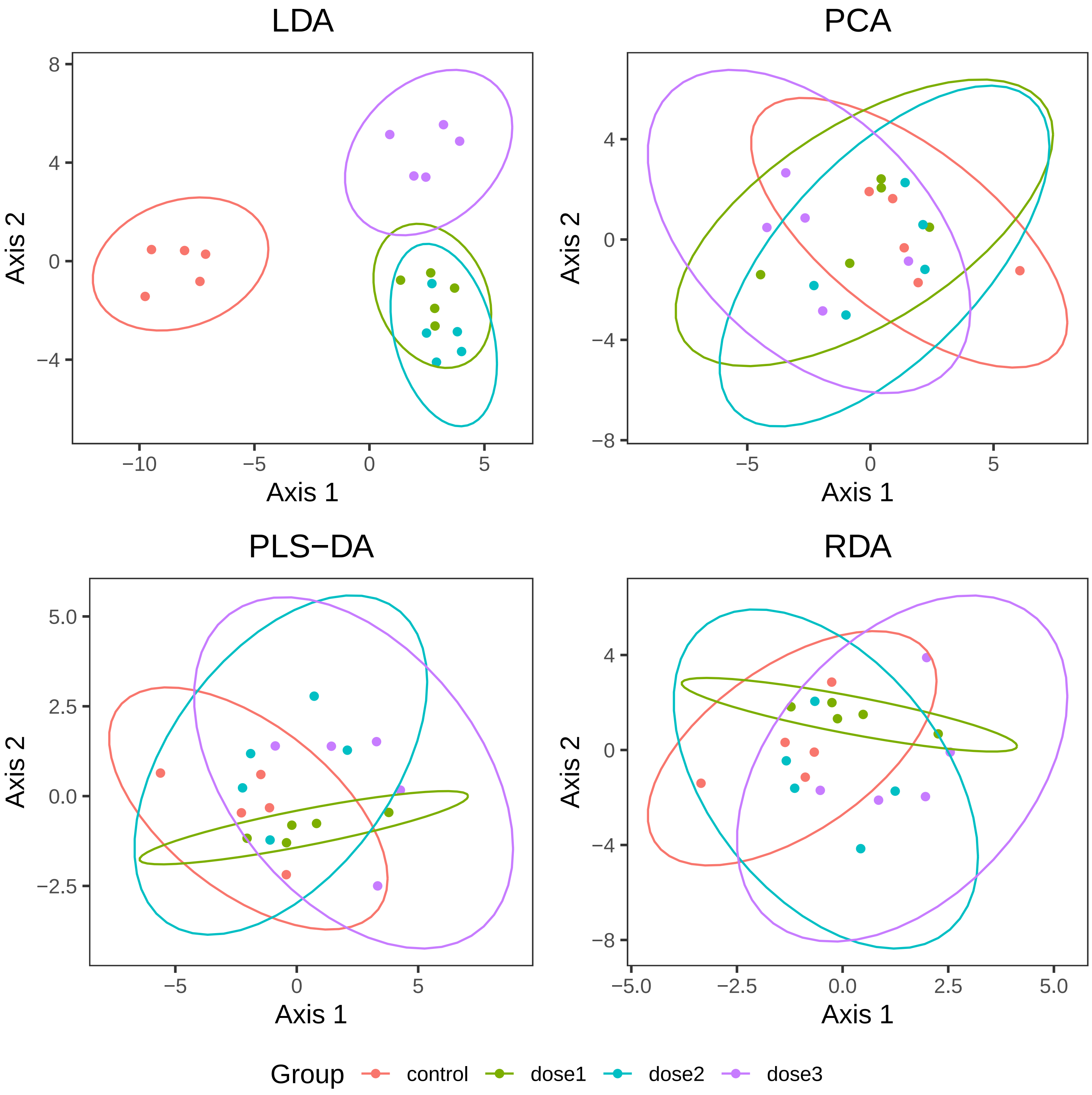
LDA
PCA
PLS−DA
RDA
Axis 1
Axis 2
Group
control
dose1
dose2
dose3

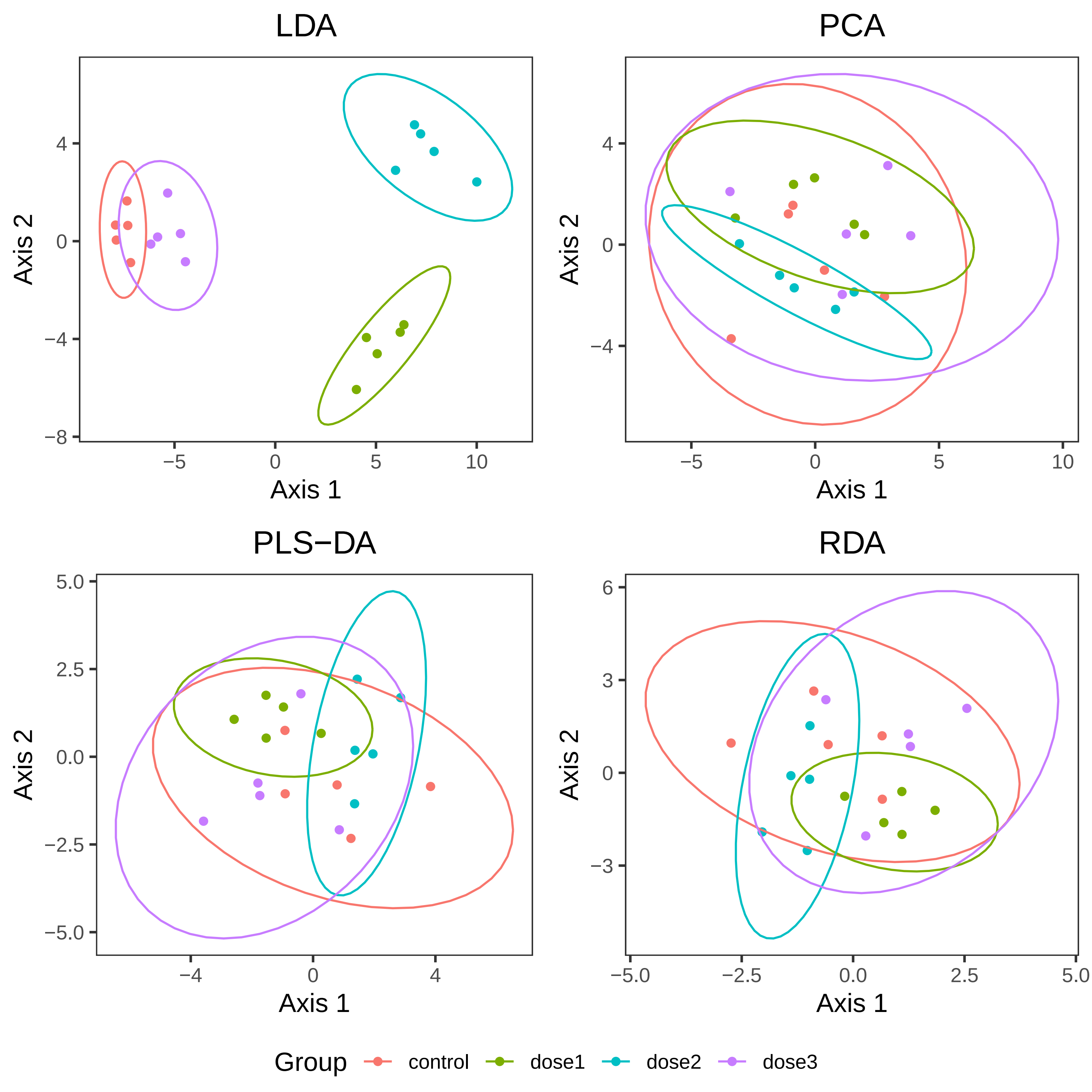
LDA
PCA
PLS−DA
RDA
Axis 1
Axis 2
Group
control
dose1
dose2
dose3

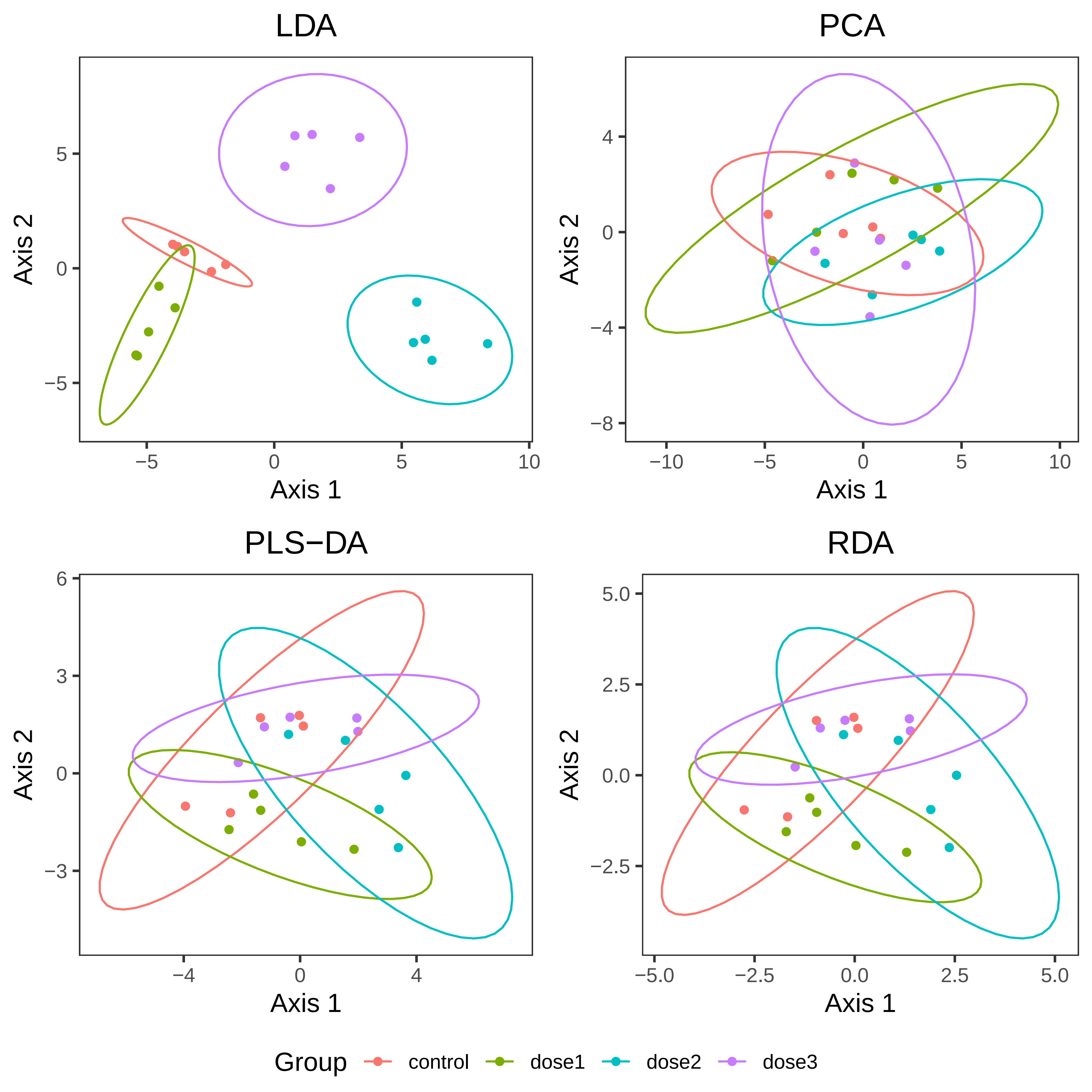
LDA
PCA
PLS−DA
RDA
Axis 1
Axis 2
5
0
−5
10
4
−4
−8
−10
6
3
−3
5.0
2.5
0.0
−2.5
−5.0
Group
control
dose1
dose2
dose3

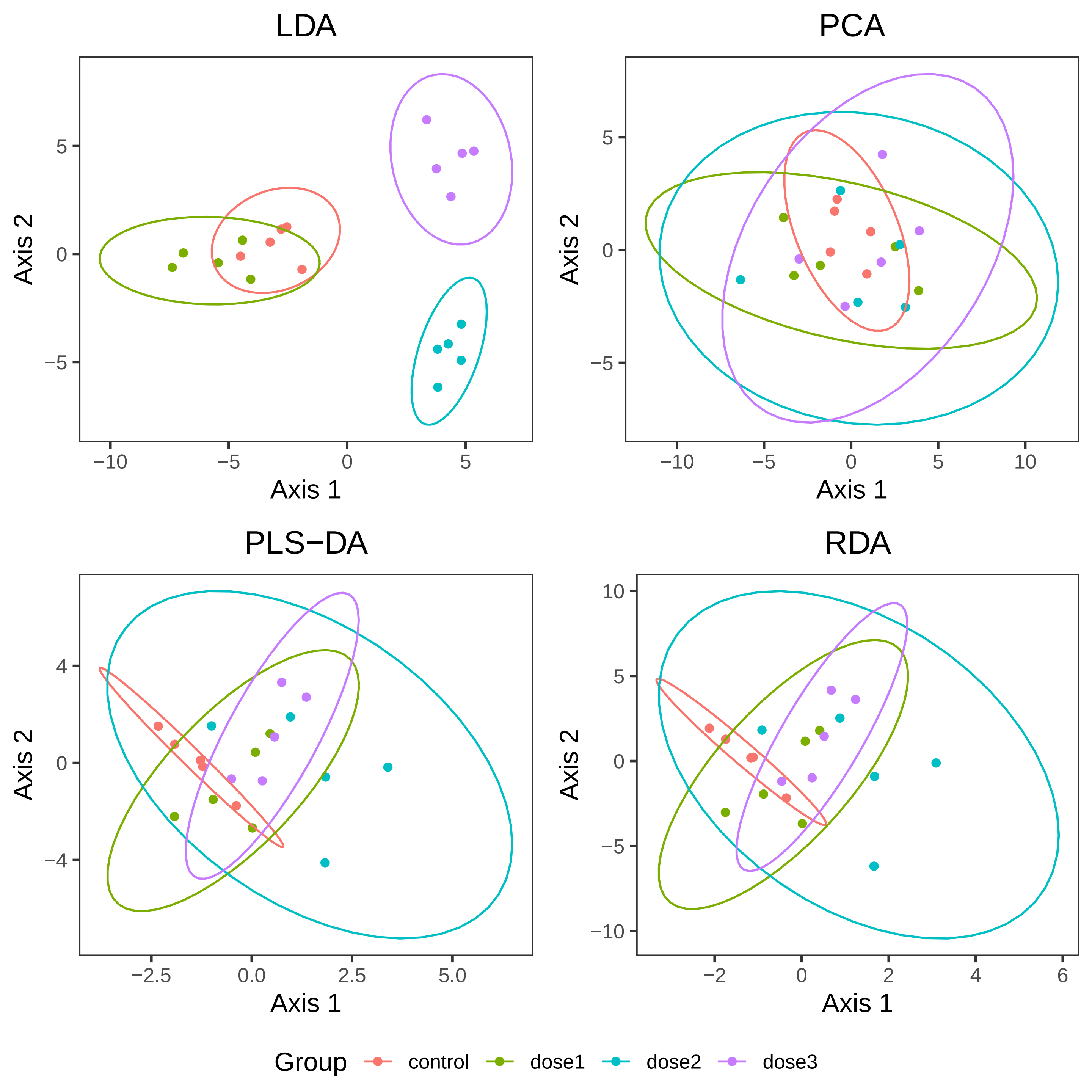

LDA
PCA
PLS−DA
RDA
Axis 1
Axis 2
Group
control
dose1
dose2
dose3

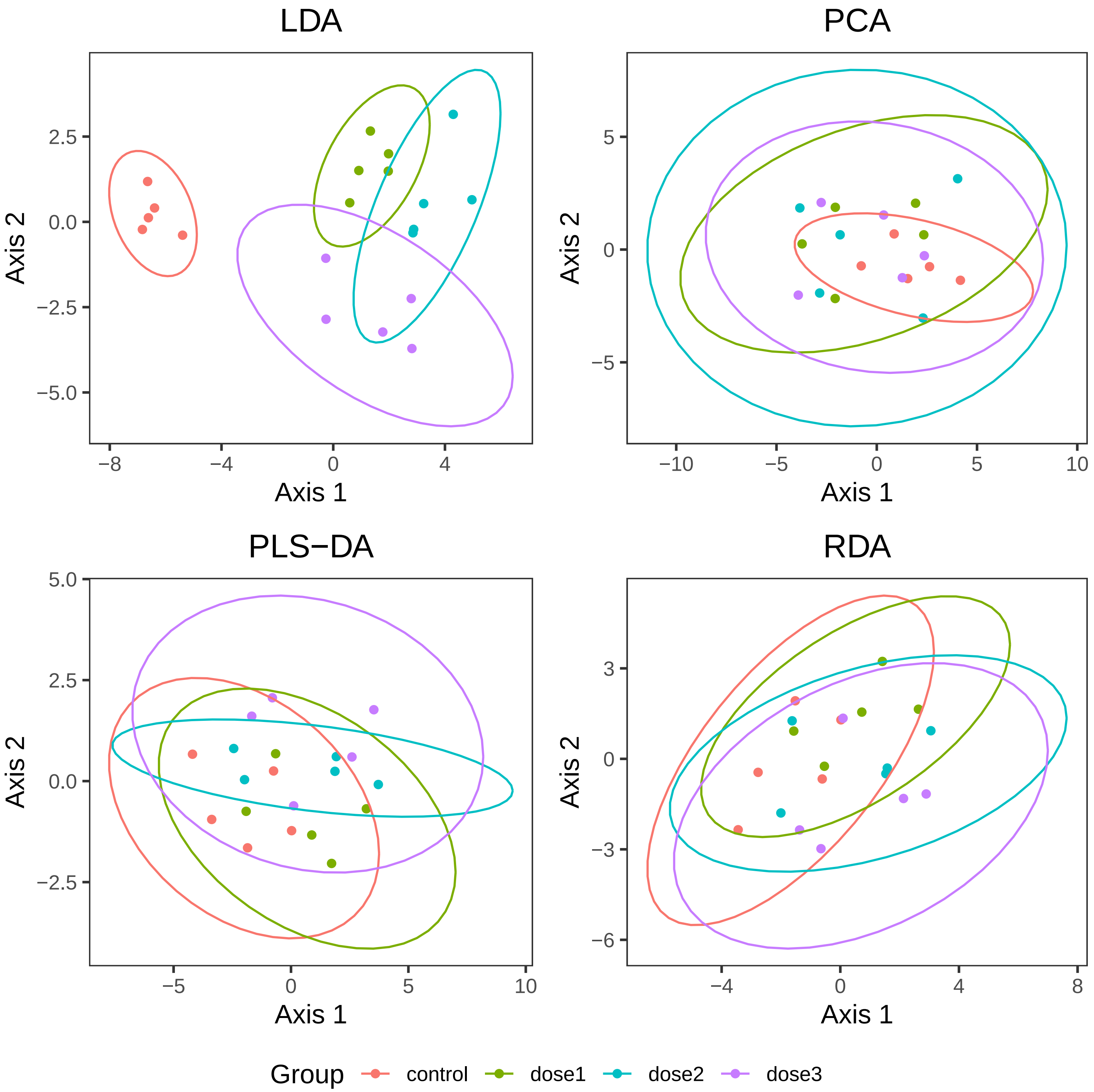
LDA
PCA
PLS−DA
RDA
Axis 1
Axis 2
2.5
0.0
−2.5
−5.0
−8
−4
0
4
5
−5
−10
10
5.0
3
−3
−6
8
Group
control
dose1
dose2
dose3

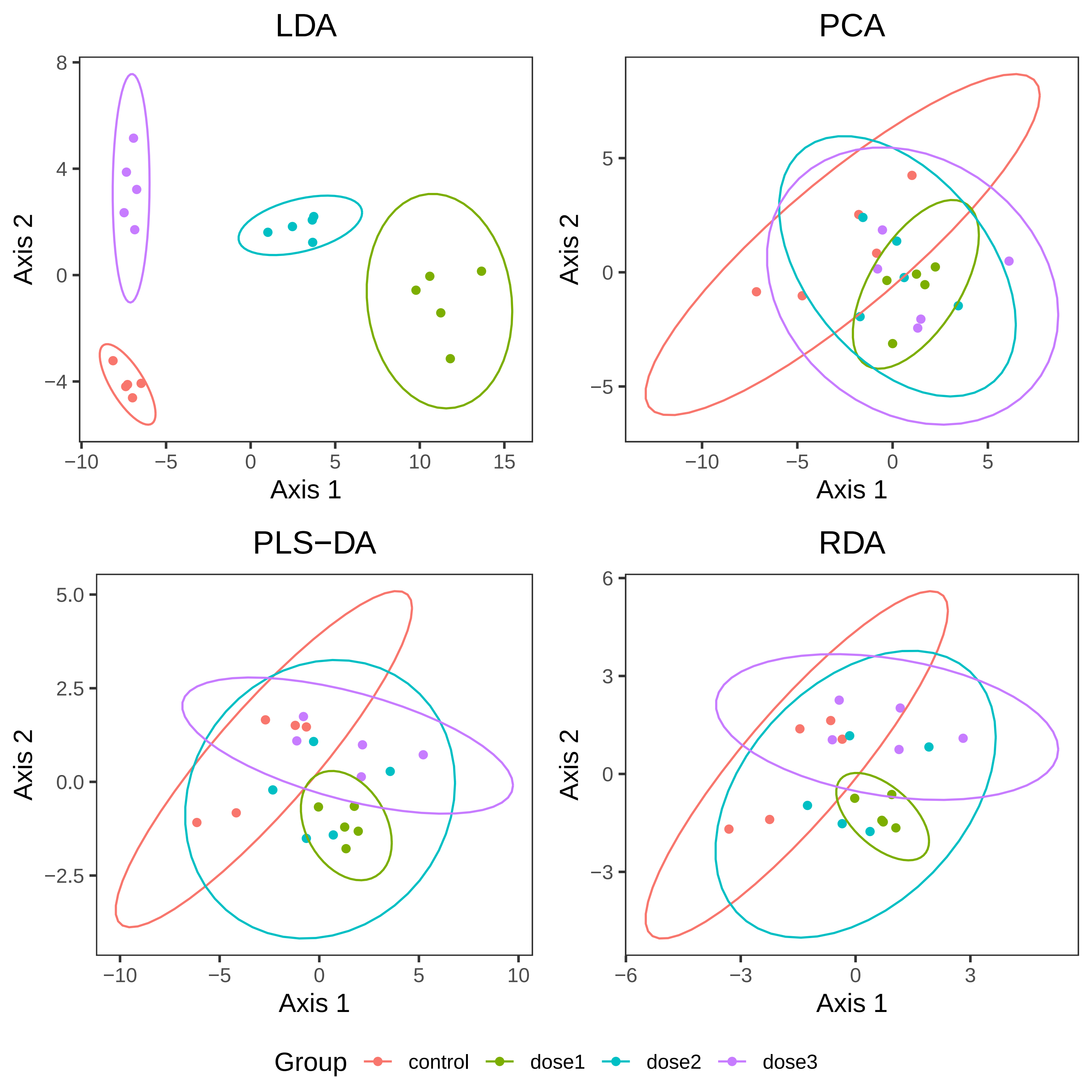

LDA
PCA
PLS−DA
RDA
Axis 1
Axis 2
Group
control
dose1
dose2
dose3

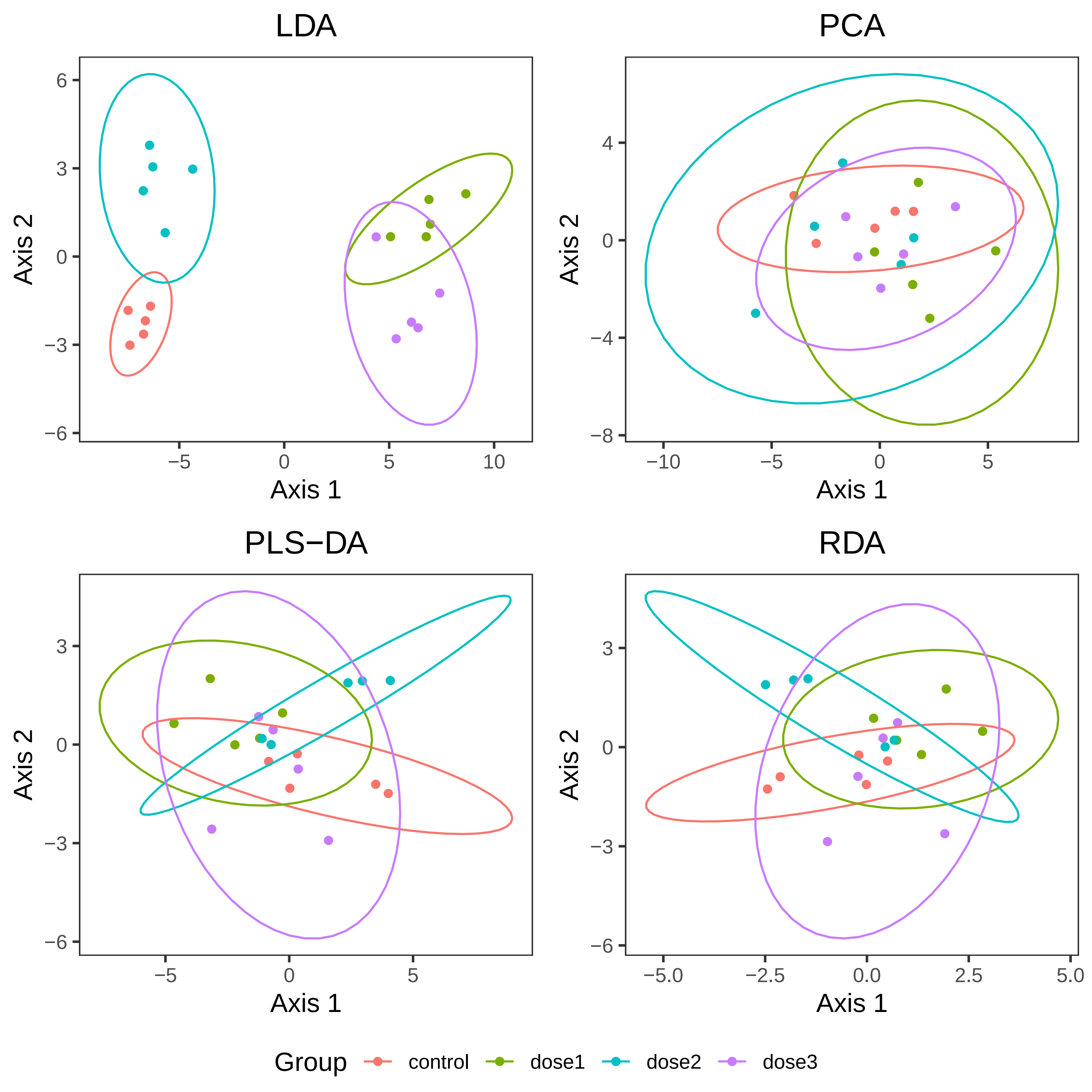

LDA
PCA
PLS−DA
RDA
Axis 1
Axis 2
6
3
0
−3
−6
−5
0
5
10
4
−4
−8
−10
−2.5
−5.0
0.0
2.5
5.0
Group
control
dose1
dose2
dose3

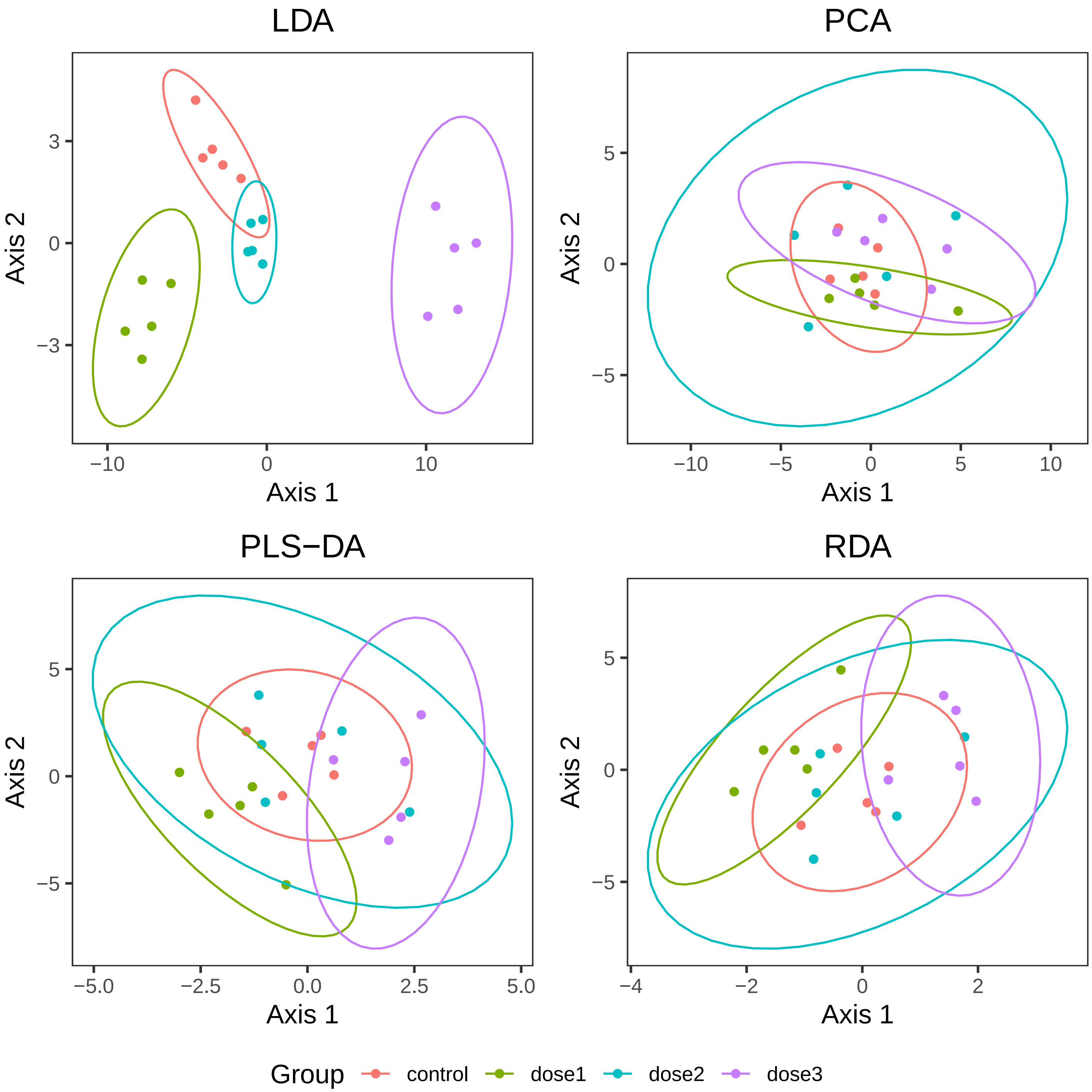

LDA
PCA
PLS−DA
RDA
Axis 1
Axis 2
−10
0
10
3
−3
−5
5
−5.0
−2.5
0.0
2.5
5.0
−4
−2
2
Group
control
dose1
dose2
dose3

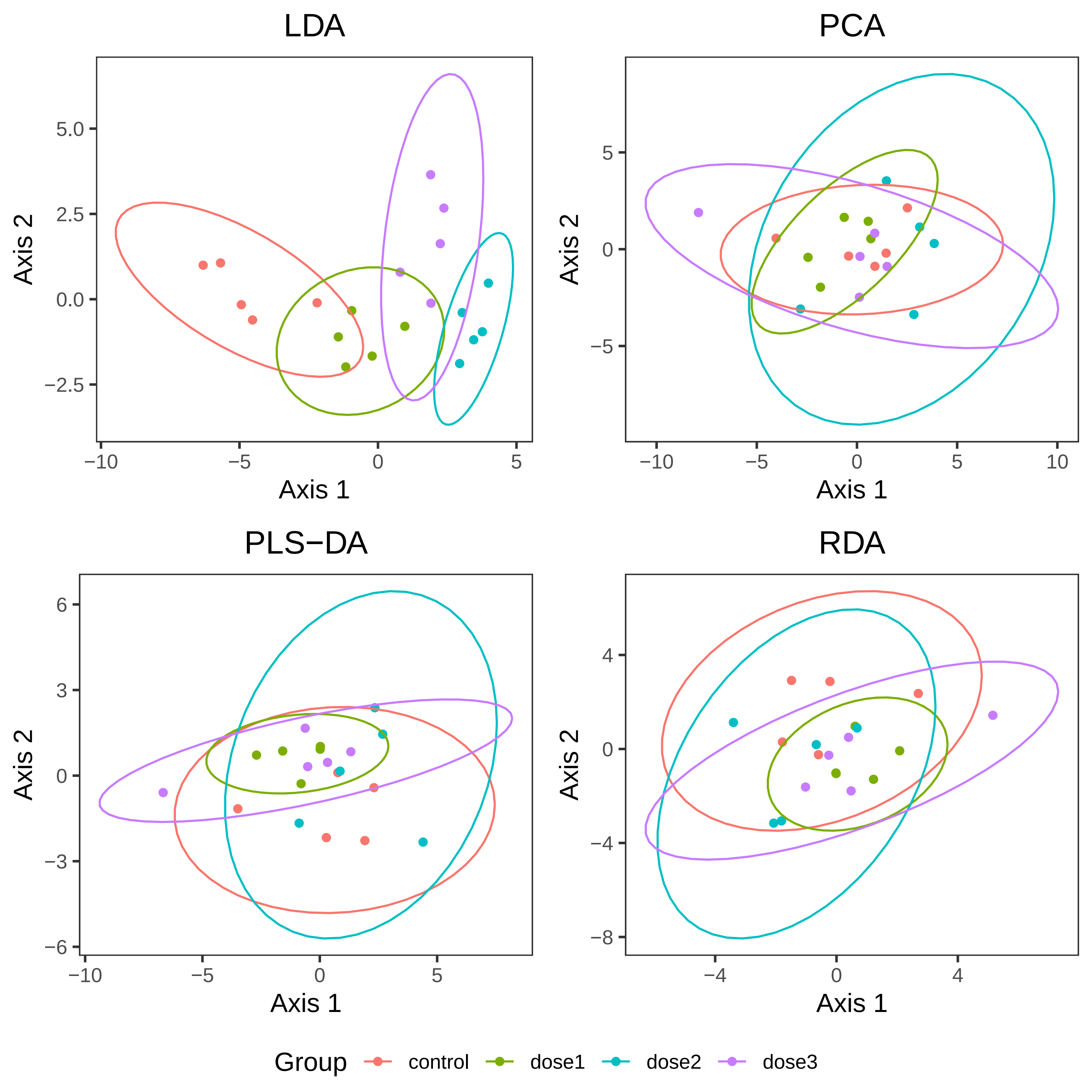
LDA
PCA
PLS−DA
RDA
Axis 1
Axis 2
Group
control
dose1
dose2
dose3

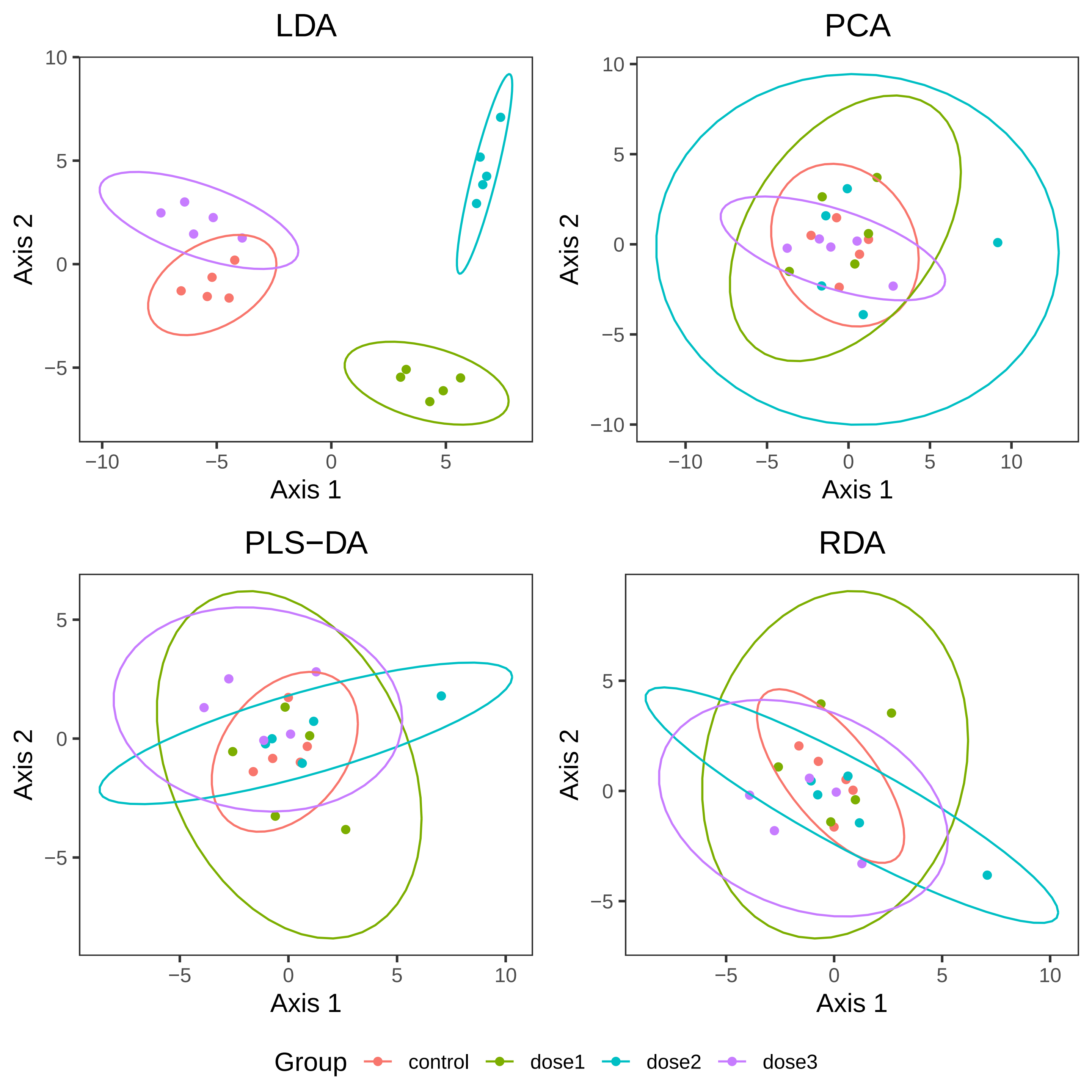
LDA
PCA
PLS−DA
RDA
Axis 1
Axis 2
Group
control
dose1
dose2
dose3

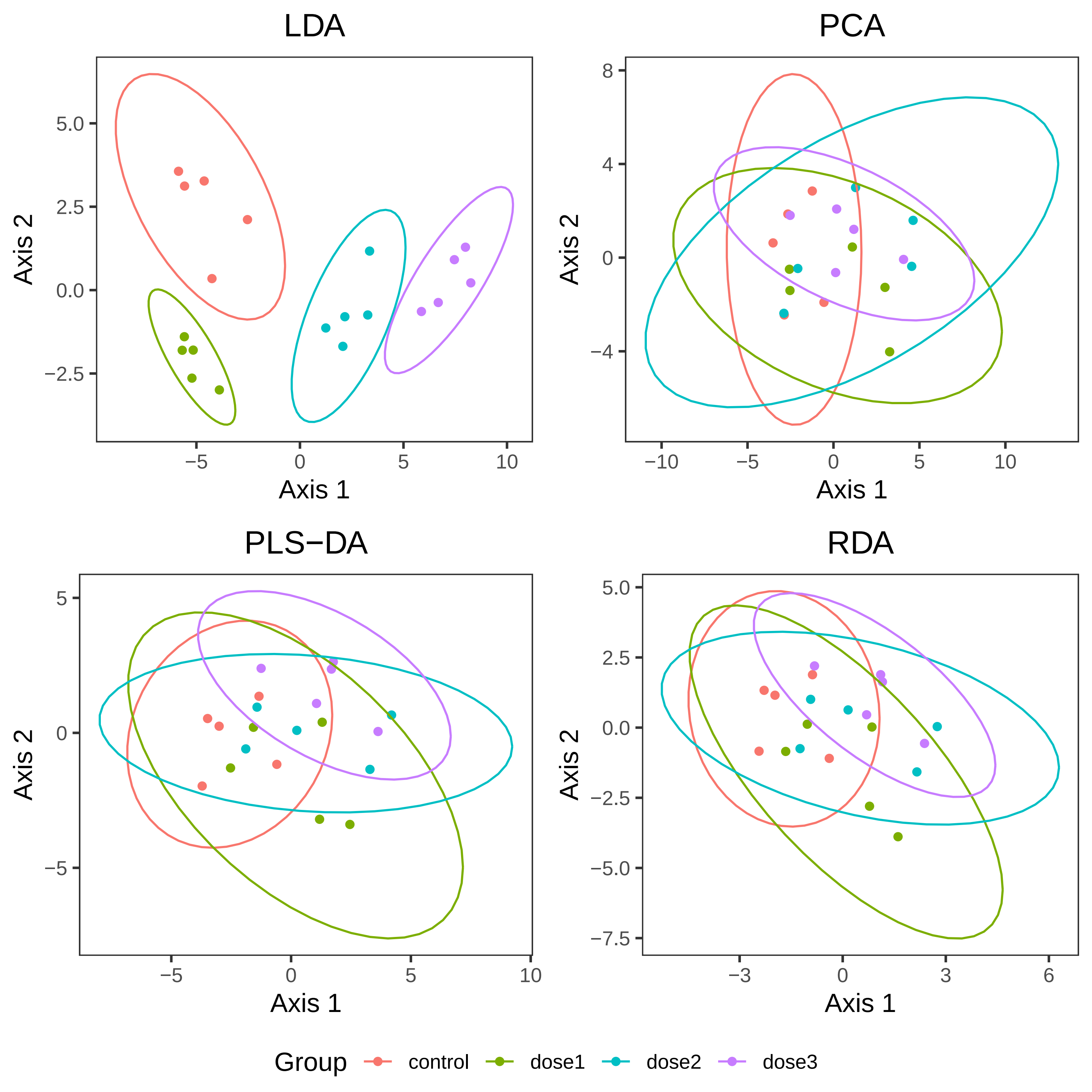

LDA
PCA
PLS−DA
RDA
Axis 1
Axis 2
5.0
2.5
0.0
−2.5
−5
0
5
10
8
4
−4
−10
−7.5
−5.0
−3
3
6
Group
control
dose1
dose2
dose3

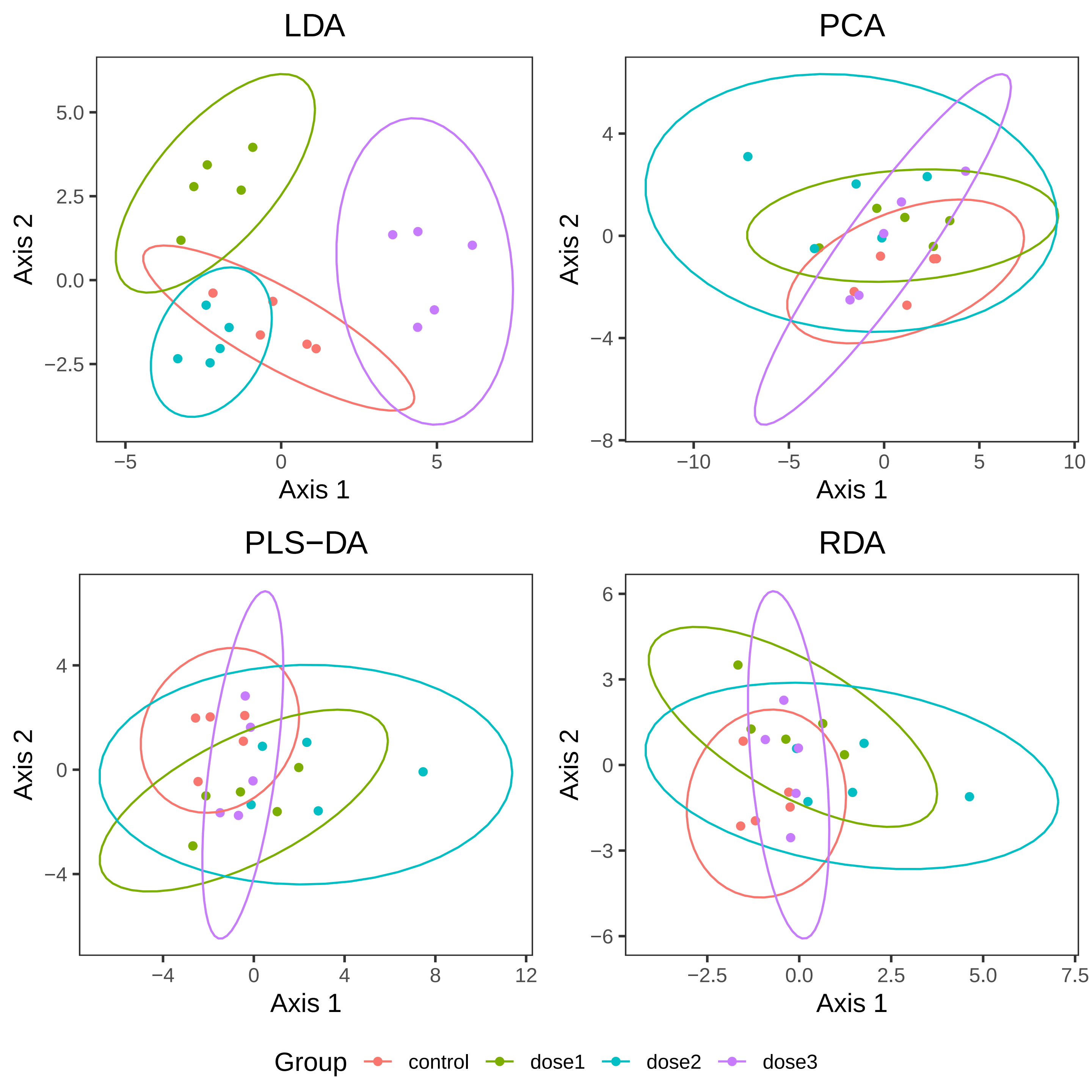

LDA
PCA
PLS−DA
RDA
Axis 1
Axis 2
Group
control
dose1
dose2
dose3

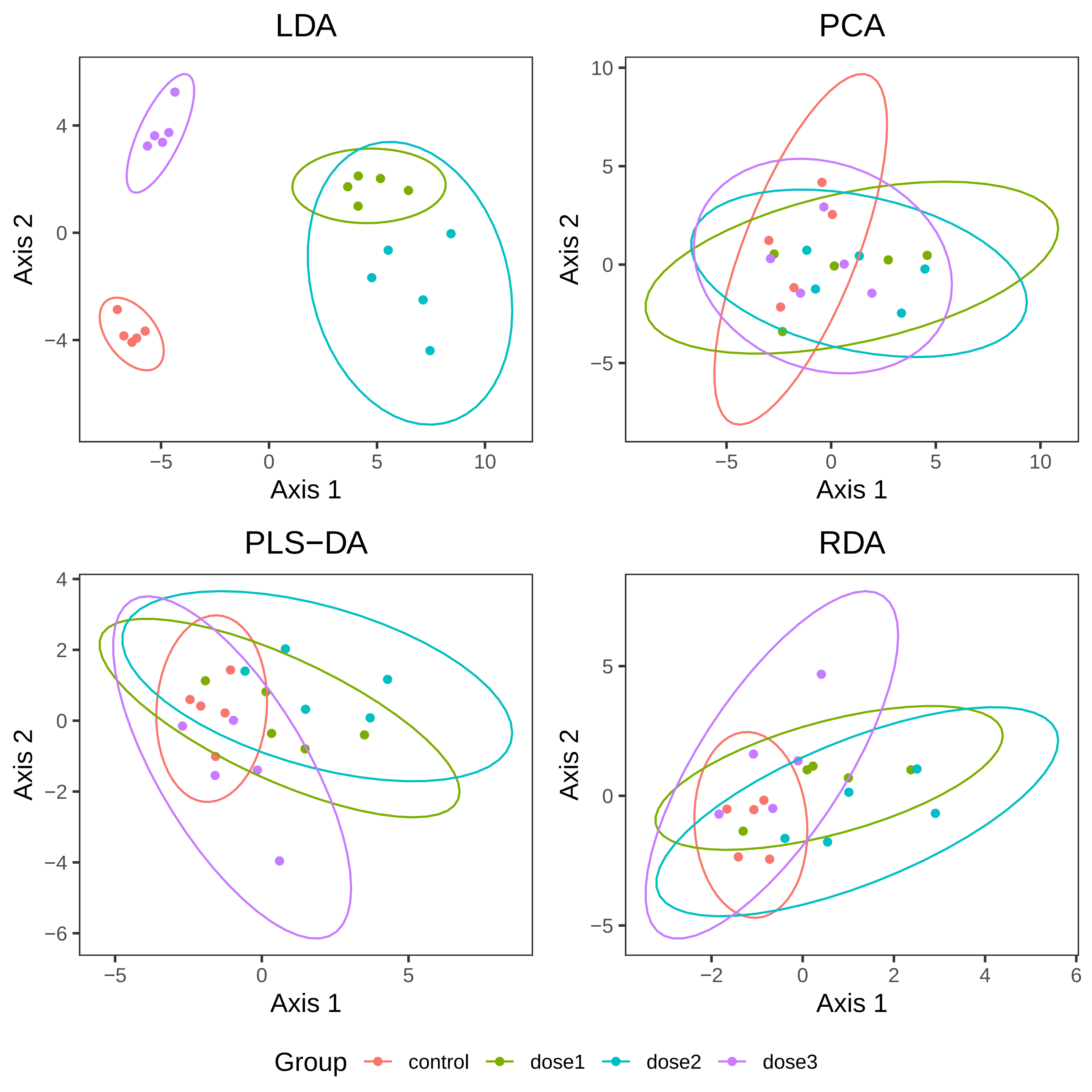
LDA
PCA
PLS−DA
RDA
Axis 1
Axis 2
Group
control
dose1
dose2
dose3

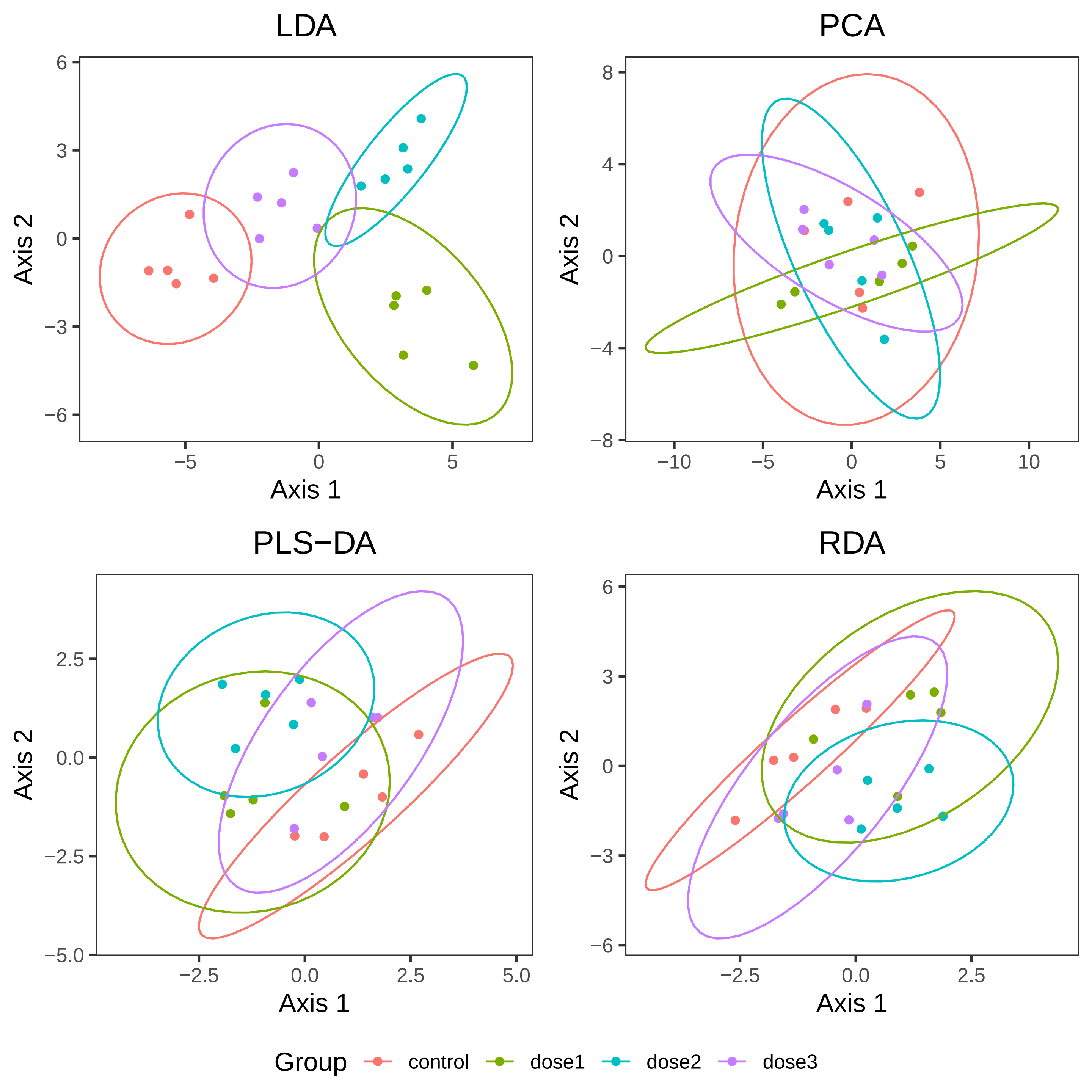
LDA
PCA
PLS−DA
RDA
Axis 1
Axis 2
6
3
0
−3
−6
−5
5
8
4
−4
−8
−10
10
2.5
0.0
−2.5
−5.0
5.0
Group
control
dose1
dose2
dose3

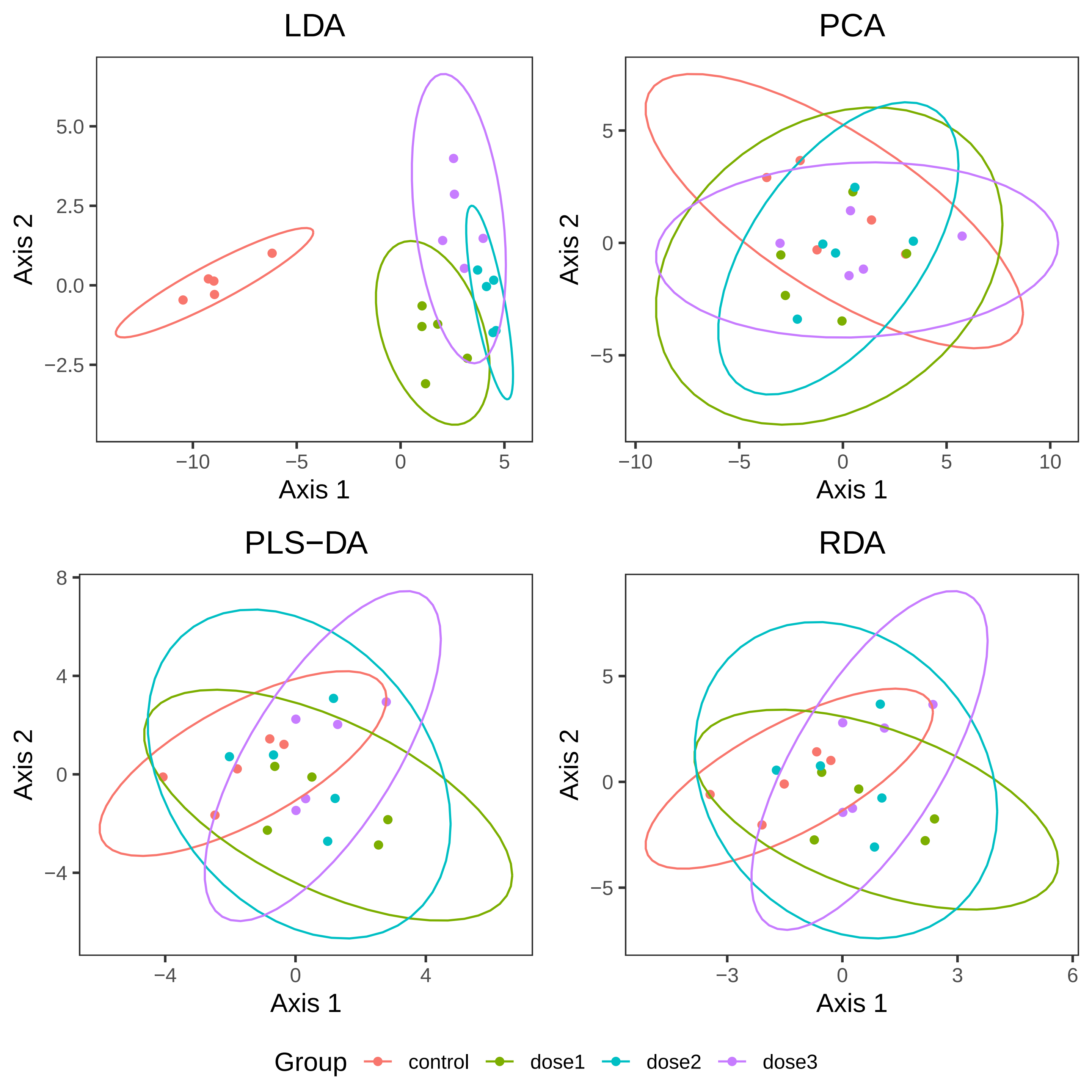

LDA
PCA
PLS−DA
RDA
Axis 1
Axis 2
−10
−5
0
5
10
5.0
2.5
0.0
−2.5
8
4
−4
−3
3
6
Group
control
dose1
dose2
dose3

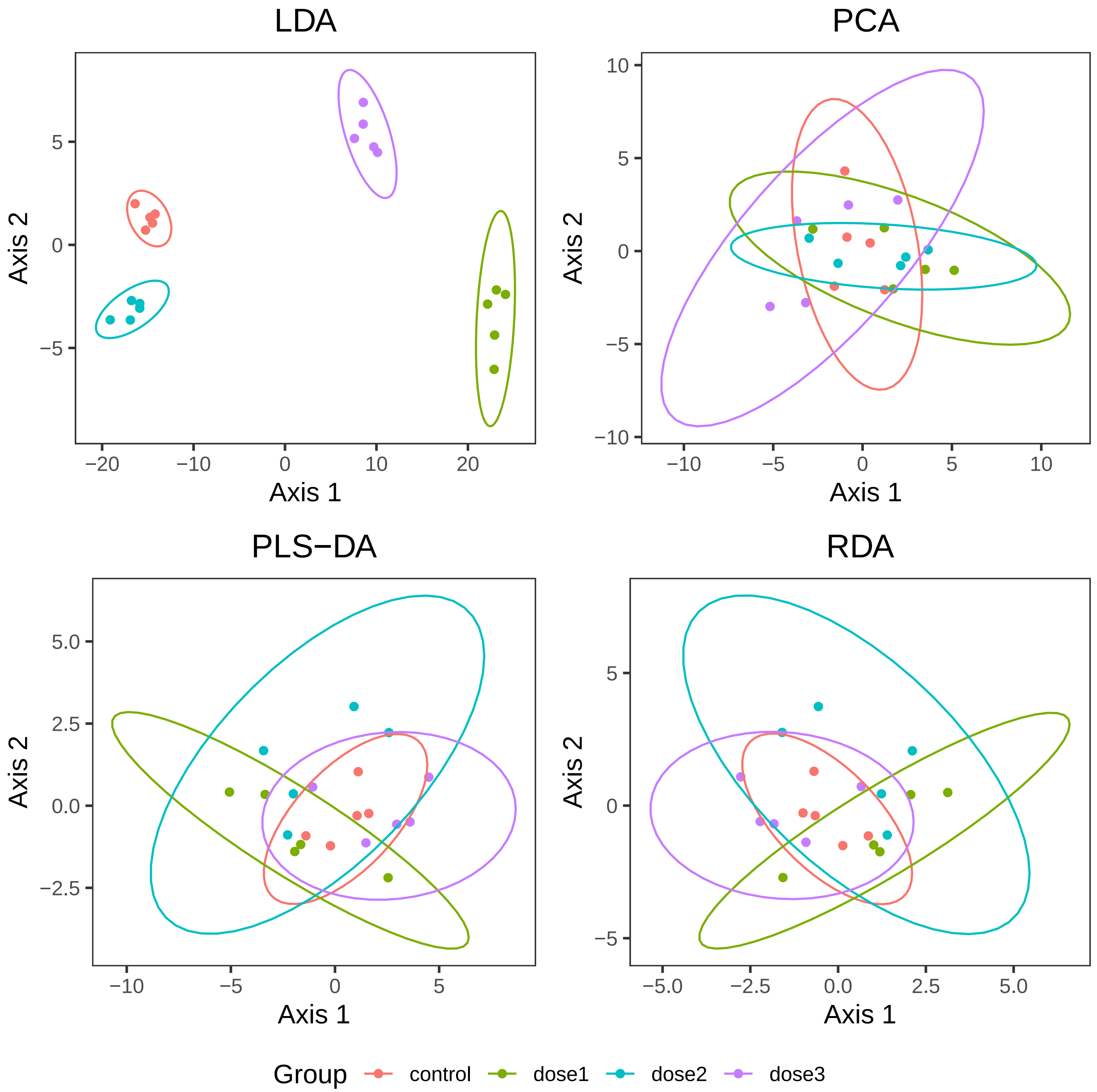

LDA
PCA
PLS−DA
RDA
Axis 1
Axis 2
−20
−10
0
10
20
5
0
−5
−10
−5
0
5
10
10
5
0
−5
−10
−10
−5
0
5
5.0
2.5
0.0
−2.5
−5.0
−2.5
0.0
2.5
5.0
5
0
−5
Group
control
dose1
dose2
dose3

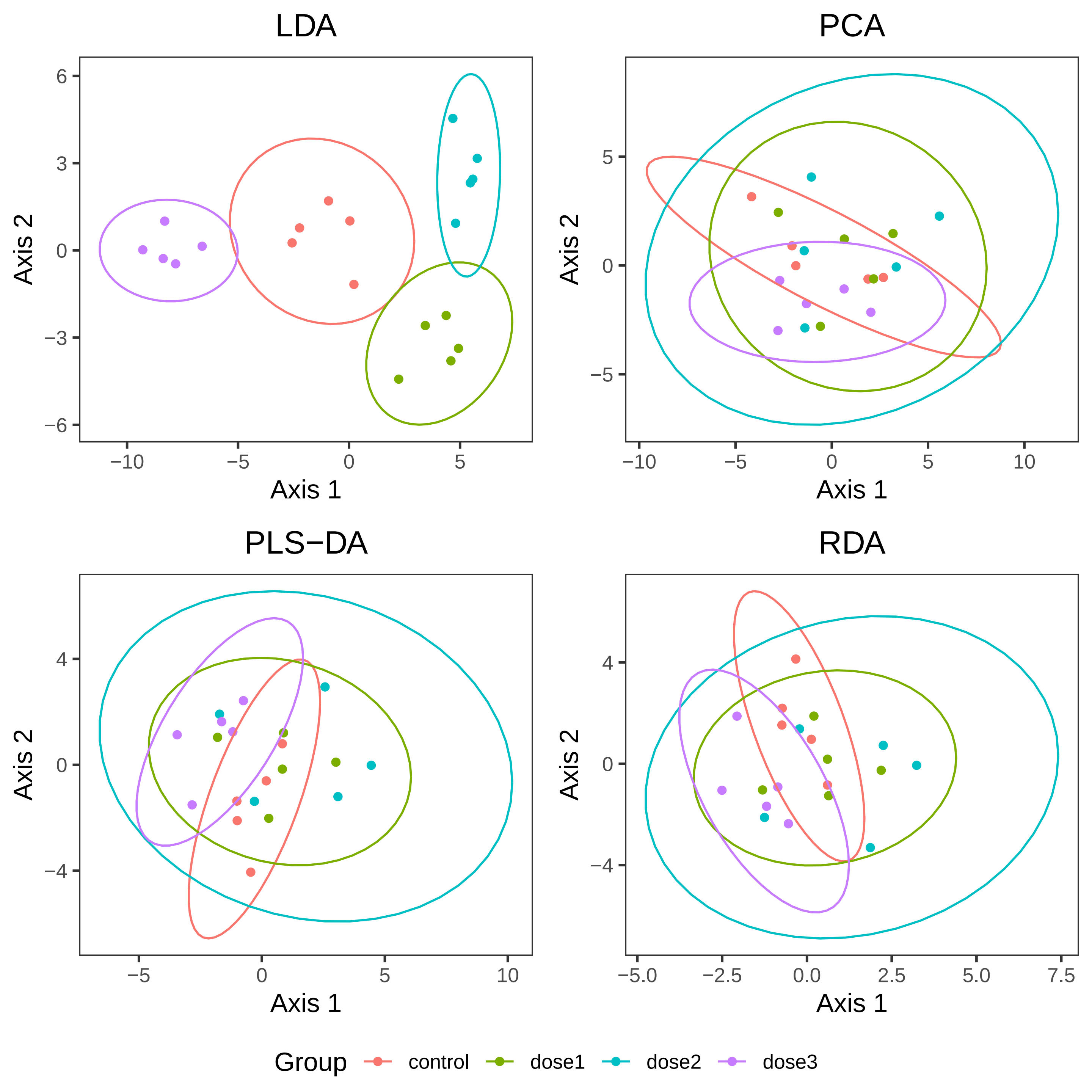
LDA
PCA
PLS−DA
RDA
Axis 1
Axis 2
6
3
0
−3
−6
−10
−5
5
10
4
−4
−5.0
−2.5
0.0
2.5
5.0
7.5
Group
control
dose1
dose2
dose3

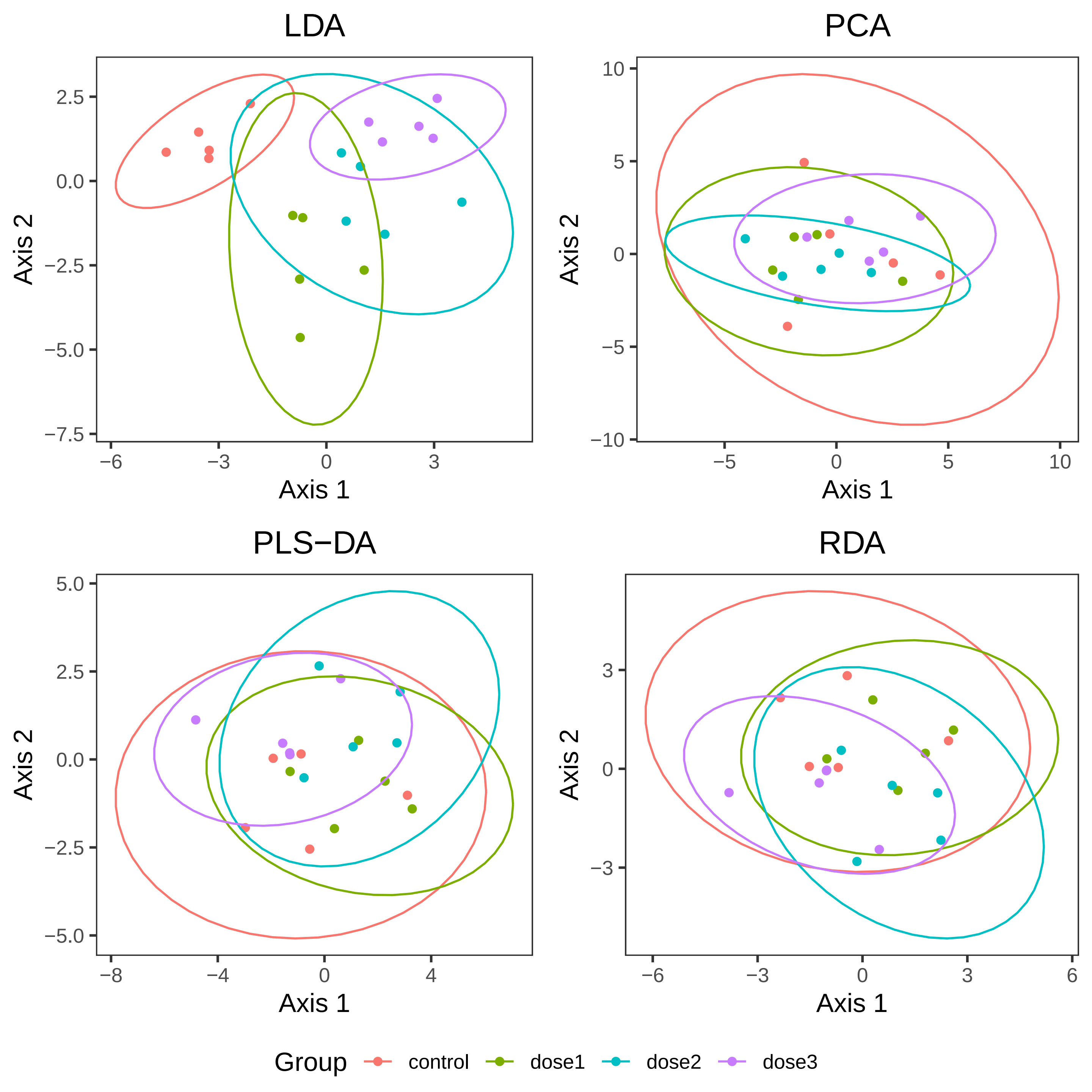
LDA
PCA
PLS−DA
RDA
Axis 1
Axis 2
Group
control
dose1
dose2
dose3

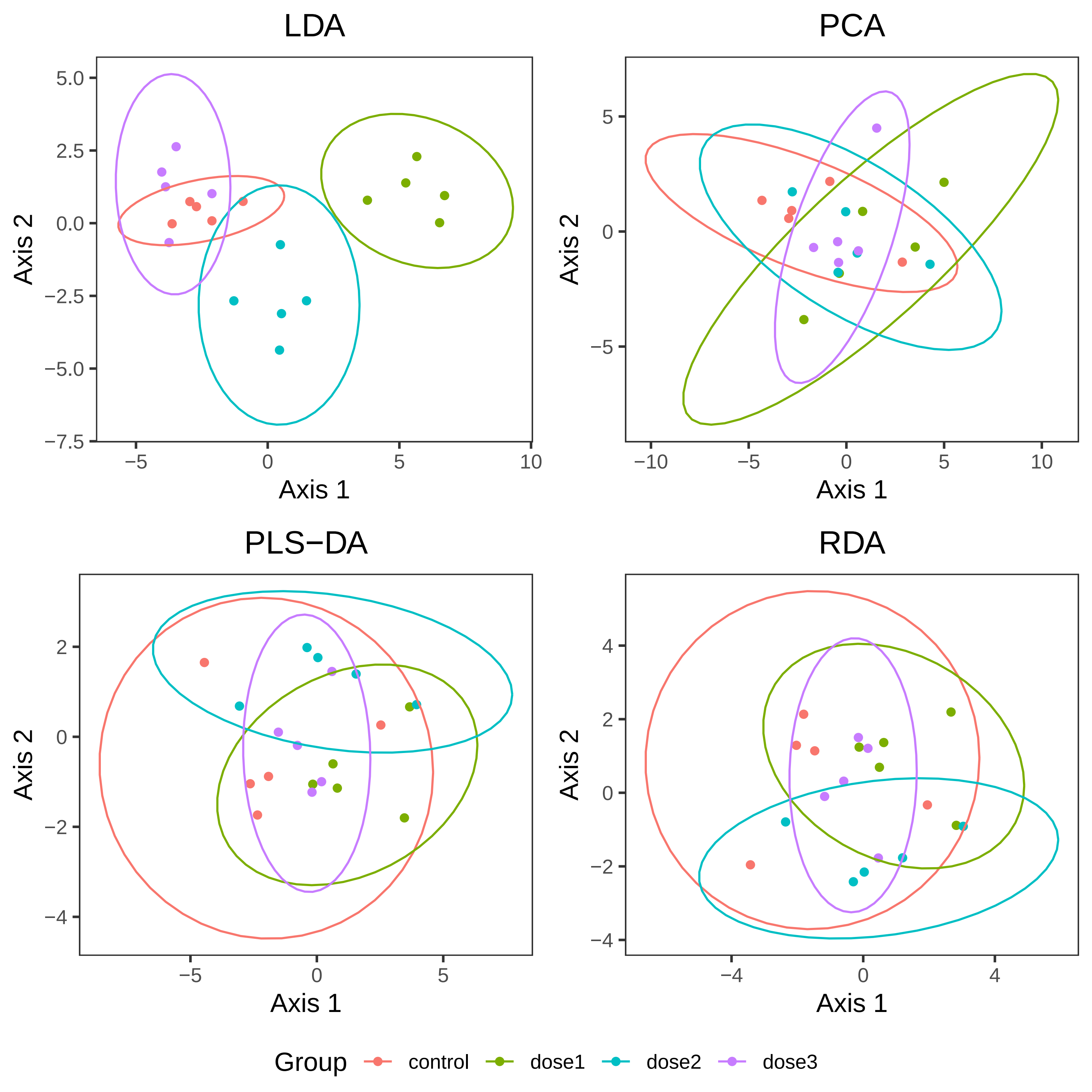

LDA
PCA
PLS−DA
RDA
Axis 1
Axis 2
5.0
2.5
0.0
−2.5
−5.0
−7.5
−5
0
5
10
−10
−4
−2
2
4
Group
control
dose1
dose2
dose3

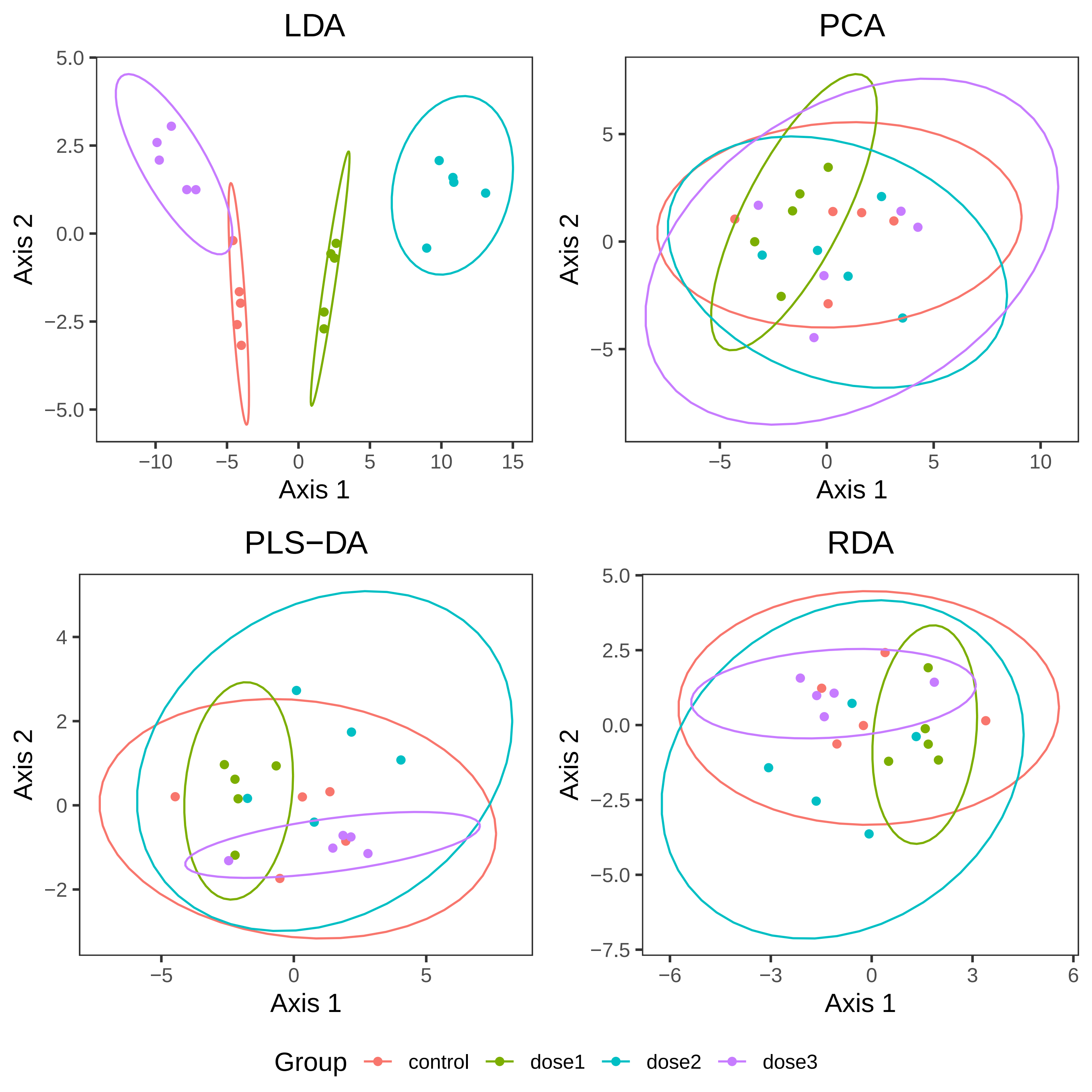

LDA
PCA
PLS−DA
RDA
Axis 1
Axis 2
5.0
2.5
0.0
−2.5
−5.0
−7.5
−10
−5
0
5
10
15
−6
−3
3
6
2
4
−2
Group
control
dose1
dose2
dose3

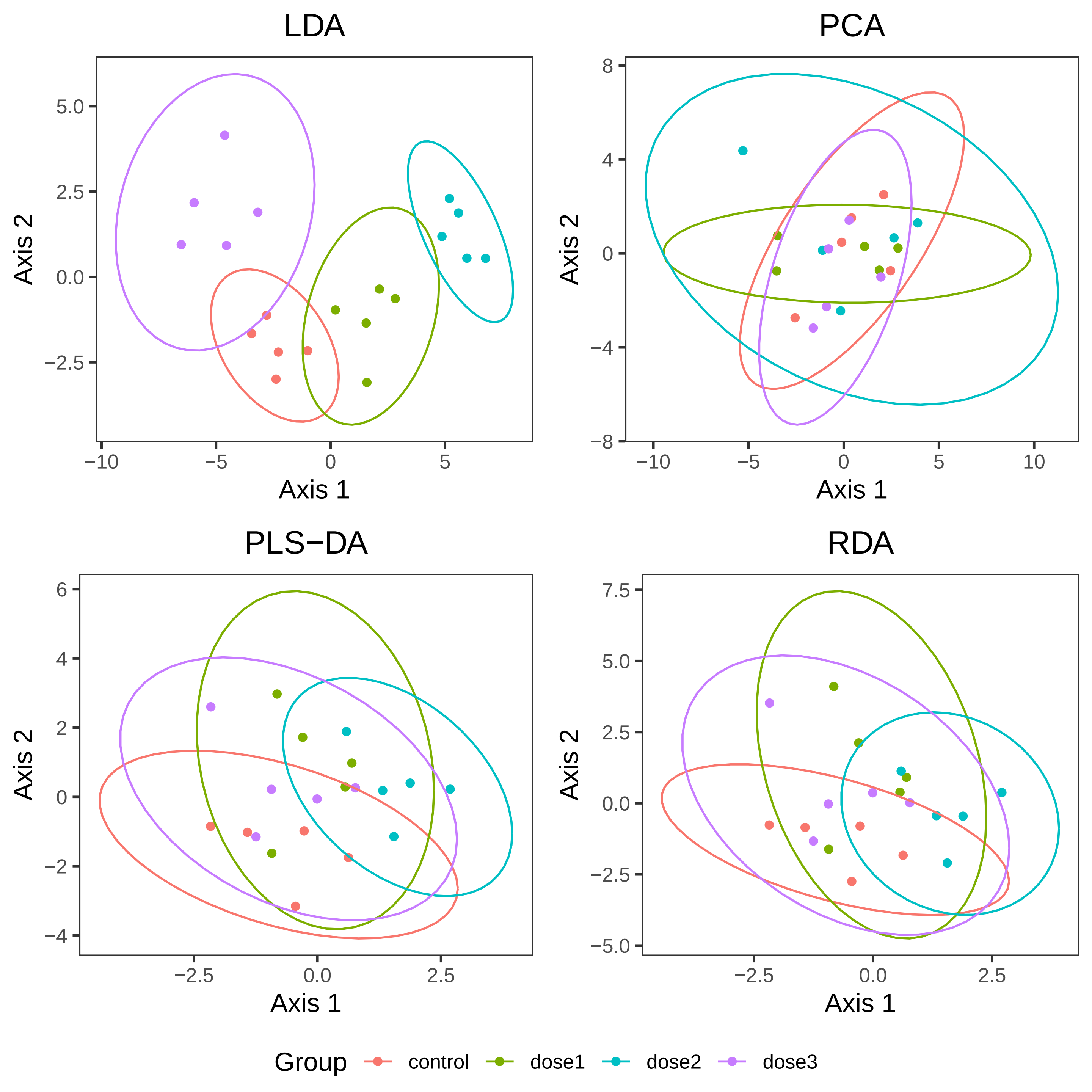

LDA
PCA
PLS−DA
RDA
Axis 1
Axis 2
Group
control
dose1
dose2
dose3

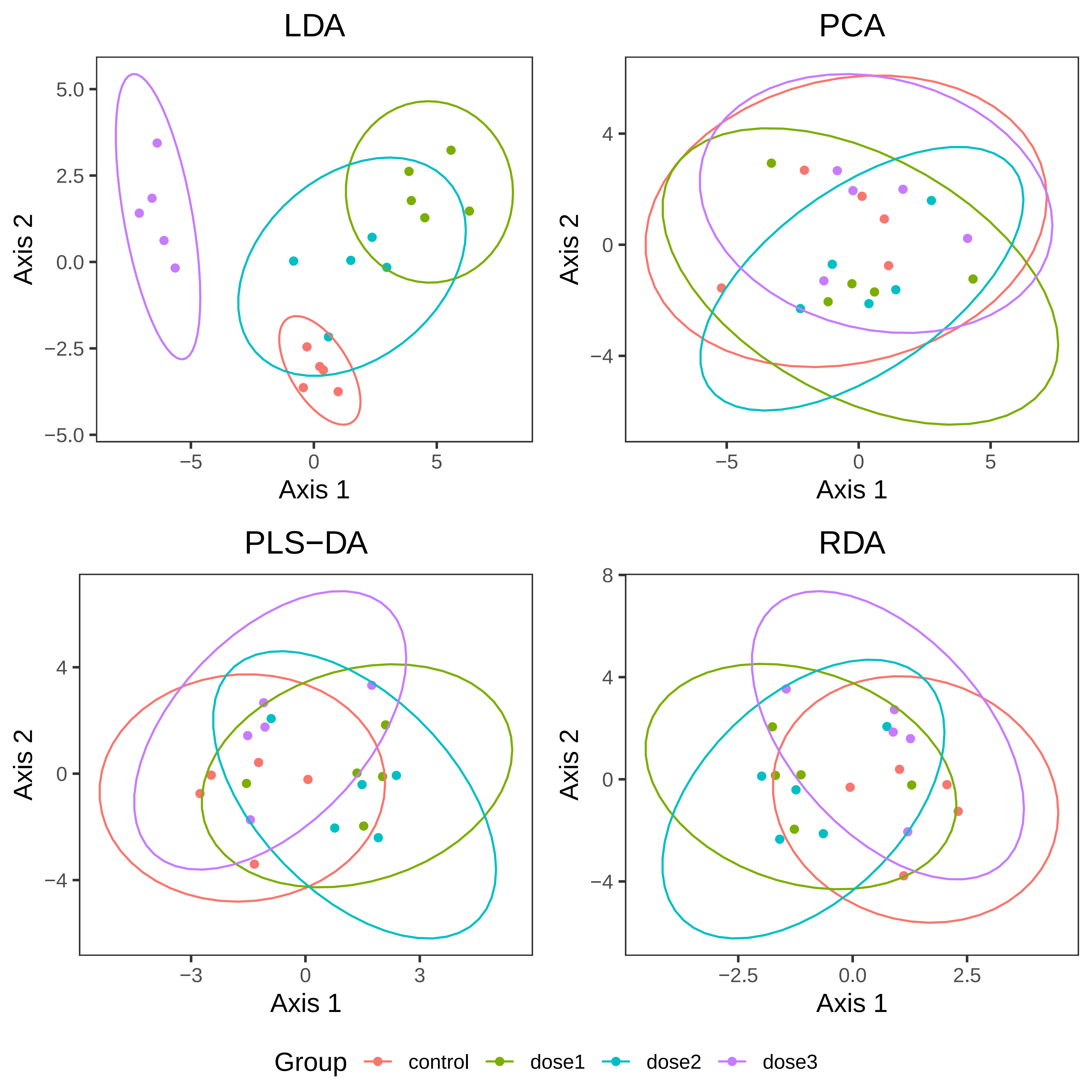

LDA
PCA
PLS−DA
RDA
Axis 1
Axis 2
5.0
2.5
0.0
−2.5
−5.0
−5
0
5
4
−4
−3
3
8
−2.5
0.0
2.5
Group
control
dose1
dose2
dose3

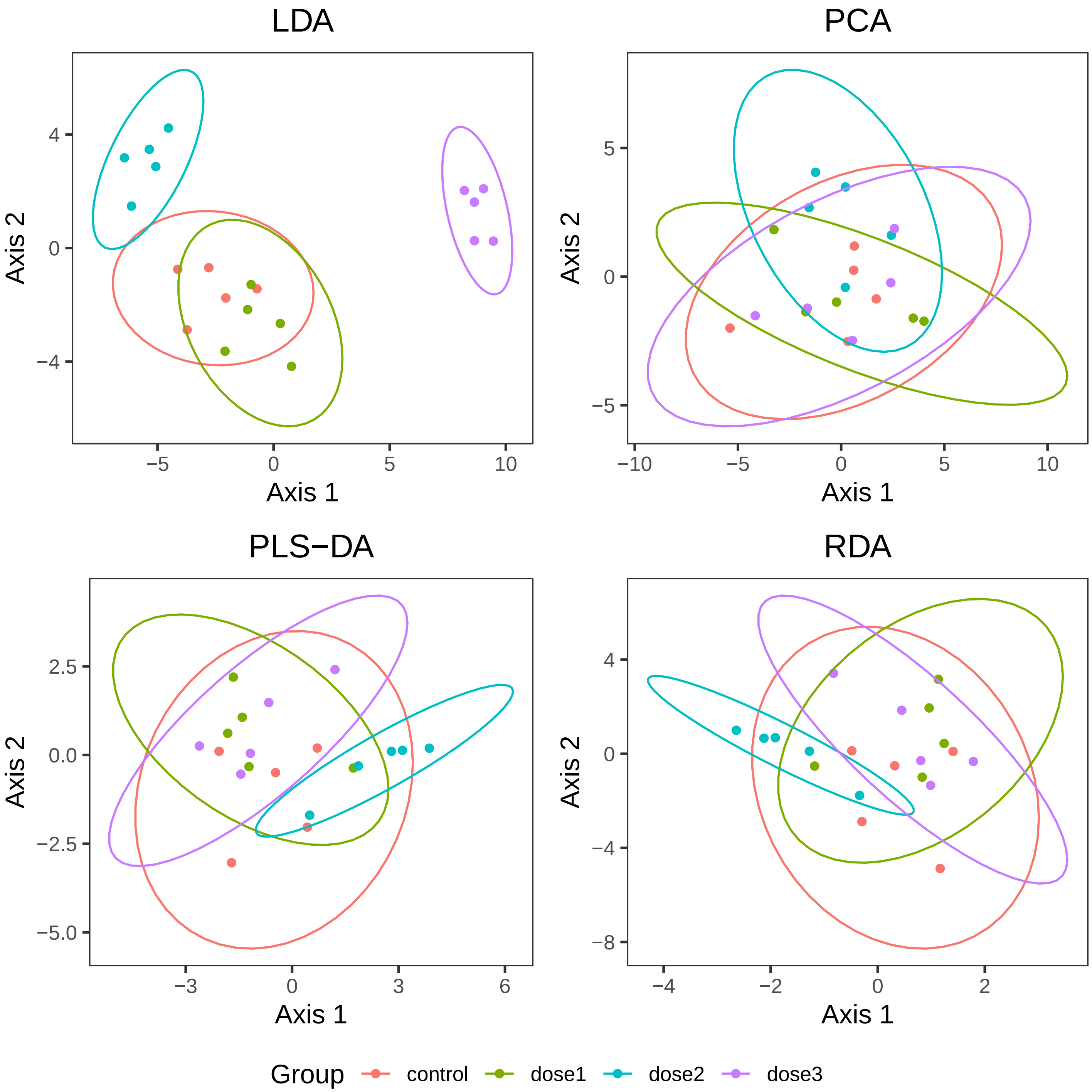

LDA
PCA
PLS−DA
RDA
Axis 1
Axis 2
Group
control
dose1
dose2
dose3

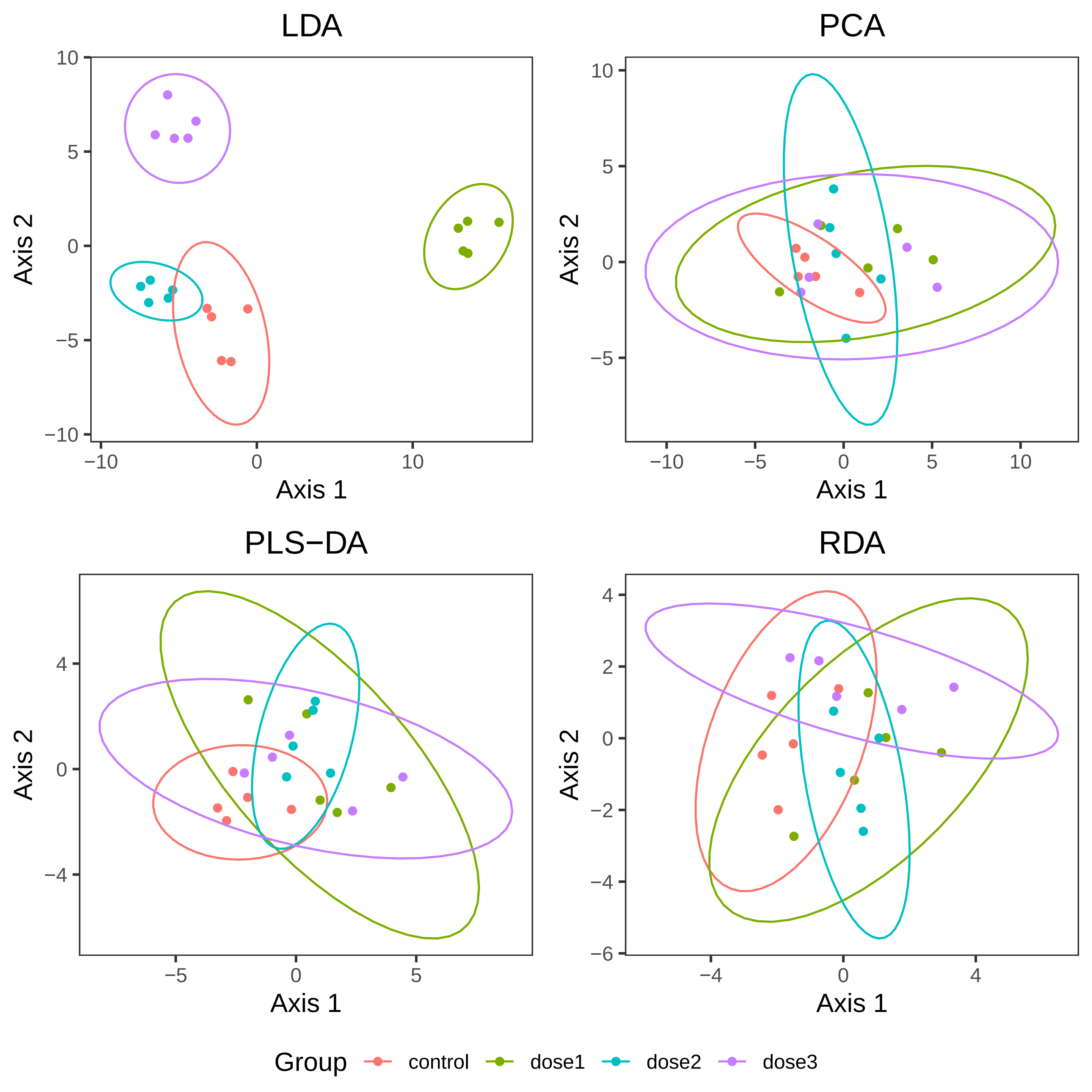

LDA
PCA
PLS−DA
RDA
Axis 1
Axis 2
10
5
0
−5
−10
−10
0
10
−5
Group
control
dose1
dose2
dose3

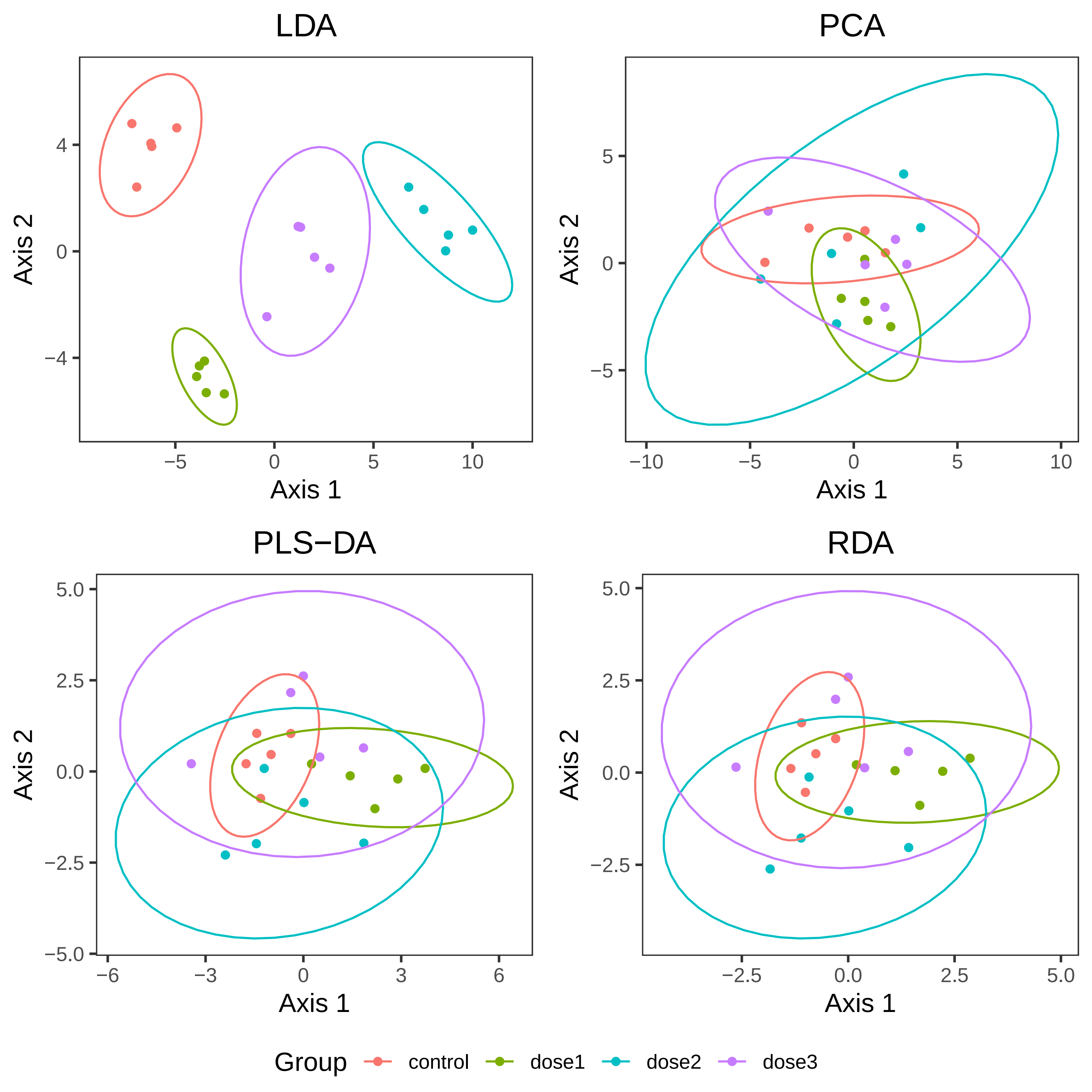

LDA
PCA
PLS−DA
RDA
Axis 1
Axis 2
Group
control
dose1
dose2
dose3

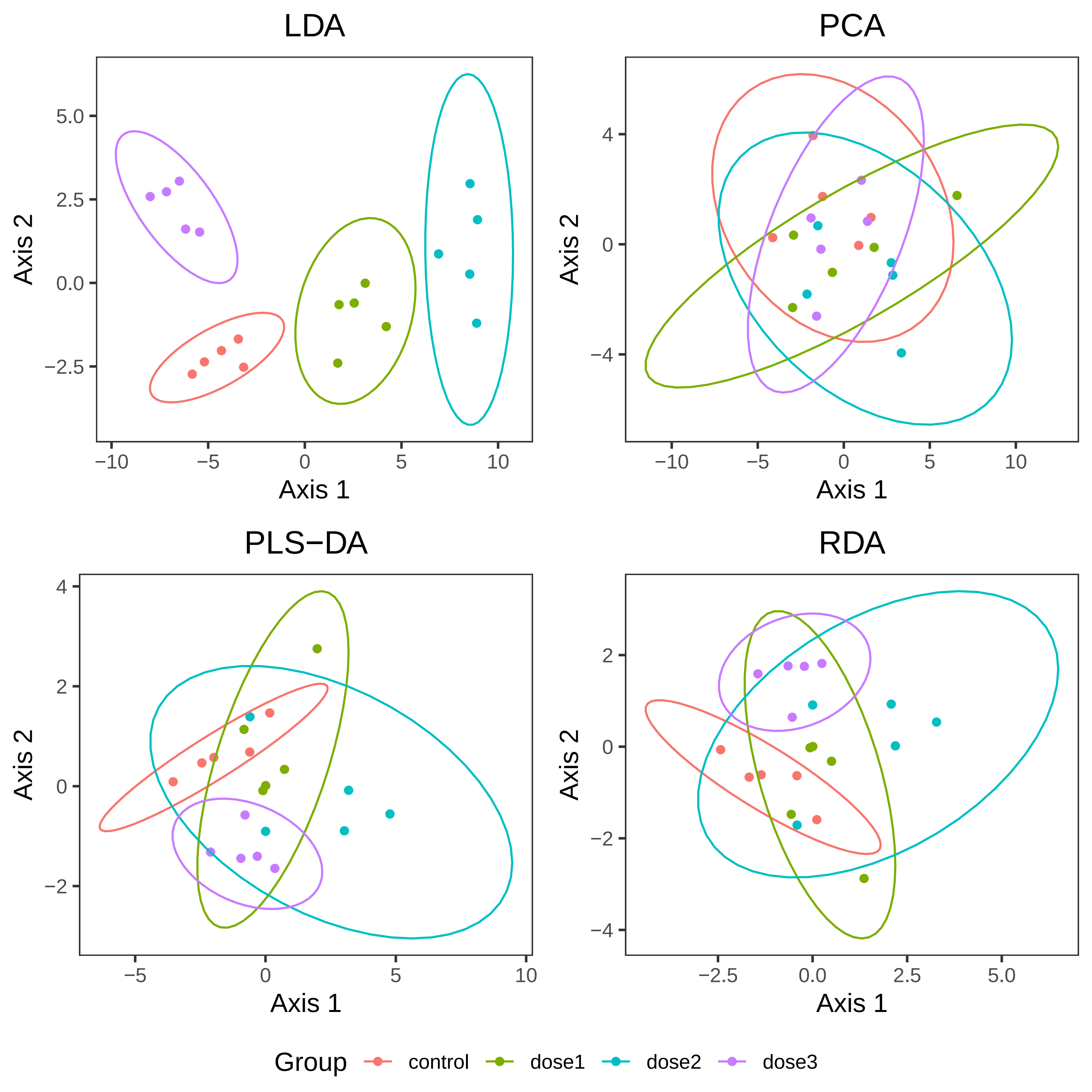

LDA
PCA
PLS−DA
RDA
Axis 1
Axis 2
Group
control
dose1
dose2
dose3

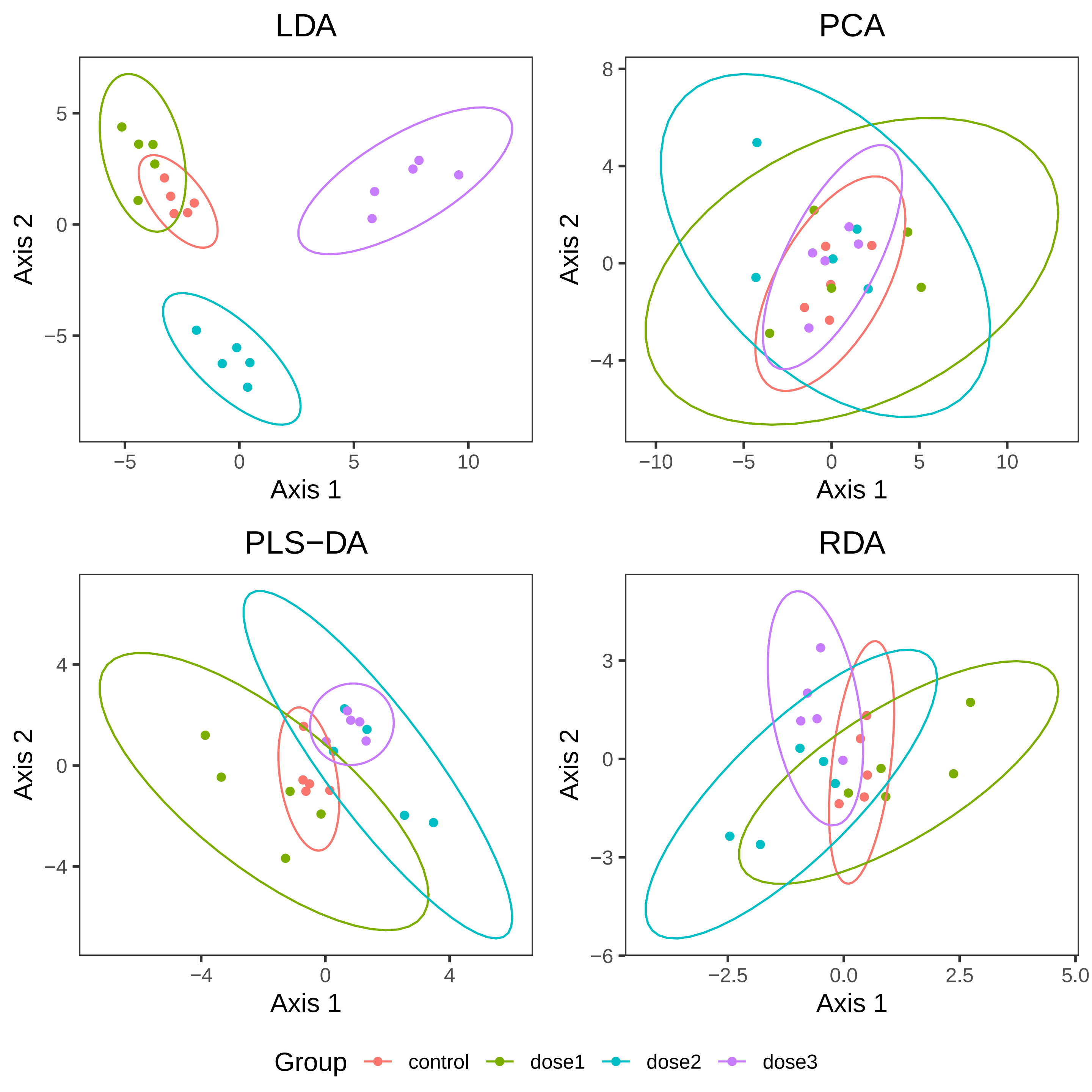
LDA
PCA
PLS−DA
RDA
Axis 1
Axis 2
Group
control
dose1
dose2
dose3

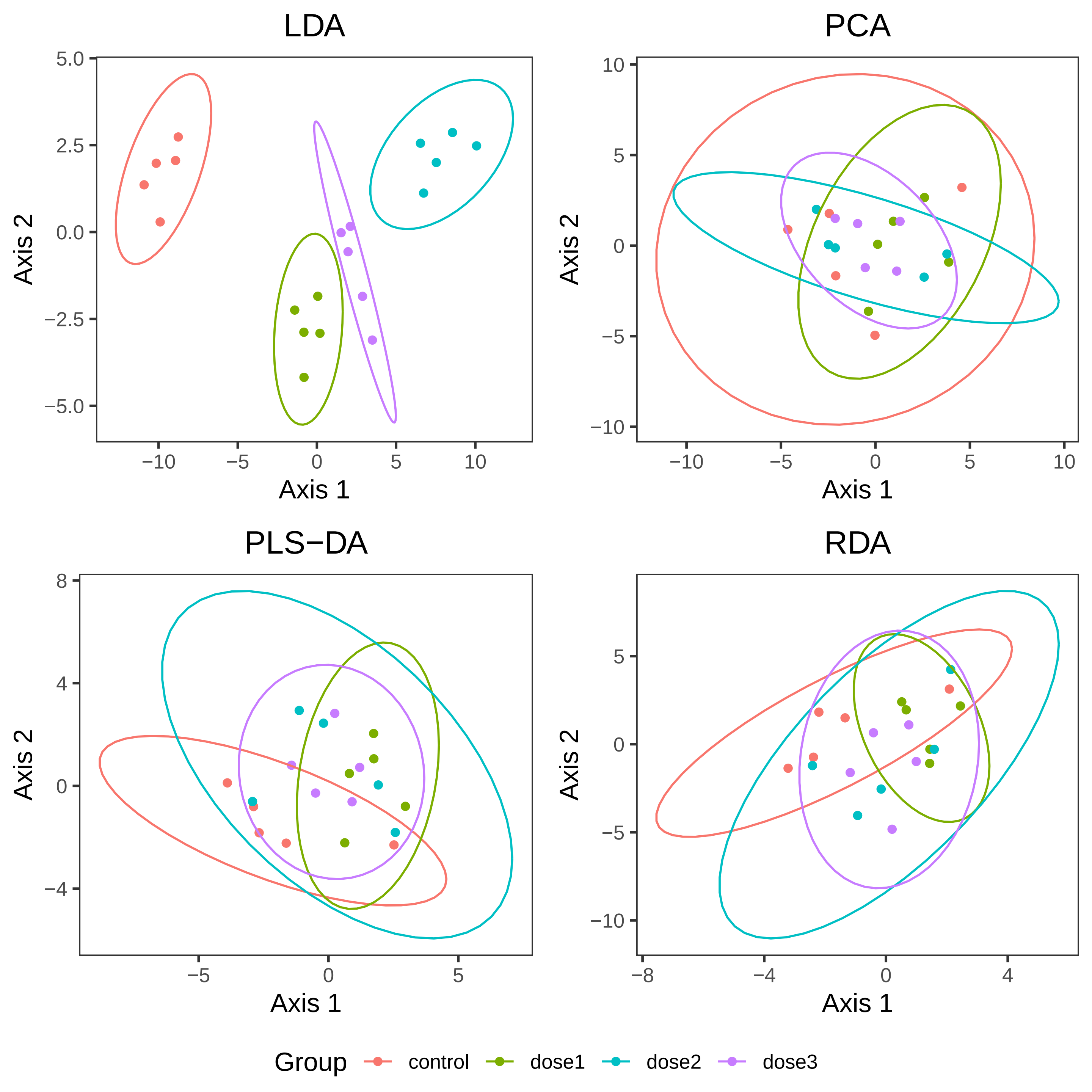
LDA
PCA
PLS−DA
RDA
Axis 1
Axis 2
Group
control
dose1
dose2
dose3

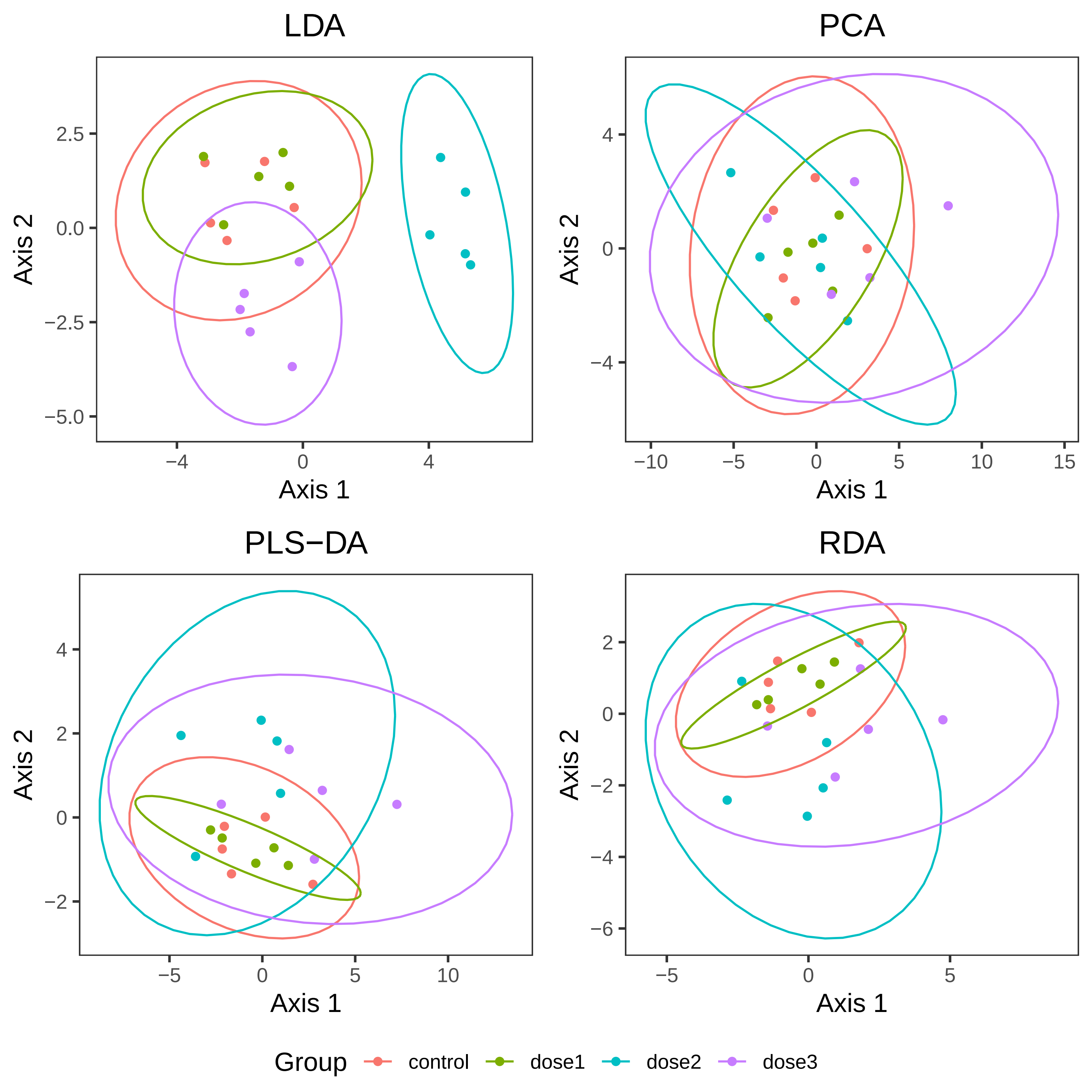
LDA
PCA
PLS−DA
RDA
Axis 1
Axis 2
Group
control
dose1
dose2
dose3

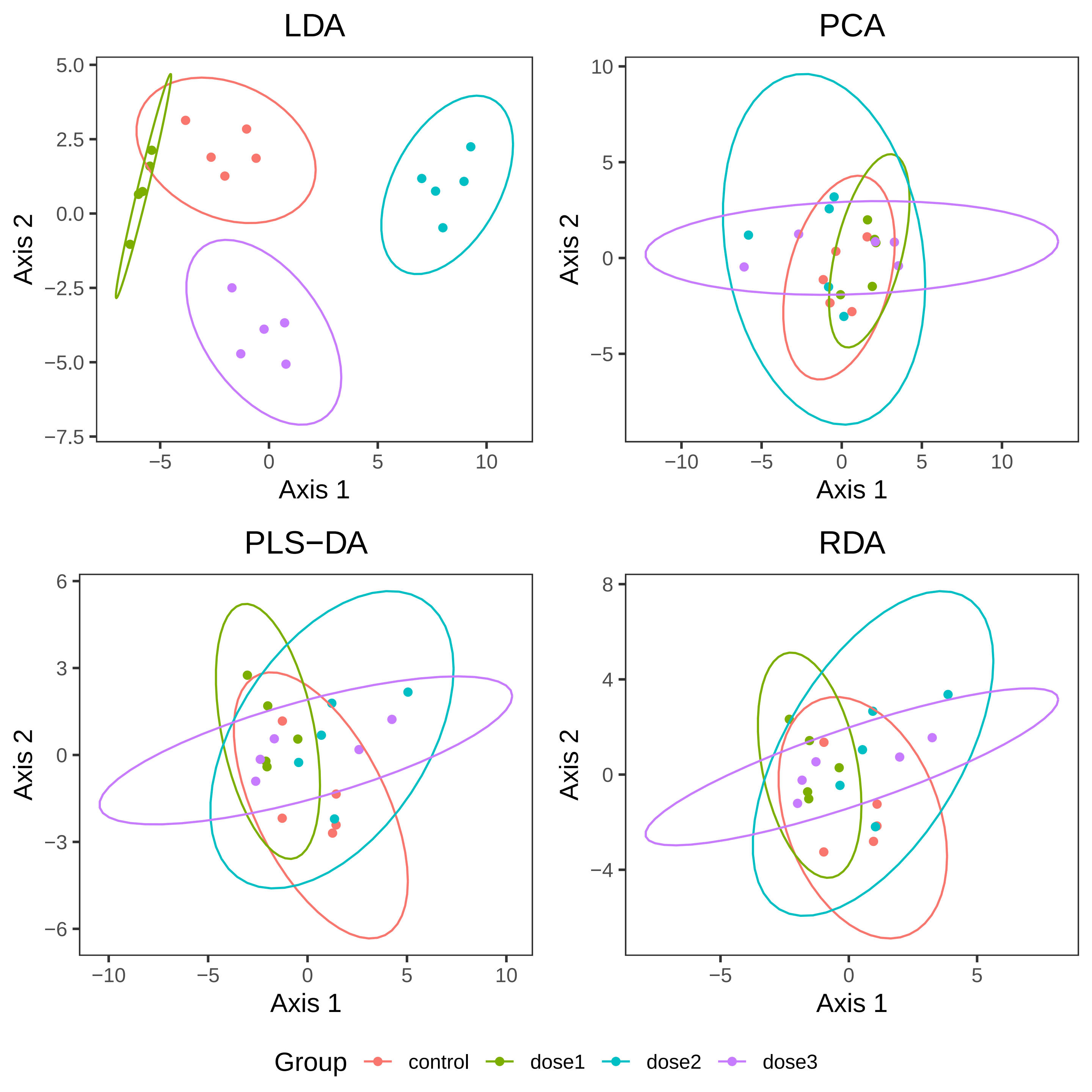

LDA
PCA
PLS−DA
RDA
Axis 1
Axis 2
5.0
2.5
0.0
−2.5
−5.0
−7.5
−5
0
5
10
−10
−6
−3
3
6
8
4
−4
Group
control
dose1
dose2
dose3

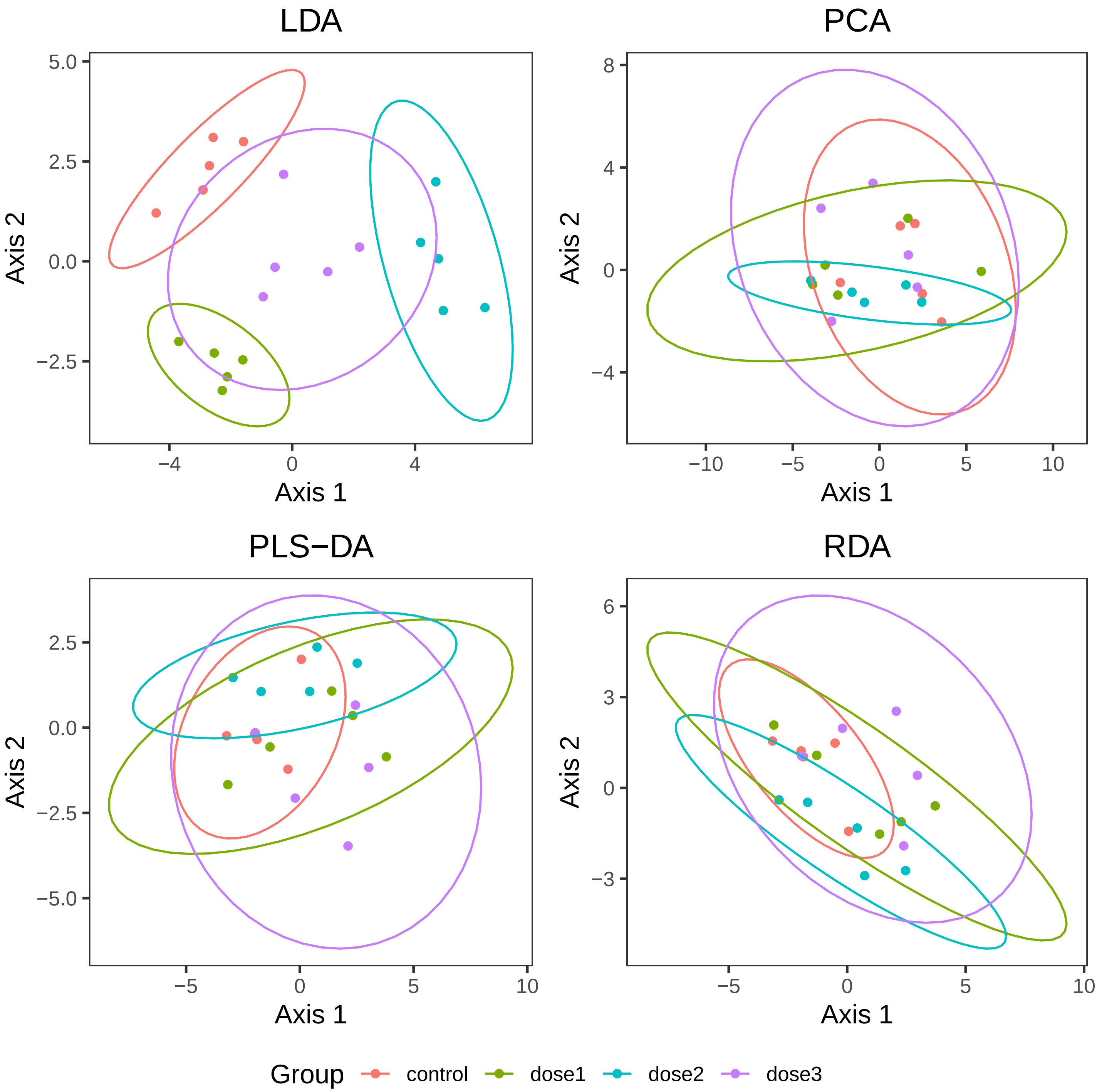
LDA
PCA
PLS−DA
RDA
Axis 1
Axis 2
5.0
2.5
0.0
−2.5
−4
0
4
8
−10
−5
5
10
−5.0
6
3
−3
Group
control
dose1
dose2
dose3

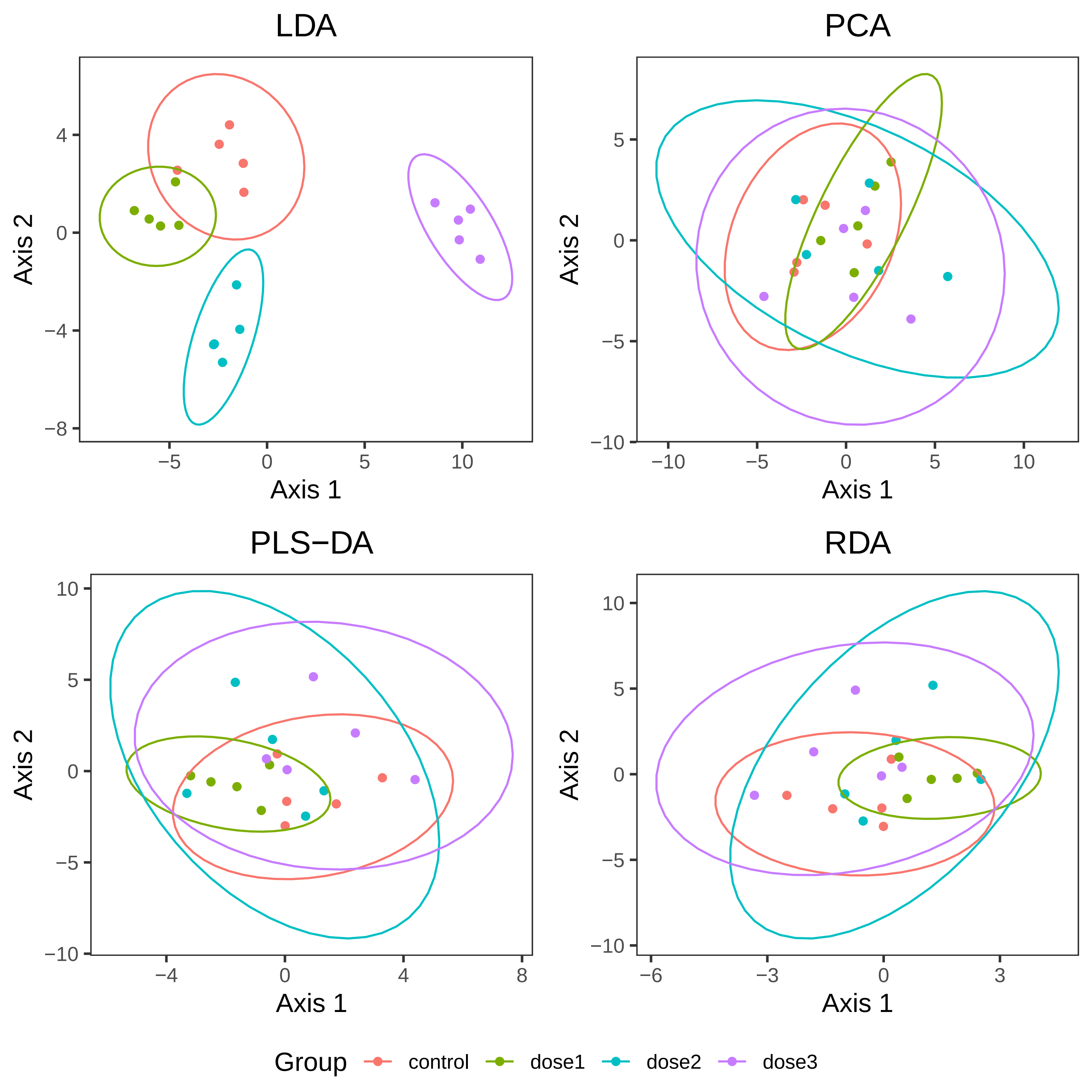
LDA
PCA
PLS−DA
RDA
Axis 1
Axis 2
Group
control
dose1
dose2
dose3

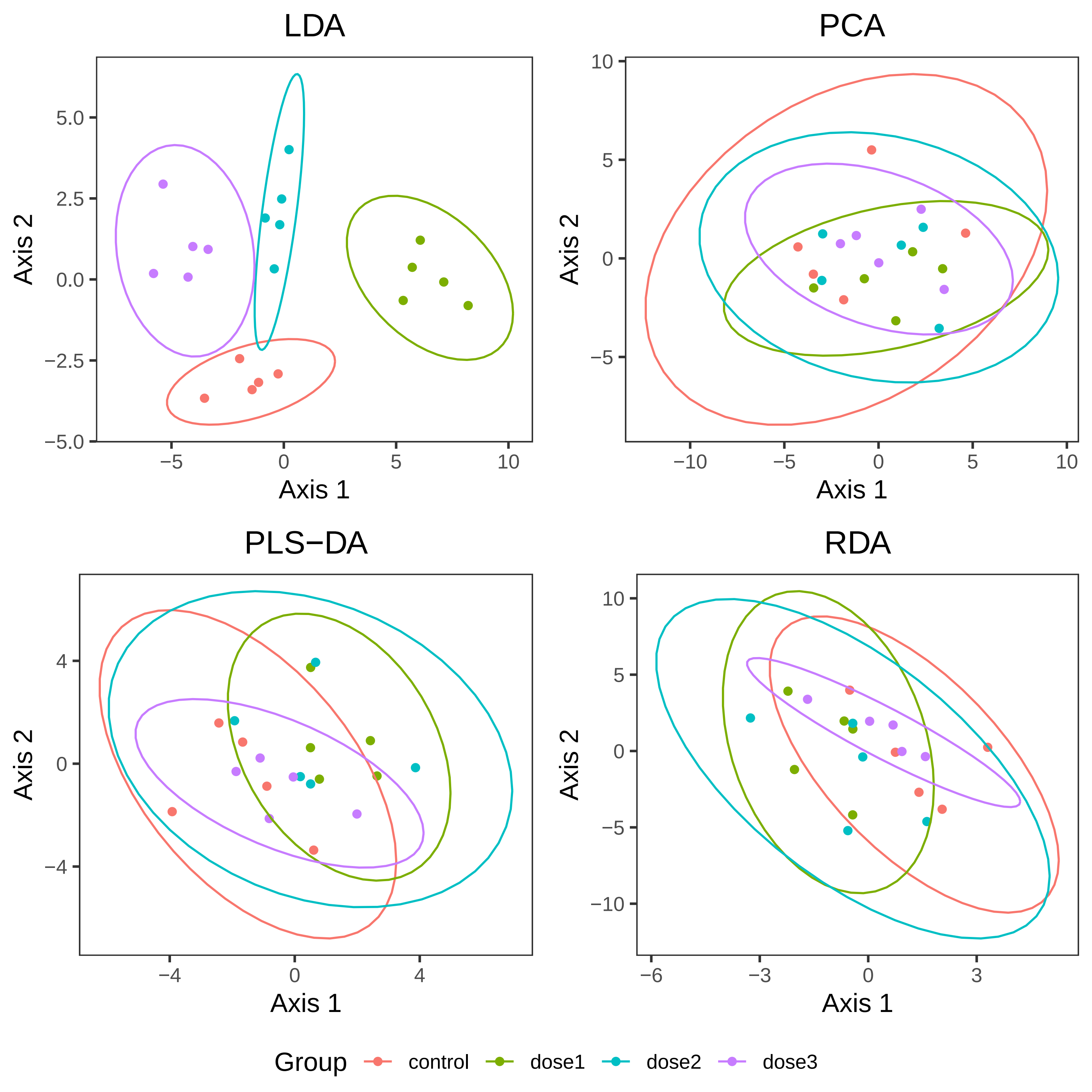

LDA
PCA
PLS−DA
RDA
Axis 1
Axis 2
Group
control
dose1
dose2
dose3

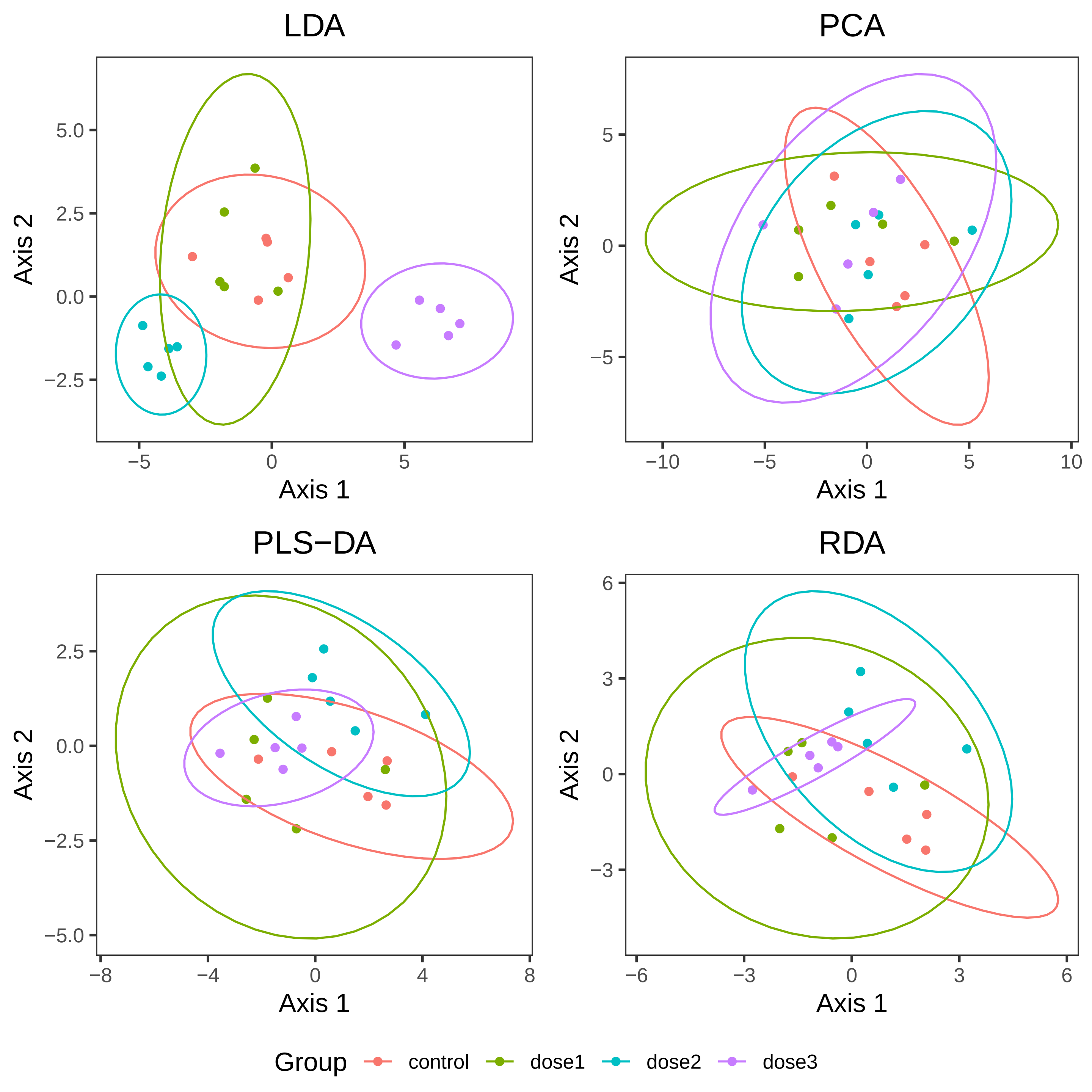
LDA
PCA
PLS−DA
RDA
Axis 1
Axis 2
Group
control
dose1
dose2
dose3

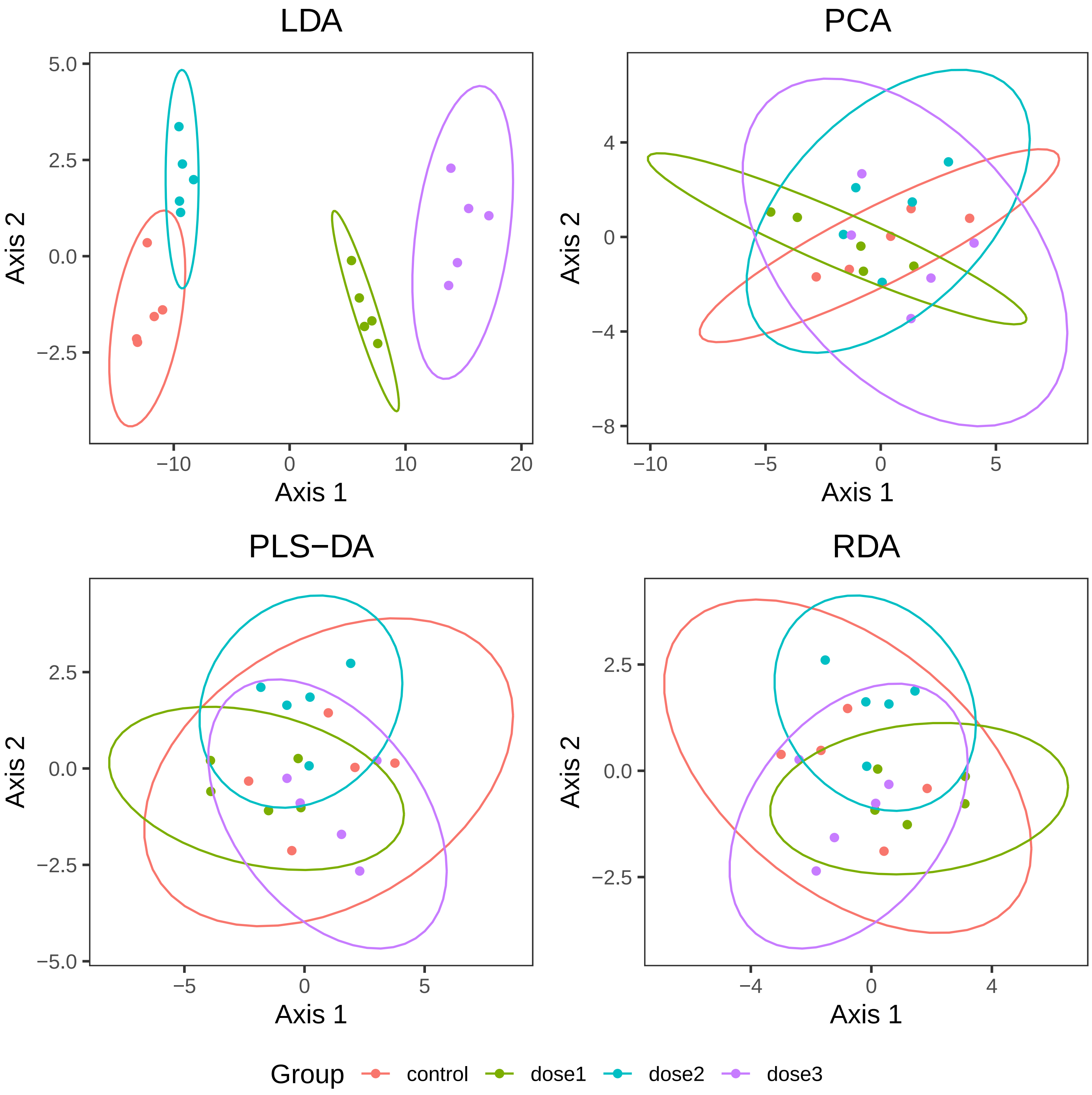

LDA
PCA
PLS−DA
RDA
Axis 1
Axis 2
5.0
2.5
0.0
−2.5
−5.0
−10
0
10
20
4
−4
−8
−5
5
Group
control
dose1
dose2
dose3

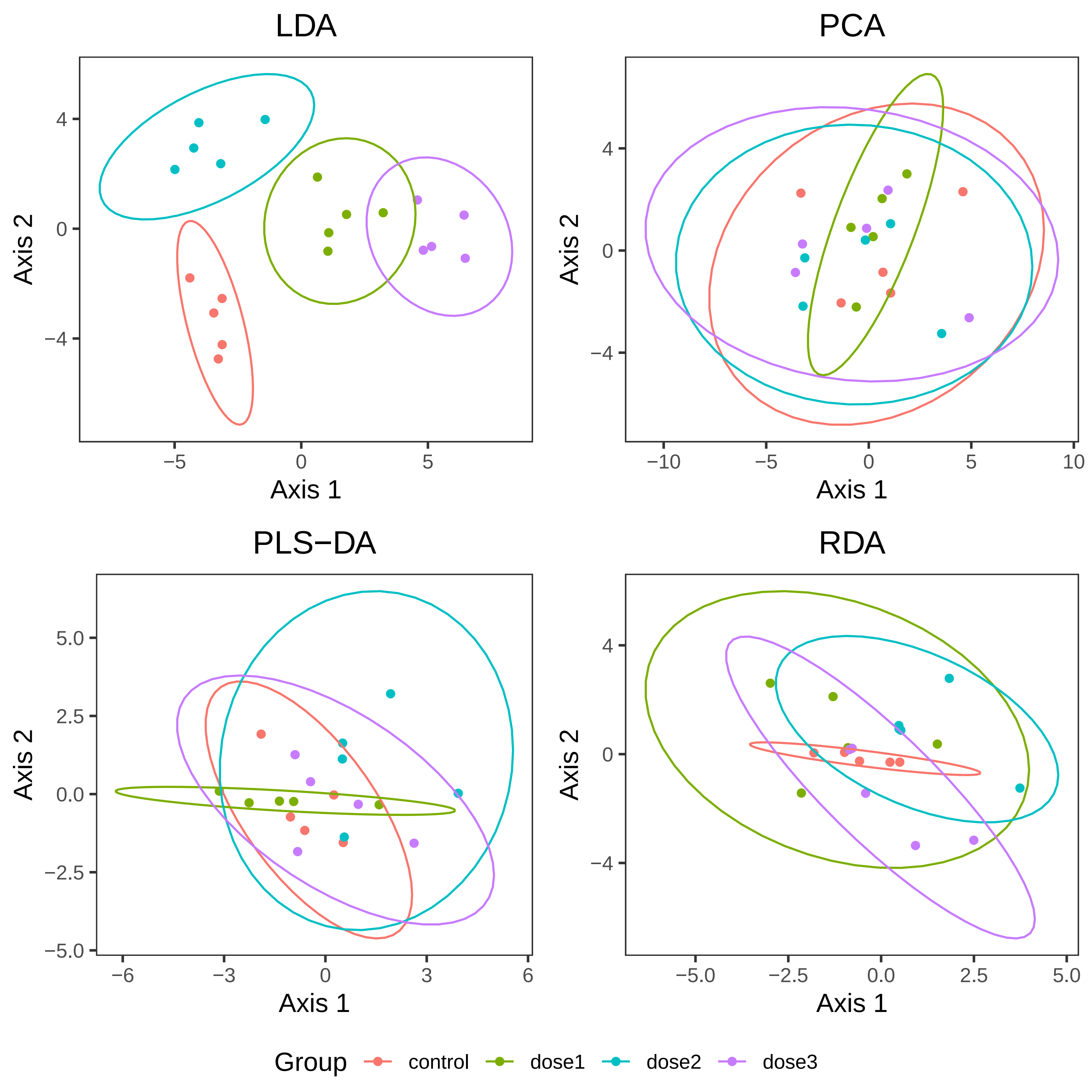

LDA
PCA
PLS−DA
RDA
Axis 1
Axis 2
Group
control
dose1
dose2
dose3

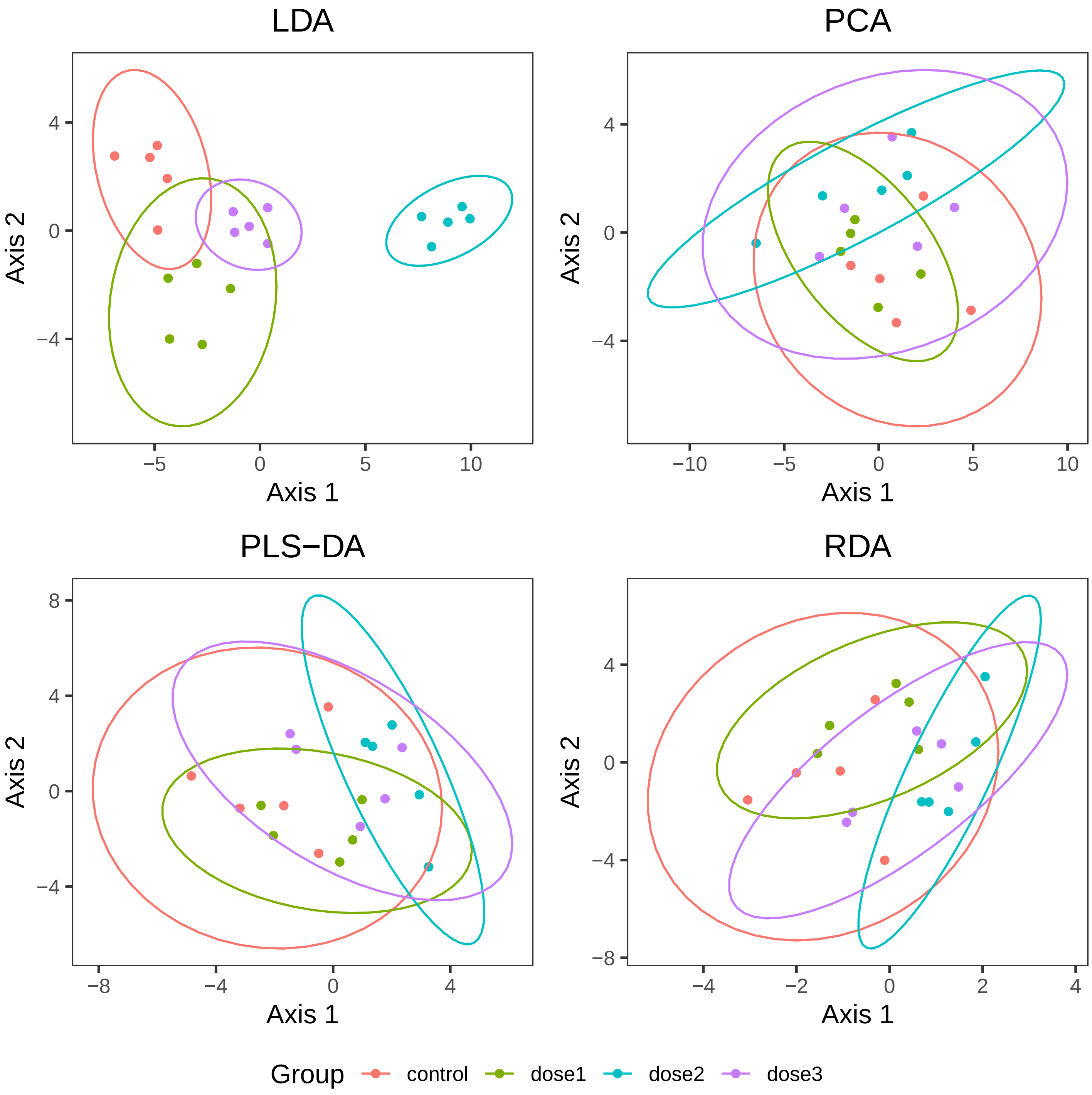

LDA
PCA
PLS−DA
RDA
Axis 1
Axis 2
−5
0
5
10
4
−4
−10
8
−8
−2
2
Group
control
dose1
dose2
dose3

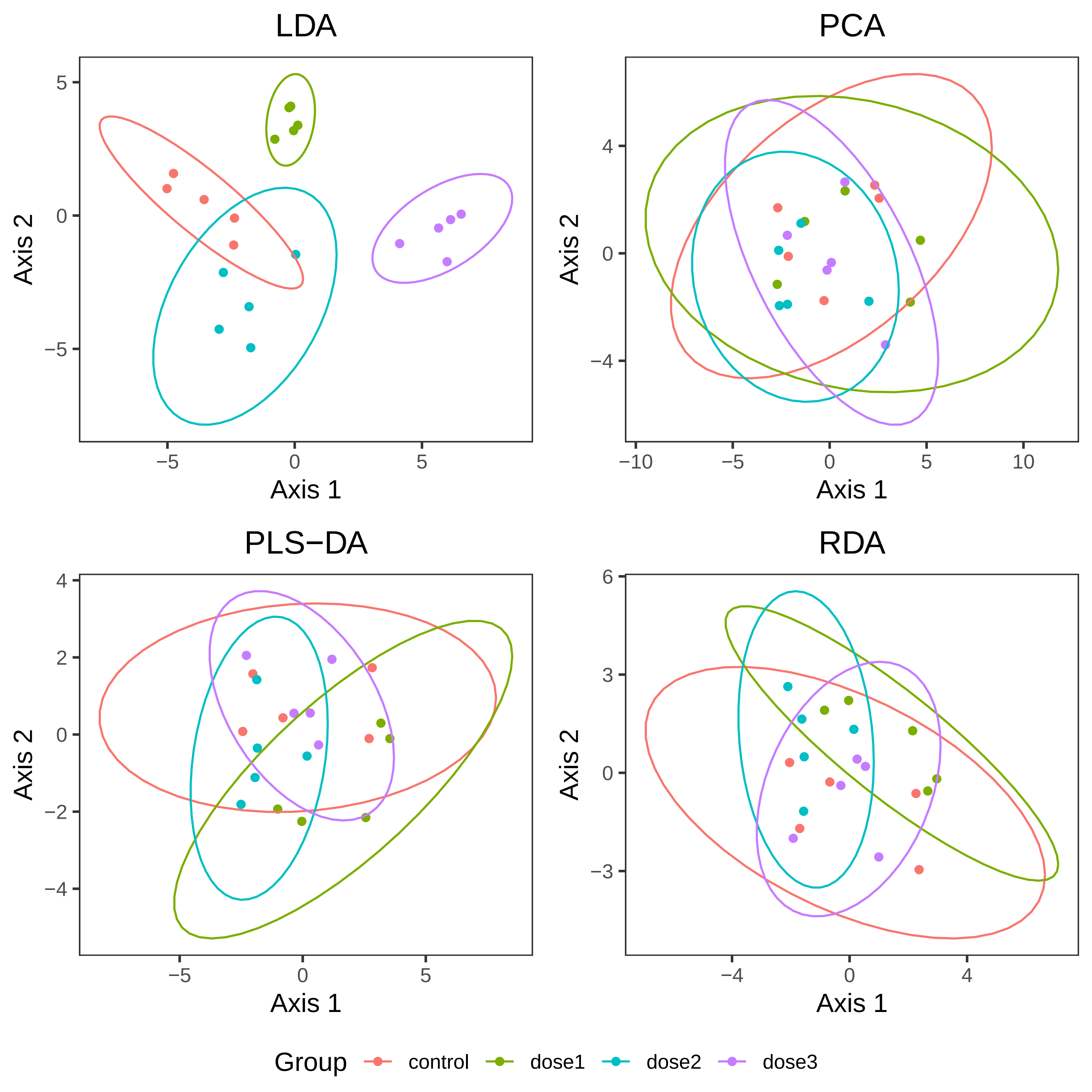

LDA
PCA
PLS−DA
RDA
Axis 1
Axis 2
Group
control
dose1
dose2
dose3

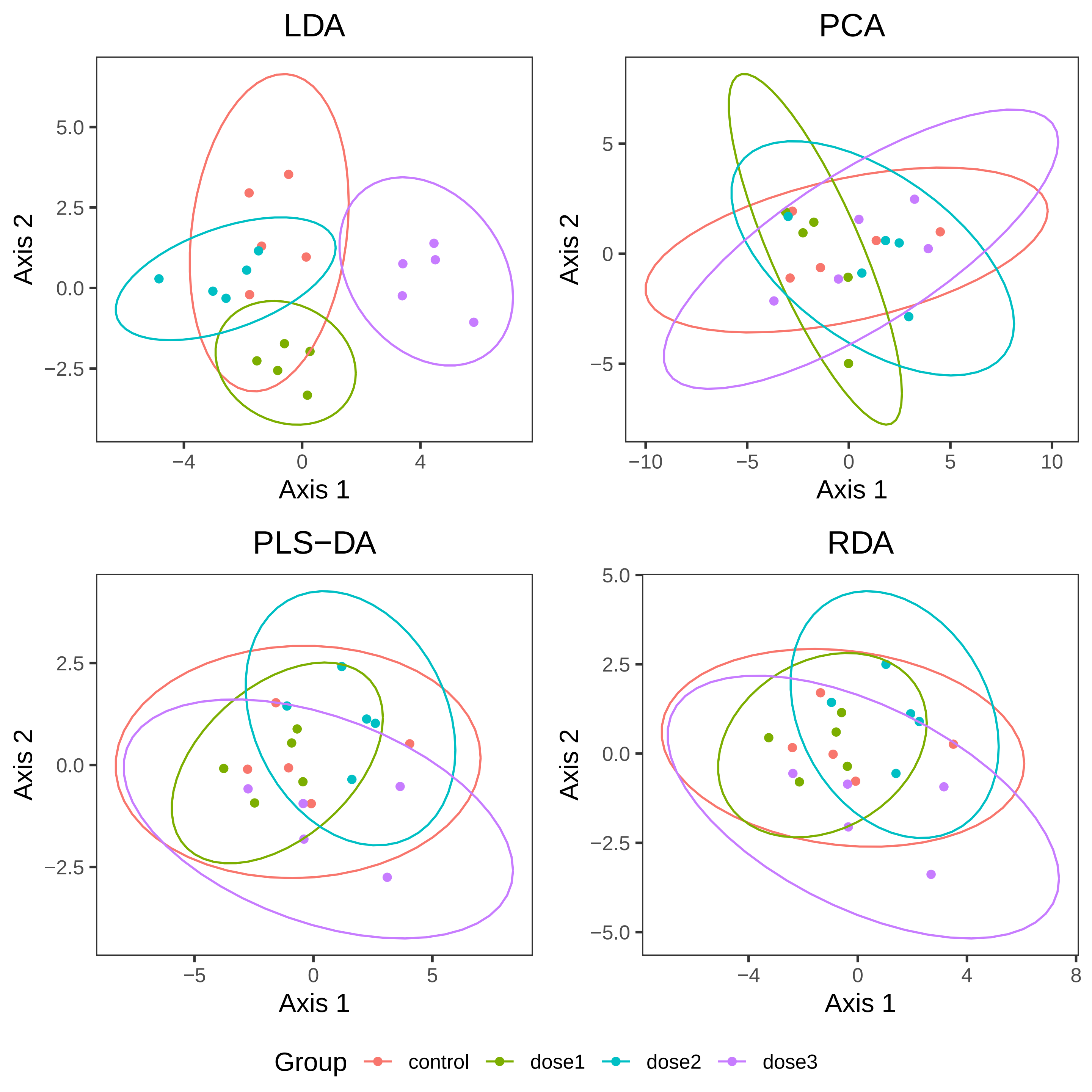
LDA
PCA
PLS−DA
RDA
Axis 1
Axis 2
Group
control
dose1
dose2
dose3

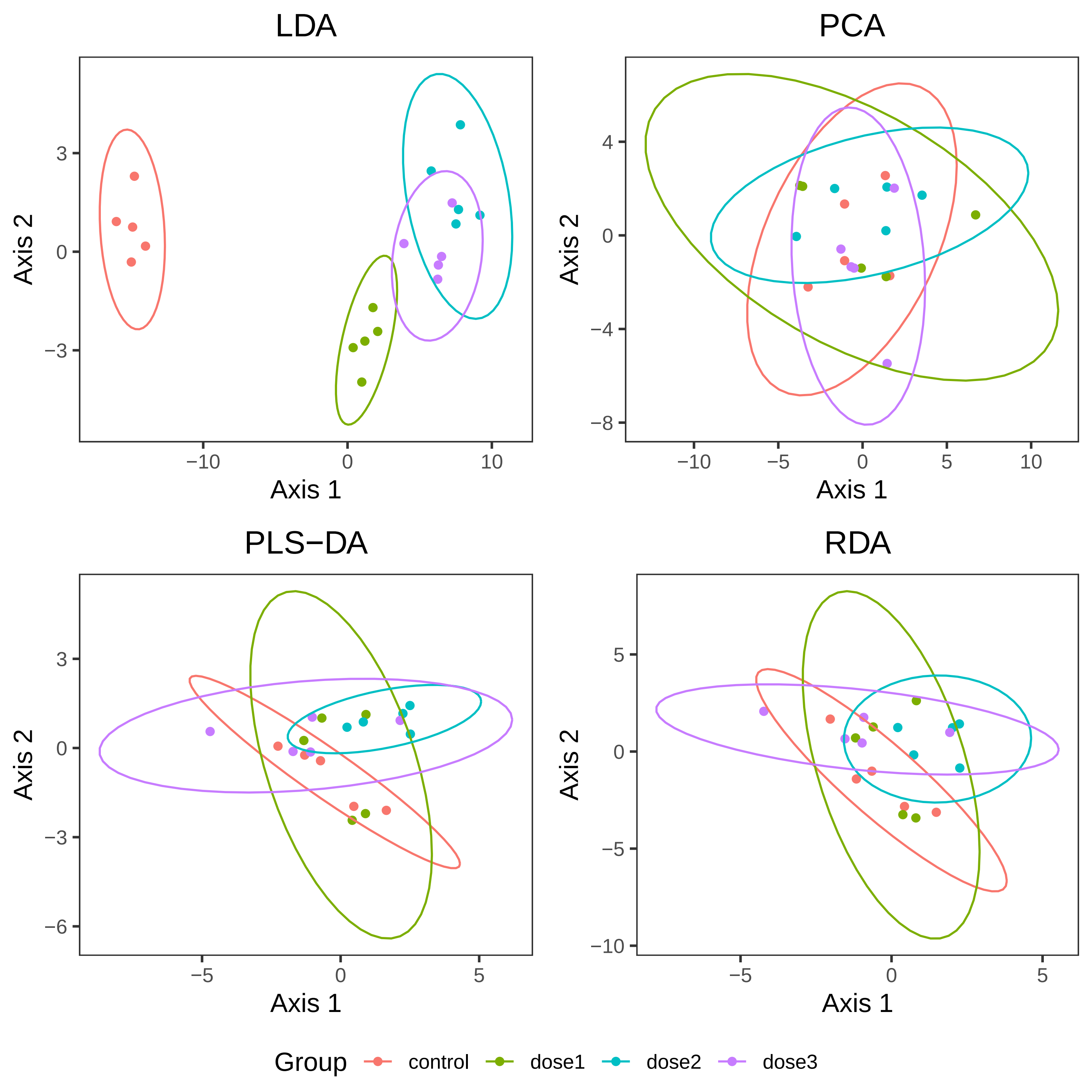

LDA
PCA
PLS−DA
RDA
Axis 1
Axis 2
−10
0
10
3
−3
−5
5
4
−4
−8
−6
−10
Group
control
dose1
dose2
dose3

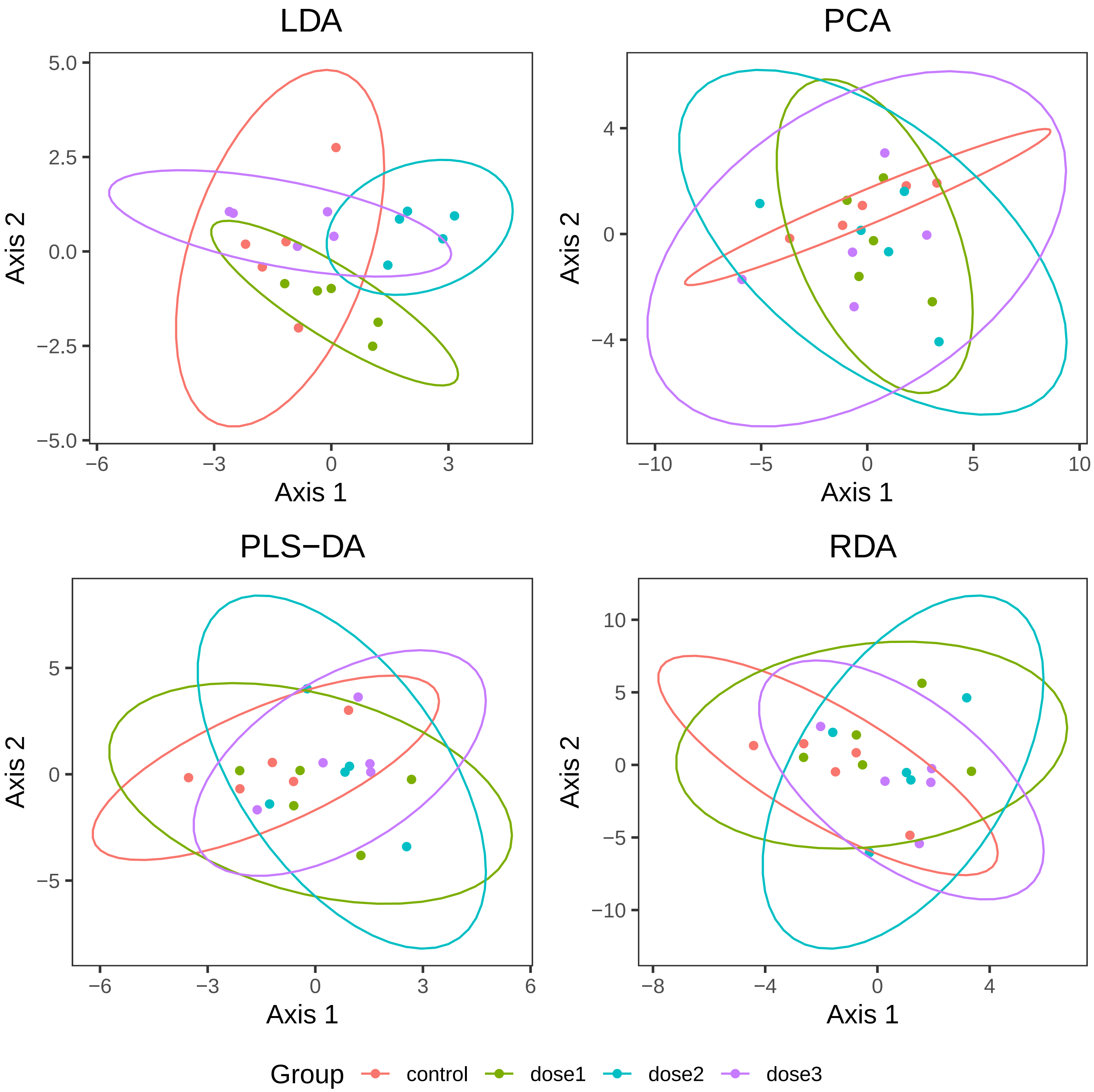

LDA
PCA
PLS−DA
RDA
Axis 1
Axis 2
5.0
2.5
0.0
−2.5
−5.0
−6
−3
0
3
4
−4
−10
−5
5
10
6
−8
Group
control
dose1
dose2
dose3

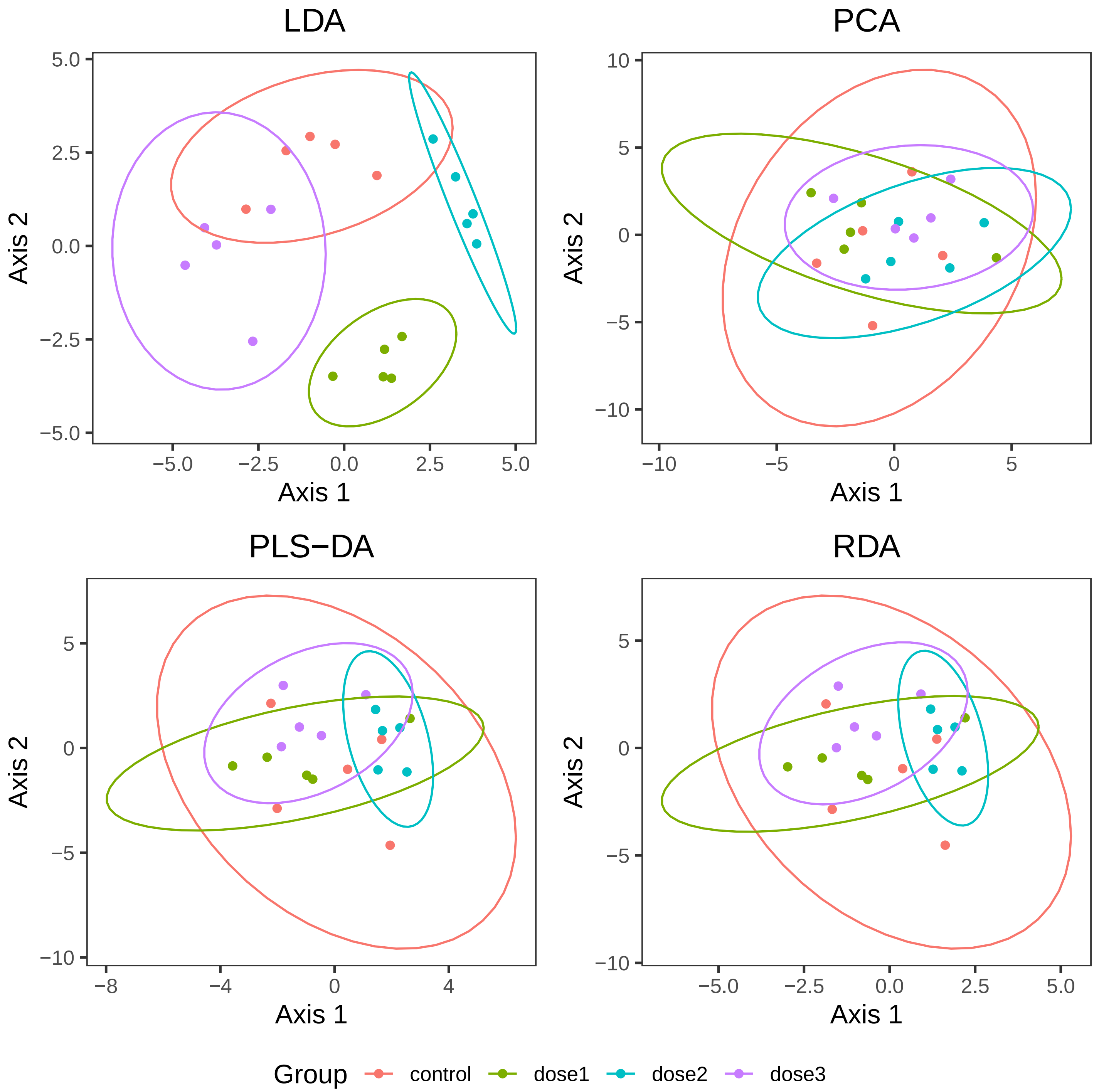
LDA
PCA
PLS−DA
RDA
Axis 1
Axis 2
5.0
2.5
0.0
−2.5
−5.0
10
5
0
−5
−10
−8
−4
4
Group
control
dose1
dose2
dose3

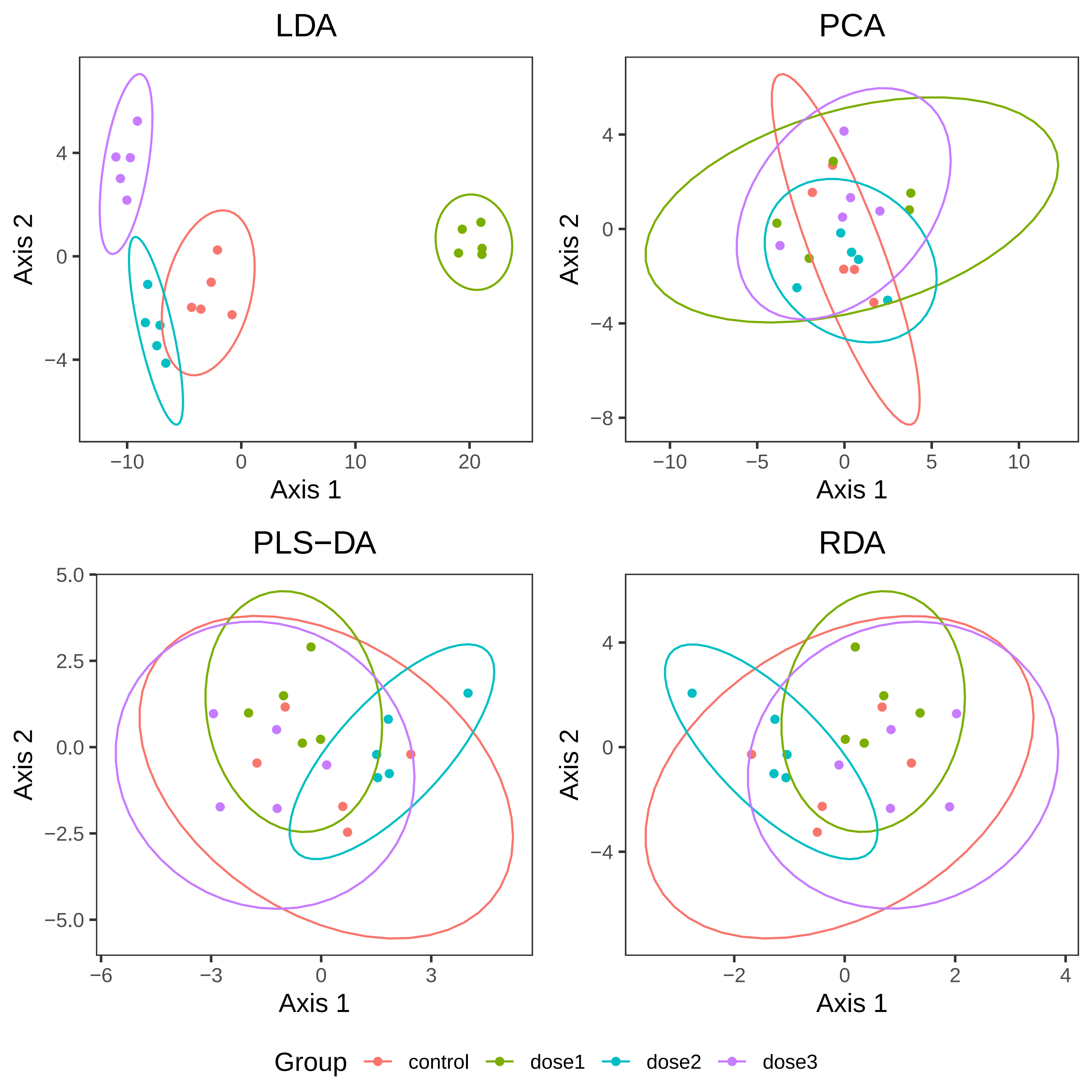

LDA
PCA
PLS−DA
RDA
Axis 1
Axis 2
Group
control
dose1
dose2
dose3

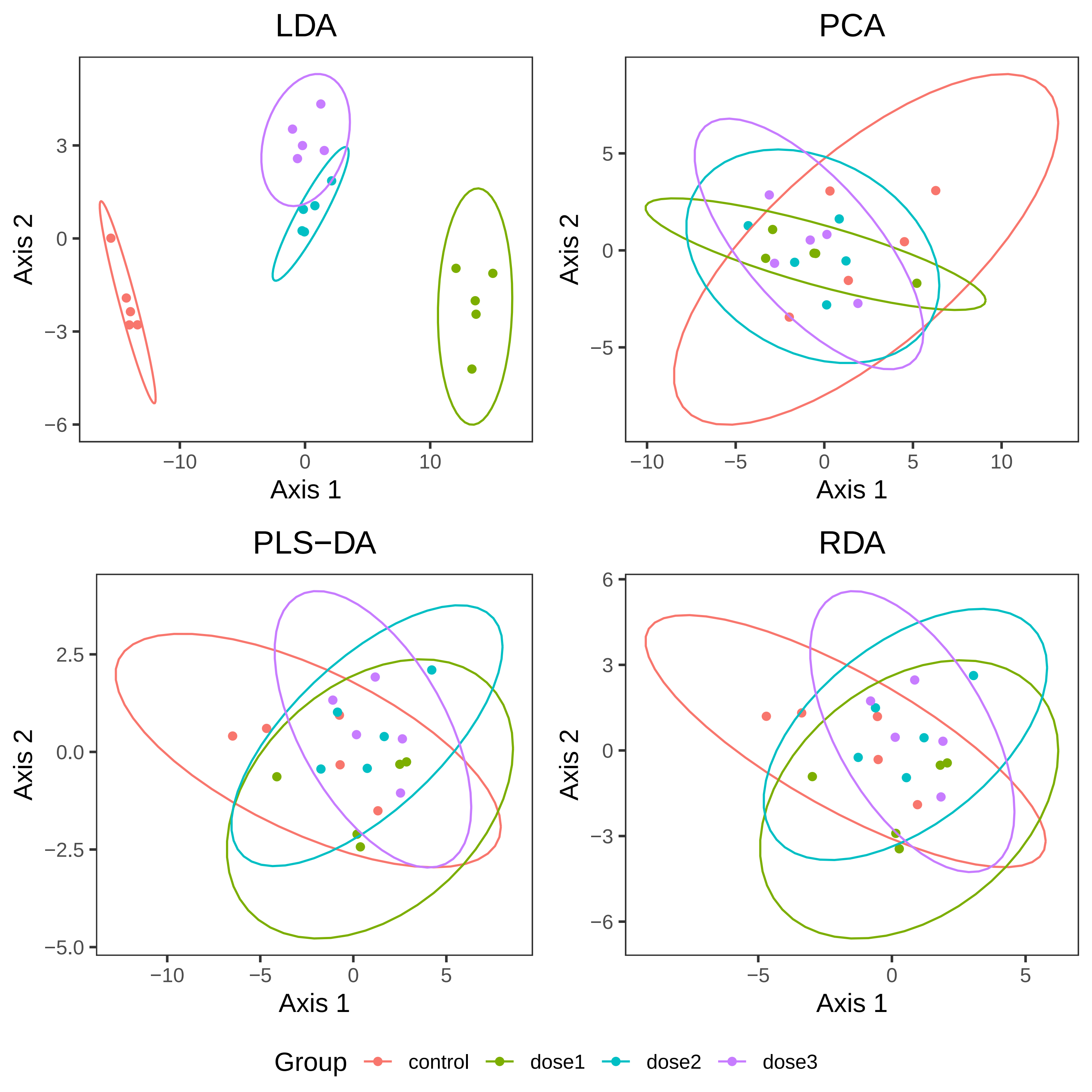

LDA
PCA
PLS−DA
RDA
Axis 1
Axis 2
3
0
−3
−6
−10
0
10
5
−5
2.5
0.0
−2.5
−5.0
6
Group
control
dose1
dose2
dose3

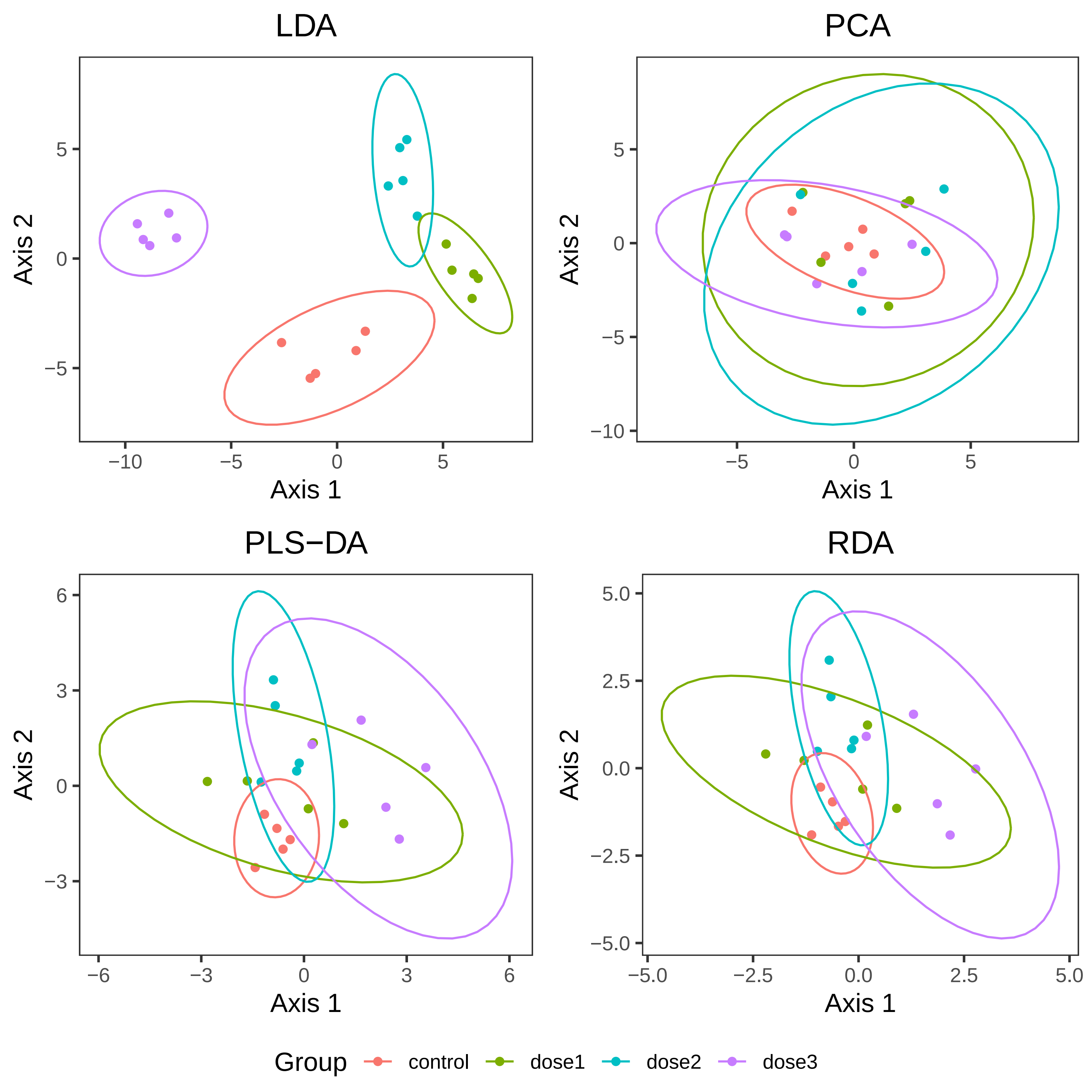

LDA
PCA
PLS−DA
RDA
Axis 1
Axis 2
Group
control
dose1
dose2
dose3

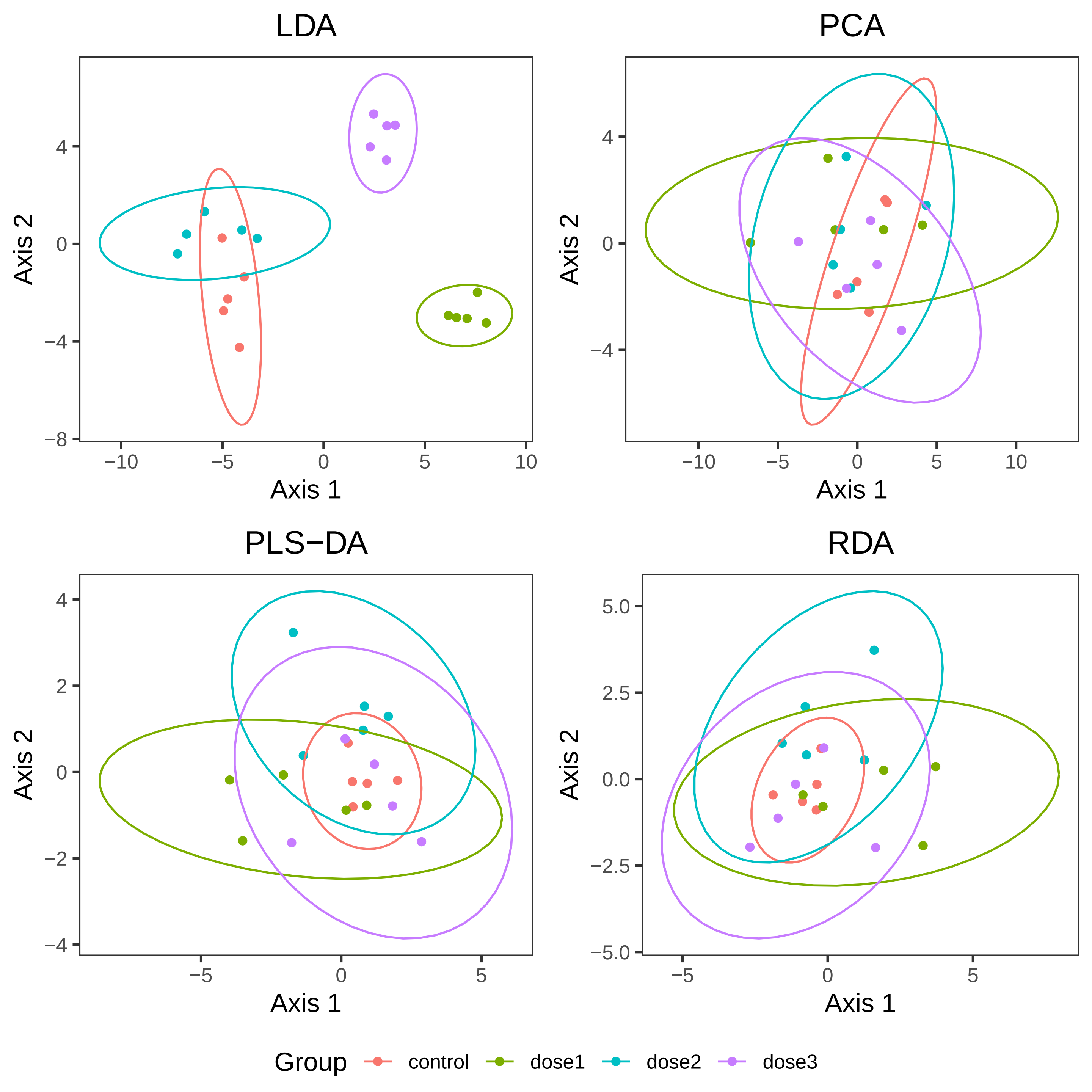

LDA
PCA
PLS−DA
RDA
Axis 1
Axis 2
Group
control
dose1
dose2
dose3

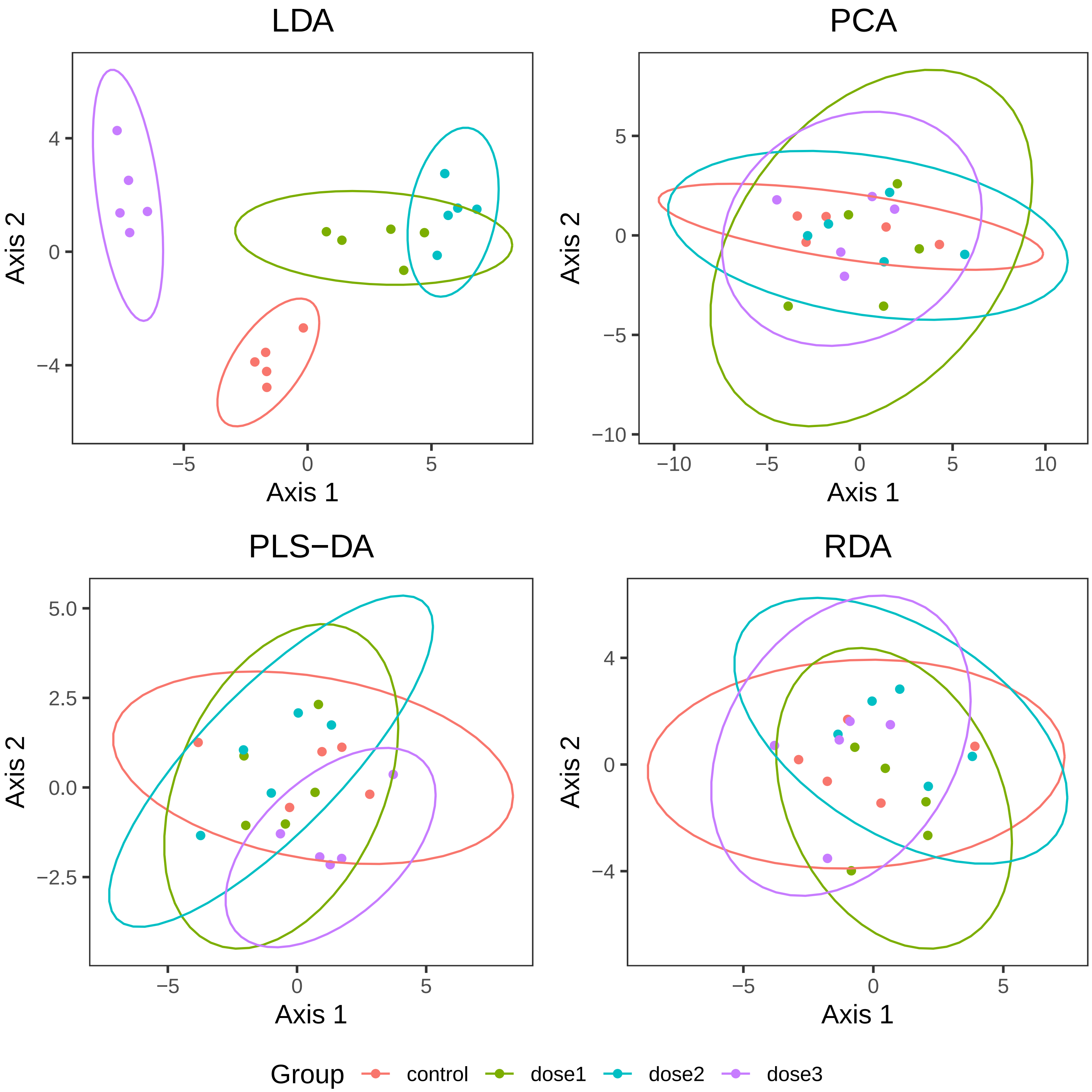
LDA
PCA
PLS−DA
RDA
Axis 1
Axis 2
Group
control
dose1
dose2
dose3

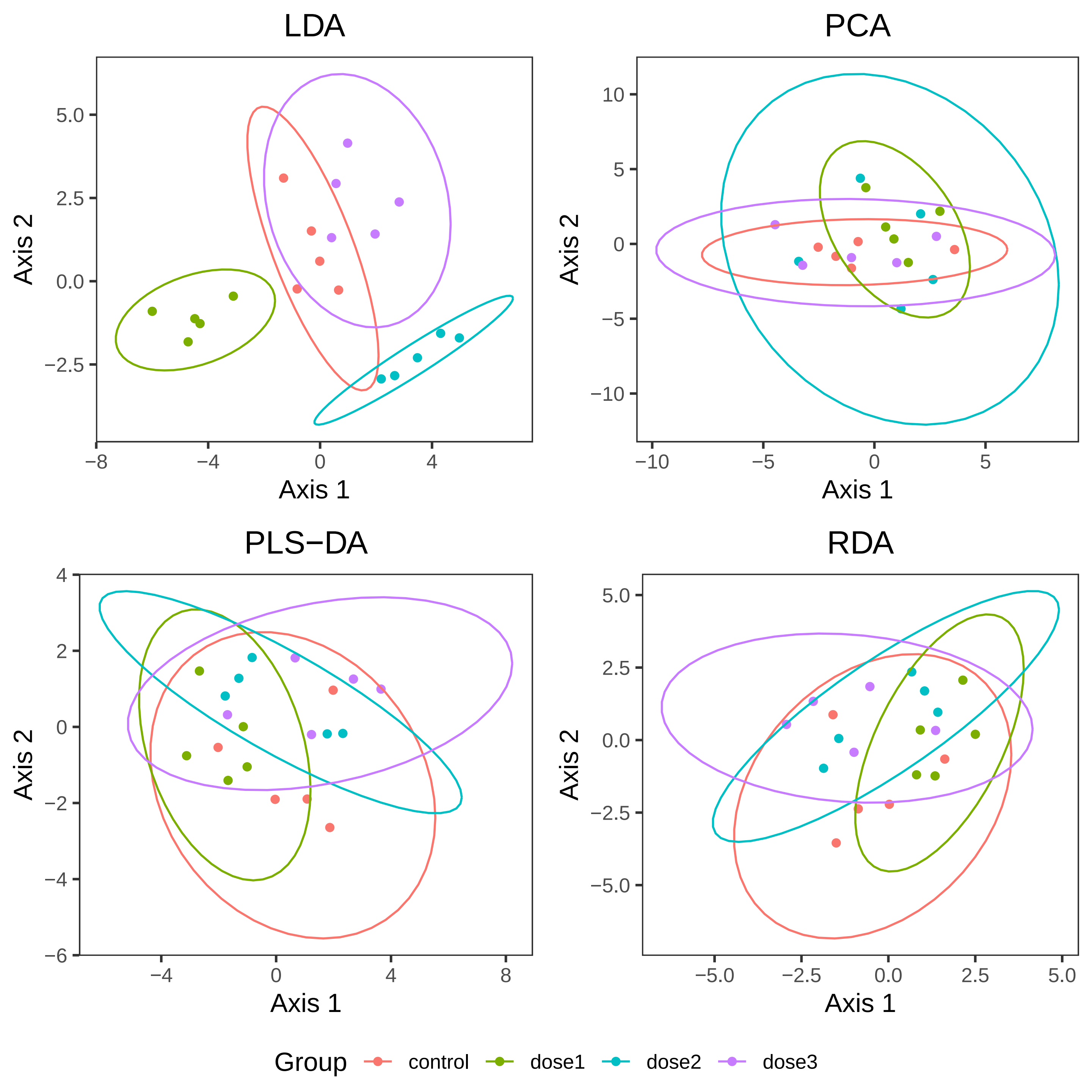
LDA
PCA
PLS−DA
RDA
Axis 1
Axis 2
Group
control
dose1
dose2
dose3

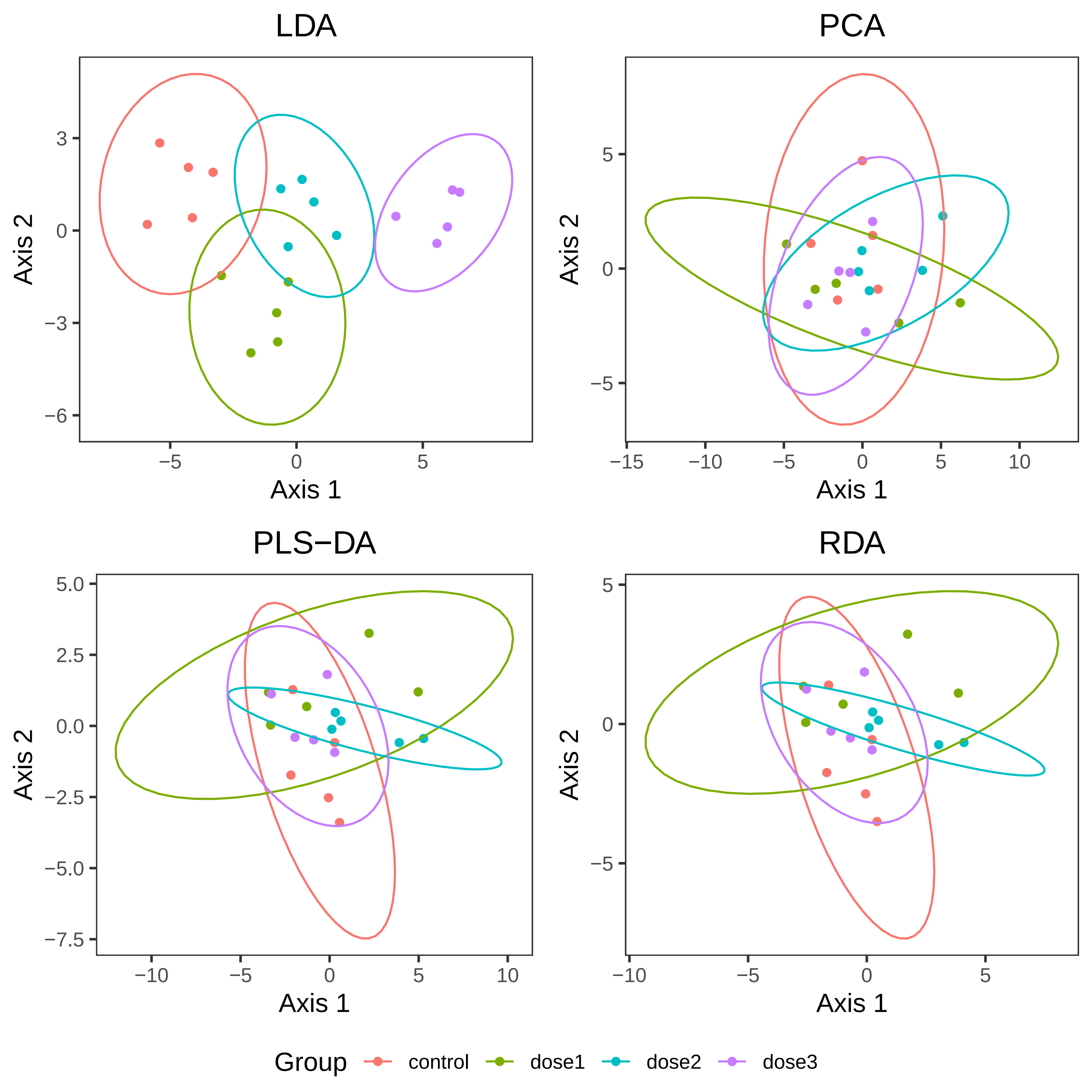
LDA
PCA
PLS−DA
RDA
Axis 1
Axis 2
Group
control
dose1
dose2
dose3

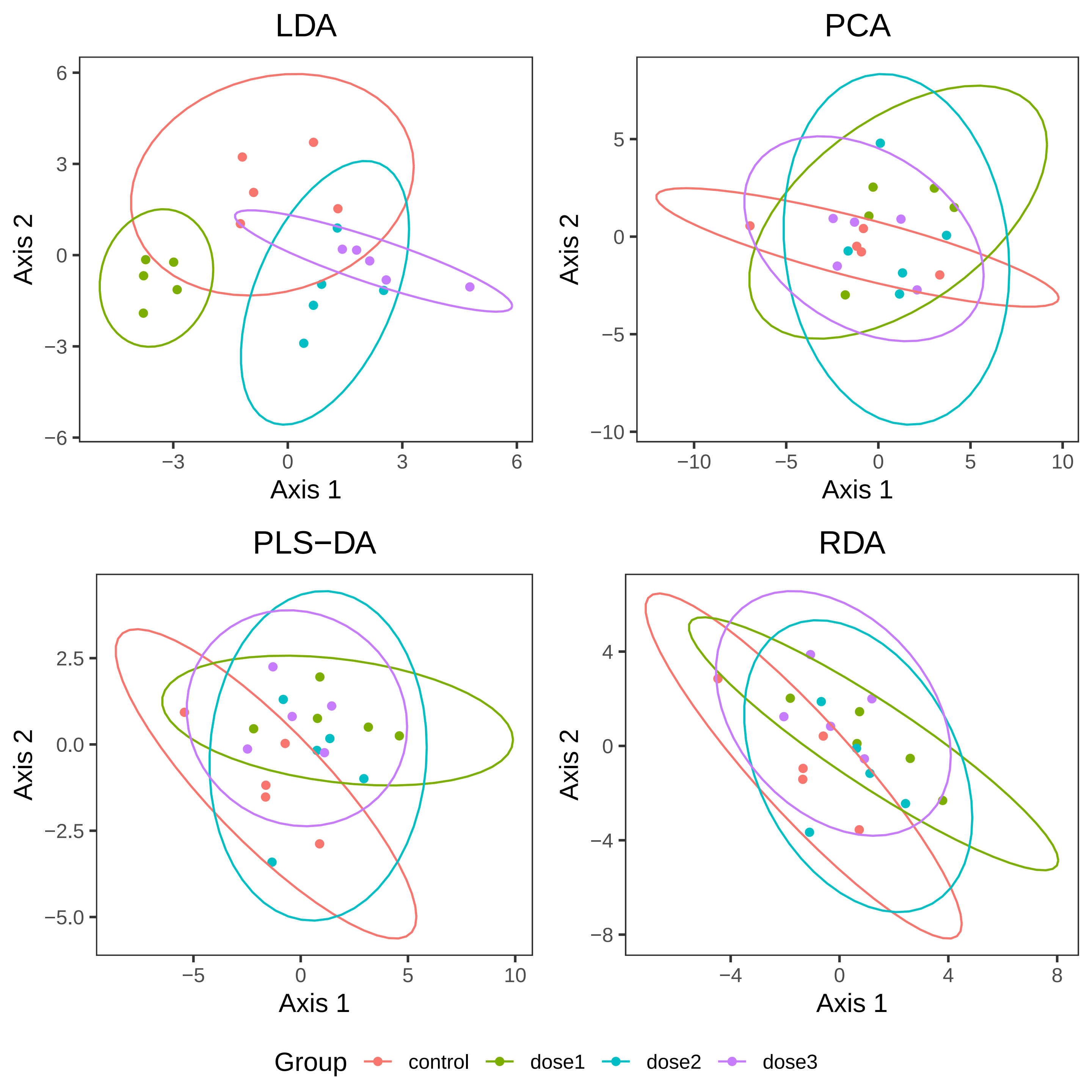

LDA
PCA
PLS−DA
RDA
Axis 1
Axis 2
6
3
0
−3
−6
5
−5
−10
10
2.5
0.0
−2.5
−5.0
4
−4
−8
8
Group
control
dose1
dose2
dose3

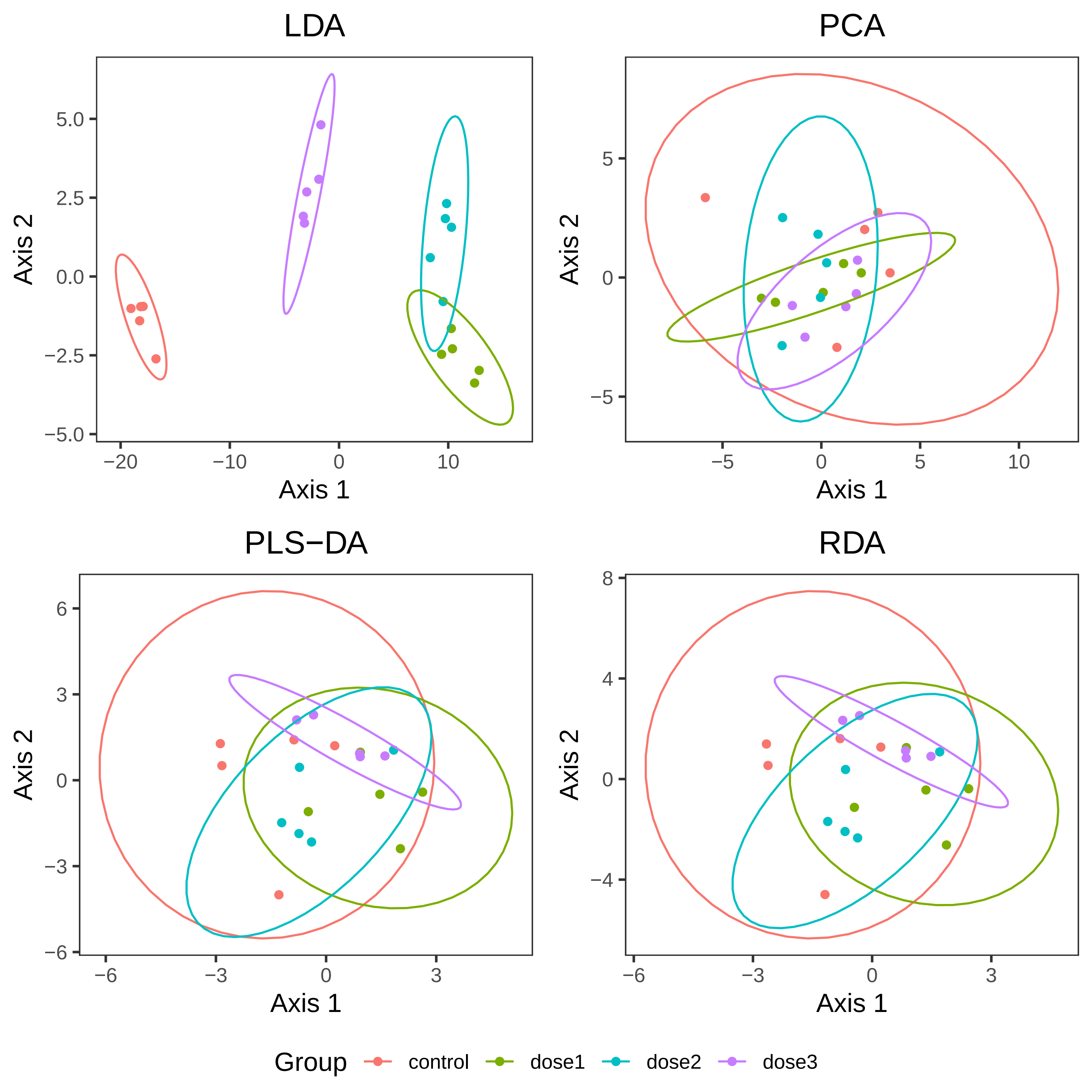

LDA
PCA
PLS−DA
RDA
Axis 1
Axis 2
5.0
2.5
0.0
−2.5
−5.0
−20
−10
0
10
5
−5
6
3
−3
−6
8
4
−4
Group
control
dose1
dose2
dose3

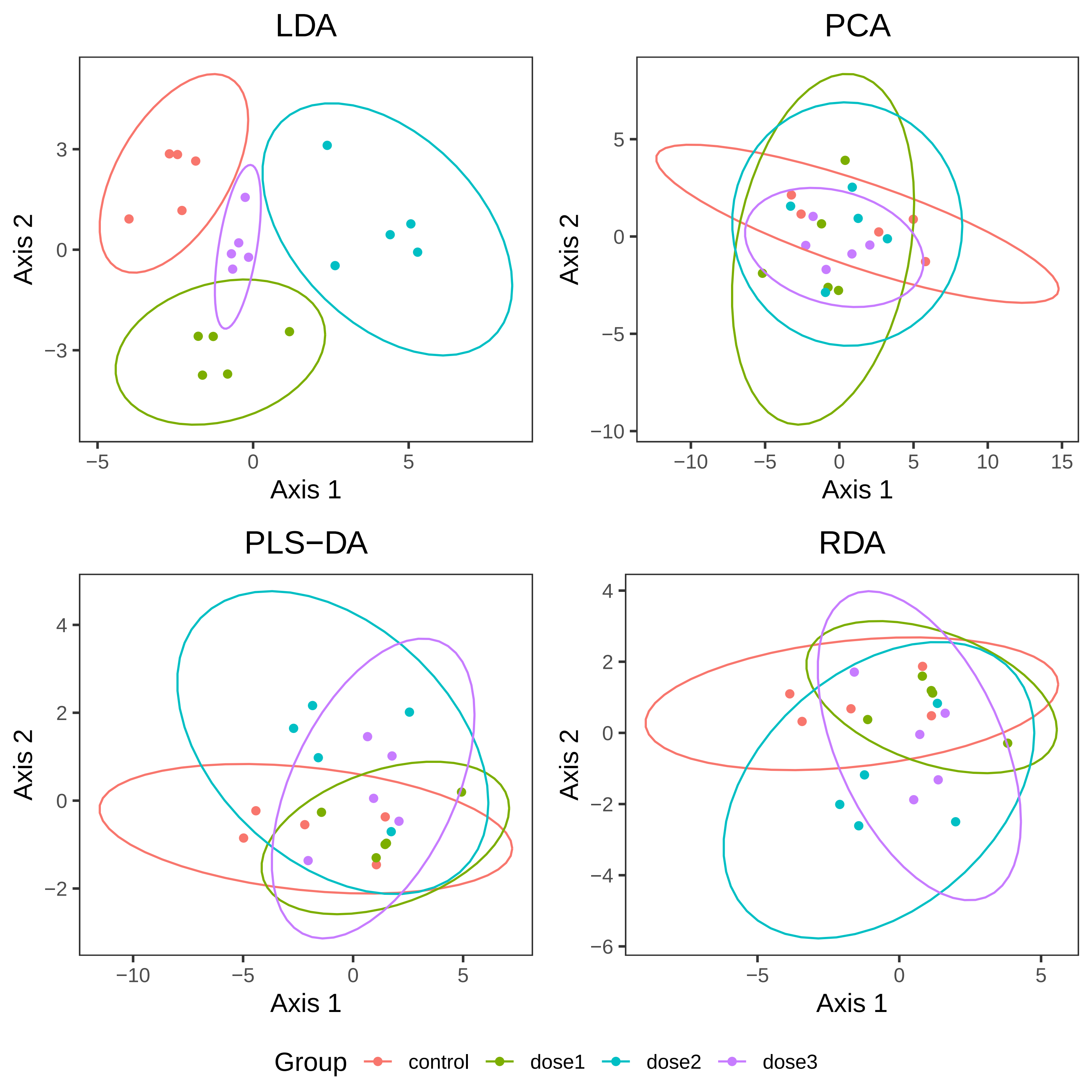
LDA
PCA
PLS−DA
RDA
Axis 1
Axis 2
Group
control
dose1
dose2
dose3

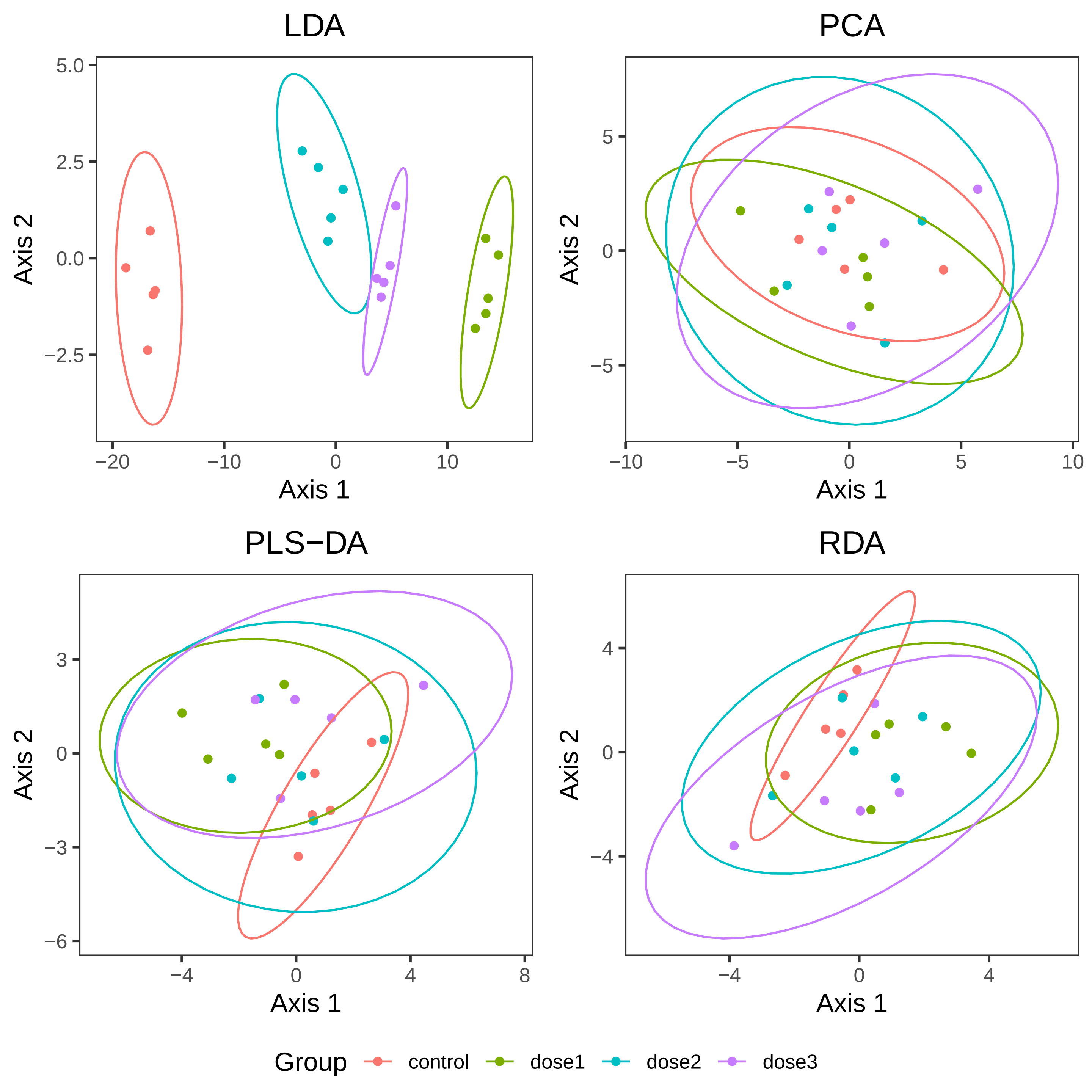

LDA
PCA
PLS−DA
RDA
Axis 1
Axis 2
Group
control
dose1
dose2
dose3

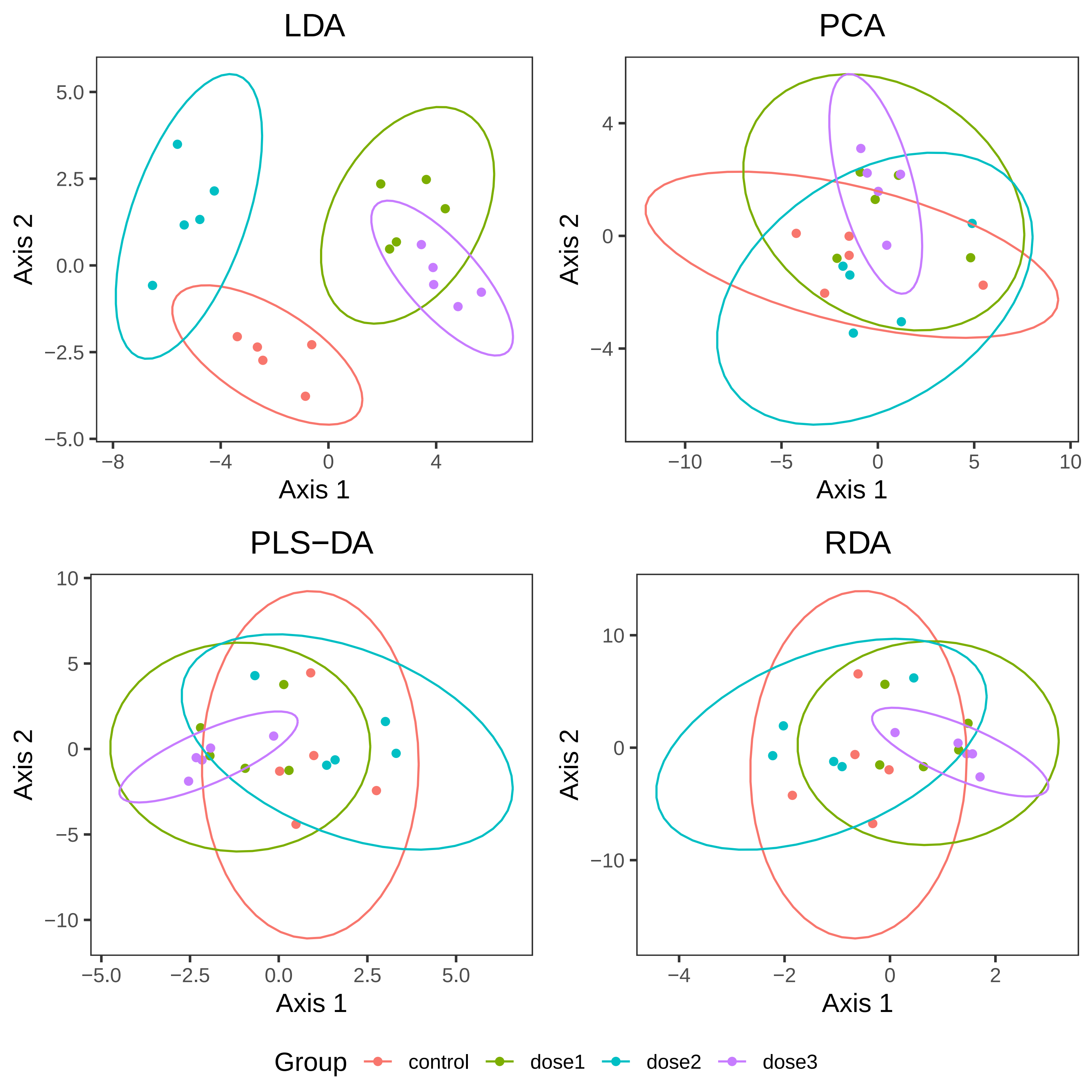

LDA
PCA
PLS−DA
RDA
Axis 1
Axis 2
5.0
2.5
0.0
−2.5
−5.0
−8
−4
0
4
−10
−5
5
10
Group
control
dose1
dose2
dose3

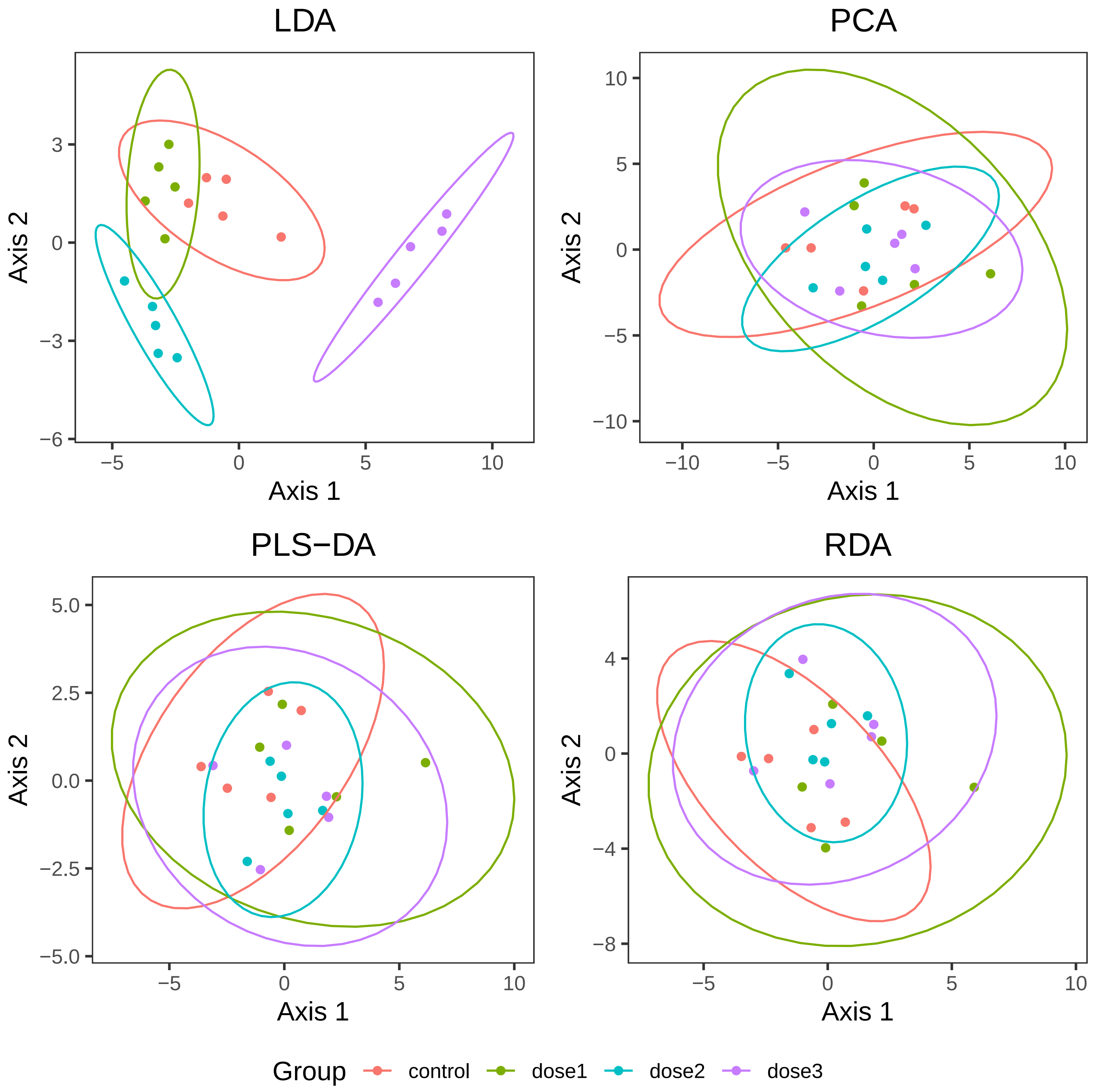
LDA
PCA
PLS−DA
RDA
Axis 1
Axis 2
3
0
−3
−6
−5
0
5
10
−10
2.5
5.0
0.0
−2.5
−5.0
4
−4
−8
Group
control
dose1
dose2
dose3

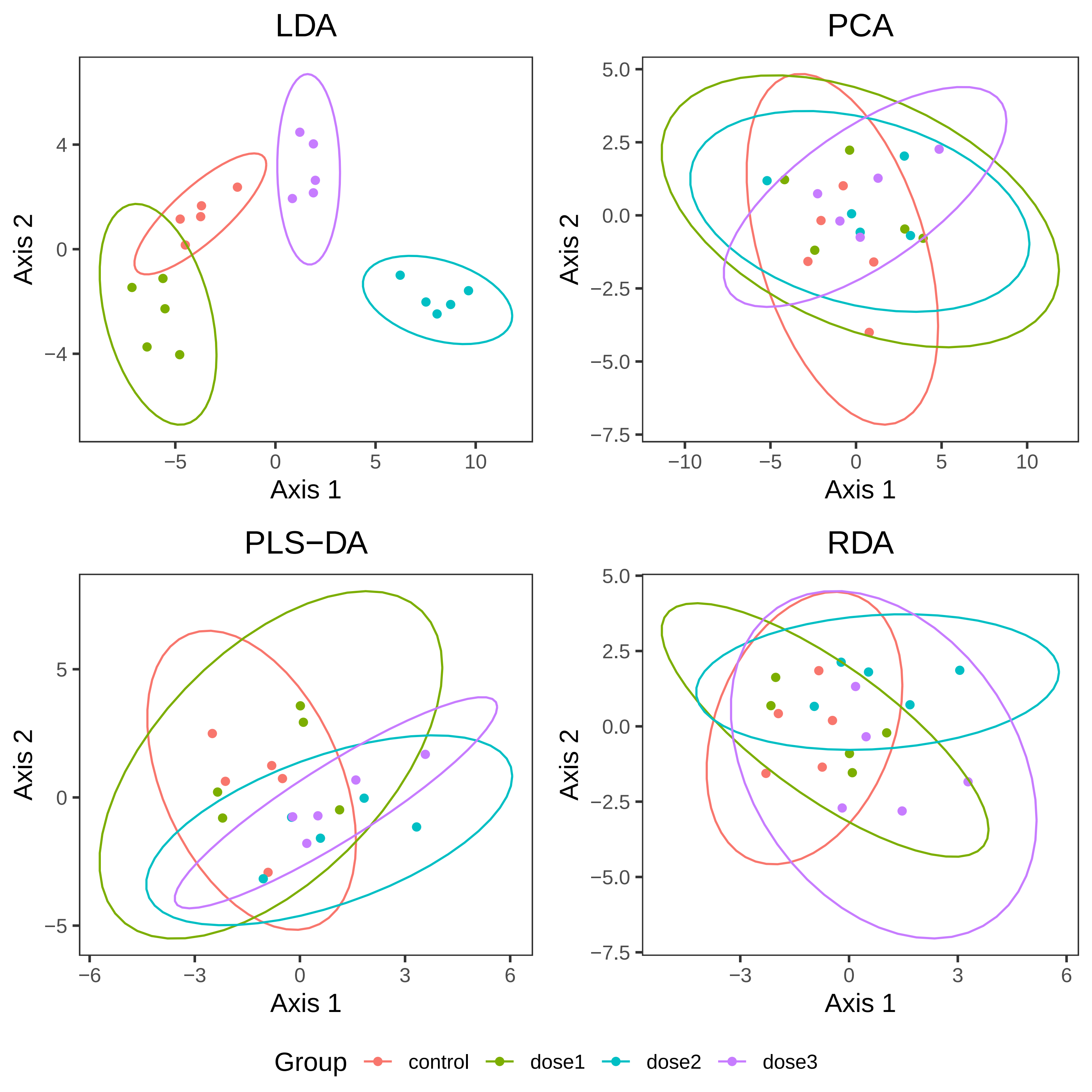
LDA
PCA
PLS−DA
RDA
Axis 1
Axis 2
Group
control
dose1
dose2
dose3

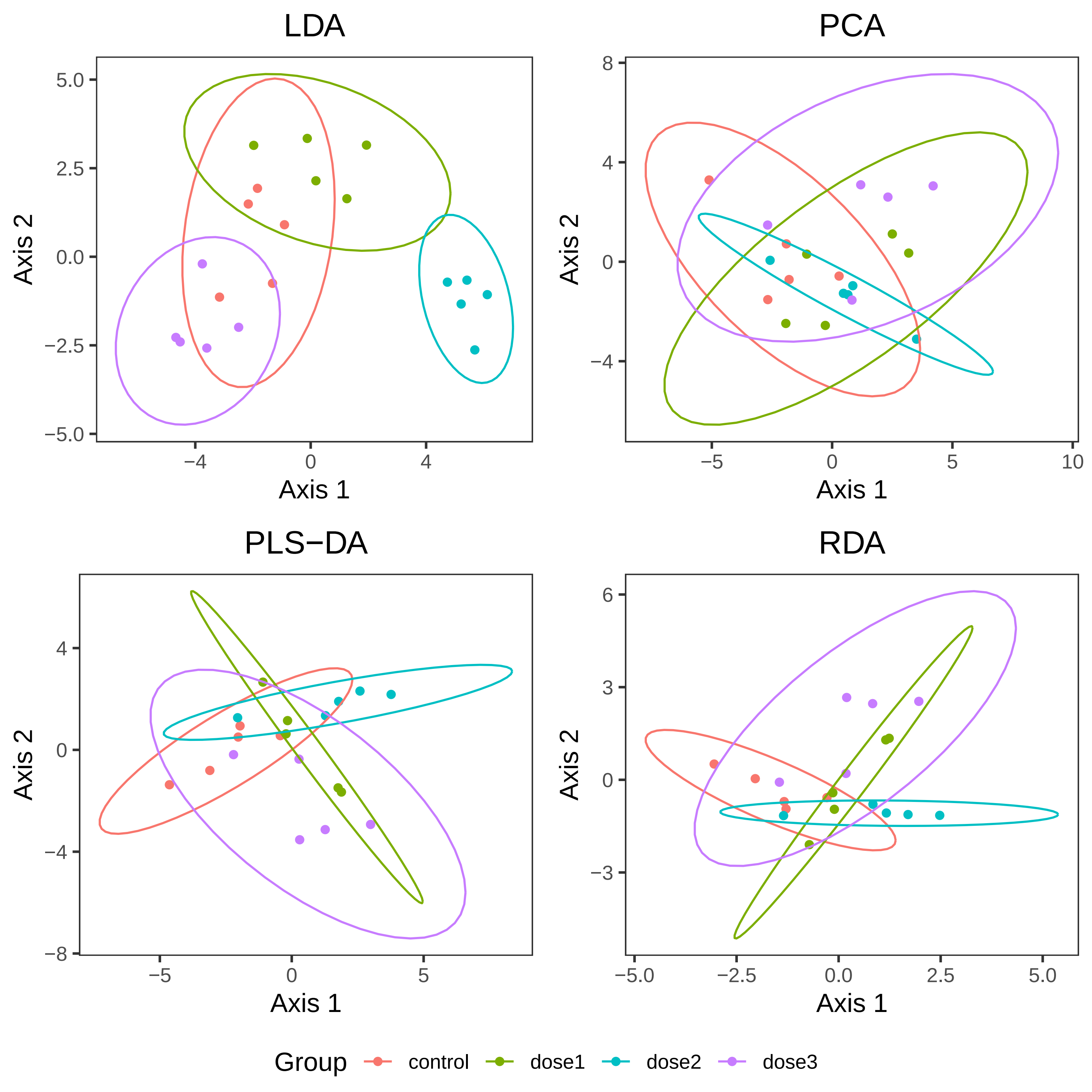
LDA
PCA
PLS−DA
RDA
Axis 1
Axis 2
5.0
2.5
0.0
−2.5
−5.0
−4
0
4
8
−5
5
10
6
3
−3
−8
−2.5
2.5
Group
control
dose1
dose2
dose3

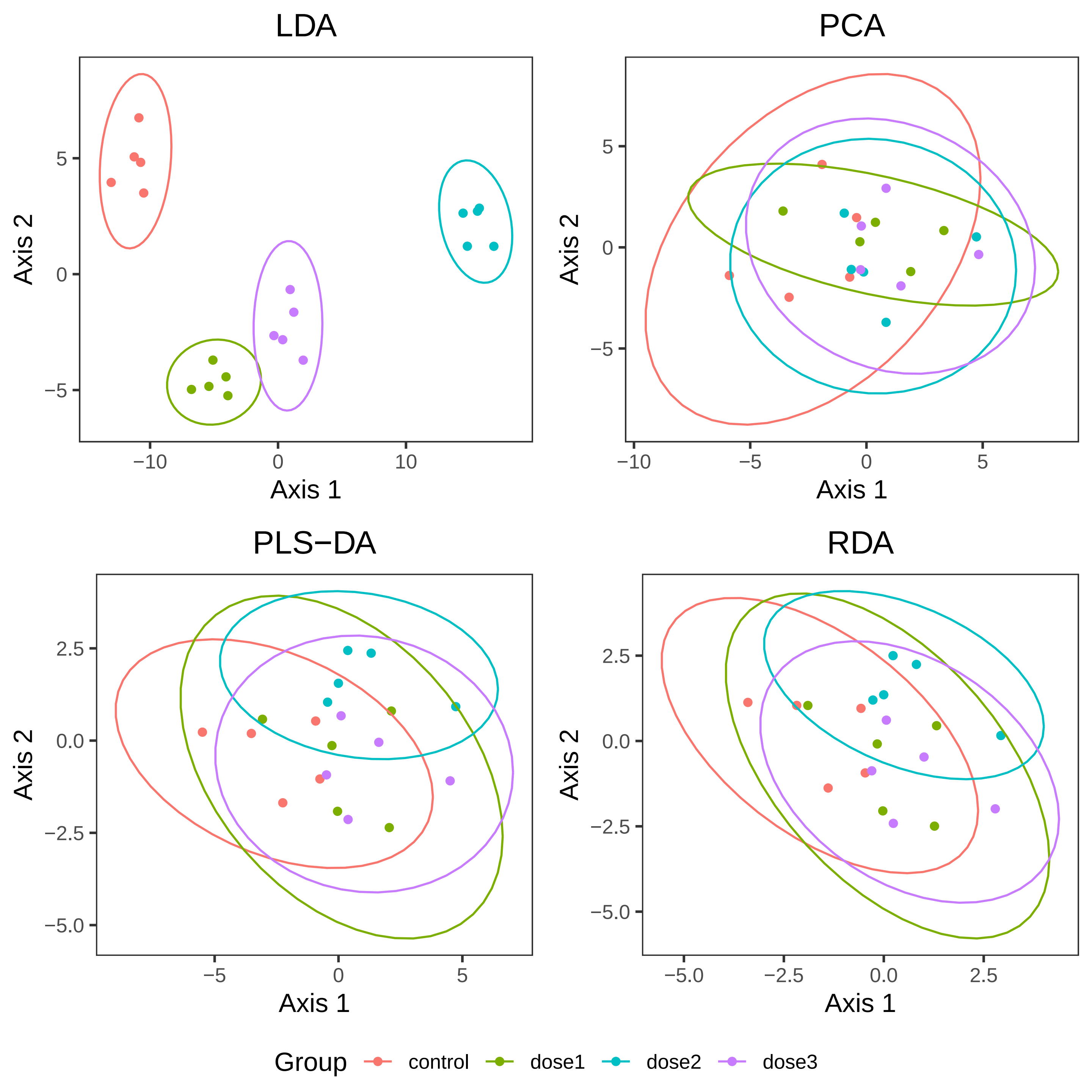

LDA
PCA
PLS−DA
RDA
Axis 1
Axis 2
Group
control
dose1
dose2
dose3

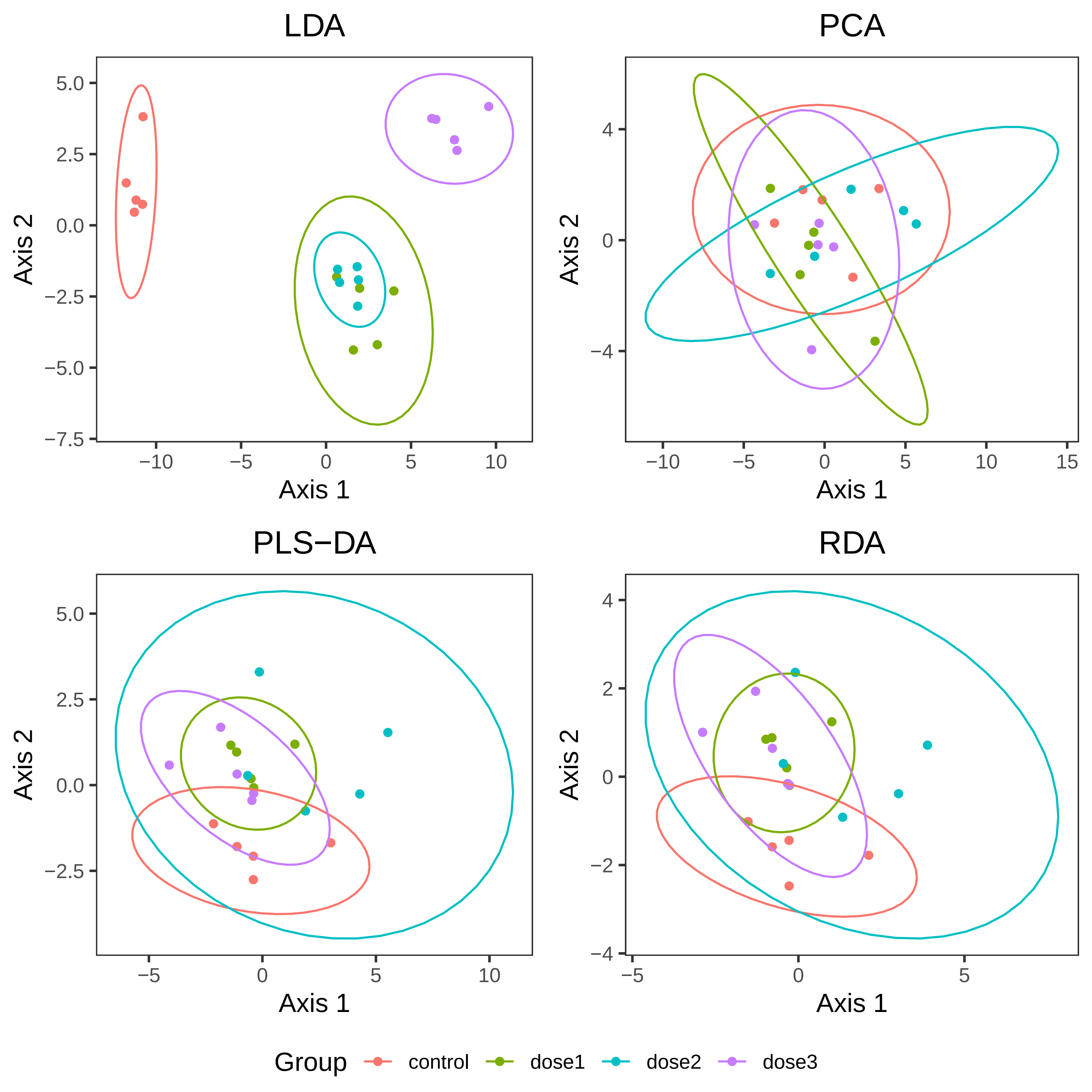

LDA
PCA
PLS−DA
RDA
Axis 1
Axis 2
Group
control
dose1
dose2
dose3

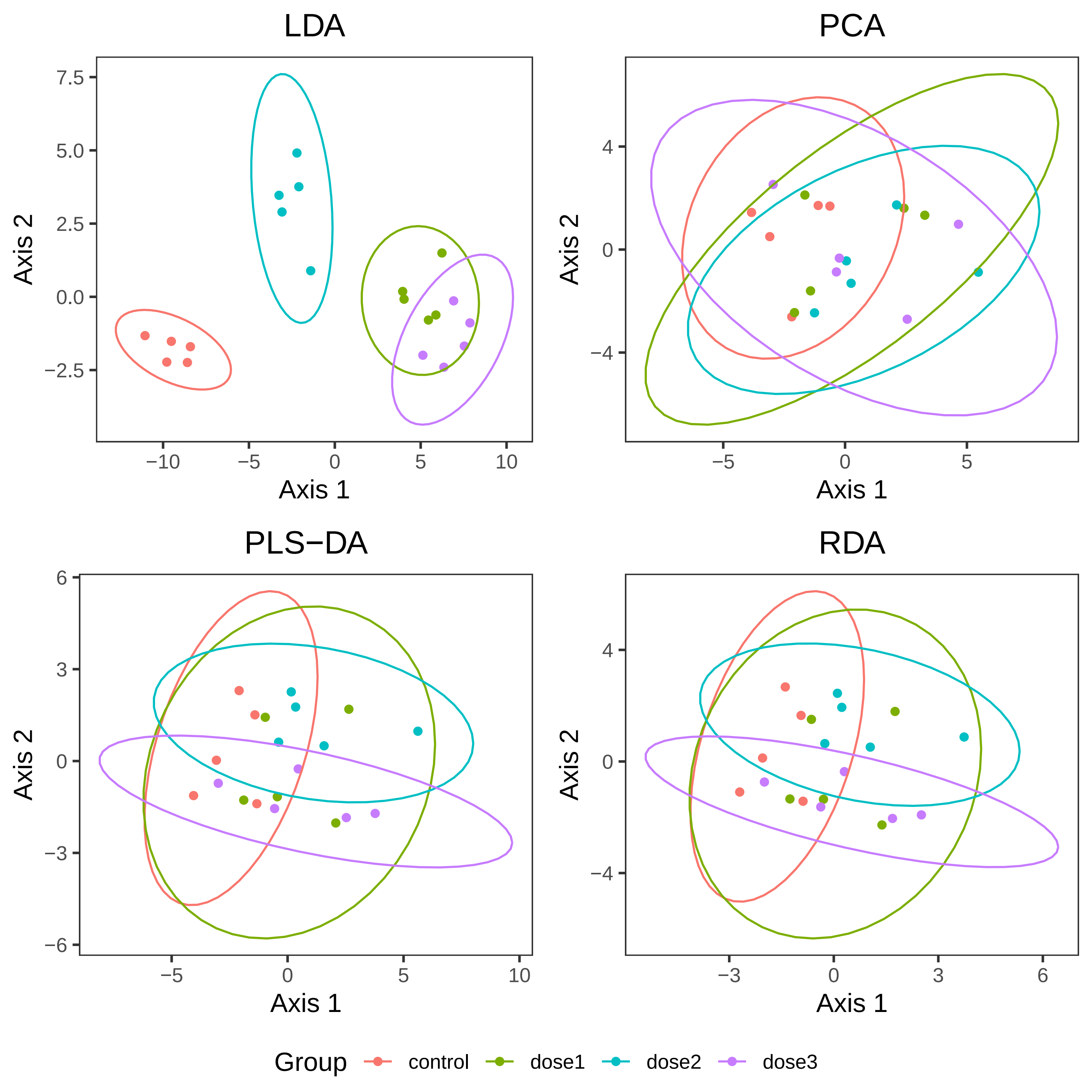
LDA
PCA
PLS−DA
RDA
Axis 1
Axis 2
7.5
5.0
2.5
0.0
−2.5
−10
−5
0
5
10
4
0
−4
6
3
−3
−6
Group
control
dose1
dose2
dose3

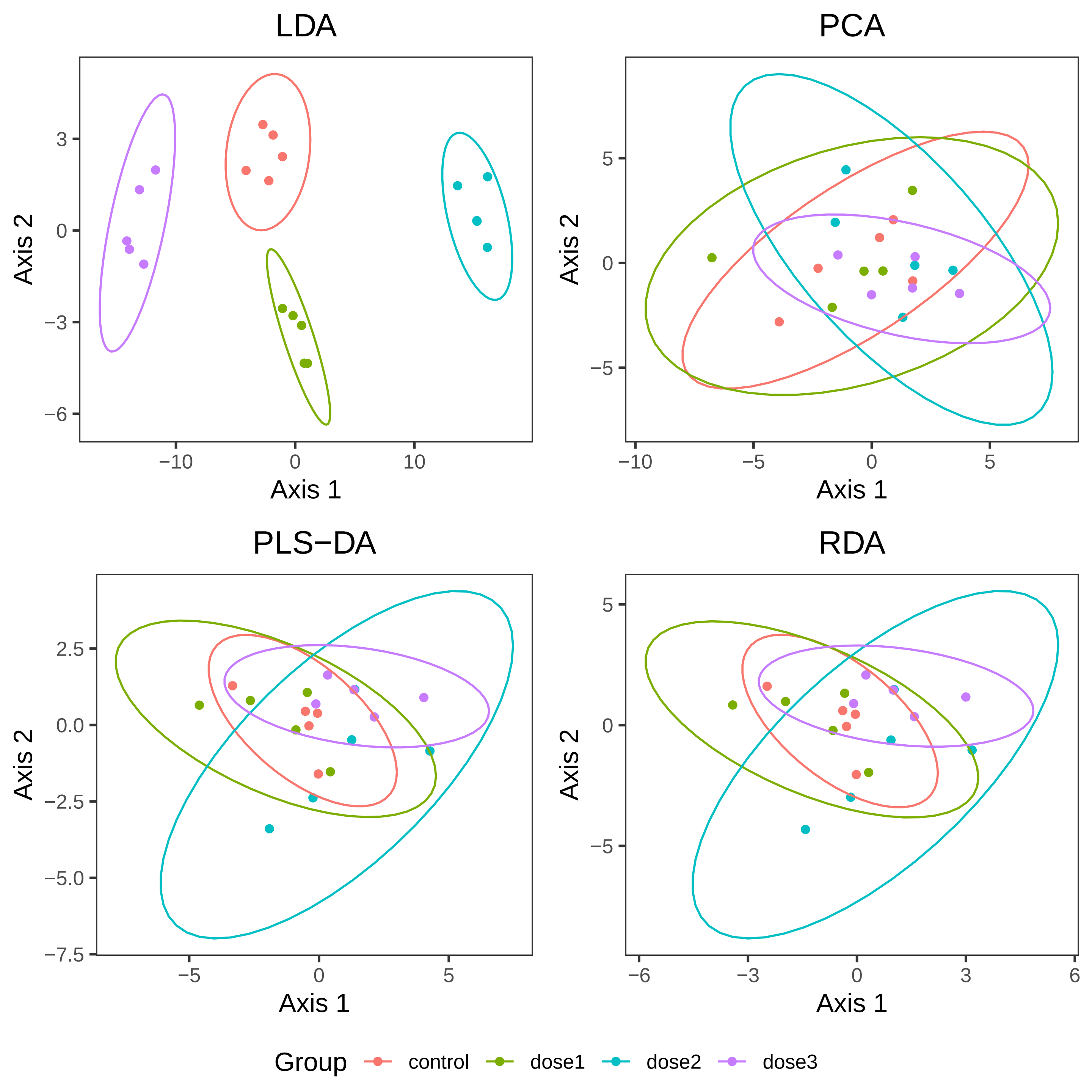

LDA
PCA
PLS−DA
RDA
Axis 1
Axis 2
3
0
−3
−6
−10
10
5
−5
2.5
0.0
−2.5
−5.0
−7.5
−6
6
Group
control
dose1
dose2
dose3

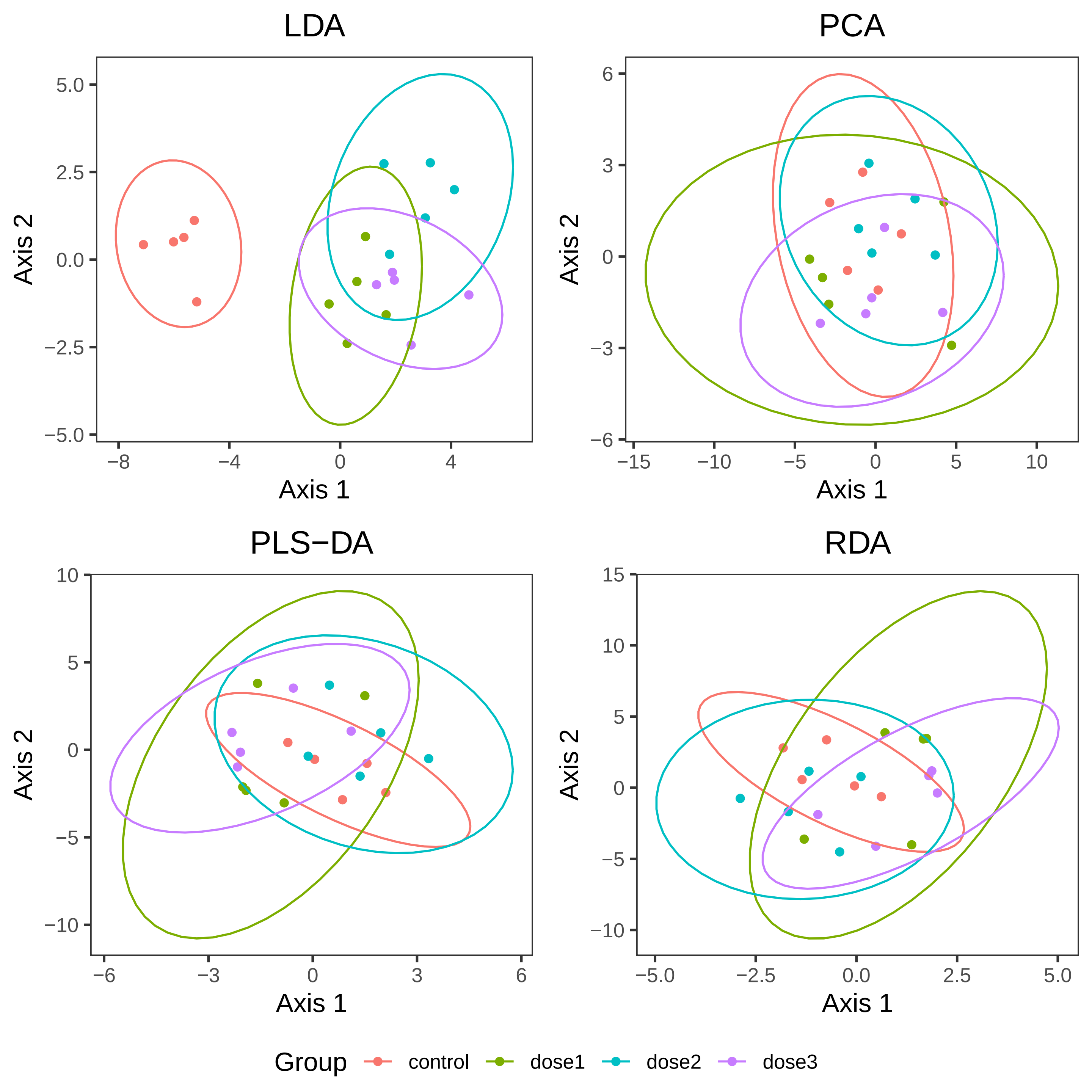
LDA
PCA
PLS−DA
RDA
Axis 1
Axis 2
5.0
2.5
0.0
−2.5
−5.0
−8
−4
0
4
6
3
0
−3
−6
−15
−10
−5
0
5
10
10
5
0
−5
−10
−6
−3
0
3
6
15
10
5
0
−5
−10
−5.0
−2.5
0.0
2.5
5.0
Group
control
dose1
dose2
dose3

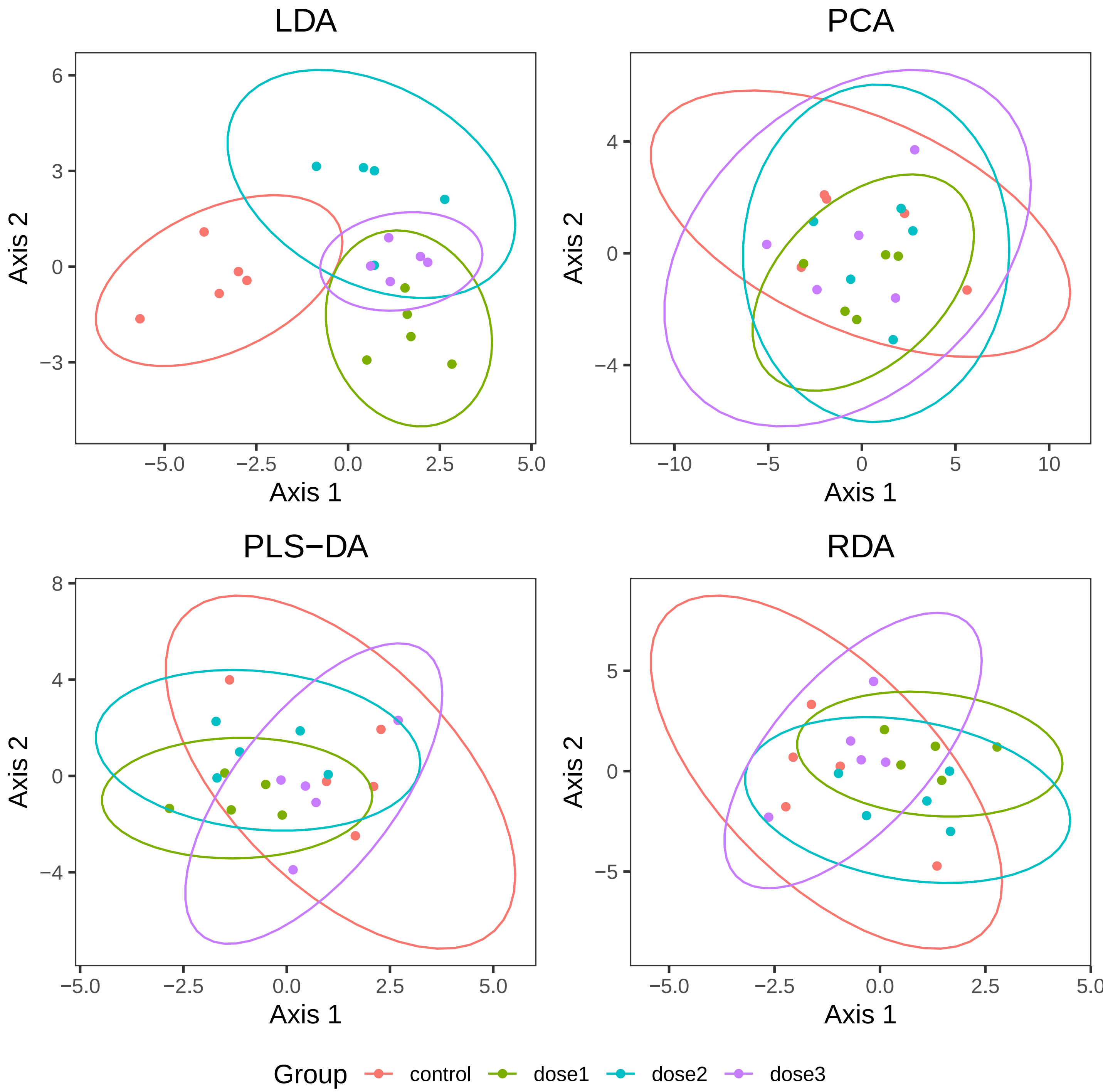

LDA
PCA
PLS−DA
RDA
Axis 1
Axis 2
Group
control
dose1
dose2
dose3

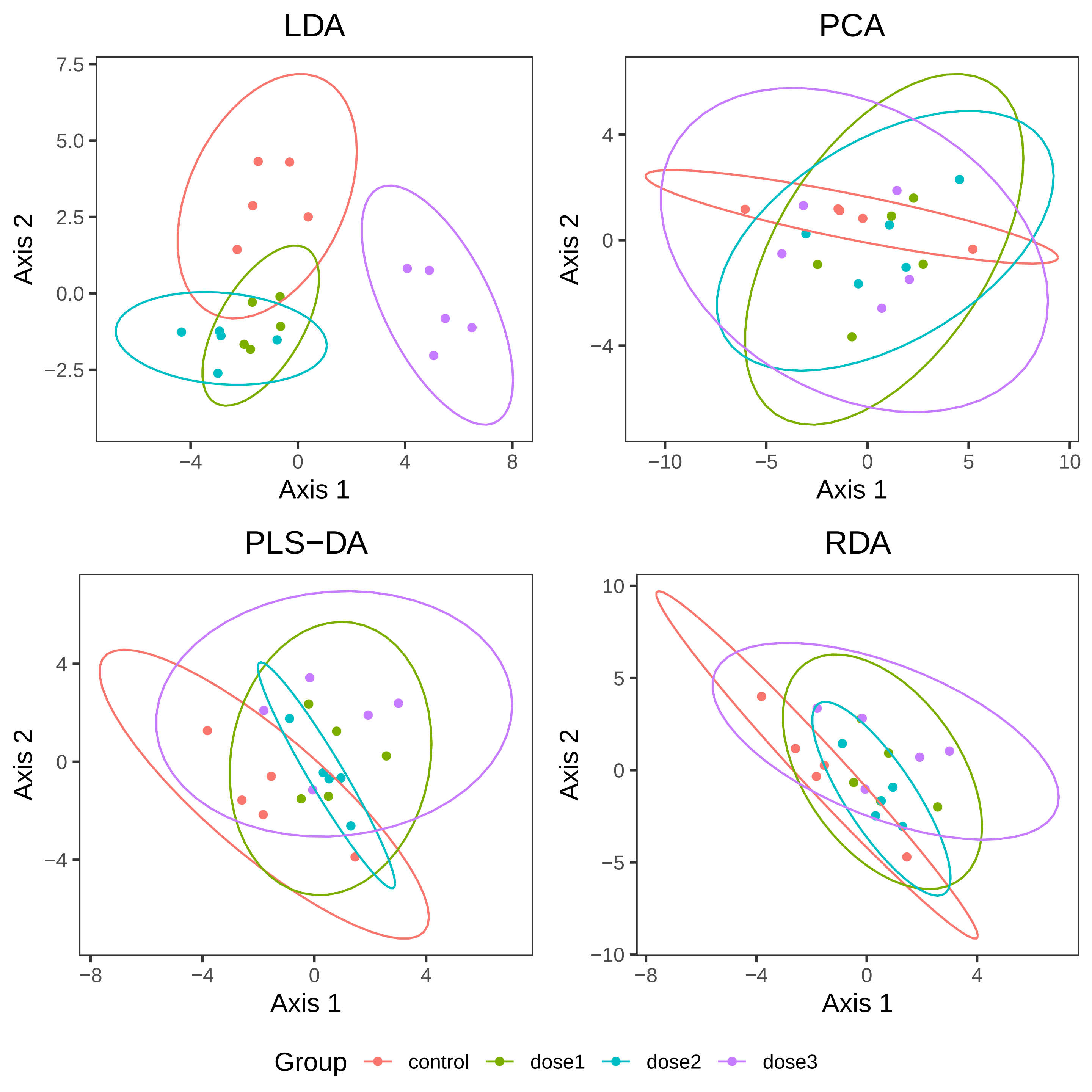
LDA
PCA
PLS−DA
RDA
Axis 1
Axis 2
7.5
5.0
2.5
0.0
−2.5
−4
0
4
8
−10
−5
5
10
Group
control
dose1
dose2
dose3

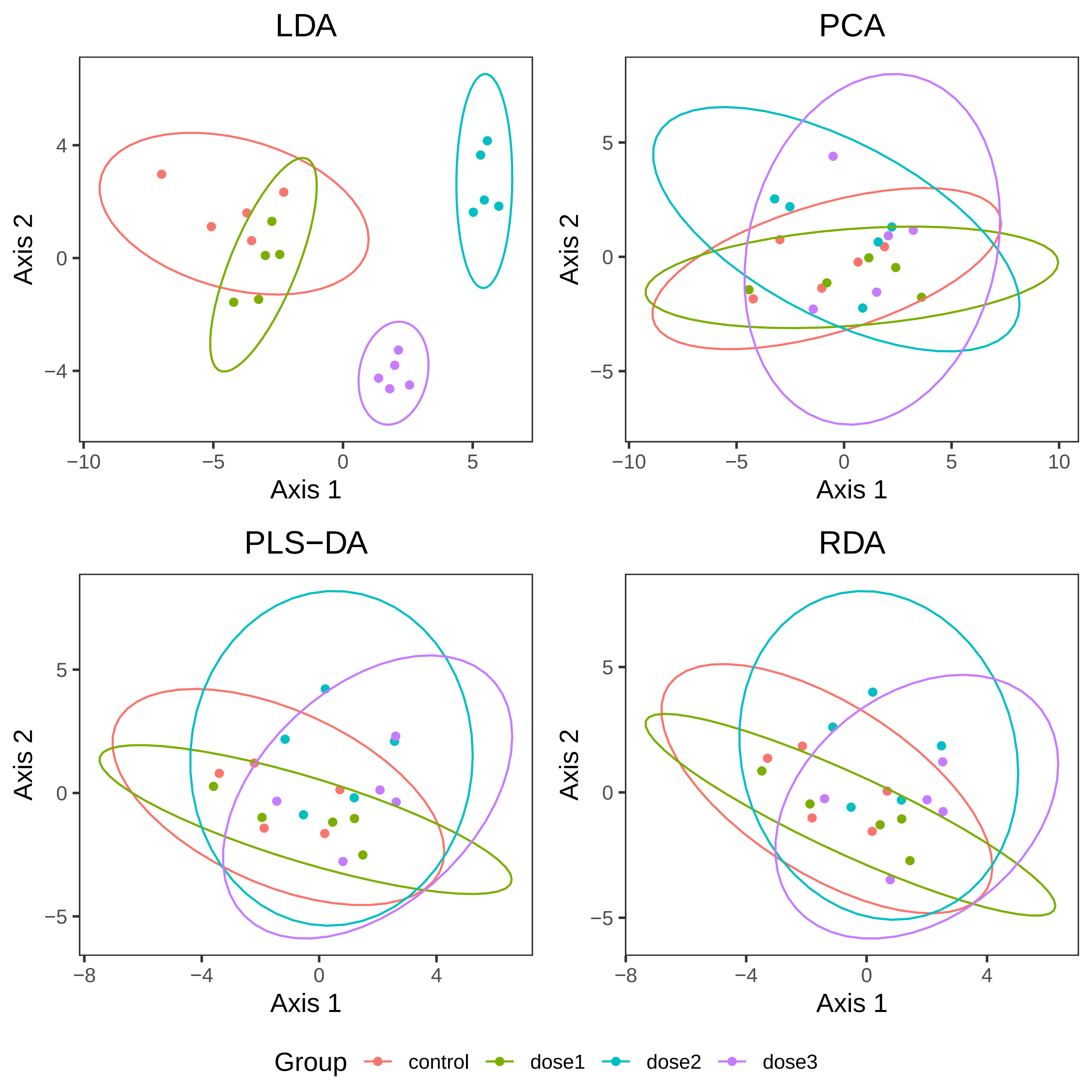
LDA
PCA
PLS−DA
RDA
Axis 1
Axis 2
Group
control
dose1
dose2
dose3

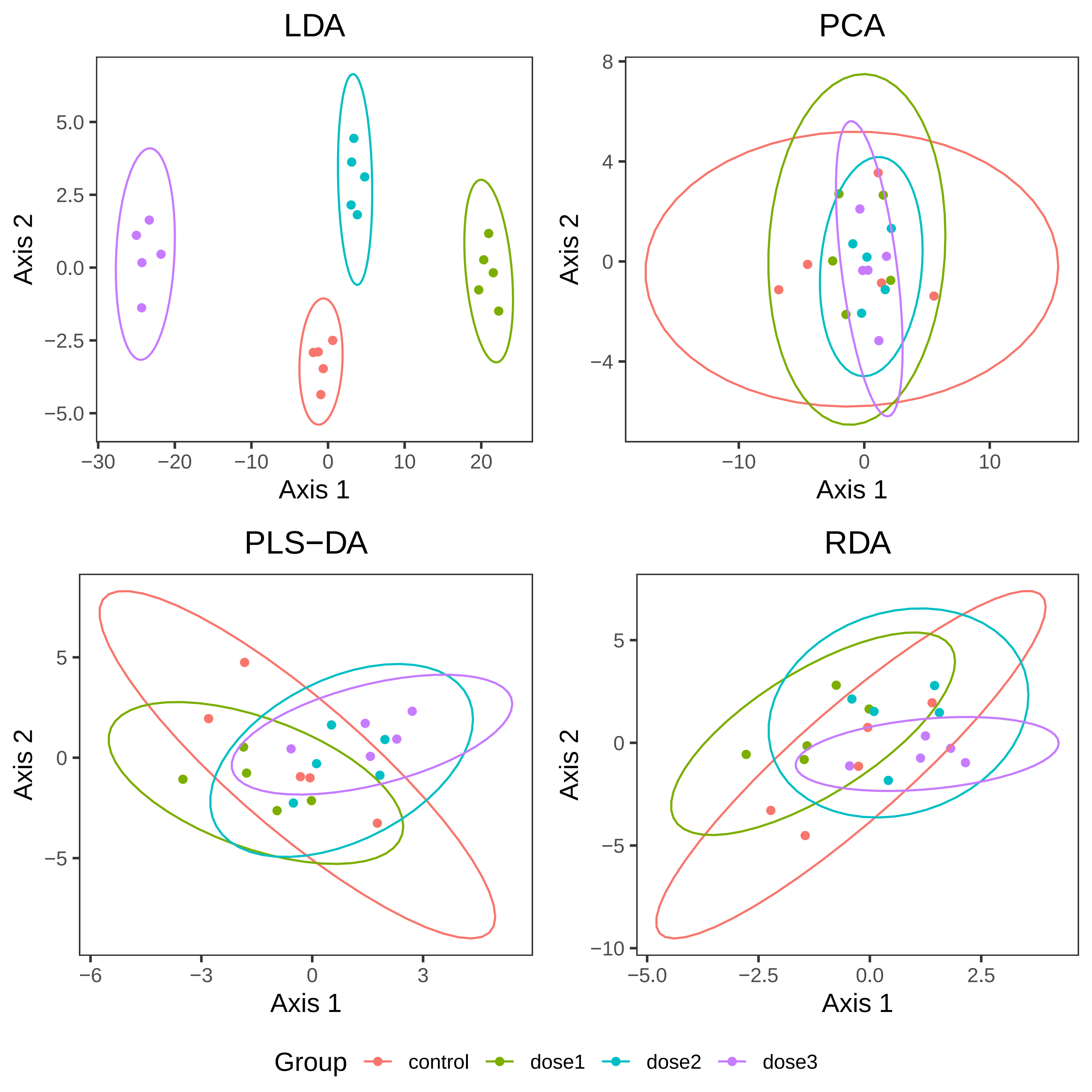
LDA
PCA
PLS−DA
RDA
Axis 1
Axis 2
−30
−20
−10
0
10
20
5.0
2.5
0.0
−2.5
−5.0
8
4
−4
−6
−3
3
5
−5
−5.0
−2.5
0.0
2.5
−10
Group
control
dose1
dose2
dose3

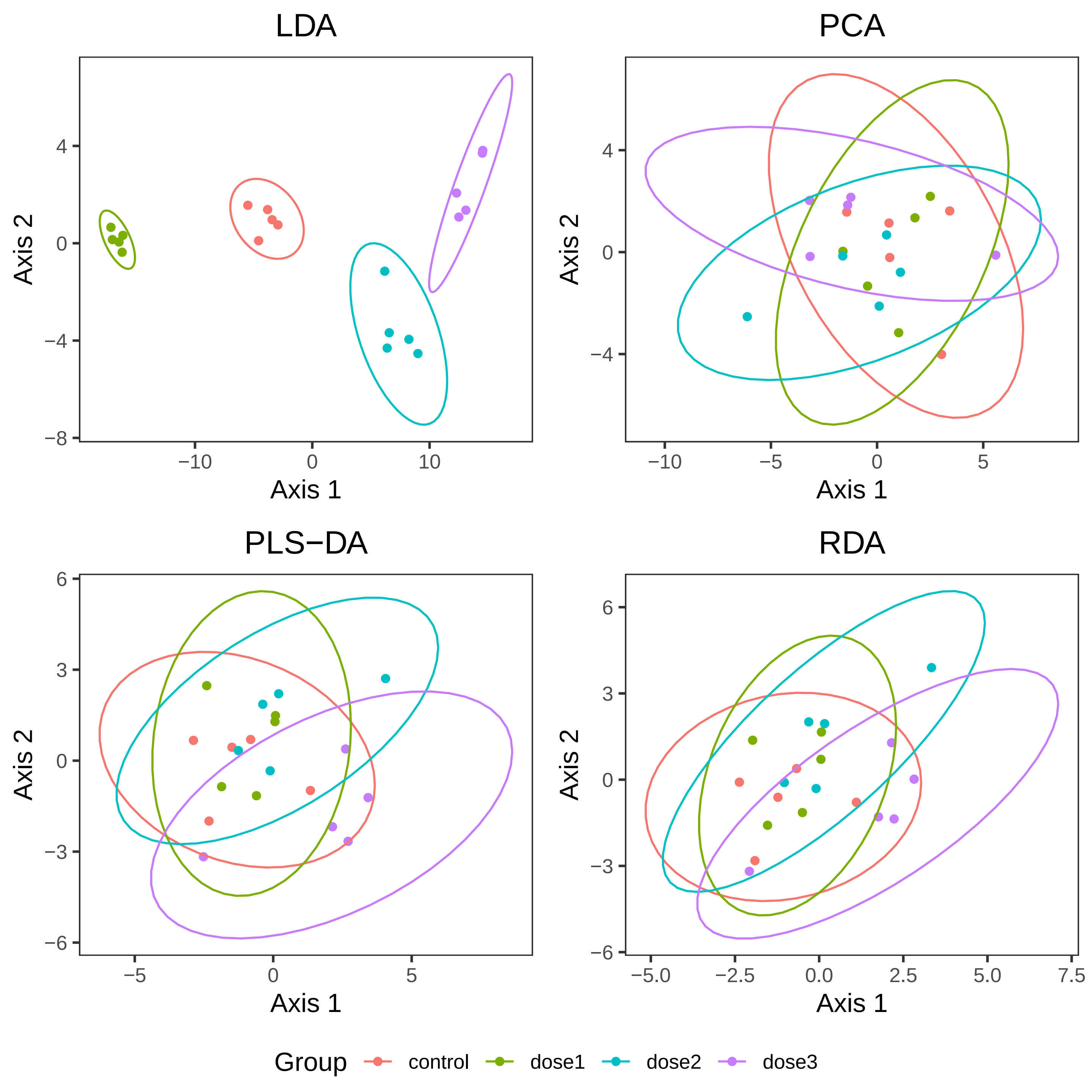
LDA
PCA
PLS−DA
RDA
Axis 1
Axis 2
Group
control
dose1
dose2
dose3

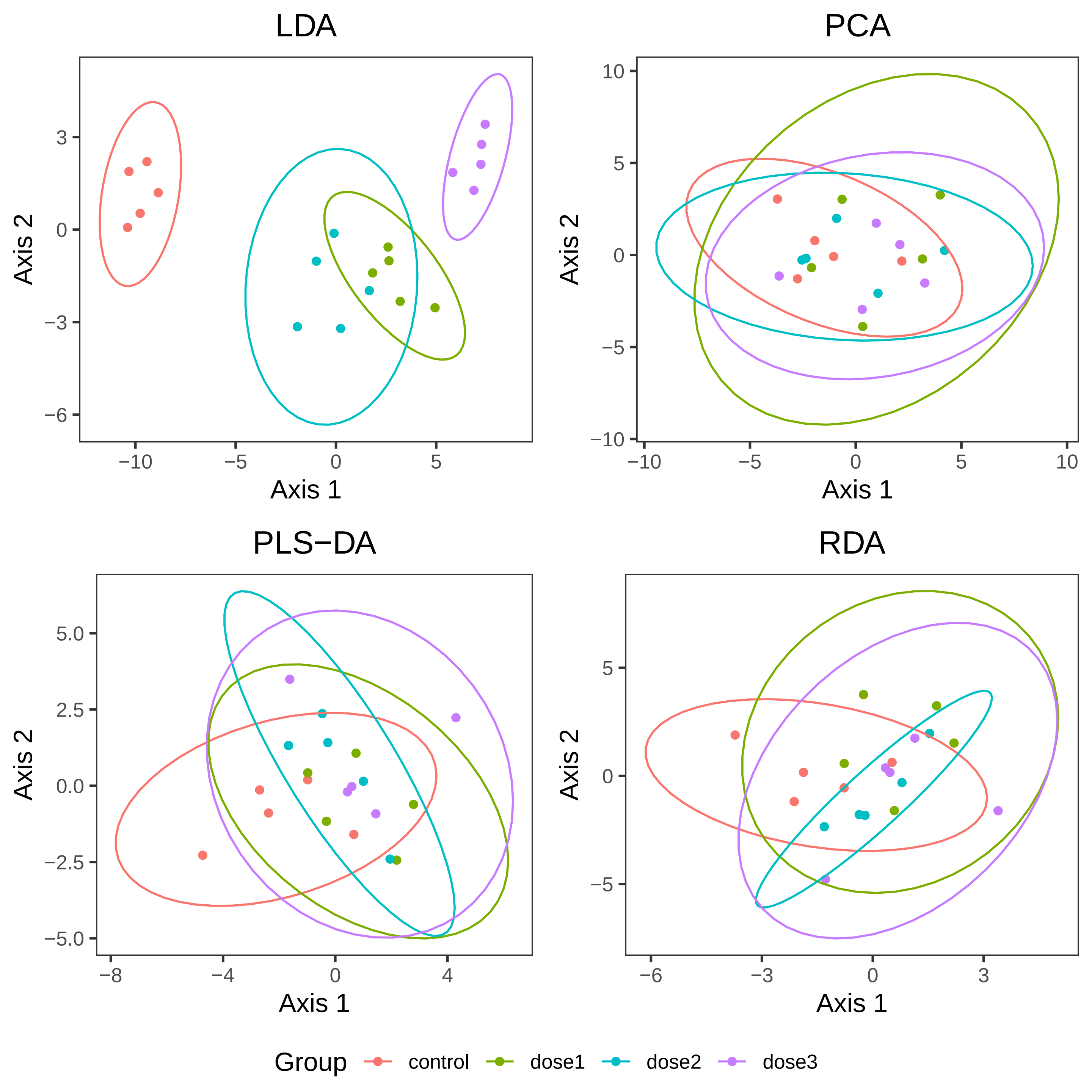
LDA
PCA
PLS−DA
RDA
Axis 1
Axis 2
Group
control
dose1
dose2
dose3

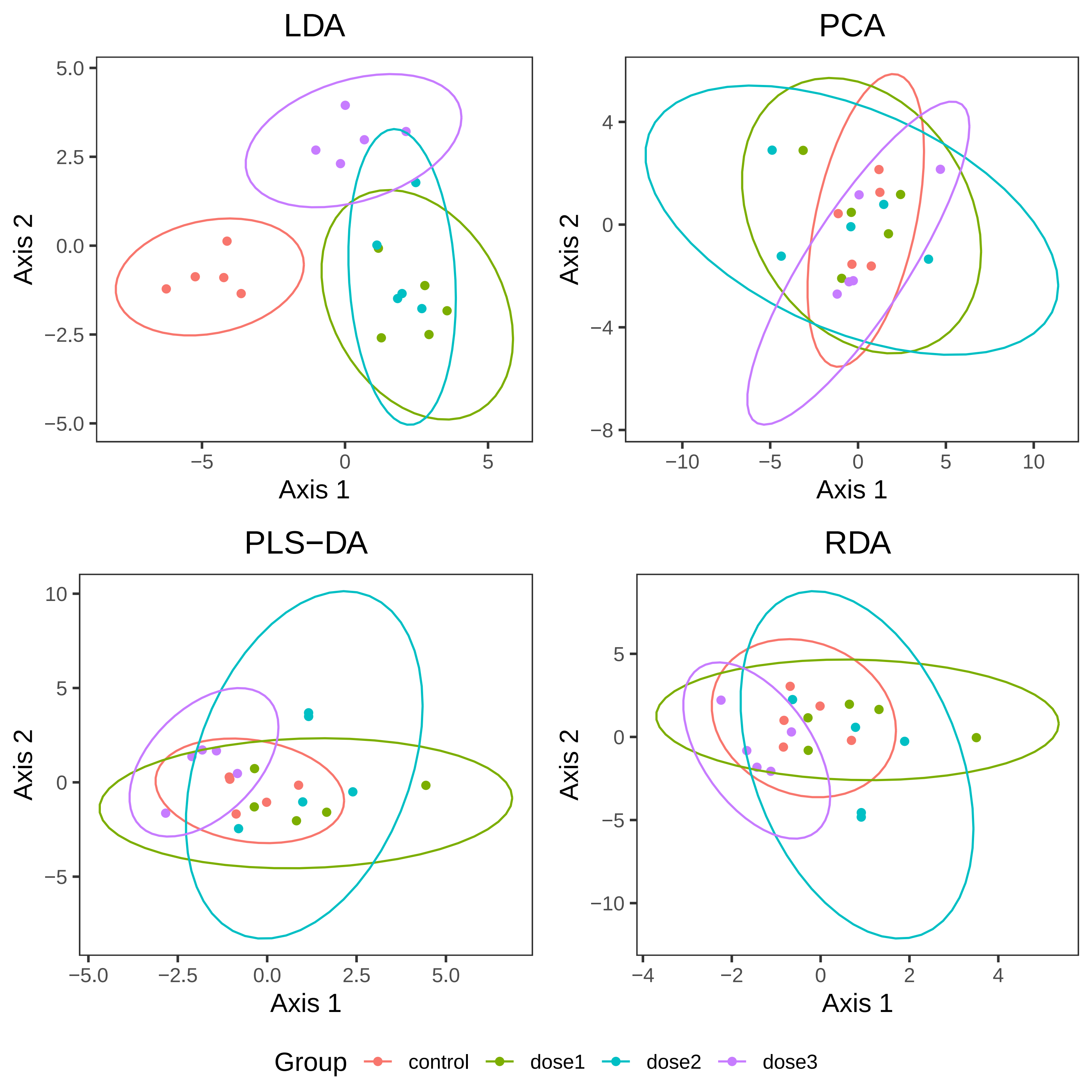

LDA
PCA
PLS−DA
RDA
Axis 1
Axis 2
Group
control
dose1
dose2
dose3

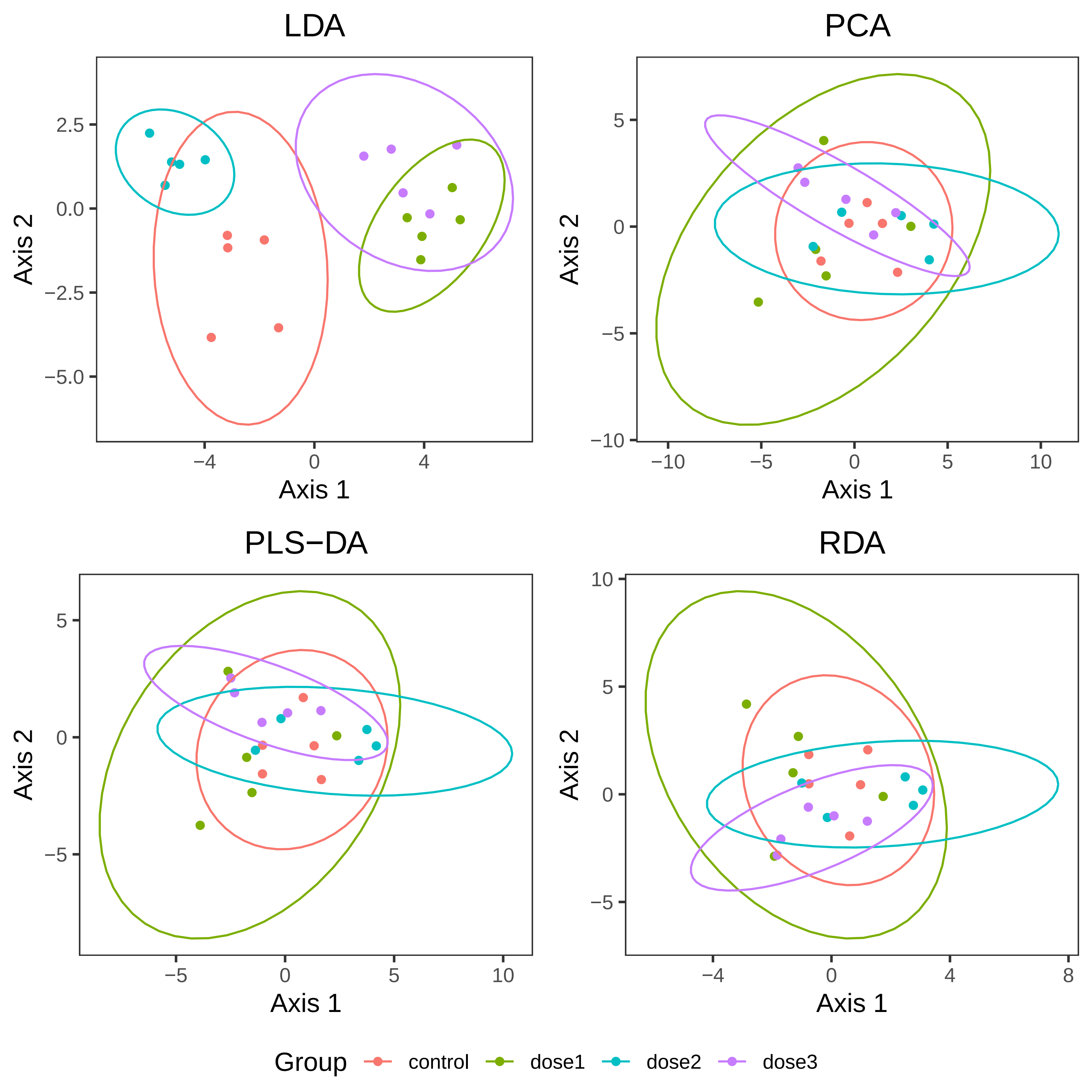

LDA
PCA
PLS−DA
RDA
Axis 1
Axis 2
Group
control
dose1
dose2
dose3

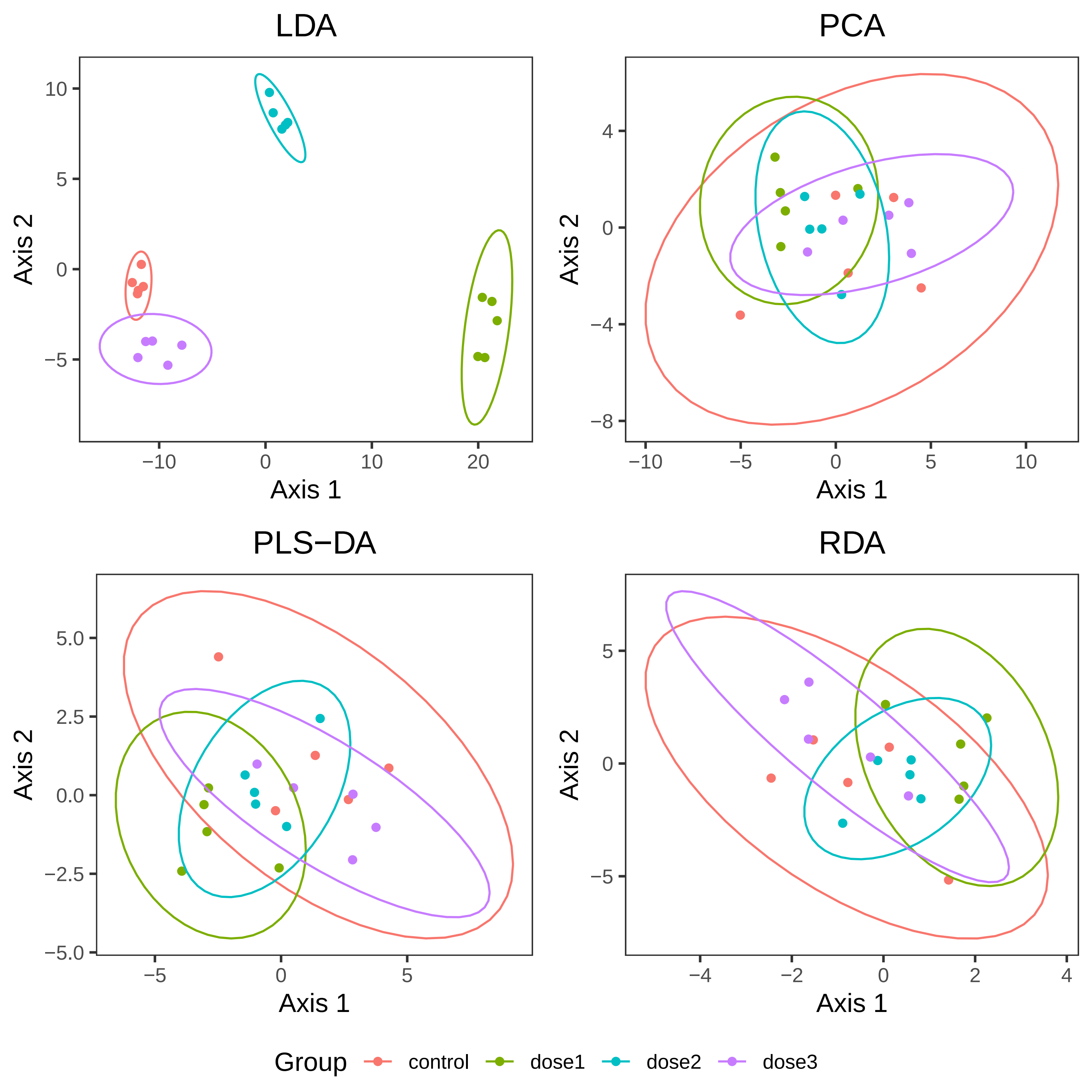

LDA
PCA
PLS−DA
RDA
Axis 1
Axis 2
Group
control
dose1
dose2
dose3

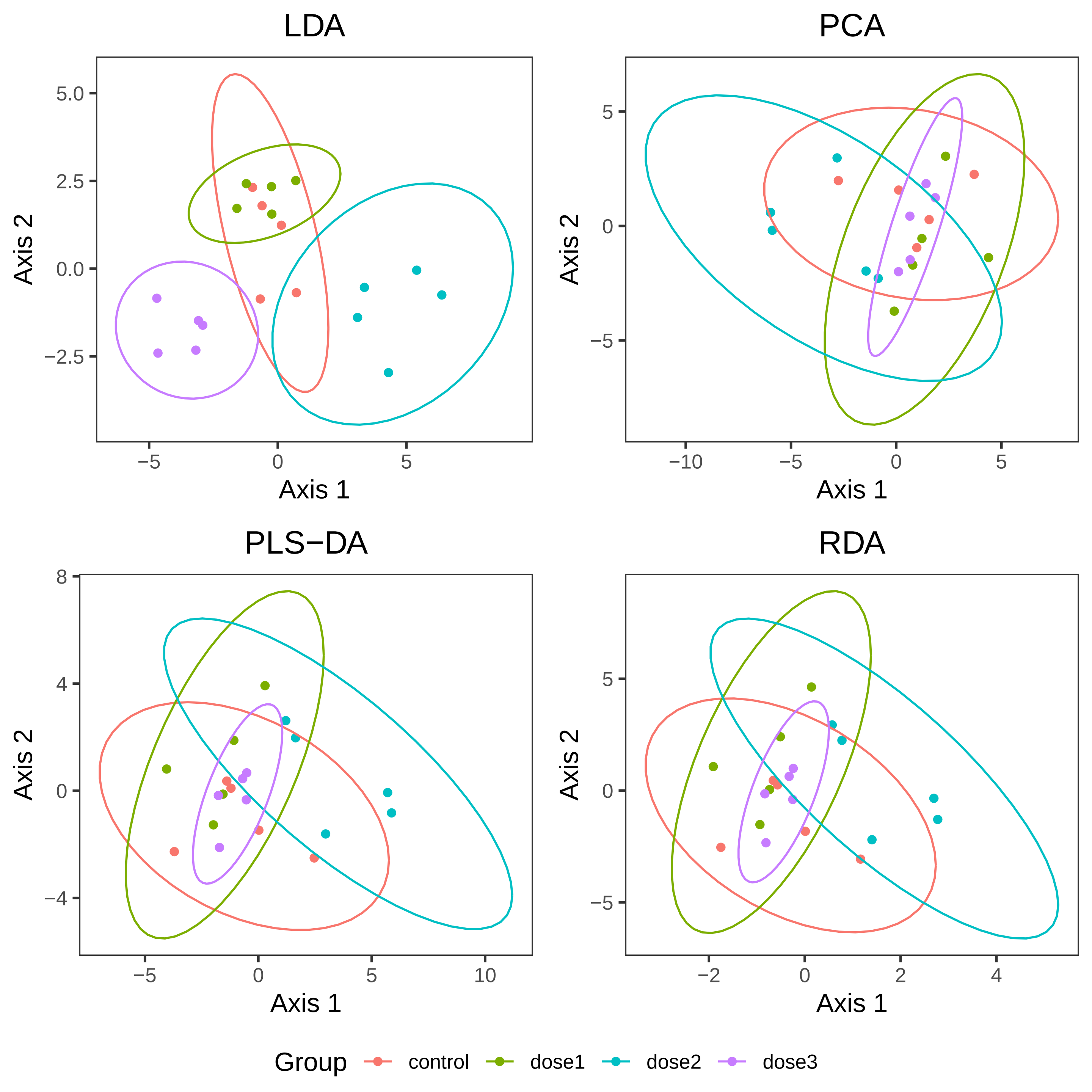
LDA
PCA
PLS−DA
RDA
Axis 1
Axis 2
5.0
2.5
0.0
−2.5
−5
0
5
−10
8
4
−4
10
−2
2
Group
control
dose1
dose2
dose3

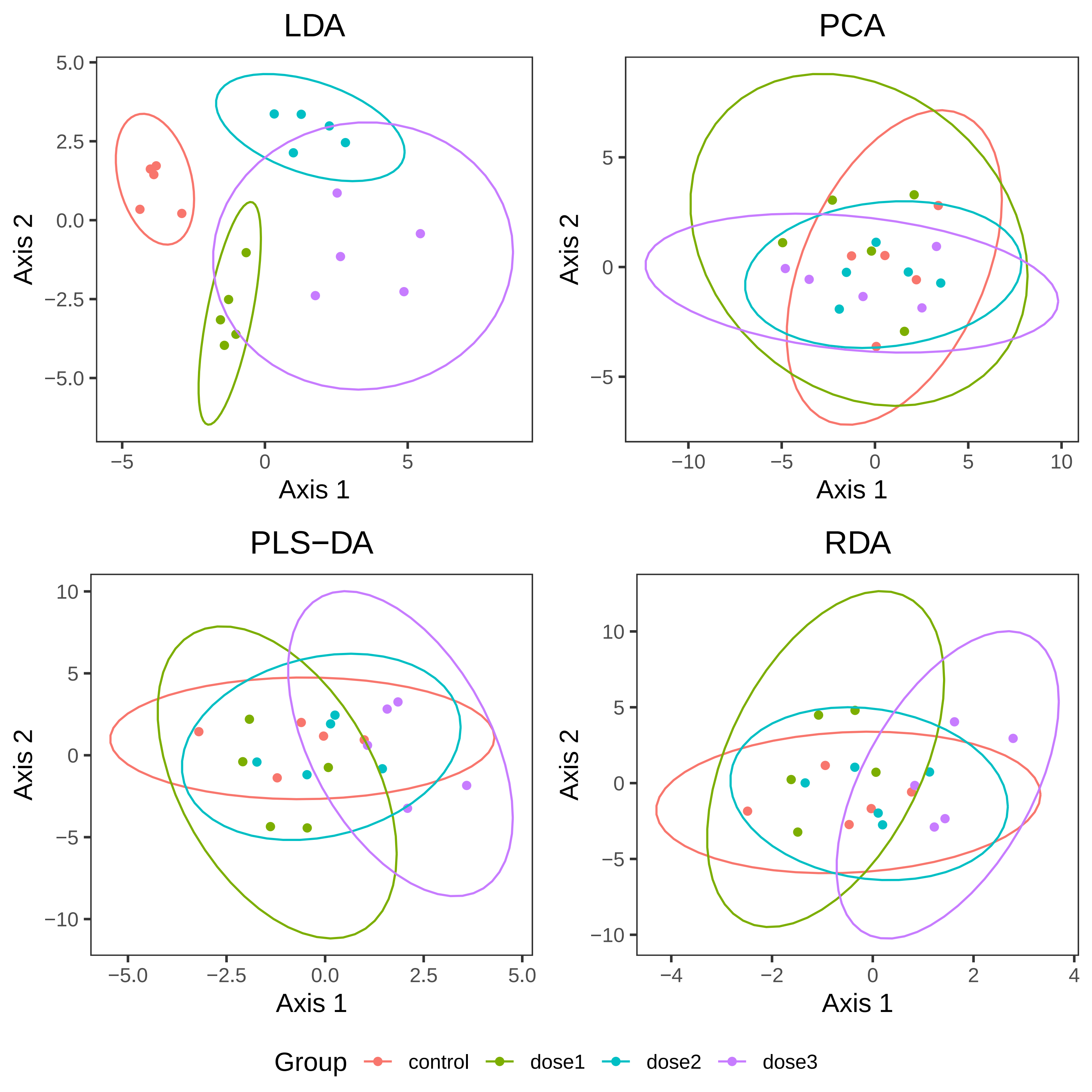

LDA
PCA
PLS−DA
RDA
Axis 1
Axis 2
Group
control
dose1
dose2
dose3

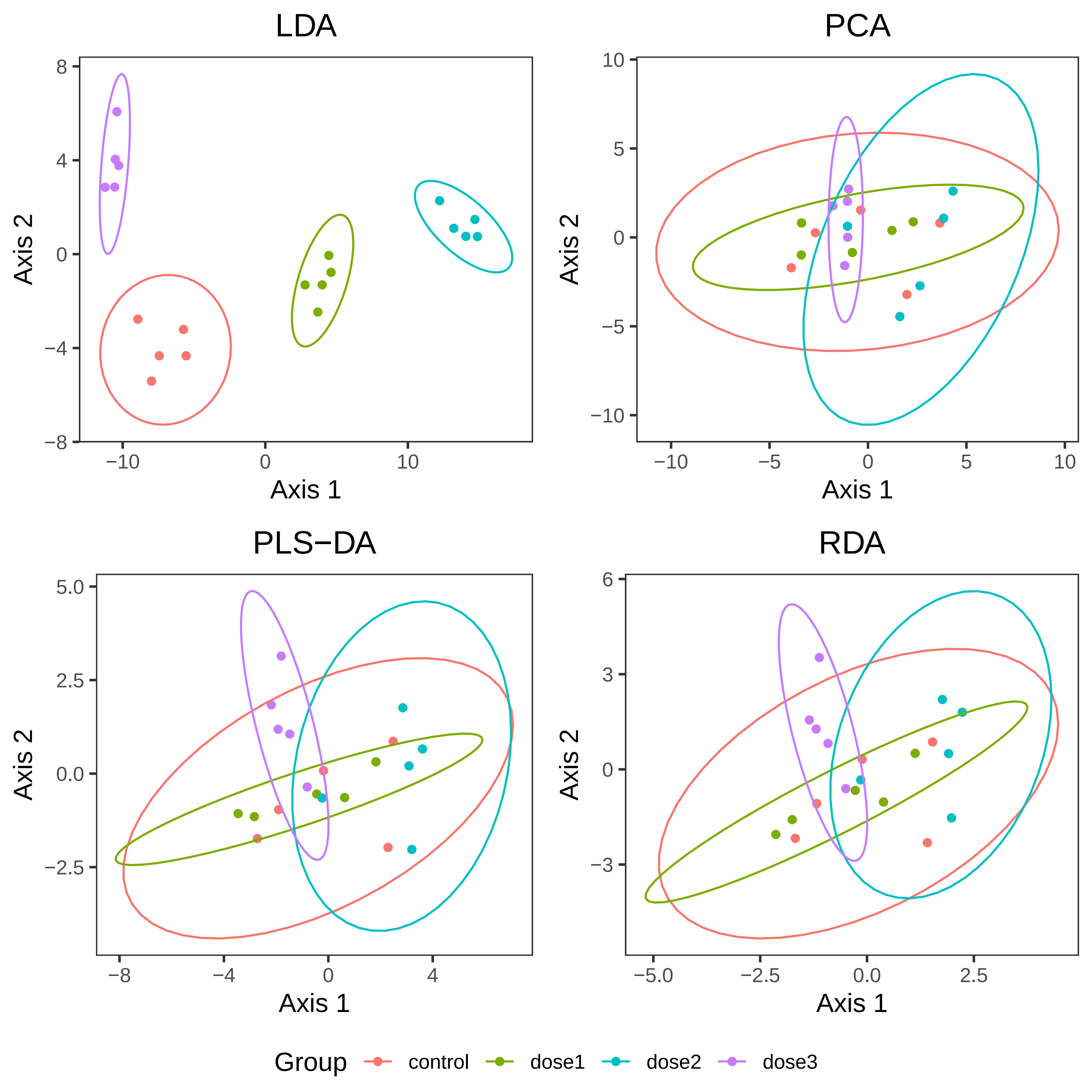
LDA
PCA
PLS−DA
RDA
Axis 1
Axis 2
Group
control
dose1
dose2
dose3

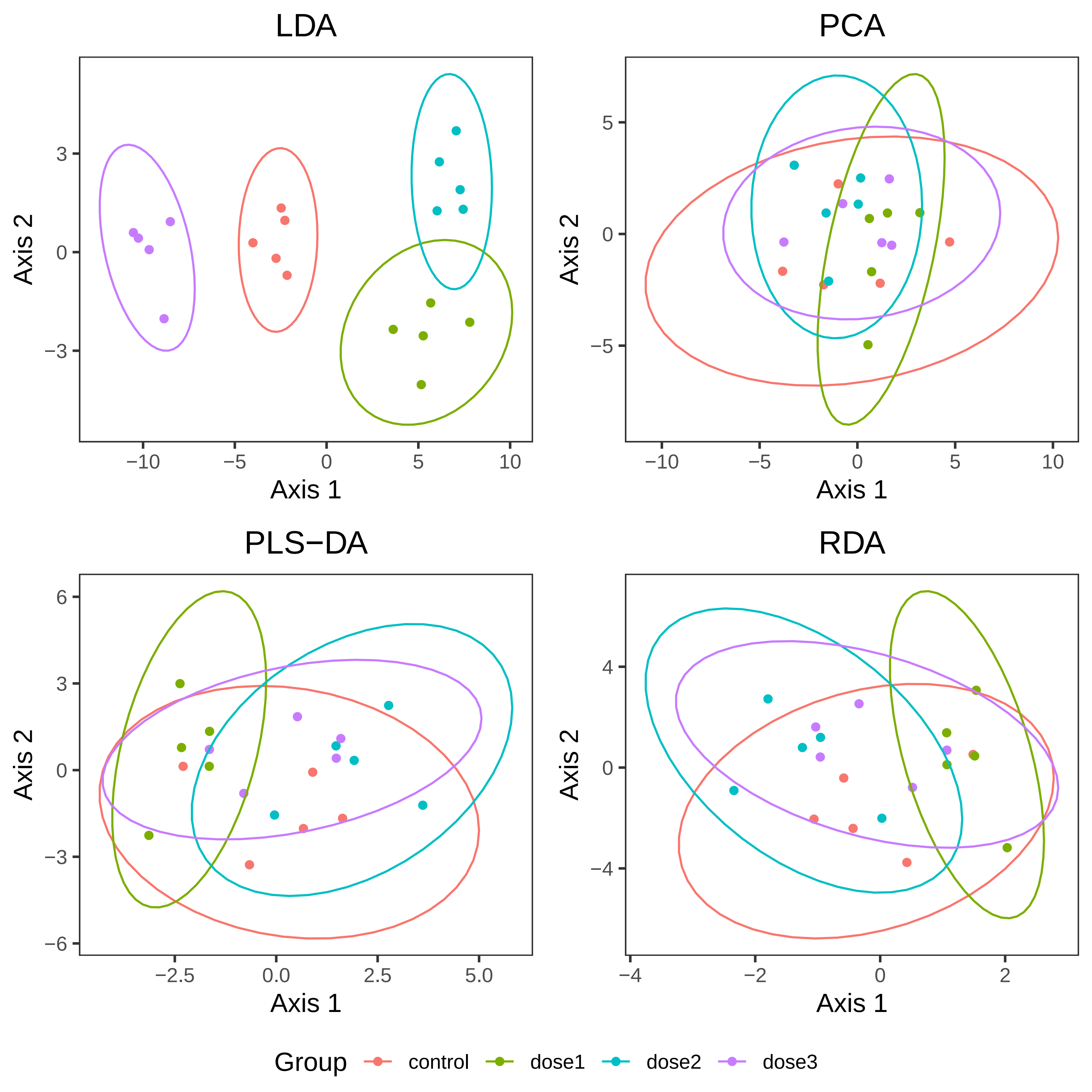
LDA
PCA
PLS−DA
RDA
Axis 1
Axis 2
Group
control
dose1
dose2
dose3

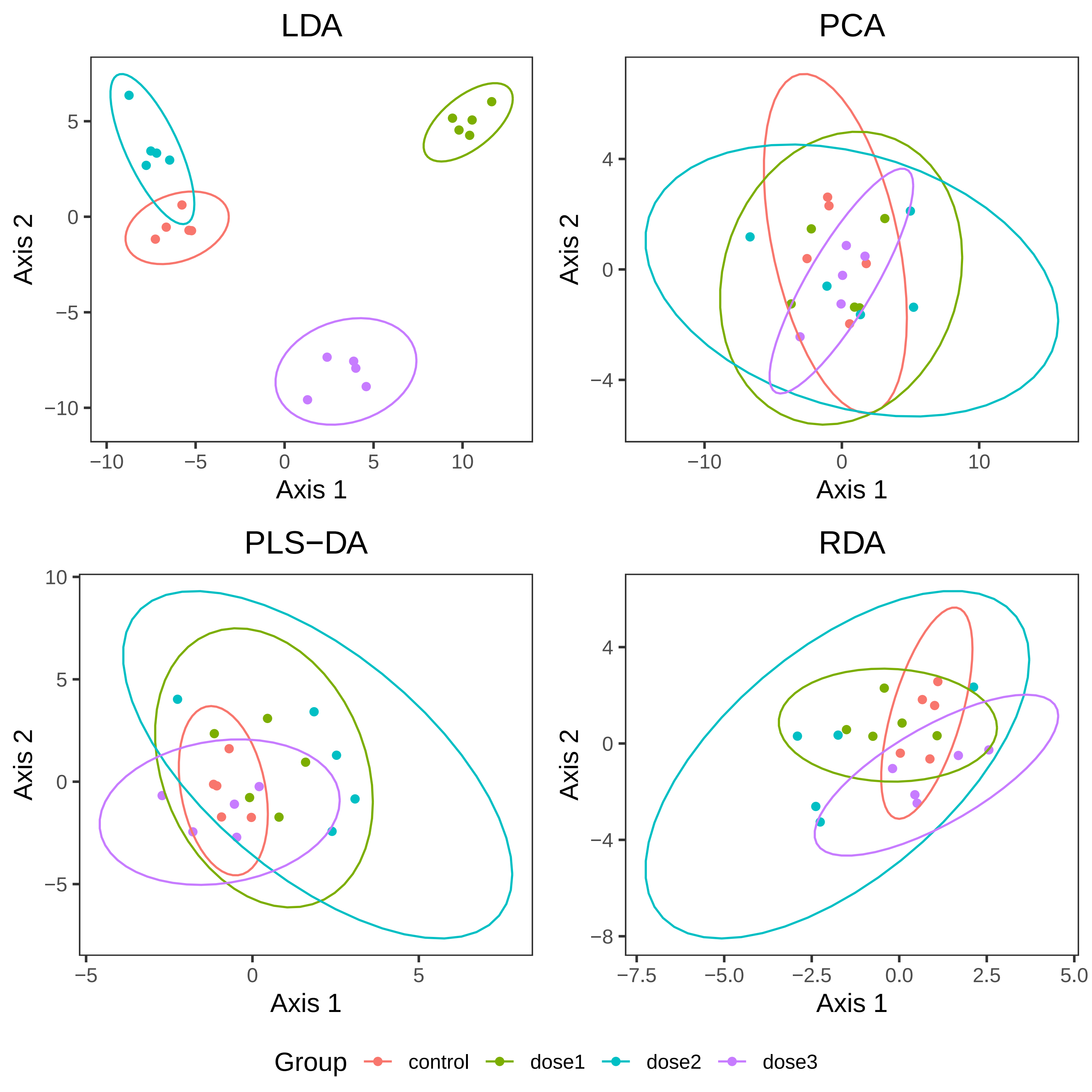

LDA
PCA
PLS−DA
RDA
Axis 1
Axis 2
Group
control
dose1
dose2
dose3

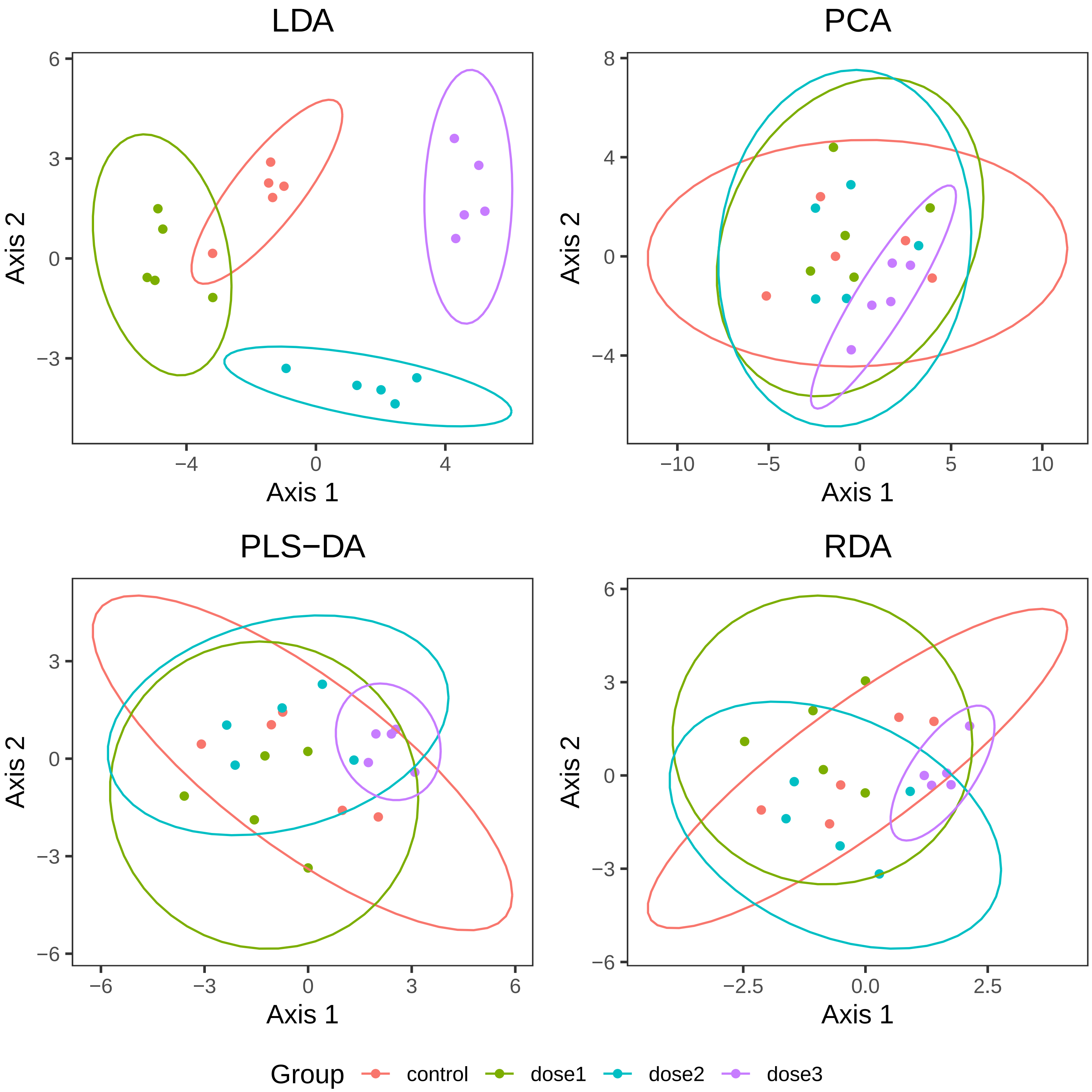
LDA
PCA
PLS−DA
RDA
Axis 1
Axis 2
Group
control
dose1
dose2
dose3

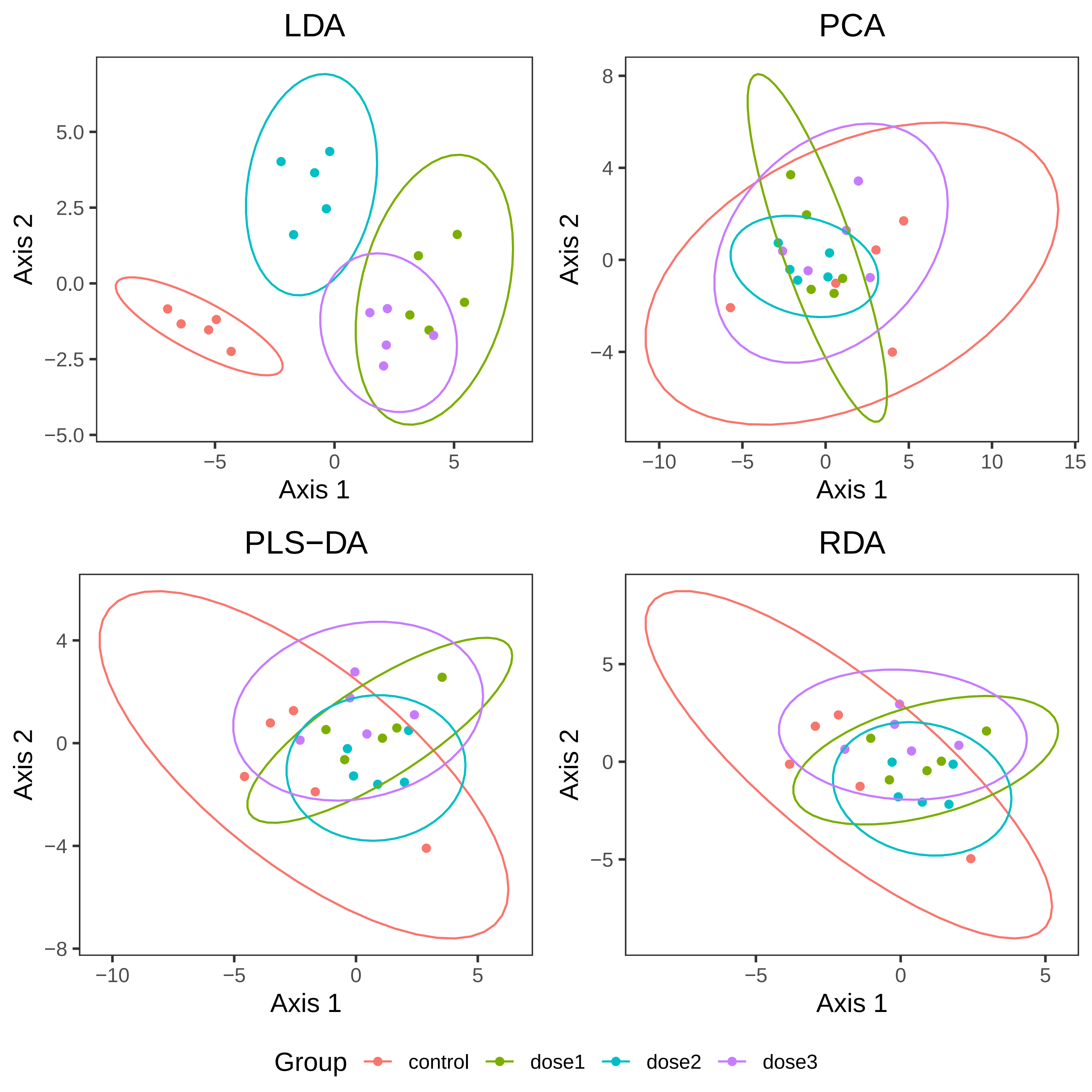
LDA
PCA
PLS−DA
RDA
Axis 1
Axis 2
Group
control
dose1
dose2
dose3

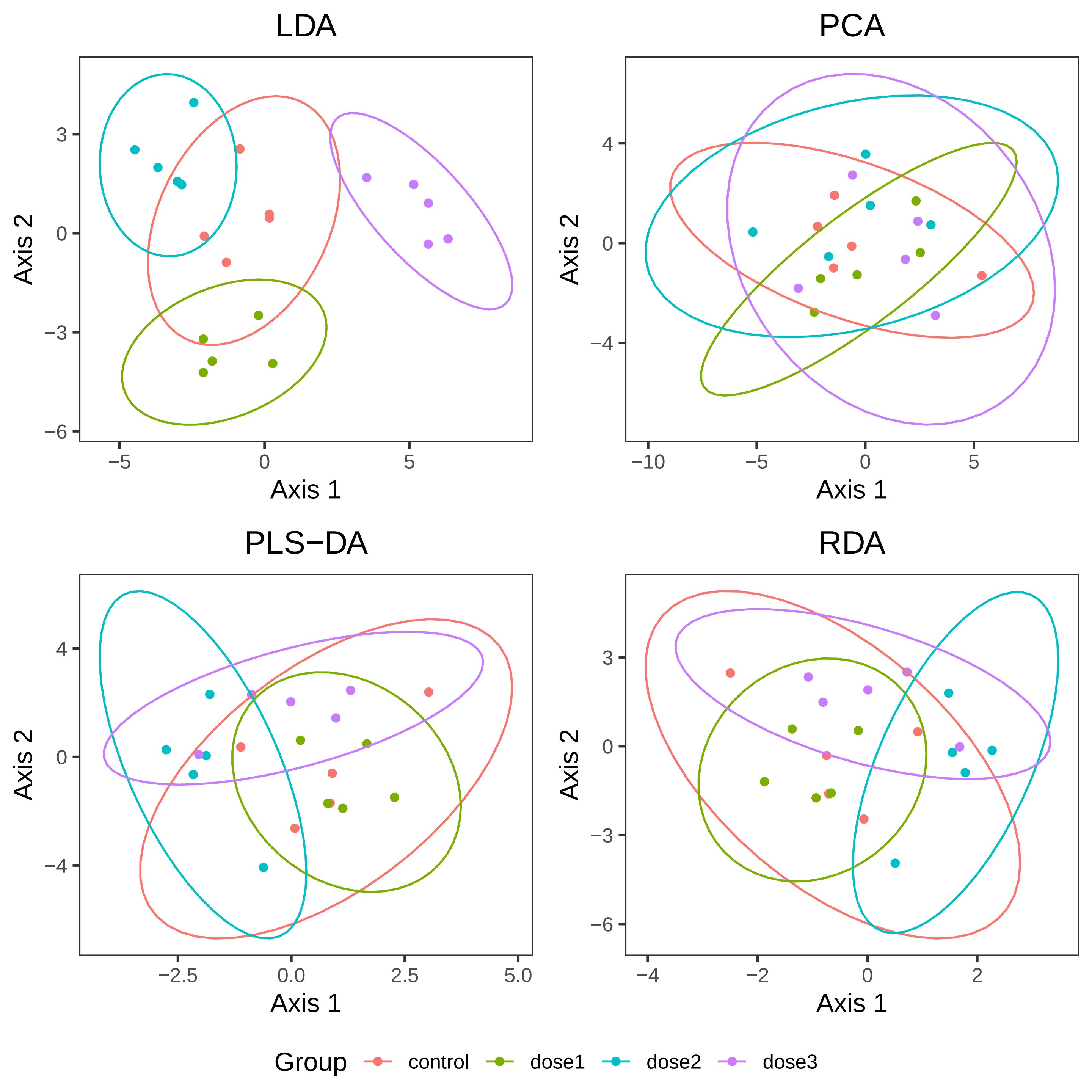

LDA
PCA
PLS−DA
RDA
Axis 1
Axis 2
Group
control
dose1
dose2
dose3

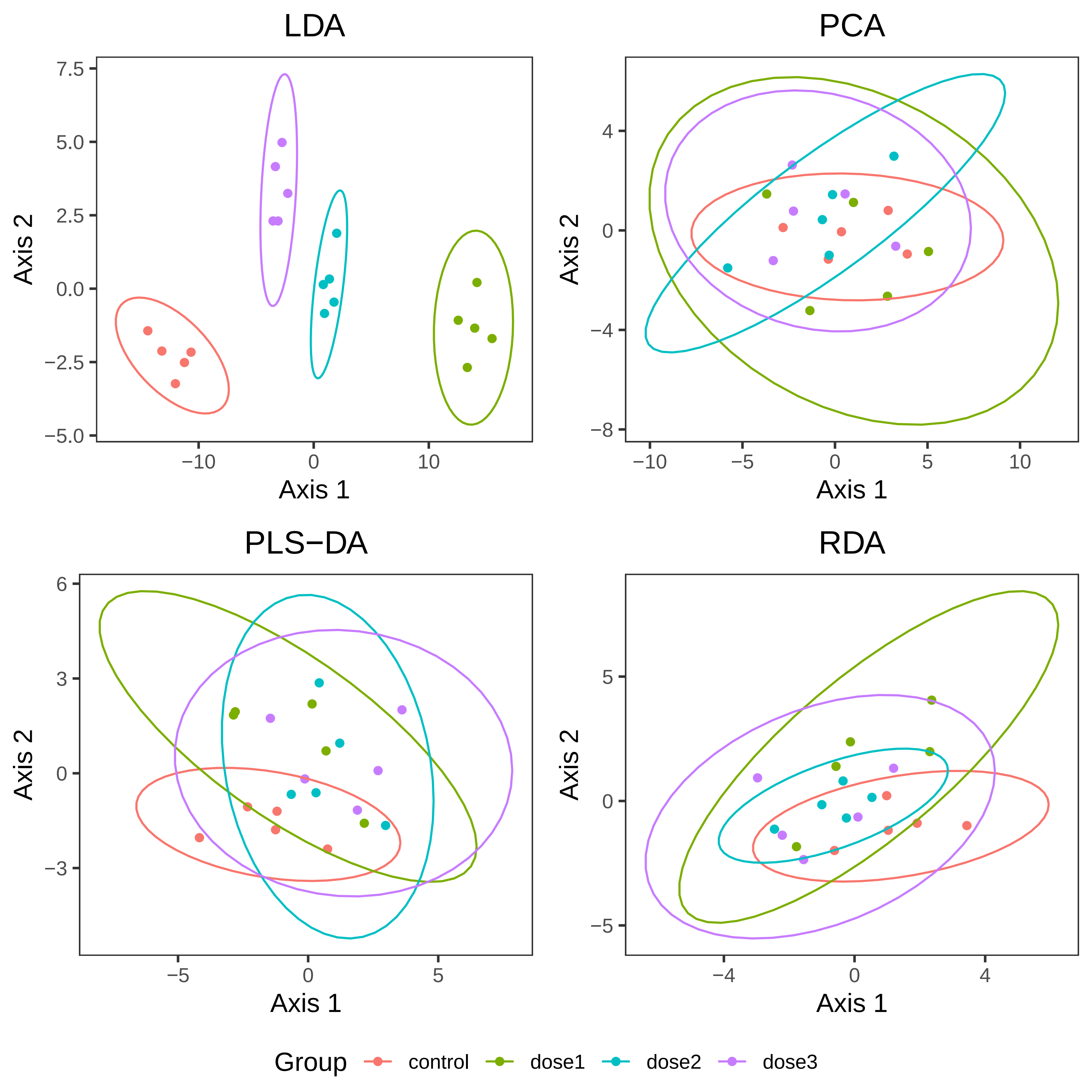

LDA
PCA
PLS−DA
RDA
Axis 1
Axis 2
7.5
5.0
2.5
0.0
−2.5
−5.0
−10
0
10
4
0
−4
−8
−5
5
6
3
−3
Group
control
dose1
dose2
dose3

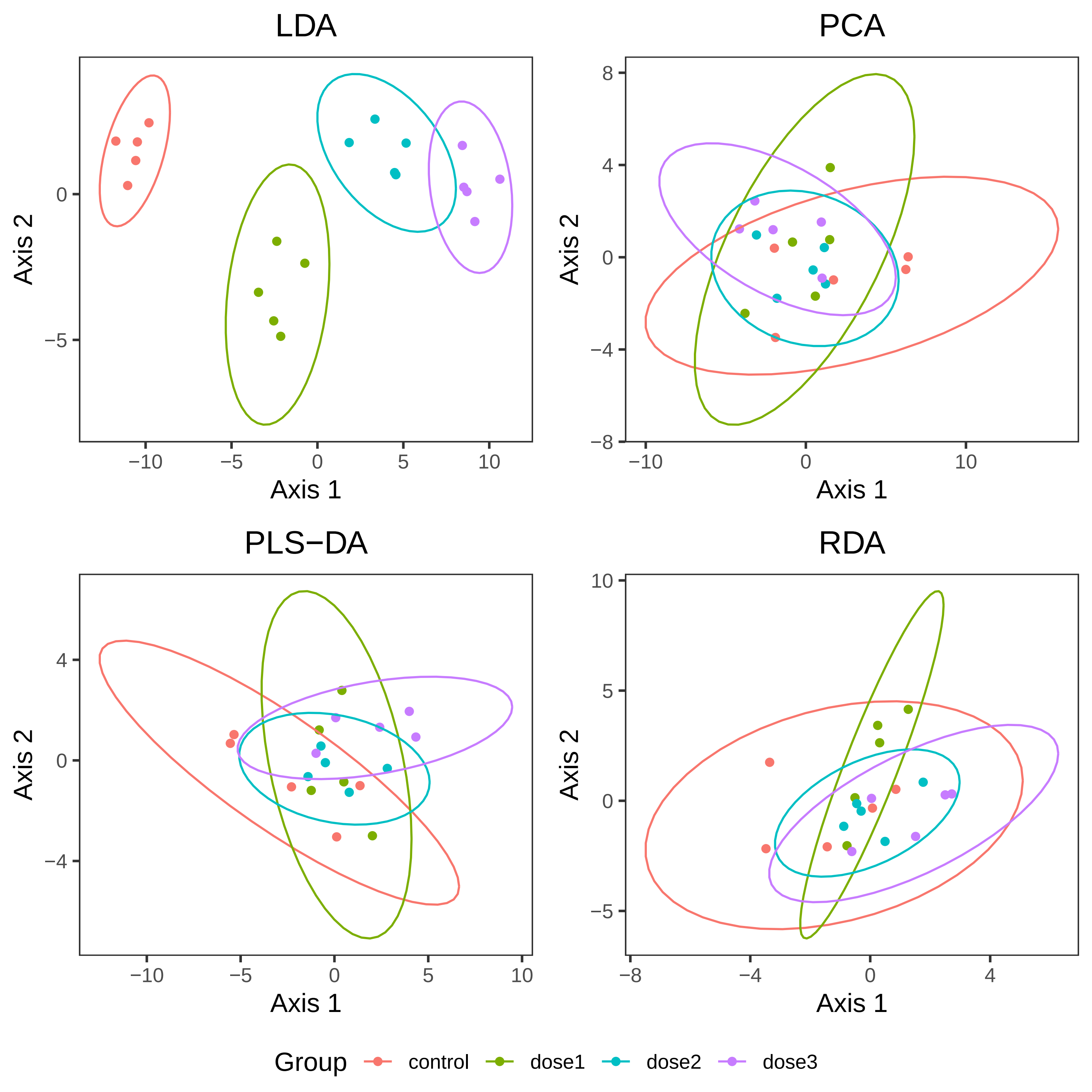

LDA
PCA
PLS−DA
RDA
Axis 1
Axis 2
−10
−5
0
5
10
8
4
−4
−8
−8
−4
Group
control
dose1
dose2
dose3

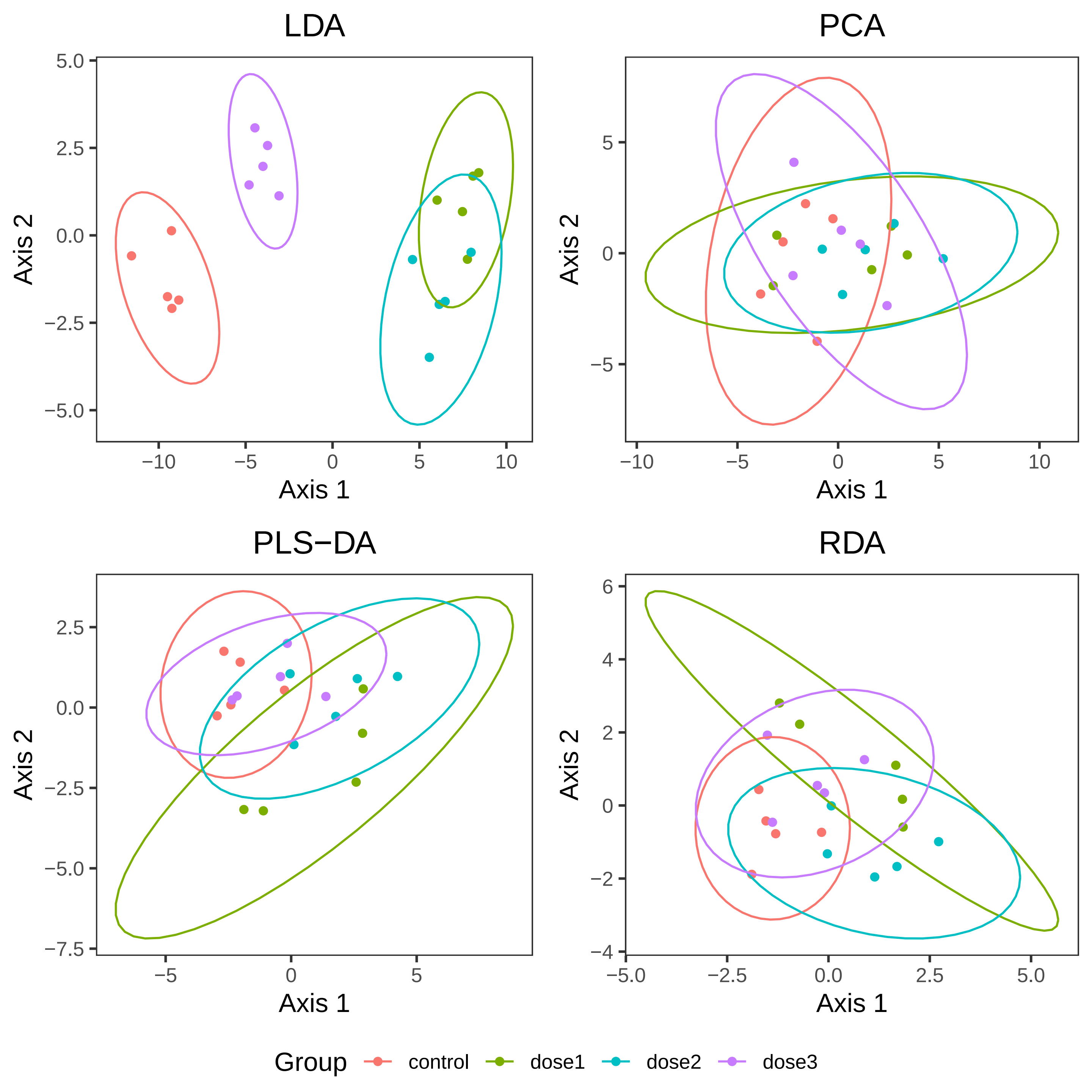

LDA
PCA
PLS−DA
RDA
Axis 1
Axis 2
5.0
2.5
0.0
−2.5
−5.0
−10
−5
0
5
10
−7.5
6
4
2
−2
−4
Group
control
dose1
dose2
dose3

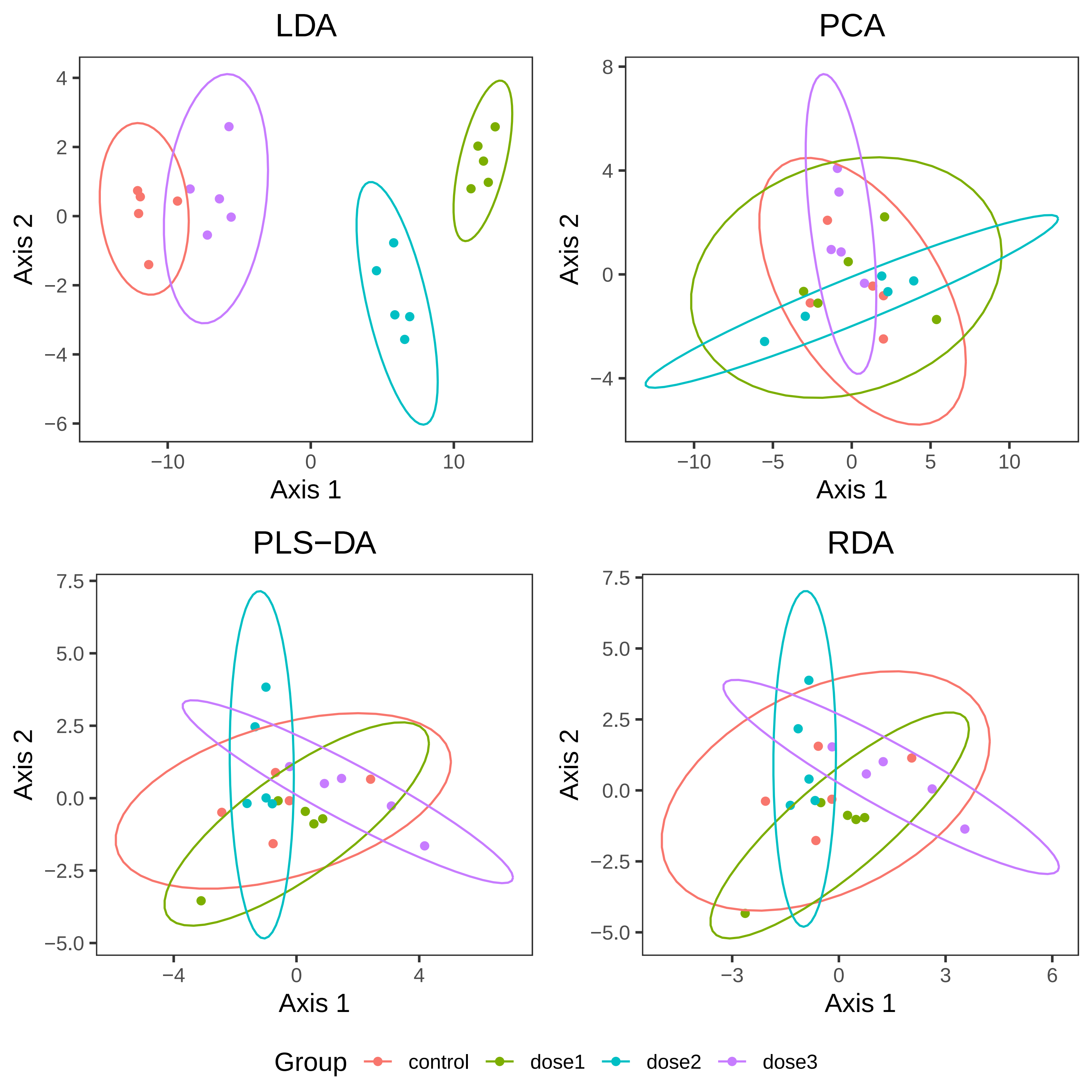
LDA
PCA
PLS−DA
RDA
Axis 1
Axis 2
Group
control
dose1
dose2
dose3

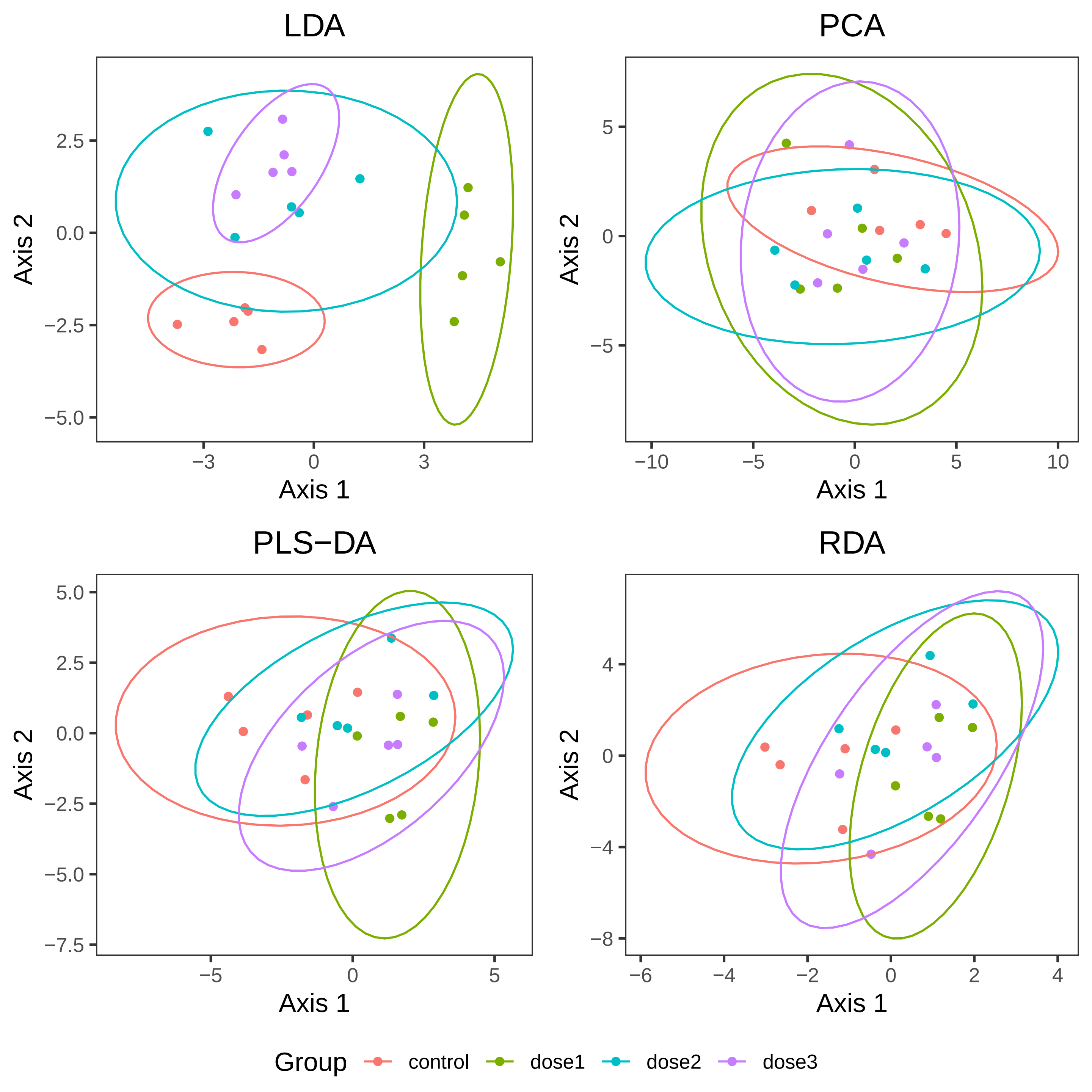

LDA
PCA
PLS−DA
RDA
Axis 1
Axis 2
Group
control
dose1
dose2
dose3

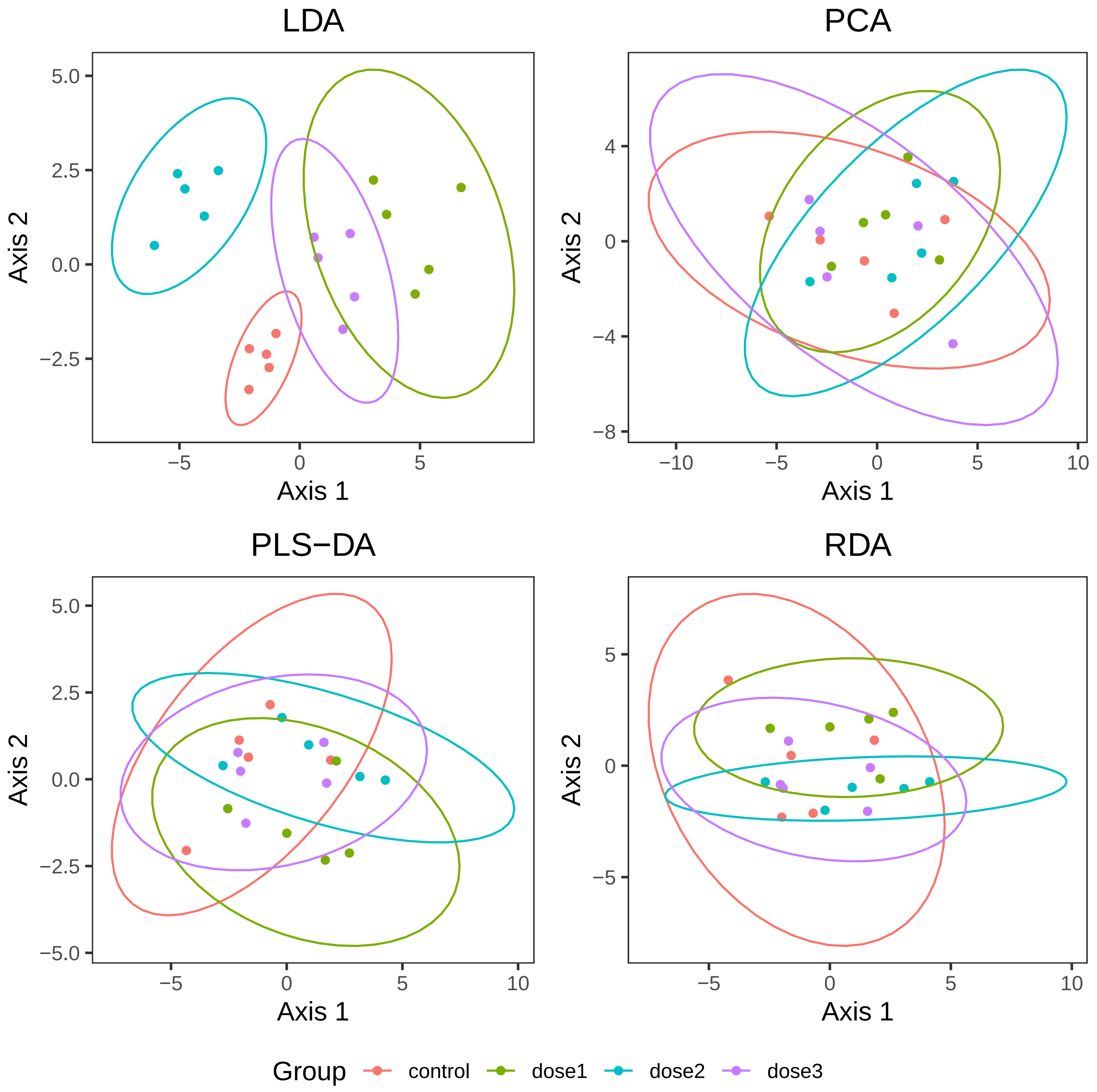

LDA
PCA
PLS−DA
RDA
Axis 1
Axis 2
Group
control
dose1
dose2
dose3

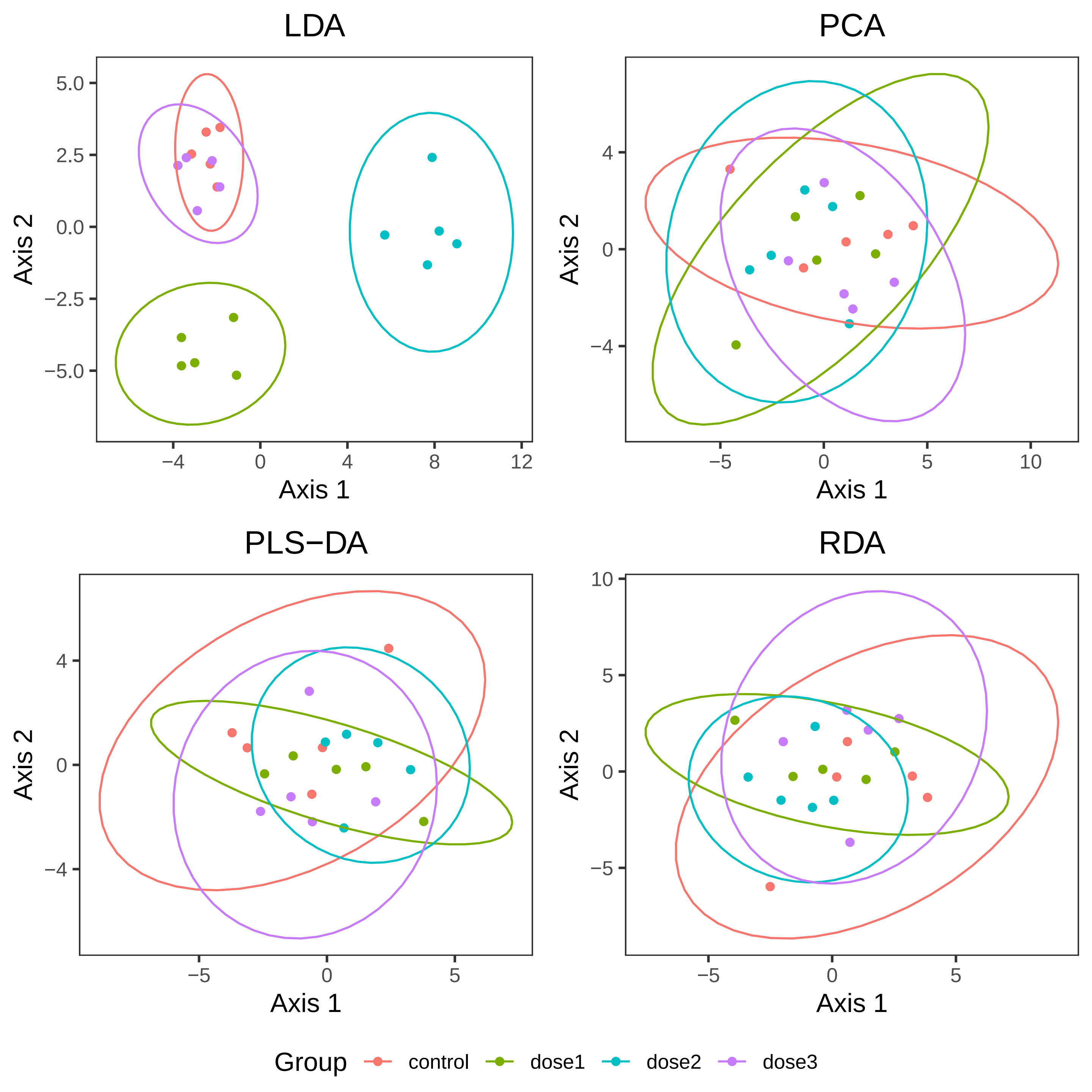

LDA
PCA
PLS−DA
RDA
Axis 1
Axis 2
5.0
2.5
0.0
−2.5
−5.0
−4
0
4
8
12
−5
5
10
−4
Group
control
dose1
dose2
dose3

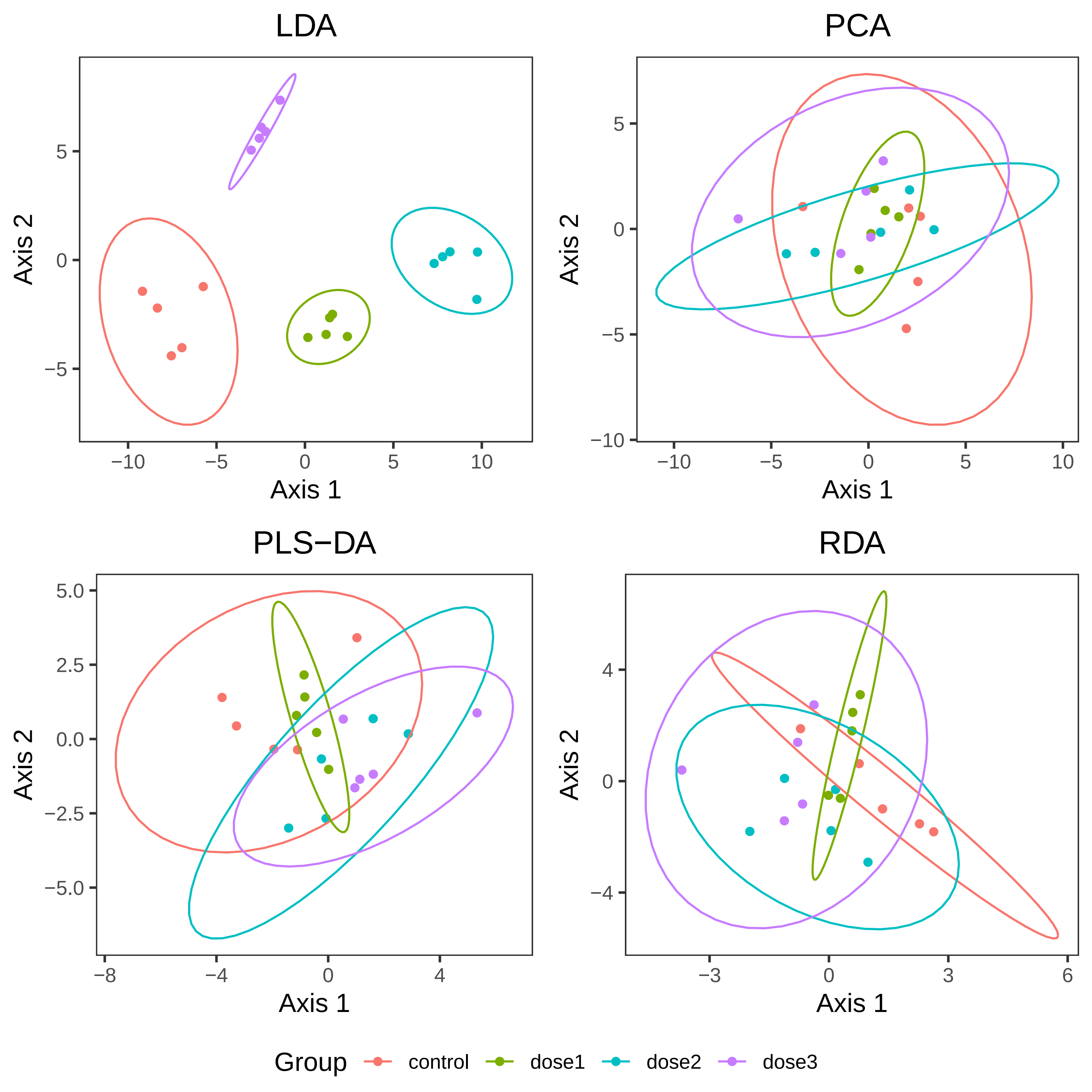
LDA
PCA
PLS−DA
RDA
Axis 1
Axis 2
−10
−5
0
5
10
5.0
2.5
0.0
−2.5
−5.0
−8
−4
4
−3
3
6
Group
control
dose1
dose2
dose3

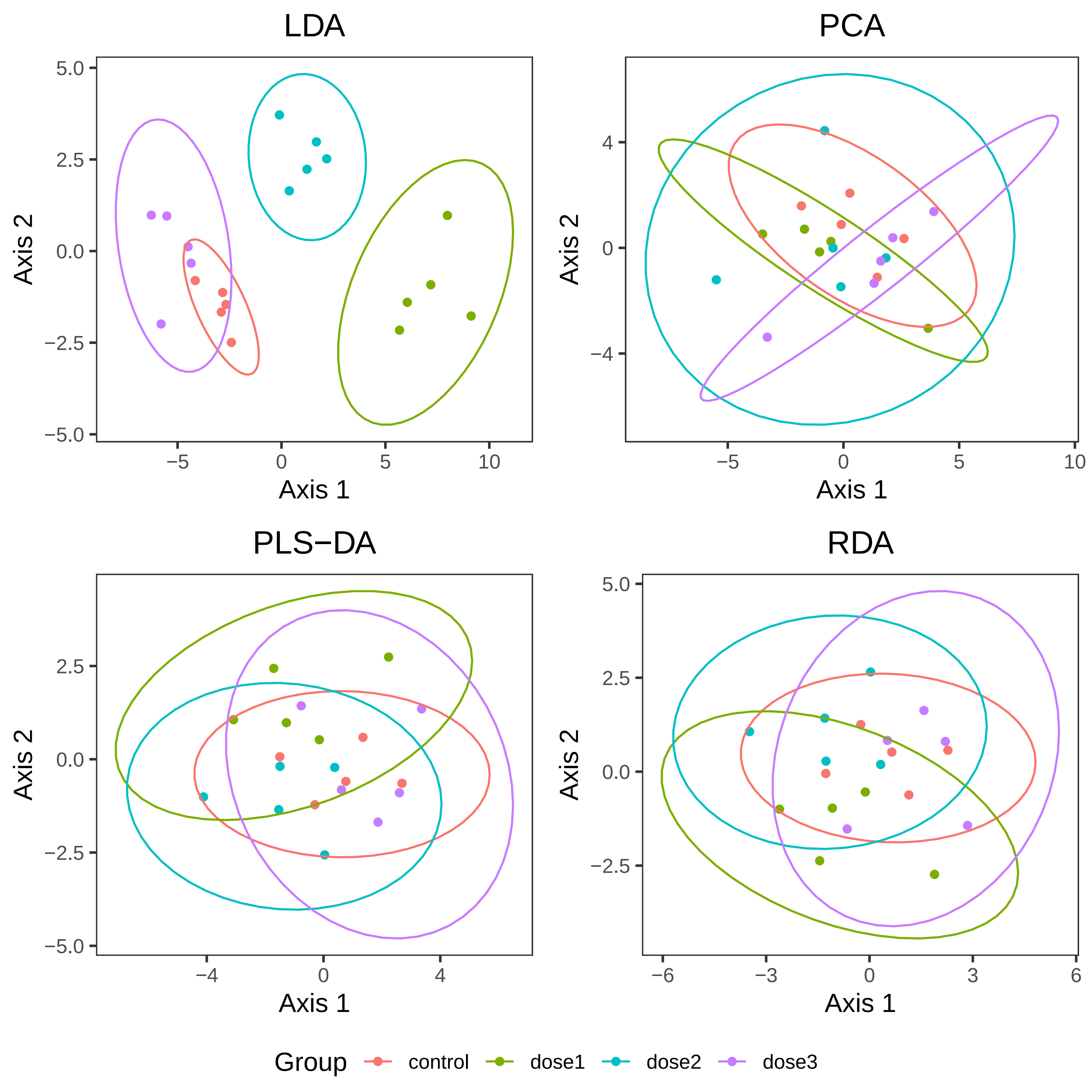

LDA
PCA
PLS−DA
RDA
Axis 1
Axis 2
5.0
2.5
0.0
−2.5
−5.0
−5
0
5
10
4
0
−4
−6
−3
3
6
Group
control
dose1
dose2
dose3

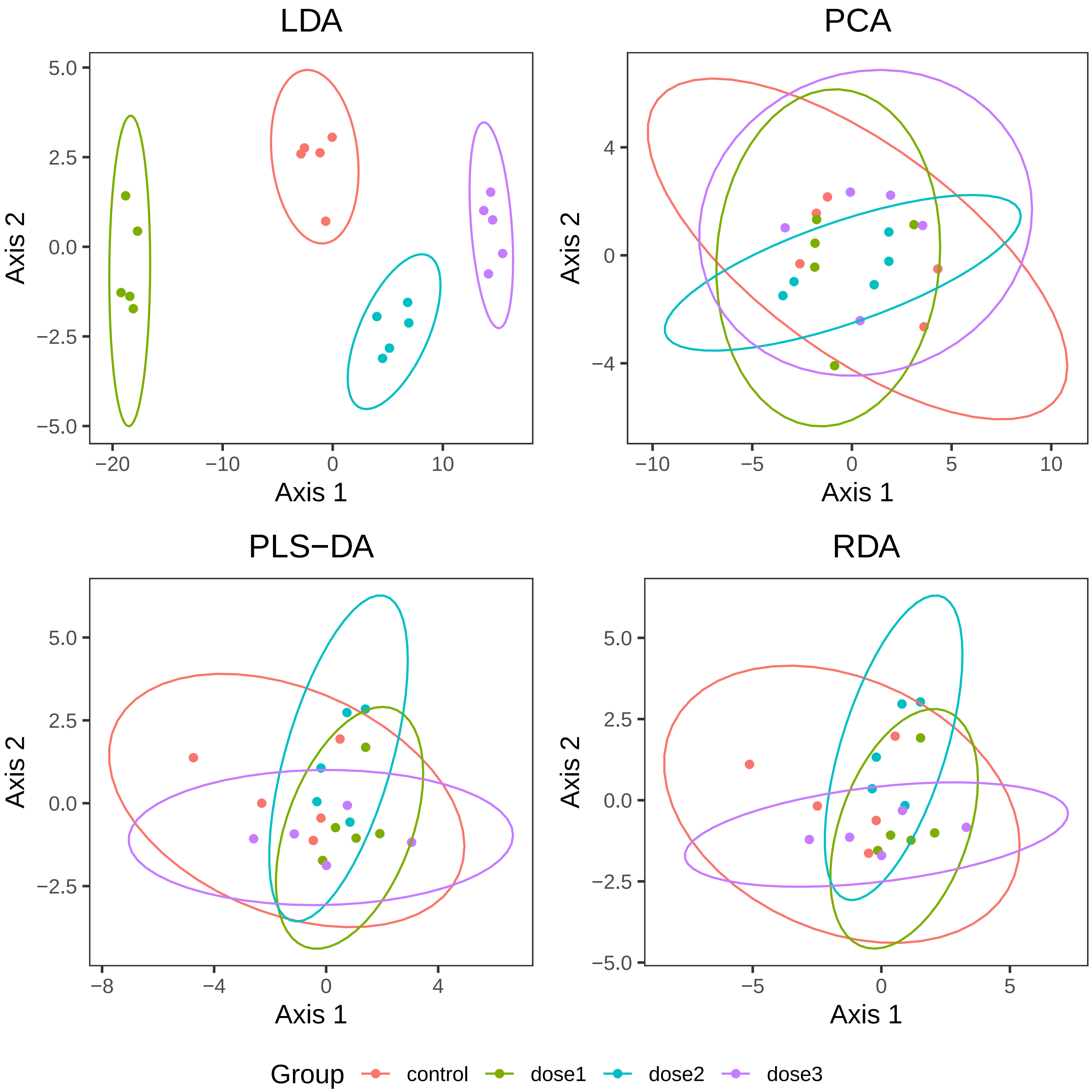

LDA
PCA
PLS−DA
RDA
Axis 1
Axis 2
5.0
2.5
0.0
−2.5
−5.0
−20
−10
0
10
4
−4
−5
5
−8
Group
control
dose1
dose2
dose3

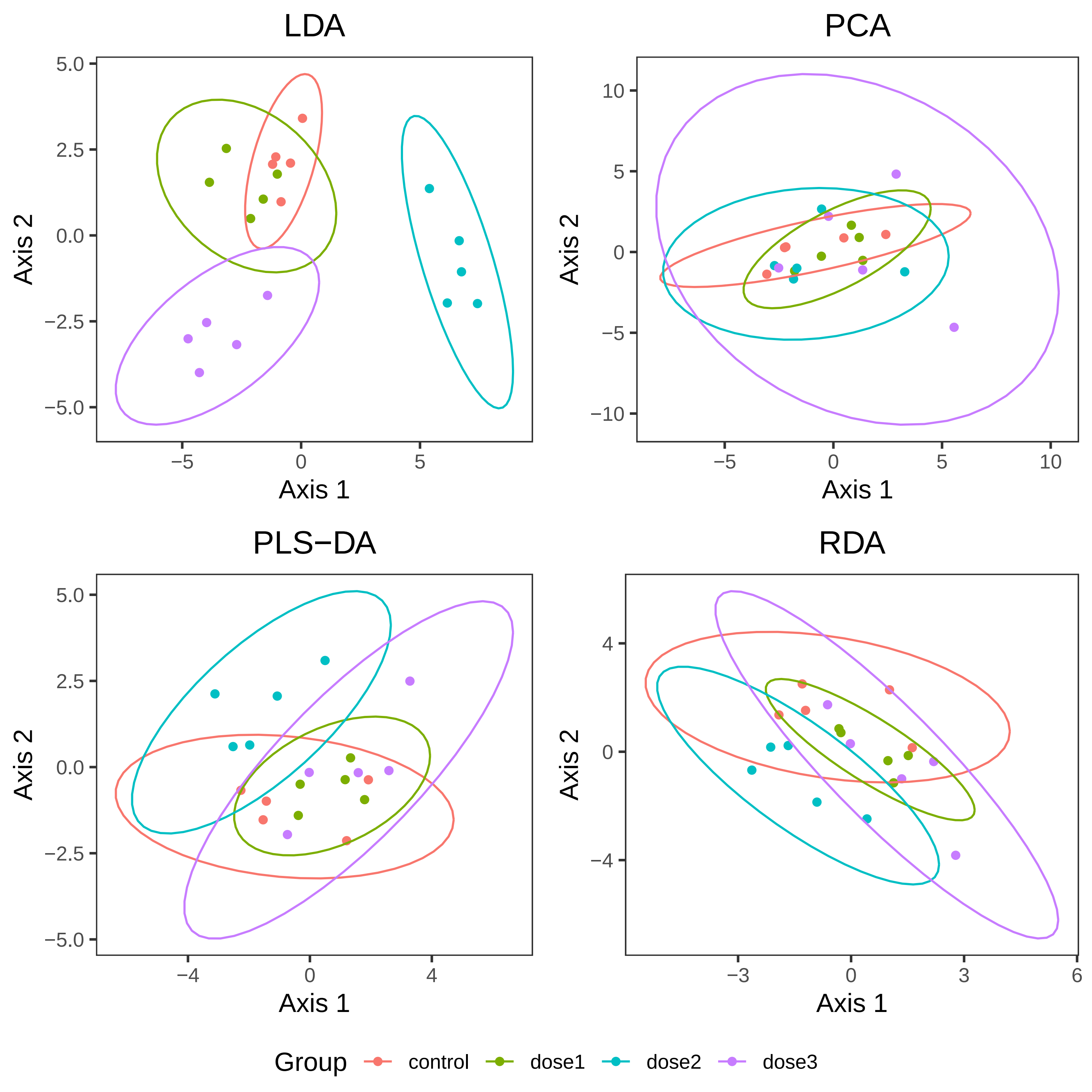

LDA
PCA
PLS−DA
RDA
Axis 1
Axis 2
5.0
2.5
0.0
−2.5
−5.0
−5
0
5
10
−10
−4
4
−3
3
6
Group
control
dose1
dose2
dose3

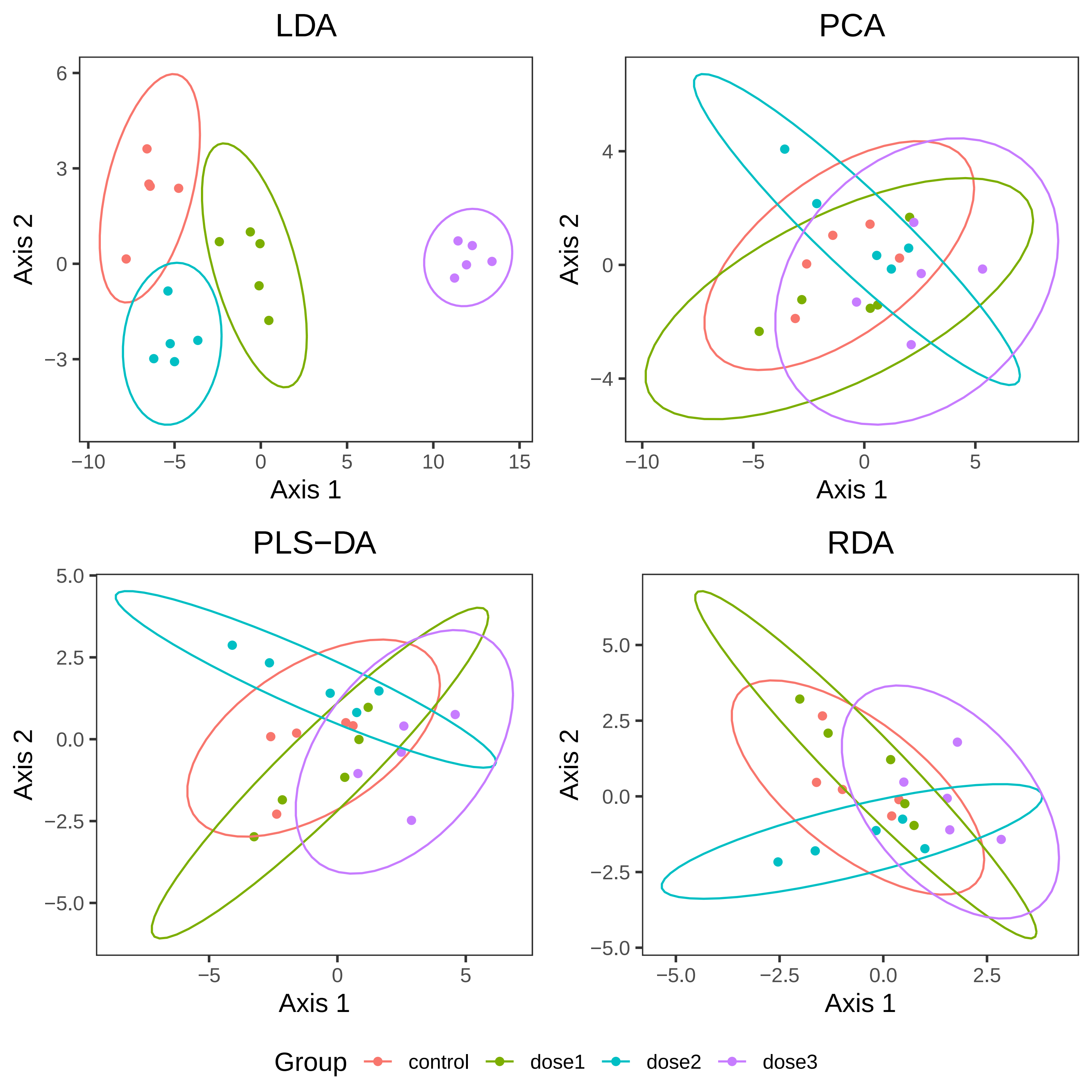
LDA
PCA
PLS−DA
RDA
Axis 1
Axis 2
Group
control
dose1
dose2
dose3

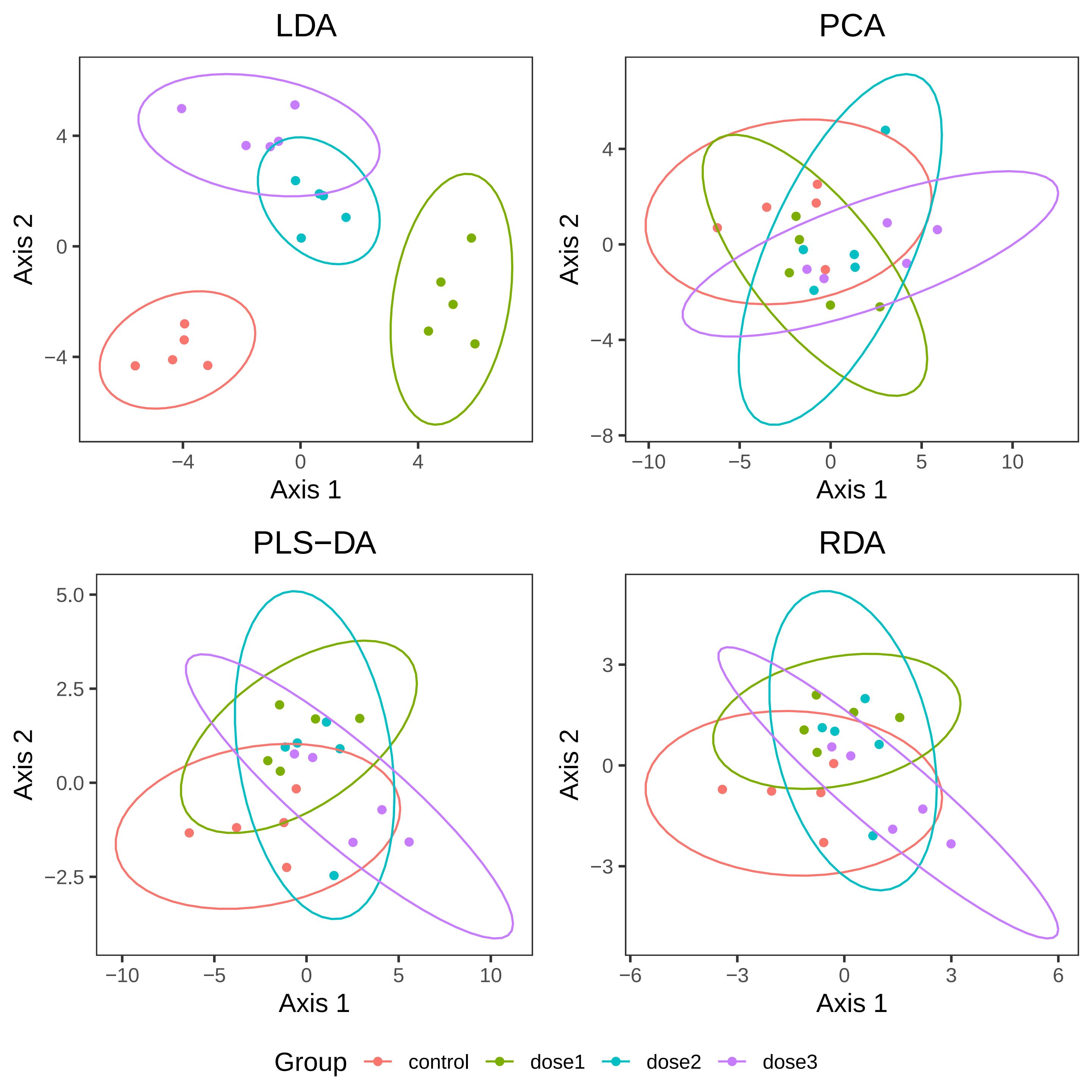

LDA
PCA
PLS−DA
RDA
Axis 1
Axis 2
Group
control
dose1
dose2
dose3

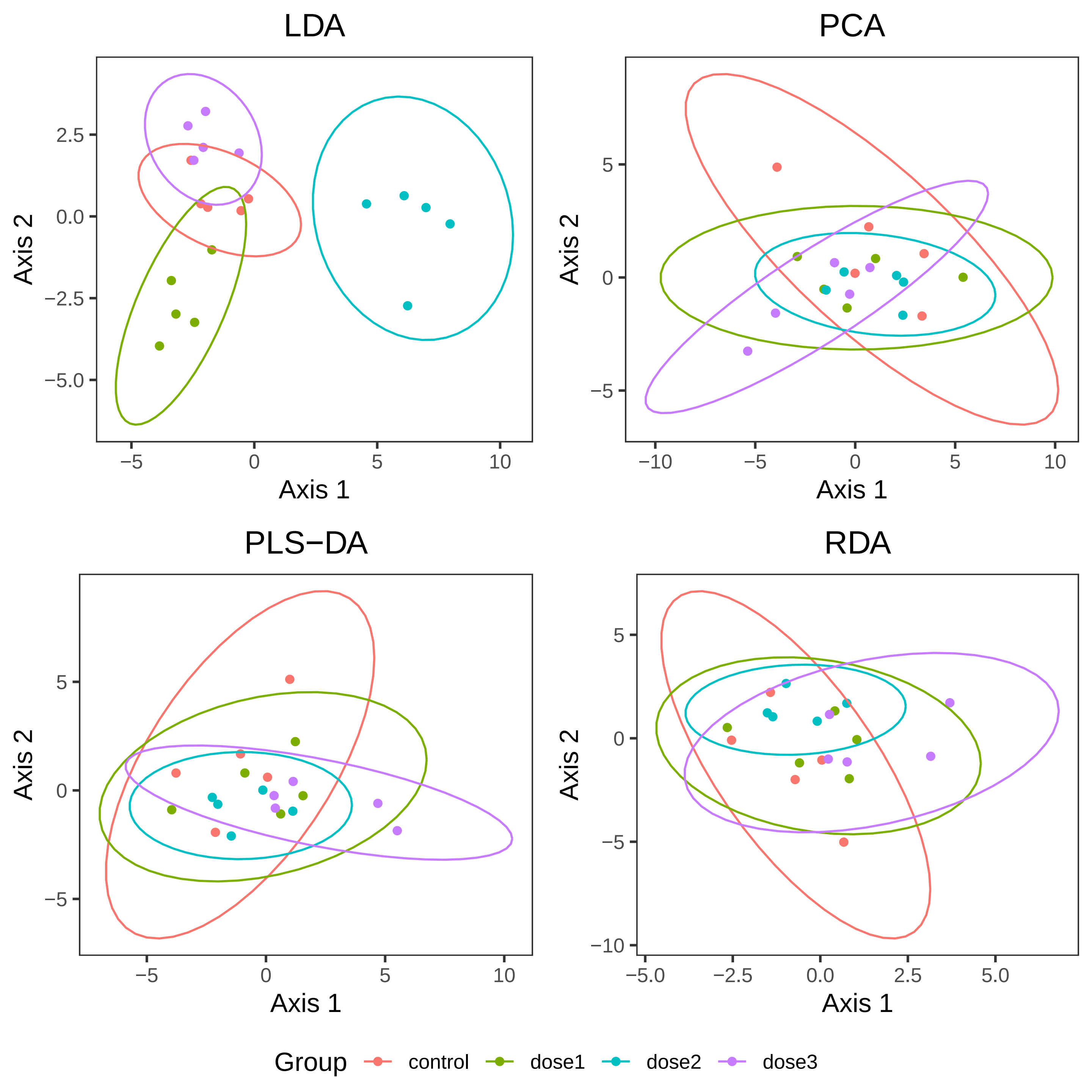

LDA
PCA
PLS−DA
RDA
Axis 1
Axis 2
Group
control
dose1
dose2
dose3

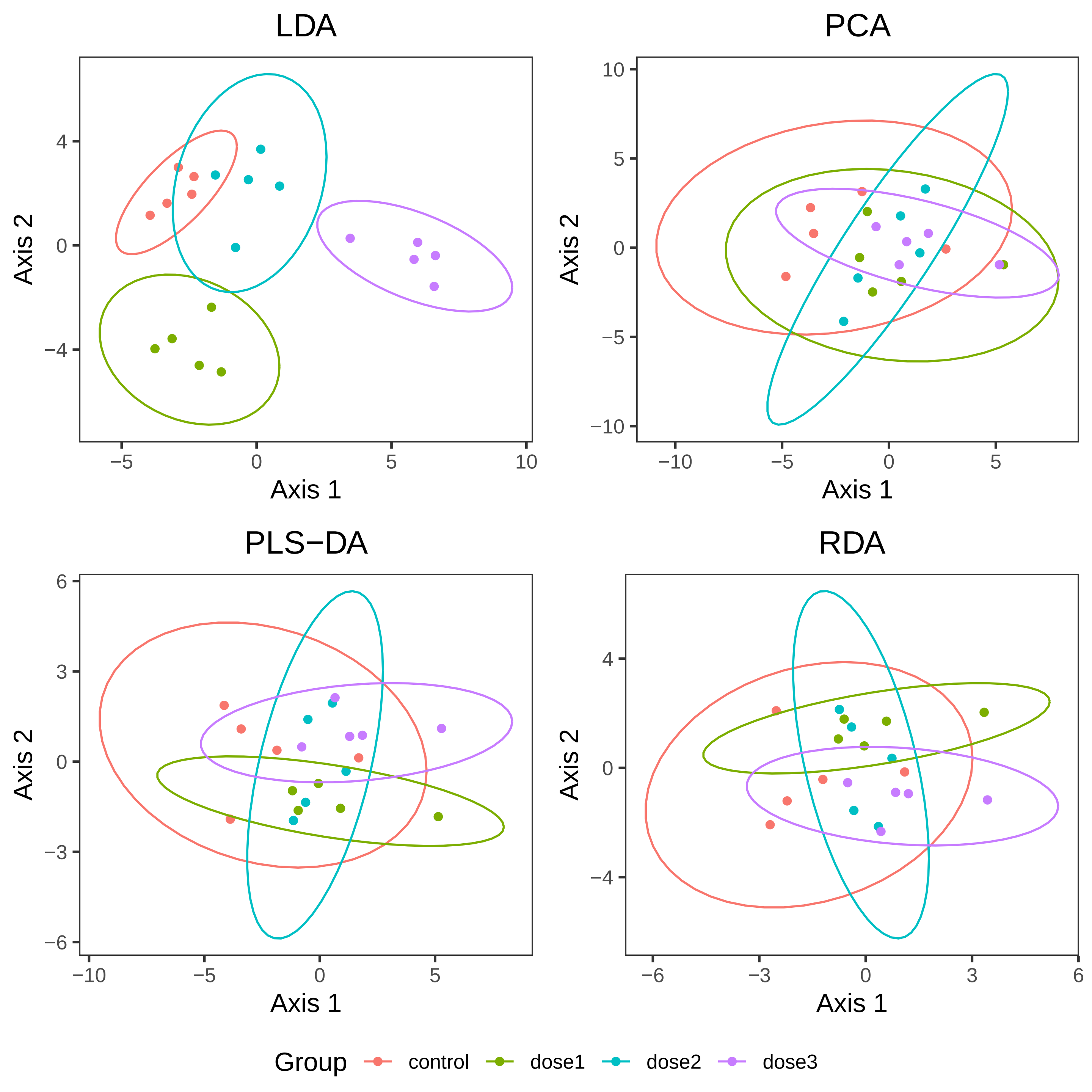

LDA
PCA
PLS−DA
RDA
Axis 1
Axis 2
Group
control
dose1
dose2
dose3

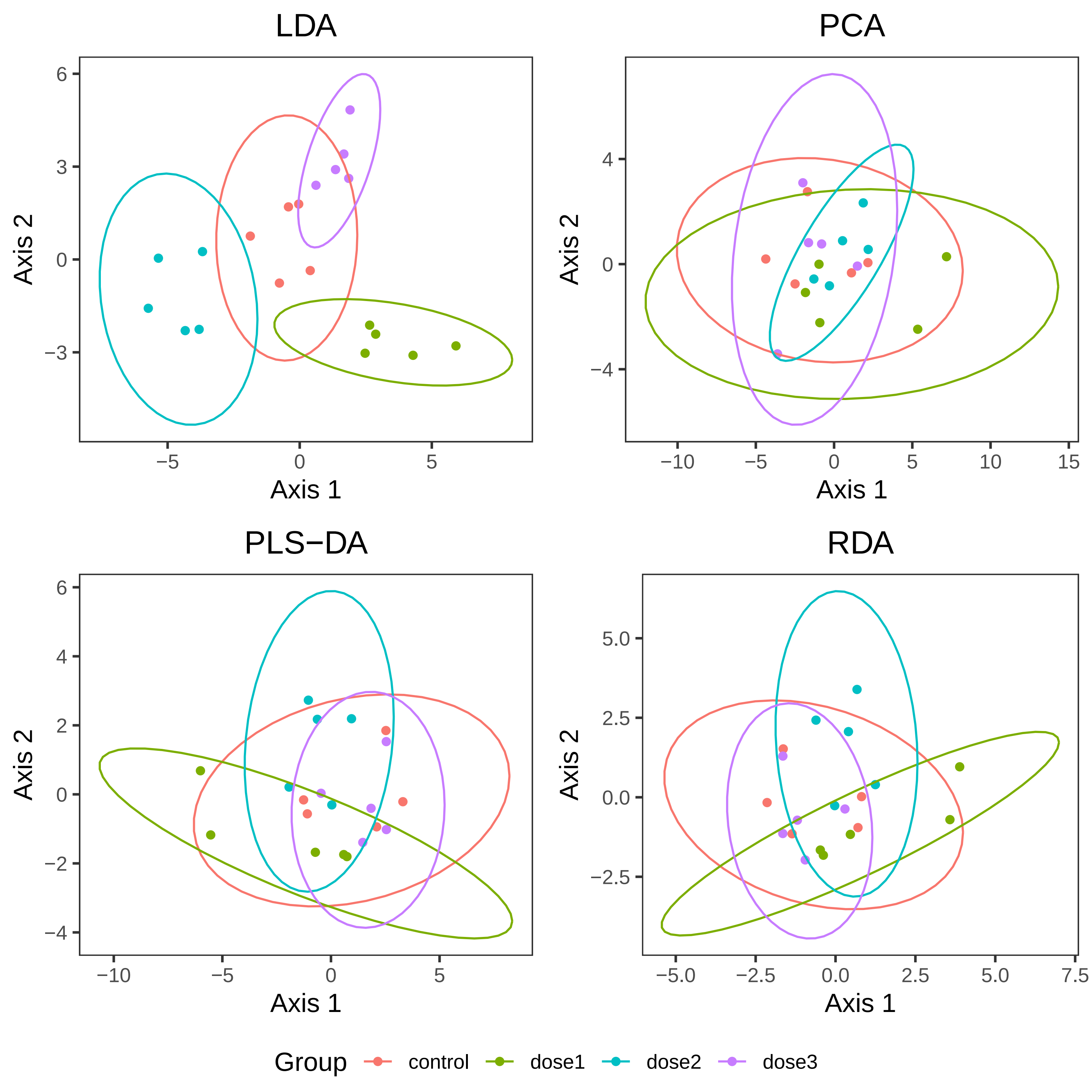

LDA
PCA
PLS−DA
RDA
Axis 1
Axis 2
Group
control
dose1
dose2
dose3

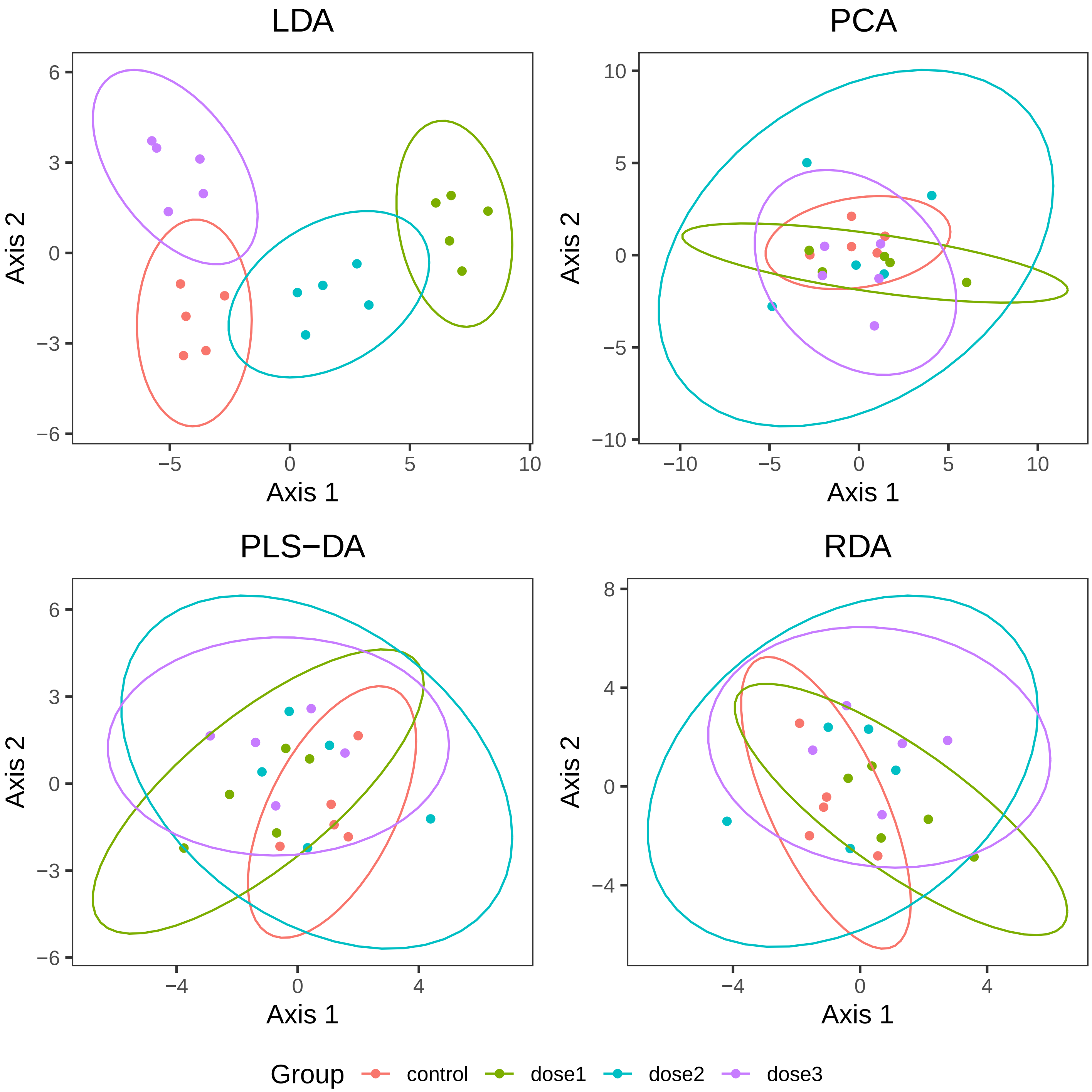

LDA
PCA
PLS−DA
RDA
Axis 1
Axis 2
6
3
0
−3
−6
−5
0
5
10
−10
−5
0
5
10
6
3
0
−3
−6
−4
0
4
8
4
0
−4
−4
0
4
Group
control
dose1
dose2
dose3

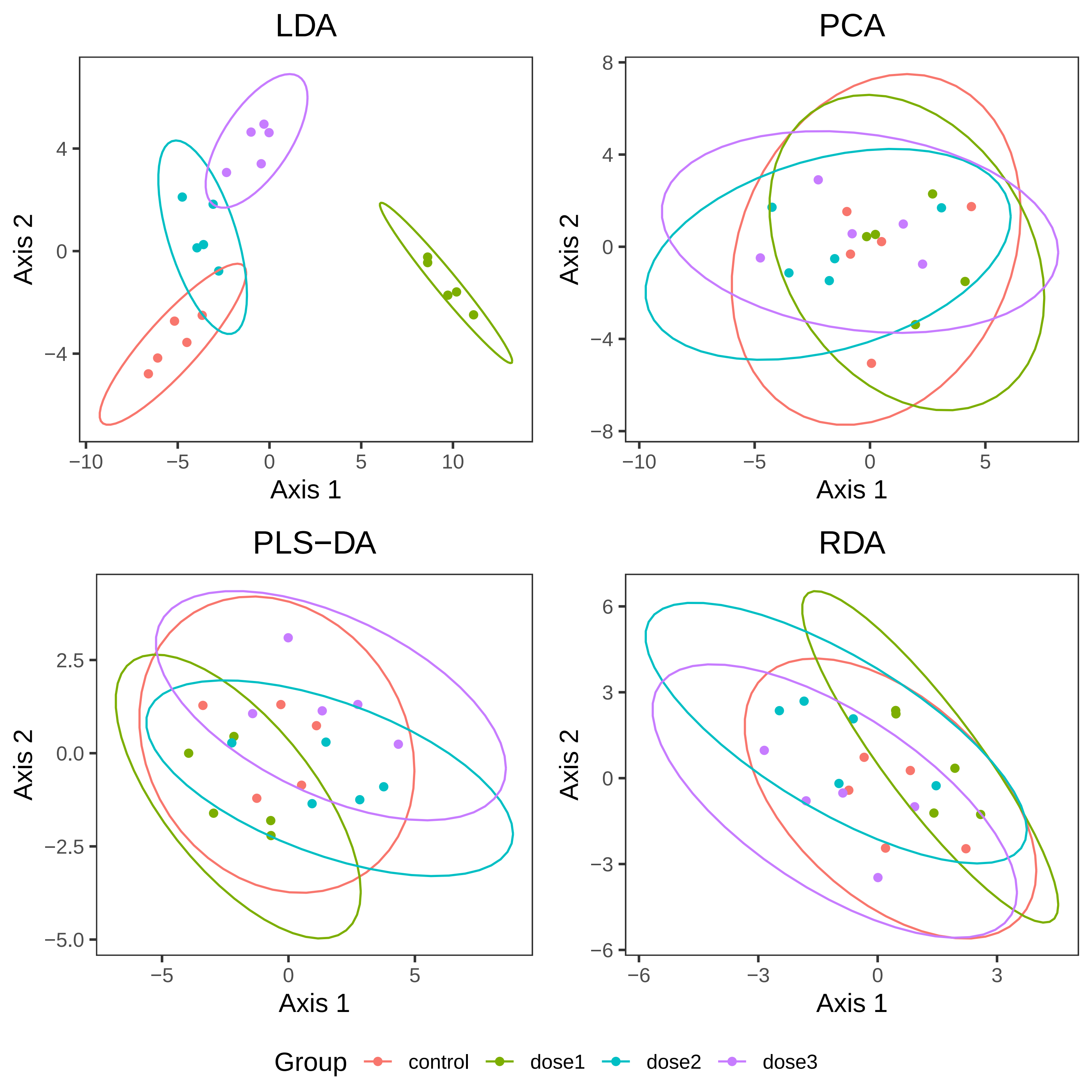
LDA
PCA
PLS−DA
RDA
Axis 1
Axis 2
Group
control
dose1
dose2
dose3

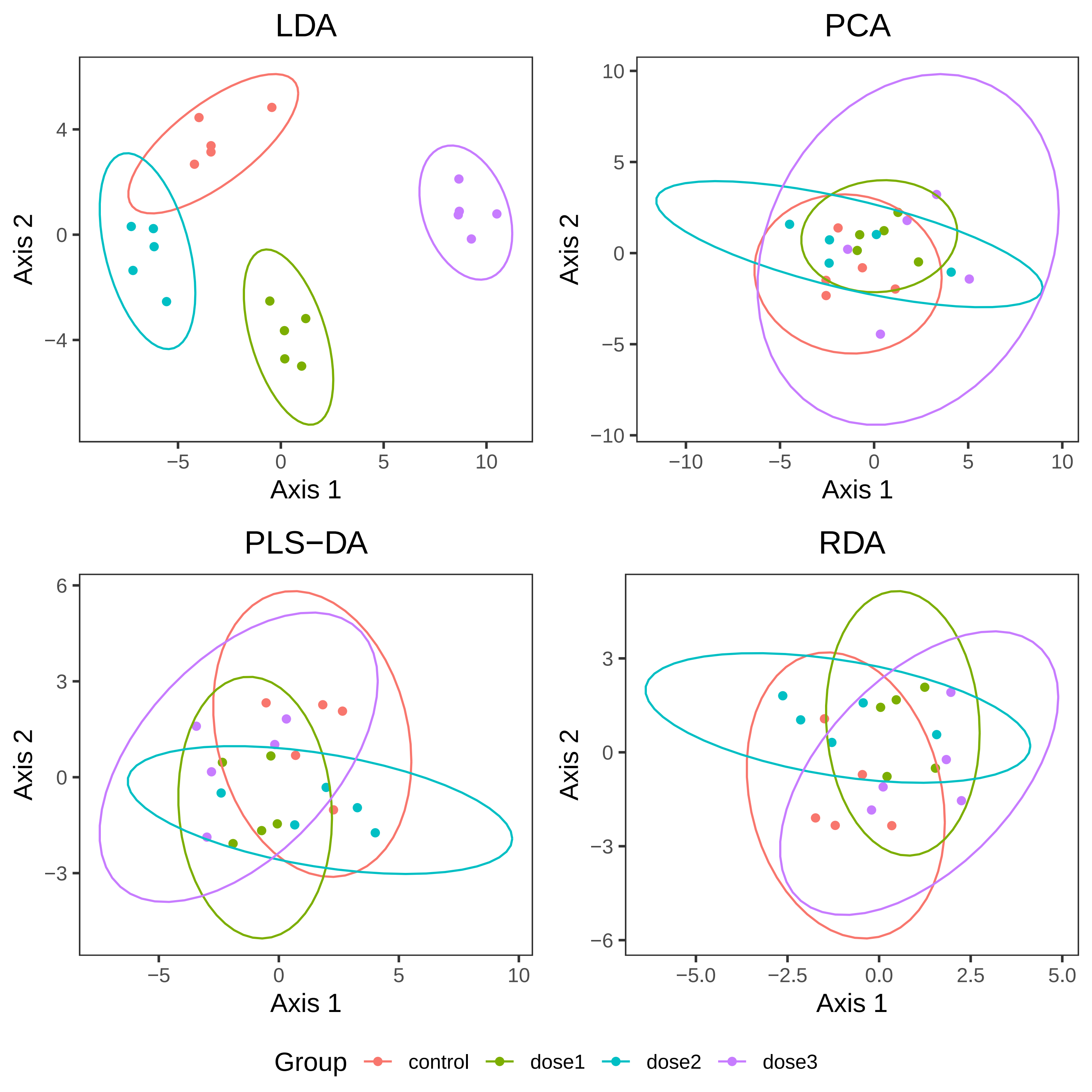

LDA
PCA
PLS−DA
RDA
Axis 1
Axis 2
Group
control
dose1
dose2
dose3

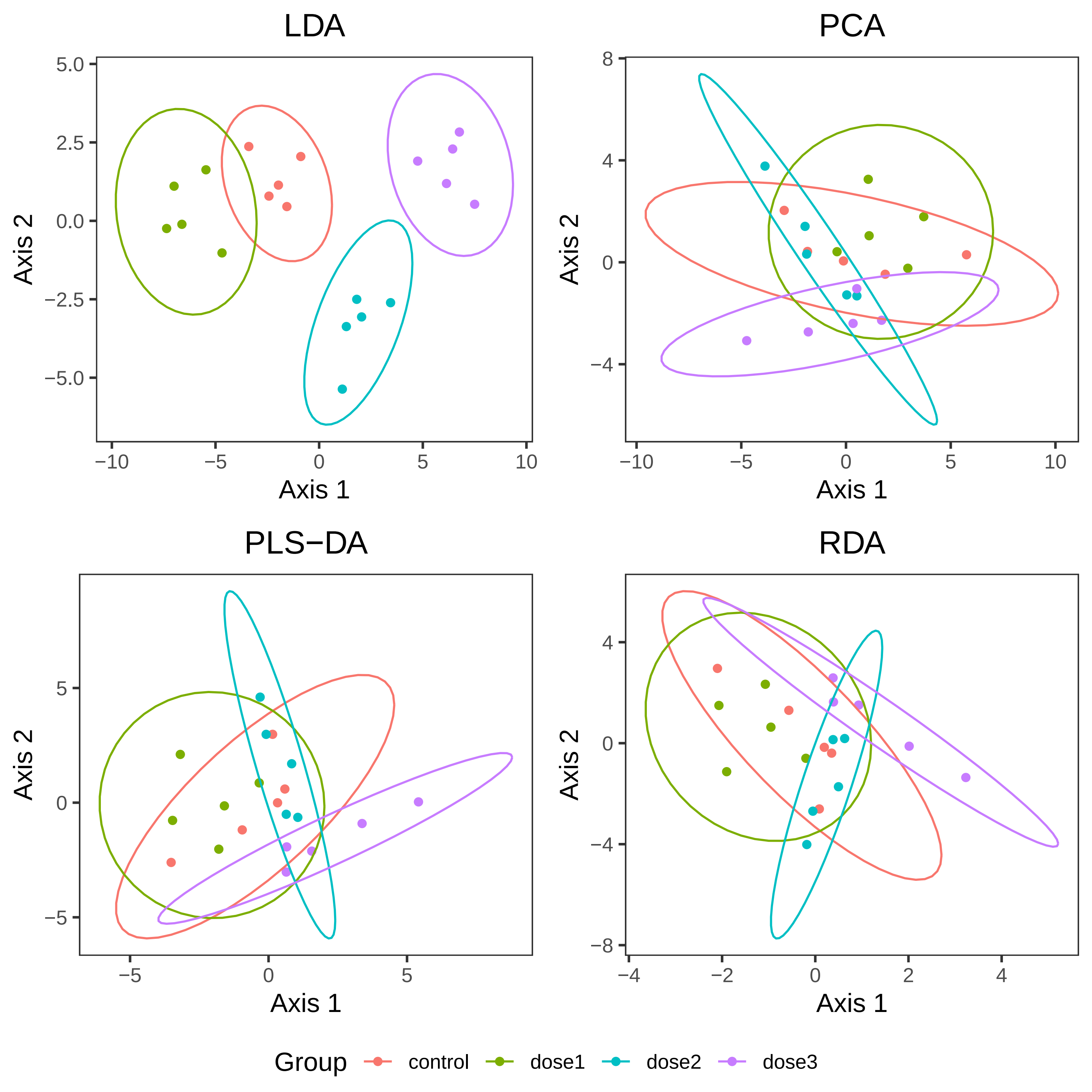
LDA
PCA
PLS−DA
RDA
Axis 1
Axis 2
5.0
2.5
0.0
−2.5
−5.0
−10
−5
0
5
10
8
4
−4
−8
−2
2
Group
control
dose1
dose2
dose3

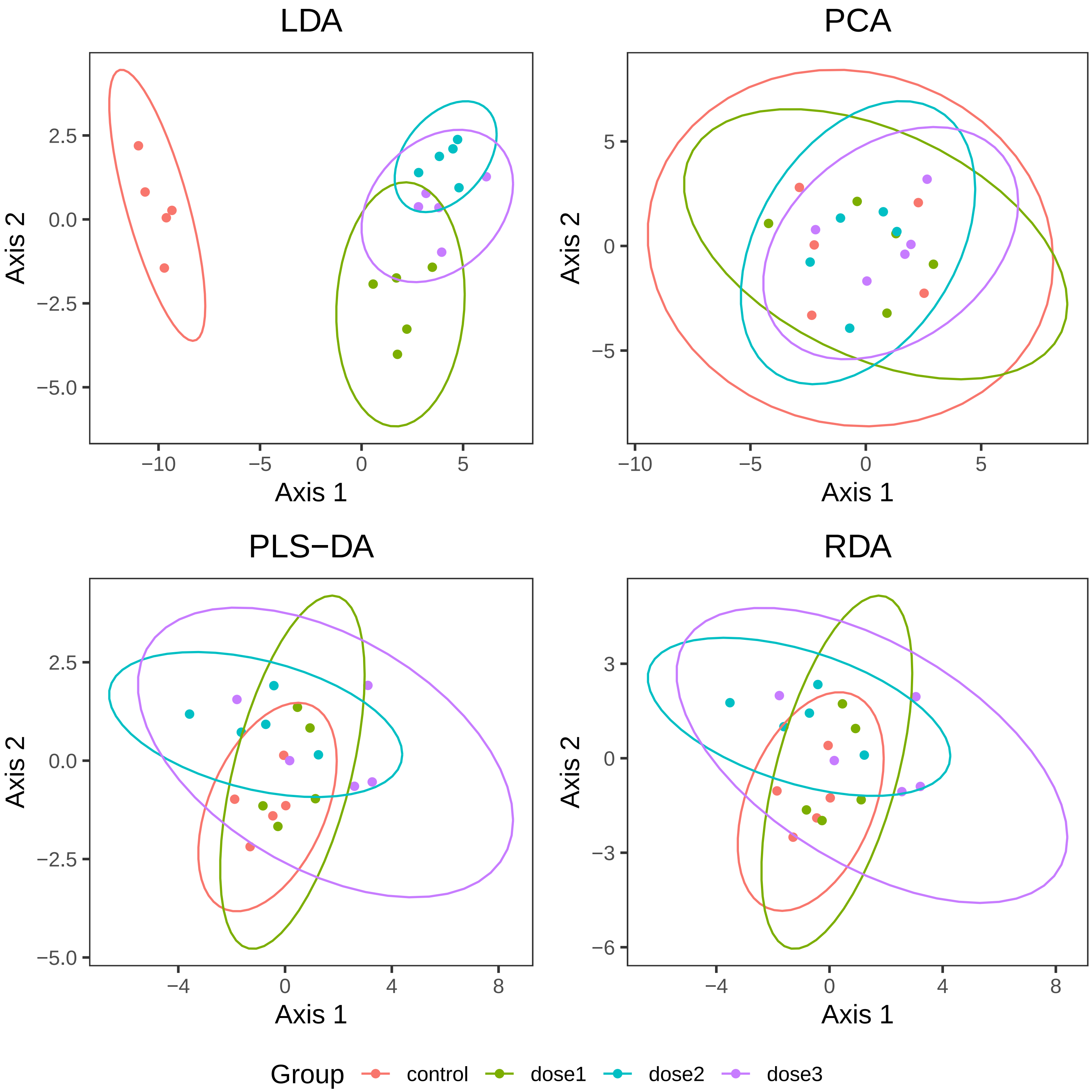

LDA
PCA
PLS−DA
RDA
Axis 1
Axis 2
2.5
0.0
−2.5
−5.0
−10
−5
0
5
−10
−5
0
5
5
0
−5
−4
0
4
8
3
0
−3
−6
Group
control
dose1
dose2
dose3

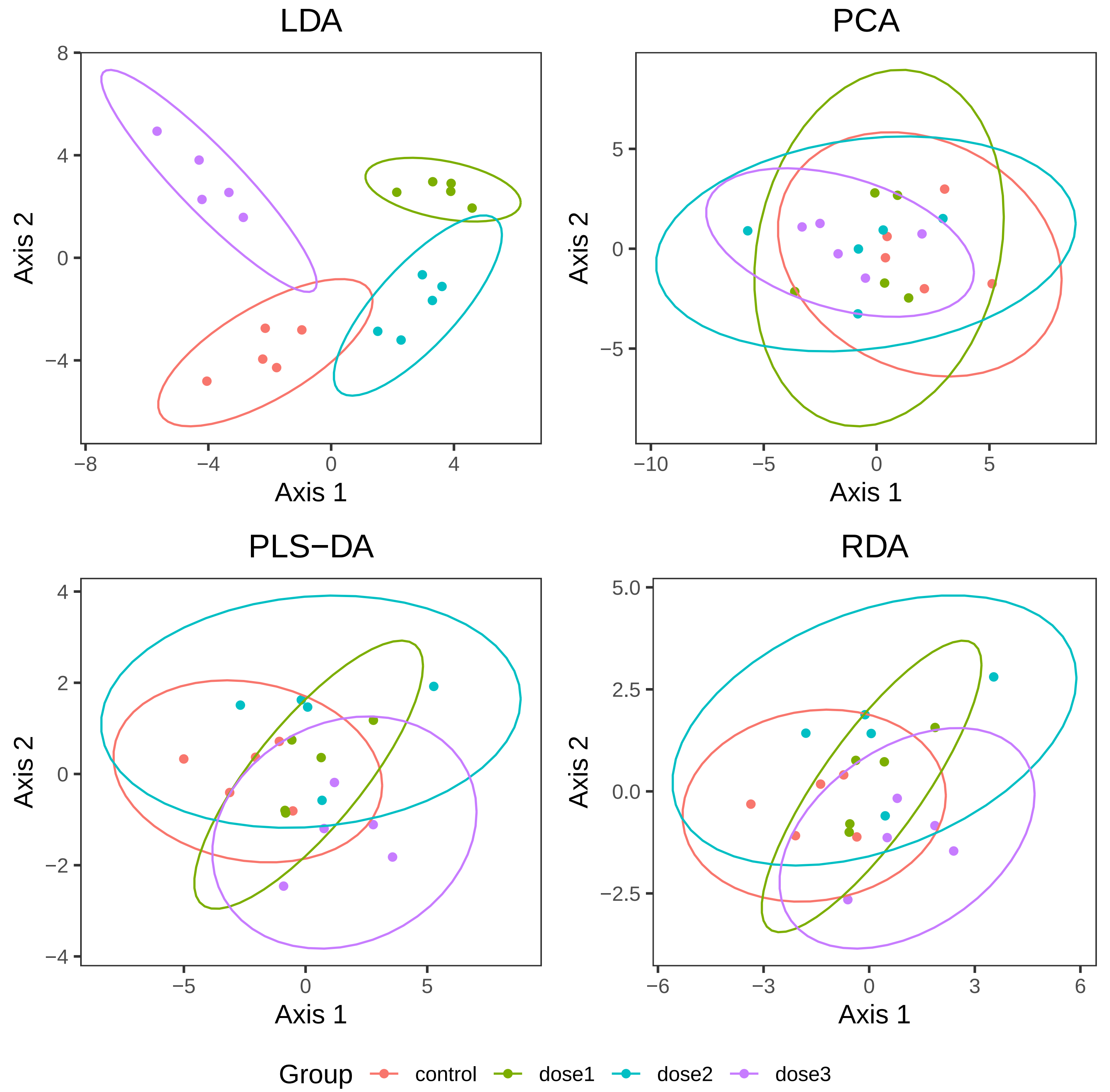
LDA
PCA
PLS−DA
RDA
Axis 1
Axis 2
8
4
0
−4
−8
5
−5
−10
2
−2
−4
5.0
2.5
0.0
−2.5
−6
−3
3
6
Group
control
dose1
dose2
dose3

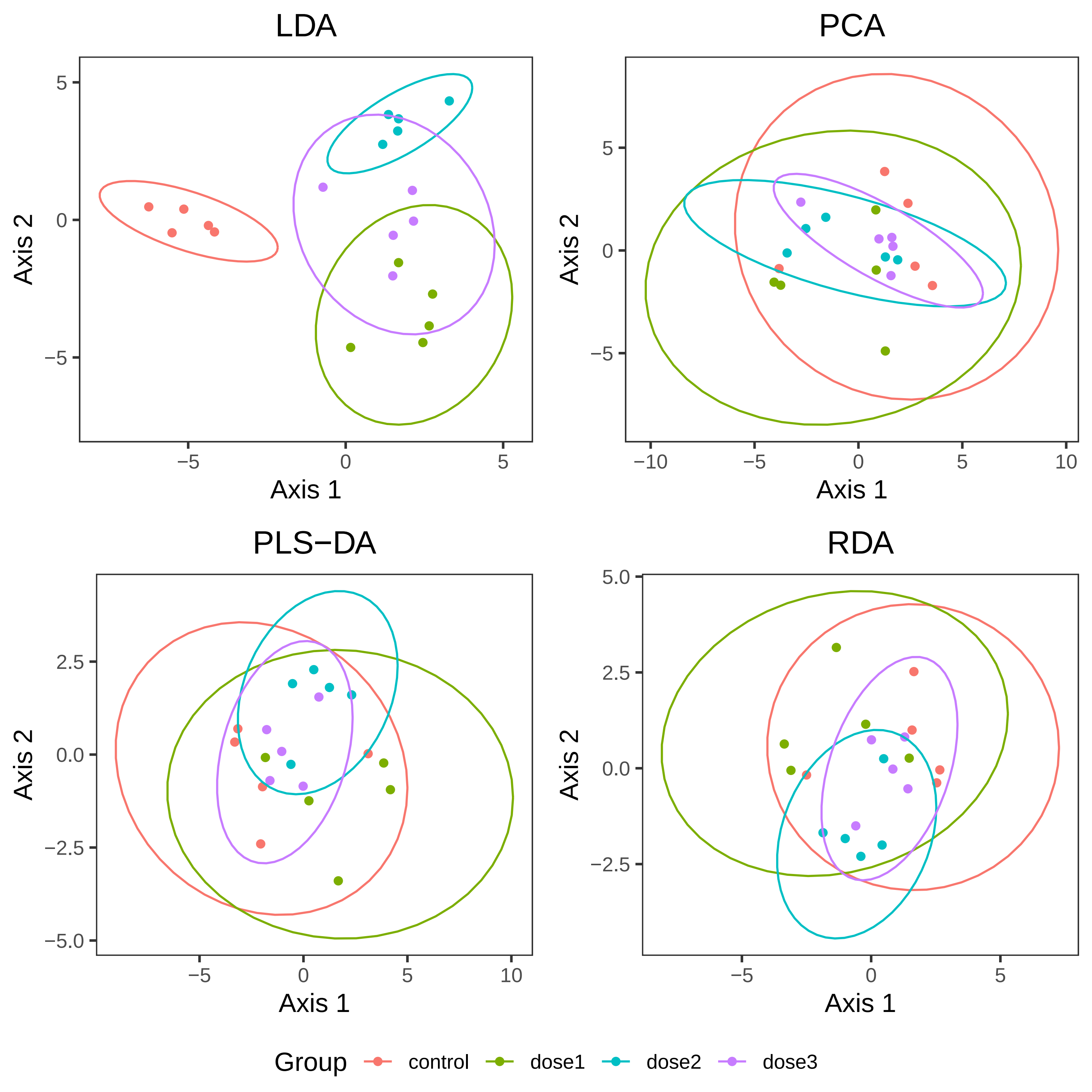
LDA
PCA
PLS−DA
RDA
Axis 1
Axis 2
Group
control
dose1
dose2
dose3

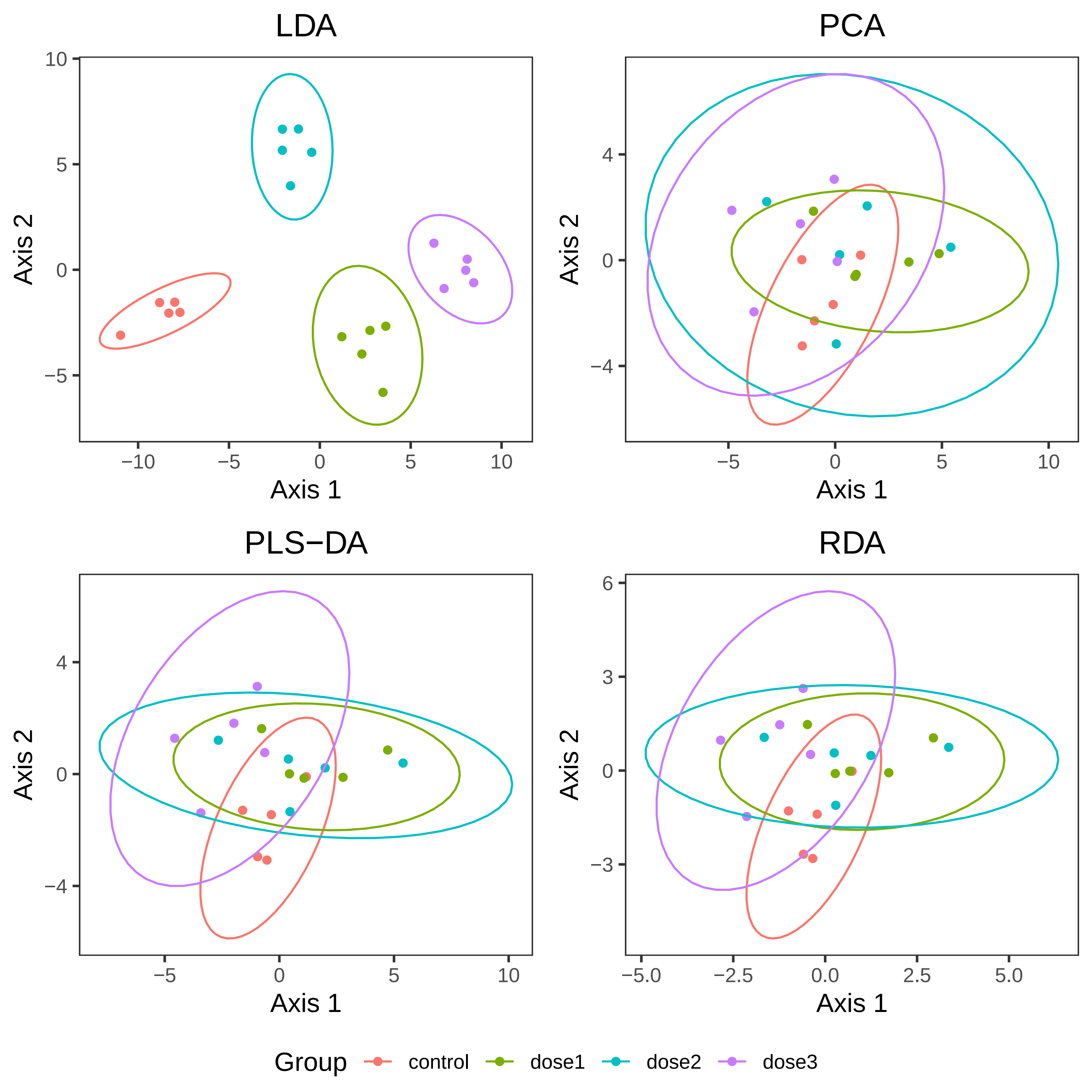
LDA
PCA
PLS−DA
RDA
Axis 1
Axis 2
Group
control
dose1
dose2
dose3

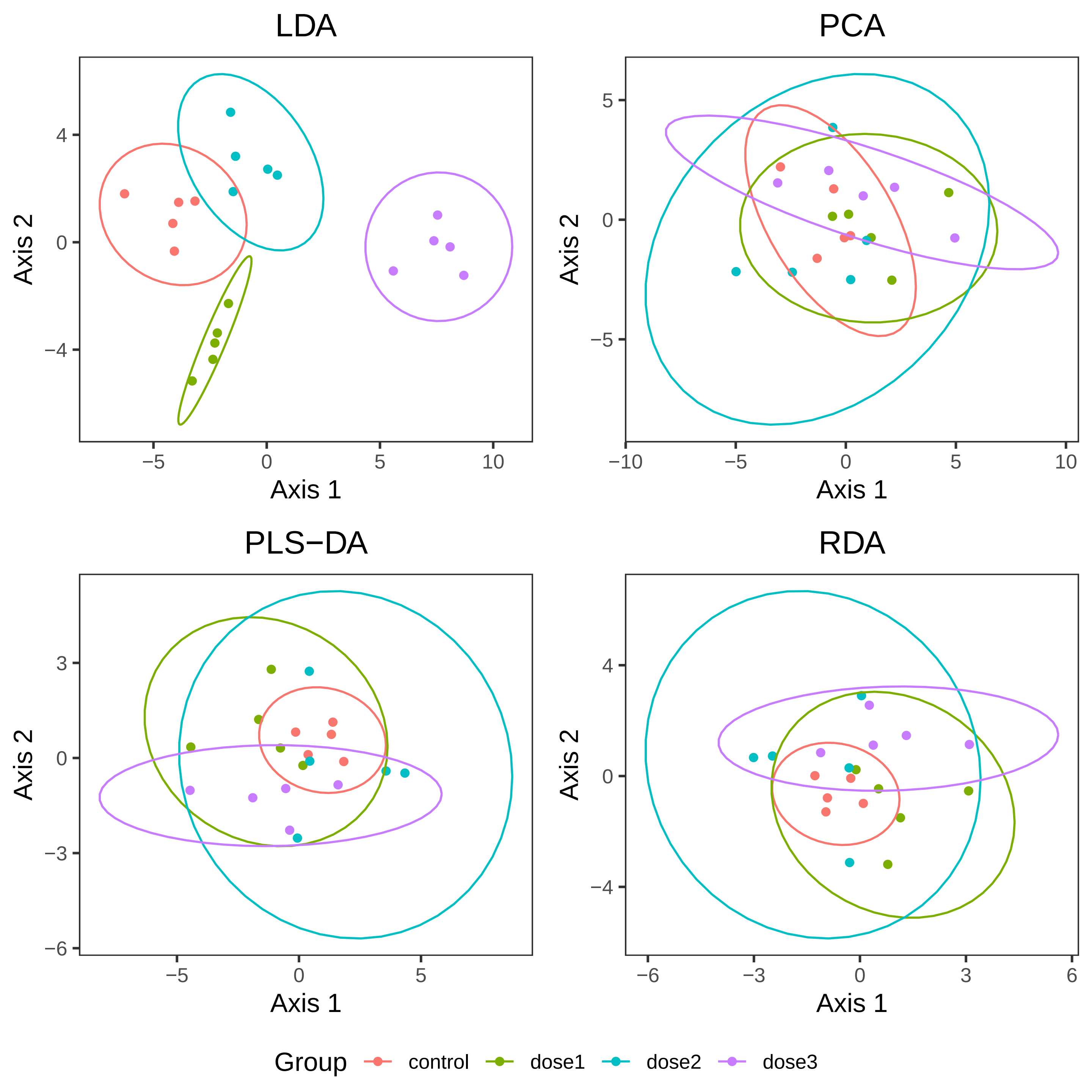

LDA
PCA
PLS−DA
RDA
Axis 1
Axis 2
Group
control
dose1
dose2
dose3

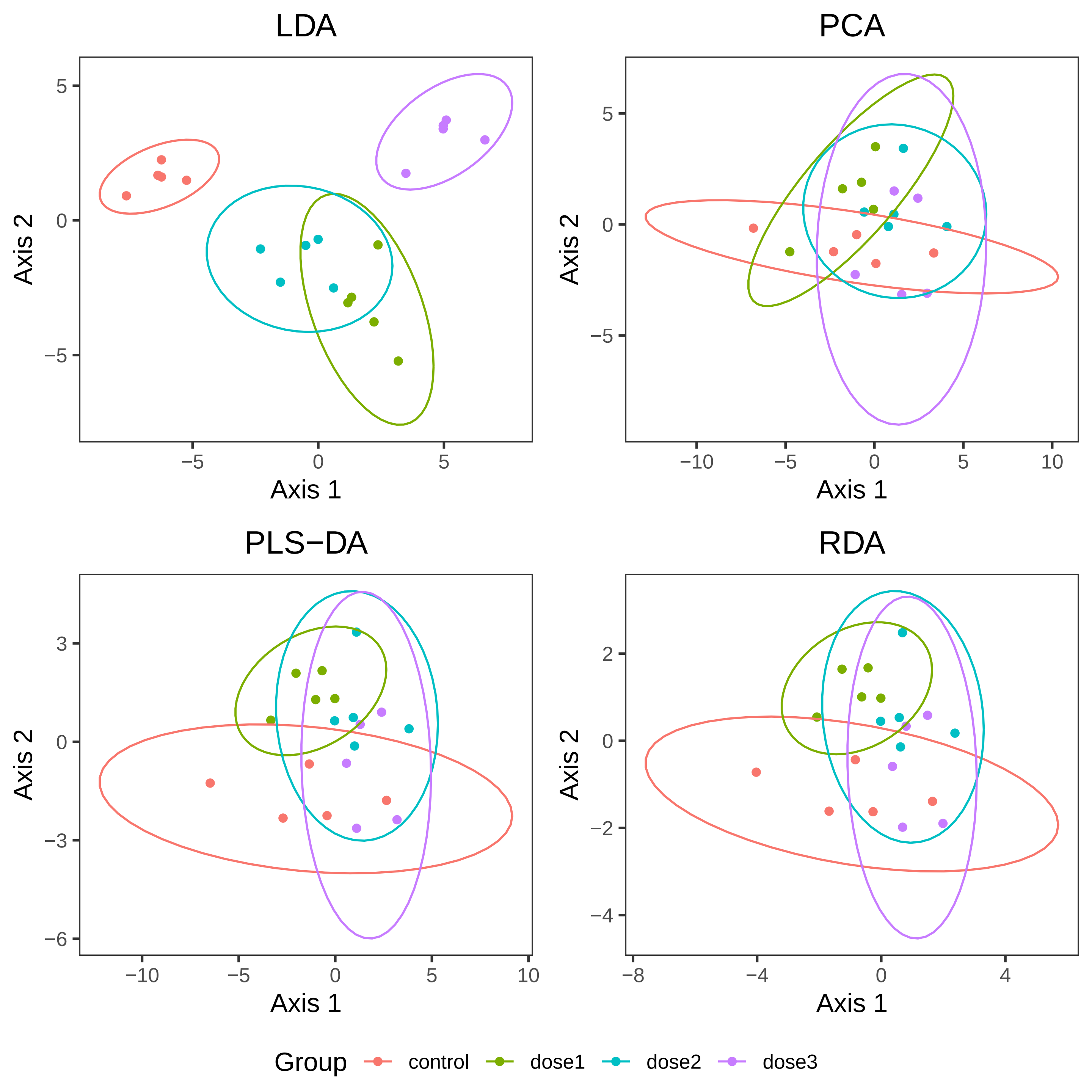
LDA
PCA
PLS−DA
RDA
Axis 1
Axis 2
5
0
−5
−10
10
3
−3
−6
−8
−4
4
2
−2
Group
control
dose1
dose2
dose3

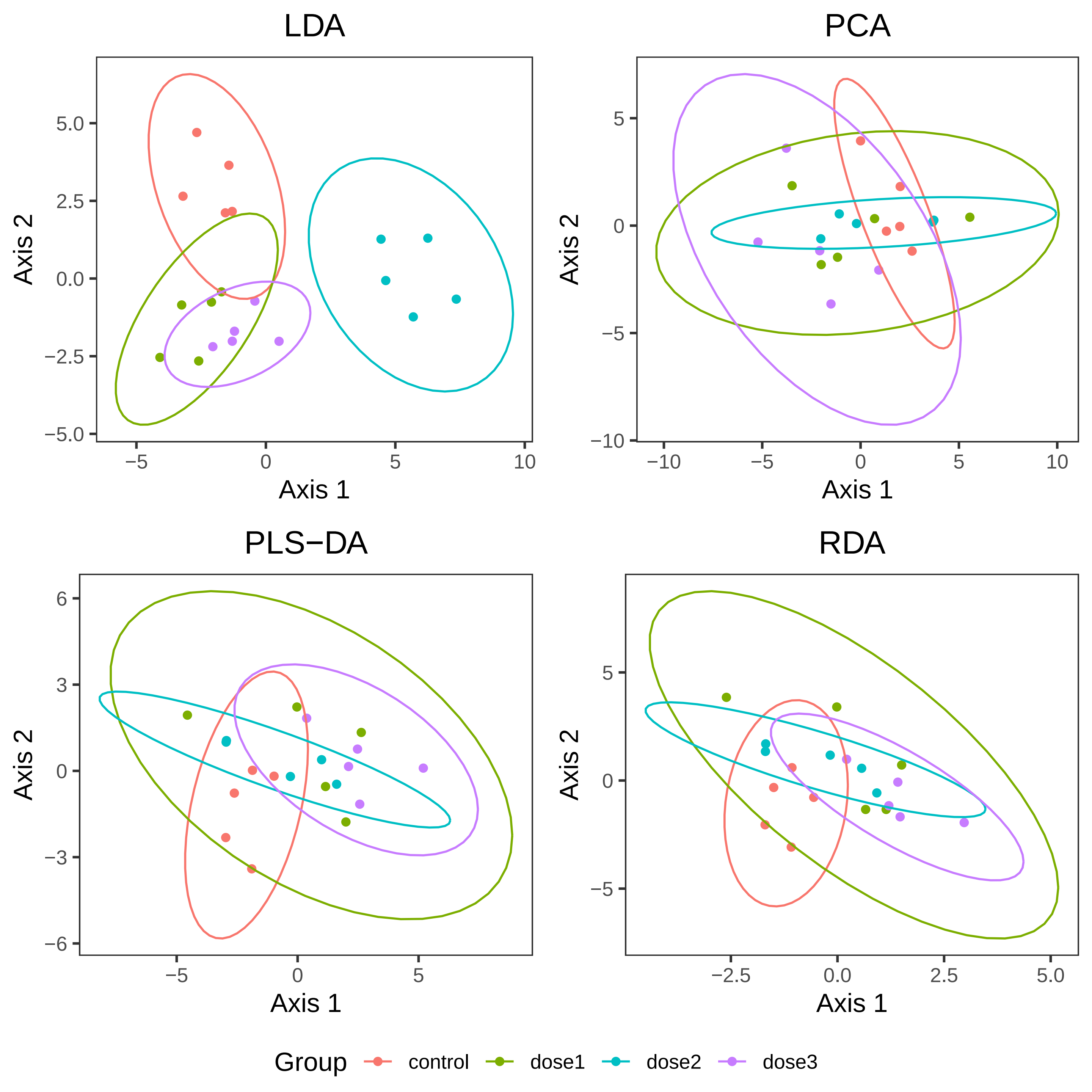

LDA
PCA
PLS−DA
RDA
Axis 1
Axis 2
5.0
2.5
0.0
−2.5
−5.0
−5
0
5
10
−10
Group
control
dose1
dose2
dose3

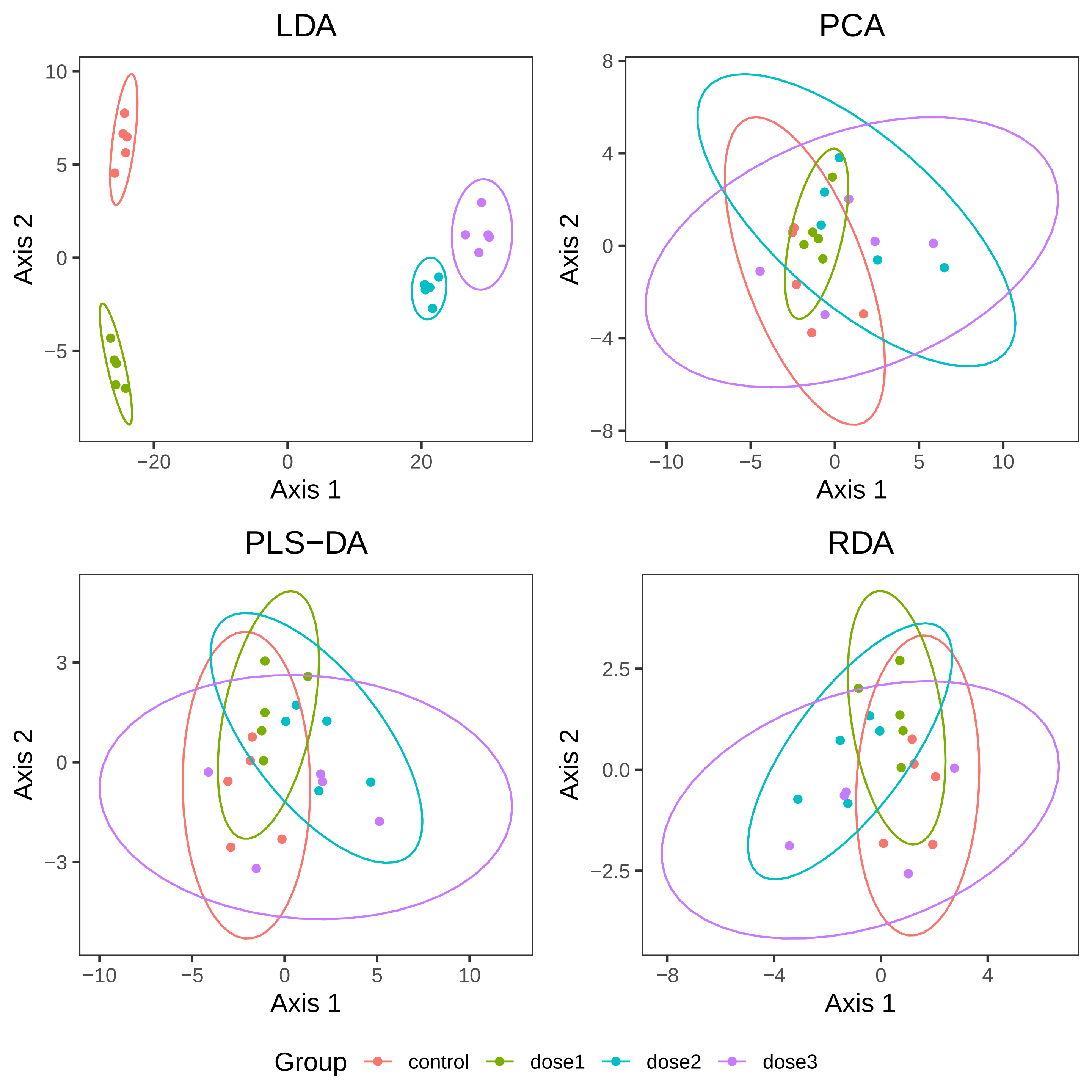
LDA
PCA
PLS−DA
RDA
Axis 1
Axis 2
Group
control
dose1
dose2
dose3

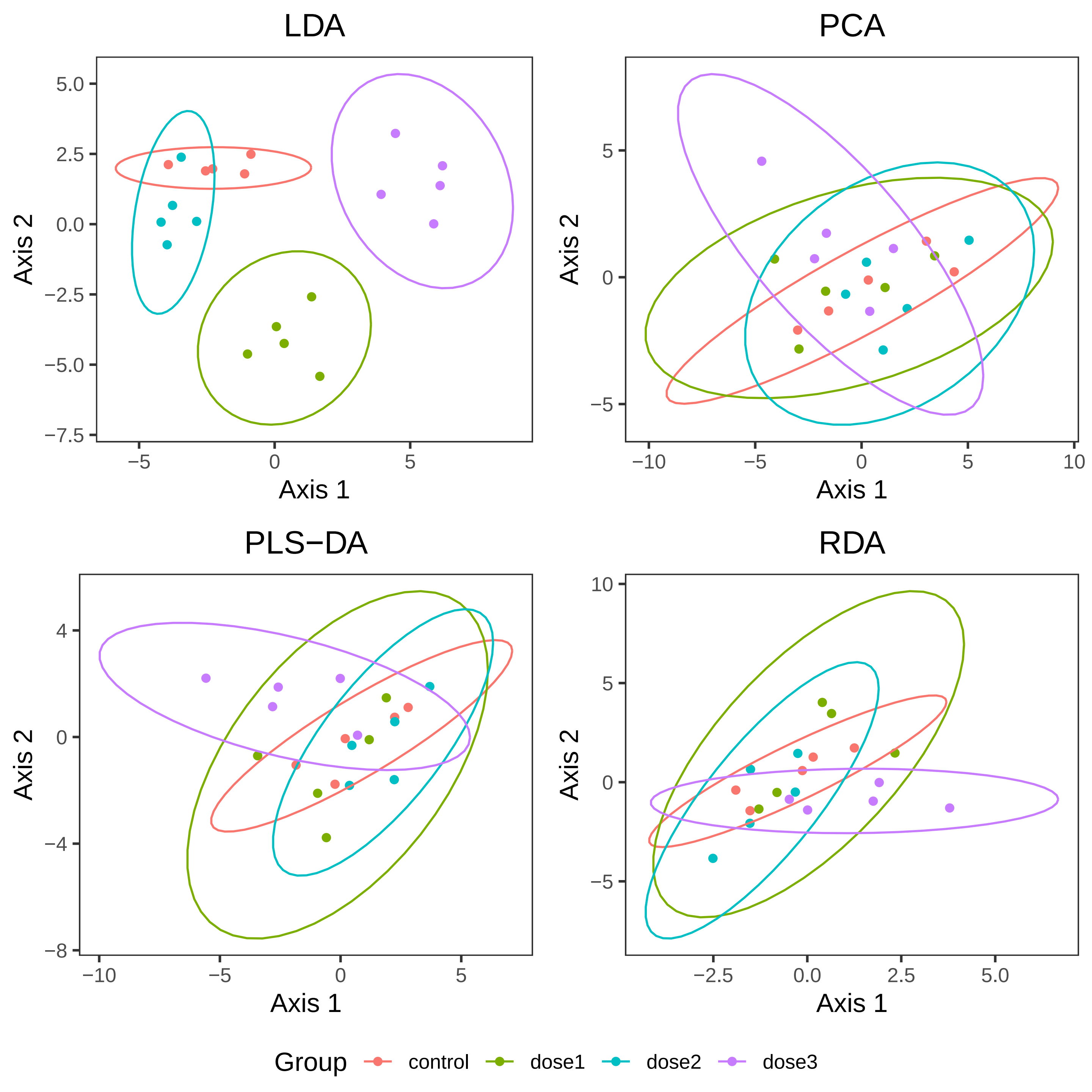
LDA
PCA
PLS−DA
RDA
Axis 1
Axis 2
5.0
2.5
0.0
−2.5
−5.0
−7.5
−5
0
5
10
−10
4
−4
−8
Group
control
dose1
dose2
dose3

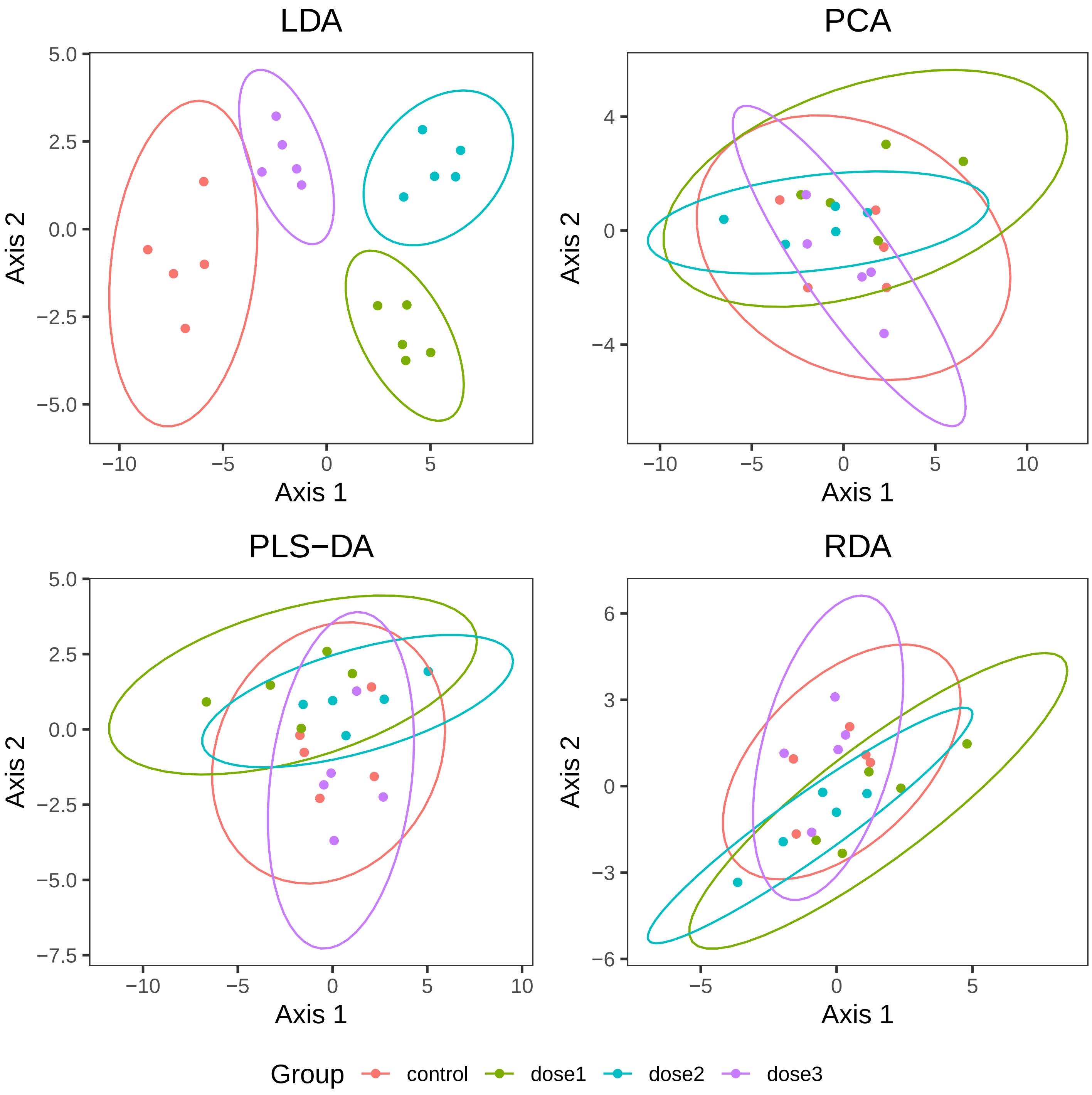

LDA
PCA
PLS−DA
RDA
Axis 1
Axis 2
5.0
2.5
0.0
−2.5
−5.0
−7.5
−10
−5
0
5
10
4
−4
6
3
−3
−6
Group
control
dose1
dose2
dose3

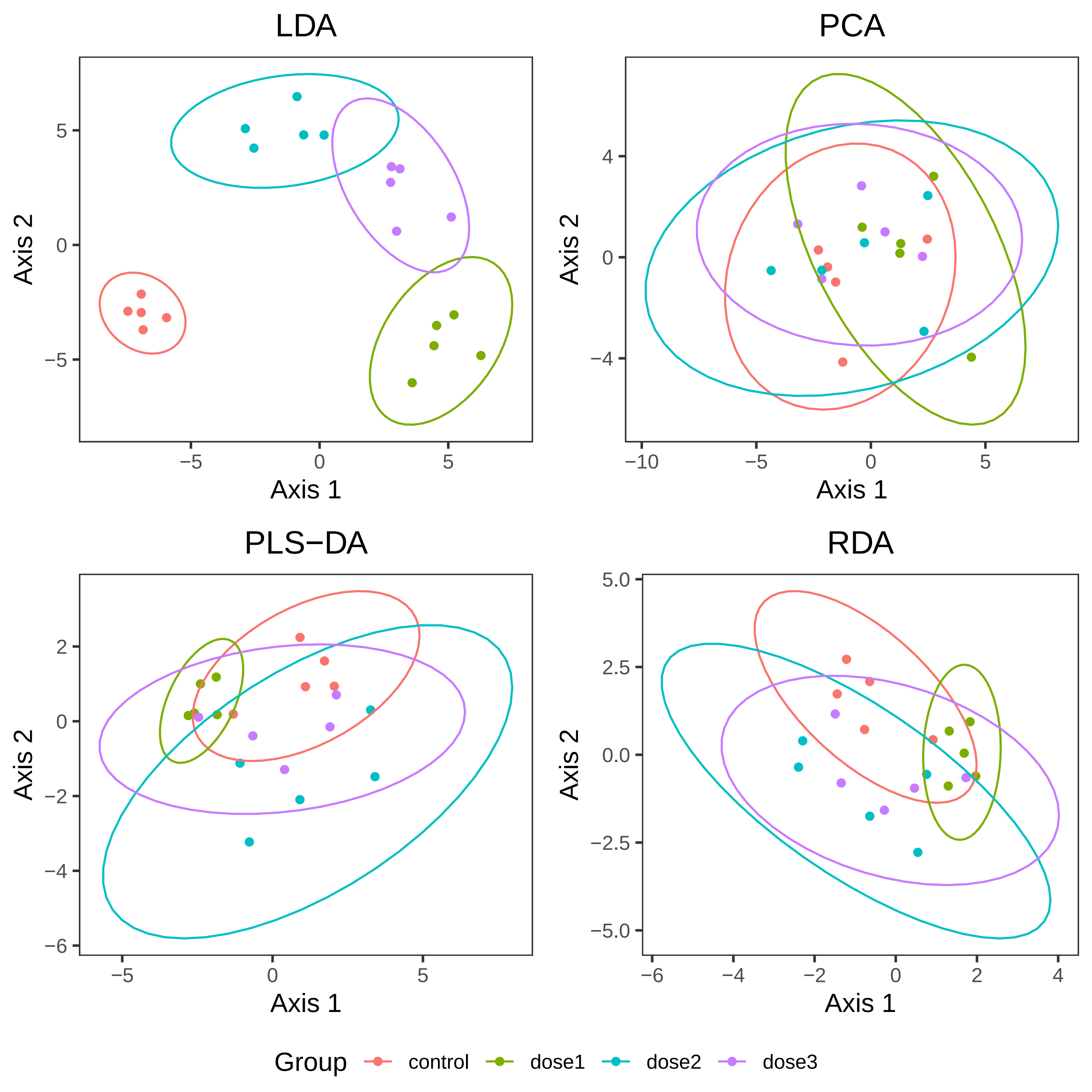

LDA
PCA
PLS−DA
RDA
Axis 1
Axis 2
Group
control
dose1
dose2
dose3

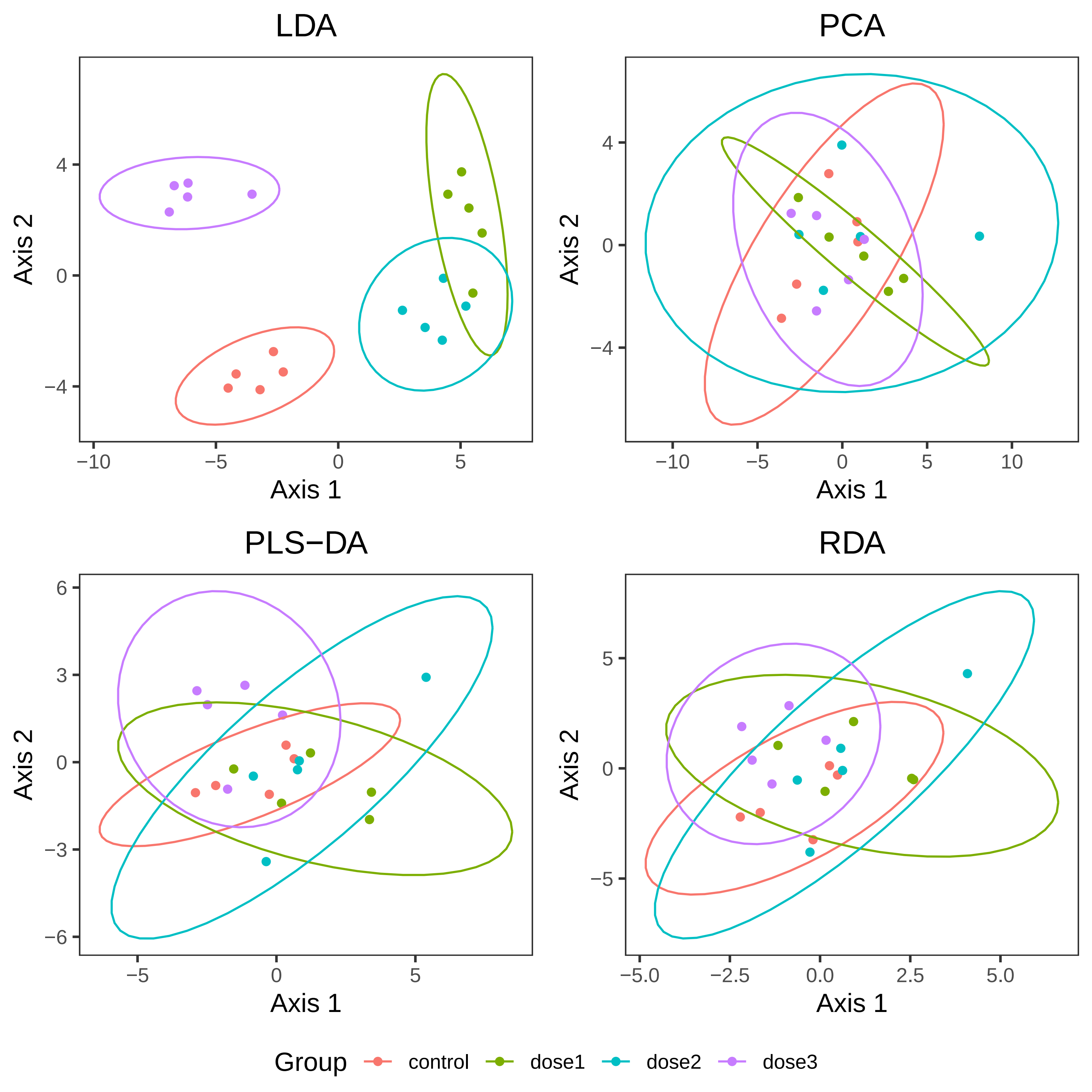
LDA
PCA
PLS−DA
RDA
Axis 1
Axis 2
Group
control
dose1
dose2
dose3

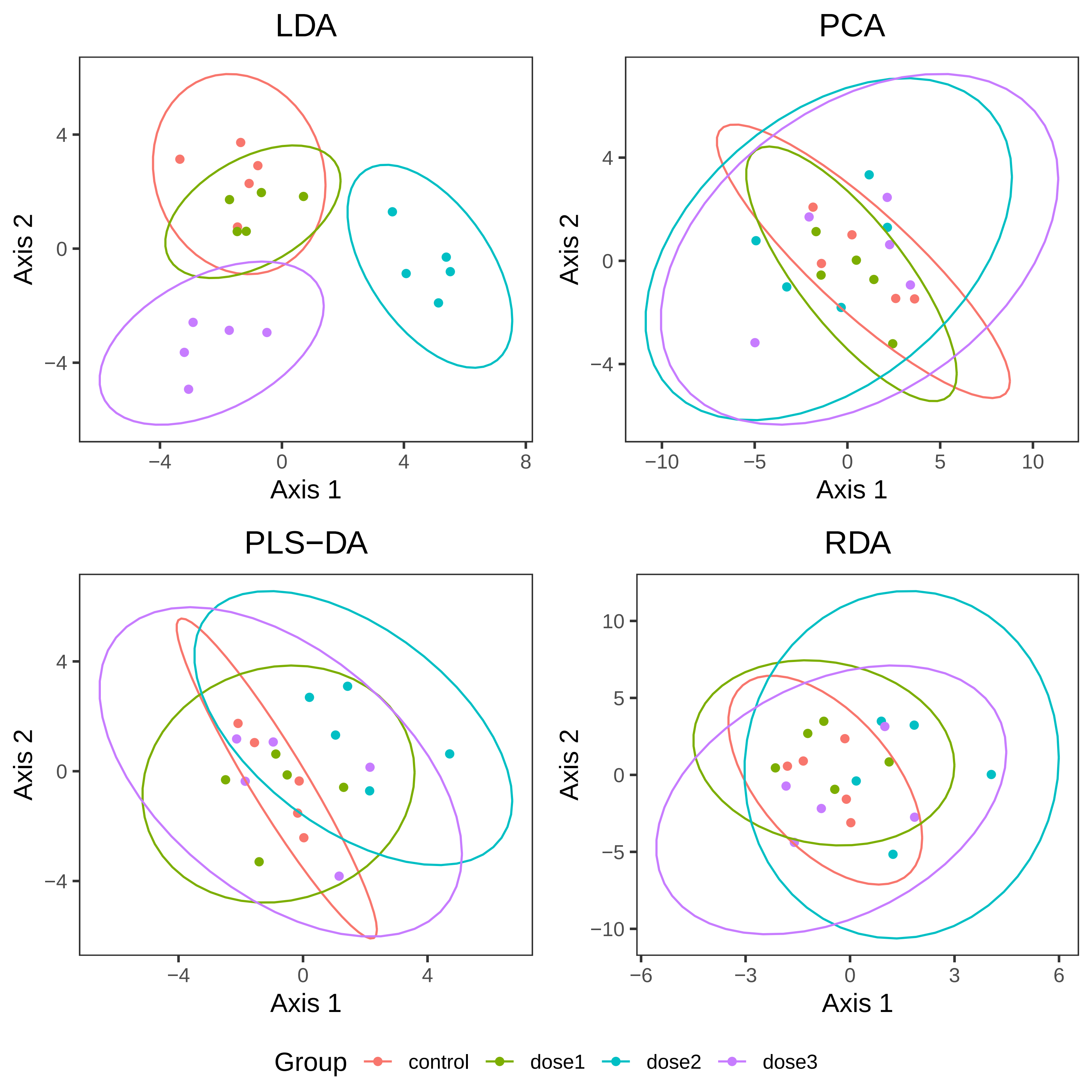
LDA
PCA
PLS−DA
RDA
Axis 1
Axis 2
4
0
−4
−4
0
4
8
−10
−5
0
5
10
10
5
0
−5
−10
−6
−3
0
3
6
Group
control
dose1
dose2
dose3

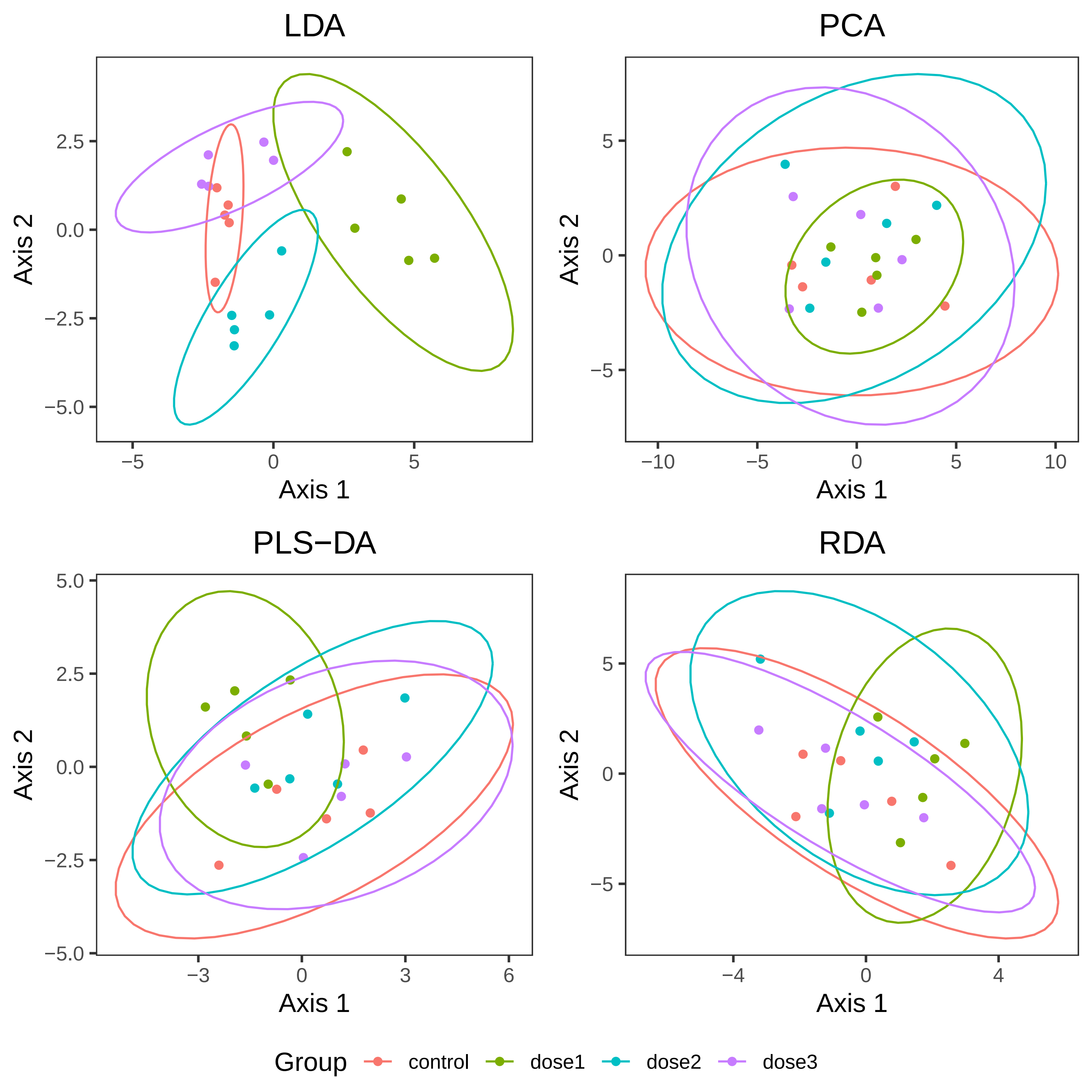

LDA
PCA
PLS−DA
RDA
Axis 1
Axis 2
2.5
0.0
−2.5
−5.0
−5
0
5
−10
10
5.0
−3
3
6
−4
4
Group
control
dose1
dose2
dose3

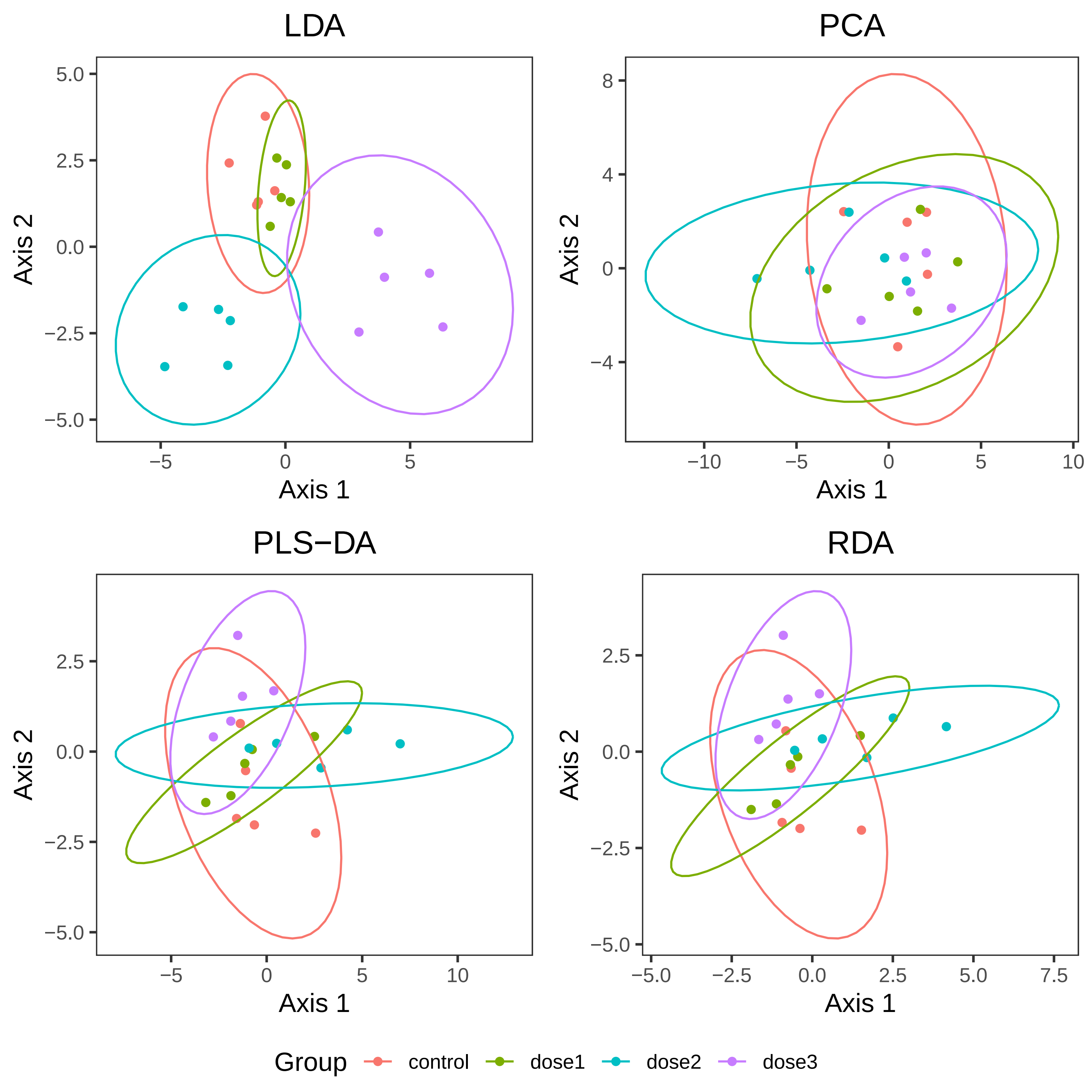
LDA
PCA
PLS−DA
RDA
Axis 1
Axis 2
Group
control
dose1
dose2
dose3

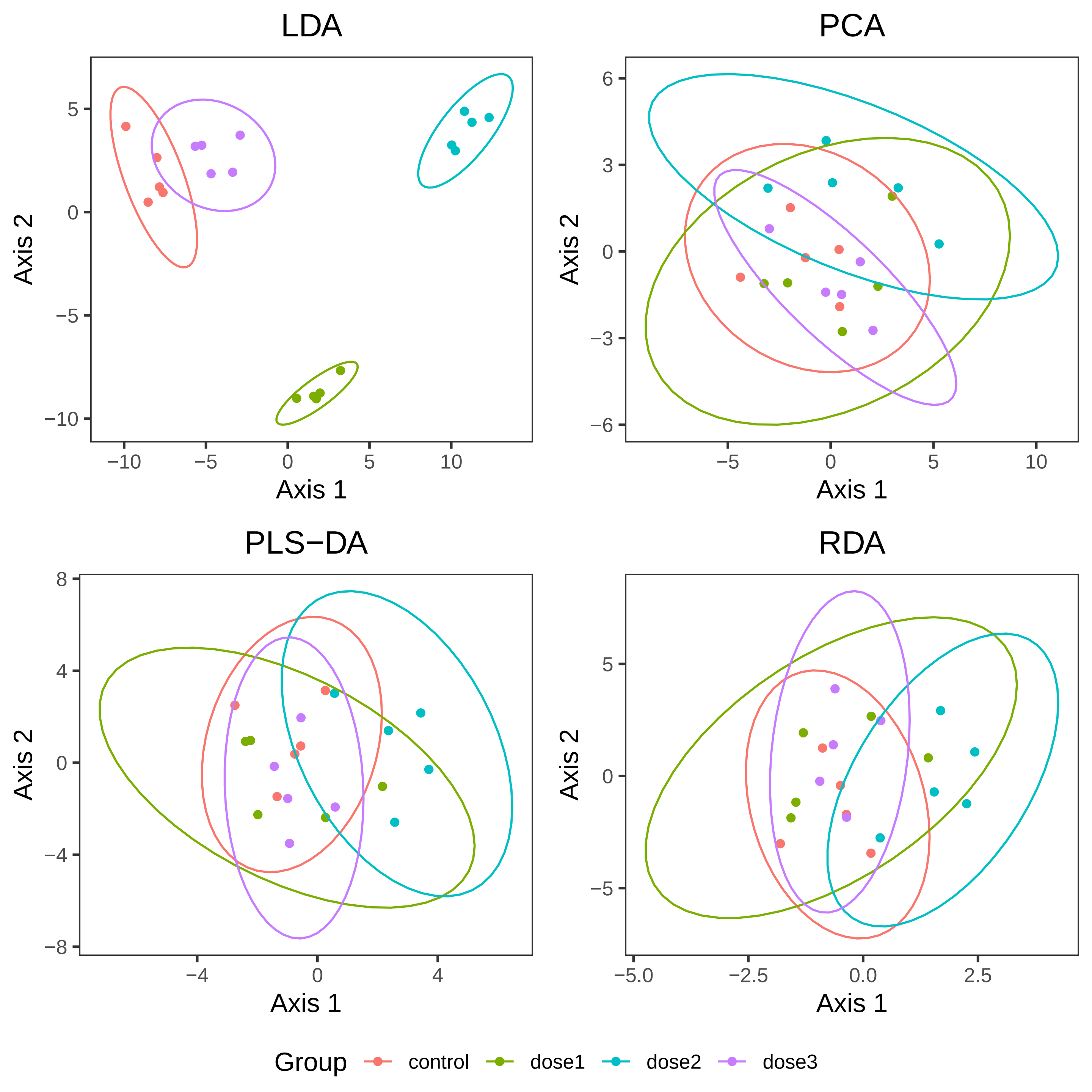

LDA
PCA
PLS−DA
RDA
Axis 1
Axis 2
Group
control
dose1
dose2
dose3

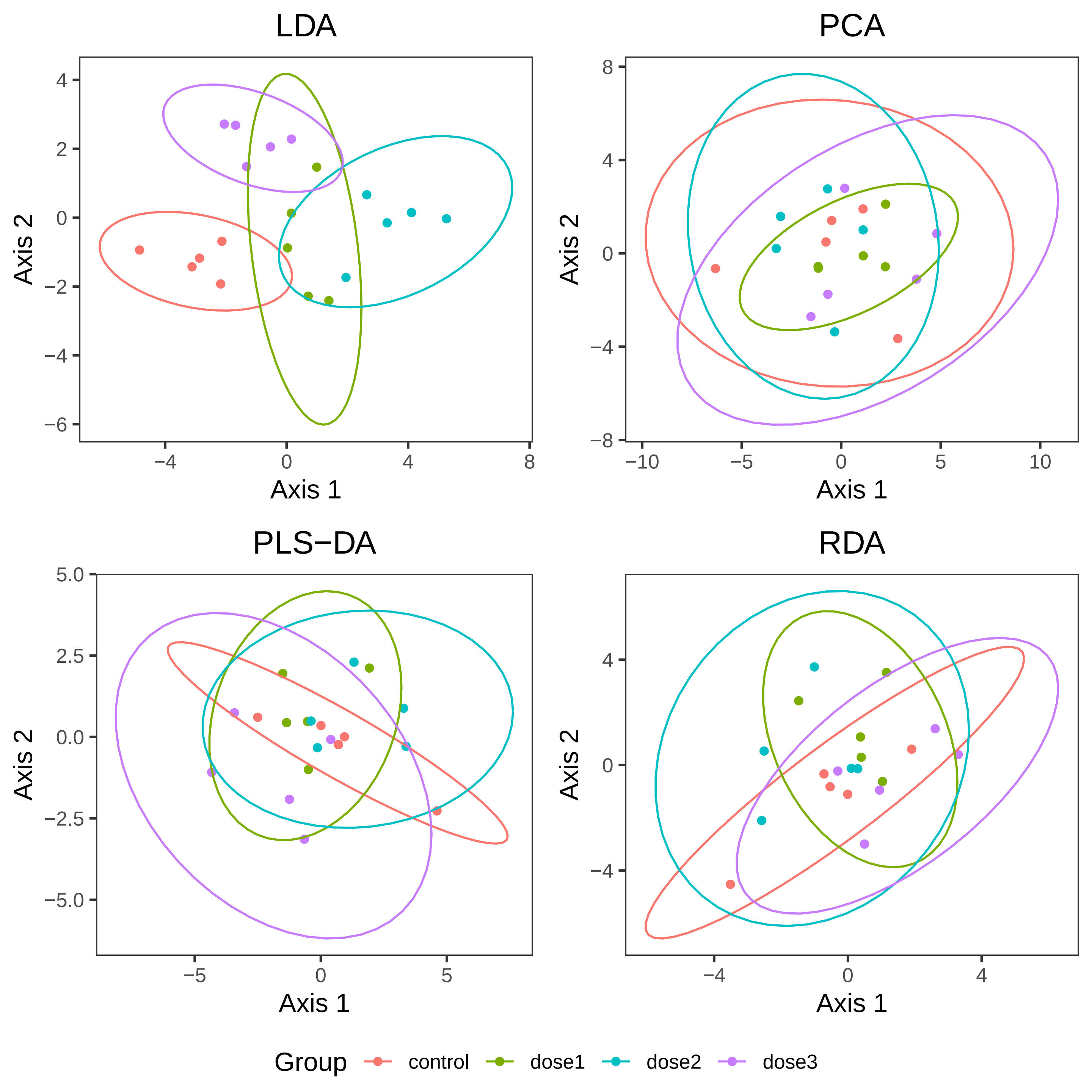
LDA
PCA
PLS−DA
RDA
Axis 1
Axis 2
Group
control
dose1
dose2
dose3

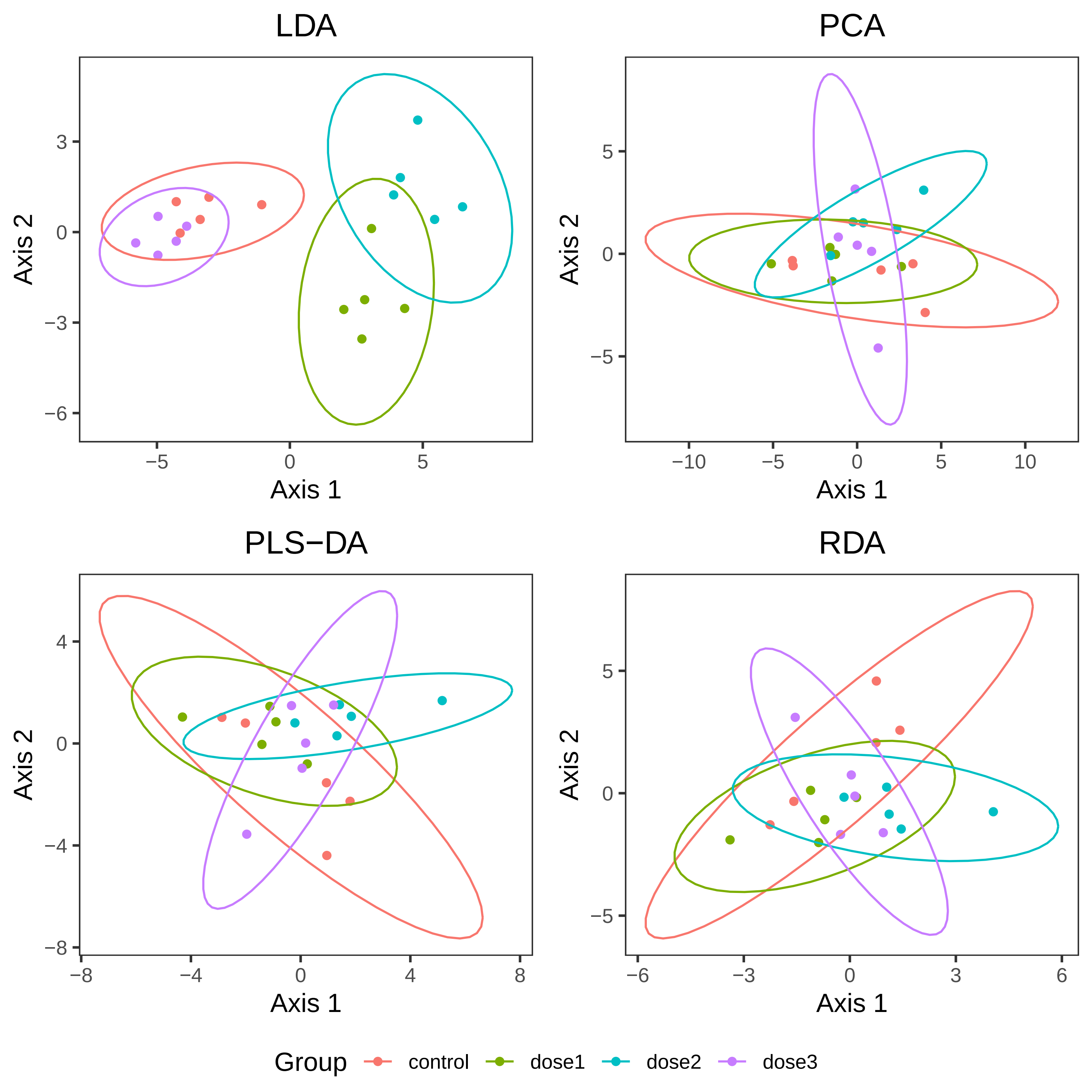

LDA
PCA
PLS−DA
RDA
Axis 1
Axis 2
3
0
−3
−6
−5
5
−10
10
4
−4
−8
8
6
Group
control
dose1
dose2
dose3

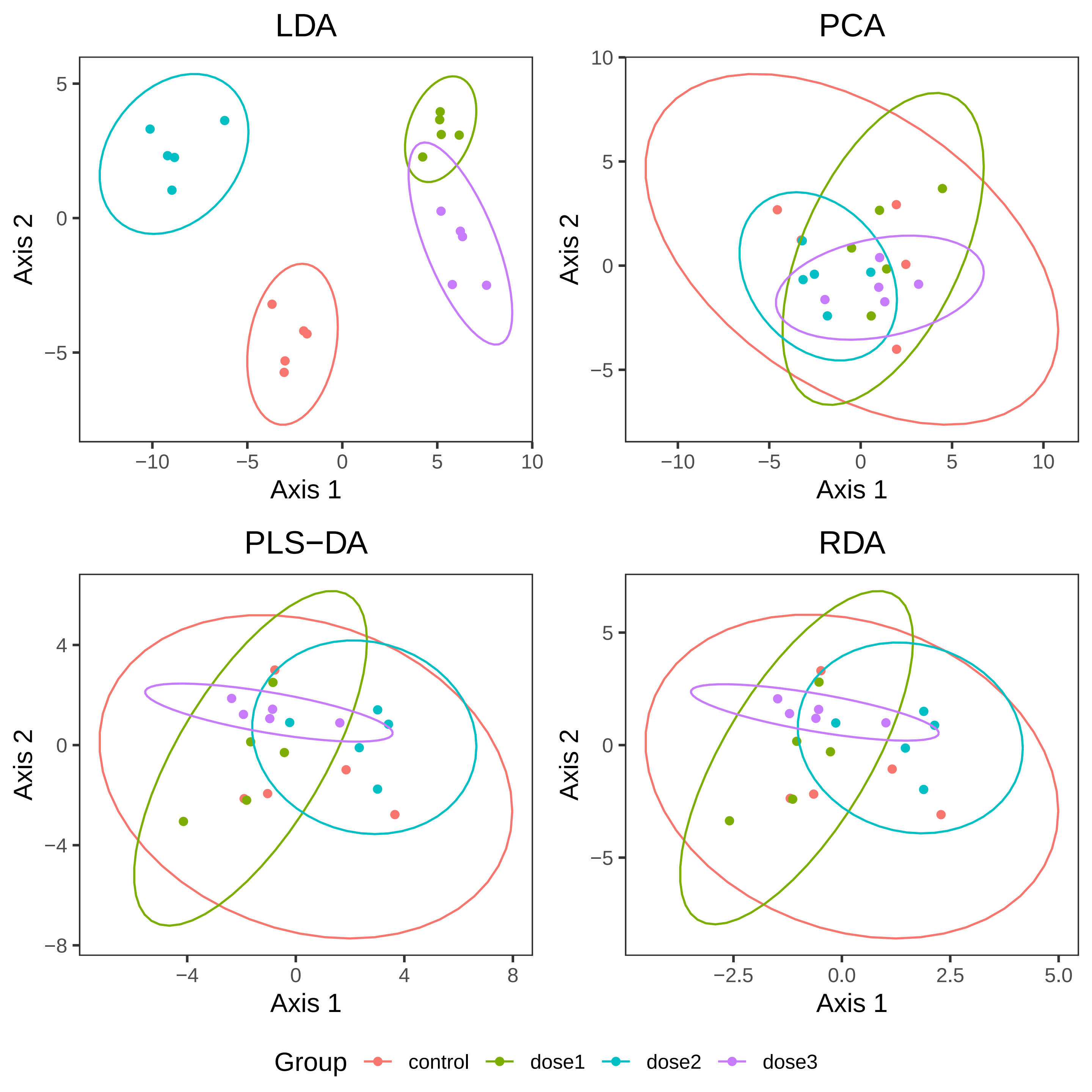

LDA
PCA
PLS−DA
RDA
Axis 1
Axis 2
Group
control
dose1
dose2
dose3

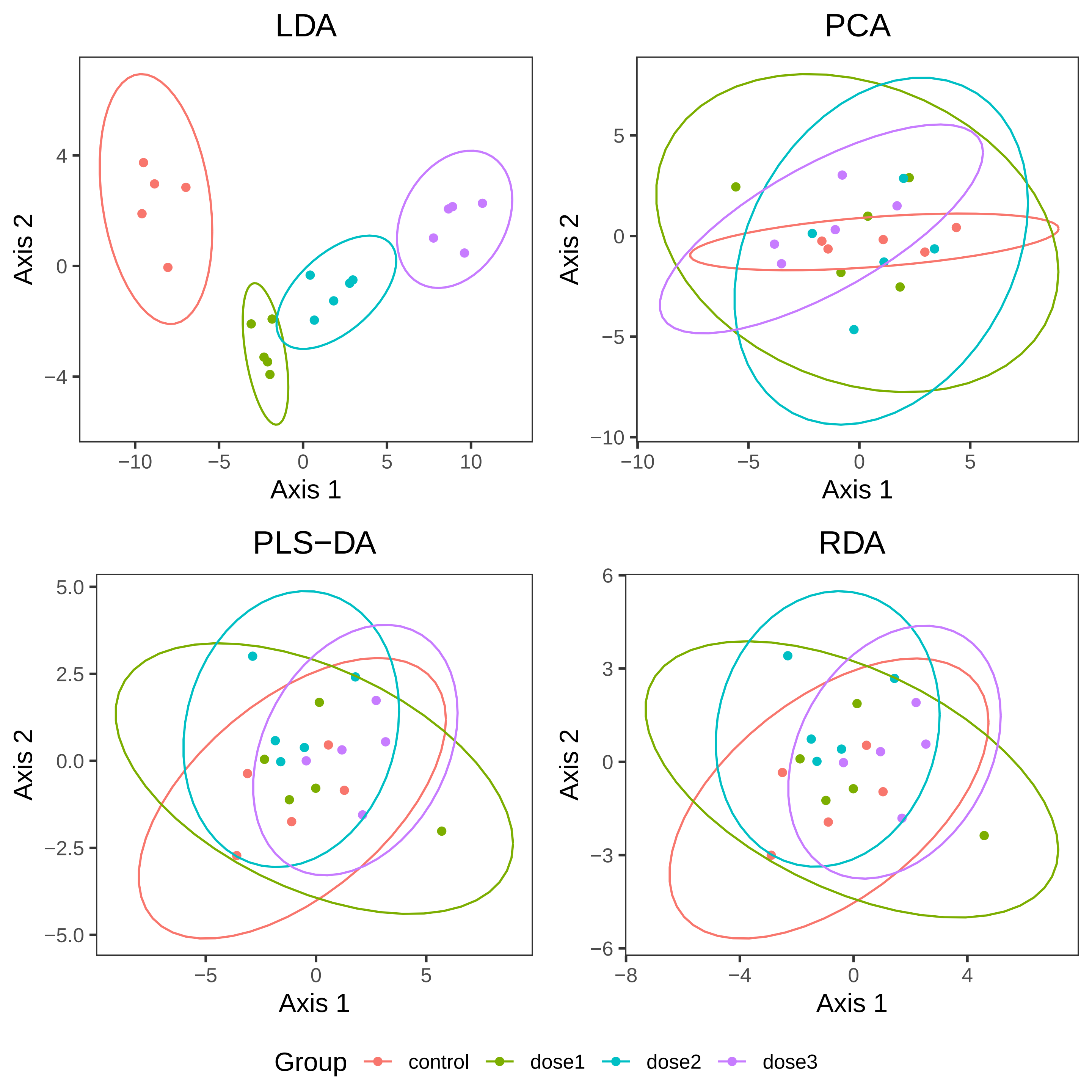

LDA
PCA
PLS−DA
RDA
Axis 1
Axis 2
Group
control
dose1
dose2
dose3

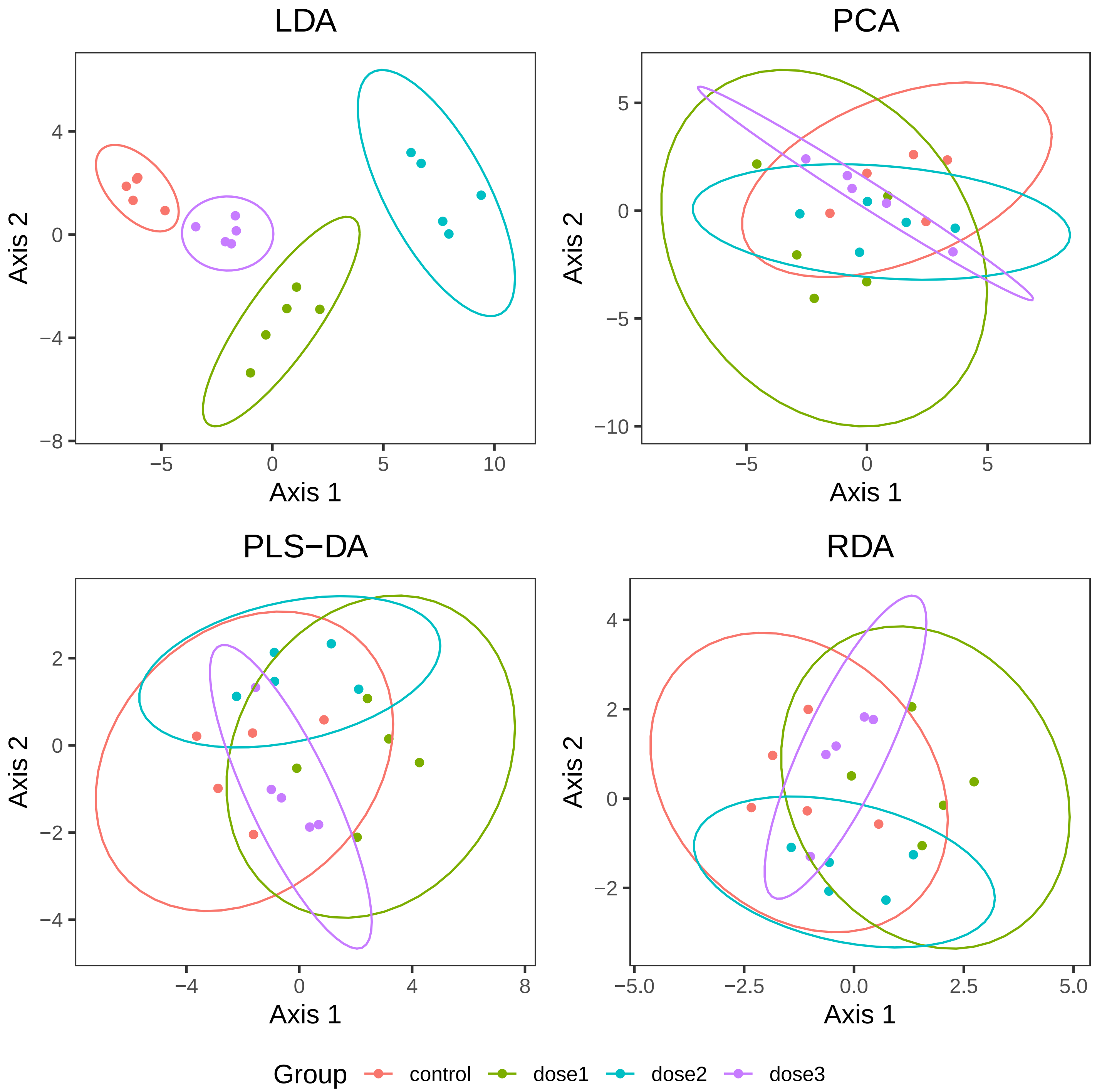
LDA
PCA
PLS−DA
RDA
Axis 1
Axis 2
Group
control
dose1
dose2
dose3

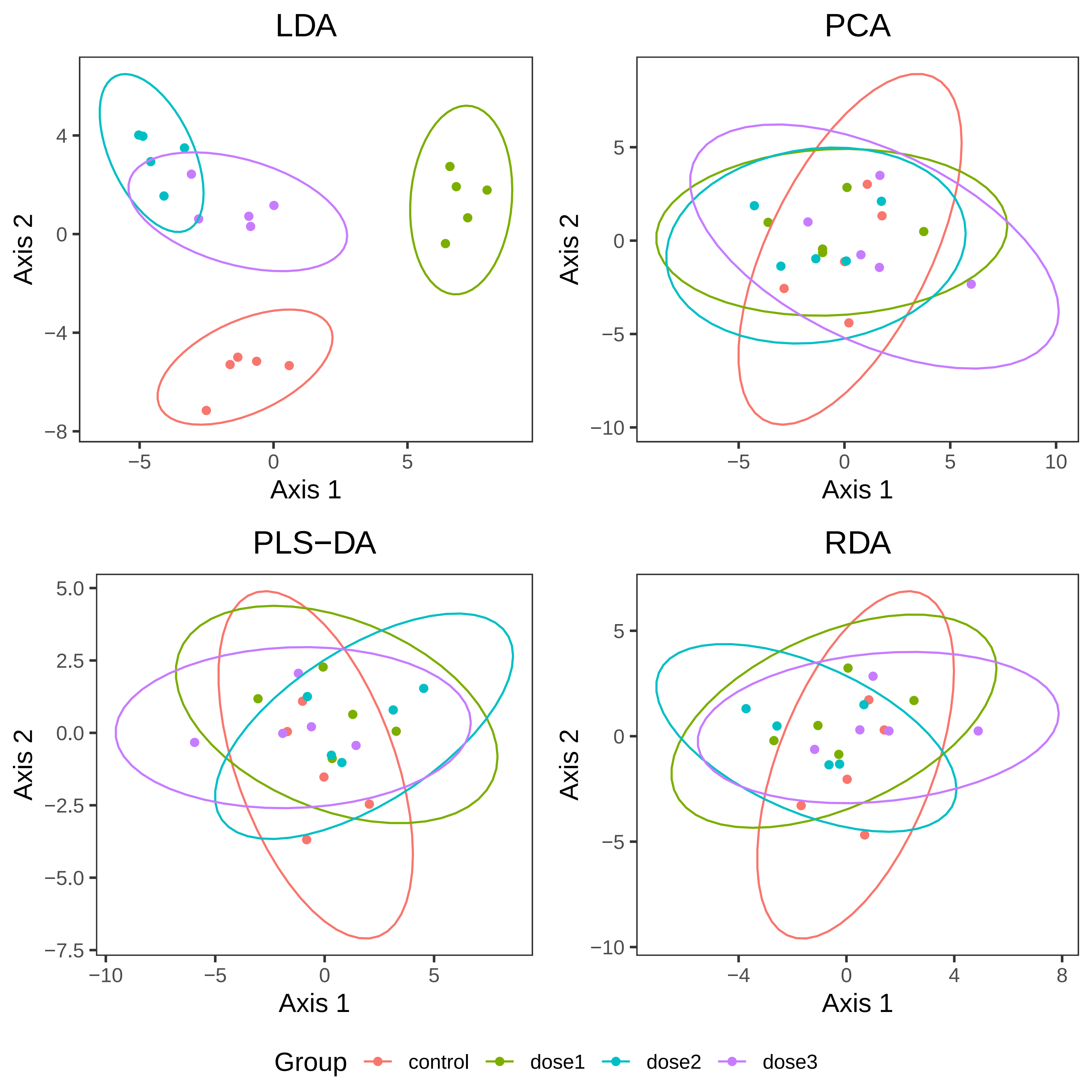
LDA
PCA
PLS−DA
RDA
Axis 1
Axis 2
Group
control
dose1
dose2
dose3

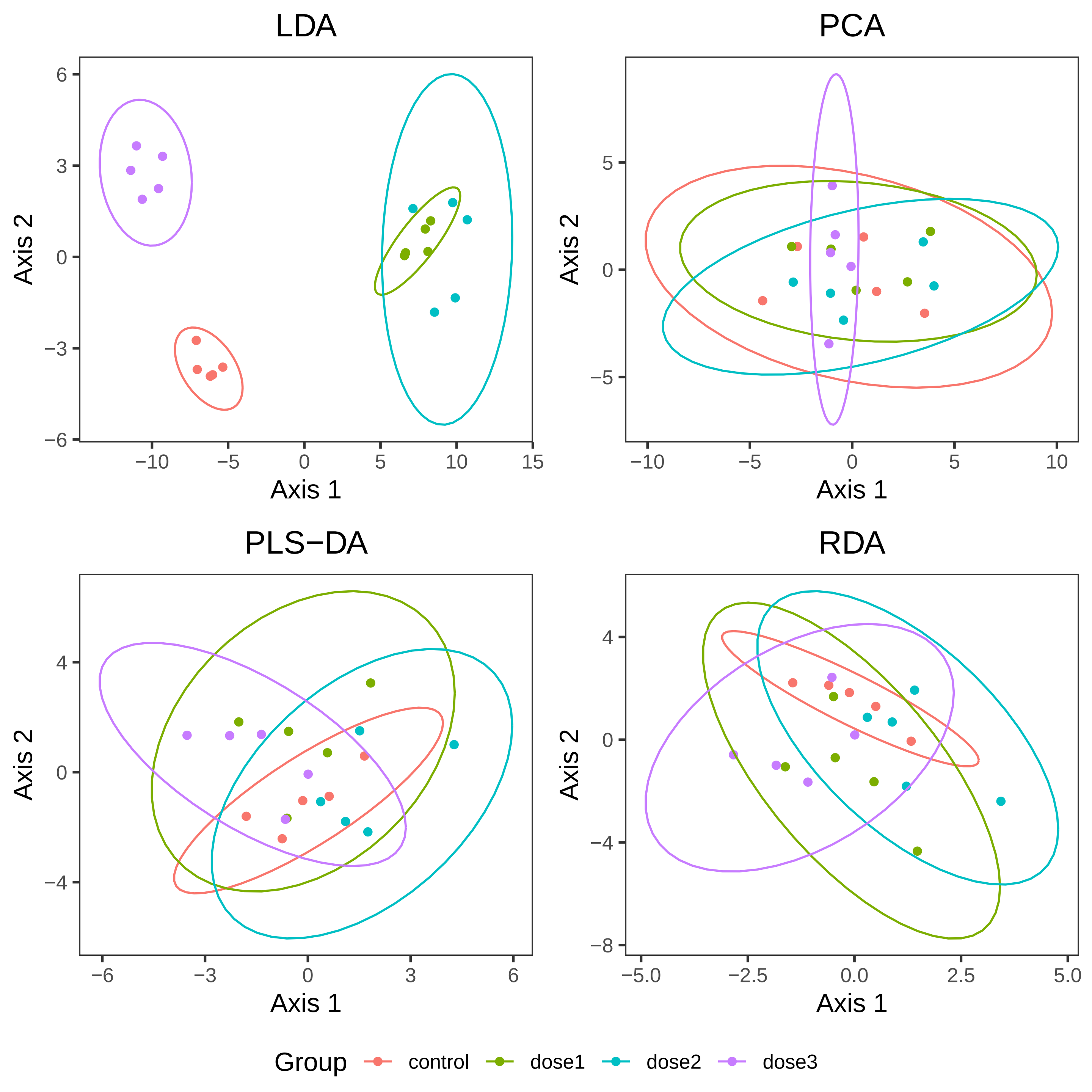
LDA
PCA
PLS−DA
RDA
Axis 1
Axis 2
Group
control
dose1
dose2
dose3

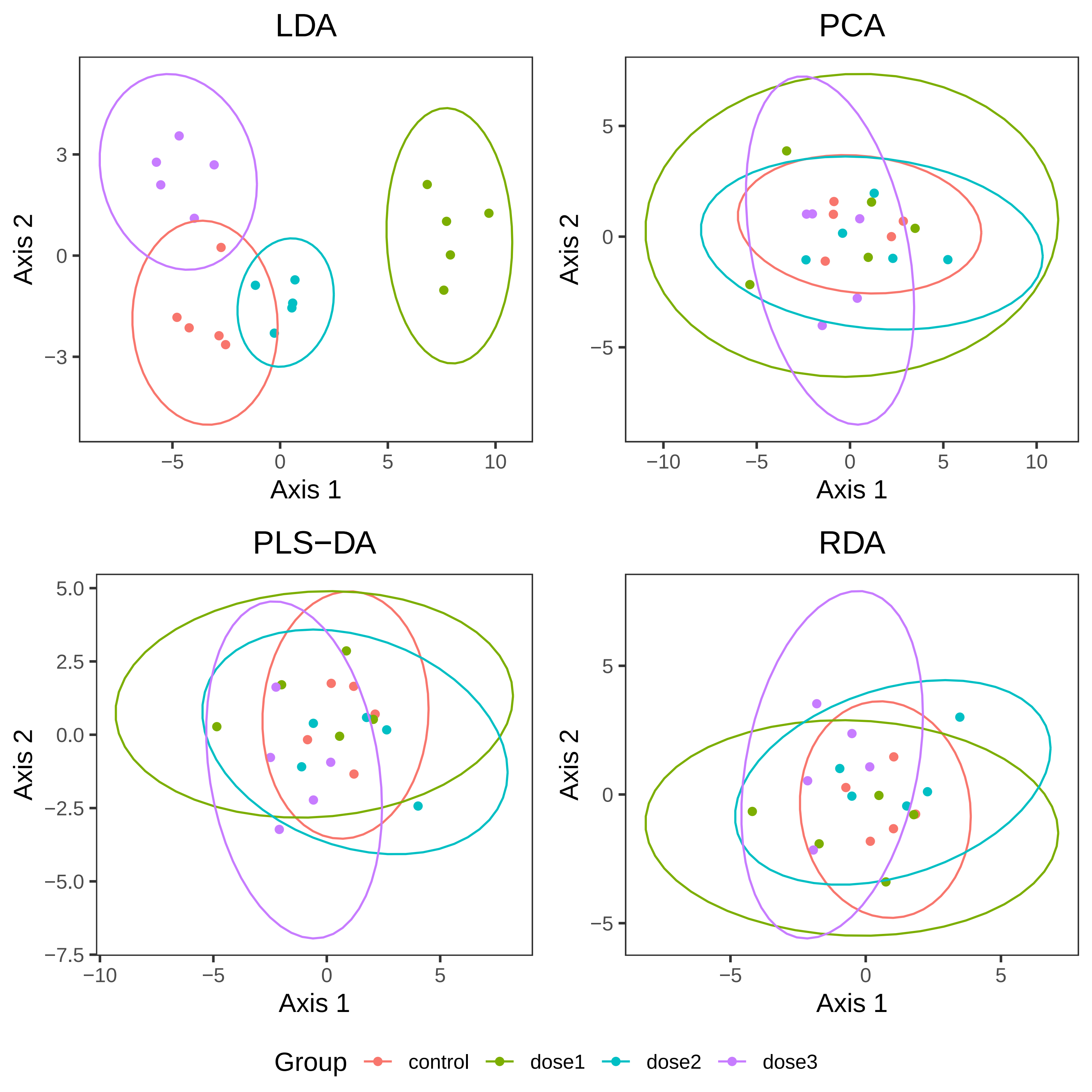

LDA
PCA
PLS−DA
RDA
Axis 1
Axis 2
3
0
−3
−5
0
5
10
−10
−5
0
5
10
5
0
−5
5.0
2.5
0.0
−2.5
−5.0
−7.5
−10
−5
0
5
5
0
−5
−5
0
5
Group
control
dose1
dose2
dose3

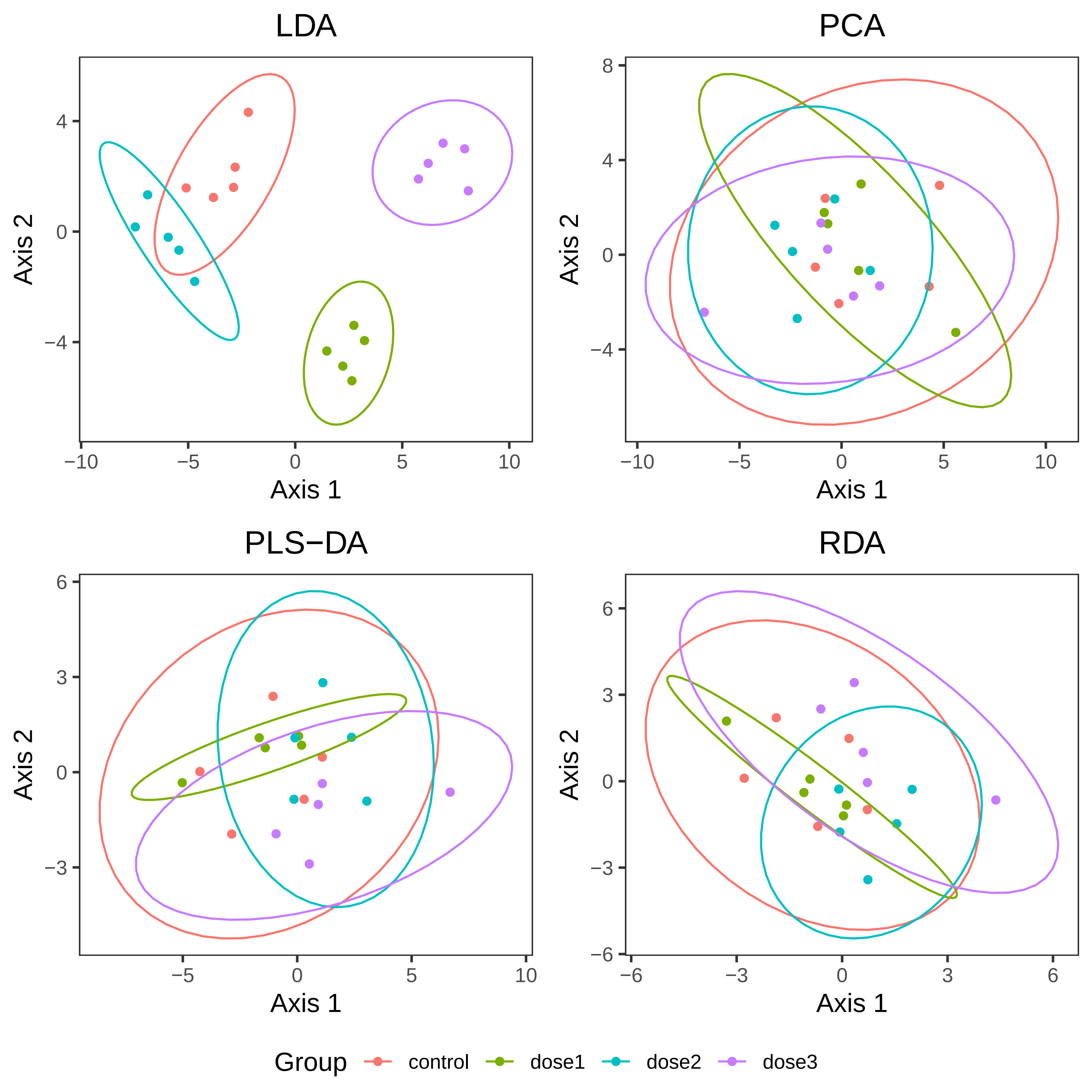

LDA
PCA
PLS−DA
RDA
Axis 1
Axis 2
Group
control
dose1
dose2
dose3

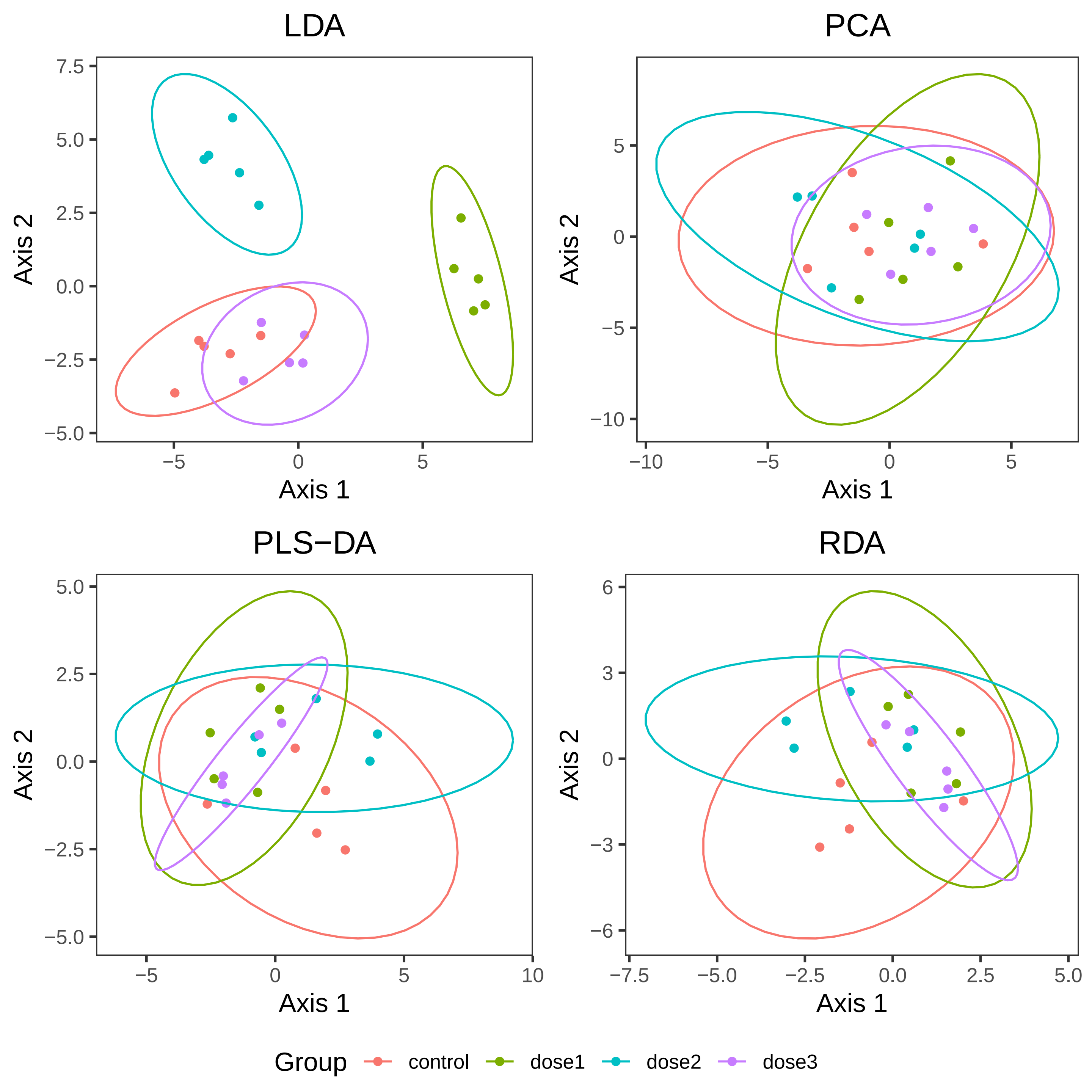

LDA
PCA
PLS−DA
RDA
Axis 1
Axis 2
7.5
5.0
2.5
0.0
−2.5
−5.0
−5
0
5
10
−10
6
3
0
−3
−6
−7.5
Group
control
dose1
dose2
dose3

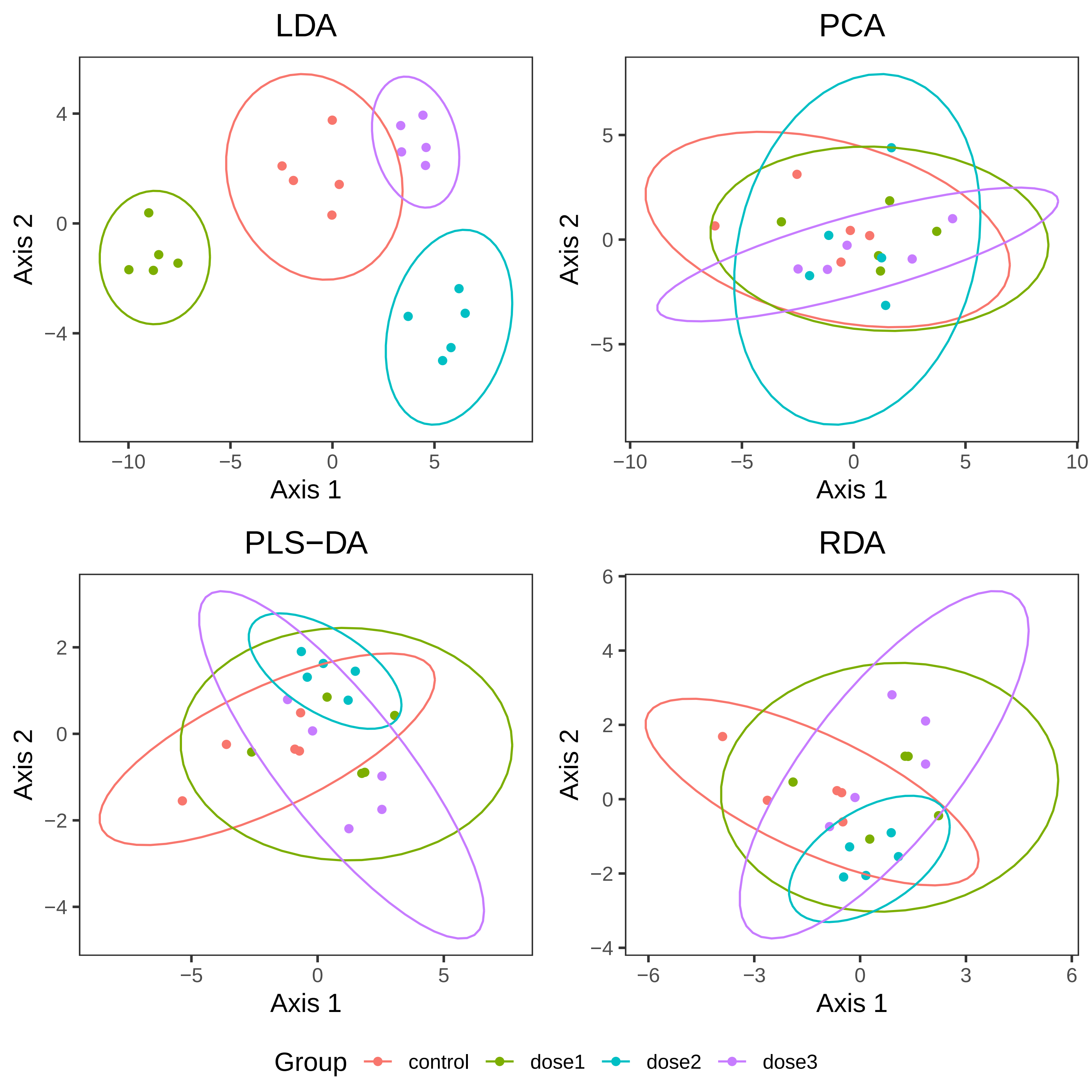

LDA
PCA
PLS−DA
RDA
Axis 1
Axis 2
4
0
−4
−10
−5
0
5
5
0
−5
−10
−5
0
5
10
2
0
−2
−4
−5
0
5
6
4
2
0
−2
−4
−6
−3
0
3
6
Group
control
dose1
dose2
dose3

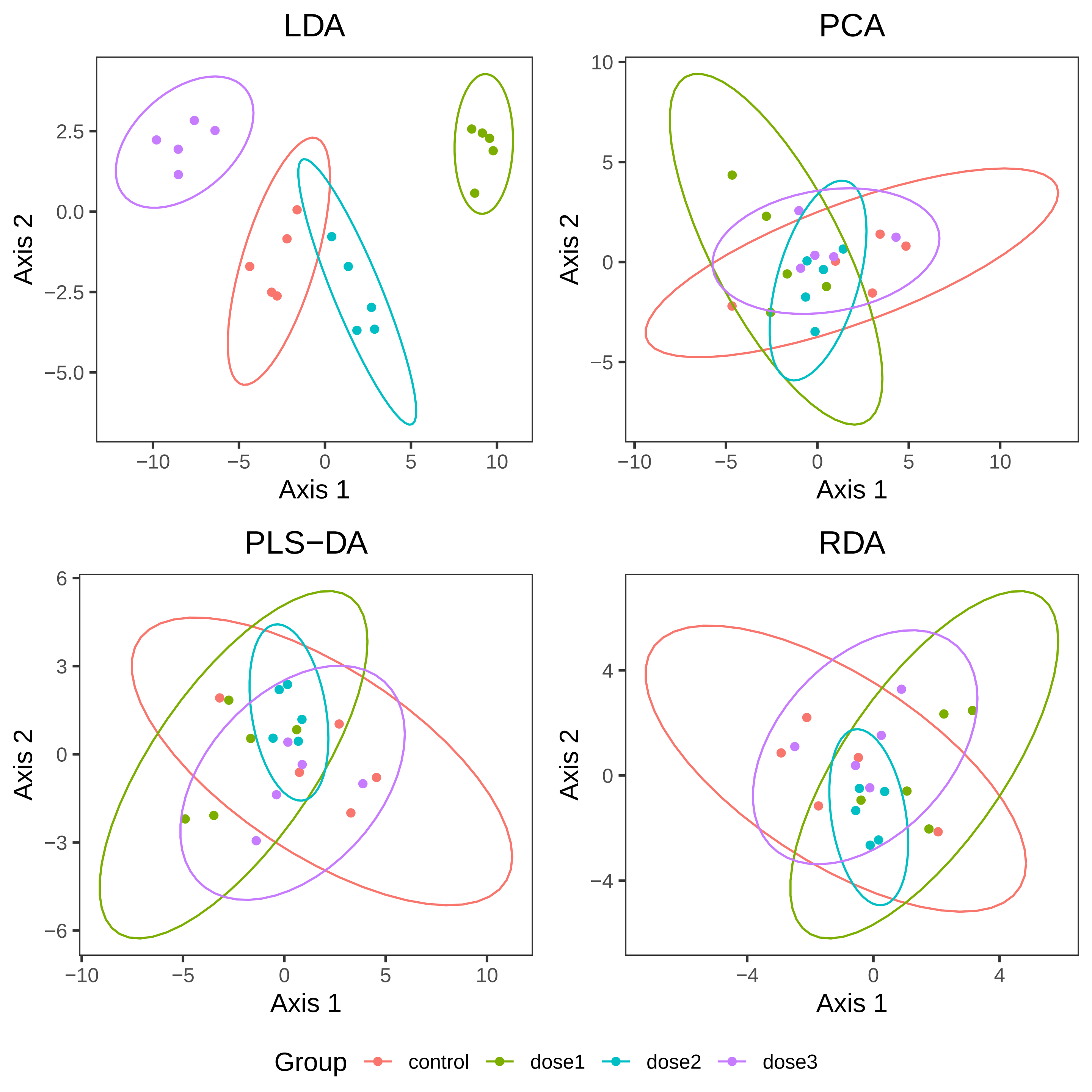

LDA
PCA
PLS−DA
RDA
Axis 1
Axis 2
Group
control
dose1
dose2
dose3

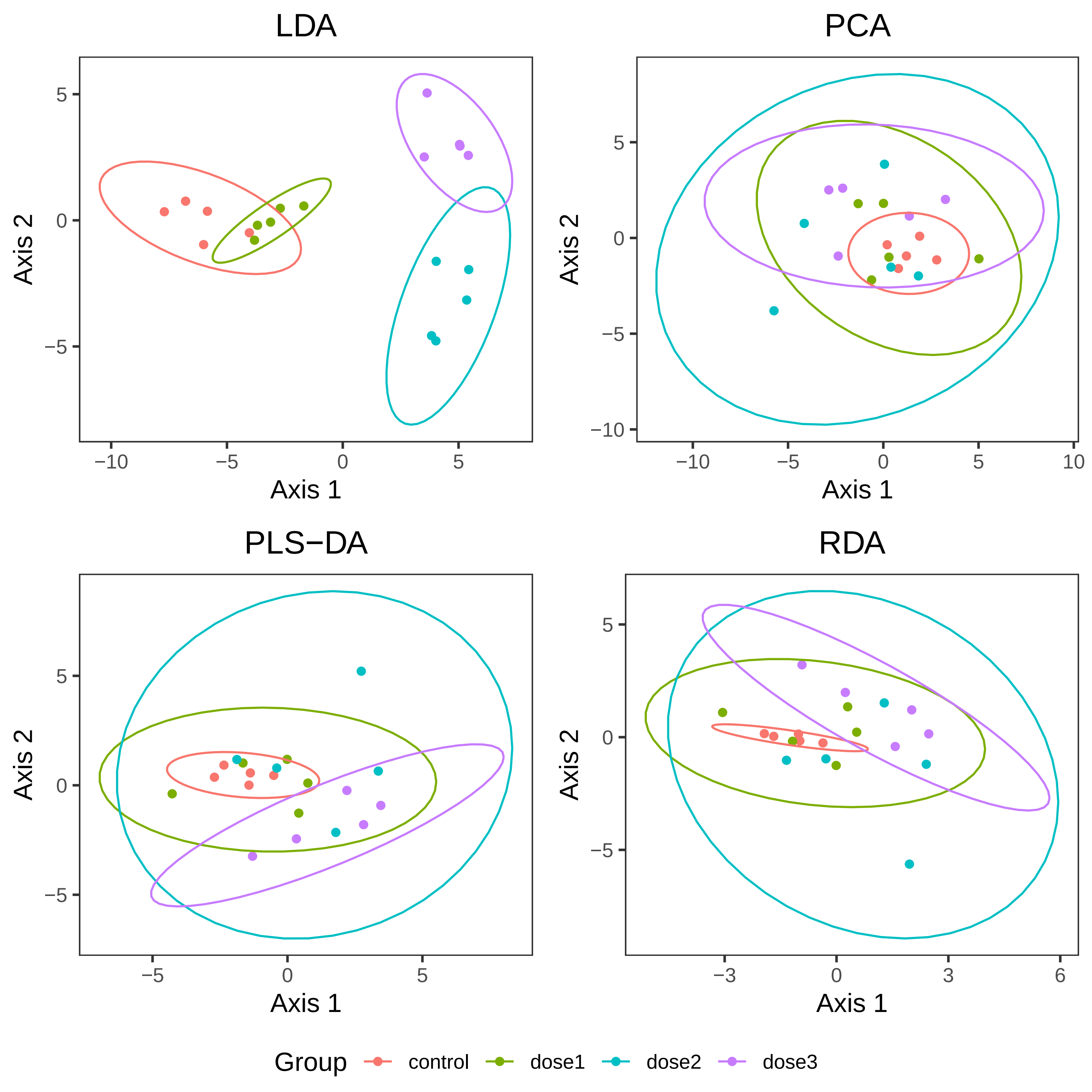

LDA
PCA
PLS−DA
RDA
Axis 1
Axis 2
5
0
−5
−10
10
−3
3
6
Group
control
dose1
dose2
dose3

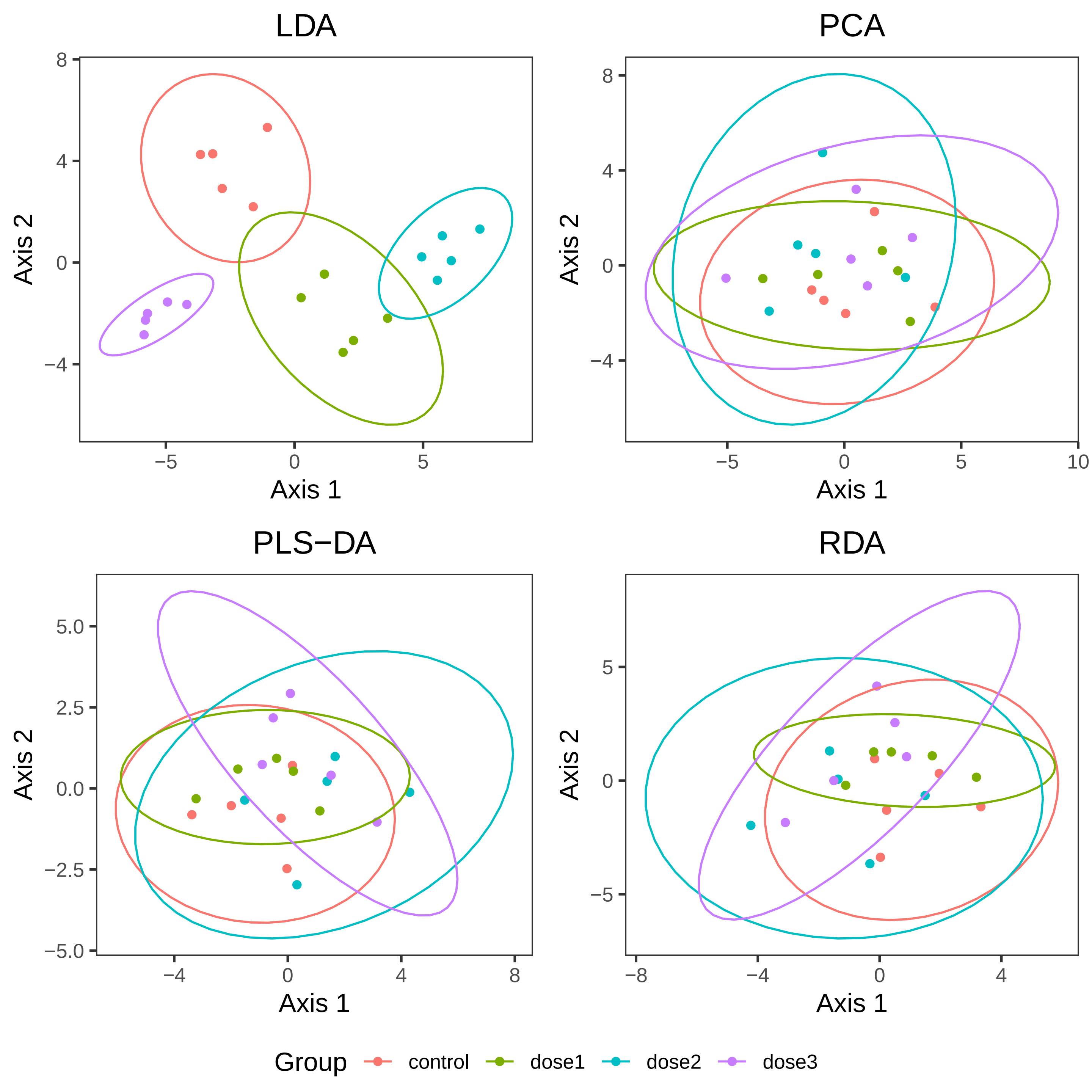
LDA
PCA
PLS−DA
RDA
Axis 1
Axis 2
Group
control
dose1
dose2
dose3

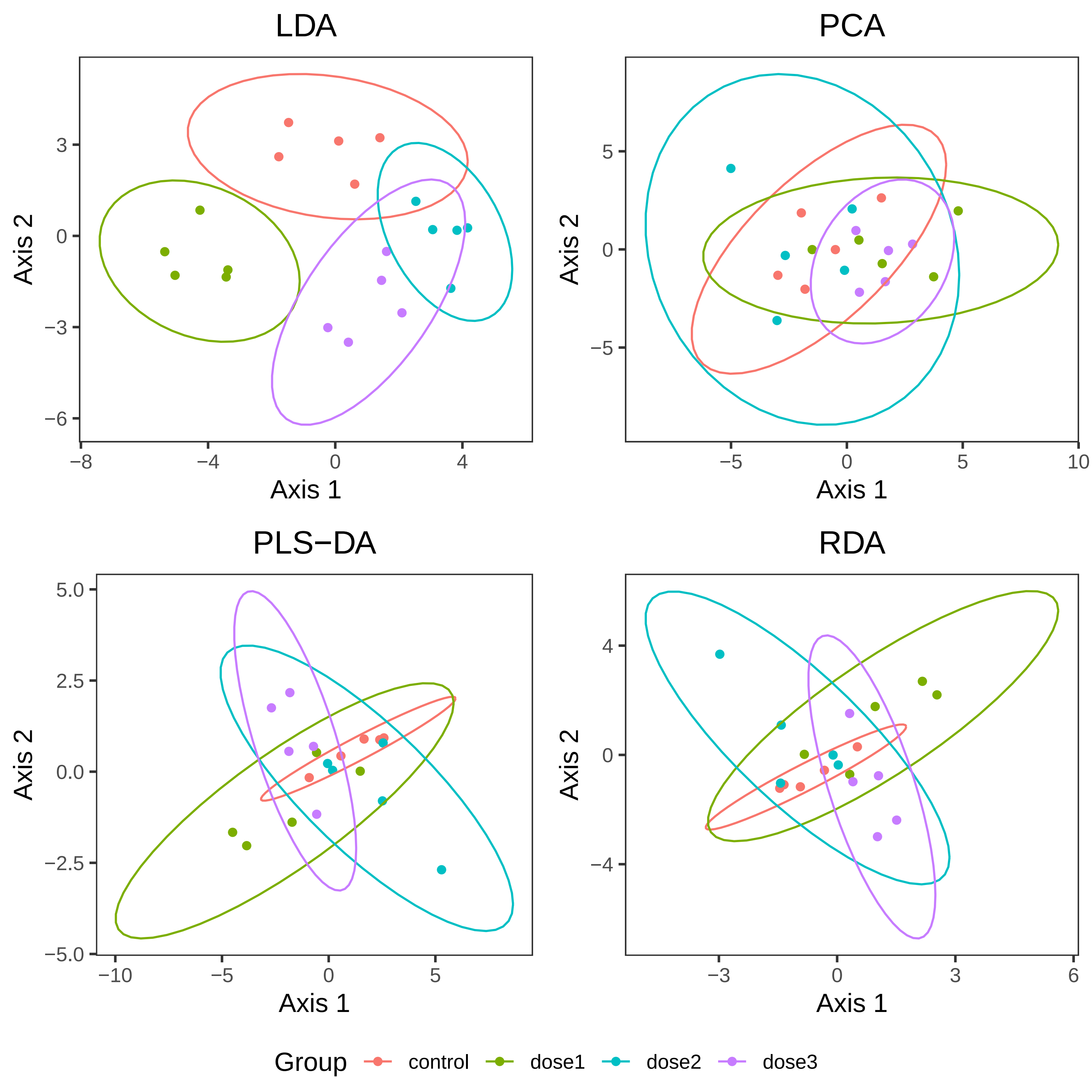

LDA
PCA
PLS−DA
RDA
Axis 1
Axis 2
3
0
−3
−6
−8
−4
0
4
5
0
−5
−5
0
5
10
5.0
2.5
0.0
−2.5
−5.0
−10
−5
0
5
4
0
−4
−3
0
3
6
Group
control
dose1
dose2
dose3

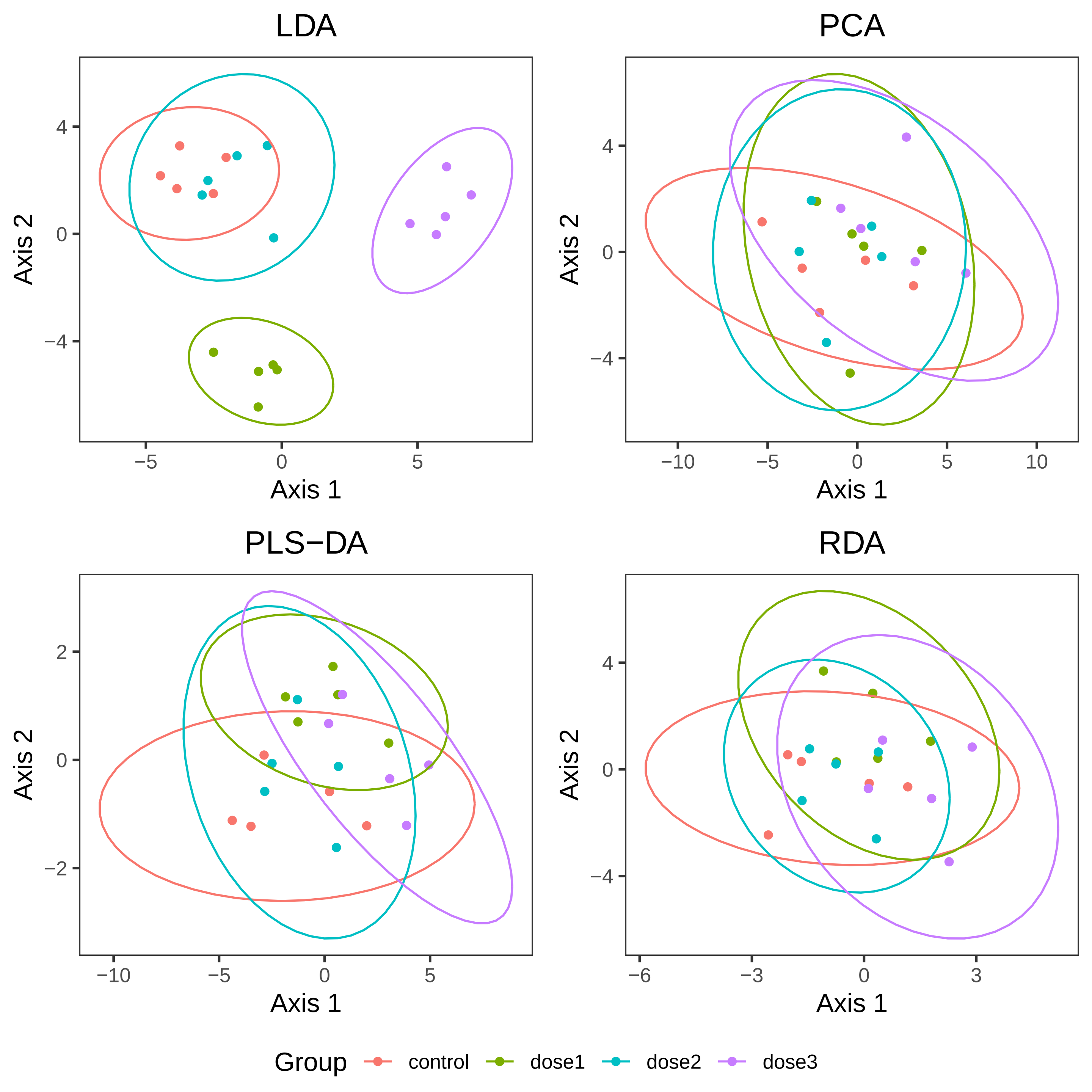
LDA
PCA
PLS−DA
RDA
Axis 1
Axis 2
4
0
−4
−5
0
5
−10
−5
0
5
10
2
0
−2
−6
−3
0
3
Group
control
dose1
dose2
dose3

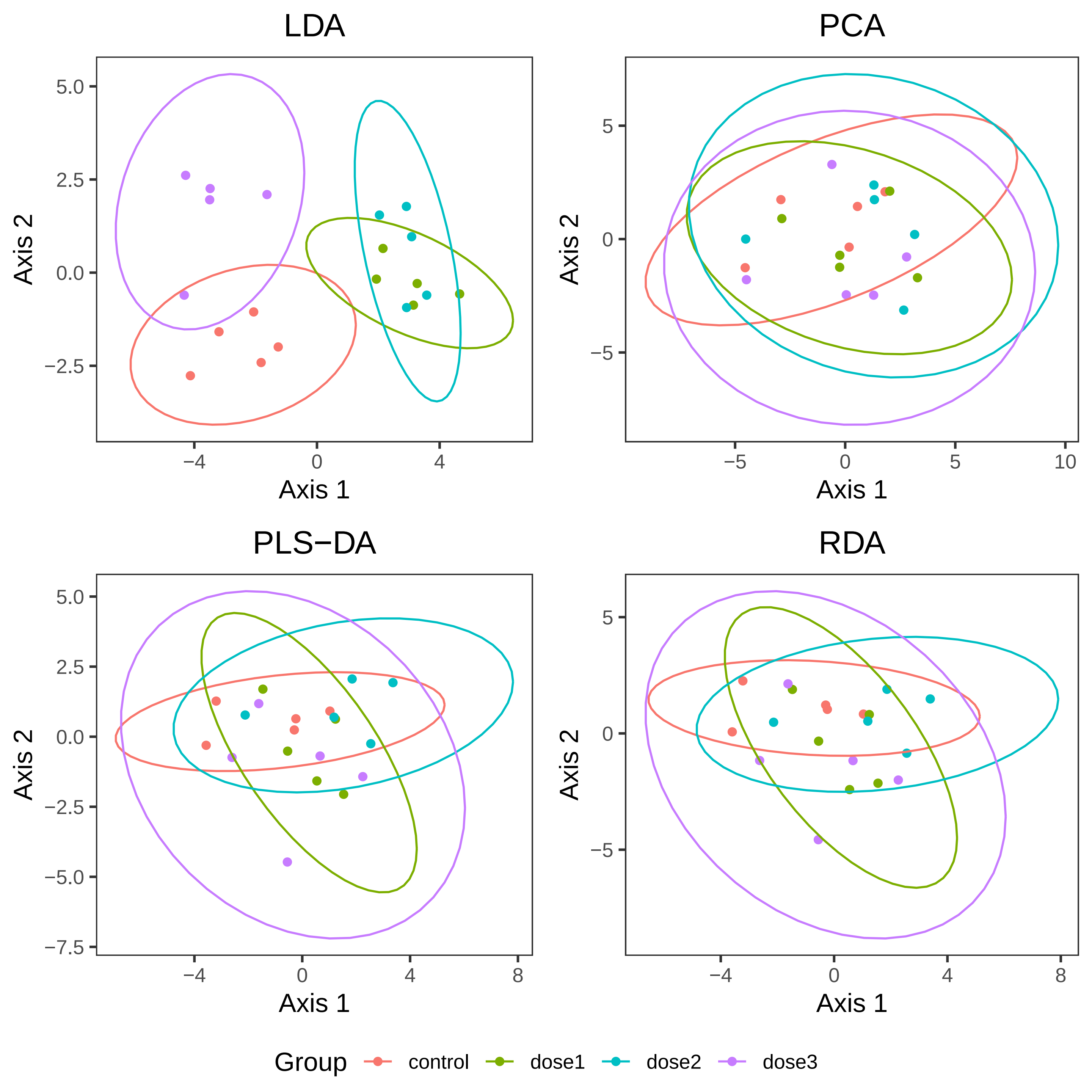

LDA
PCA
PLS−DA
RDA
Axis 1
Axis 2
5.0
2.5
0.0
−2.5
−5.0
−7.5
−4
0
4
8
5
−5
10
Group
control
dose1
dose2
dose3

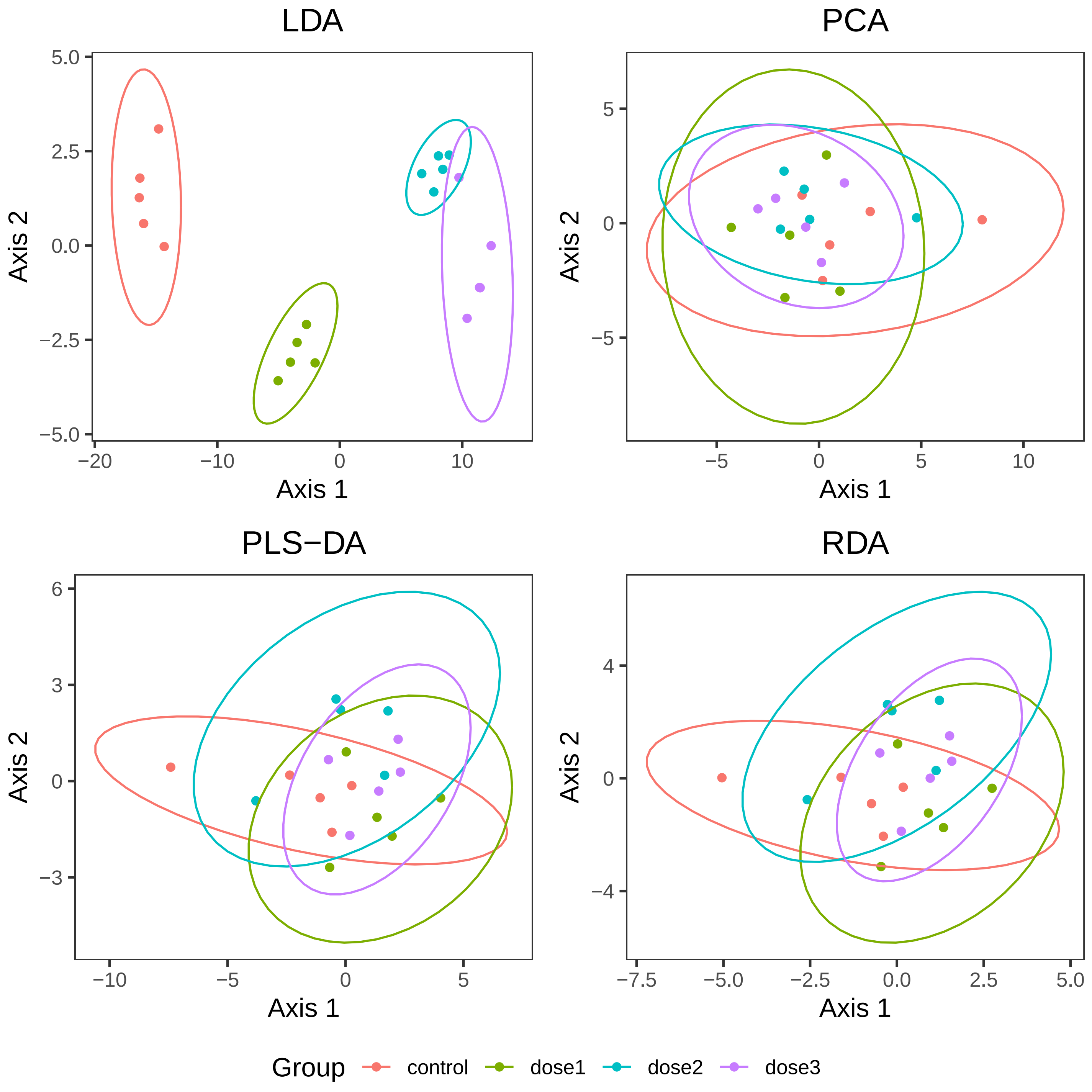
LDA
PCA
PLS−DA
RDA
Axis 1
Axis 2
Group
control
dose1
dose2
dose3

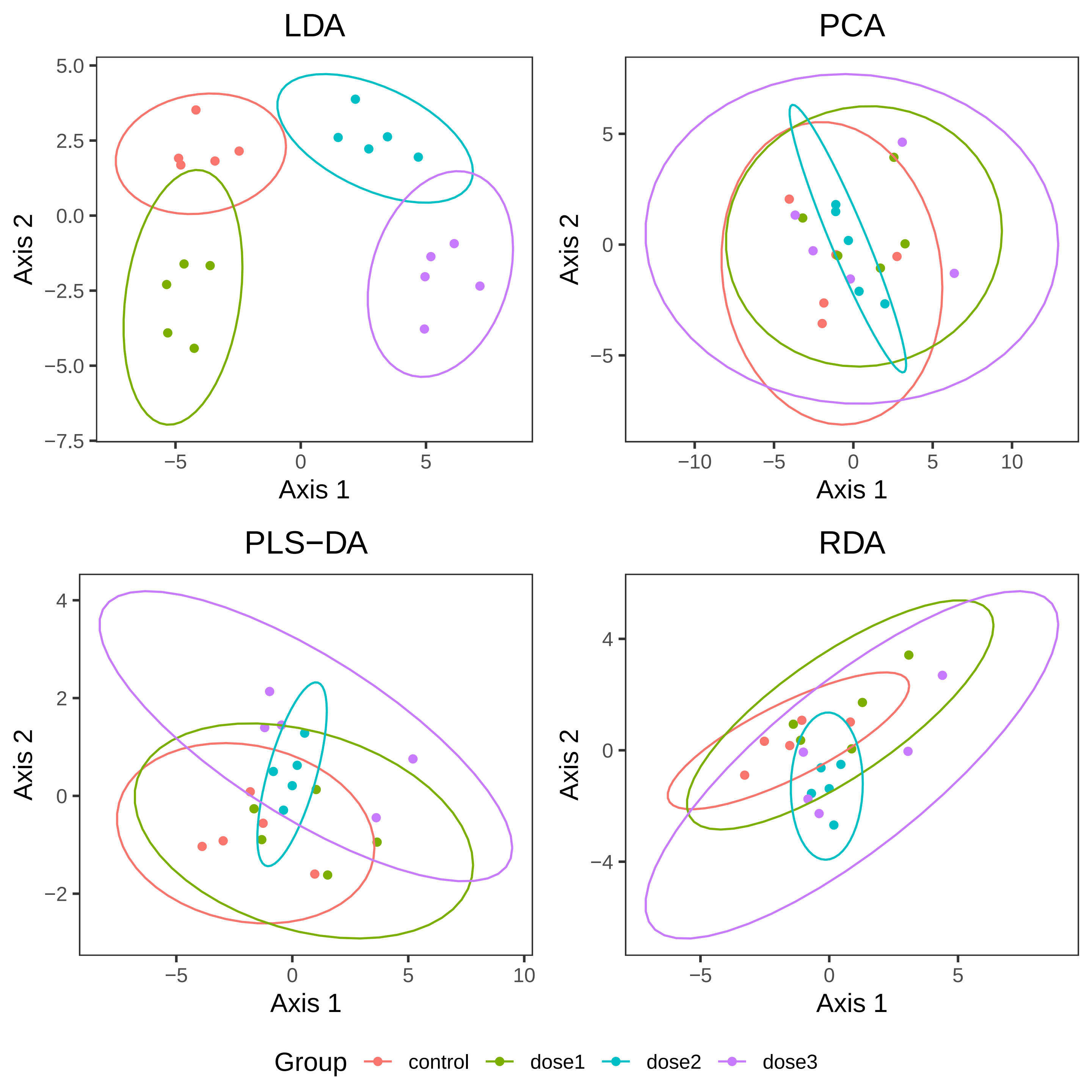
LDA
PCA
PLS−DA
RDA
Axis 1
Axis 2
Group
control
dose1
dose2
dose3

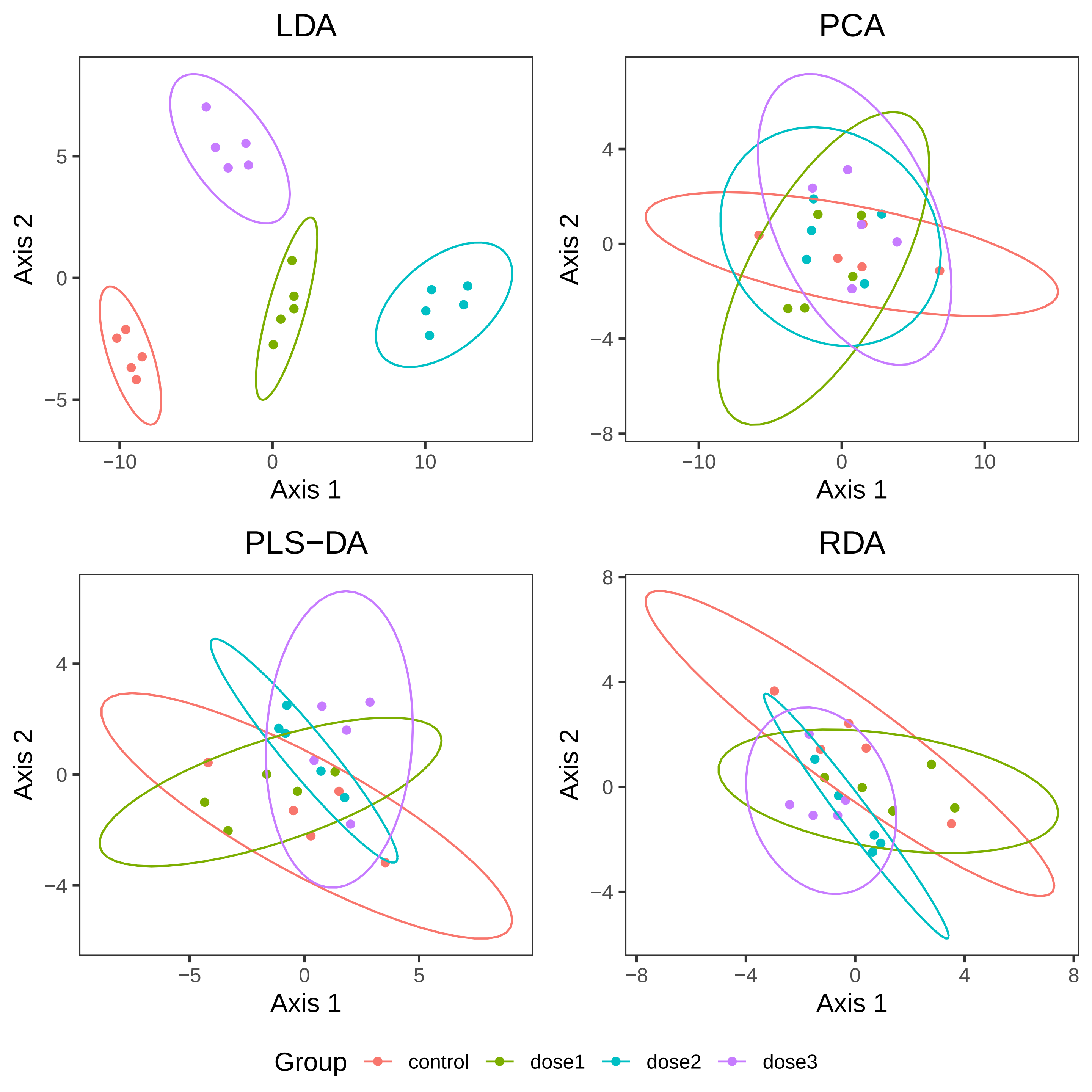

LDA
PCA
PLS−DA
RDA
Axis 1
Axis 2
Group
control
dose1
dose2
dose3

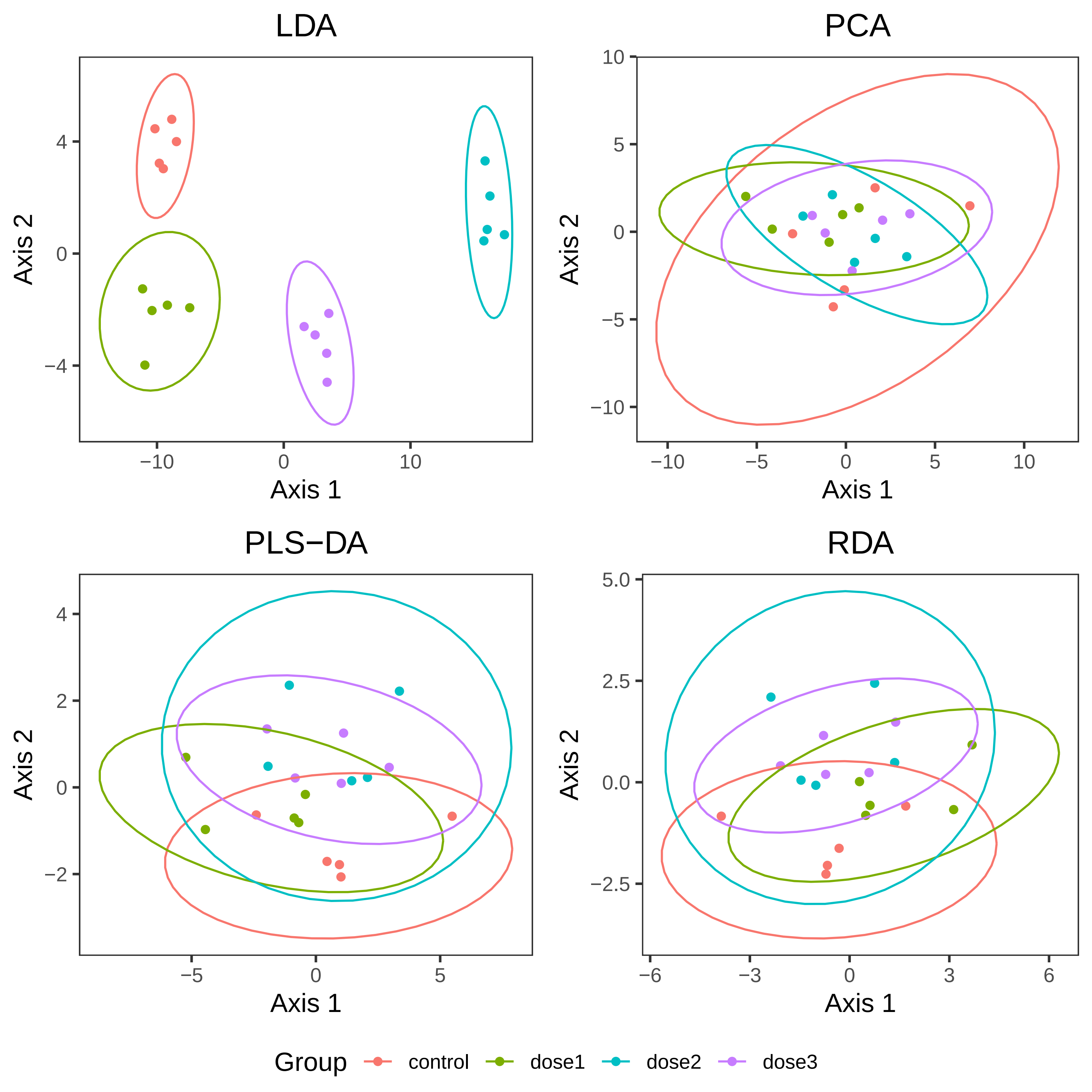
LDA
PCA
PLS−DA
RDA
Axis 1
Axis 2
Group
control
dose1
dose2
dose3

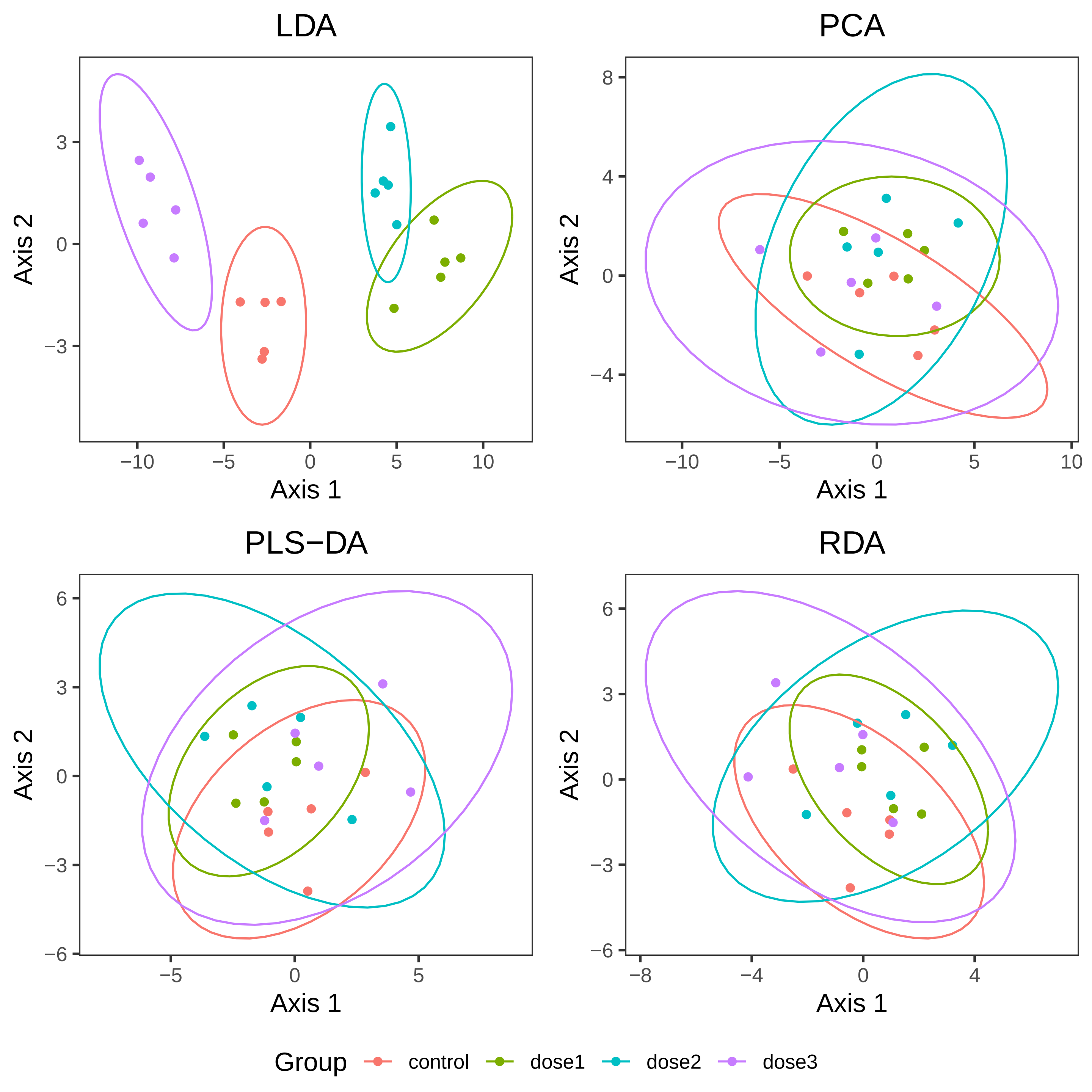
LDA
PCA
PLS−DA
RDA
Axis 1
Axis 2
−10
−5
0
5
10
3
−3
8
4
−4
6
−6
−8
Group
control
dose1
dose2
dose3

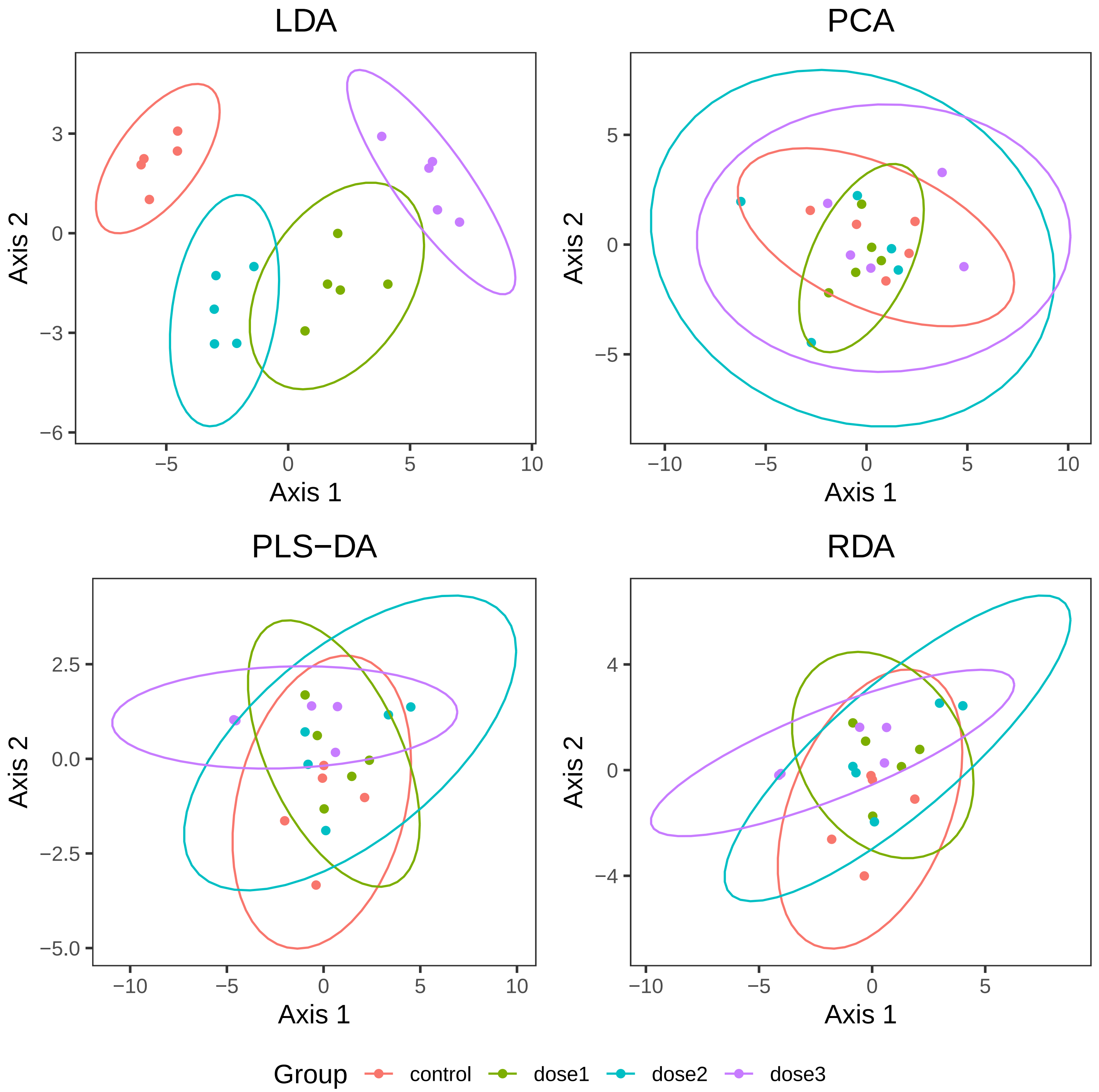
LDA
PCA
PLS−DA
RDA
Axis 1
Axis 2
3
0
−3
−6
−5
0
5
10
−10
2.5
0.0
−2.5
−5.0
4
−4
Group
control
dose1
dose2
dose3

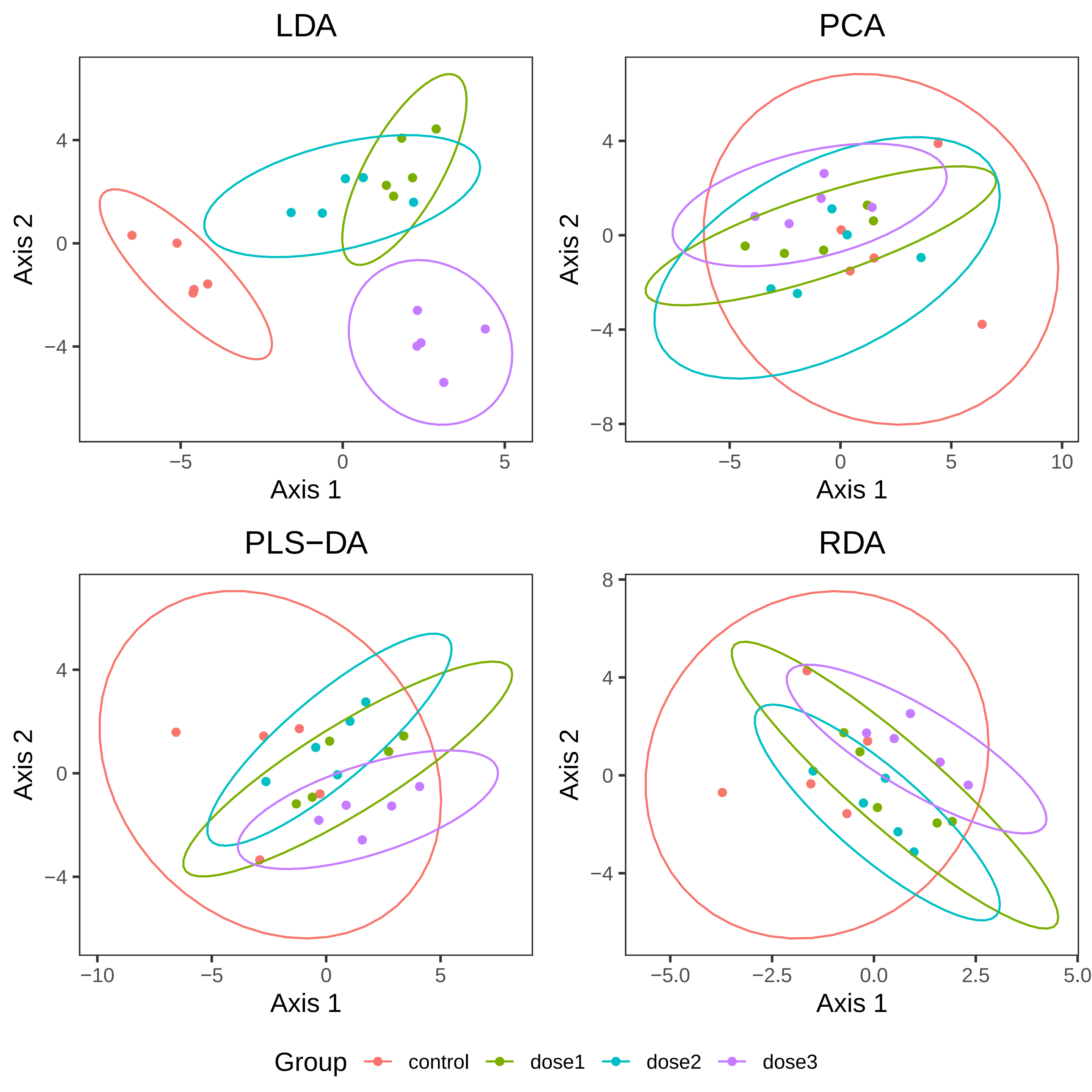

LDA
PCA
PLS−DA
RDA
Axis 1
Axis 2
Group
control
dose1
dose2
dose3

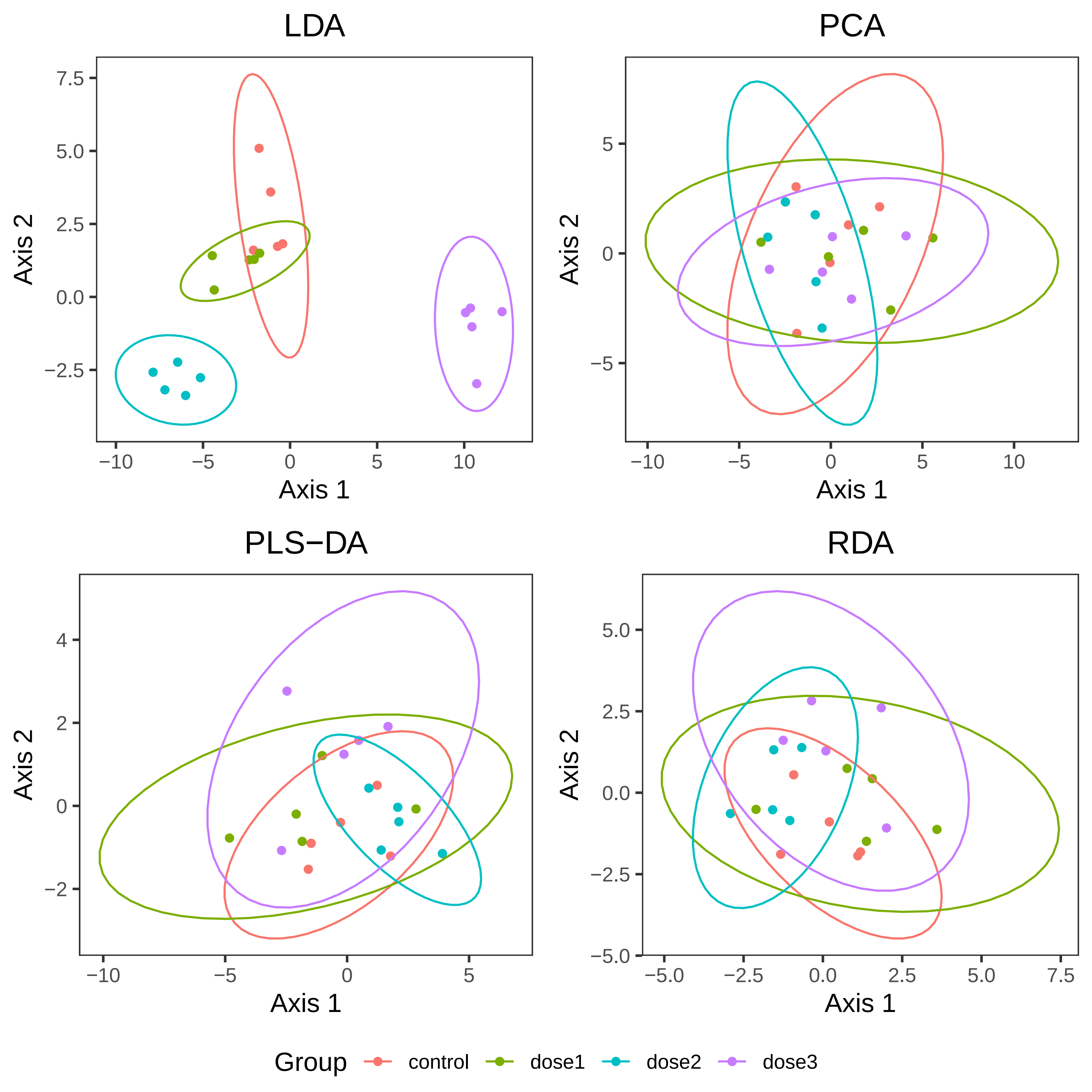

LDA
PCA
PLS−DA
RDA
Axis 1
Axis 2
−10
−5
0
5
10
7.5
5.0
2.5
0.0
−2.5
−5.0
Group
control
dose1
dose2
dose3

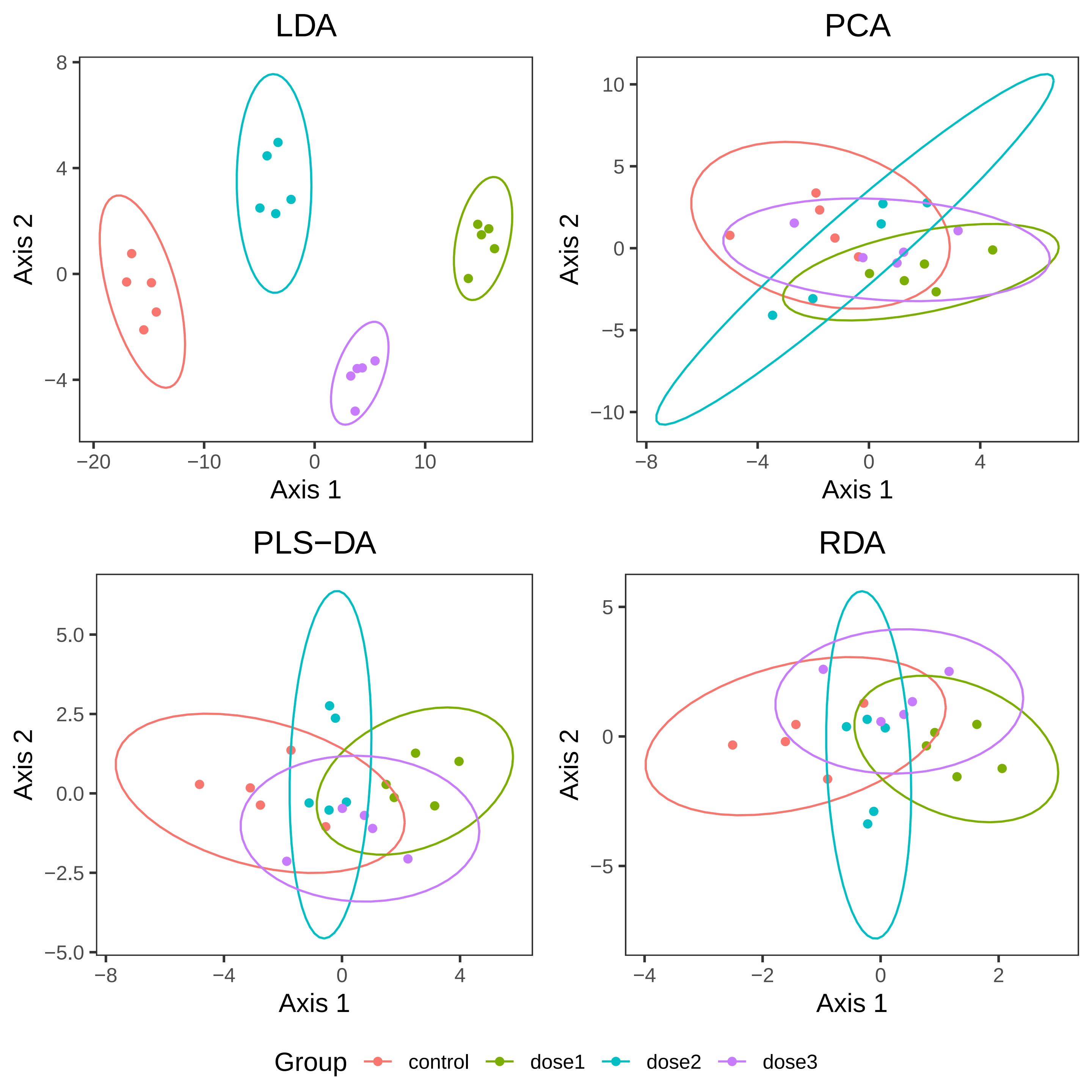
LDA
PCA
PLS−DA
RDA
Axis 1
Axis 2
Group
control
dose1
dose2
dose3

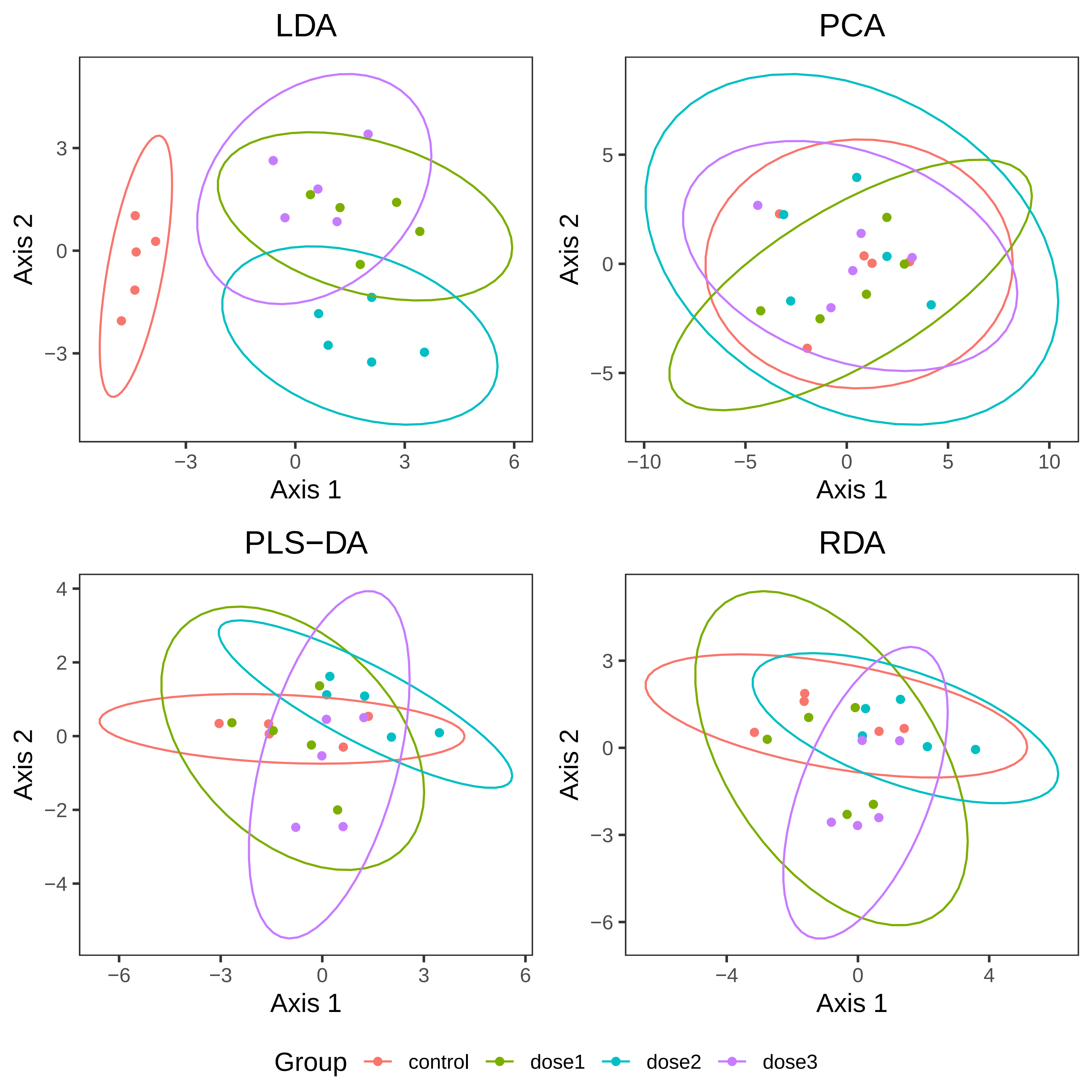

LDA
PCA
PLS−DA
RDA
Axis 1
Axis 2
Group
control
dose1
dose2
dose3

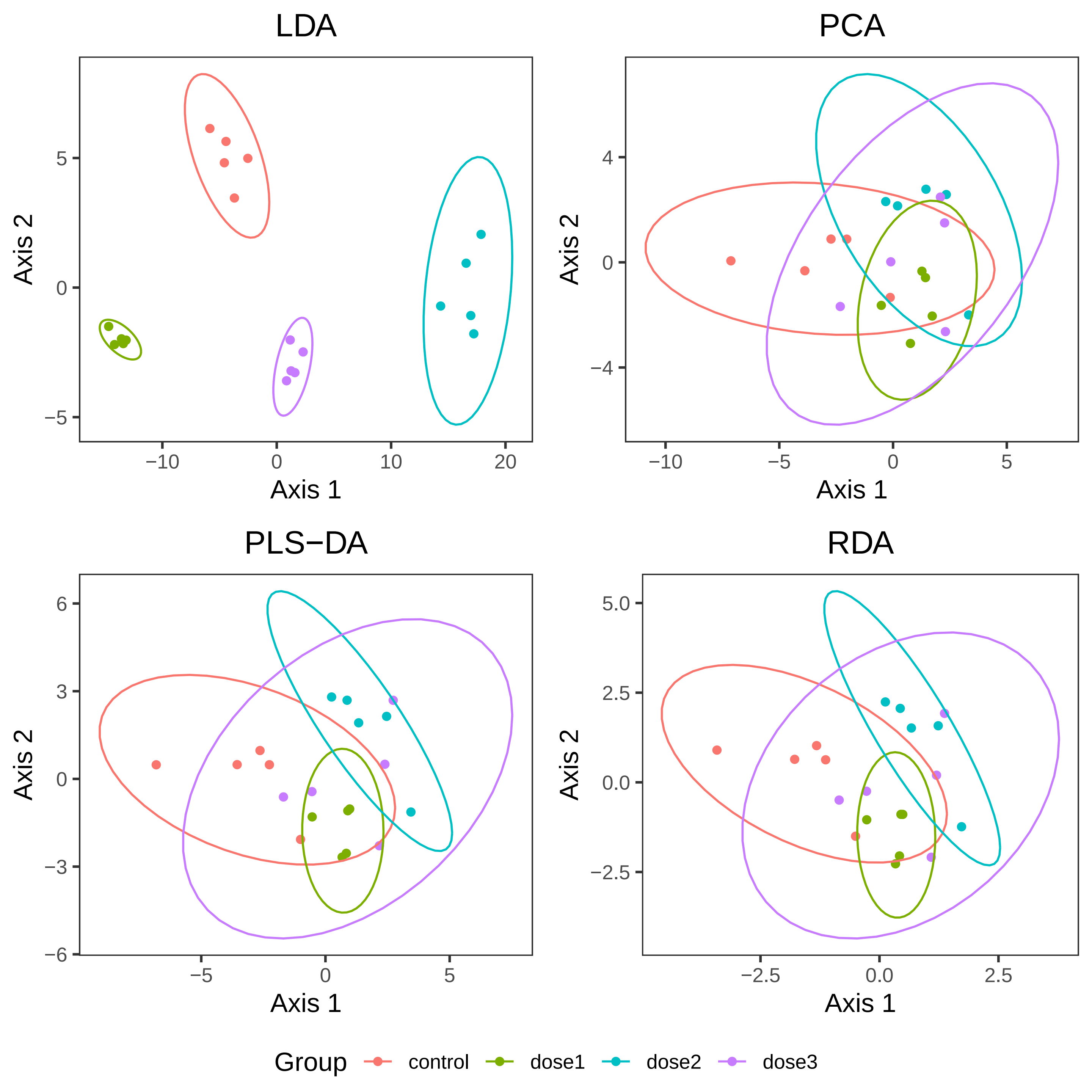

LDA
PCA
PLS−DA
RDA
Axis 1
Axis 2
5
0
−5
−10
0
10
20
4
0
−4
−10
−5
0
5
6
3
0
−3
−6
−5
0
5
5.0
2.5
0.0
−2.5
−2.5
0.0
2.5
Group
control
dose1
dose2
dose3

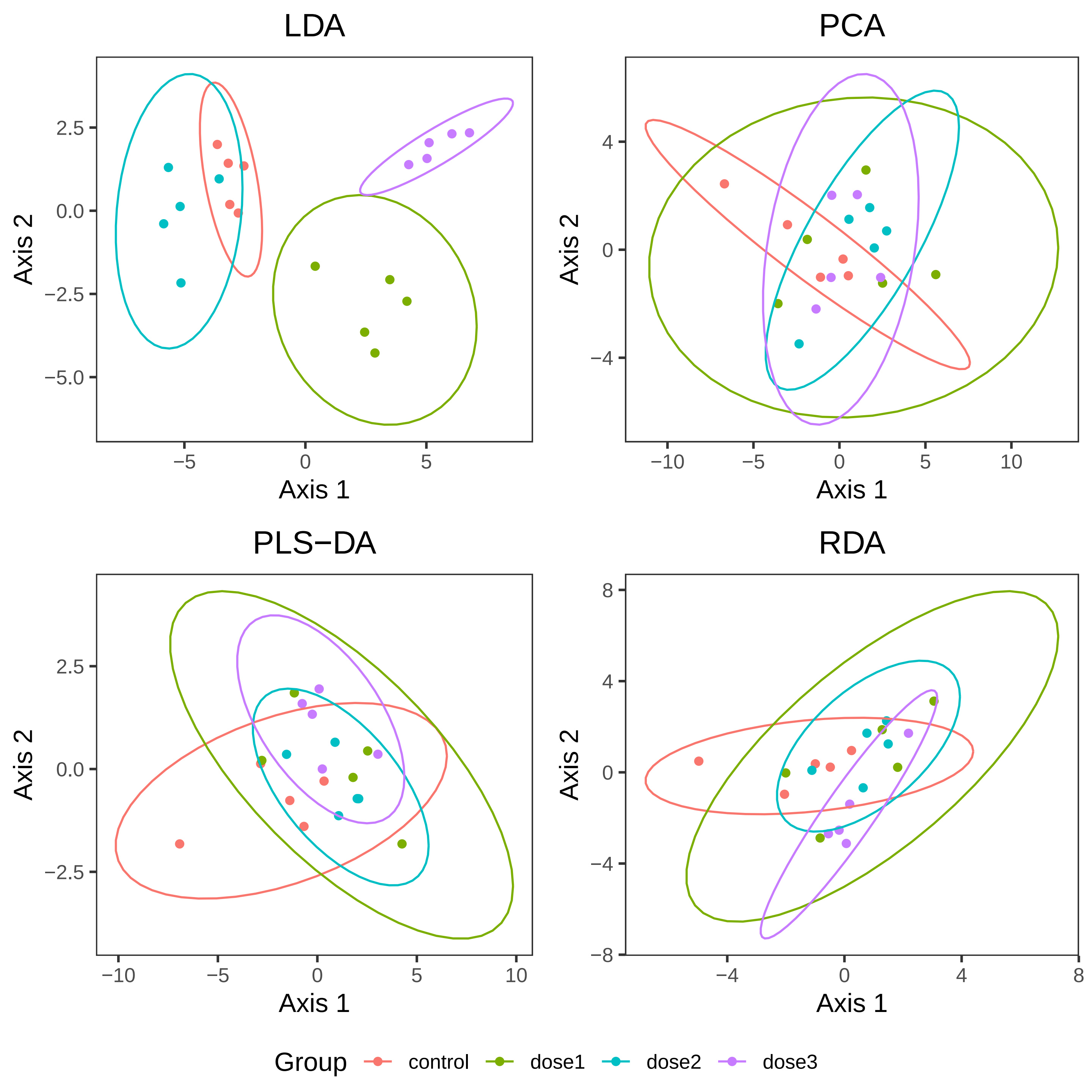

LDA
PCA
PLS−DA
RDA
Axis 1
Axis 2
Group
control
dose1
dose2
dose3

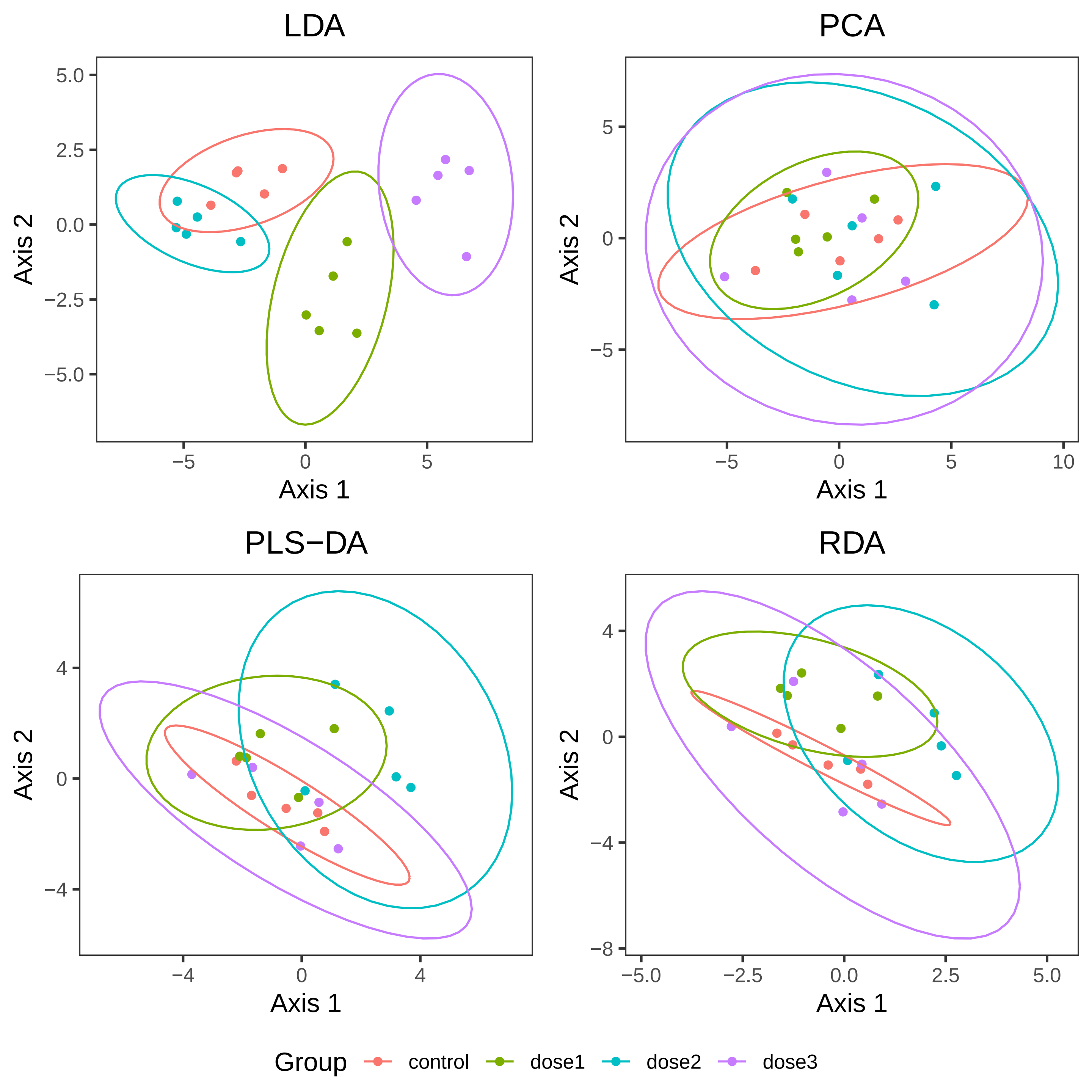

LDA
PCA
PLS−DA
RDA
Axis 1
Axis 2
5.0
2.5
0.0
−2.5
−5.0
−5
0
5
10
4
−4
−8
Group
control
dose1
dose2
dose3

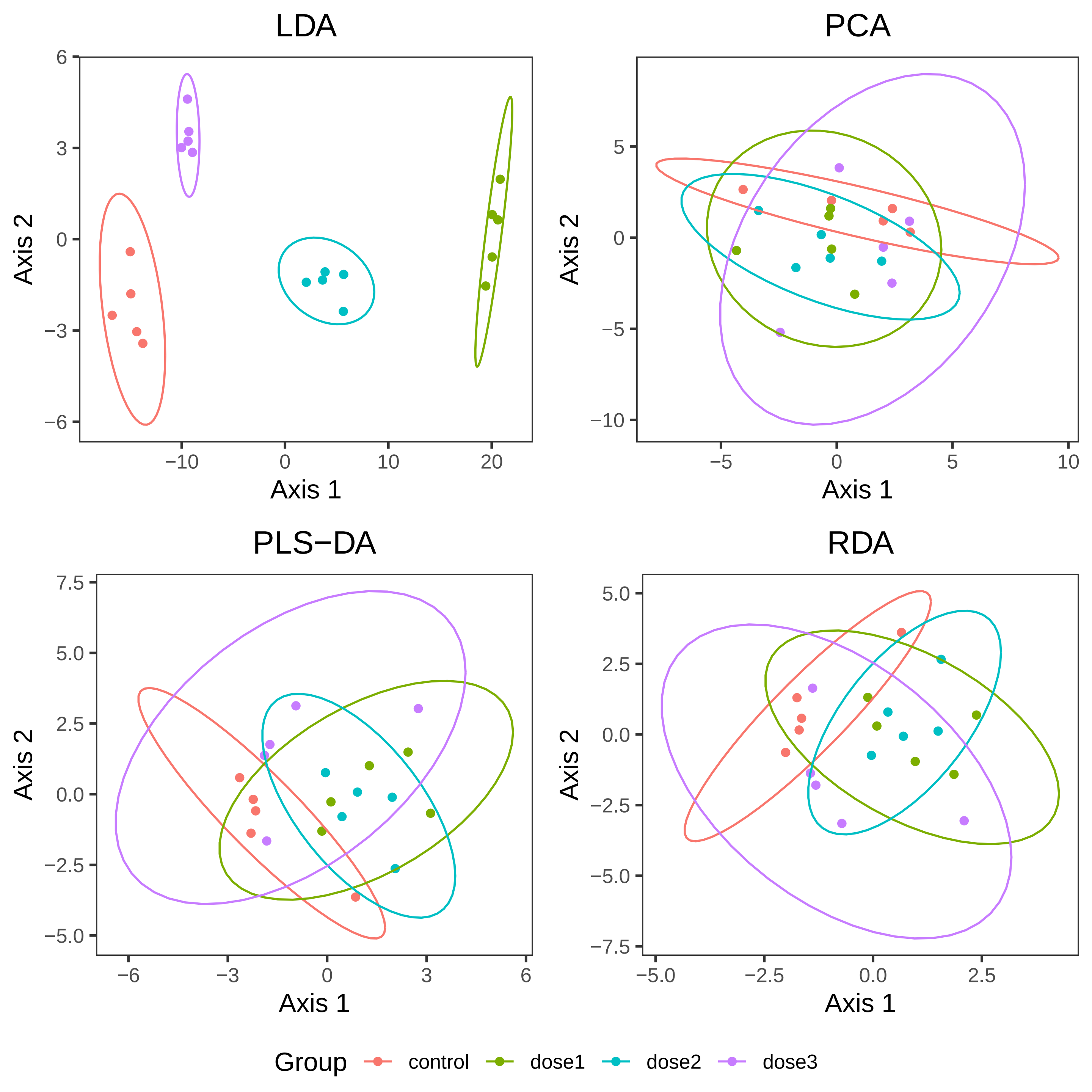

LDA
PCA
PLS−DA
RDA
Axis 1
Axis 2
Group
control
dose1
dose2
dose3

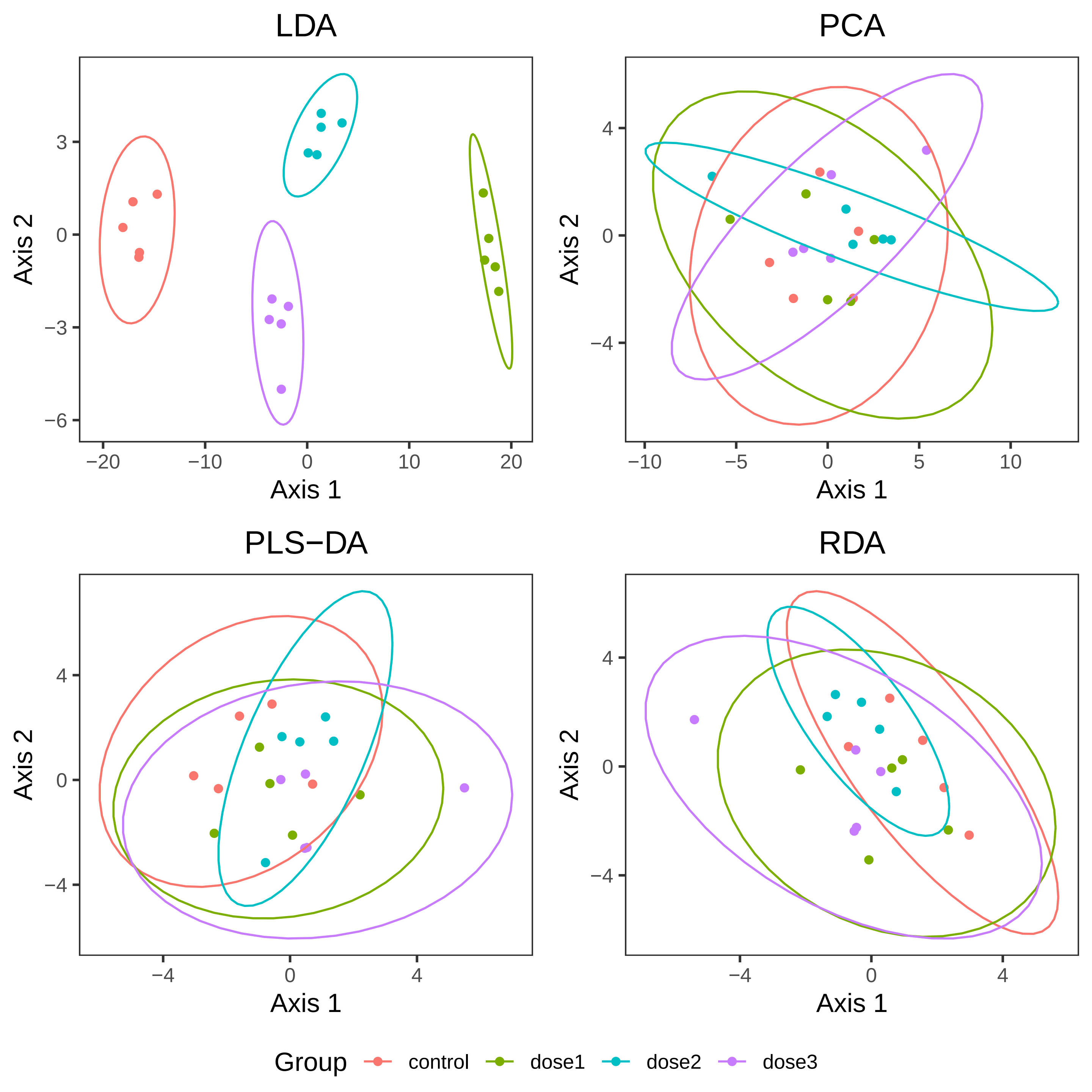

LDA
PCA
PLS−DA
RDA
Axis 1
Axis 2
Group
control
dose1
dose2
dose3

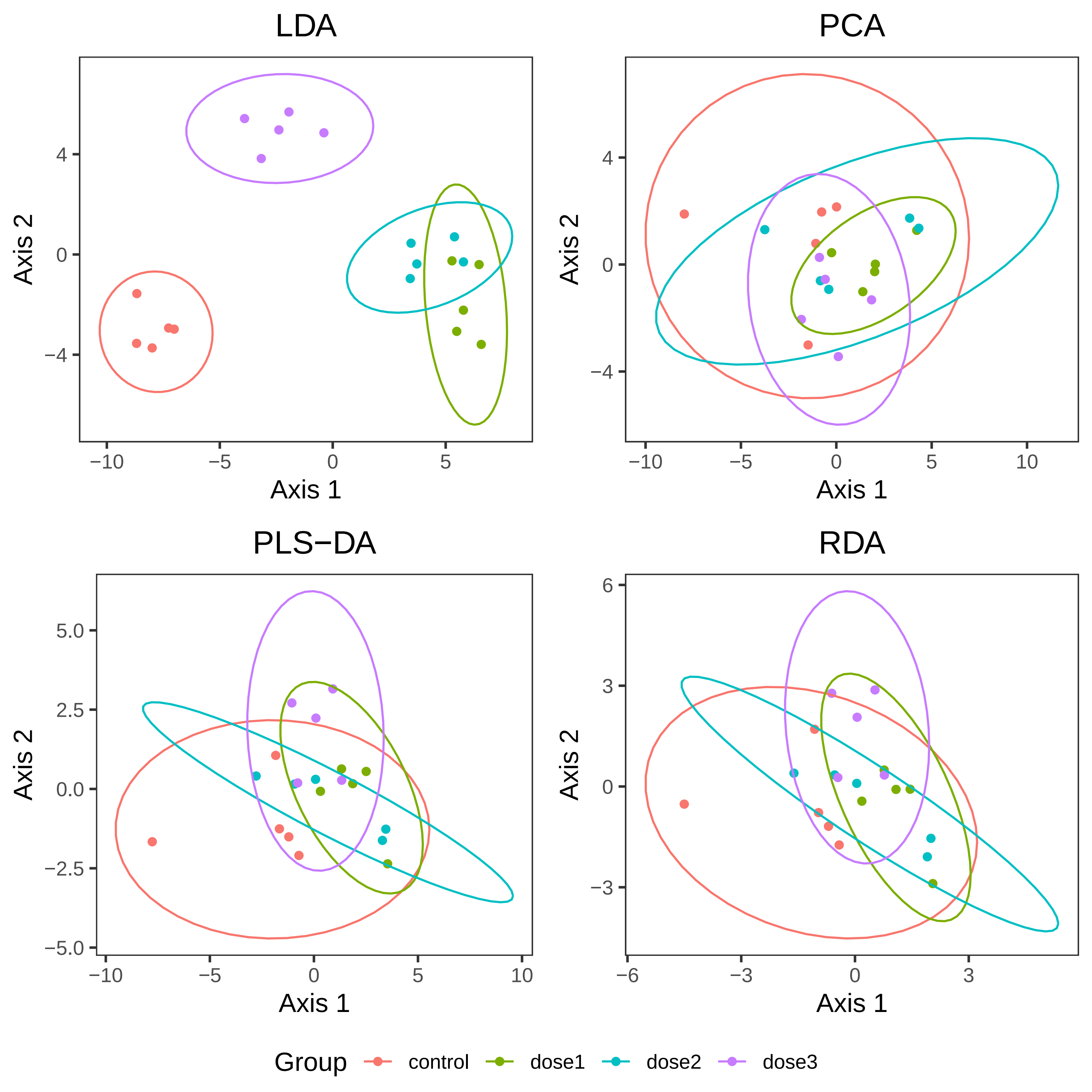

LDA
PCA
PLS−DA
RDA
Axis 1
Axis 2
Group
control
dose1
dose2
dose3

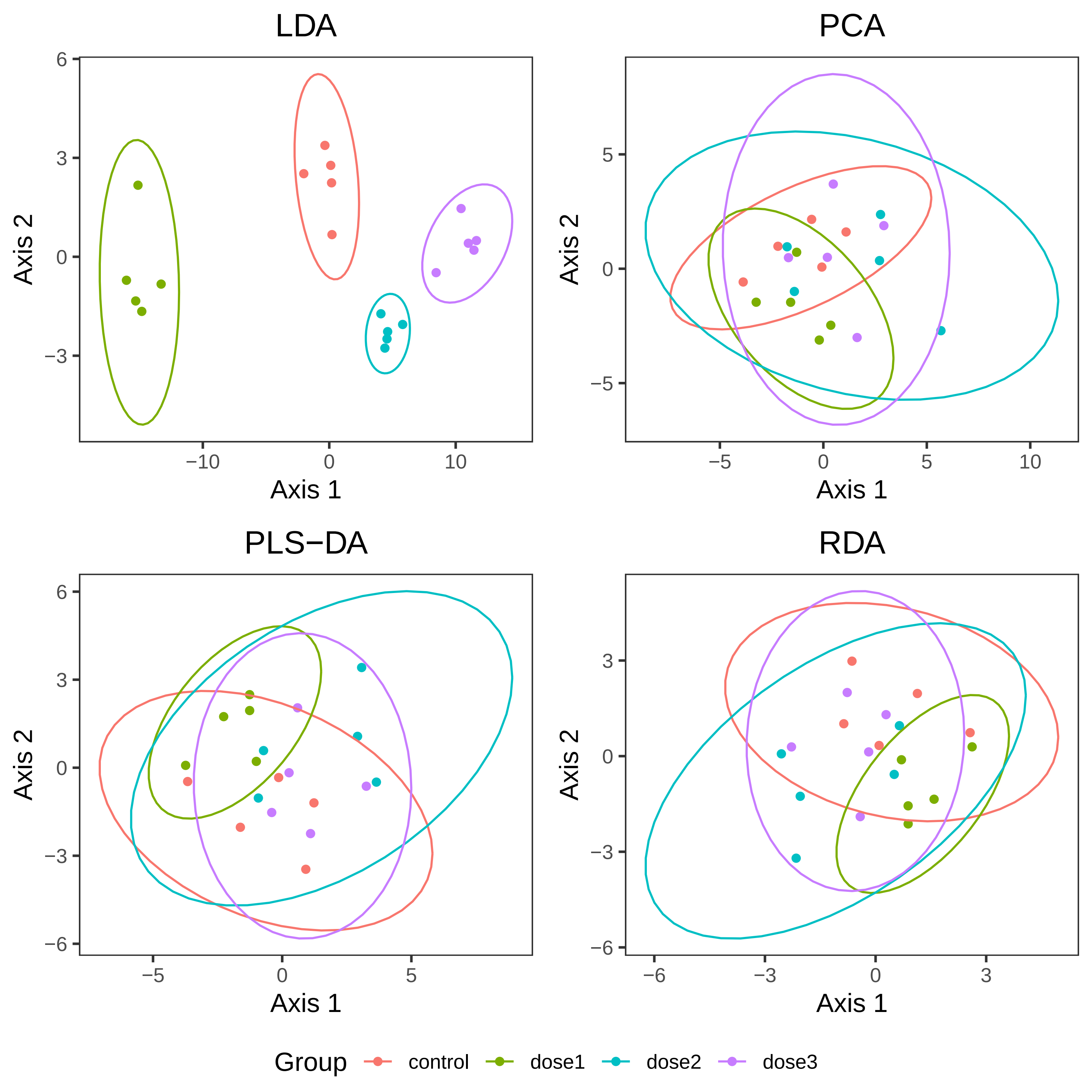
LDA
PCA
PLS−DA
RDA
Axis 1
Axis 2
6
3
0
−3
−6
−10
0
10
−5
5
Group
control
dose1
dose2
dose3

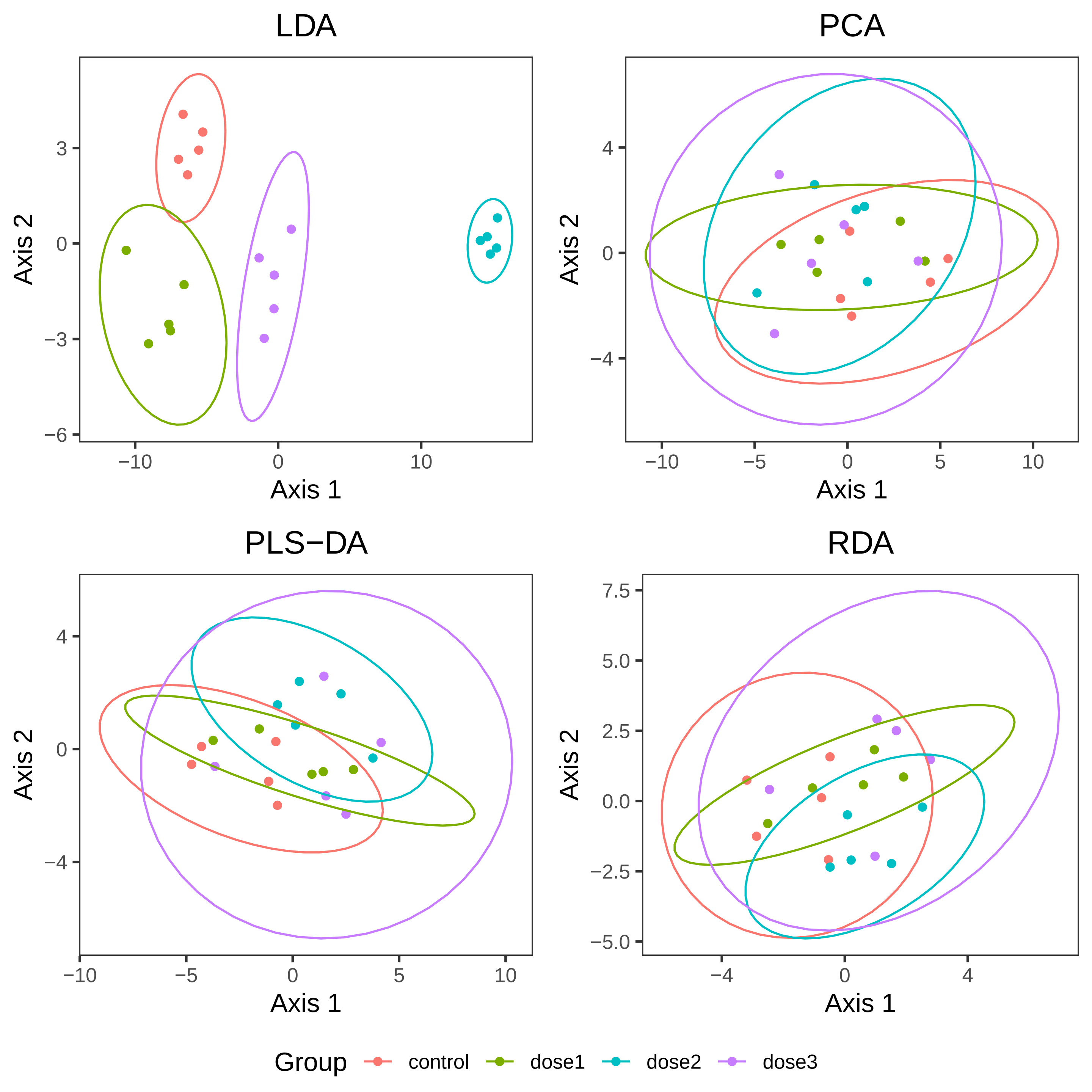

LDA
PCA
PLS−DA
RDA
Axis 1
Axis 2
3
0
−3
−6
−10
0
10
4
0
−4
−10
−5
0
5
10
7.5
5.0
2.5
0.0
−2.5
−5.0
−4
0
4
Group
control
dose1
dose2
dose3

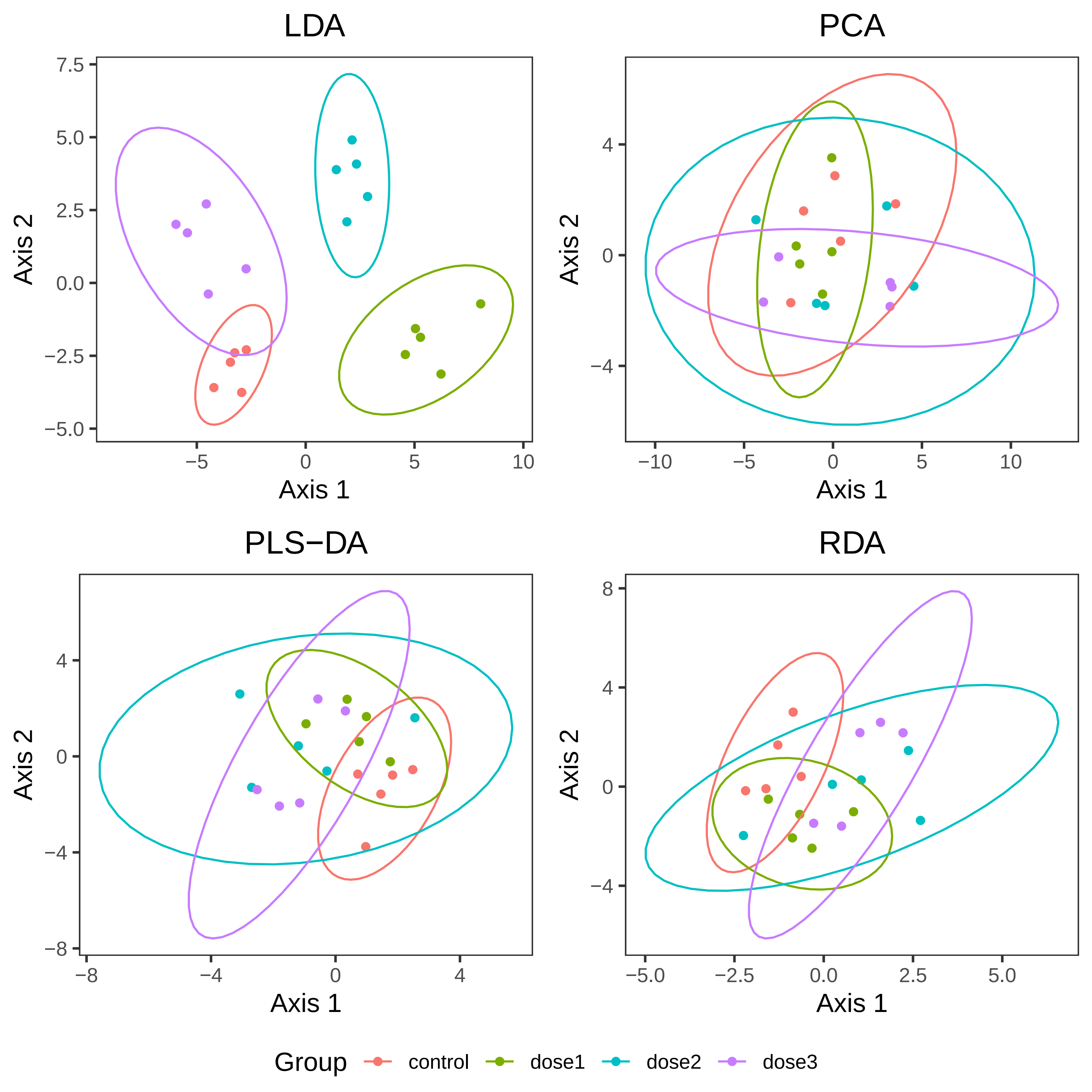
LDA
PCA
PLS−DA
RDA
Axis 1
Axis 2
Group
control
dose1
dose2
dose3

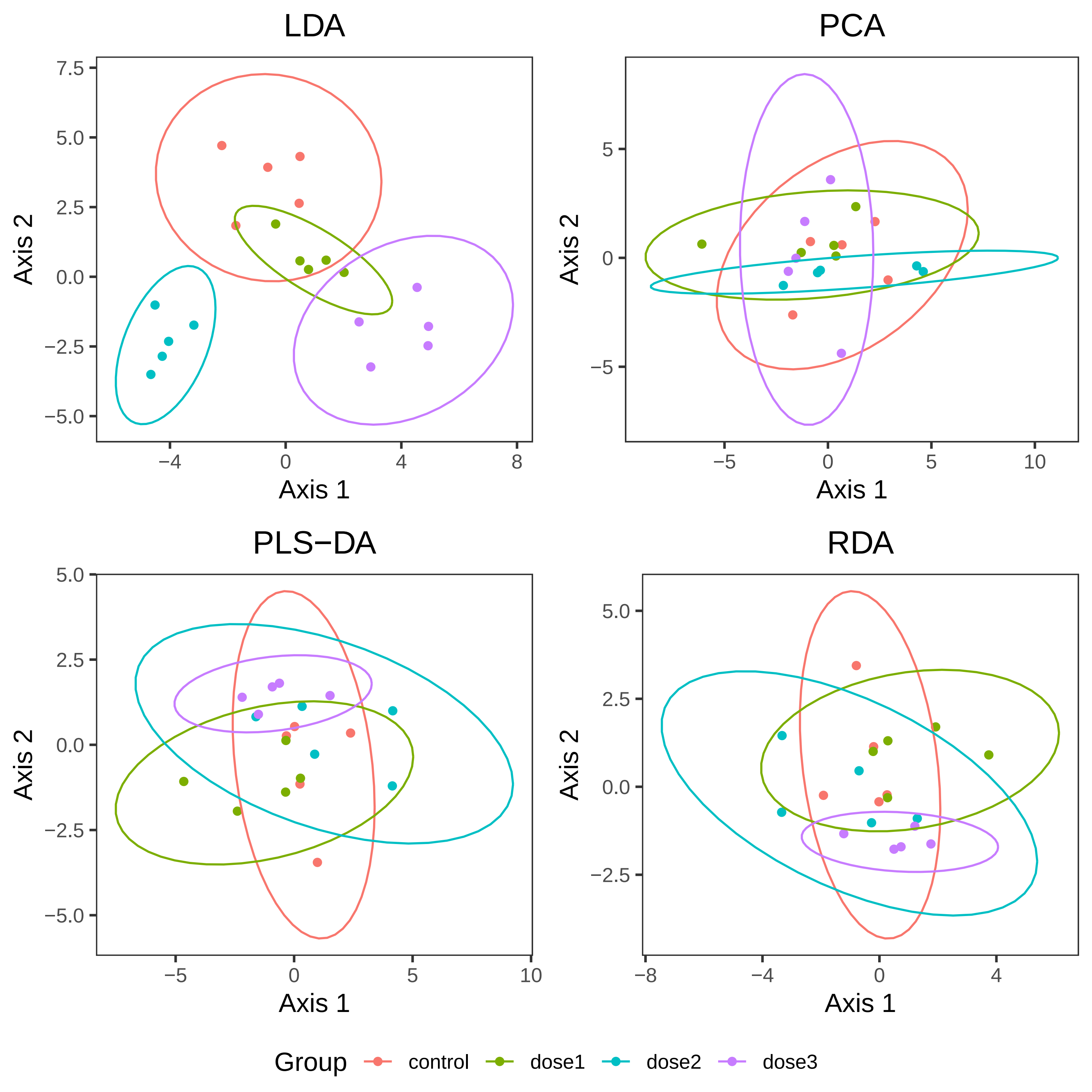

LDA
PCA
PLS−DA
RDA
Axis 1
Axis 2
7.5
5.0
2.5
0.0
−2.5
−5.0
−4
0
4
8
5
−5
10
−8
Group
control
dose1
dose2
dose3

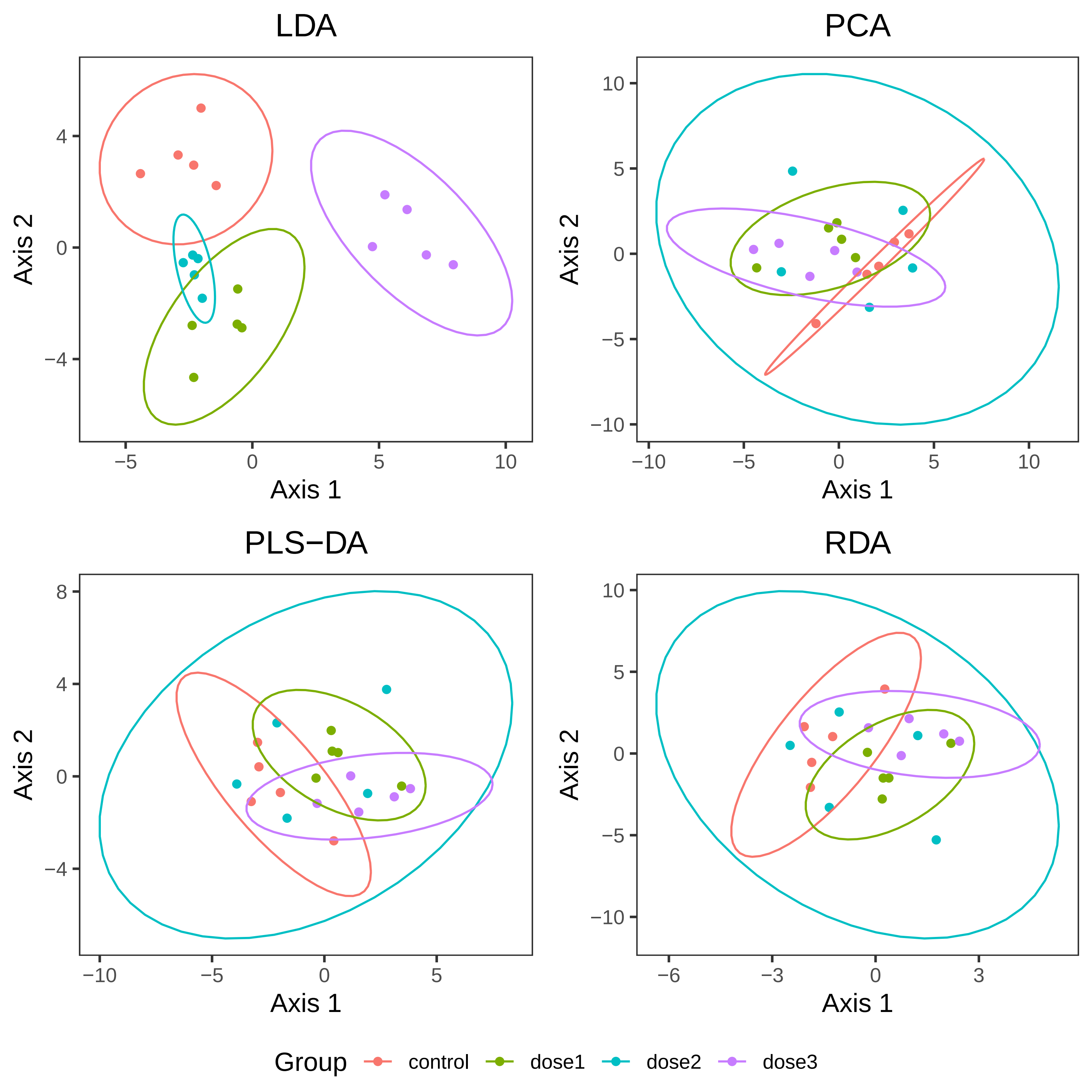

LDA
PCA
PLS−DA
RDA
Axis 1
Axis 2
Group
control
dose1
dose2
dose3

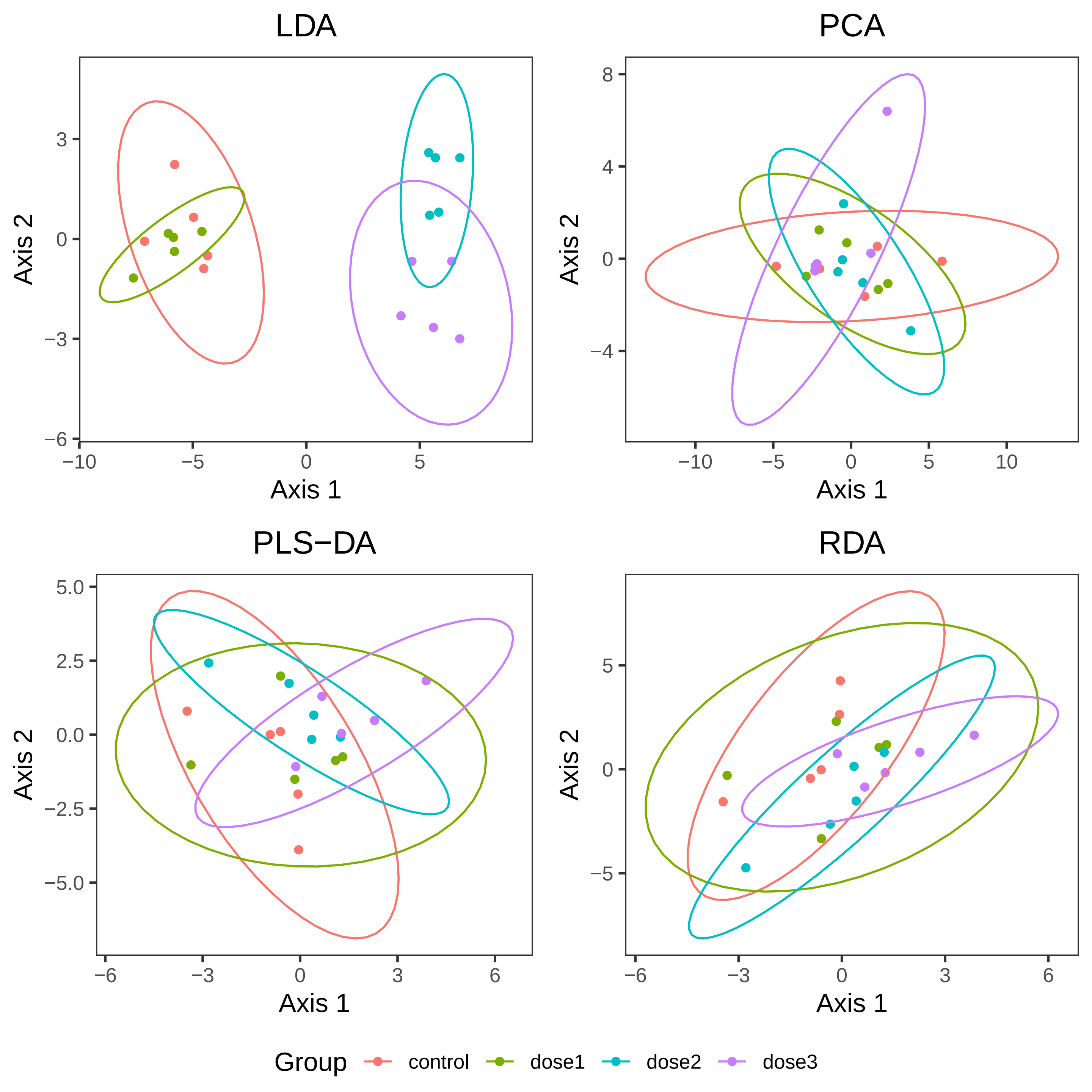
LDA
PCA
PLS−DA
RDA
Axis 1
Axis 2
Group
control
dose1
dose2
dose3
−10
−5
0
5
10
3
−3
−6
8
4
−4
5.0
2.5
0.0
−2.5
−5.0
−6
6

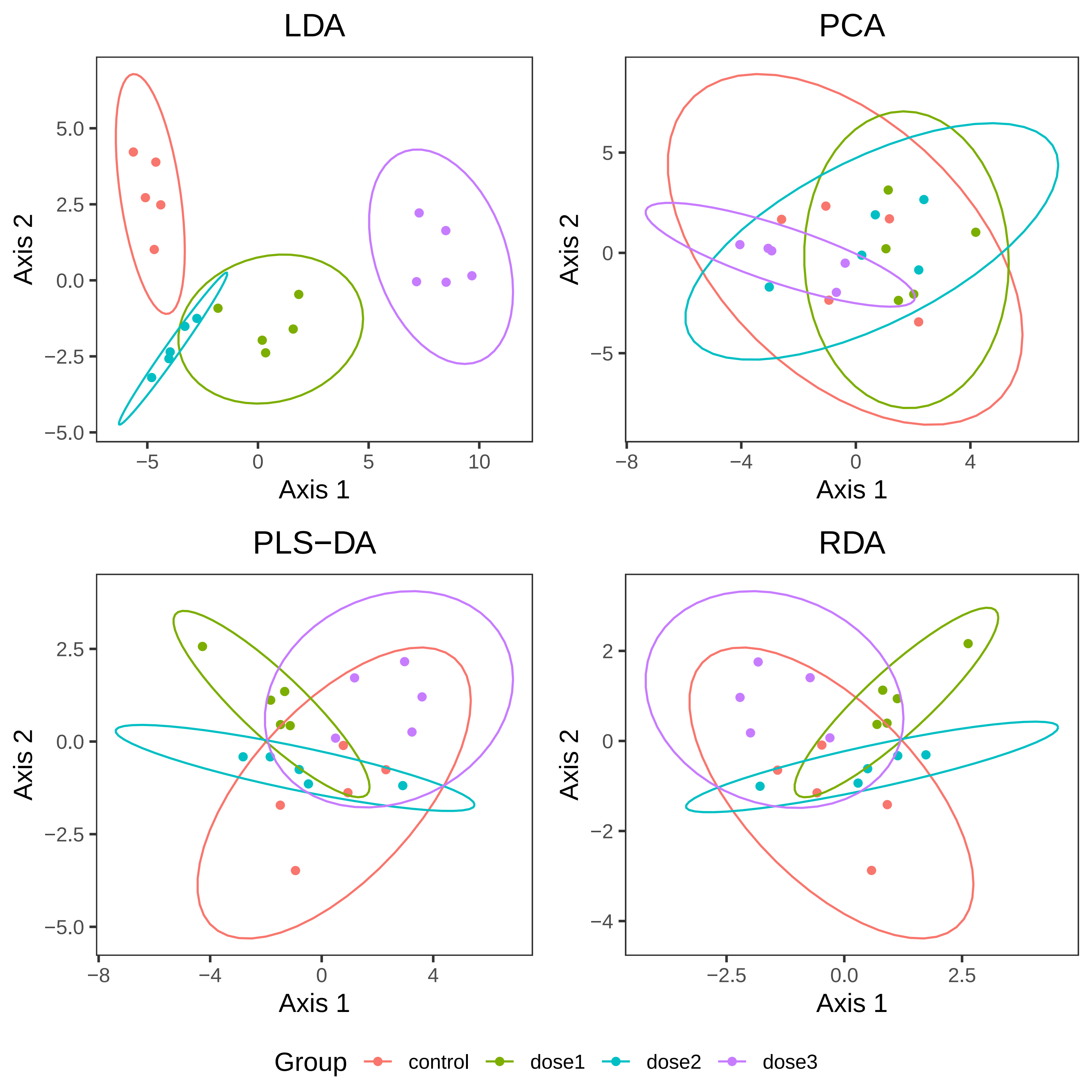
LDA
PCA
PLS−DA
RDA
Axis 1
Axis 2
Group
control
dose1
dose2
dose3

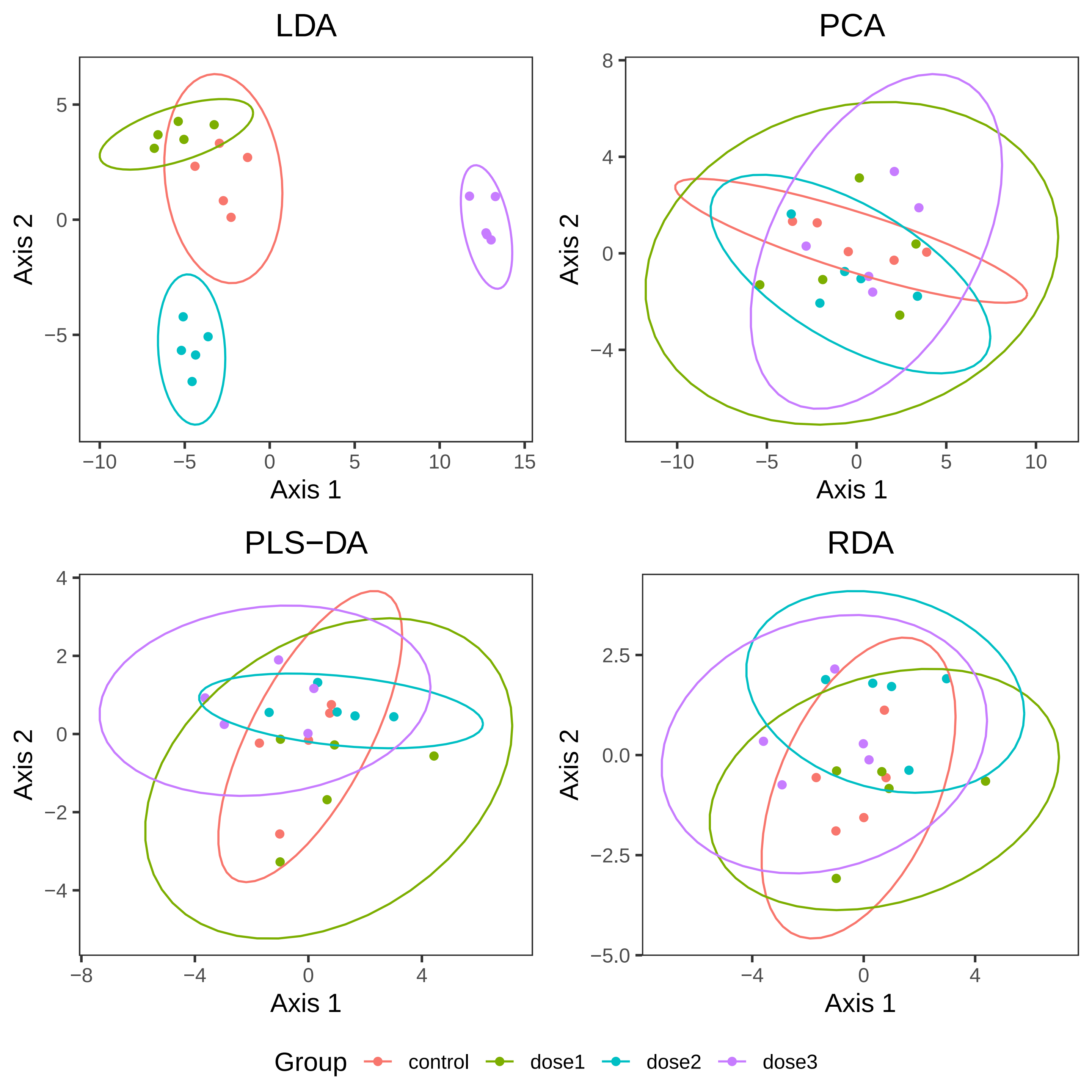

LDA
PCA
PLS−DA
RDA
Axis 1
Axis 2
Group
control
dose1
dose2
dose3

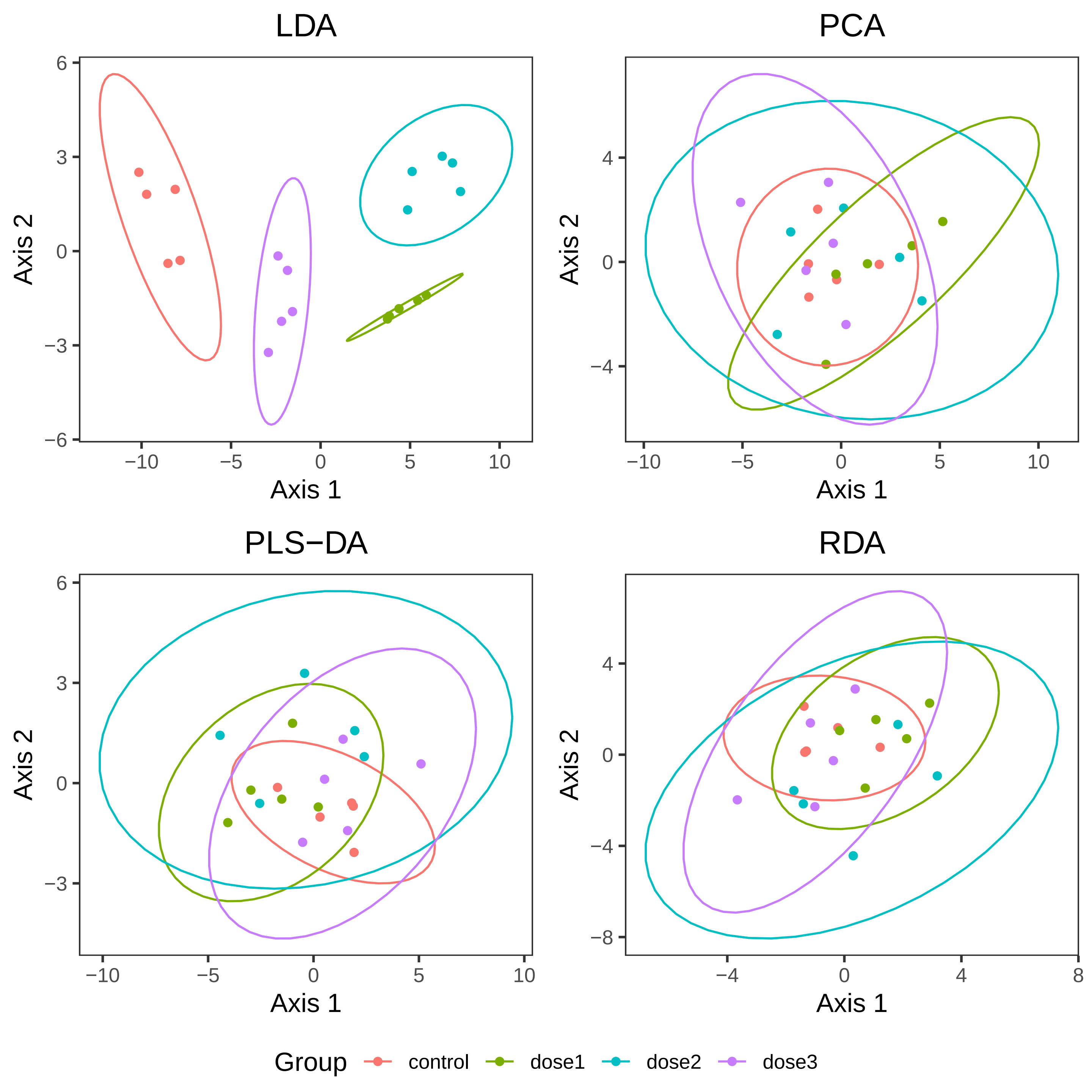

LDA
PCA
PLS−DA
RDA
Axis 1
Axis 2
Group
control
dose1
dose2
dose3

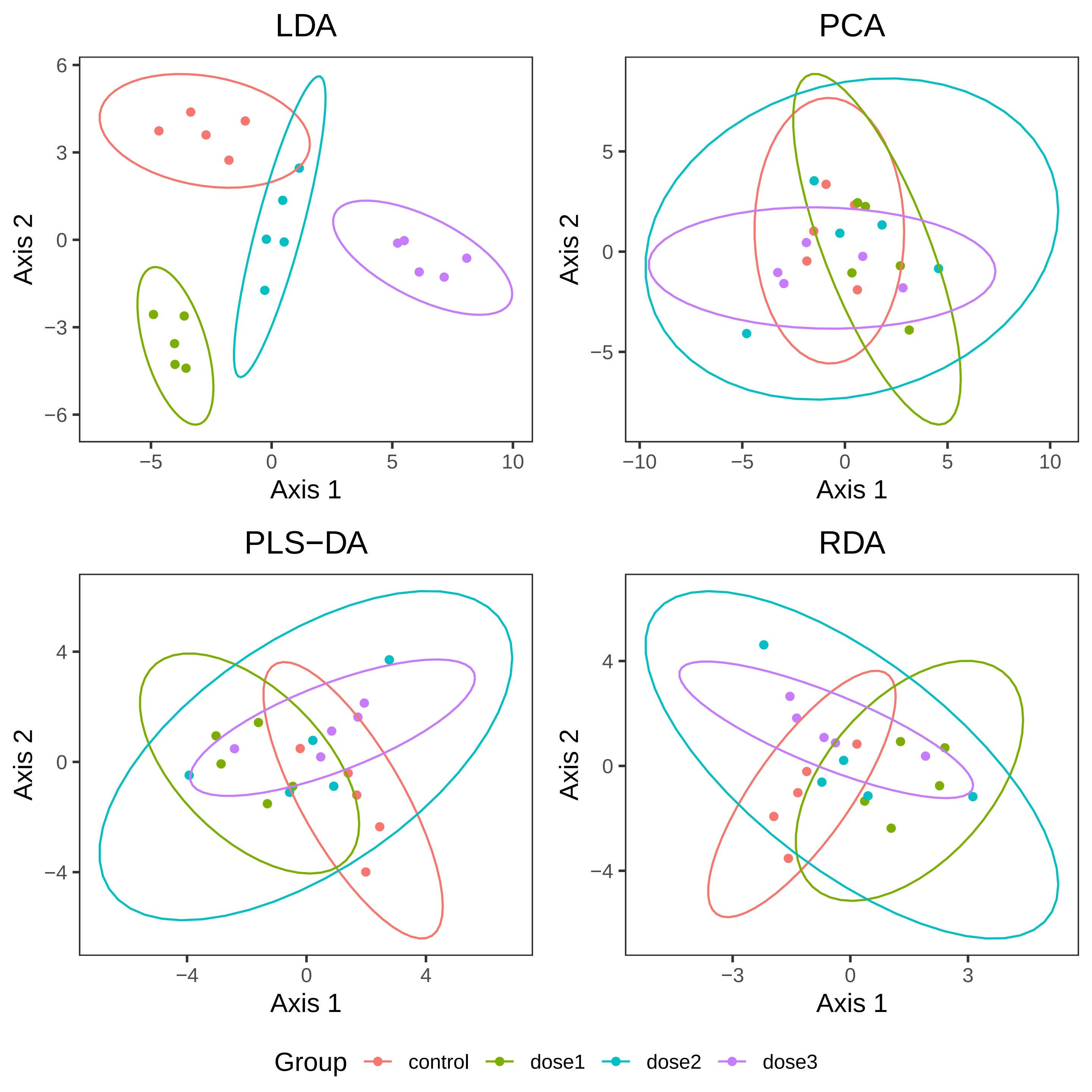

LDA
PCA
PLS−DA
RDA
Axis 1
Axis 2
Group
control
dose1
dose2
dose3

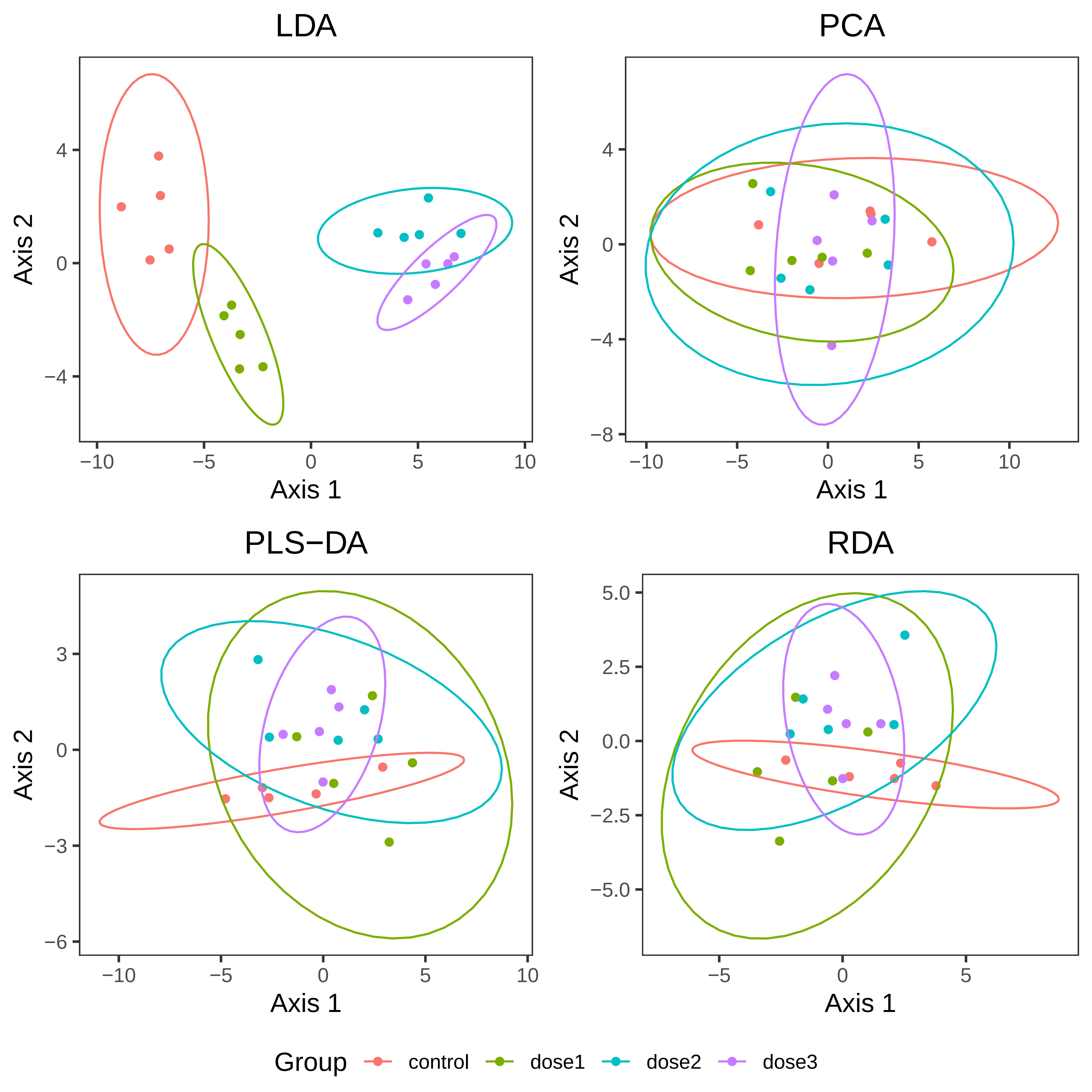
LDA
PCA
PLS−DA
RDA
Axis 1
Axis 2
Group
control
dose1
dose2
dose3

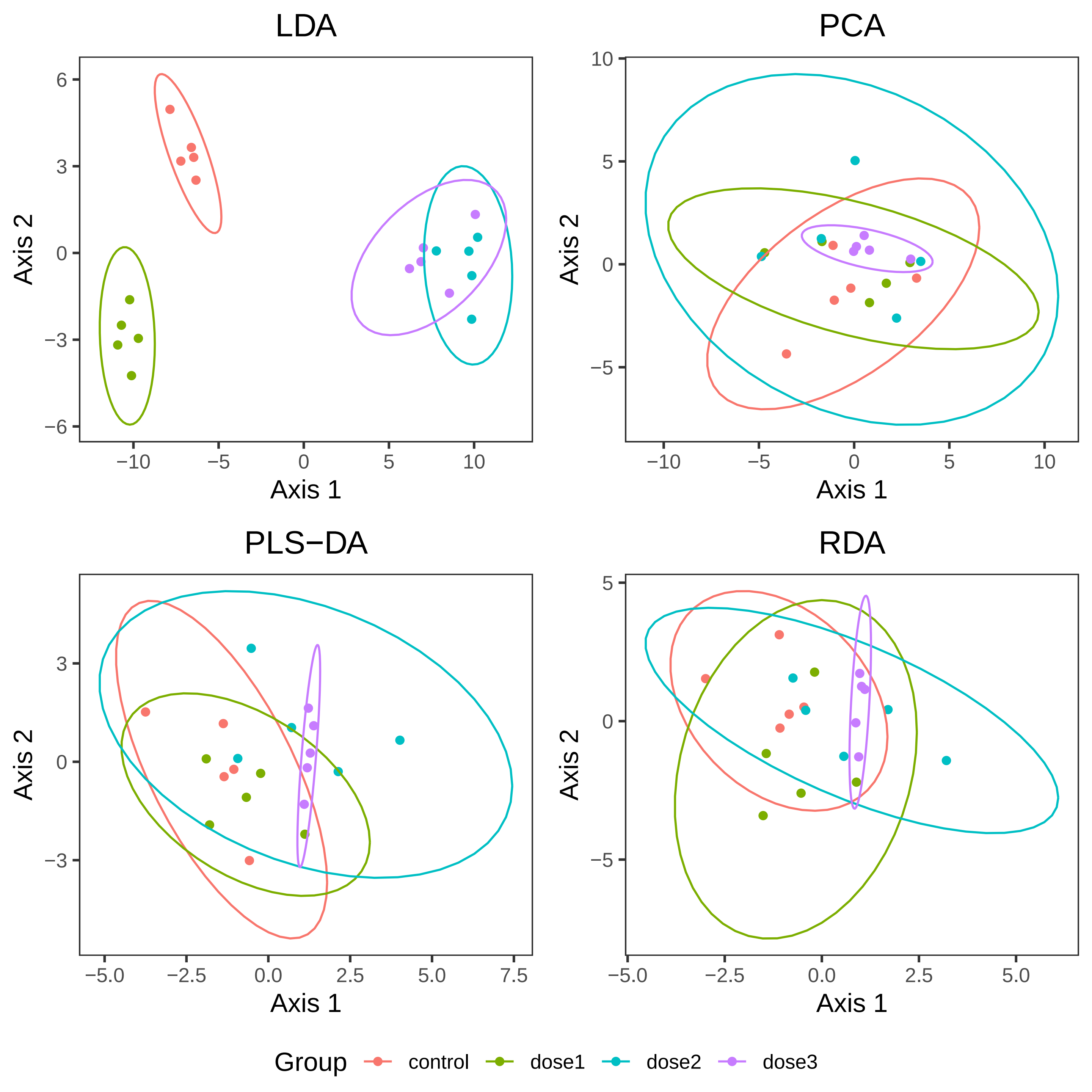

LDA
PCA
PLS−DA
RDA
Axis 1
Axis 2
Group
control
dose1
dose2
dose3

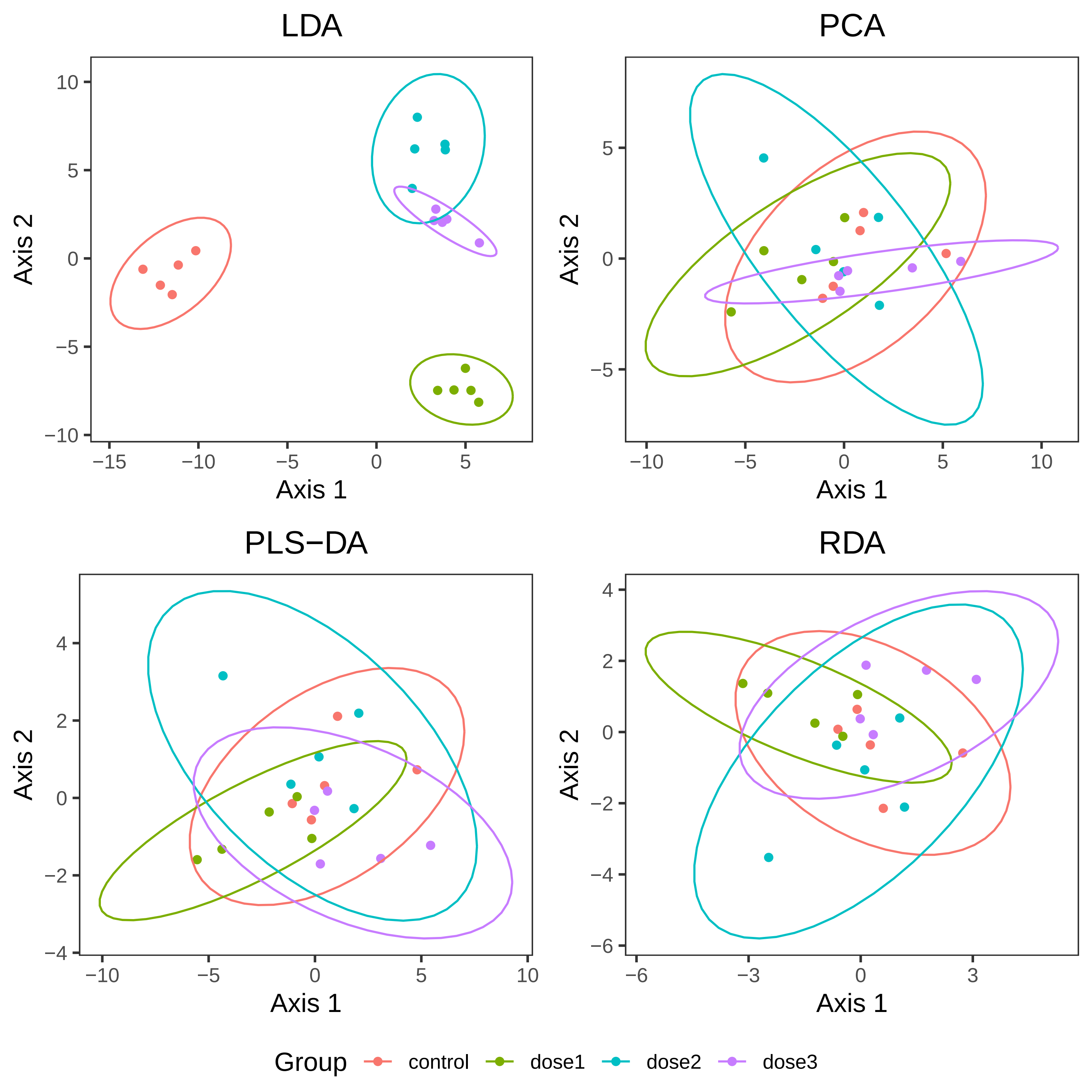

LDA
PCA
PLS−DA
RDA
Axis 1
Axis 2
Group
control
dose1
dose2
dose3

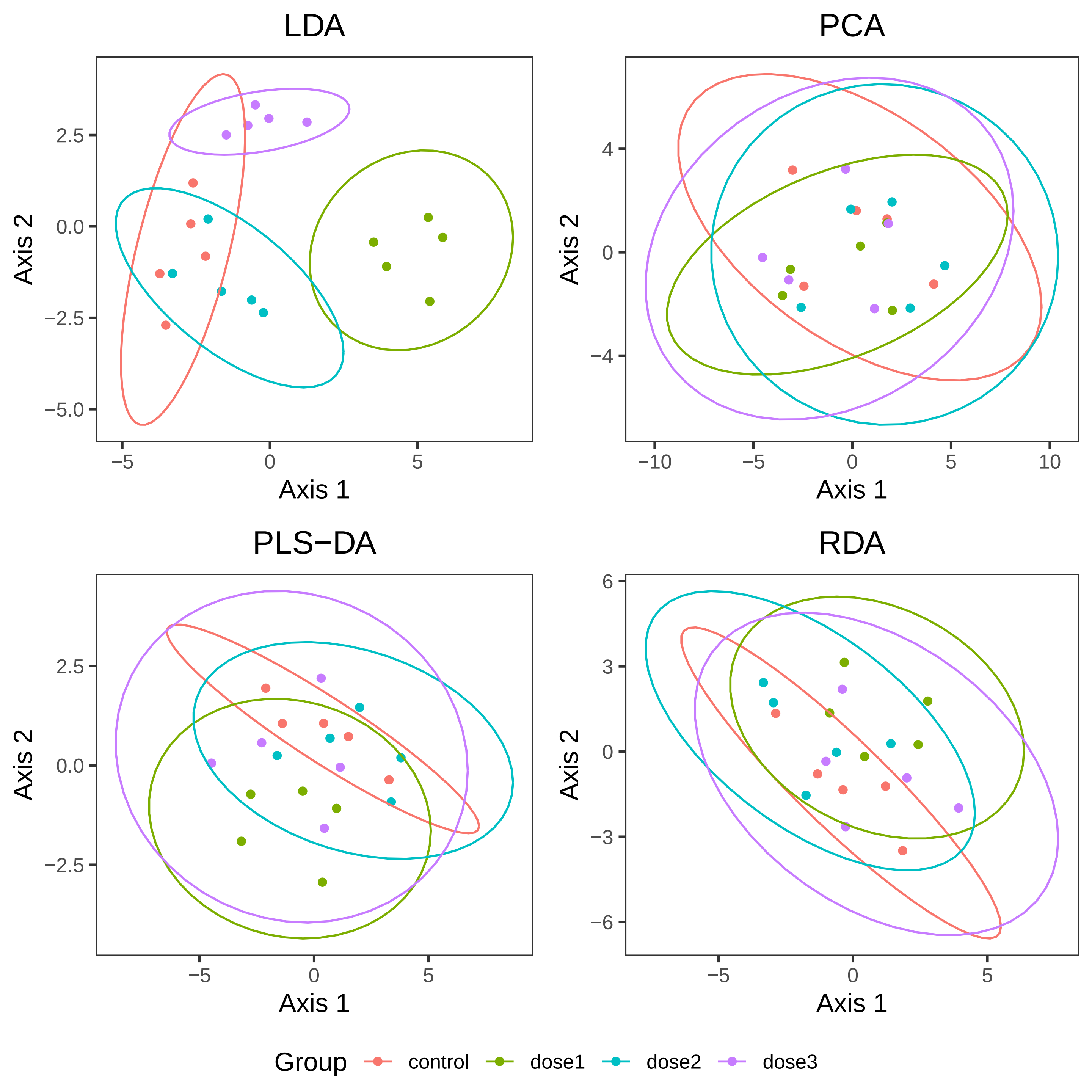

LDA
PCA
PLS−DA
RDA
Axis 1
Axis 2
Group
control
dose1
dose2
dose3

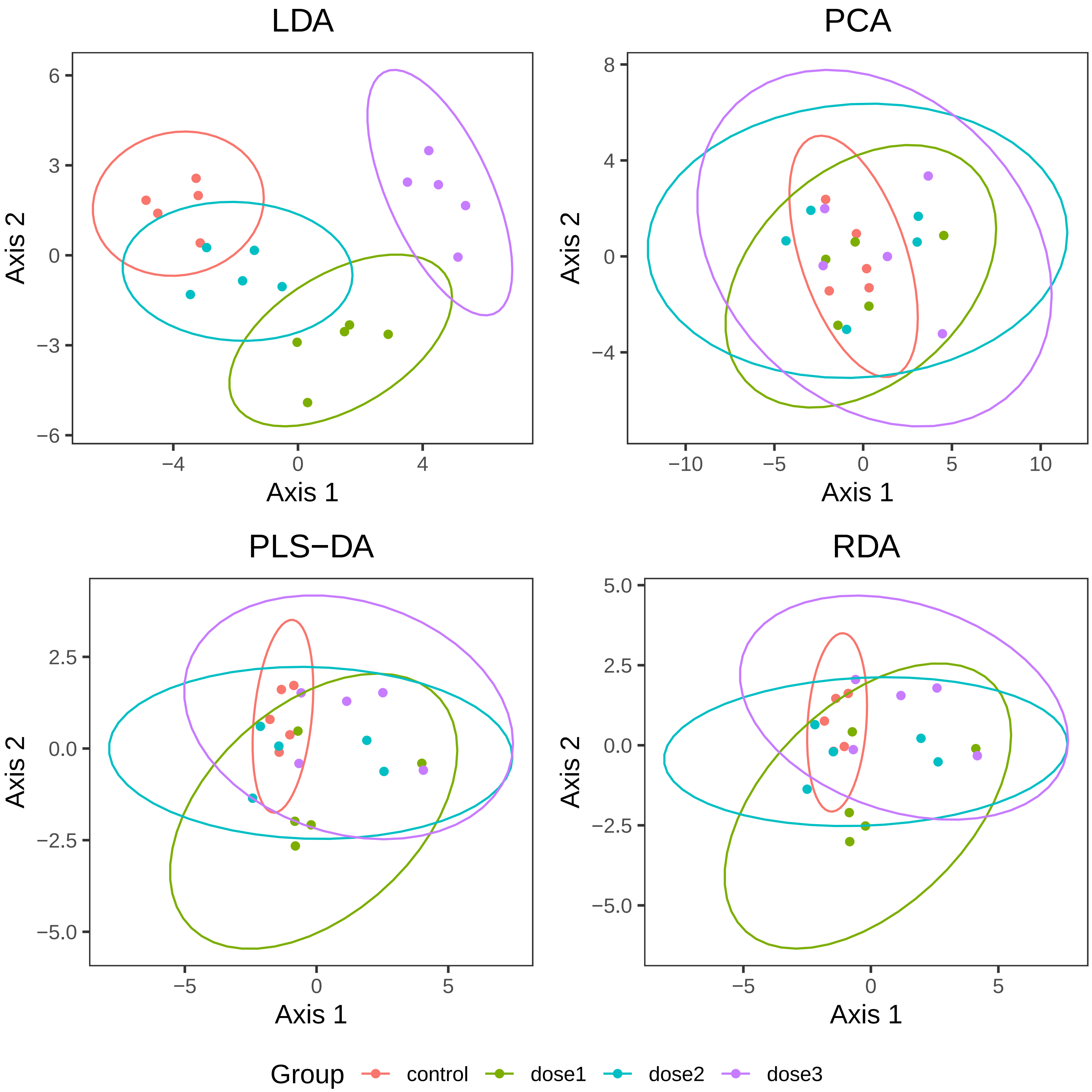
LDA
PCA
PLS−DA
RDA
Axis 1
Axis 2
6
3
0
−3
−6
−4
0
4
8
4
0
−4
−10
−5
0
5
10
2.5
0.0
−2.5
−5.0
−5
0
5
5.0
2.5
0.0
−2.5
−5.0
−5
0
5
Group
control
dose1
dose2
dose3

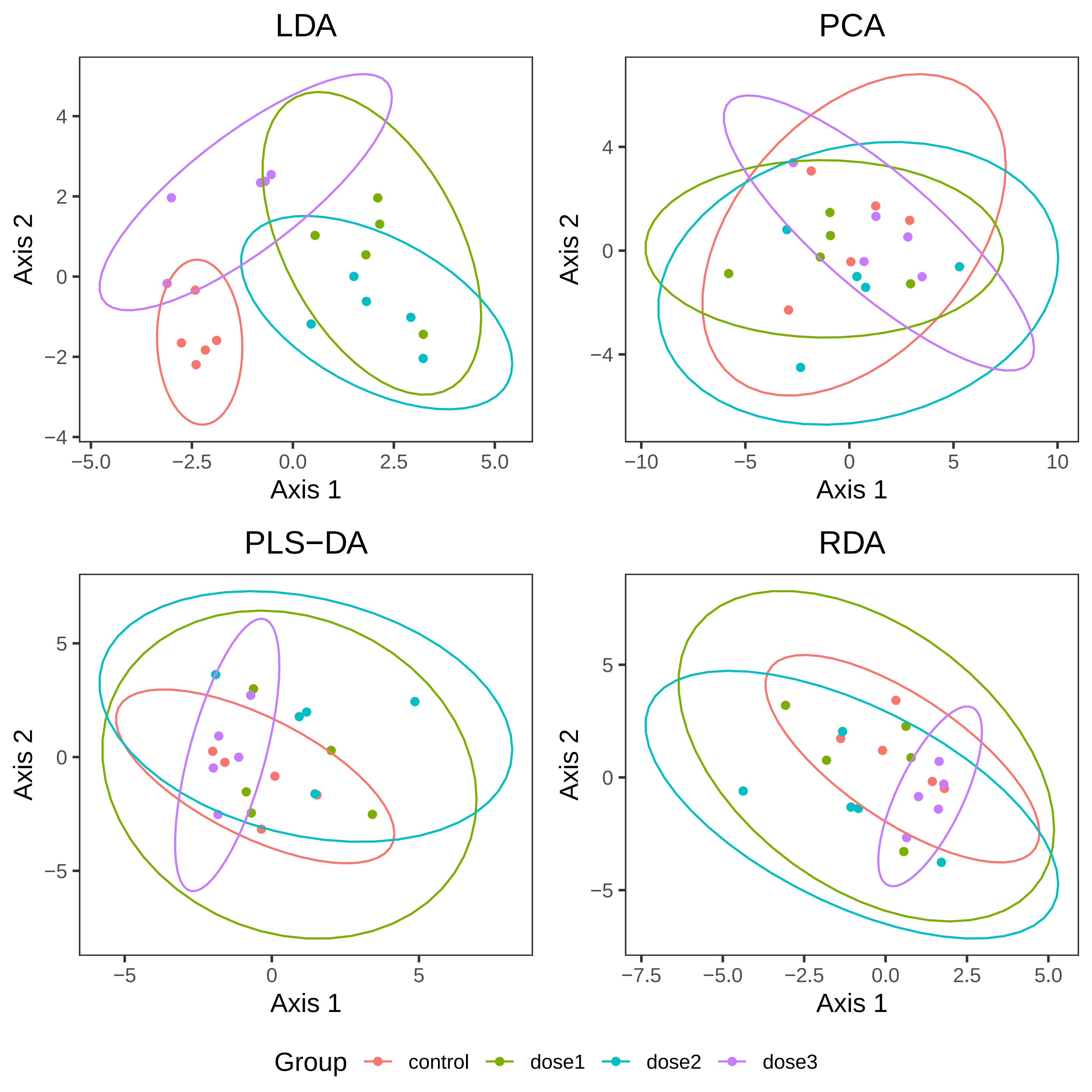
LDA
PCA
PLS−DA
RDA
Axis 1
Axis 2
Group
control
dose1
dose2
dose3

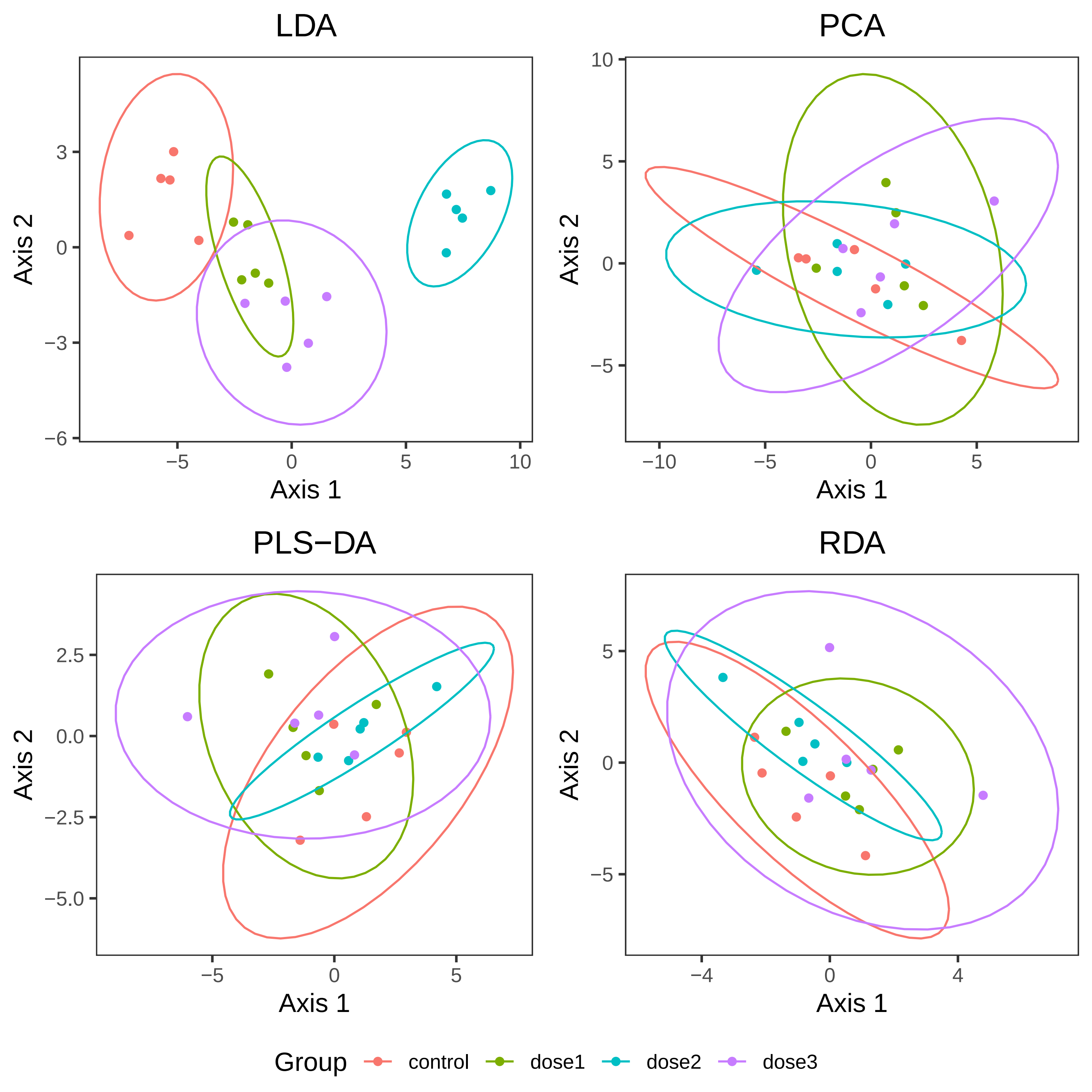

LDA
PCA
PLS−DA
RDA
Axis 1
Axis 2
3
0
−3
−6
−5
0
5
10
−10
−5
0
5
10
5
0
−5
2.5
0.0
−2.5
−5.0
−4
0
4
Group
control
dose1
dose2
dose3

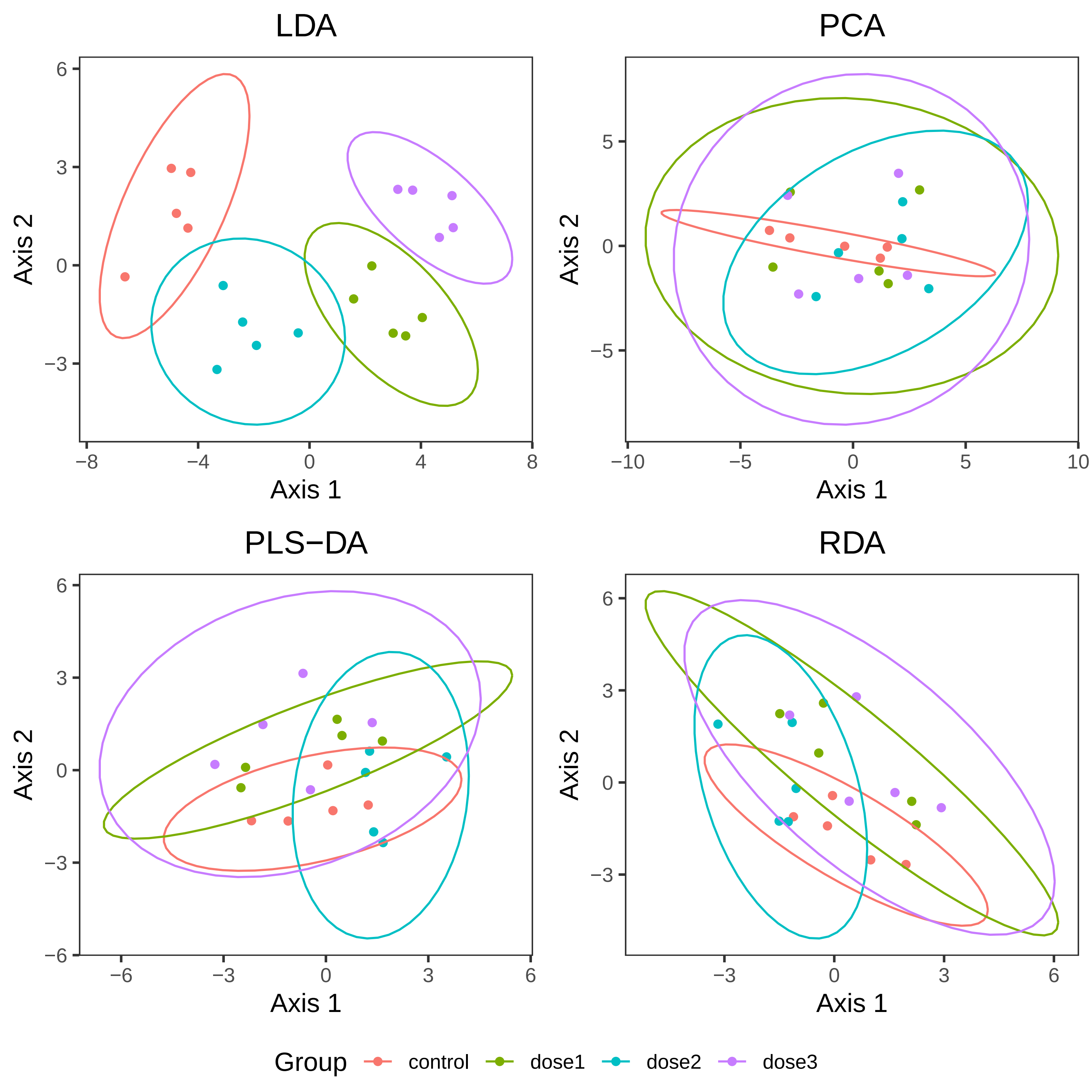

LDA
PCA
PLS−DA
RDA
Axis 1
Axis 2
6
3
0
−3
−6
−8
−4
0
4
8
5
−5
−10
10
Group
control
dose1
dose2
dose3

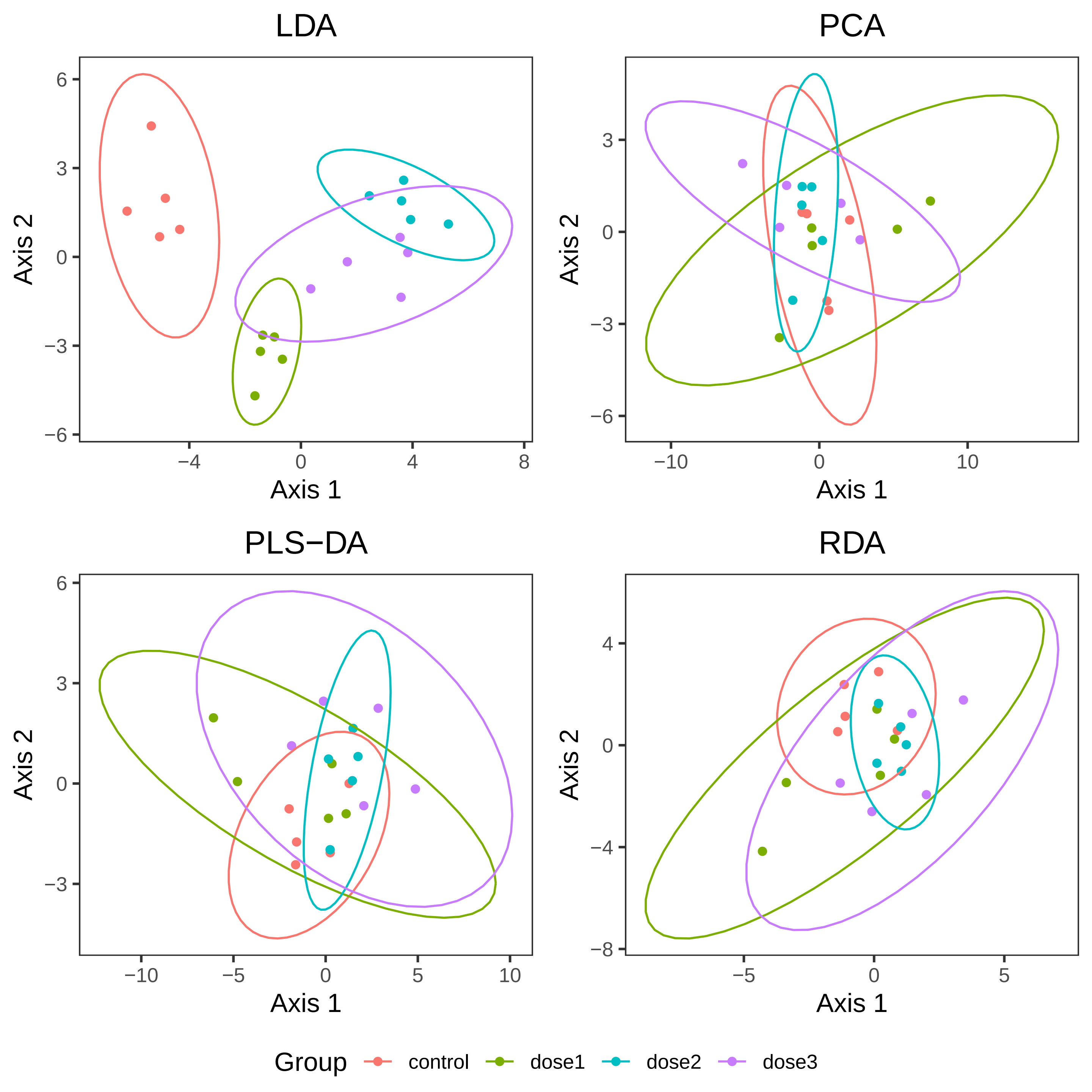
LDA
PCA
PLS−DA
RDA
Axis 1
Axis 2
Group
control
dose1
dose2
dose3

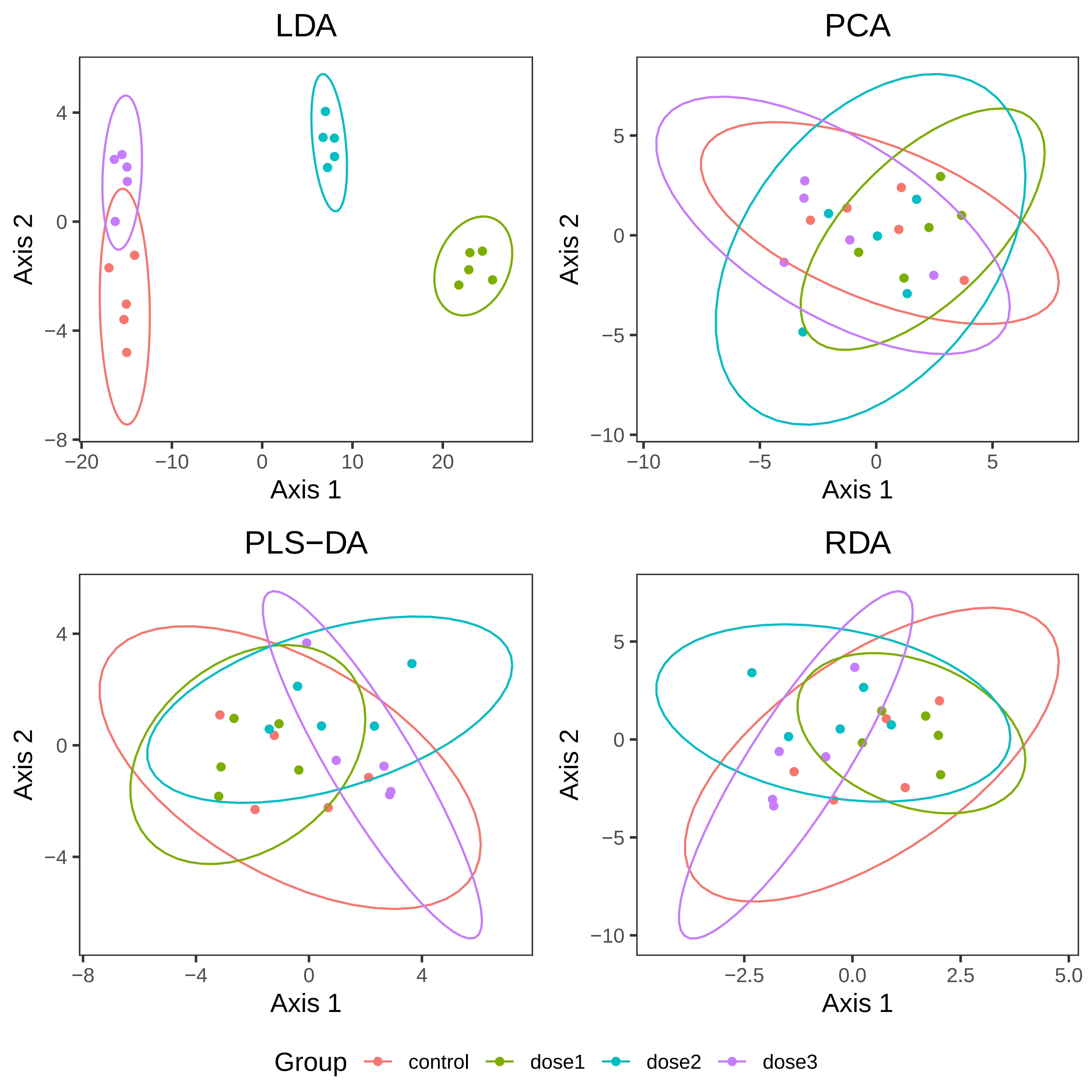
LDA
PCA
PLS−DA
RDA
Axis 1
Axis 2
−20
−10
0
10
20
4
0
−4
−8
−10
−5
5
−8
2.5
−2.5
0.0
5.0
Group
control
dose1
dose2
dose3

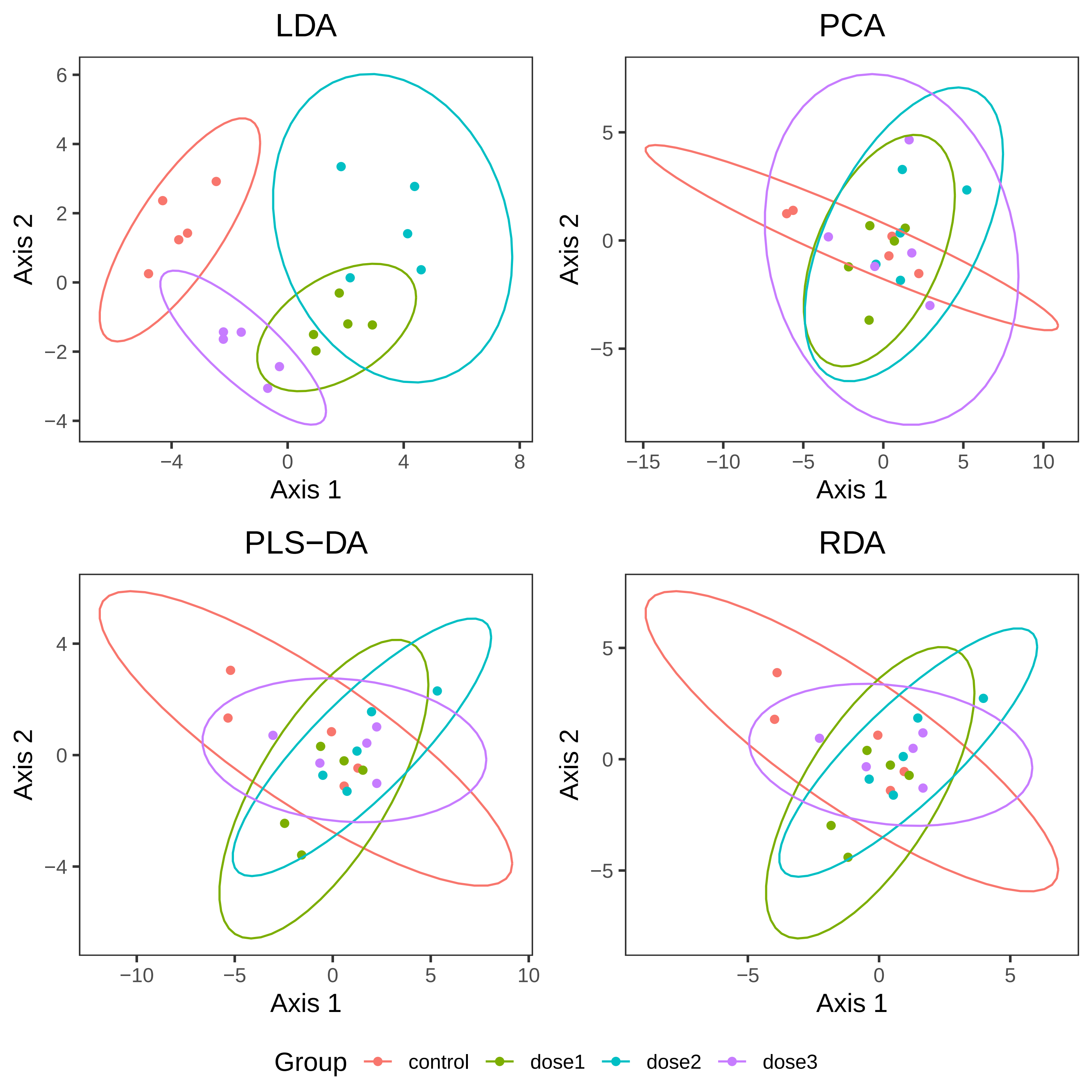

LDA
PCA
PLS−DA
RDA
Axis 1
Axis 2
Group
control
dose1
dose2
dose3

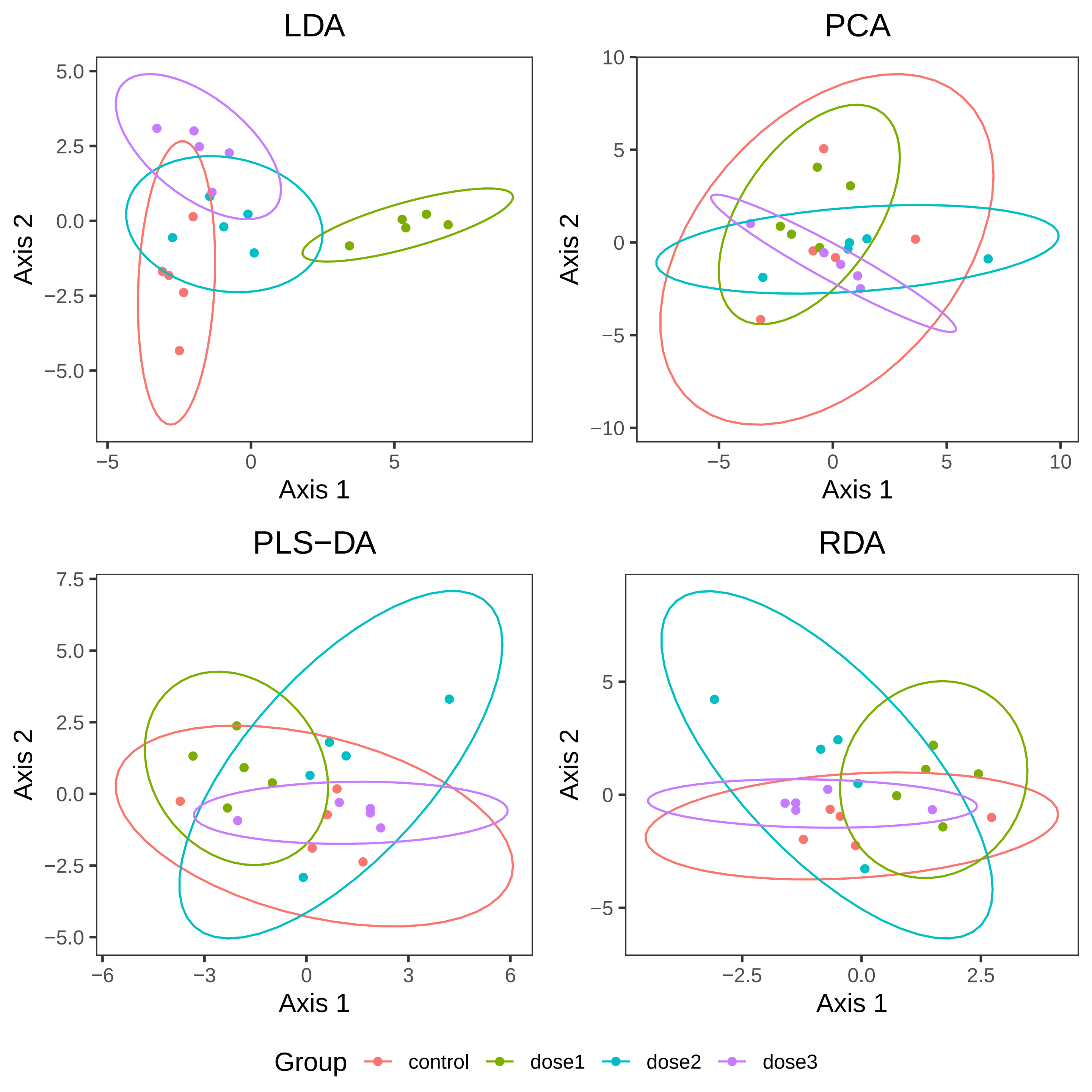
LDA
PCA
PLS−DA
RDA
Axis 1
Axis 2
5.0
2.5
0.0
−2.5
−5.0
−5
0
5
10
−10
7.5
−6
−3
3
6
−2.5
0.0
2.5
Group
control
dose1
dose2
dose3

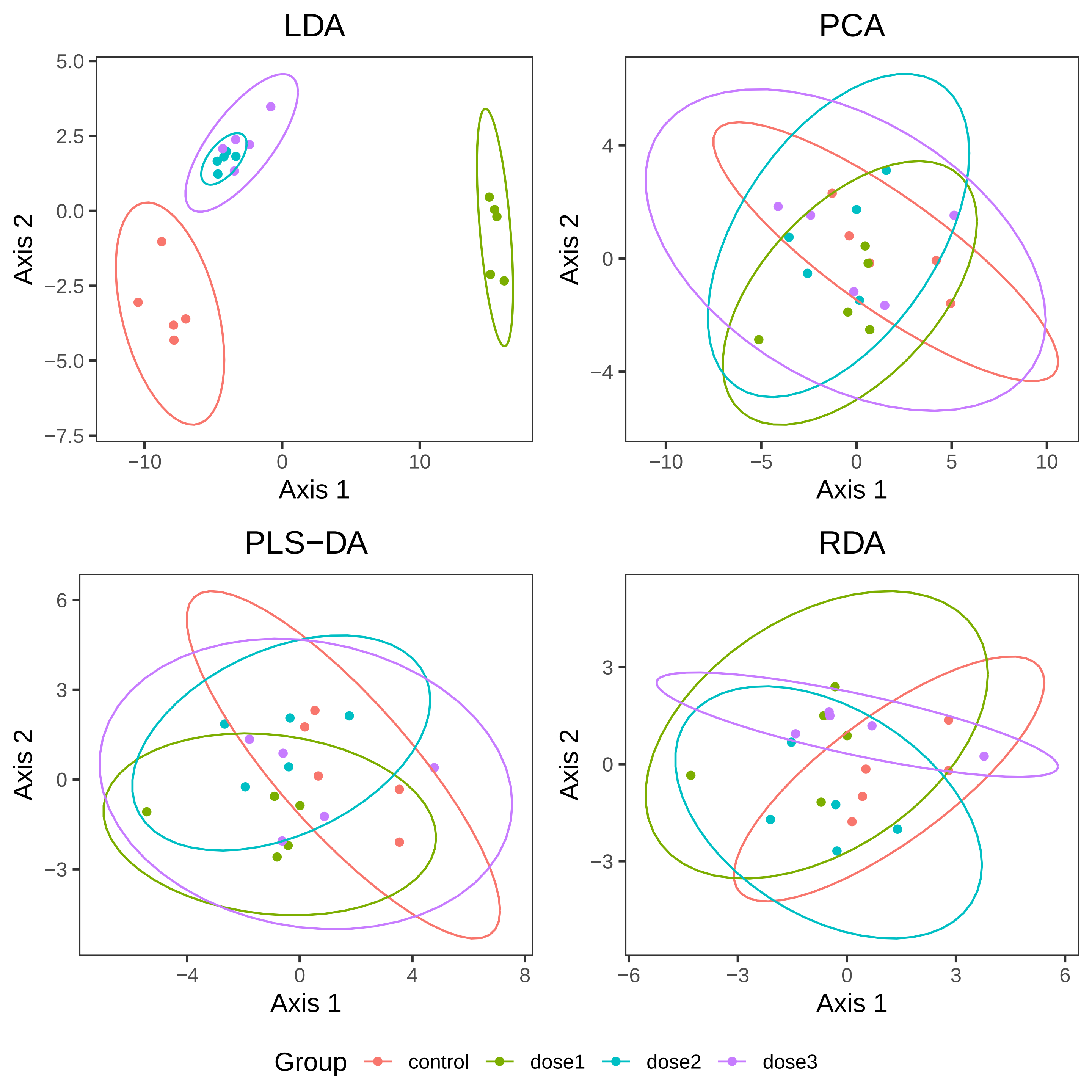

LDA
PCA
PLS−DA
RDA
Axis 1
Axis 2
Group
control
dose1
dose2
dose3

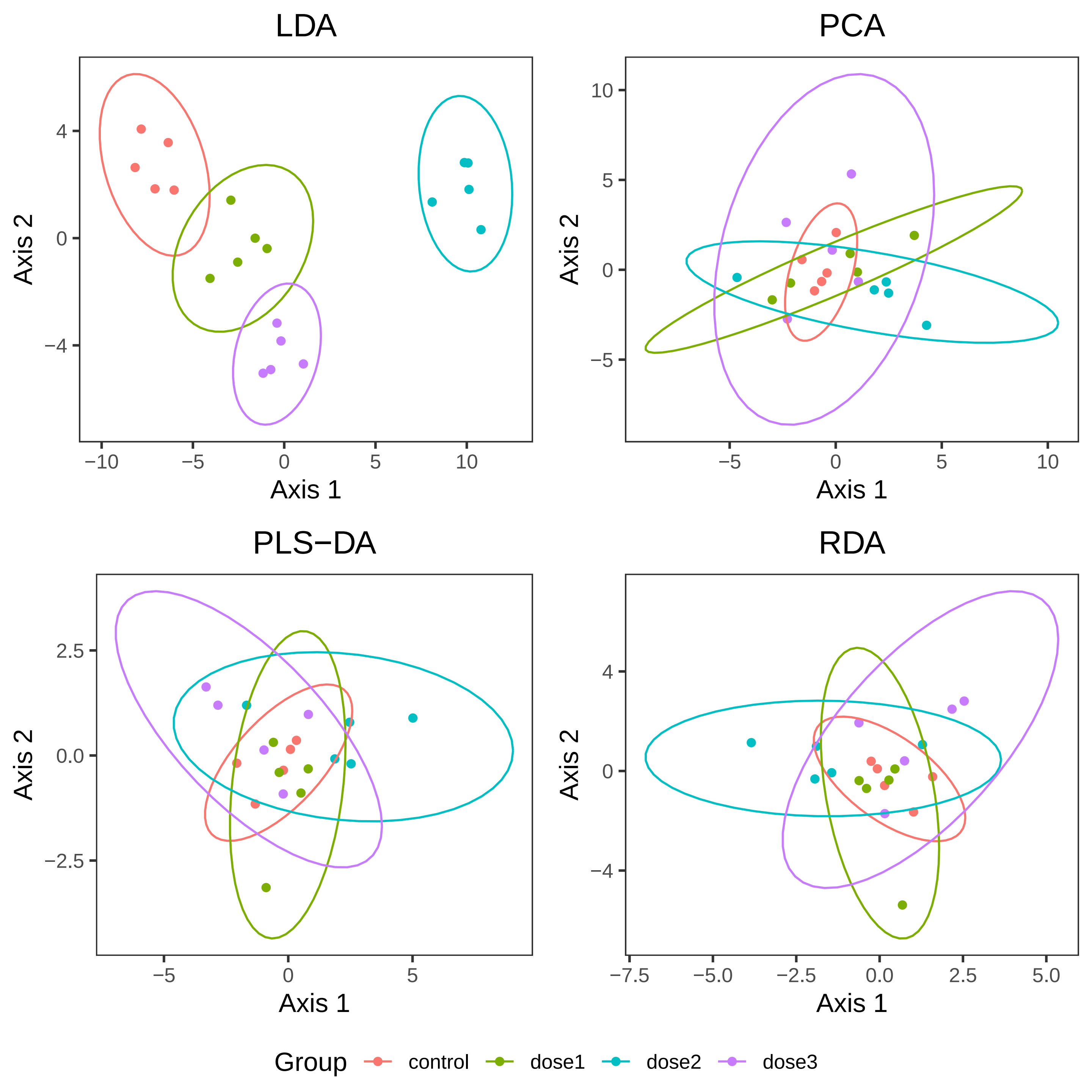
LDA
PCA
PLS−DA
RDA
Axis 1
Axis 2
−10
−5
0
5
10
4
0
−4
−2.5
0.0
2.5
−7.5
−5.0
−2.5
0.0
2.5
5.0
Group
control
dose1
dose2
dose3

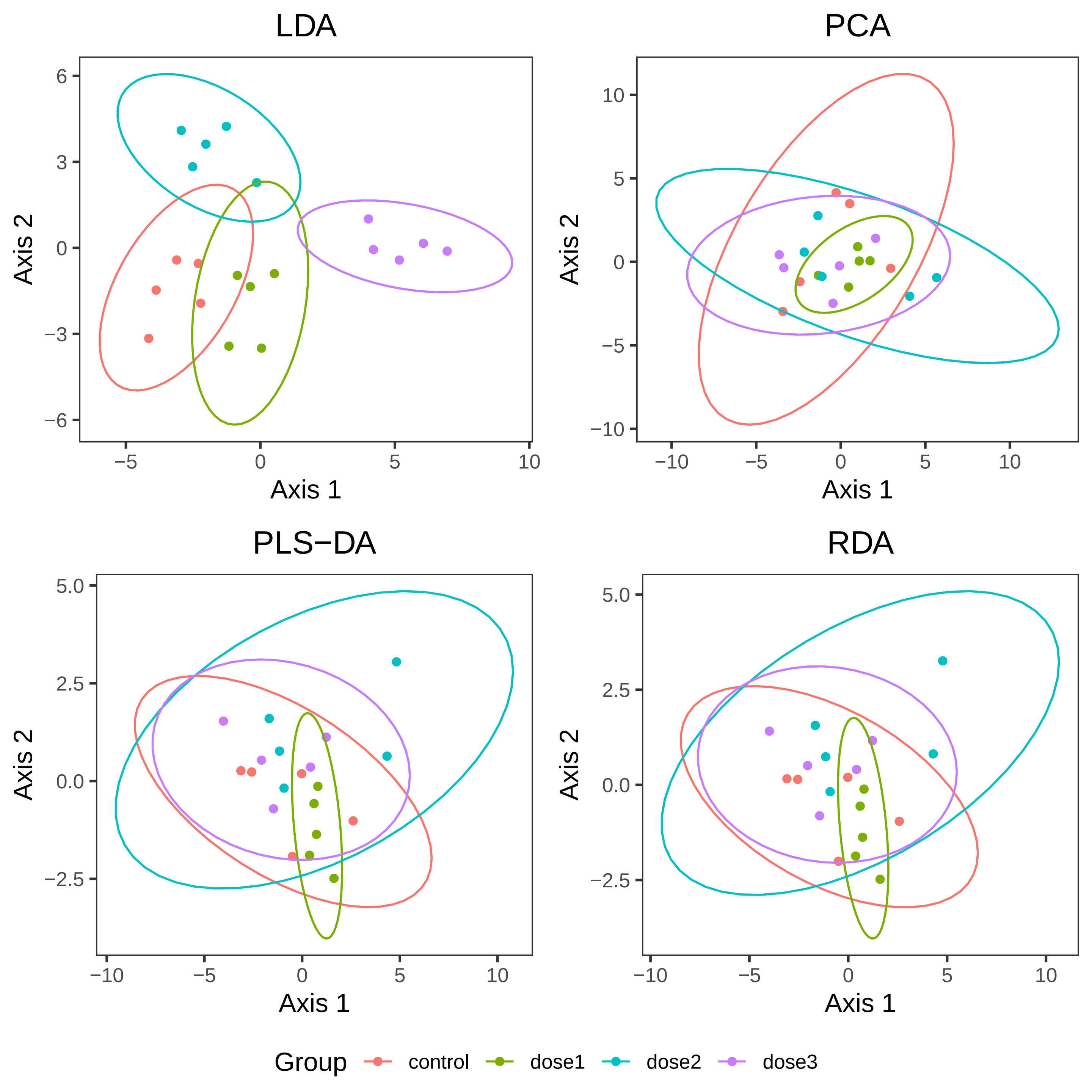
LDA
PCA
PLS−DA
RDA
Axis 1
Axis 2
Group
control
dose1
dose2
dose3

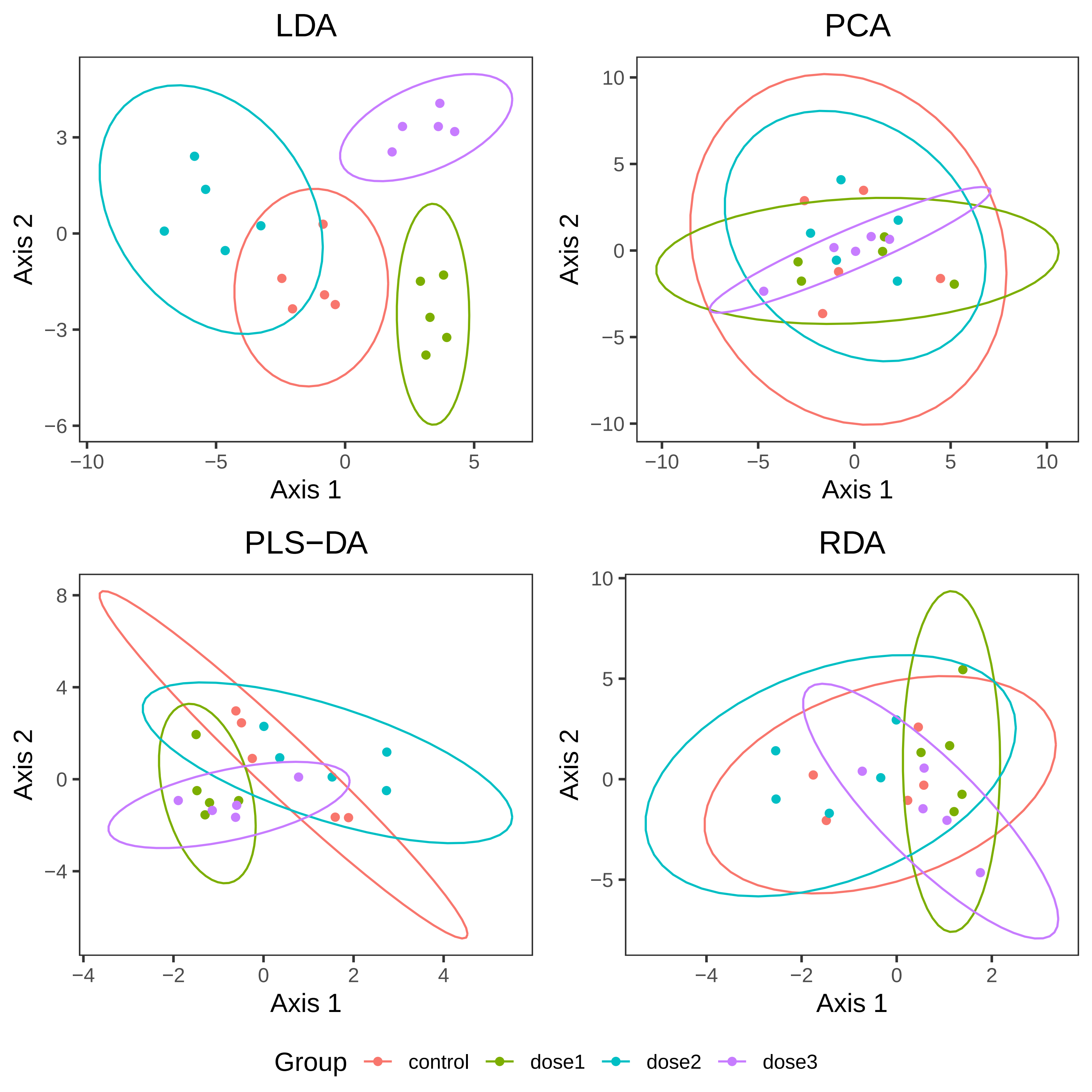

LDA
PCA
PLS−DA
RDA
Axis 1
Axis 2
3
0
−3
−6
−10
−5
0
5
10
8
4
−4
−2
2
Group
control
dose1
dose2
dose3

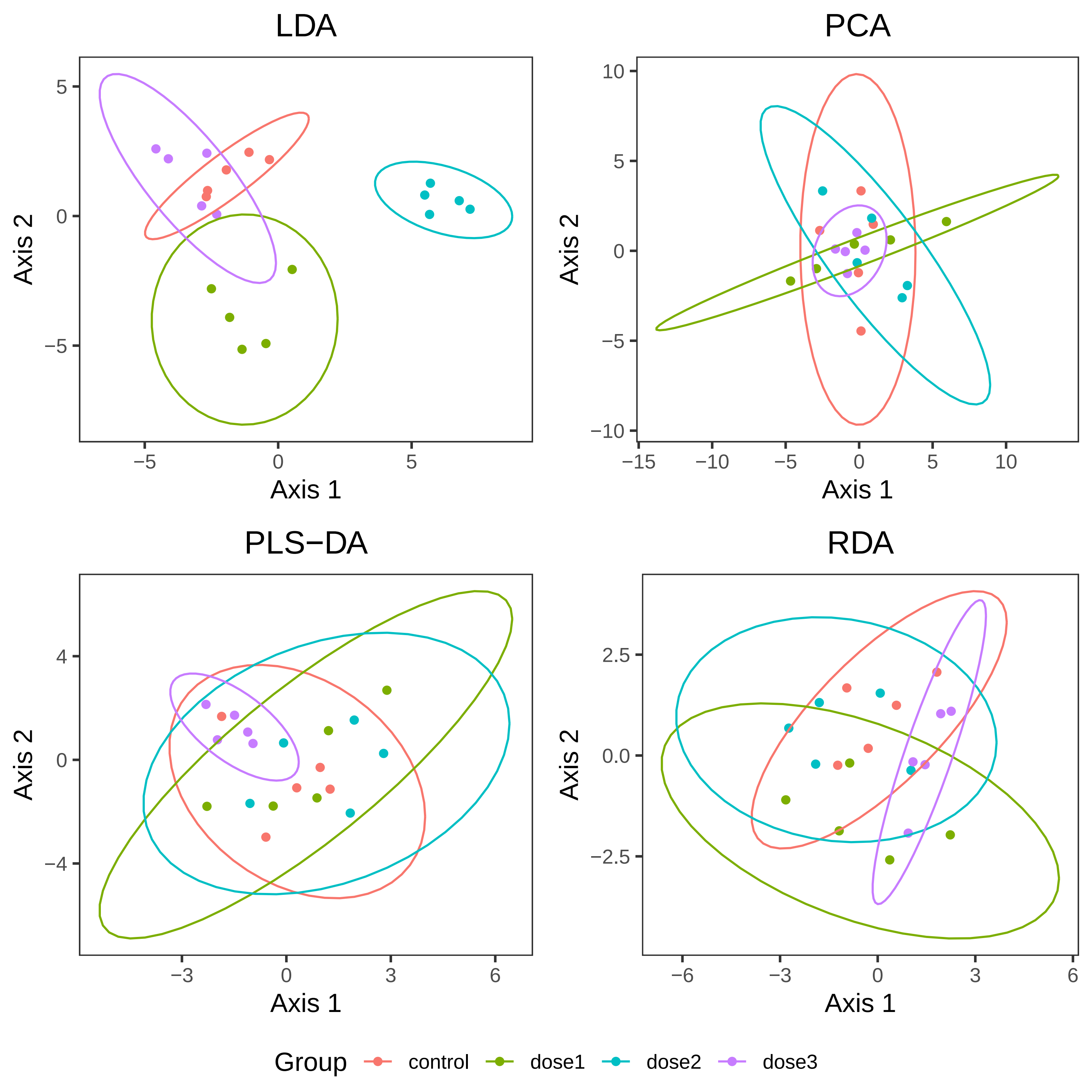
LDA
PCA
PLS−DA
RDA
Axis 1
Axis 2
Group
control
dose1
dose2
dose3

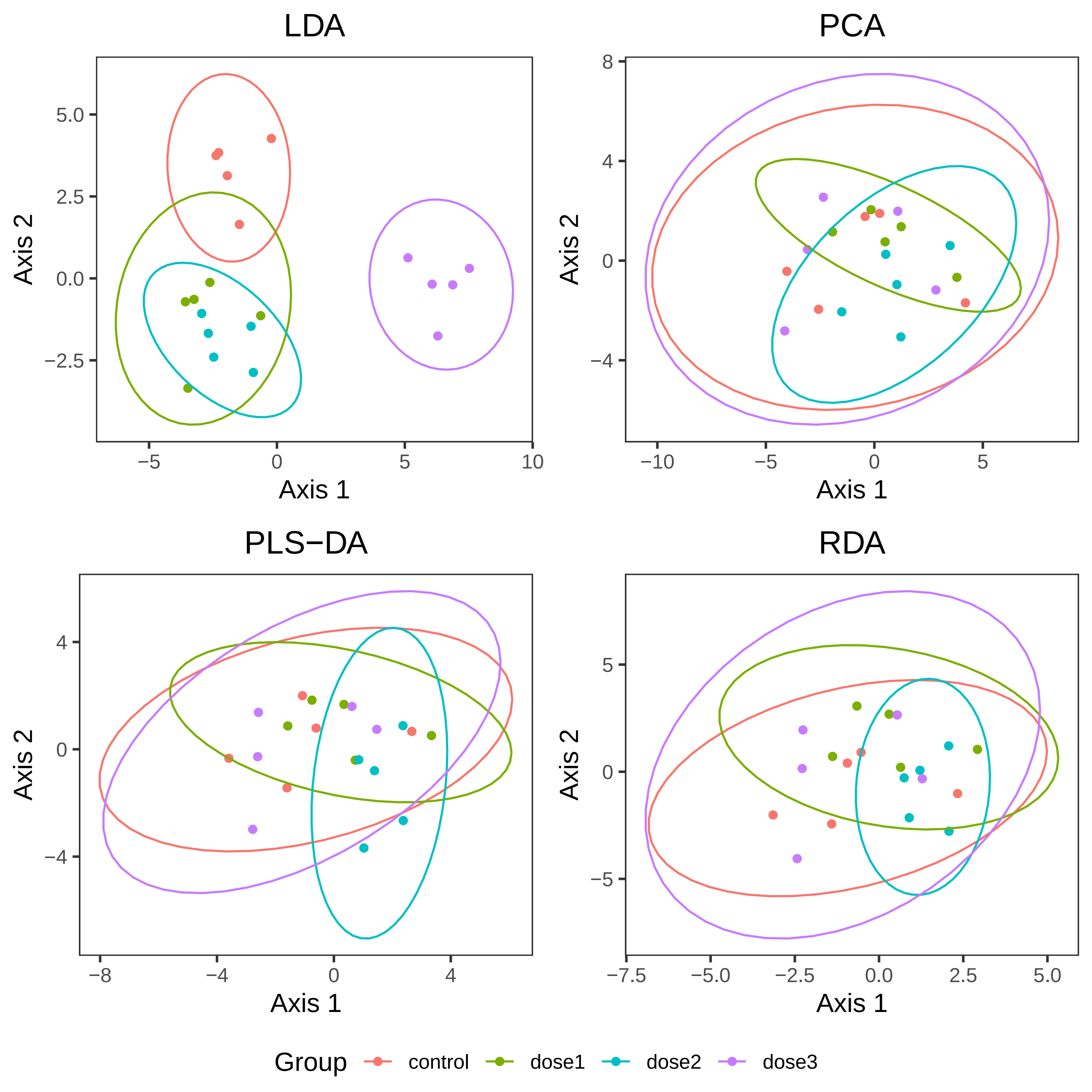
LDA
PCA
PLS−DA
RDA
Axis 1
Axis 2
5.0
2.5
0.0
−2.5
−5
0
5
10
8
4
−4
−10
−8
−7.5
−5.0
−2.5
Group
control
dose1
dose2
dose3

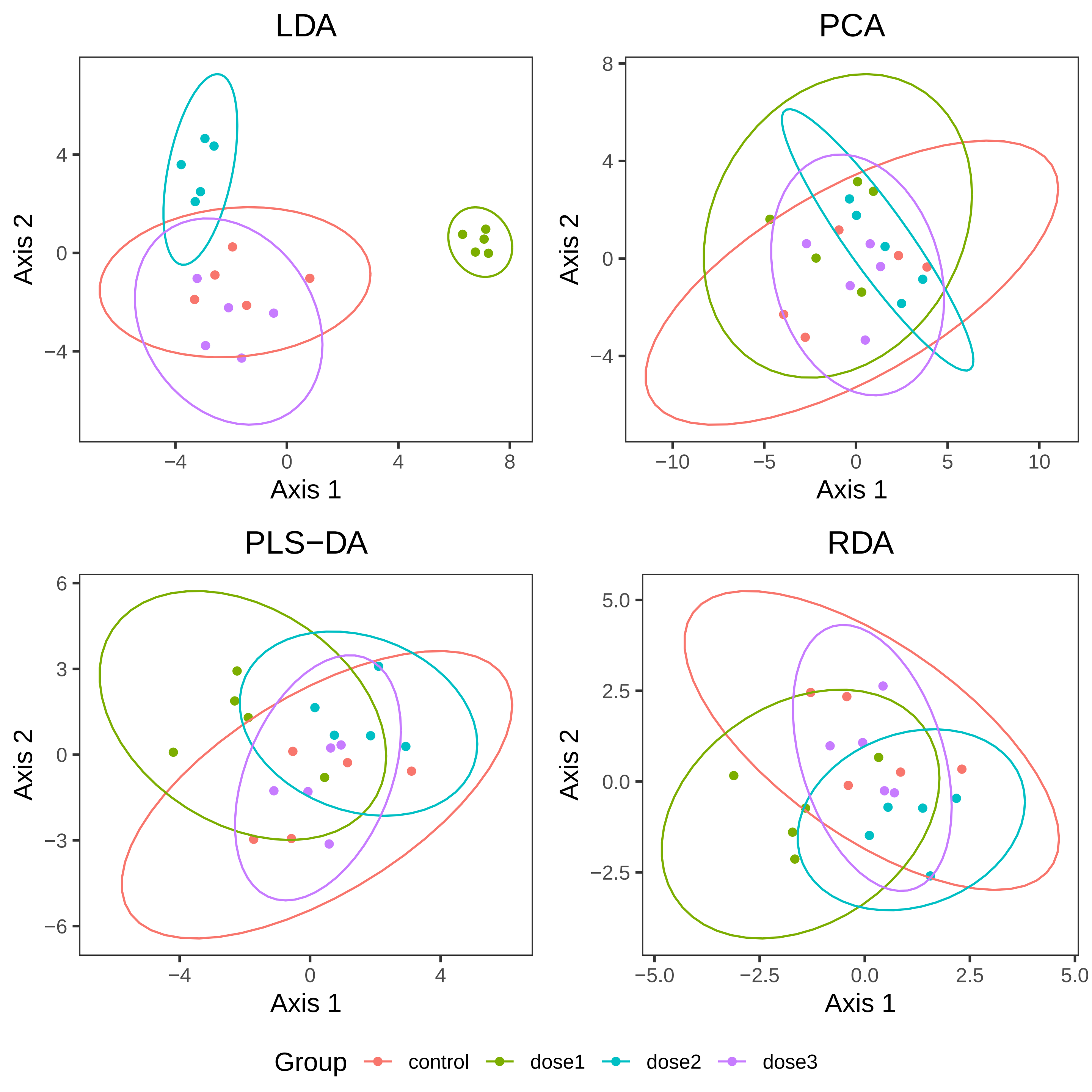

LDA
PCA
PLS−DA
RDA
Axis 1
Axis 2
Group
control
dose1
dose2
dose3

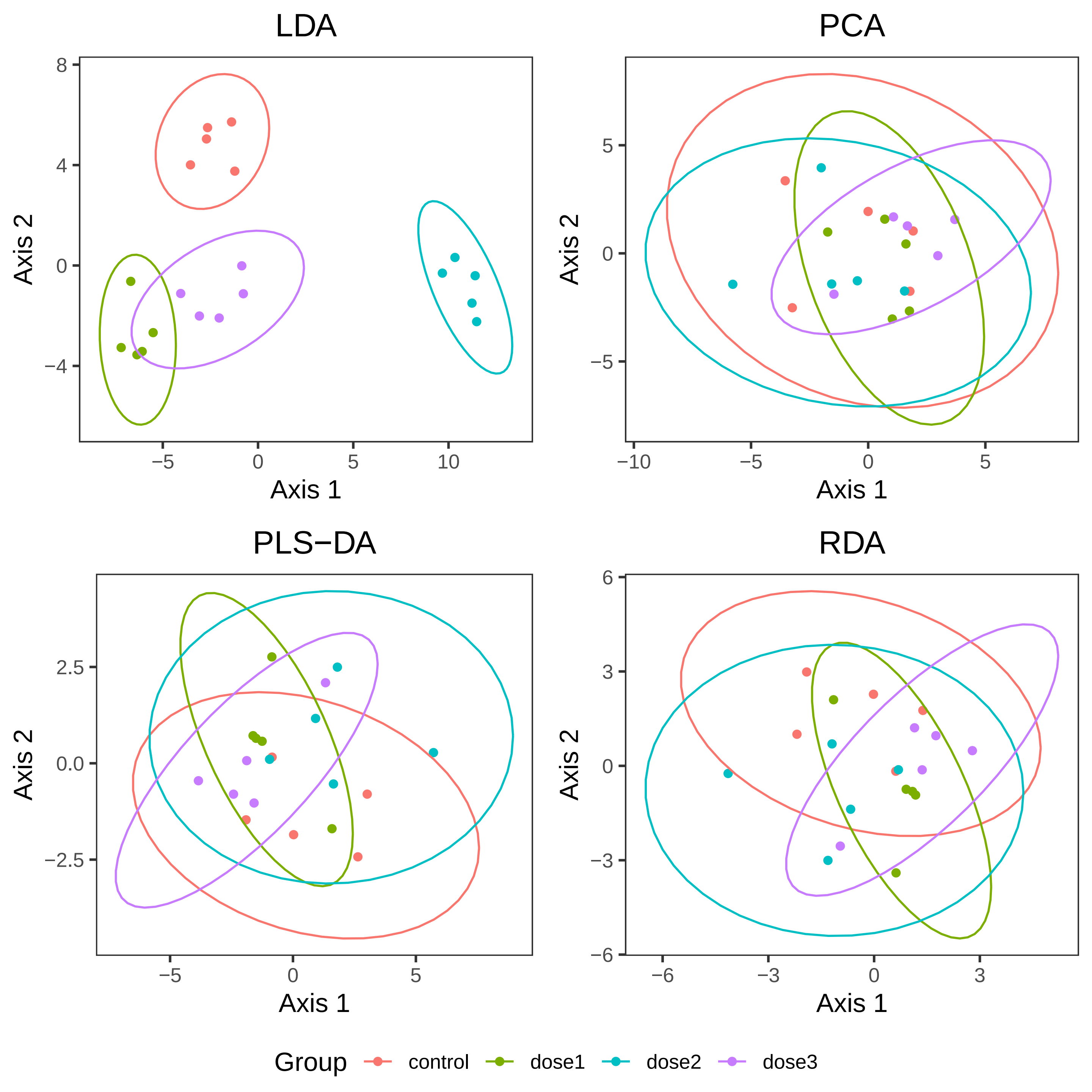
LDA
PCA
PLS−DA
RDA
Axis 1
Axis 2
Group
control
dose1
dose2
dose3

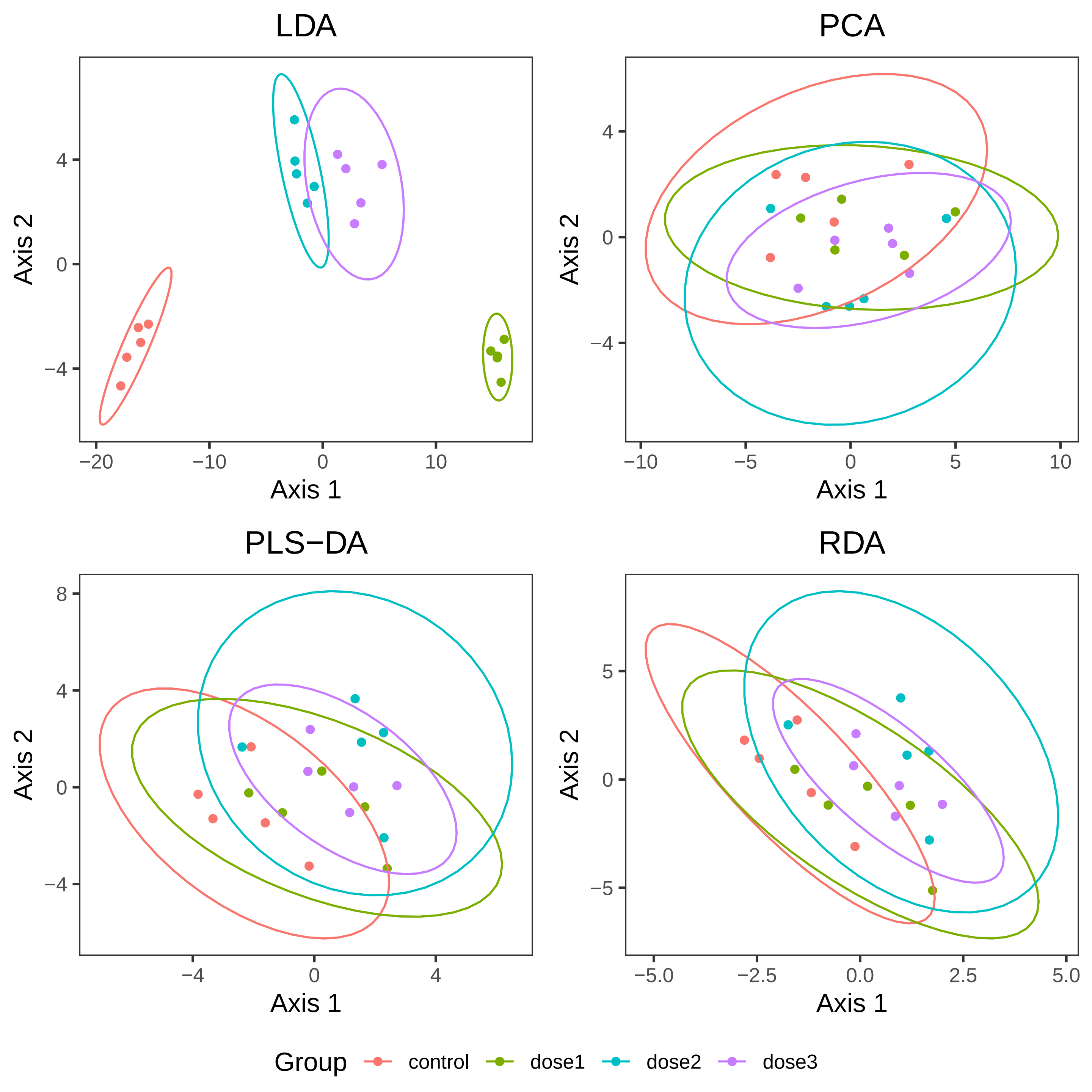

LDA
PCA
PLS−DA
RDA
Axis 1
Axis 2
−20
−10
0
10
4
0
−4
−10
−5
0
5
10
8
4
0
−4
−5.0
−2.5
0.0
2.5
5.0
5
0
−5
Group
control
dose1
dose2
dose3

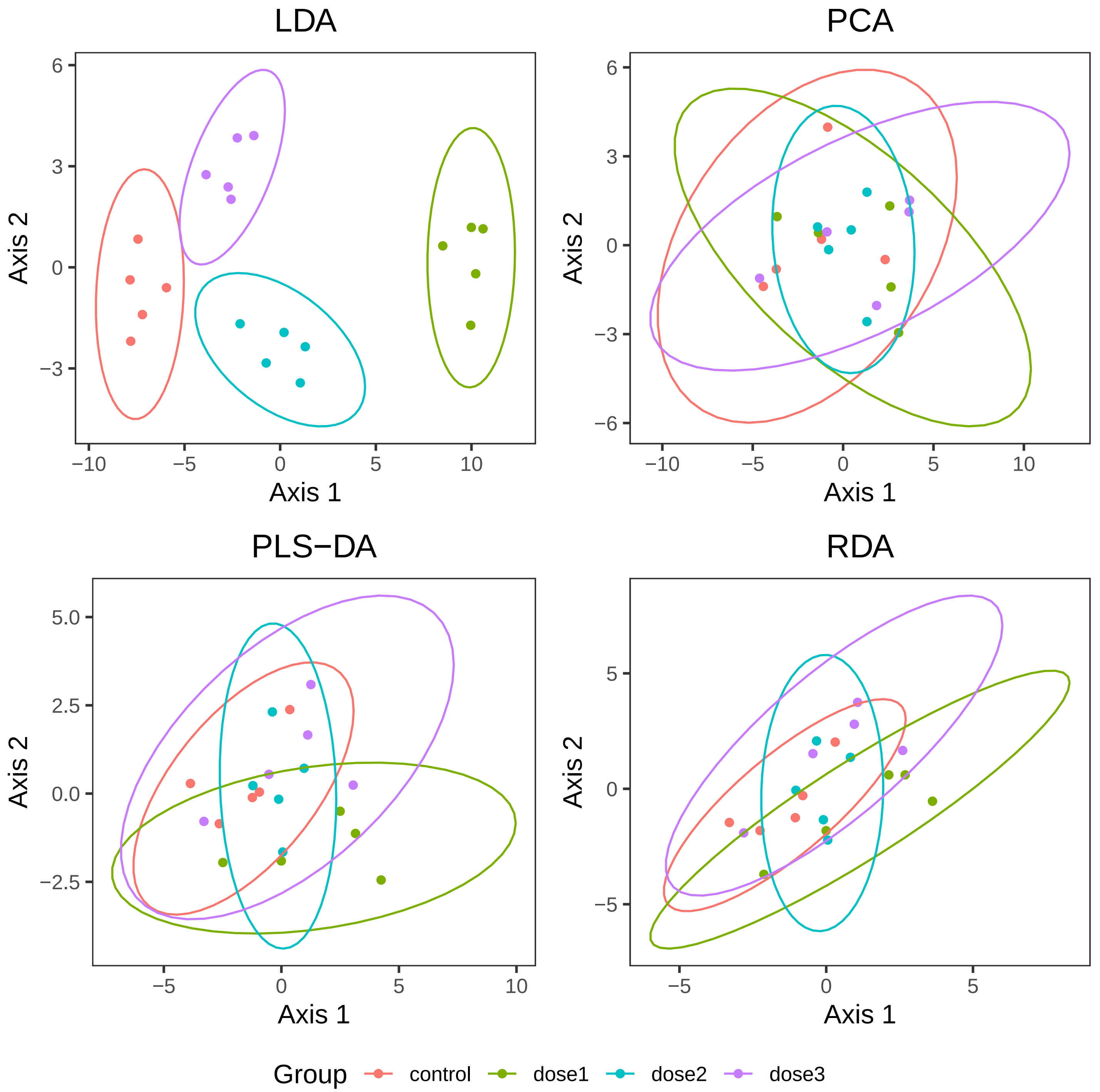
LDA
PCA
PLS−DA
RDA
Axis 1
Axis 2
Group
control
dose1
dose2
dose3

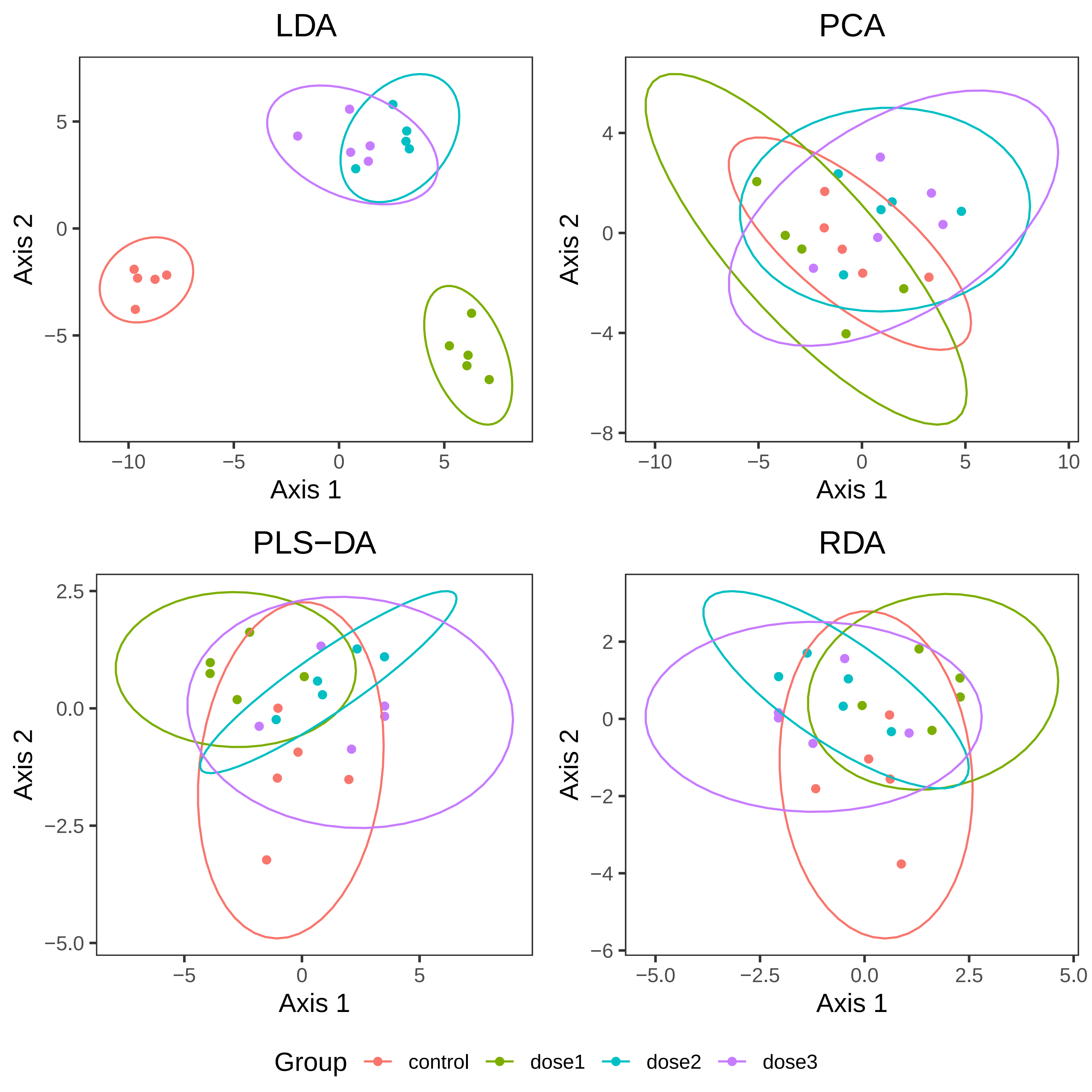
LDA
PCA
PLS−DA
RDA
Axis 1
Axis 2
Group
control
dose1
dose2
dose3

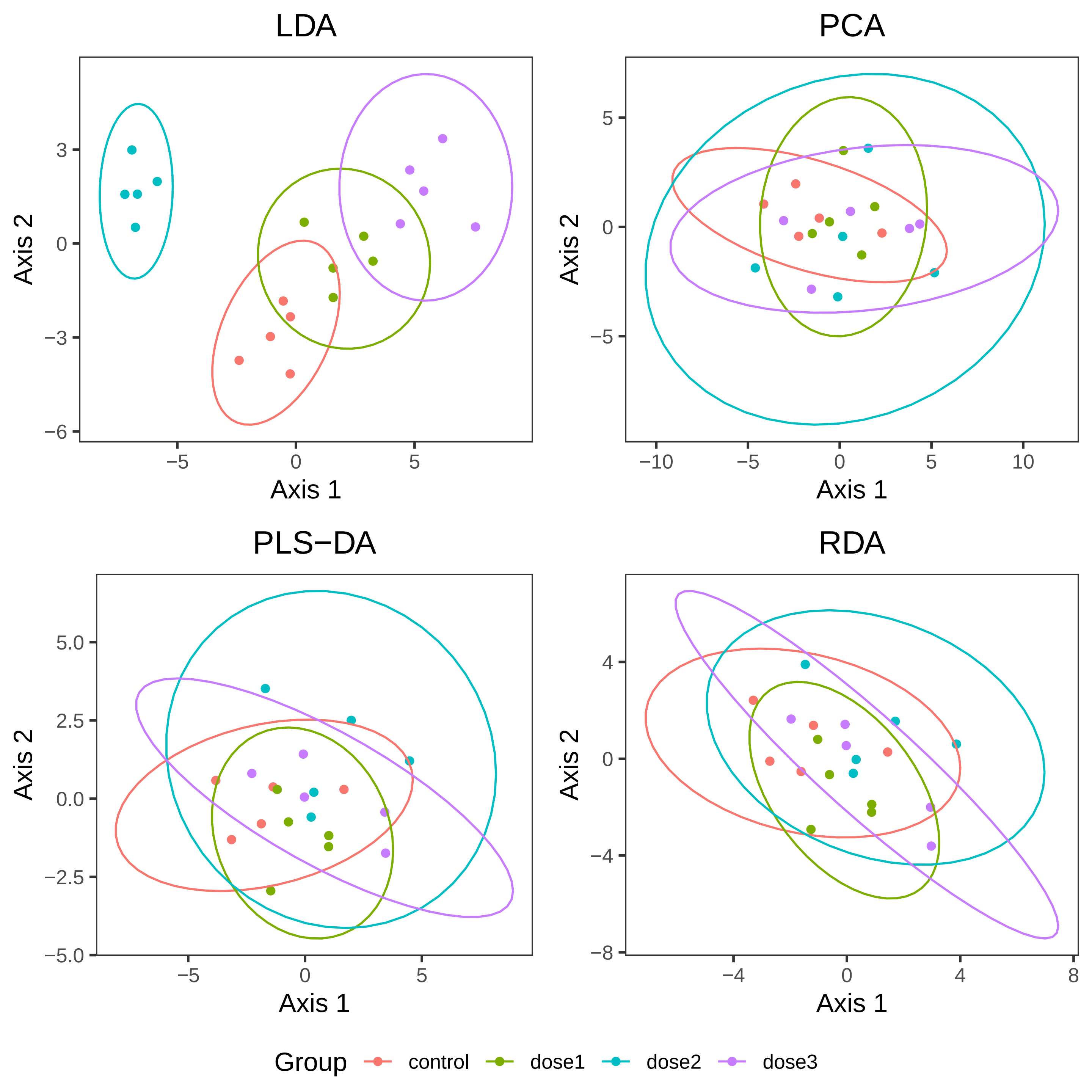
LDA
PCA
PLS−DA
RDA
Axis 1
Axis 2
−5
0
5
10
−10
3
−3
−6
2.5
5.0
0.0
−2.5
−5.0
−4
4
8
−8
Group
control
dose1
dose2
dose3

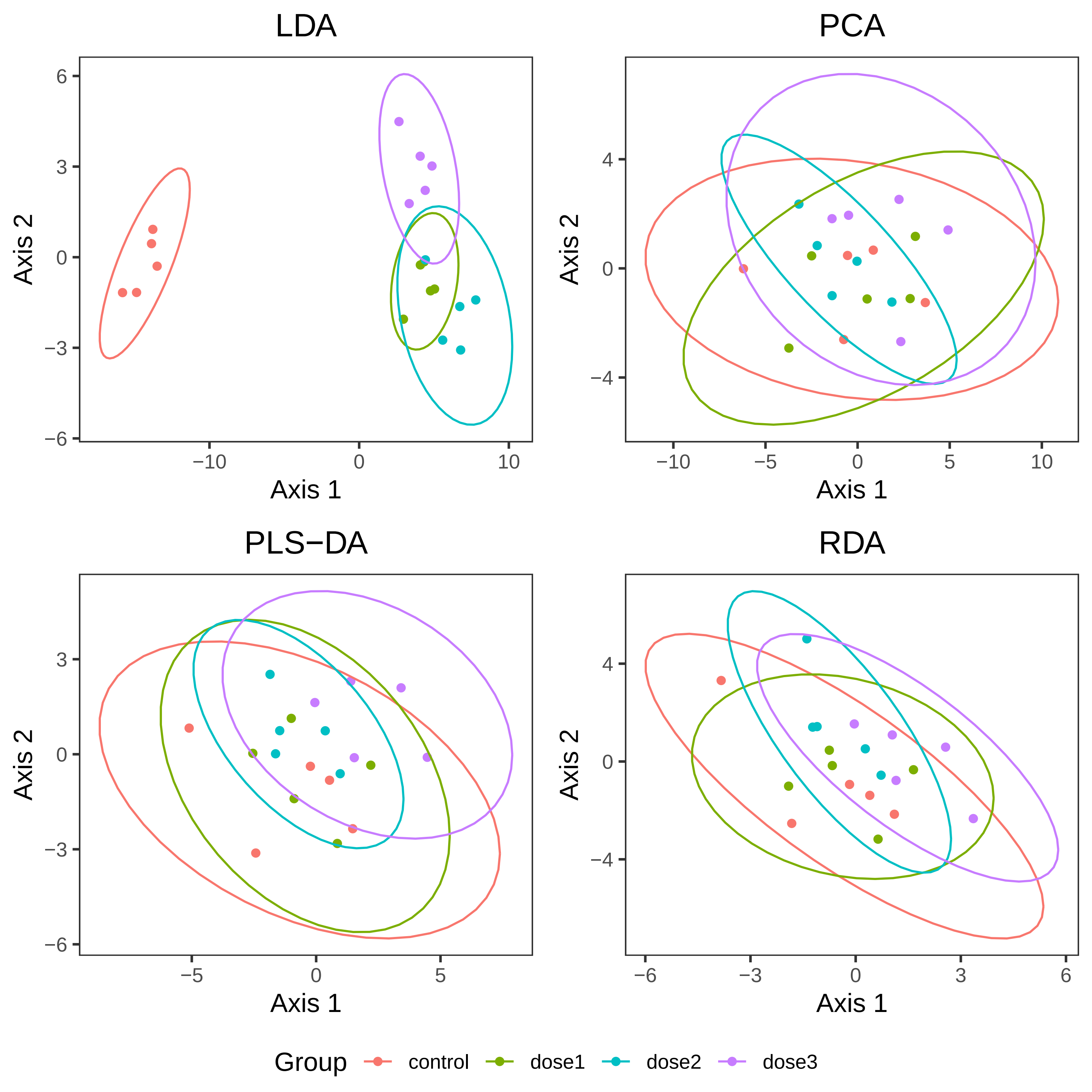

LDA
PCA
PLS−DA
RDA
Axis 1
Axis 2
6
3
0
−3
−6
−10
0
10
4
0
−4
−10
−5
0
5
10
3
0
−3
−6
−5
0
5
4
0
−4
−6
−3
0
3
6
Group
control
dose1
dose2
dose3

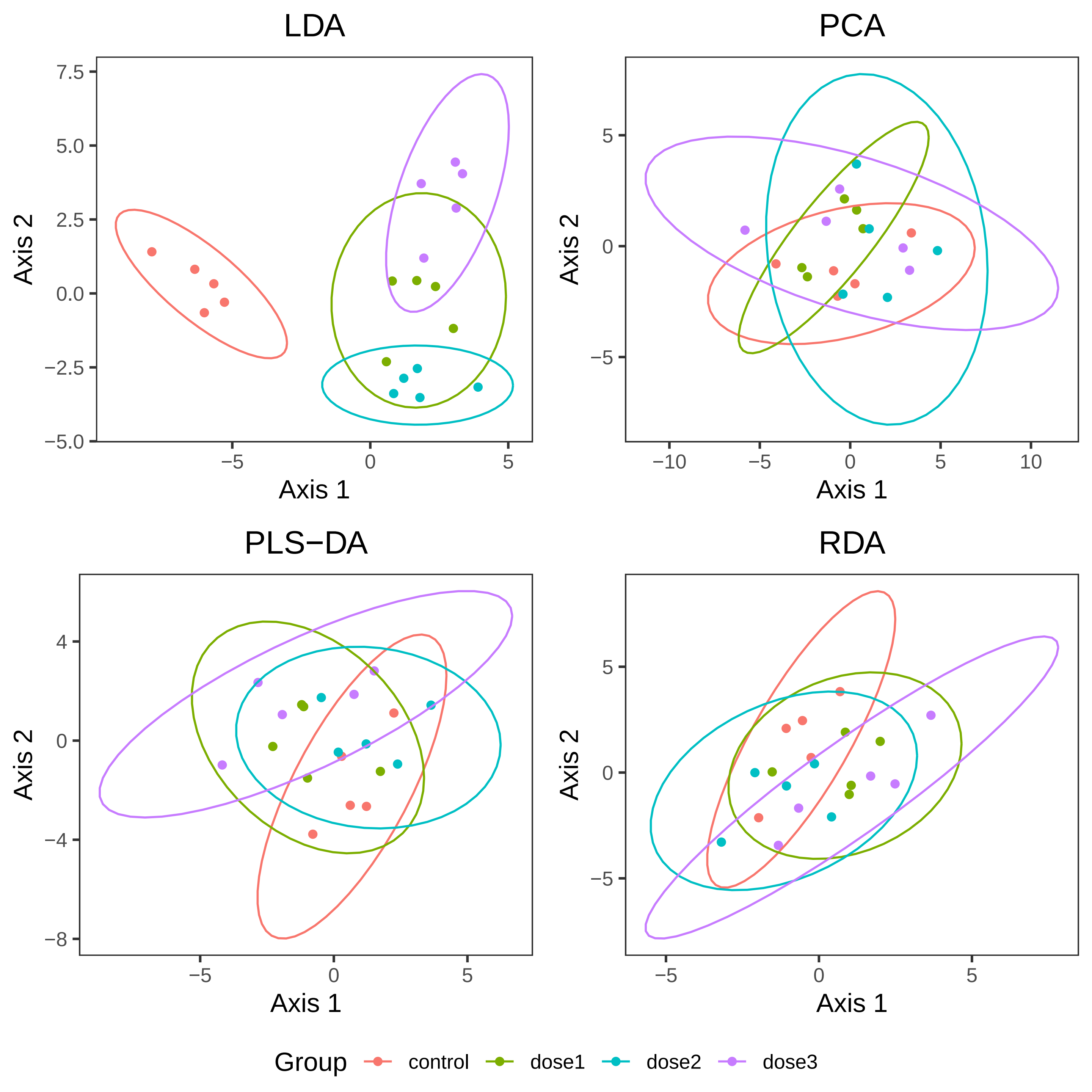

LDA
PCA
PLS−DA
RDA
Axis 1
Axis 2
Group
control
dose1
dose2
dose3

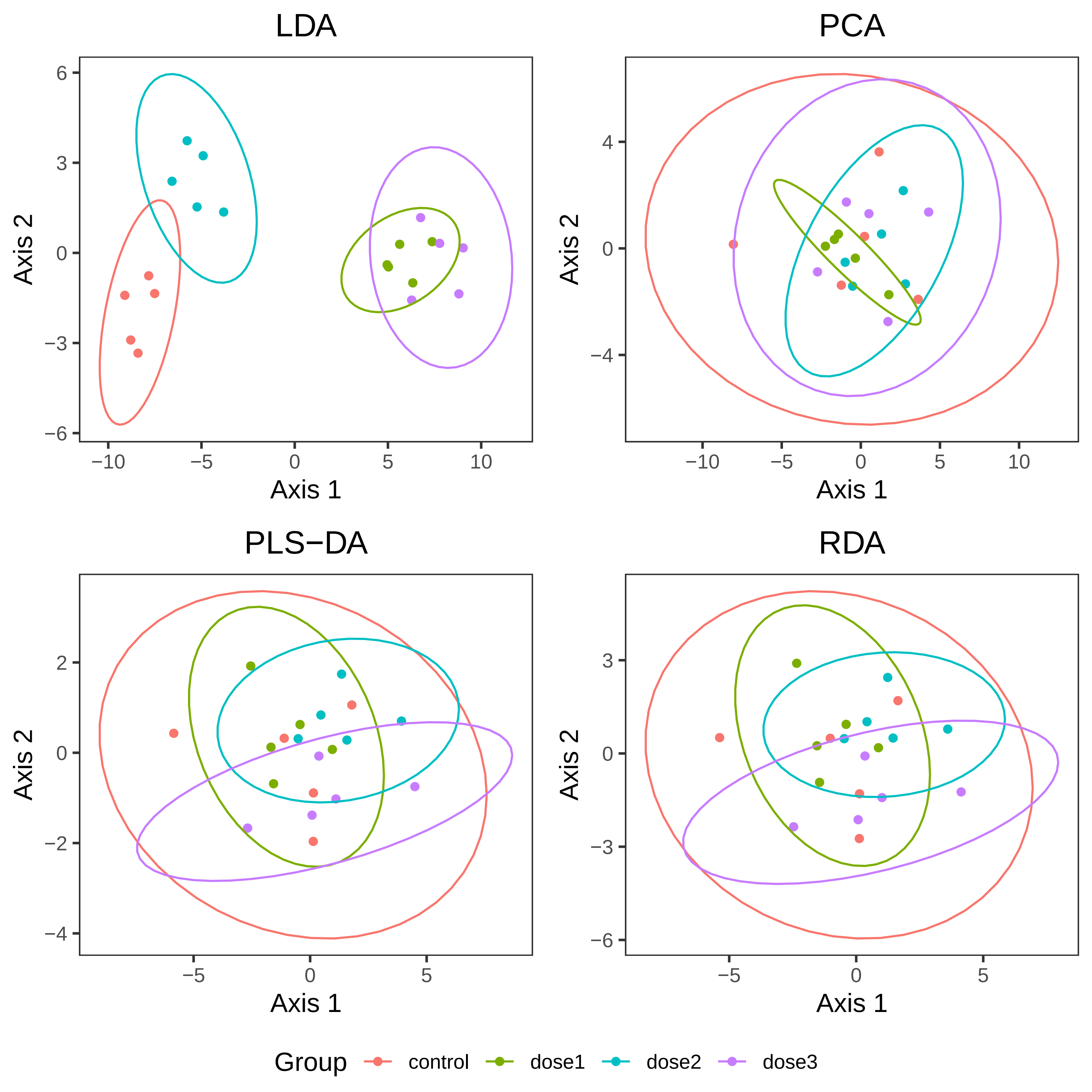

LDA
PCA
PLS−DA
RDA
Axis 1
Axis 2
6
3
0
−3
−6
−10
−5
0
5
10
4
−4
2
−2
Group
control
dose1
dose2
dose3

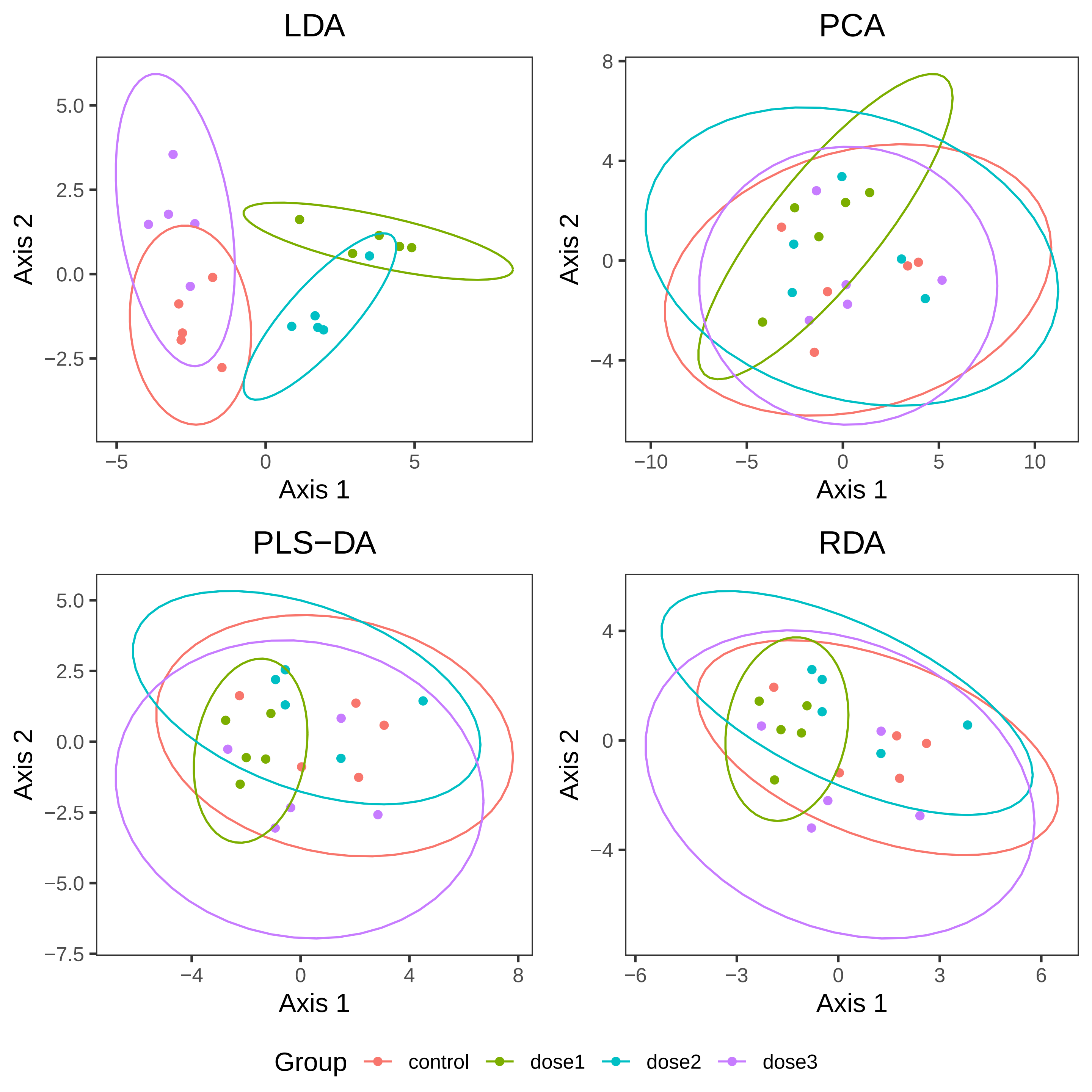

LDA
PCA
PLS−DA
RDA
Axis 1
Axis 2
5.0
2.5
0.0
−2.5
−5
0
5
8
4
0
−4
−10
−5
0
5
10
5.0
2.5
0.0
−2.5
−5.0
−7.5
−4
0
4
8
4
0
−4
−6
−3
0
3
6
Group
control
dose1
dose2
dose3

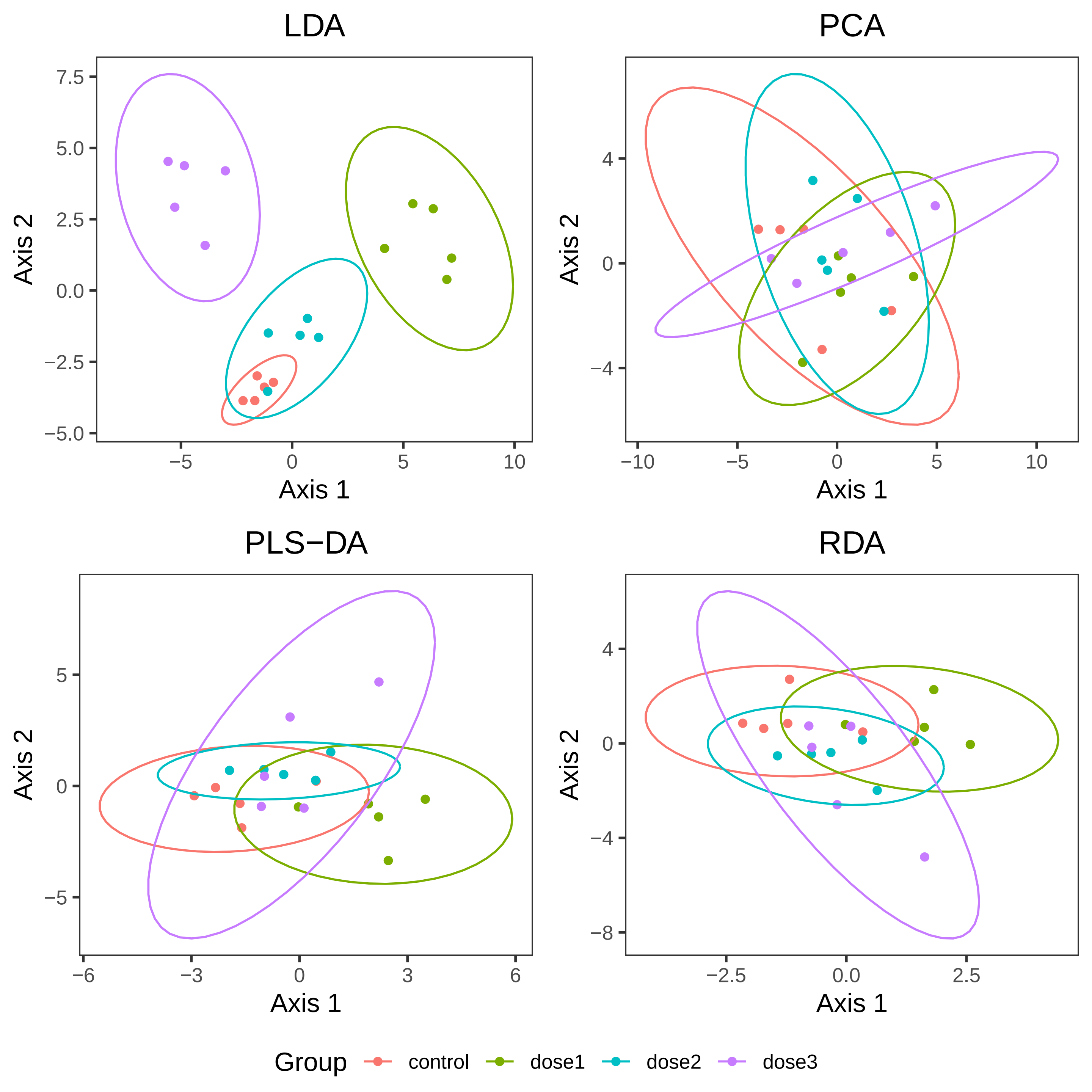

LDA
PCA
PLS−DA
RDA
Axis 1
Axis 2
7.5
5.0
2.5
0.0
−2.5
−5.0
−5
0
5
10
4
0
−4
−10
−6
−3
3
6
−8
−2.5
0.0
2.5
Group
control
dose1
dose2
dose3

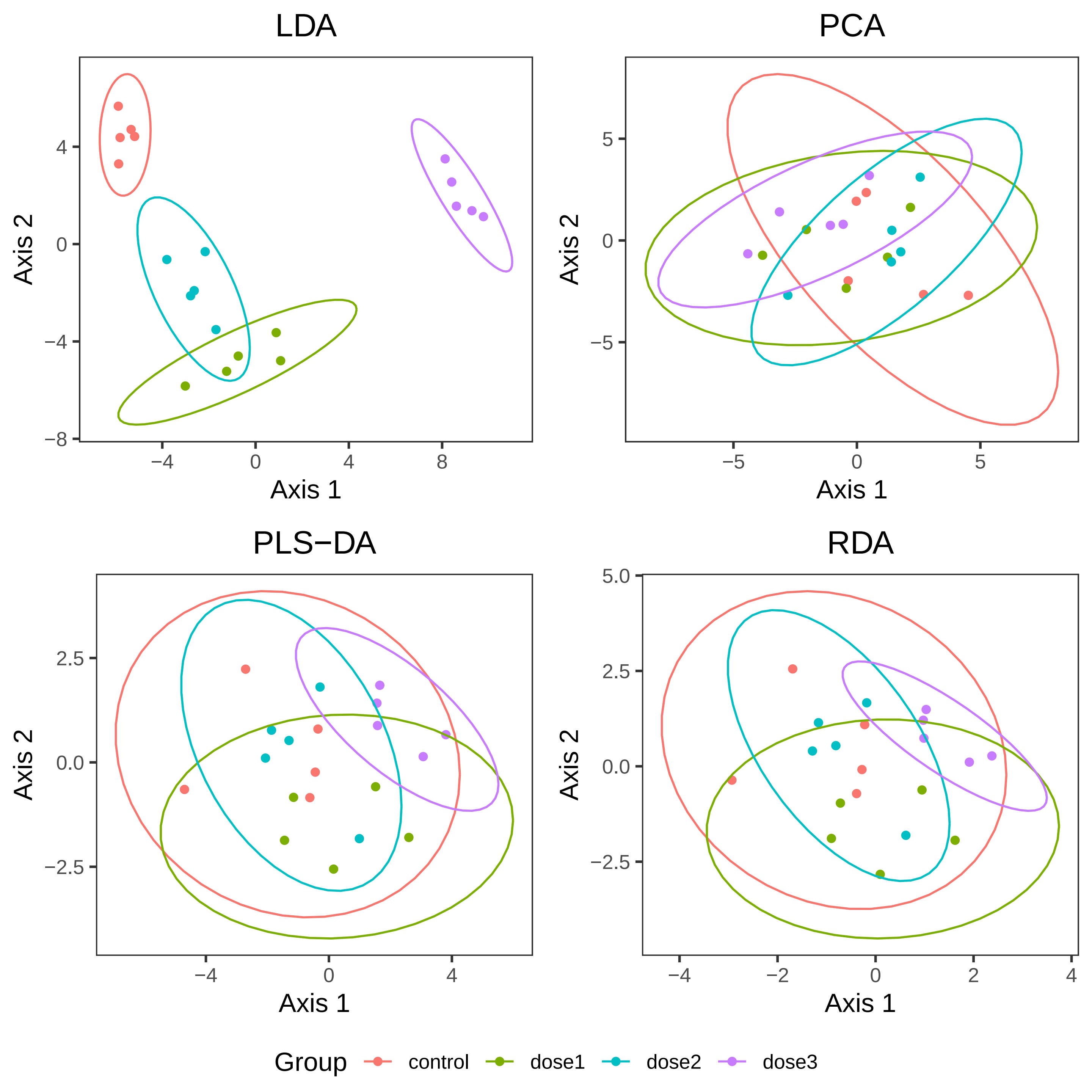
LDA
PCA
PLS−DA
RDA
Axis 1
Axis 2
Group
control
dose1
dose2
dose3

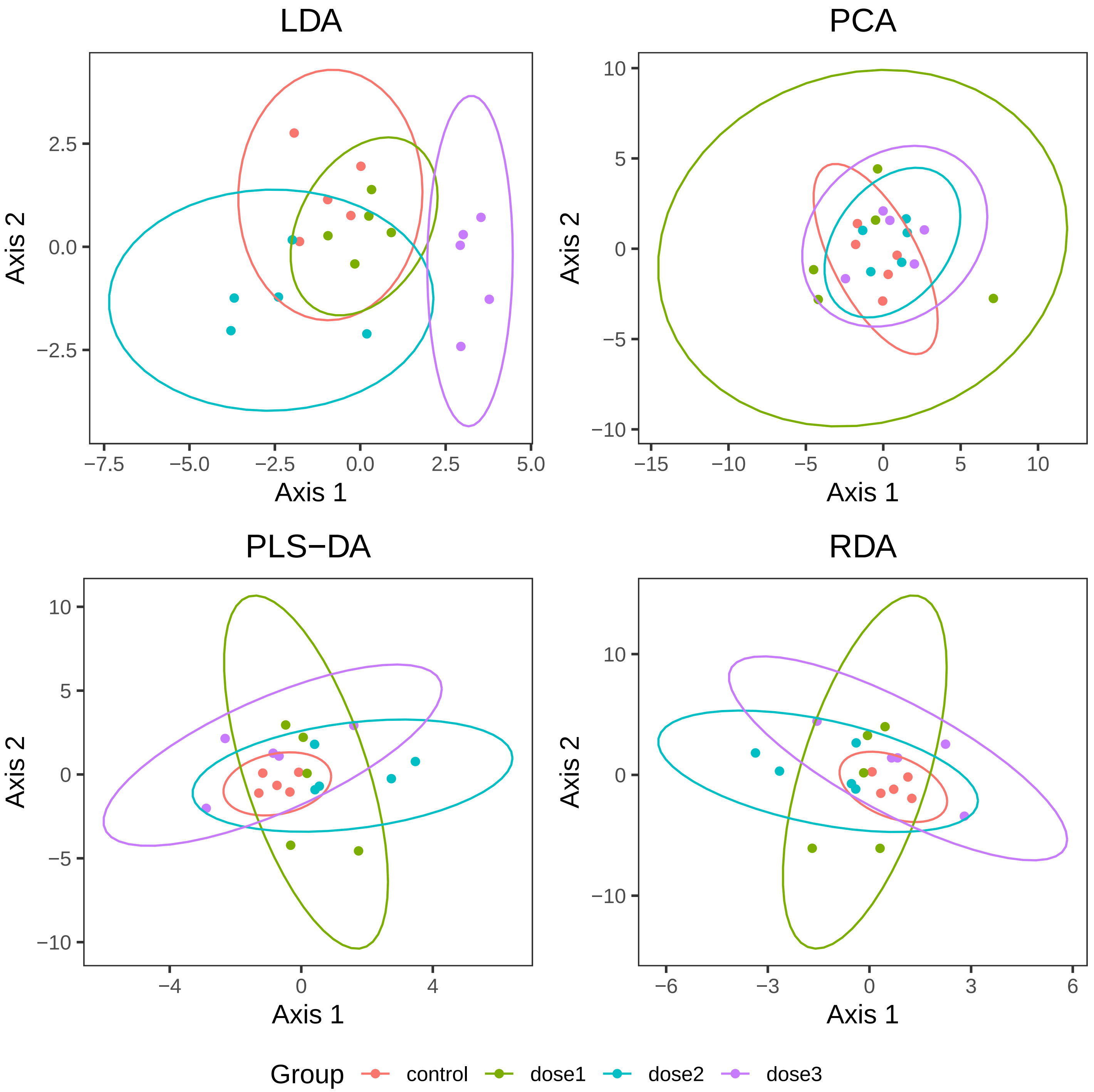
LDA
PCA
PLS−DA
RDA
Axis 1
Axis 2
2.5
0.0
−2.5
−7.5
−5.0
5.0
10
5
0
−5
−10
−15
−4
4
−6
−3
3
6
Group
control
dose1
dose2
dose3

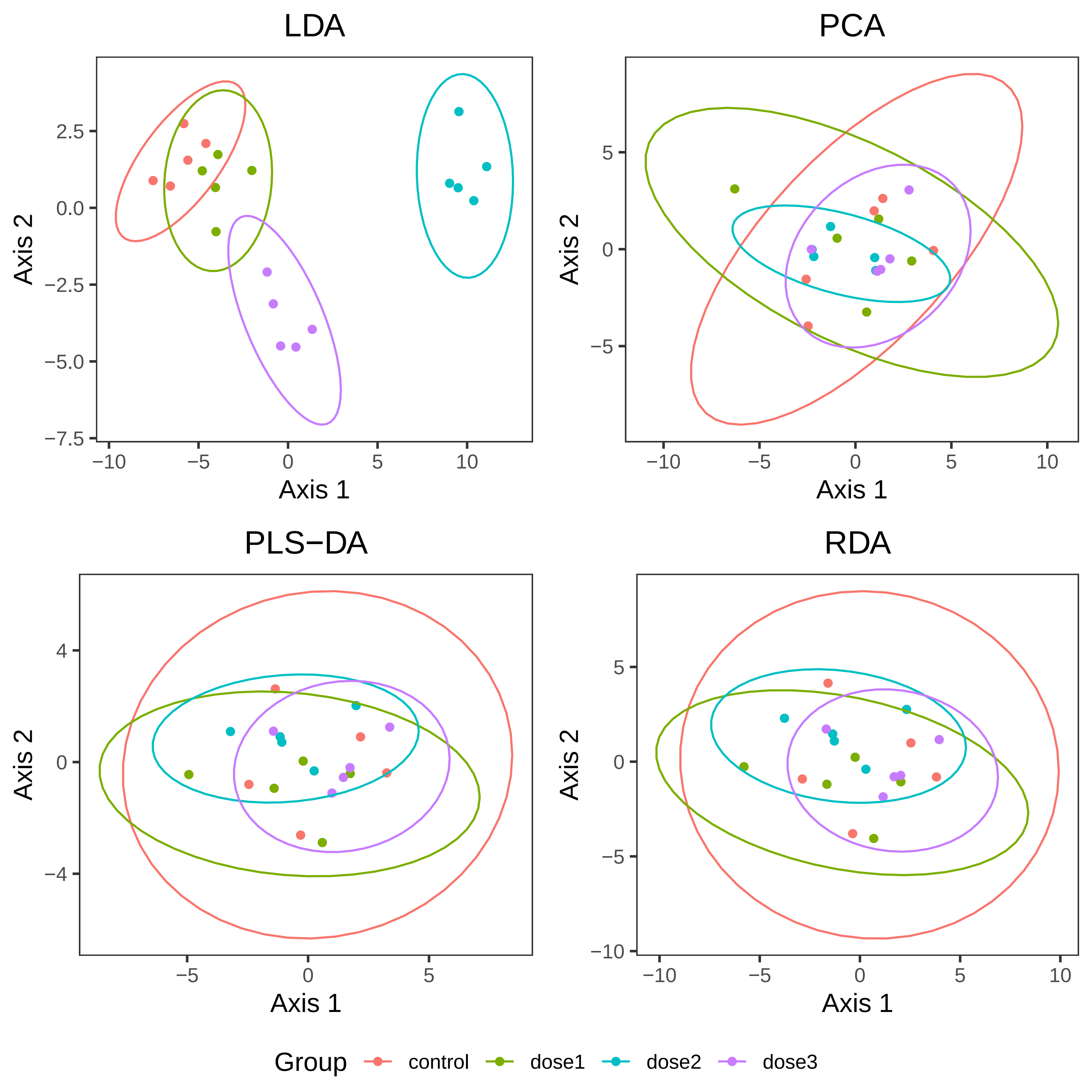

LDA
PCA
PLS−DA
RDA
Axis 1
Axis 2
2.5
0.0
−2.5
−5.0
−7.5
−10
−5
0
5
10
4
−4
Group
control
dose1
dose2
dose3

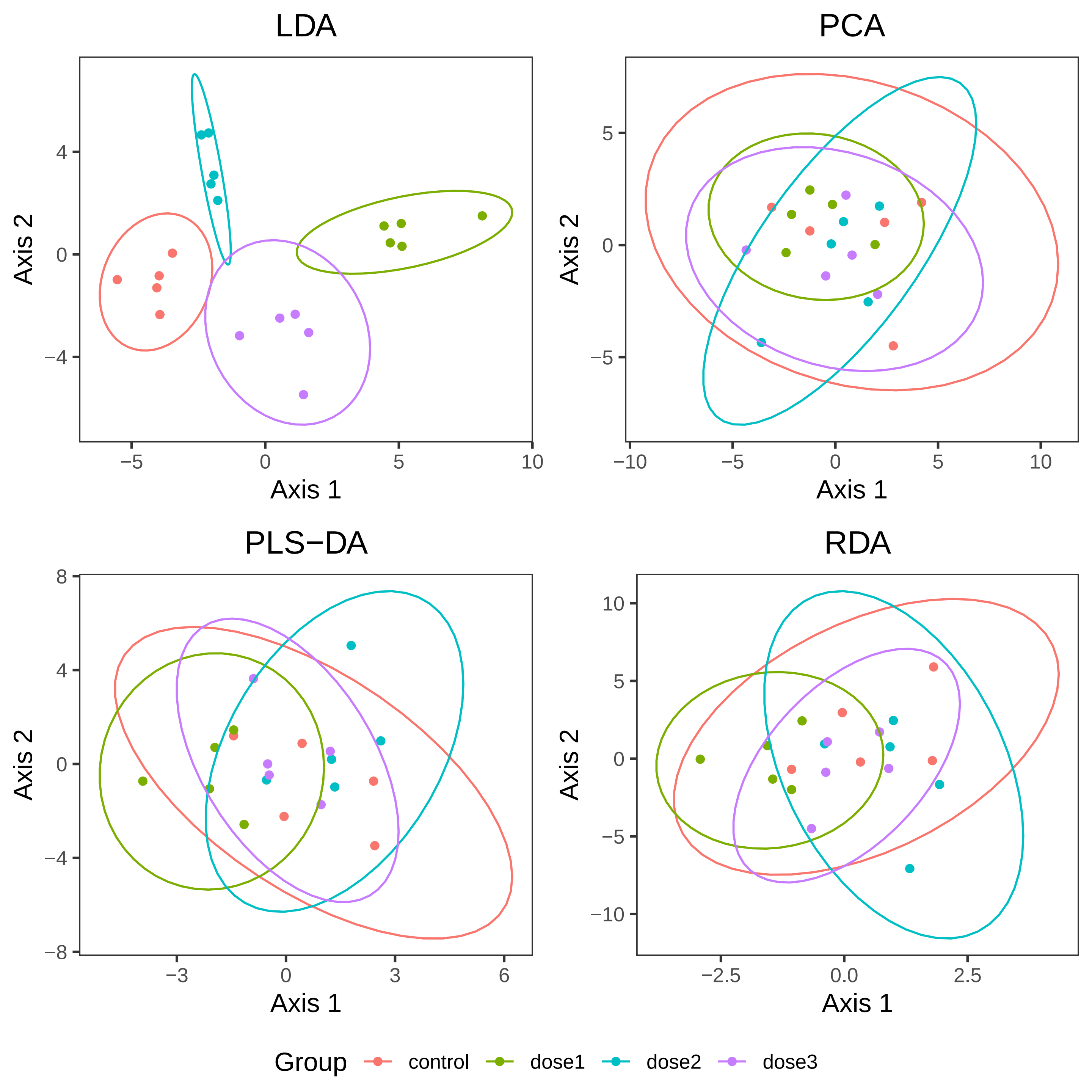
LDA
PCA
PLS−DA
RDA
Axis 1
Axis 2
−5
0
5
10
4
0
−4
−10
−5
0
5
10
5
0
−5
−3
0
3
6
8
4
0
−4
−8
−2.5
0.0
2.5
10
5
0
−5
−10
Group
control
dose1
dose2
dose3

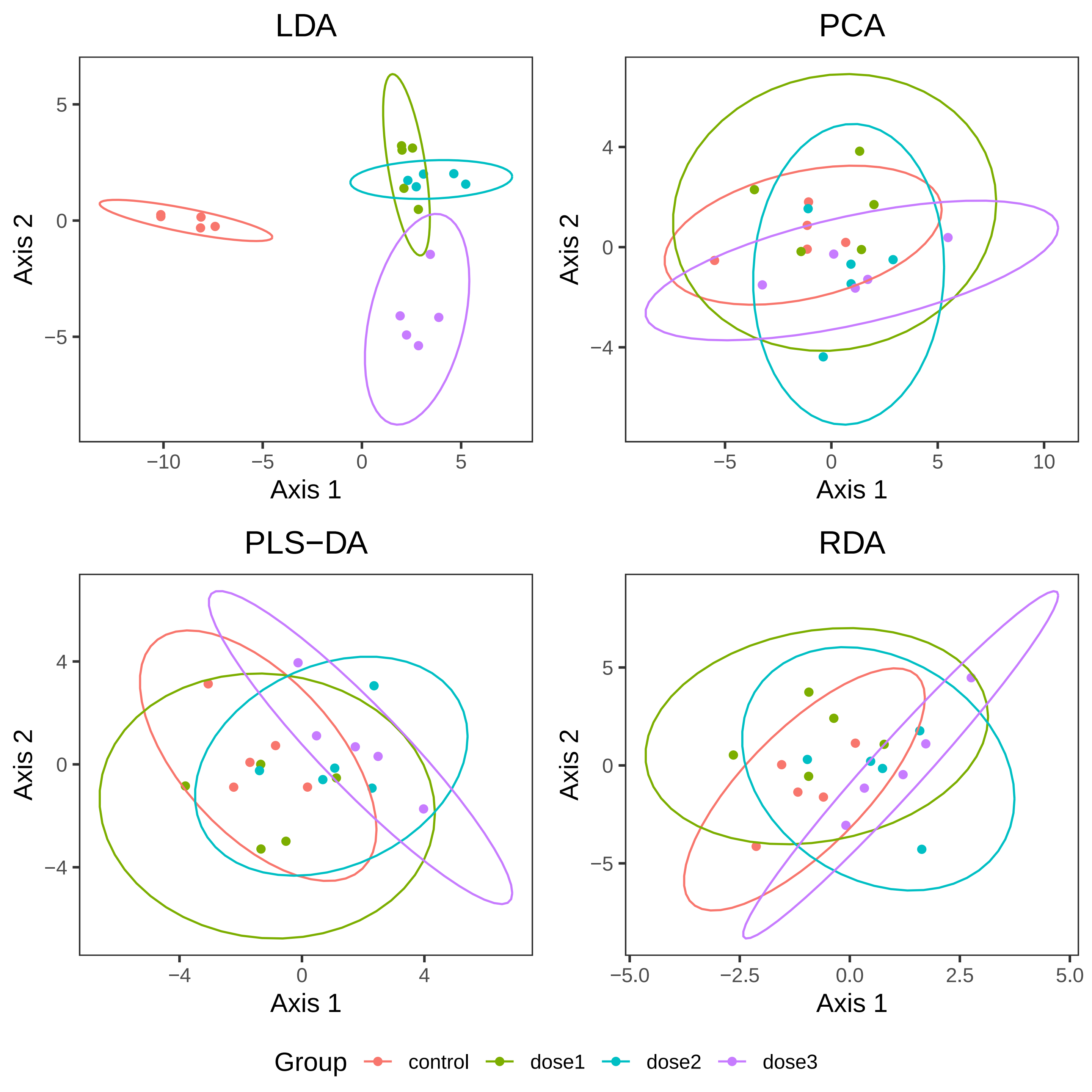

LDA
PCA
PLS−DA
RDA
Axis 1
Axis 2
Group
control
dose1
dose2
dose3

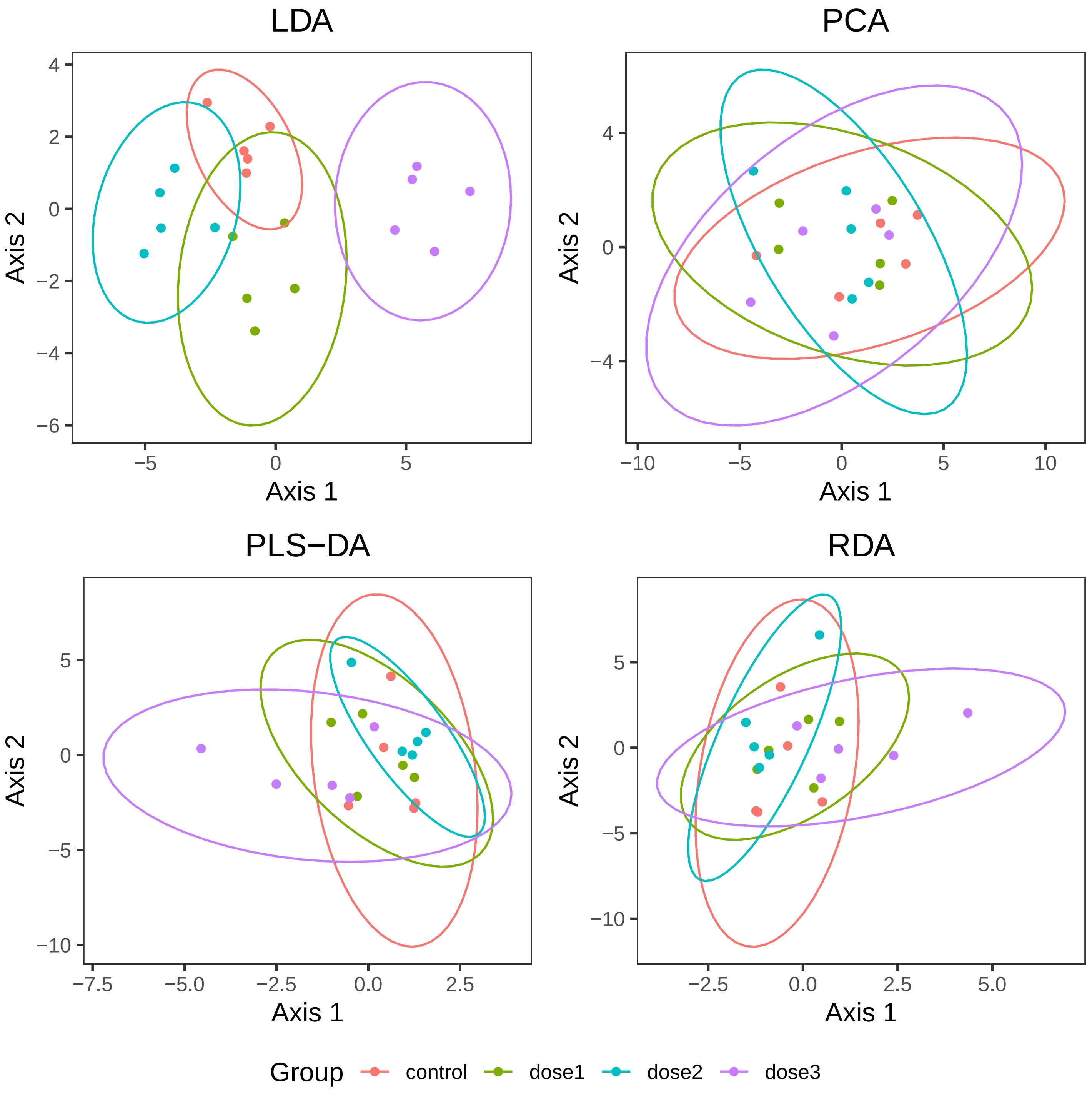

LDA
PCA
PLS−DA
RDA
Axis 1
Axis 2
Group
control
dose1
dose2
dose3

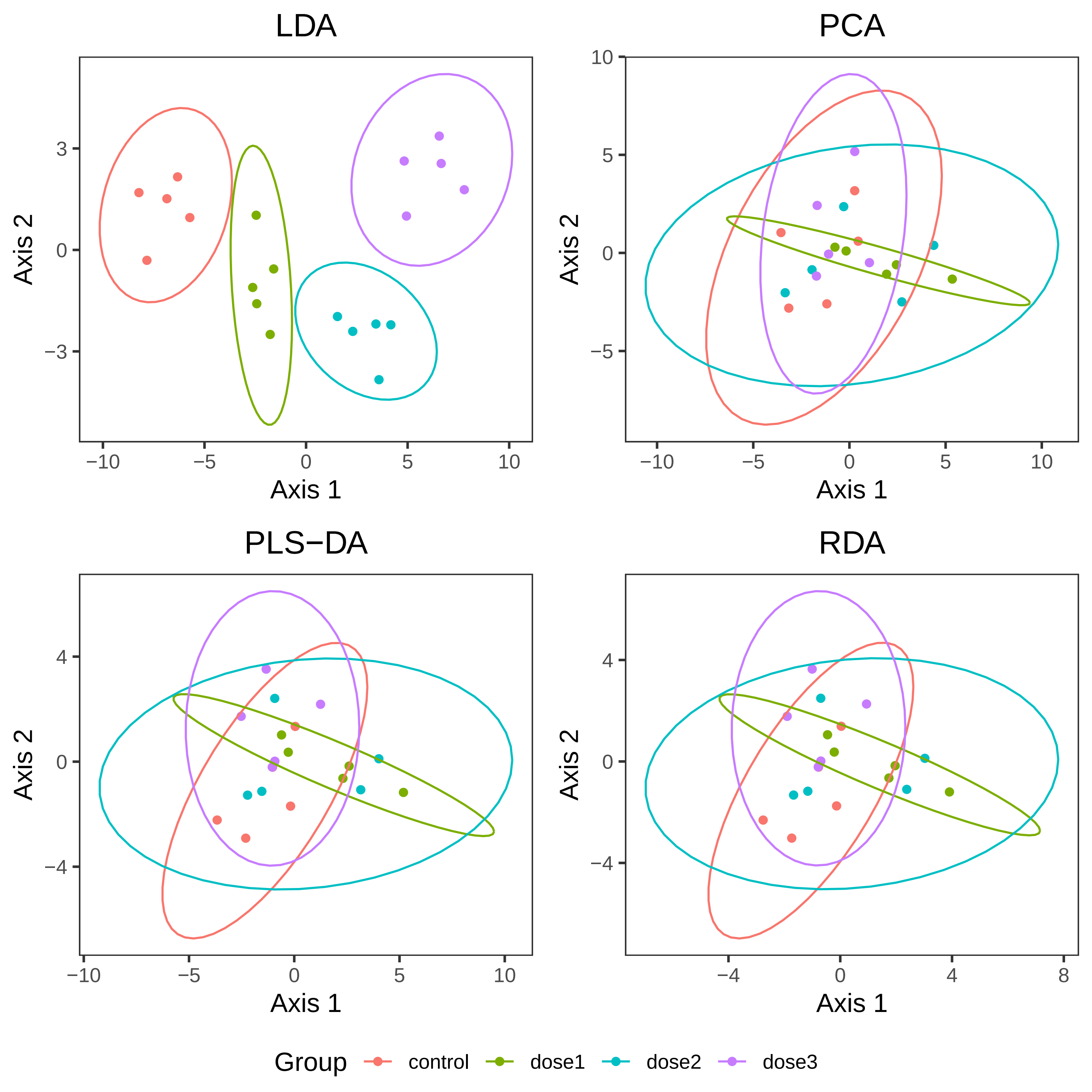

LDA
PCA
PLS−DA
RDA
Axis 1
Axis 2
Group
control
dose1
dose2
dose3

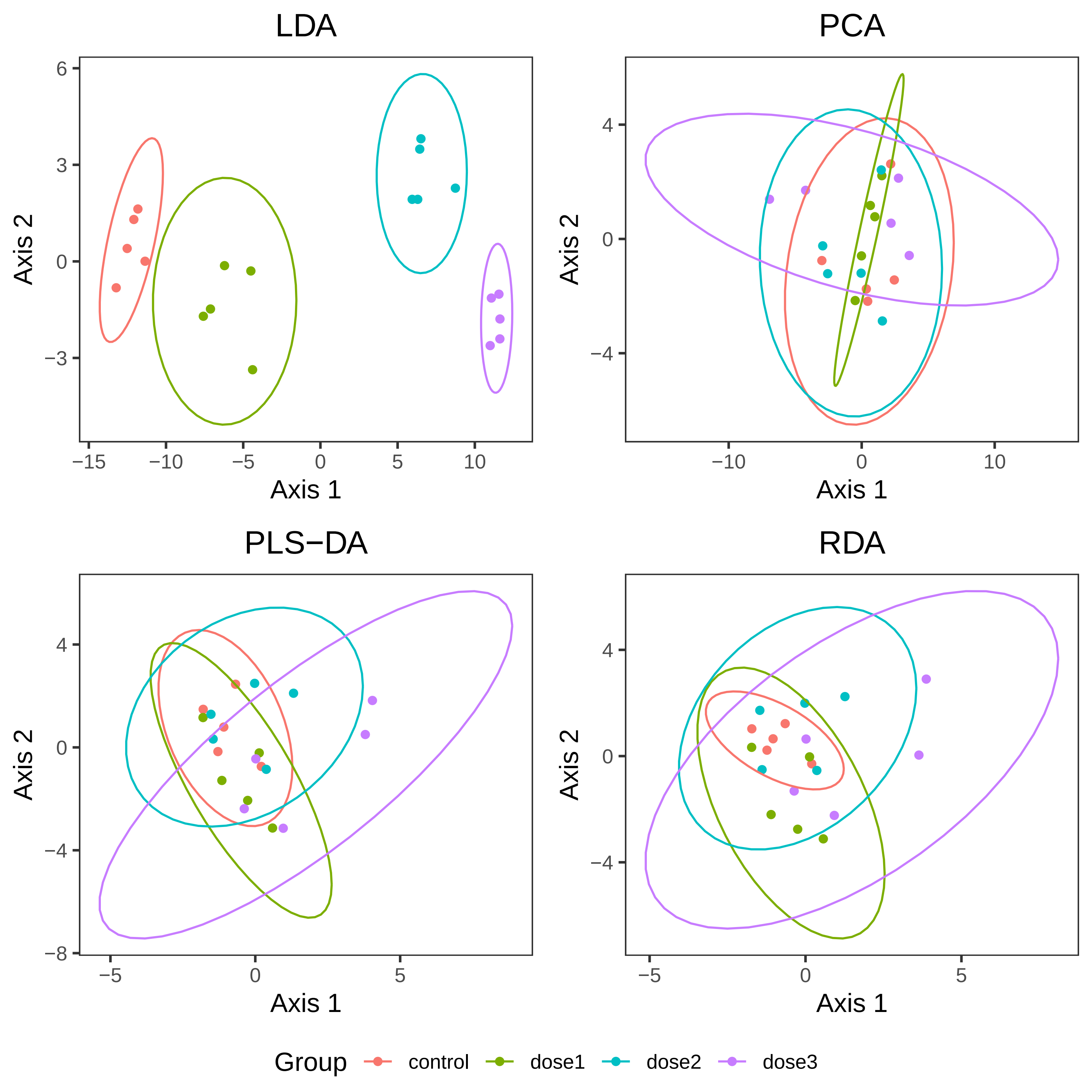

LDA
PCA
PLS−DA
RDA
Axis 1
Axis 2
−15
−10
−5
0
5
10
6
3
−3
4
−4
−8
Group
control
dose1
dose2
dose3

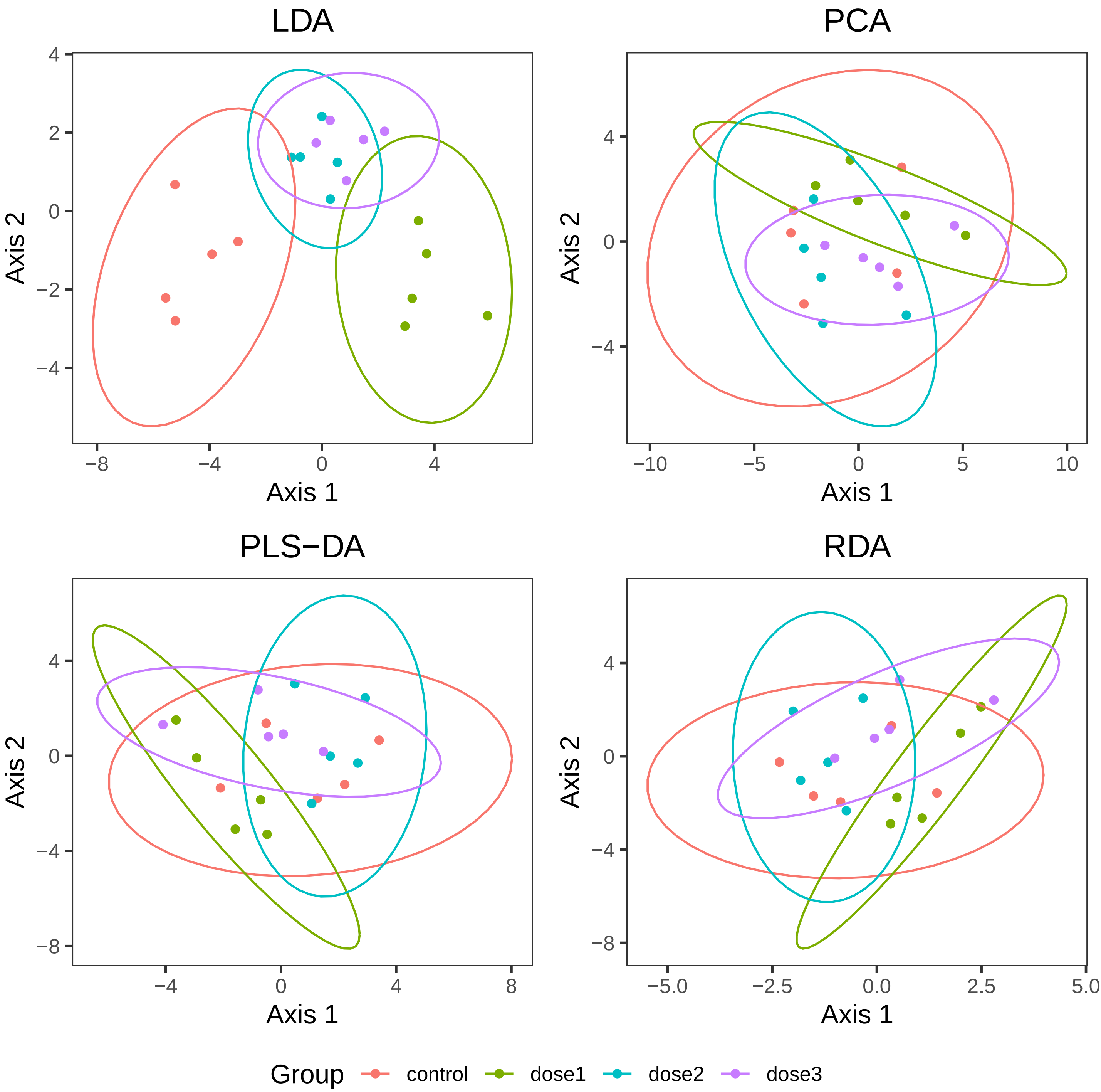
LDA
PCA
PLS−DA
RDA
Axis 1
Axis 2
Group
control
dose1
dose2
dose3

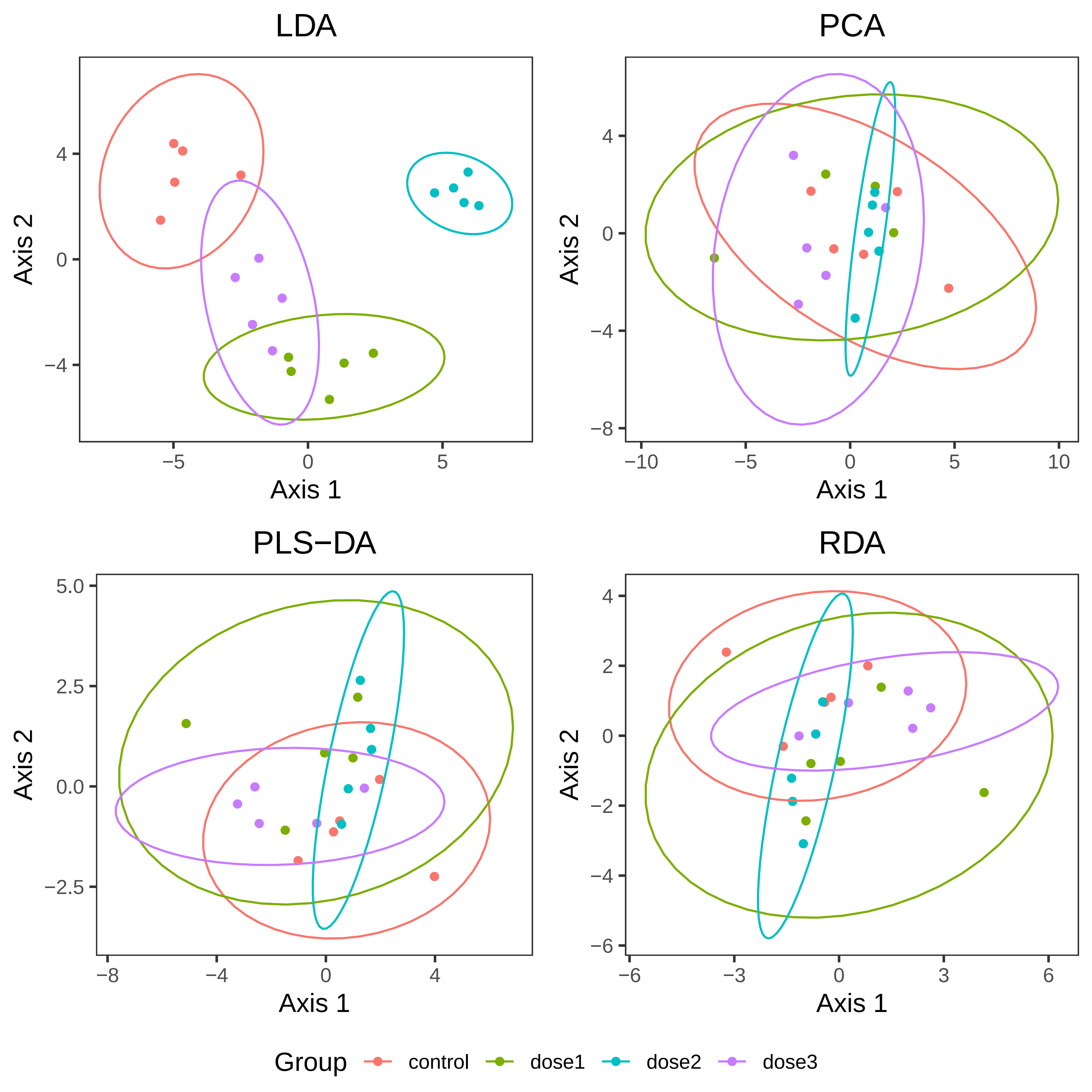
LDA
PCA
PLS−DA
RDA
Axis 1
Axis 2
Group
control
dose1
dose2
dose3

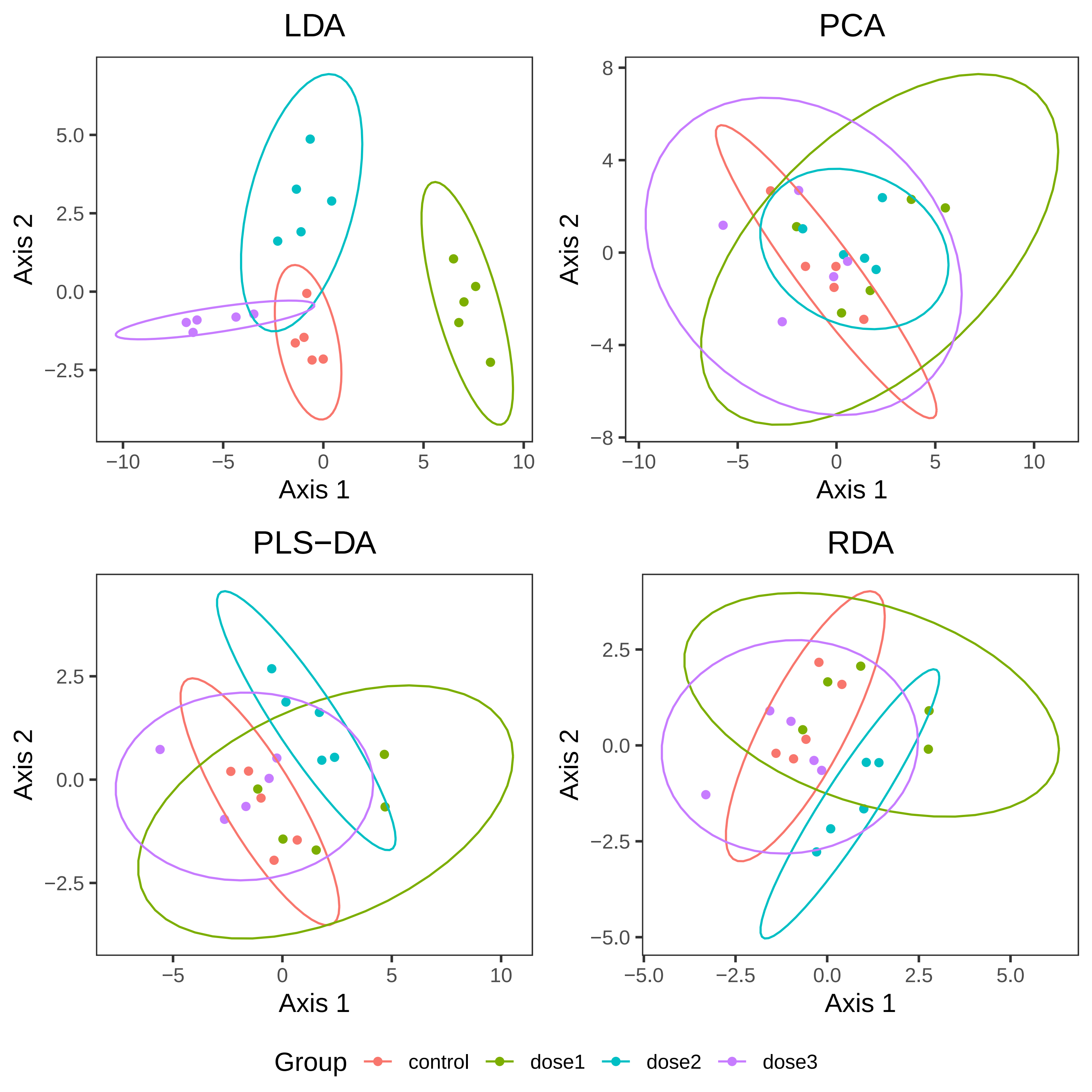

LDA
PCA
PLS−DA
RDA
Axis 1
Axis 2
Group
control
dose1
dose2
dose3

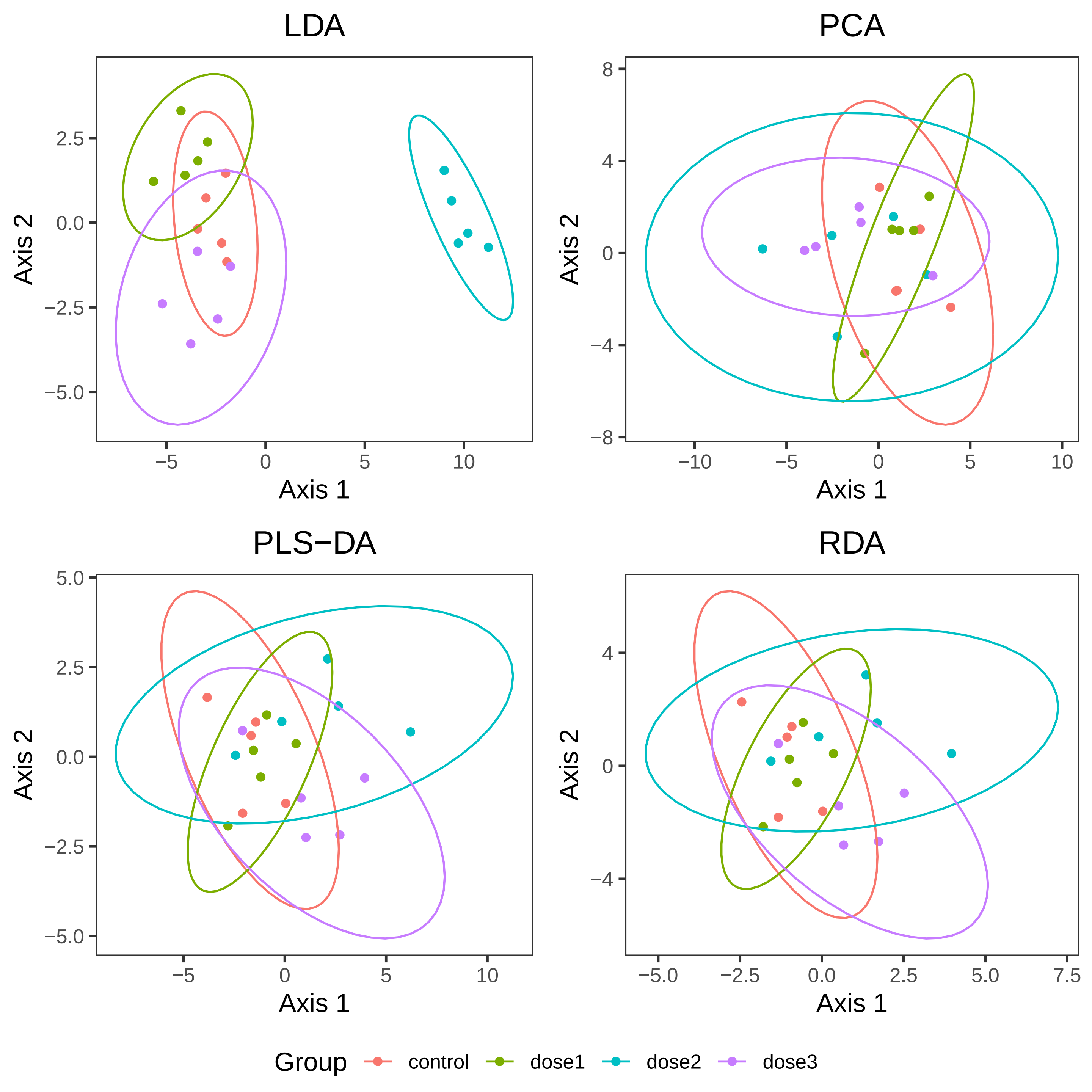
LDA
PCA
PLS−DA
RDA
Axis 1
Axis 2
Group
control
dose1
dose2
dose3

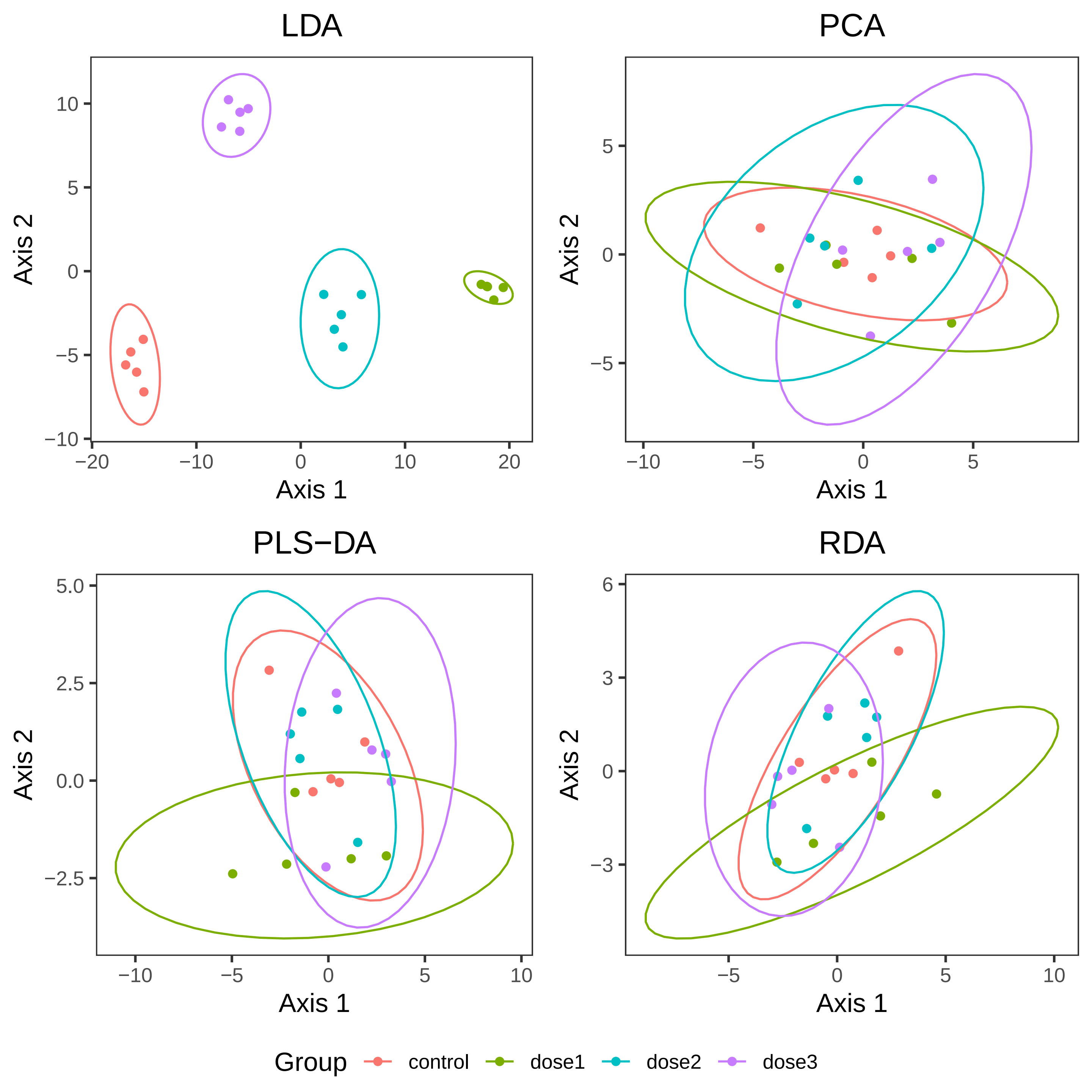

LDA
PCA
PLS−DA
RDA
Axis 1
Axis 2
Group
control
dose1
dose2
dose3

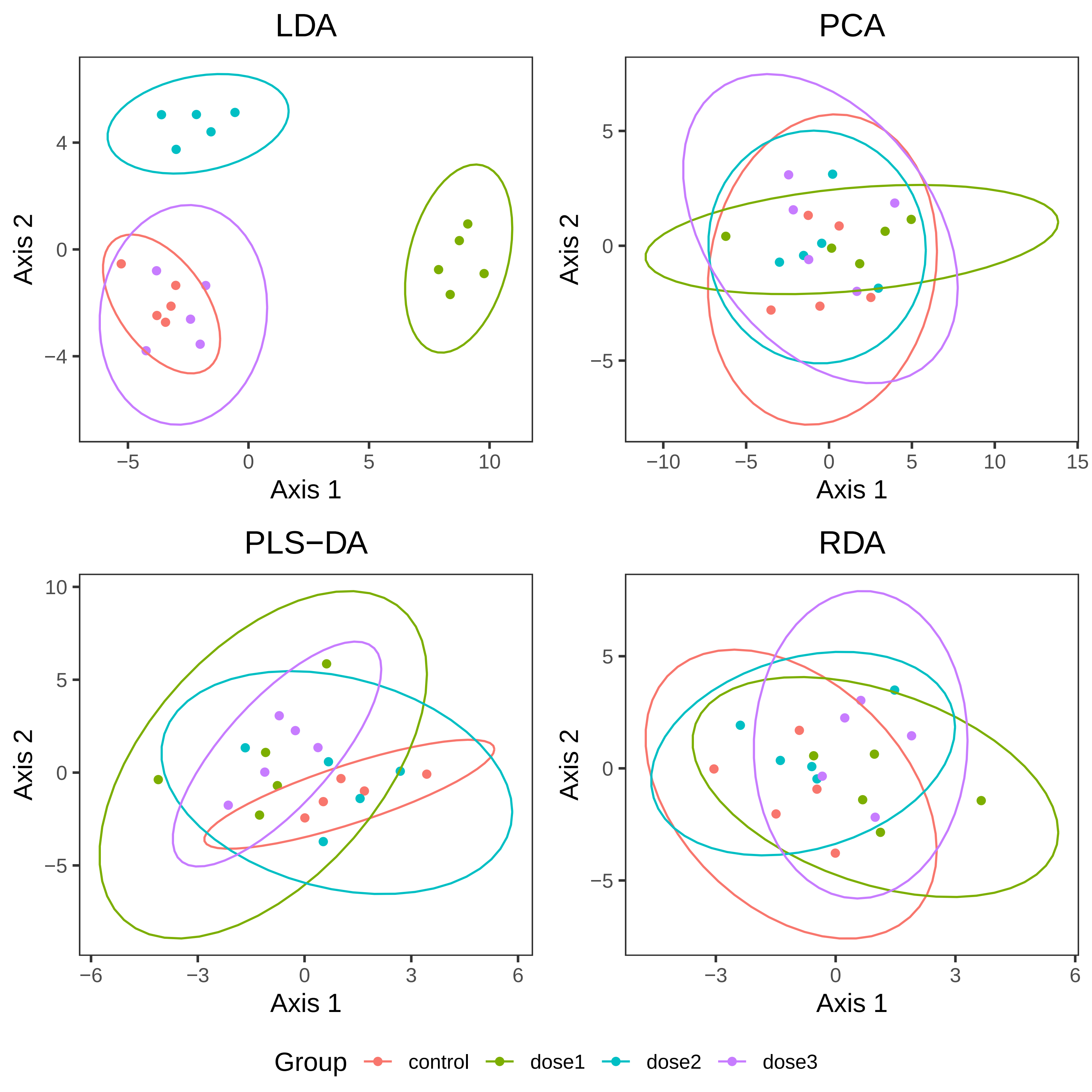

LDA
PCA
PLS−DA
RDA
Axis 1
Axis 2
Group
control
dose1
dose2
dose3

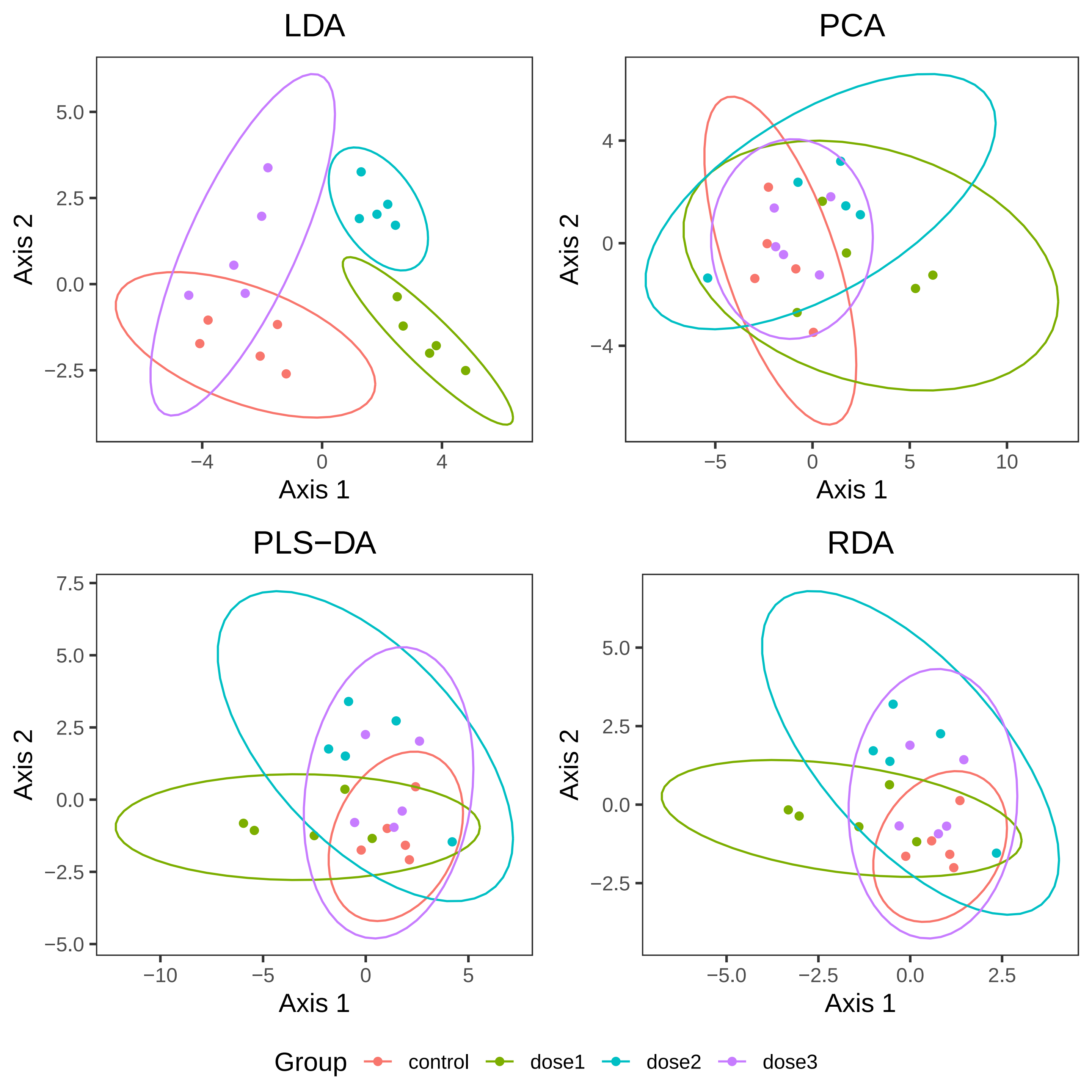
LDA
PCA
PLS−DA
RDA
Axis 1
Axis 2
Group
control
dose1
dose2
dose3

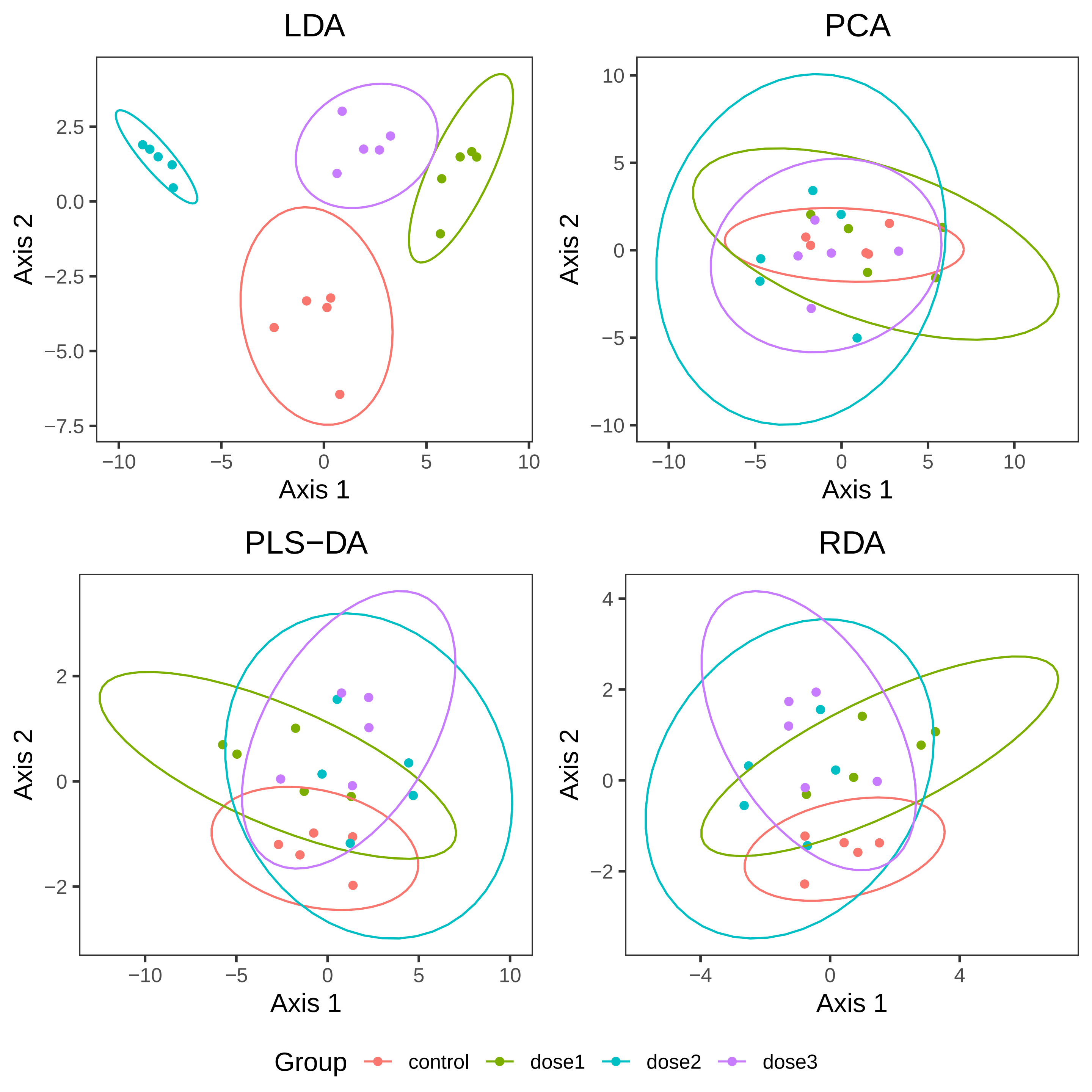
LDA
PCA
PLS−DA
RDA
Axis 1
Axis 2
2.5
0.0
−2.5
−5.0
−7.5
−10
−5
0
5
10
Group
control
dose1
dose2
dose3

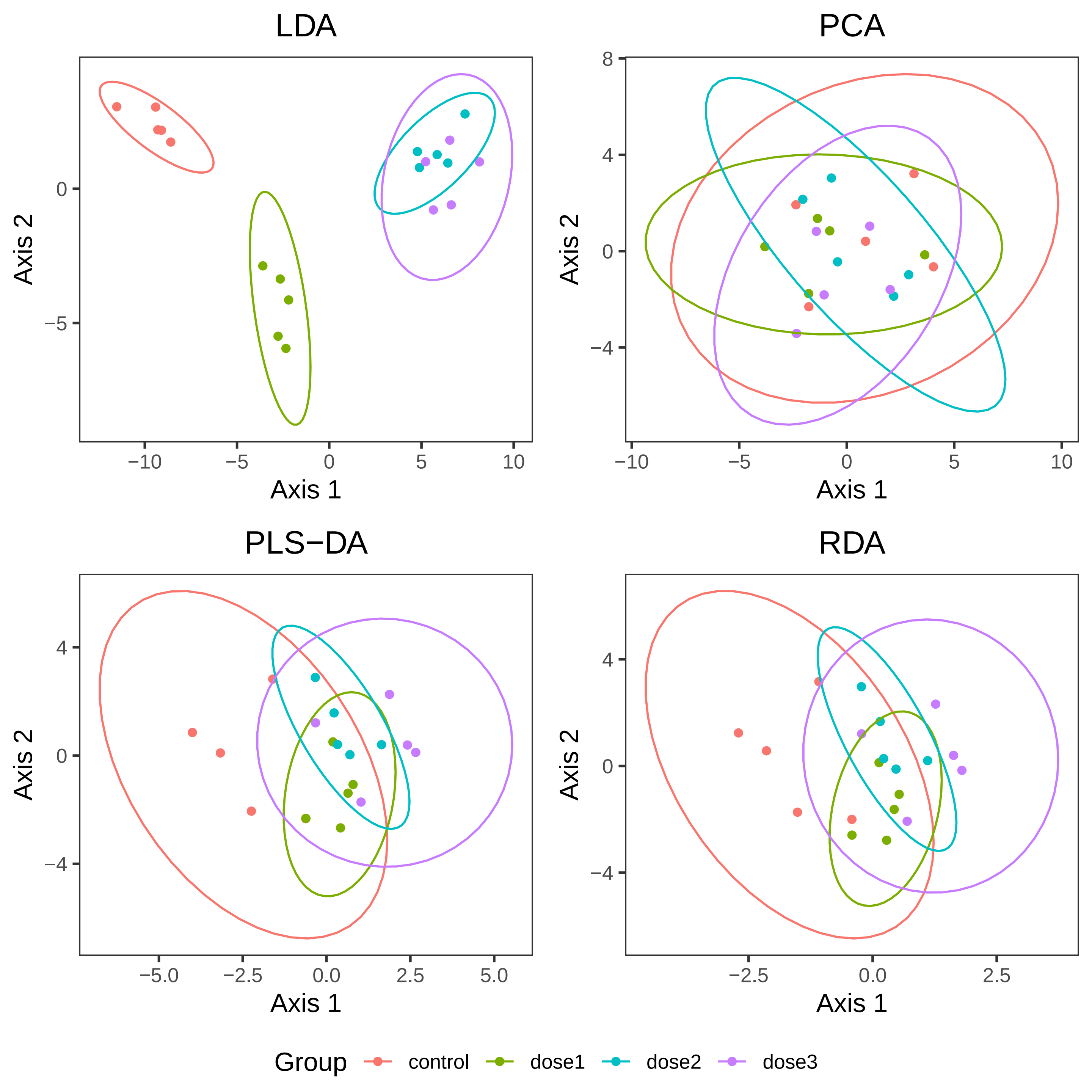
LDA
PCA
PLS−DA
RDA
Axis 1
Axis 2
Group
control
dose1
dose2
dose3

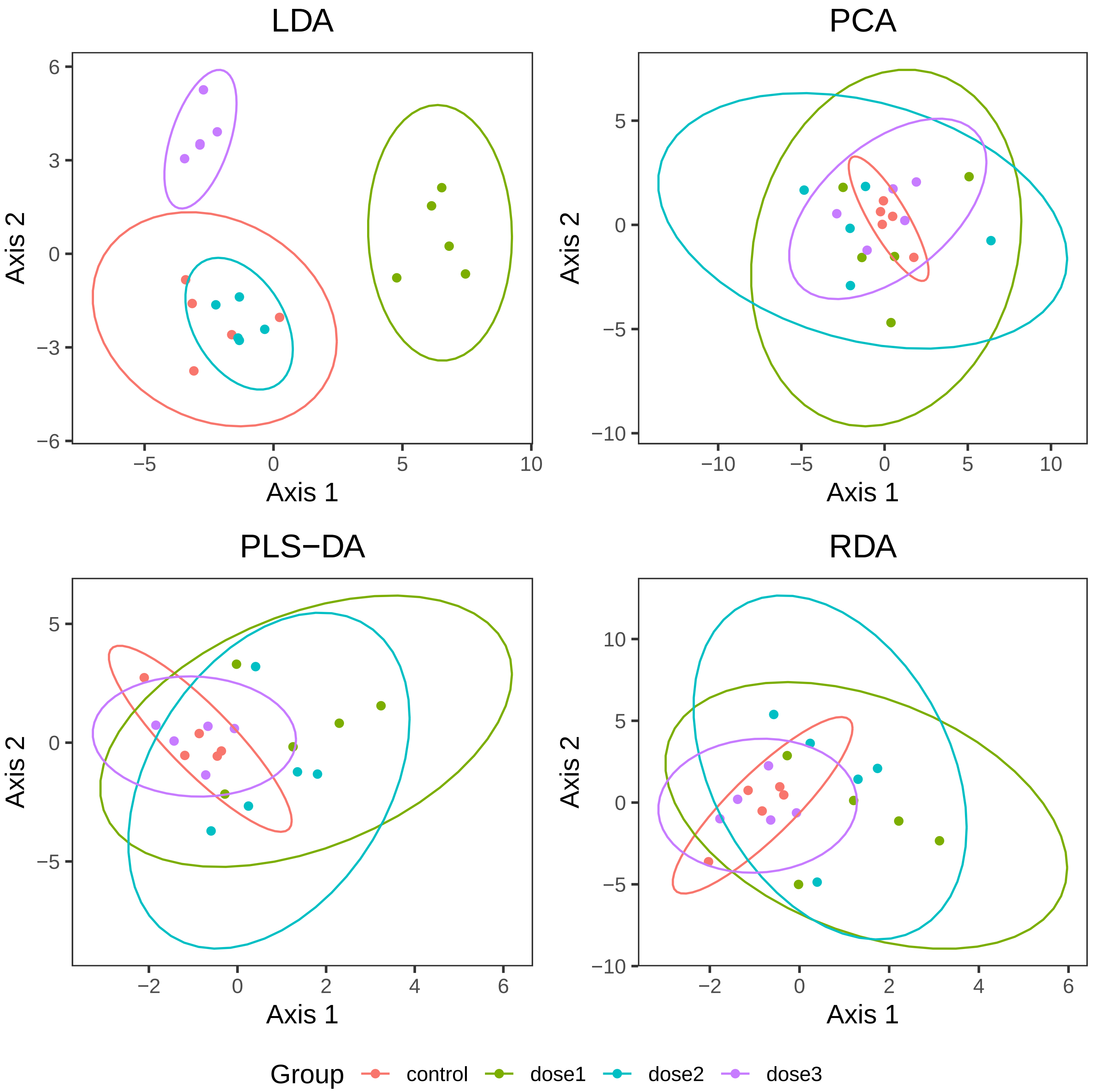

LDA
PCA
PLS−DA
RDA
Axis 1
Axis 2
Group
control
dose1
dose2
dose3

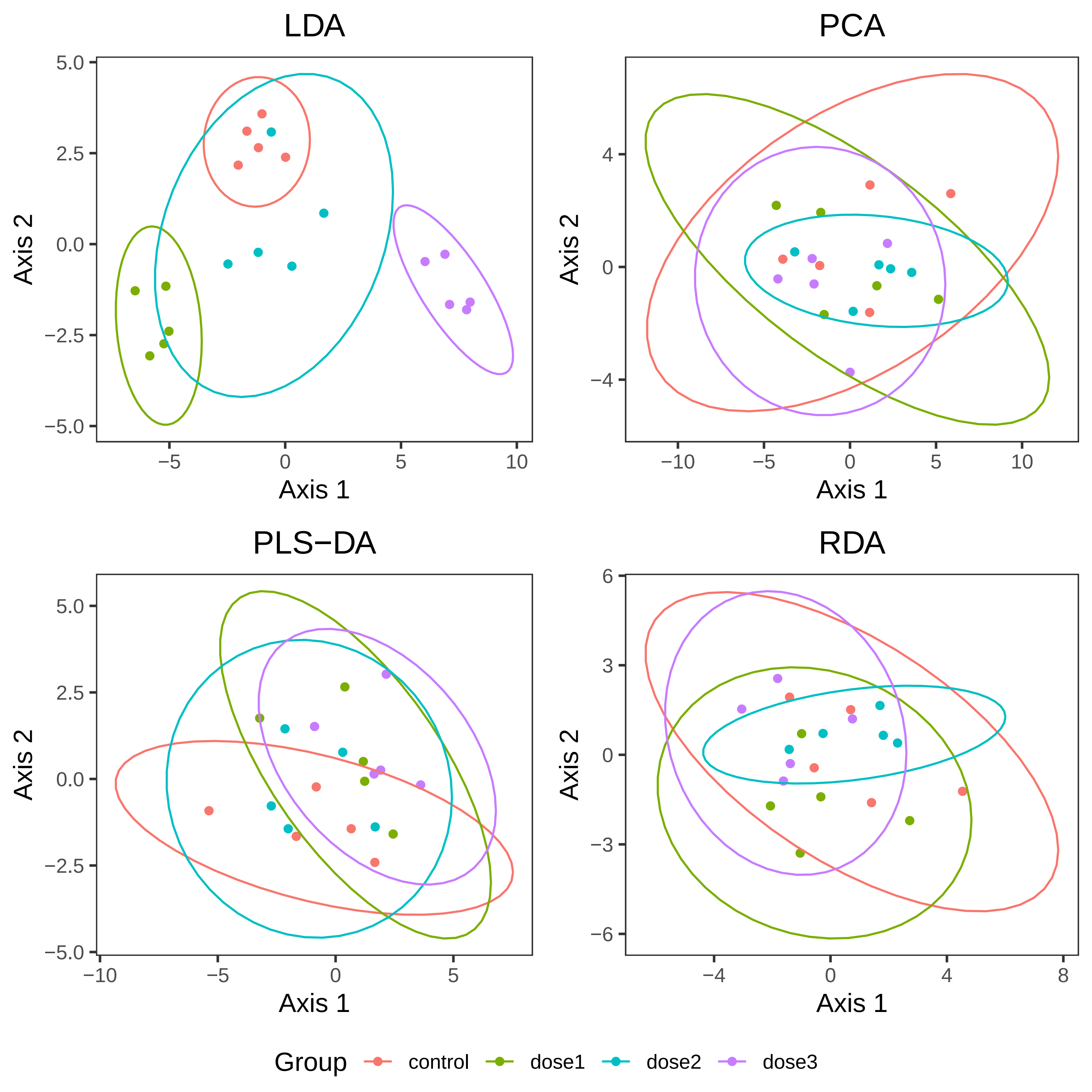

LDA
PCA
PLS−DA
RDA
Axis 1
Axis 2
Group
control
dose1
dose2
dose3

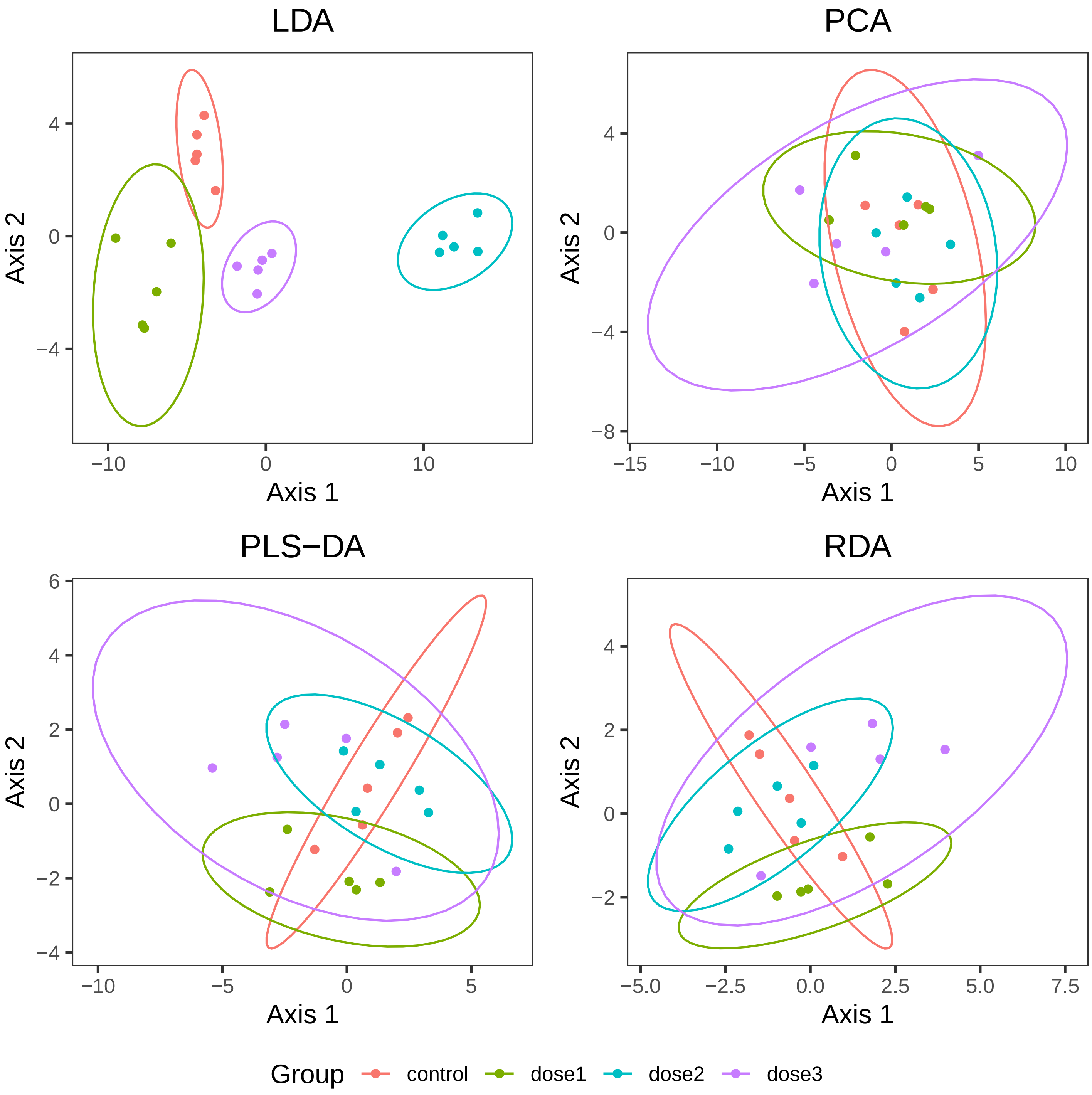
LDA
PCA
PLS−DA
RDA
Axis 1
Axis 2
Group
control
dose1
dose2
dose3

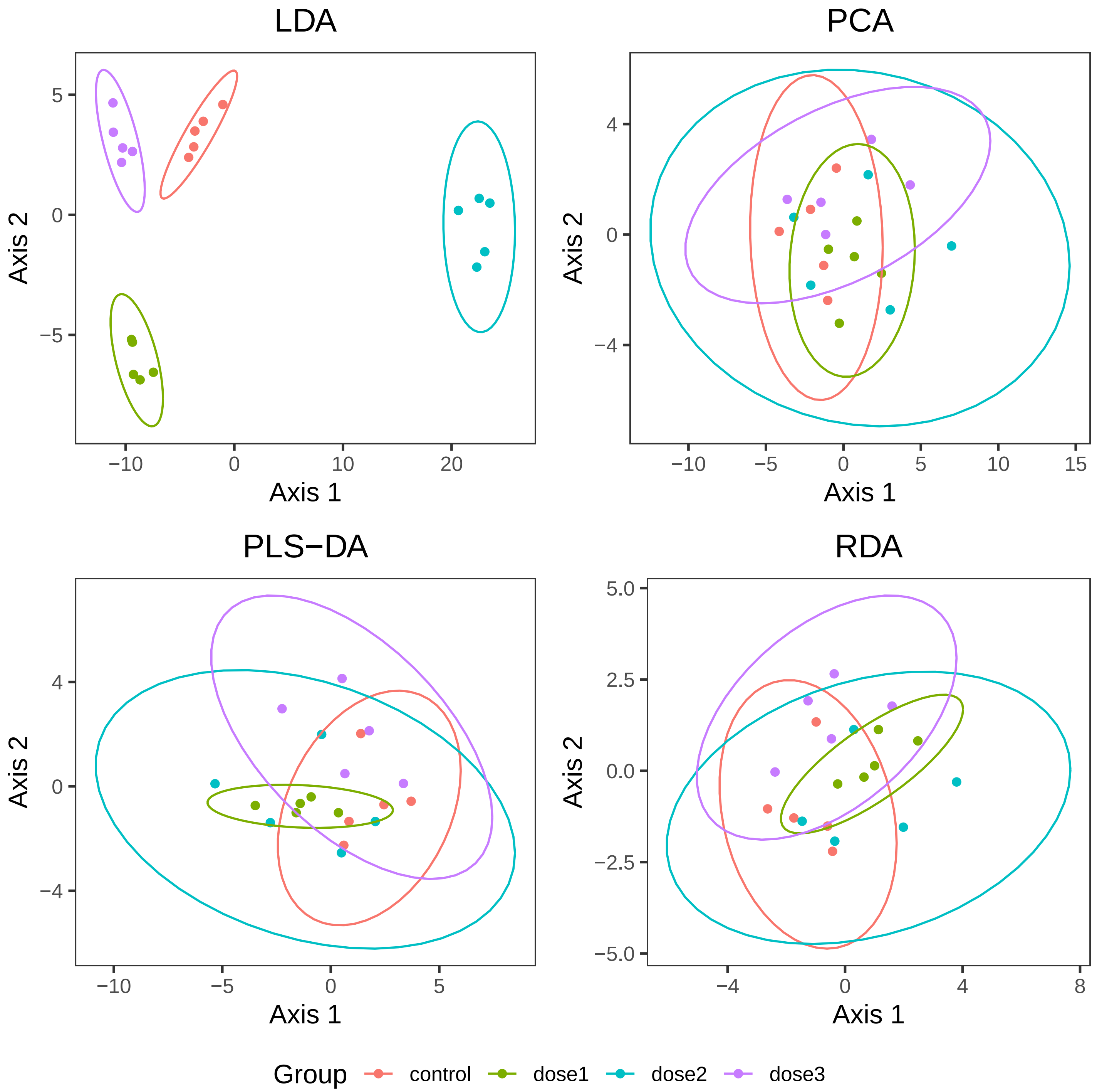

LDA
PCA
PLS−DA
RDA
Axis 1
Axis 2
Group
control
dose1
dose2
dose3

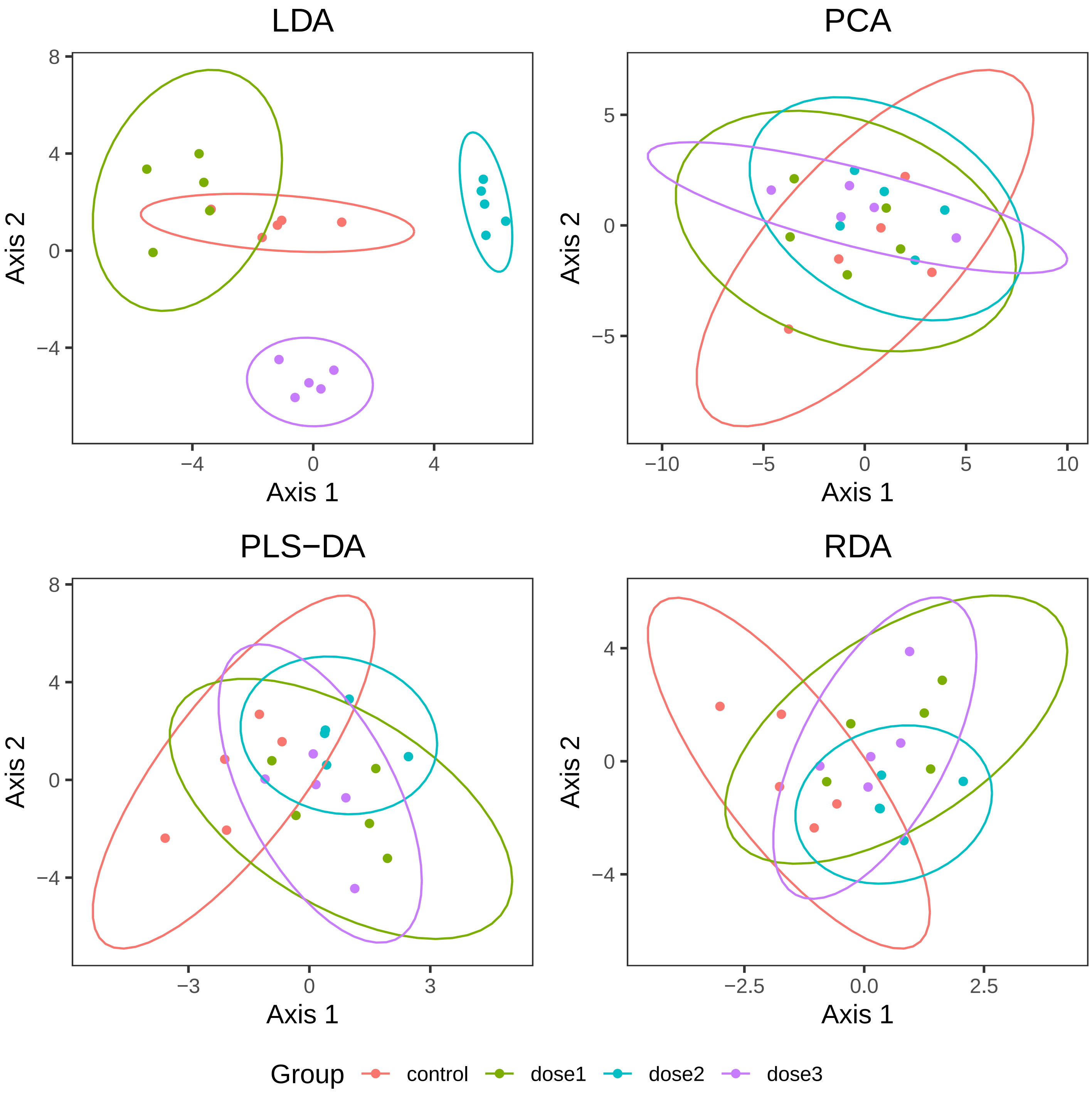

LDA
PCA
PLS−DA
RDA
Axis 1
Axis 2
8
4
0
−4
−10
−5
5
10
−3
3
−2.5
0.0
2.5
Group
control
dose1
dose2
dose3

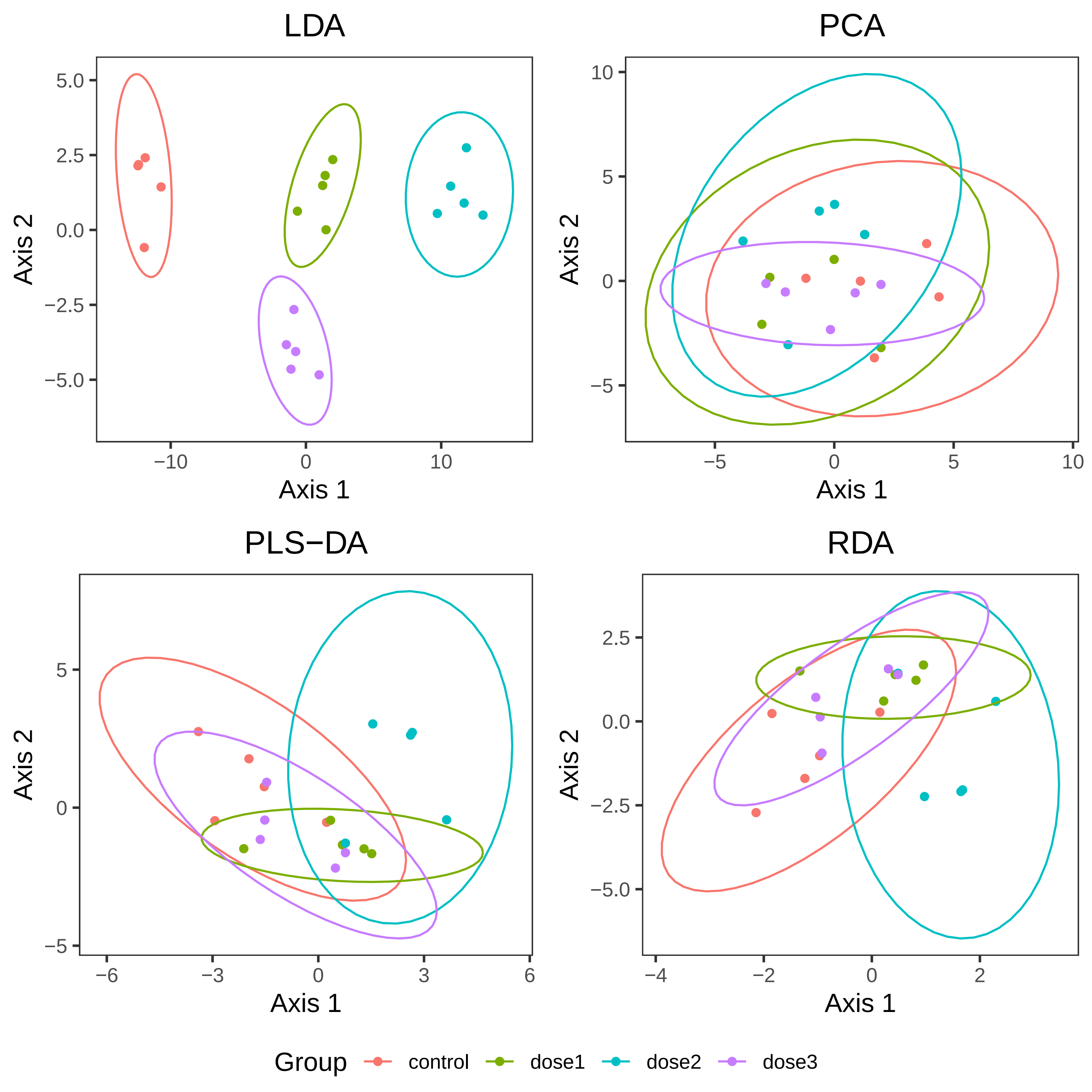

LDA
PCA
PLS−DA
RDA
Axis 1
Axis 2
5.0
2.5
0.0
−2.5
−5.0
−10
0
10
−5
5
−6
−3
3
6
−4
−2
2
Group
control
dose1
dose2
dose3

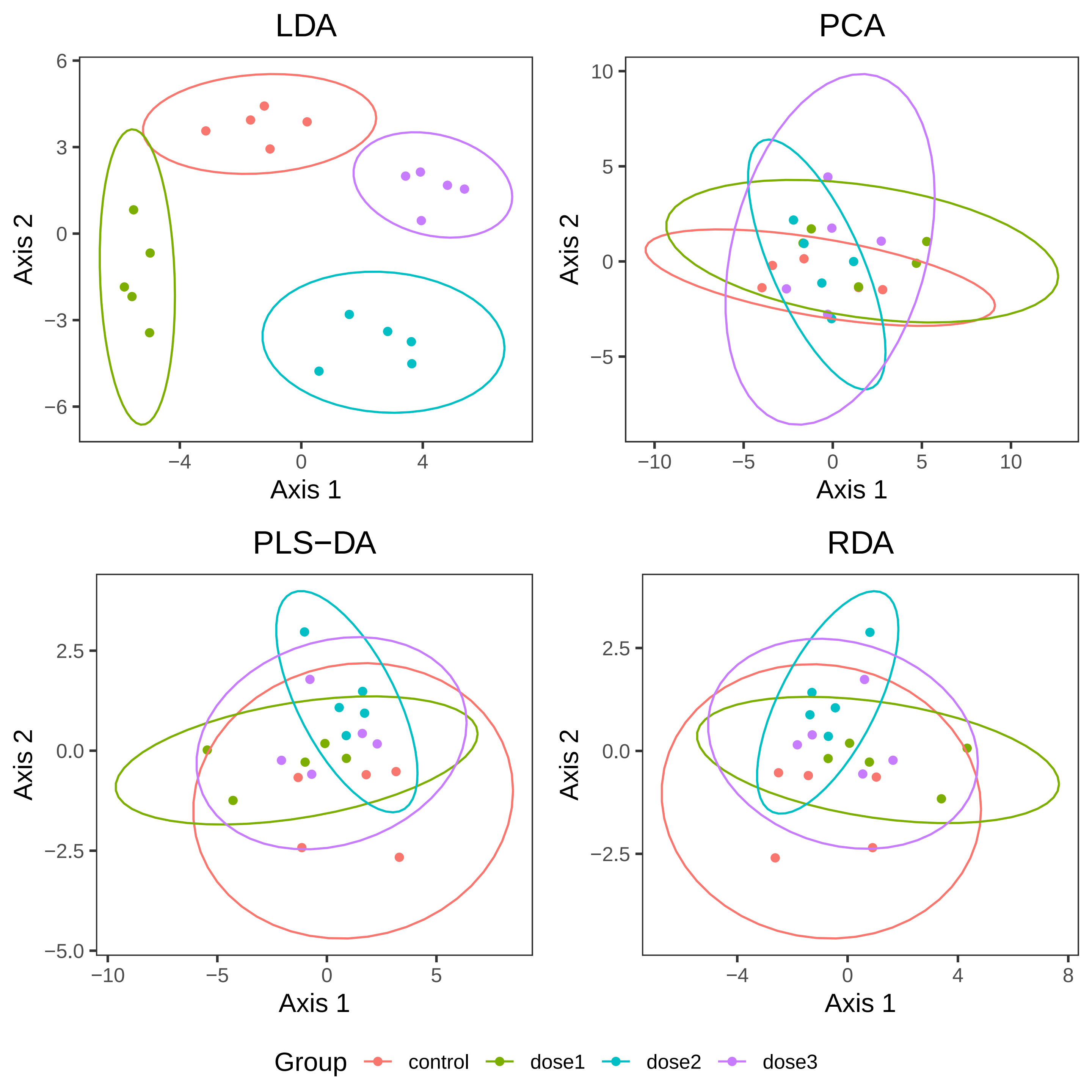

LDA
PCA
PLS−DA
RDA
Axis 1
Axis 2
Group
control
dose1
dose2
dose3

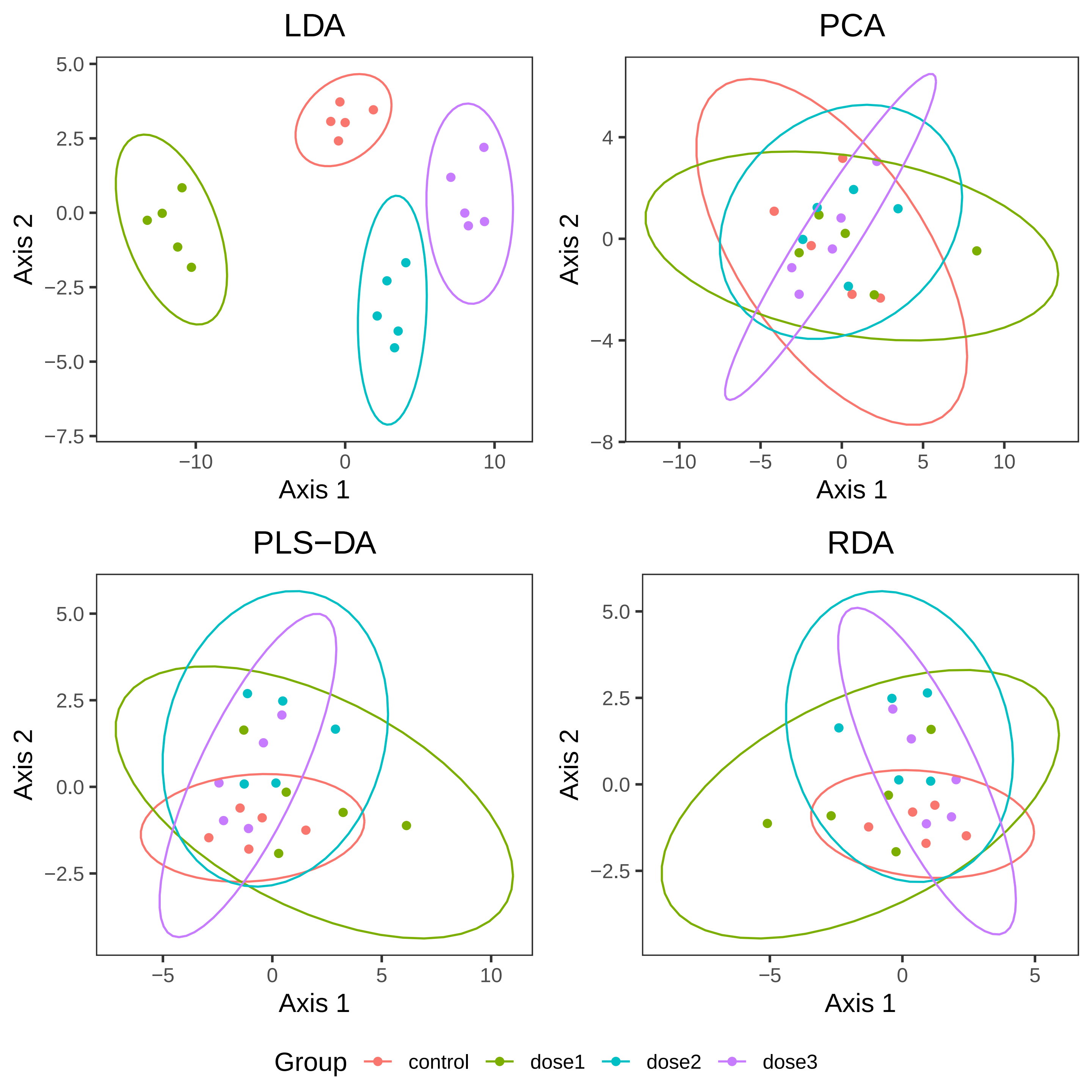

LDA
PCA
PLS−DA
RDA
Axis 1
Axis 2
Group
control
dose1
dose2
dose3

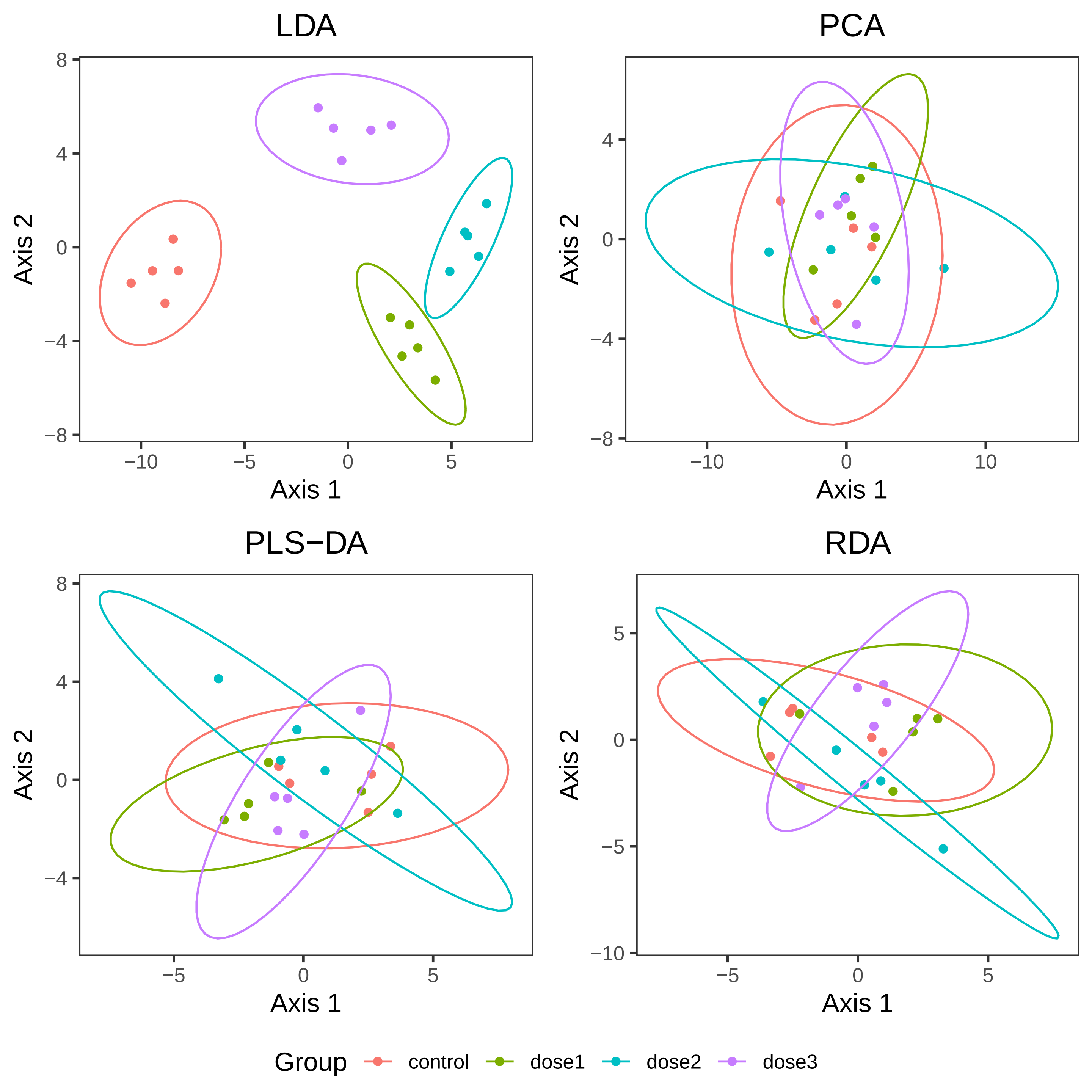

LDA
PCA
PLS−DA
RDA
Axis 1
Axis 2
8
4
0
−4
−8
−10
−5
0
5
10
Group
control
dose1
dose2
dose3

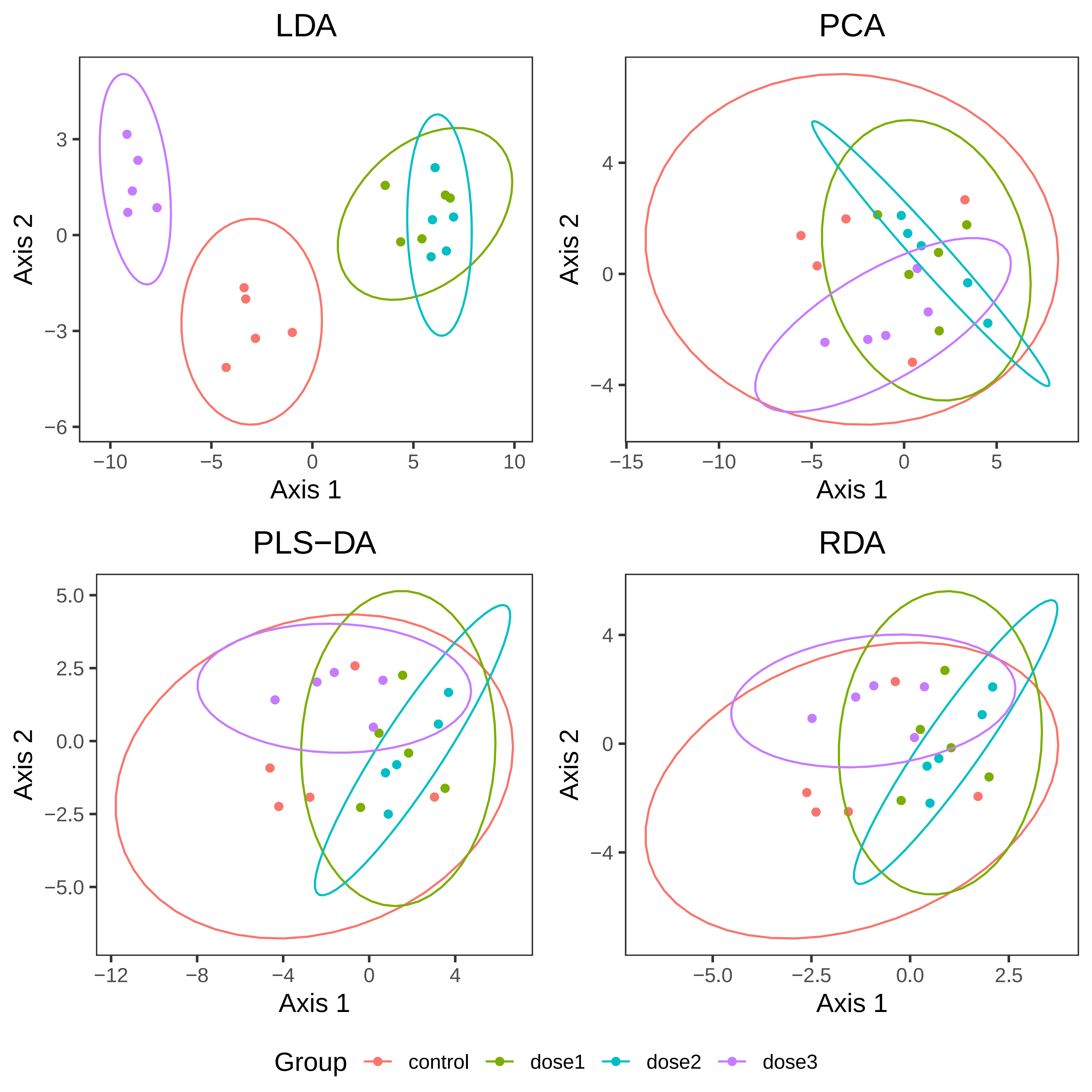

LDA
PCA
PLS−DA
RDA
Axis 1
Axis 2
Group
control
dose1
dose2
dose3

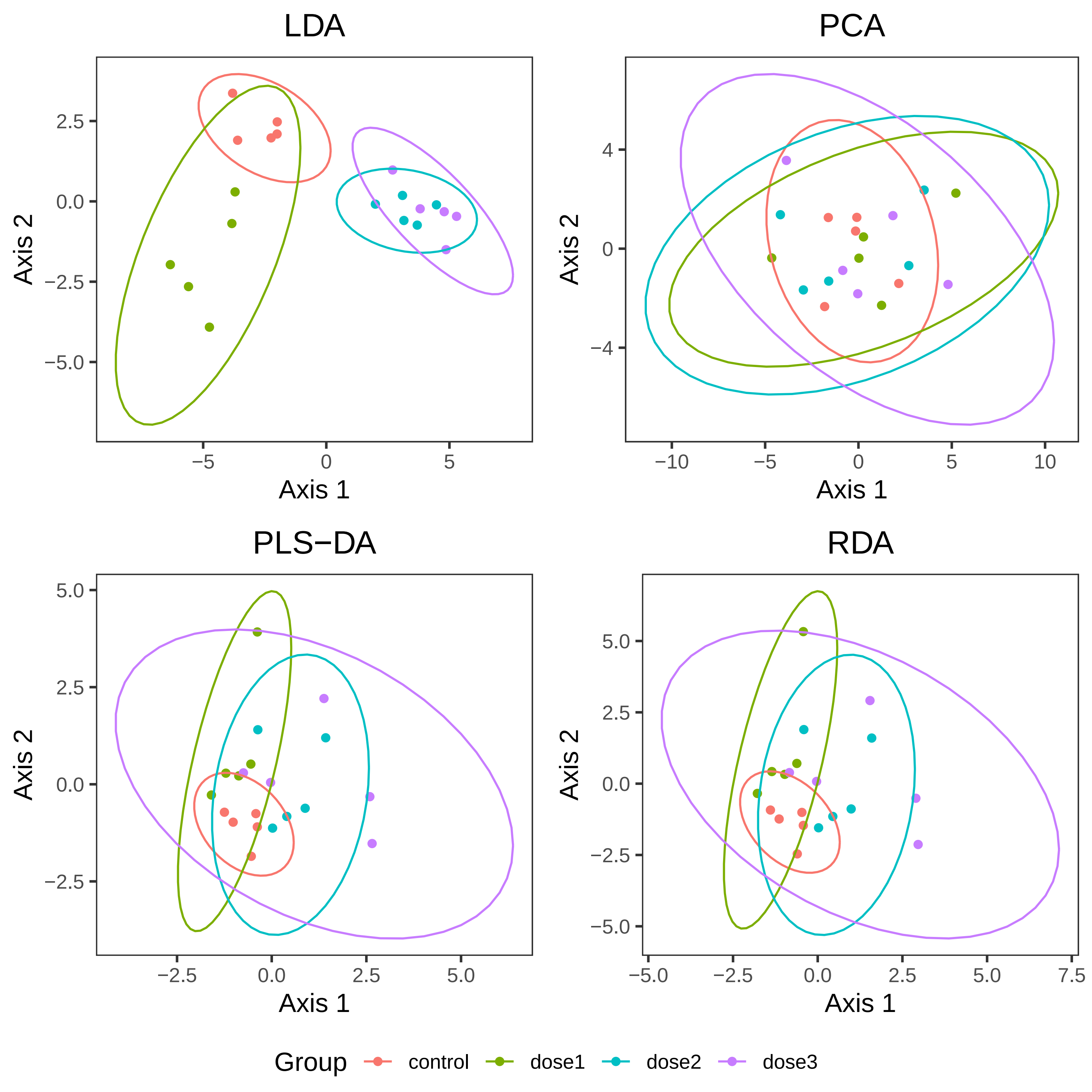

LDA
PCA
PLS−DA
RDA
Axis 1
Axis 2
Group
control
dose1
dose2
dose3

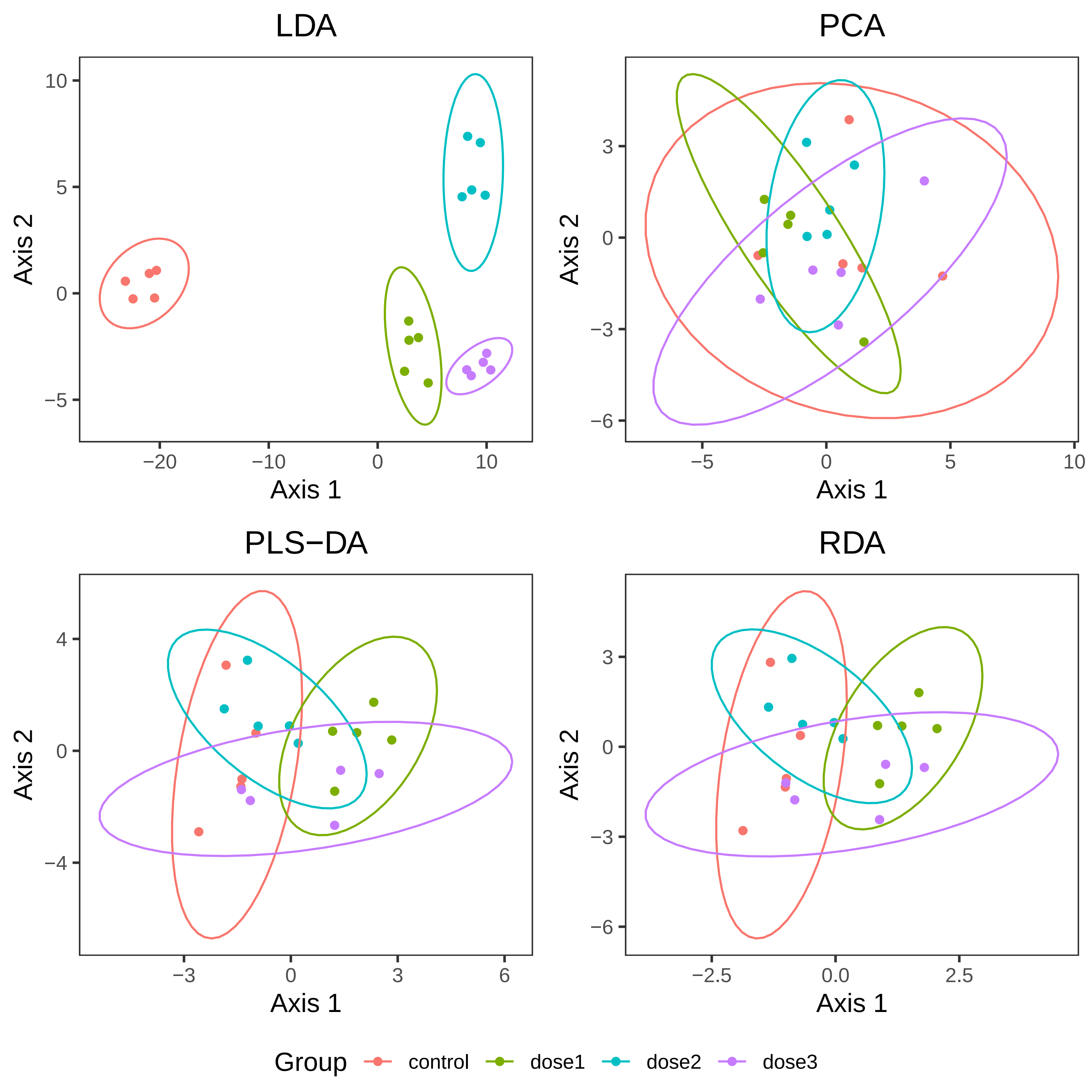

LDA
PCA
PLS−DA
RDA
Axis 1
Axis 2
Group
control
dose1
dose2
dose3

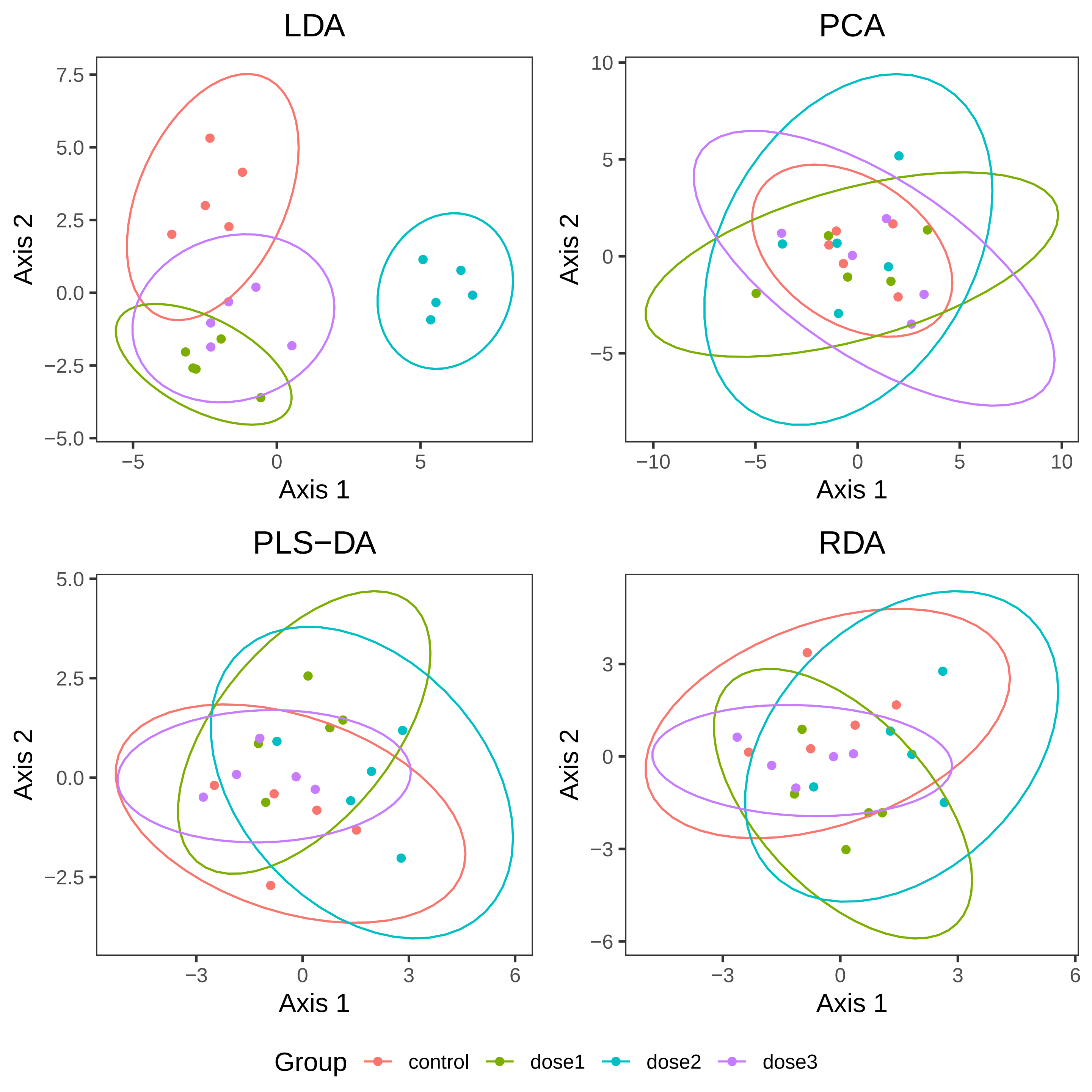

LDA
PCA
PLS−DA
RDA
Axis 1
Axis 2
Group
control
dose1
dose2
dose3

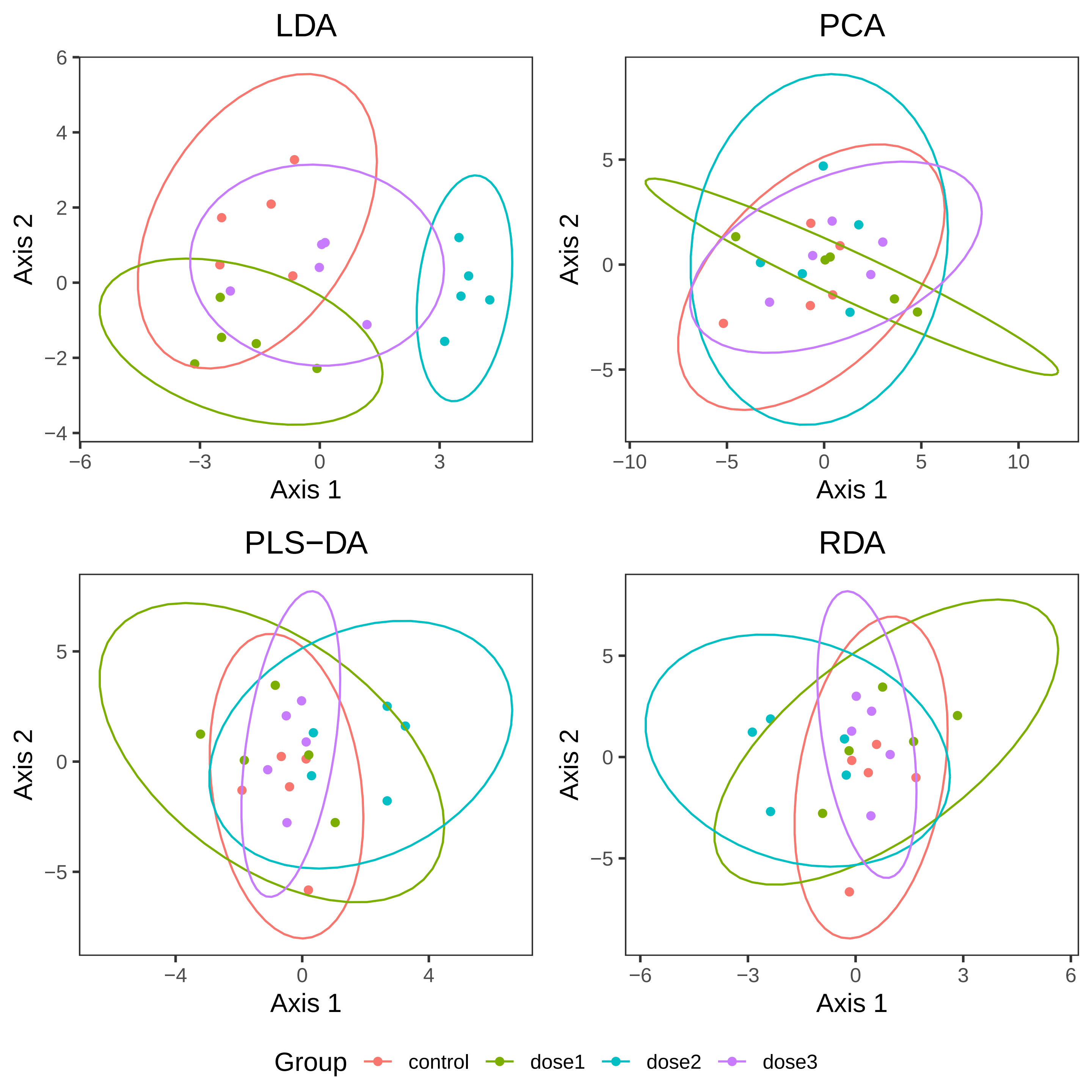
LDA
PCA
PLS−DA
RDA
Axis 1
Axis 2
Group
control
dose1
dose2
dose3

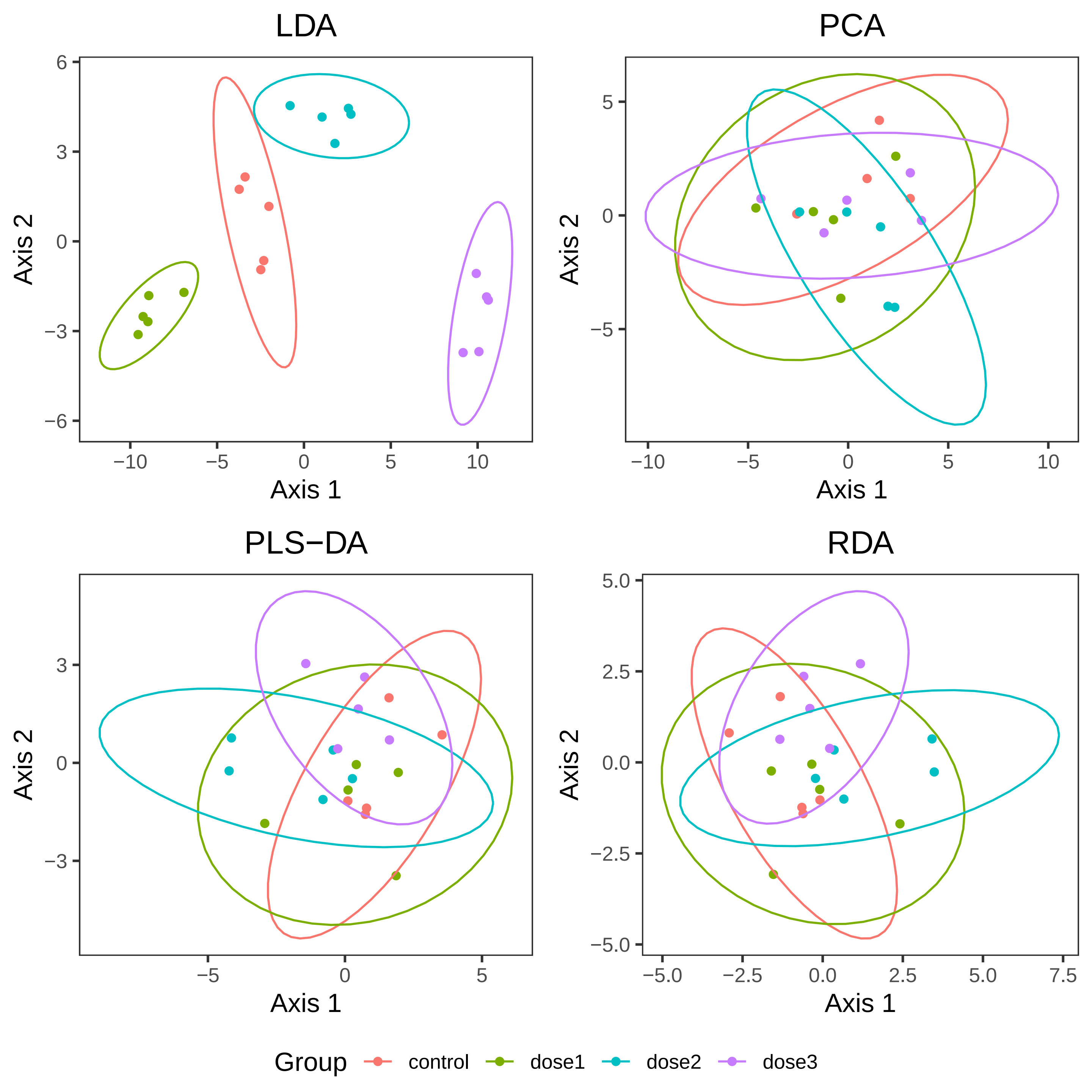

LDA
PCA
PLS−DA
RDA
Axis 1
Axis 2
Group
control
dose1
dose2
dose3

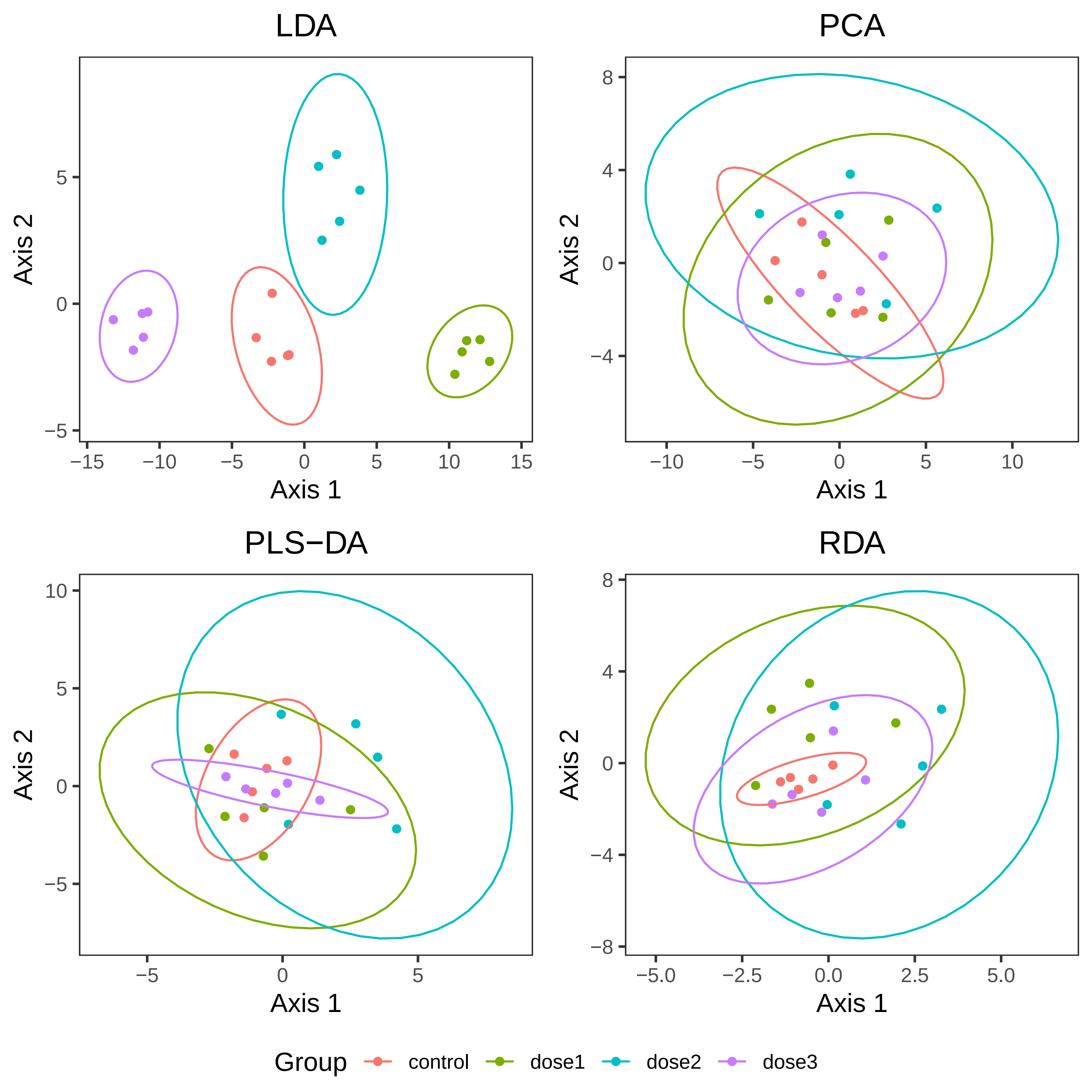
LDA
PCA
PLS−DA
RDA
Axis 1
Axis 2
Group
control
dose1
dose2
dose3

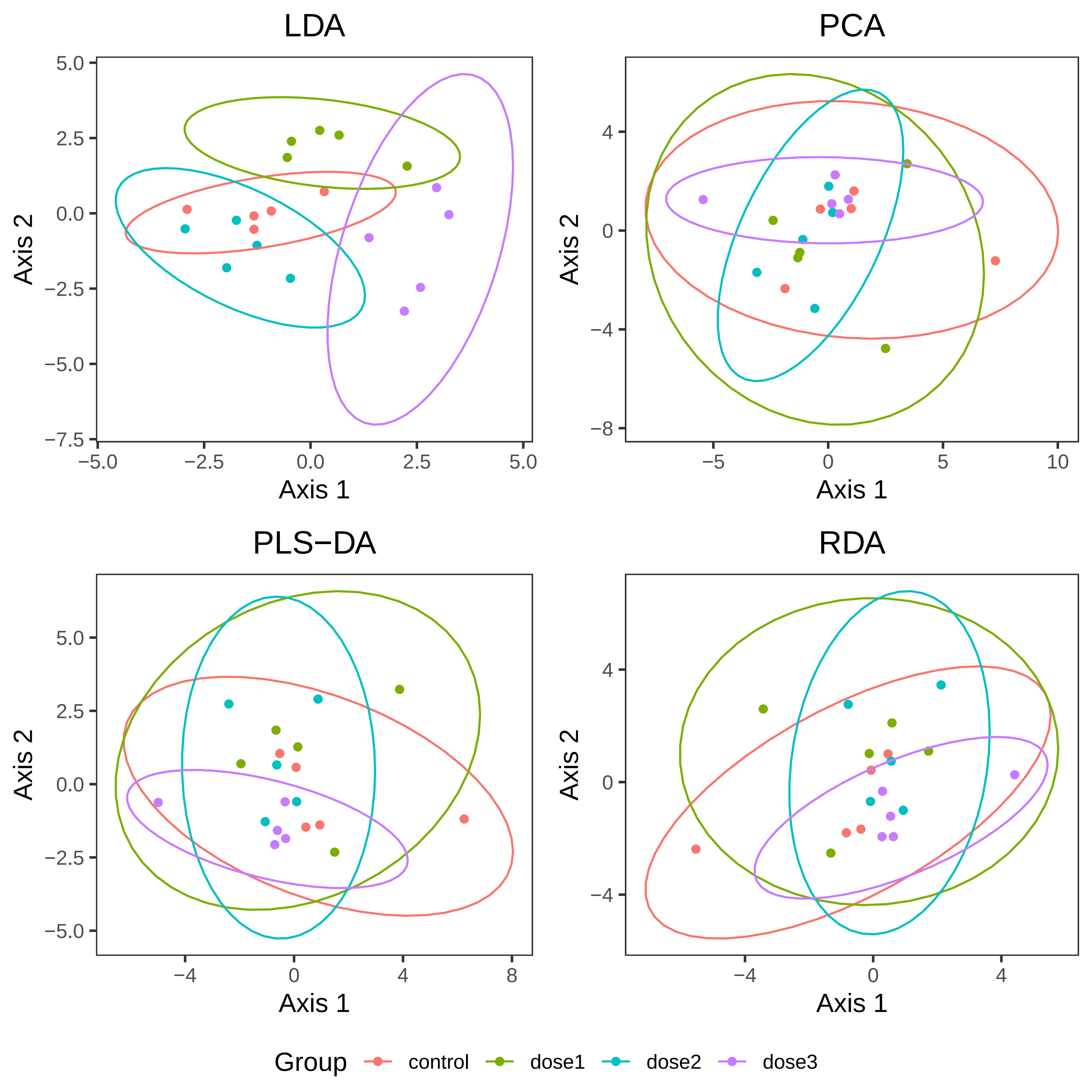

LDA
PCA
PLS−DA
RDA
Axis 1
Axis 2
Group
control
dose1
dose2
dose3

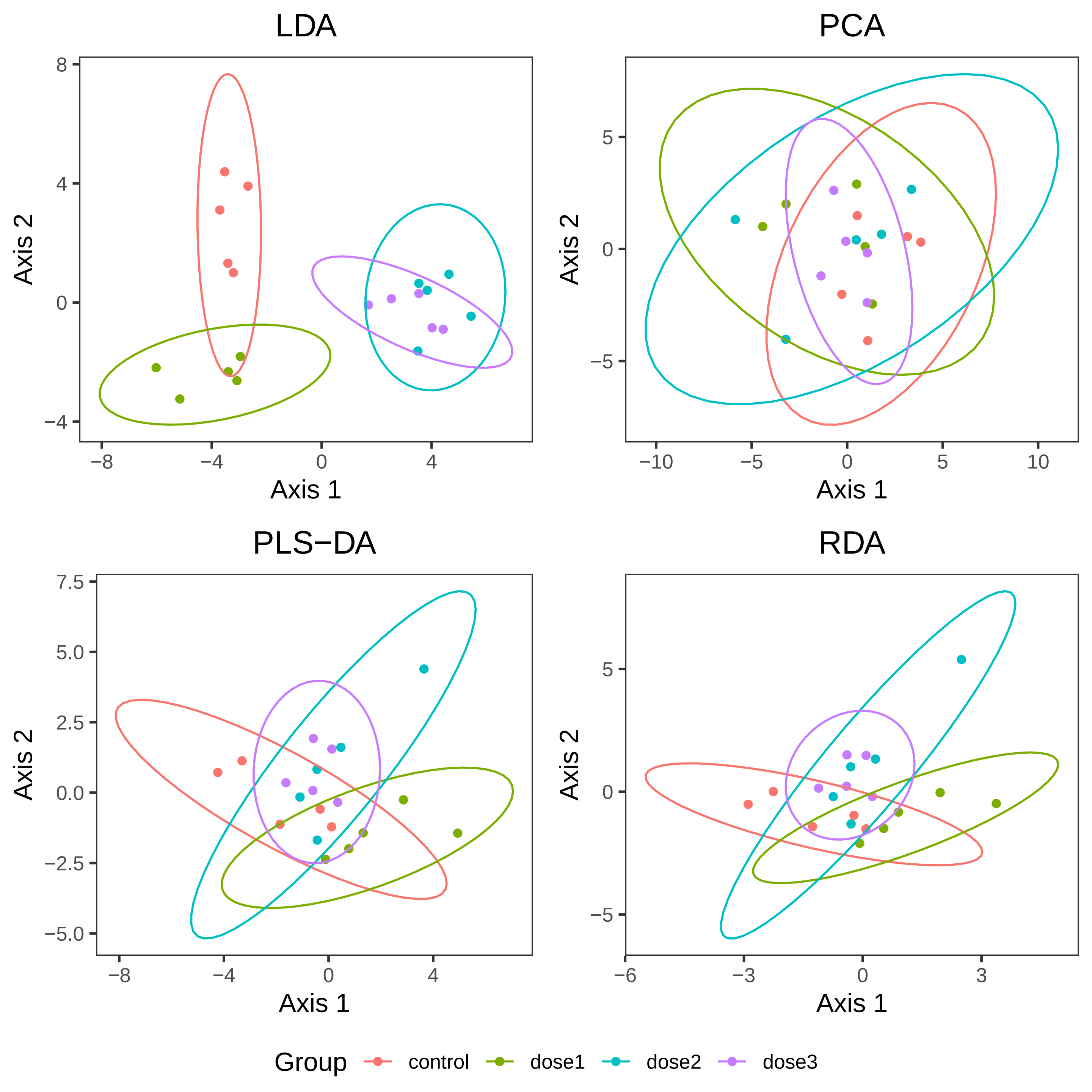

LDA
PCA
PLS−DA
RDA
Axis 1
Axis 2
8
4
0
−4
−8
−4
0
4
5
0
−5
−10
−5
0
5
10
7.5
5.0
2.5
0.0
−2.5
−5.0
−8
−4
0
4
5
0
−5
−6
−3
0
3
Group
control
dose1
dose2
dose3

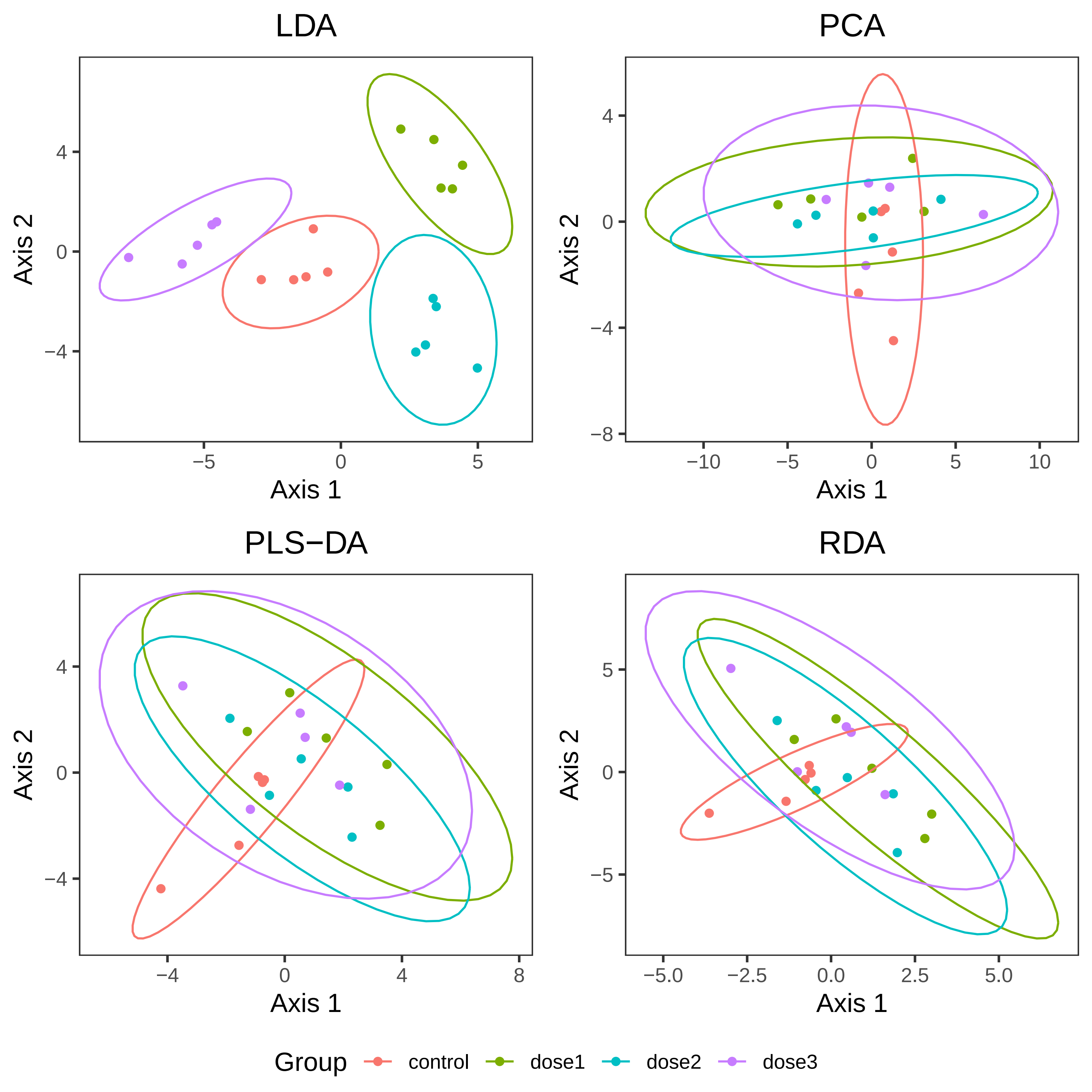

LDA
PCA
PLS−DA
RDA
Axis 1
Axis 2
−5
0
5
4
−4
−10
10
−8
8
−5.0
−2.5
0.0
2.5
5.0
Group
control
dose1
dose2
dose3

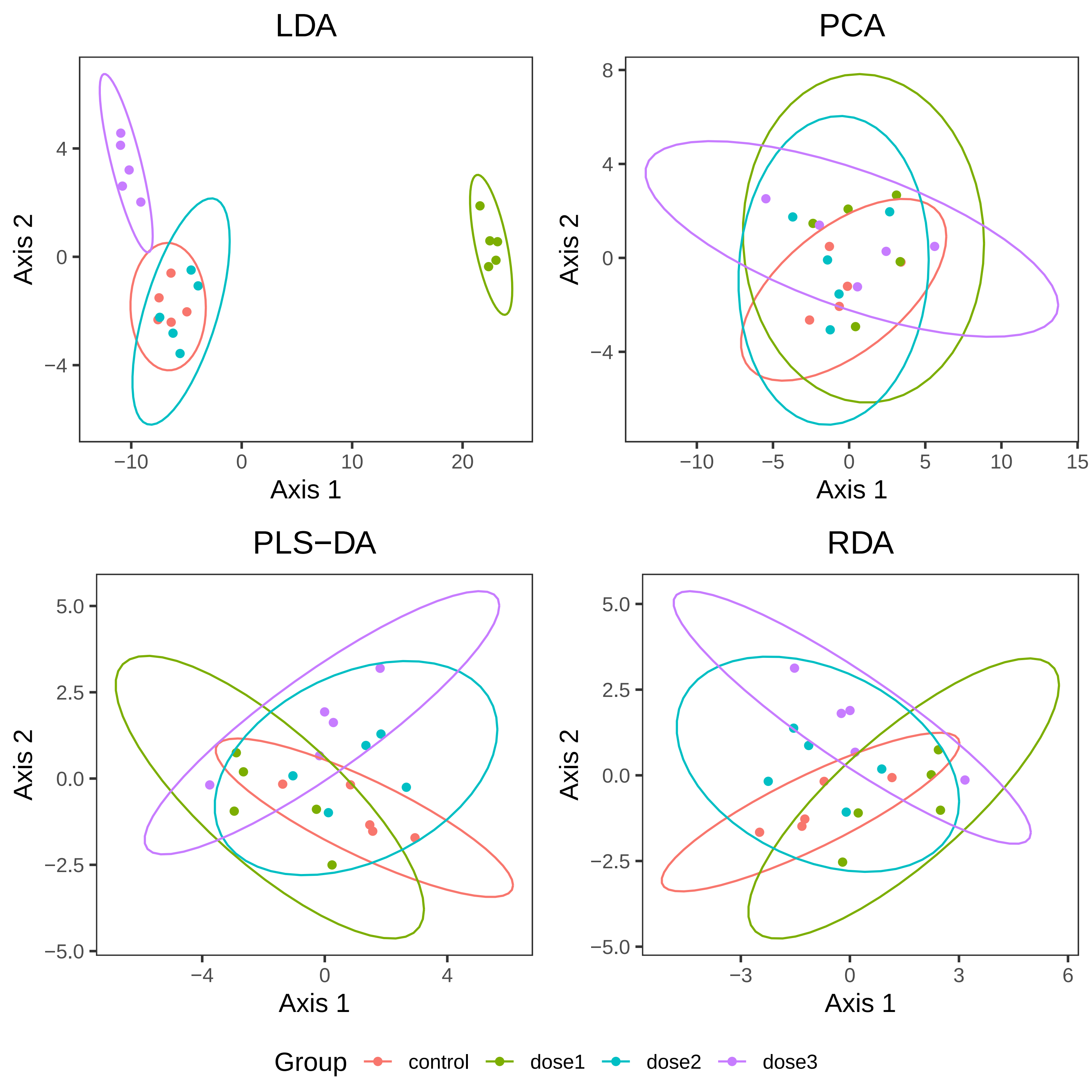

LDA
PCA
PLS−DA
RDA
Axis 1
Axis 2
Group
control
dose1
dose2
dose3

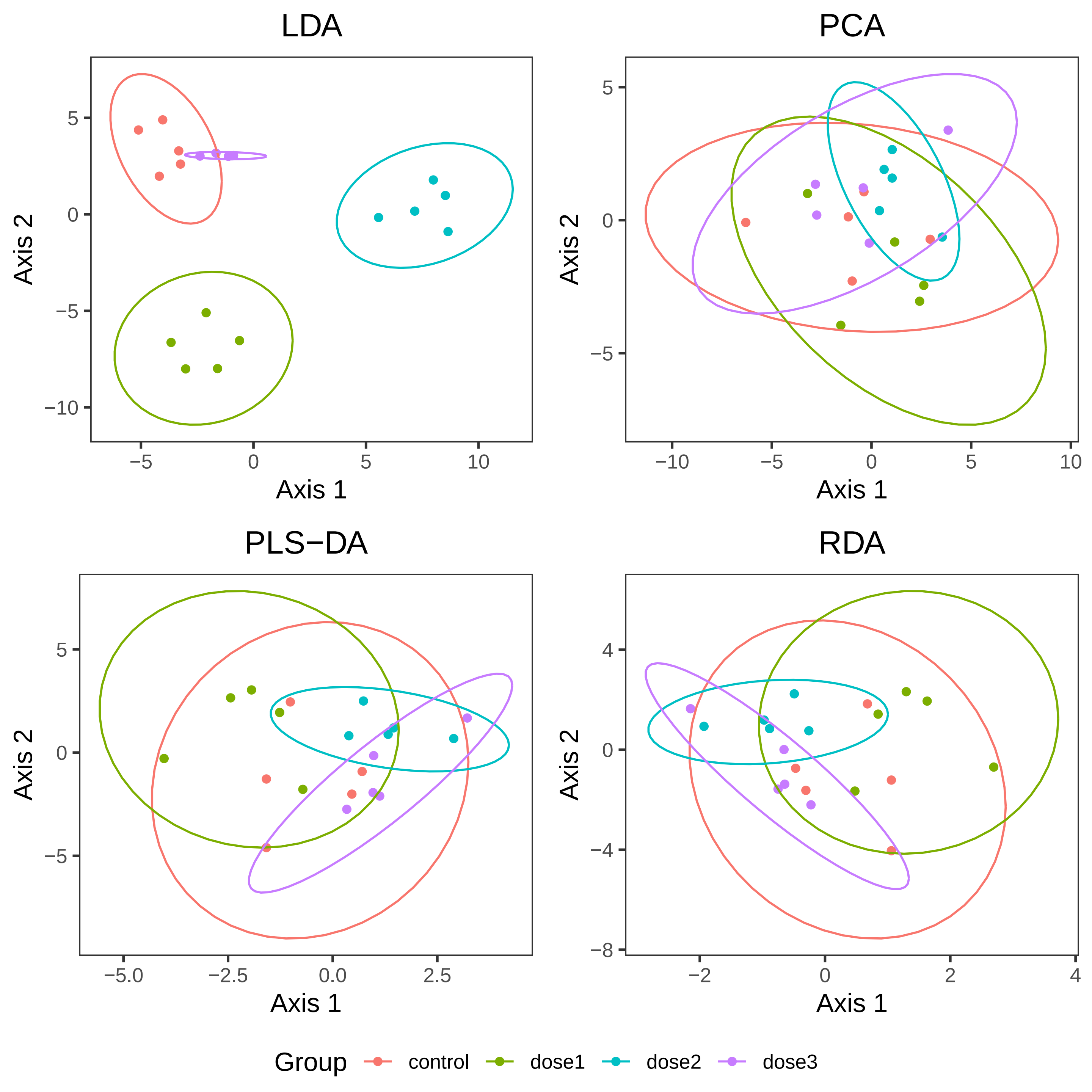

LDA
PCA
PLS−DA
RDA
Axis 1
Axis 2
Group
control
dose1
dose2
dose3

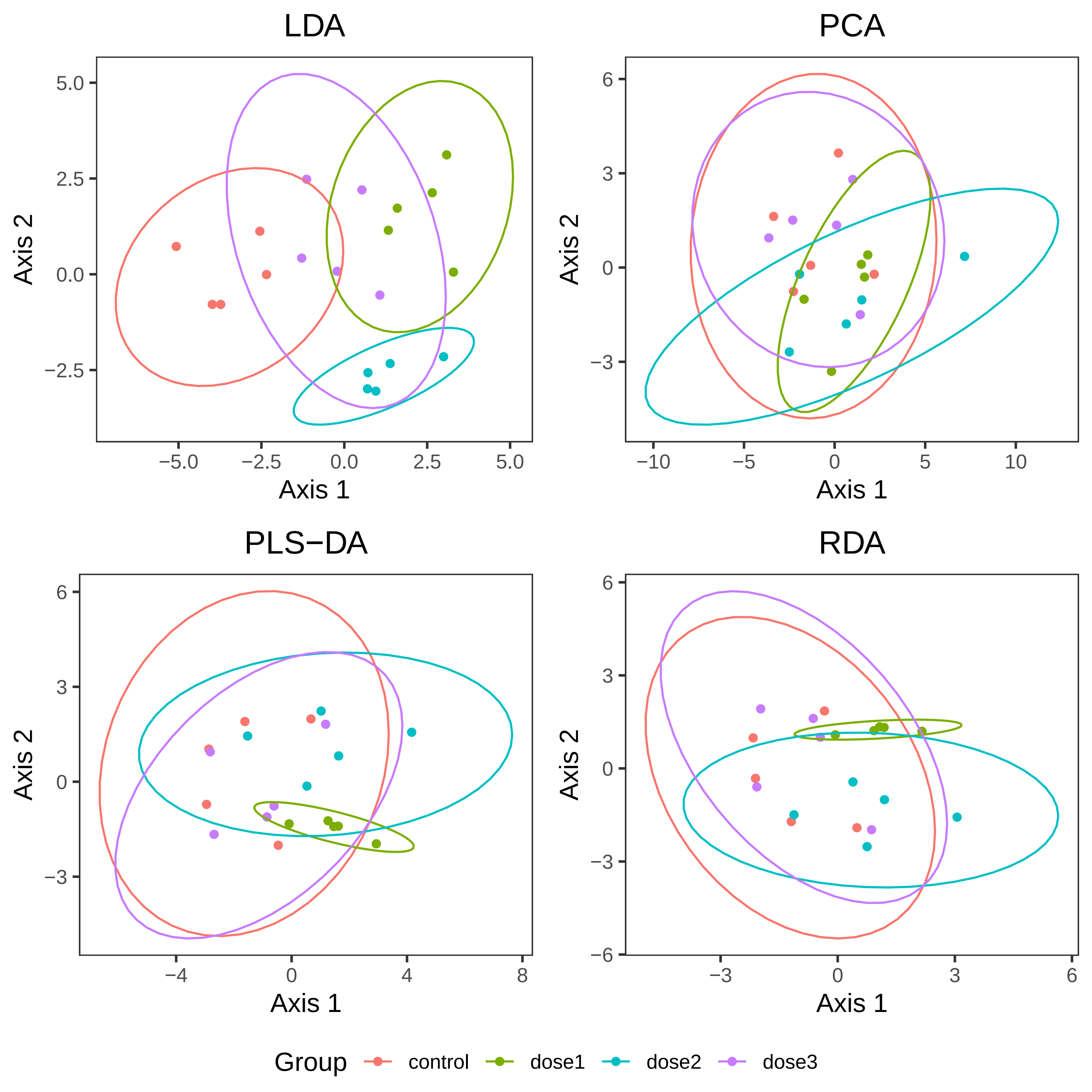

LDA
PCA
PLS−DA
RDA
Axis 1
Axis 2
5.0
2.5
0.0
−2.5
−5.0
6
3
0
−3
−6
−4
4
8
−10
−5
5
10
Group
control
dose1
dose2
dose3

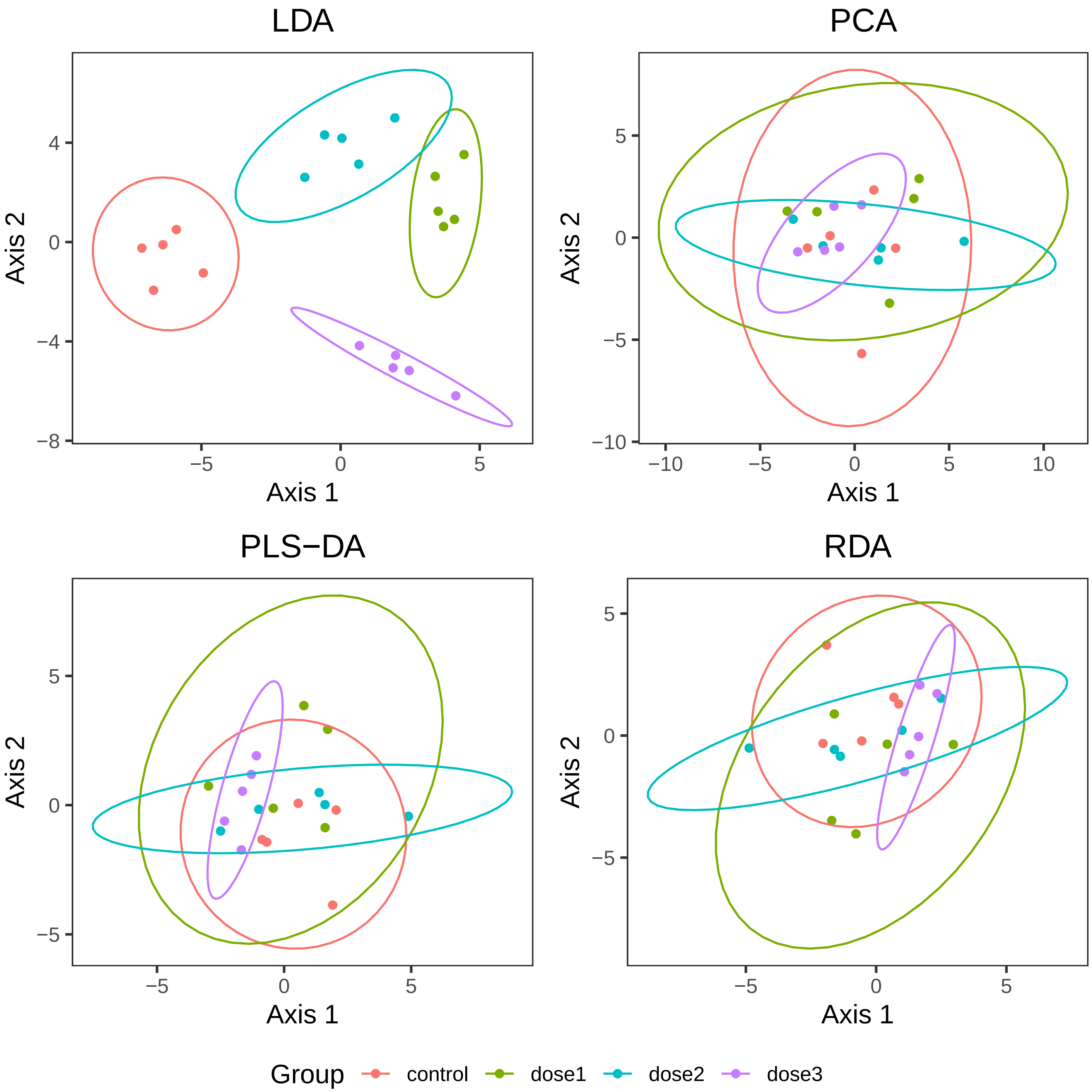
LDA
PCA
PLS−DA
RDA
Axis 1
Axis 2
Group
control
dose1
dose2
dose3

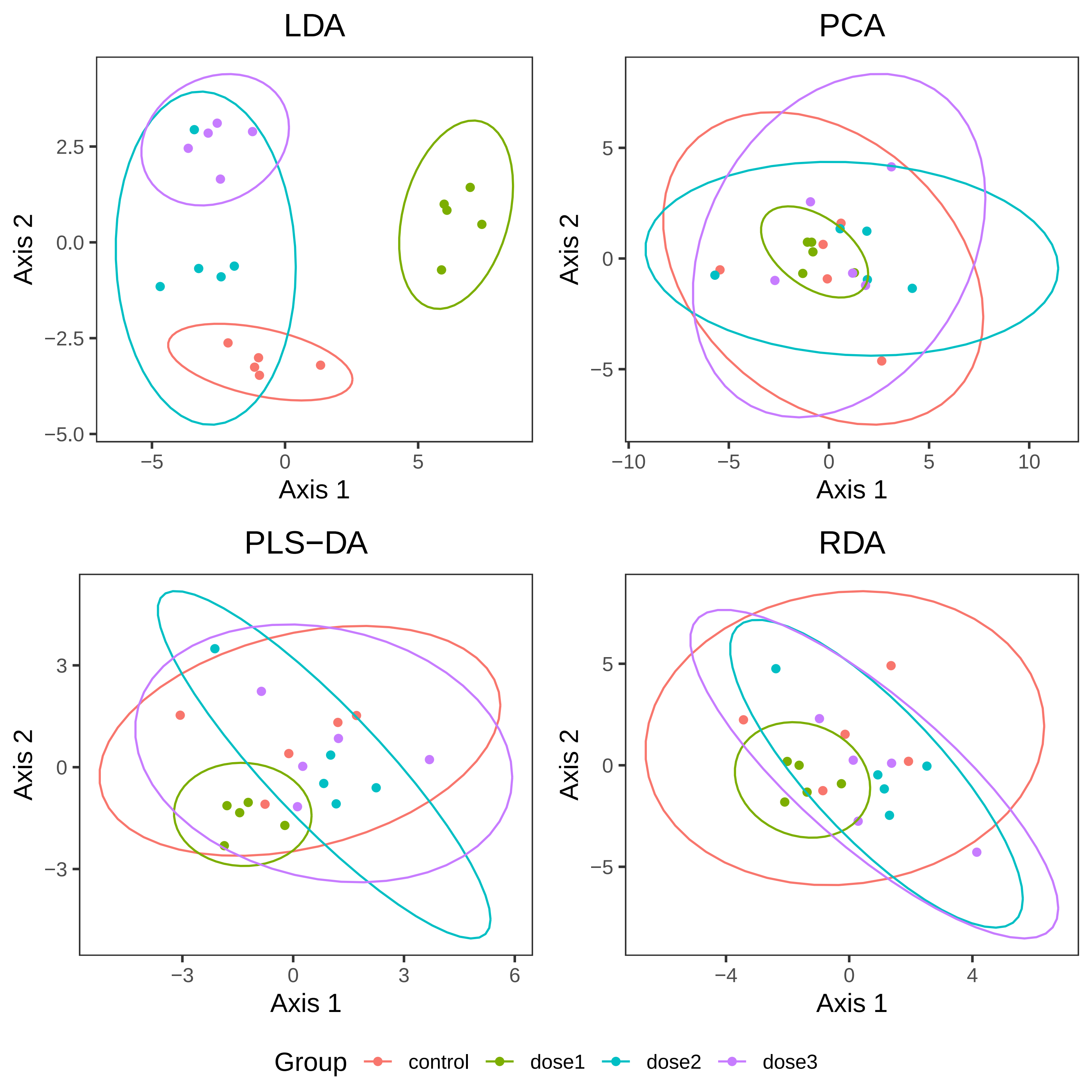
LDA
PCA
PLS−DA
RDA
Axis 1
Axis 2
2.5
0.0
−2.5
−5.0
−5
0
5
10
−10
3
−3
6
−4
4
Group
control
dose1
dose2
dose3

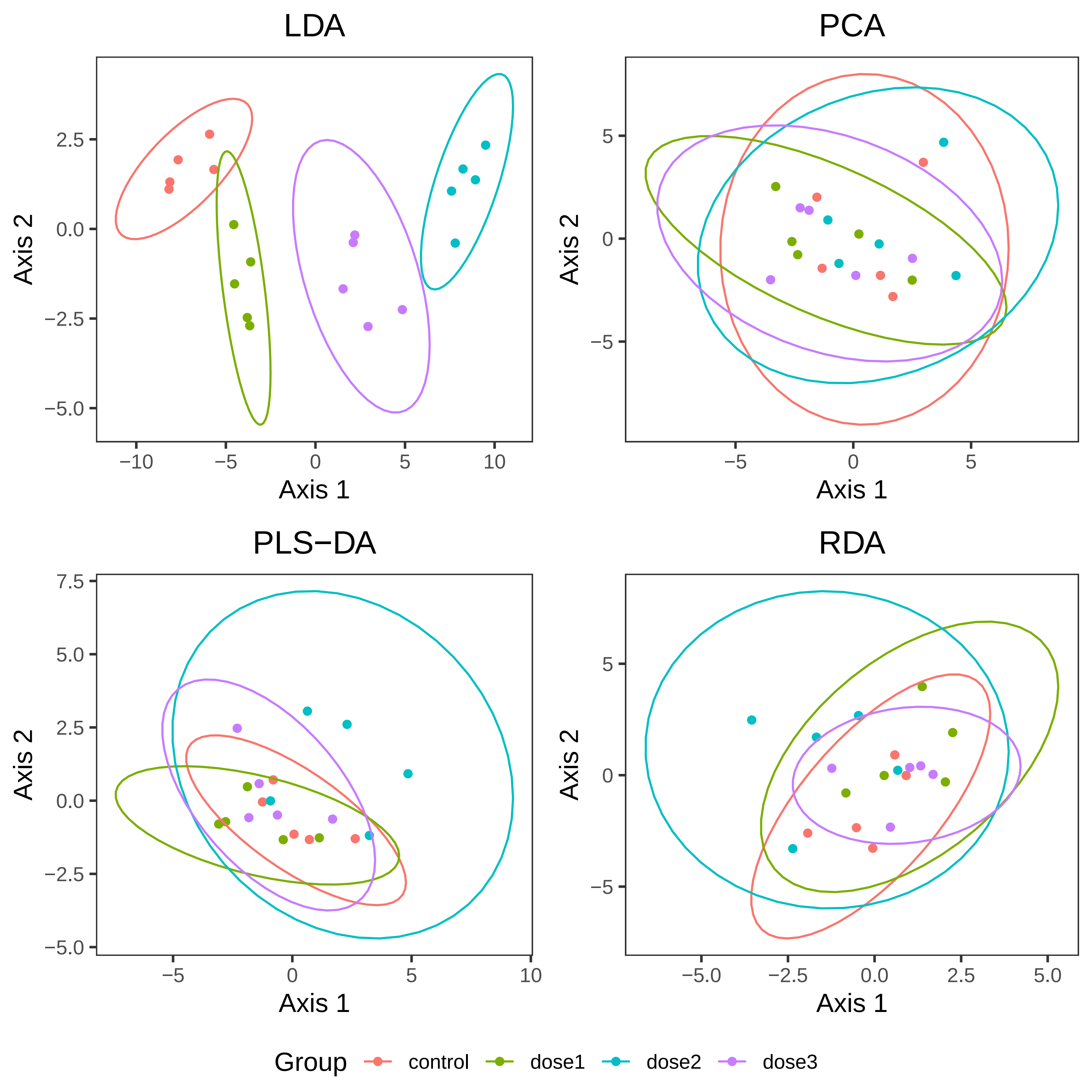

LDA
PCA
PLS−DA
RDA
Axis 1
Axis 2
Group
control
dose1
dose2
dose3

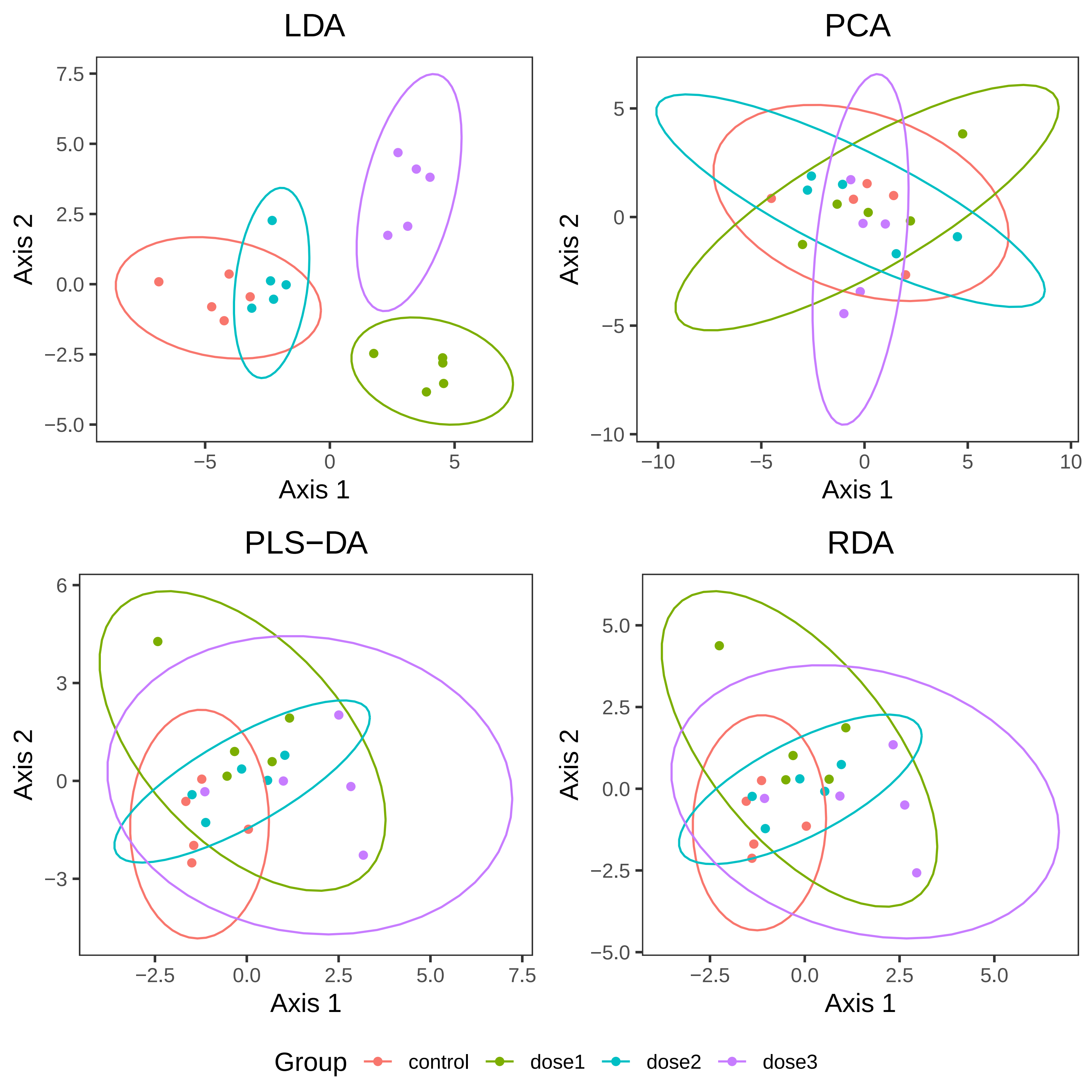
LDA
PCA
PLS−DA
RDA
Axis 1
Axis 2
Group
control
dose1
dose2
dose3

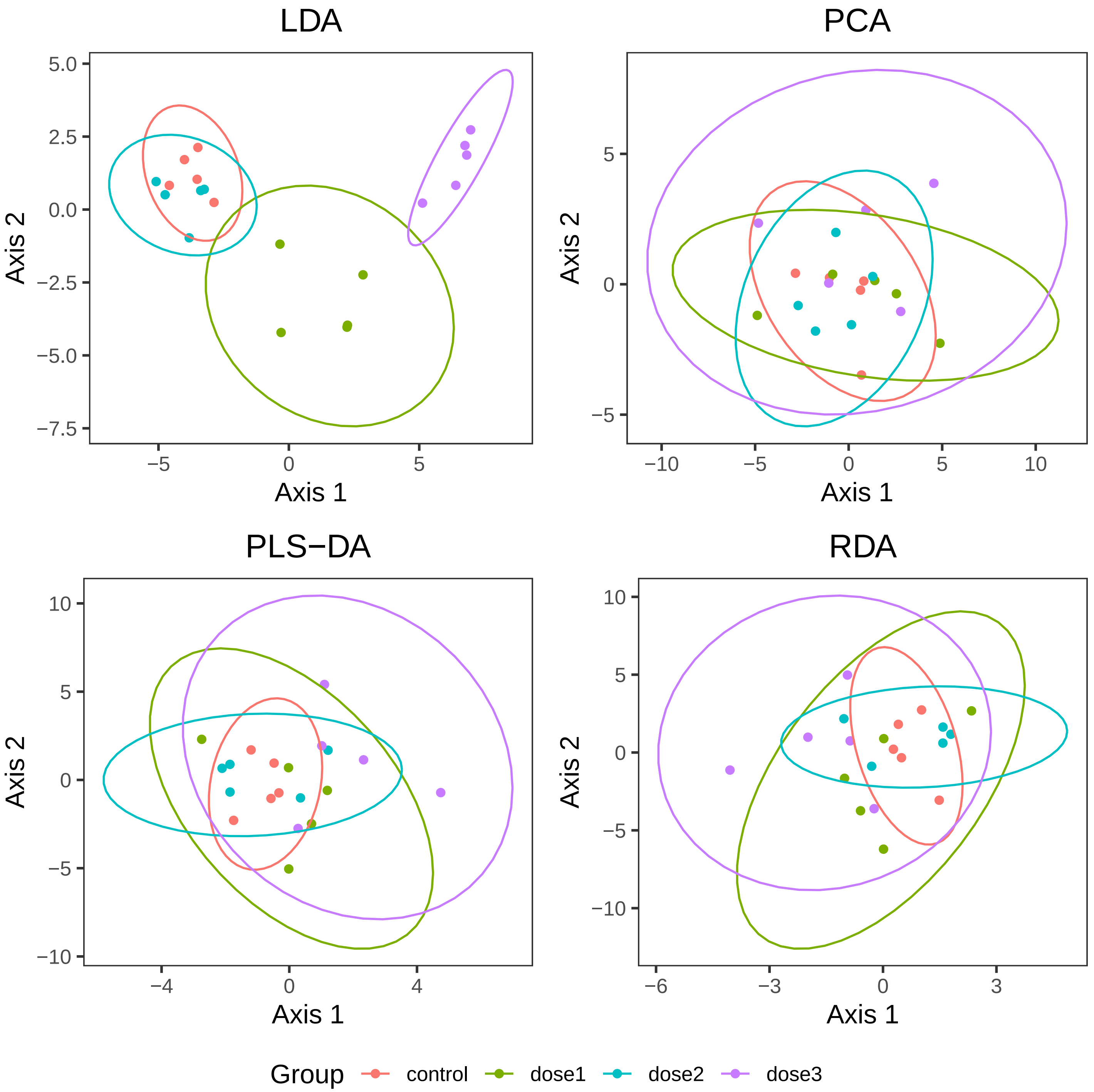
LDA
PCA
PLS−DA
RDA
Axis 1
Axis 2
5.0
2.5
0.0
−2.5
−5.0
−7.5
−5
0
5
−10
10
−4
4
−6
−3
3
Group
control
dose1
dose2
dose3

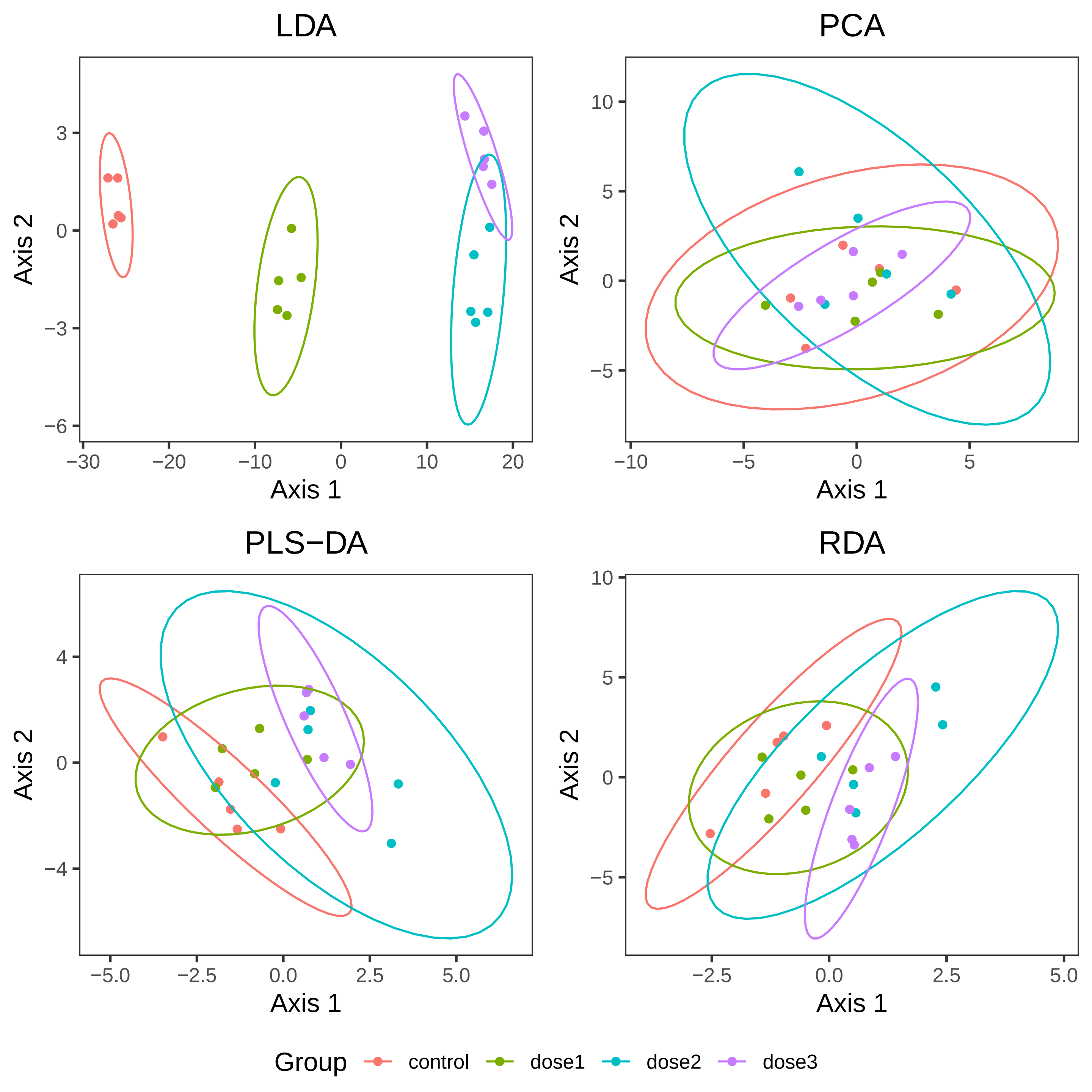

LDA
PCA
PLS−DA
RDA
Axis 1
Axis 2
3
0
−3
−6
−30
−20
−10
0
10
20
10
5
0
−5
−10
−5
0
5
4
0
−4
−5.0
−2.5
0.0
2.5
5.0
10
5
0
−5
−2.5
0.0
2.5
5.0
Group
control
dose1
dose2
dose3

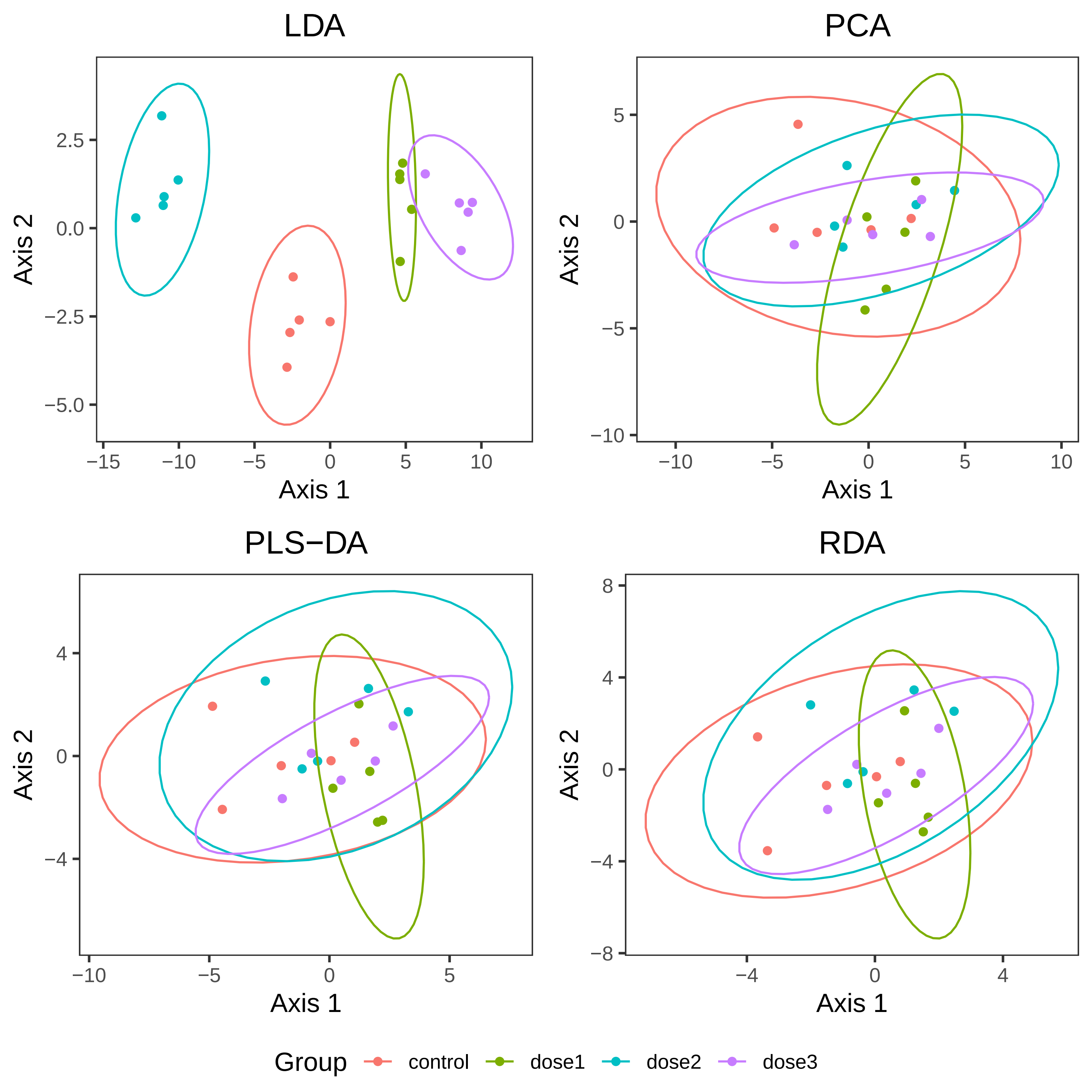
LDA
PCA
PLS−DA
RDA
Axis 1
Axis 2
Group
control
dose1
dose2
dose3

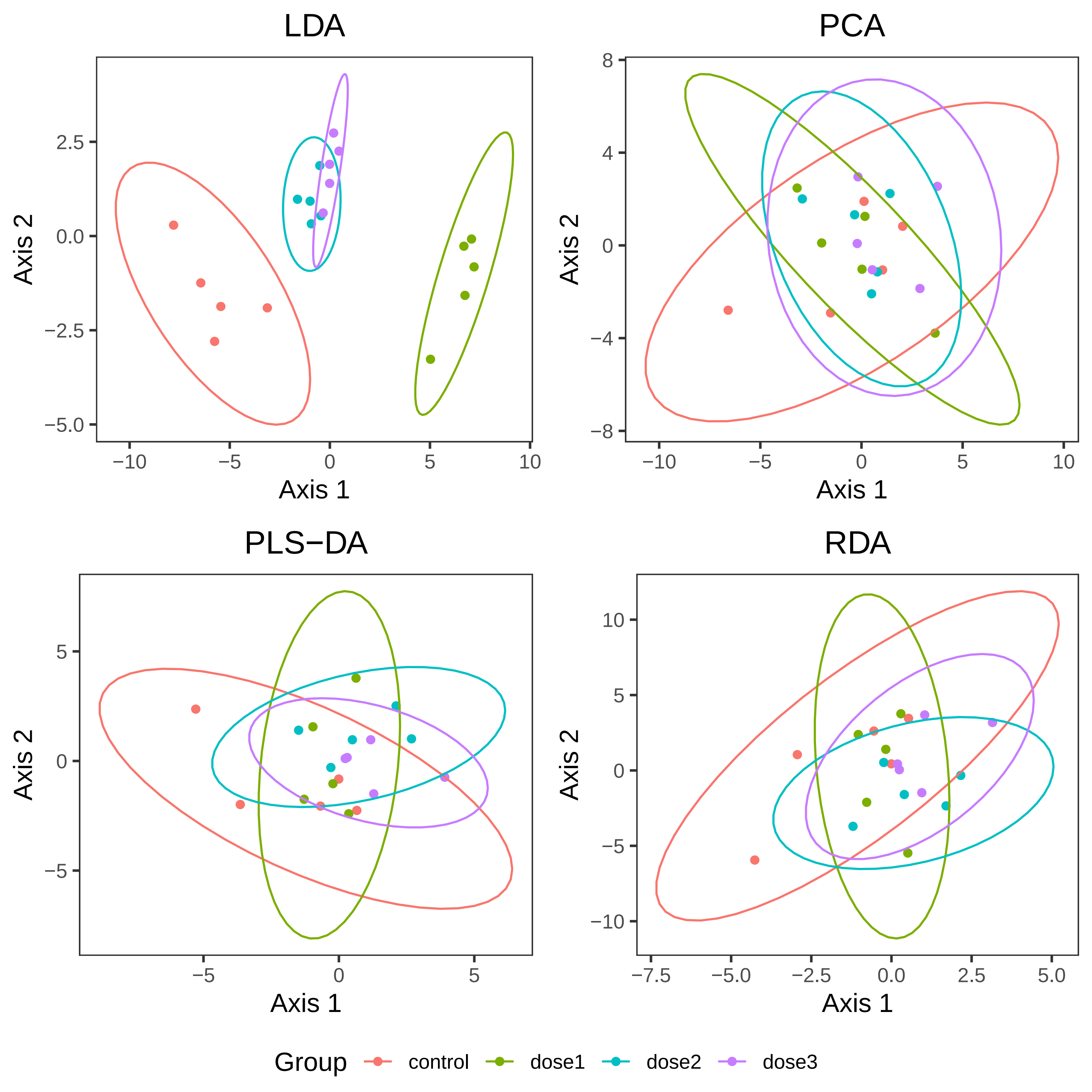

LDA
PCA
PLS−DA
RDA
Axis 1
Axis 2
Group
control
dose1
dose2
dose3

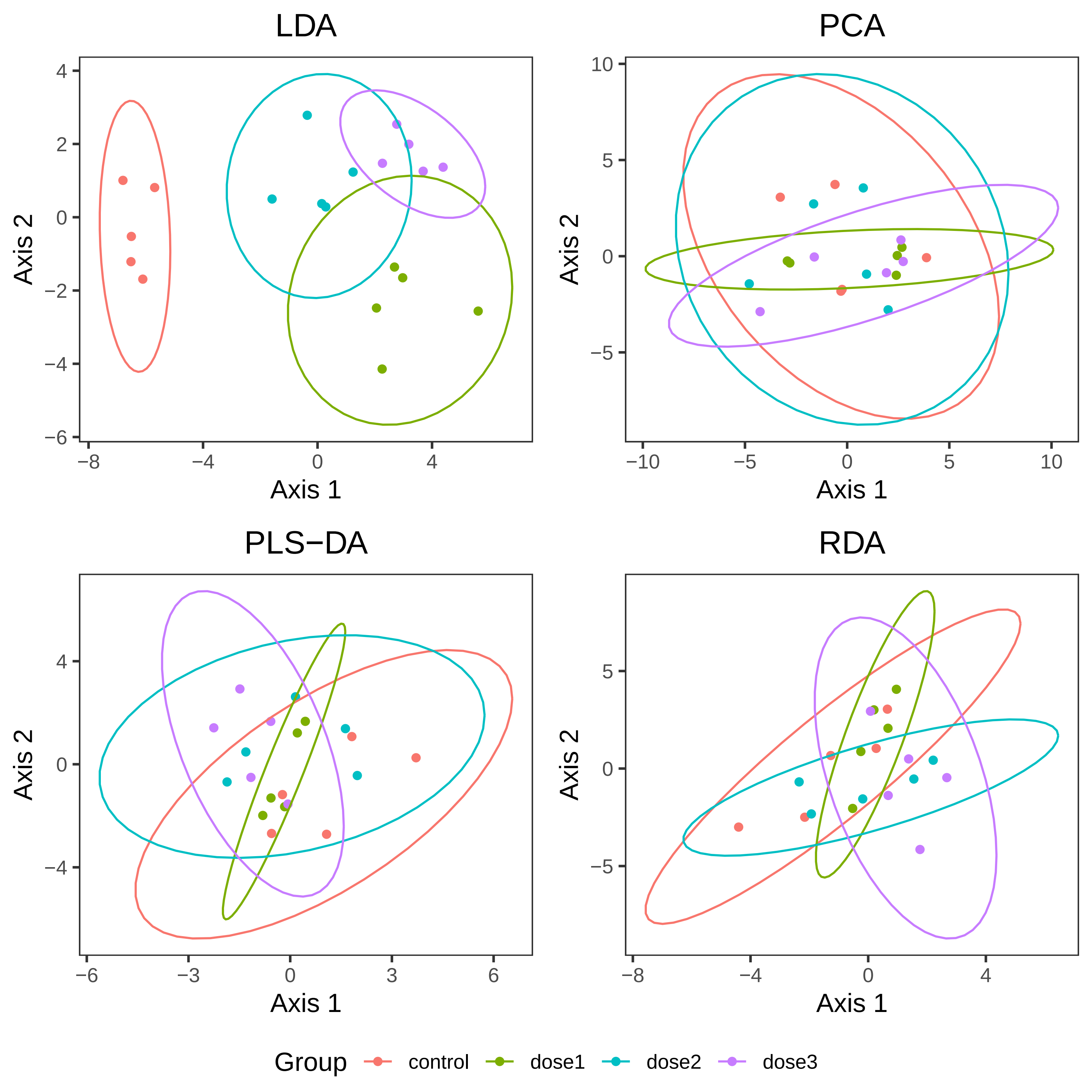

LDA
PCA
PLS−DA
RDA
Axis 1
Axis 2
Group
control
dose1
dose2
dose3

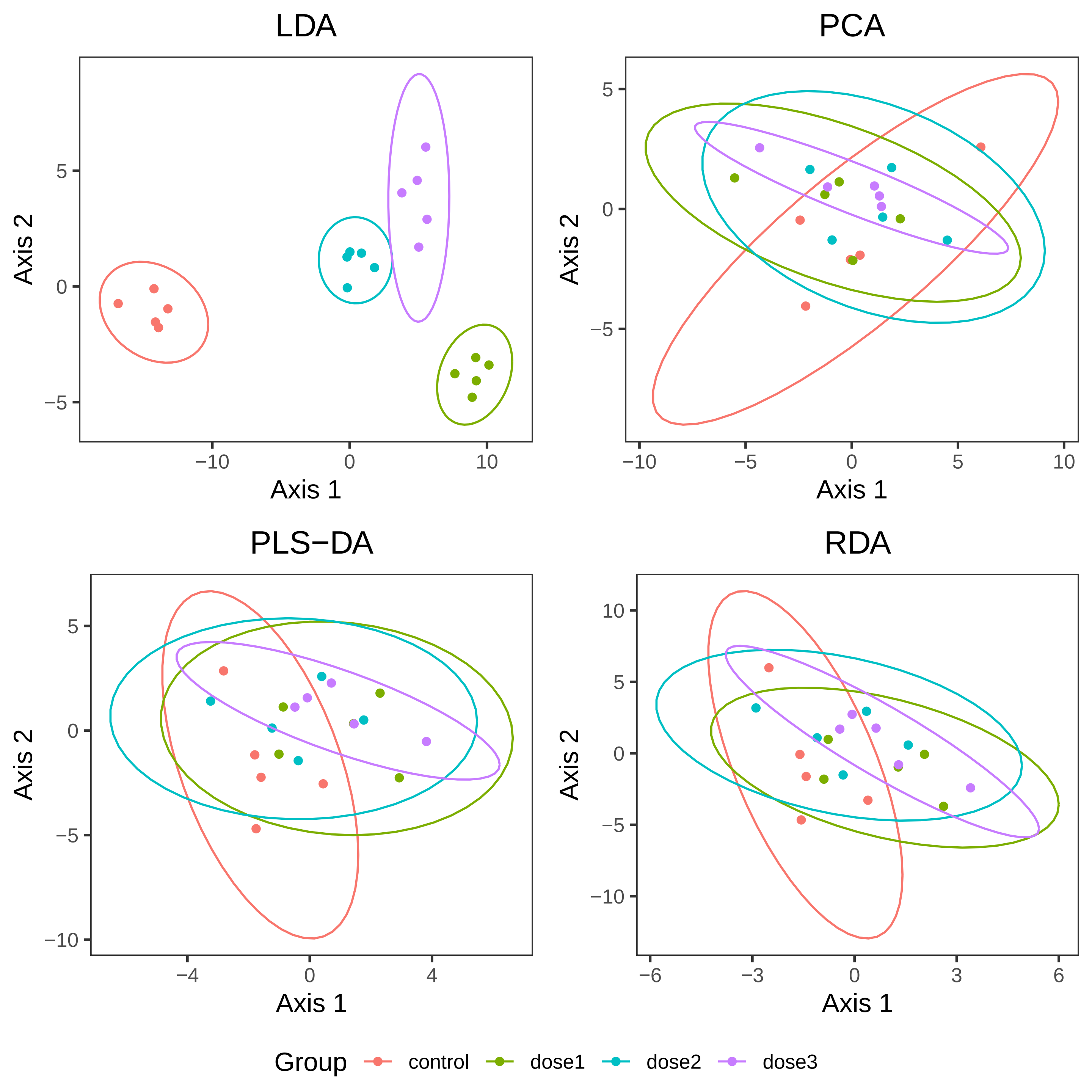
LDA
PCA
PLS−DA
RDA
Axis 1
Axis 2
Group
control
dose1
dose2
dose3

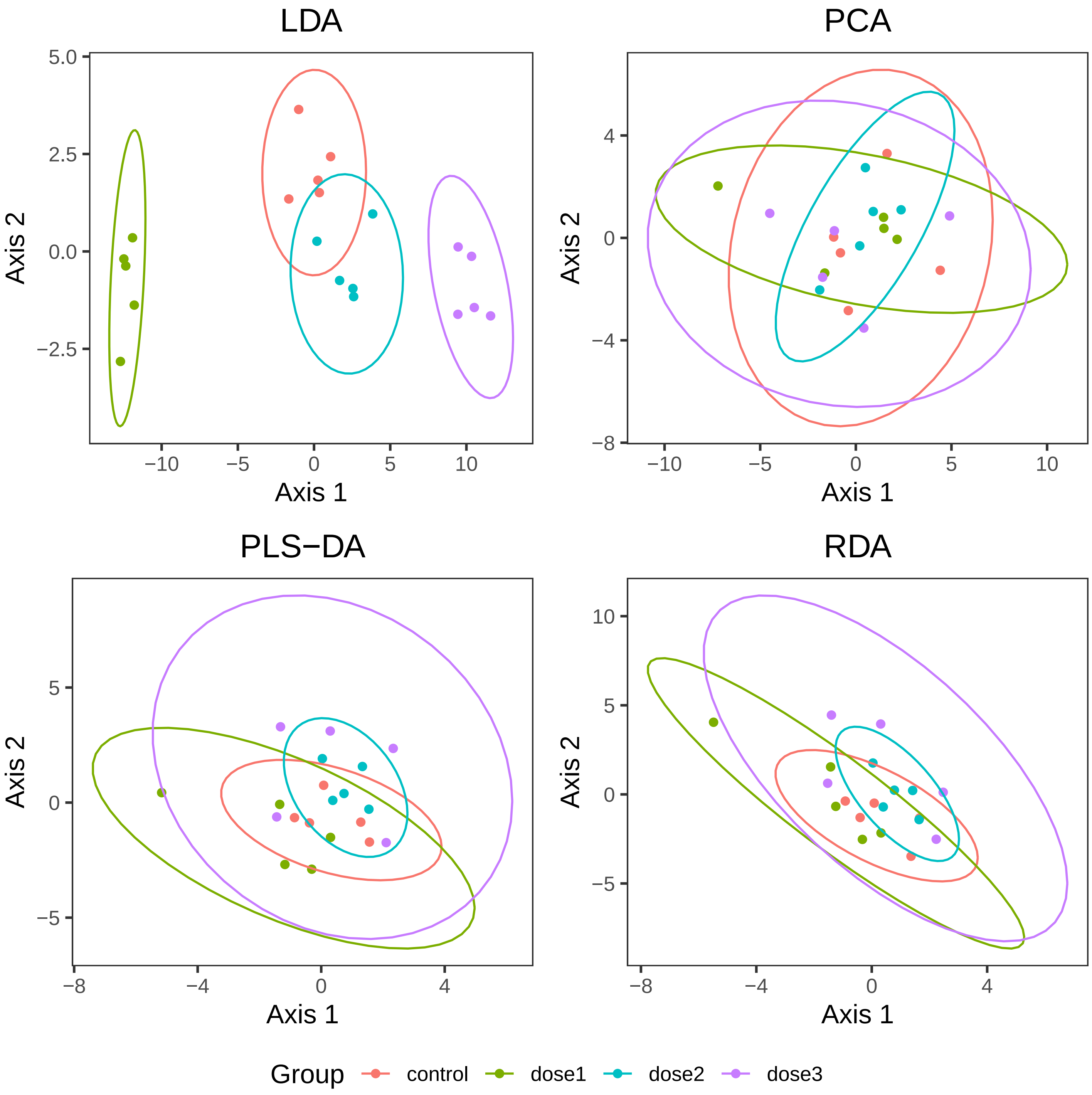

LDA
PCA
PLS−DA
RDA
Axis 1
Axis 2
5.0
2.5
0.0
−2.5
−10
−5
0
5
10
4
0
−4
−8
−8
−4
0
4
5
0
−5
10
Group
control
dose1
dose2
dose3

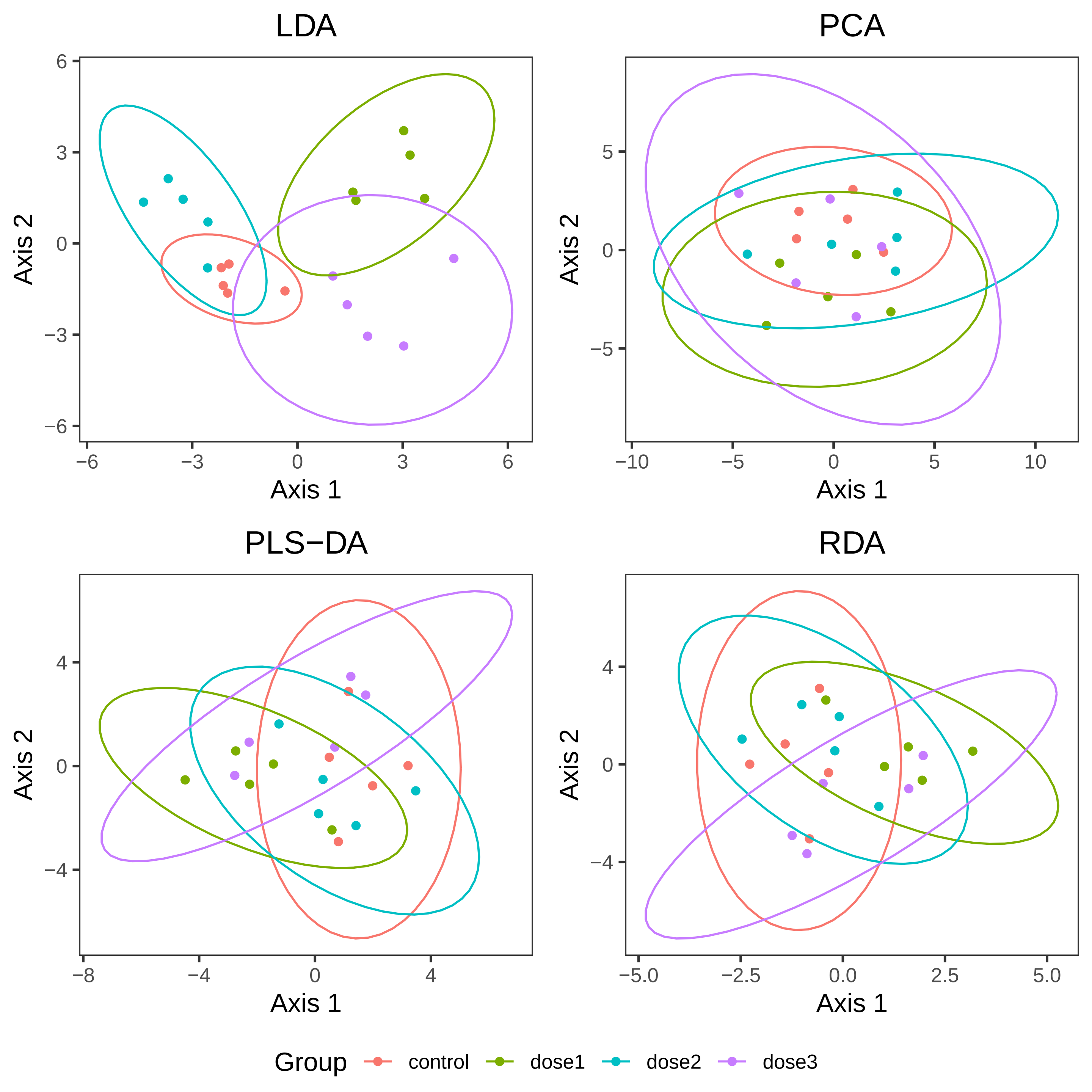

LDA
PCA
PLS−DA
RDA
Axis 1
Axis 2
Group
control
dose1
dose2
dose3

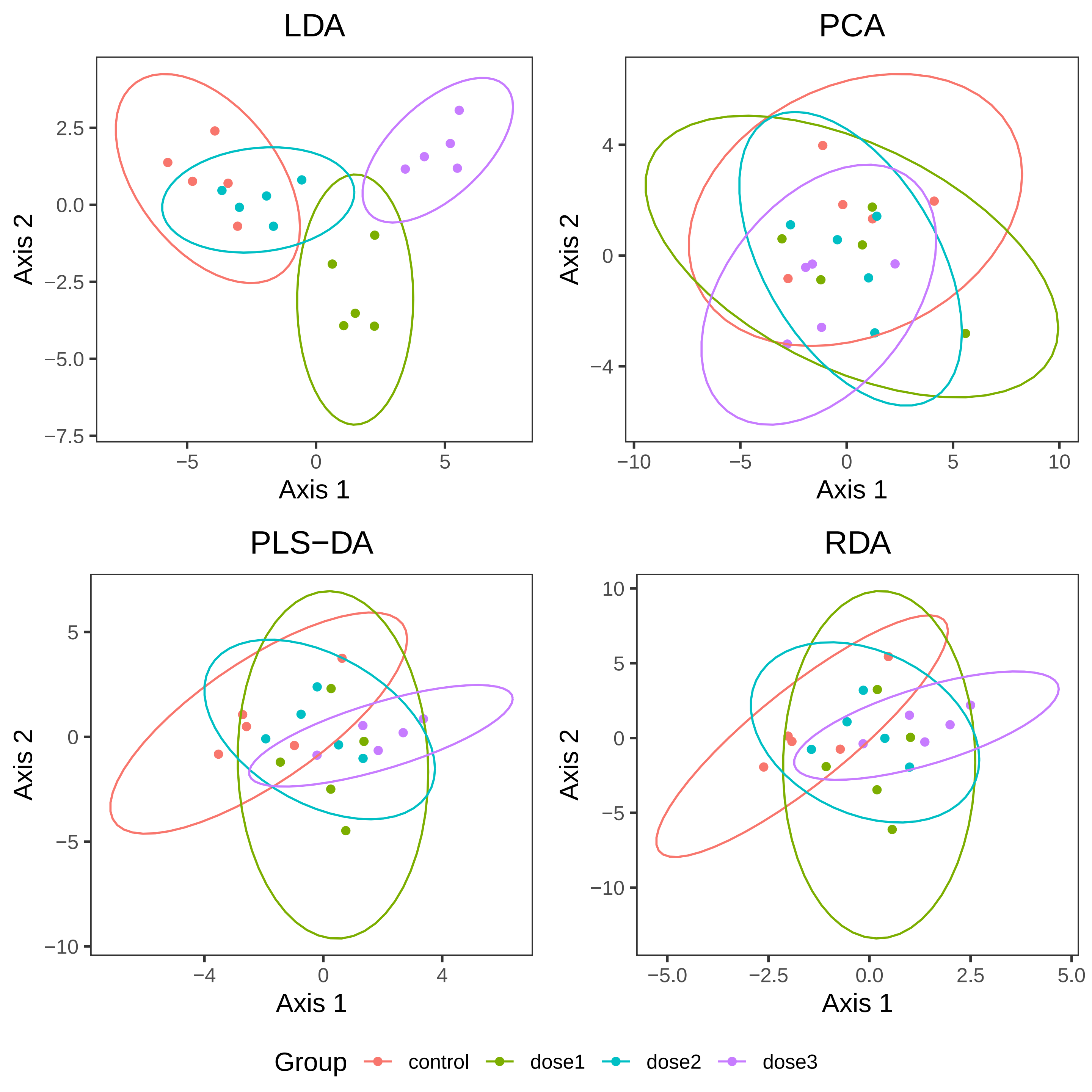
LDA
PCA
PLS−DA
RDA
Axis 1
Axis 2
2.5
0.0
−2.5
−5.0
−7.5
−5
0
5
4
0
−4
−10
10
−10
5
0
−5
−10
−4
0
4
10
5
0
−5
−10
−5.0
−2.5
0.0
2.5
5.0
Group
control
dose1
dose2
dose3

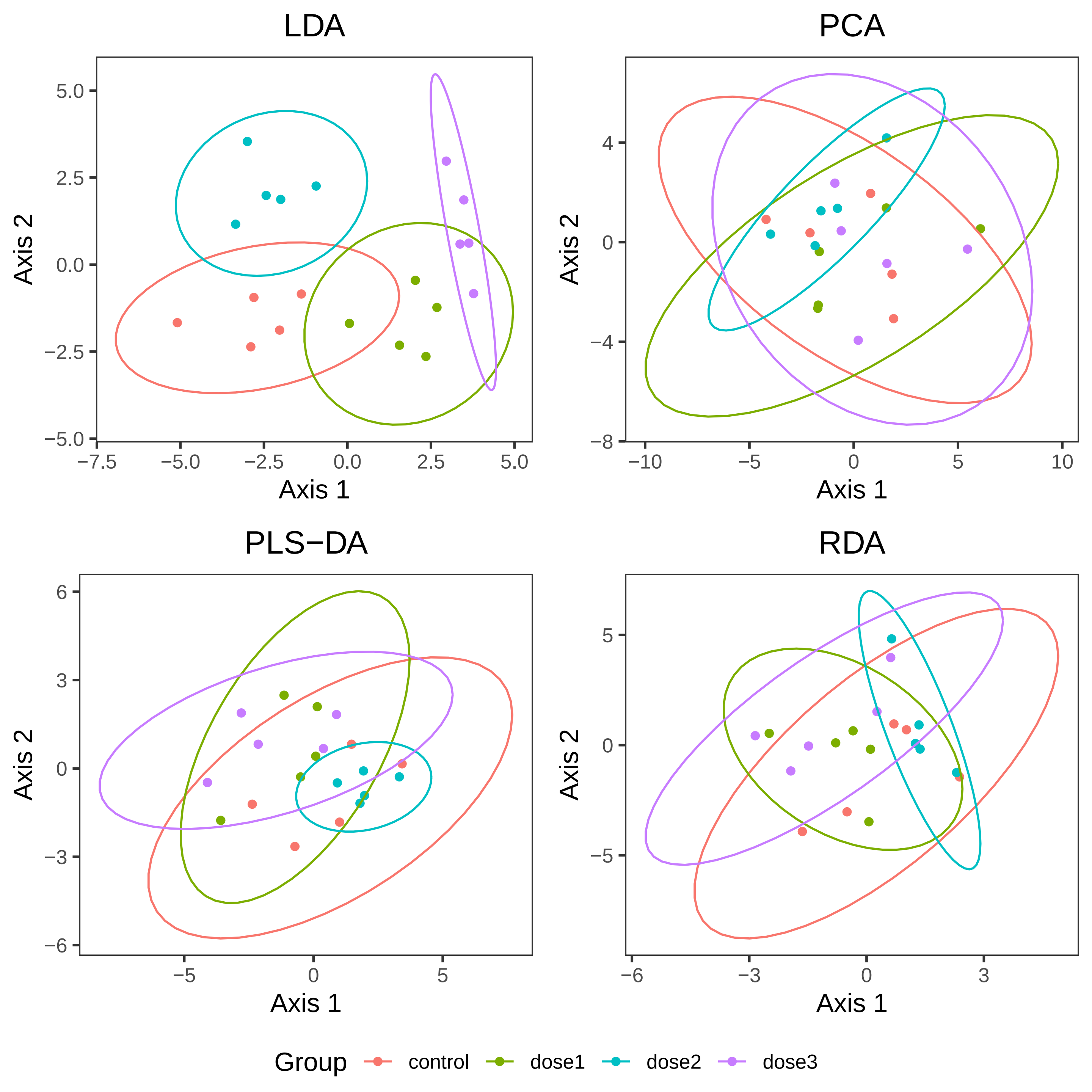

LDA
PCA
PLS−DA
RDA
Axis 1
Axis 2
5.0
2.5
0.0
−2.5
−5.0
−7.5
4
0
−4
−8
−10
−5
5
10
6
3
−3
−6
Group
control
dose1
dose2
dose3

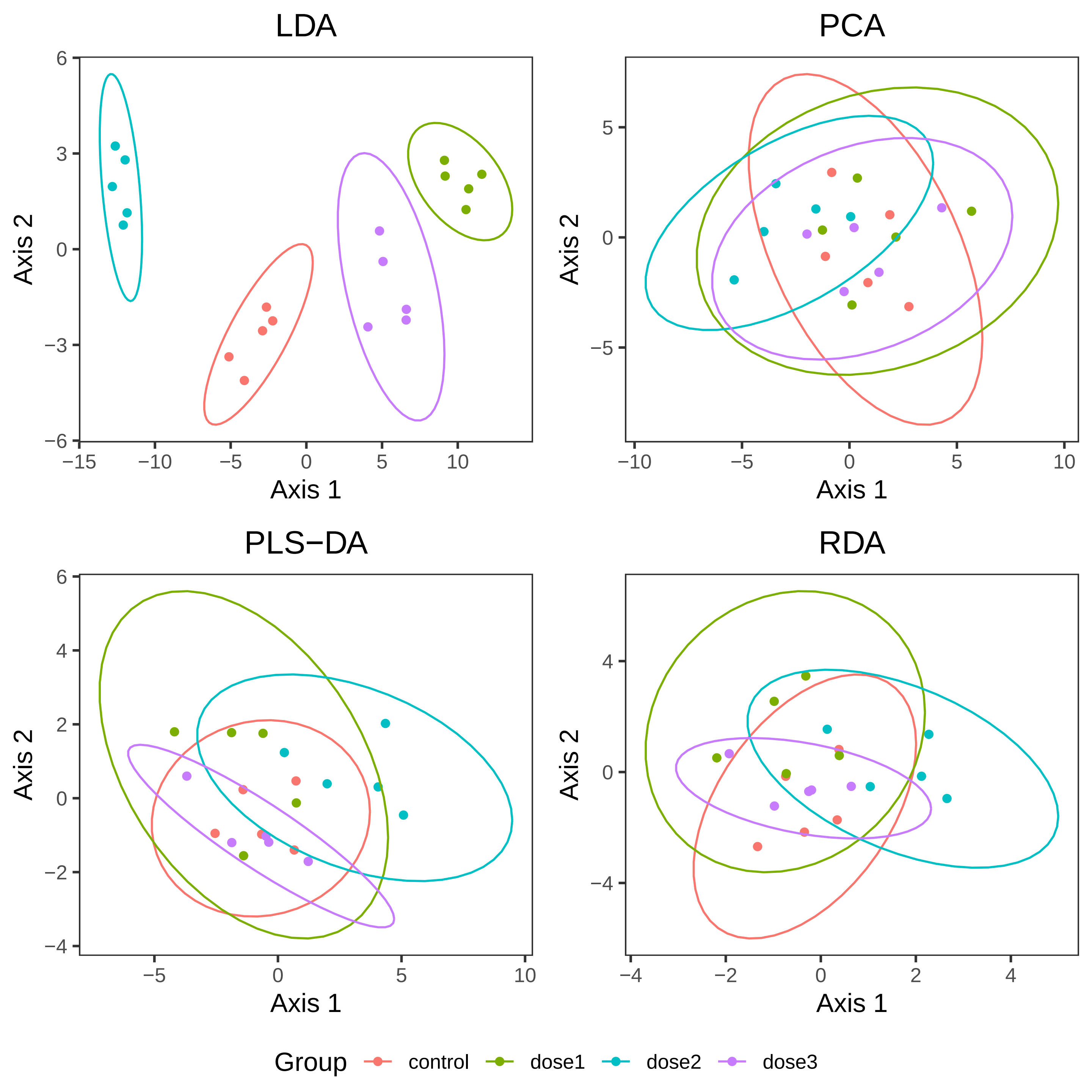

LDA
PCA
PLS−DA
RDA
Axis 1
Axis 2
Group
control
dose1
dose2
dose3

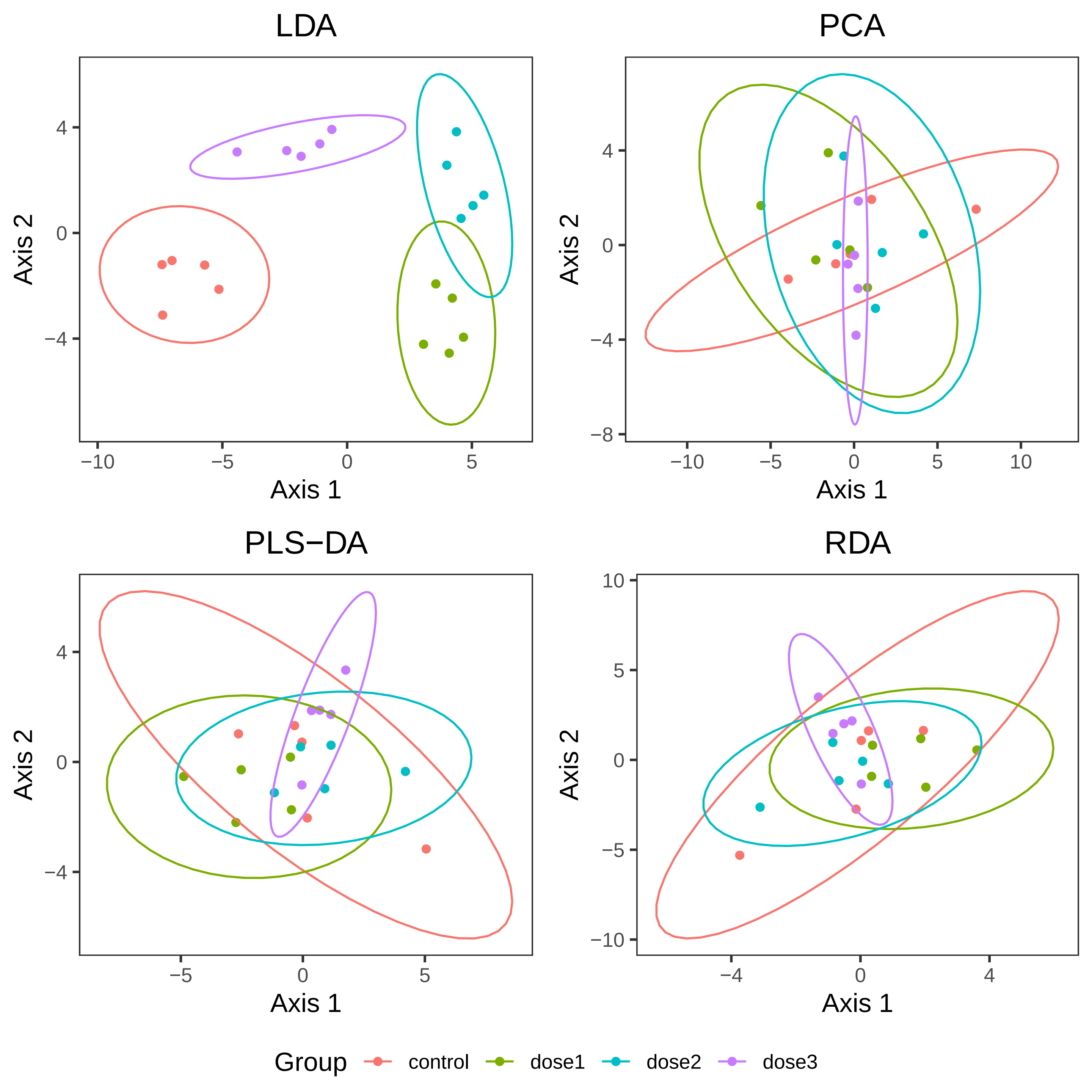
LDA
PCA
PLS−DA
RDA
Axis 1
Axis 2
Group
control
dose1
dose2
dose3

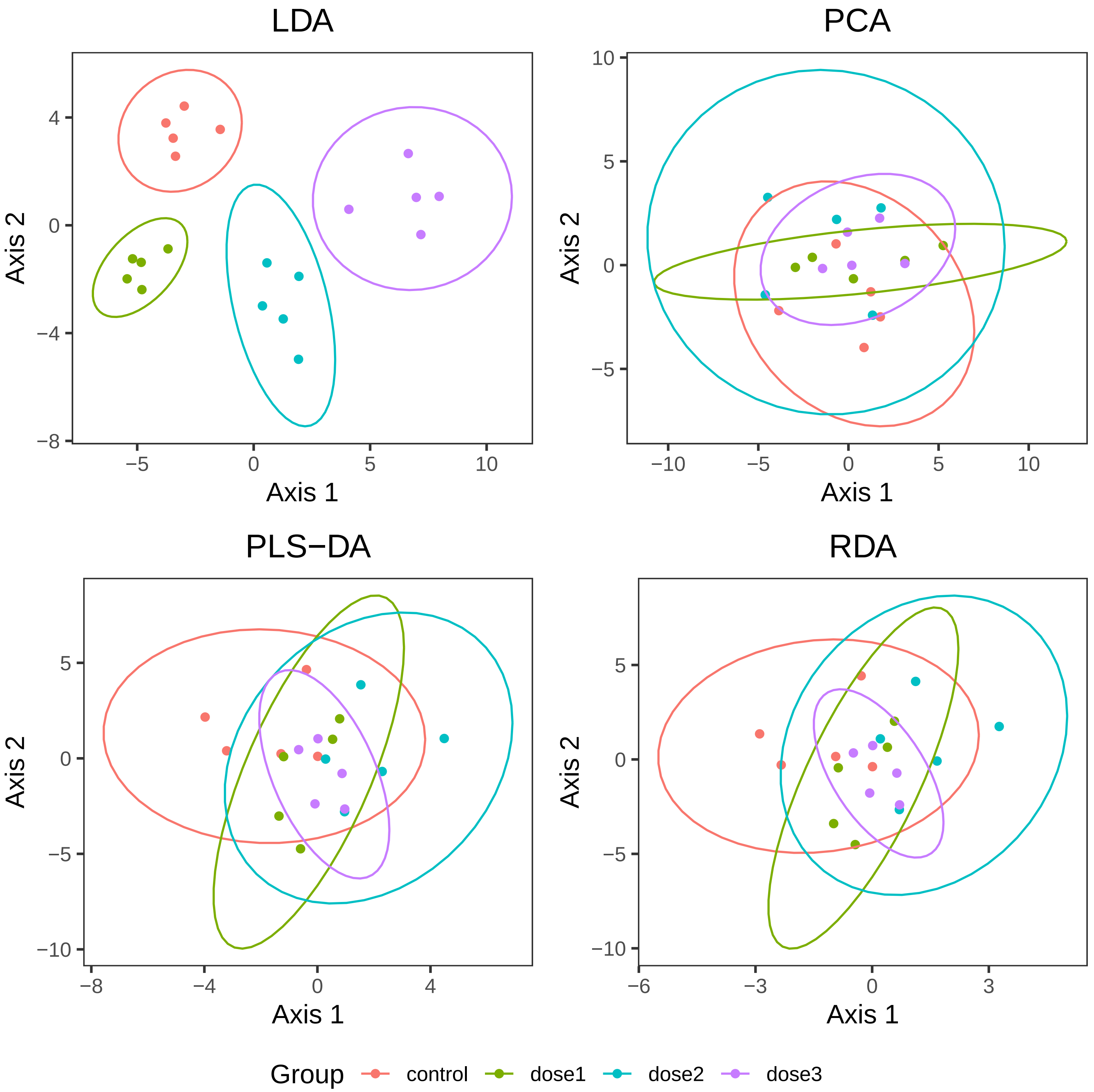
LDA
PCA
PLS−DA
RDA
Axis 1
Axis 2
Group
control
dose1
dose2
dose3

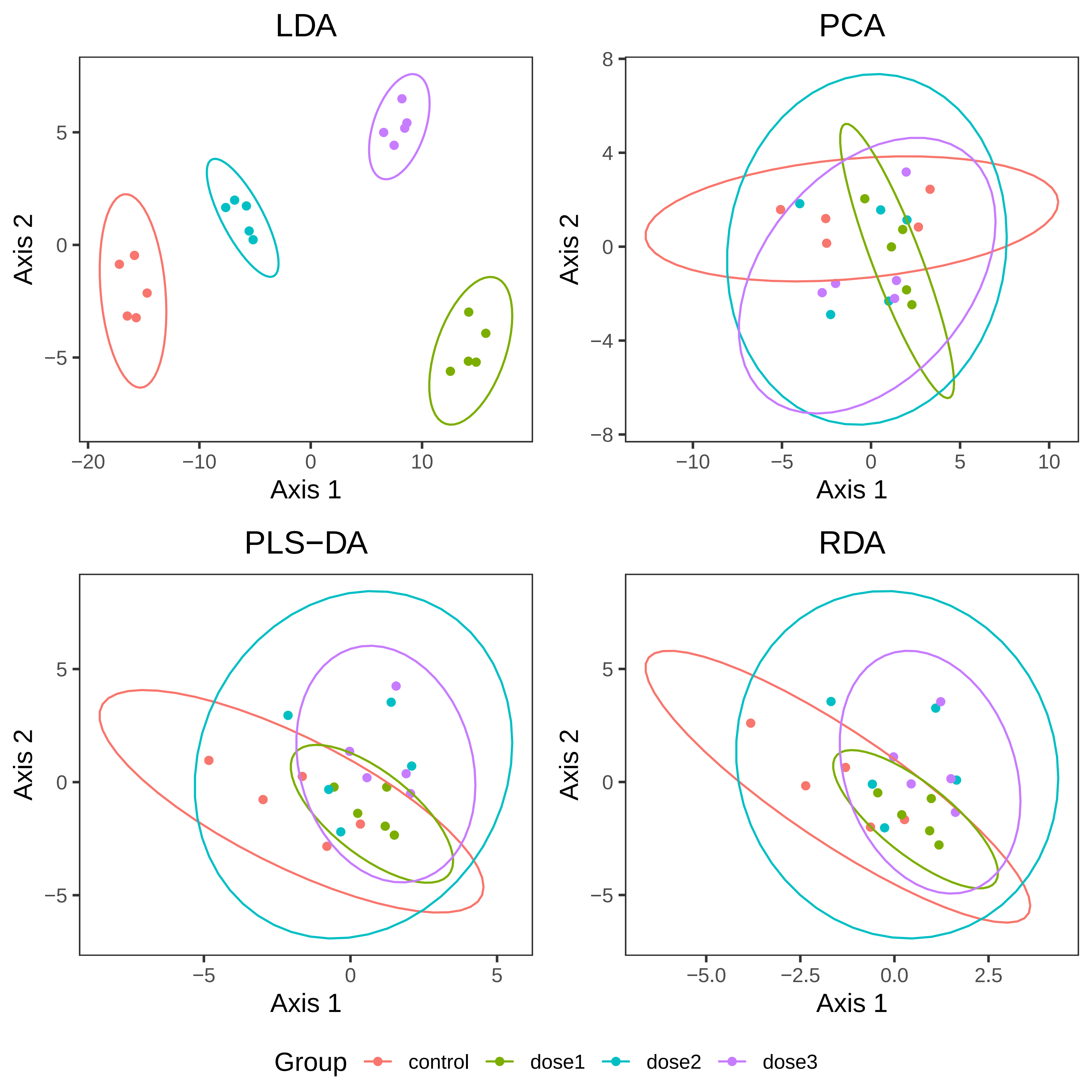
LDA
PCA
PLS−DA
RDA
Axis 1
Axis 2
Group
control
dose1
dose2
dose3

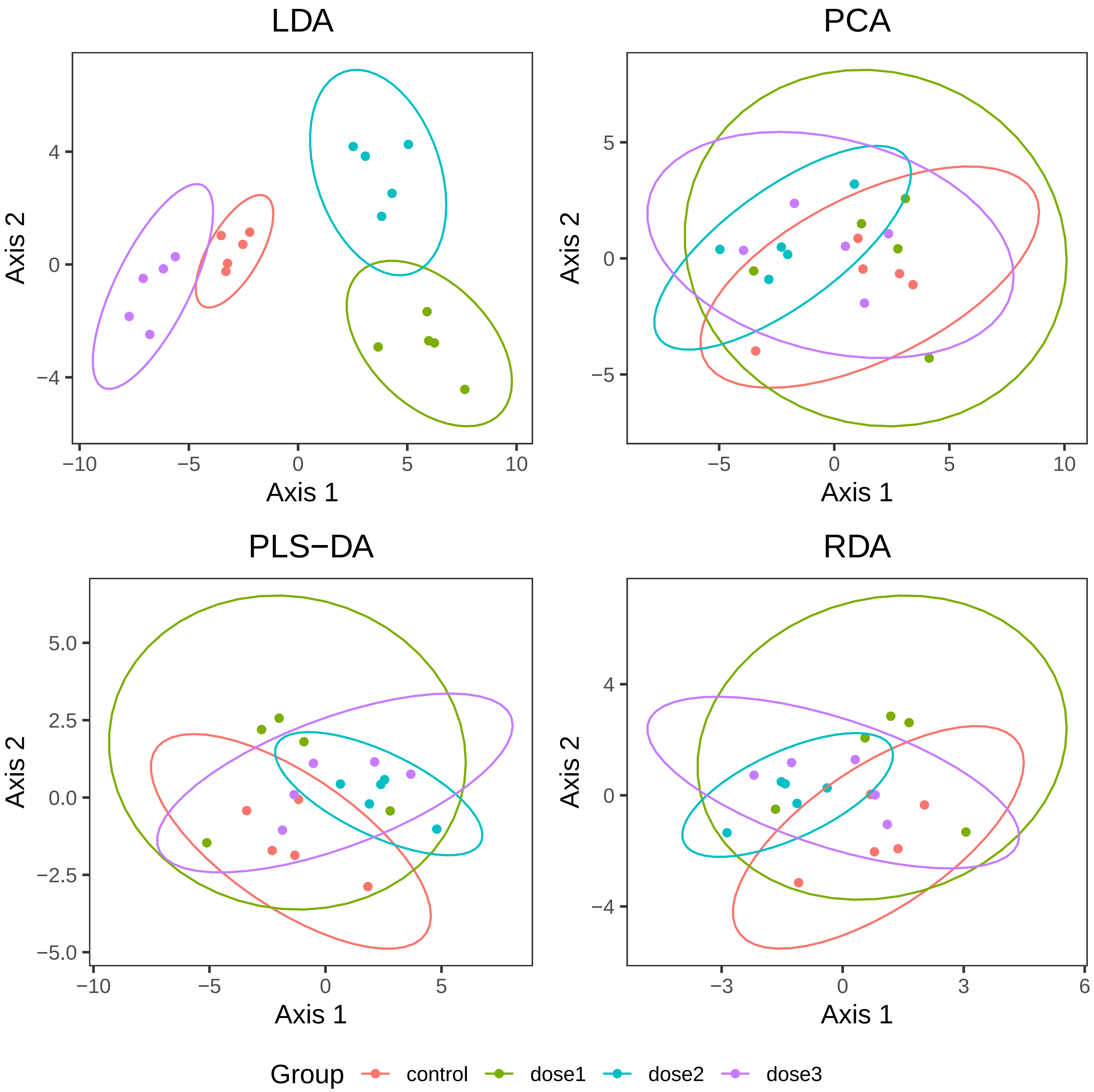
LDA
PCA
PLS−DA
RDA
Axis 1
Axis 2
Group
control
dose1
dose2
dose3

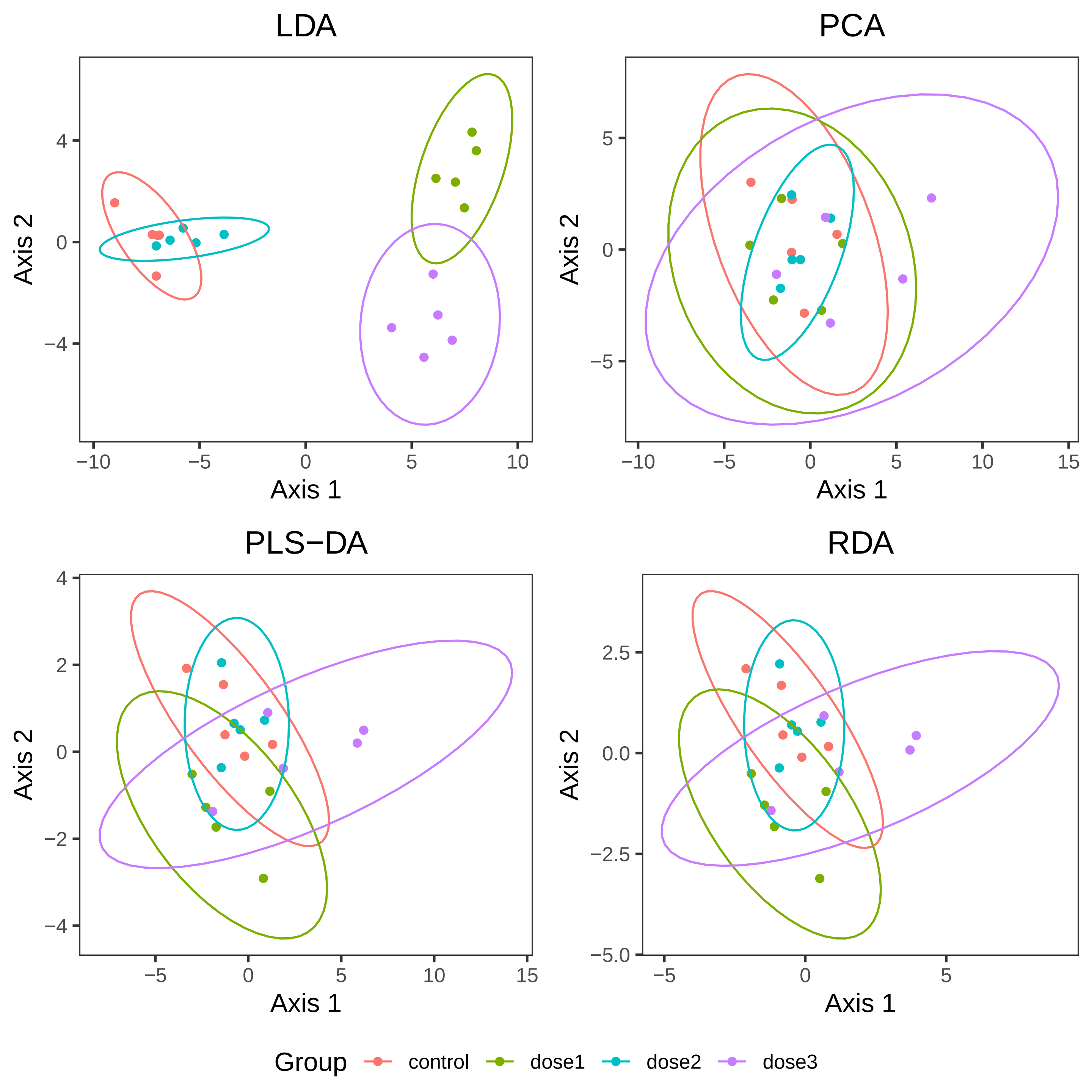
LDA
PCA
PLS−DA
RDA
Axis 1
Axis 2
4
0
−4
−10
−5
0
5
10
5
0
−5
15
2
−2
2.5
0.0
−2.5
−5.0
Group
control
dose1
dose2
dose3

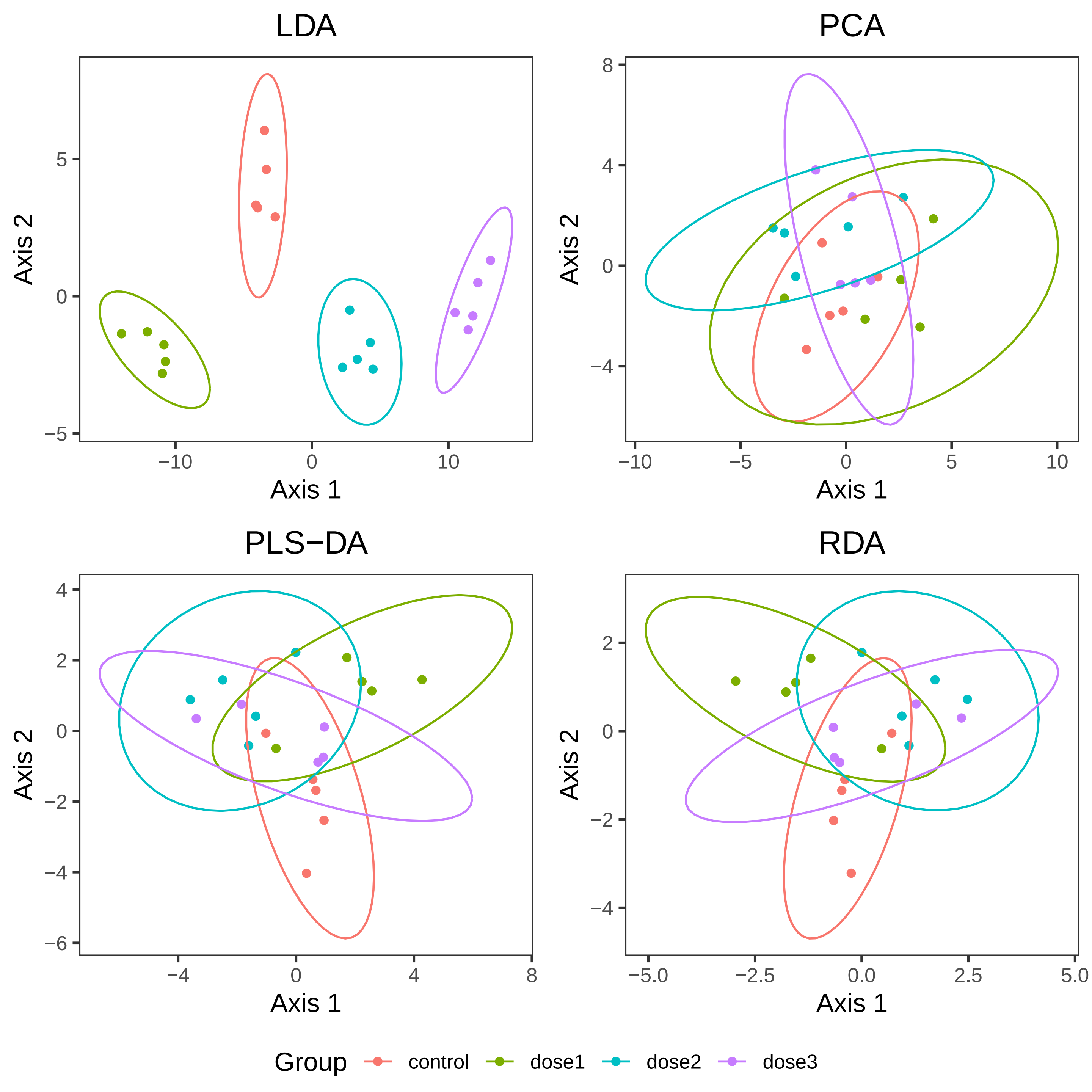
LDA
PCA
PLS−DA
RDA
Axis 1
Axis 2
Group
control
dose1
dose2
dose3

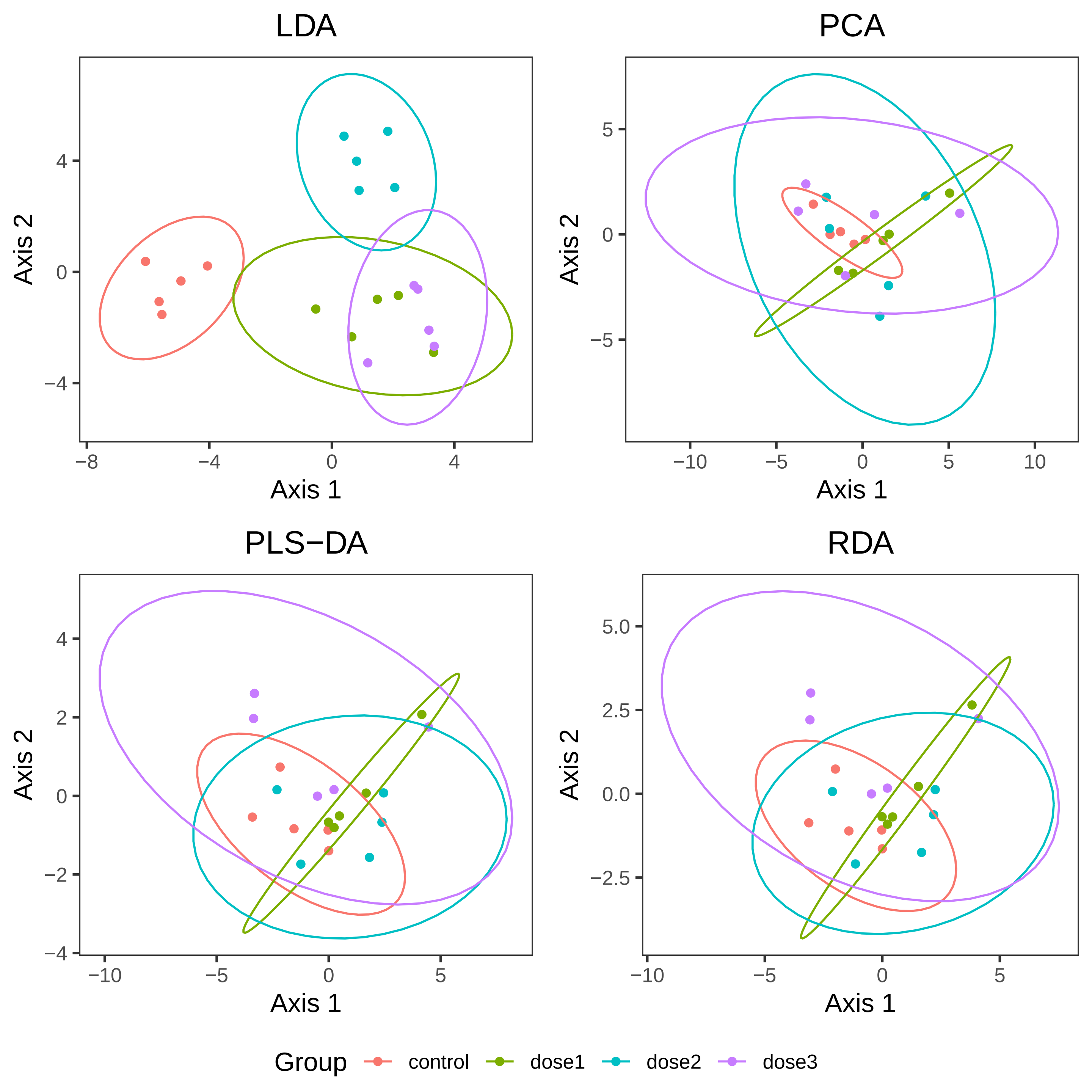
LDA
PCA
PLS−DA
RDA
Axis 1
Axis 2
Group
control
dose1
dose2
dose3

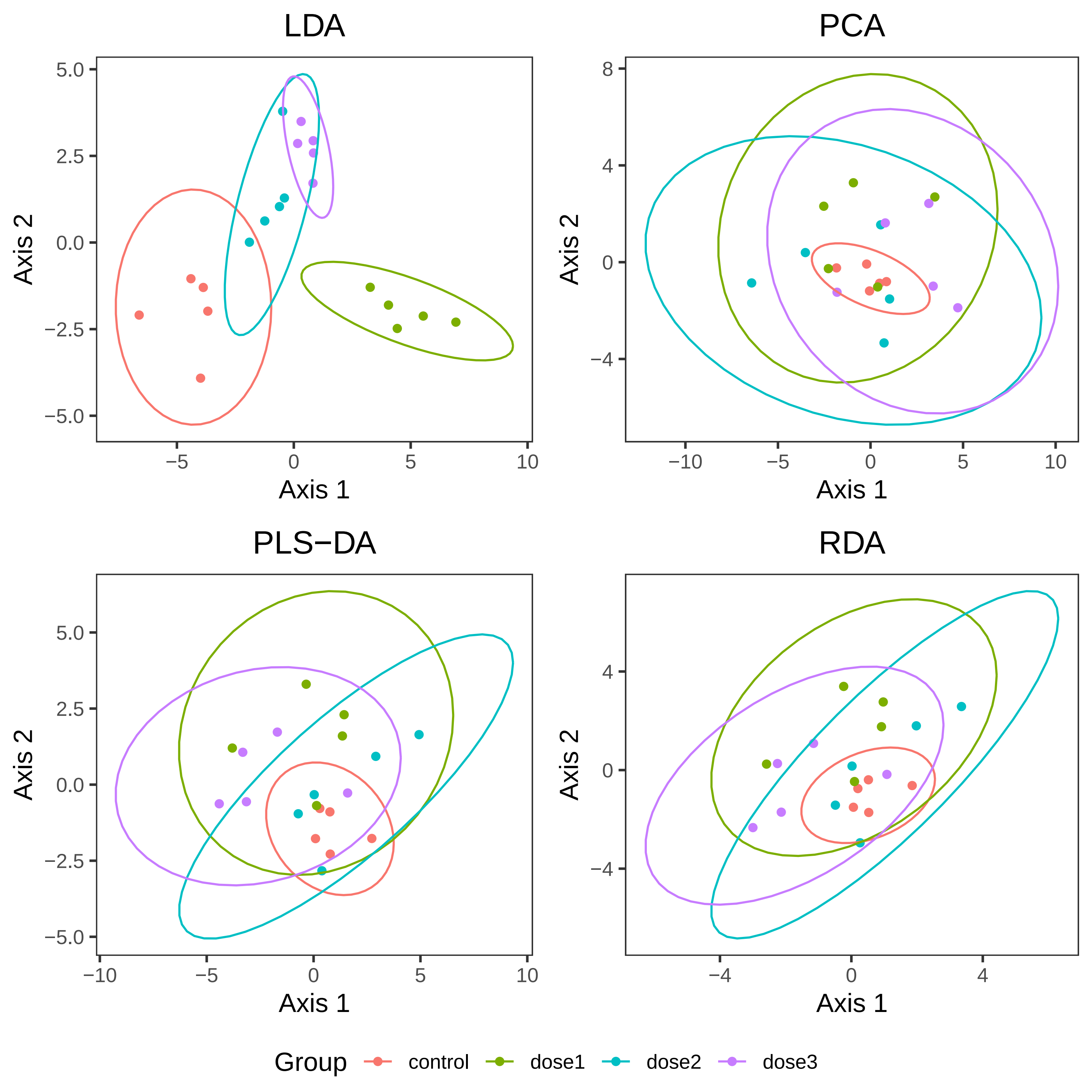
LDA
PCA
PLS−DA
RDA
Axis 1
Axis 2
Group
control
dose1
dose2
dose3

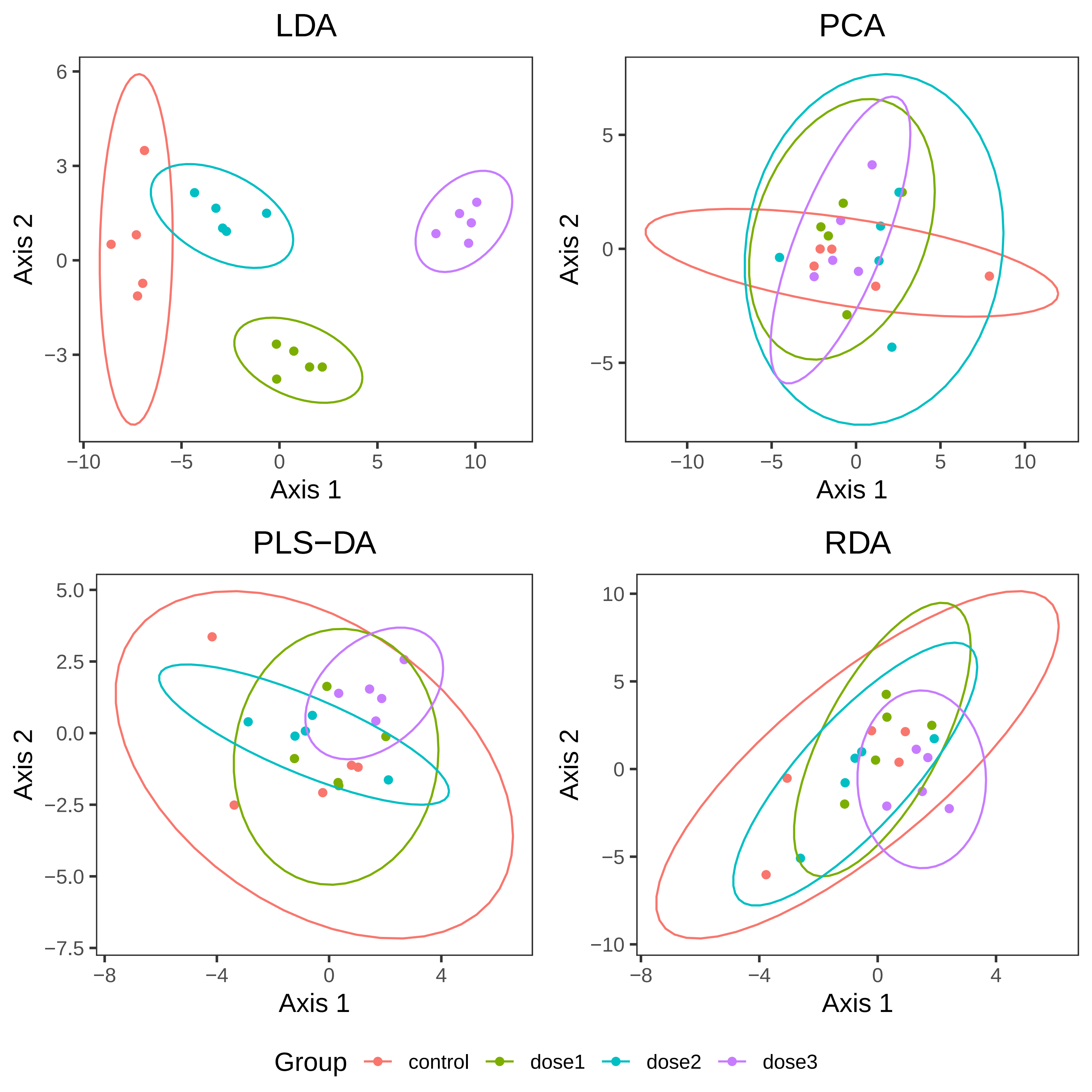
LDA
PCA
PLS−DA
RDA
Axis 1
Axis 2
Group
control
dose1
dose2
dose3

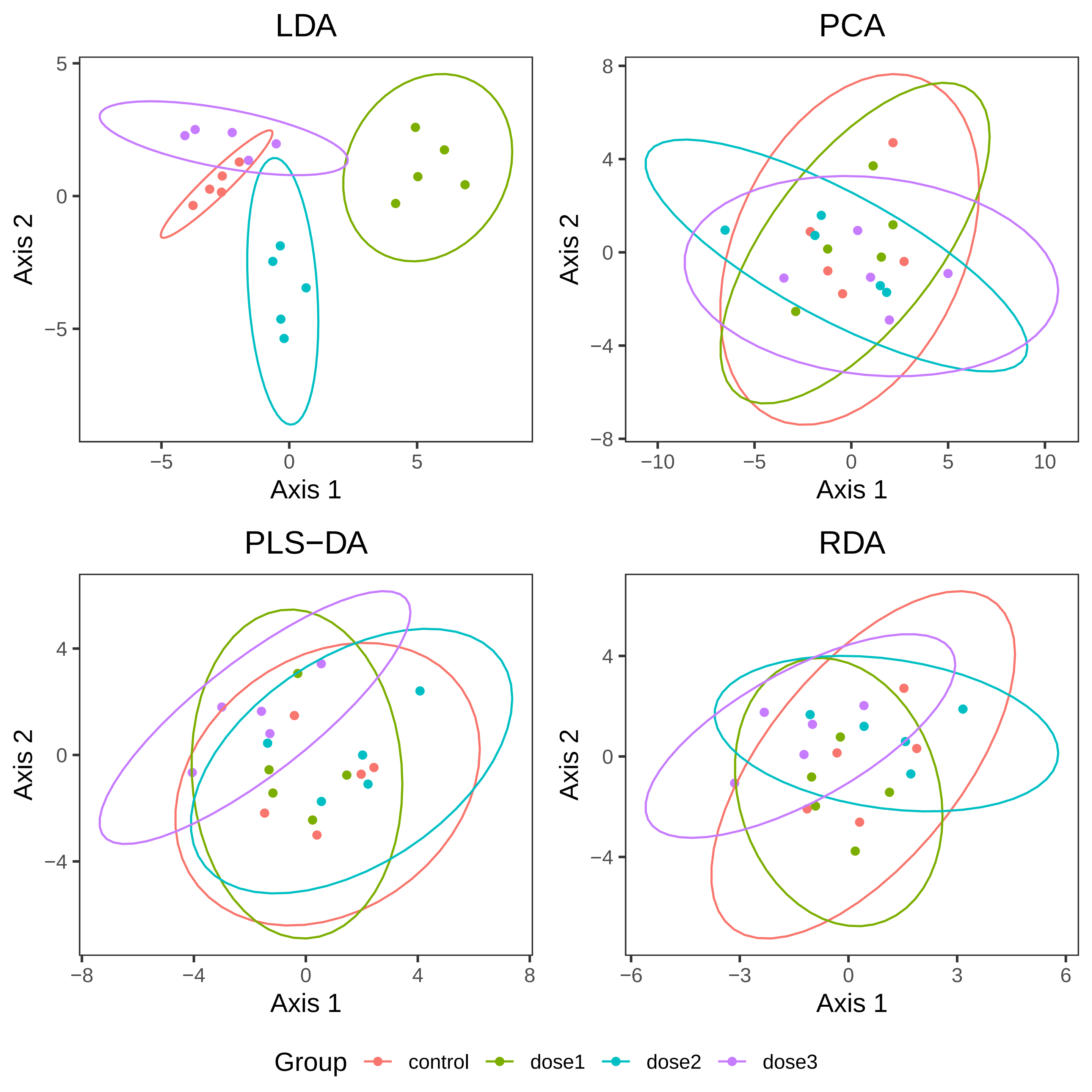

LDA
PCA
PLS−DA
RDA
Axis 1
Axis 2
Group
control
dose1
dose2
dose3

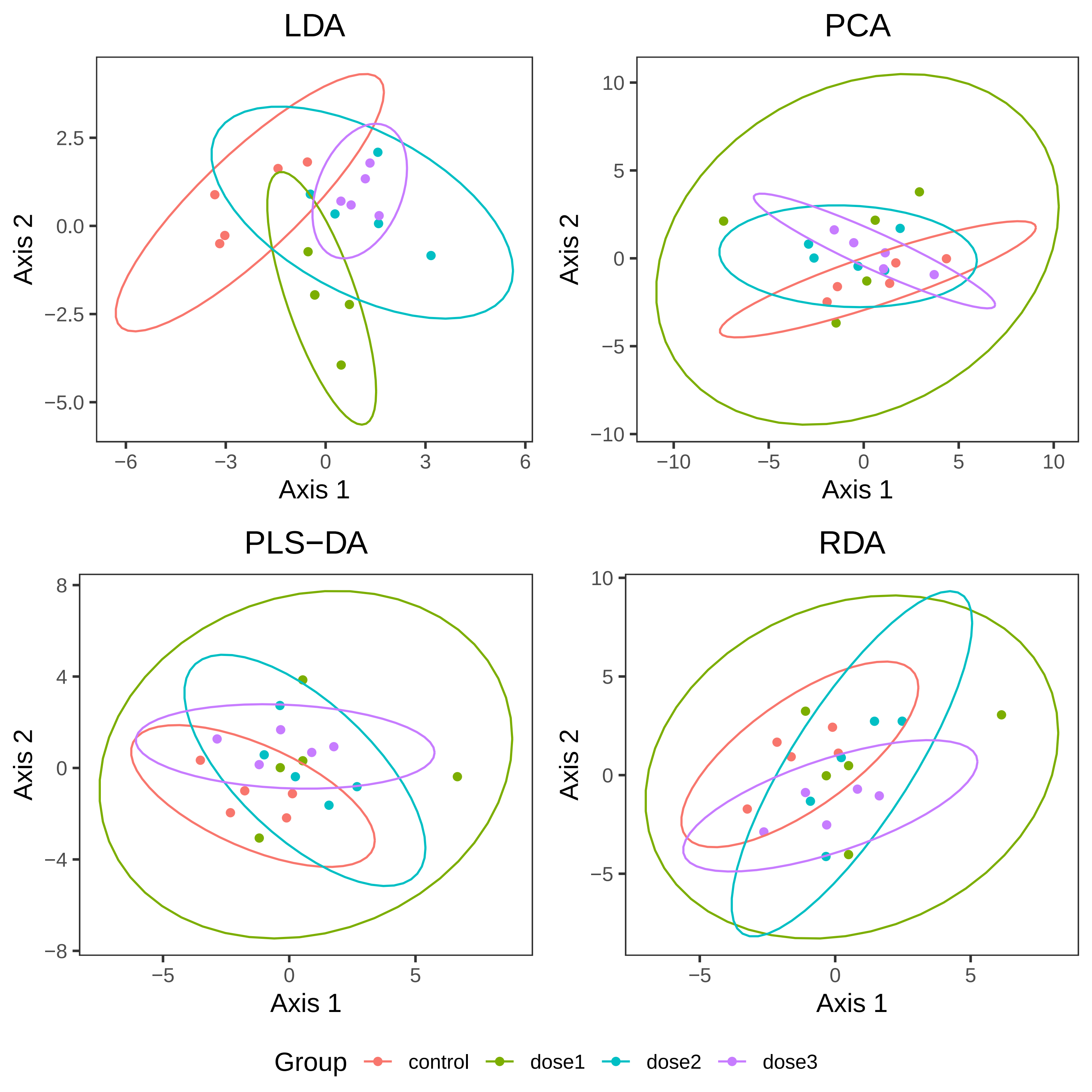
LDA
PCA
PLS−DA
RDA
Axis 1
Axis 2
−6
−3
0
3
6
2.5
0.0
−2.5
−5.0
−10
−5
0
5
10
8
4
−4
−8
Group
control
dose1
dose2
dose3

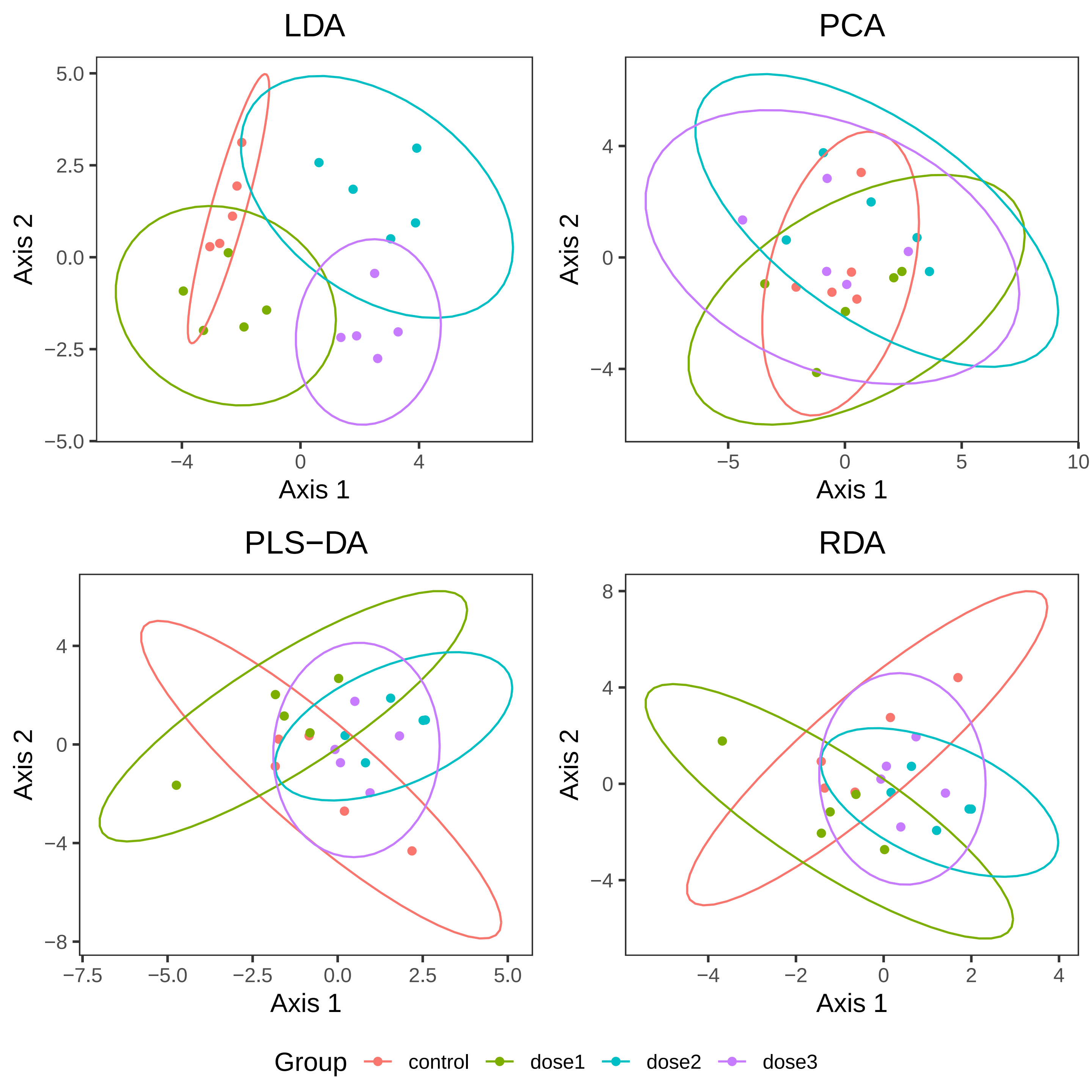

LDA
PCA
PLS−DA
RDA
Axis 1
Axis 2
5.0
2.5
0.0
−2.5
−5.0
−4
0
4
4
−4
−5
0
5
10
−8
−7.5
−5.0
−2.5
0.0
2.5
5.0
8
−2
2
Group
control
dose1
dose2
dose3

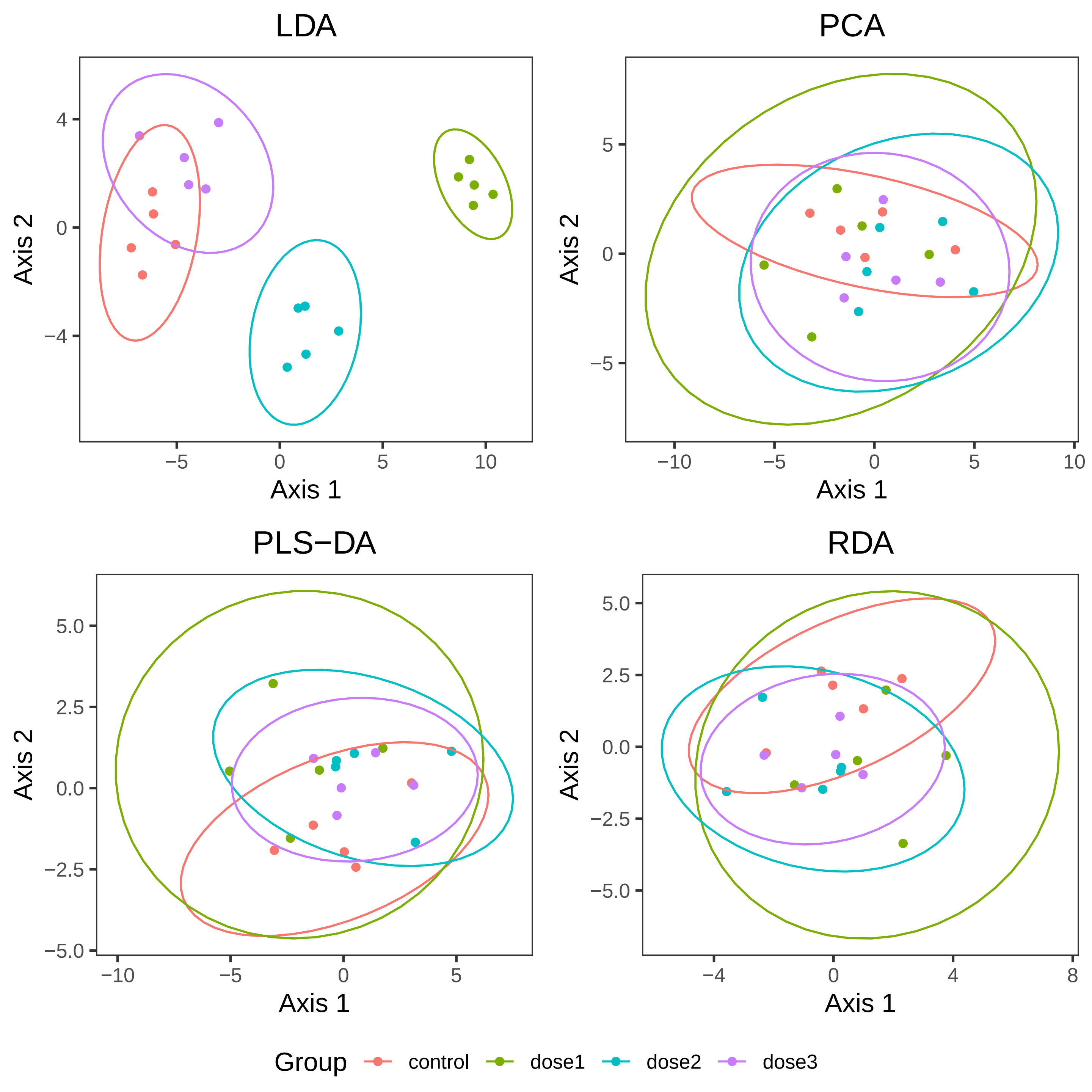

LDA
PCA
PLS−DA
RDA
Axis 1
Axis 2
Group
control
dose1
dose2
dose3

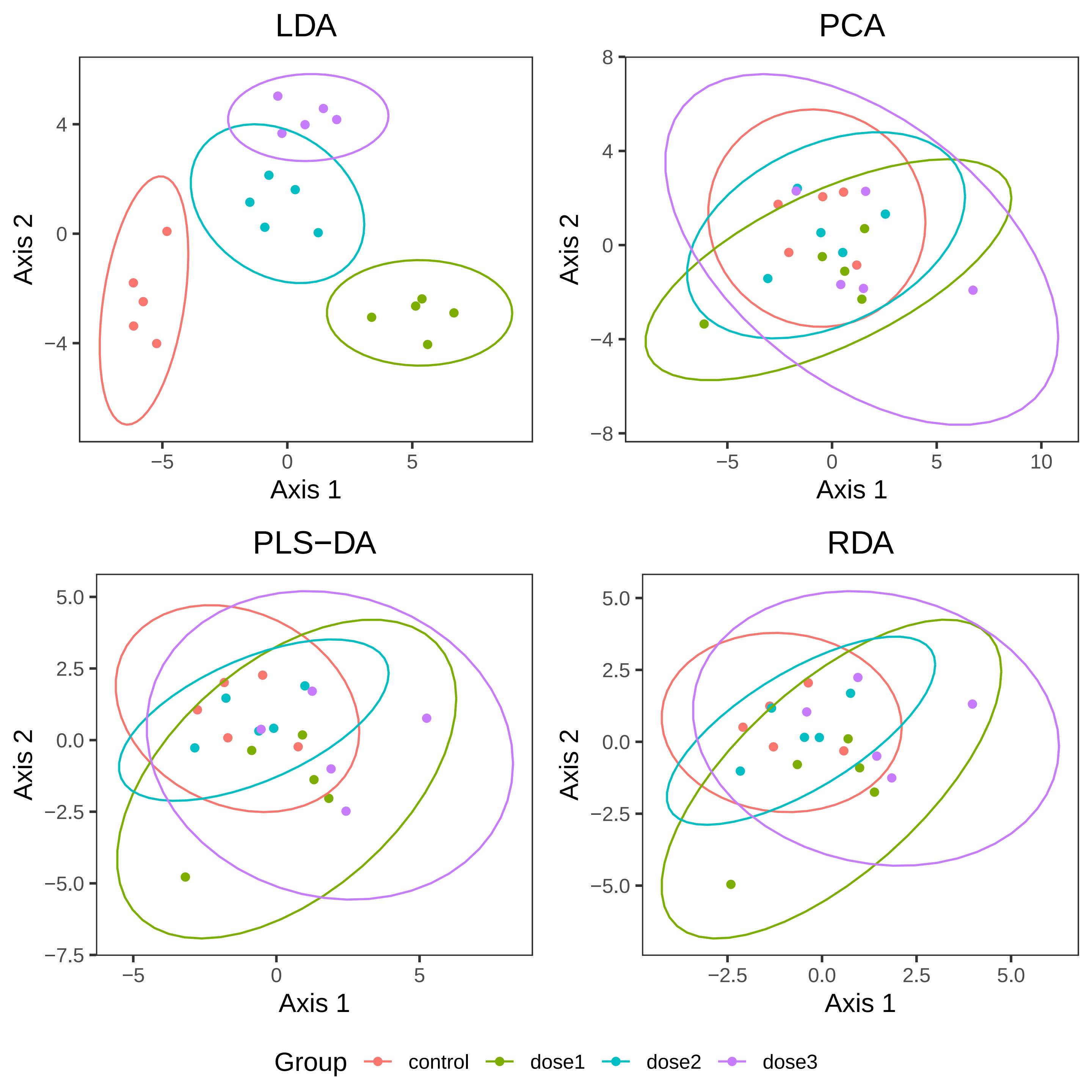

LDA
PCA
PLS−DA
RDA
Axis 1
Axis 2
Group
control
dose1
dose2
dose3

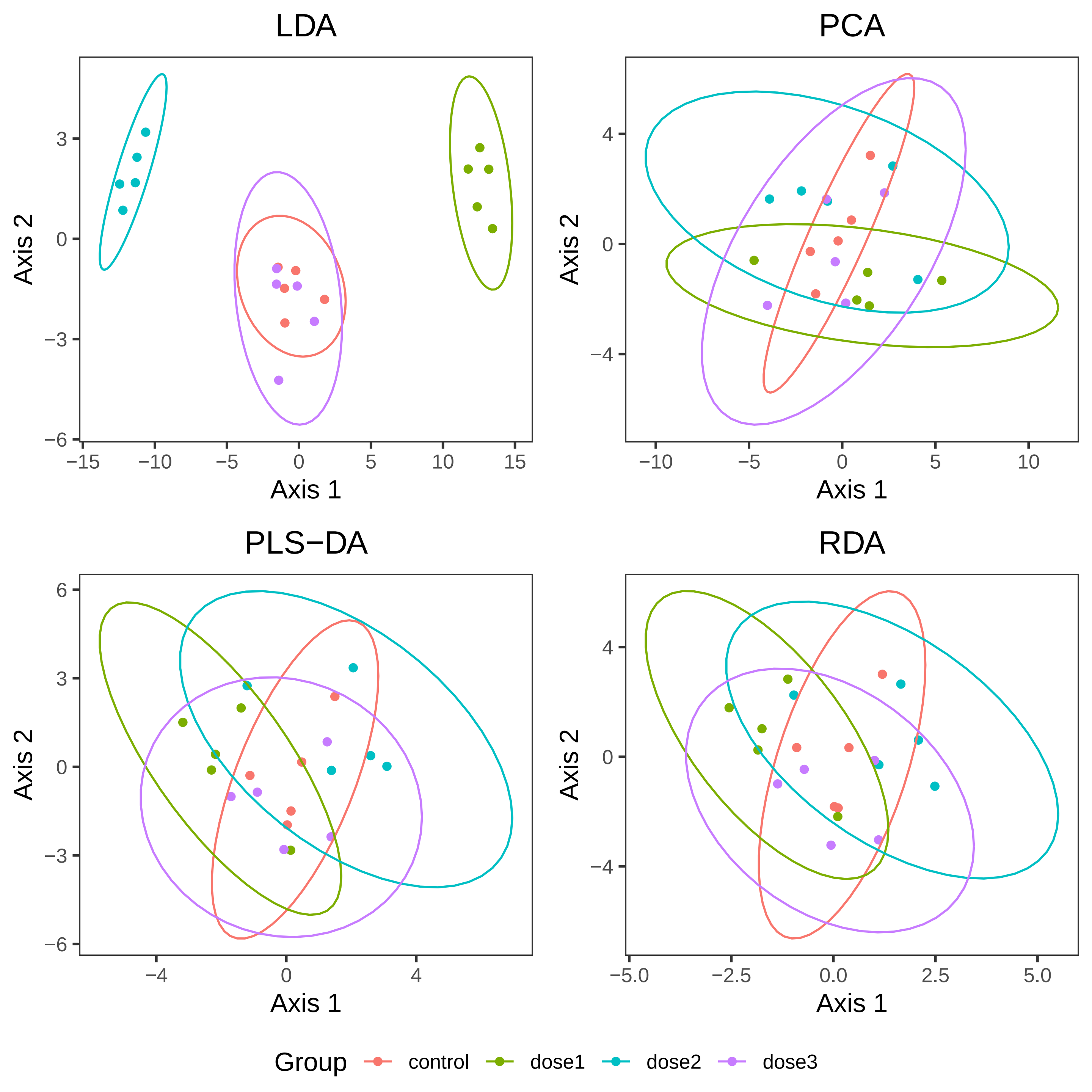
LDA
PCA
PLS−DA
RDA
Axis 1
Axis 2
Group
control
dose1
dose2
dose3
