# Supplementary material for: Applying univariate vs. multivariate statistics to investigate therapeutic efficacy in (pre)clinical trials: A Monte Carlo simulation study on the example of a controlled preclinical neurotrauma trial
Source: PLoS One. 2020 Mar 26;15(3):e0230798. doi: 10.1371/journal.pone.0230798 (PMC7098614; doi:10.1371/journal.pone.0230798)
Supplement: S3 Appendix — The file contains results from 1000 simulated data sets with 5 measurements per group and data obtained from a multivariate normal distribution with equal variance in all groups. PCA, RDA, or PLS-DA were considered to correctly capture a treatment effect pattern if the 95% confidence ellipse around the control group did not overlap with the confidence ellipses of the treatment groups (dose 1 to dose3). (PDF) [file pone.0230798.s003.pdf]

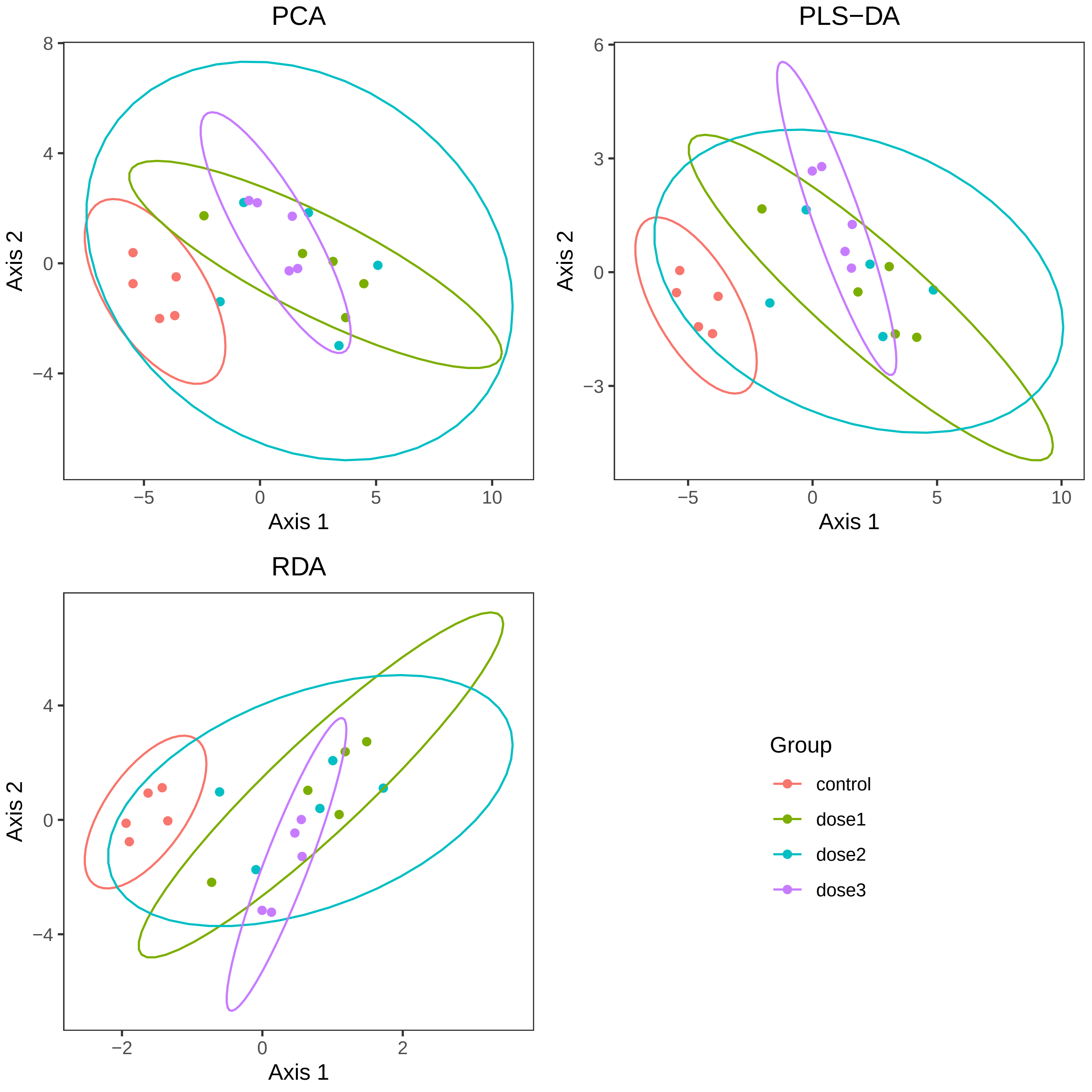

PCA
PLS−DA
RDA
Axis 1
Axis 2
8
4
0
−4
−5
5
10
6
3
−3
2
−2
Group
control
dose1
dose2
dose3

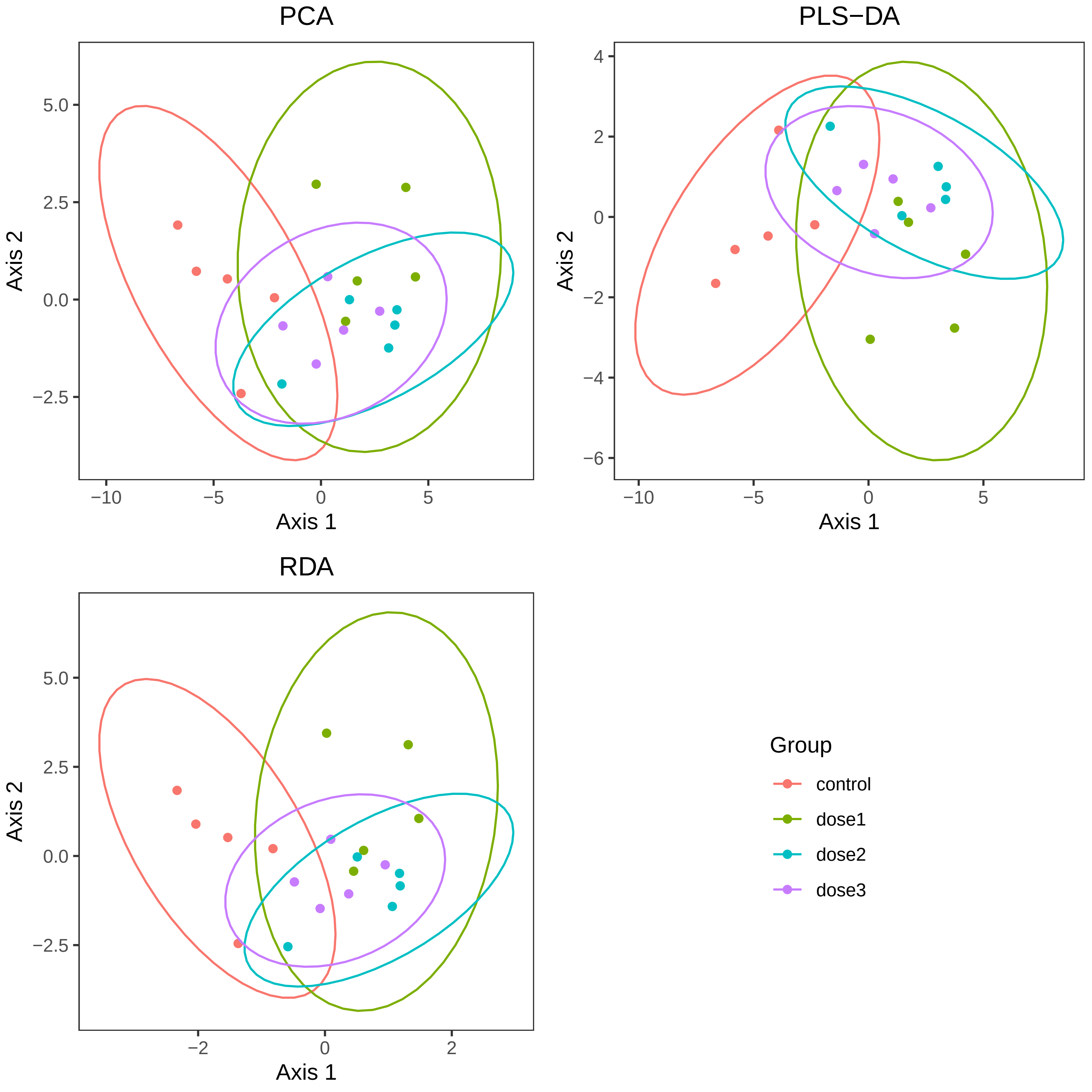

PCA
PLS−DA
RDA
Axis 1
Axis 2
5.0
2.5
0.0
−2.5
−10
−5
0
5
4
2
−2
−4
−6
Group
control
dose1
dose2
dose3

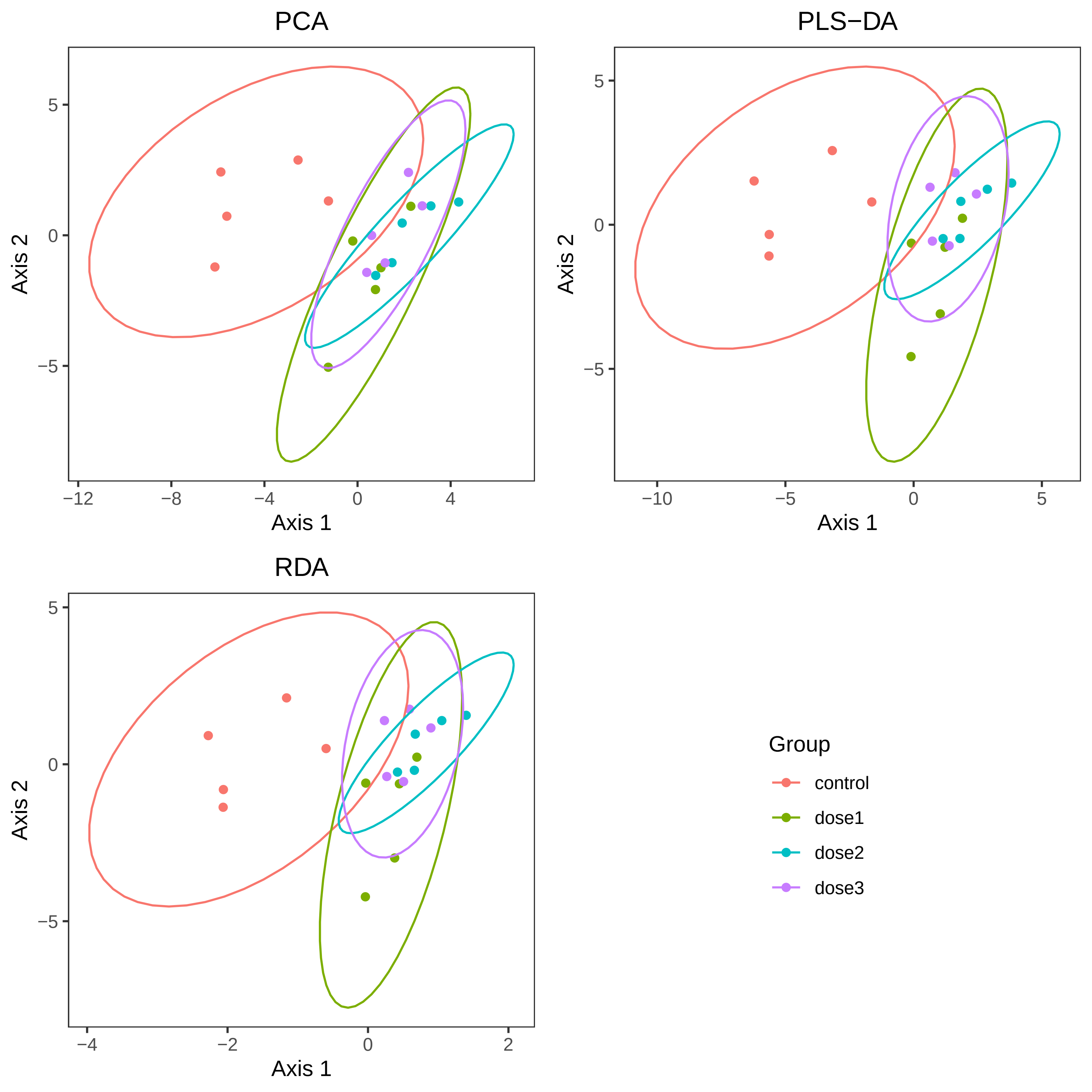

PCA
PLS−DA
RDA
Axis 1
Axis 2
−12
−8
−4
0
4
−10
−5
5
−2
2
Group
control
dose1
dose2
dose3

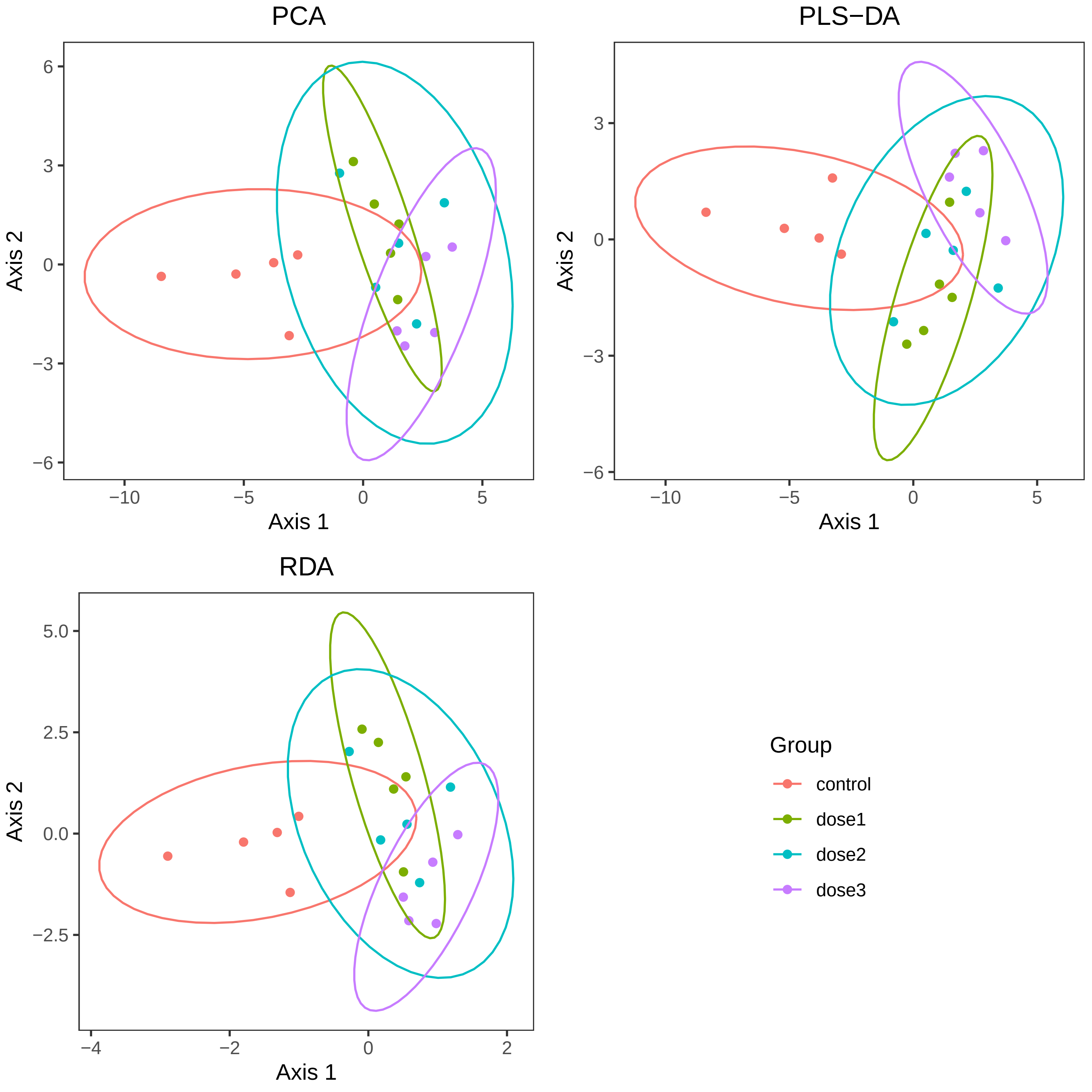
PCA
PLS−DA
RDA
Axis 1
Axis 2
Group
control
dose1
dose2
dose3

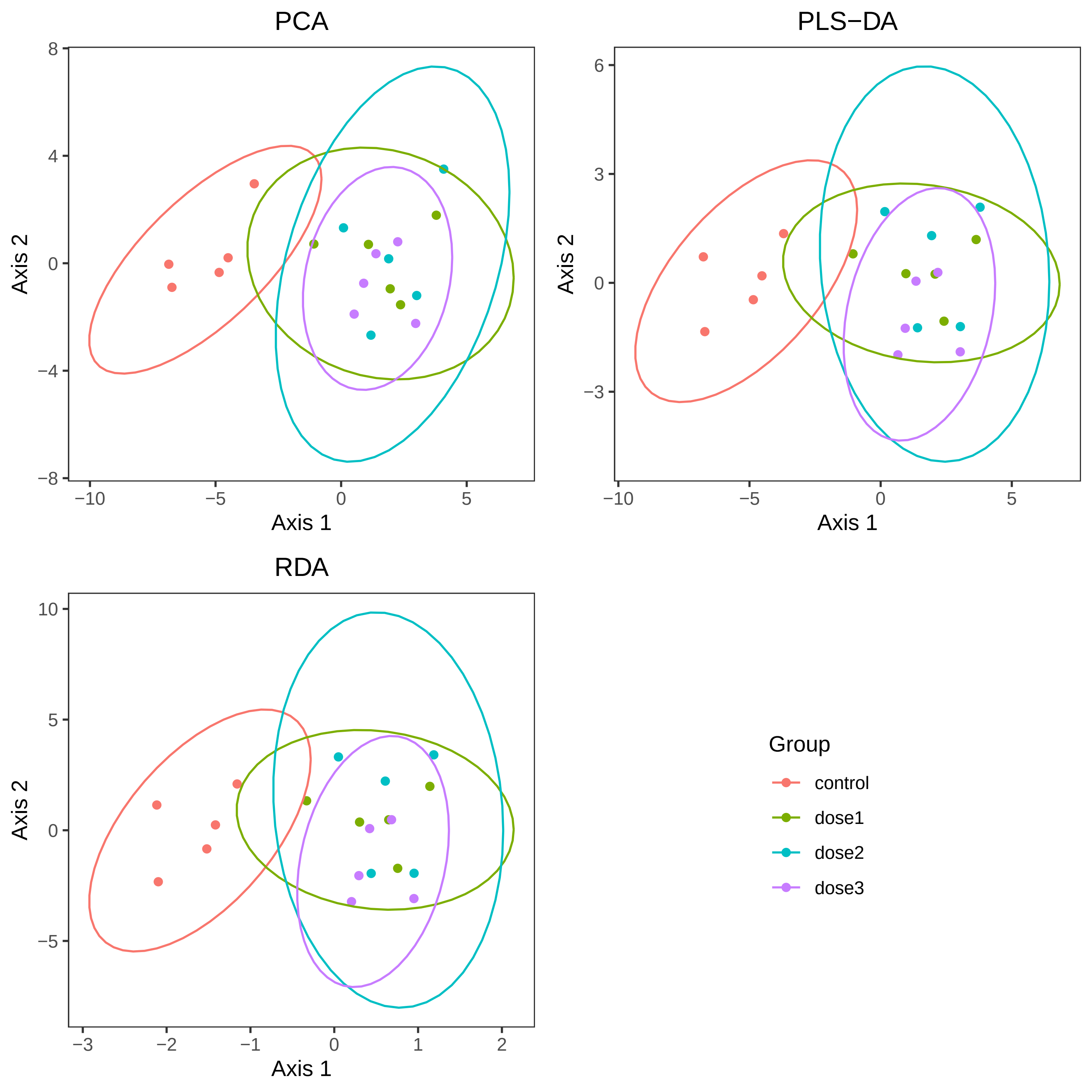

PCA
PLS−DA
RDA
Axis 1
Axis 2
Group
control
dose1
dose2
dose3

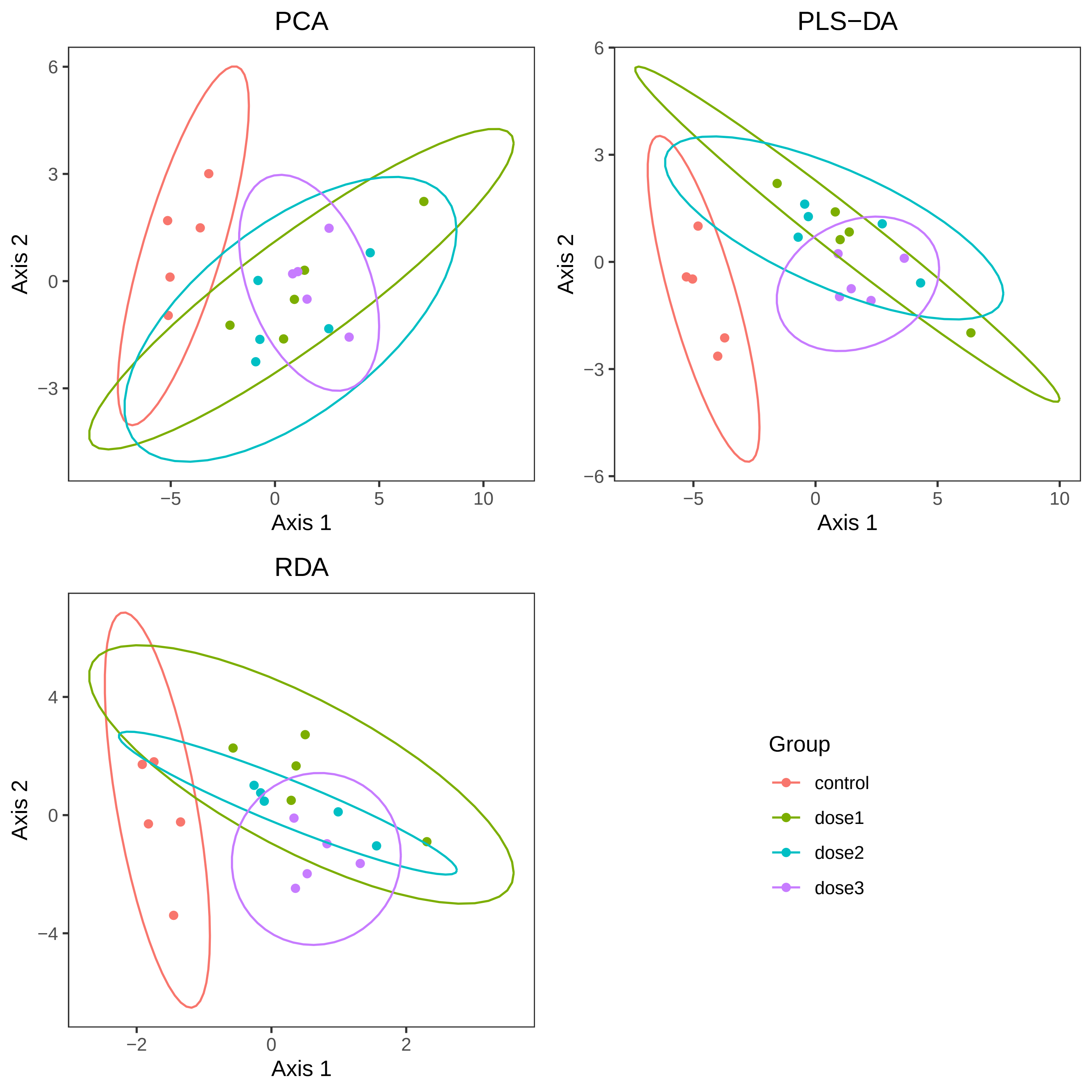
PCA
PLS−DA
RDA
Axis 1
Axis 2
Group
control
dose1
dose2
dose3

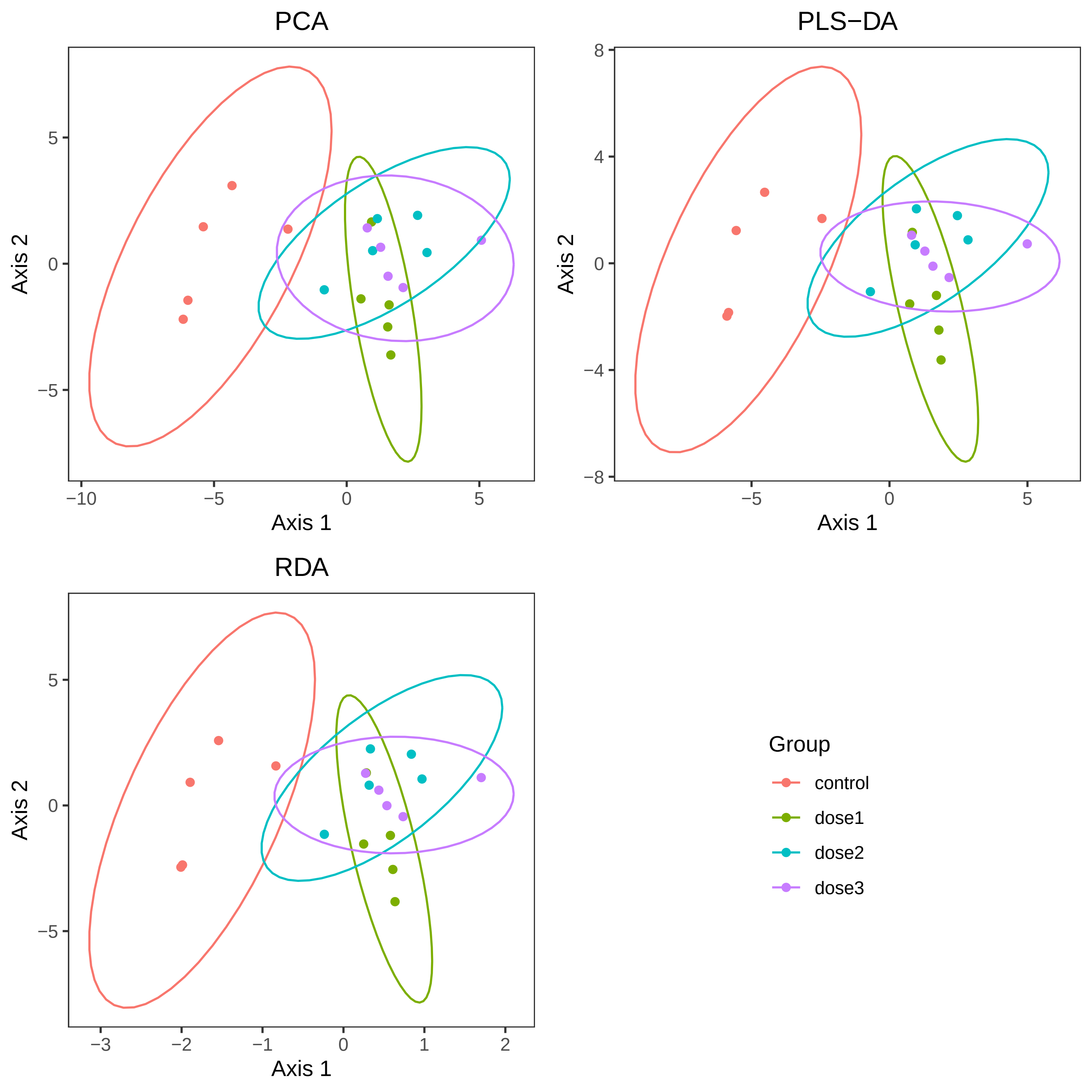

PCA
PLS−DA
RDA
Axis 1
Axis 2
−10
−5
0
5
8
4
−4
−8
−3
−2
−1
1
2
Group
control
dose1
dose2
dose3

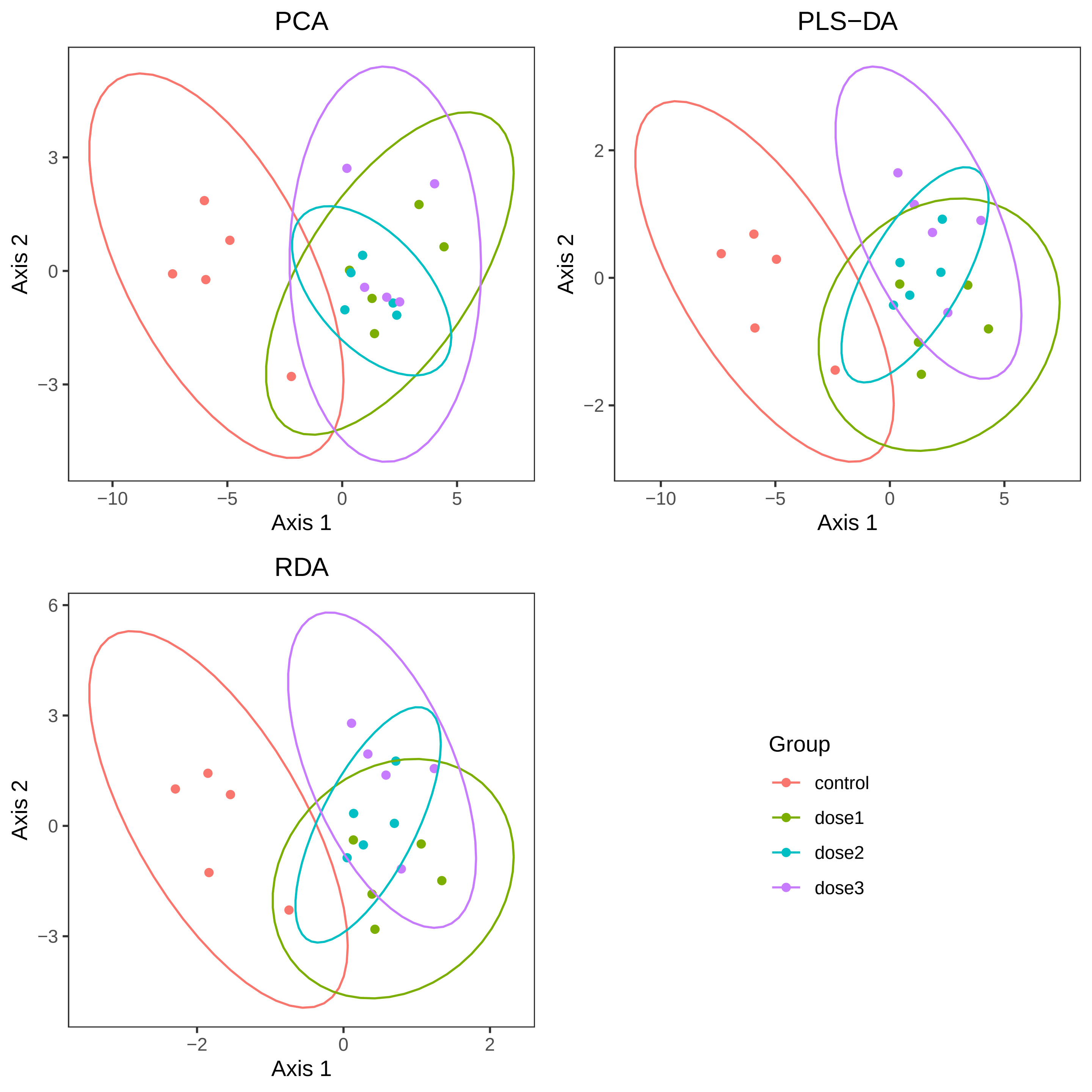
PCA
PLS−DA
RDA
Axis 1
Axis 2
−10
−5
0
5
3
0
−3
2
−2
6
Group
control
dose1
dose2
dose3

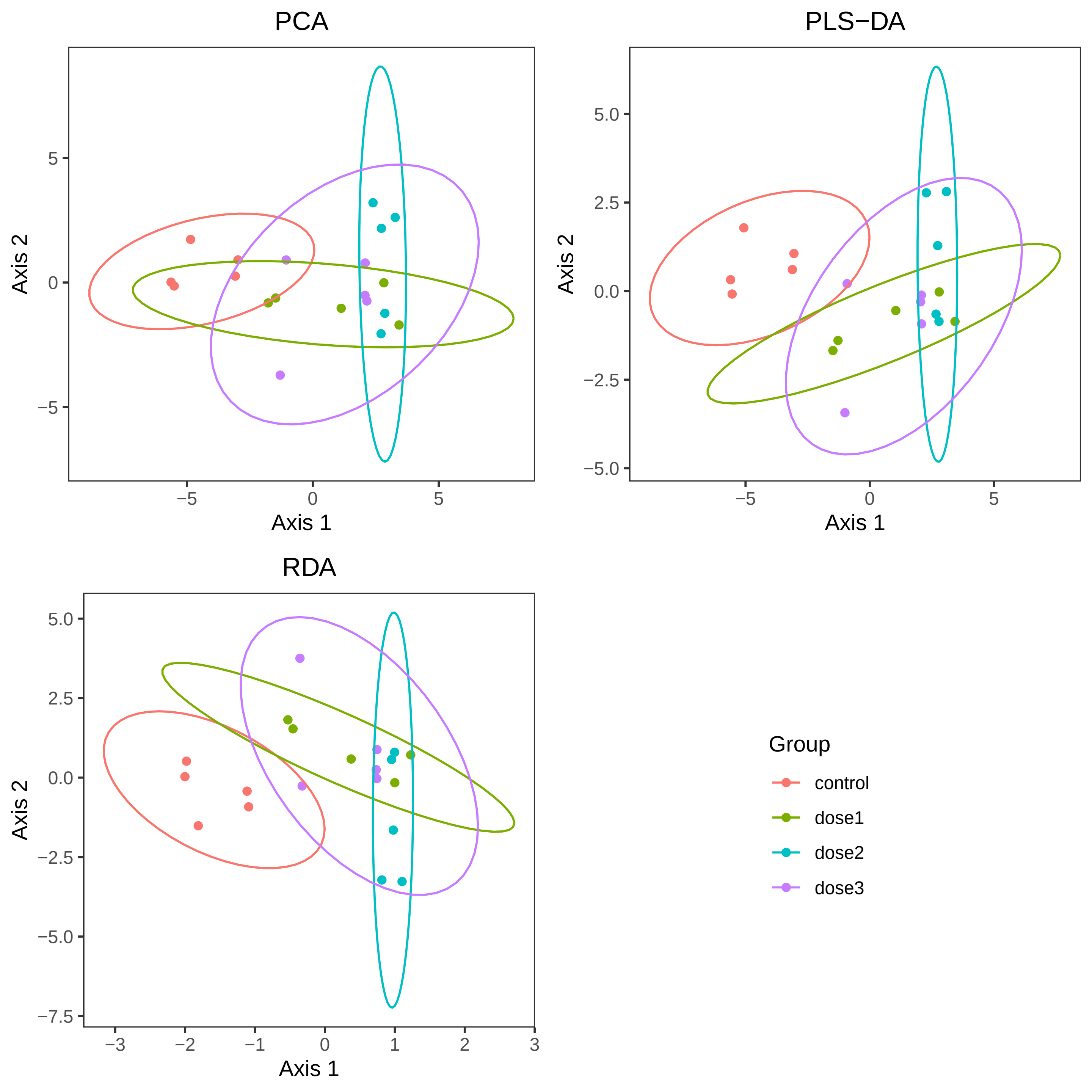

PCA
PLS−DA
RDA
Axis 1
Axis 2
5
0
−5
5.0
2.5
0.0
−2.5
−5.0
−7.5
−3
−2
−1
1
2
3
Group
control
dose1
dose2
dose3

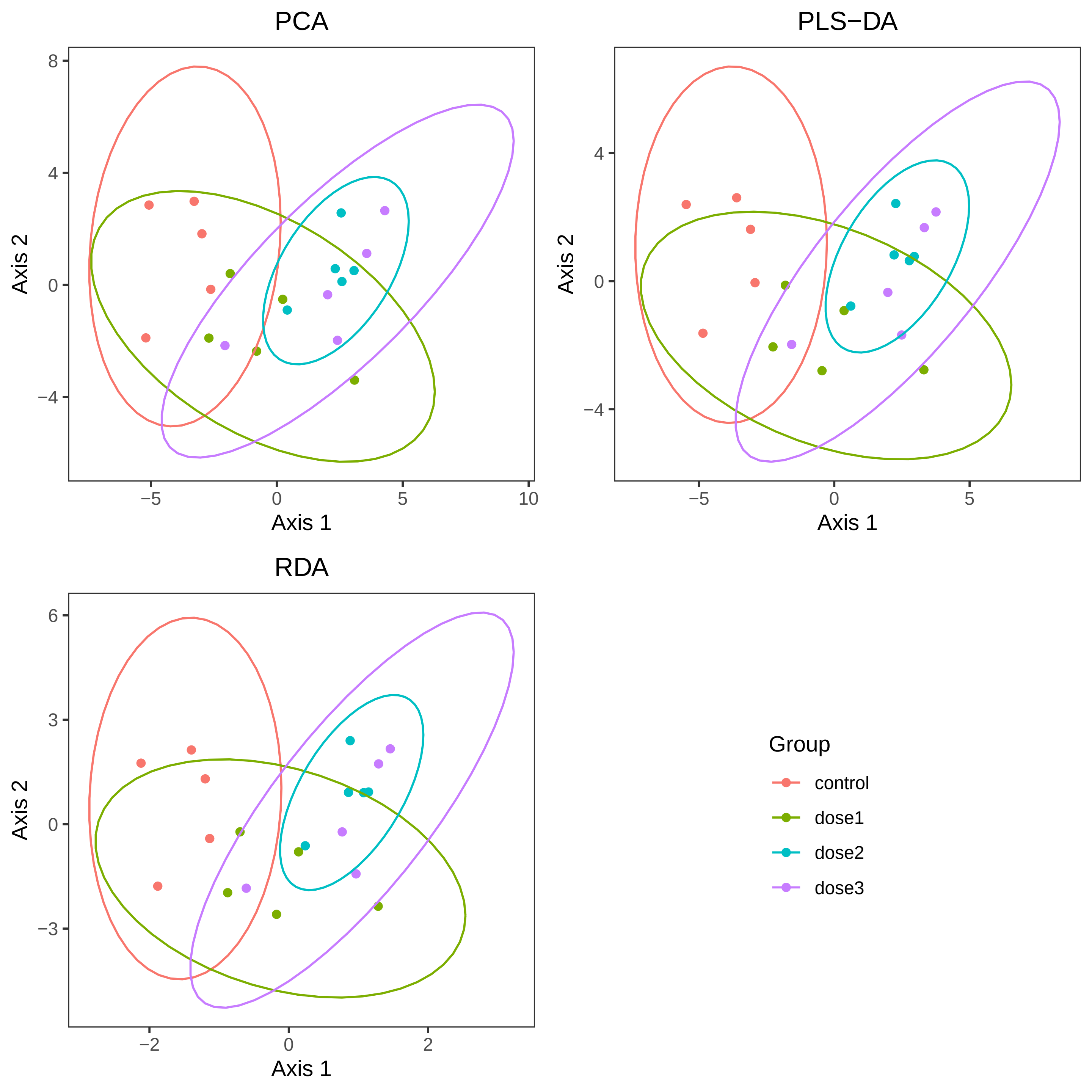

PCA
PLS−DA
RDA
Axis 1
Axis 2
8
4
0
−4
−5
0
5
10
−3
3
6
−2
2
Group
control
dose1
dose2
dose3

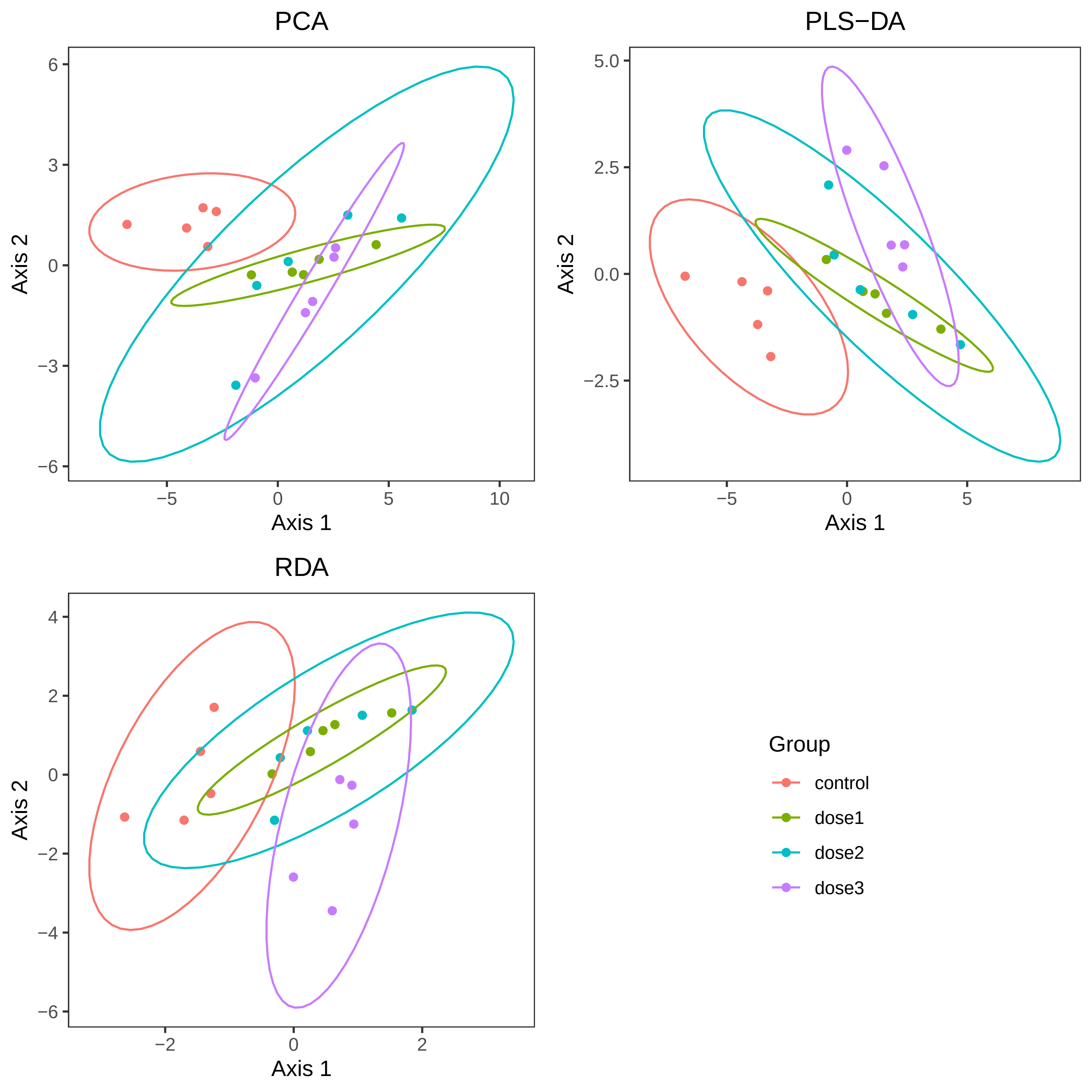
PCA
PLS−DA
RDA
Axis 1
Axis 2
Group
control
dose1
dose2
dose3

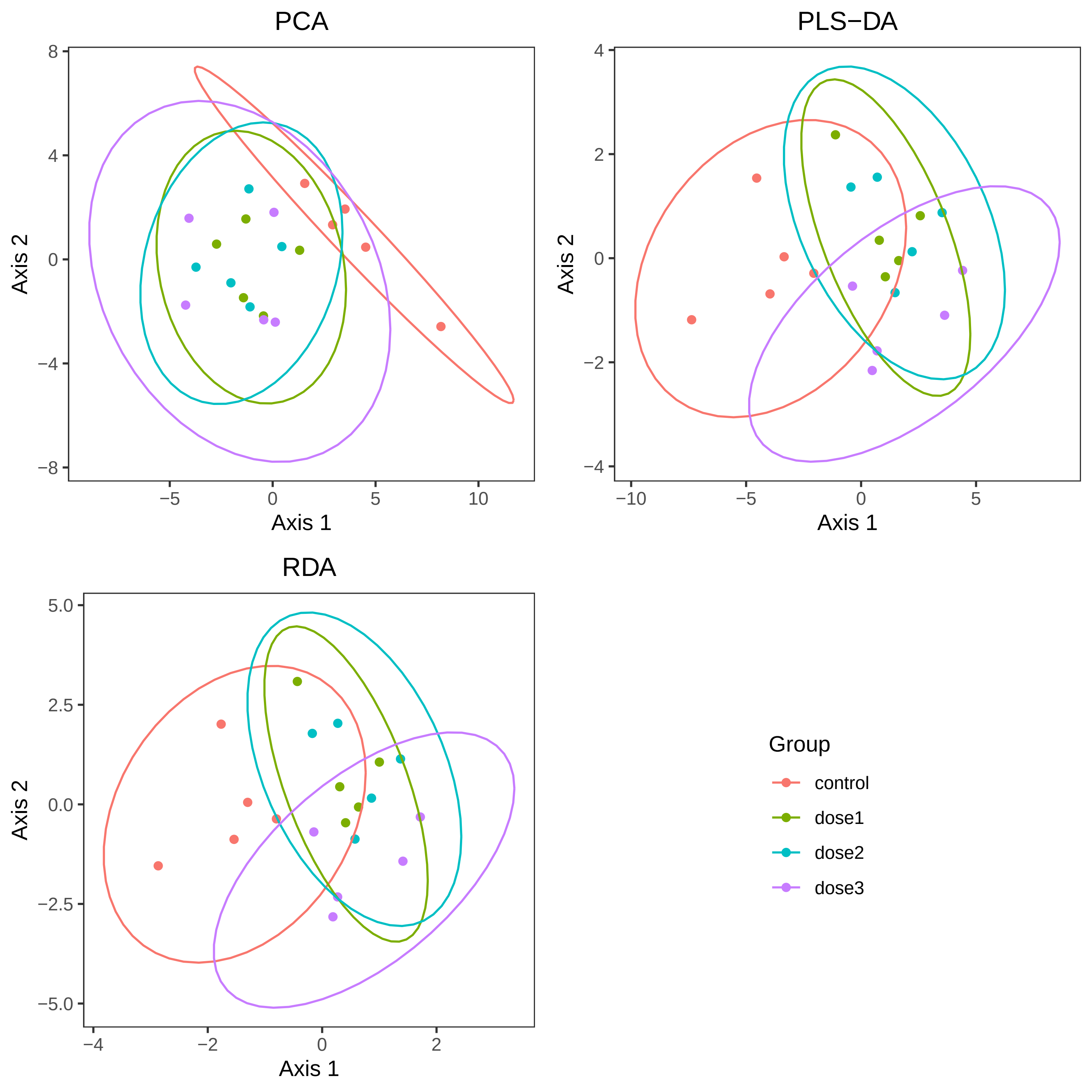

PCA
PLS−DA
RDA
Axis 1
Axis 2
Group
control
dose1
dose2
dose3

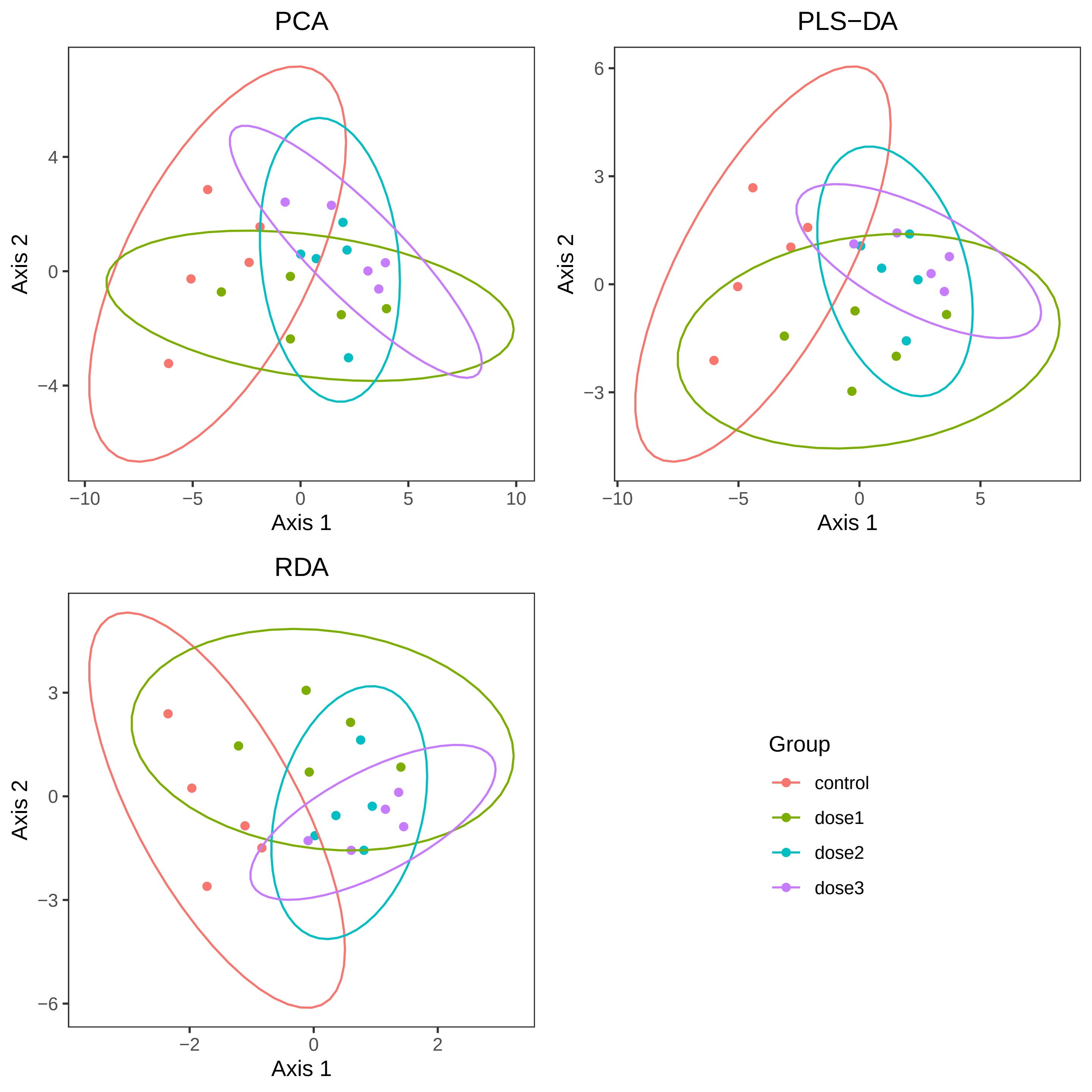

PCA
Axis 2
4
0
−4
−10
−5
0
5
10
Axis 1
PLS−DA
Axis 2
6
3
0
−3
−10
−5
0
5
Axis 1
RDA
Axis 2
3
0
−3
−6
−2
0
2
Axis 1
Group
control
dose1
dose2
dose3

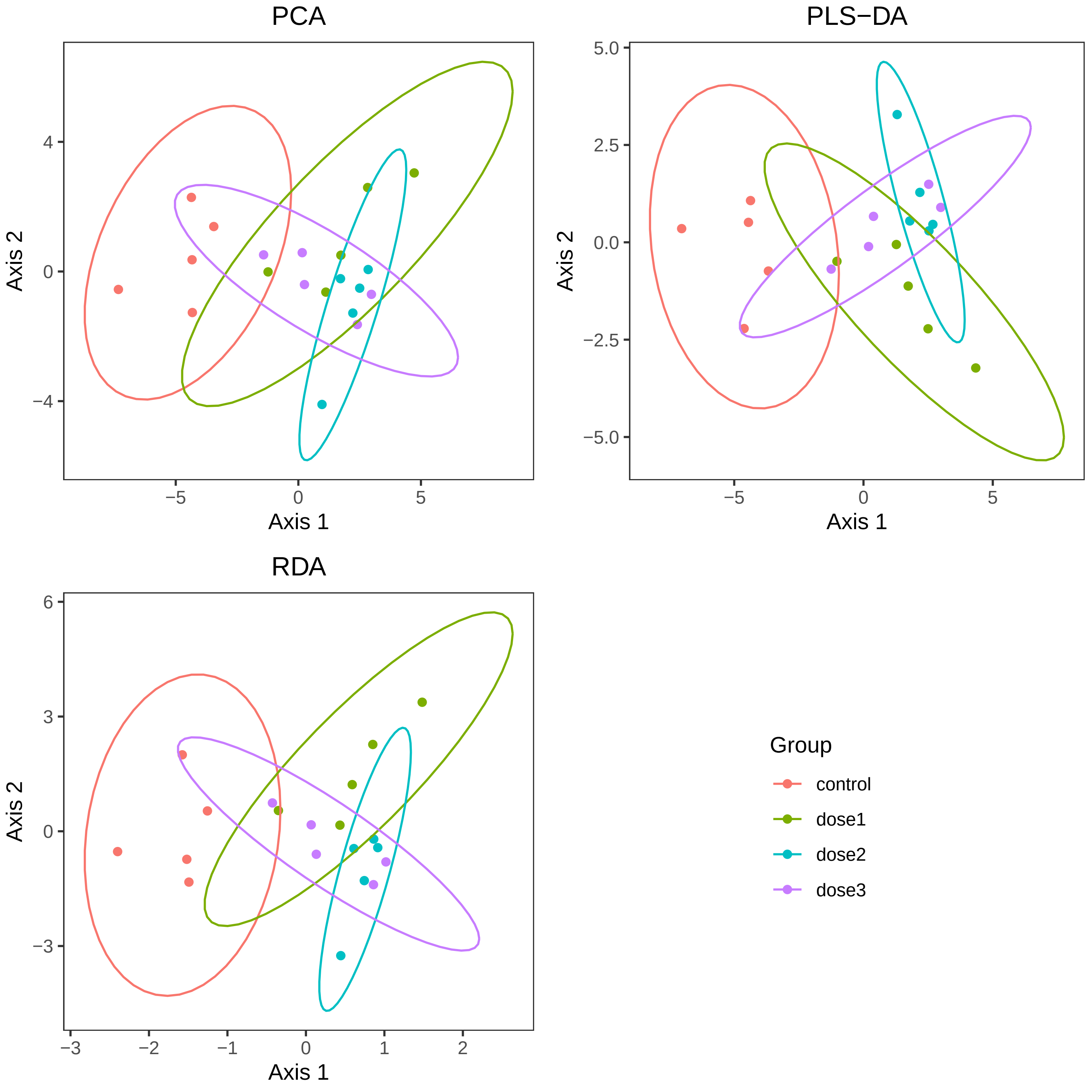
PCA
PLS−DA
RDA
Axis 1
Axis 2
Group
control
dose1
dose2
dose3

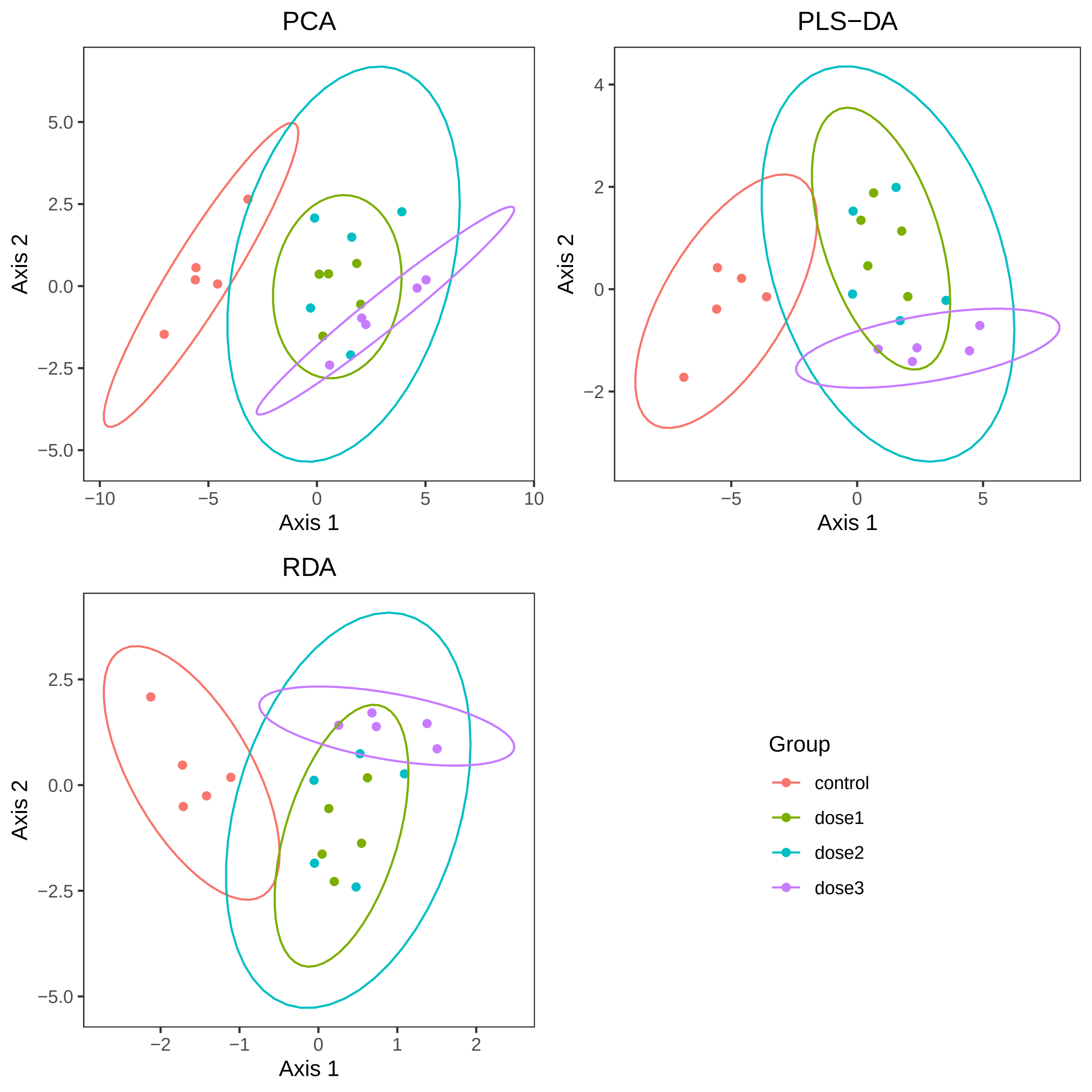

PCA
PLS−DA
RDA
Axis 1
Axis 2
5.0
2.5
0.0
−2.5
−5.0
−10
−5
0
5
10
4
2
0
−2
−2
−1
1
Group
control
dose1
dose2
dose3

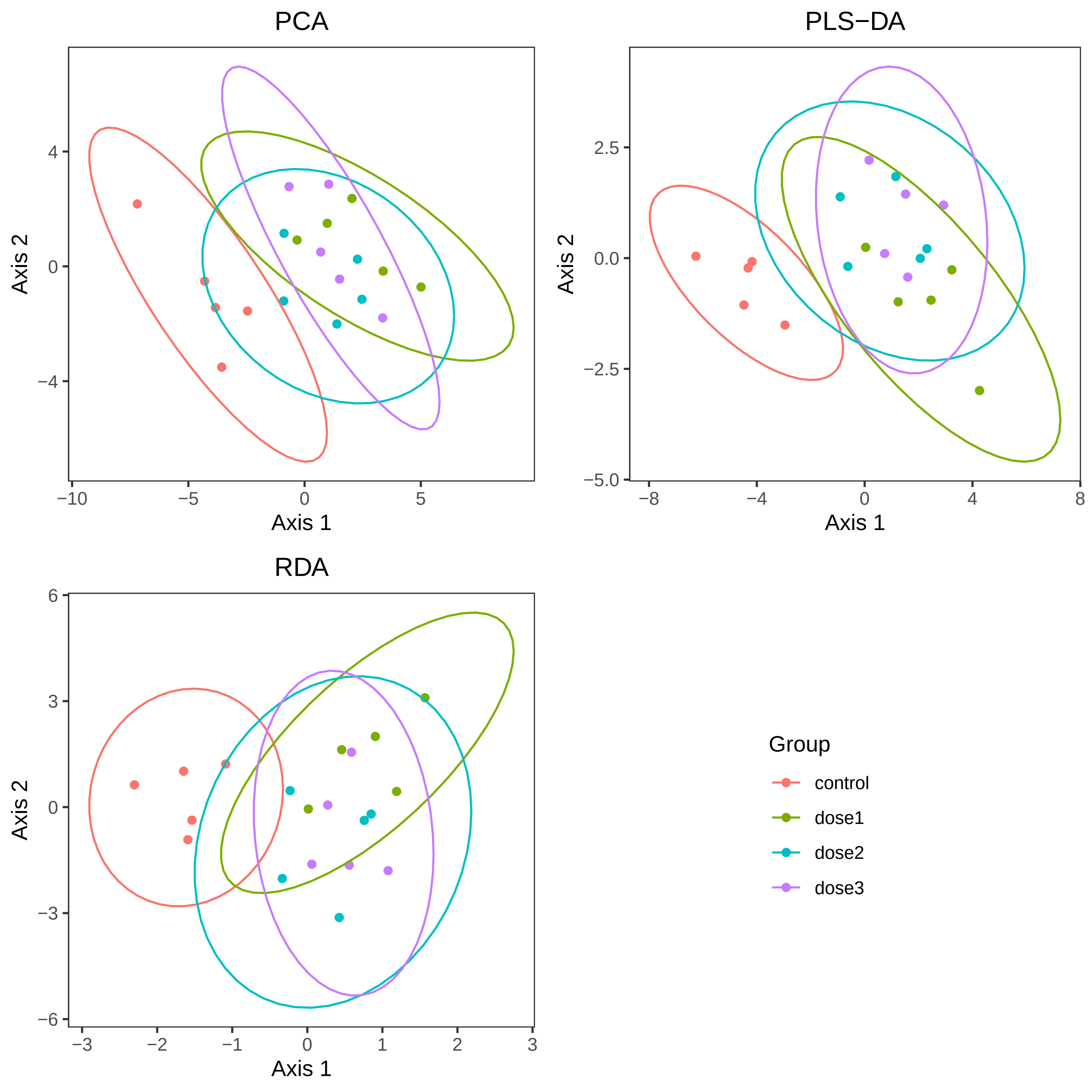

PCA
PLS−DA
RDA
Axis 1
Axis 2
Group
control
dose1
dose2
dose3

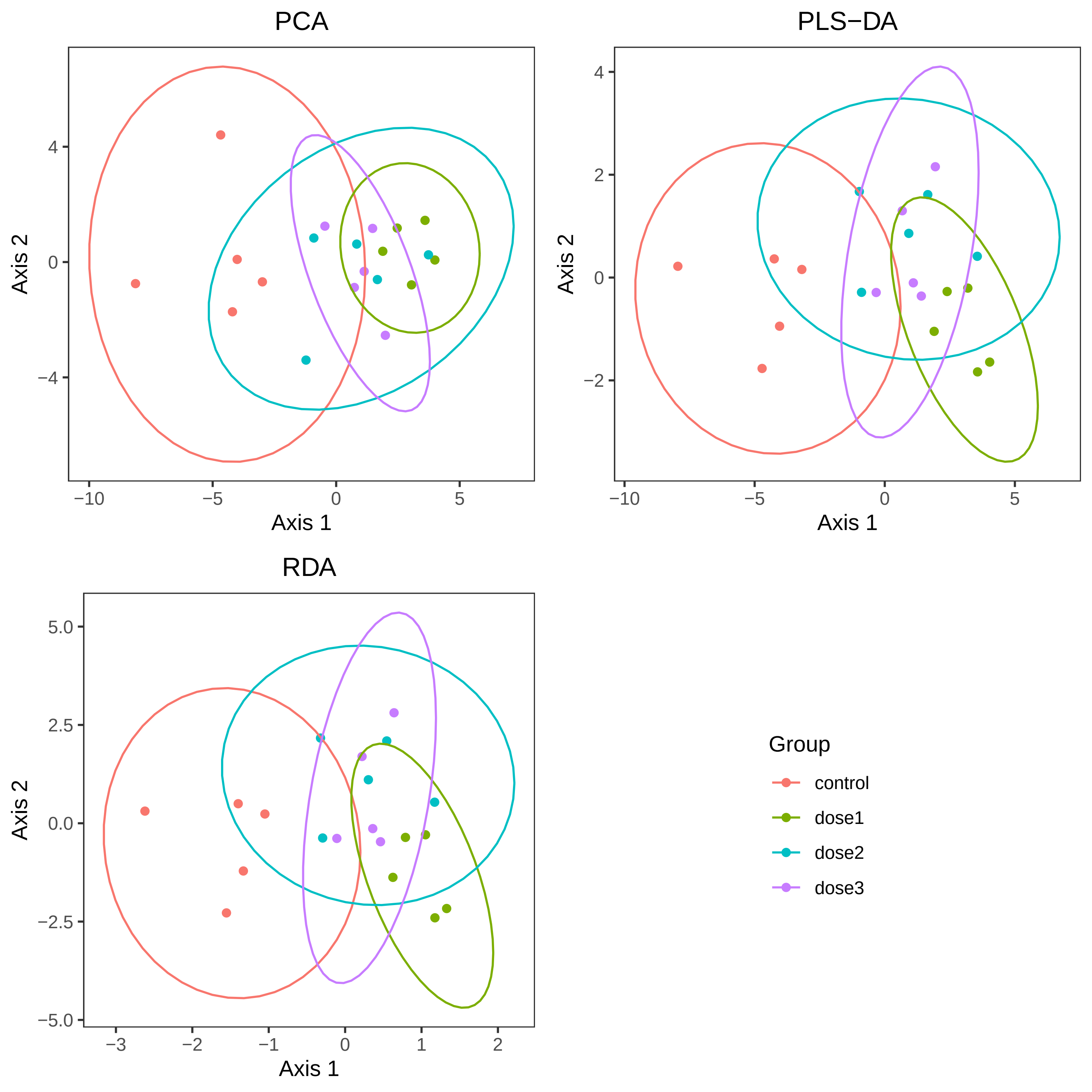
PCA
PLS−DA
RDA
Axis 1
Axis 2
−10
−5
0
5
4
0
−4
2
−2
−3
−2
−1
1
2
5.0
2.5
0.0
−2.5
−5.0
Group
control
dose1
dose2
dose3

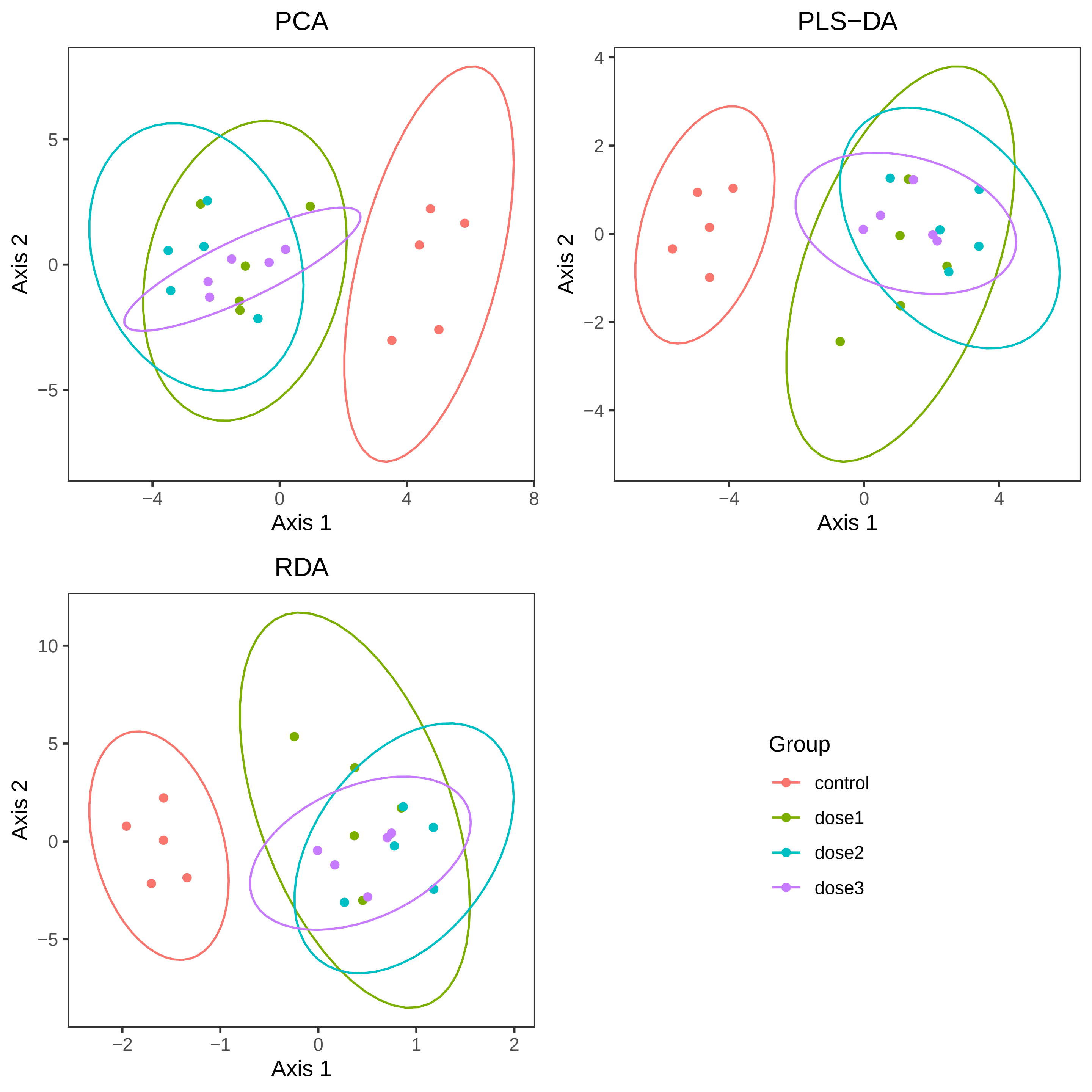
PCA
PLS−DA
RDA
Axis 1
Axis 2
−4
0
4
8
5
0
−5
2
−2
−4
10
−2
−1
1
Group
control
dose1
dose2
dose3

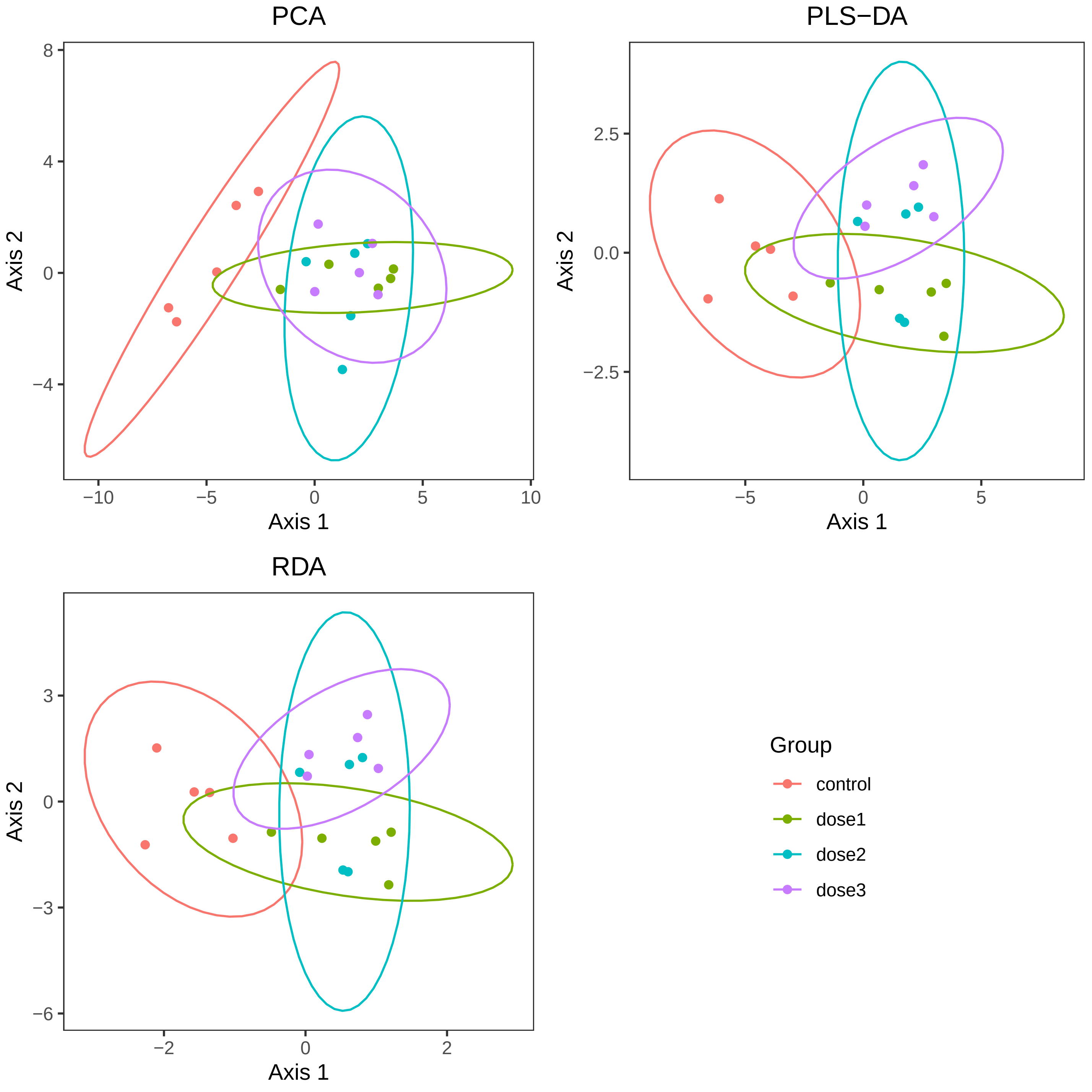
PCA
PLS−DA
RDA
Axis 1
Axis 2
Group
control
dose1
dose2
dose3

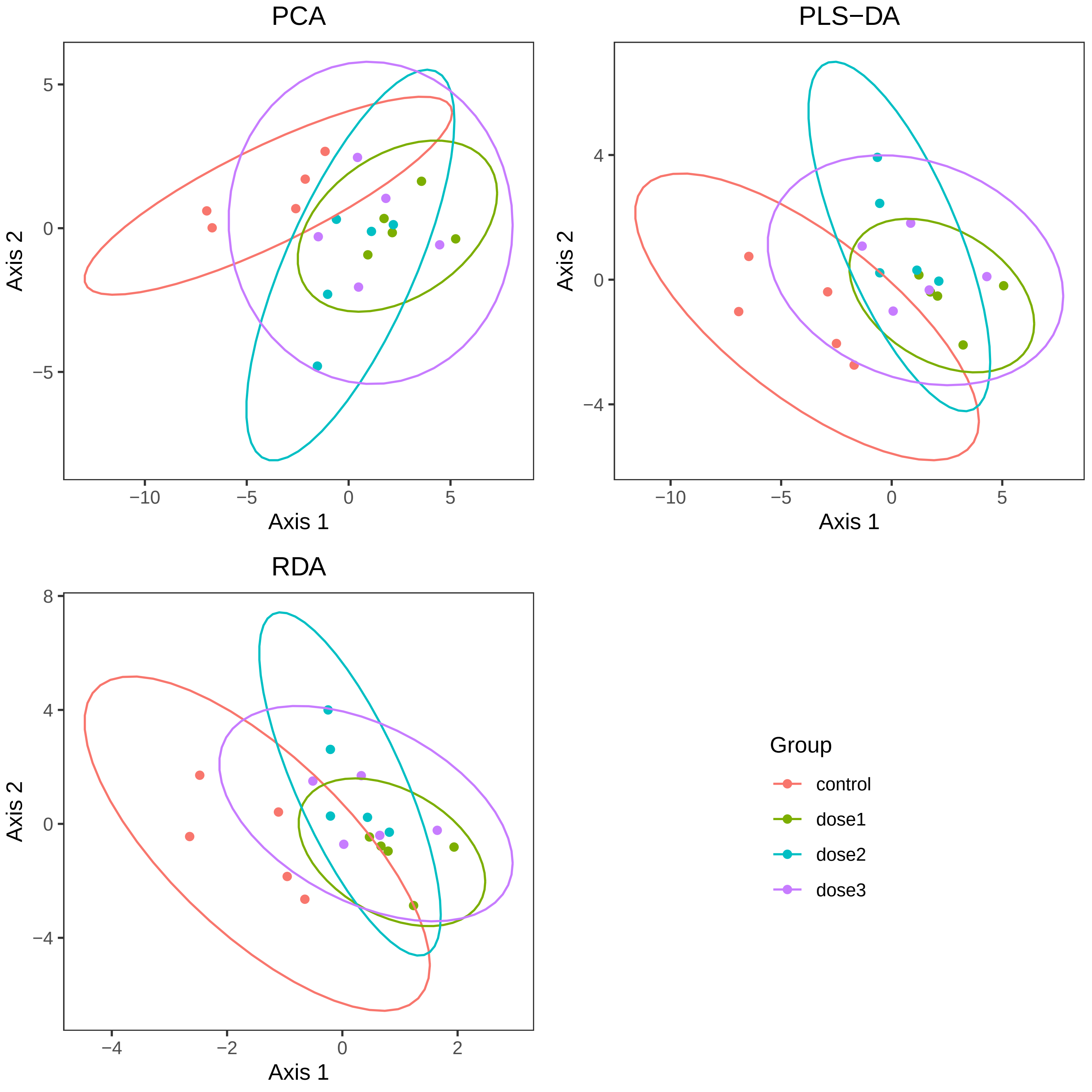
PCA
PLS−DA
RDA
Axis 1
Axis 2
Group
control
dose1
dose2
dose3

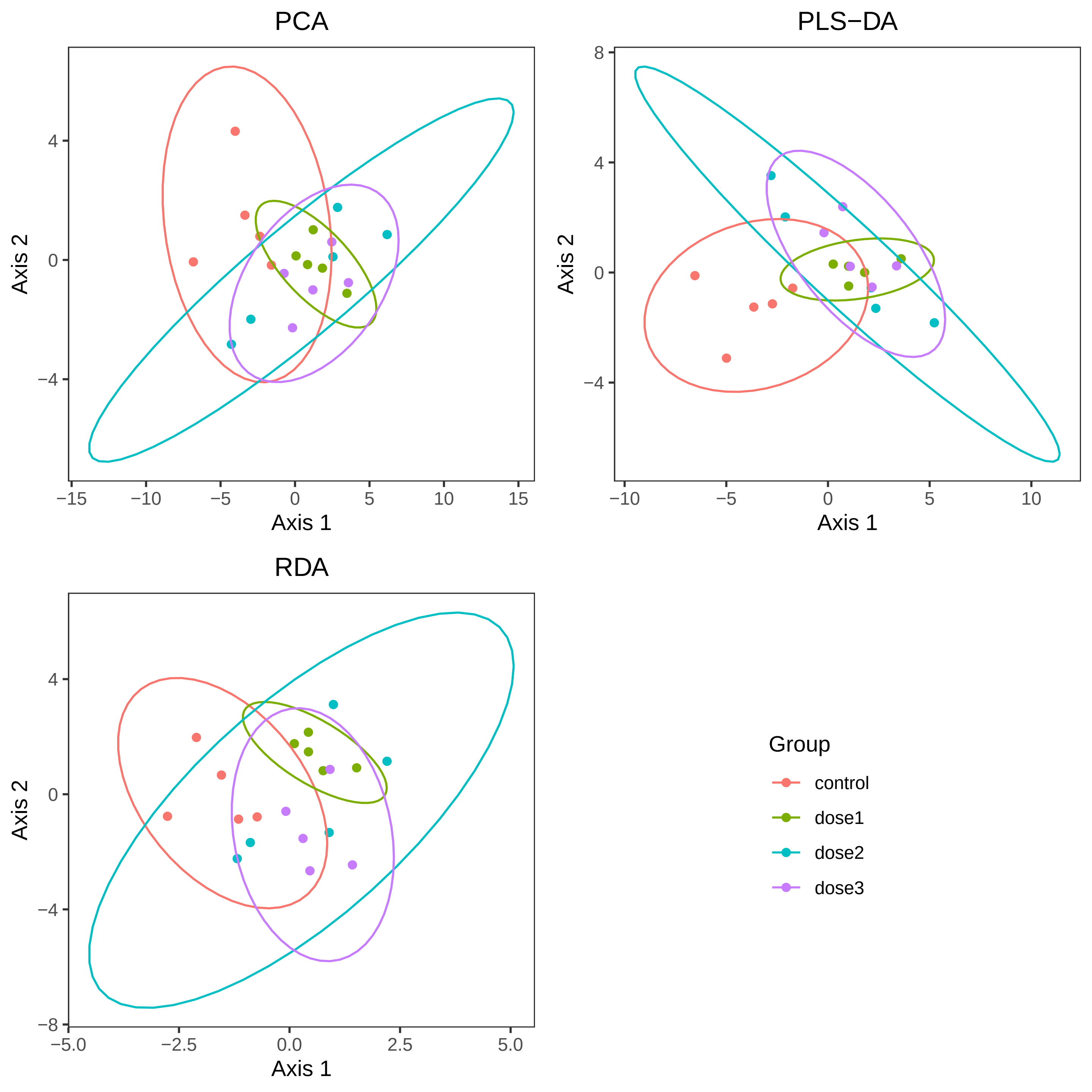
PCA
PLS−DA
RDA
Axis 1
Axis 2
Group
control
dose1
dose2
dose3

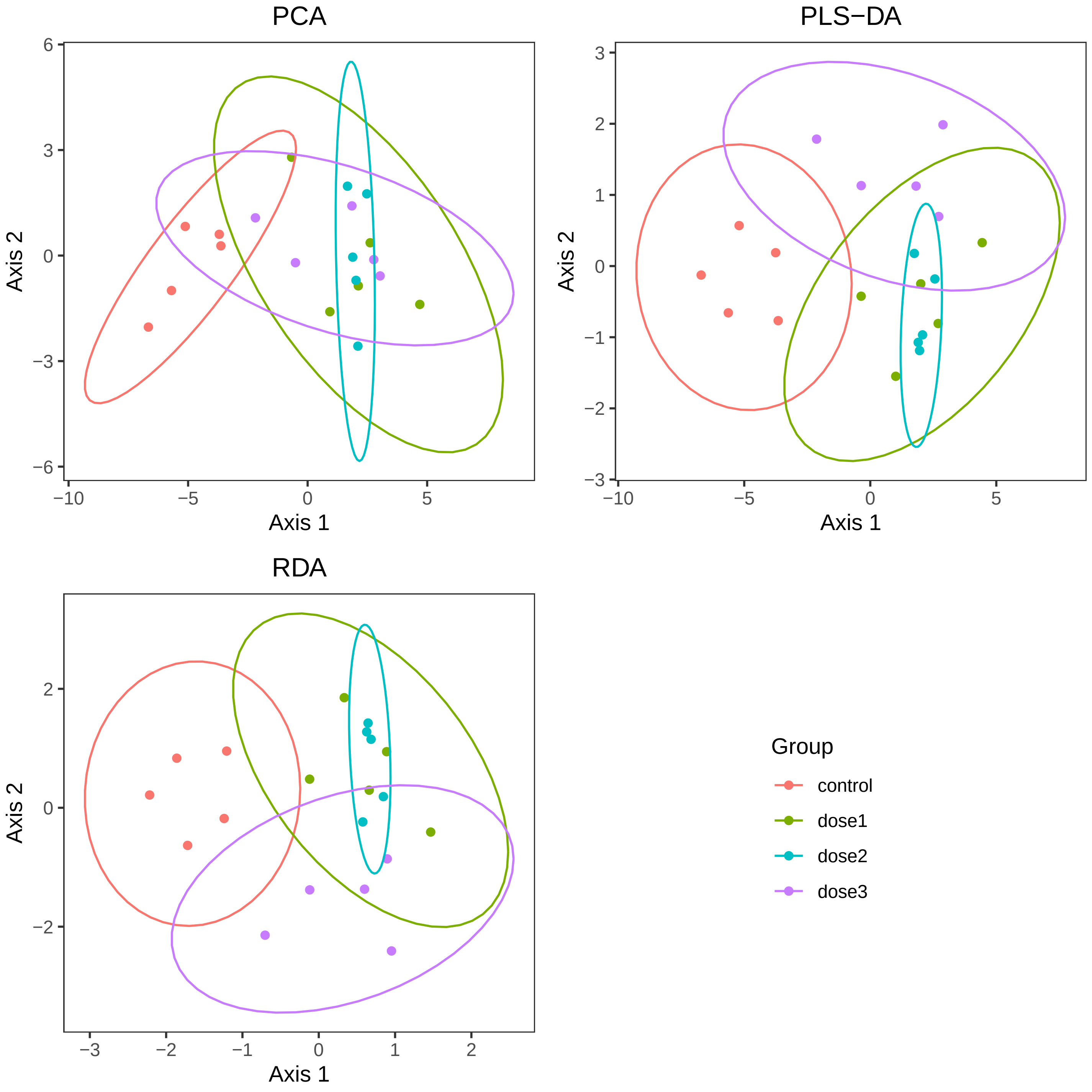
PCA
PLS−DA
RDA
Axis 1
Axis 2
Group
control
dose1
dose2
dose3

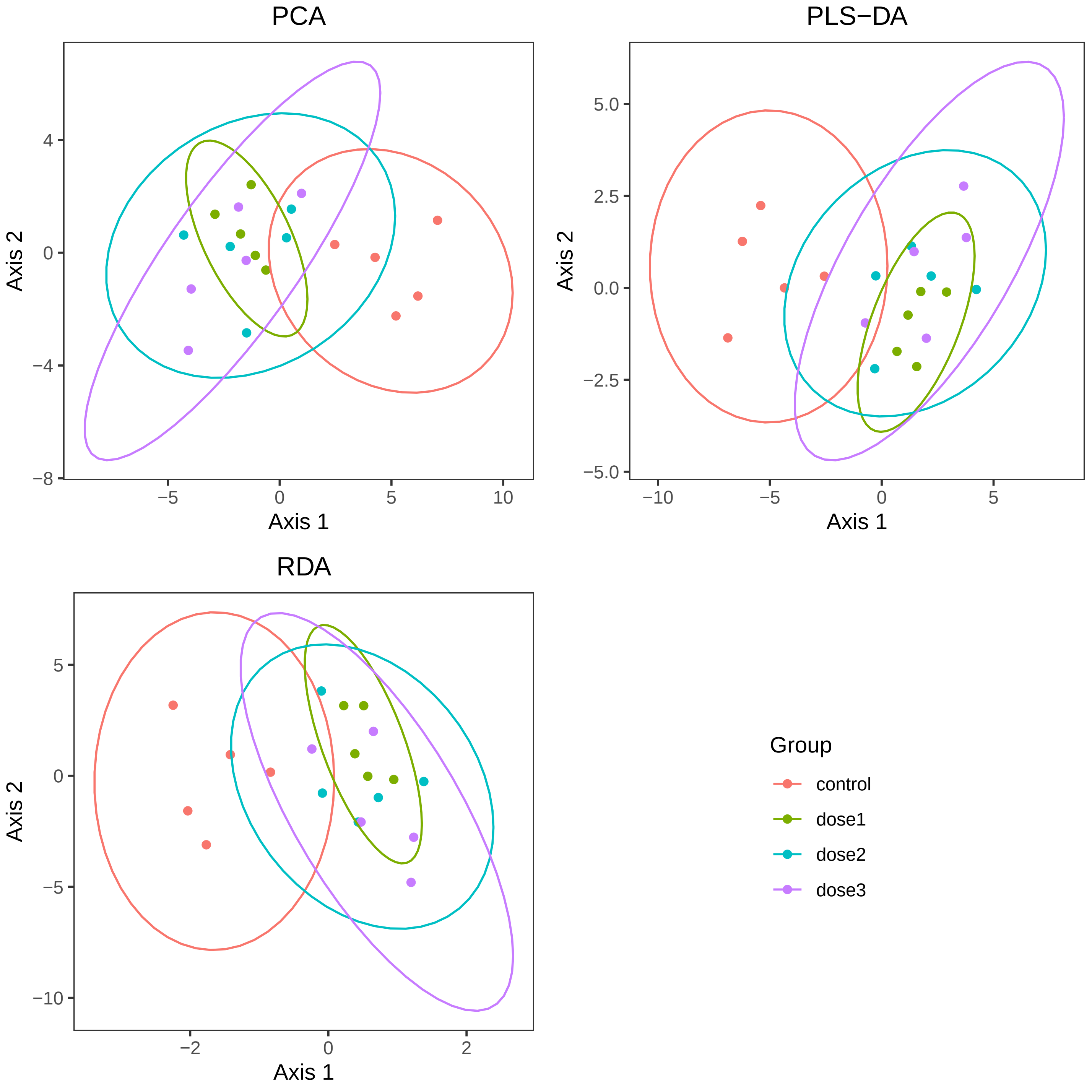

PCA
PLS−DA
RDA
Axis 1
Axis 2
Group
control
dose1
dose2
dose3

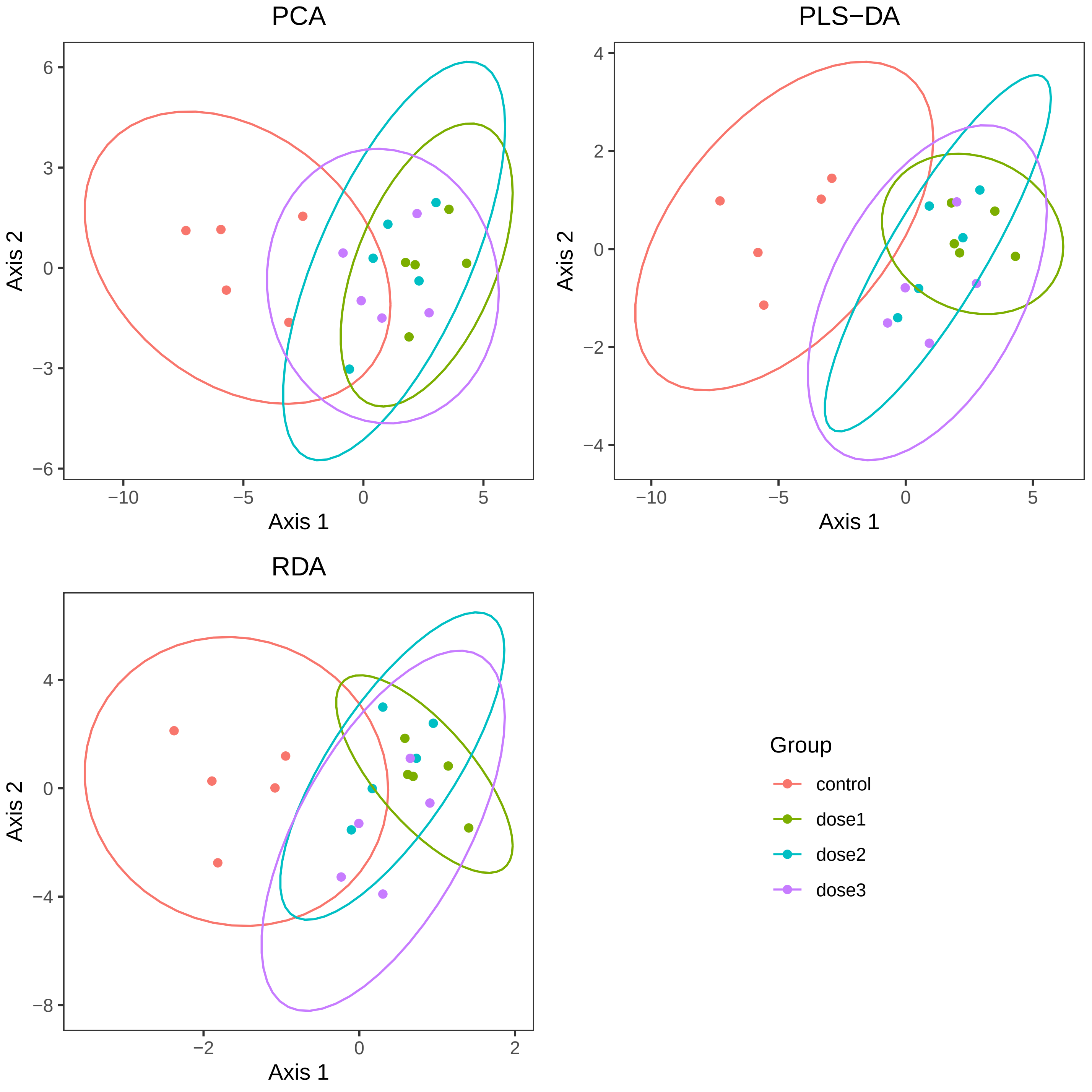

PCA
PLS−DA
RDA
Axis 1
Axis 2
−10
−5
0
5
6
3
−3
−6
4
2
−2
−4
−8
Group
control
dose1
dose2
dose3

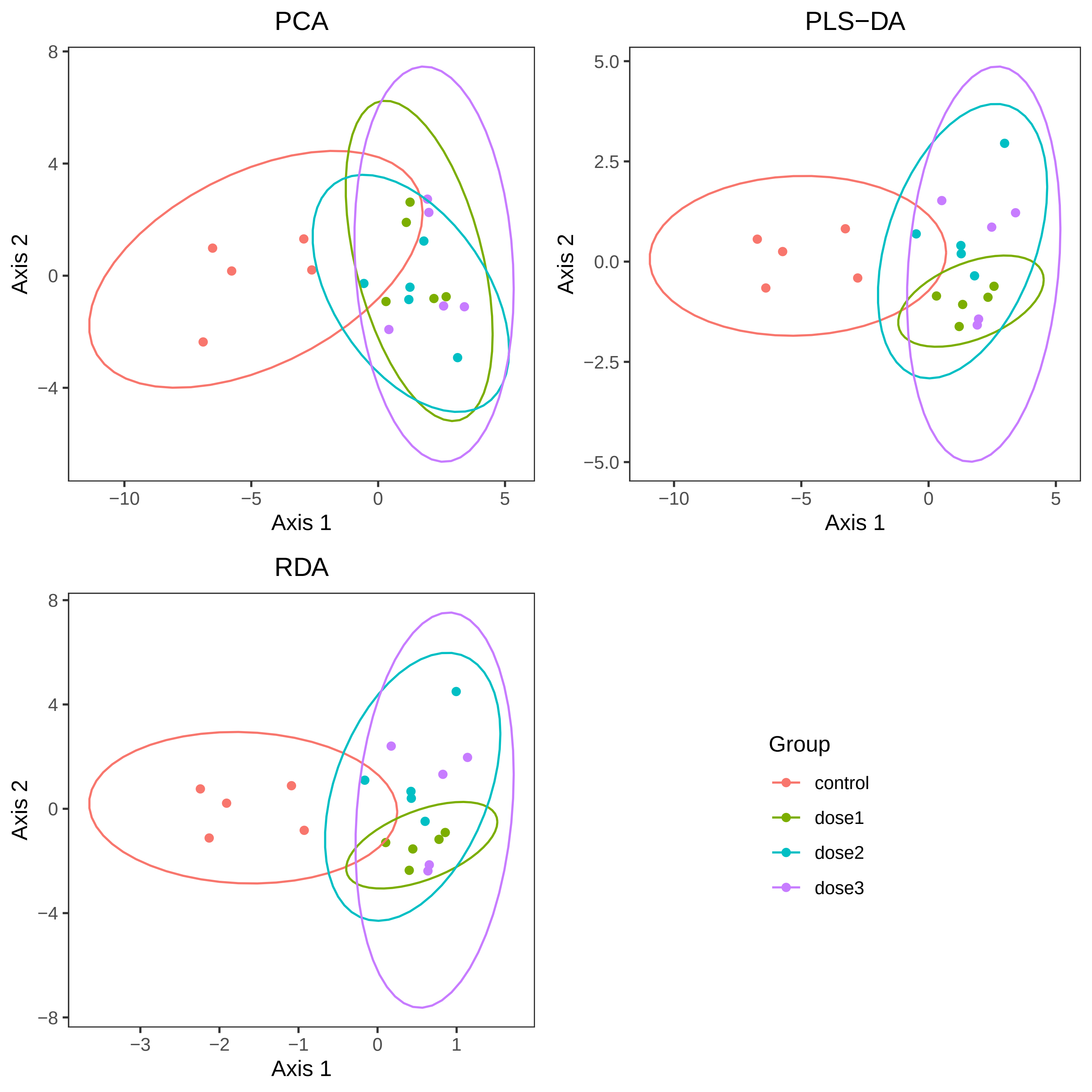

PCA
PLS−DA
RDA
Axis 1
Axis 2
8
4
0
−4
−8
5.0
2.5
0.0
−2.5
−5.0
−10
−5
0
5
−3
−2
−1
1
Group
control
dose1
dose2
dose3

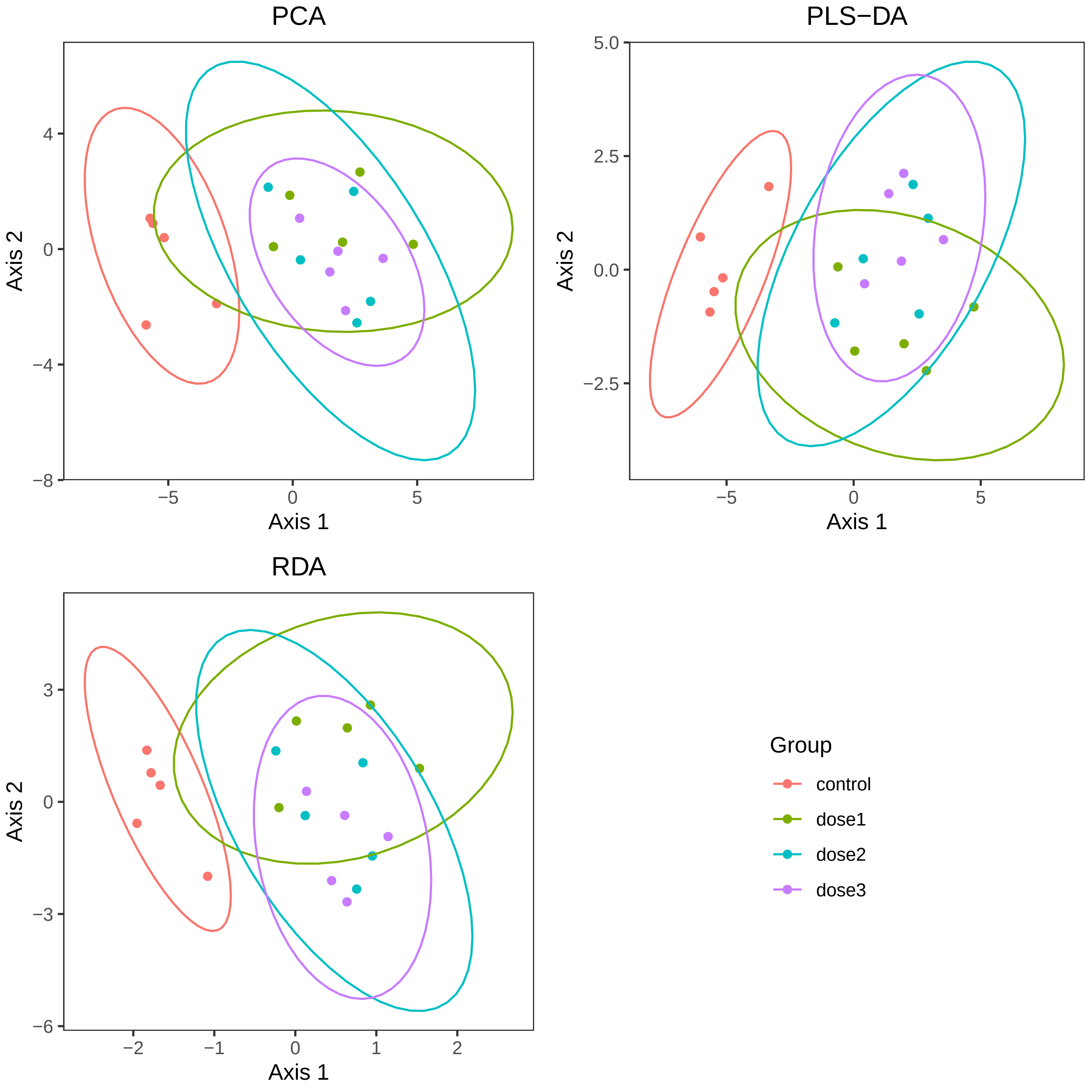

PCA
PLS−DA
RDA
Axis 1
Axis 2
Group
control
dose1
dose2
dose3

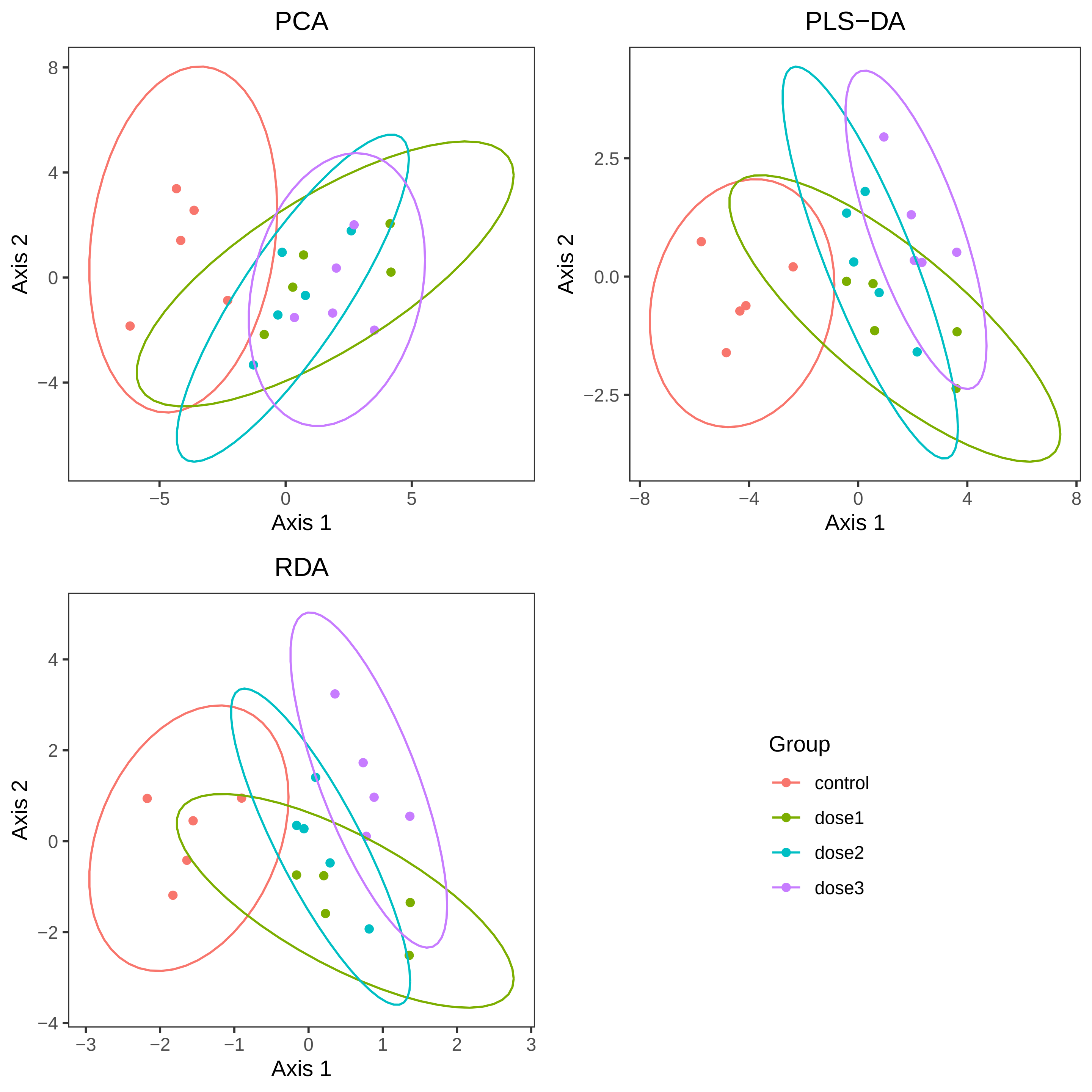
PCA
PLS−DA
RDA
Axis 1
Axis 2
Group
control
dose1
dose2
dose3

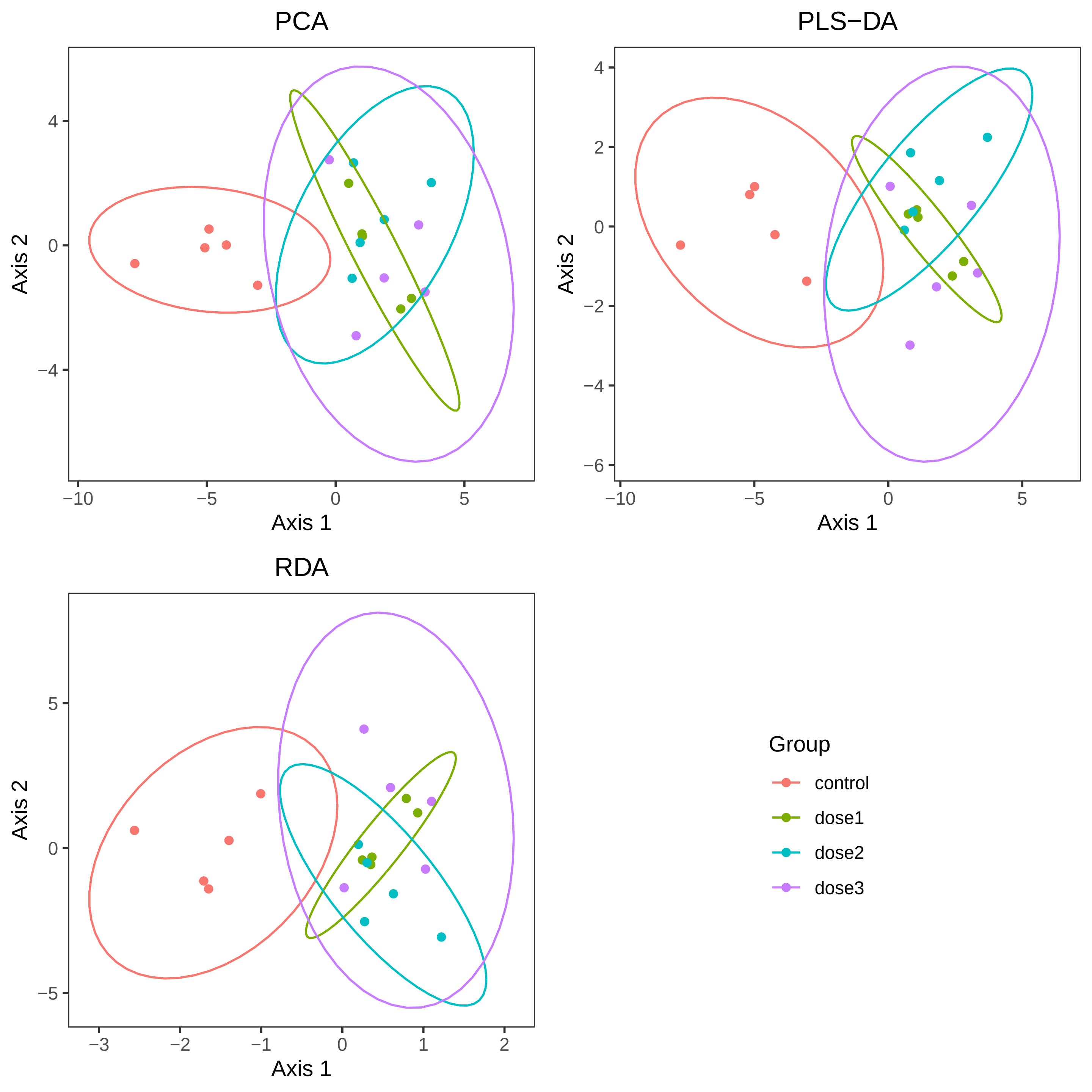

PCA
PLS−DA
RDA
Axis 1
Axis 2
Group
control
dose1
dose2
dose3

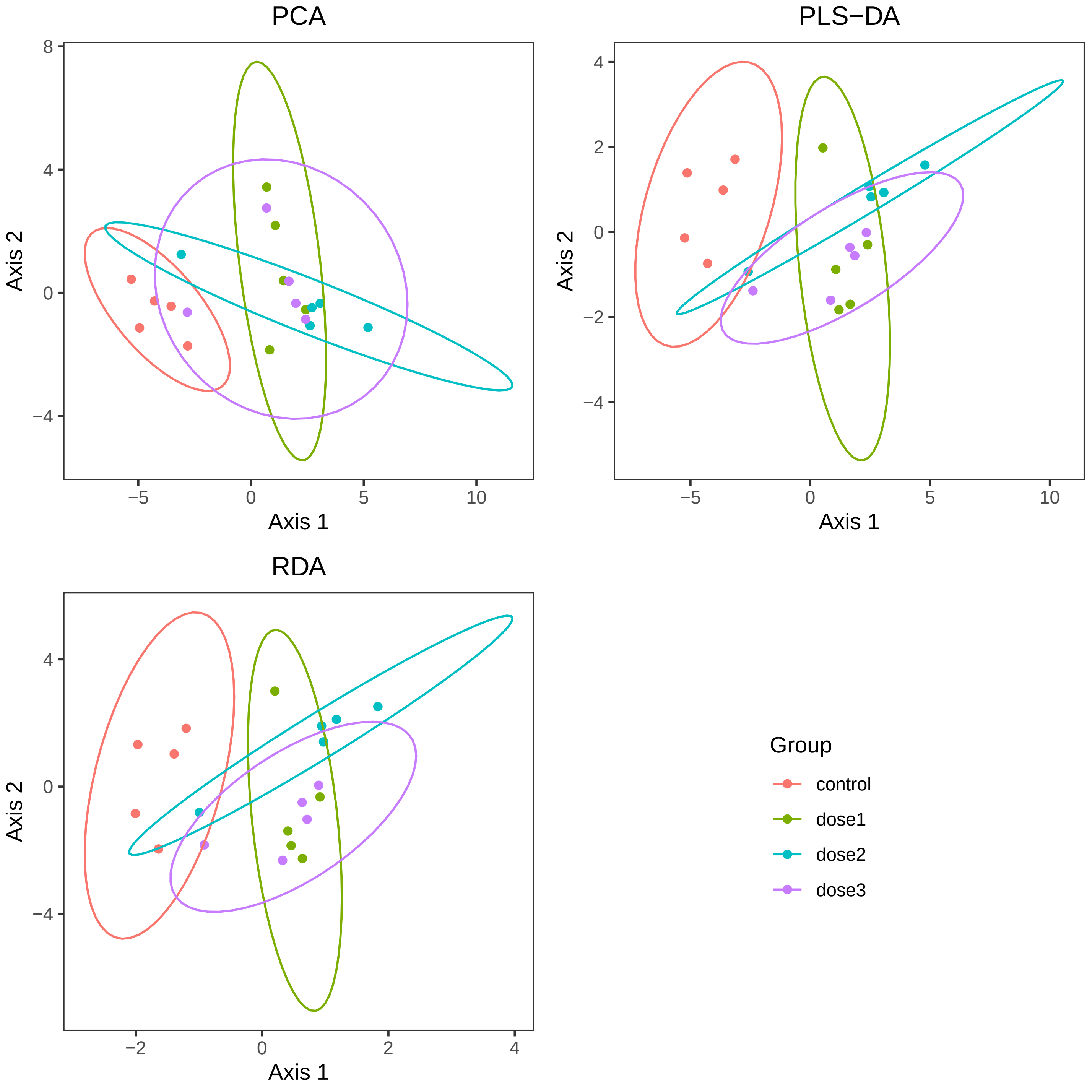

PCA
PLS−DA
RDA
Axis 1
Axis 2
Group
control
dose1
dose2
dose3

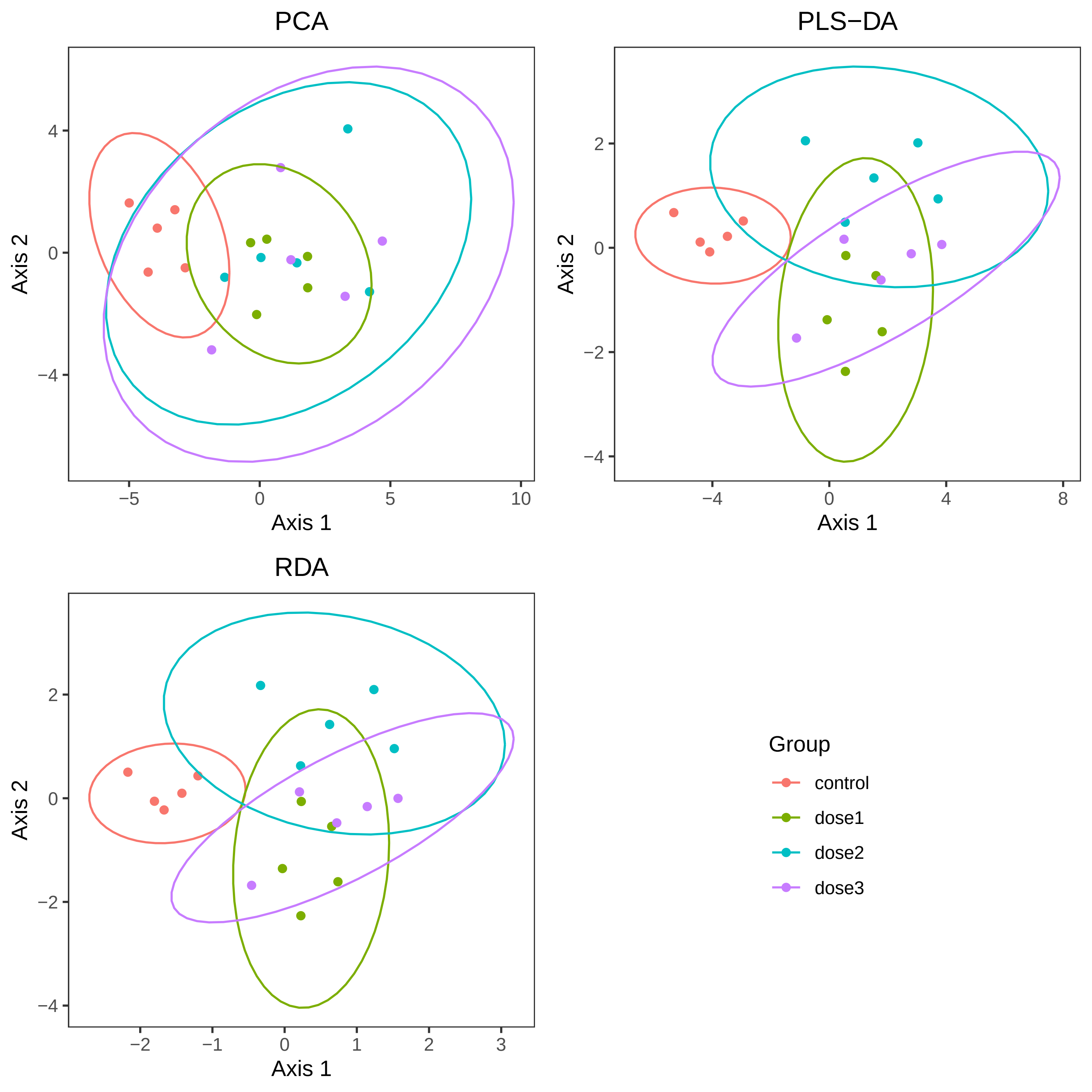

PCA
PLS−DA
RDA
Axis 1
Axis 2
Group
control
dose1
dose2
dose3

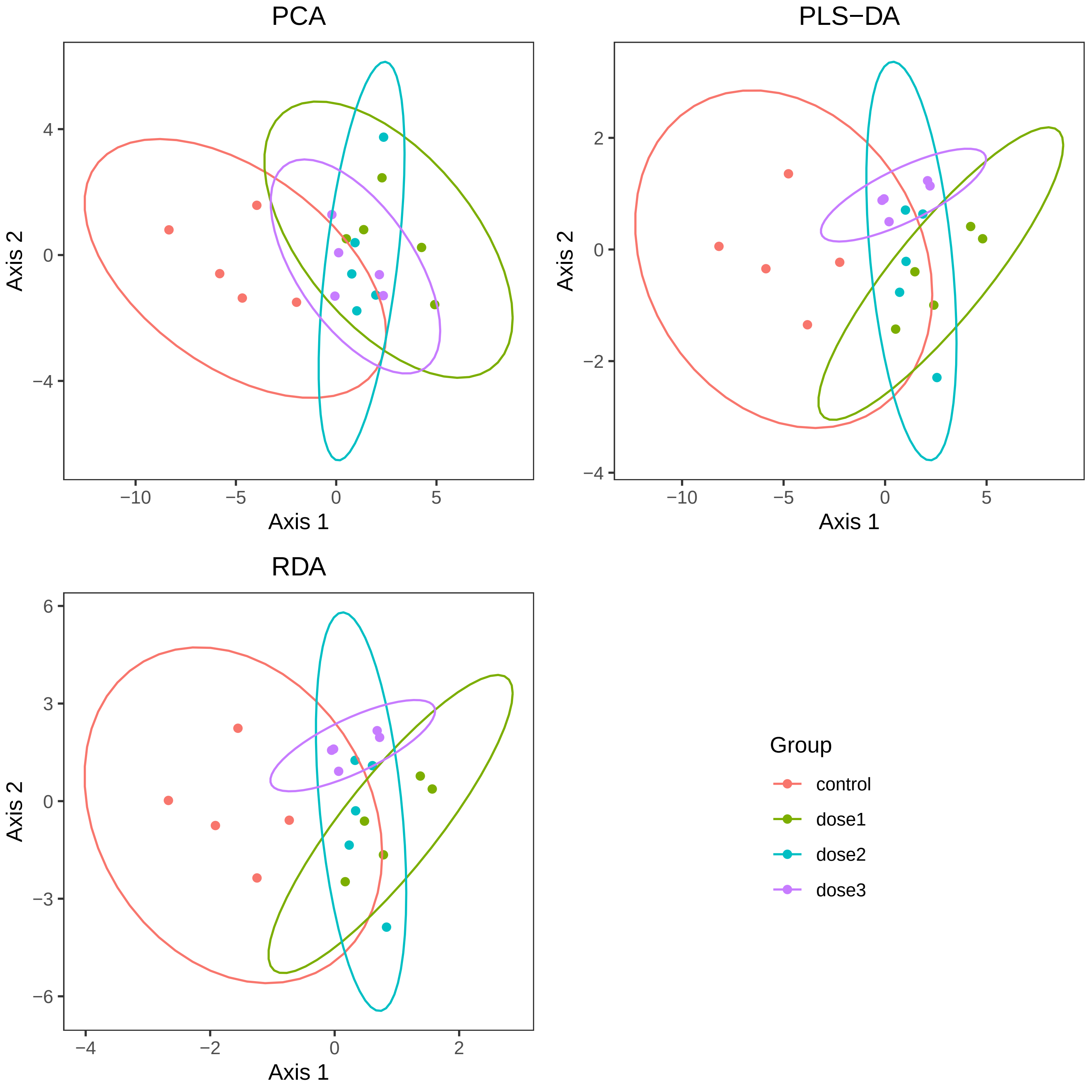
PCA
PLS−DA
RDA
Axis 1
Axis 2
Group
control
dose1
dose2
dose3

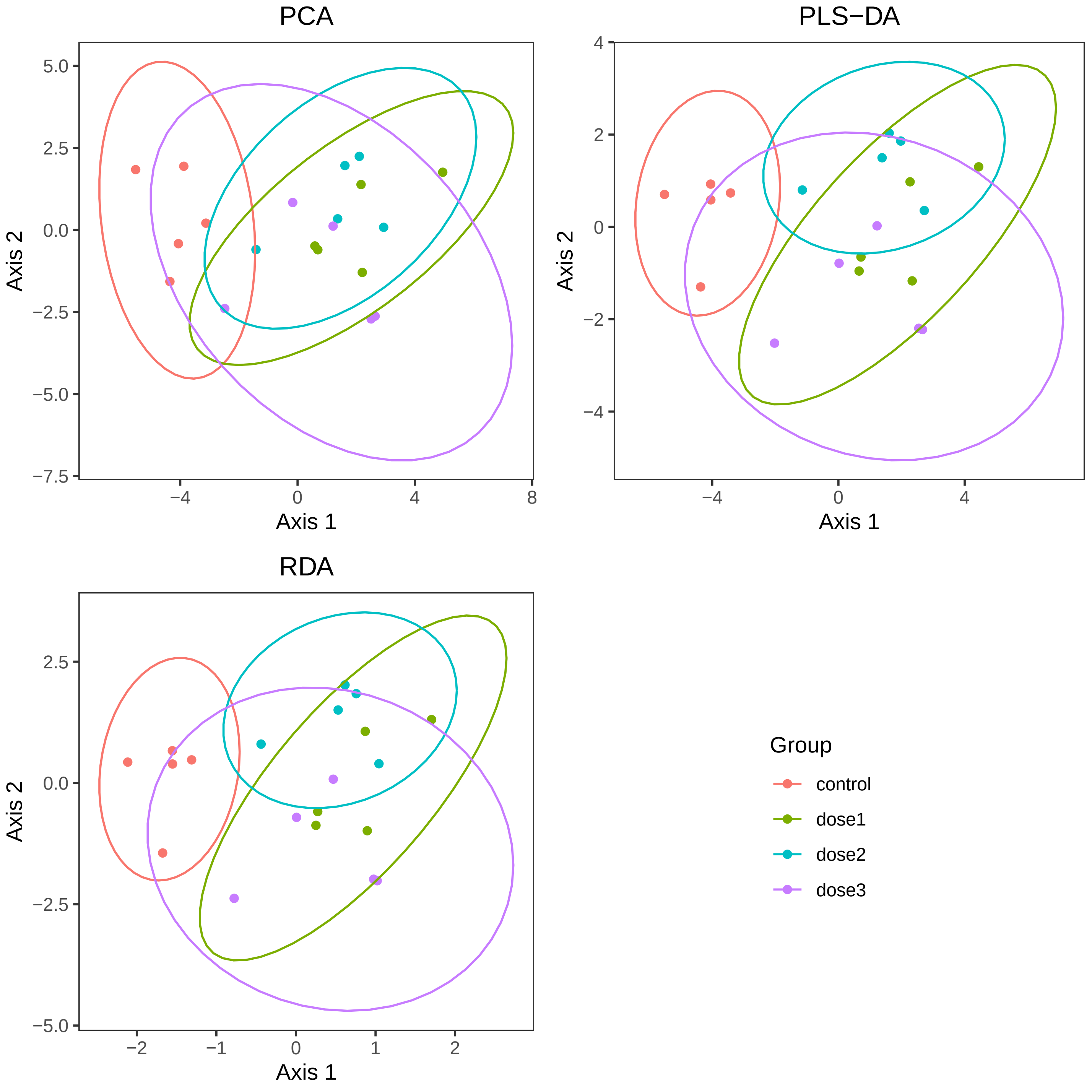
PCA
PLS−DA
RDA
Axis 1
Axis 2
Group
control
dose1
dose2
dose3

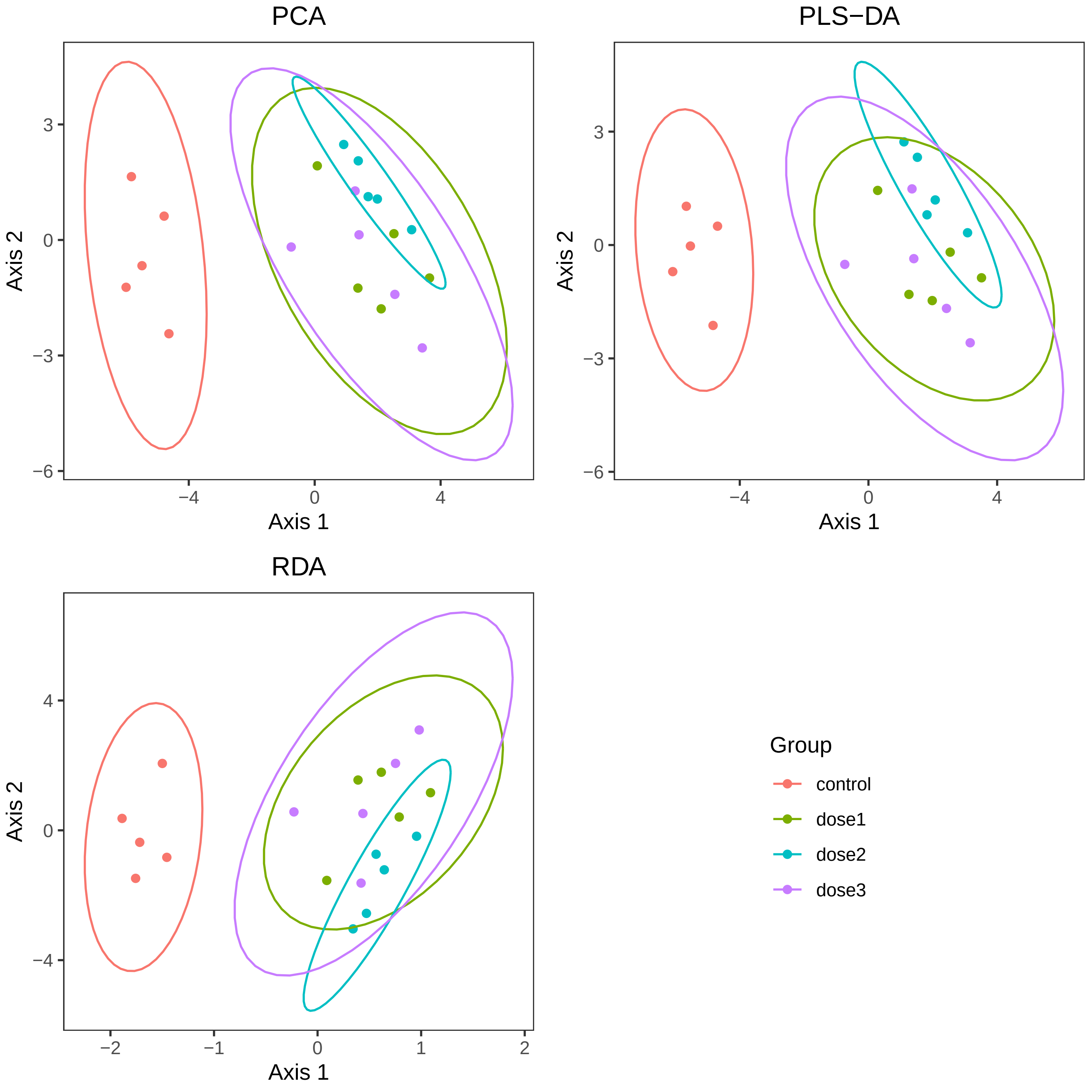

PCA
PLS−DA
RDA
Axis 1
Axis 2
Group
control
dose1
dose2
dose3

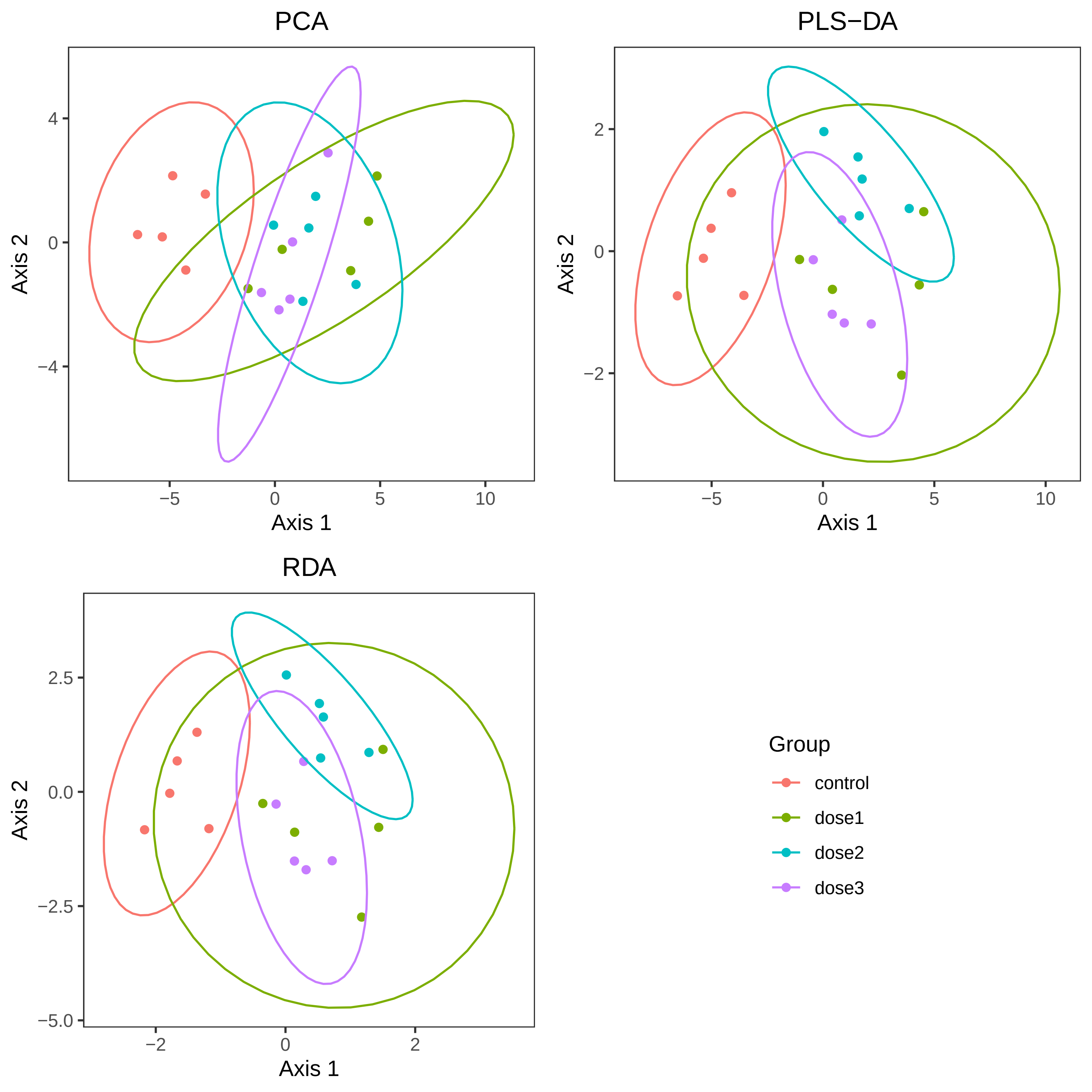

PCA
PLS−DA
RDA
Axis 1
Axis 2
Group
control
dose1
dose2
dose3

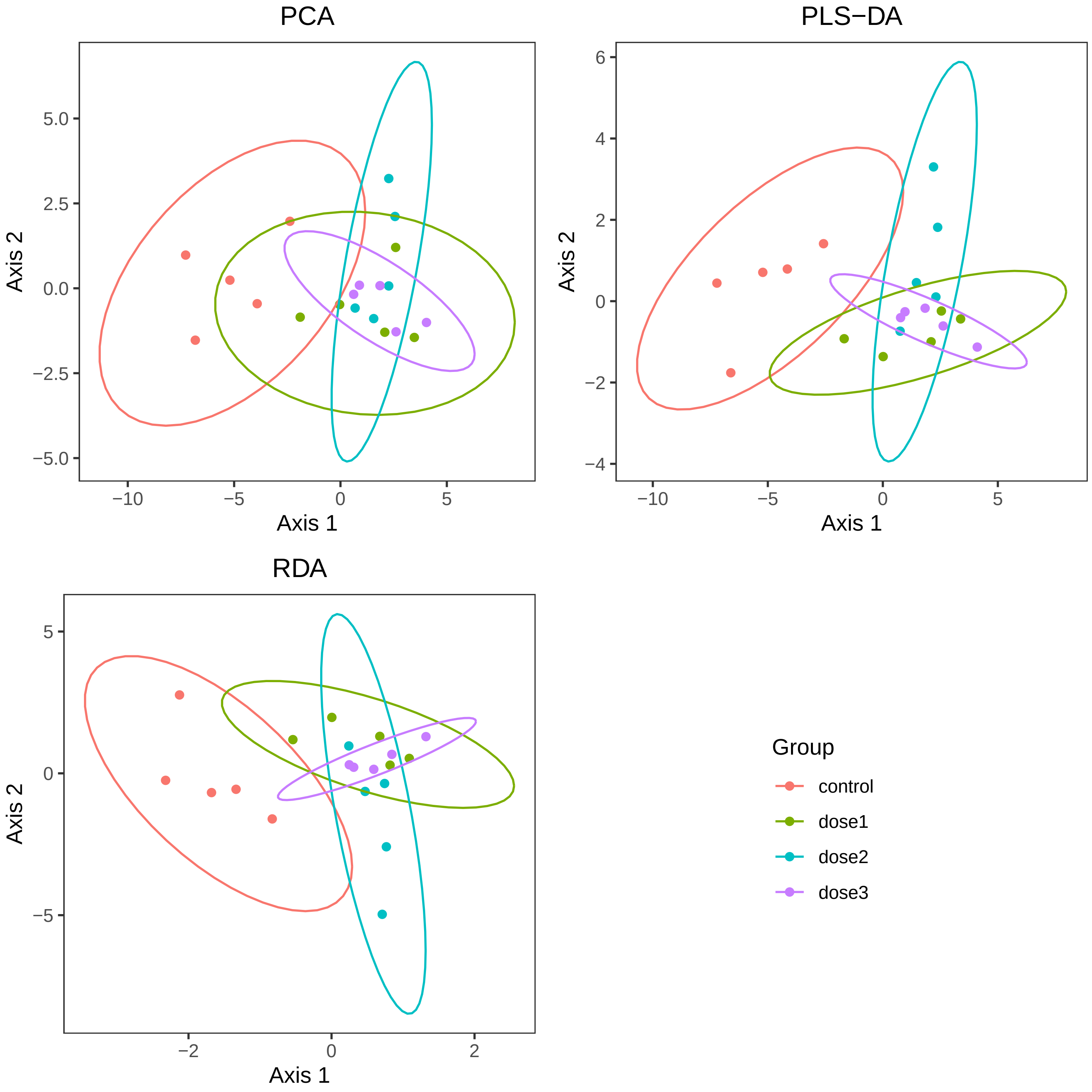
PCA
PLS−DA
RDA
Axis 1
Axis 2
Group
control
dose1
dose2
dose3

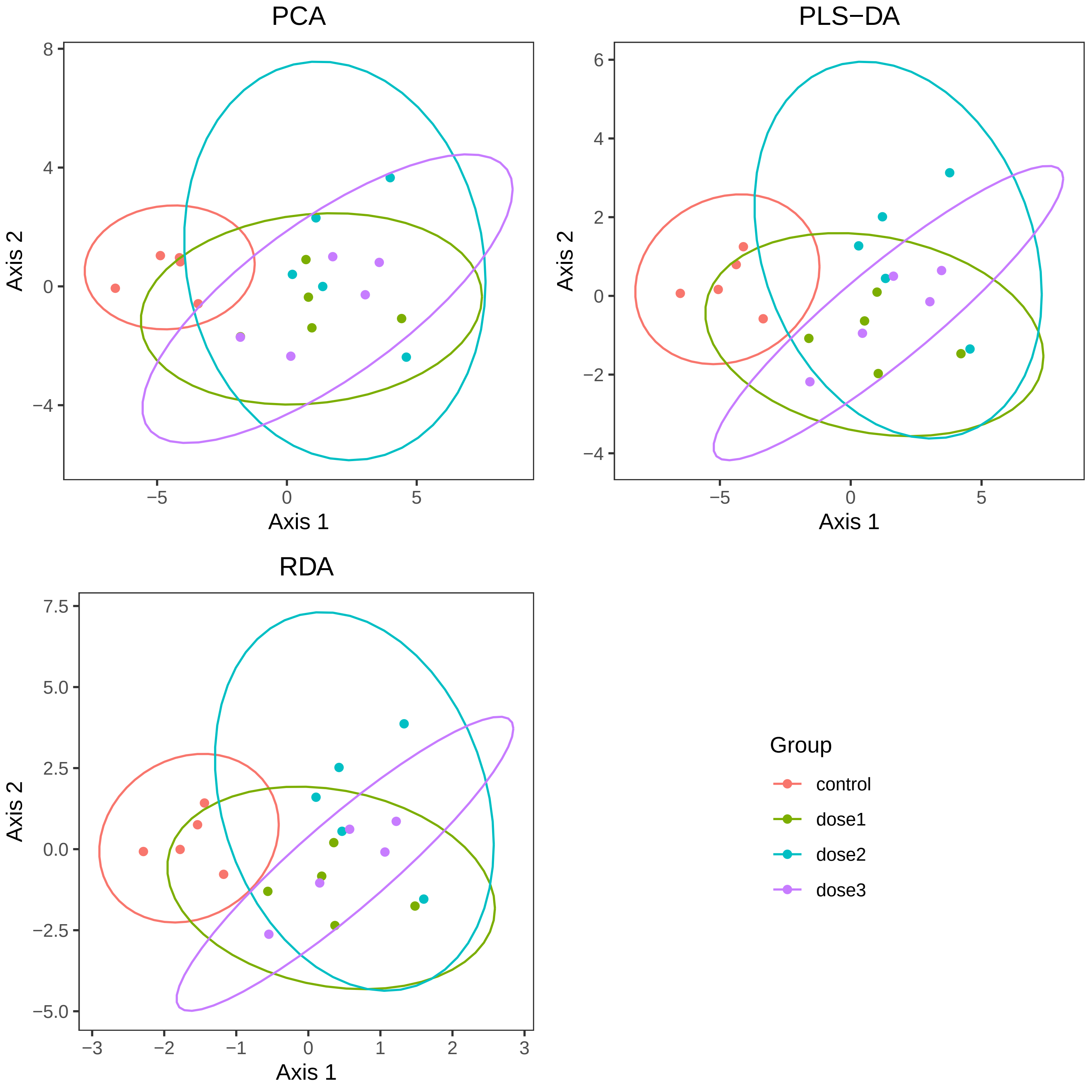

PCA
PLS−DA
RDA
Axis 1
Axis 2
Group
control
dose1
dose2
dose3

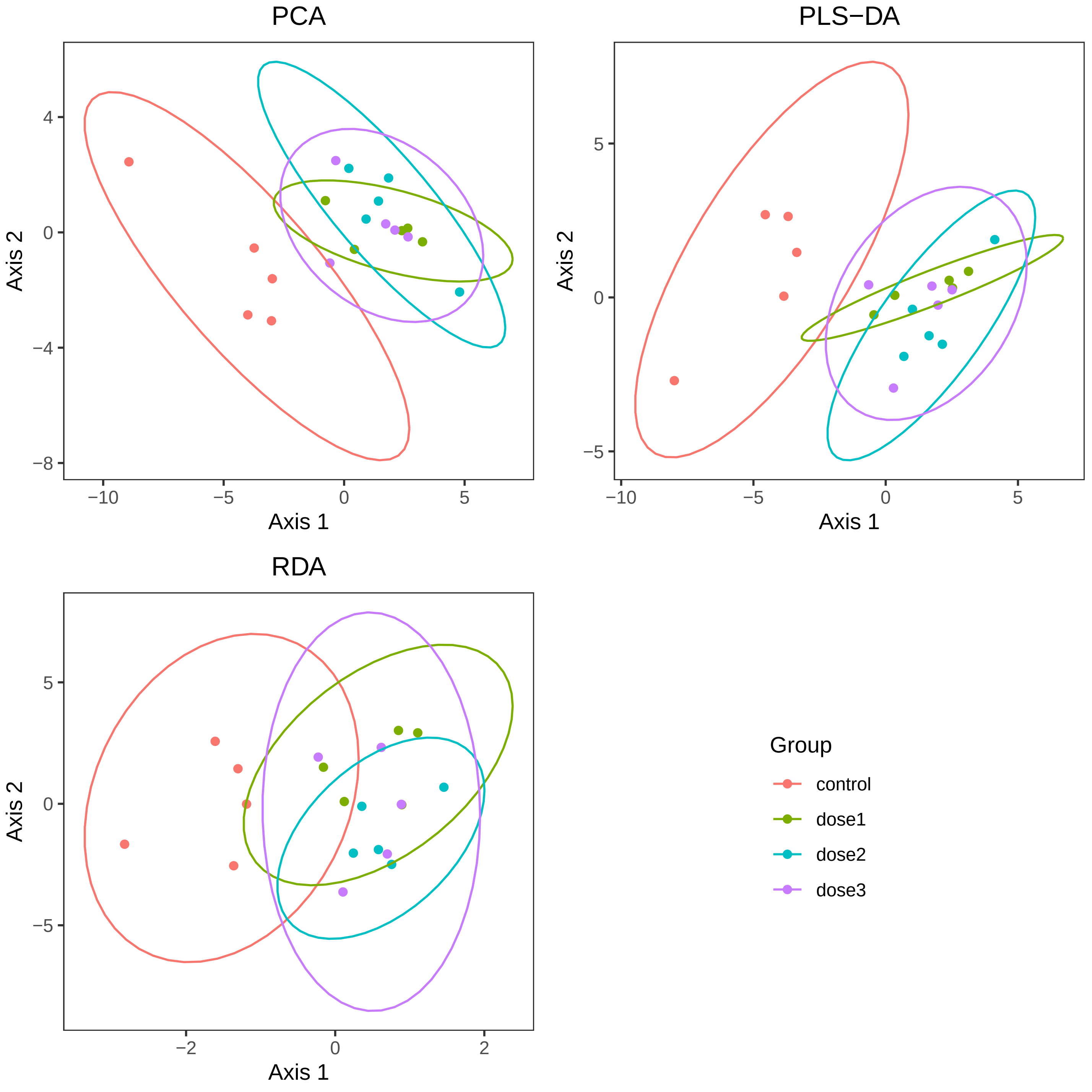
PCA
PLS−DA
RDA
Axis 1
Axis 2
Group
control
dose1
dose2
dose3

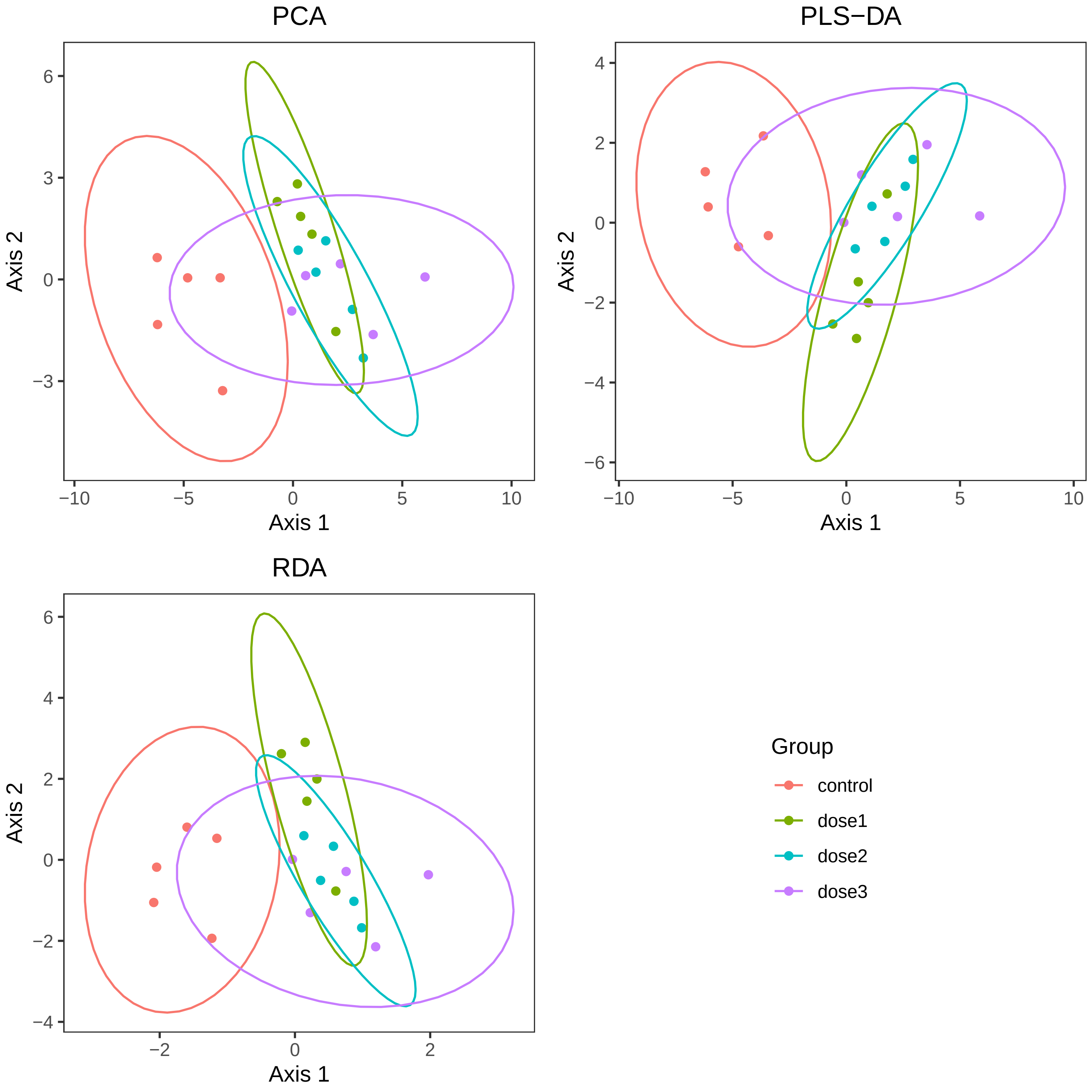

PCA
PLS−DA
RDA
Axis 1
Axis 2
Group
control
dose1
dose2
dose3

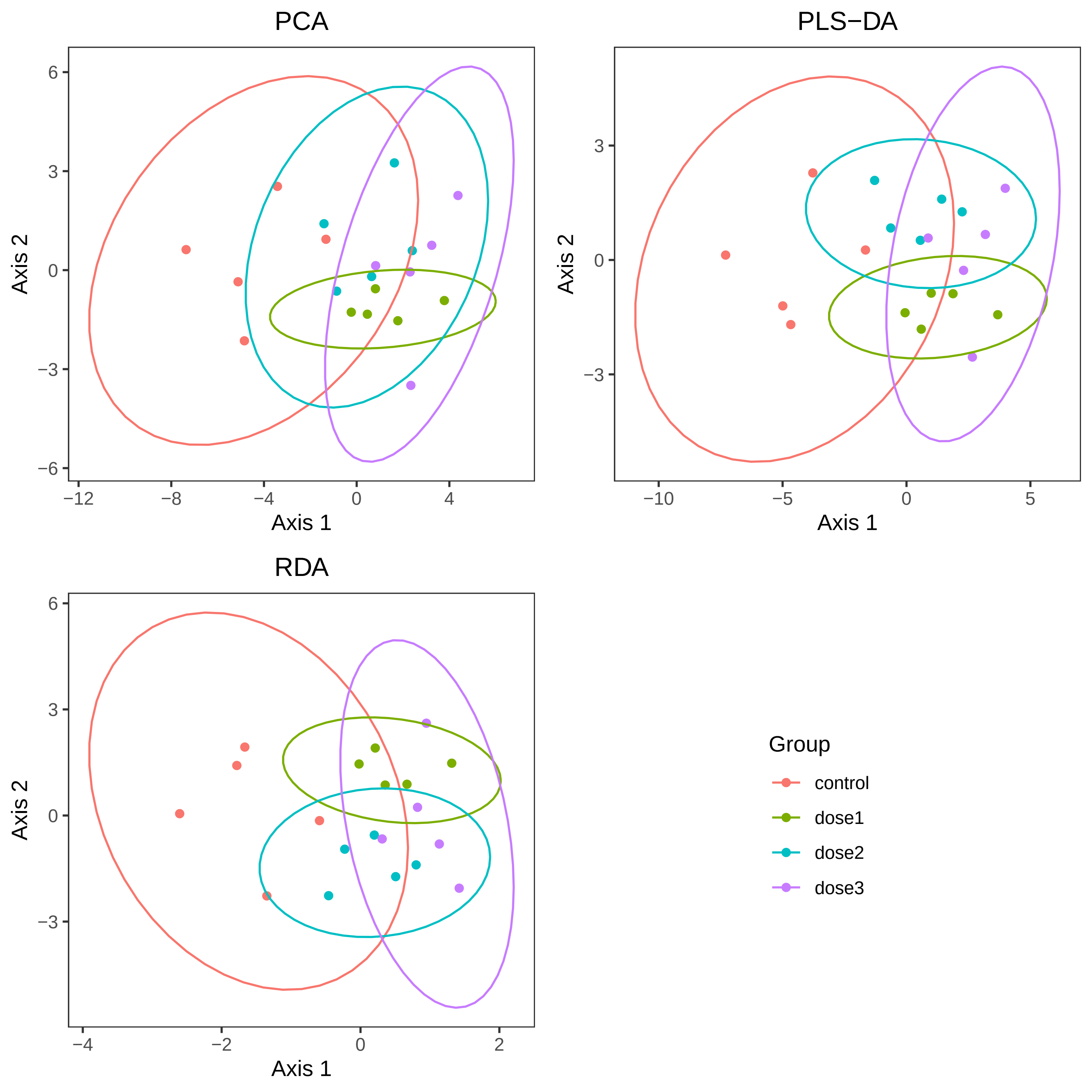
PCA
PLS−DA
RDA
Axis 1
Axis 2
Group
control
dose1
dose2
dose3

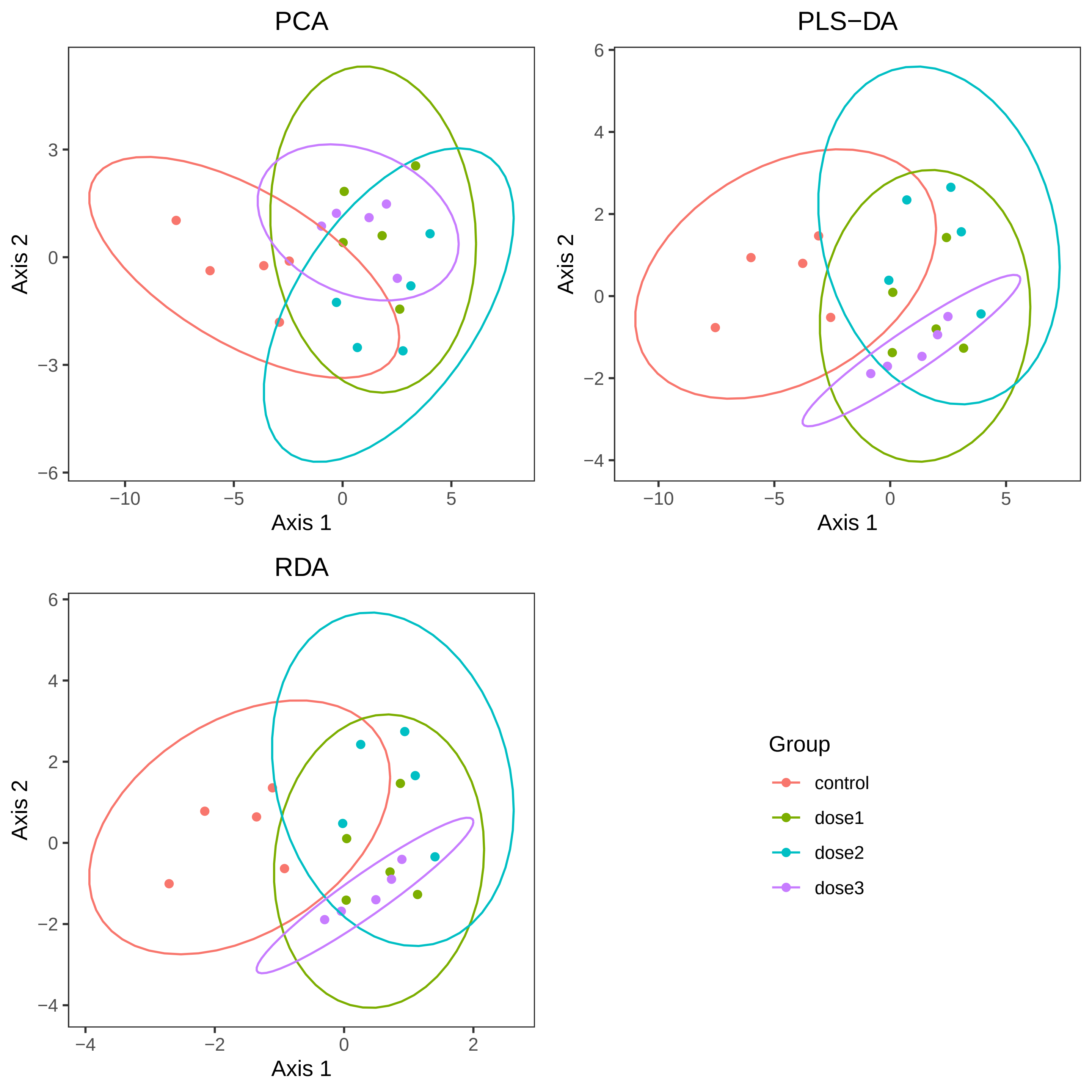
PCA
PLS−DA
RDA
Axis 1
Axis 2
Group
control
dose1
dose2
dose3

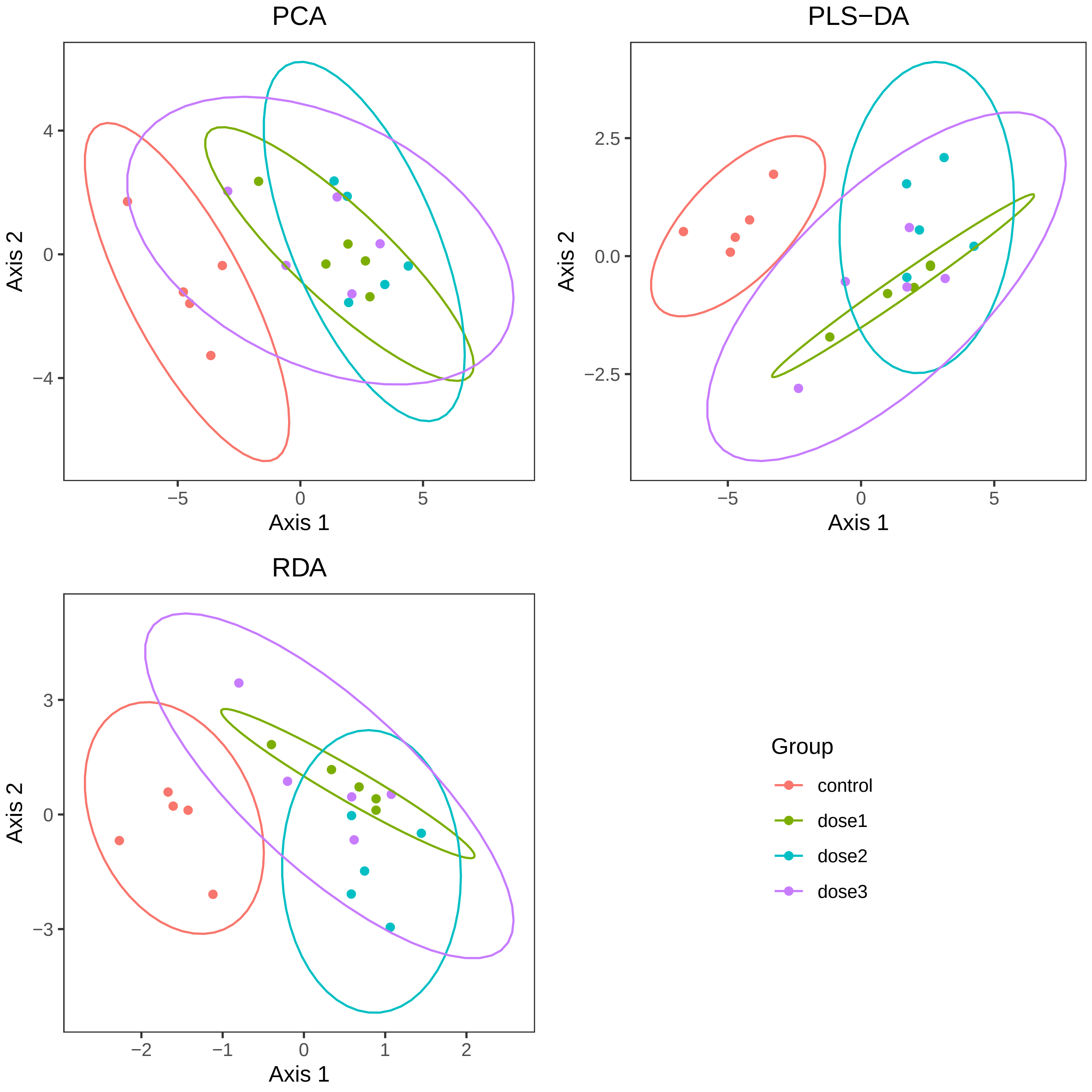

PCA
PLS−DA
RDA
Axis 1
Axis 2
Group
control
dose1
dose2
dose3

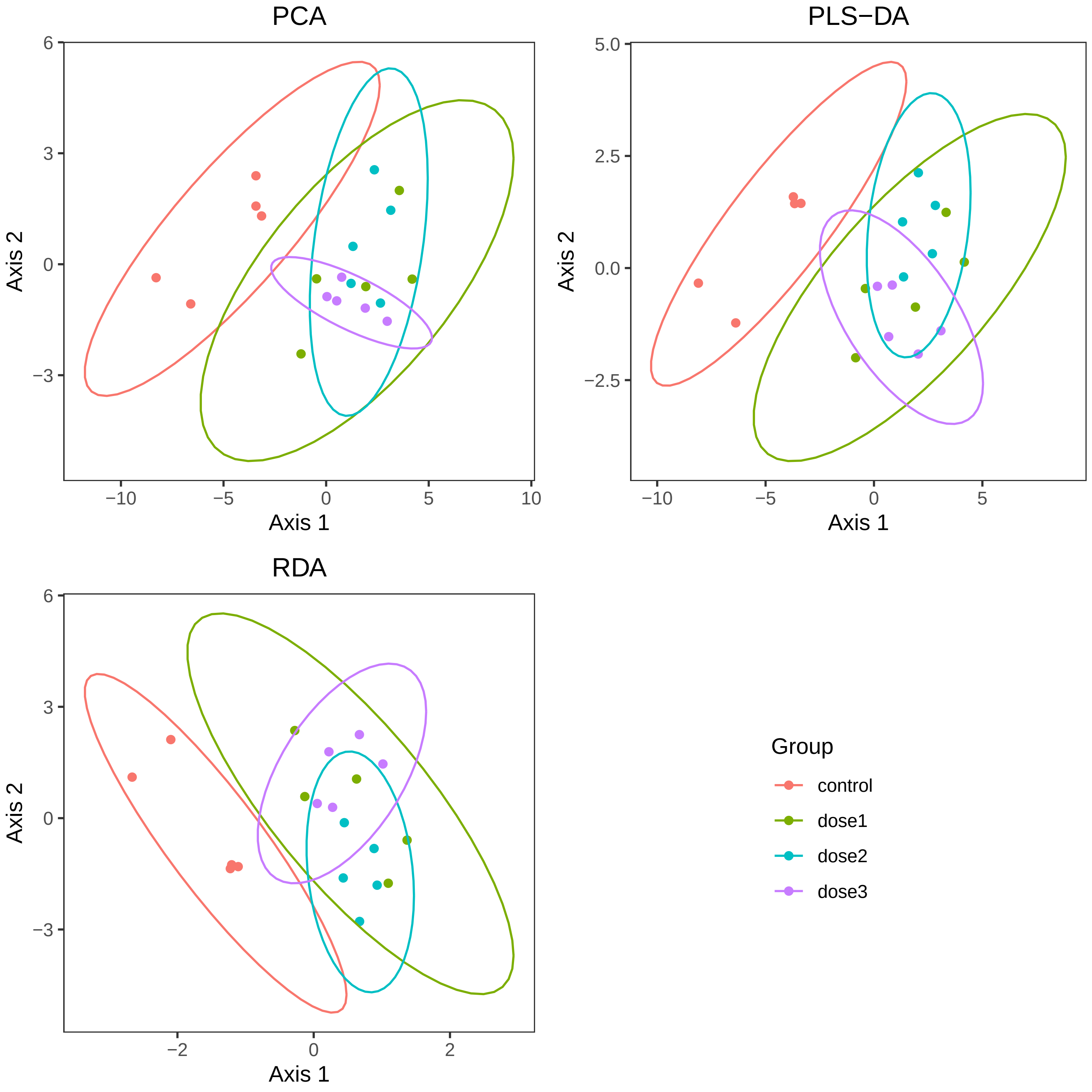
PCA
PLS−DA
RDA
Axis 1
Axis 2
Group
control
dose1
dose2
dose3
−10
−5
0
5
10
6
3
0
−3
5.0
2.5
0.0
−2.5
−2
2

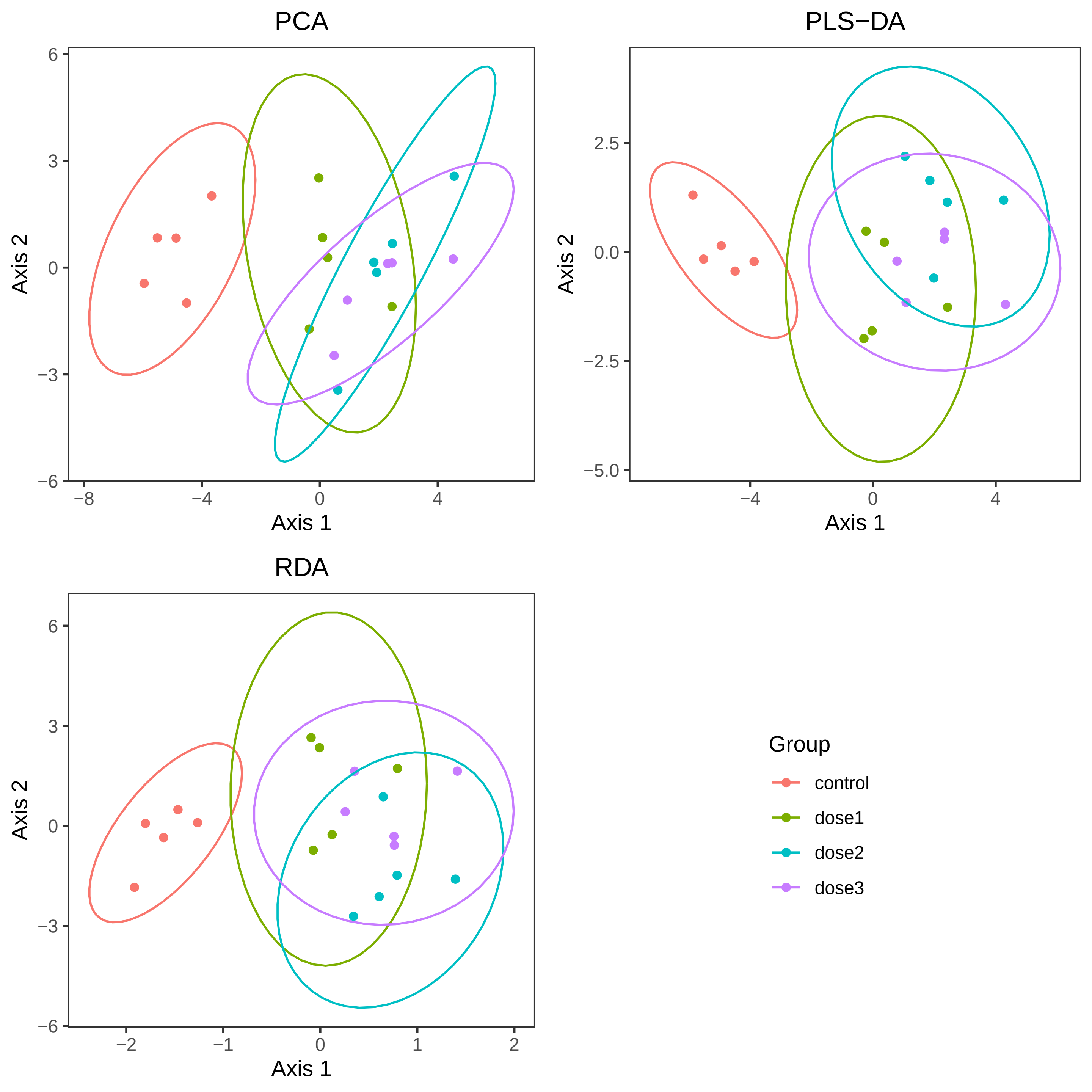

PCA
PLS−DA
RDA
Axis 1
Axis 2
Group
control
dose1
dose2
dose3

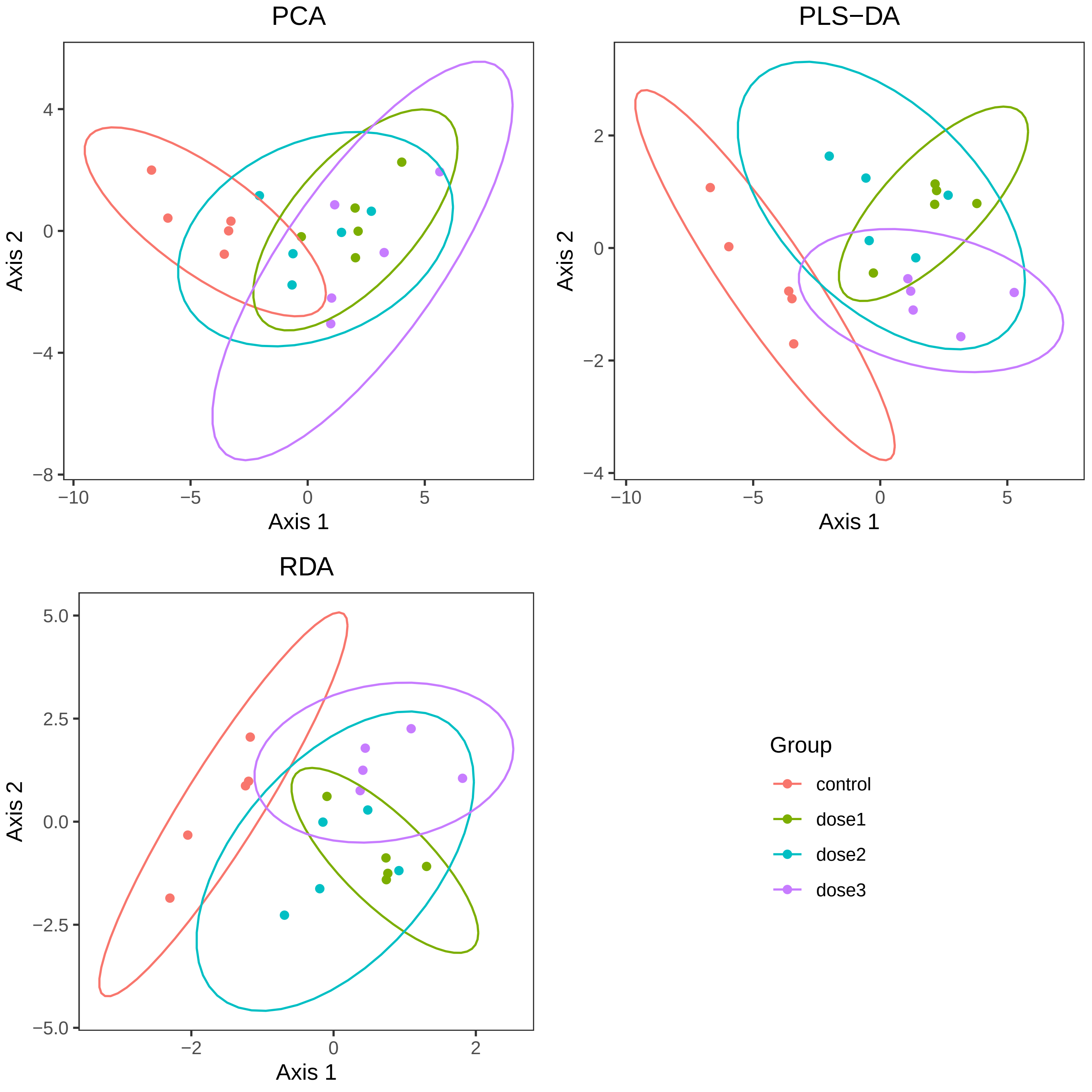
PCA
PLS−DA
RDA
Axis 1
Axis 2
Group
control
dose1
dose2
dose3

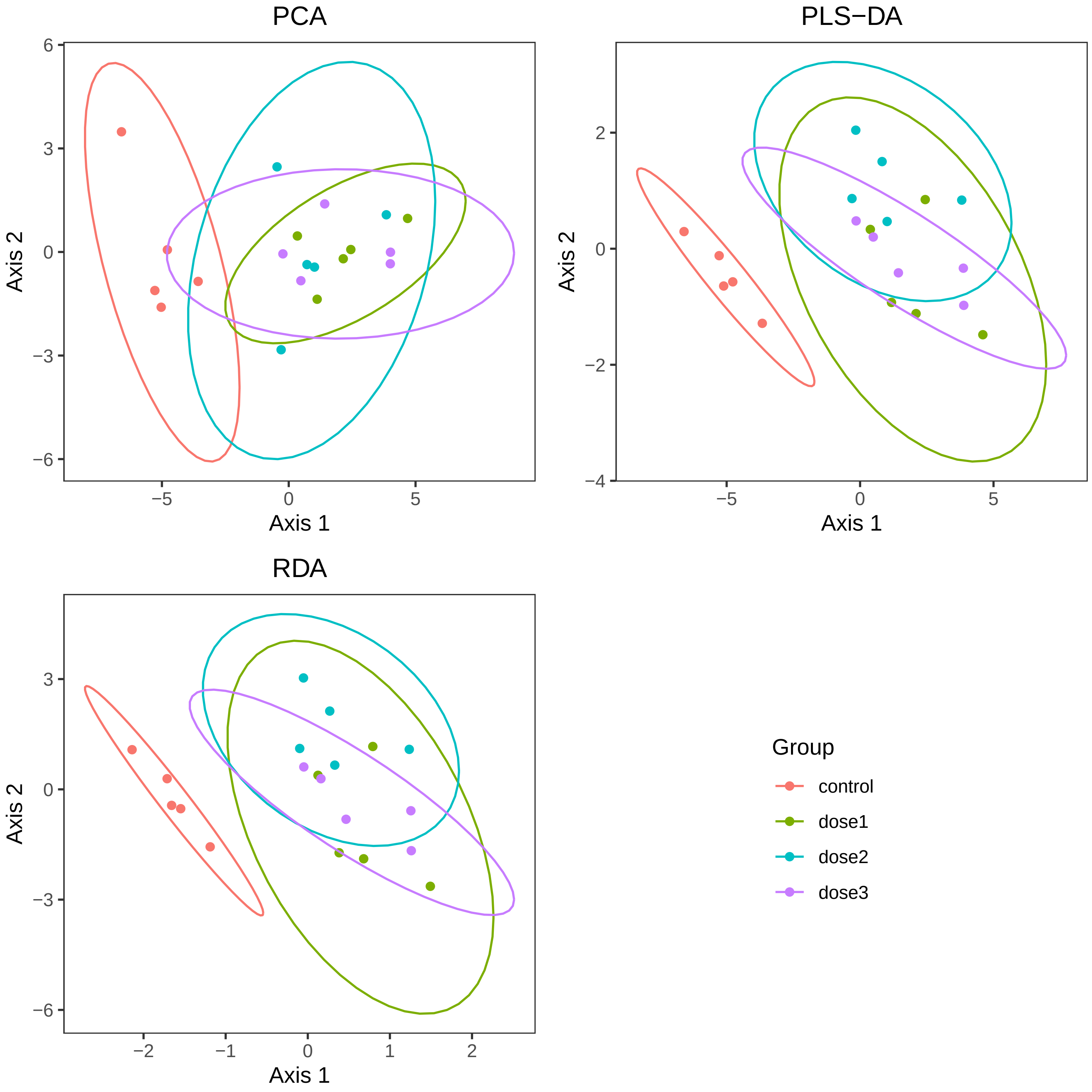

PCA
PLS−DA
RDA
Axis 1
Axis 2
Group
control
dose1
dose2
dose3

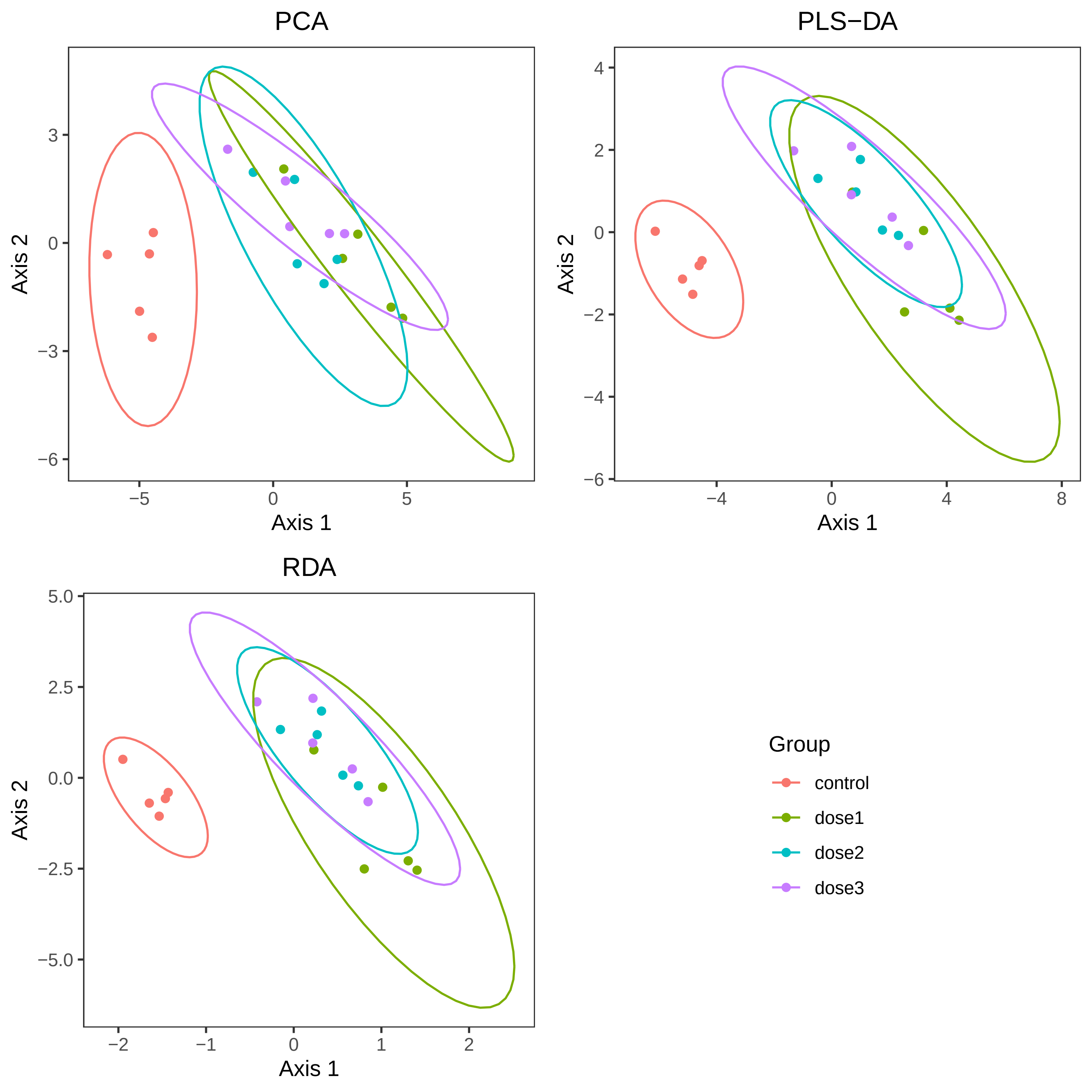

PCA
PLS−DA
RDA
Axis 1
Axis 2
Group
control
dose1
dose2
dose3

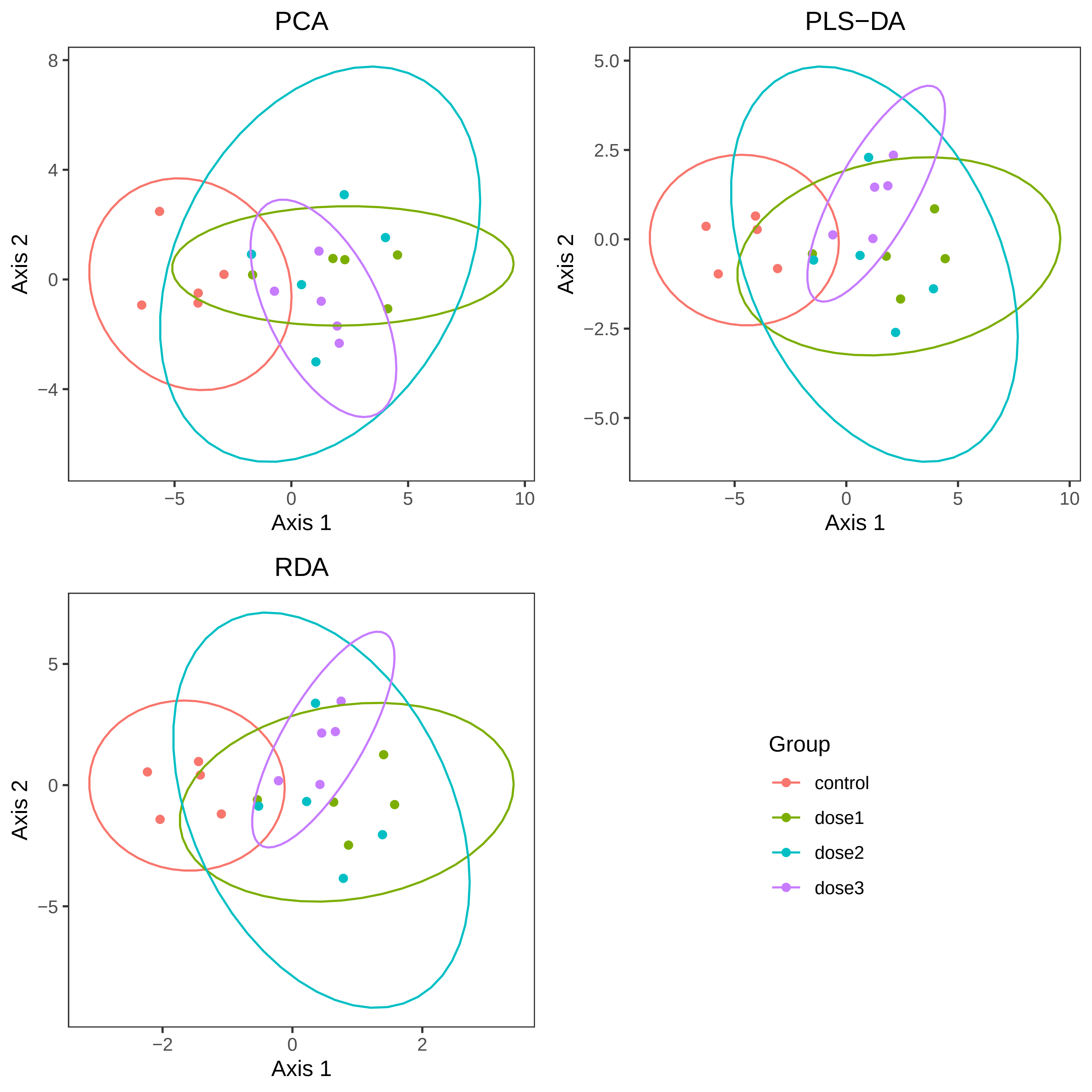

PCA
PLS−DA
RDA
Axis 1
Axis 2
8
4
0
−4
−5
0
5
10
5.0
2.5
0.0
−2.5
−5.0
5
0
−5
−2
0
2
Group
control
dose1
dose2
dose3

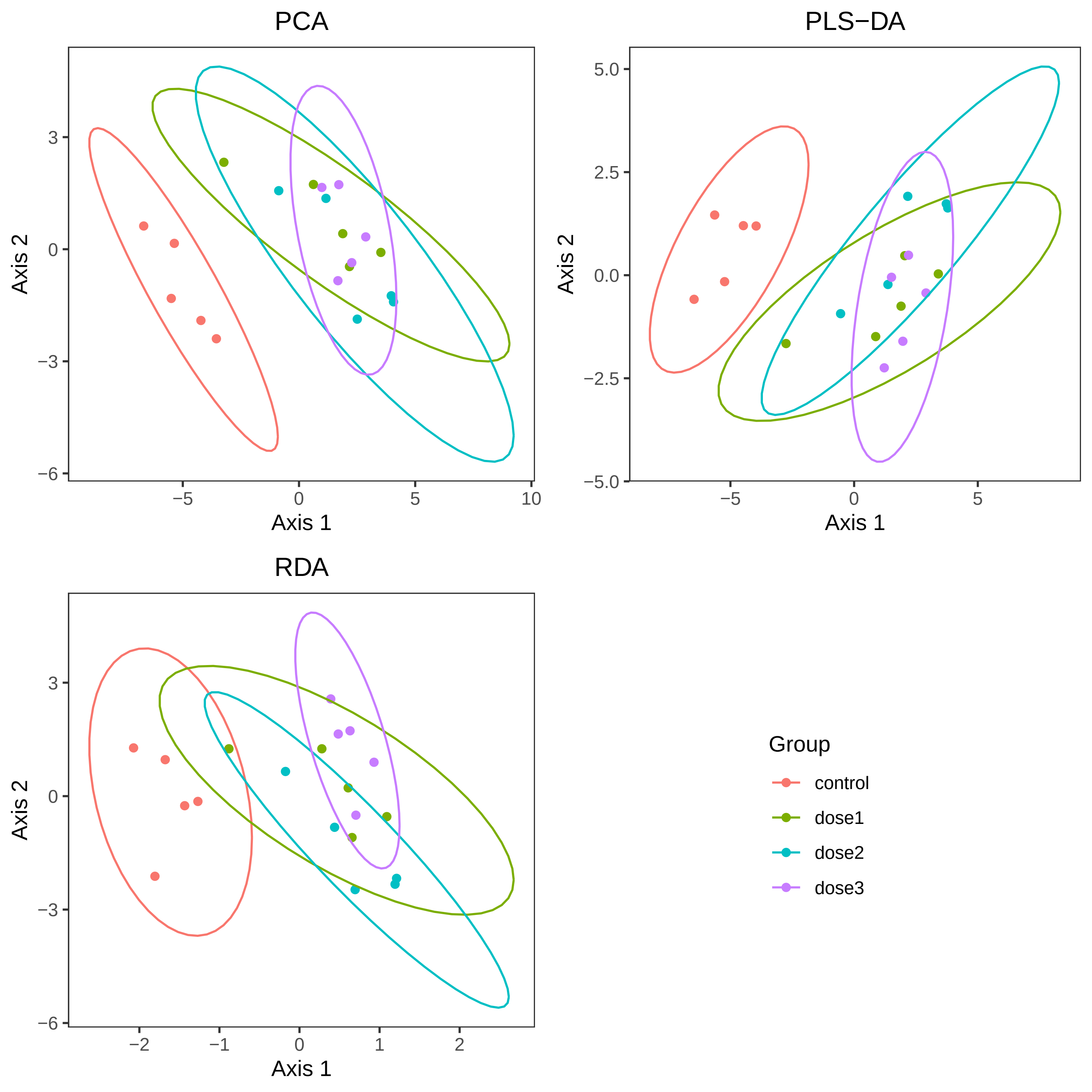

PCA
PLS−DA
RDA
Axis 1
Axis 2
Group
control
dose1
dose2
dose3

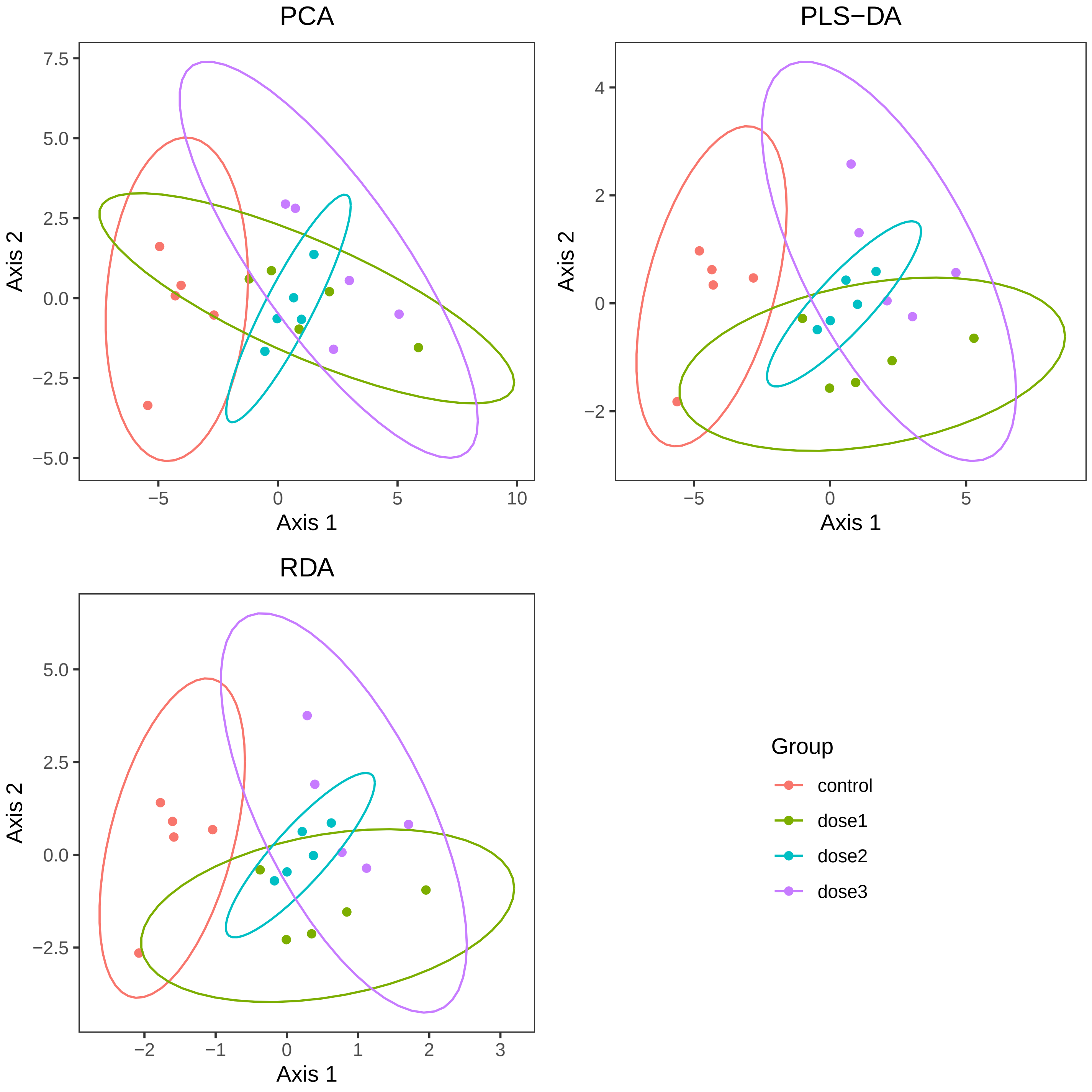
PCA
PLS−DA
RDA
Axis 1
Axis 2
Group
control
dose1
dose2
dose3

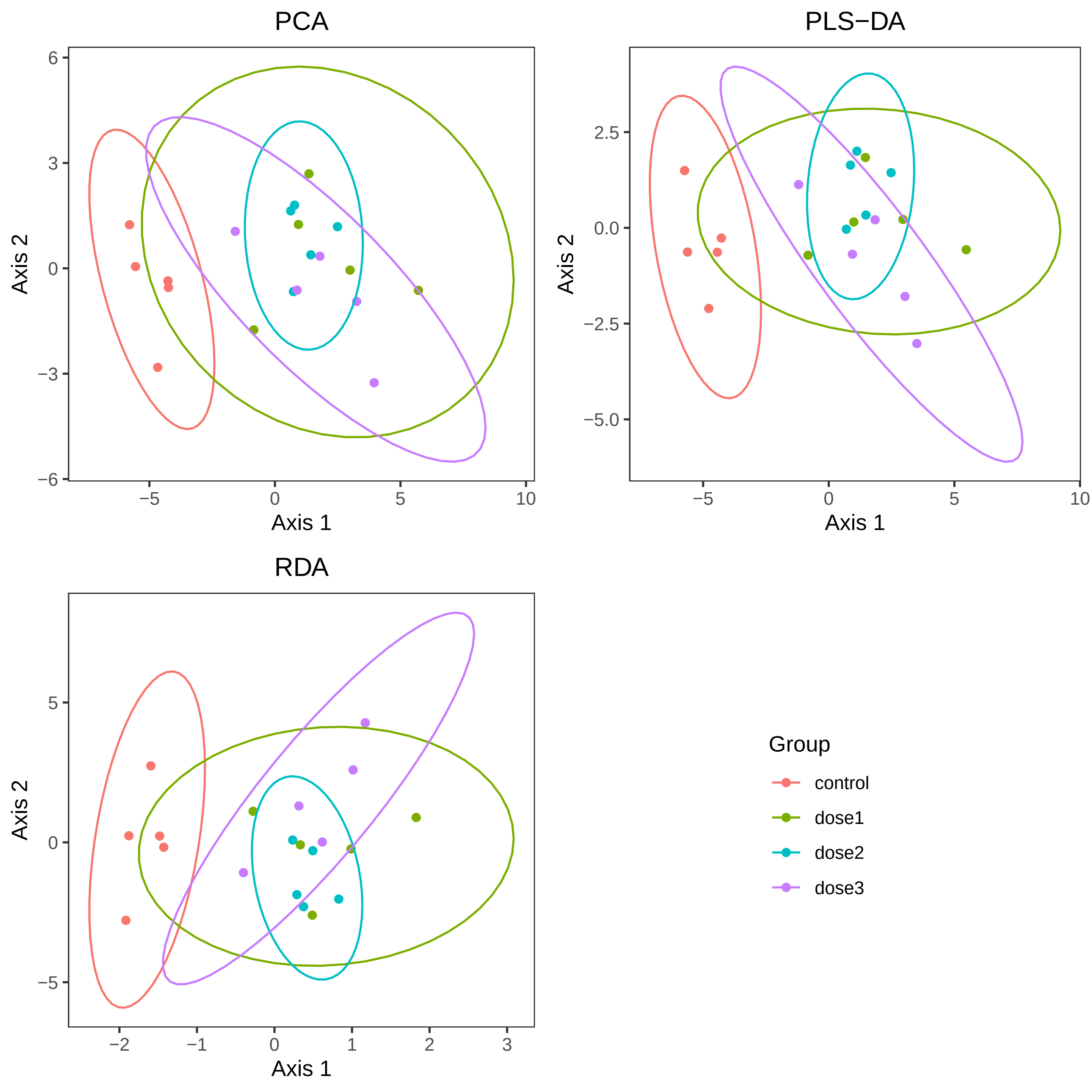
PCA
PLS−DA
RDA
Axis 1
Axis 2
Group
control
dose1
dose2
dose3

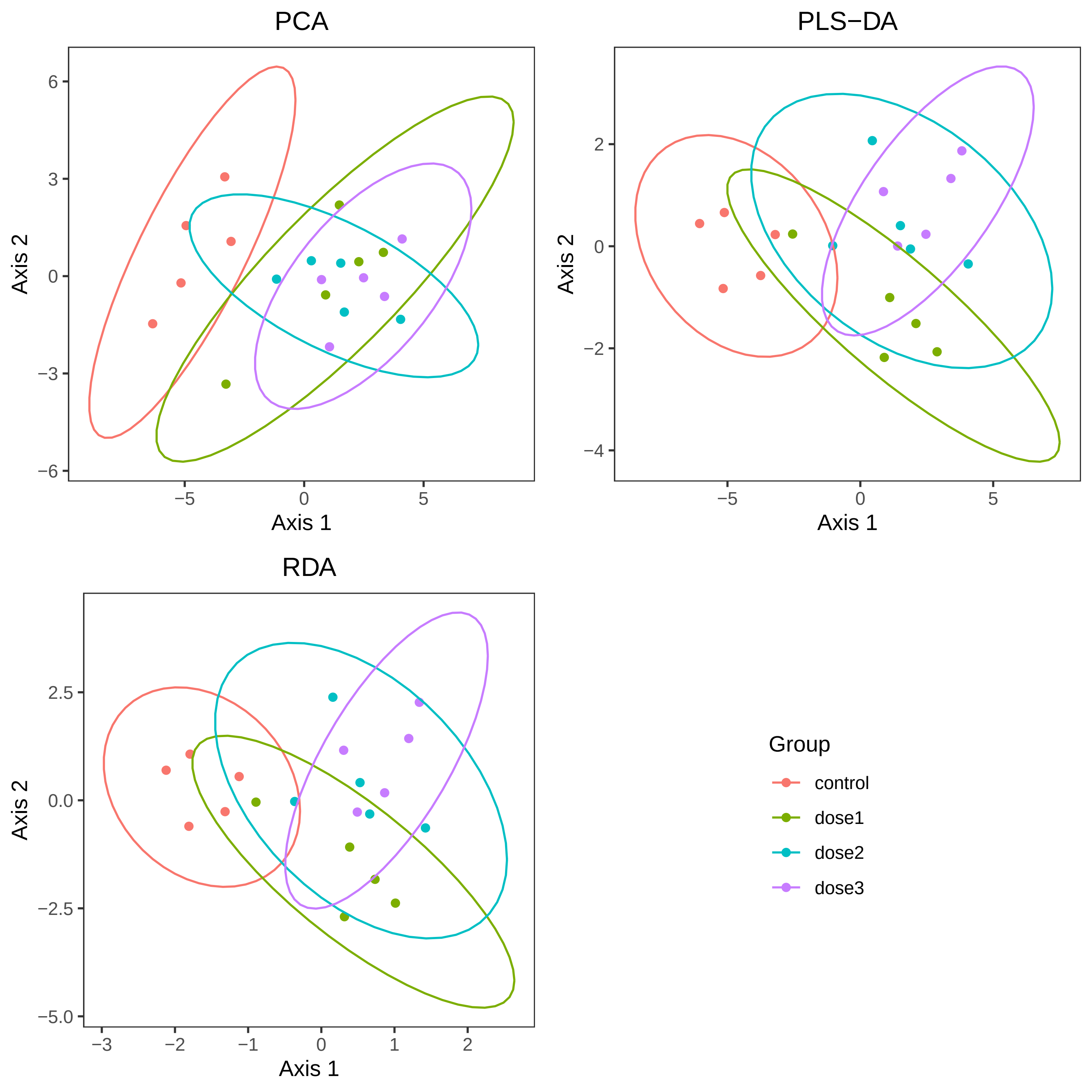
PCA
PLS−DA
RDA
Axis 1
Axis 2
Group
control
dose1
dose2
dose3

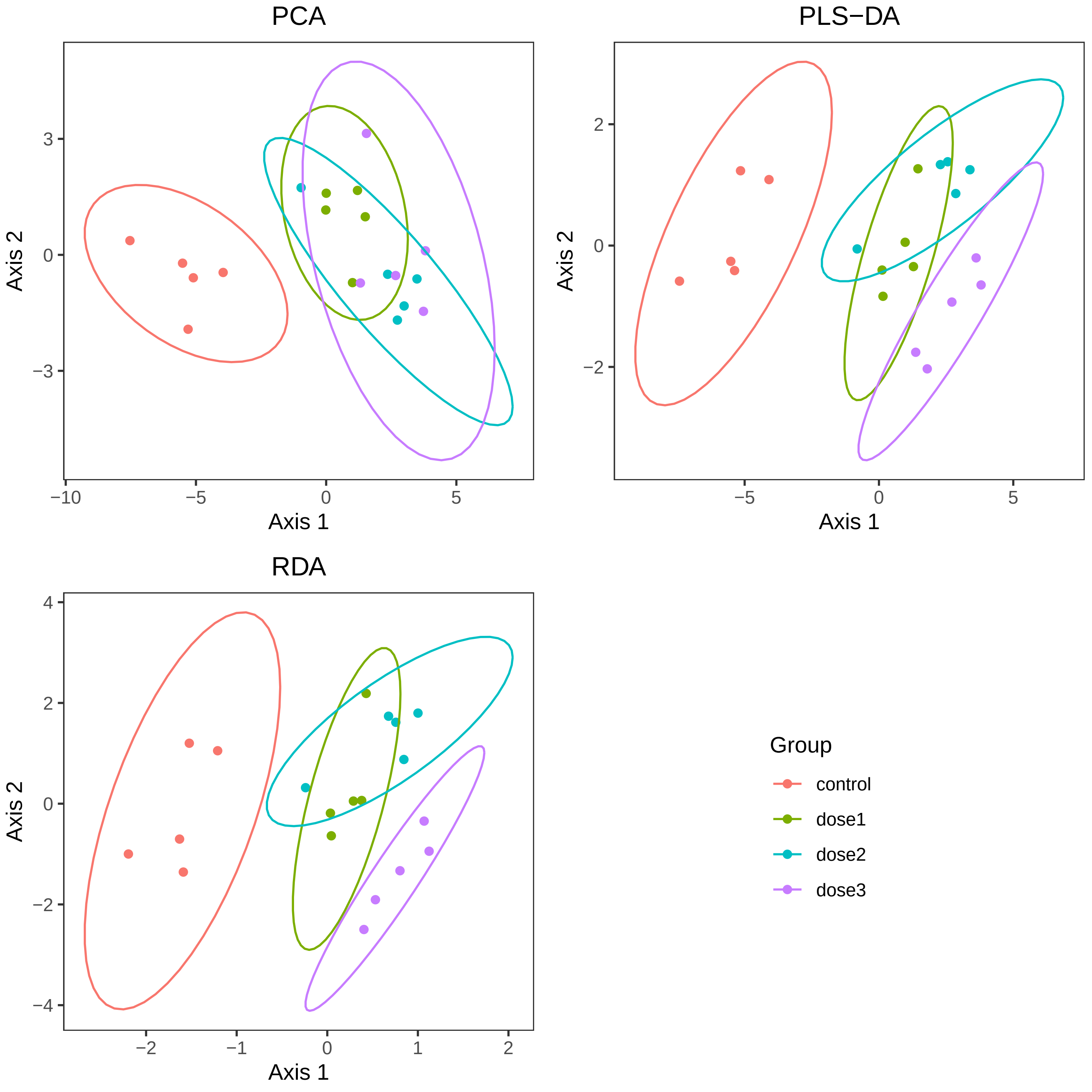
PCA
PLS−DA
RDA
Axis 1
Axis 2
Group
control
dose1
dose2
dose3

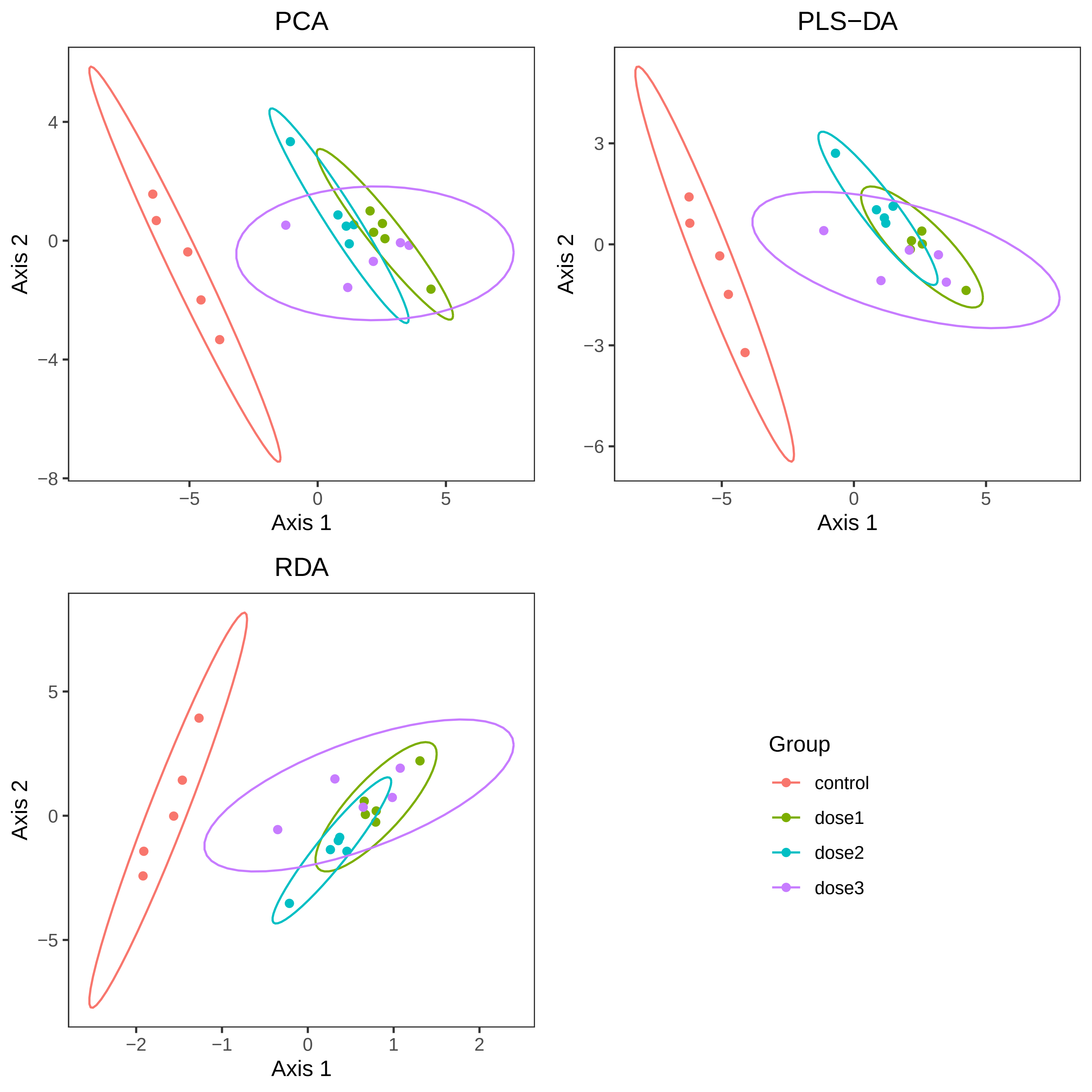
PCA
PLS−DA
RDA
Axis 1
Axis 2
4
0
−4
−8
−5
0
5
3
−3
−6
−2
−1
1
2
Group
control
dose1
dose2
dose3

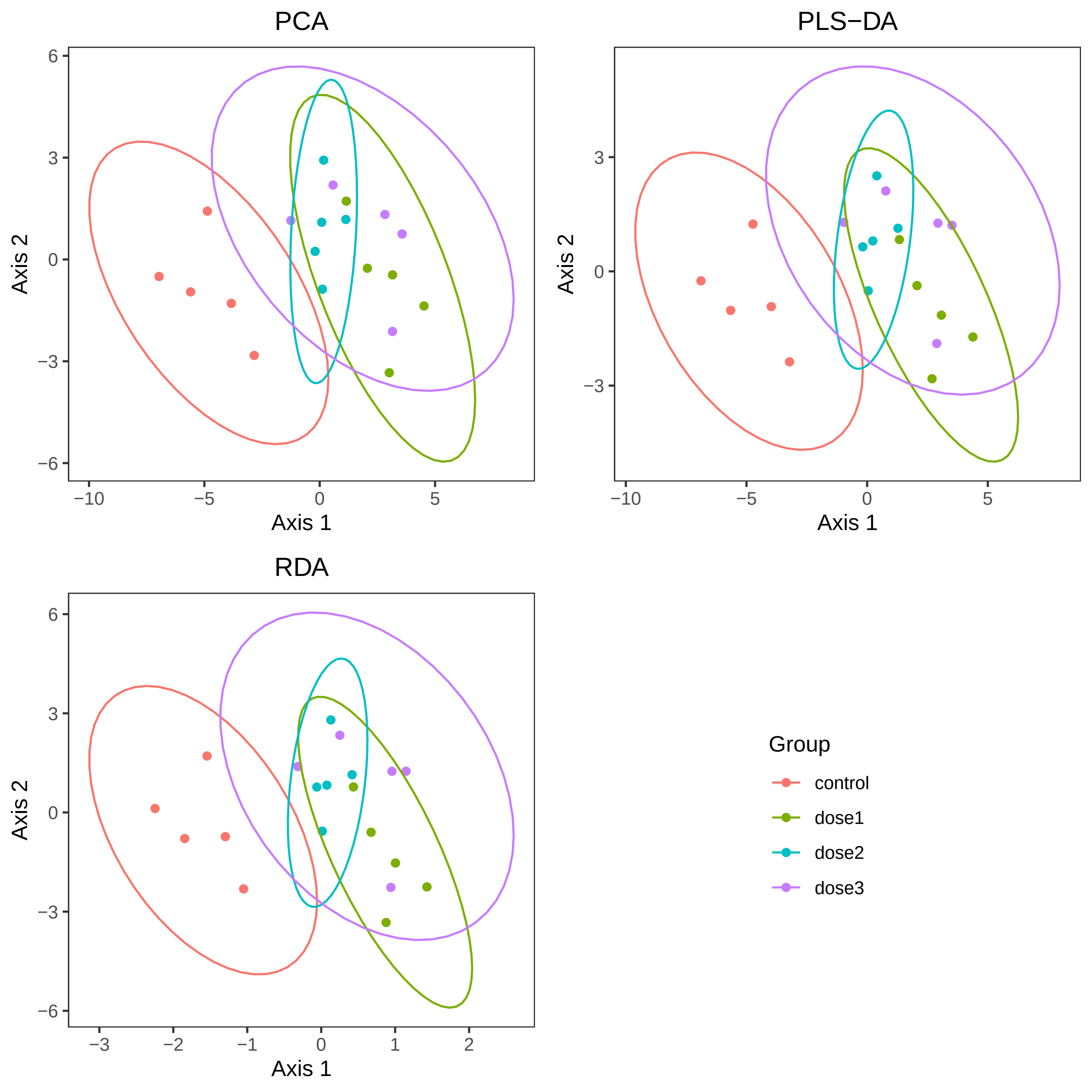
PCA
PLS−DA
RDA
Axis 1
Axis 2
Group
control
dose1
dose2
dose3

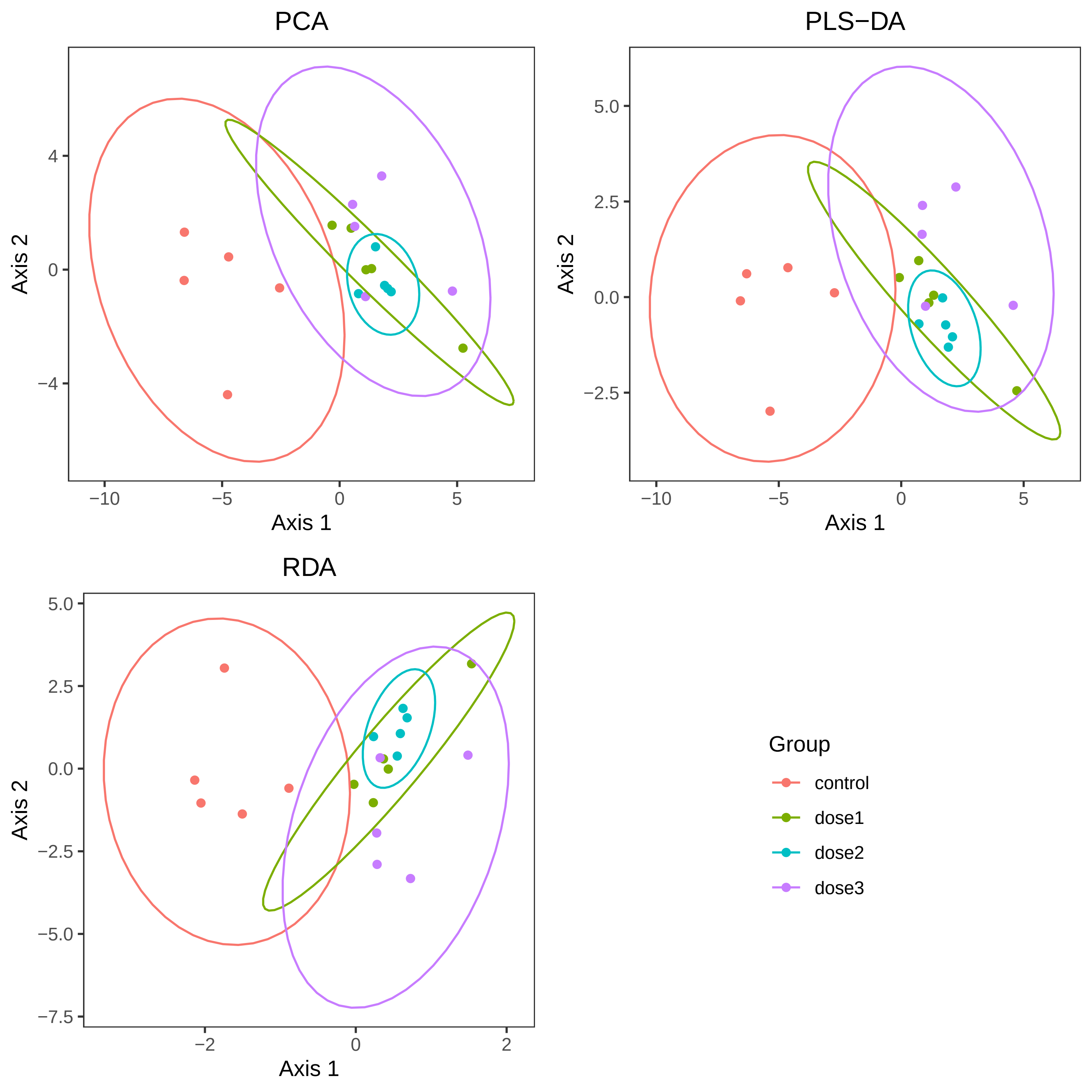
PCA
PLS−DA
RDA
Axis 1
Axis 2
−10
−5
0
5
4
−4
5.0
2.5
0.0
−2.5
−7.5
−5.0
−2
2
Group
control
dose1
dose2
dose3

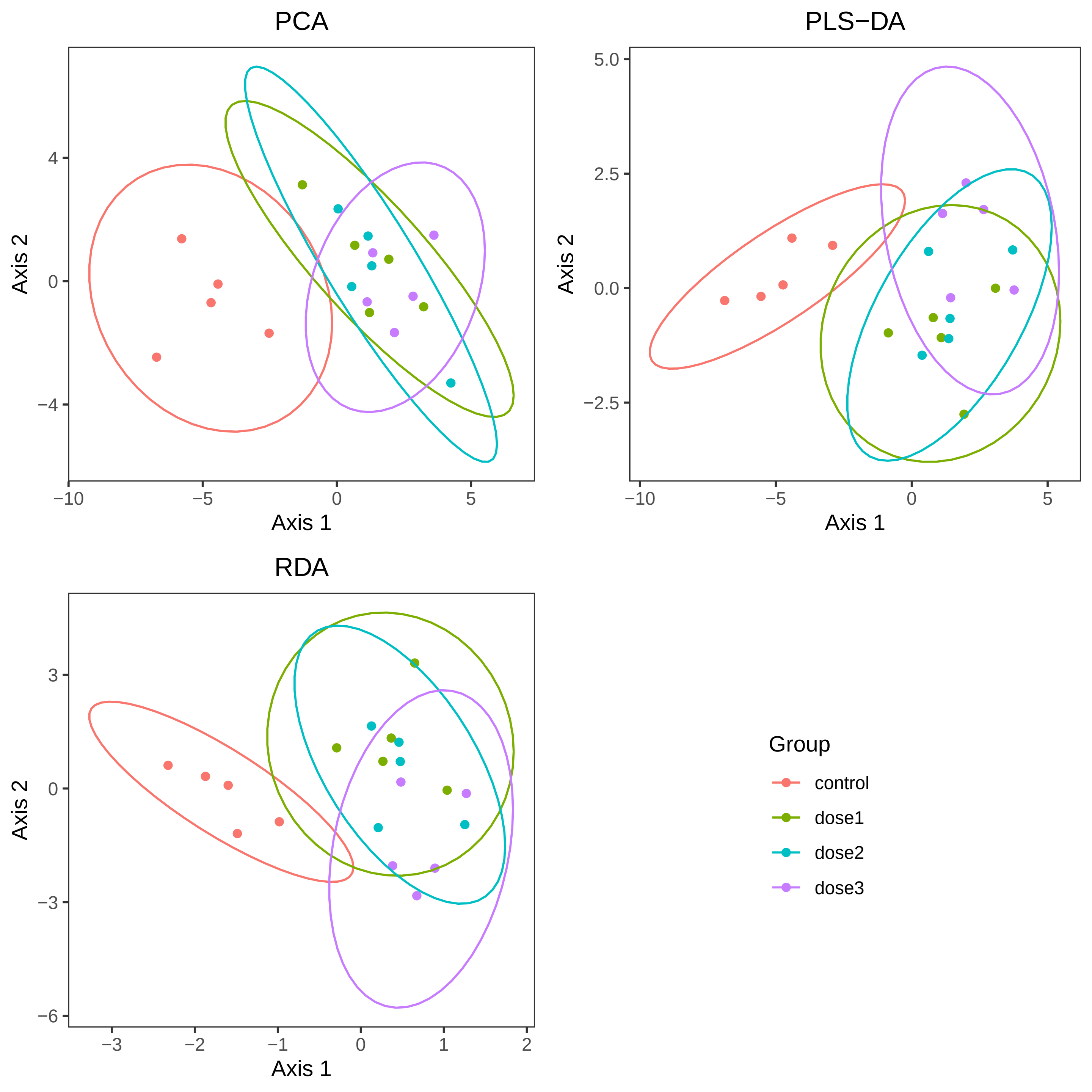
PCA
PLS−DA
RDA
Axis 1
Axis 2
Group
control
dose1
dose2
dose3

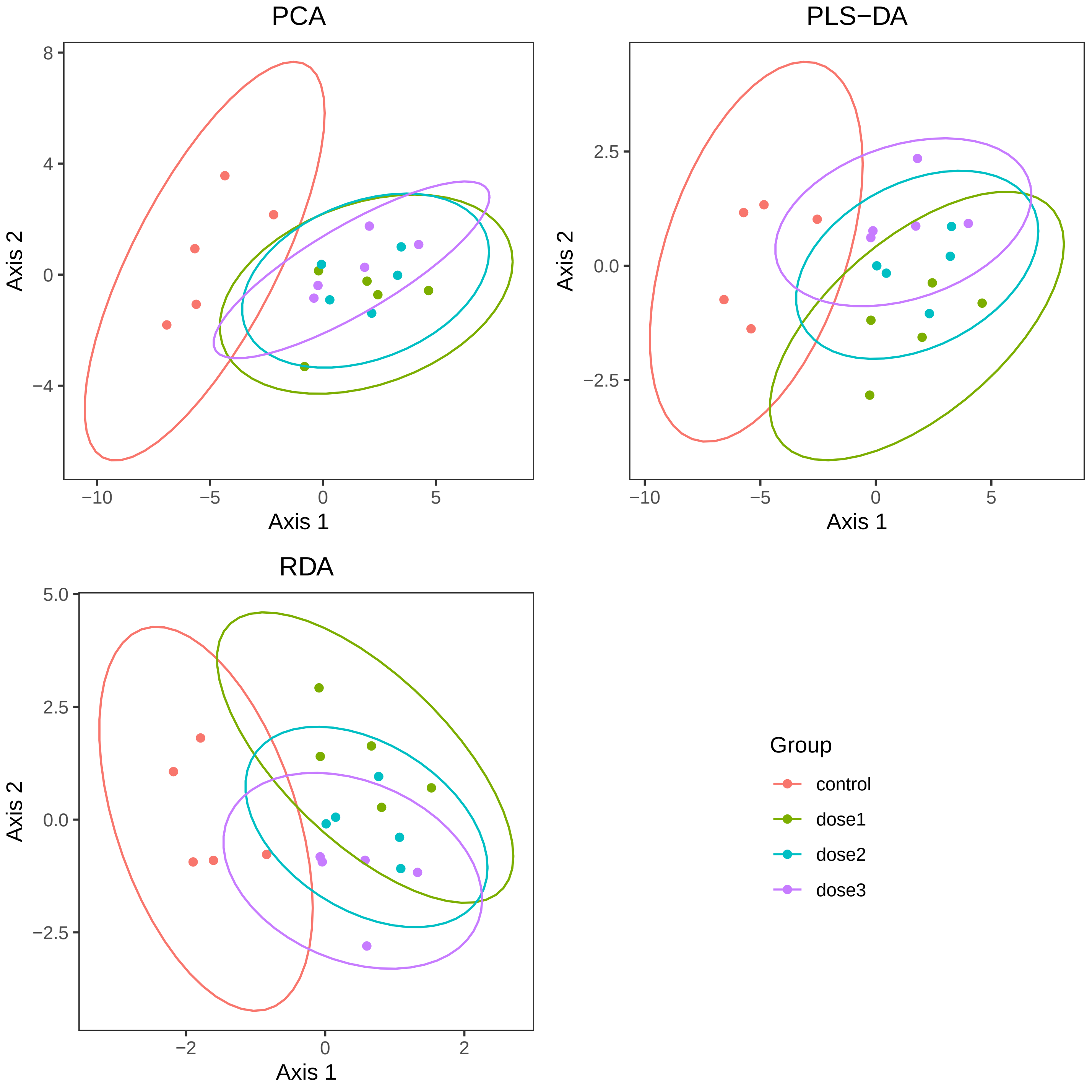
PCA
PLS−DA
RDA
Axis 1
Axis 2
Group
control
dose1
dose2
dose3

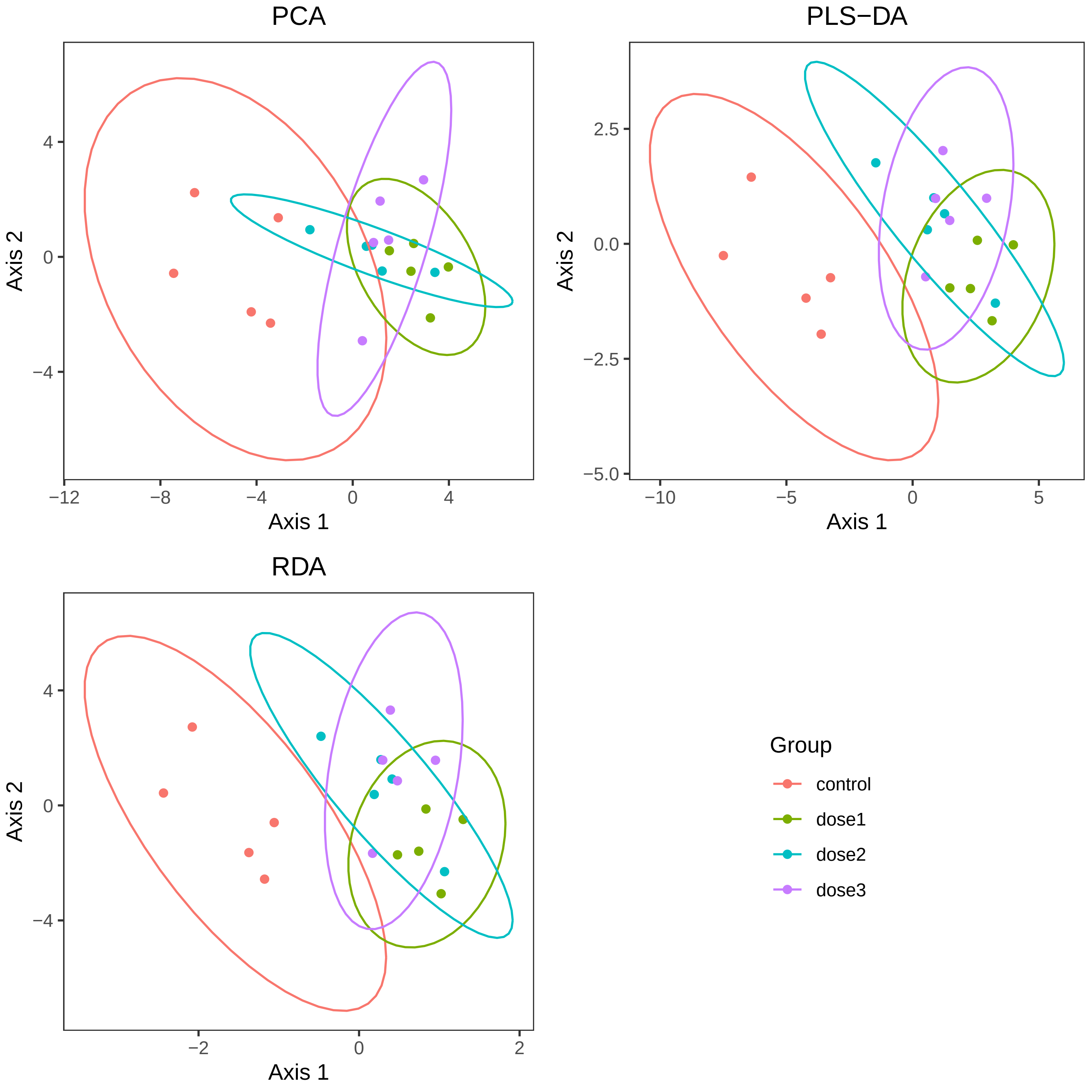

PCA
PLS−DA
RDA
Axis 1
Axis 2
Group
control
dose1
dose2
dose3

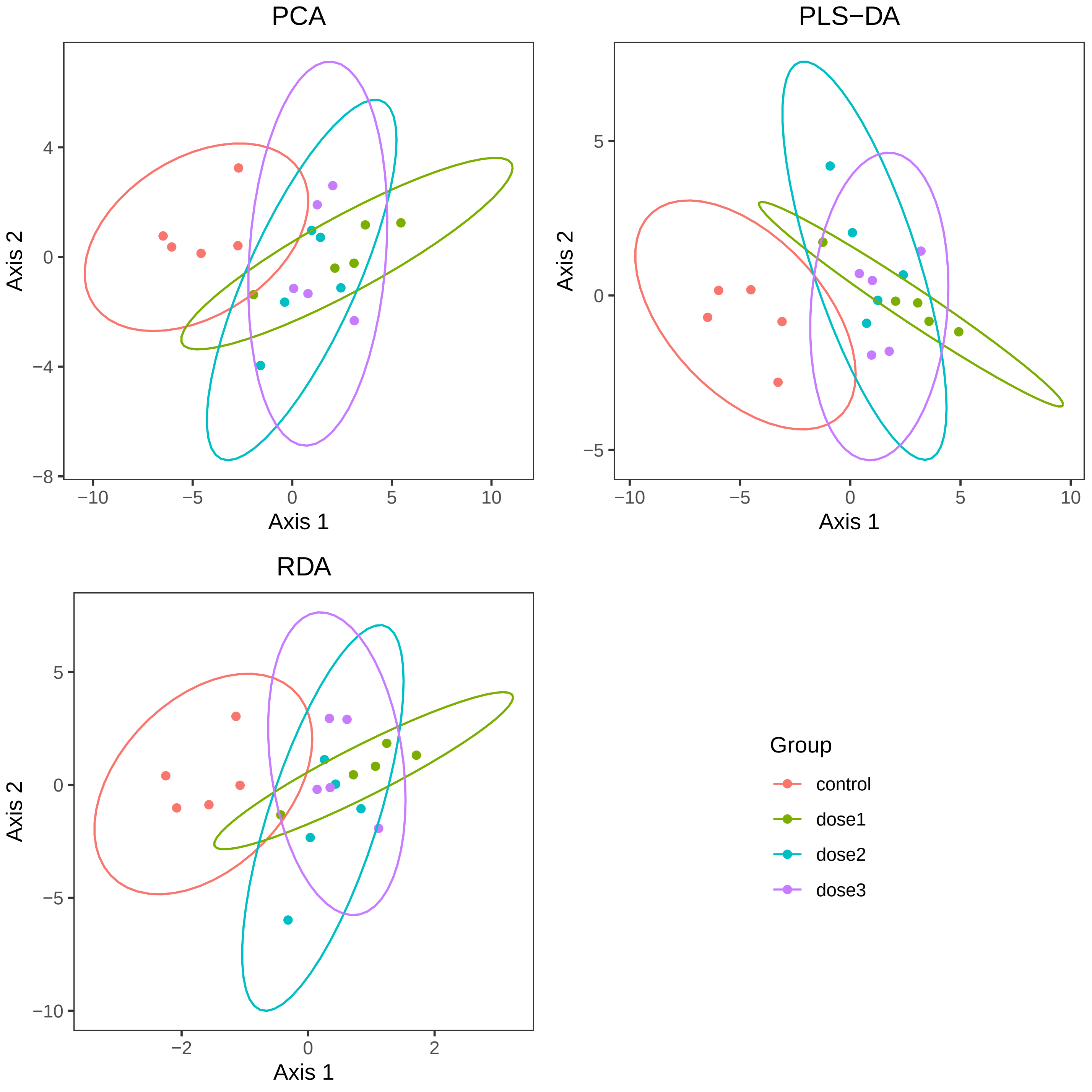
PCA
PLS−DA
RDA
Axis 1
Axis 2
Group
control
dose1
dose2
dose3

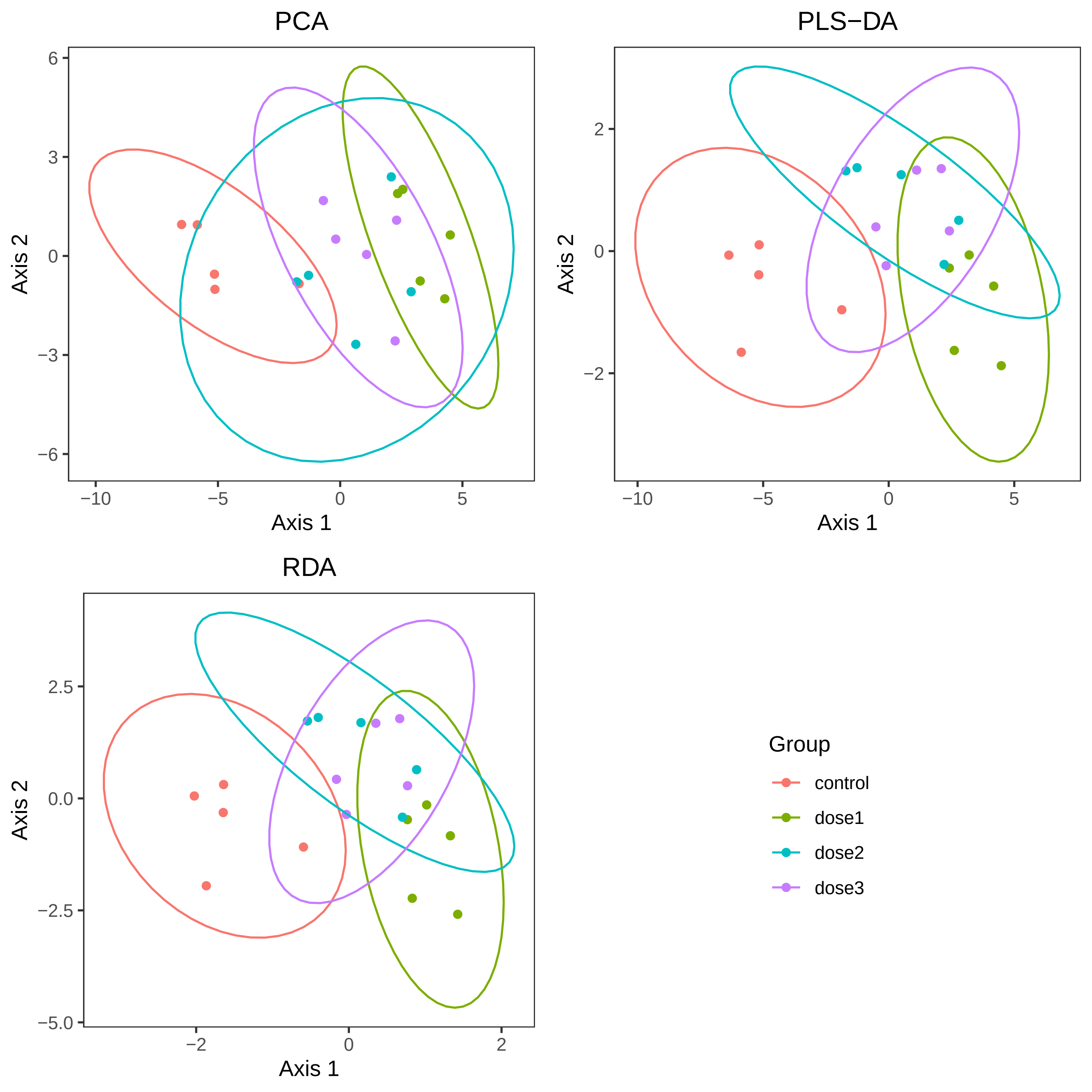
PCA
PLS−DA
RDA
Axis 1
Axis 2
Group
control
dose1
dose2
dose3

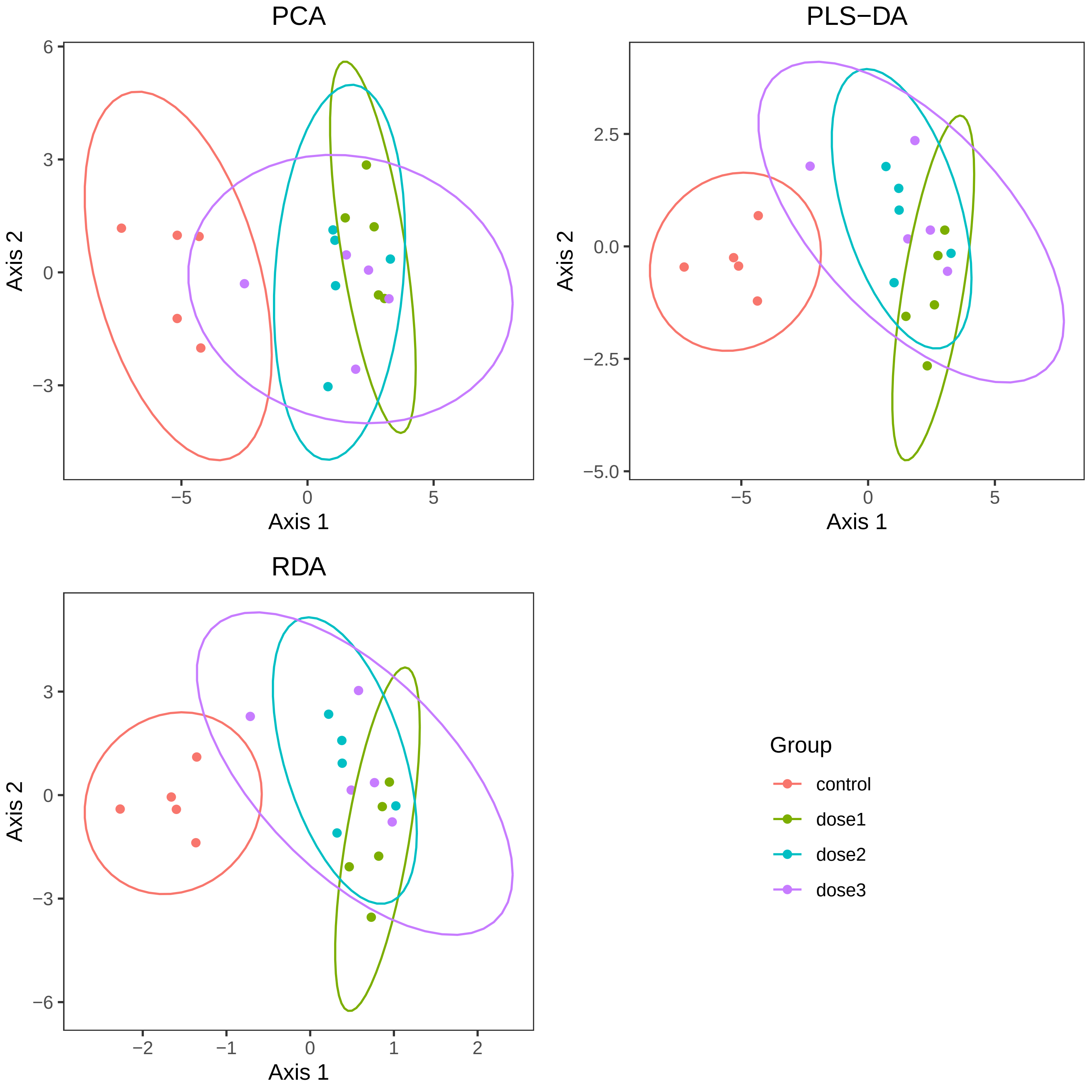

PCA
PLS−DA
RDA
Axis 1
Axis 2
Group
control
dose1
dose2
dose3

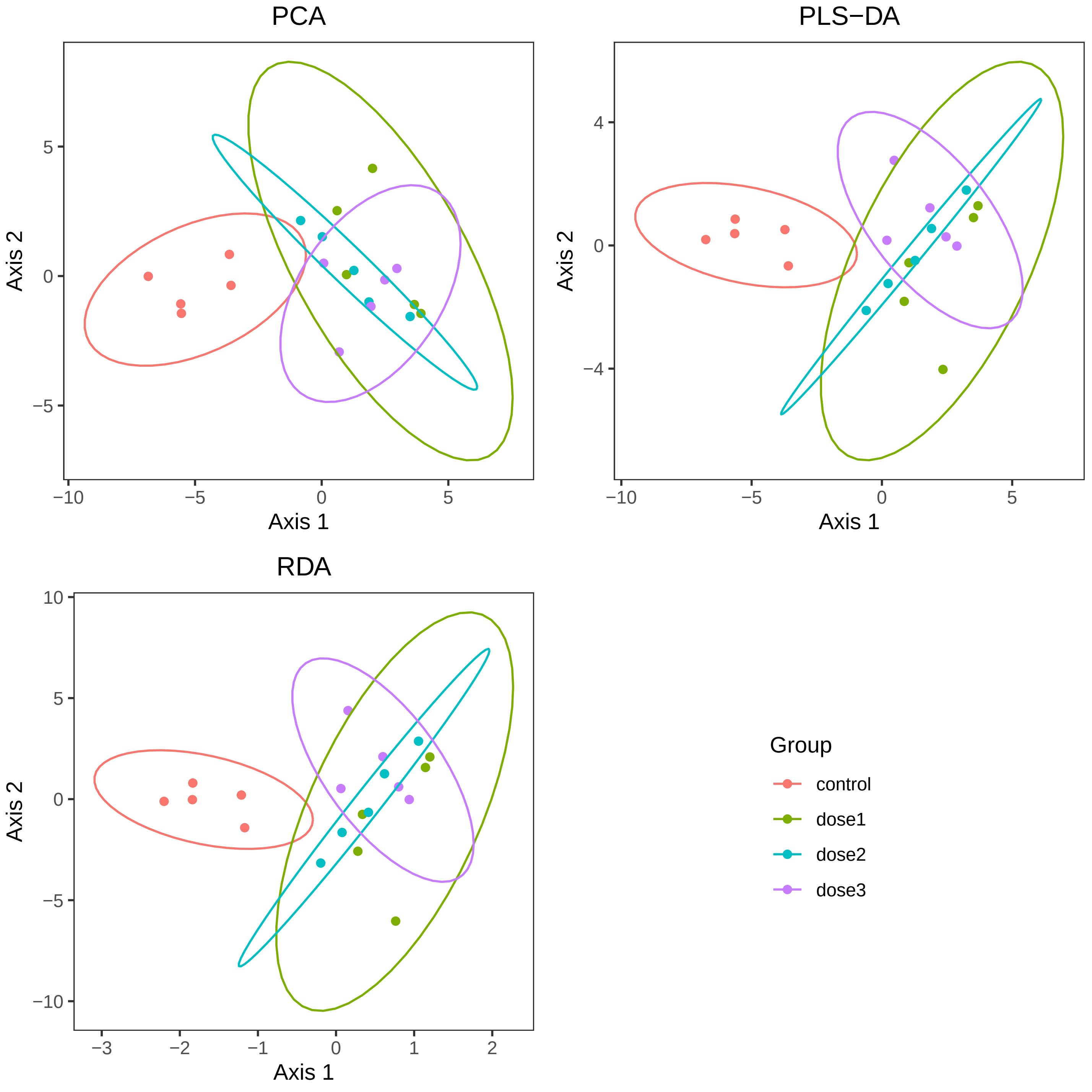

PCA
PLS−DA
RDA
Axis 1
Axis 2
−10
−5
0
5
4
−4
10
−3
−2
−1
1
2
Group
control
dose1
dose2
dose3

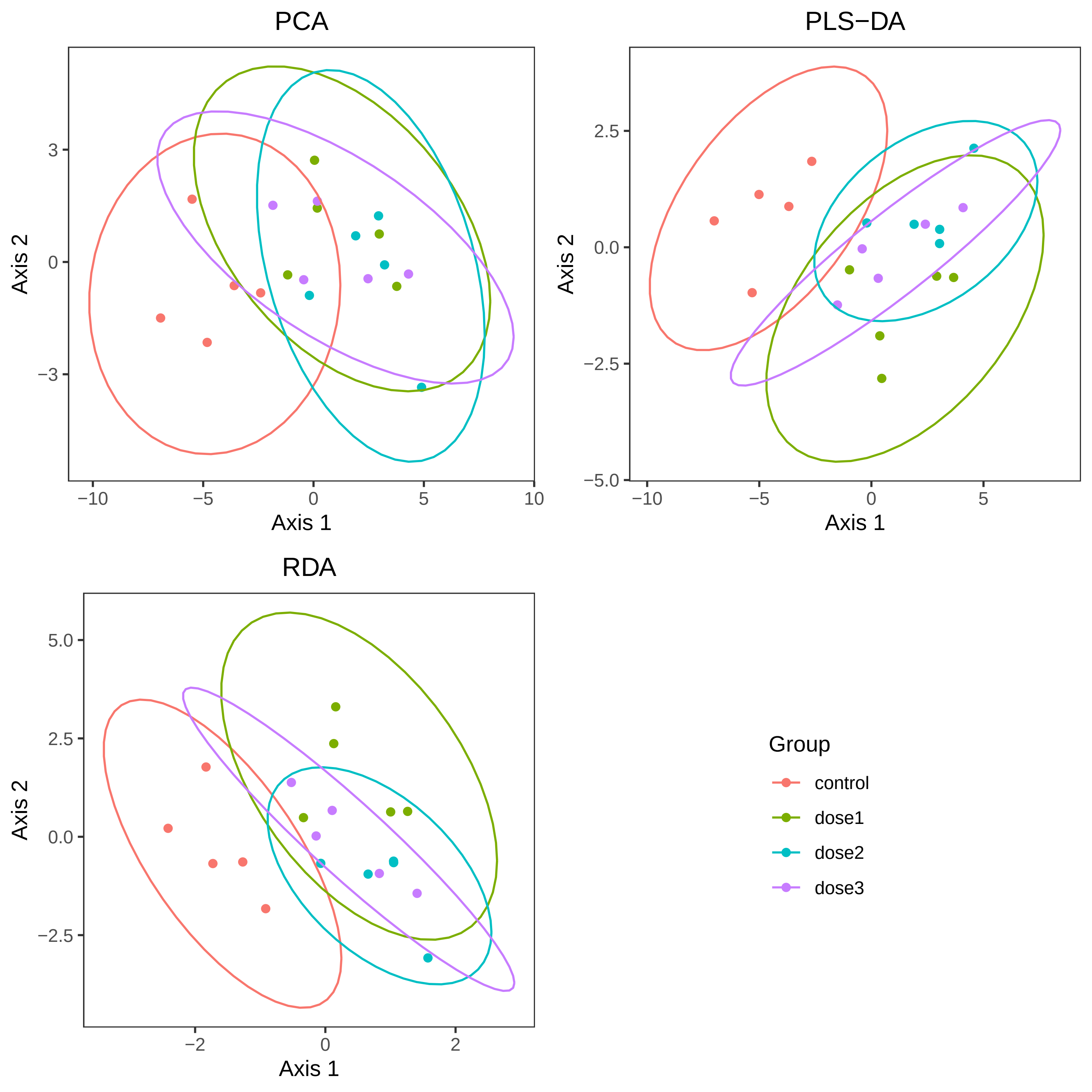

PCA
PLS−DA
RDA
Axis 1
Axis 2
Group
control
dose1
dose2
dose3

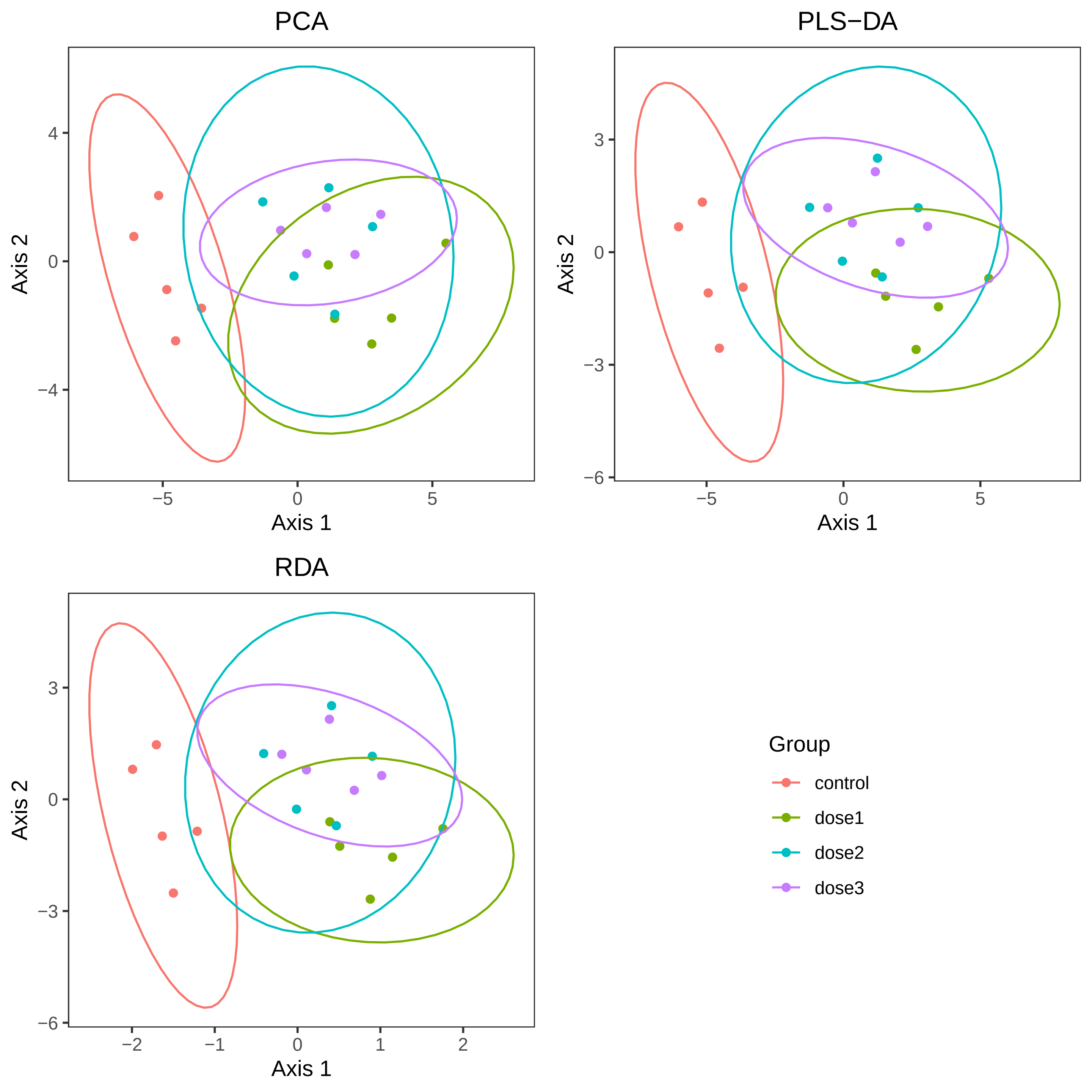
PCA
PLS−DA
RDA
Axis 1
Axis 2
Group
control
dose1
dose2
dose3

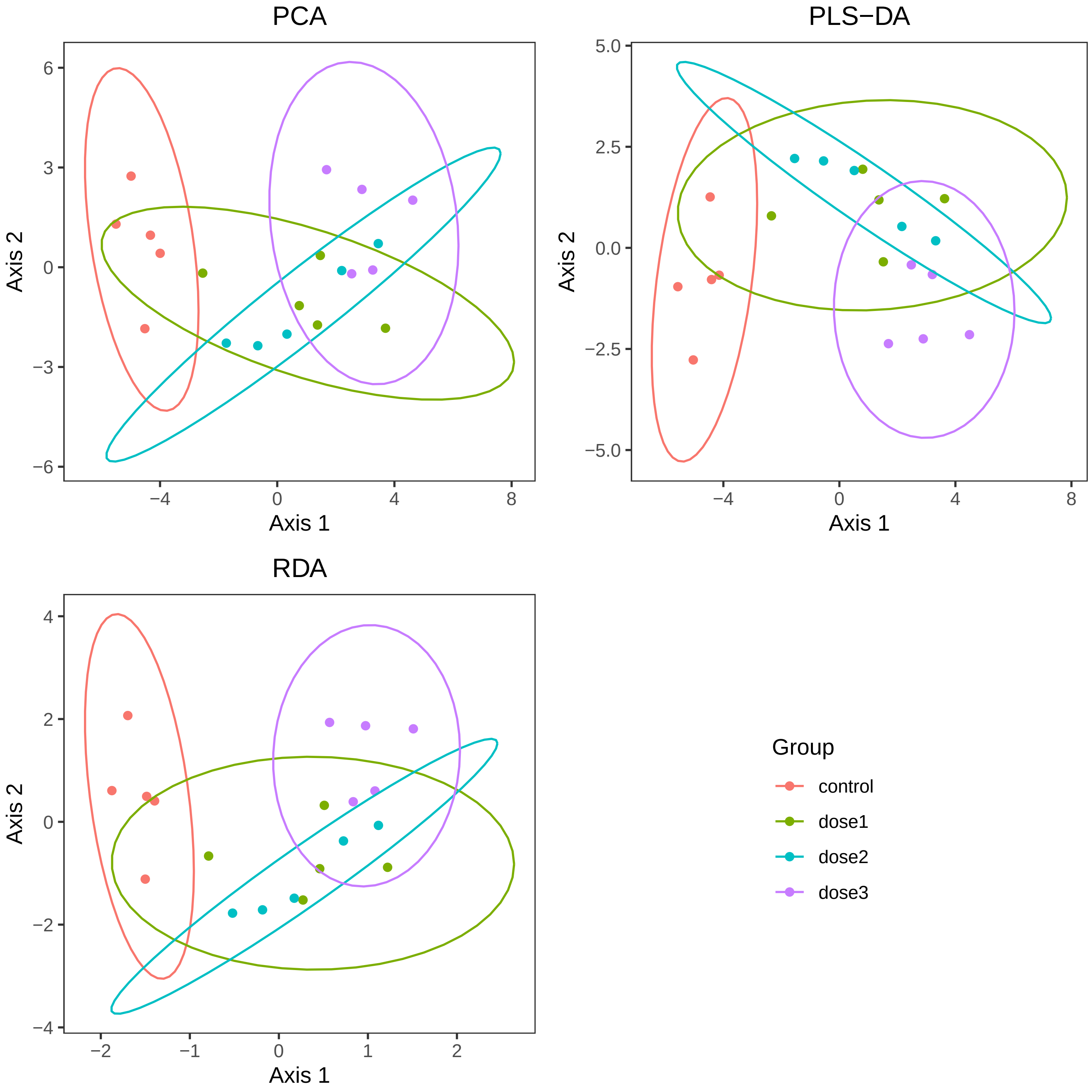
PCA
PLS−DA
RDA
Axis 1
Axis 2
Group
control
dose1
dose2
dose3

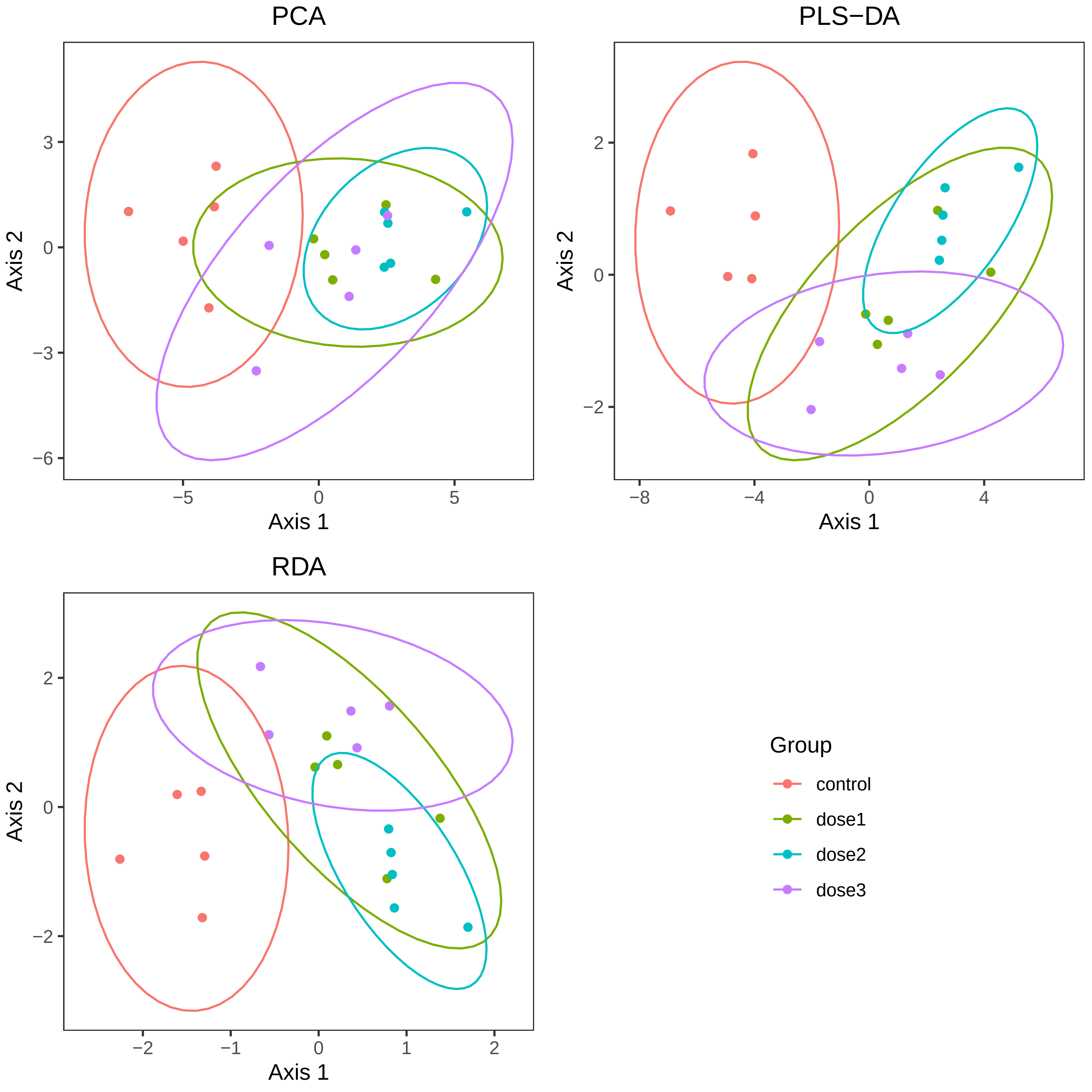
PCA
PLS−DA
RDA
Axis 1
Axis 2
Group
control
dose1
dose2
dose3

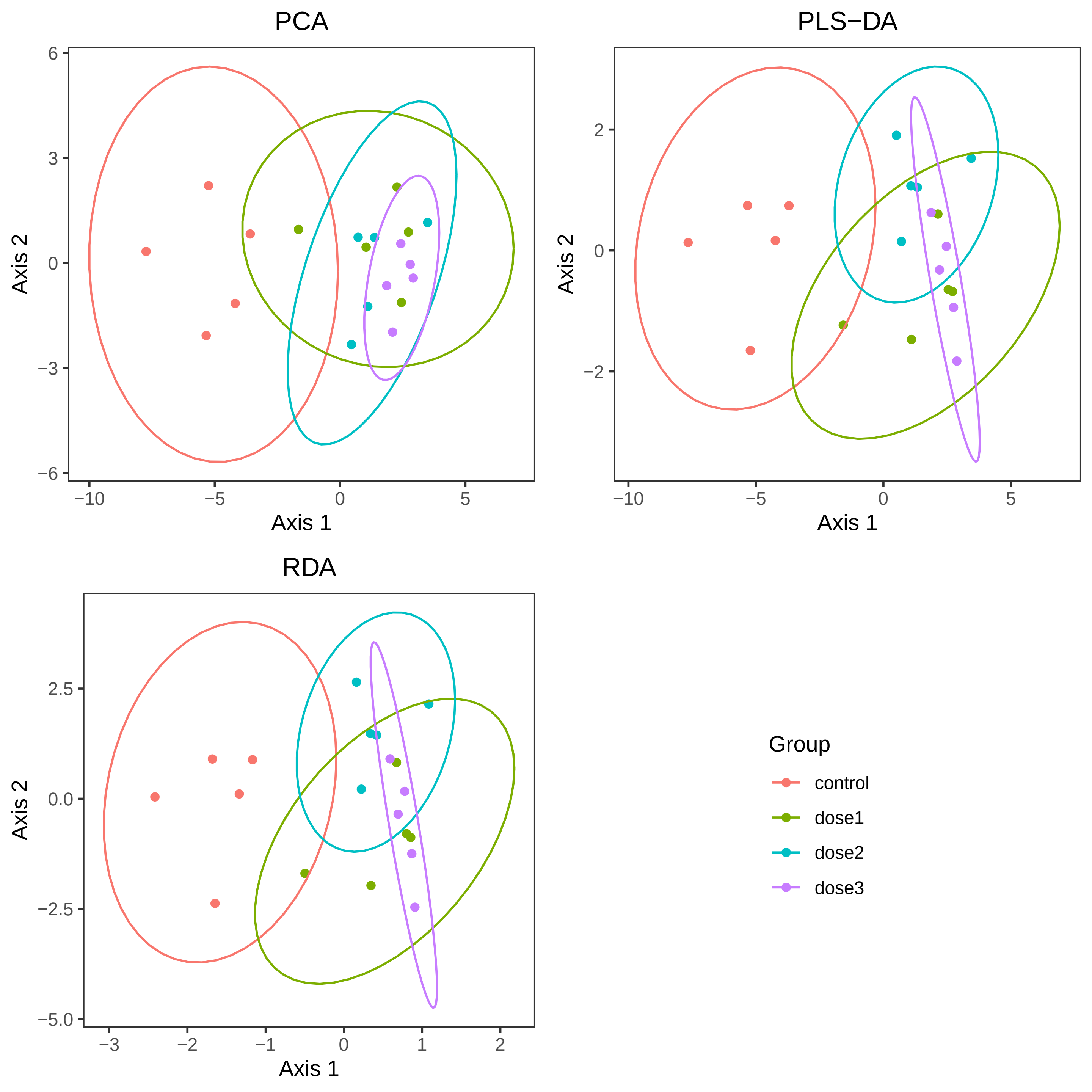

PCA
PLS−DA
RDA
Axis 1
Axis 2
Group
control
dose1
dose2
dose3

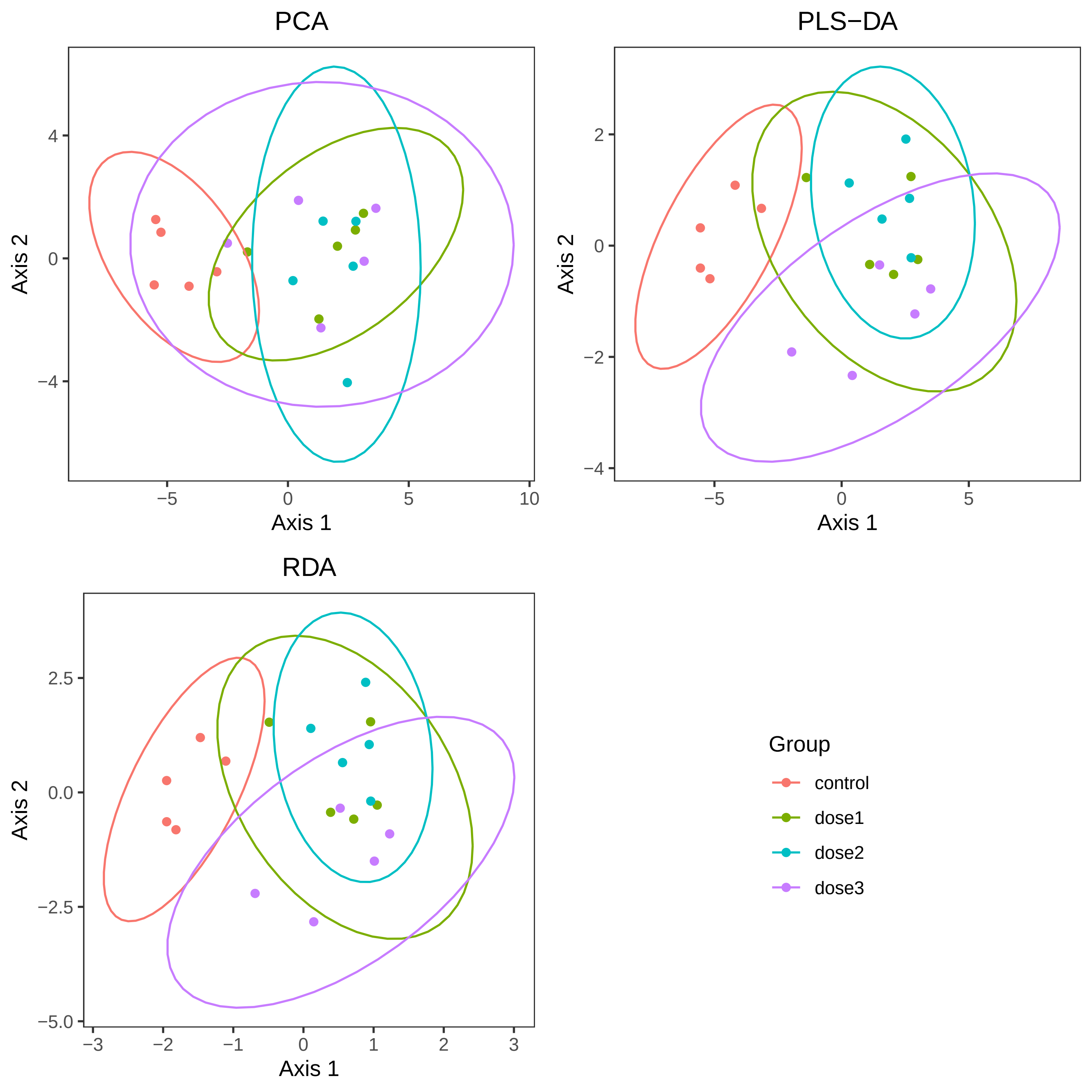
PCA
PLS−DA
RDA
Axis 1
Axis 2
Group
control
dose1
dose2
dose3

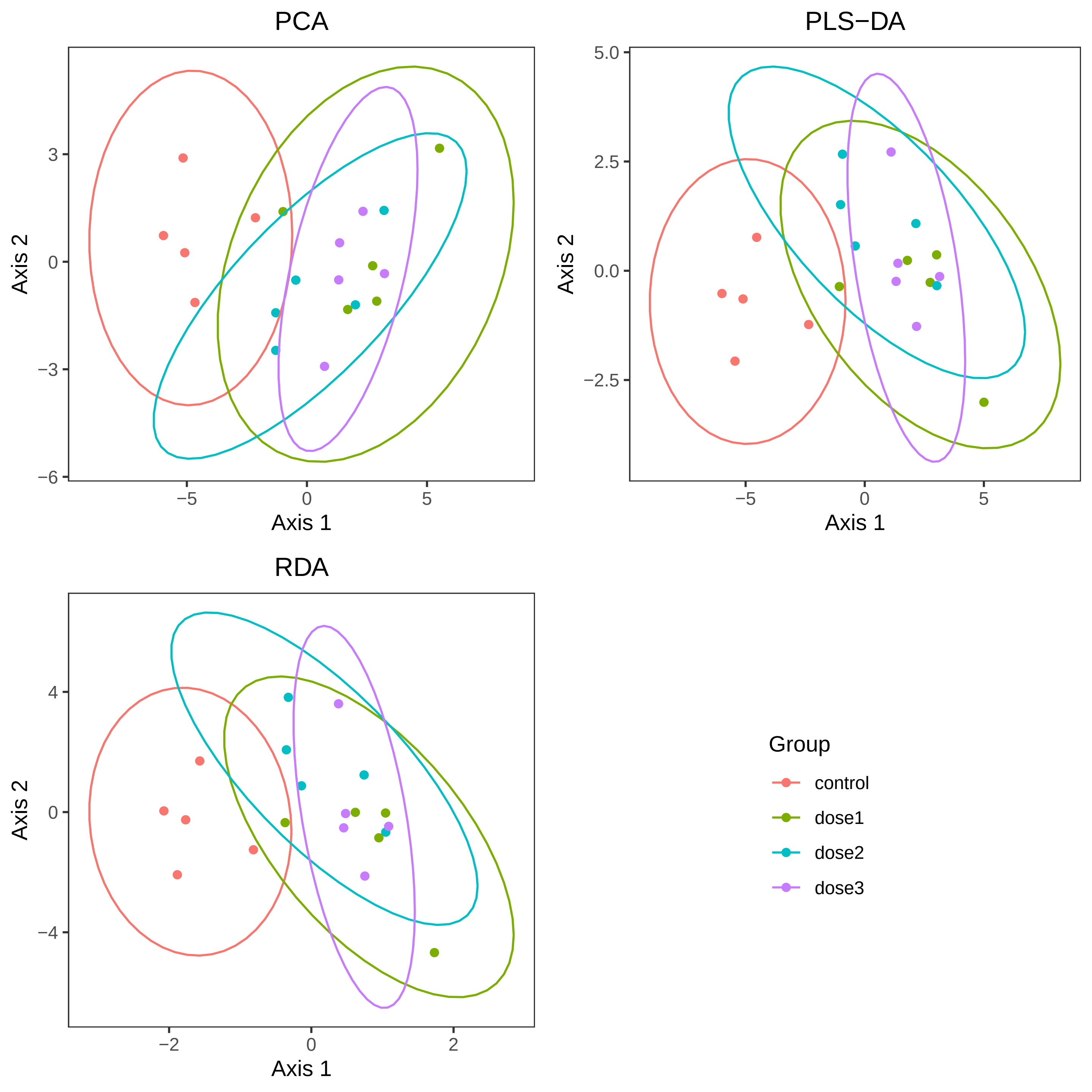

PCA
PLS−DA
RDA
Axis 1
Axis 2
Group
control
dose1
dose2
dose3

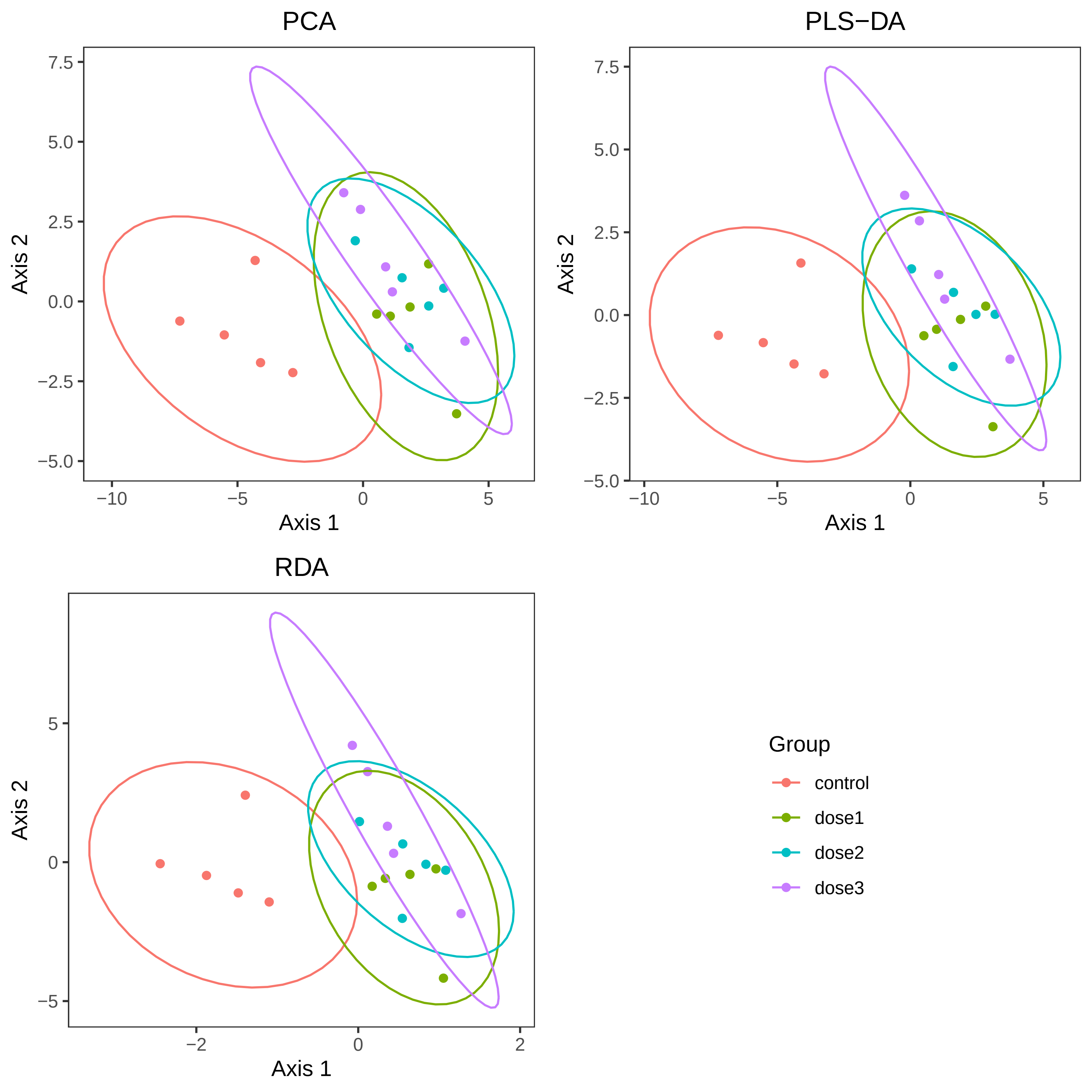

PCA
PLS−DA
RDA
Axis 1
Axis 2
7.5
5.0
2.5
0.0
−2.5
−5.0
−10
−5
0
5
2
−2
Group
control
dose1
dose2
dose3

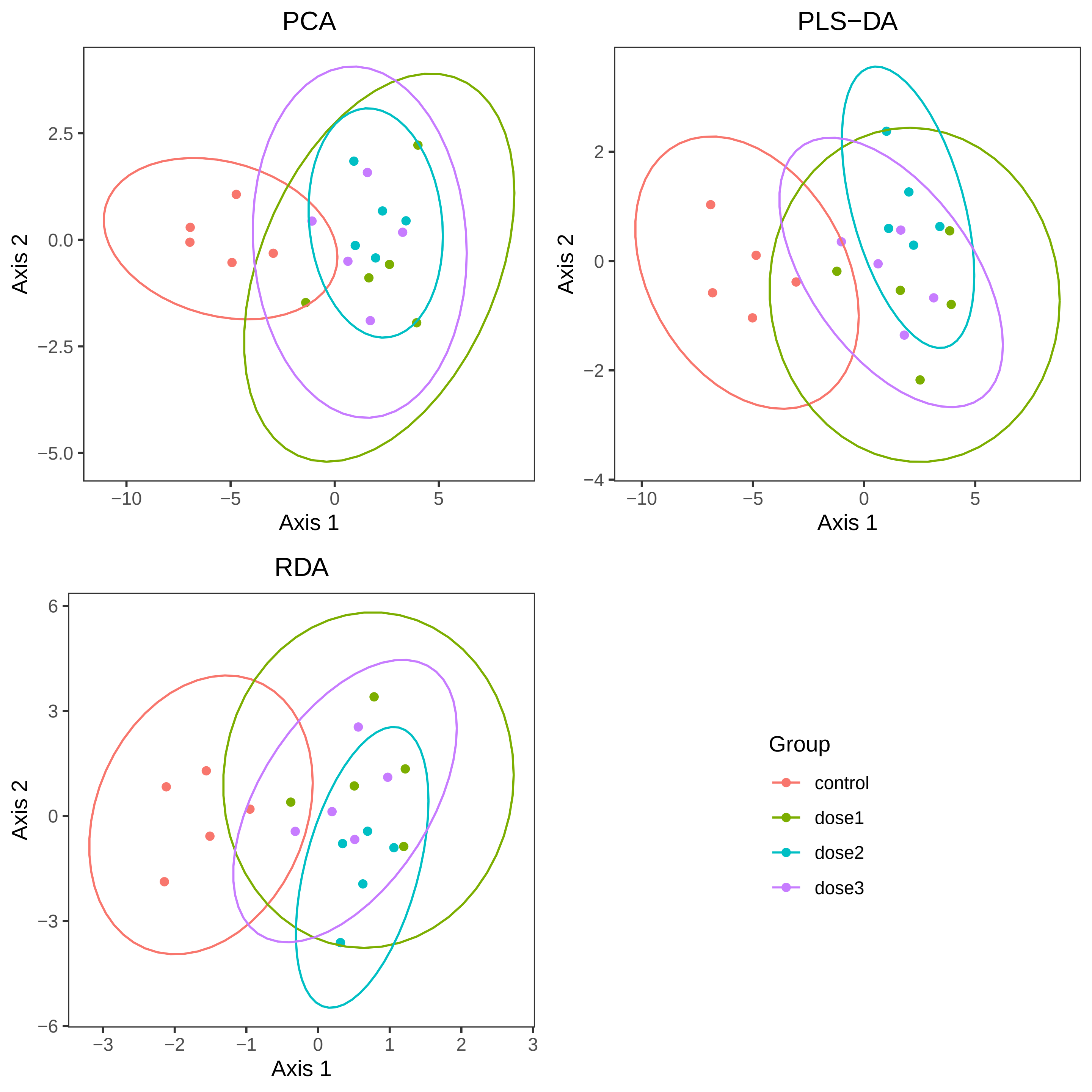

PCA
PLS−DA
RDA
Axis 1
Axis 2
2.5
0.0
−2.5
−5.0
−10
−5
0
5
2
0
−2
−4
6
3
−3
−6
−3
−2
−1
1
2
3
Group
control
dose1
dose2
dose3

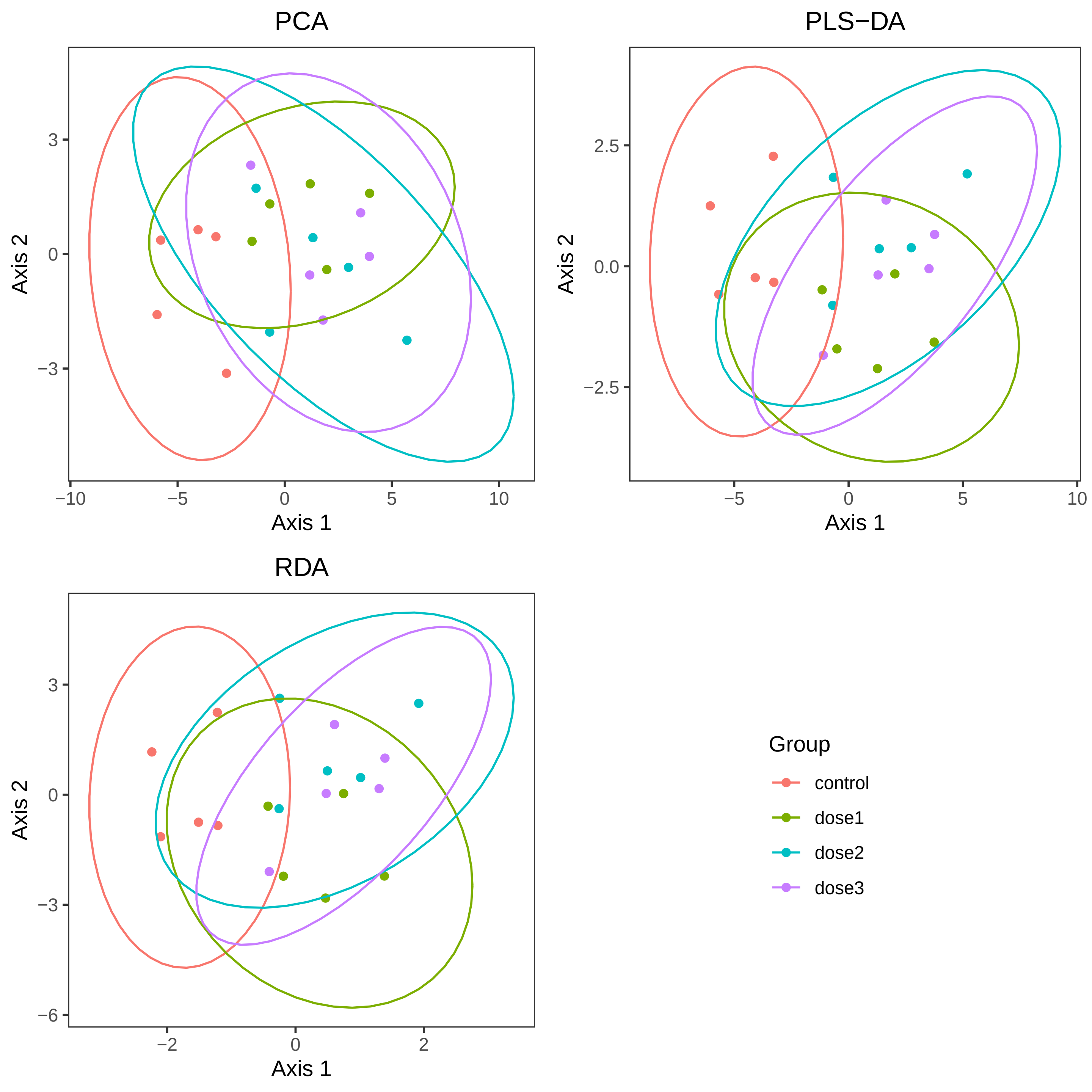
PCA
PLS−DA
RDA
Axis 1
Axis 2
−10
−5
0
5
10
3
0
−3
2.5
0.0
−2.5
−2
2
−6
Group
control
dose1
dose2
dose3

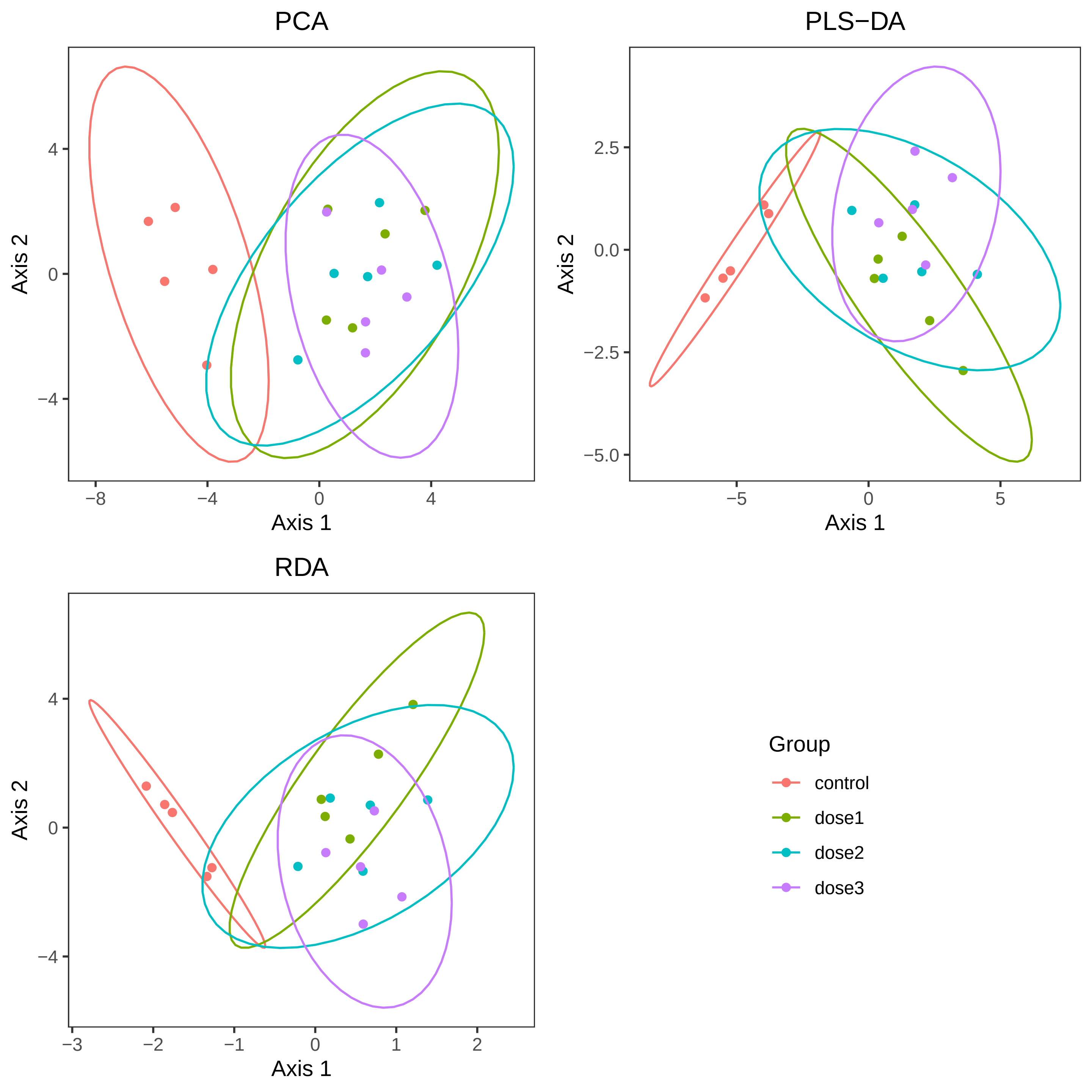

PCA
PLS−DA
RDA
Axis 1
Axis 2
Group
control
dose1
dose2
dose3

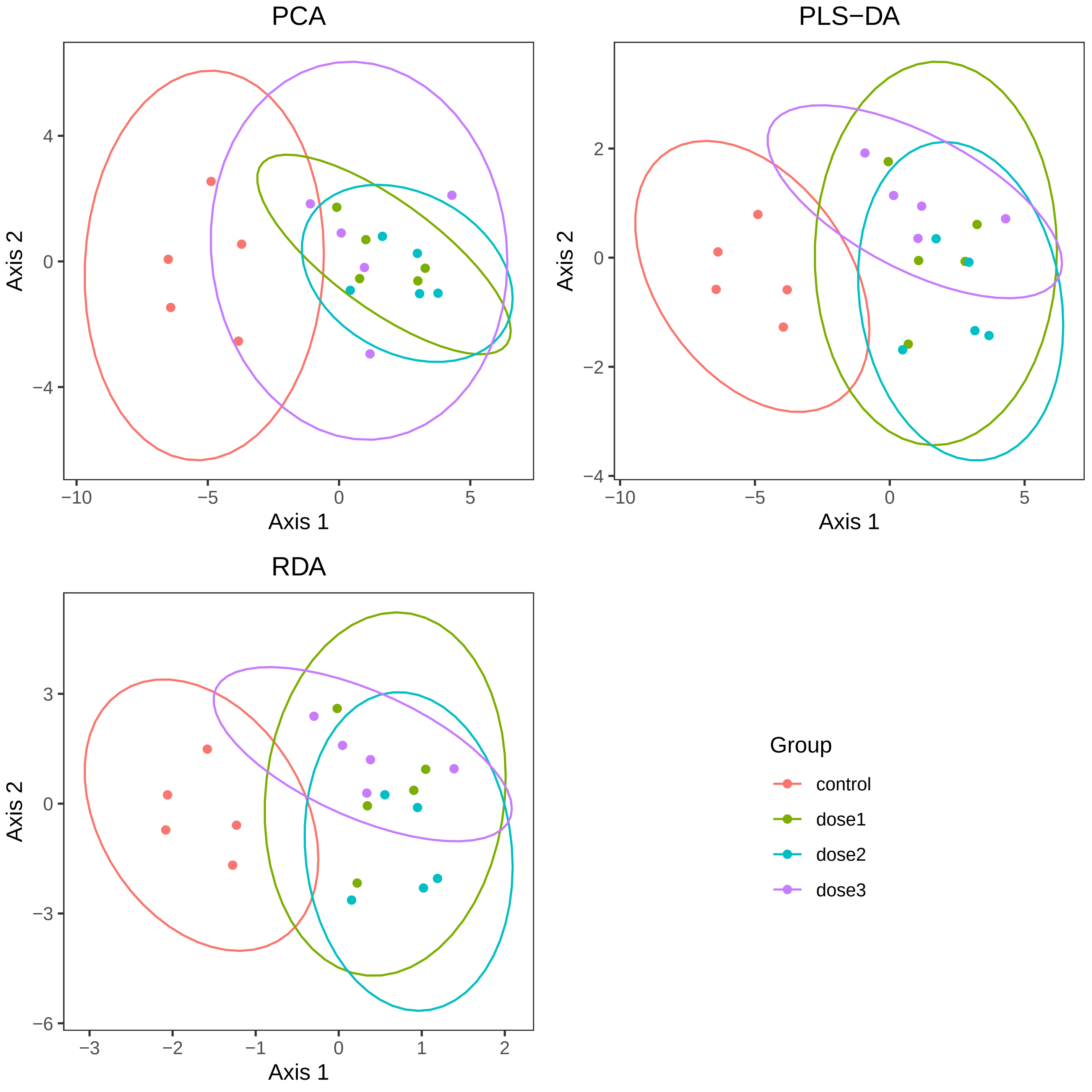
PCA
PLS−DA
RDA
Axis 1
Axis 2
Group
control
dose1
dose2
dose3

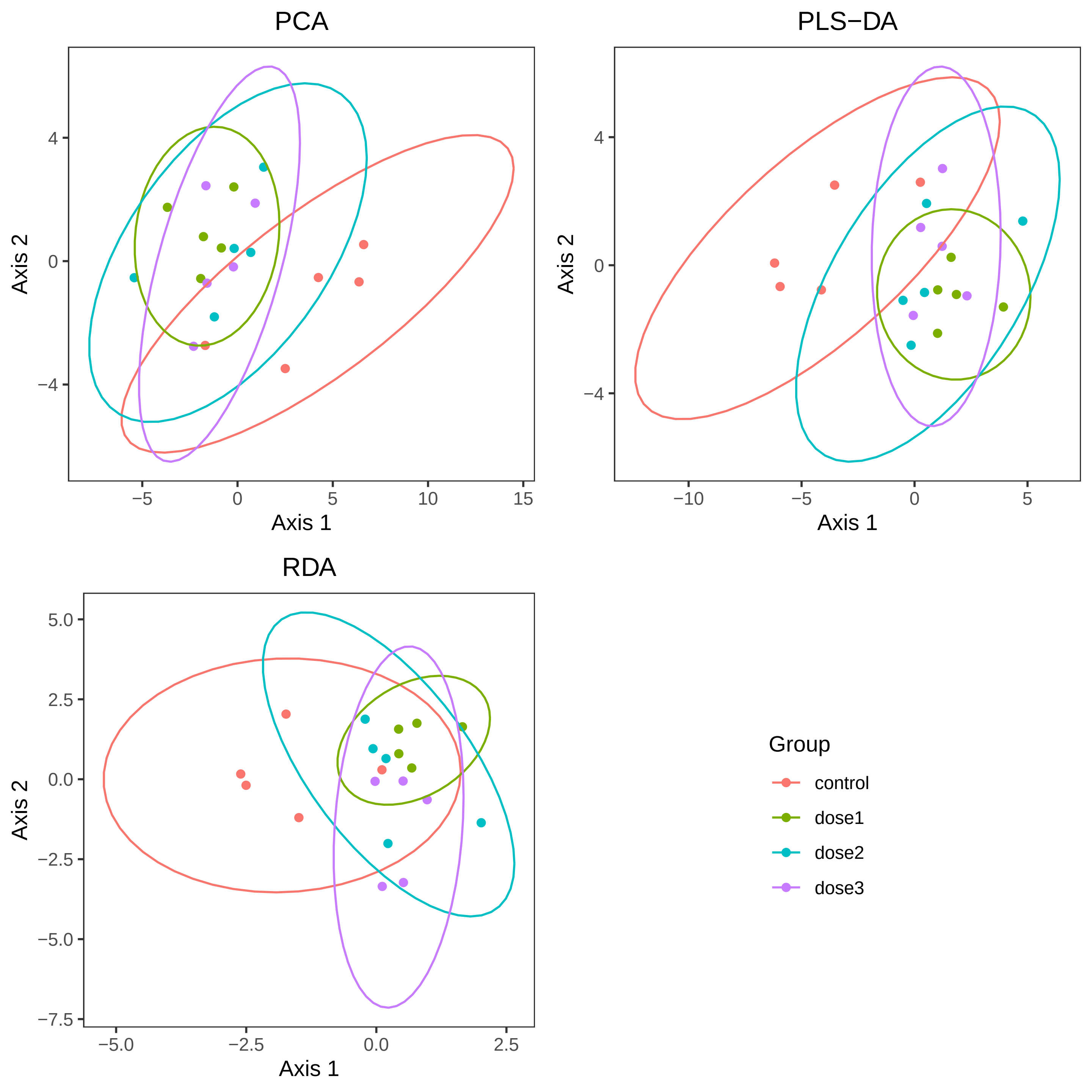

PCA
PLS−DA
RDA
Axis 1
Axis 2
Group
control
dose1
dose2
dose3

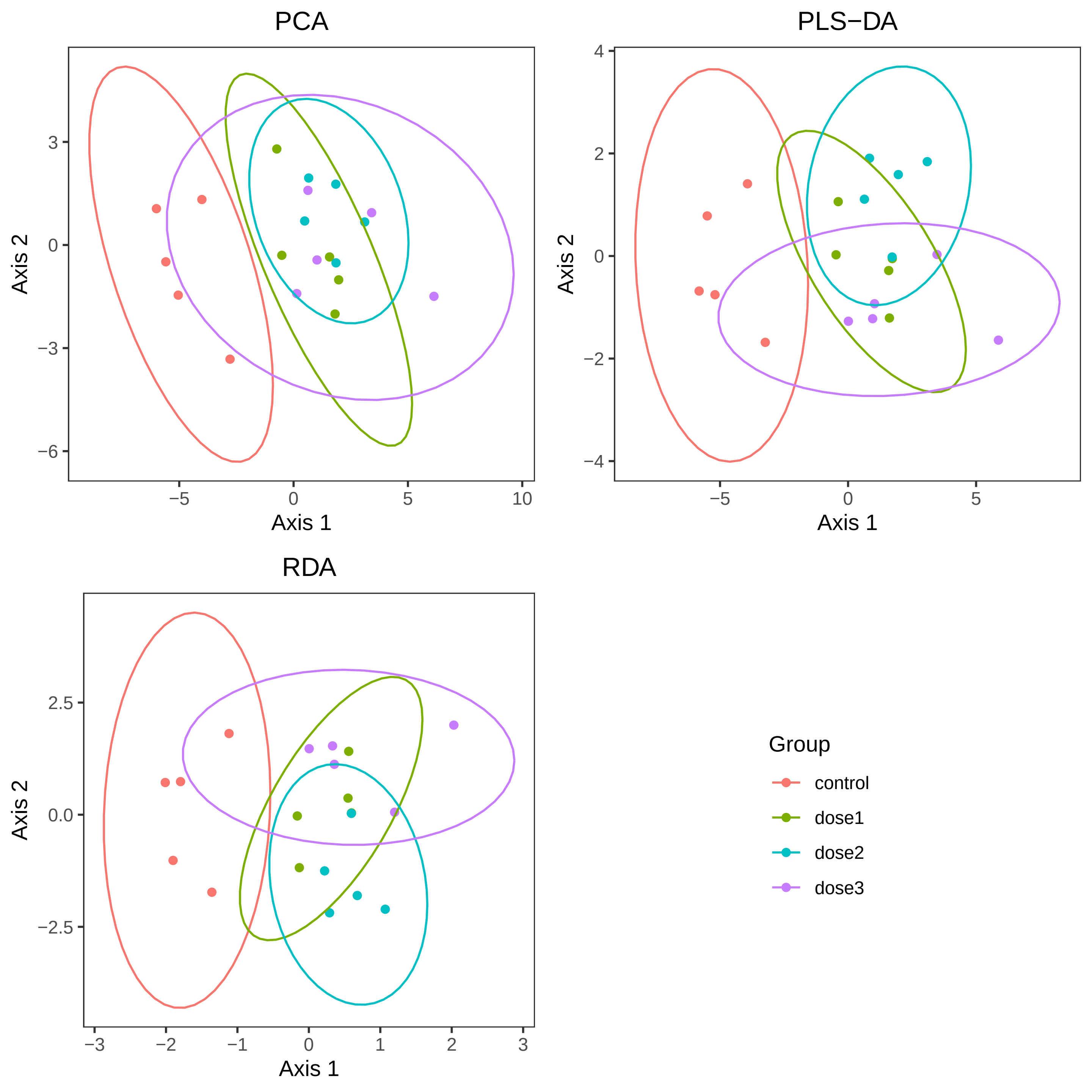
PCA
PLS−DA
RDA
Axis 1
Axis 2
3
0
−3
−6
−5
0
5
10
4
2
−2
−4
2.5
0.0
−2.5
−3
−2
−1
1
Group
control
dose1
dose2
dose3

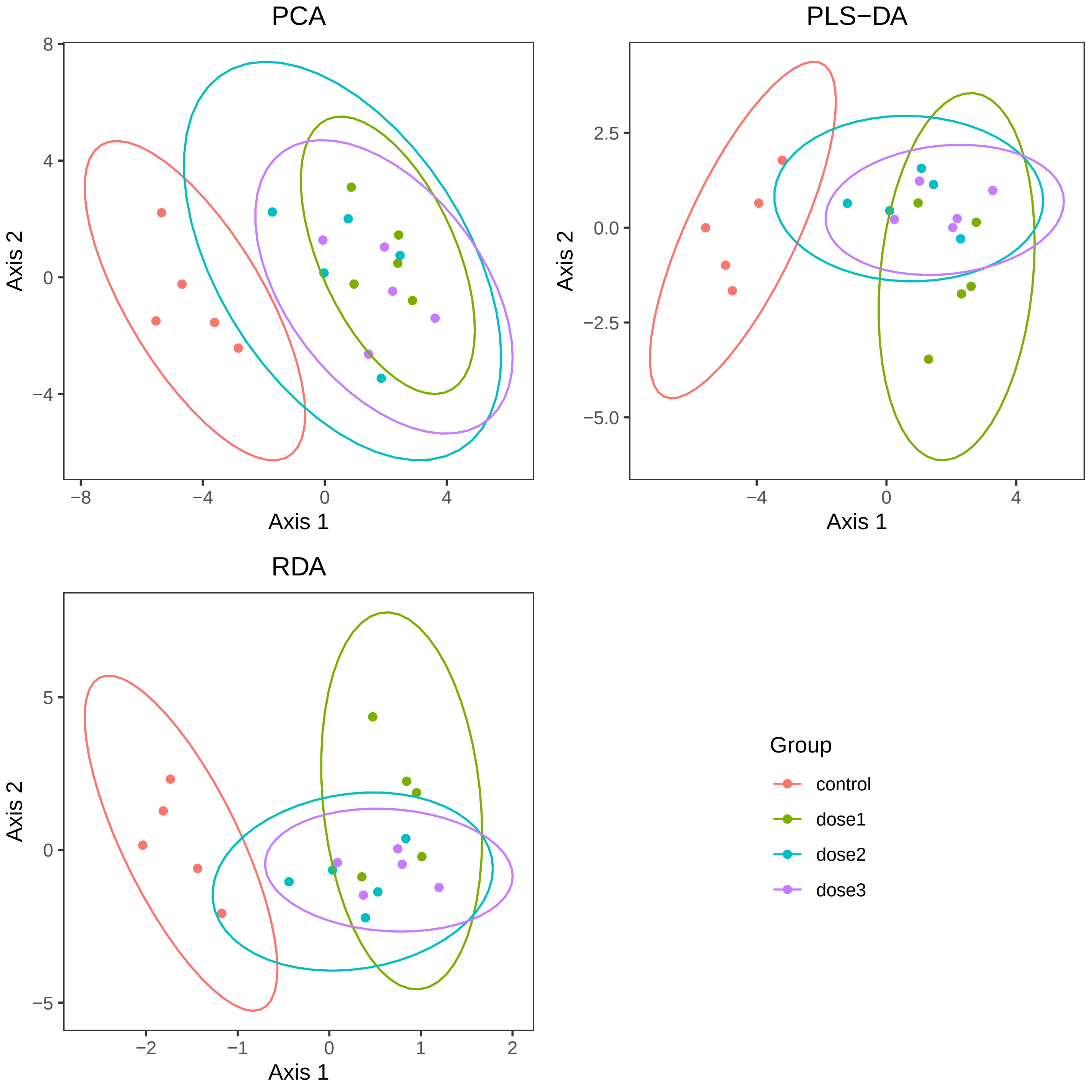
PCA
PLS−DA
RDA
Axis 1
Axis 2
Group
control
dose1
dose2
dose3
−8
−4
0
4
8
2.5
0.0
−2.5
−5.0
5
−5
−2
−1
1
2

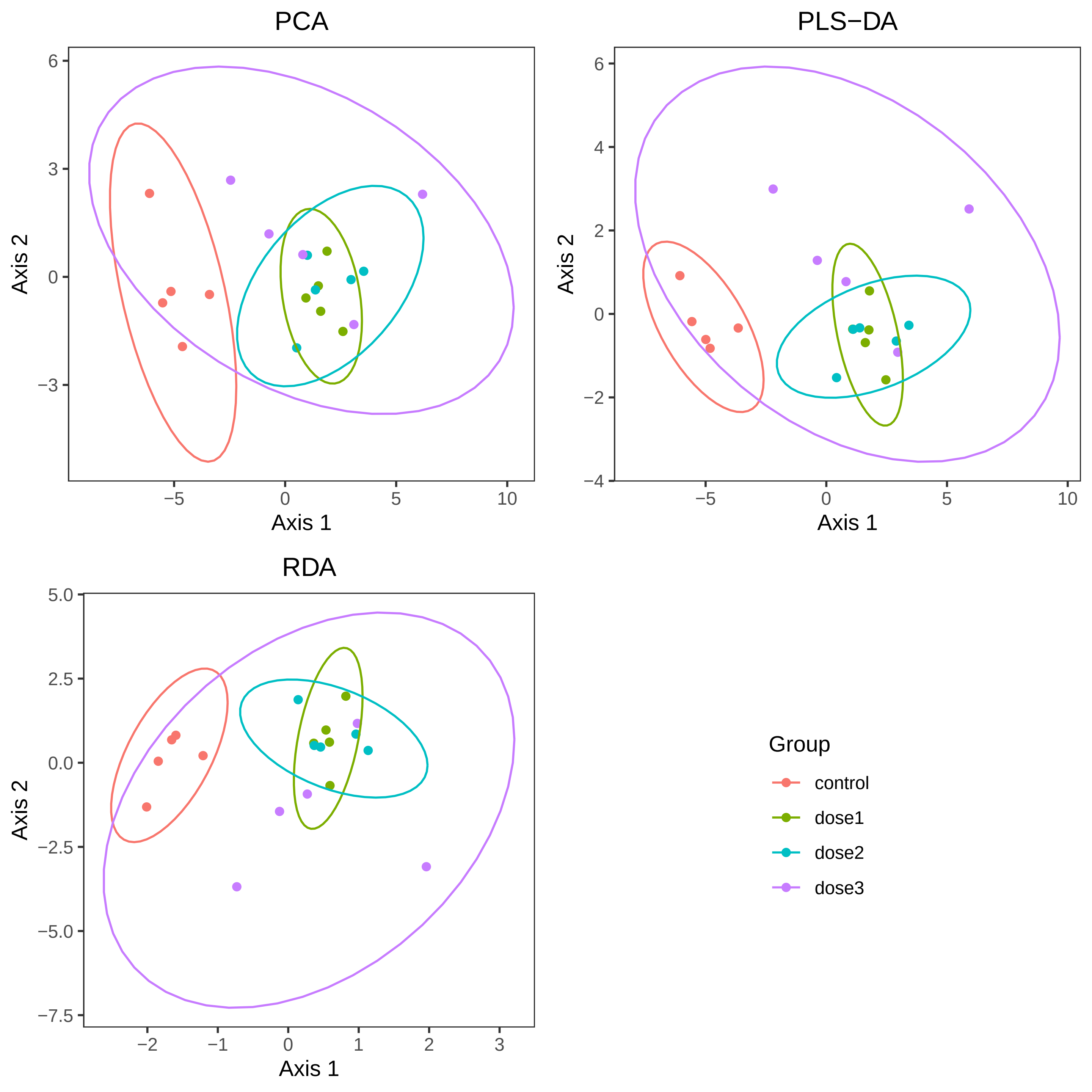

PCA
PLS−DA
RDA
Axis 1
Axis 2
Group
control
dose1
dose2
dose3

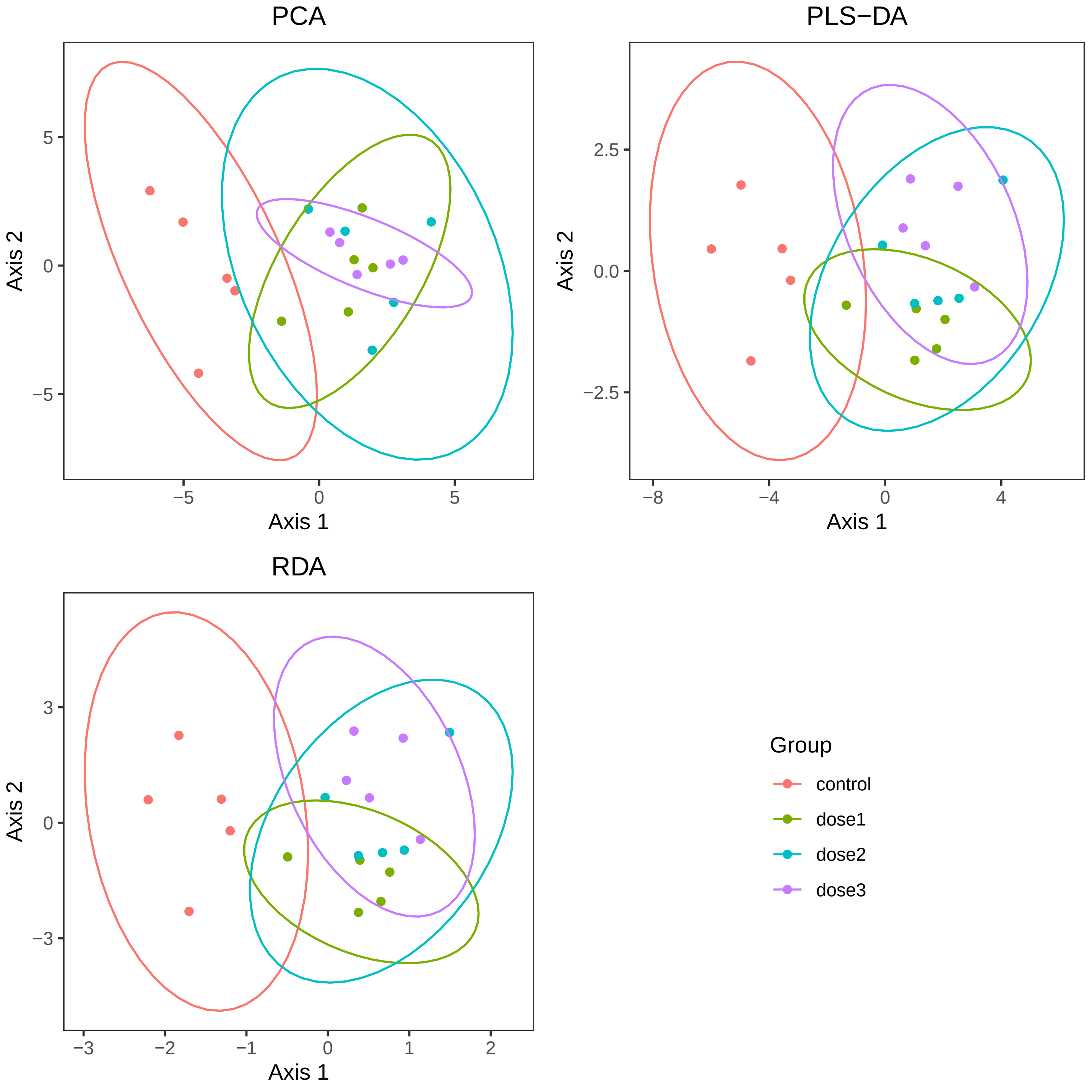

PCA
Axis 1
Axis 2
−5
0
5
PLS−DA
Axis 1
Axis 2
−8
−4
0
4
2.5
0.0
−2.5
RDA
Axis 1
Axis 2
−3
−2
−1
0
1
2
3
Group
control
dose1
dose2
dose3

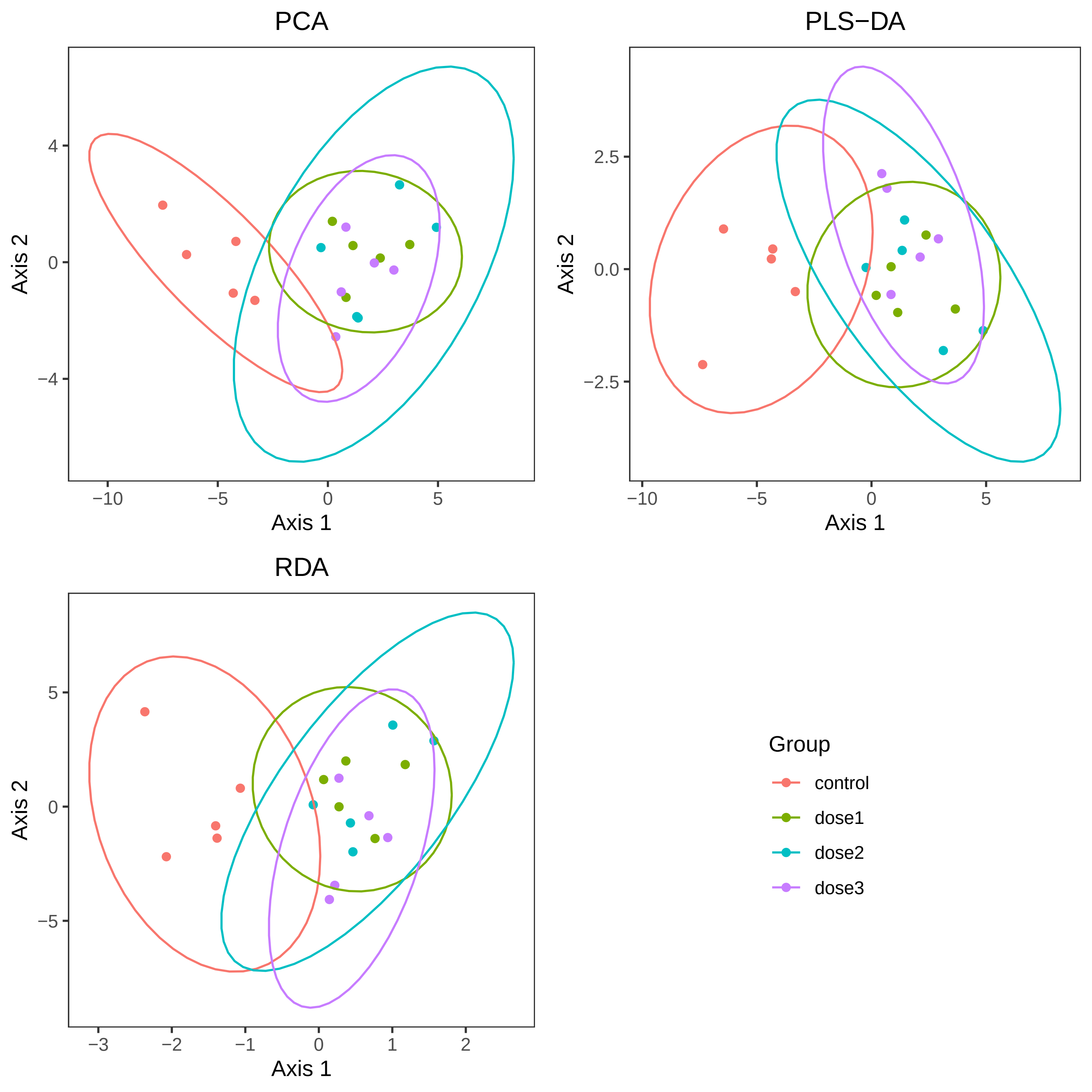
PCA
PLS−DA
RDA
Axis 1
Axis 2
Group
control
dose1
dose2
dose3

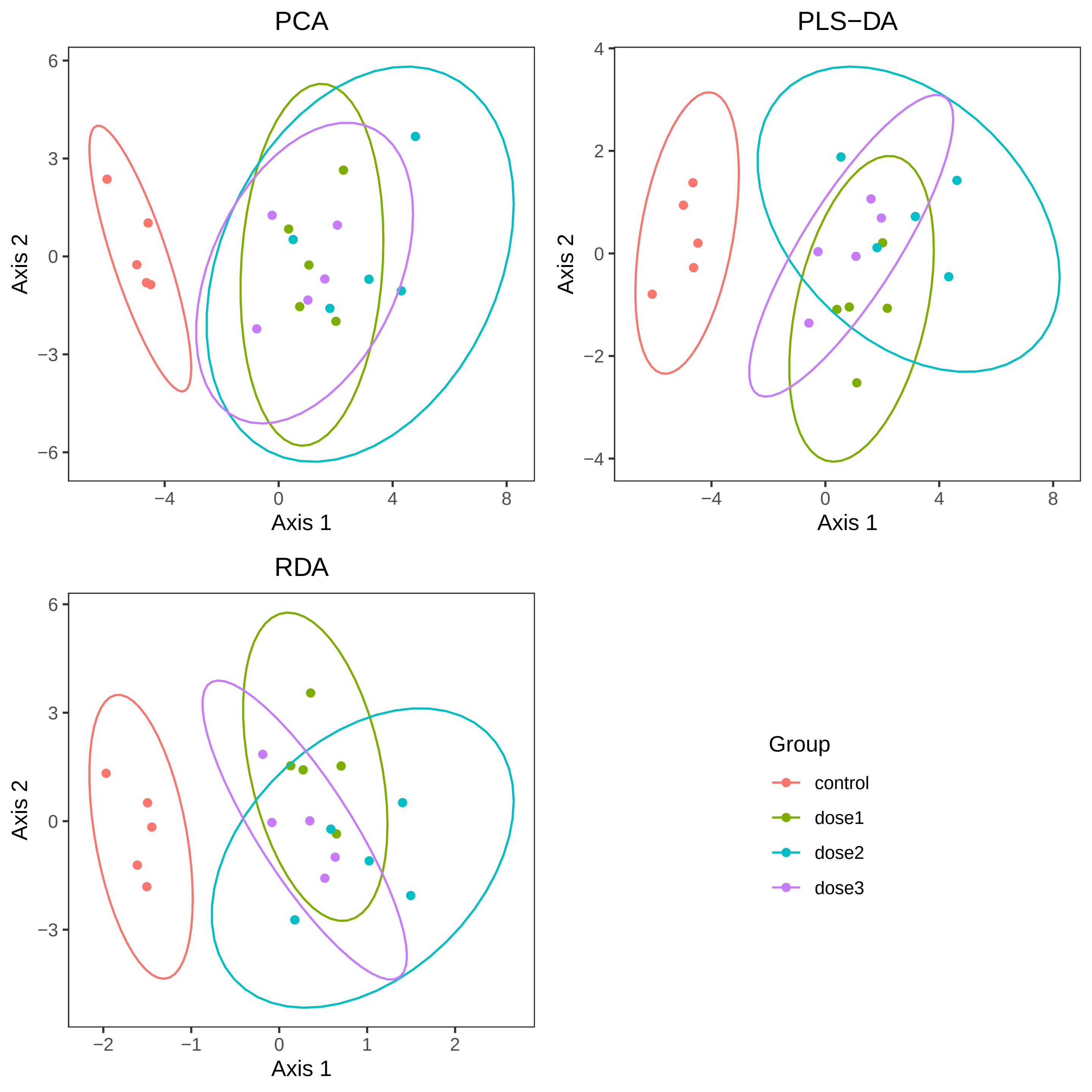
PCA
PLS−DA
RDA
Axis 1
Axis 2
Group
control
dose1
dose2
dose3

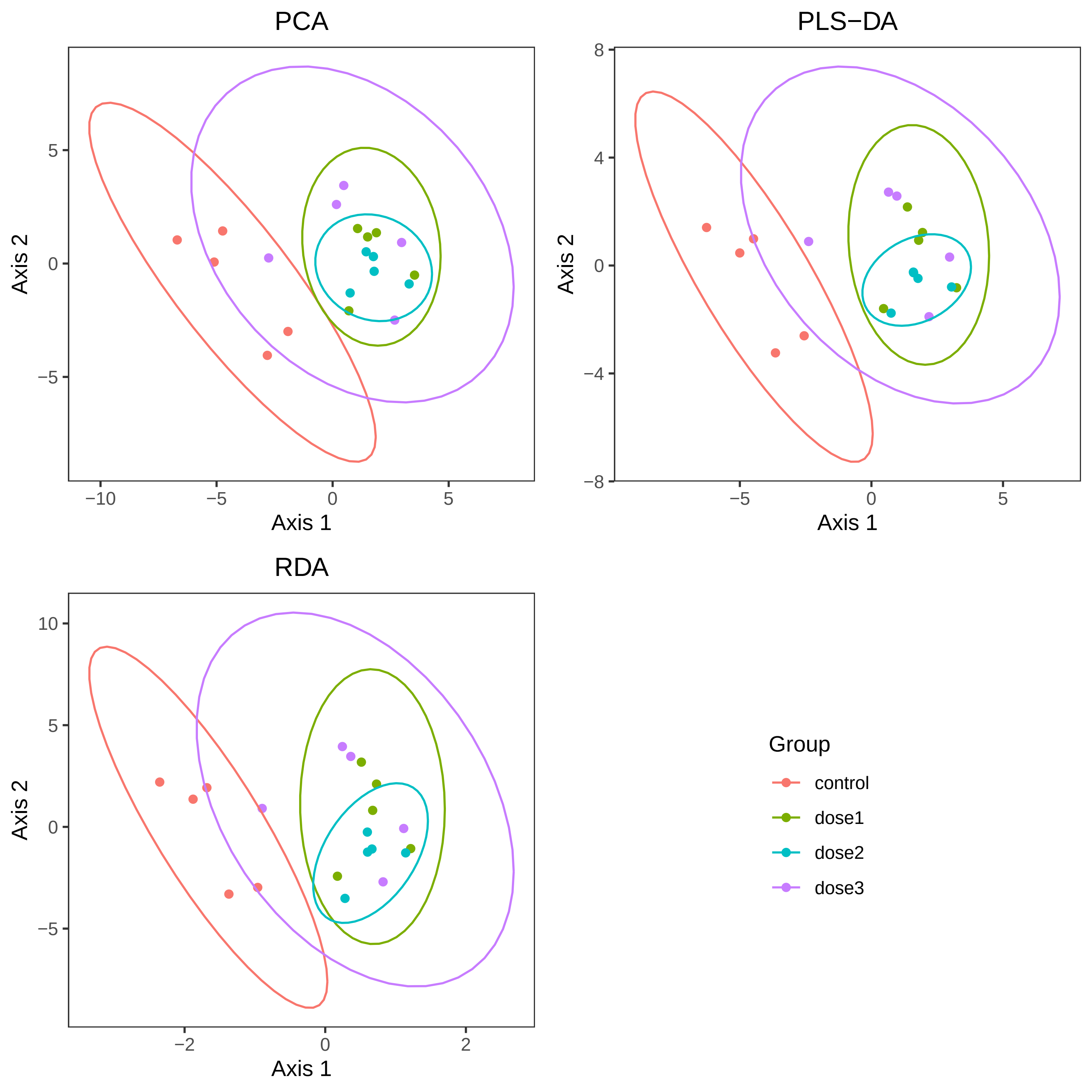
PCA
PLS−DA
RDA
Axis 1
Axis 2
−10
−5
0
5
8
4
−4
−8
10
−2
2
Group
control
dose1
dose2
dose3

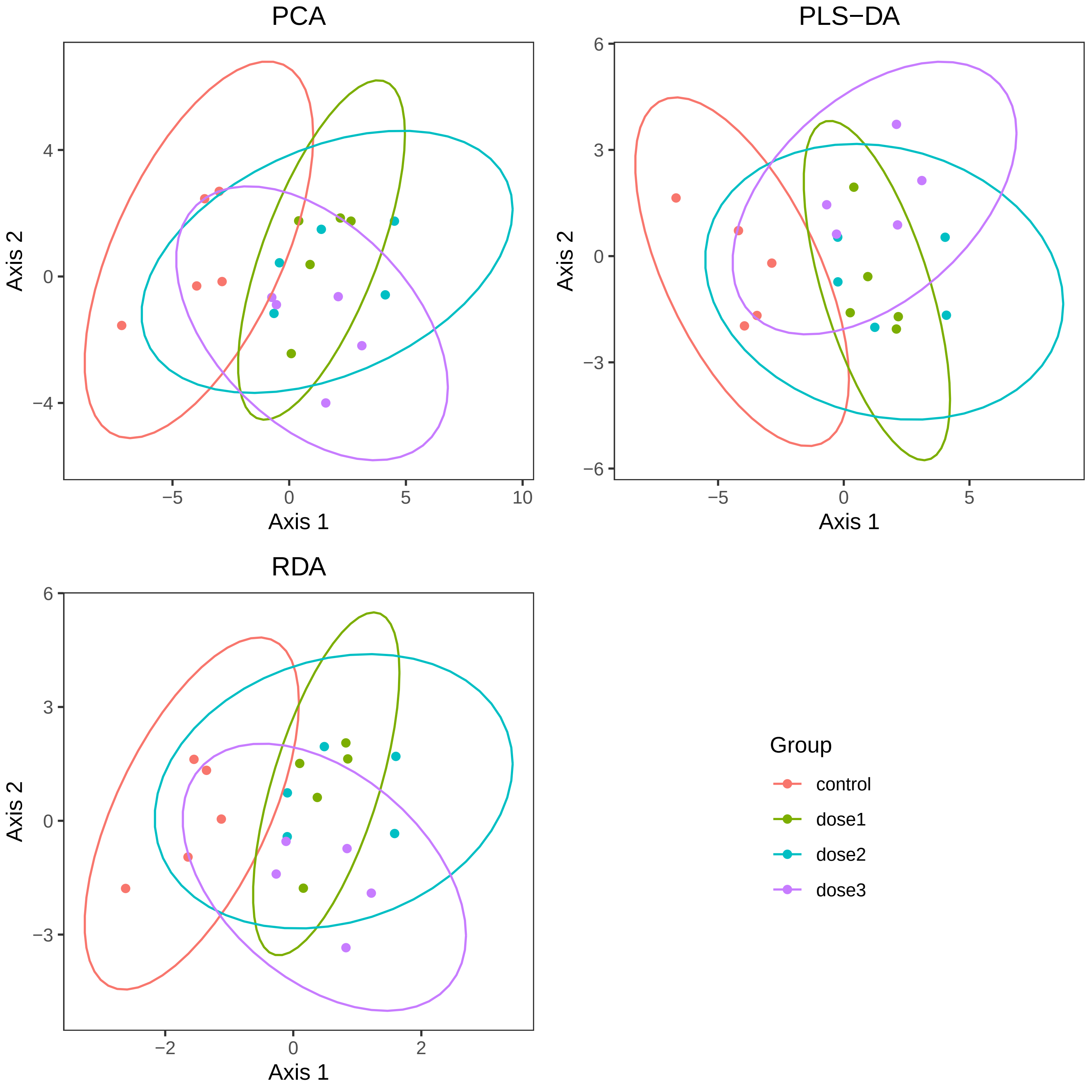

PCA
PLS−DA
RDA
Axis 1
Axis 2
Group
control
dose1
dose2
dose3

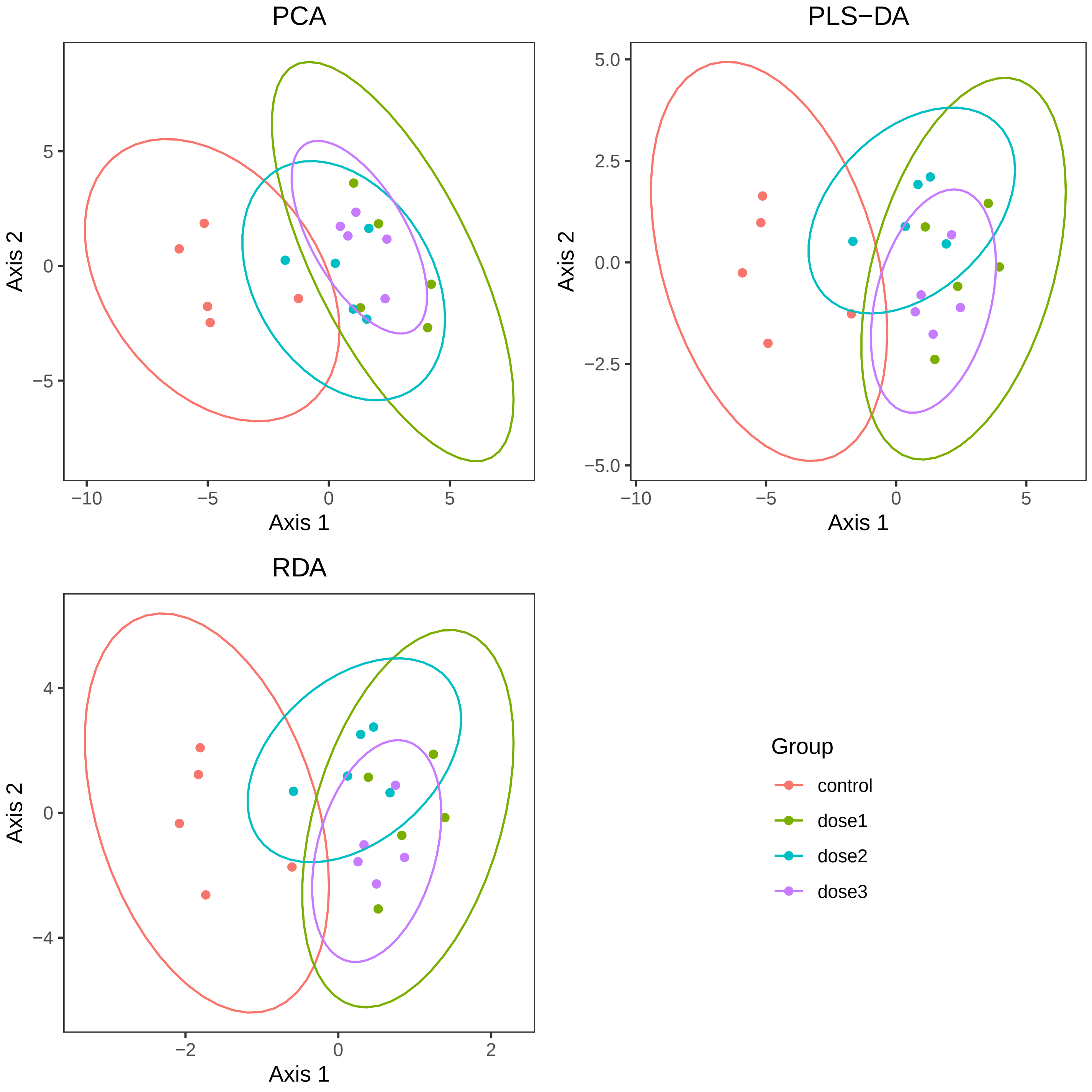
PCA
PLS−DA
RDA
Axis 1
Axis 2
Group
control
dose1
dose2
dose3

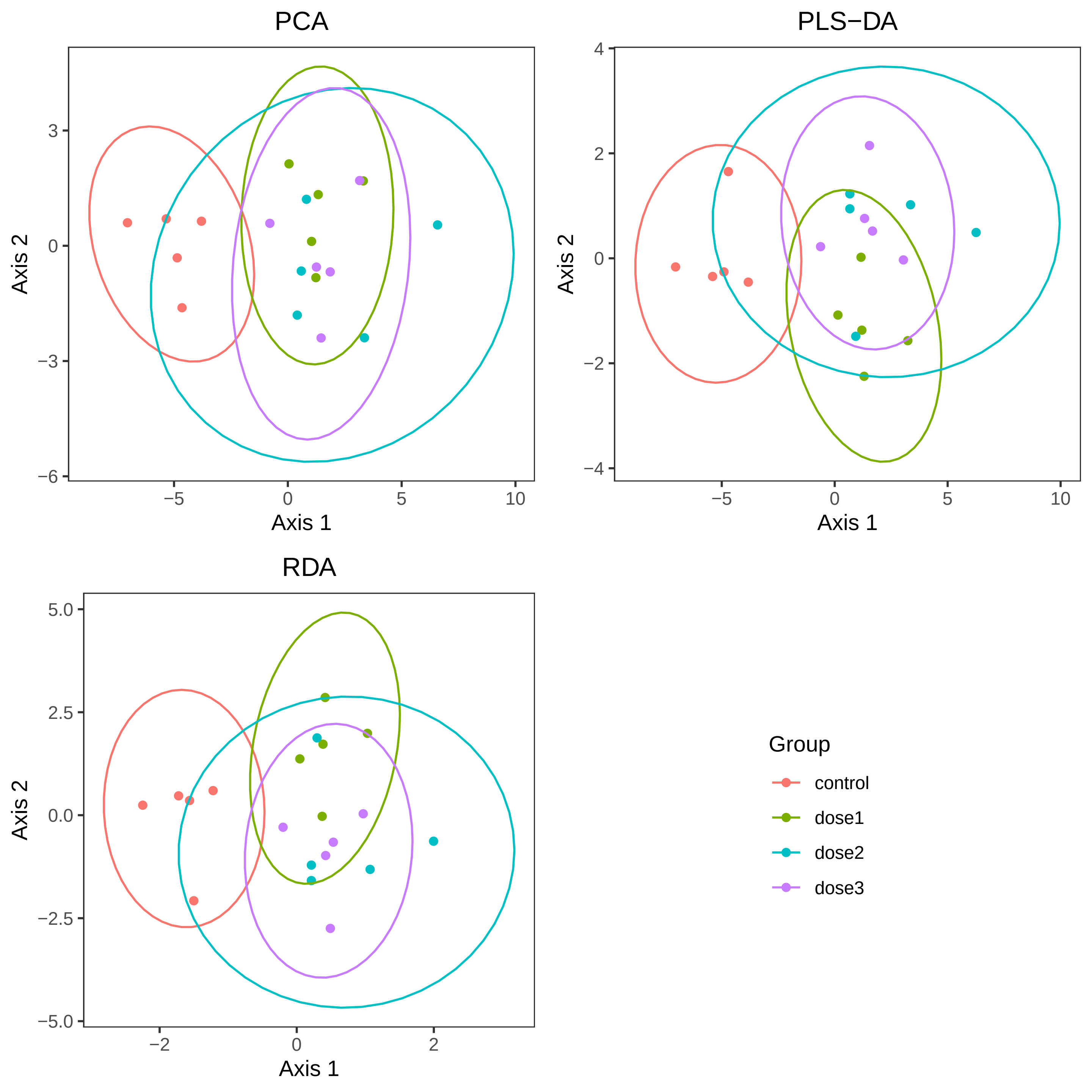
PCA
PLS−DA
RDA
Axis 1
Axis 2
Group
control
dose1
dose2
dose3

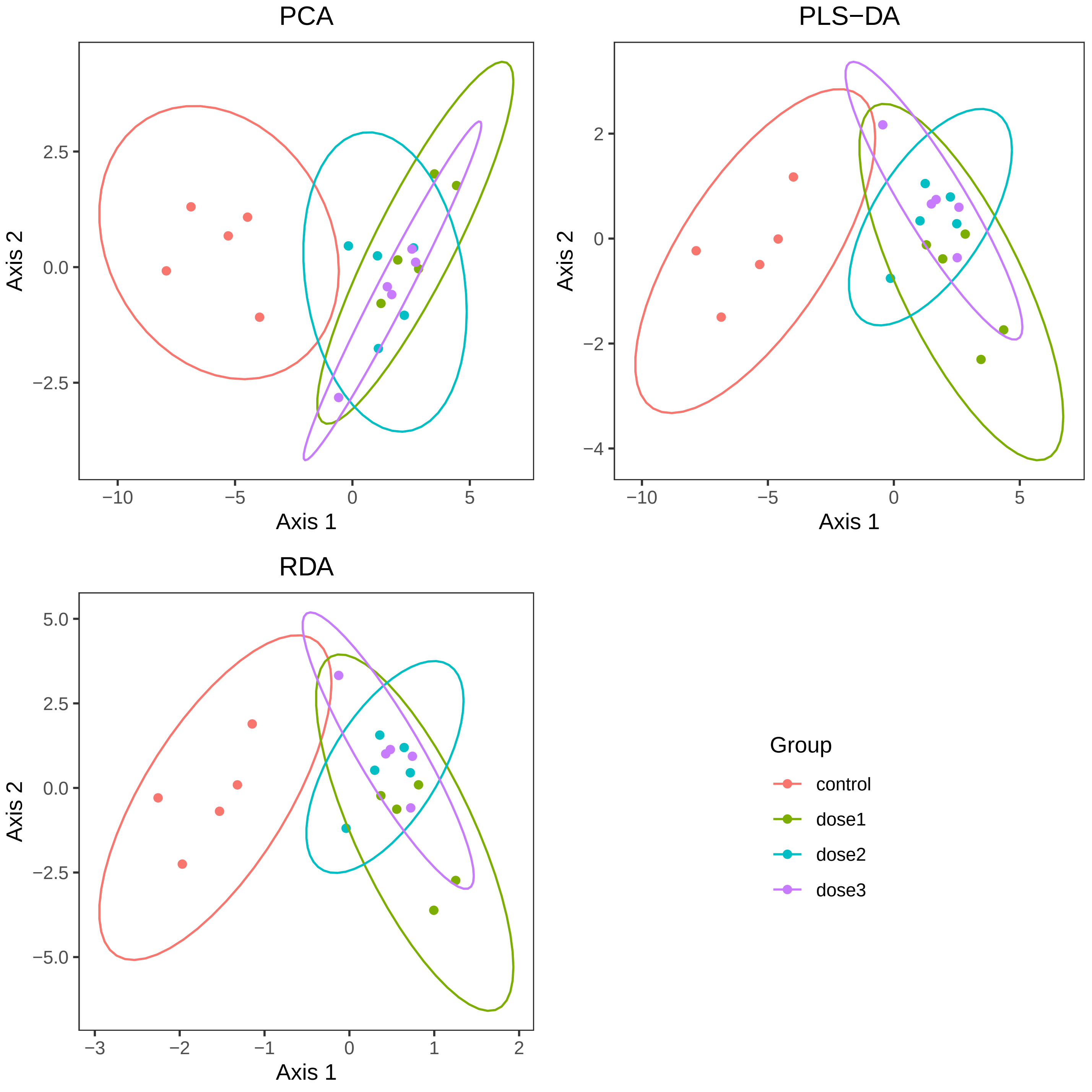

PCA
PLS−DA
RDA
Axis 1
Axis 2
2.5
0.0
−2.5
−10
−5
0
5
2
0
−2
−4
5.0
2.5
0.0
−2.5
−5.0
−3
−2
−1
0
1
2
Group
control
dose1
dose2
dose3

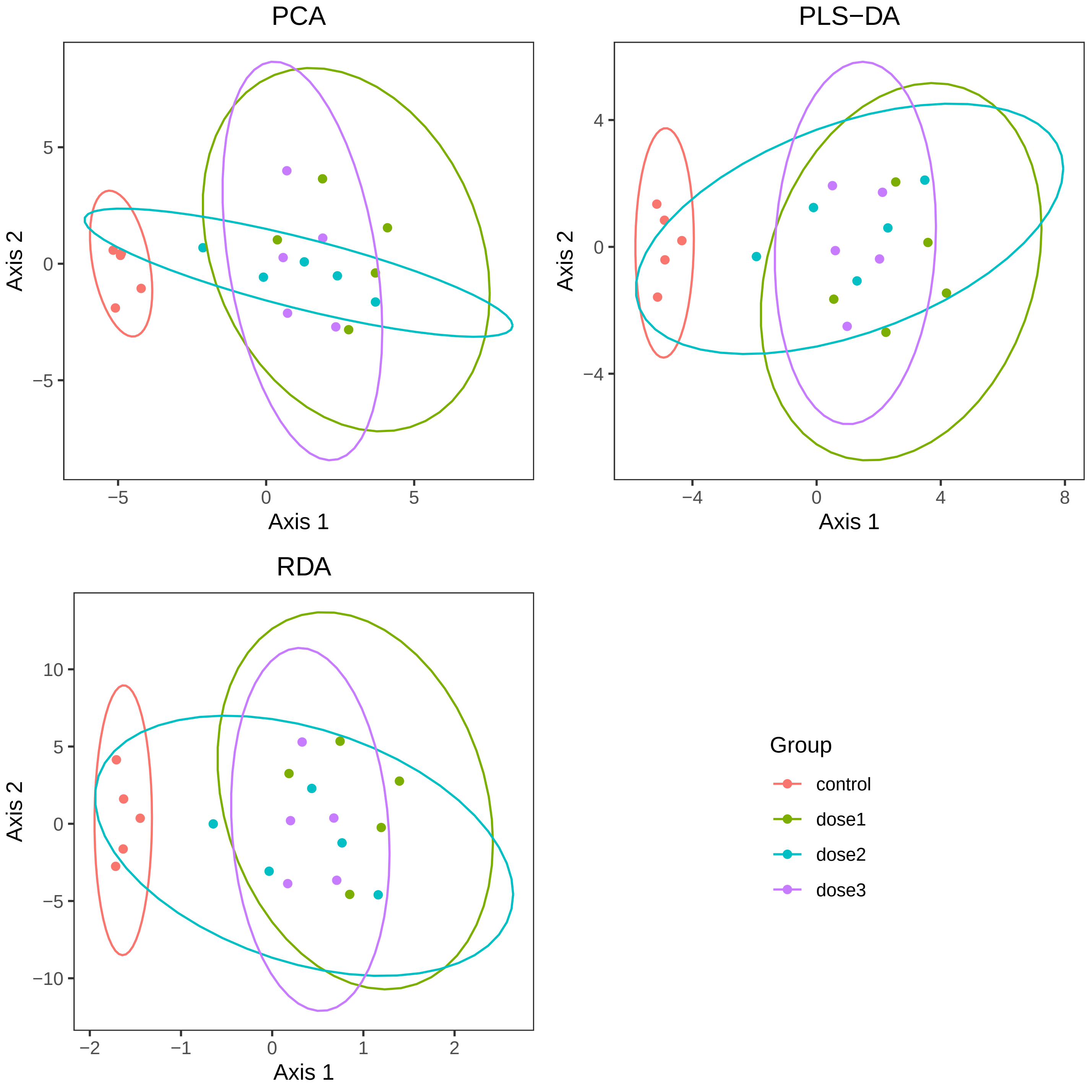
PCA
PLS−DA
RDA
Axis 1
Axis 2
Group
control
dose1
dose2
dose3

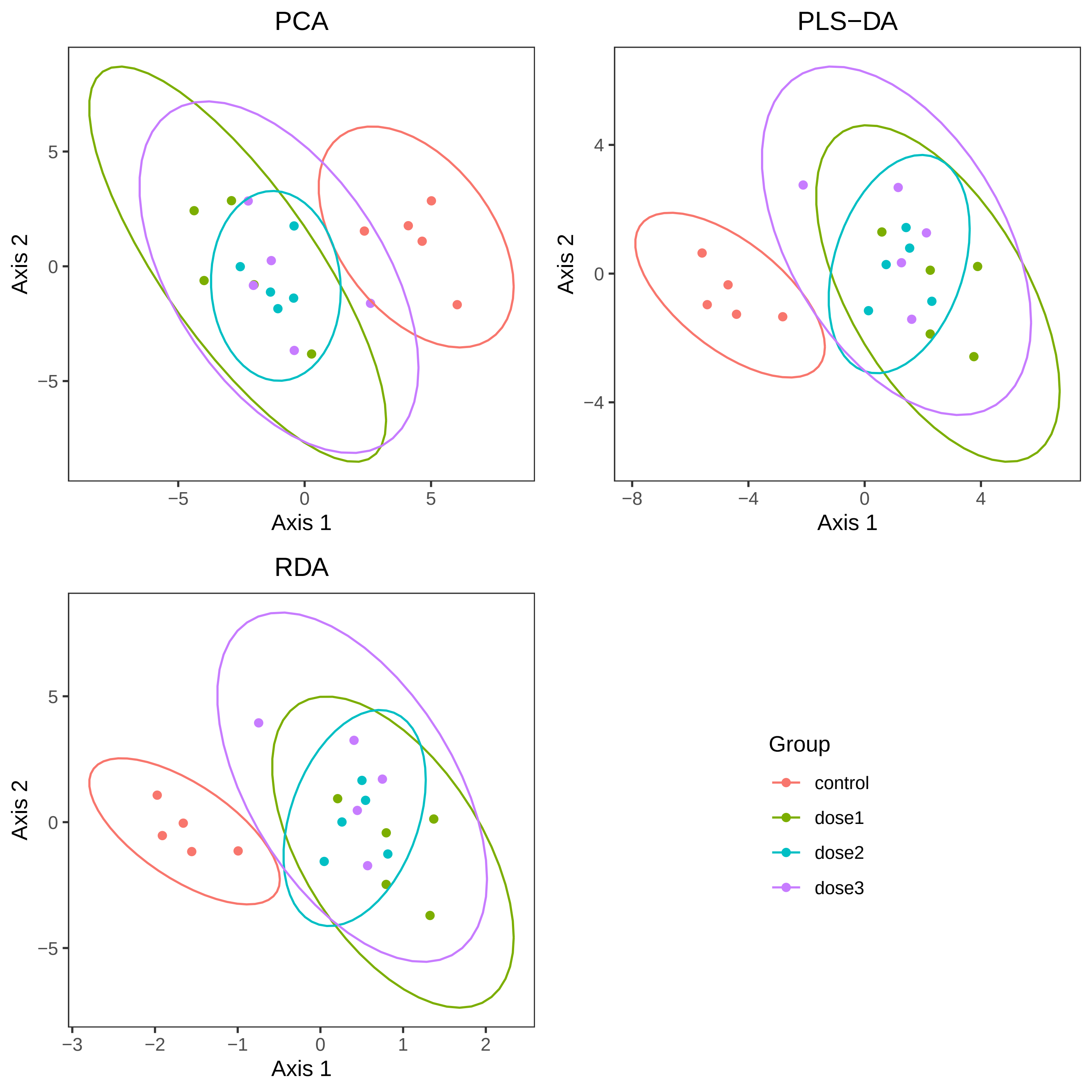
PCA
PLS−DA
RDA
Axis 1
Axis 2
Group
control
dose1
dose2
dose3

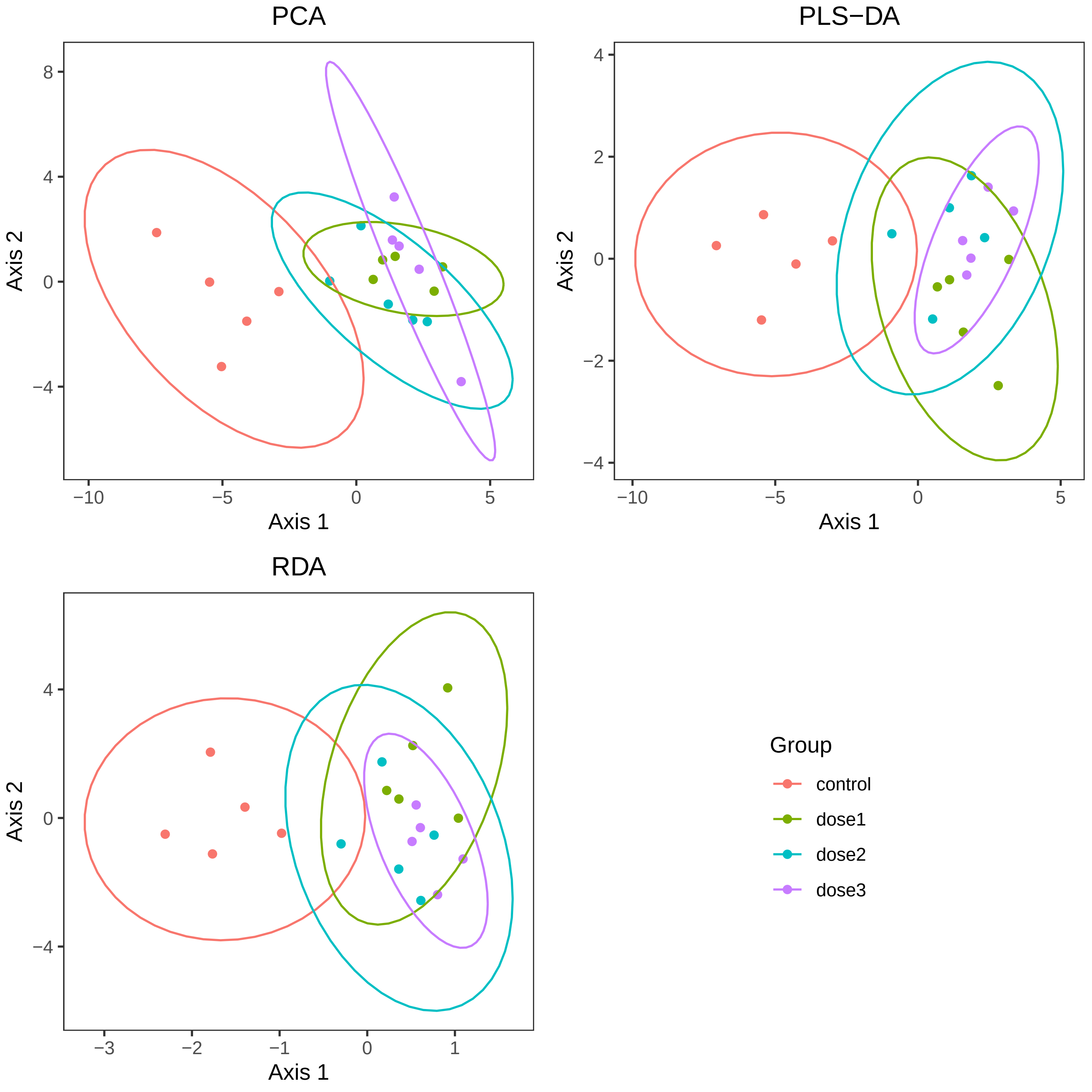

PCA
PLS−DA
RDA
Axis 1
Axis 2
Group
control
dose1
dose2
dose3

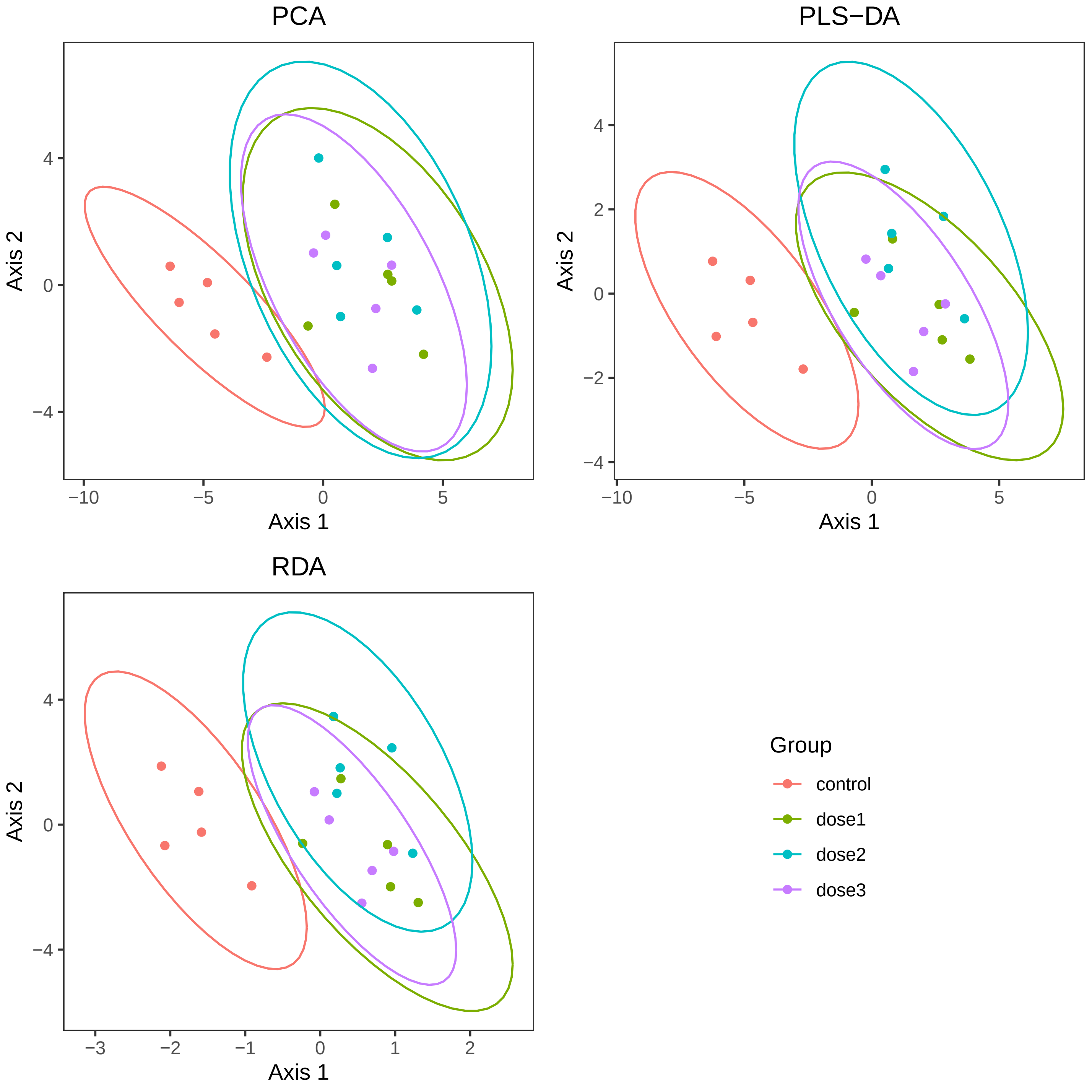

PCA
PLS−DA
RDA
Axis 1
Axis 2
Group
control
dose1
dose2
dose3

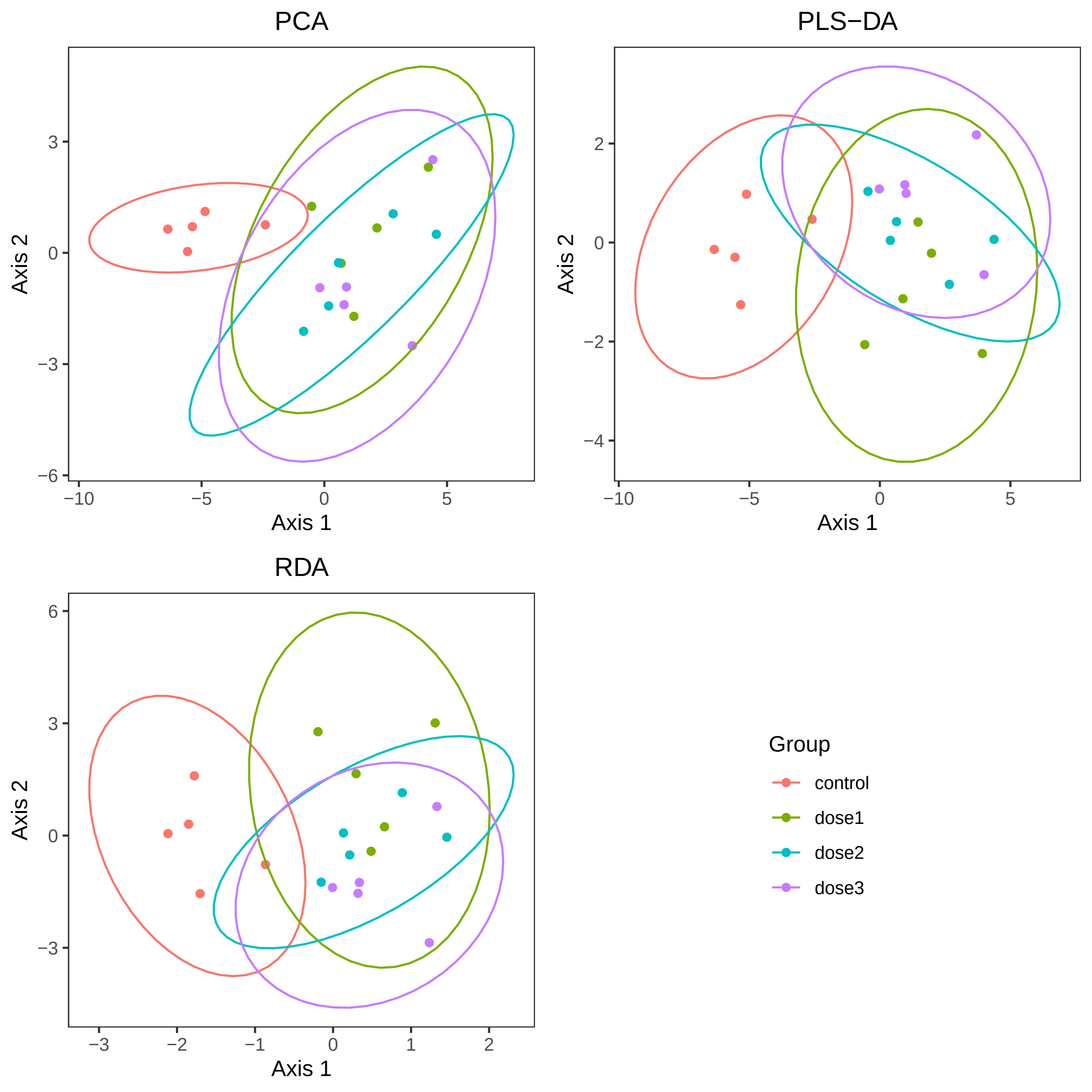
PCA
PLS−DA
RDA
Axis 1
Axis 2
Group
control
dose1
dose2
dose3

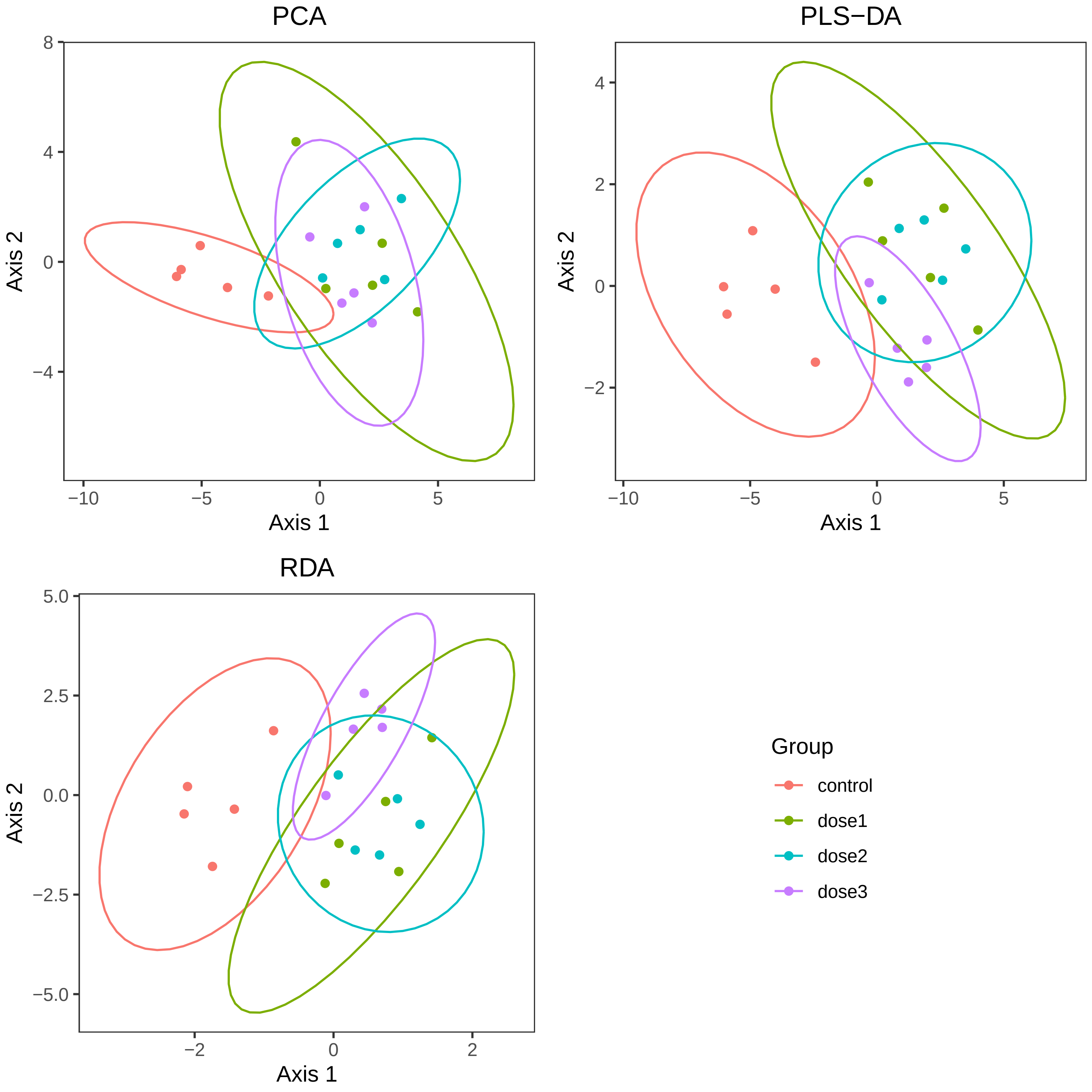

PCA
PLS−DA
RDA
Axis 1
Axis 2
−10
−5
0
5
8
4
0
−4
2
−2
5.0
2.5
0.0
−2.5
−5.0
Group
control
dose1
dose2
dose3

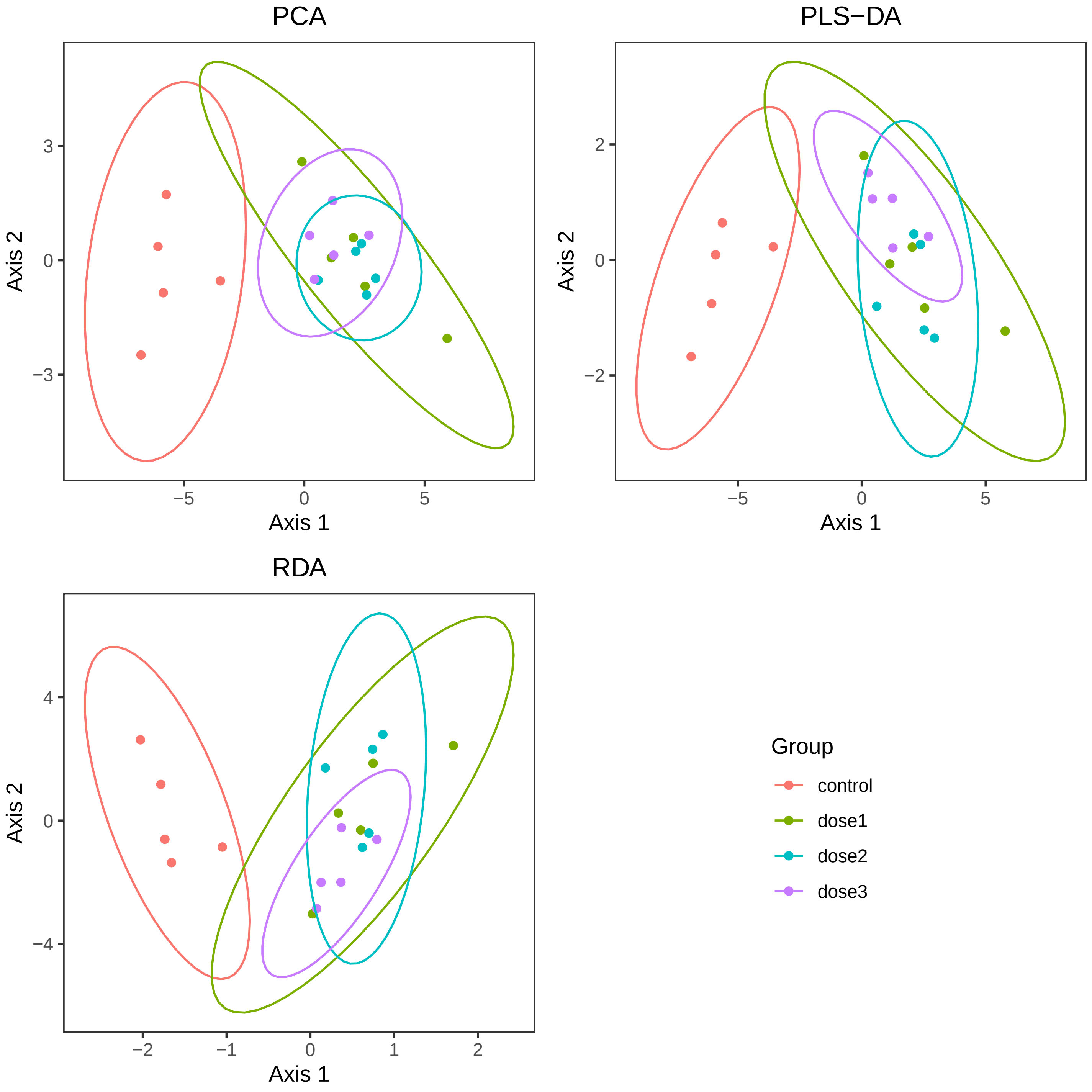

PCA
PLS−DA
RDA
Axis 1
Axis 2
−5
0
5
3
−3
2
−2
−2
−1
1
4
−4
Group
control
dose1
dose2
dose3

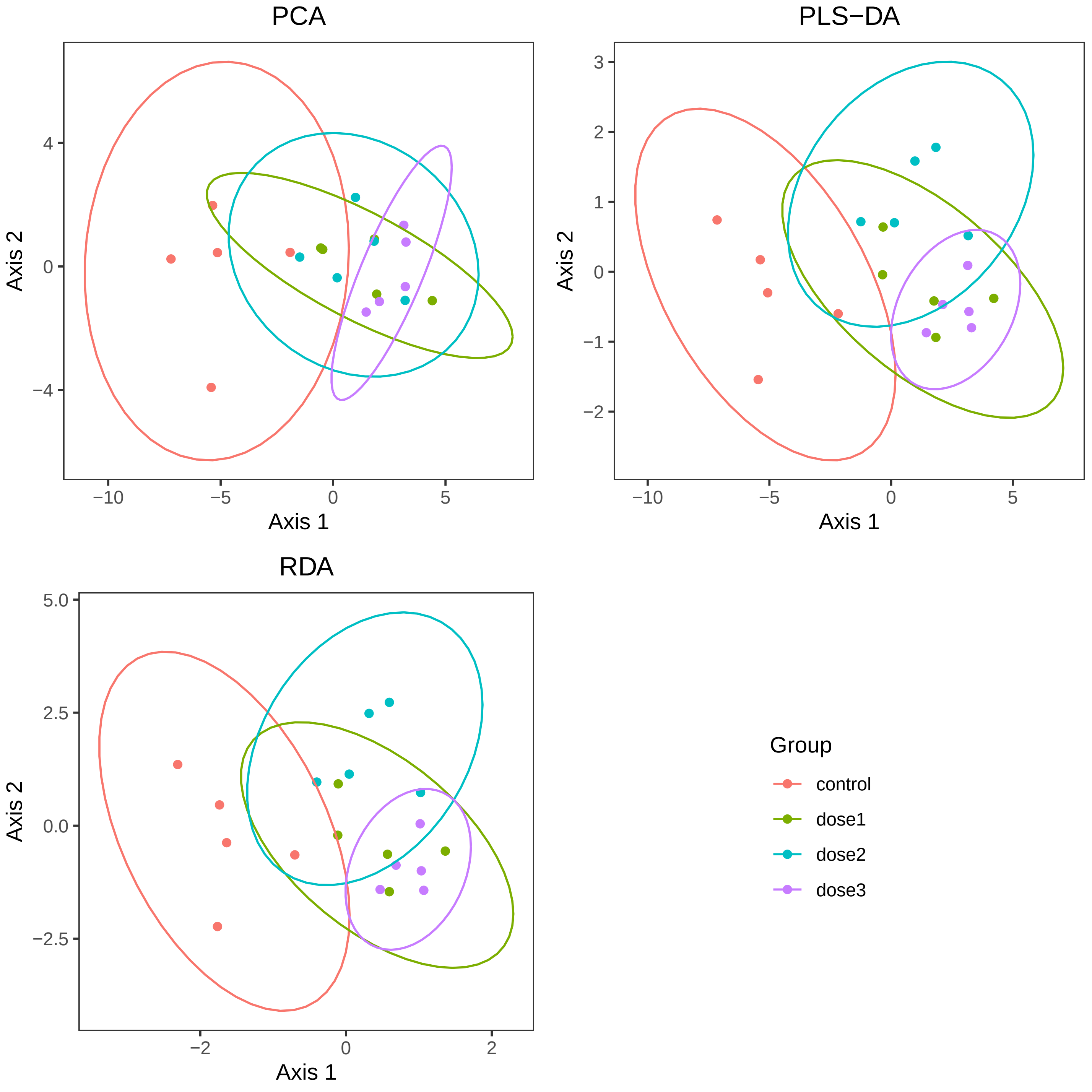

PCA
PLS−DA
RDA
Axis 1
Axis 2
Group
control
dose1
dose2
dose3

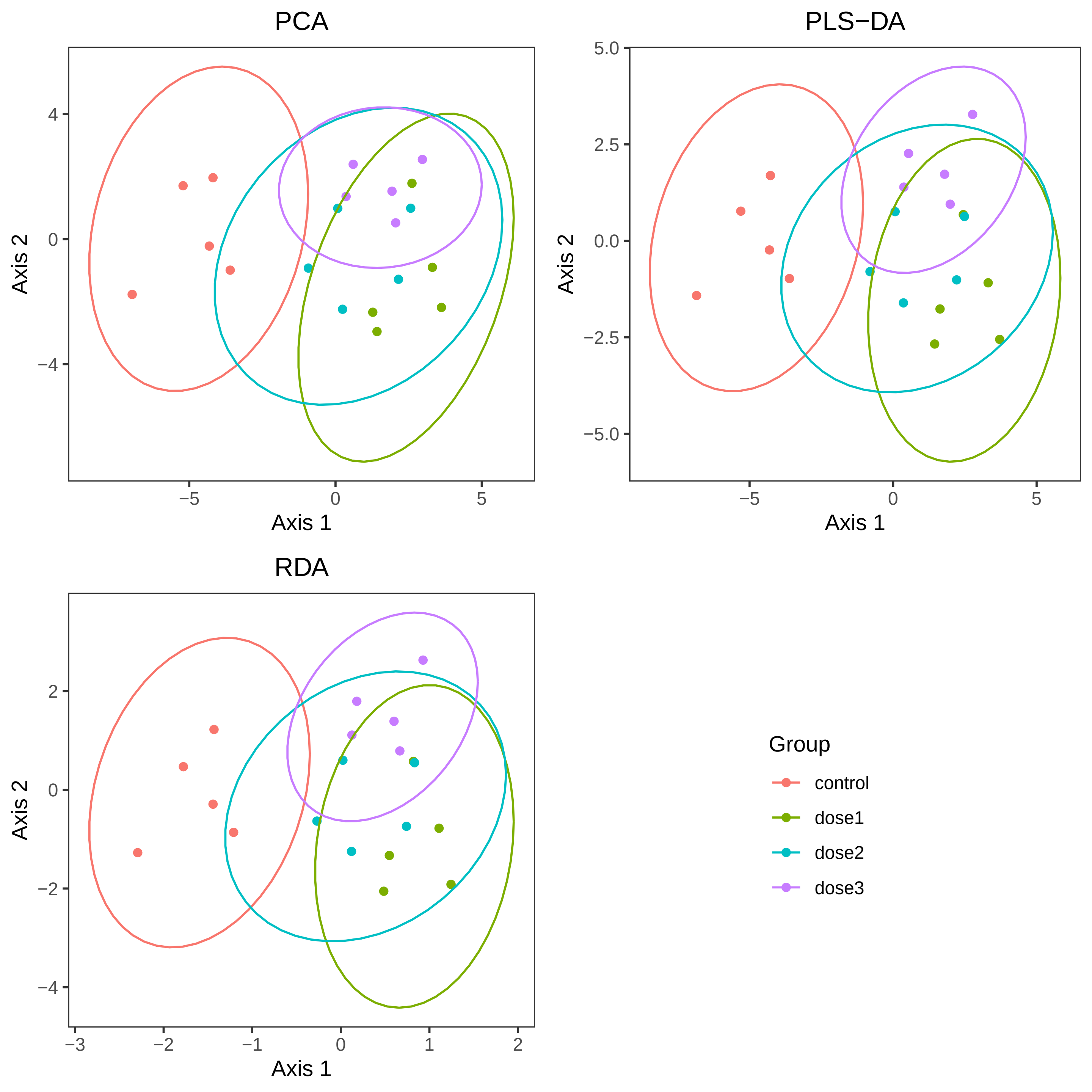

PCA
PLS−DA
RDA
Axis 1
Axis 2
Group
control
dose1
dose2
dose3

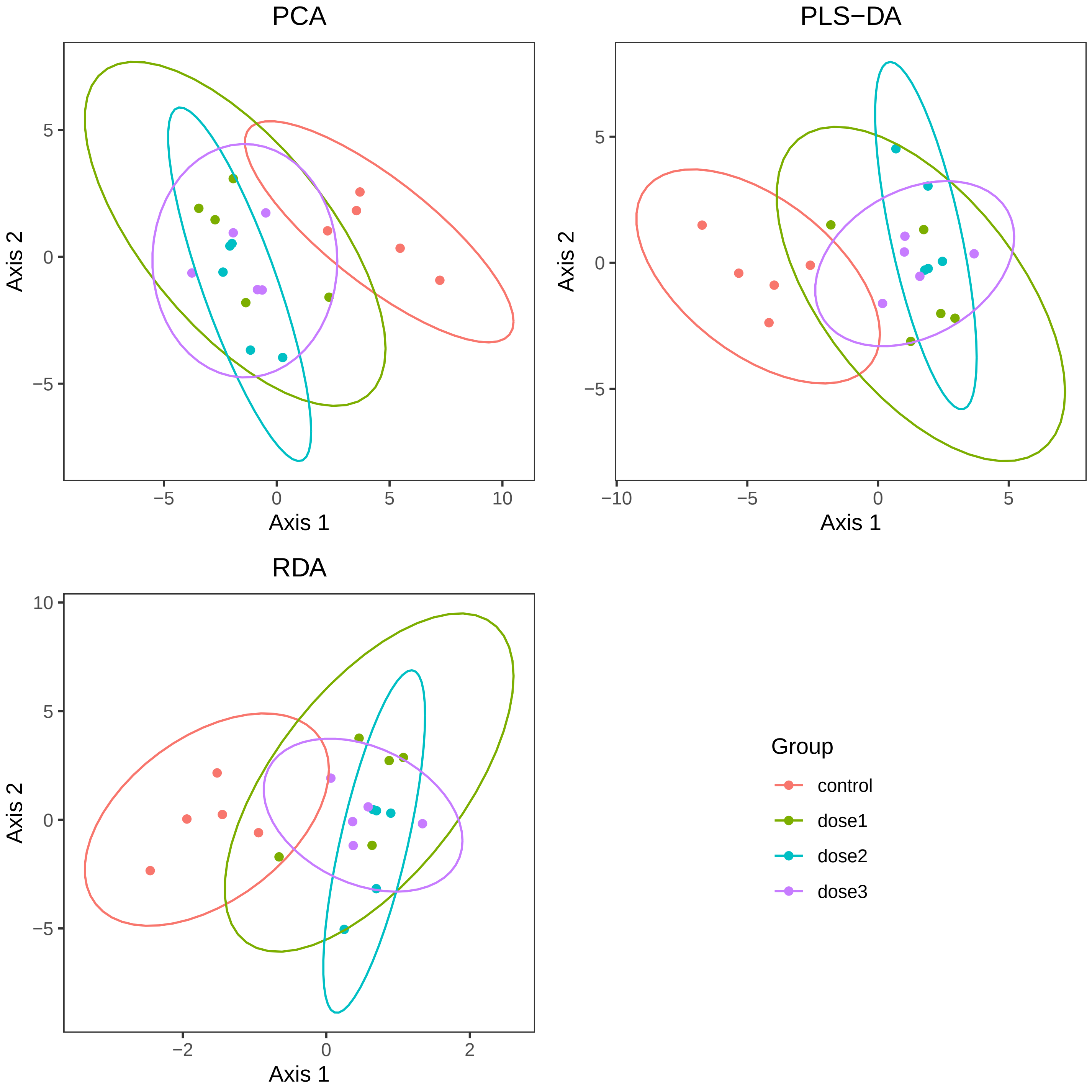
PCA
PLS−DA
RDA
Axis 1
Axis 2
Group
control
dose1
dose2
dose3

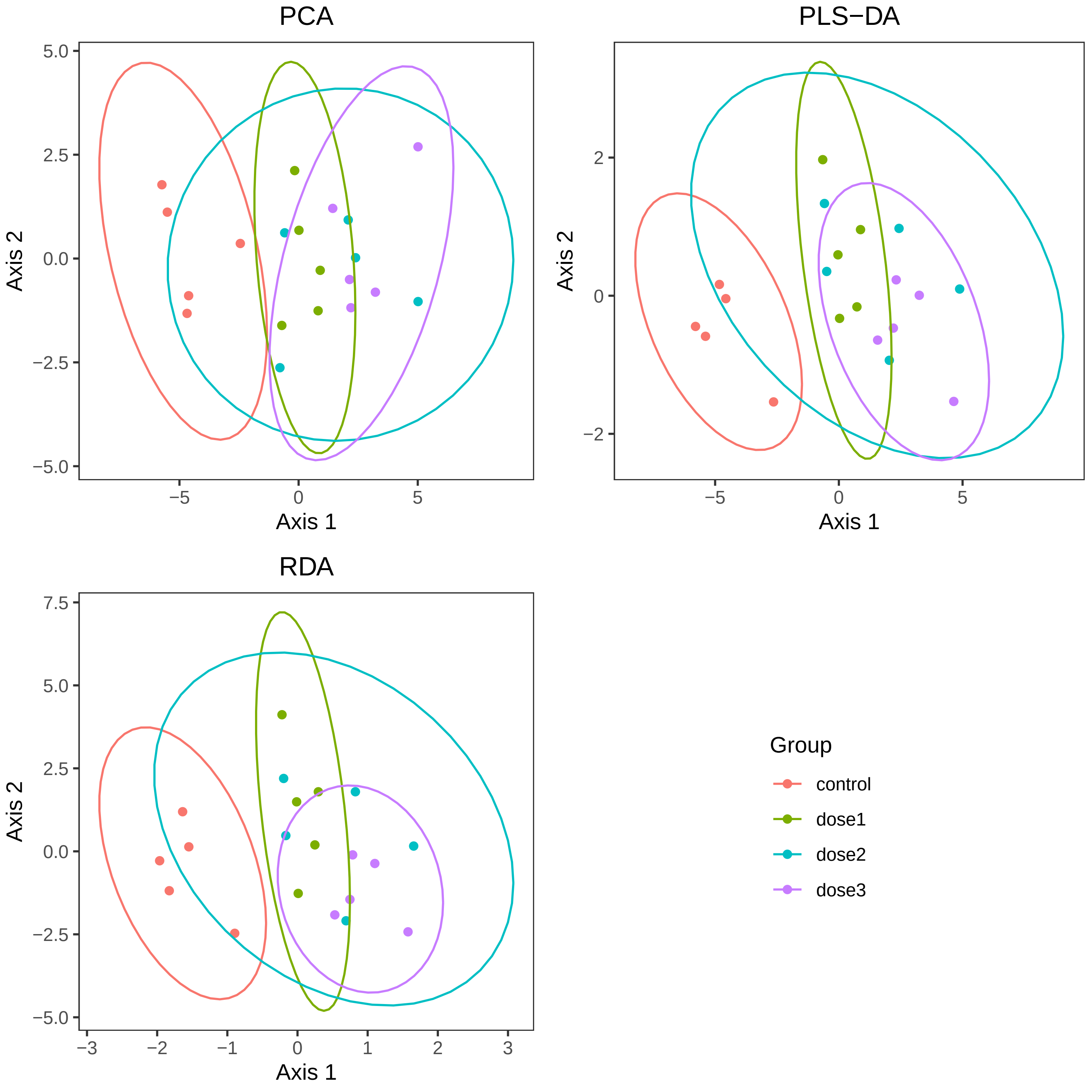

PCA
PLS−DA
RDA
Axis 1
Axis 2
Group
control
dose1
dose2
dose3

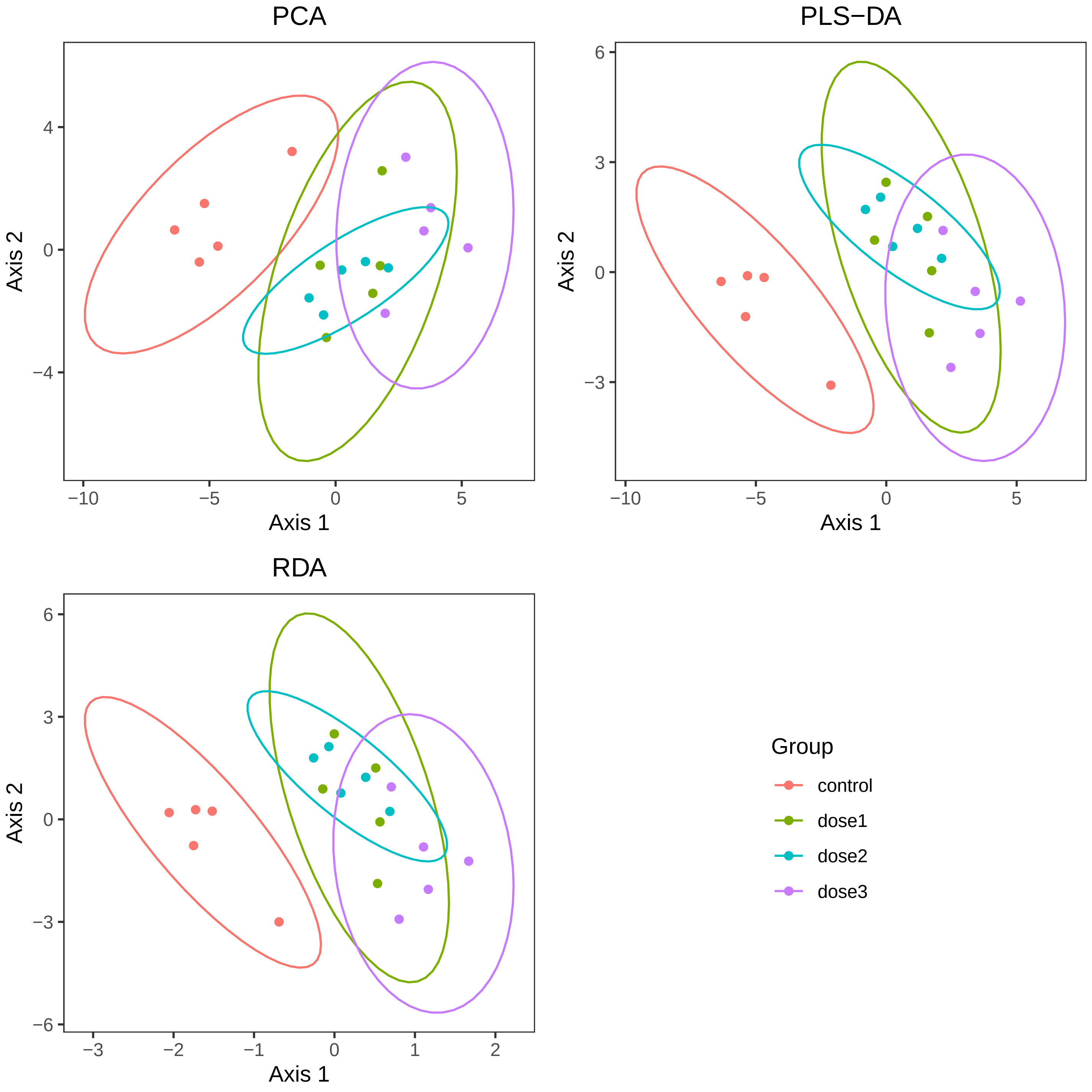

PCA
PLS−DA
RDA
Axis 1
Axis 2
Group
control
dose1
dose2
dose3

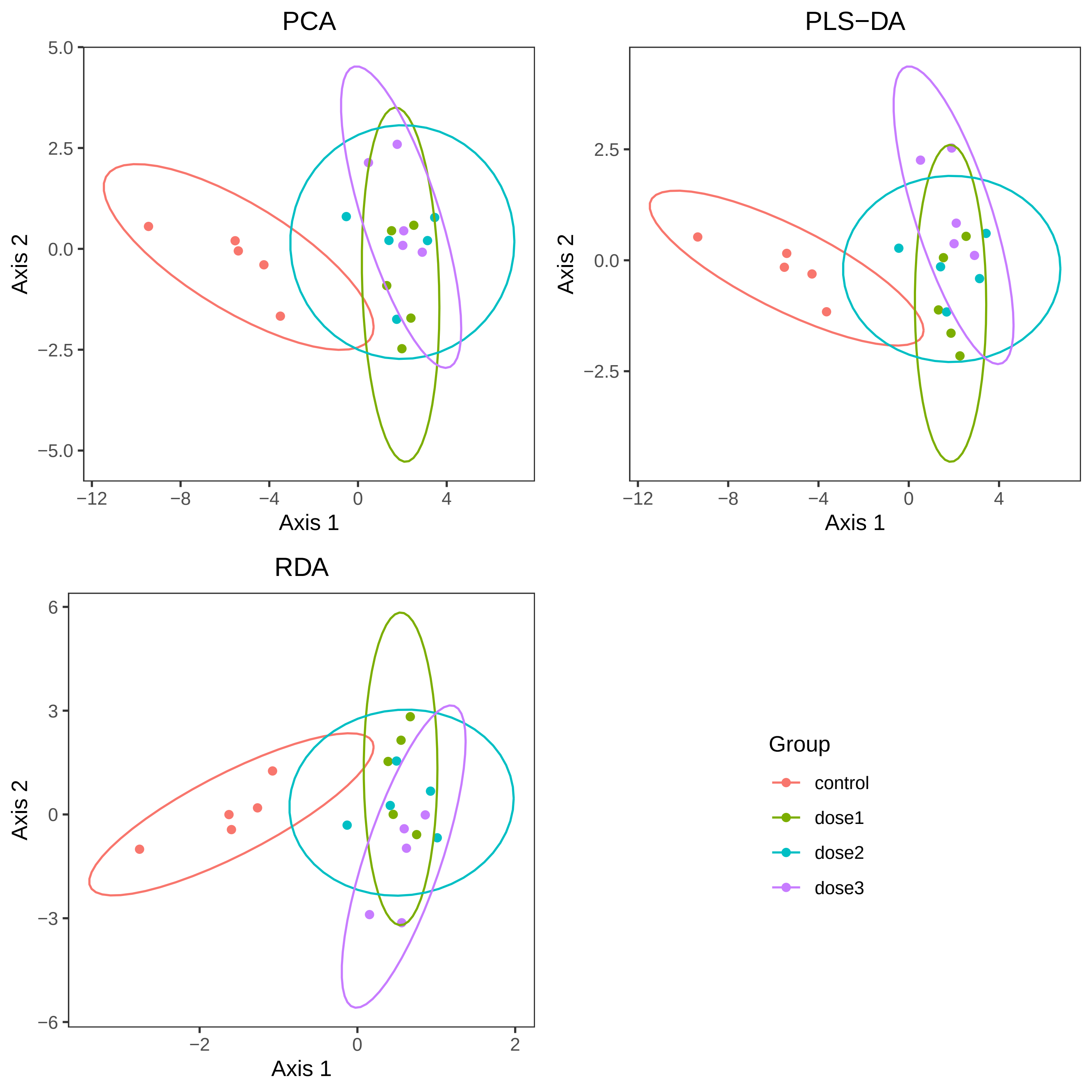

PCA
PLS−DA
RDA
Axis 1
Axis 2
5.0
2.5
0.0
−2.5
−5.0
−12
−8
−4
0
4
2
6
3
−3
−6
−2
Group
control
dose1
dose2
dose3

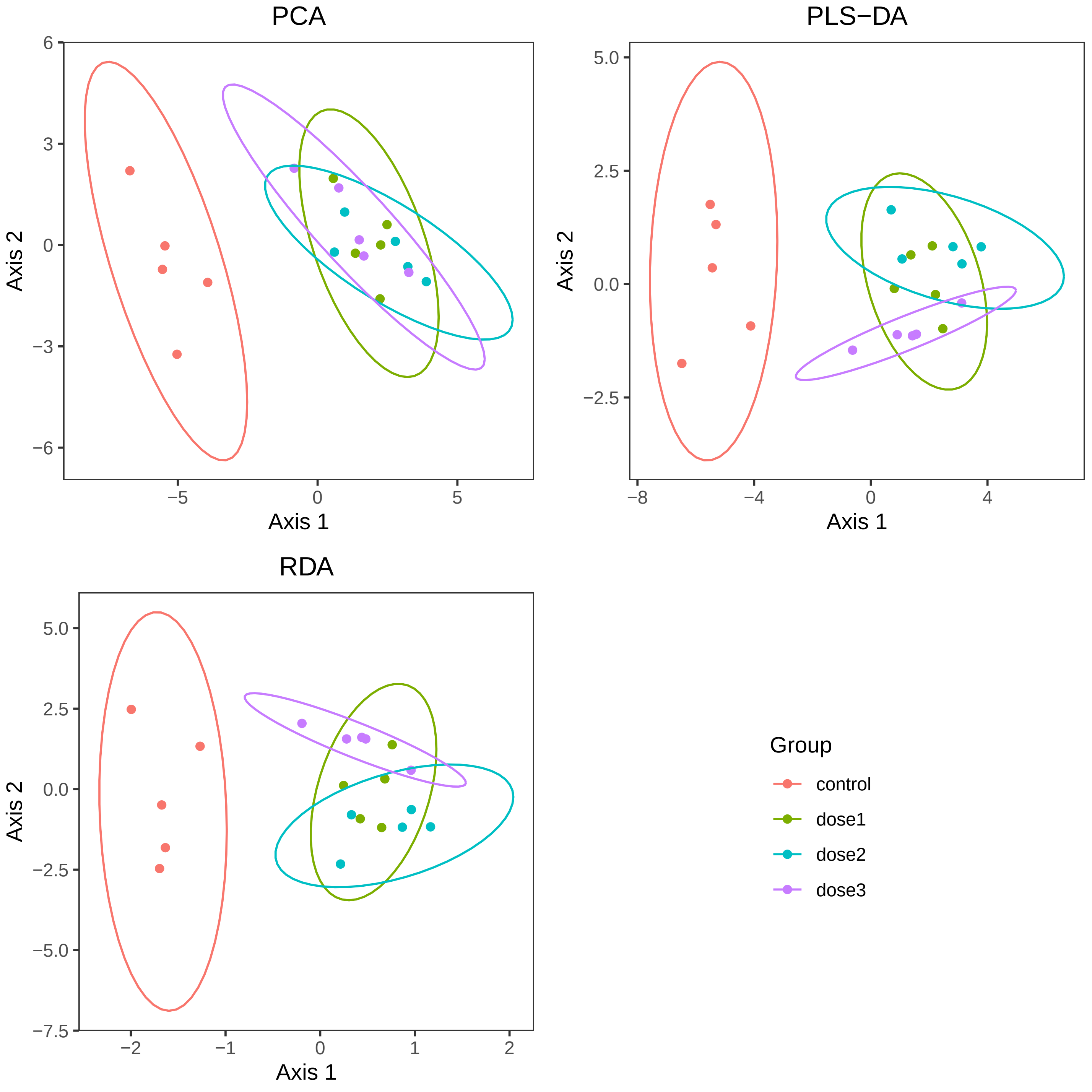
PCA
PLS−DA
RDA
Axis 1
Axis 2
Group
control
dose1
dose2
dose3

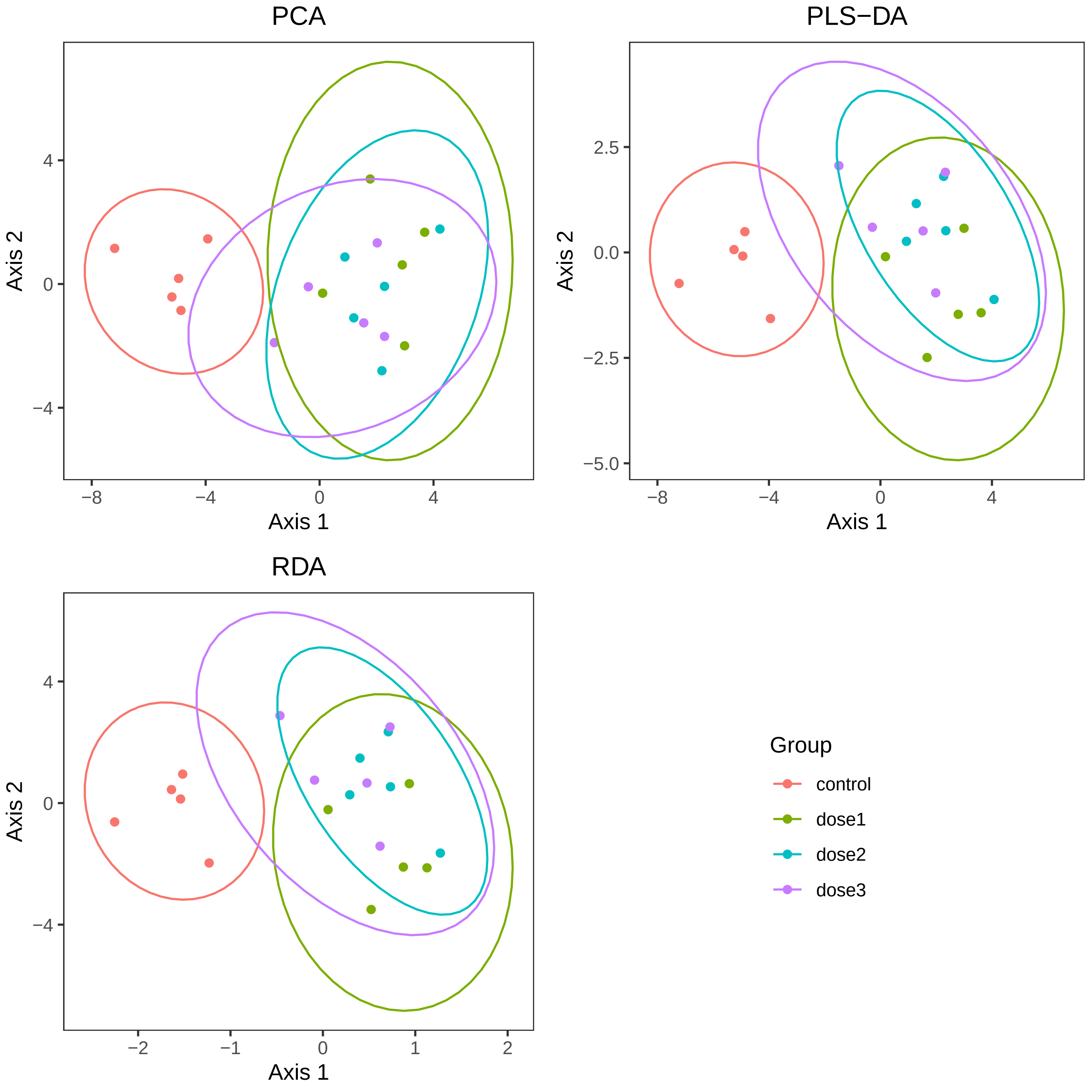

PCA
PLS−DA
RDA
Axis 1
Axis 2
4
0
−4
−8
−4
0
4
2.5
0.0
−2.5
−5.0
−2
−1
0
1
2
Group
control
dose1
dose2
dose3

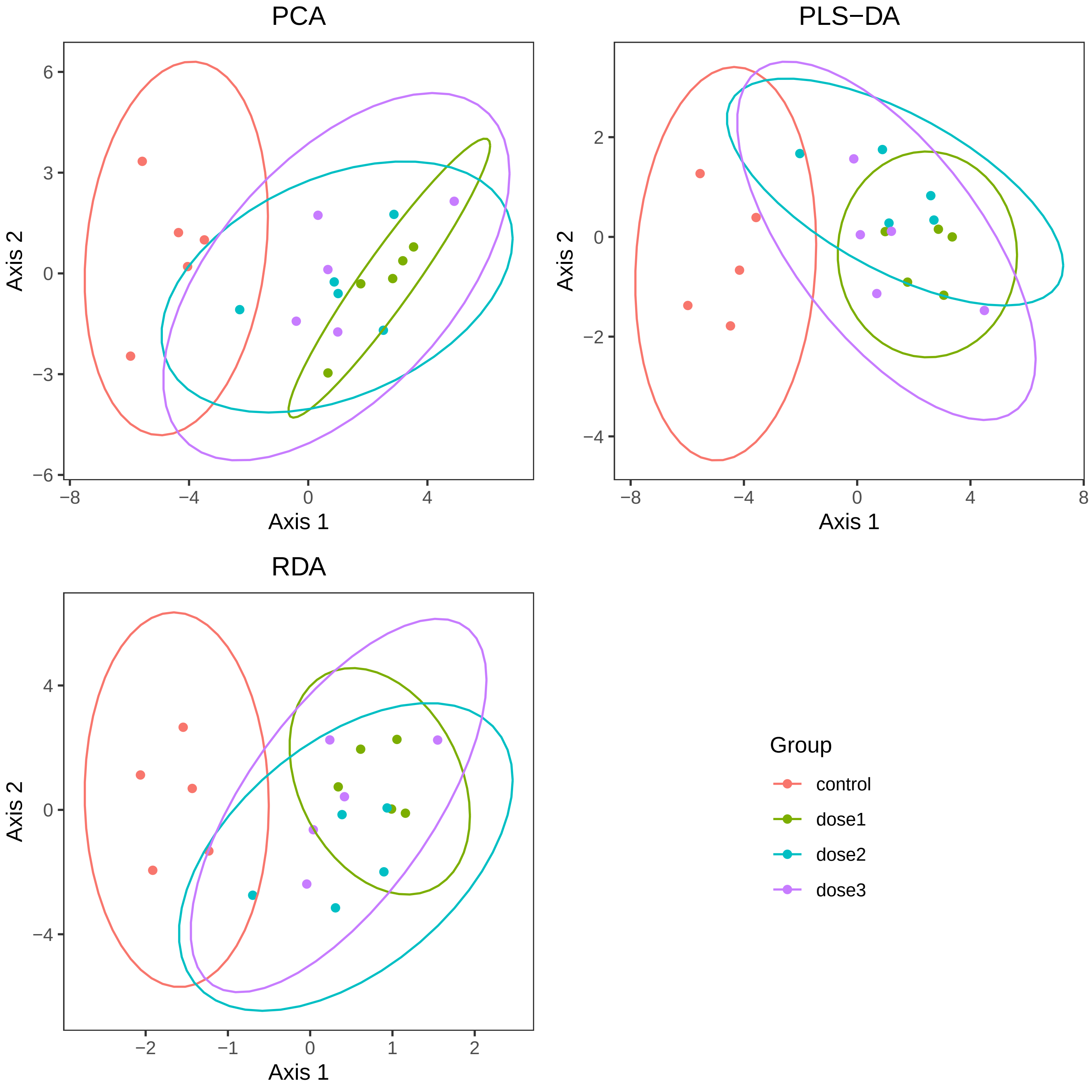
PCA
PLS−DA
RDA
Axis 1
Axis 2
Group
control
dose1
dose2
dose3

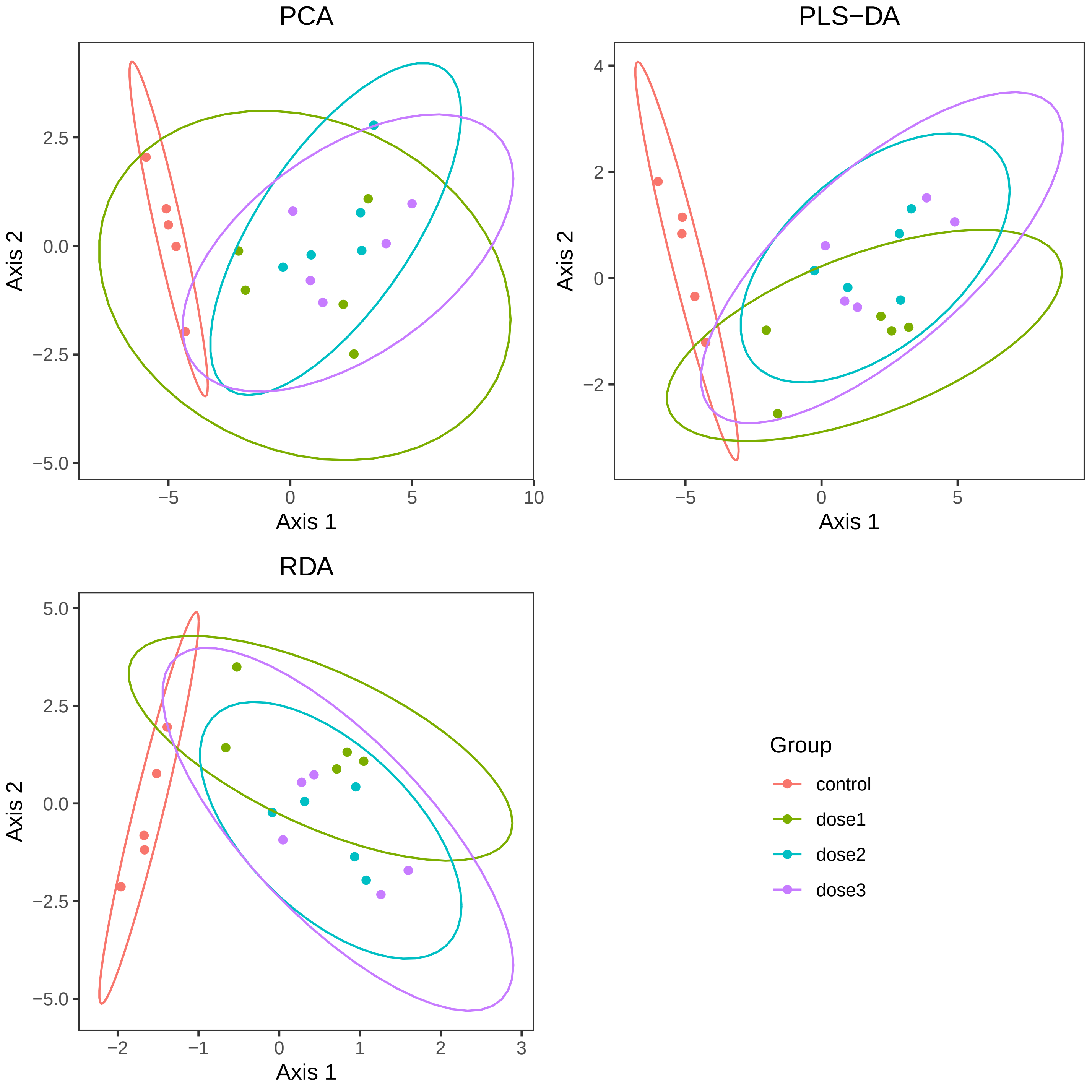

PCA
PLS−DA
RDA
Axis 1
Axis 2
Group
control
dose1
dose2
dose3

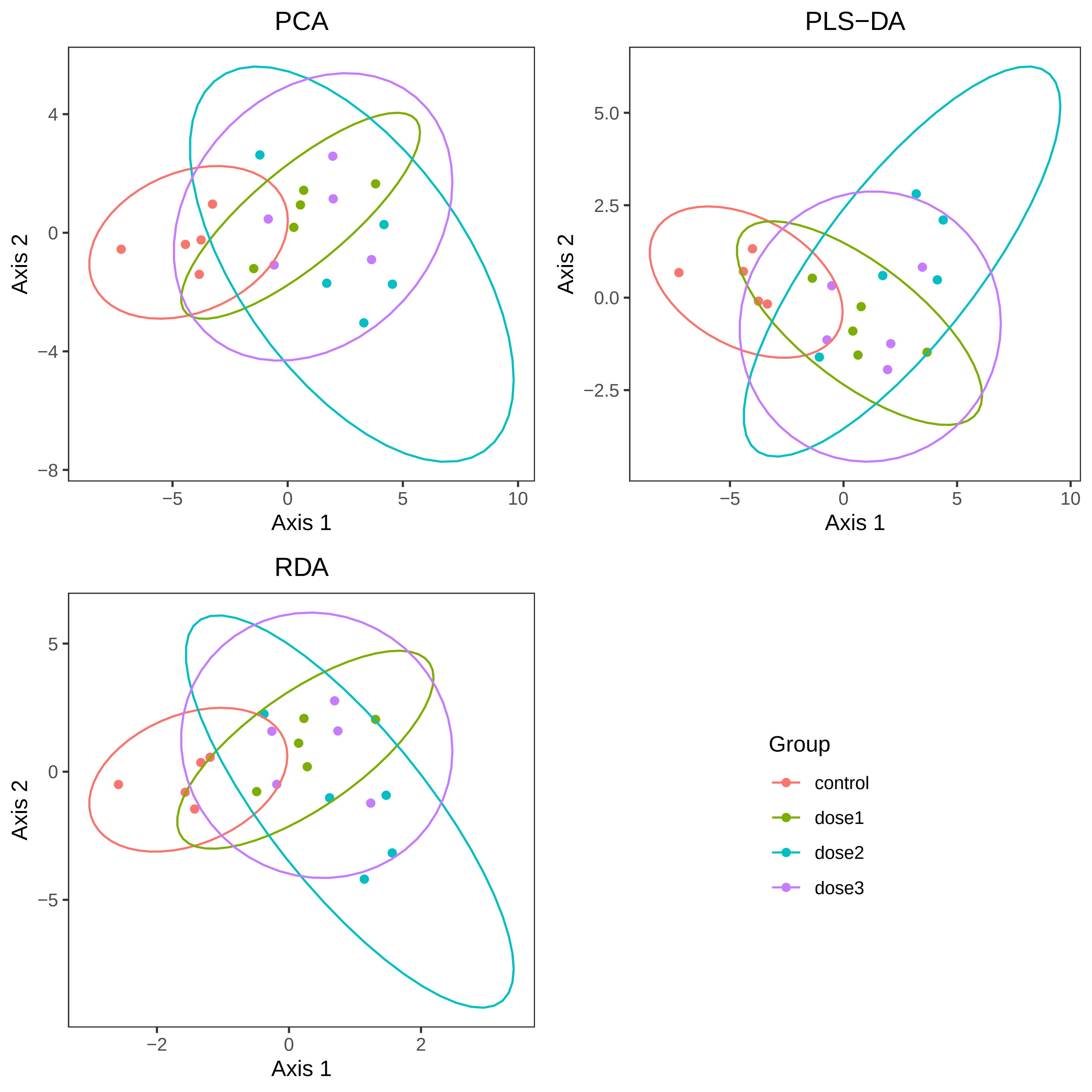
PCA
Axis 2
Axis 1
4
0
−4
−8
−5
0
5
10
PLS−DA
5.0
2.5
0.0
−2.5
RDA
5
−5
−2
2
Group
control
dose1
dose2
dose3

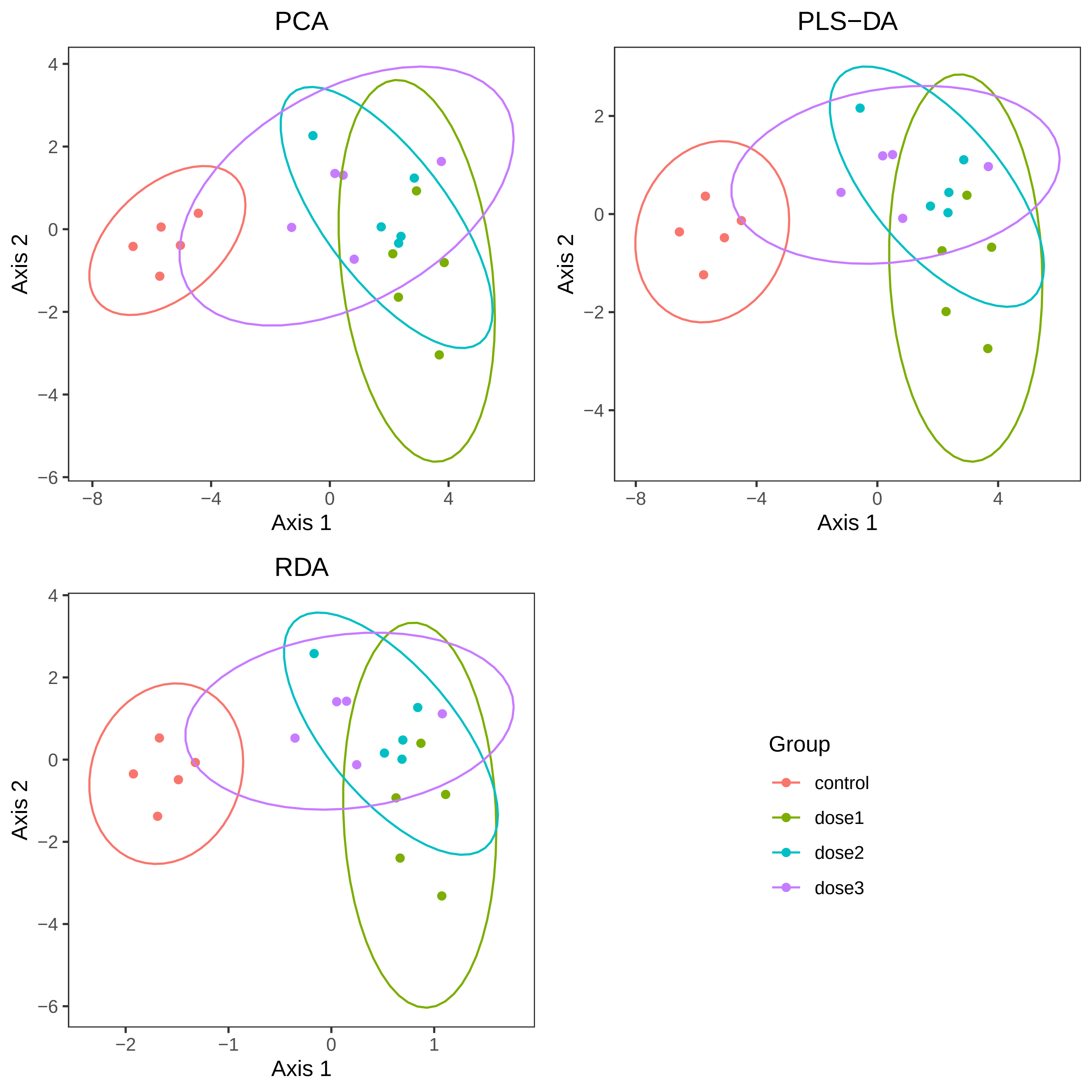

PCA
PLS−DA
RDA
Axis 1
Axis 2
Group
control
dose1
dose2
dose3

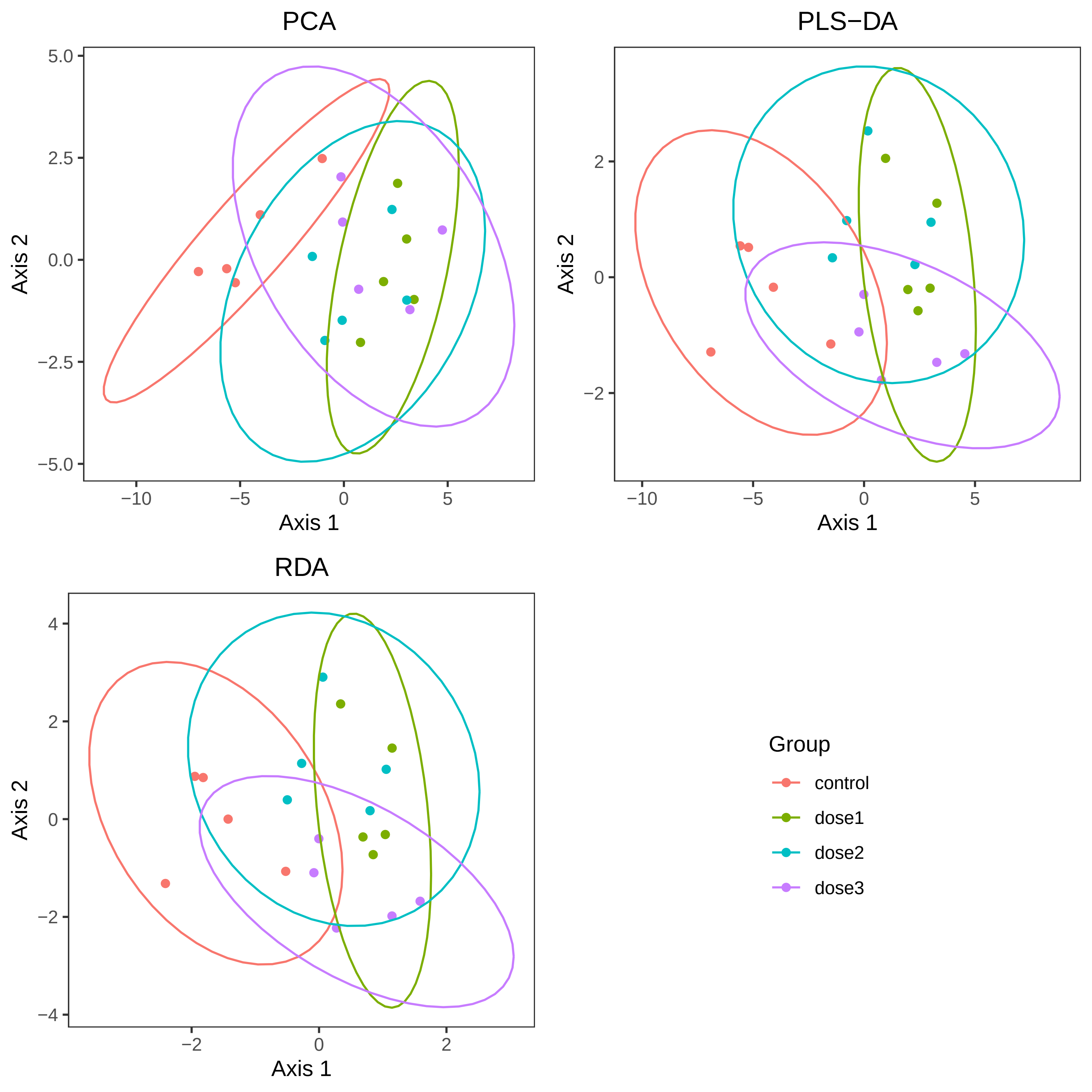

PCA
PLS−DA
RDA
Axis 1
Axis 2
Group
control
dose1
dose2
dose3

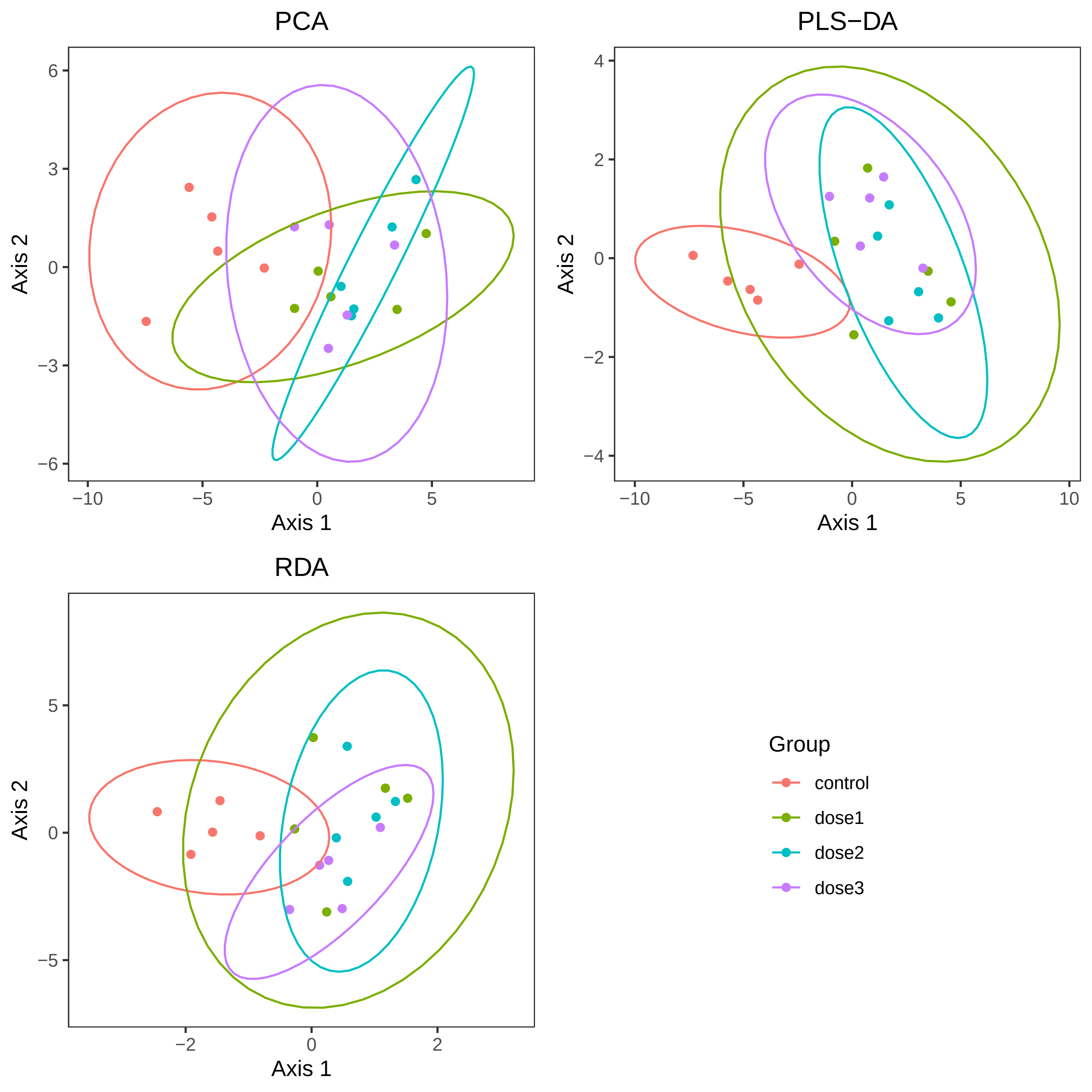

PCA
PLS−DA
RDA
Axis 1
Axis 2
Group
control
dose1
dose2
dose3

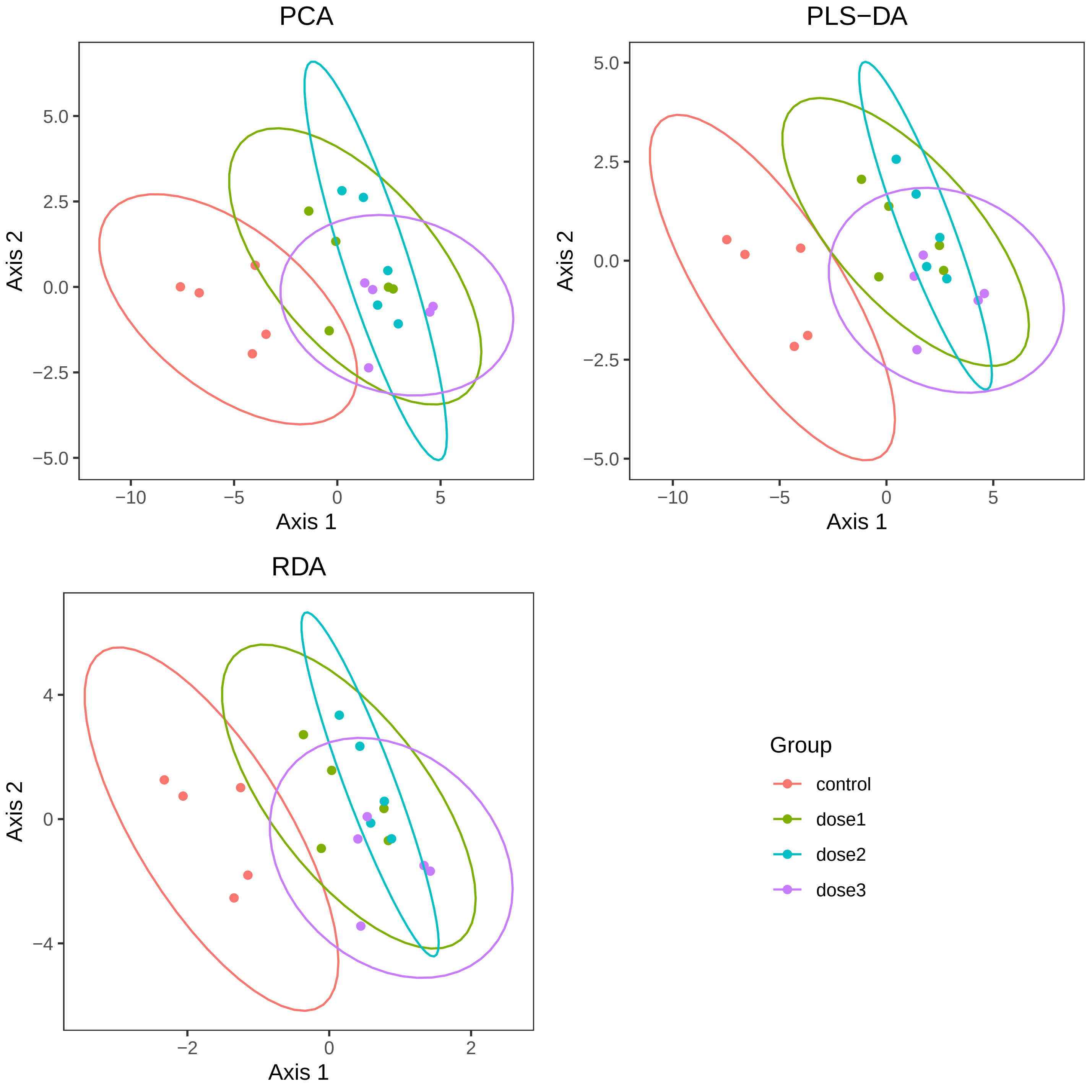
PCA
PLS−DA
RDA
Axis 1
Axis 2
Group
control
dose1
dose2
dose3

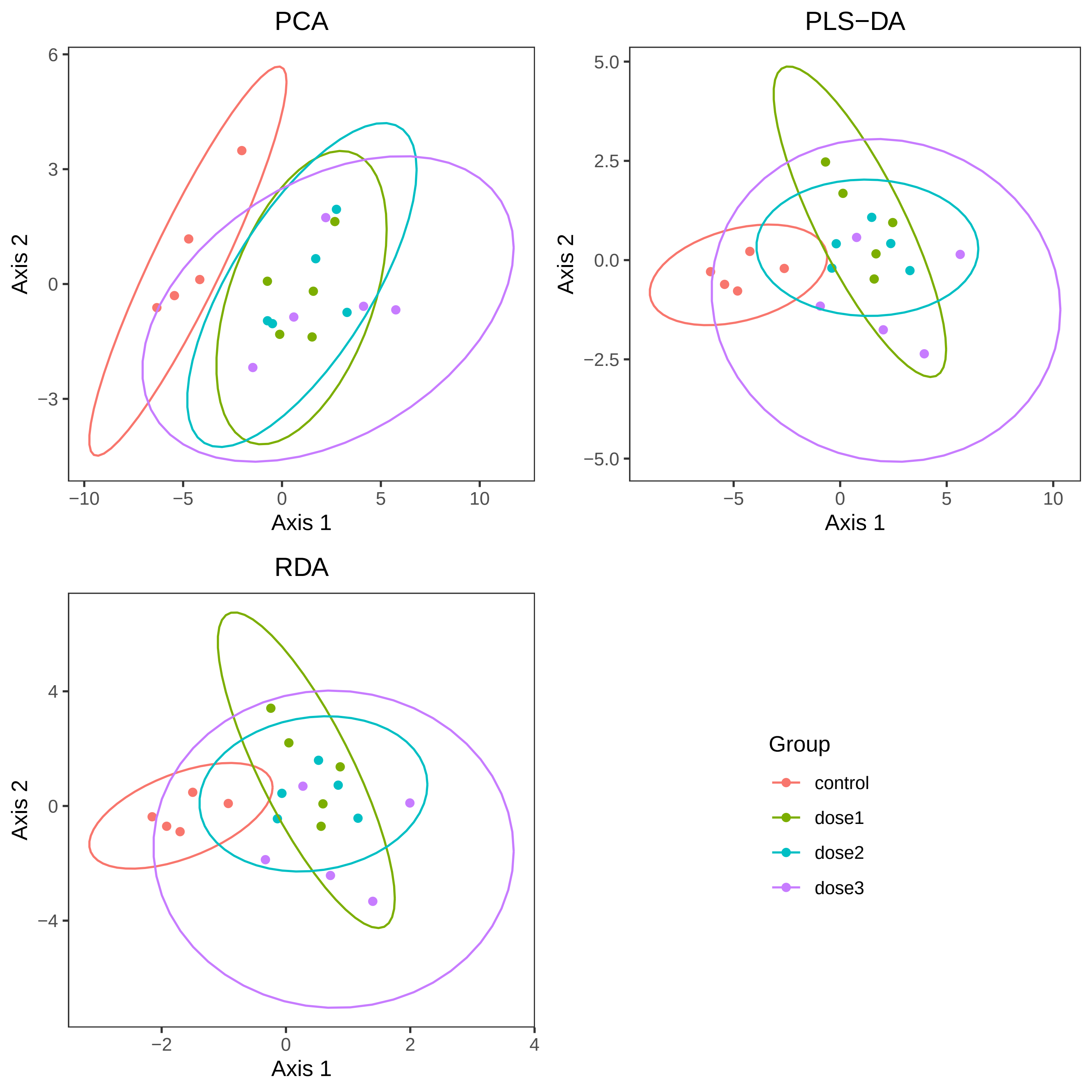
PCA
PLS−DA
RDA
Axis 1
Axis 2
Group
control
dose1
dose2
dose3

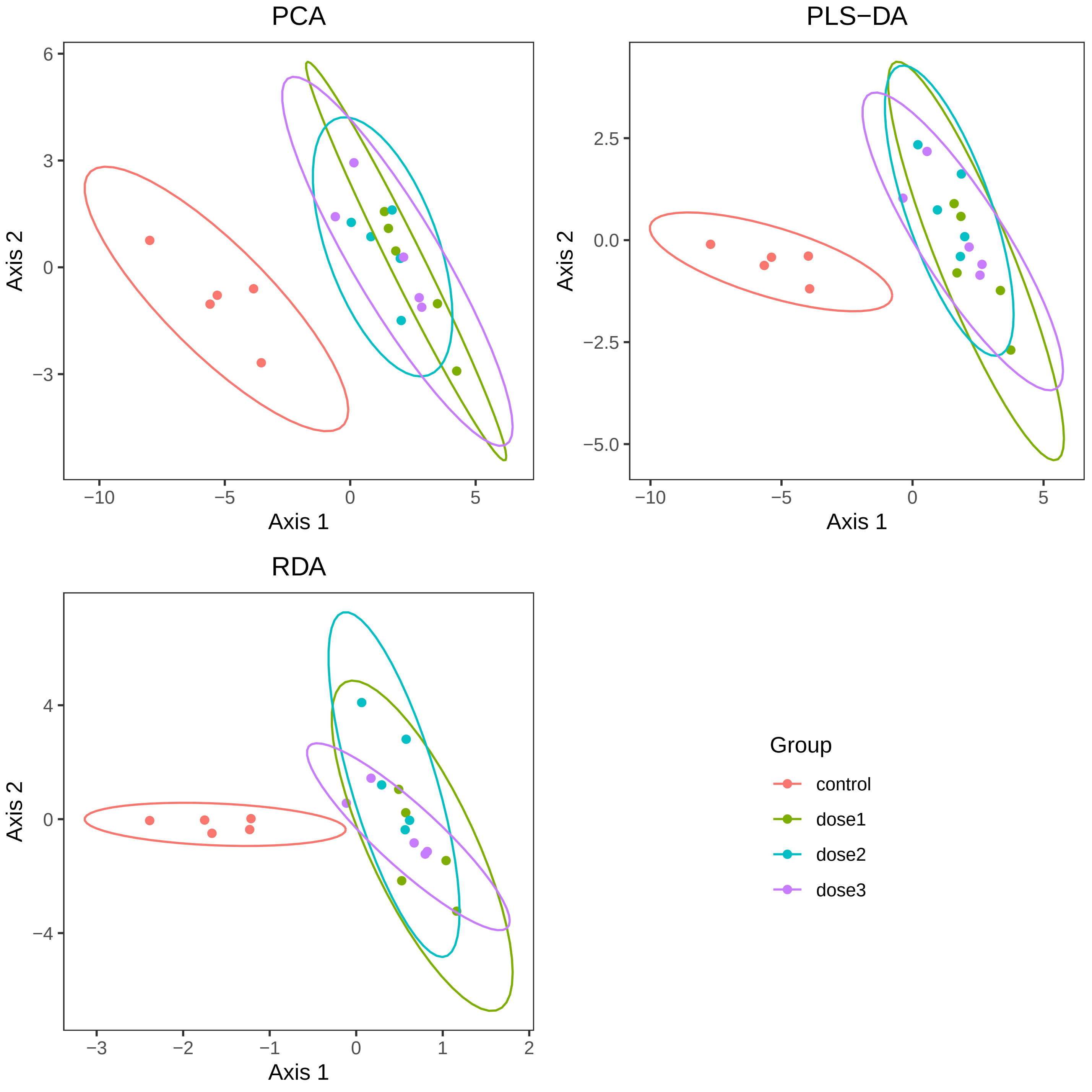

PCA
PLS−DA
RDA
Axis 1
Axis 2
Group
control
dose1
dose2
dose3

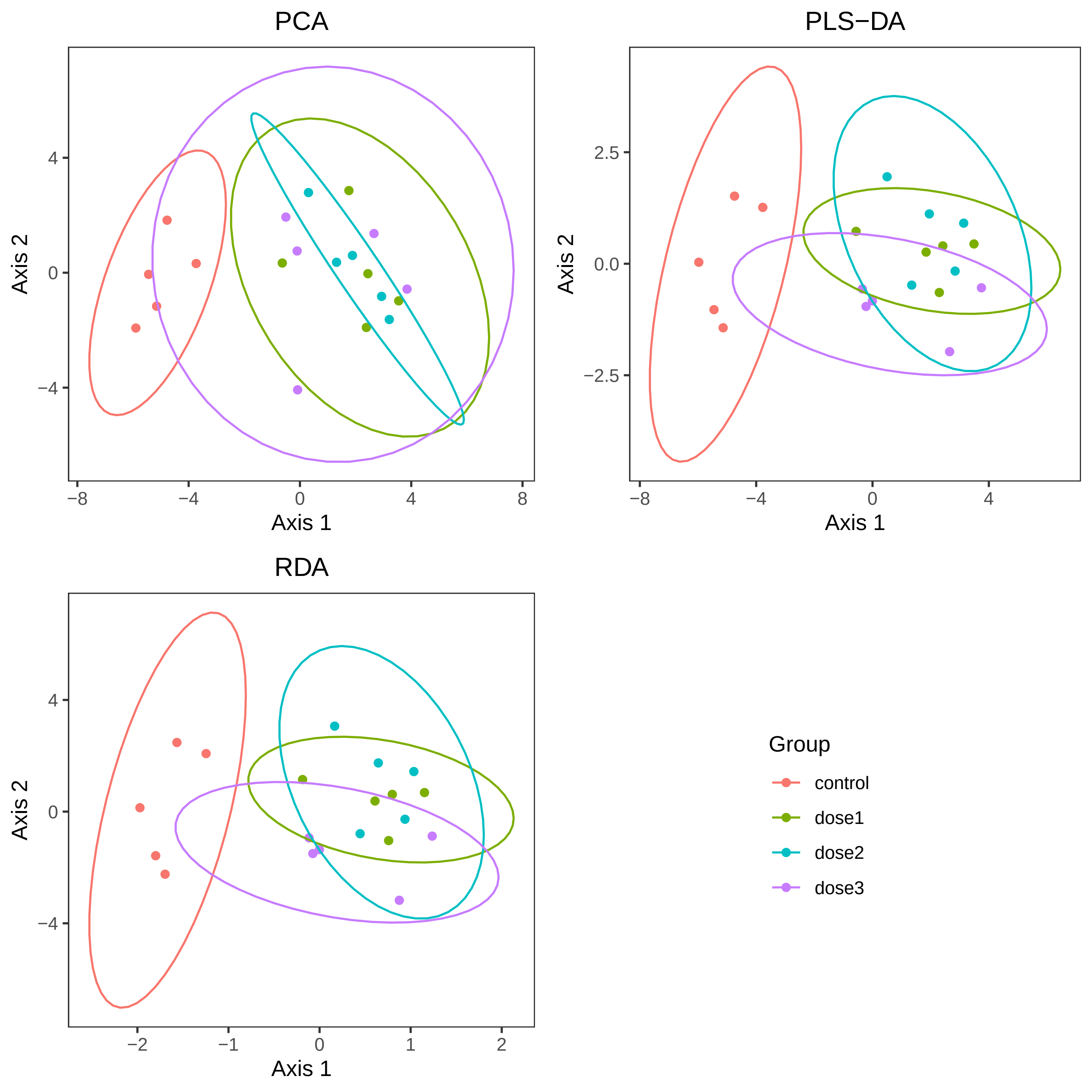

PCA
PLS−DA
RDA
Axis 1
Axis 2
−8
−4
0
4
8
2.5
0.0
−2.5
−2
−1
1
2
Group
control
dose1
dose2
dose3

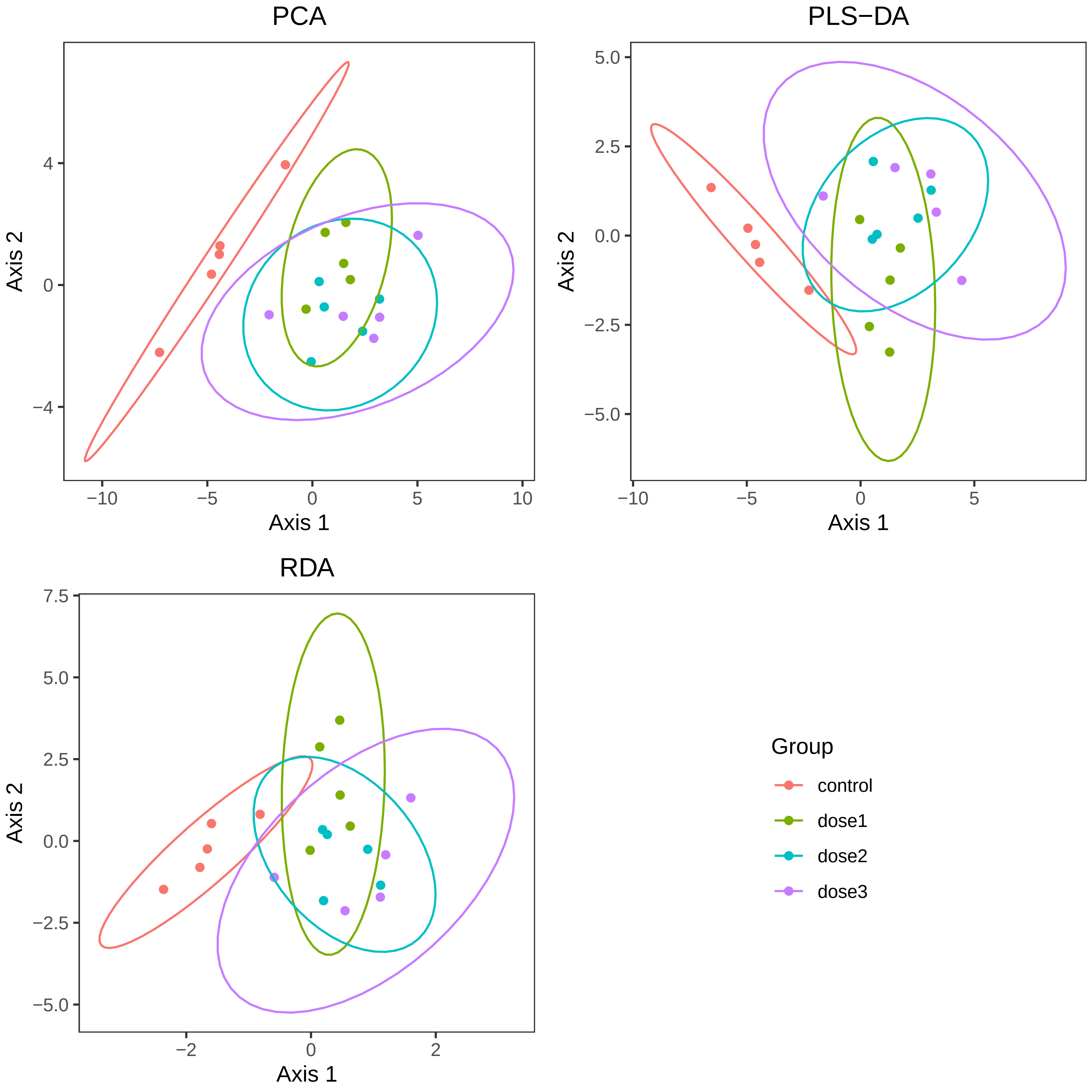
PCA
PLS−DA
RDA
Axis 1
Axis 2
Group
control
dose1
dose2
dose3

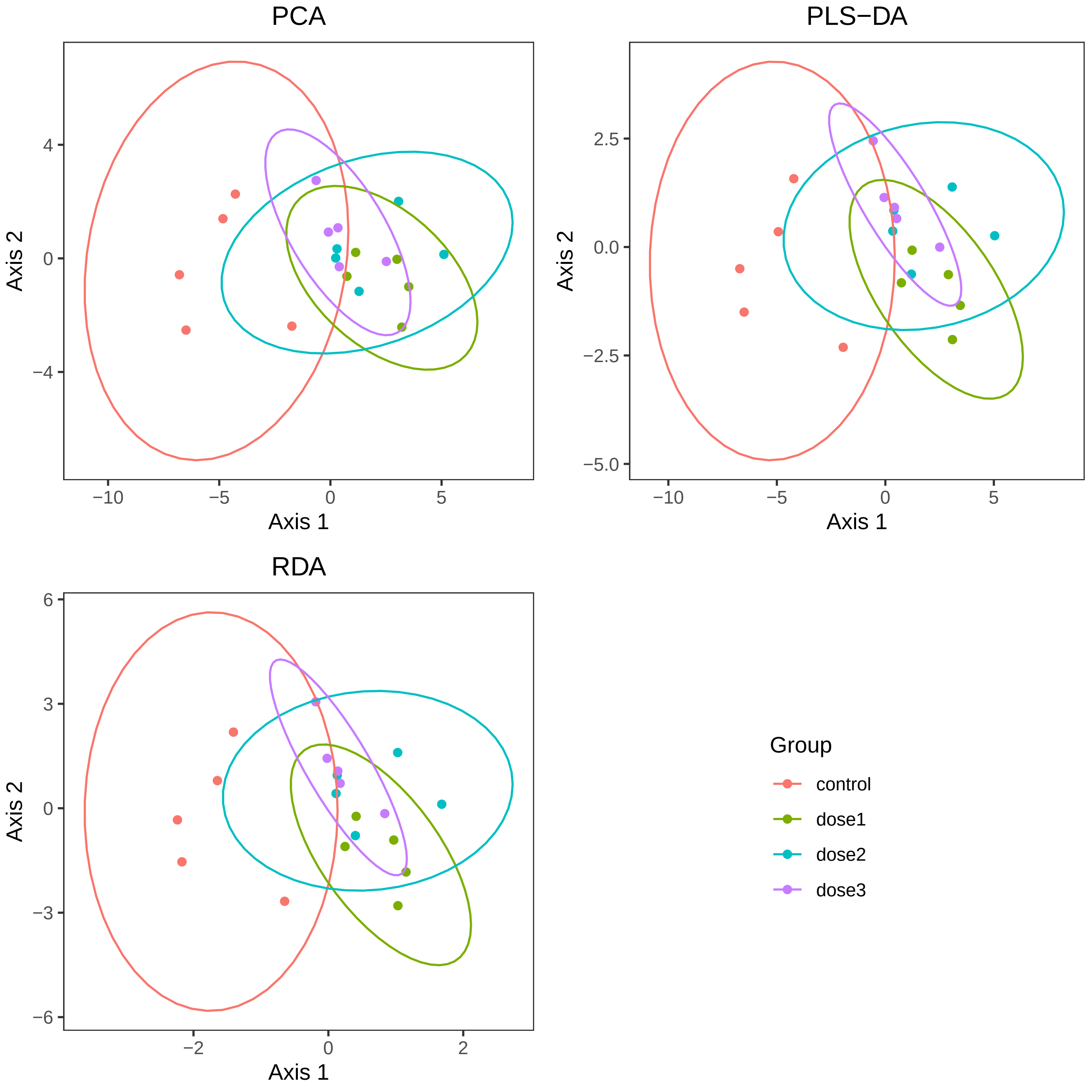

PCA
PLS−DA
RDA
Axis 1
Axis 2
−10
−5
0
5
4
−4
2.5
0.0
−2.5
−5.0
−2
2
6
3
−3
−6
Group
control
dose1
dose2
dose3

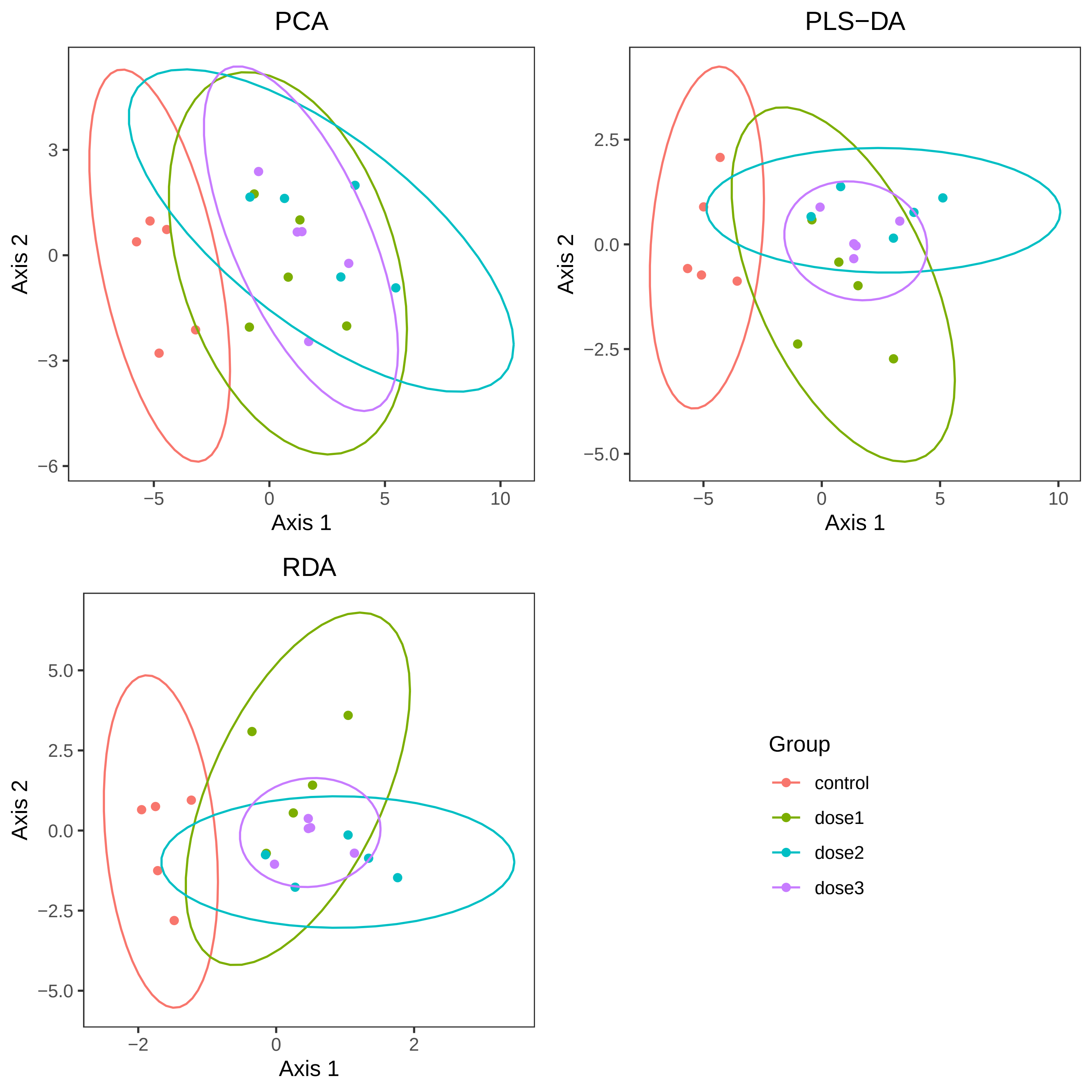
PCA
PLS−DA
RDA
Axis 1
Axis 2
−5
0
5
10
3
0
−3
−6
2.5
0.0
−2.5
−5.0
−2
2
5.0
Group
control
dose1
dose2
dose3

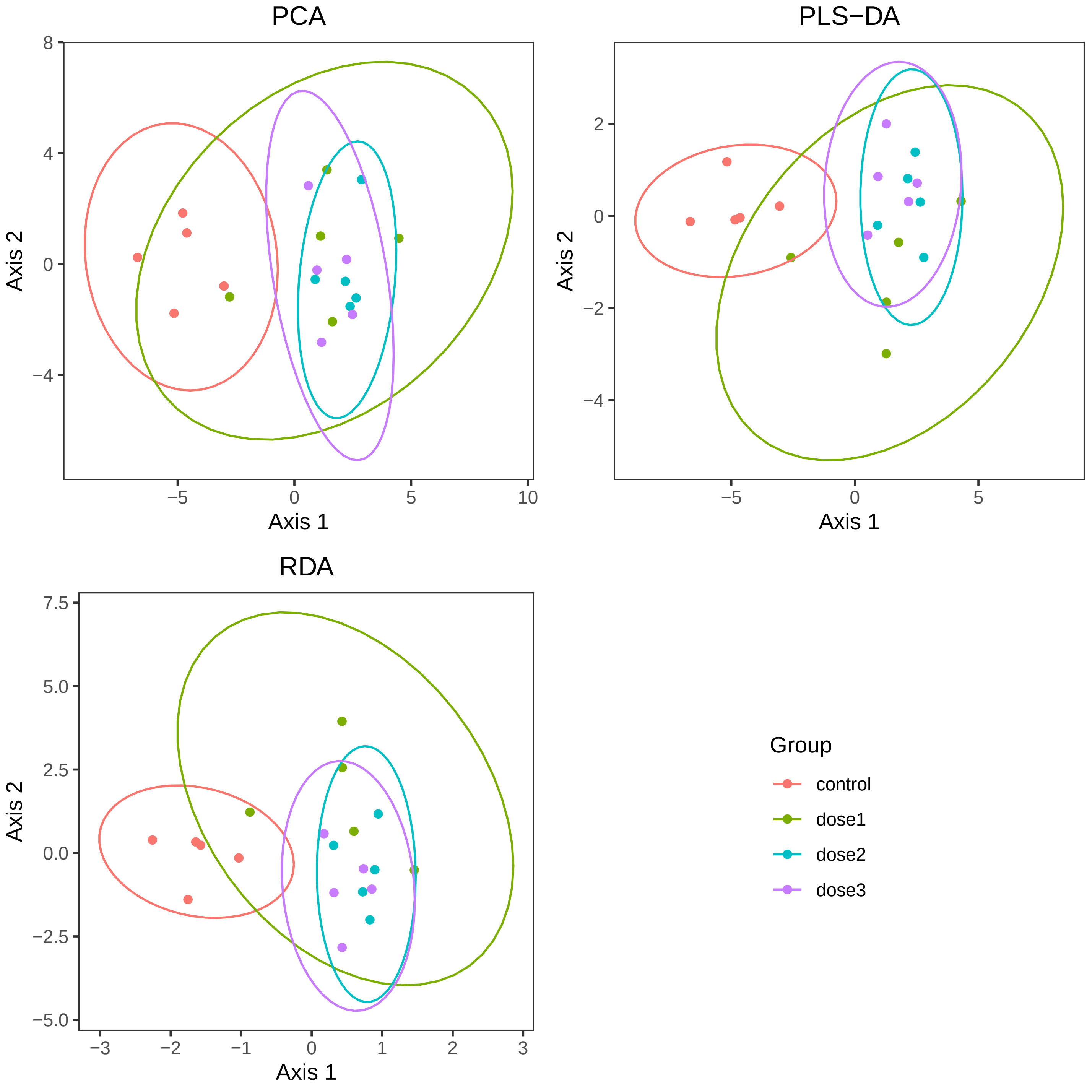

PCA
PLS−DA
RDA
Axis 1
Axis 2
Group
control
dose1
dose2
dose3

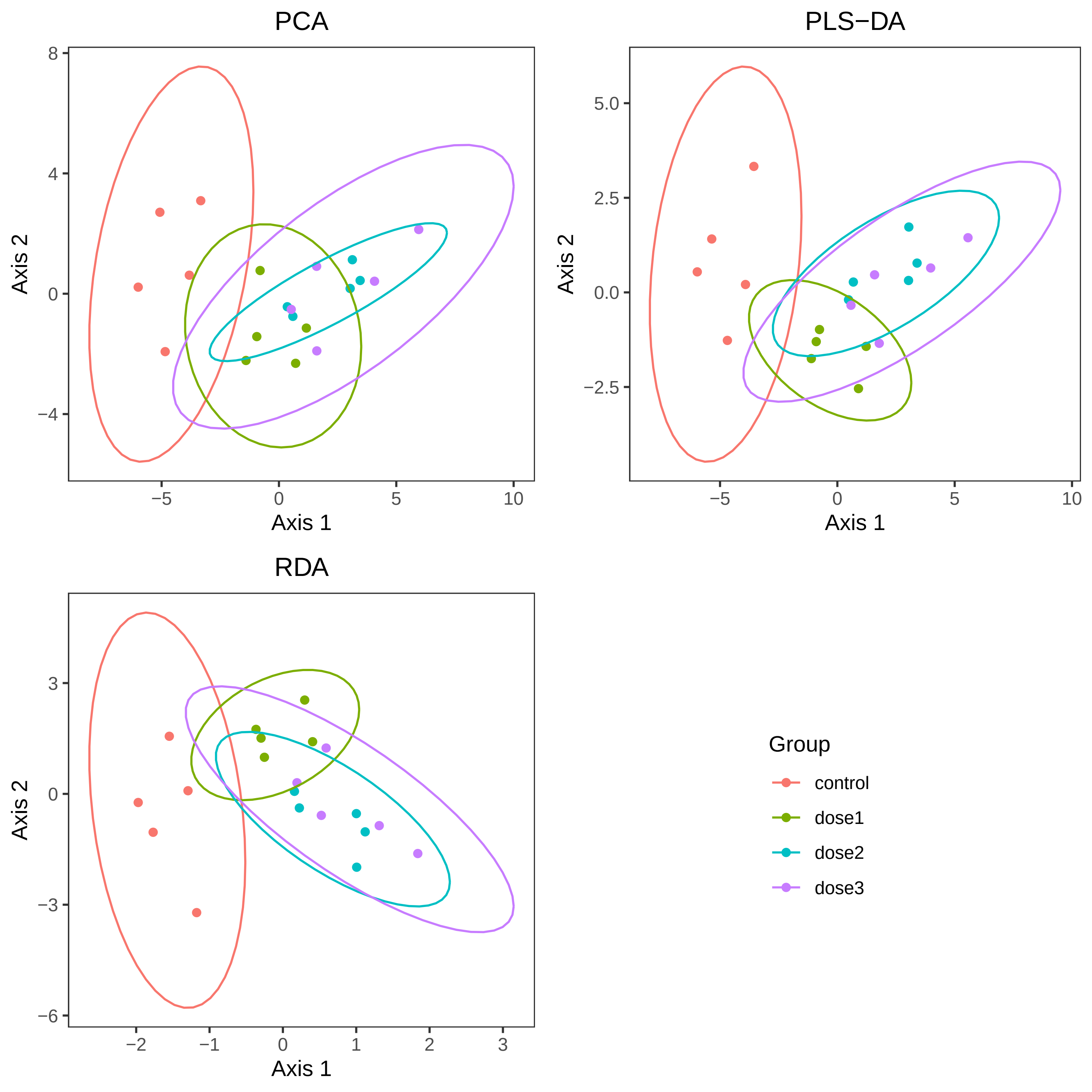
PCA
PLS−DA
RDA
Axis 1
Axis 2
Group
control
dose1
dose2
dose3

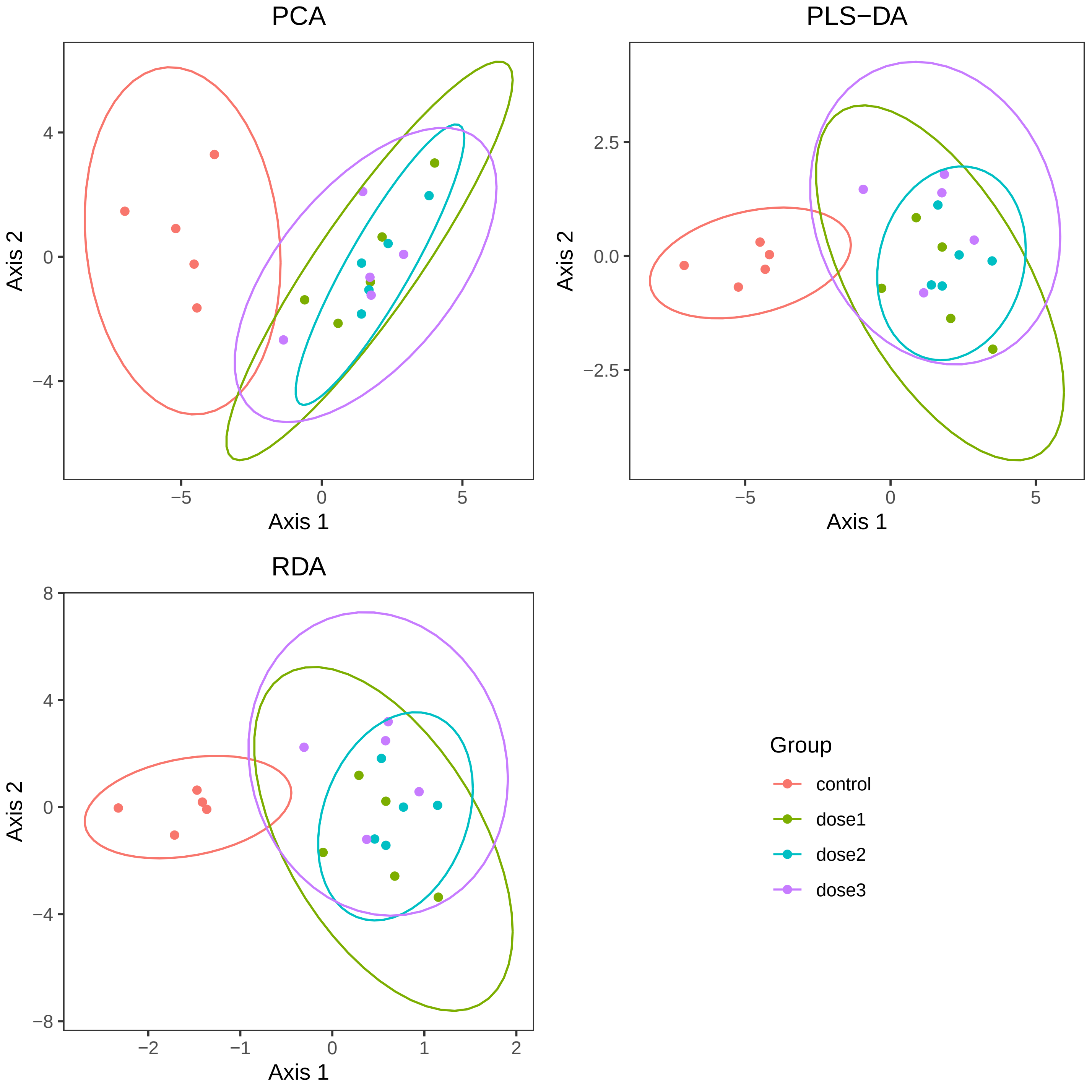

PCA
PLS−DA
RDA
Axis 1
Axis 2
−5
0
5
4
0
−4
2.5
0.0
−2.5
8
−8
−2
−1
1
2
Group
control
dose1
dose2
dose3

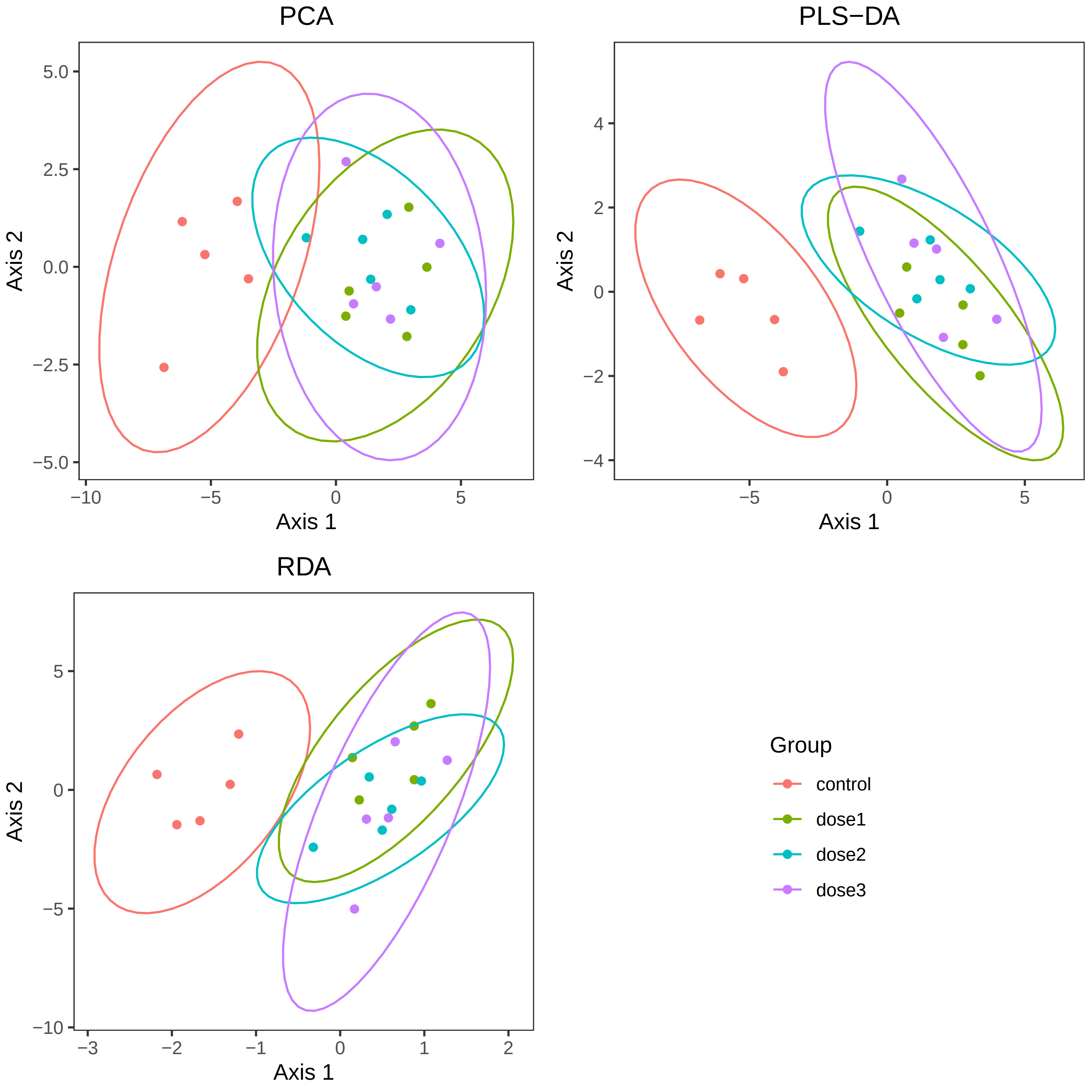

PCA
PLS−DA
RDA
Axis 1
Axis 2
Group
control
dose1
dose2
dose3

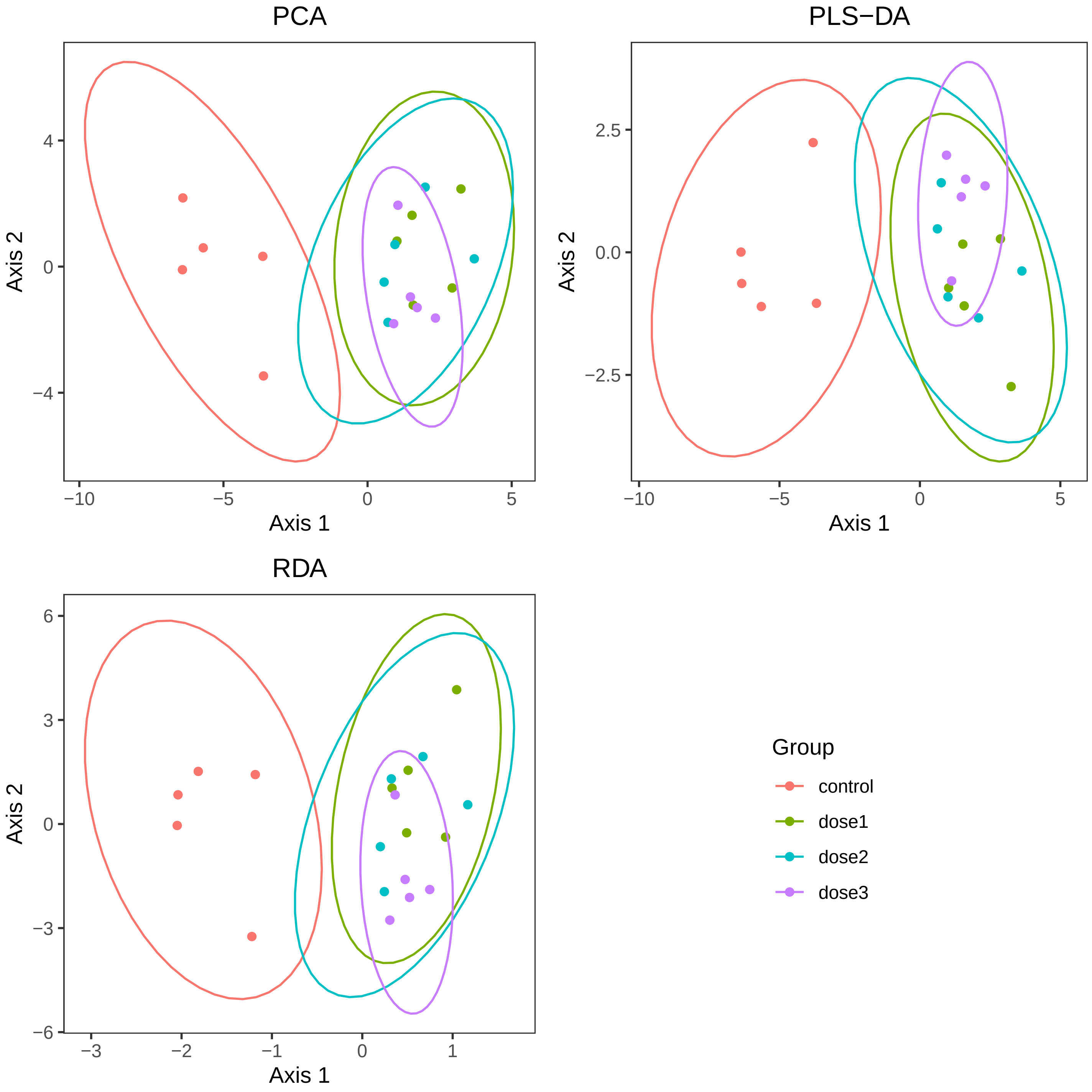

PCA
PLS−DA
RDA
Axis 1
Axis 2
Group
control
dose1
dose2
dose3

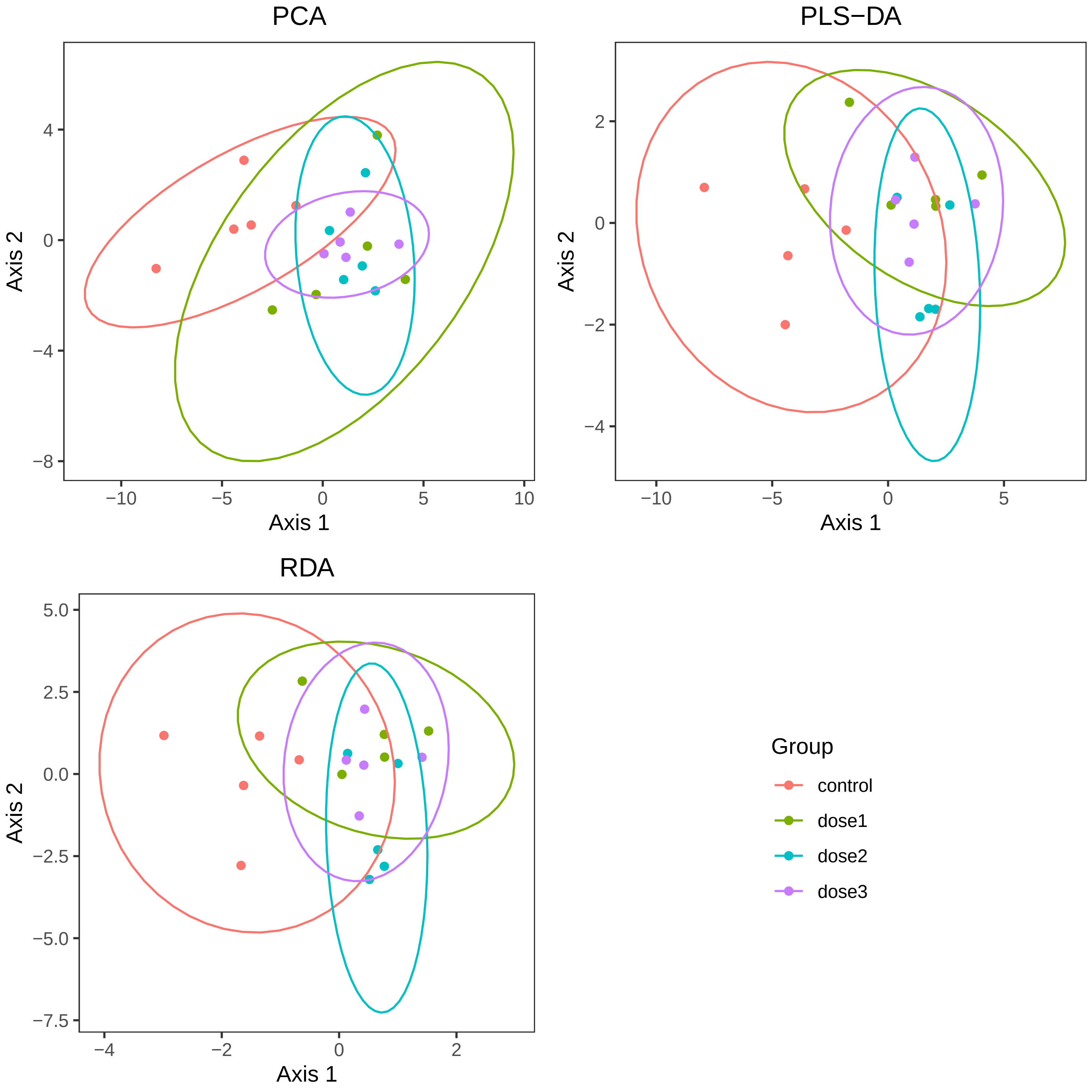
PCA
PLS−DA
RDA
Axis 1
Axis 2
−10
−5
0
5
10
4
0
−4
−8
2
−2
5.0
2.5
0.0
−2.5
−5.0
−7.5
−4
−2
0
2
Group
control
dose1
dose2
dose3

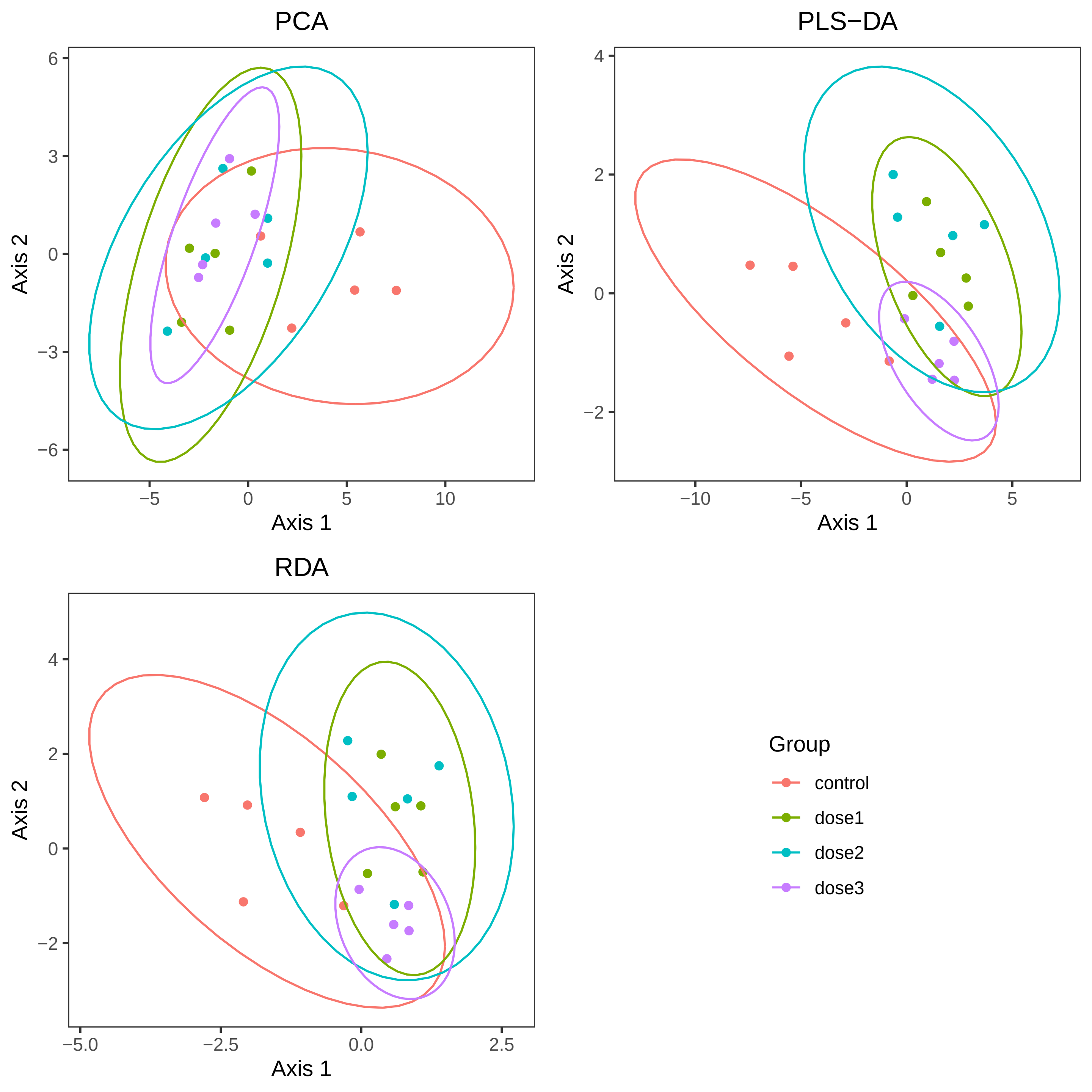

PCA
PLS−DA
RDA
Axis 1
Axis 2
Group
control
dose1
dose2
dose3

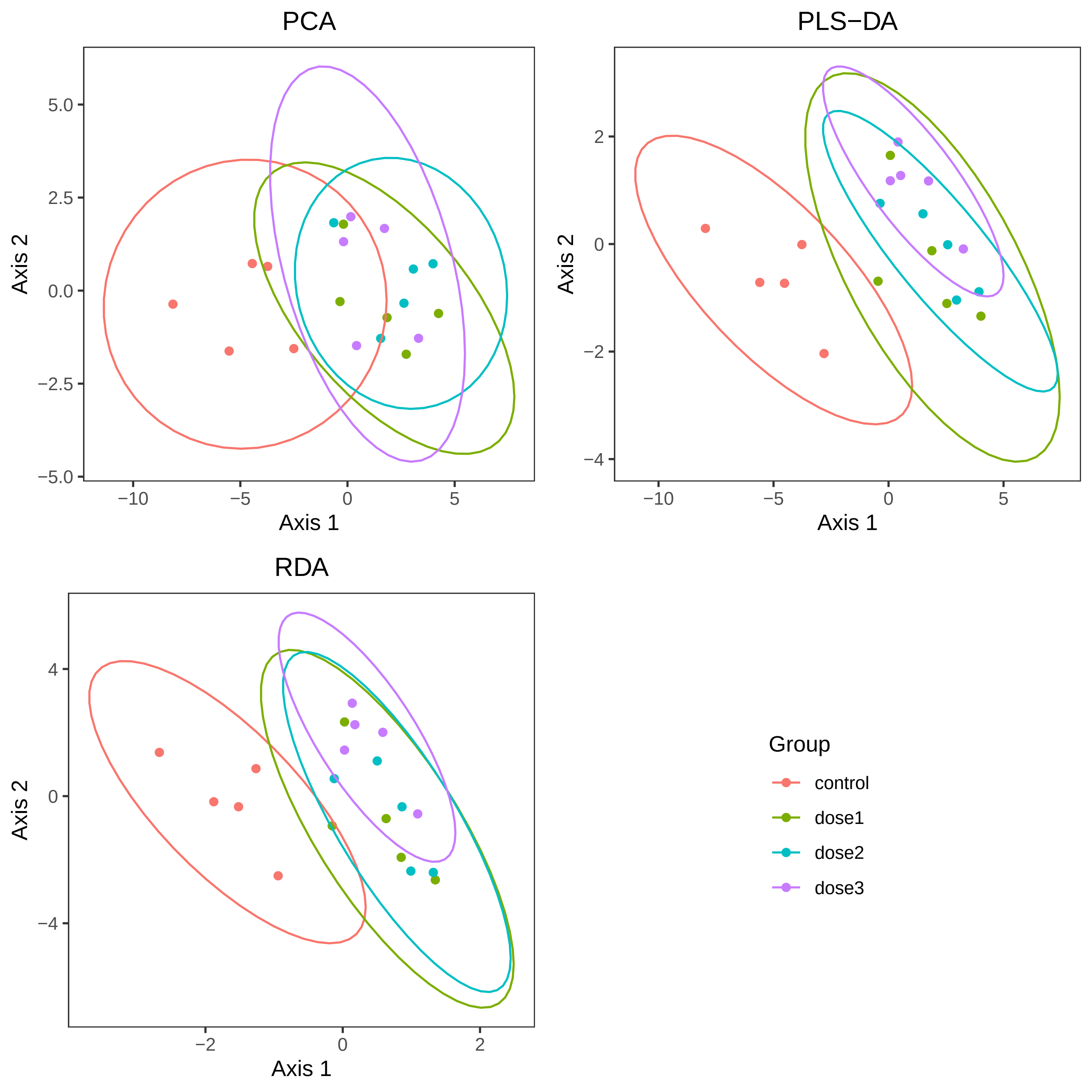
PCA
PLS−DA
RDA
Axis 1
Axis 2
Group
control
dose1
dose2
dose3

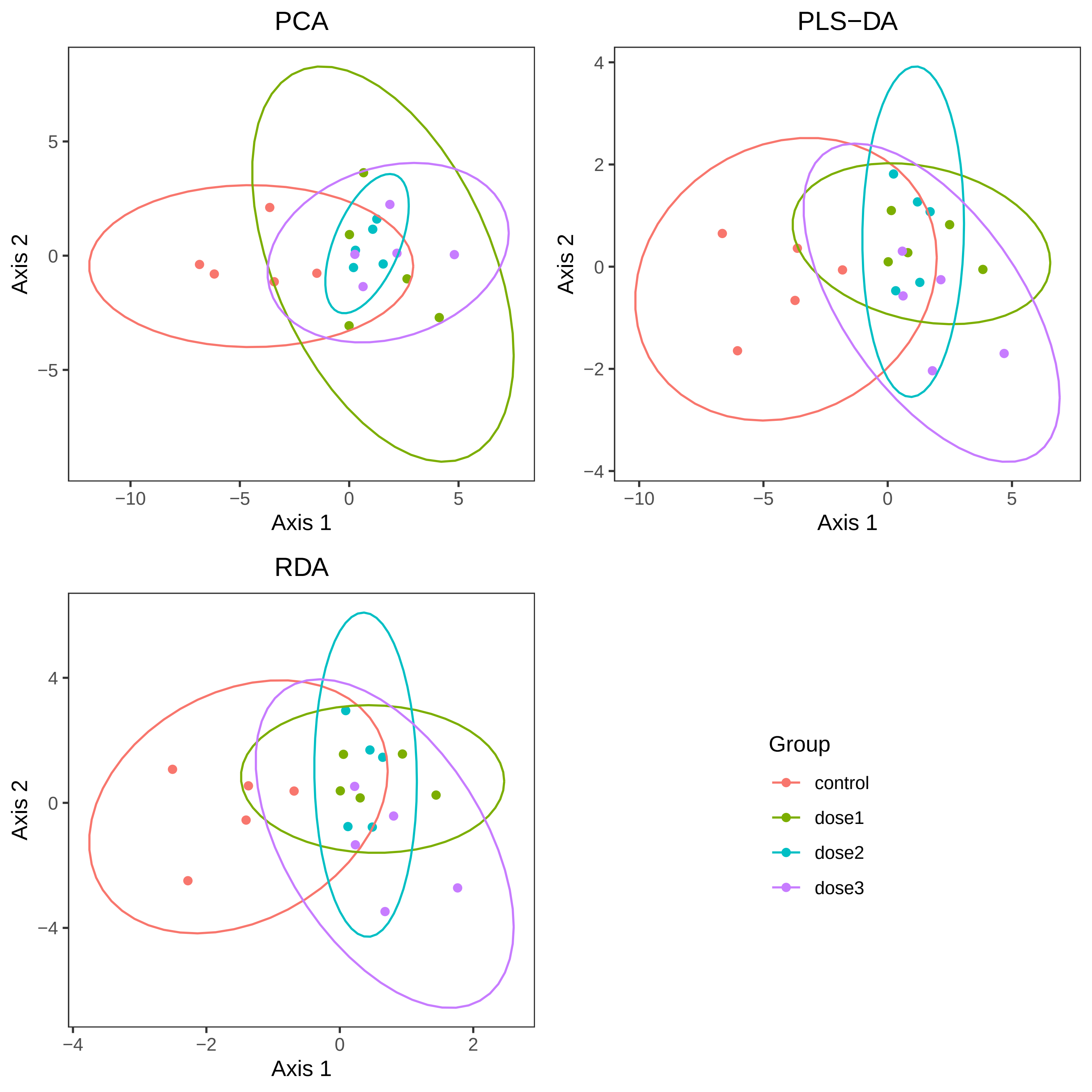
PCA
PLS−DA
RDA
Axis 1
Axis 2
Group
control
dose1
dose2
dose3

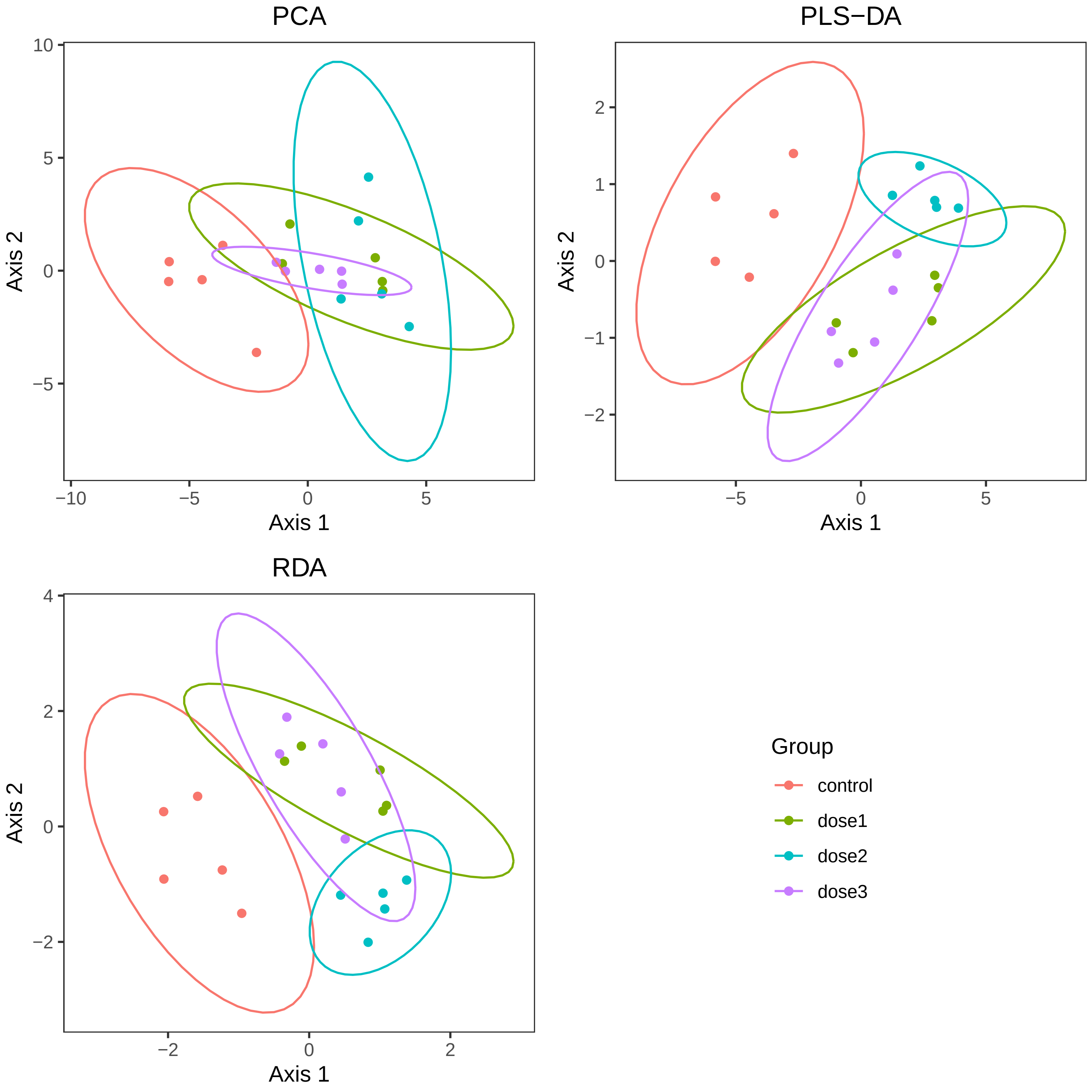

PCA
PLS−DA
RDA
Axis 1
Axis 2
Group
control
dose1
dose2
dose3

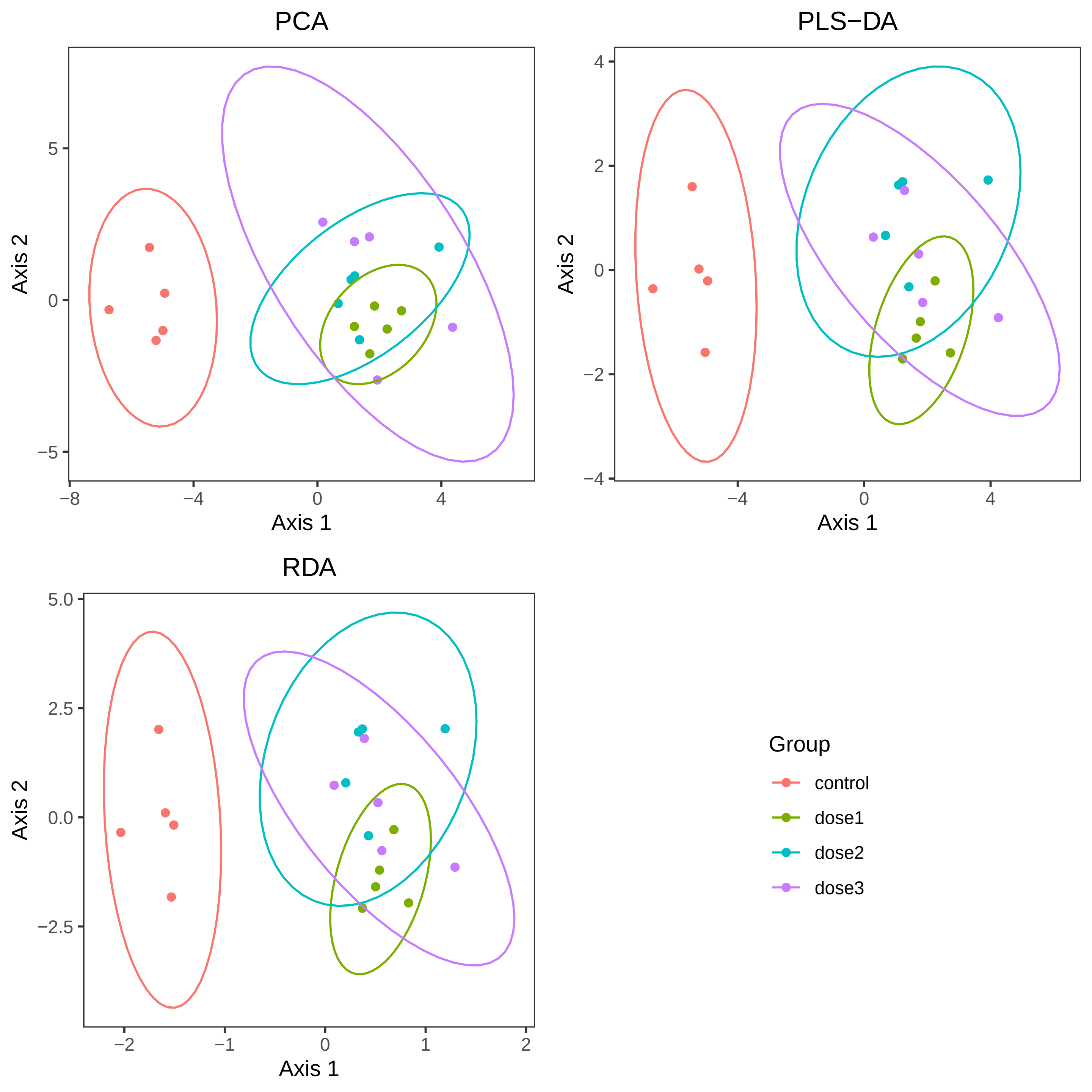

PCA
PLS−DA
RDA
Axis 1
Axis 2
Group
control
dose1
dose2
dose3

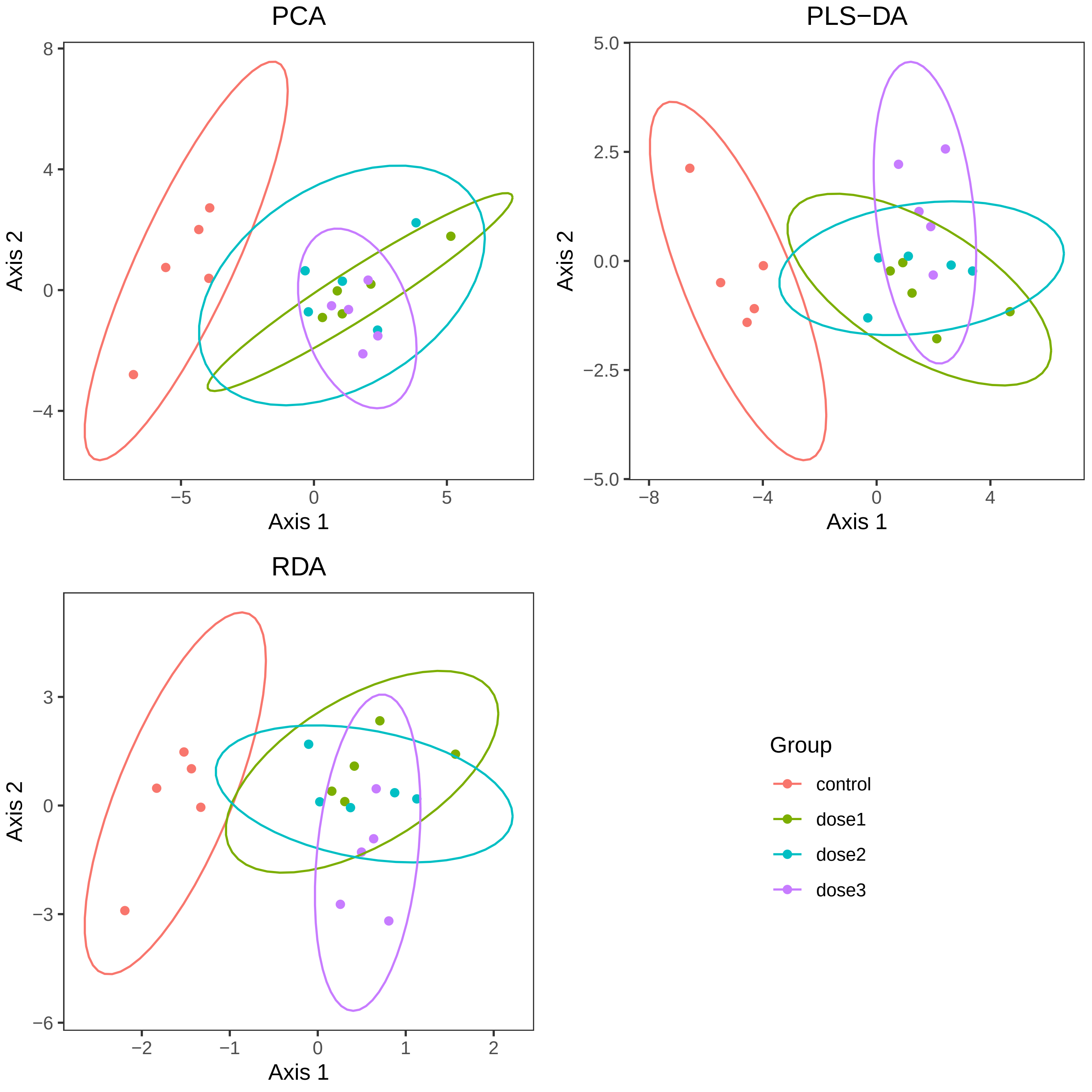

PCA
PLS−DA
RDA
Axis 1
Axis 2
Group
control
dose1
dose2
dose3

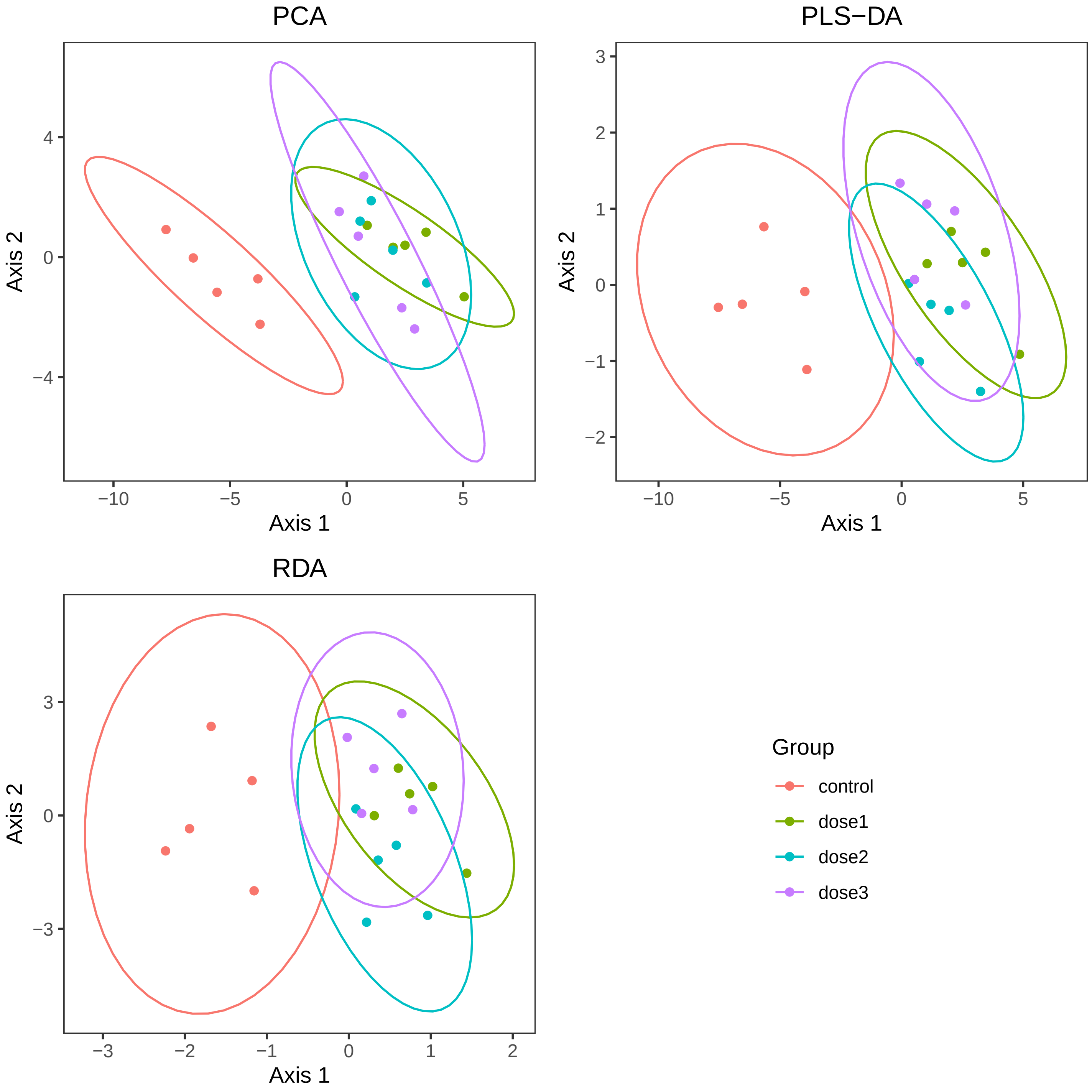

PCA
PLS−DA
RDA
Axis 1
Axis 2
Group
control
dose1
dose2
dose3

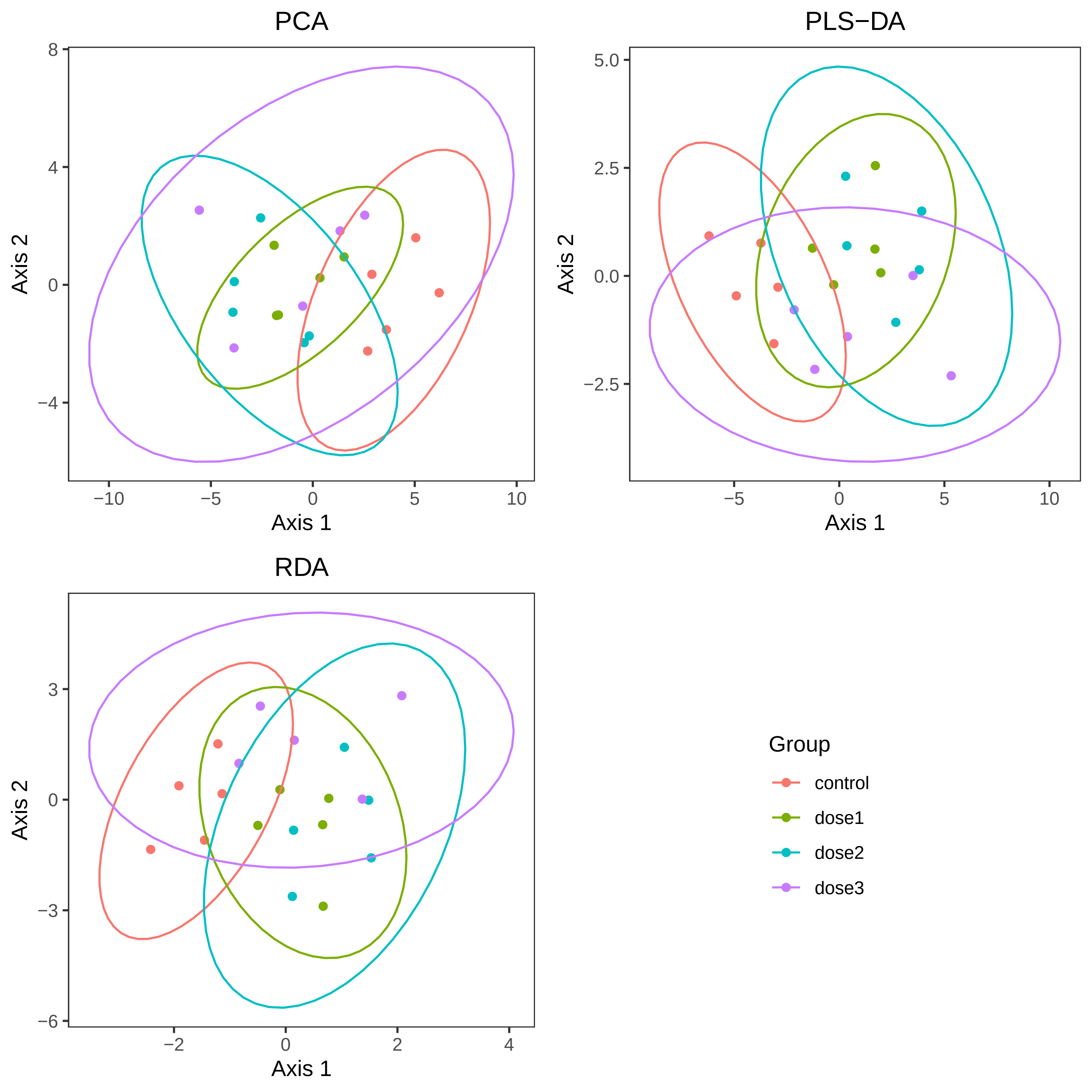

PCA
PLS−DA
RDA
Axis 1
Axis 2
Group
control
dose1
dose2
dose3

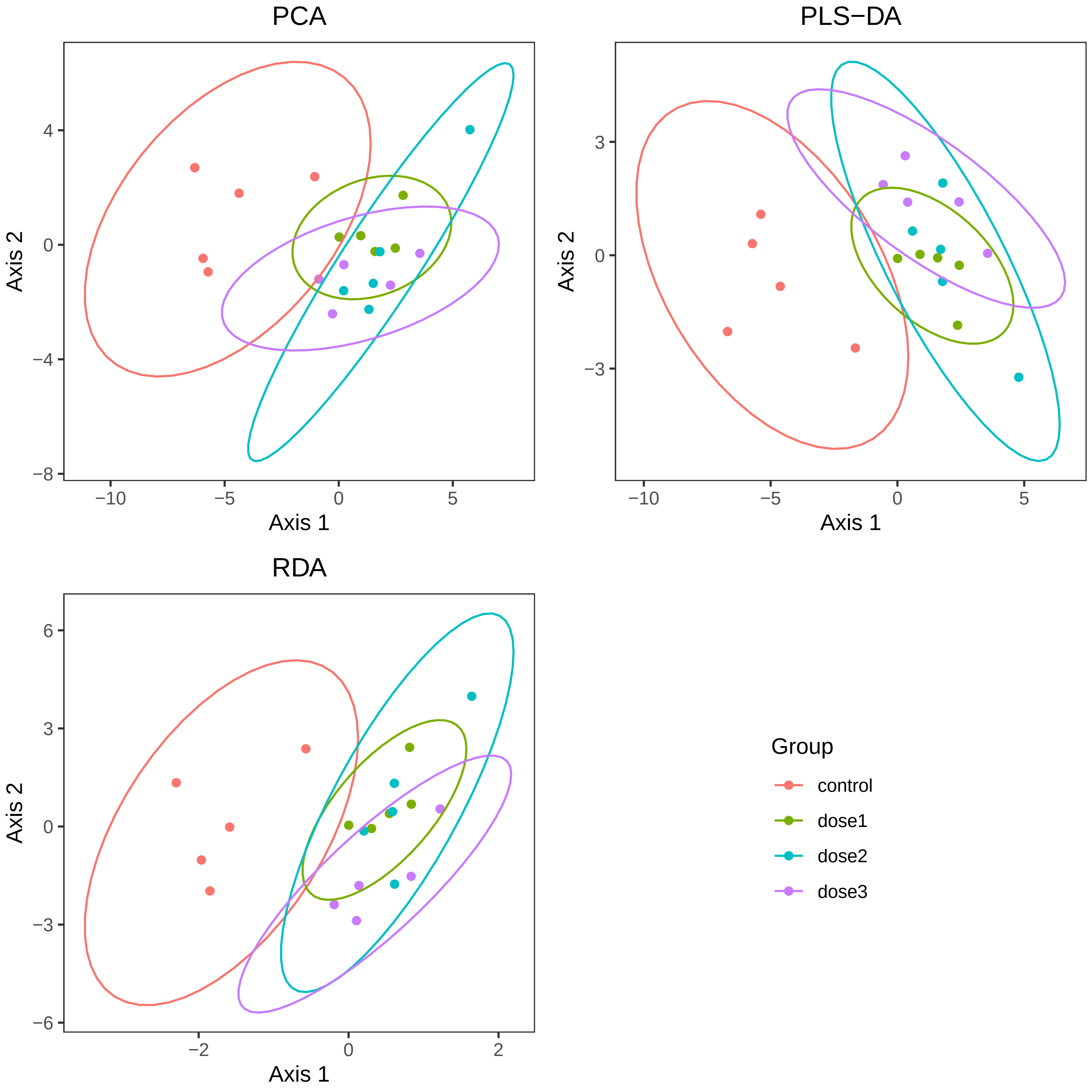

PCA
PLS−DA
RDA
Axis 1
Axis 2
Group
control
dose1
dose2
dose3

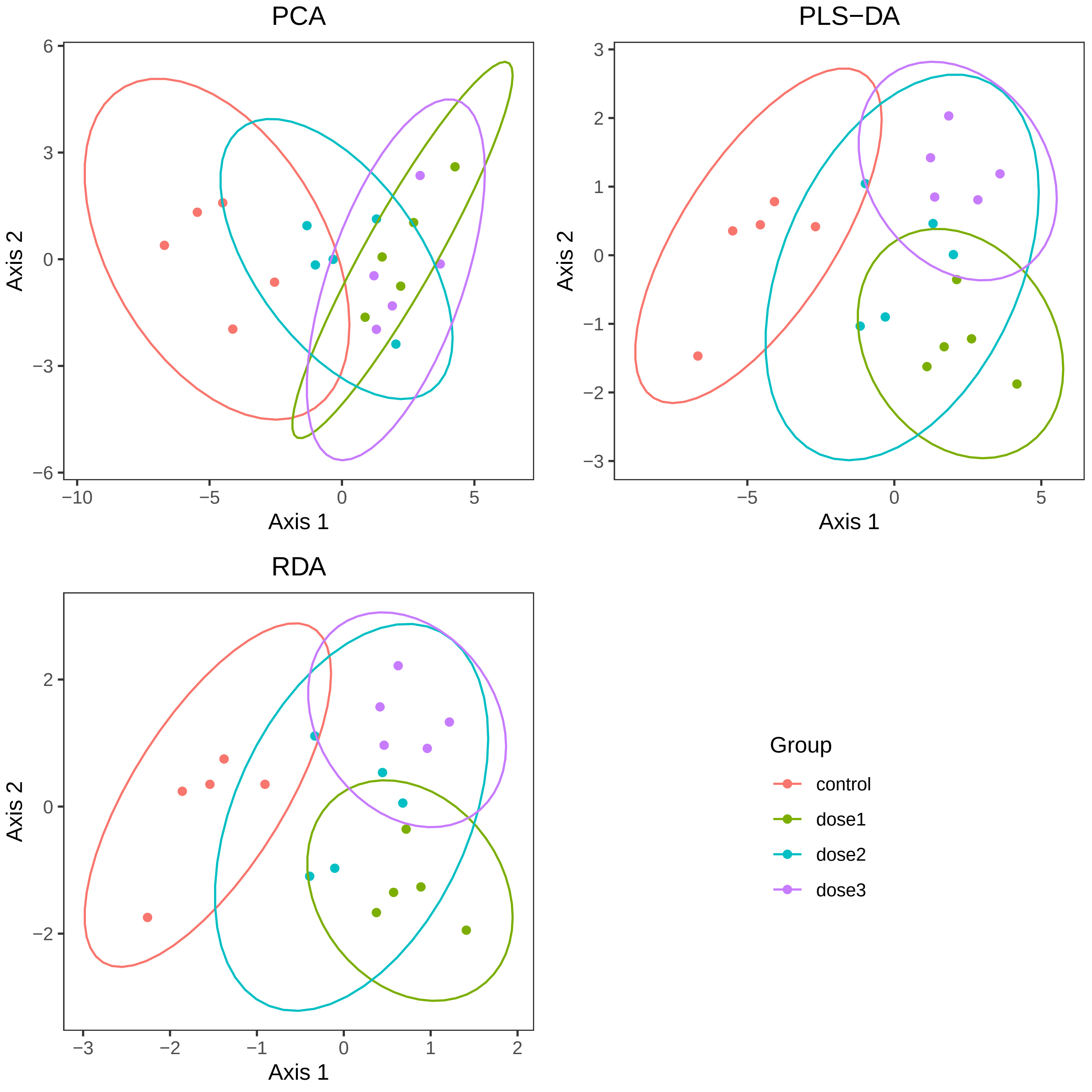

PCA
PLS−DA
RDA
Axis 1
Axis 2
Group
control
dose1
dose2
dose3

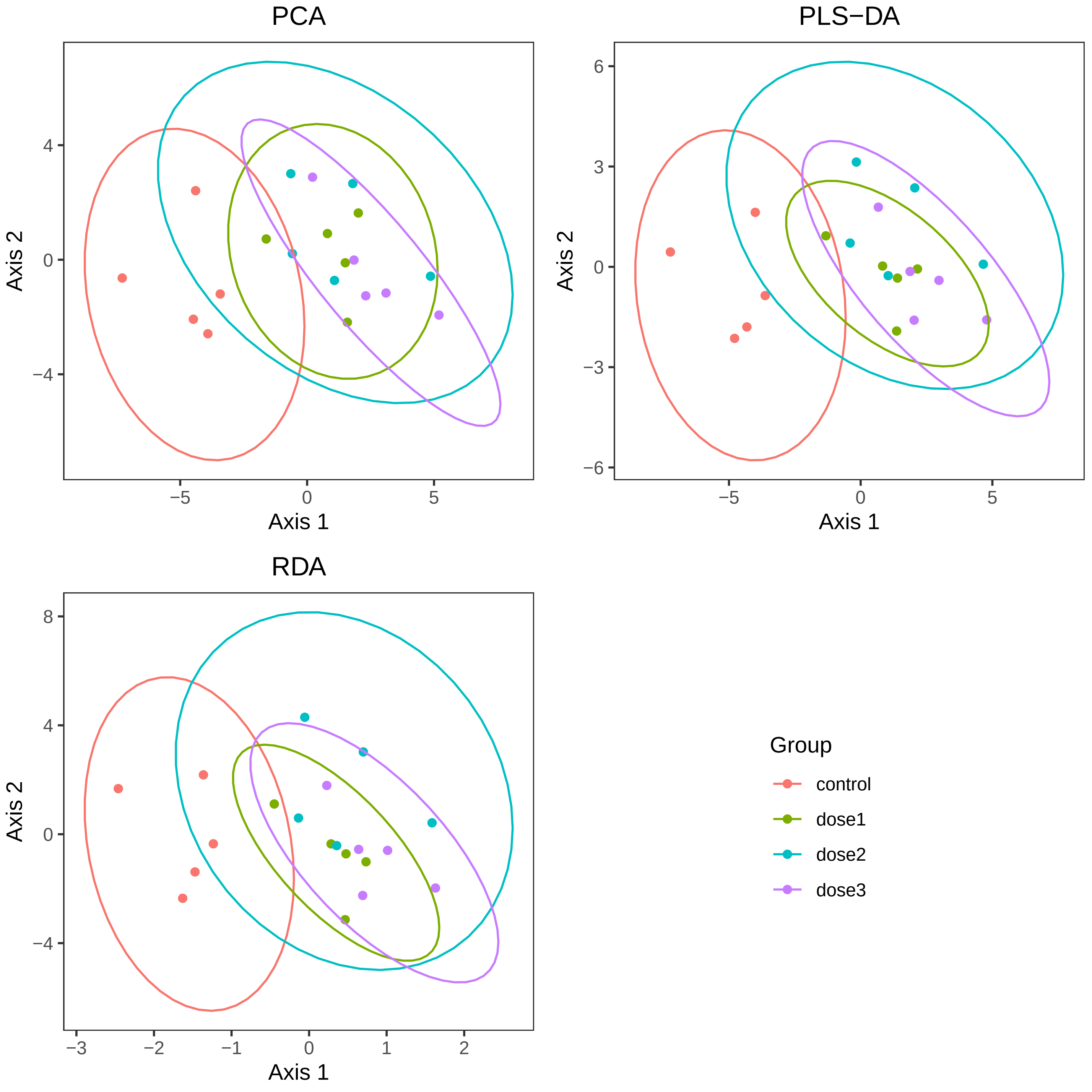
PCA
PLS−DA
RDA
Axis 1
Axis 2
Group
control
dose1
dose2
dose3

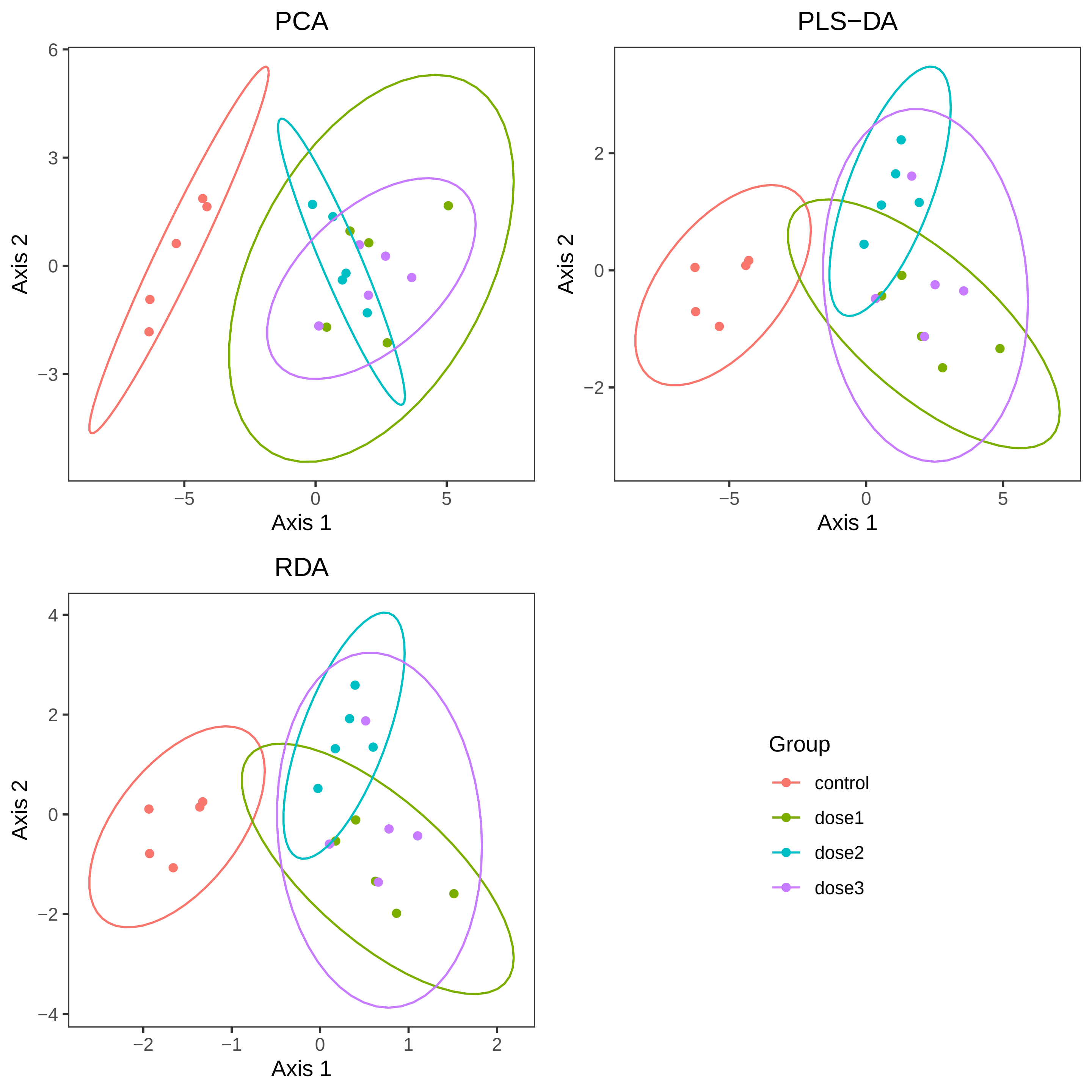

PCA
PLS−DA
RDA
Axis 1
Axis 2
−5
0
5
6
3
0
−3
2
−2
−2
−1
1
4
−4
Group
control
dose1
dose2
dose3

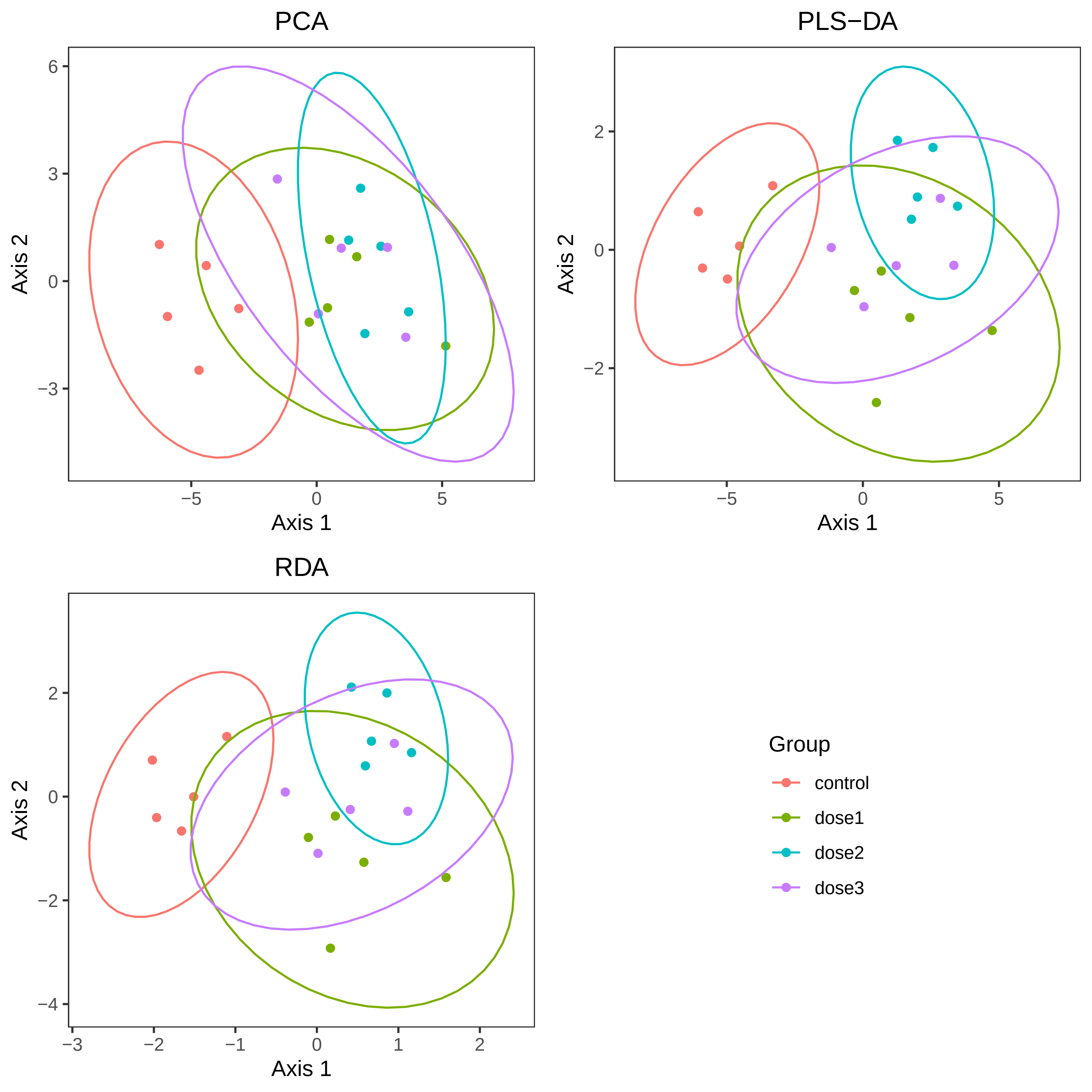
PCA
PLS−DA
RDA
Axis 1
Axis 2
Group
control
dose1
dose2
dose3

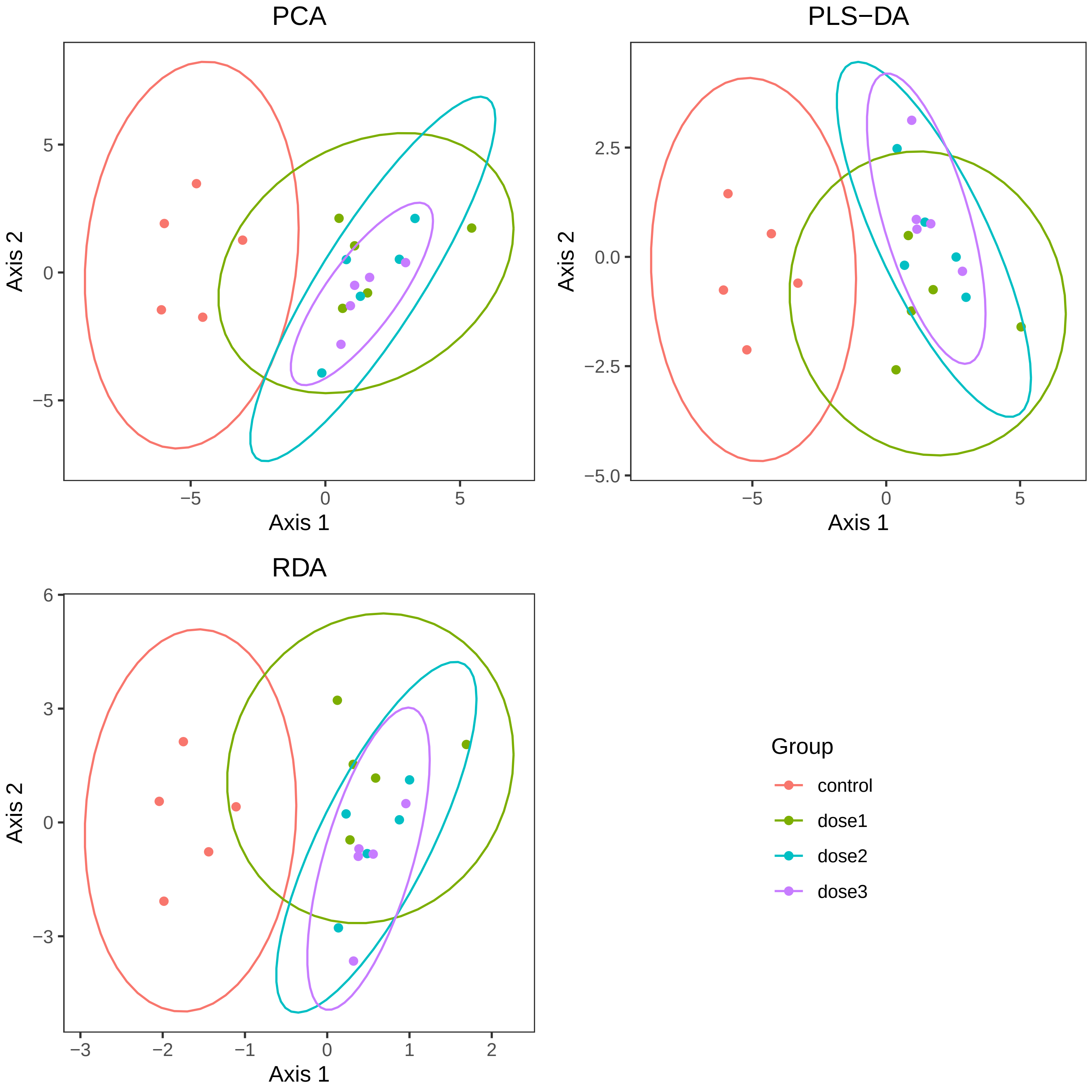

PCA
PLS−DA
RDA
Axis 1
Axis 2
Group
control
dose1
dose2
dose3

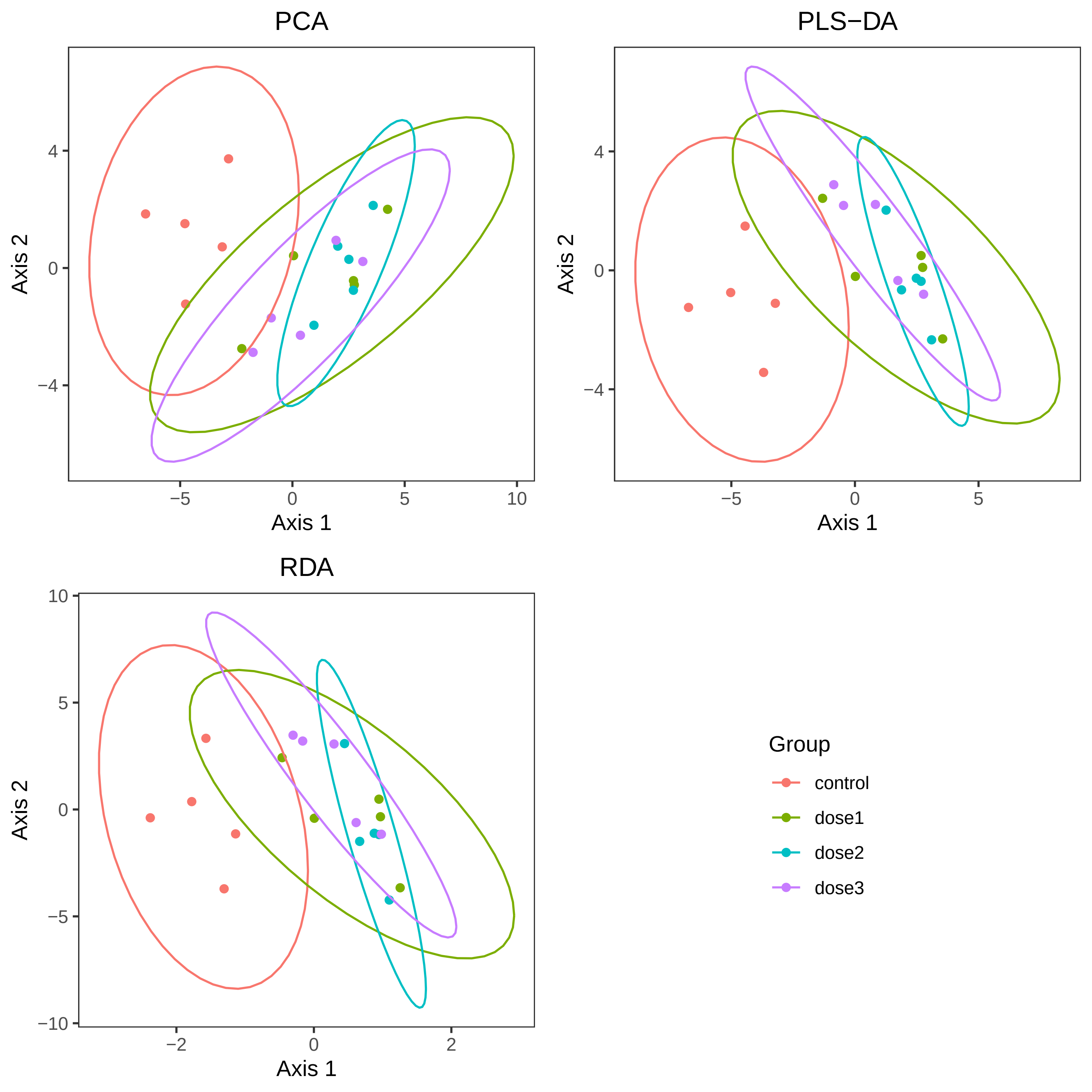

PCA
PLS−DA
RDA
Axis 1
Axis 2
−5
0
5
10
4
0
−4
−2
2
−10
Group
control
dose1
dose2
dose3

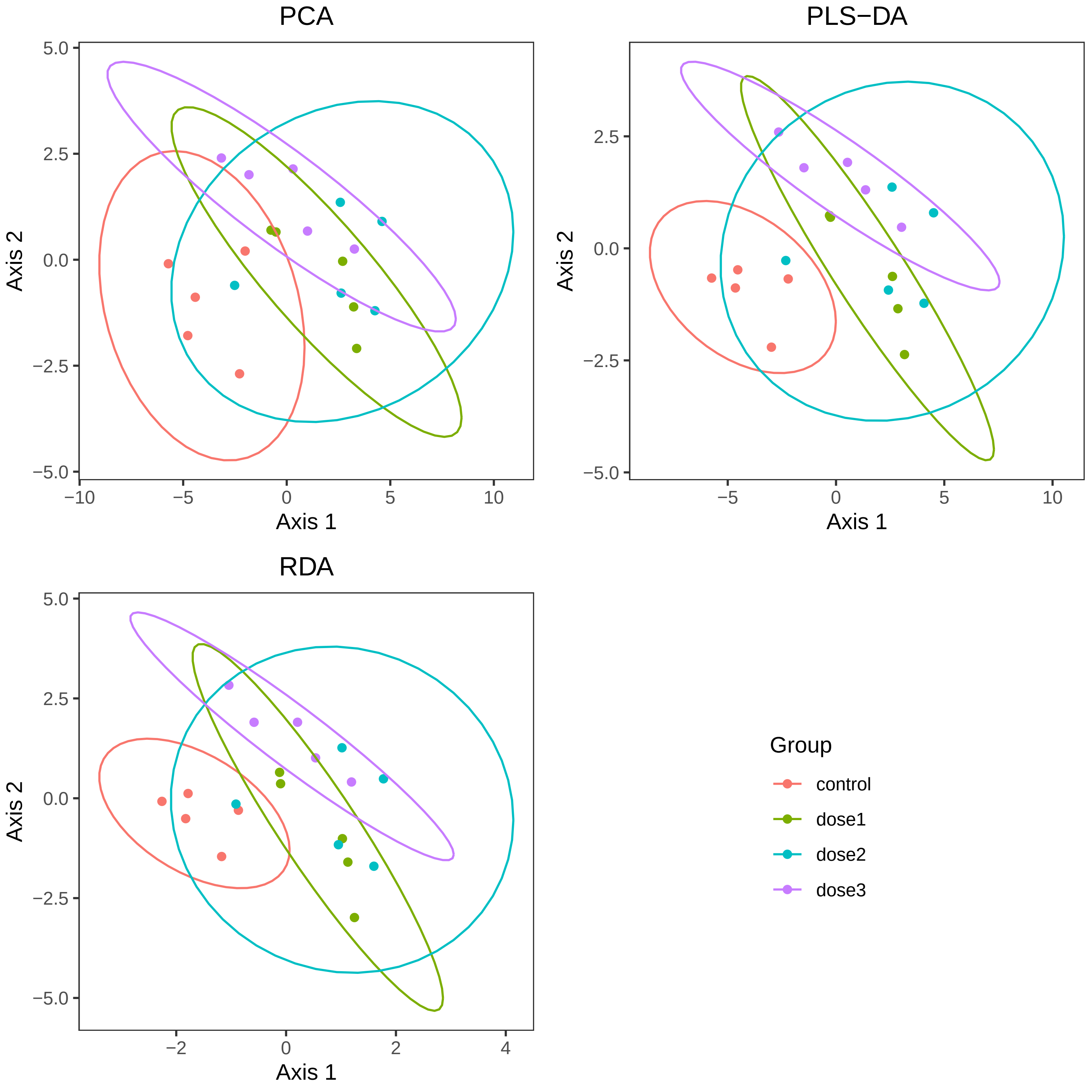

PCA
PLS−DA
RDA
Axis 1
Axis 2
Group
control
dose1
dose2
dose3

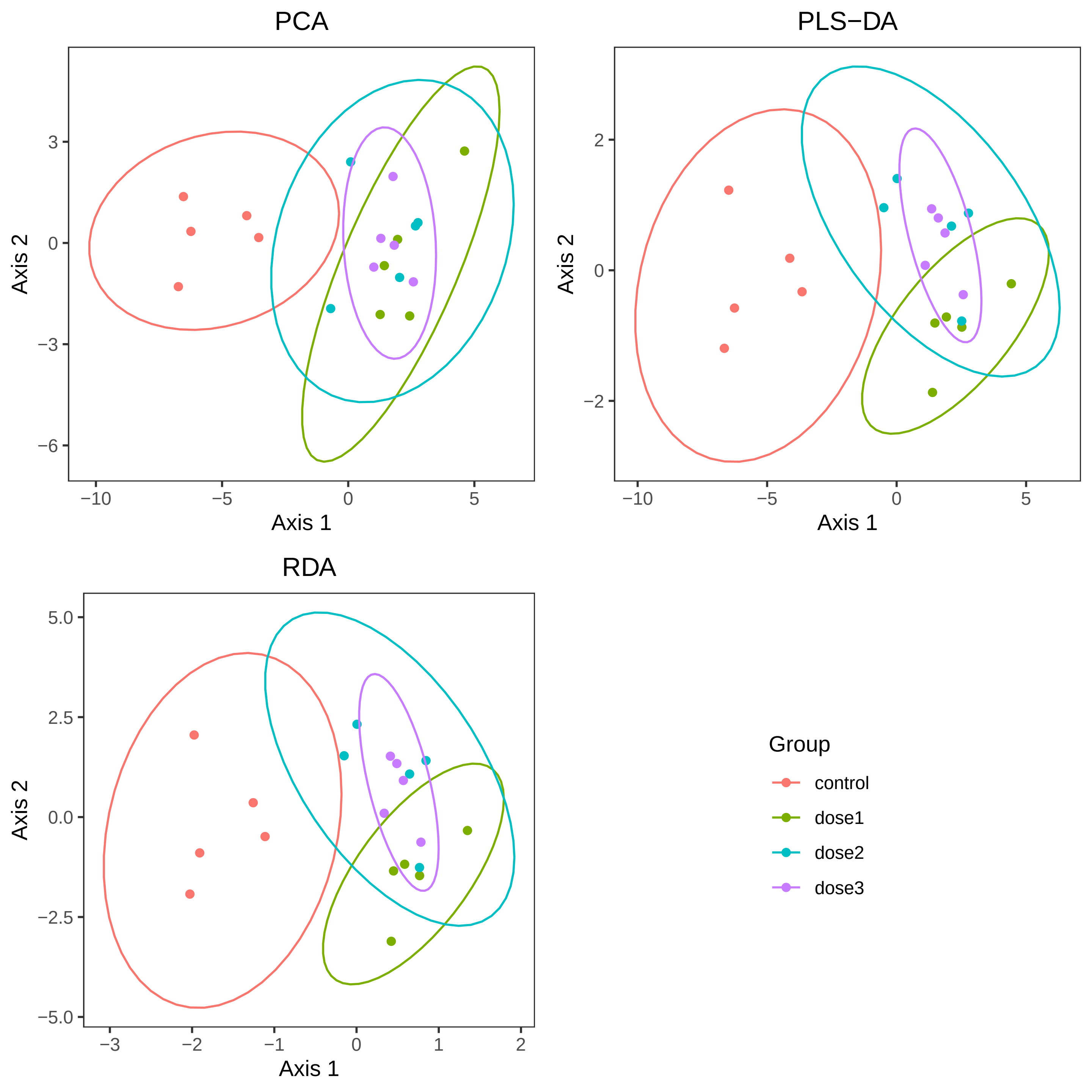
PCA
PLS−DA
RDA
Axis 1
Axis 2
Group
control
dose1
dose2
dose3

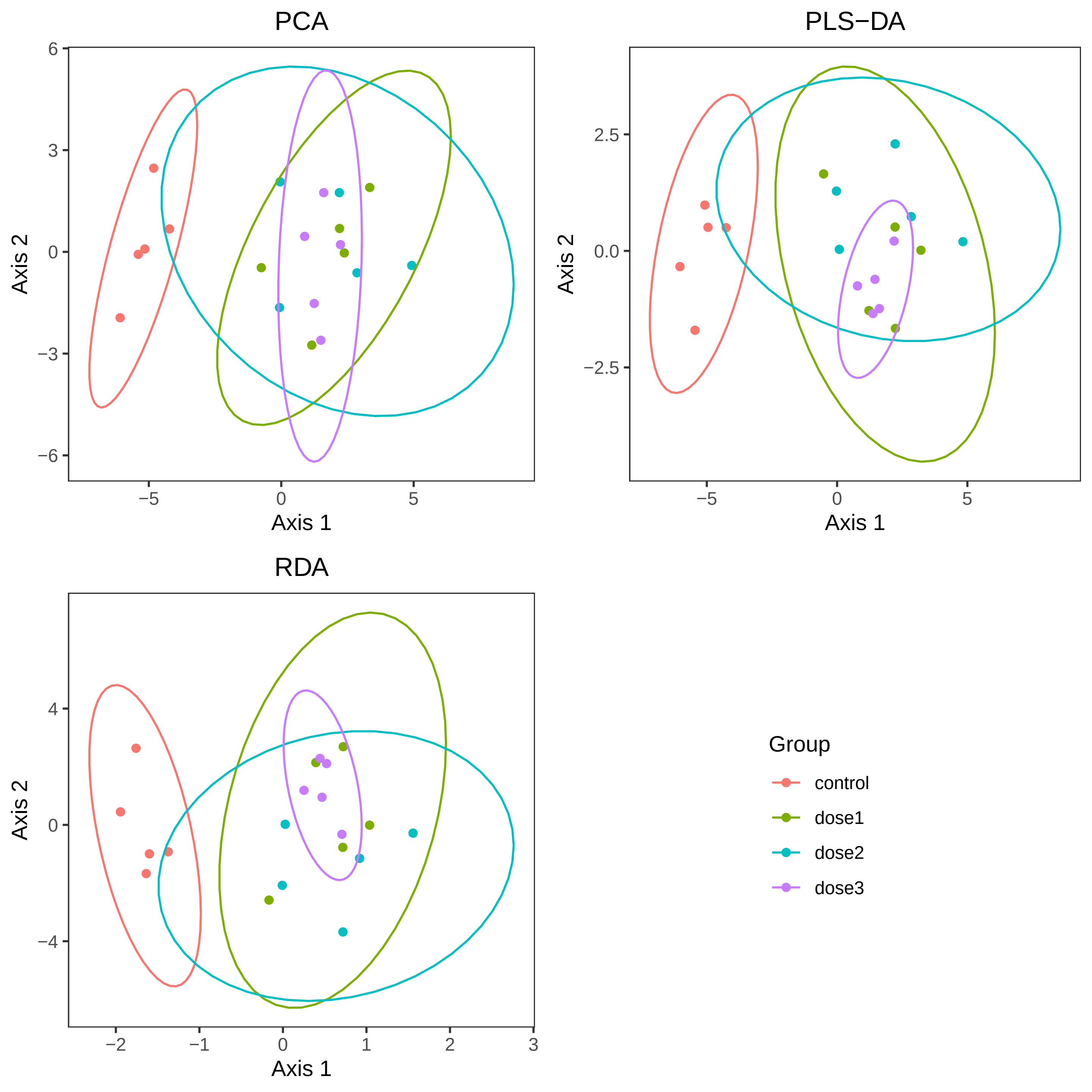
PCA
PLS−DA
RDA
Axis 1
Axis 2
Group
control
dose1
dose2
dose3

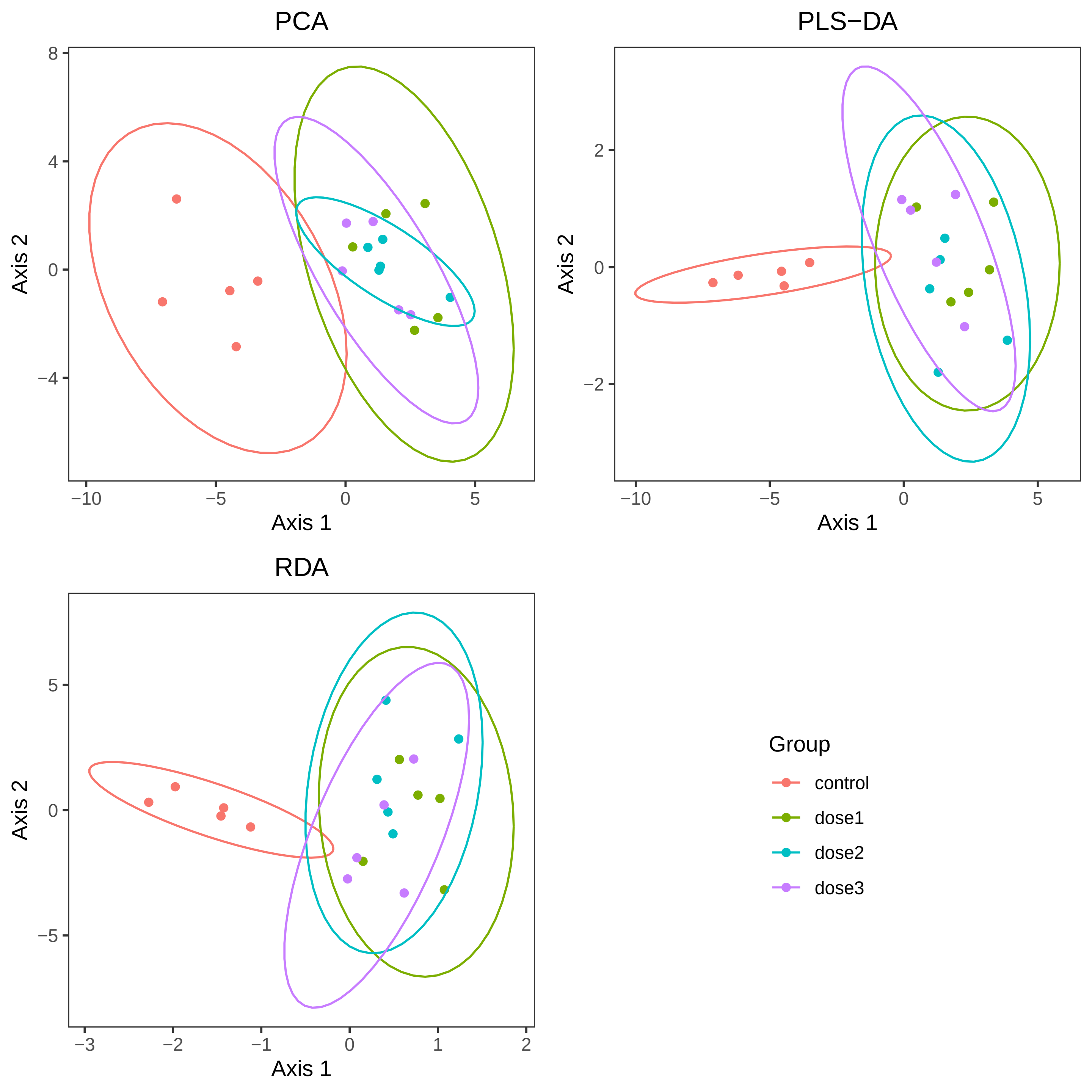
PCA
PLS−DA
RDA
Axis 1
Axis 2
Group
control
dose1
dose2
dose3

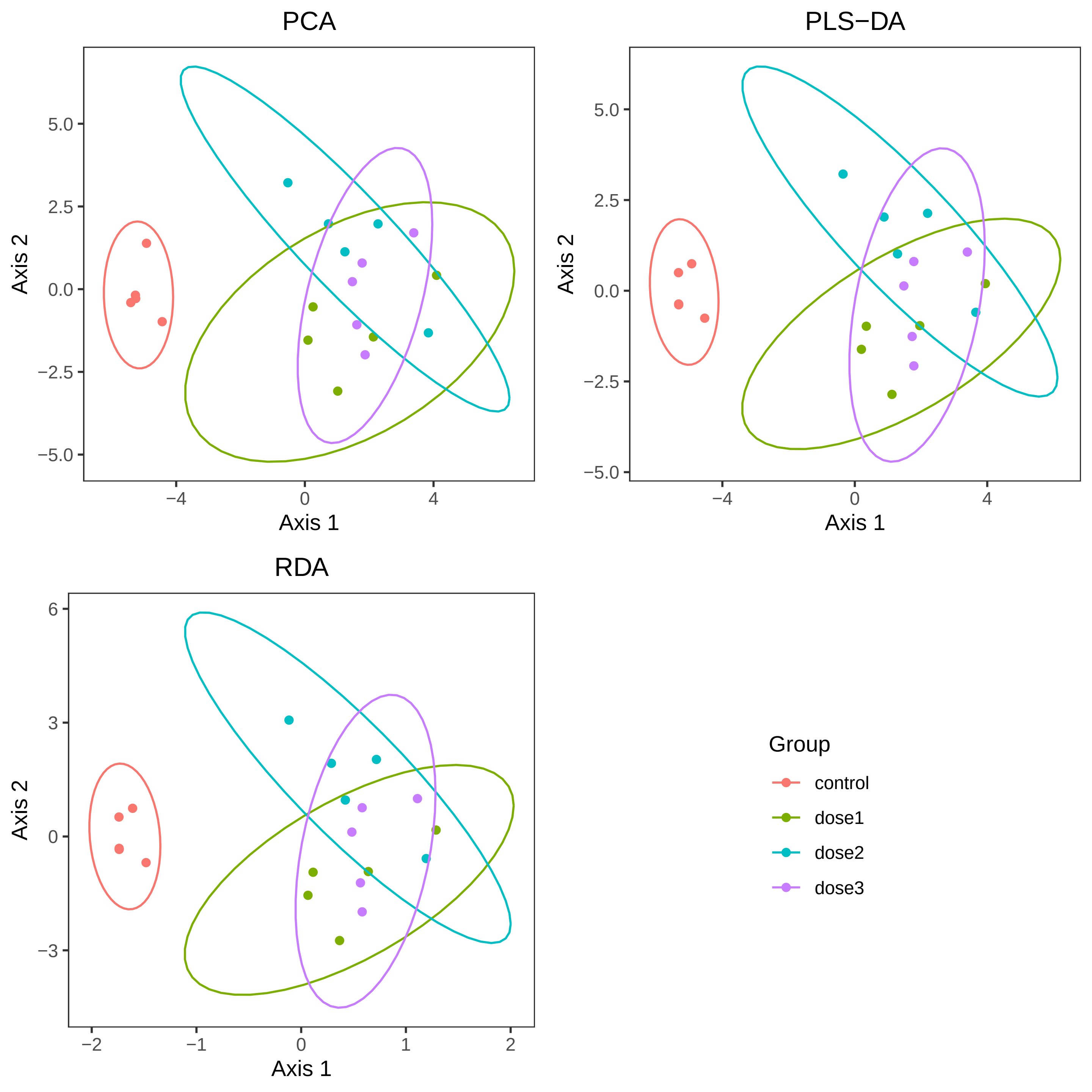
PCA
PLS−DA
RDA
Axis 1
Axis 2
5.0
2.5
0.0
−2.5
−5.0
−4
0
4
6
3
0
−3
−2
−1
1
2
Group
control
dose1
dose2
dose3

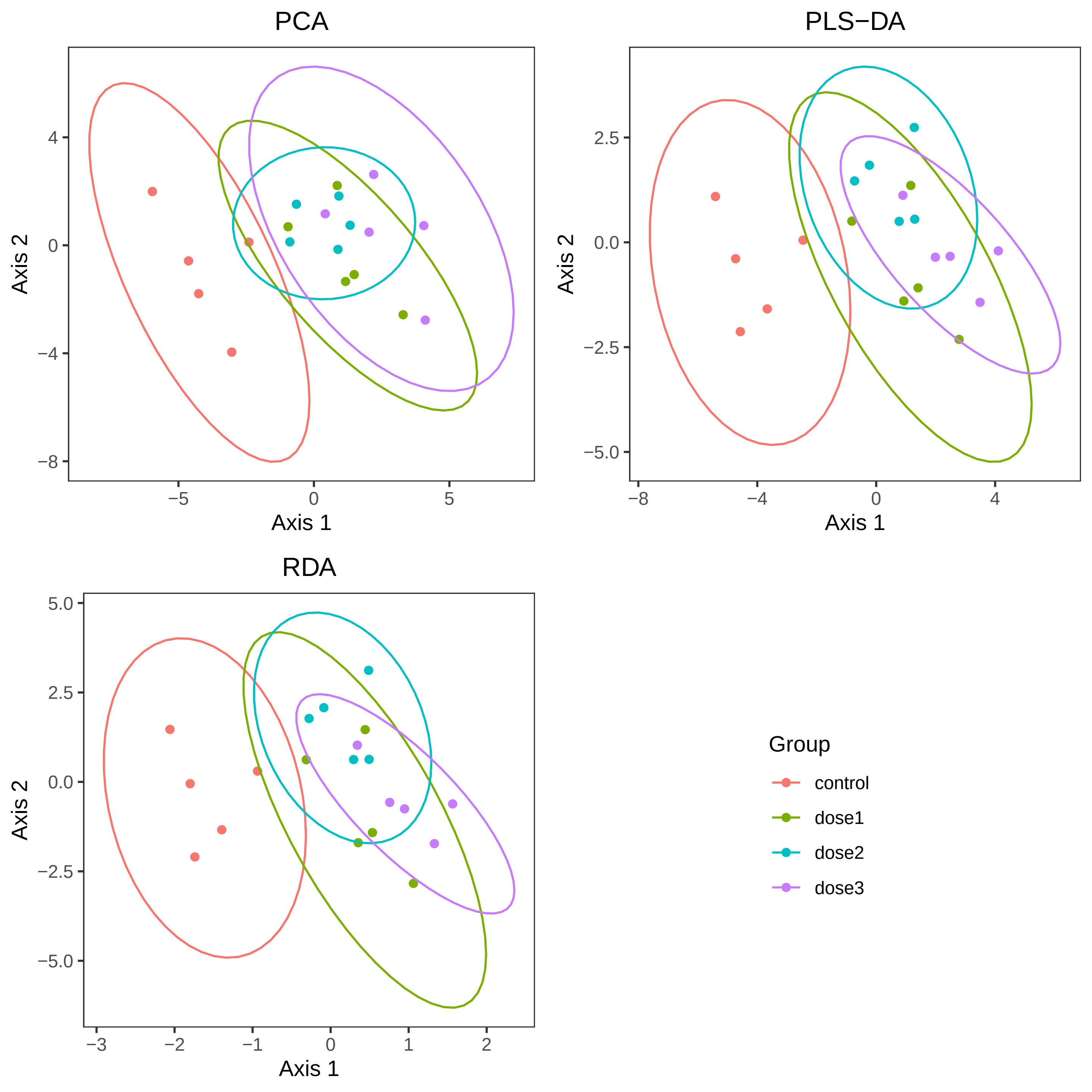

PCA
PLS−DA
RDA
Axis 1
Axis 2
Group
control
dose1
dose2
dose3

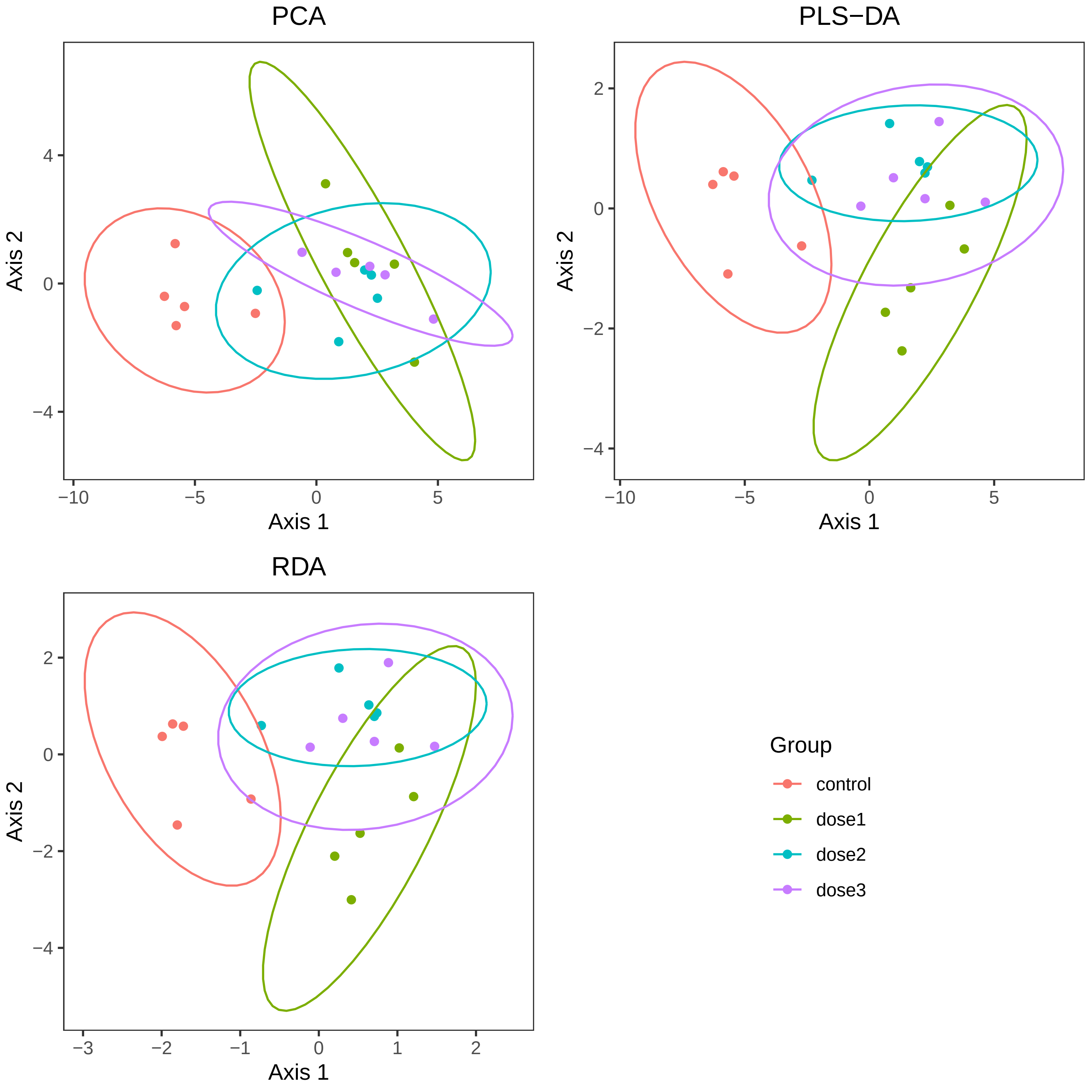

PCA
PLS−DA
RDA
Axis 1
Axis 2
−10
−5
0
5
4
0
−4
−3
−2
−1
1
2
Group
control
dose1
dose2
dose3

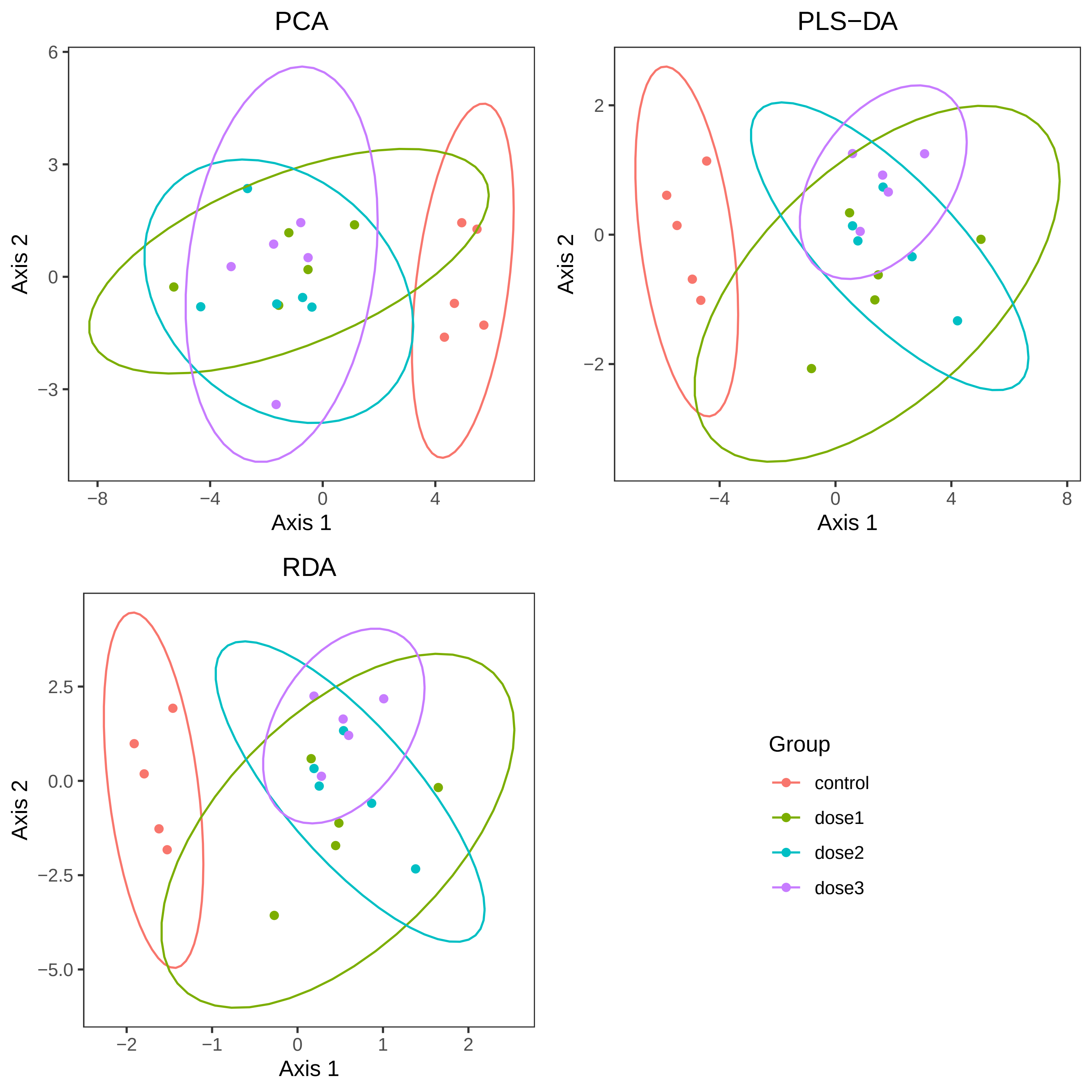
PCA
PLS−DA
RDA
Axis 1
Axis 2
6
3
0
−3
−8
−4
0
4
2
0
−2
−4
0
4
8
2.5
0.0
−2.5
−5.0
−2
−1
0
1
2
Group
control
dose1
dose2
dose3

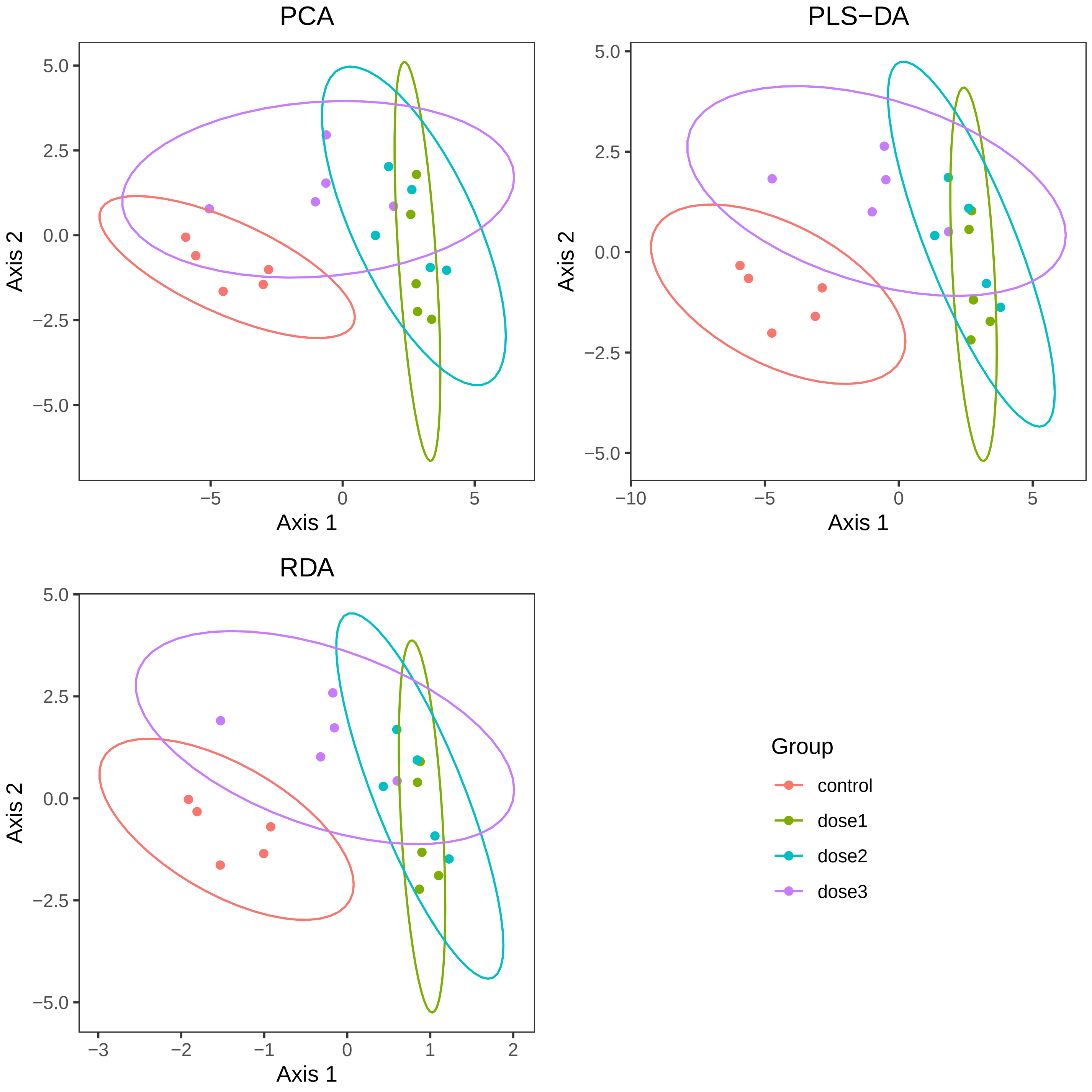
PCA
PLS−DA
RDA
Axis 1
Axis 2
5.0
2.5
0.0
−2.5
−5.0
−10
−5
0
5
−3
−2
−1
1
2
Group
control
dose1
dose2
dose3

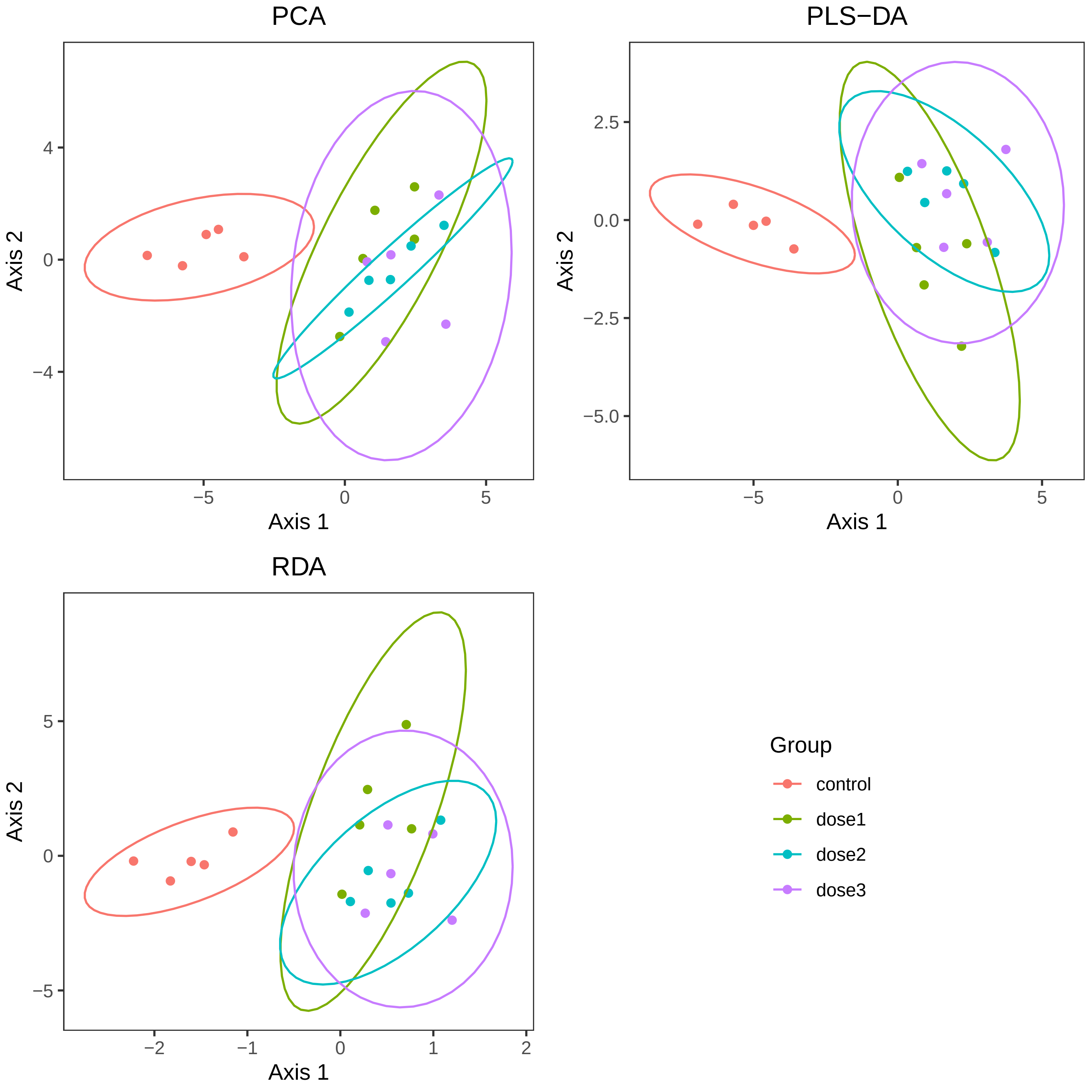
PCA
Axis 2
4
0
−4
−5
0
5
Axis 1
PLS−DA
Axis 2
2.5
0.0
−2.5
−5.0
−5
0
5
Axis 1
RDA
Axis 2
5
0
−5
−2
−1
0
1
2
Axis 1
Group
control
dose1
dose2
dose3

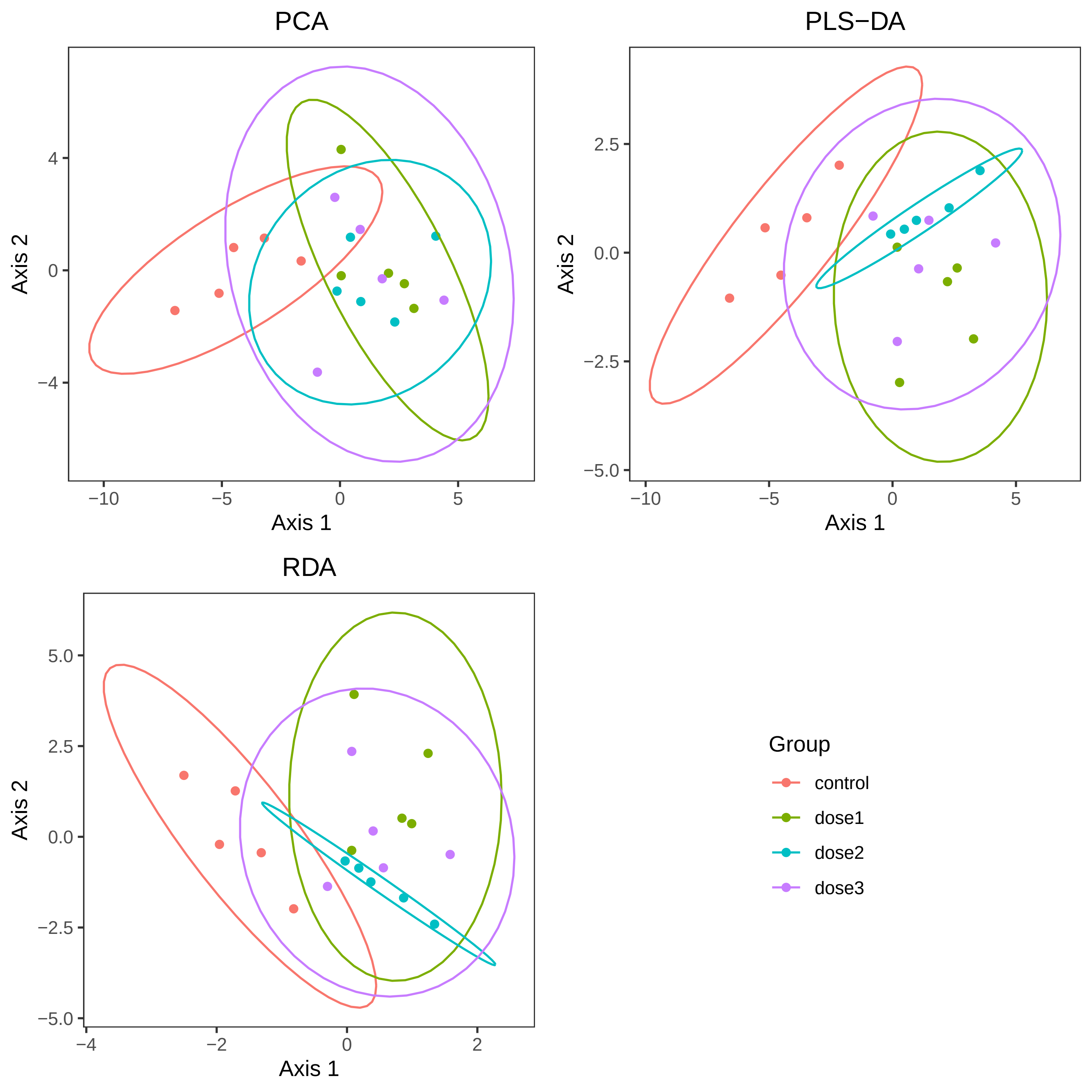

PCA
Axis 2
Axis 1
4
0
−4
−10
−5
0
5
PLS−DA
Axis 2
Axis 1
2.5
0.0
−2.5
−5.0
−10
−5
0
5
RDA
Axis 2
Axis 1
5.0
2.5
0.0
−2.5
−5.0
−4
−2
0
2
Group
control
dose1
dose2
dose3

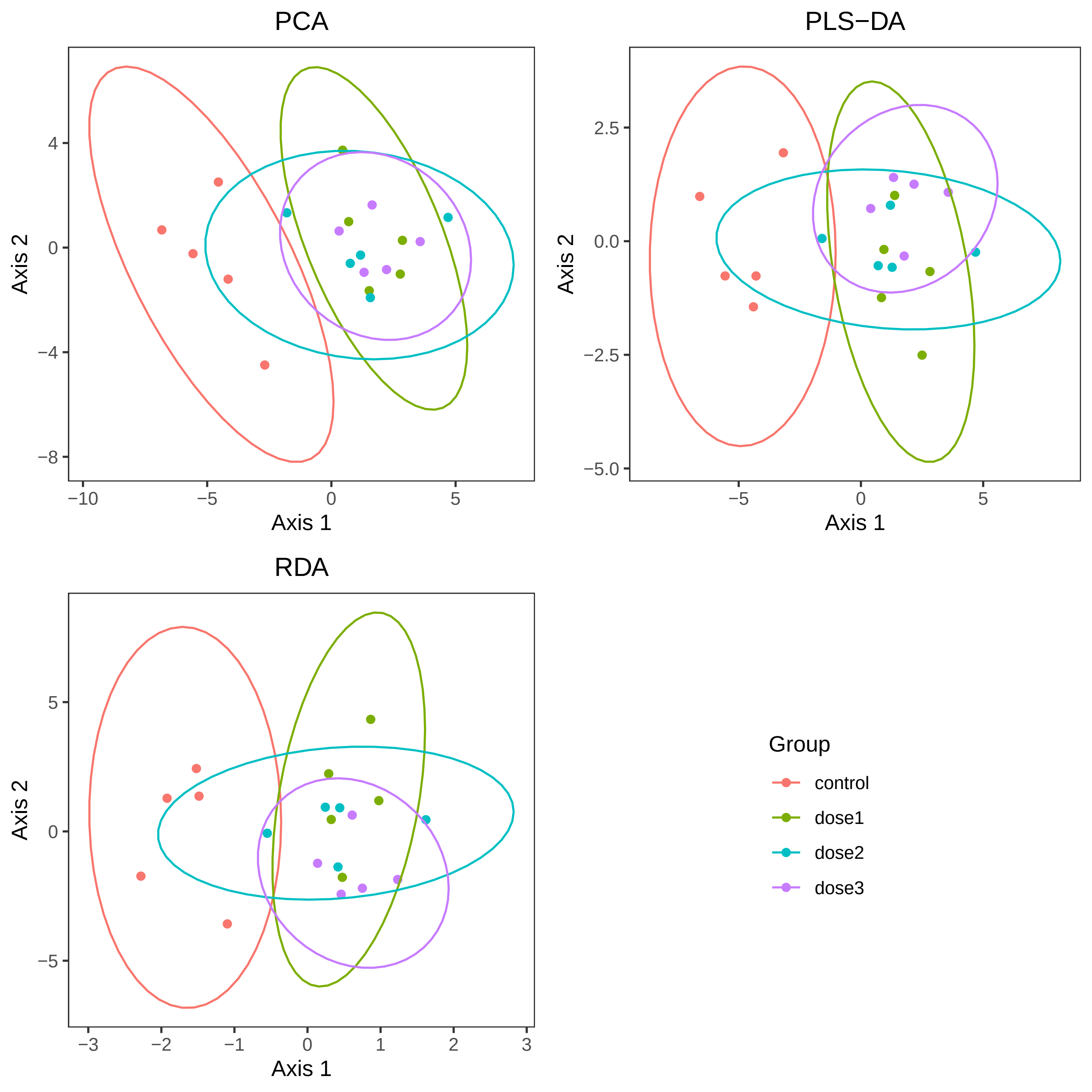

PCA
PLS−DA
RDA
Axis 1
Axis 2
Group
control
dose1
dose2
dose3

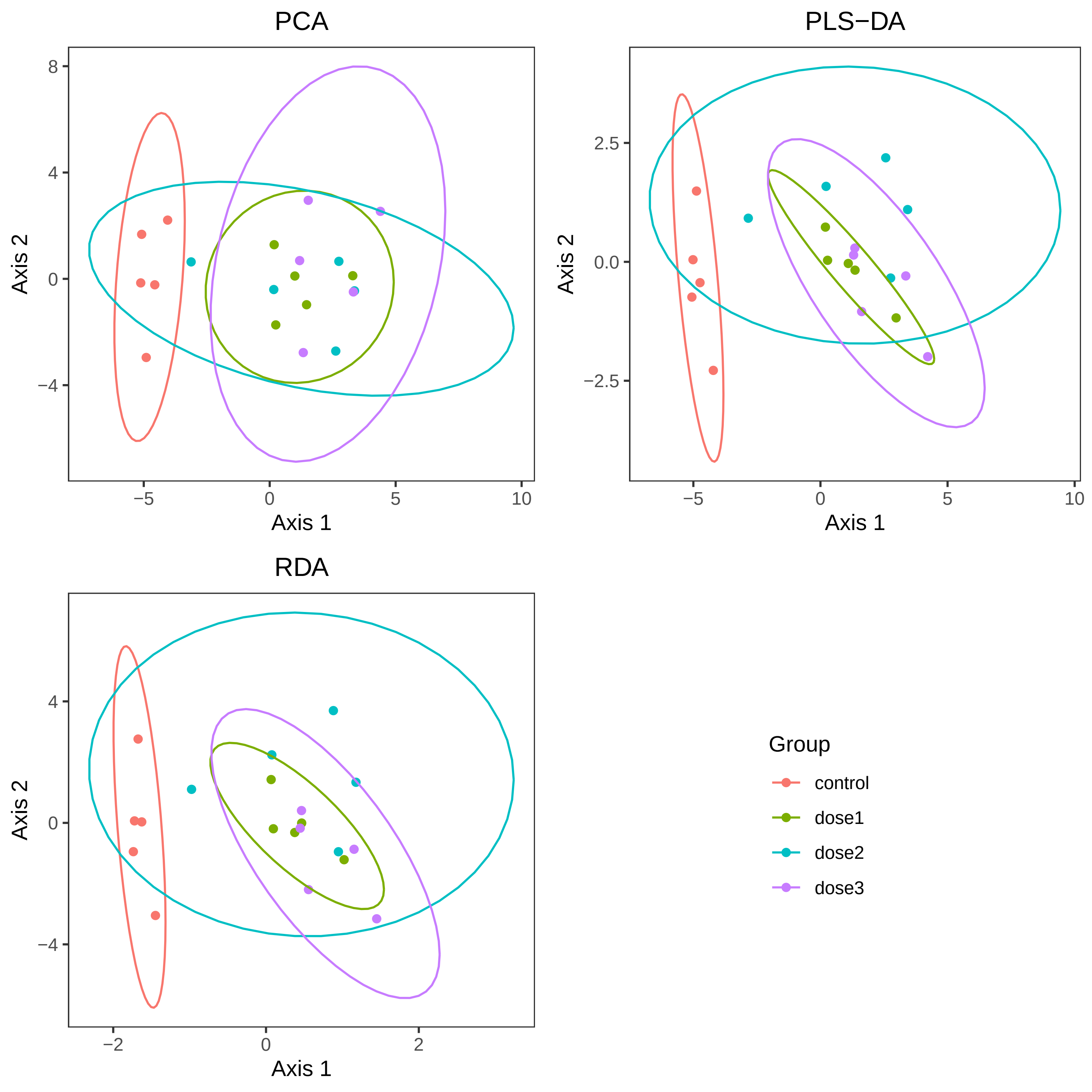

PCA
PLS−DA
RDA
Axis 1
Axis 2
8
4
0
−4
−5
0
5
10
2.5
0.0
−2.5
−2
2
Group
control
dose1
dose2
dose3

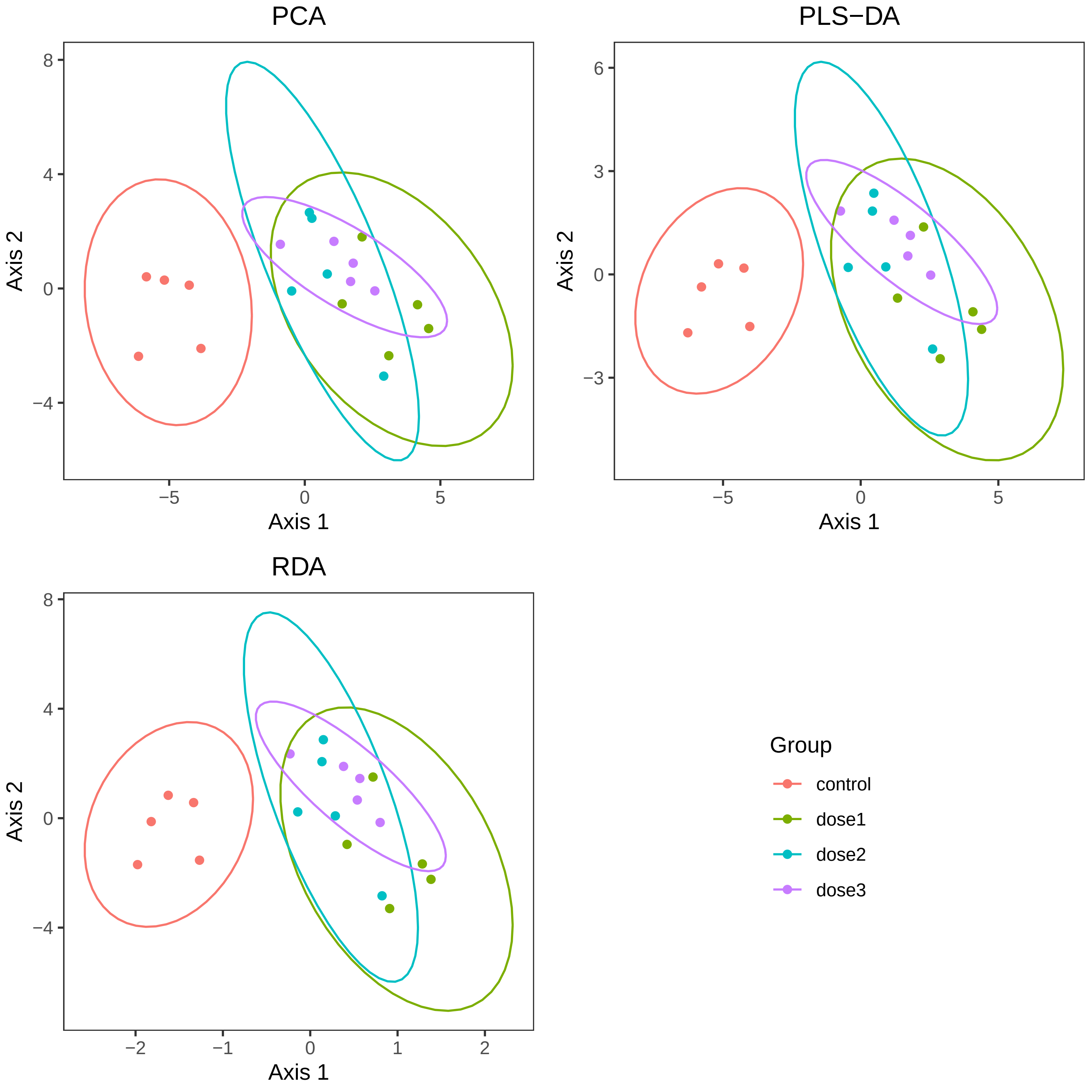

PCA
PLS−DA
RDA
Axis 1
Axis 2
Group
control
dose1
dose2
dose3

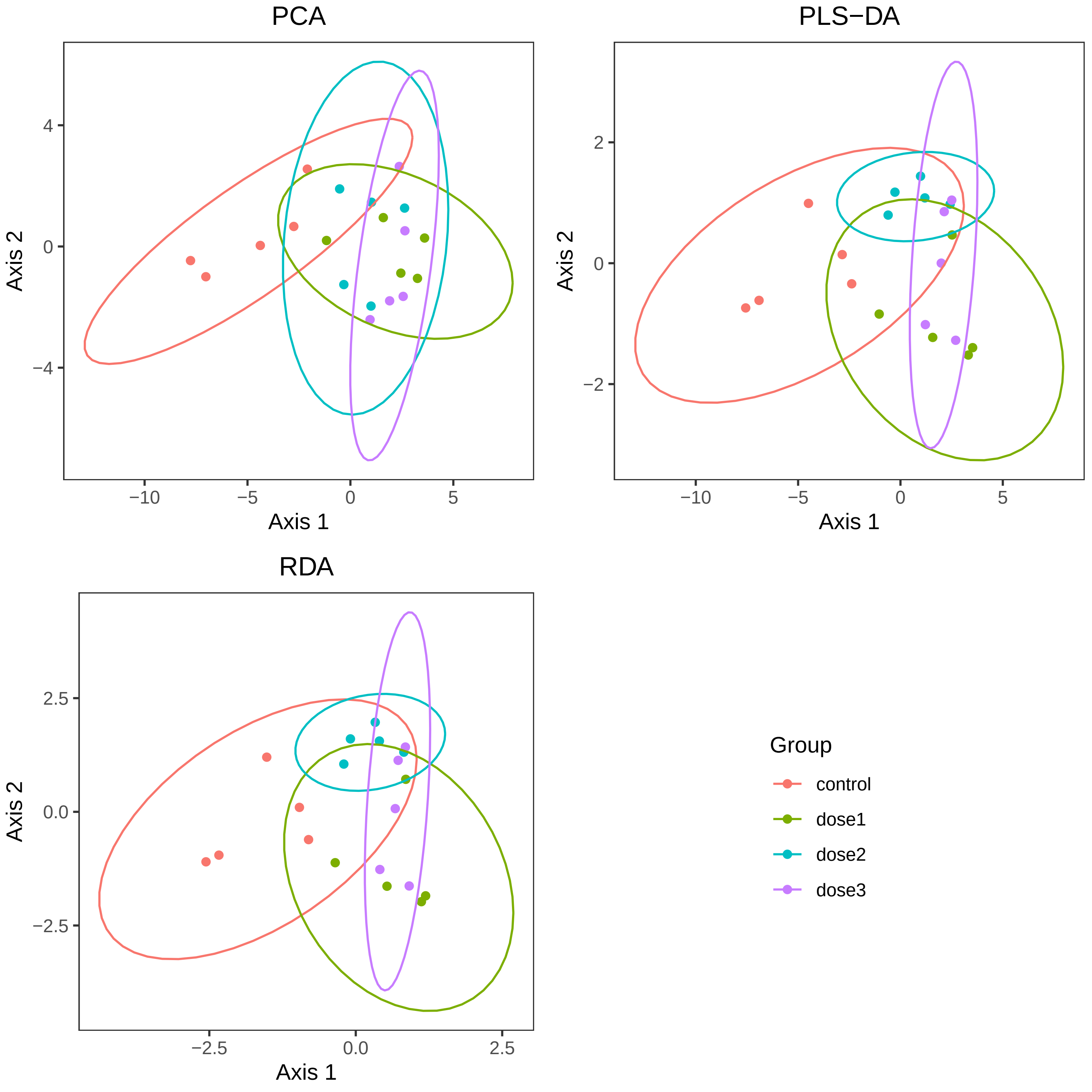
PCA
PLS−DA
RDA
Axis 1
Axis 2
Group
control
dose1
dose2
dose3

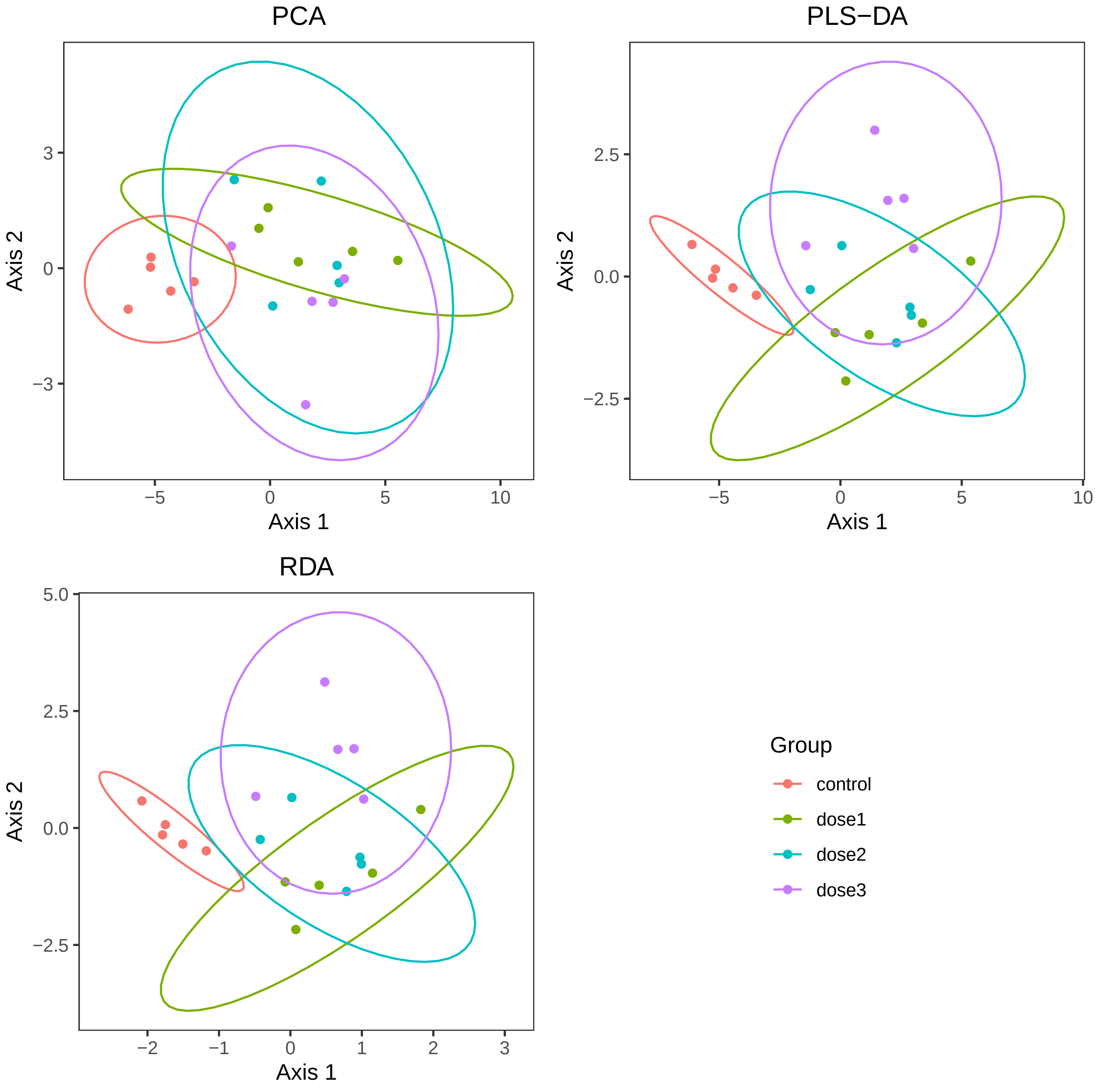

PCA
PLS−DA
RDA
Axis 1
Axis 2
3
0
−3
−5
0
5
10
2.5
0.0
−2.5
5.0
−2
−1
1
2
Group
control
dose1
dose2
dose3

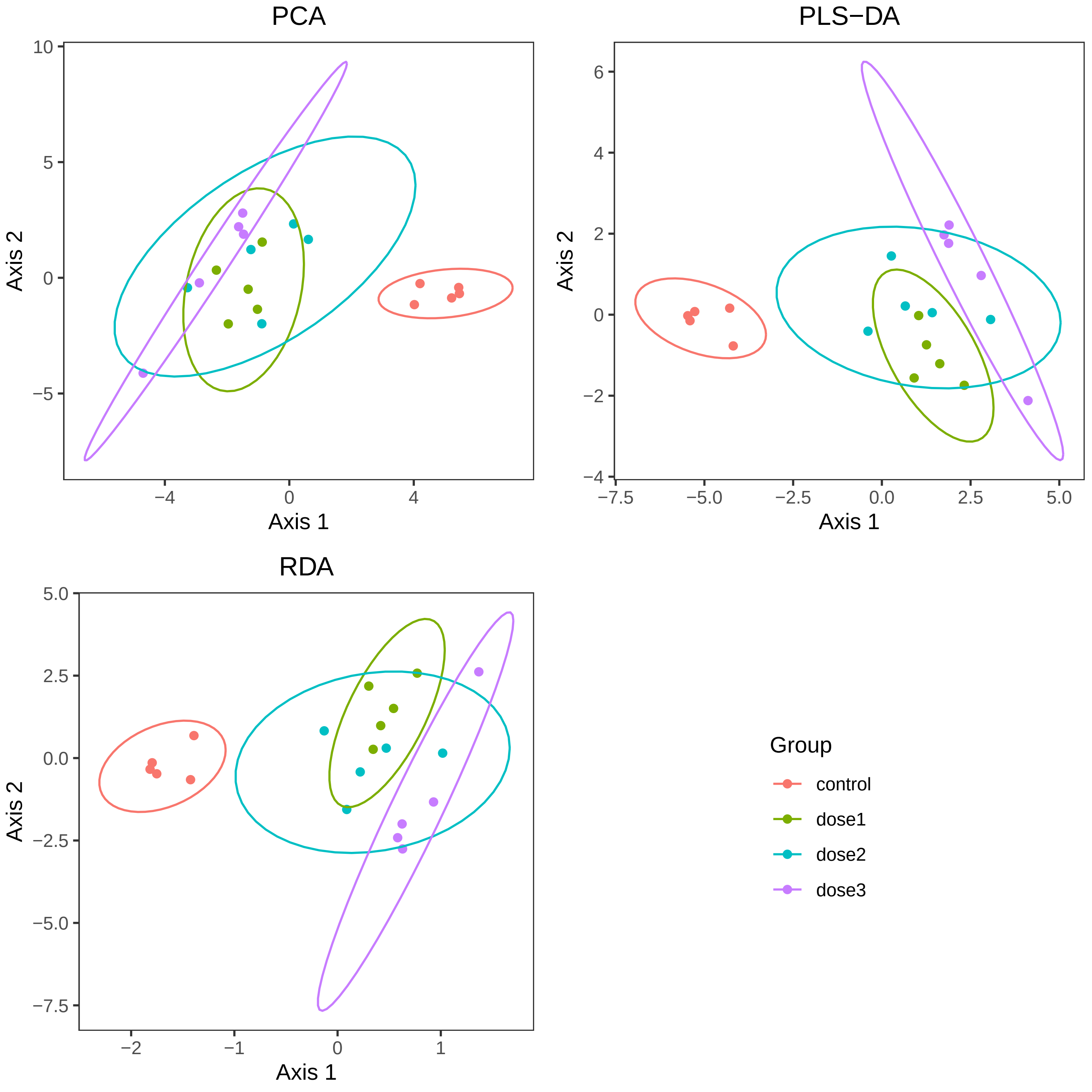

PCA
PLS−DA
RDA
Axis 1
Axis 2
Group
control
dose1
dose2
dose3

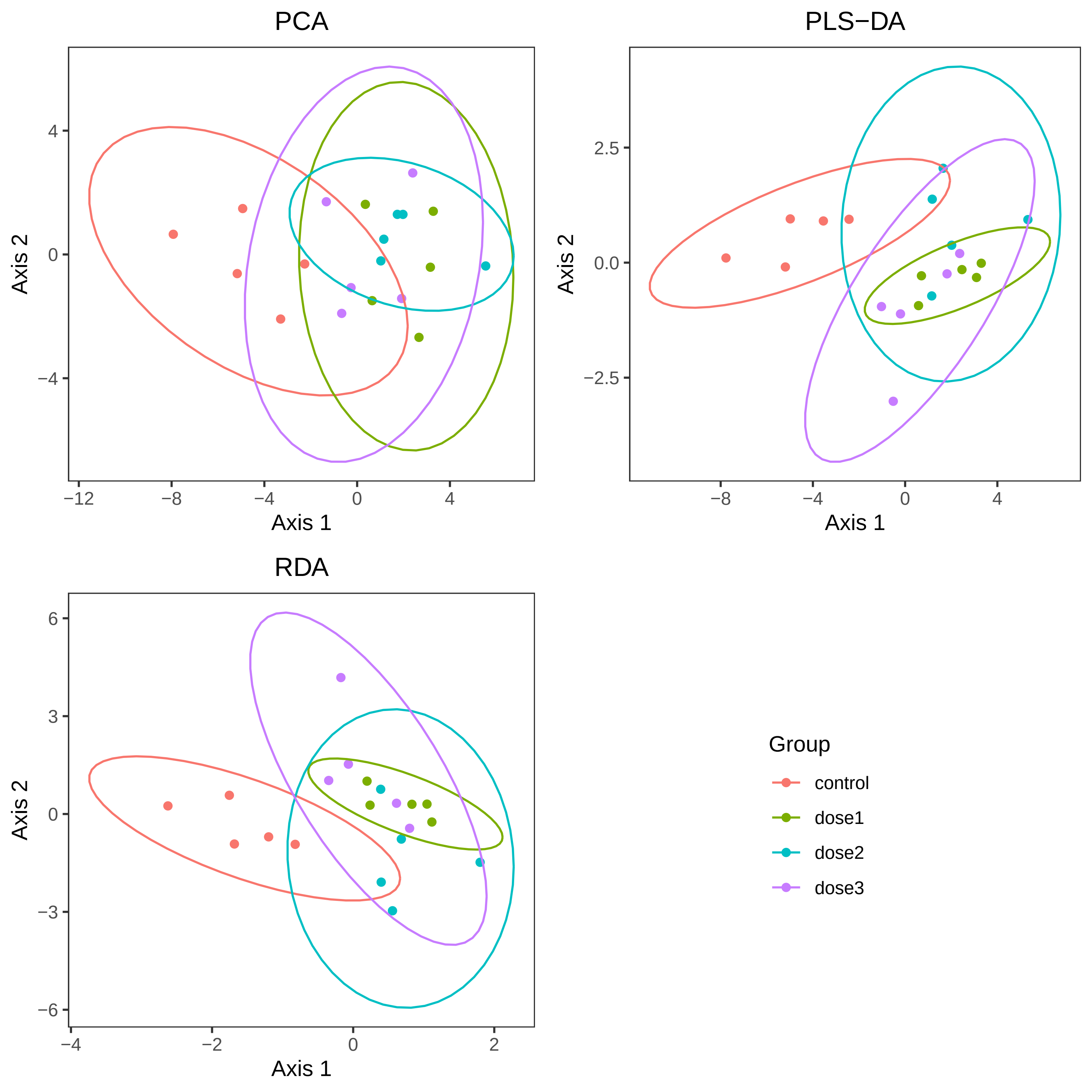
PCA
PLS−DA
RDA
Axis 1
Axis 2
4
0
−4
−12
−8
−4
0
4
2.5
0.0
−2.5
−8
−4
0
4
6
3
0
−3
−6
−4
−2
0
2
Group
control
dose1
dose2
dose3

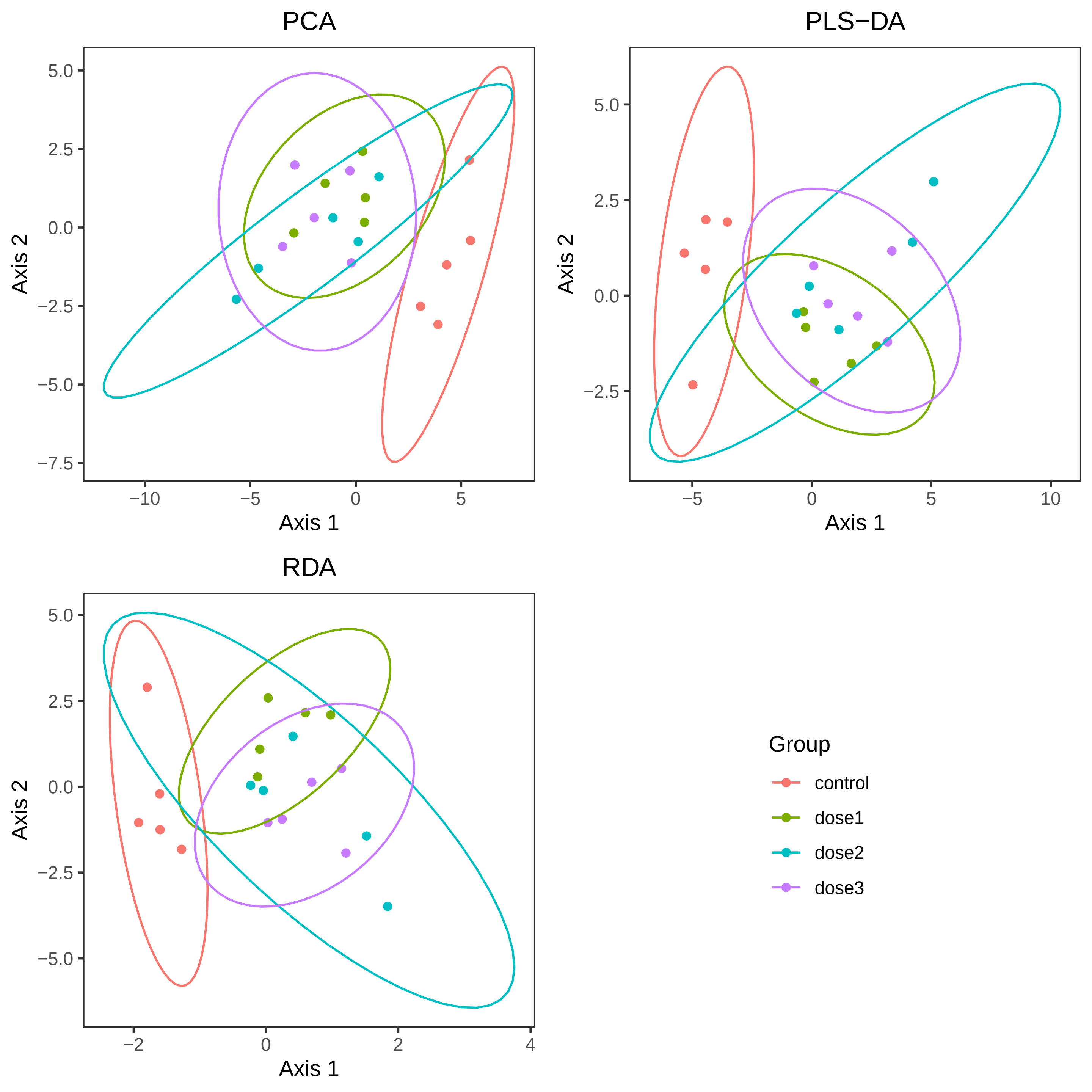
PCA
PLS−DA
RDA
Axis 1
Axis 2
Group
control
dose1
dose2
dose3

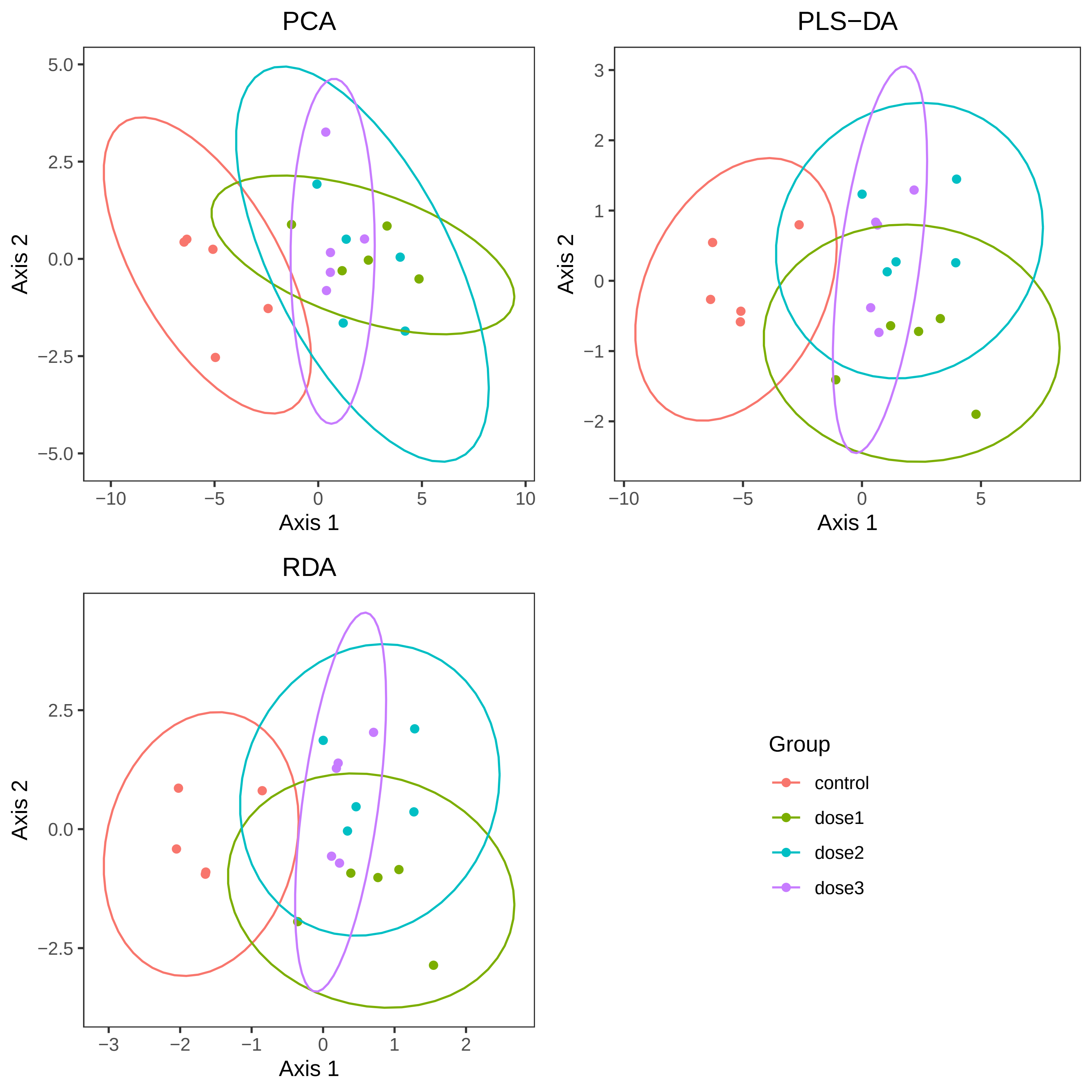

PCA
PLS−DA
RDA
Axis 1
Axis 2
Group
control
dose1
dose2
dose3

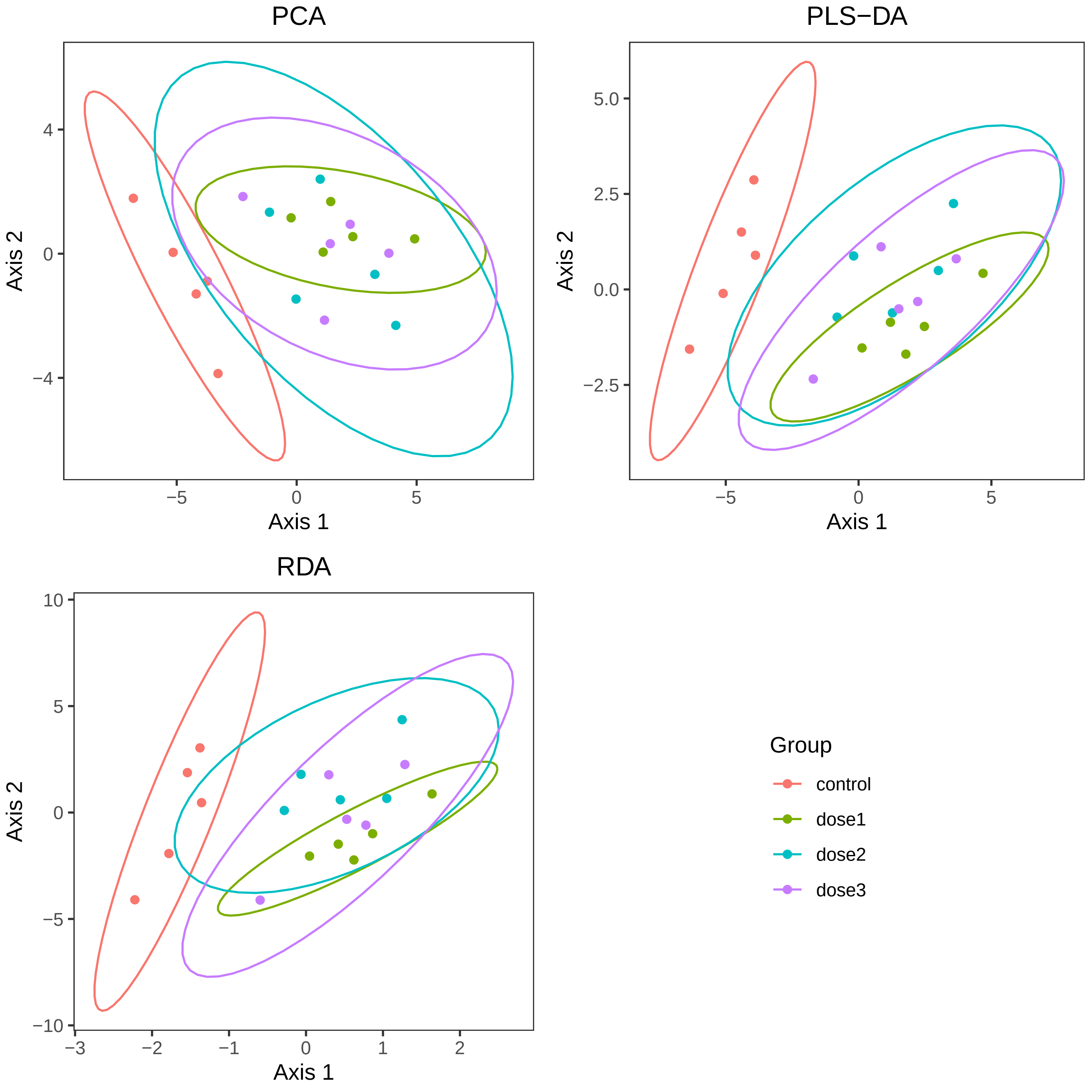
PCA
PLS−DA
RDA
Axis 1
Axis 2
Group
control
dose1
dose2
dose3

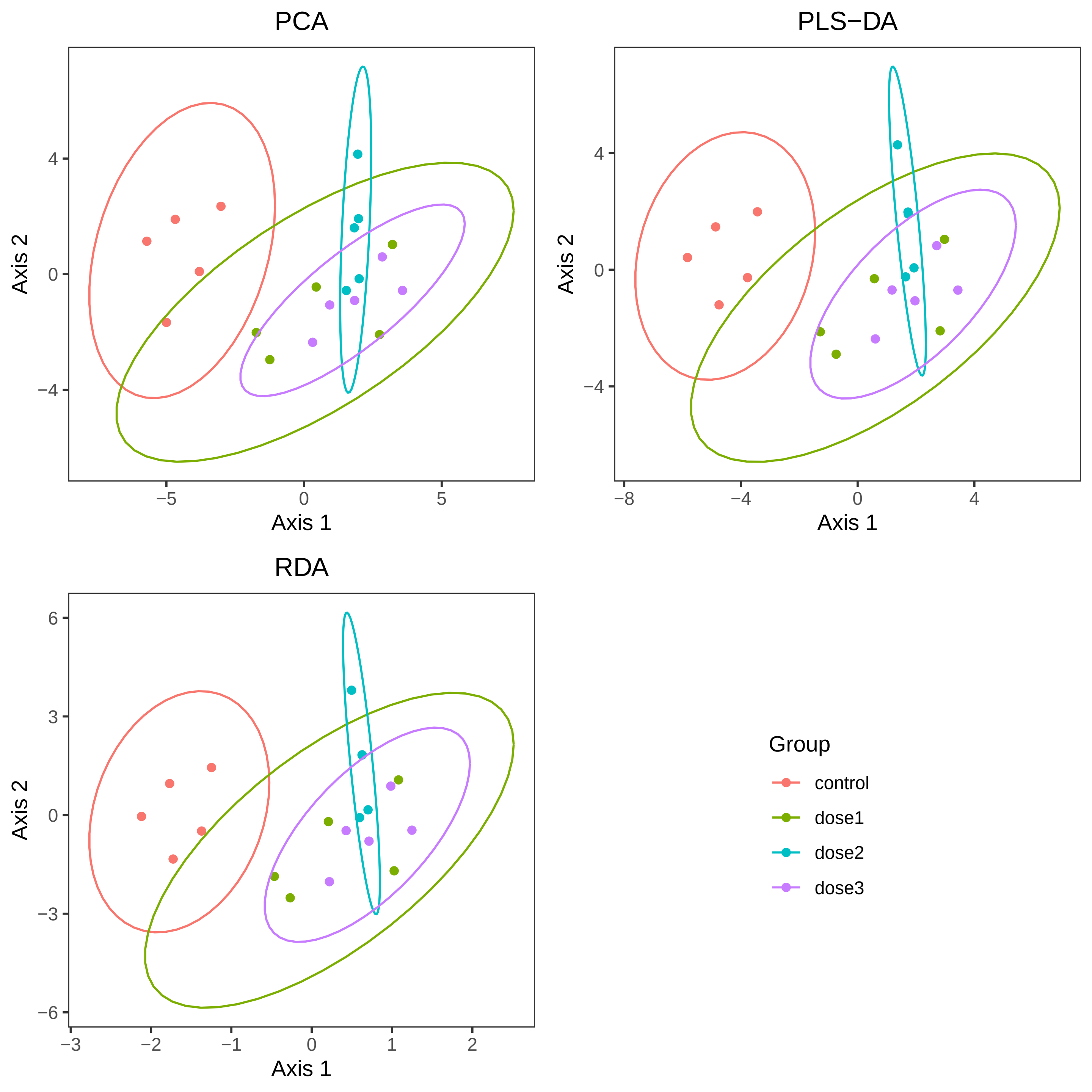
PCA
PLS−DA
RDA
Axis 1
Axis 2
Group
control
dose1
dose2
dose3

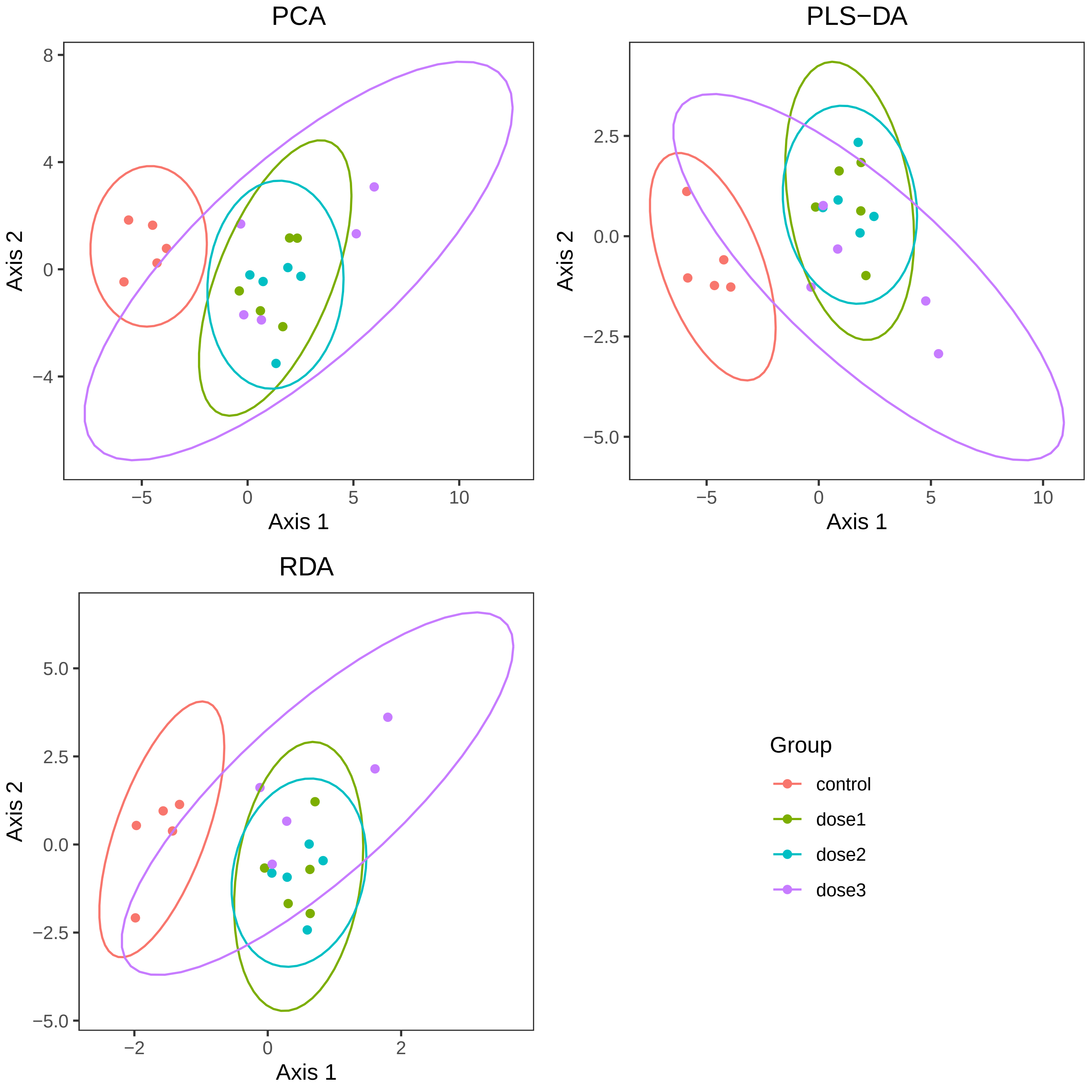

PCA
PLS−DA
RDA
Axis 1
Axis 2
8
4
0
−4
−5
0
5
10
2.5
0.0
−2.5
−5.0
5.0
−2
2
Group
control
dose1
dose2
dose3

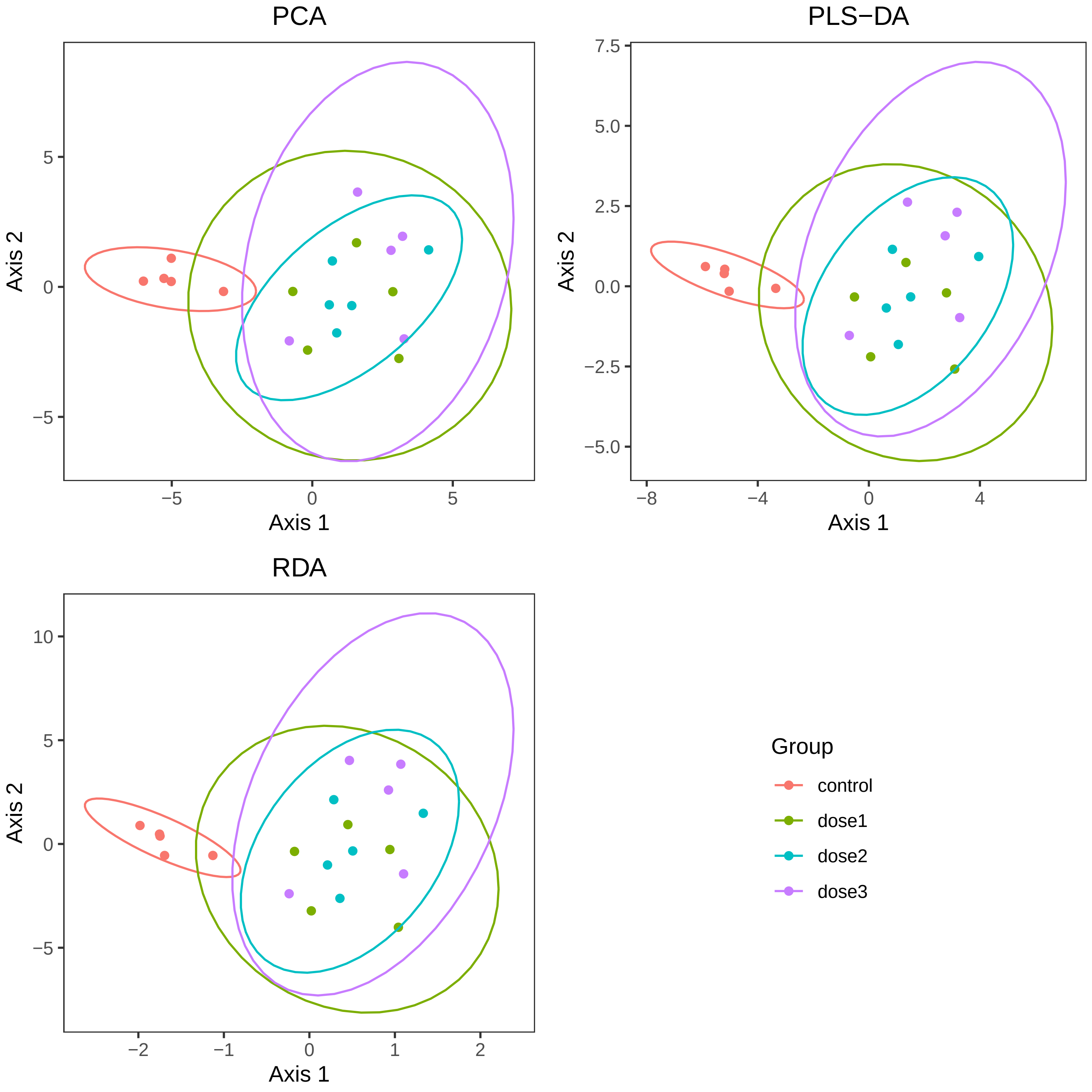
PCA
PLS−DA
RDA
Axis 1
Axis 2
Group
control
dose1
dose2
dose3

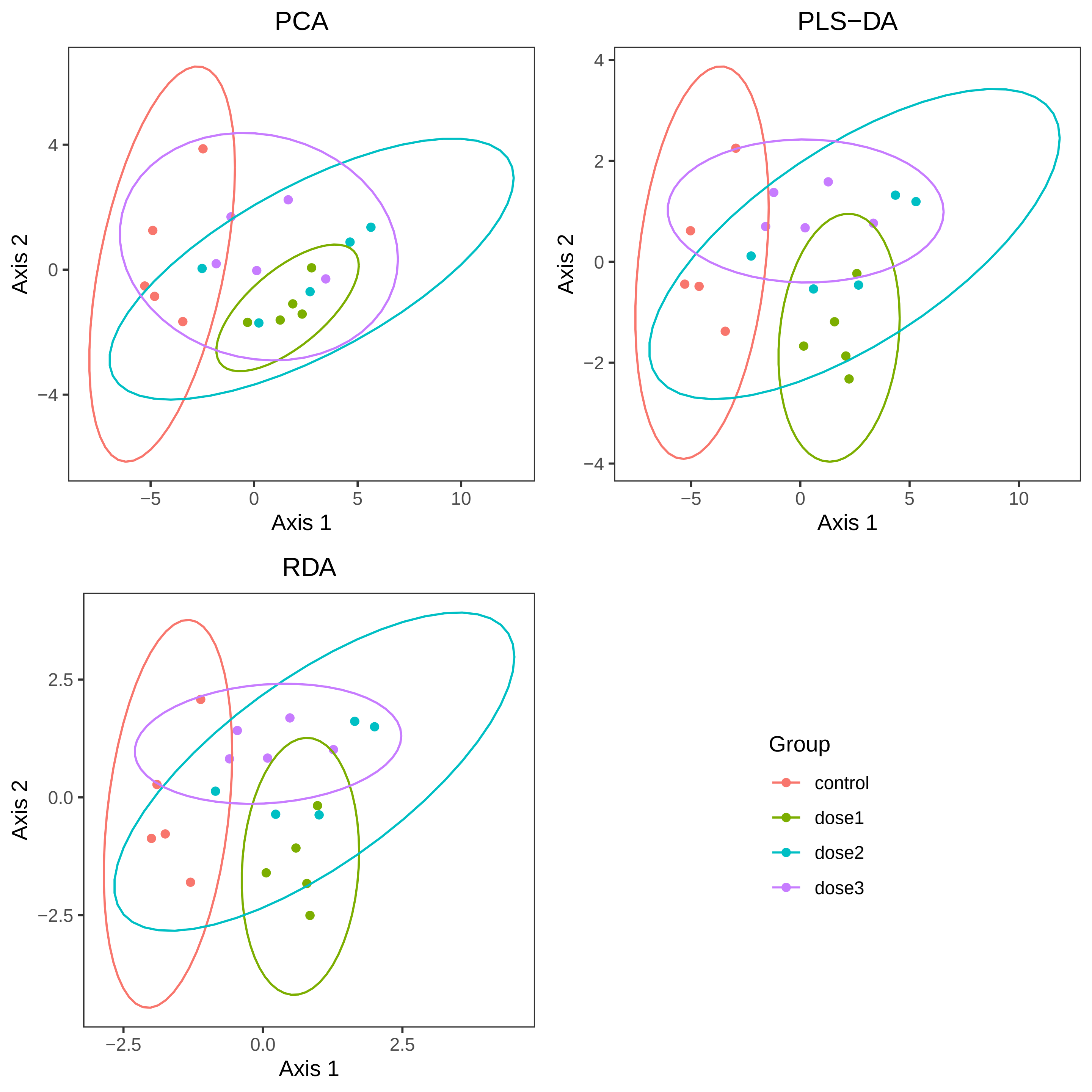
PCA
PLS−DA
RDA
Axis 1
Axis 2
Group
control
dose1
dose2
dose3

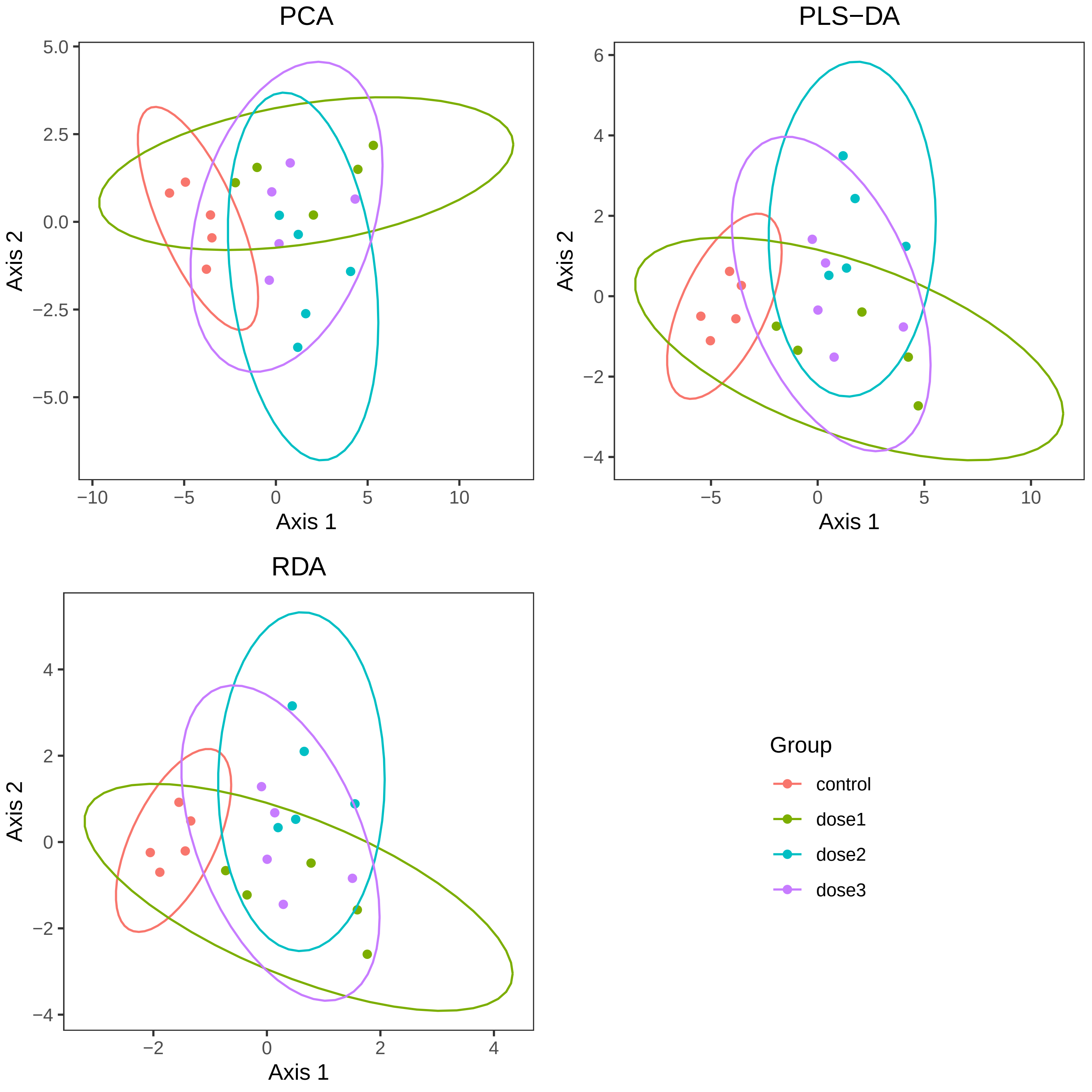
PCA
PLS−DA
RDA
Axis 1
Axis 2
5.0
2.5
0.0
−2.5
−5.0
−10
−5
0
5
10
6
4
2
0
−2
−4
Group
control
dose1
dose2
dose3

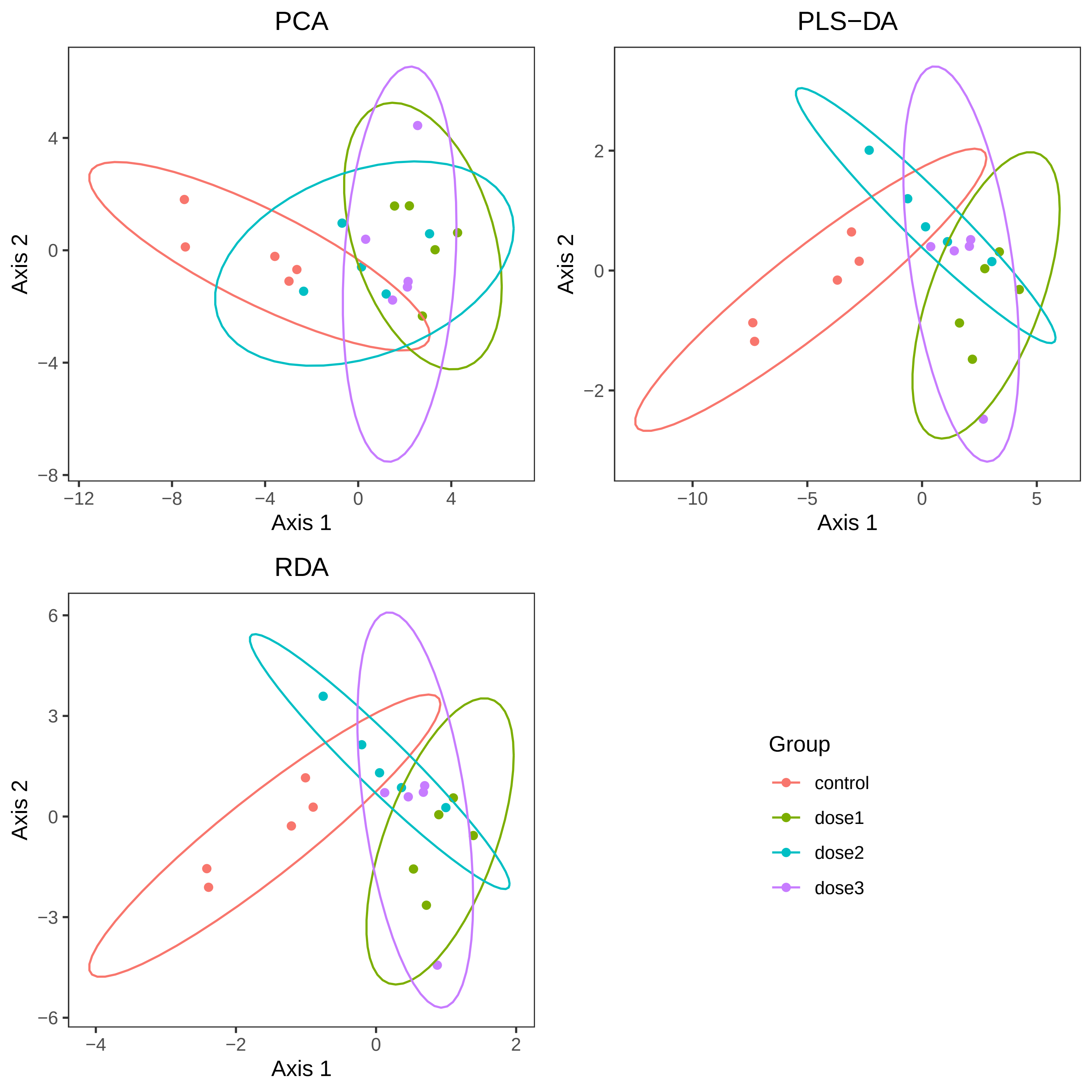

PCA
PLS−DA
RDA
Axis 1
Axis 2
−12
−8
−4
0
4
−10
−5
5
2
−2
6
3
−3
−6
−4
−8
Group
control
dose1
dose2
dose3

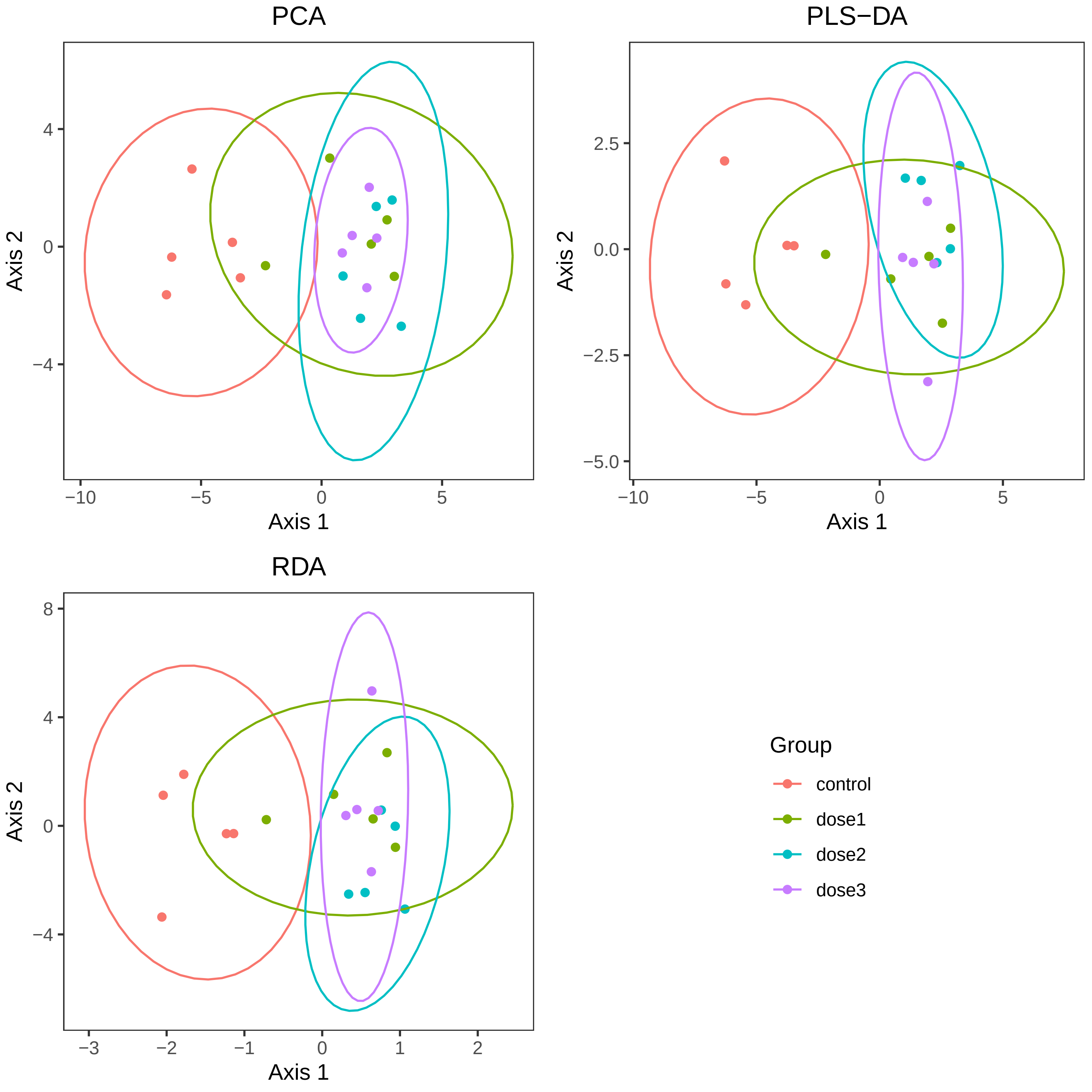
PCA
PLS−DA
RDA
Axis 1
Axis 2
Group
control
dose1
dose2
dose3

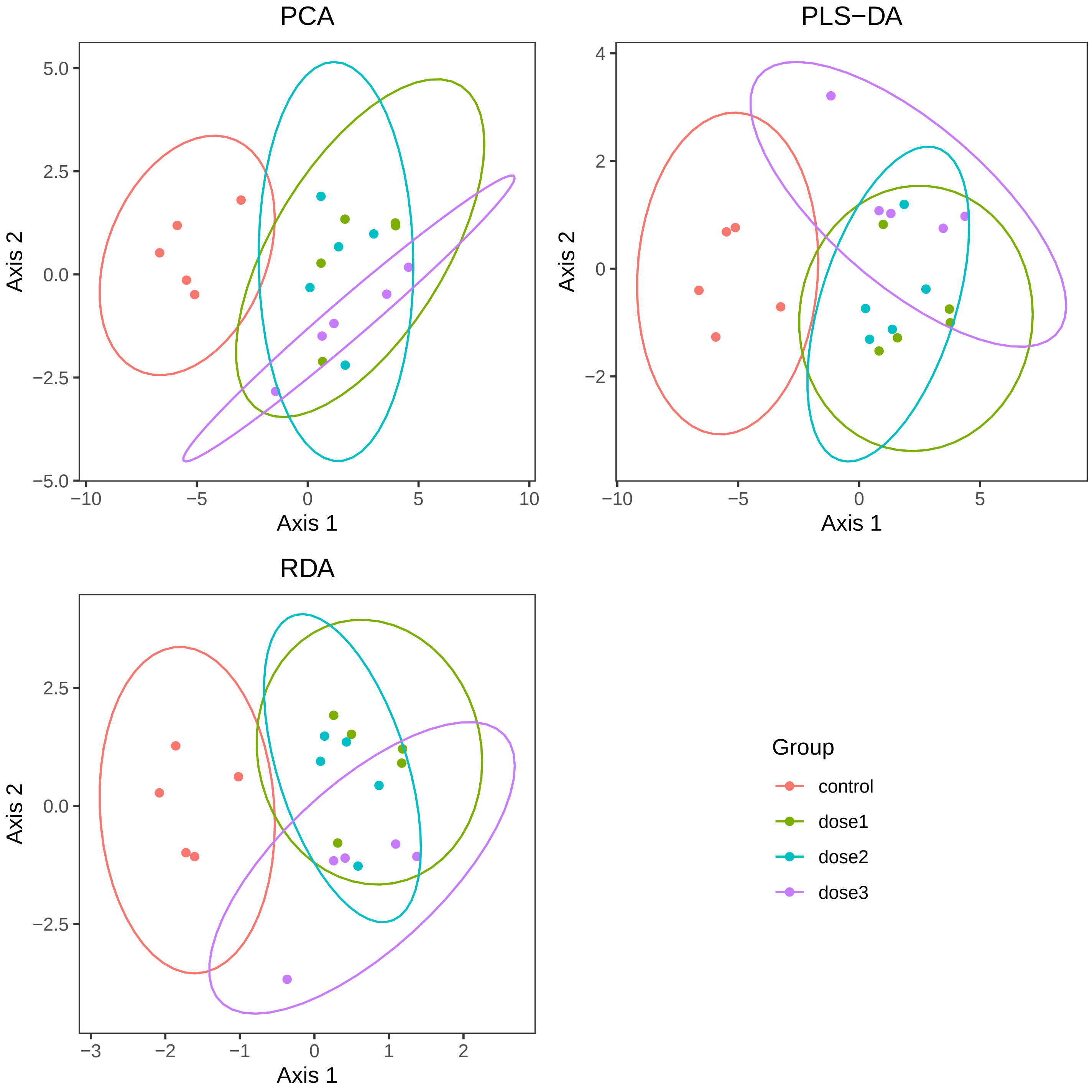
PCA
PLS−DA
RDA
Axis 1
Axis 2
Group
control
dose1
dose2
dose3

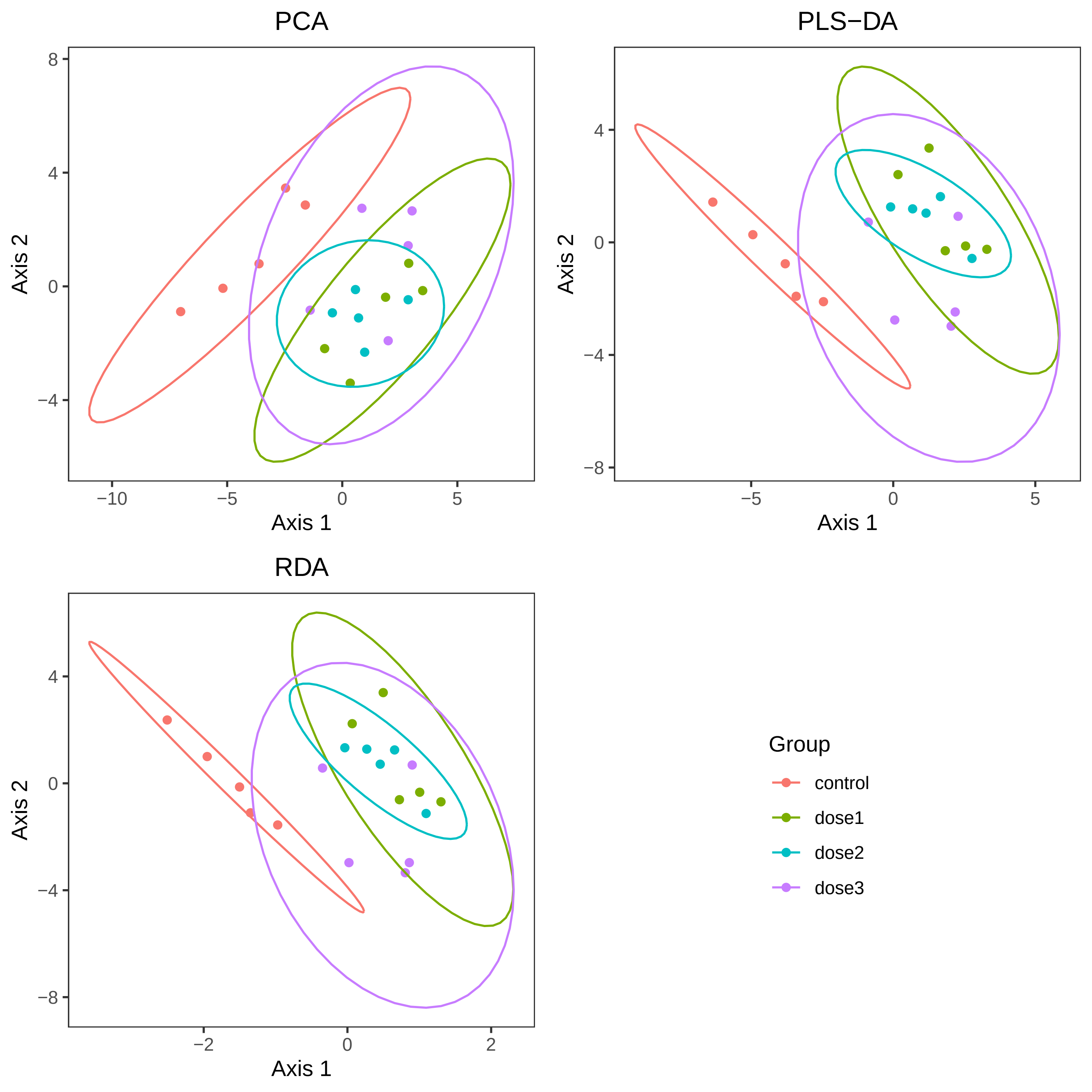

PCA
PLS−DA
RDA
Axis 1
Axis 2
Group
control
dose1
dose2
dose3

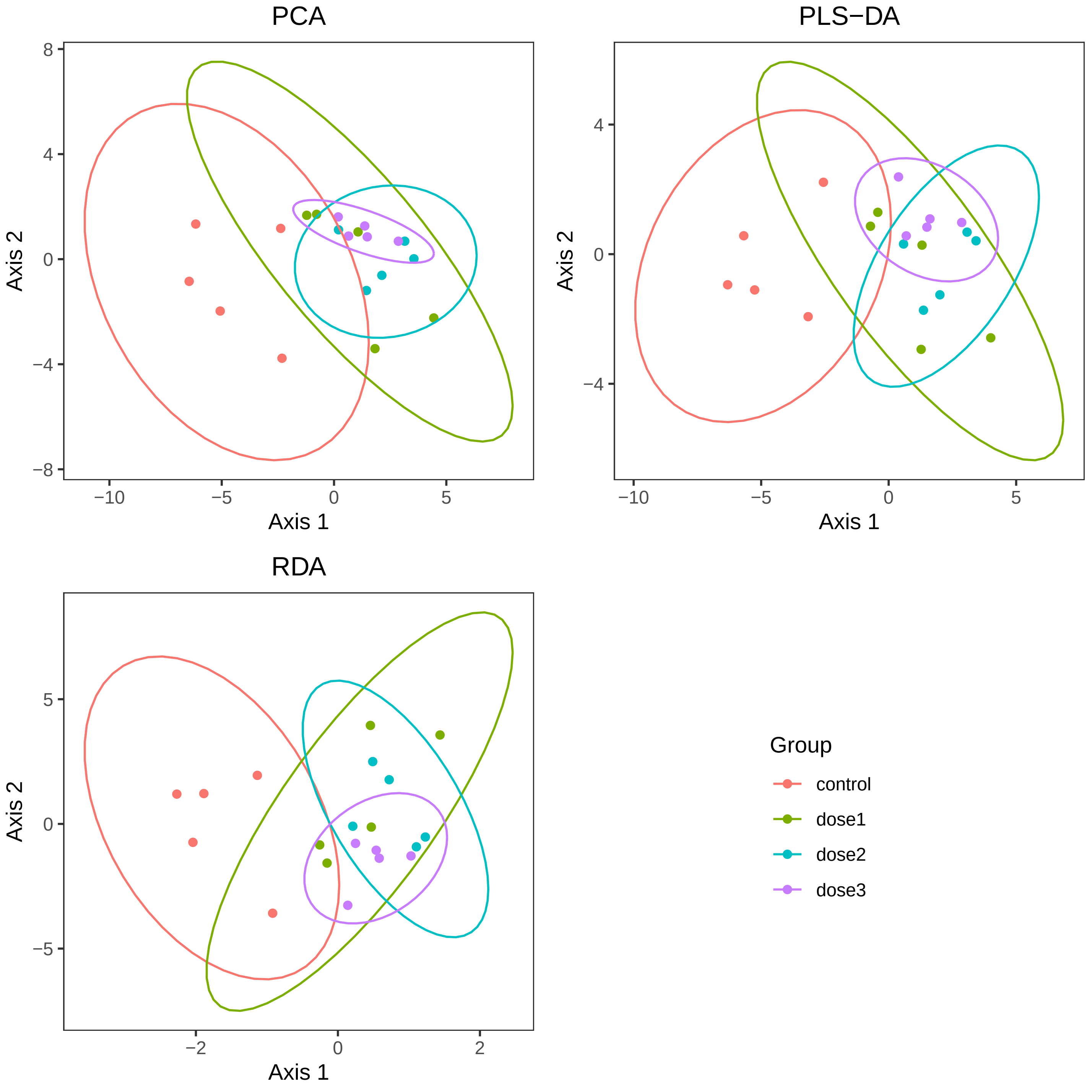
PCA
PLS−DA
RDA
Axis 1
Axis 2
Group
control
dose1
dose2
dose3

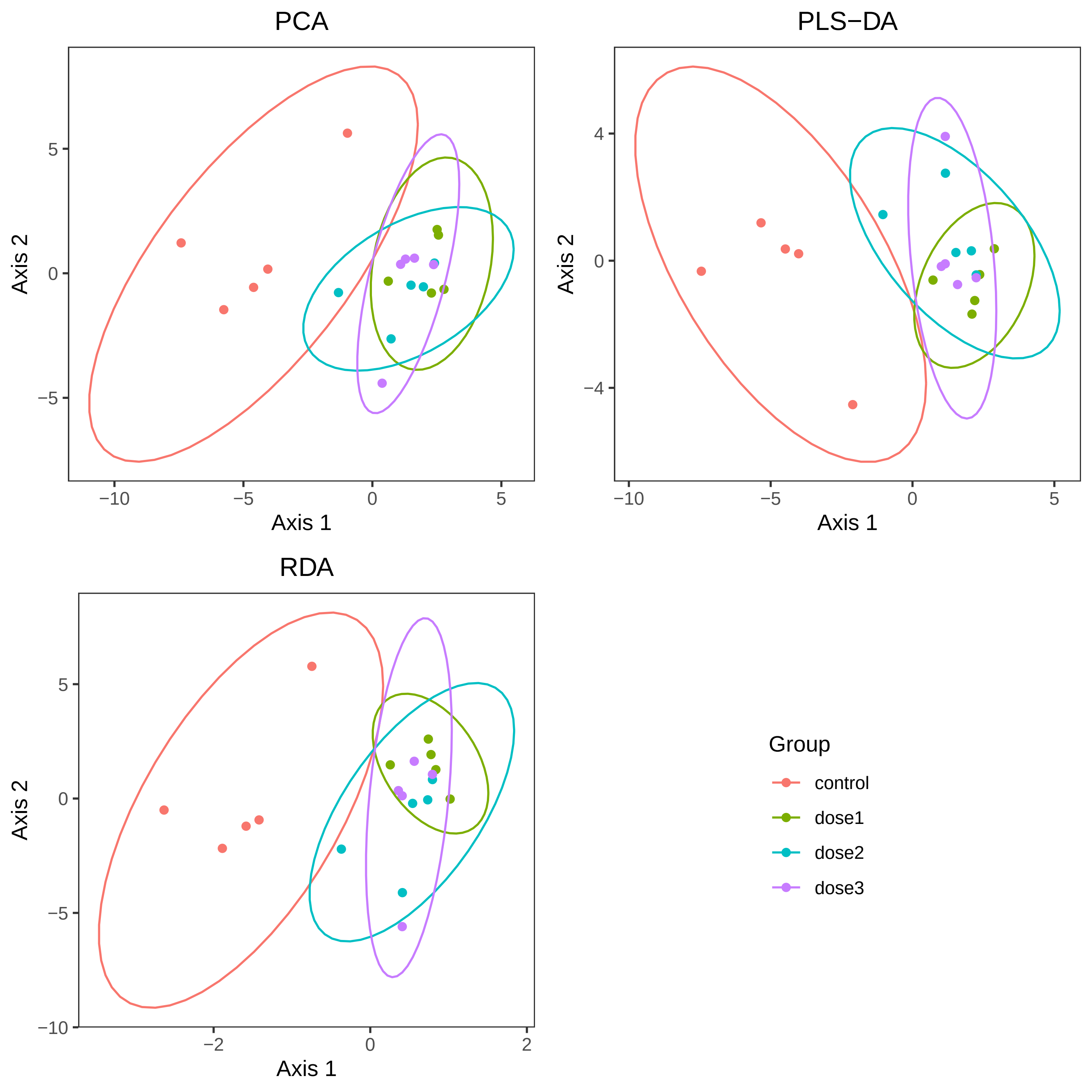

PCA
PLS−DA
RDA
Axis 1
Axis 2
Group
control
dose1
dose2
dose3

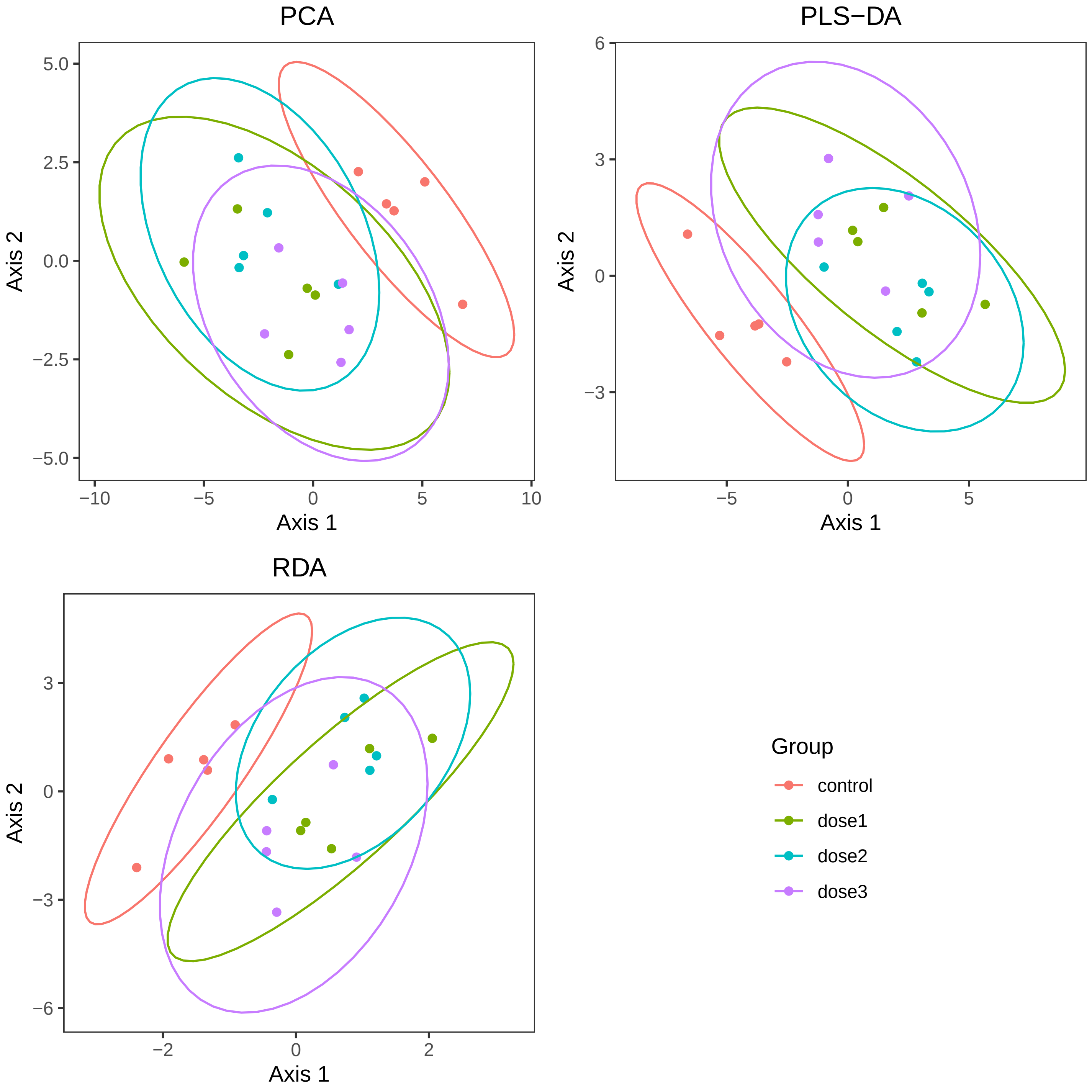

PCA
PLS−DA
RDA
Axis 1
Axis 2
−10
−5
0
5
10
5.0
2.5
0.0
−2.5
−5.0
6
3
0
−3
−2
2
−6
Group
control
dose1
dose2
dose3

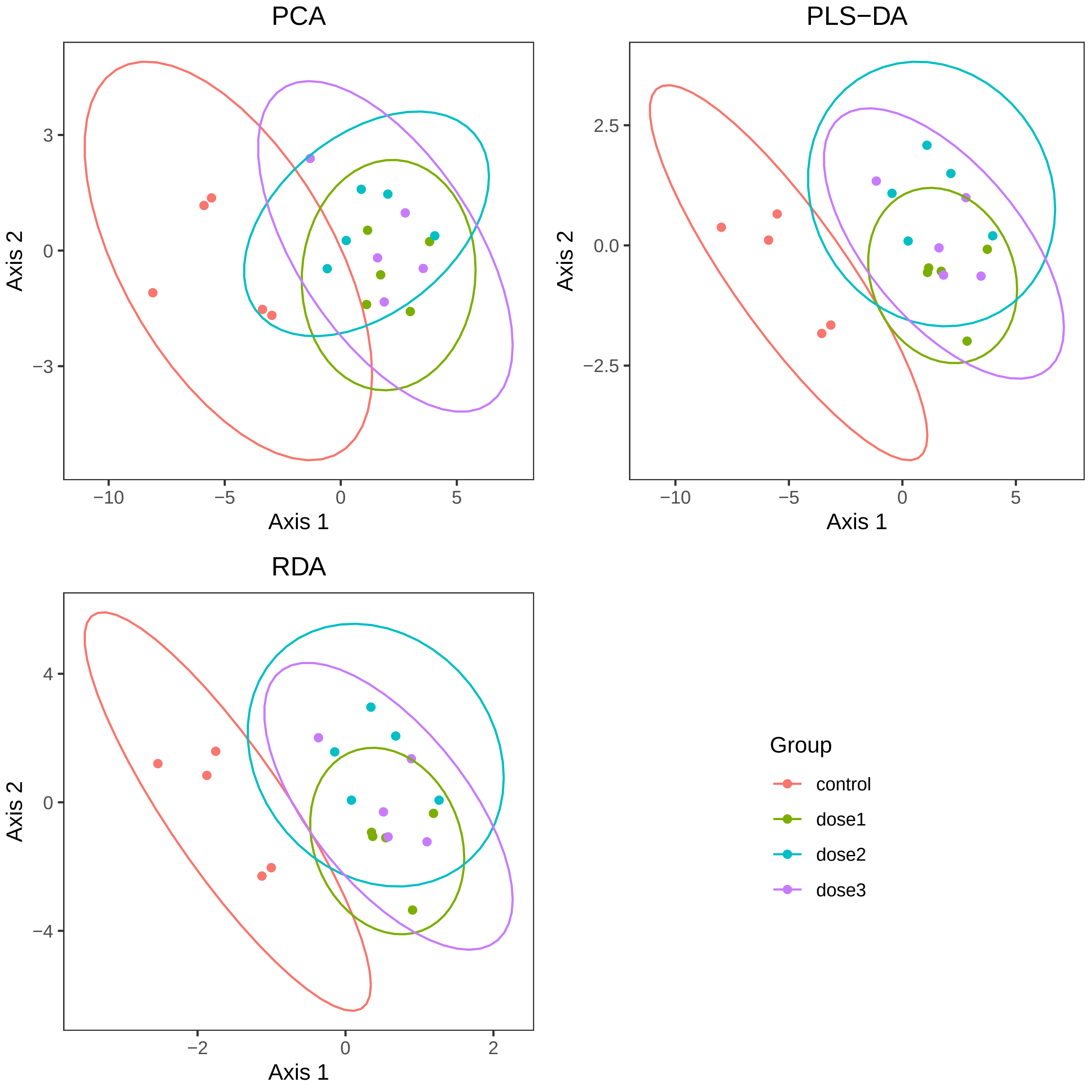

PCA
PLS−DA
RDA
Axis 1
Axis 2
Group
control
dose1
dose2
dose3

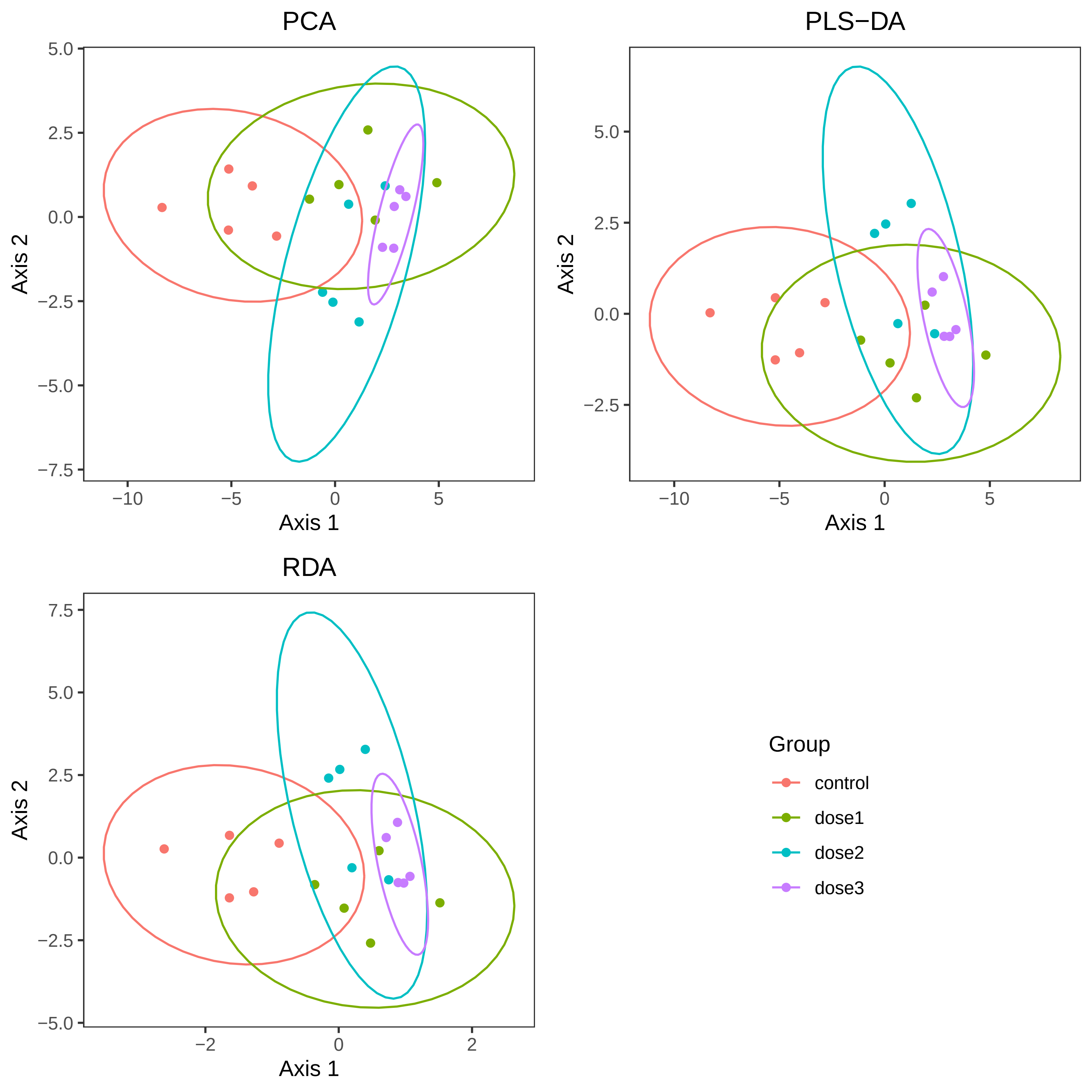
PCA
PLS−DA
RDA
Axis 1
Axis 2
Group
control
dose1
dose2
dose3

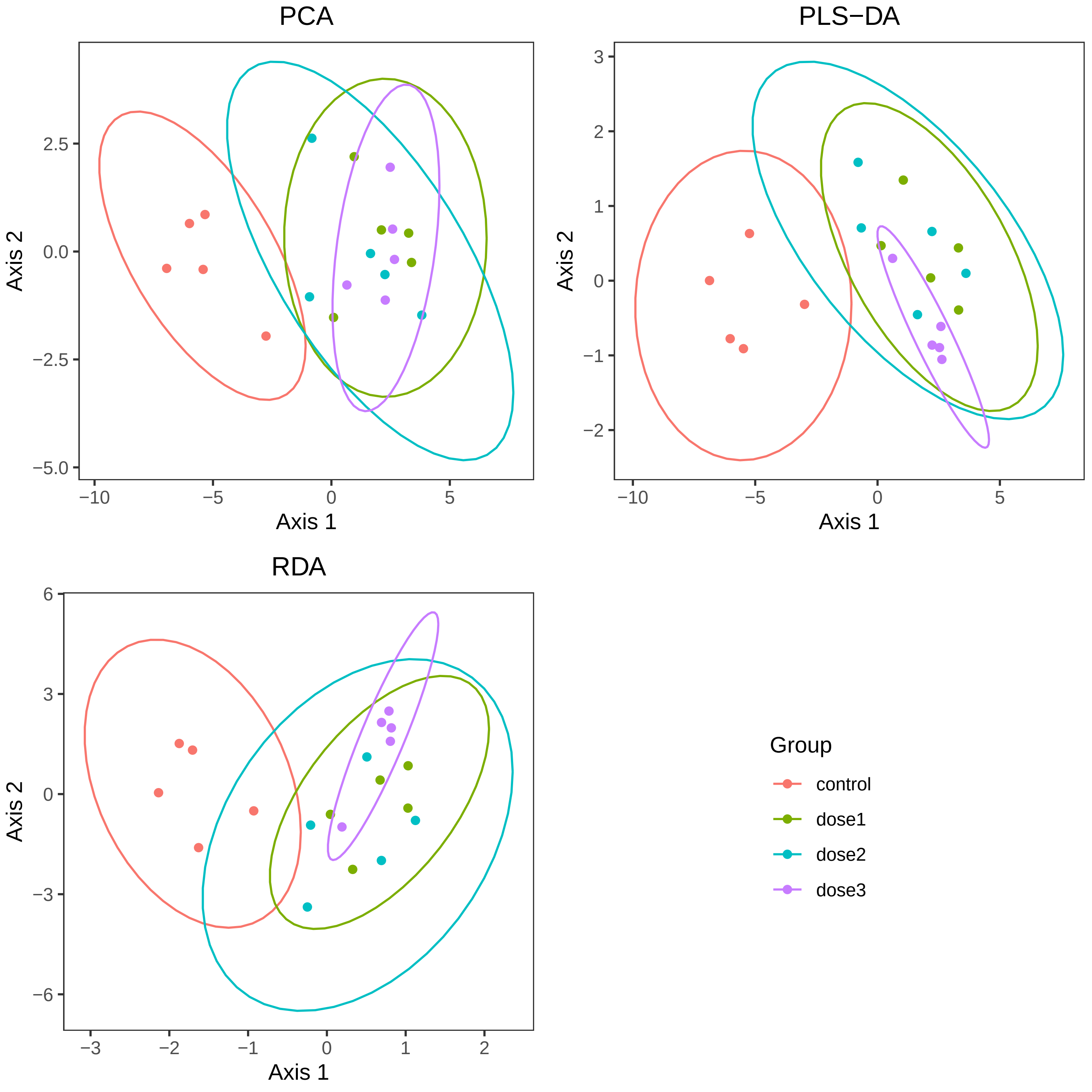
PCA
PLS−DA
RDA
Axis 1
Axis 2
−10
−5
0
5
2.5
0.0
−2.5
−5.0
3
2
1
0
−1
−2
6
−3
−6
−3
−2
−1
0
1
2
Group
control
dose1
dose2
dose3

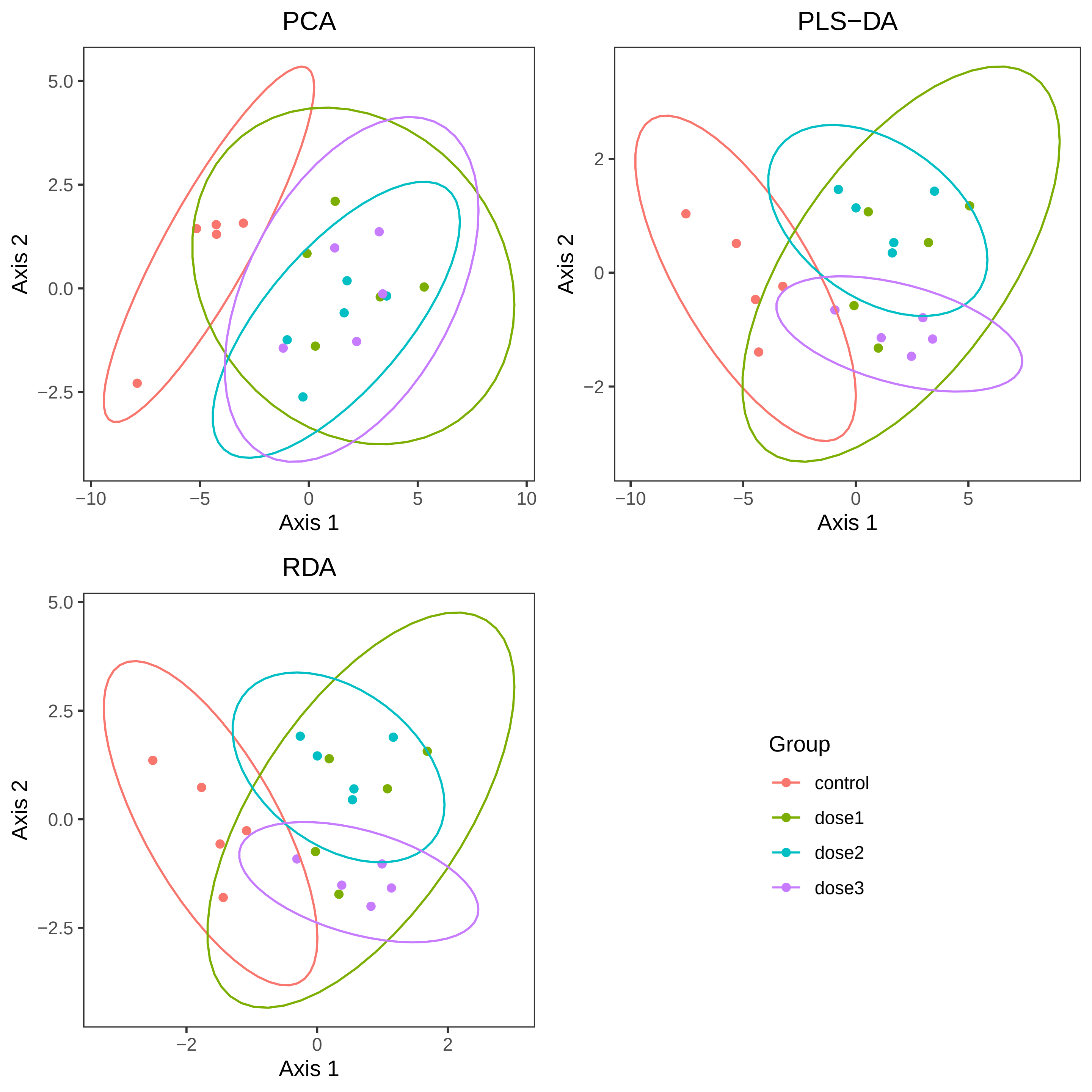

PCA
PLS−DA
RDA
Axis 1
Axis 2
5.0
2.5
0.0
−2.5
−10
−5
0
5
10
2
−2
Group
control
dose1
dose2
dose3

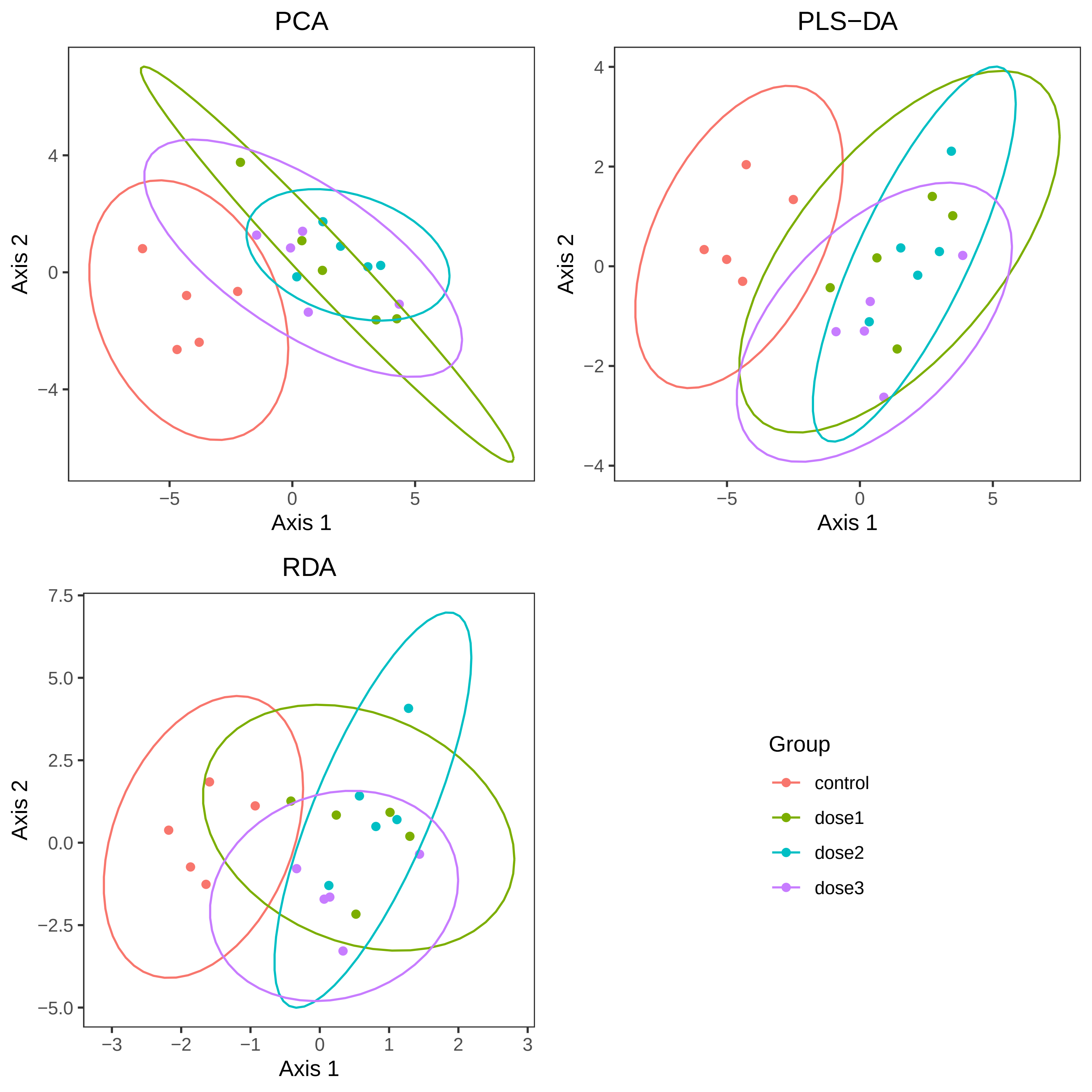

PCA
PLS−DA
RDA
Axis 1
Axis 2
Group
control
dose1
dose2
dose3

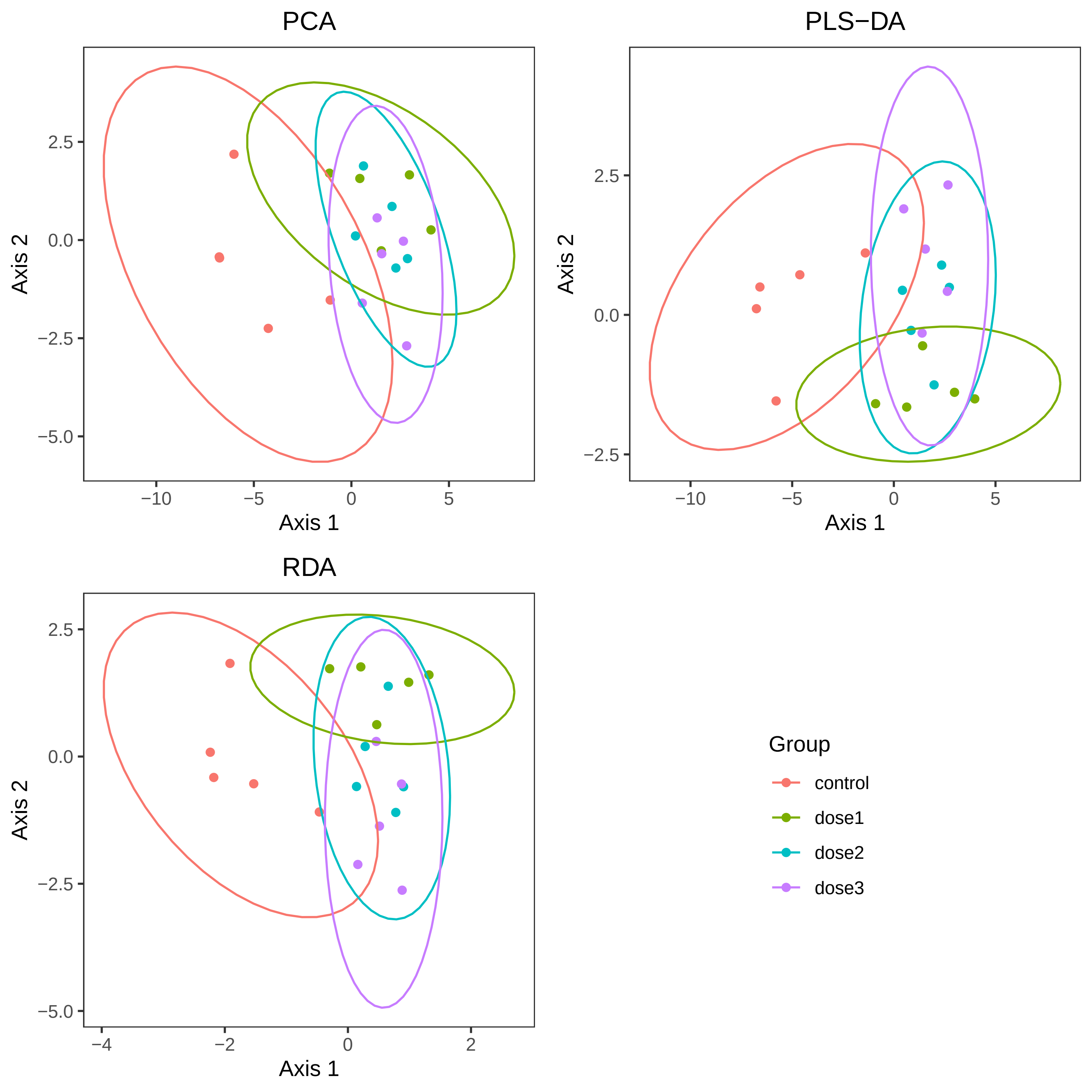
PCA
PLS−DA
RDA
Axis 1
Axis 2
Group
control
dose1
dose2
dose3

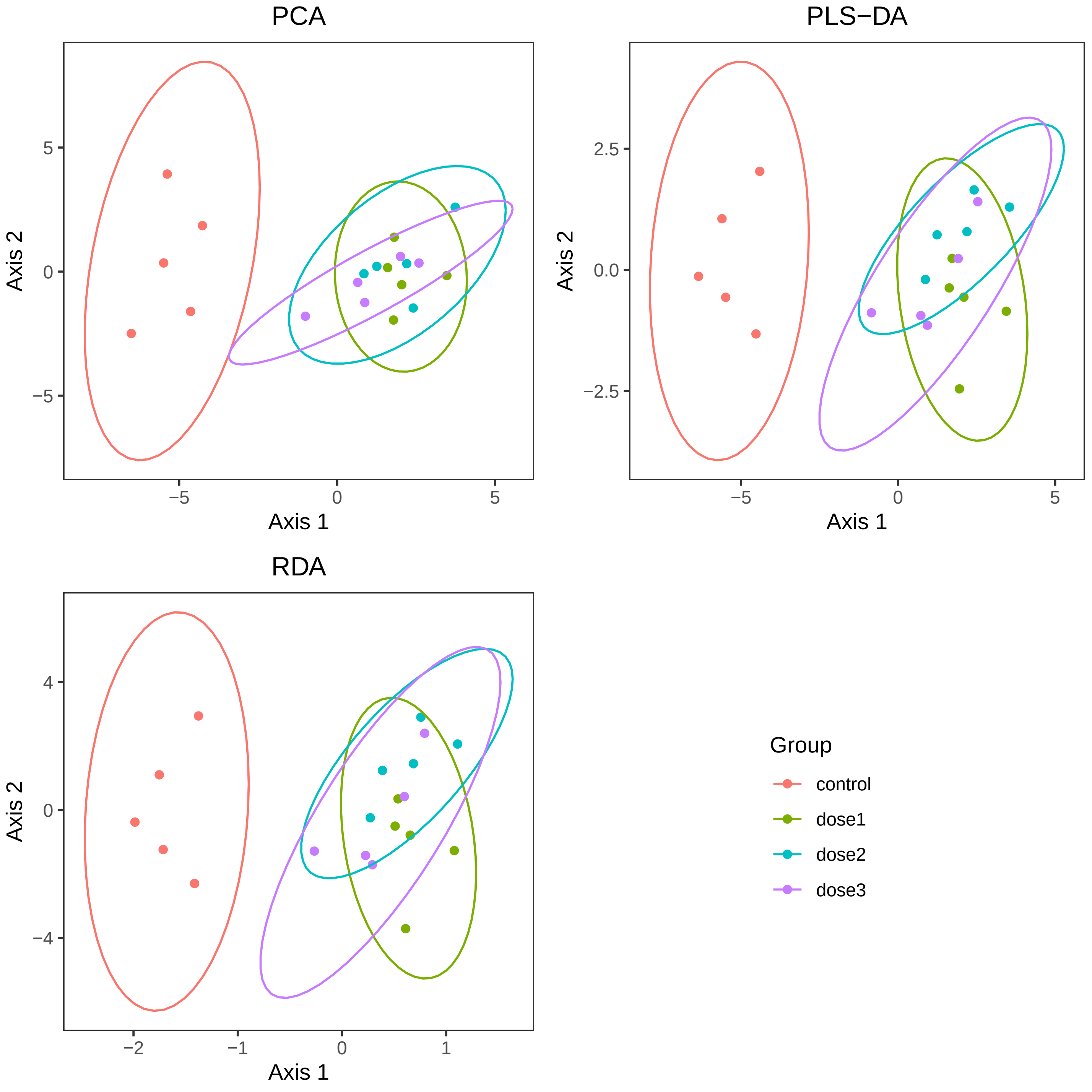
PCA
PLS−DA
RDA
Axis 1
Axis 2
Group
control
dose1
dose2
dose3

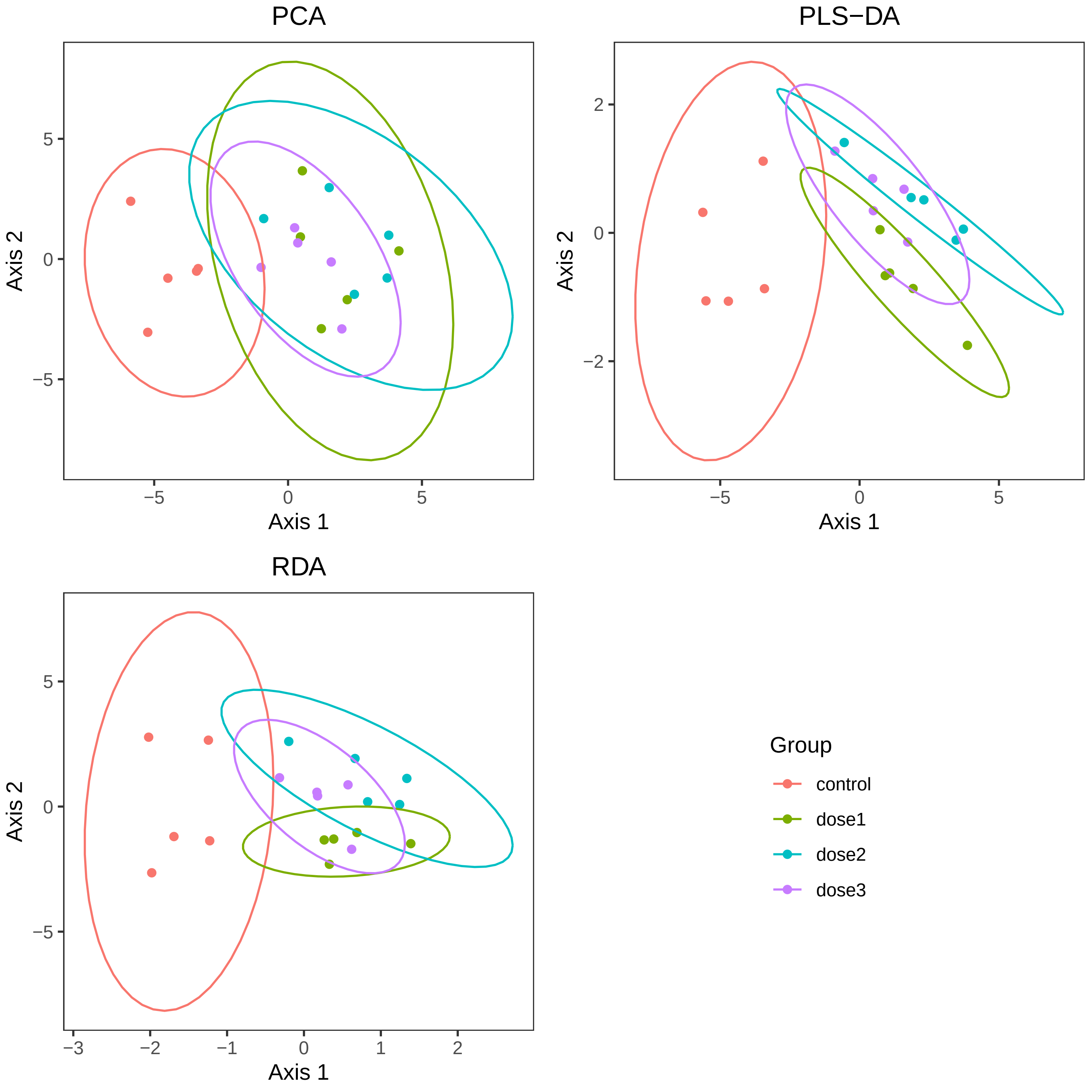
PCA
PLS−DA
RDA
Axis 1
Axis 2
−5
0
5
2
−2
−3
−2
−1
1
Group
control
dose1
dose2
dose3

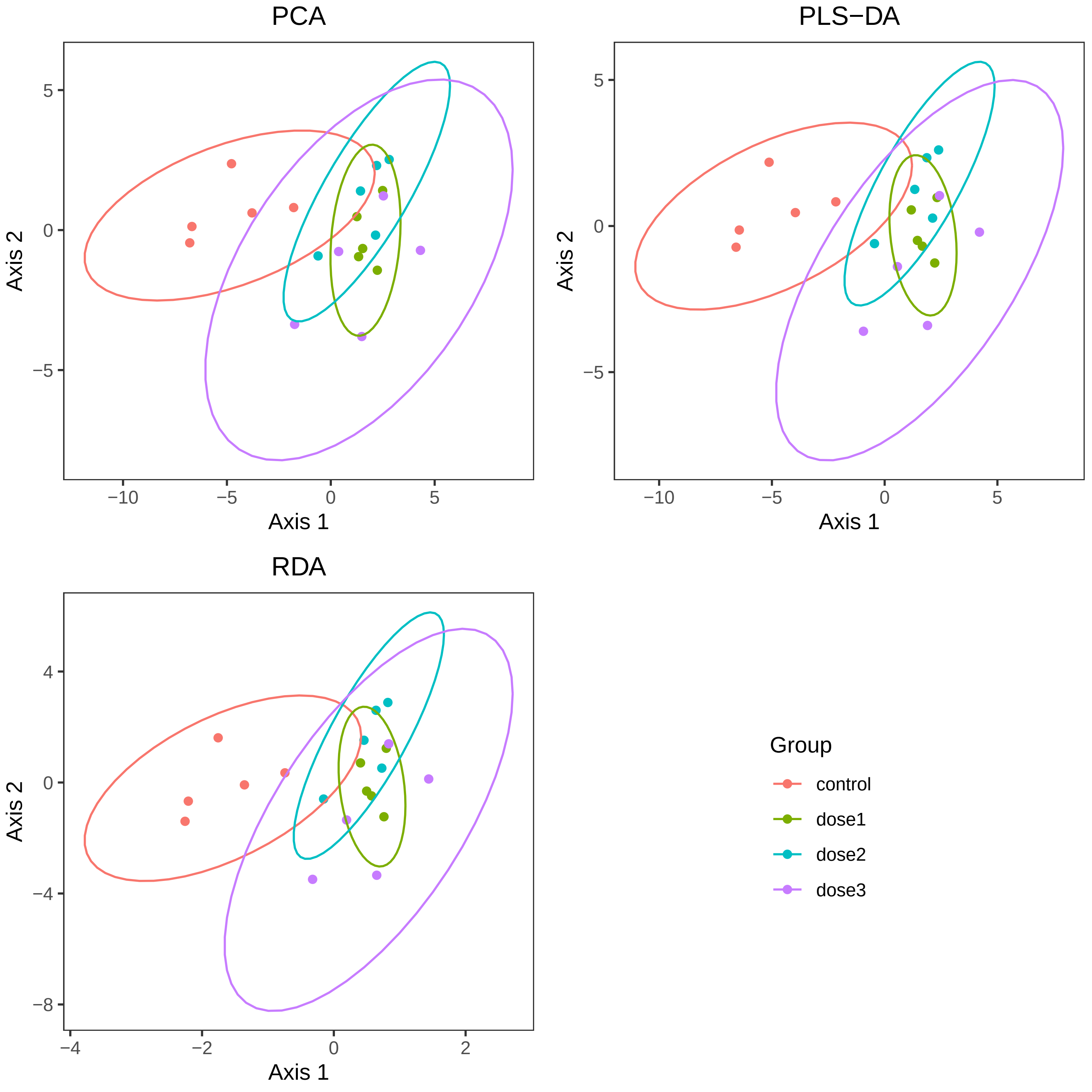

PCA
PLS−DA
RDA
Axis 1
Axis 2
−10
−5
0
5
4
−4
−8
−2
2
Group
control
dose1
dose2
dose3

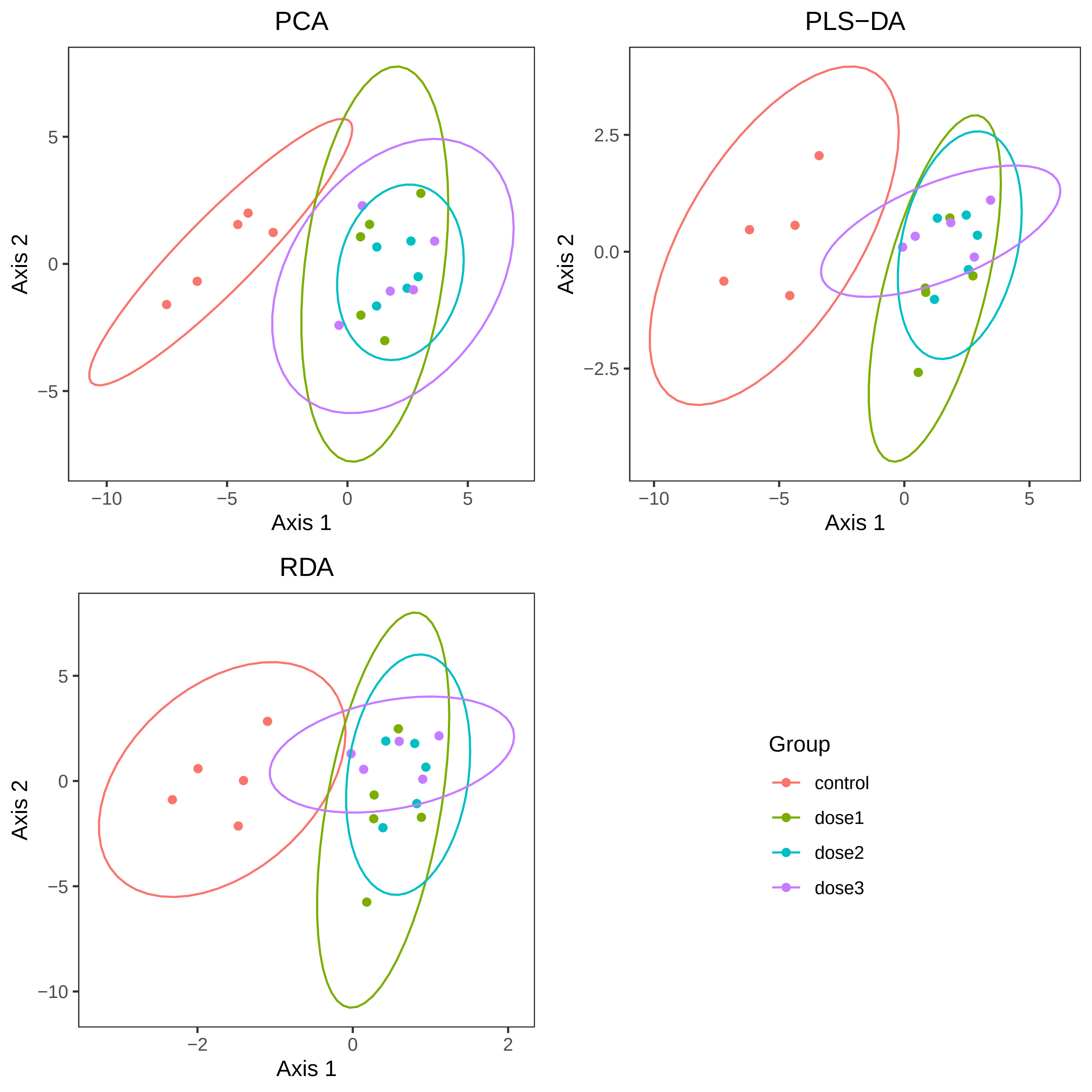

PCA
PLS−DA
RDA
Axis 1
Axis 2
Group
control
dose1
dose2
dose3

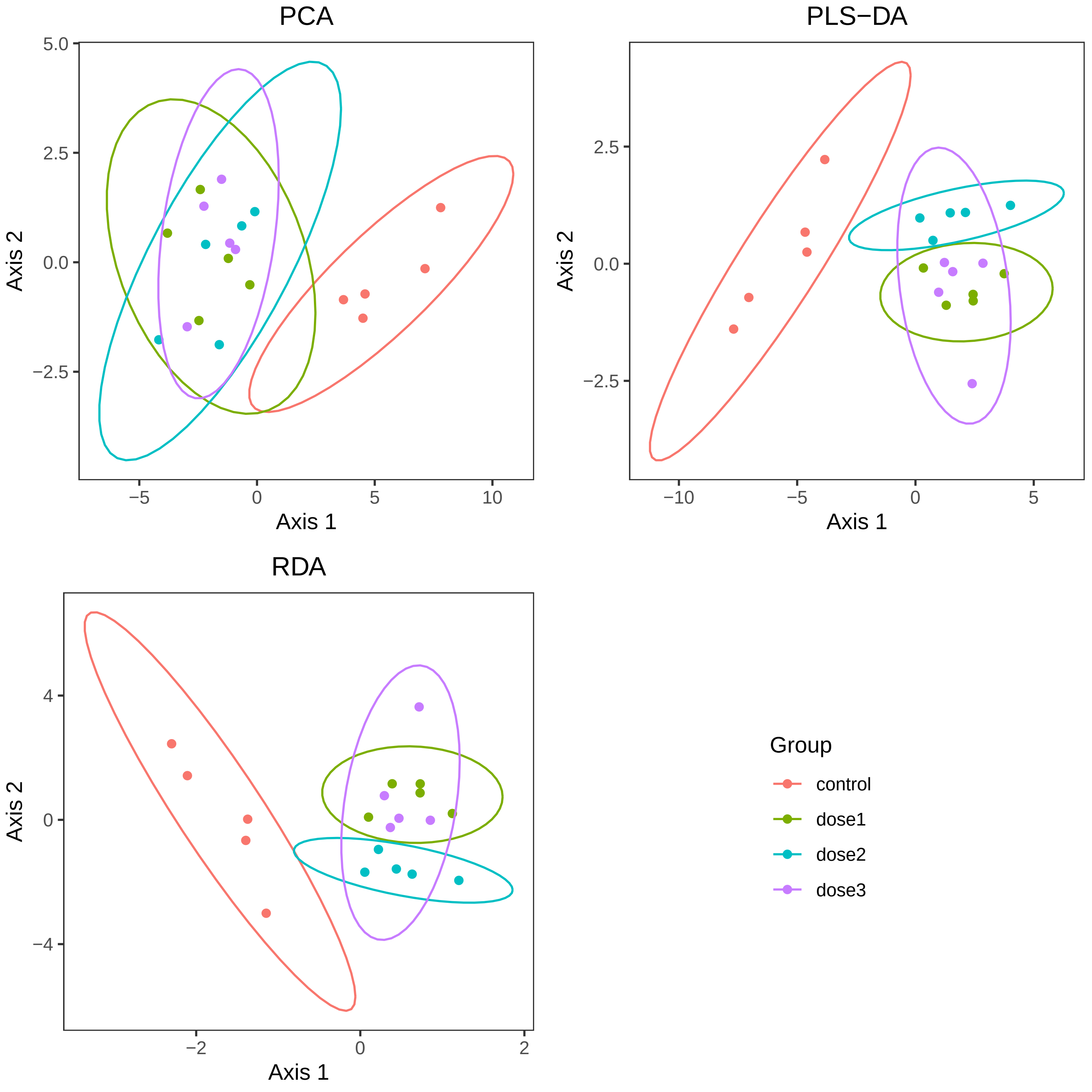

PCA
PLS−DA
RDA
Axis 1
Axis 2
5.0
2.5
0.0
−2.5
−5
0
5
10
−10
−4
−2
2
4
Group
control
dose1
dose2
dose3

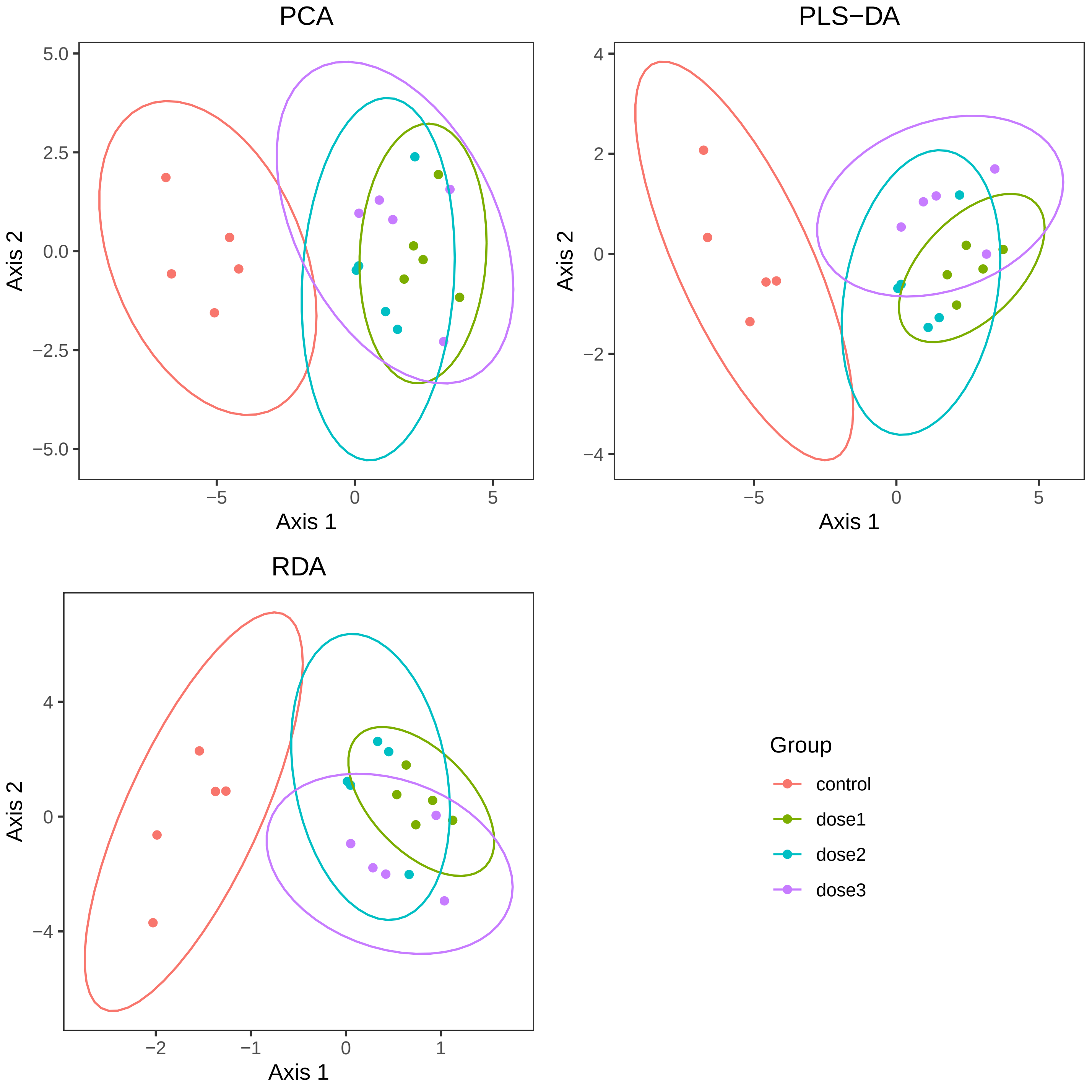

PCA
PLS−DA
RDA
Axis 1
Axis 2
Group
control
dose1
dose2
dose3

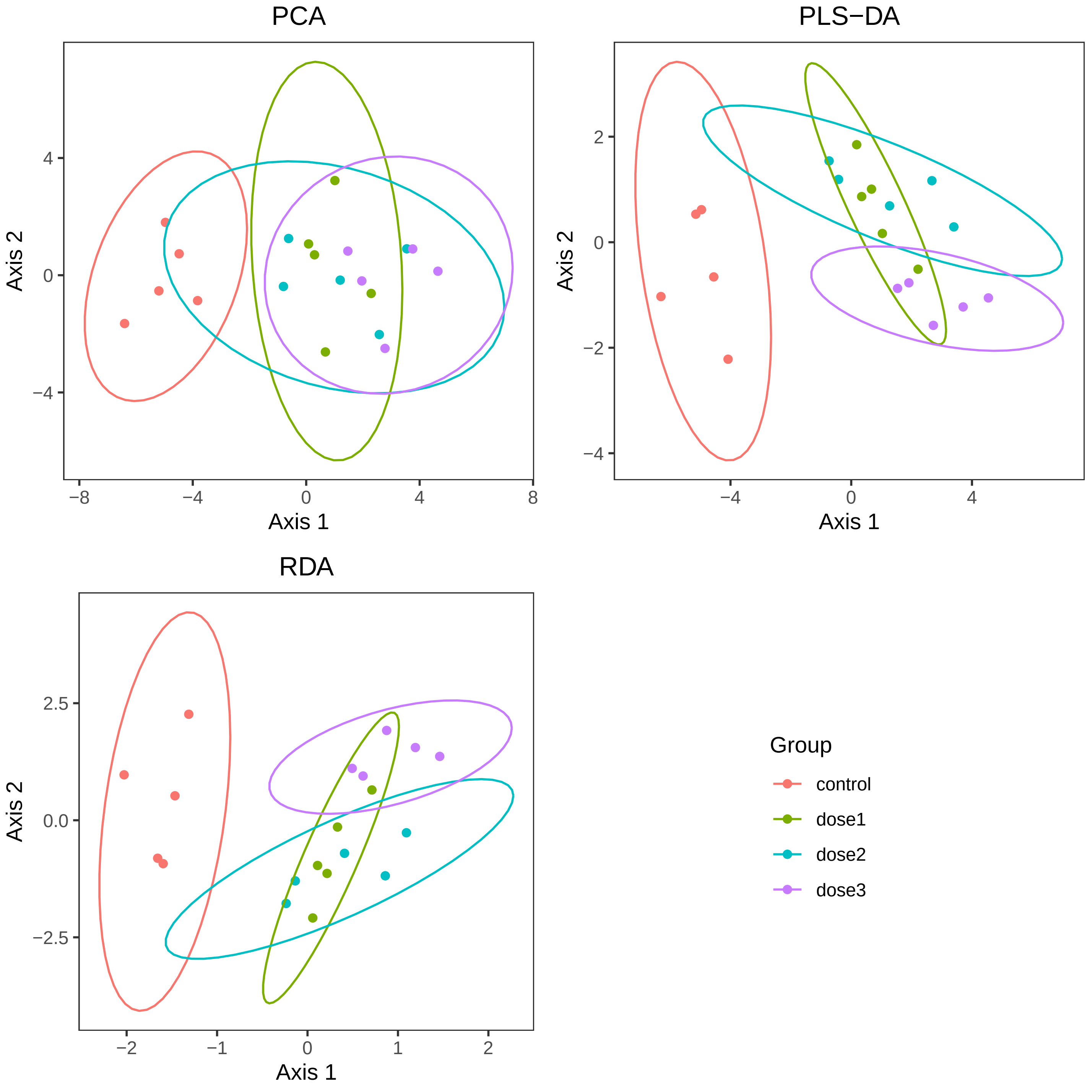
PCA
PLS−DA
RDA
Axis 1
Axis 2
Group
control
dose1
dose2
dose3

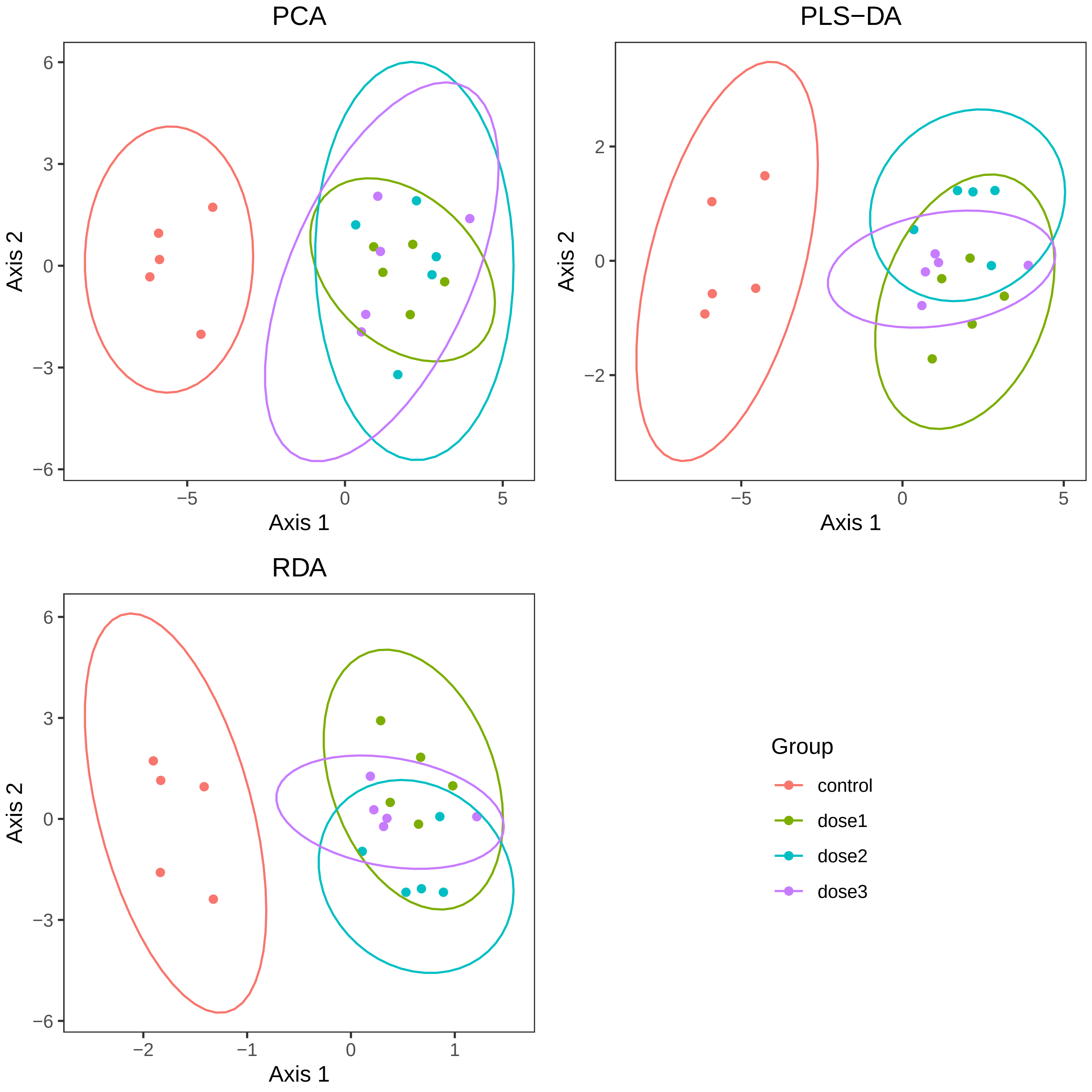
PCA
PLS−DA
RDA
Axis 1
Axis 2
Group
control
dose1
dose2
dose3

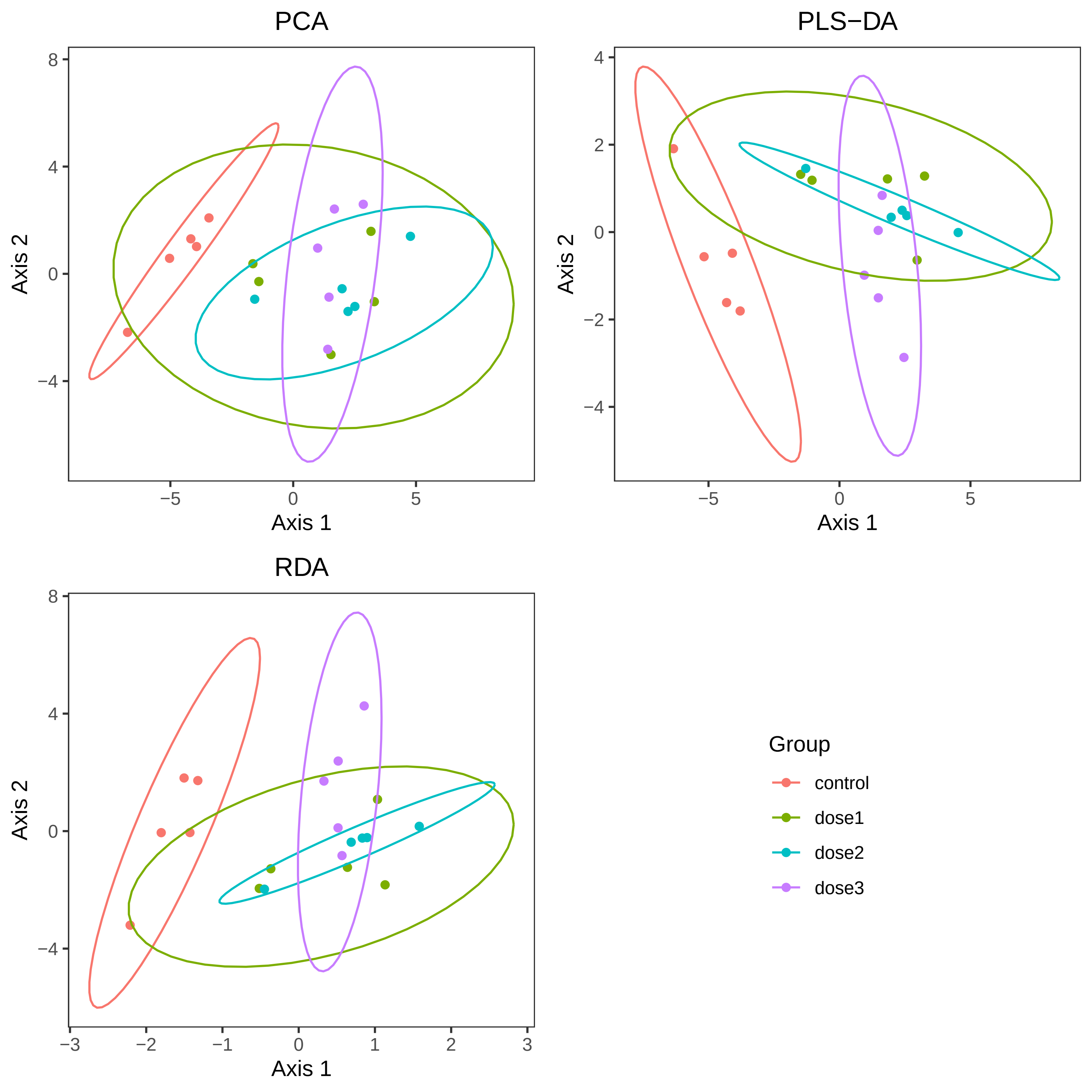

PCA
PLS−DA
RDA
Axis 1
Axis 2
Group
control
dose1
dose2
dose3

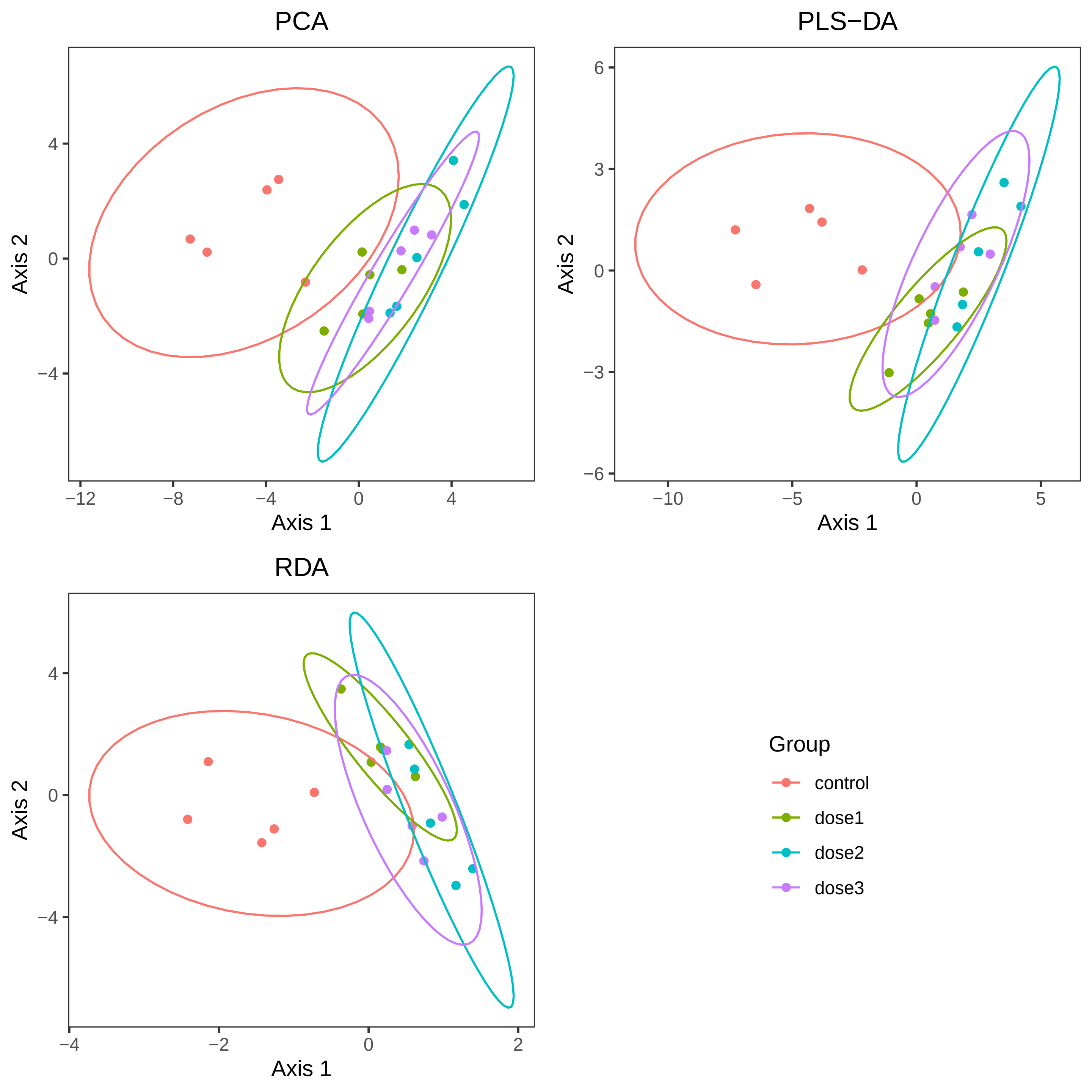
PCA
Axis 2
Axis 1
4
0
−4
−12
−8
−4
0
4
PLS−DA
Axis 2
Axis 1
6
3
0
−3
−6
−10
−5
0
5
RDA
Axis 2
Axis 1
4
0
−4
−4
−2
0
2
Group
control
dose1
dose2
dose3

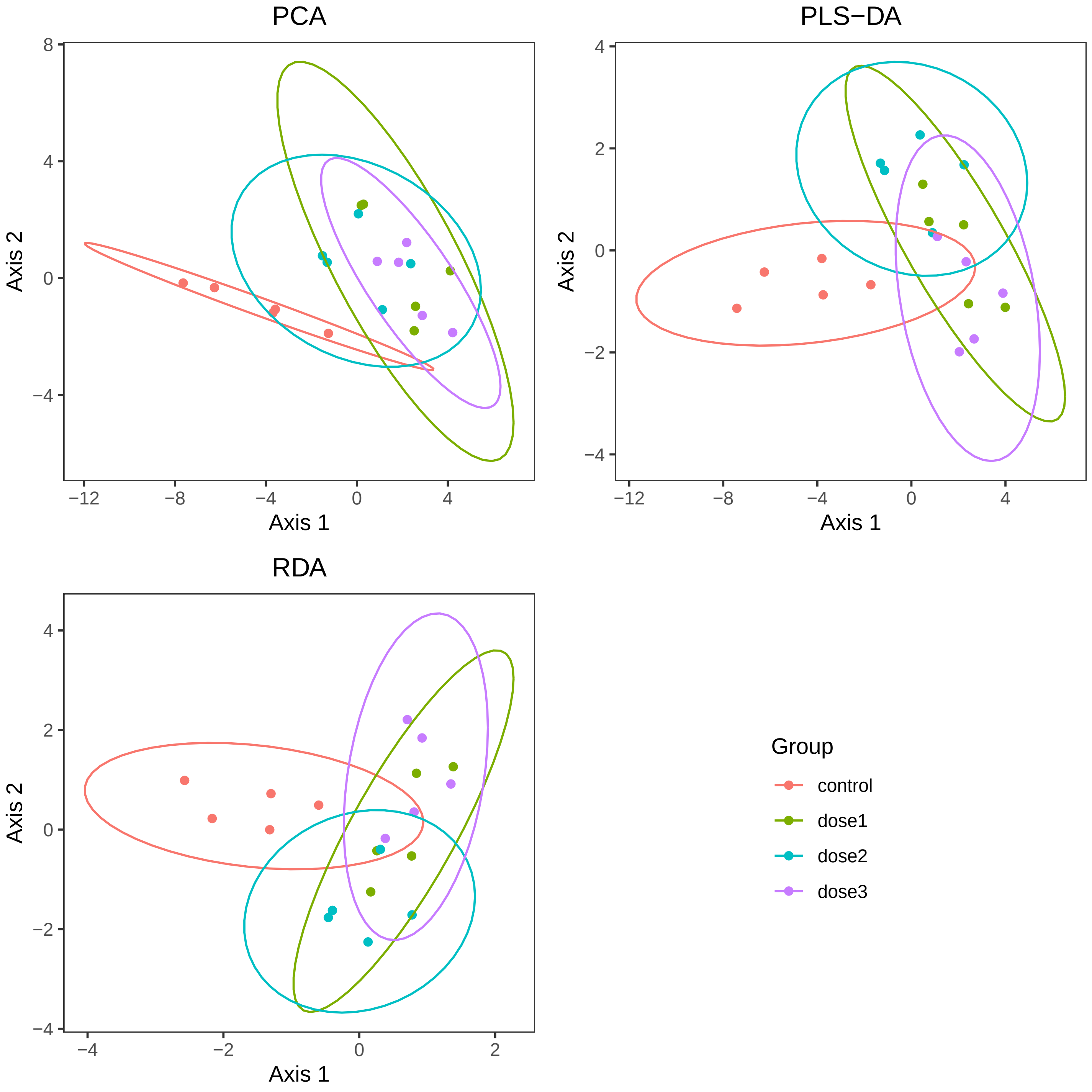

PCA
PLS−DA
RDA
Axis 1
Axis 2
−12
−8
−4
0
4
8
2
−2
Group
control
dose1
dose2
dose3

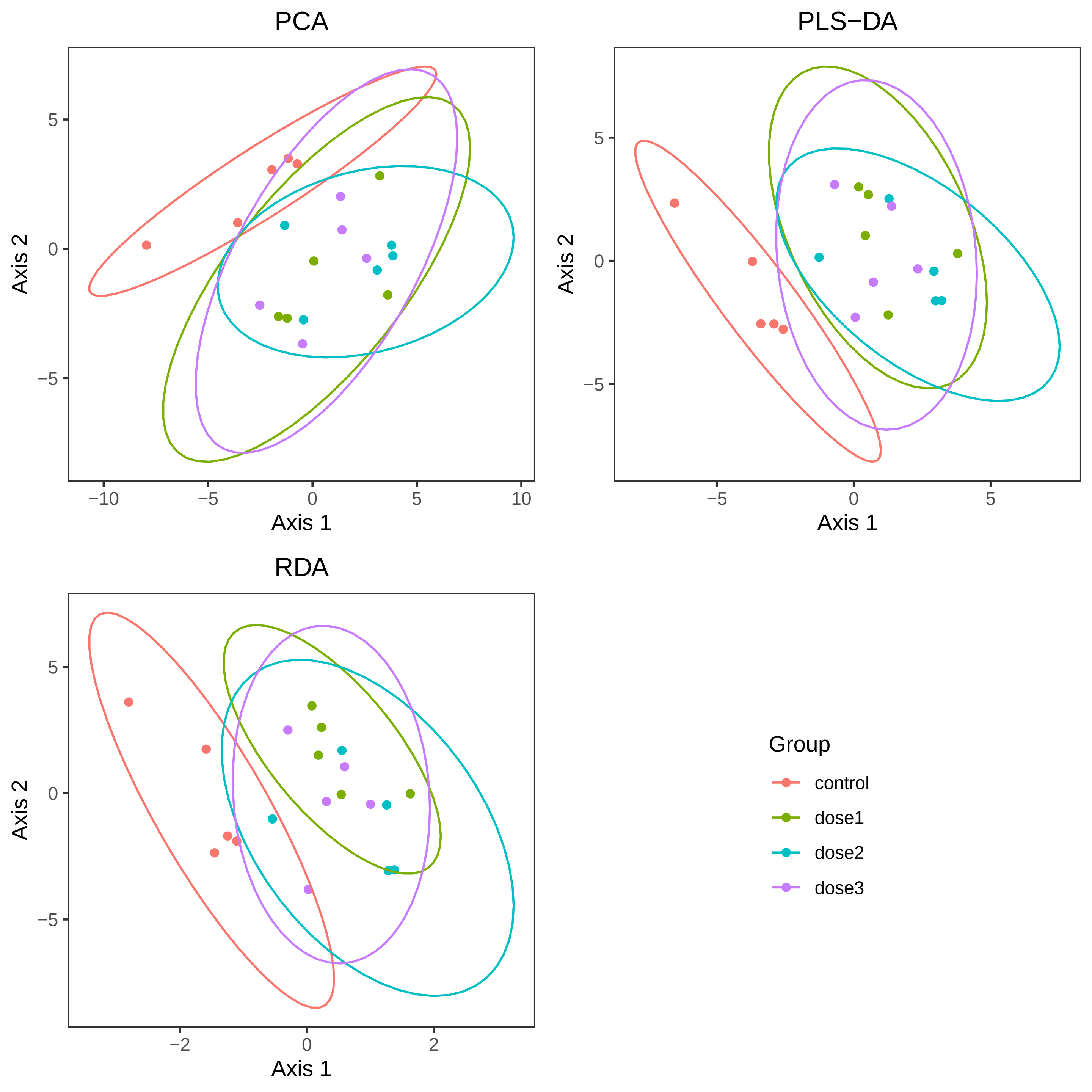

PCA
PLS−DA
RDA
Axis 1
Axis 2
−10
−5
0
5
10
Group
control
dose1
dose2
dose3

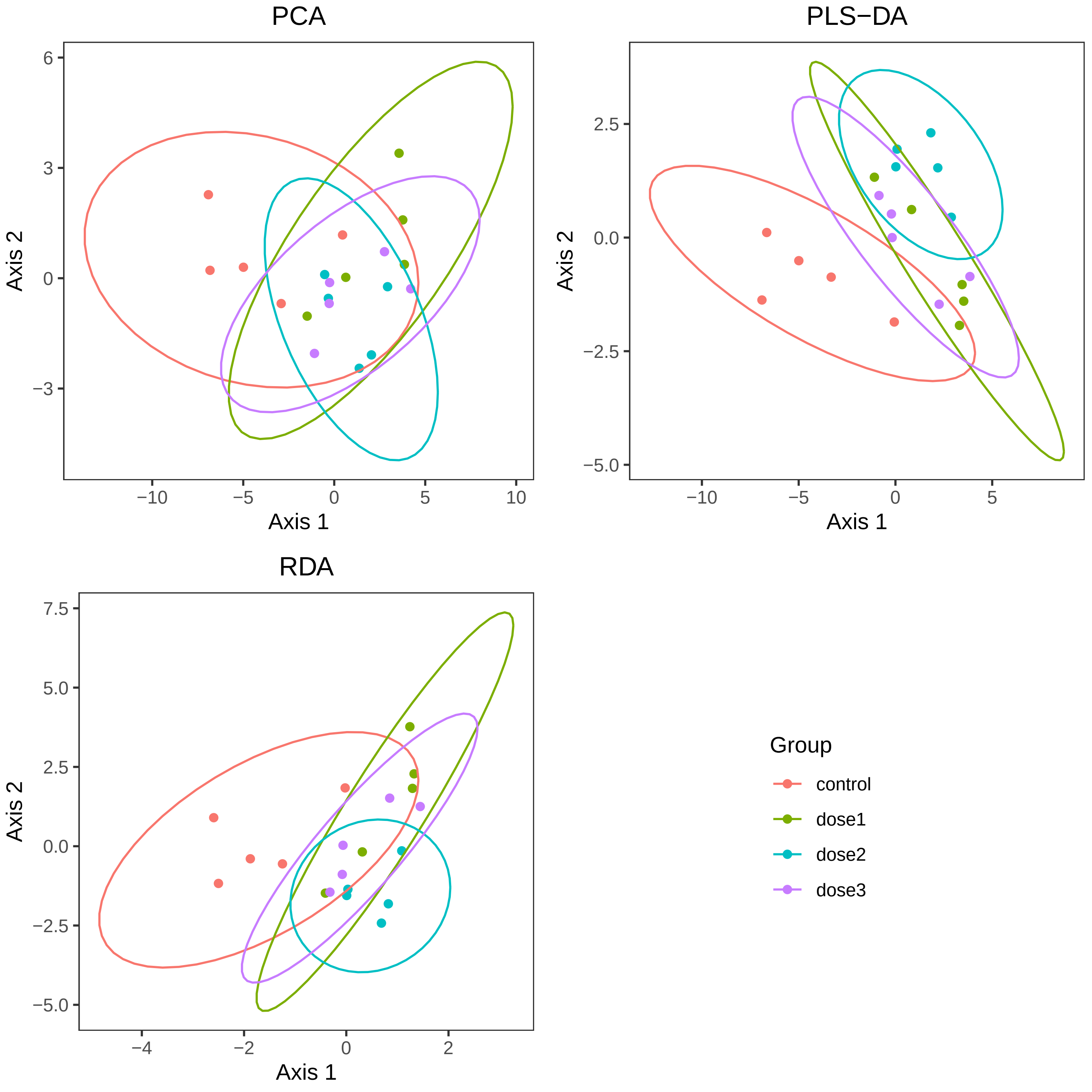

PCA
PLS−DA
RDA
Axis 1
Axis 2
−10
−5
0
5
10
6
3
0
−3
2.5
0.0
−2.5
−5.0
−4
−2
2
7.5
5.0
Group
control
dose1
dose2
dose3

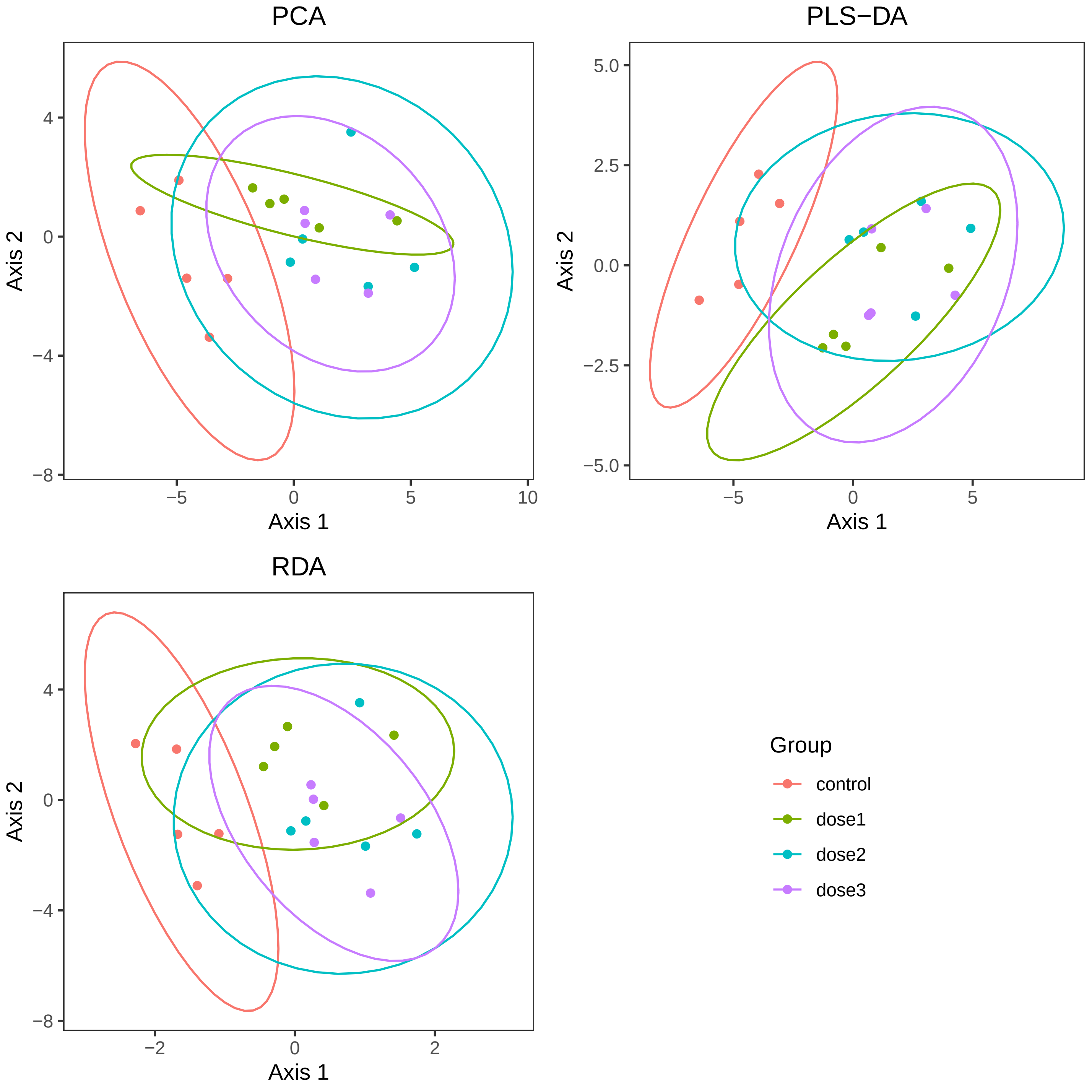

PCA
PLS−DA
RDA
Axis 1
Axis 2
Group
control
dose1
dose2
dose3

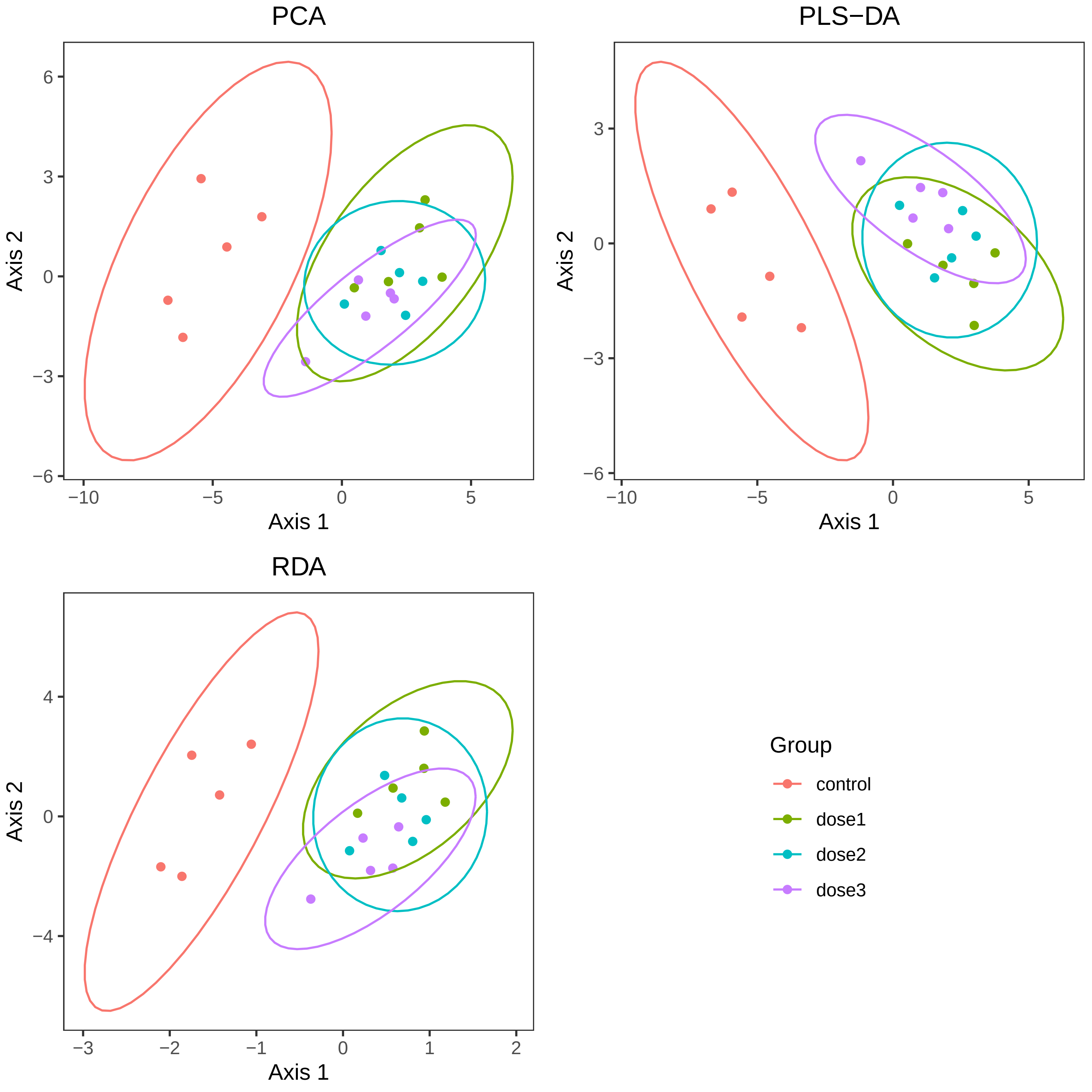

PCA
PLS−DA
RDA
Axis 1
Axis 2
Group
control
dose1
dose2
dose3

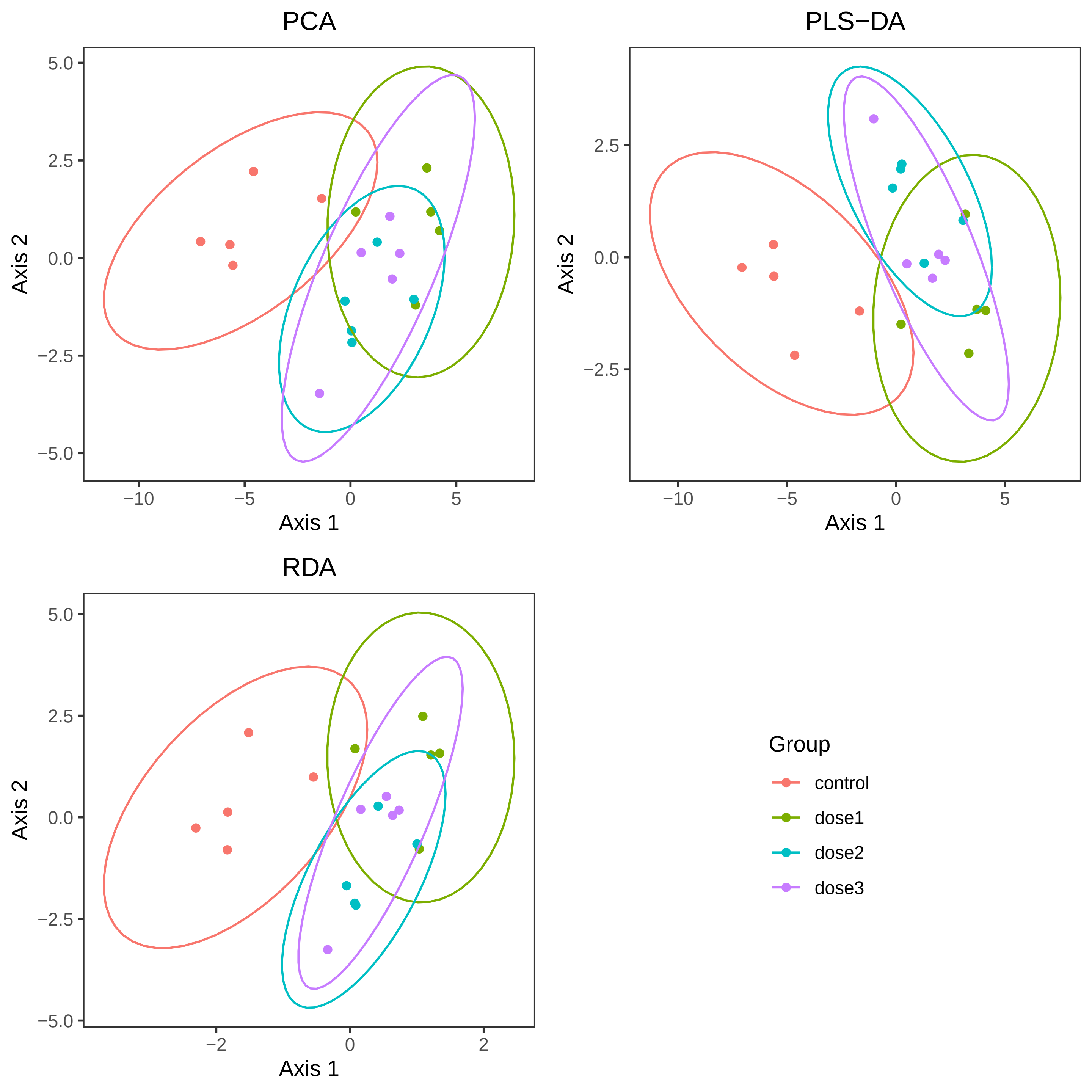

PCA
PLS−DA
RDA
Axis 1
Axis 2
5.0
2.5
0.0
−2.5
−5.0
−10
−5
0
5
−2
2
Group
control
dose1
dose2
dose3

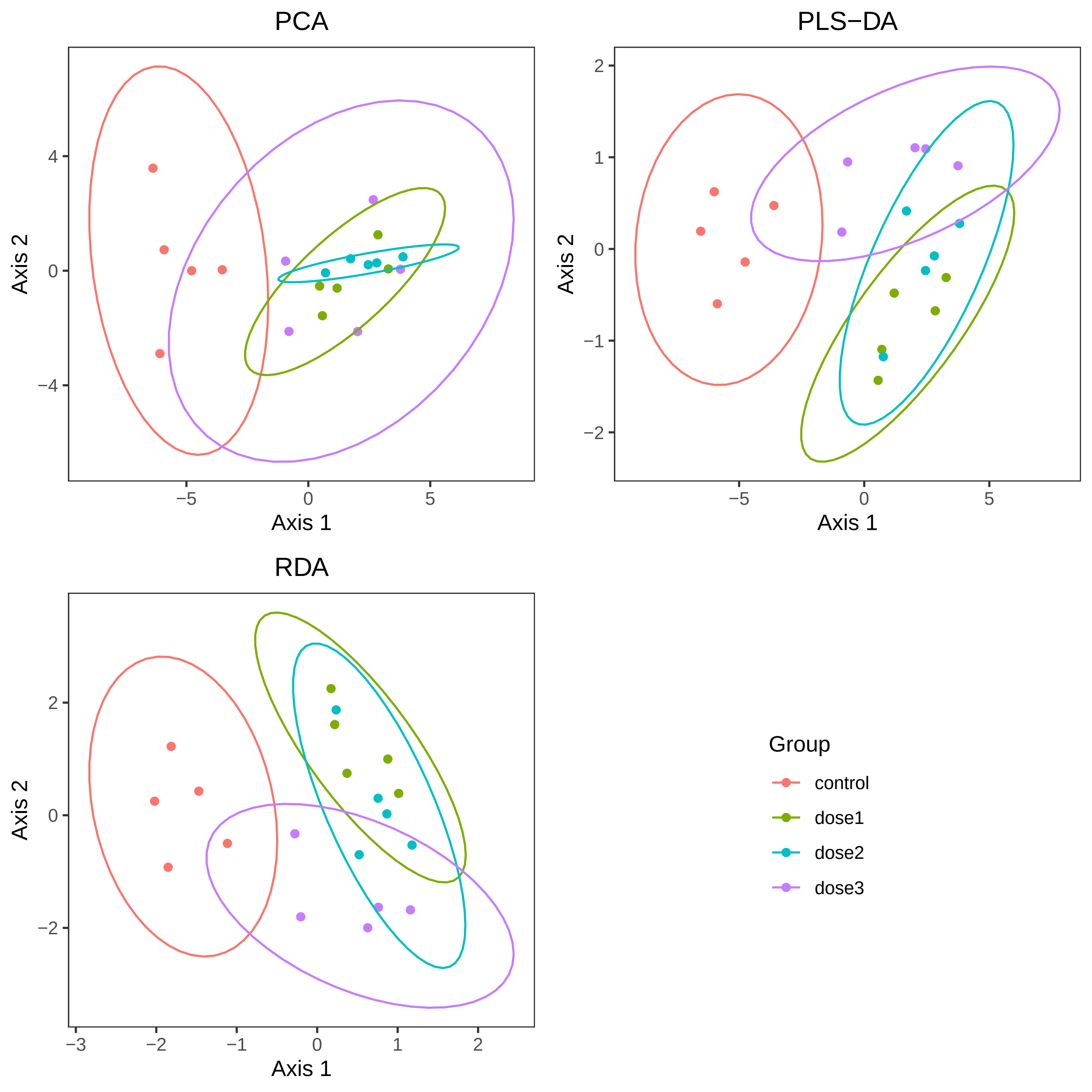

PCA
PLS−DA
RDA
Axis 1
Axis 2
Group
control
dose1
dose2
dose3

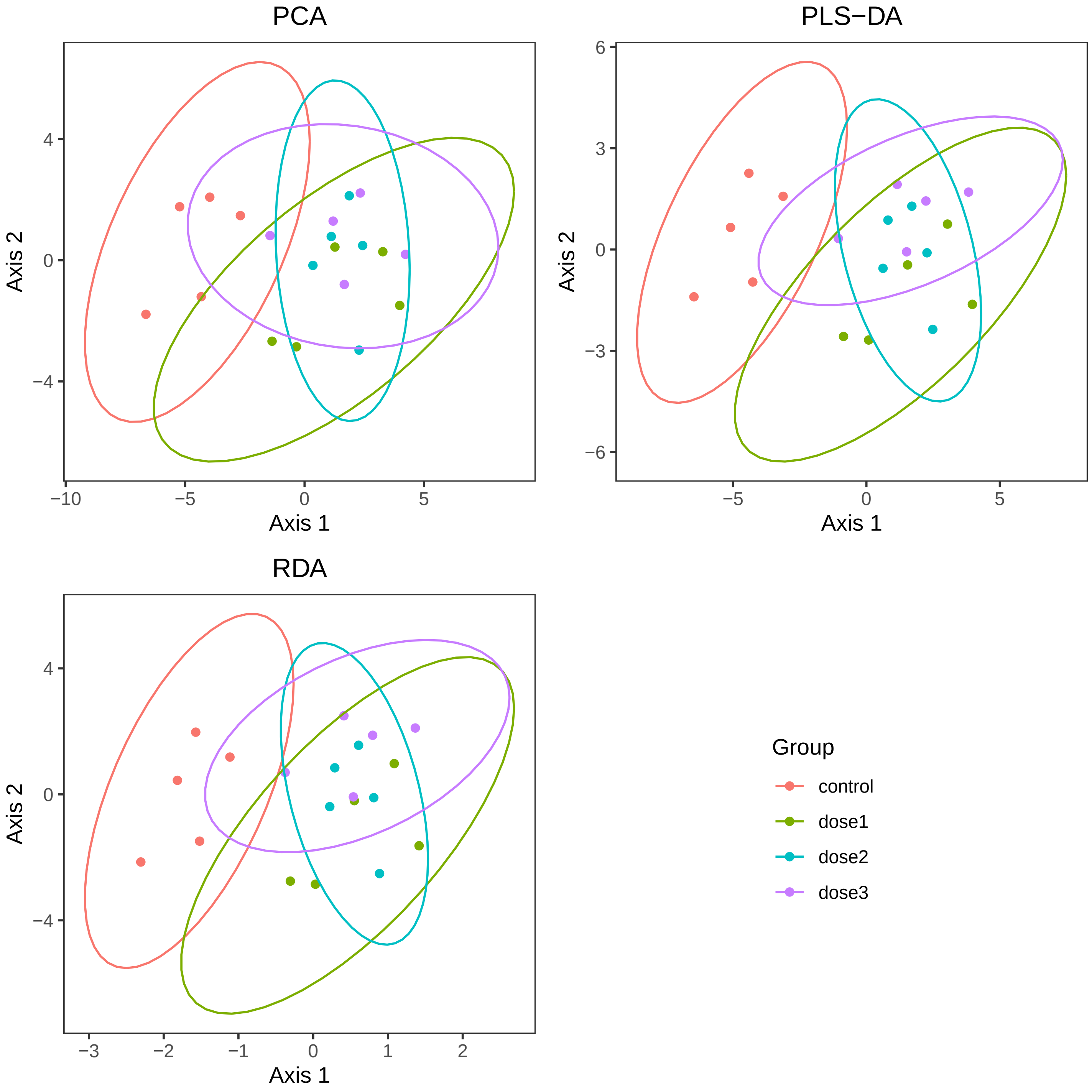
PCA
PLS−DA
RDA
Axis 1
Axis 2
Group
control
dose1
dose2
dose3

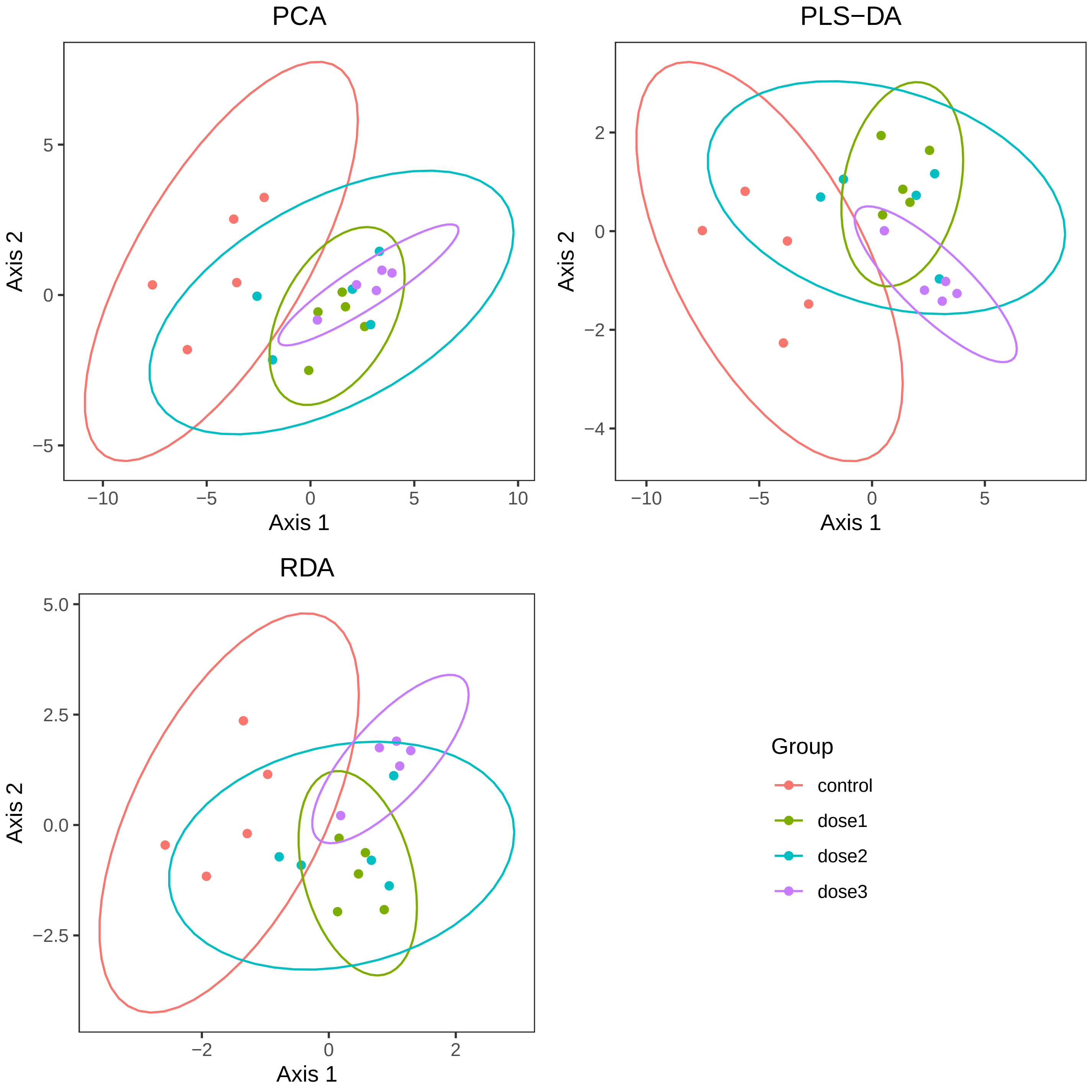

PCA
PLS−DA
RDA
Axis 1
Axis 2
Group
control
dose1
dose2
dose3

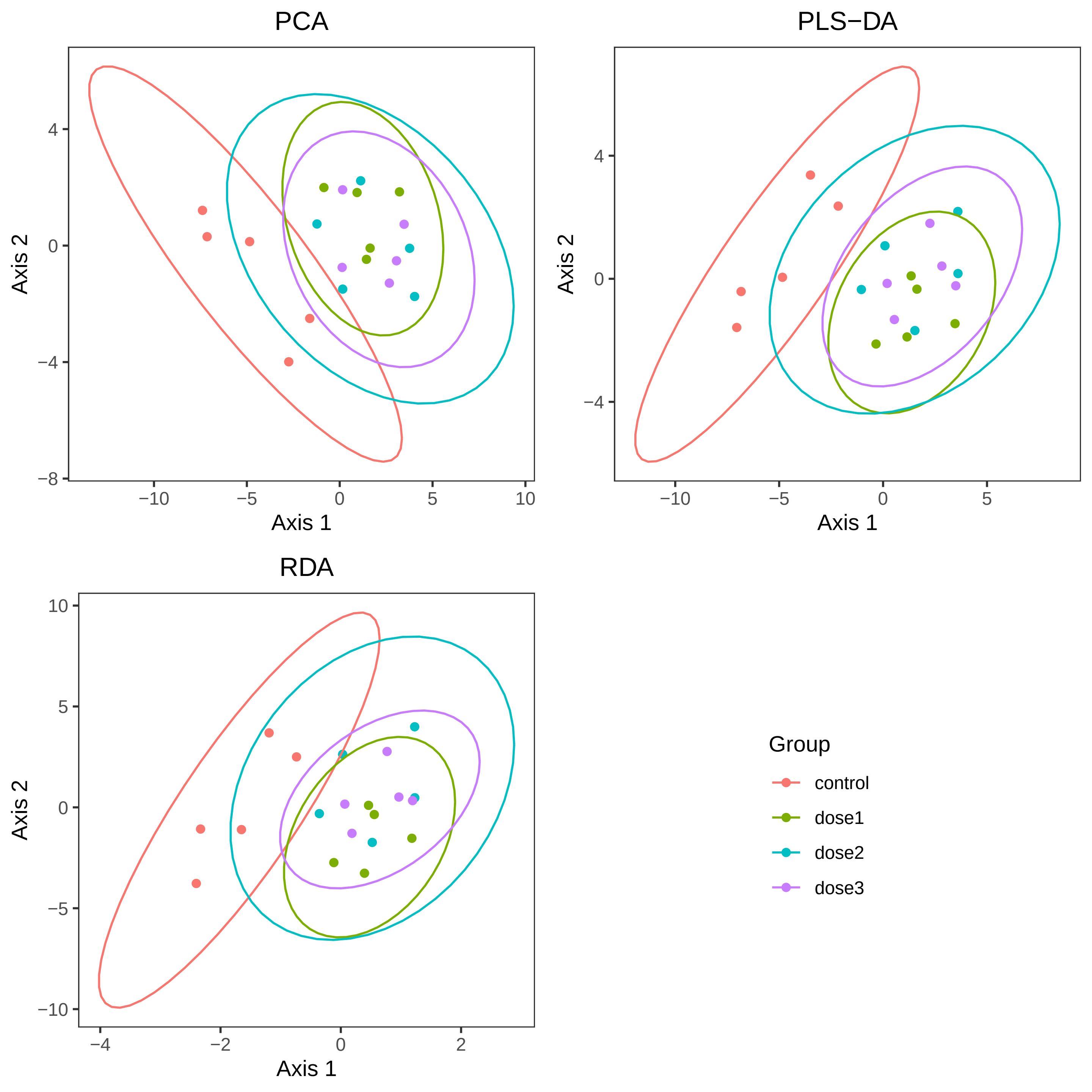

PCA
PLS−DA
RDA
Axis 1
Axis 2
Group
control
dose1
dose2
dose3

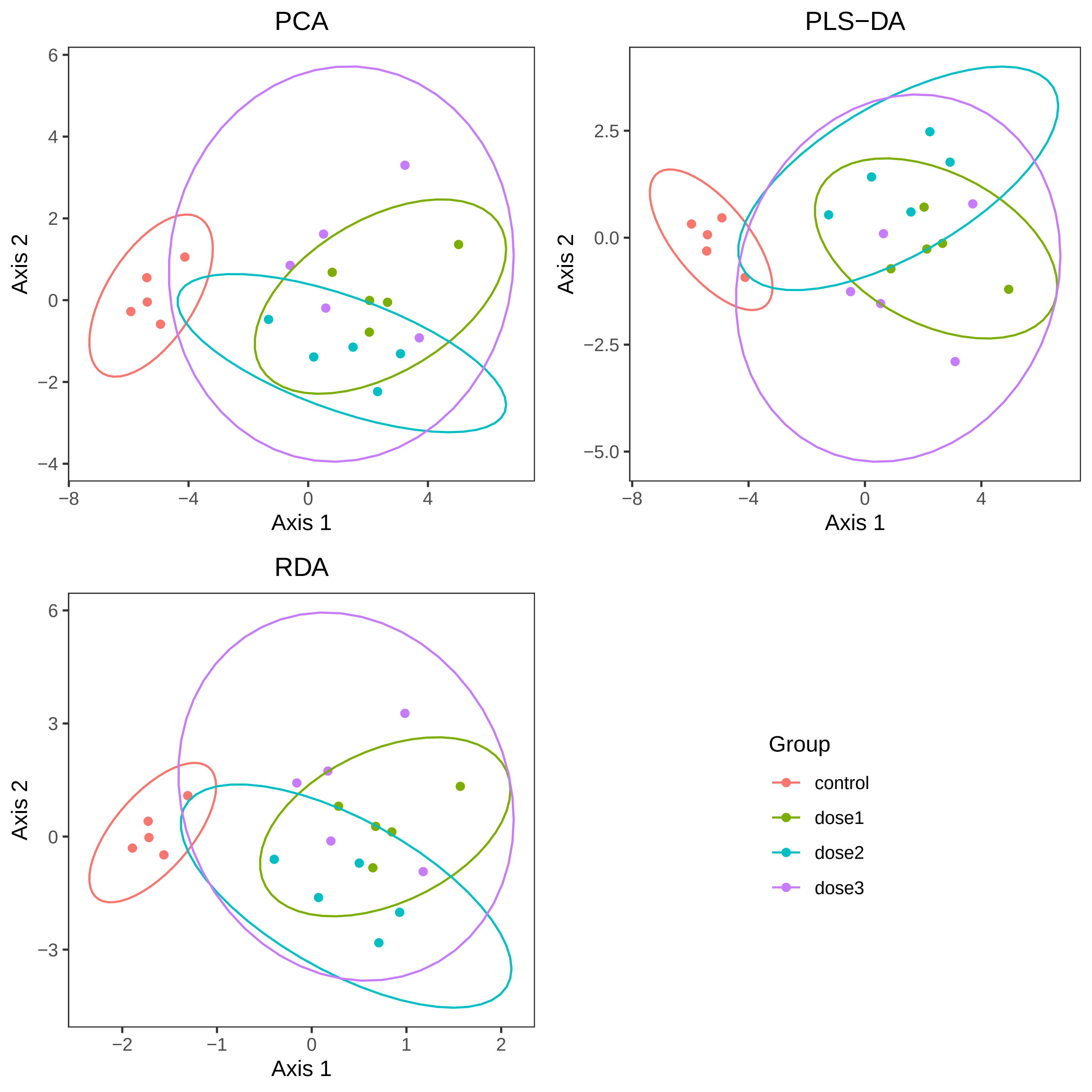
PCA
PLS−DA
RDA
Axis 1
Axis 2
Group
control
dose1
dose2
dose3

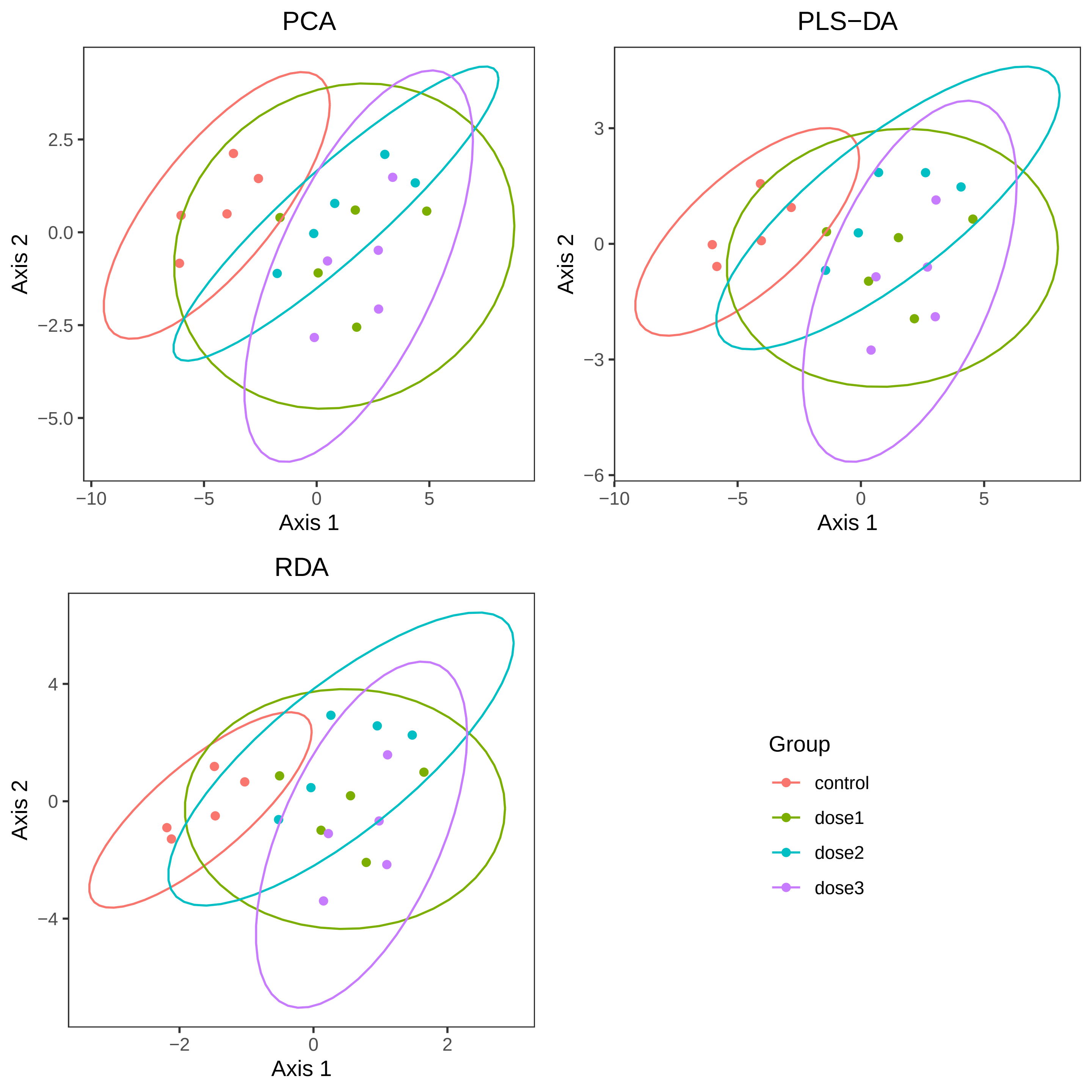

PCA
PLS−DA
RDA
Axis 1
Axis 2
2.5
0.0
−2.5
−5.0
−10
−5
0
5
3
0
−3
−6
4
0
−4
−2
2
Group
control
dose1
dose2
dose3

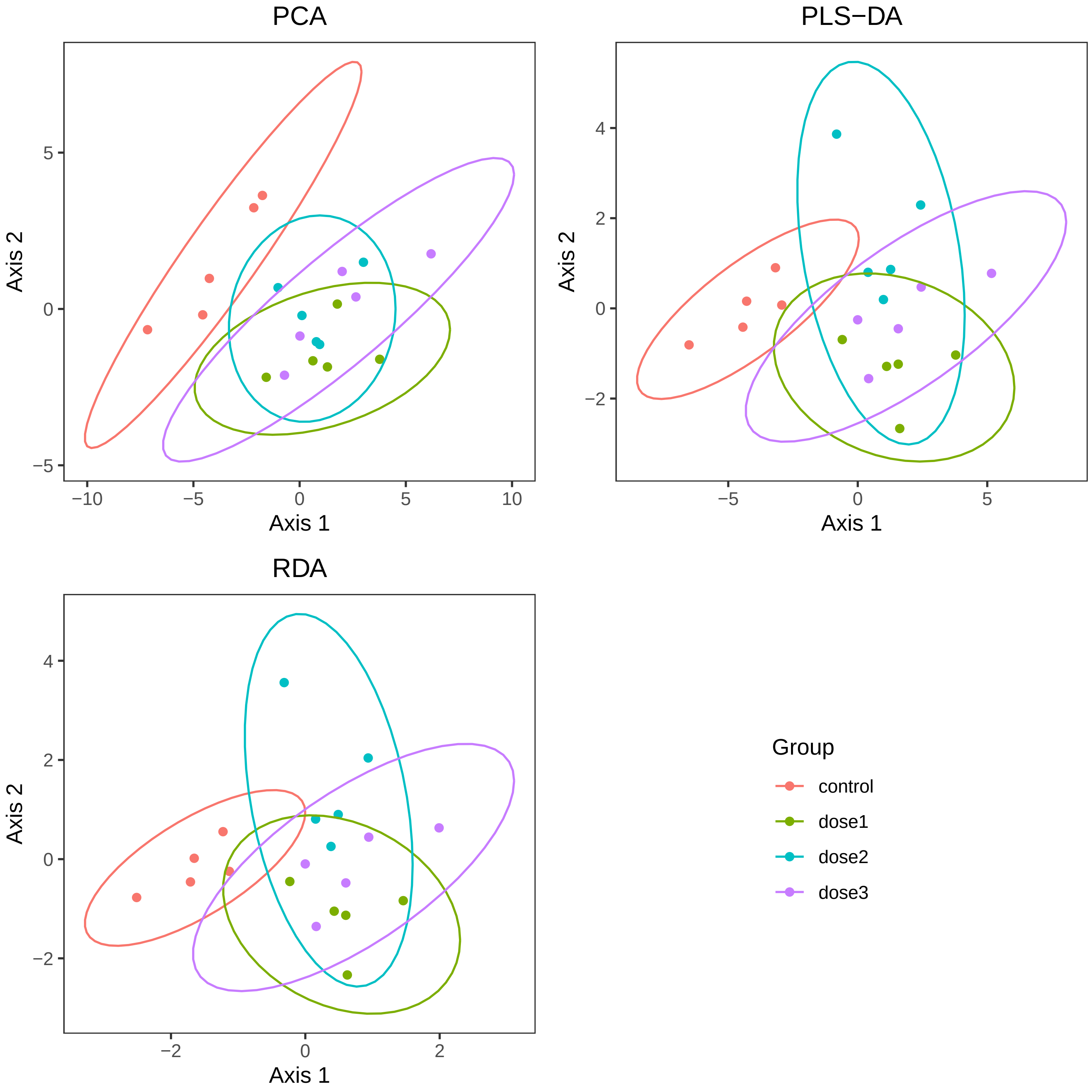
PCA
PLS−DA
RDA
Axis 1
Axis 2
Group
control
dose1
dose2
dose3

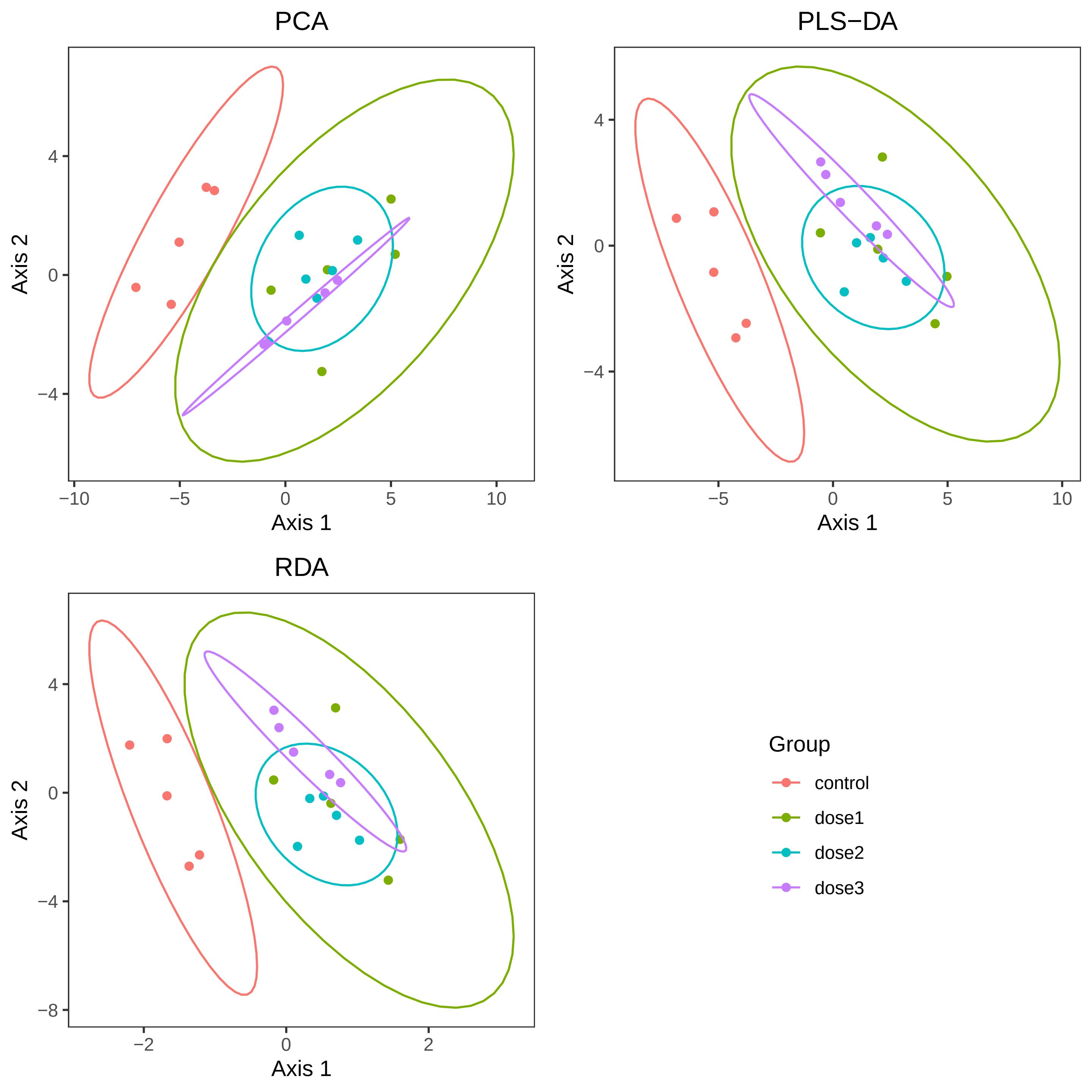

PCA
PLS−DA
RDA
Axis 1
Axis 2
Group
control
dose1
dose2
dose3

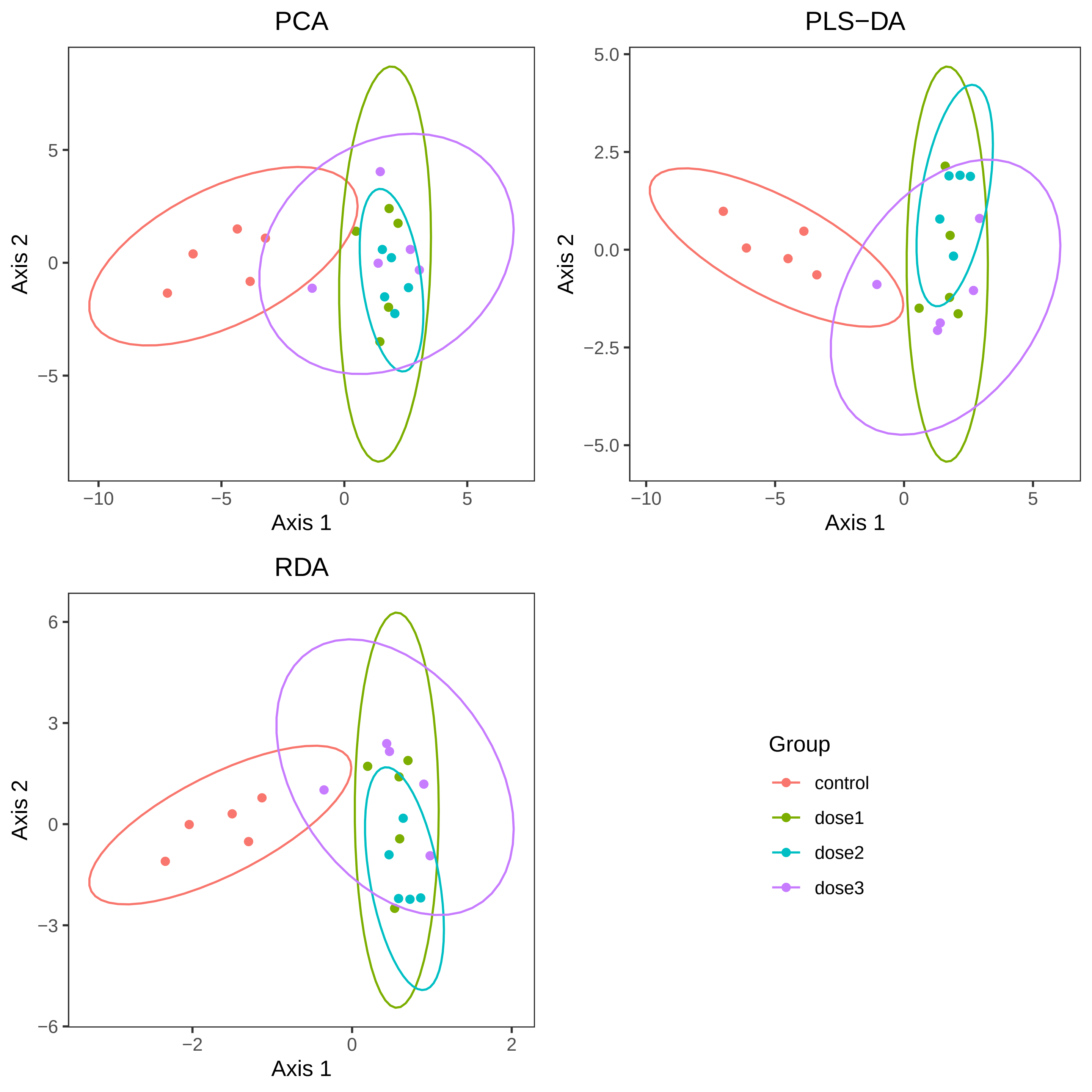
PCA
PLS−DA
RDA
Axis 1
Axis 2
Group
control
dose1
dose2
dose3

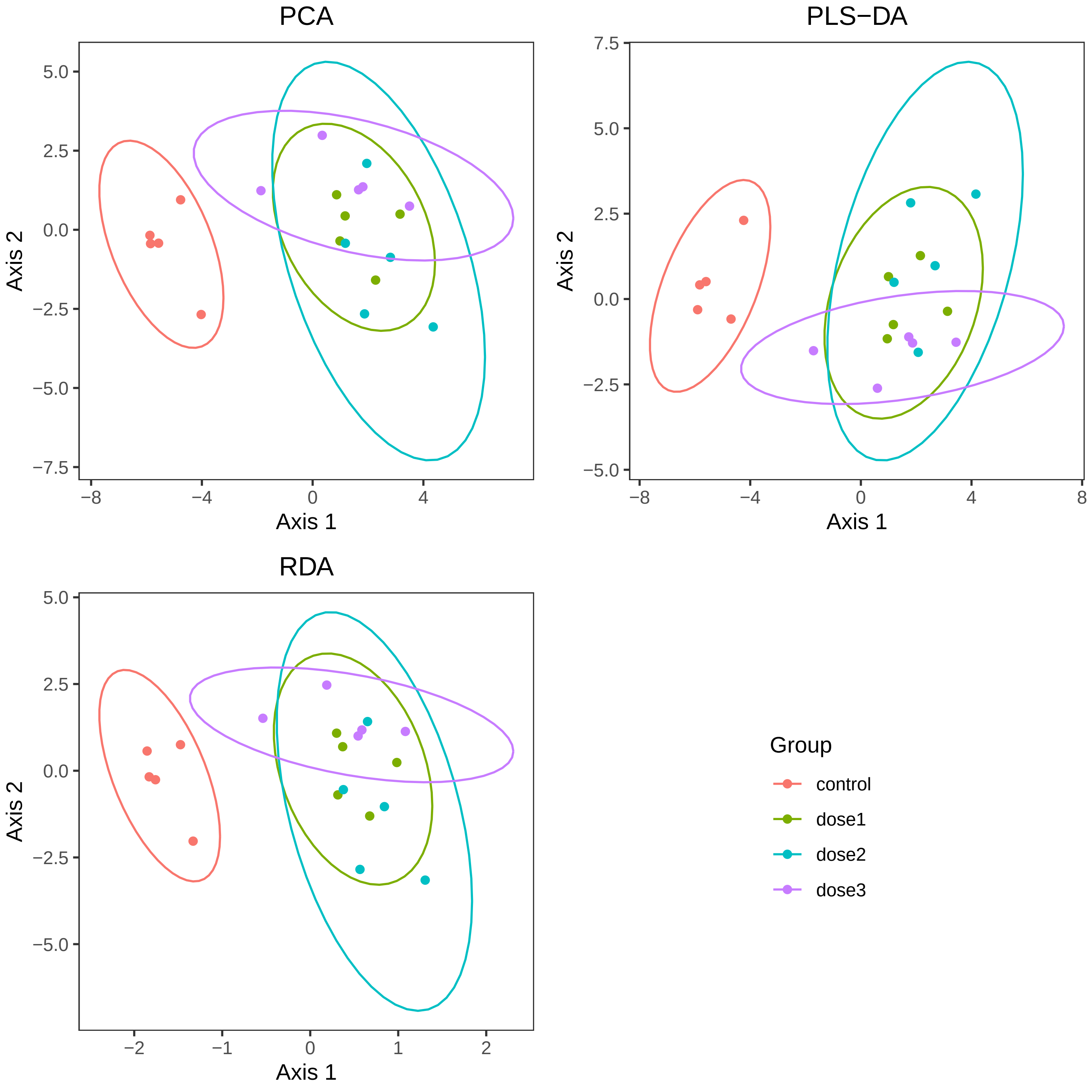
PCA
PLS−DA
RDA
Axis 1
Axis 2
5.0
2.5
0.0
−2.5
−5.0
−7.5
7.5
−8
−4
0
4
8
−2
−1
1
2
Group
control
dose1
dose2
dose3

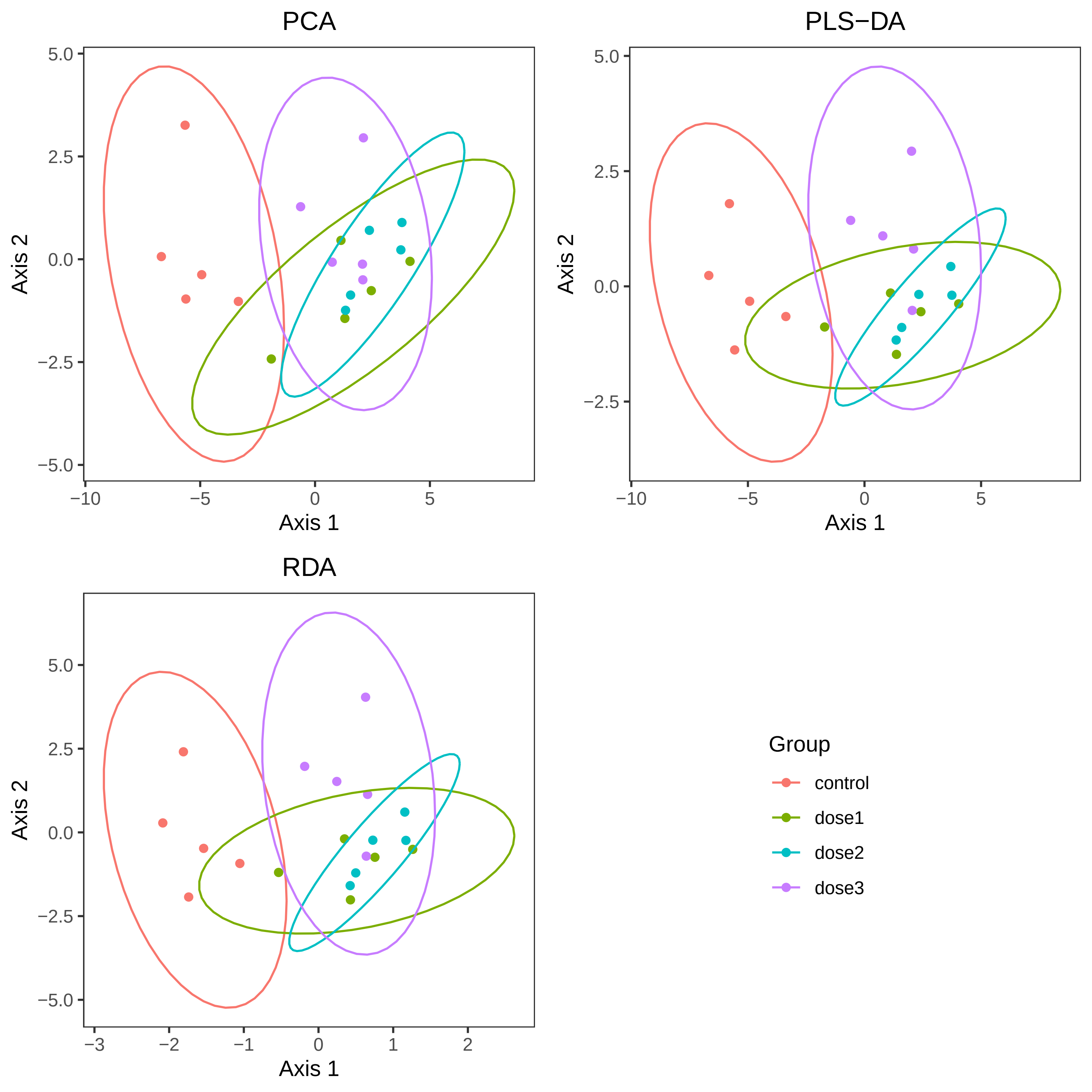
PCA
PLS−DA
RDA
Axis 1
Axis 2
Group
control
dose1
dose2
dose3

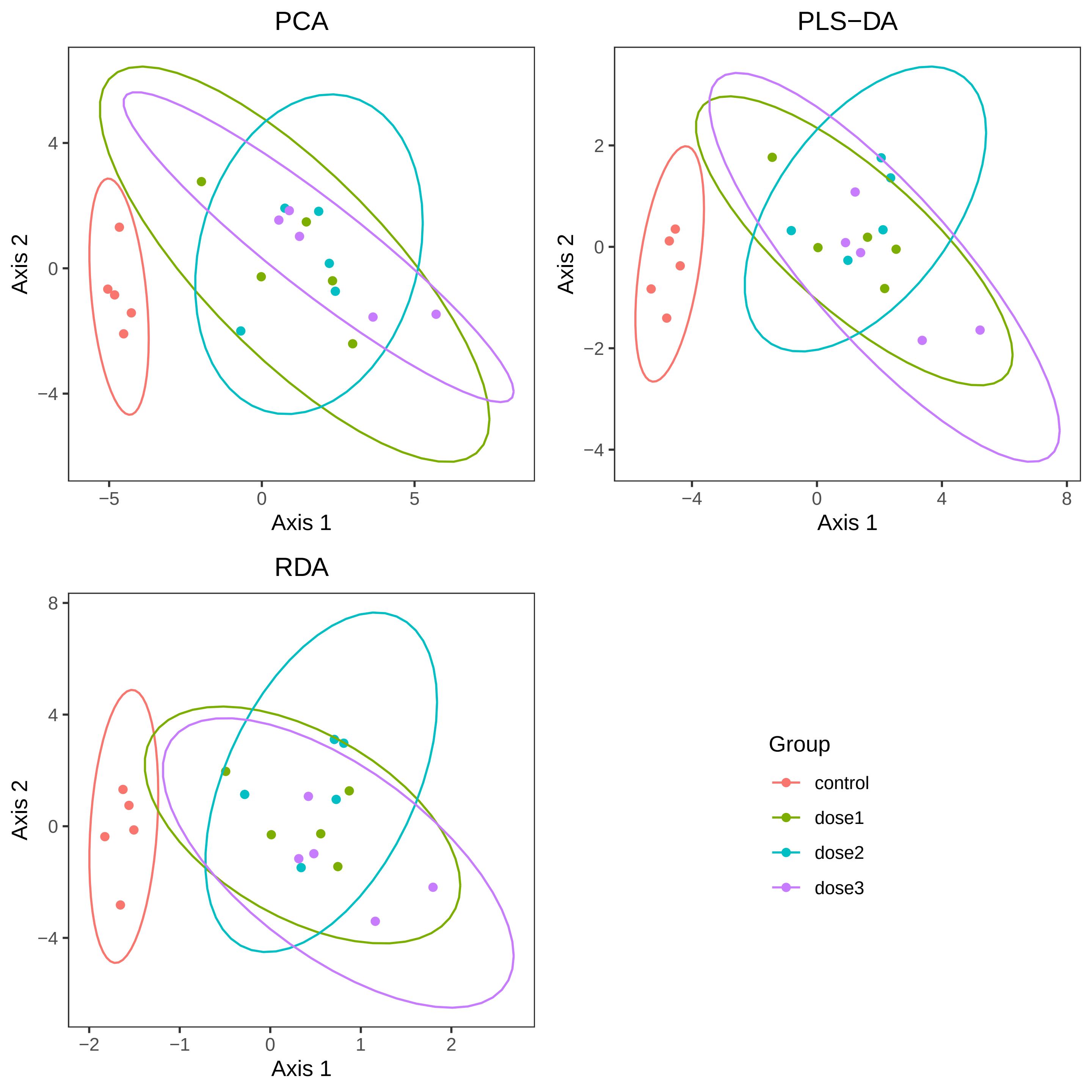
PCA
PLS−DA
RDA
Axis 1
Axis 2
Group
control
dose1
dose2
dose3

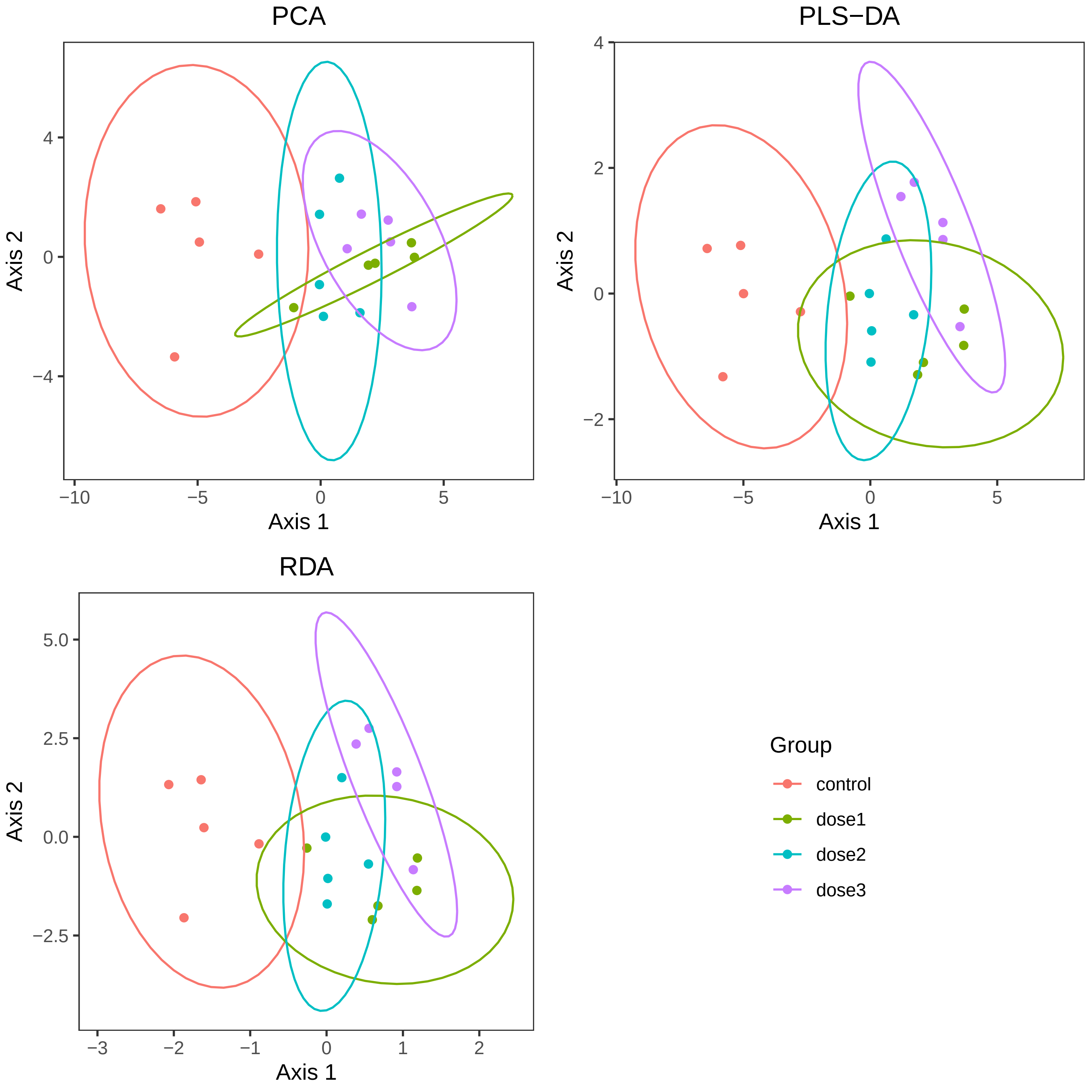
PCA
PLS−DA
RDA
Axis 1
Axis 2
Group
control
dose1
dose2
dose3

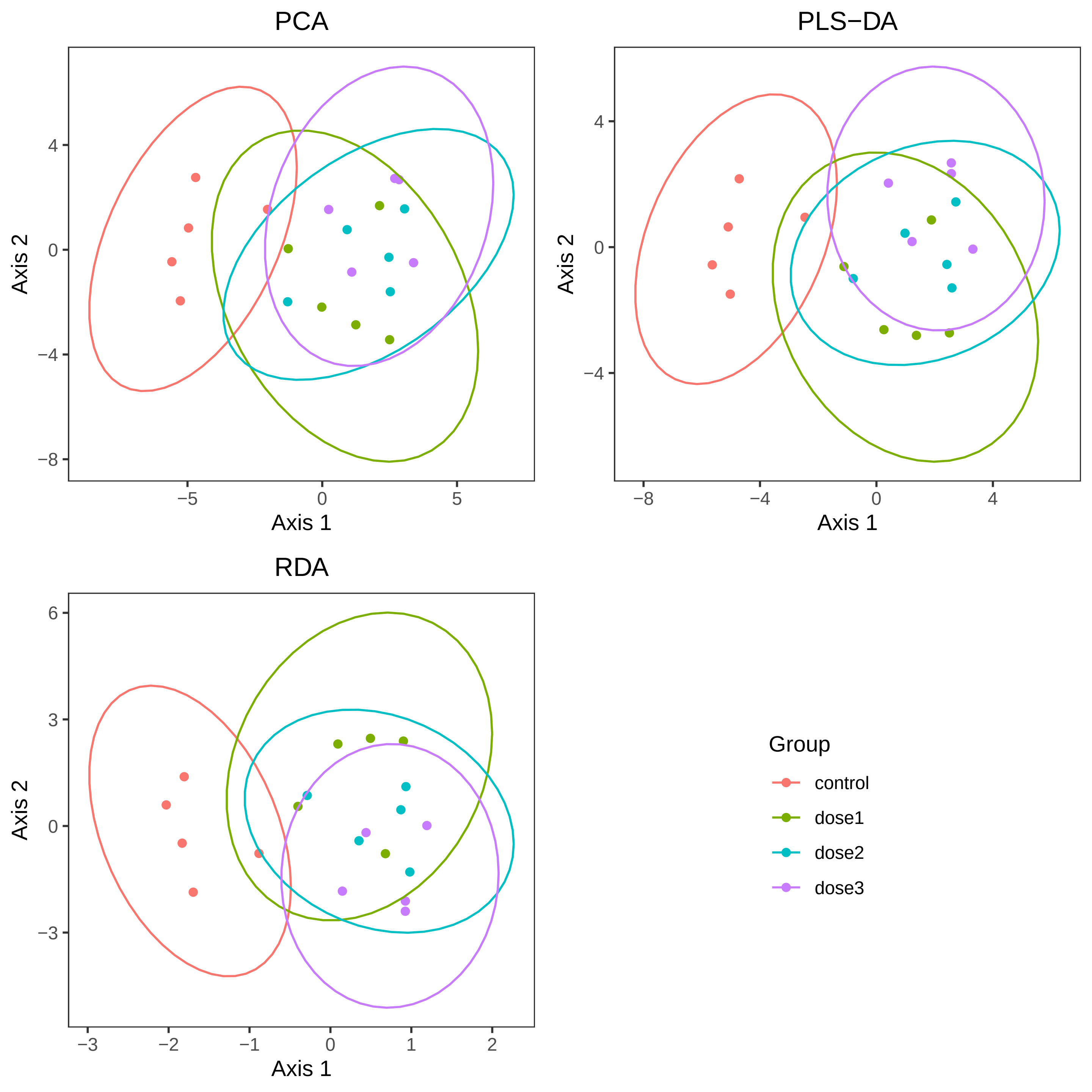
PCA
PLS−DA
RDA
Axis 1
Axis 2
−5
0
5
4
0
−4
−8
−8
−4
0
4
−3
−2
−1
0
1
2
6
3
0
−3
Group
control
dose1
dose2
dose3

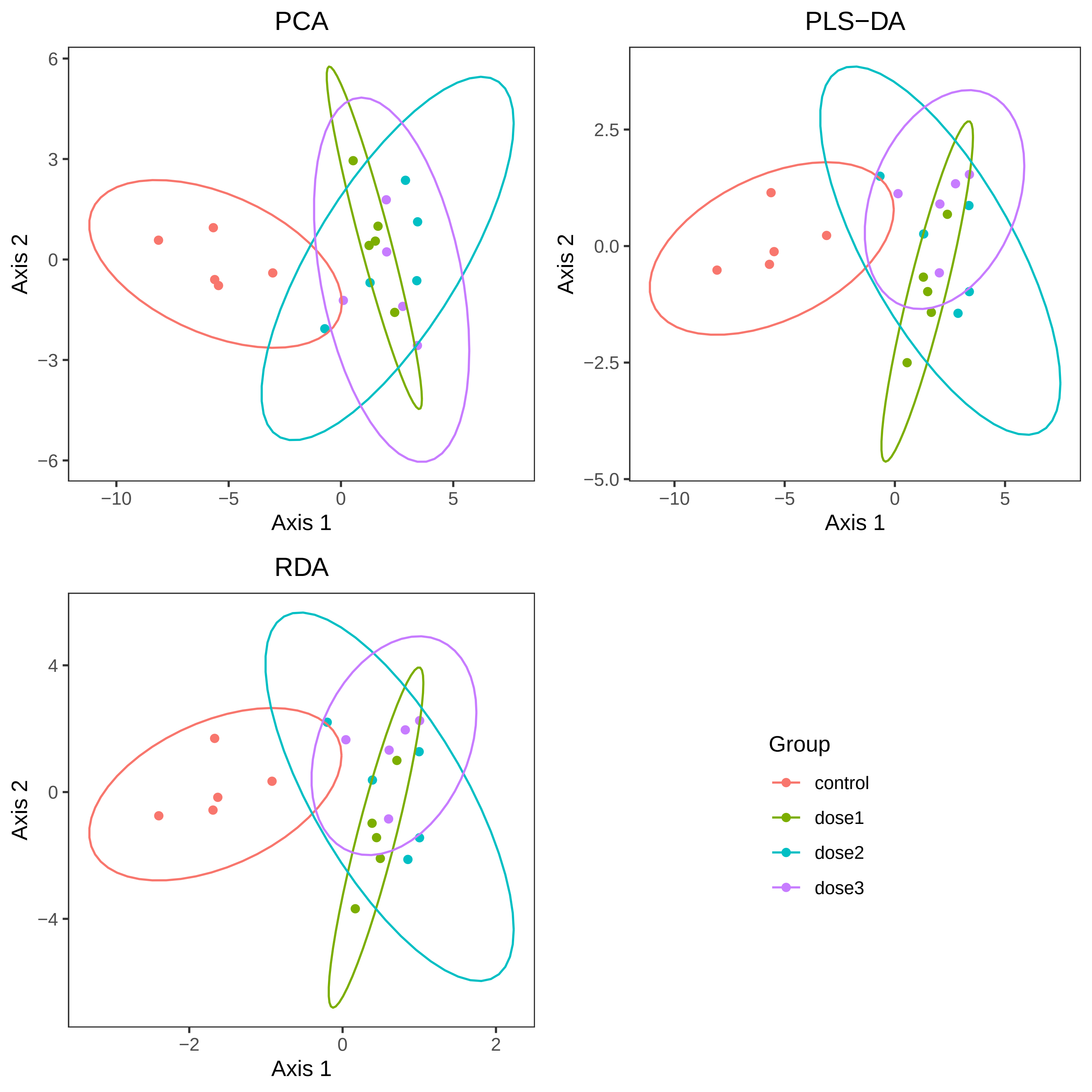
PCA
PLS−DA
RDA
Axis 1
Axis 2
Group
control
dose1
dose2
dose3

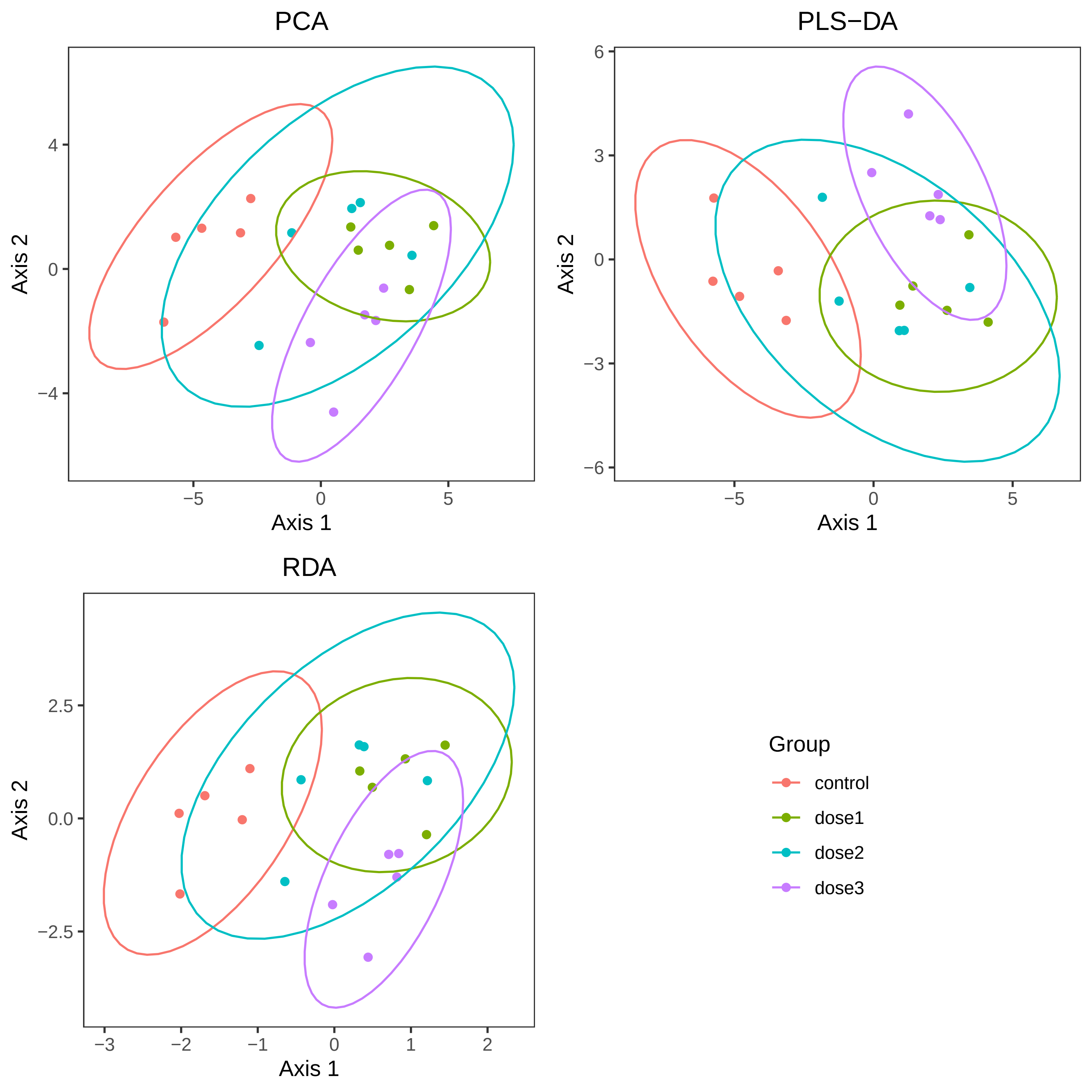
PCA
PLS−DA
RDA
Axis 1
Axis 2
Group
control
dose1
dose2
dose3

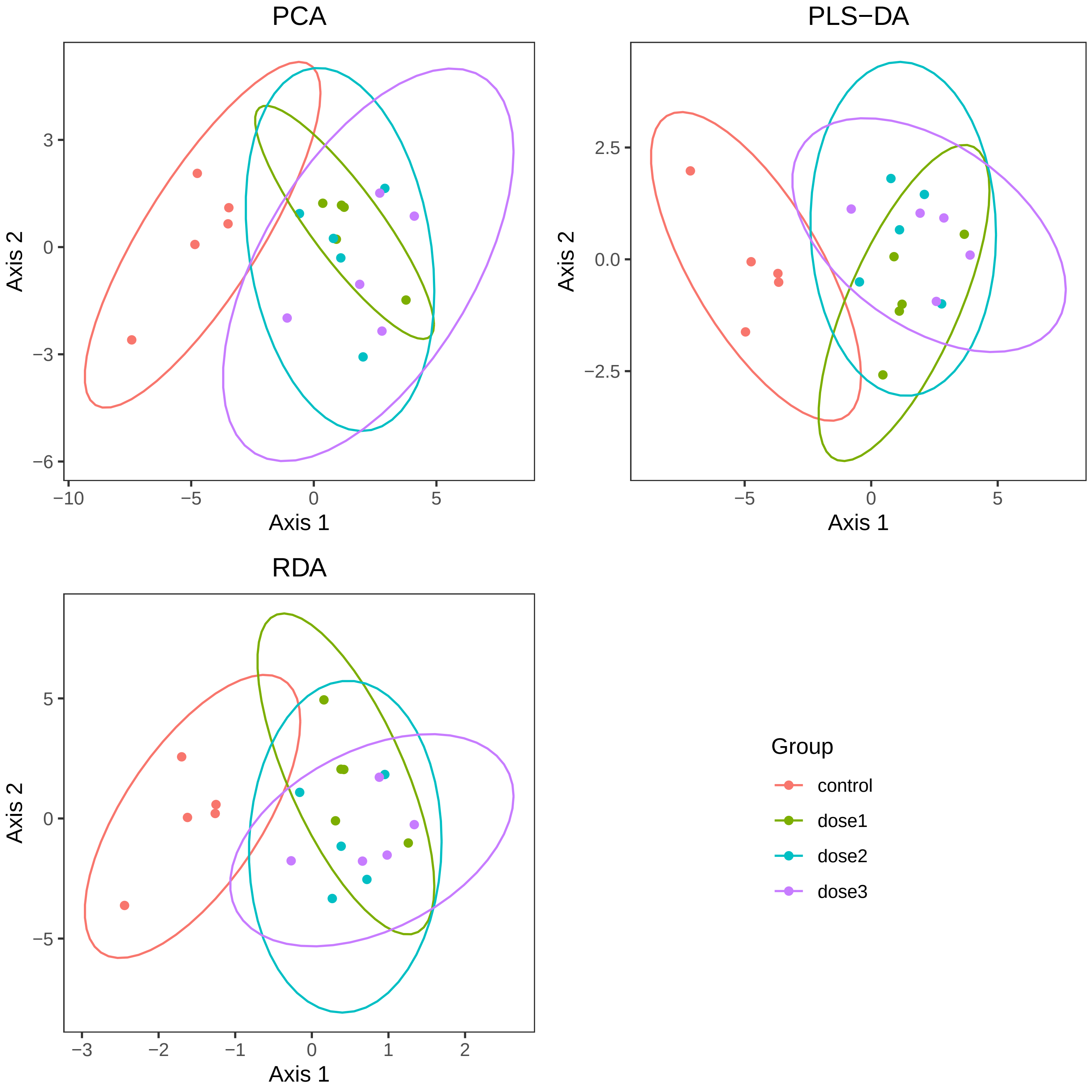

PCA
PLS−DA
RDA
Axis 1
Axis 2
Group
control
dose1
dose2
dose3

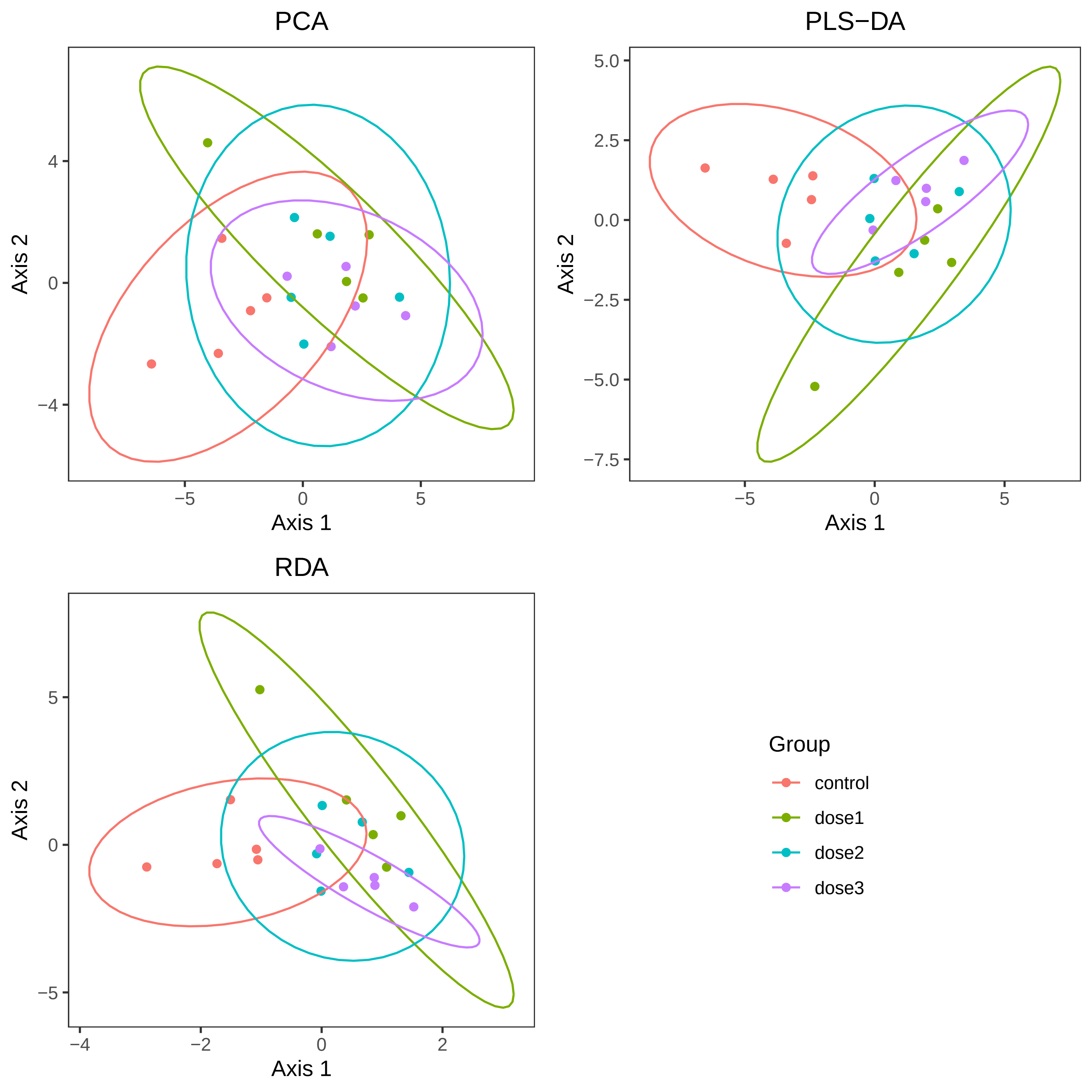
PCA
PLS−DA
RDA
Axis 1
Axis 2
Group
control
dose1
dose2
dose3

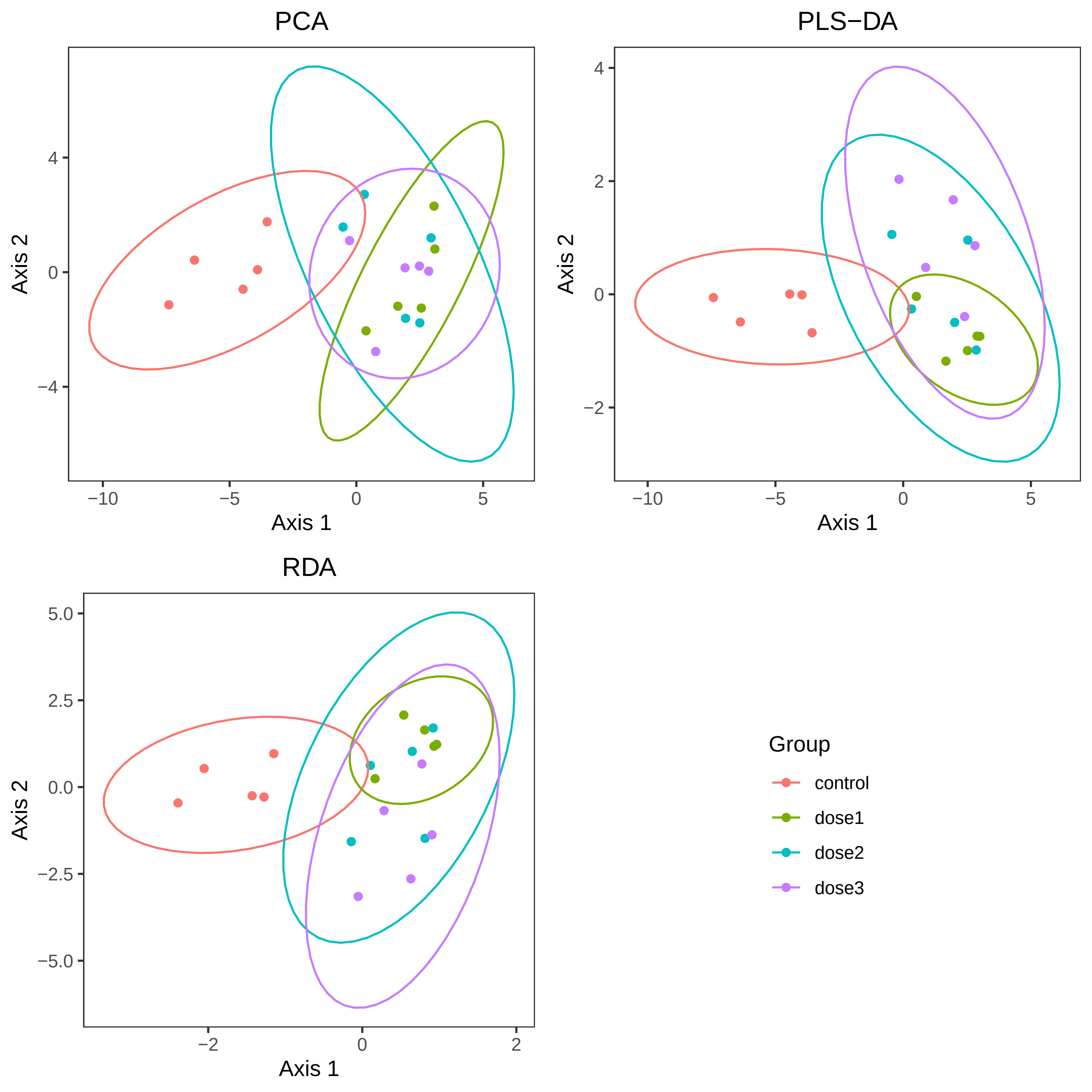

PCA
Axis 2
Axis 1
4
0
−4
−10
−5
0
5
PLS−DA
Axis 2
Axis 1
4
2
0
−2
−10
−5
0
5
RDA
Axis 2
Axis 1
5.0
2.5
0.0
−2.5
−5.0
−2
0
2
Group
control
dose1
dose2
dose3

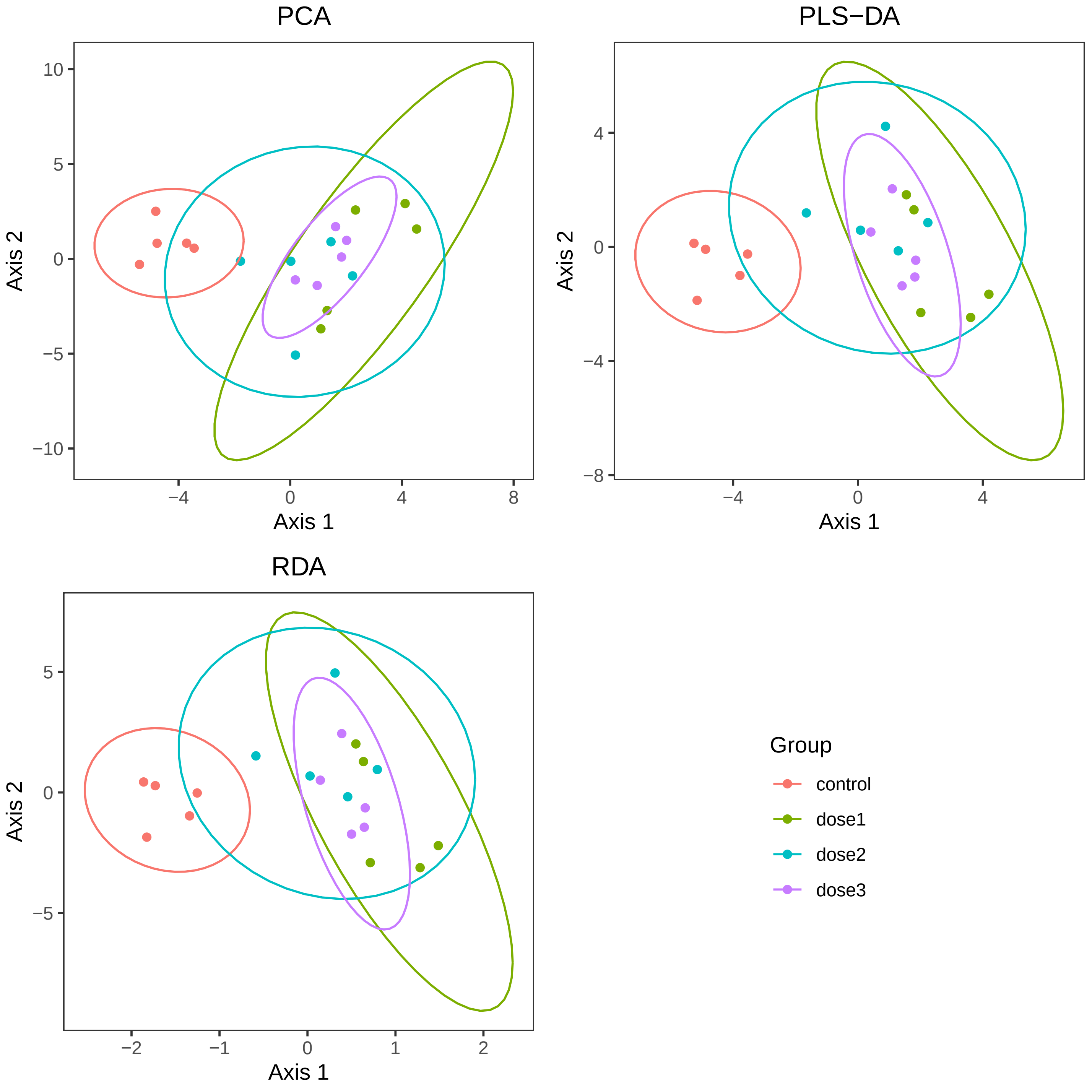
PCA
PLS−DA
RDA
Axis 1
Axis 2
Group
control
dose1
dose2
dose3

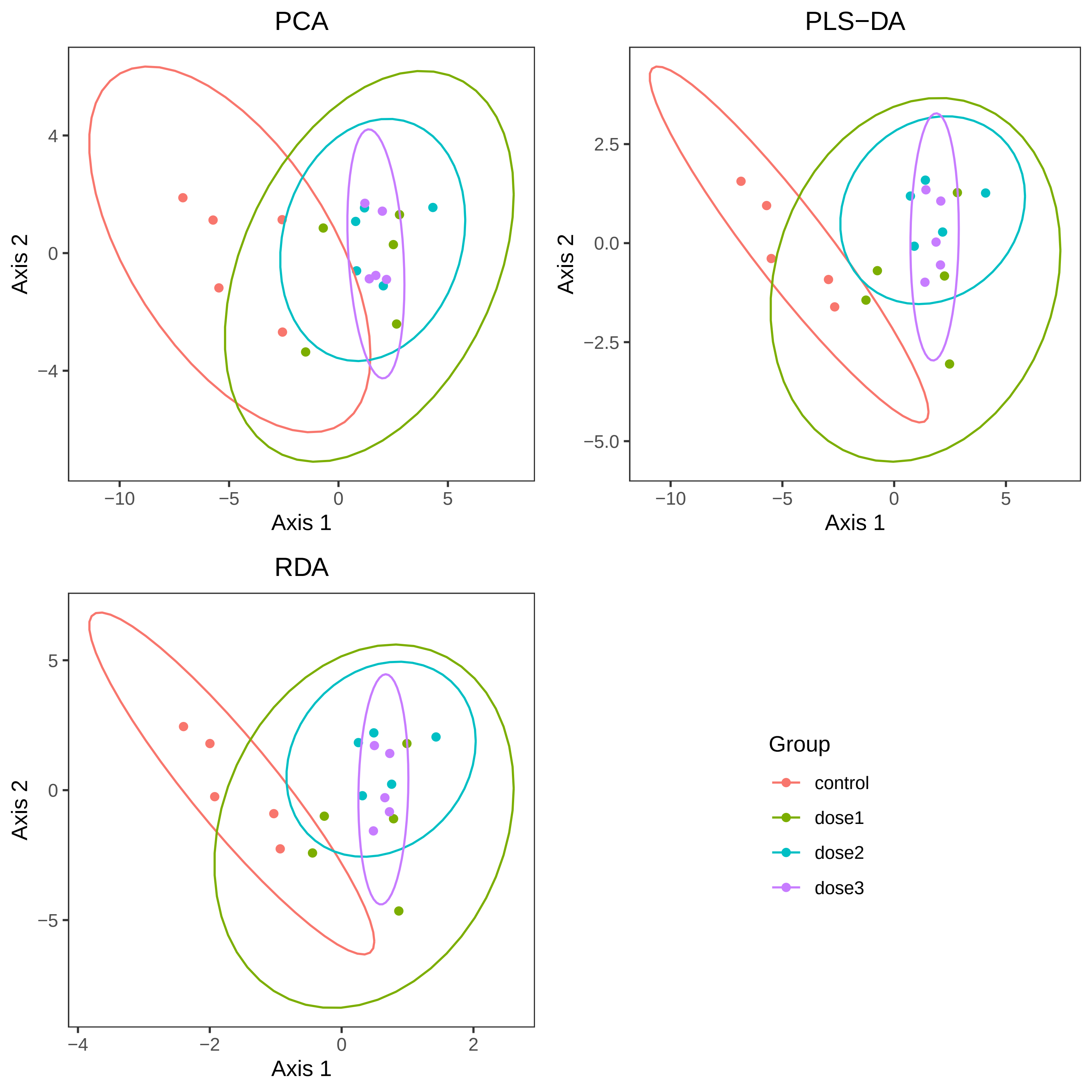
PCA
PLS−DA
RDA
Axis 1
Axis 2
−10
−5
0
5
4
−4
2.5
0.0
−2.5
−5.0
−4
−2
2
Group
control
dose1
dose2
dose3

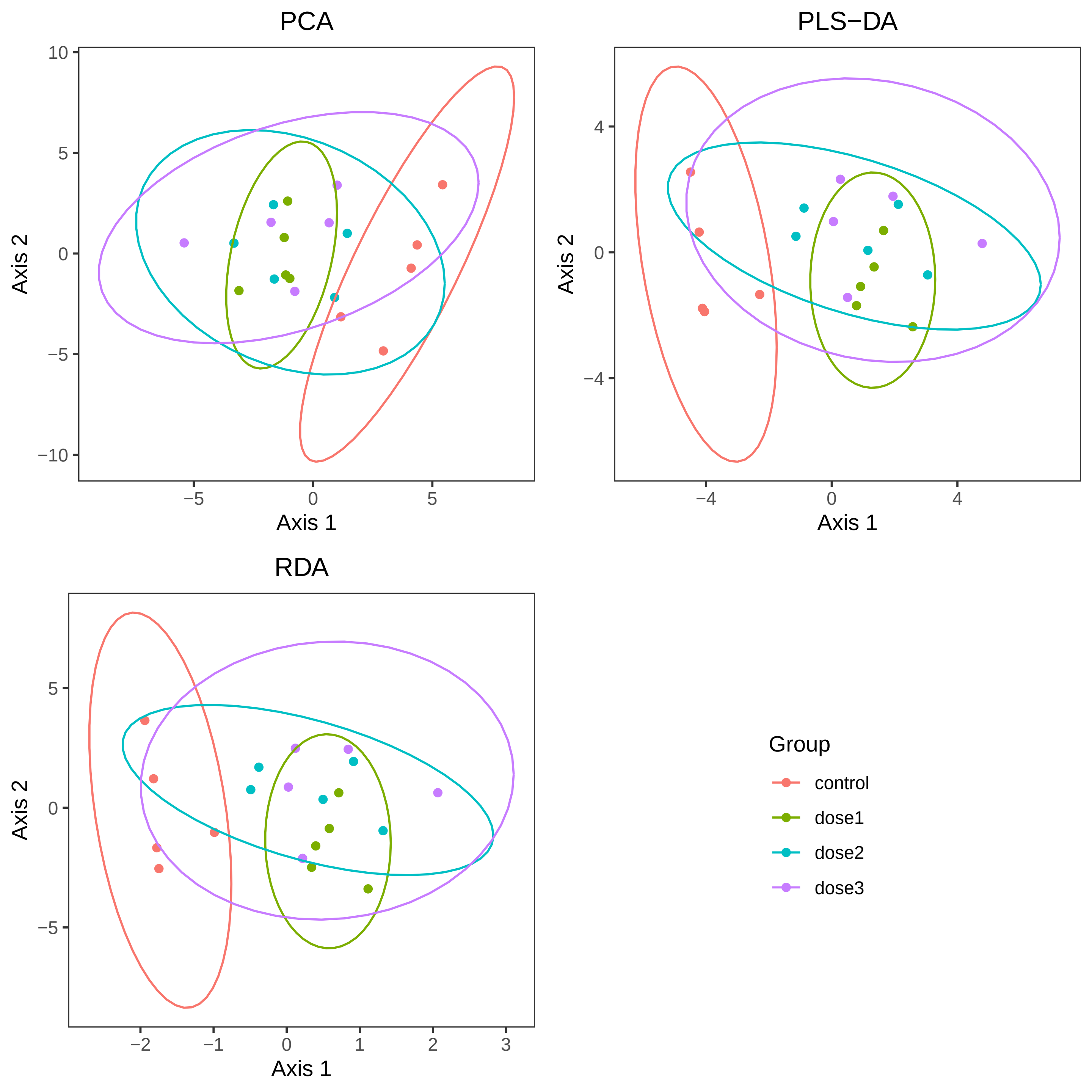
PCA
Axis 1
Axis 2
PLS−DA
Axis 1
Axis 2
RDA
Axis 1
Axis 2
Group
control
dose1
dose2
dose3

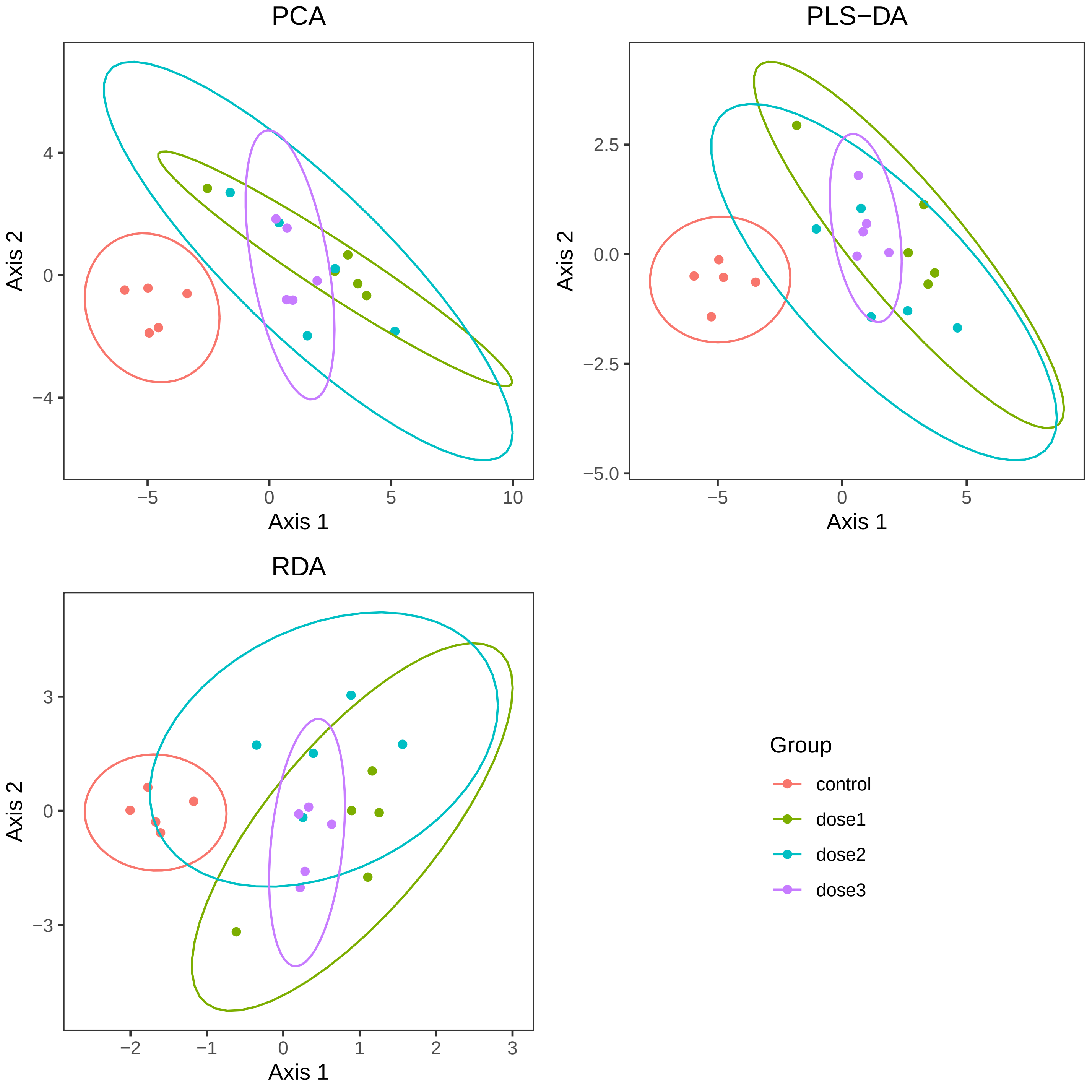
PCA
PLS−DA
RDA
Axis 1
Axis 2
−5
0
5
10
4
0
−4
2.5
0.0
−2.5
−5.0
−2
−1
1
2
3
−3
Group
control
dose1
dose2
dose3

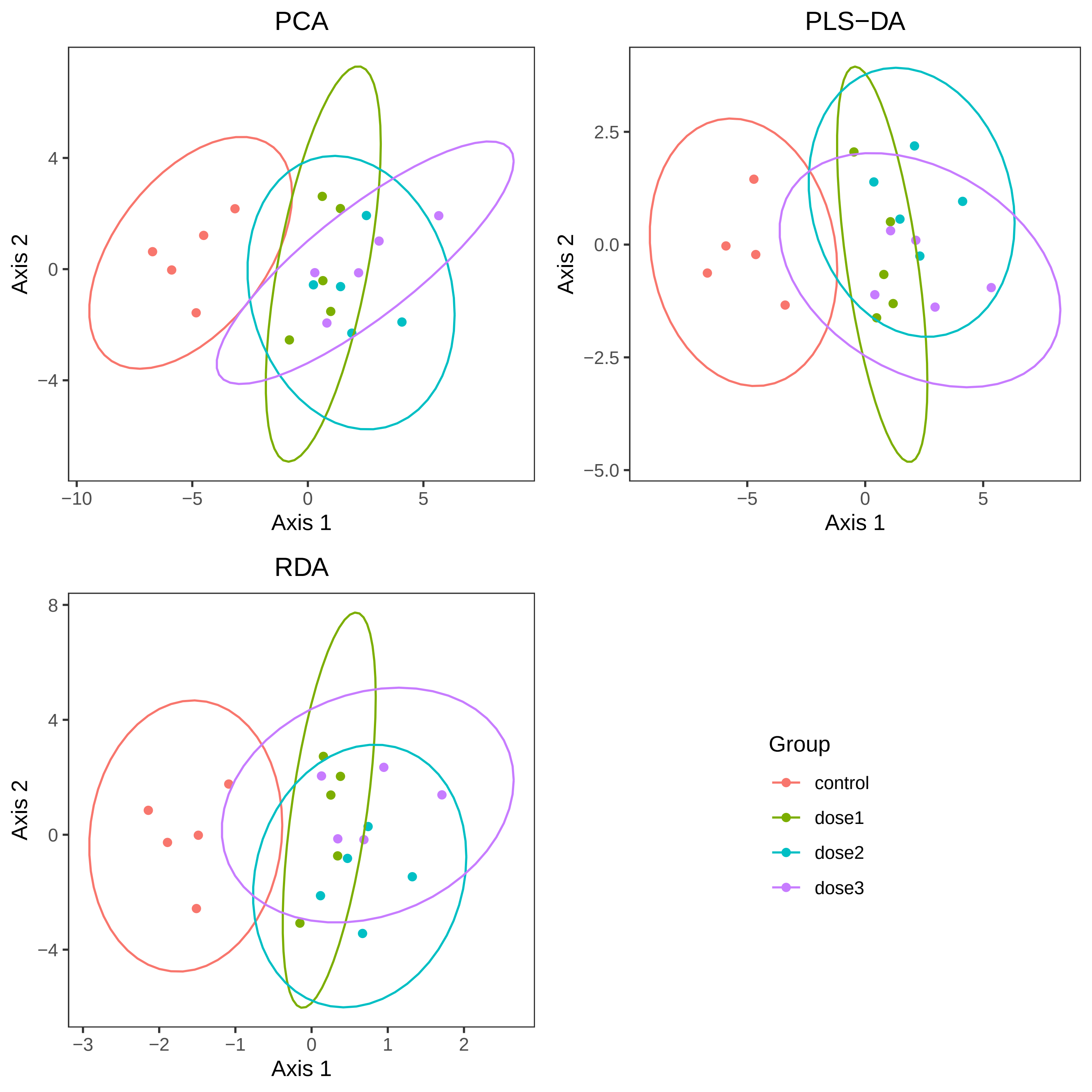
PCA
PLS−DA
RDA
Axis 1
Axis 2
Group
control
dose1
dose2
dose3

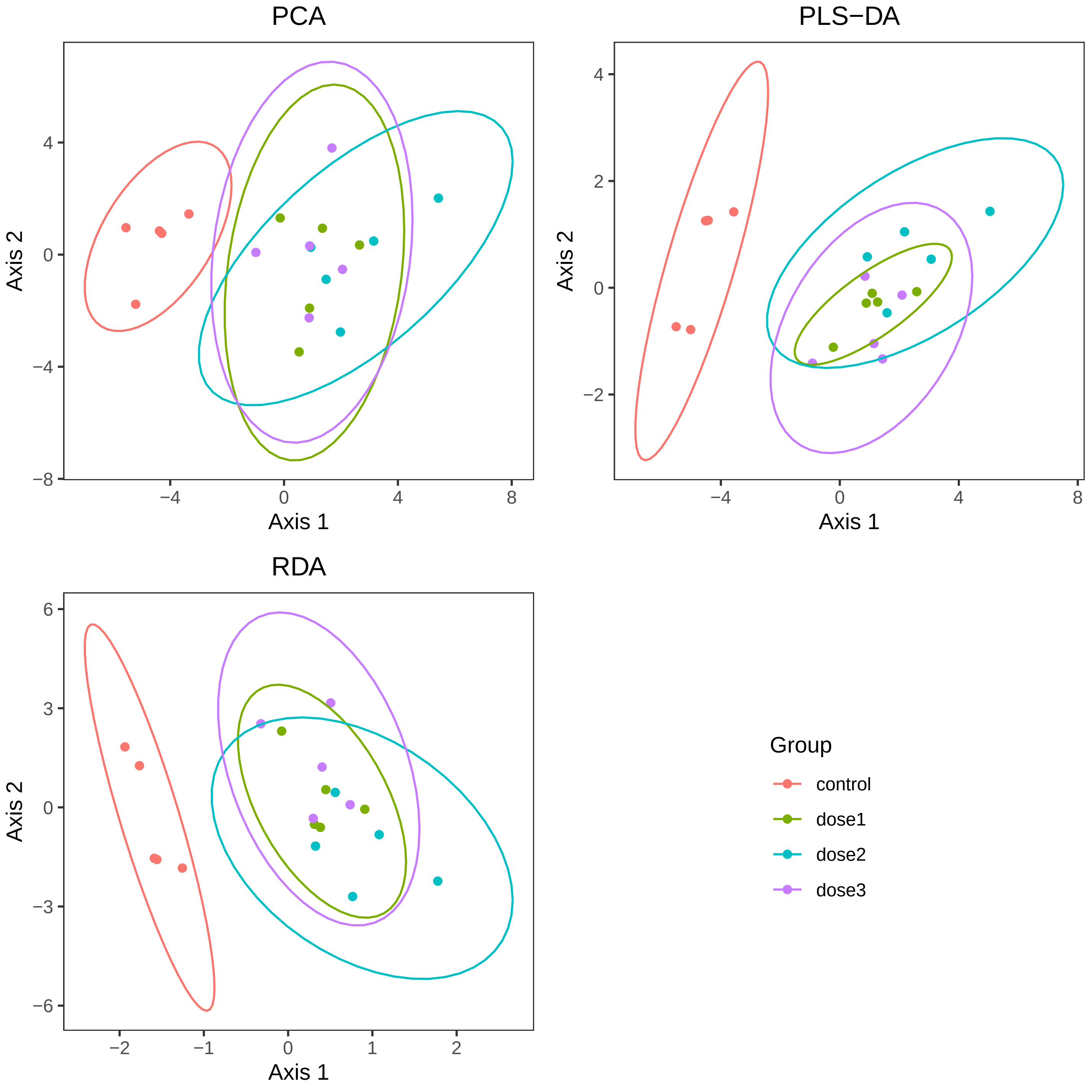

PCA
PLS−DA
RDA
Axis 1
Axis 2
Group
control
dose1
dose2
dose3

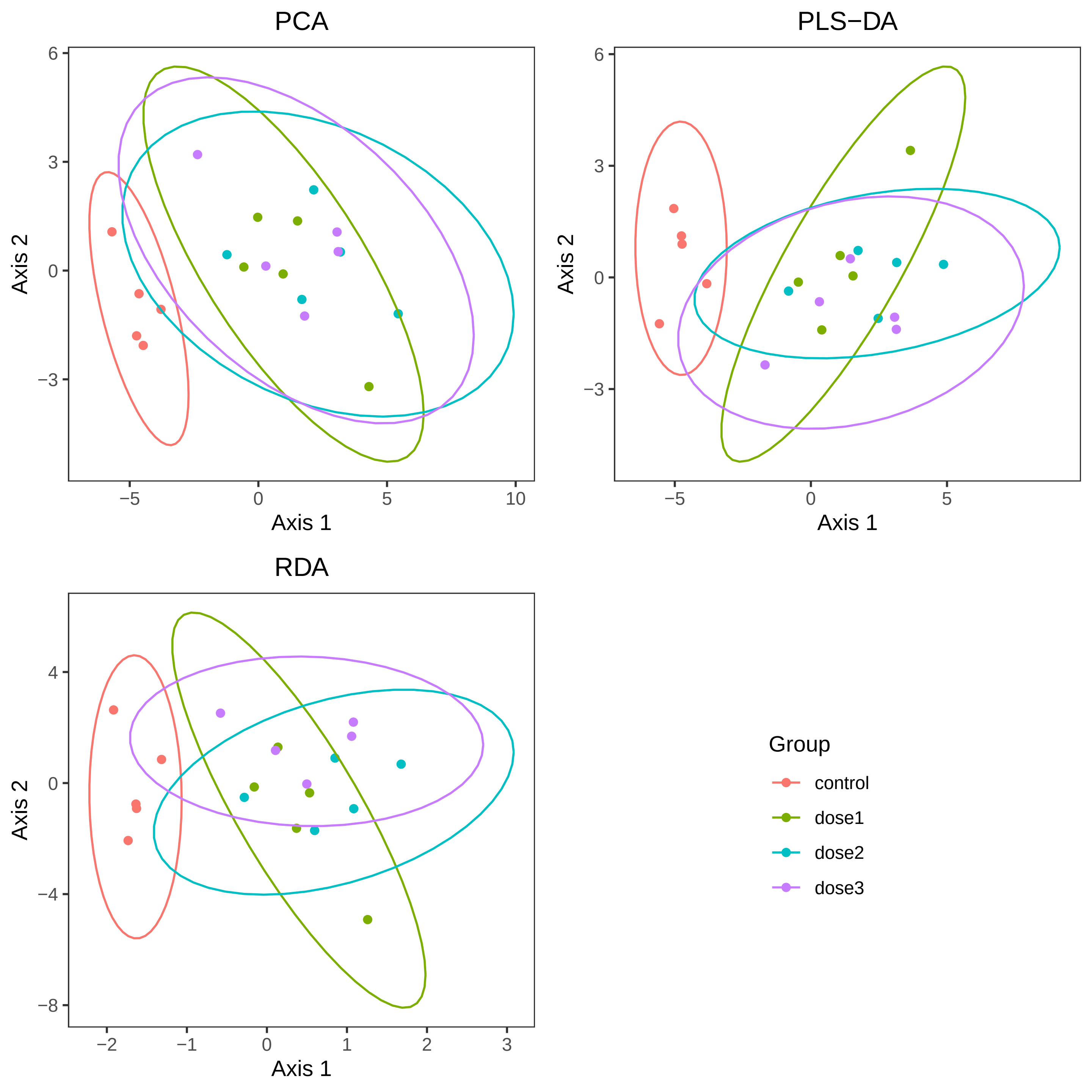

PCA
PLS−DA
RDA
Axis 1
Axis 2
Group
control
dose1
dose2
dose3

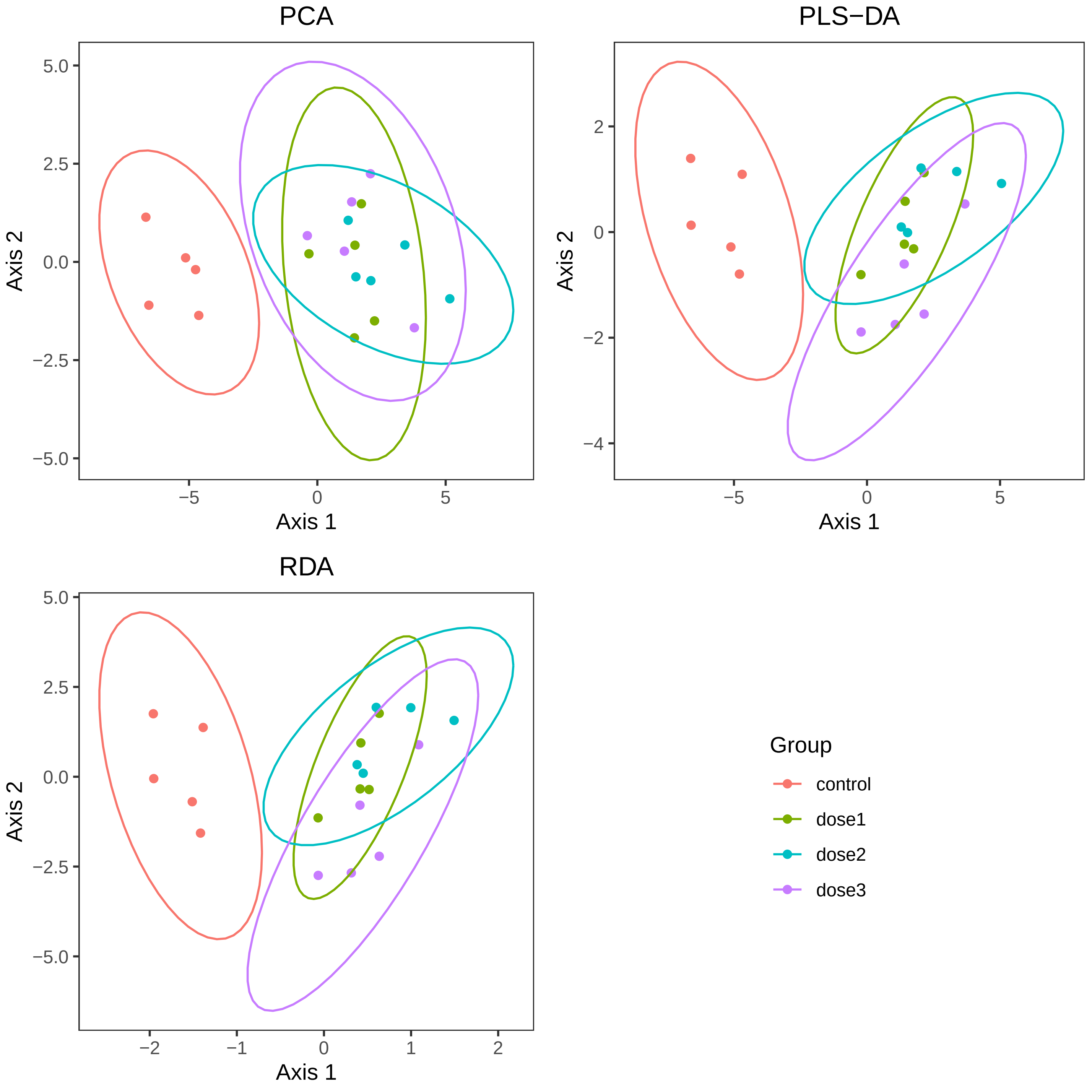

PCA
PLS−DA
RDA
Axis 1
Axis 2
Group
control
dose1
dose2
dose3

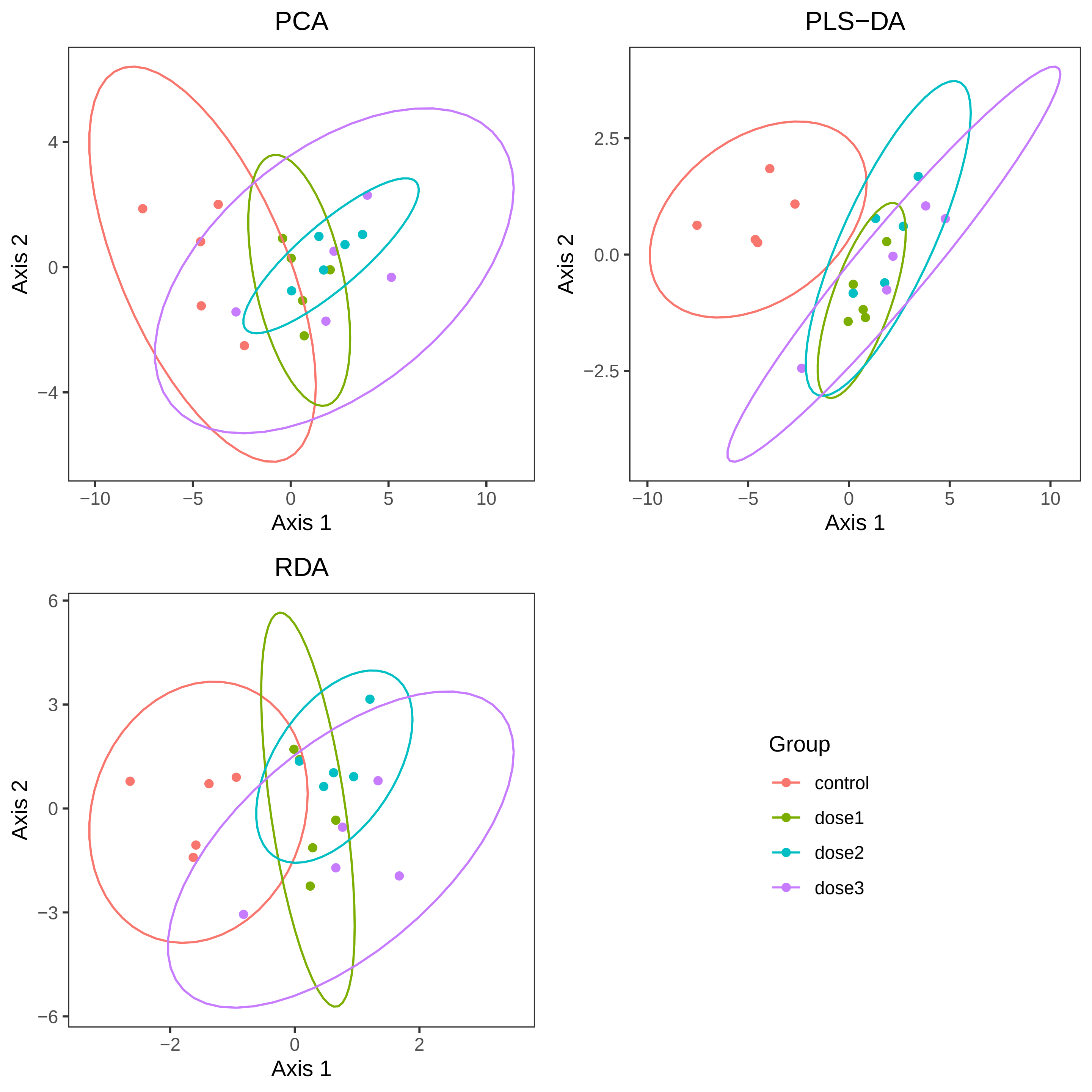
PCA
PLS−DA
RDA
Axis 1
Axis 2
−10
−5
0
5
10
4
−4
2.5
0.0
−2.5
−2
2
6
3
−3
−6
Group
control
dose1
dose2
dose3

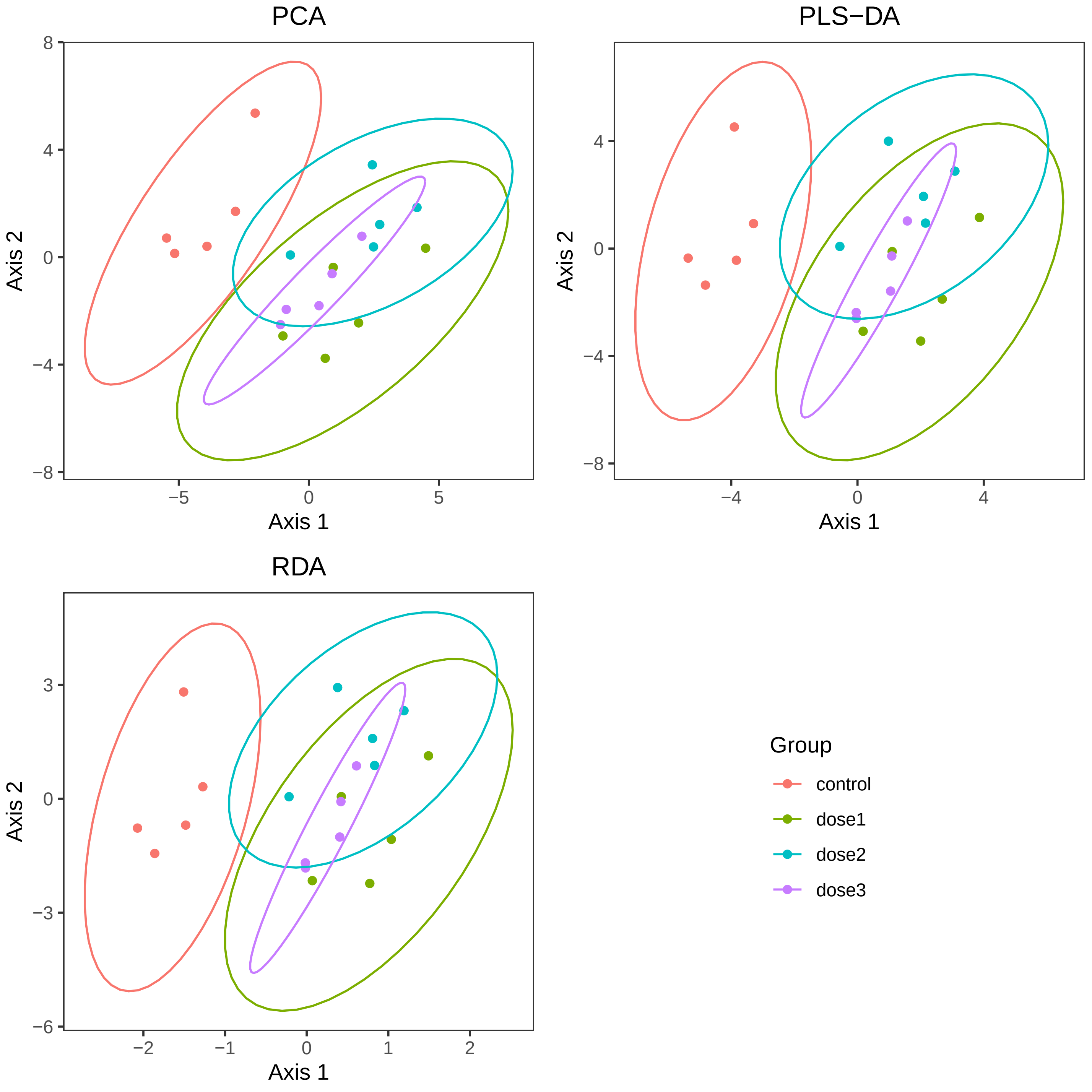

PCA
PLS−DA
RDA
Axis 1
Axis 2
8
4
0
−4
−8
−5
0
5
−4
0
4
3
−3
−6
−2
−1
1
2
Group
control
dose1
dose2
dose3

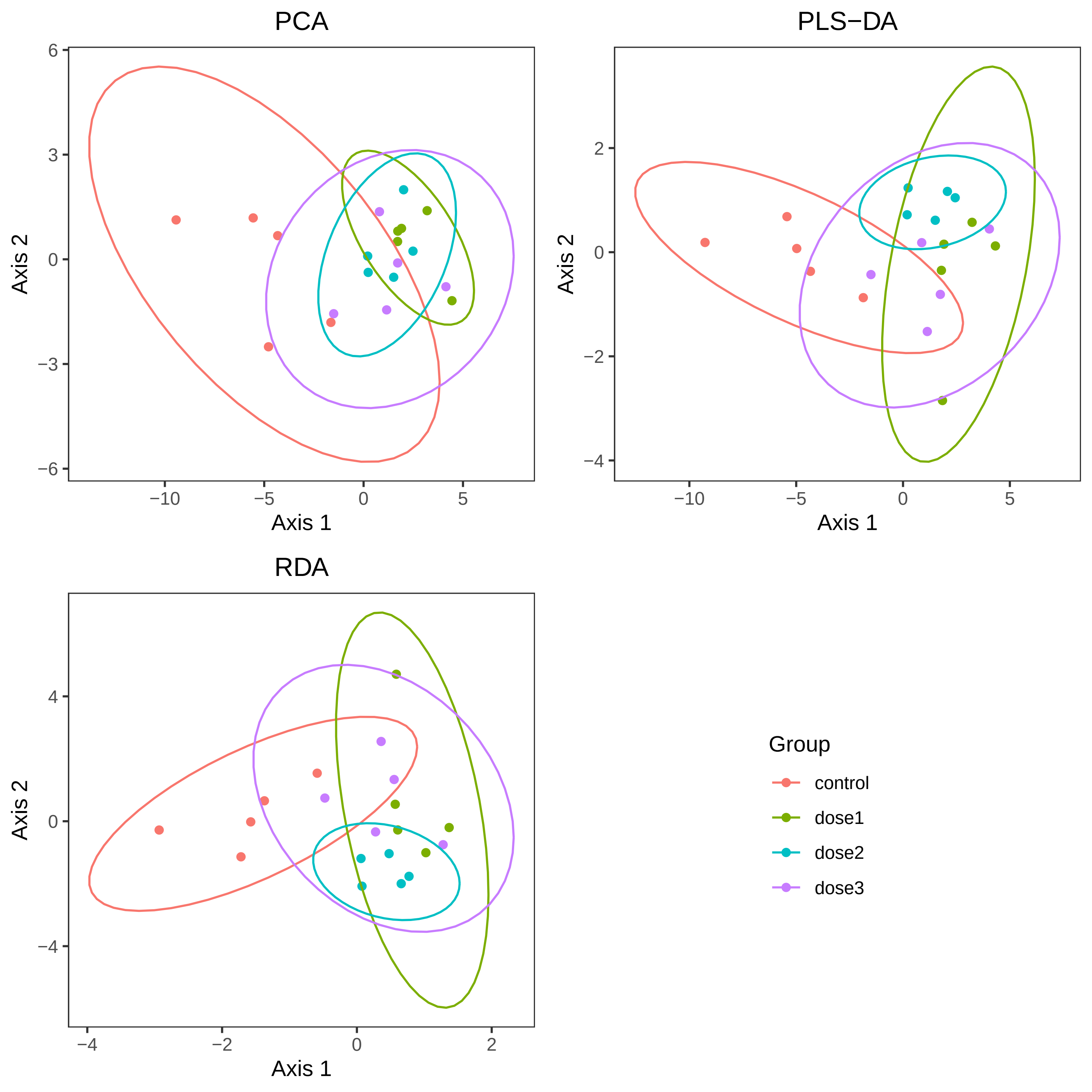
PCA
PLS−DA
RDA
Axis 1
Axis 2
Group
control
dose1
dose2
dose3

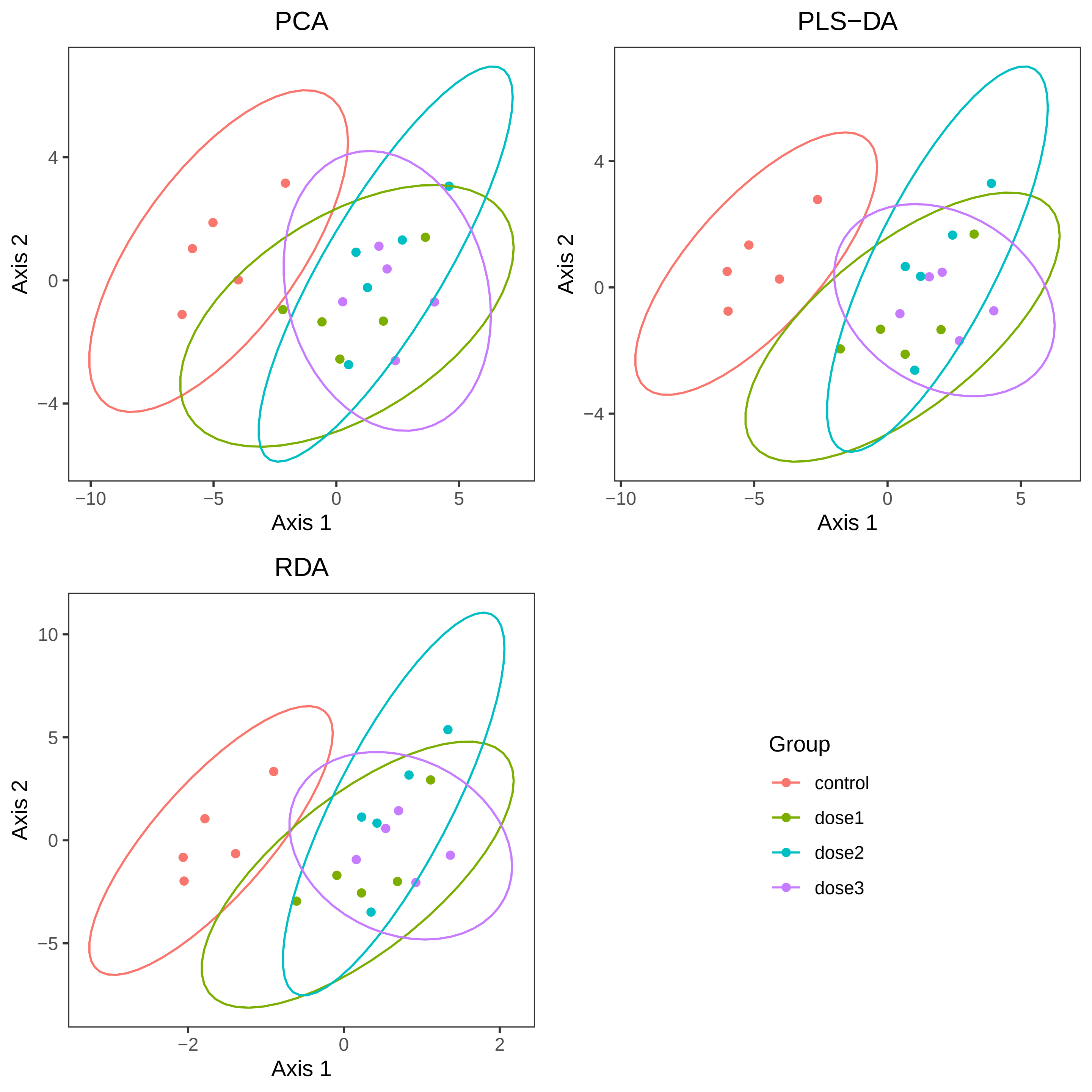

PCA
PLS−DA
RDA
Axis 1
Axis 2
−10
−5
0
5
4
−4
10
−2
2
Group
control
dose1
dose2
dose3

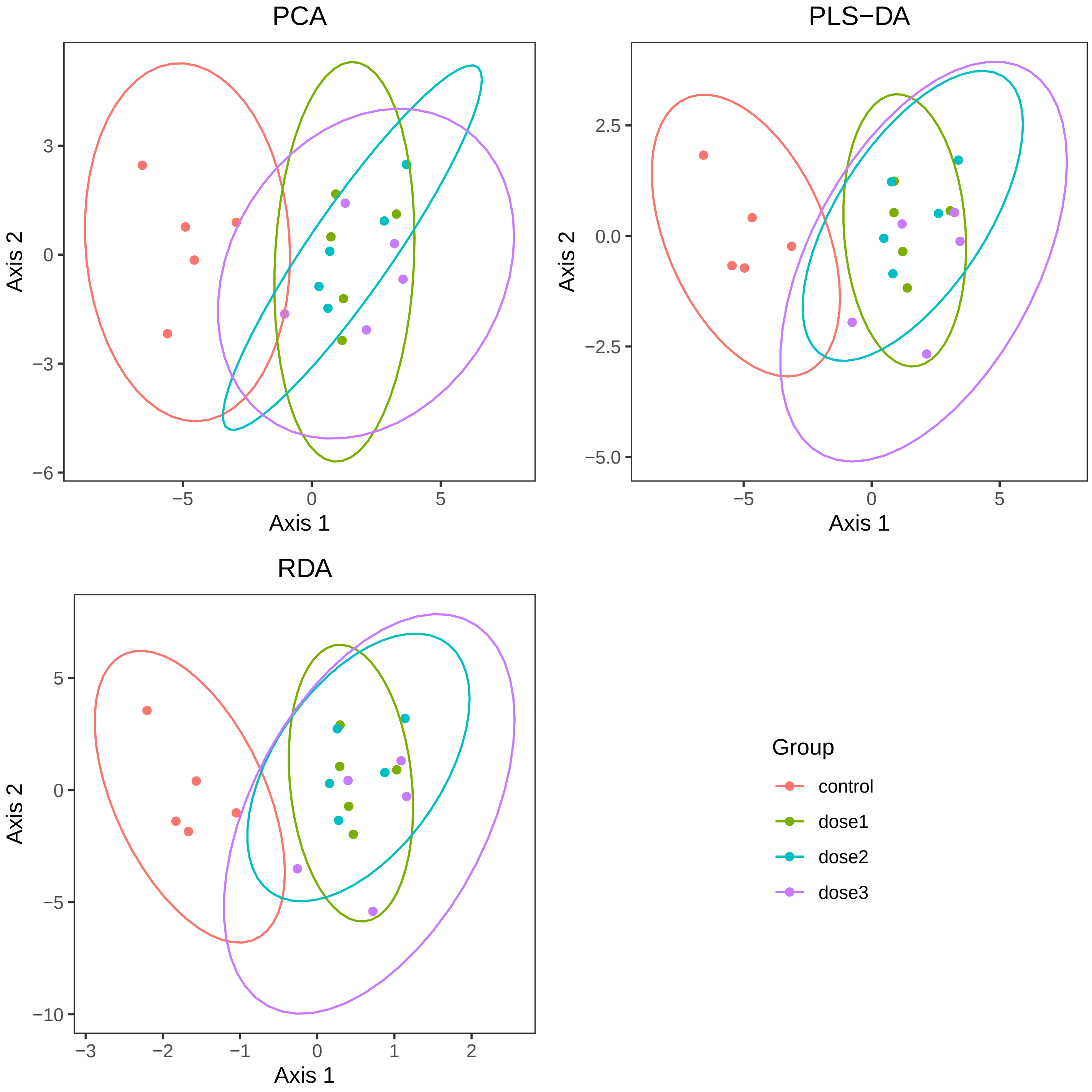
PCA
PLS−DA
RDA
Axis 1
Axis 2
Group
control
dose1
dose2
dose3

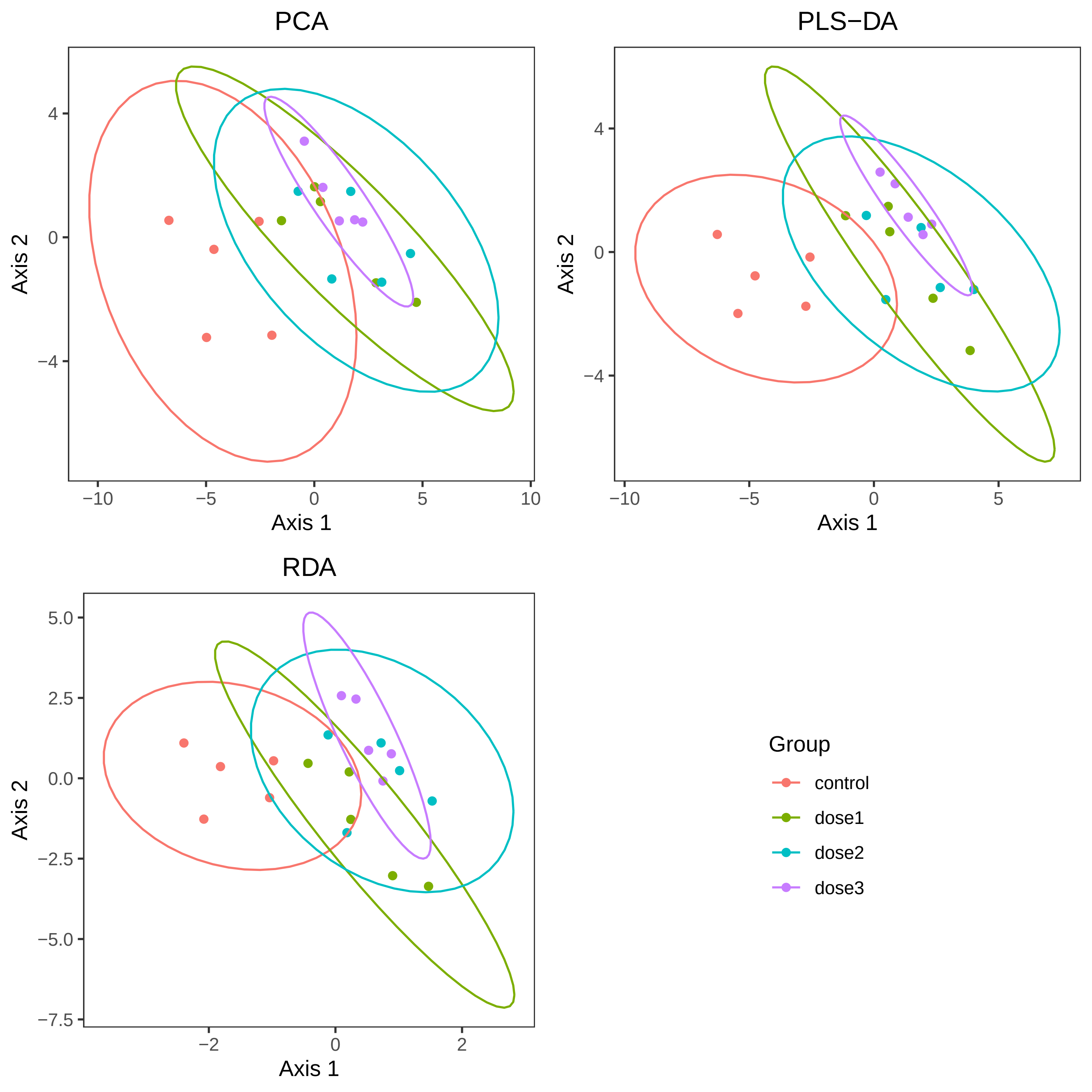
PCA
PLS−DA
RDA
Axis 1
Axis 2
Group
control
dose1
dose2
dose3

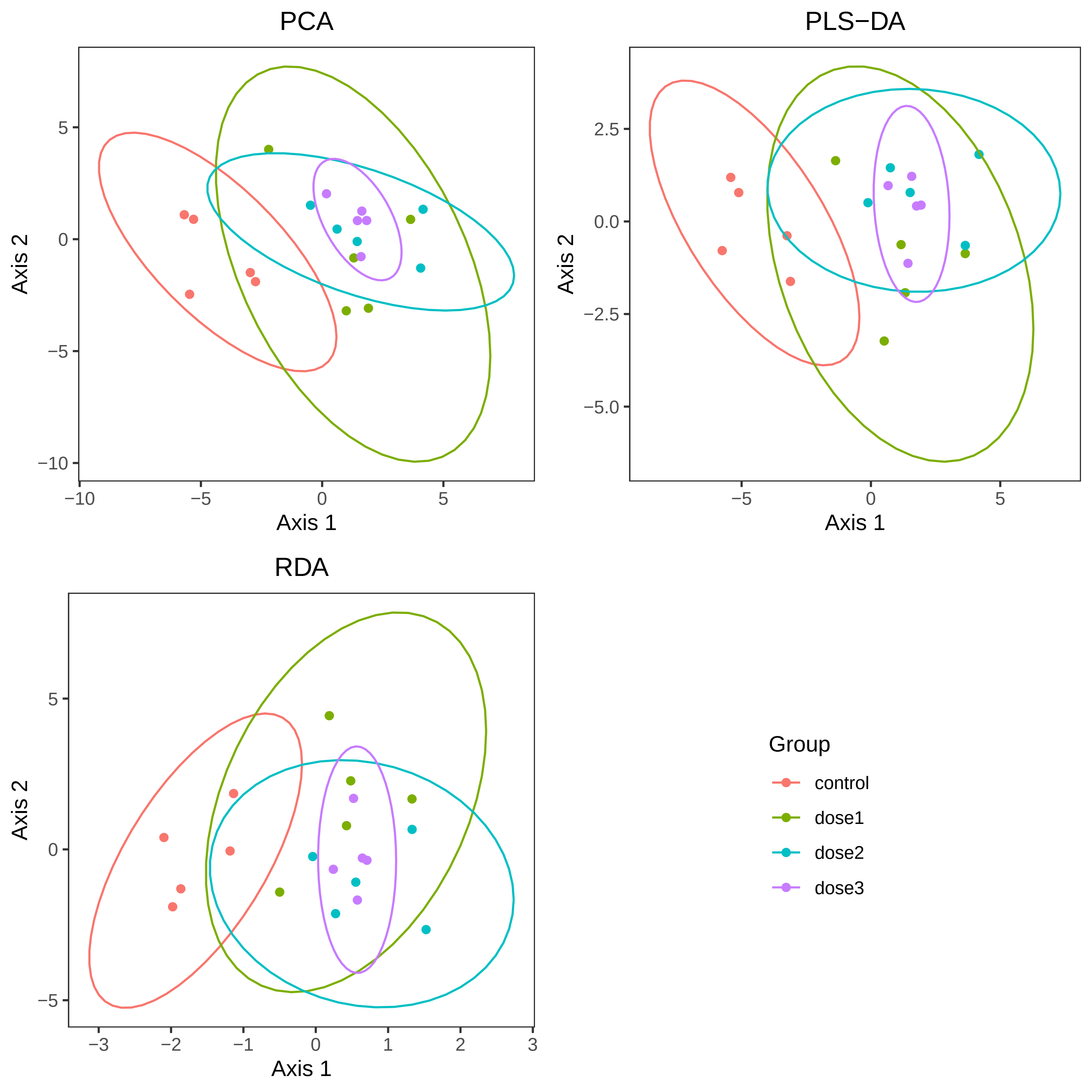
PCA
PLS−DA
RDA
Axis 1
Axis 2
Group
control
dose1
dose2
dose3

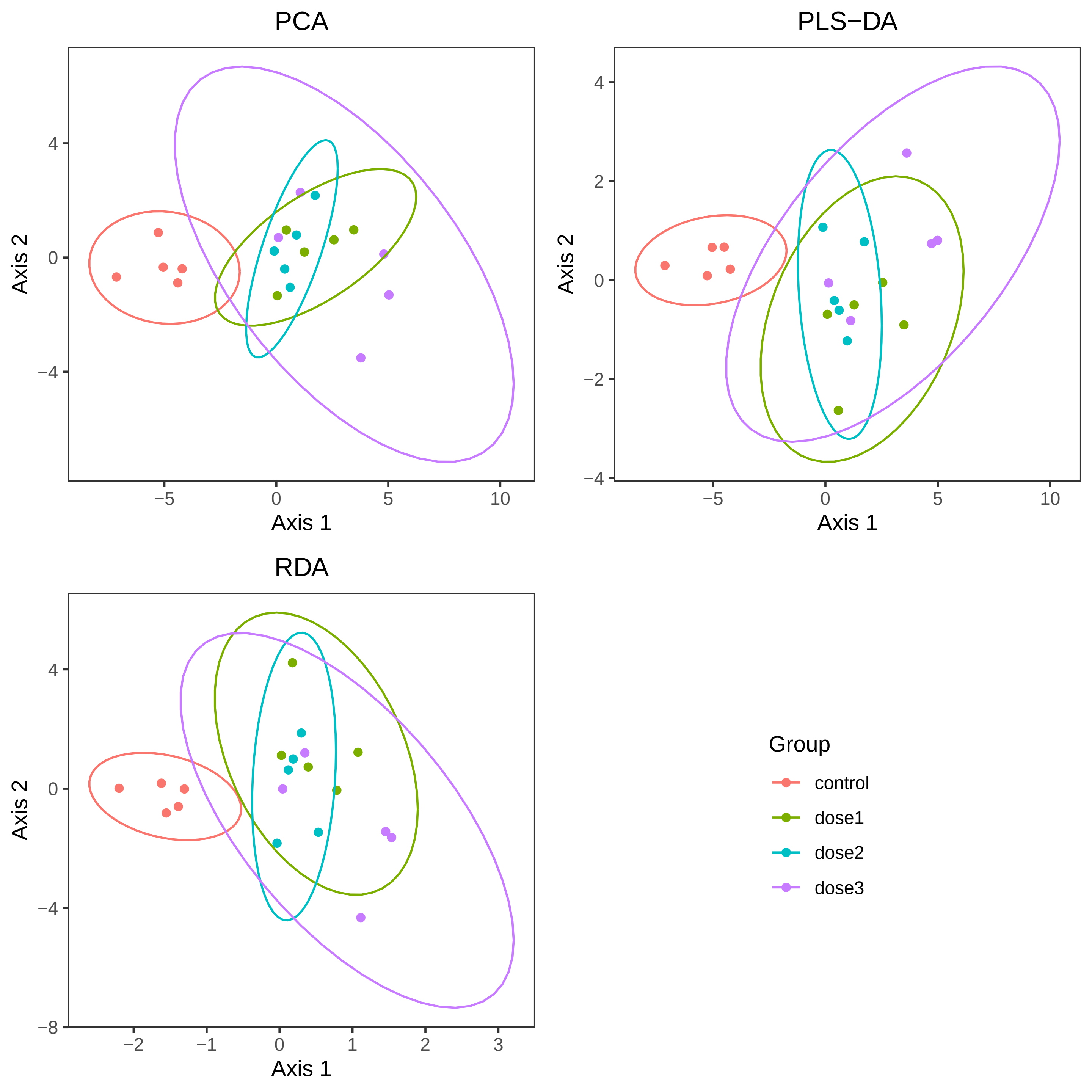
PCA
PLS−DA
RDA
Axis 1
Axis 2
Group
control
dose1
dose2
dose3

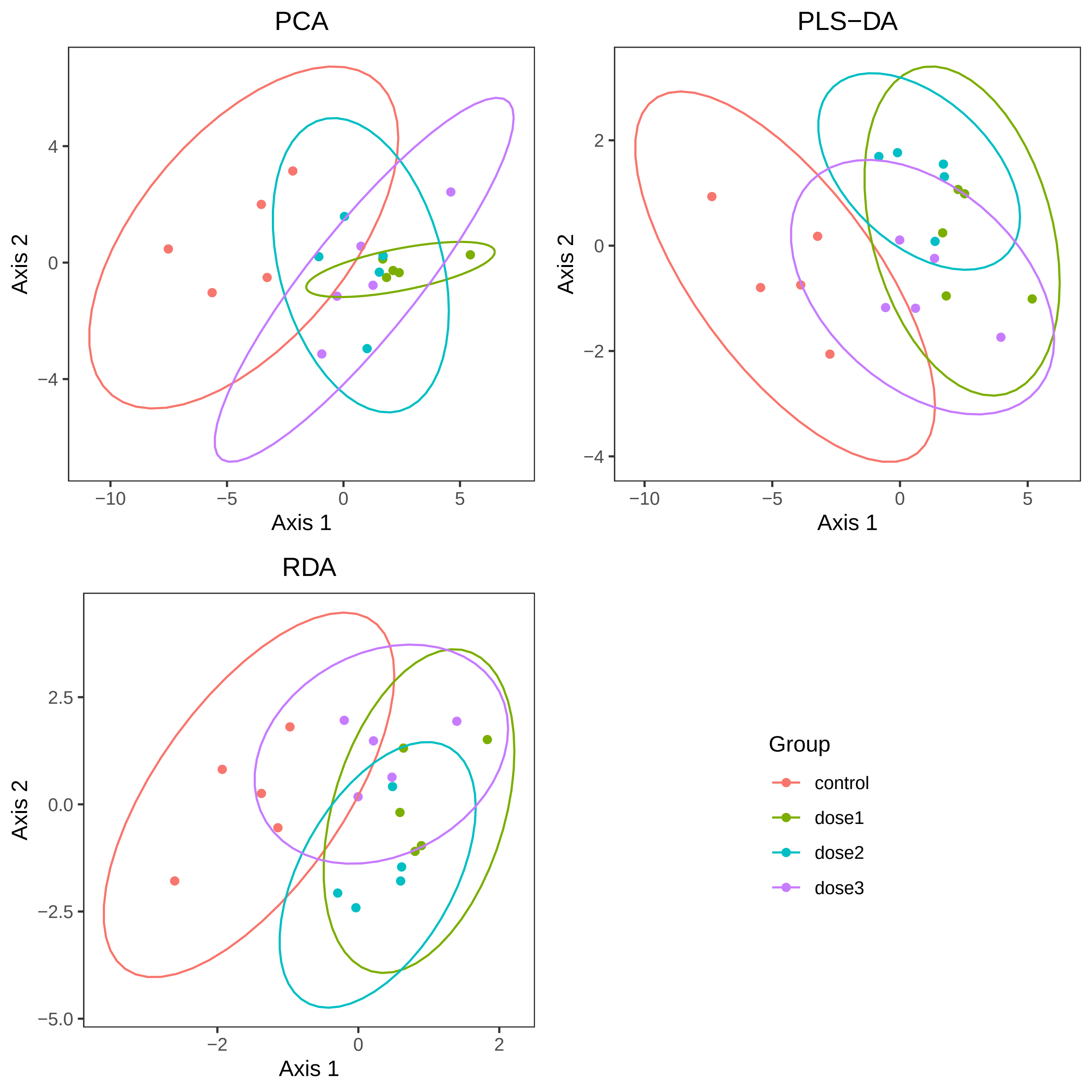

PCA
Axis 2
4
0
−4
−10
−5
0
5
Axis 1
PLS−DA
Axis 2
2
0
−2
−4
−10
−5
0
5
Axis 1
RDA
Axis 2
2.5
0.0
−2.5
−5.0
−2
0
2
Axis 1
Group
control
dose1
dose2
dose3

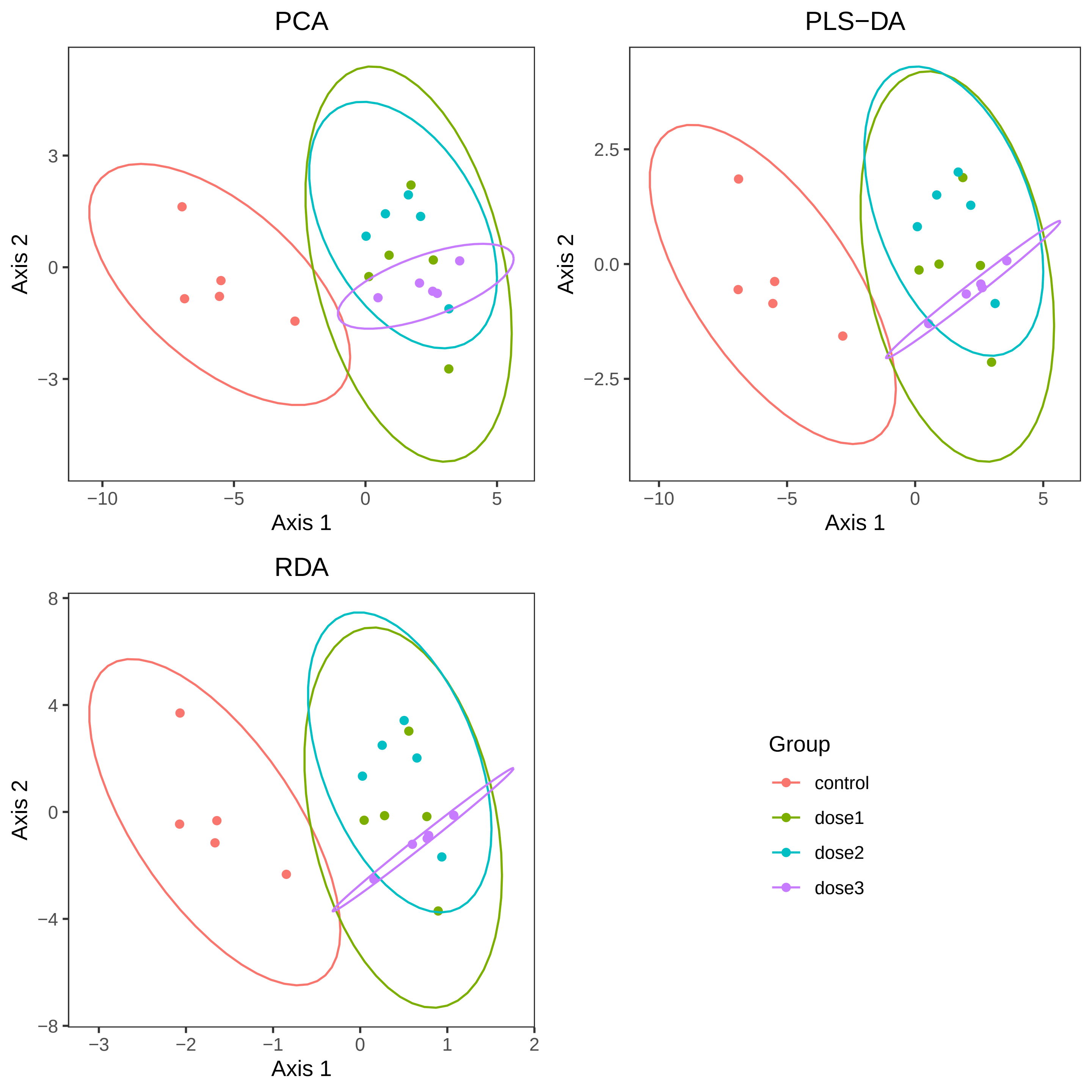

PCA
PLS−DA
RDA
Axis 1
Axis 2
Group
control
dose1
dose2
dose3

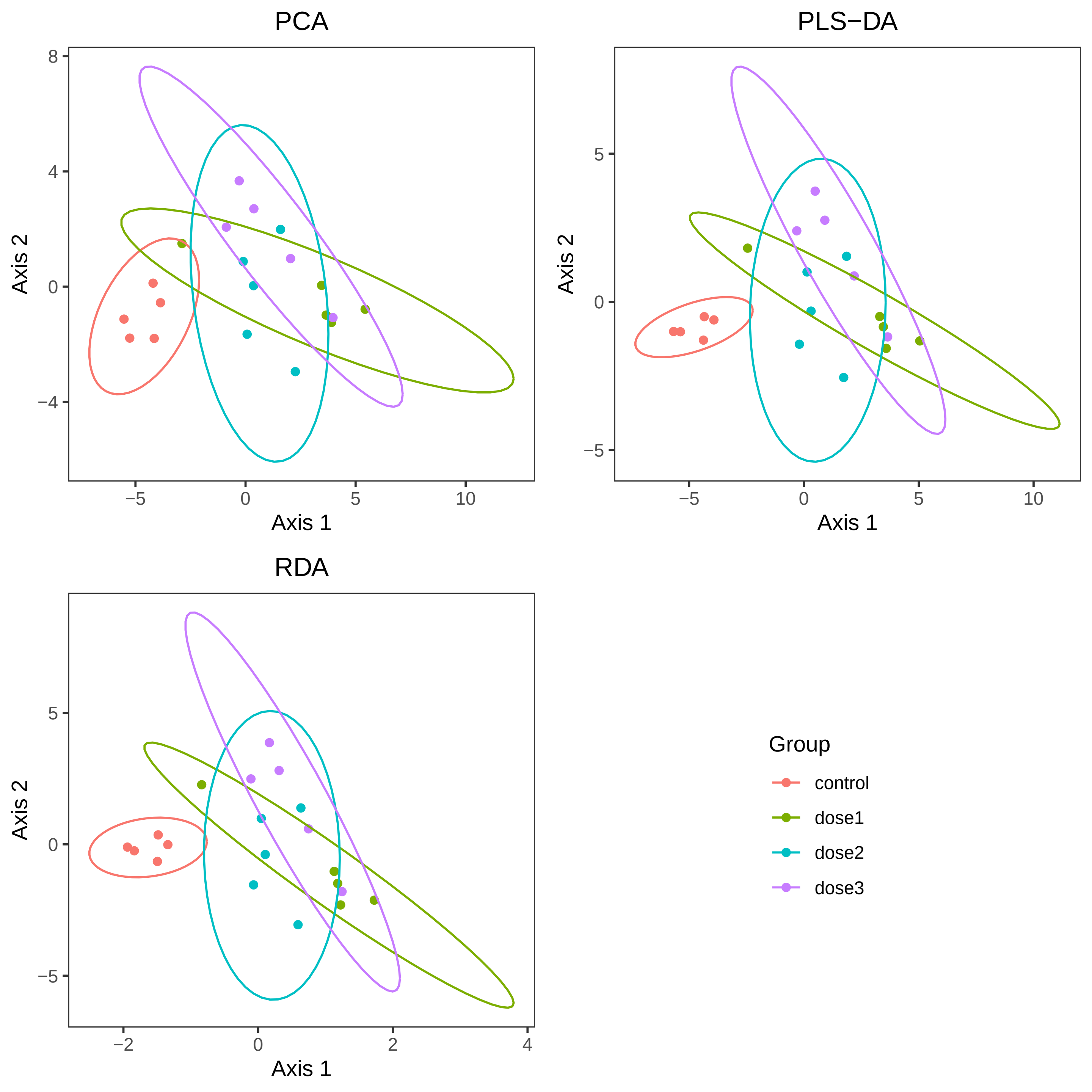
PCA
PLS−DA
RDA
Axis 1
Axis 2
Group
control
dose1
dose2
dose3

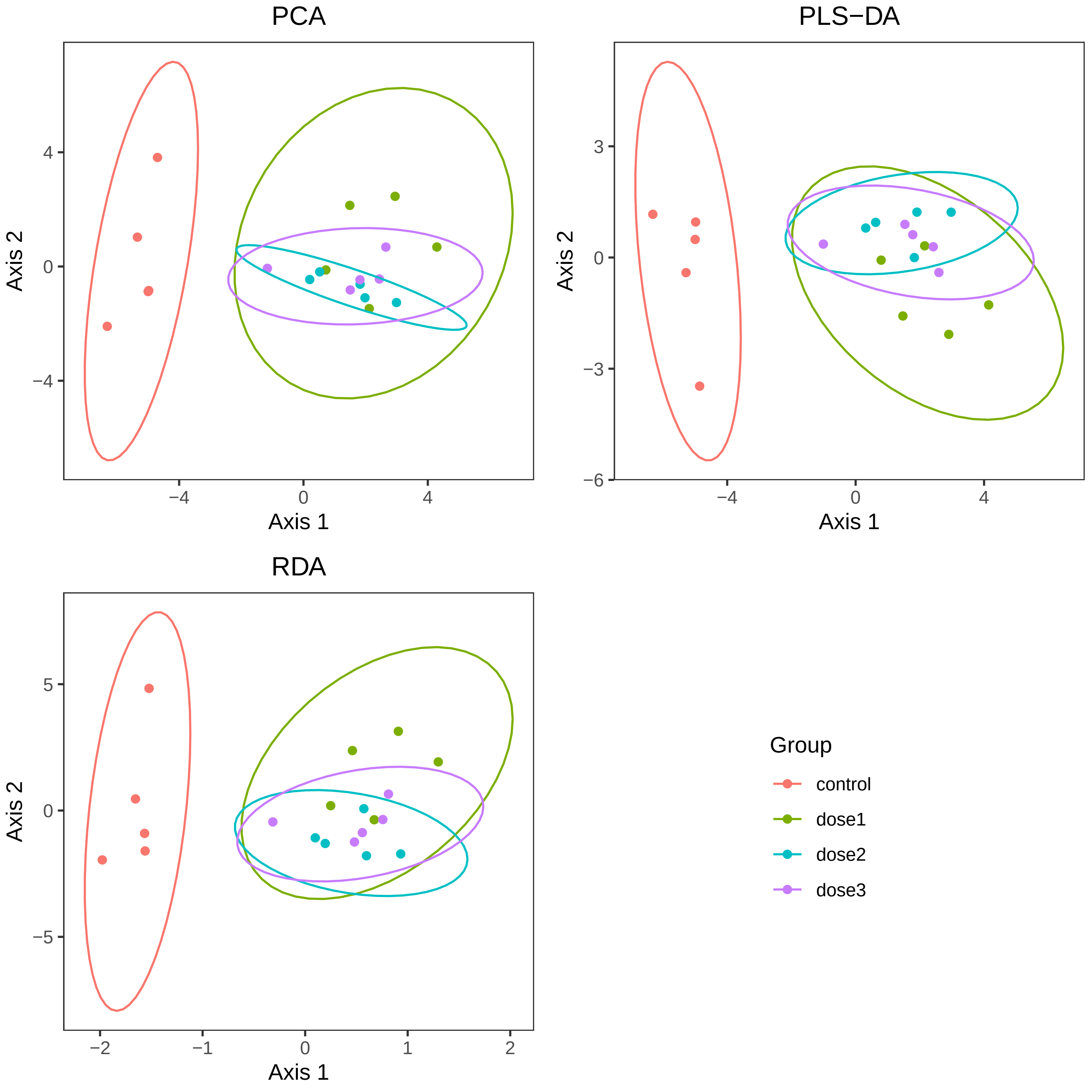
PCA
PLS−DA
RDA
Axis 1
Axis 2
Group
control
dose1
dose2
dose3

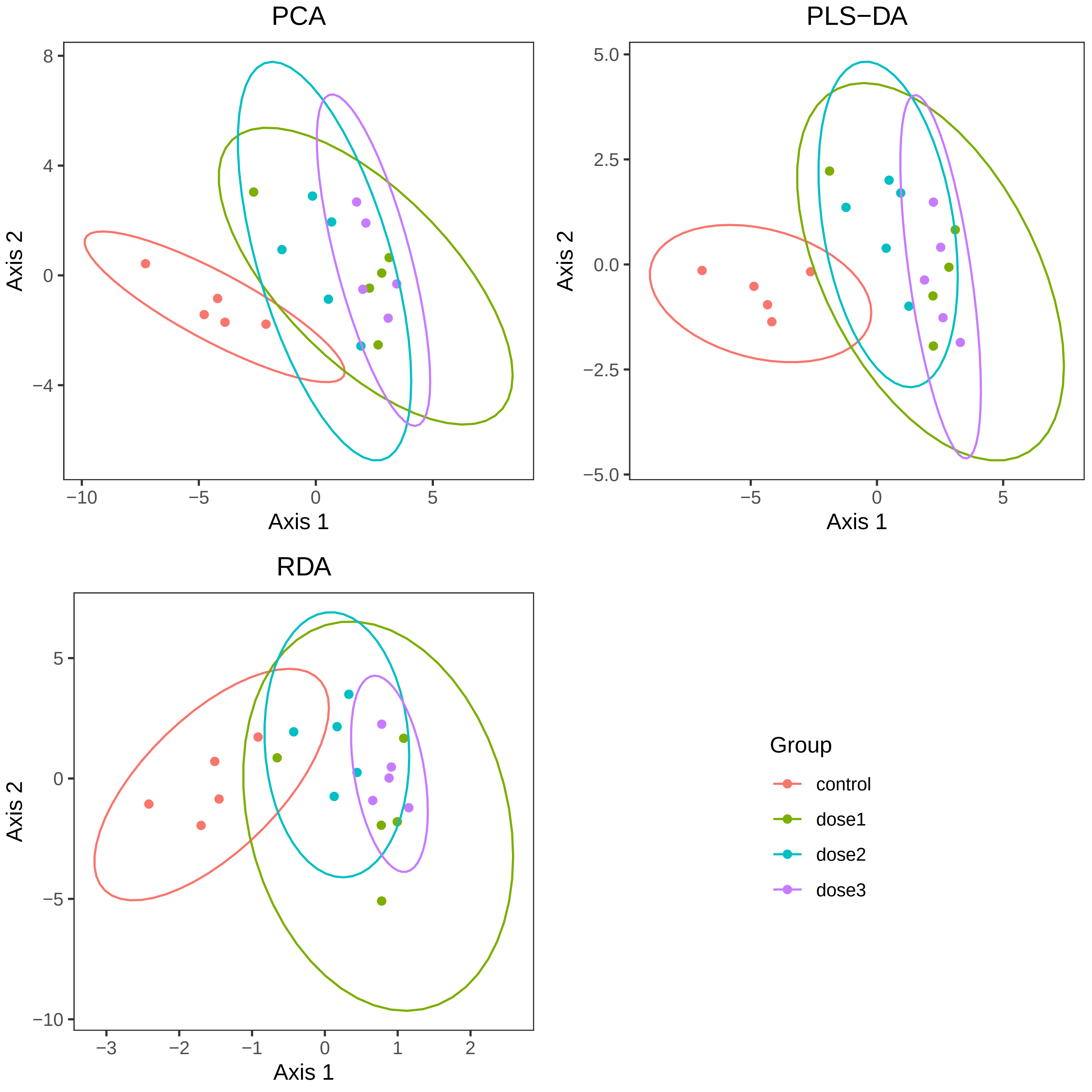
PCA
PLS−DA
RDA
Axis 1
Axis 2
Group
control
dose1
dose2
dose3

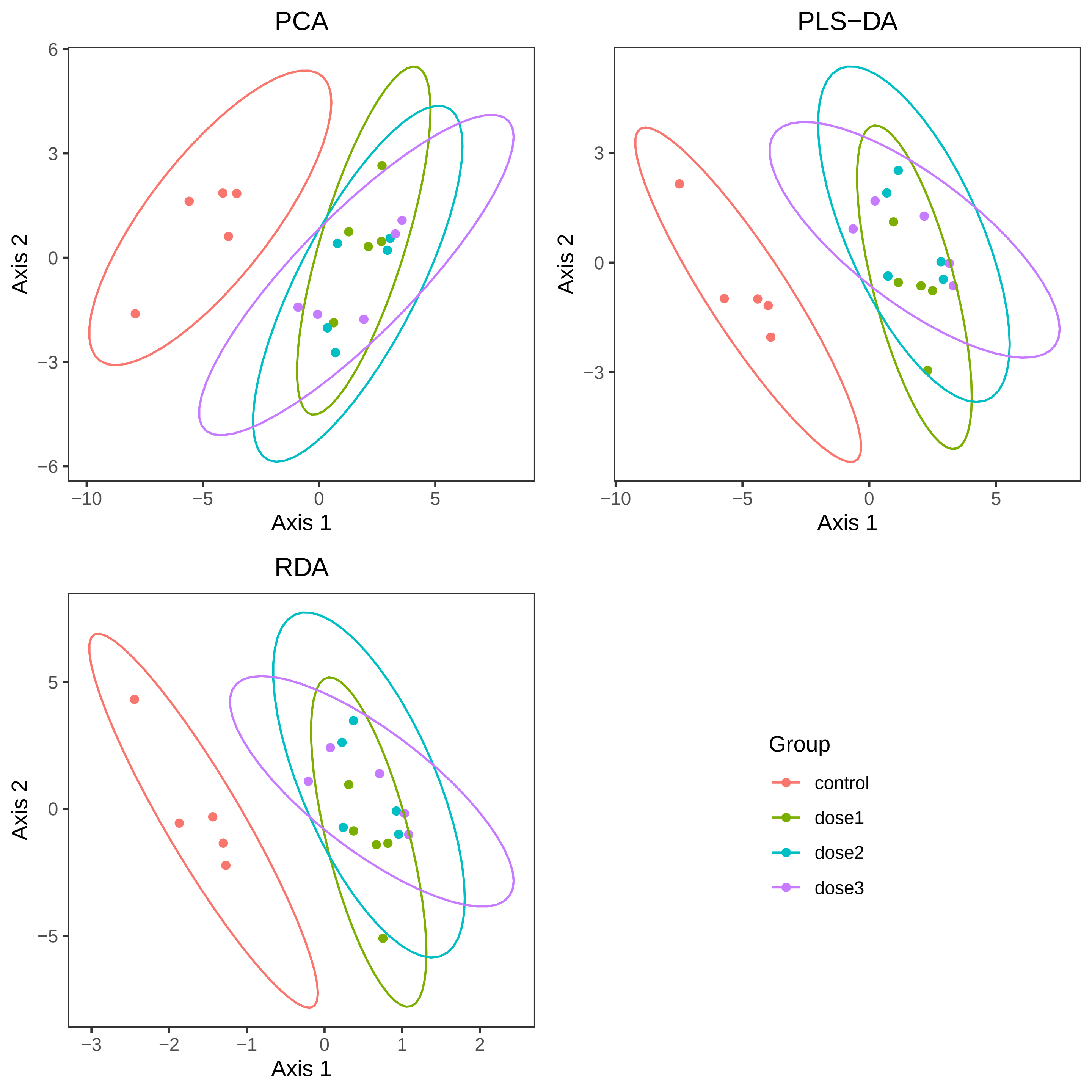

PCA
PLS−DA
RDA
Axis 1
Axis 2
6
3
0
−3
−6
−10
−5
0
5
−3
−2
−1
1
2
Group
control
dose1
dose2
dose3

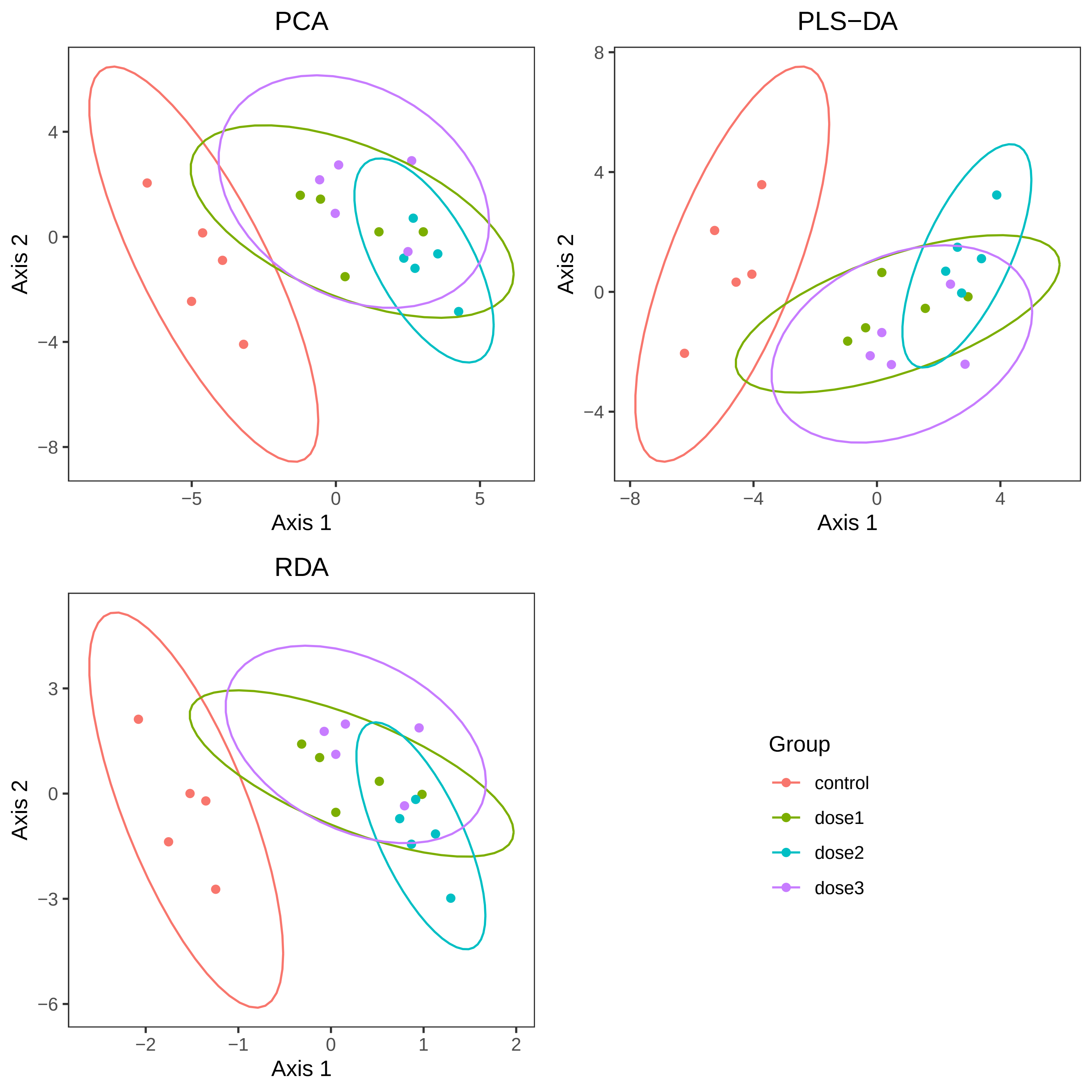
PCA
PLS−DA
RDA
Axis 1
Axis 2
−5
0
5
4
0
−4
−8
8
4
0
−4
−8
−4
0
4
3
0
−3
−6
−2
−1
0
1
2
Group
control
dose1
dose2
dose3

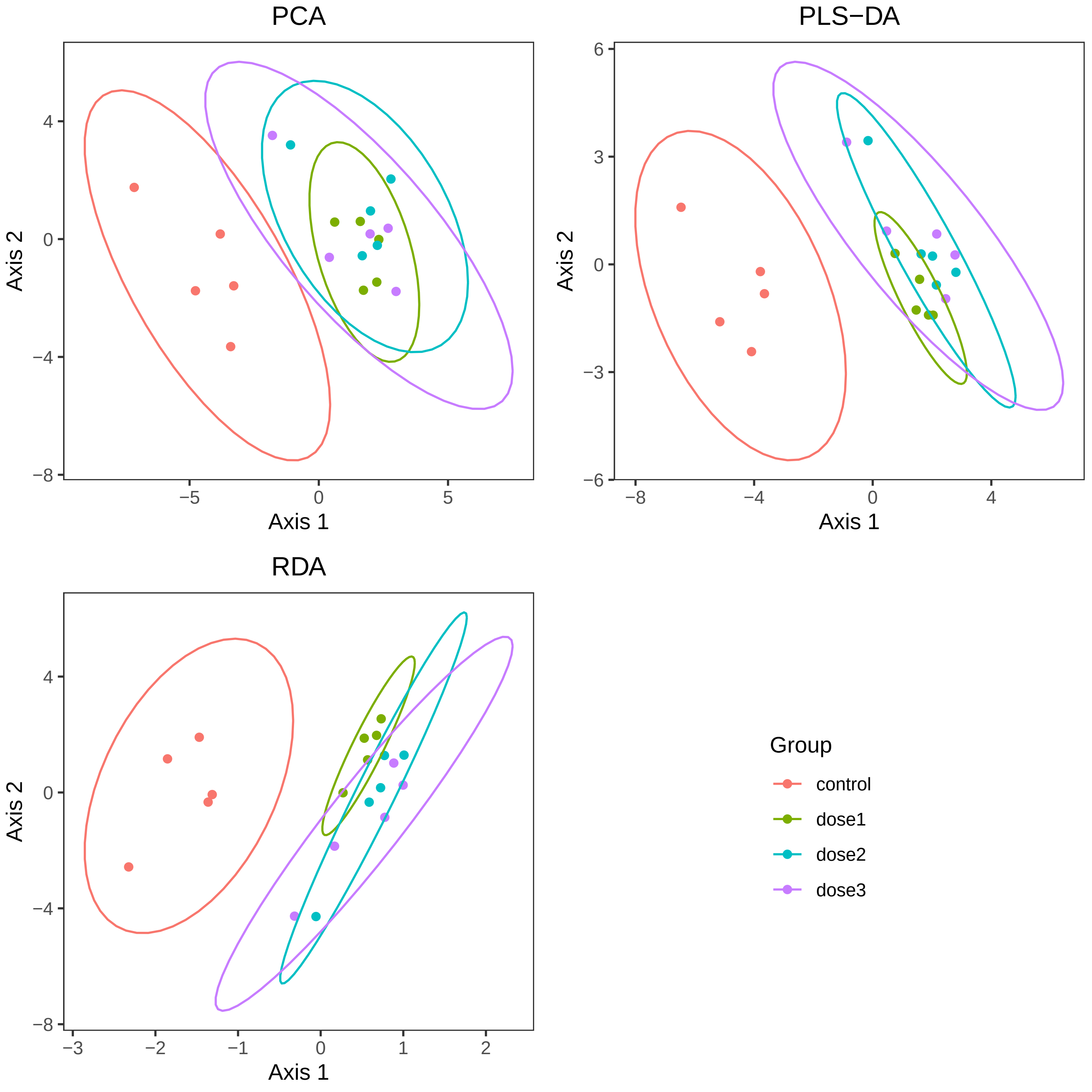
PCA
PLS−DA
RDA
Axis 1
Axis 2
Group
control
dose1
dose2
dose3

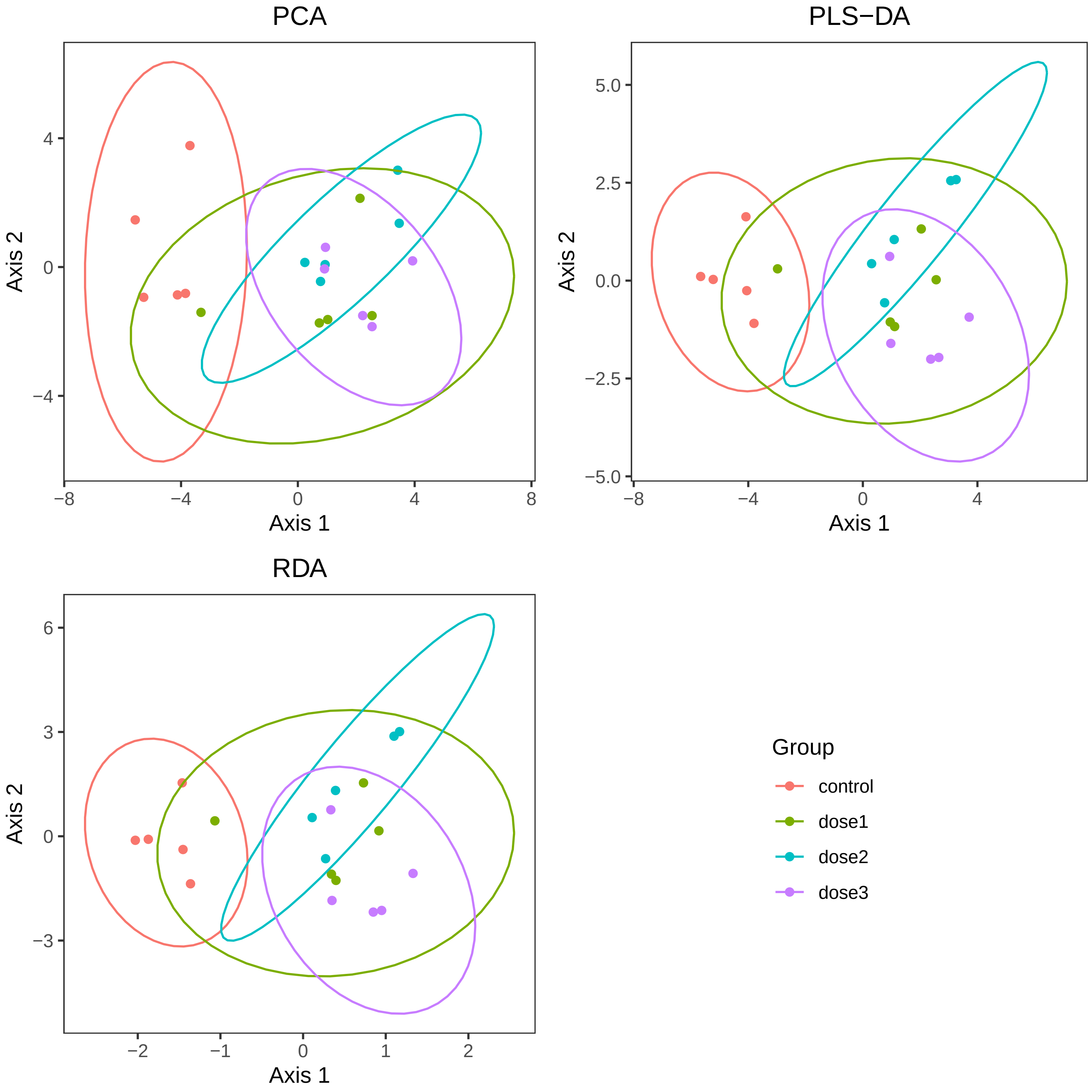

PCA
PLS−DA
RDA
Axis 1
Axis 2
Group
control
dose1
dose2
dose3

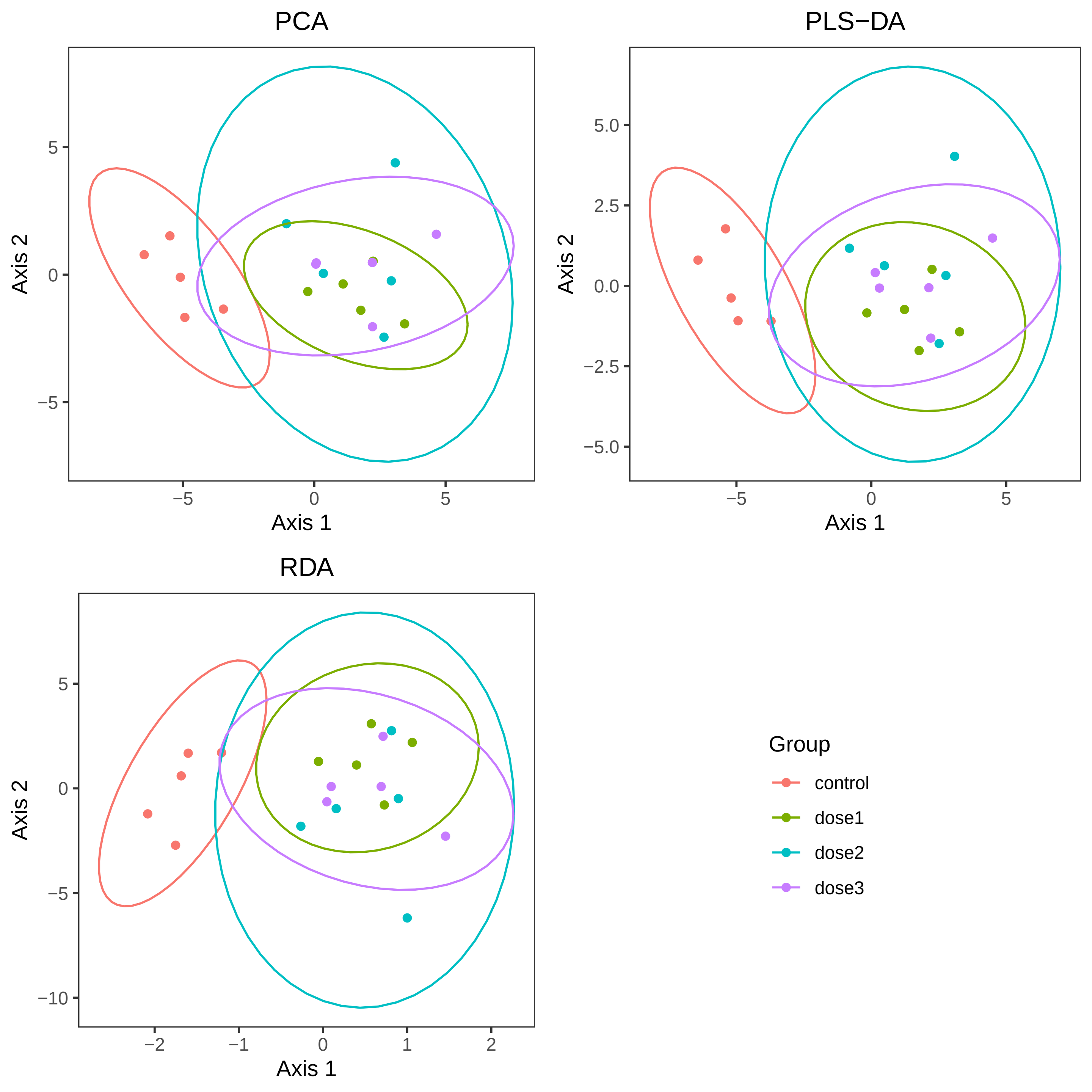
PCA
PLS−DA
RDA
Axis 1
Axis 2
5
0
−5
−10
5.0
2.5
0.0
−2.5
−5.0
−2
−1
1
2
Group
control
dose1
dose2
dose3

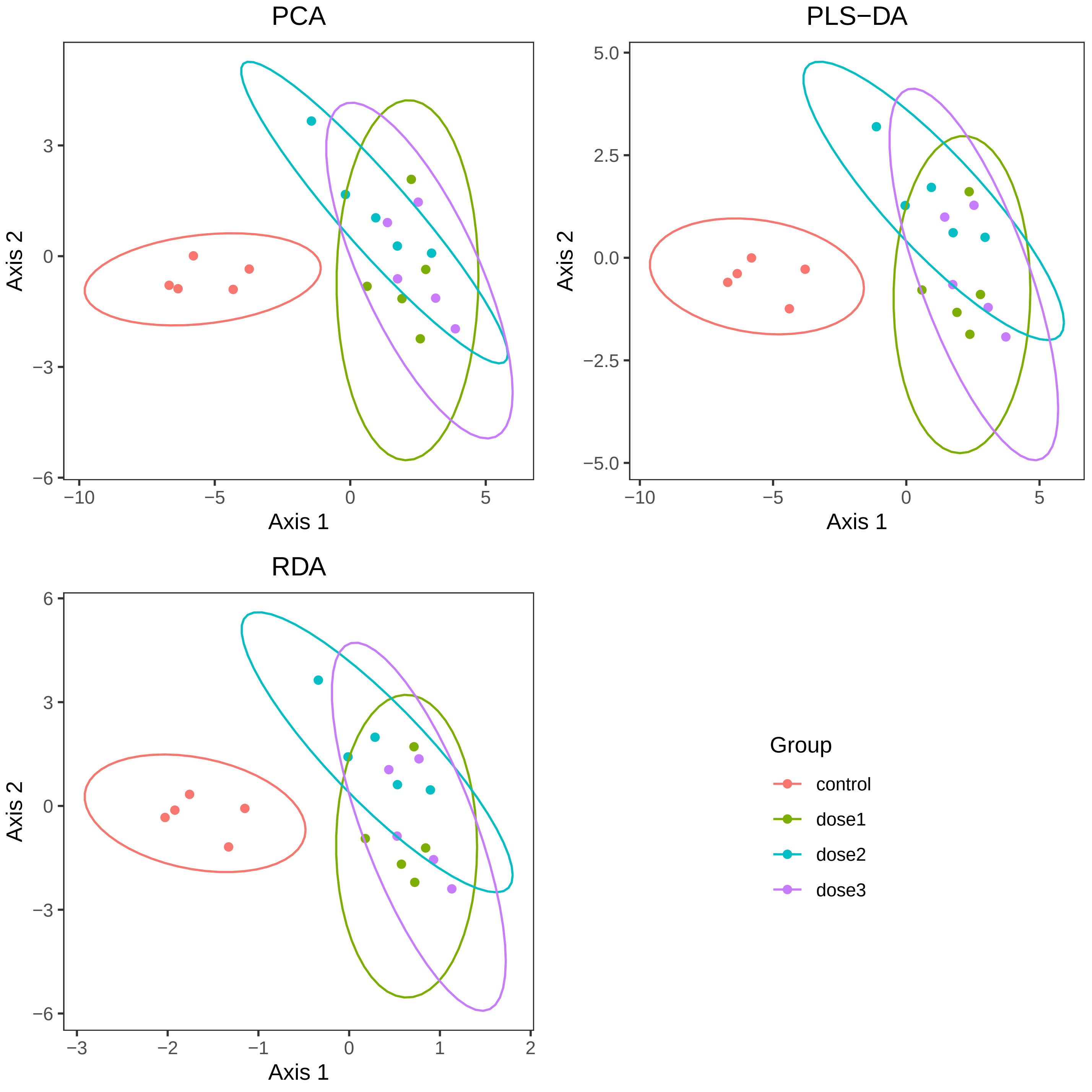
PCA
PLS−DA
RDA
Axis 1
Axis 2
Group
control
dose1
dose2
dose3

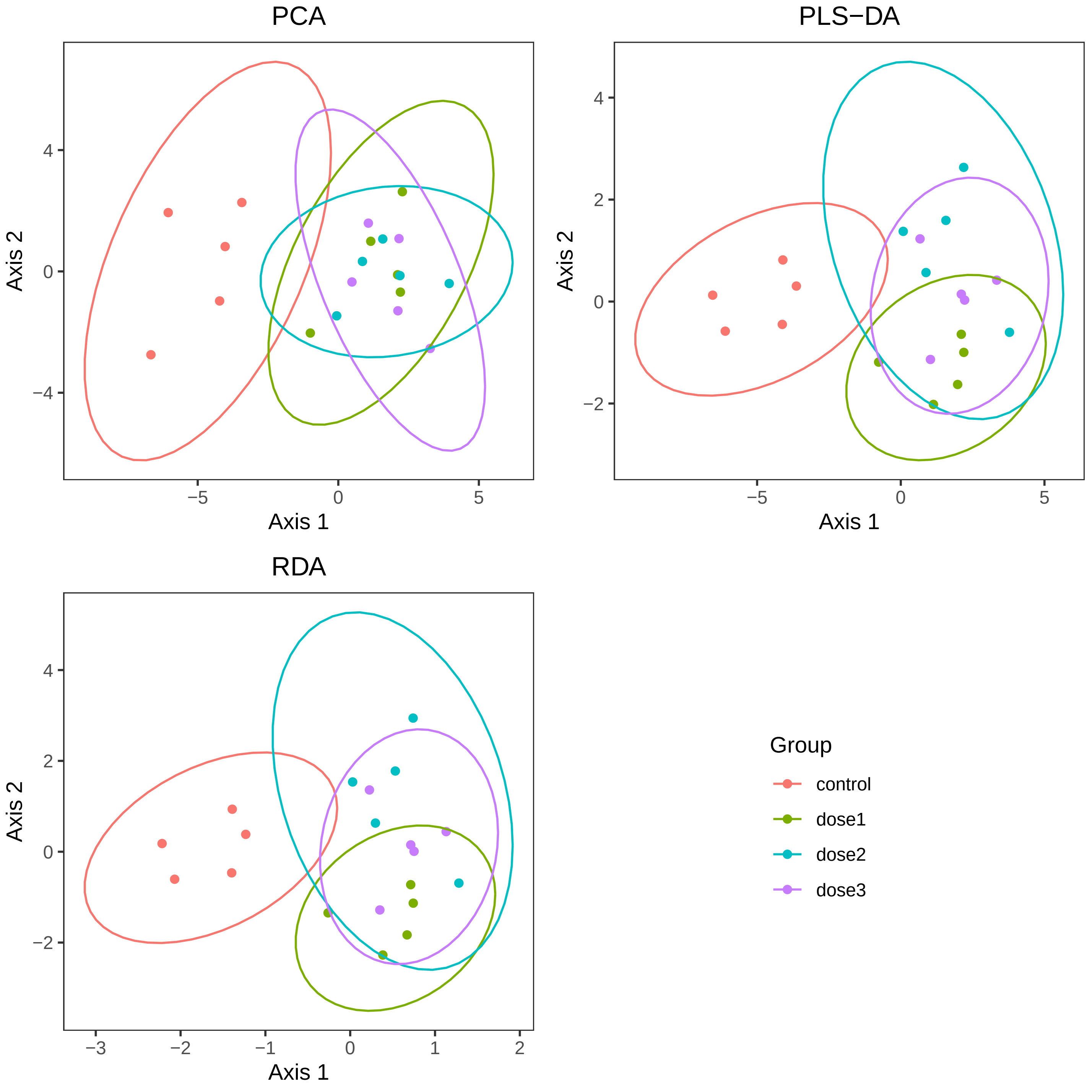

PCA
PLS−DA
RDA
Axis 1
Axis 2
Group
control
dose1
dose2
dose3

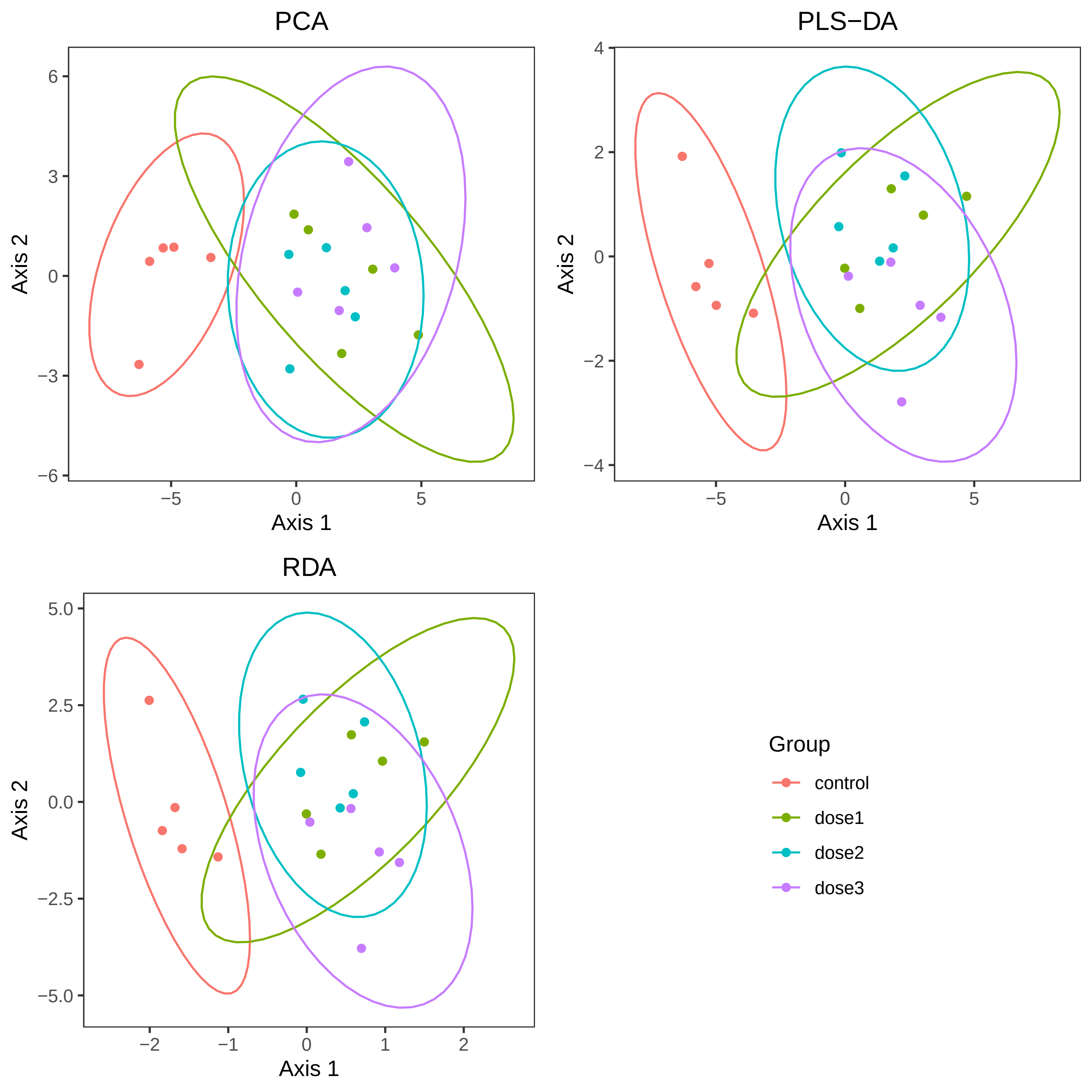
PCA
PLS−DA
RDA
Axis 1
Axis 2
Group
control
dose1
dose2
dose3

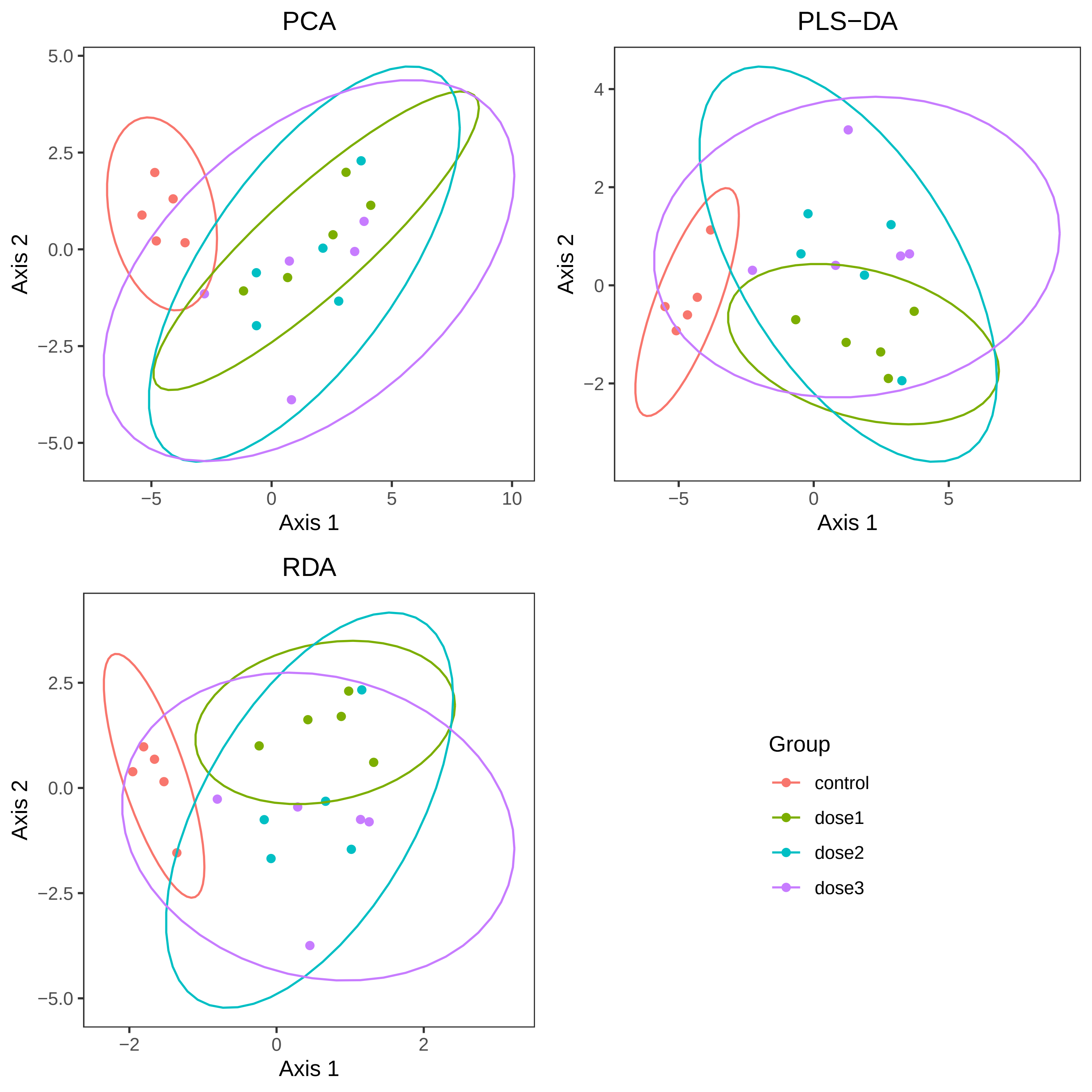

PCA
PLS−DA
RDA
Axis 1
Axis 2
Group
control
dose1
dose2
dose3

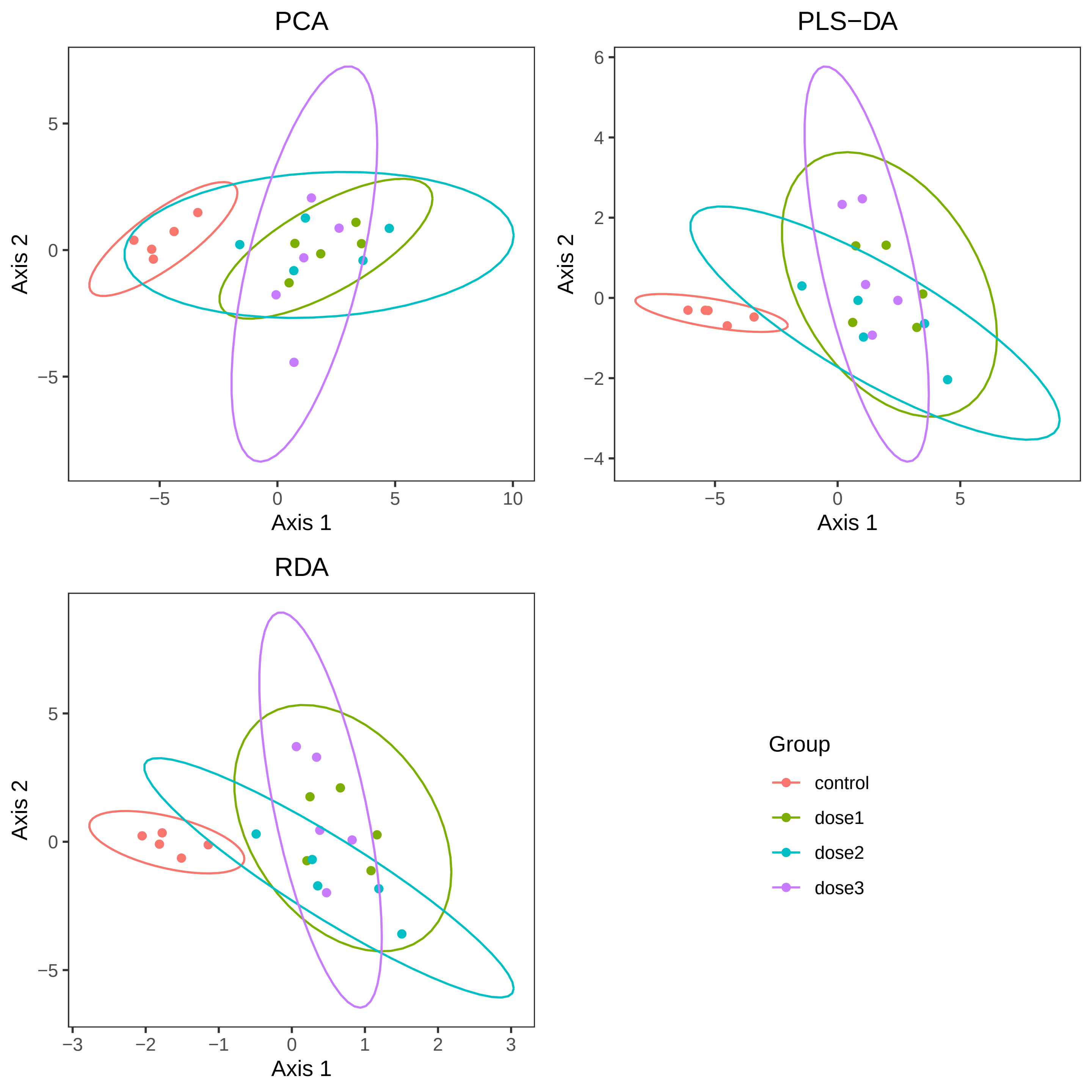
PCA
PLS−DA
RDA
Axis 1
Axis 2
Group
control
dose1
dose2
dose3

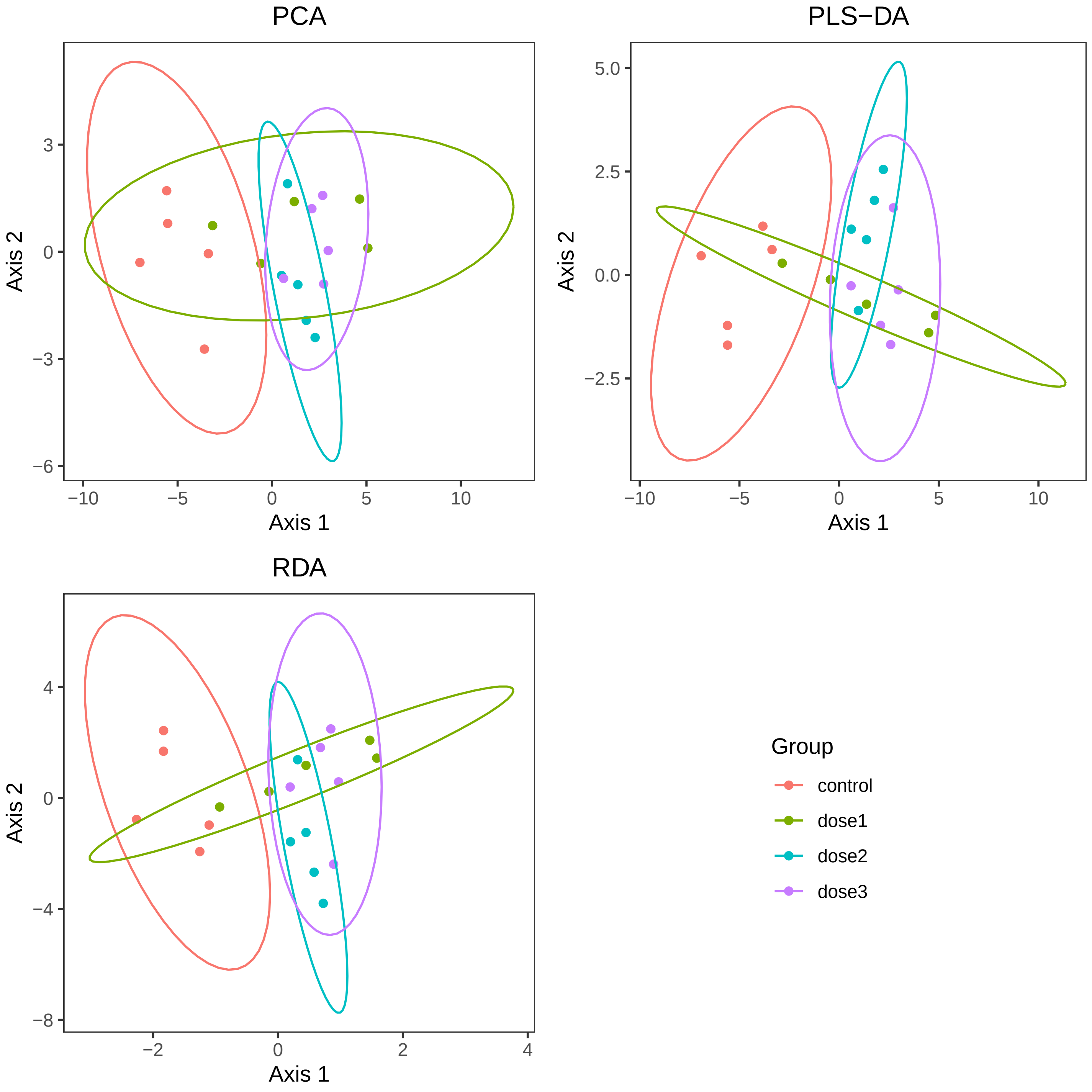
PCA
Axis 2
Axis 1
3
0
−3
−6
−10
−5
0
5
10
PLS−DA
Axis 2
Axis 1
5.0
2.5
0.0
−2.5
−10
−5
0
5
10
RDA
Axis 2
Axis 1
4
0
−4
−8
−2
0
2
4
Group
control
dose1
dose2
dose3

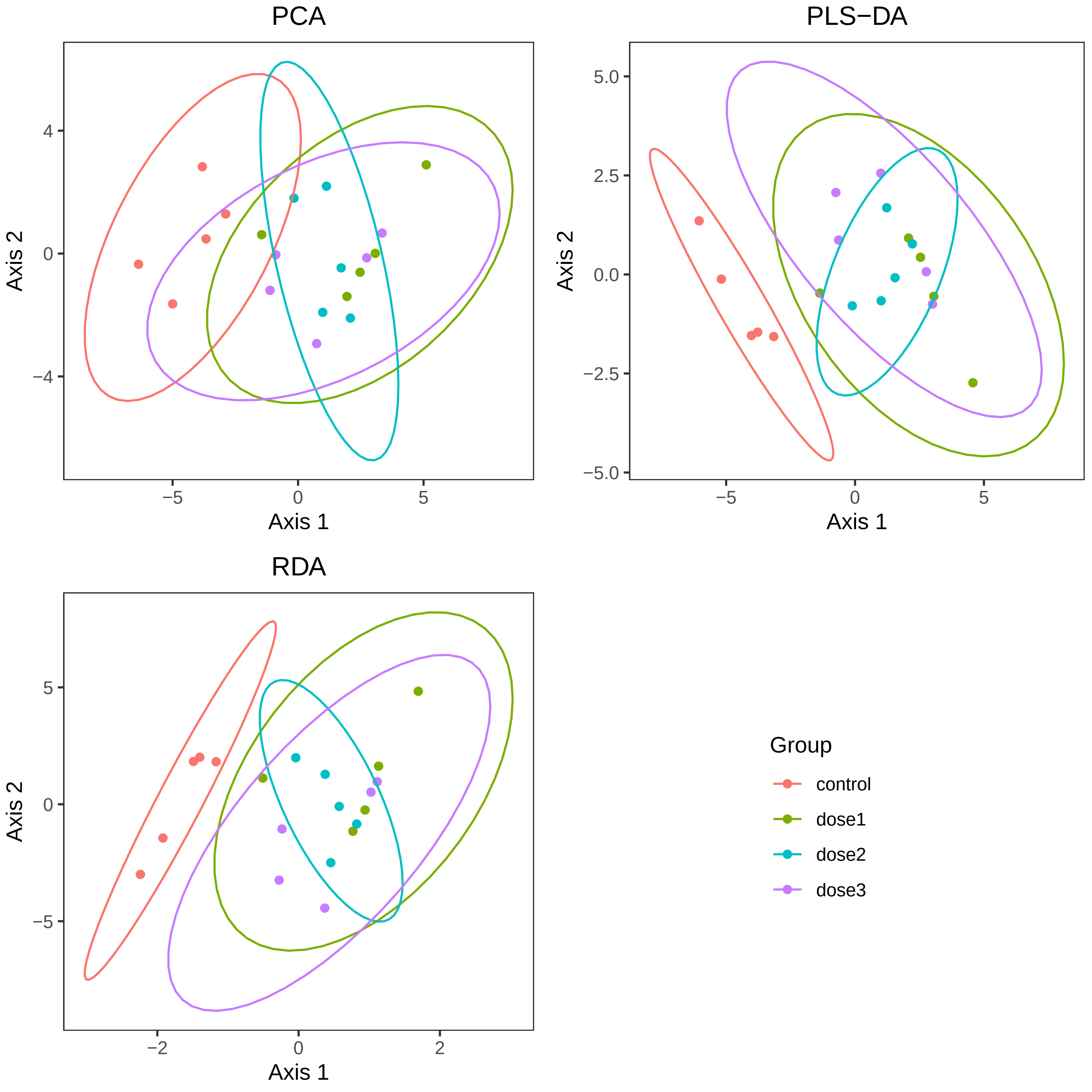
PCA
PLS−DA
RDA
Axis 1
Axis 2
4
0
−4
−5
5
5.0
2.5
0.0
−2.5
−5.0
−2
2
Group
control
dose1
dose2
dose3

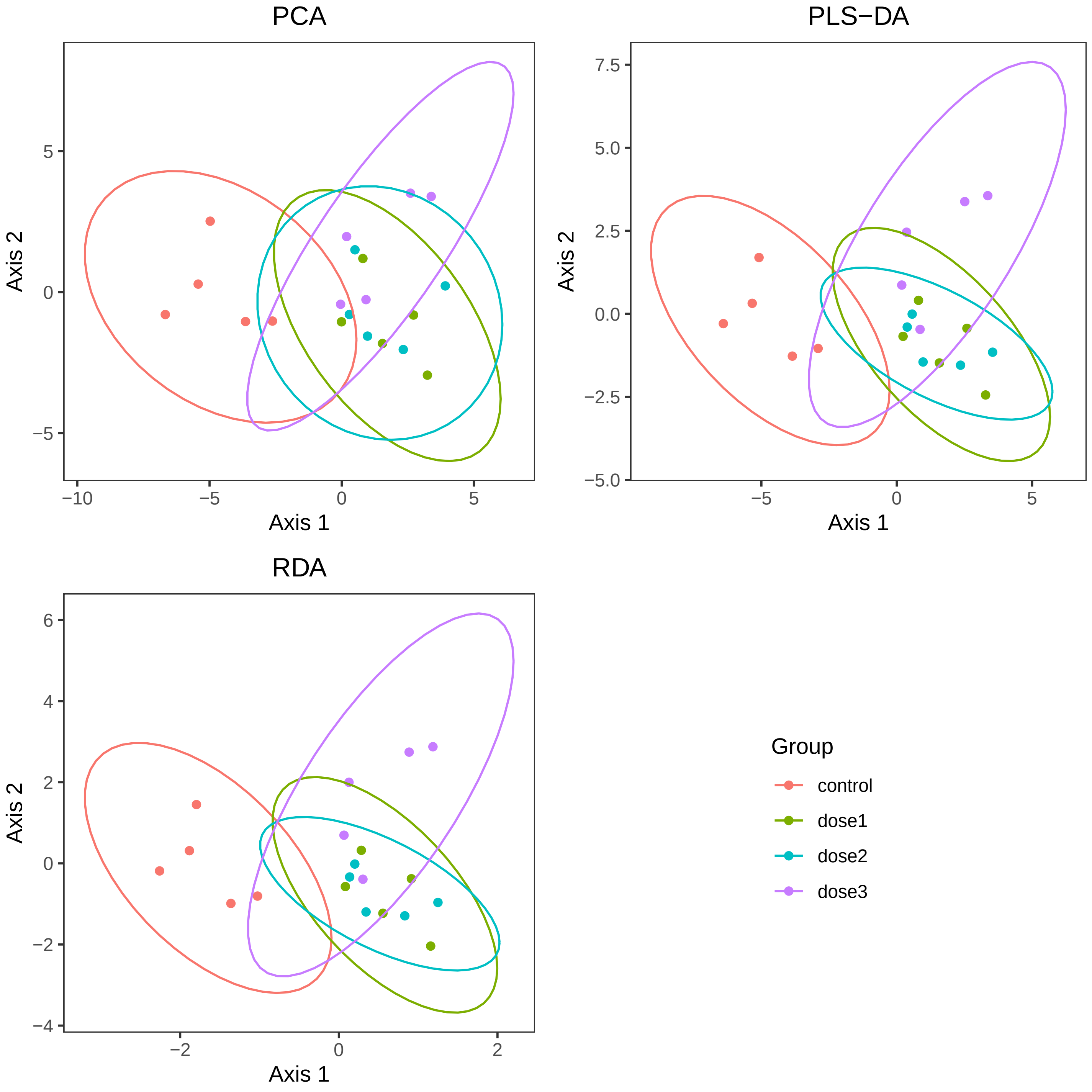

PCA
PLS−DA
RDA
Axis 1
Axis 2
Group
control
dose1
dose2
dose3

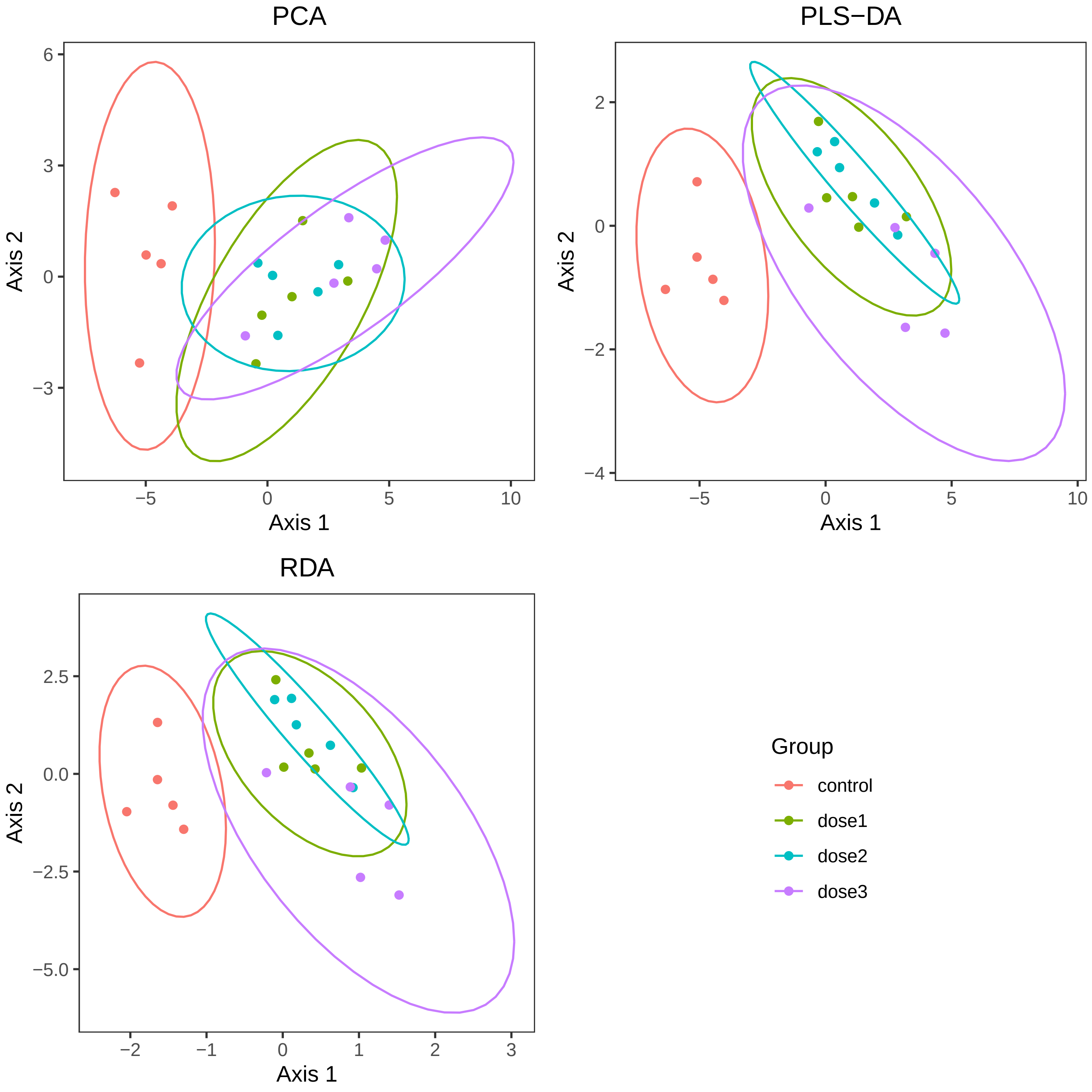

PCA
PLS−DA
RDA
Axis 1
Axis 2
Group
control
dose1
dose2
dose3

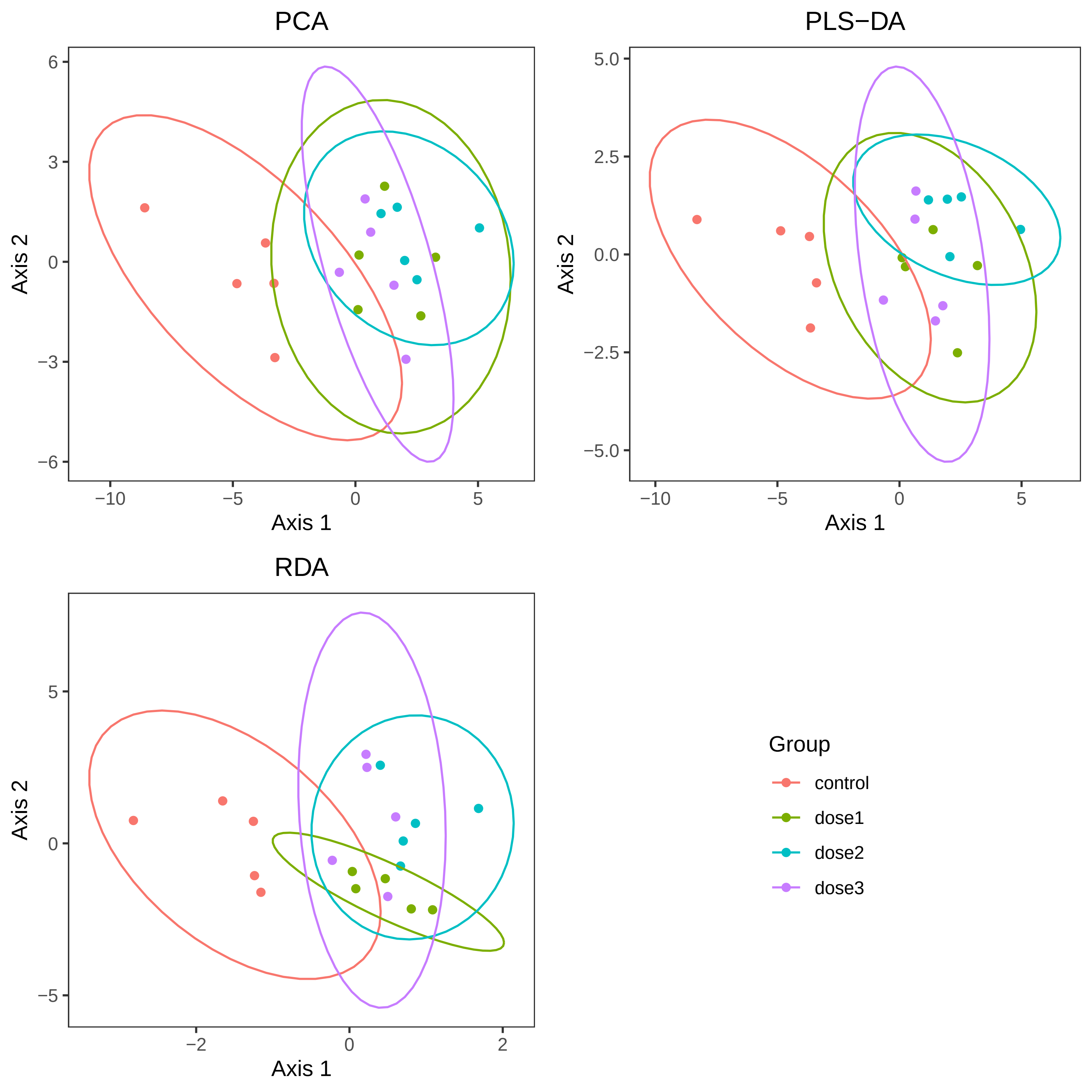

PCA
PLS−DA
RDA
Axis 1
Axis 2
Group
control
dose1
dose2
dose3

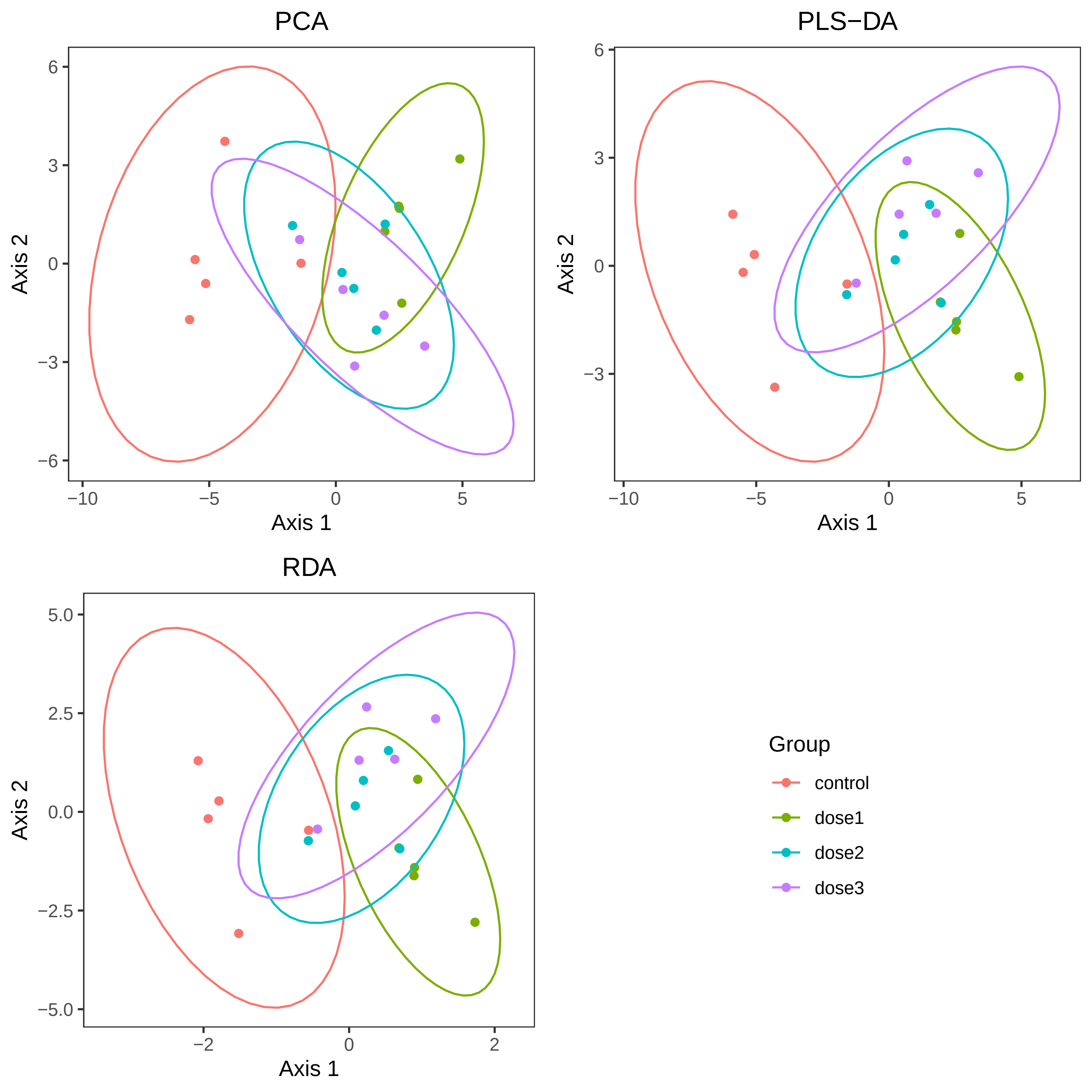
PCA
PLS−DA
RDA
Axis 1
Axis 2
Group
control
dose1
dose2
dose3

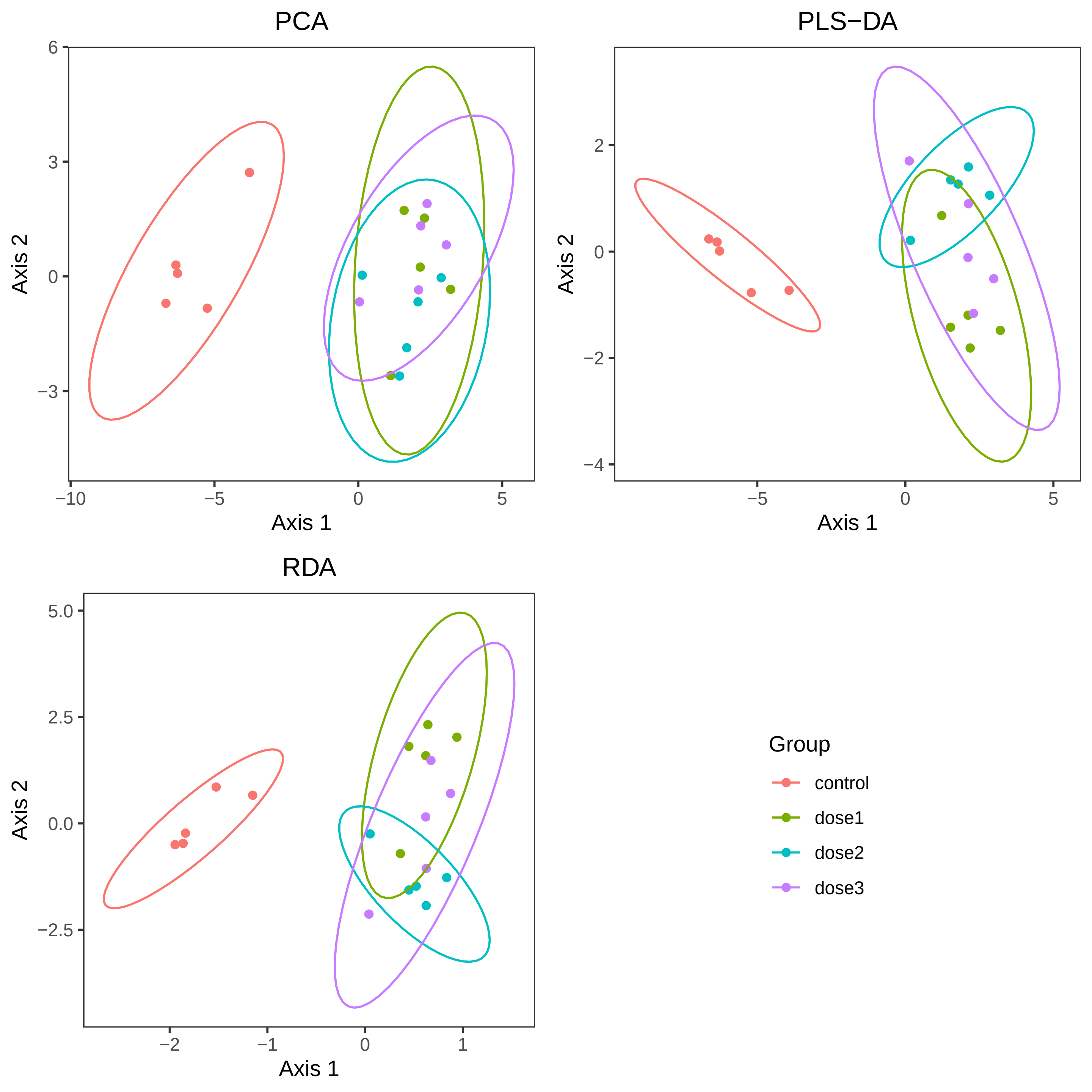
PCA
PLS−DA
RDA
Axis 1
Axis 2
Group
control
dose1
dose2
dose3

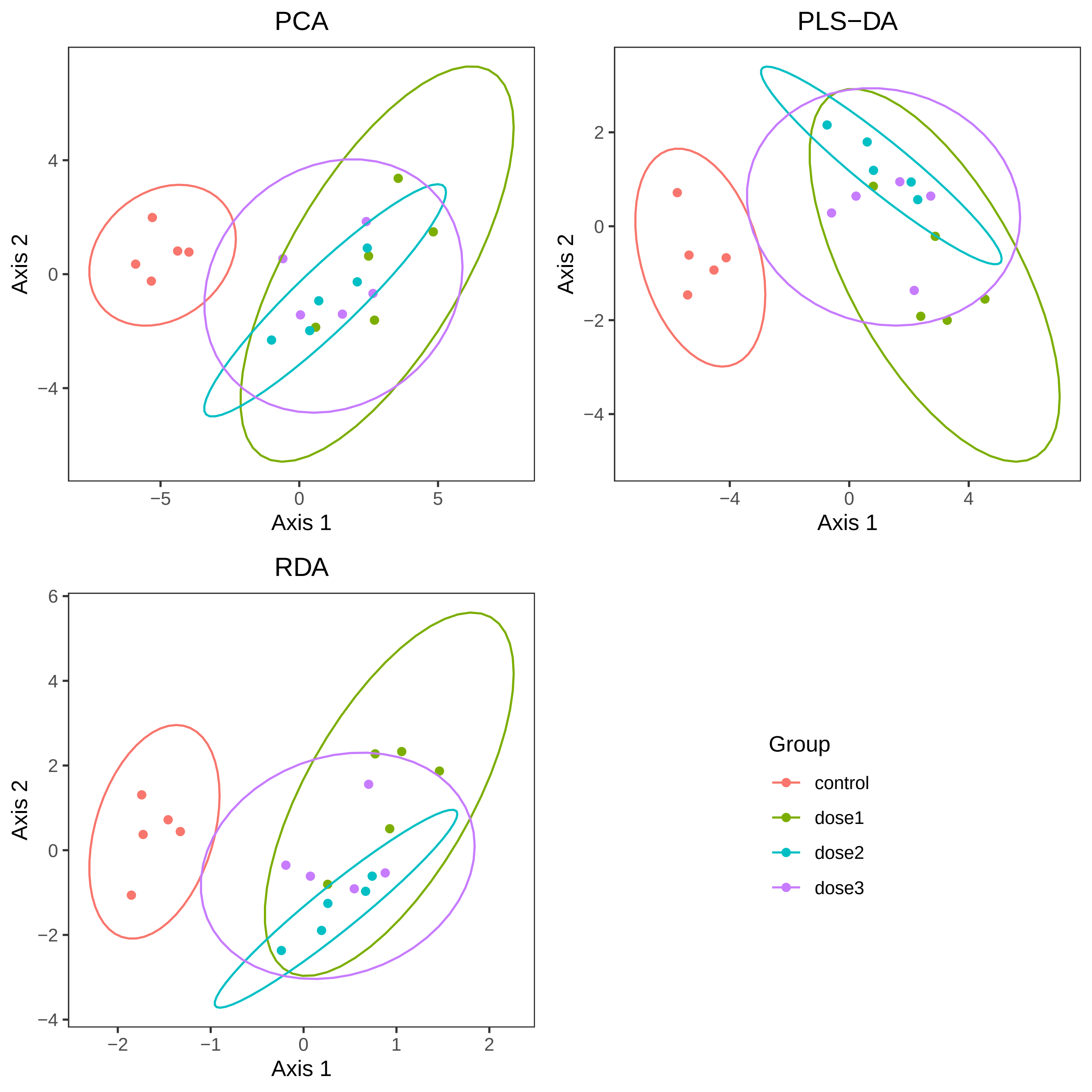

PCA
PLS−DA
RDA
Axis 1
Axis 2
Group
control
dose1
dose2
dose3

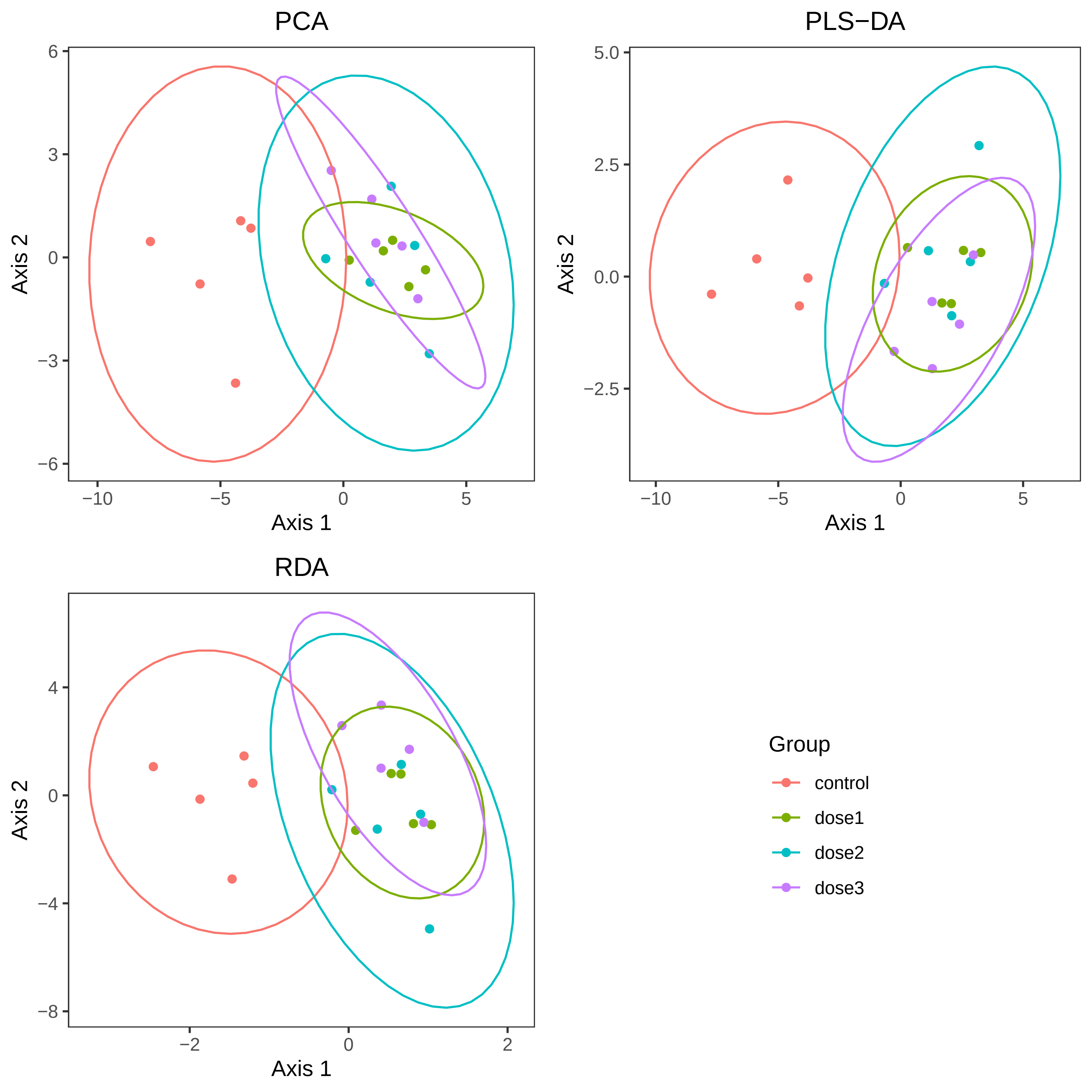
PCA
PLS−DA
RDA
Axis 1
Axis 2
Group
control
dose1
dose2
dose3

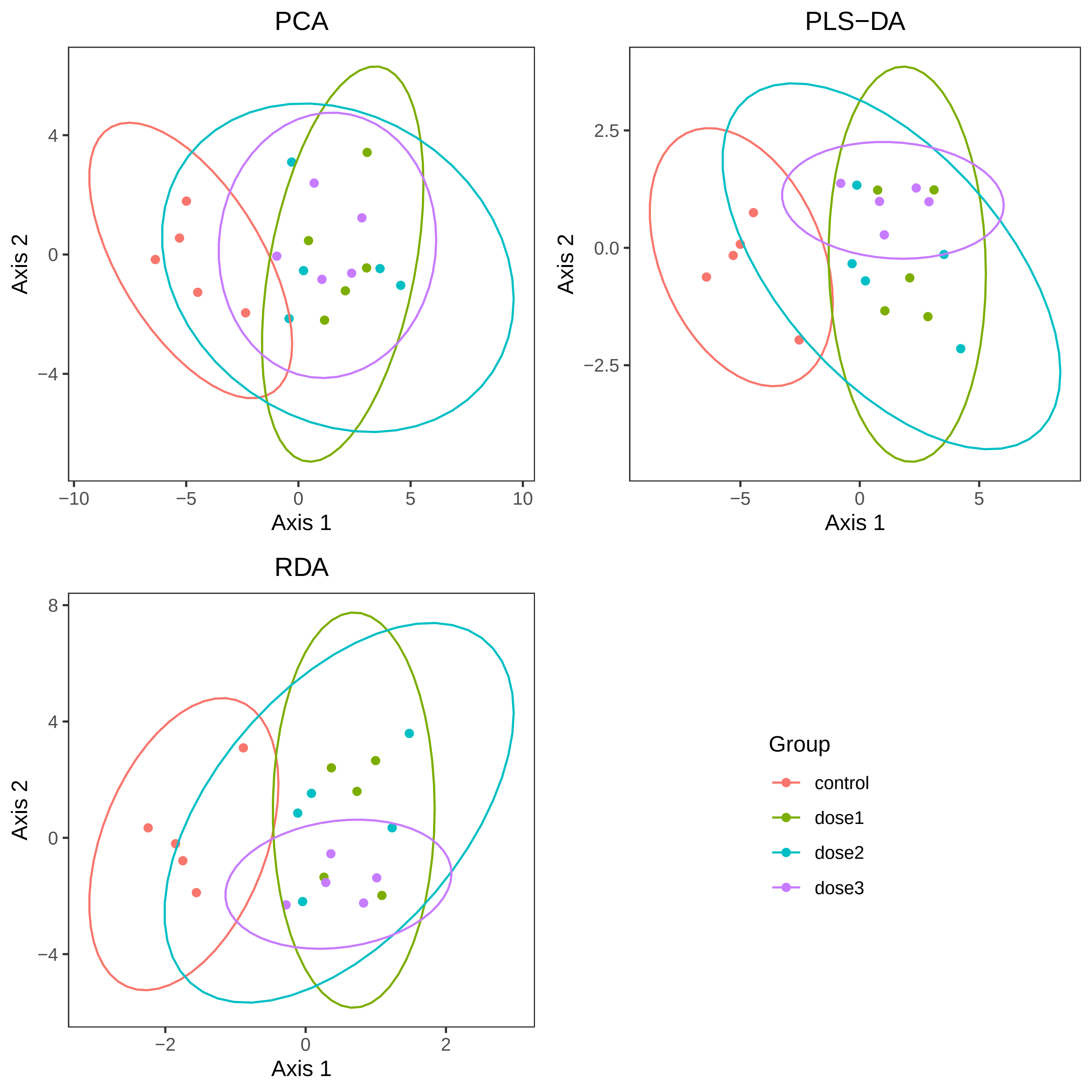
PCA
Axis 2
Axis 1
4
0
−4
−10
−5
0
5
10
PLS−DA
2.5
0.0
−2.5
RDA
8
−2
2
Group
control
dose1
dose2
dose3

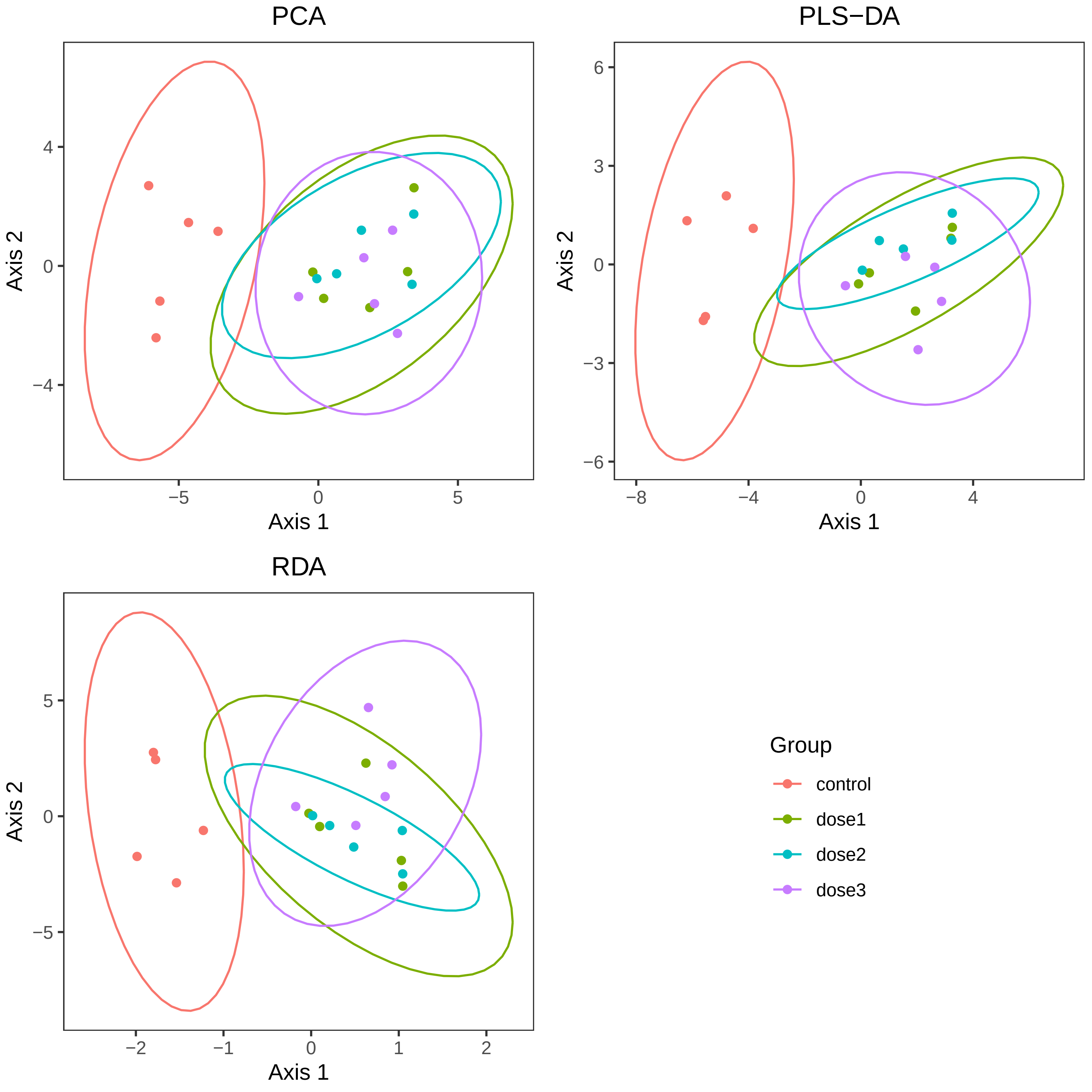
PCA
PLS−DA
RDA
Axis 1
Axis 2
Group
control
dose1
dose2
dose3

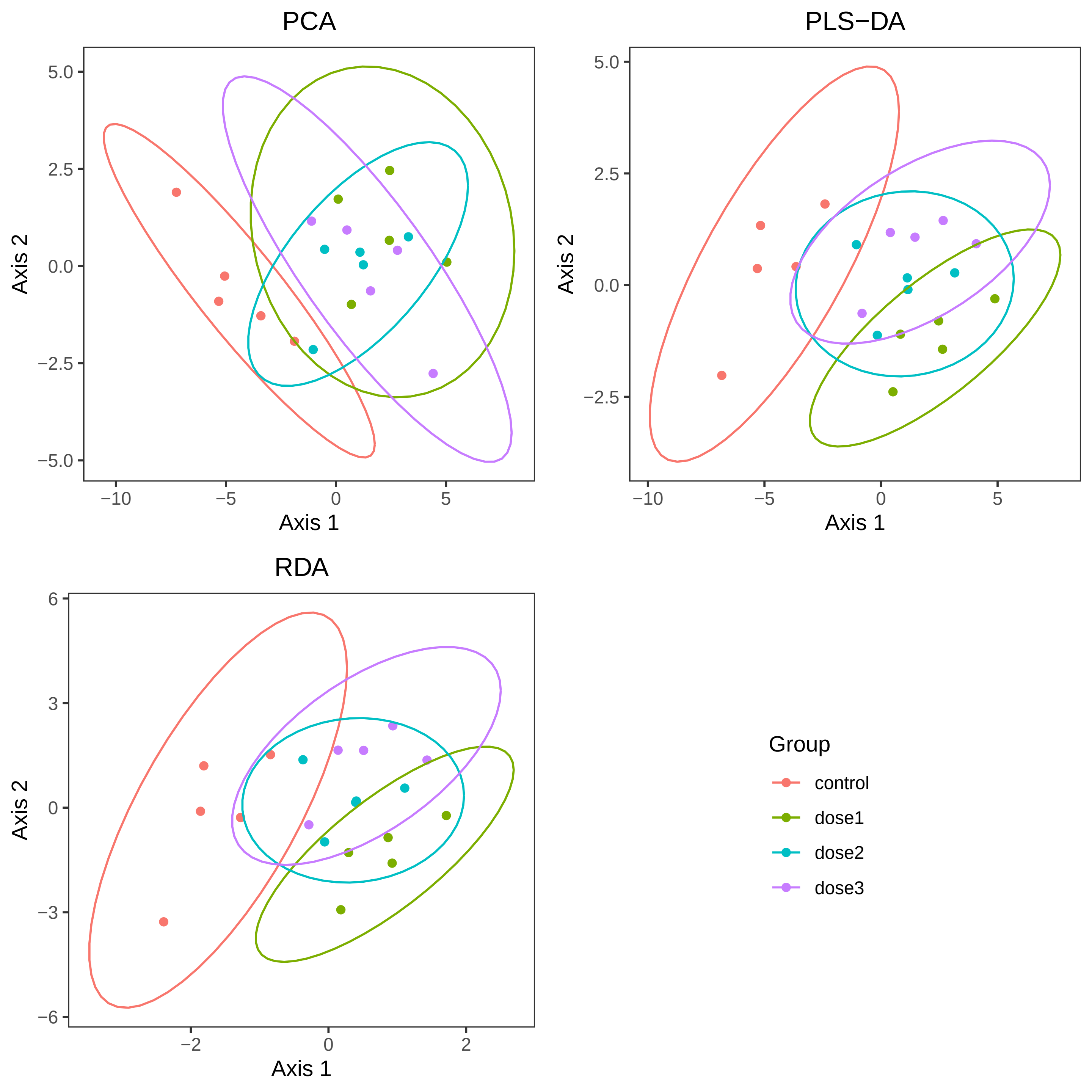
PCA
PLS−DA
RDA
Axis 1
Axis 2
5.0
2.5
0.0
−2.5
−5.0
−10
−5
0
5
6
3
−3
−6
−2
2
Group
control
dose1
dose2
dose3

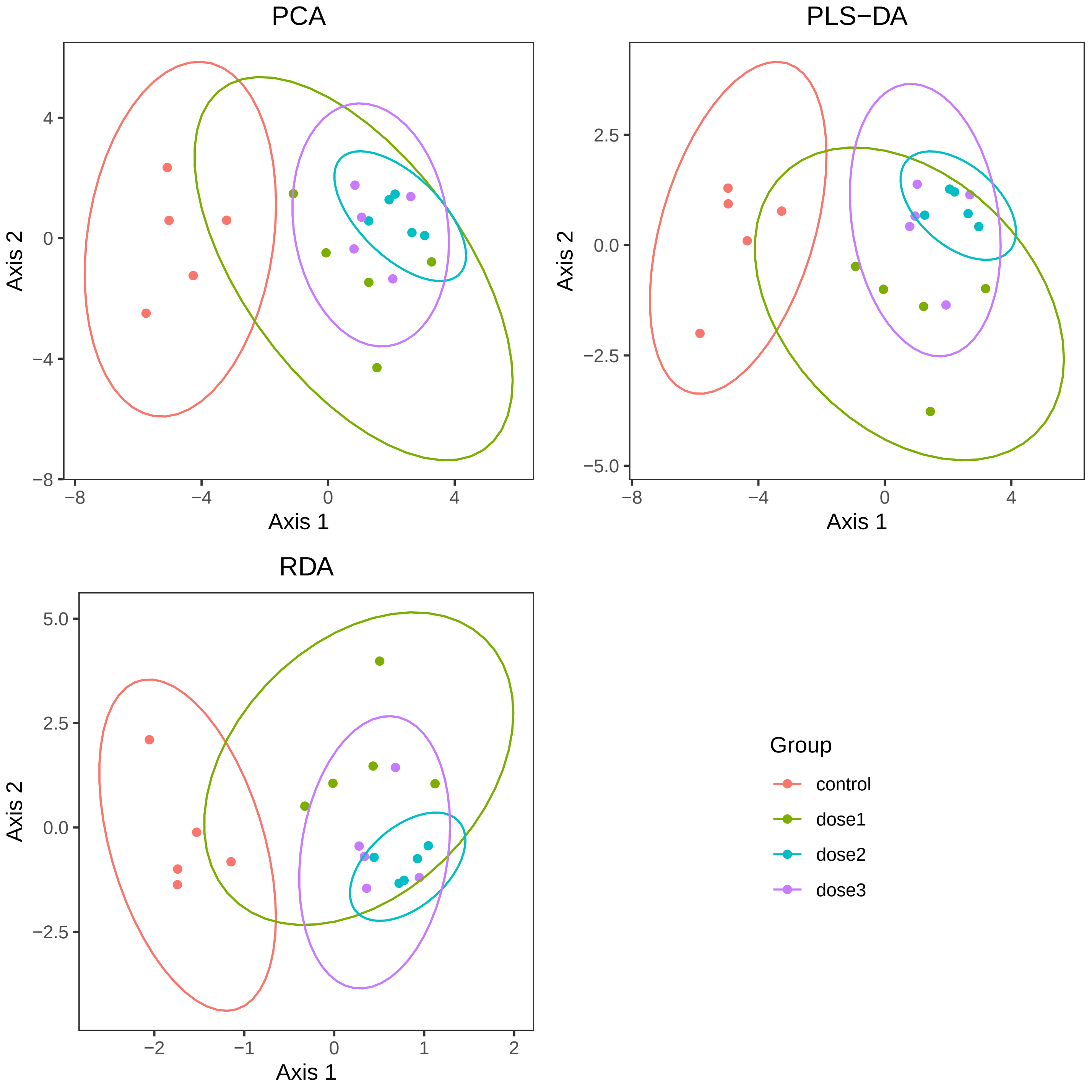
PCA
PLS−DA
RDA
Axis 1
Axis 2
Group
control
dose1
dose2
dose3

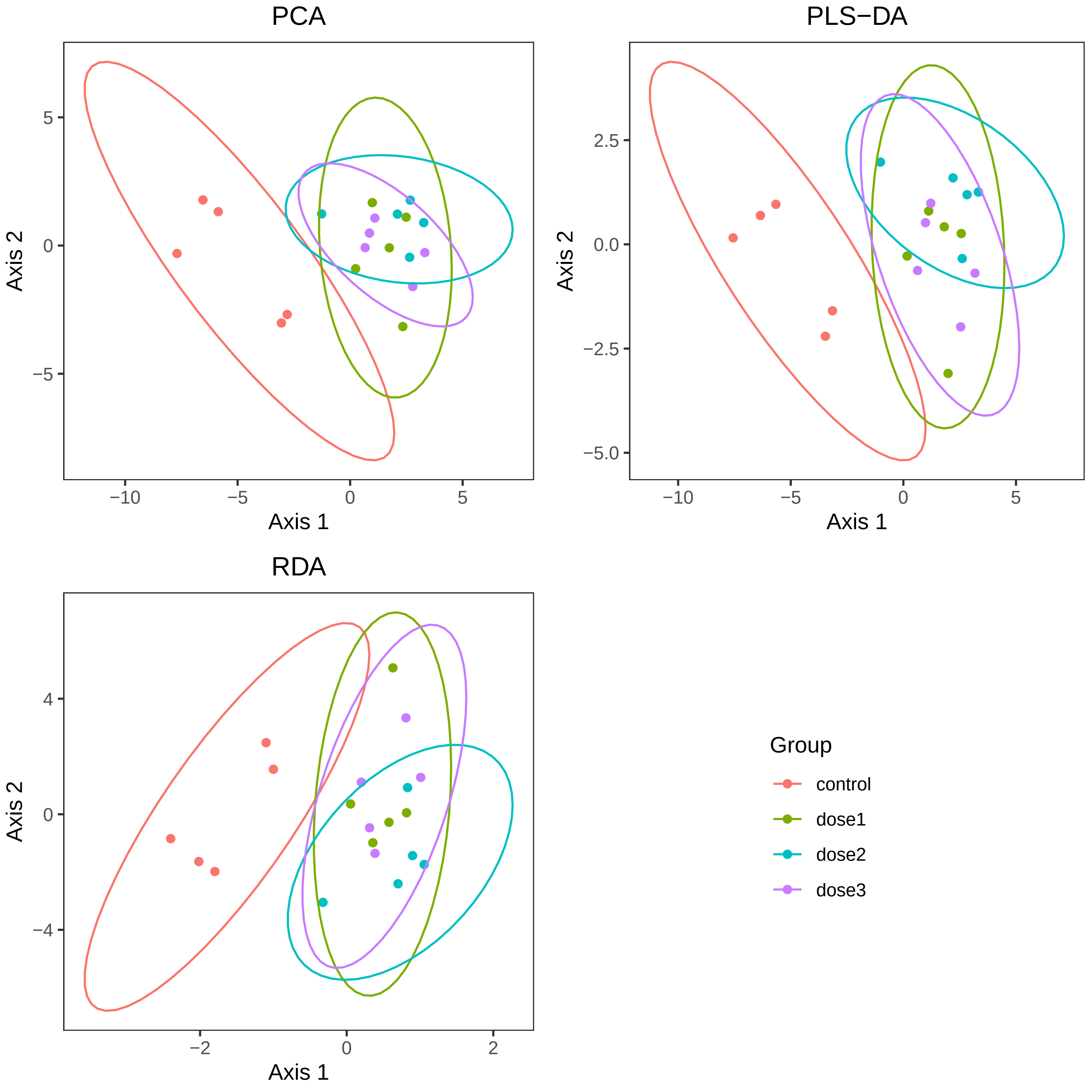
PCA
PLS−DA
RDA
Axis 1
Axis 2
Group
control
dose1
dose2
dose3

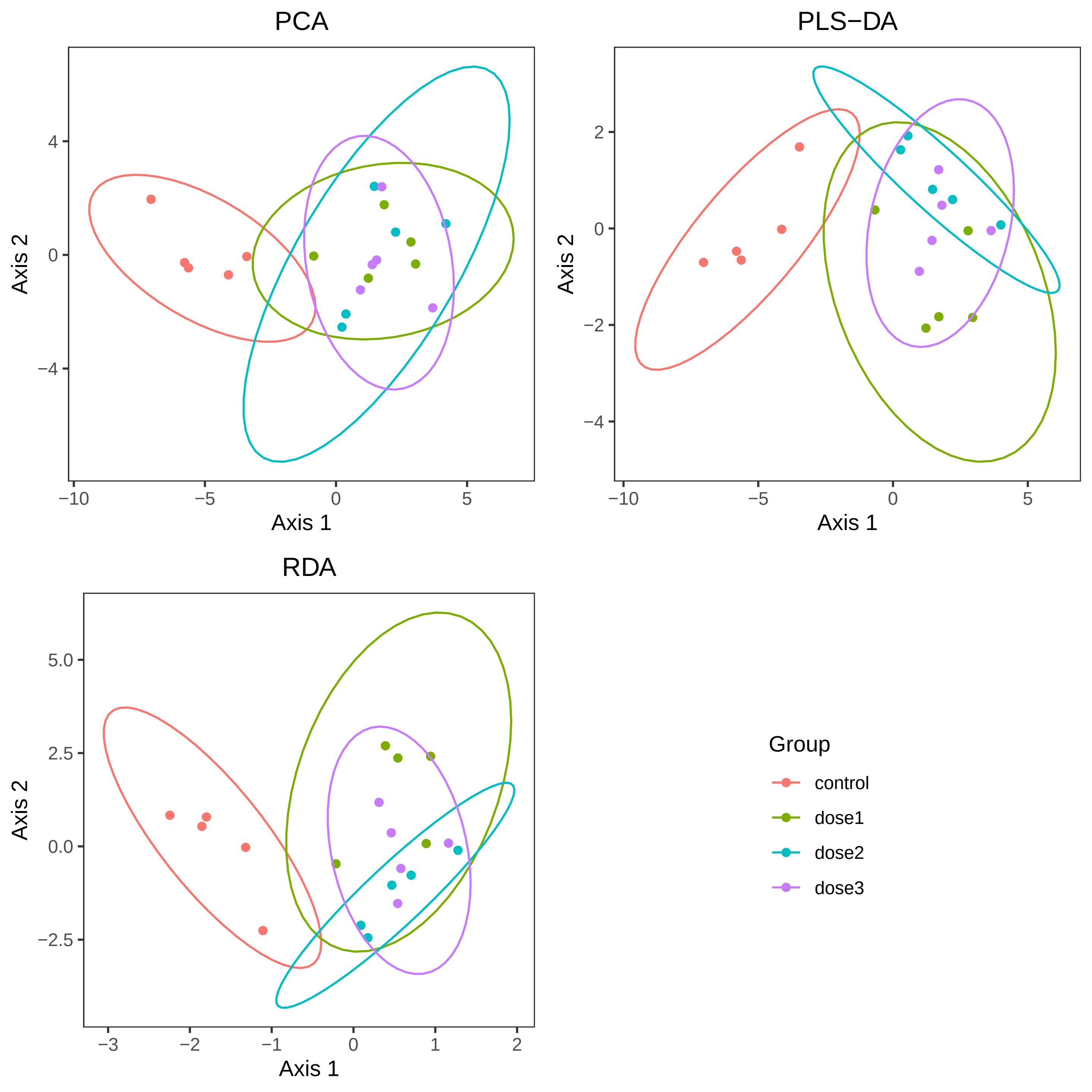

PCA
PLS−DA
RDA
Axis 1
Axis 2
Group
control
dose1
dose2
dose3

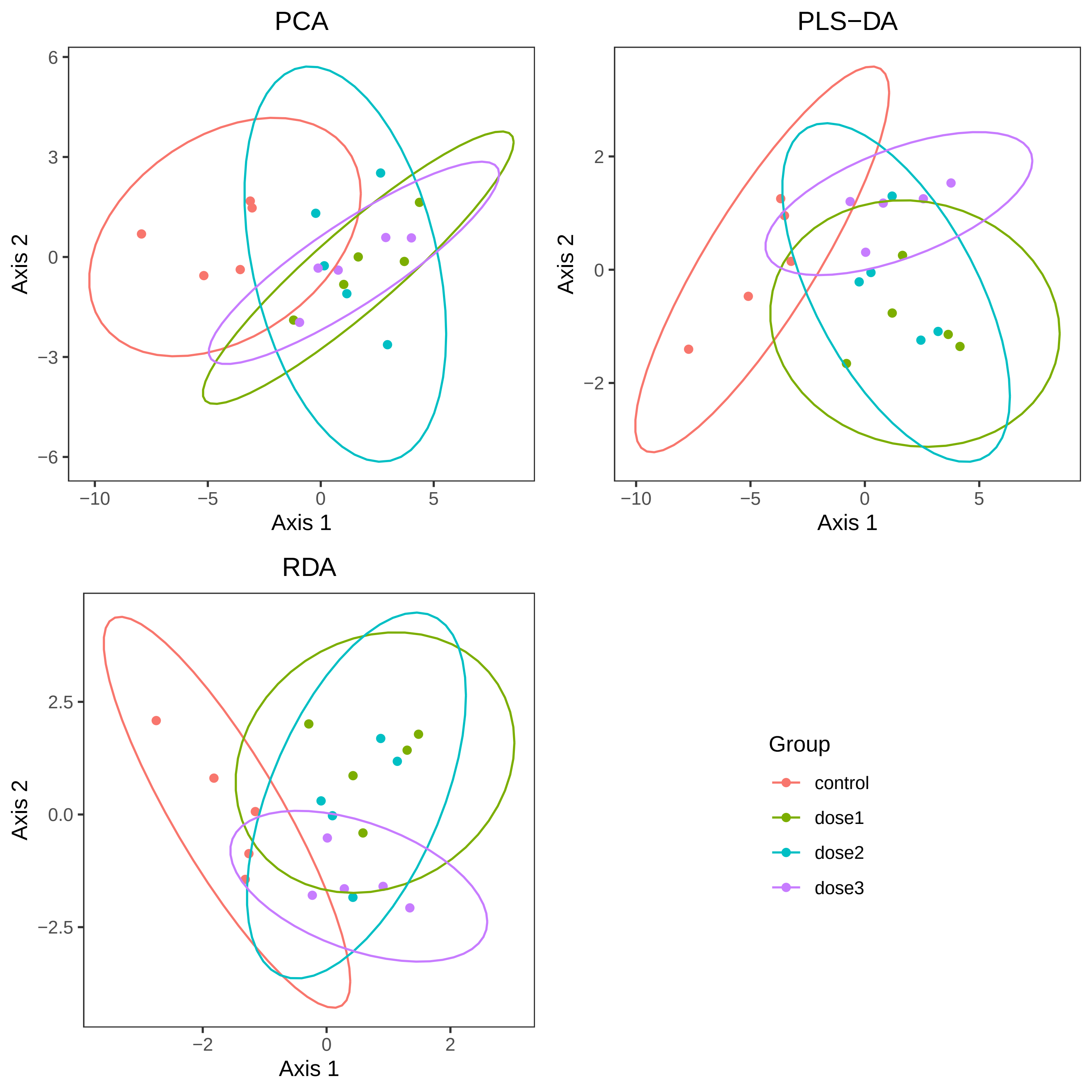

PCA
PLS−DA
RDA
Axis 1
Axis 2
6
3
0
−3
−6
−10
−5
0
5
2
−2
2.5
0.0
−2.5
Group
control
dose1
dose2
dose3

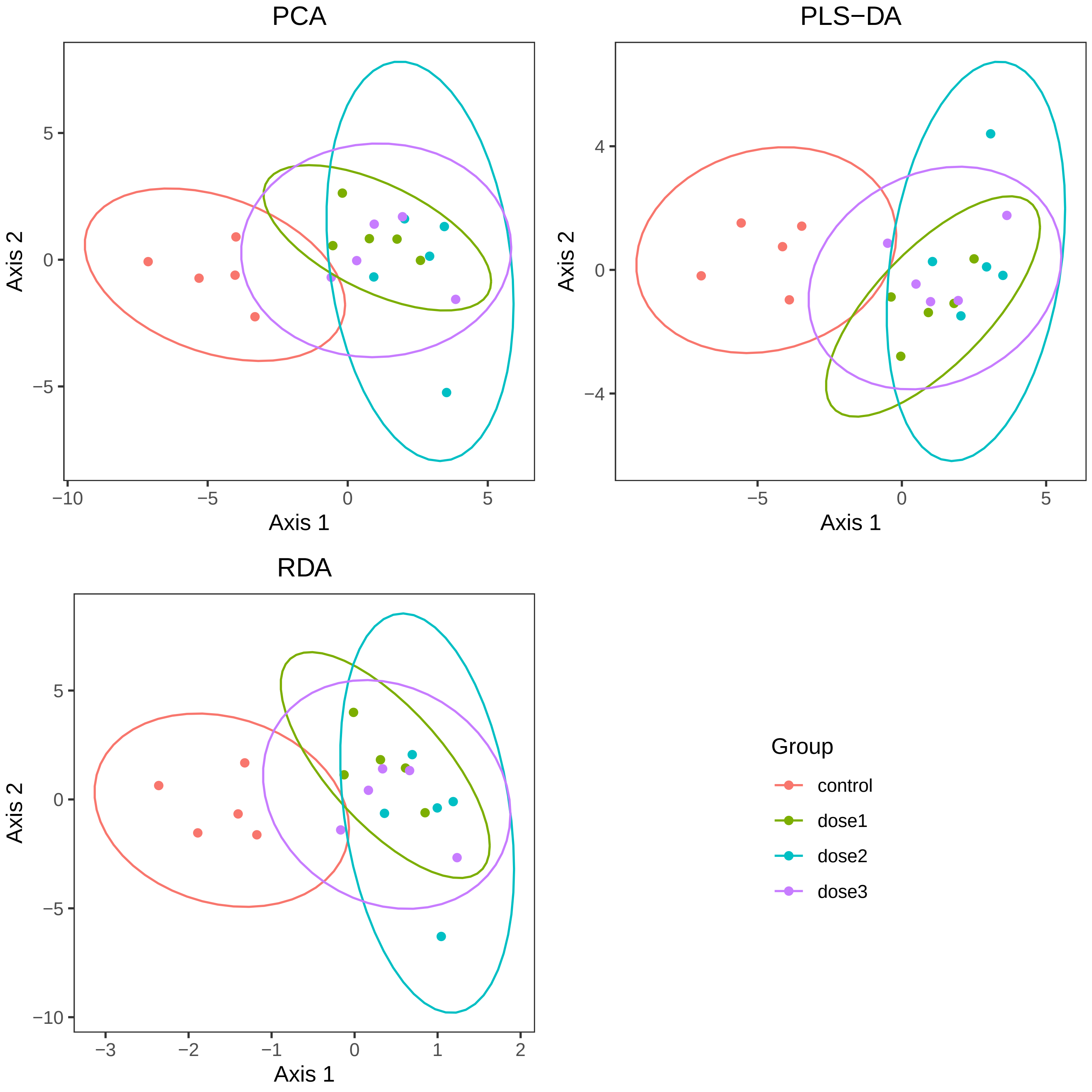

PCA
PLS−DA
RDA
Axis 1
Axis 2
Group
control
dose1
dose2
dose3

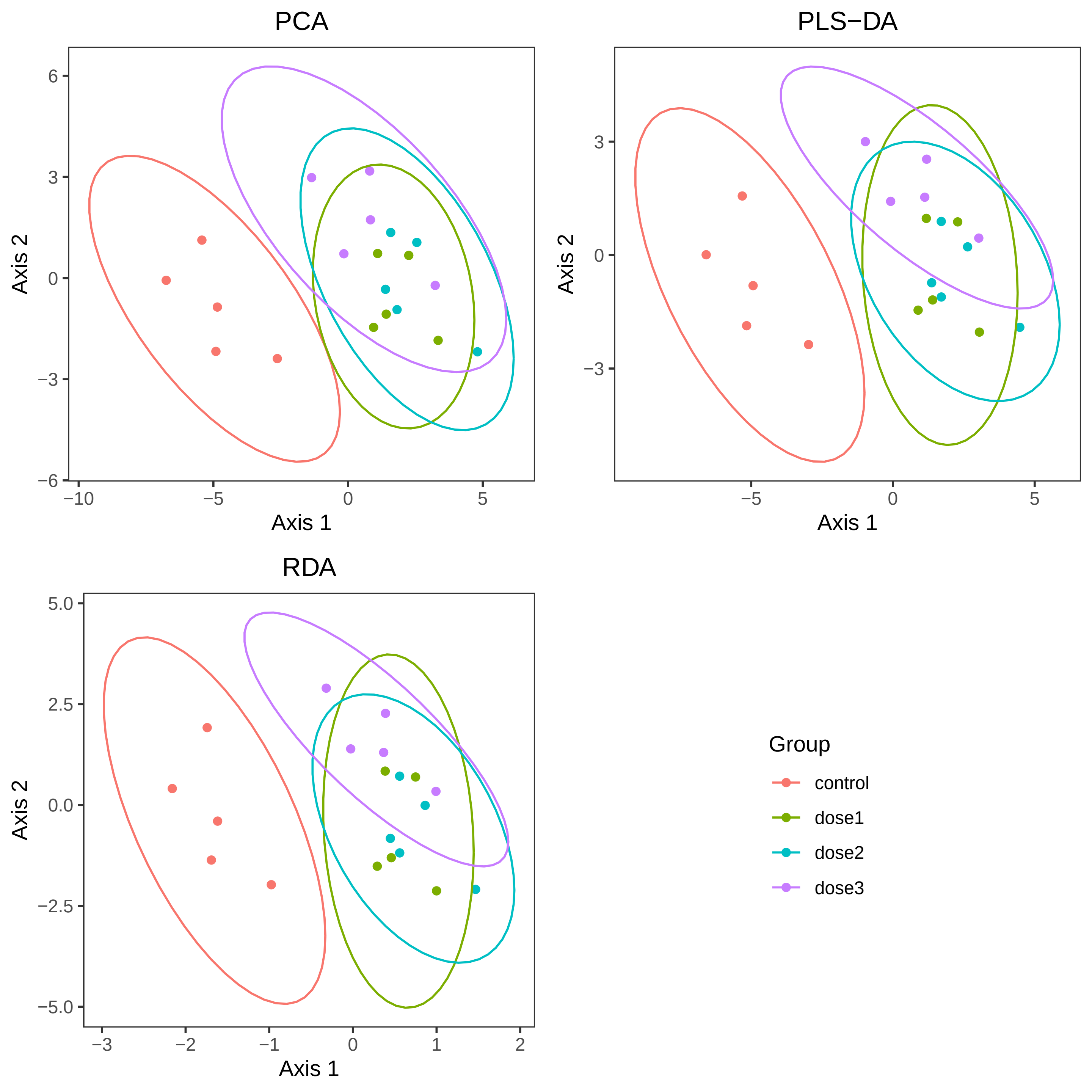

PCA
PLS−DA
RDA
Axis 1
Axis 2
6
3
0
−3
−6
−10
−5
0
5
5.0
2.5
0.0
−2.5
−5.0
−3
−2
−1
1
2
Group
control
dose1
dose2
dose3

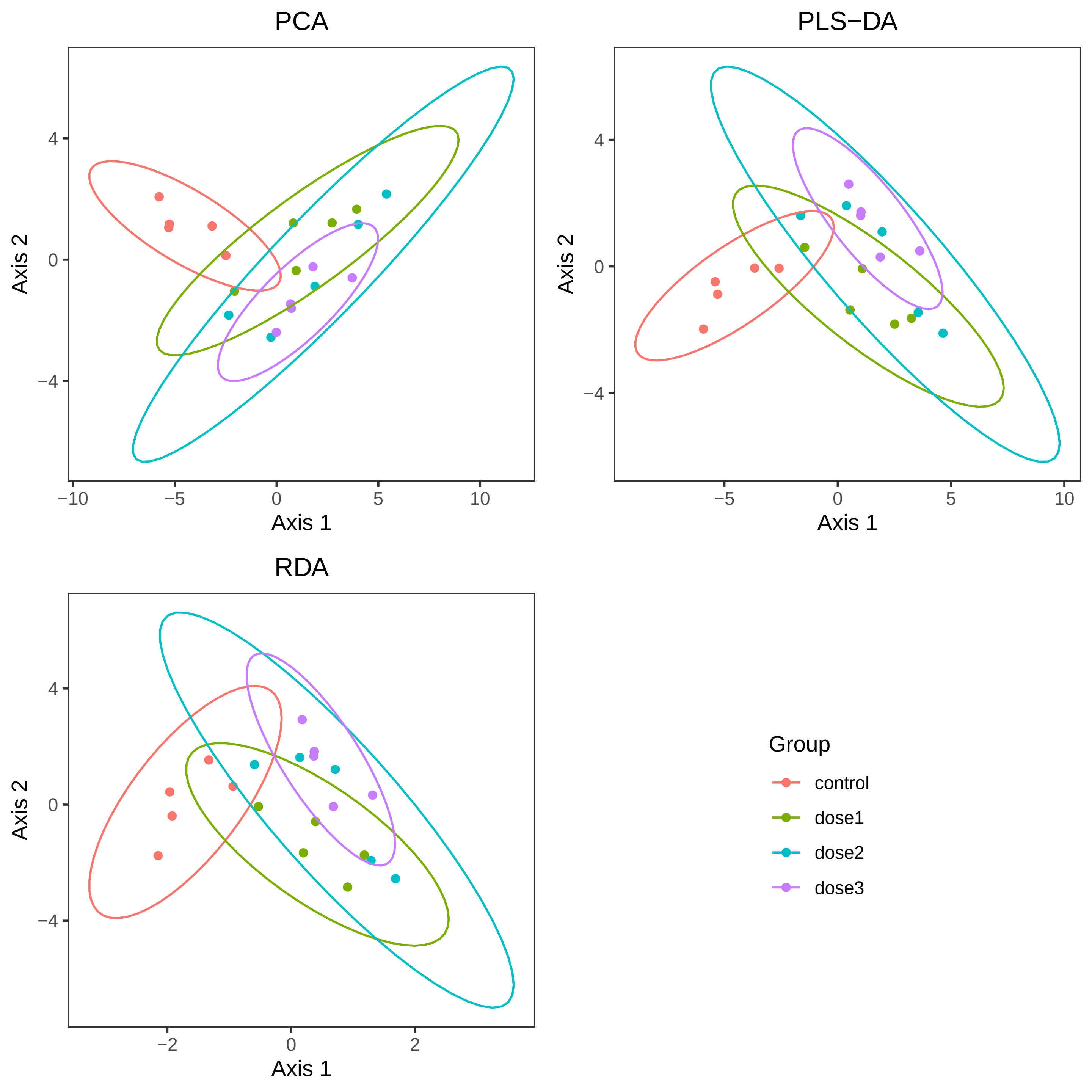

PCA
PLS−DA
RDA
Axis 1
Axis 2
Group
control
dose1
dose2
dose3

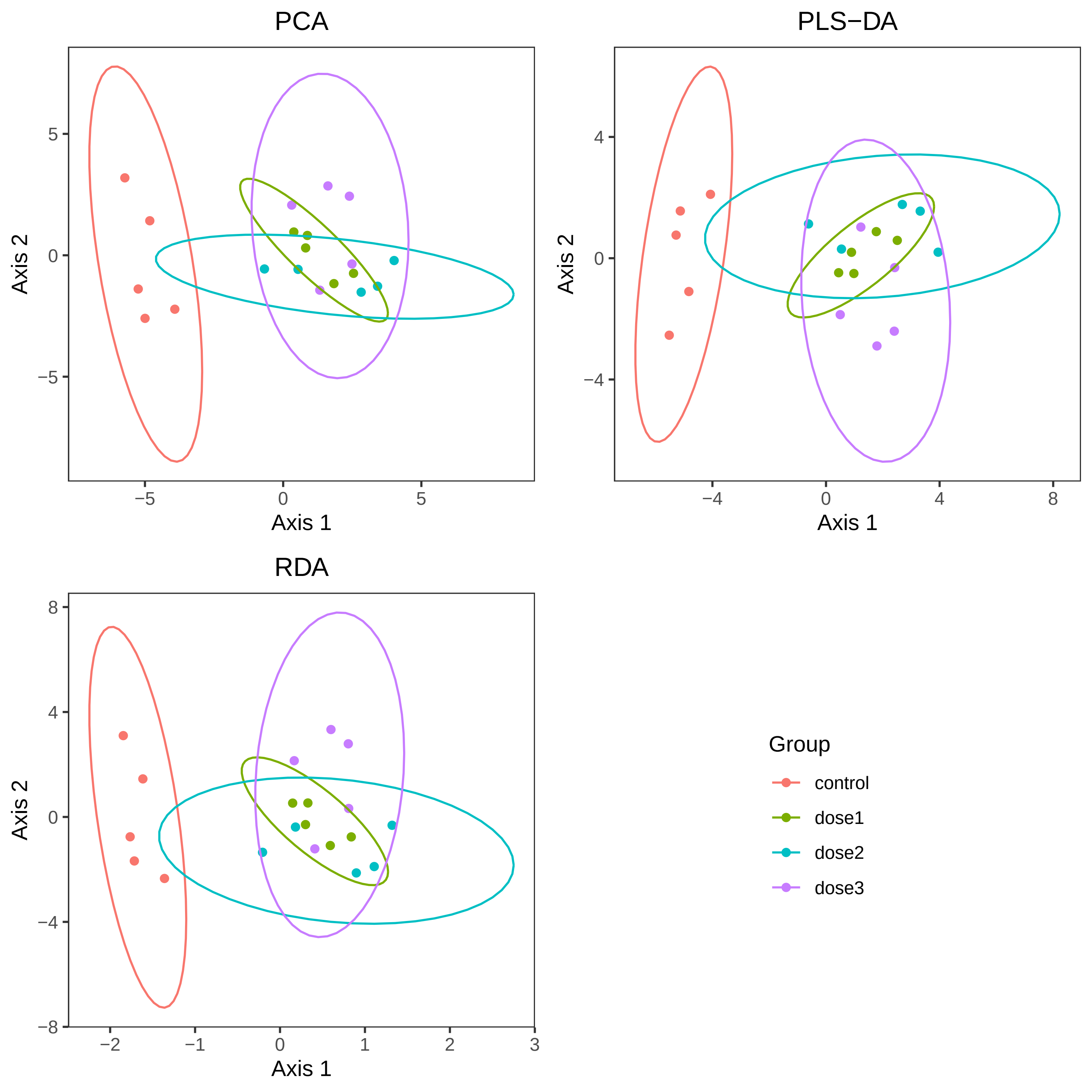
PCA
PLS−DA
RDA
Axis 1
Axis 2
Group
control
dose1
dose2
dose3

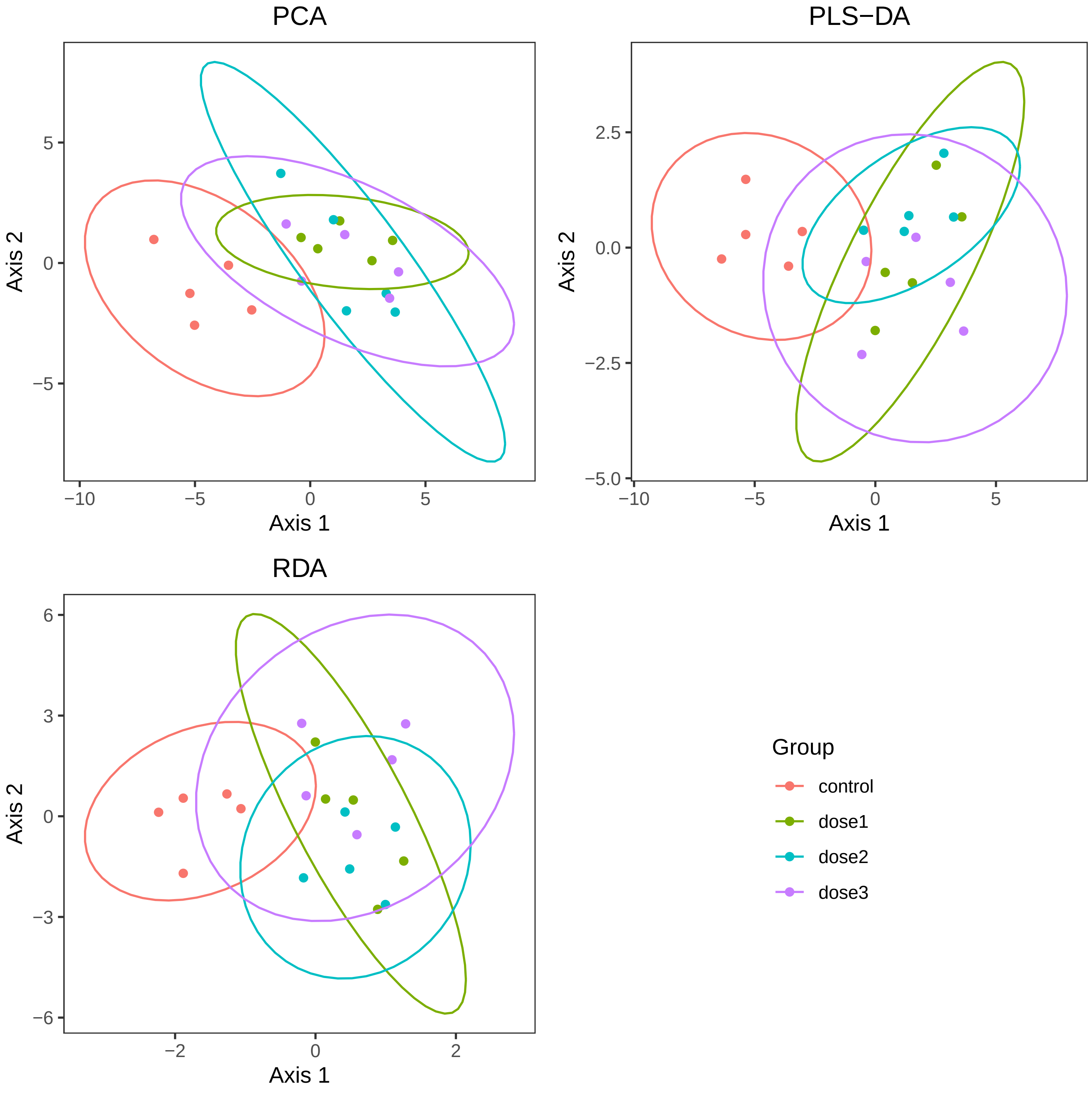
PCA
PLS−DA
RDA
Axis 1
Axis 2
Group
control
dose1
dose2
dose3

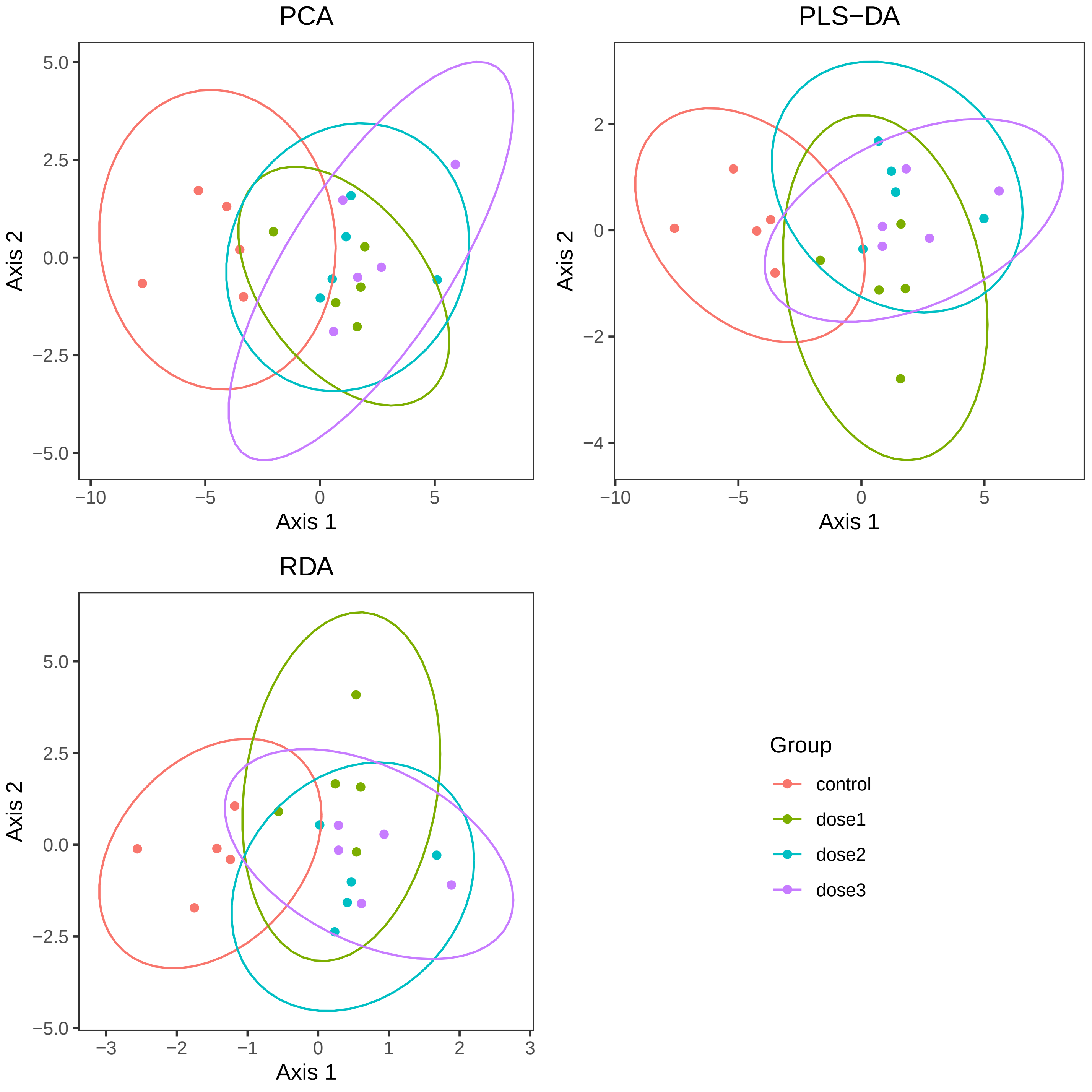
PCA
PLS−DA
RDA
Axis 1
Axis 2
Group
control
dose1
dose2
dose3

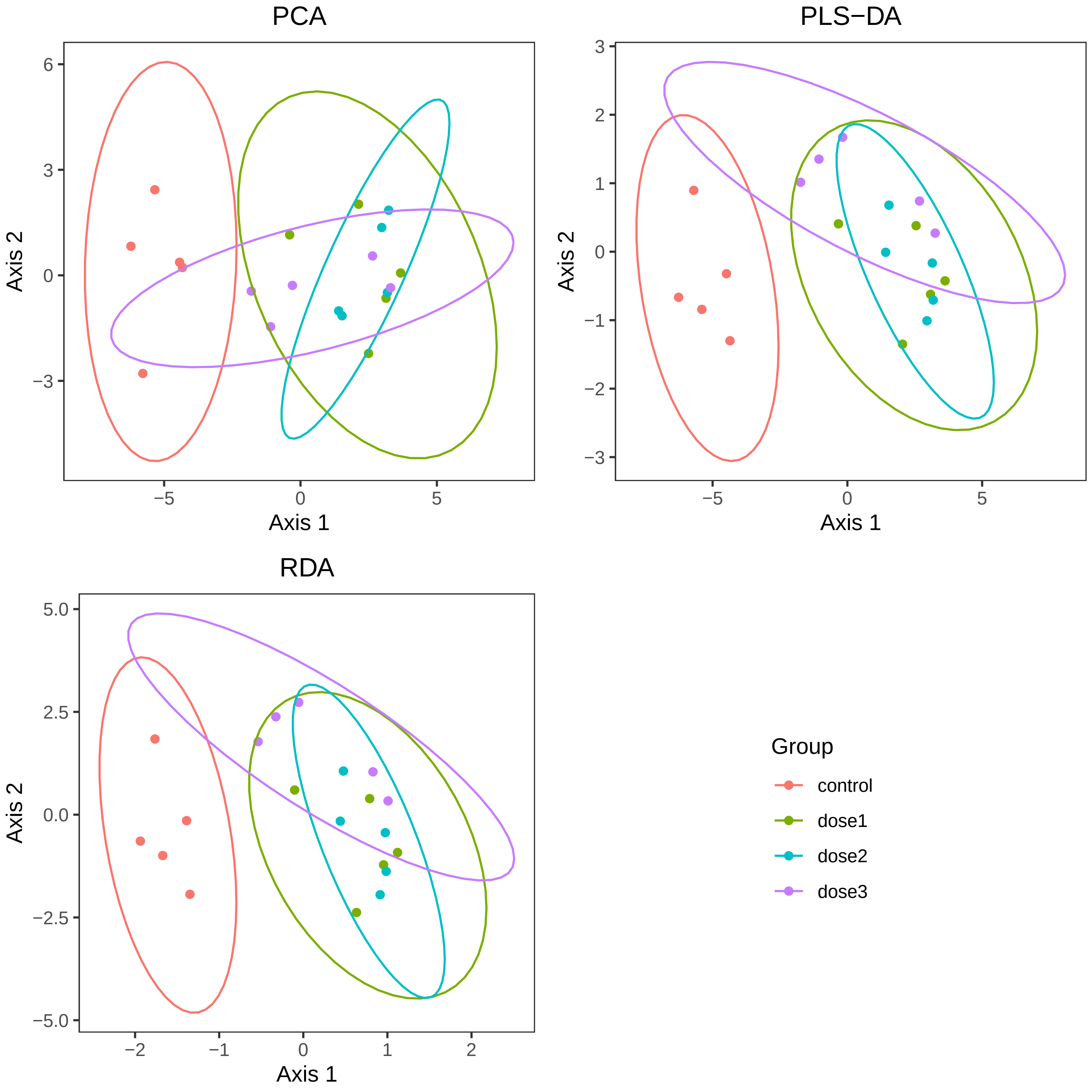
PCA
PLS−DA
RDA
Axis 1
Axis 2
Group
control
dose1
dose2
dose3

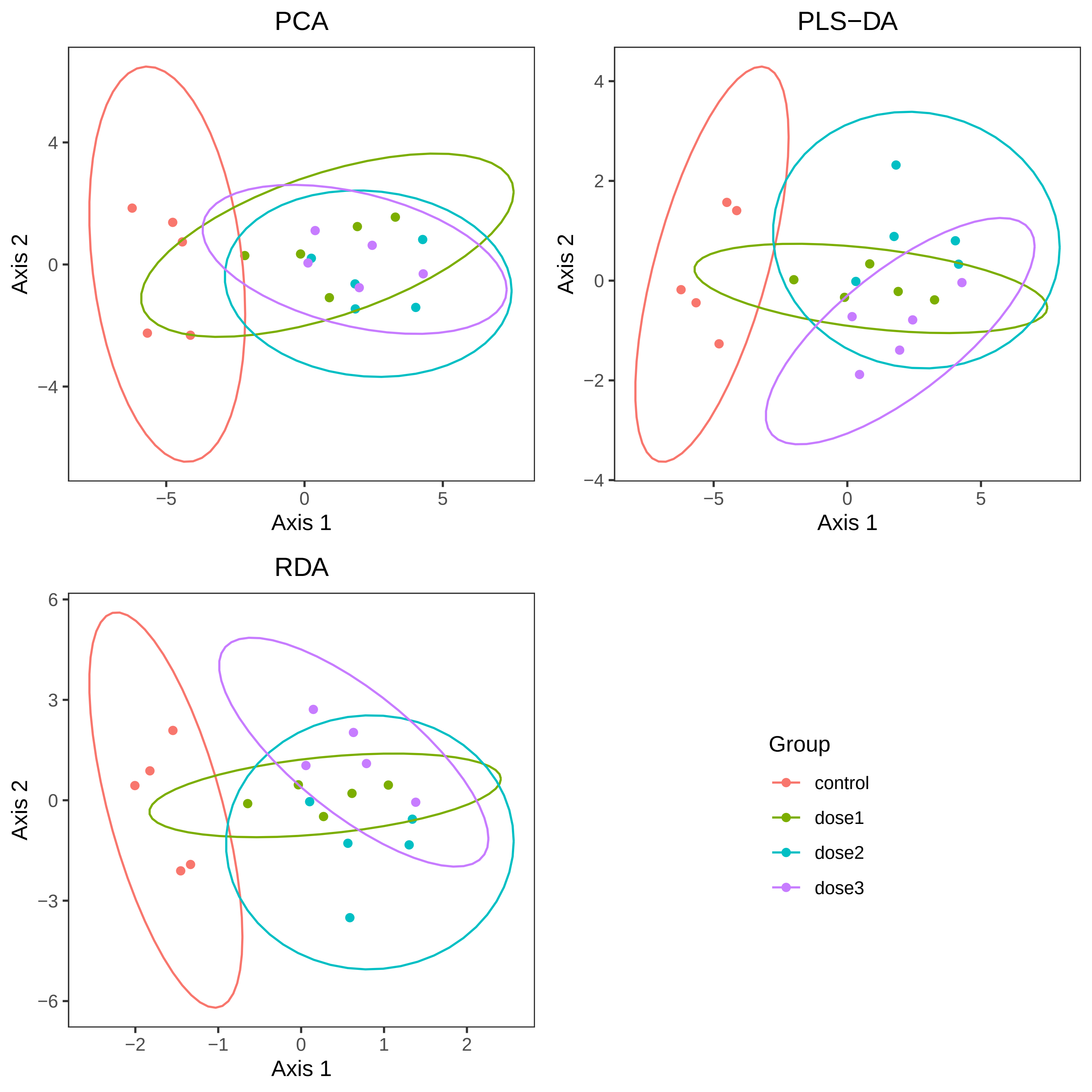
PCA
PLS−DA
RDA
Axis 1
Axis 2
Group
control
dose1
dose2
dose3

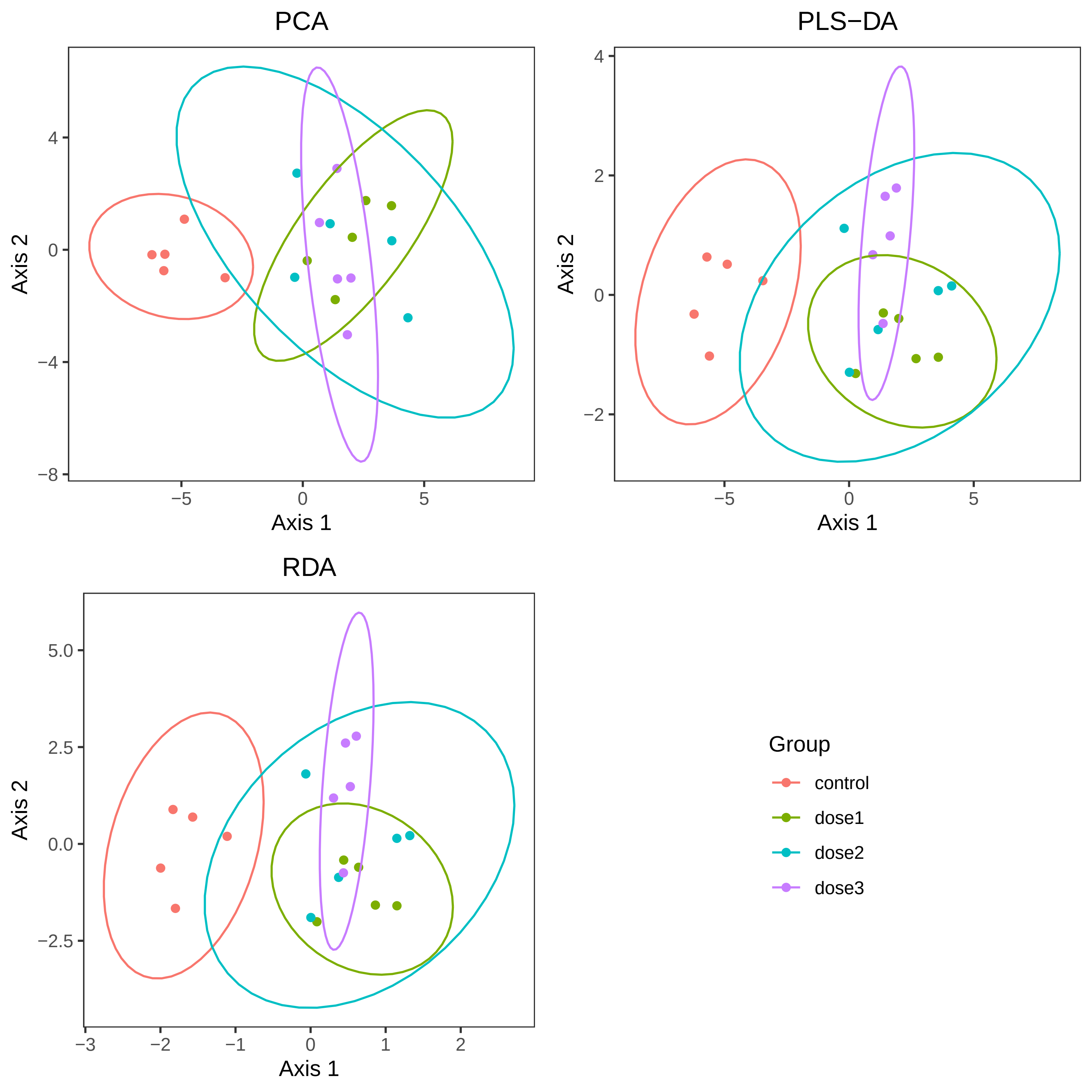
PCA
PLS−DA
RDA
Axis 1
Axis 2
Group
control
dose1
dose2
dose3

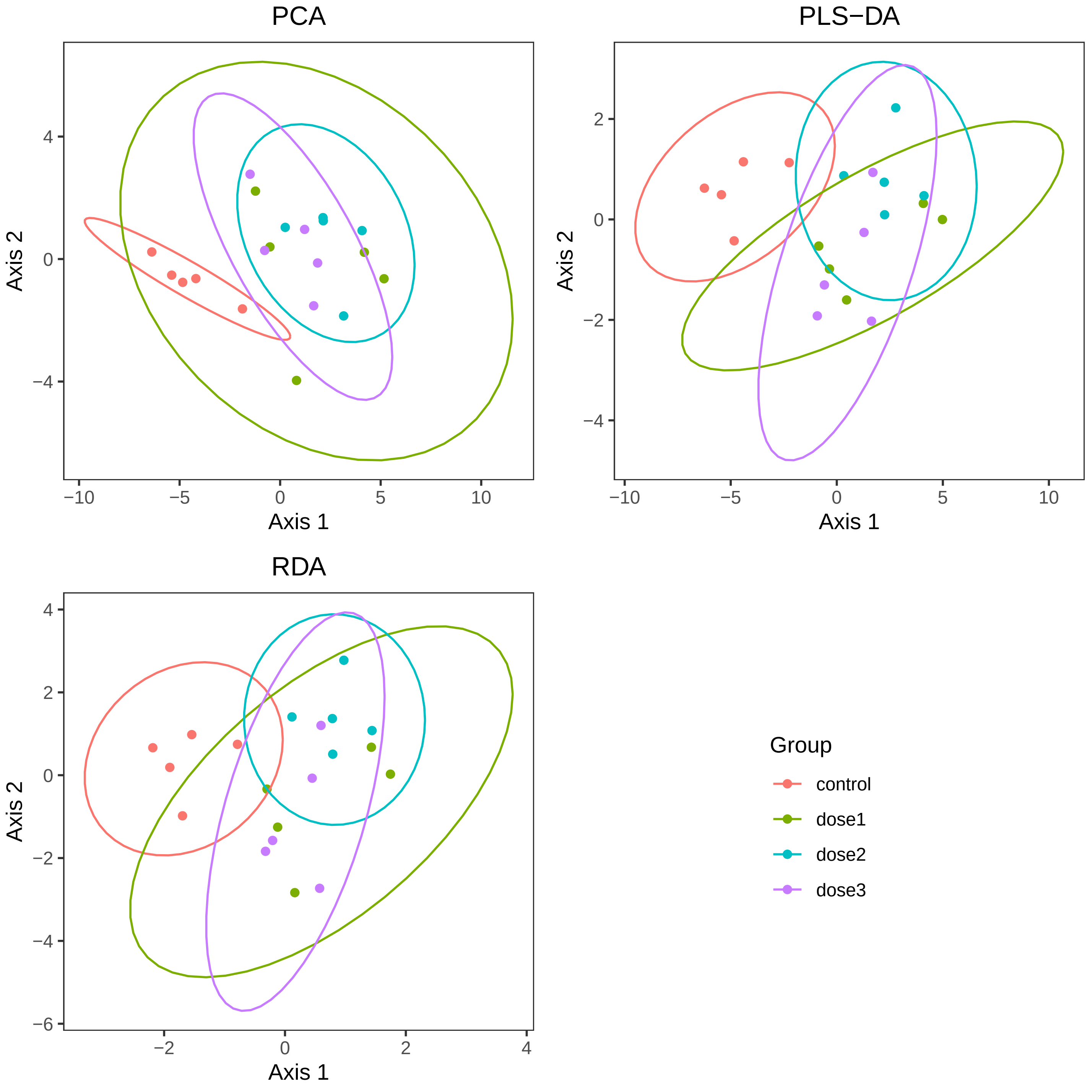
PCA
PLS−DA
RDA
Axis 1
Axis 2
Group
control
dose1
dose2
dose3

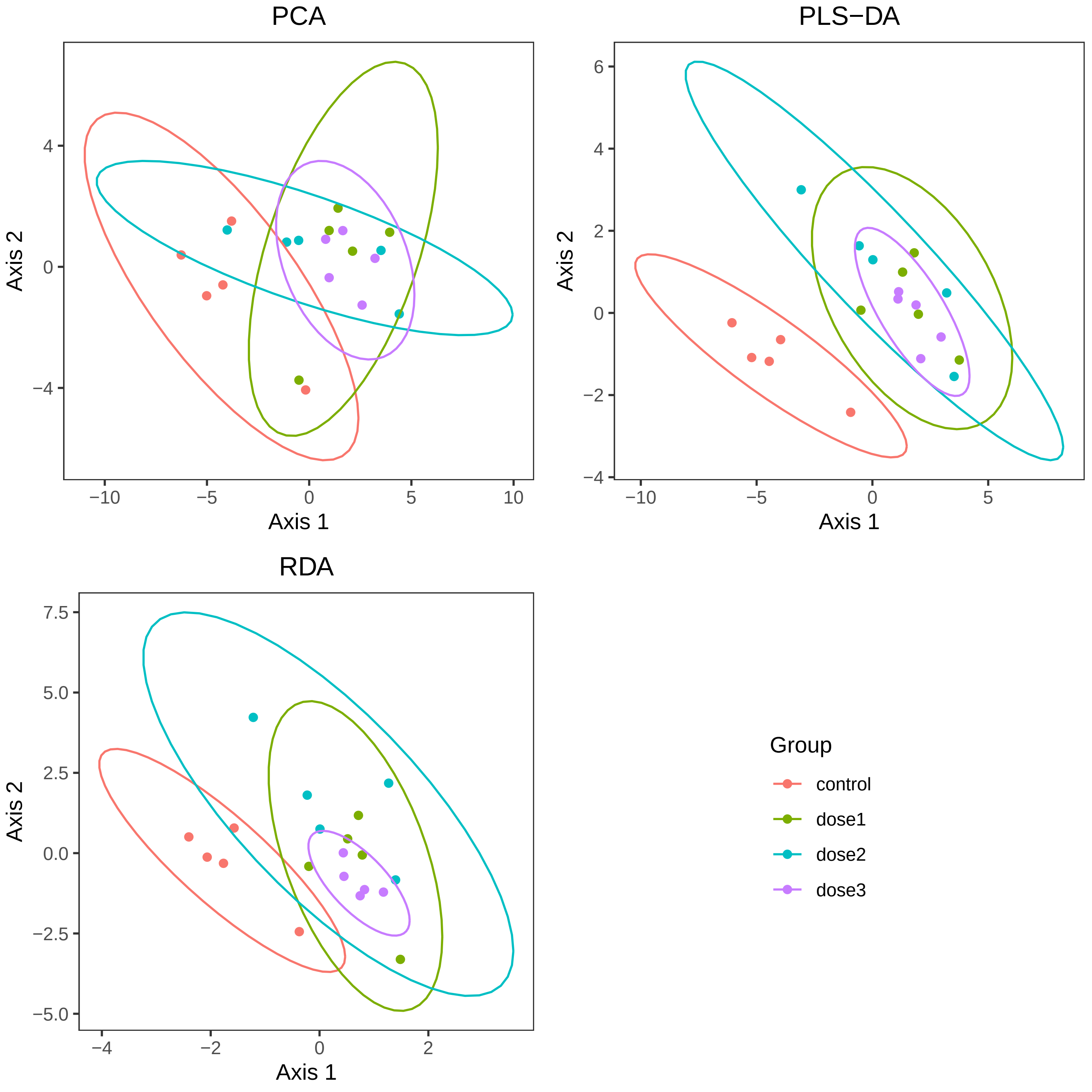

PCA
PLS−DA
RDA
Axis 1
Axis 2
Group
control
dose1
dose2
dose3

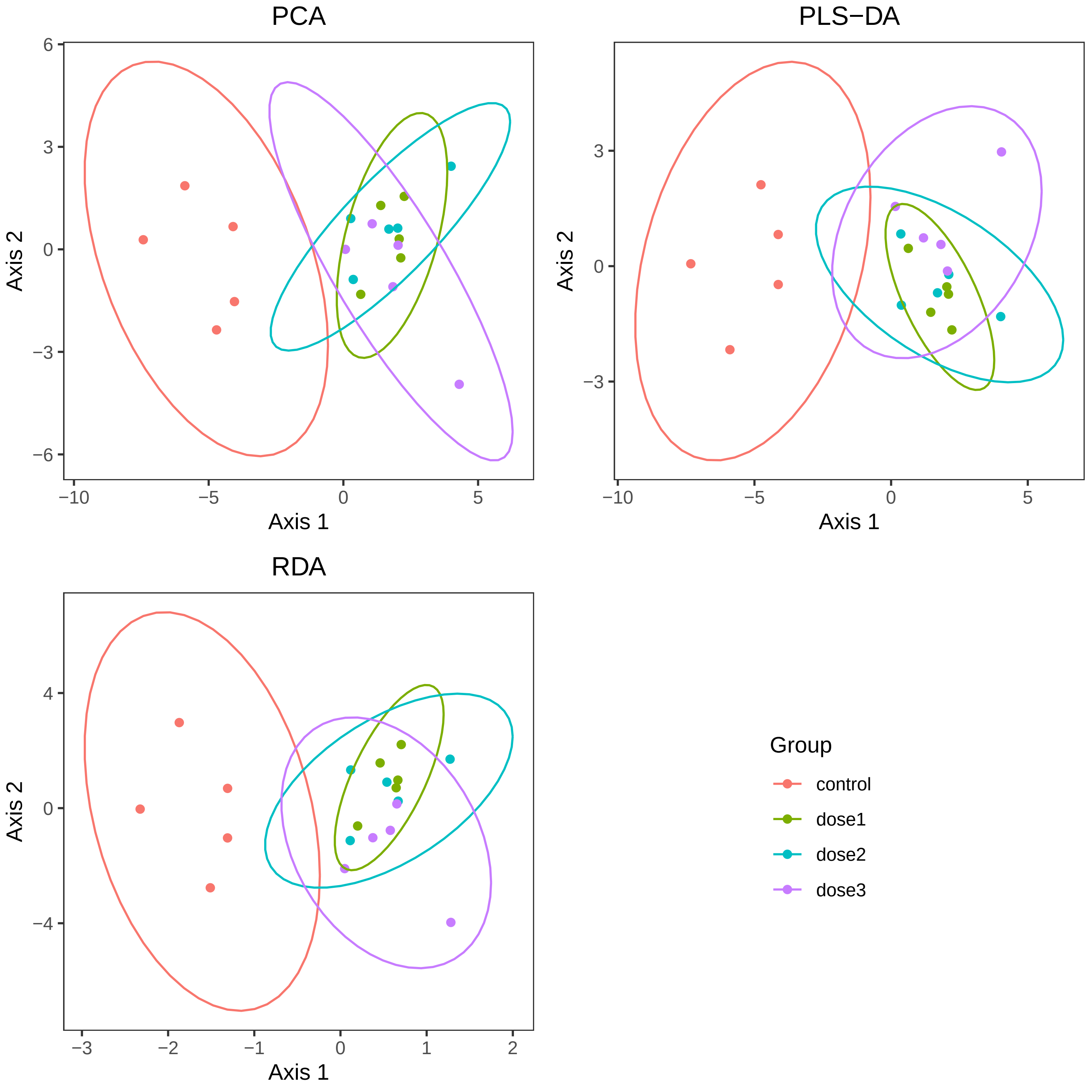
PCA
PLS−DA
RDA
Axis 1
Axis 2
Group
control
dose1
dose2
dose3

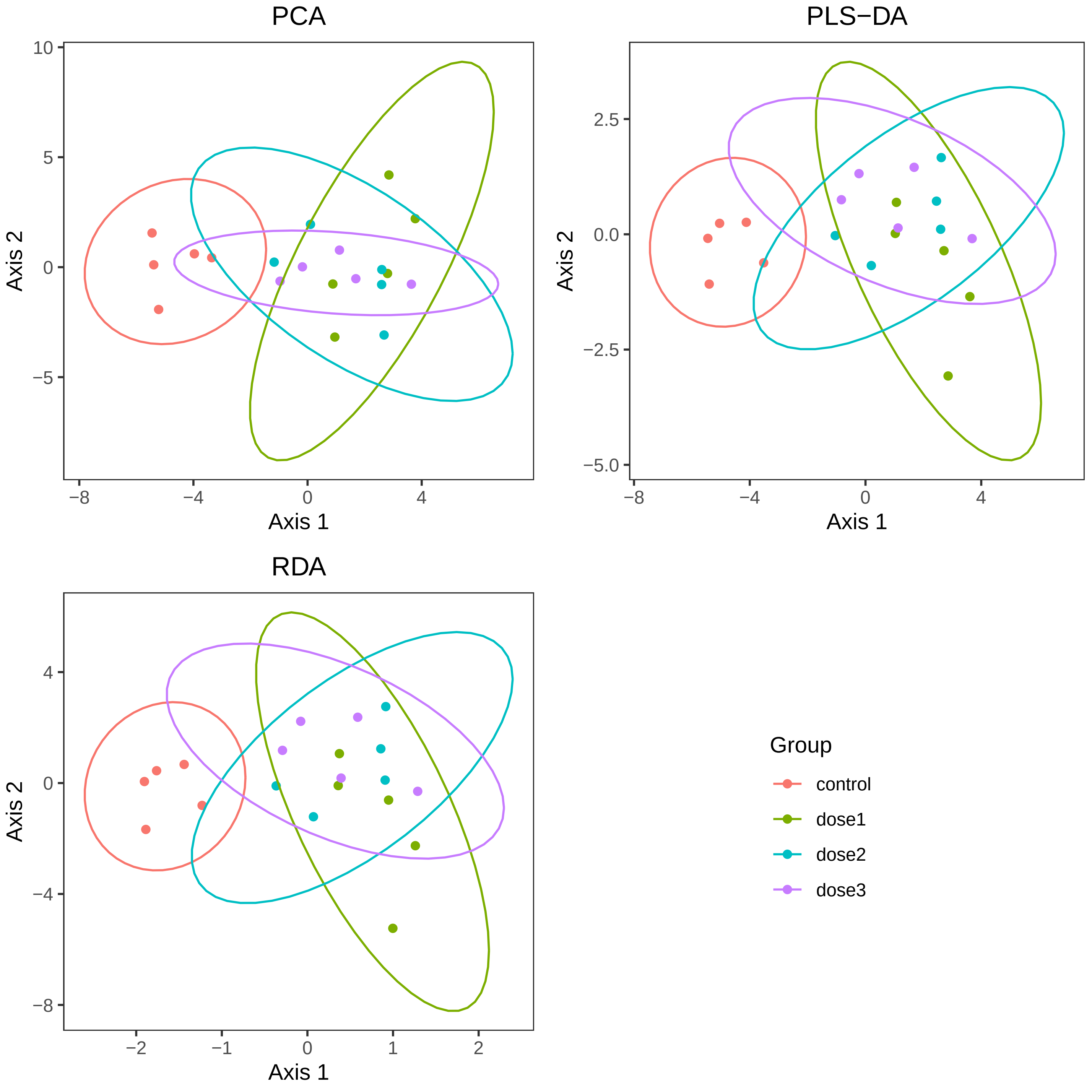

PCA
PLS−DA
RDA
Axis 1
Axis 2
Group
control
dose1
dose2
dose3

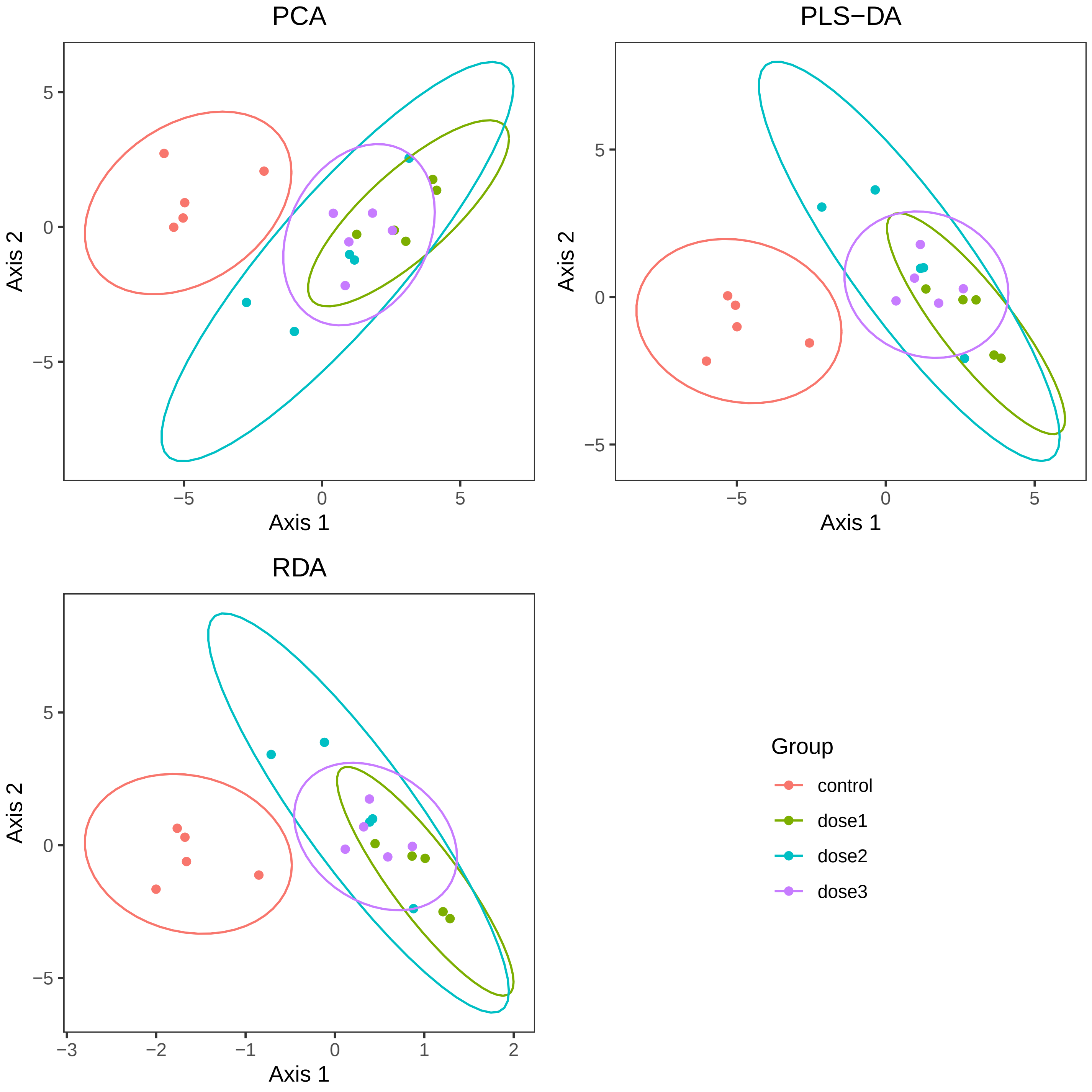

PCA
PLS−DA
RDA
Axis 1
Axis 2
5
0
−5
Group
control
dose1
dose2
dose3

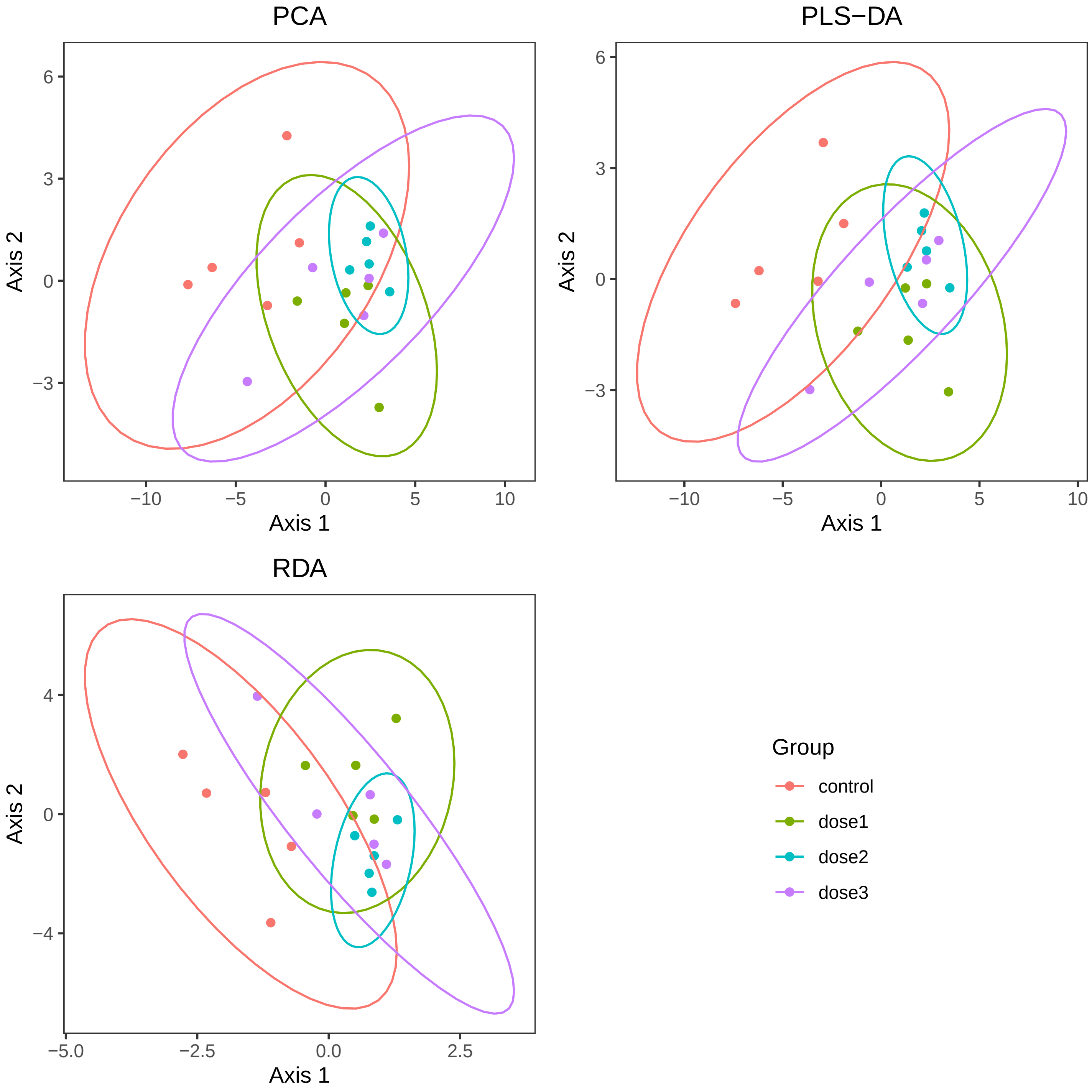
PCA
PLS−DA
RDA
Axis 1
Axis 2
Group
control
dose1
dose2
dose3

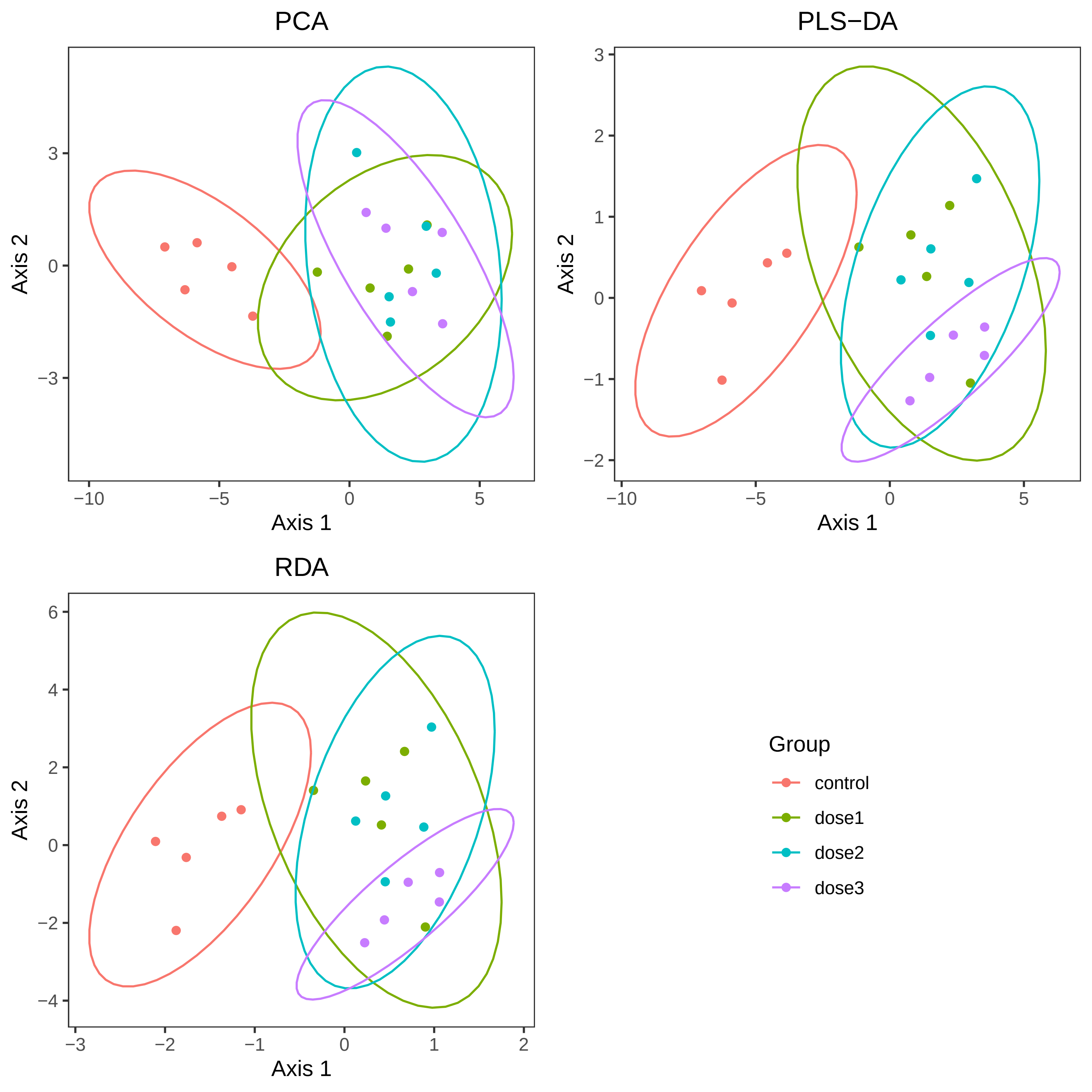
PCA
PLS−DA
RDA
Axis 1
Axis 2
Group
control
dose1
dose2
dose3

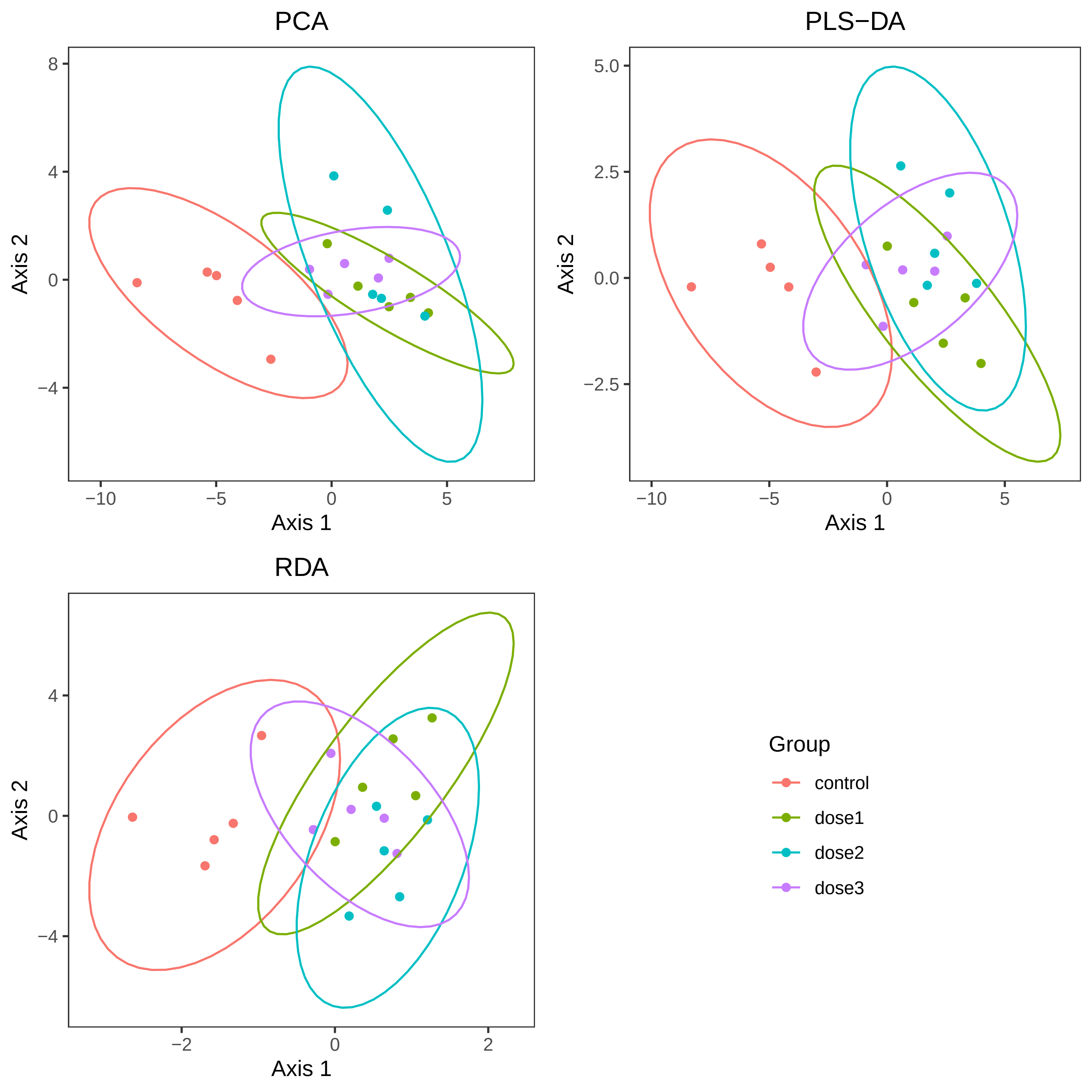

PCA
PLS−DA
RDA
Axis 1
Axis 2
Group
control
dose1
dose2
dose3

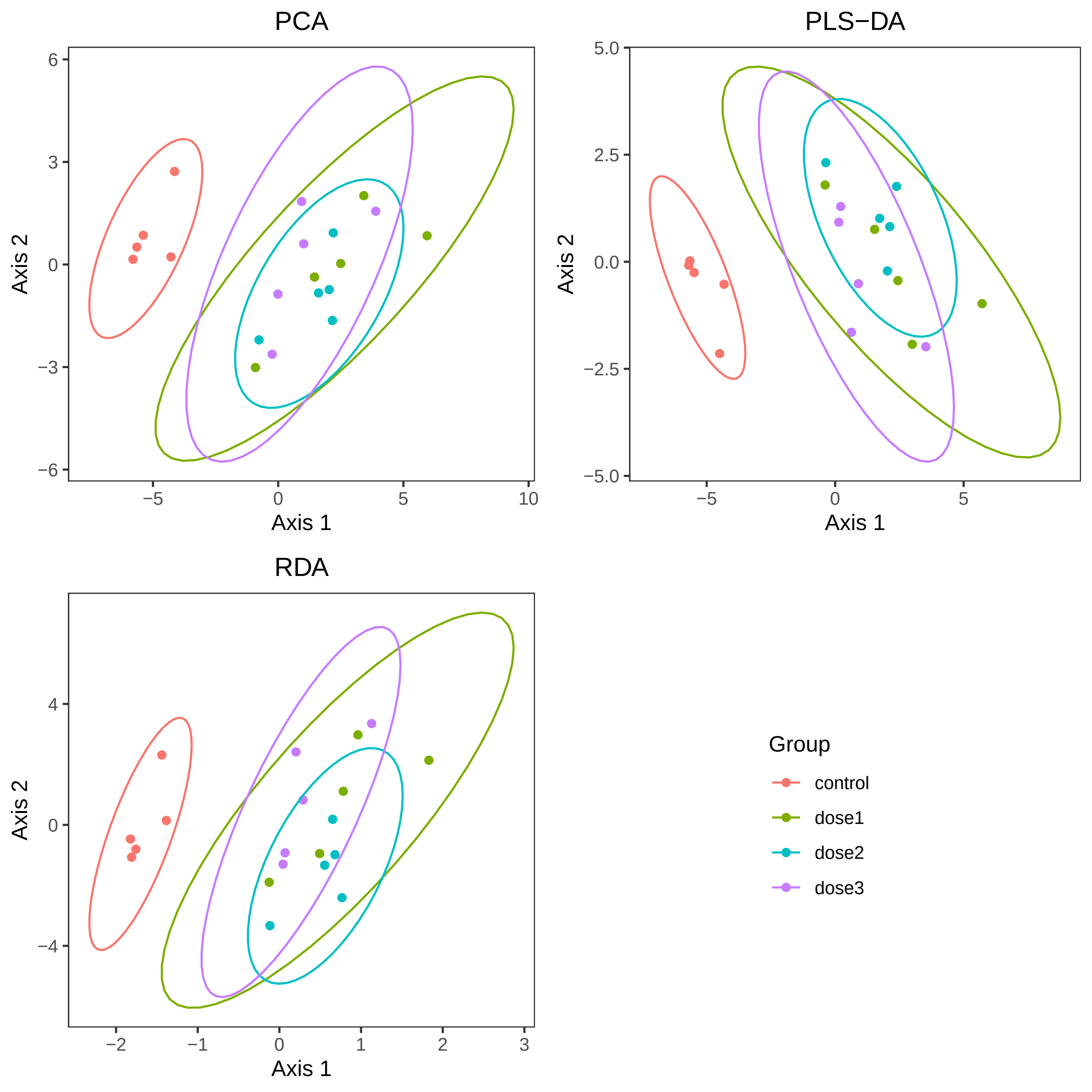

PCA
PLS−DA
RDA
Axis 1
Axis 2
Group
control
dose1
dose2
dose3

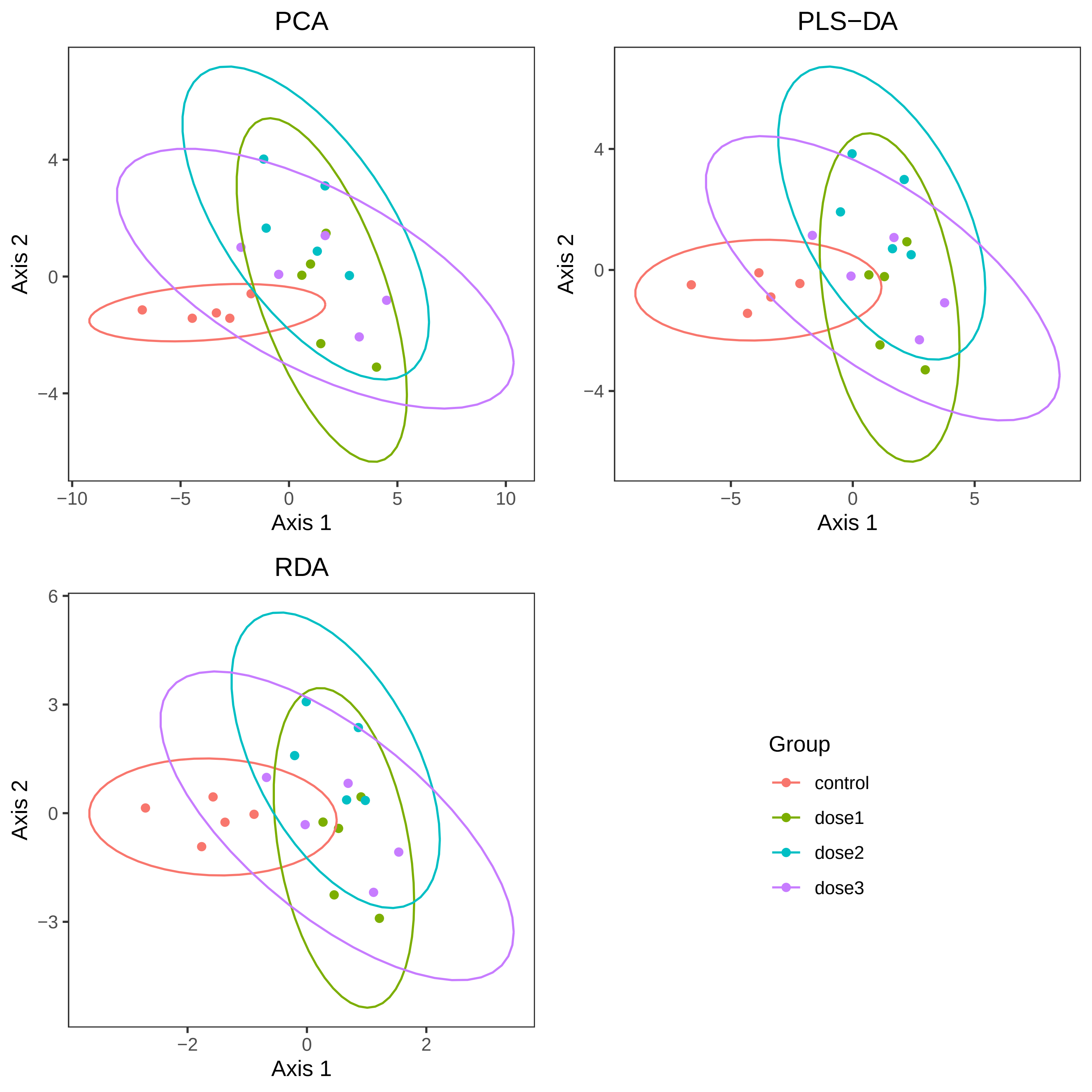

PCA
PLS−DA
RDA
Axis 1
Axis 2
−10
−5
0
5
10
4
0
−4
2
−2
6
3
−3
Group
control
dose1
dose2
dose3

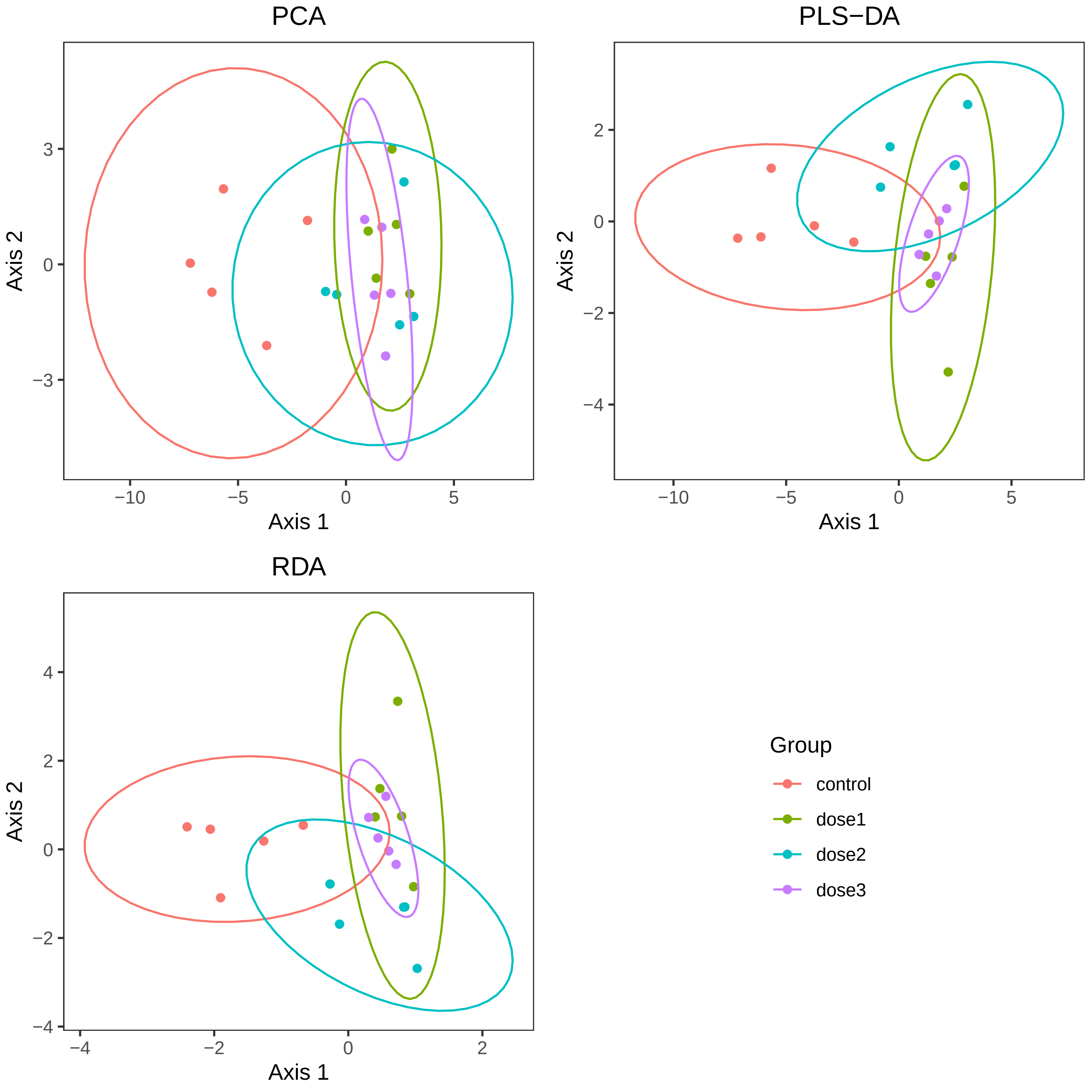

PCA
PLS−DA
RDA
Axis 1
Axis 2
Group
control
dose1
dose2
dose3

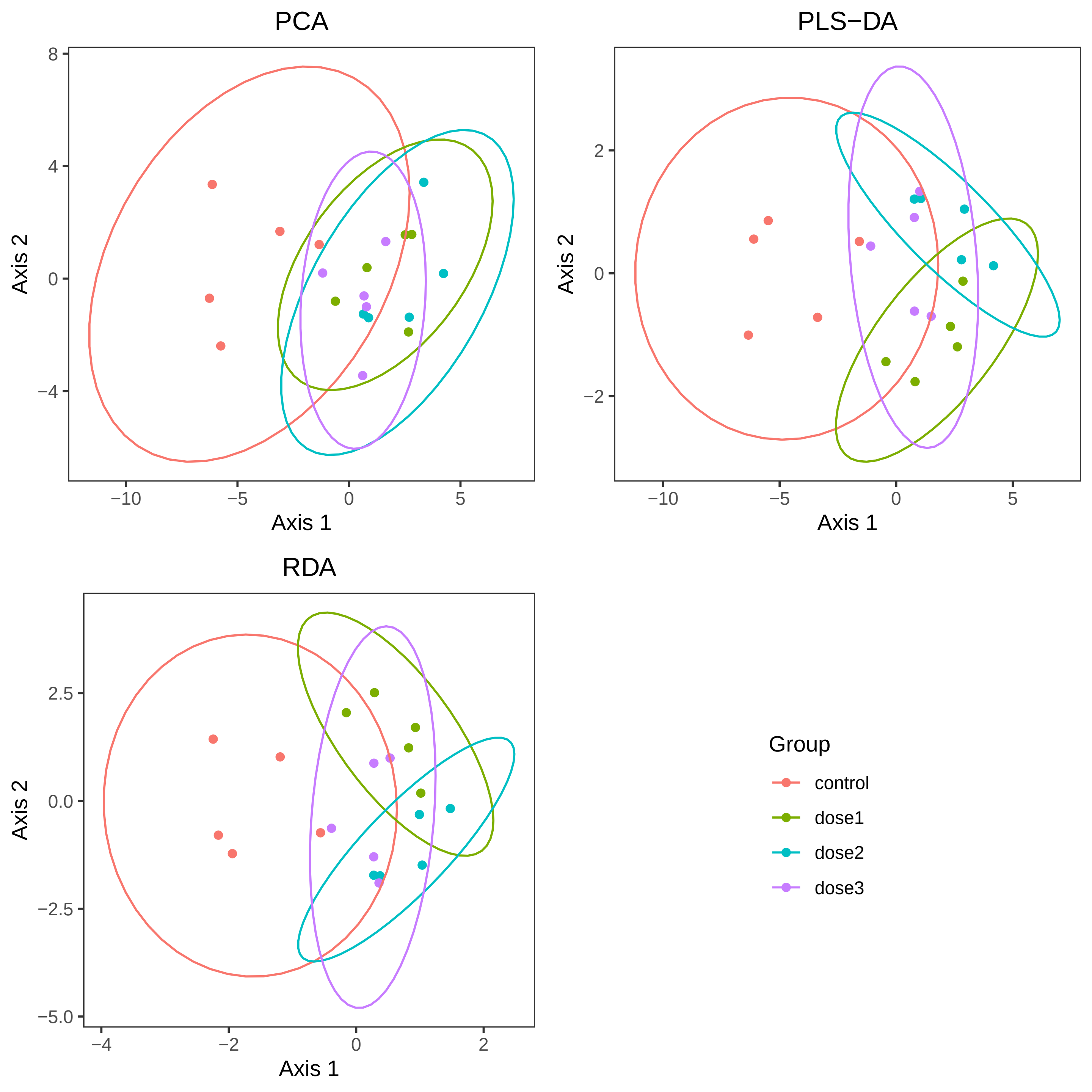
PCA
PLS−DA
RDA
Axis 1
Axis 2
8
4
0
−4
−10
−5
0
5
2
0
−2
2.5
0.0
−2.5
−5.0
−4
−2
0
2
Group
control
dose1
dose2
dose3

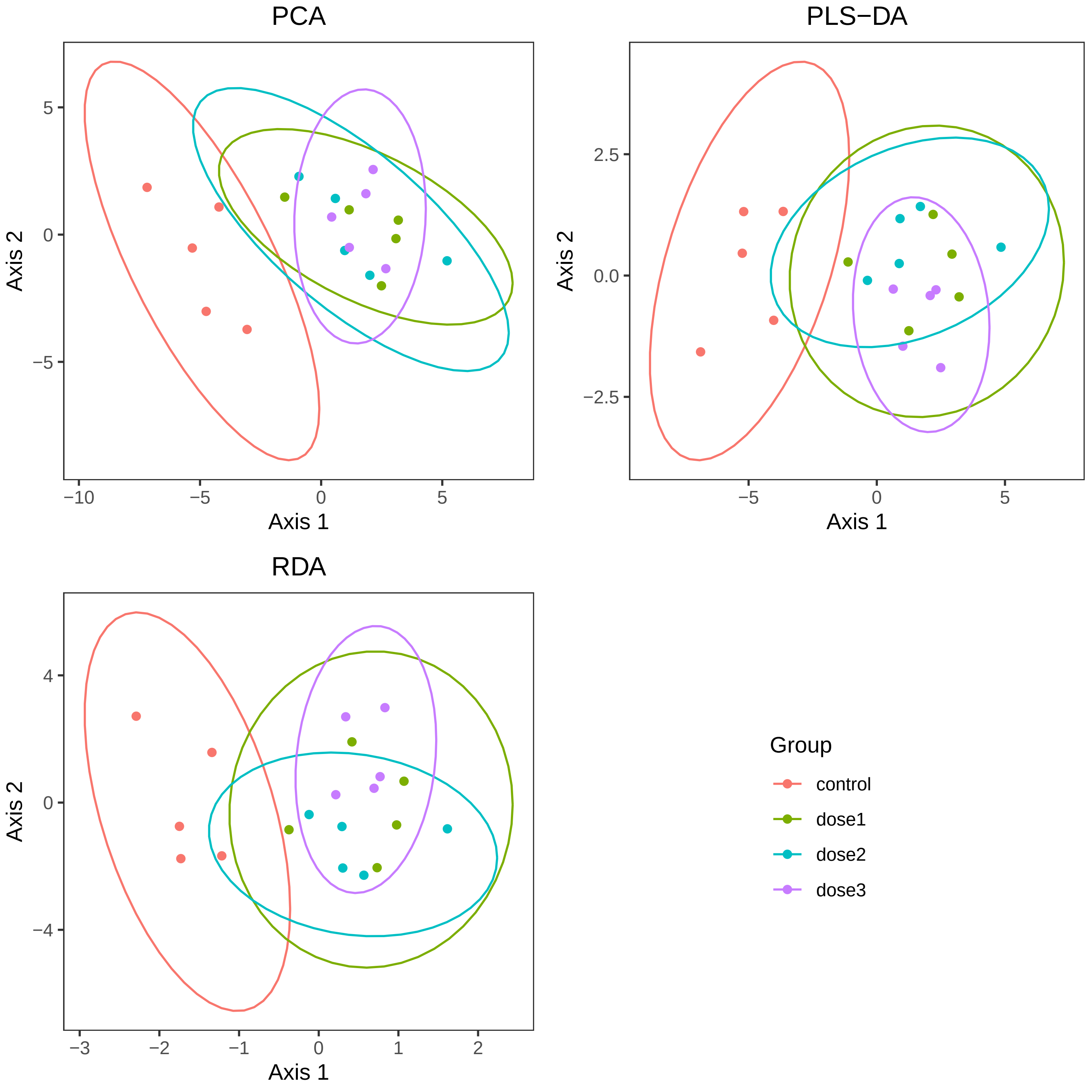

PCA
PLS−DA
RDA
Axis 1
Axis 2
Group
control
dose1
dose2
dose3

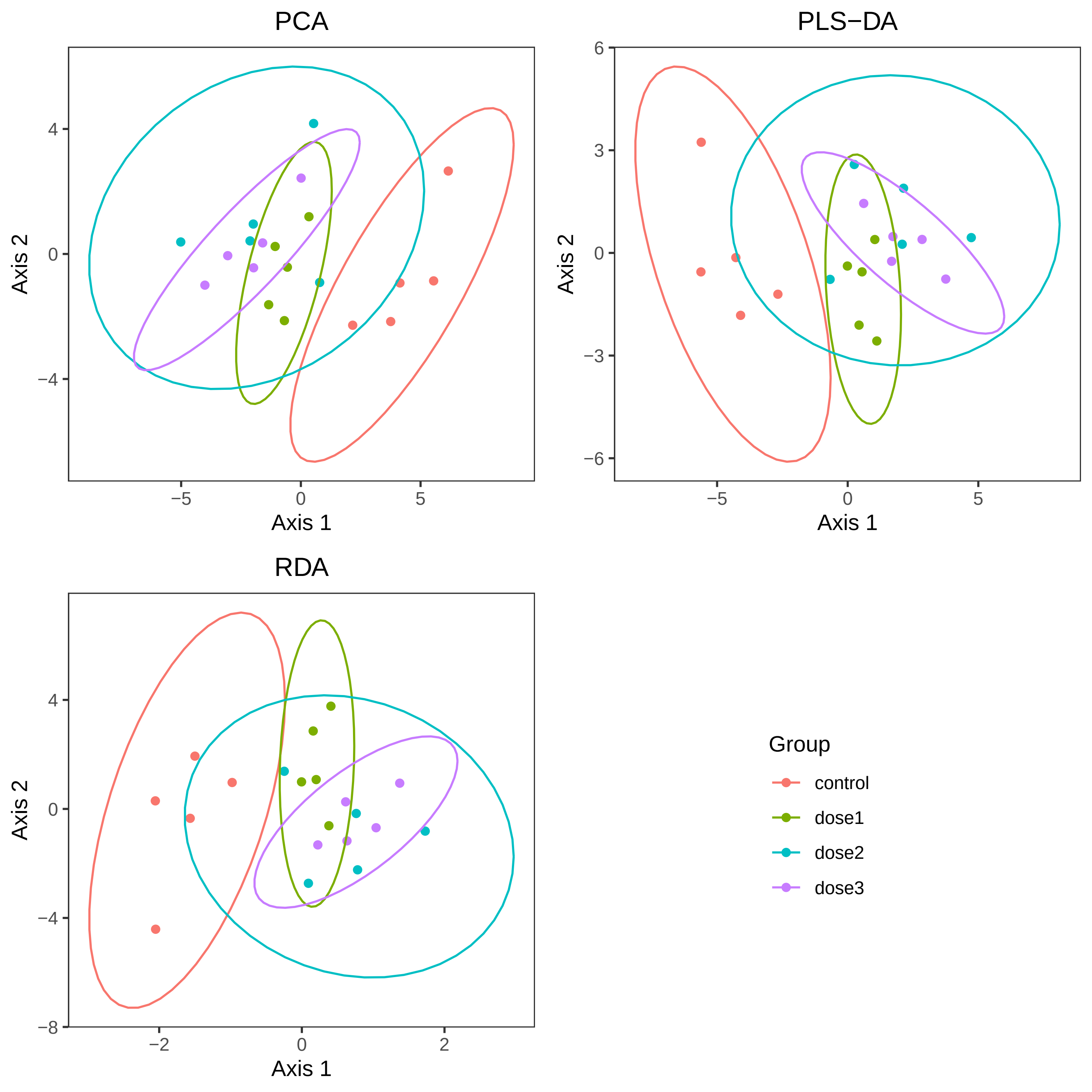
PCA
PLS−DA
RDA
Axis 1
Axis 2
Group
control
dose1
dose2
dose3

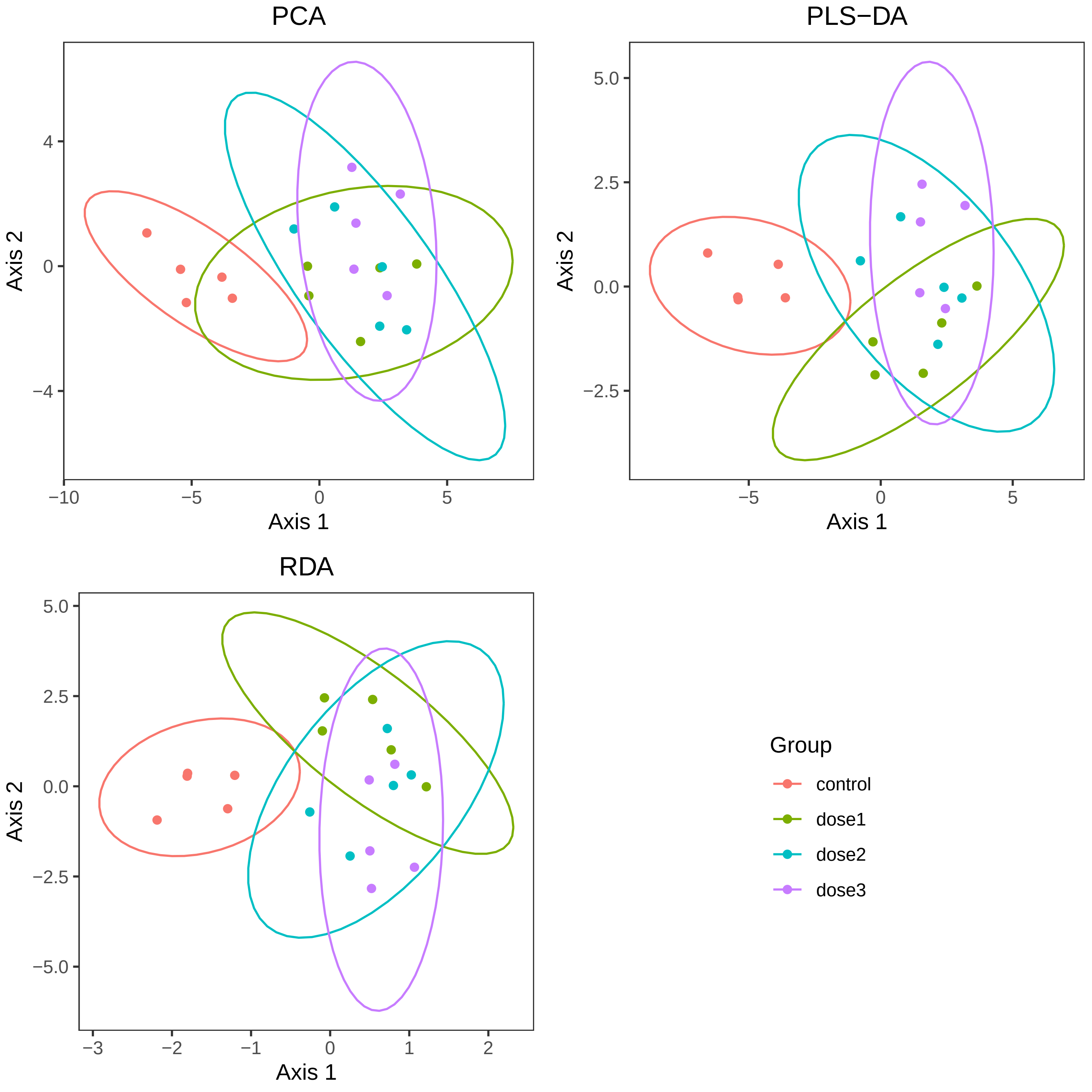
PCA
Axis 1
Axis 2
PLS−DA
Axis 1
Axis 2
RDA
Axis 1
Axis 2
Group
control
dose1
dose2
dose3

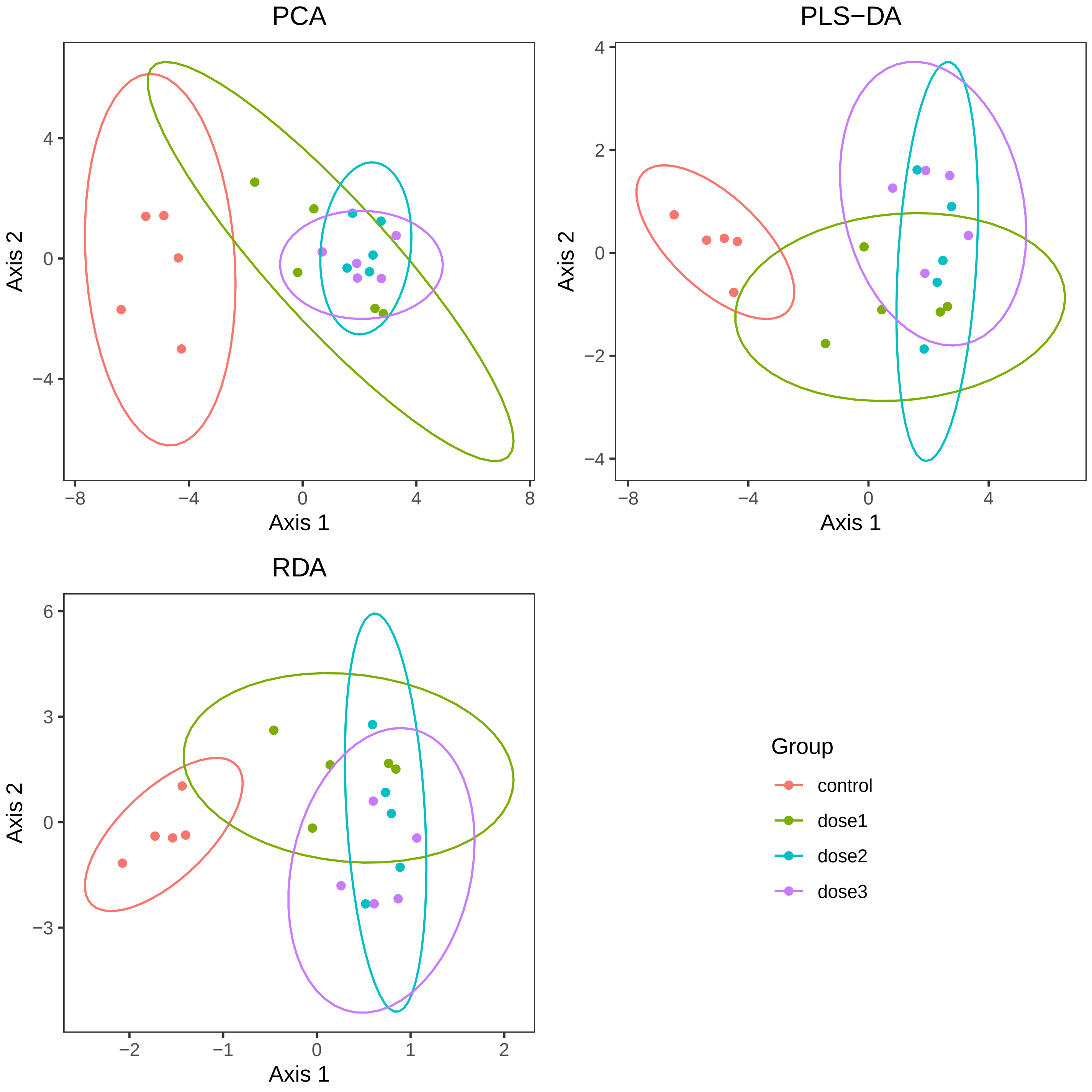
PCA
PLS−DA
RDA
Axis 1
Axis 2
Group
control
dose1
dose2
dose3

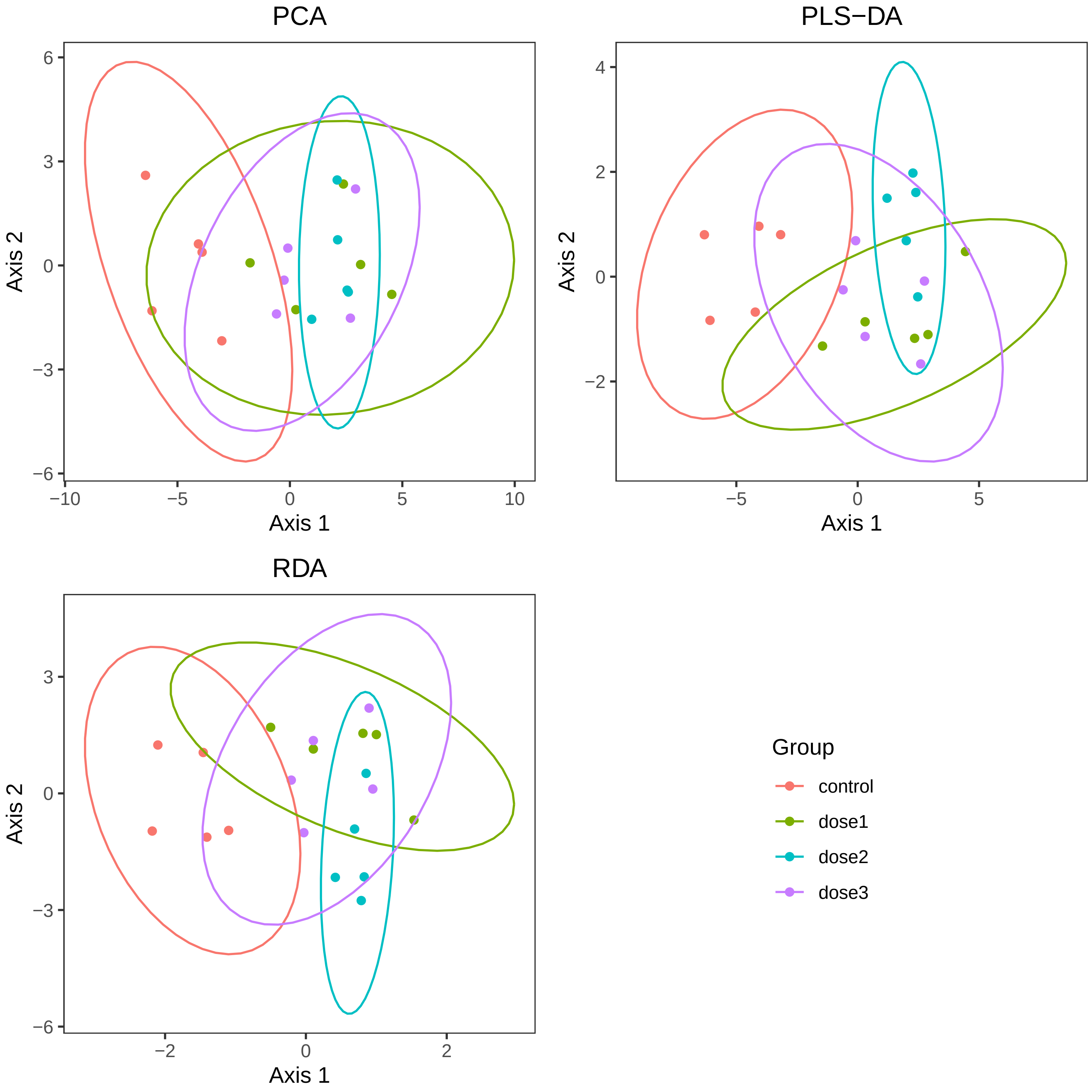

PCA
PLS−DA
RDA
Axis 1
Axis 2
Group
control
dose1
dose2
dose3

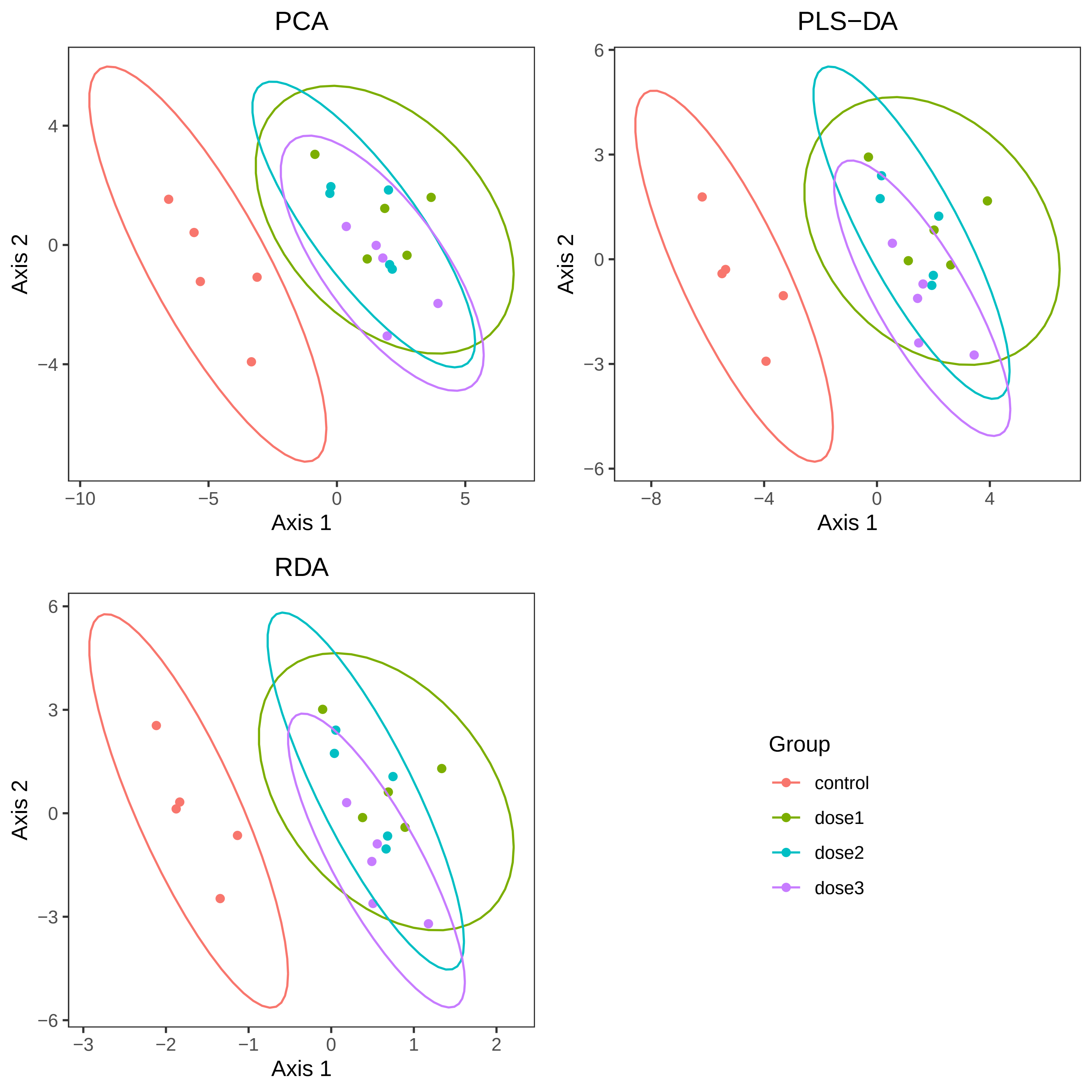

PCA
PLS−DA
RDA
Axis 1
Axis 2
Group
control
dose1
dose2
dose3

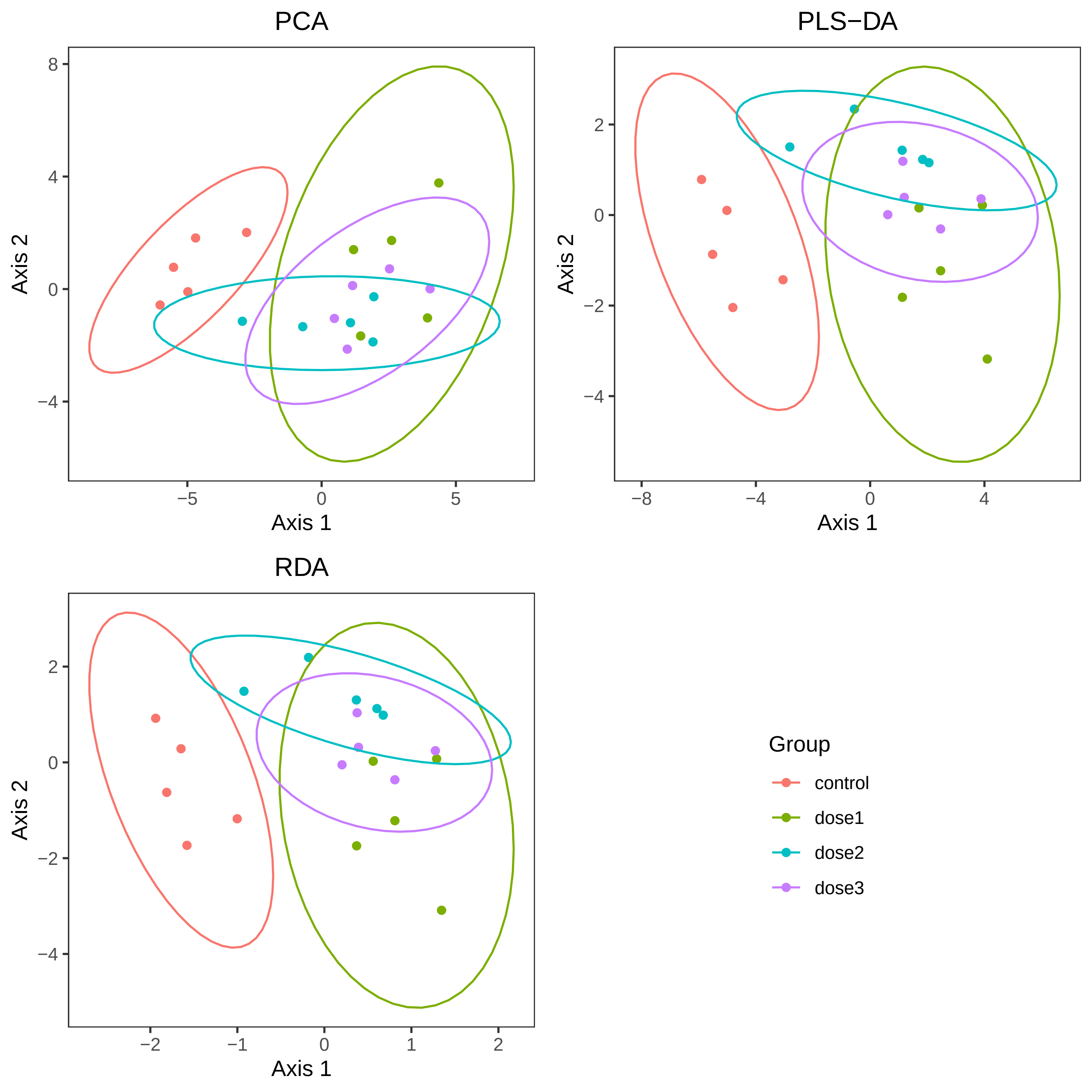
PCA
PLS−DA
RDA
Axis 1
Axis 2
Group
control
dose1
dose2
dose3

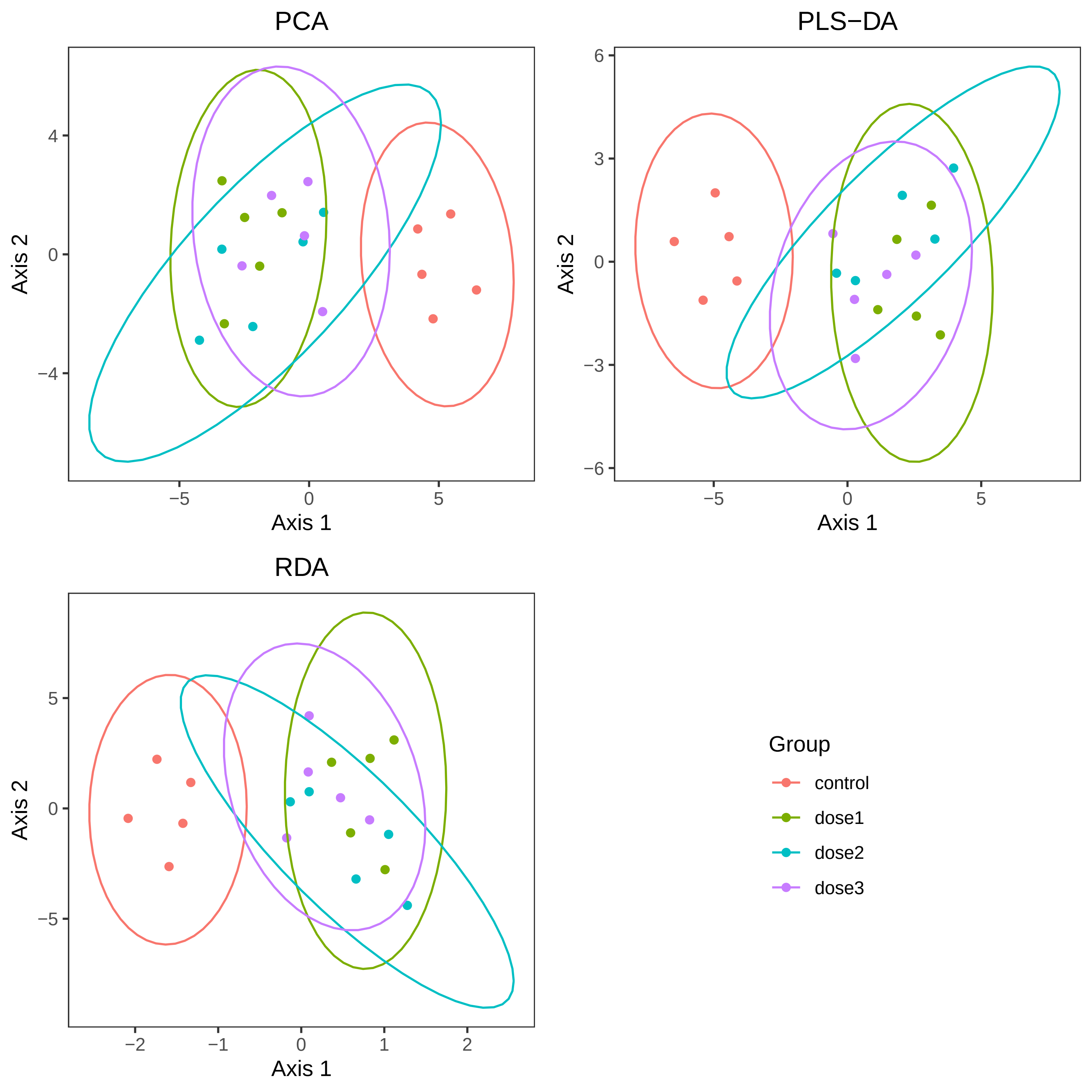
PCA
PLS−DA
RDA
Axis 1
Axis 2
−5
0
5
4
−4
6
3
−3
−6
−2
−1
1
2
Group
control
dose1
dose2
dose3

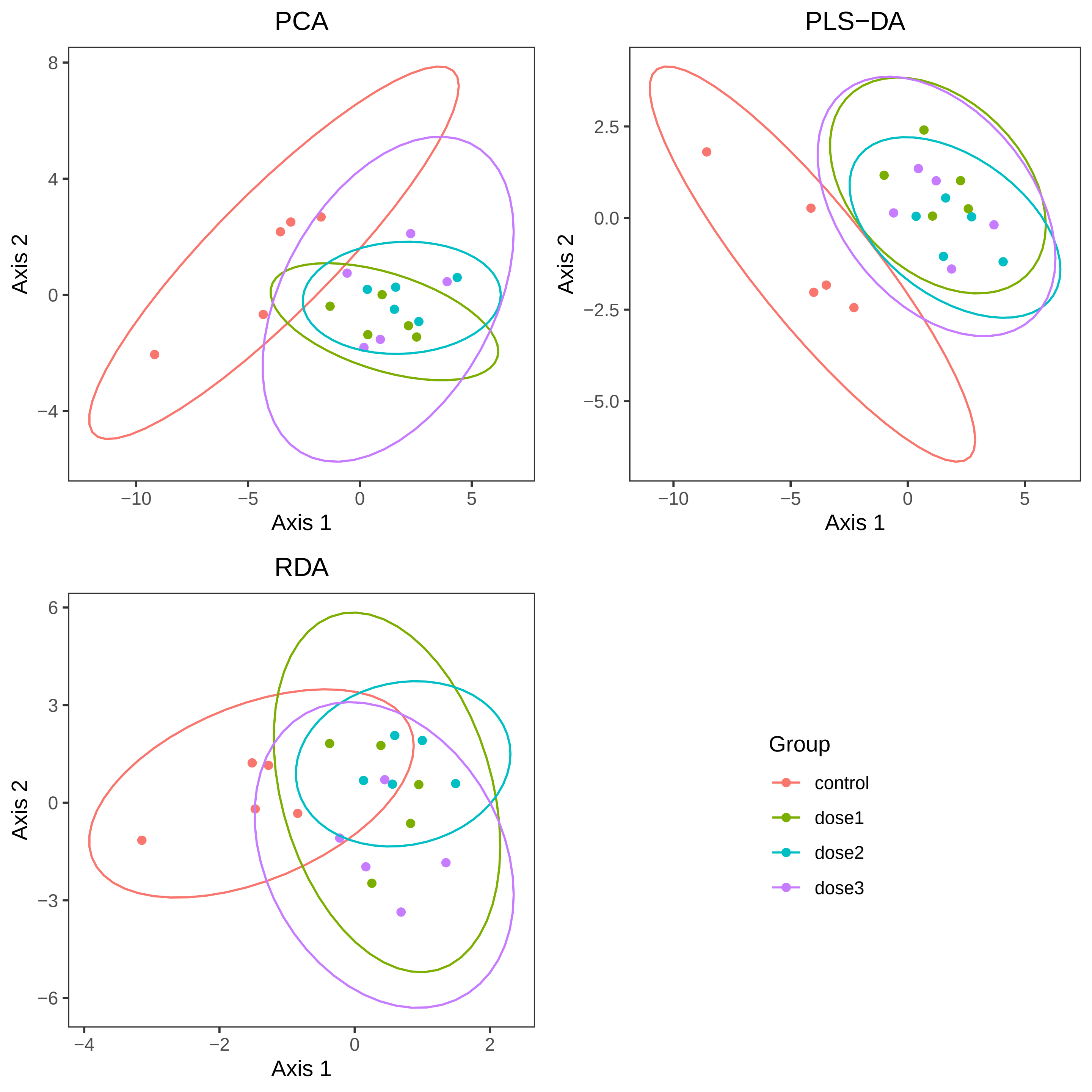

PCA
PLS−DA
RDA
Axis 1
Axis 2
Group
control
dose1
dose2
dose3

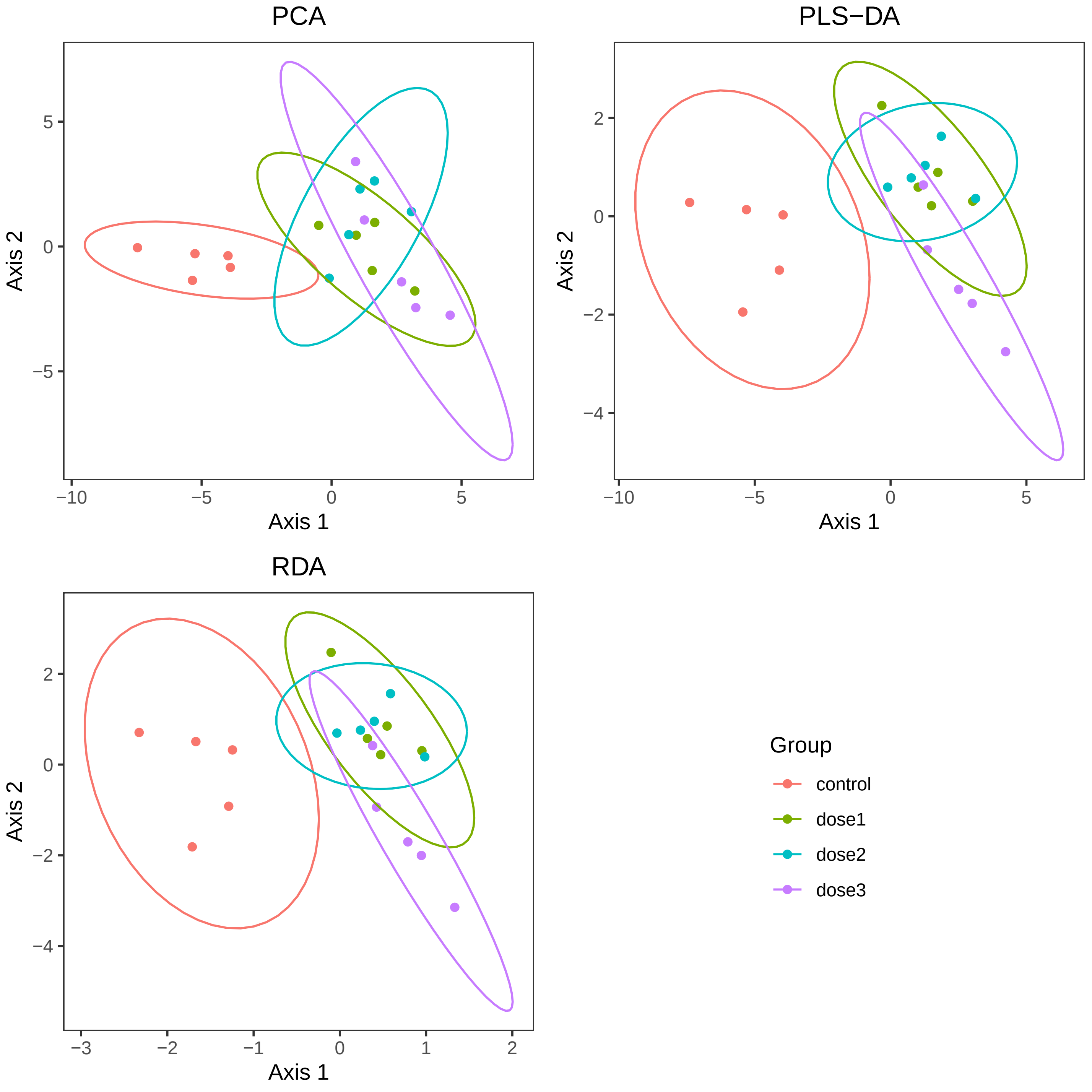

PCA
PLS−DA
RDA
Axis 1
Axis 2
−10
−5
0
5
−4
−2
2
−3
−1
1
Group
control
dose1
dose2
dose3

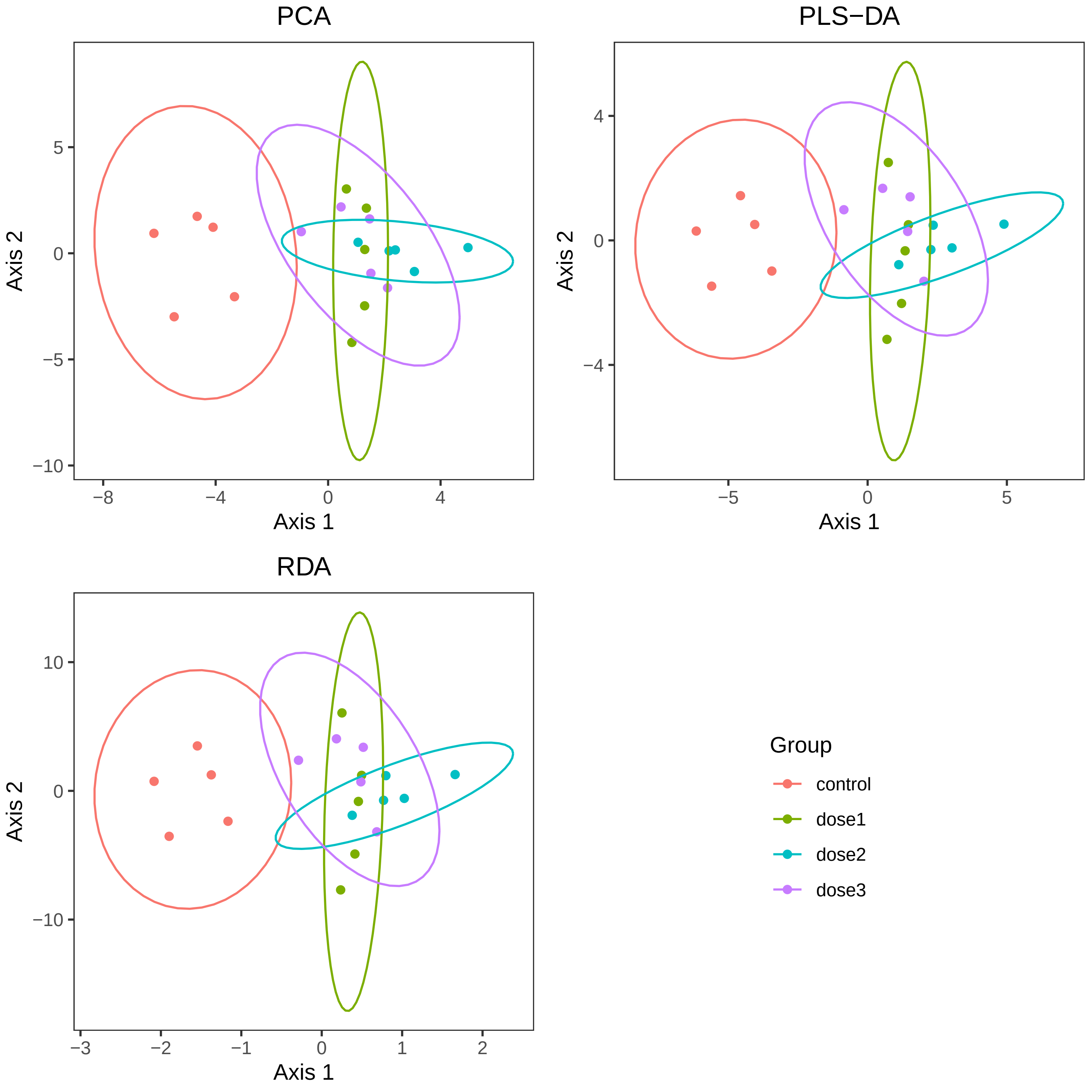

PCA
PLS−DA
RDA
Axis 1
Axis 2
Group
control
dose1
dose2
dose3

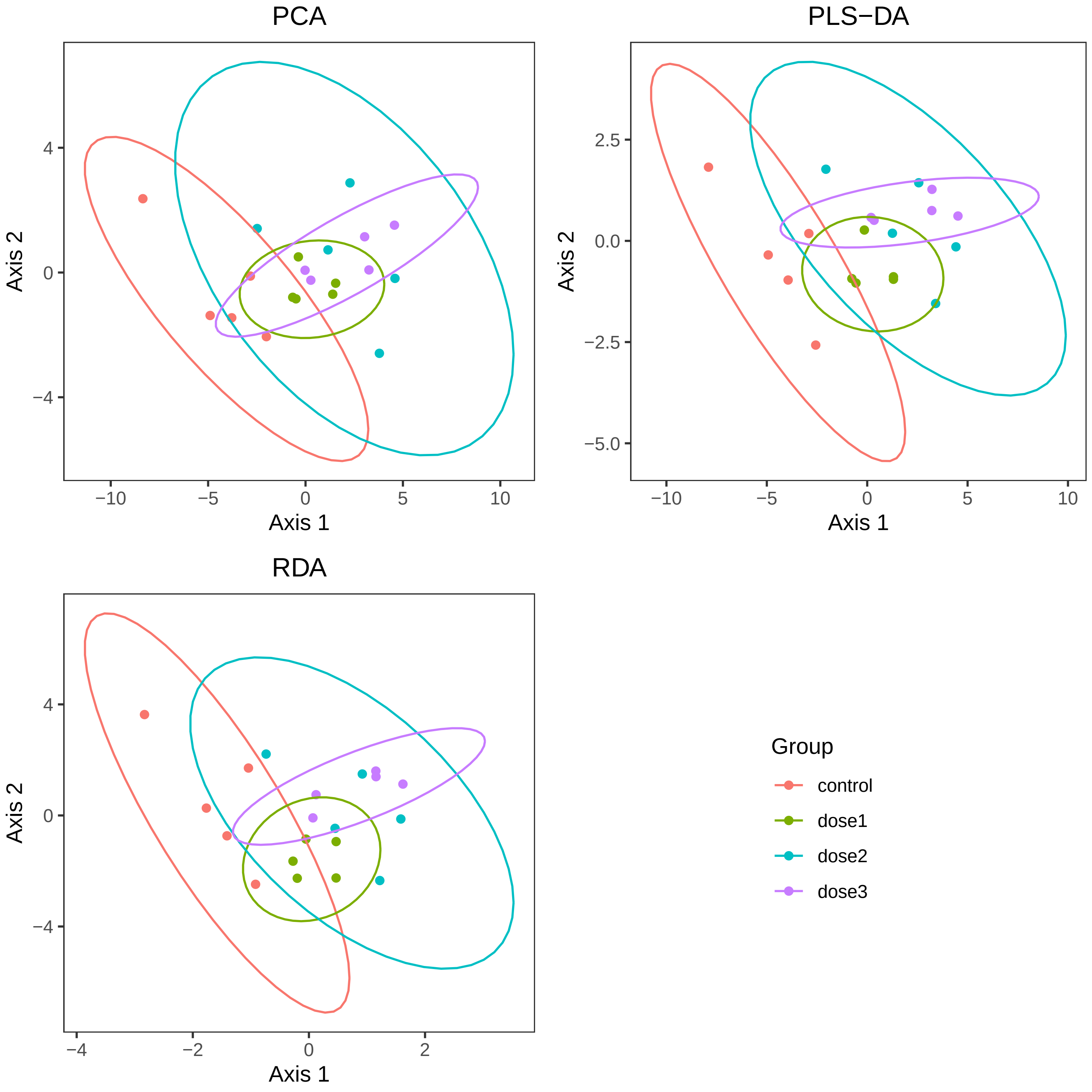

PCA
PLS−DA
RDA
Axis 1
Axis 2
Group
control
dose1
dose2
dose3

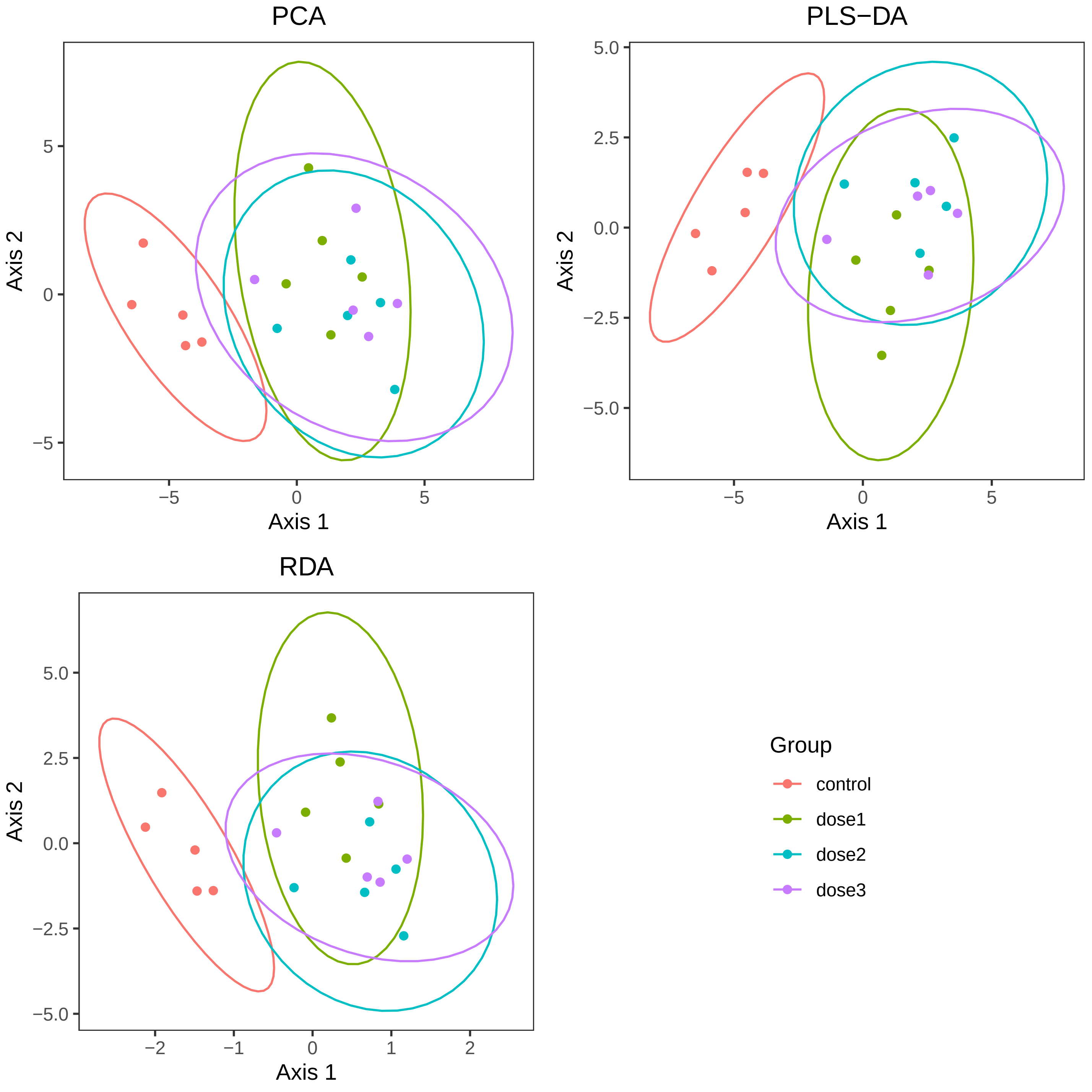
PCA
PLS−DA
RDA
Axis 1
Axis 2
Group
control
dose1
dose2
dose3
−5
0
5
5.0
2.5
0.0
−2.5
−5.0
−2
−1
1
2

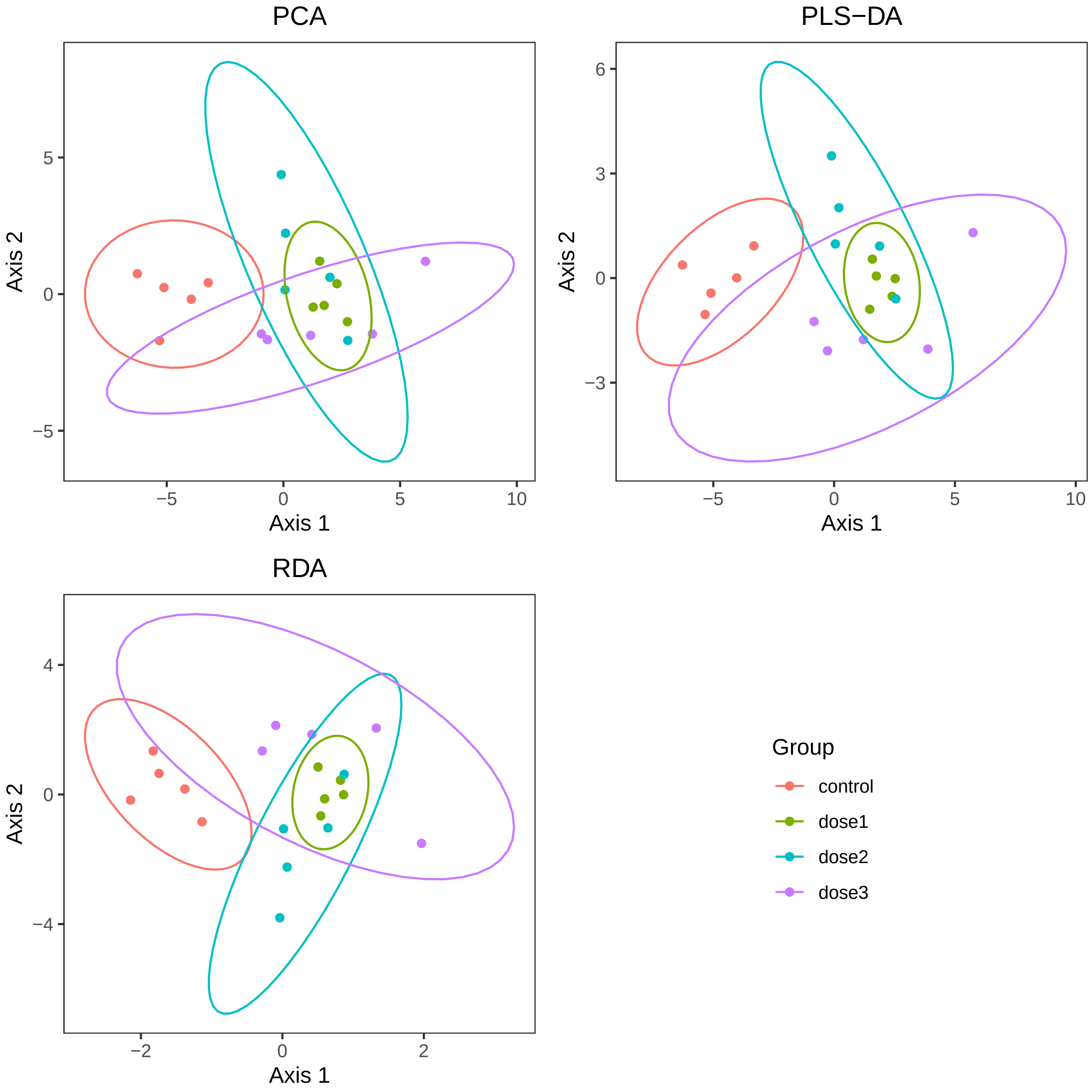

PCA
PLS−DA
RDA
Axis 1
Axis 2
Group
control
dose1
dose2
dose3

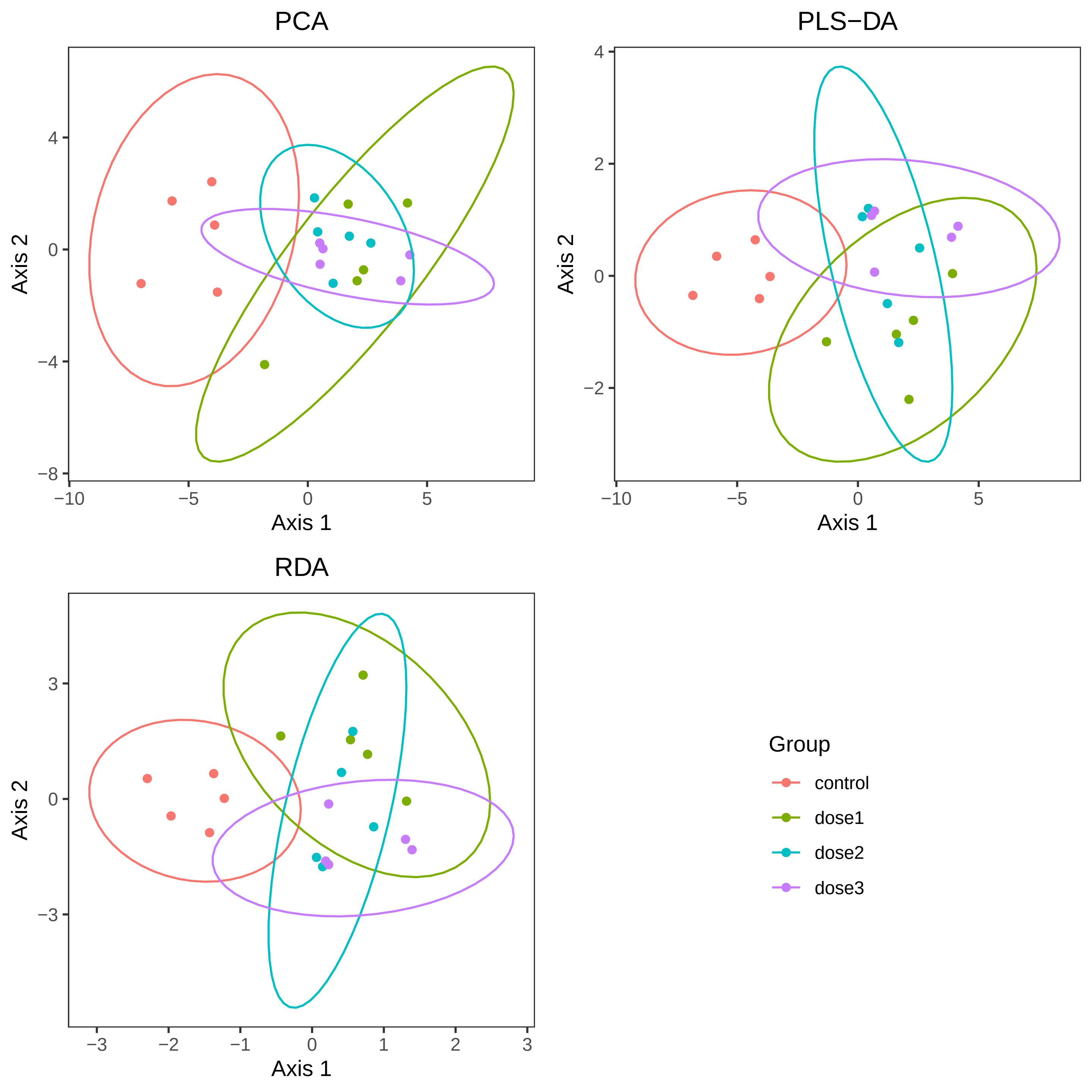

PCA
PLS−DA
RDA
Axis 1
Axis 2
Group
control
dose1
dose2
dose3

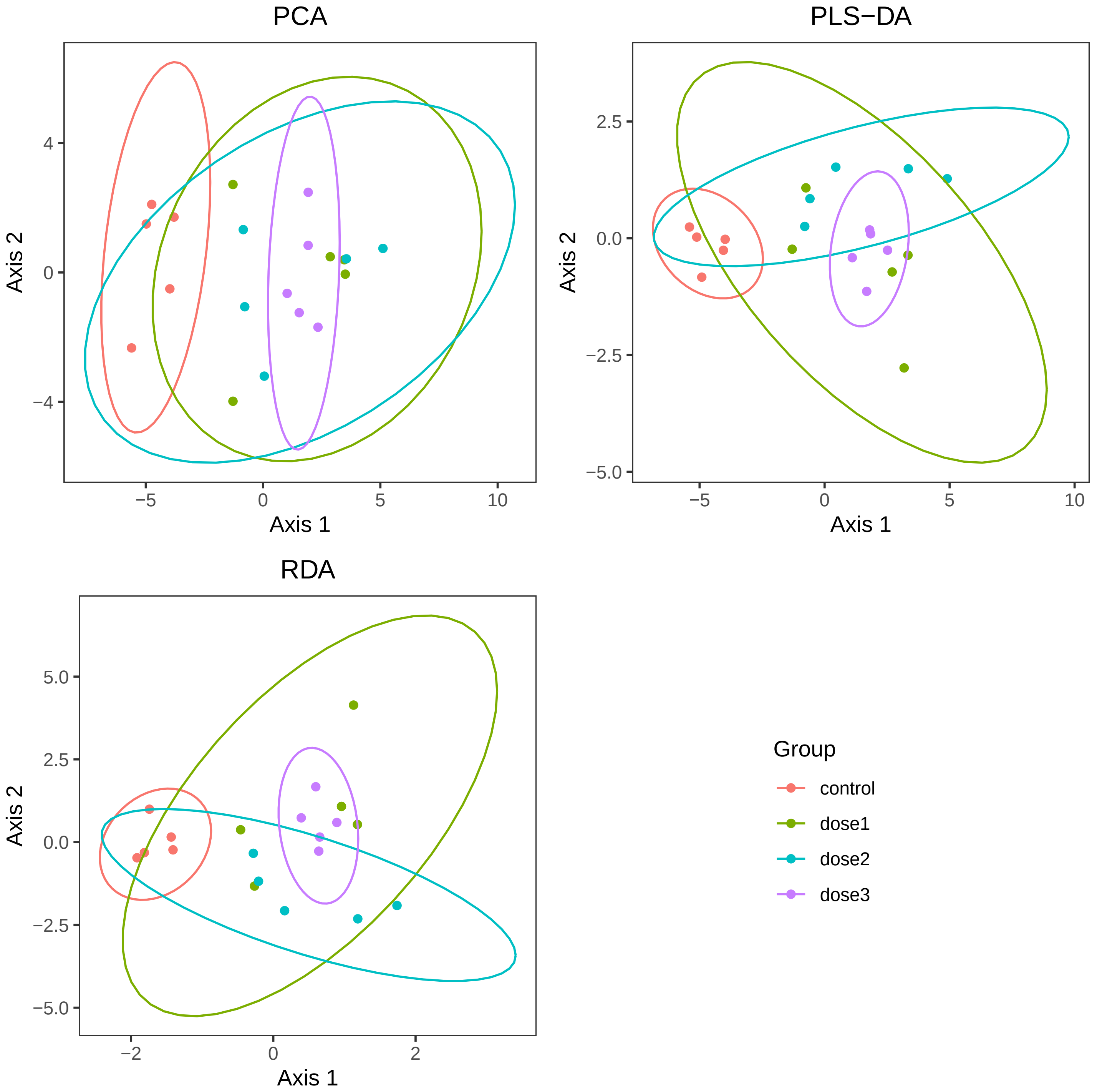

PCA
PLS−DA
RDA
Axis 1
Axis 2
Group
control
dose1
dose2
dose3

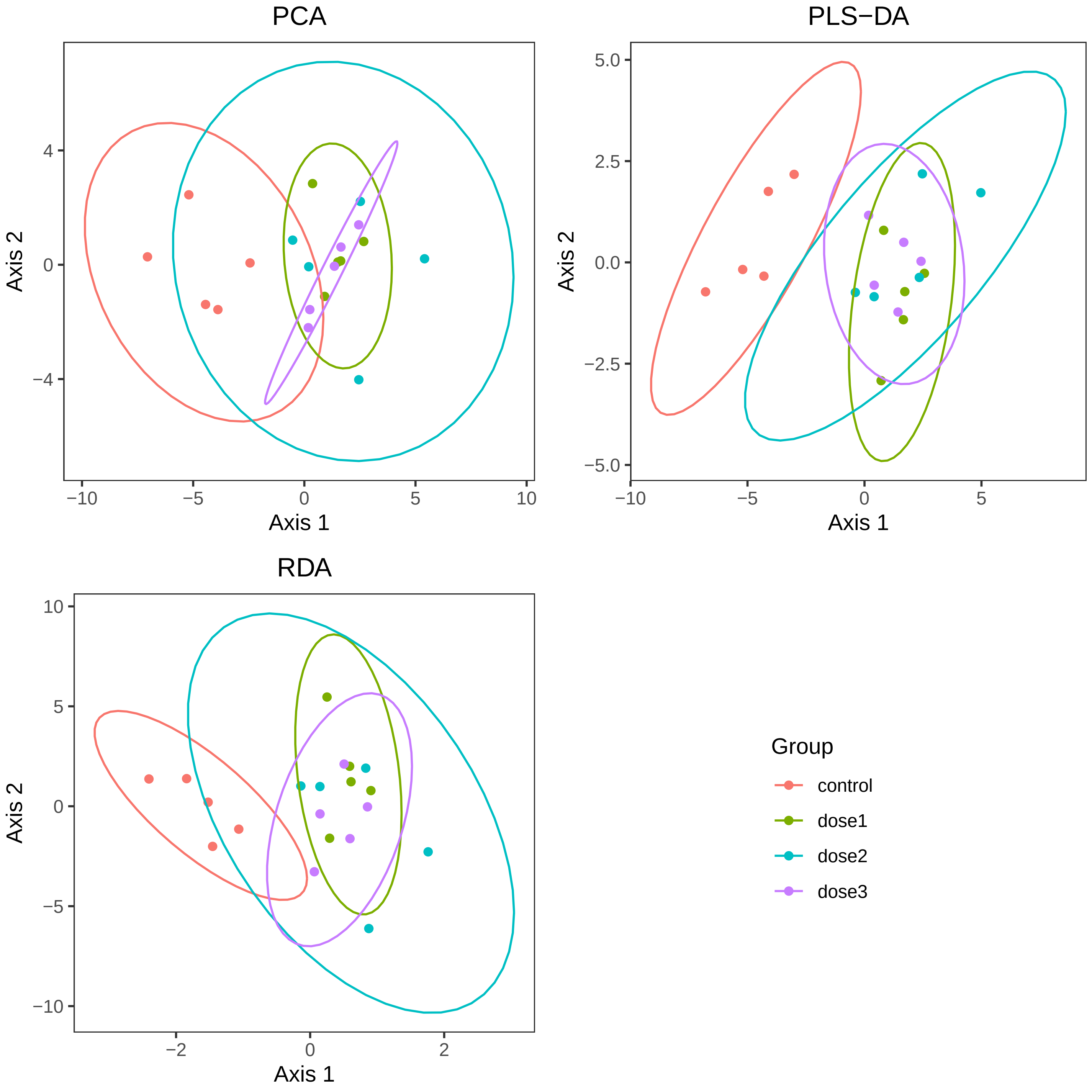

PCA
PLS−DA
RDA
Axis 1
Axis 2
Group
control
dose1
dose2
dose3

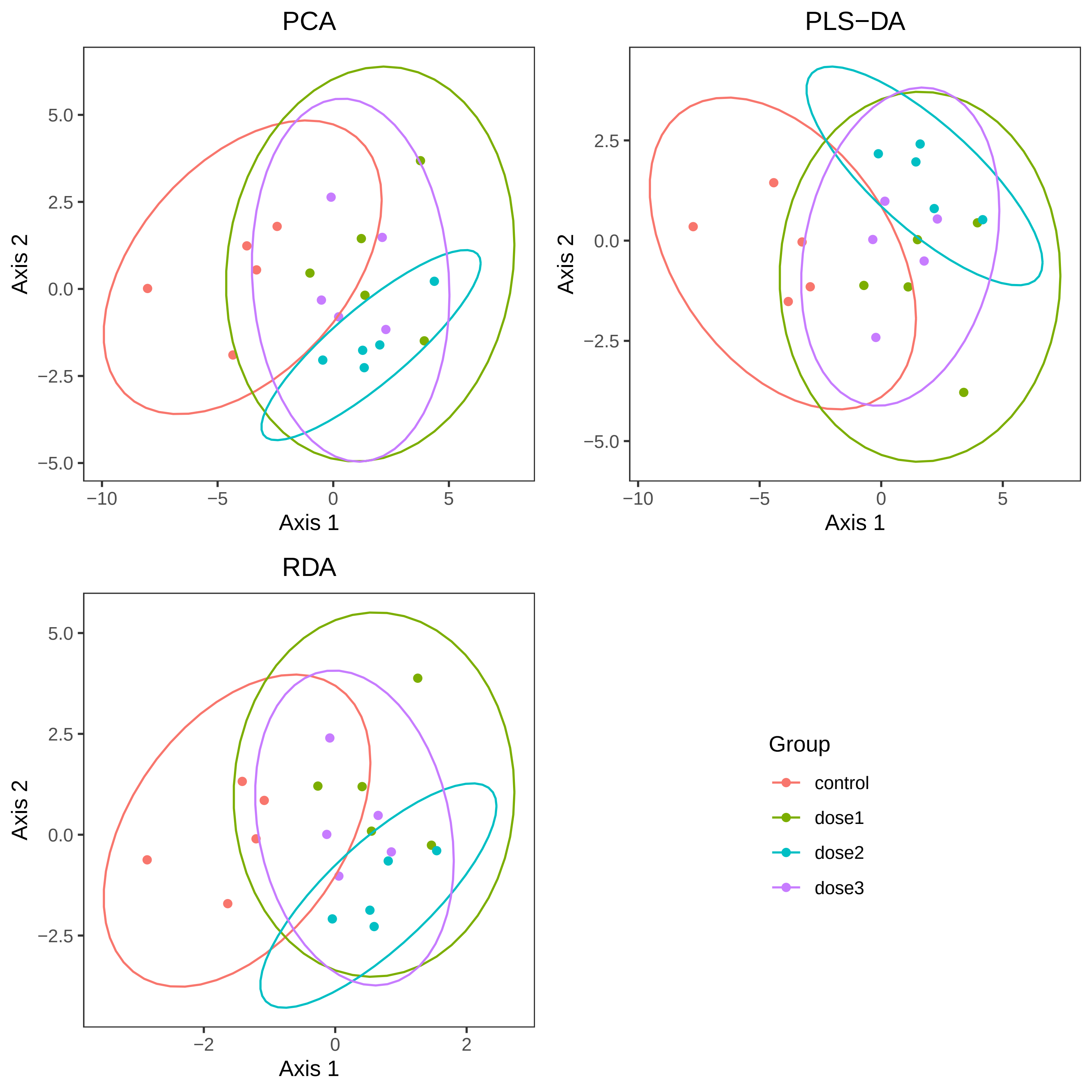

PCA
PLS−DA
RDA
Axis 1
Axis 2
Group
control
dose1
dose2
dose3

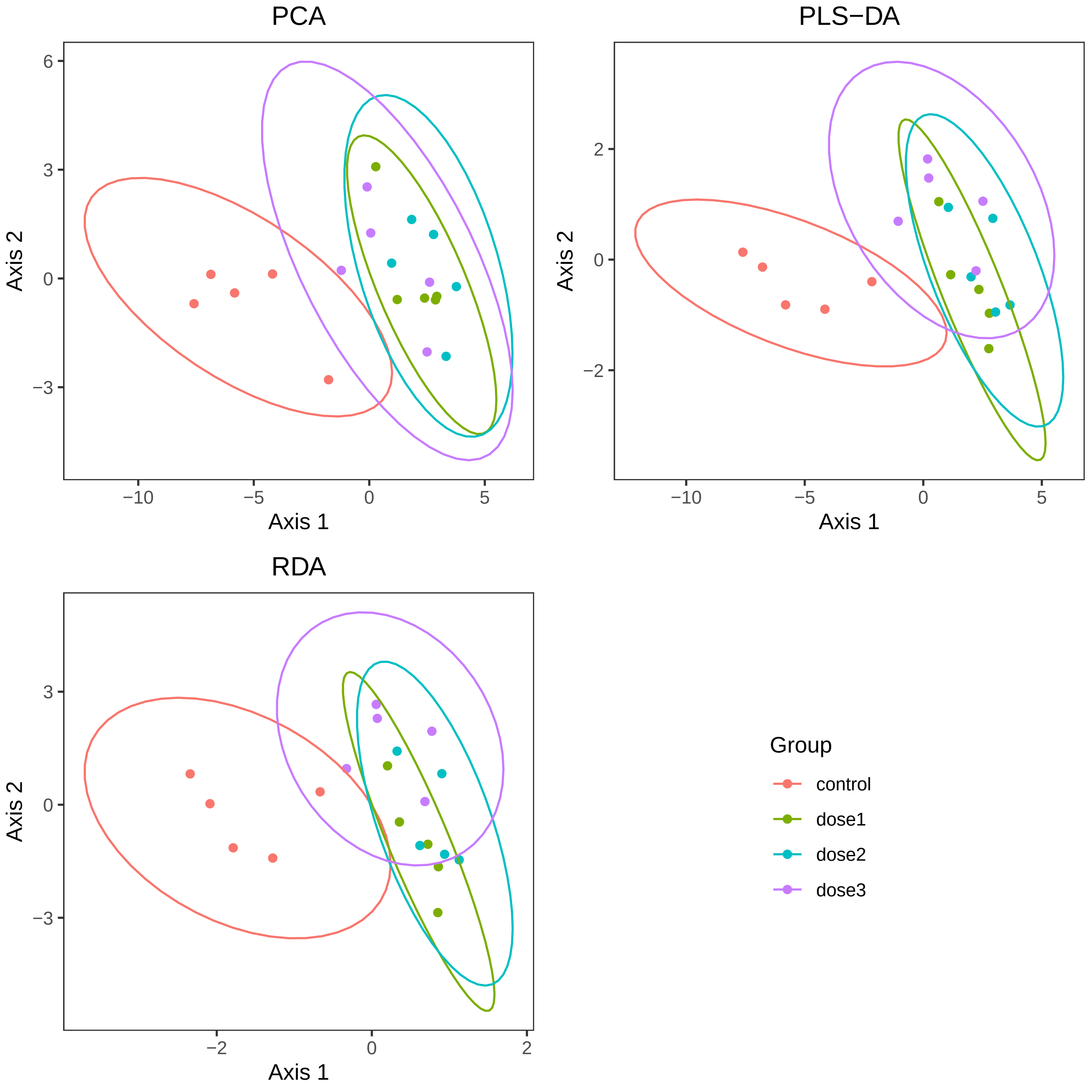

PCA
PLS−DA
RDA
Axis 1
Axis 2
Group
control
dose1
dose2
dose3

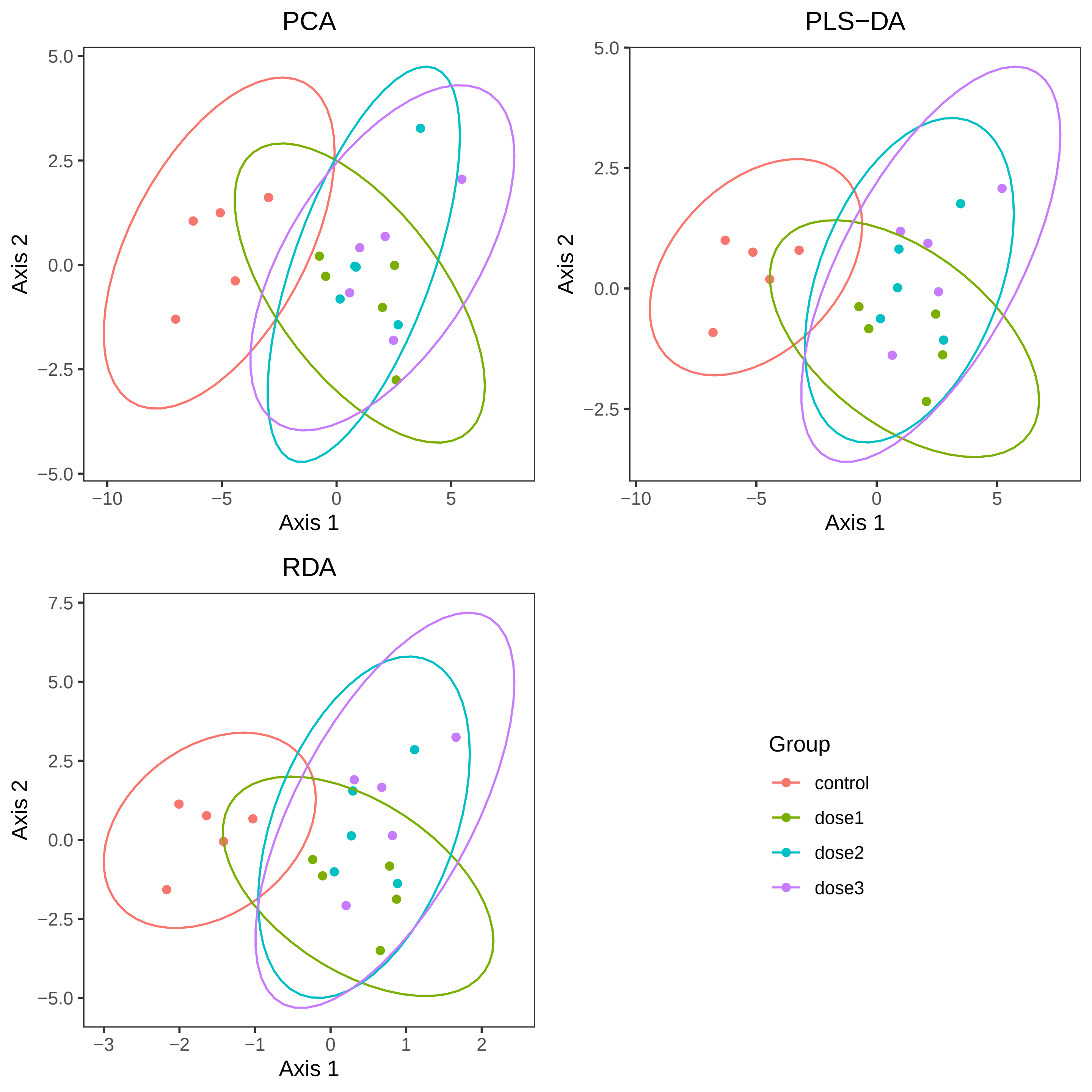
PCA
PLS−DA
RDA
Axis 1
Axis 2
Group
control
dose1
dose2
dose3

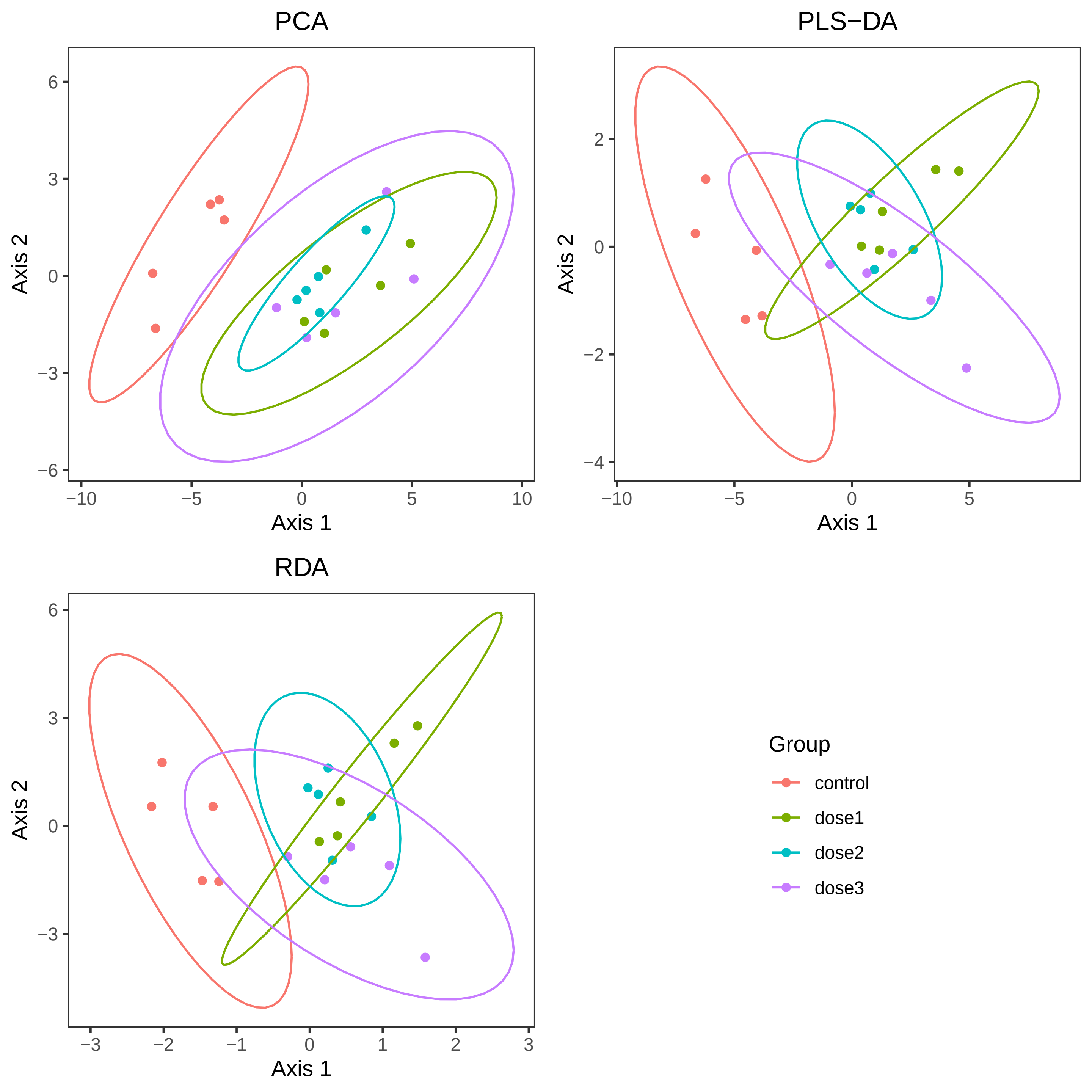
PCA
PLS−DA
RDA
Axis 1
Axis 2
Group
control
dose1
dose2
dose3

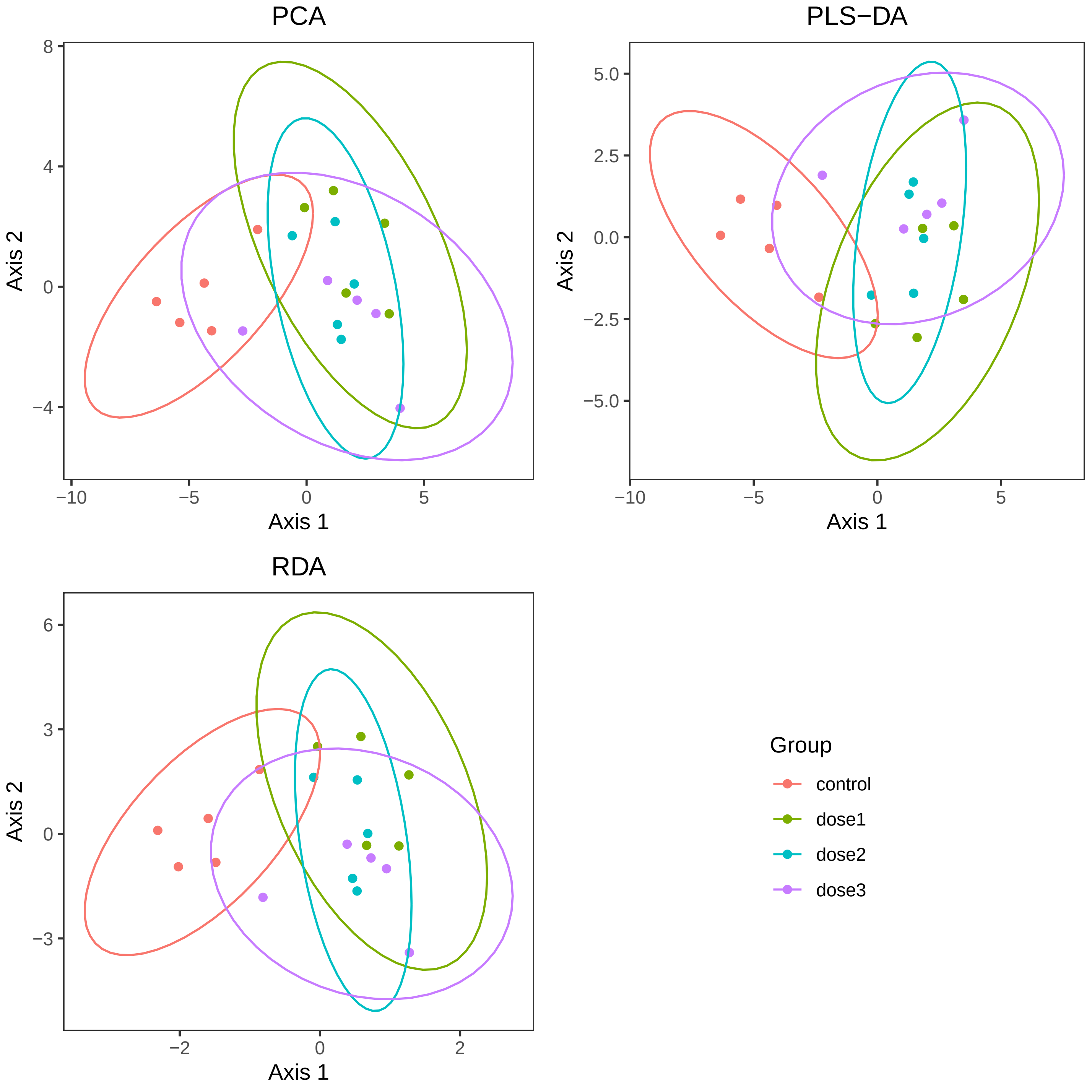
PCA
PLS−DA
RDA
Axis 1
Axis 2
Group
control
dose1
dose2
dose3

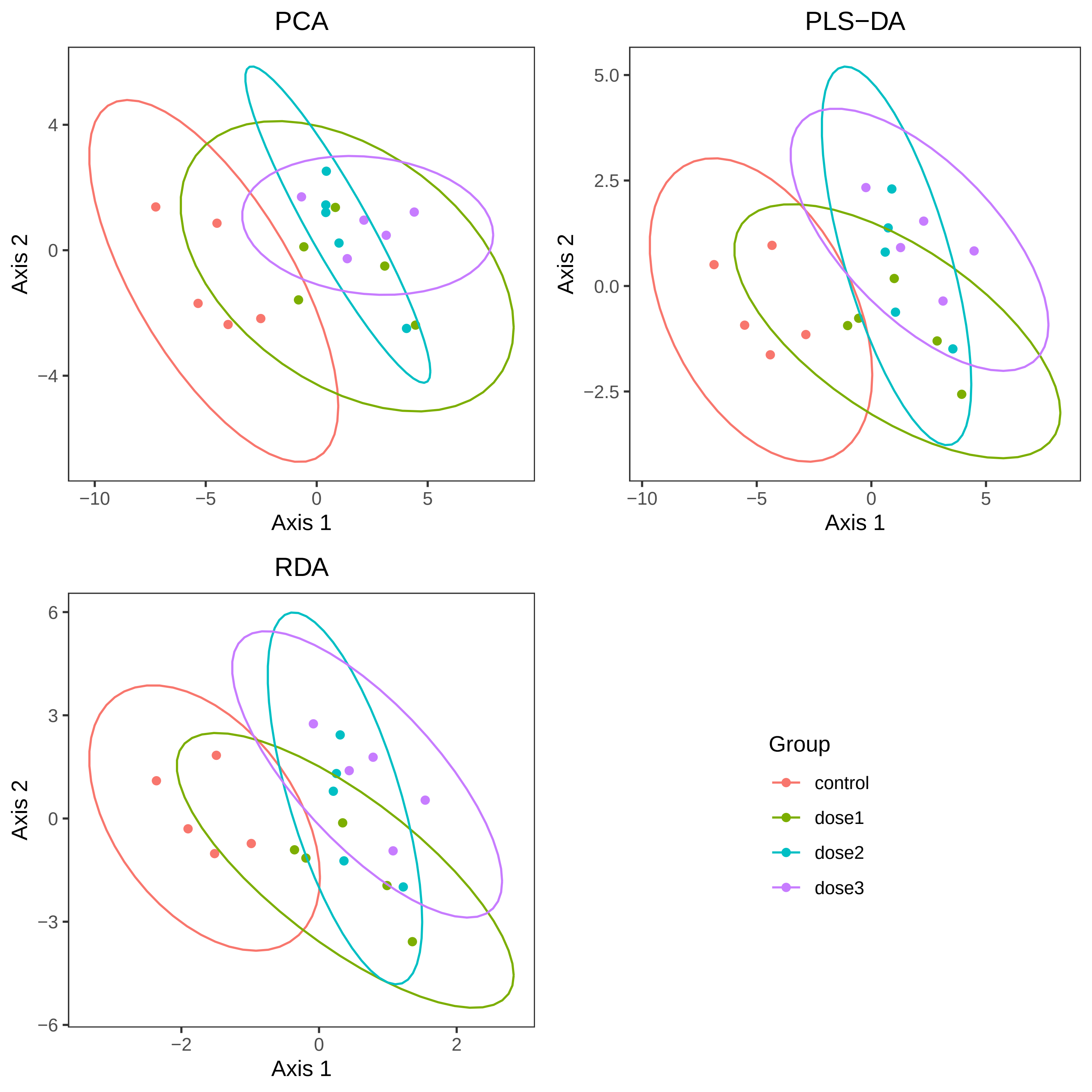
PCA
PLS−DA
RDA
Axis 1
Axis 2
−10
−5
0
5
4
−4
5.0
2.5
0.0
−2.5
6
3
−3
−6
−2
2
Group
control
dose1
dose2
dose3

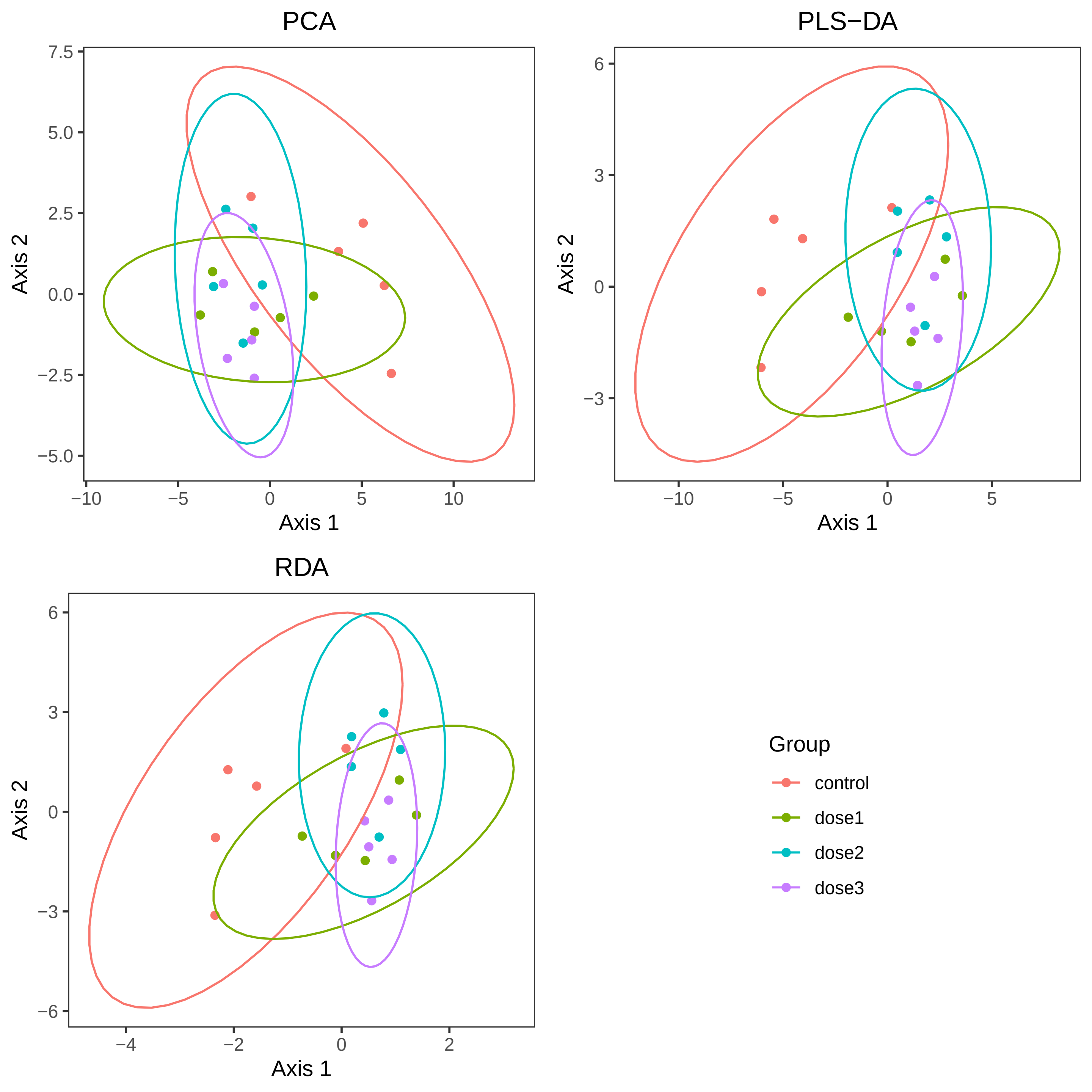
PCA
PLS−DA
RDA
Axis 1
Axis 2
7.5
5.0
2.5
0.0
−2.5
−5.0
−10
−5
0
5
10
6
3
0
−3
−6
−4
−2
2
Group
control
dose1
dose2
dose3

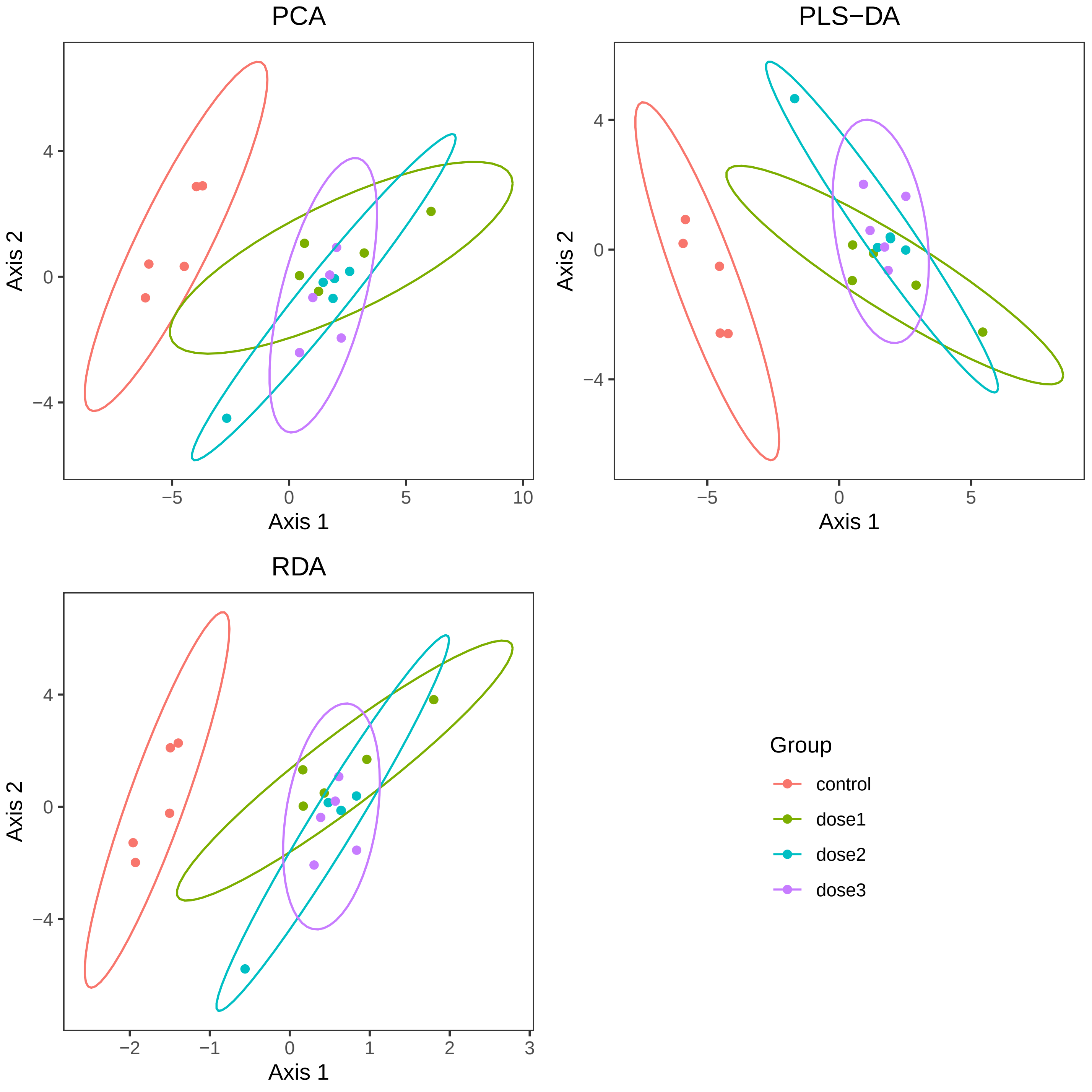

PCA
PLS−DA
RDA
Axis 1
Axis 2
Group
control
dose1
dose2
dose3

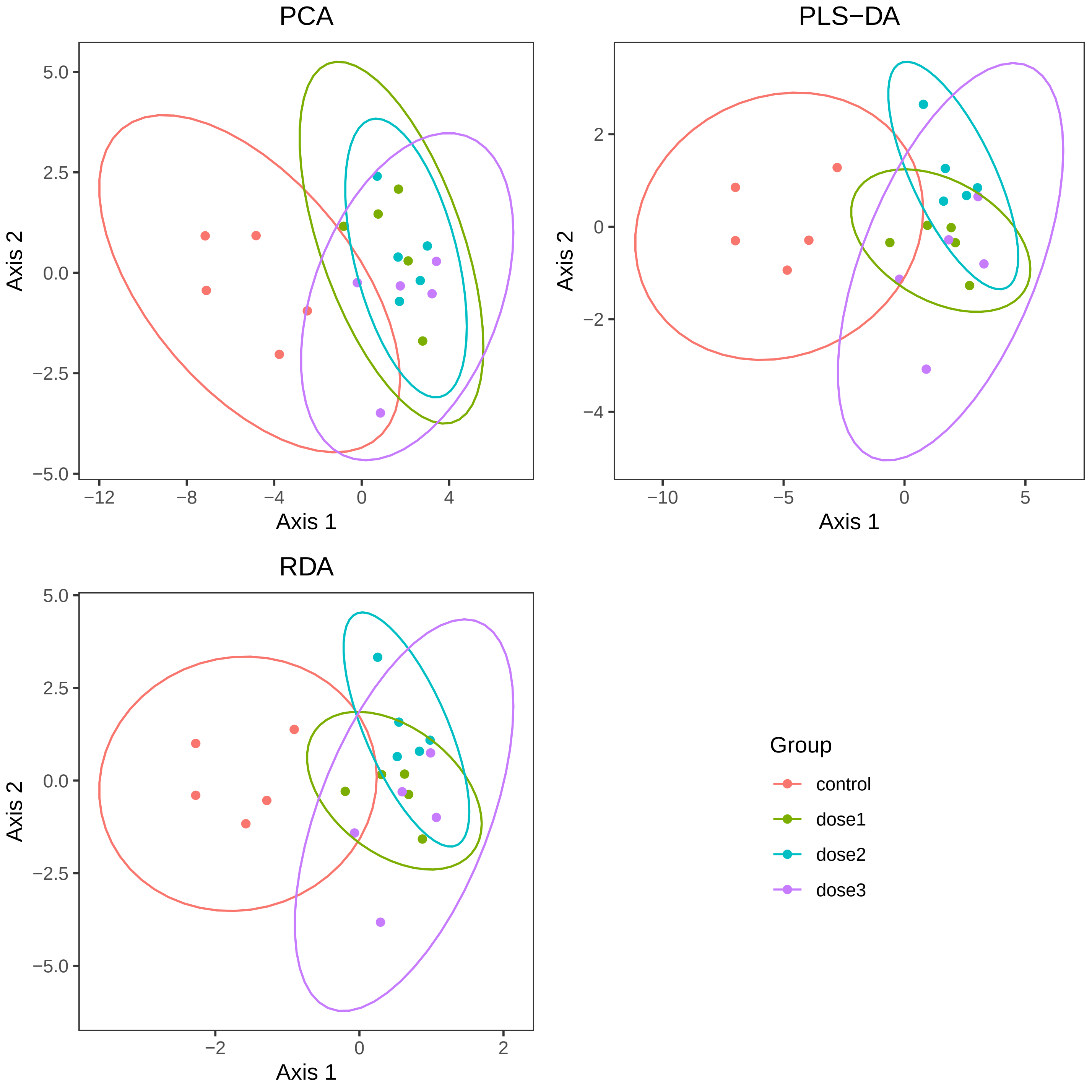
PCA
PLS−DA
RDA
Axis 1
Axis 2
Group
control
dose1
dose2
dose3

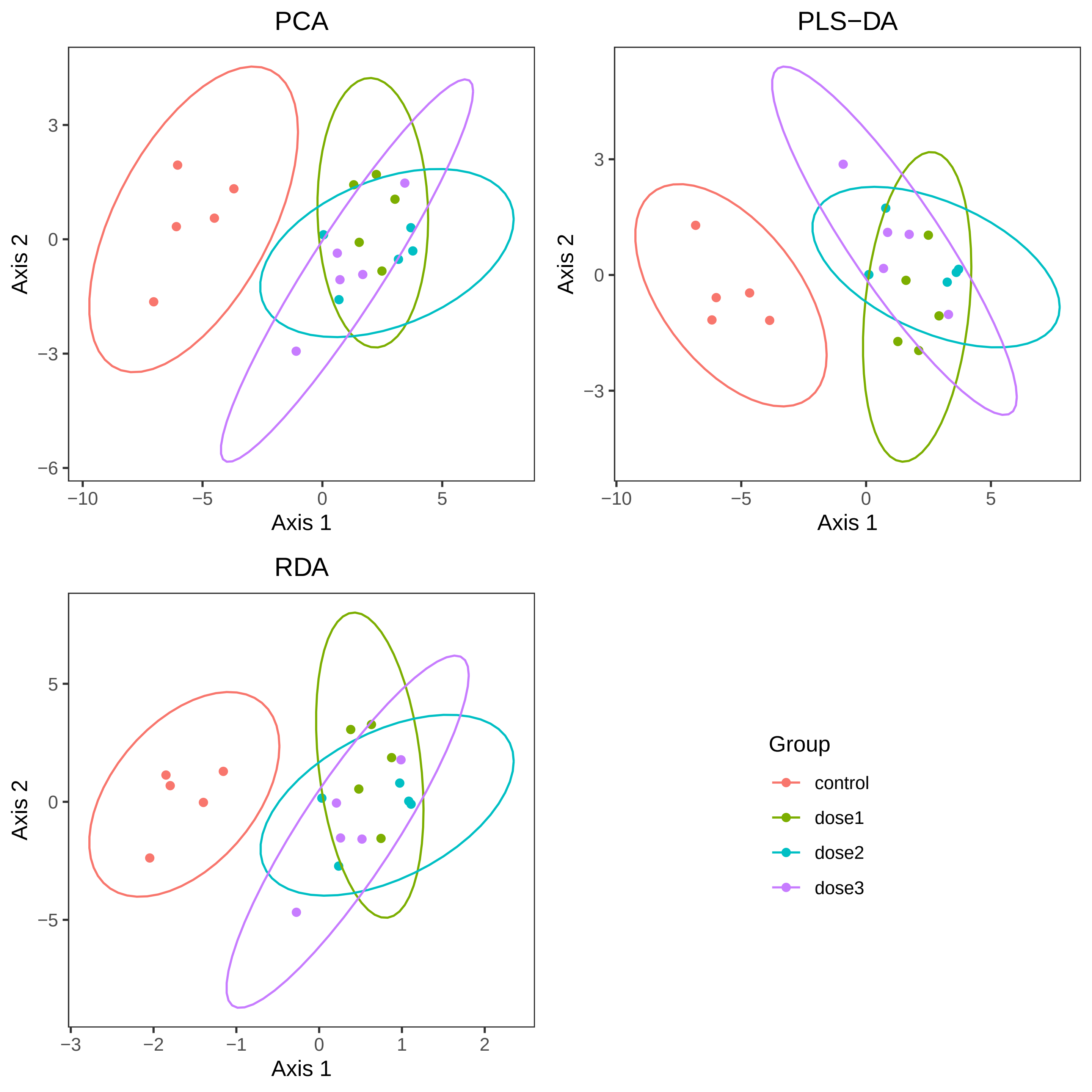
PCA
PLS−DA
RDA
Axis 1
Axis 2
Group
control
dose1
dose2
dose3

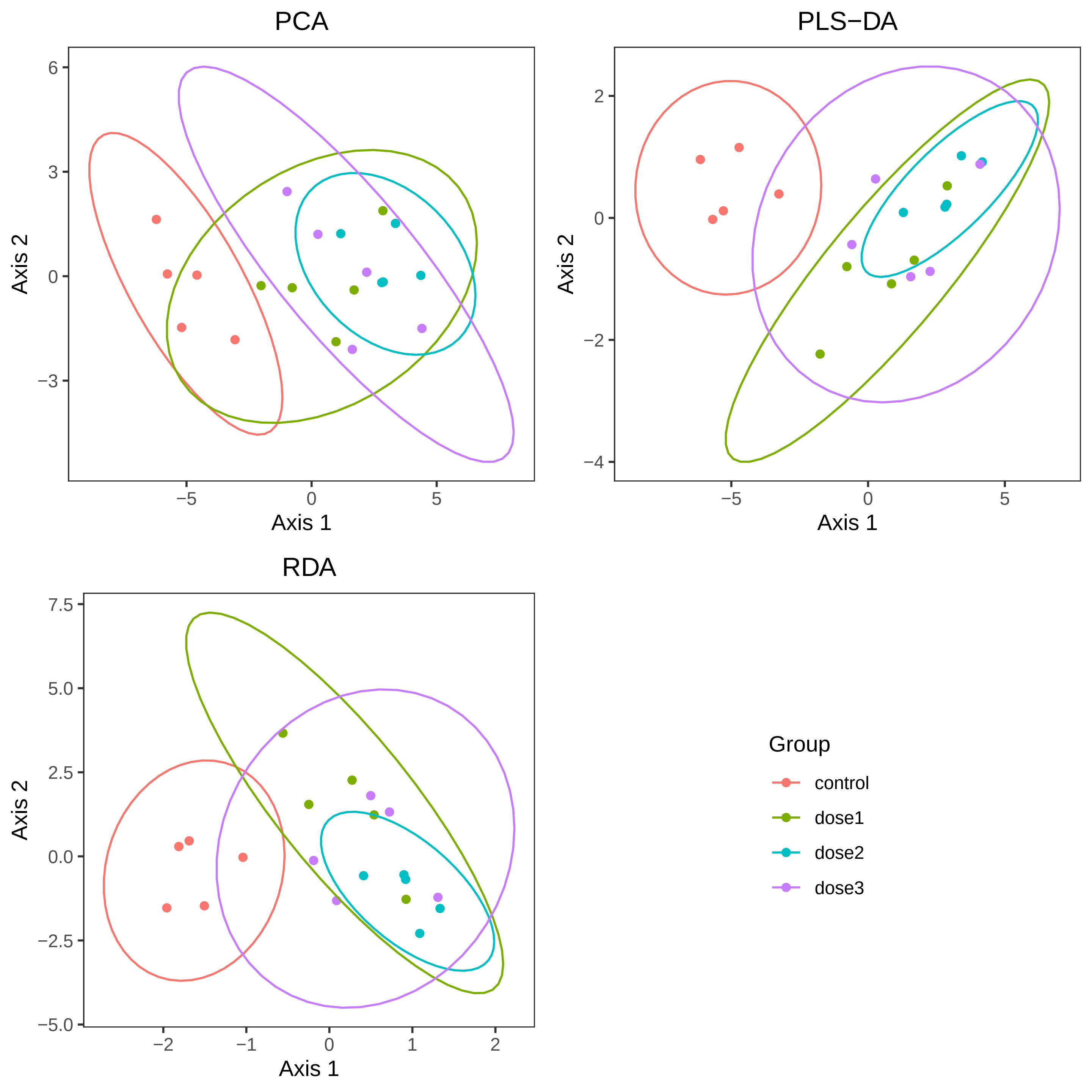

PCA
PLS−DA
RDA
Axis 1
Axis 2
Group
control
dose1
dose2
dose3

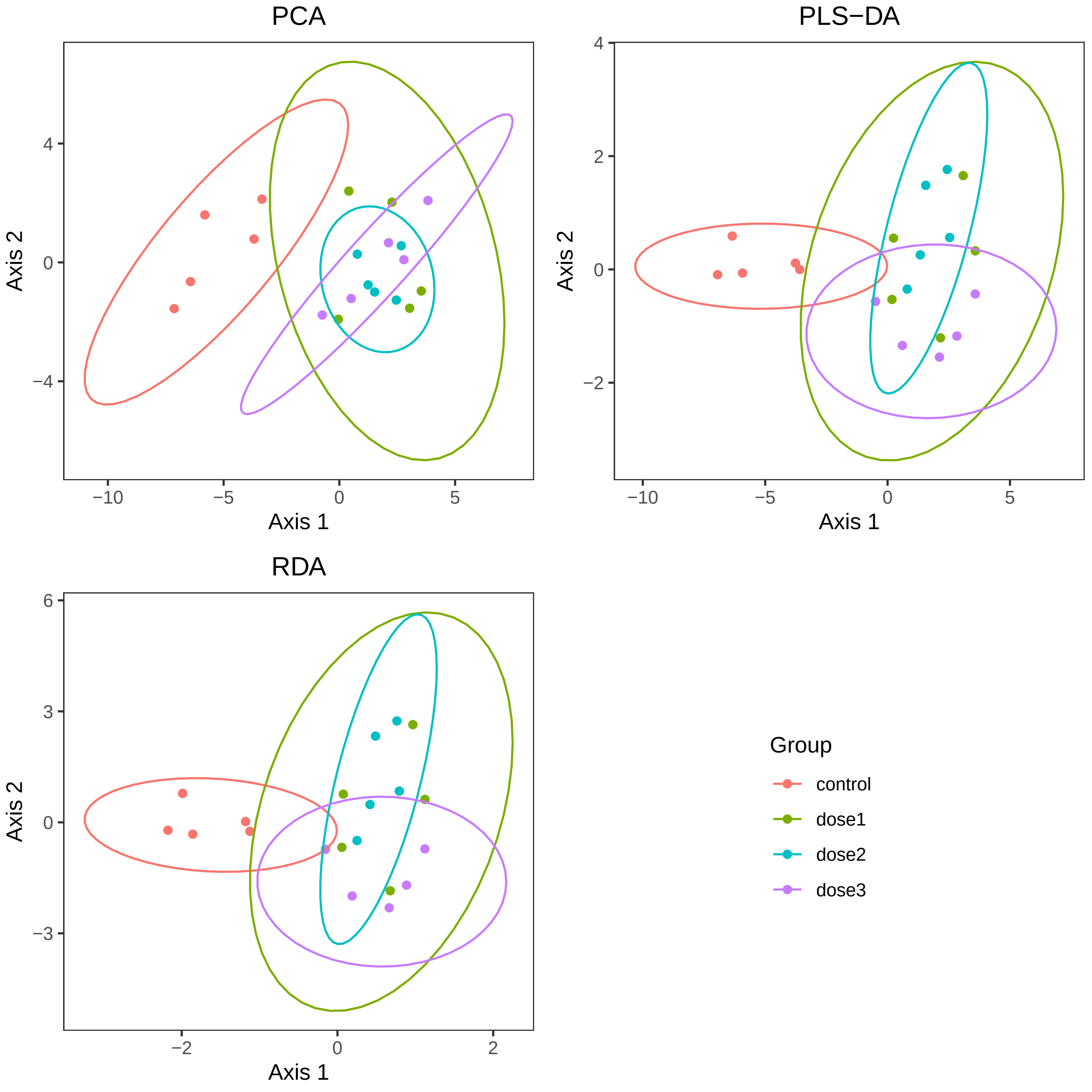

PCA
PLS−DA
RDA
Axis 1
Axis 2
Group
control
dose1
dose2
dose3

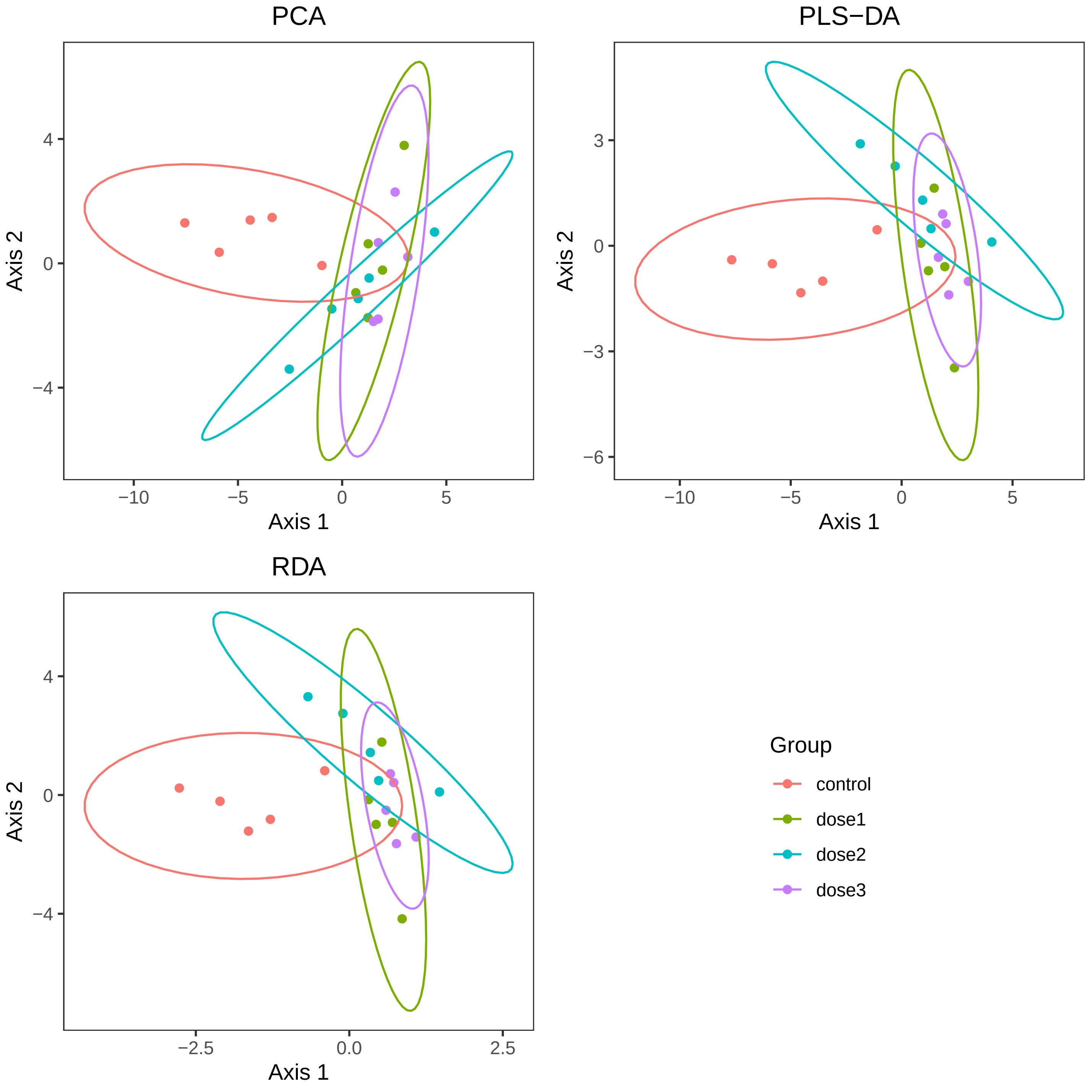
PCA
PLS−DA
RDA
Axis 1
Axis 2
Group
control
dose1
dose2
dose3

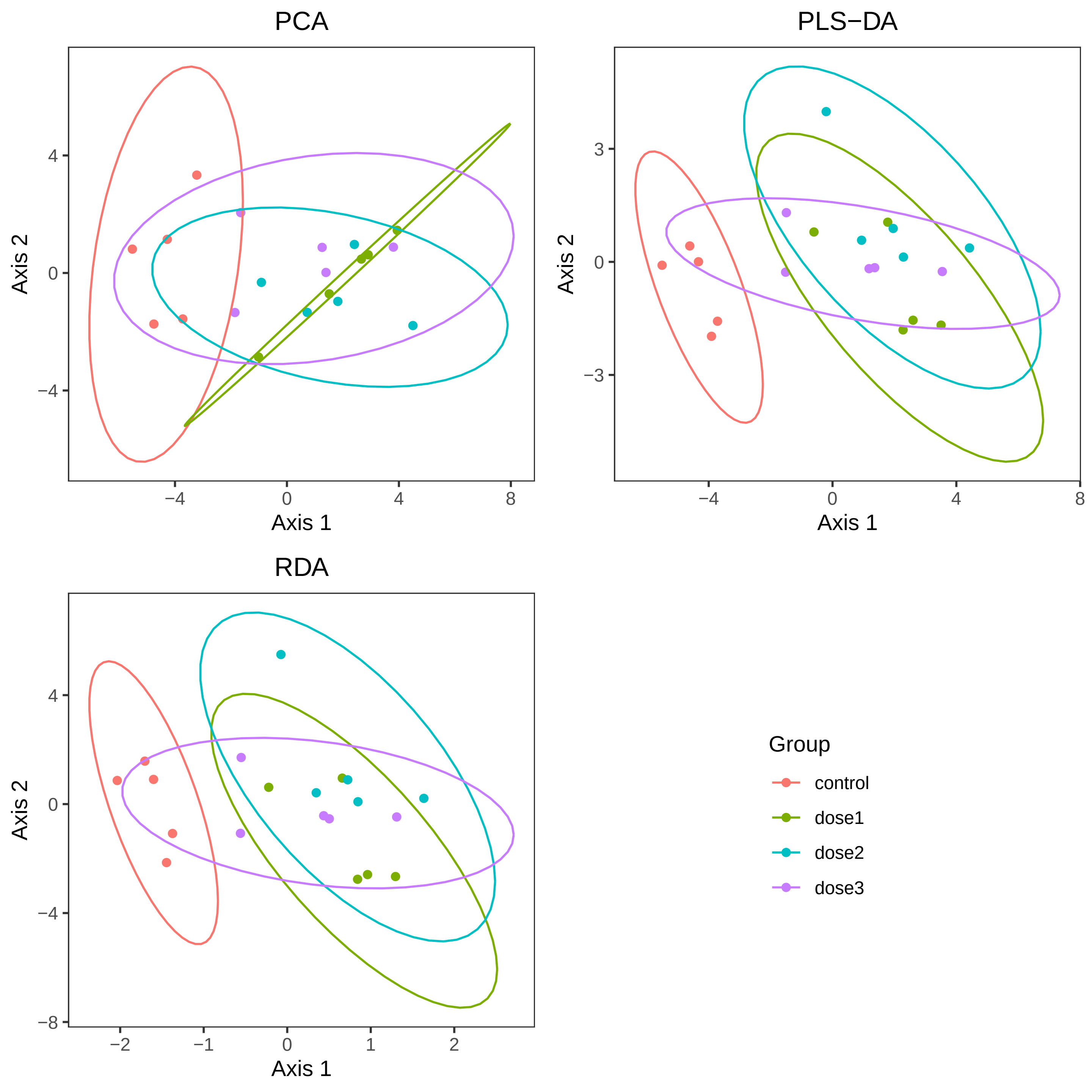
PCA
PLS−DA
RDA
Axis 1
Axis 2
Group
control
dose1
dose2
dose3

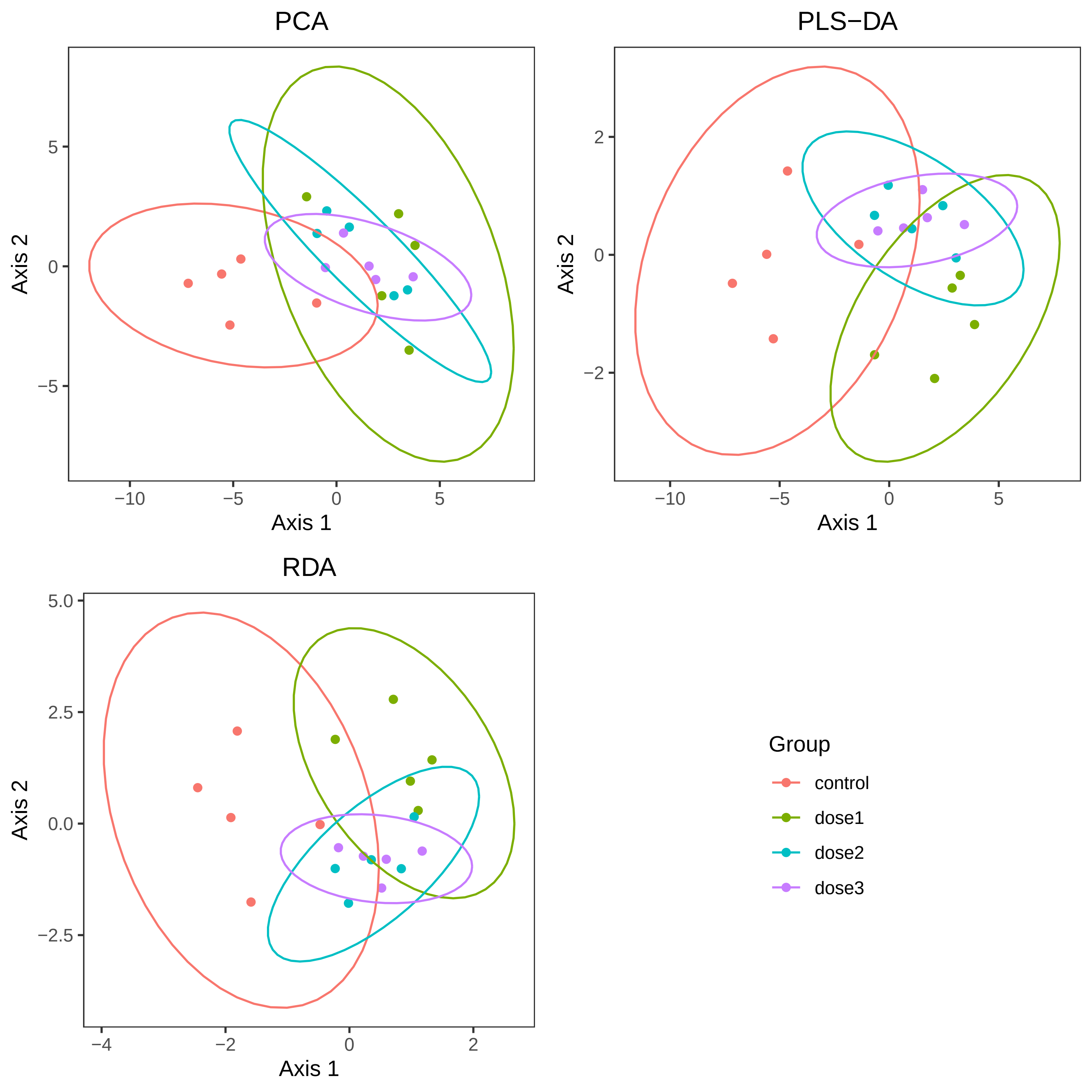

PCA
PLS−DA
RDA
Axis 1
Axis 2
−10
−5
0
5
−2
2
−4
−2.5
0.0
2.5
5.0
Group
control
dose1
dose2
dose3

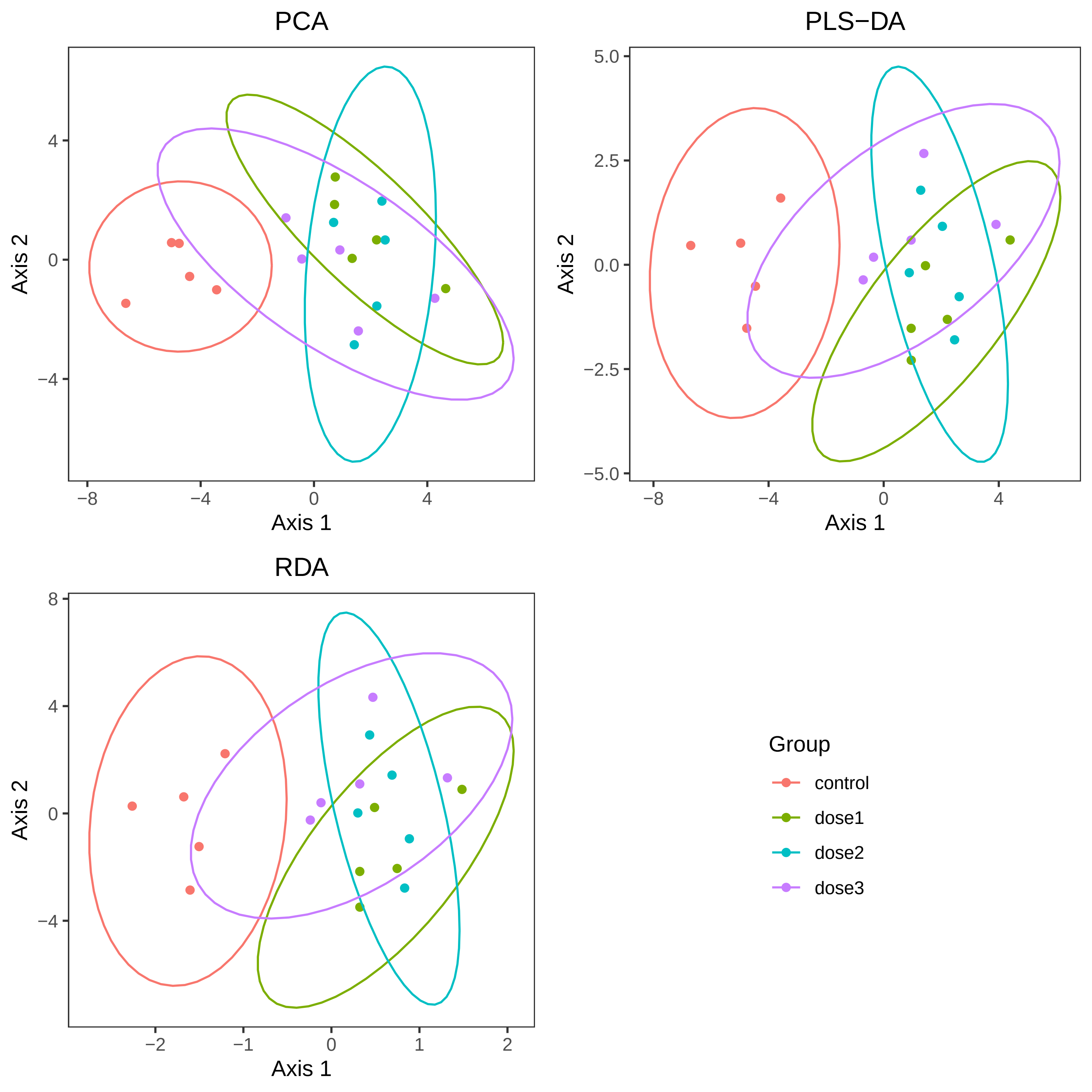

PCA
PLS−DA
RDA
Axis 1
Axis 2
−8
−4
0
4
5.0
2.5
0.0
−2.5
−5.0
8
−2
−1
1
2
Group
control
dose1
dose2
dose3

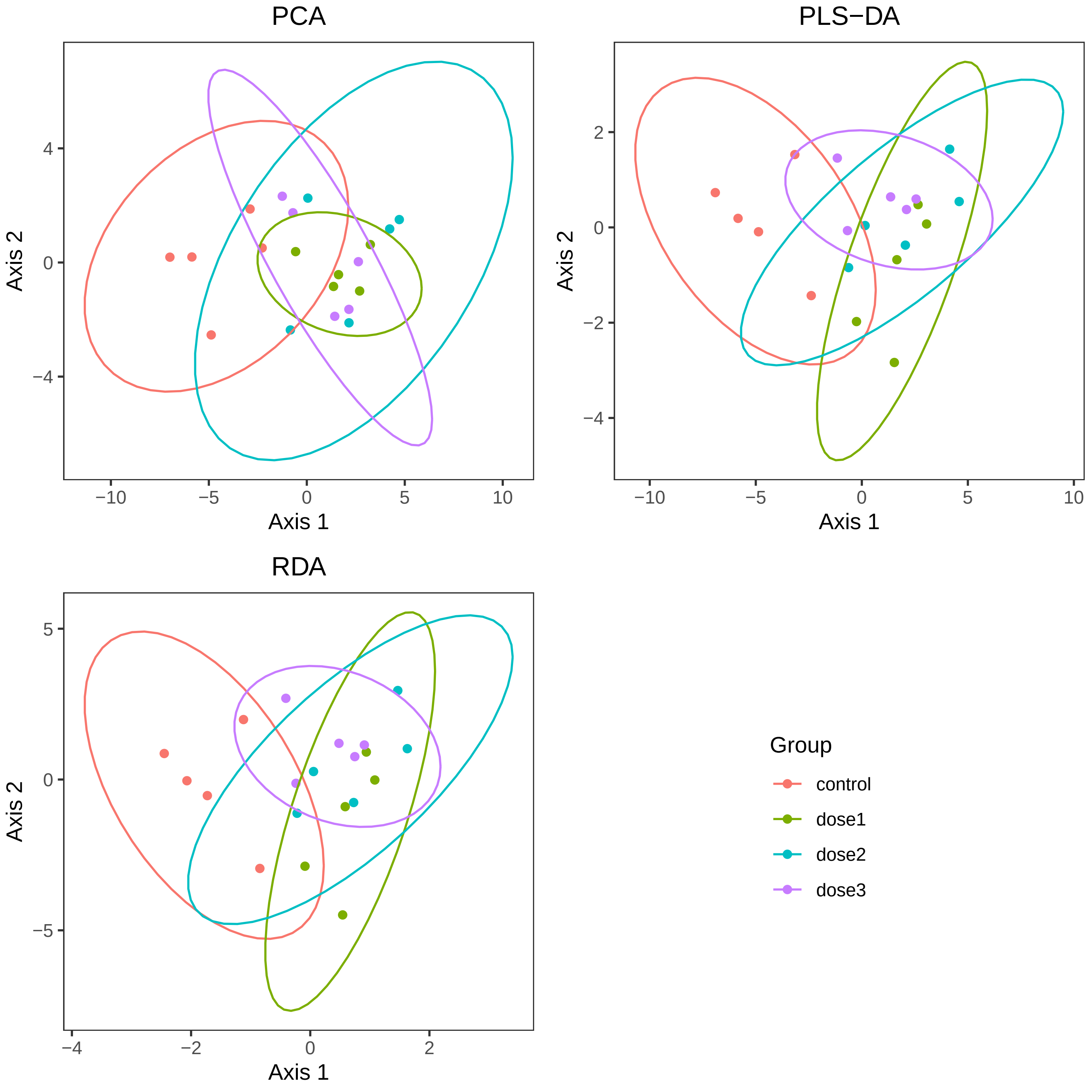

PCA
PLS−DA
RDA
Axis 1
Axis 2
Group
control
dose1
dose2
dose3

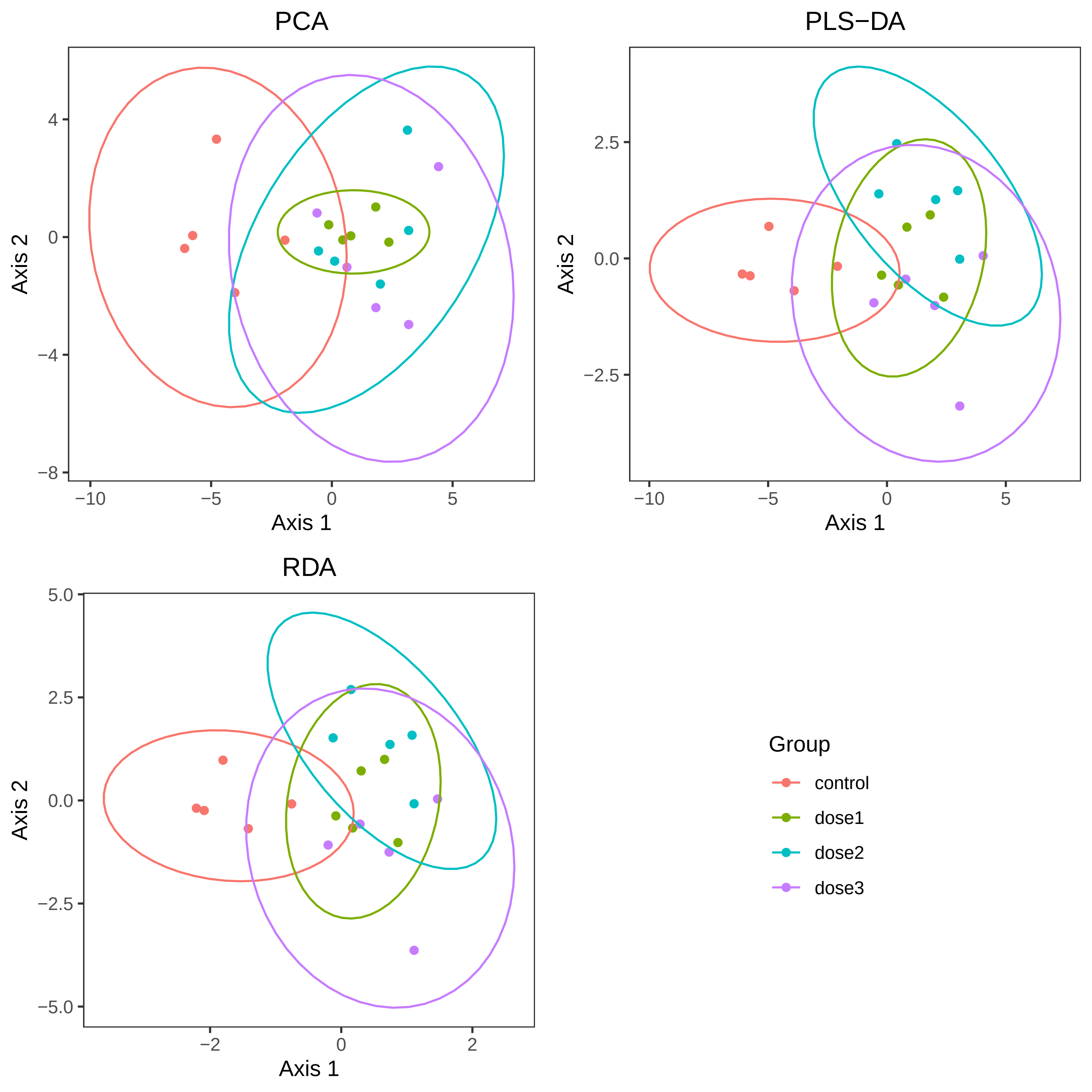

PCA
PLS−DA
RDA
Axis 1
Axis 2
Group
control
dose1
dose2
dose3

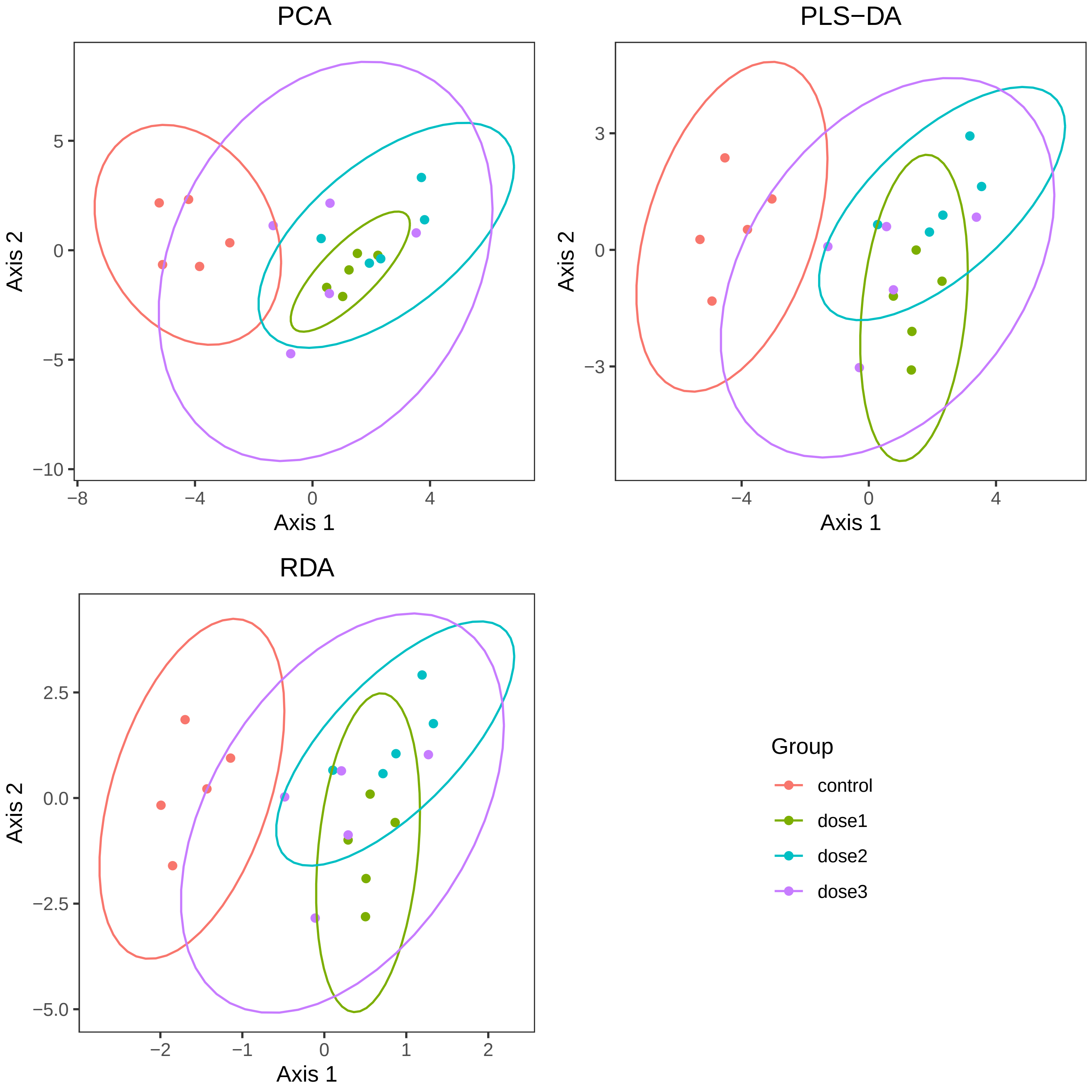
PCA
PLS−DA
RDA
Axis 1
Axis 2
5
0
−5
−10
−8
−4
0
4
3
0
−3
−4
0
4
2.5
0.0
−2.5
−5.0
−2
−1
0
1
2
Group
control
dose1
dose2
dose3

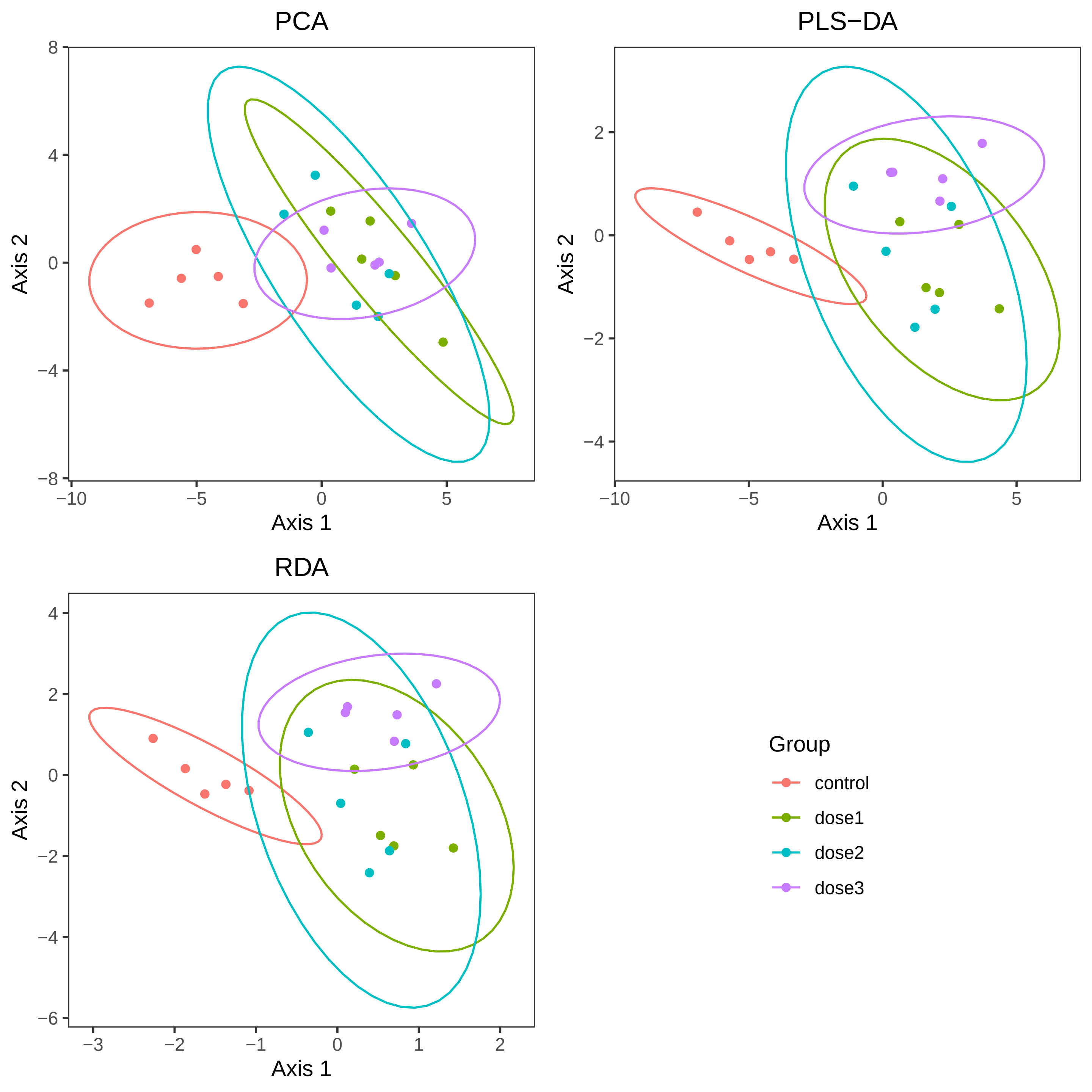

PCA
PLS−DA
RDA
Axis 1
Axis 2
Group
control
dose1
dose2
dose3

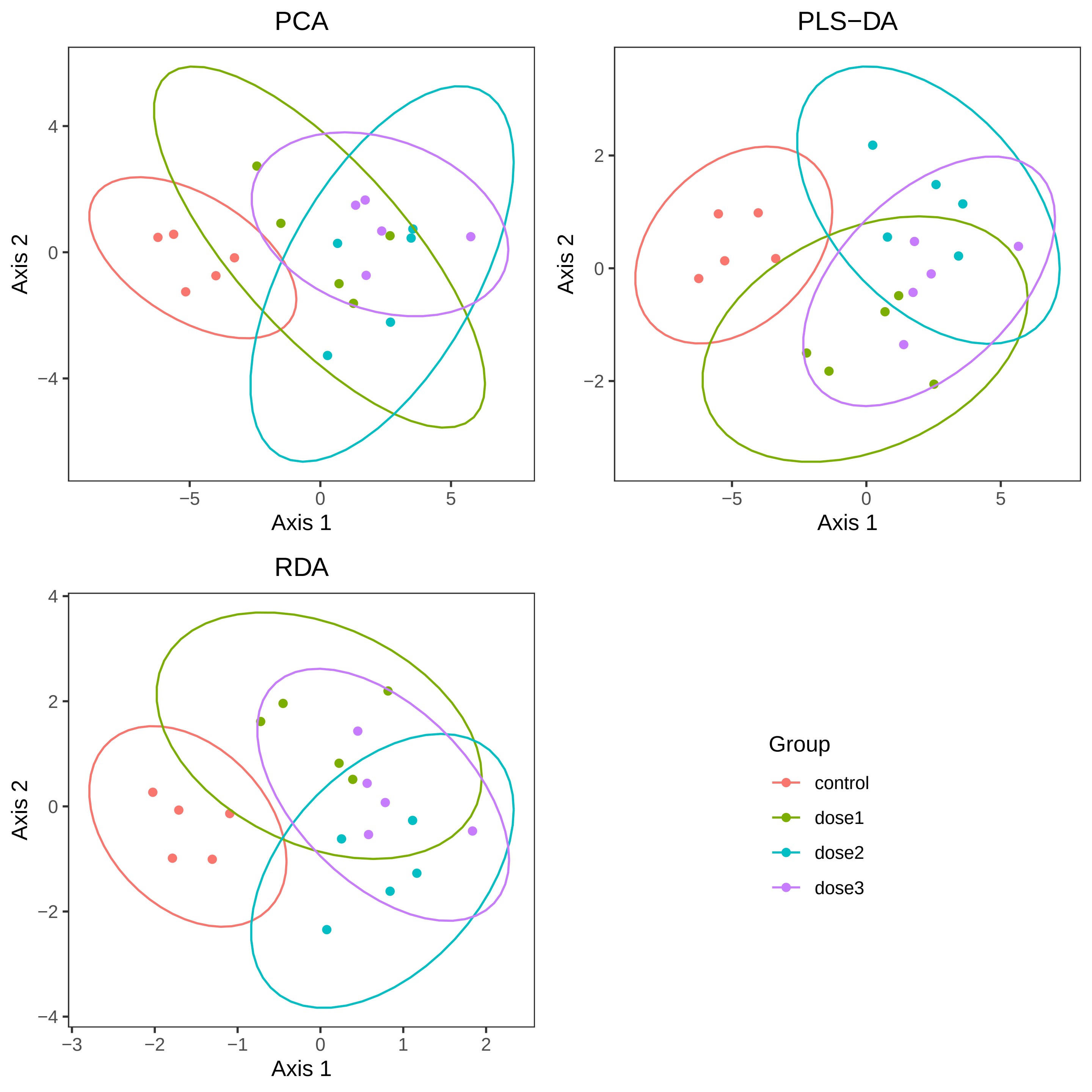

PCA
PLS−DA
RDA
Axis 1
Axis 2
−5
0
5
4
0
−4
2
−2
−3
−2
−1
1
Group
control
dose1
dose2
dose3

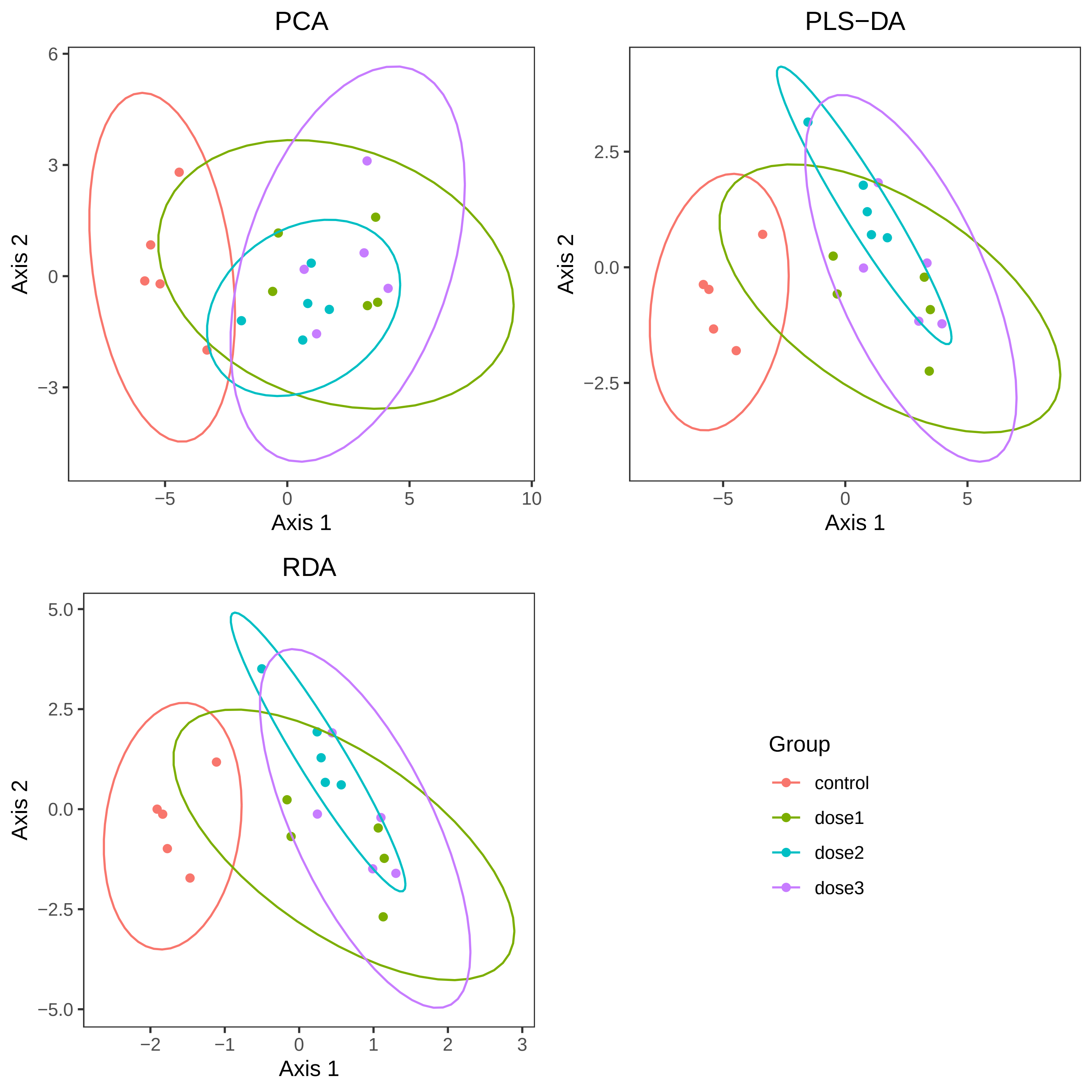
PCA
PLS−DA
RDA
Axis 1
Axis 2
Group
control
dose1
dose2
dose3

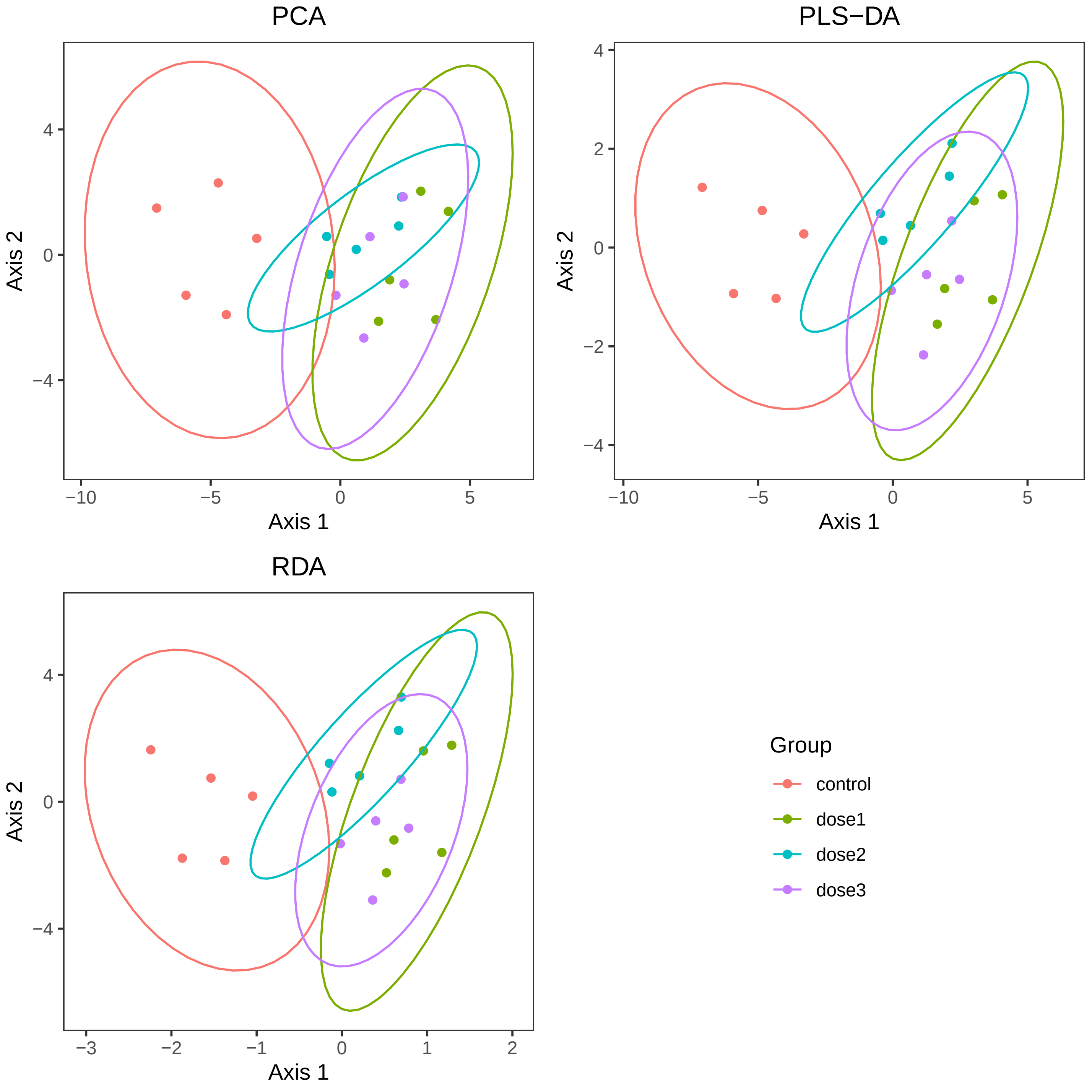
PCA
PLS−DA
RDA
Axis 1
Axis 2
Group
control
dose1
dose2
dose3

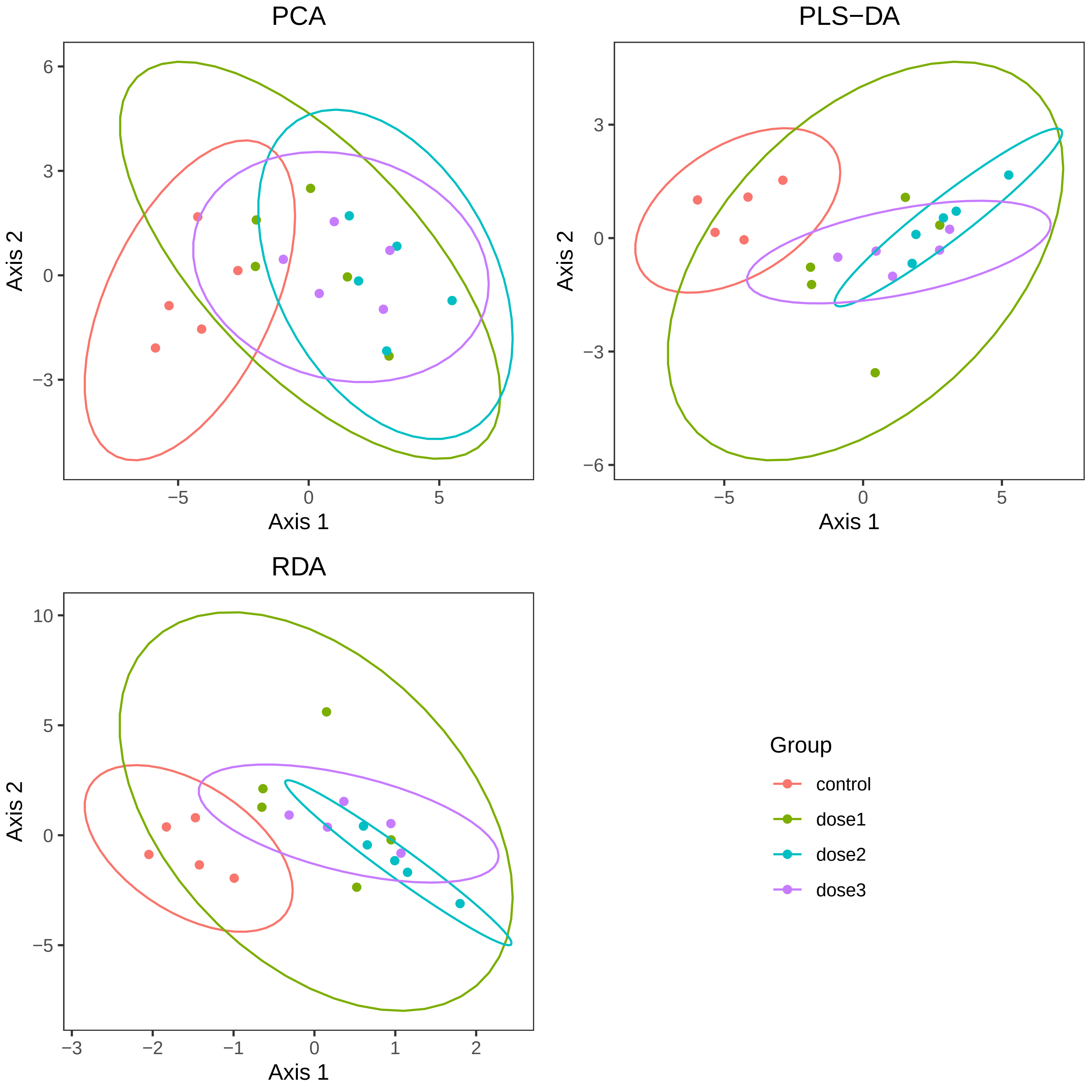

PCA
PLS−DA
RDA
Axis 1
Axis 2
Group
control
dose1
dose2
dose3

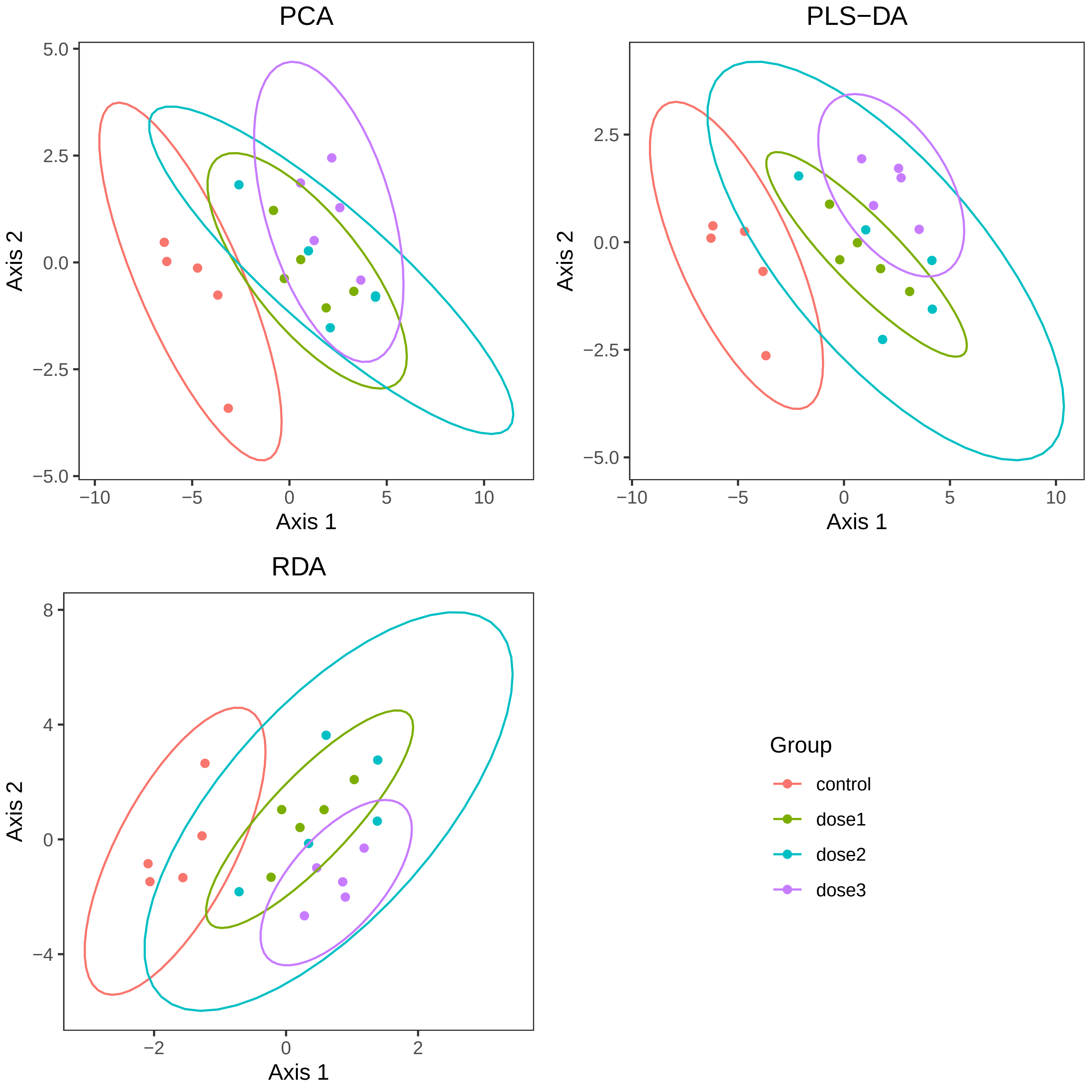
PCA
PLS−DA
RDA
Axis 1
Axis 2
Group
control
dose1
dose2
dose3

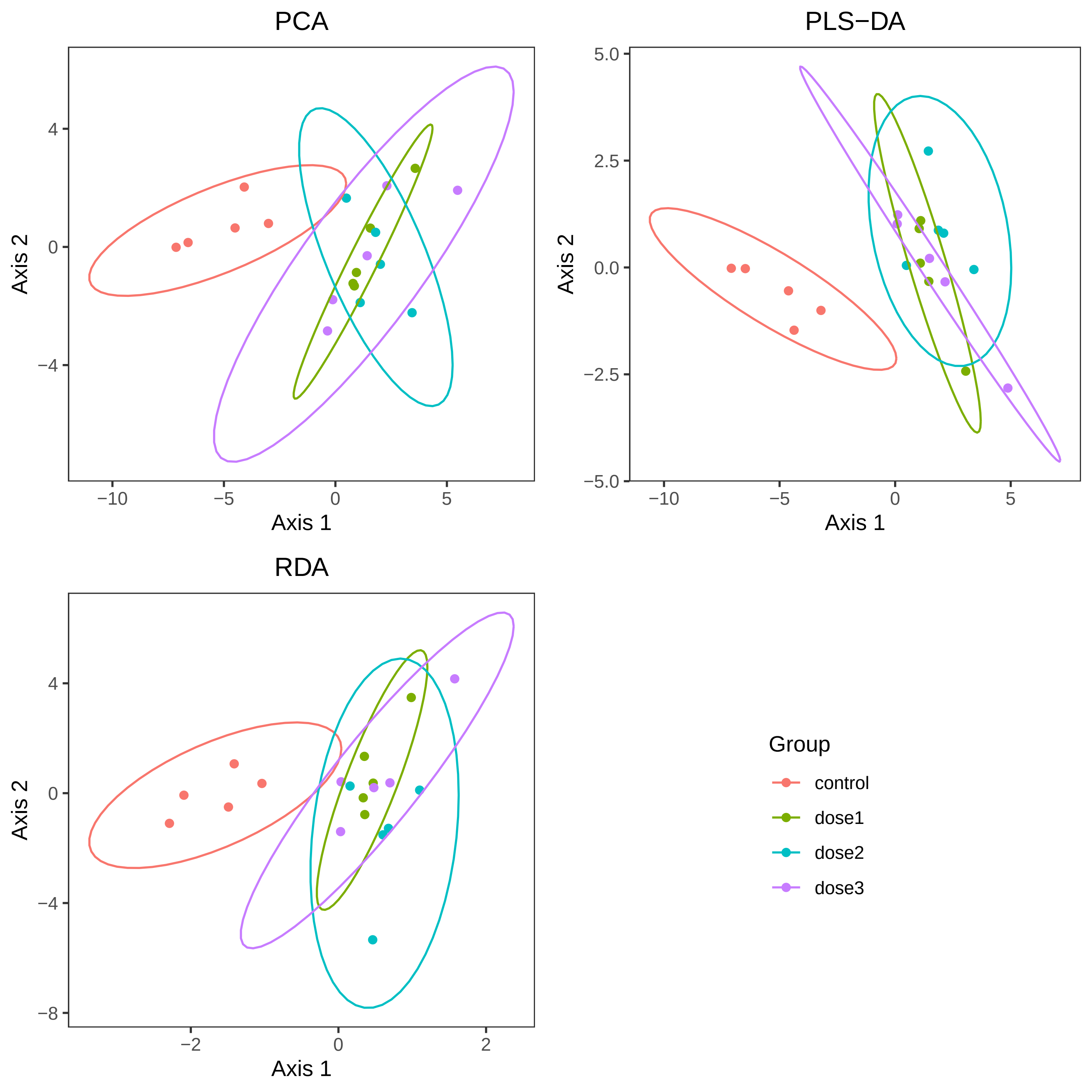
PCA
PLS−DA
RDA
Axis 1
Axis 2
Group
control
dose1
dose2
dose3

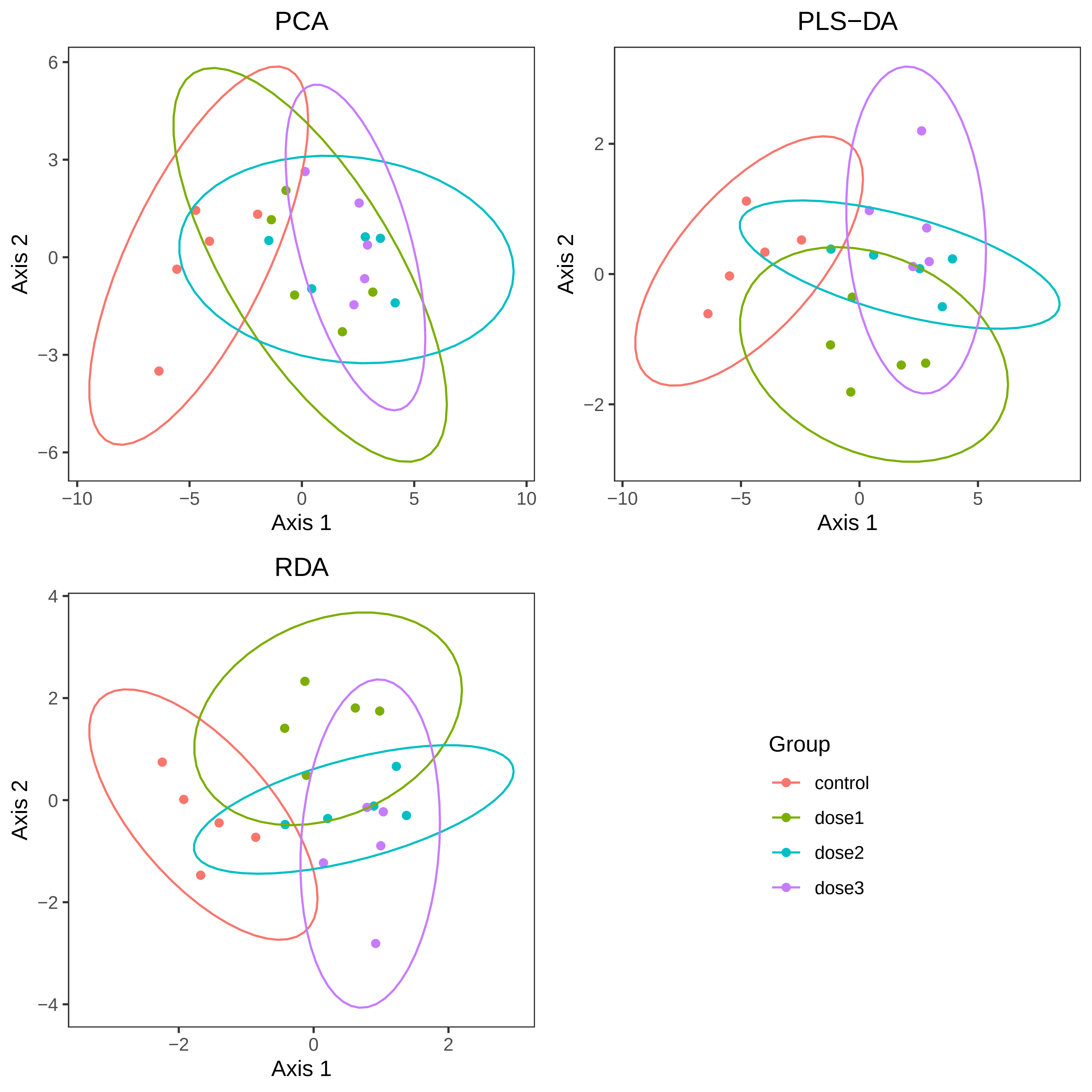

PCA
PLS−DA
RDA
Axis 1
Axis 2
Group
control
dose1
dose2
dose3

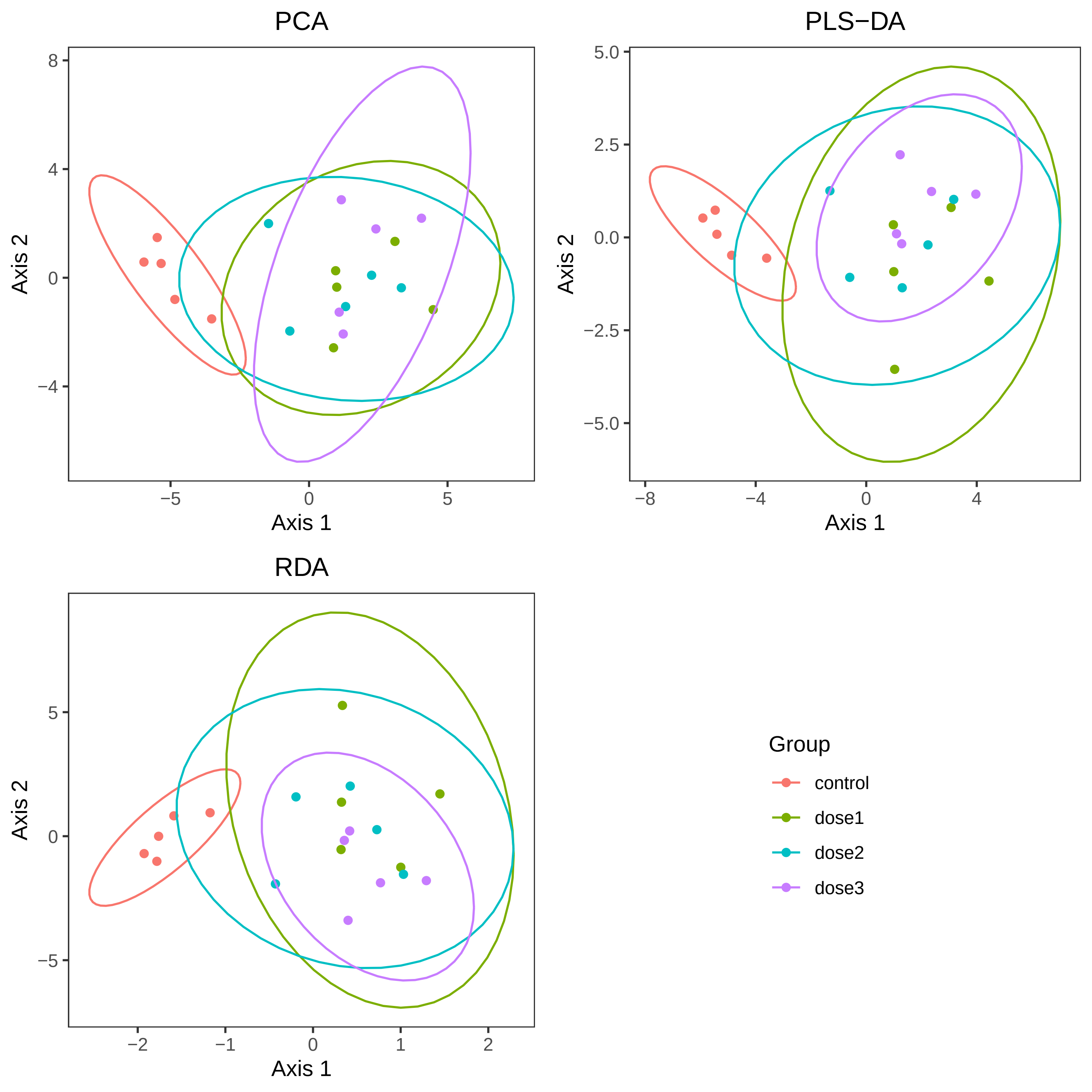

PCA
PLS−DA
RDA
Axis 1
Axis 2
8
4
0
−4
−5
0
5
5.0
2.5
0.0
−2.5
−5.0
−8
−4
0
4
5
0
−5
−2
−1
0
1
2
Group
control
dose1
dose2
dose3

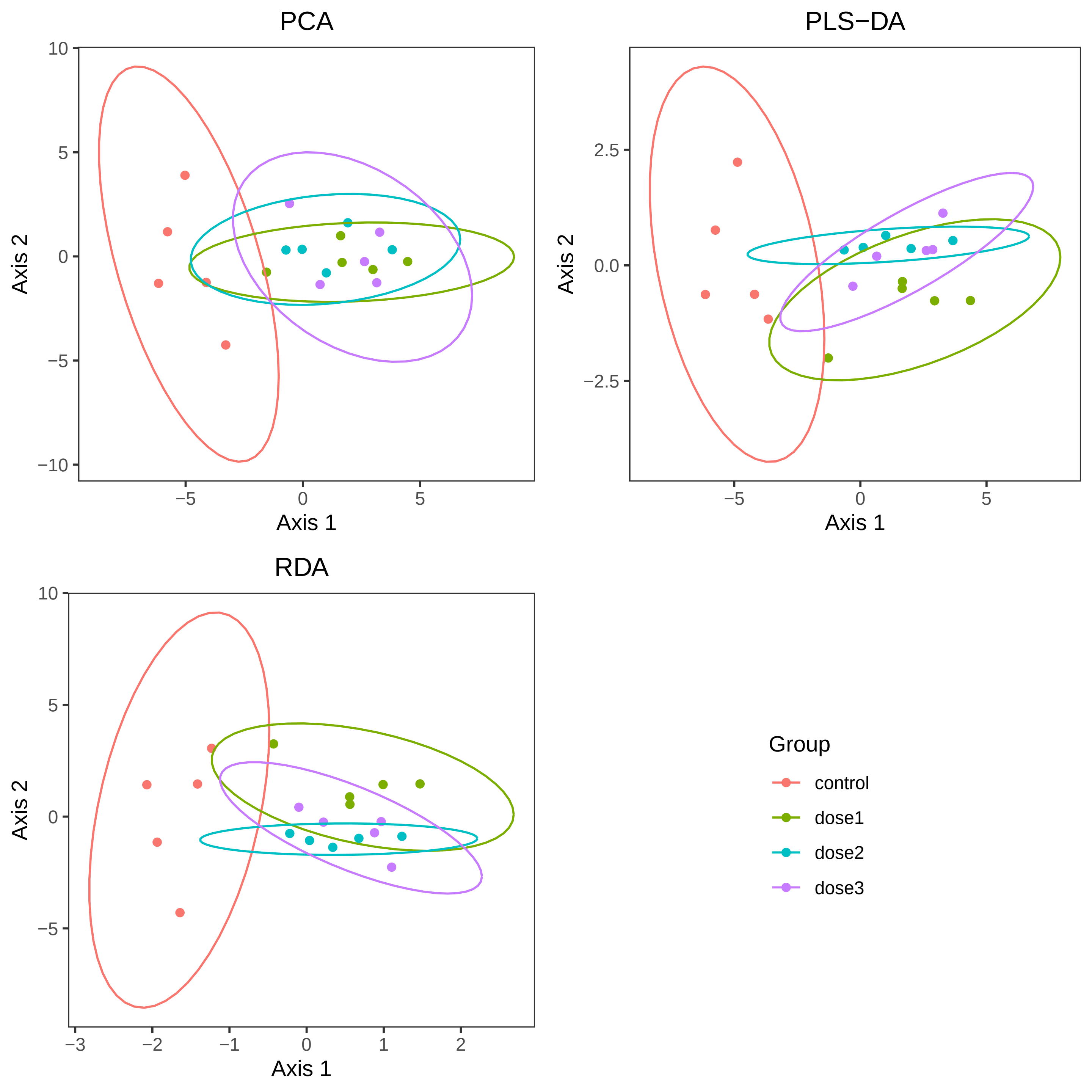

PCA
PLS−DA
RDA
Axis 1
Axis 2
10
5
0
−5
−10
2.5
0.0
−2.5
−3
−2
−1
1
2
Group
control
dose1
dose2
dose3

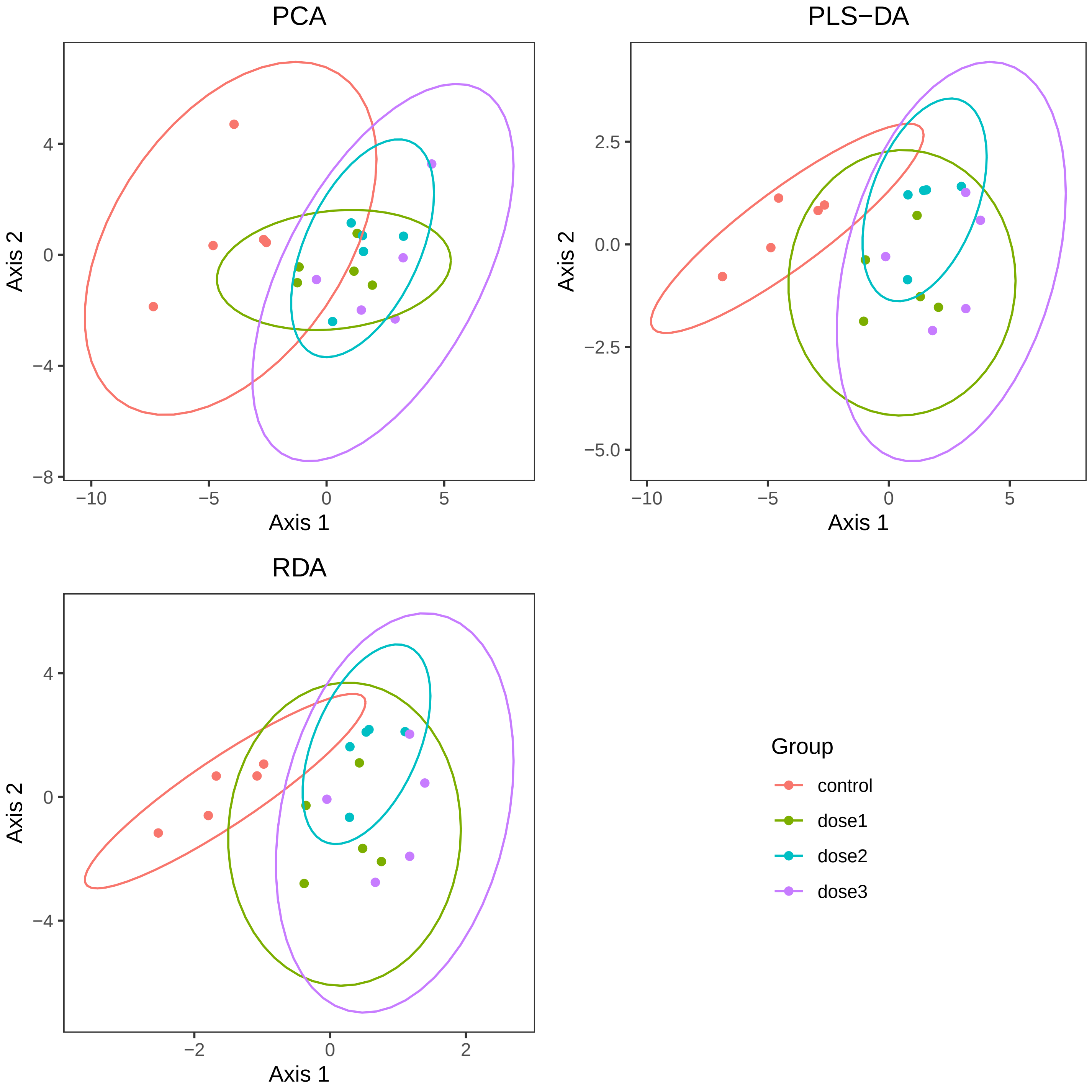

PCA
PLS−DA
RDA
Axis 1
Axis 2
Group
control
dose1
dose2
dose3

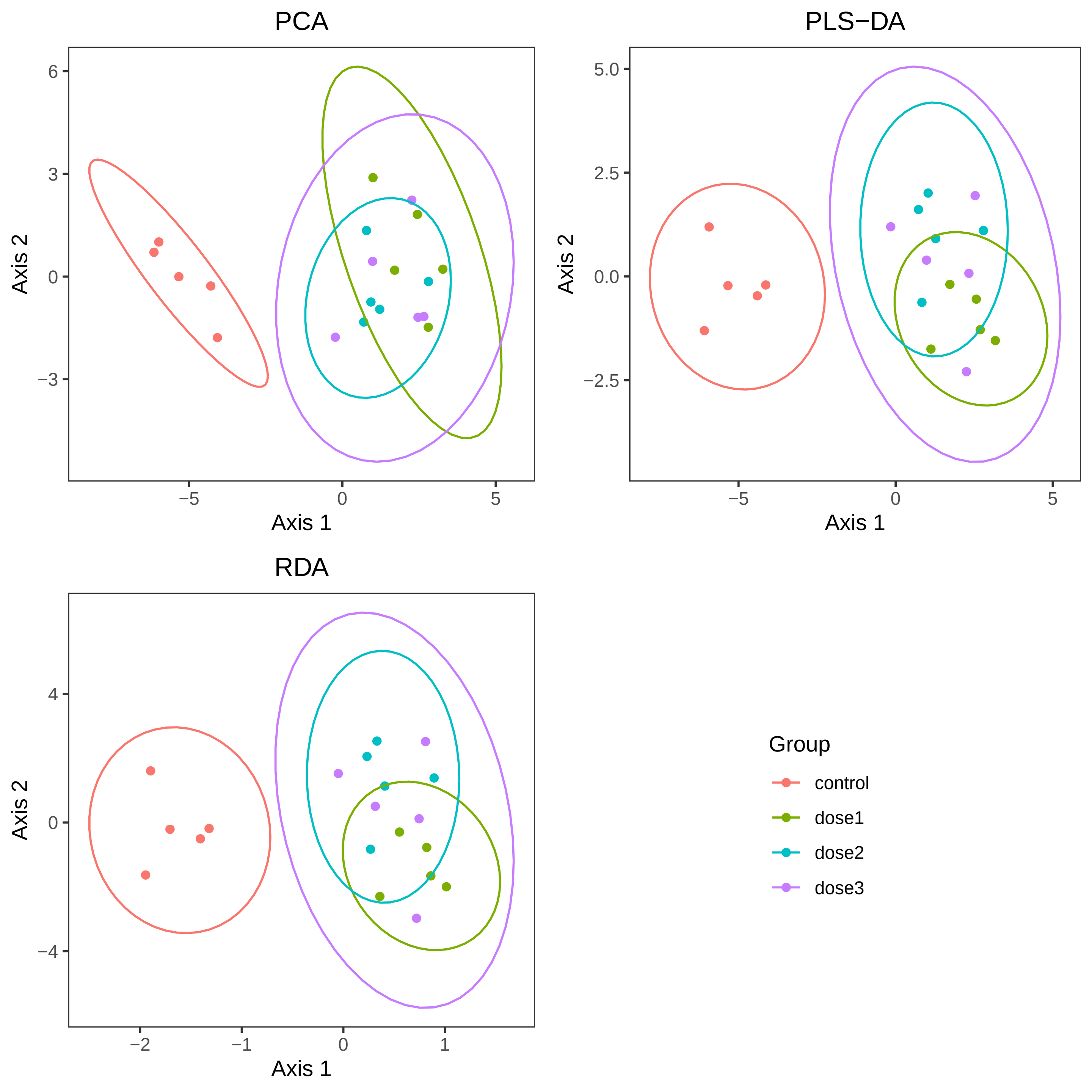
PCA
PLS−DA
RDA
Axis 1
Axis 2
Group
control
dose1
dose2
dose3

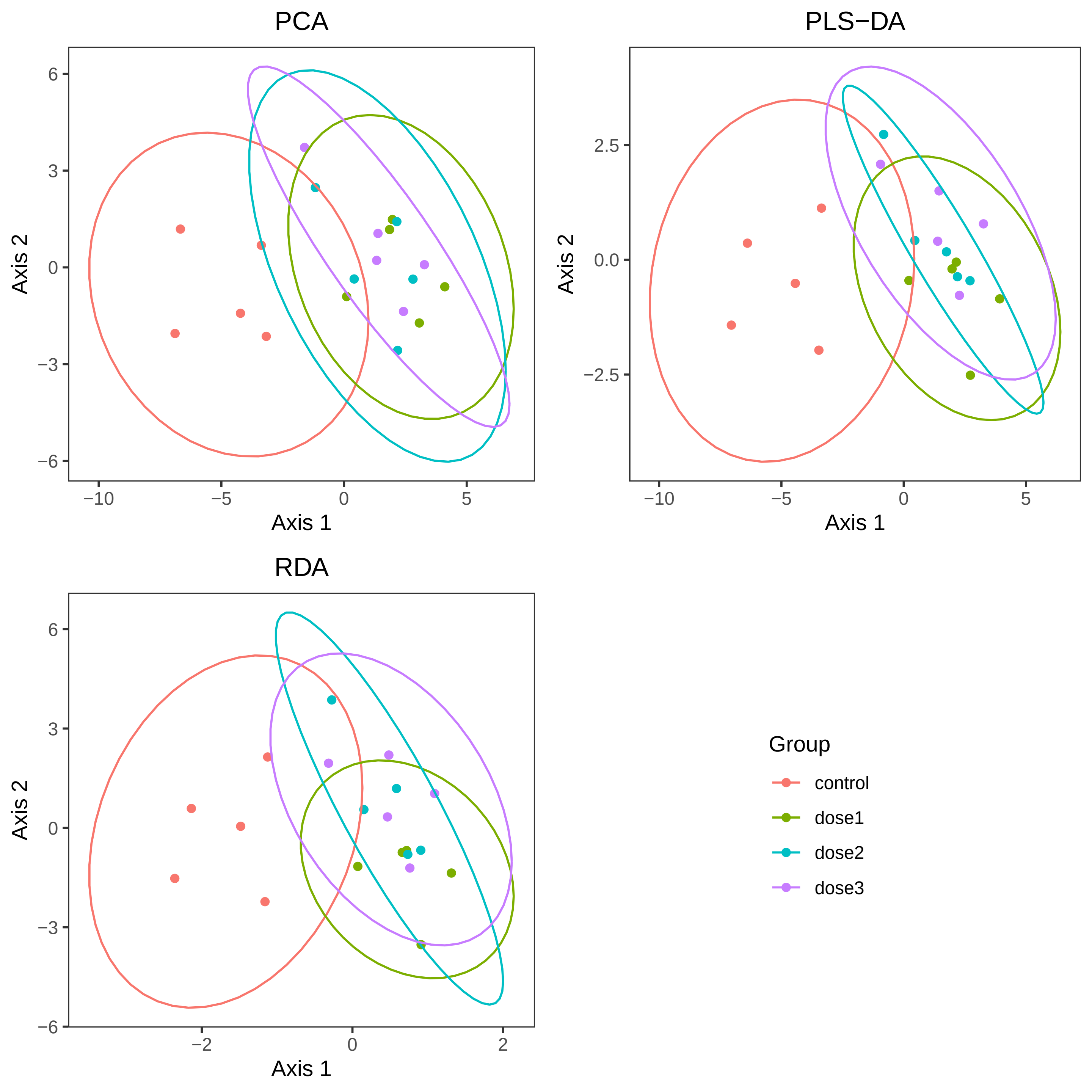

PCA
PLS−DA
RDA
Axis 1
Axis 2
−10
−5
0
5
6
3
0
−3
−6
2.5
0.0
−2.5
−2
2
Group
control
dose1
dose2
dose3

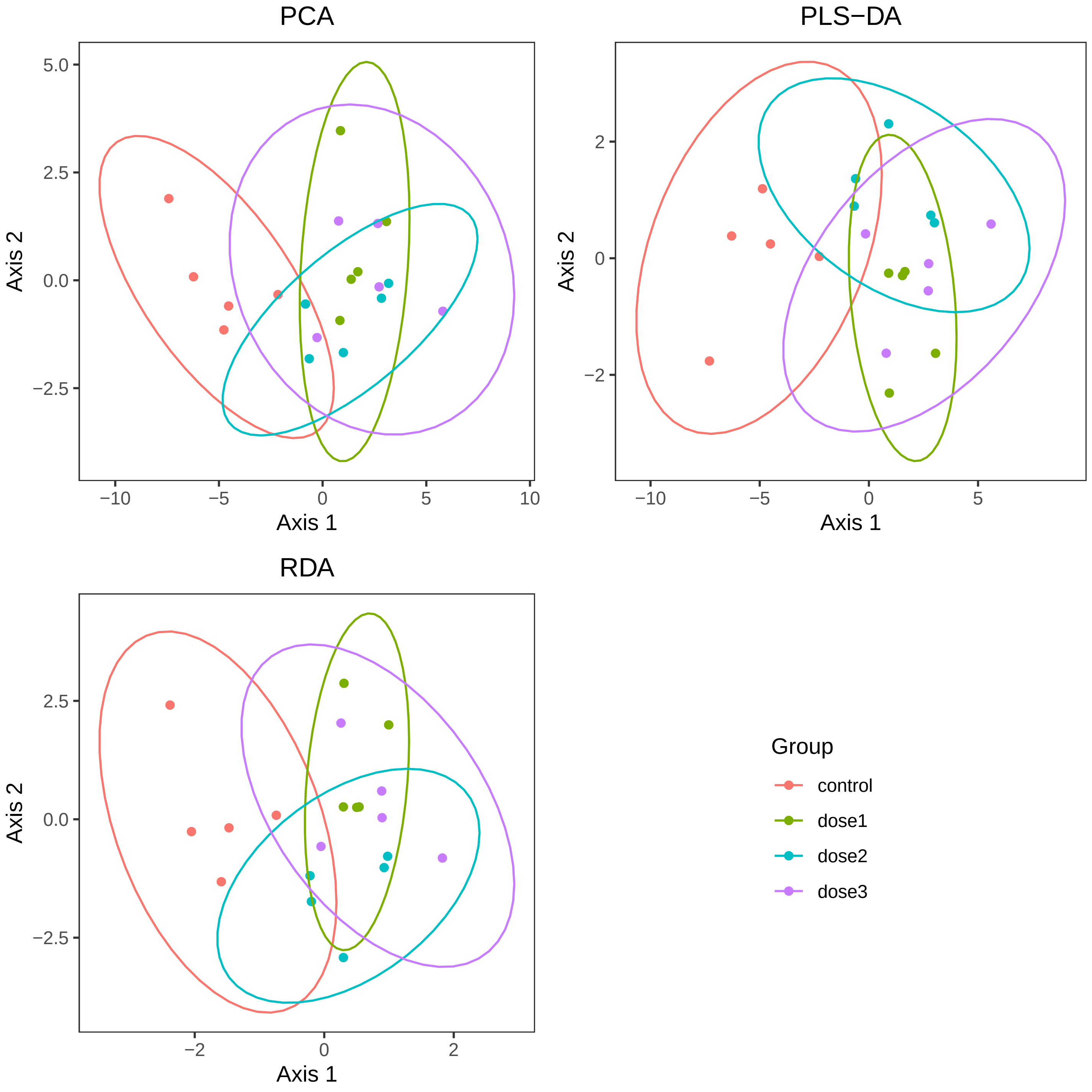

PCA
PLS−DA
RDA
Axis 1
Axis 2
Group
control
dose1
dose2
dose3

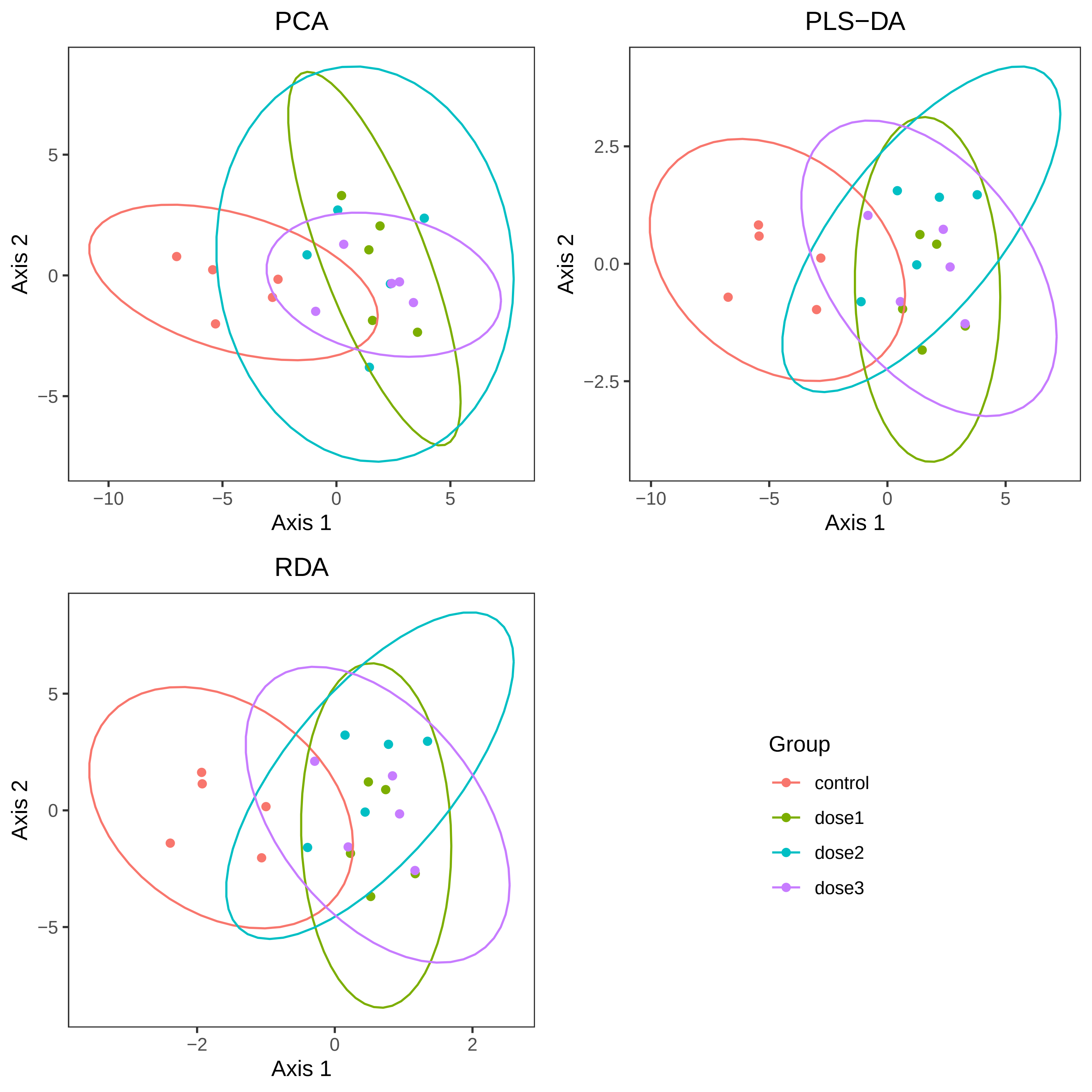

PCA
PLS−DA
RDA
Axis 1
Axis 2
Group
control
dose1
dose2
dose3

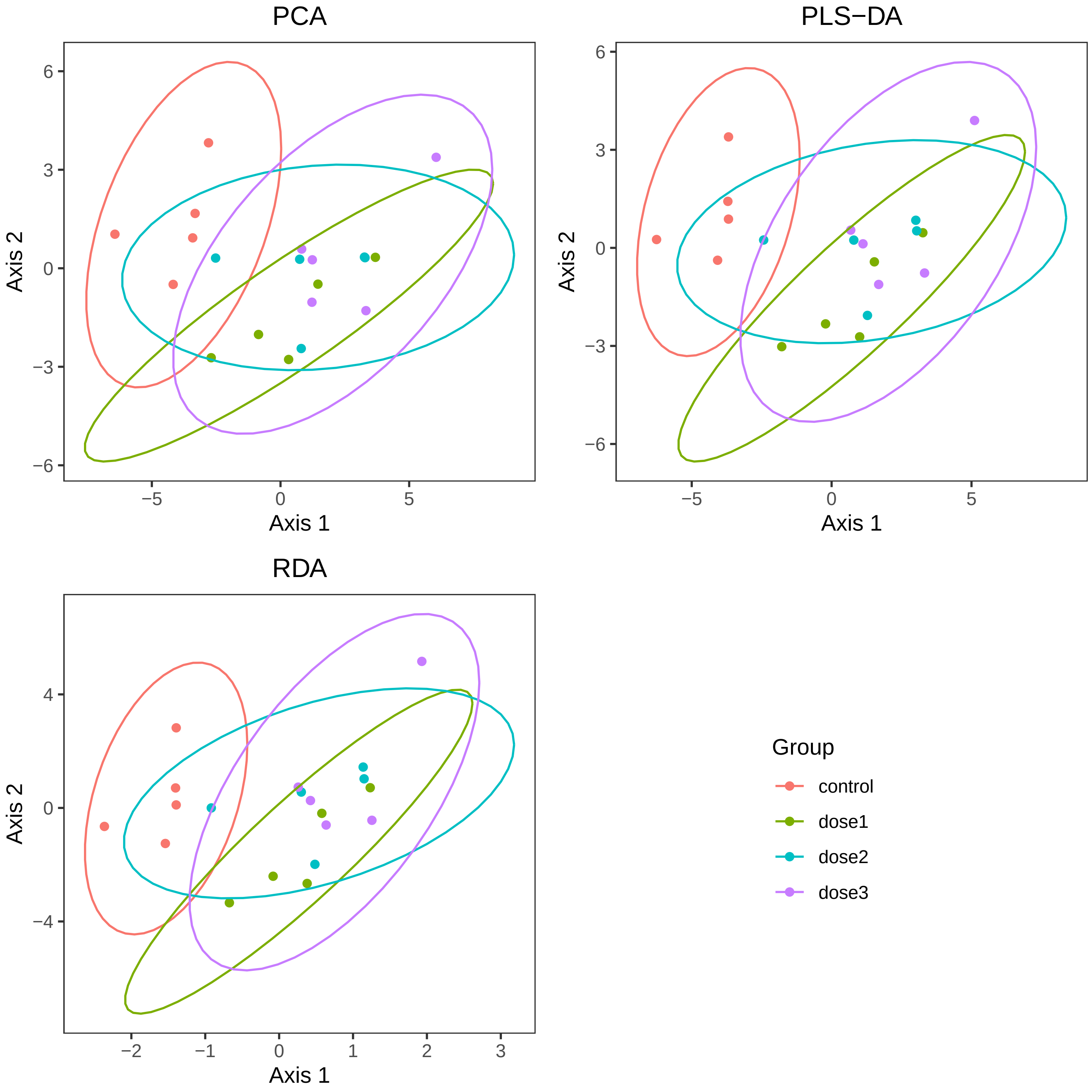
PCA
PLS−DA
RDA
Axis 1
Axis 2
Group
control
dose1
dose2
dose3

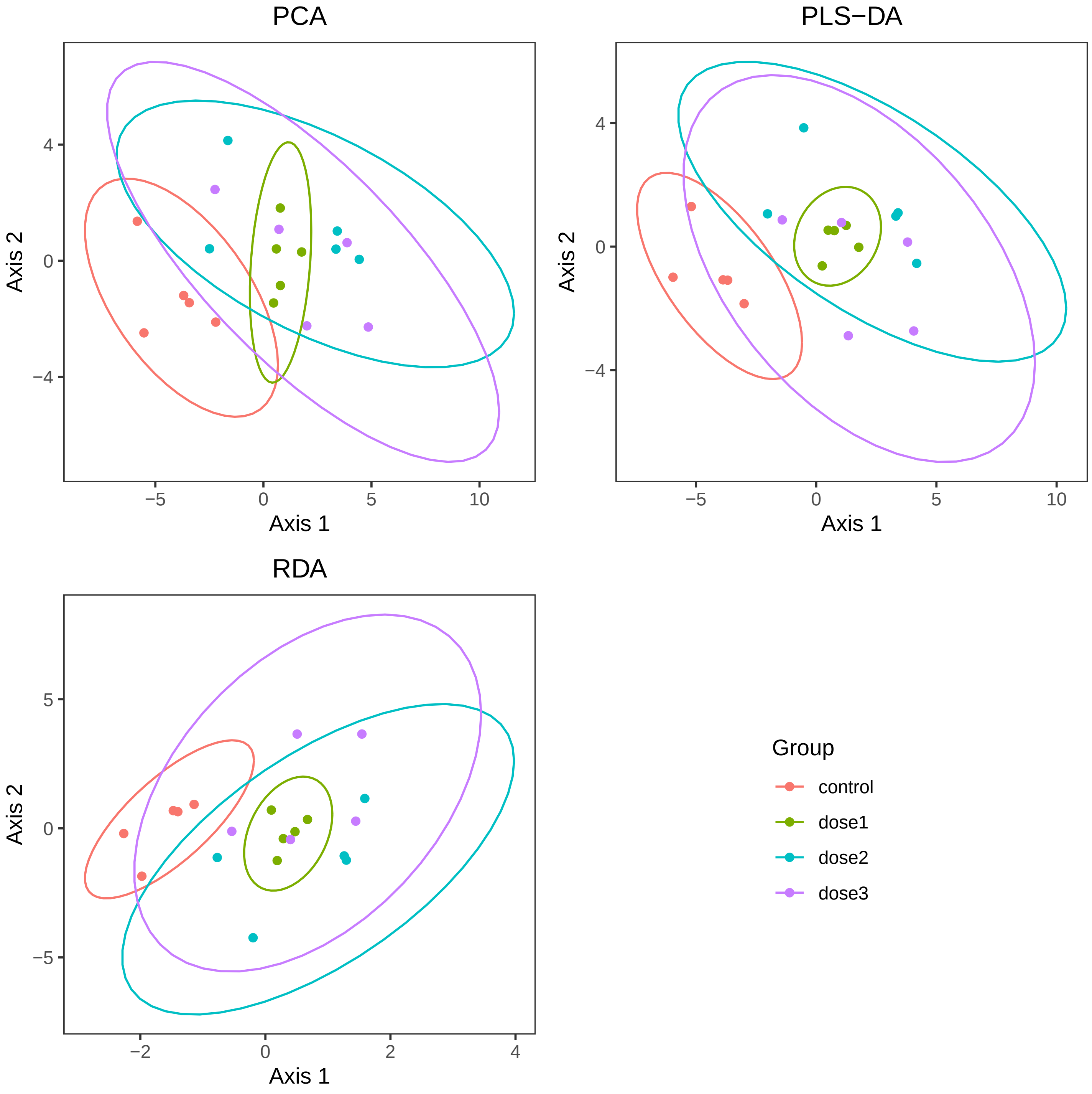
PCA
PLS−DA
RDA
Axis 1
Axis 2
Group
control
dose1
dose2
dose3

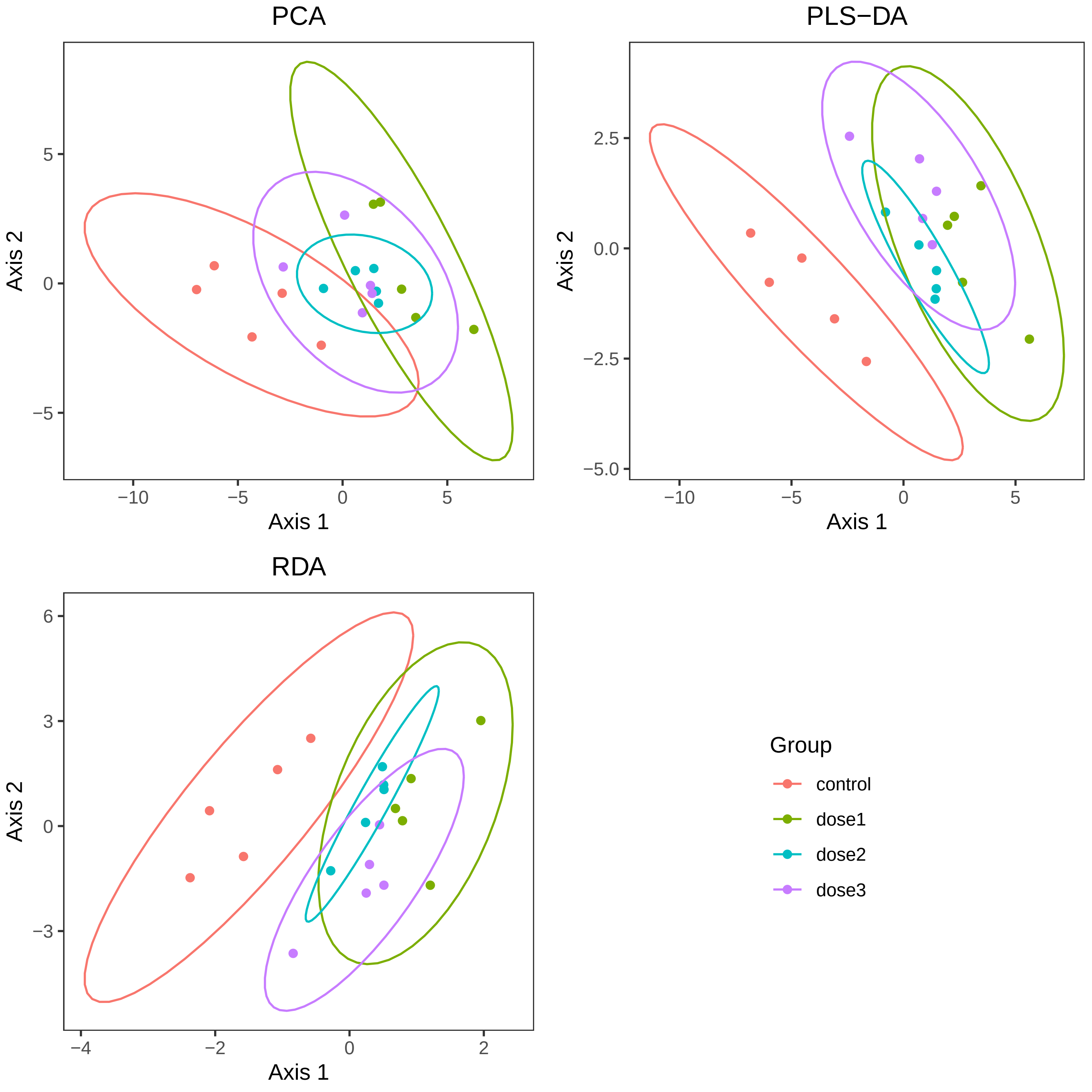
PCA
PLS−DA
RDA
Axis 1
Axis 2
Group
control
dose1
dose2
dose3

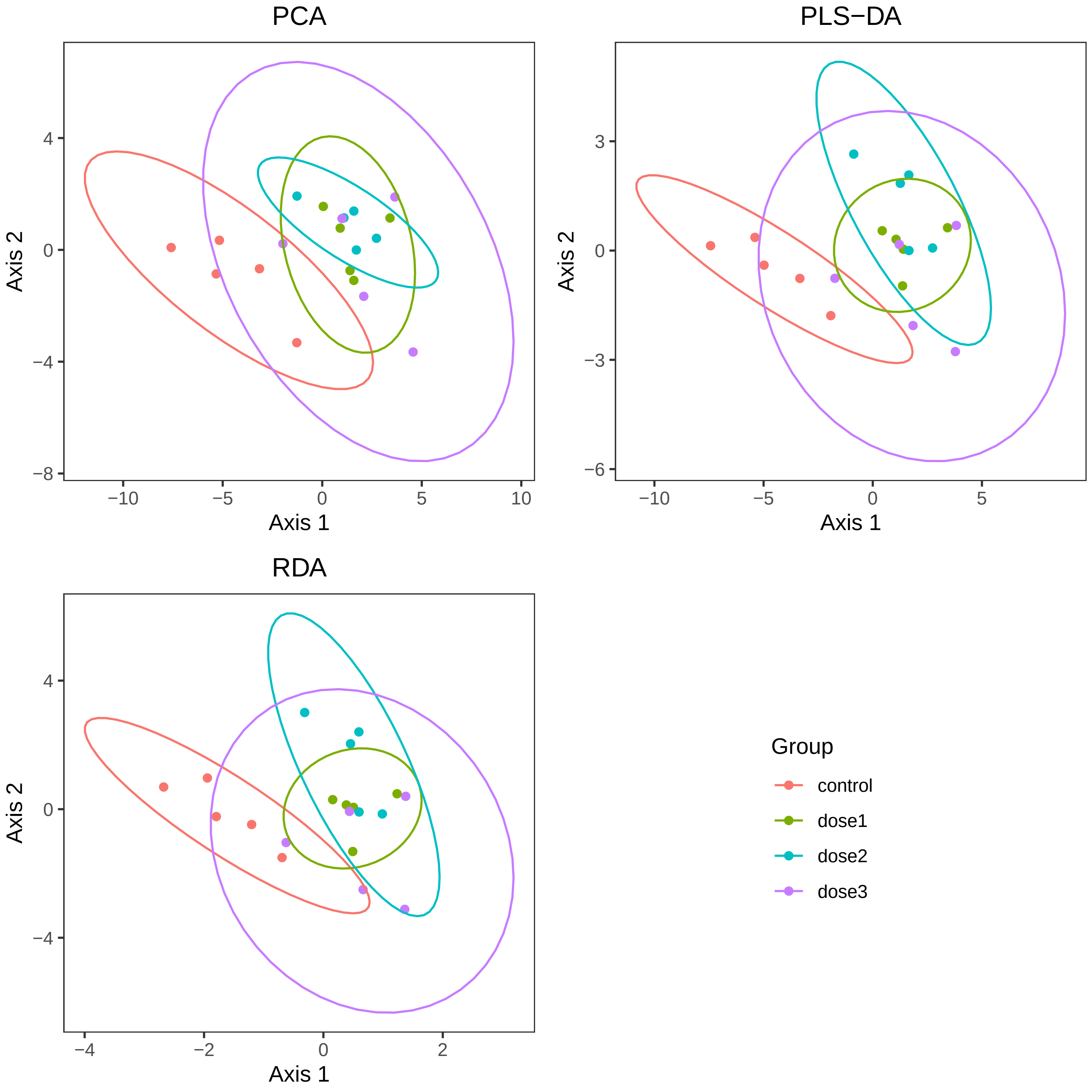
PCA
PLS−DA
RDA
Axis 1
Axis 2
Group
control
dose1
dose2
dose3

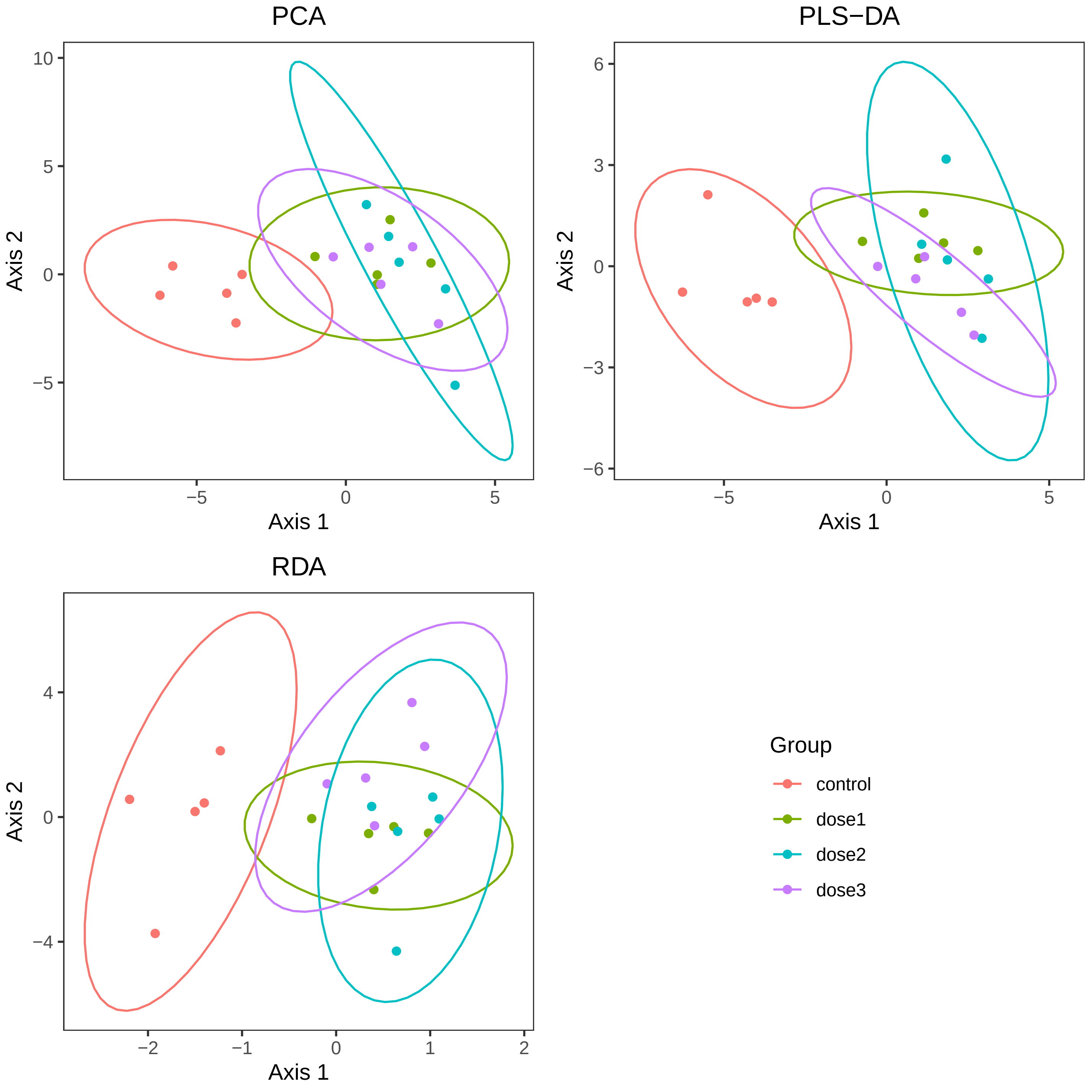
PCA
PLS−DA
RDA
Axis 1
Axis 2
Group
control
dose1
dose2
dose3

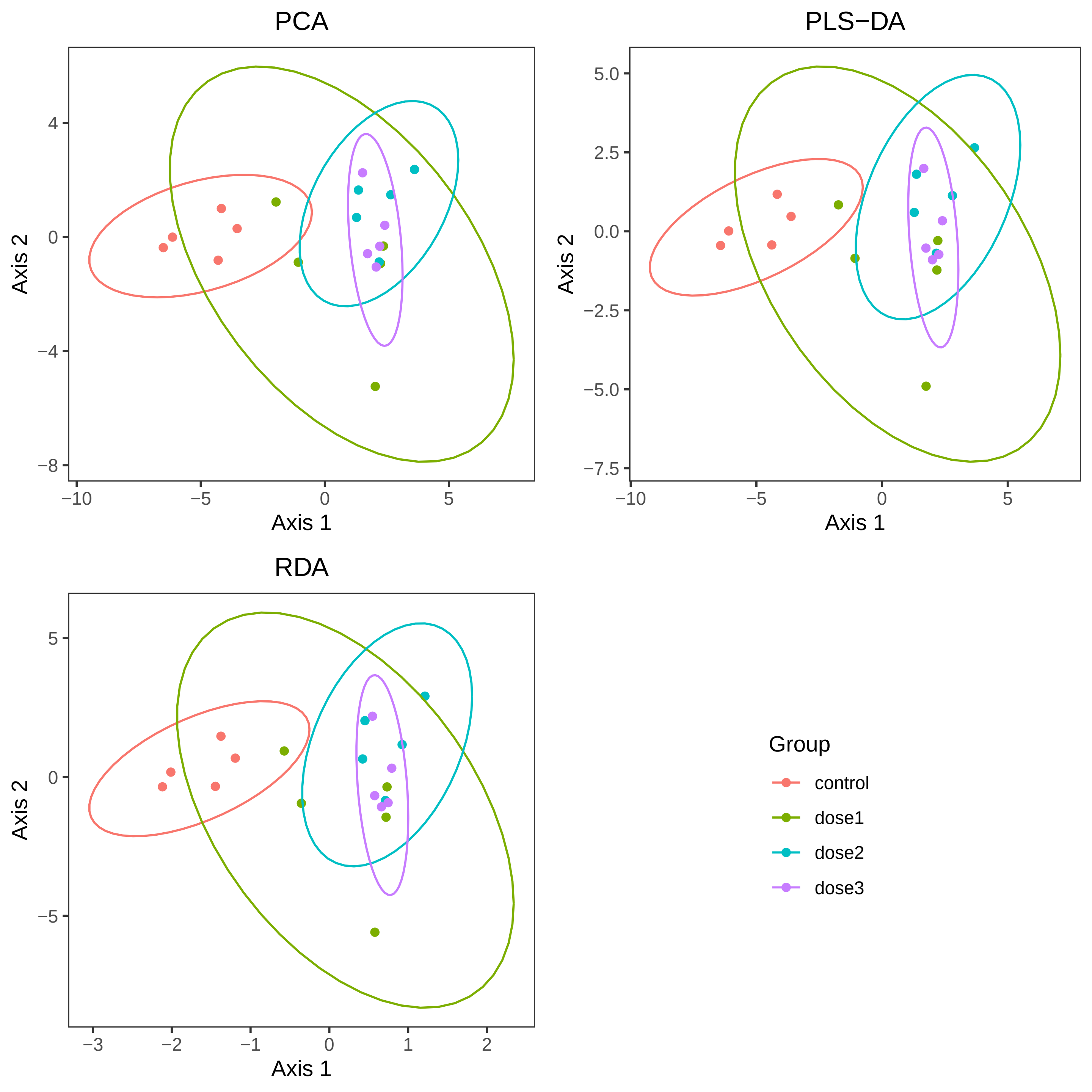
PCA
Axis 1
Axis 2
PLS−DA
Axis 1
Axis 2
RDA
Axis 1
Axis 2
Group
control
dose1
dose2
dose3

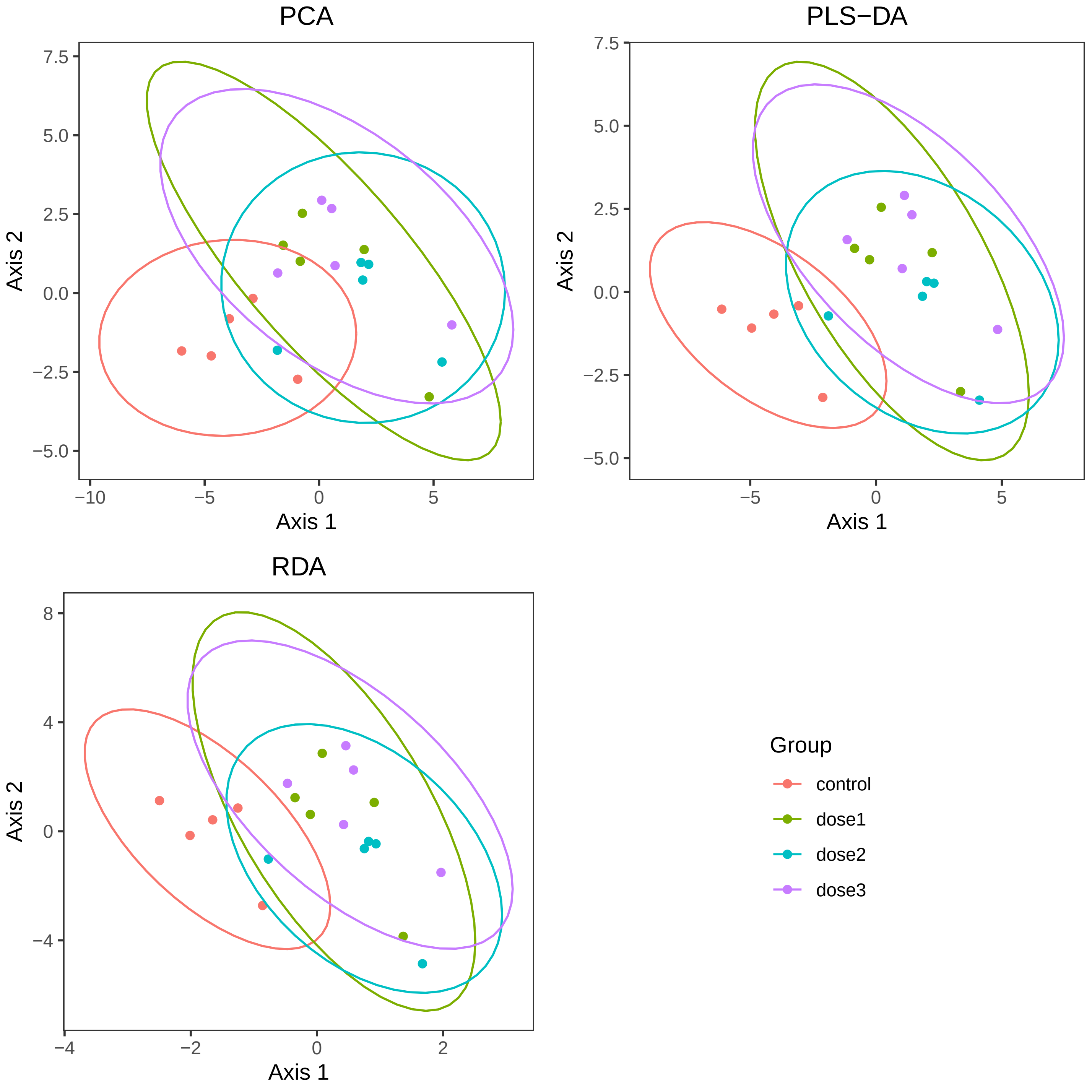
PCA
PLS−DA
RDA
Axis 1
Axis 2
Group
control
dose1
dose2
dose3

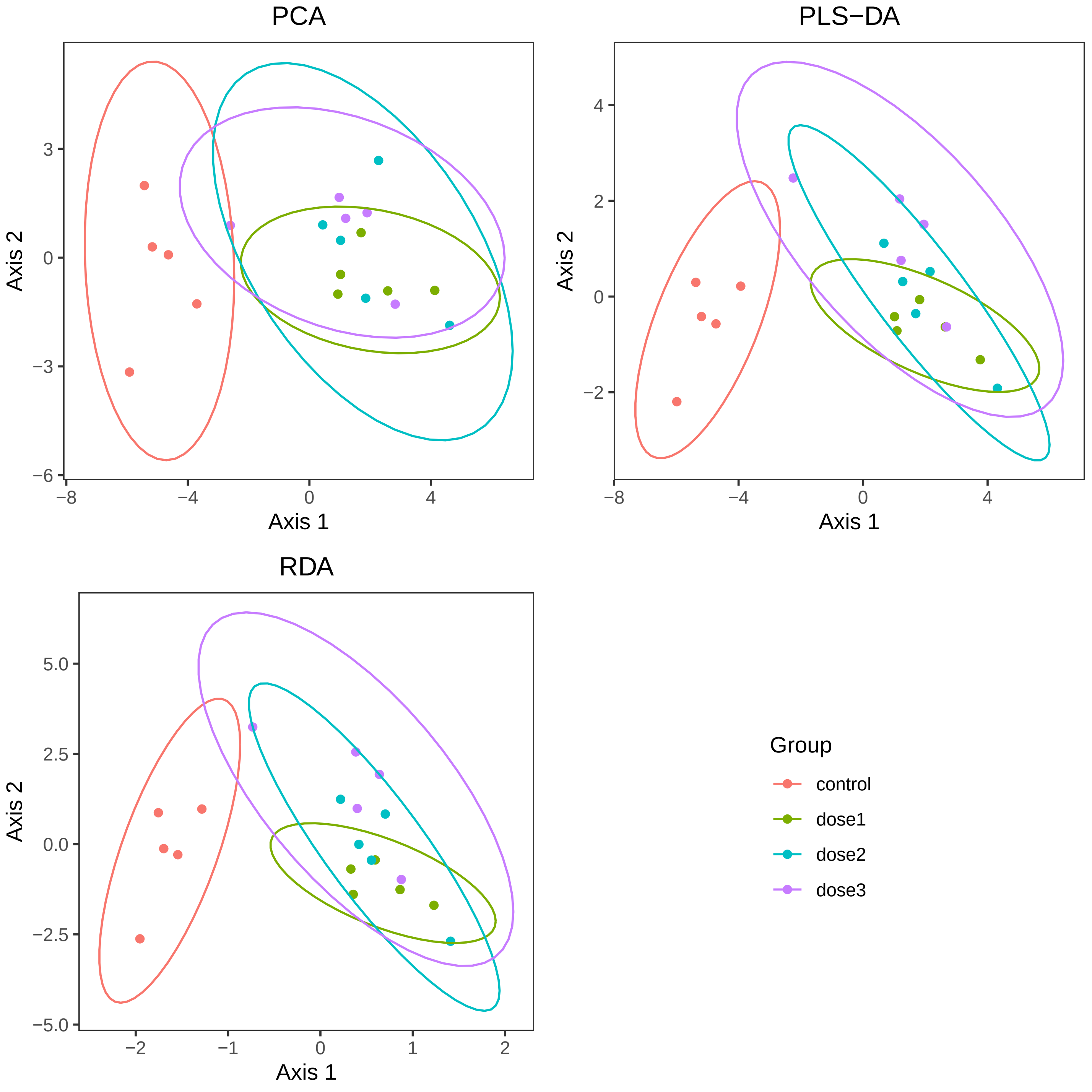

PCA
PLS−DA
RDA
Axis 1
Axis 2
−8
−4
0
4
3
0
−3
−6
2
−2
5.0
2.5
0.0
−2.5
−5.0
−1
1
Group
control
dose1
dose2
dose3

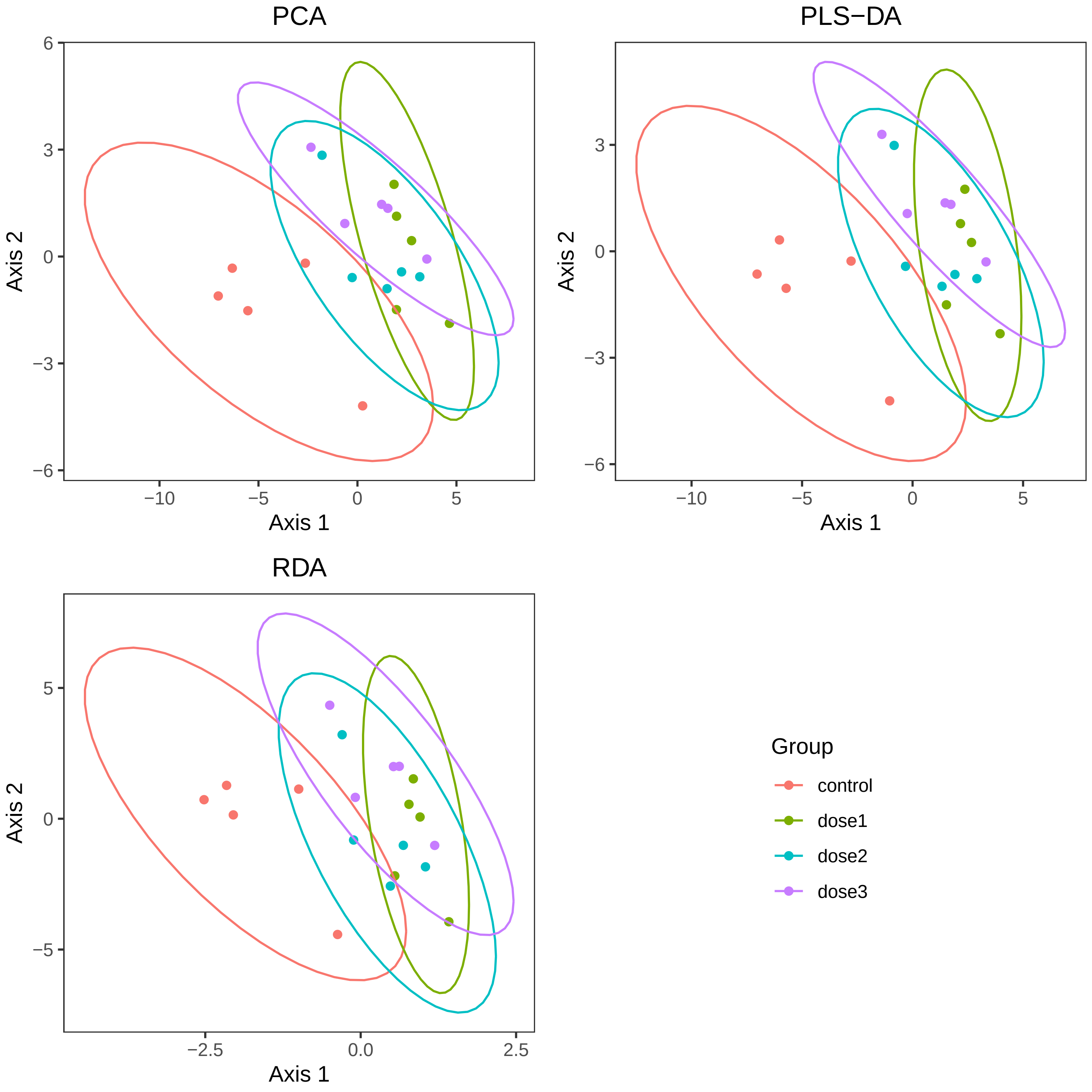
PCA
PLS−DA
RDA
Axis 1
Axis 2
6
3
0
−3
−6
−10
−5
0
5
−2.5
0.0
2.5
Group
control
dose1
dose2
dose3

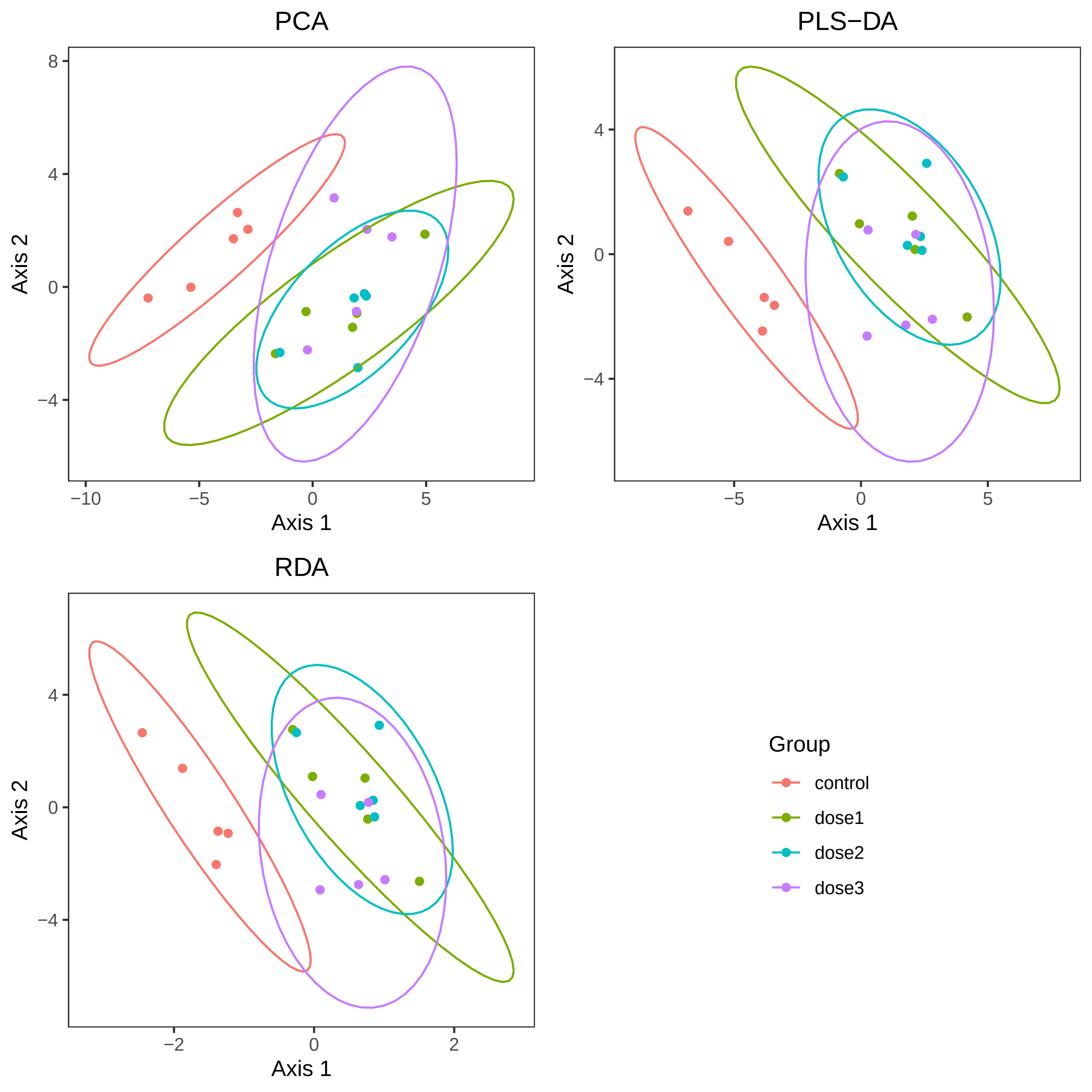

PCA
PLS−DA
RDA
Axis 1
Axis 2
−10
−5
0
5
8
4
−4
Group
control
dose1
dose2
dose3

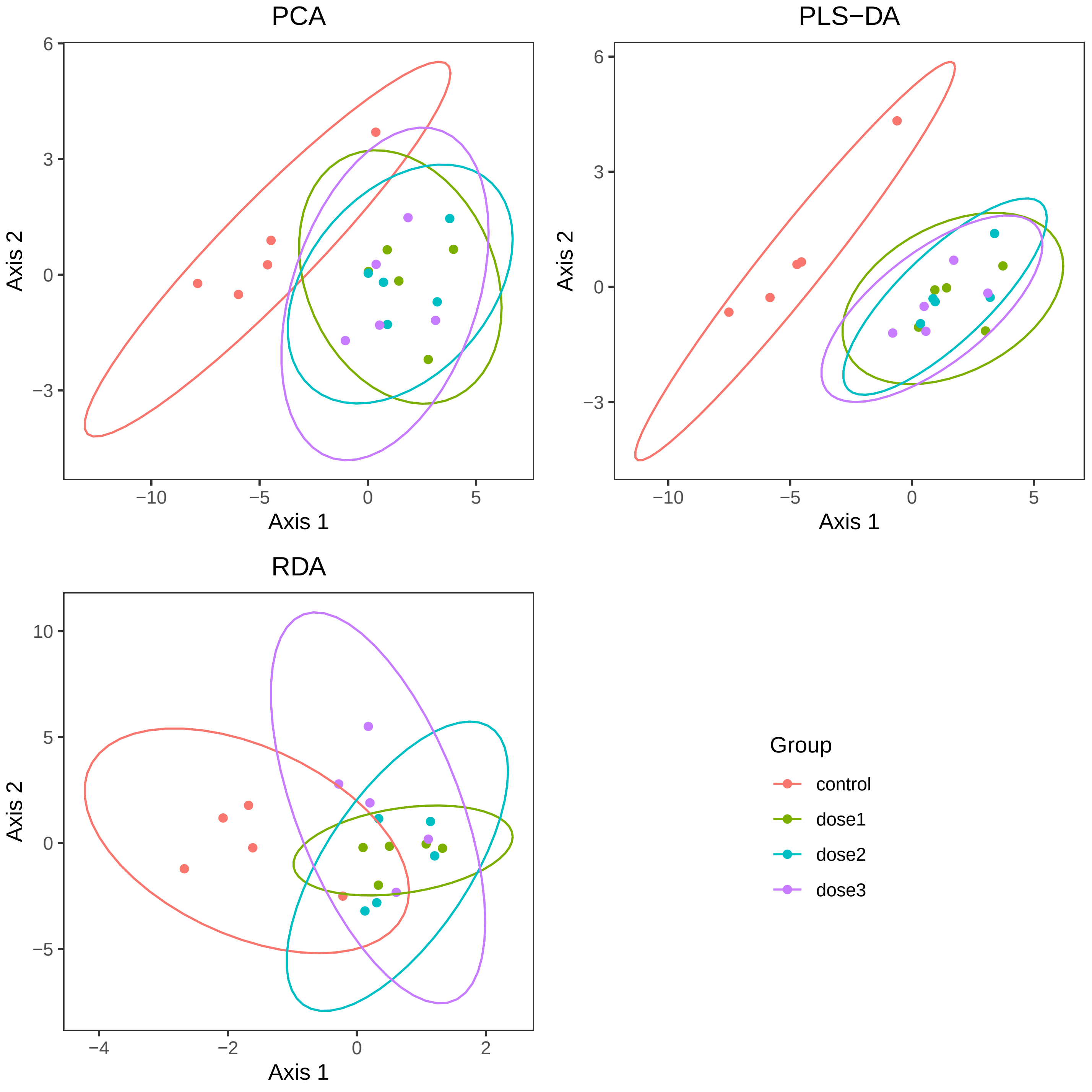

PCA
PLS−DA
RDA
Axis 1
Axis 2
6
3
0
−3
−10
−5
0
5
10
5
0
−5
−4
−2
0
2
Group
control
dose1
dose2
dose3

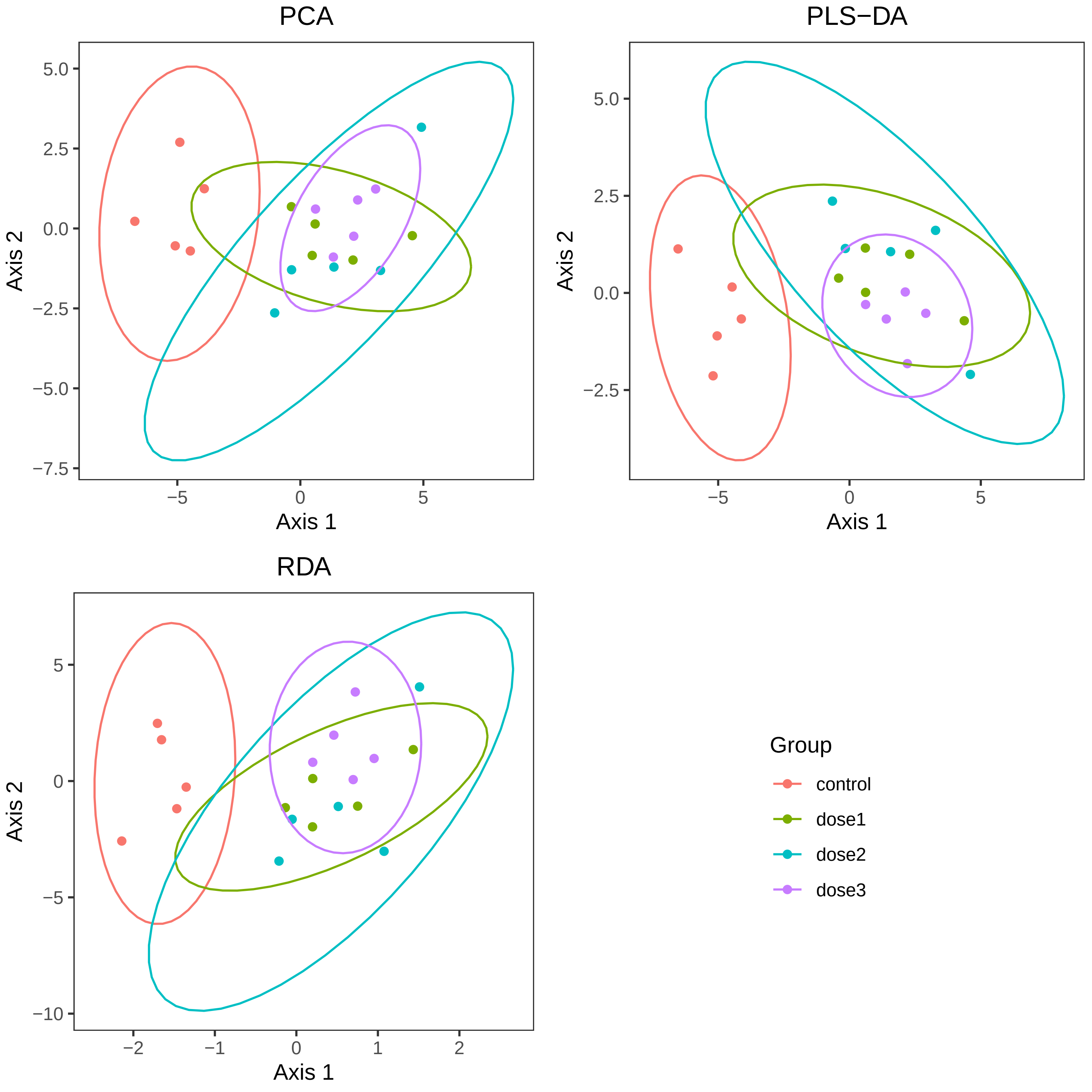

PCA
PLS−DA
RDA
Axis 1
Axis 2
5.0
2.5
0.0
−2.5
−5.0
−7.5
−5
0
5
−2
−1
1
2
−10
Group
control
dose1
dose2
dose3

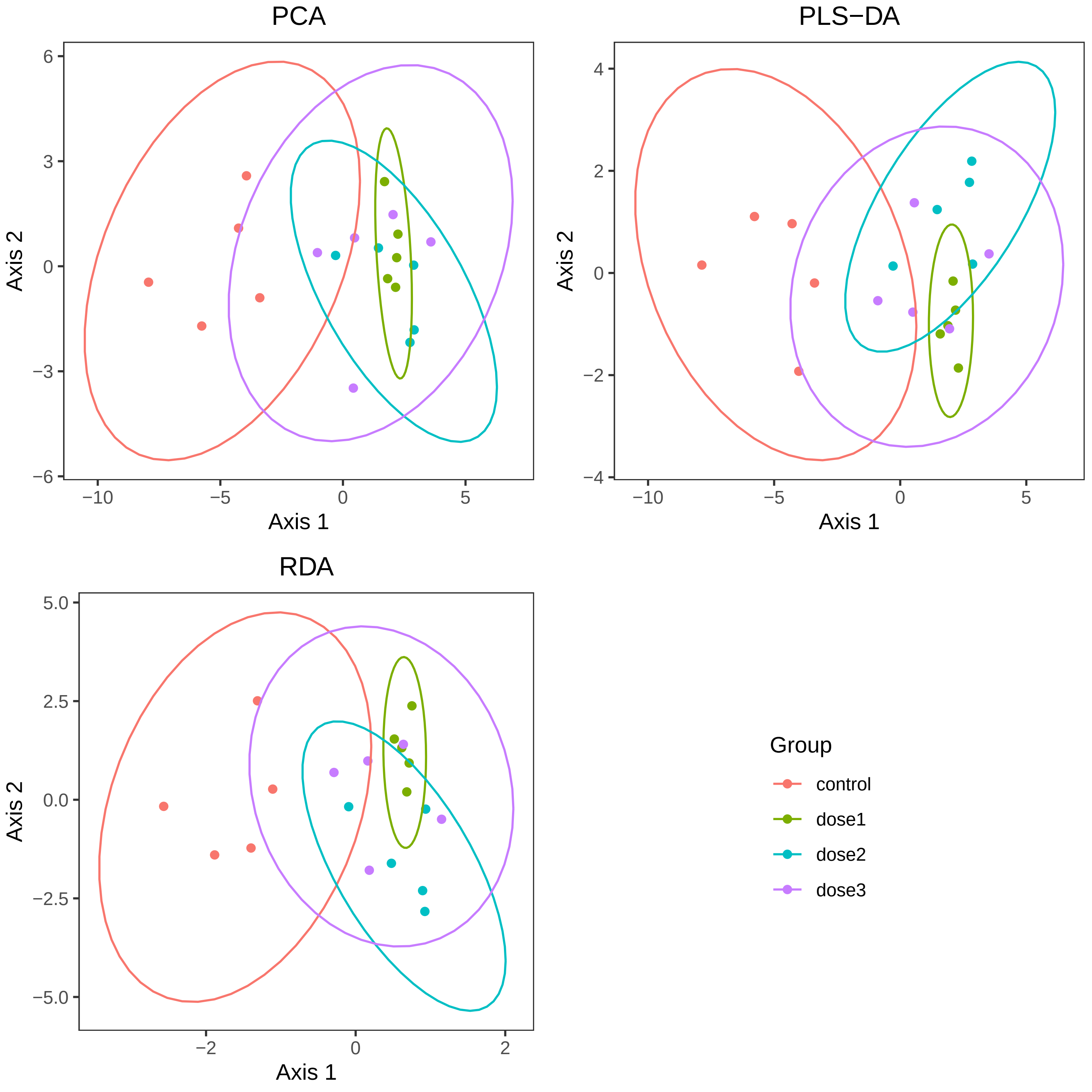
PCA
PLS−DA
RDA
Axis 1
Axis 2
Group
control
dose1
dose2
dose3

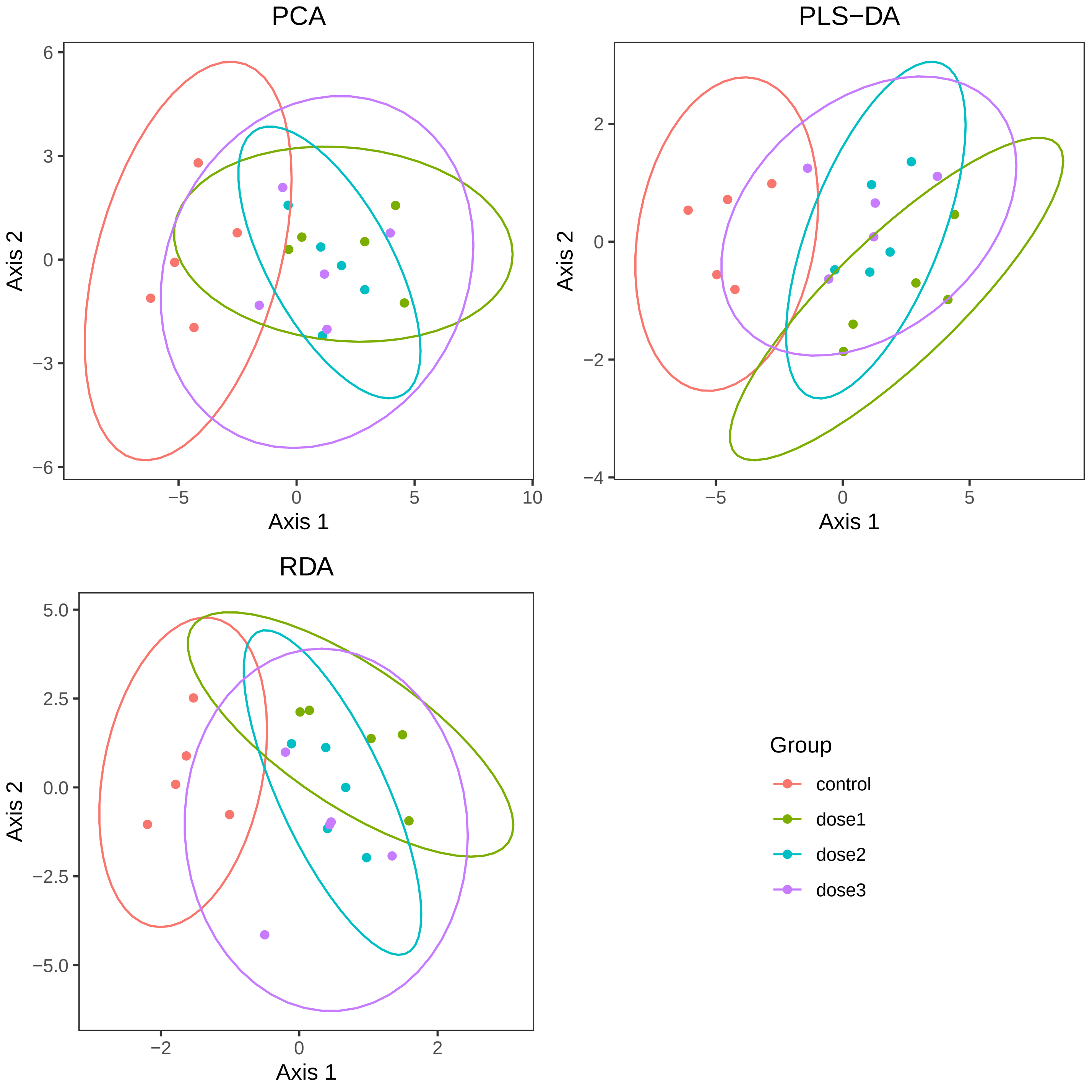
PCA
PLS−DA
RDA
Axis 1
Axis 2
Group
control
dose1
dose2
dose3

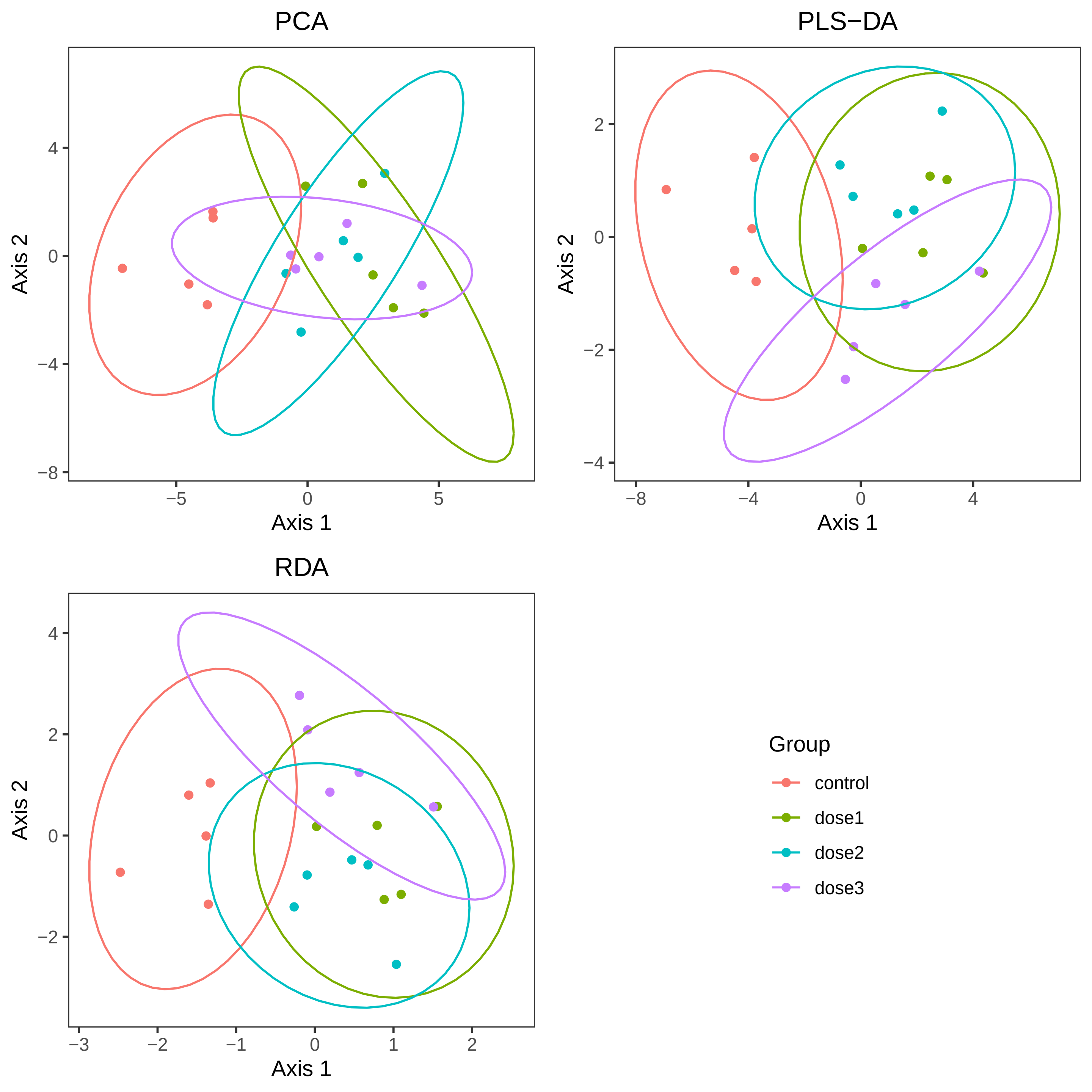

PCA
PLS−DA
RDA
Axis 1
Axis 2
Group
control
dose1
dose2
dose3

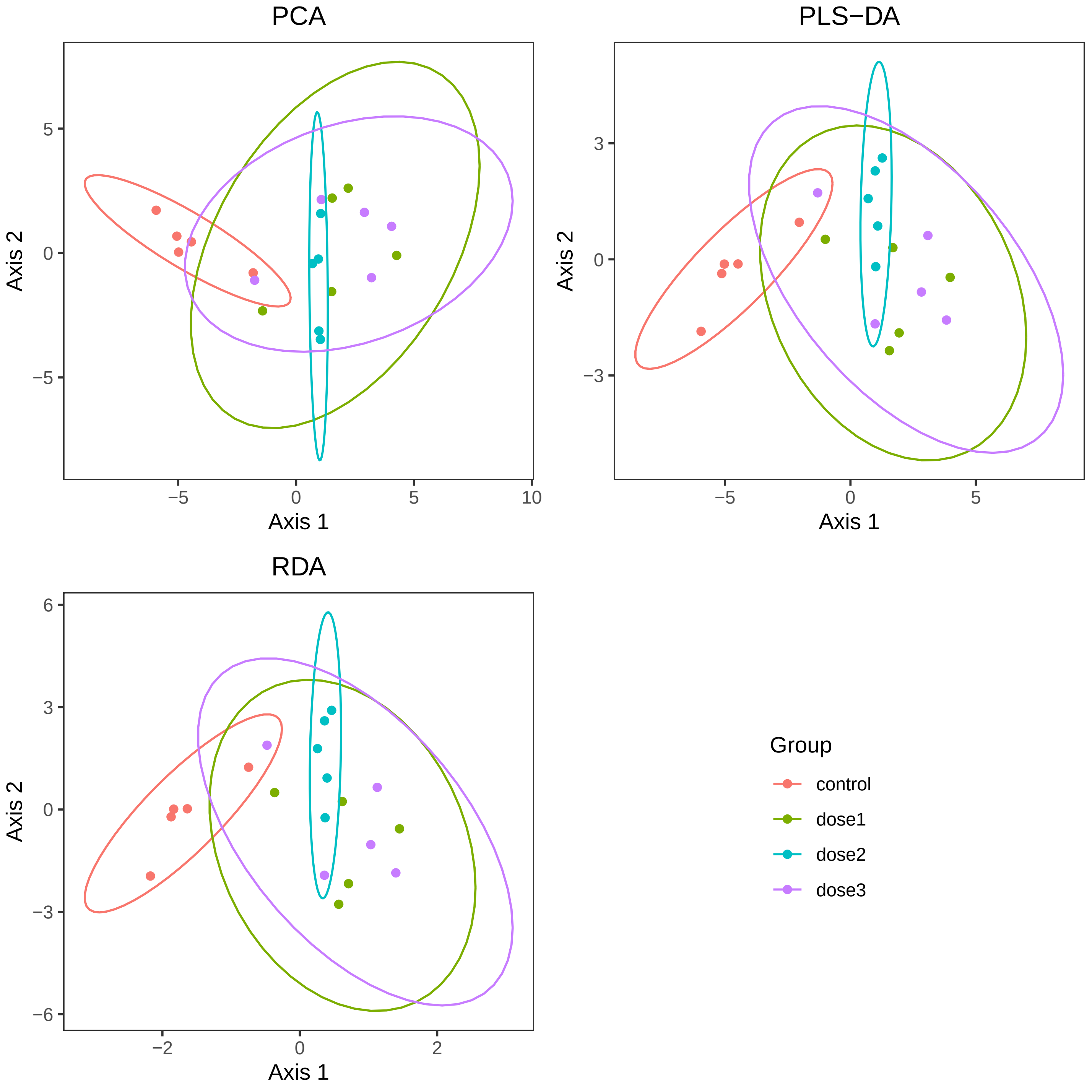
PCA
PLS−DA
RDA
Axis 1
Axis 2
Group
control
dose1
dose2
dose3

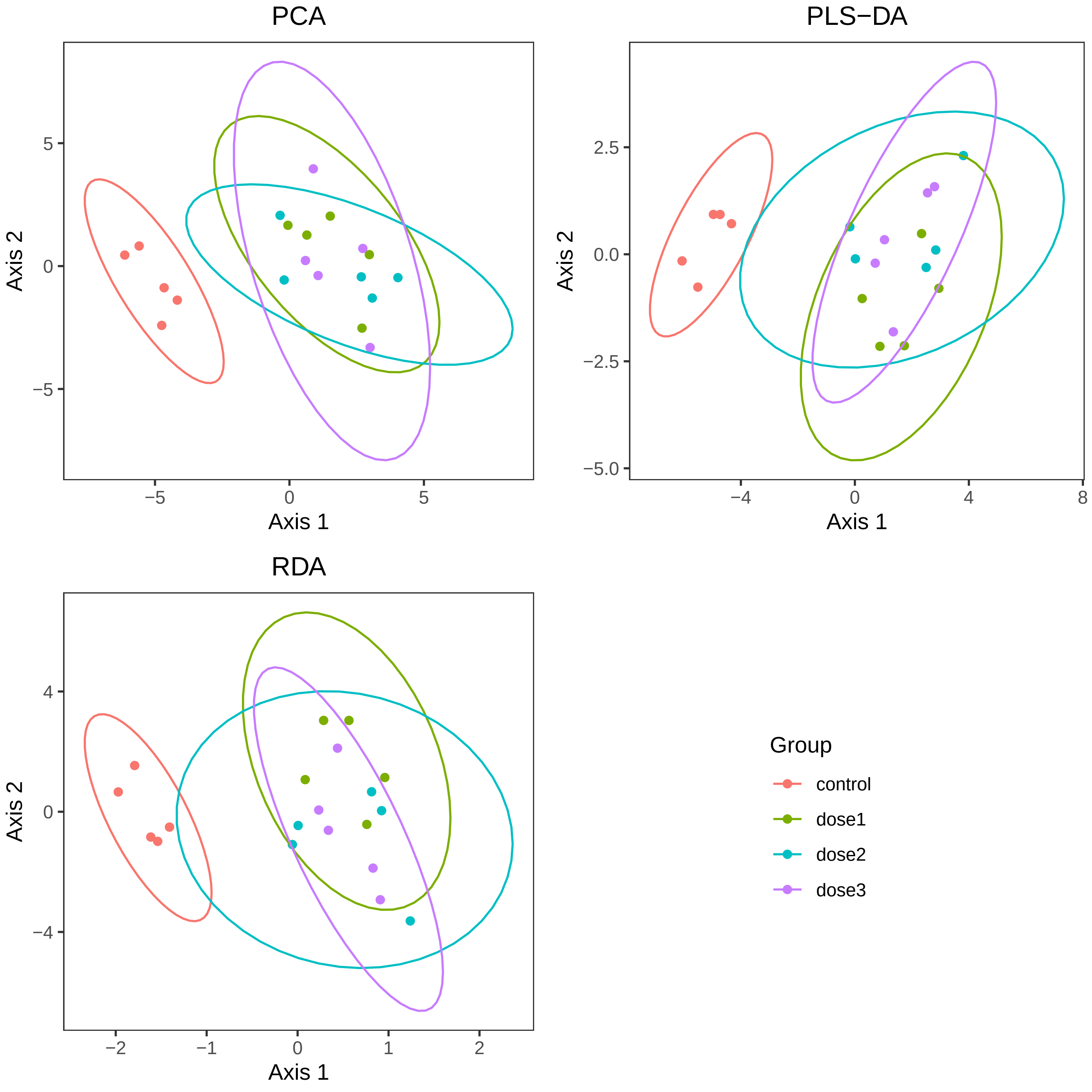

PCA
PLS−DA
RDA
Axis 1
Axis 2
−5
0
5
−4
4
8
2.5
0.0
−2.5
−5.0
−2
−1
1
2
Group
control
dose1
dose2
dose3

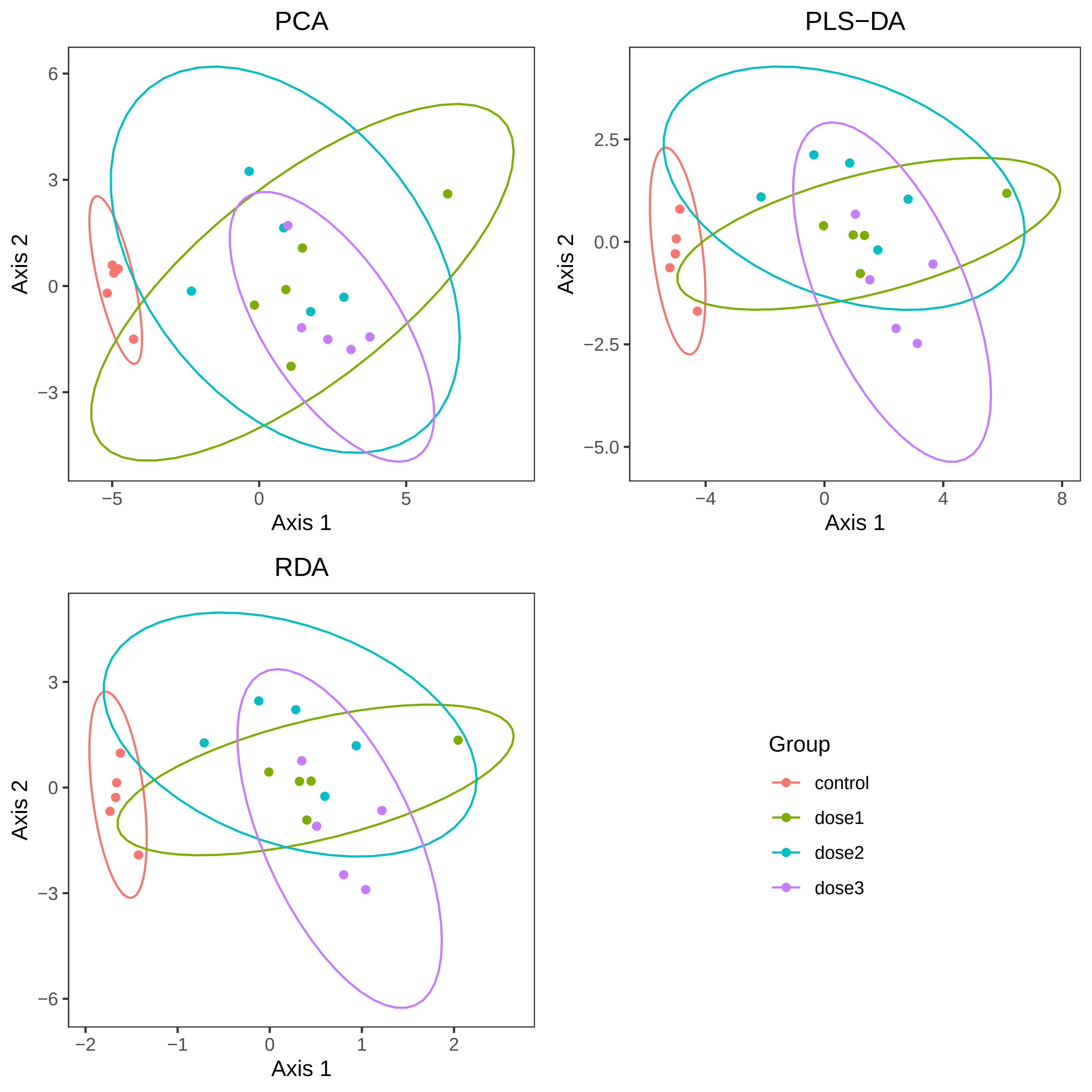
PCA
PLS−DA
RDA
Axis 1
Axis 2
6
3
0
−3
−5
5
2.5
0.0
−2.5
−5.0
−4
4
8
−6
−2
−1
1
2
Group
control
dose1
dose2
dose3

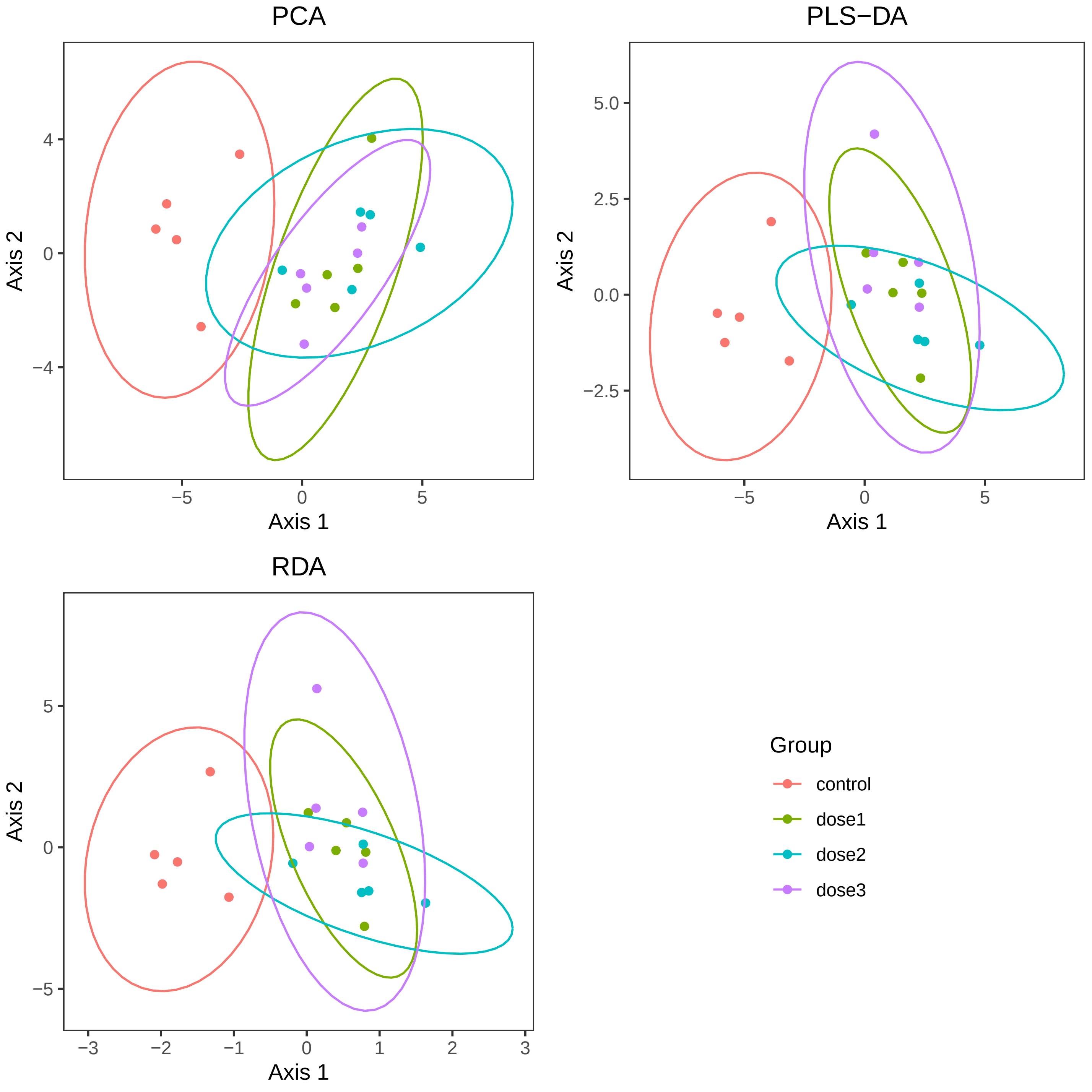
PCA
PLS−DA
RDA
Axis 1
Axis 2
Group
control
dose1
dose2
dose3

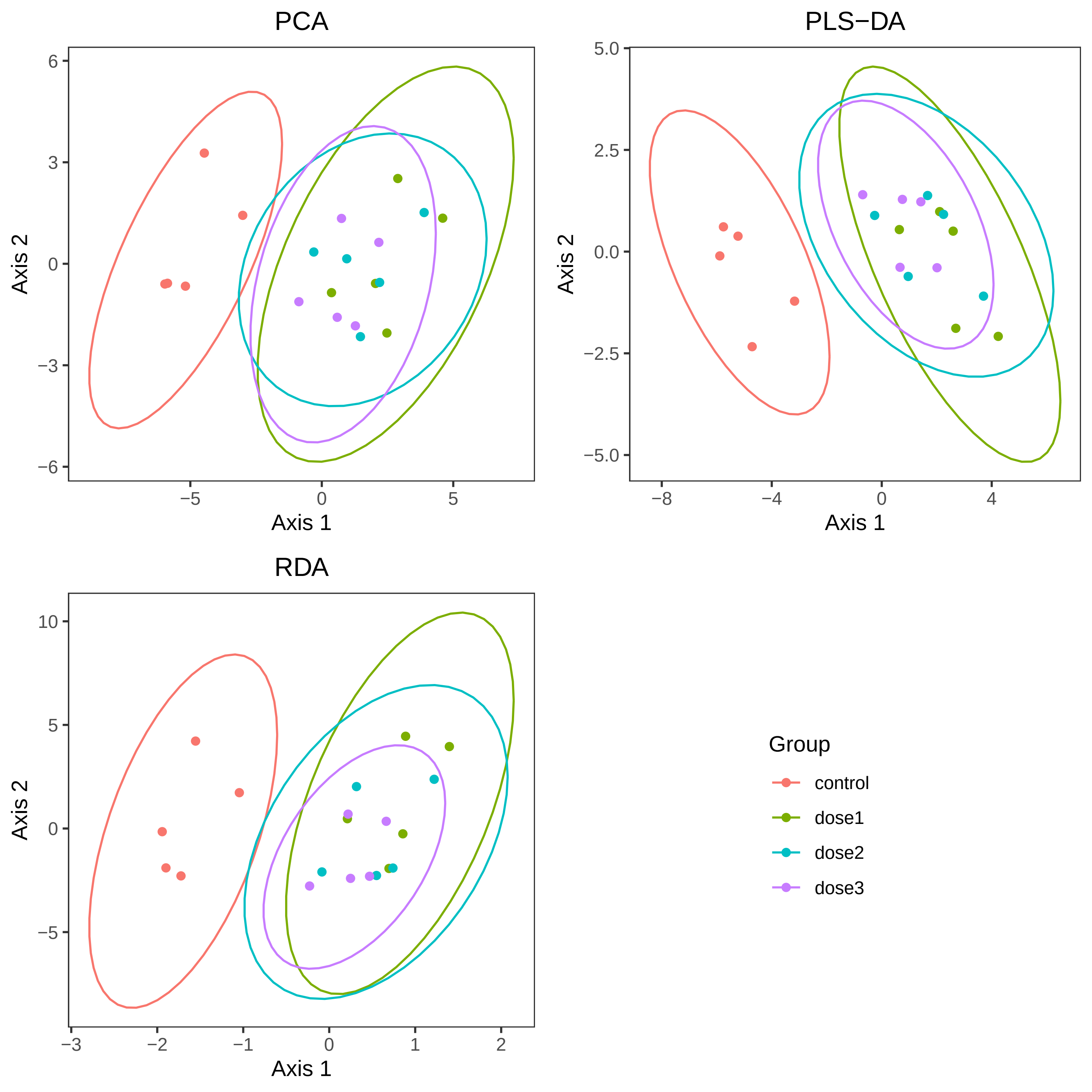

PCA
PLS−DA
RDA
Axis 1
Axis 2
Group
control
dose1
dose2
dose3

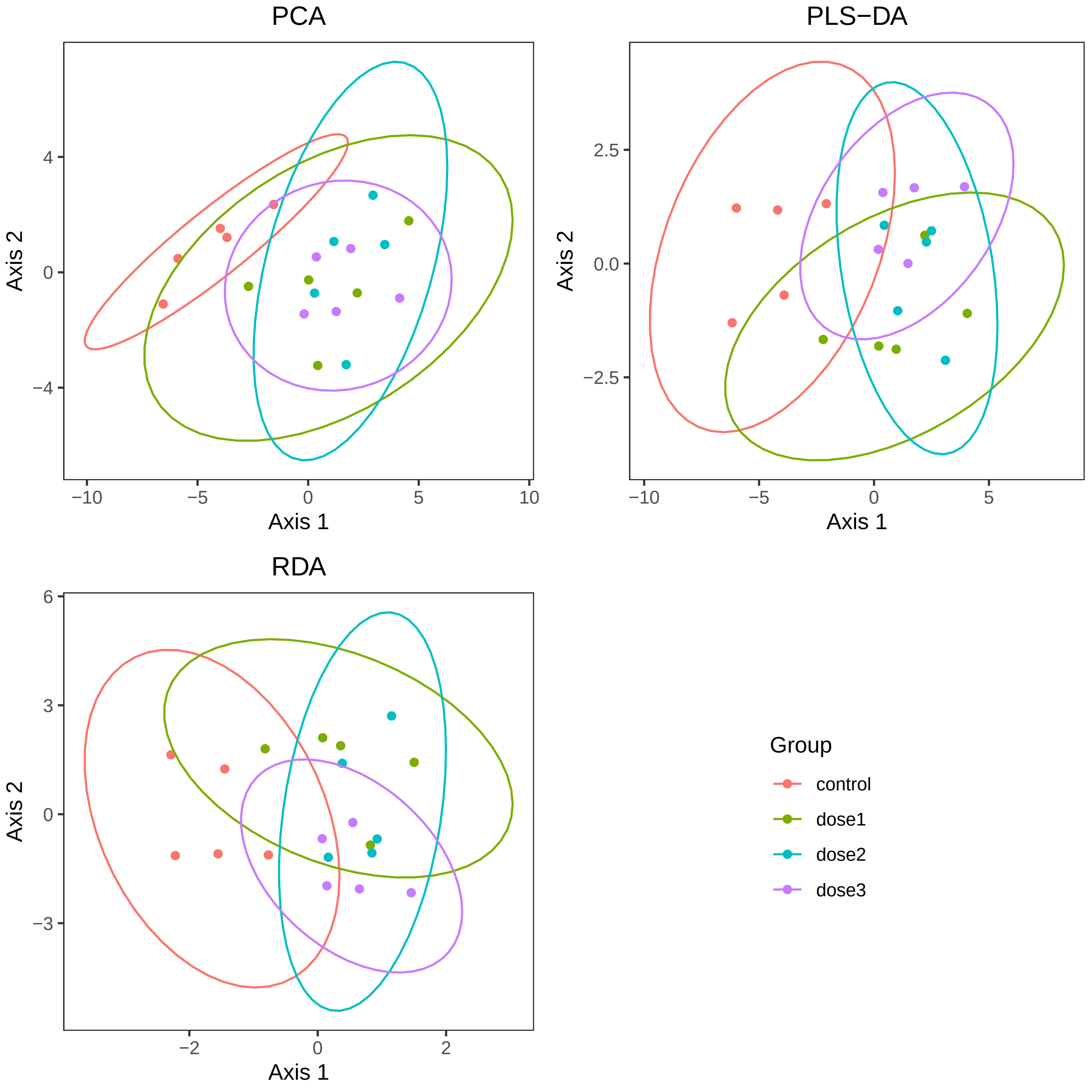
PCA
PLS−DA
RDA
Axis 1
Axis 2
Group
control
dose1
dose2
dose3

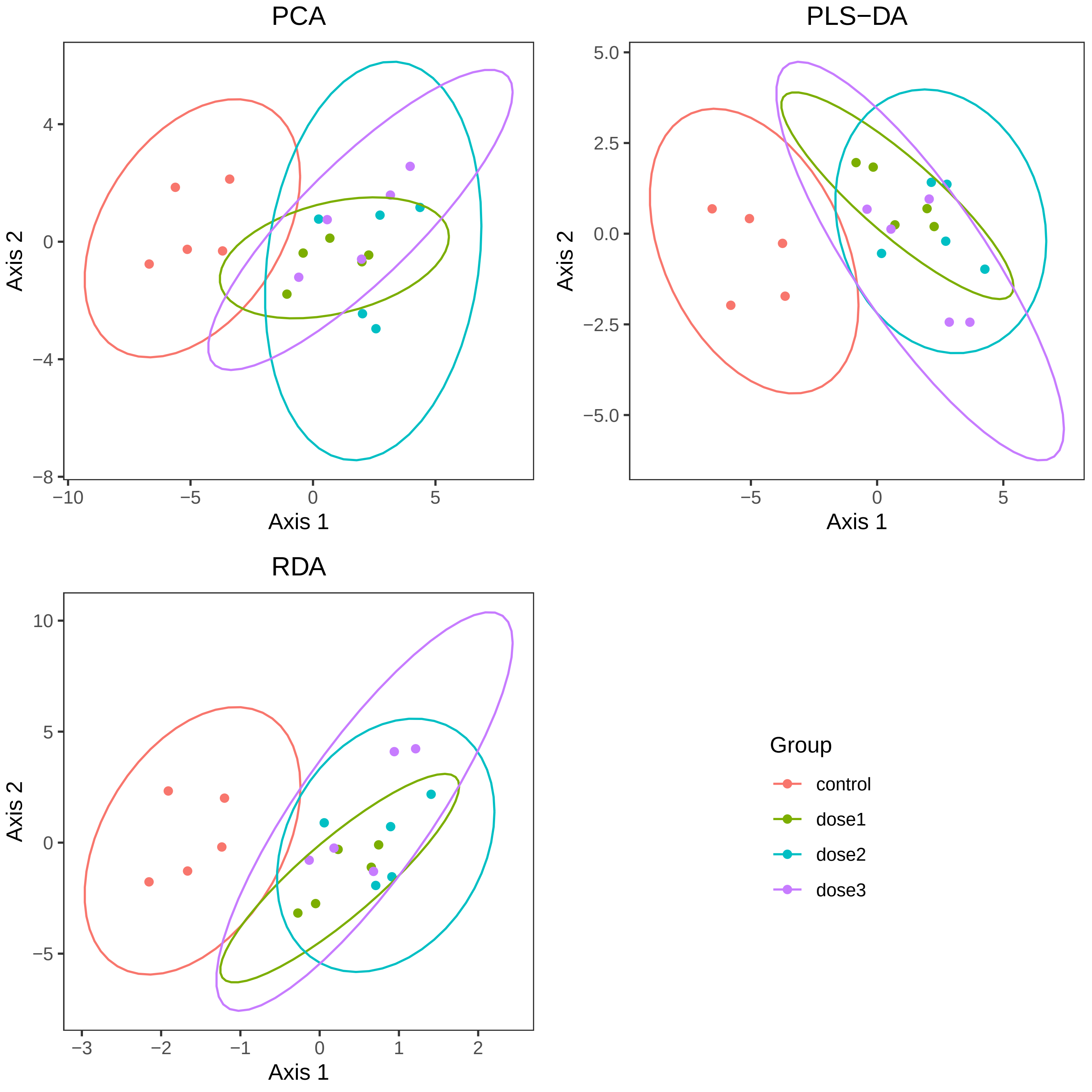
PCA
PLS−DA
RDA
Axis 1
Axis 2
Group
control
dose1
dose2
dose3

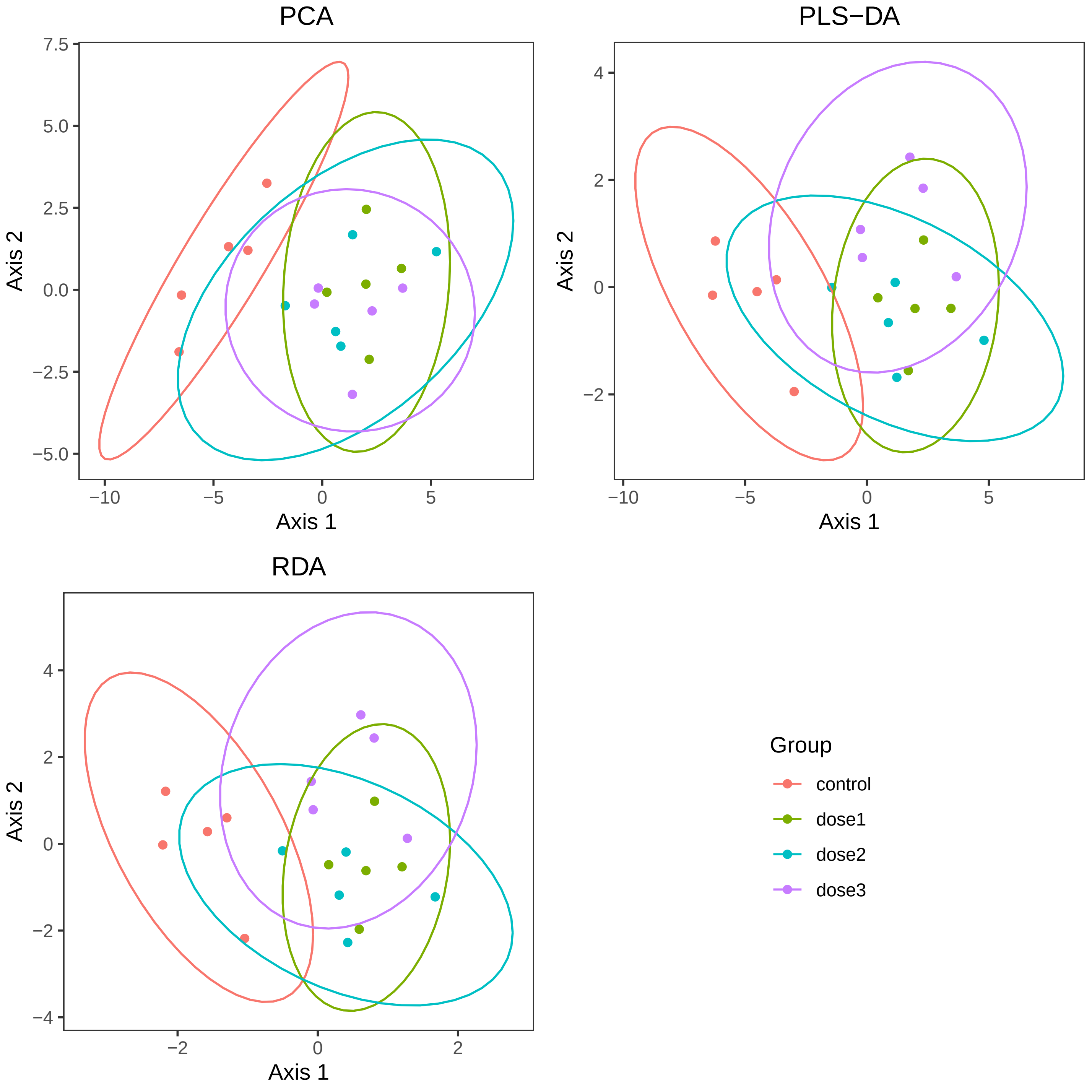

PCA
PLS−DA
RDA
Axis 1
Axis 2
Group
control
dose1
dose2
dose3

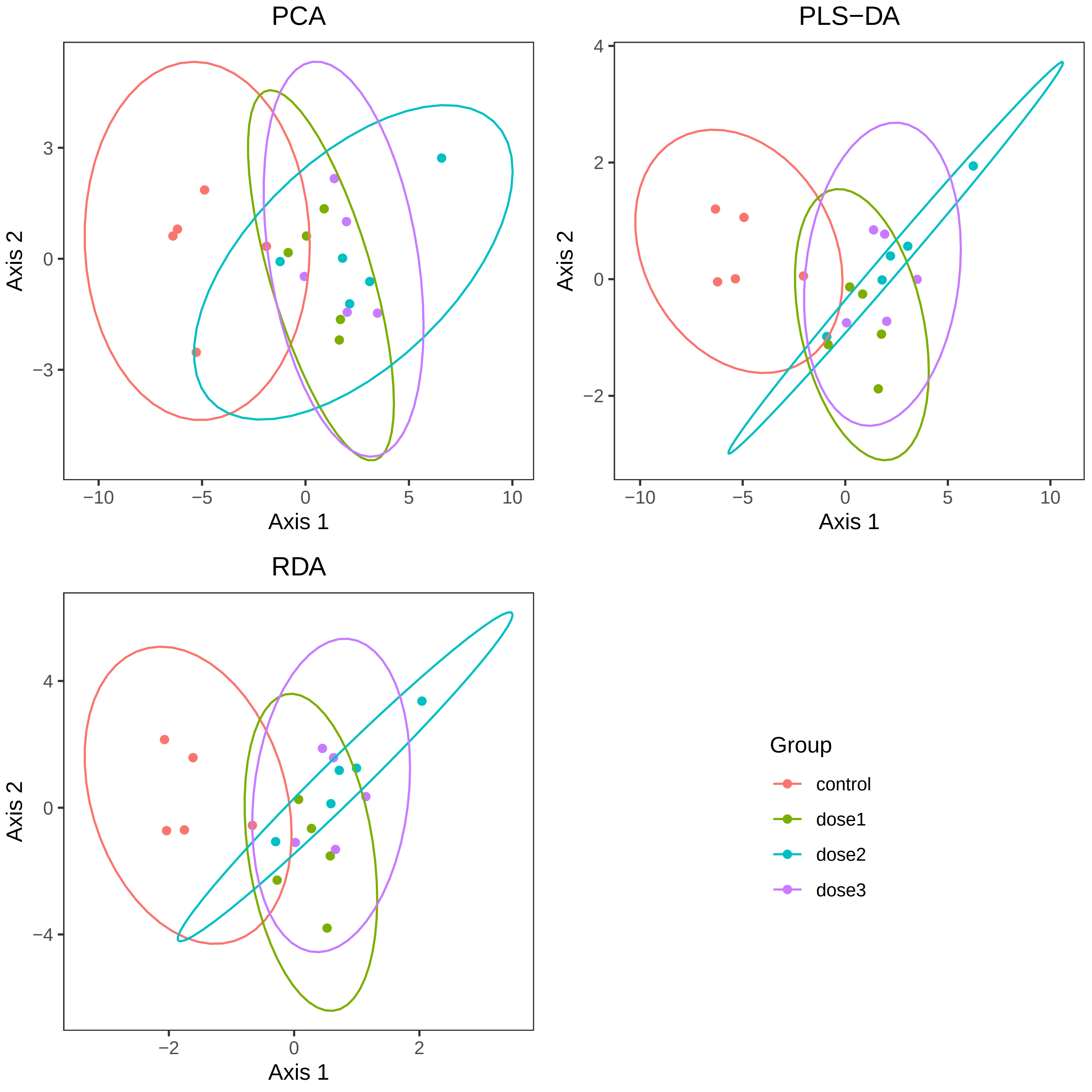
PCA
PLS−DA
RDA
Axis 1
Axis 2
−10
−5
0
5
10
3
0
−3
4
2
−2
−4
Group
control
dose1
dose2
dose3

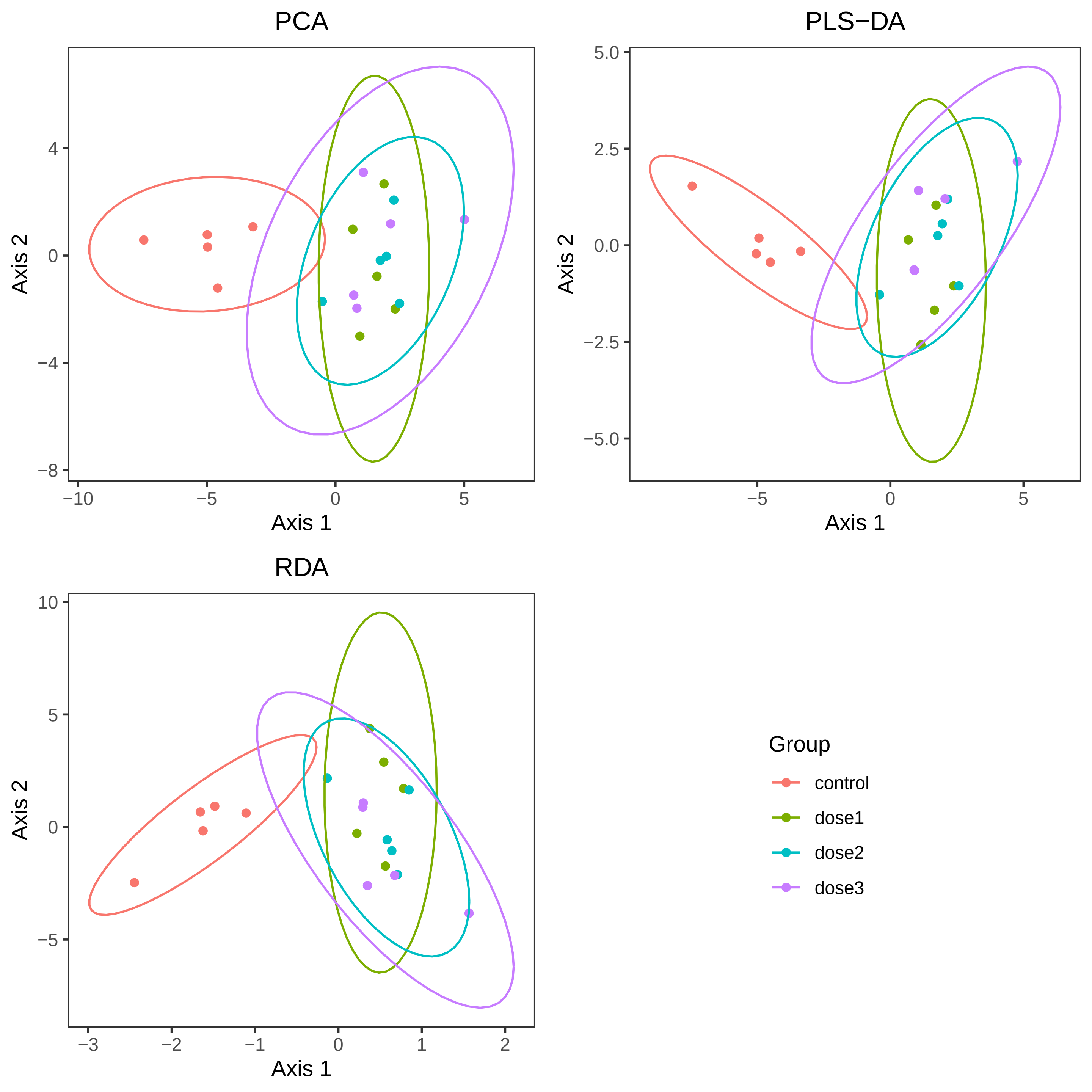
PCA
PLS−DA
RDA
Axis 1
Axis 2
Group
control
dose1
dose2
dose3

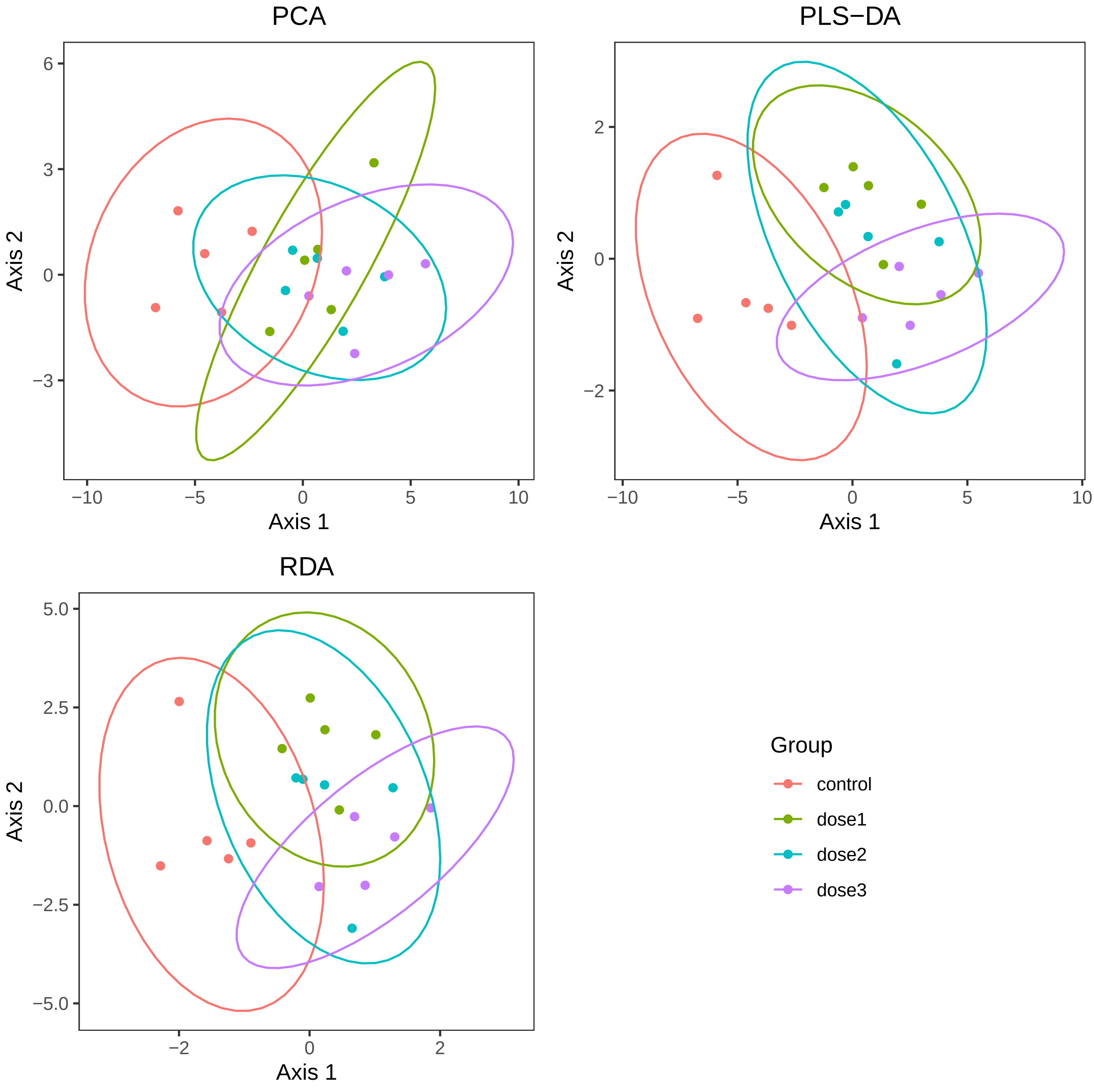
PCA
PLS−DA
RDA
Axis 1
Axis 2
−10
−5
0
5
10
6
3
0
−3
2
−2
5.0
2.5
0.0
−2.5
−5.0
Group
control
dose1
dose2
dose3

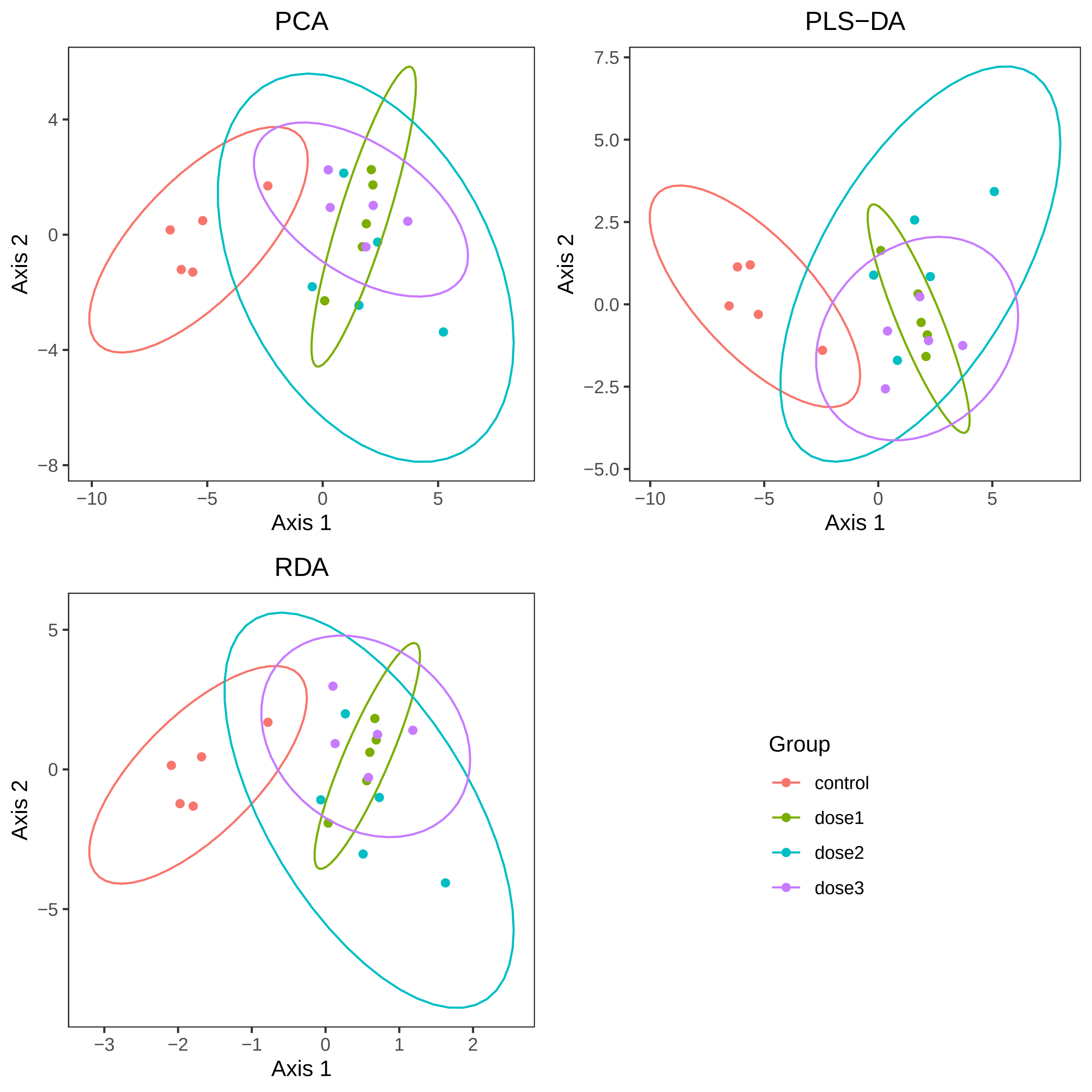

PCA
PLS−DA
RDA
Axis 1
Axis 2
Group
control
dose1
dose2
dose3

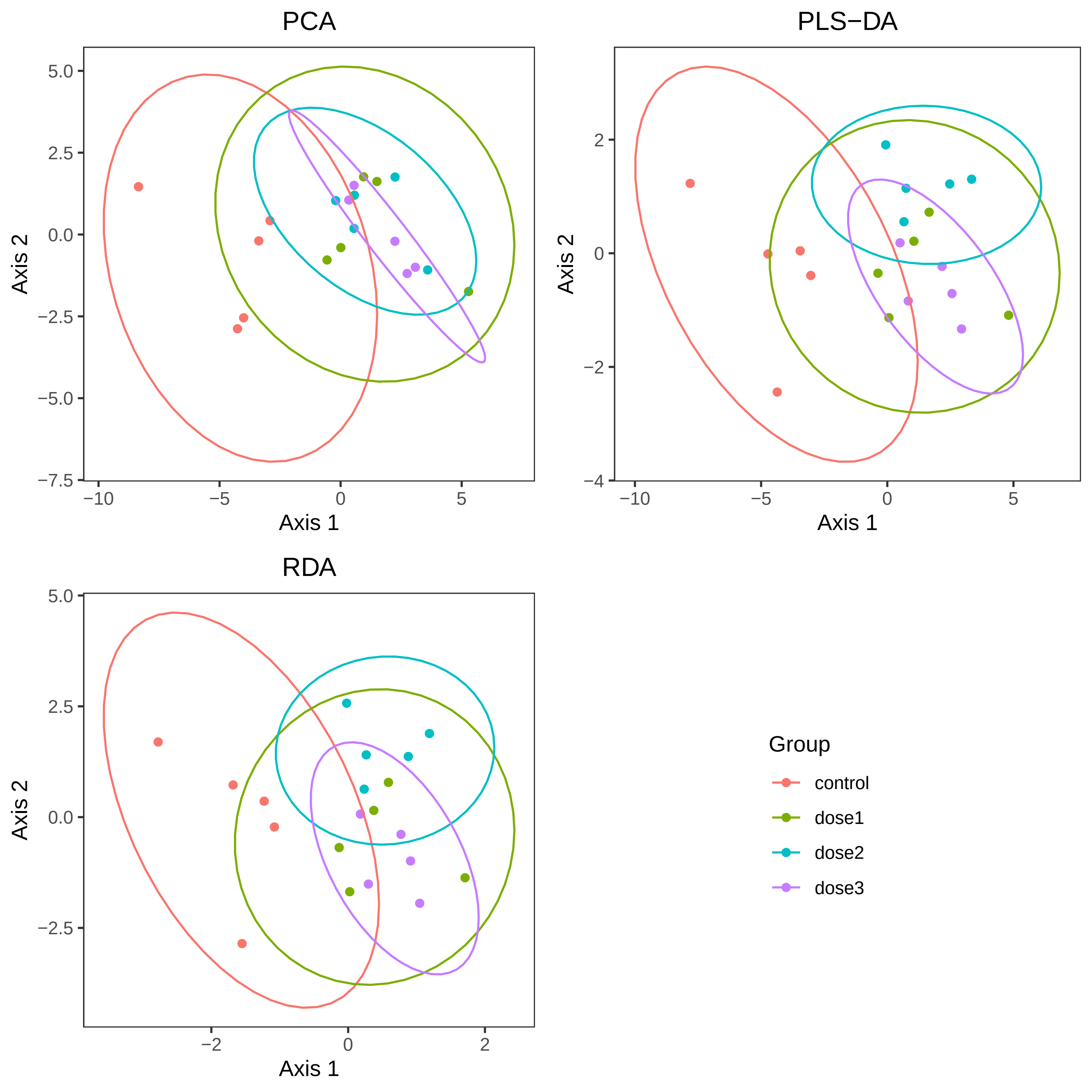

PCA
PLS−DA
RDA
Axis 1
Axis 2
Group
control
dose1
dose2
dose3

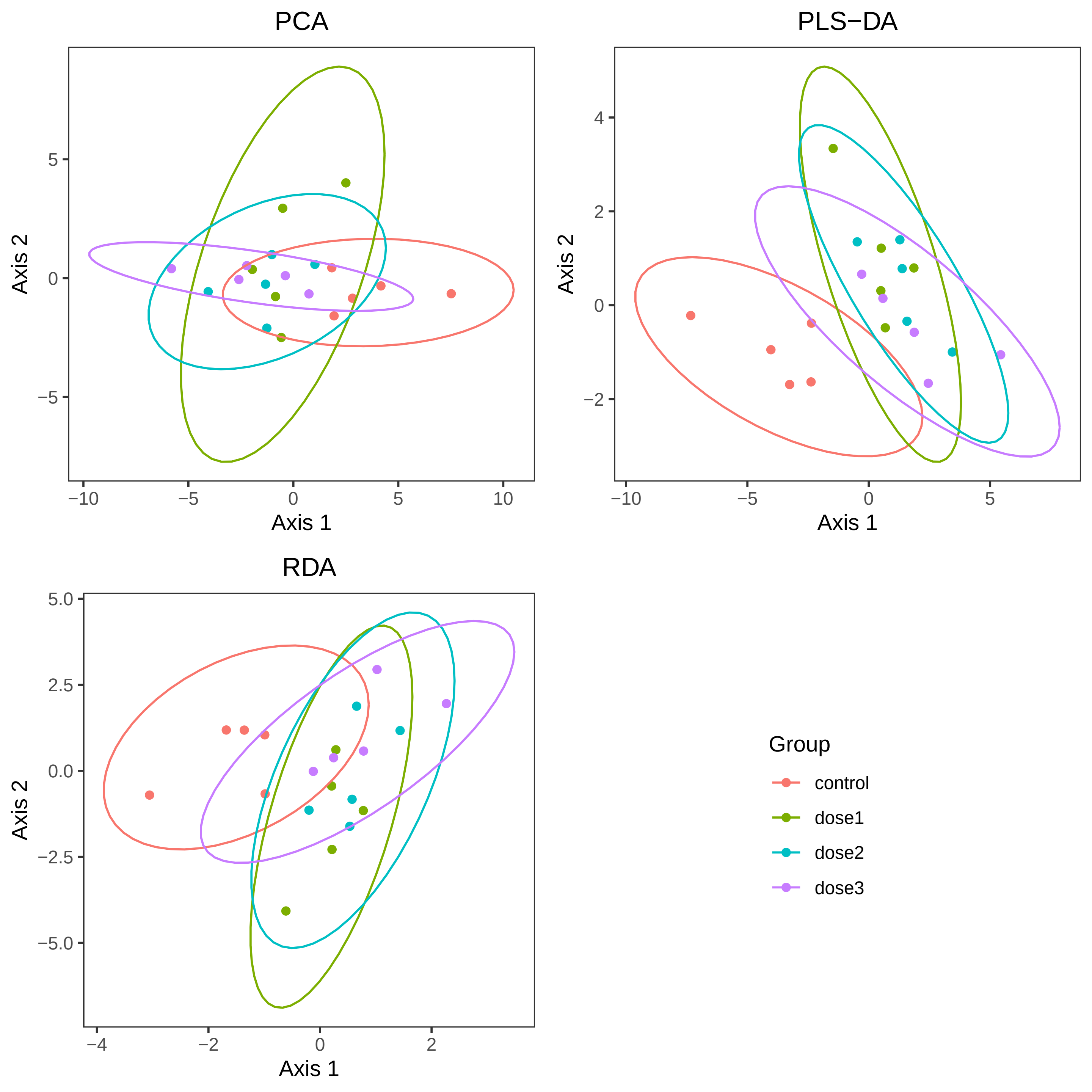
PCA
PLS−DA
RDA
Axis 1
Axis 2
Group
control
dose1
dose2
dose3

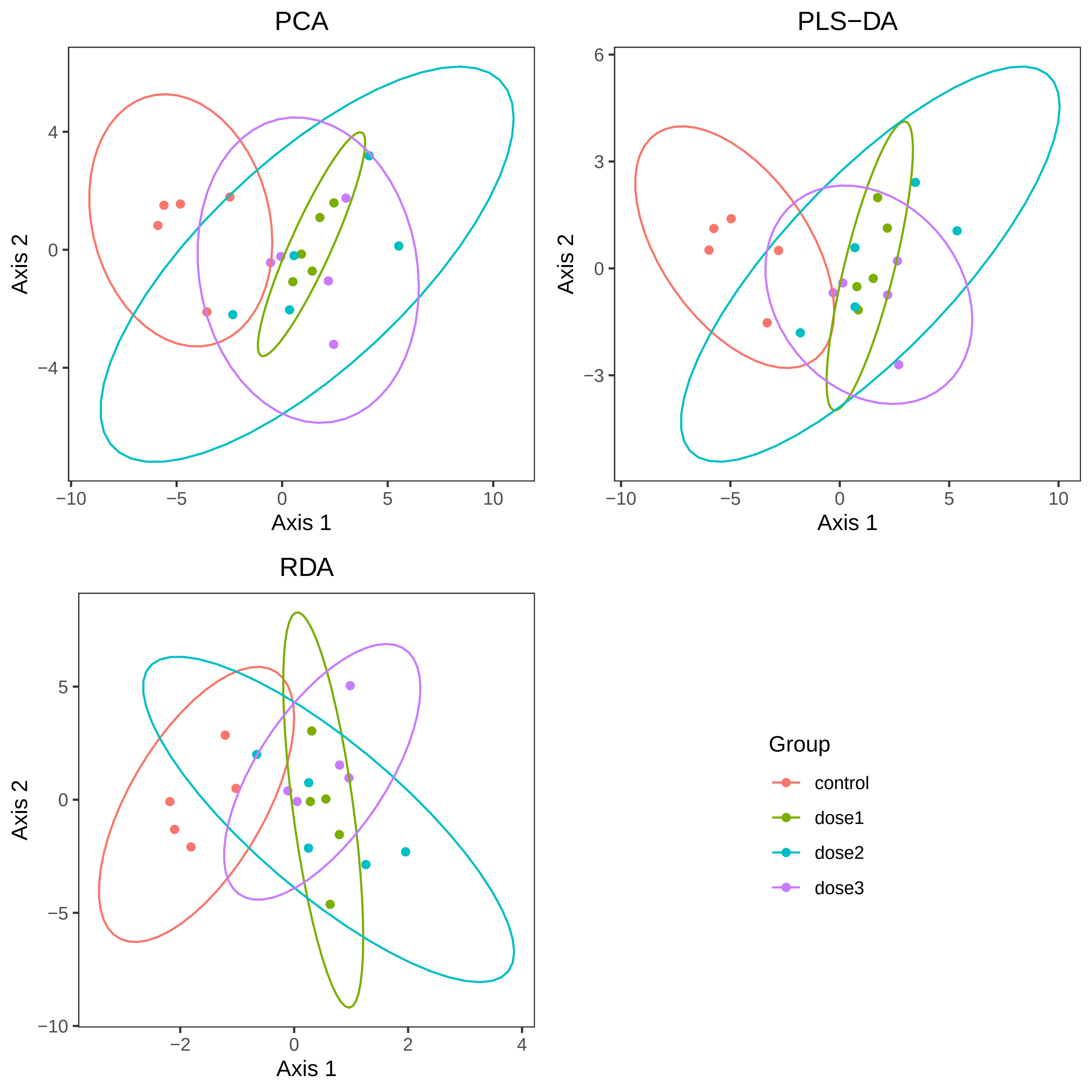
PCA
PLS−DA
RDA
Axis 1
Axis 2
Group
control
dose1
dose2
dose3

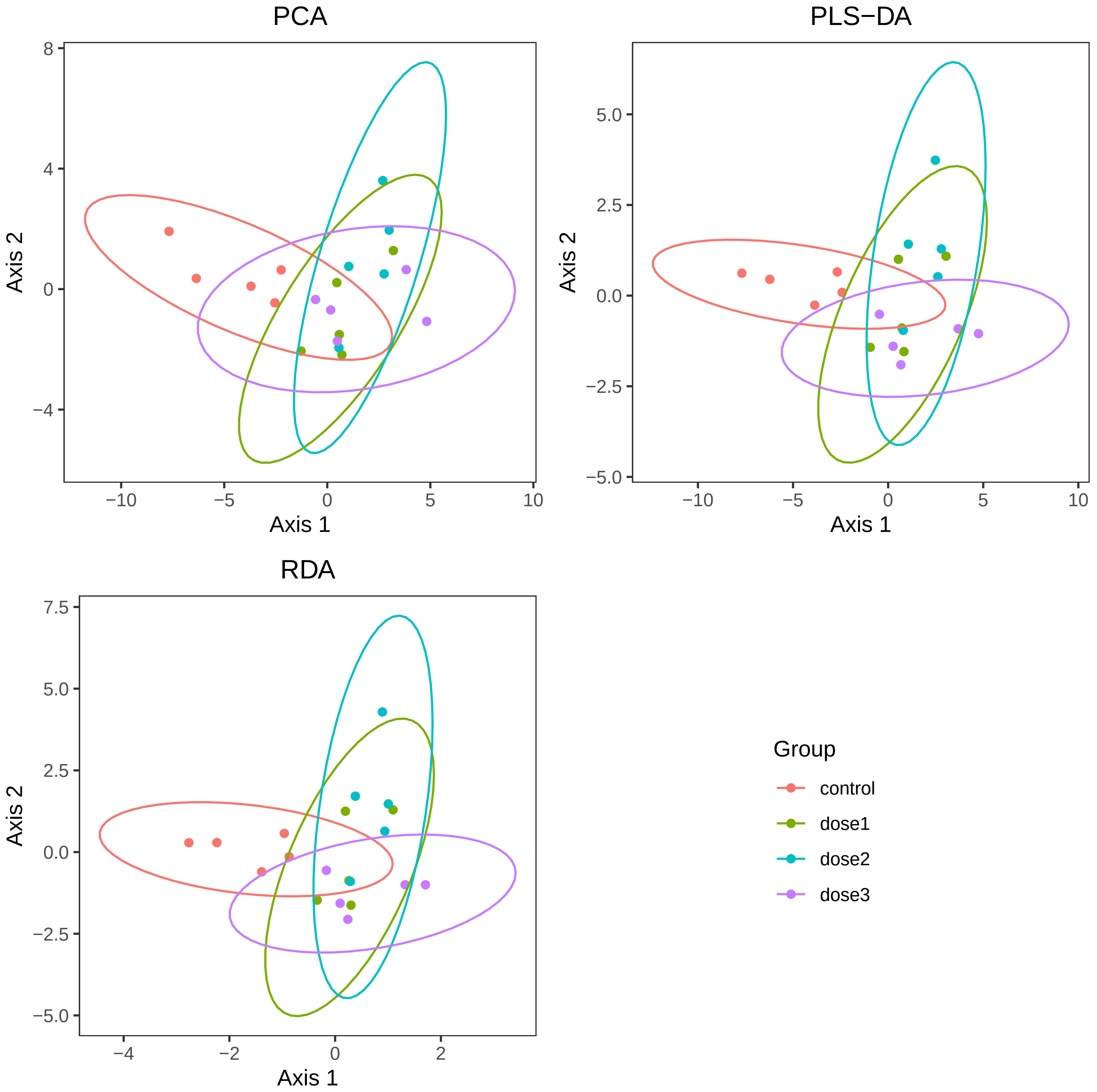
PCA
PLS−DA
RDA
Axis 1
Axis 2
Group
control
dose1
dose2
dose3

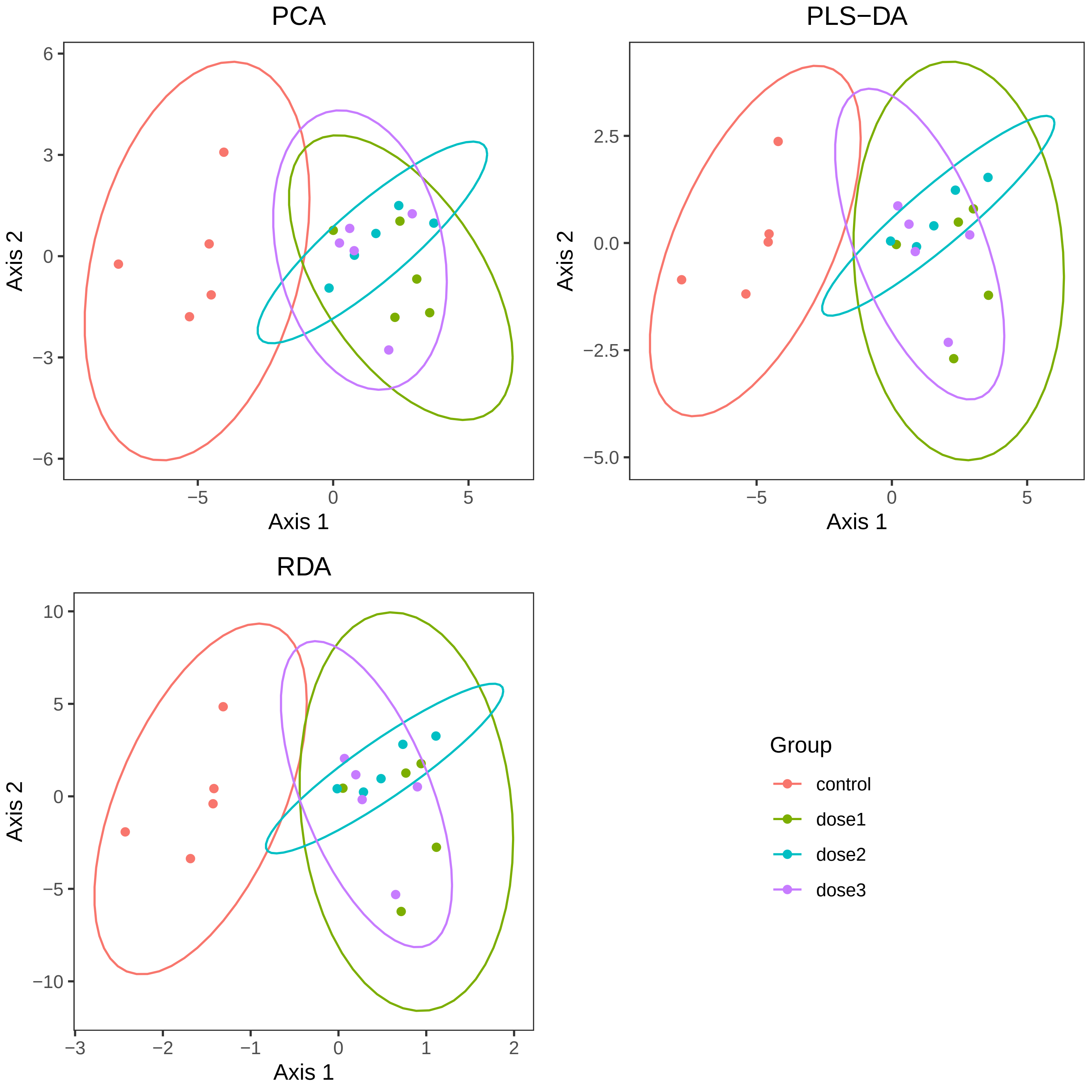
PCA
PLS−DA
RDA
Axis 1
Axis 2
6
3
0
−3
−6
−5
0
5
2.5
0.0
−2.5
−5.0
10
−10
−3
−2
−1
1
2
Group
control
dose1
dose2
dose3

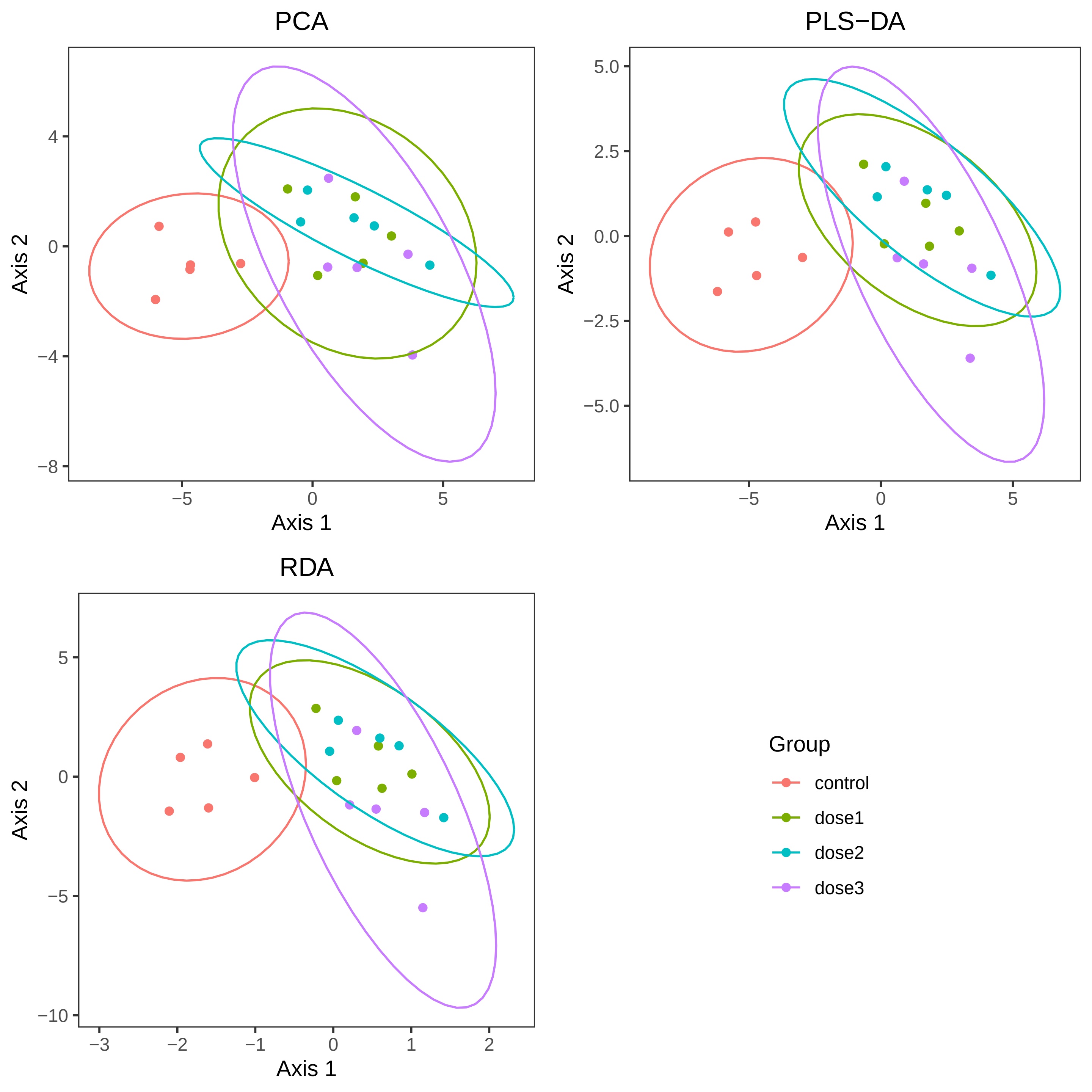

PCA
PLS−DA
RDA
Axis 1
Axis 2
4
0
−4
−8
−5
0
5
5.0
2.5
0.0
−2.5
−5.0
−10
−3
−2
−1
1
2
Group
control
dose1
dose2
dose3

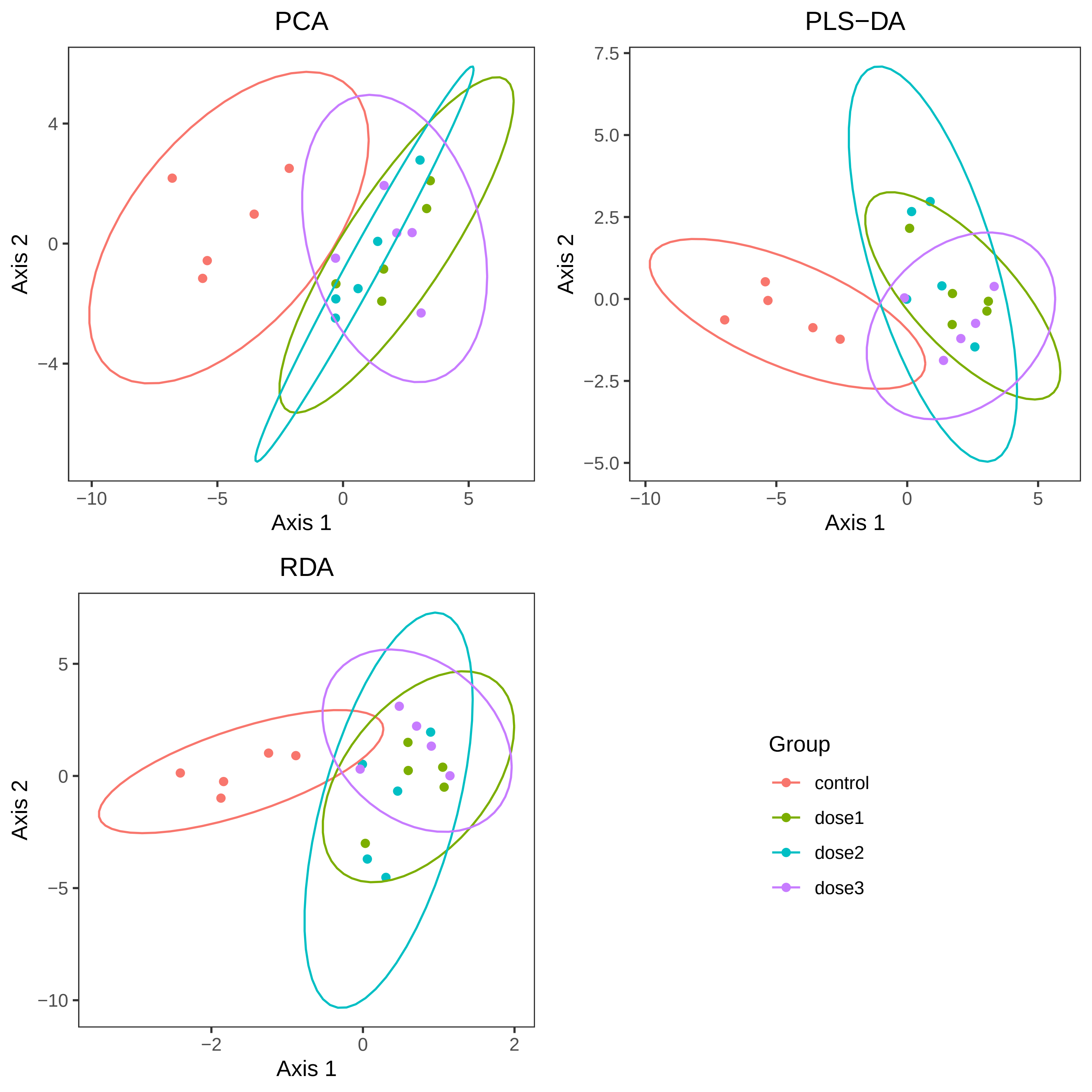
PCA
PLS−DA
RDA
Axis 1
Axis 2
Group
control
dose1
dose2
dose3

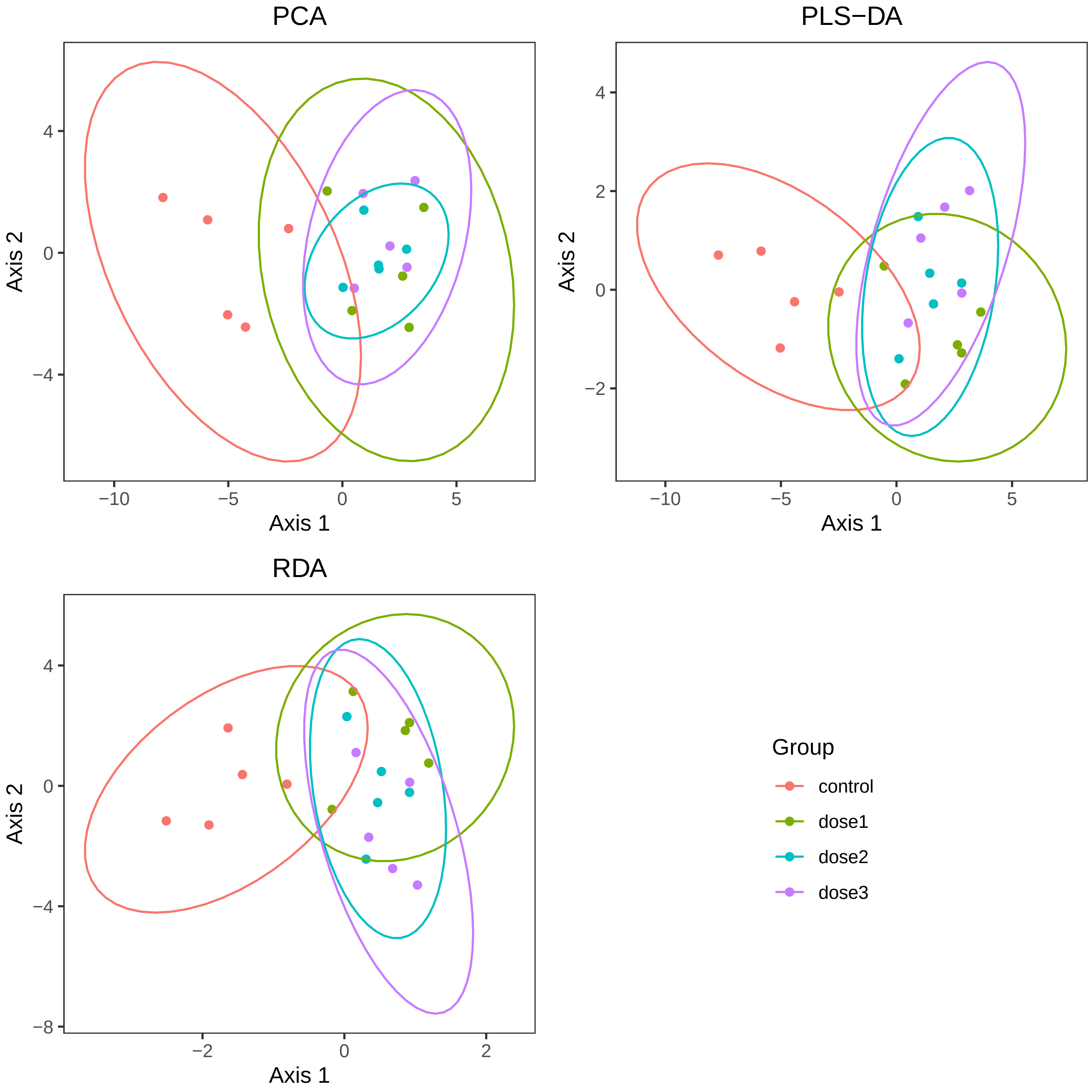

PCA
PLS−DA
RDA
Axis 1
Axis 2
4
0
−4
−8
−10
−5
0
5
2
−2
Group
control
dose1
dose2
dose3

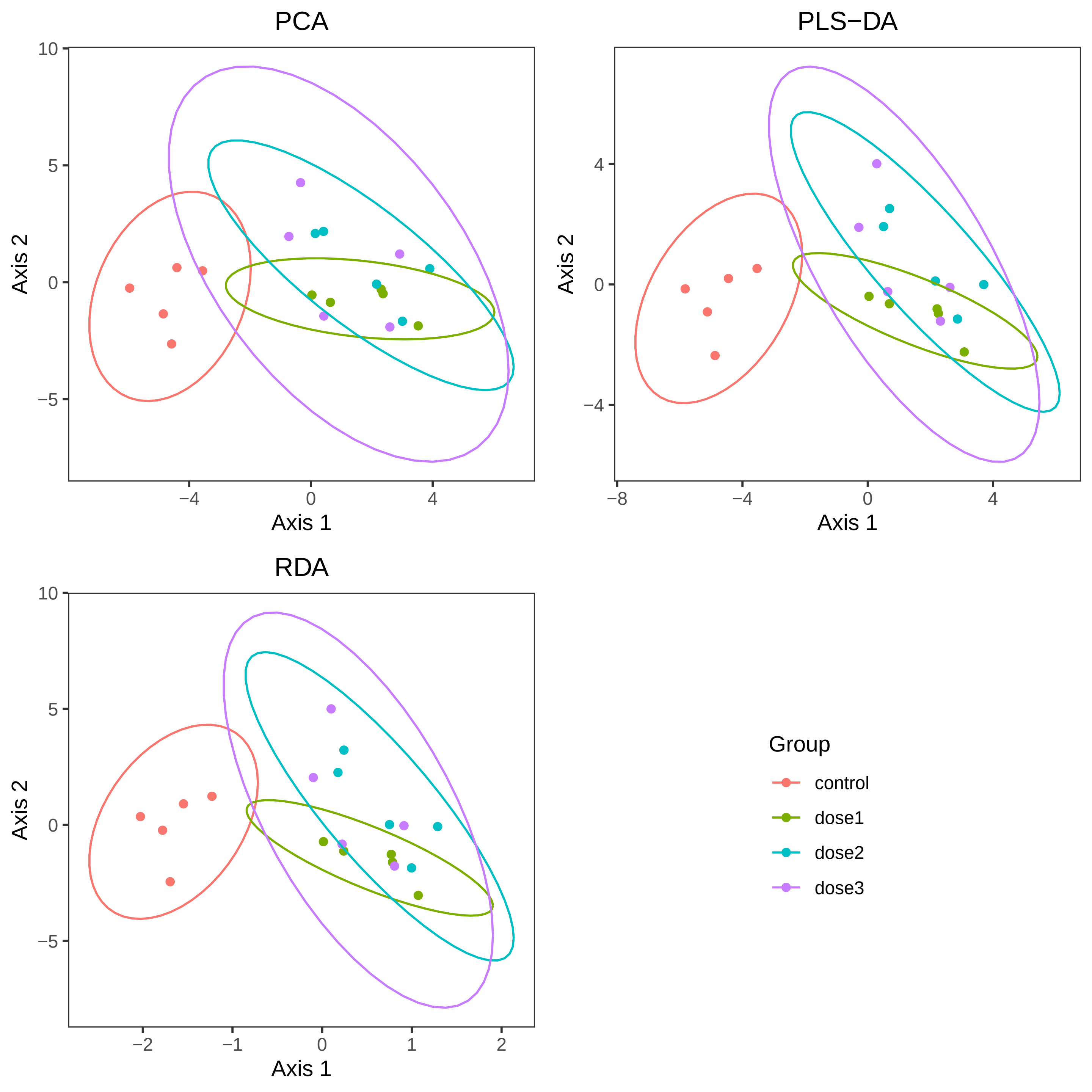
PCA
PLS−DA
RDA
Axis 1
Axis 2
10
5
0
−5
−4
4
−8
2
1
−1
−2
Group
control
dose1
dose2
dose3

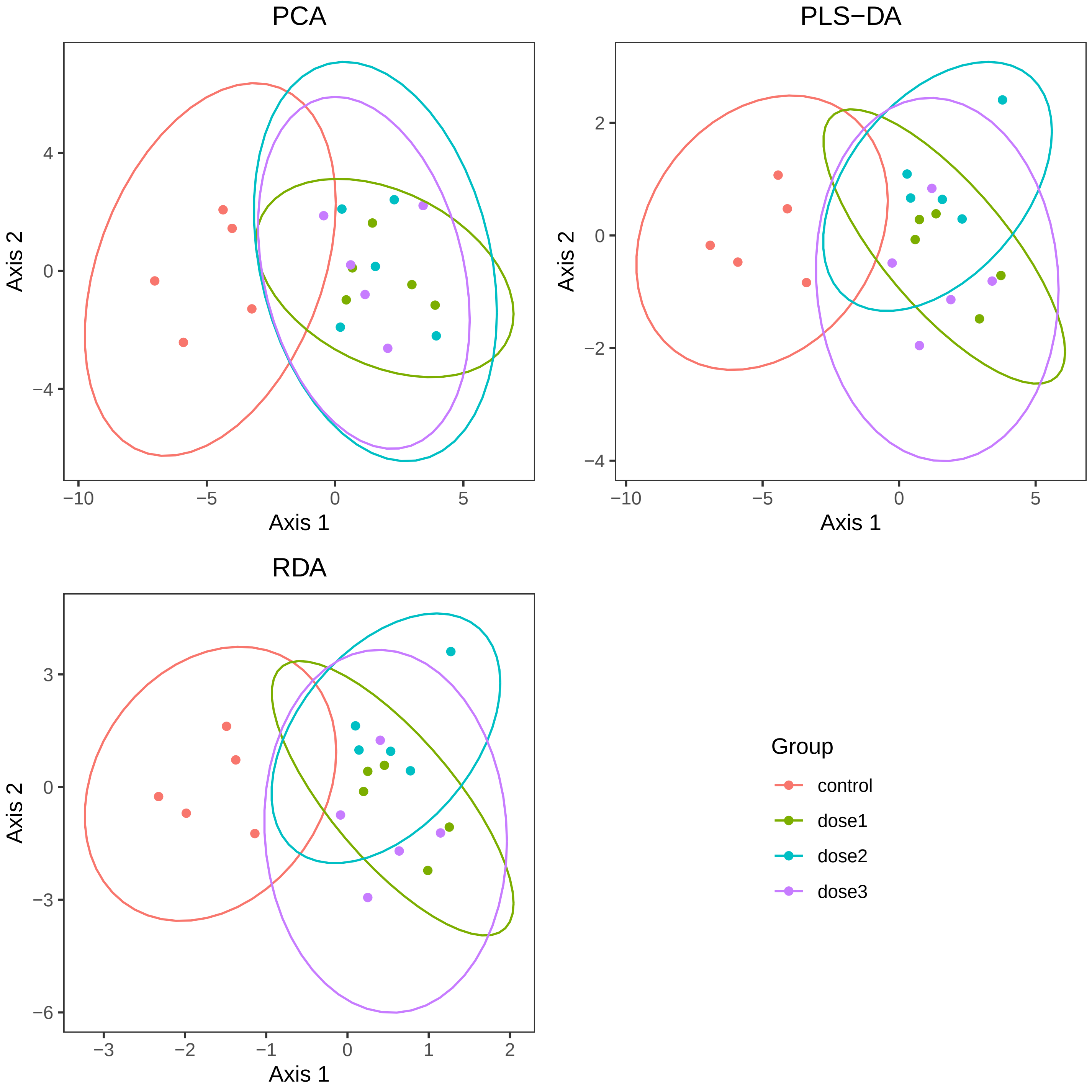
PCA
PLS−DA
RDA
Axis 1
Axis 2
Group
control
dose1
dose2
dose3

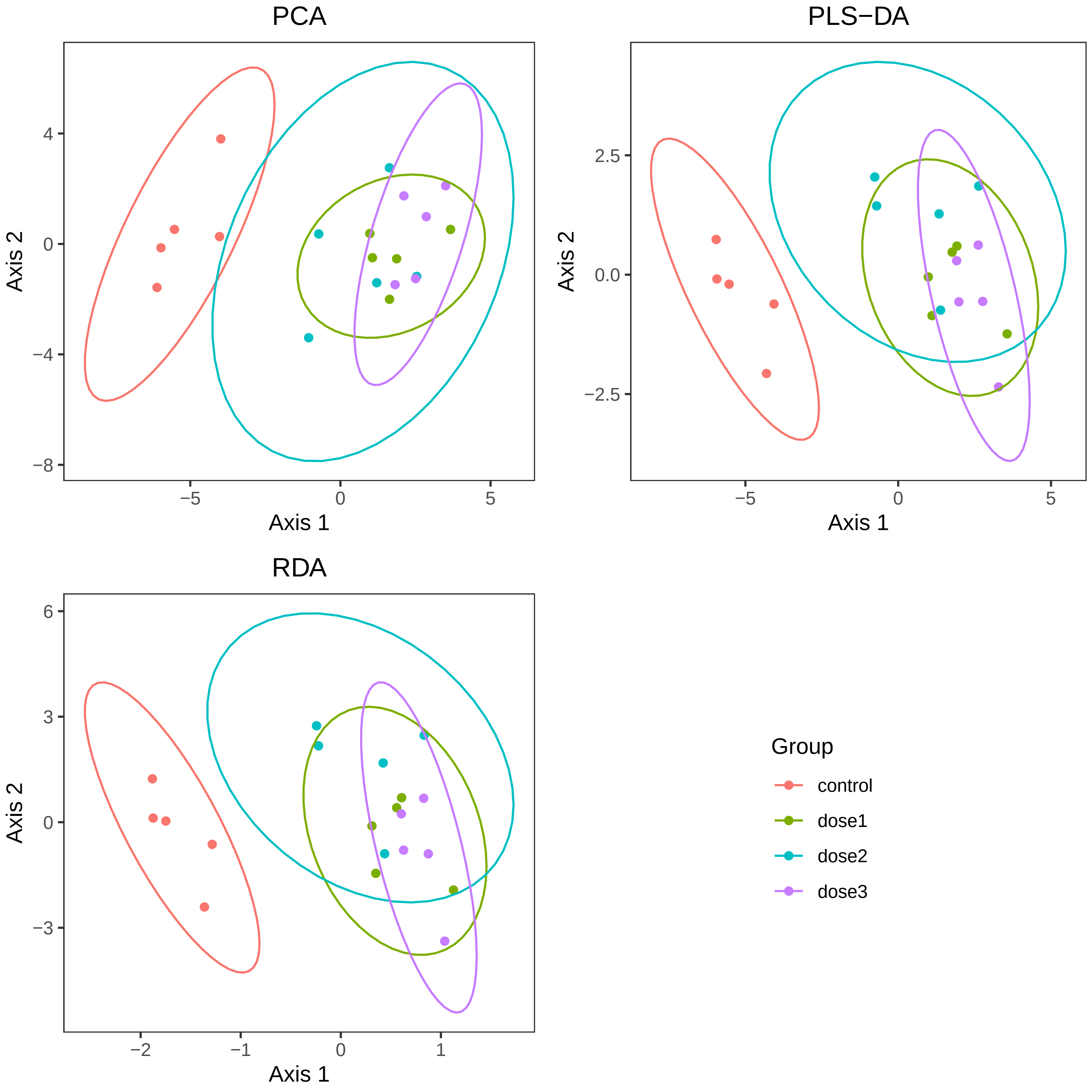

PCA
PLS−DA
RDA
Axis 1
Axis 2
Group
control
dose1
dose2
dose3

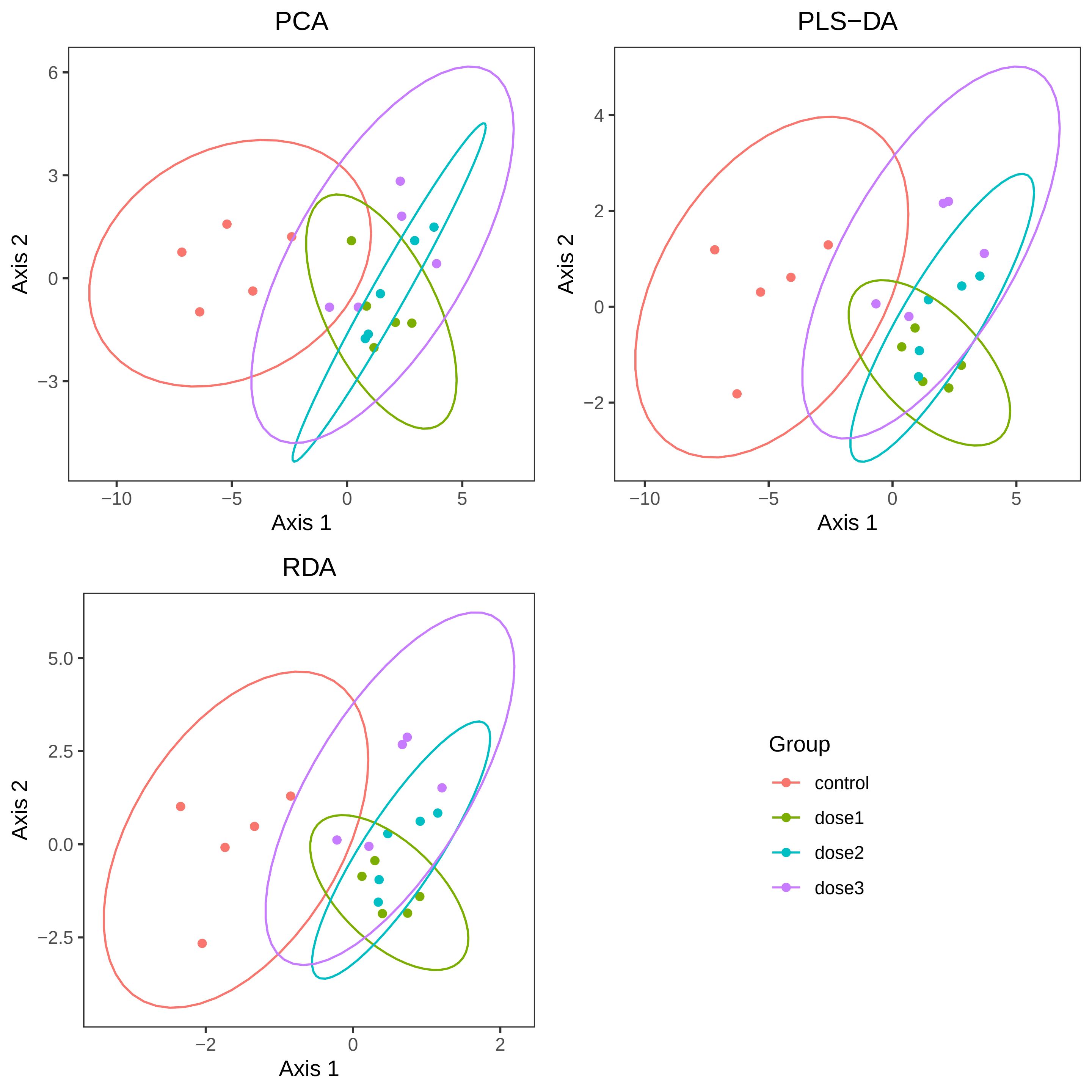

PCA
Axis 2
Axis 1
6
3
0
−3
−10
−5
0
5
PLS−DA
Axis 2
Axis 1
4
2
0
−2
−10
−5
0
5
RDA
Axis 2
Axis 1
5.0
2.5
0.0
−2.5
−2
0
2
Group
control
dose1
dose2
dose3

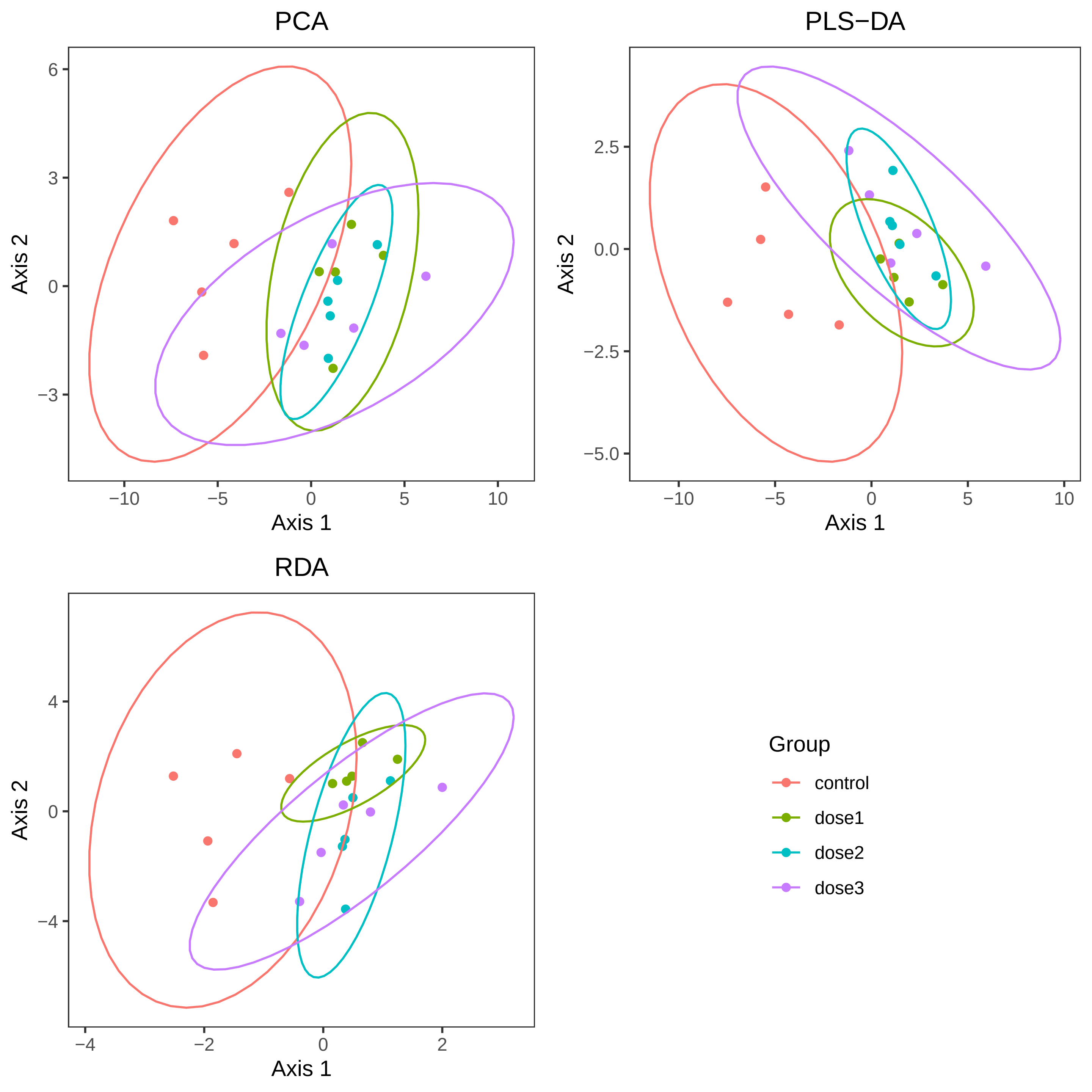

PCA
PLS−DA
RDA
Axis 1
Axis 2
Group
control
dose1
dose2
dose3

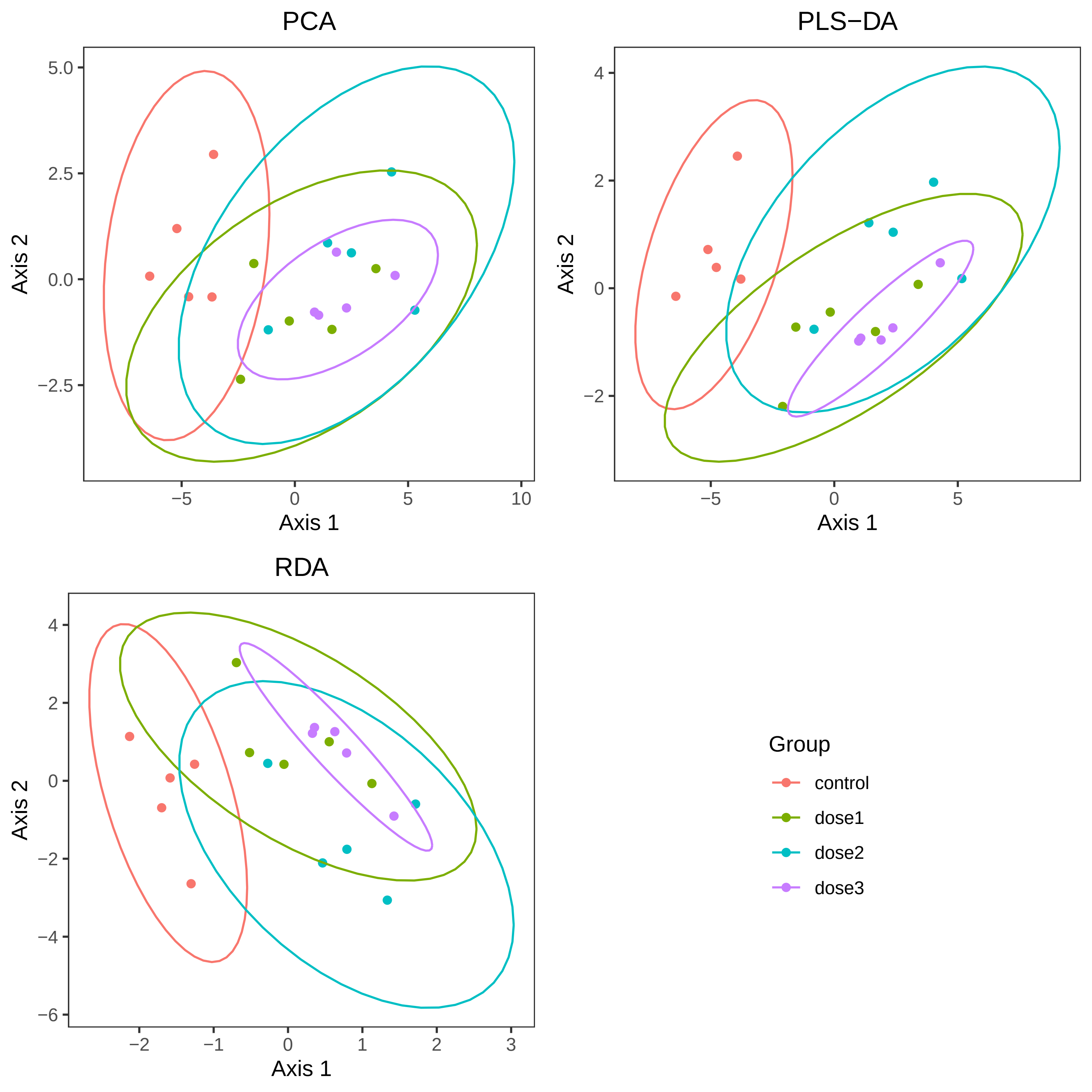
PCA
PLS−DA
RDA
Axis 1
Axis 2
Group
control
dose1
dose2
dose3

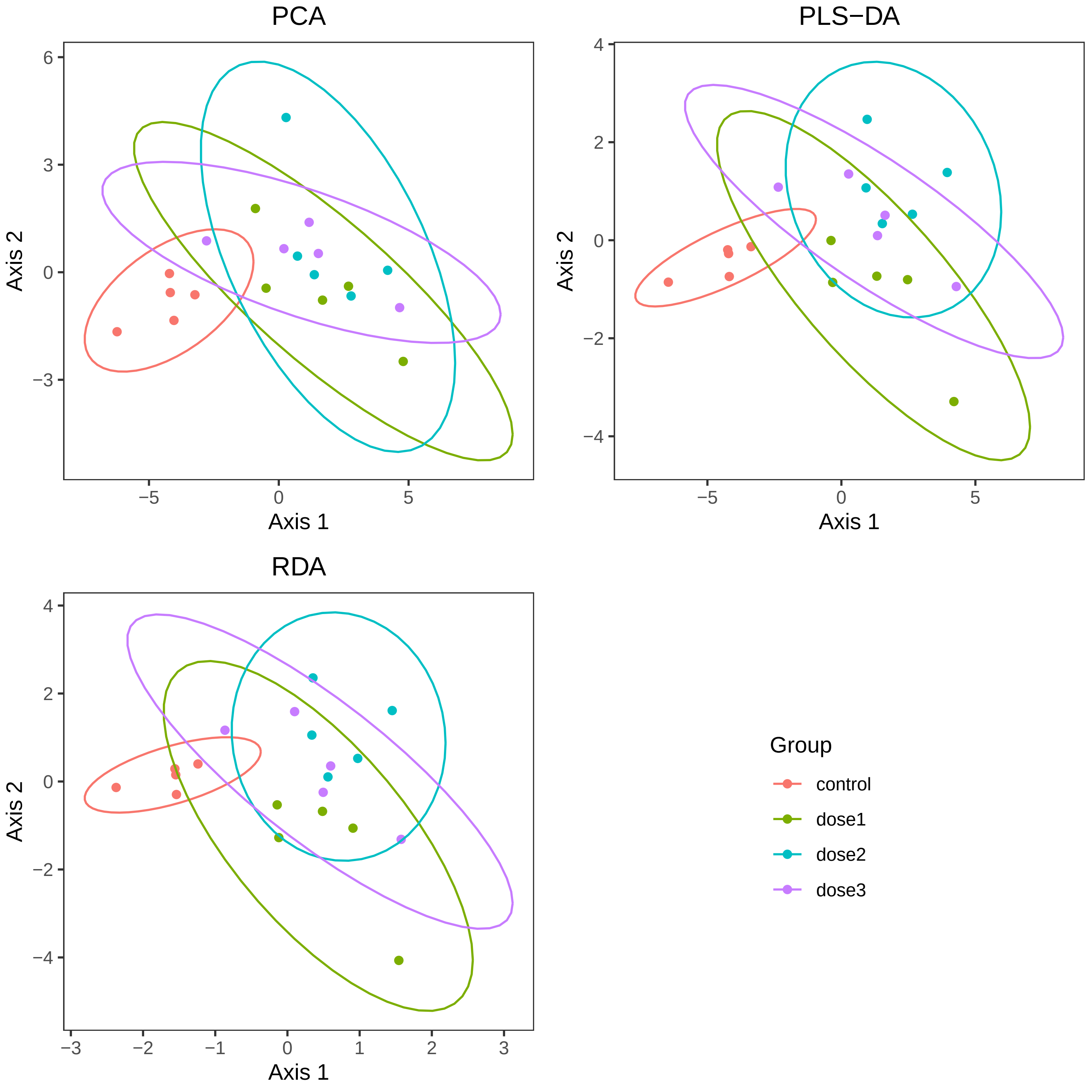

PCA
PLS−DA
RDA
Axis 1
Axis 2
Group
control
dose1
dose2
dose3

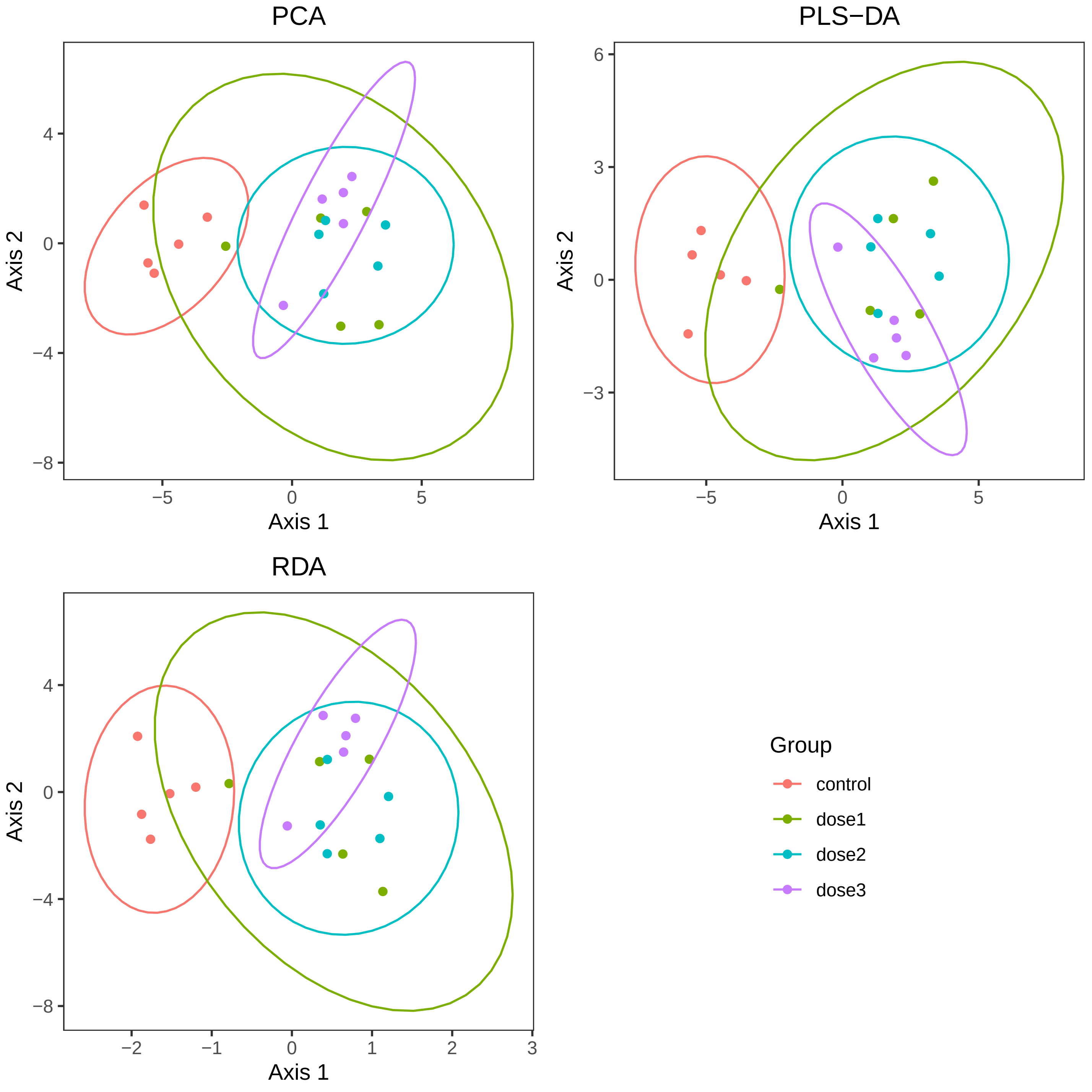
PCA
PLS−DA
RDA
Axis 1
Axis 2
Group
control
dose1
dose2
dose3

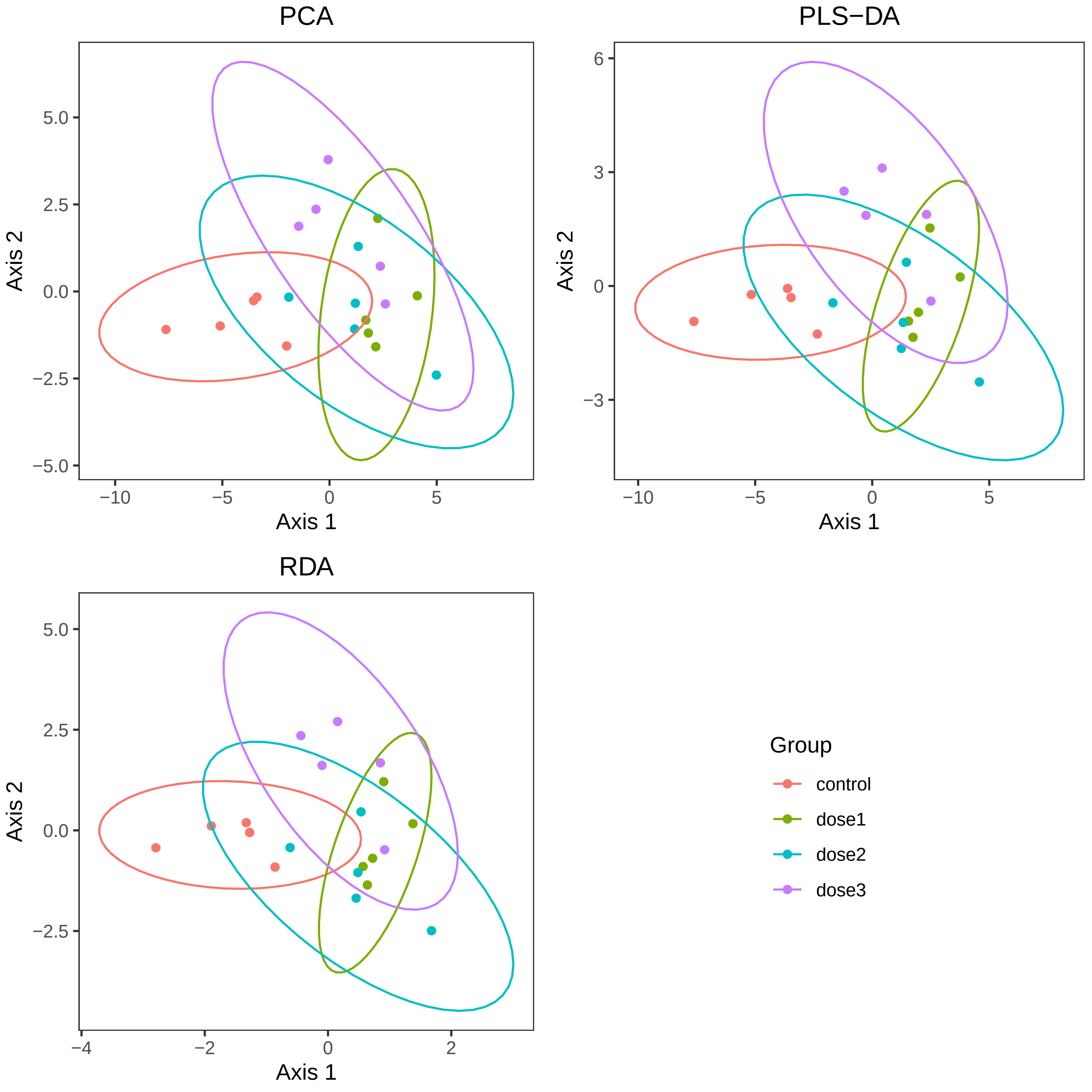

PCA
PLS−DA
RDA
Axis 1
Axis 2
Group
control
dose1
dose2
dose3

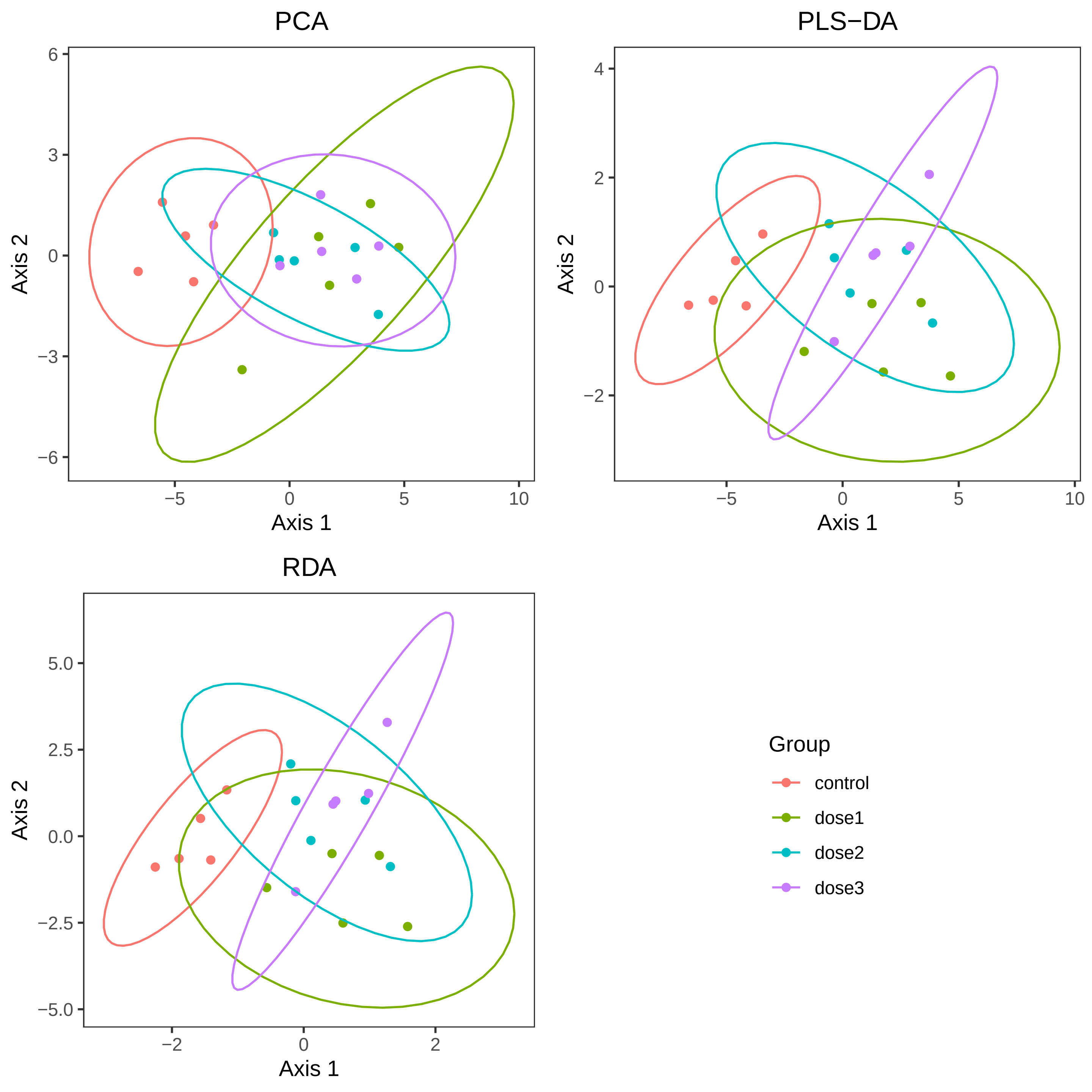

PCA
PLS−DA
RDA
Axis 1
Axis 2
6
3
0
−3
−6
−5
0
5
10
4
2
0
−2
5.0
2.5
0.0
−2.5
−5.0
−2
0
2
Group
control
dose1
dose2
dose3

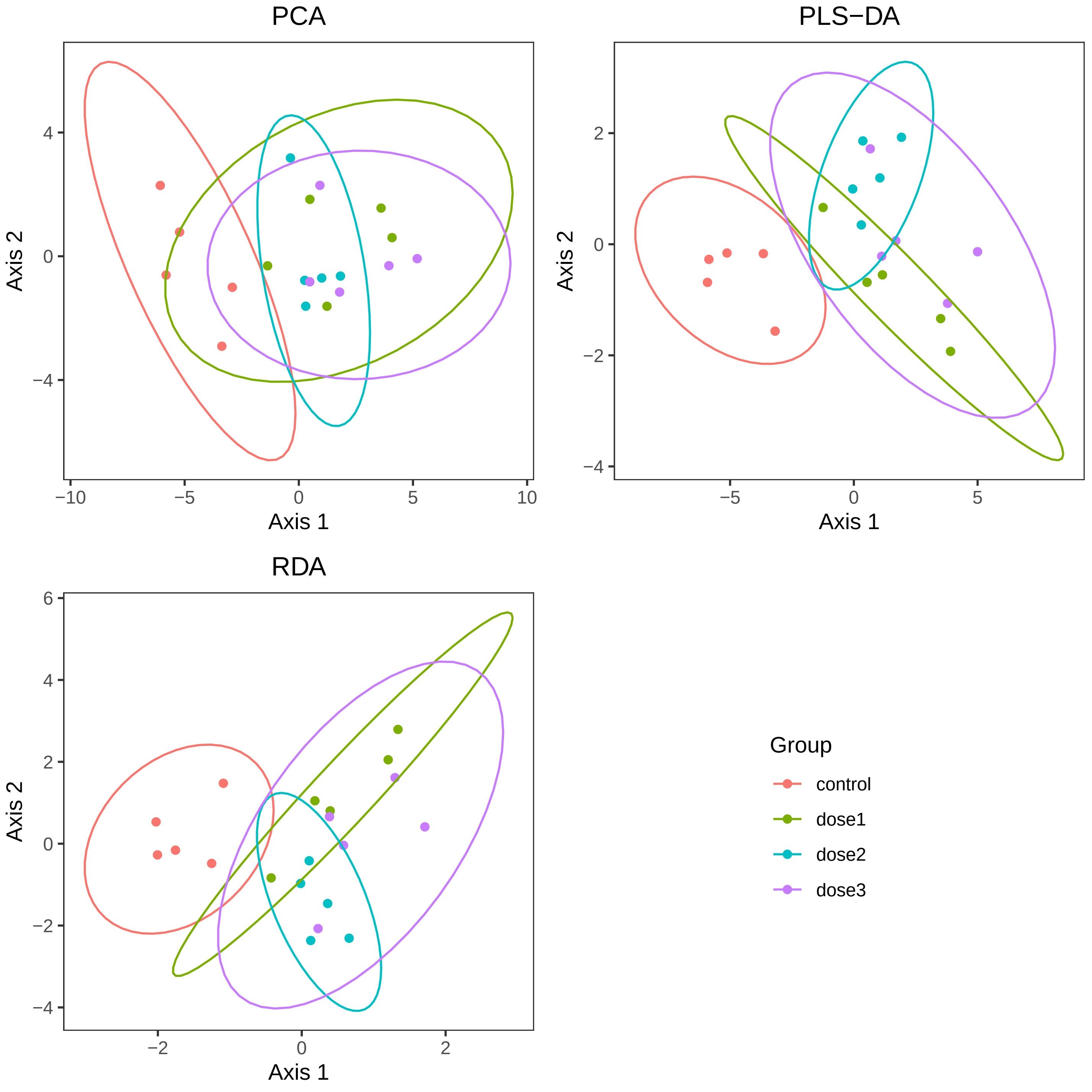

PCA
PLS−DA
RDA
Axis 1
Axis 2
Group
control
dose1
dose2
dose3

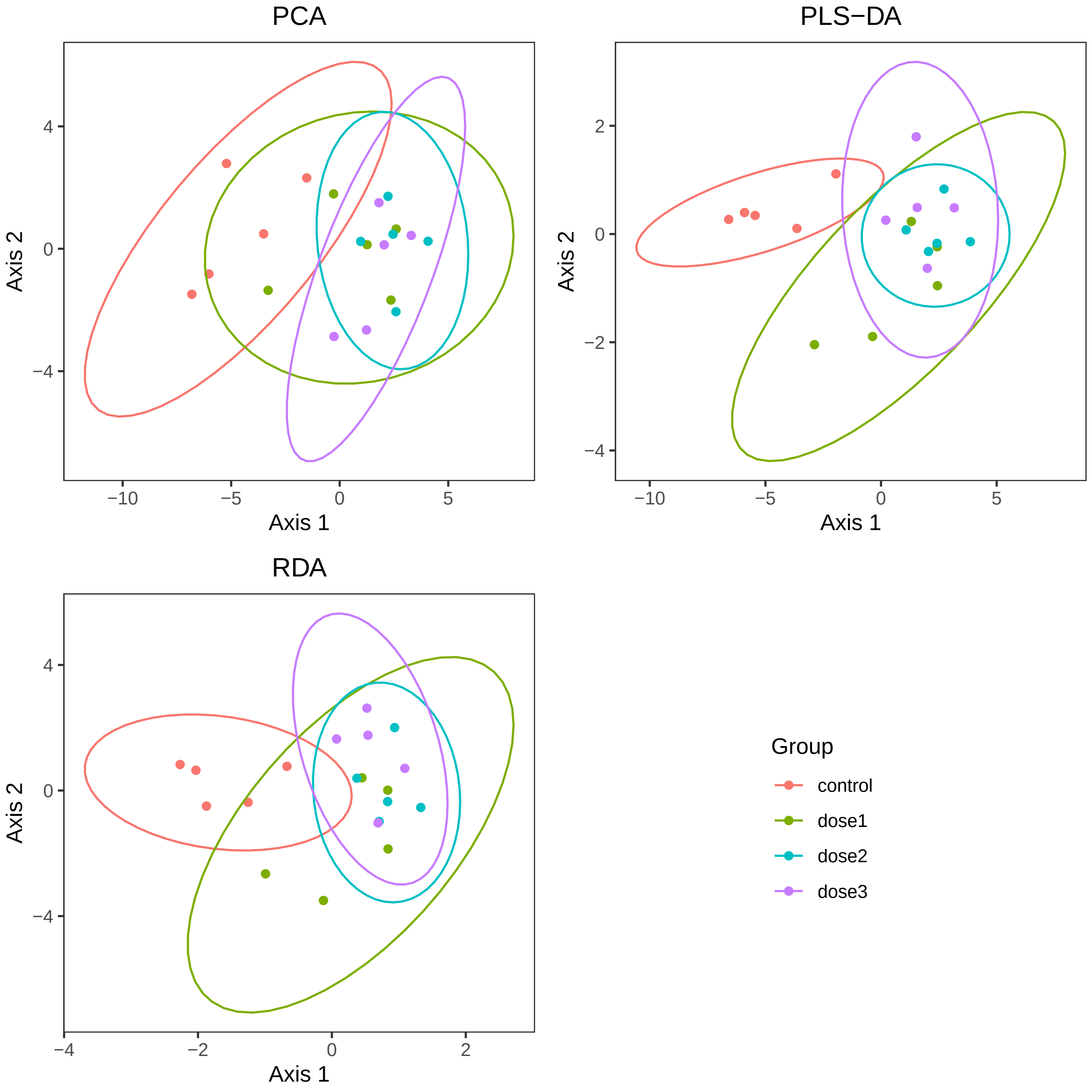

PCA
PLS−DA
RDA
Axis 1
Axis 2
−10
−5
0
5
4
0
−4
2
−2
Group
control
dose1
dose2
dose3

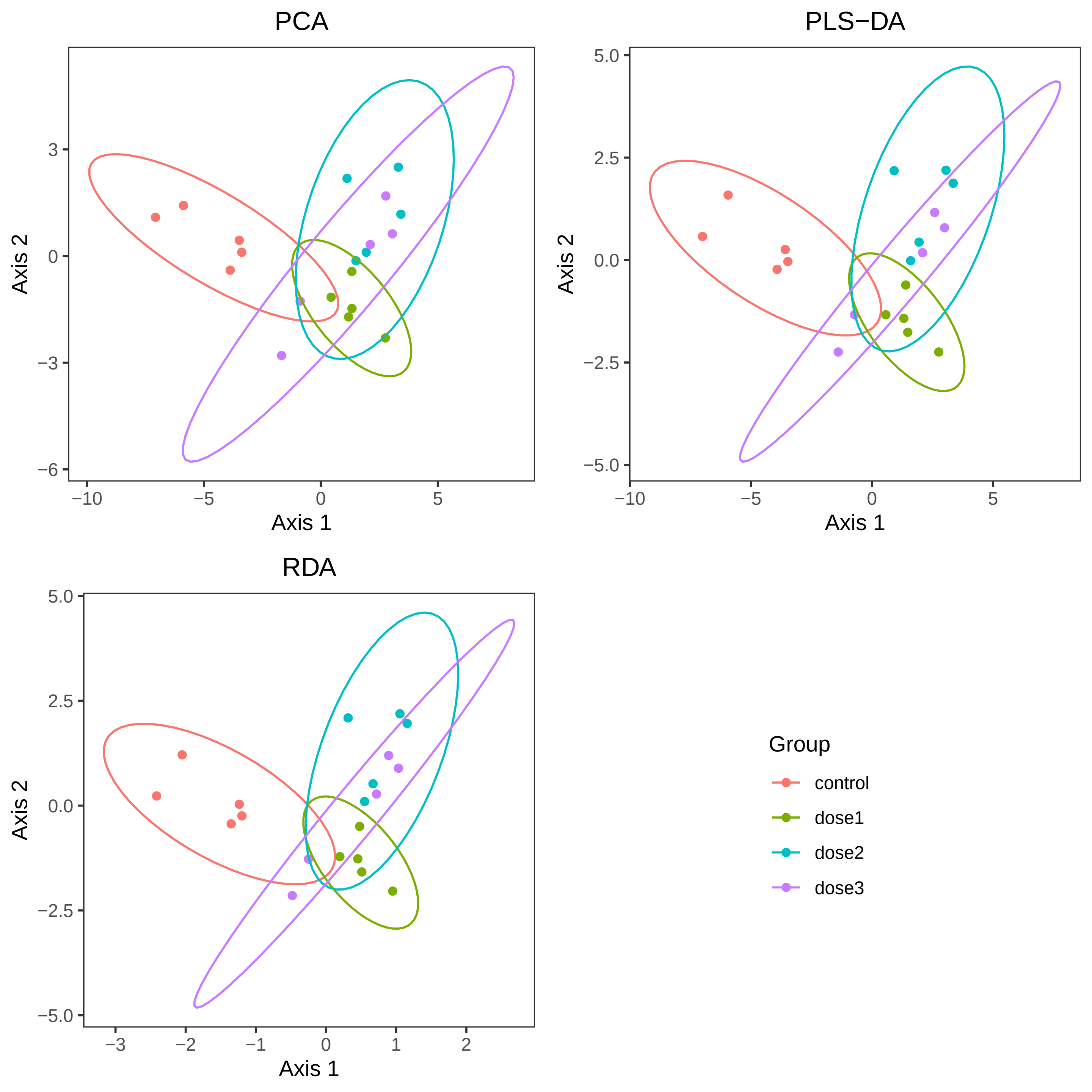

PCA
PLS−DA
RDA
Axis 1
Axis 2
Group
control
dose1
dose2
dose3

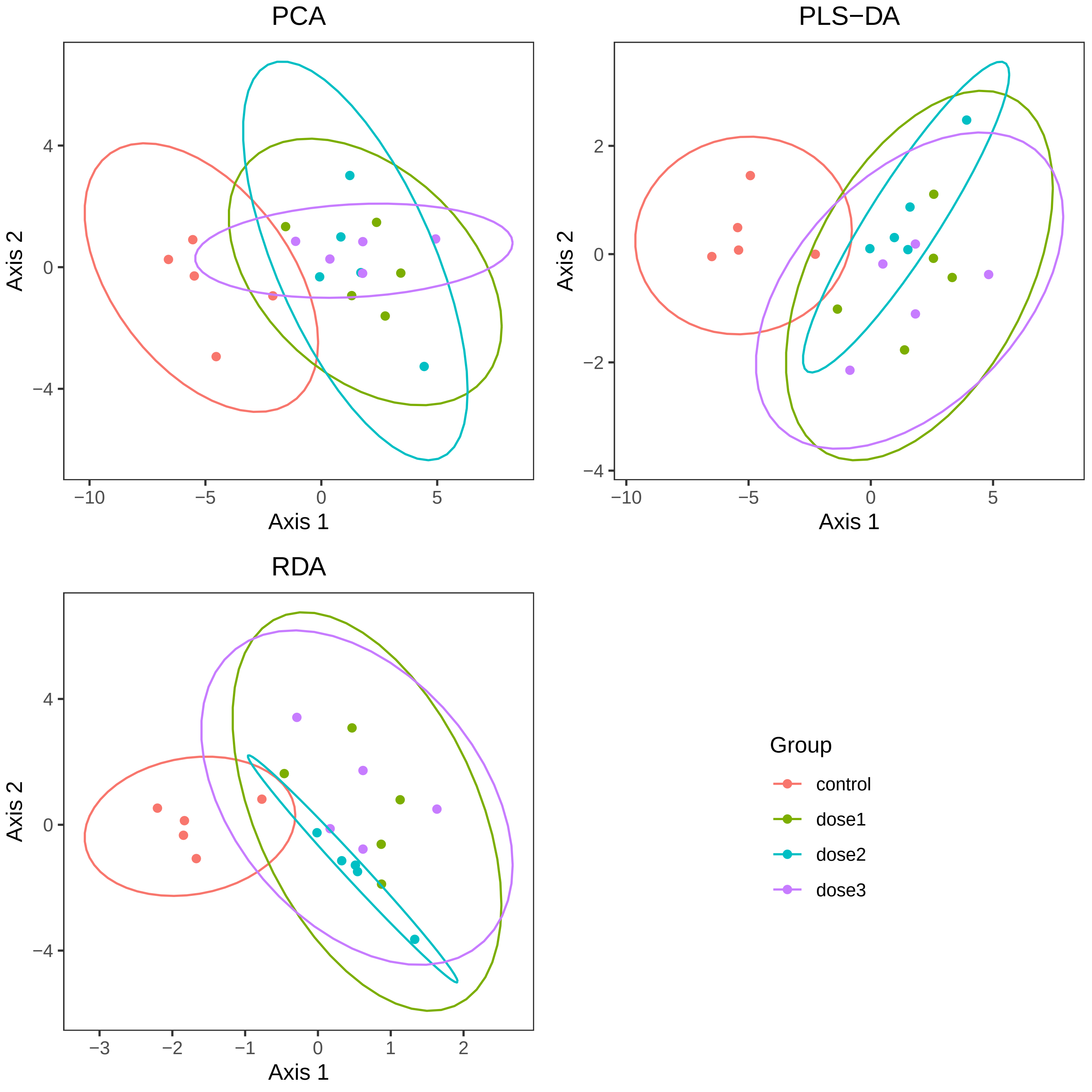
PCA
PLS−DA
RDA
Axis 1
Axis 2
Group
control
dose1
dose2
dose3

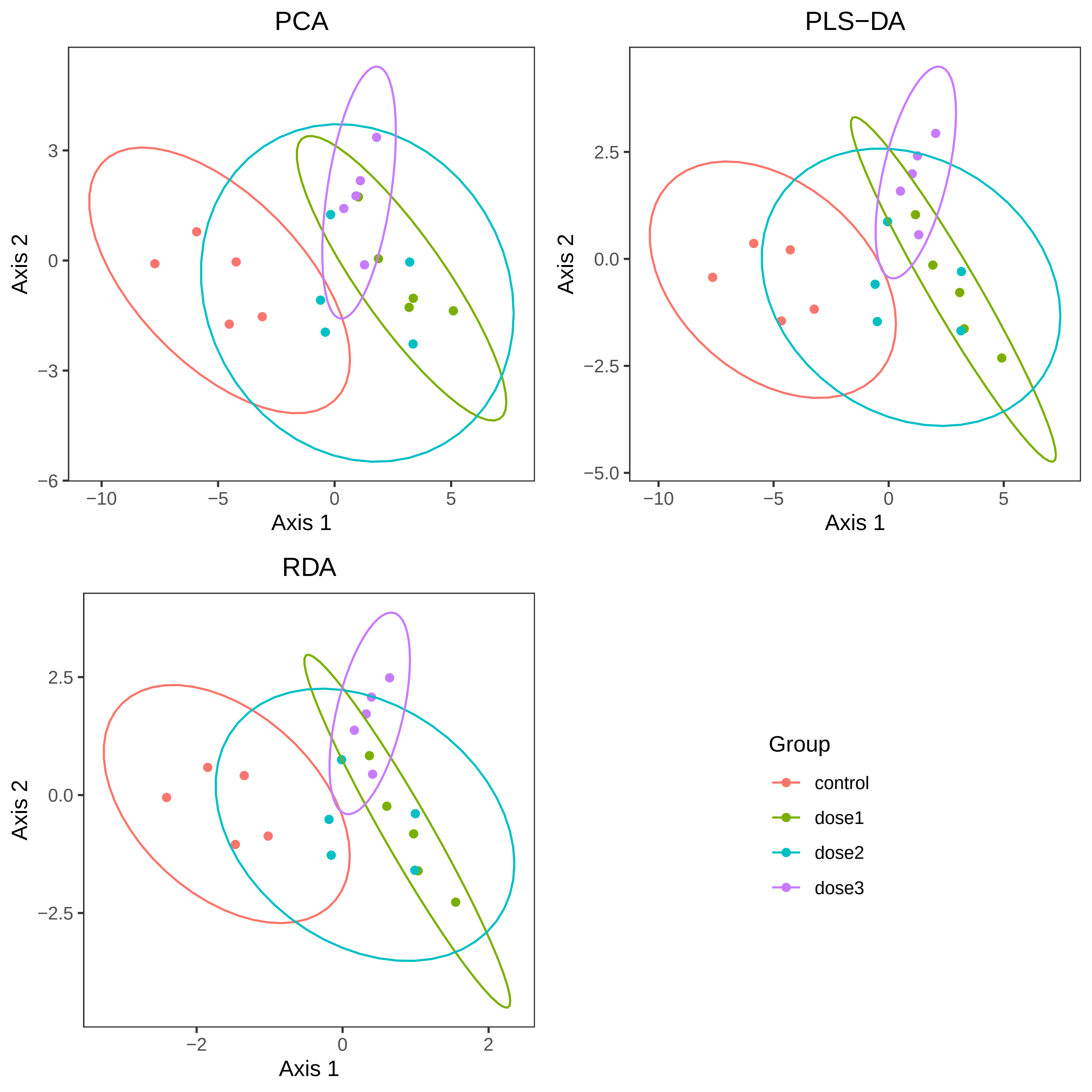

PCA
PLS−DA
RDA
Axis 1
Axis 2
Group
control
dose1
dose2
dose3

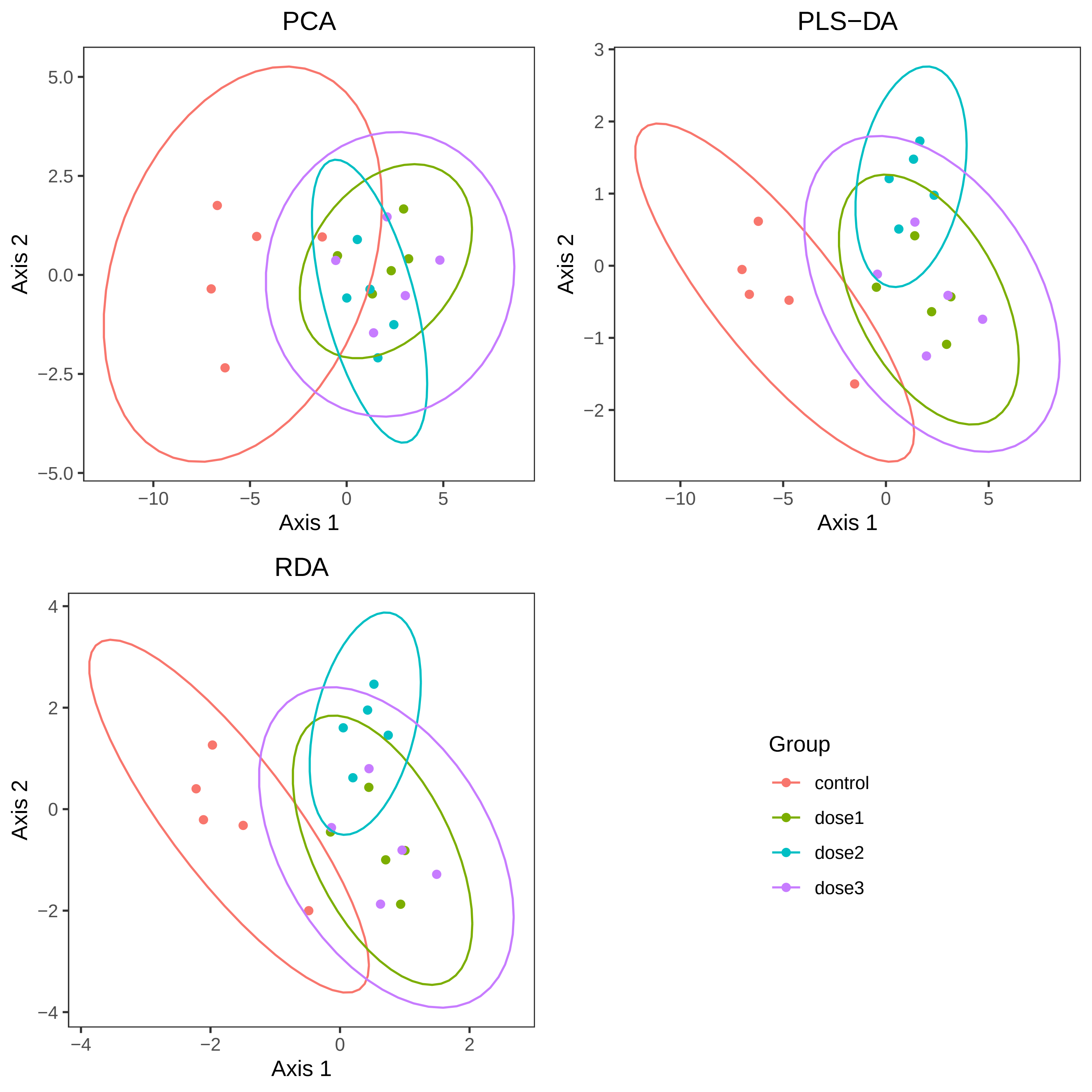

PCA
Axis 2
Axis 1
PLS−DA
Axis 2
Axis 1
RDA
Axis 2
Axis 1
Group
control
dose1
dose2
dose3

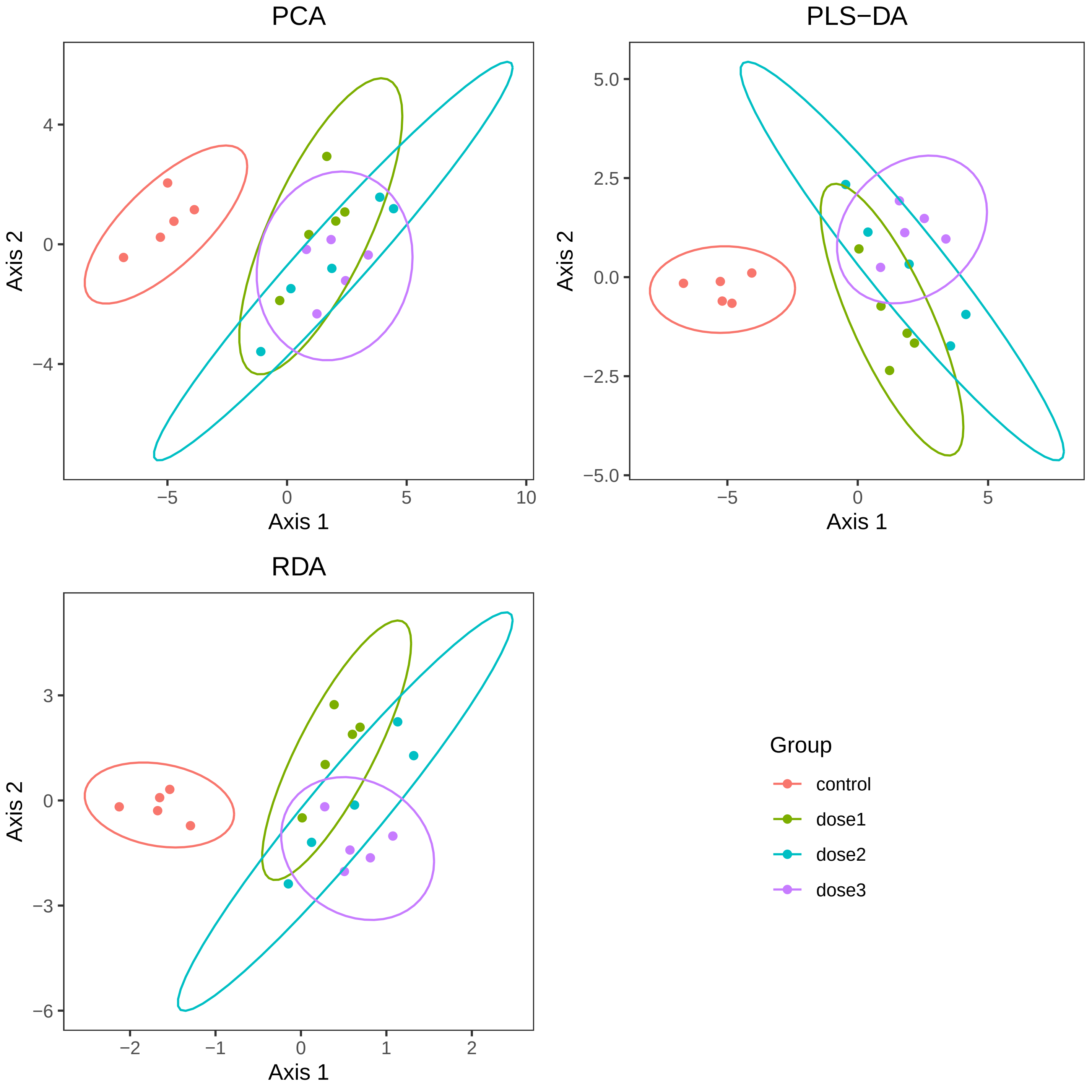

PCA
PLS−DA
RDA
Axis 1
Axis 2
4
0
−4
−5
0
5
10
5.0
2.5
0.0
−2.5
−5.0
3
−3
−6
−2
−1
1
2
Group
control
dose1
dose2
dose3

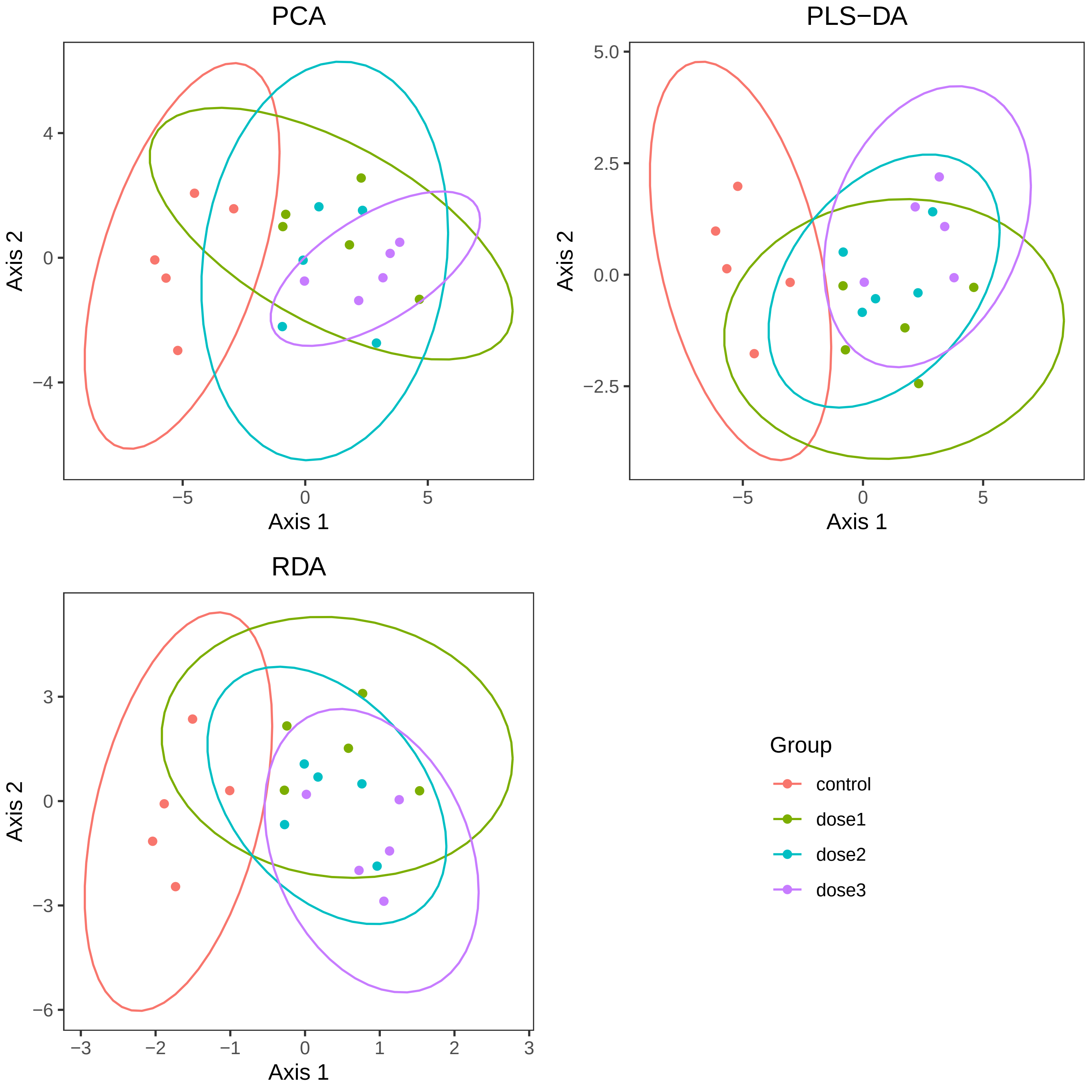

PCA
Axis 2
Axis 1
4
0
−4
−5
0
5
PLS−DA
5.0
2.5
0.0
−2.5
RDA
3
−3
−6
−3
−2
−1
1
2
Group
control
dose1
dose2
dose3

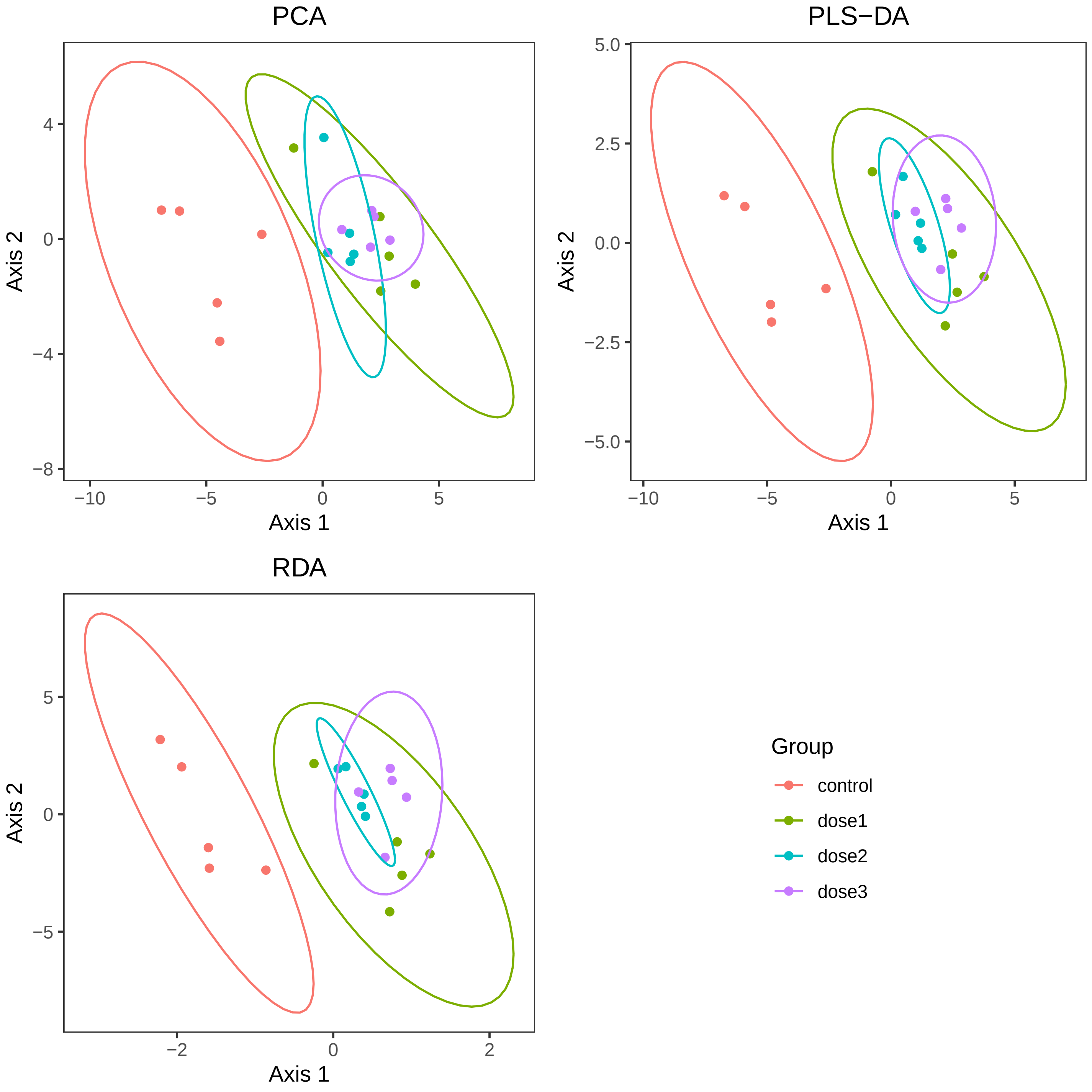

PCA
PLS−DA
RDA
Axis 1
Axis 2
Group
control
dose1
dose2
dose3

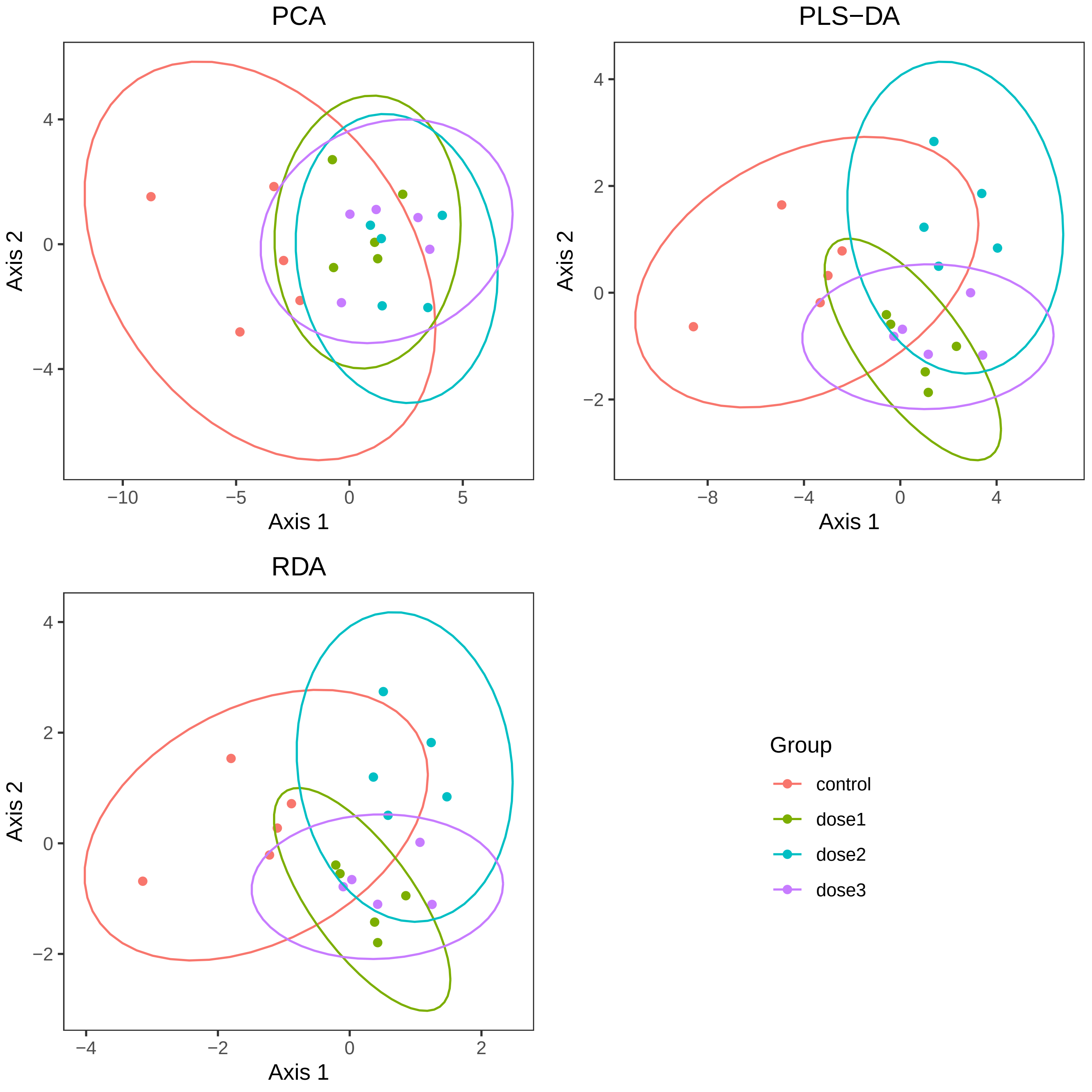

PCA
PLS−DA
RDA
Axis 1
Axis 2
Group
control
dose1
dose2
dose3

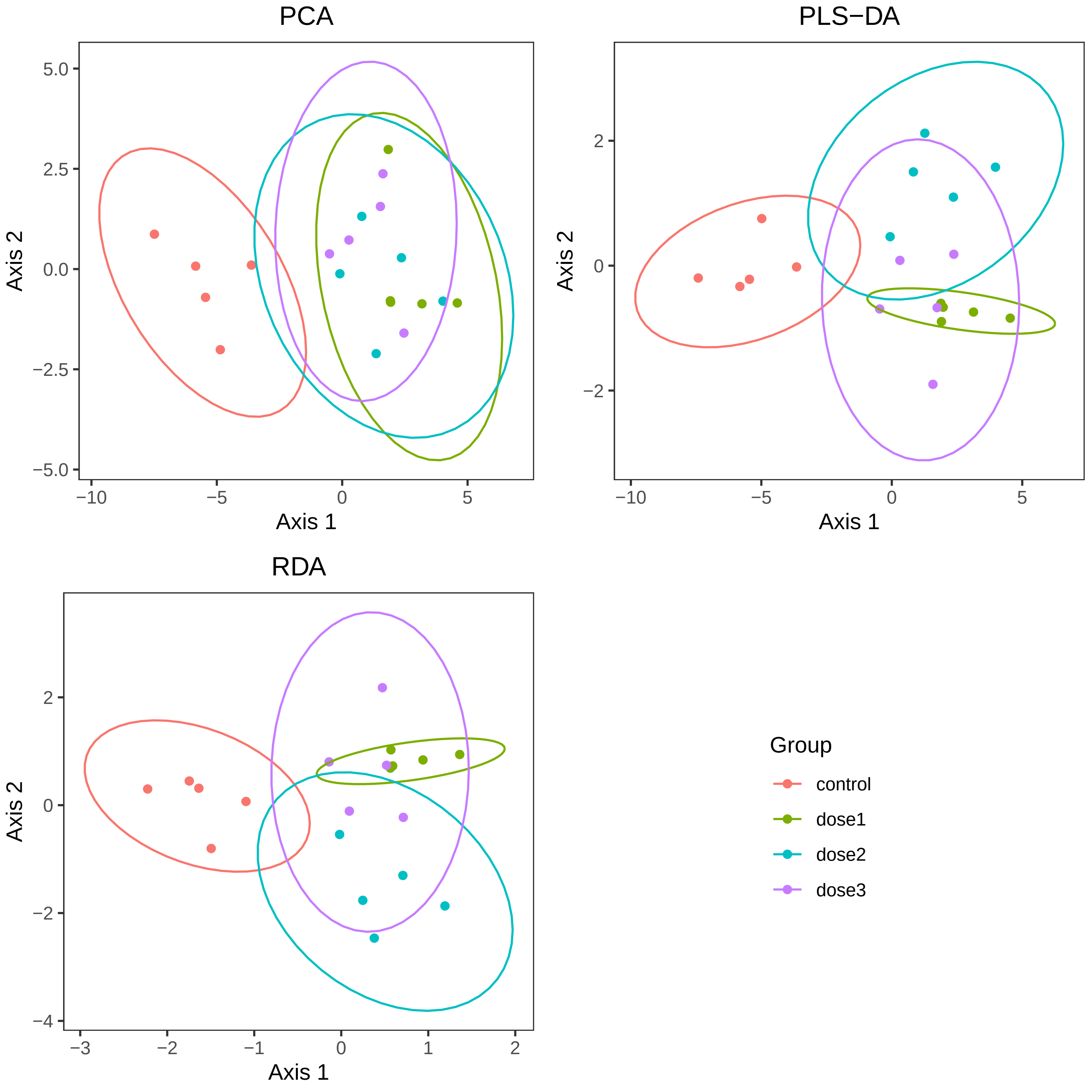
PCA
PLS−DA
RDA
Axis 1
Axis 2
5.0
2.5
0.0
−2.5
−5.0
−10
−5
0
5
2
0
−2
−4
−3
−1
1
Group
control
dose1
dose2
dose3

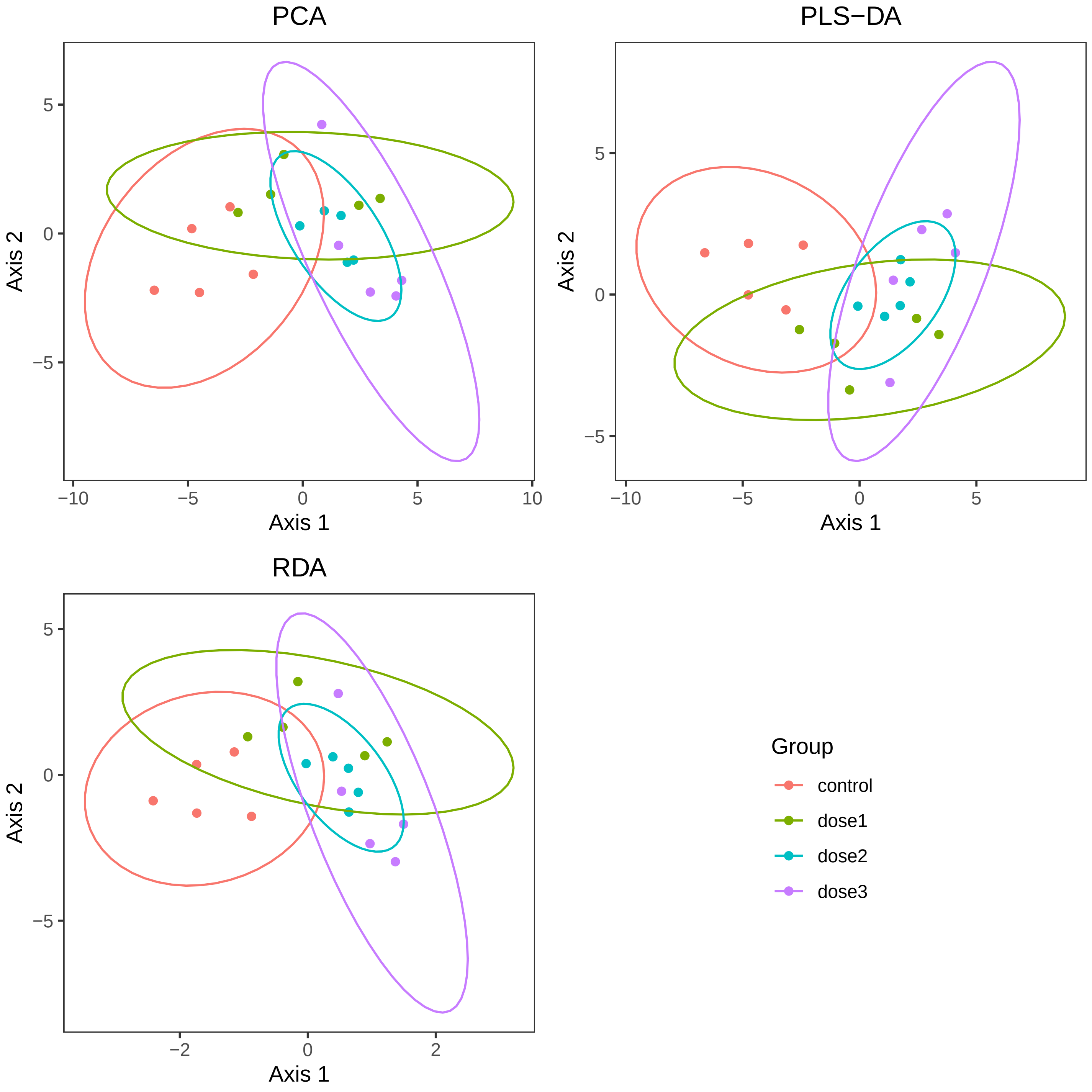
PCA
PLS−DA
RDA
Axis 1
Axis 2
Group
control
dose1
dose2
dose3

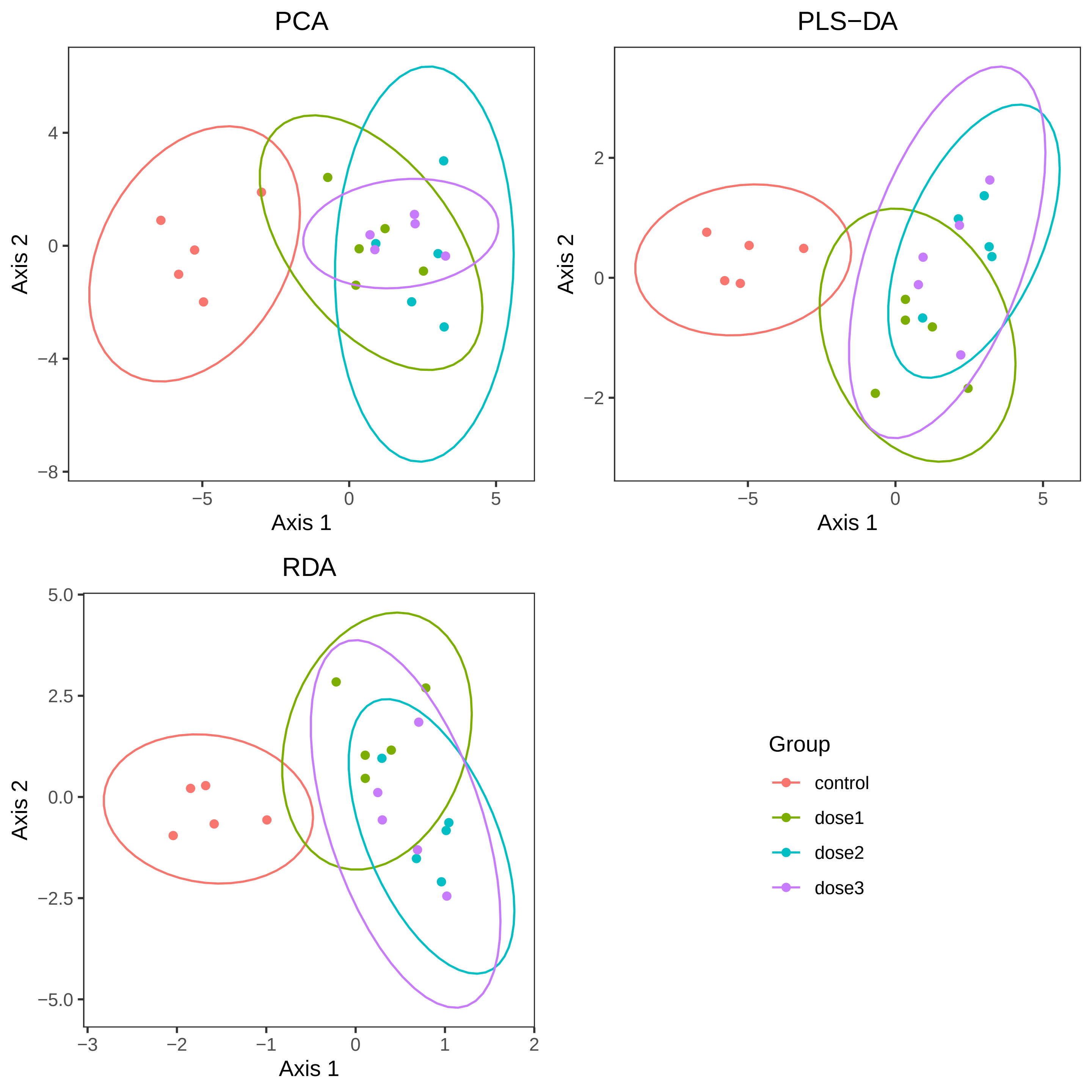
PCA
PLS−DA
RDA
Axis 1
Axis 2
Group
control
dose1
dose2
dose3

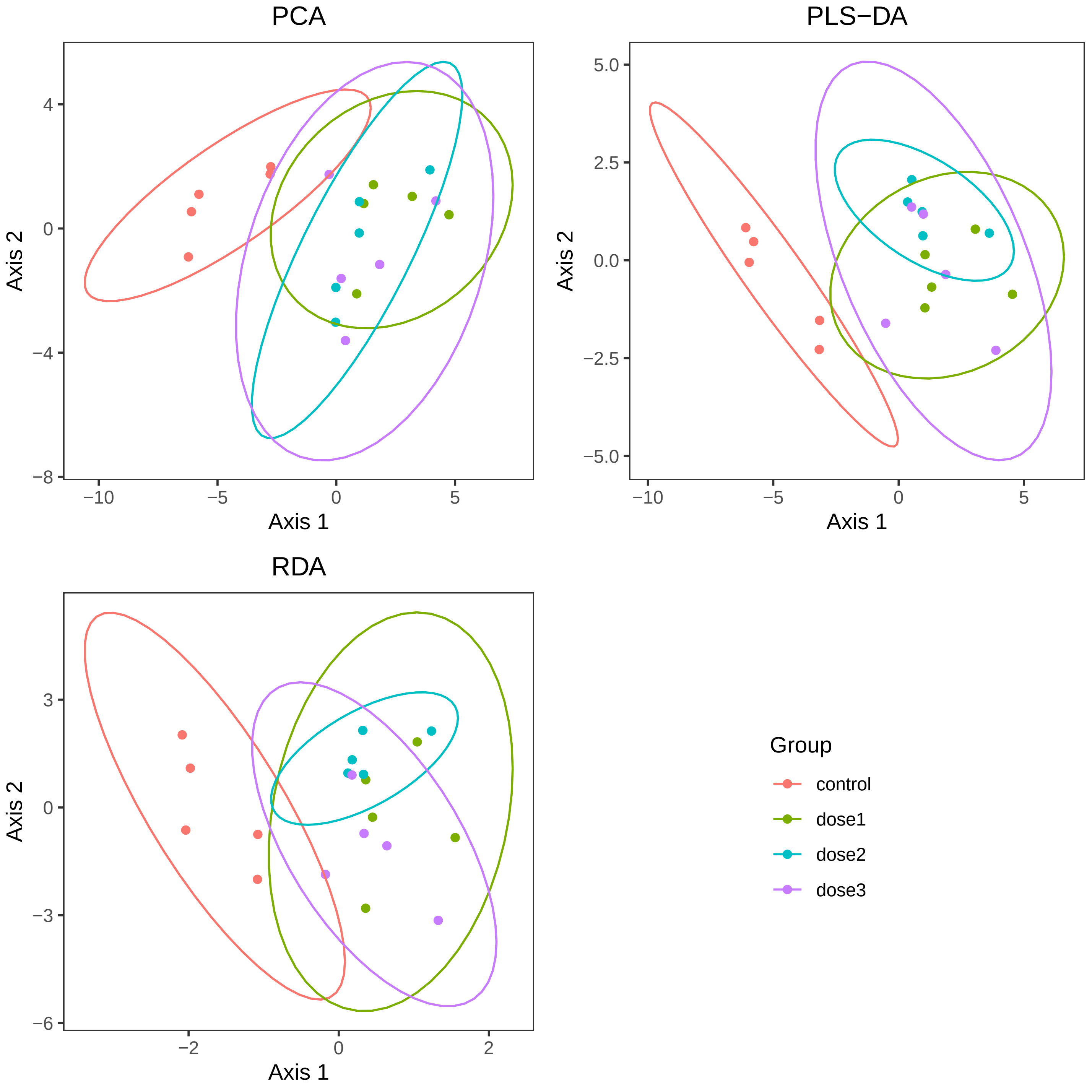
PCA
PLS−DA
RDA
Axis 1
Axis 2
Group
control
dose1
dose2
dose3

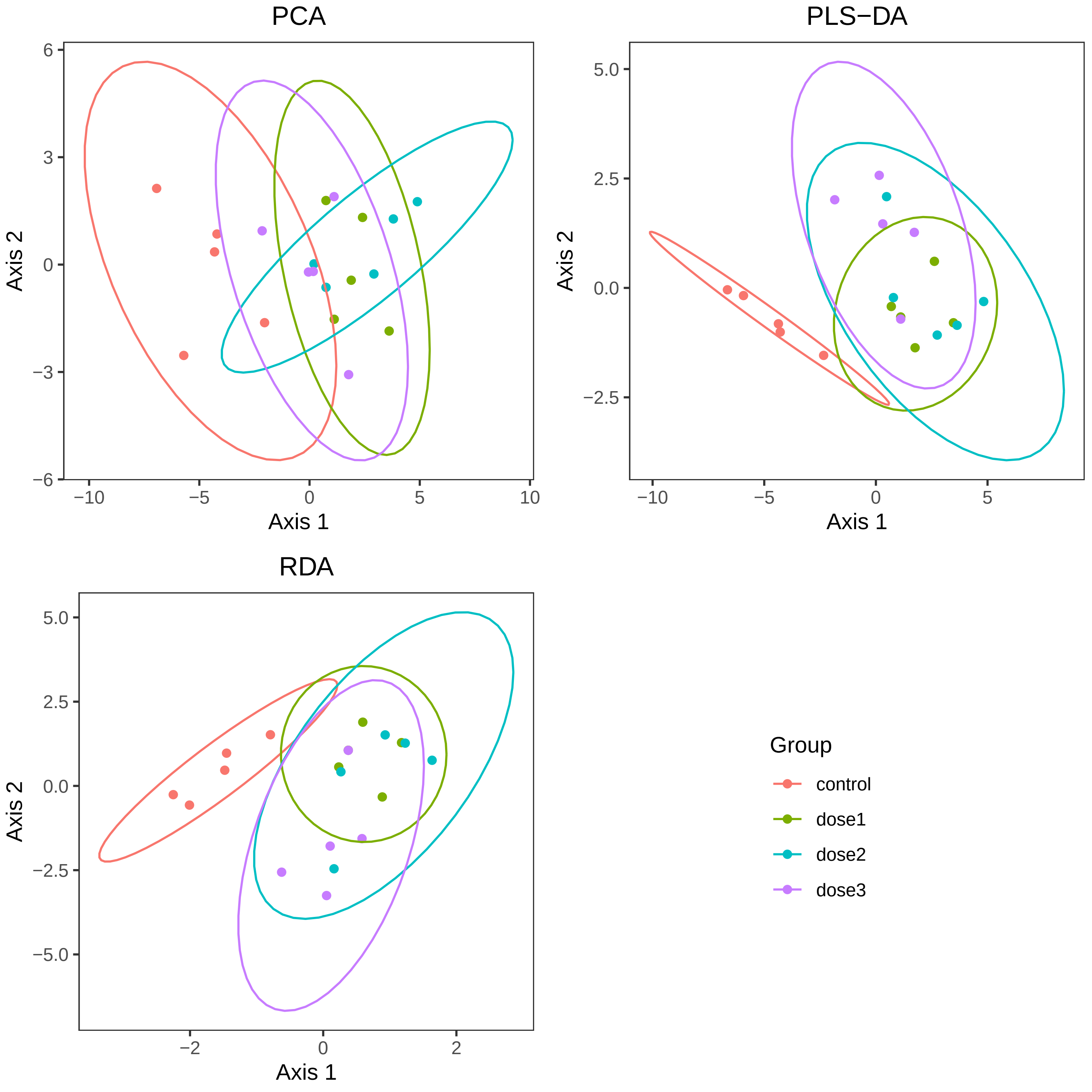

PCA
PLS−DA
RDA
Axis 1
Axis 2
Group
control
dose1
dose2
dose3

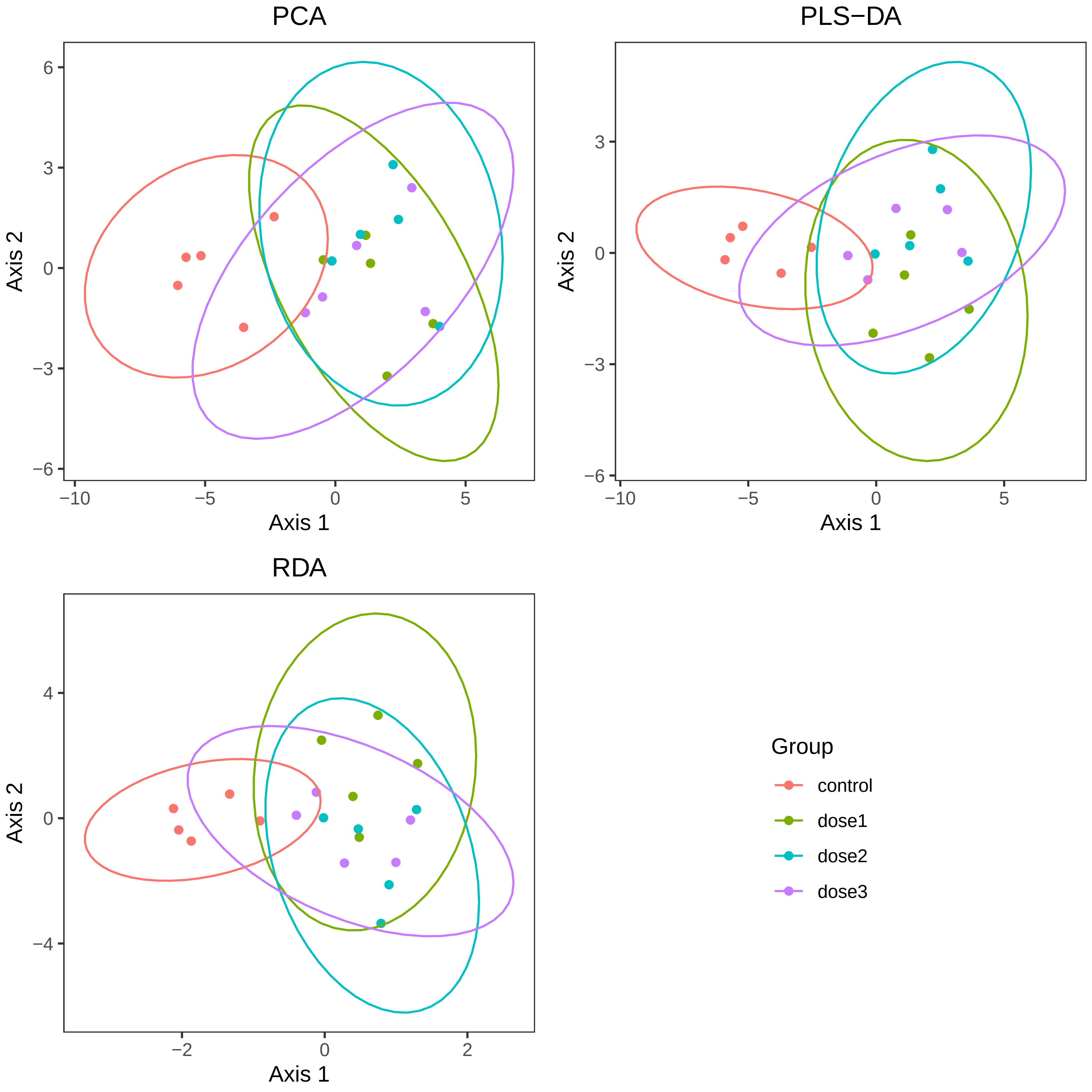

PCA
PLS−DA
RDA
Axis 1
Axis 2
Group
control
dose1
dose2
dose3

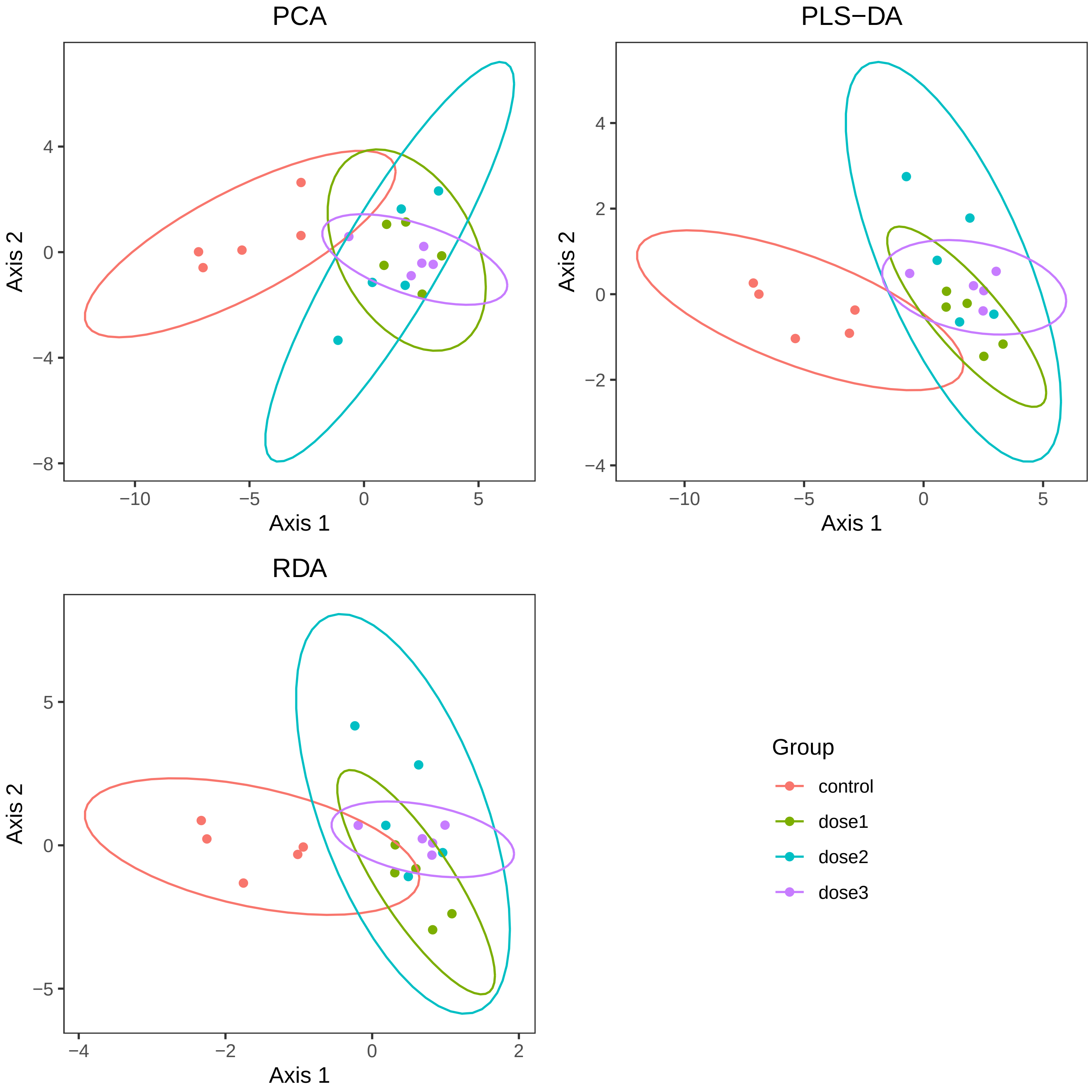
PCA
PLS−DA
RDA
Axis 1
Axis 2
−10
−5
0
5
4
0
−4
−8
2
−2
−4
5
0
−5
Group
control
dose1
dose2
dose3

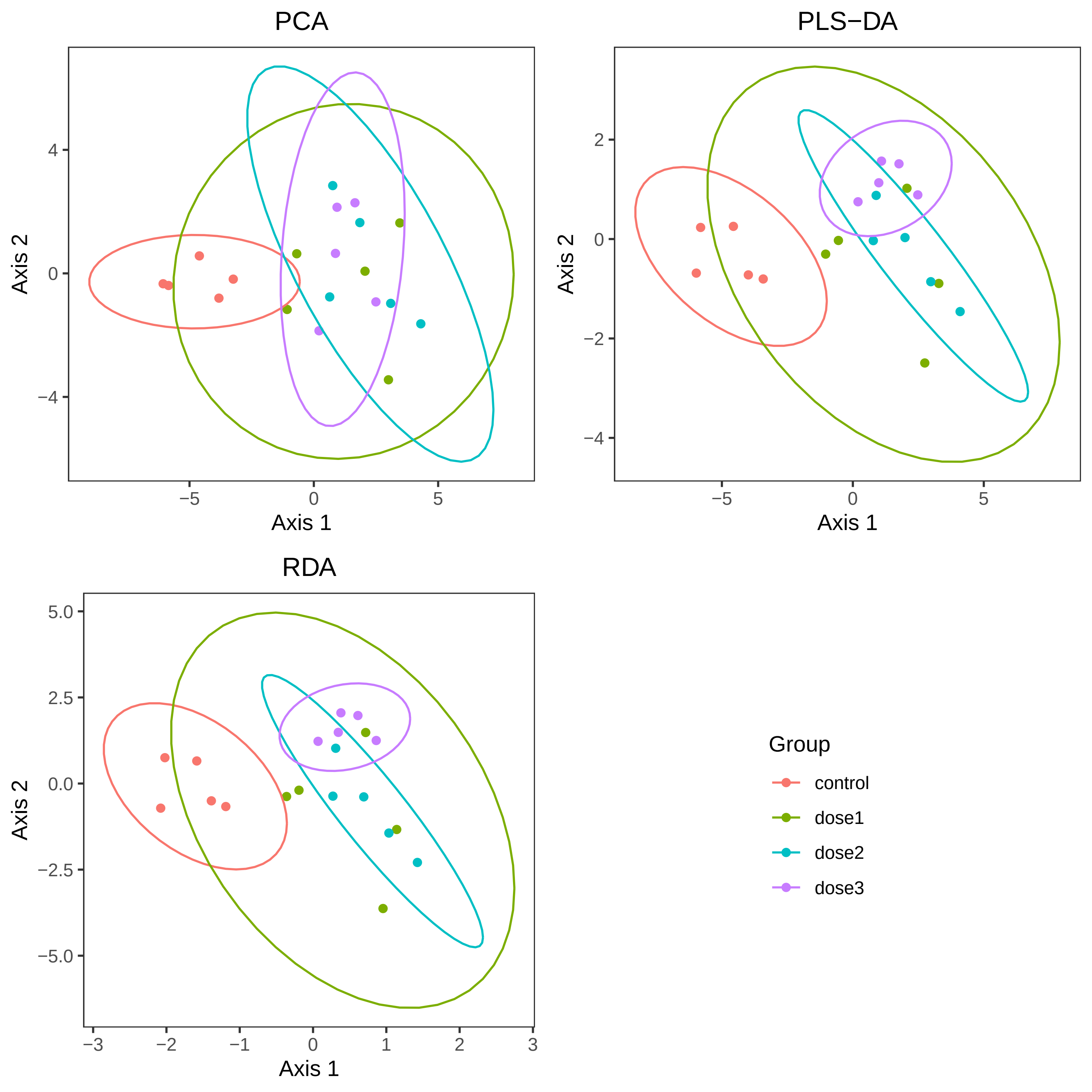

PCA
PLS−DA
RDA
Axis 1
Axis 2
Group
control
dose1
dose2
dose3

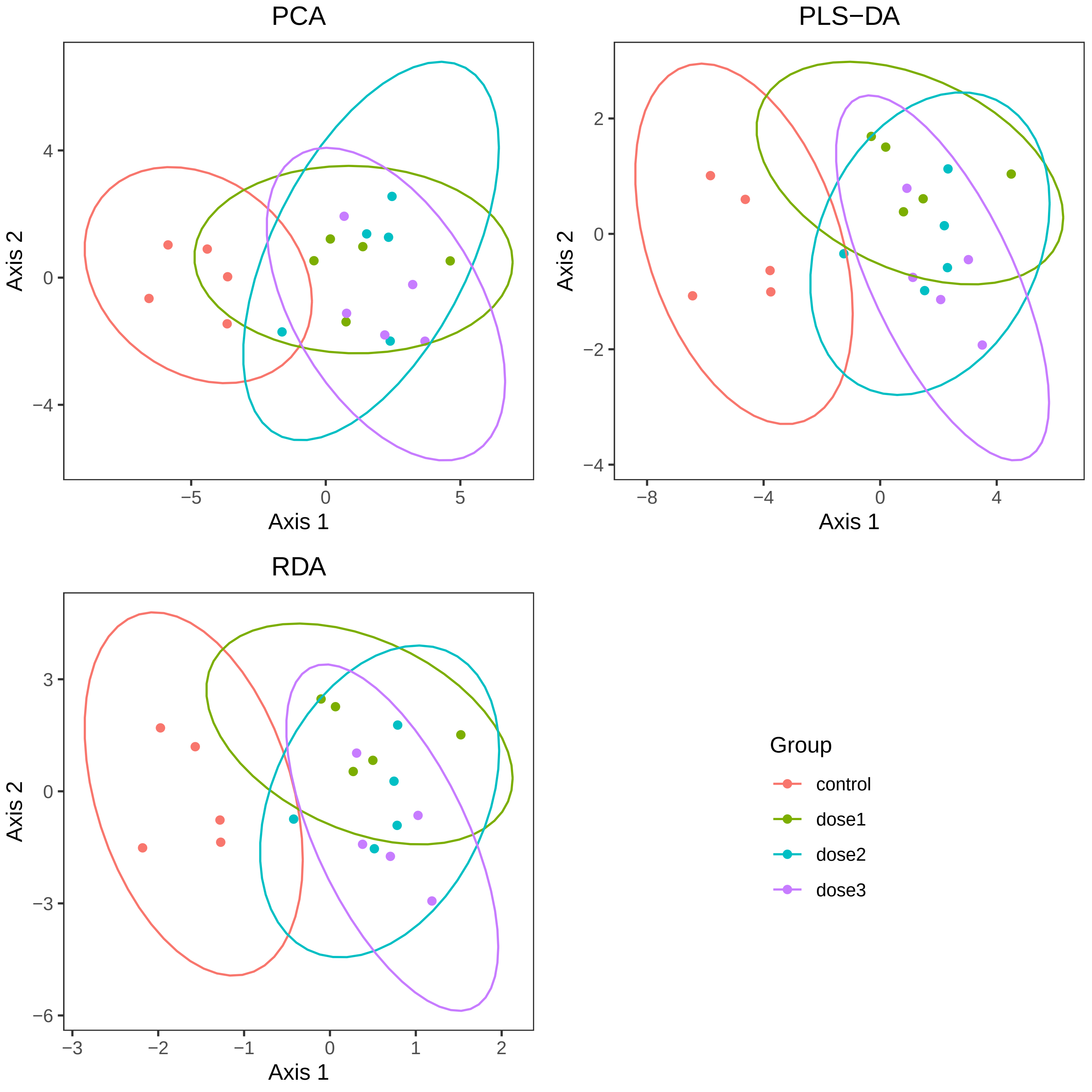

PCA
PLS−DA
RDA
Axis 1
Axis 2
Group
control
dose1
dose2
dose3

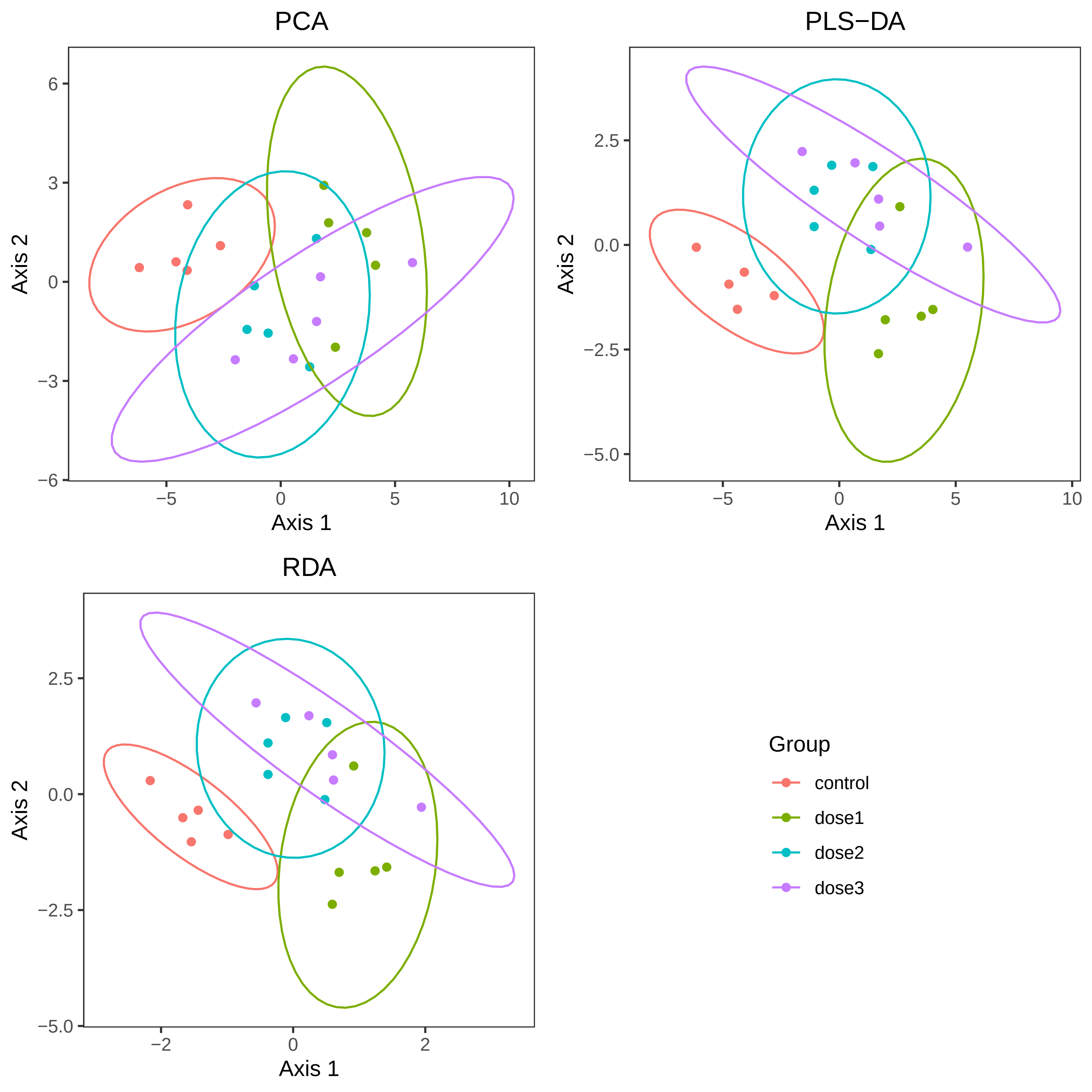

PCA
PLS−DA
RDA
Axis 1
Axis 2
6
3
0
−3
−6
−5
0
5
10
2.5
0.0
−2.5
−5.0
−2
2
Group
control
dose1
dose2
dose3

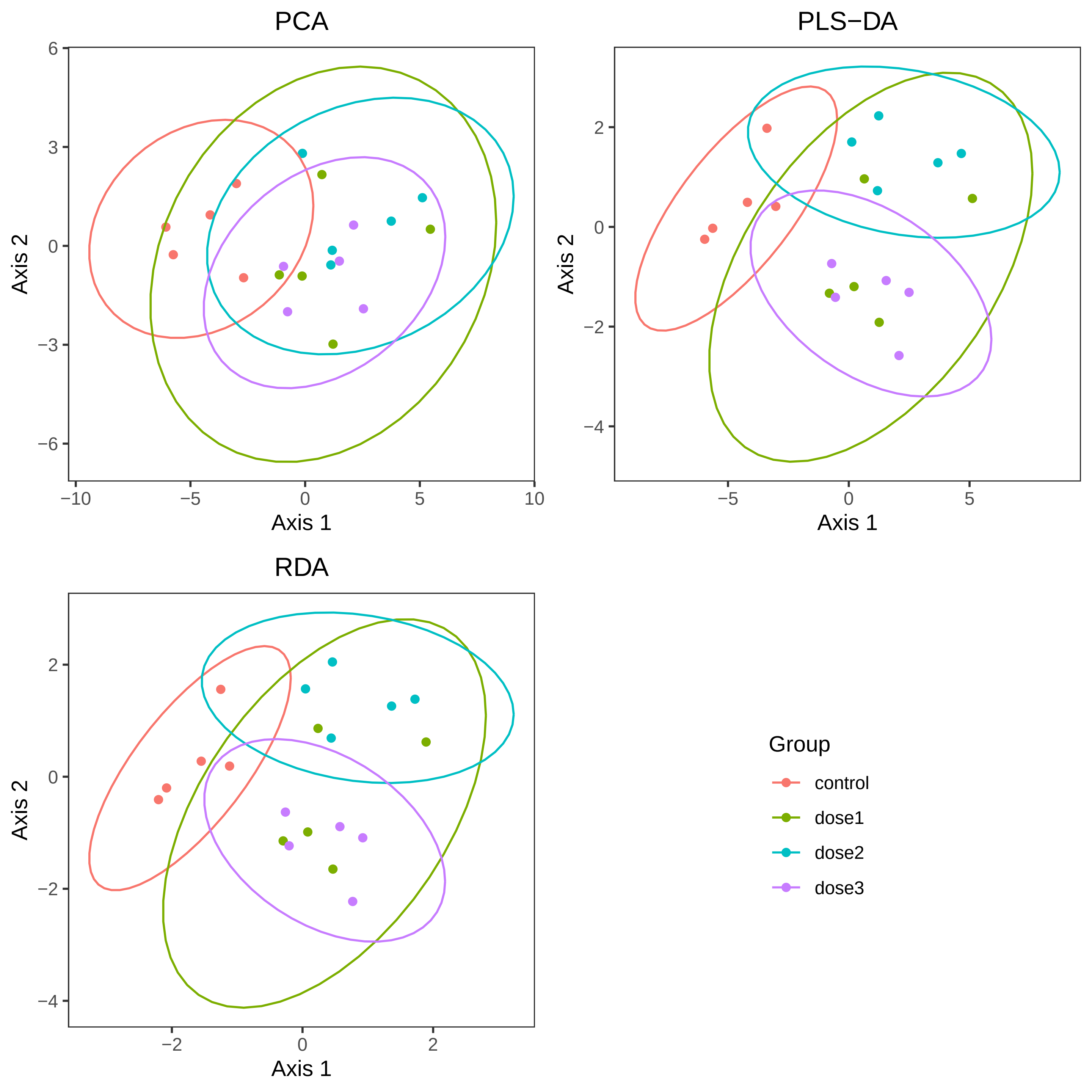

PCA
PLS−DA
RDA
Axis 1
Axis 2
Group
control
dose1
dose2
dose3

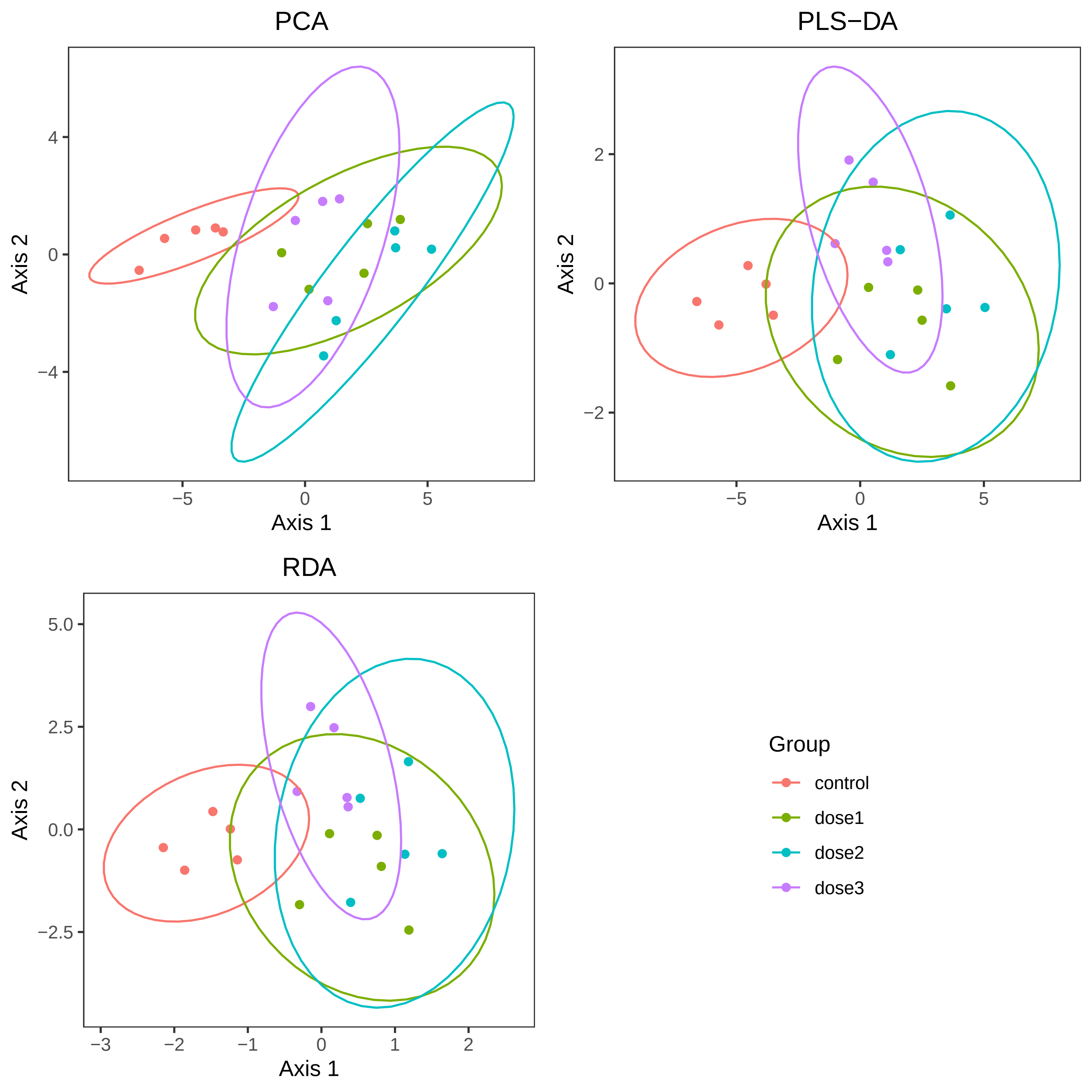
PCA
PLS−DA
RDA
Axis 1
Axis 2
Group
control
dose1
dose2
dose3

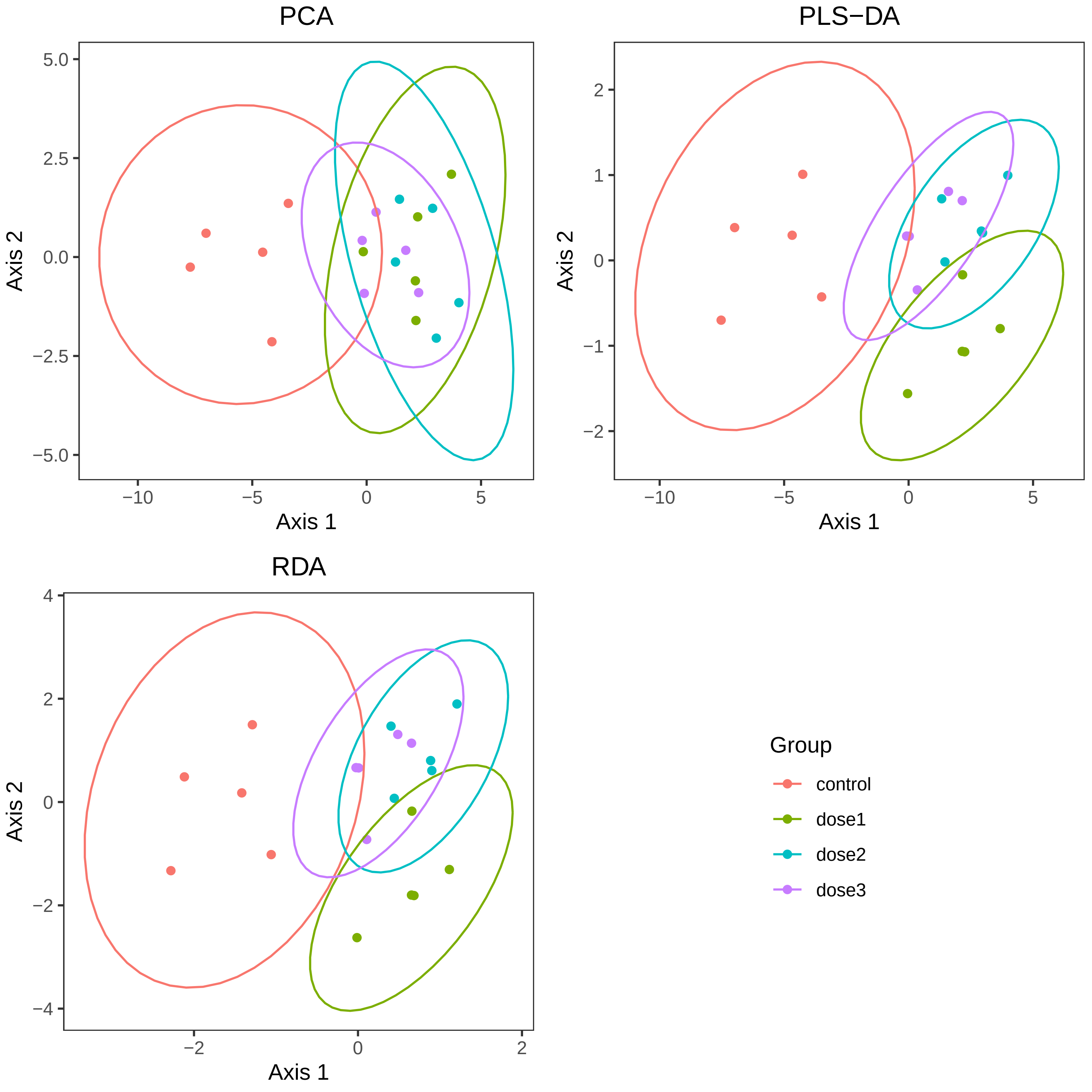
PCA
PLS−DA
RDA
Axis 1
Axis 2
Group
control
dose1
dose2
dose3

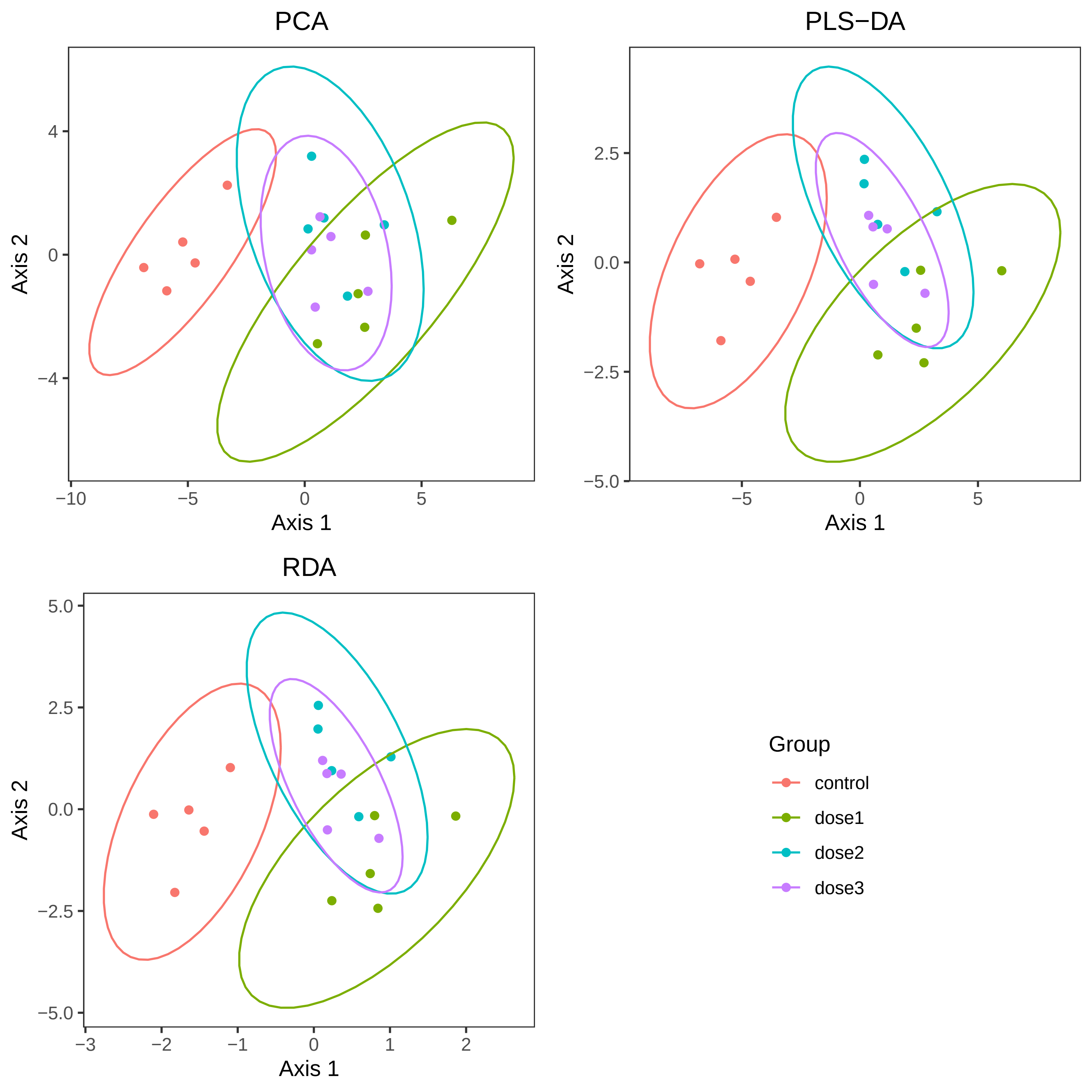

PCA
PLS−DA
RDA
Axis 1
Axis 2
Group
control
dose1
dose2
dose3

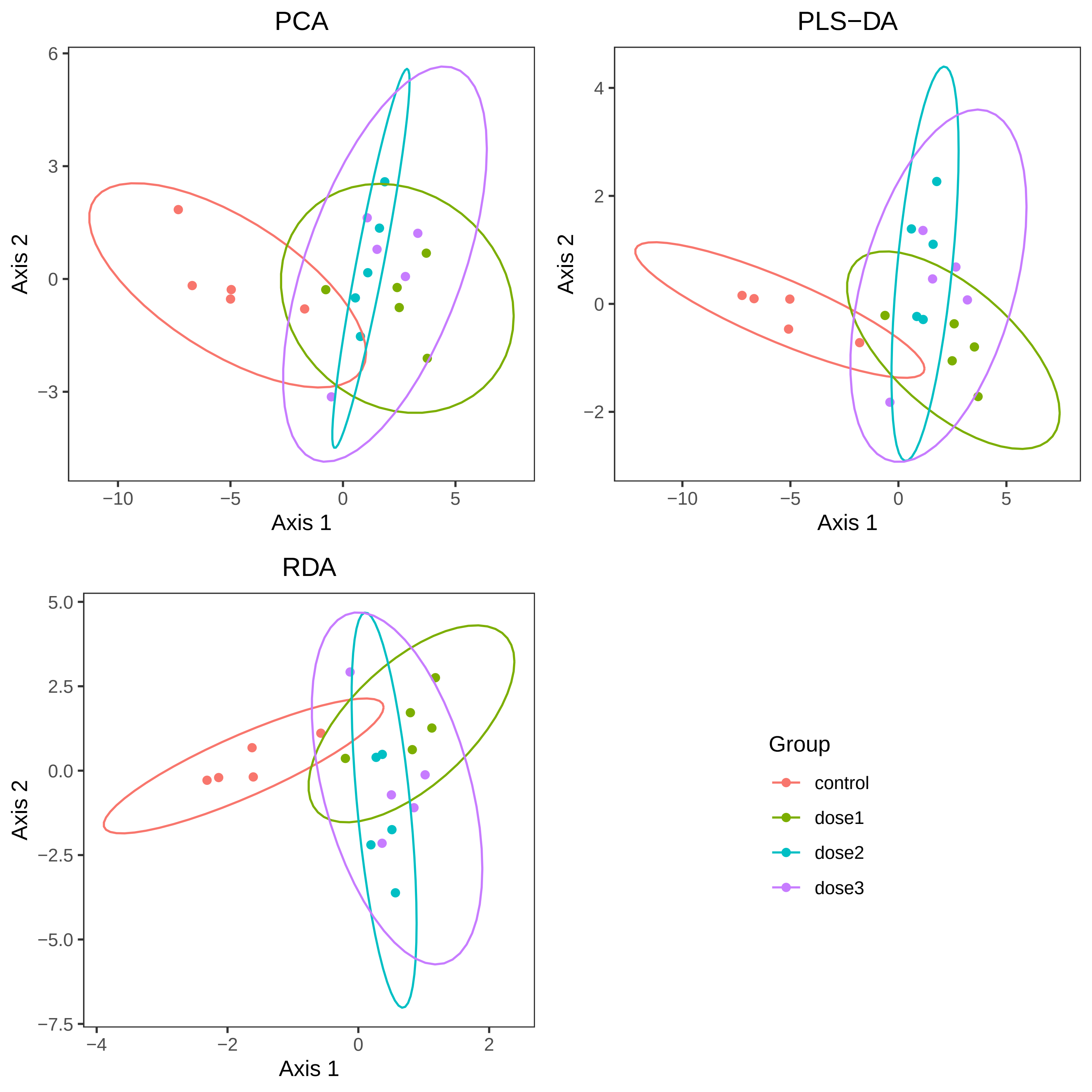
PCA
PLS−DA
RDA
Axis 1
Axis 2
Group
control
dose1
dose2
dose3

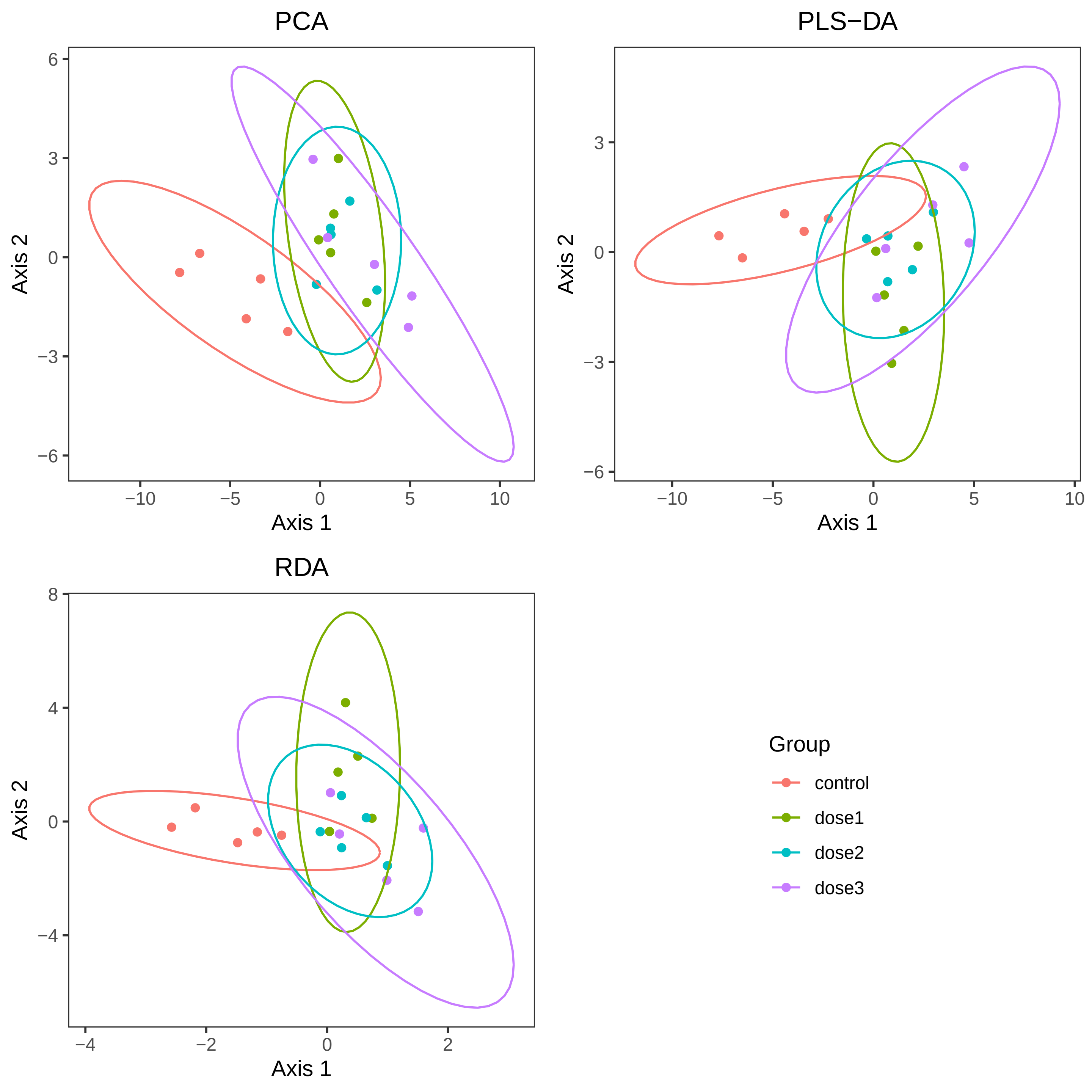
PCA
PLS−DA
RDA
Axis 1
Axis 2
Group
control
dose1
dose2
dose3

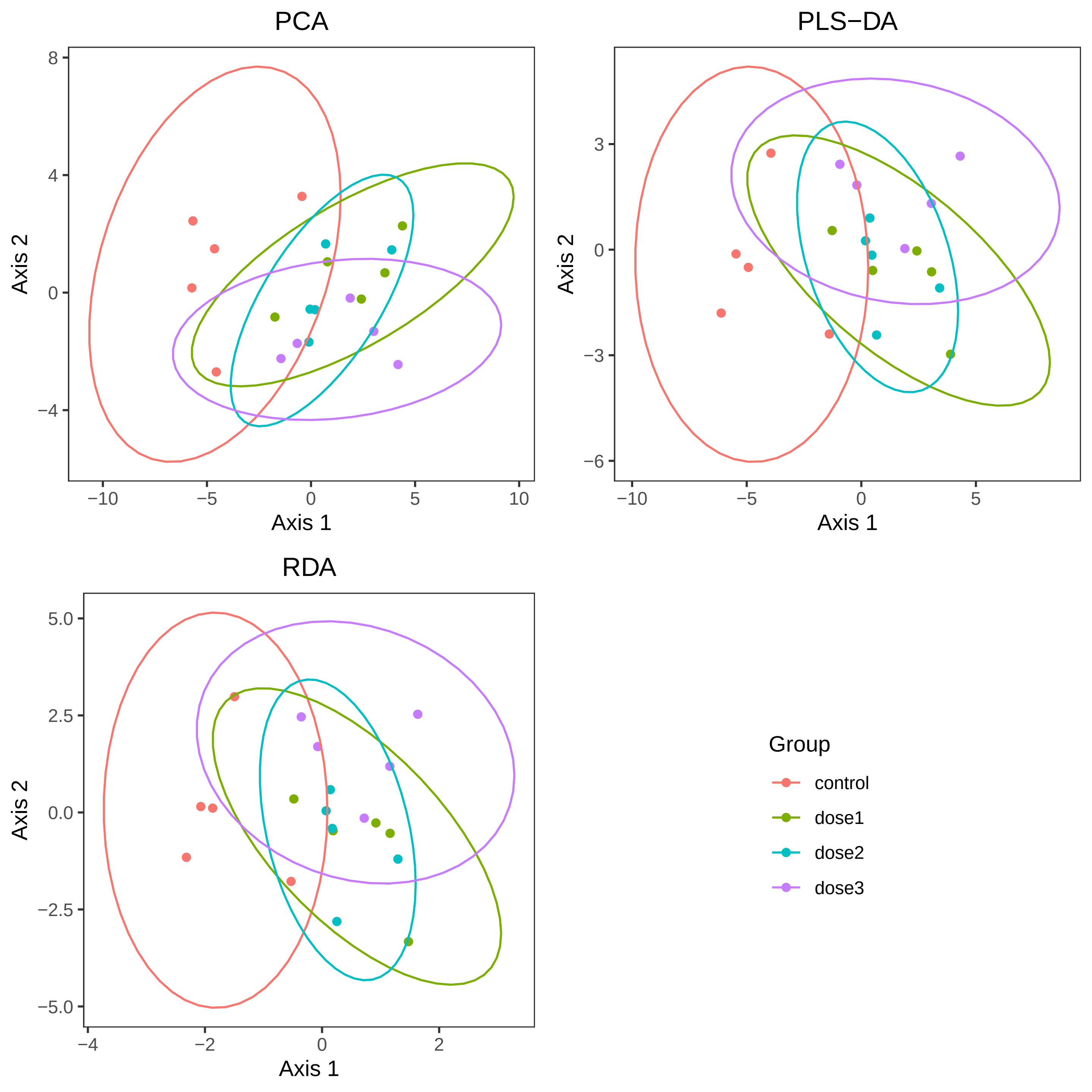

PCA
PLS−DA
RDA
Axis 1
Axis 2
Group
control
dose1
dose2
dose3

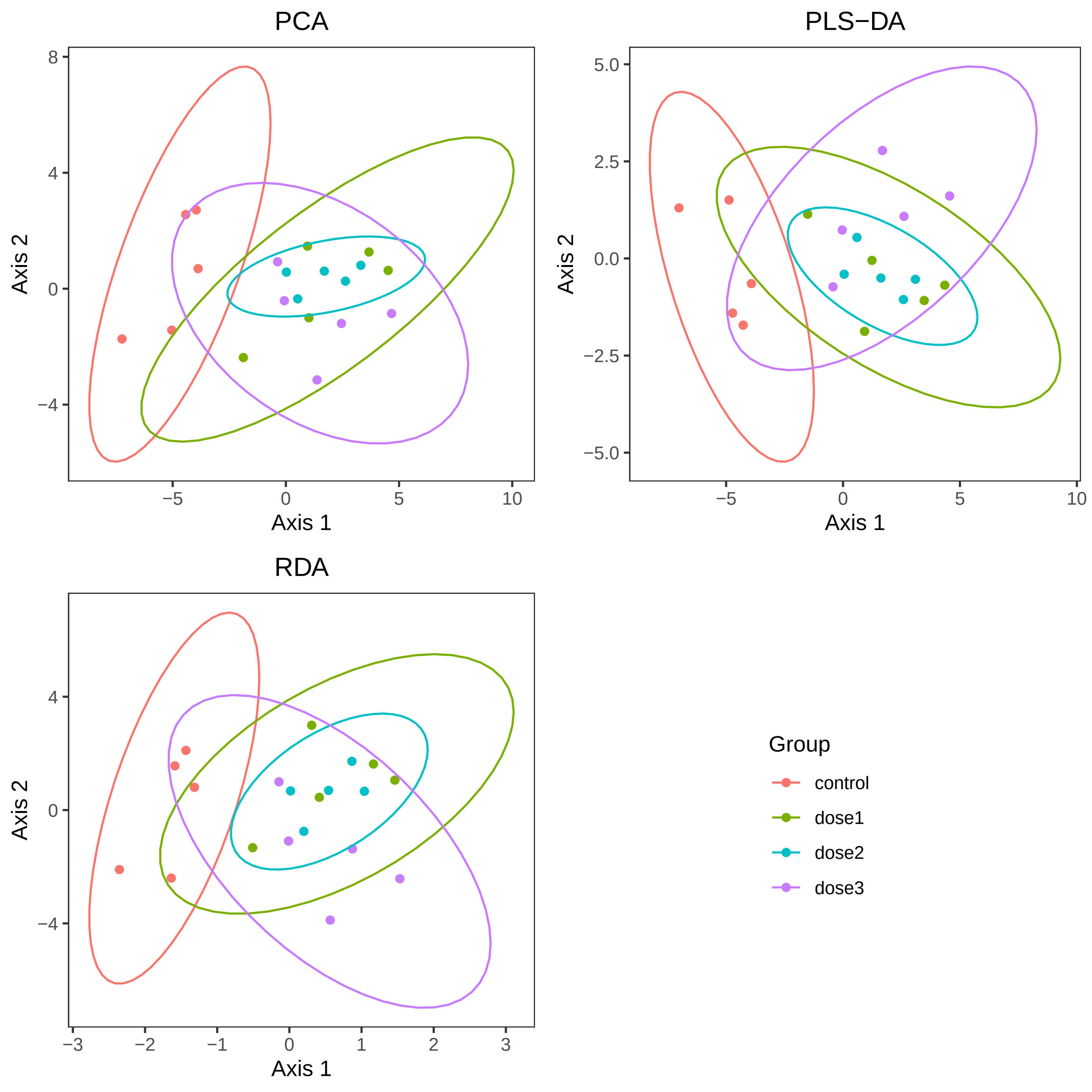

PCA
PLS−DA
RDA
Axis 1
Axis 2
8
4
0
−4
−5
5
10
5.0
2.5
0.0
−2.5
−5.0
−3
−2
−1
1
2
3
Group
control
dose1
dose2
dose3

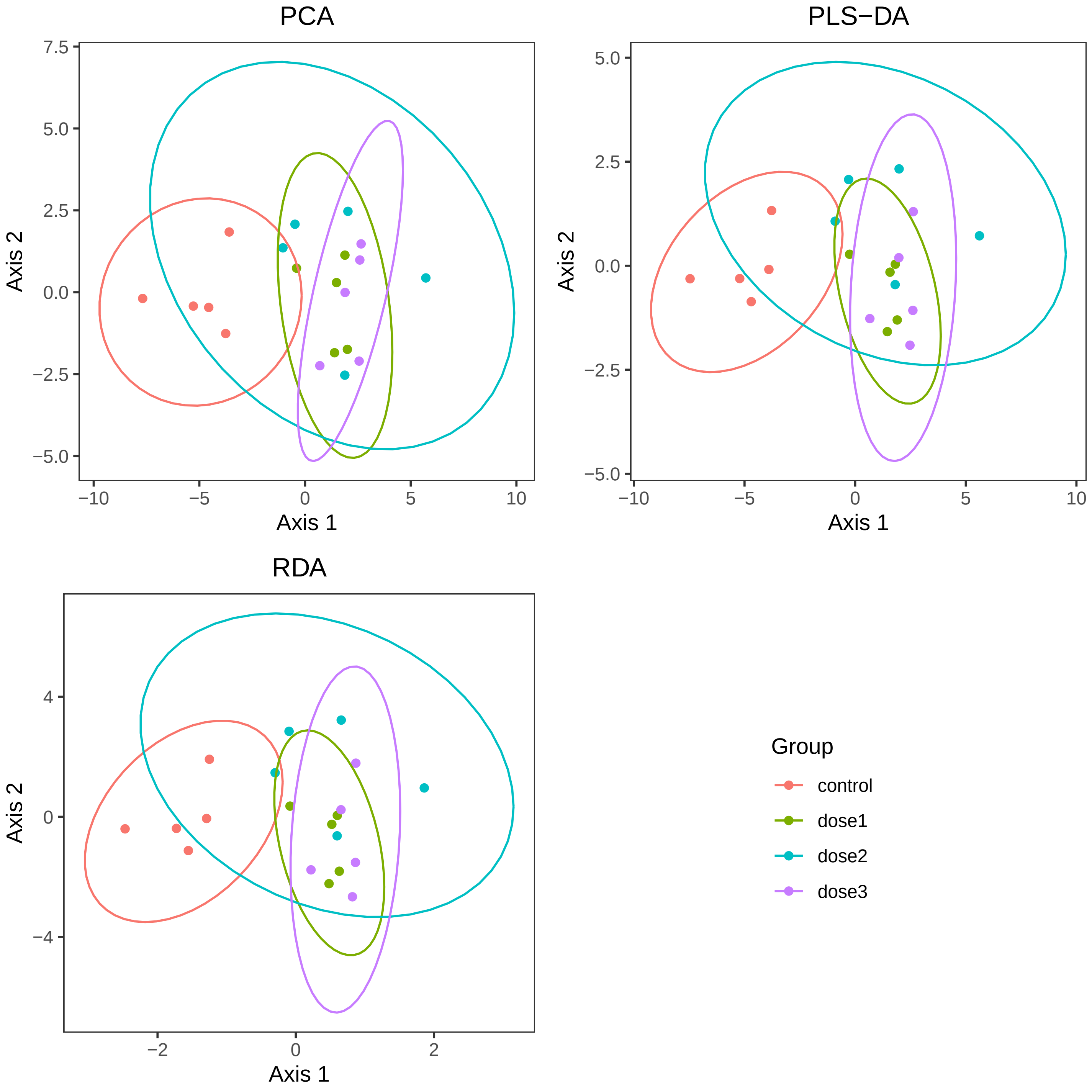

PCA
PLS−DA
RDA
Axis 1
Axis 2
Group
control
dose1
dose2
dose3

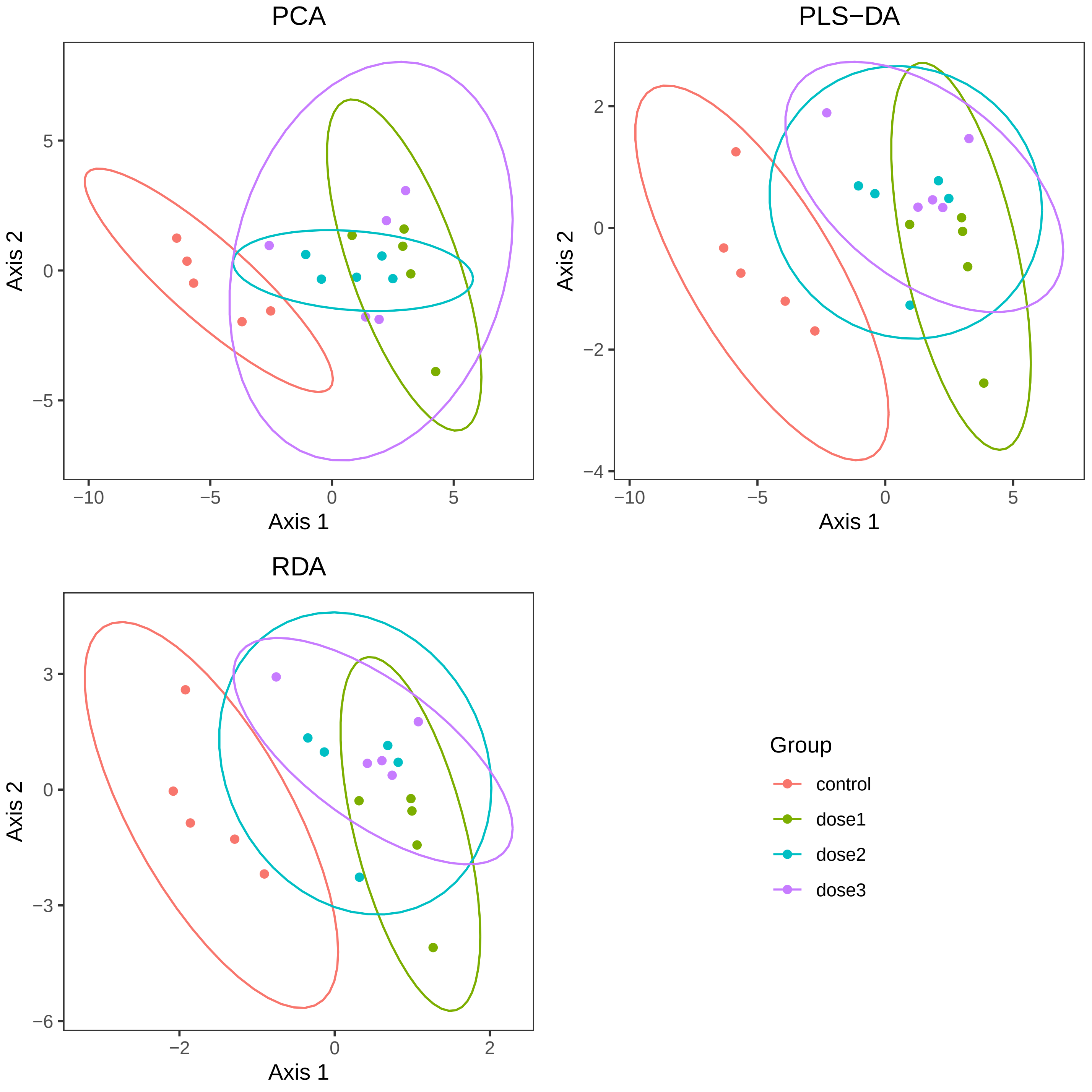
PCA
PLS−DA
RDA
Axis 1
Axis 2
Group
control
dose1
dose2
dose3

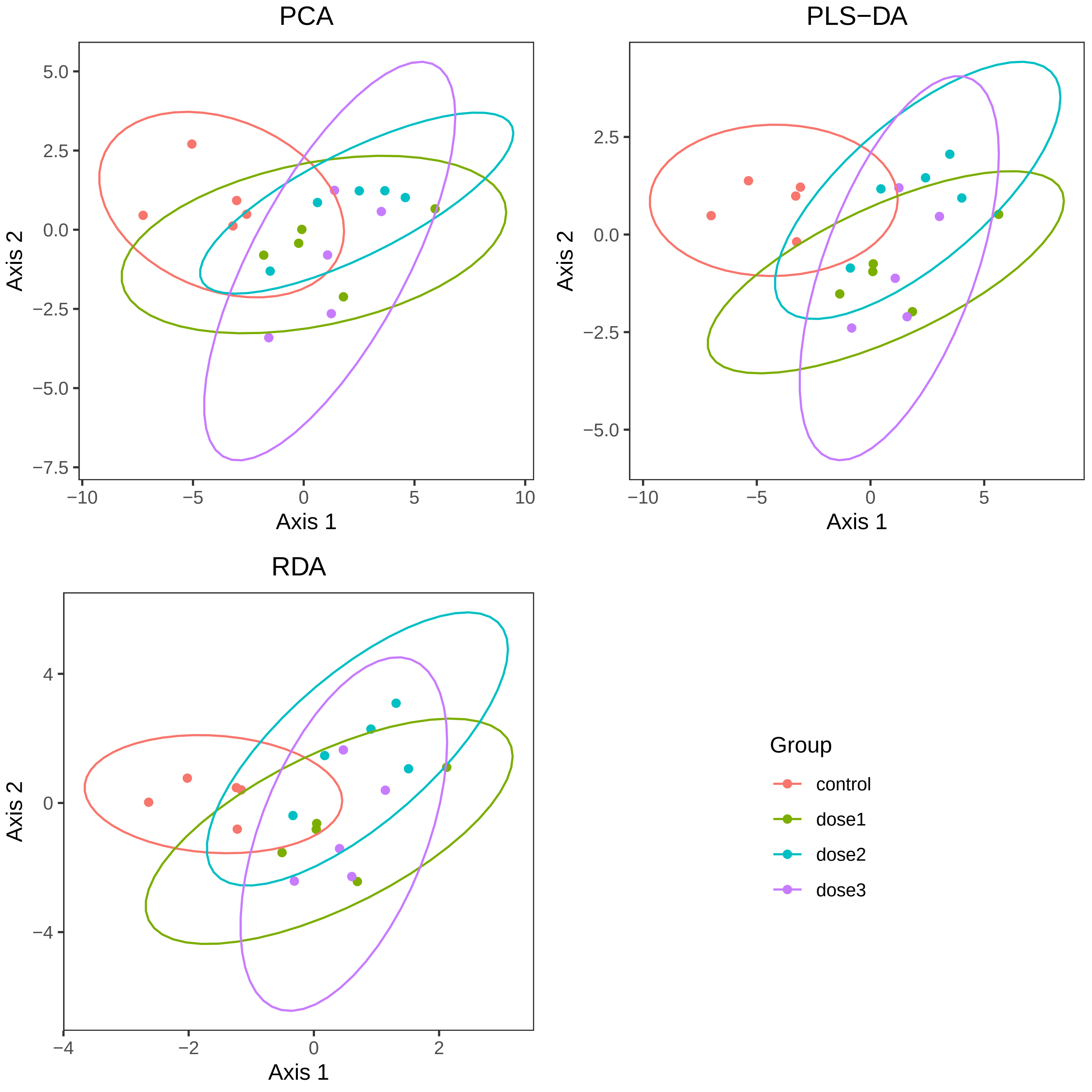

PCA
PLS−DA
RDA
Axis 1
Axis 2
5.0
2.5
0.0
−2.5
−5.0
−7.5
−10
−5
0
5
10
4
−4
−2
2
Group
control
dose1
dose2
dose3

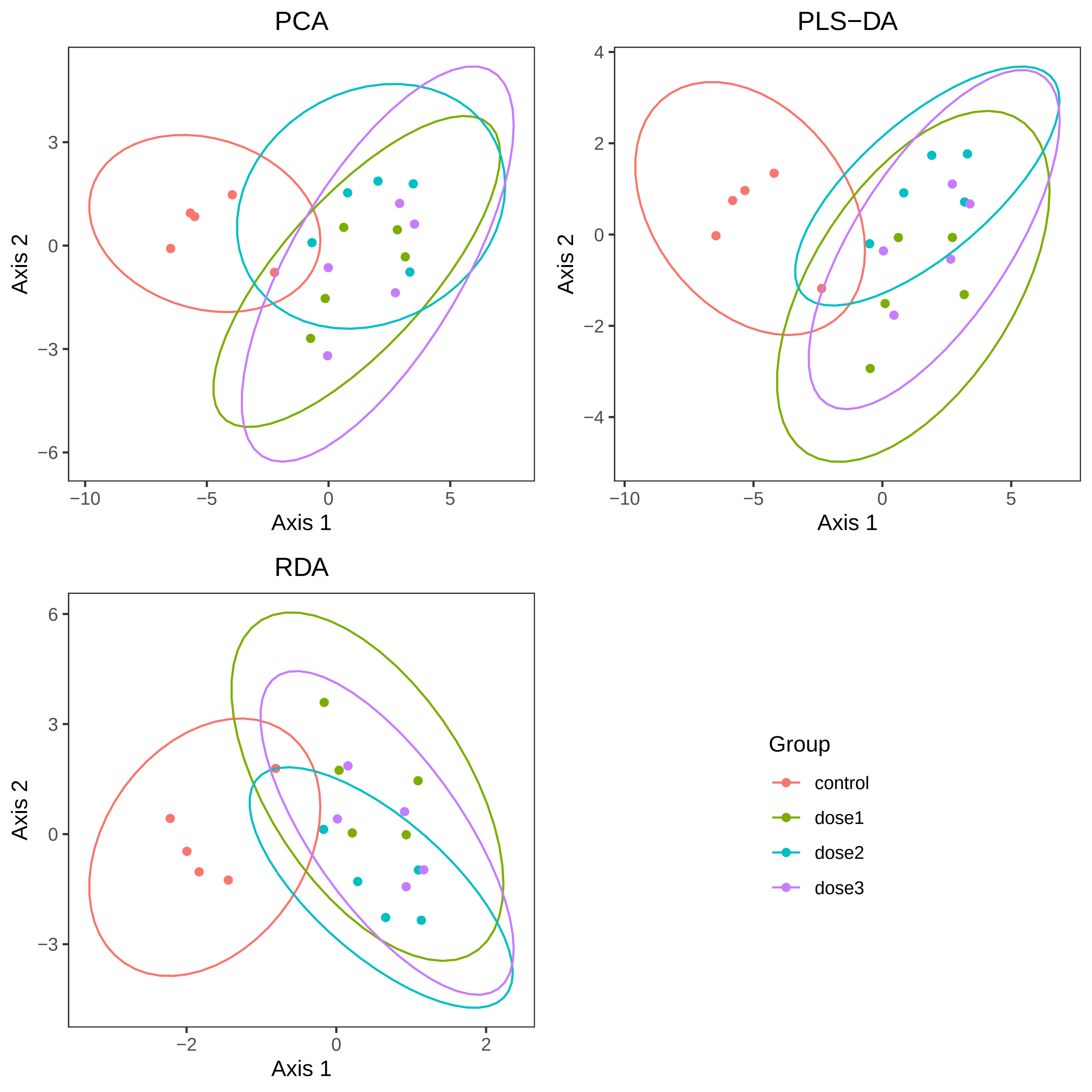
PCA
PLS−DA
RDA
Axis 1
Axis 2
Group
control
dose1
dose2
dose3

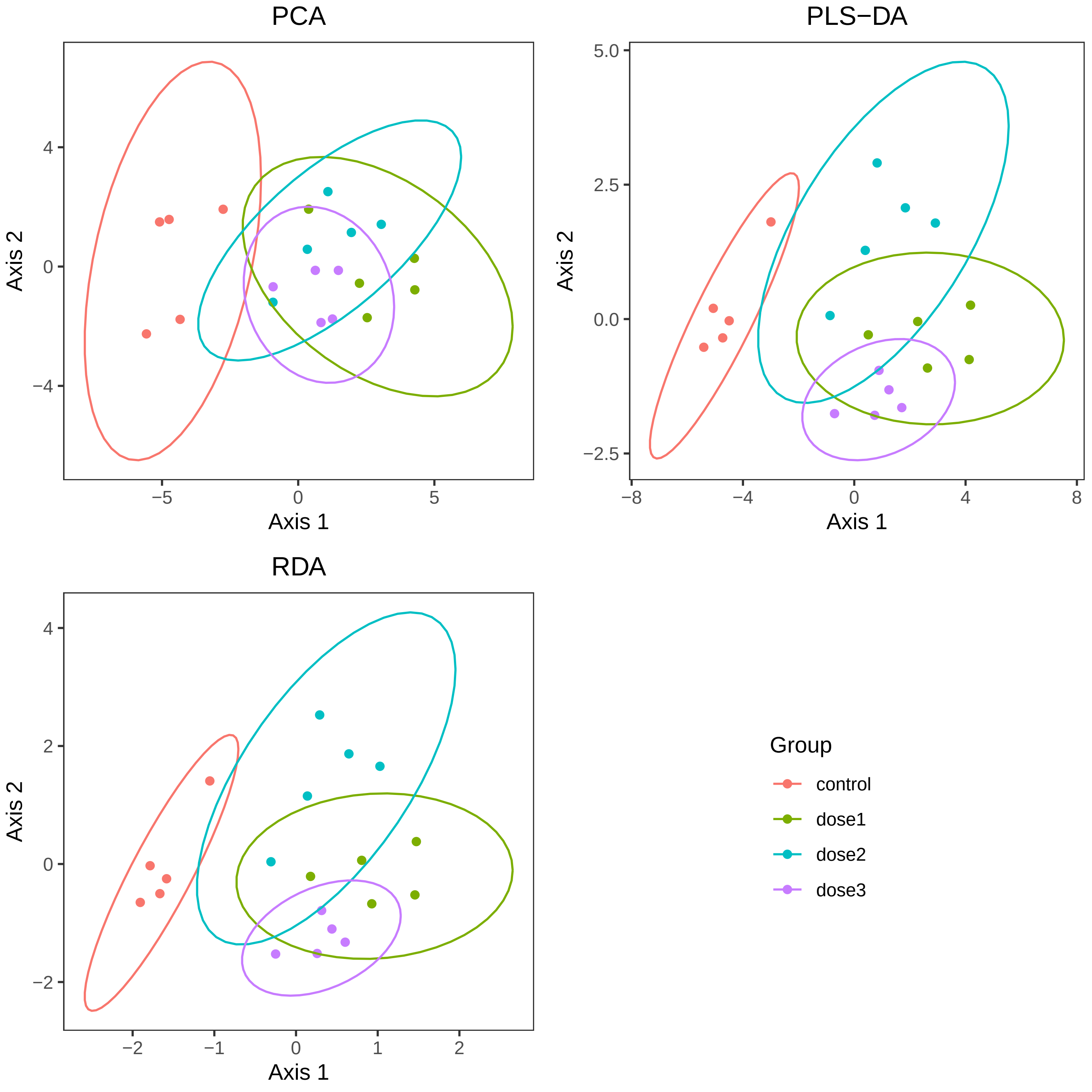

PCA
PLS−DA
RDA
Axis 1
Axis 2
Group
control
dose1
dose2
dose3

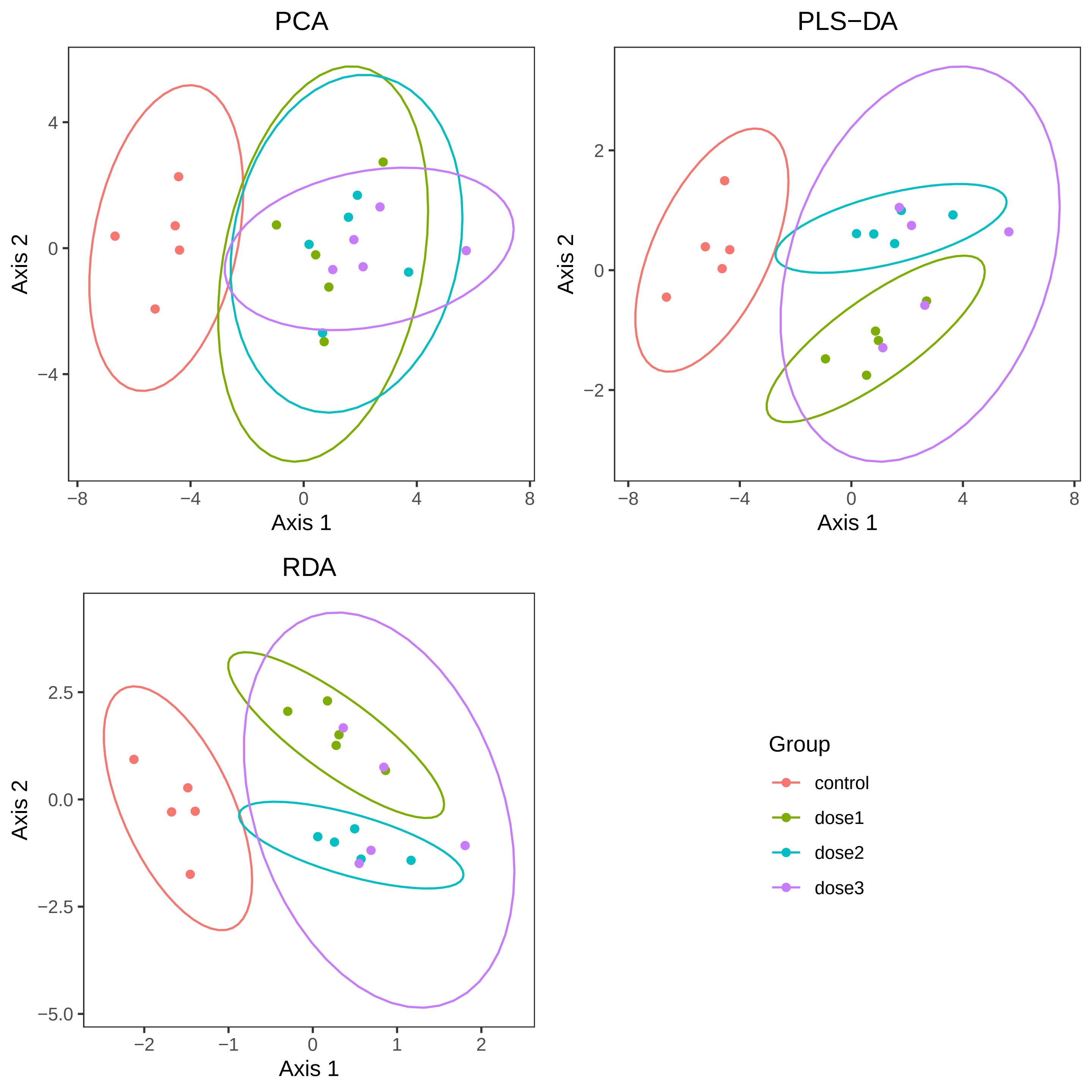

PCA
PLS−DA
RDA
Axis 1
Axis 2
−8
−4
0
4
8
2
−2
2.5
0.0
−2.5
−5.0
−2
−1
1
Group
control
dose1
dose2
dose3

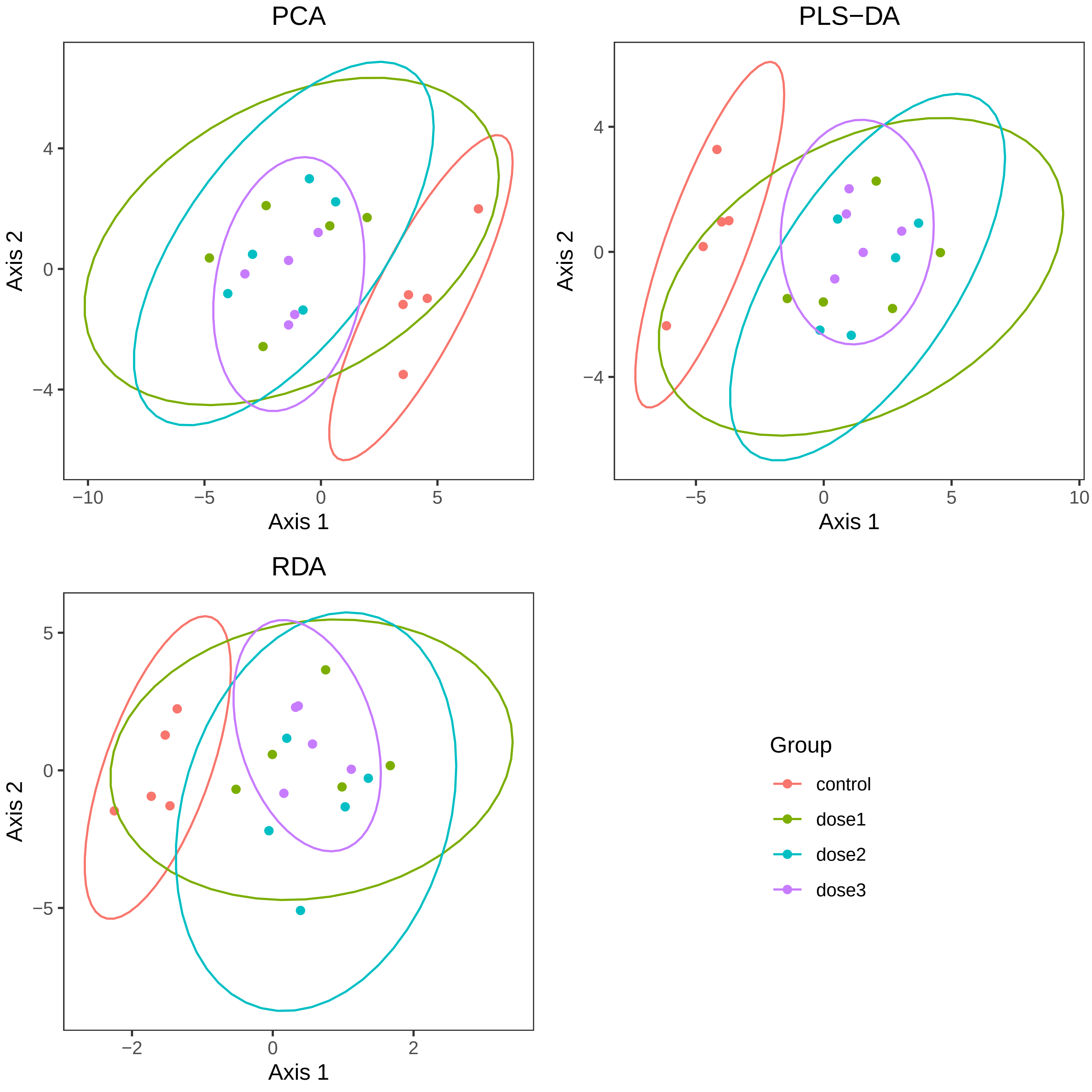
PCA
PLS−DA
RDA
Axis 1
Axis 2
Group
control
dose1
dose2
dose3

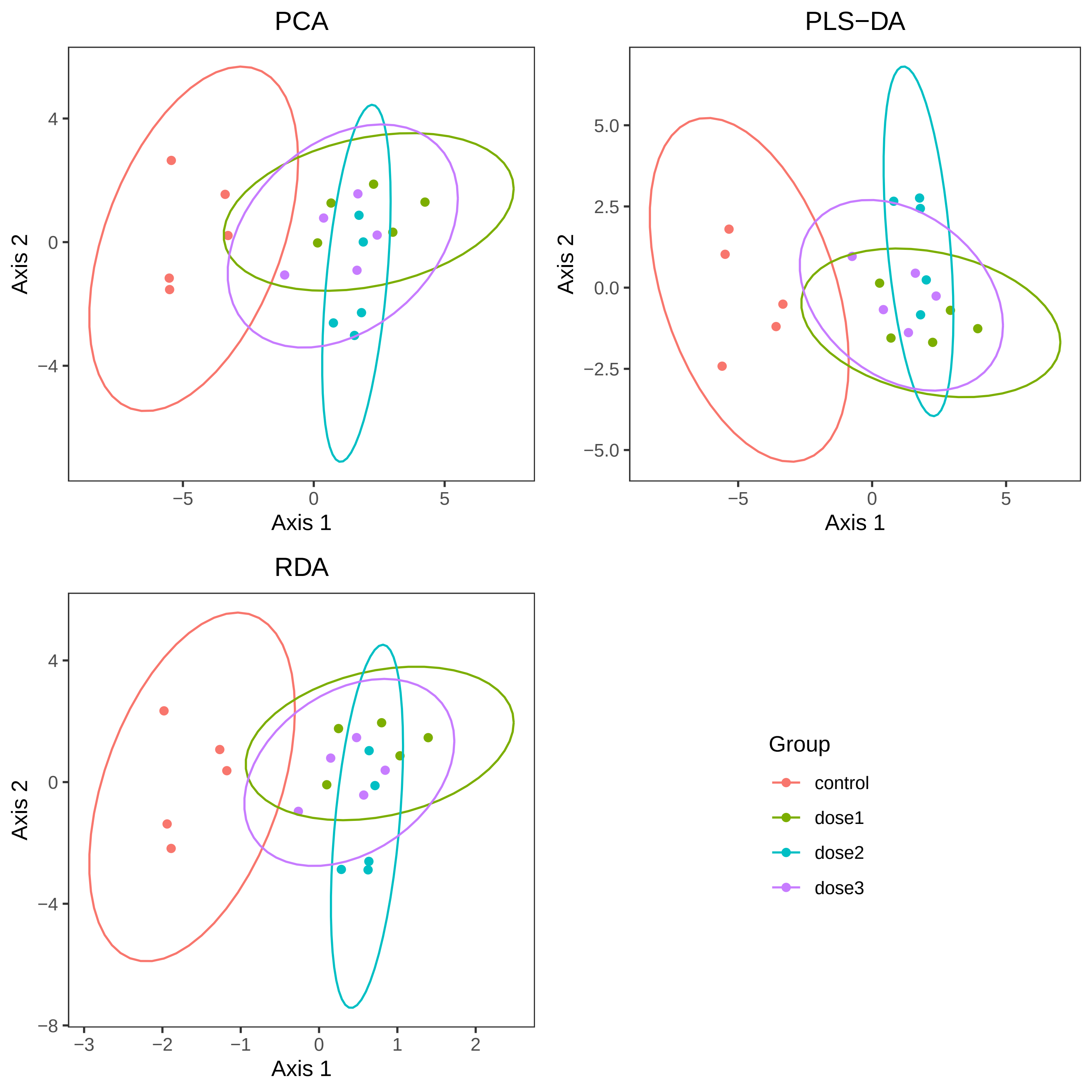

PCA
PLS−DA
RDA
Axis 1
Axis 2
Group
control
dose1
dose2
dose3

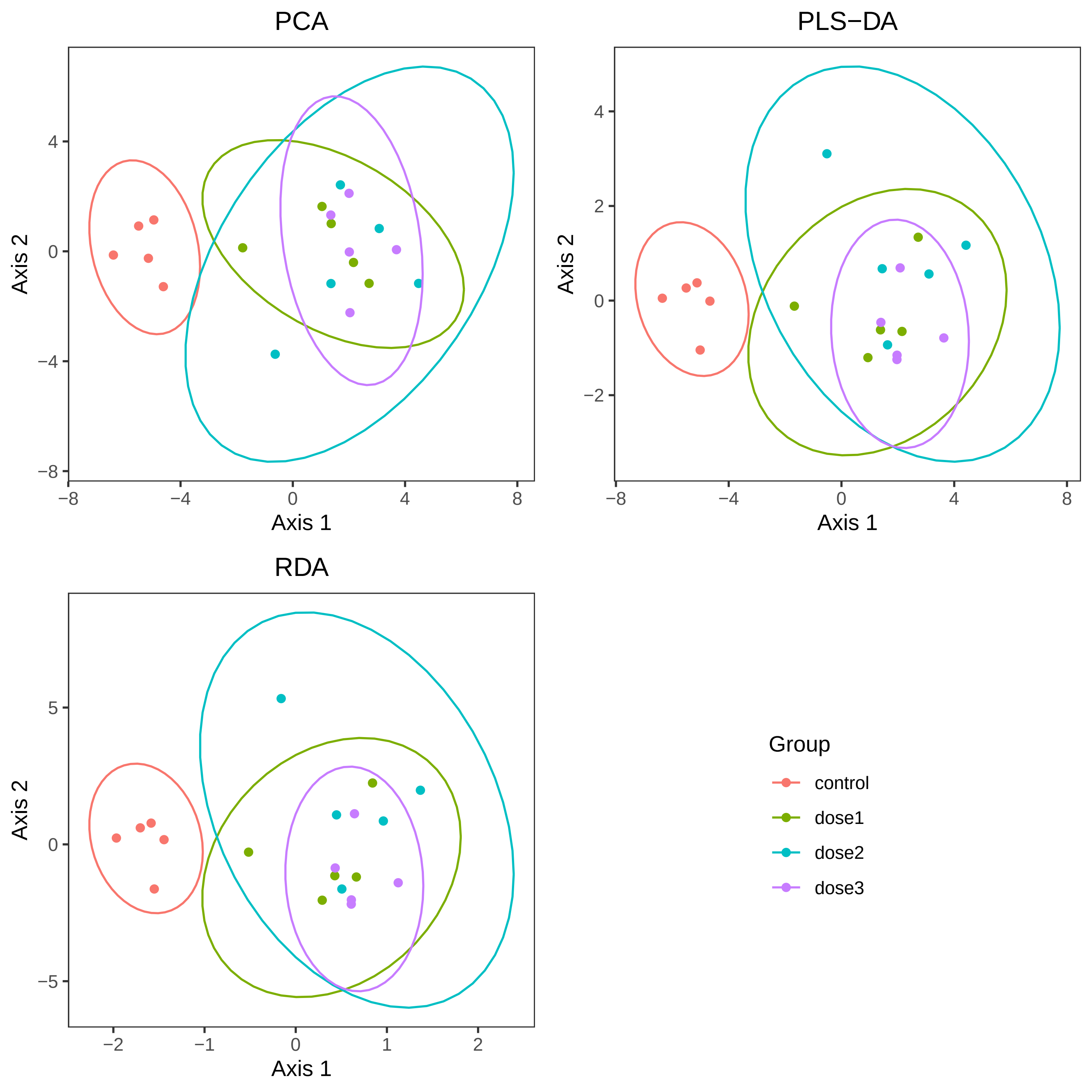

PCA
PLS−DA
RDA
Axis 1
Axis 2
Group
control
dose1
dose2
dose3

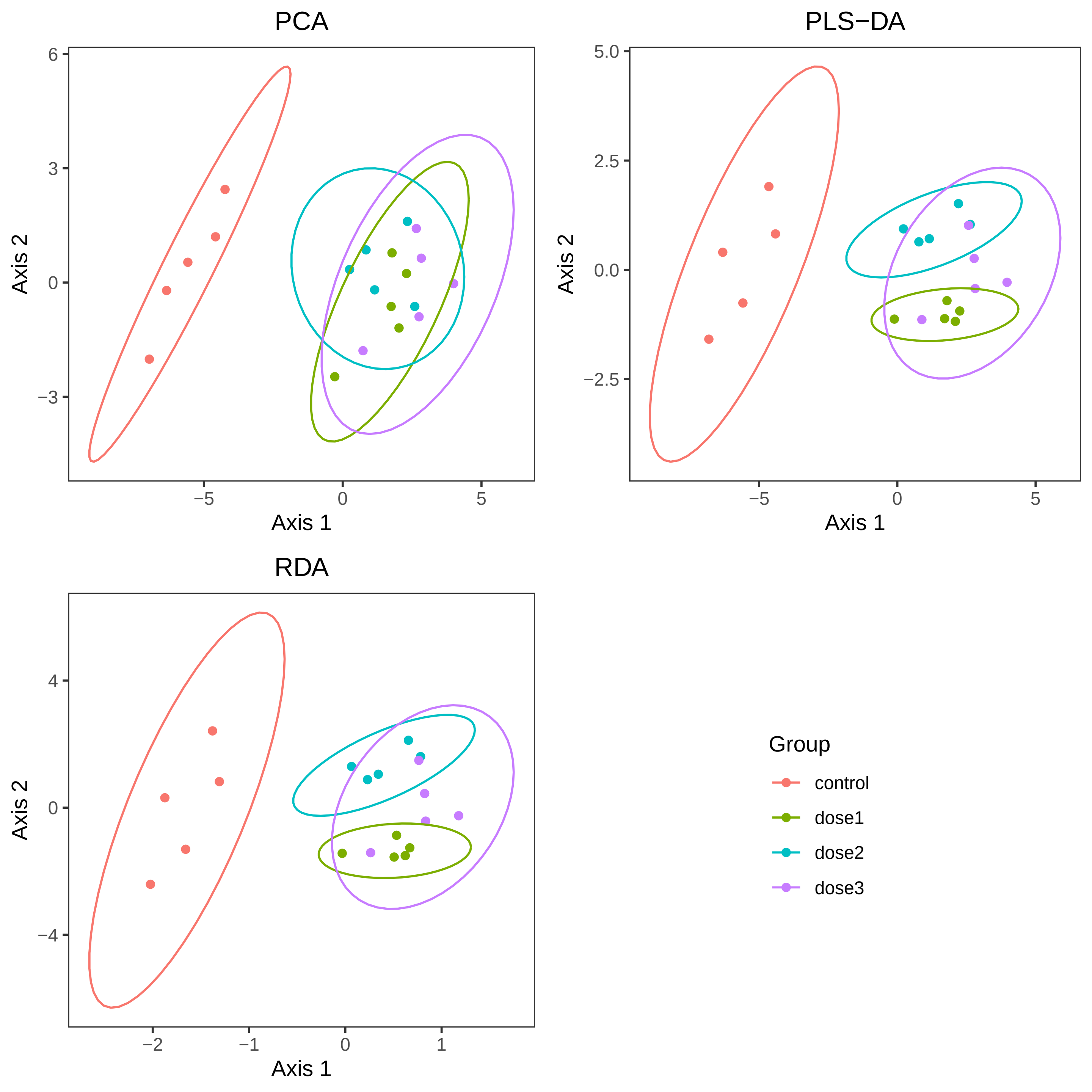

PCA
PLS−DA
RDA
Axis 1
Axis 2
Group
control
dose1
dose2
dose3

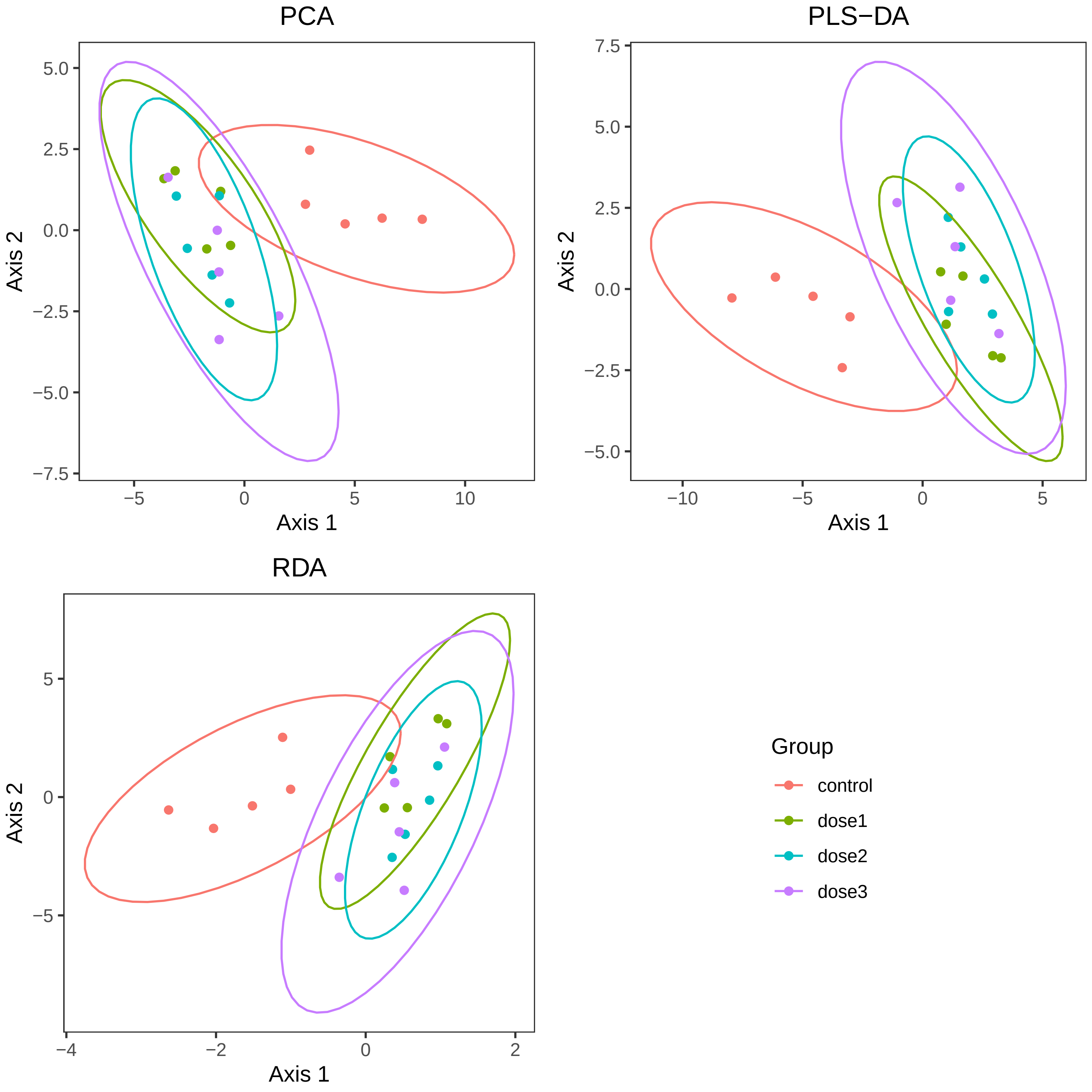
PCA
PLS−DA
RDA
Axis 1
Axis 2
Group
control
dose1
dose2
dose3
5.0
2.5
0.0
−2.5
−5.0
−7.5
−5
0
5
10
7.5
−10
−4
−2
2

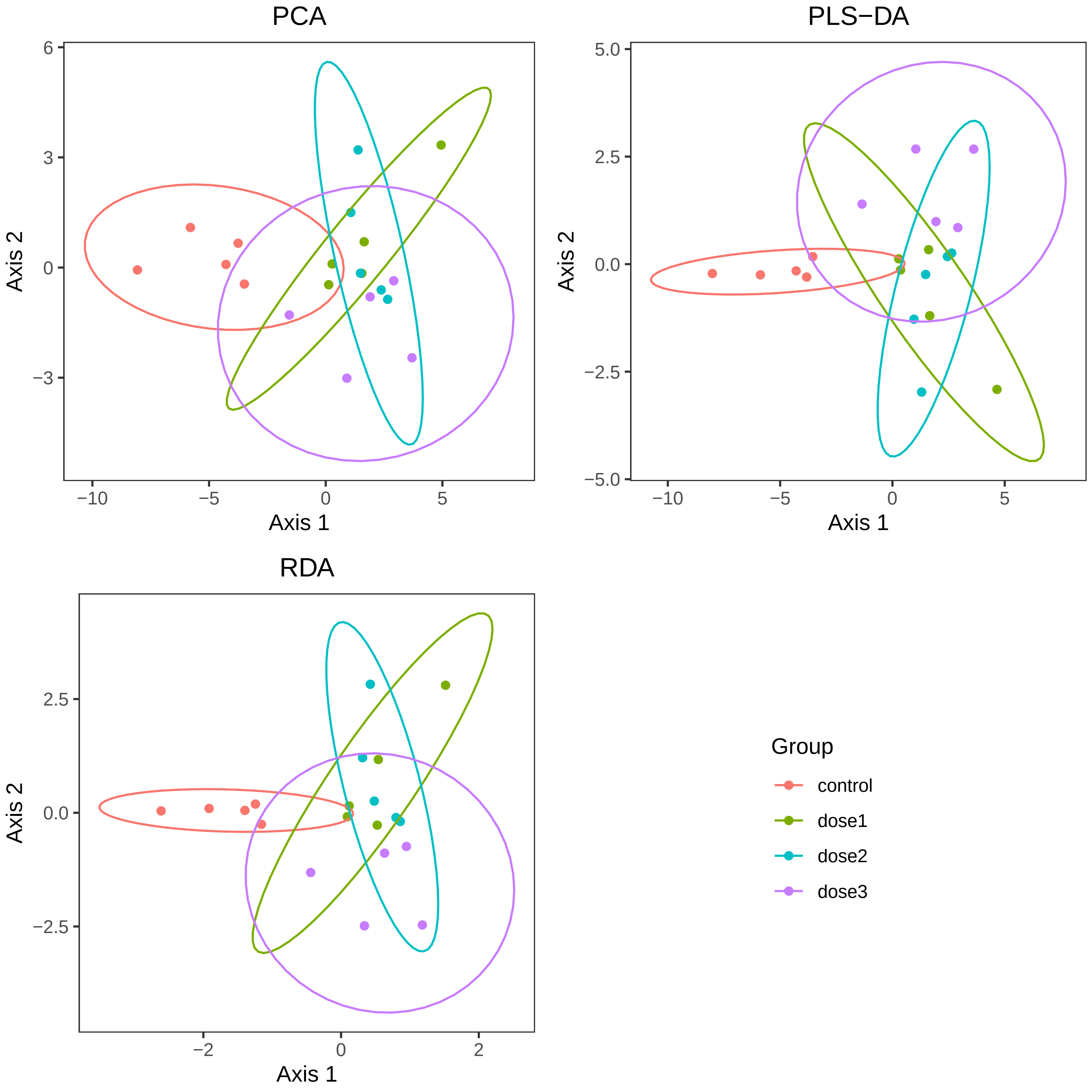

PCA
PLS−DA
RDA
Axis 1
Axis 2
6
3
0
−3
−10
−5
0
5
5.0
2.5
0.0
−2.5
−5.0
−2
2
Group
control
dose1
dose2
dose3

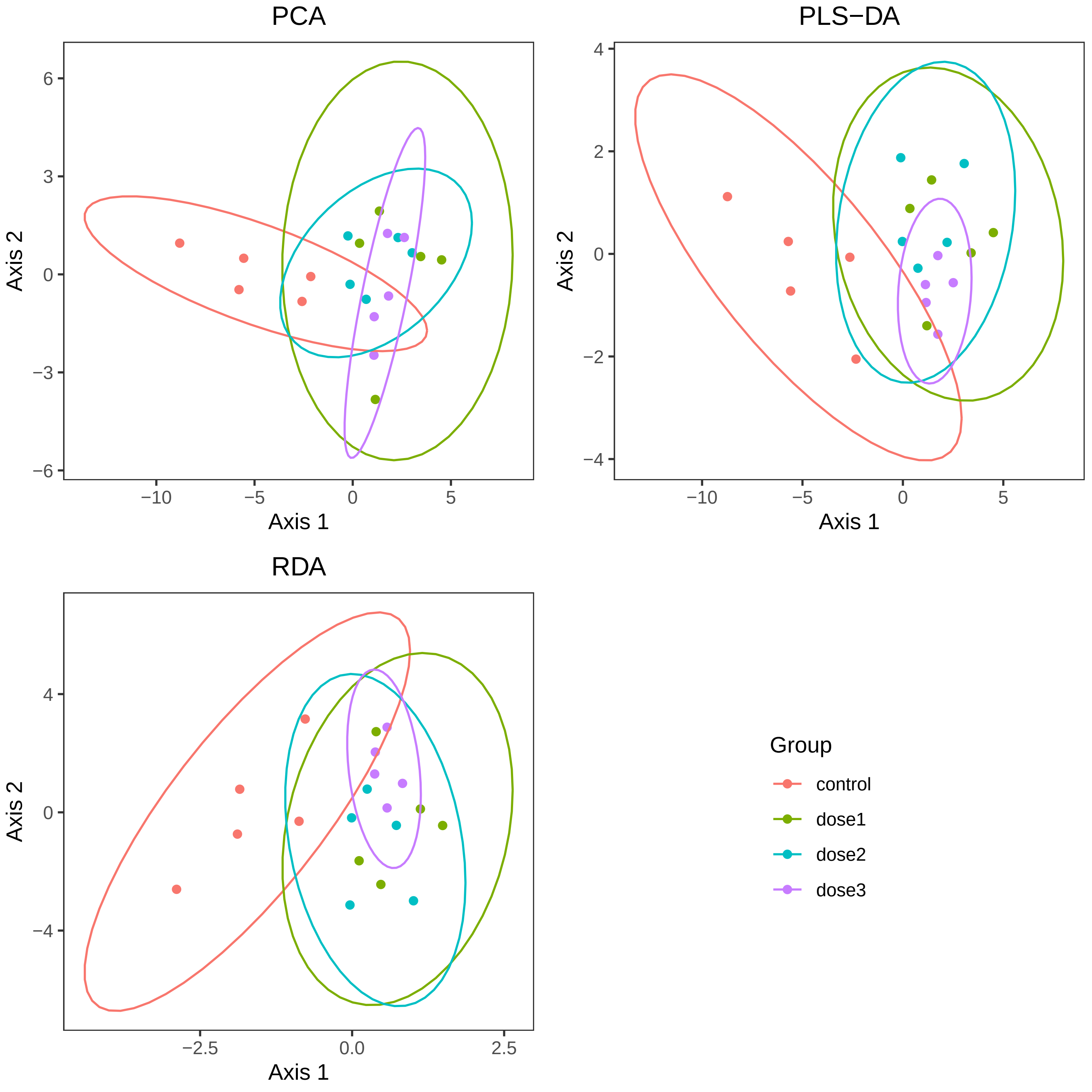
PCA
PLS−DA
RDA
Axis 1
Axis 2
6
3
0
−3
−6
−10
−5
0
5
4
2
0
−2
−4
4
0
−4
−2.5
0.0
2.5
Group
control
dose1
dose2
dose3

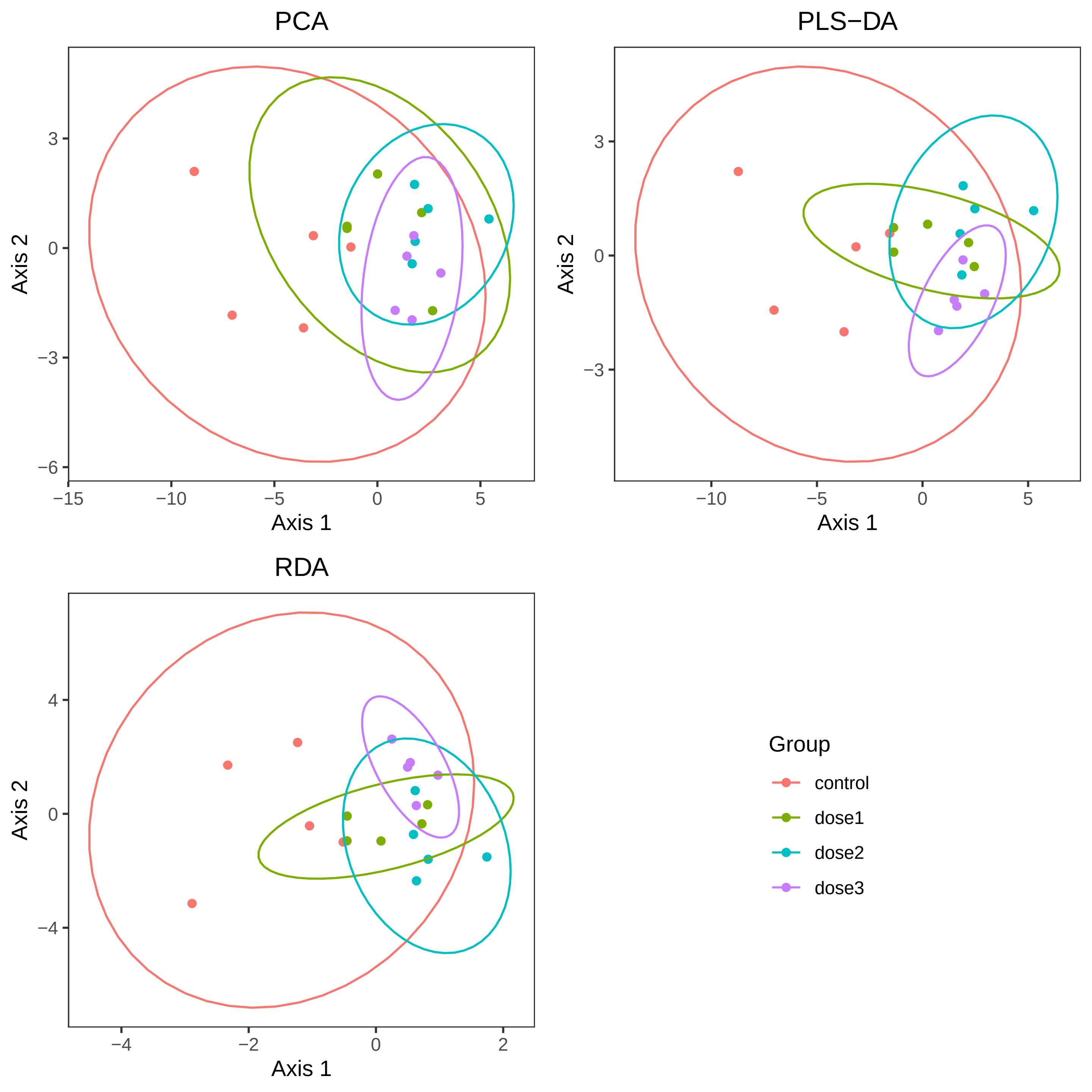

PCA
PLS−DA
RDA
Axis 1
Axis 2
Group
control
dose1
dose2
dose3

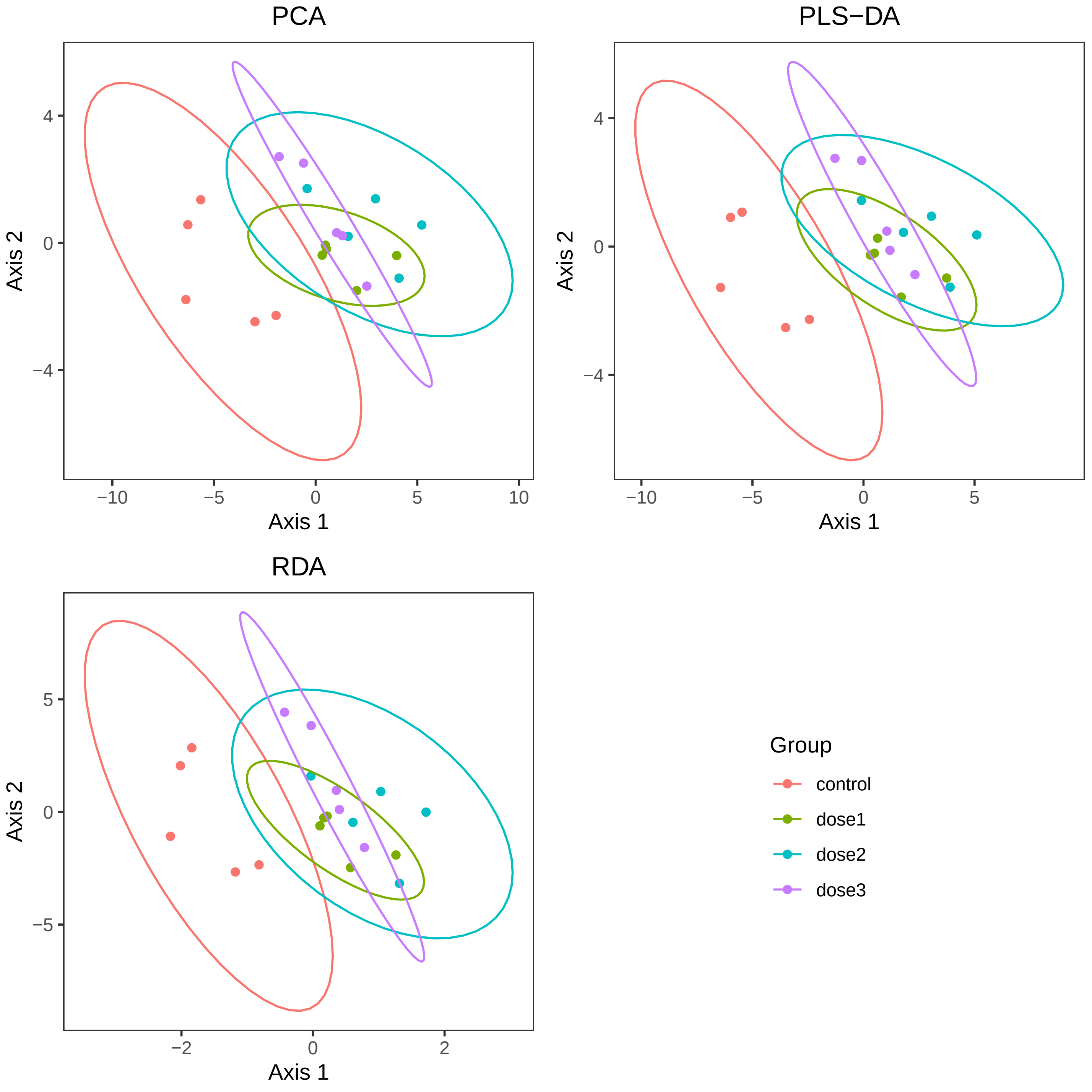

PCA
PLS−DA
RDA
Axis 1
Axis 2
4
0
−4
−10
−5
0
5
10
5
−5
−2
2
Group
control
dose1
dose2
dose3

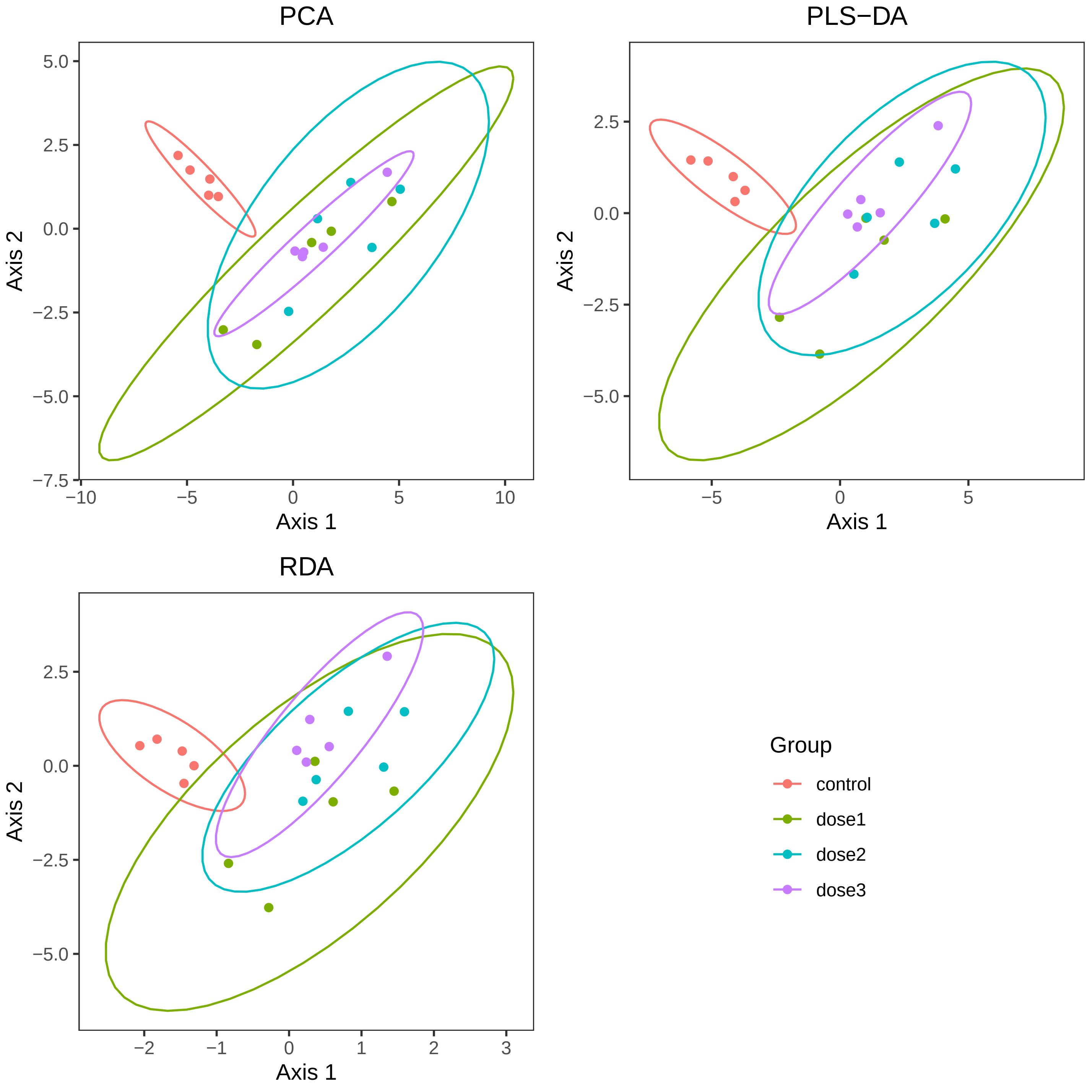
PCA
PLS−DA
RDA
Axis 1
Axis 2
Group
control
dose1
dose2
dose3

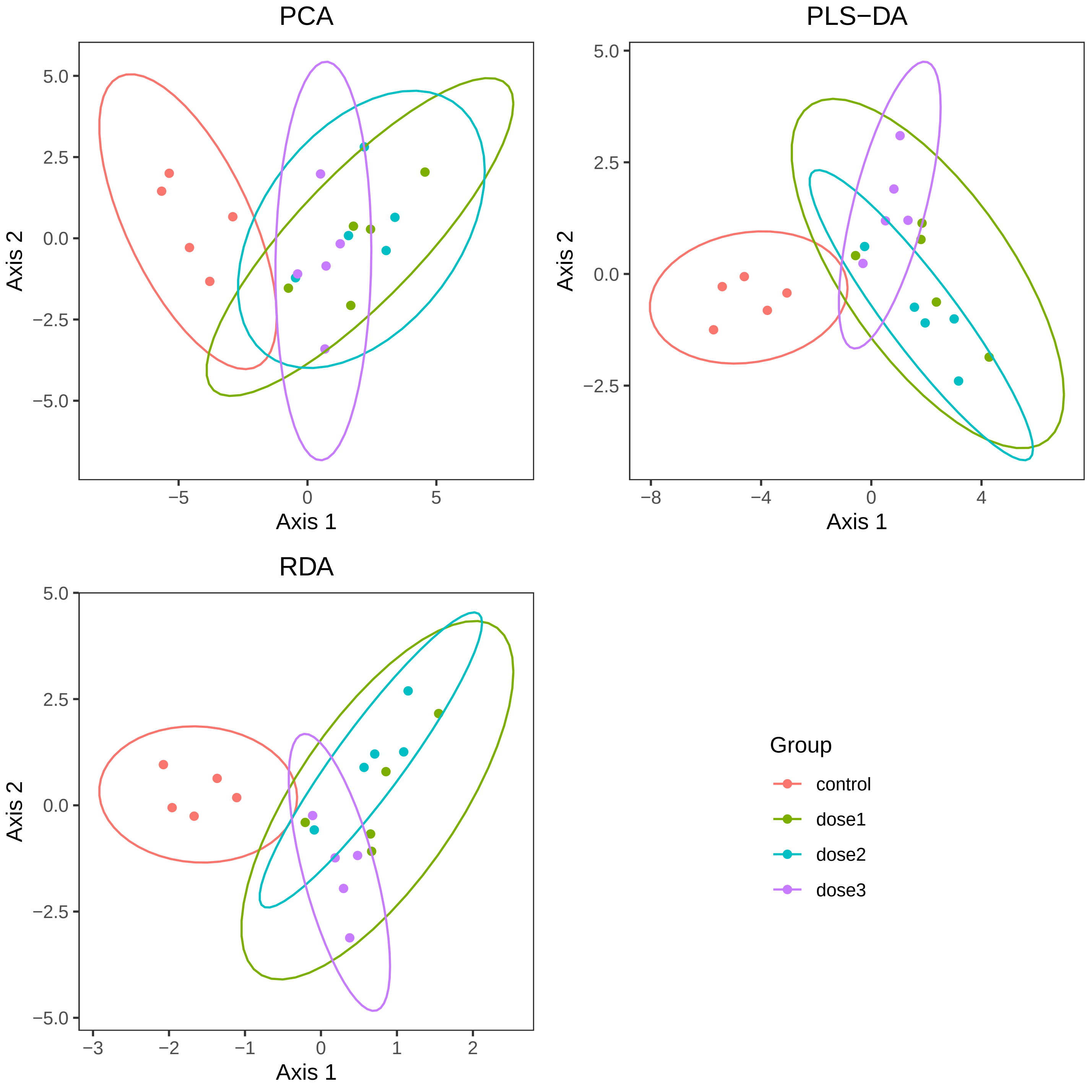

PCA
PLS−DA
RDA
Axis 1
Axis 2
5.0
2.5
0.0
−2.5
−5.0
−5
0
5
−8
−4
4
−3
−2
−1
1
2
Group
control
dose1
dose2
dose3

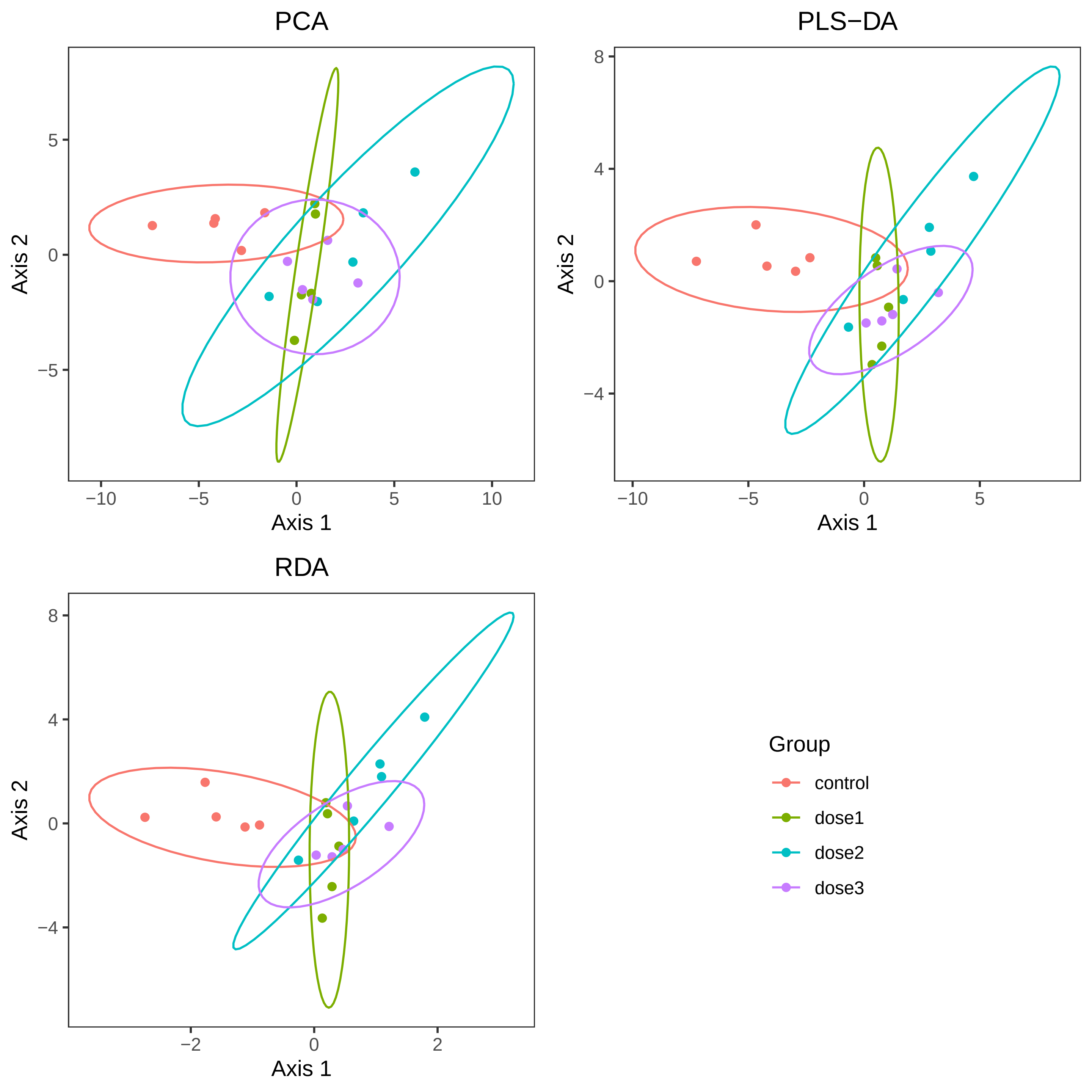
PCA
PLS−DA
RDA
Axis 1
Axis 2
Group
control
dose1
dose2
dose3

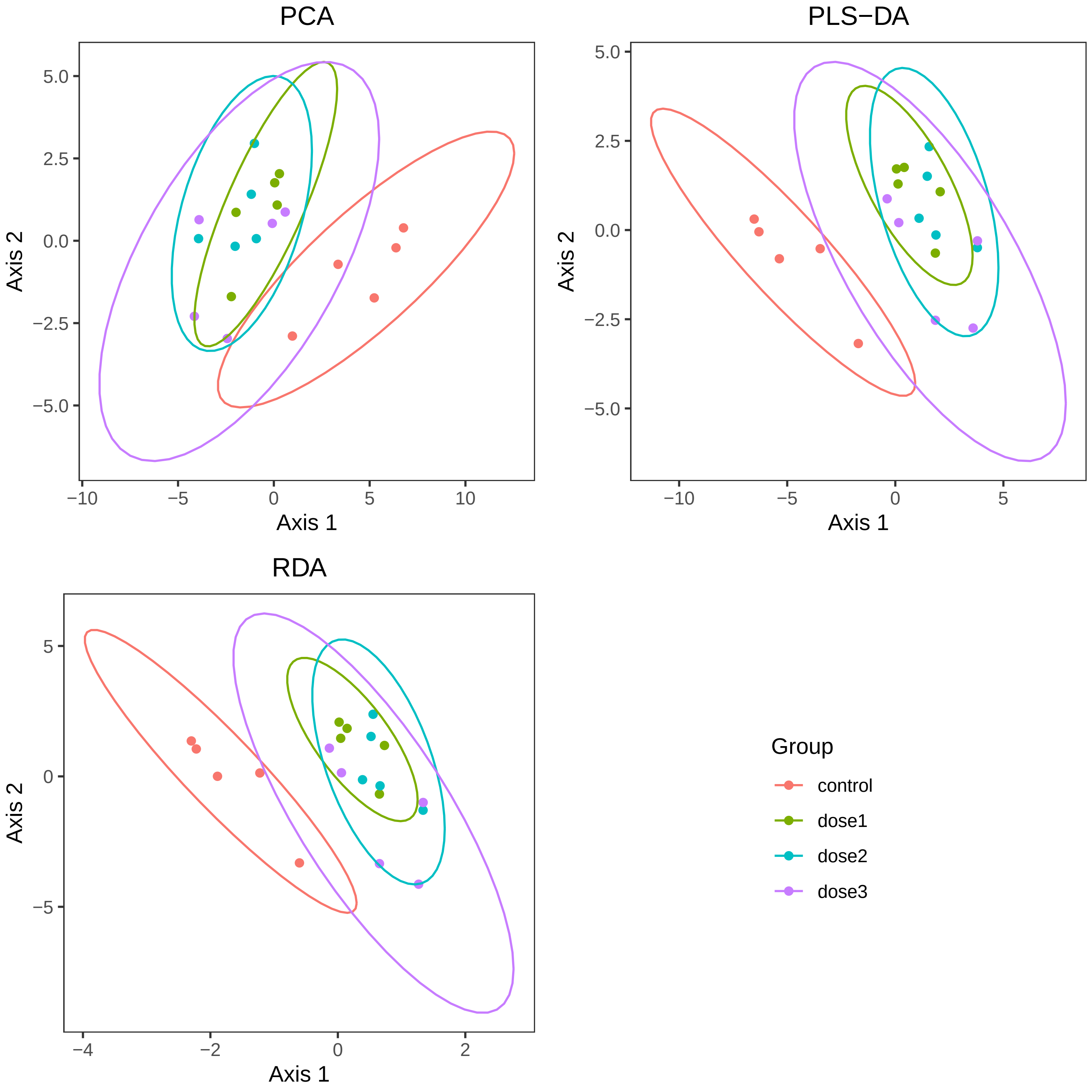

PCA
PLS−DA
RDA
Axis 1
Axis 2
Group
control
dose1
dose2
dose3

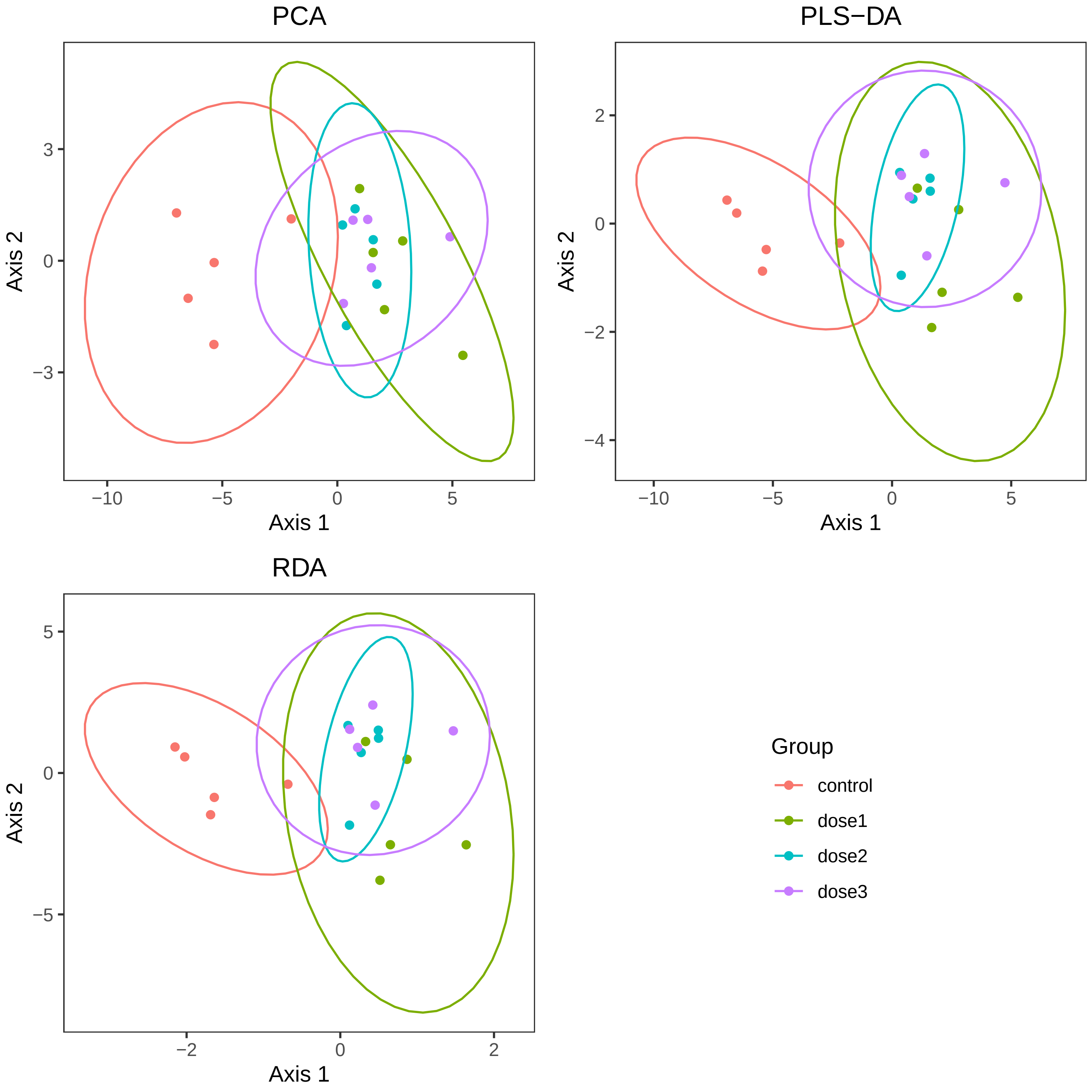
PCA
PLS−DA
RDA
Axis 1
Axis 2
Group
control
dose1
dose2
dose3
−10
−5
0
5
3
−3
2
−2
−4
−2
0
2
5
−5

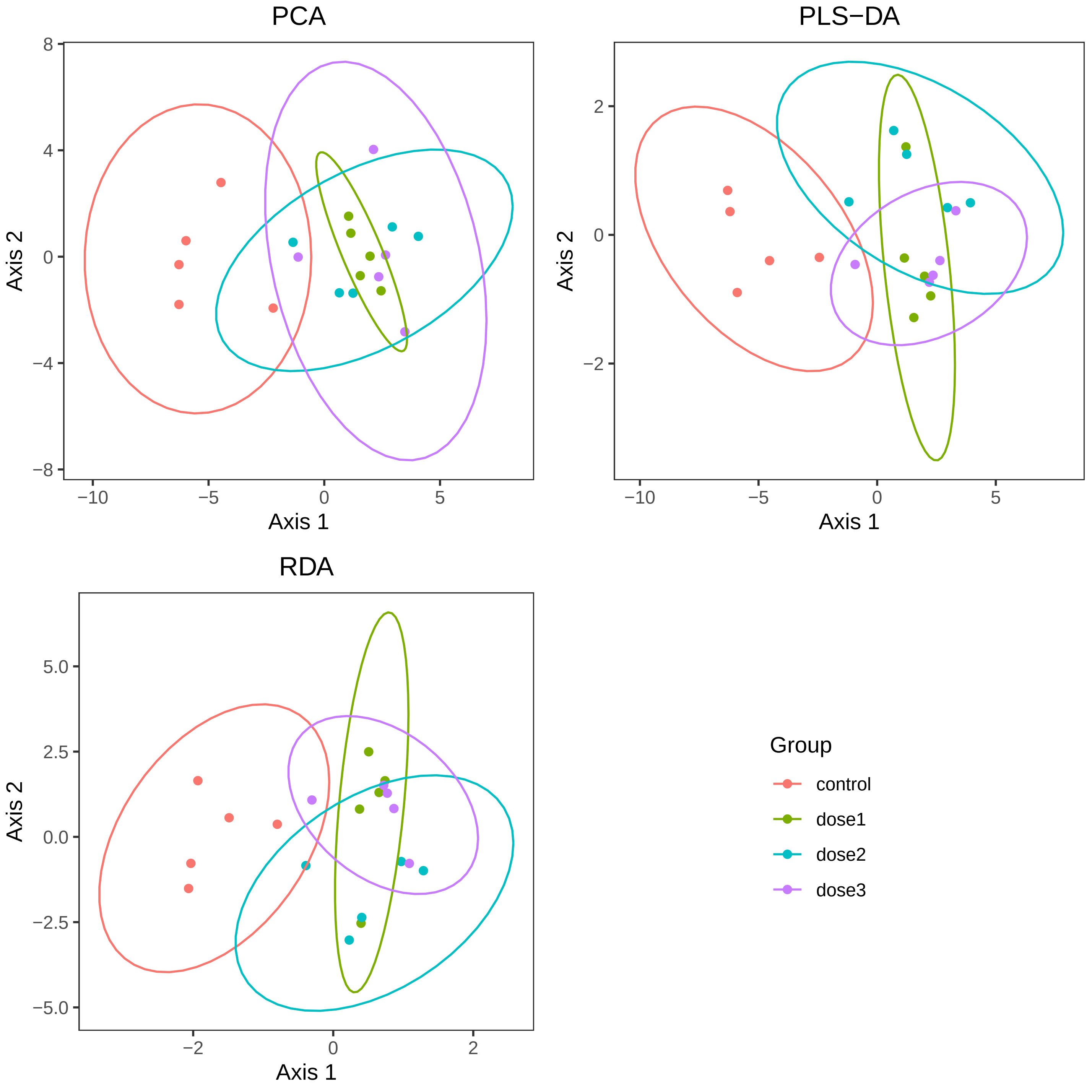
PCA
PLS−DA
RDA
Axis 1
Axis 2
Group
control
dose1
dose2
dose3

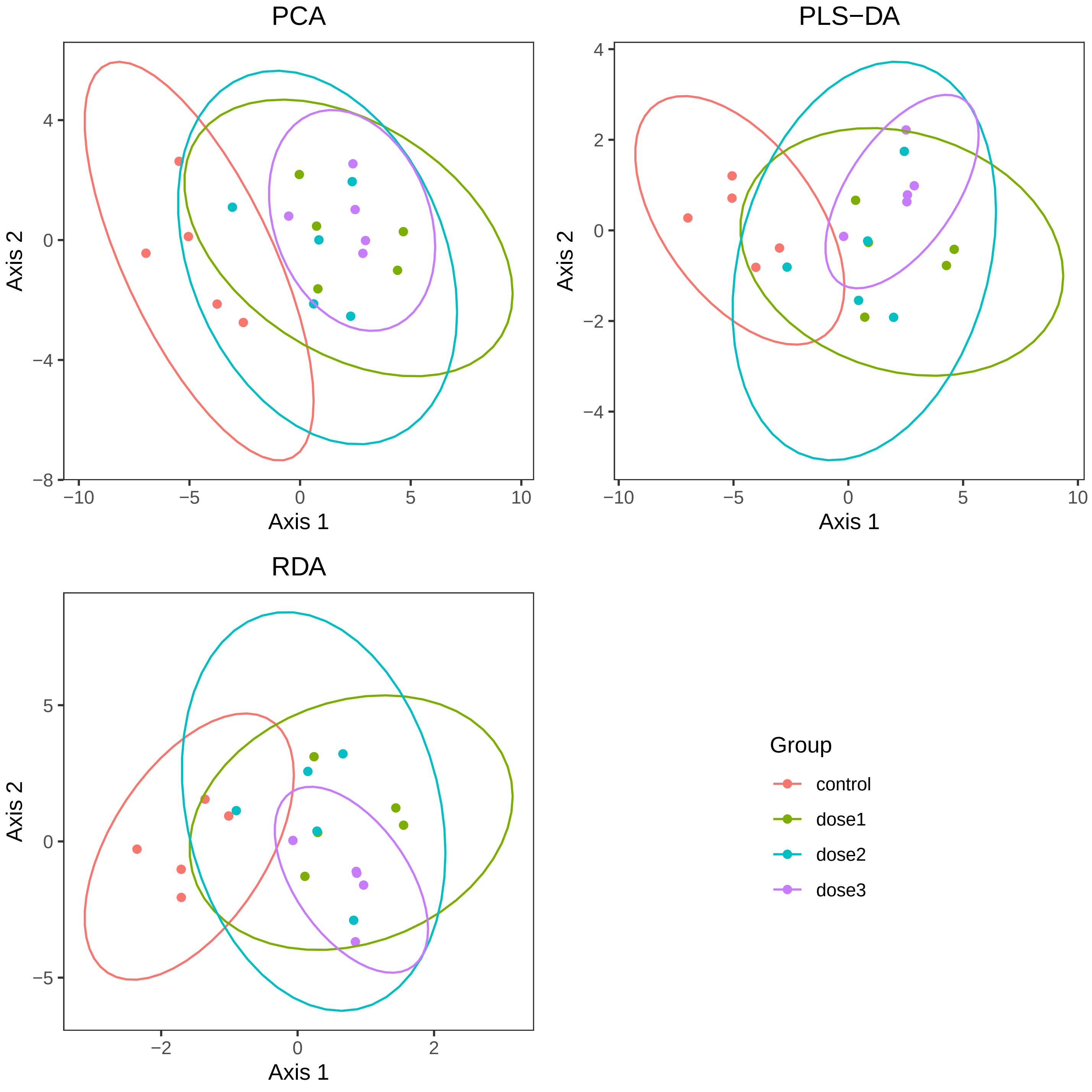

PCA
PLS−DA
RDA
Axis 1
Axis 2
Group
control
dose1
dose2
dose3

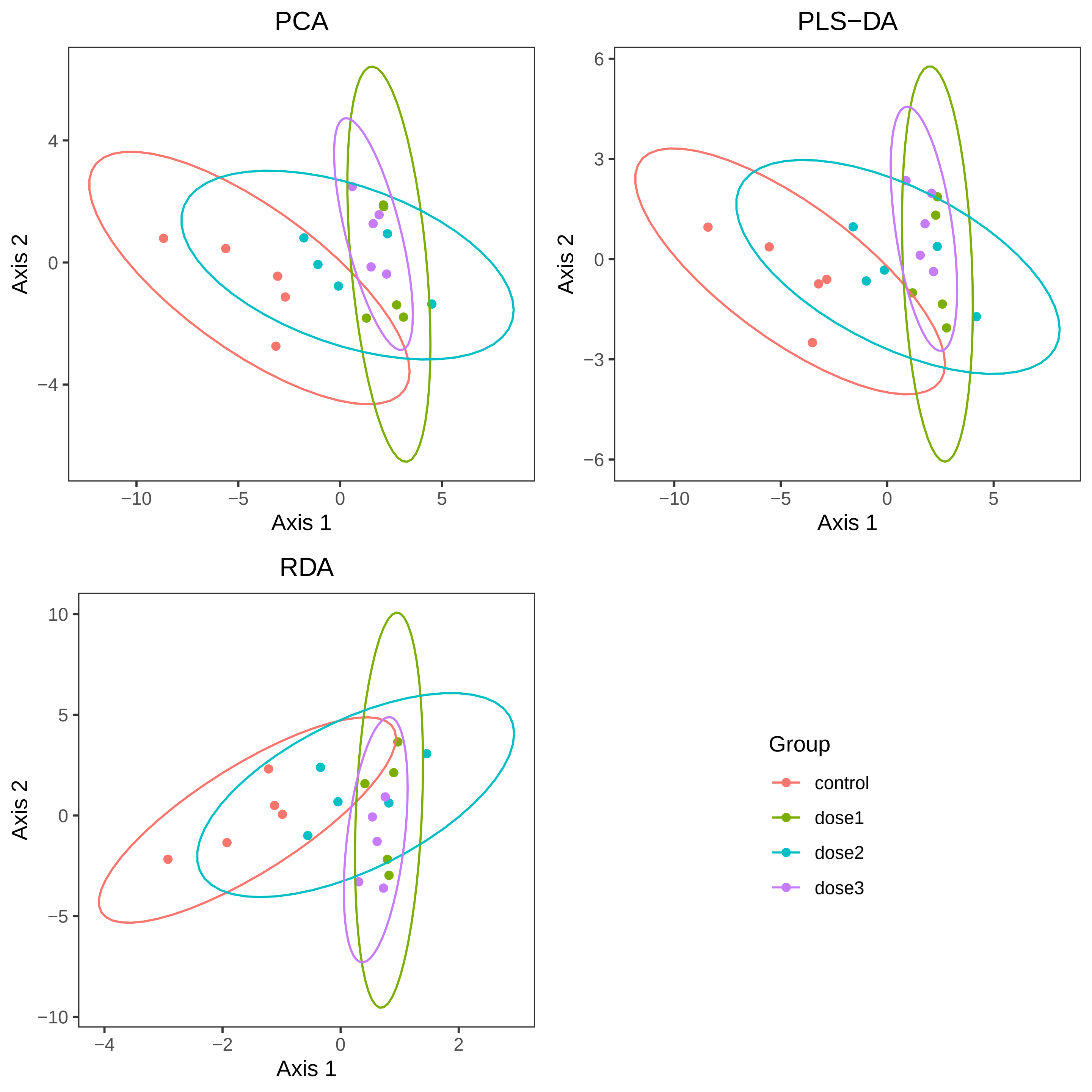

PCA
PLS−DA
RDA
Axis 1
Axis 2
−10
−5
0
5
4
−4
6
3
−3
−6
10
−10
−4
−2
2
Group
control
dose1
dose2
dose3

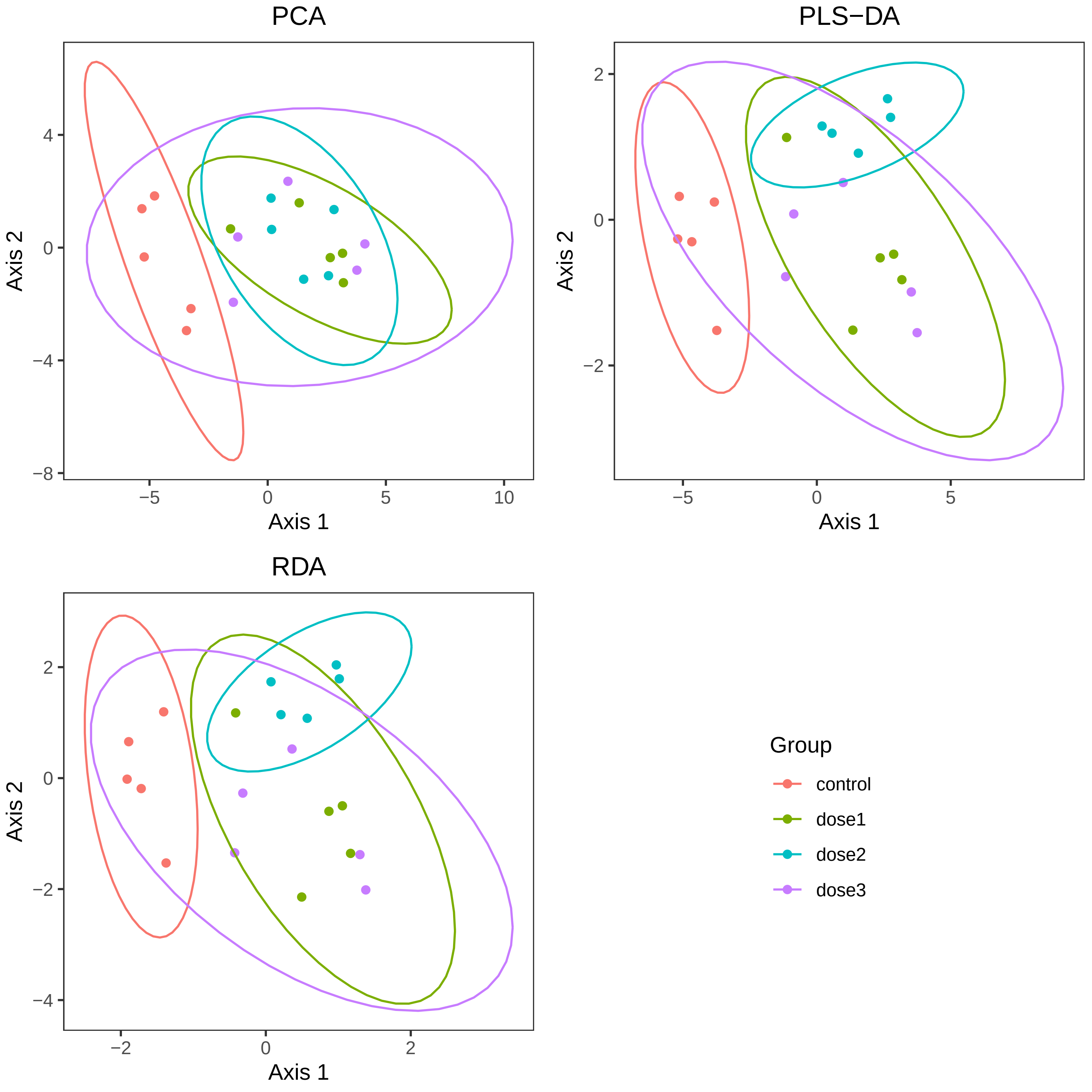

PCA
PLS−DA
RDA
Axis 1
Axis 2
−5
0
5
10
4
0
−4
−8
2
−2
Group
control
dose1
dose2
dose3

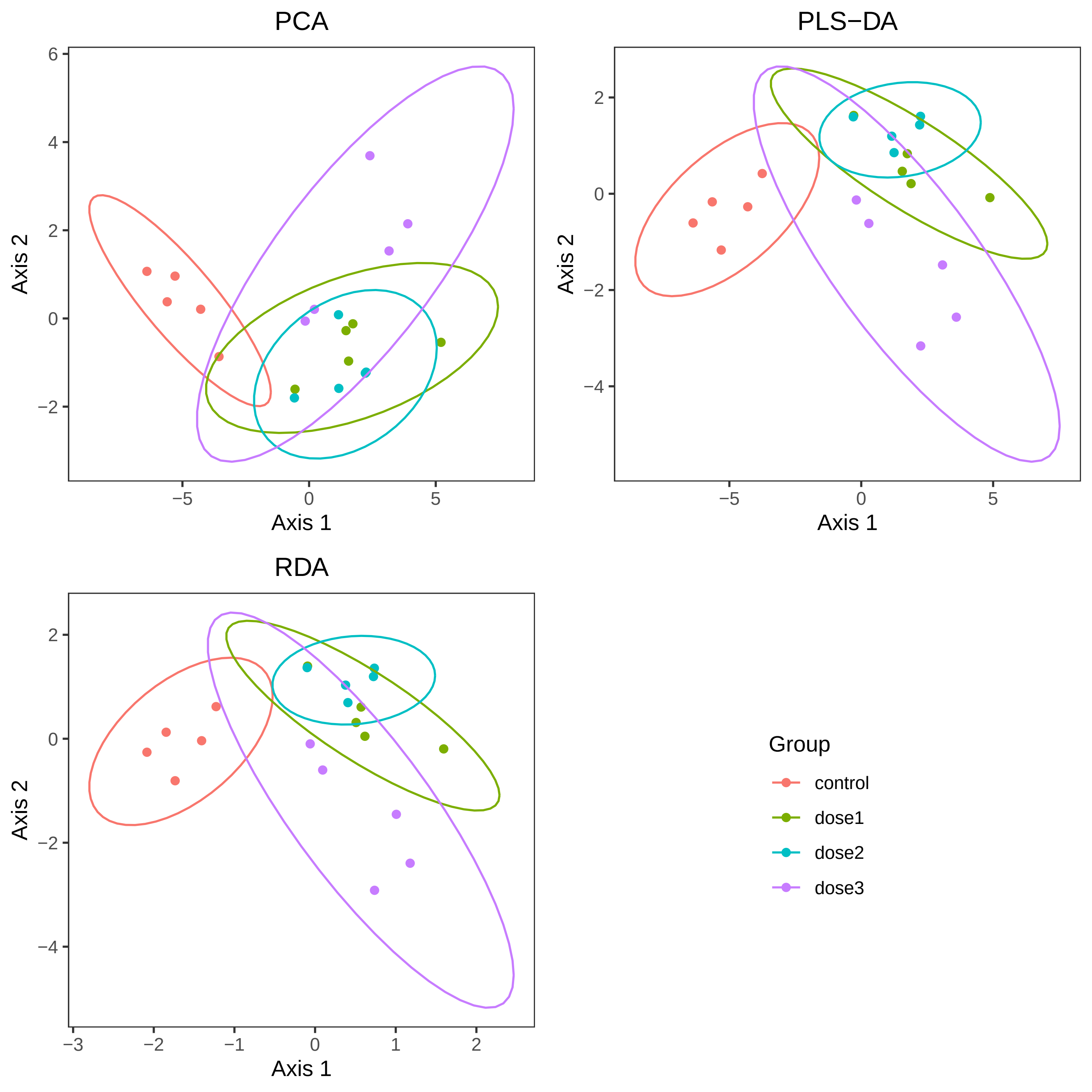

PCA
PLS−DA
RDA
Axis 1
Axis 2
Group
control
dose1
dose2
dose3

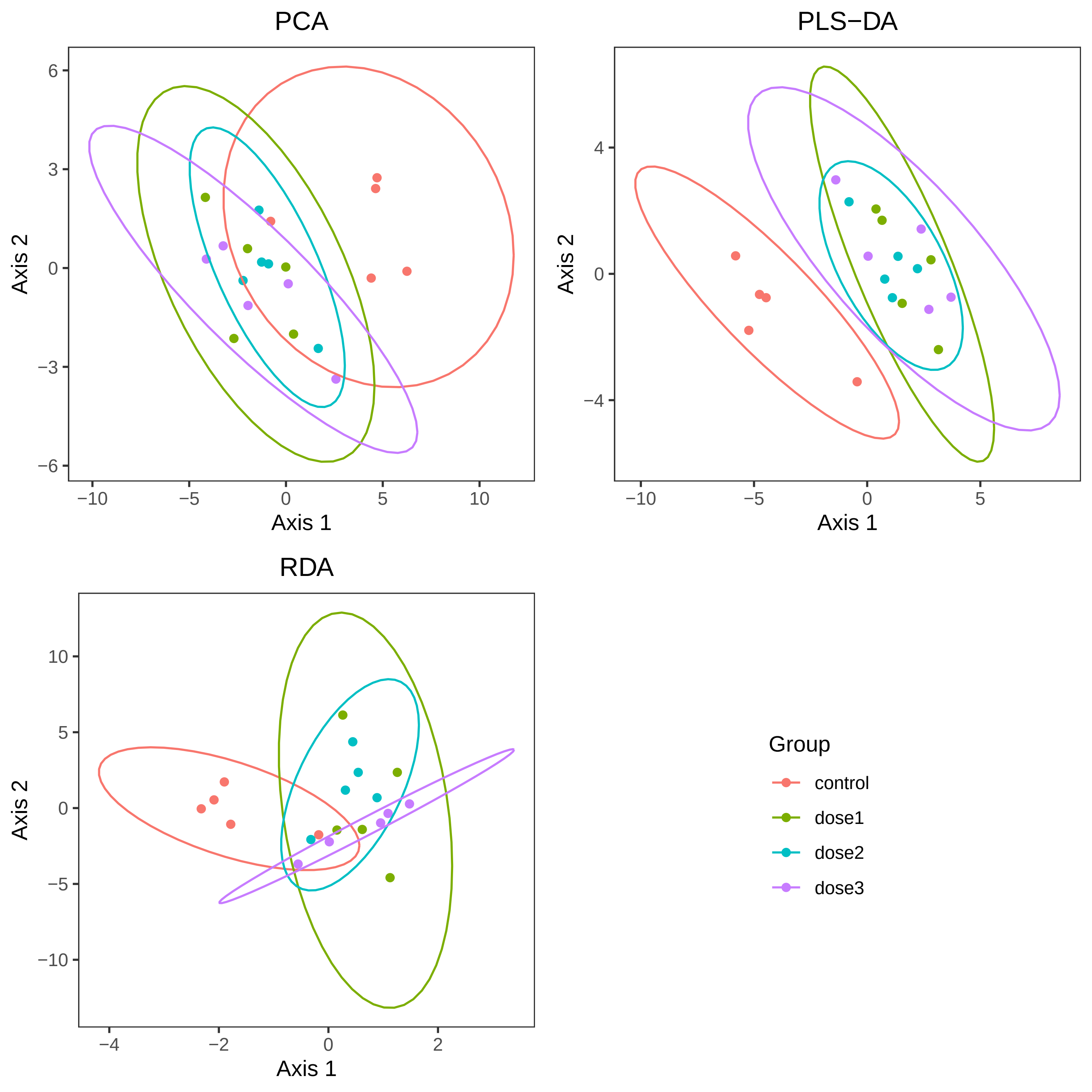
PCA
Axis 1
Axis 2
PLS−DA
Axis 1
Axis 2
RDA
Axis 1
Axis 2
Group
control
dose1
dose2
dose3

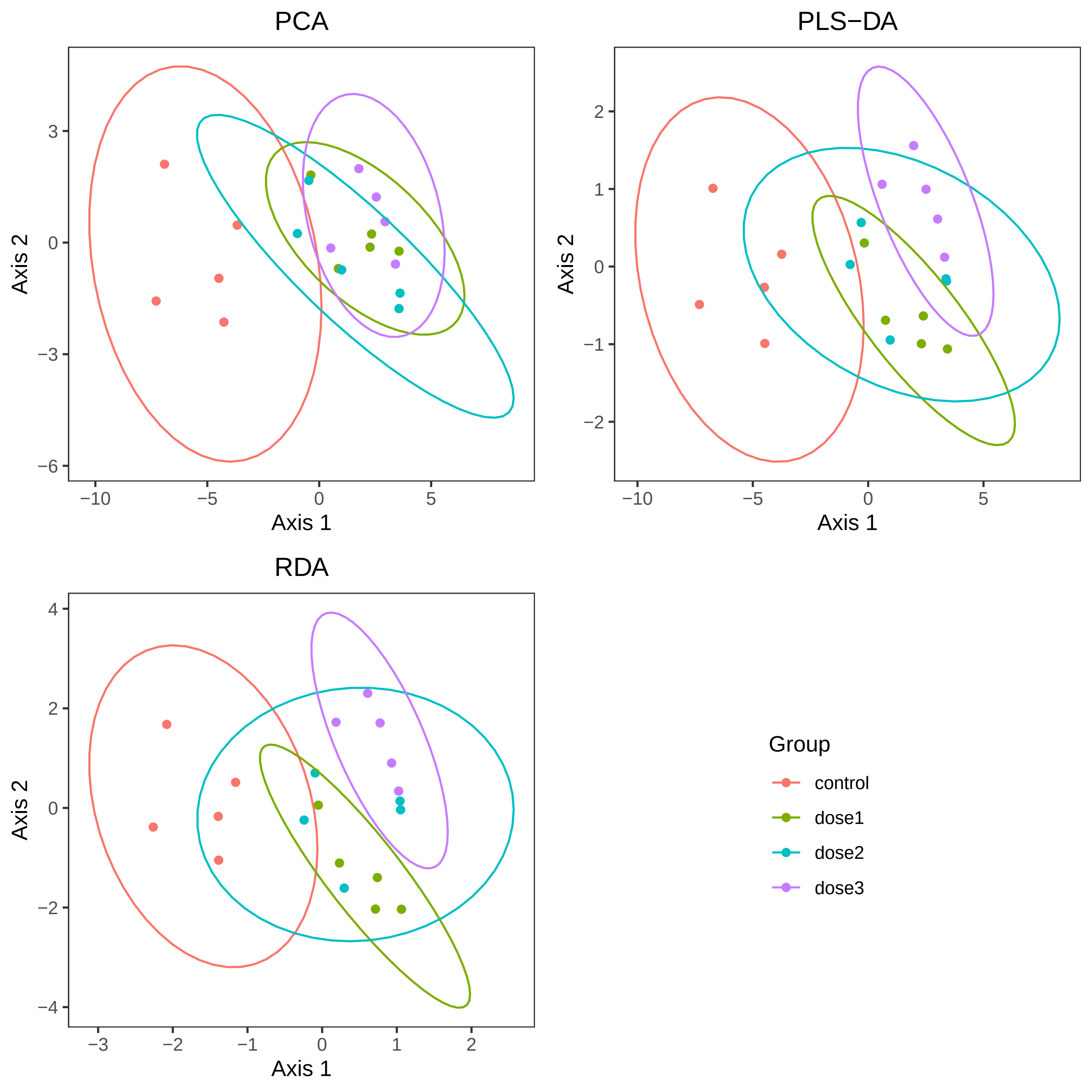

PCA
PLS−DA
RDA
Axis 1
Axis 2
Group
control
dose1
dose2
dose3

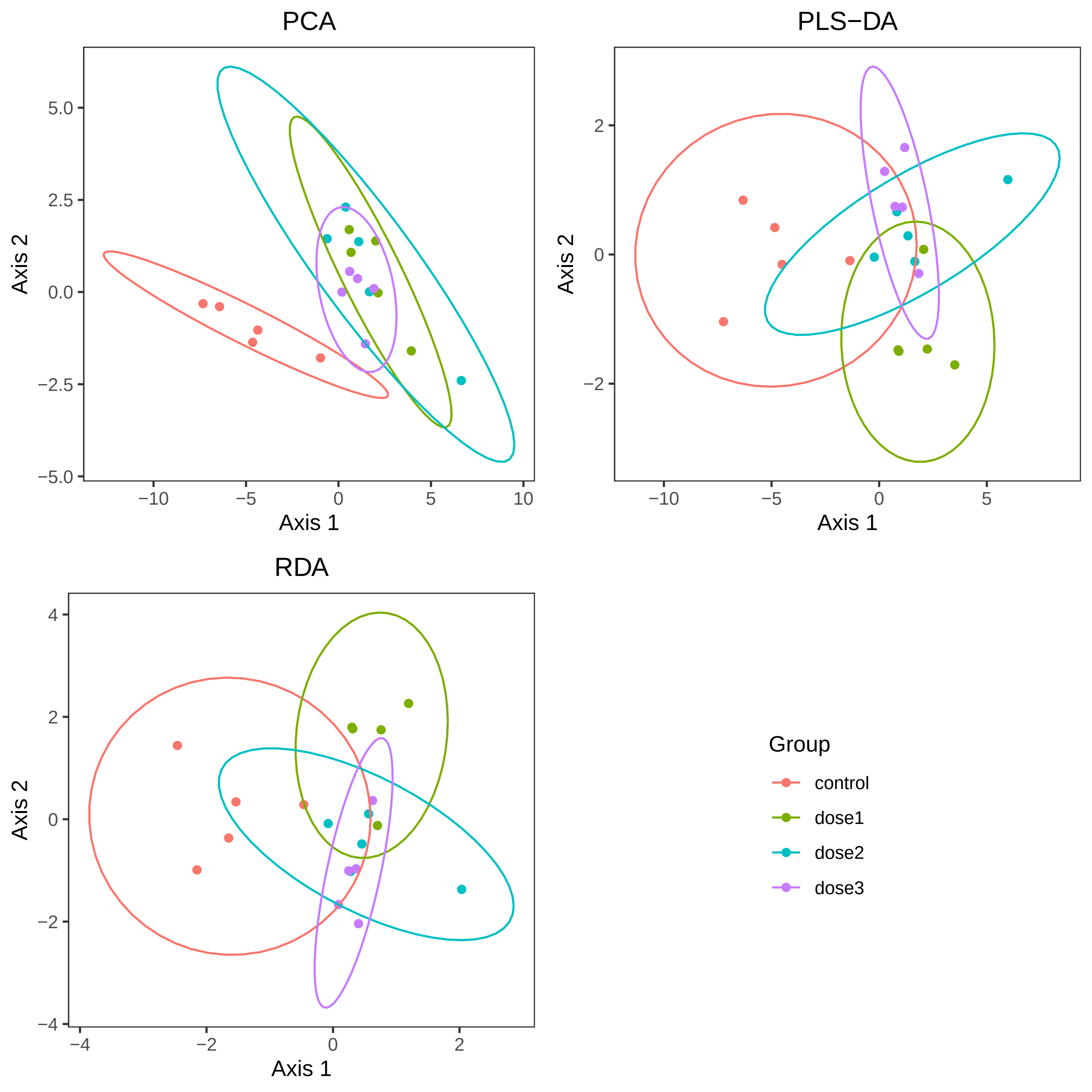

PCA
PLS−DA
RDA
Axis 1
Axis 2
Group
control
dose1
dose2
dose3

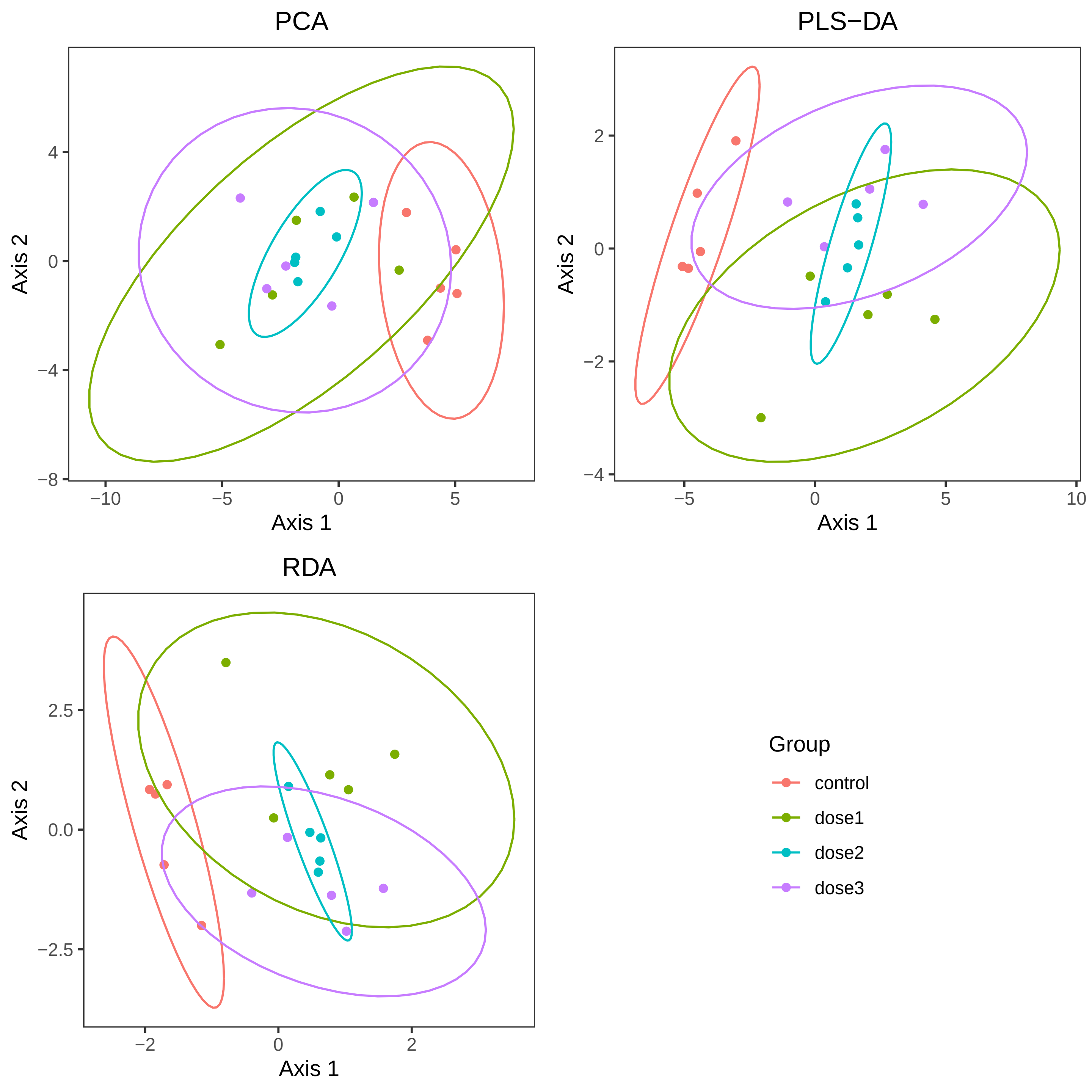
PCA
PLS−DA
RDA
Axis 1
Axis 2
Group
control
dose1
dose2
dose3

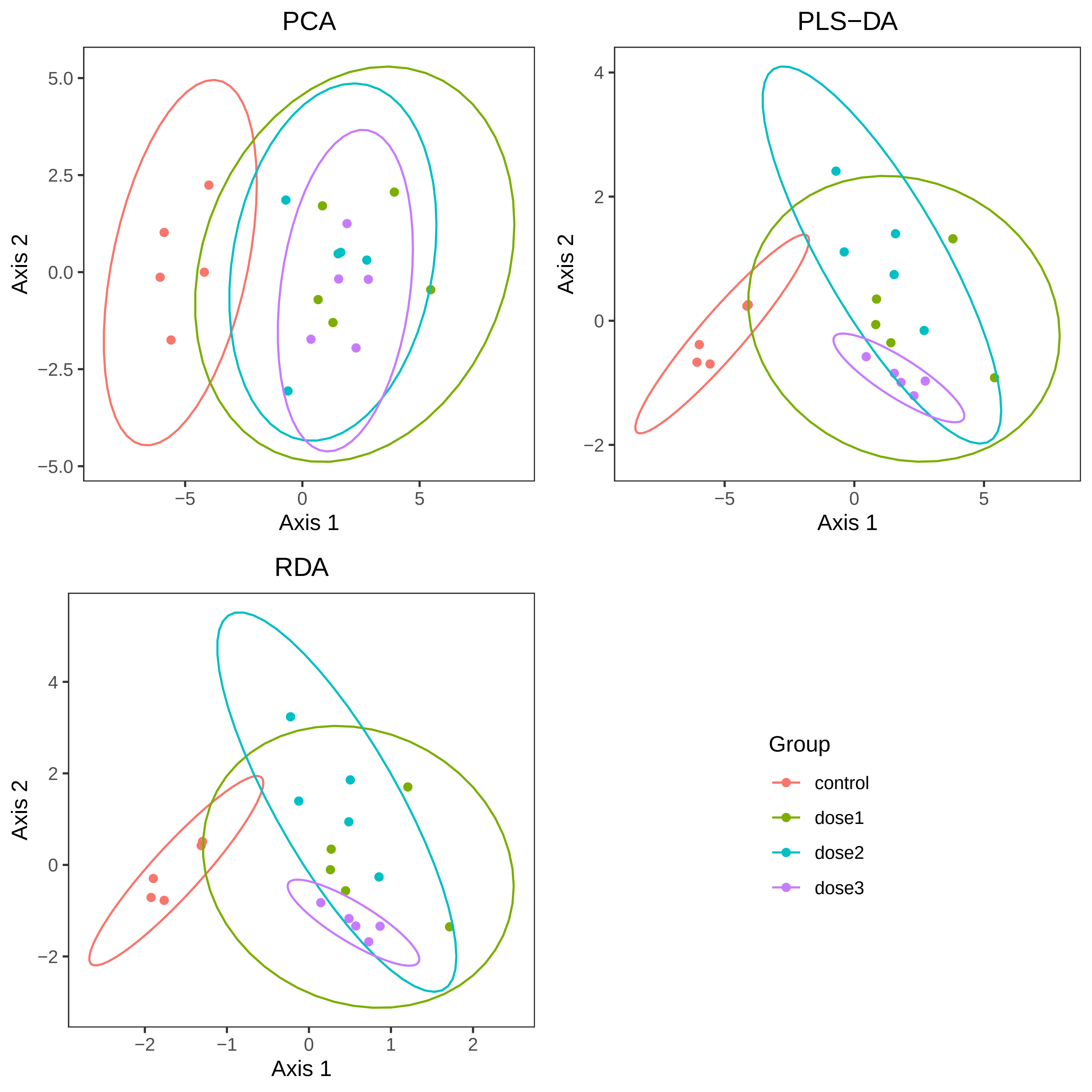

PCA
PLS−DA
RDA
Axis 1
Axis 2
Group
control
dose1
dose2
dose3

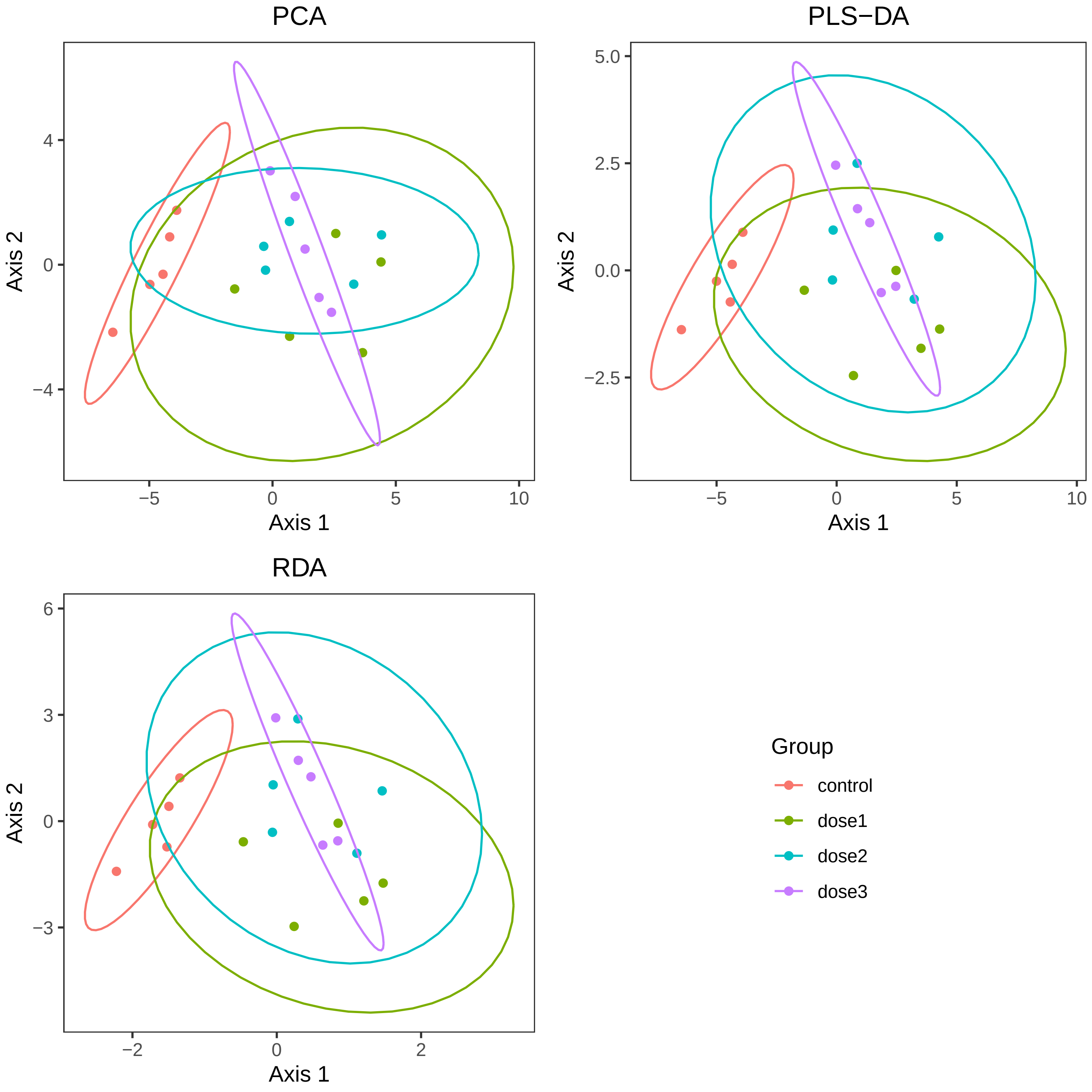
PCA
PLS−DA
RDA
Axis 1
Axis 2
Group
control
dose1
dose2
dose3

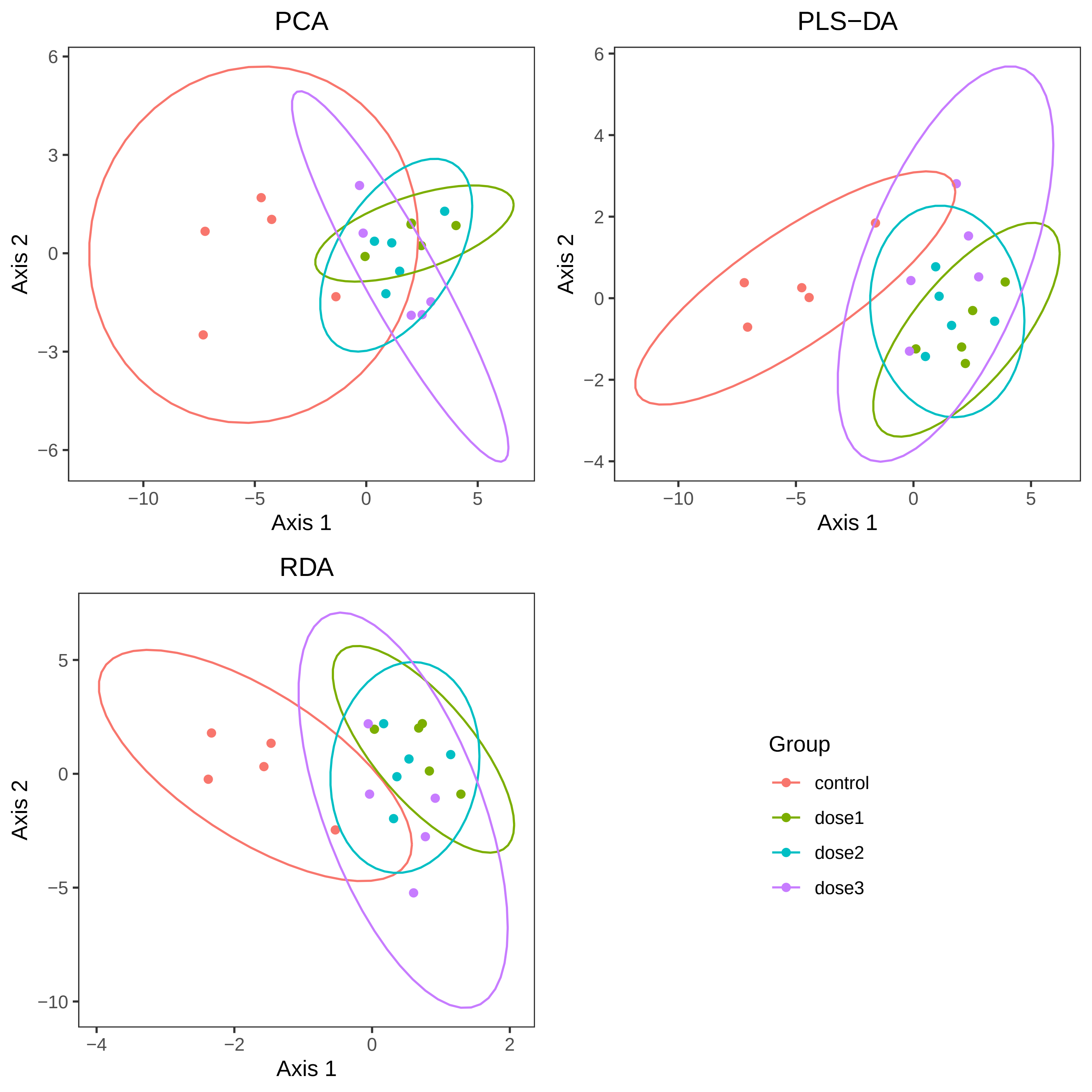
PCA
PLS−DA
RDA
Axis 1
Axis 2
Group
control
dose1
dose2
dose3

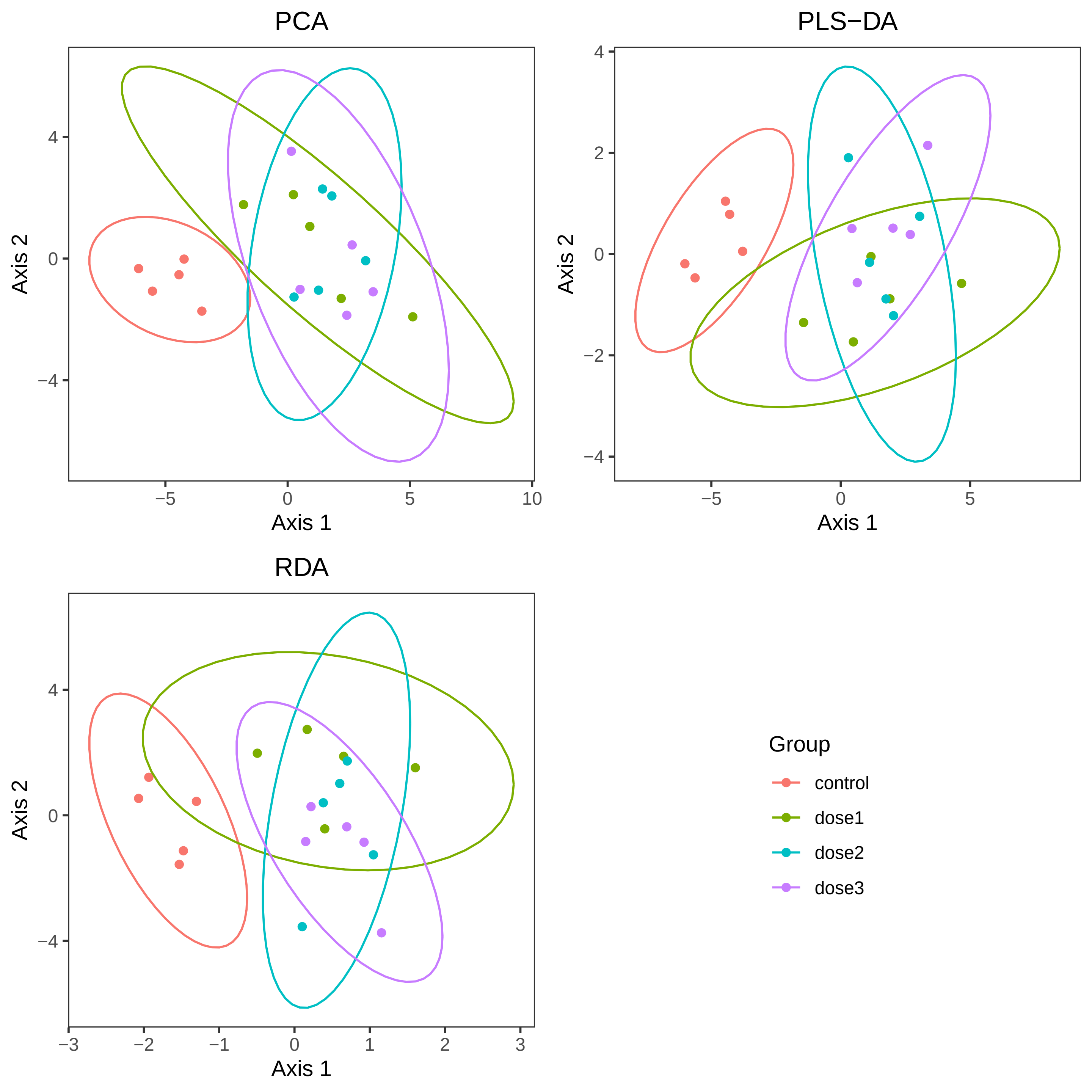
PCA
PLS−DA
RDA
Axis 1
Axis 2
Group
control
dose1
dose2
dose3

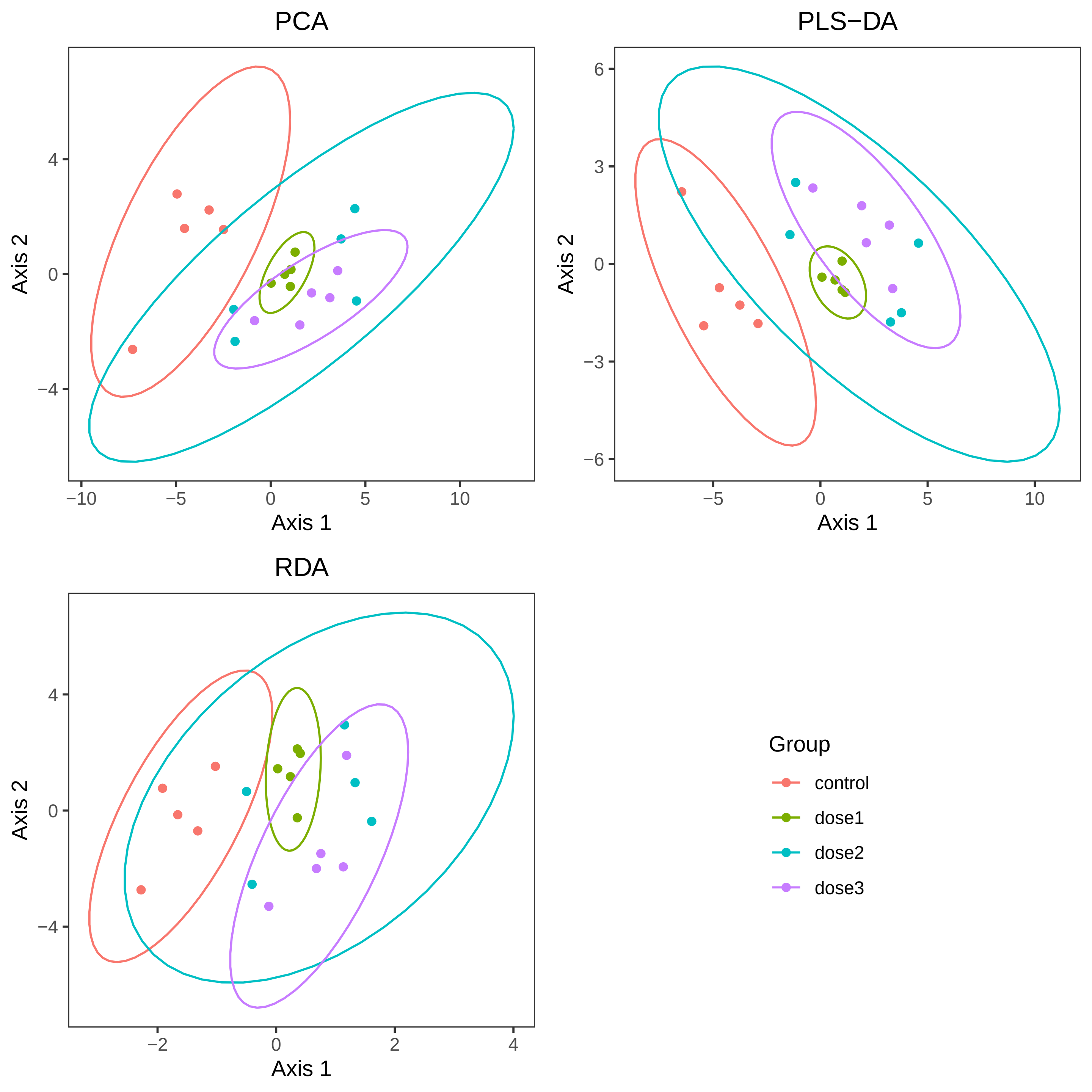
PCA
PLS−DA
RDA
Axis 1
Axis 2
Group
control
dose1
dose2
dose3

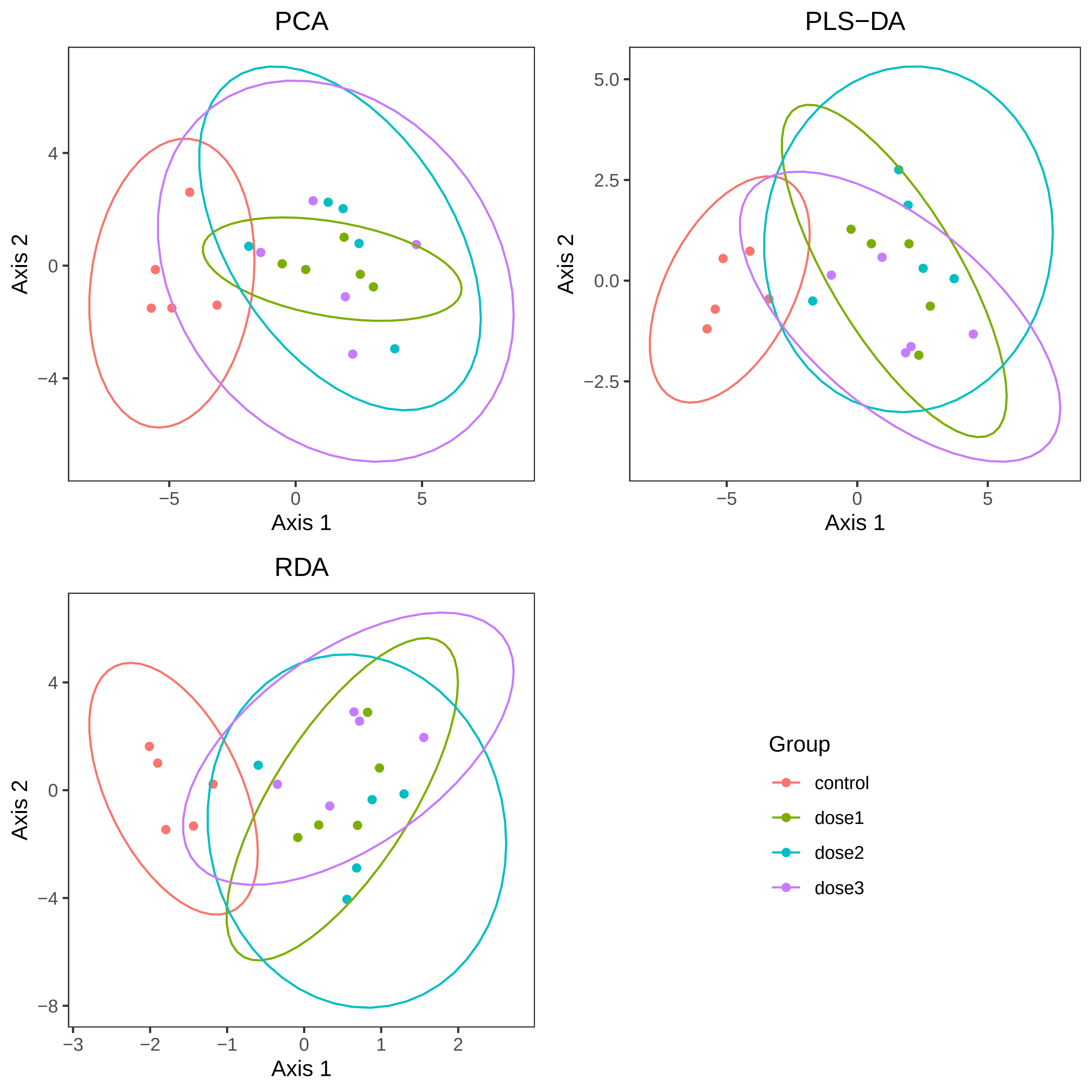

PCA
PLS−DA
RDA
Axis 1
Axis 2
4
0
−4
−5
0
5
5.0
2.5
0.0
−2.5
−8
−3
−2
−1
1
2
Group
control
dose1
dose2
dose3

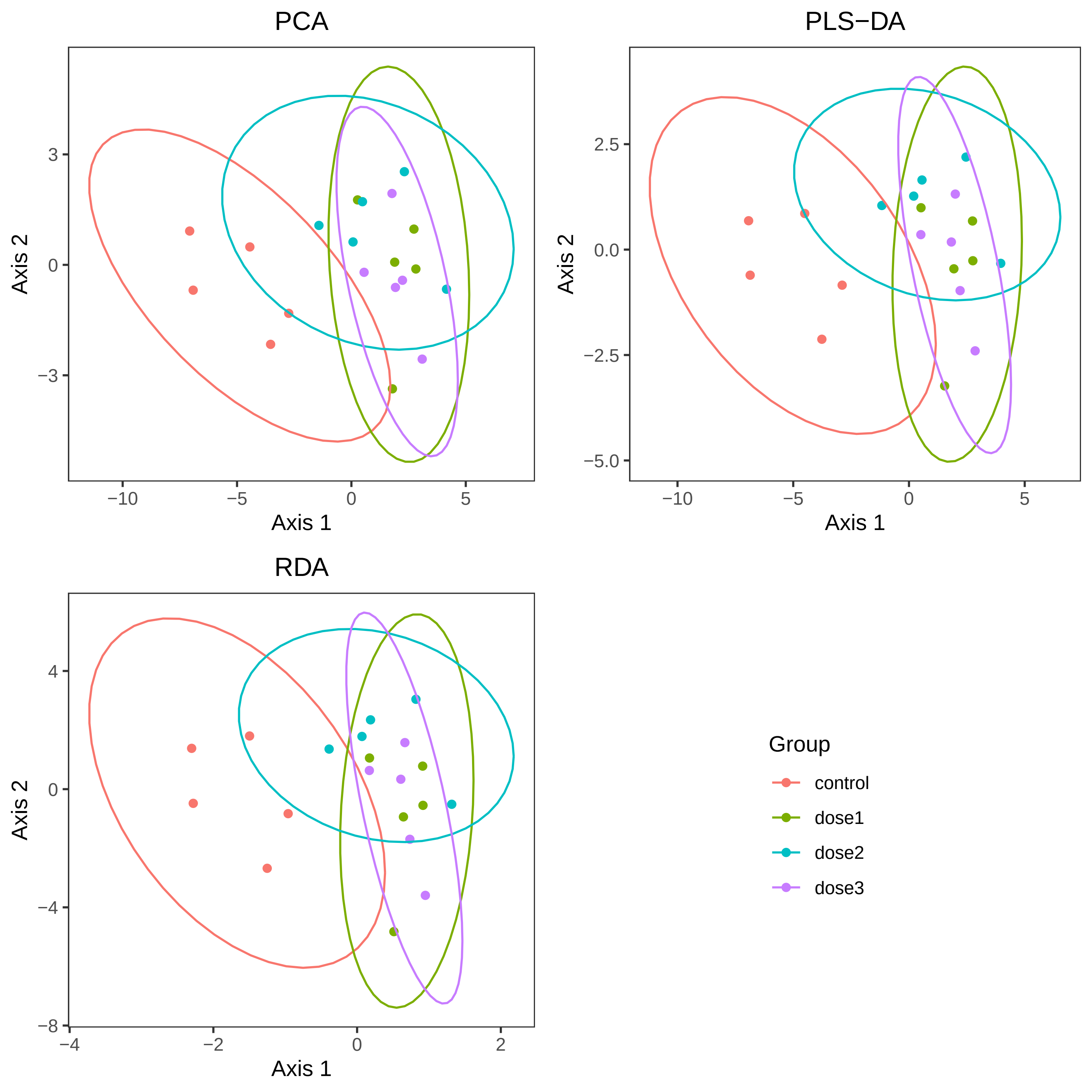
PCA
PLS−DA
RDA
Axis 1
Axis 2
3
0
−3
−10
−5
0
5
2.5
0.0
−2.5
−5.0
4
0
−4
−8
−4
−2
2
Group
control
dose1
dose2
dose3

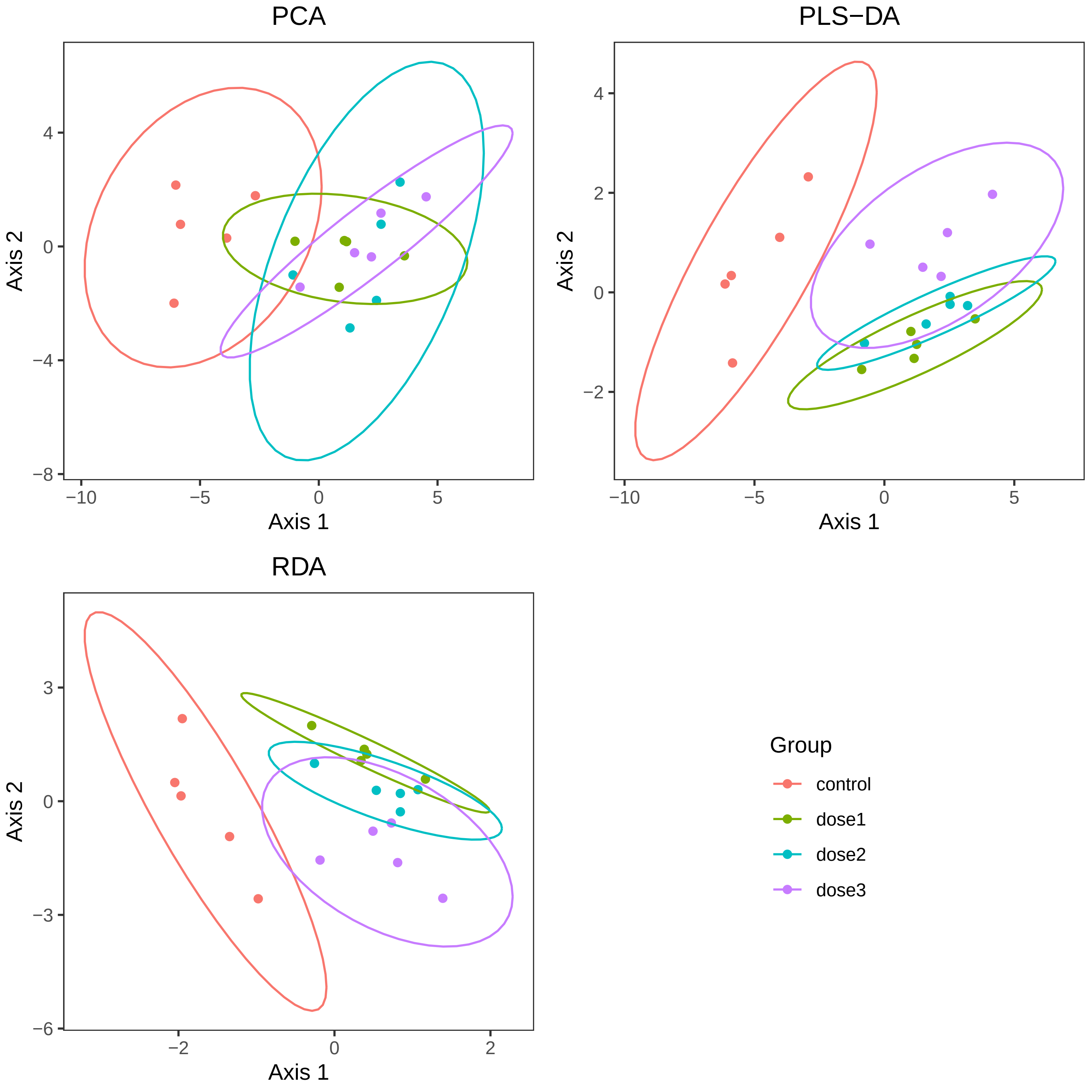
PCA
PLS−DA
RDA
Axis 1
Axis 2
Group
control
dose1
dose2
dose3

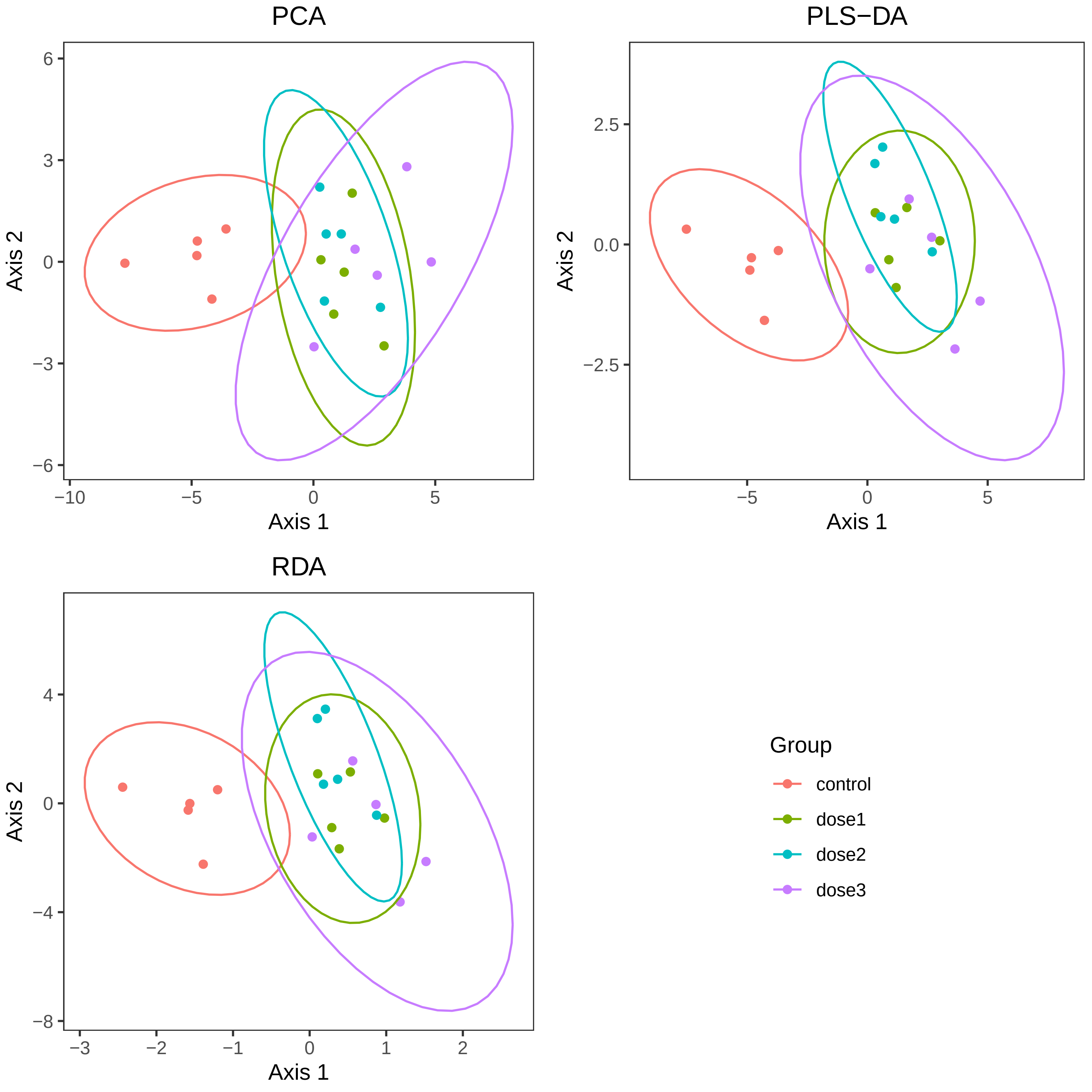
PCA
PLS−DA
RDA
Axis 1
Axis 2
Group
control
dose1
dose2
dose3

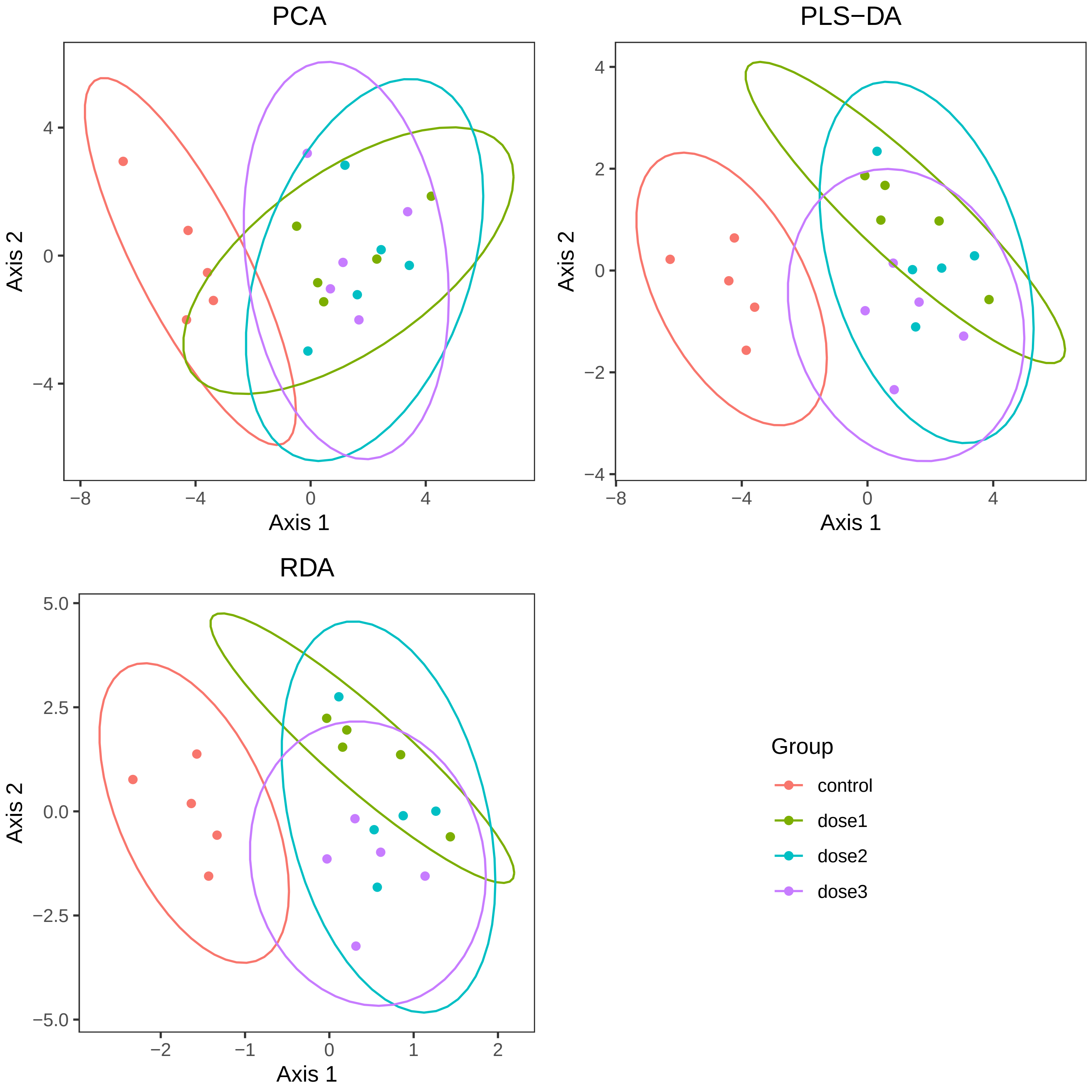
PCA
PLS−DA
RDA
Axis 1
Axis 2
Group
control
dose1
dose2
dose3

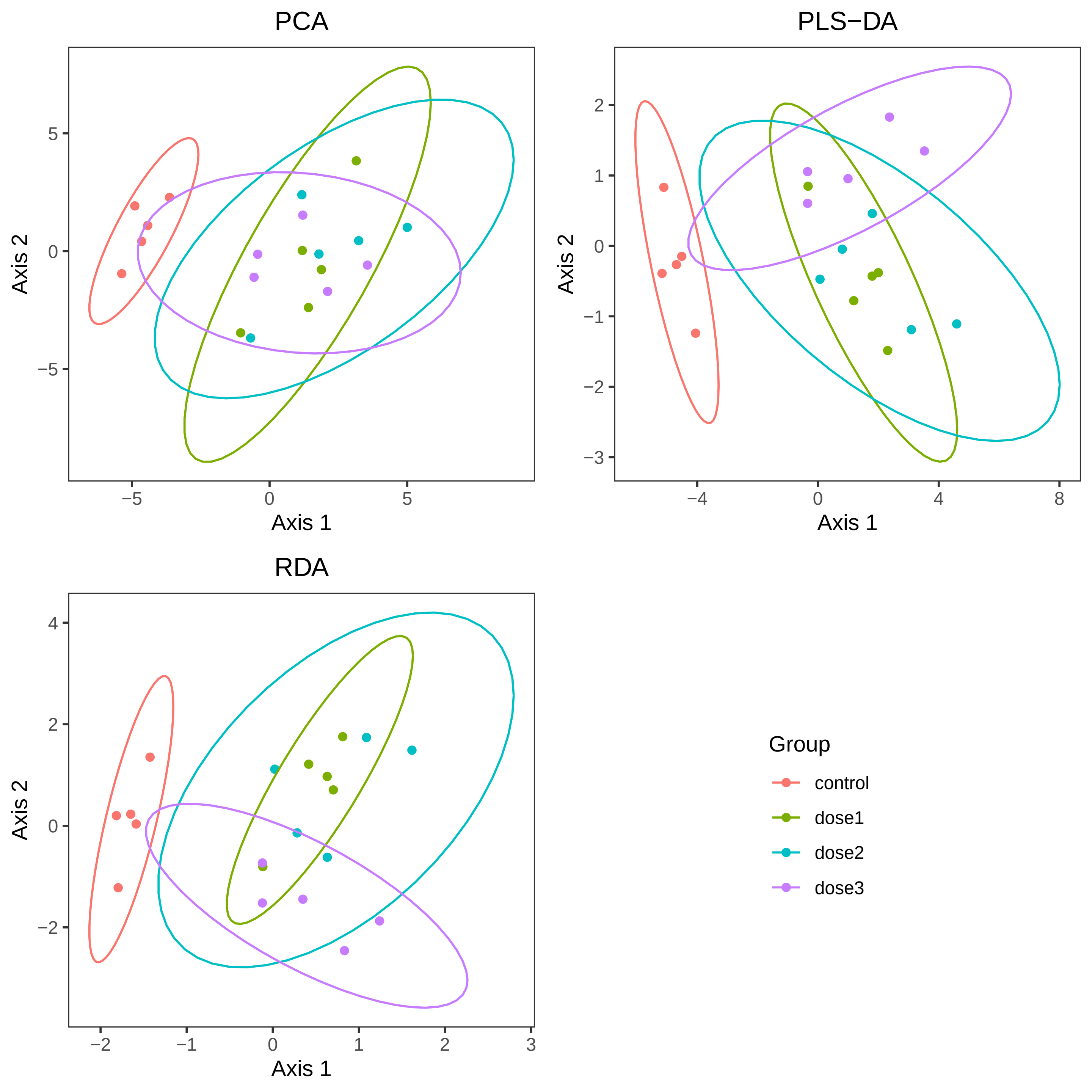
PCA
PLS−DA
RDA
Axis 1
Axis 2
Group
control
dose1
dose2
dose3

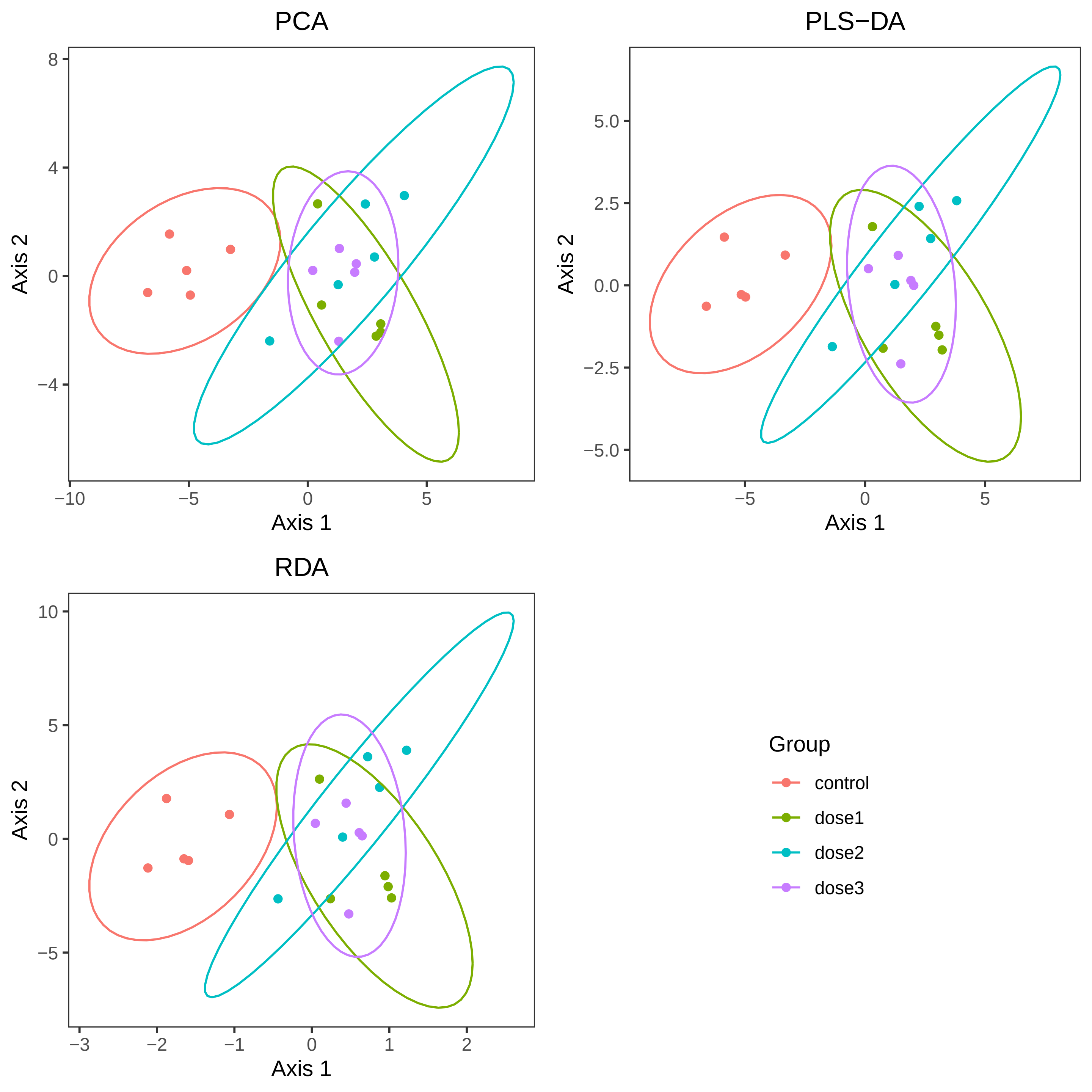

PCA
PLS−DA
RDA
Axis 1
Axis 2
8
4
0
−4
−10
−5
0
5
5.0
2.5
0.0
−2.5
−5.0
10
−3
−2
−1
1
2
Group
control
dose1
dose2
dose3

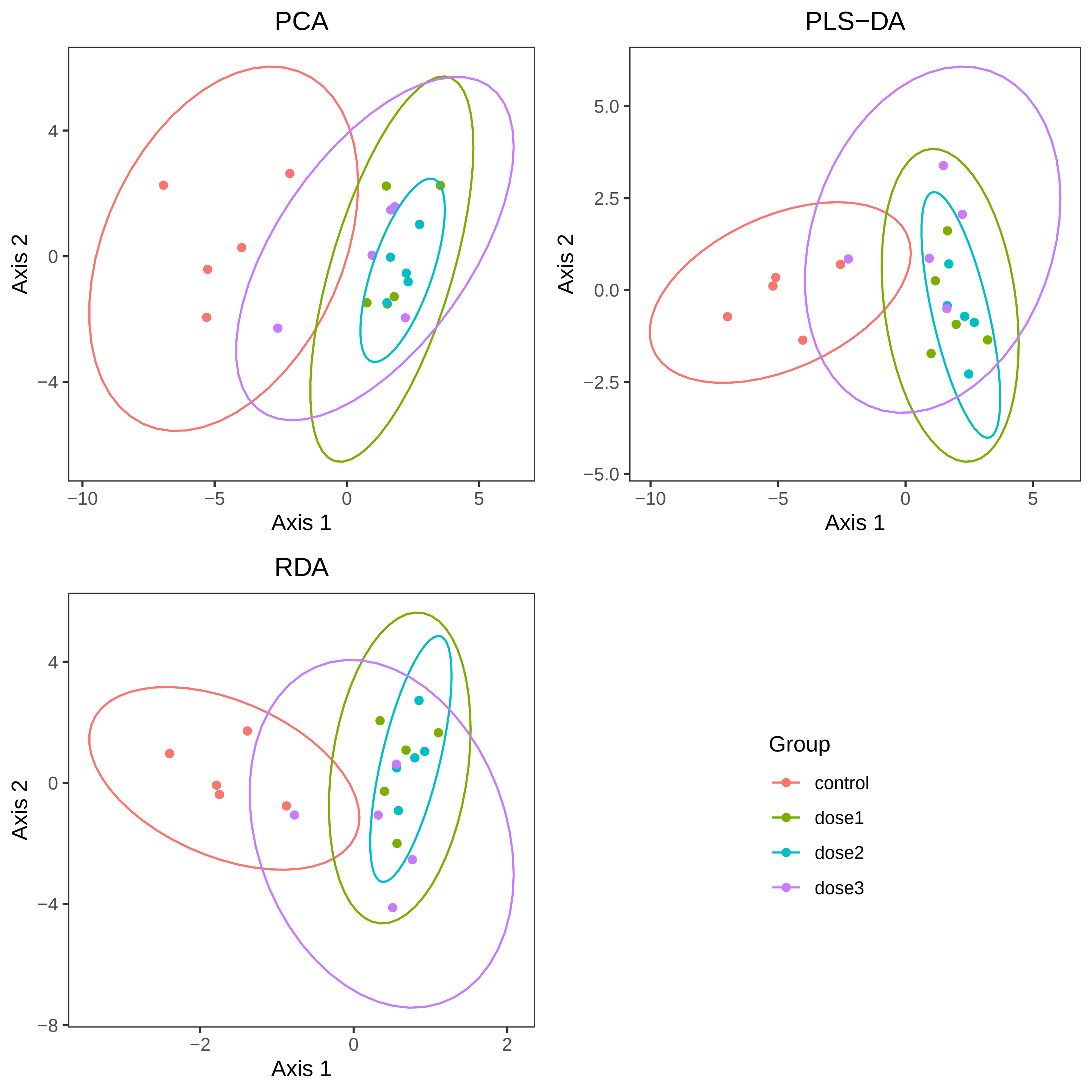

PCA
PLS−DA
RDA
Axis 1
Axis 2
Group
control
dose1
dose2
dose3

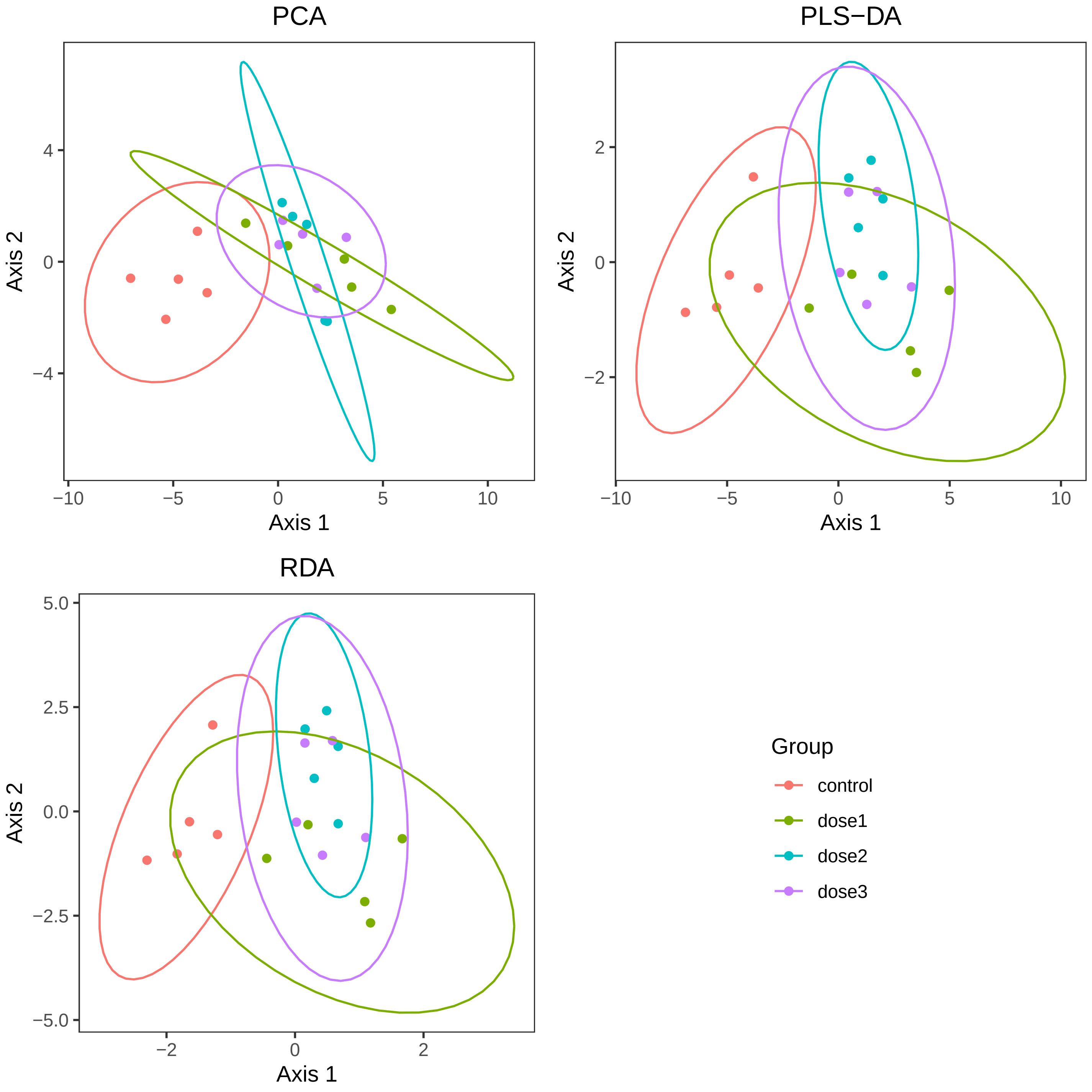
PCA
PLS−DA
RDA
Axis 1
Axis 2
−10
−5
0
5
10
4
0
−4
2
−2
5.0
2.5
0.0
−2.5
−5.0
Group
control
dose1
dose2
dose3

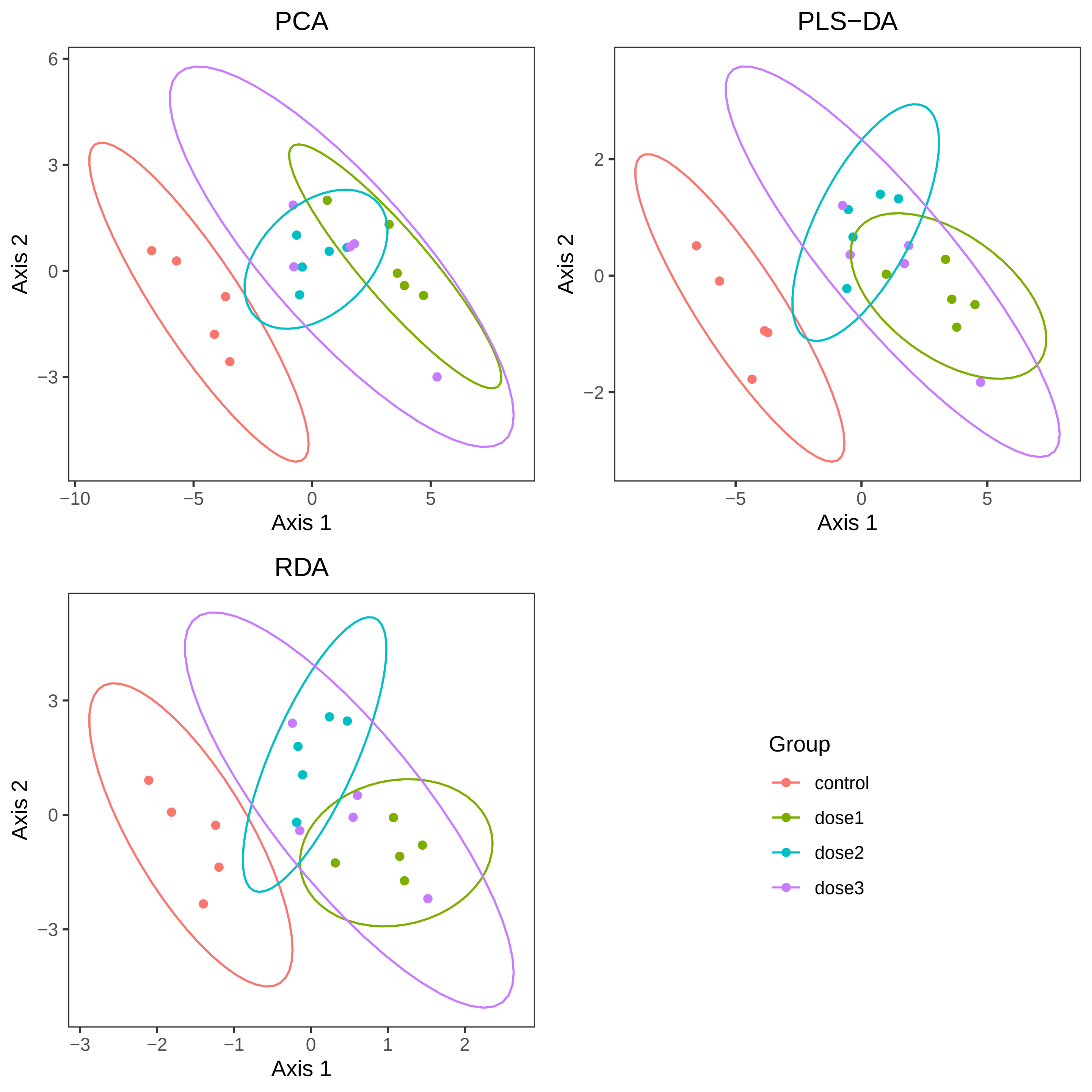
PCA
PLS−DA
RDA
Axis 1
Axis 2
−10
−5
0
5
6
3
−3
2
−2
−1
1
Group
control
dose1
dose2
dose3

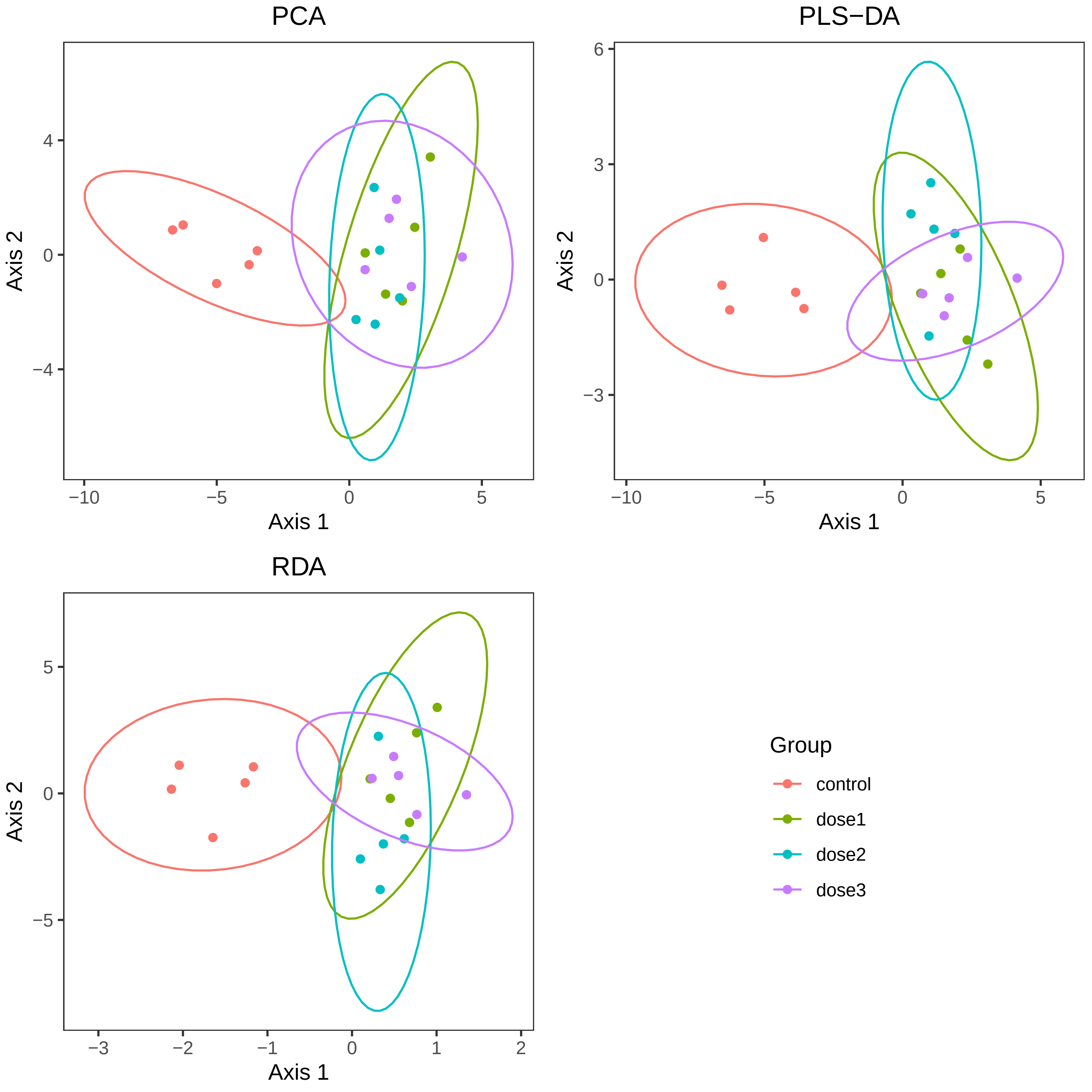
PCA
PLS−DA
RDA
Axis 1
Axis 2
Group
control
dose1
dose2
dose3

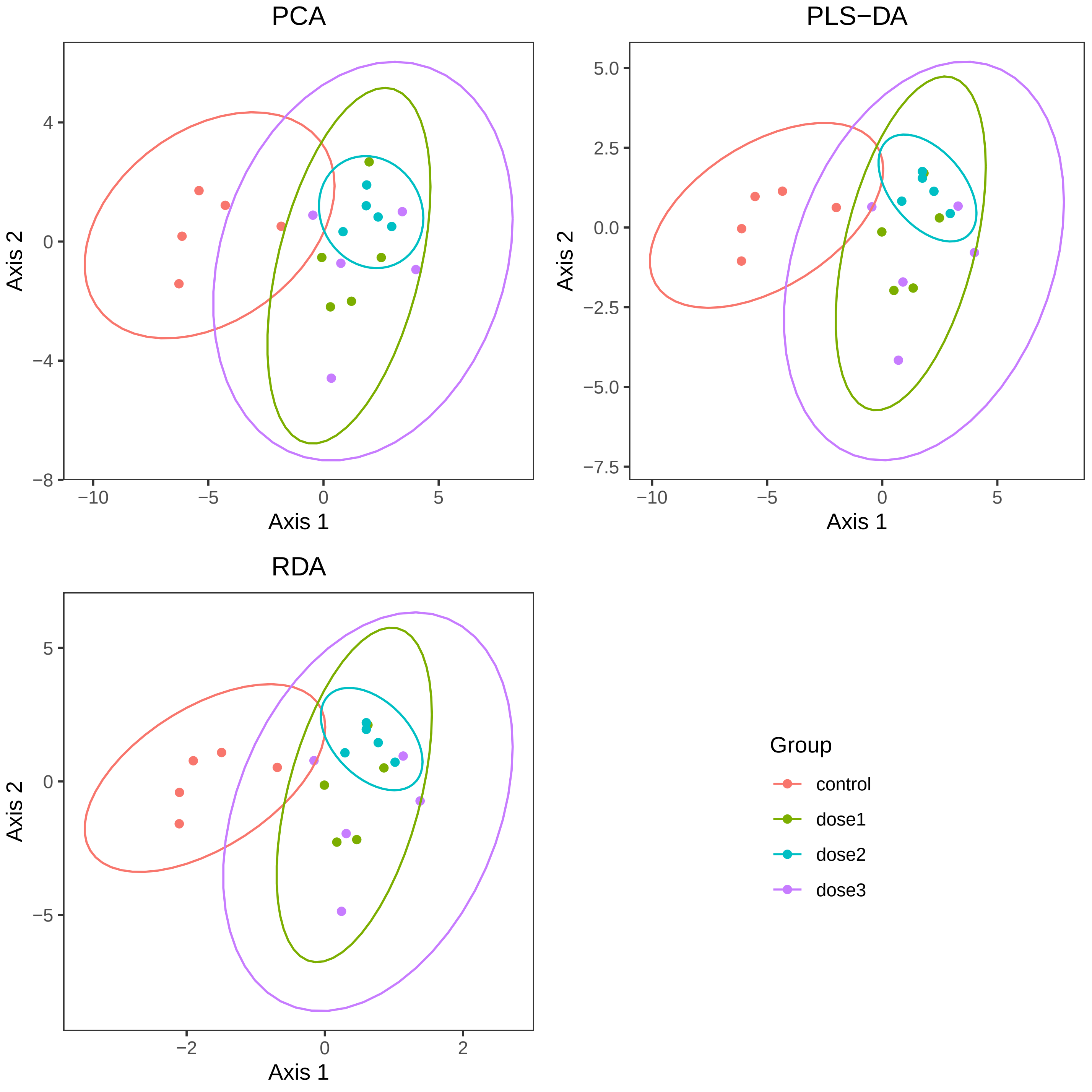

PCA
PLS−DA
RDA
Axis 1
Axis 2
−10
−5
0
5
4
−4
−8
5.0
2.5
0.0
−2.5
−5.0
−7.5
−2
2
Group
control
dose1
dose2
dose3

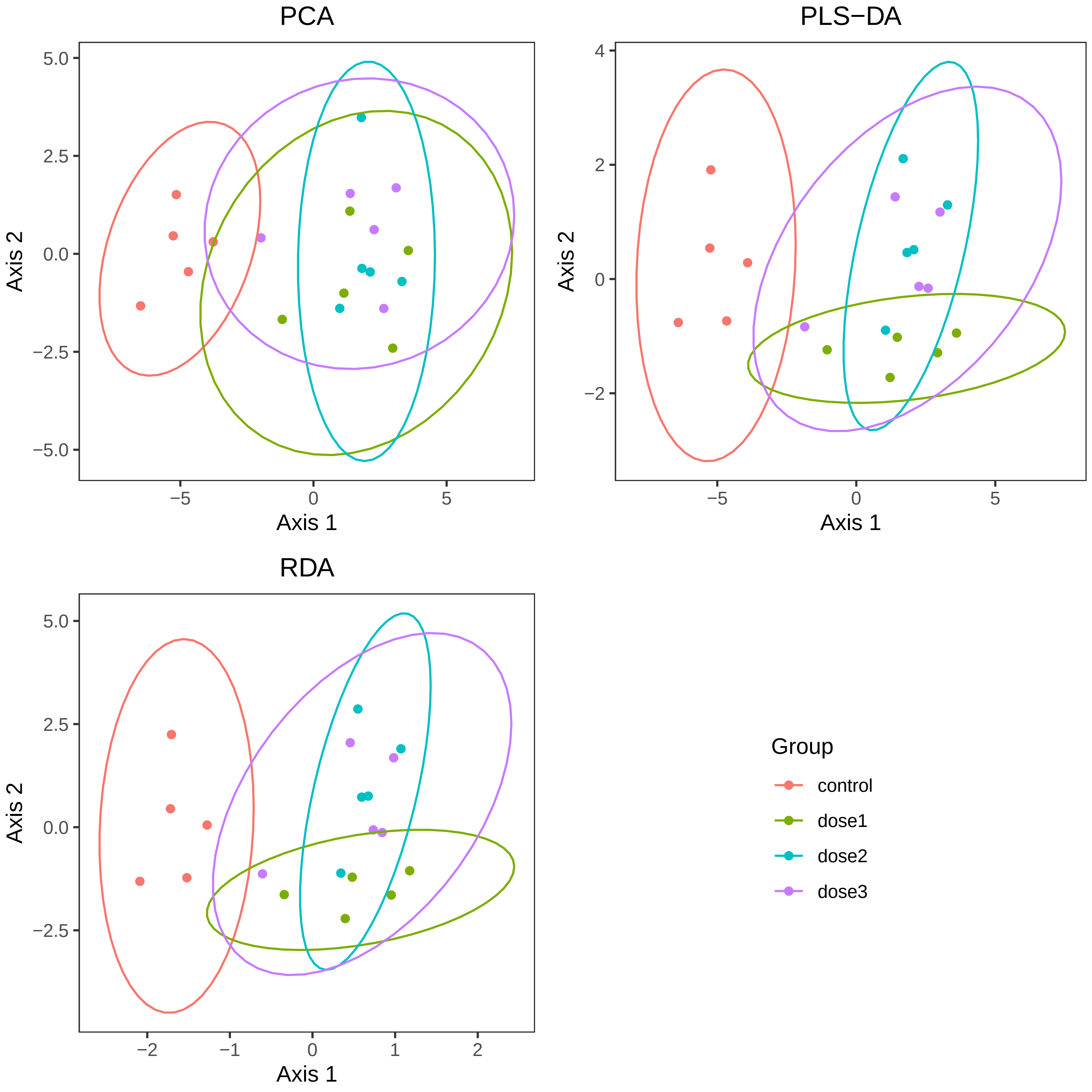
PCA
PLS−DA
RDA
Axis 1
Axis 2
Group
control
dose1
dose2
dose3

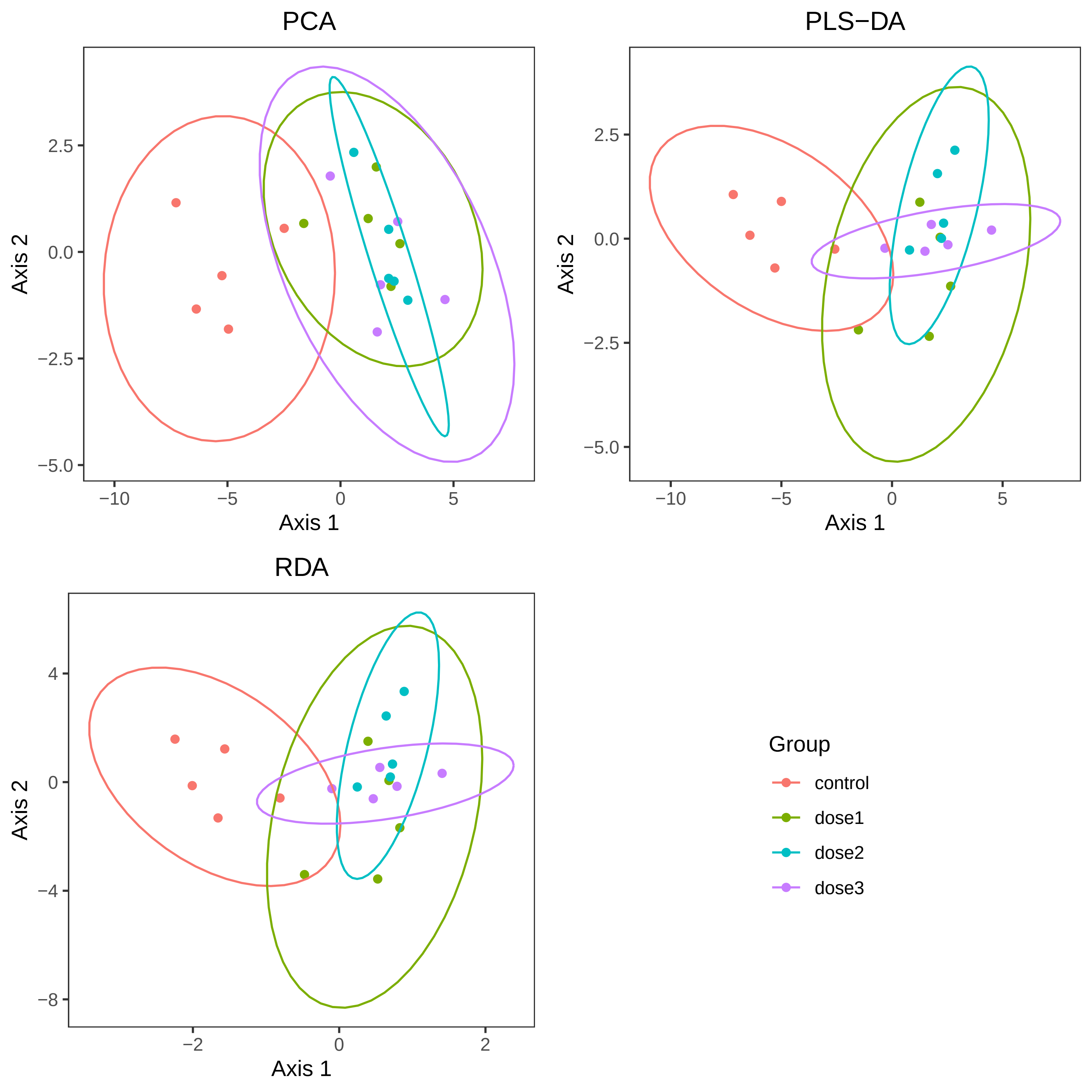
PCA
PLS−DA
RDA
Axis 1
Axis 2
−10
−5
0
5
2.5
0.0
−2.5
−5.0
−2
2
4
−4
−8
Group
control
dose1
dose2
dose3

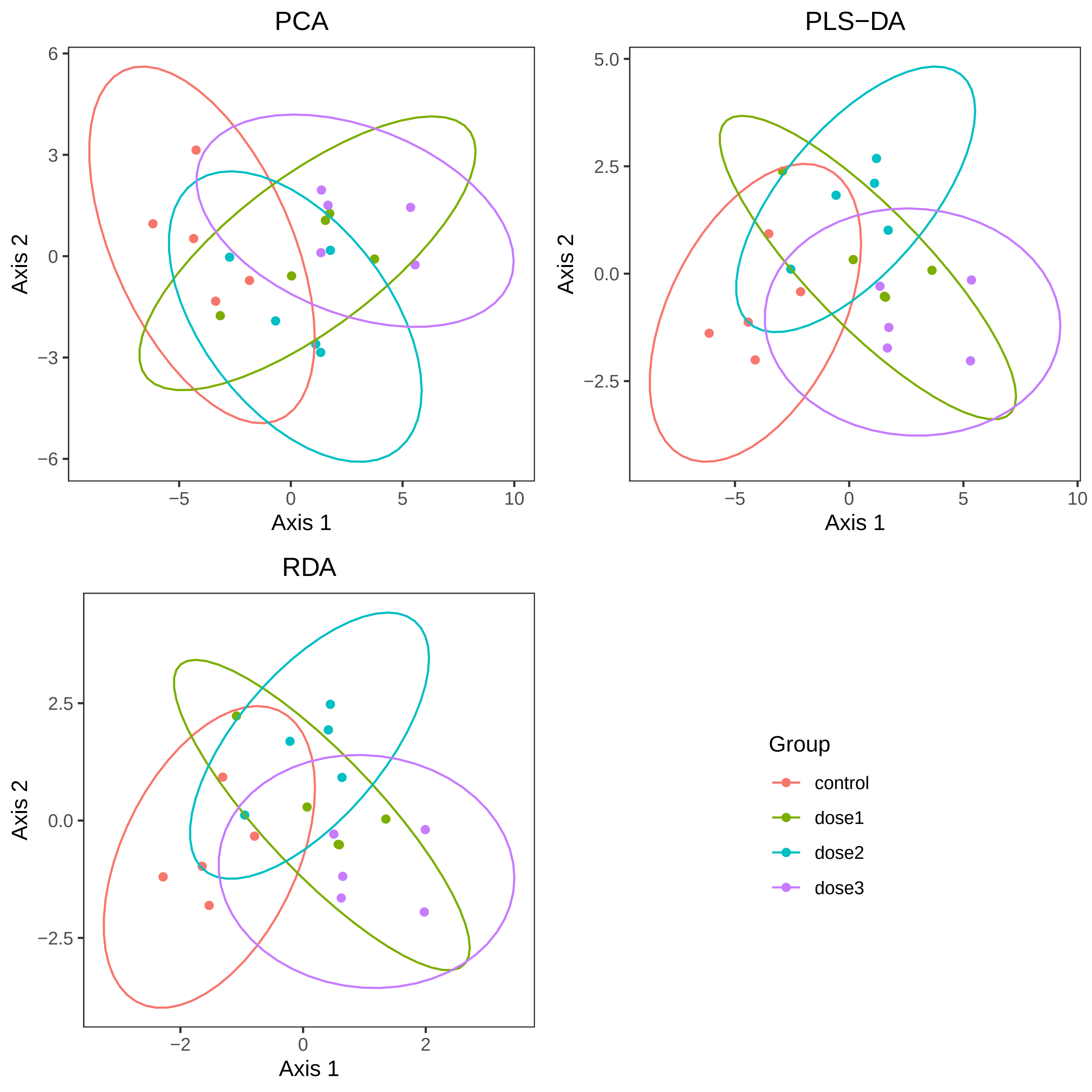

PCA
PLS−DA
RDA
Axis 1
Axis 2
6
3
0
−3
−6
−5
0
5
10
5.0
2.5
0.0
−2.5
2.5
0.0
−2.5
−2
0
2
Group
control
dose1
dose2
dose3

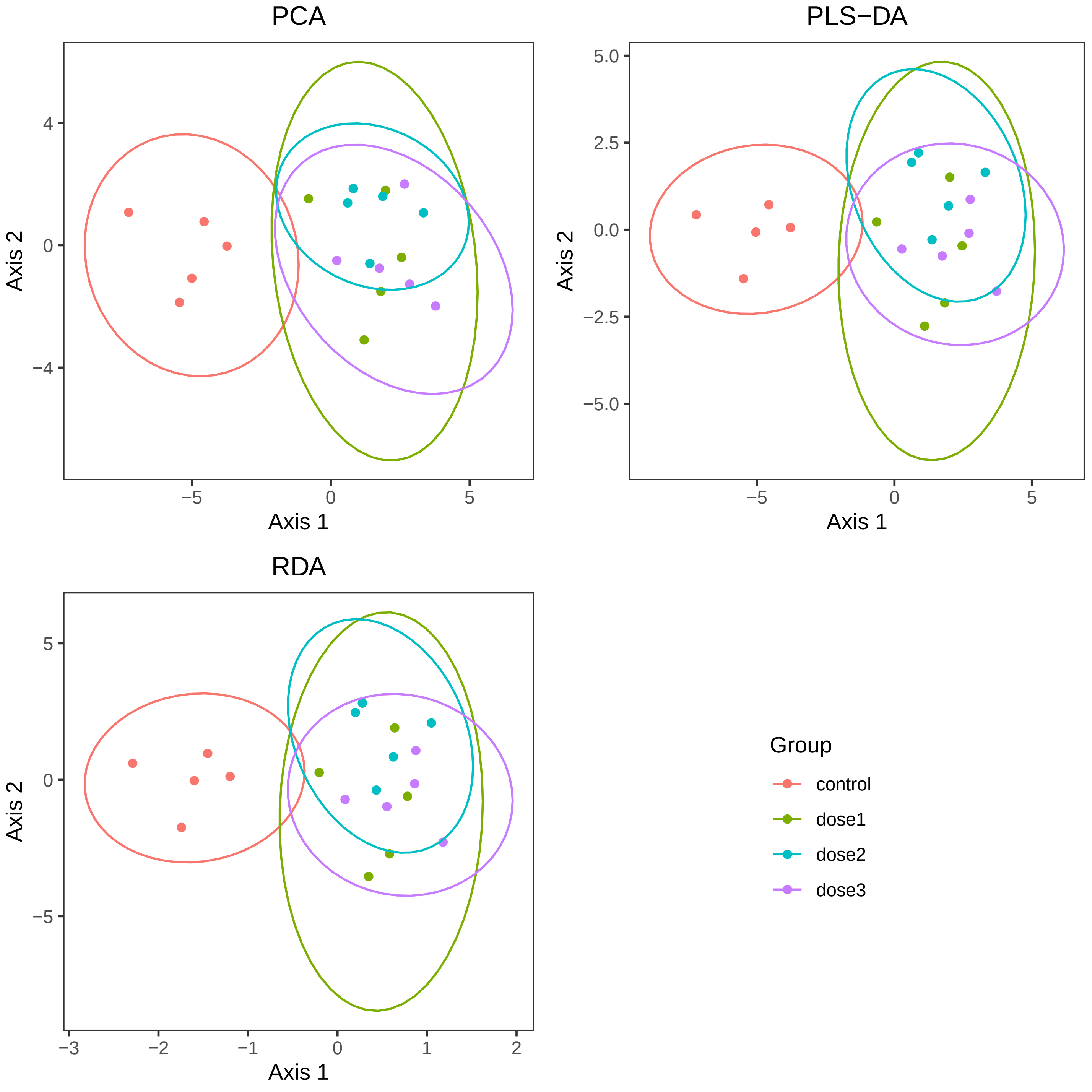

PCA
PLS−DA
RDA
Axis 1
Axis 2
Group
control
dose1
dose2
dose3

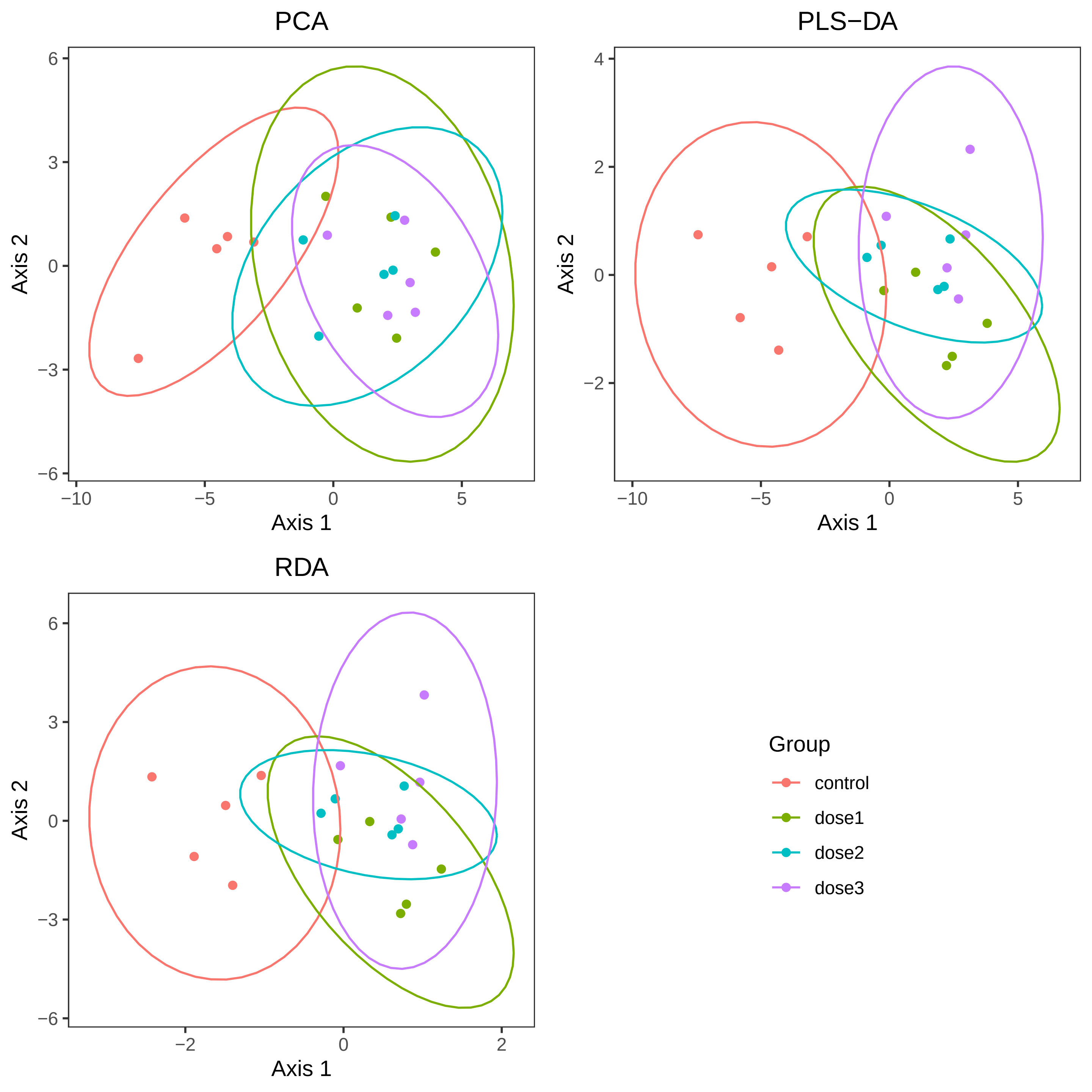
PCA
PLS−DA
RDA
Axis 1
Axis 2
Group
control
dose1
dose2
dose3

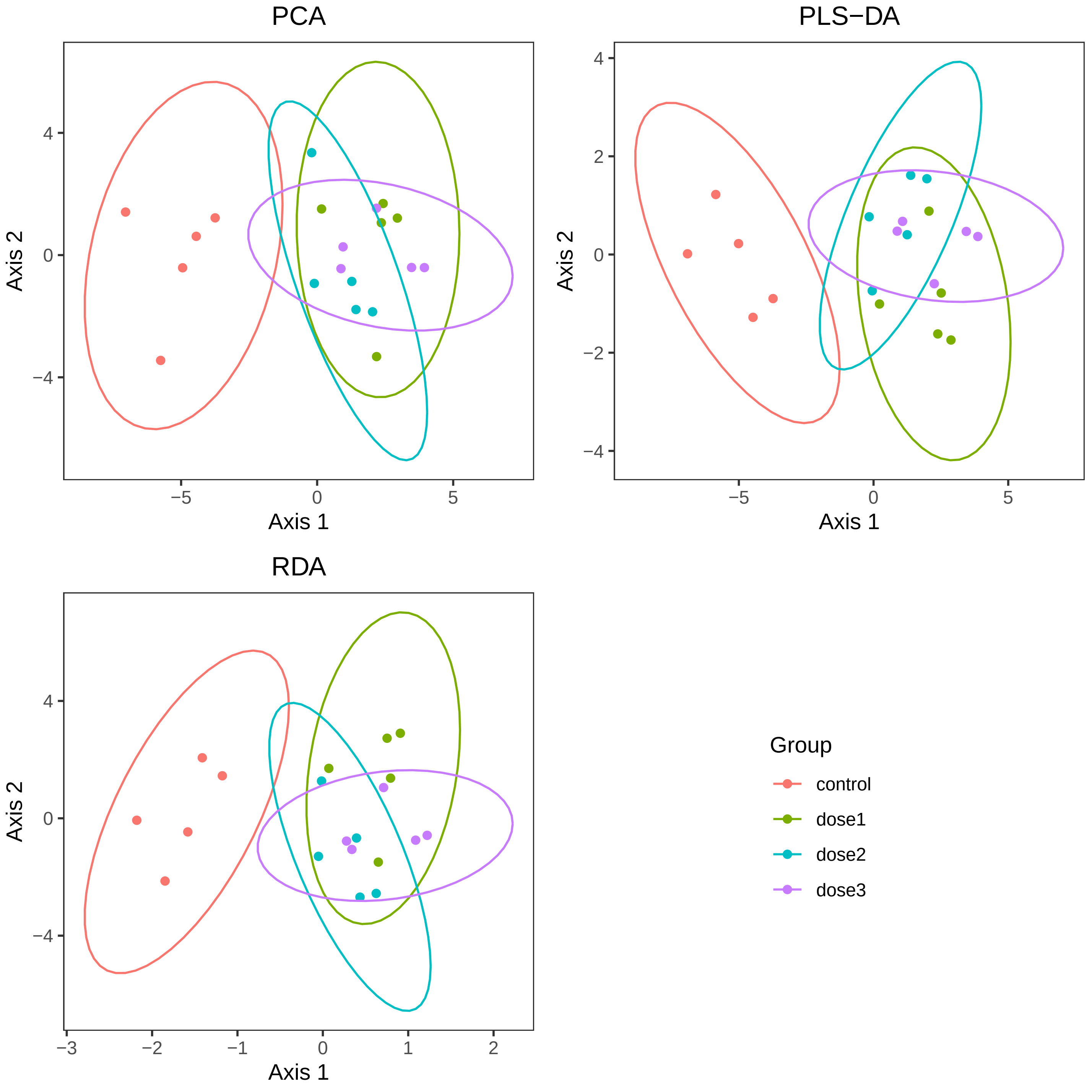
PCA
PLS−DA
RDA
Axis 1
Axis 2
−5
0
5
4
0
−4
2
−2
−3
−2
−1
1
Group
control
dose1
dose2
dose3

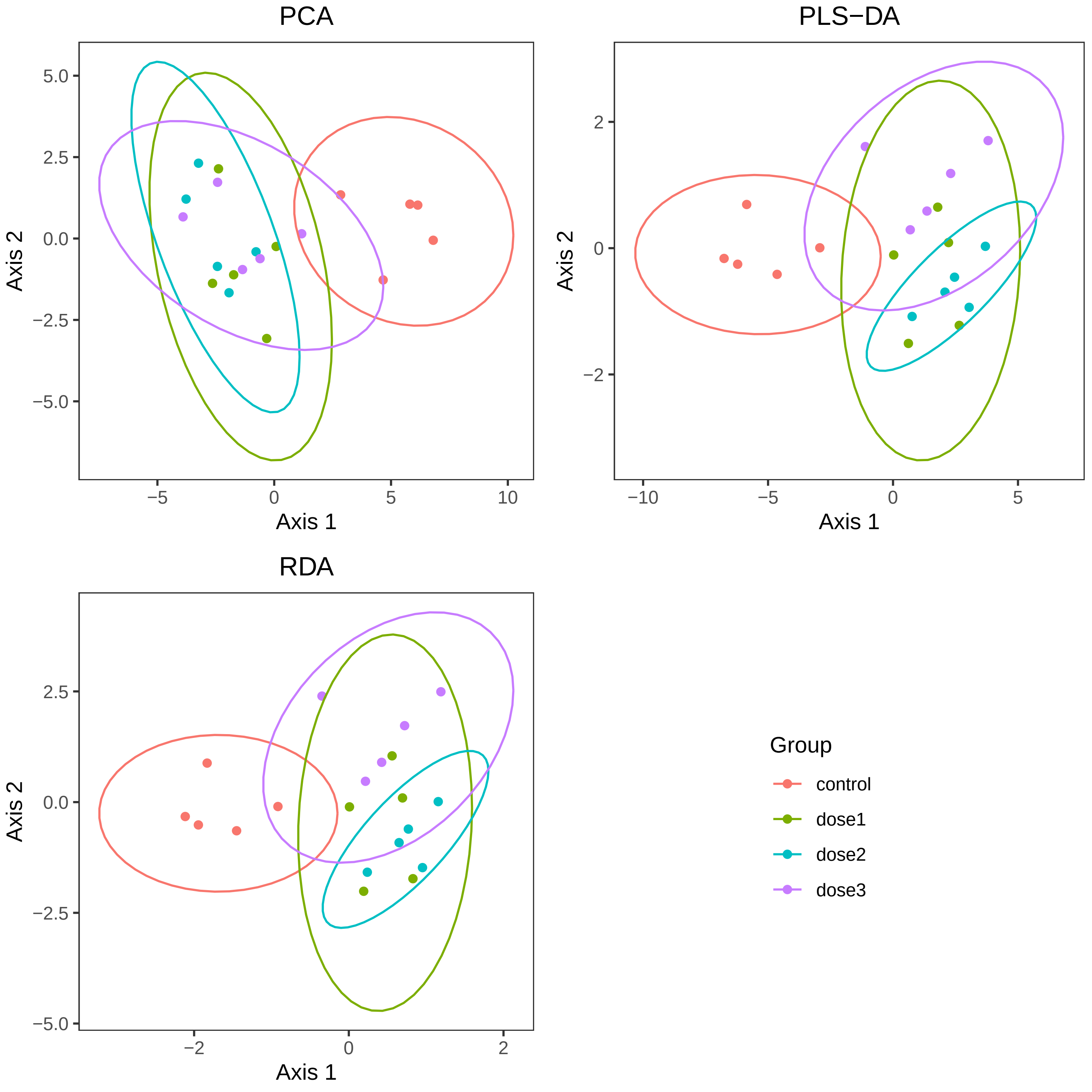

PCA
Axis 2
Axis 1
5.0
2.5
0.0
−2.5
−5.0
−5
0
5
10
PLS−DA
Axis 2
Axis 1
2
0
−2
−10
−5
0
5
RDA
Axis 2
Axis 1
2.5
0.0
−2.5
−5.0
−2
0
2
Group
control
dose1
dose2
dose3

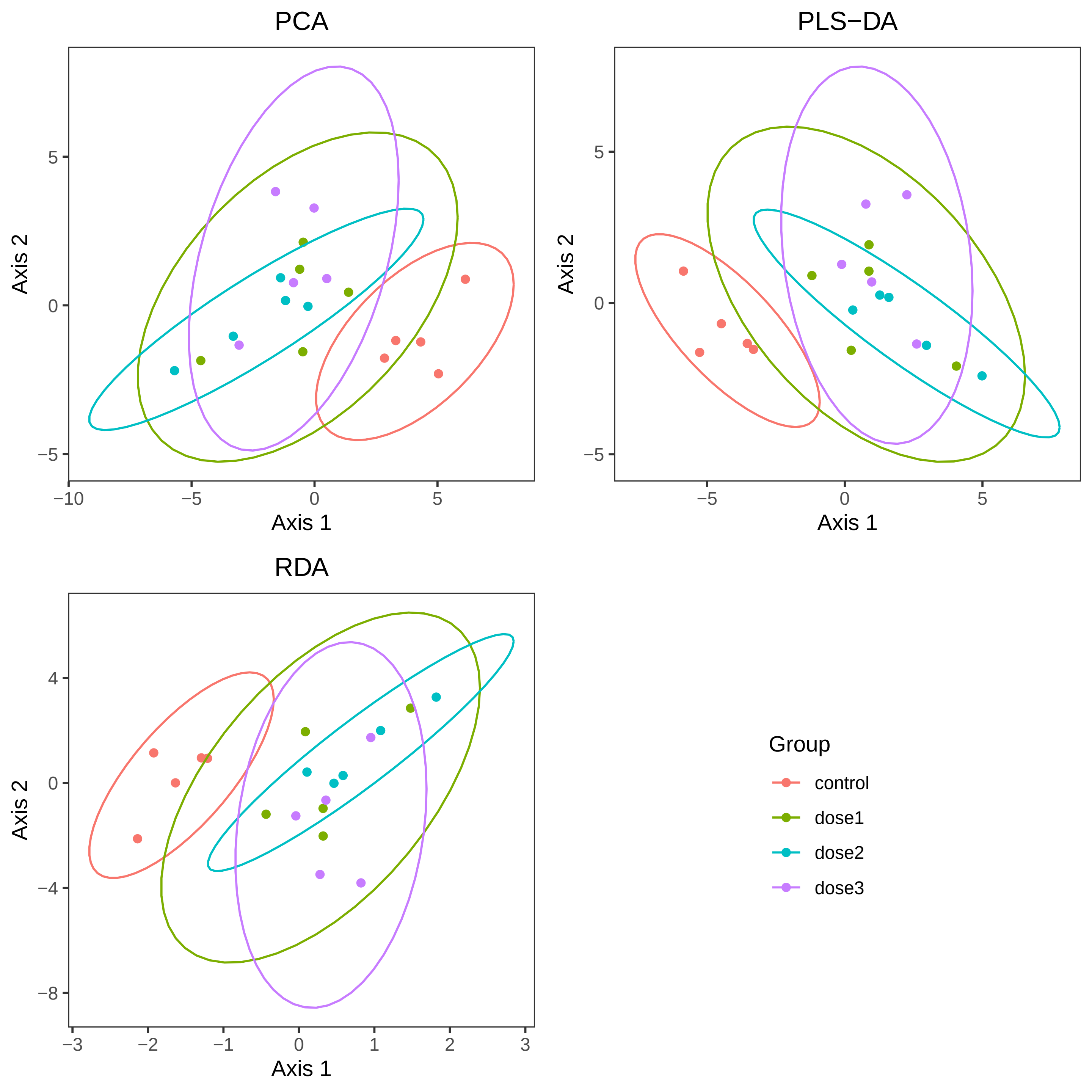
PCA
PLS−DA
RDA
Axis 1
Axis 2
−10
−5
0
5
4
−4
−8
−3
−2
−1
1
2
3
Group
control
dose1
dose2
dose3

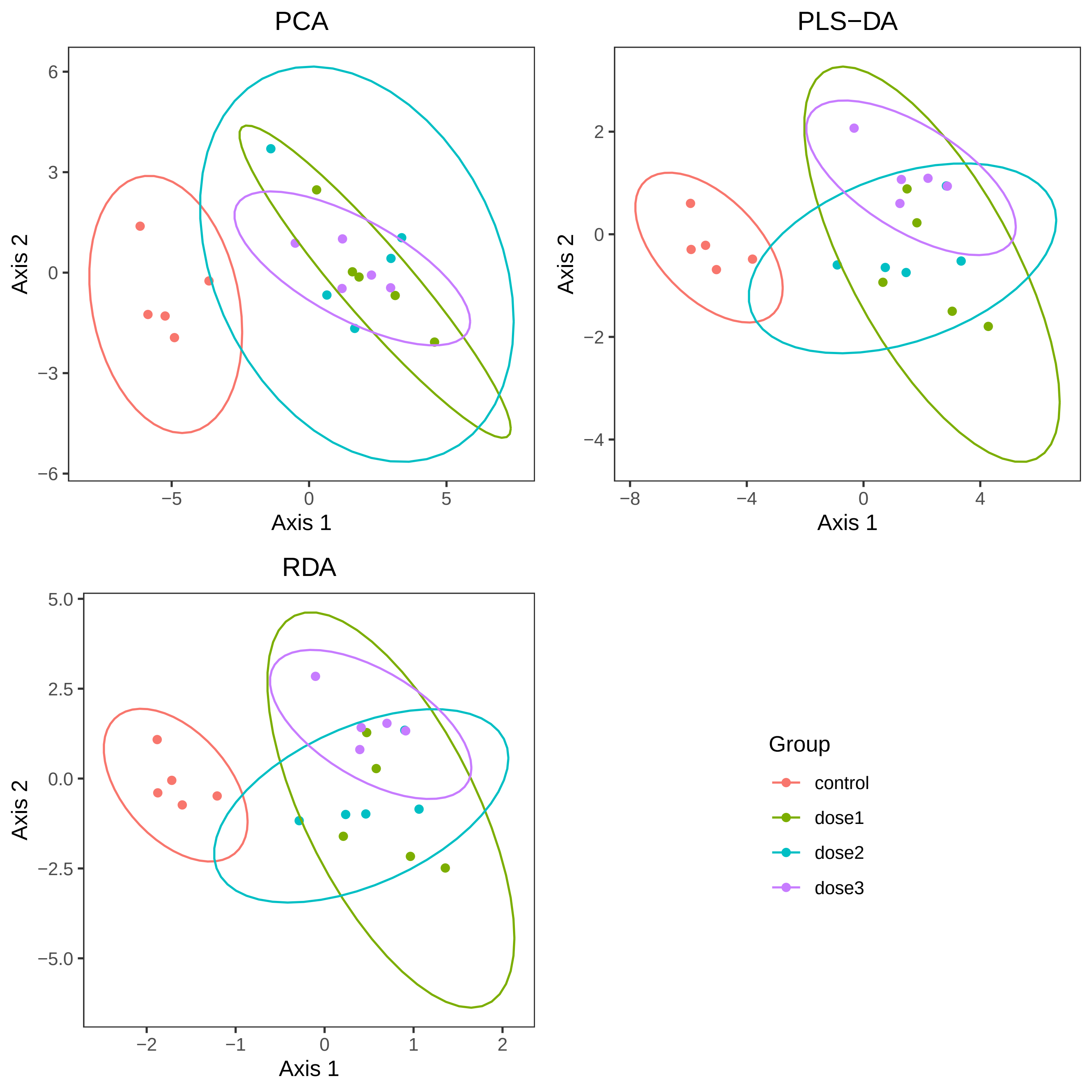
PCA
PLS−DA
RDA
Axis 1
Axis 2
Group
control
dose1
dose2
dose3

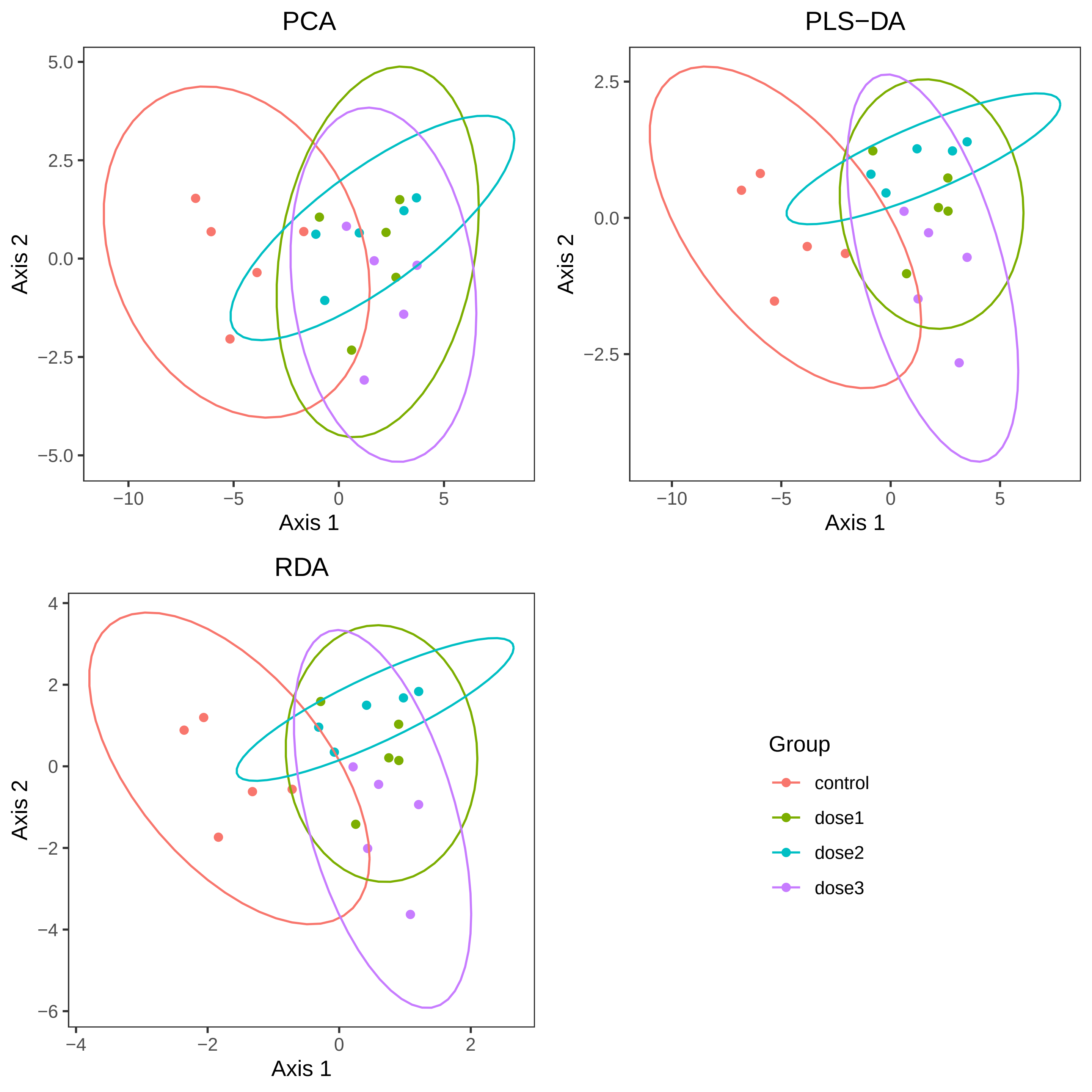
PCA
PLS−DA
RDA
Axis 1
Axis 2
Group
control
dose1
dose2
dose3

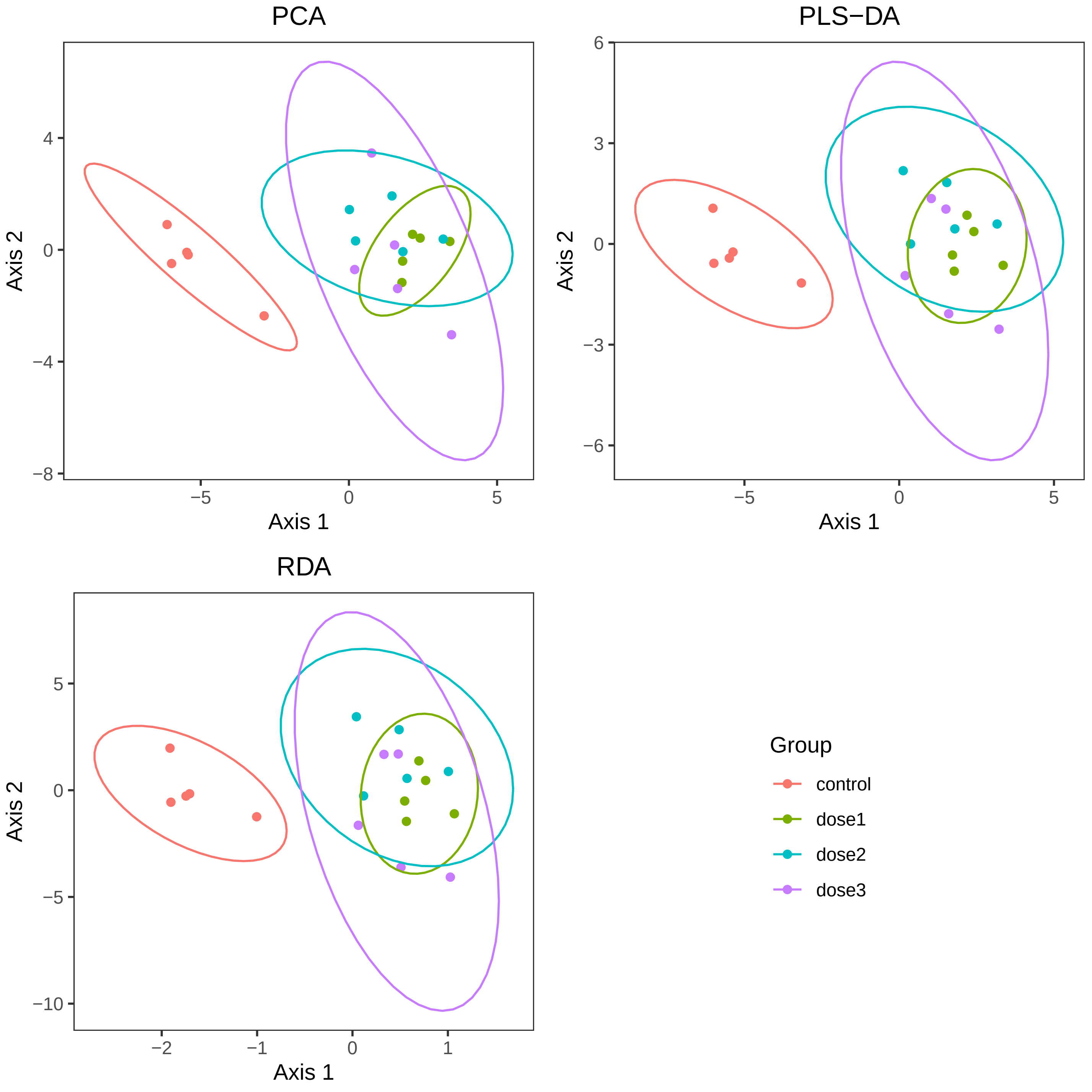
PCA
Axis 2
Axis 1
4
0
−4
−8
−5
0
5
PLS−DA
6
3
0
−3
−6
RDA
5
0
−5
−10
−2
−1
1
Group
control
dose1
dose2
dose3

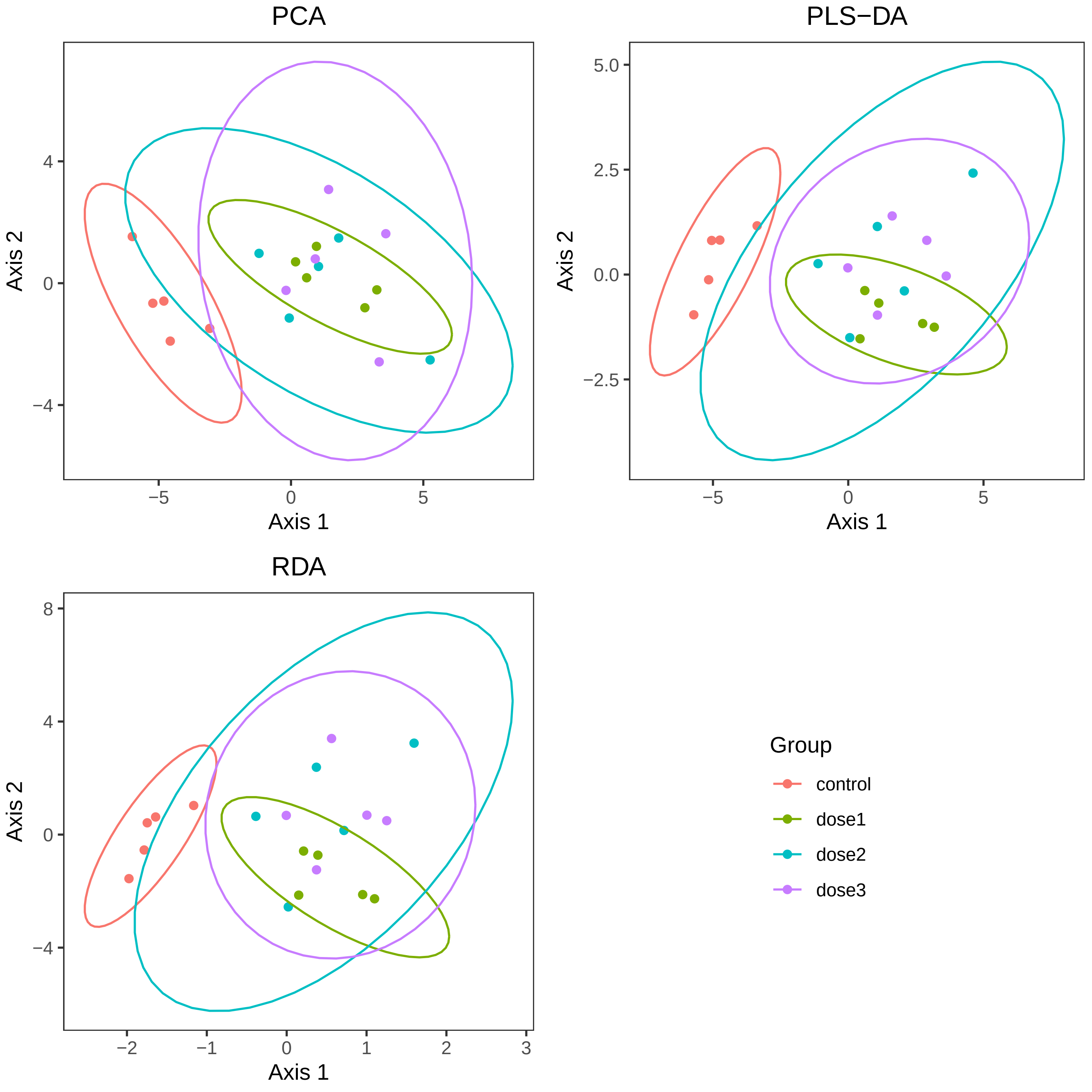
PCA
PLS−DA
RDA
Axis 1
Axis 2
Group
control
dose1
dose2
dose3

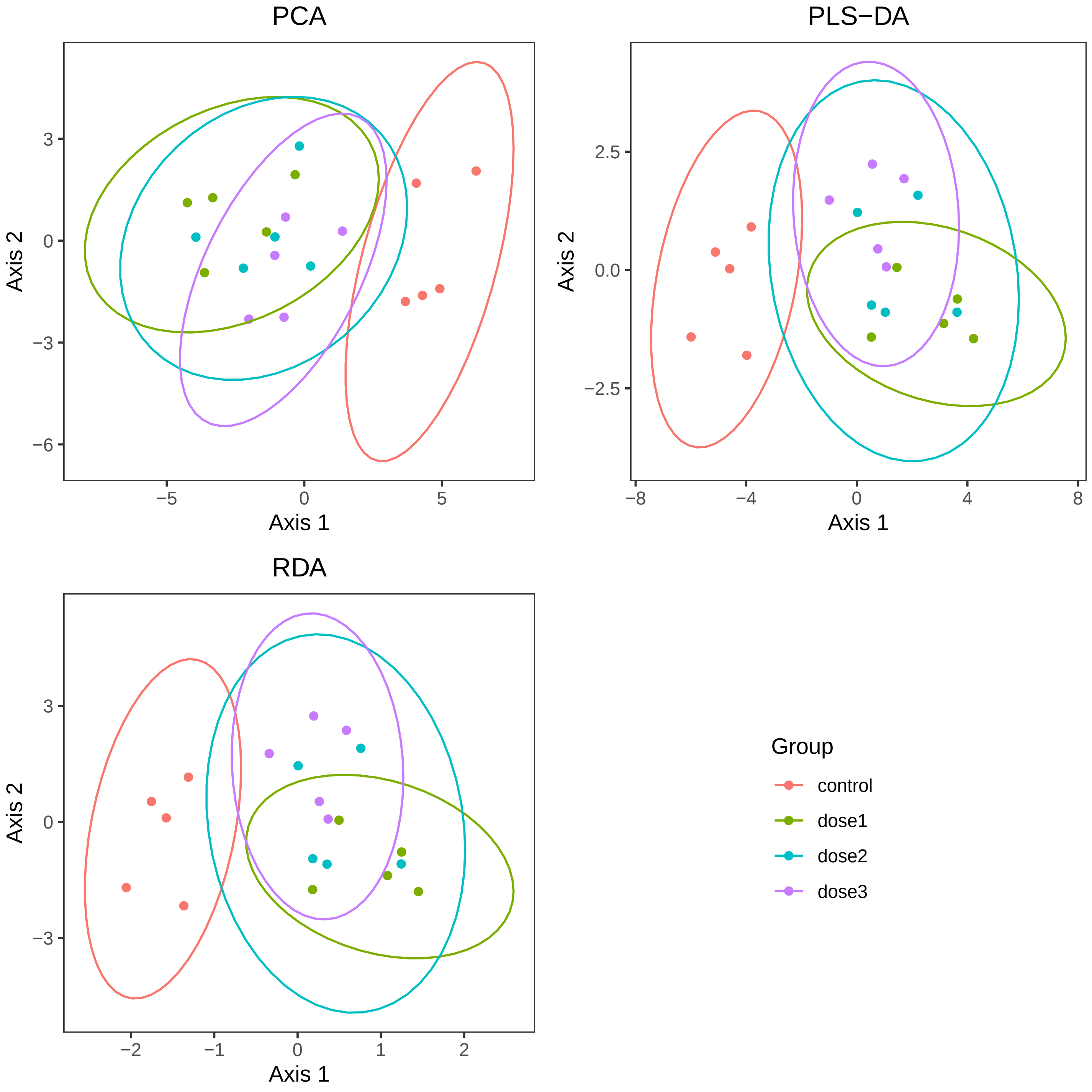

PCA
PLS−DA
RDA
Axis 1
Axis 2
Group
control
dose1
dose2
dose3

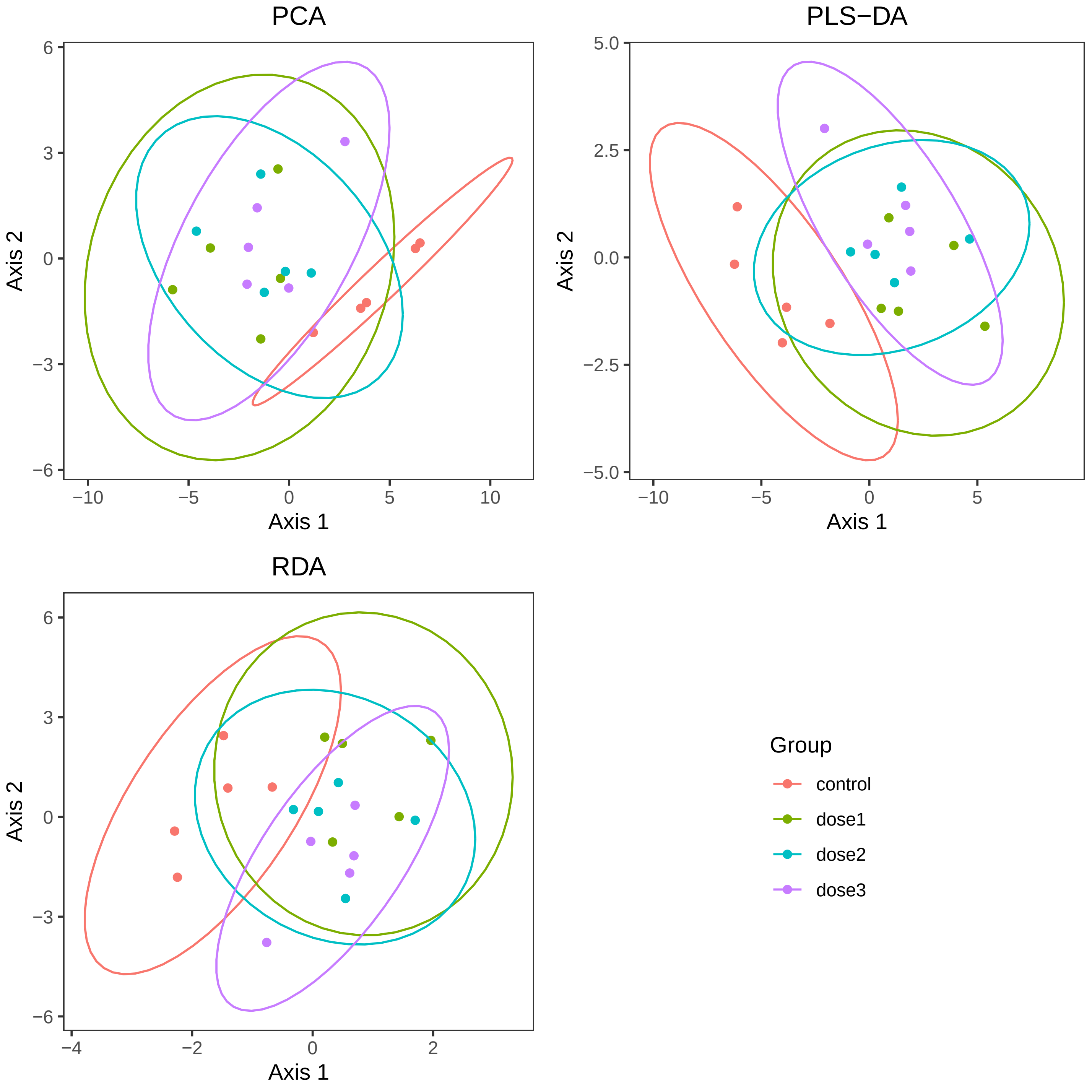

PCA
PLS−DA
RDA
Axis 1
Axis 2
Group
control
dose1
dose2
dose3

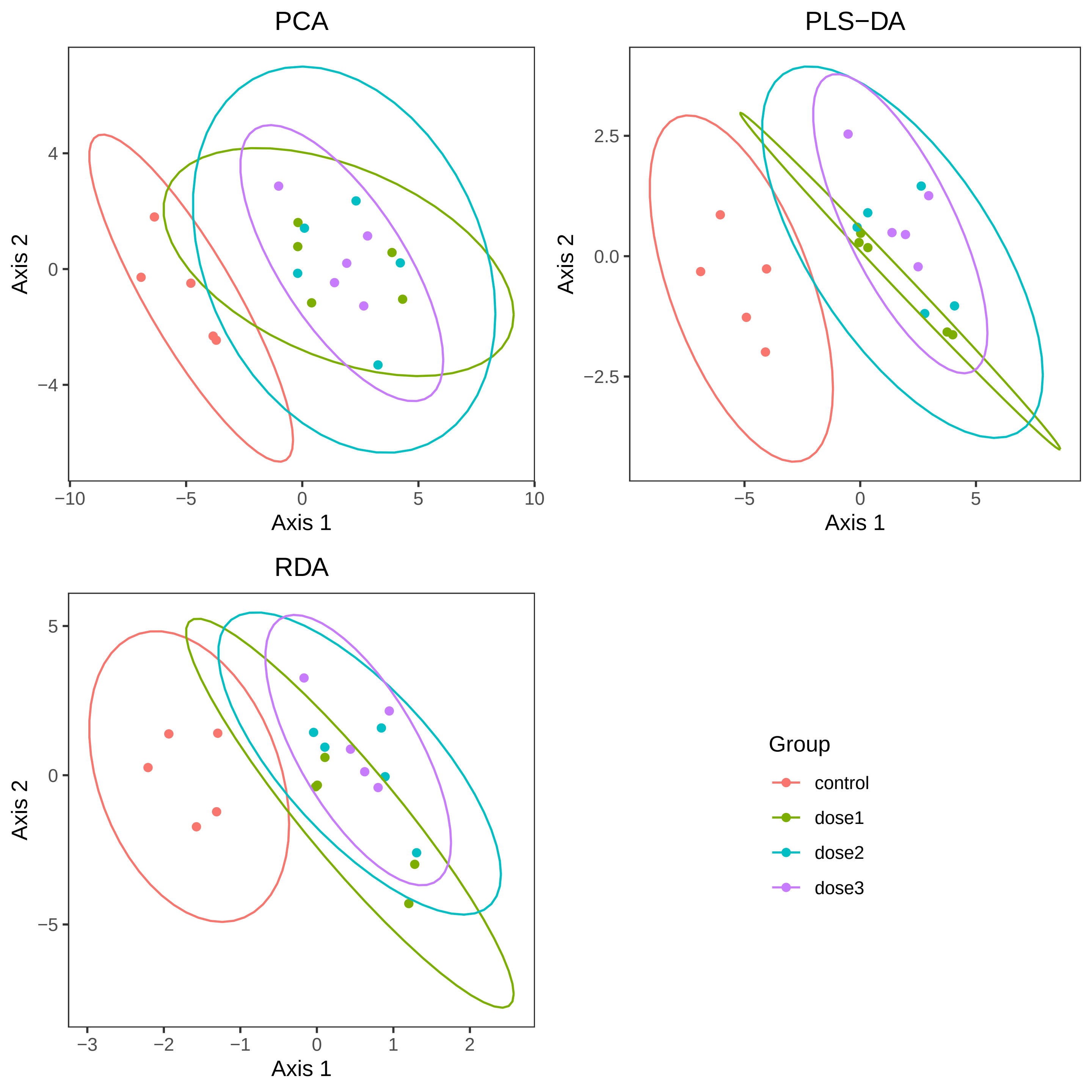
PCA
PLS−DA
RDA
Axis 1
Axis 2
Group
control
dose1
dose2
dose3

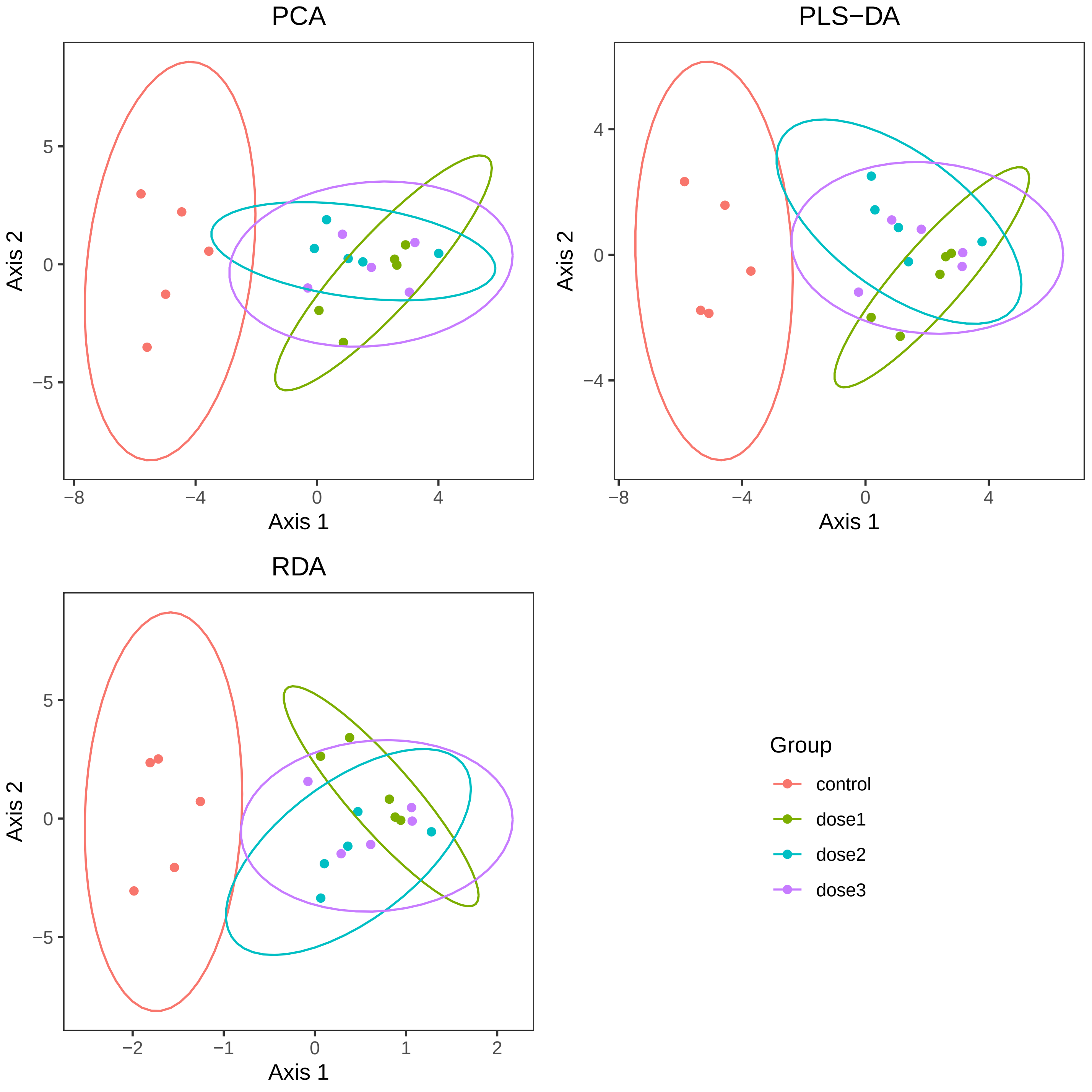
PCA
PLS−DA
RDA
Axis 1
Axis 2
Group
control
dose1
dose2
dose3

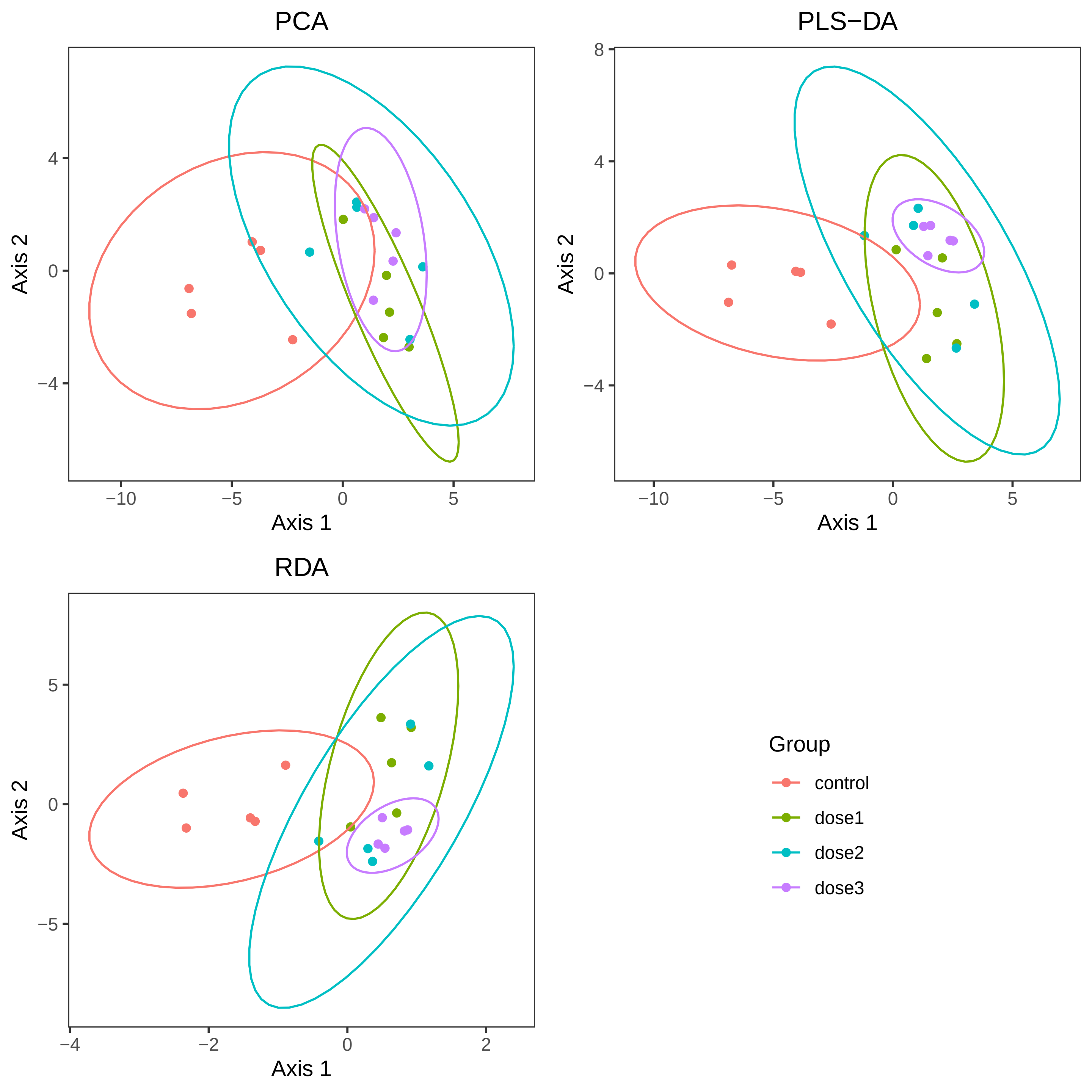

PCA
PLS−DA
RDA
Axis 1
Axis 2
Group
control
dose1
dose2
dose3

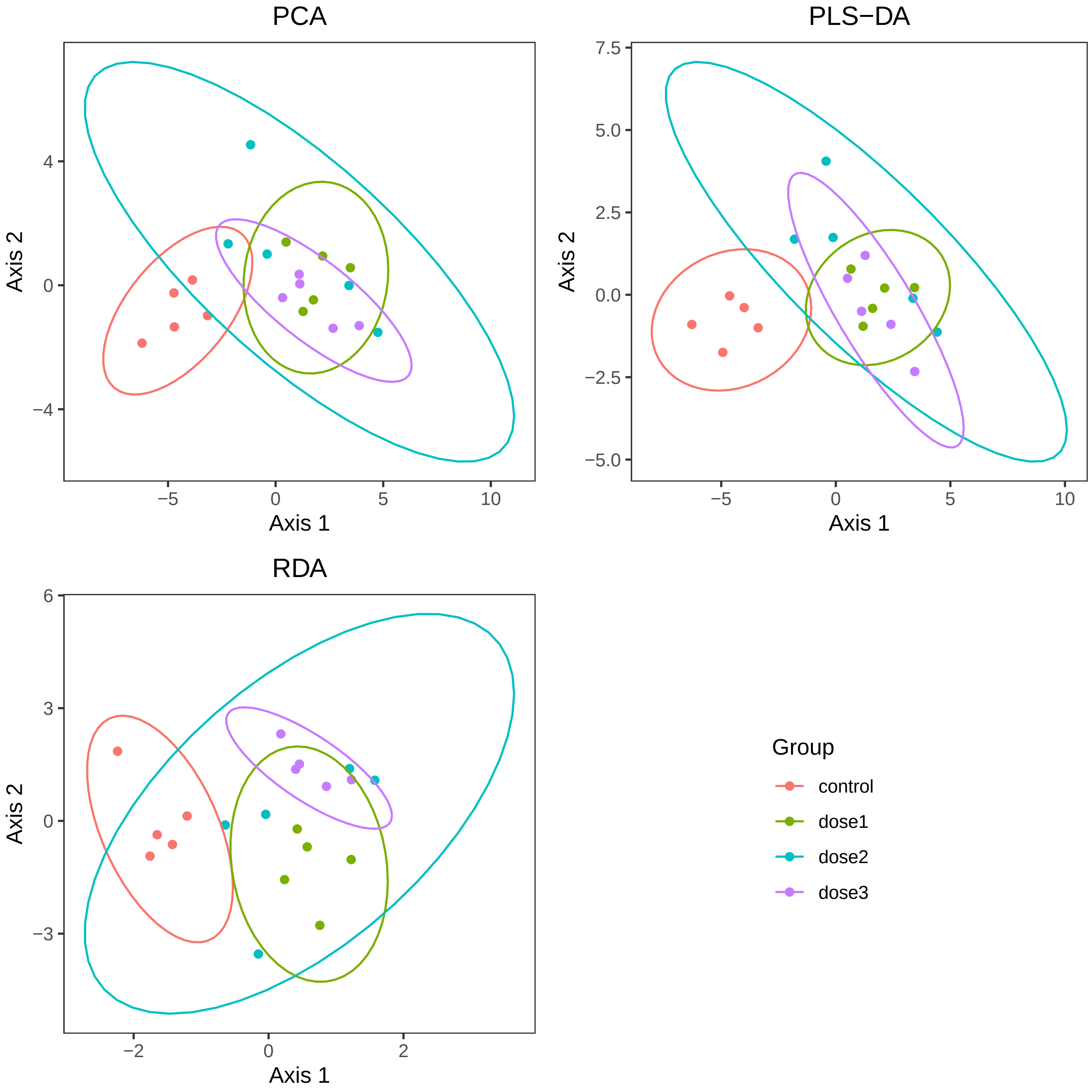

PCA
PLS−DA
RDA
Axis 1
Axis 2
Group
control
dose1
dose2
dose3

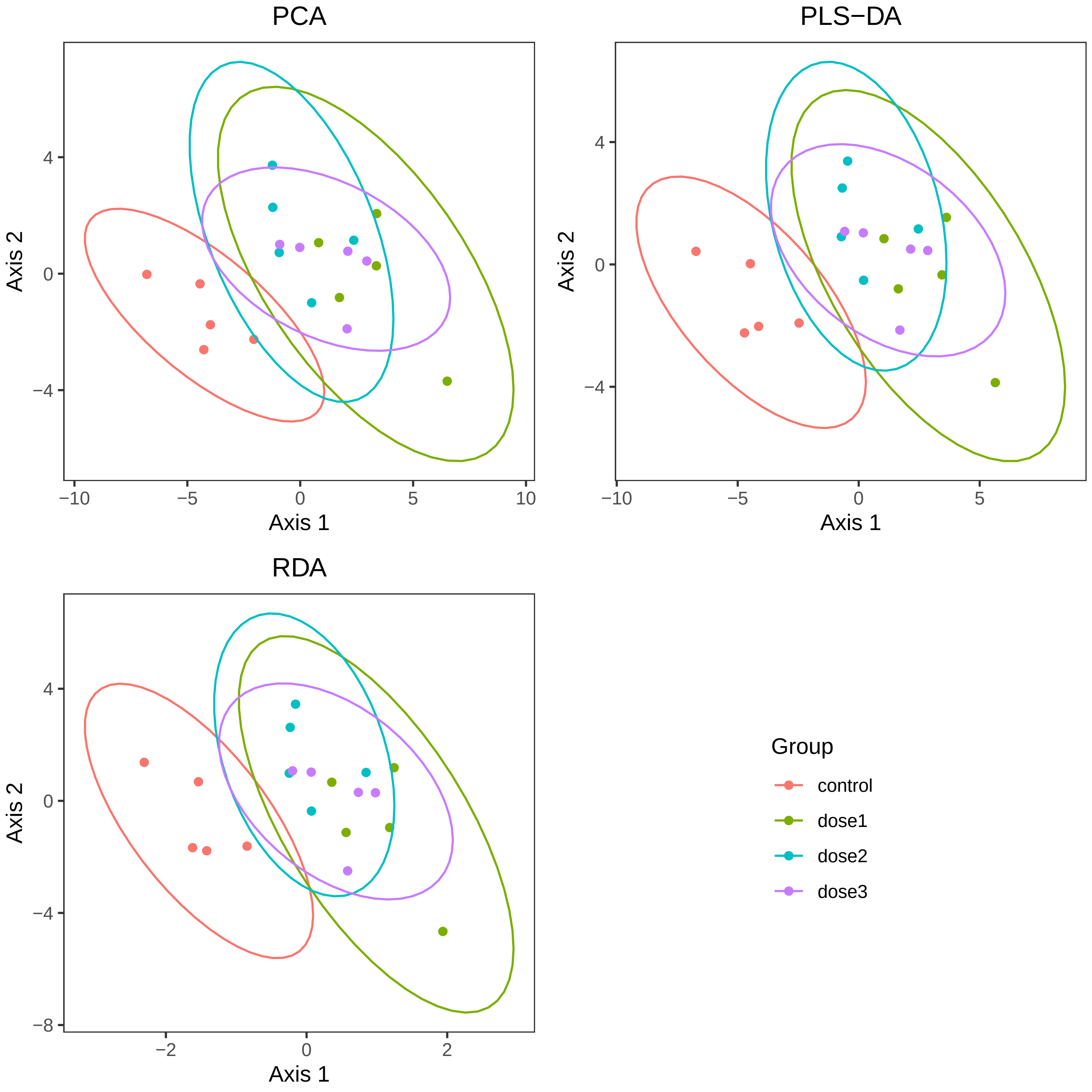

PCA
PLS−DA
RDA
Axis 1
Axis 2
−10
−5
0
5
10
4
0
−4
−8
−2
2
Group
control
dose1
dose2
dose3

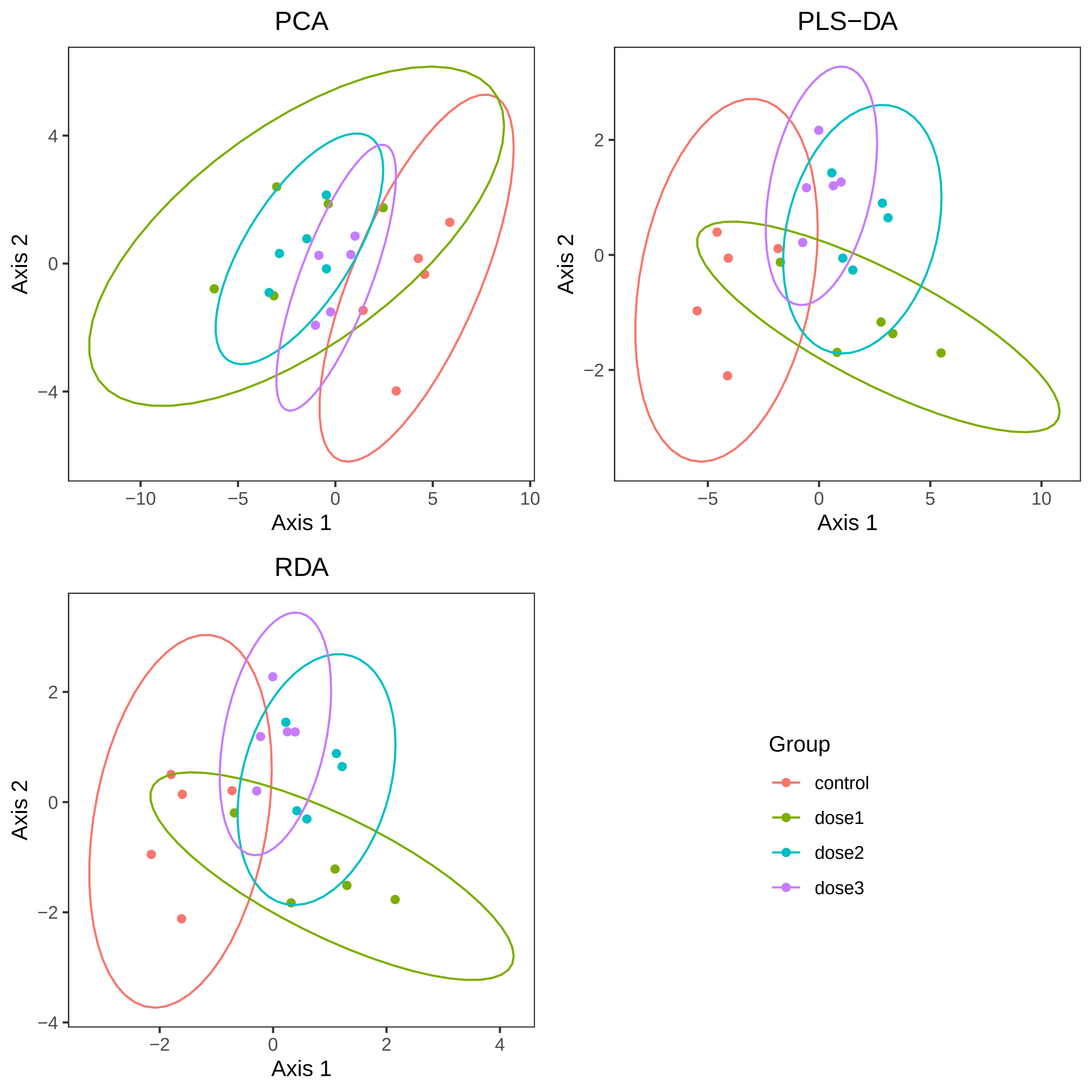

PCA
PLS−DA
RDA
Axis 1
Axis 2
Group
control
dose1
dose2
dose3

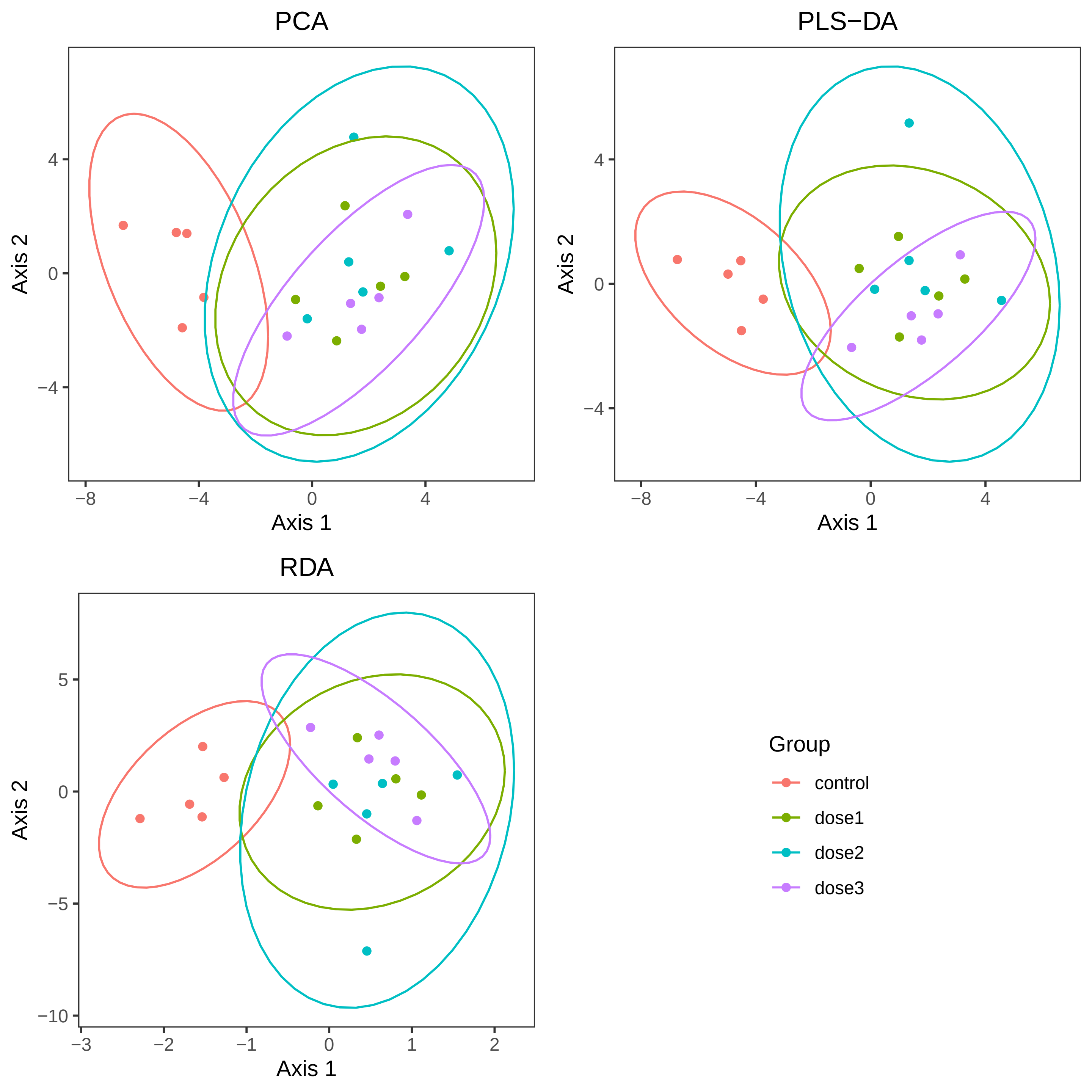

PCA
PLS−DA
RDA
Axis 1
Axis 2
Group
control
dose1
dose2
dose3

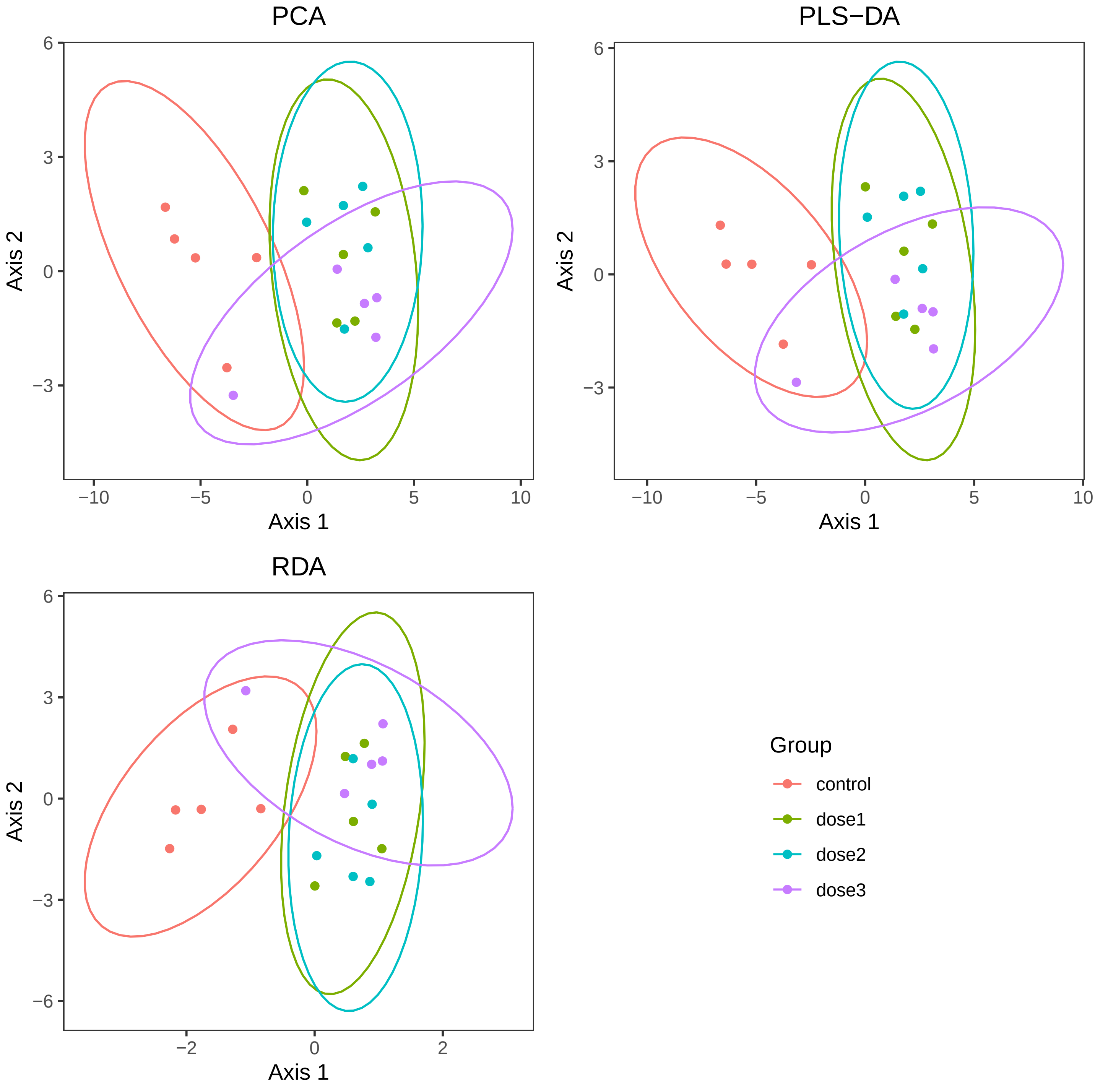

PCA
PLS−DA
RDA
Axis 1
Axis 2
−10
−5
0
5
10
6
3
0
−3
−6
−2
2
Group
control
dose1
dose2
dose3

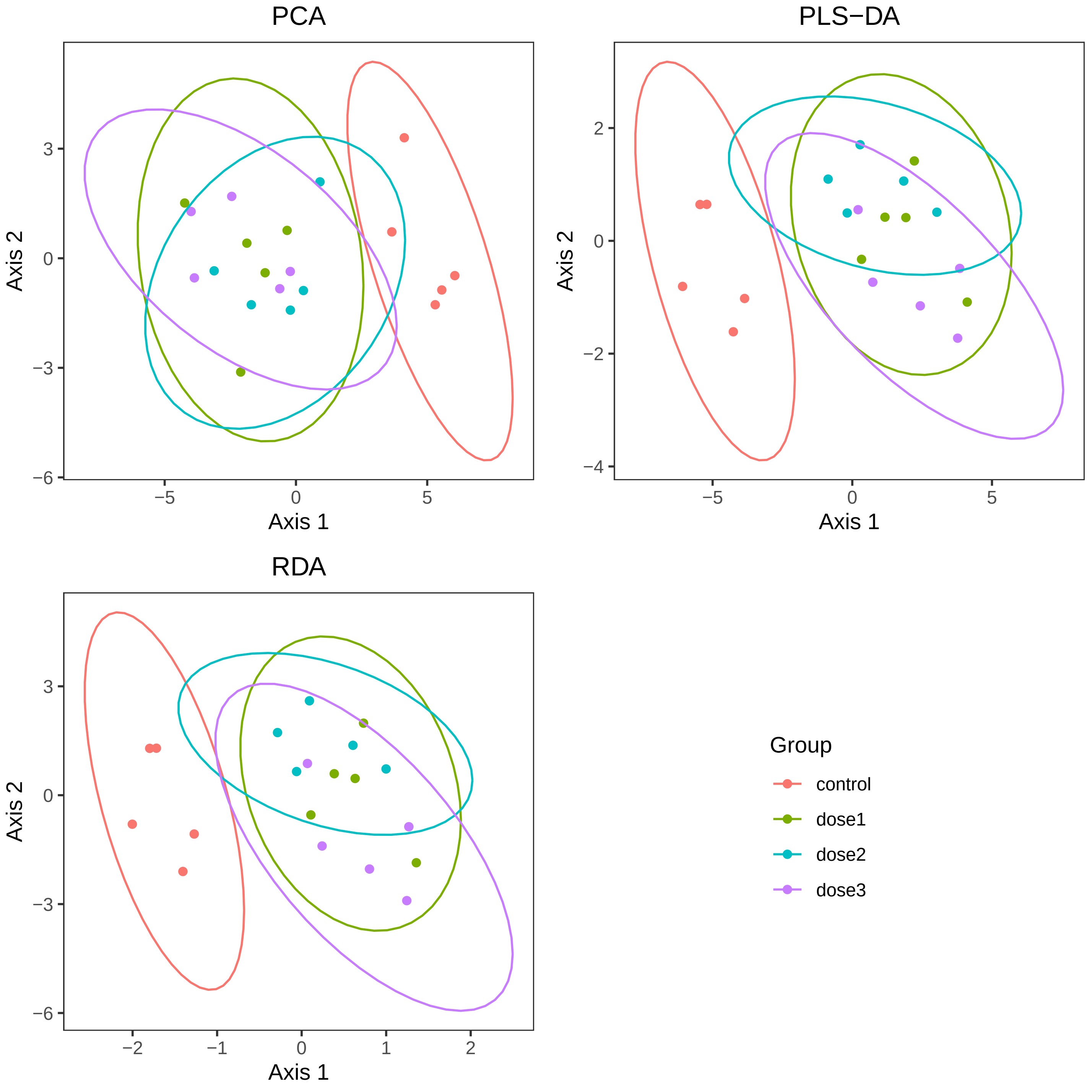
PCA
PLS−DA
RDA
Axis 1
Axis 2
3
0
−3
−6
−5
0
5
2
0
−2
−4
−2
−1
1
2
Group
control
dose1
dose2
dose3

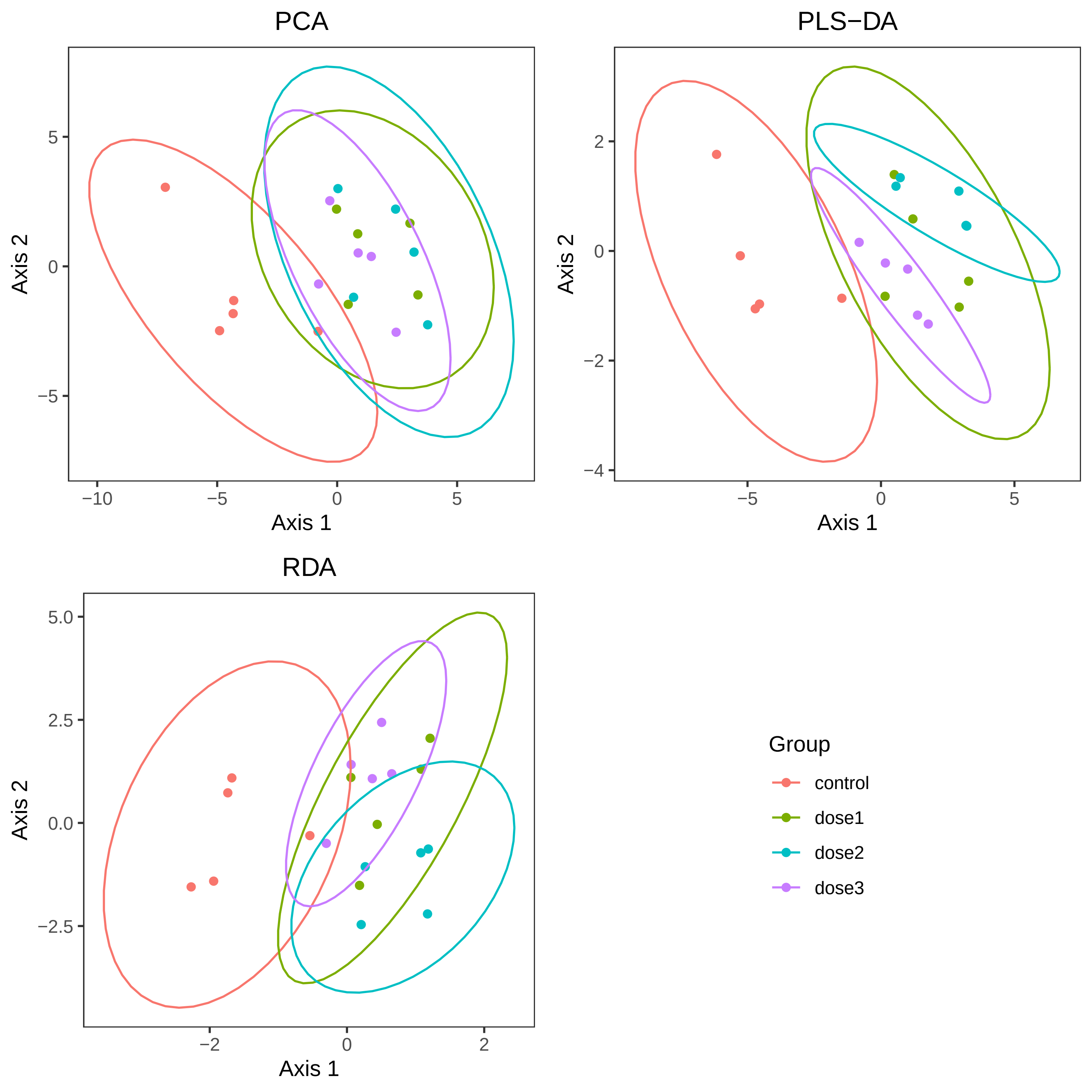

PCA
PLS−DA
RDA
Axis 1
Axis 2
Group
control
dose1
dose2
dose3

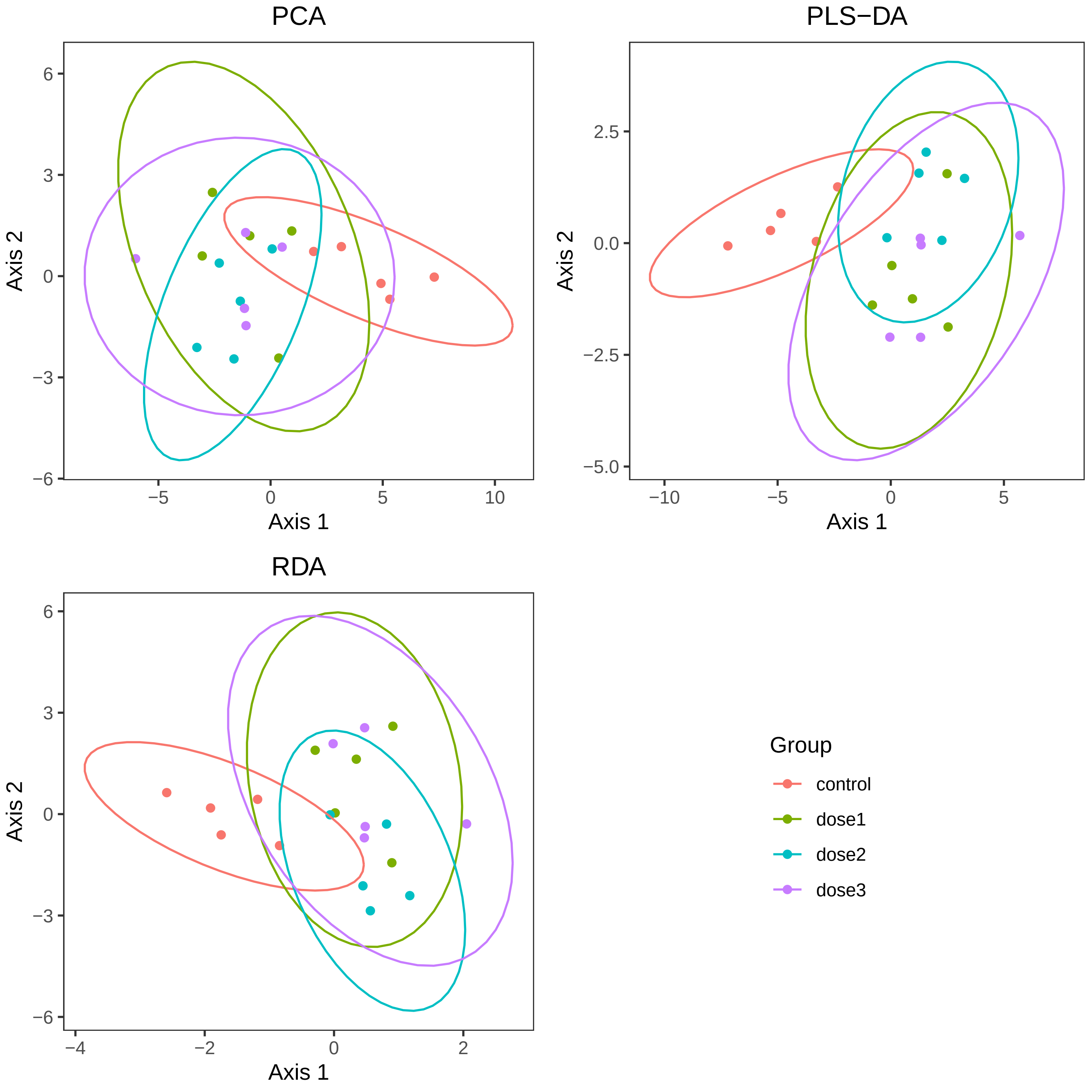

PCA
PLS−DA
RDA
Axis 1
Axis 2
Group
control
dose1
dose2
dose3

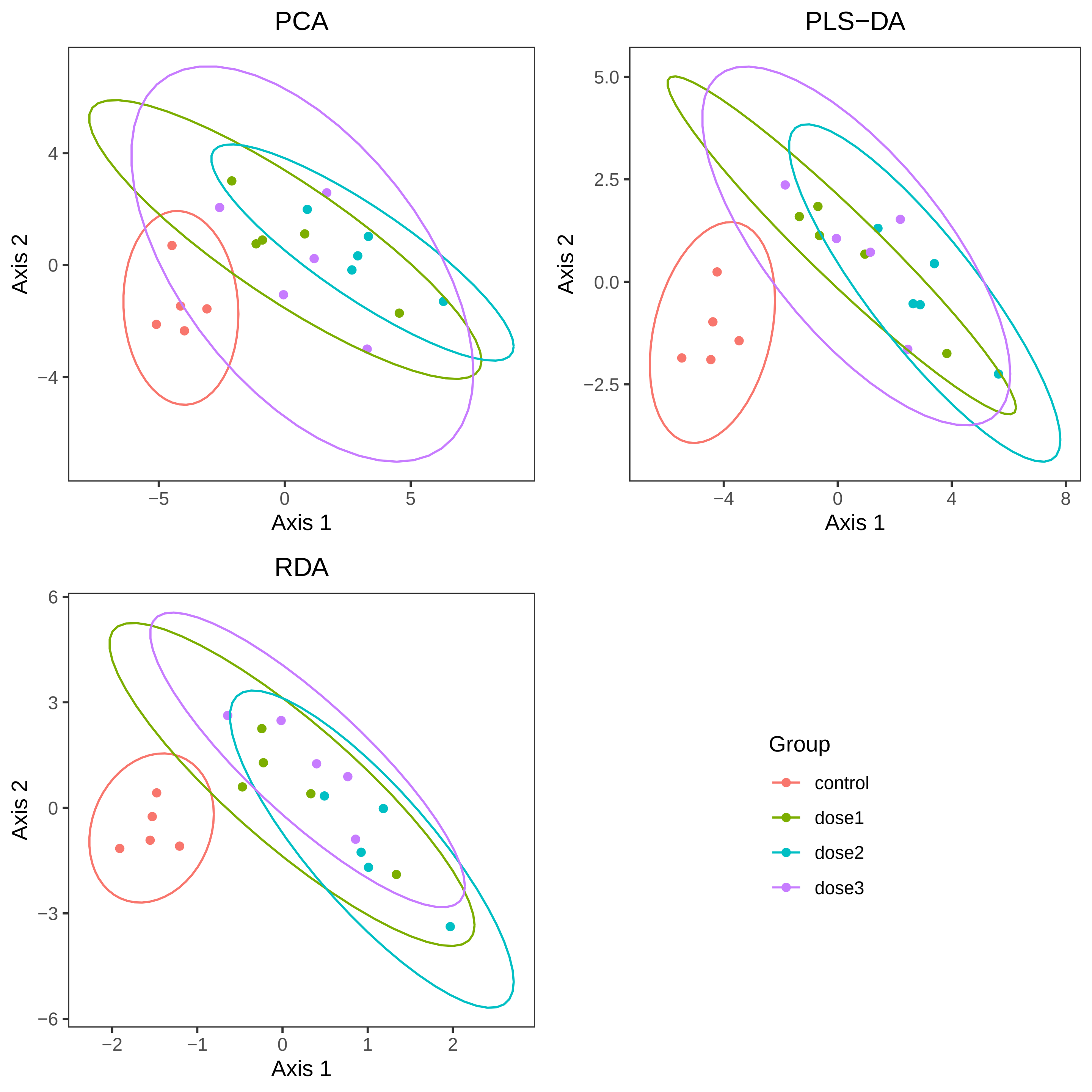

PCA
PLS−DA
RDA
Axis 1
Axis 2
4
0
−4
−5
5
5.0
2.5
0.0
−2.5
8
6
3
−3
−6
−2
−1
1
2
Group
control
dose1
dose2
dose3

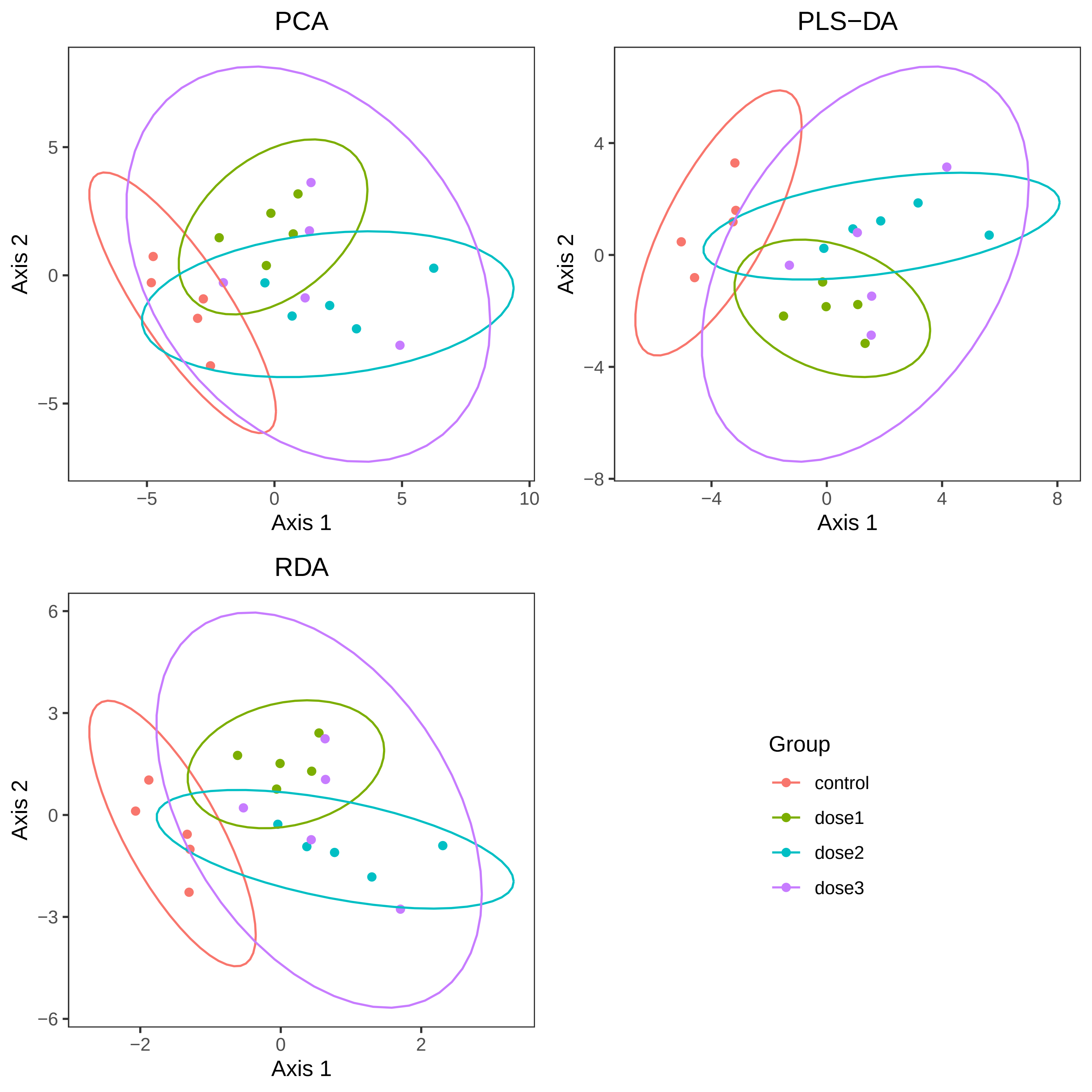

PCA
PLS−DA
RDA
Axis 1
Axis 2
Group
control
dose1
dose2
dose3

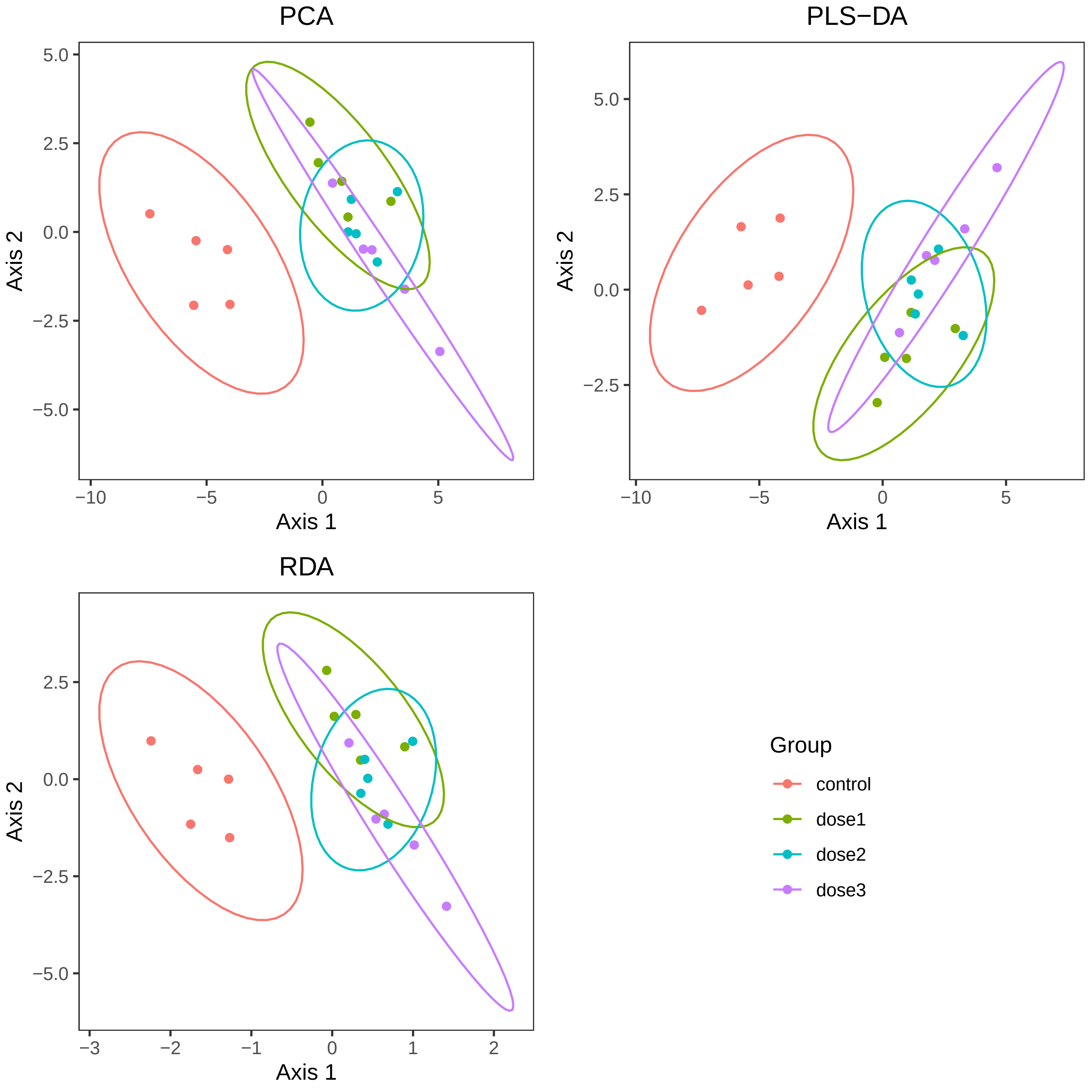

PCA
PLS−DA
RDA
Axis 1
Axis 2
−10
−5
0
5
5.0
2.5
0.0
−2.5
−5.0
−3
−2
−1
1
2
Group
control
dose1
dose2
dose3

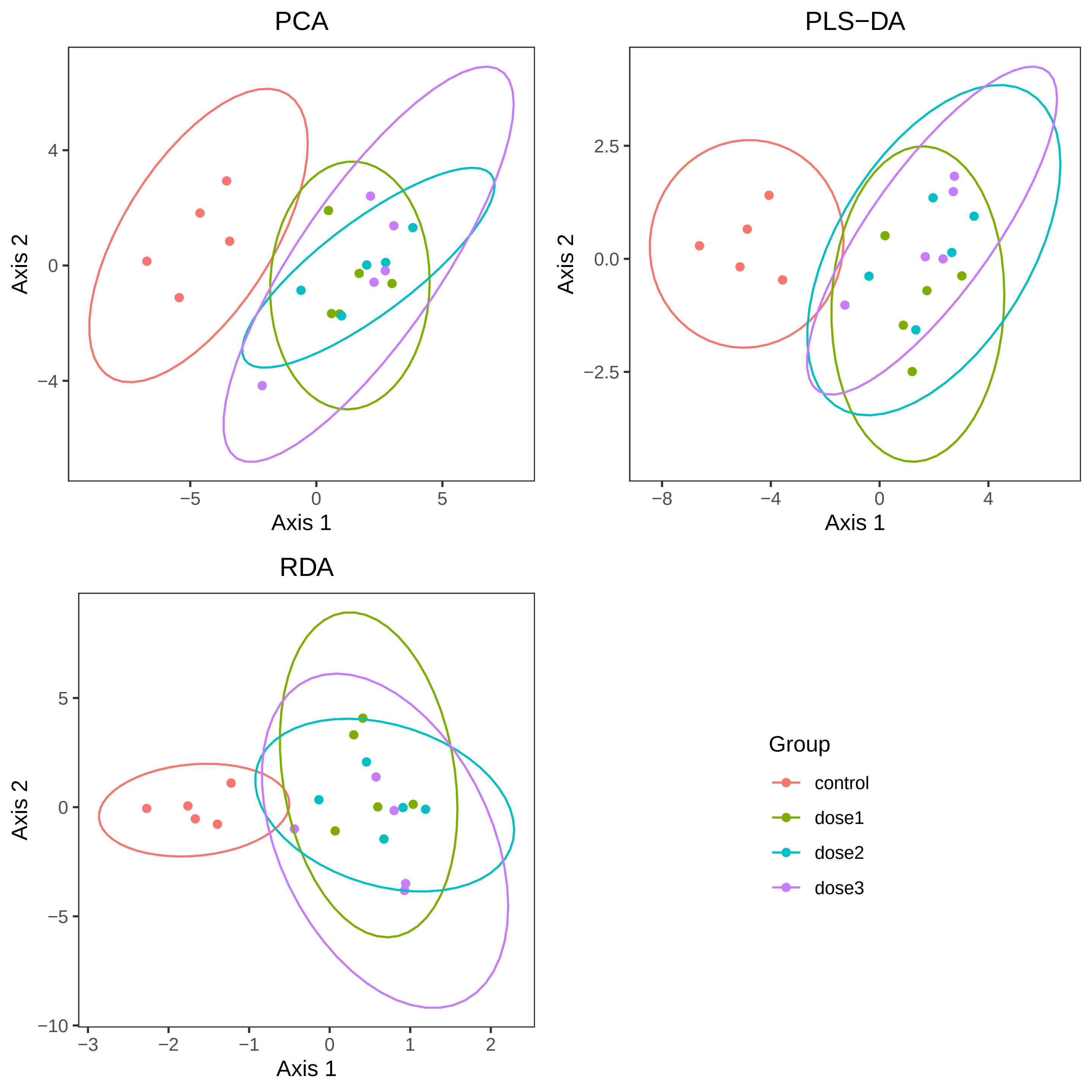

PCA
PLS−DA
RDA
Axis 1
Axis 2
Group
control
dose1
dose2
dose3

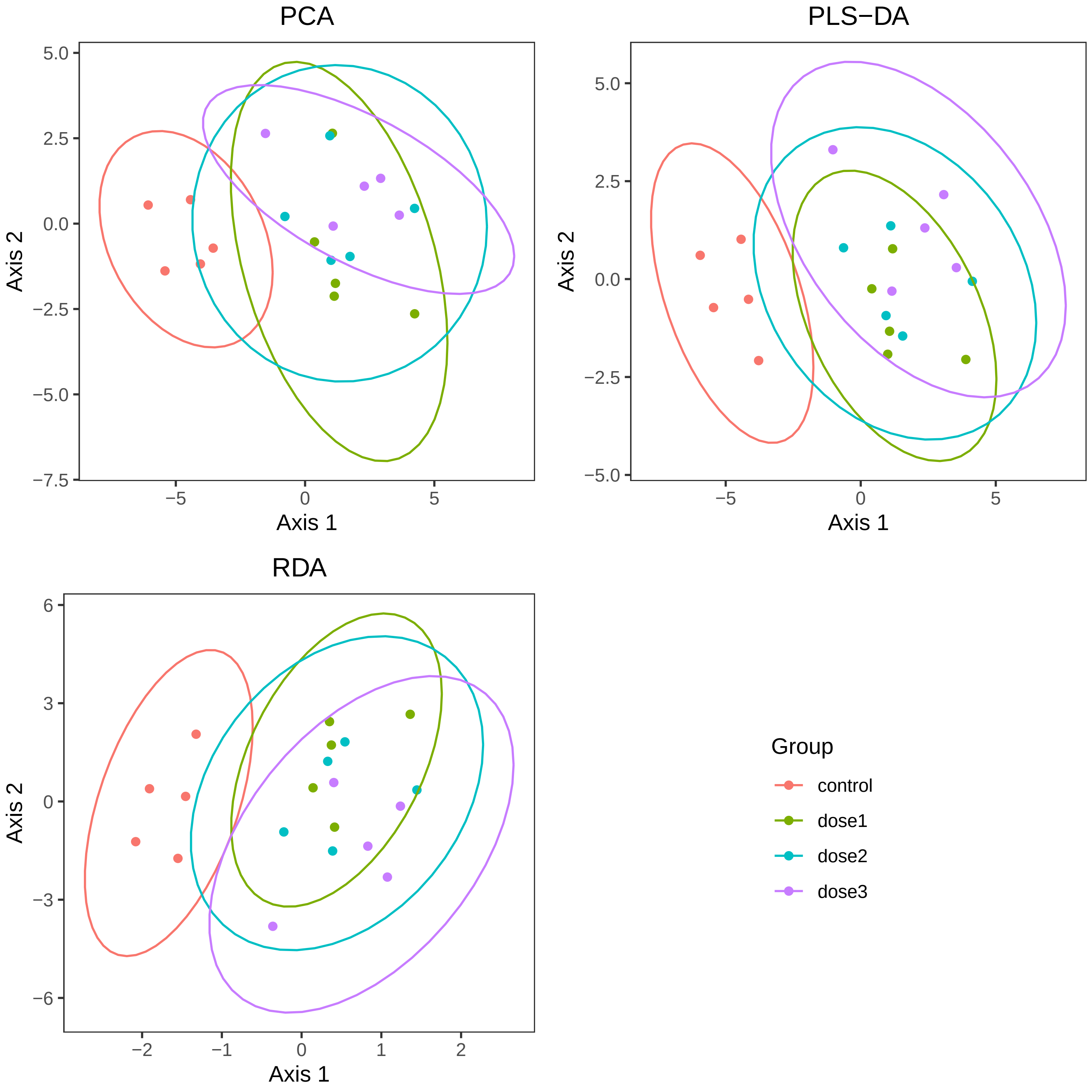
PCA
PLS−DA
RDA
Axis 1
Axis 2
5.0
2.5
0.0
−2.5
−5.0
−7.5
−5
0
5
−6
−3
0
3
6
−2
−1
1
2
Group
control
dose1
dose2
dose3

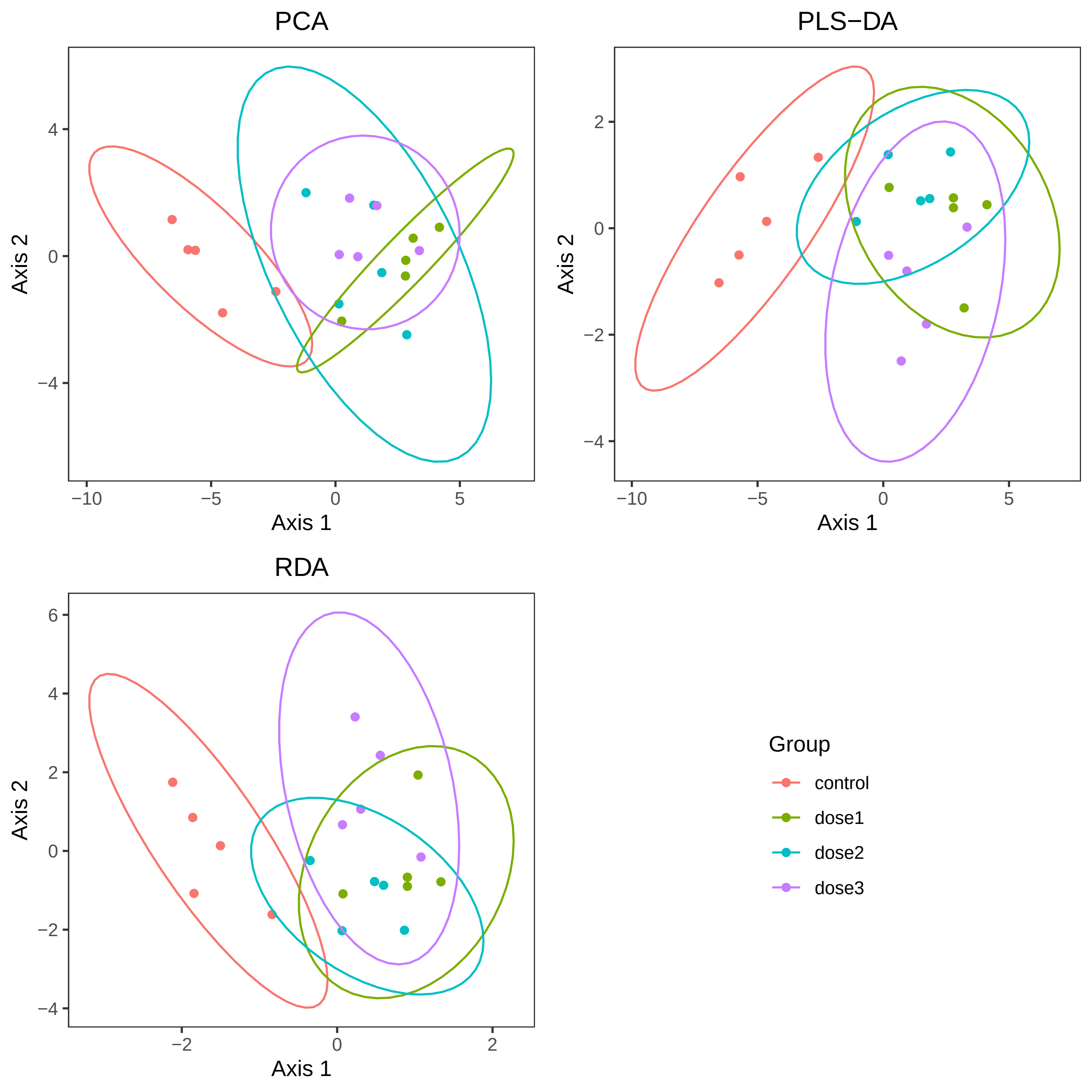

PCA
PLS−DA
RDA
Axis 1
Axis 2
Group
control
dose1
dose2
dose3

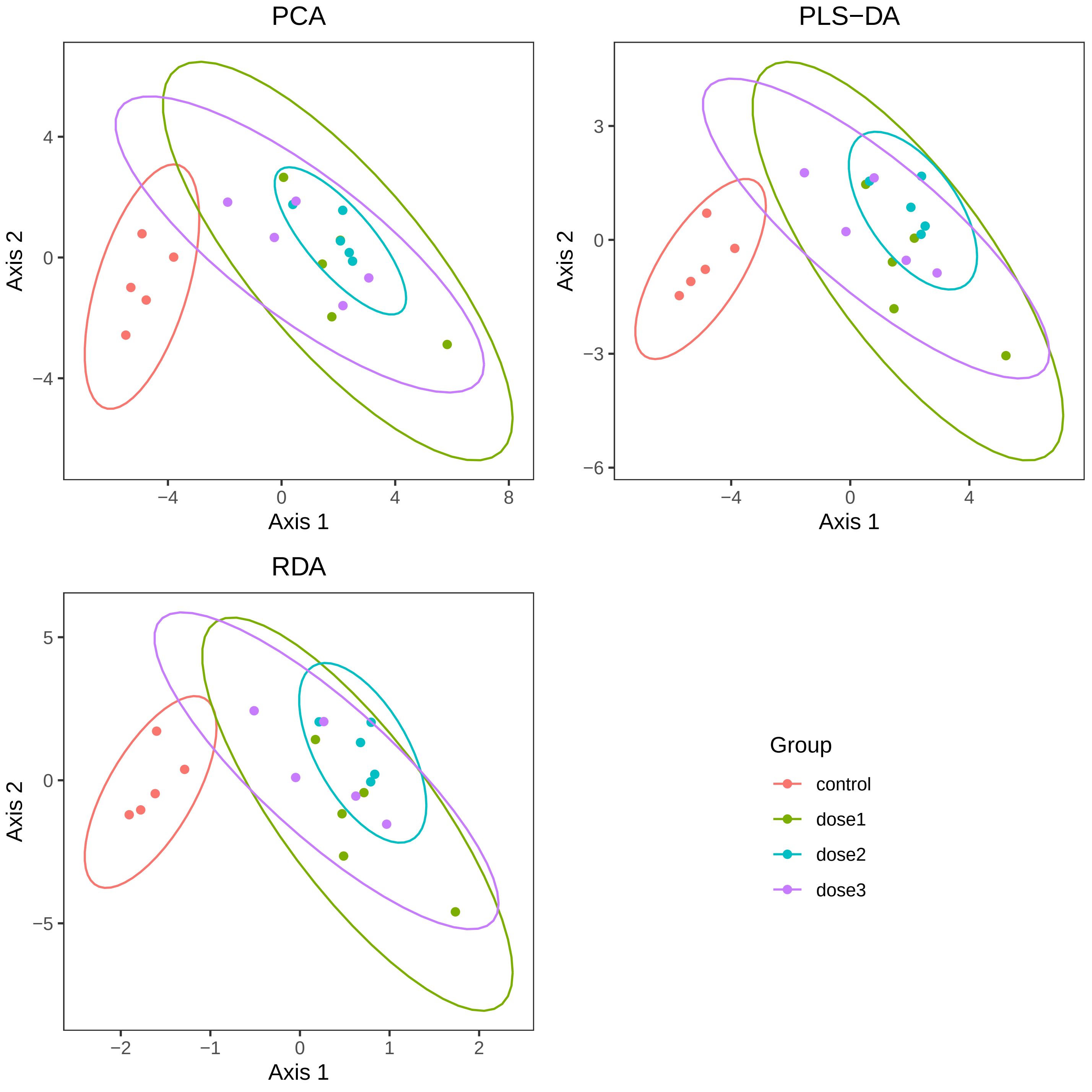

PCA
PLS−DA
RDA
Axis 1
Axis 2
Group
control
dose1
dose2
dose3

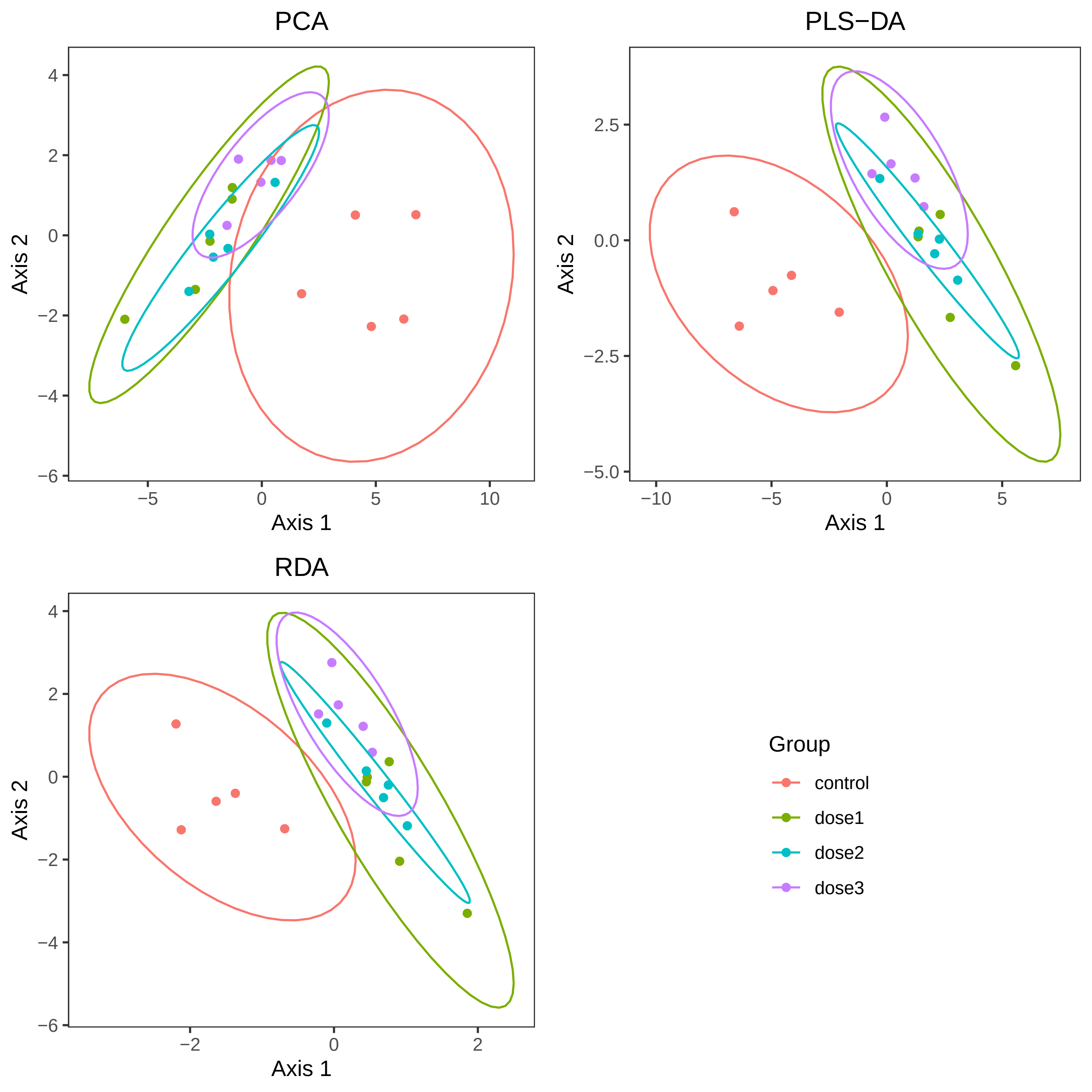
PCA
PLS−DA
RDA
Axis 1
Axis 2
Group
control
dose1
dose2
dose3

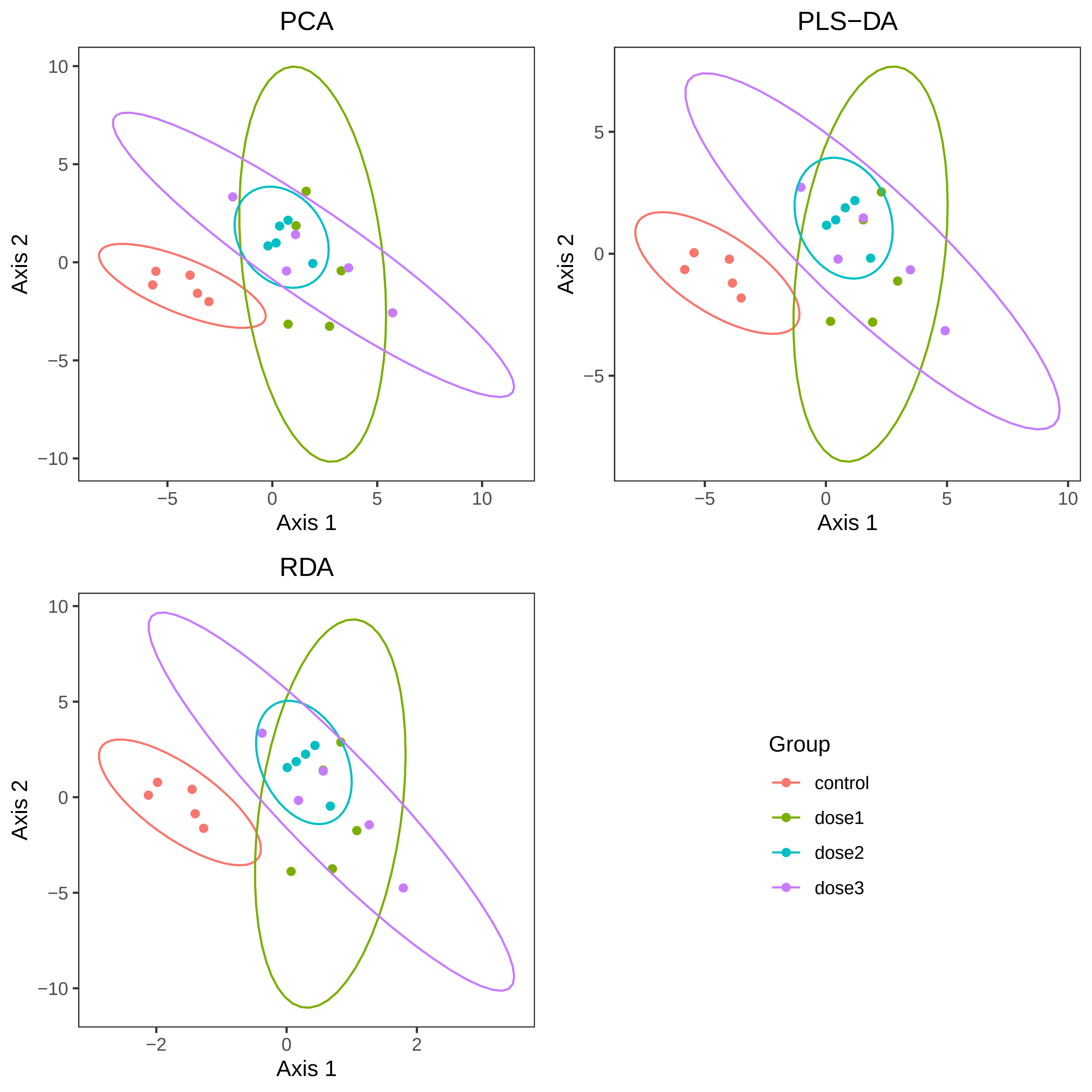
PCA
PLS−DA
RDA
Axis 1
Axis 2
10
5
0
−5
−10
−2
2
Group
control
dose1
dose2
dose3

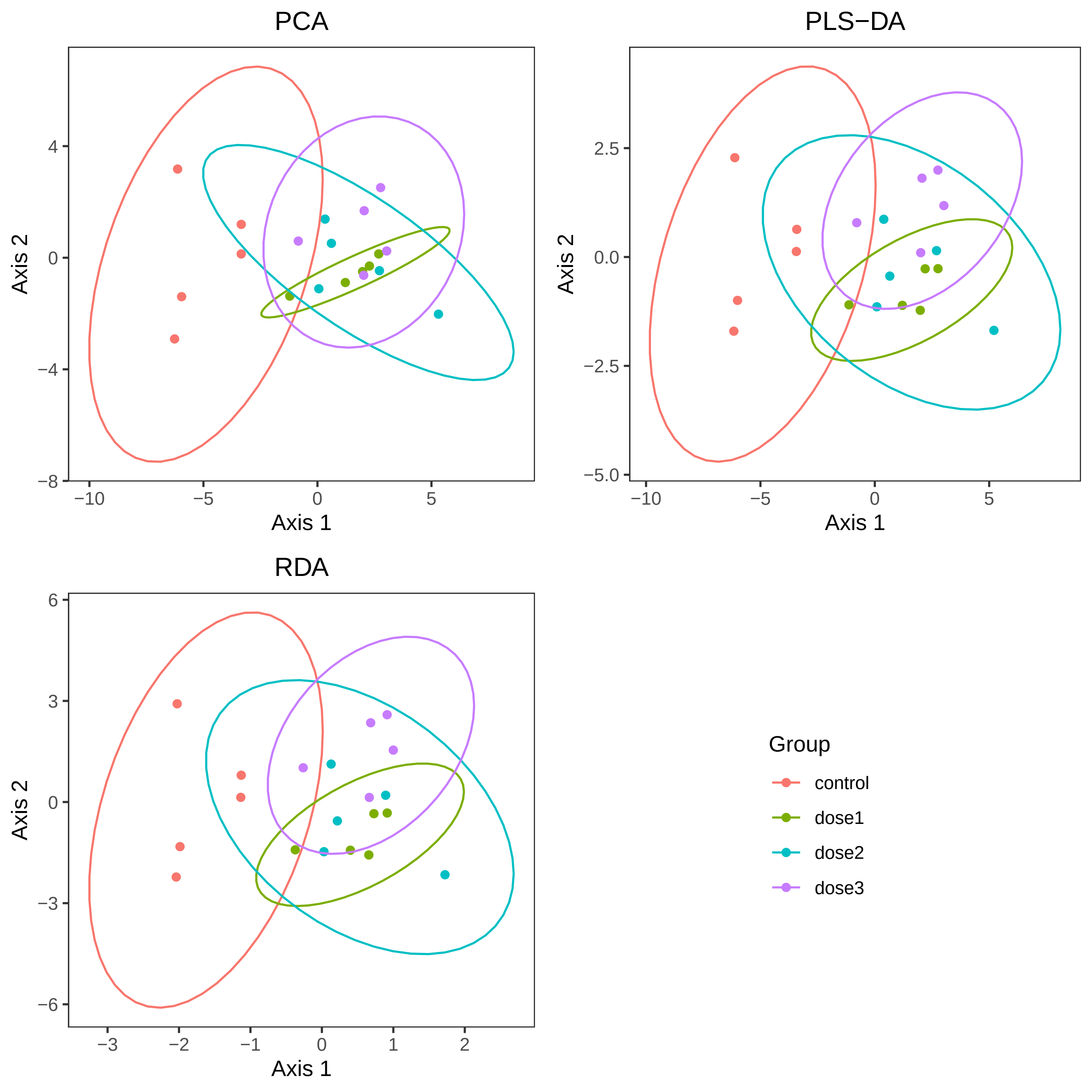
PCA
PLS−DA
RDA
Axis 1
Axis 2
Group
control
dose1
dose2
dose3

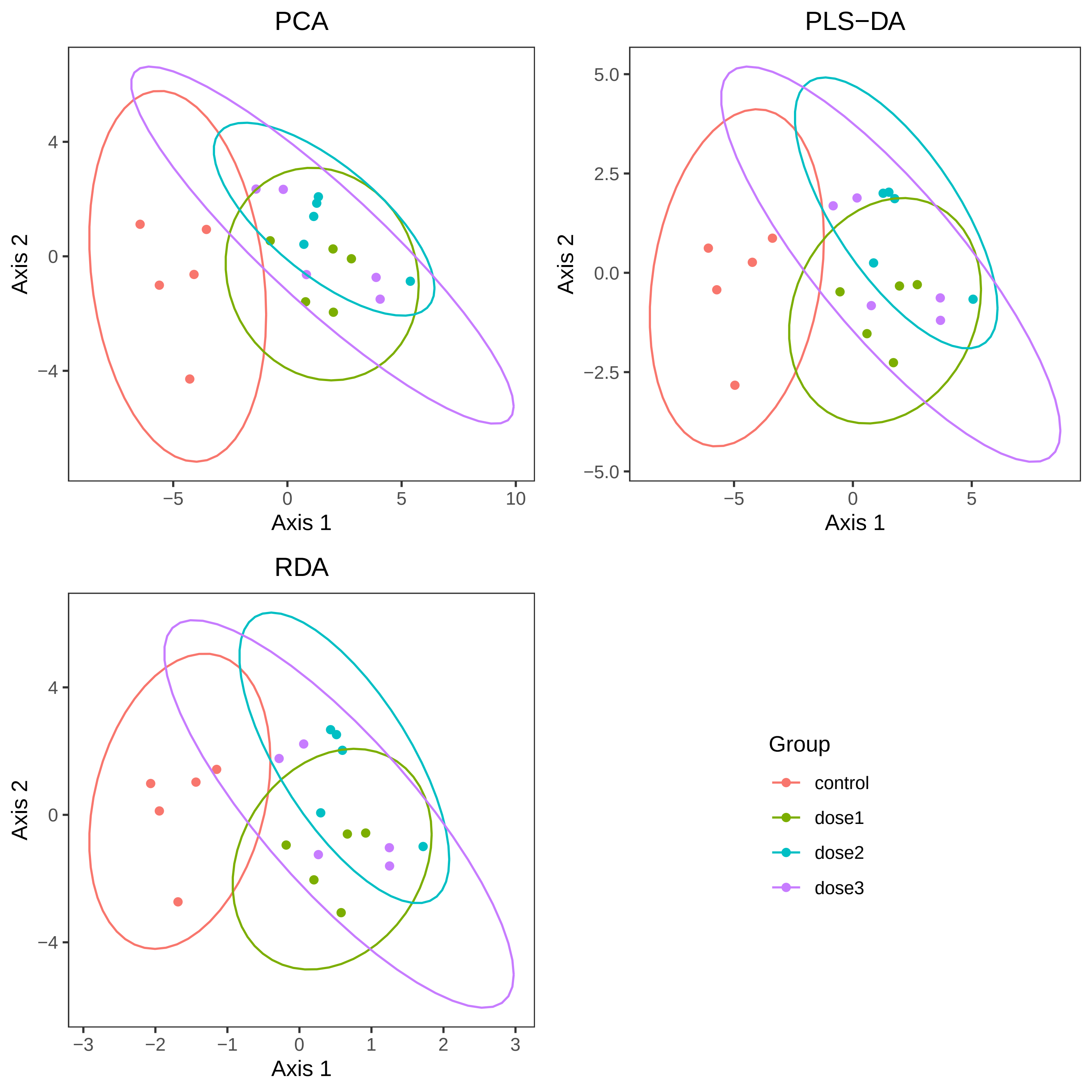
PCA
PLS−DA
RDA
Axis 1
Axis 2
−5
0
5
10
4
0
−4
5.0
2.5
0.0
−2.5
−5.0
−3
−2
−1
1
2
3
Group
control
dose1
dose2
dose3

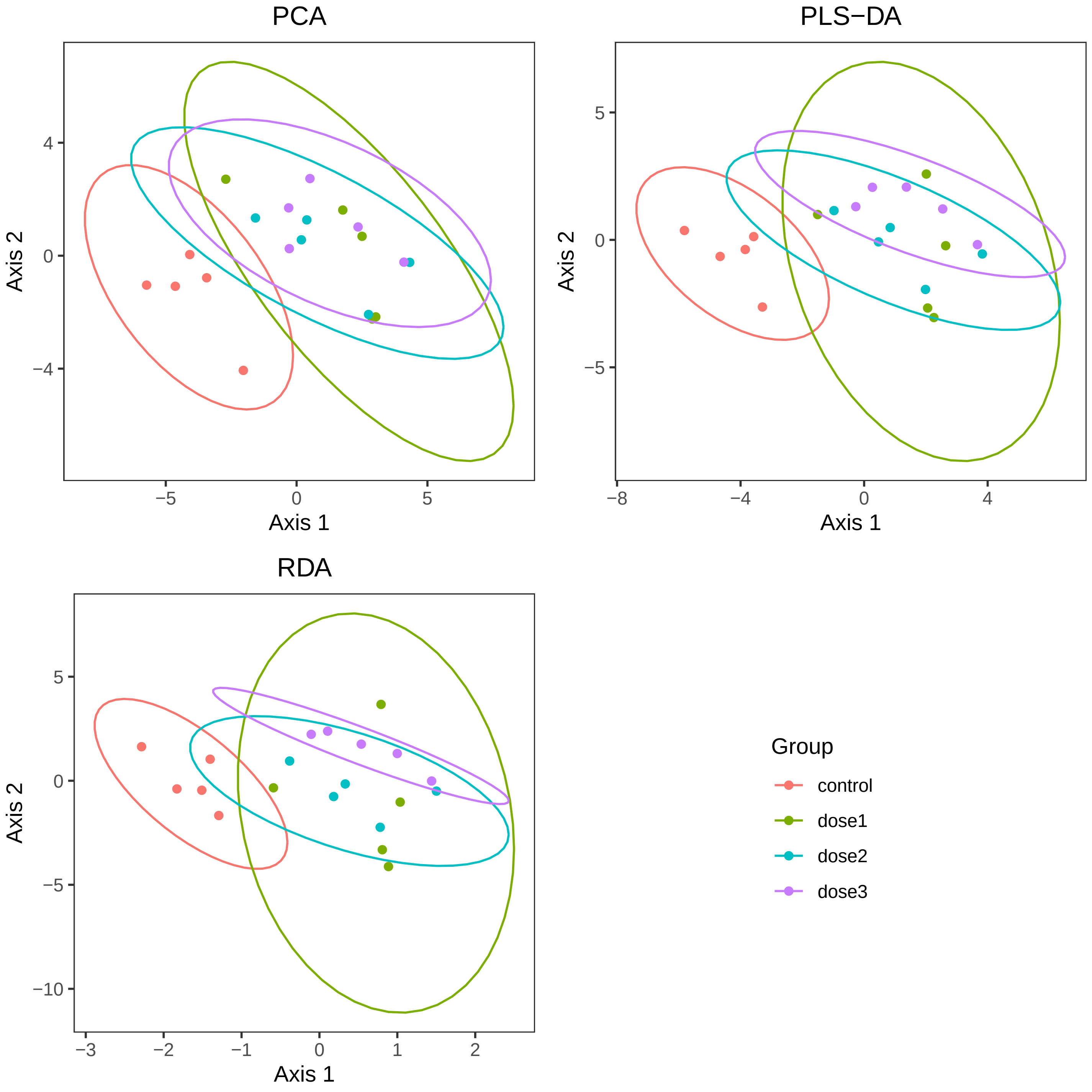

PCA
PLS−DA
RDA
Axis 1
Axis 2
Group
control
dose1
dose2
dose3

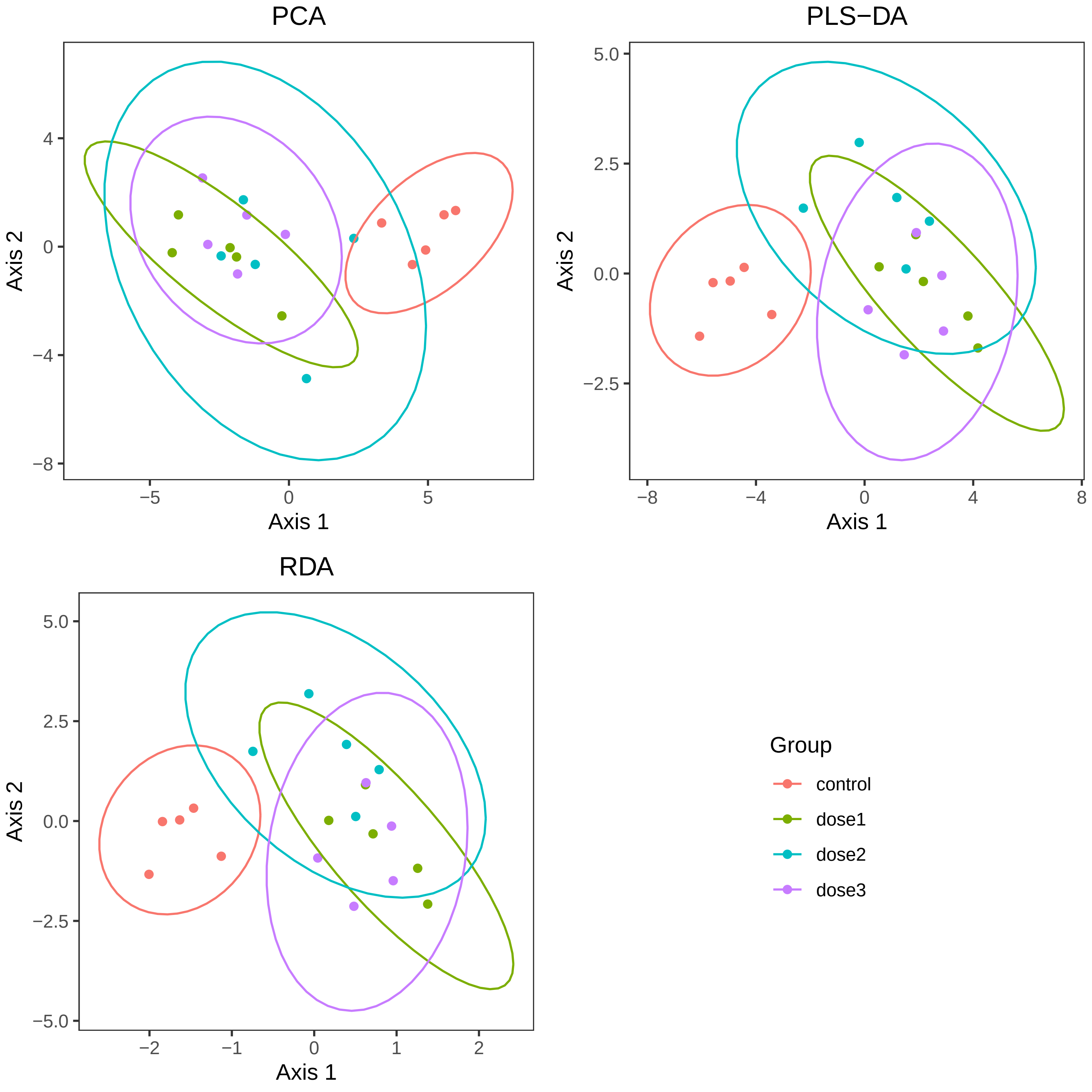

PCA
PLS−DA
RDA
Axis 1
Axis 2
Group
control
dose1
dose2
dose3

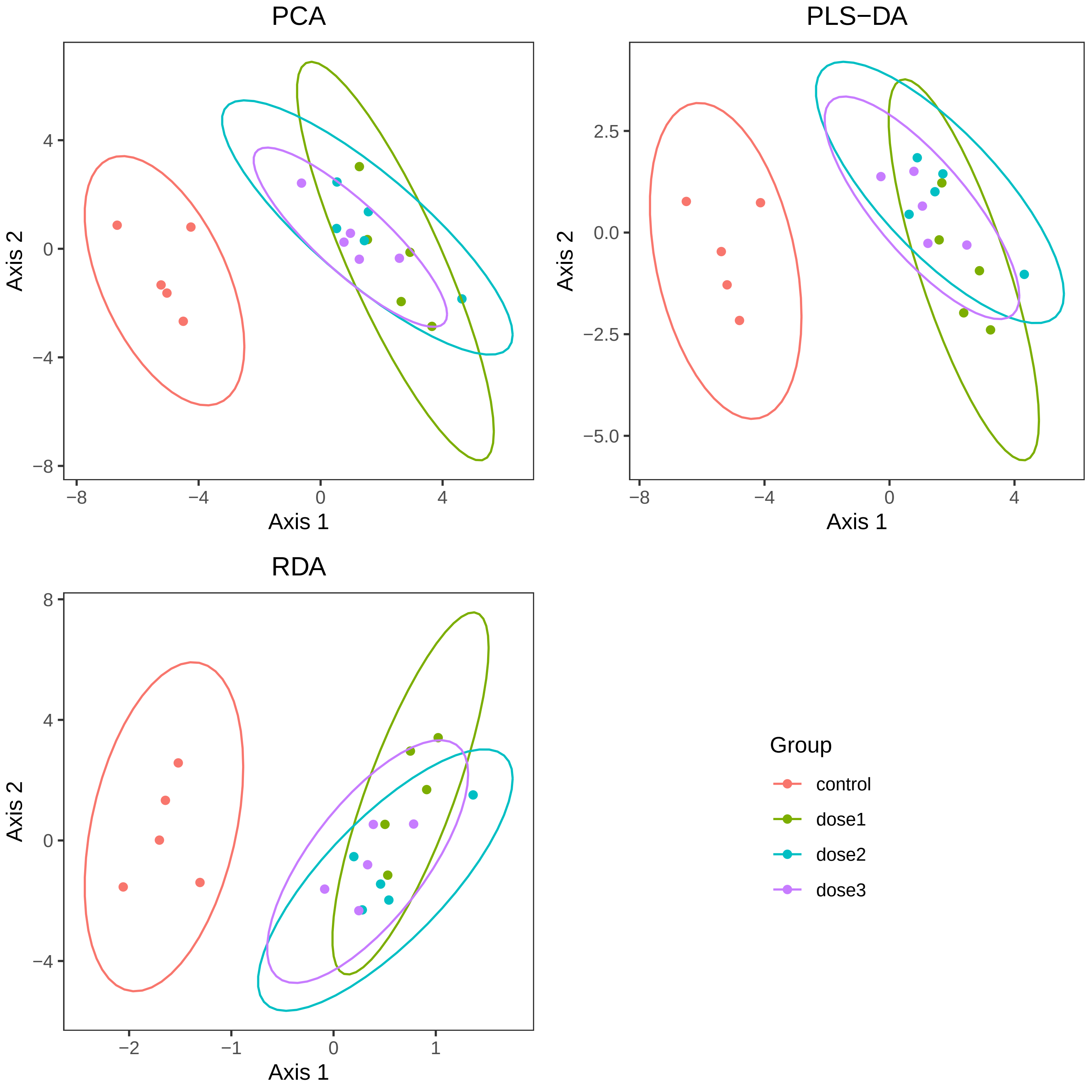

PCA
PLS−DA
RDA
Axis 1
Axis 2
Group
control
dose1
dose2
dose3

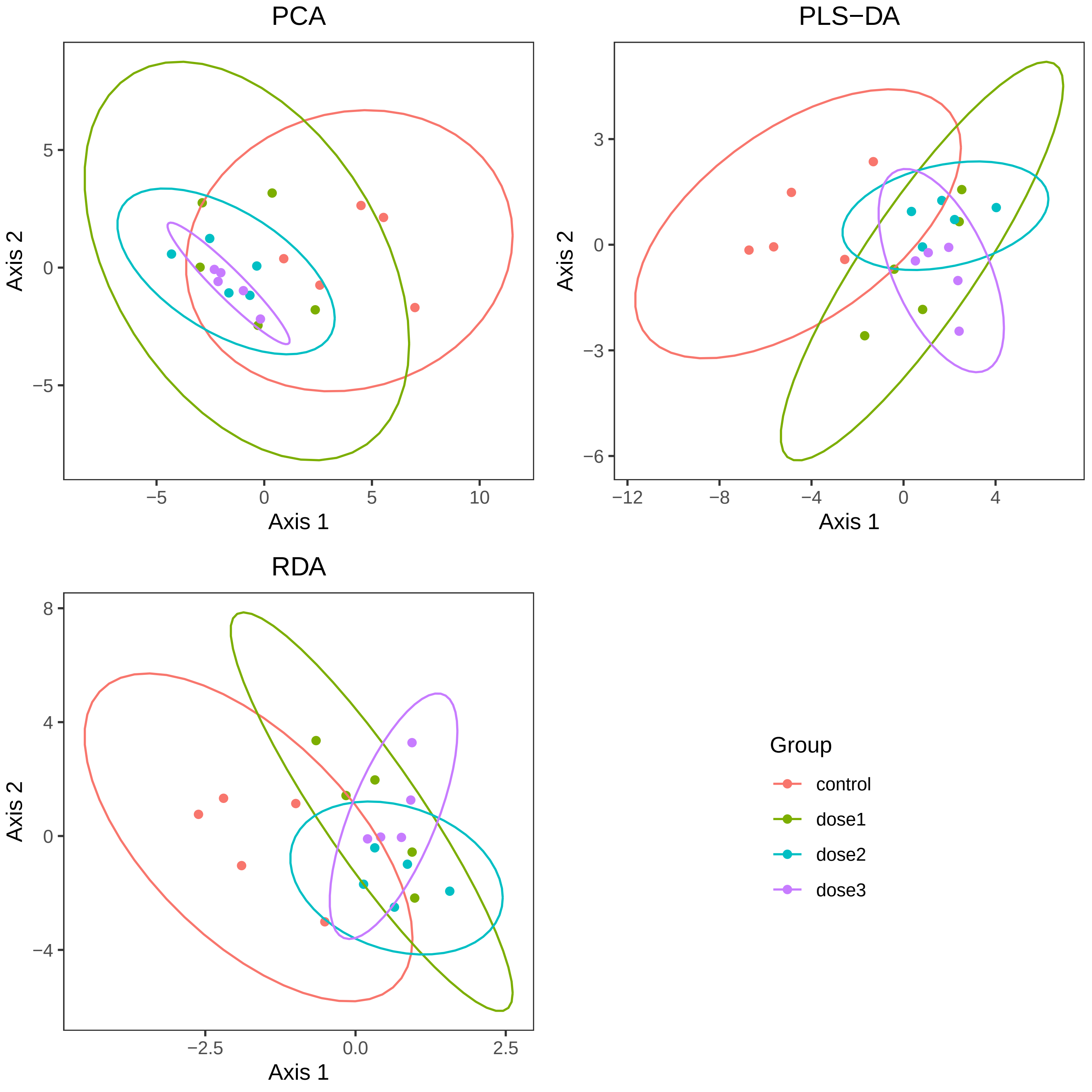

PCA
PLS−DA
RDA
Axis 1
Axis 2
Group
control
dose1
dose2
dose3

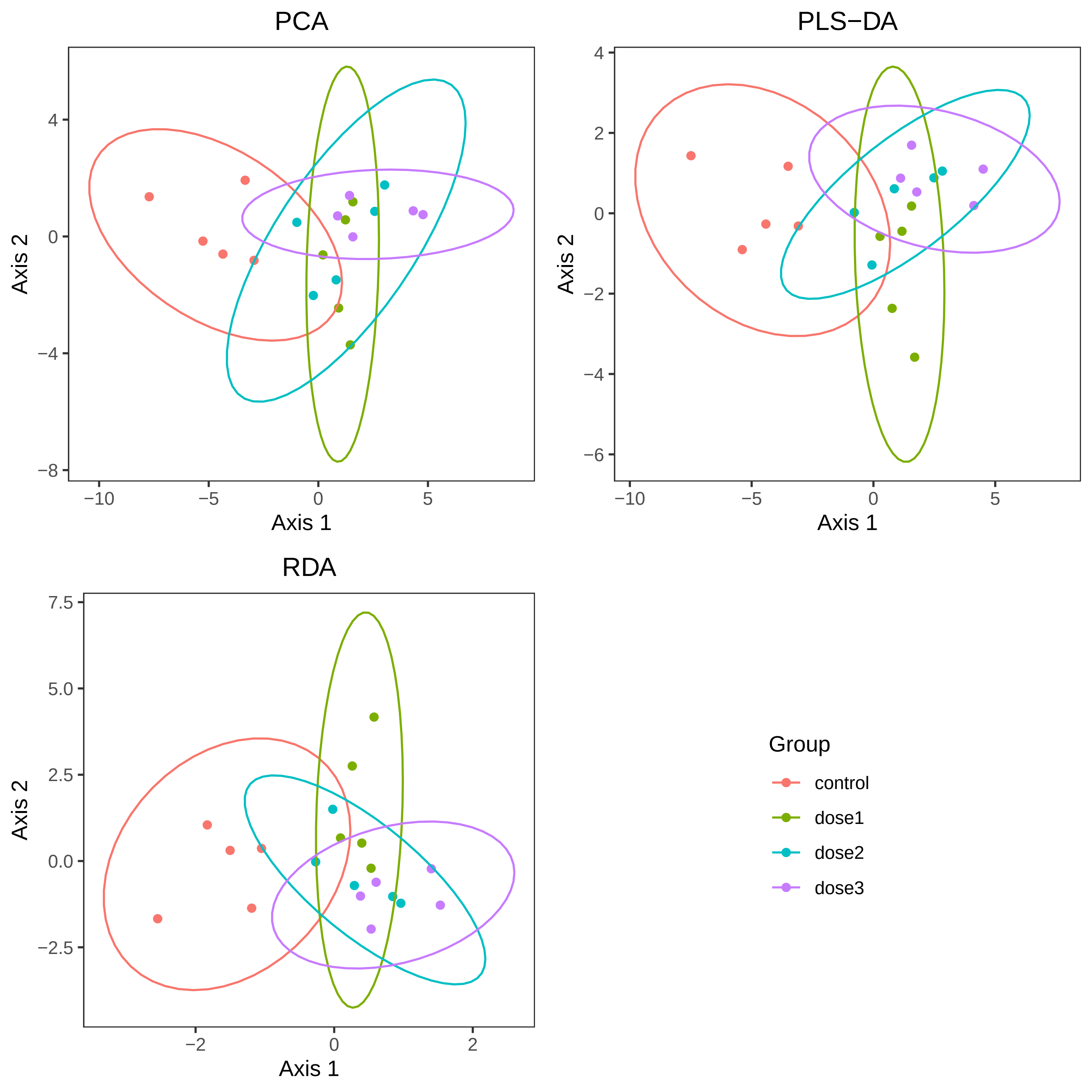

PCA
PLS−DA
RDA
Axis 1
Axis 2
Group
control
dose1
dose2
dose3

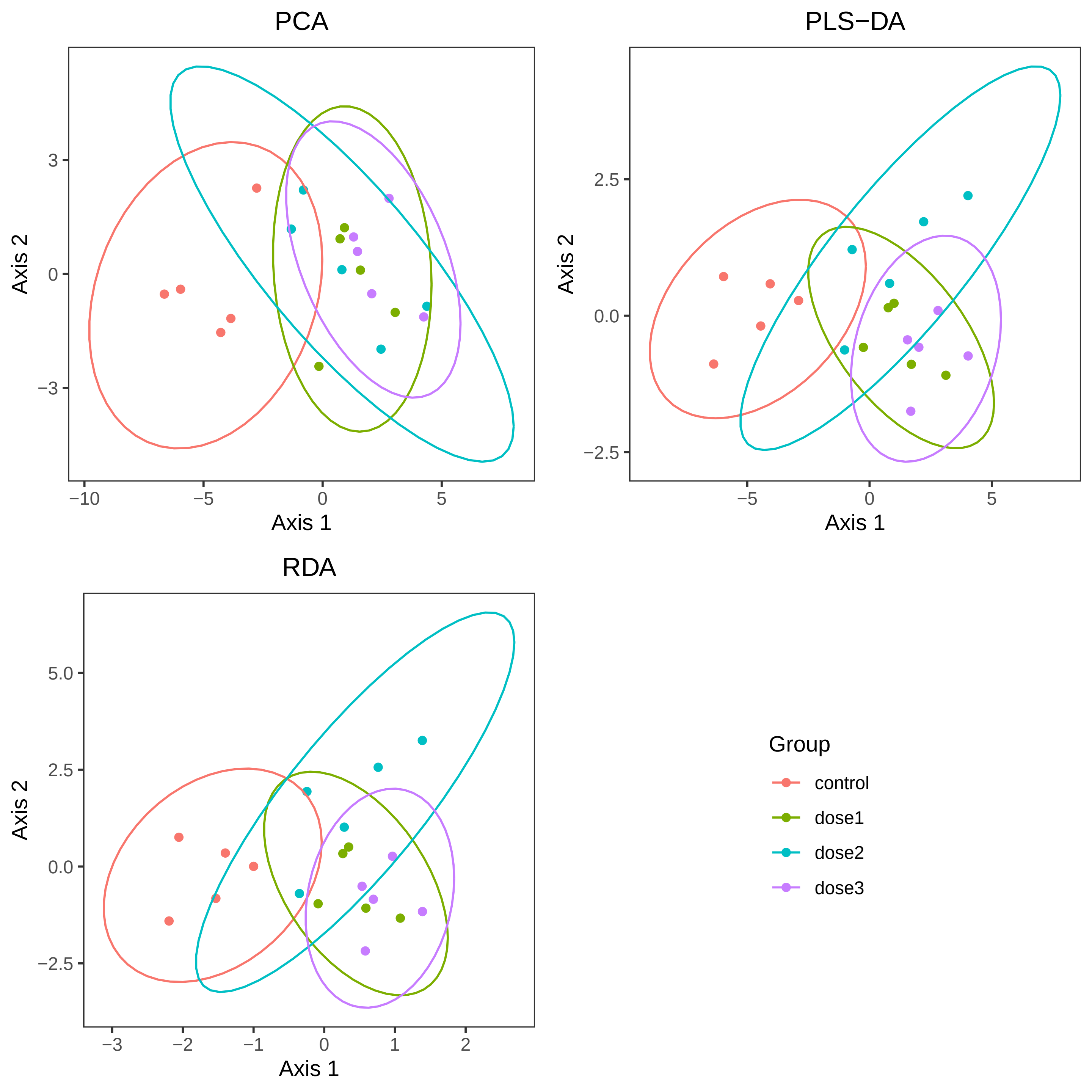
PCA
PLS−DA
RDA
Axis 1
Axis 2
Group
control
dose1
dose2
dose3

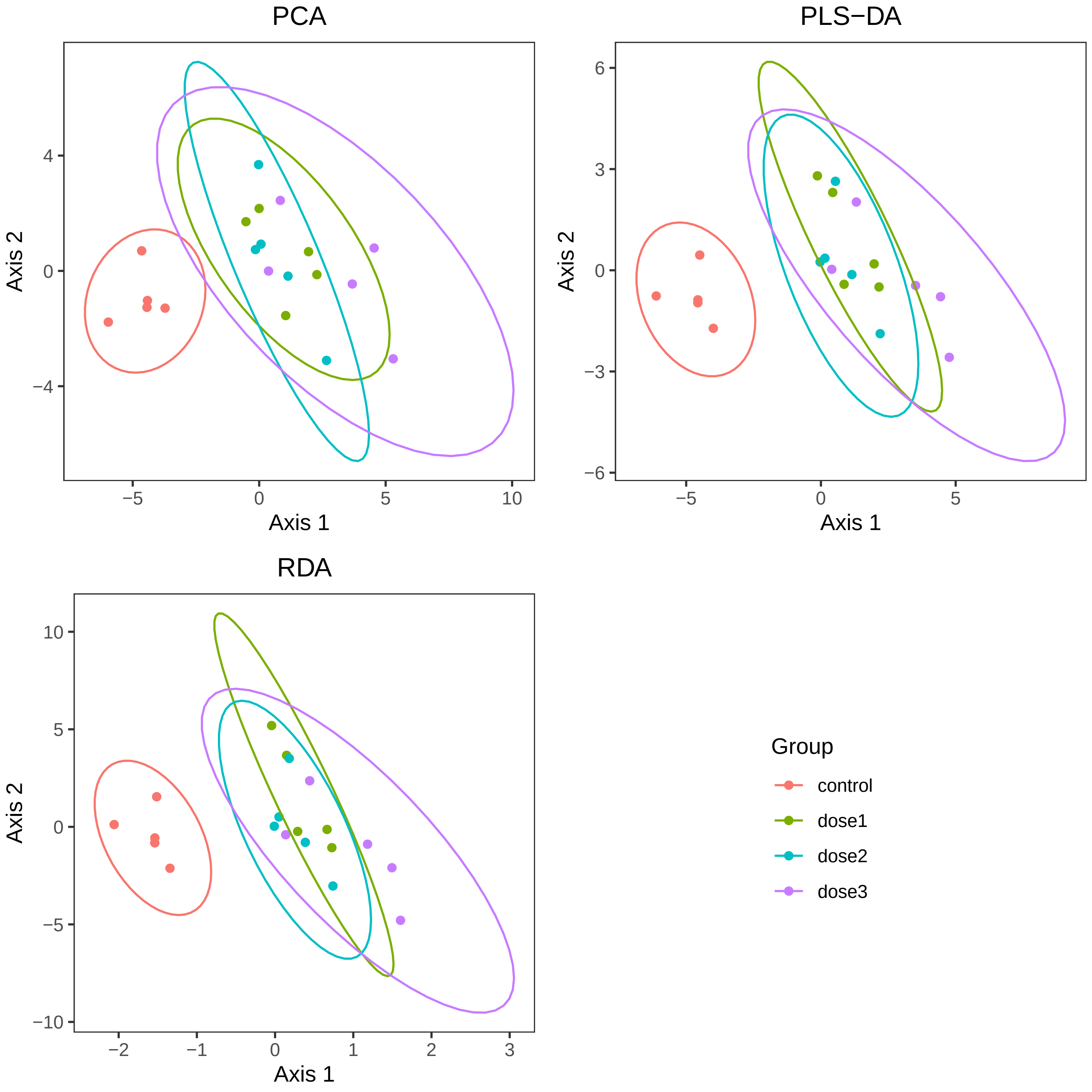
PCA
PLS−DA
RDA
Axis 1
Axis 2
Group
control
dose1
dose2
dose3

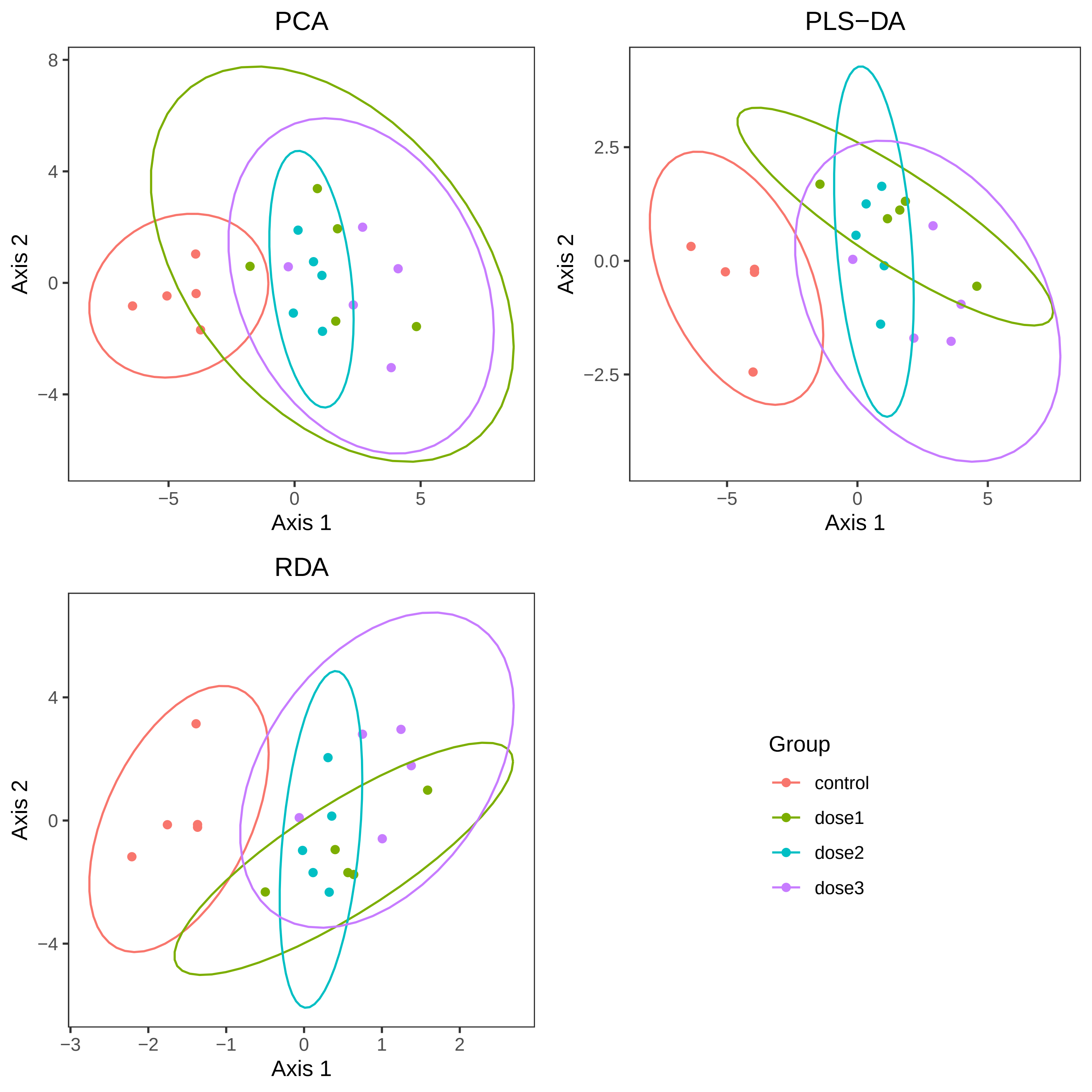

PCA
PLS−DA
RDA
Axis 1
Axis 2
8
4
0
−4
−5
0
5
2.5
0.0
−2.5
−3
−2
−1
1
2
Group
control
dose1
dose2
dose3

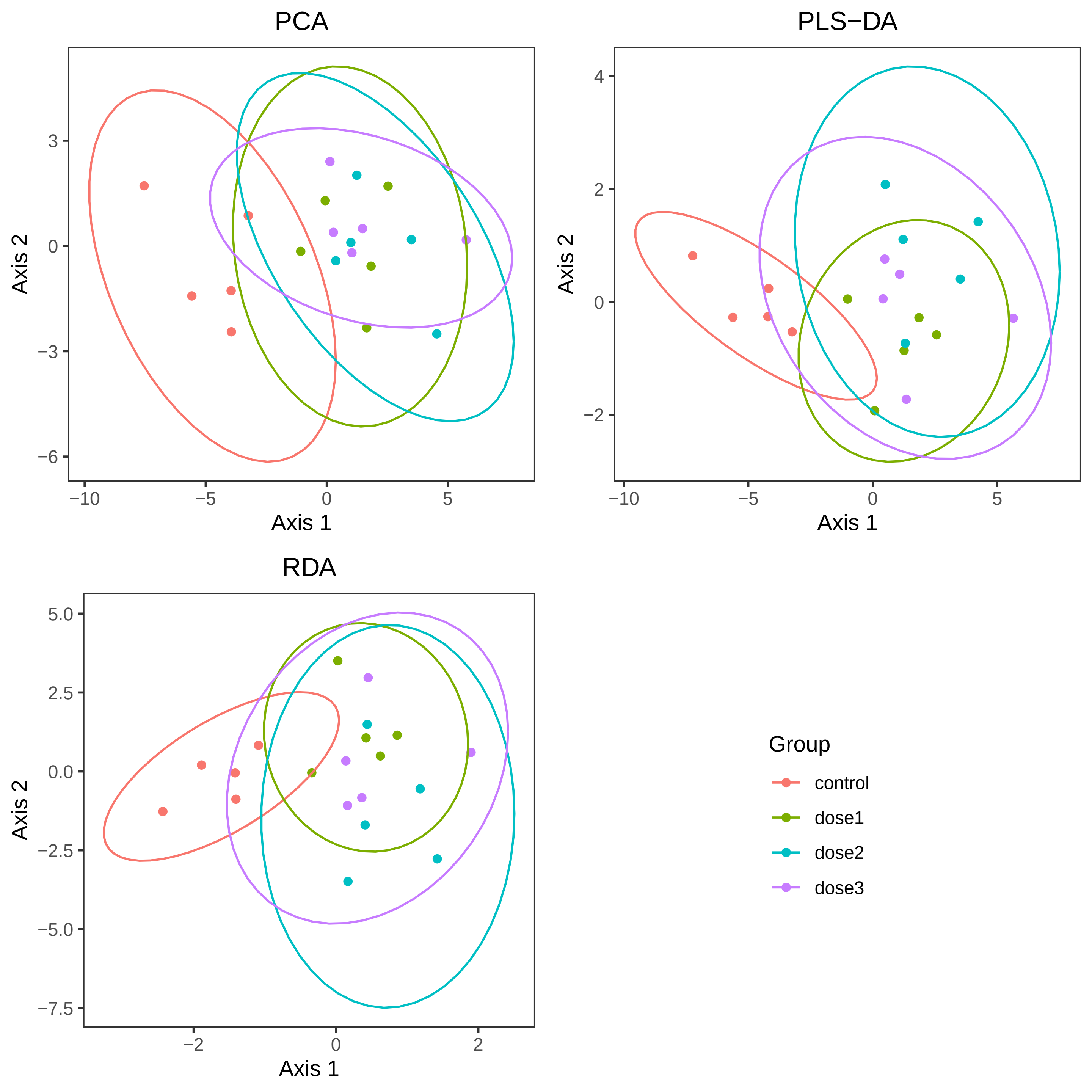

PCA
PLS−DA
RDA
Axis 1
Axis 2
3
0
−3
−6
−10
−5
0
5
4
2
0
−2
5.0
2.5
0.0
−2.5
−5.0
−7.5
−2
0
2
Group
control
dose1
dose2
dose3

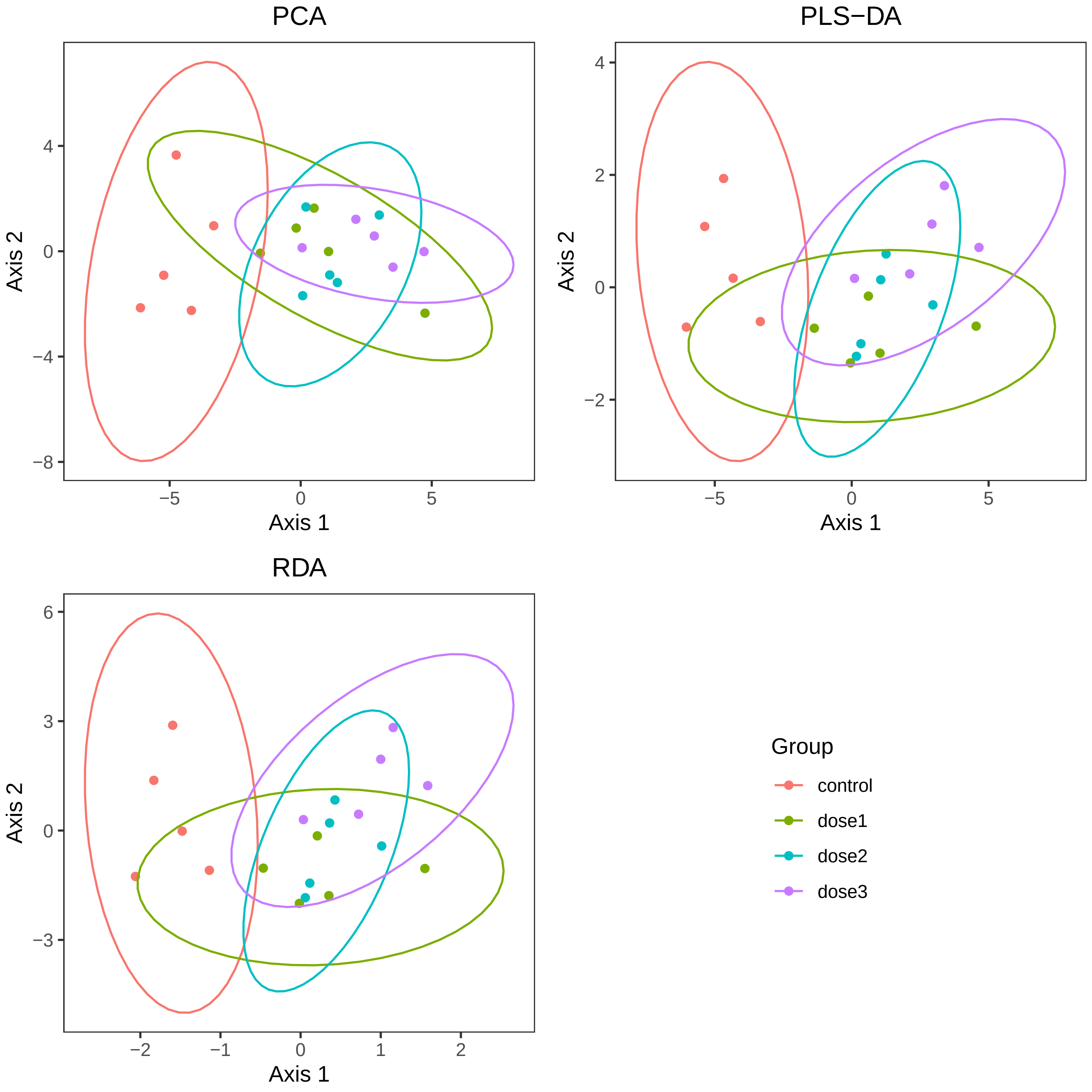
PCA
PLS−DA
RDA
Axis 1
Axis 2
Group
control
dose1
dose2
dose3

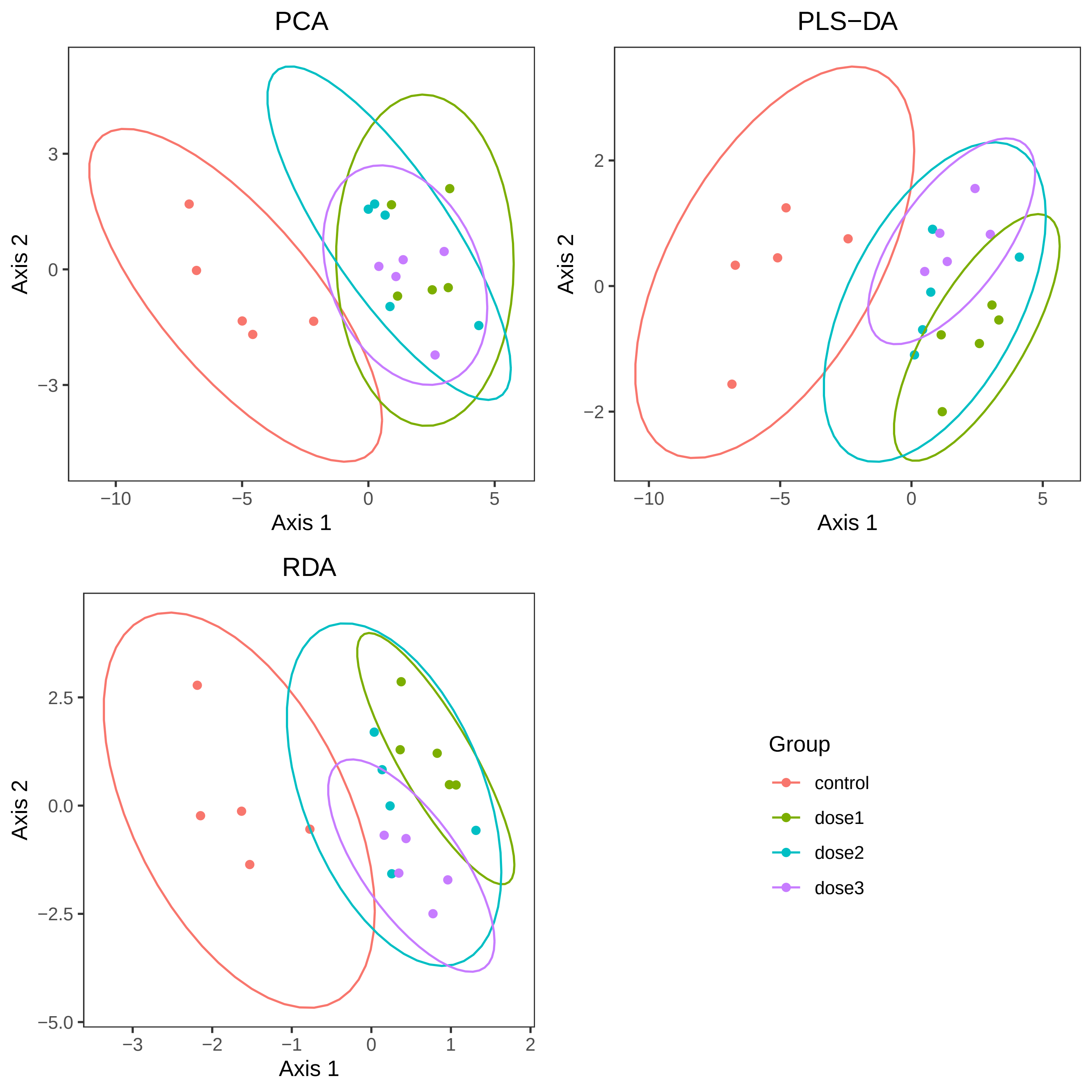
PCA
PLS−DA
RDA
Axis 1
Axis 2
Group
control
dose1
dose2
dose3

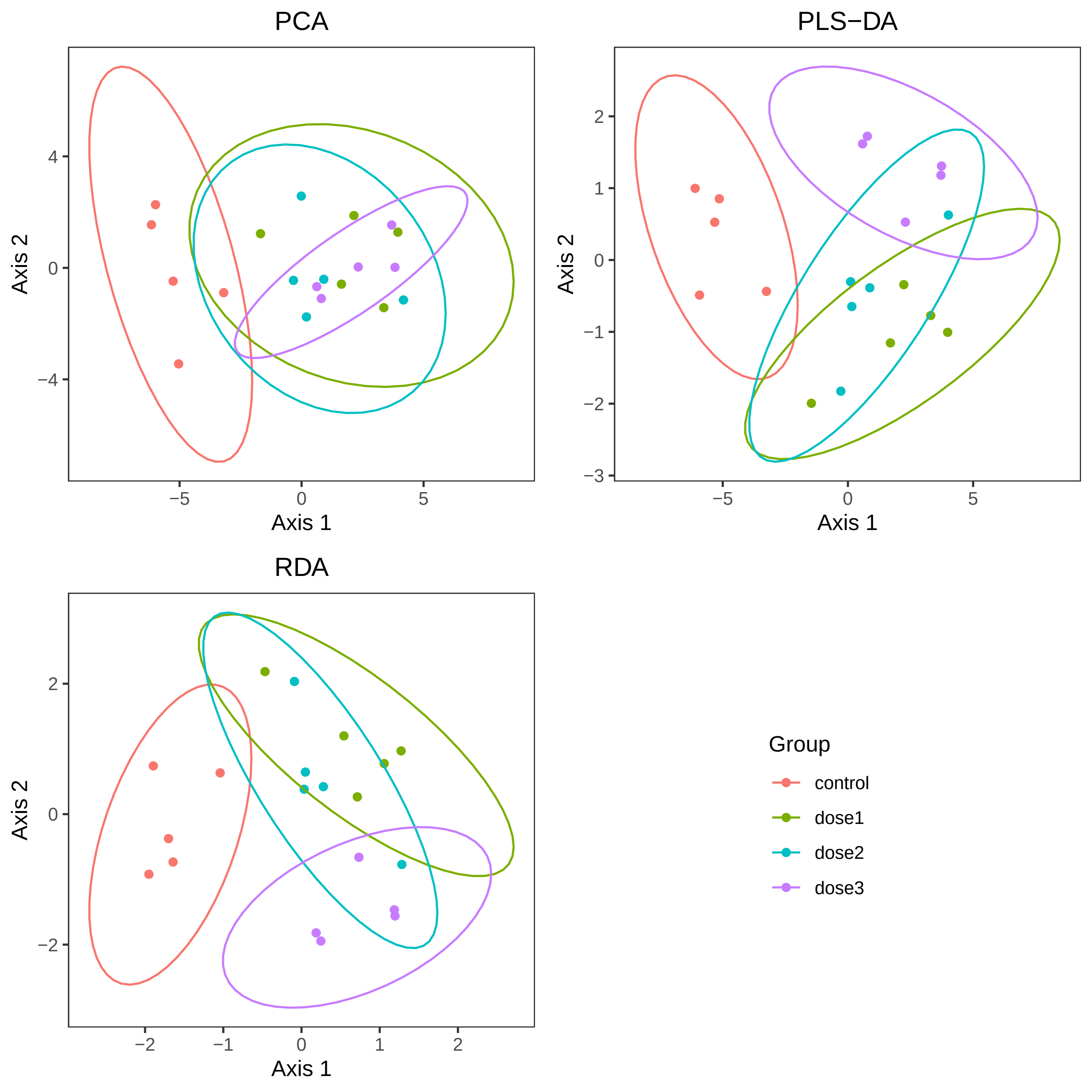
PCA
PLS−DA
RDA
Axis 1
Axis 2
Group
control
dose1
dose2
dose3

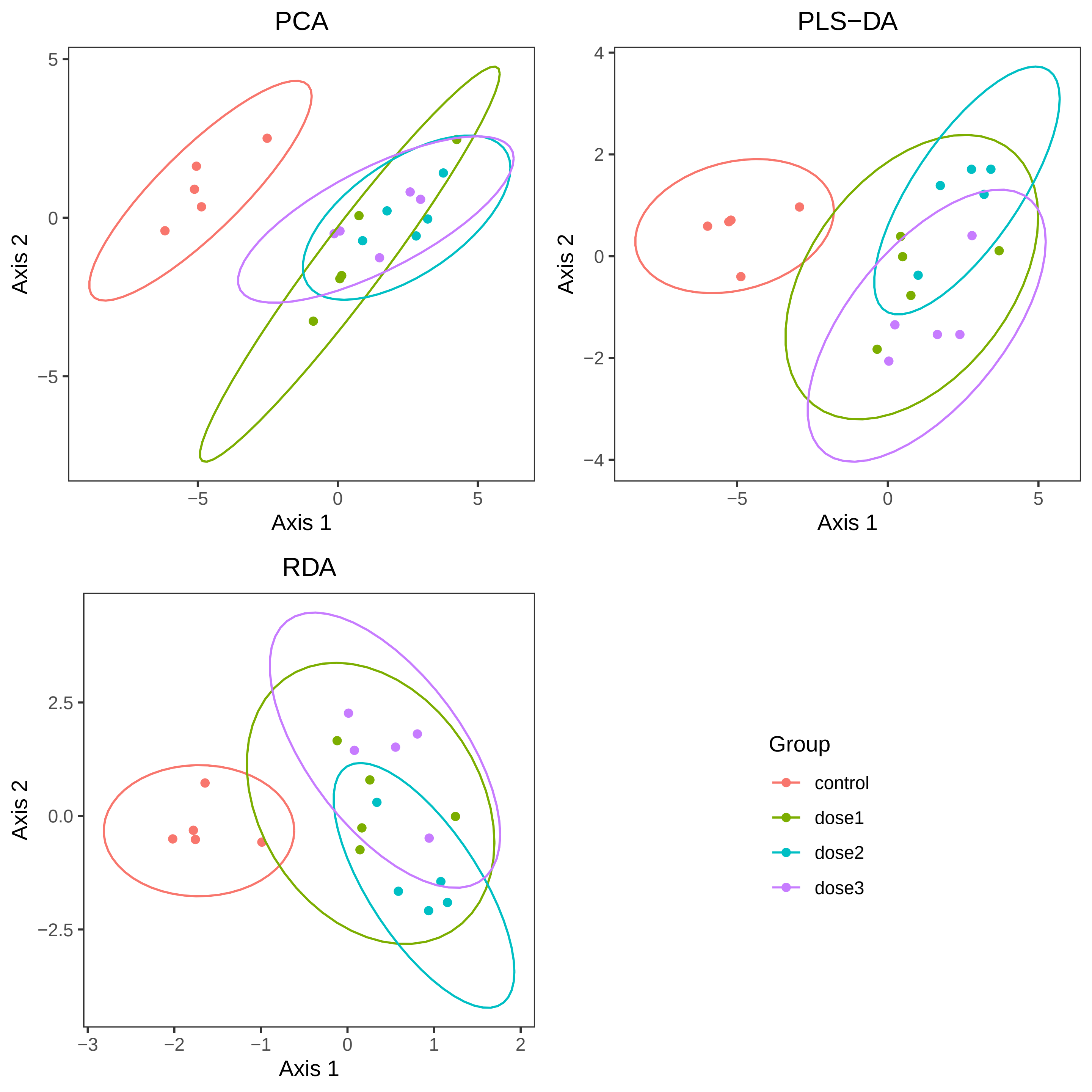
PCA
PLS−DA
RDA
Axis 1
Axis 2
Group
control
dose1
dose2
dose3

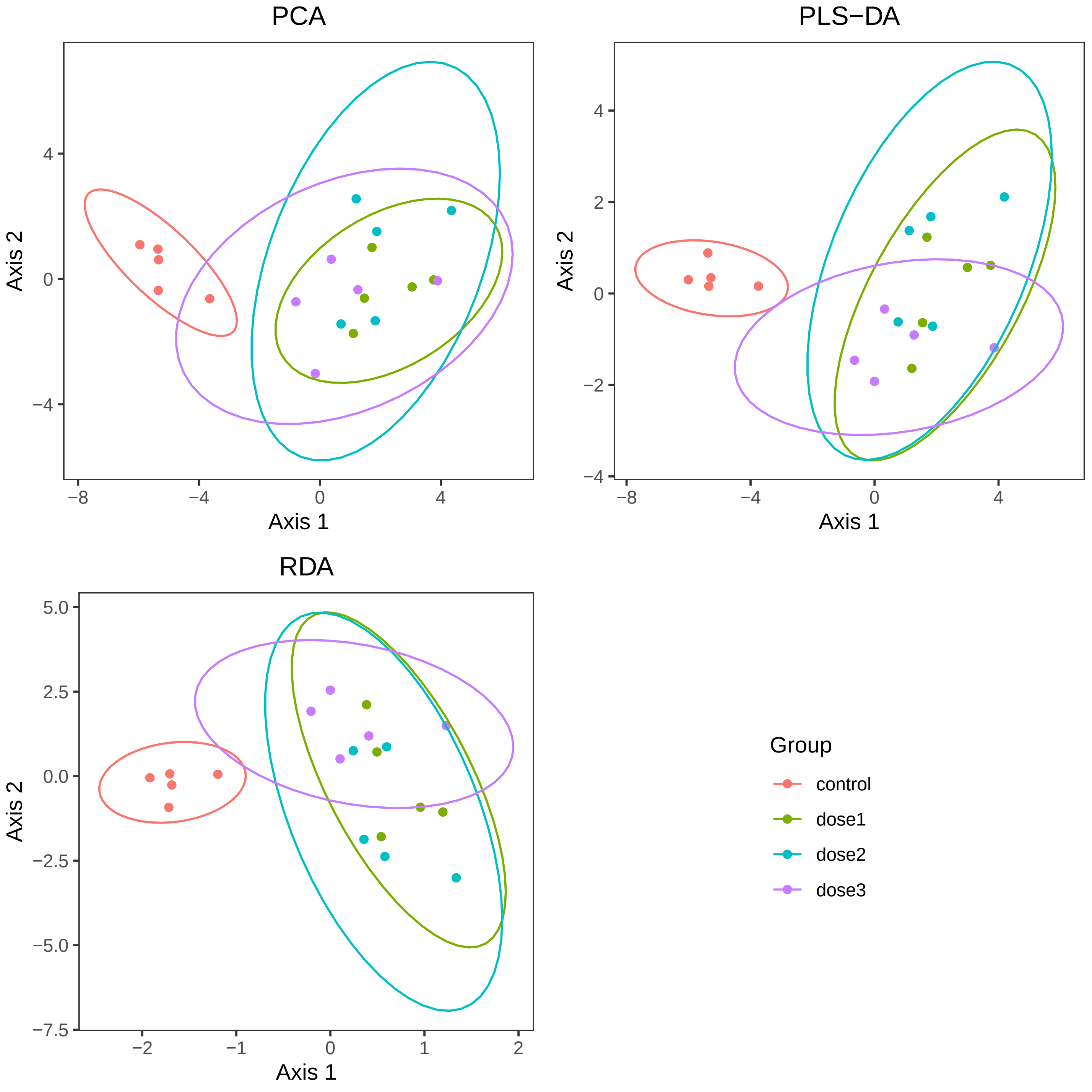

PCA
PLS−DA
RDA
Axis 1
Axis 2
Group
control
dose1
dose2
dose3

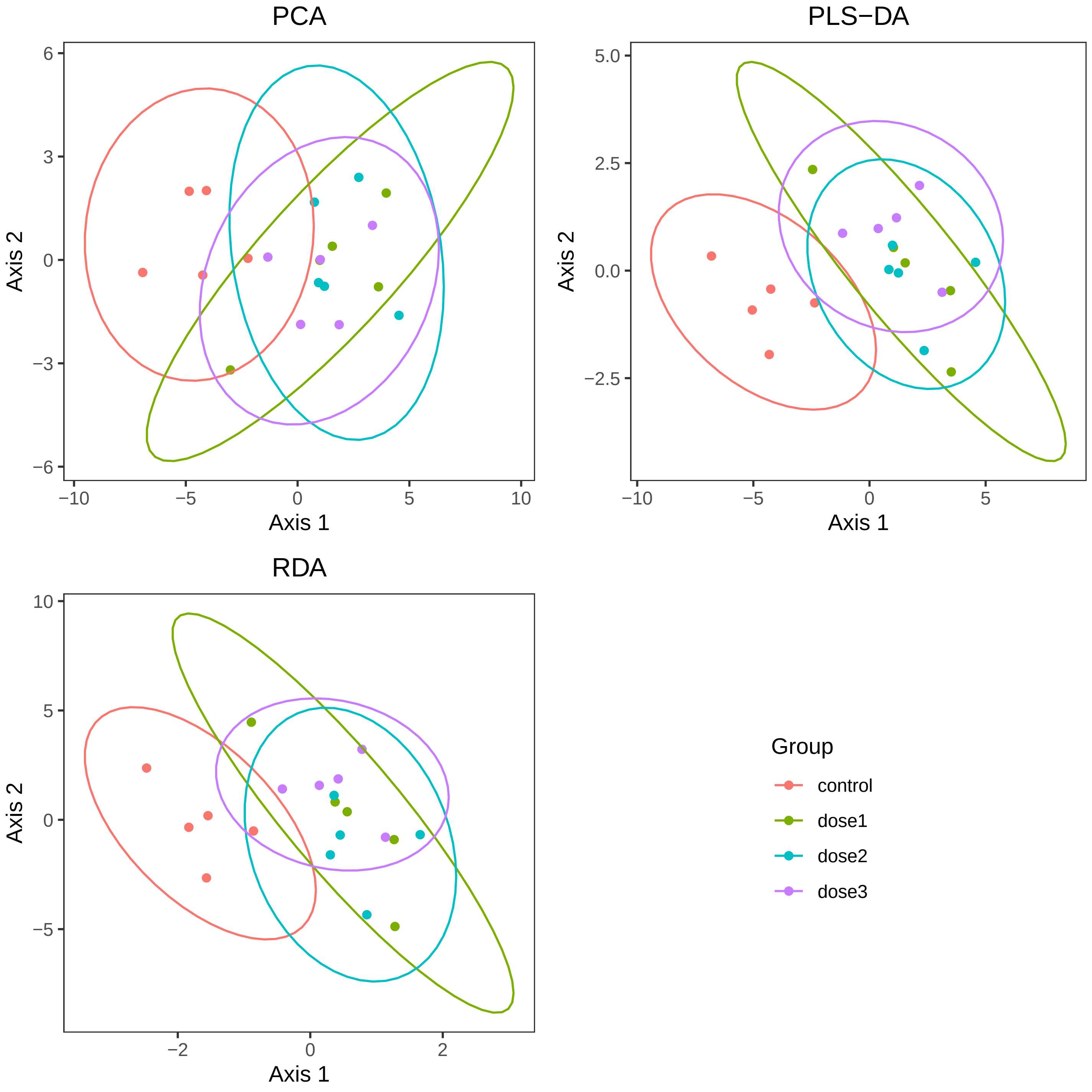

PCA
PLS−DA
RDA
Axis 1
Axis 2
6
3
0
−3
−6
−10
−5
0
5
10
5.0
2.5
0.0
−2.5
10
5
0
−5
−2
2
Group
control
dose1
dose2
dose3

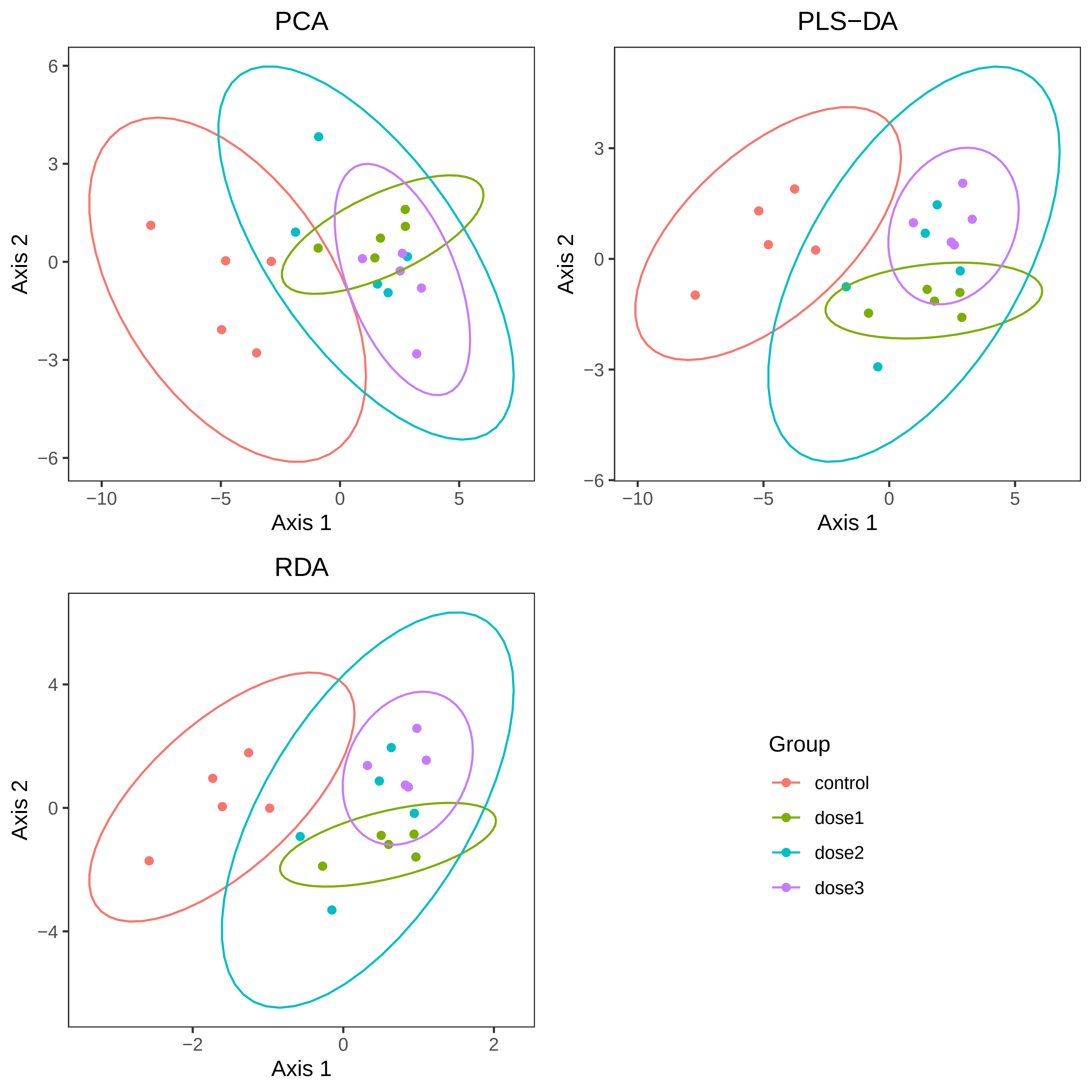
PCA
PLS−DA
RDA
Axis 1
Axis 2
Group
control
dose1
dose2
dose3

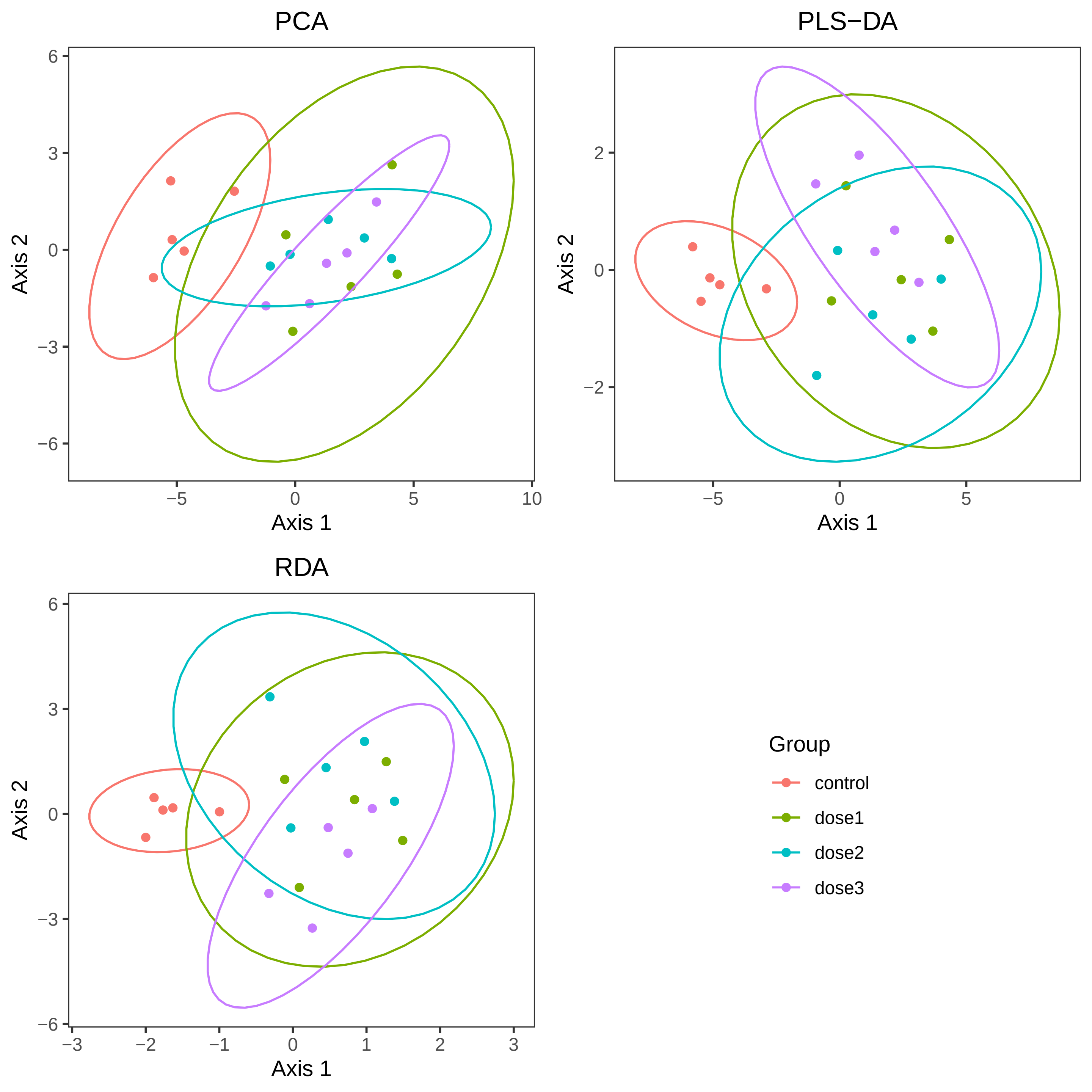

PCA
PLS−DA
RDA
Axis 1
Axis 2
Group
control
dose1
dose2
dose3

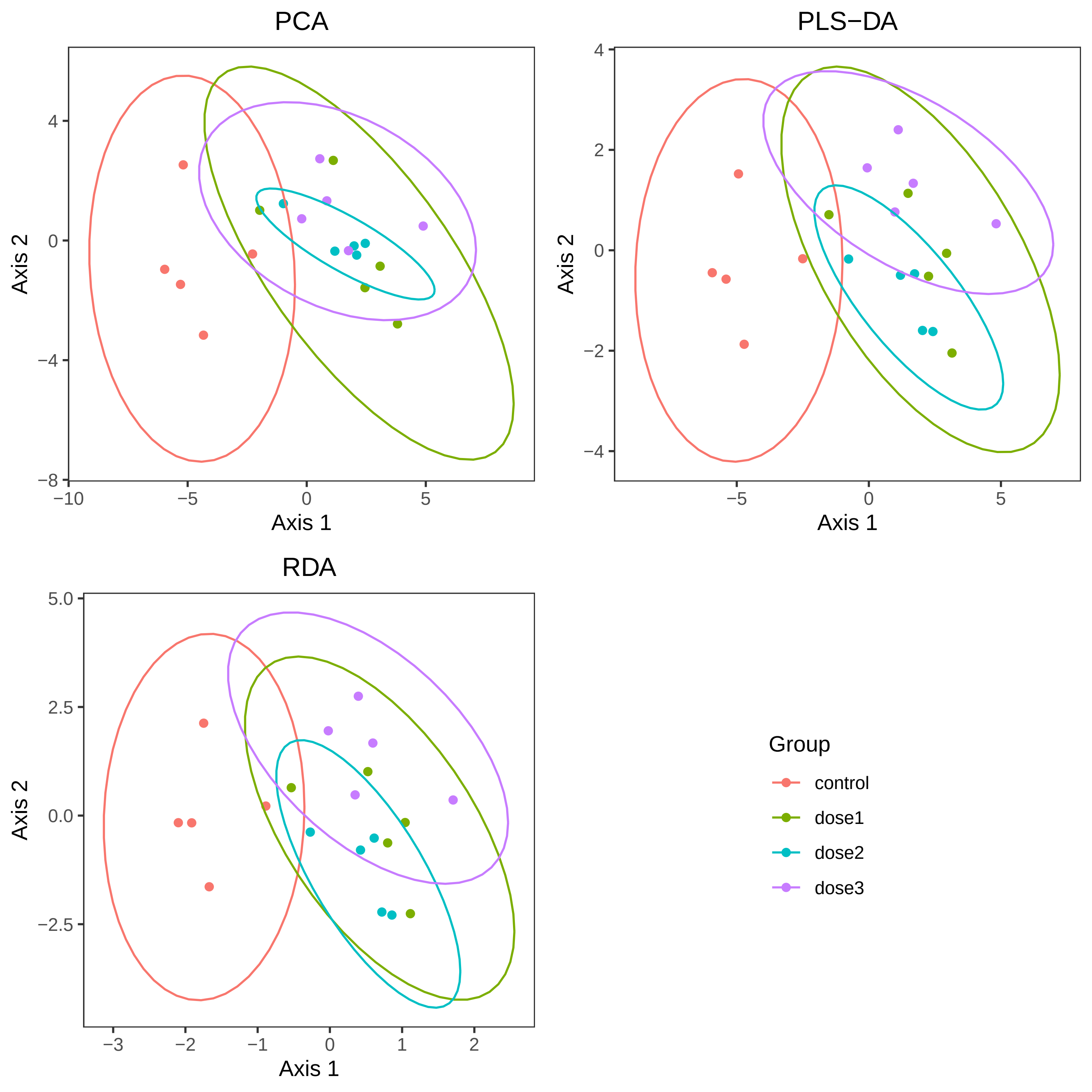
PCA
PLS−DA
RDA
Axis 1
Axis 2
−10
−5
0
5
4
0
−4
−8
2
−2
5.0
2.5
0.0
−2.5
−3
−1
1
Group
control
dose1
dose2
dose3

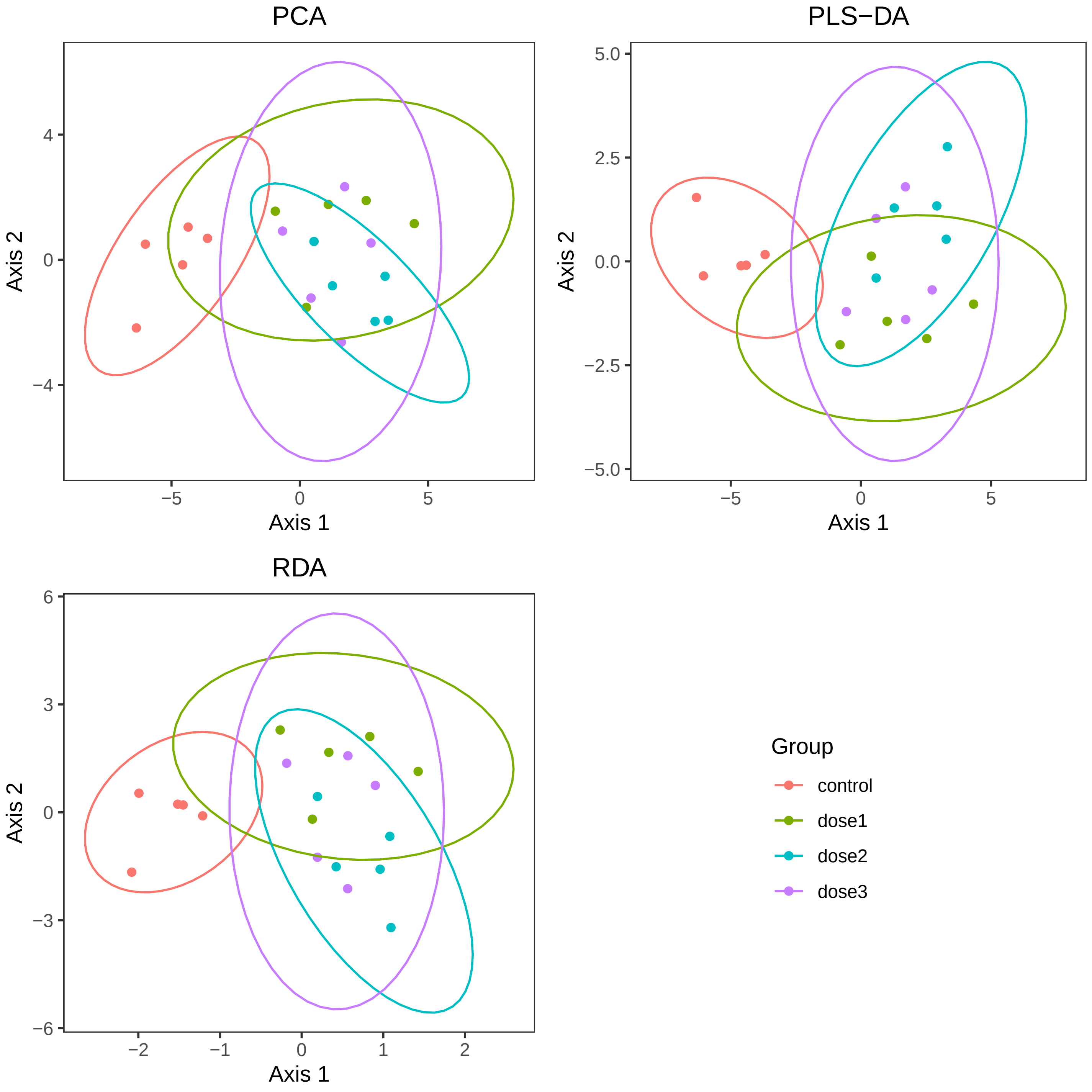
PCA
PLS−DA
RDA
Axis 1
Axis 2
Group
control
dose1
dose2
dose3

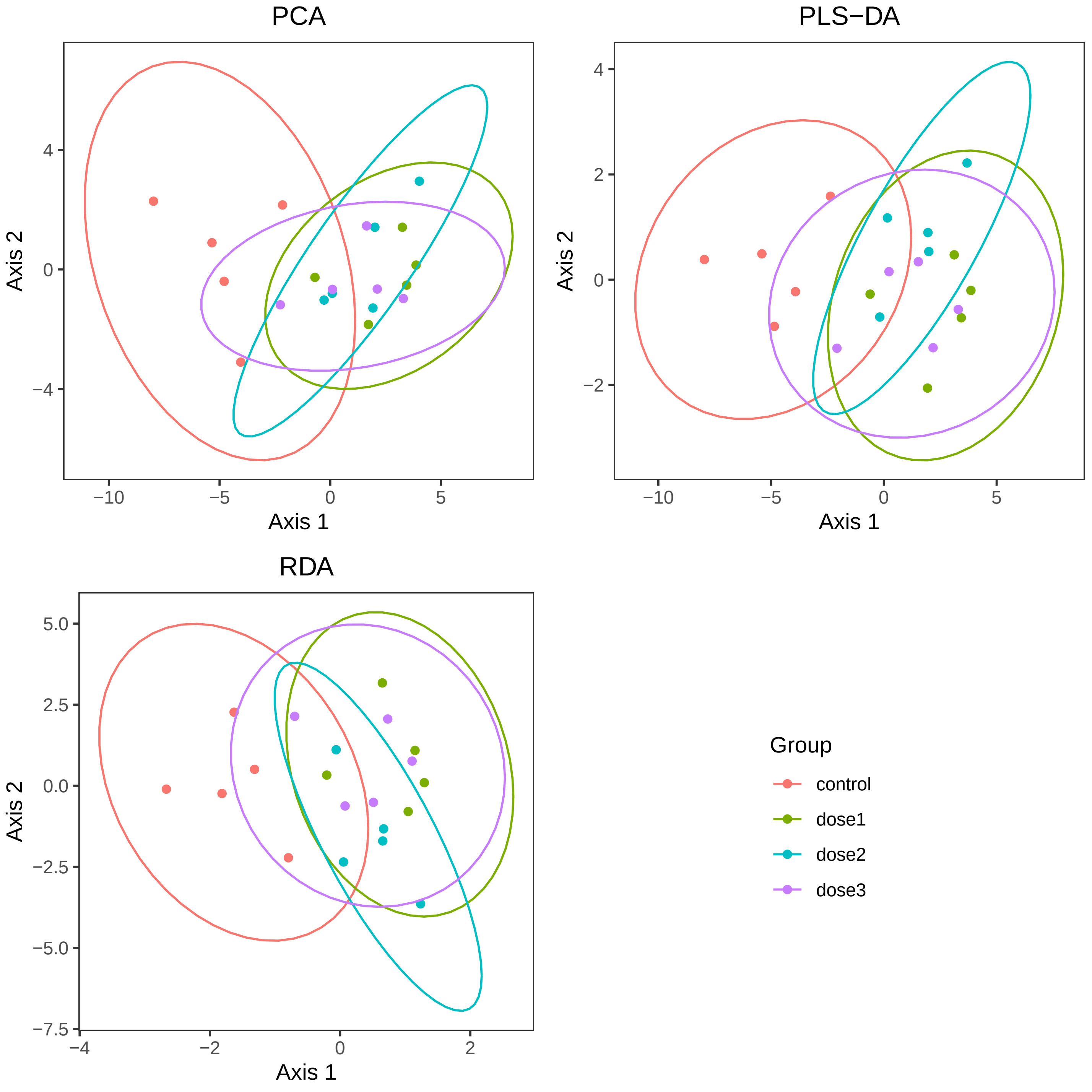
PCA
PLS−DA
RDA
Axis 1
Axis 2
Group
control
dose1
dose2
dose3

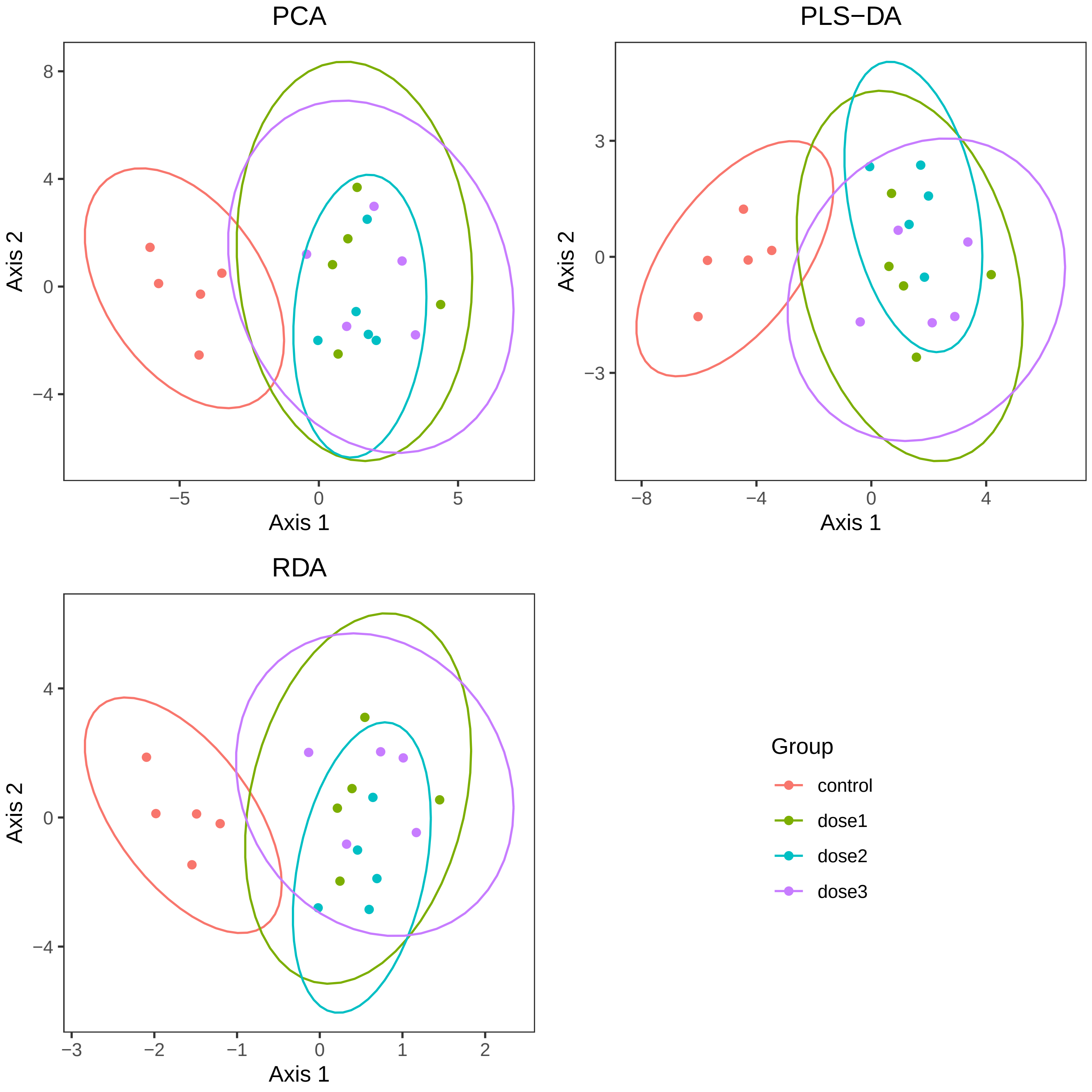

PCA
PLS−DA
RDA
Axis 1
Axis 2
Group
control
dose1
dose2
dose3

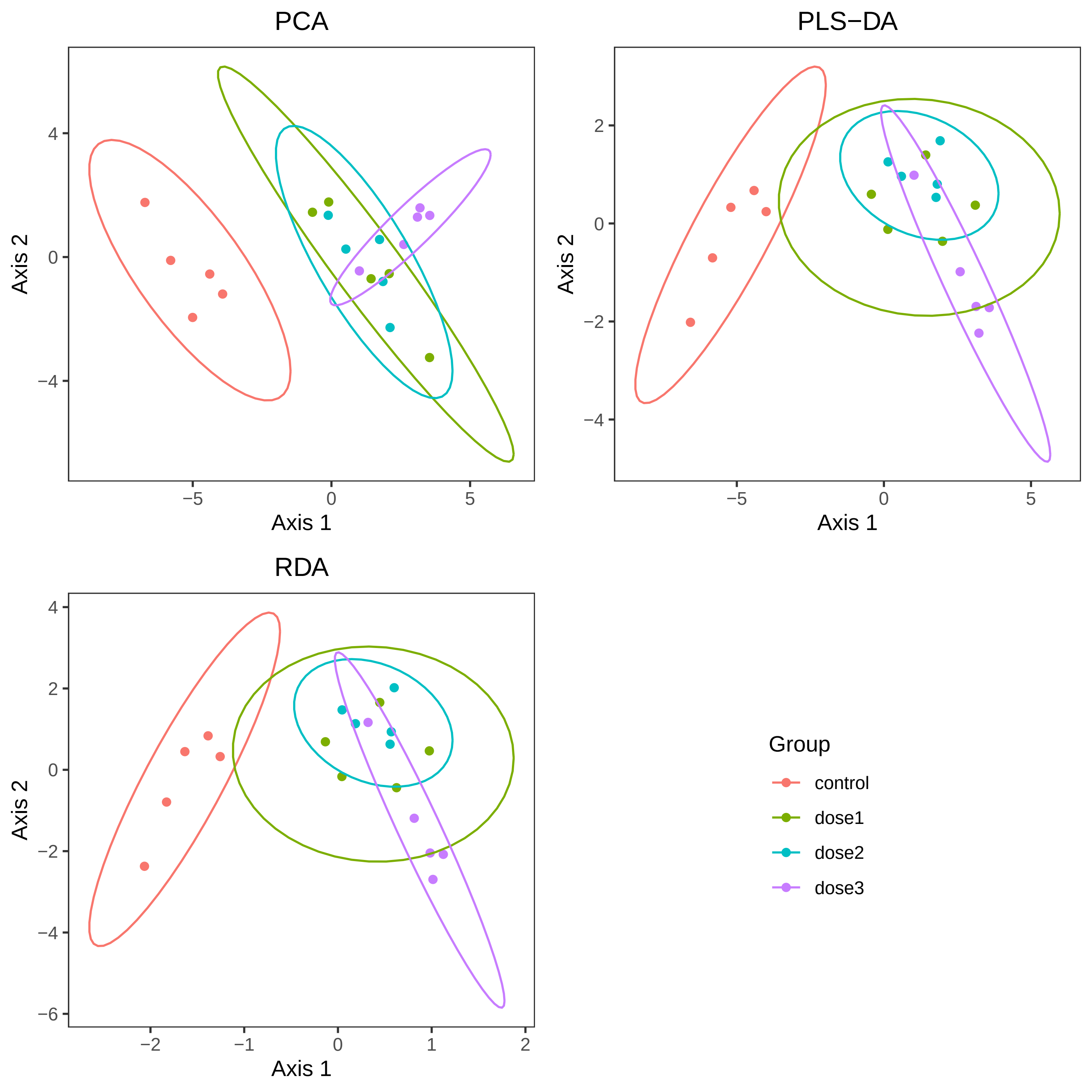
PCA
PLS−DA
RDA
Axis 1
Axis 2
Group
control
dose1
dose2
dose3

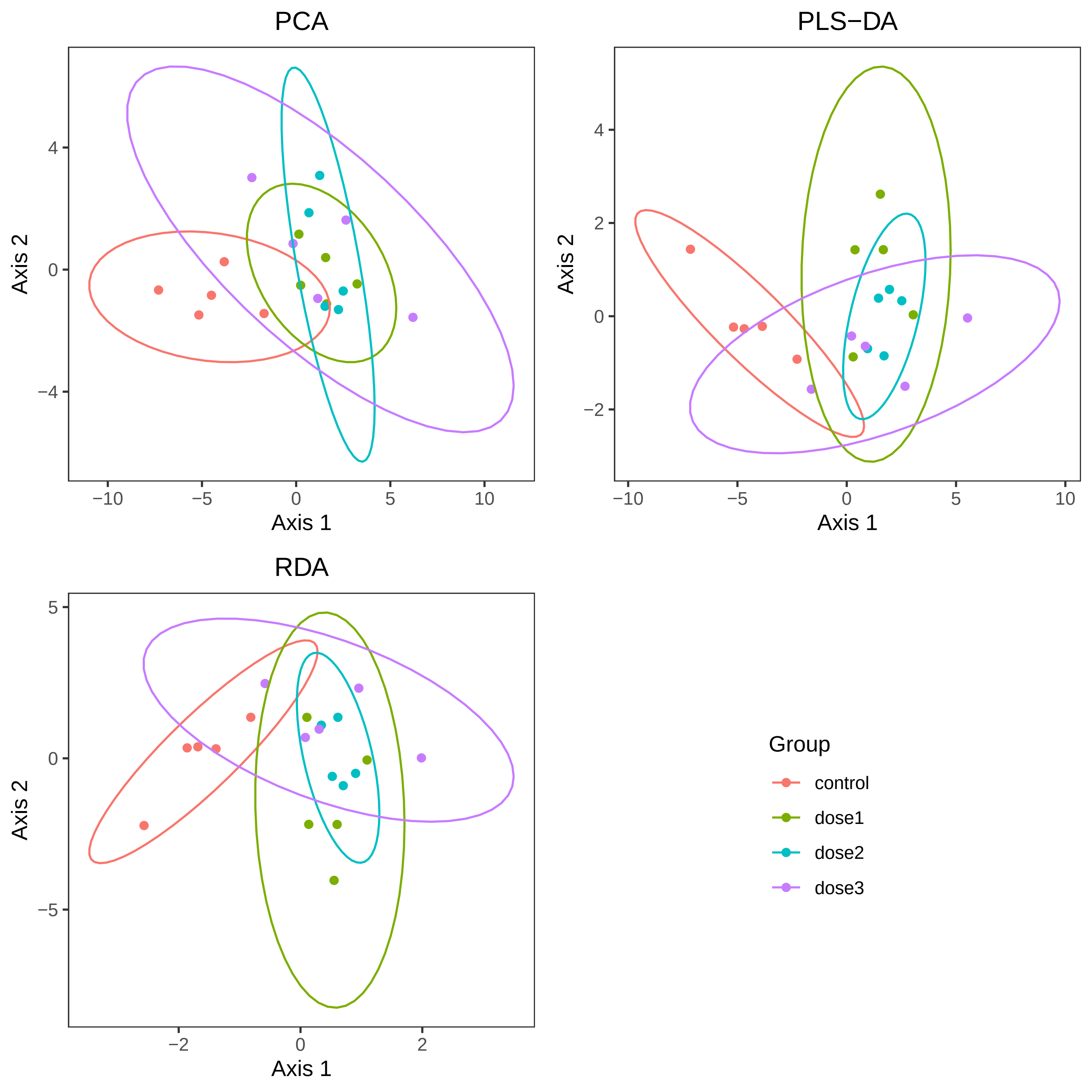

PCA
PLS−DA
RDA
Axis 1
Axis 2
Group
control
dose1
dose2
dose3

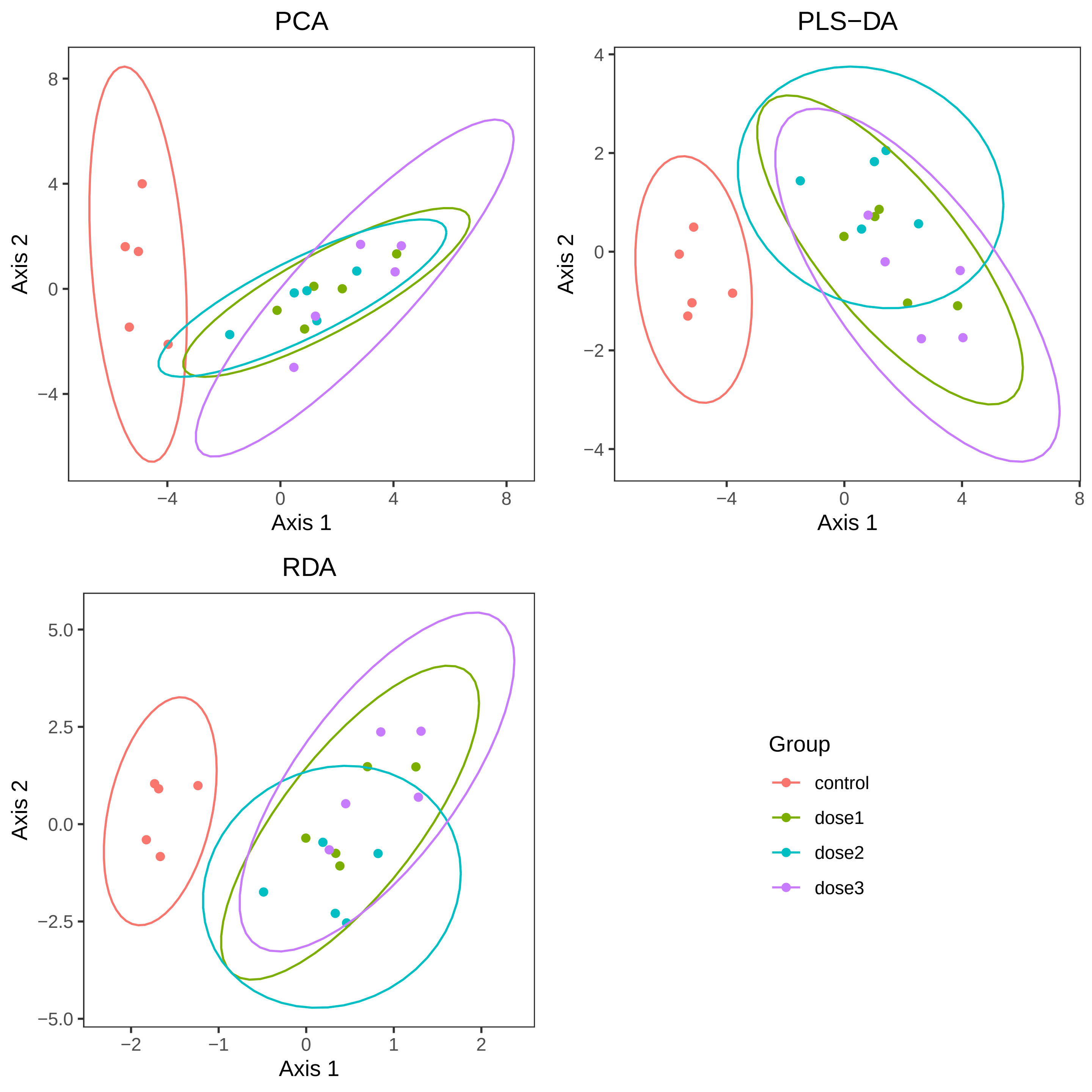
PCA
PLS−DA
RDA
Axis 1
Axis 2
Group
control
dose1
dose2
dose3

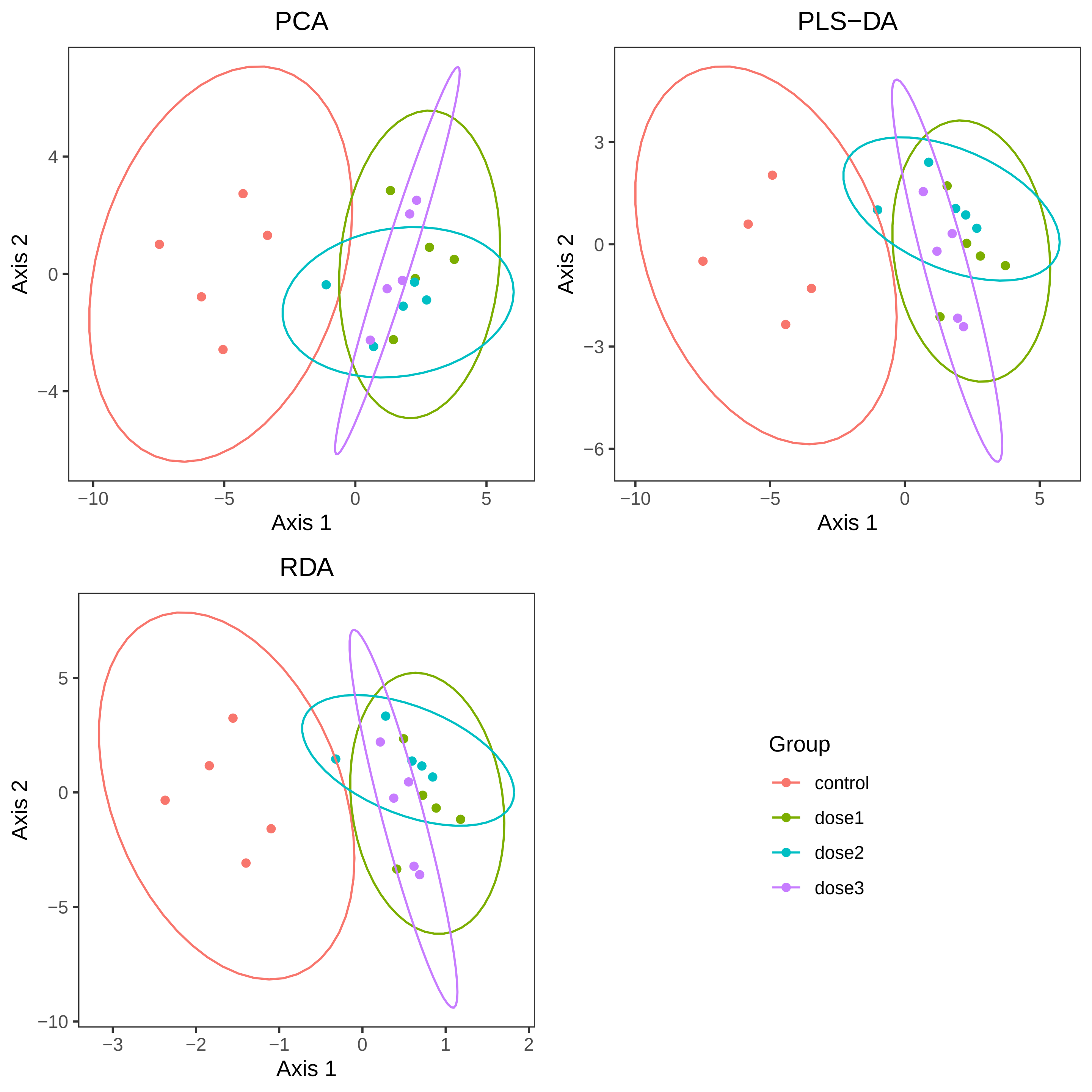
PCA
Axis 2
4
0
−4
−10
−5
0
5
Axis 1
PLS−DA
3
0
−3
−6
RDA
5
−5
−10
−3
−2
−1
1
2
Group
control
dose1
dose2
dose3

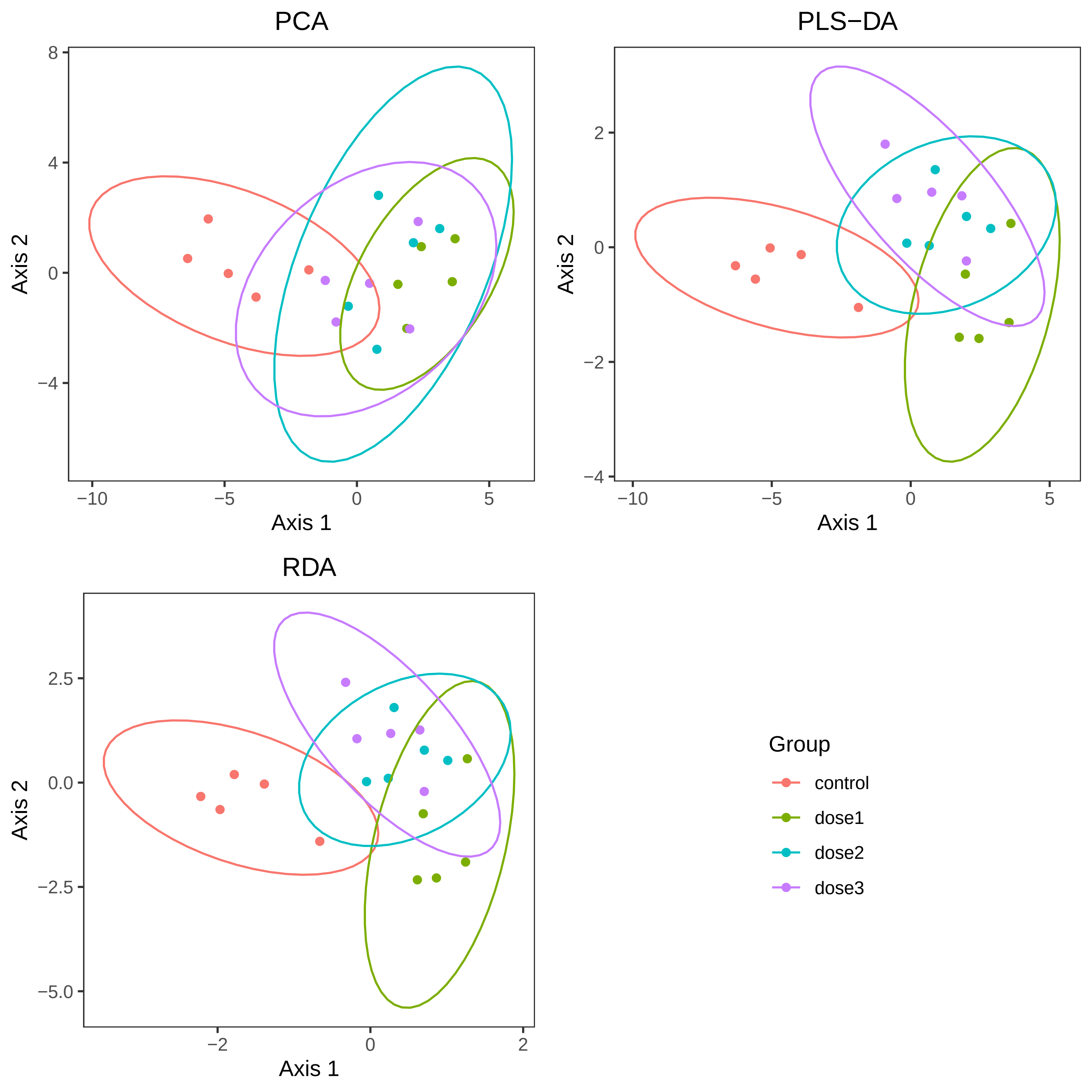

PCA
PLS−DA
RDA
Axis 1
Axis 2
Group
control
dose1
dose2
dose3

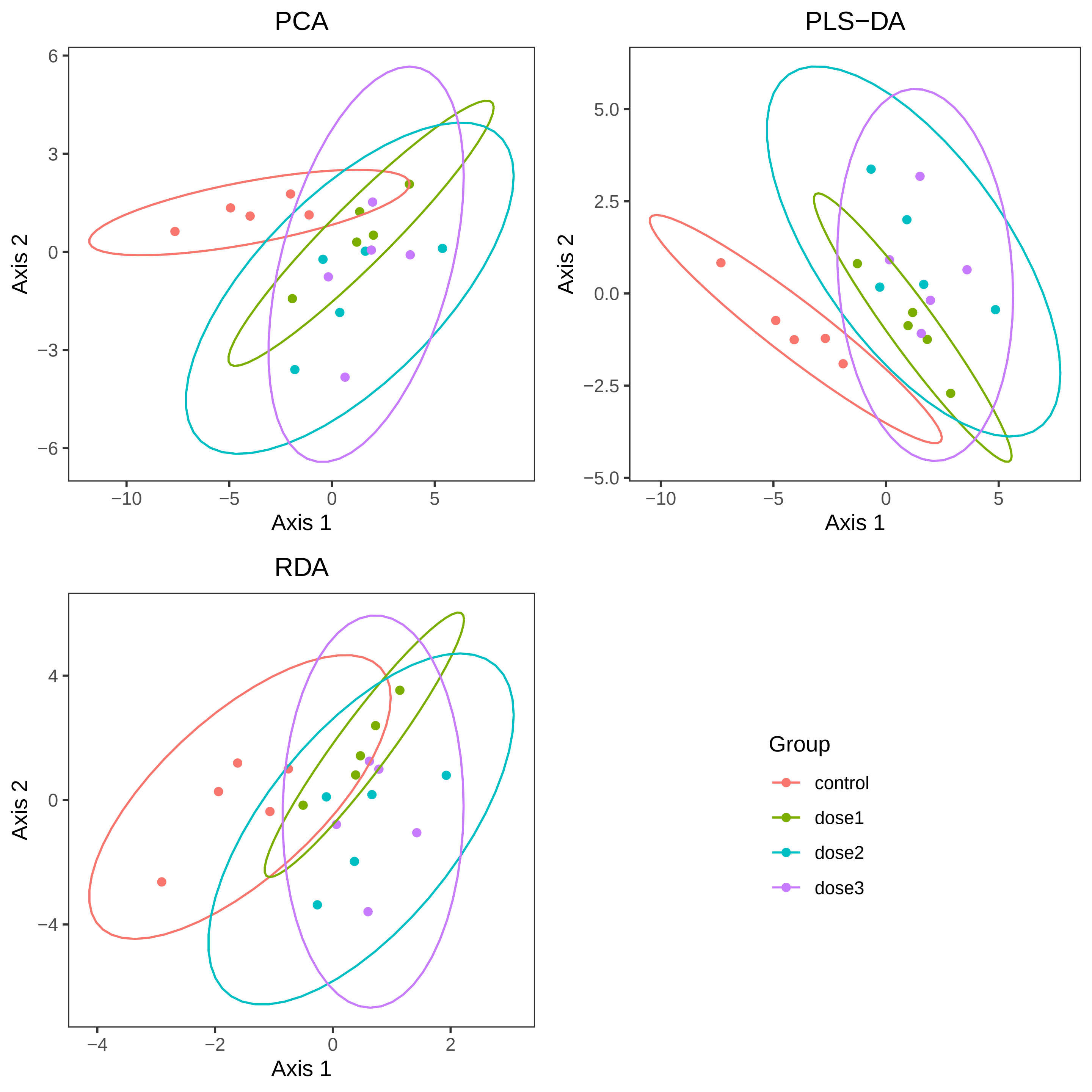

PCA
PLS−DA
RDA
Axis 1
Axis 2
Group
control
dose1
dose2
dose3

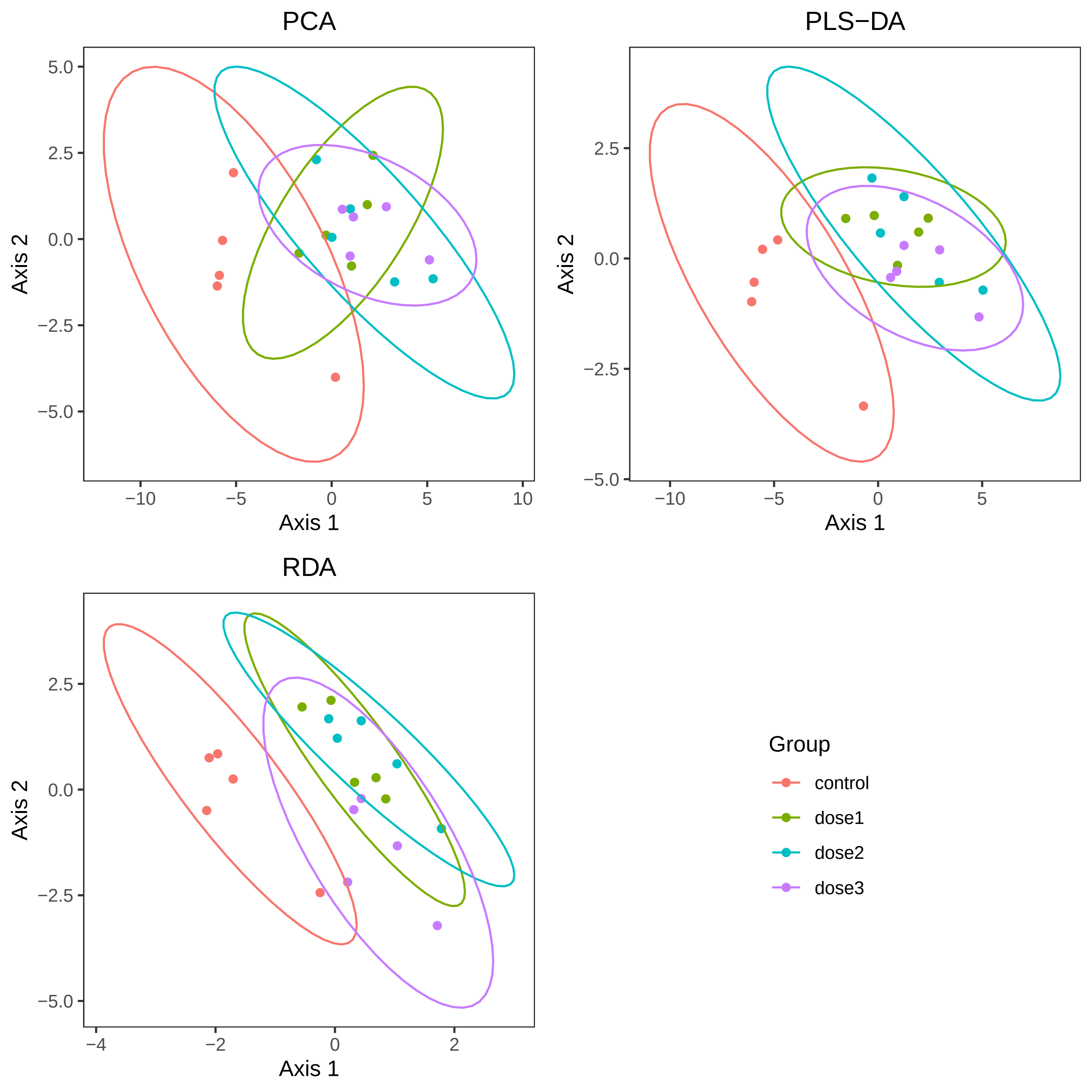

PCA
PLS−DA
RDA
Axis 1
Axis 2
5.0
2.5
0.0
−2.5
−5.0
−10
−5
0
5
10
−4
−2
2
Group
control
dose1
dose2
dose3

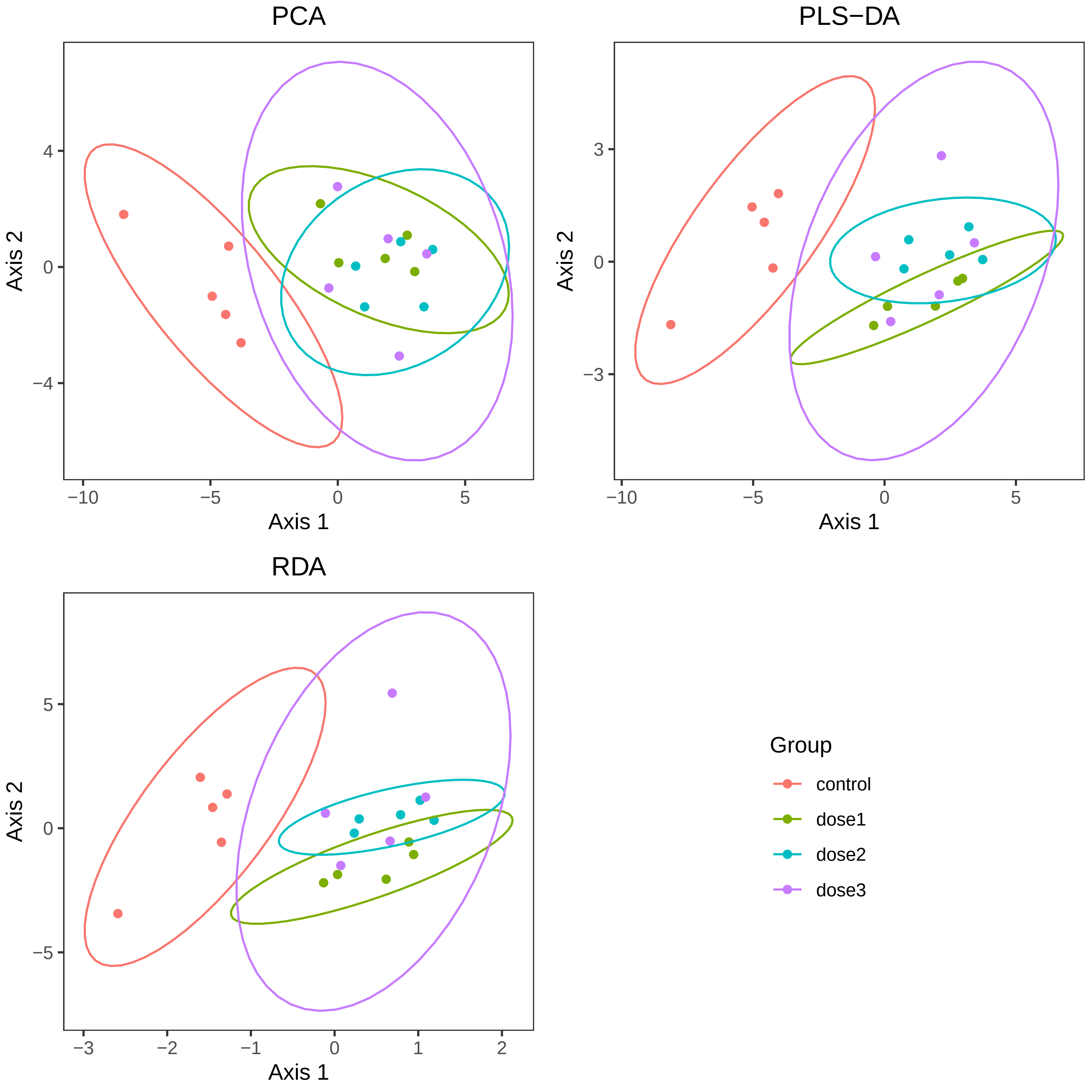

PCA
PLS−DA
RDA
Axis 1
Axis 2
Group
control
dose1
dose2
dose3

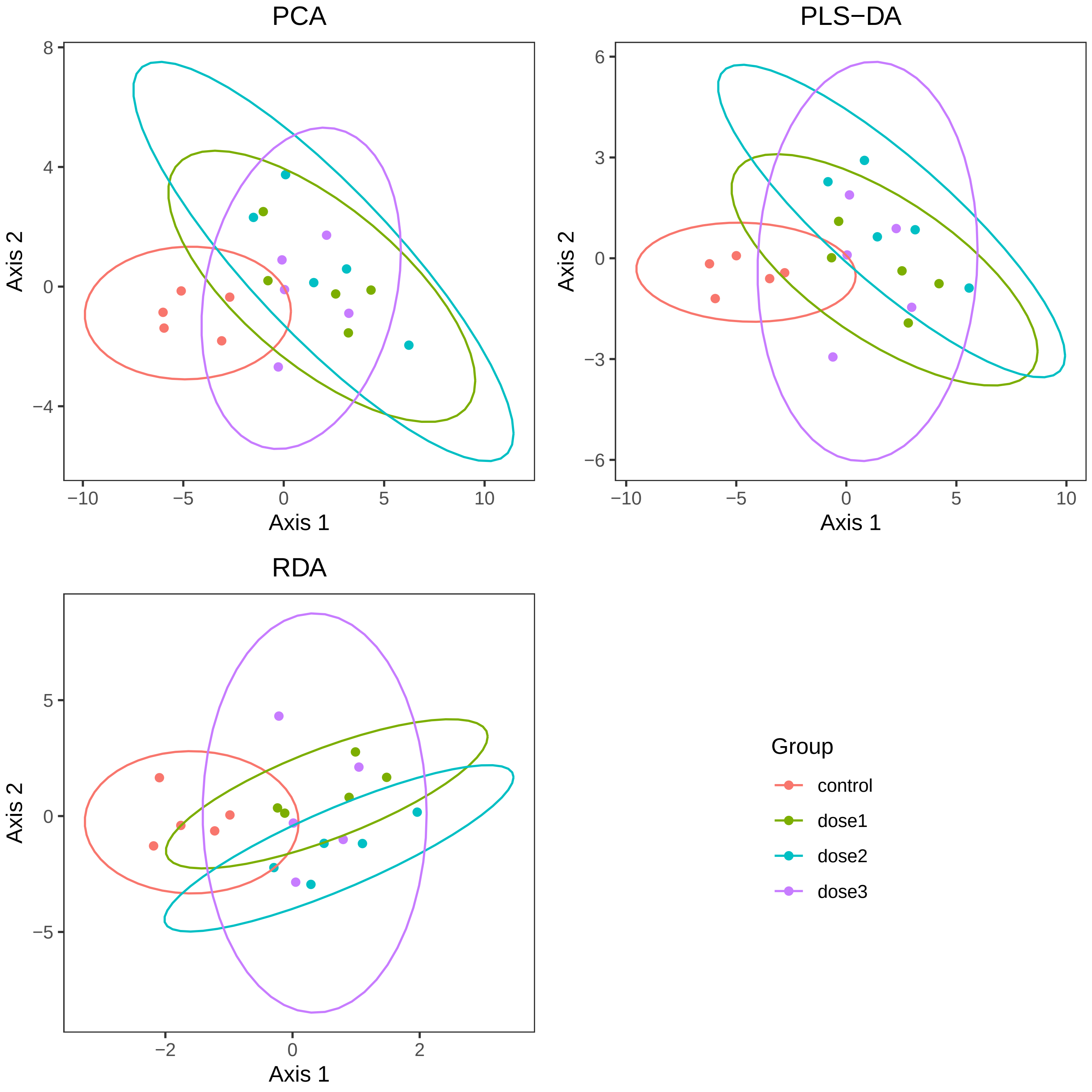

PCA
PLS−DA
RDA
Axis 1
Axis 2
Group
control
dose1
dose2
dose3

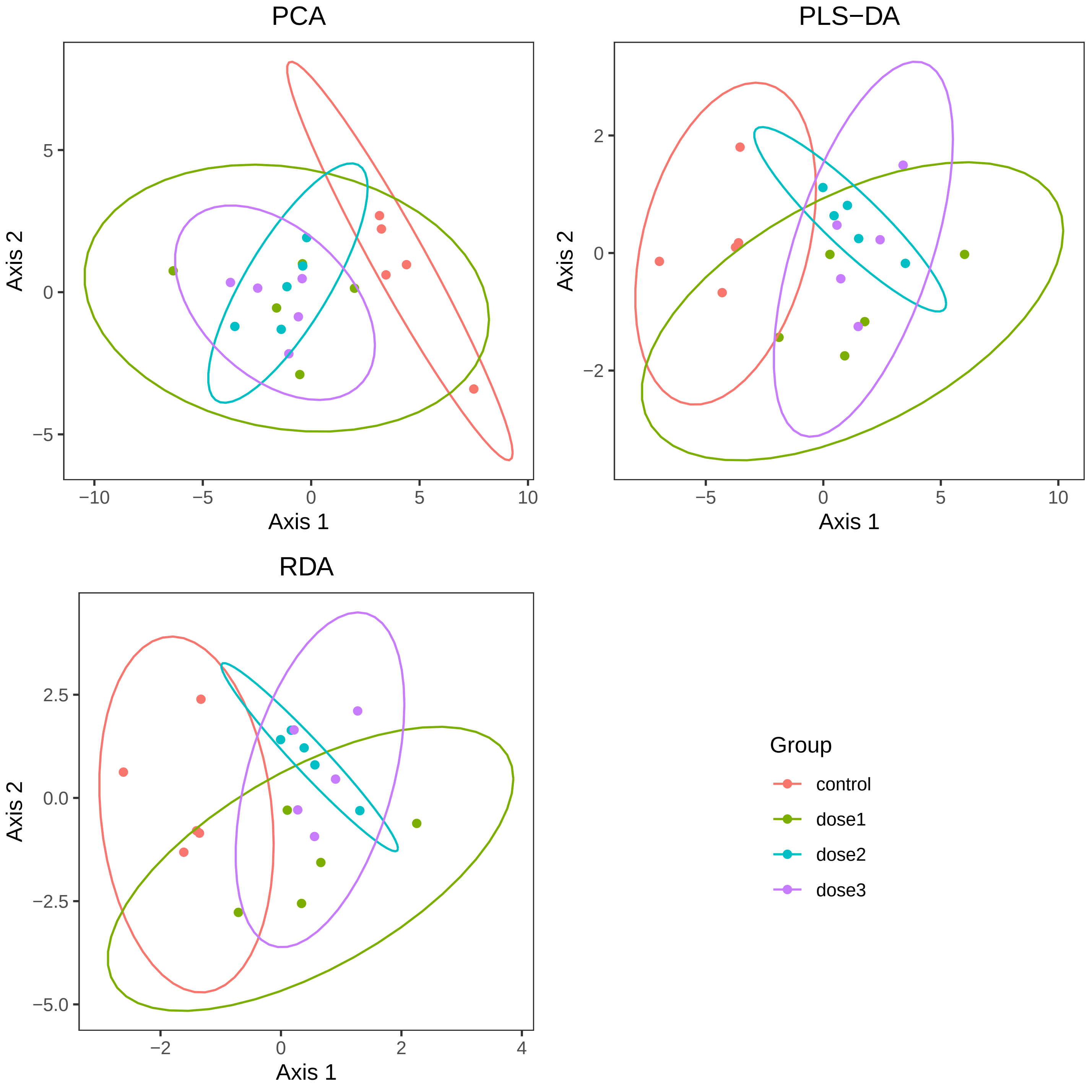

PCA
PLS−DA
RDA
Axis 1
Axis 2
Group
control
dose1
dose2
dose3

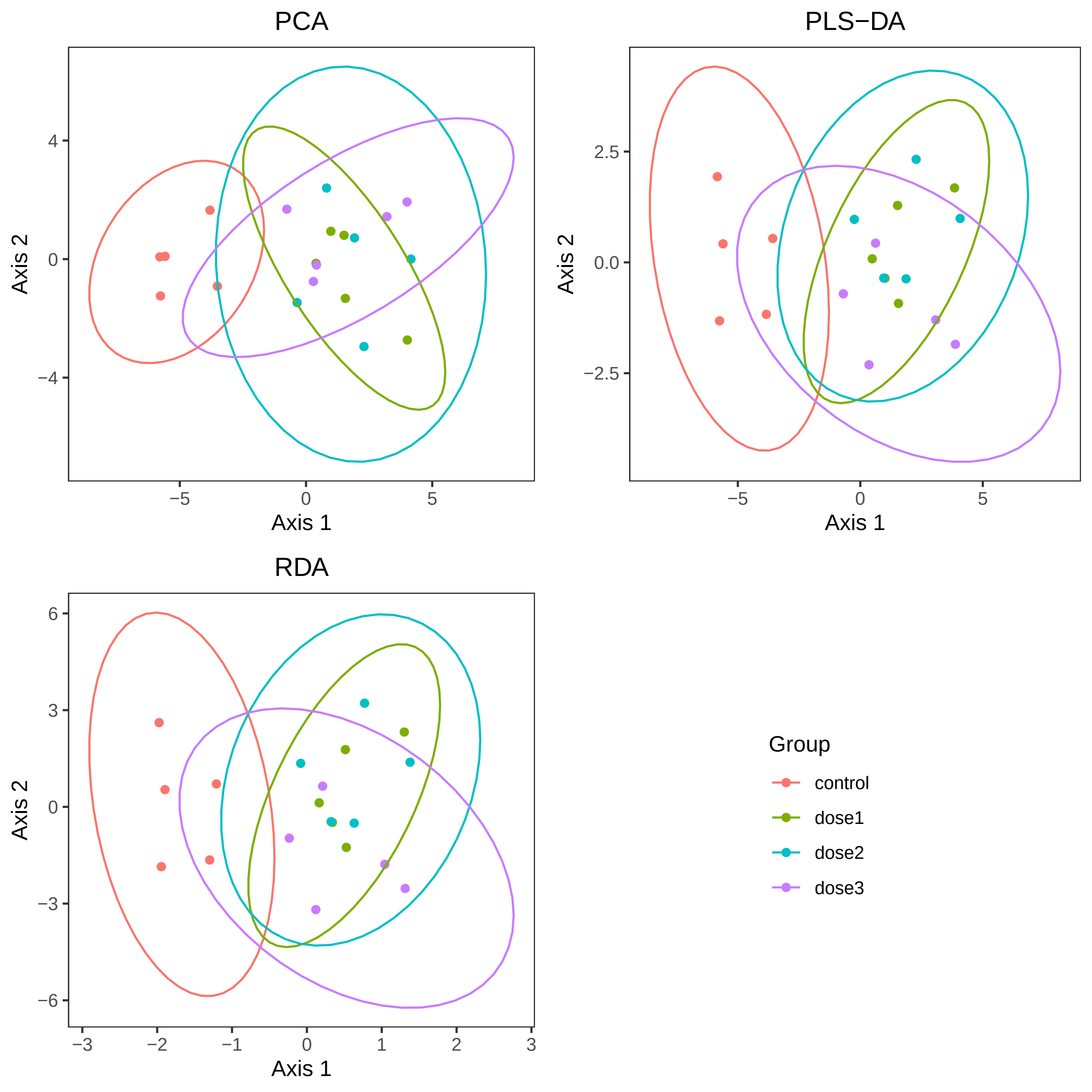

PCA
PLS−DA
RDA
Axis 1
Axis 2
Group
control
dose1
dose2
dose3

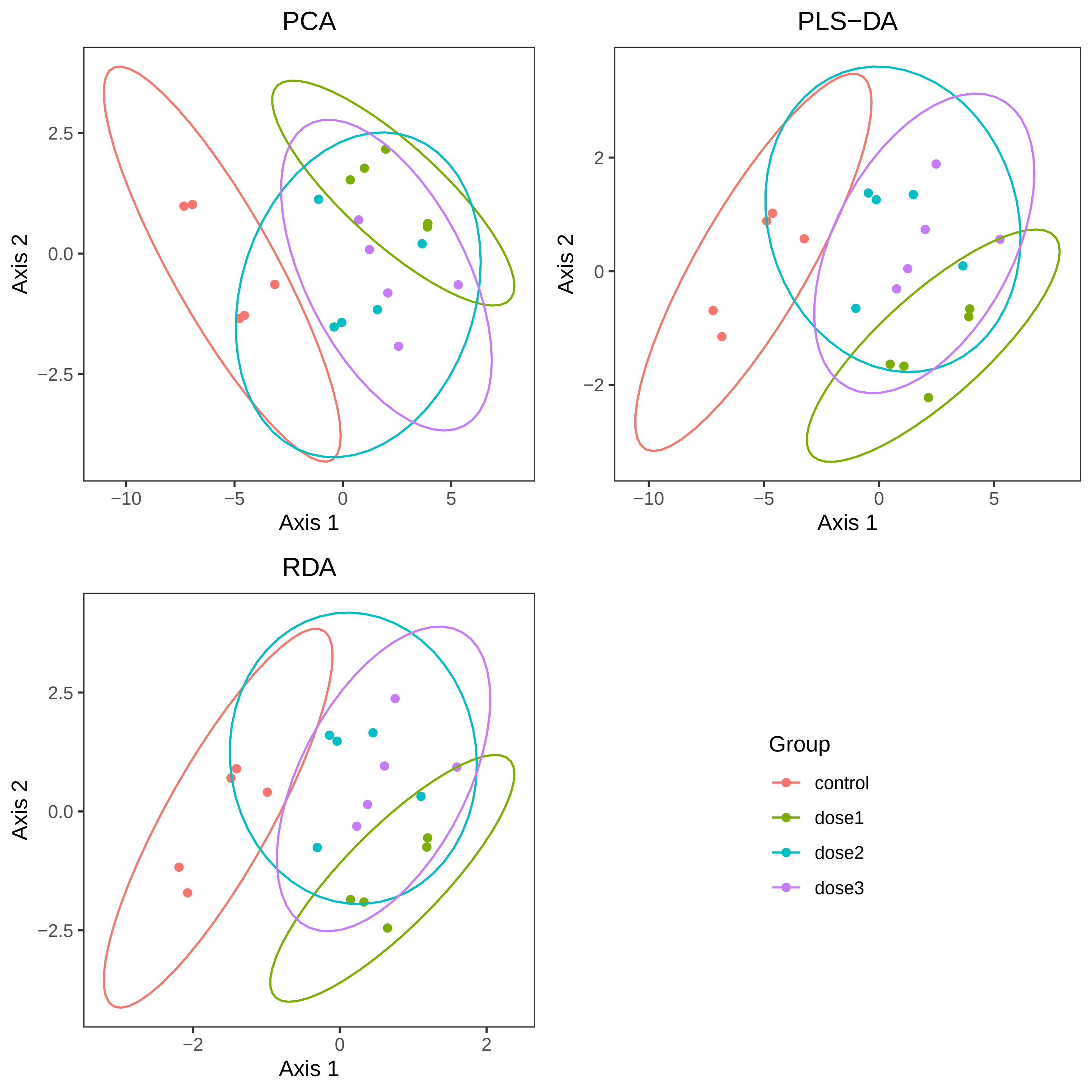

PCA
PLS−DA
RDA
Axis 1
Axis 2
−10
−5
0
5
2.5
0.0
−2.5
2
−2
Group
control
dose1
dose2
dose3

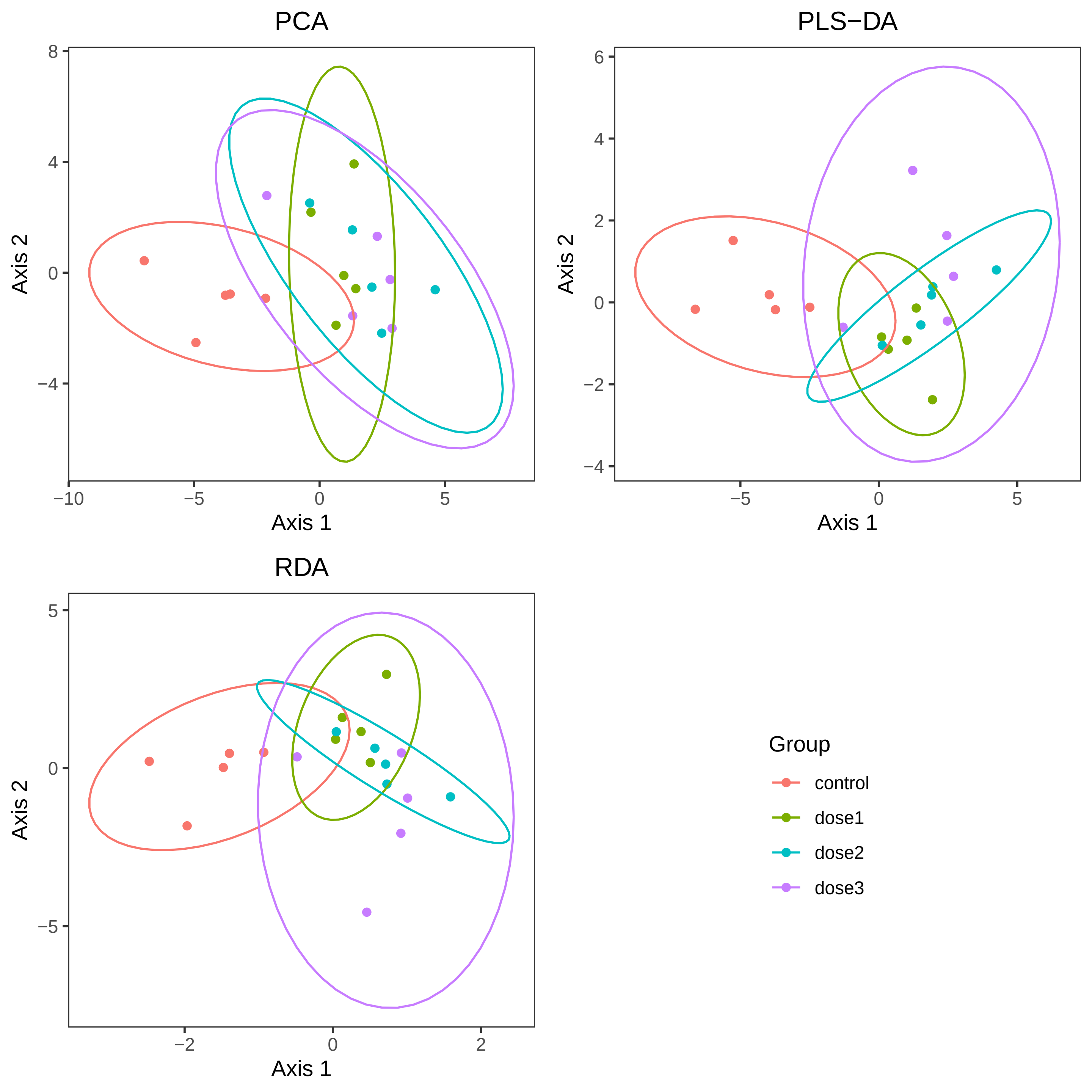

PCA
PLS−DA
RDA
Axis 1
Axis 2
Group
control
dose1
dose2
dose3

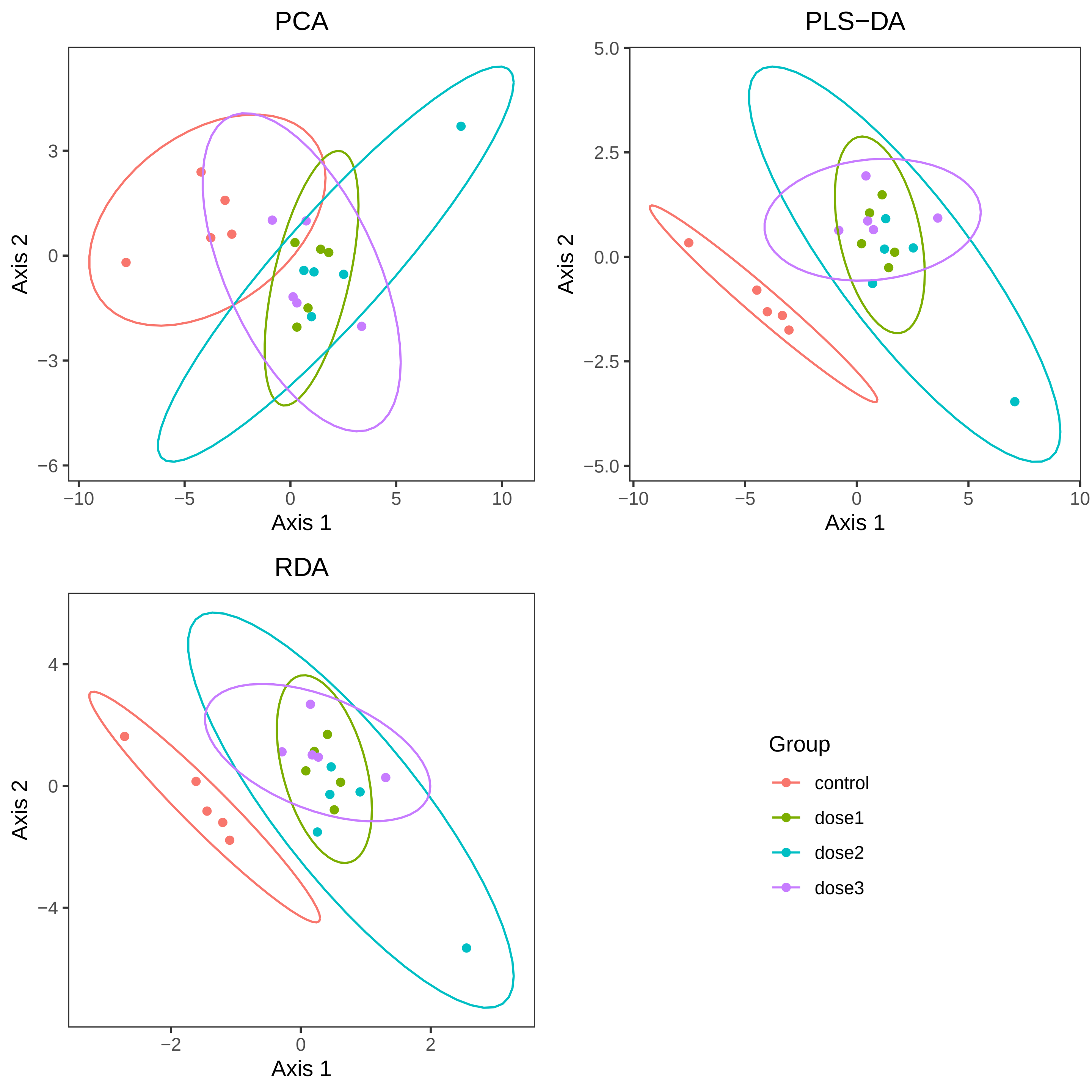

PCA
PLS−DA
RDA
Axis 1
Axis 2
Group
control
dose1
dose2
dose3

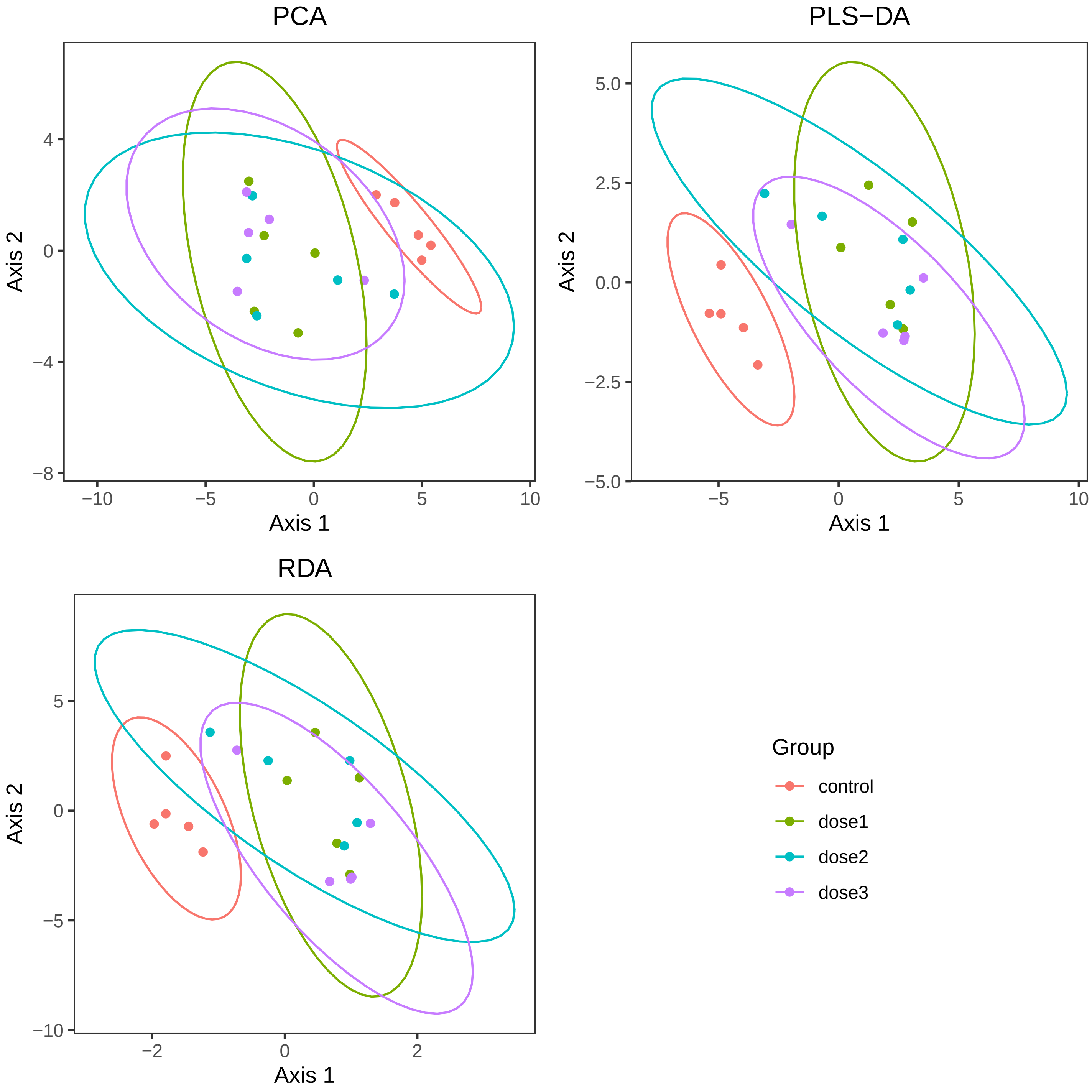

PCA
PLS−DA
RDA
Axis 1
Axis 2
Group
control
dose1
dose2
dose3

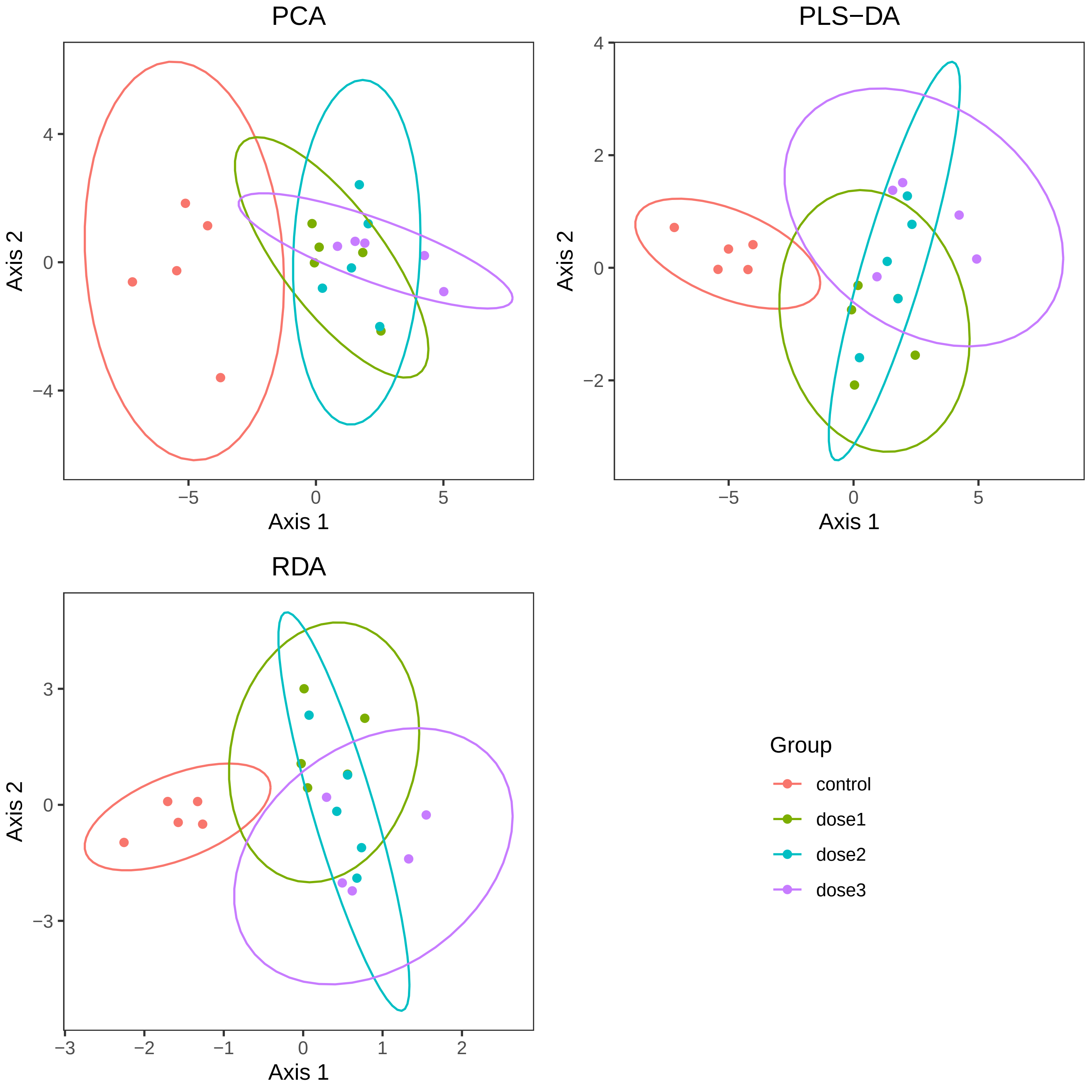

PCA
PLS−DA
RDA
Axis 1
Axis 2
4
0
−4
−5
5
2
−2
3
−3
−2
−1
1
Group
control
dose1
dose2
dose3

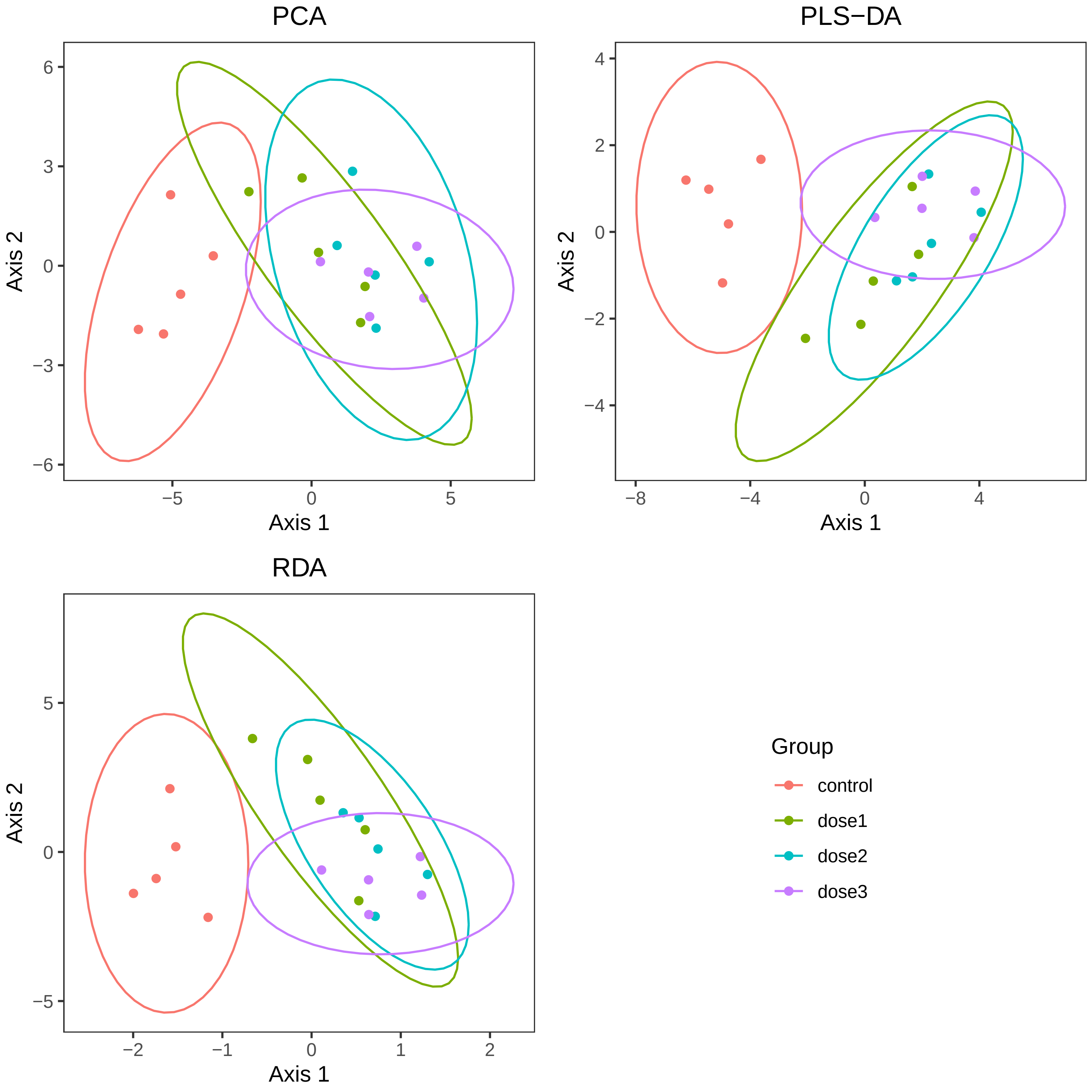

PCA
PLS−DA
RDA
Axis 1
Axis 2
6
3
0
−3
−6
−5
0
5
4
2
0
−2
−4
−8
−4
0
4
5
0
−5
−2
−1
0
1
2
Group
control
dose1
dose2
dose3

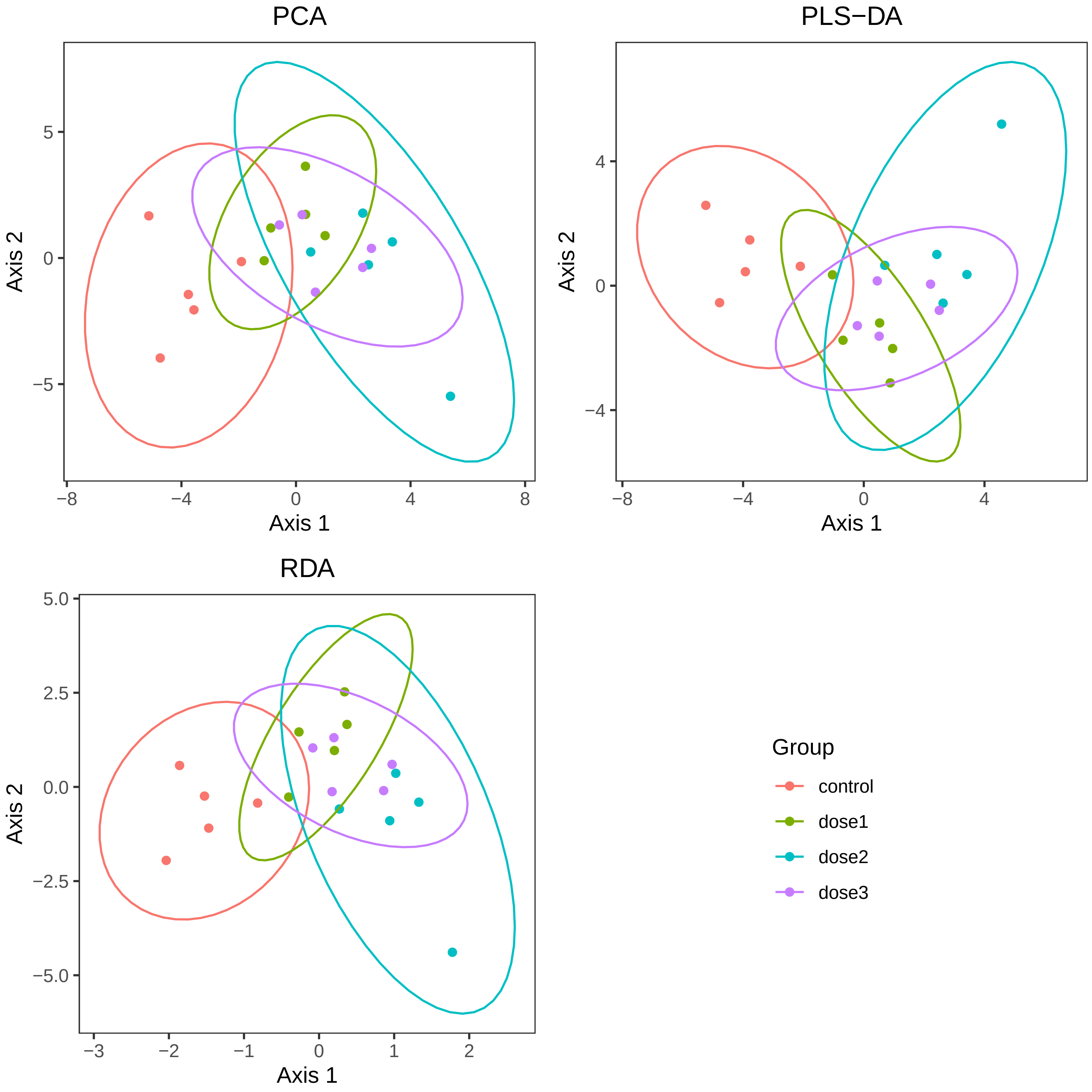
PCA
PLS−DA
RDA
Axis 1
Axis 2
Group
control
dose1
dose2
dose3

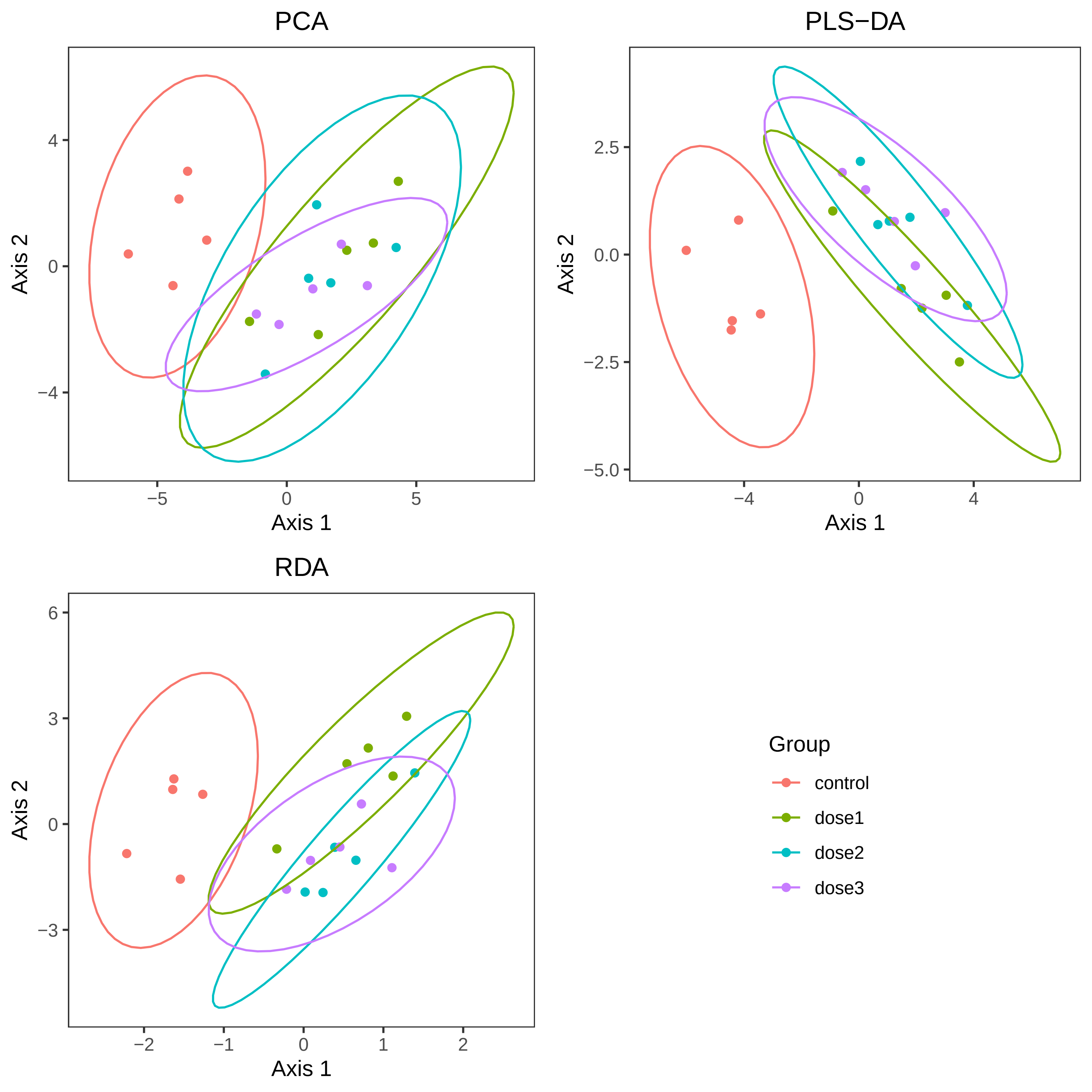
PCA
PLS−DA
RDA
Axis 1
Axis 2
Group
control
dose1
dose2
dose3

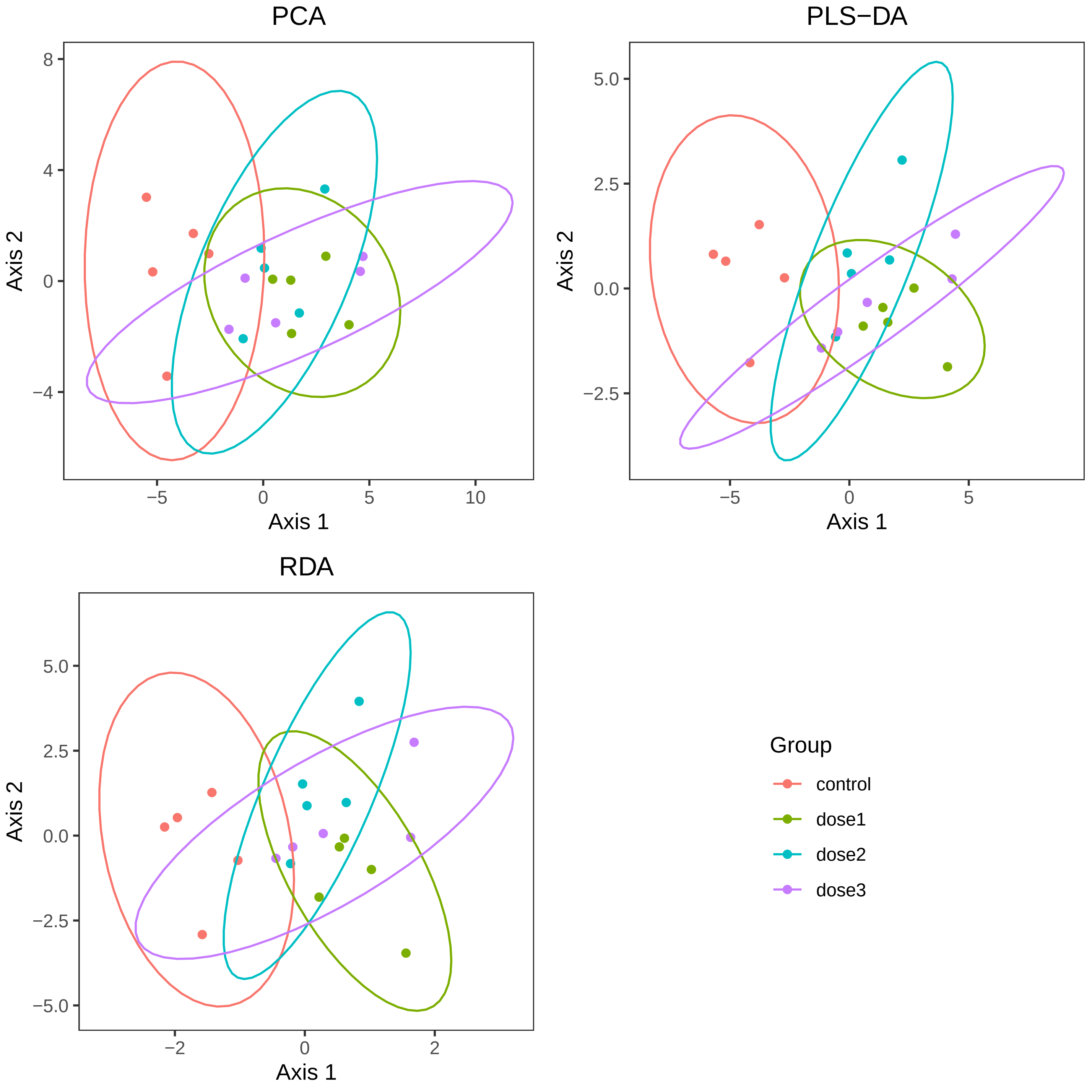

PCA
PLS−DA
RDA
Axis 1
Axis 2
Group
control
dose1
dose2
dose3

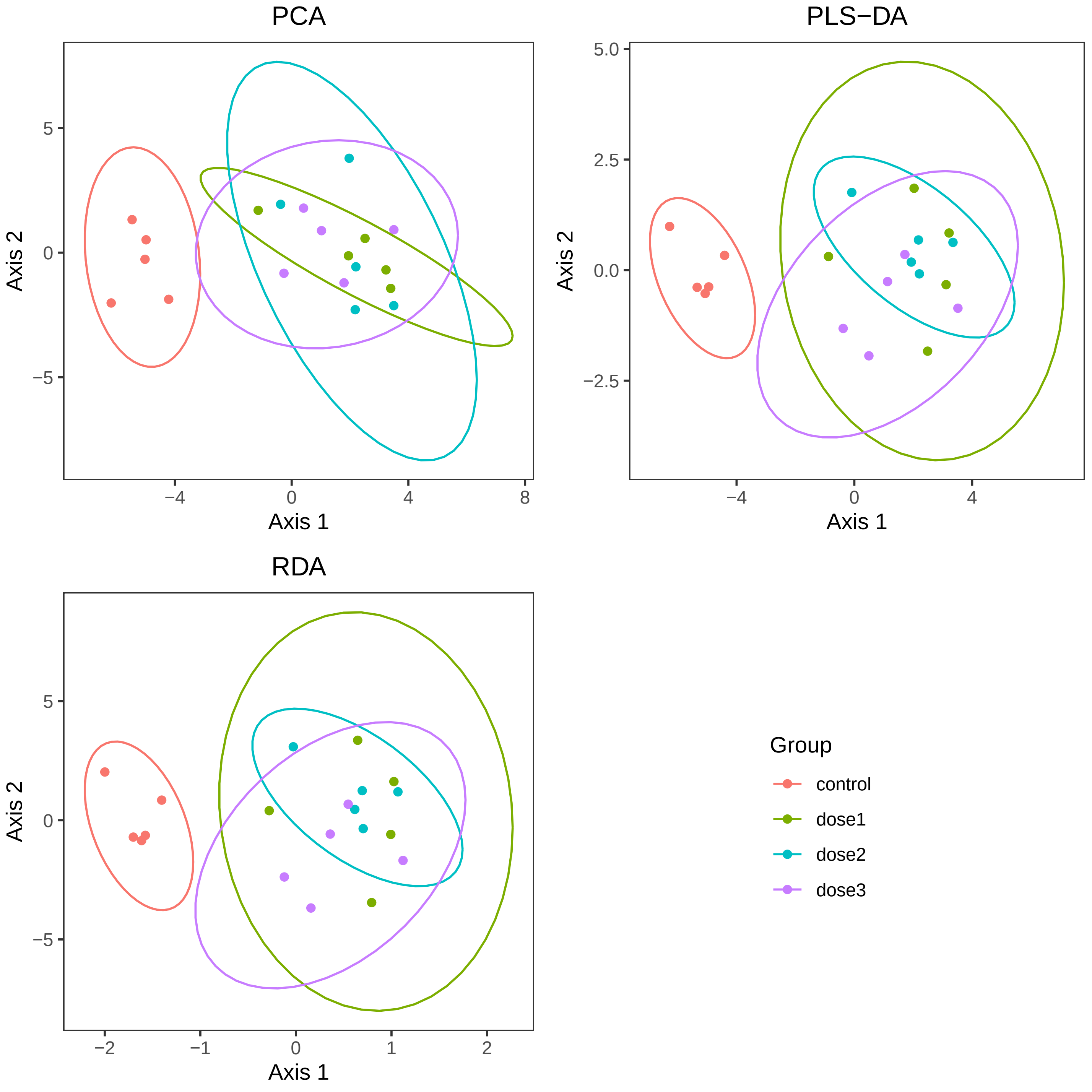

PCA
Axis 2
Axis 1
5
0
−5
−4
0
4
8
PLS−DA
5.0
2.5
0.0
−2.5
RDA
−2
−1
1
2
Group
control
dose1
dose2
dose3

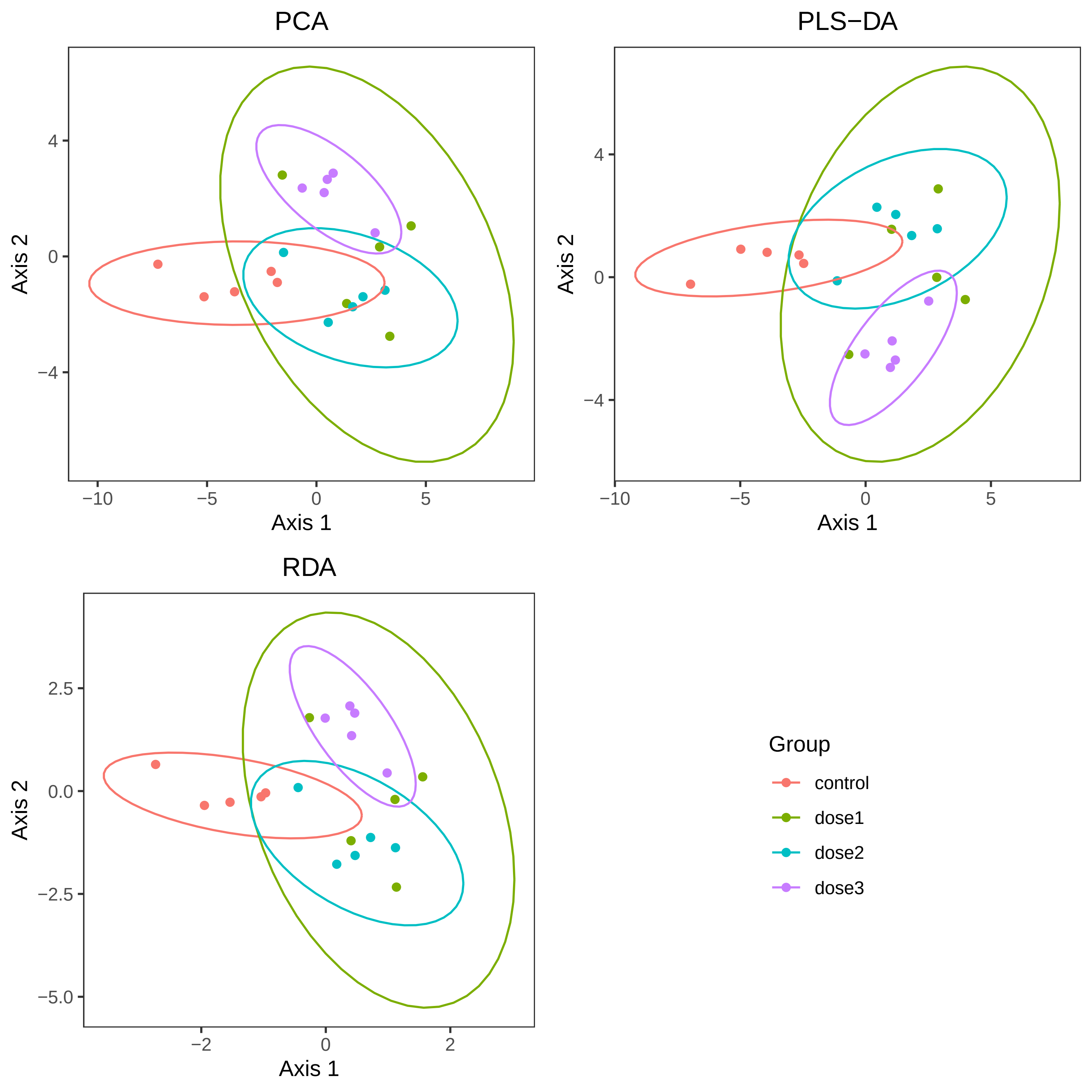
PCA
PLS−DA
RDA
Axis 1
Axis 2
Group
control
dose1
dose2
dose3

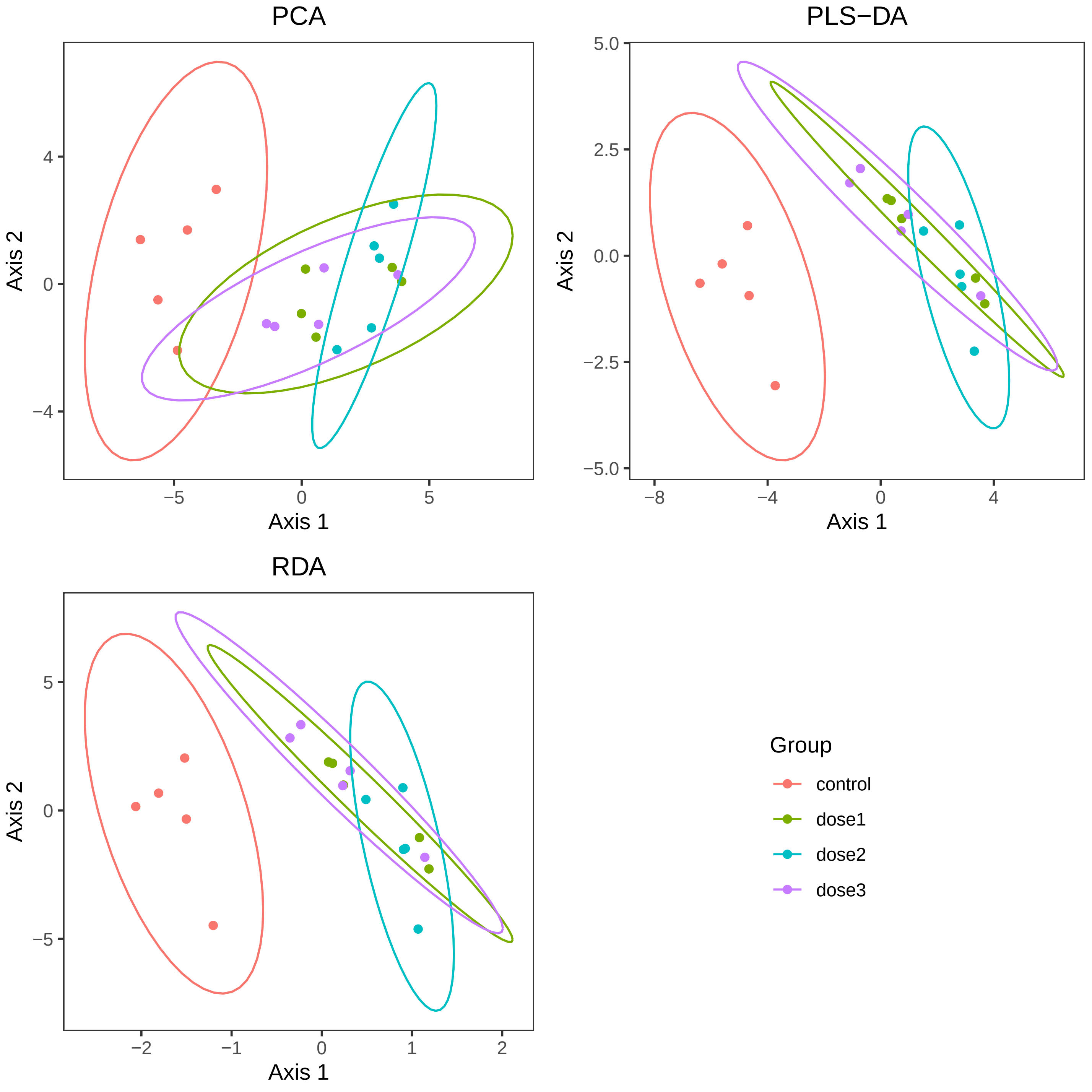

PCA
PLS−DA
RDA
Axis 1
Axis 2
Group
control
dose1
dose2
dose3

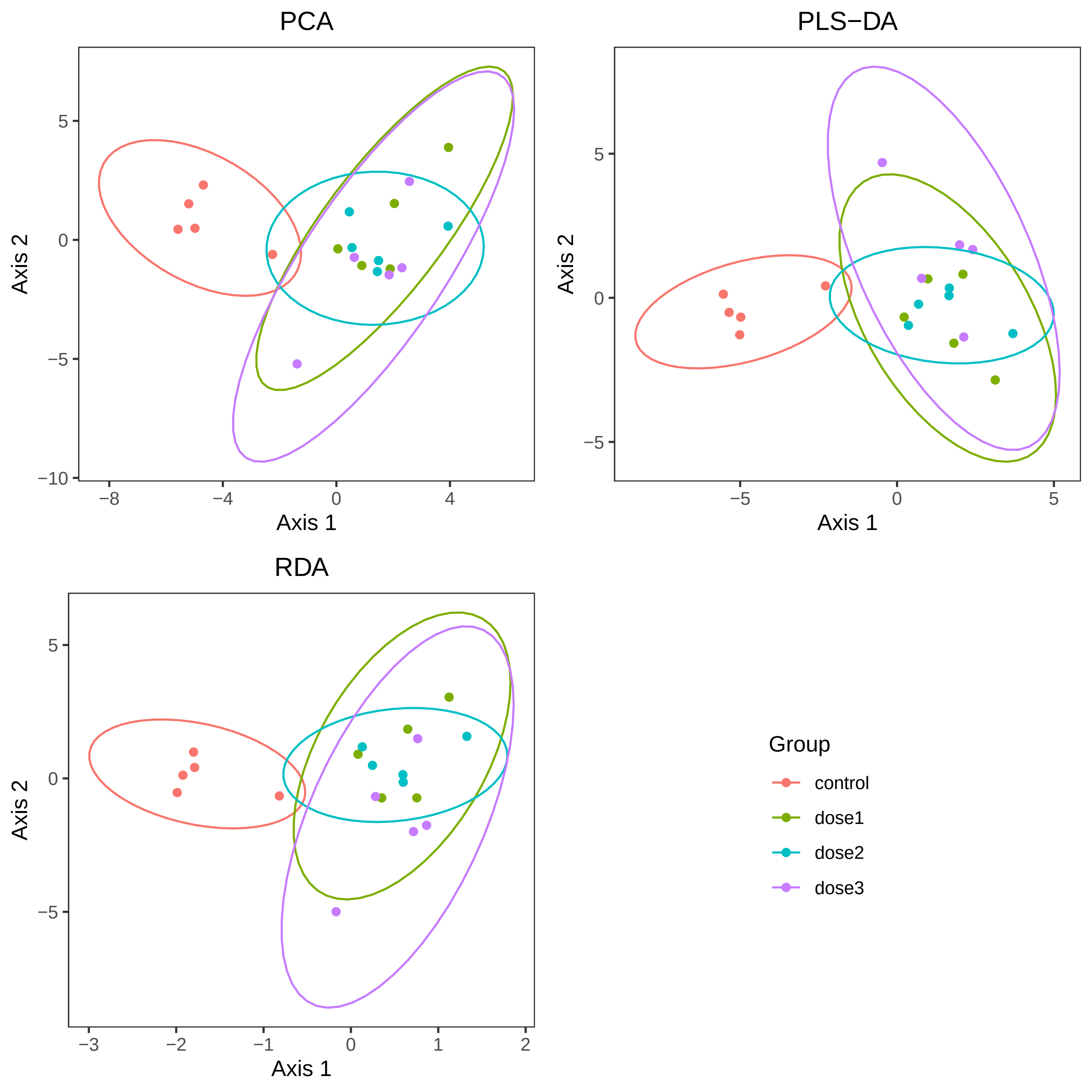
PCA
PLS−DA
RDA
Axis 1
Axis 2
Group
control
dose1
dose2
dose3

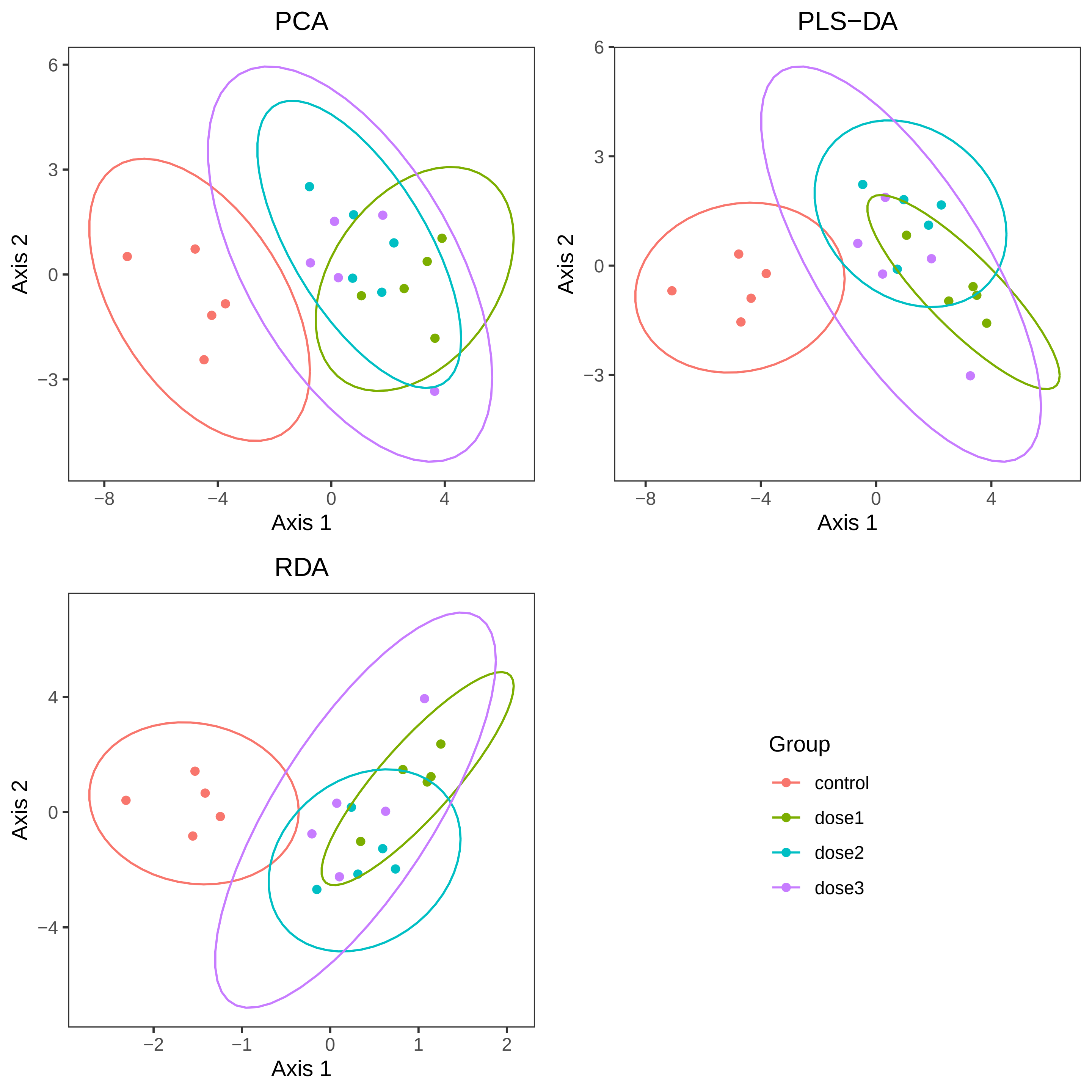
PCA
PLS−DA
RDA
Axis 1
Axis 2
−8
−4
0
4
6
3
0
−3
−2
−1
1
2
Group
control
dose1
dose2
dose3

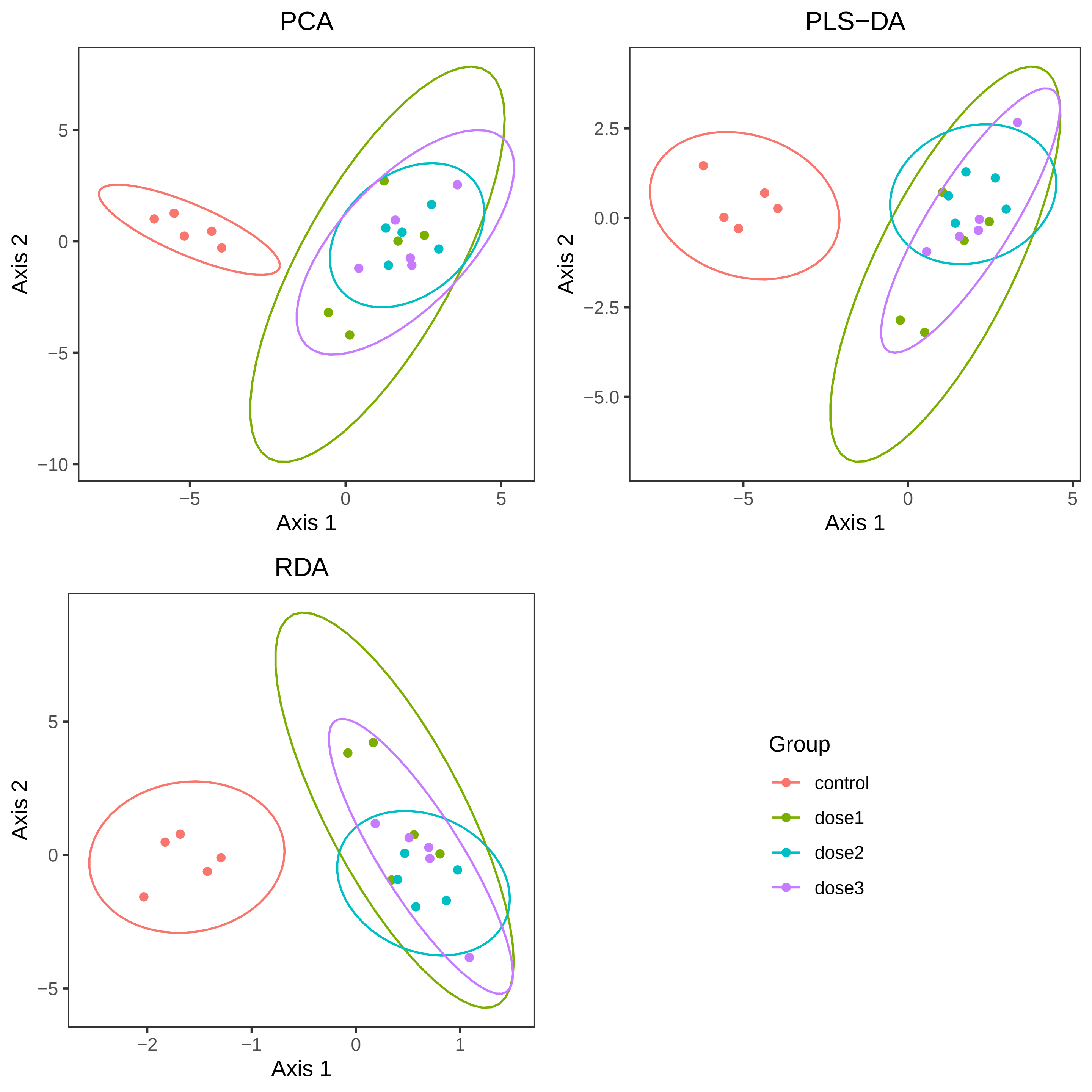

PCA
PLS−DA
RDA
Axis 1
Axis 2
Group
control
dose1
dose2
dose3

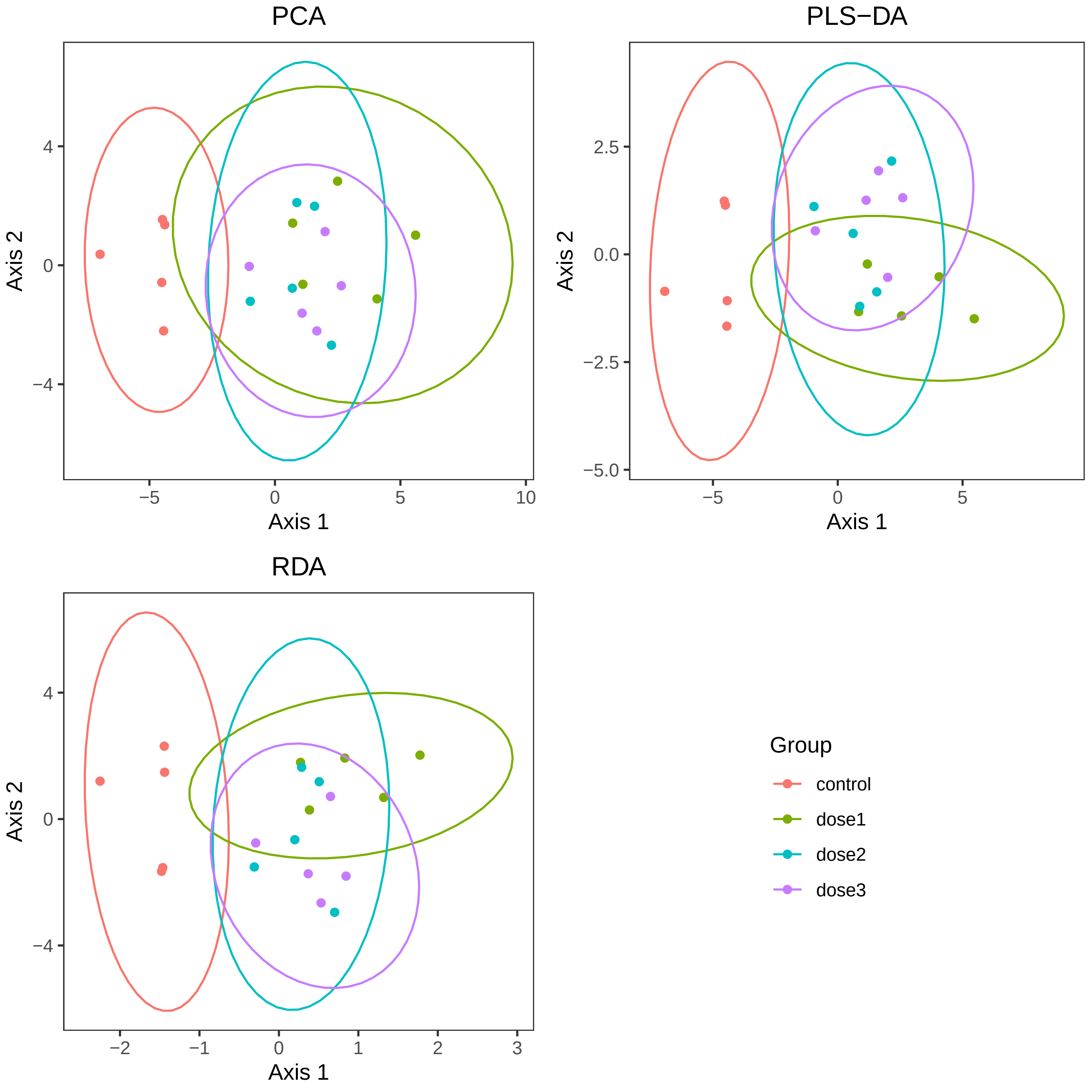
PCA
PLS−DA
RDA
Axis 1
Axis 2
Group
control
dose1
dose2
dose3

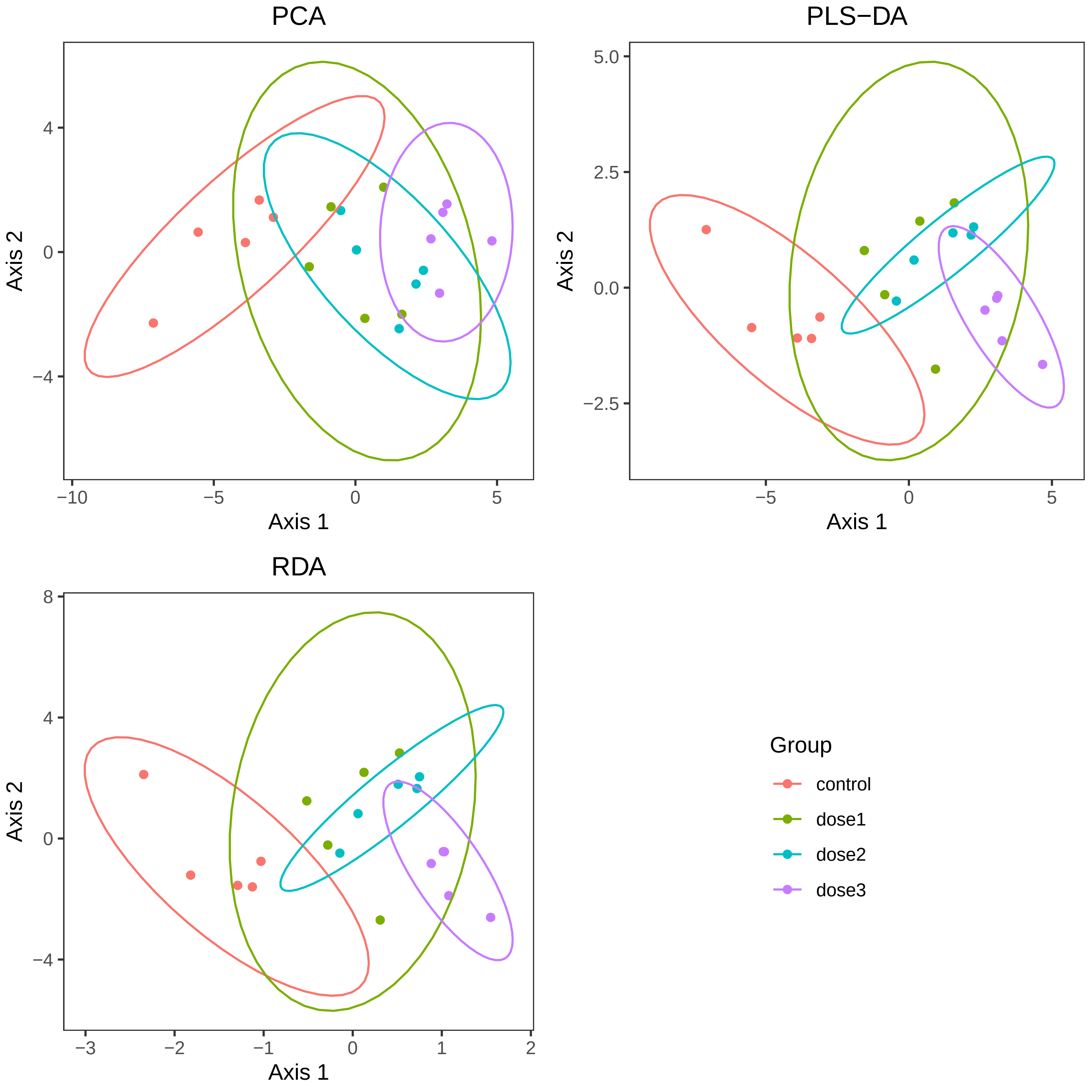
PCA
PLS−DA
RDA
Axis 1
Axis 2
Group
control
dose1
dose2
dose3

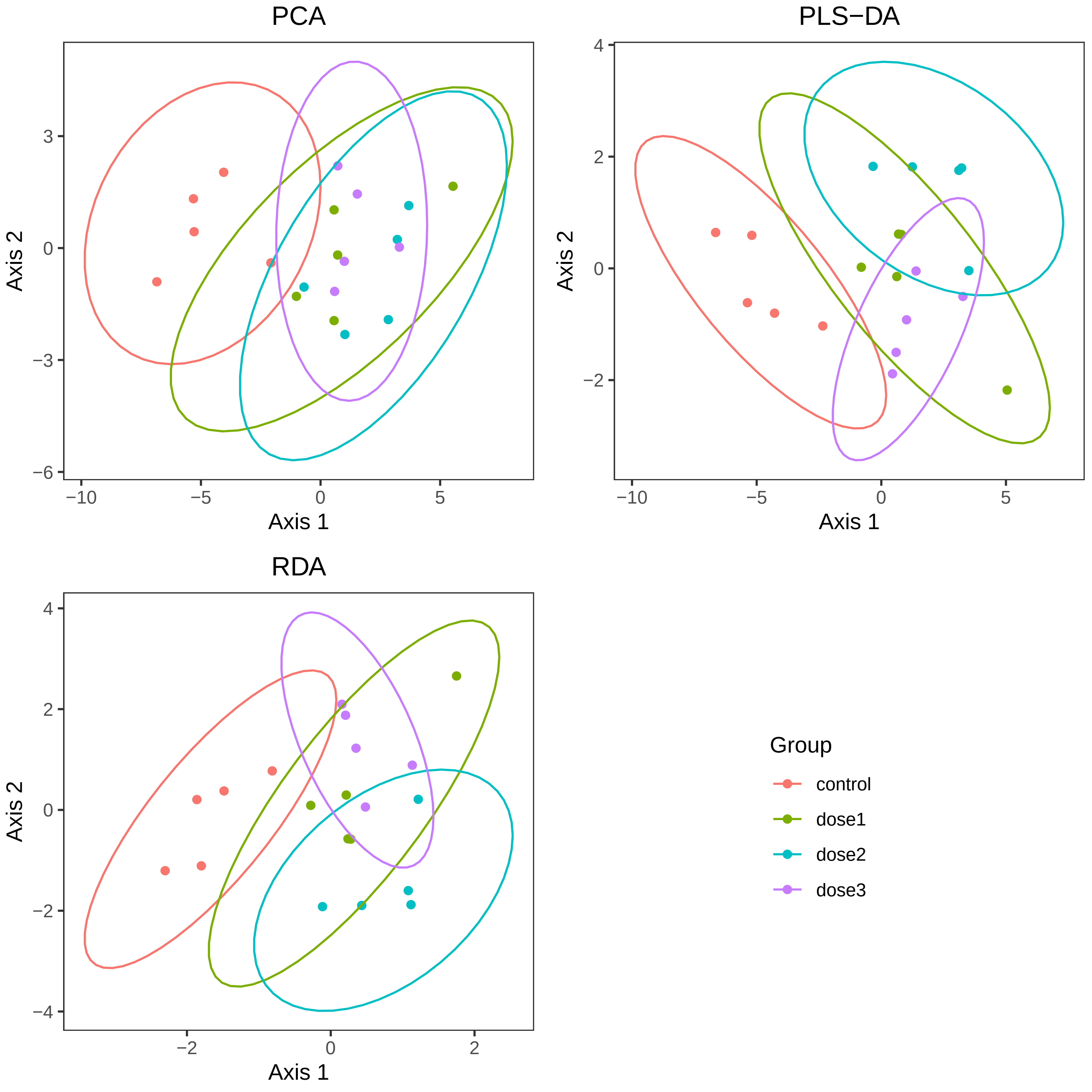

PCA
PLS−DA
RDA
Axis 1
Axis 2
Group
control
dose1
dose2
dose3

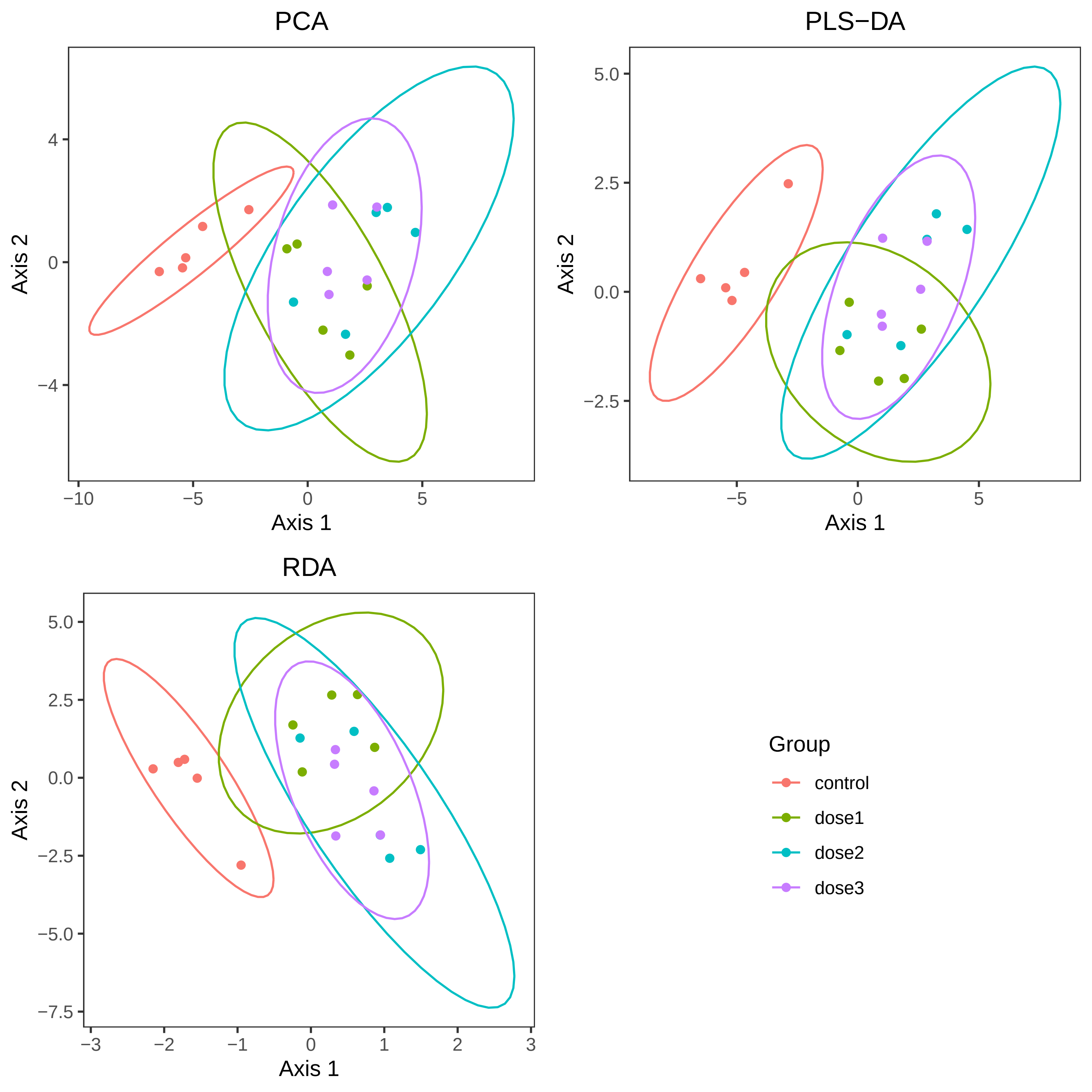

PCA
PLS−DA
RDA
Axis 1
Axis 2
Group
control
dose1
dose2
dose3

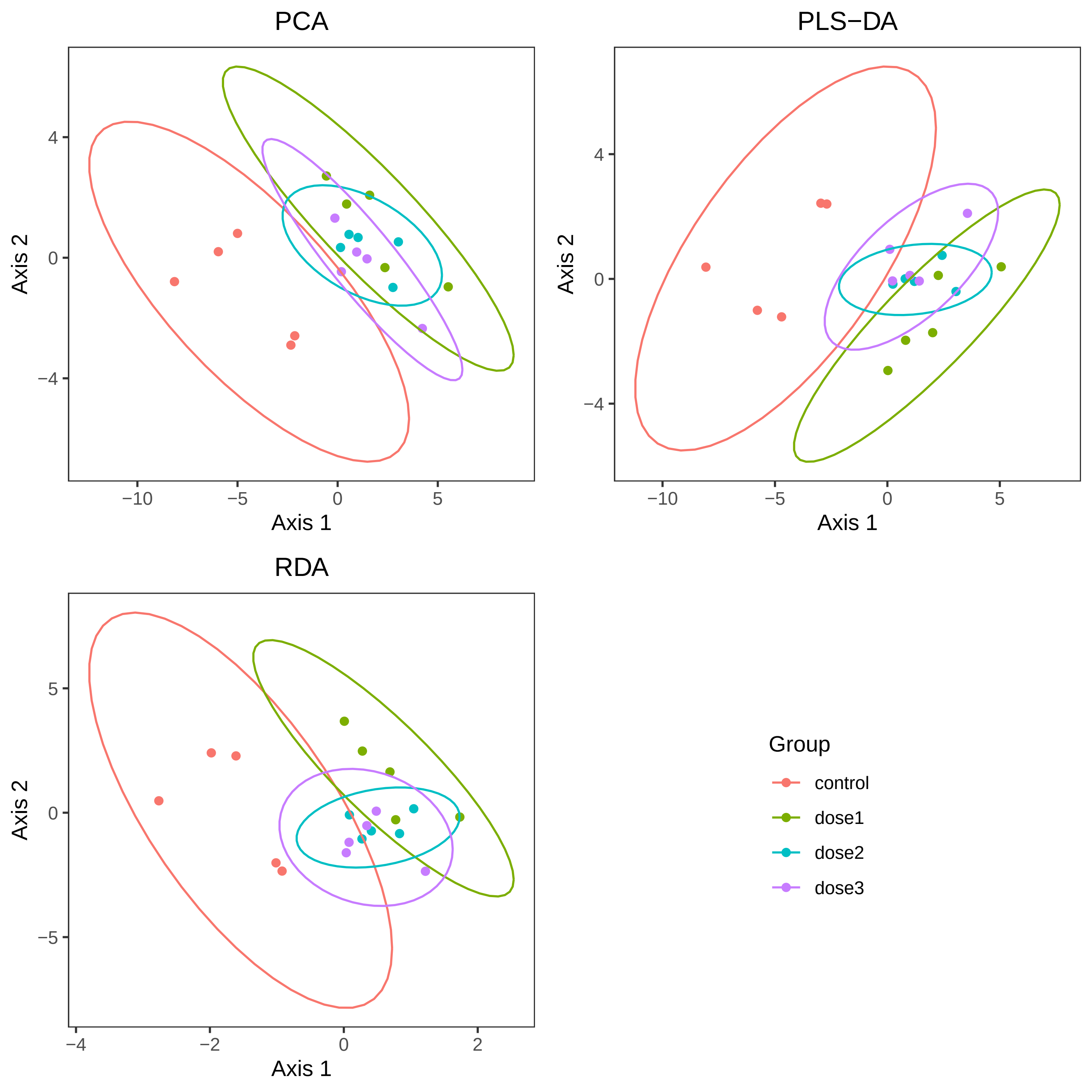

PCA
PLS−DA
RDA
Axis 1
Axis 2
−10
−5
0
5
4
−4
−2
2
Group
control
dose1
dose2
dose3

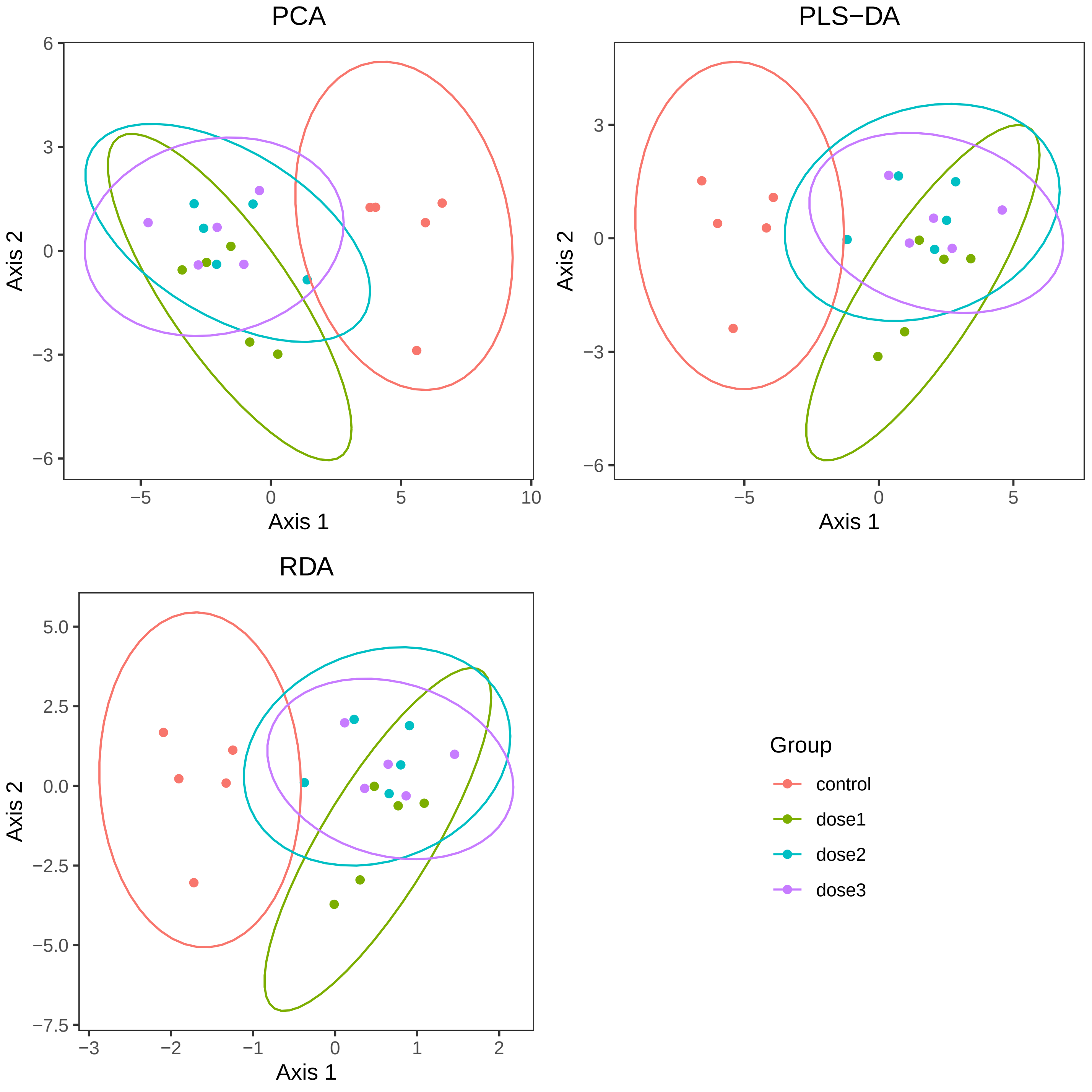
PCA
PLS−DA
RDA
Axis 1
Axis 2
Group
control
dose1
dose2
dose3

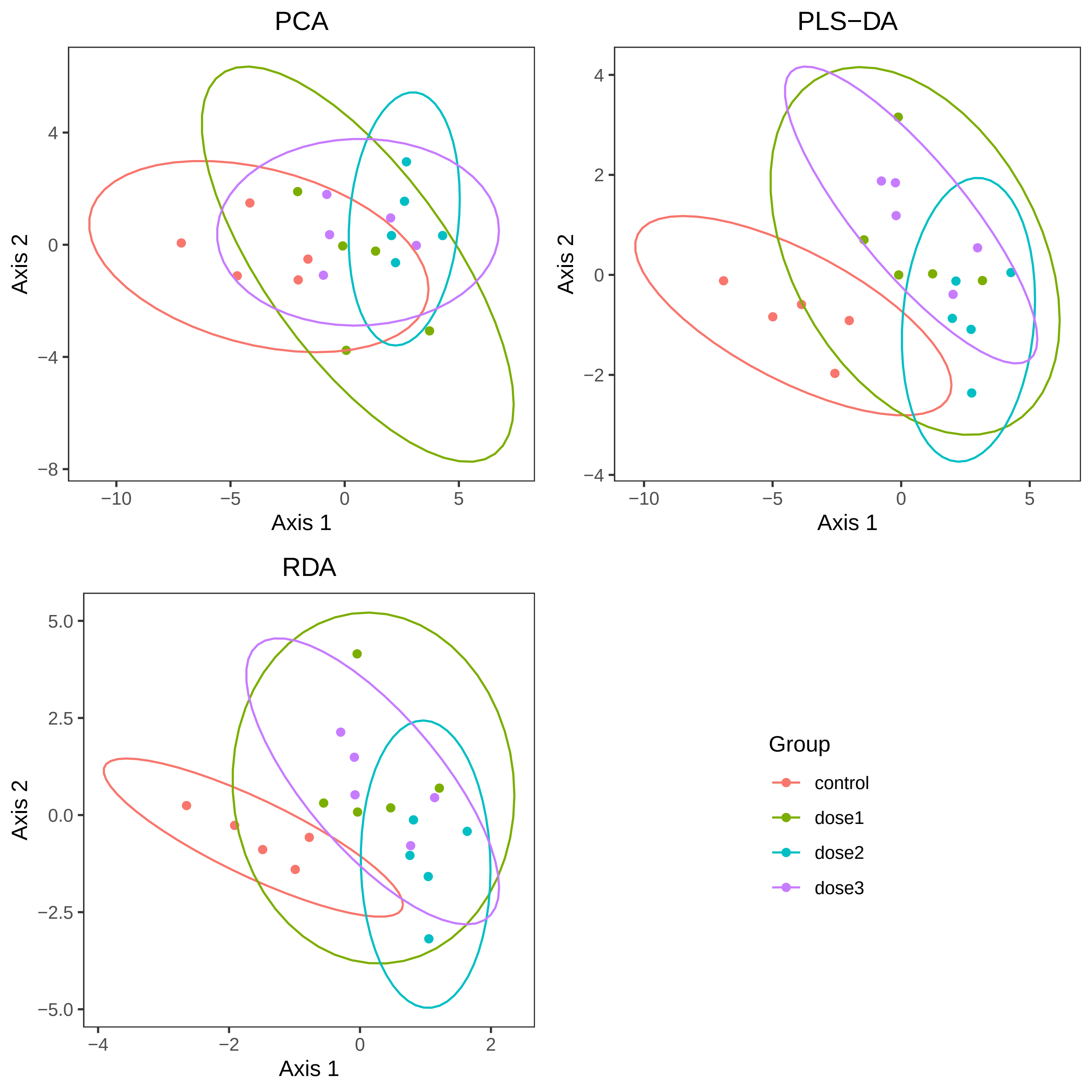
PCA
PLS−DA
RDA
Axis 1
Axis 2
Group
control
dose1
dose2
dose3

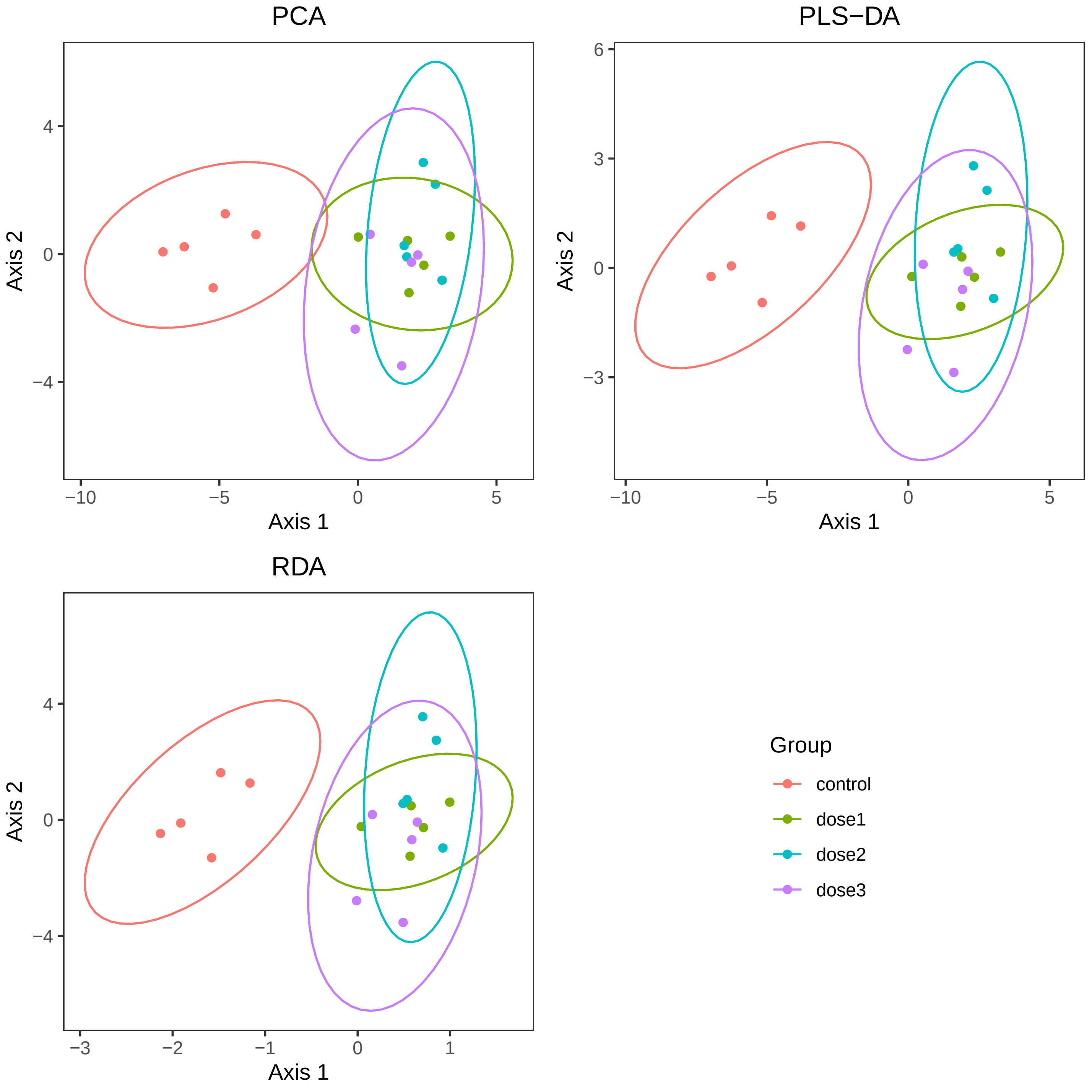
PCA
PLS−DA
RDA
Axis 1
Axis 2
−10
−5
0
5
4
−4
6
3
−3
−3
−2
−1
1
Group
control
dose1
dose2
dose3

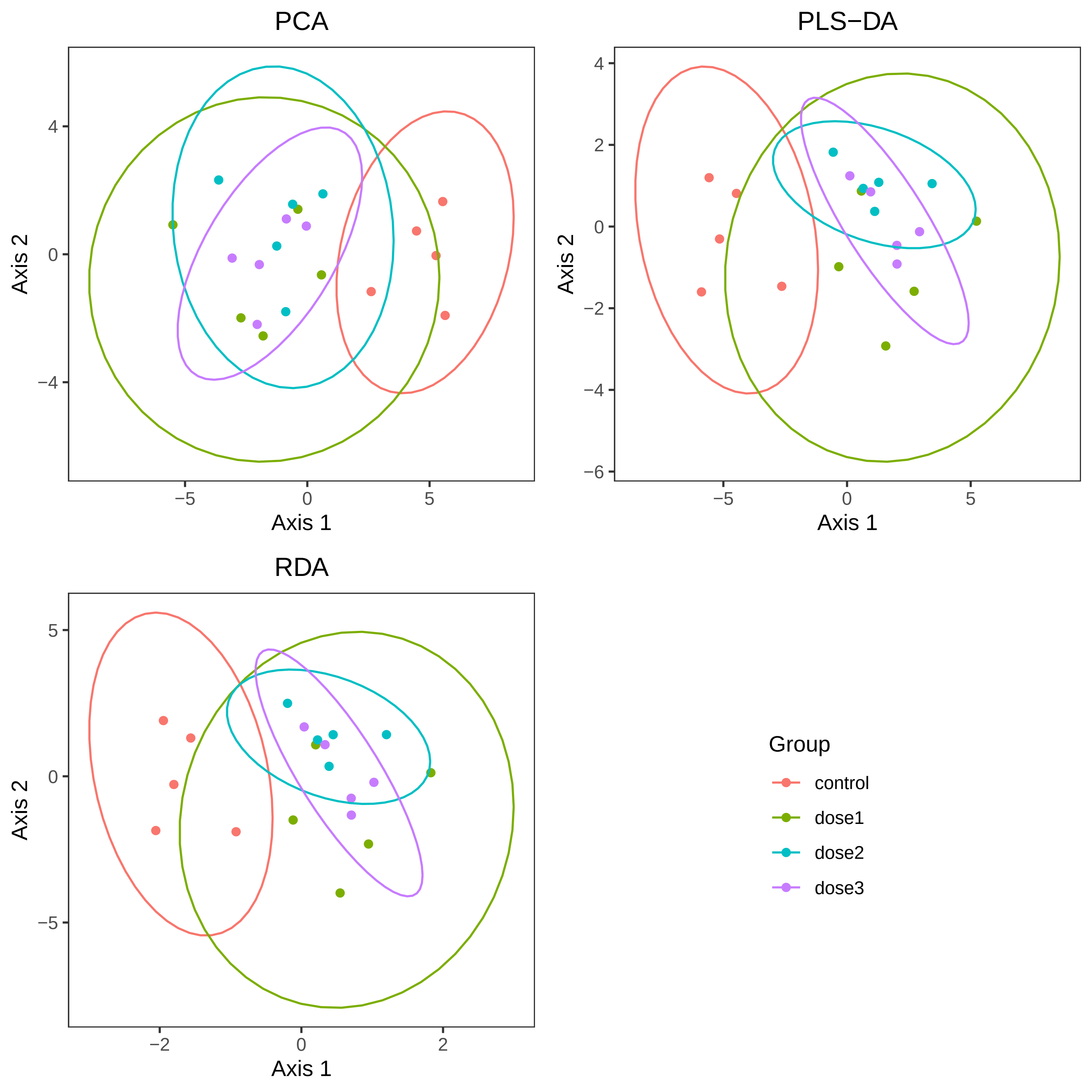
PCA
PLS−DA
RDA
Axis 1
Axis 2
Group
control
dose1
dose2
dose3

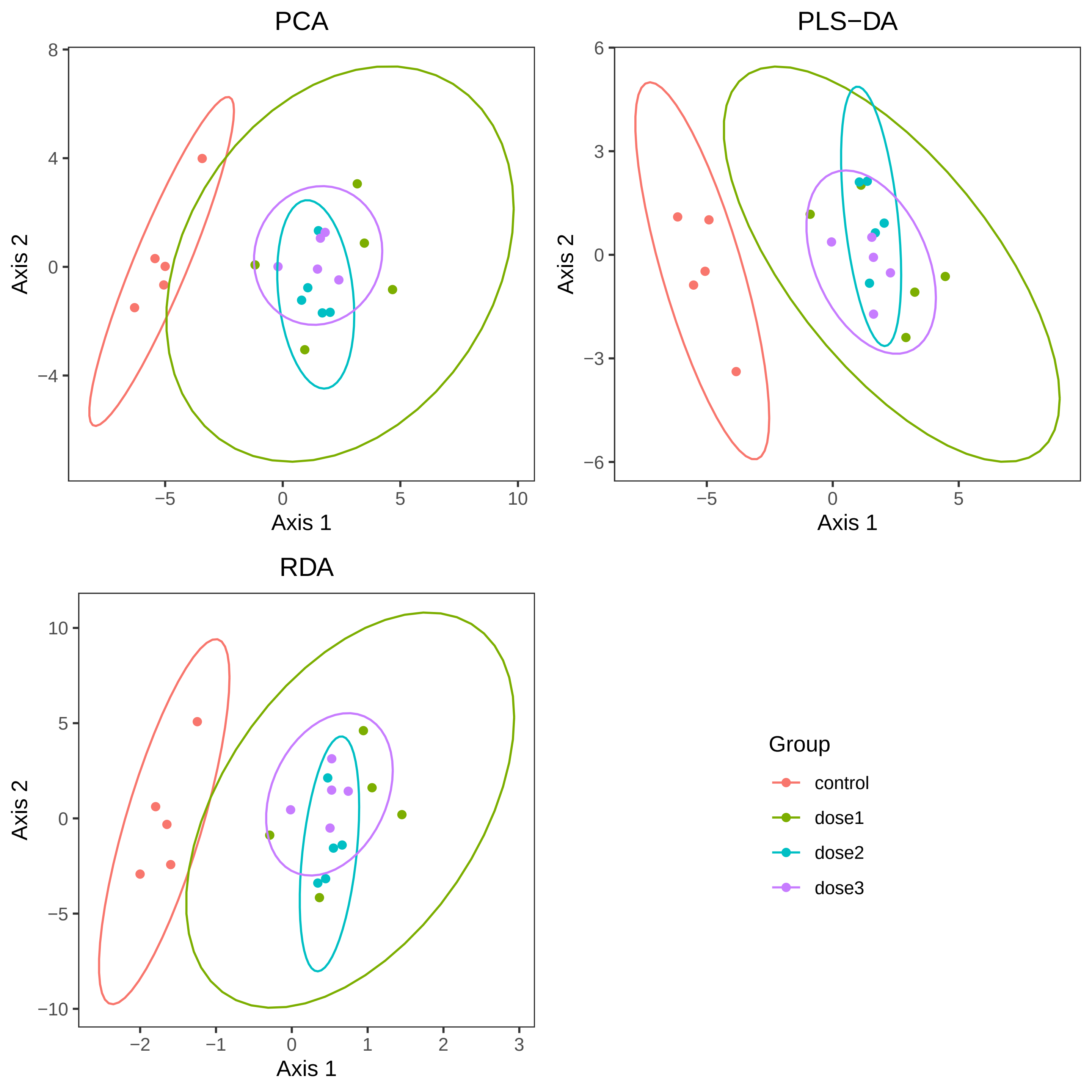

PCA
PLS−DA
RDA
Axis 1
Axis 2
Group
control
dose1
dose2
dose3

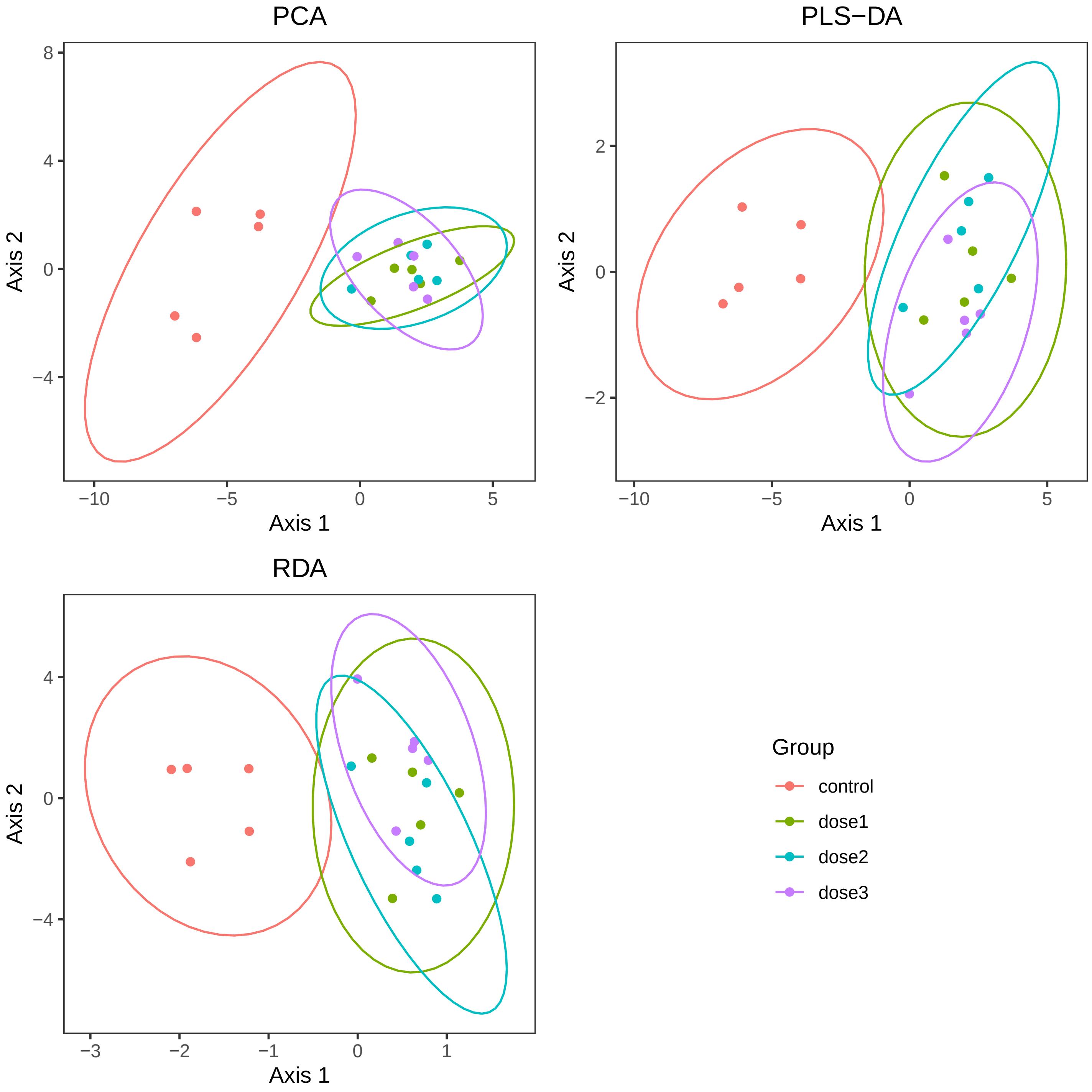
PCA
PLS−DA
RDA
Axis 1
Axis 2
8
4
0
−4
−10
−5
0
5
2
−2
−3
−2
−1
1
Group
control
dose1
dose2
dose3

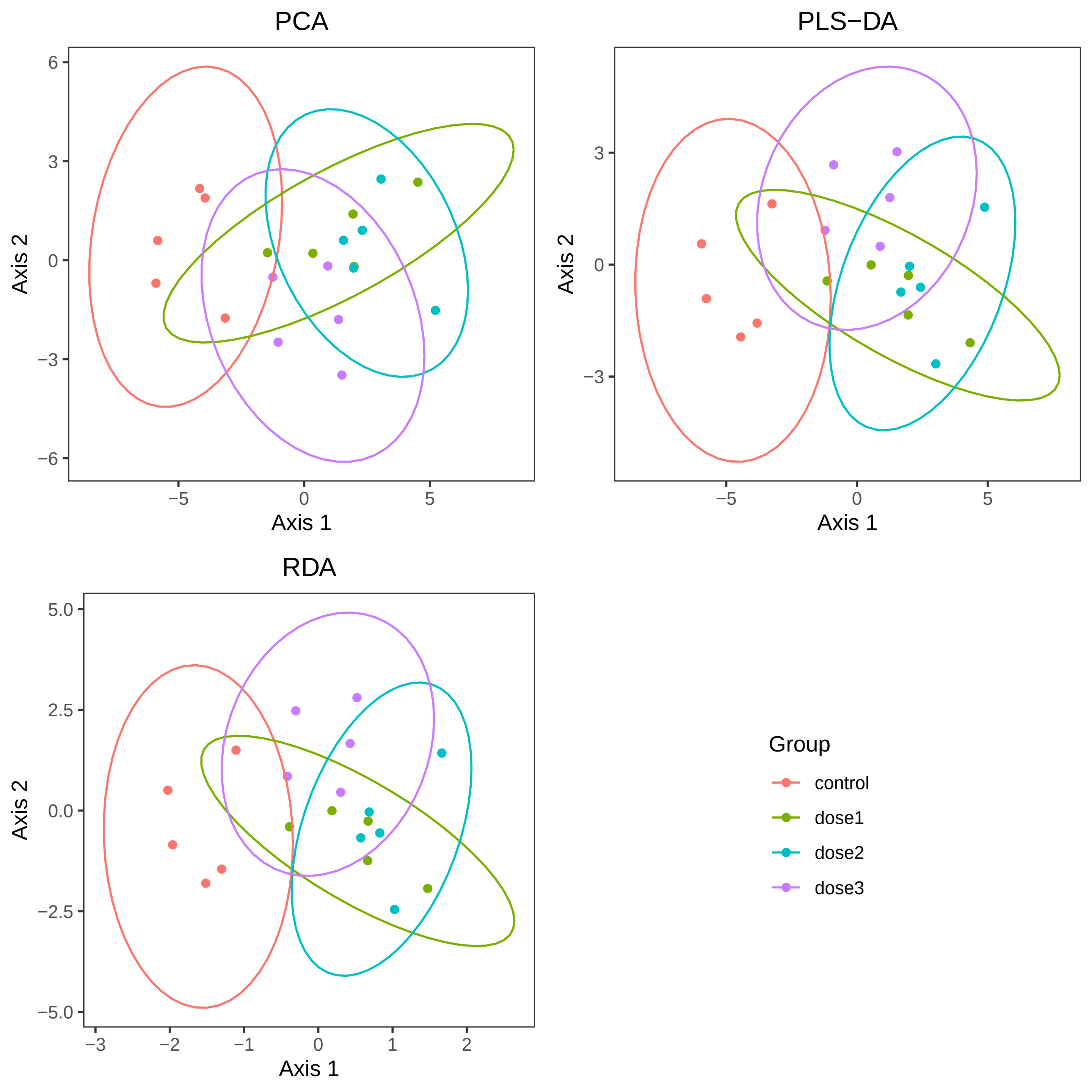
PCA
PLS−DA
RDA
Axis 1
Axis 2
Group
control
dose1
dose2
dose3

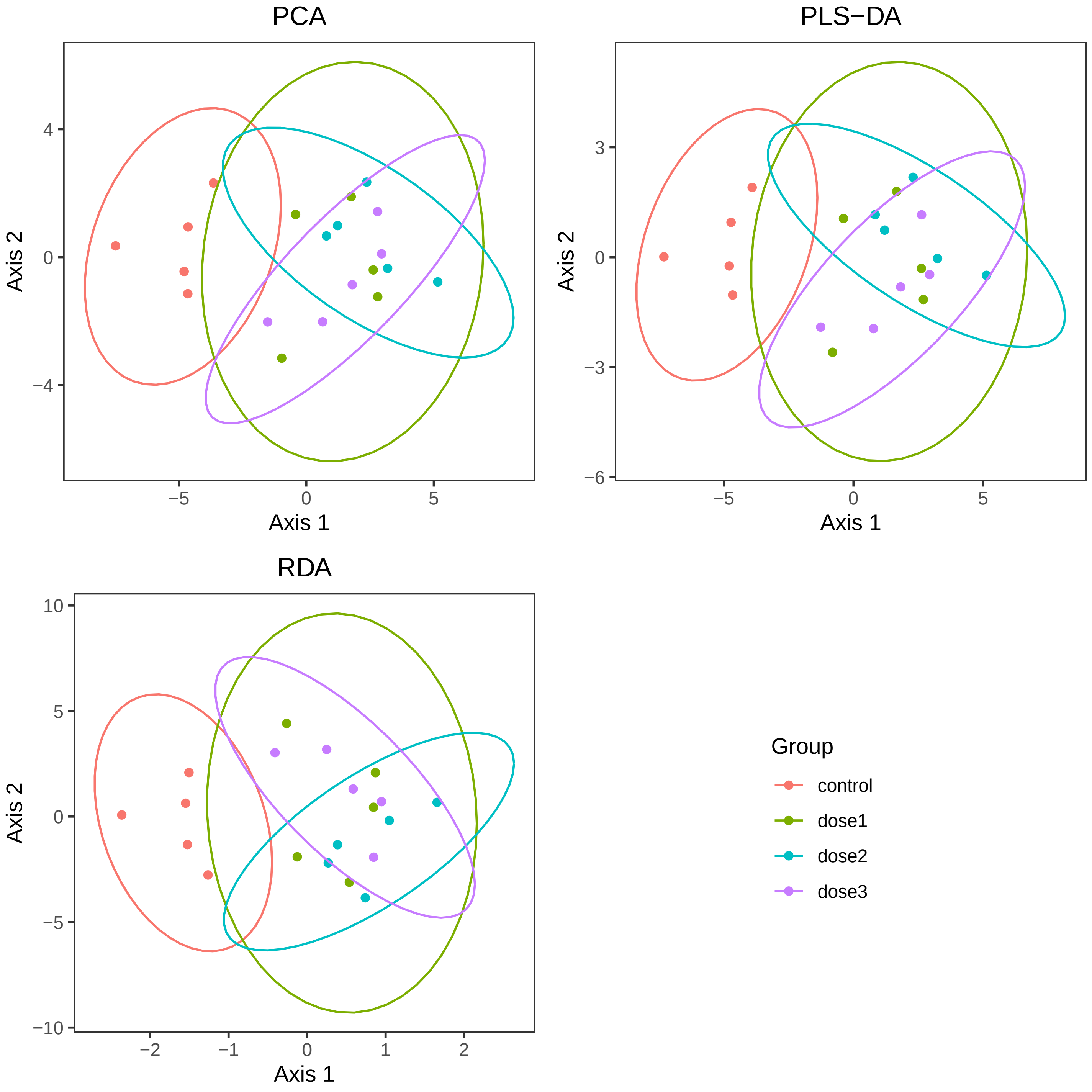

PCA
PLS−DA
RDA
Axis 1
Axis 2
Group
control
dose1
dose2
dose3

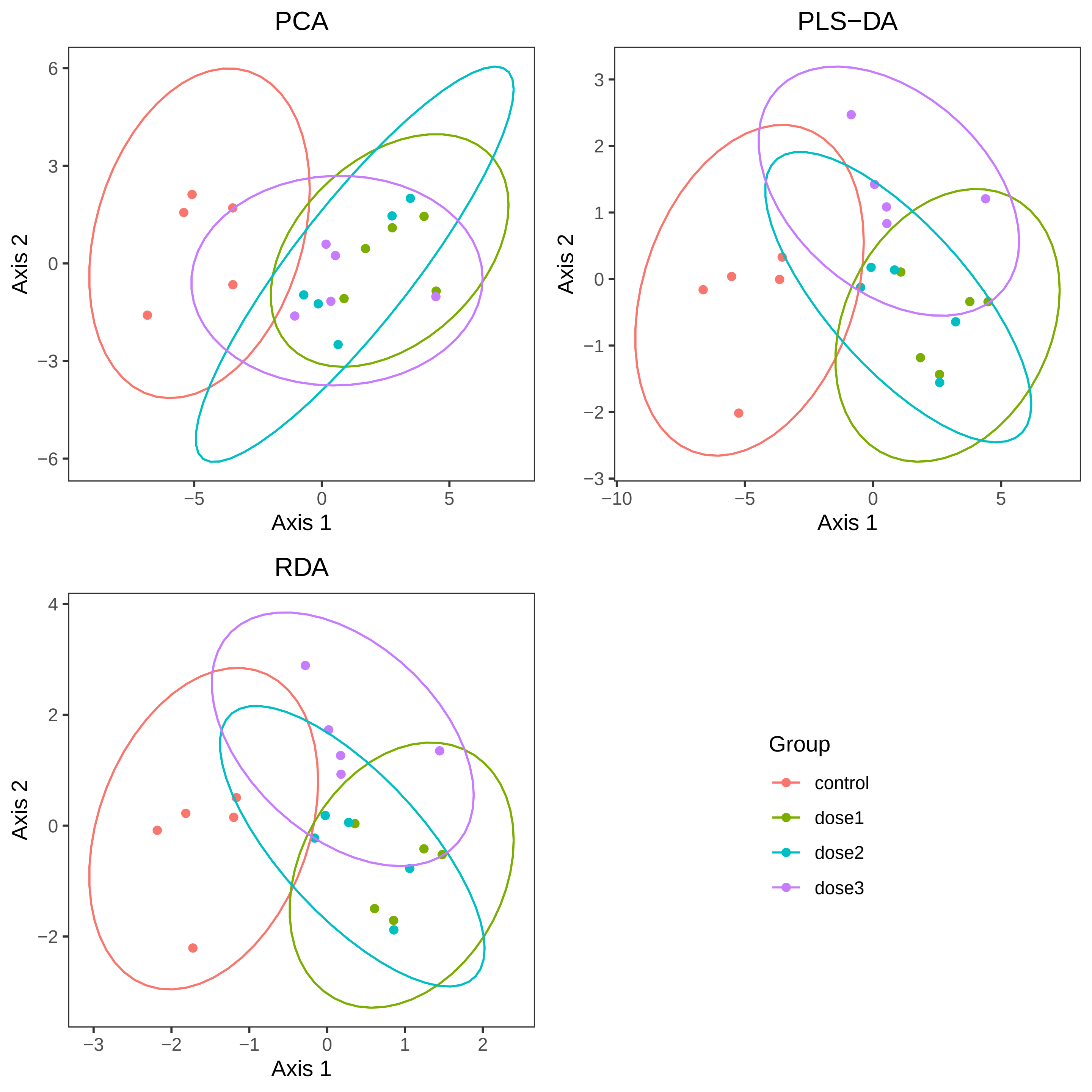

PCA
PLS−DA
RDA
Axis 1
Axis 2
Group
control
dose1
dose2
dose3

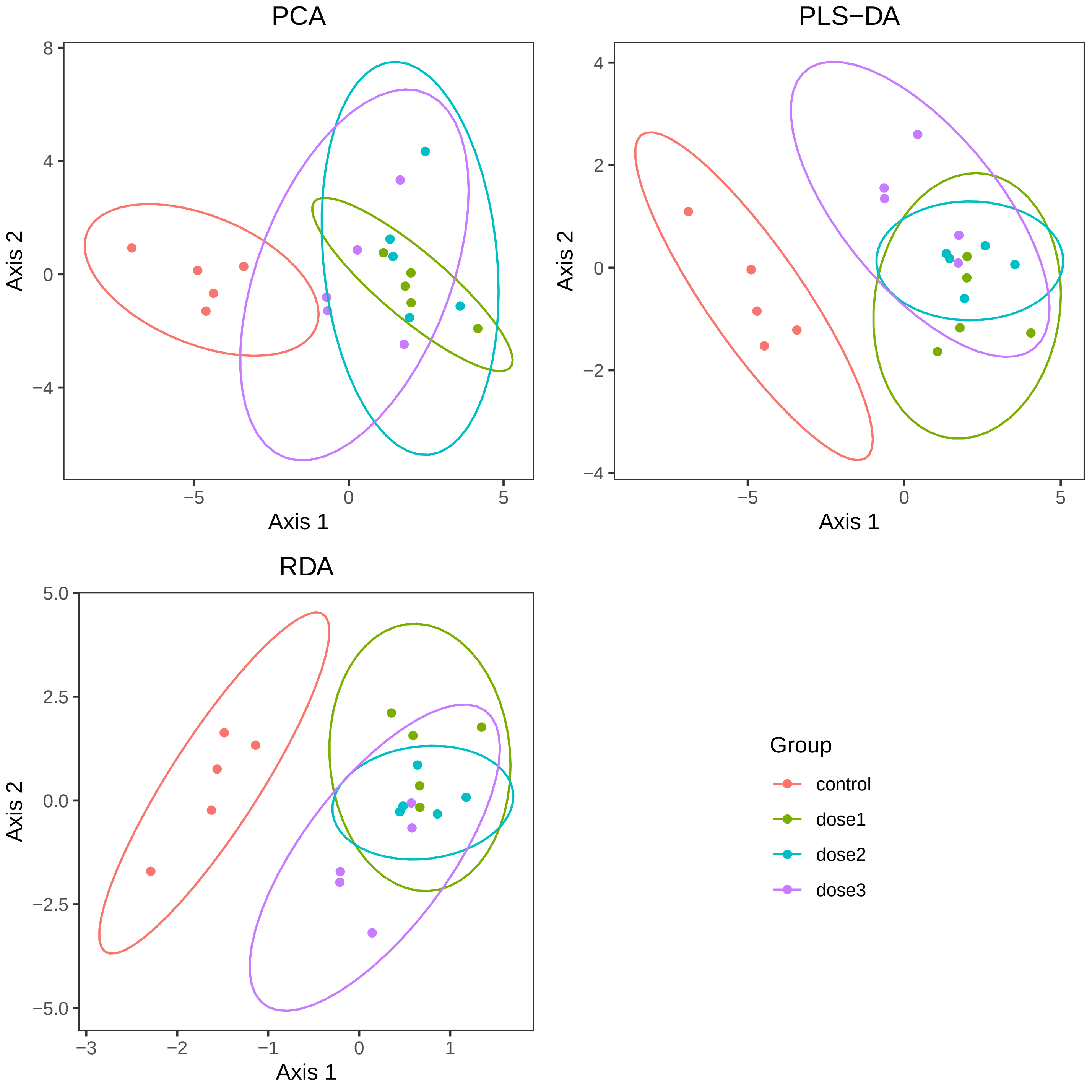

PCA
PLS−DA
RDA
Axis 1
Axis 2
Group
control
dose1
dose2
dose3

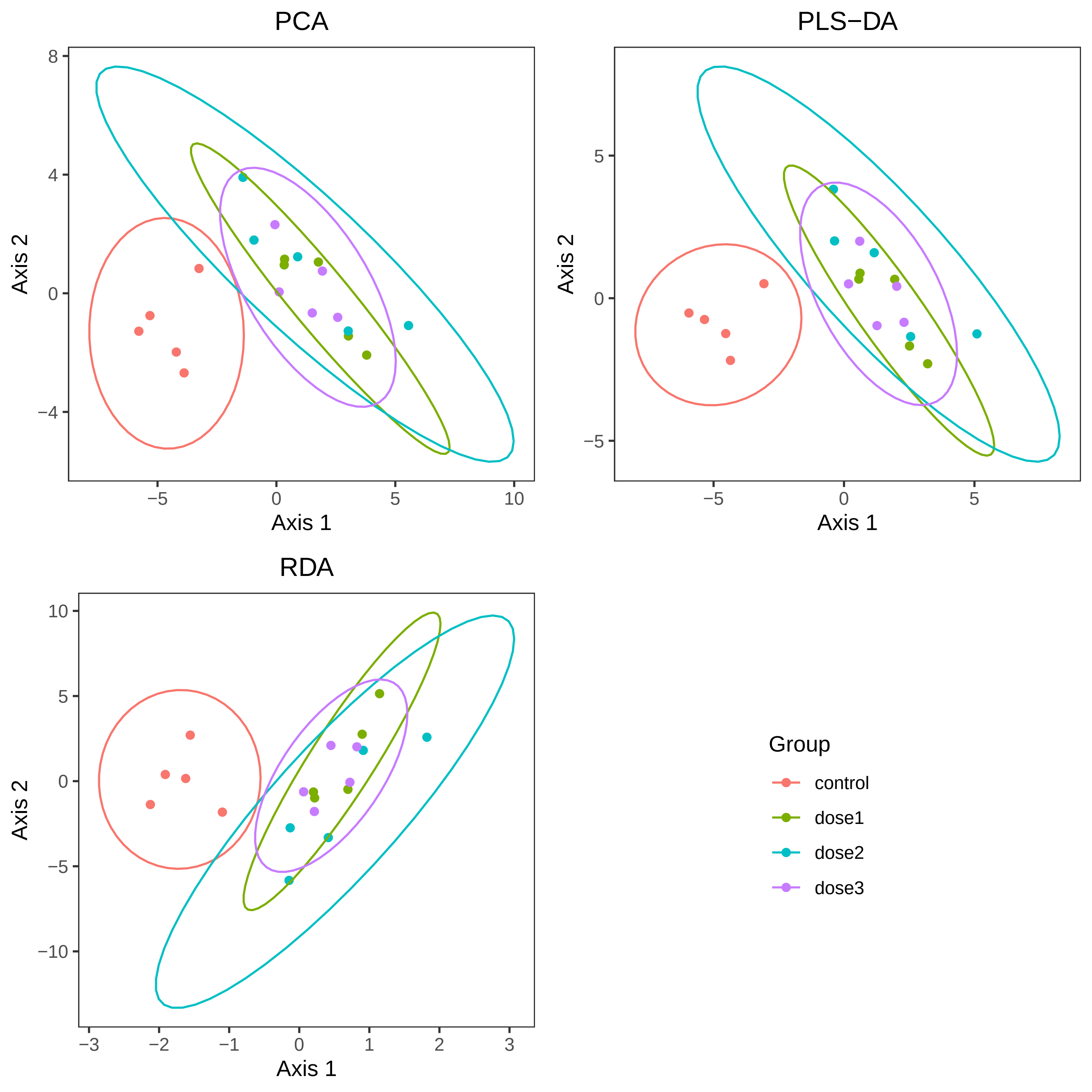
PCA
PLS−DA
RDA
Axis 1
Axis 2
Group
control
dose1
dose2
dose3

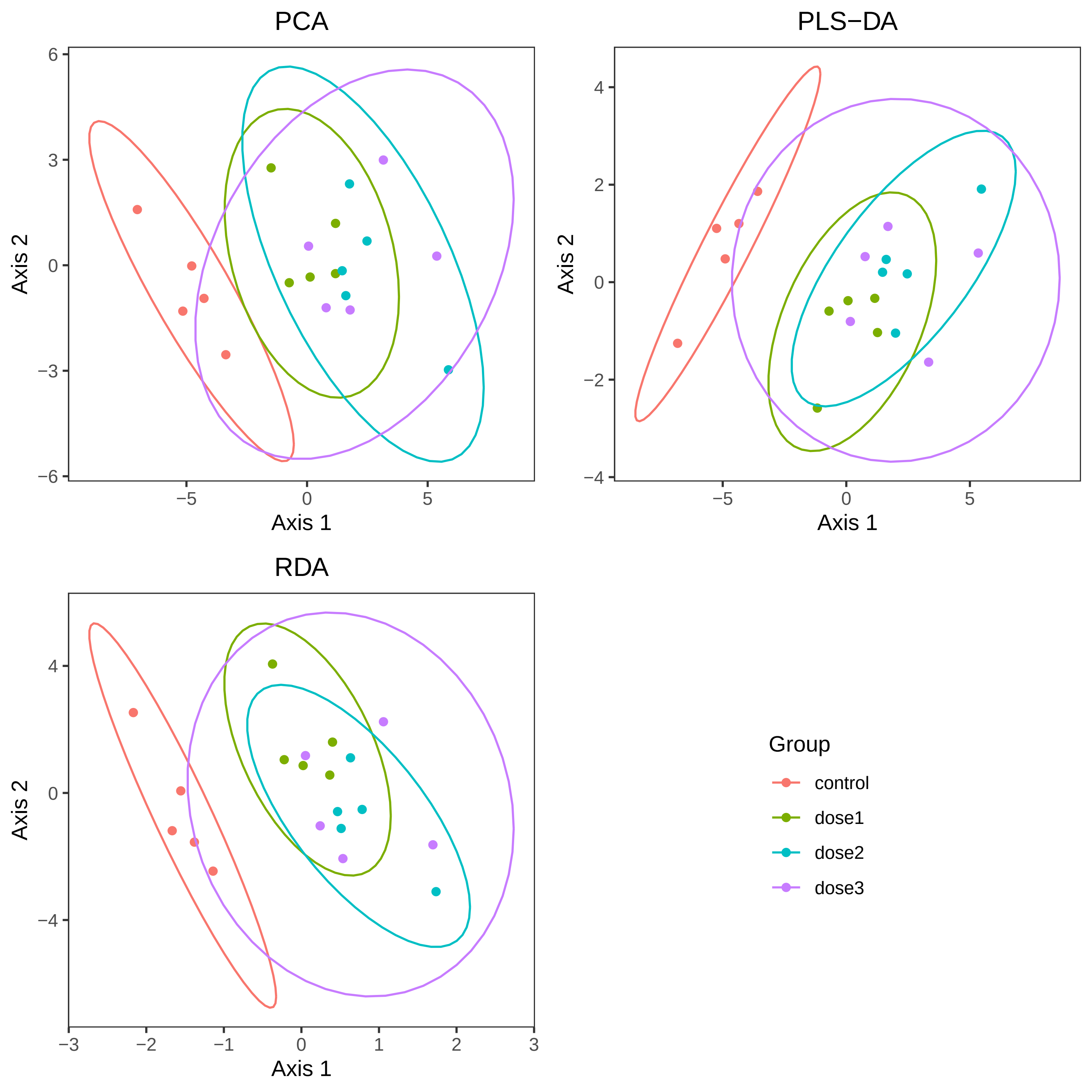
PCA
PLS−DA
RDA
Axis 1
Axis 2
Group
control
dose1
dose2
dose3

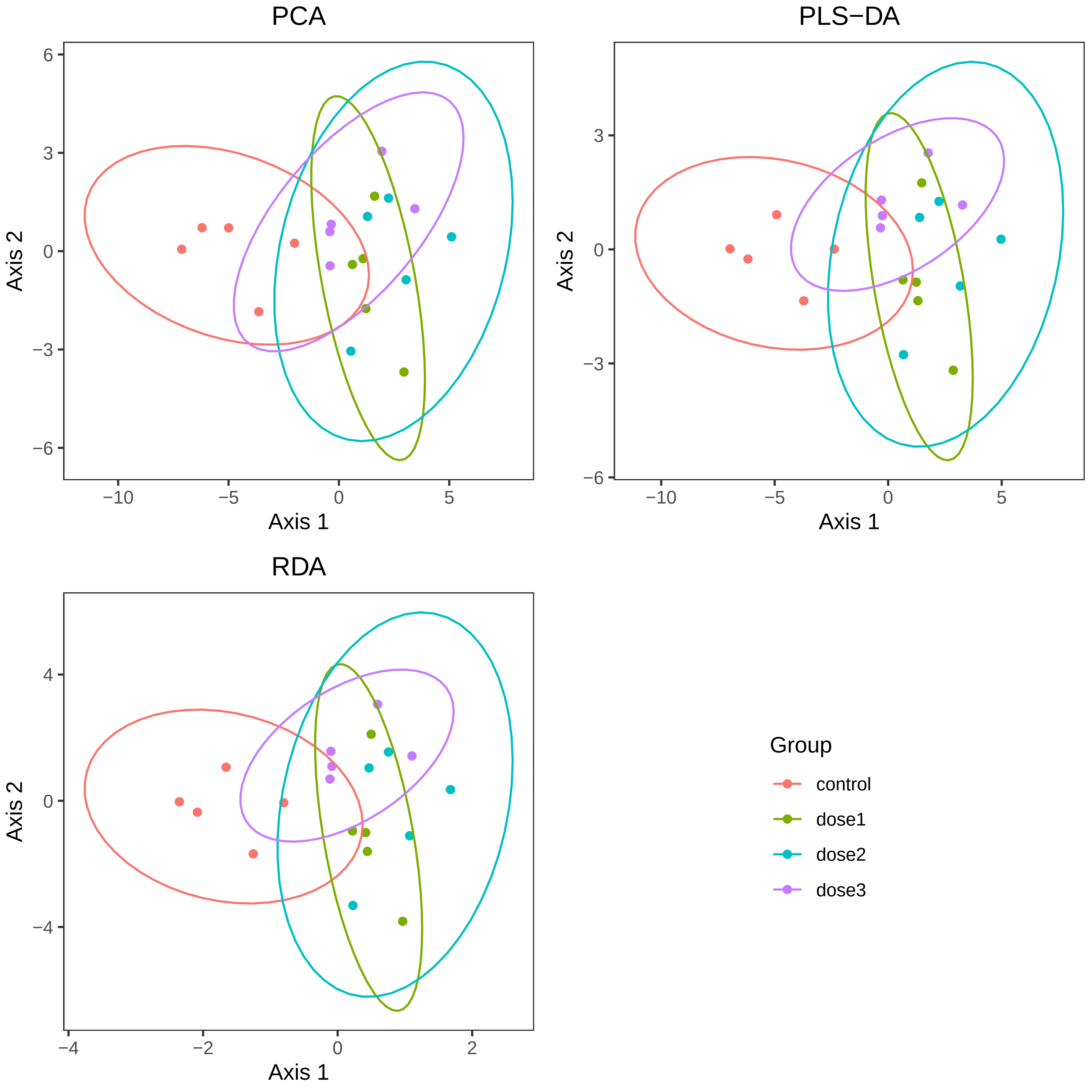

PCA
PLS−DA
RDA
Axis 1
Axis 2
Group
control
dose1
dose2
dose3

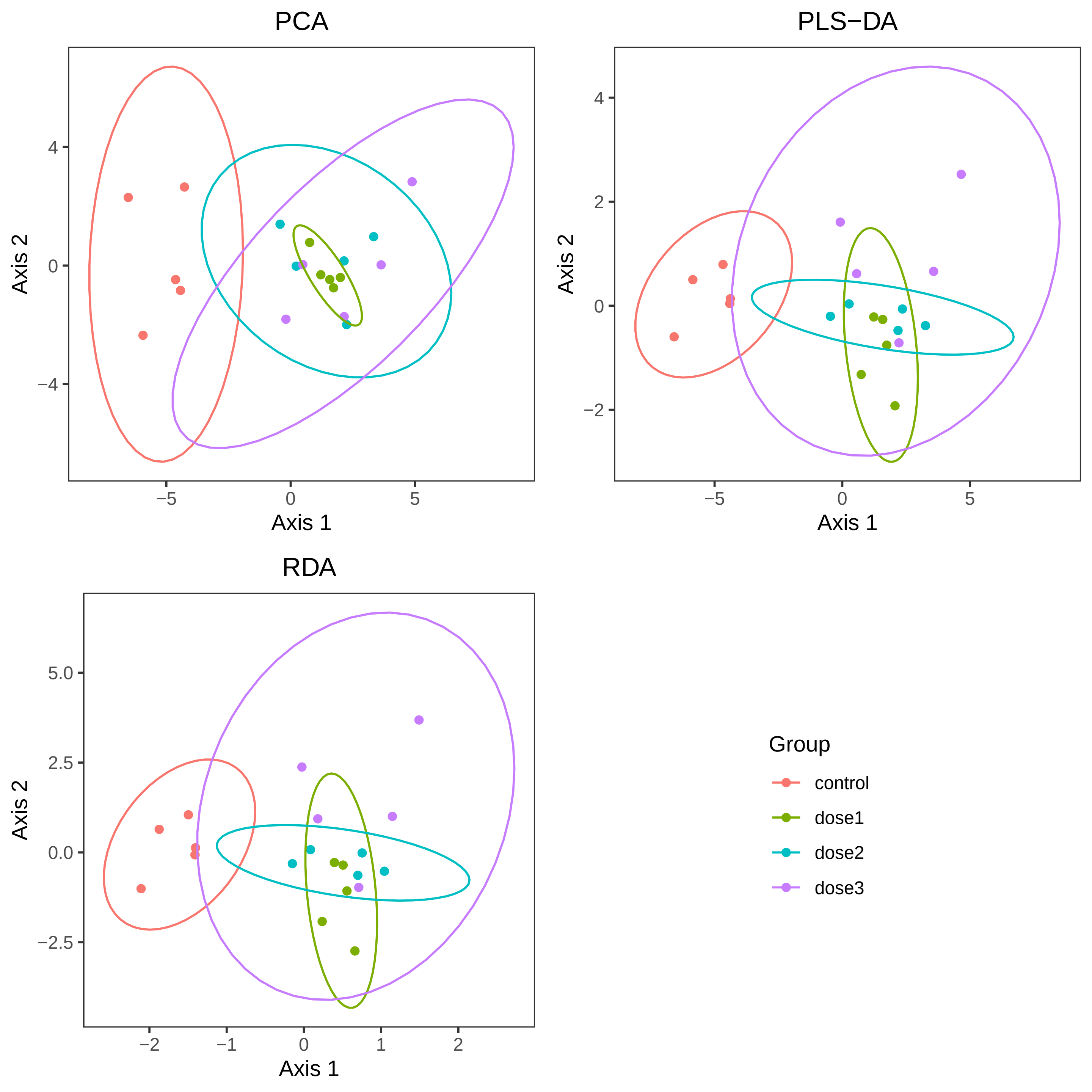

PCA
PLS−DA
RDA
Axis 1
Axis 2
Group
control
dose1
dose2
dose3

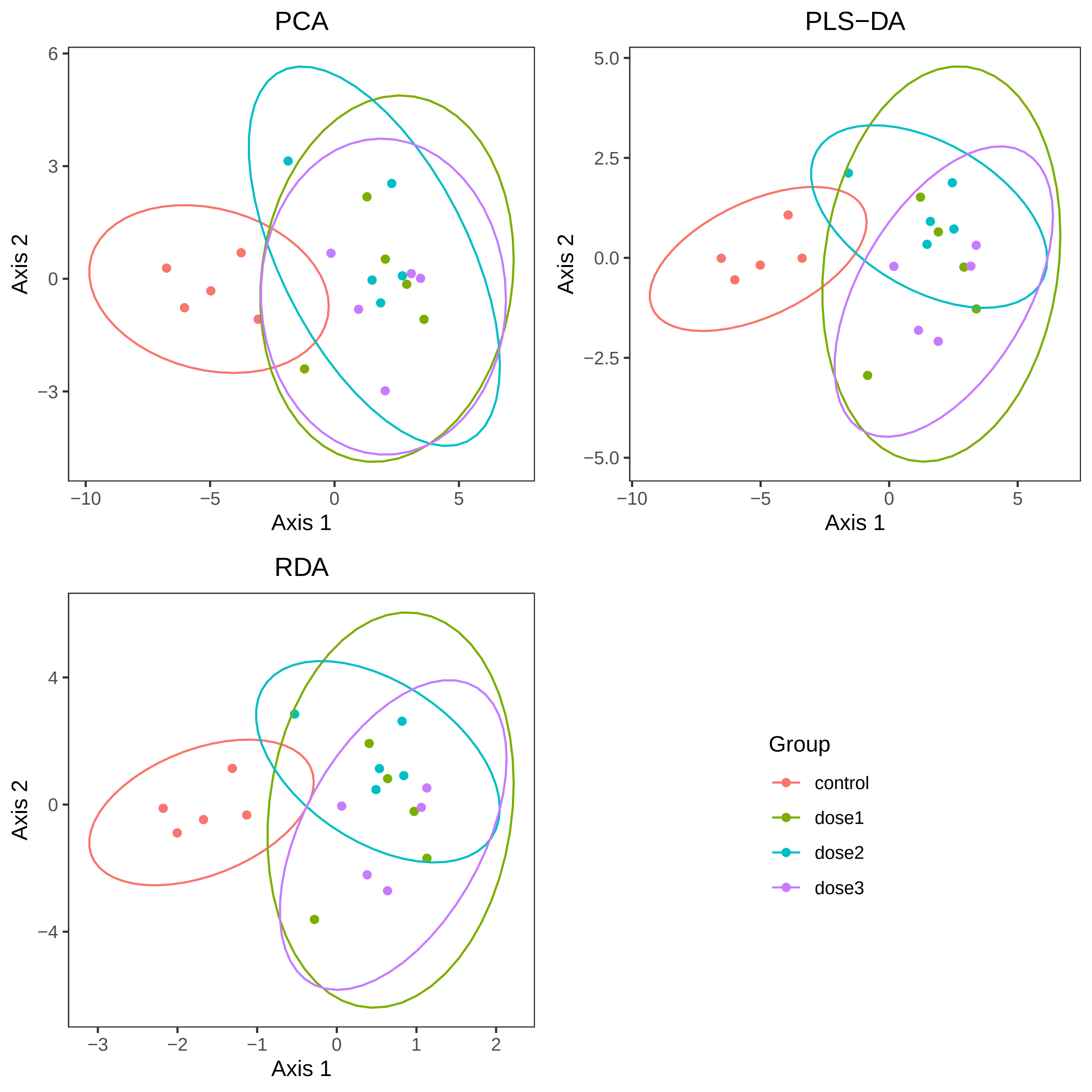
PCA
PLS−DA
RDA
Axis 1
Axis 2
Group
control
dose1
dose2
dose3

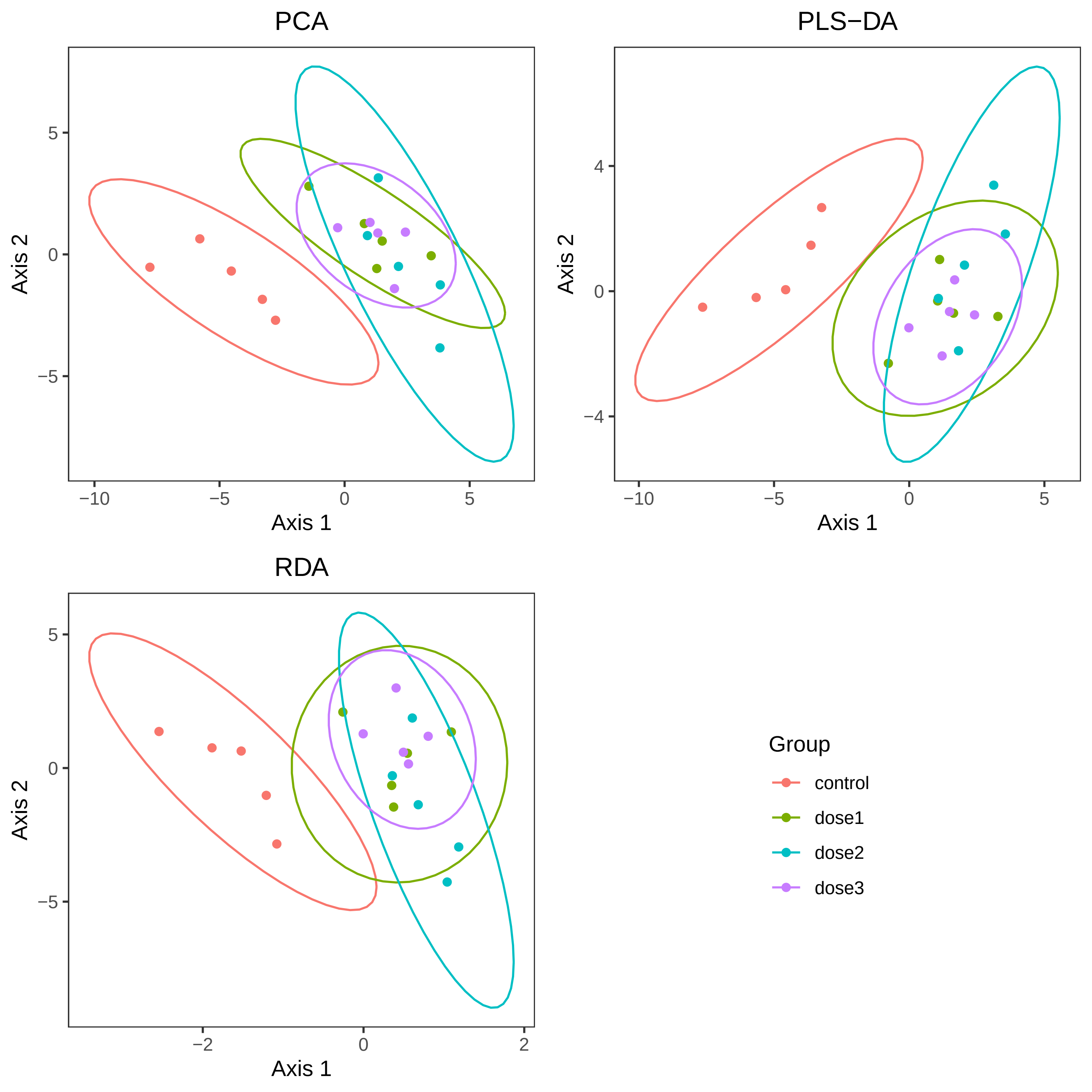

PCA
PLS−DA
RDA
Axis 1
Axis 2
Group
control
dose1
dose2
dose3

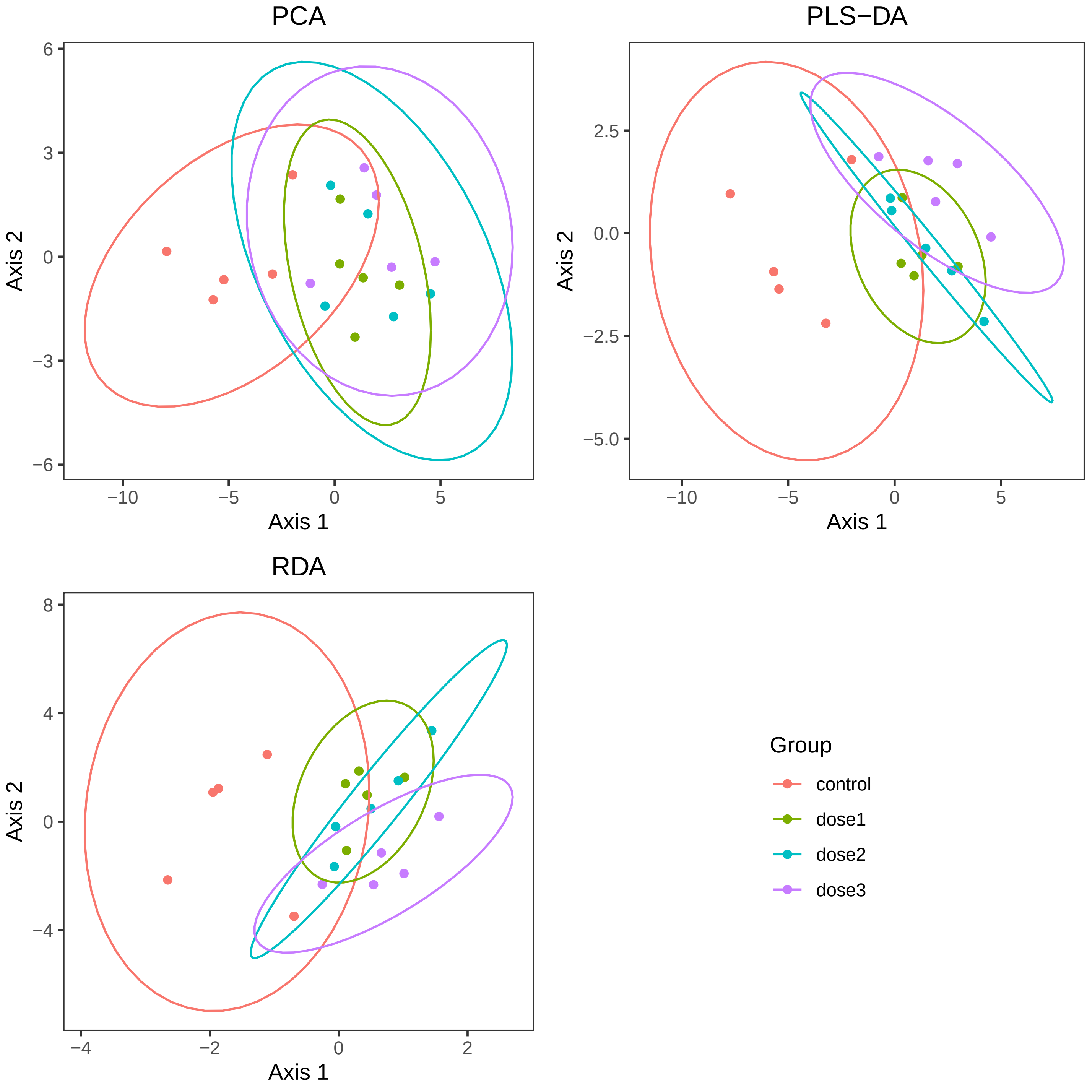

PCA
PLS−DA
RDA
Axis 1
Axis 2
Group
control
dose1
dose2
dose3

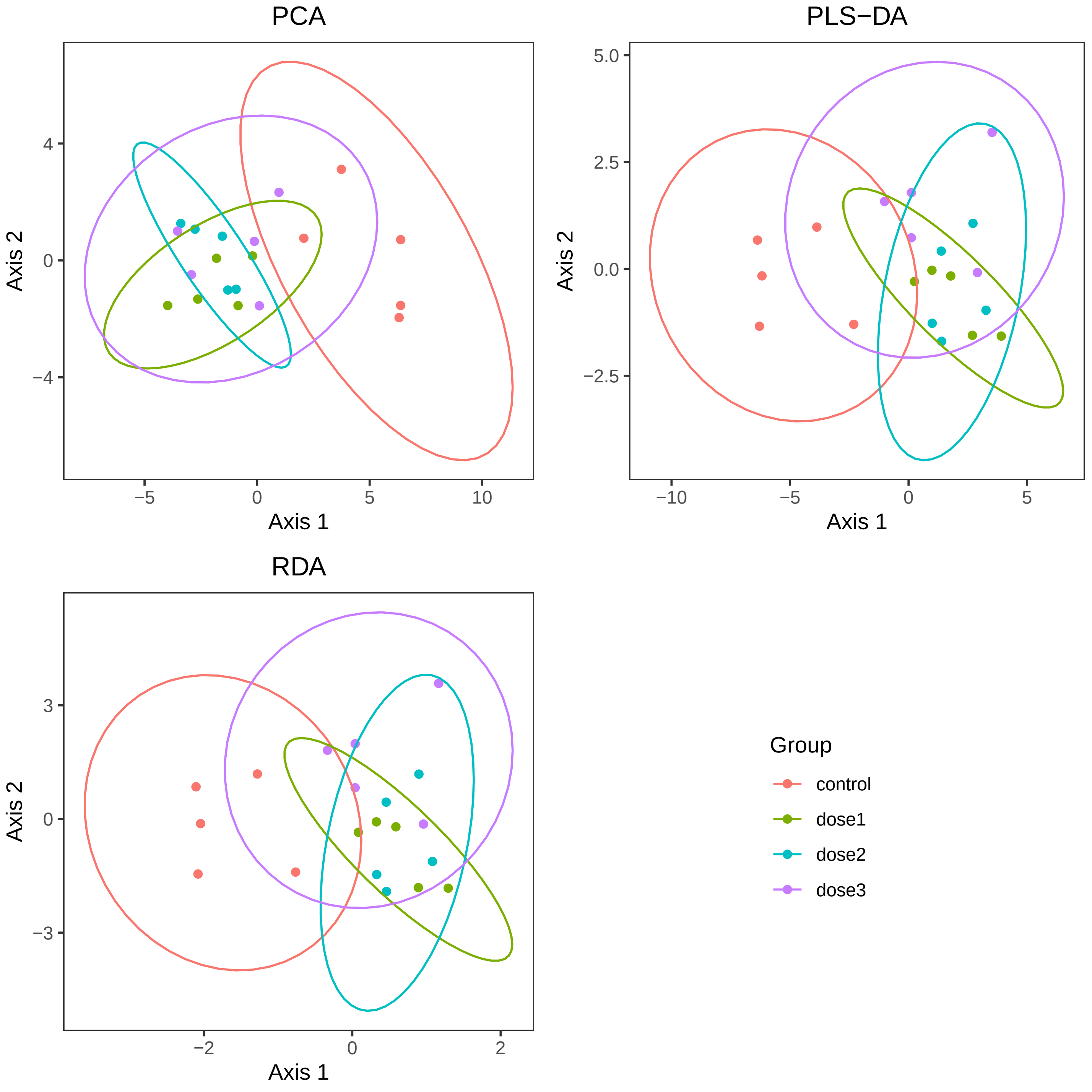

PCA
PLS−DA
RDA
Axis 1
Axis 2
−5
0
5
10
4
0
−4
−10
5.0
2.5
0.0
−2.5
−2
2
3
−3
Group
control
dose1
dose2
dose3

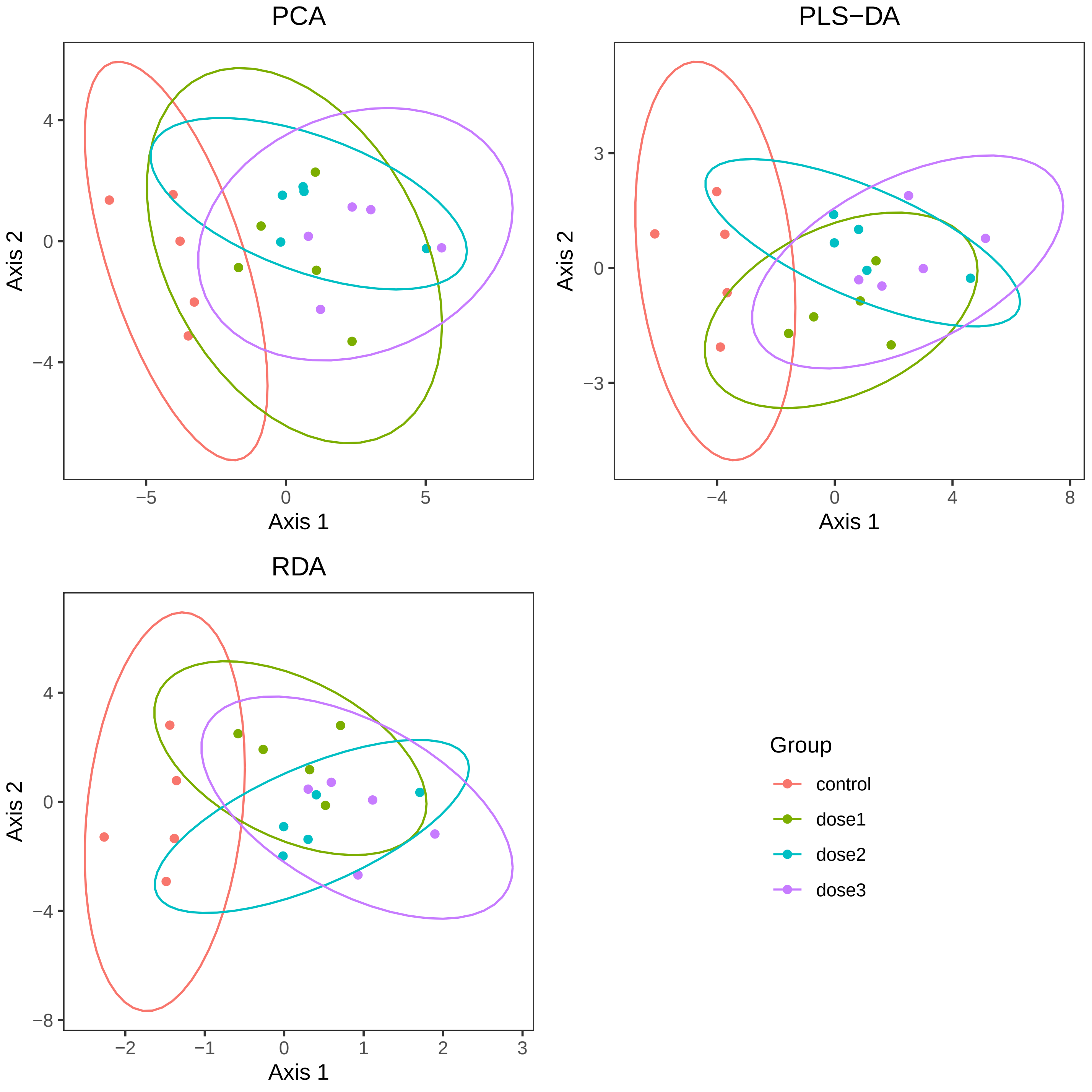
PCA
PLS−DA
RDA
Axis 1
Axis 2
Group
control
dose1
dose2
dose3

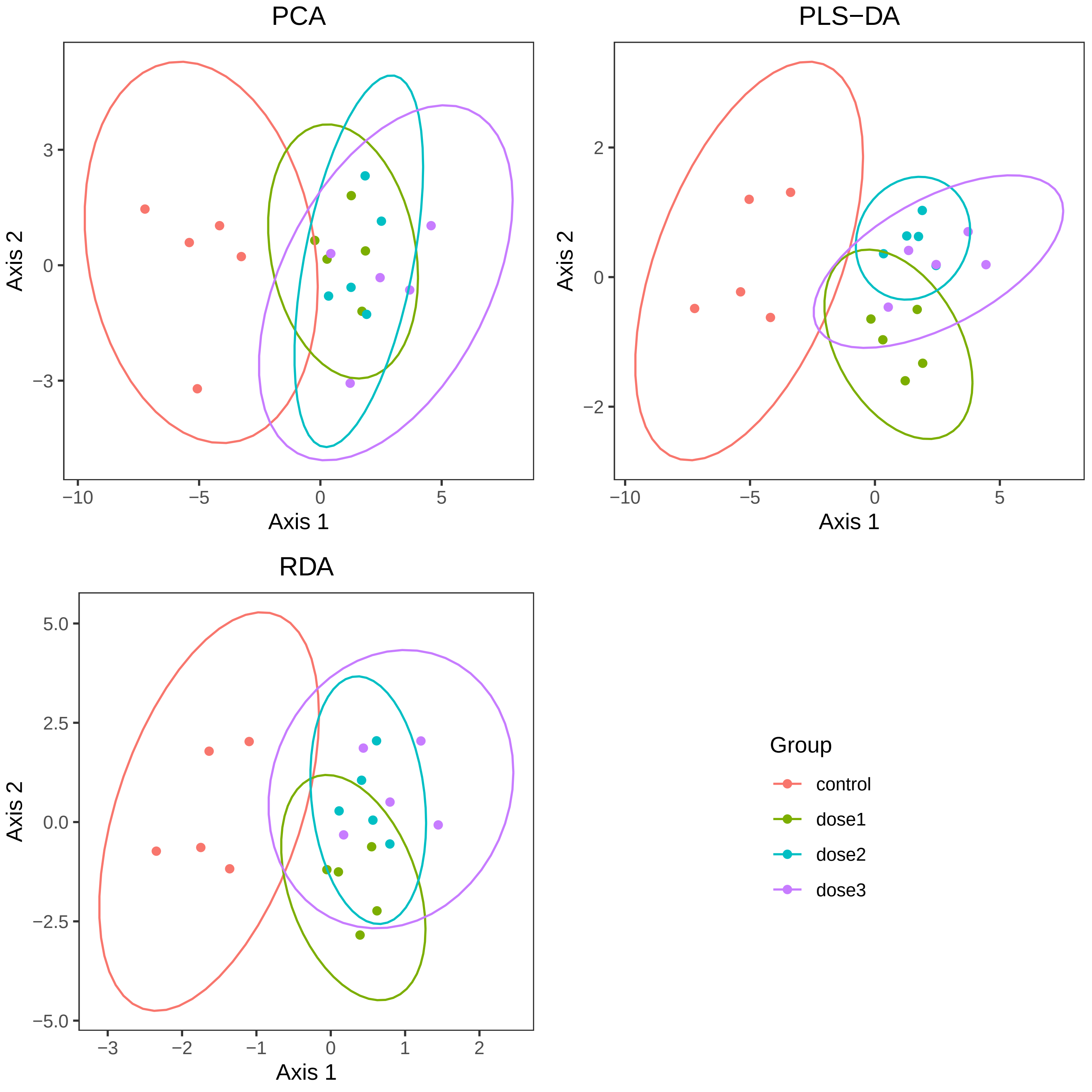

PCA
PLS−DA
RDA
Axis 1
Axis 2
Group
control
dose1
dose2
dose3

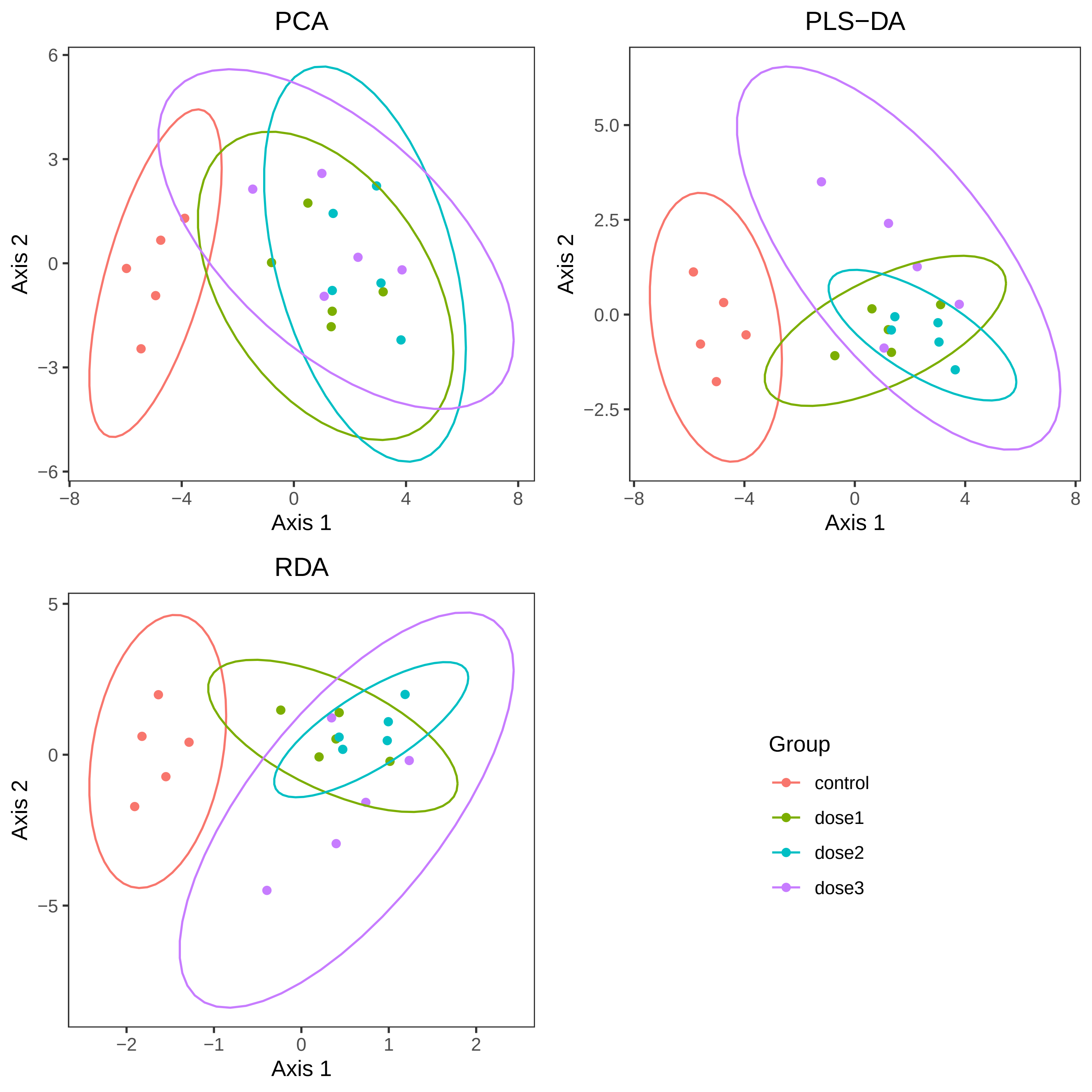

PCA
PLS−DA
RDA
Axis 1
Axis 2
6
3
0
−3
−6
−8
−4
0
4
8
5.0
2.5
0.0
−2.5
5
−5
−2
−1
1
2
Group
control
dose1
dose2
dose3

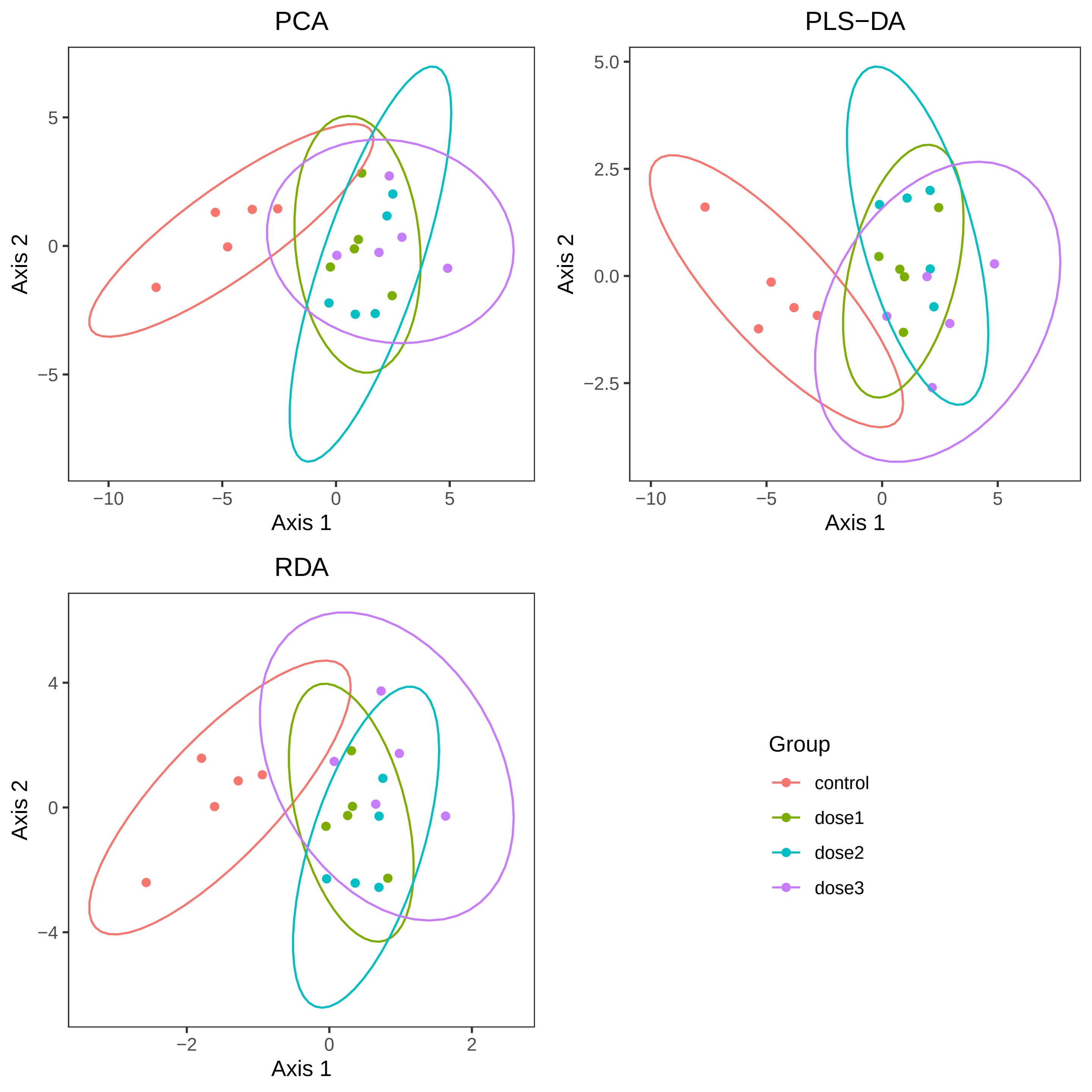

PCA
PLS−DA
RDA
Axis 1
Axis 2
Group
control
dose1
dose2
dose3

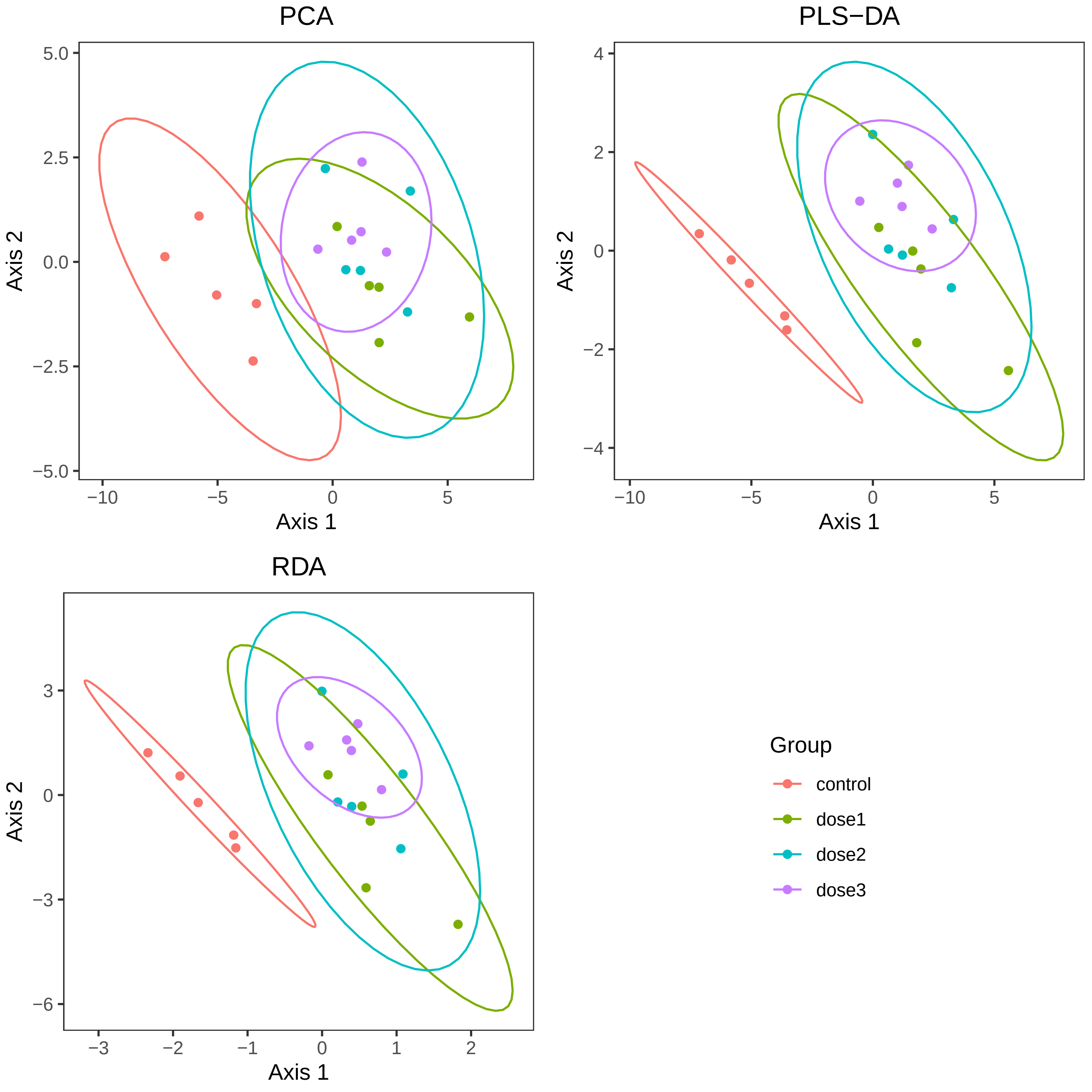

PCA
PLS−DA
RDA
Axis 1
Axis 2
Group
control
dose1
dose2
dose3

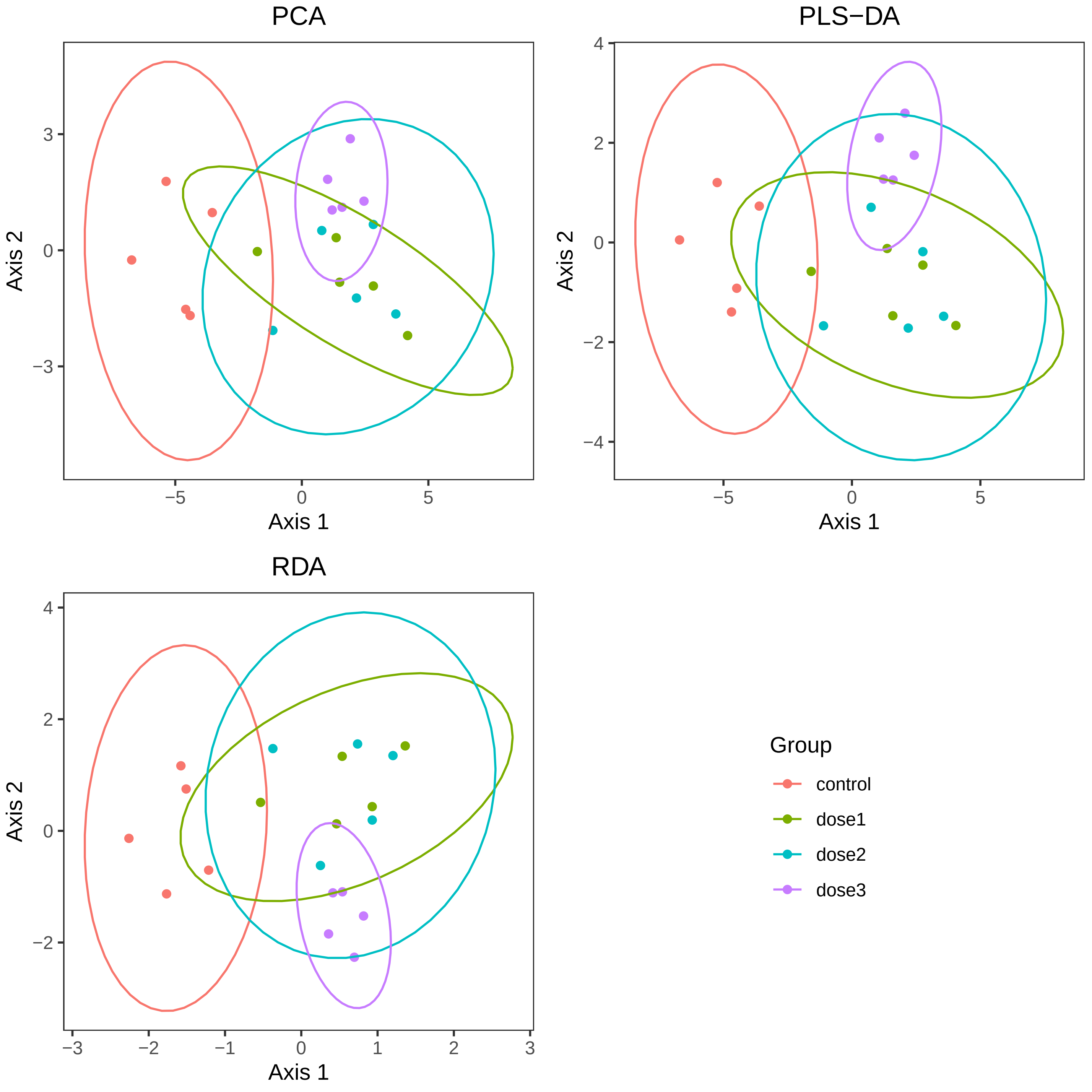
PCA
PLS−DA
RDA
Axis 1
Axis 2
Group
control
dose1
dose2
dose3

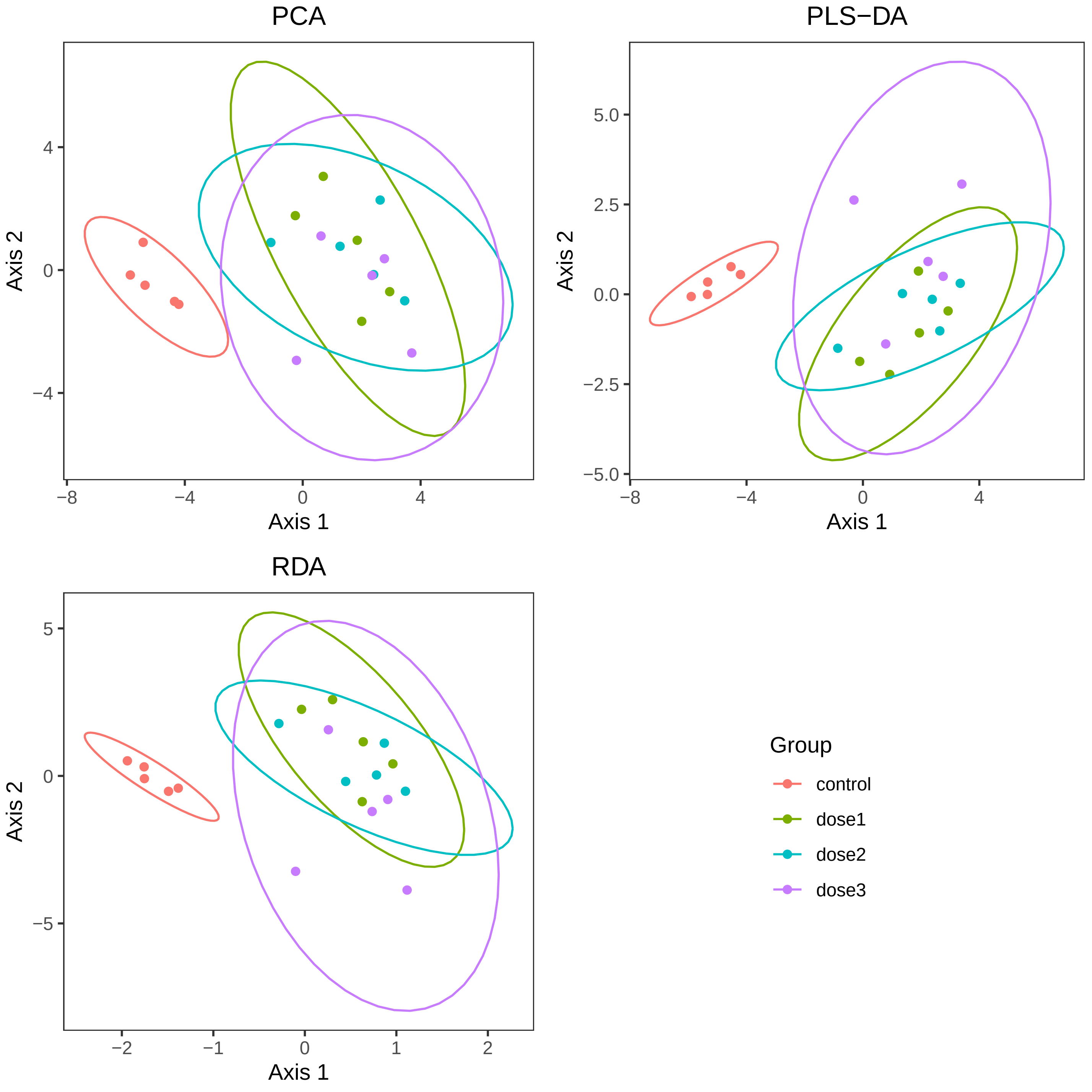
PCA
PLS−DA
RDA
Axis 1
Axis 2
Group
control
dose1
dose2
dose3
−8
−4
0
4
−2
−1
1
2
5.0
2.5
0.0
−2.5
−5.0
5
−5

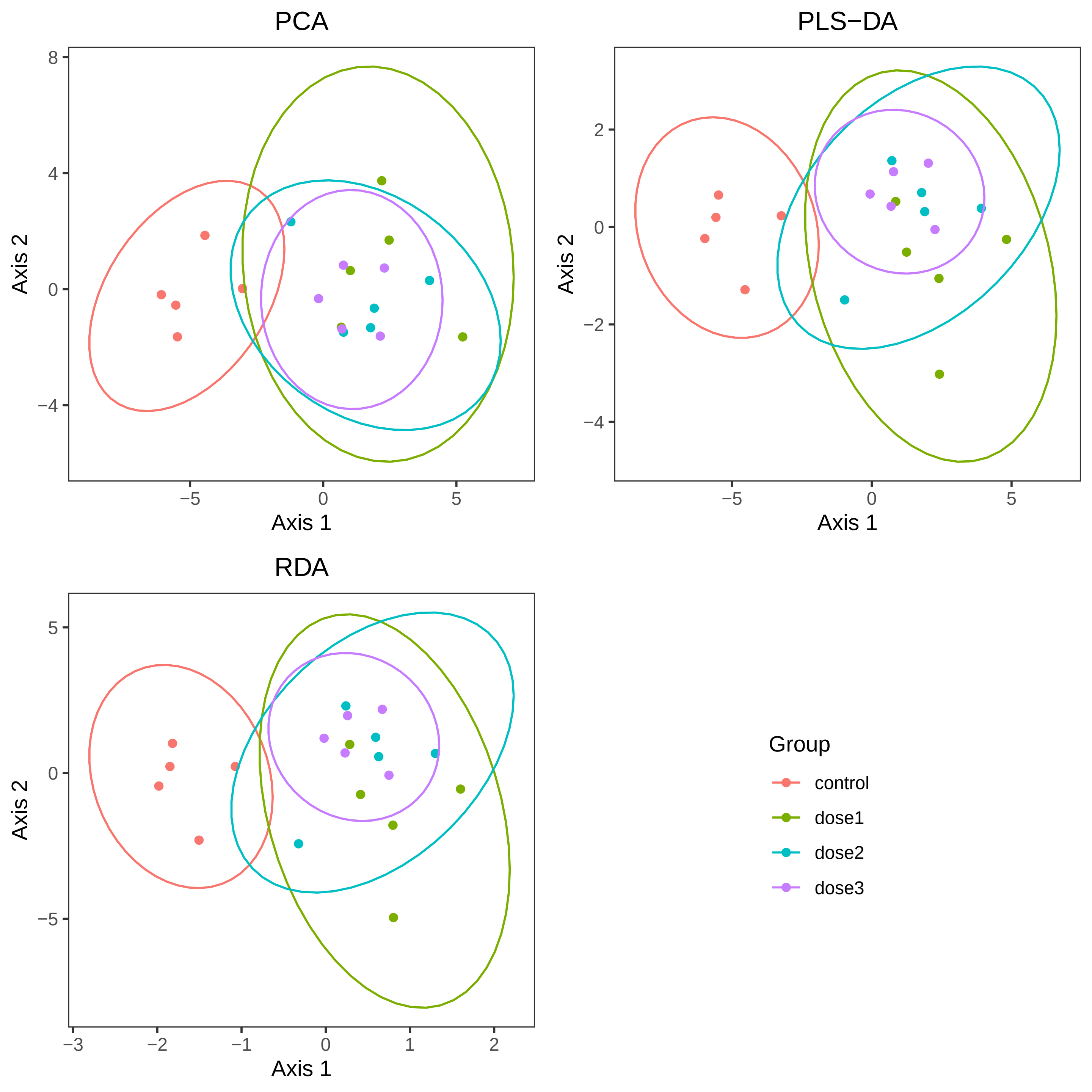
PCA
PLS−DA
RDA
Axis 1
Axis 2
Group
control
dose1
dose2
dose3

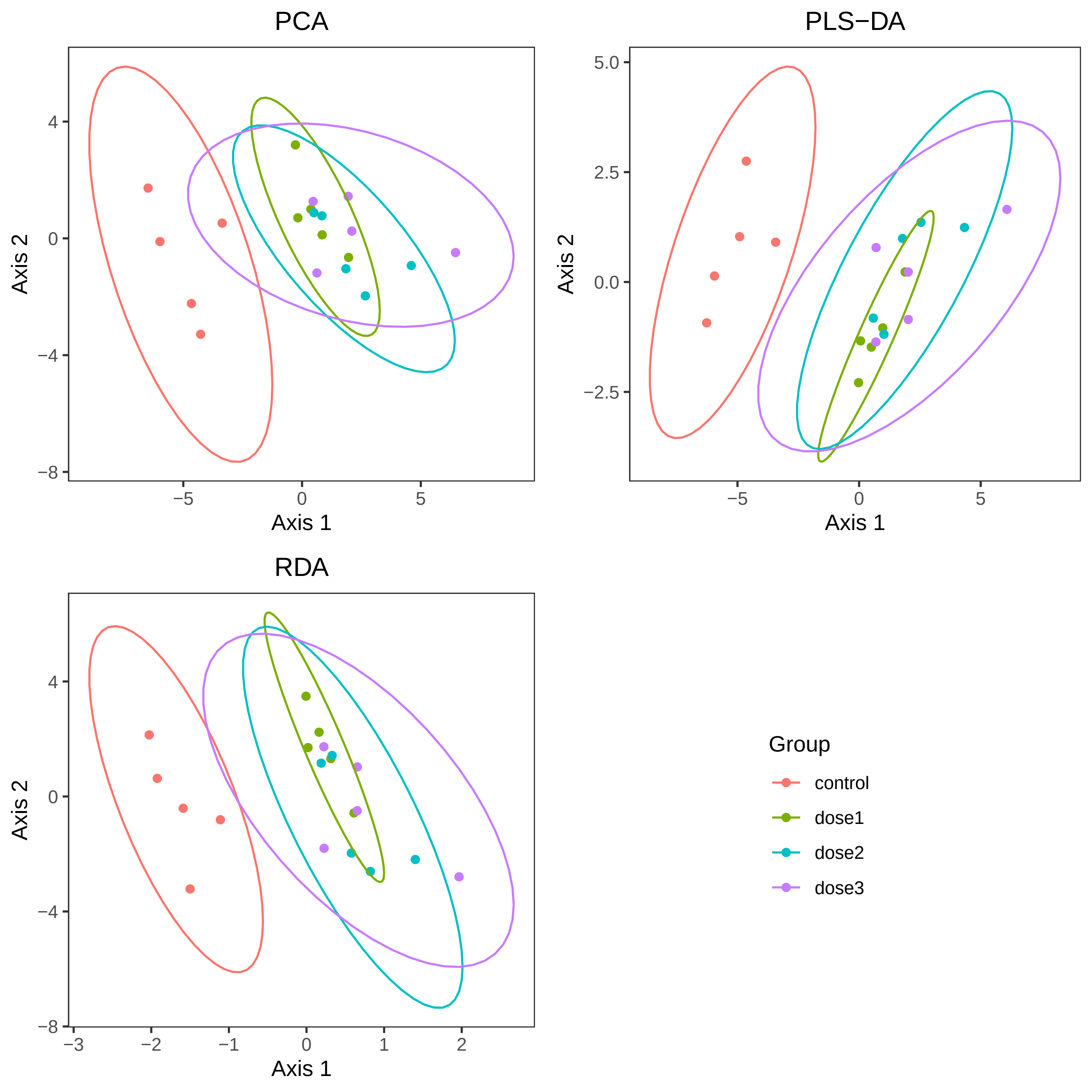
PCA
PLS−DA
RDA
Axis 1
Axis 2
4
0
−4
−8
−5
0
5
5.0
2.5
0.0
−2.5
−3
−2
−1
1
2
Group
control
dose1
dose2
dose3

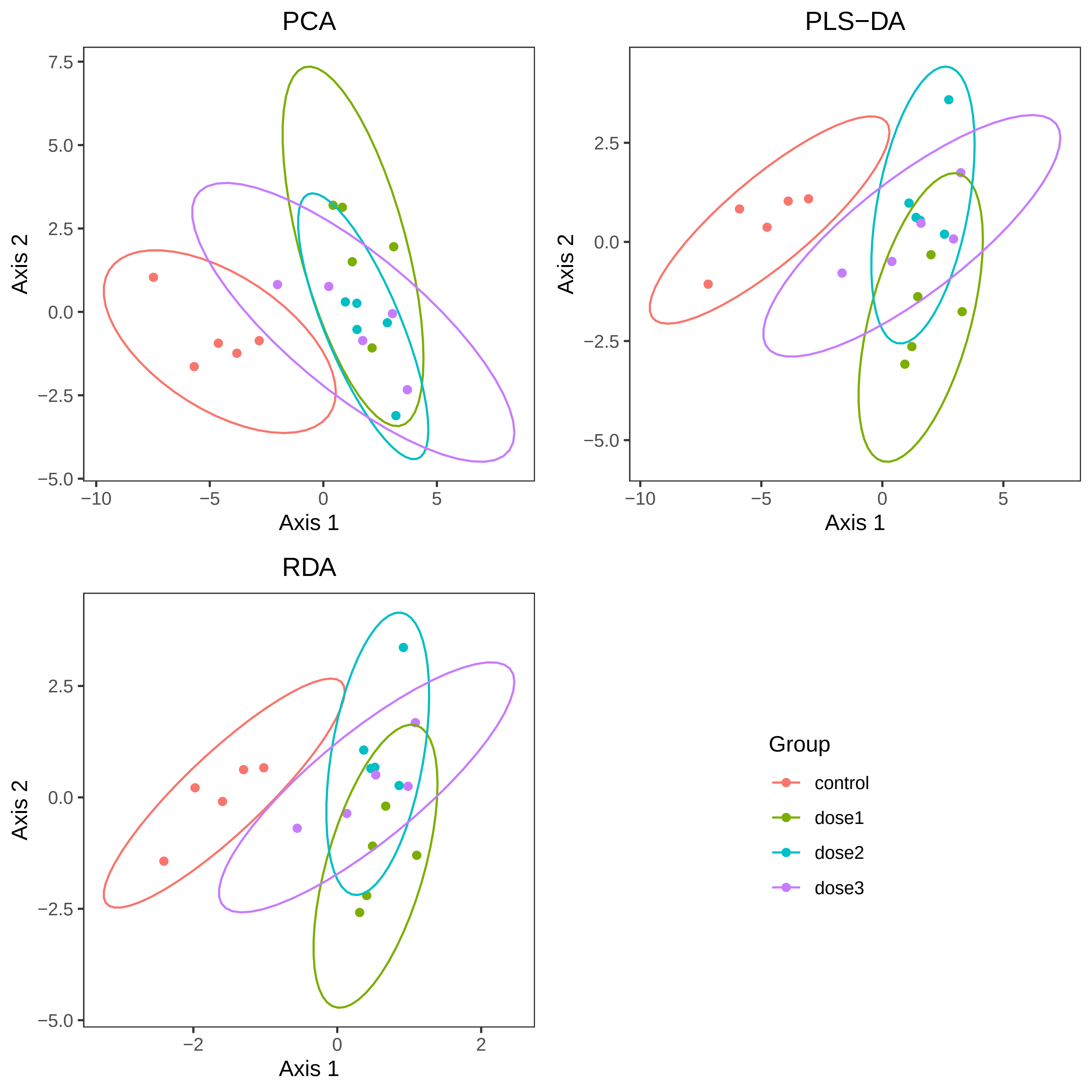

PCA
PLS−DA
RDA
Axis 1
Axis 2
7.5
5.0
2.5
0.0
−2.5
−5.0
−10
−5
0
5
−2
2
Group
control
dose1
dose2
dose3

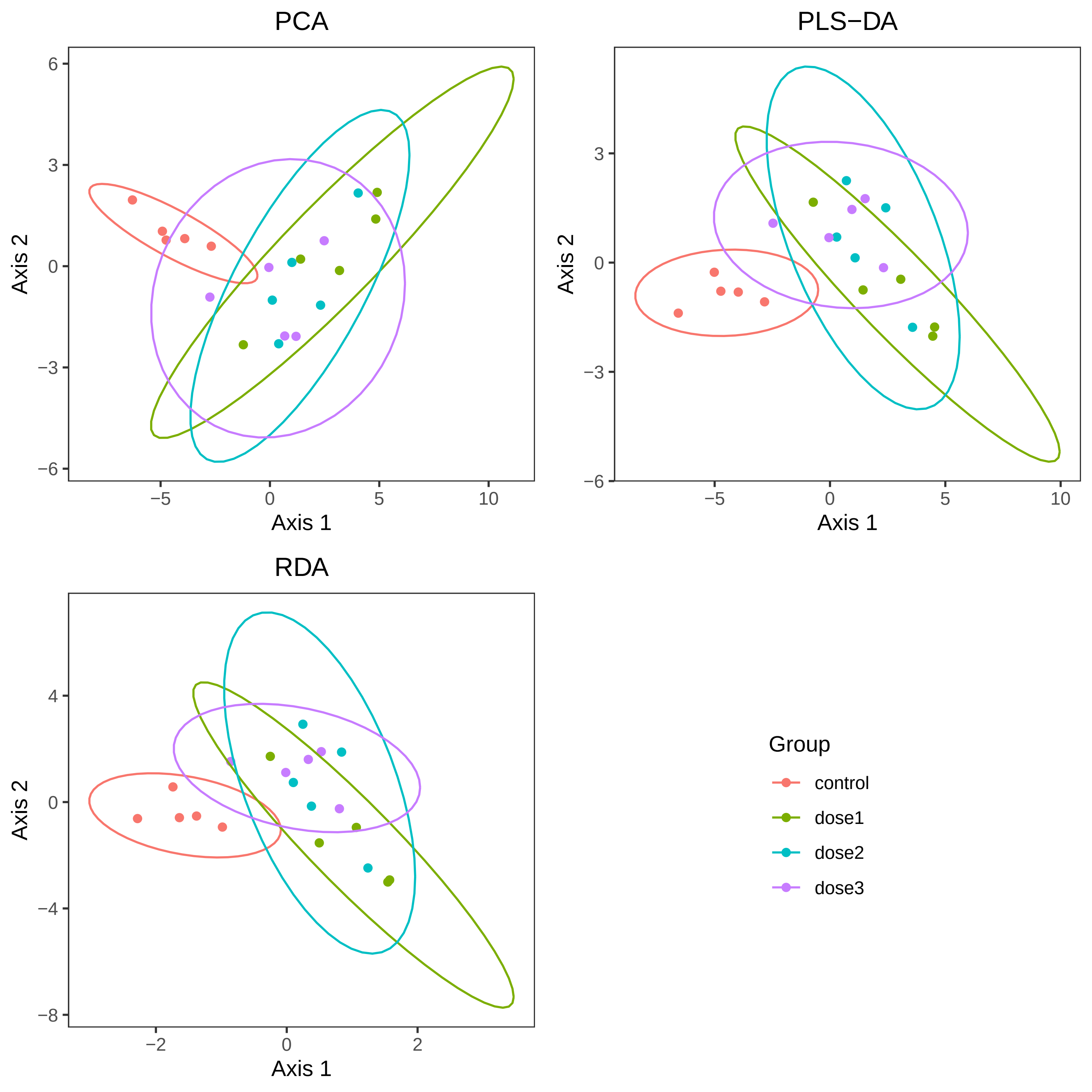
PCA
PLS−DA
RDA
Axis 1
Axis 2
Group
control
dose1
dose2
dose3

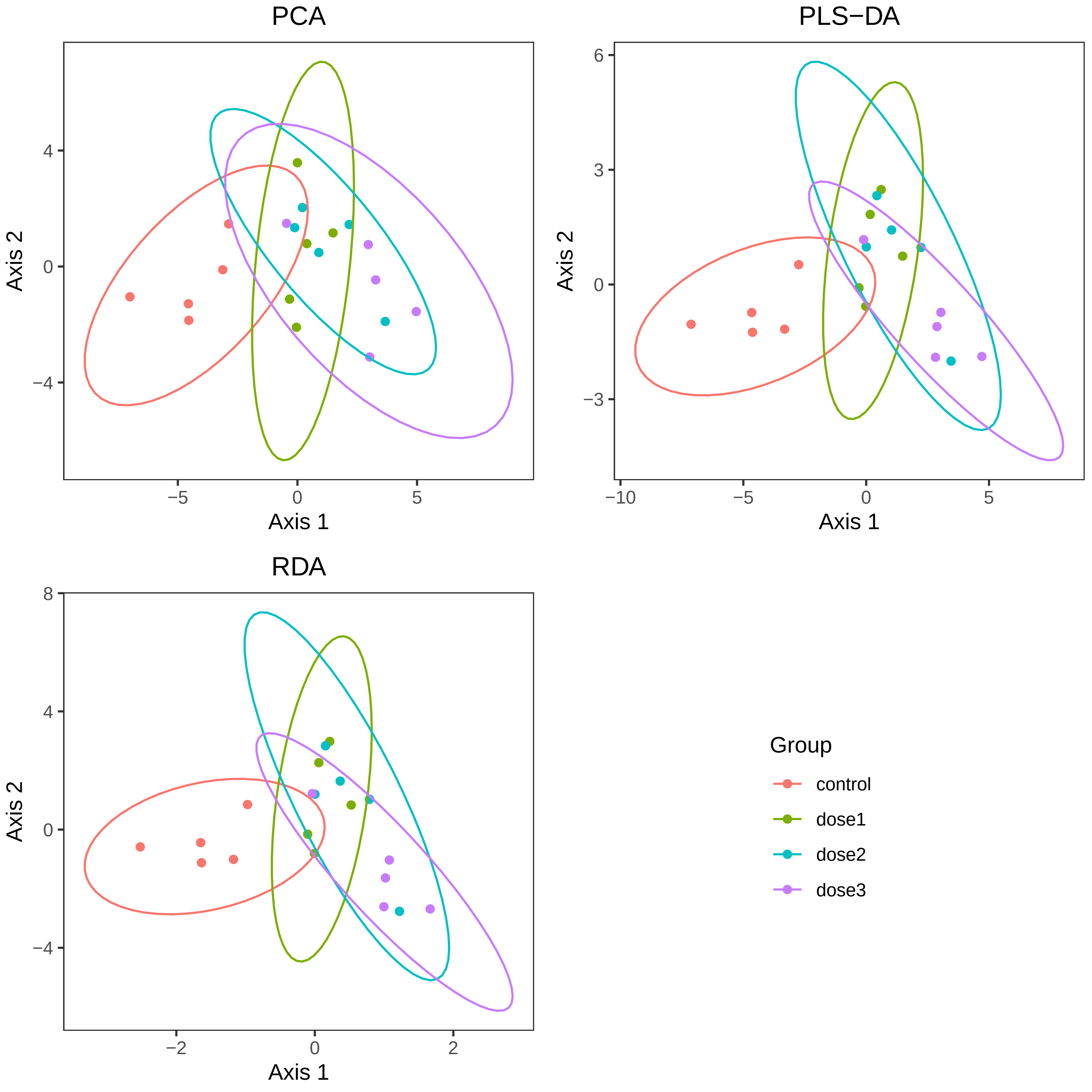

PCA
PLS−DA
RDA
Axis 1
Axis 2
Group
control
dose1
dose2
dose3

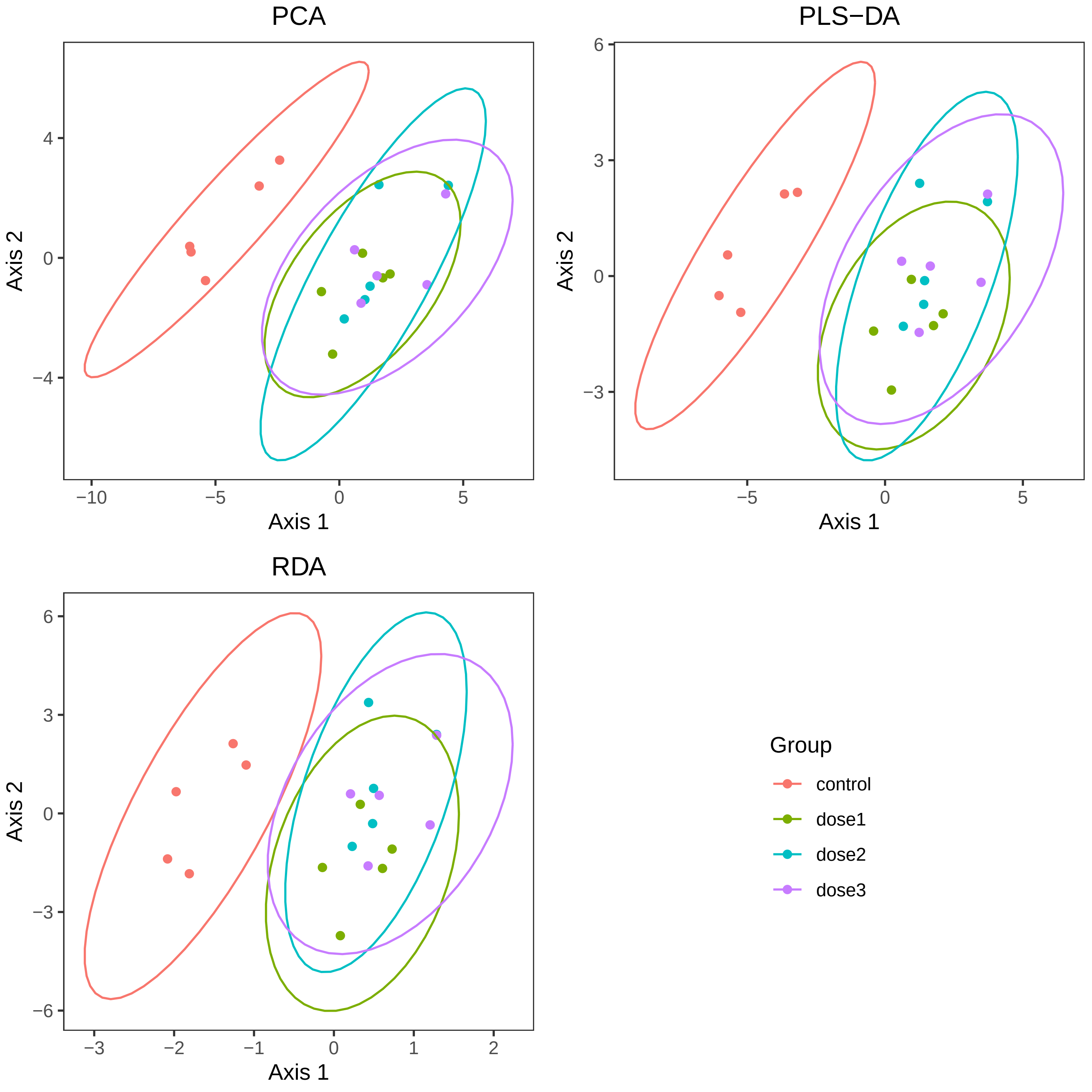
PCA
PLS−DA
RDA
Axis 1
Axis 2
Group
control
dose1
dose2
dose3

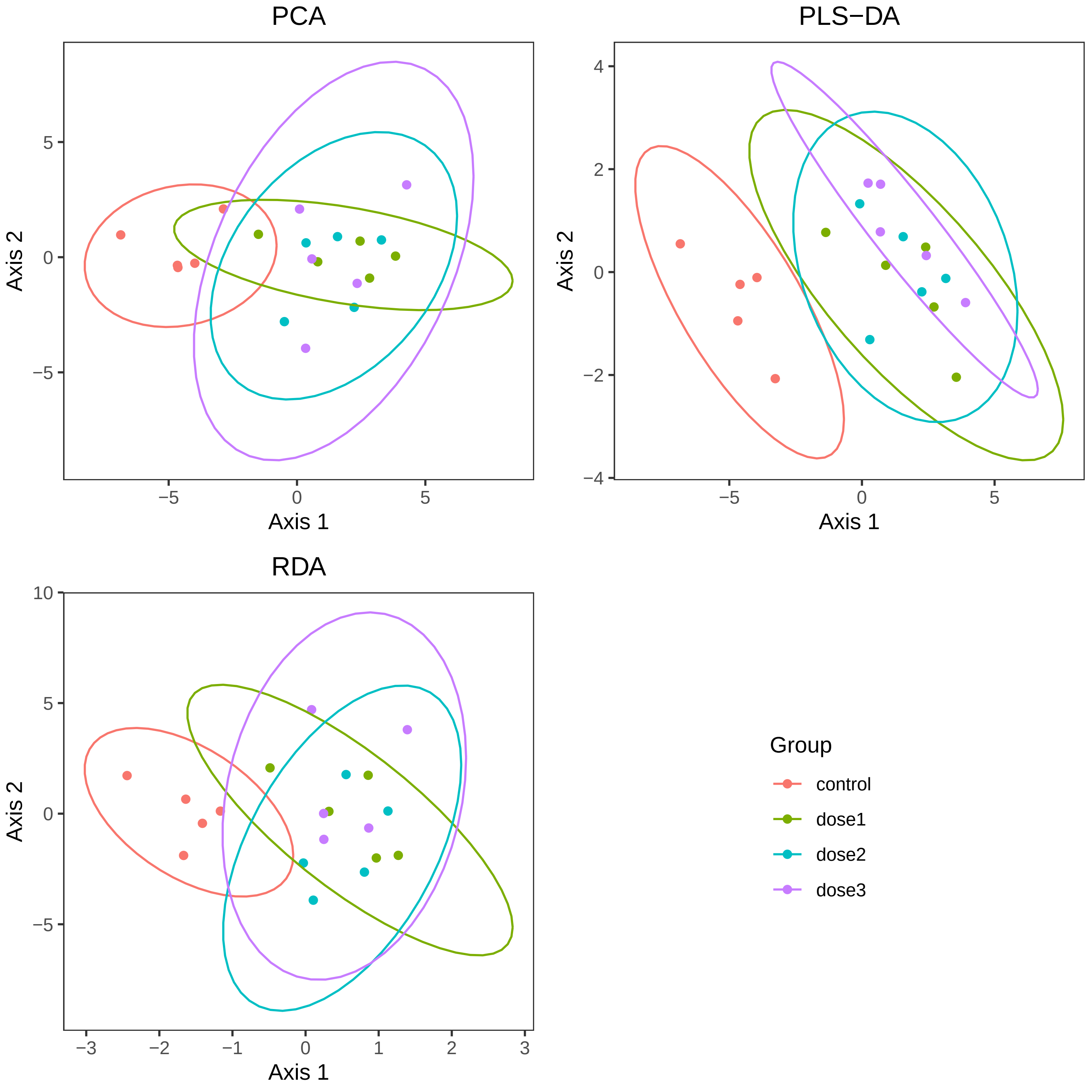
PCA
PLS−DA
RDA
Axis 1
Axis 2
Group
control
dose1
dose2
dose3

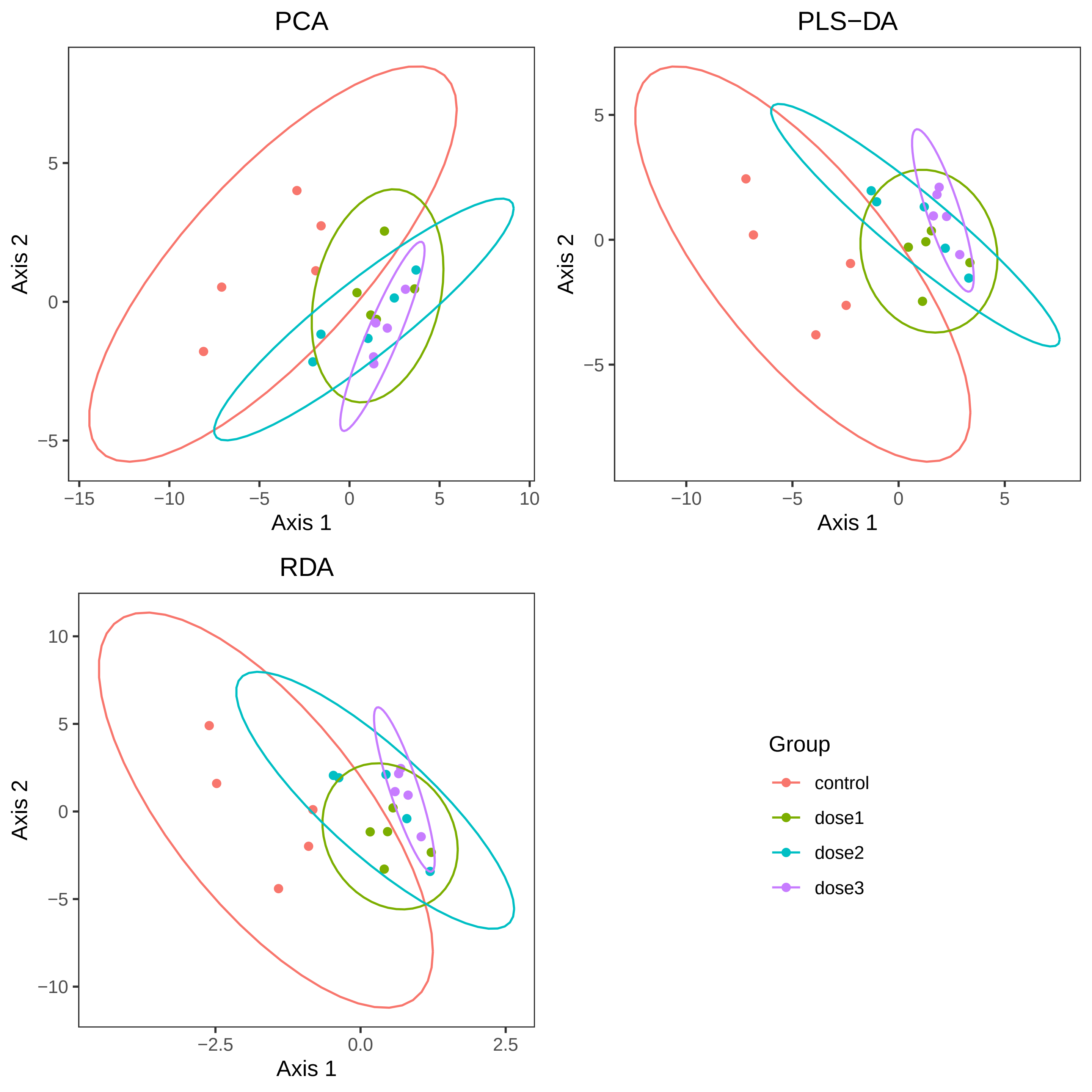

PCA
PLS−DA
RDA
Axis 1
Axis 2
Group
control
dose1
dose2
dose3

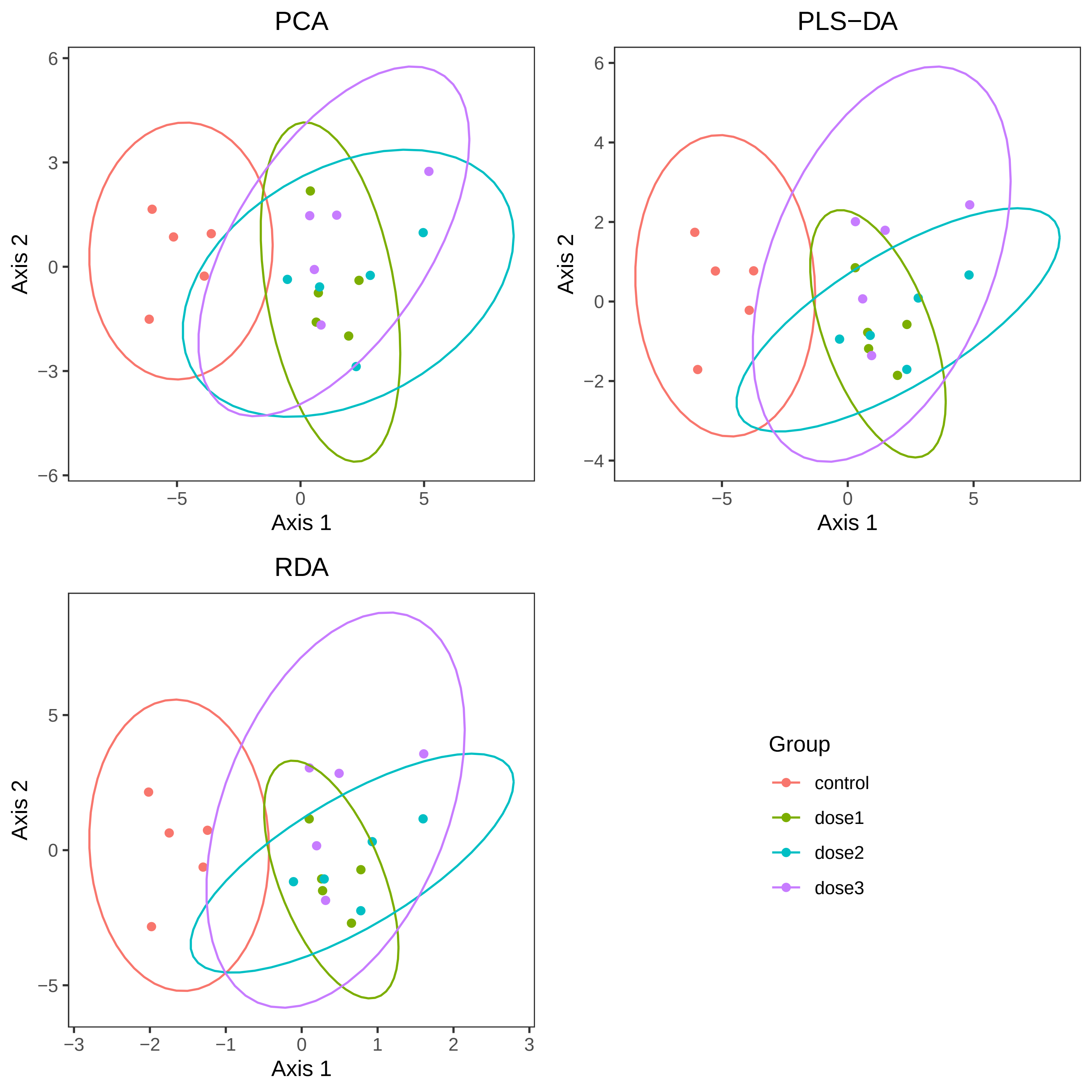
PCA
PLS−DA
RDA
Axis 1
Axis 2
Group
control
dose1
dose2
dose3

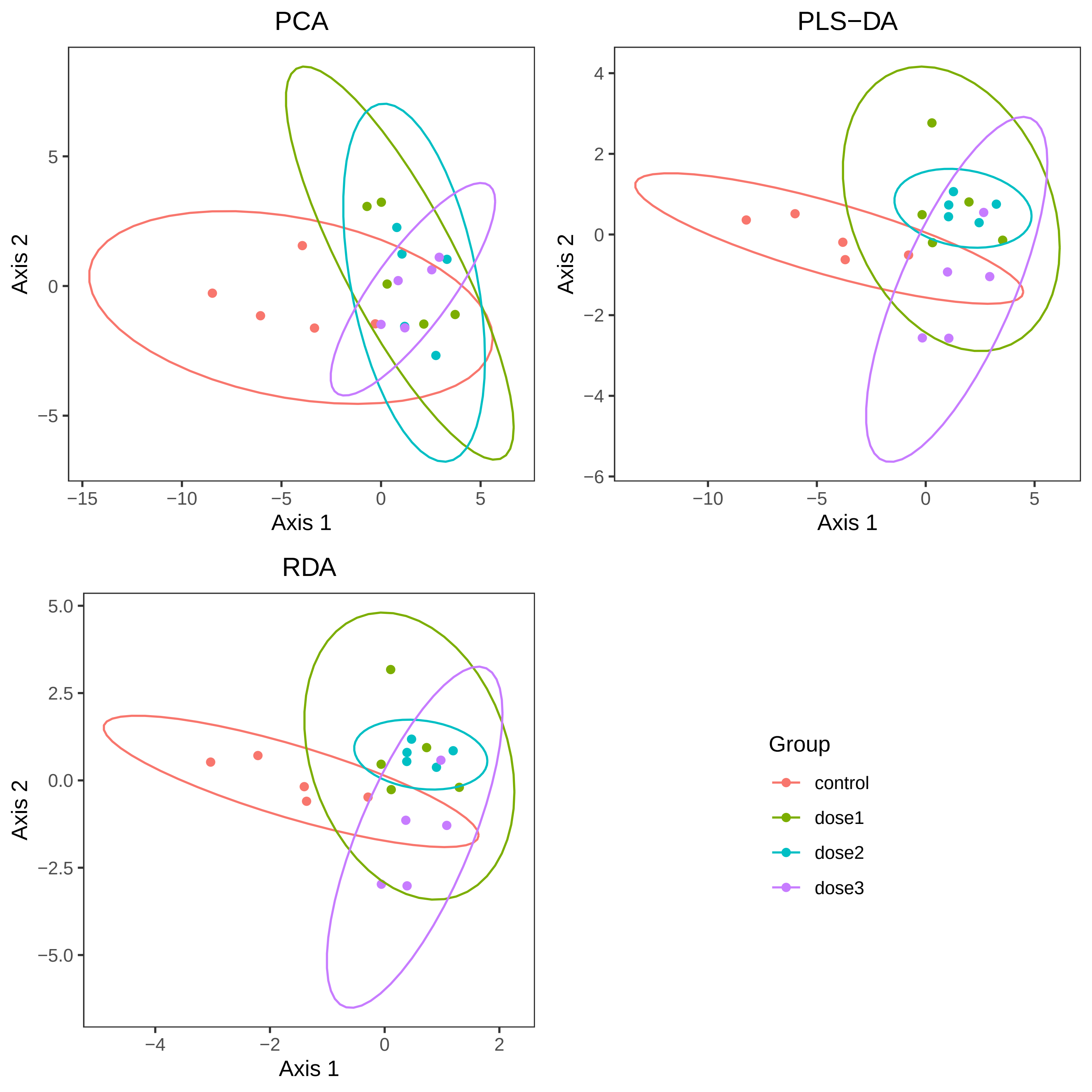
PCA
PLS−DA
RDA
Axis 1
Axis 2
Group
control
dose1
dose2
dose3

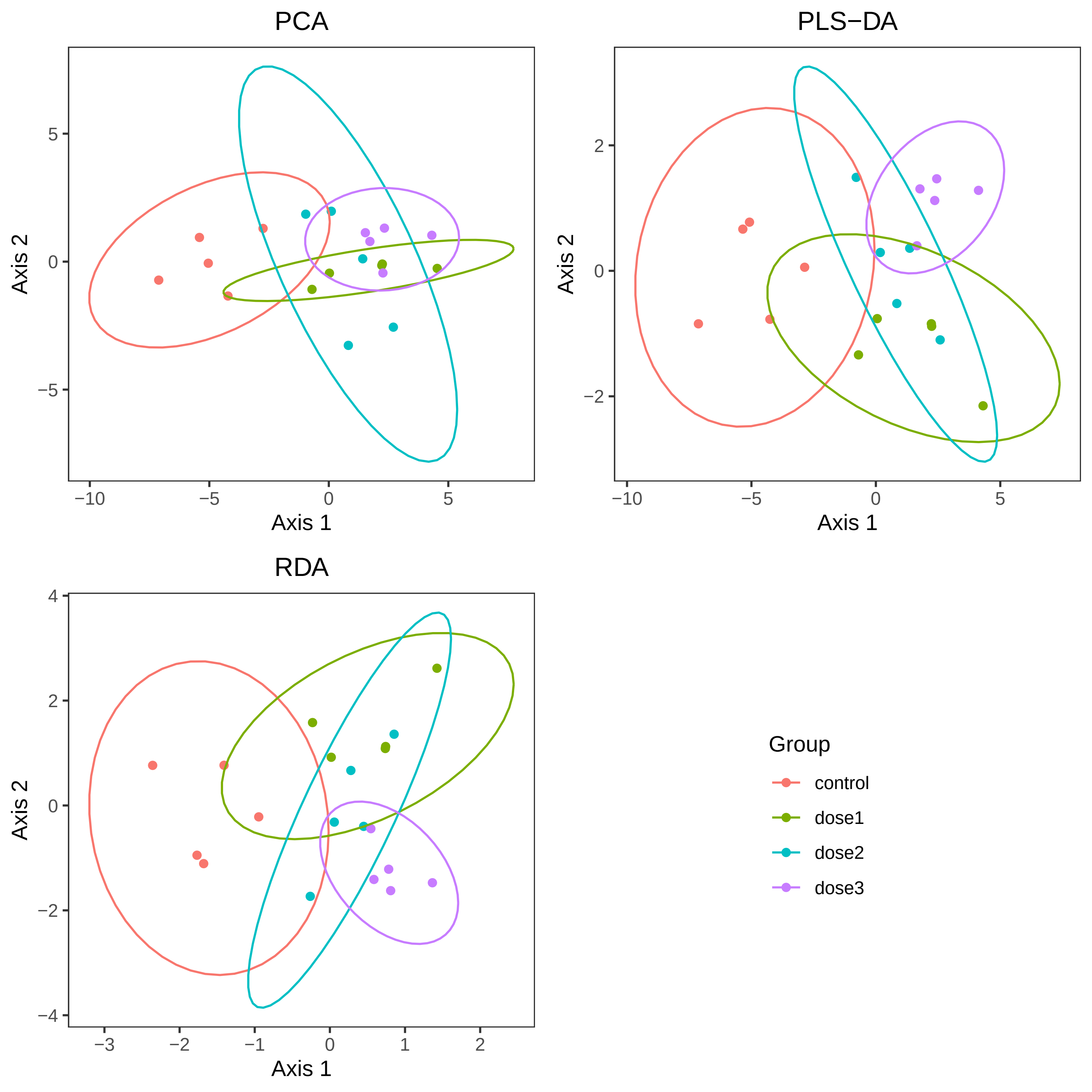

PCA
PLS−DA
RDA
Axis 1
Axis 2
−10
−5
0
5
2
−2
4
−4
−3
−1
1
Group
control
dose1
dose2
dose3

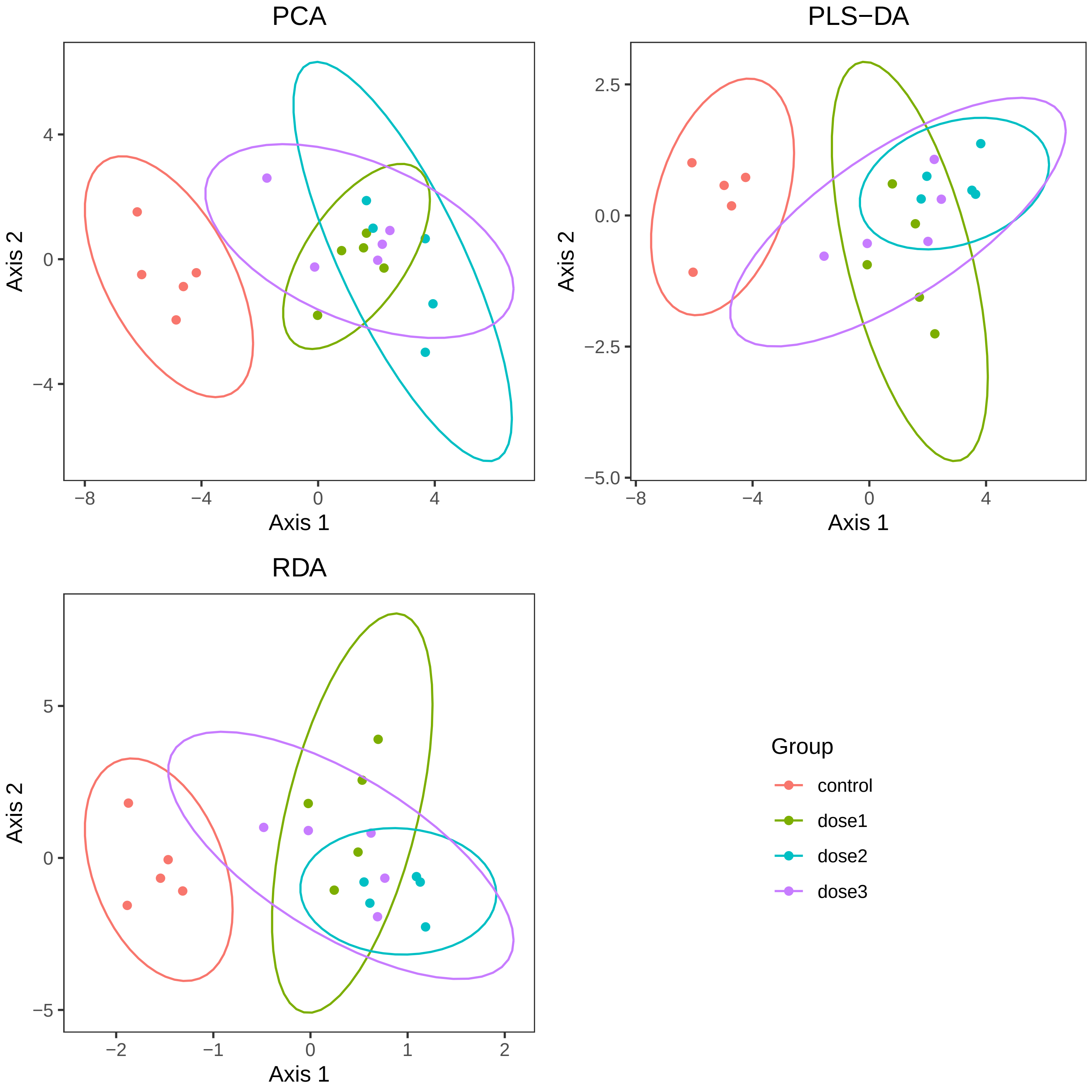
PCA
PLS−DA
RDA
Axis 1
Axis 2
Group
control
dose1
dose2
dose3

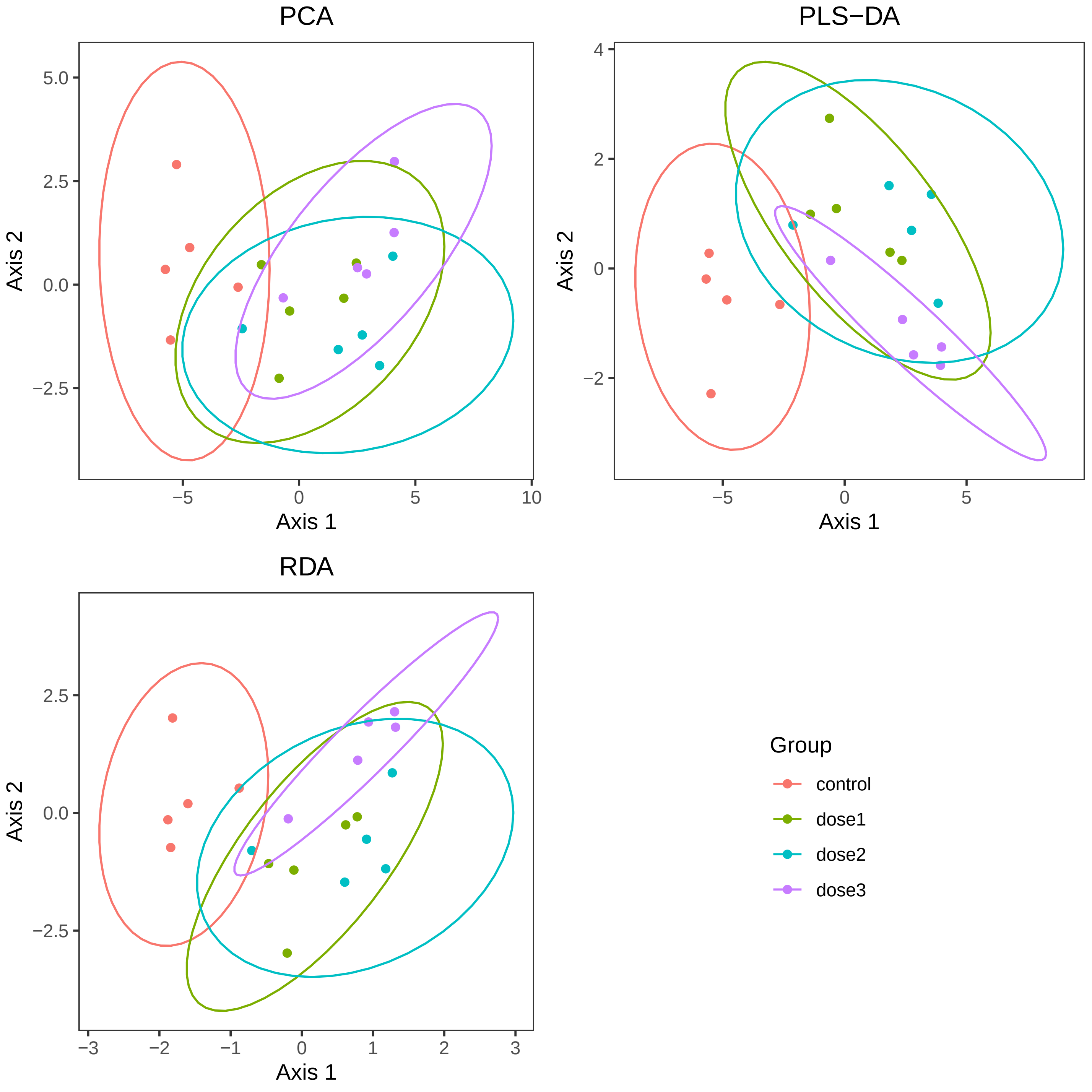
PCA
PLS−DA
RDA
Axis 1
Axis 2
Group
control
dose1
dose2
dose3

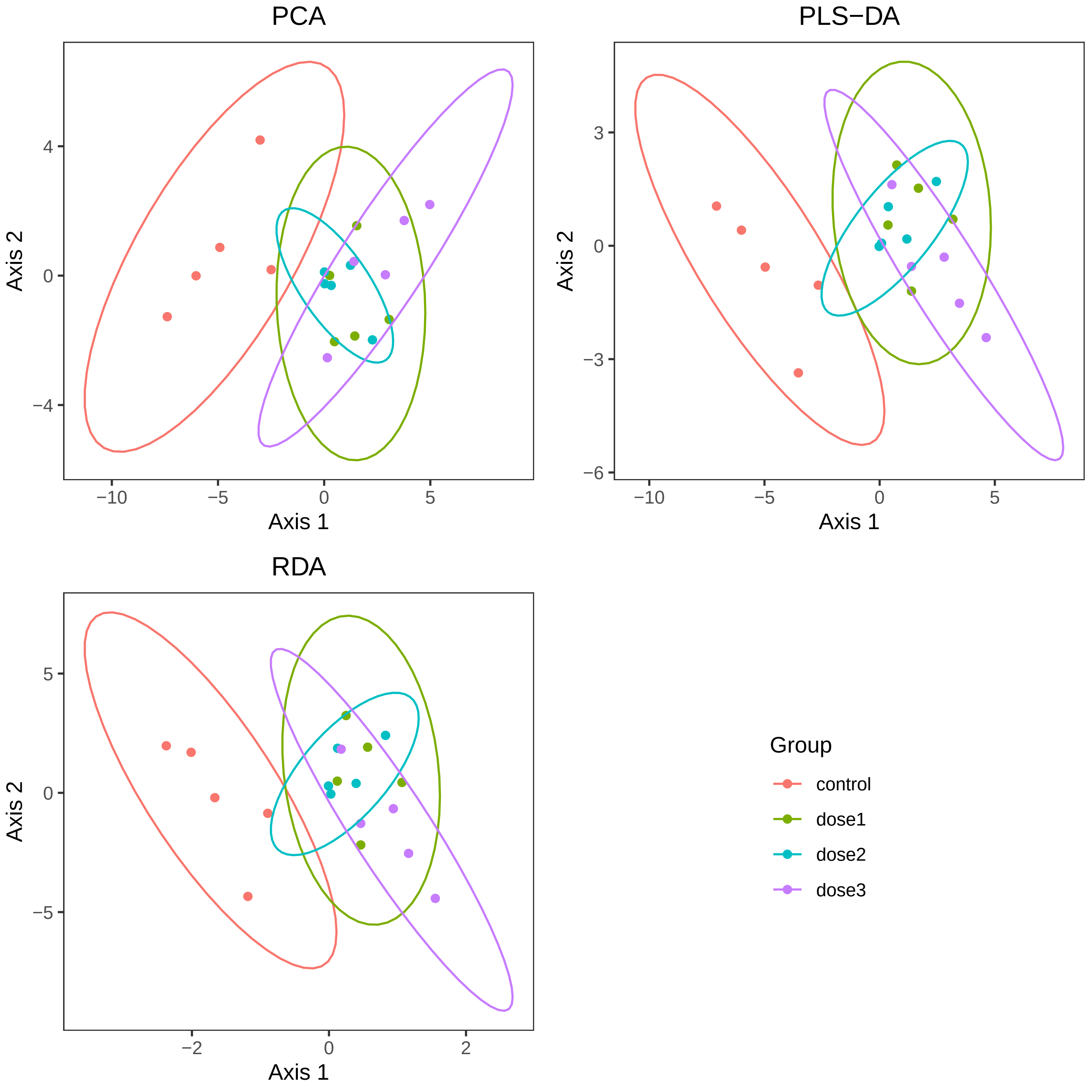

PCA
PLS−DA
RDA
Axis 1
Axis 2
Group
control
dose1
dose2
dose3

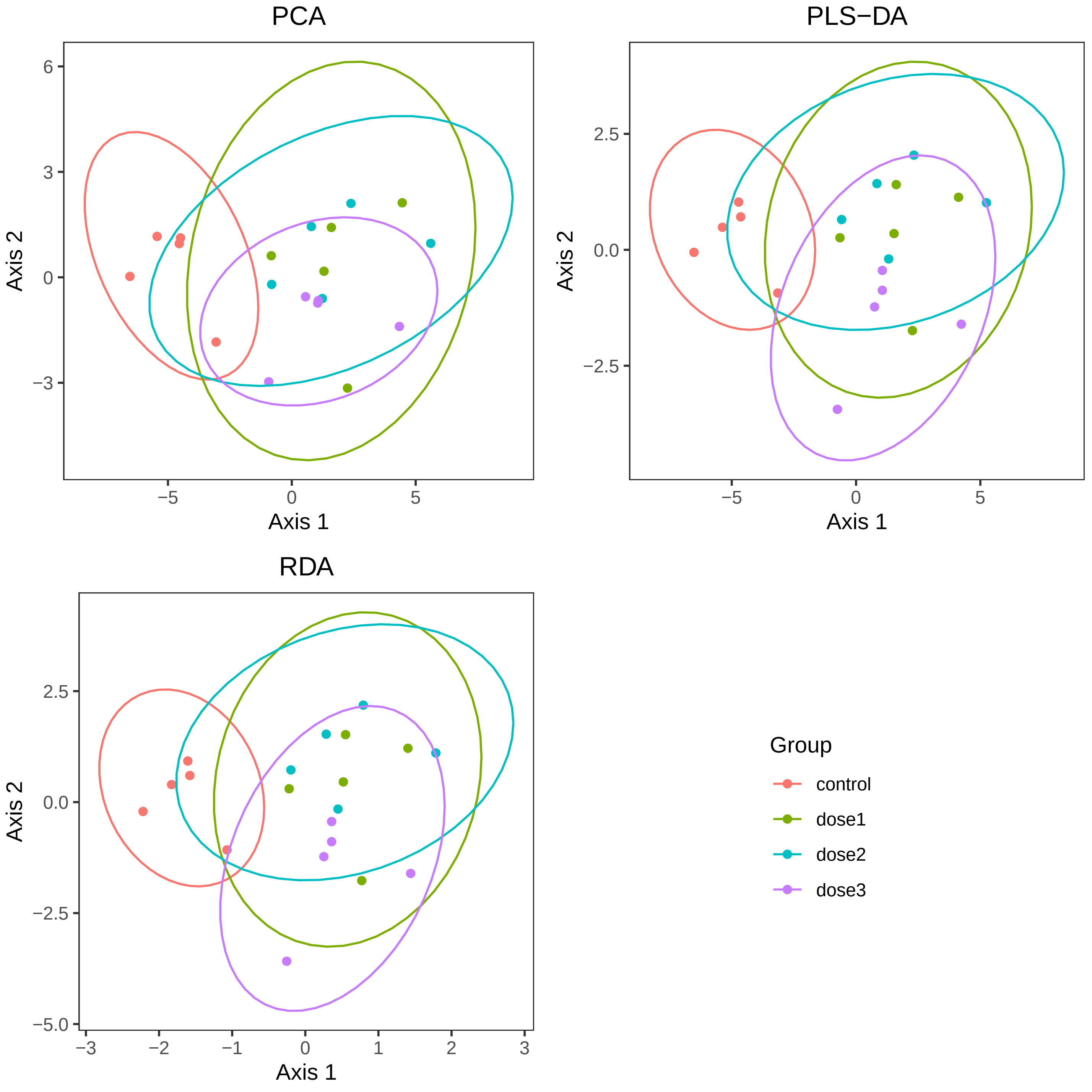

PCA
PLS−DA
RDA
Axis 1
Axis 2
Group
control
dose1
dose2
dose3

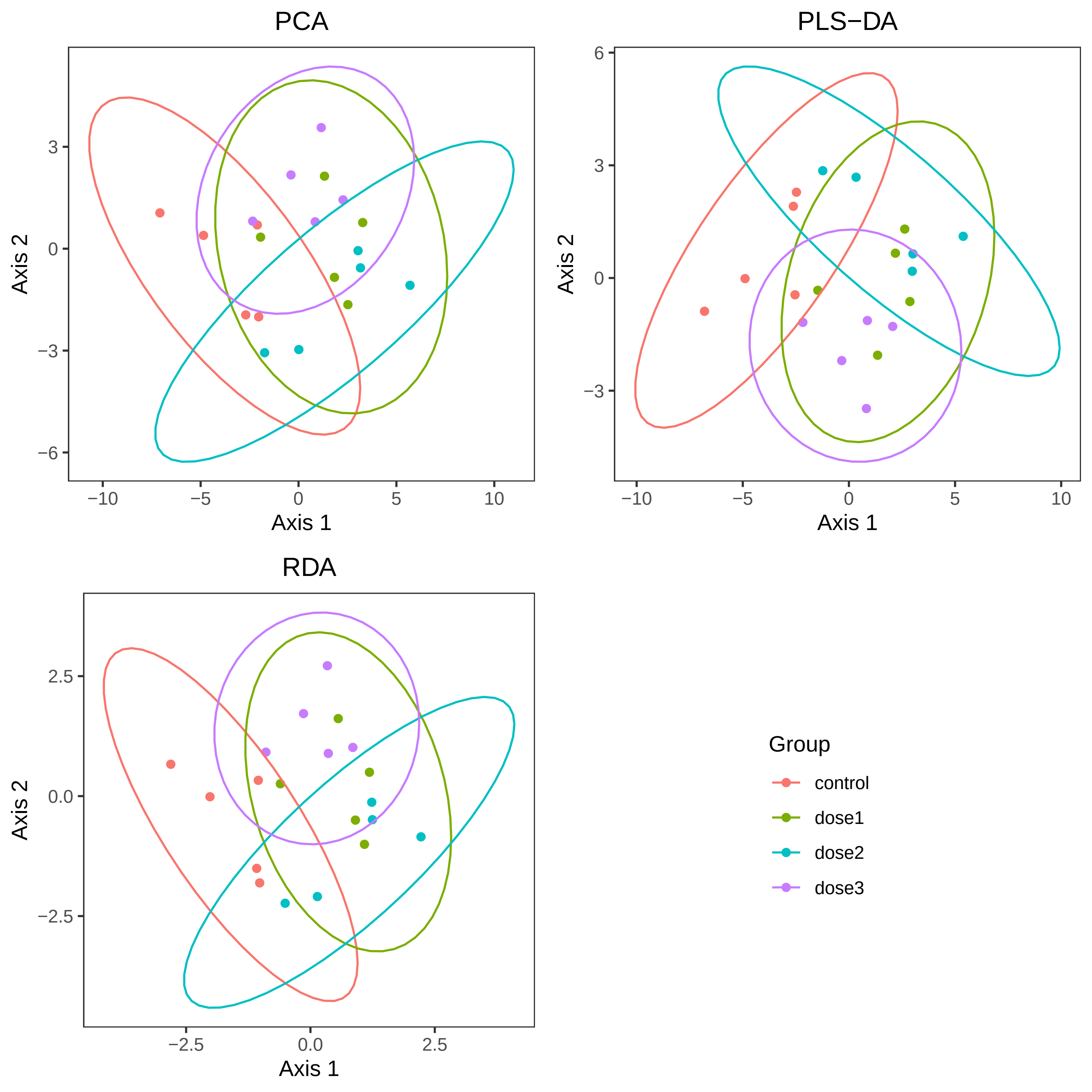
PCA
PLS−DA
RDA
Axis 1
Axis 2
Group
control
dose1
dose2
dose3

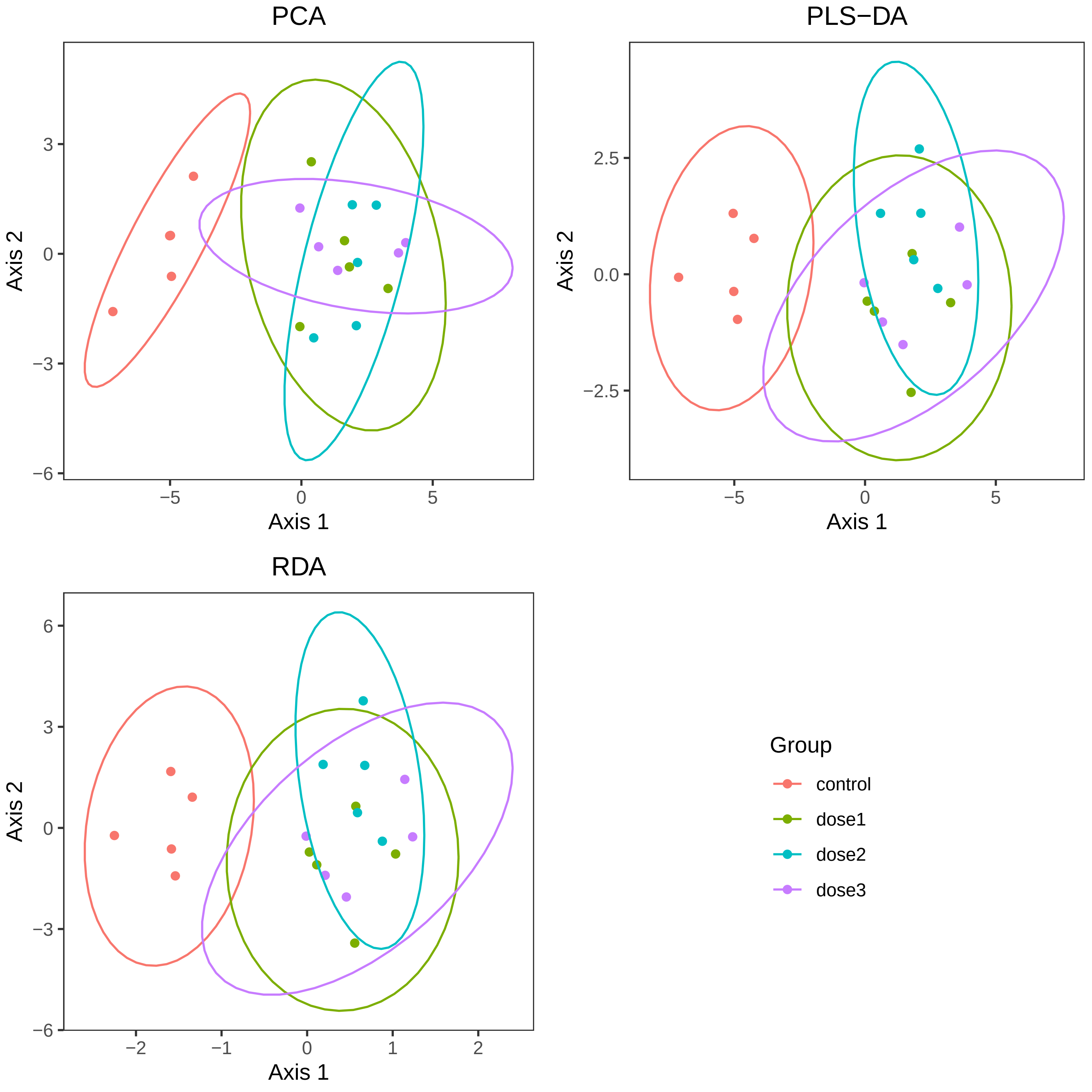
PCA
PLS−DA
RDA
Axis 1
Axis 2
Group
control
dose1
dose2
dose3

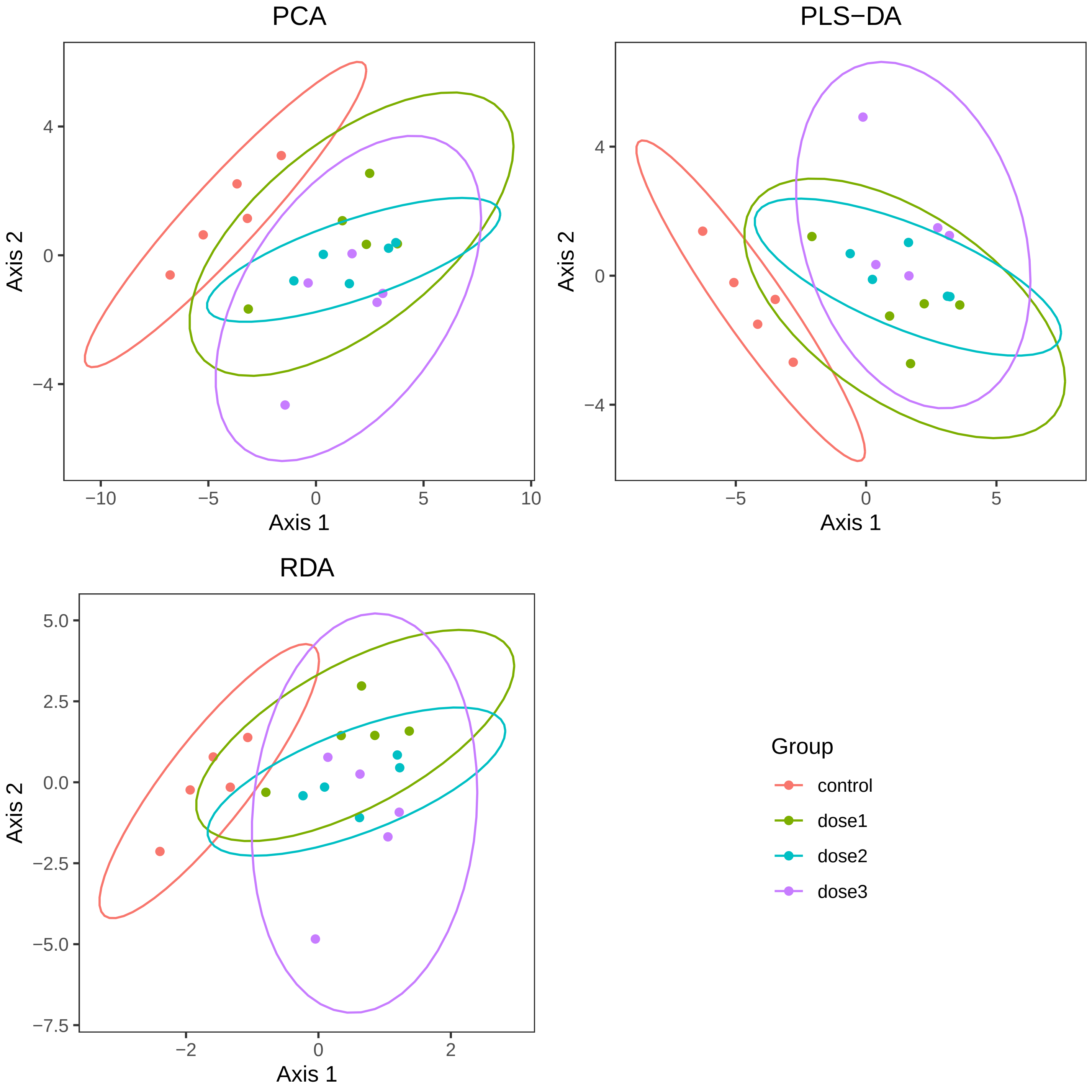
PCA
PLS−DA
RDA
Axis 1
Axis 2
Group
control
dose1
dose2
dose3

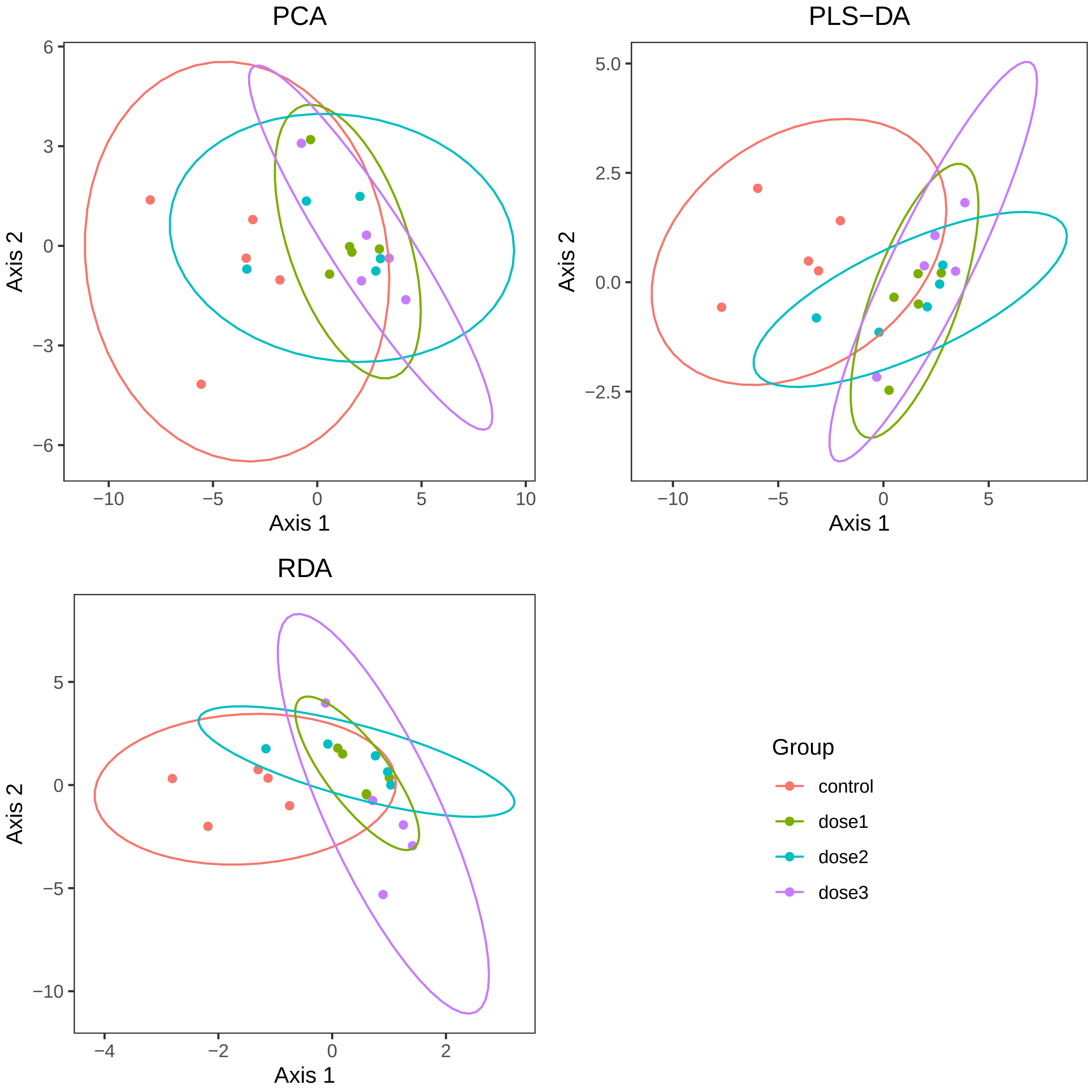
PCA
PLS−DA
RDA
Axis 1
Axis 2
Group
control
dose1
dose2
dose3

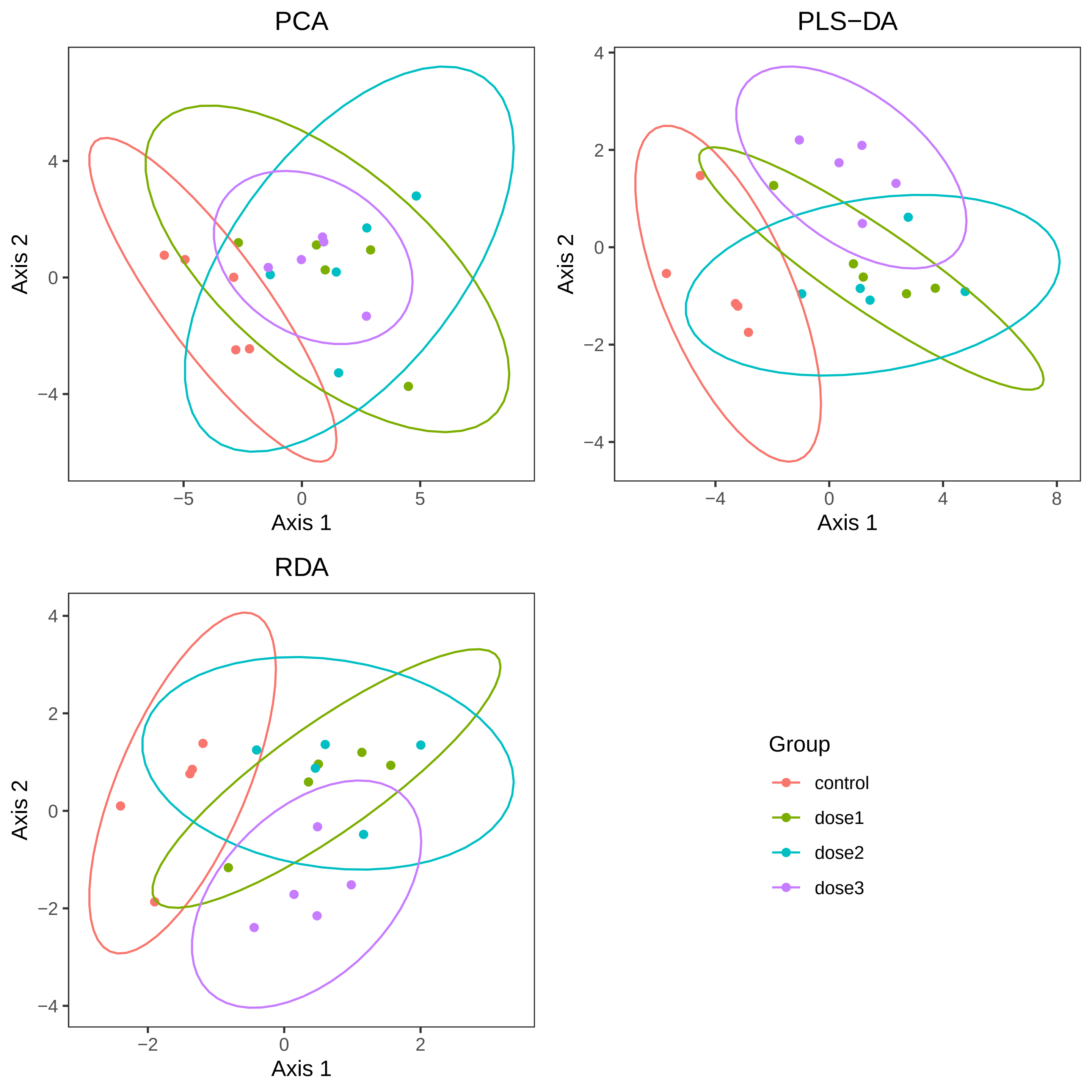
PCA
PLS−DA
RDA
Axis 1
Axis 2
Group
control
dose1
dose2
dose3

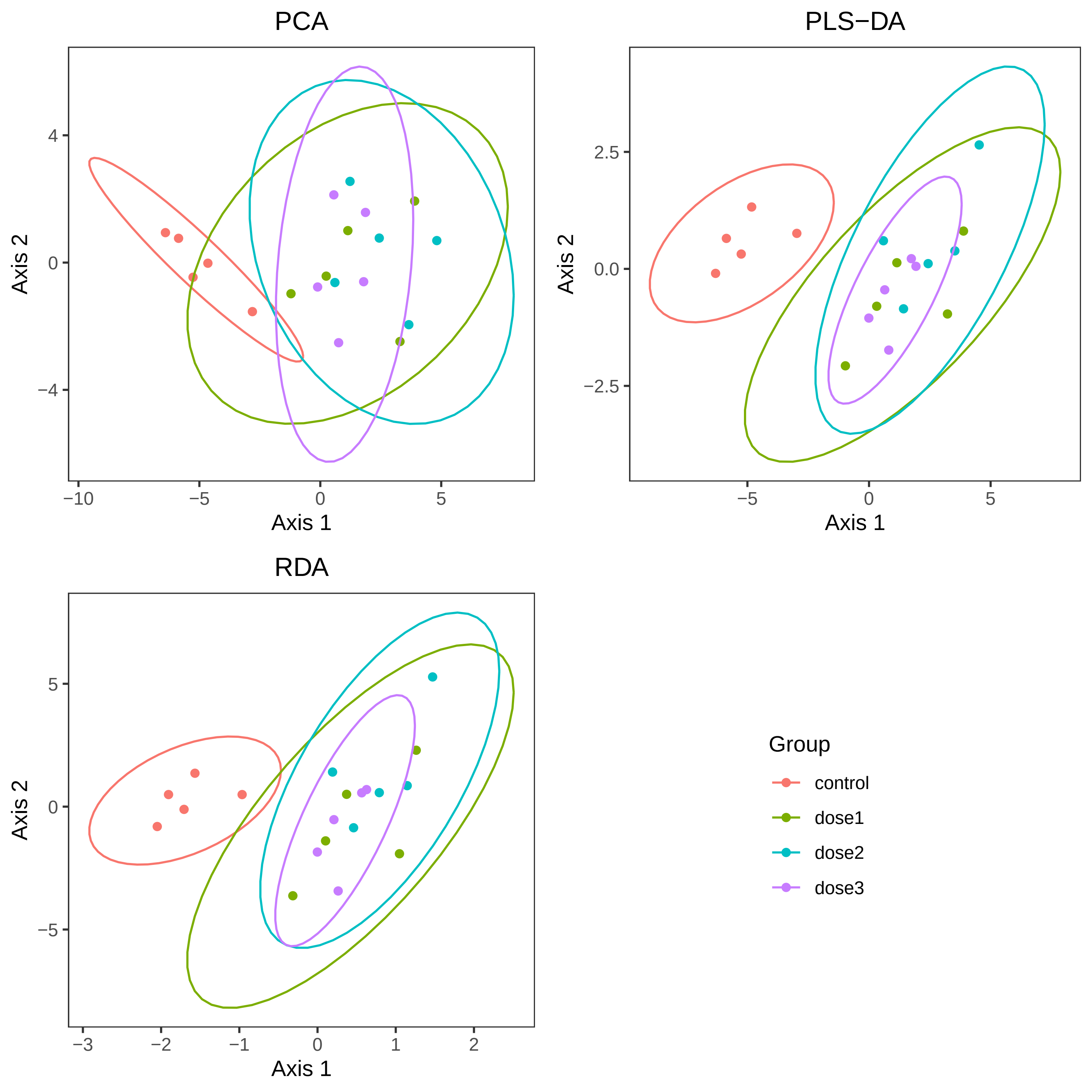
PCA
PLS−DA
RDA
Axis 1
Axis 2
Group
control
dose1
dose2
dose3

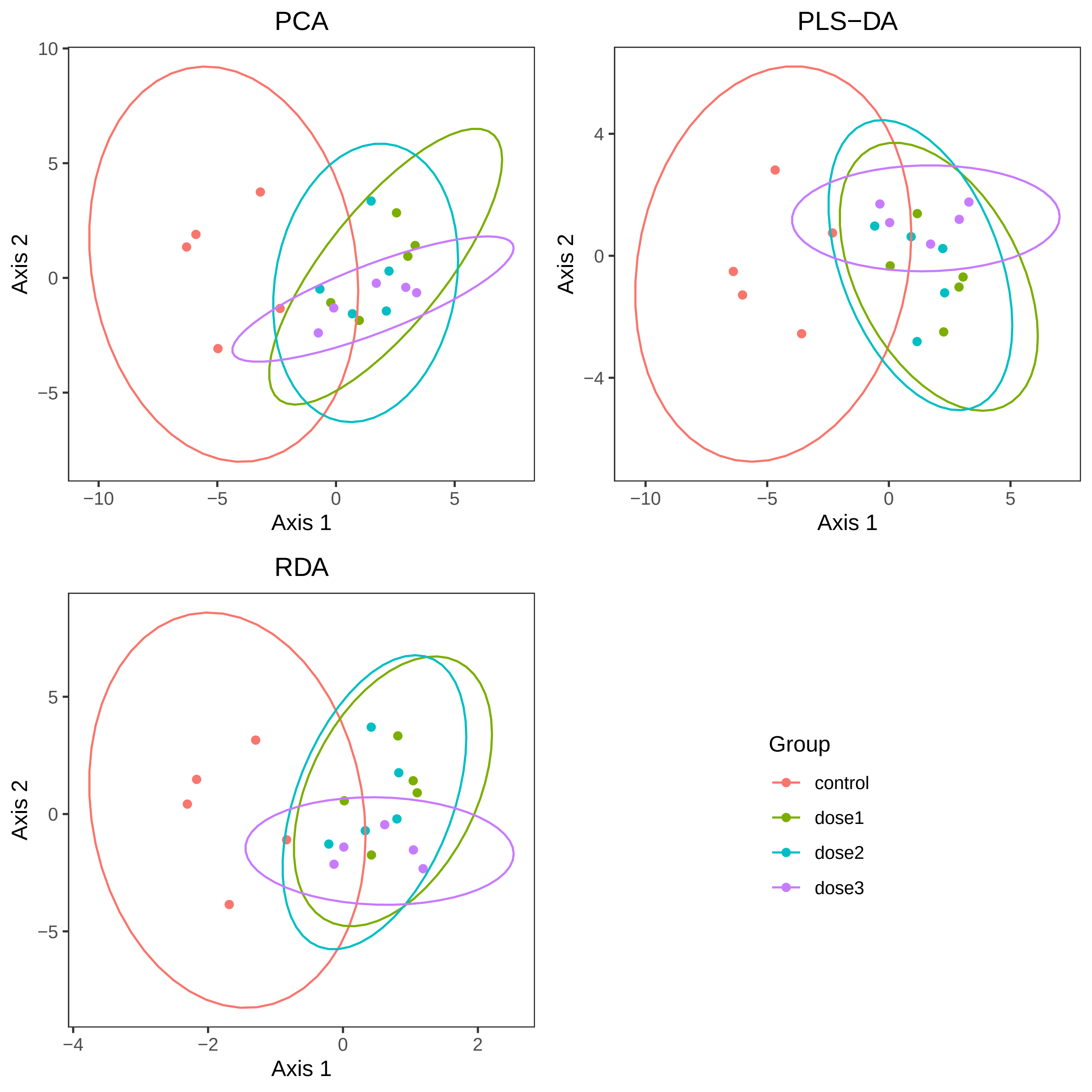
PCA
PLS−DA
RDA
Axis 1
Axis 2
10
5
0
−5
−10
4
−4
2
−2
Group
control
dose1
dose2
dose3

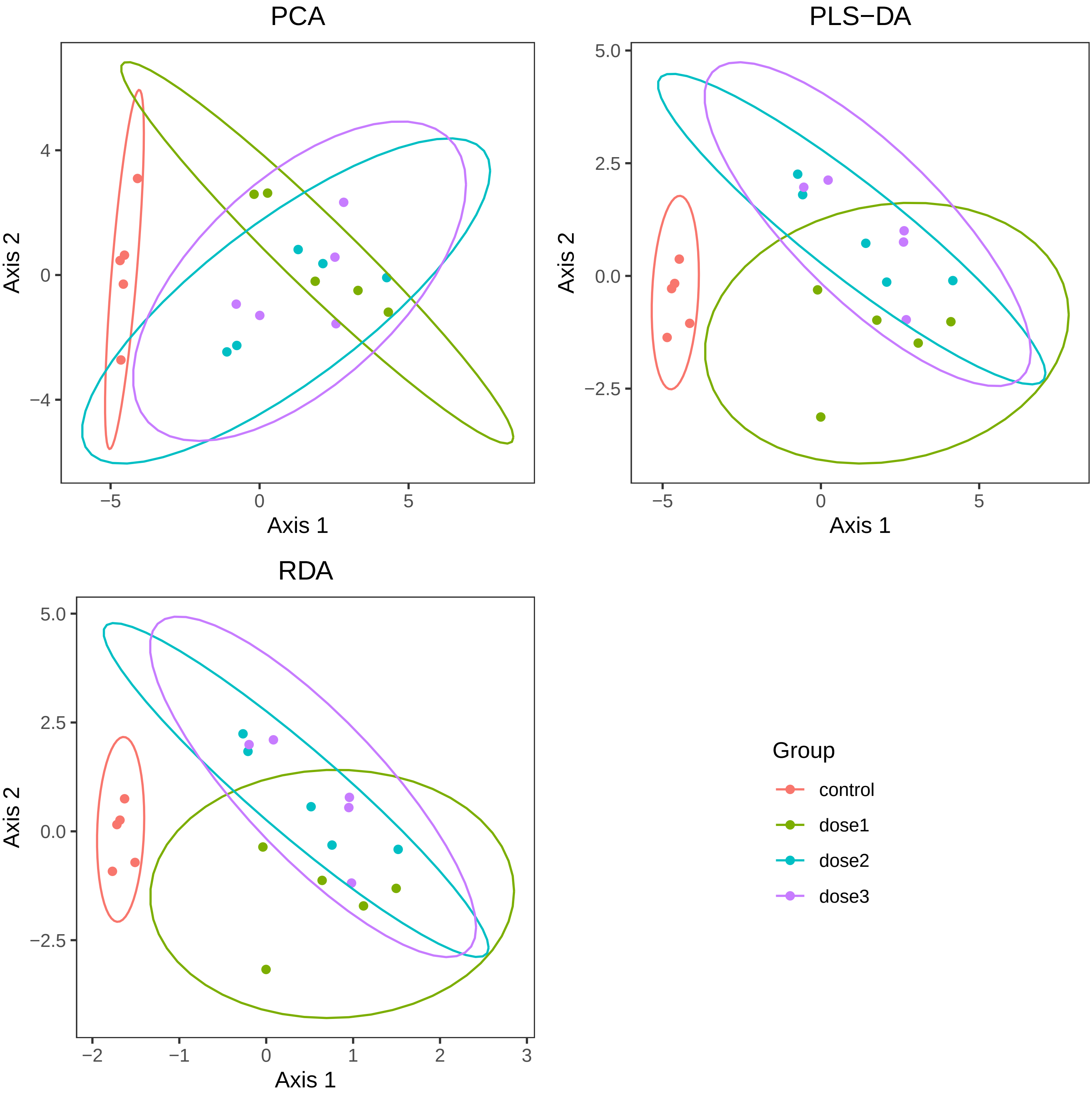
PCA
PLS−DA
RDA
Axis 1
Axis 2
Group
control
dose1
dose2
dose3

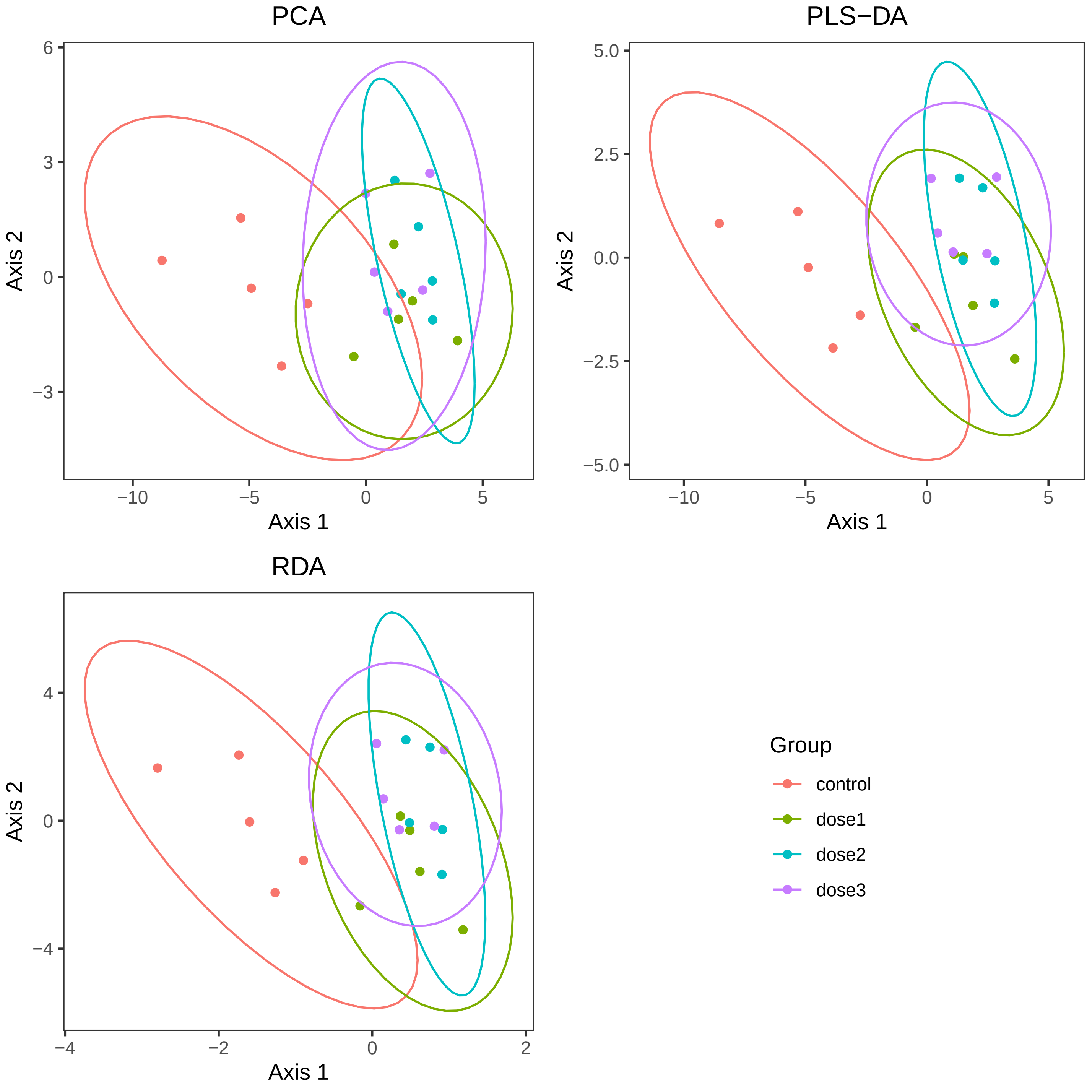
PCA
PLS−DA
RDA
Axis 1
Axis 2
Group
control
dose1
dose2
dose3

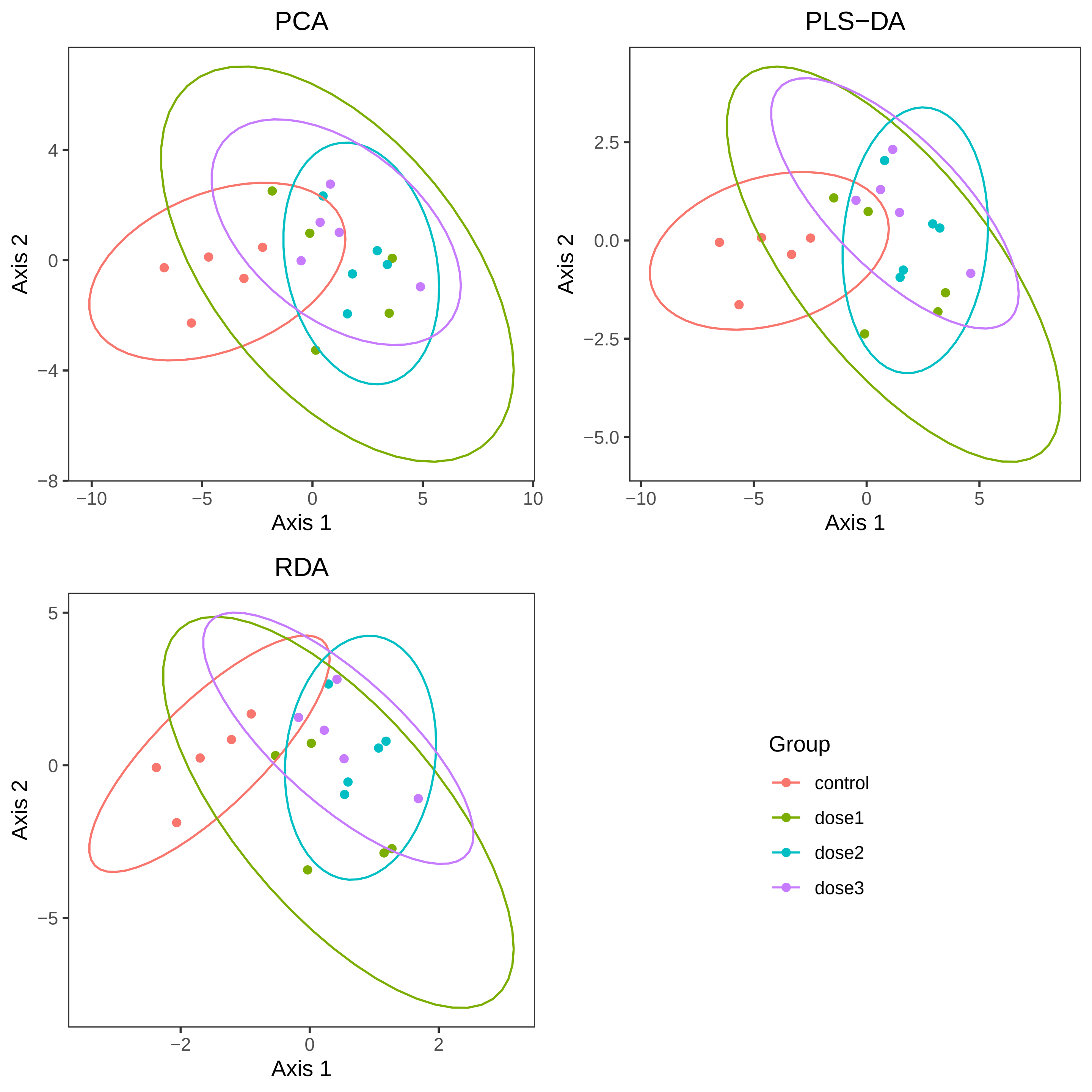
PCA
PLS−DA
RDA
Axis 1
Axis 2
Group
control
dose1
dose2
dose3

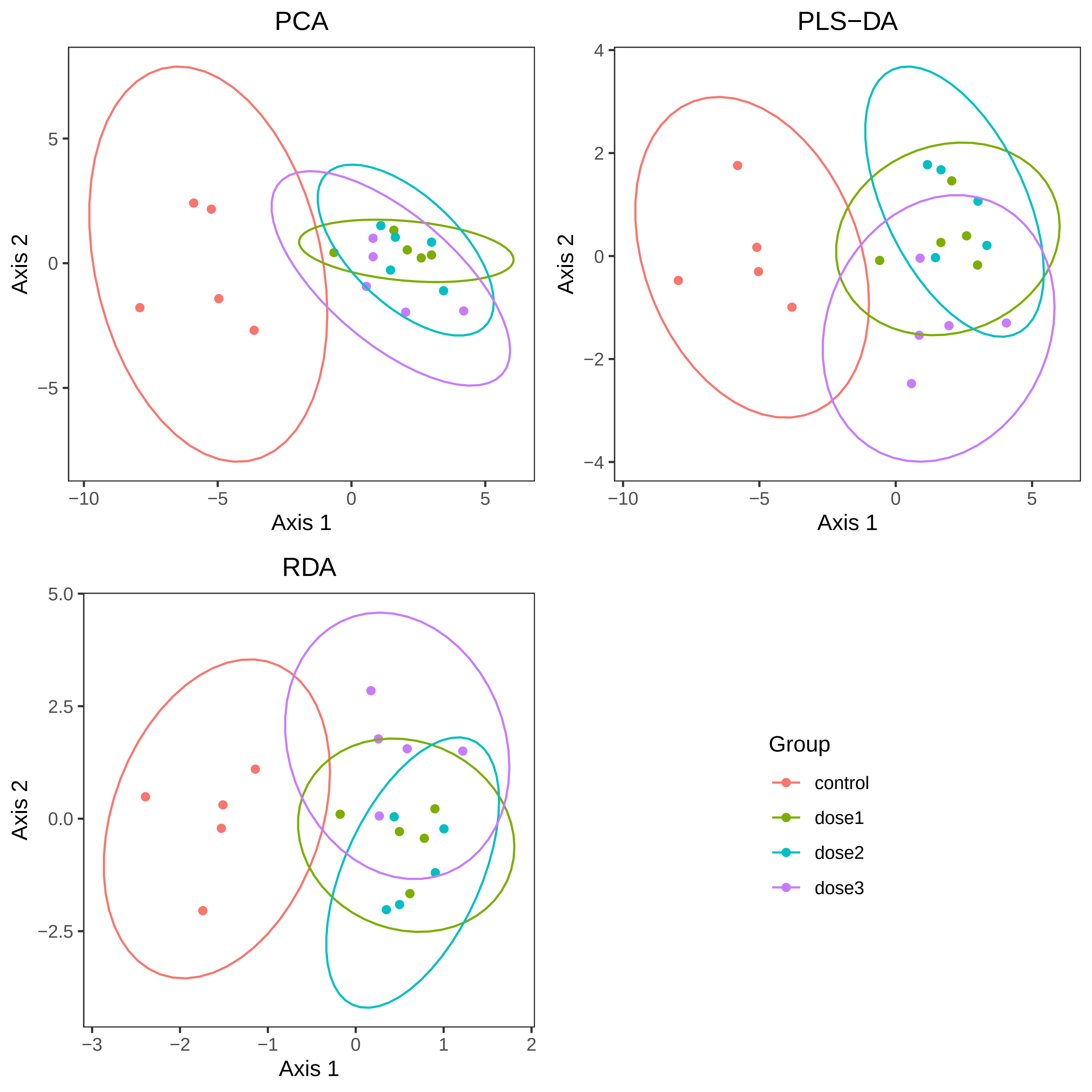
PCA
Axis 2
Axis 1
5
0
−5
−10
−5
0
5
PLS−DA
4
2
0
−2
−4
RDA
5.0
2.5
0.0
−2.5
−3
−2
−1
0
1
2
Group
control
dose1
dose2
dose3

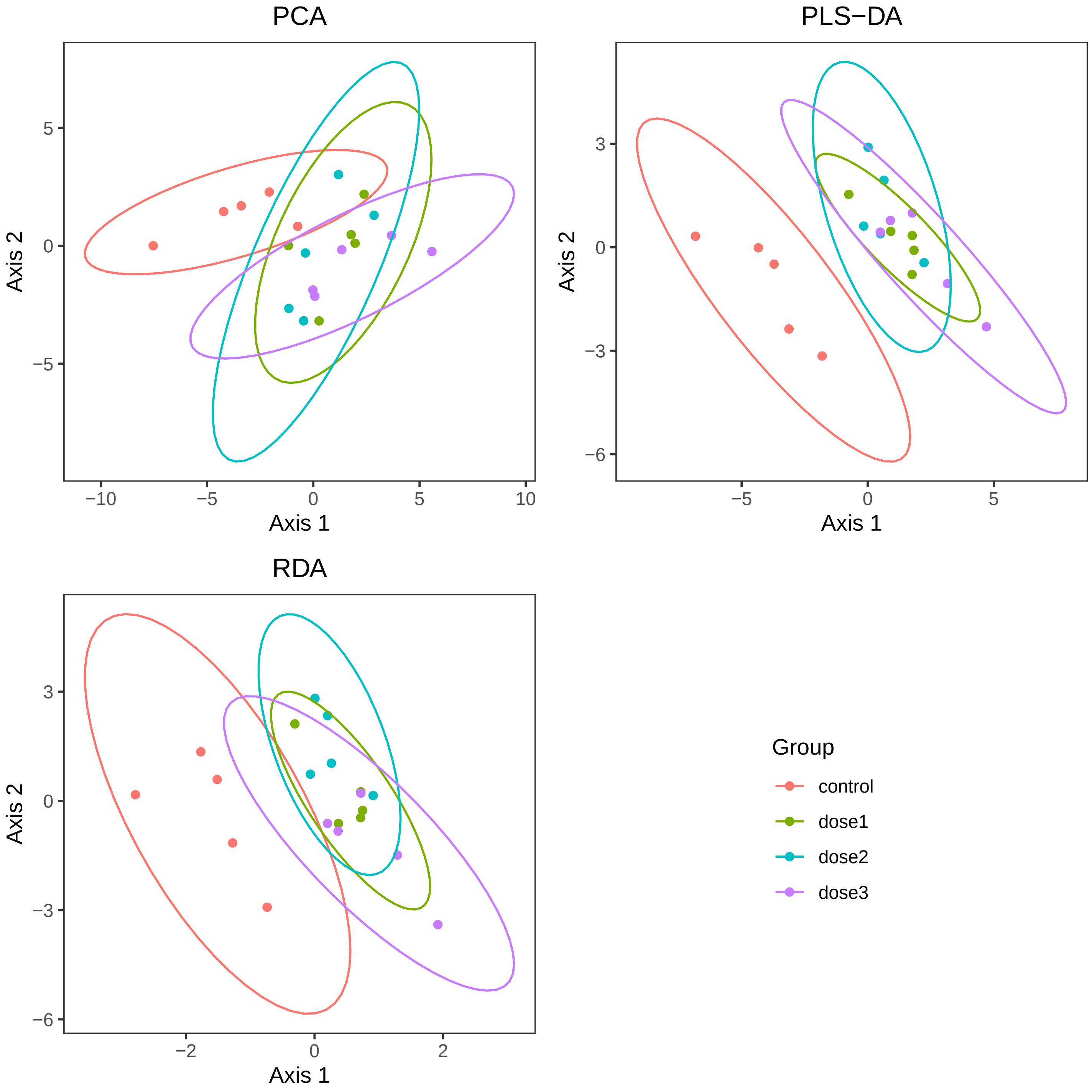
PCA
PLS−DA
RDA
Axis 1
Axis 2
Group
control
dose1
dose2
dose3

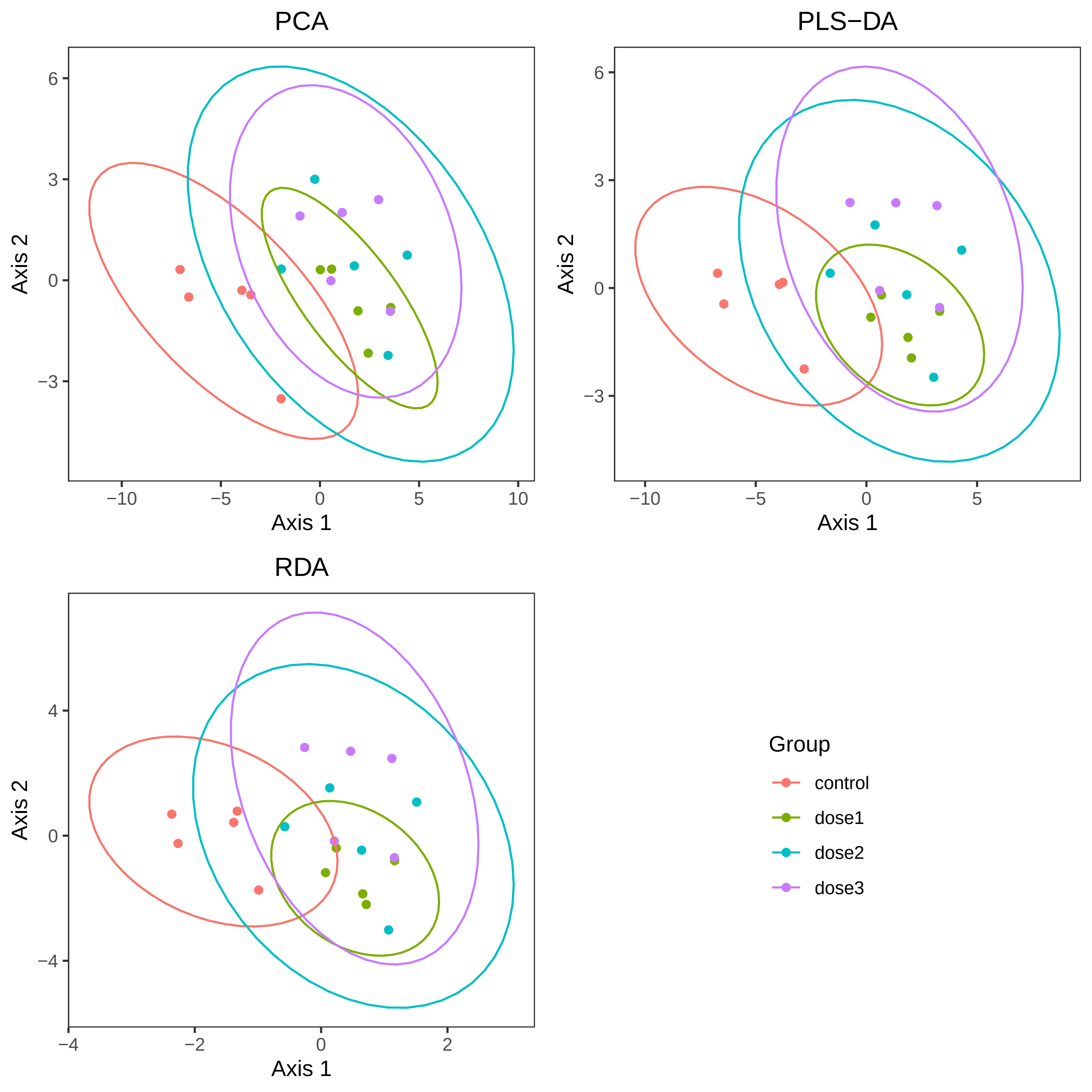
PCA
PLS−DA
RDA
Axis 1
Axis 2
Group
control
dose1
dose2
dose3

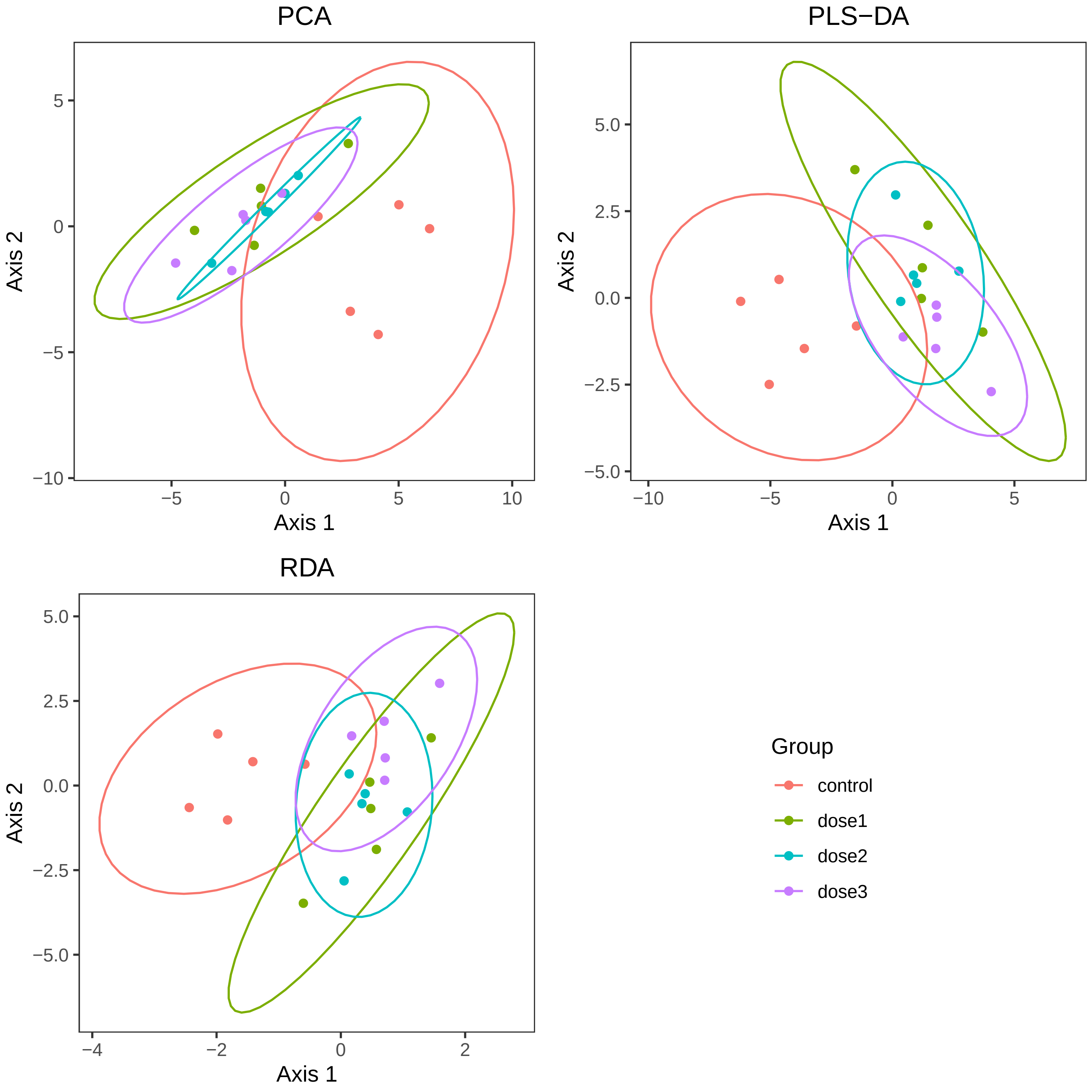
PCA
PLS−DA
RDA
Axis 1
Axis 2
Group
control
dose1
dose2
dose3

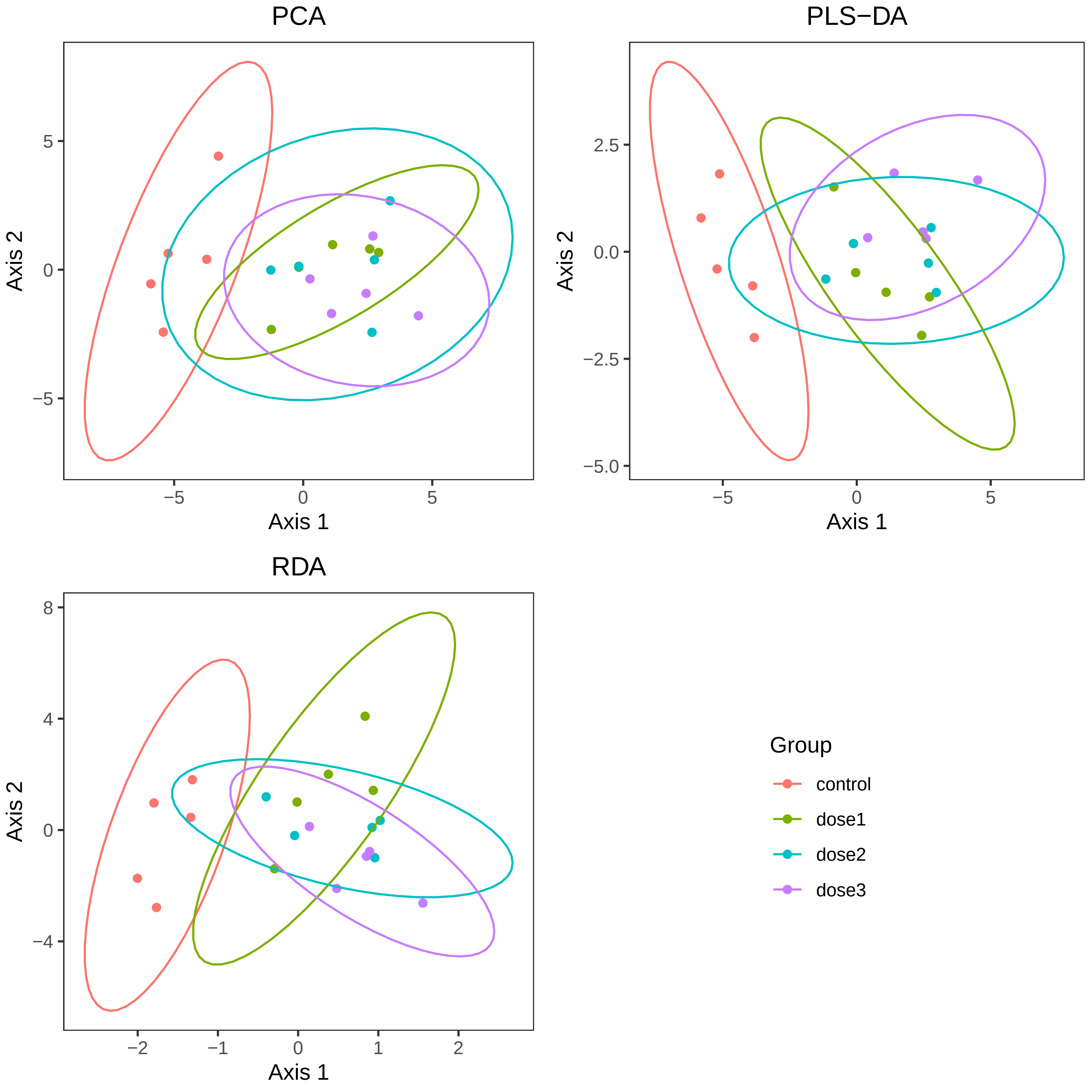
PCA
PLS−DA
RDA
Axis 1
Axis 2
Group
control
dose1
dose2
dose3

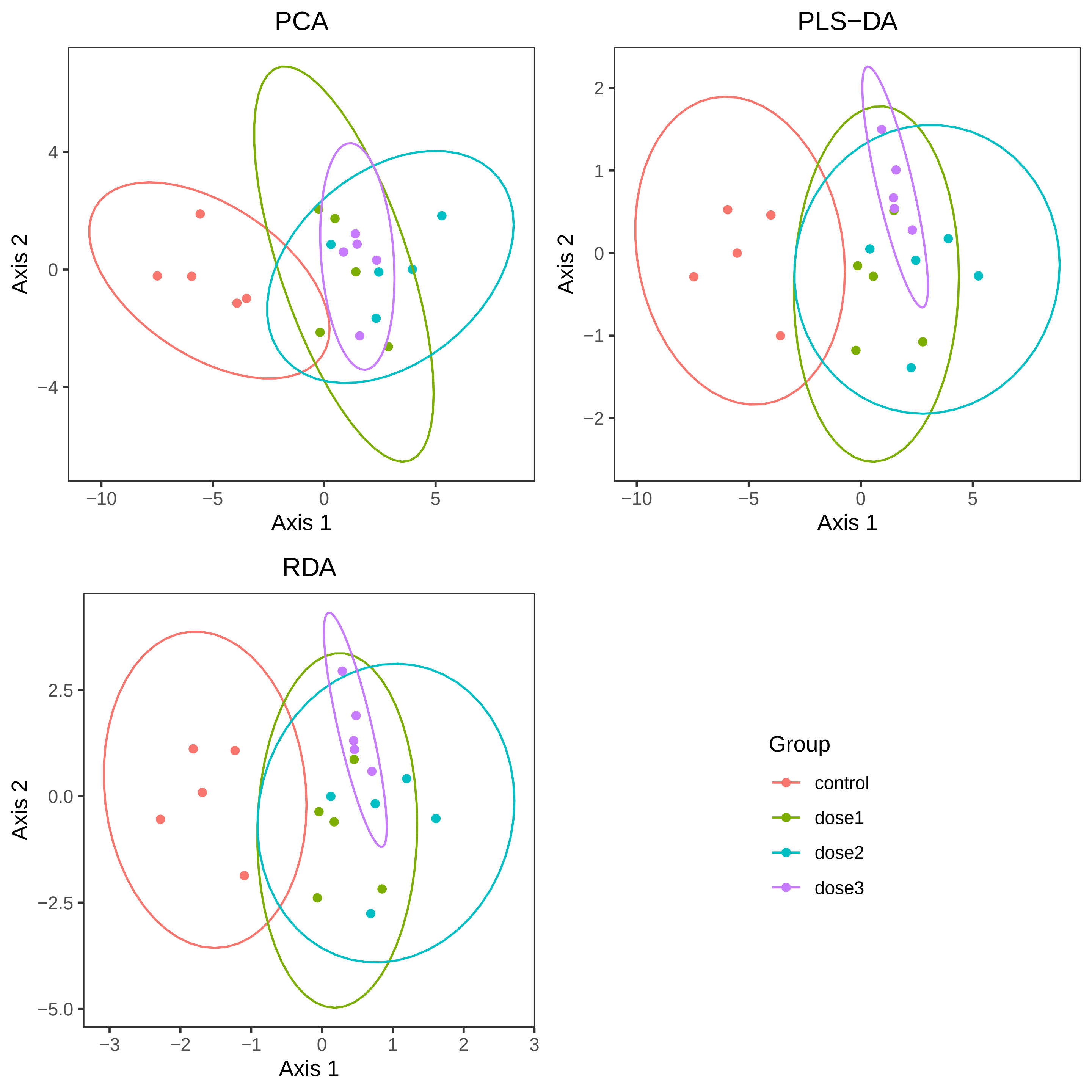
PCA
PLS−DA
RDA
Axis 1
Axis 2
Group
control
dose1
dose2
dose3

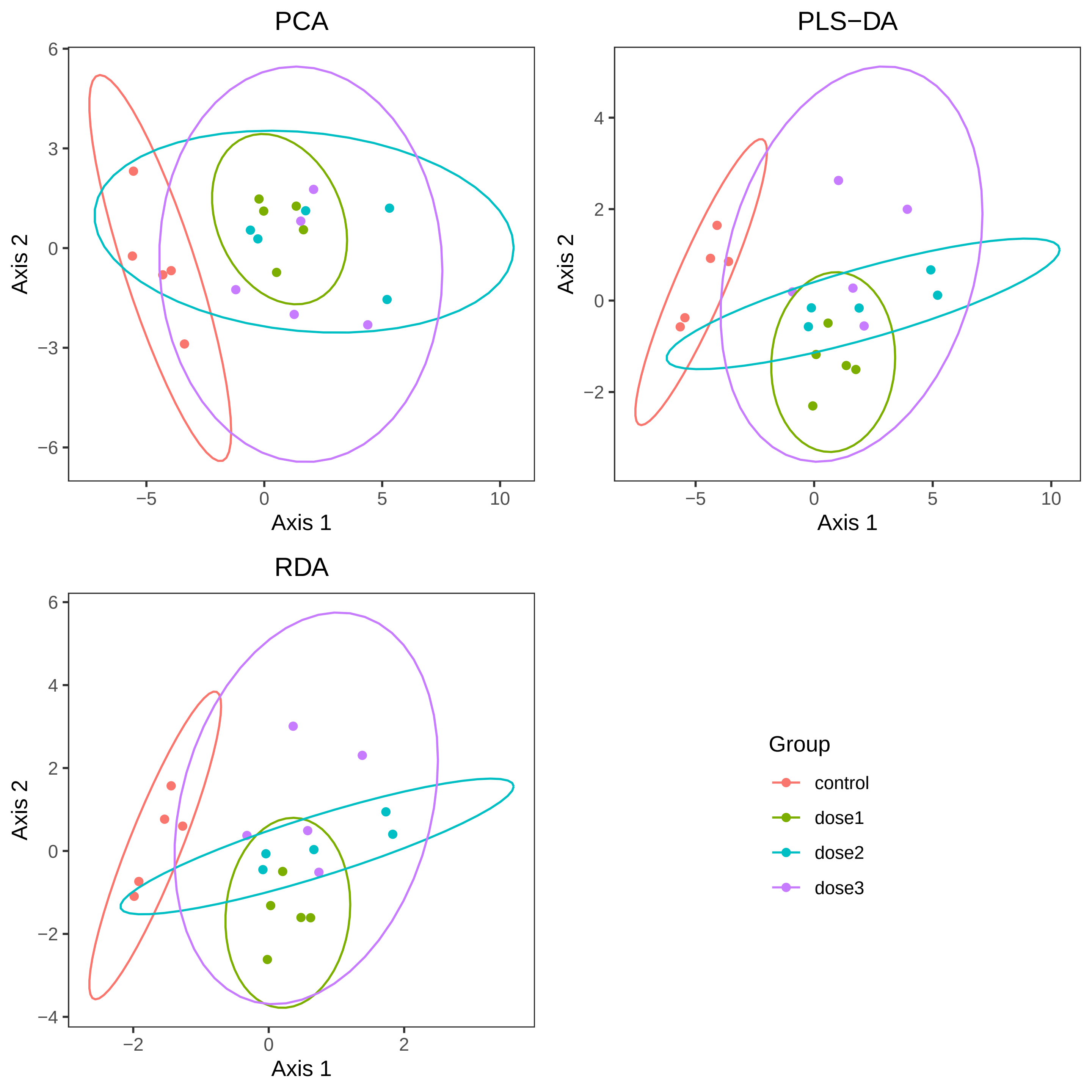
PCA
PLS−DA
RDA
Axis 1
Axis 2
Group
control
dose1
dose2
dose3

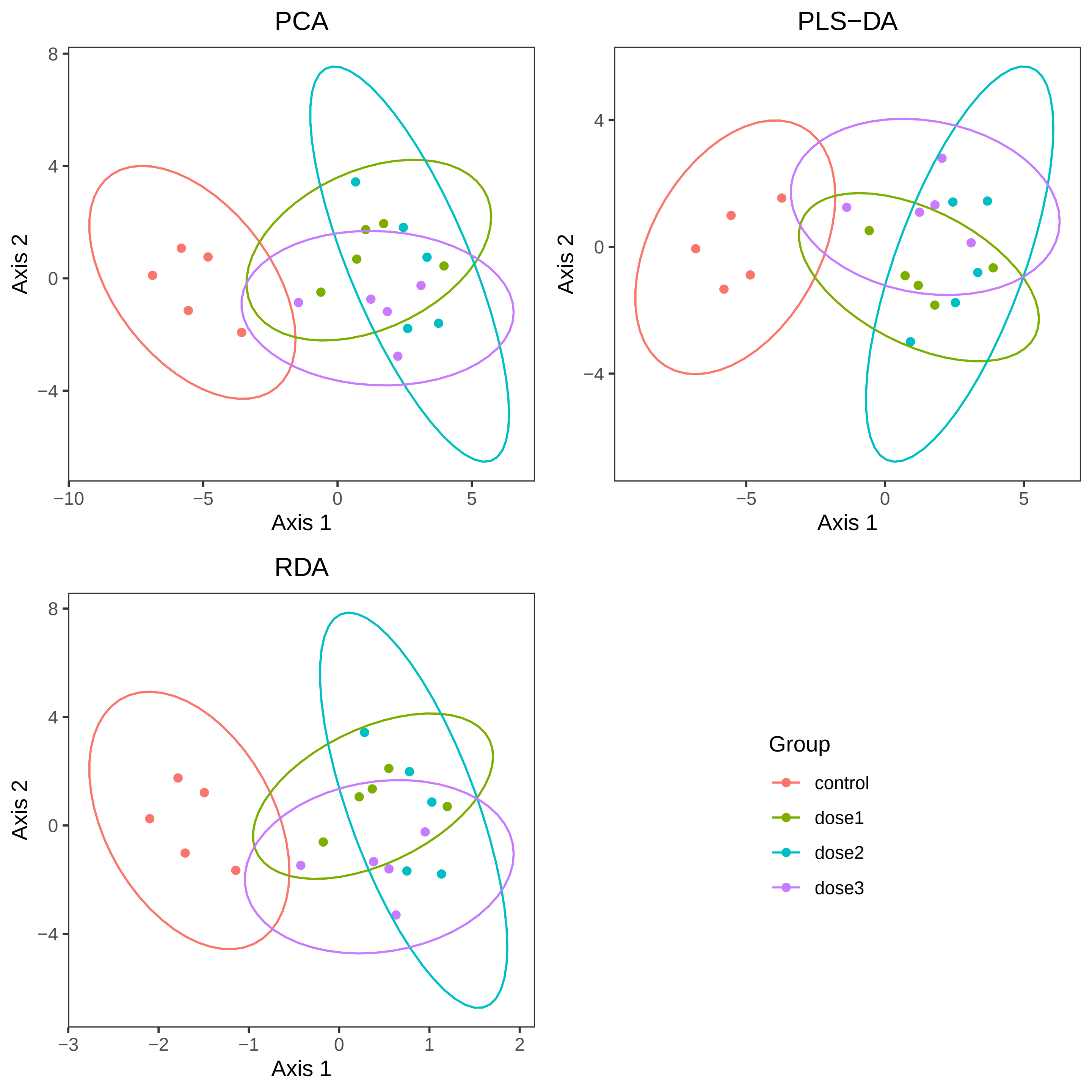
PCA
PLS−DA
RDA
Axis 1
Axis 2
Group
control
dose1
dose2
dose3

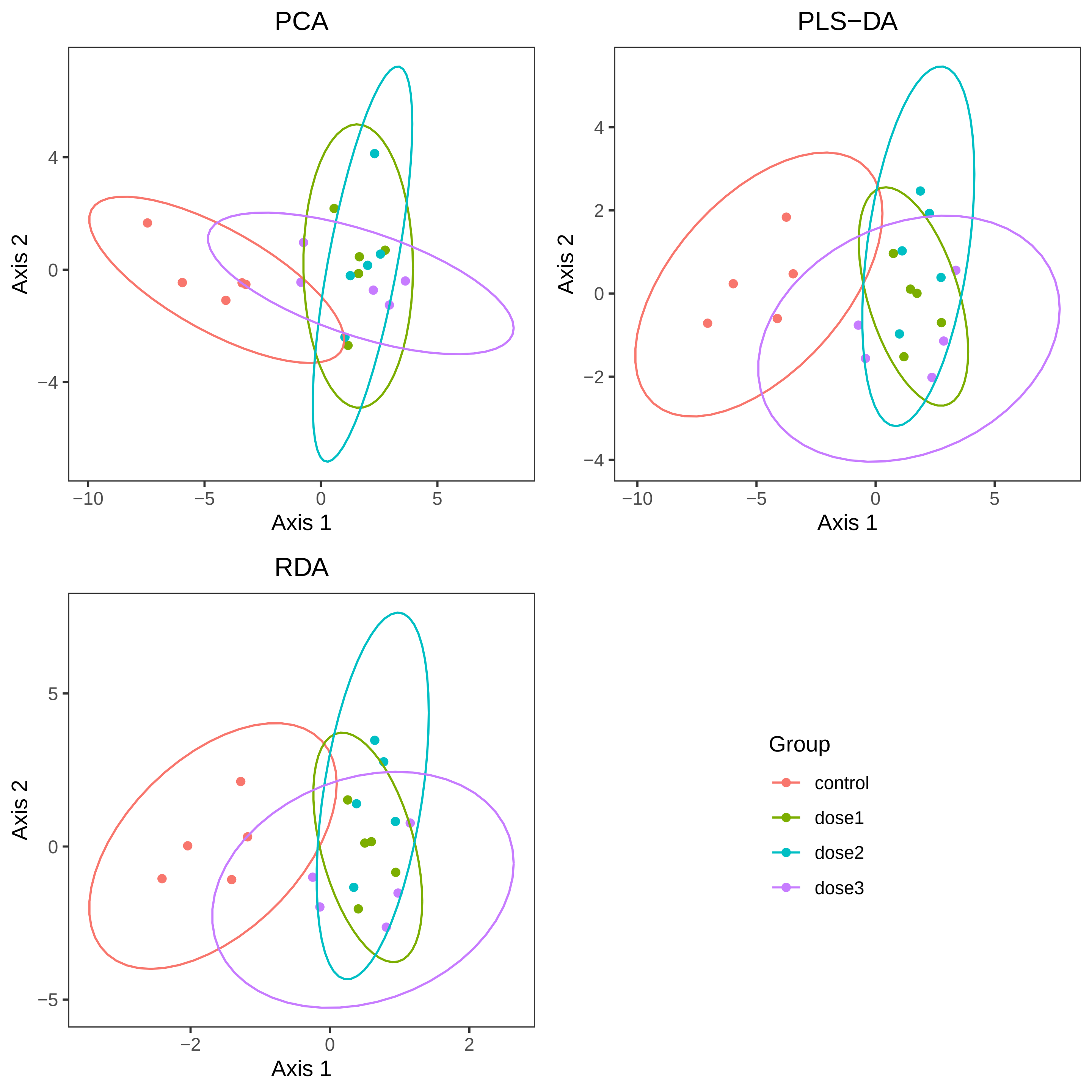
PCA
PLS−DA
RDA
Axis 1
Axis 2
−10
−5
0
5
4
0
−4
2
−2
−5
Group
control
dose1
dose2
dose3

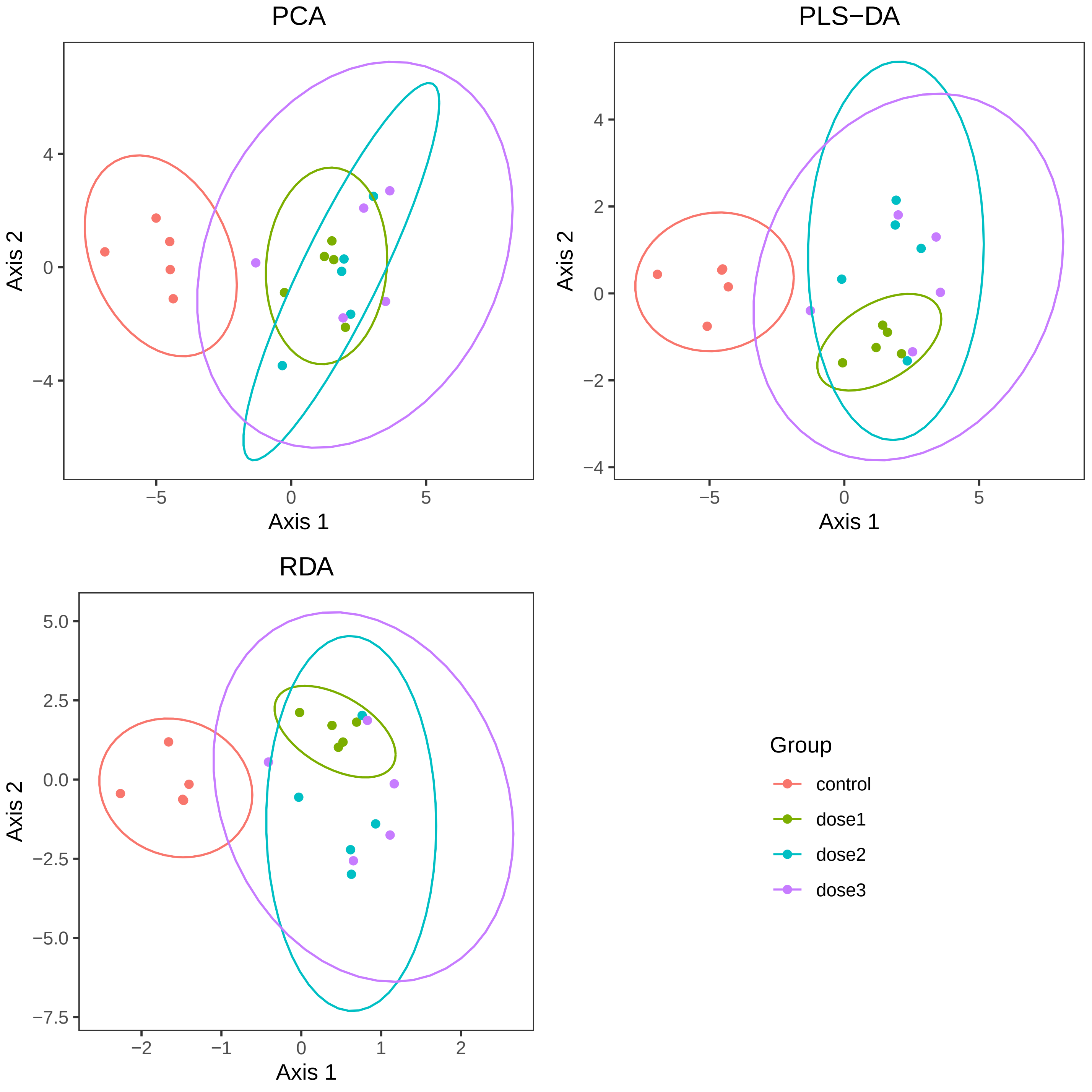
PCA
PLS−DA
RDA
Axis 1
Axis 2
Group
control
dose1
dose2
dose3

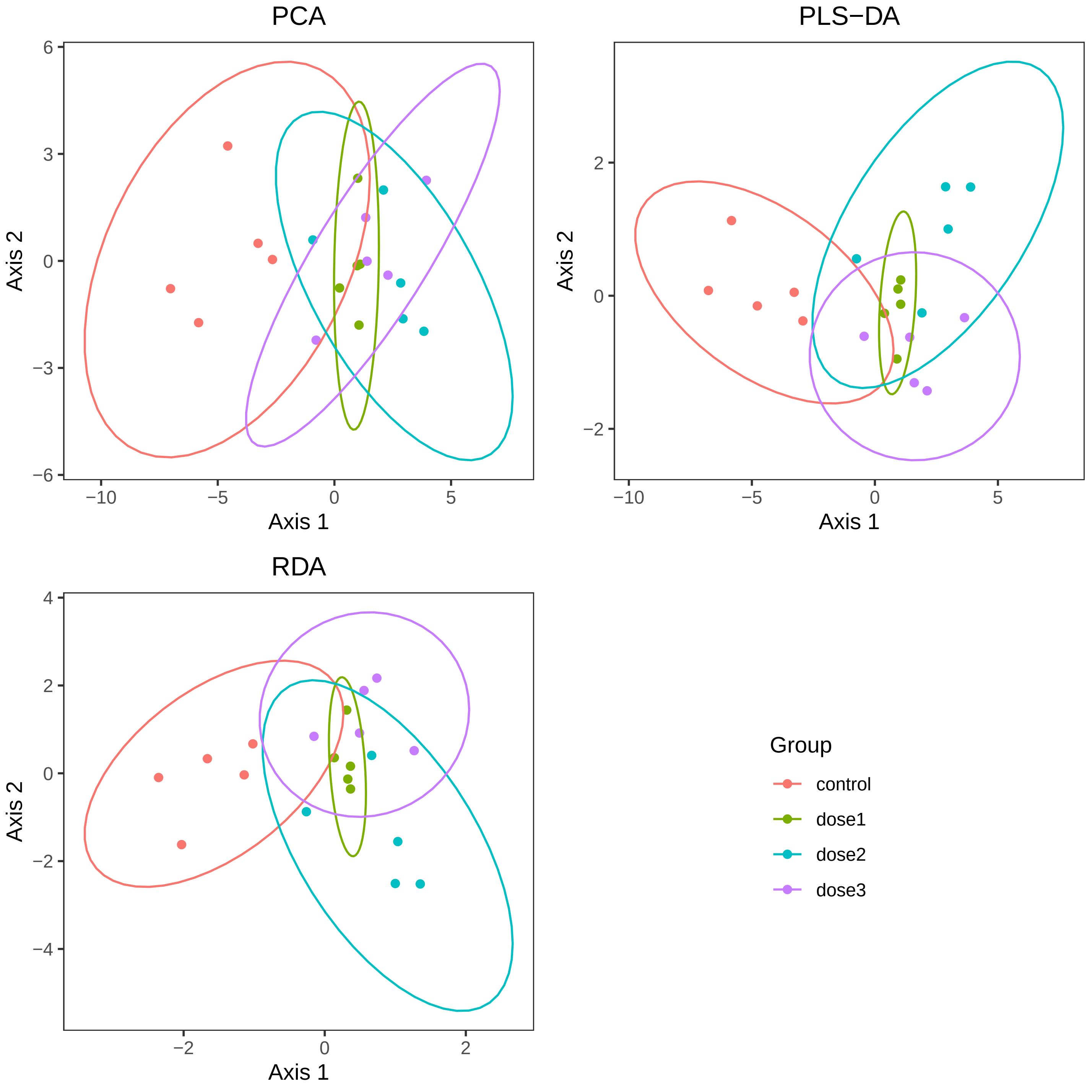

PCA
PLS−DA
RDA
Axis 1
Axis 2
Group
control
dose1
dose2
dose3

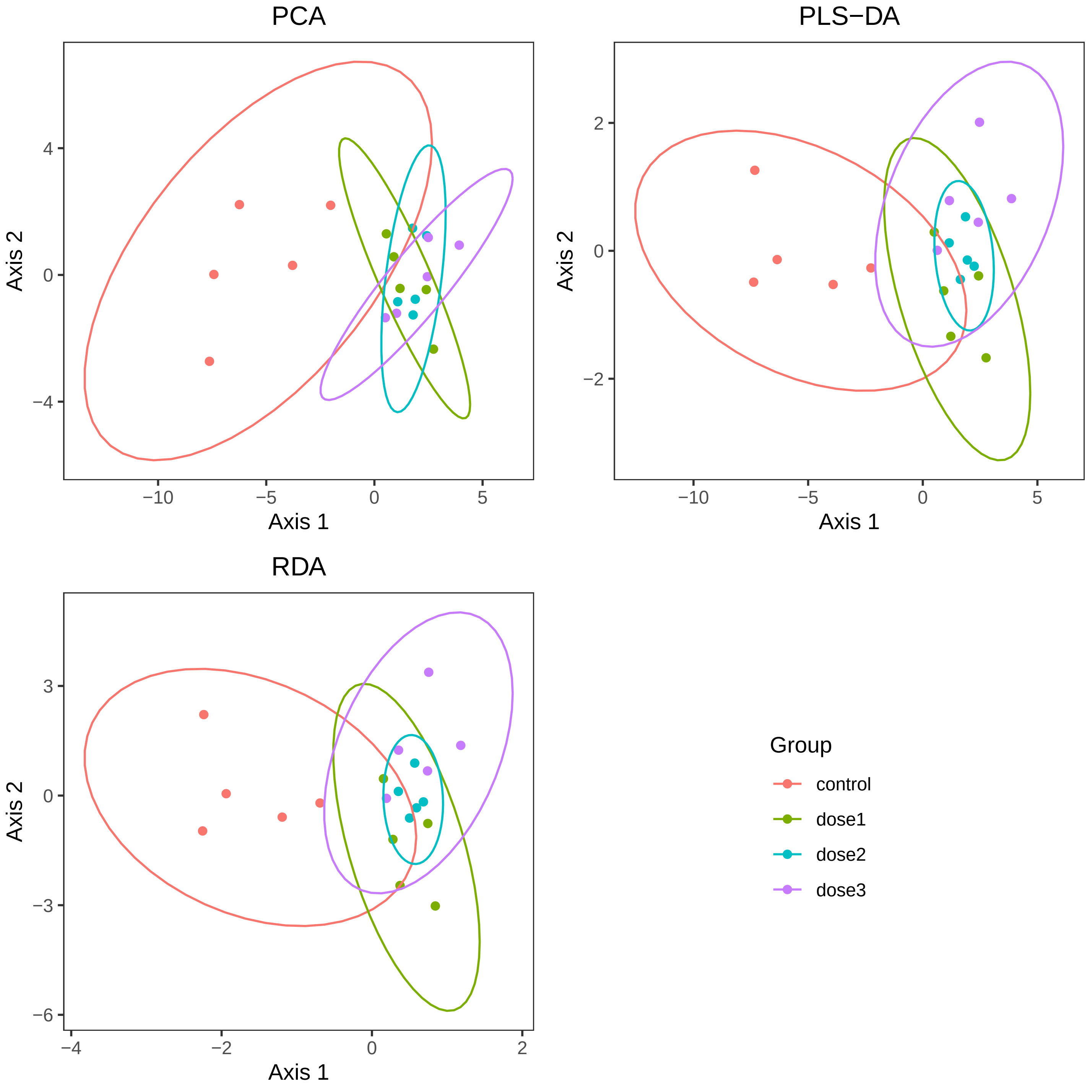
PCA
PLS−DA
RDA
Axis 1
Axis 2
−10
−5
0
5
4
0
−4
2
−2
−4
−2
0
2
3
0
−3
−6
Group
control
dose1
dose2
dose3

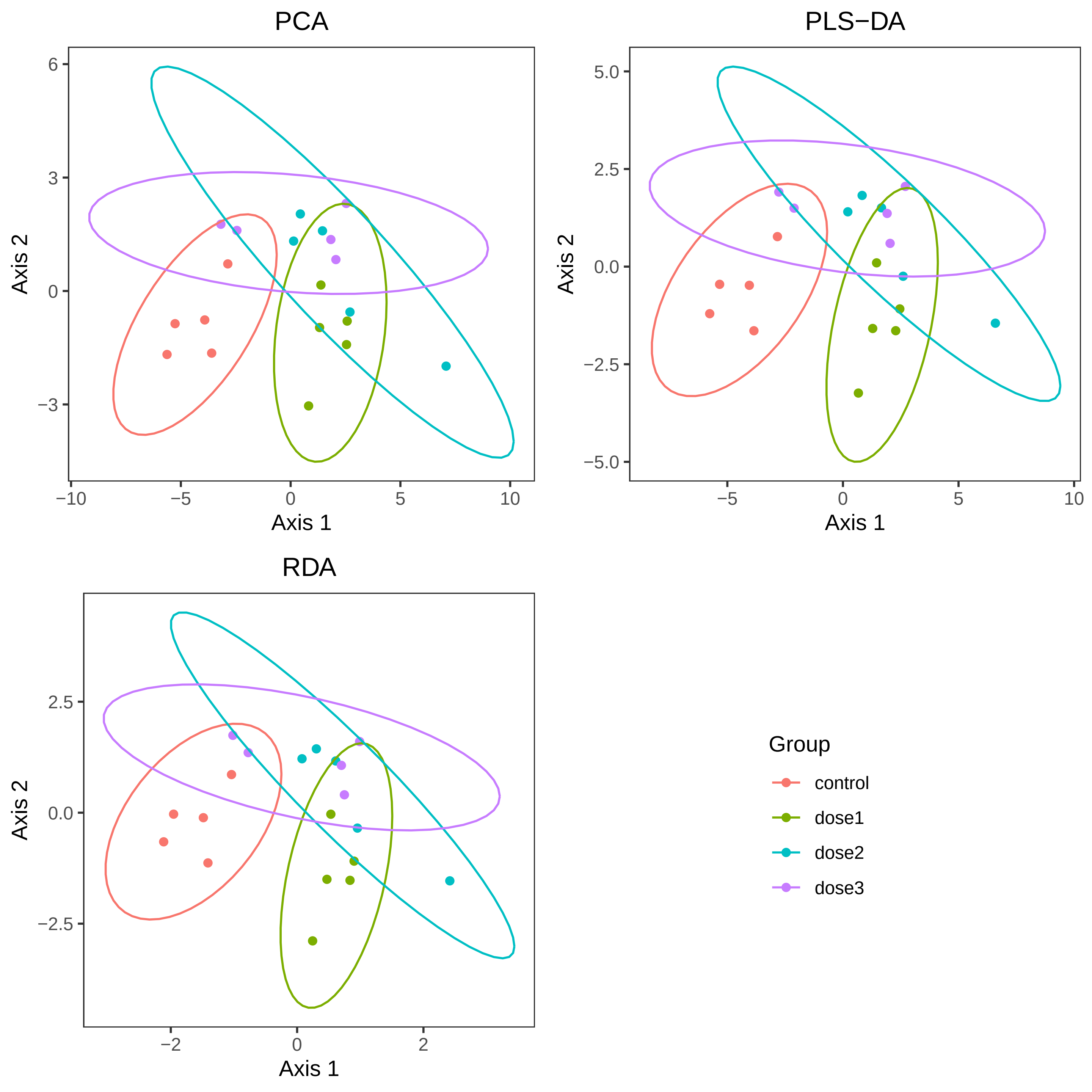
PCA
PLS−DA
RDA
Axis 1
Axis 2
Group
control
dose1
dose2
dose3

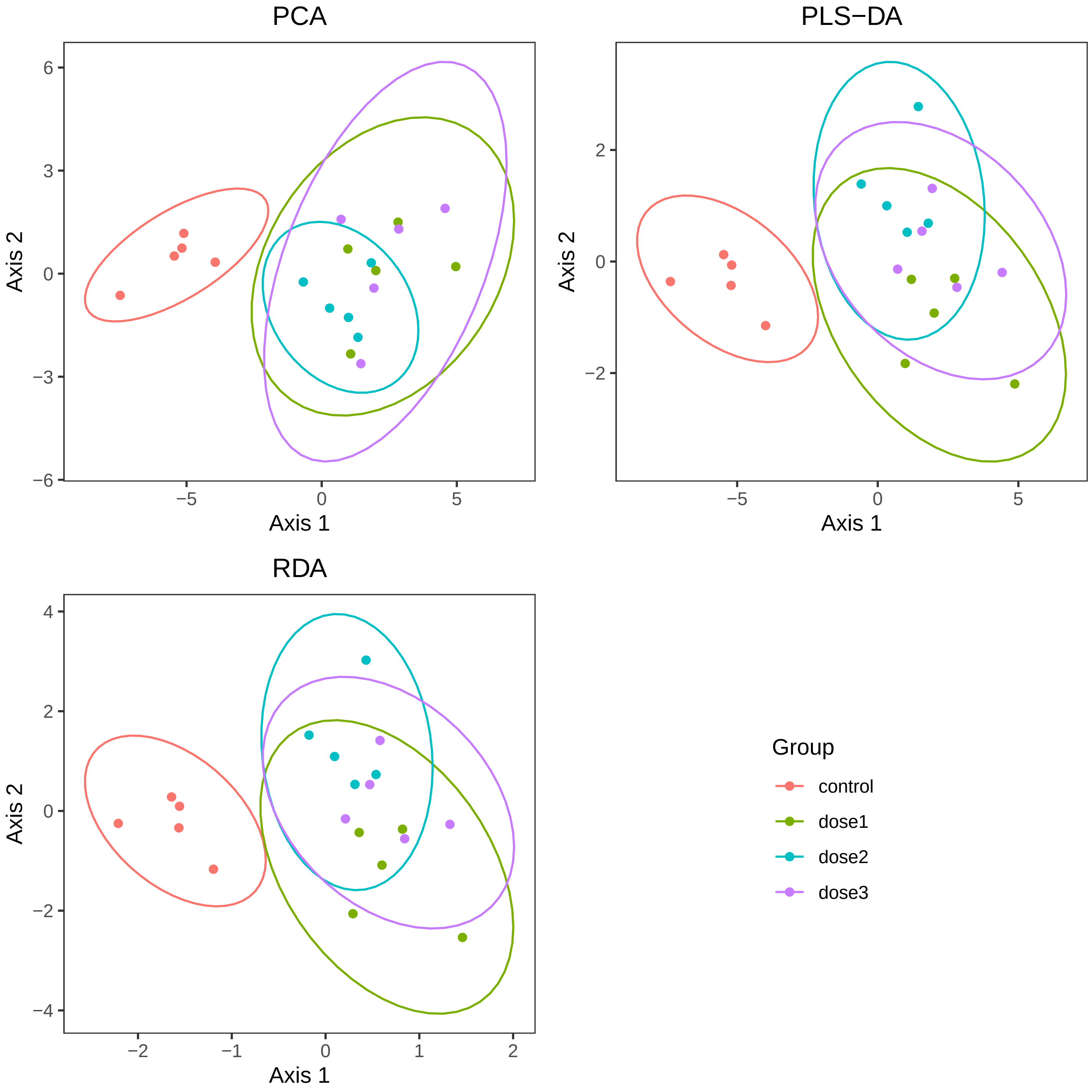
PCA
PLS−DA
RDA
Axis 1
Axis 2
Group
control
dose1
dose2
dose3

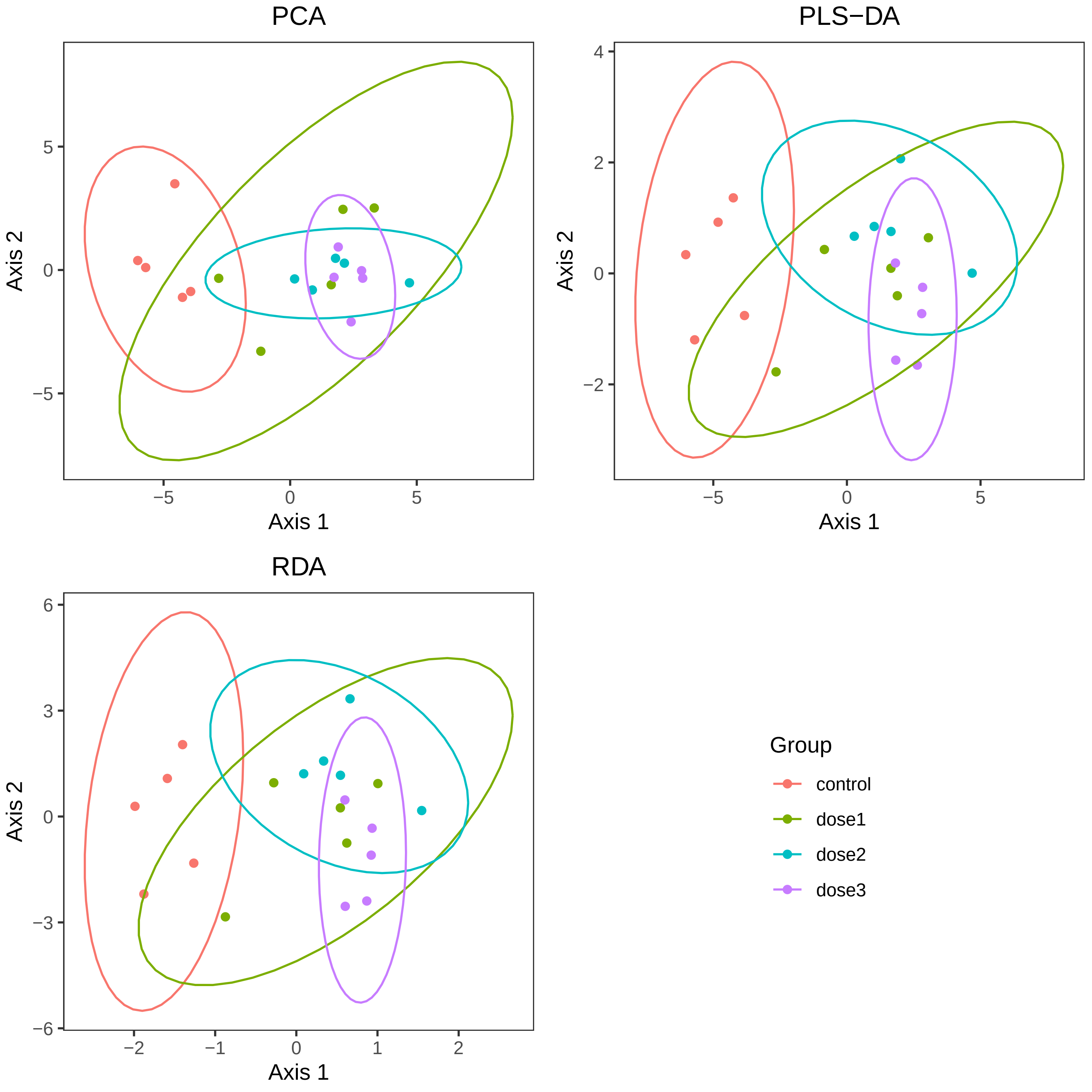

PCA
PLS−DA
RDA
Axis 1
Axis 2
Group
control
dose1
dose2
dose3

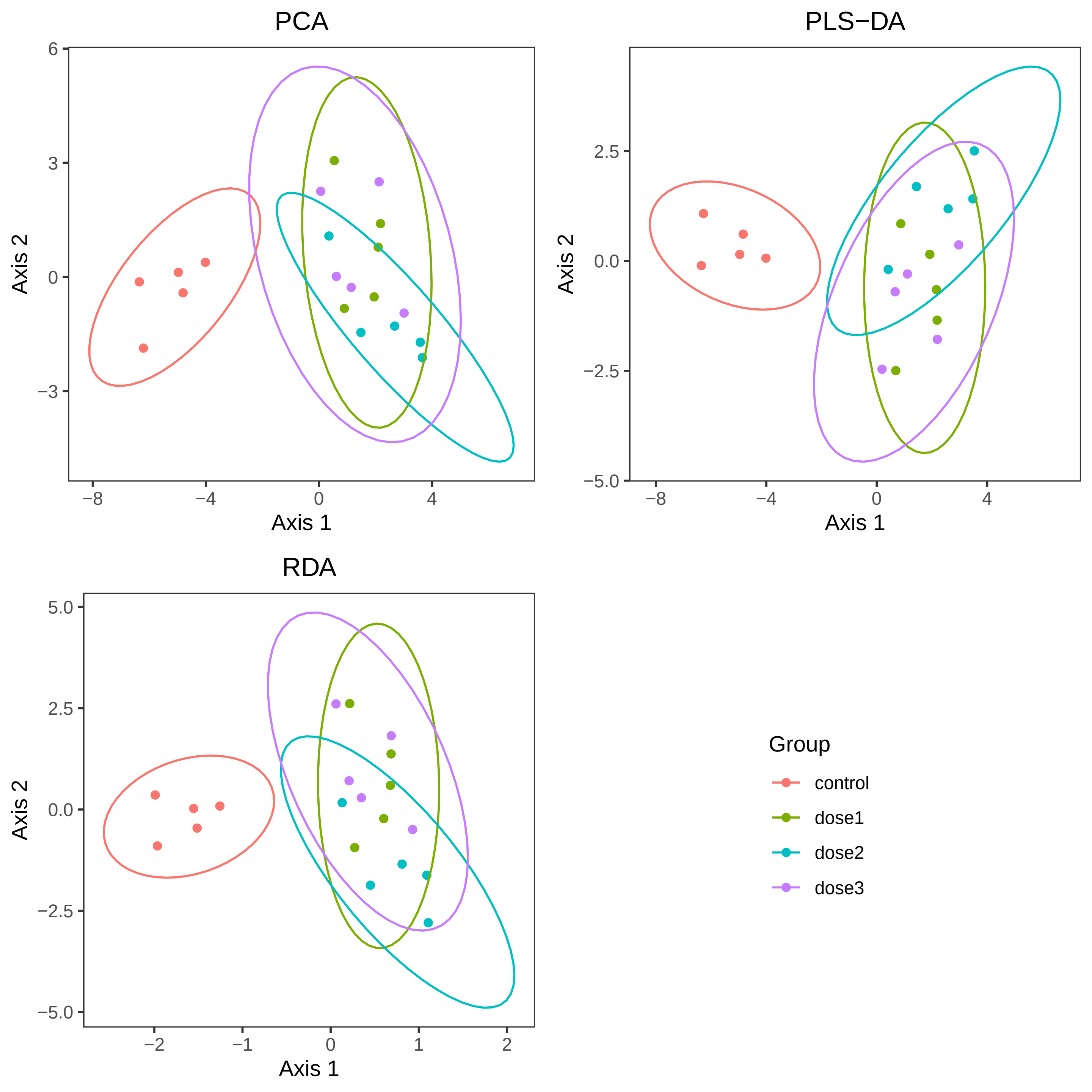
PCA
PLS−DA
RDA
Axis 1
Axis 2
6
3
0
−3
−8
−4
0
4
2.5
0.0
−2.5
−5.0
5.0
−2
−1
1
2
Group
control
dose1
dose2
dose3

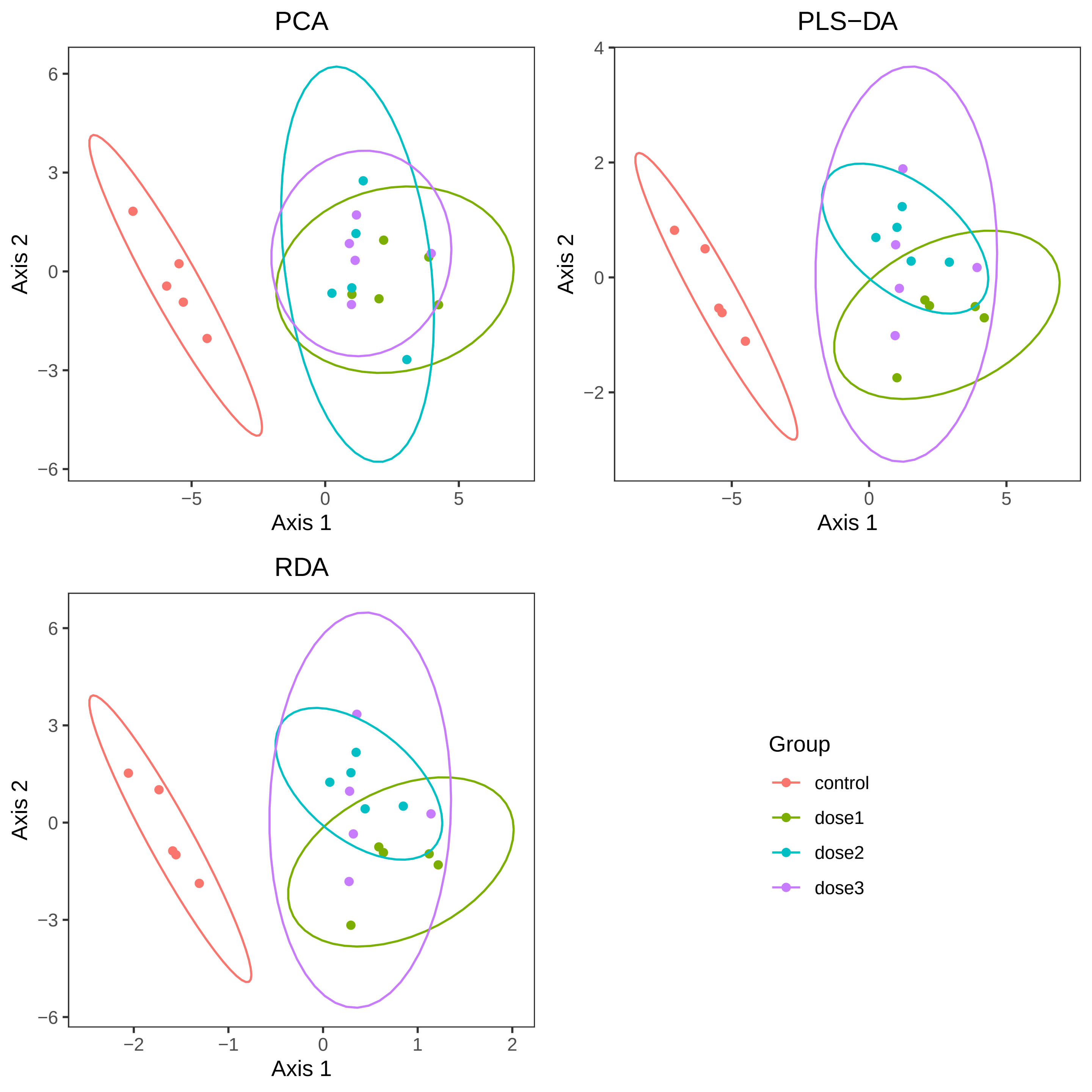

PCA
PLS−DA
RDA
Axis 1
Axis 2
−5
0
5
6
3
0
−3
−6
4
2
−2
−2
−1
1
2
Group
control
dose1
dose2
dose3

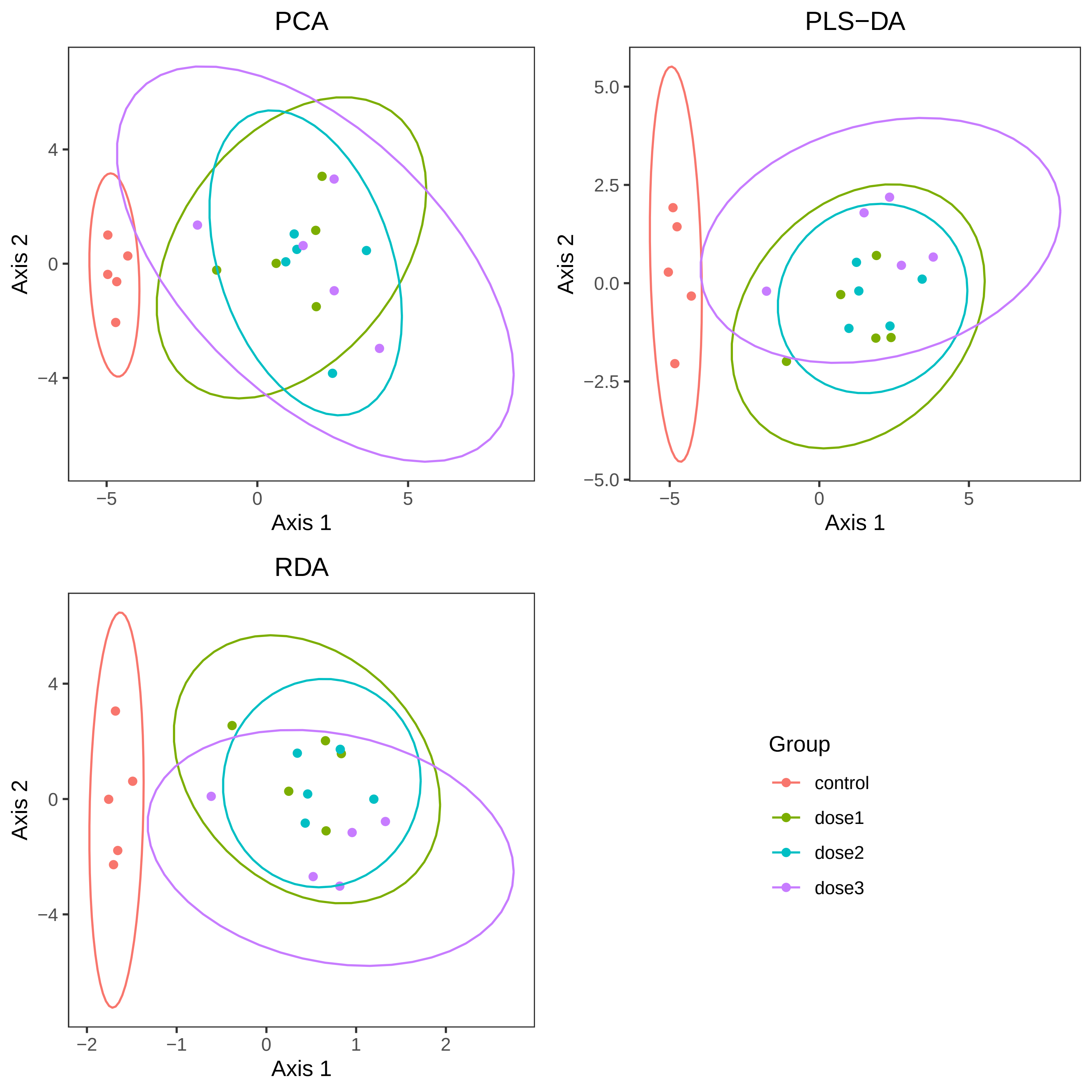

PCA
PLS−DA
RDA
Axis 1
Axis 2
Group
control
dose1
dose2
dose3

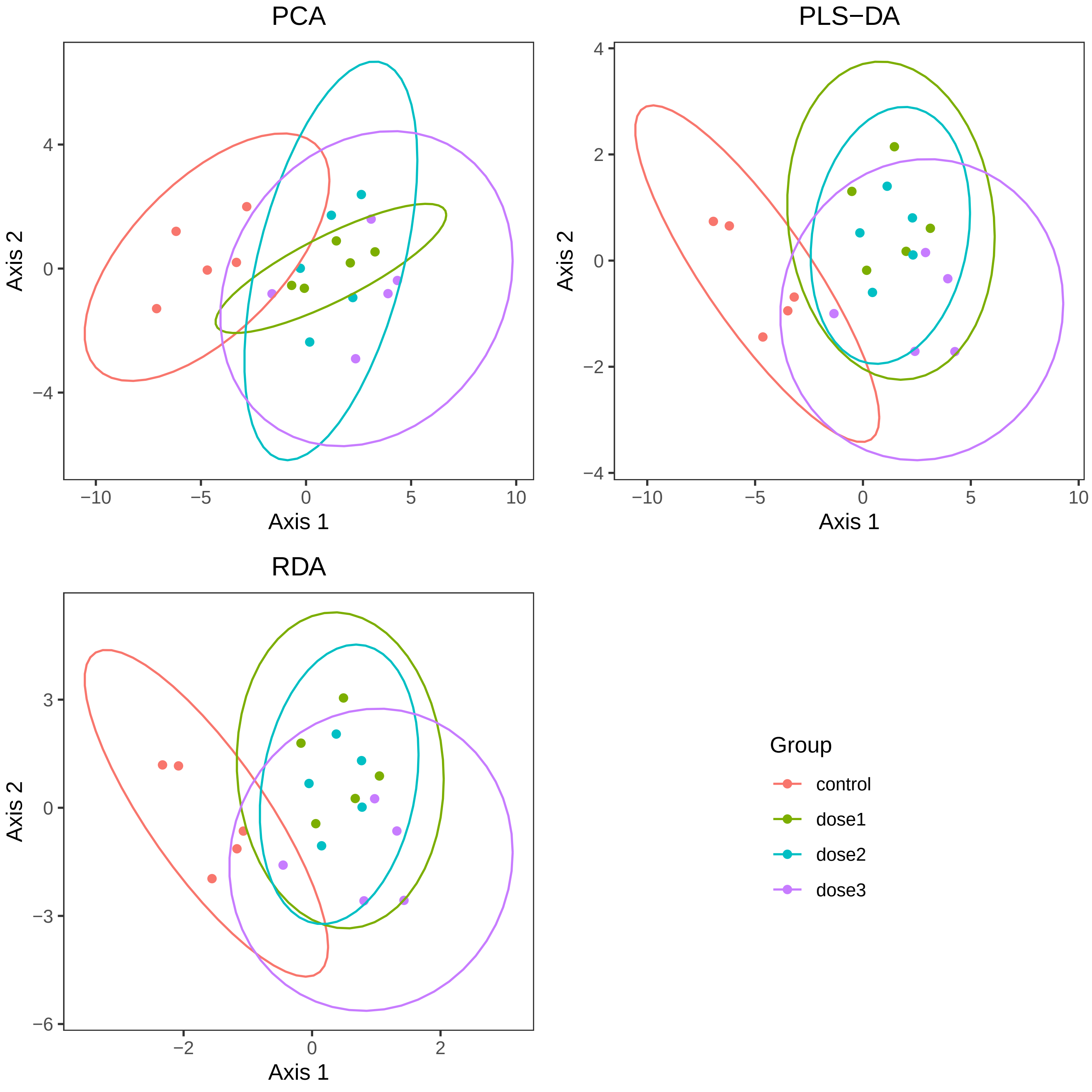

PCA
PLS−DA
RDA
Axis 1
Axis 2
Group
control
dose1
dose2
dose3

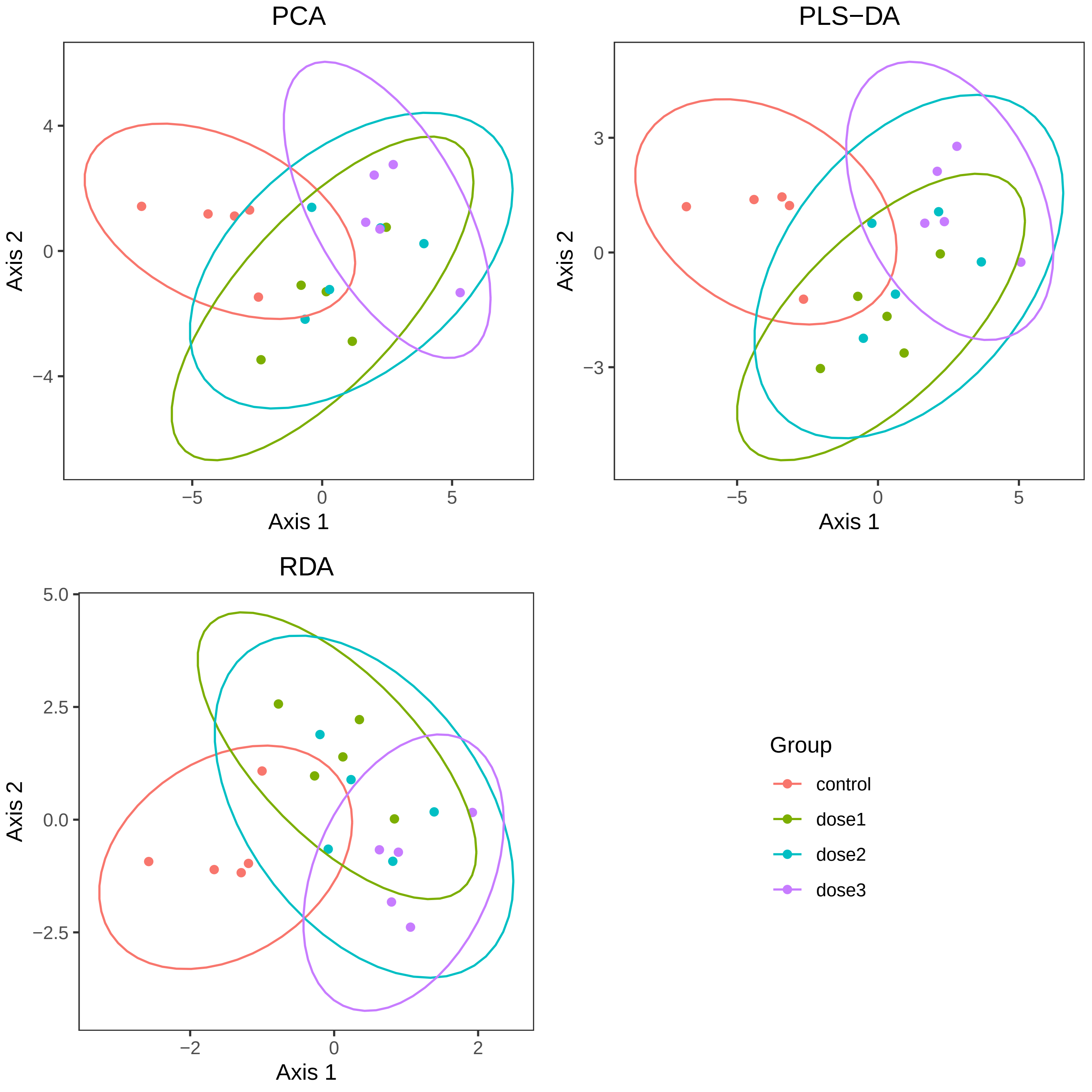

PCA
PLS−DA
RDA
Axis 1
Axis 2
Group
control
dose1
dose2
dose3

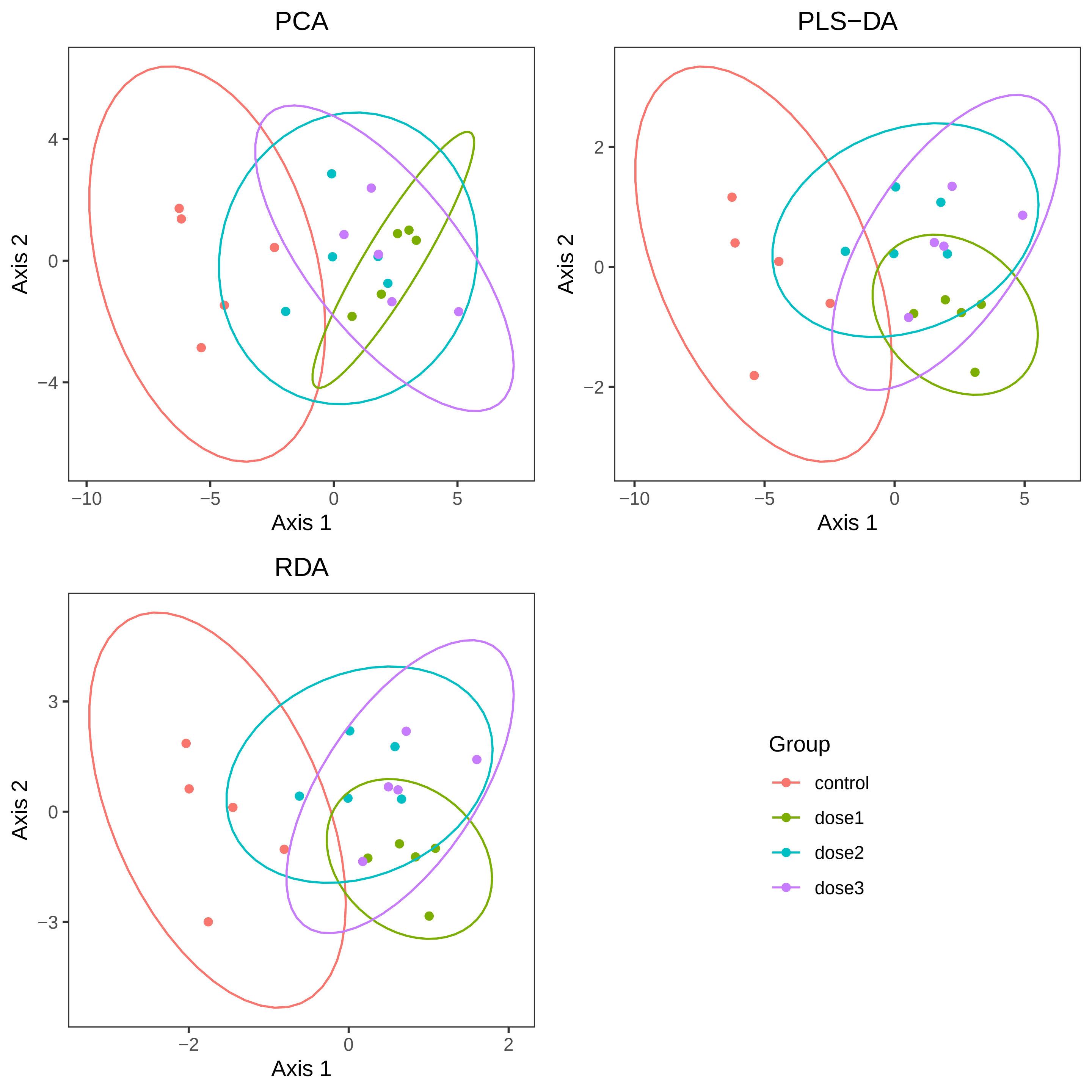
PCA
PLS−DA
RDA
Axis 1
Axis 2
Group
control
dose1
dose2
dose3

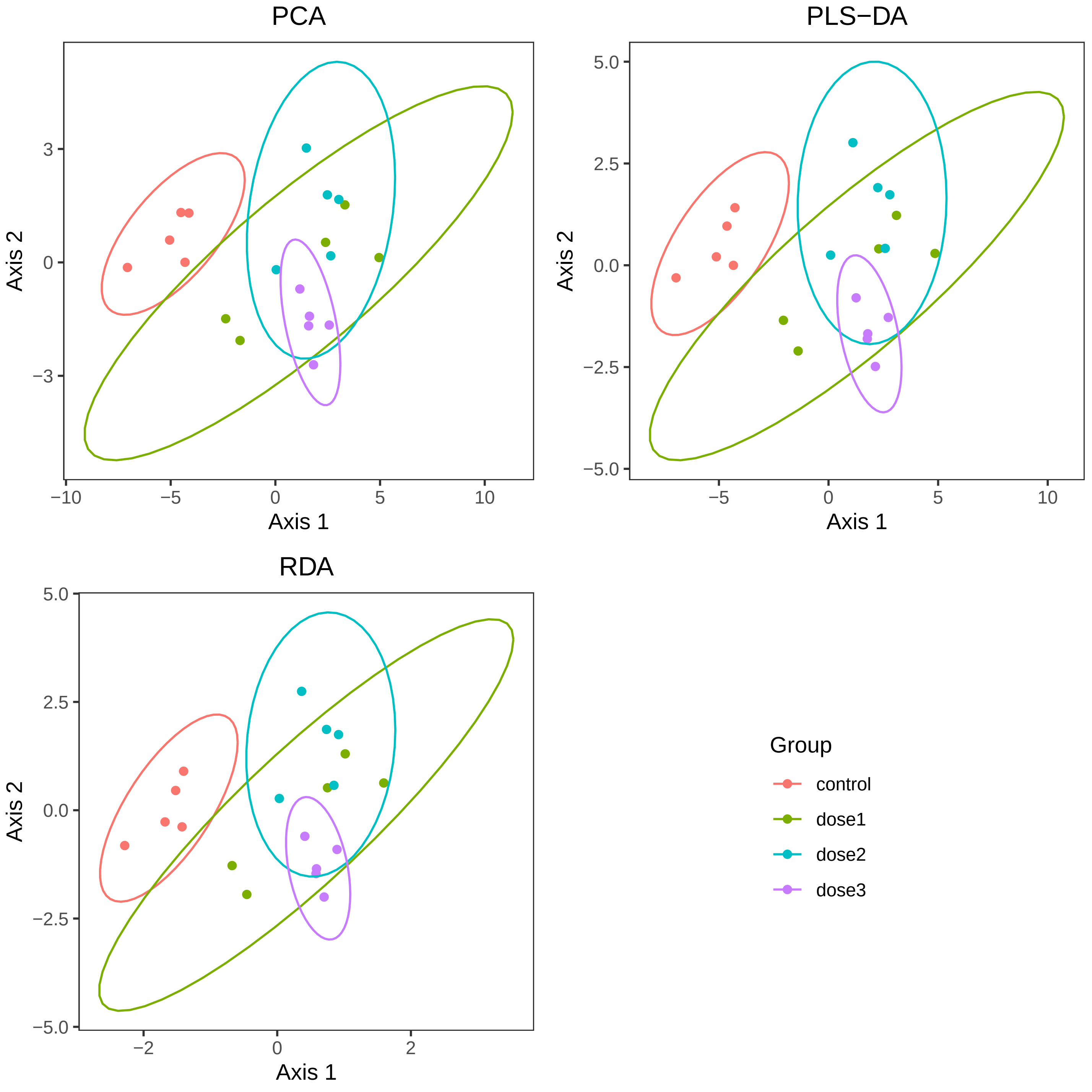
PCA
PLS−DA
RDA
Axis 1
Axis 2
Group
control
dose1
dose2
dose3
−10
−5
0
5
10
3
0
−3
5.0
2.5
0.0
−2.5
−5.0
−2
2

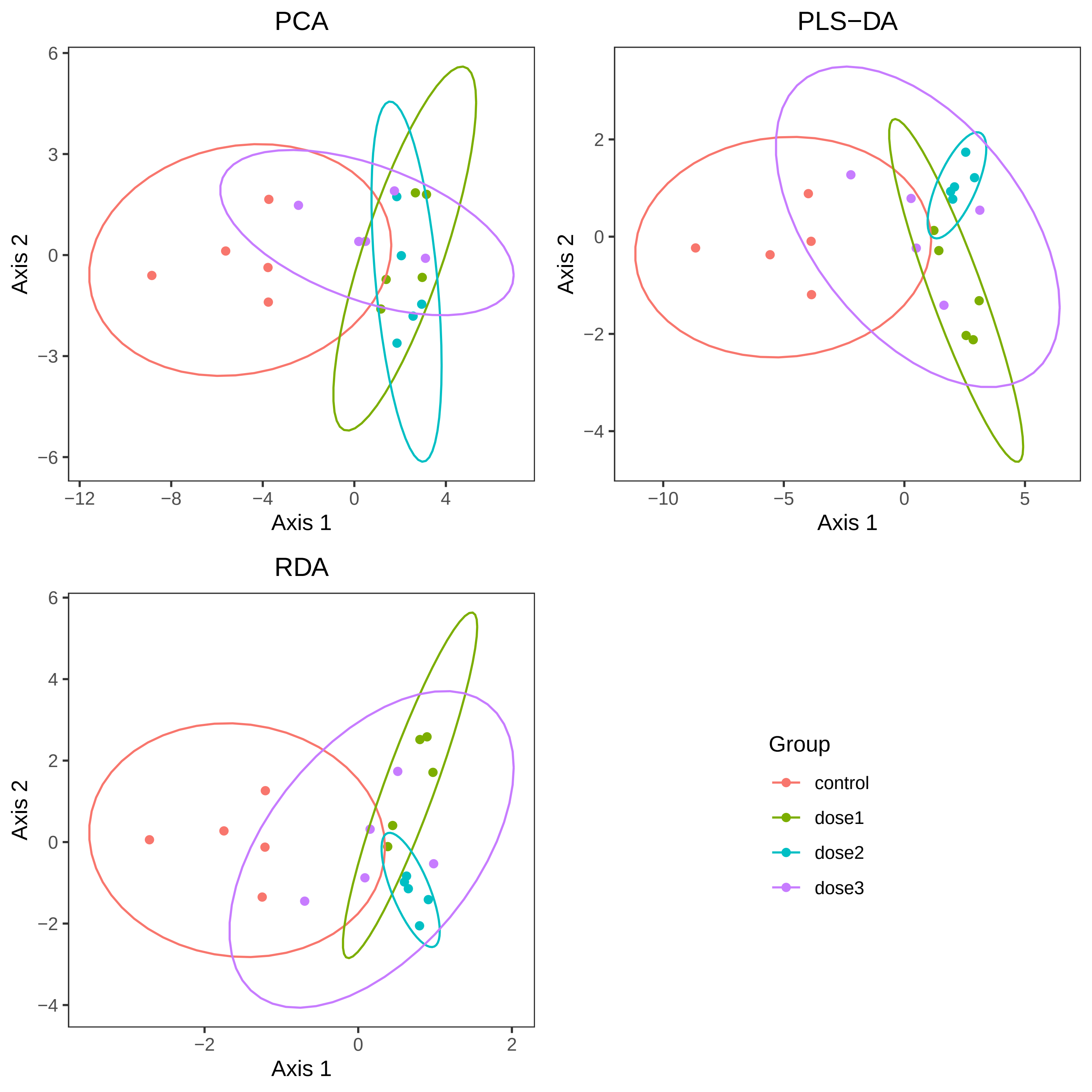
PCA
PLS−DA
RDA
Axis 1
Axis 2
Group
control
dose1
dose2
dose3

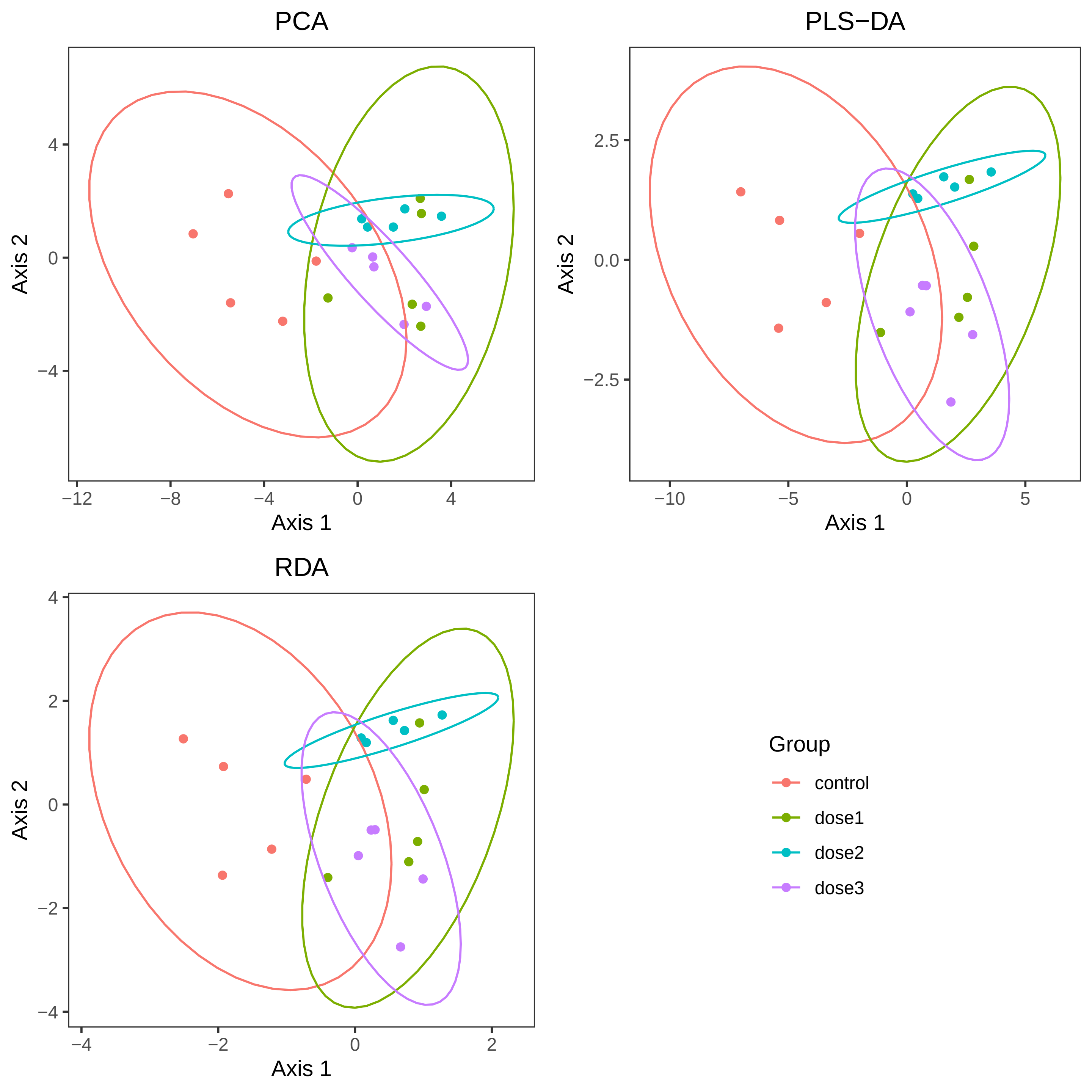
PCA
PLS−DA
RDA
Axis 1
Axis 2
−12
−8
−4
0
4
2.5
0.0
−2.5
−10
−5
5
2
−2
Group
control
dose1
dose2
dose3

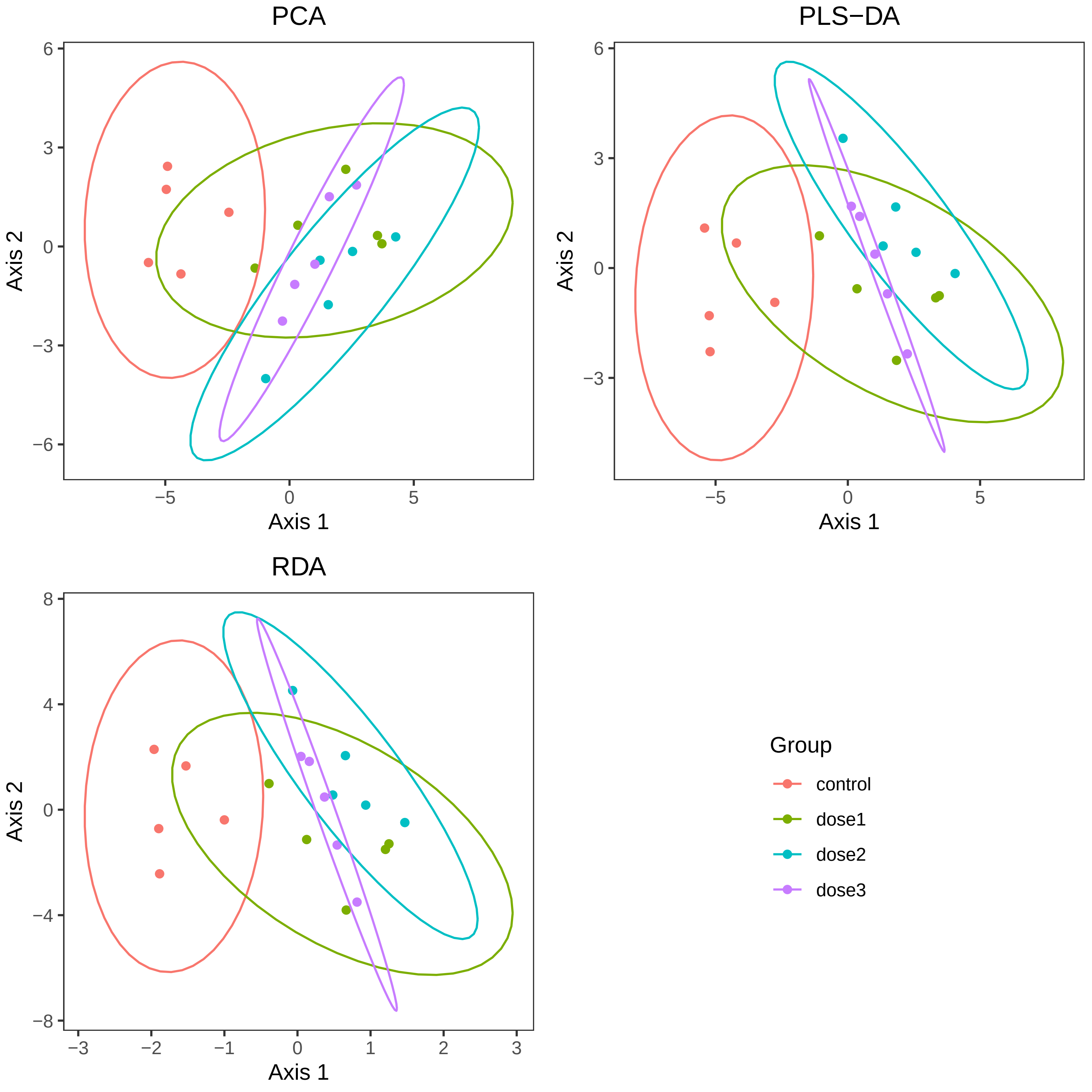

PCA
PLS−DA
RDA
Axis 1
Axis 2
Group
control
dose1
dose2
dose3

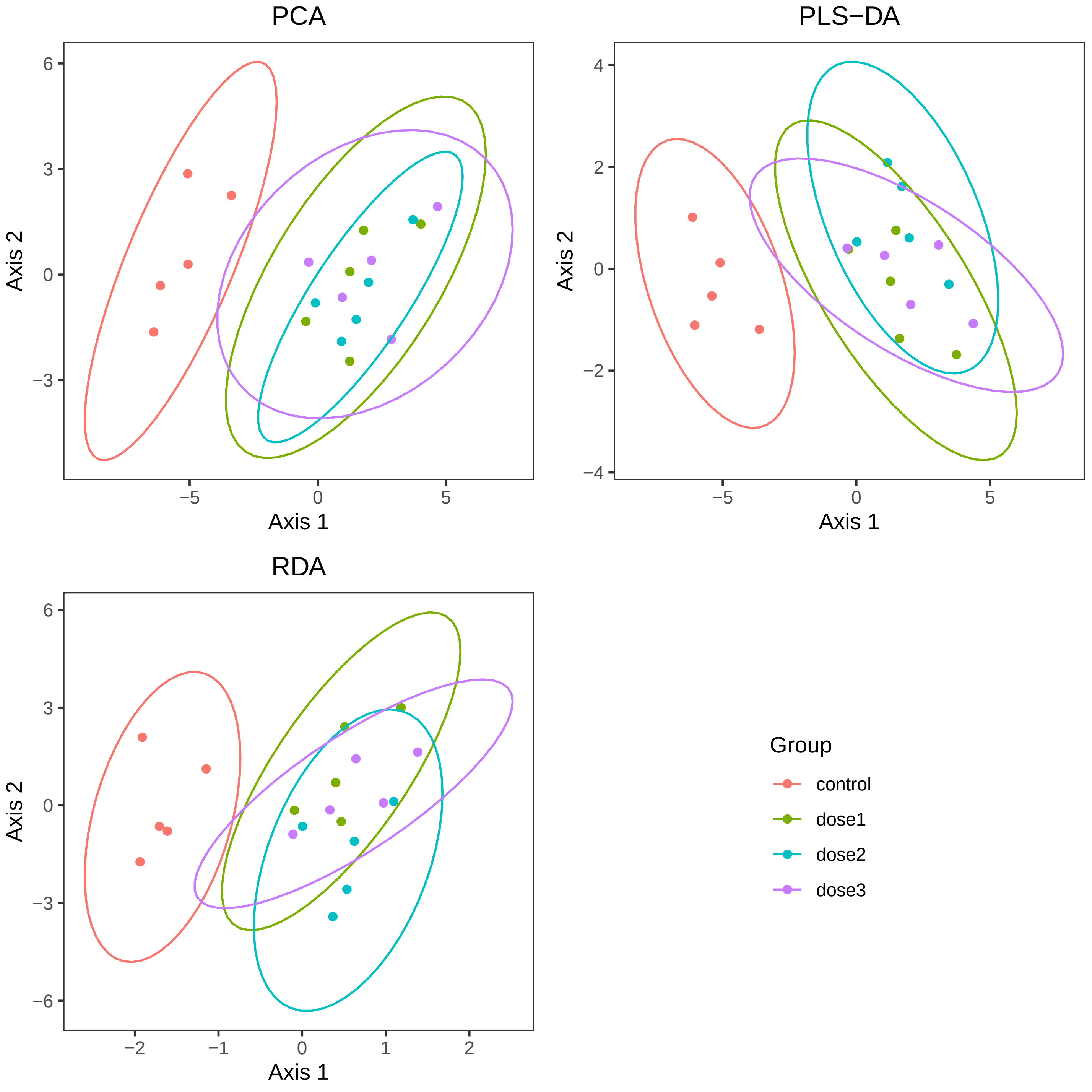
PCA
PLS−DA
RDA
Axis 1
Axis 2
6
3
0
−3
−6
4
2
−2
−4
−5
5
−2
−1
1
Group
control
dose1
dose2
dose3

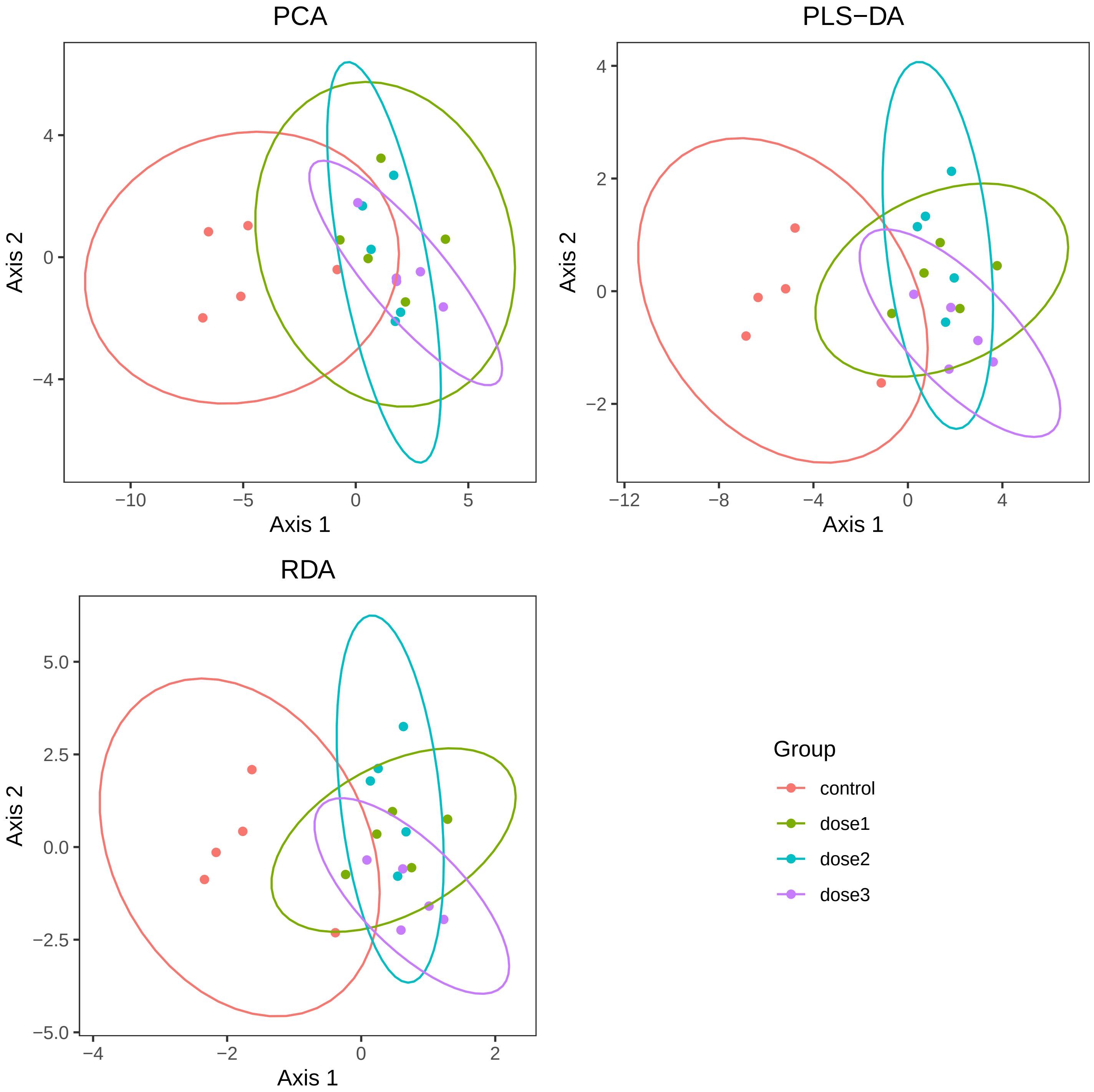
PCA
Axis 1
Axis 2
−10
−5
0
5
4
0
−4
PLS−DA
Axis 1
Axis 2
−12
−8
−4
0
4
4
2
0
−2
RDA
Axis 1
Axis 2
−4
−2
0
2
5.0
2.5
0.0
−2.5
−5.0
Group
control
dose1
dose2
dose3

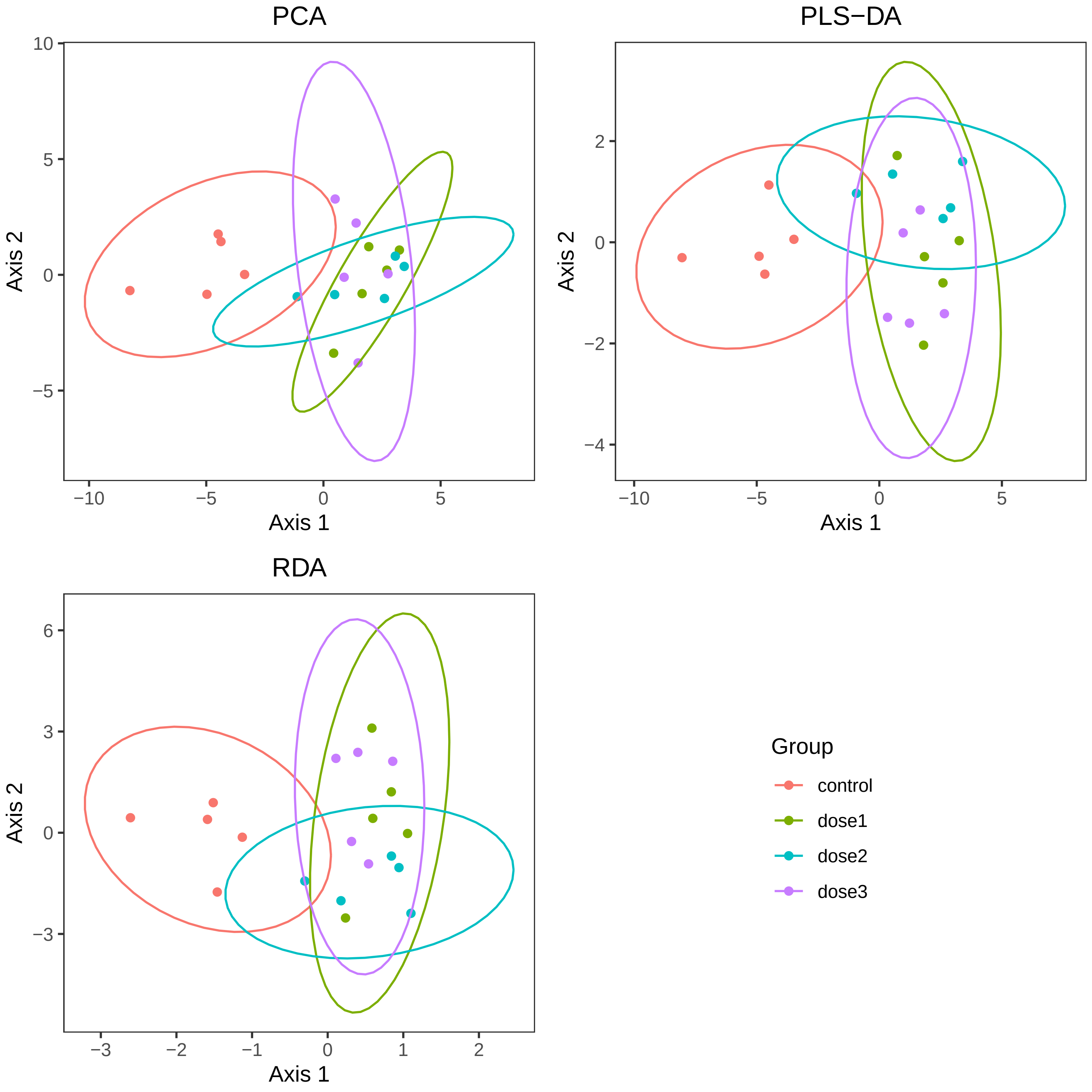
PCA
PLS−DA
RDA
Axis 1
Axis 2
Group
control
dose1
dose2
dose3

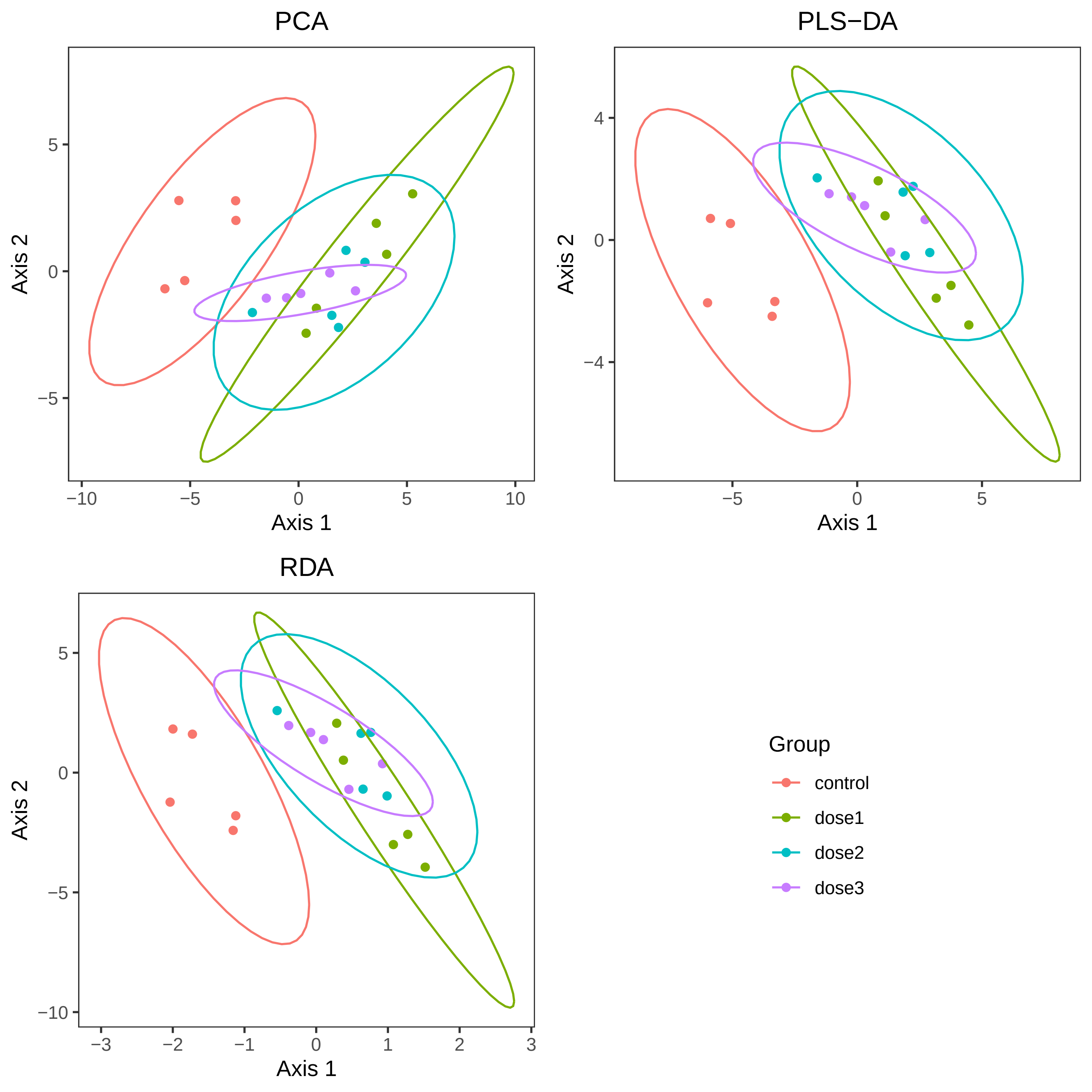
PCA
PLS−DA
RDA
Axis 1
Axis 2
Group
control
dose1
dose2
dose3

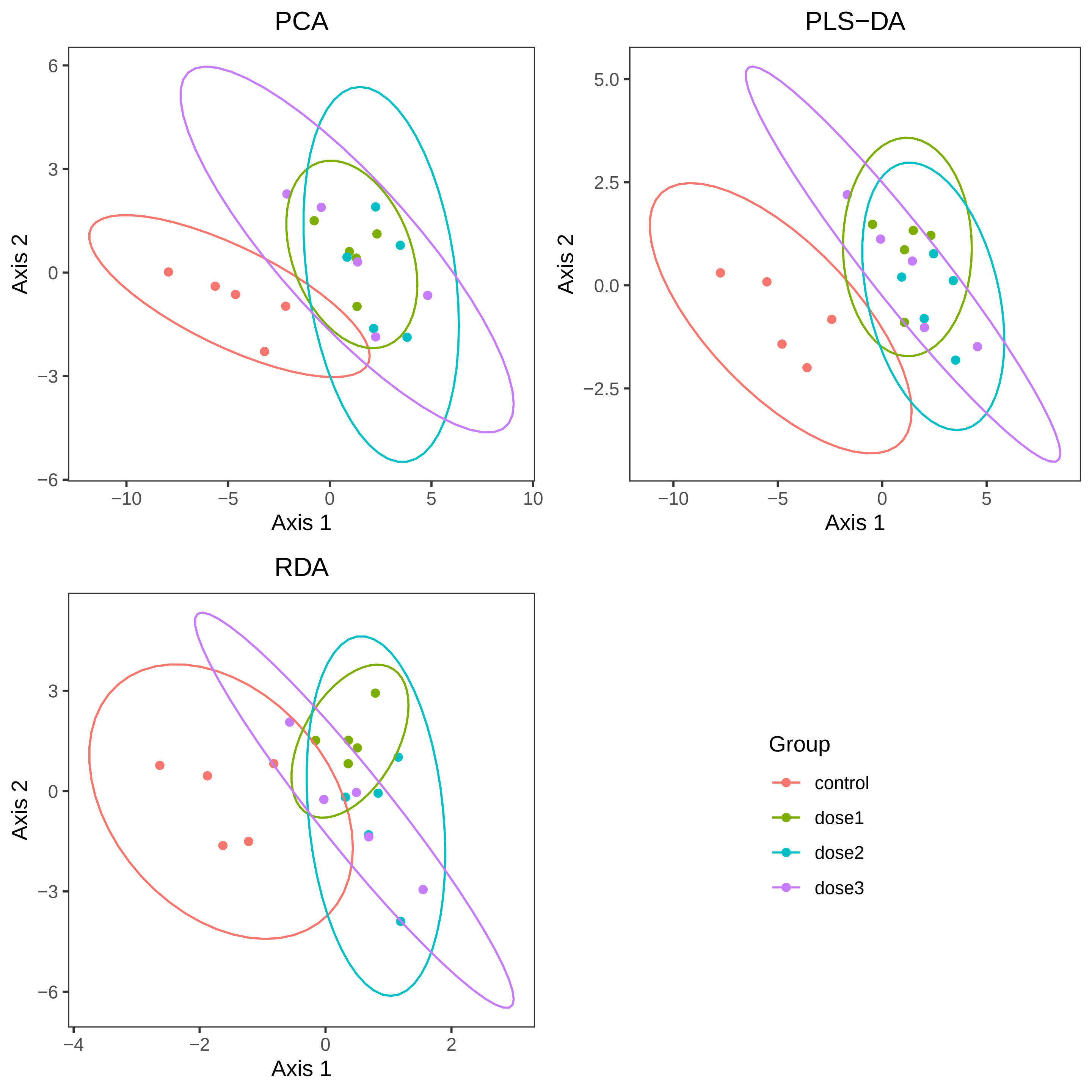
PCA
PLS−DA
RDA
Axis 1
Axis 2
Group
control
dose1
dose2
dose3

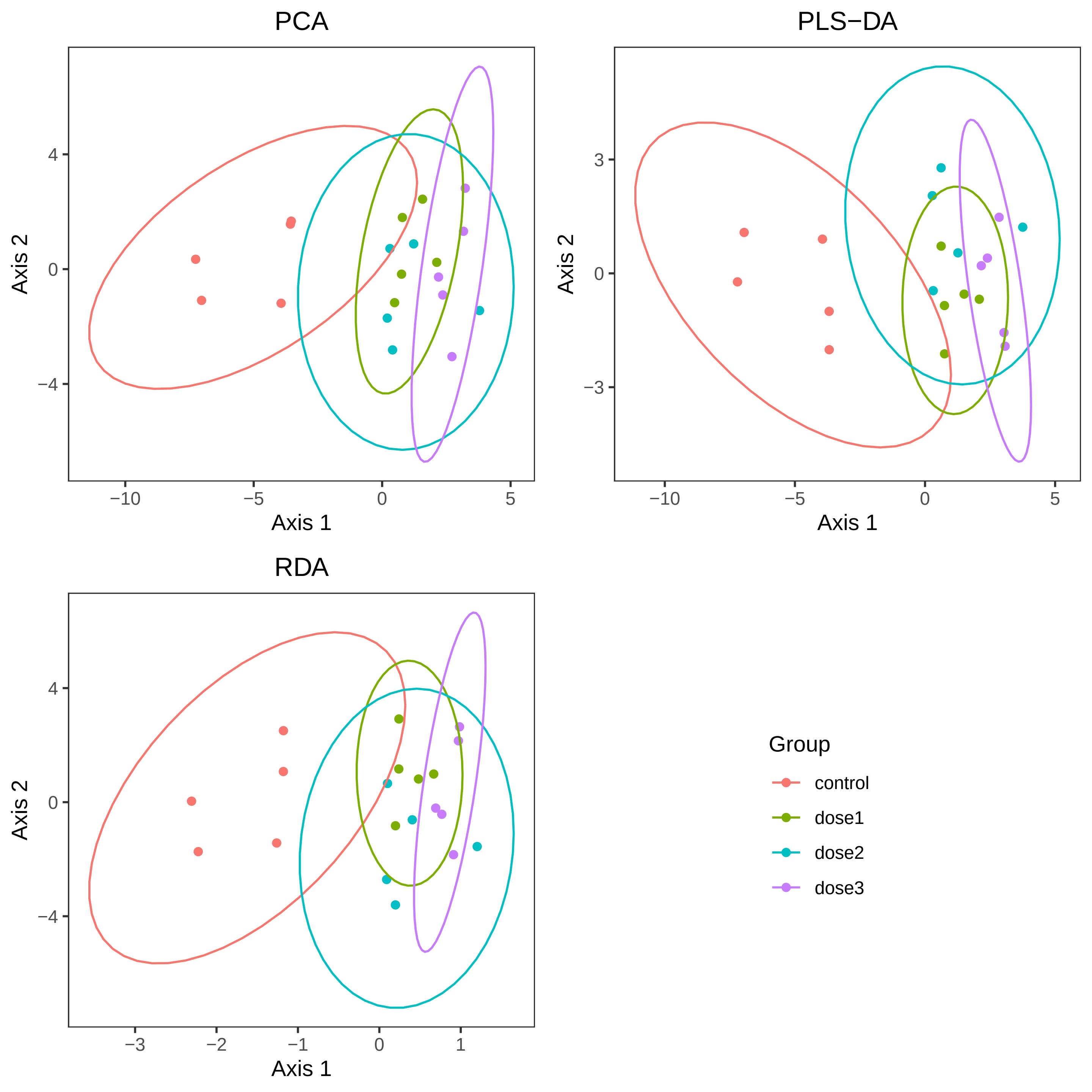
PCA
PLS−DA
RDA
Axis 1
Axis 2
−10
−5
0
5
4
−4
3
−3
−3
−2
−1
1
Group
control
dose1
dose2
dose3

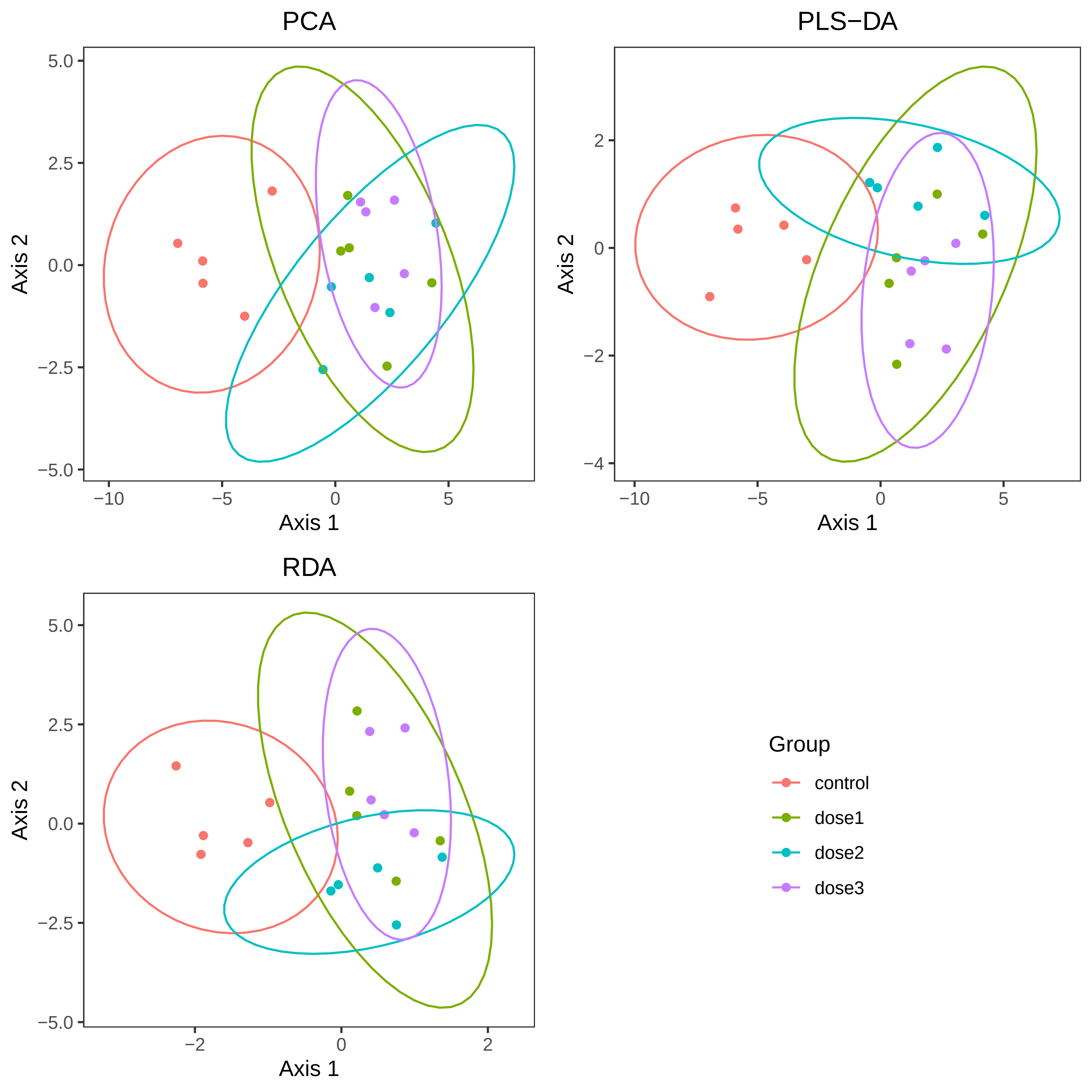
PCA
PLS−DA
RDA
Axis 1
Axis 2
Group
control
dose1
dose2
dose3

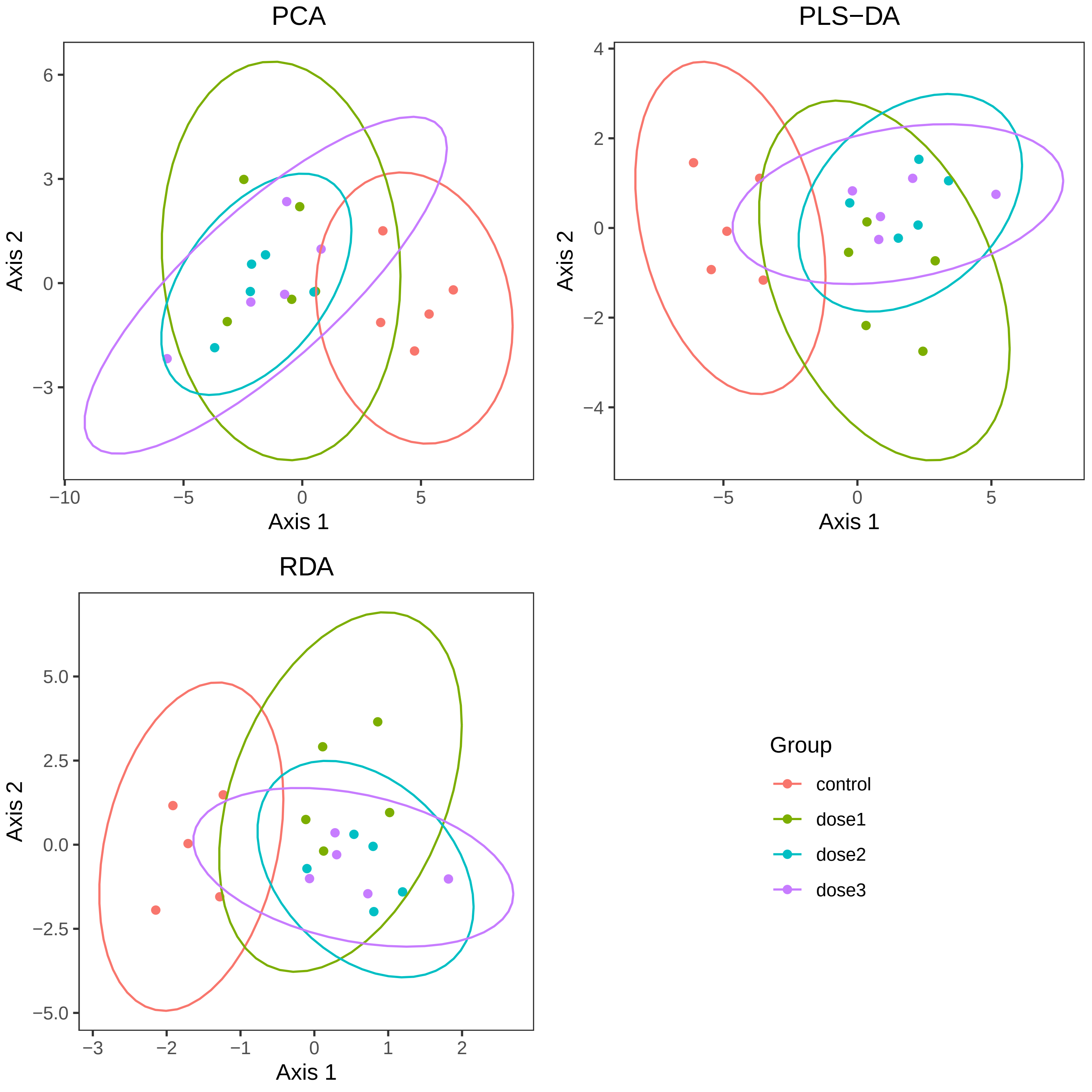
PCA
PLS−DA
RDA
Axis 1
Axis 2
Group
control
dose1
dose2
dose3

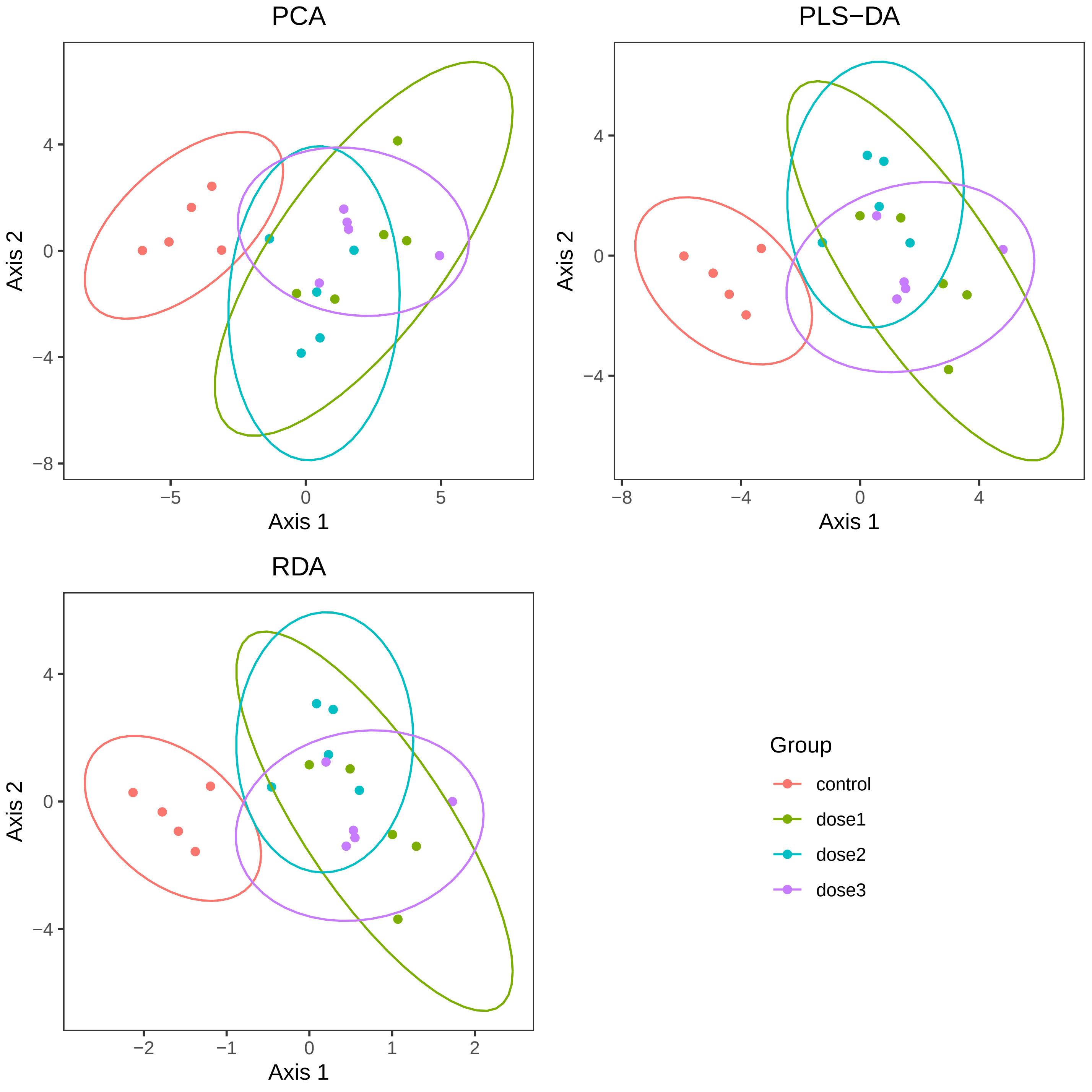

PCA
PLS−DA
RDA
Axis 1
Axis 2
Group
control
dose1
dose2
dose3

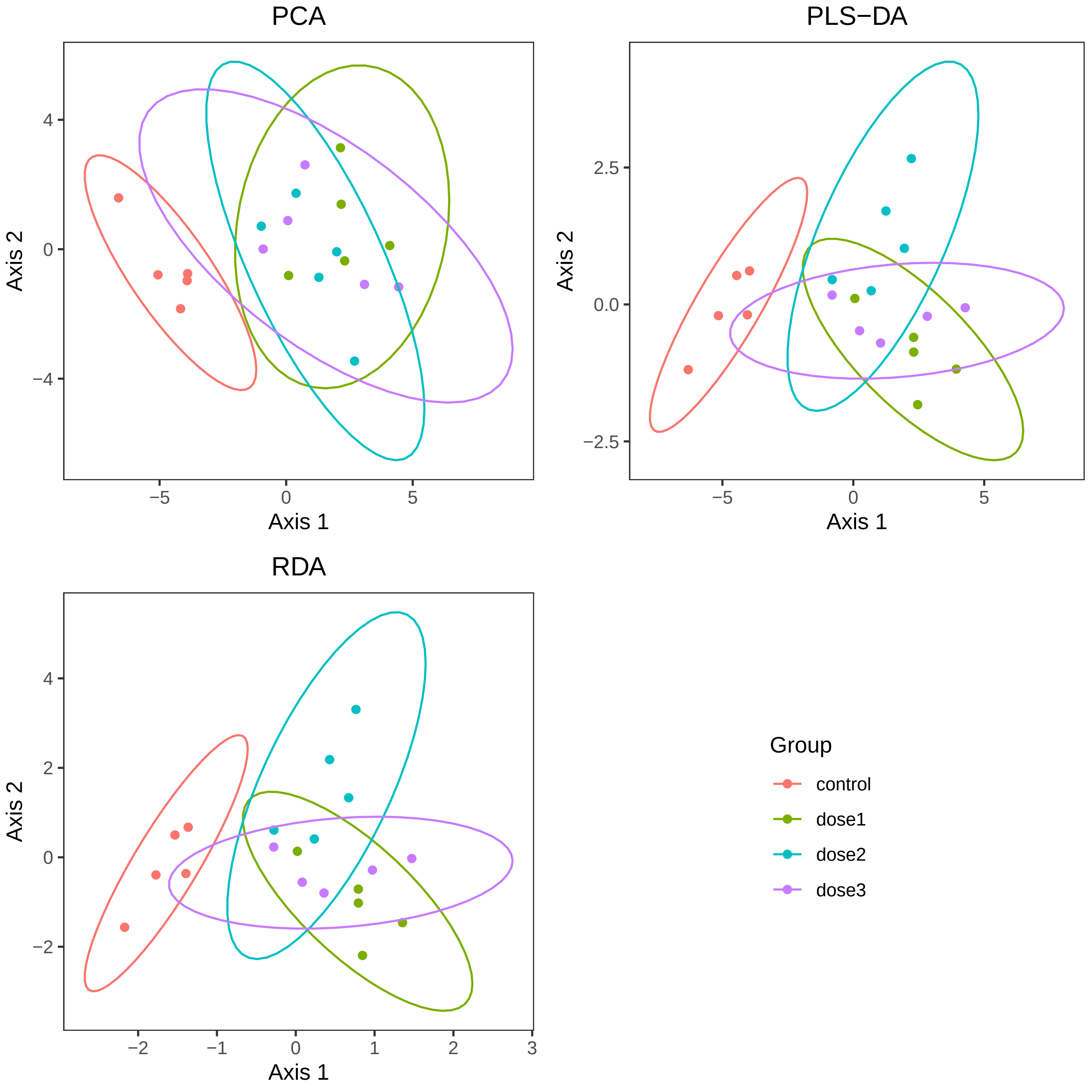
PCA
PLS−DA
RDA
Axis 1
Axis 2
Group
control
dose1
dose2
dose3

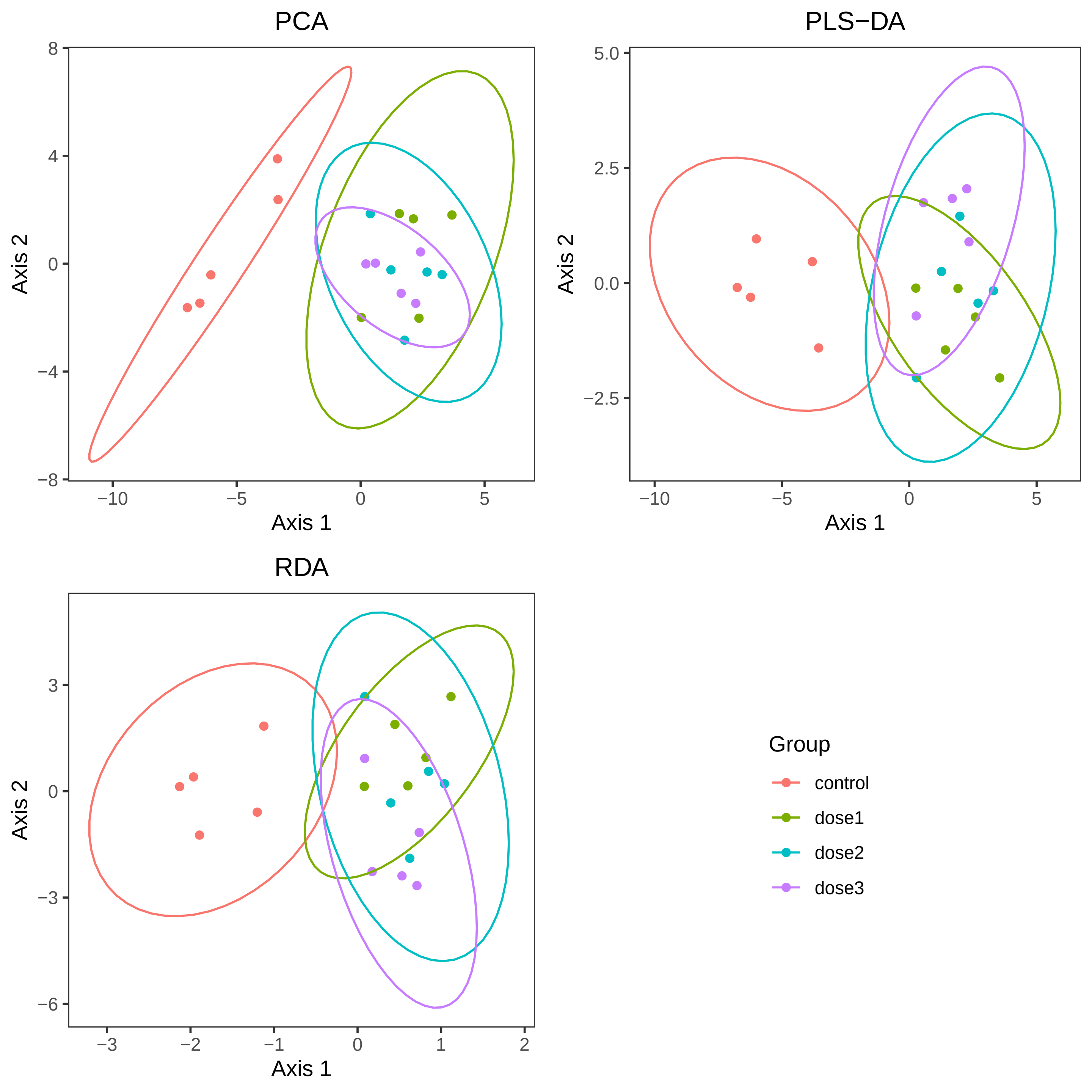

PCA
PLS−DA
RDA
Axis 1
Axis 2
8
4
0
−4
−8
−10
−5
0
5
5.0
2.5
0.0
−2.5
3
0
−3
−6
−3
−2
−1
0
1
2
Group
control
dose1
dose2
dose3

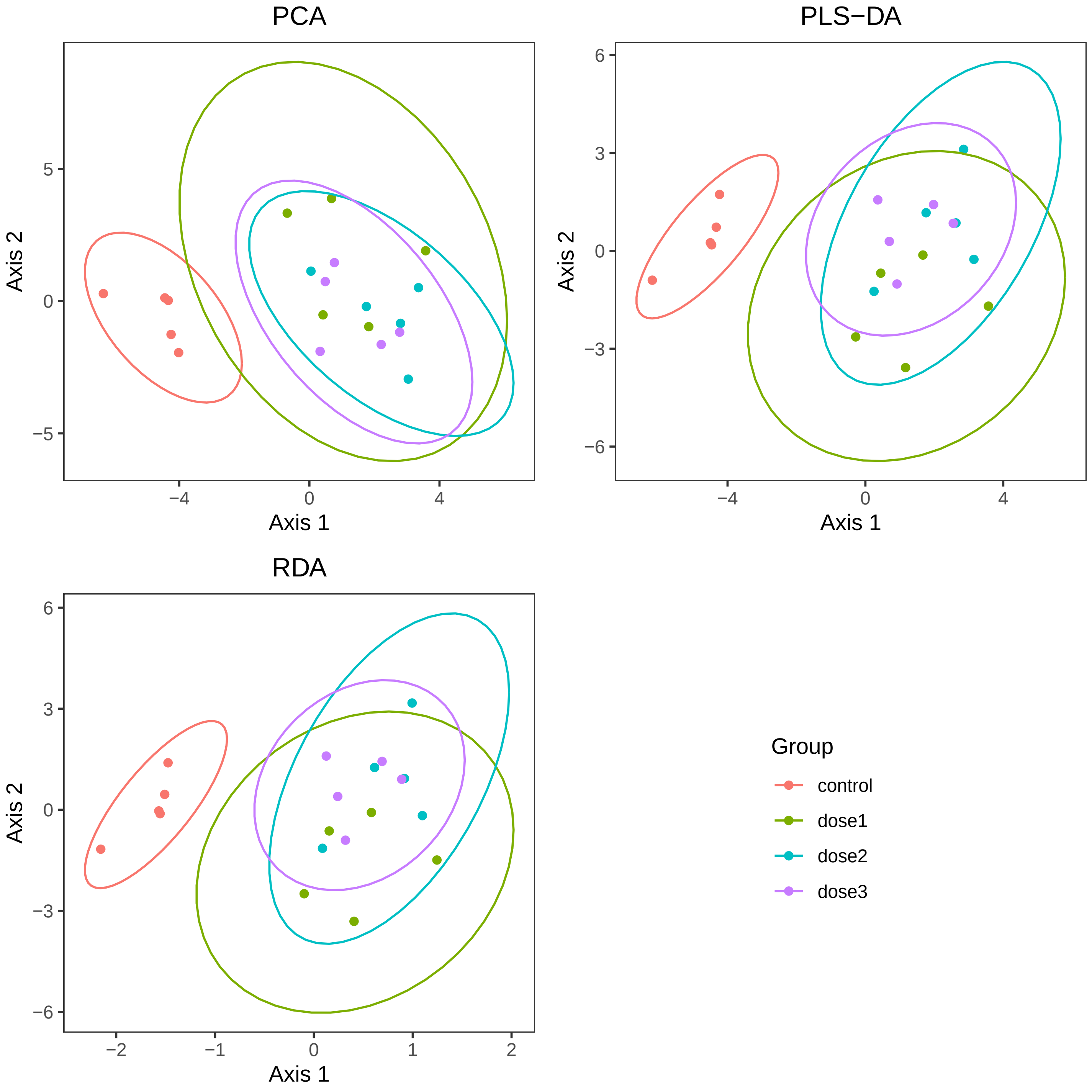

PCA
PLS−DA
RDA
Axis 1
Axis 2
Group
control
dose1
dose2
dose3

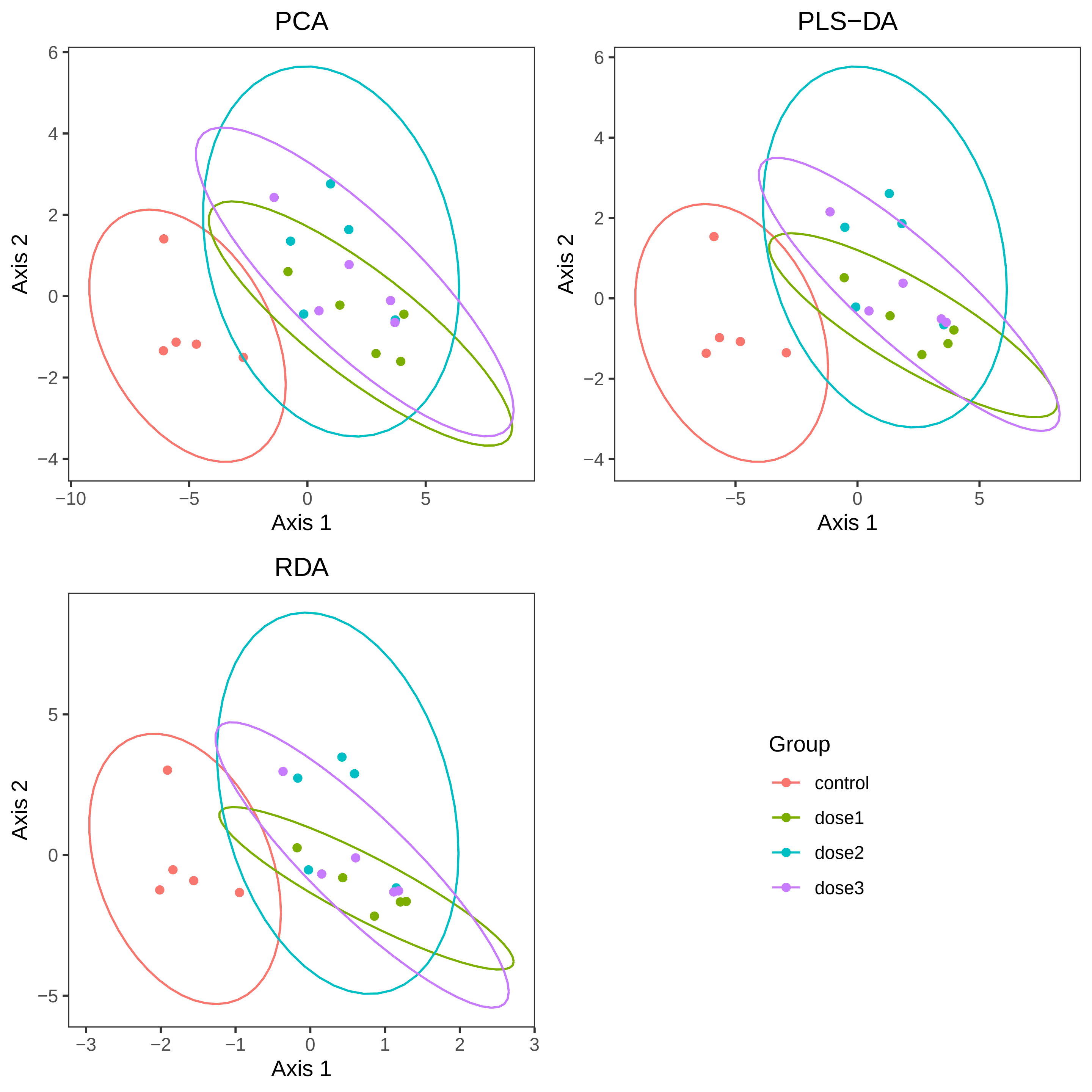
PCA
PLS−DA
RDA
Axis 1
Axis 2
Group
control
dose1
dose2
dose3

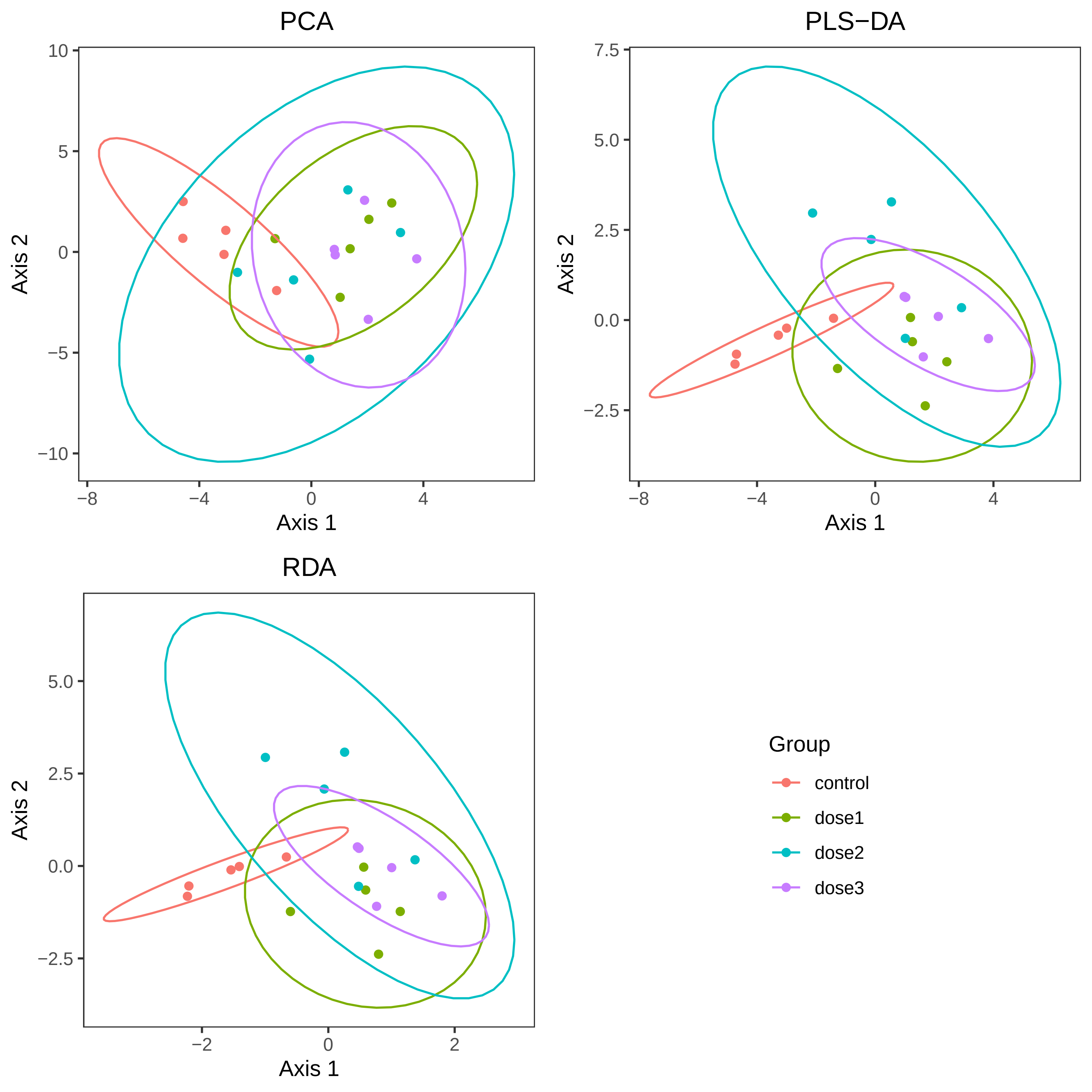

PCA
PLS−DA
RDA
Axis 1
Axis 2
Group
control
dose1
dose2
dose3

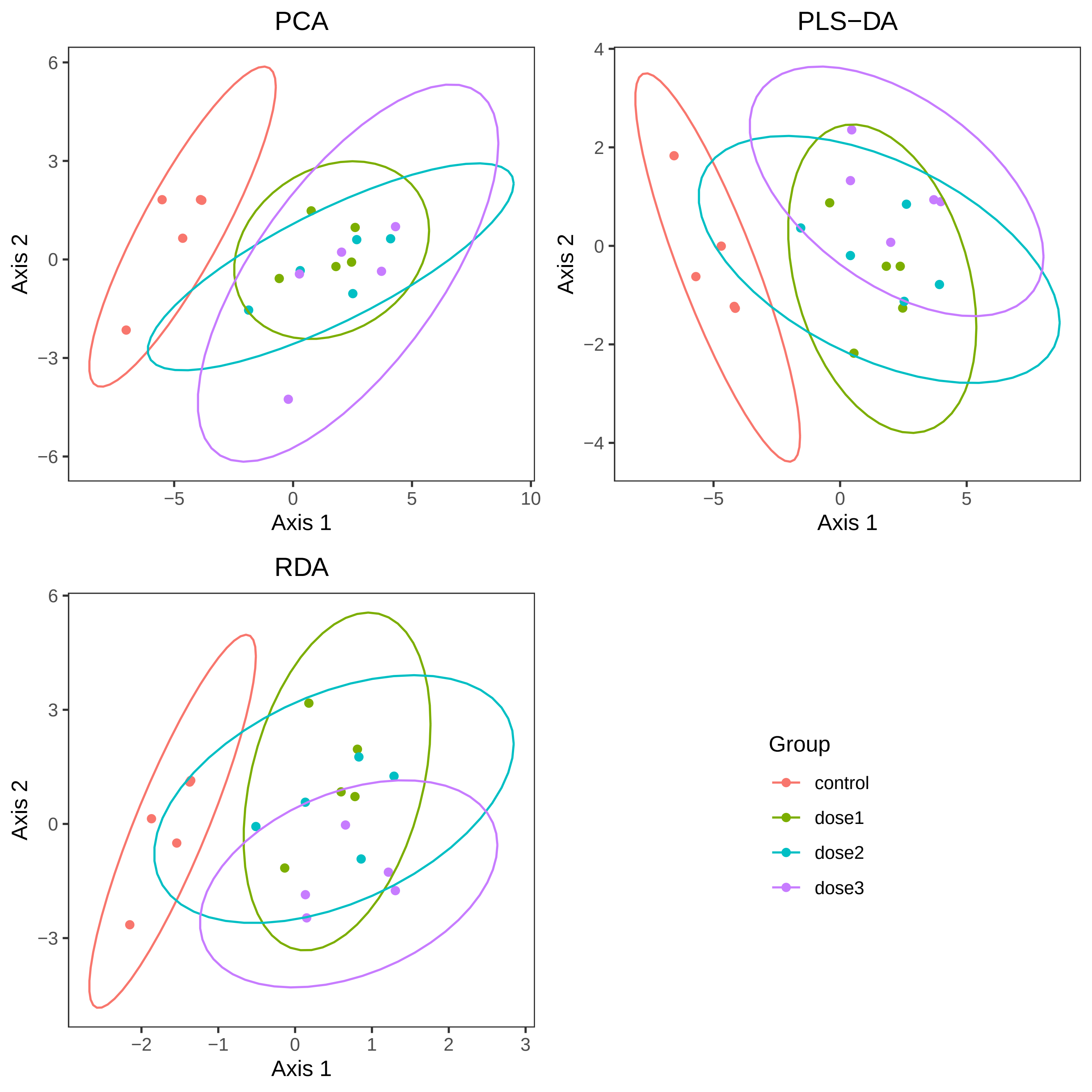
PCA
PLS−DA
RDA
Axis 1
Axis 2
Group
control
dose1
dose2
dose3

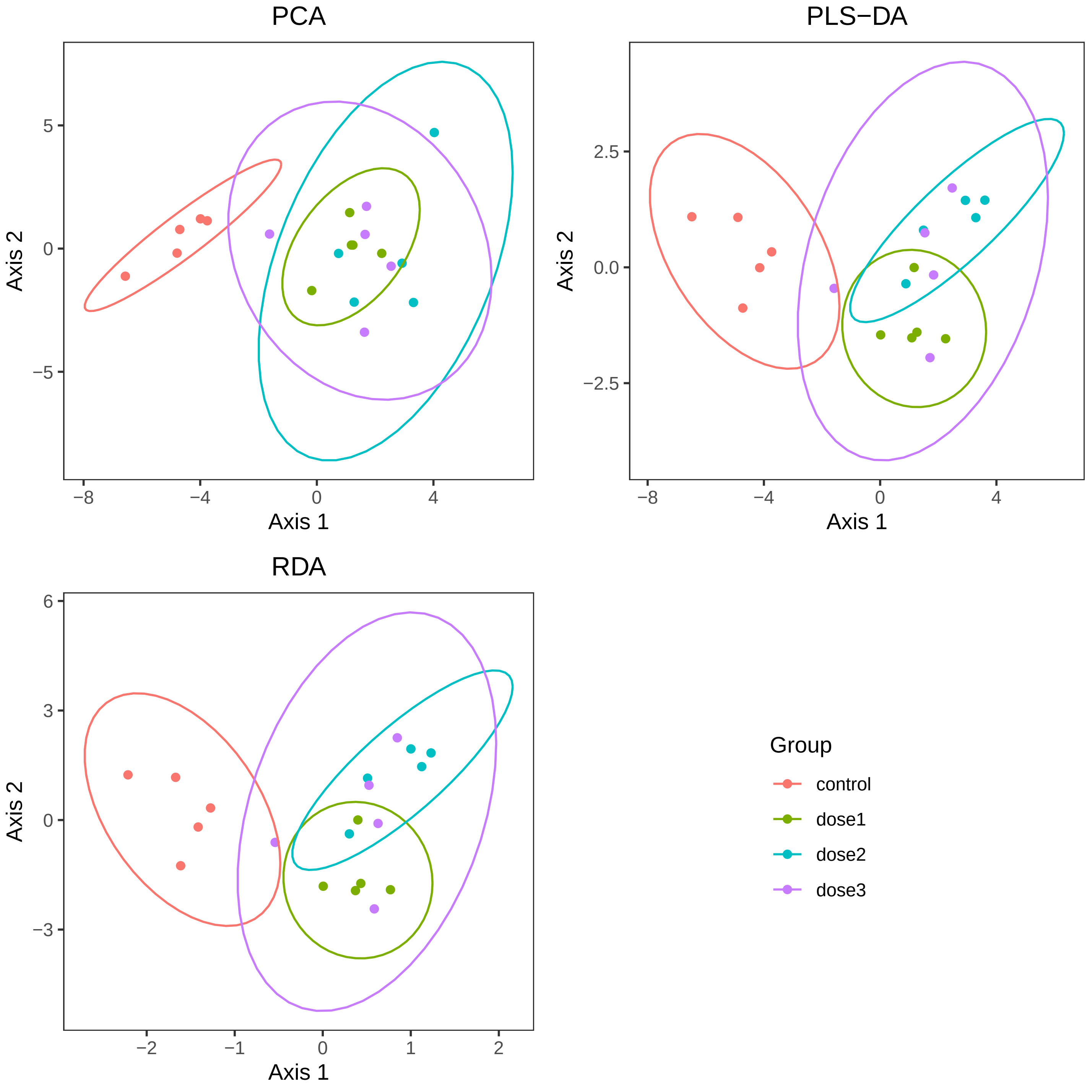
PCA
PLS−DA
RDA
Axis 1
Axis 2
Group
control
dose1
dose2
dose3

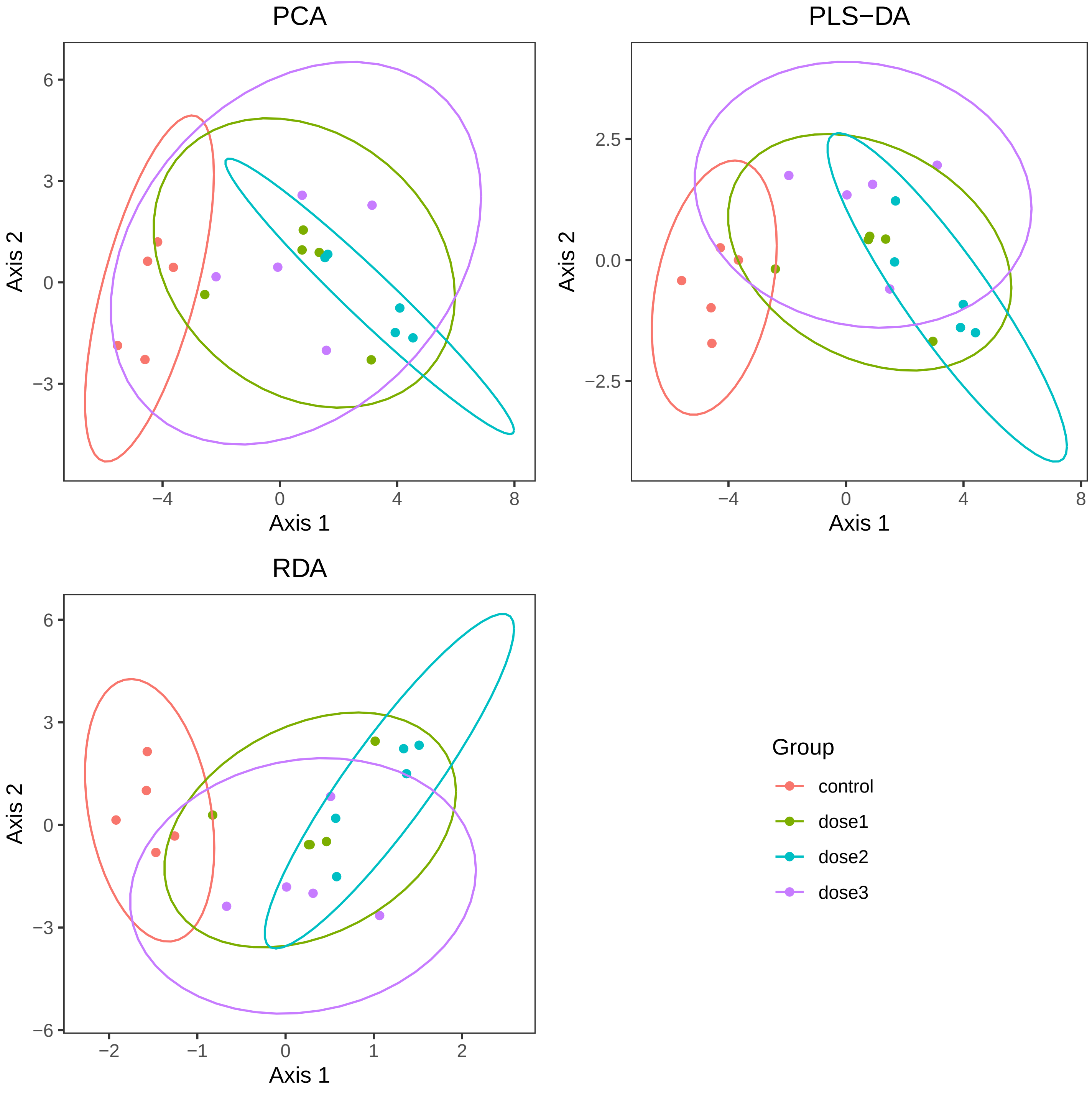
PCA
PLS−DA
RDA
Axis 1
Axis 2
Group
control
dose1
dose2
dose3

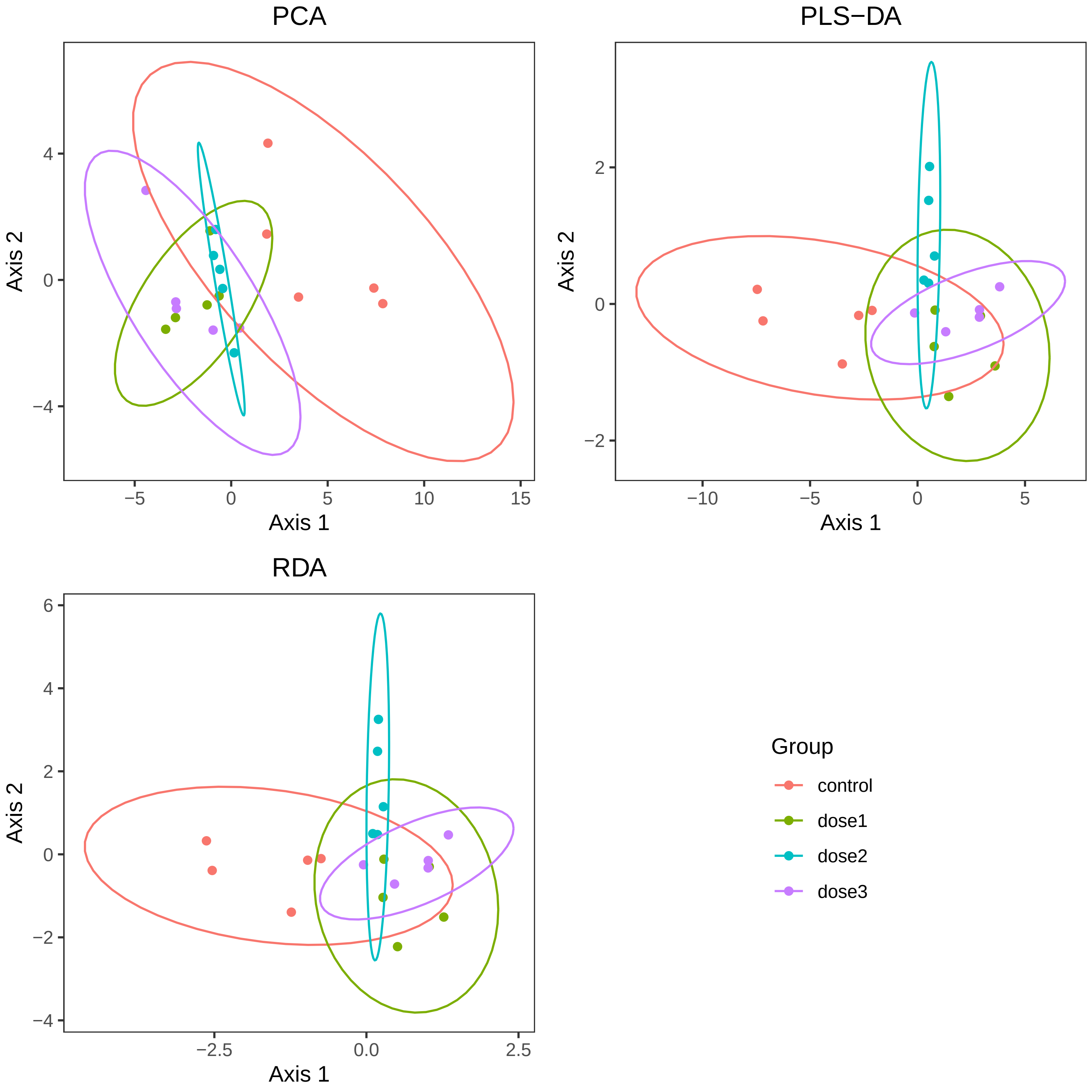

PCA
PLS−DA
RDA
Axis 1
Axis 2
Group
control
dose1
dose2
dose3

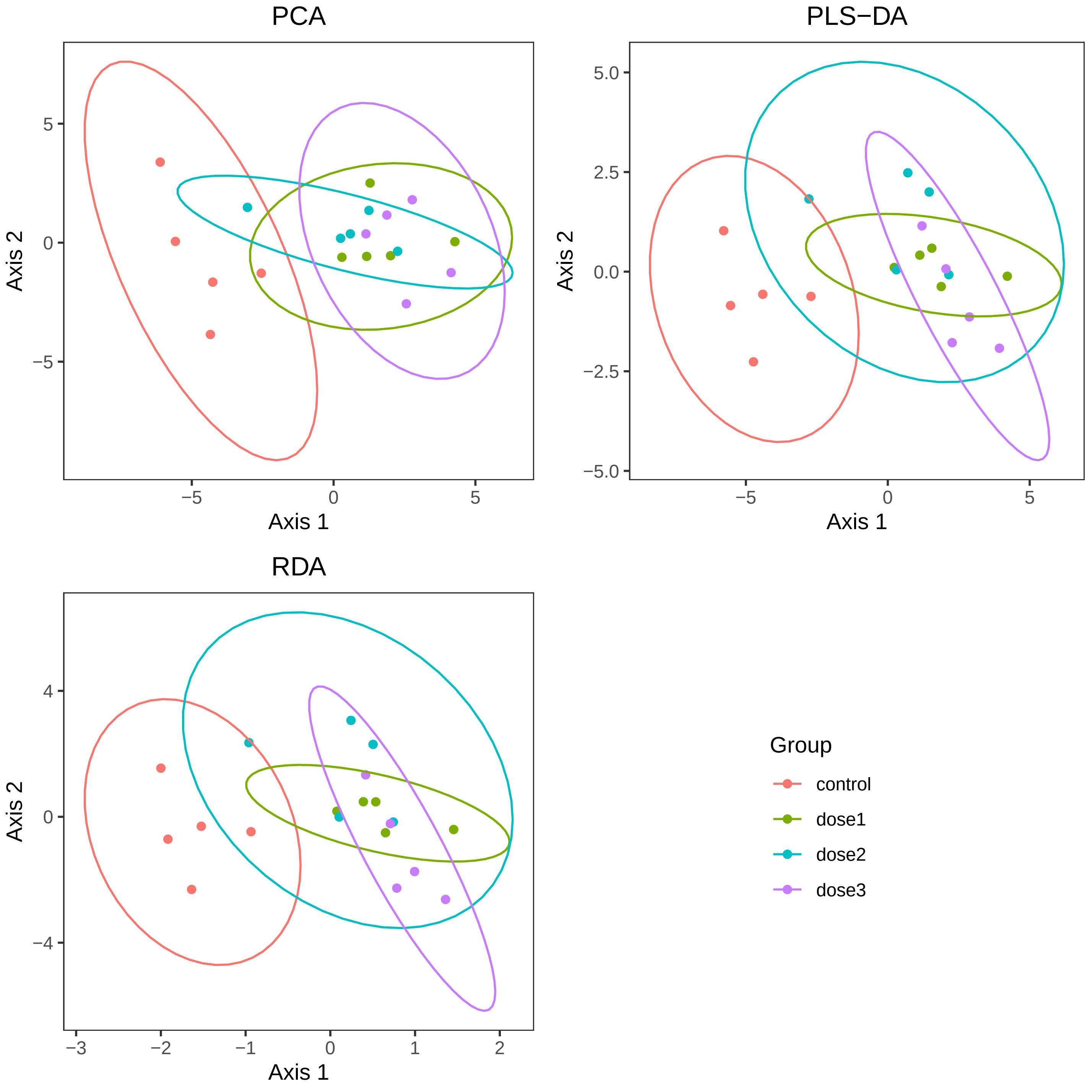
PCA
PLS−DA
RDA
Axis 1
Axis 2
Group
control
dose1
dose2
dose3

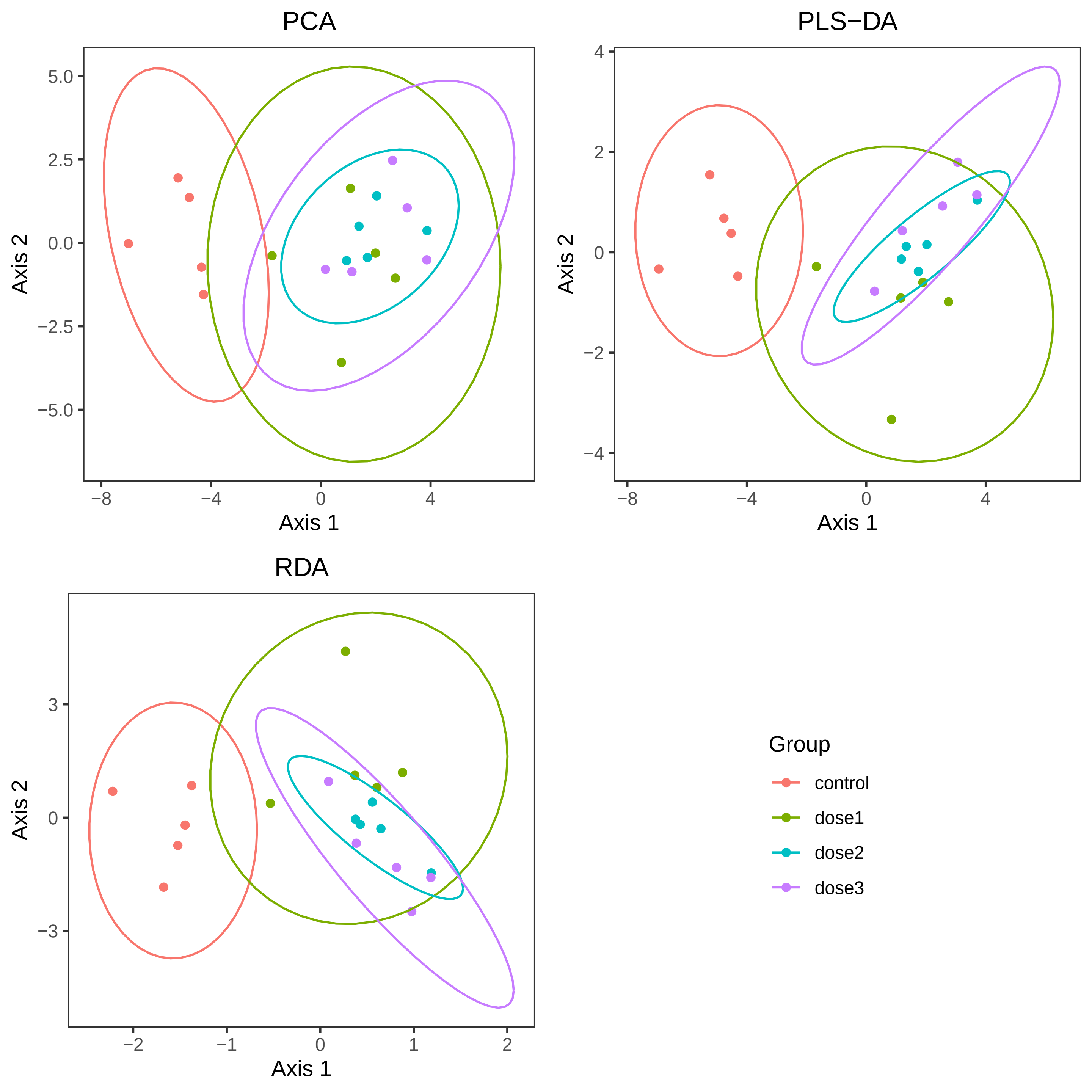
PCA
PLS−DA
RDA
Axis 1
Axis 2
5.0
2.5
0.0
−2.5
−5.0
−8
−4
0
4
2
−2
−1
1
3
−3
Group
control
dose1
dose2
dose3

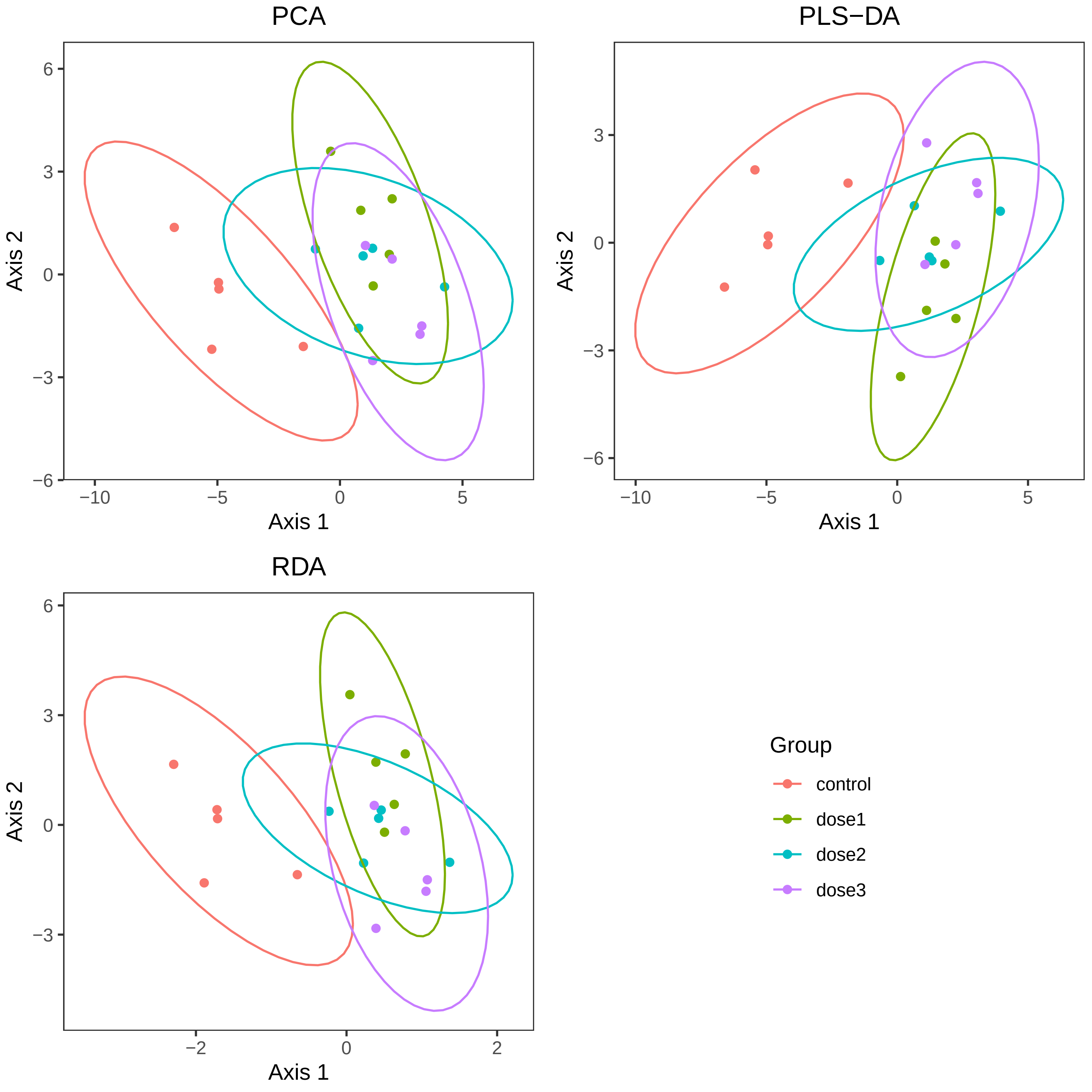
PCA
PLS−DA
RDA
Axis 1
Axis 2
Group
control
dose1
dose2
dose3

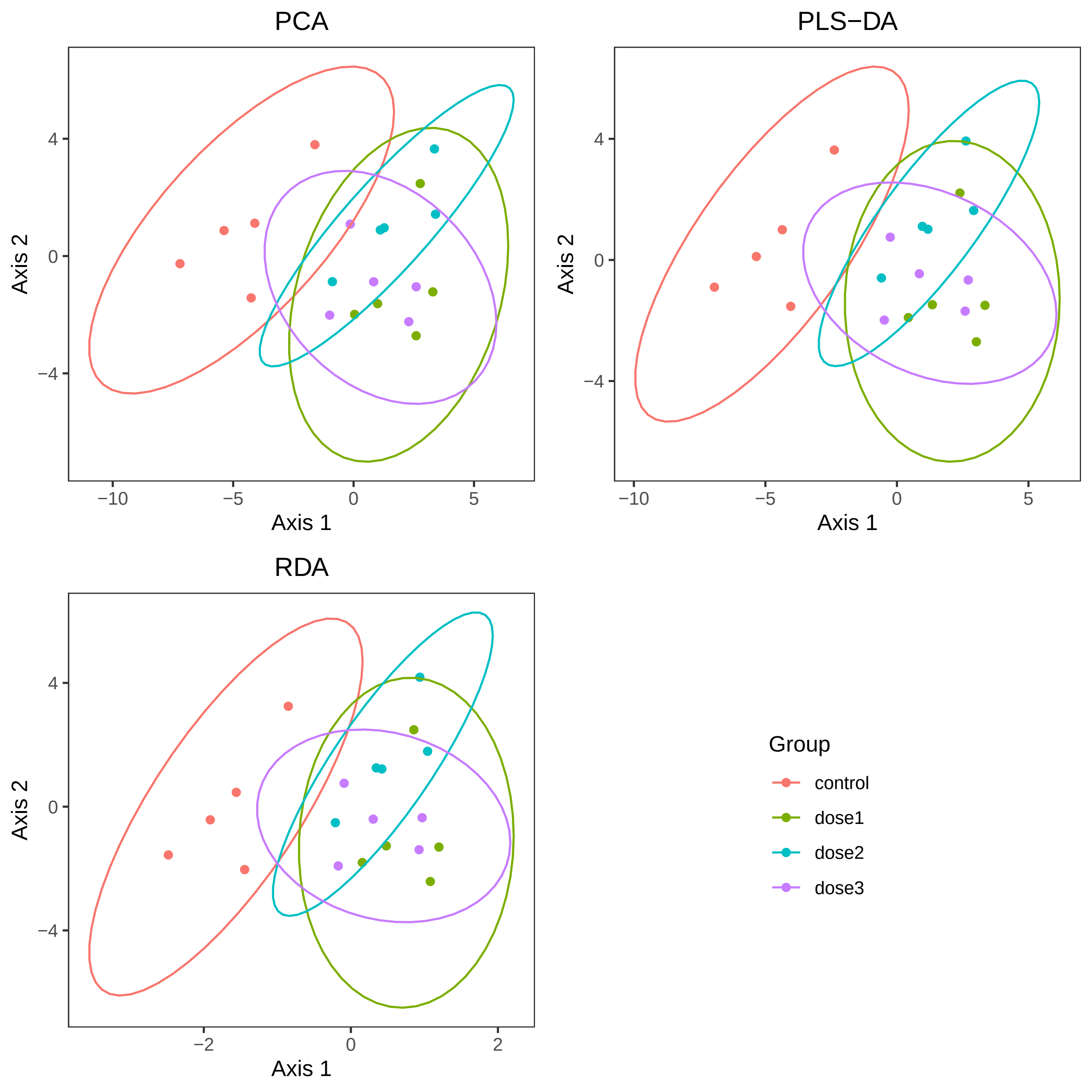

PCA
PLS−DA
RDA
Axis 1
Axis 2
−10
−5
0
5
4
0
−4
−2
2
Group
control
dose1
dose2
dose3

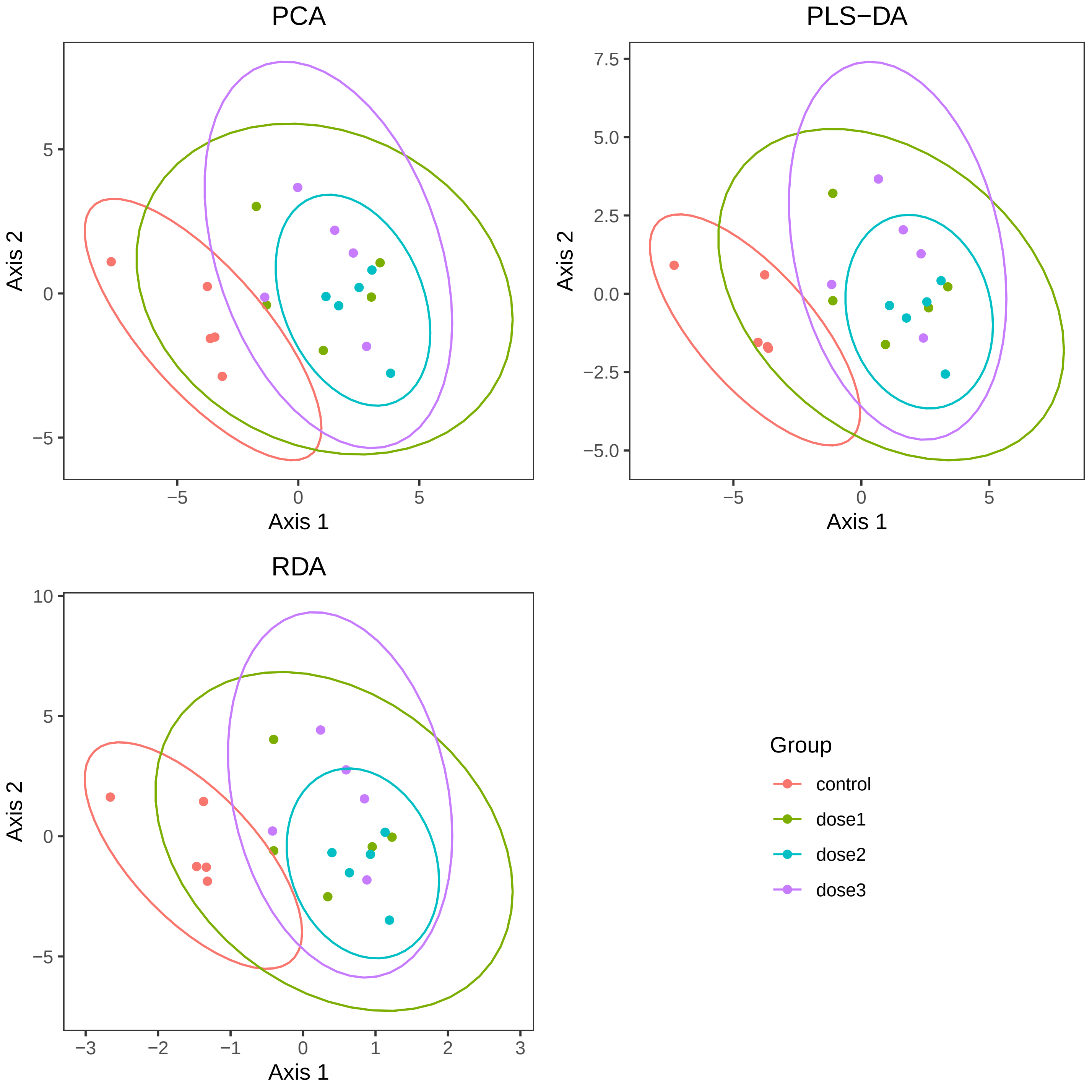

PCA
PLS−DA
RDA
Axis 1
Axis 2
Group
control
dose1
dose2
dose3

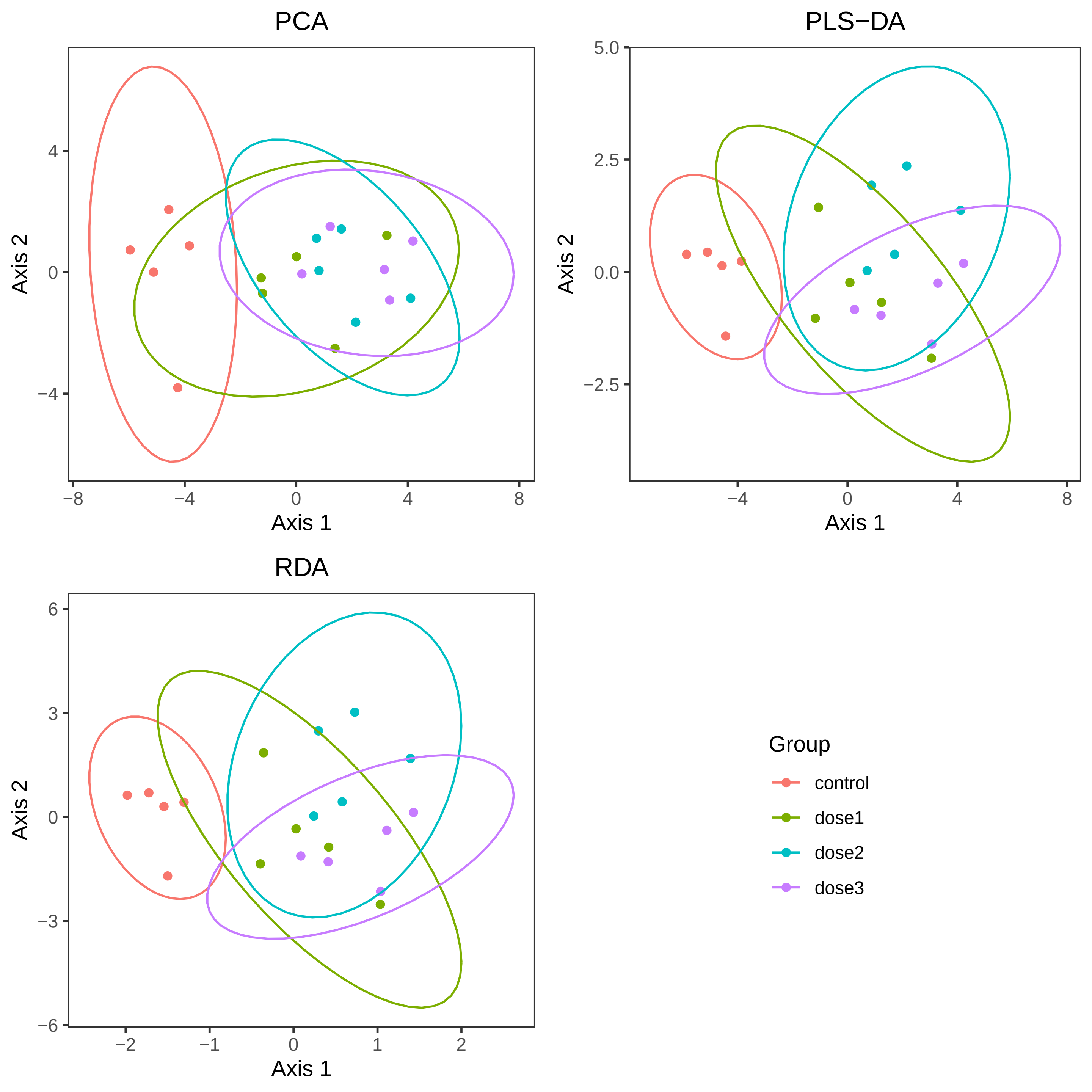
PCA
PLS−DA
RDA
Axis 1
Axis 2
Group
control
dose1
dose2
dose3

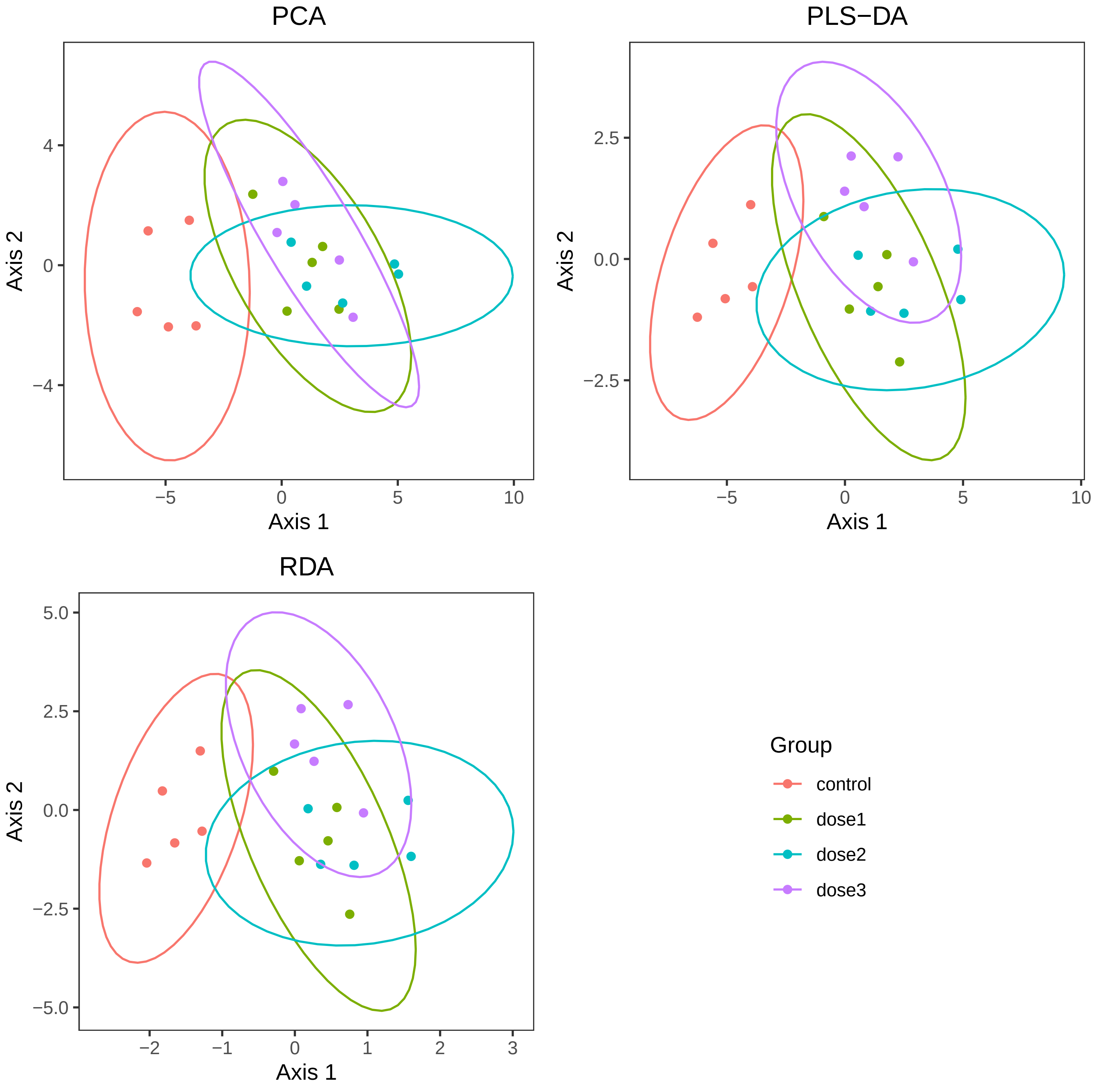
PCA
PLS−DA
RDA
Axis 1
Axis 2
Group
control
dose1
dose2
dose3

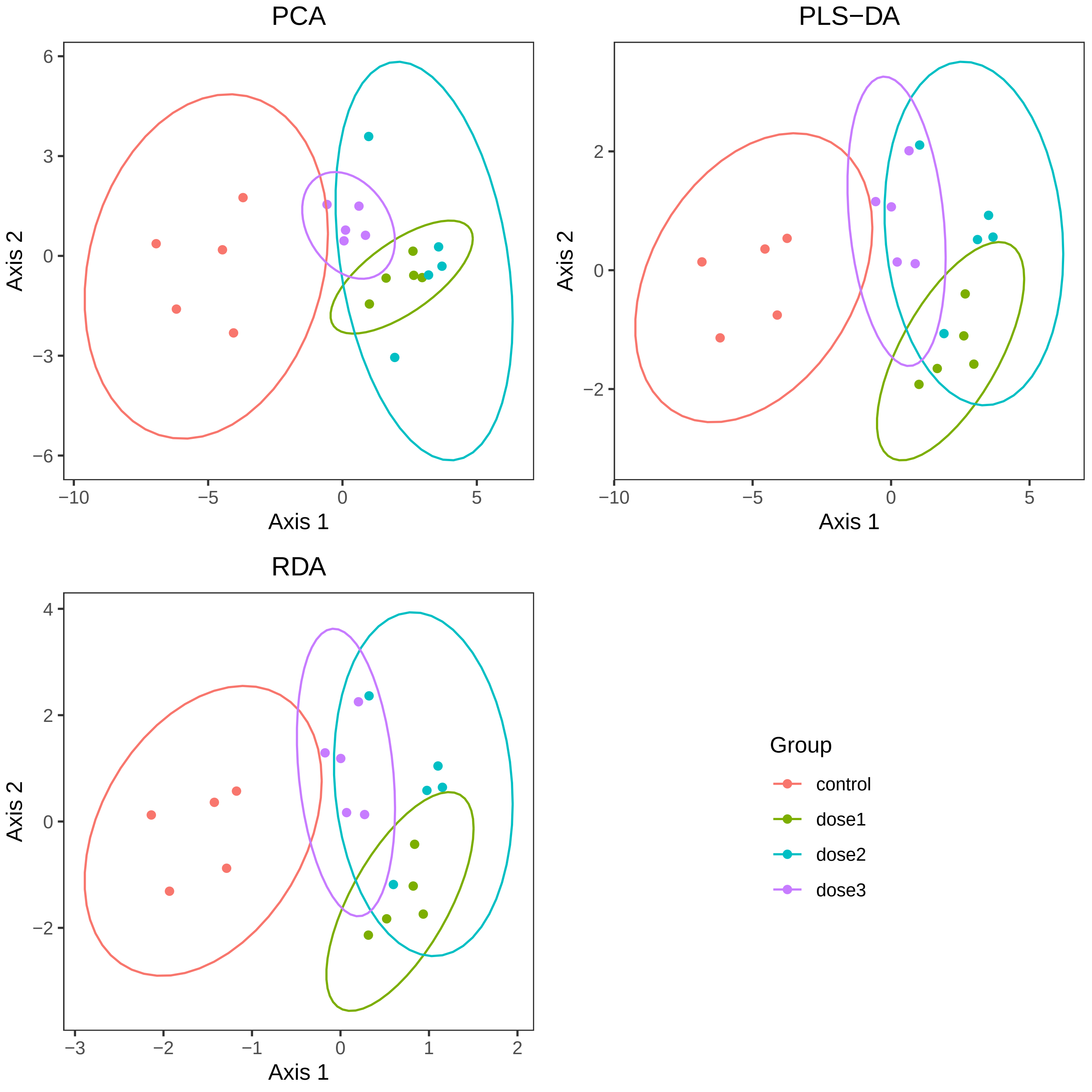

PCA
PLS−DA
RDA
Axis 1
Axis 2
Group
control
dose1
dose2
dose3

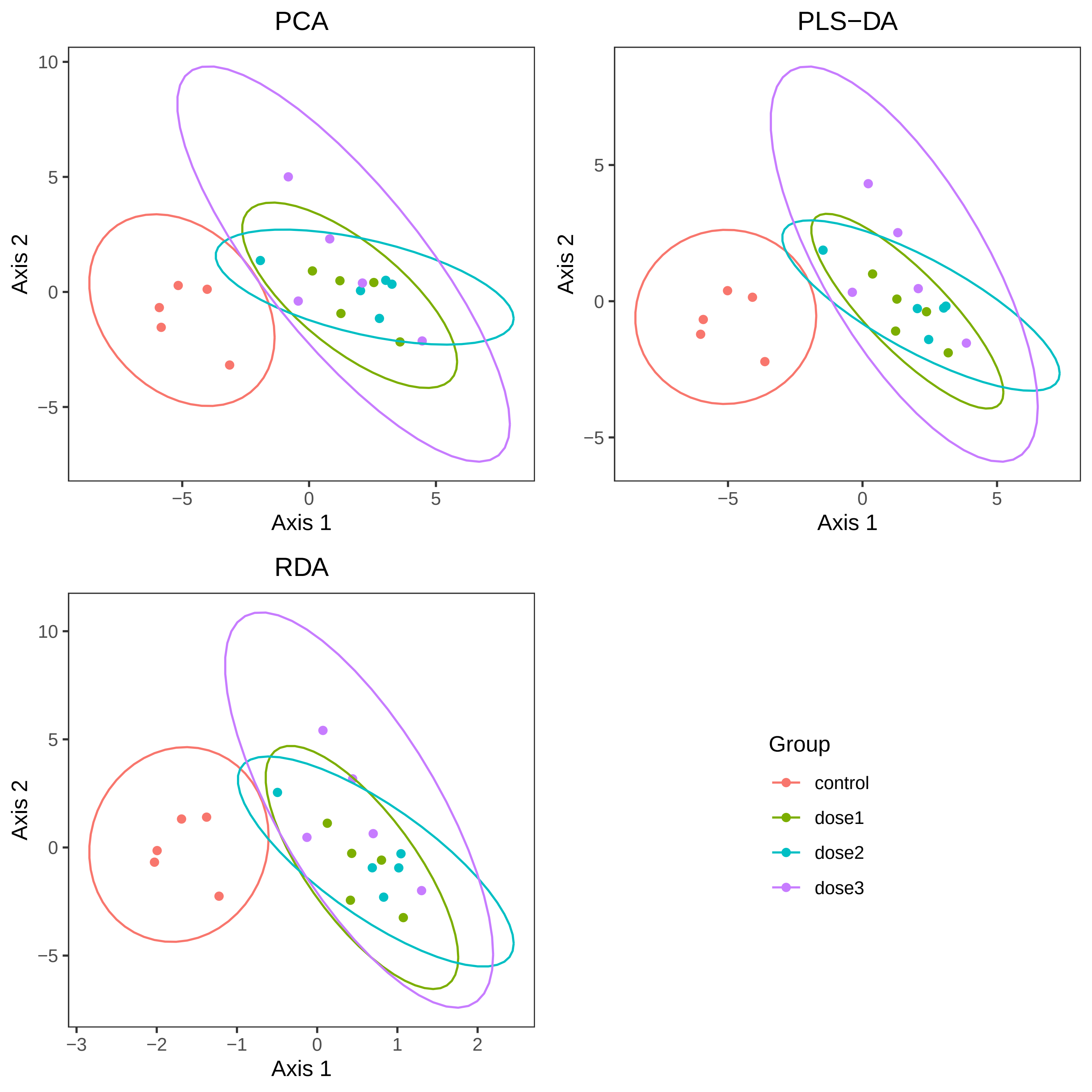
PCA
PLS−DA
RDA
Axis 1
Axis 2
Group
control
dose1
dose2
dose3

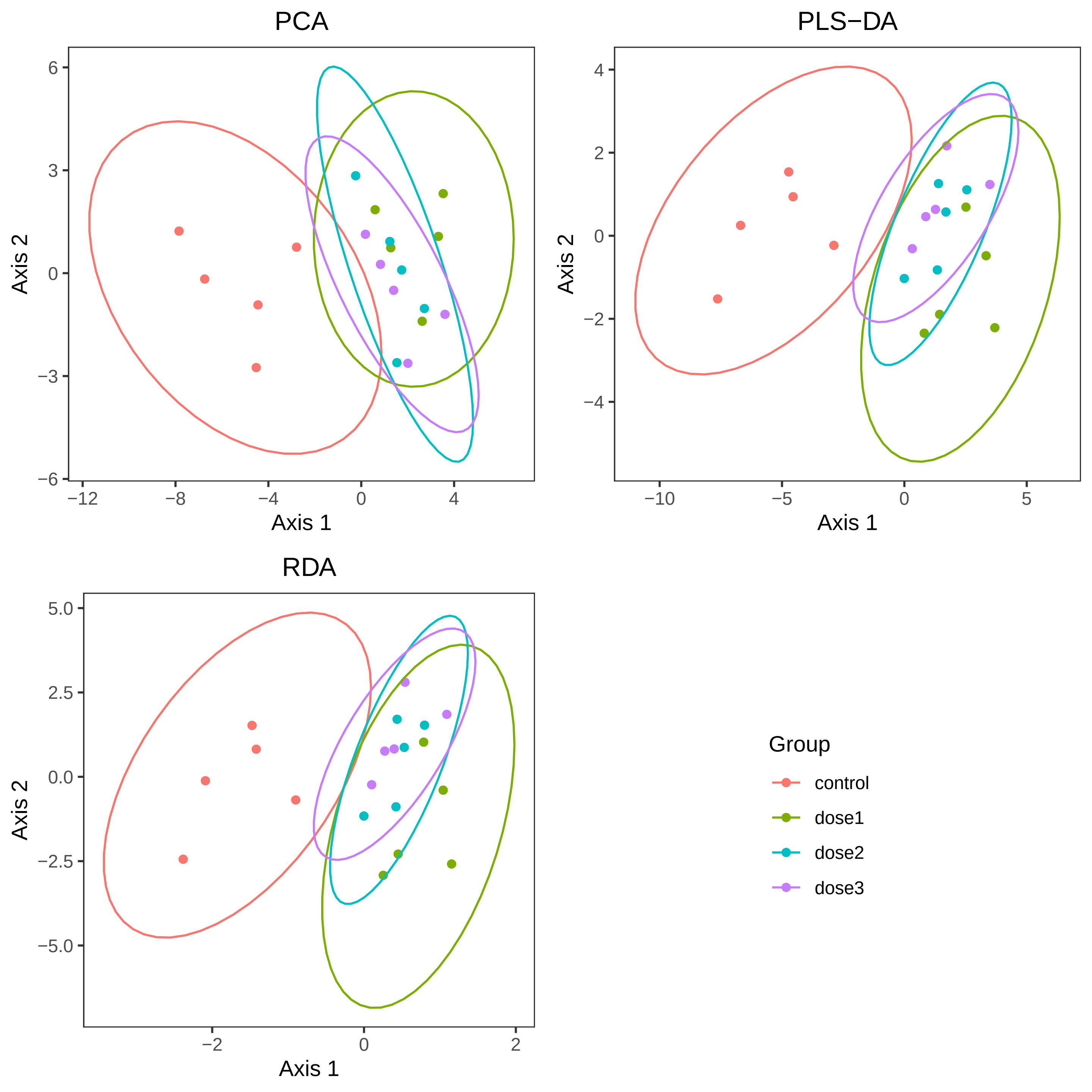

PCA
PLS−DA
RDA
Axis 1
Axis 2
Group
control
dose1
dose2
dose3

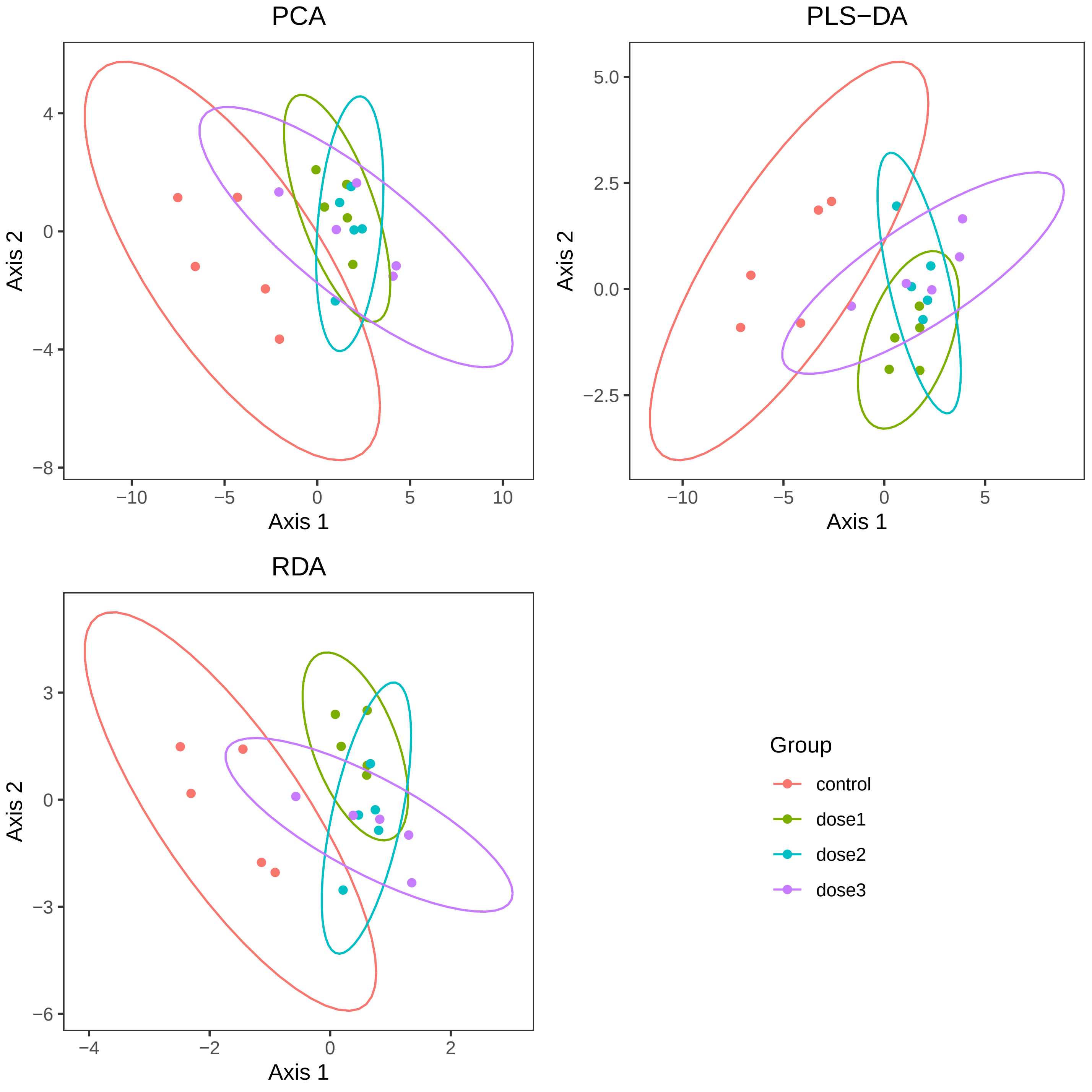
PCA
PLS−DA
RDA
Axis 1
Axis 2
Group
control
dose1
dose2
dose3

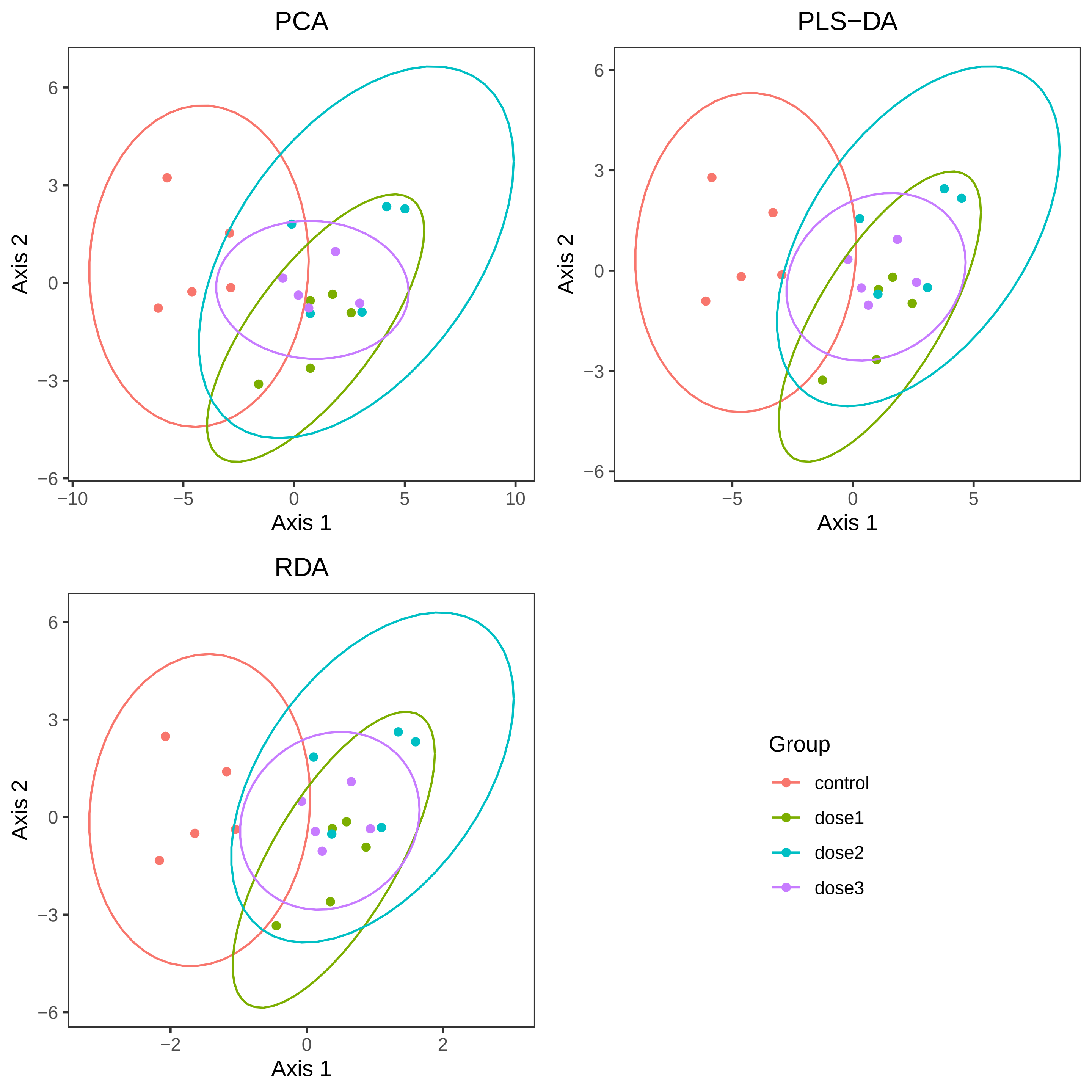
PCA
PLS−DA
RDA
Axis 1
Axis 2
Group
control
dose1
dose2
dose3

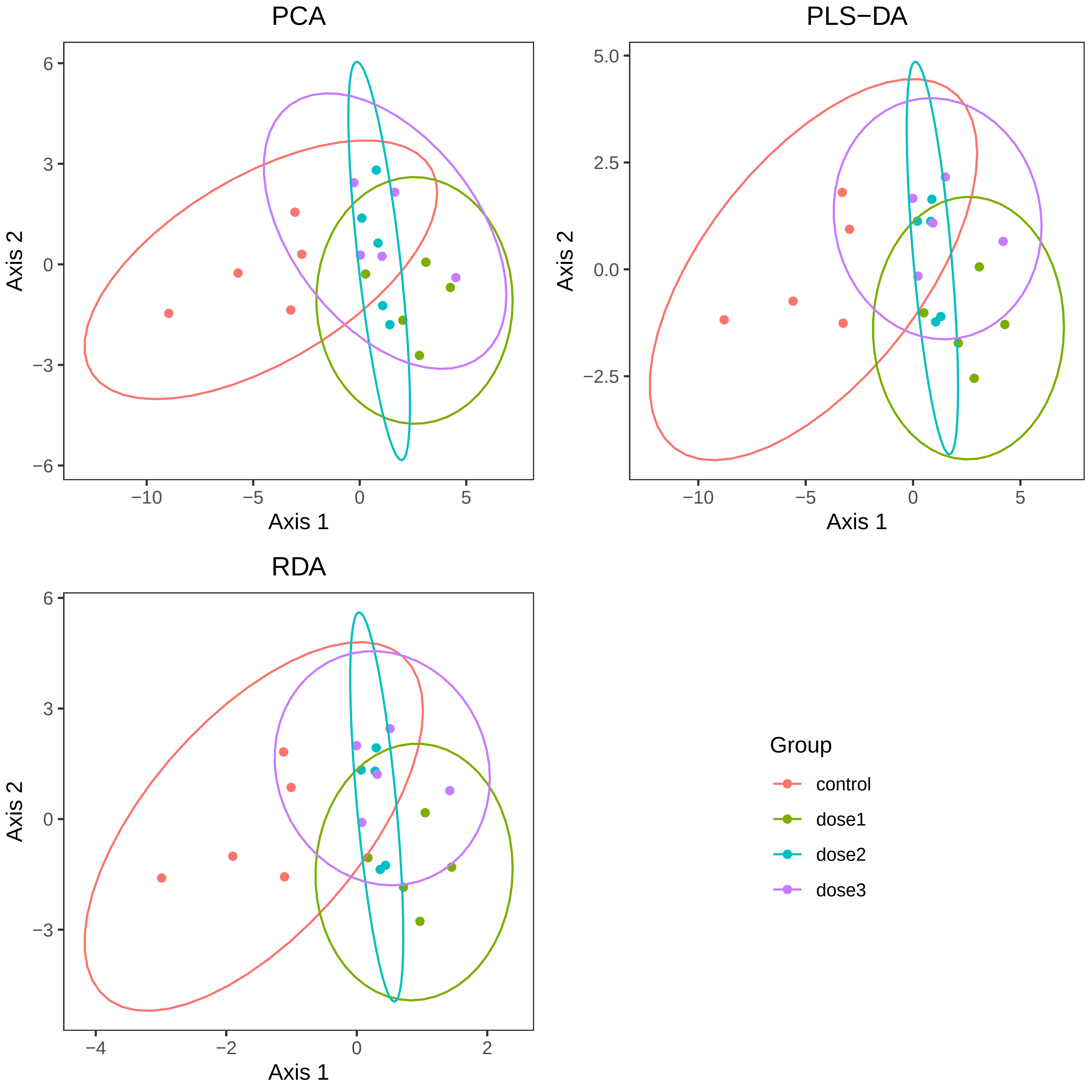

PCA
PLS−DA
RDA
Axis 1
Axis 2
Group
control
dose1
dose2
dose3

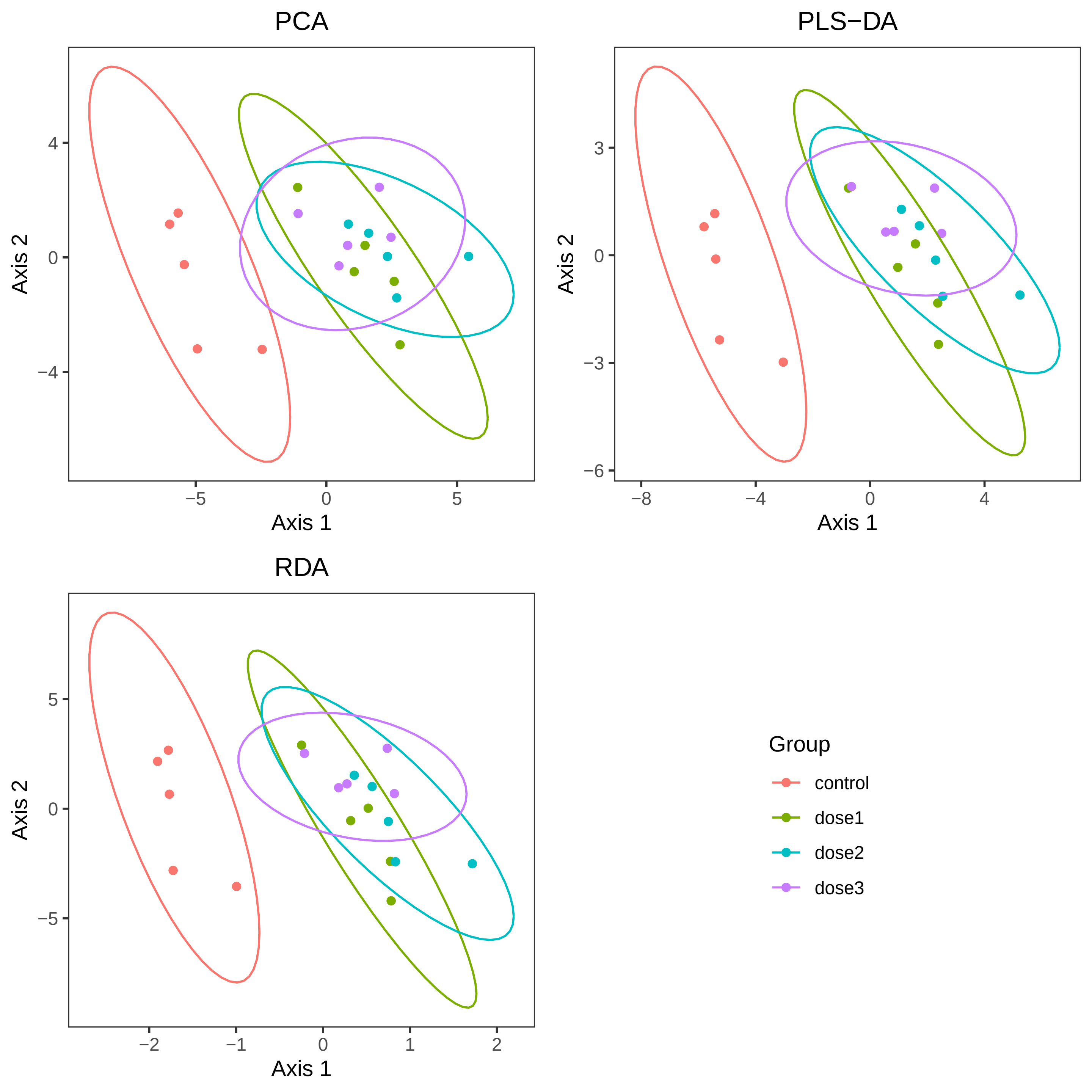
PCA
PLS−DA
RDA
Axis 1
Axis 2
4
0
−4
−5
0
5
3
0
−3
−6
−8
−4
0
4
5
0
−5
−2
−1
0
1
2
Group
control
dose1
dose2
dose3

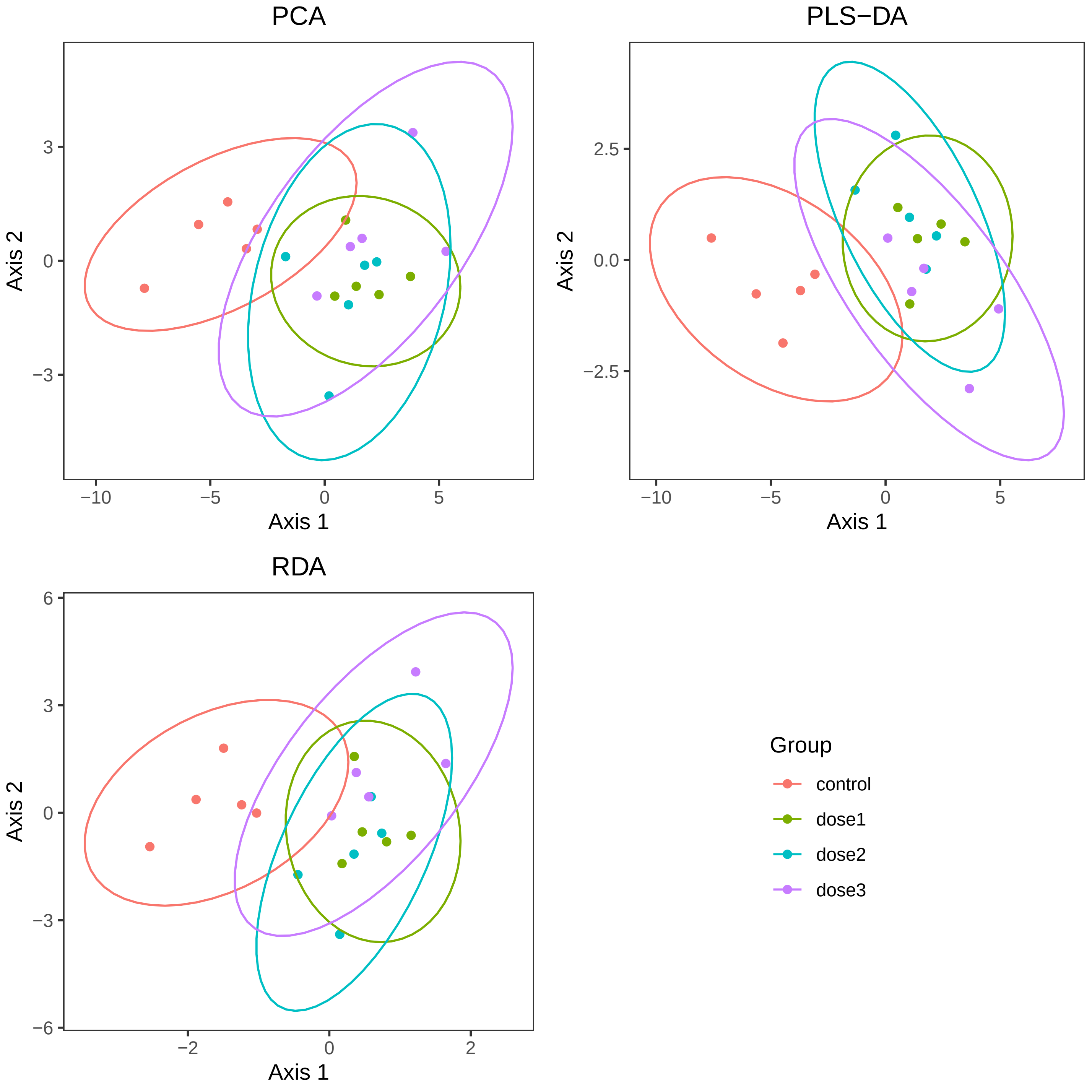
PCA
PLS−DA
RDA
Axis 1
Axis 2
3
0
−3
−10
−5
0
5
2.5
0.0
−2.5
6
−6
−2
2
Group
control
dose1
dose2
dose3

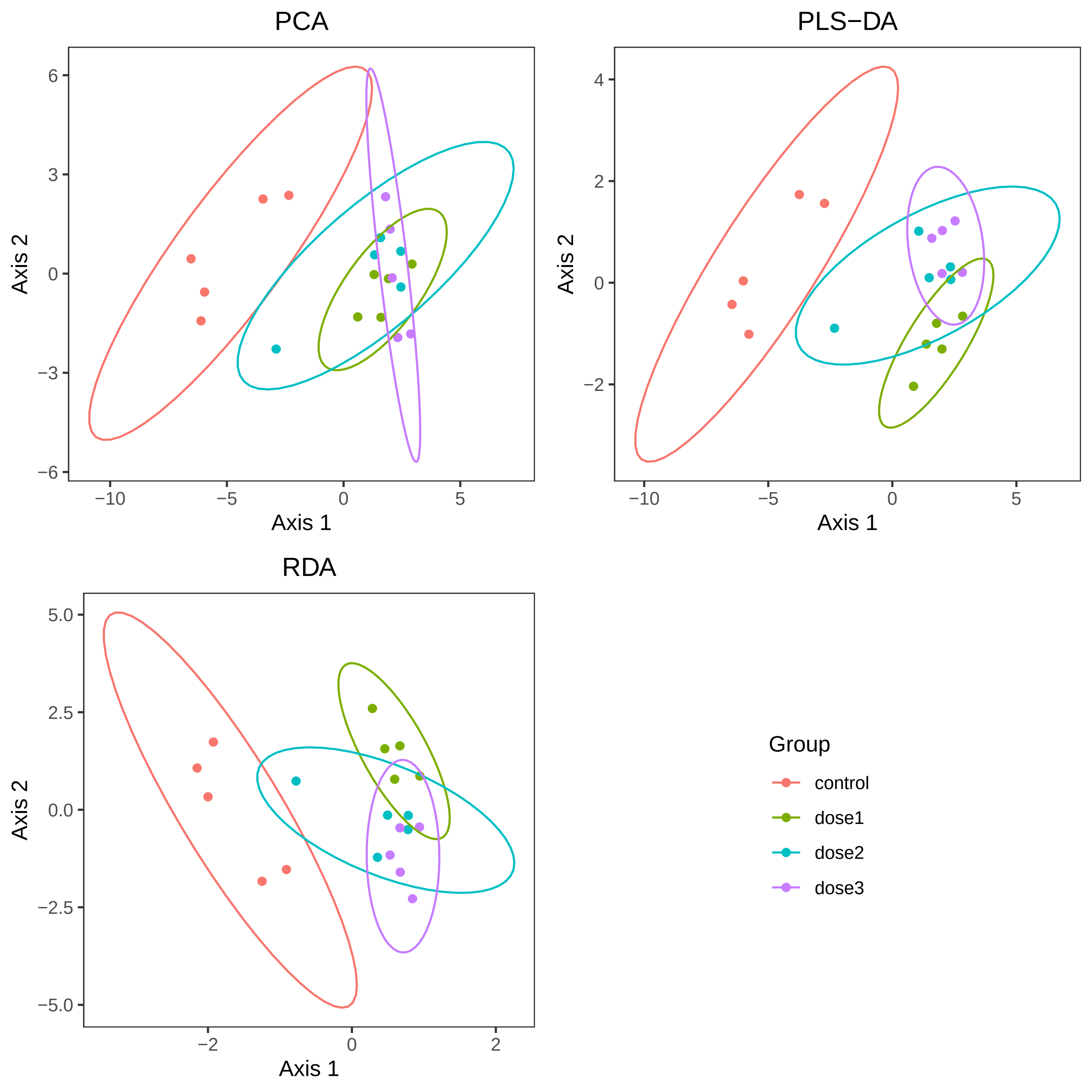

PCA
PLS−DA
RDA
Axis 1
Axis 2
6
3
0
−3
−6
−10
−5
0
5
4
2
0
−2
5.0
2.5
0.0
−2.5
−5.0
−2
0
2
Group
control
dose1
dose2
dose3

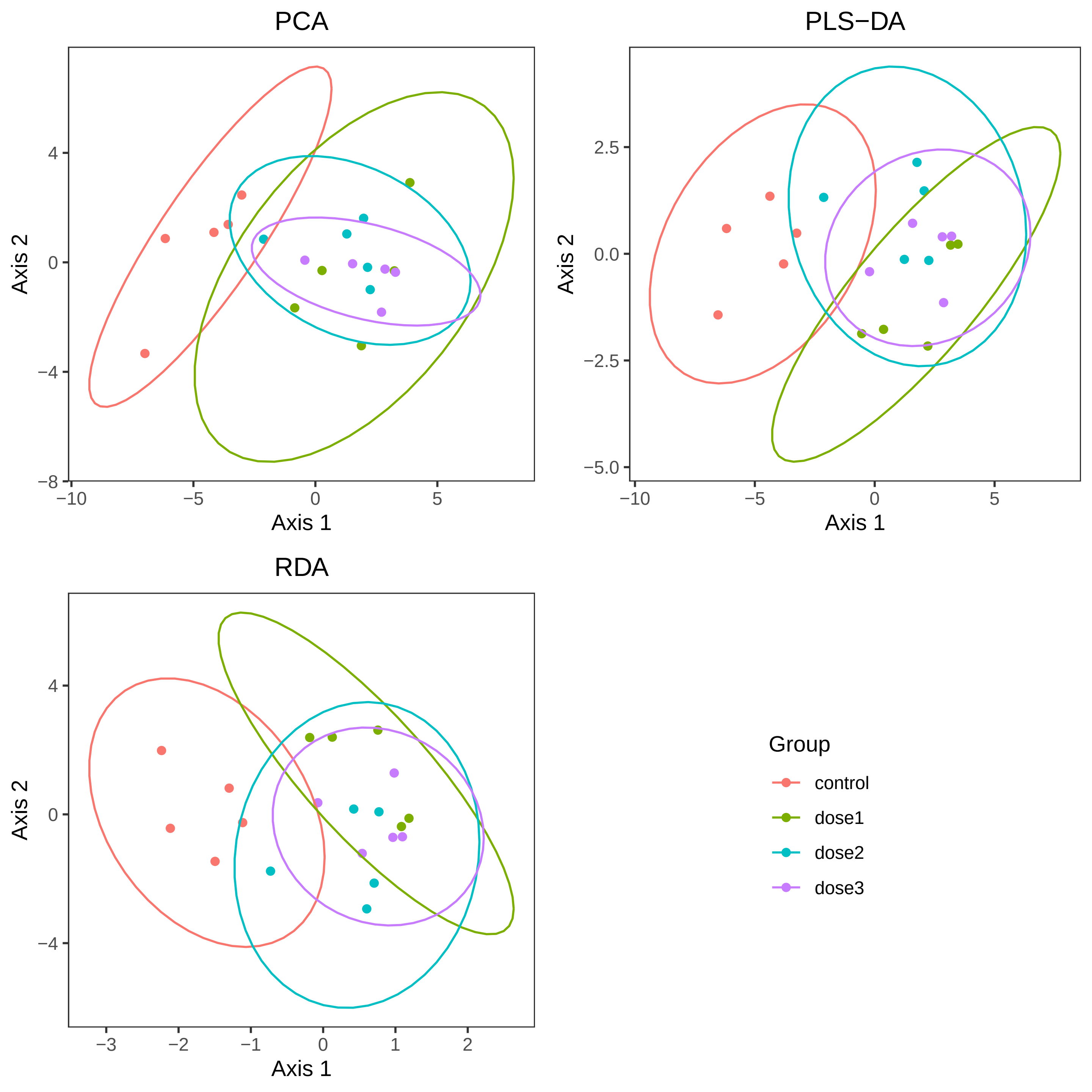
PCA
PLS−DA
RDA
Axis 1
Axis 2
−10
−5
0
5
4
0
−4
−8
2.5
0.0
−2.5
−5.0
−3
−2
−1
1
2
Group
control
dose1
dose2
dose3

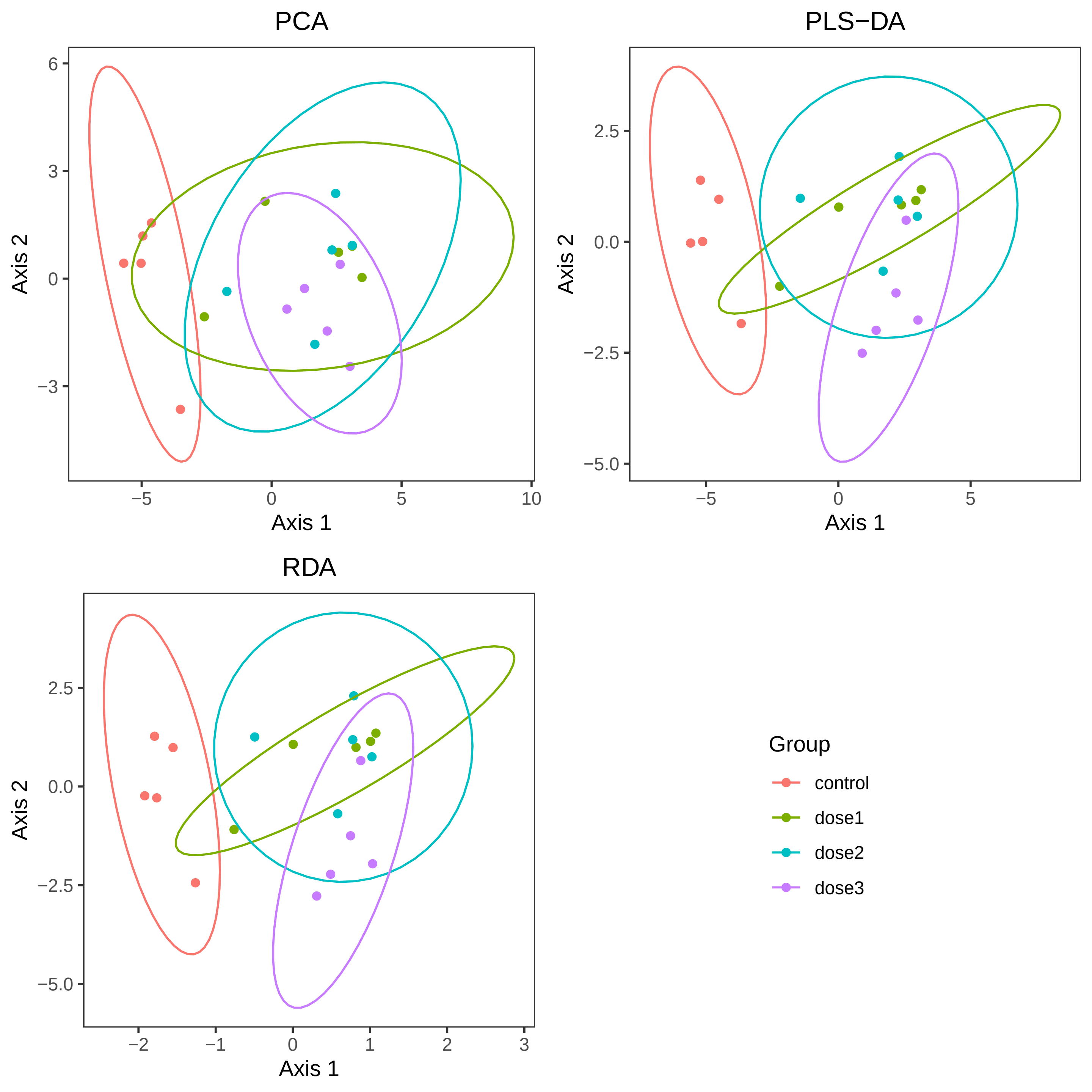

PCA
PLS−DA
RDA
Axis 1
Axis 2
Group
control
dose1
dose2
dose3

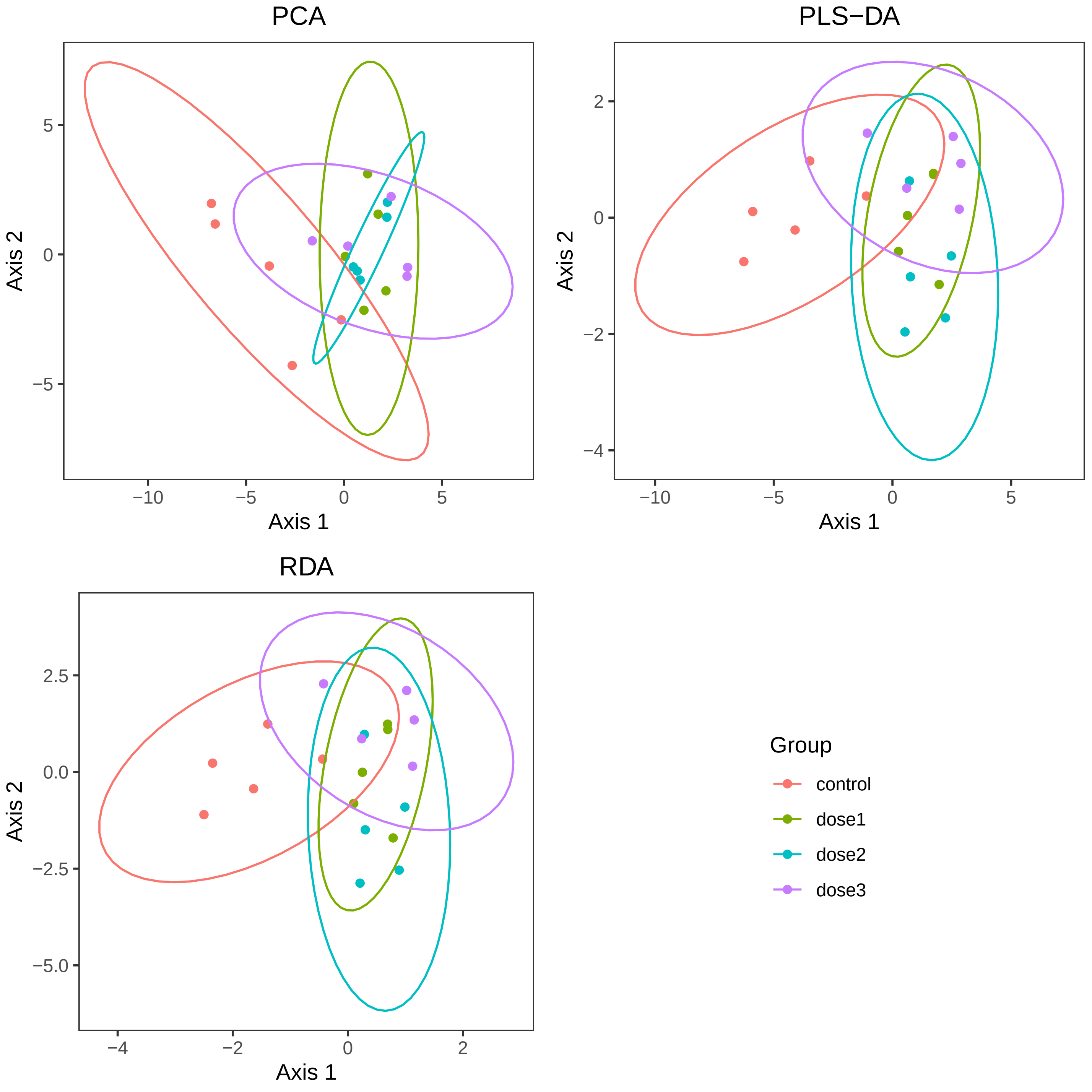

PCA
PLS−DA
RDA
Axis 1
Axis 2
Group
control
dose1
dose2
dose3

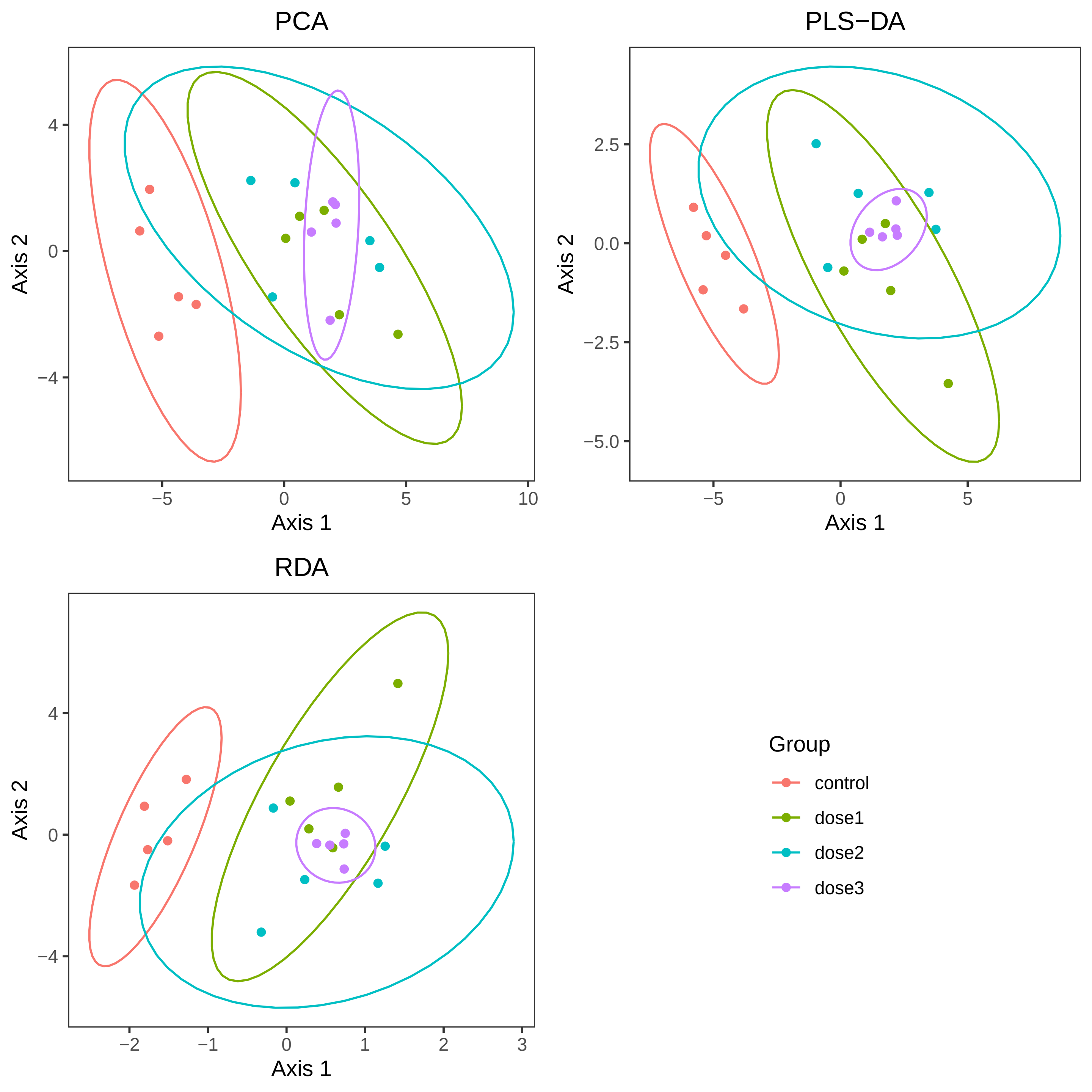

PCA
Axis 1
Axis 2
−5
0
5
10
4
0
−4
PLS−DA
Axis 1
Axis 2
−5
0
5
2.5
0.0
−2.5
−5.0
RDA
Axis 1
Axis 2
−2
−1
0
1
2
3
4
0
−4
Group
control
dose1
dose2
dose3

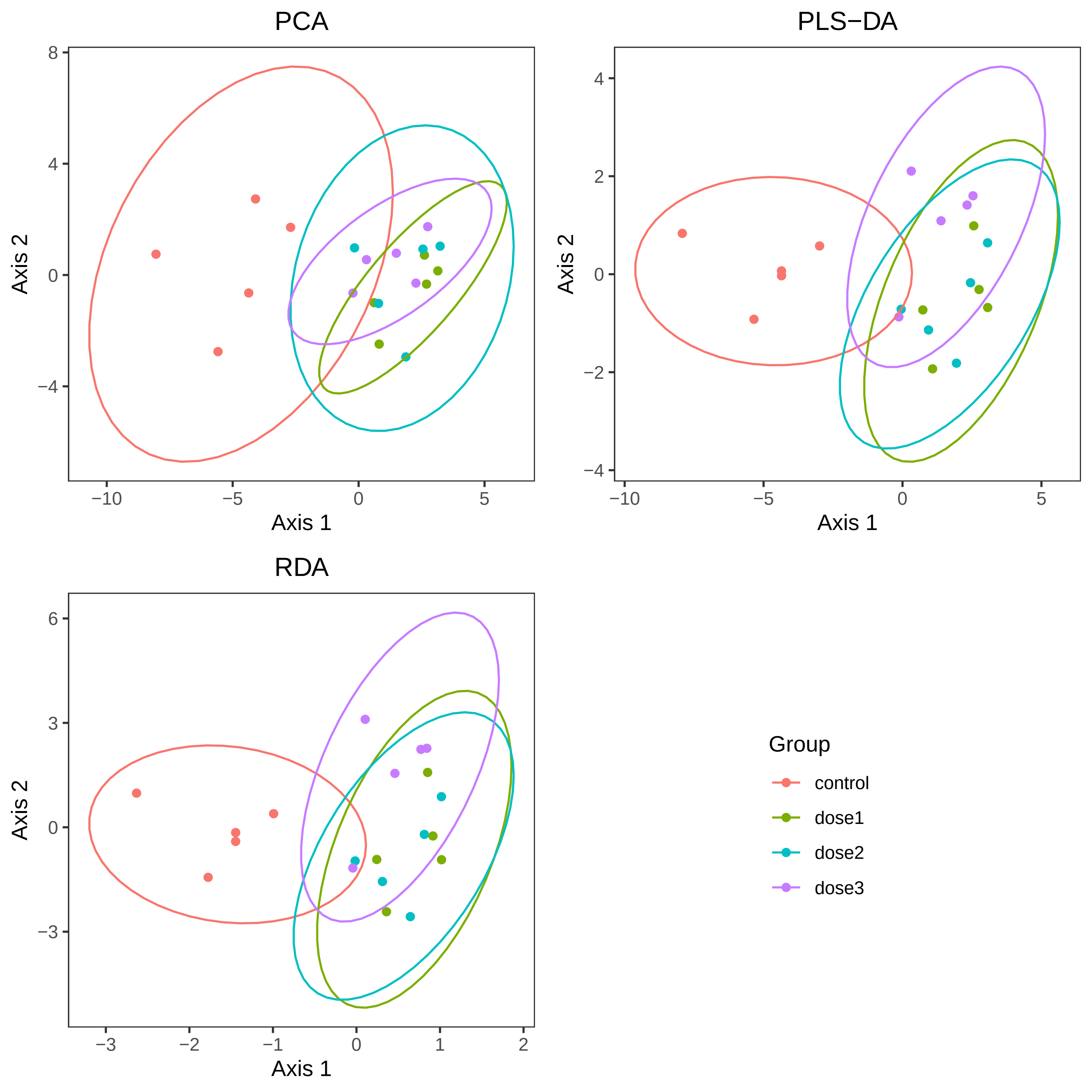
PCA
PLS−DA
RDA
Axis 1
Axis 2
Group
control
dose1
dose2
dose3

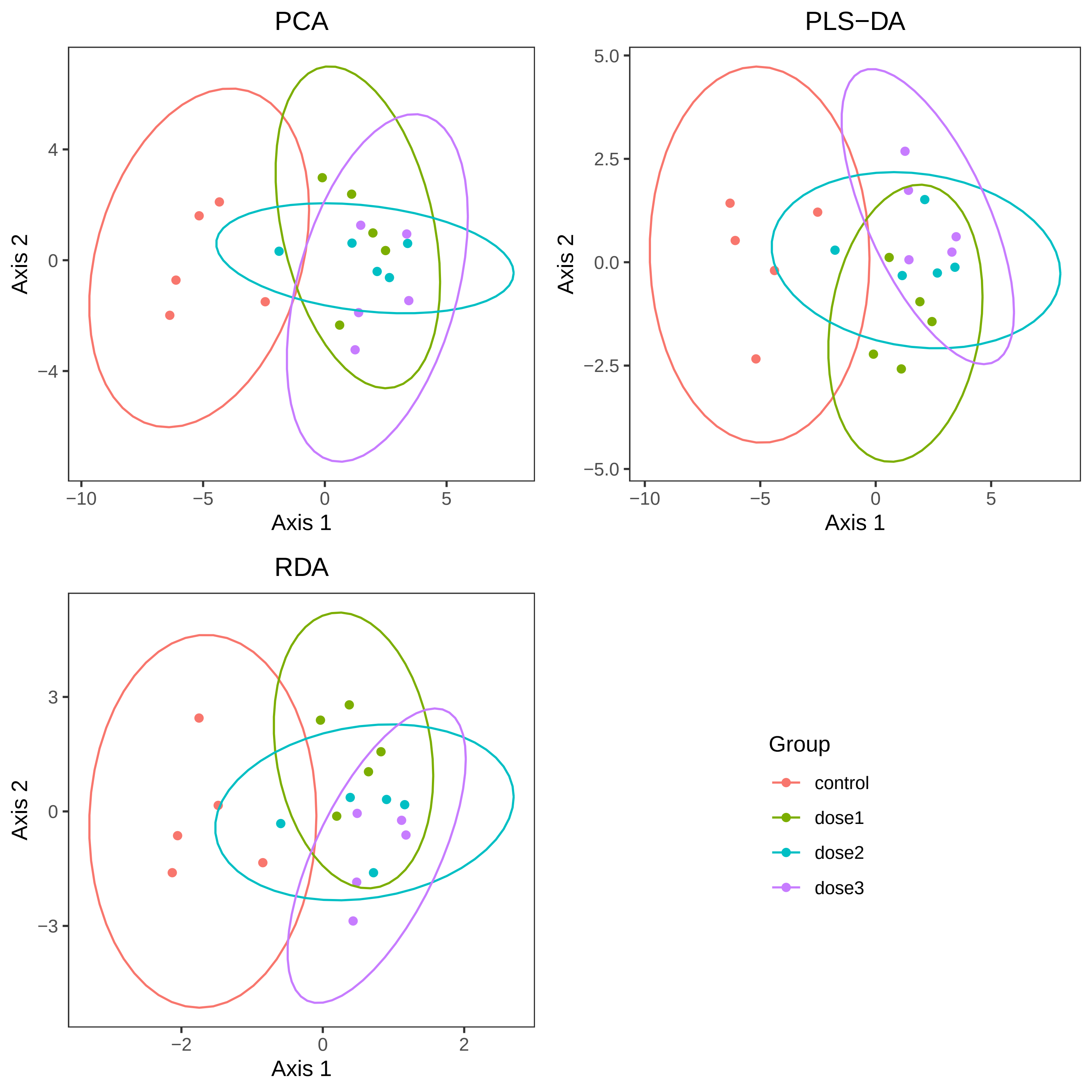

PCA
PLS−DA
RDA
Axis 1
Axis 2
Group
control
dose1
dose2
dose3

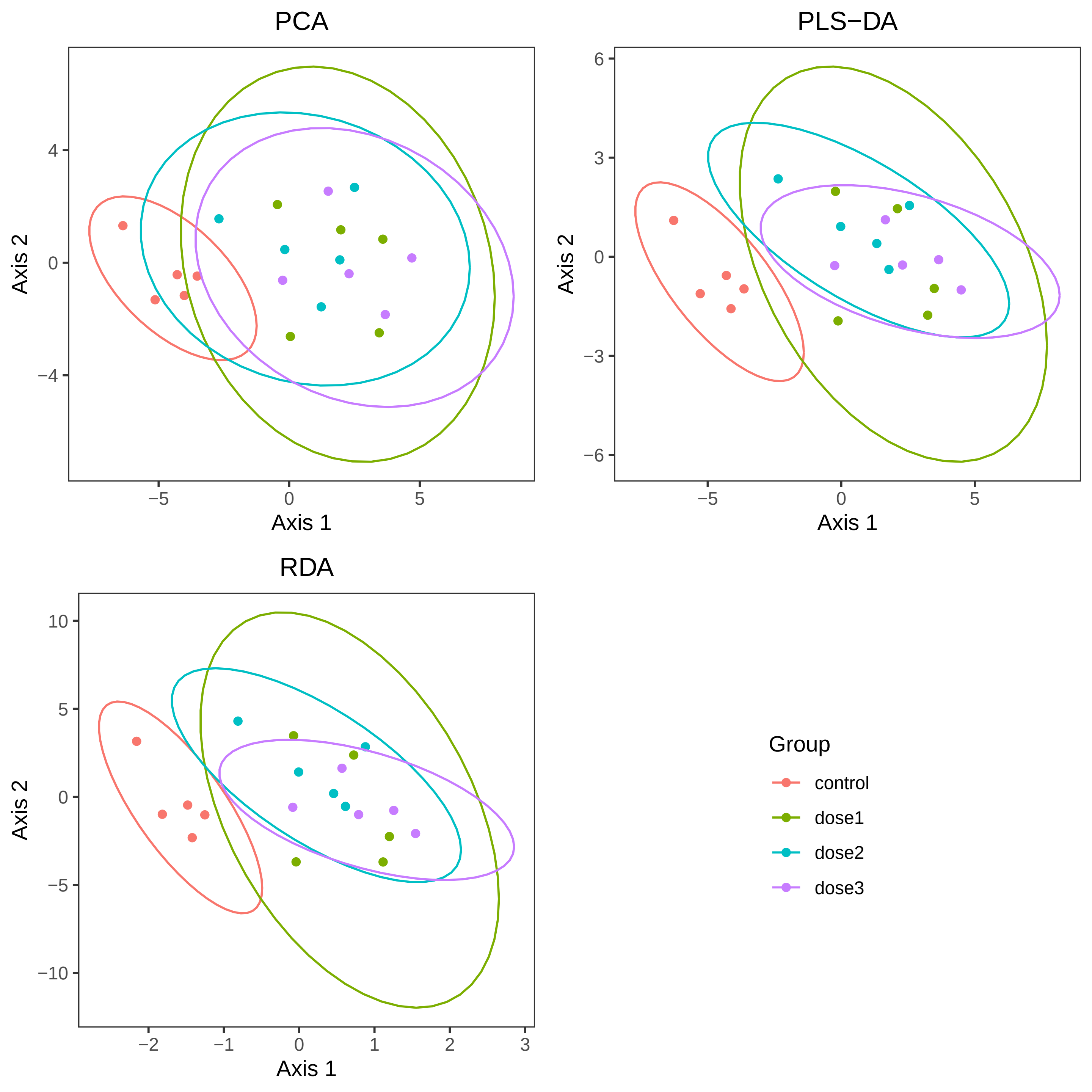

PCA
PLS−DA
RDA
Axis 1
Axis 2
Group
control
dose1
dose2
dose3

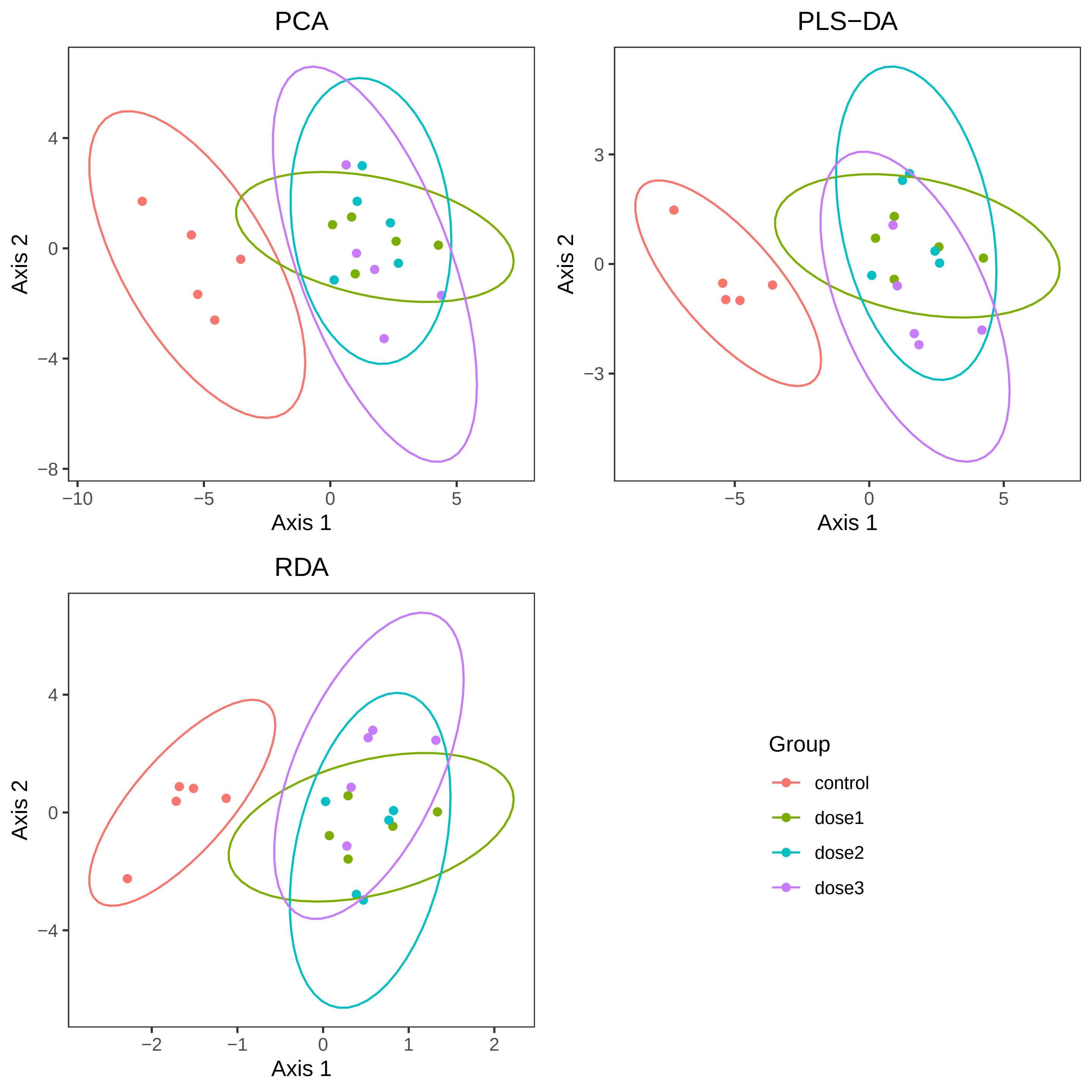
PCA
PLS−DA
RDA
Axis 1
Axis 2
Group
control
dose1
dose2
dose3

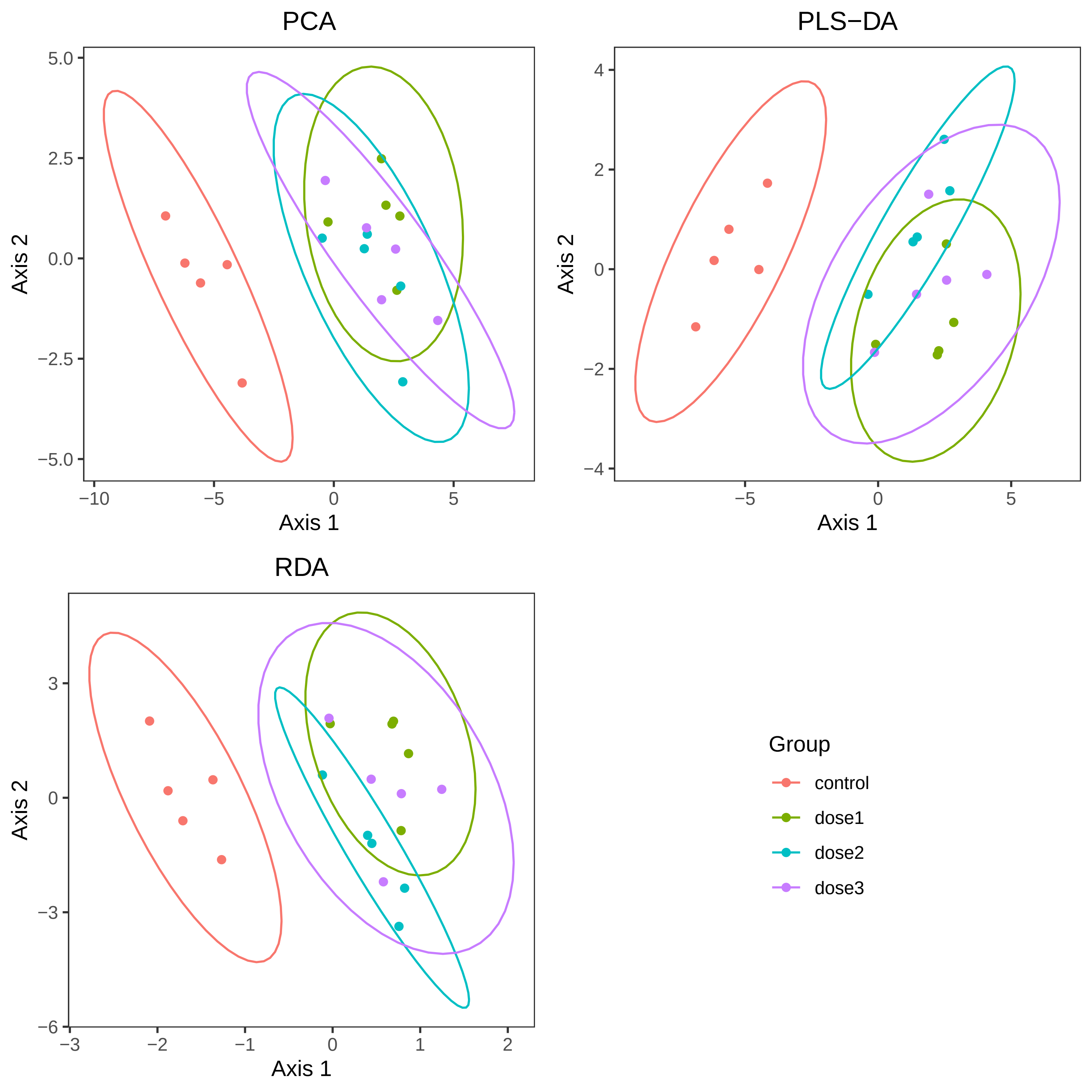

PCA
5.0
2.5
0.0
−2.5
−5.0
−10
−5
0
5
Axis 1
Axis 2
PLS−DA
4
2
0
−2
−4
−5
0
5
Axis 1
Axis 2
RDA
3
0
−3
−6
−3
−2
−1
0
1
2
Axis 1
Axis 2
Group
control
dose1
dose2
dose3

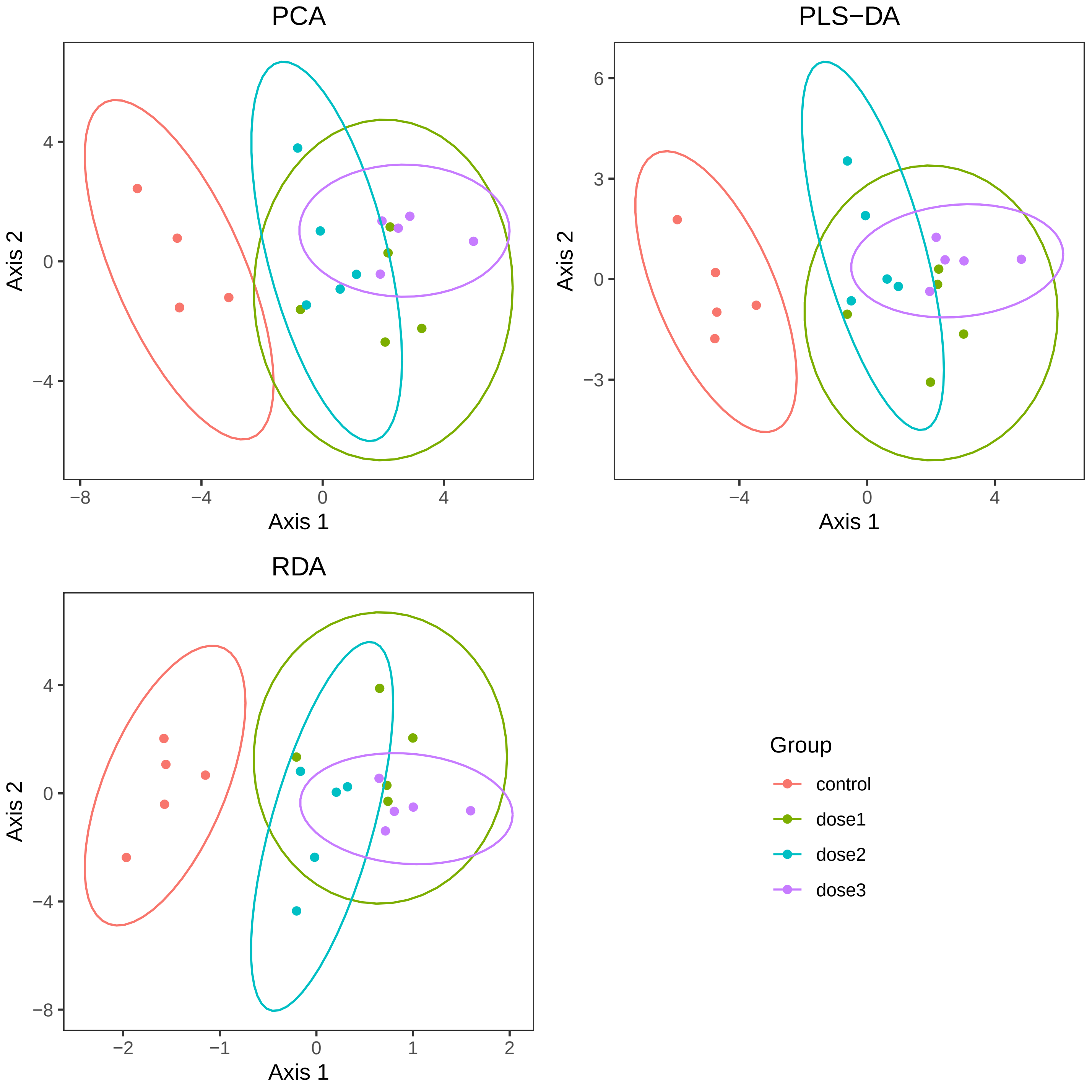

PCA
PLS−DA
RDA
Axis 1
Axis 2
Group
control
dose1
dose2
dose3

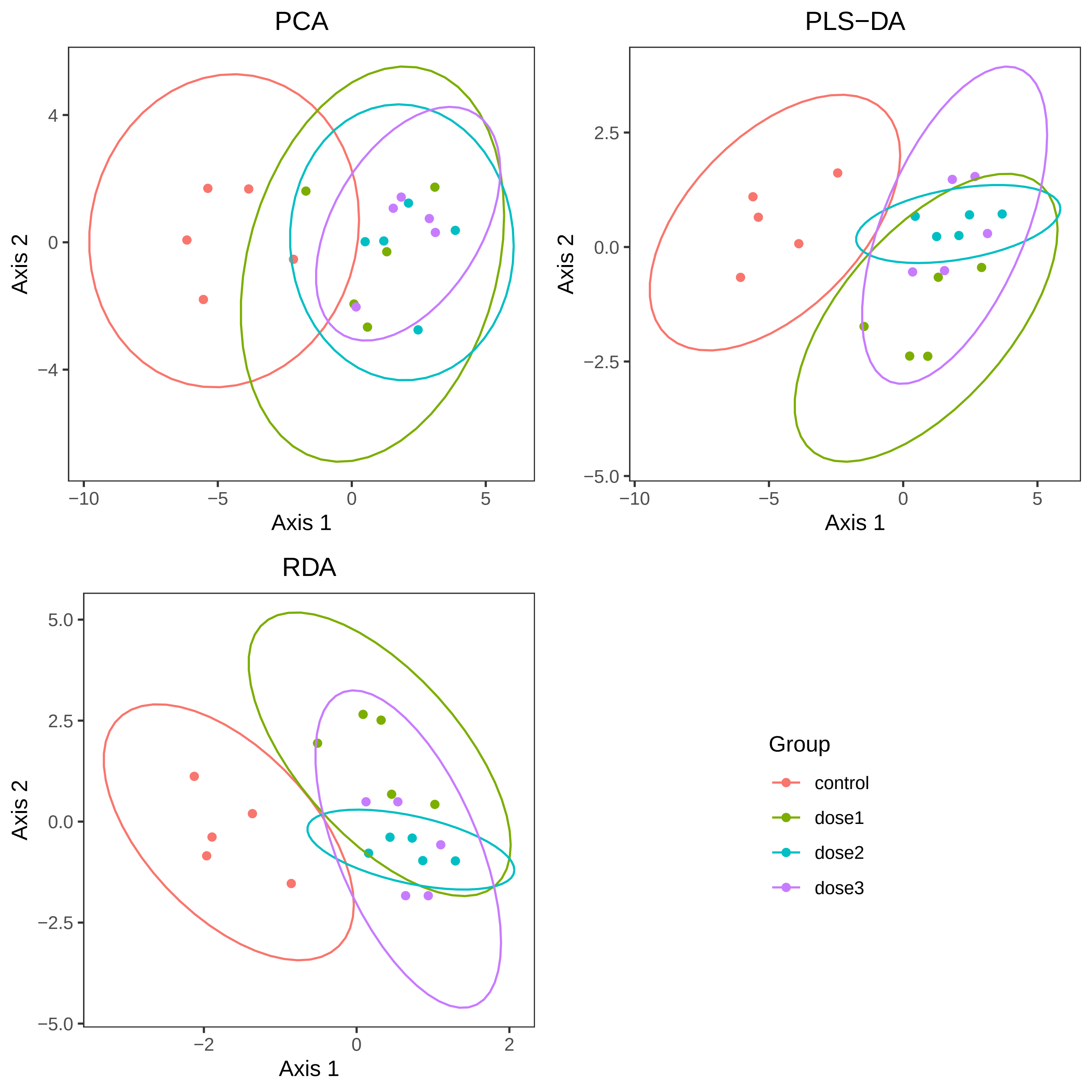
PCA
PLS−DA
RDA
Axis 1
Axis 2
4
0
−4
−10
−5
0
5
2.5
0.0
−2.5
−5.0
5.0
−2
2
Group
control
dose1
dose2
dose3

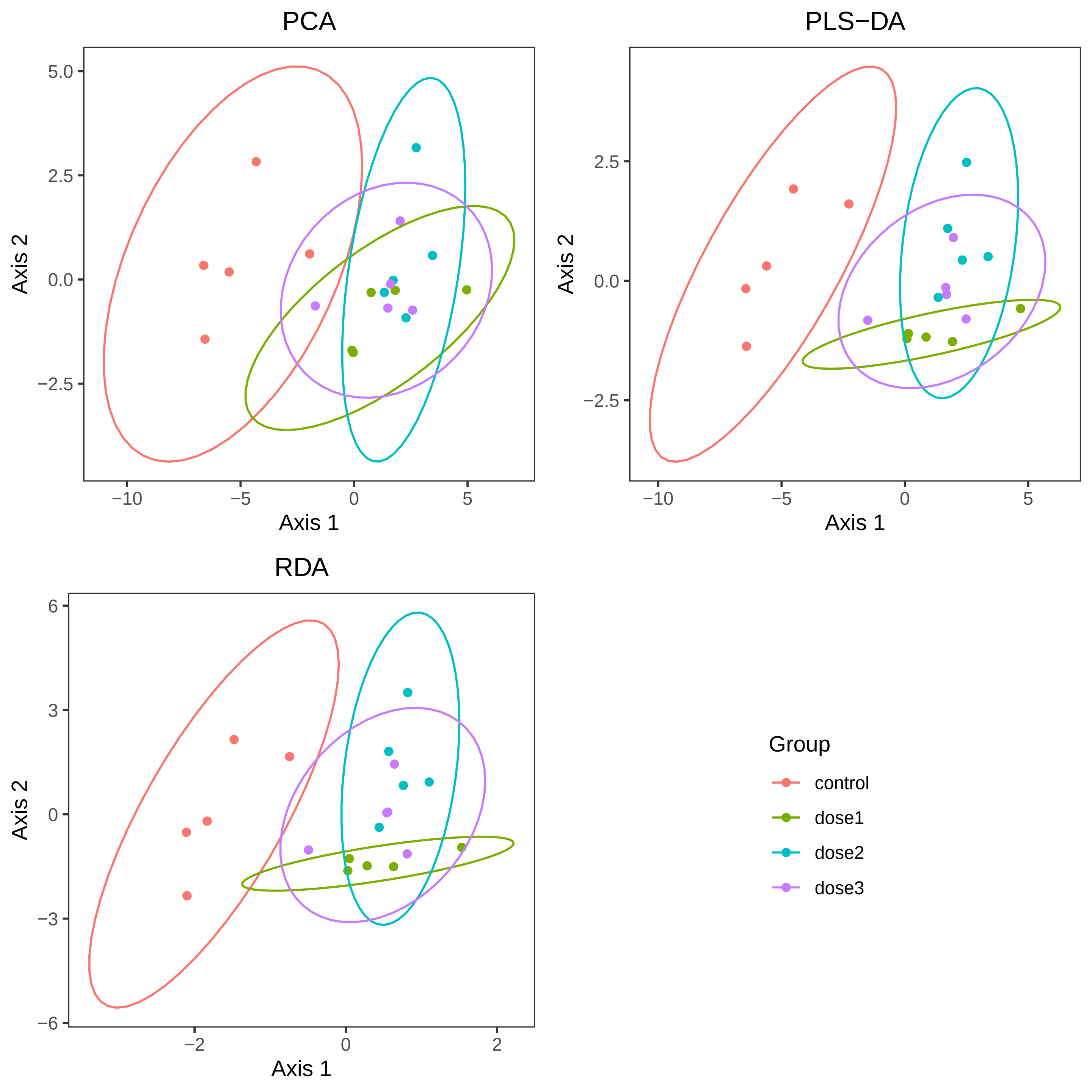

PCA
PLS−DA
RDA
Axis 1
Axis 2
5.0
2.5
0.0
−2.5
−10
−5
0
5
6
3
0
−3
−6
−2
2
Group
control
dose1
dose2
dose3

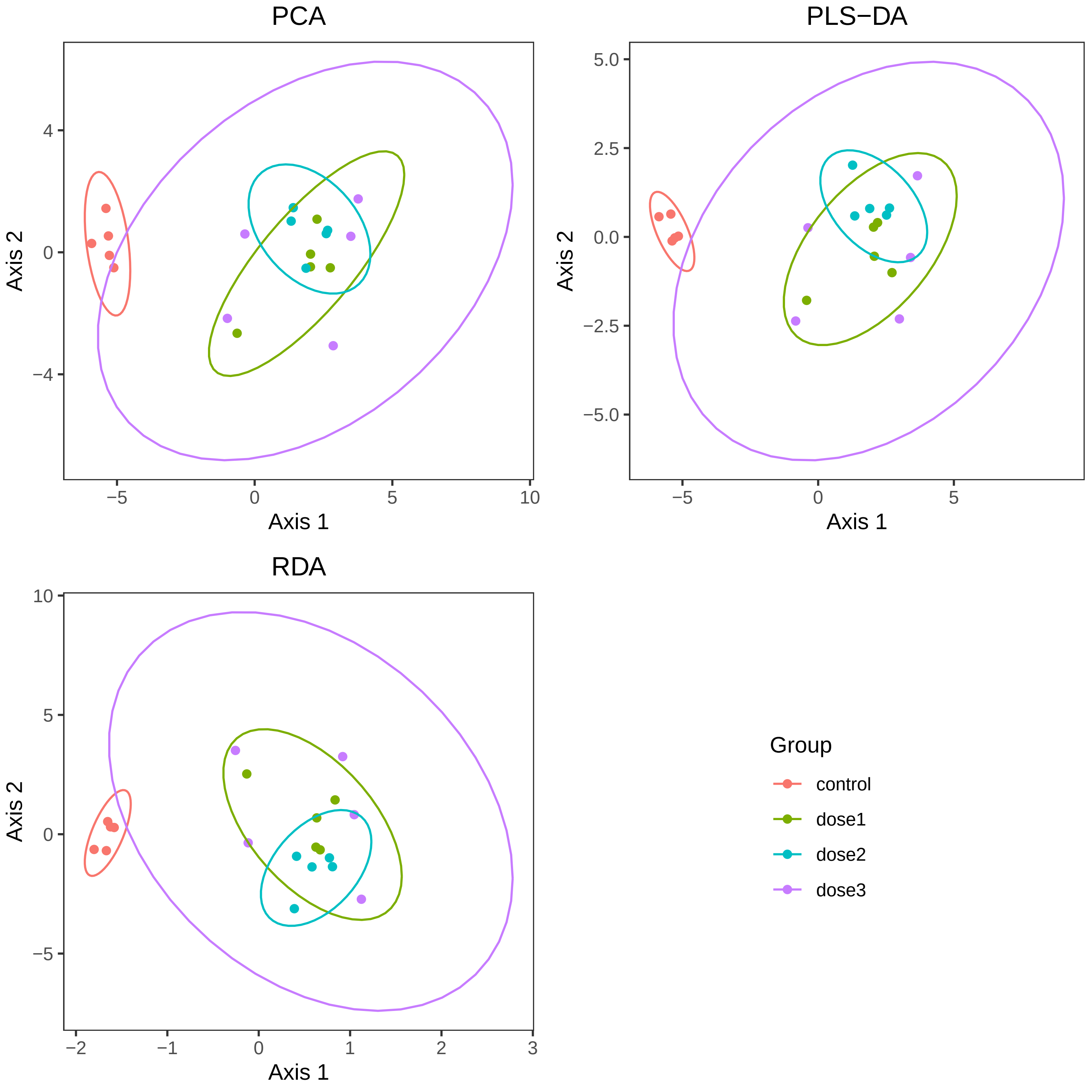
PCA
PLS−DA
RDA
Axis 1
Axis 2
Group
control
dose1
dose2
dose3

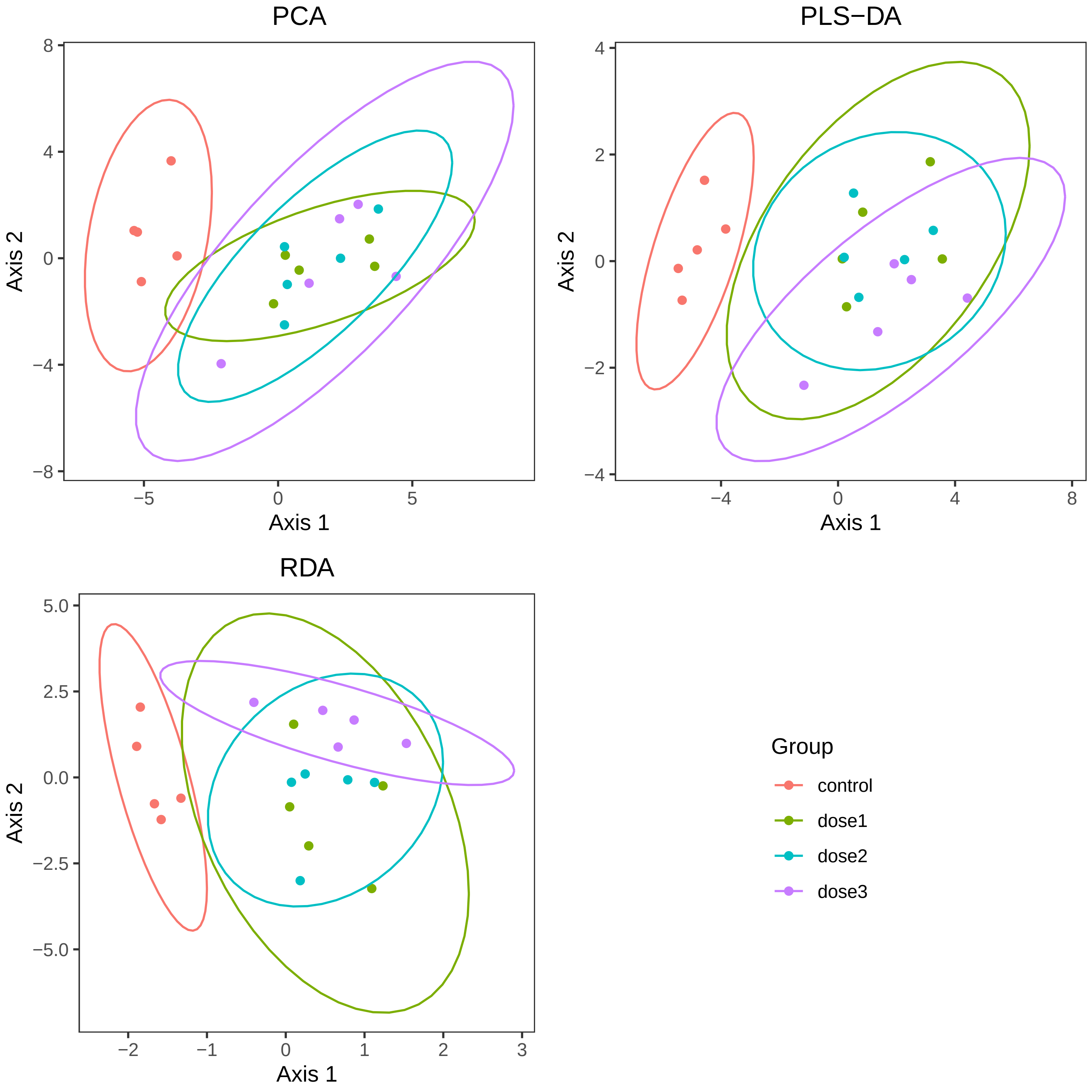

PCA
PLS−DA
RDA
Axis 1
Axis 2
Group
control
dose1
dose2
dose3

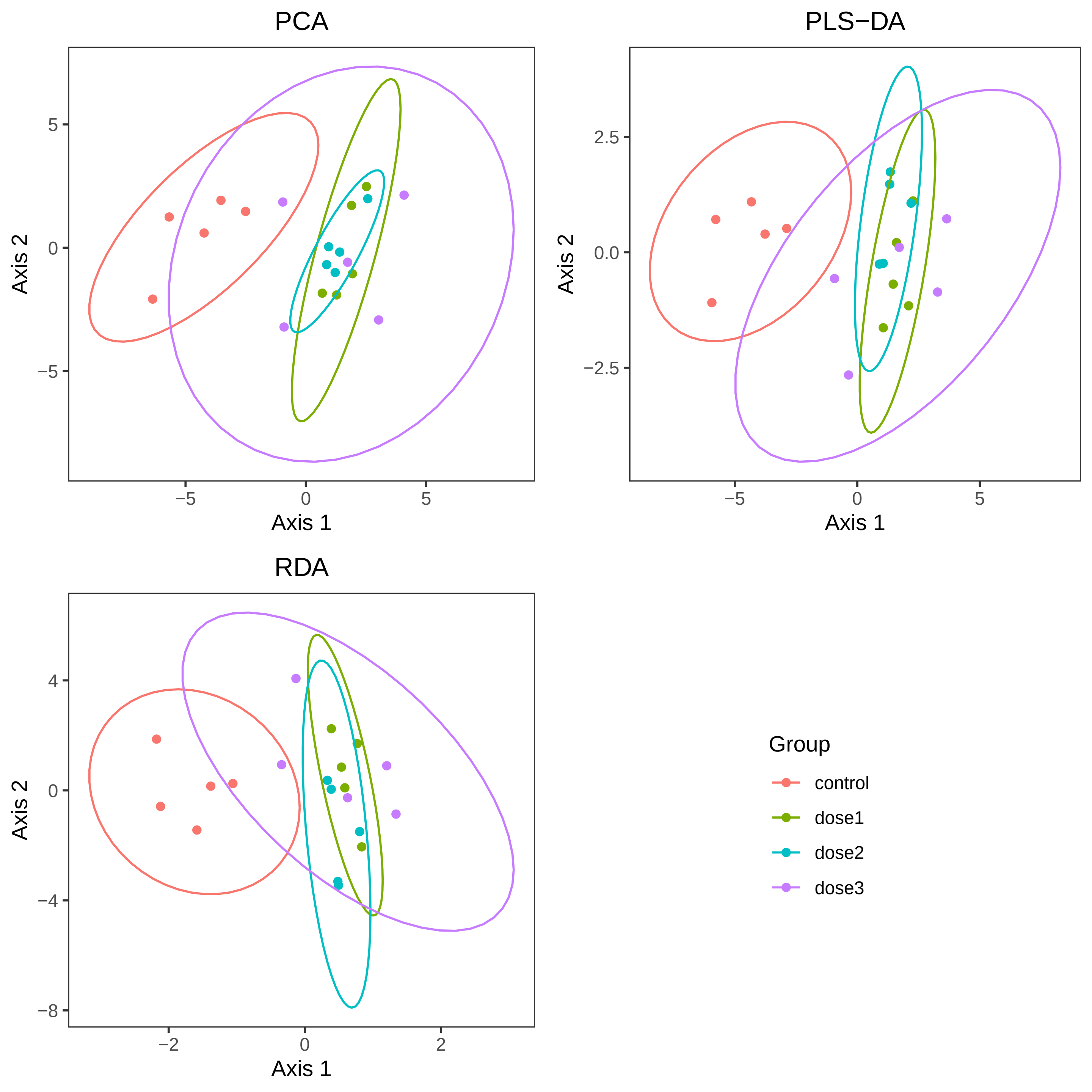
PCA
PLS−DA
RDA
Axis 1
Axis 2
Group
control
dose1
dose2
dose3

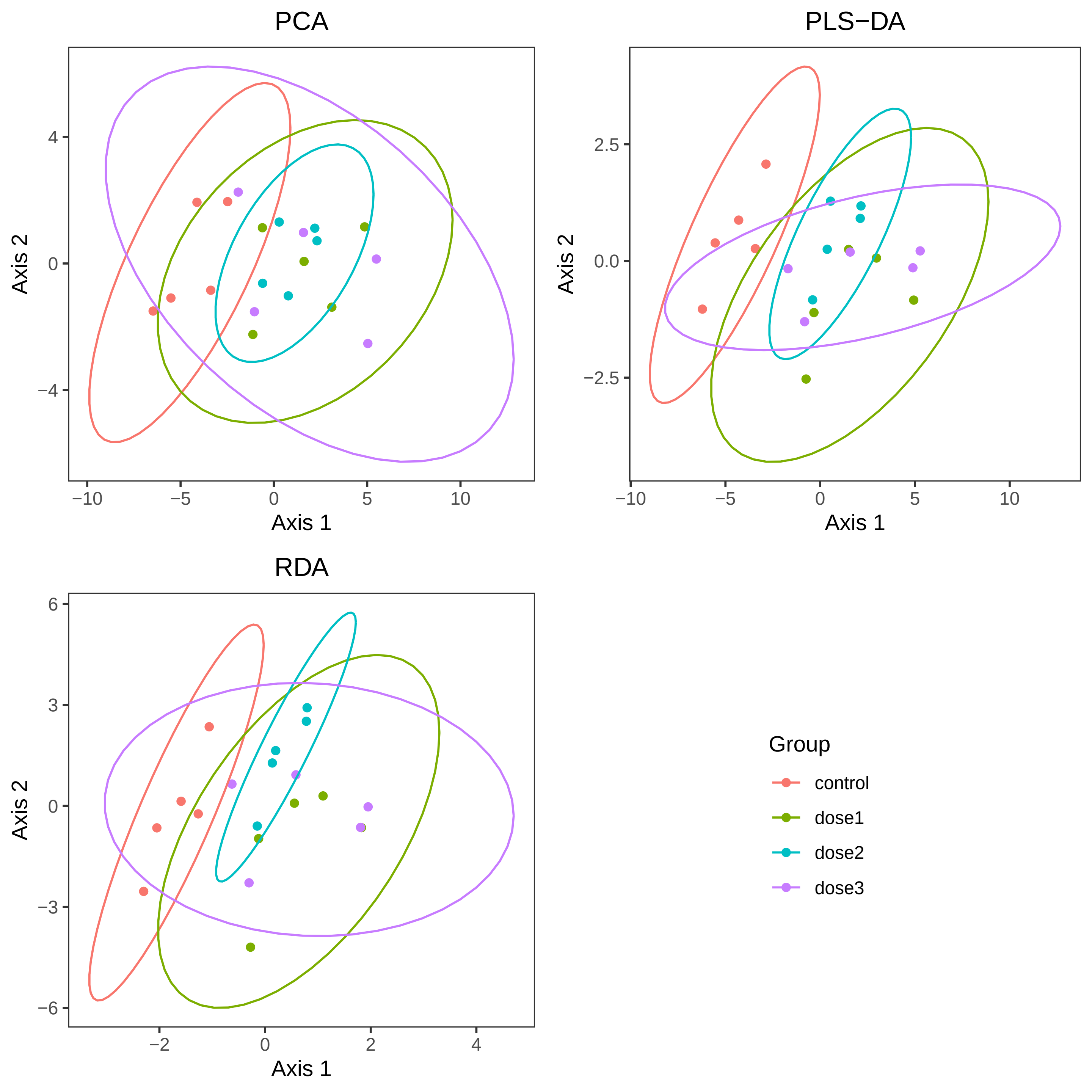

PCA
PLS−DA
RDA
Axis 1
Axis 2
−10
−5
0
5
10
4
0
−4
2.5
0.0
−2.5
−2
2
6
3
−3
−6
Group
control
dose1
dose2
dose3

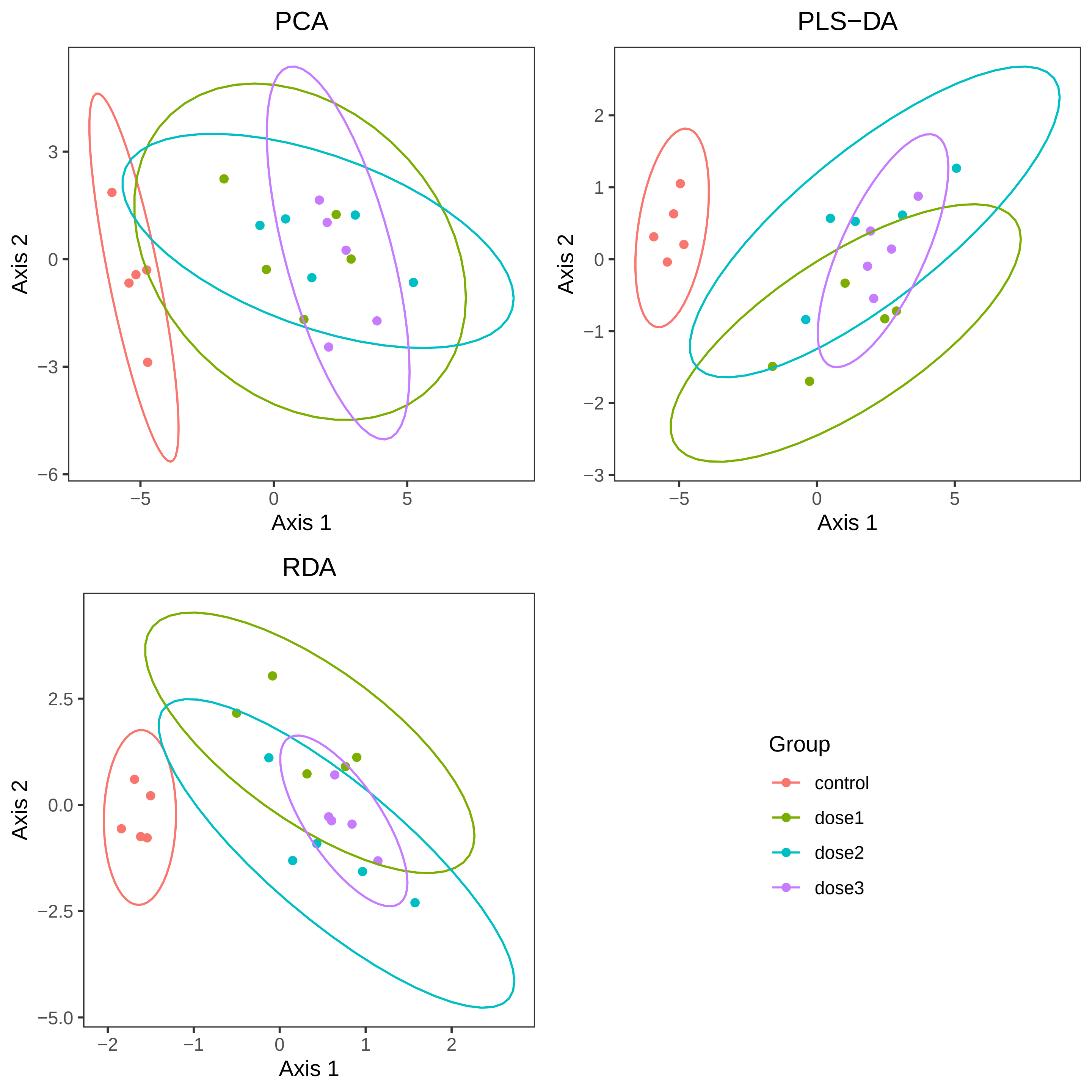

PCA
PLS−DA
RDA
Axis 1
Axis 2
Group
control
dose1
dose2
dose3

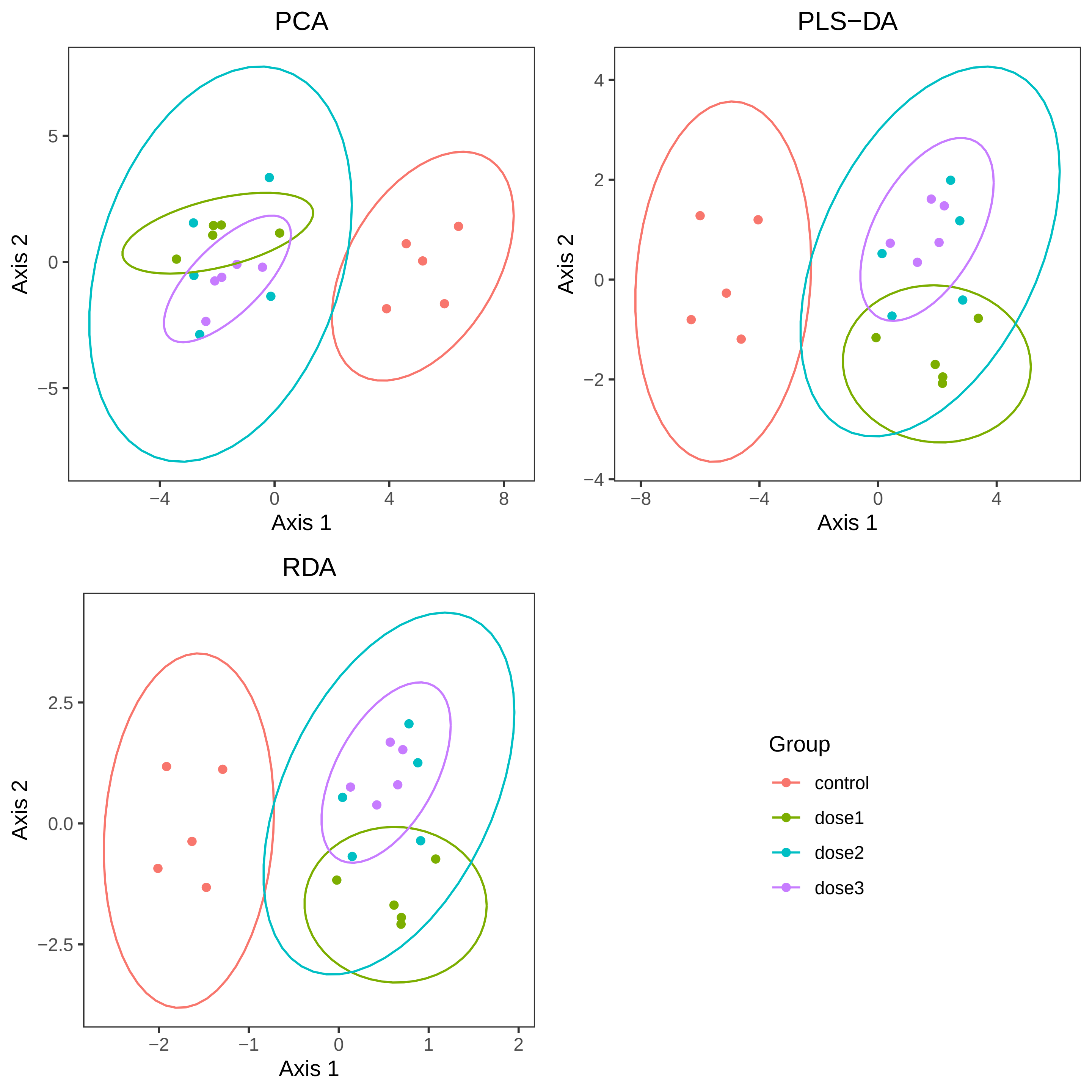

PCA
PLS−DA
RDA
Axis 1
Axis 2
Group
control
dose1
dose2
dose3

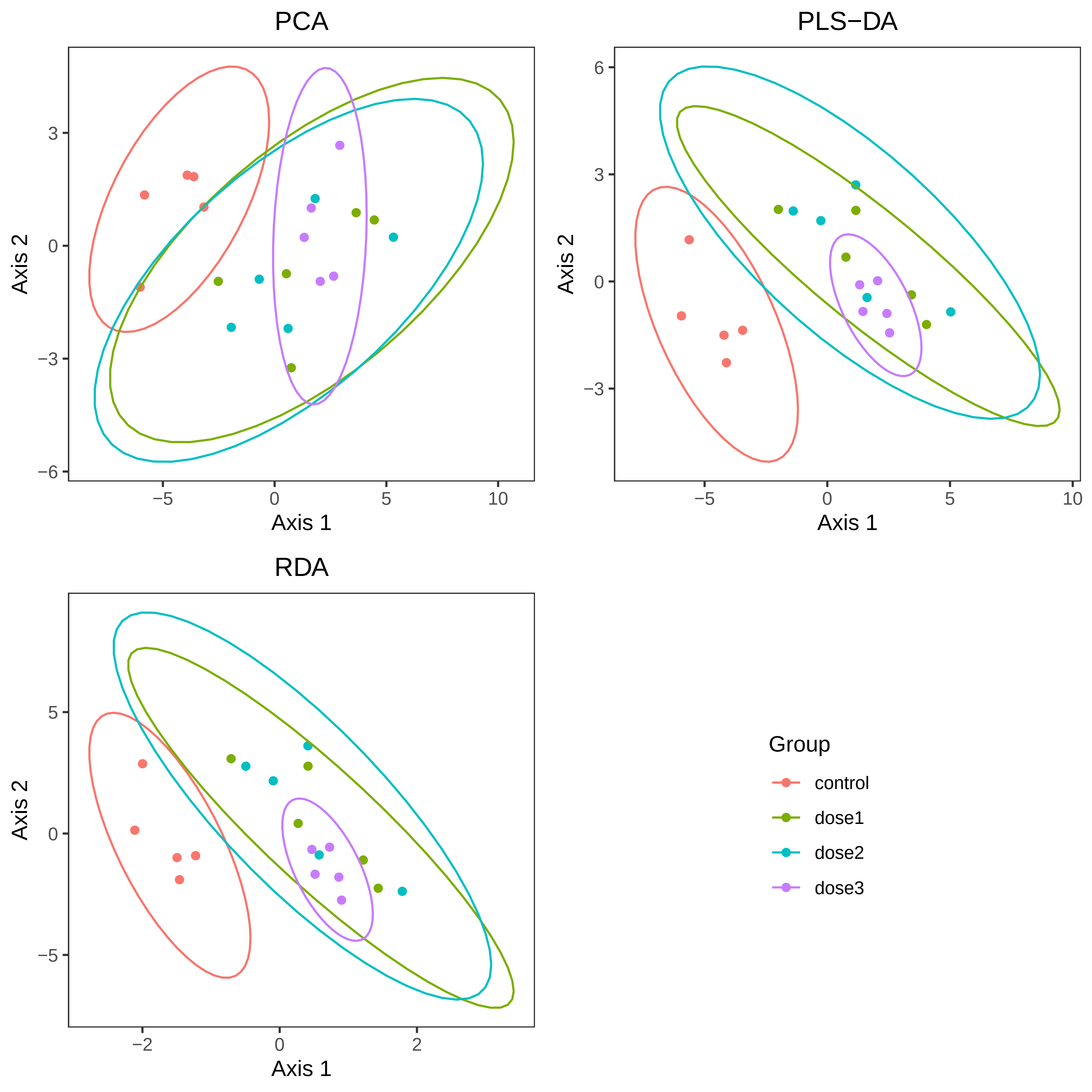

PCA
PLS−DA
RDA
Axis 1
Axis 2
Group
control
dose1
dose2
dose3

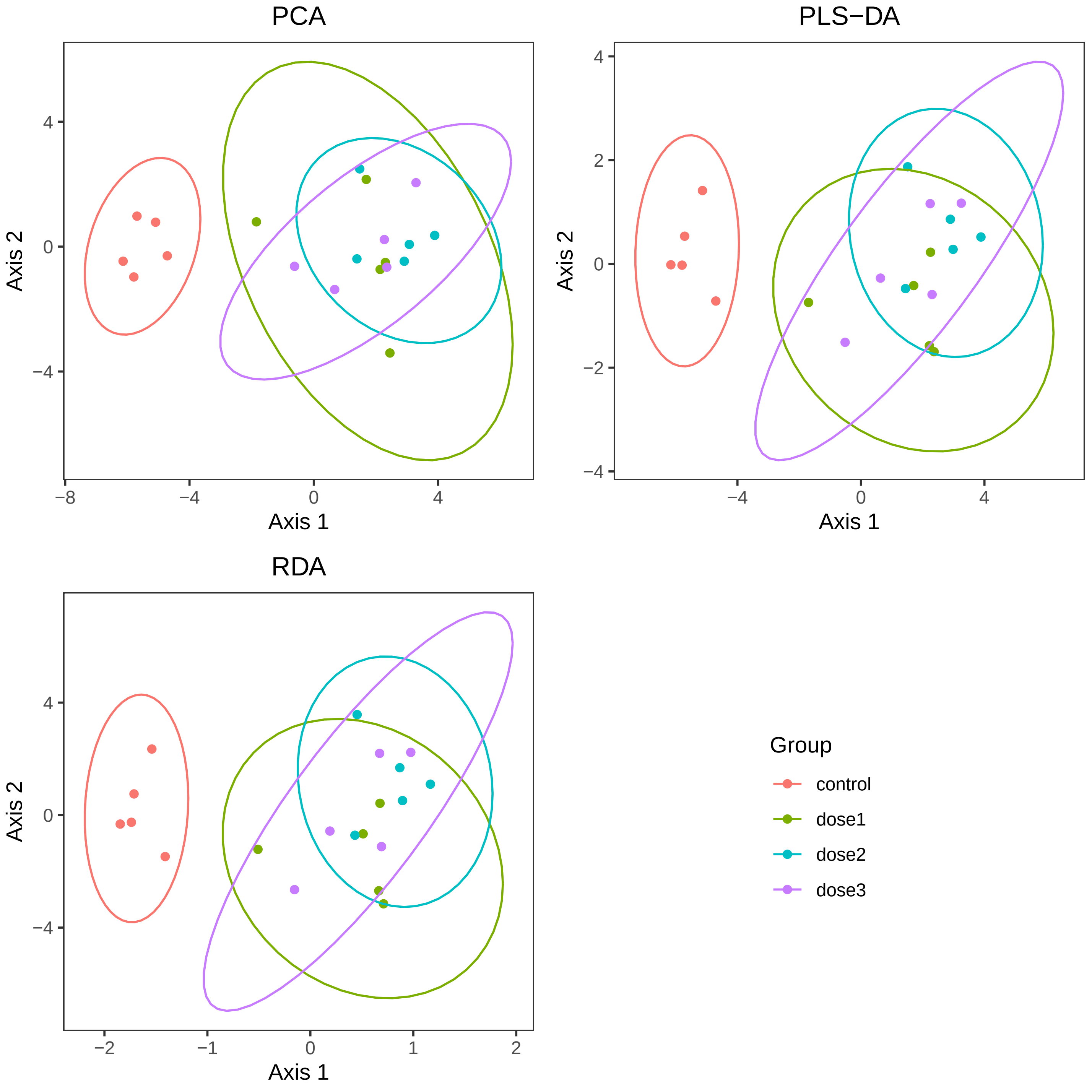
PCA
Axis 1
Axis 2
PLS−DA
Axis 1
Axis 2
RDA
Axis 1
Axis 2
Group
control
dose1
dose2
dose3

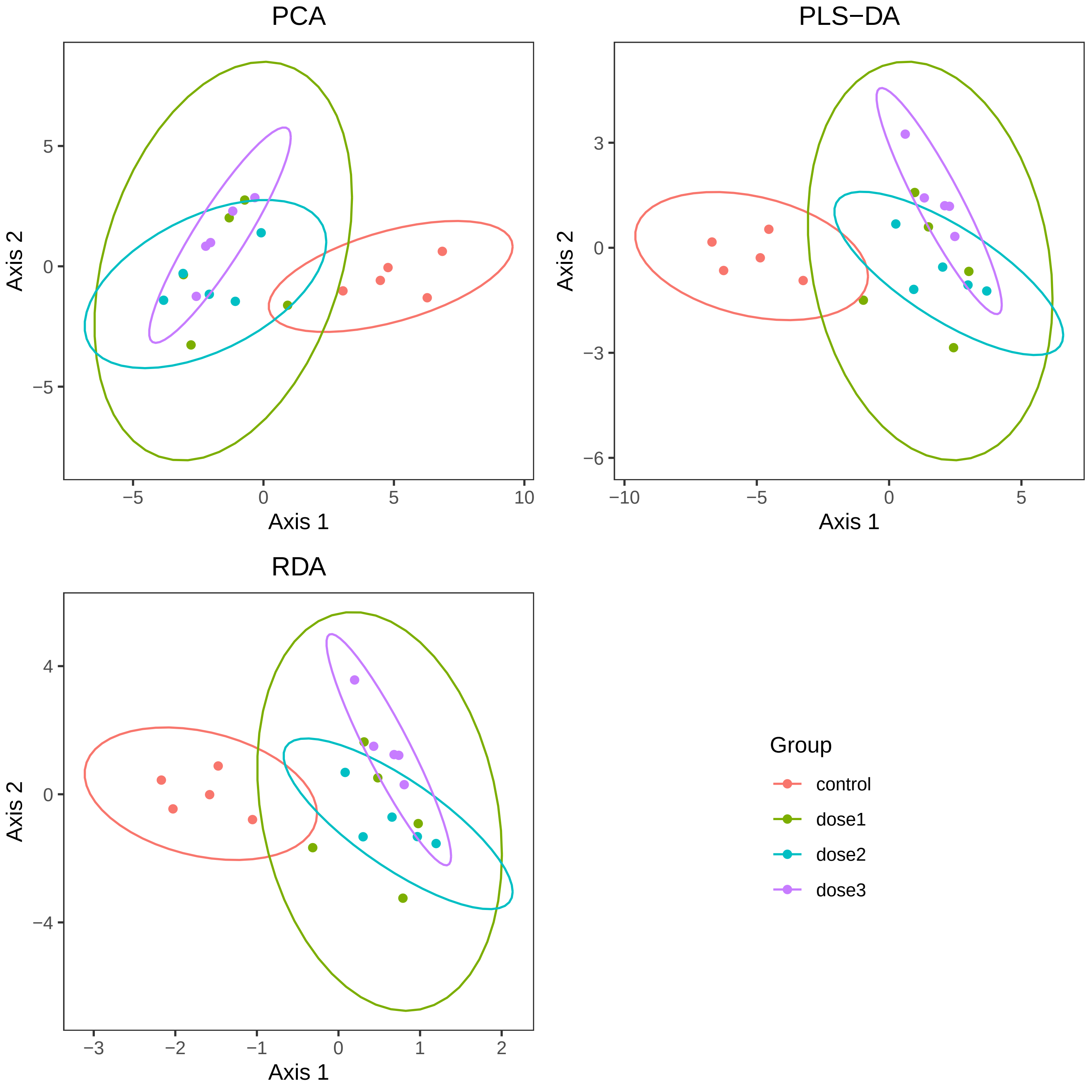

PCA
PLS−DA
RDA
Axis 1
Axis 2
Group
control
dose1
dose2
dose3

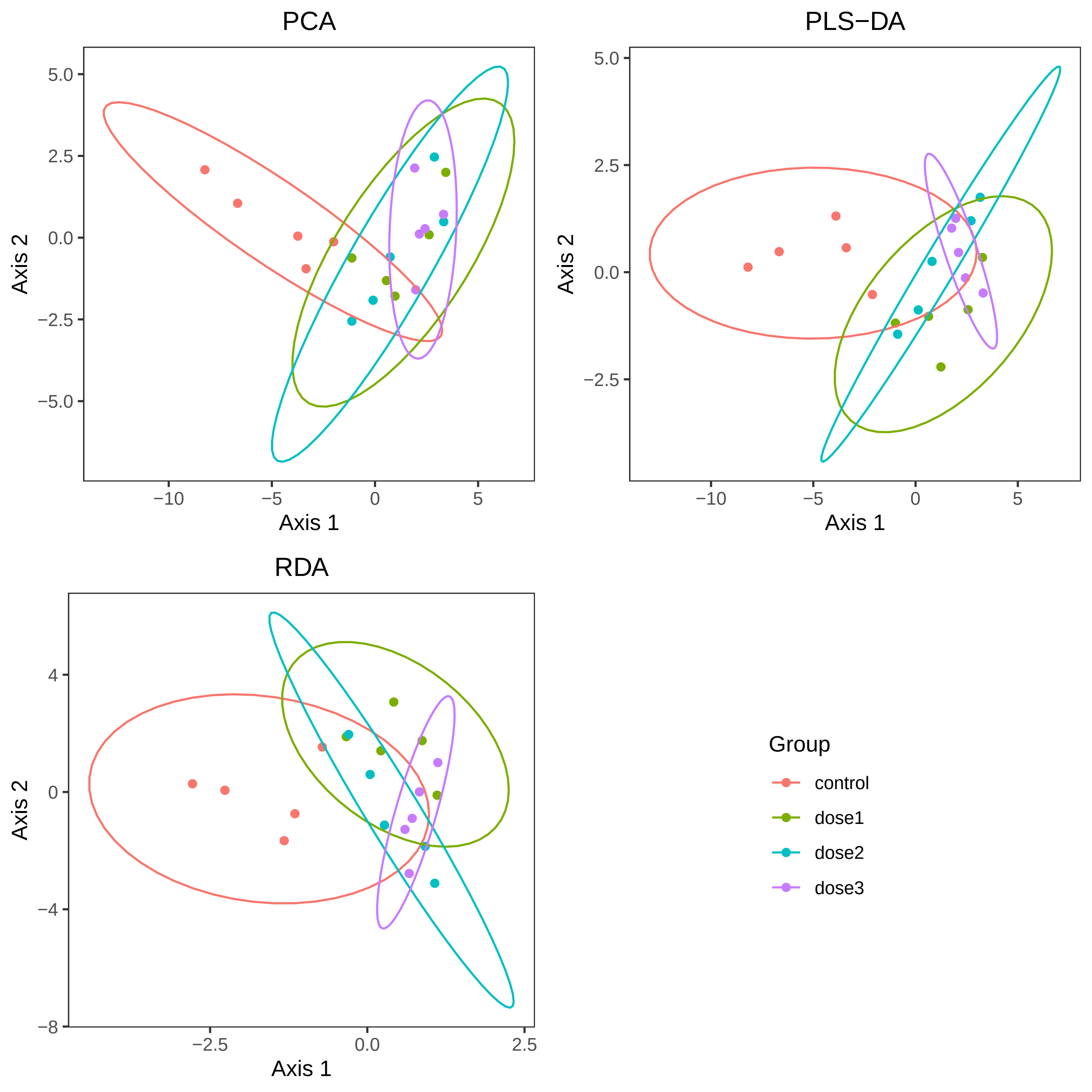

PCA
PLS−DA
RDA
Axis 1
Axis 2
5.0
2.5
0.0
−2.5
−5.0
−10
−5
0
5
4
−4
−8
2.5
Group
control
dose1
dose2
dose3

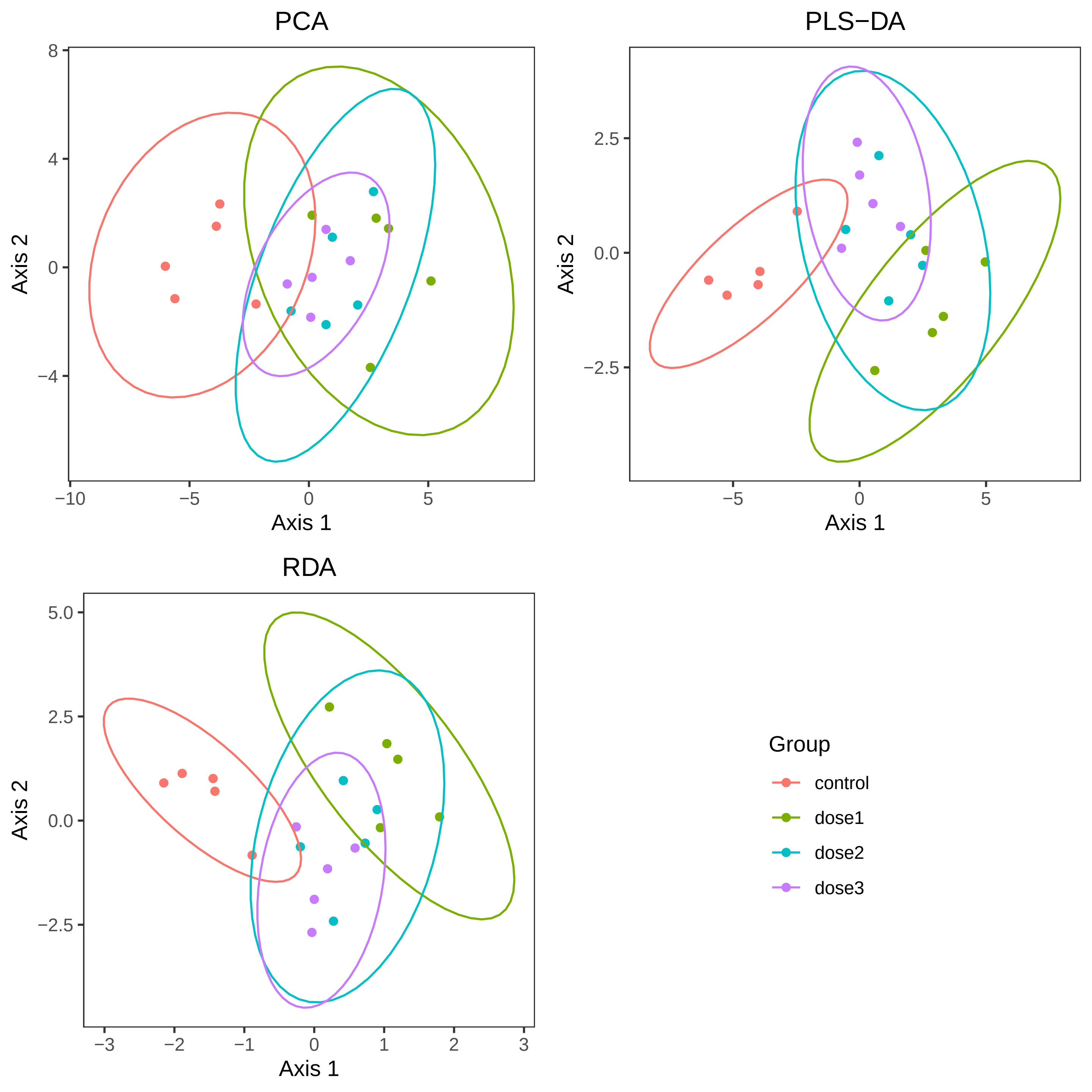
PCA
PLS−DA
RDA
Axis 1
Axis 2
Group
control
dose1
dose2
dose3

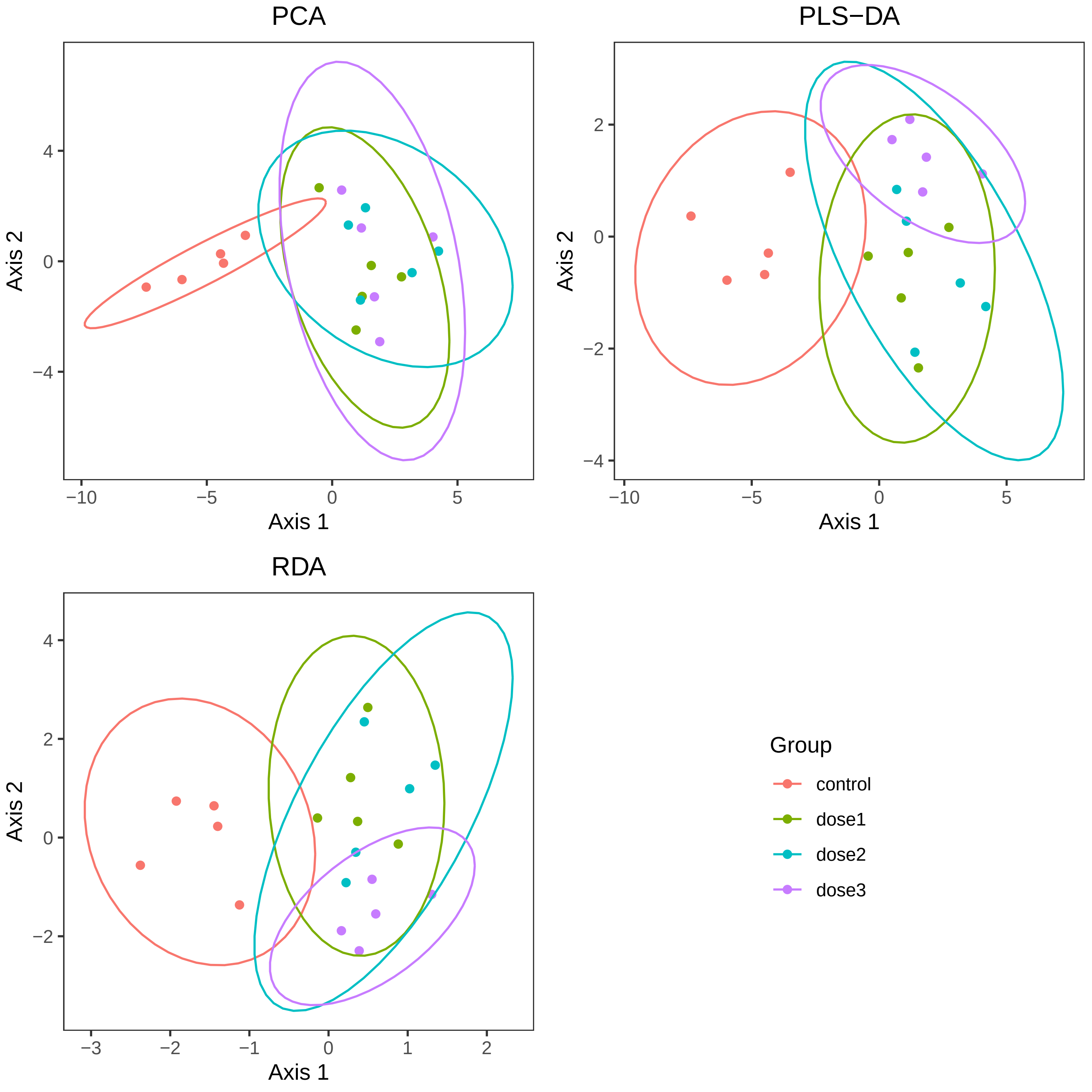

PCA
PLS−DA
RDA
Axis 1
Axis 2
Group
control
dose1
dose2
dose3

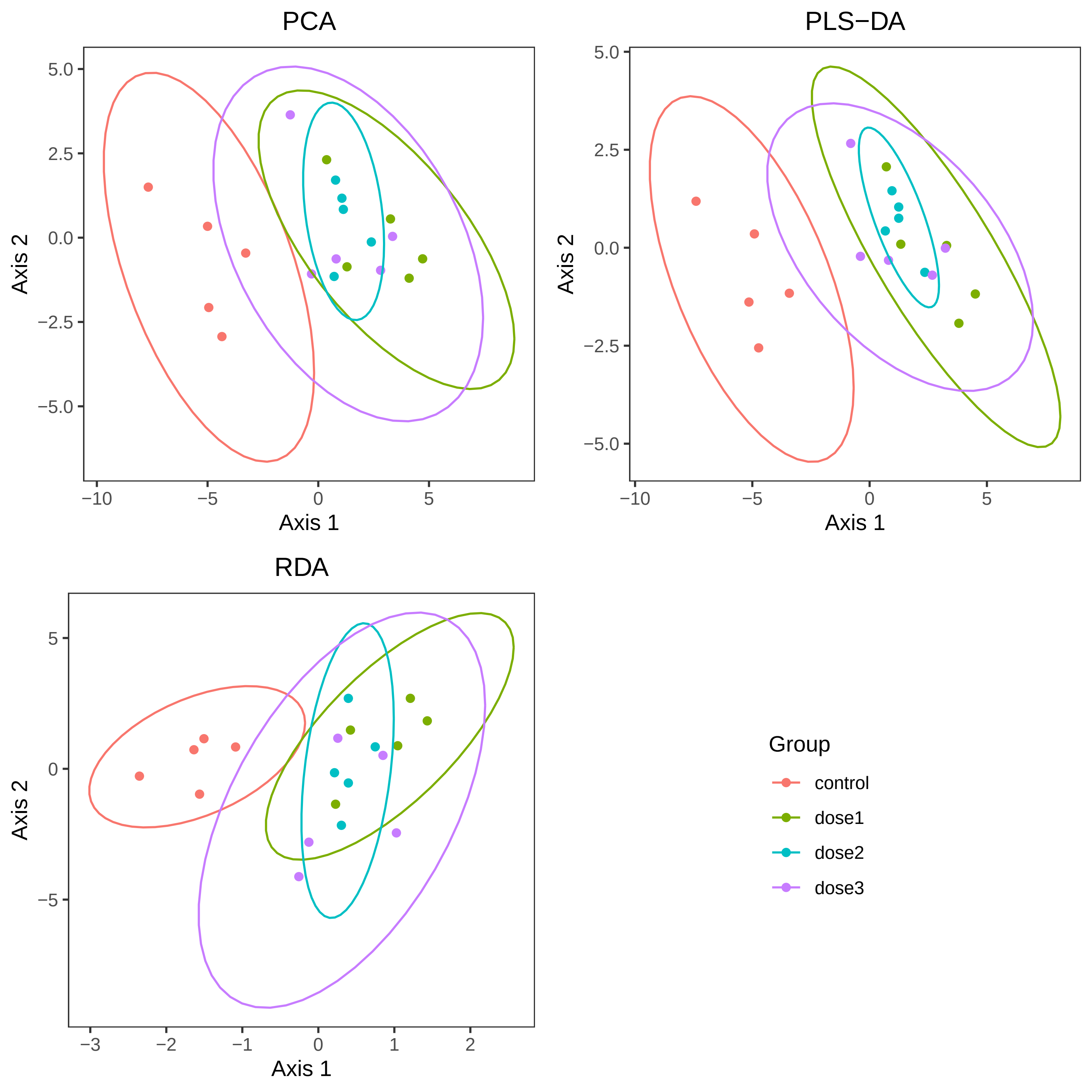
PCA
Axis 2
Axis 1
5.0
2.5
0.0
−2.5
−5.0
−10
−5
0
5
PLS−DA
Axis 2
Axis 1
5.0
2.5
0.0
−2.5
−5.0
−10
−5
0
5
RDA
Axis 2
Axis 1
5
0
−5
−3
−2
−1
0
1
2
Group
control
dose1
dose2
dose3

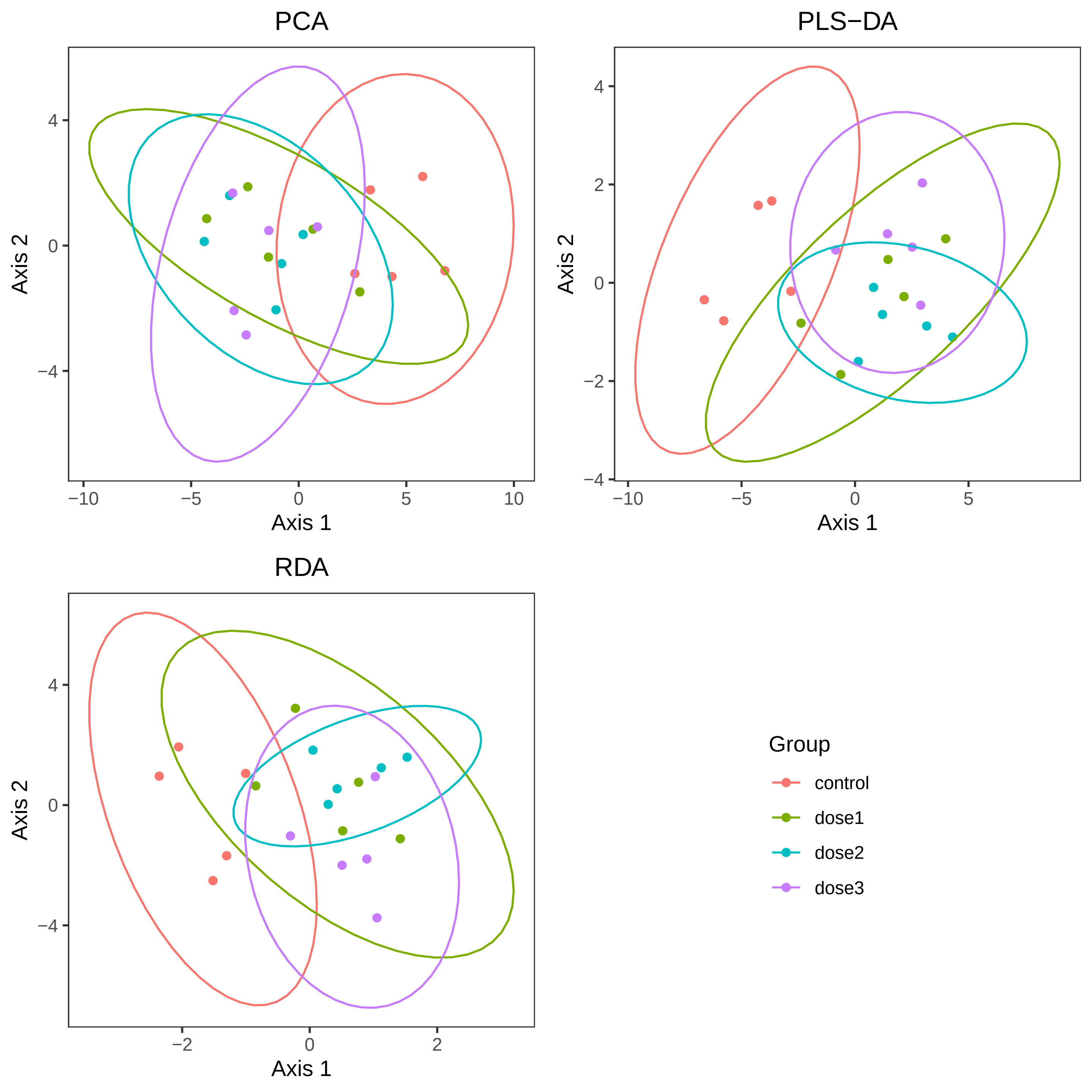

PCA
Axis 2
Axis 1
−10
−5
0
5
10
4
0
−4
PLS−DA
Axis 2
Axis 1
−10
−5
0
5
4
2
0
−2
−4
RDA
Axis 2
Axis 1
−2
0
2
4
0
−4
Group
control
dose1
dose2
dose3

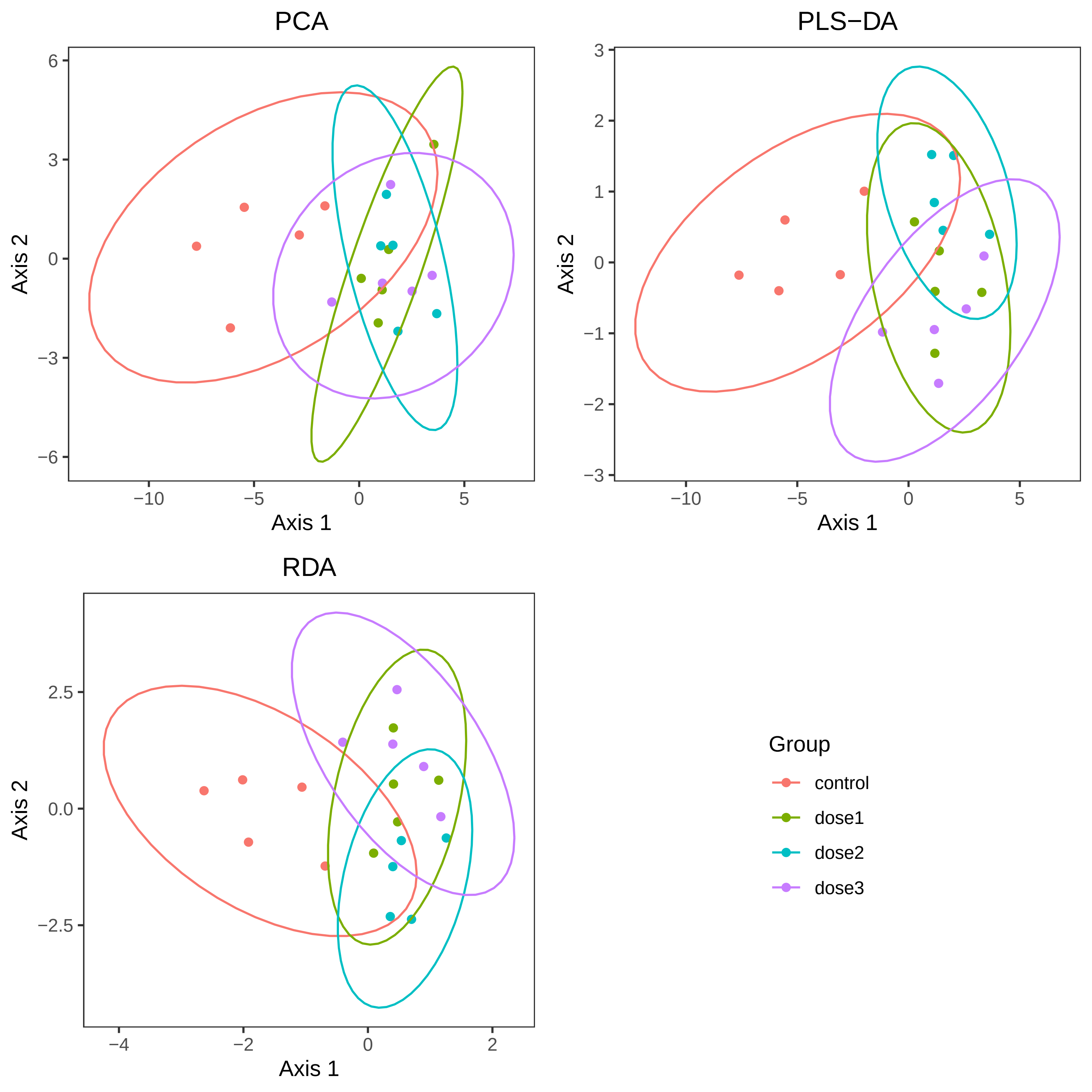

PCA
PLS−DA
RDA
Axis 1
Axis 2
Group
control
dose1
dose2
dose3

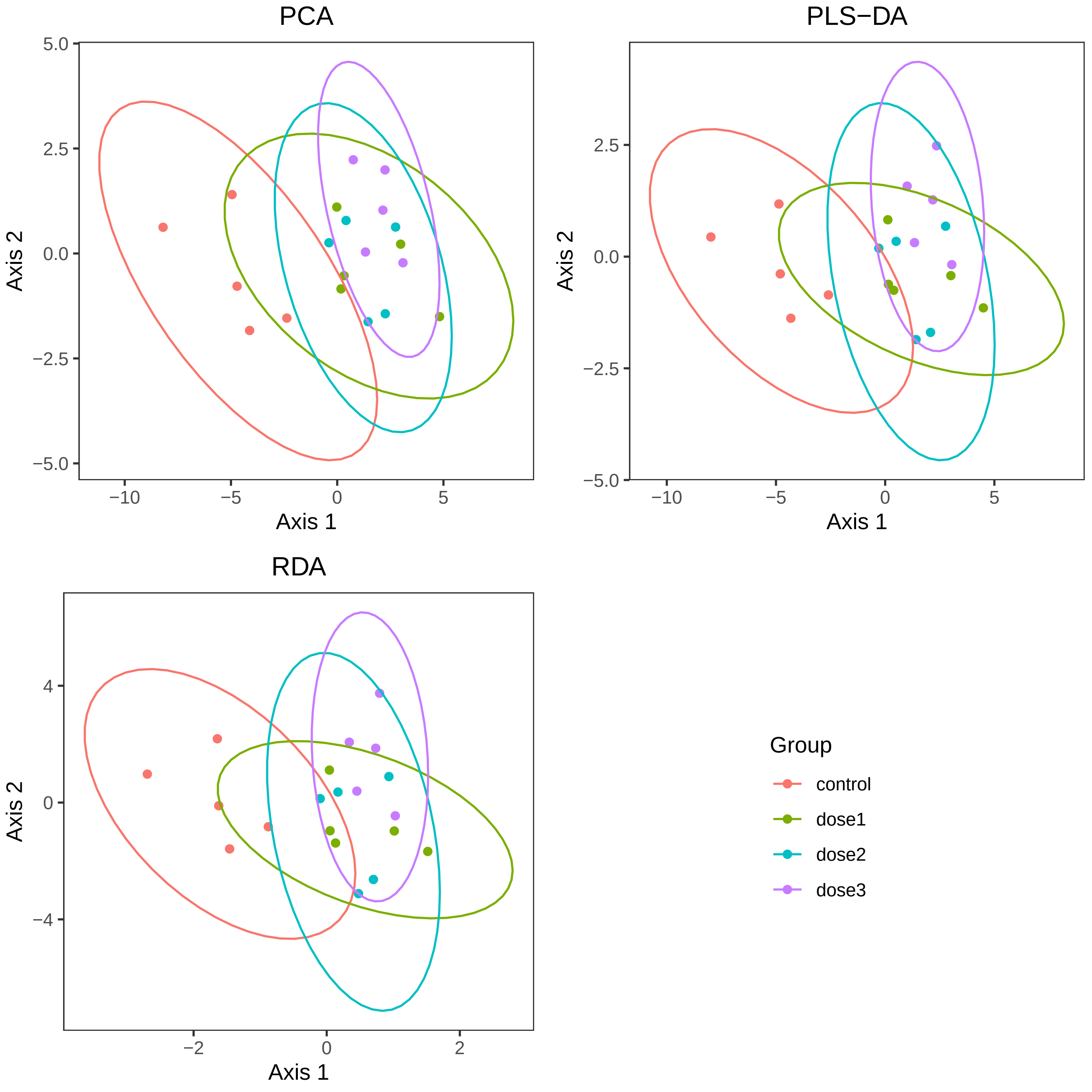

PCA
PLS−DA
RDA
Axis 1
Axis 2
Group
control
dose1
dose2
dose3

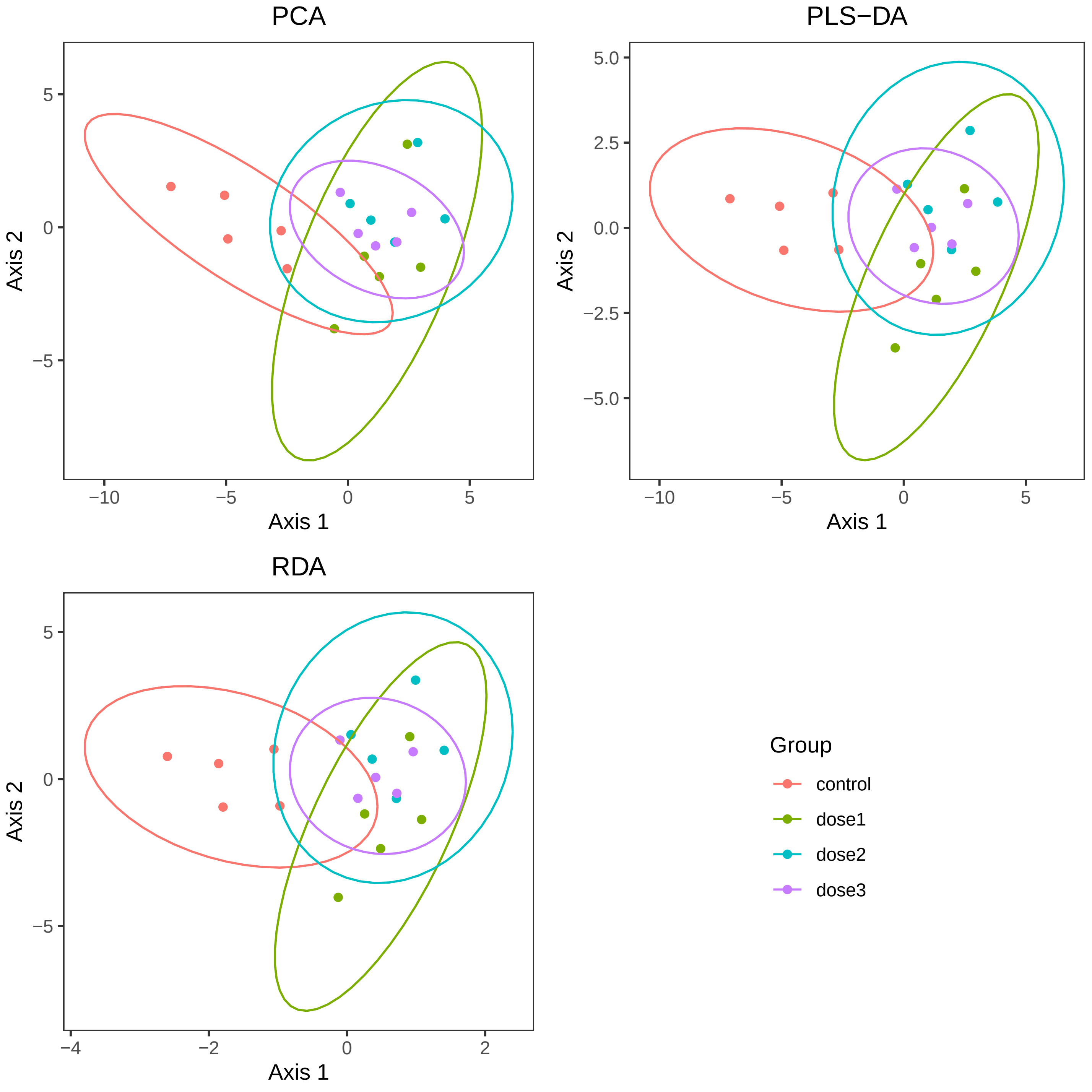
PCA
PLS−DA
RDA
Axis 1
Axis 2
Group
control
dose1
dose2
dose3

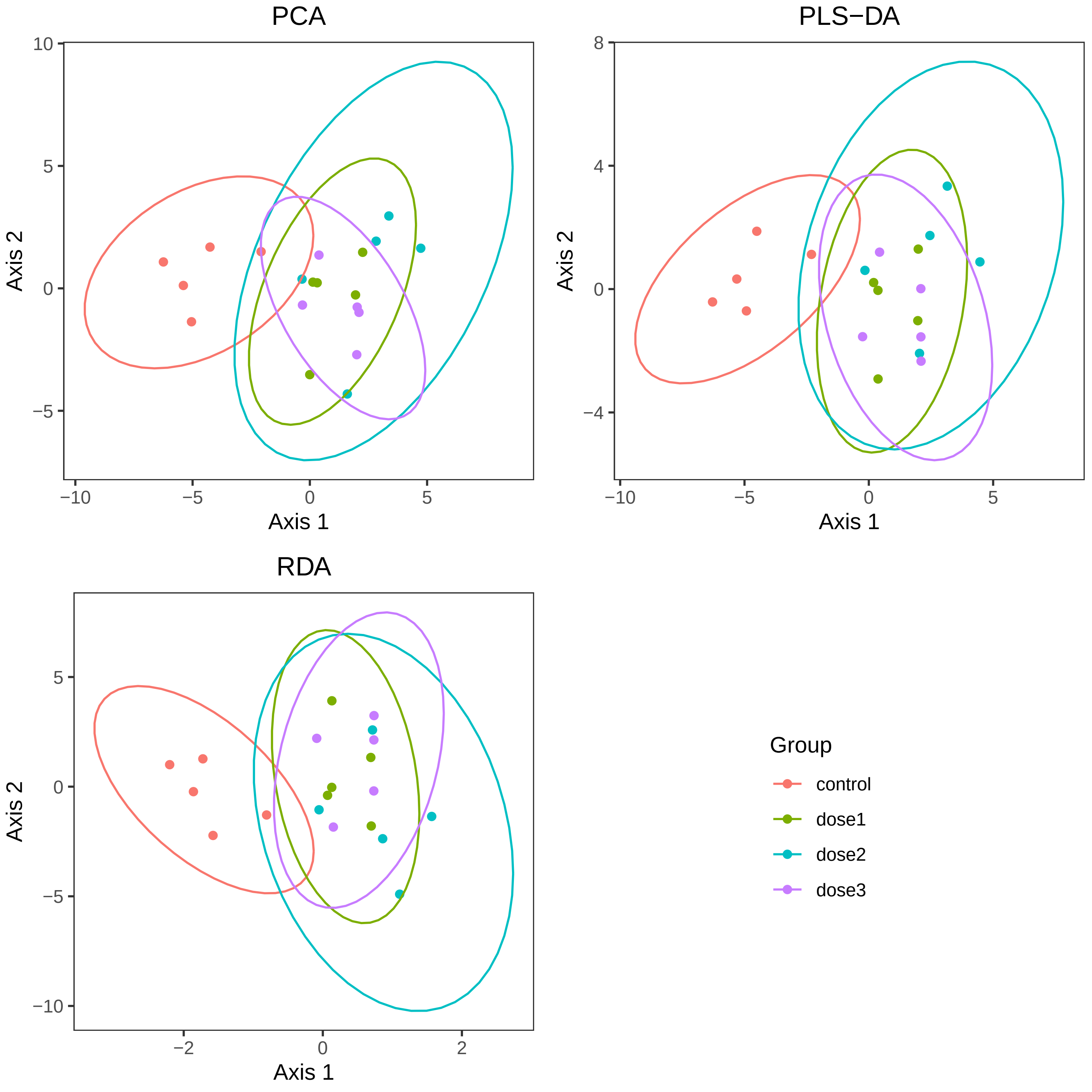

PCA
PLS−DA
RDA
Axis 1
Axis 2
10
5
0
−5
−10
8
4
−4
2
−2
Group
control
dose1
dose2
dose3

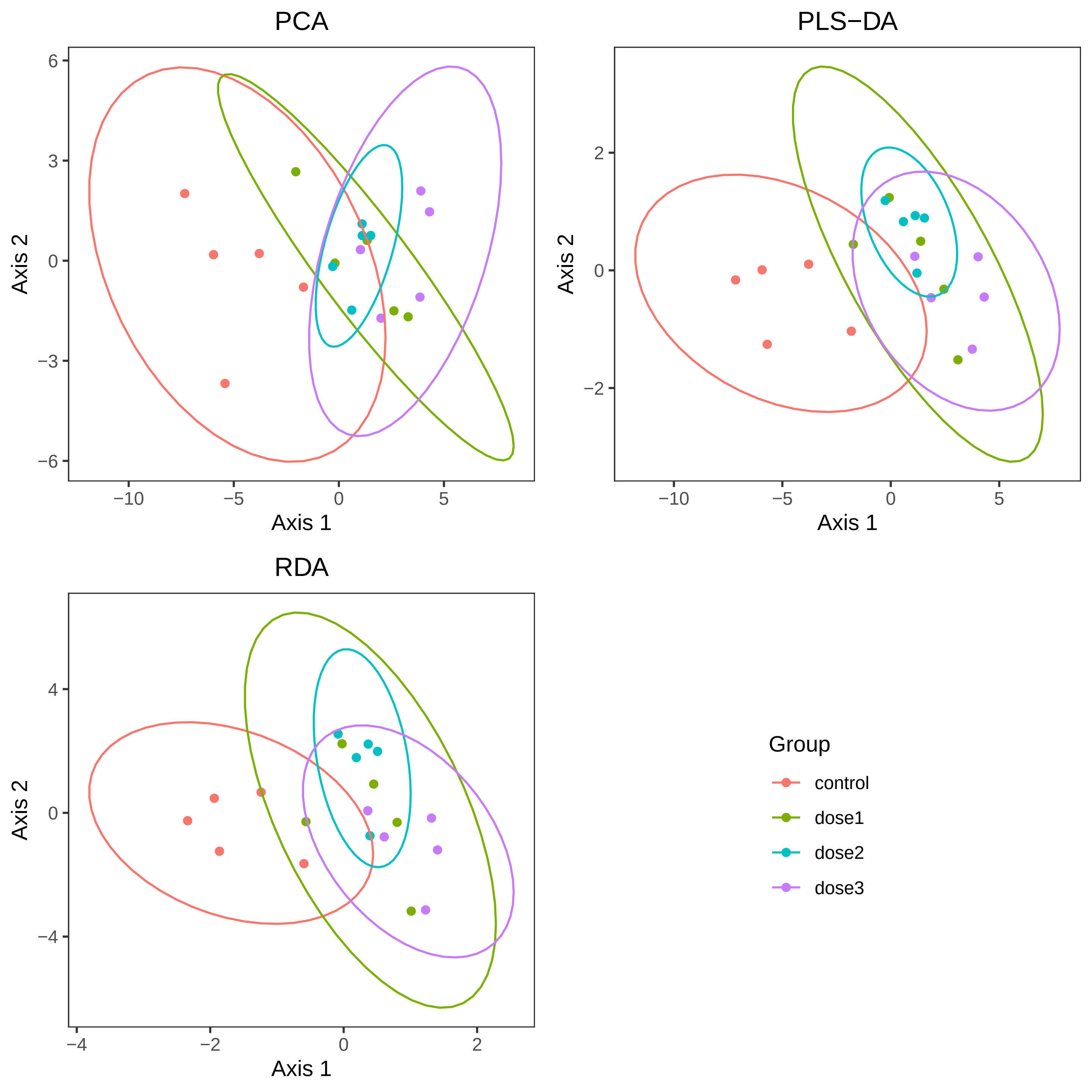
PCA
PLS−DA
RDA
Axis 1
Axis 2
−10
−5
0
5
6
3
0
−3
−6
2
−2
−4
4
Group
control
dose1
dose2
dose3

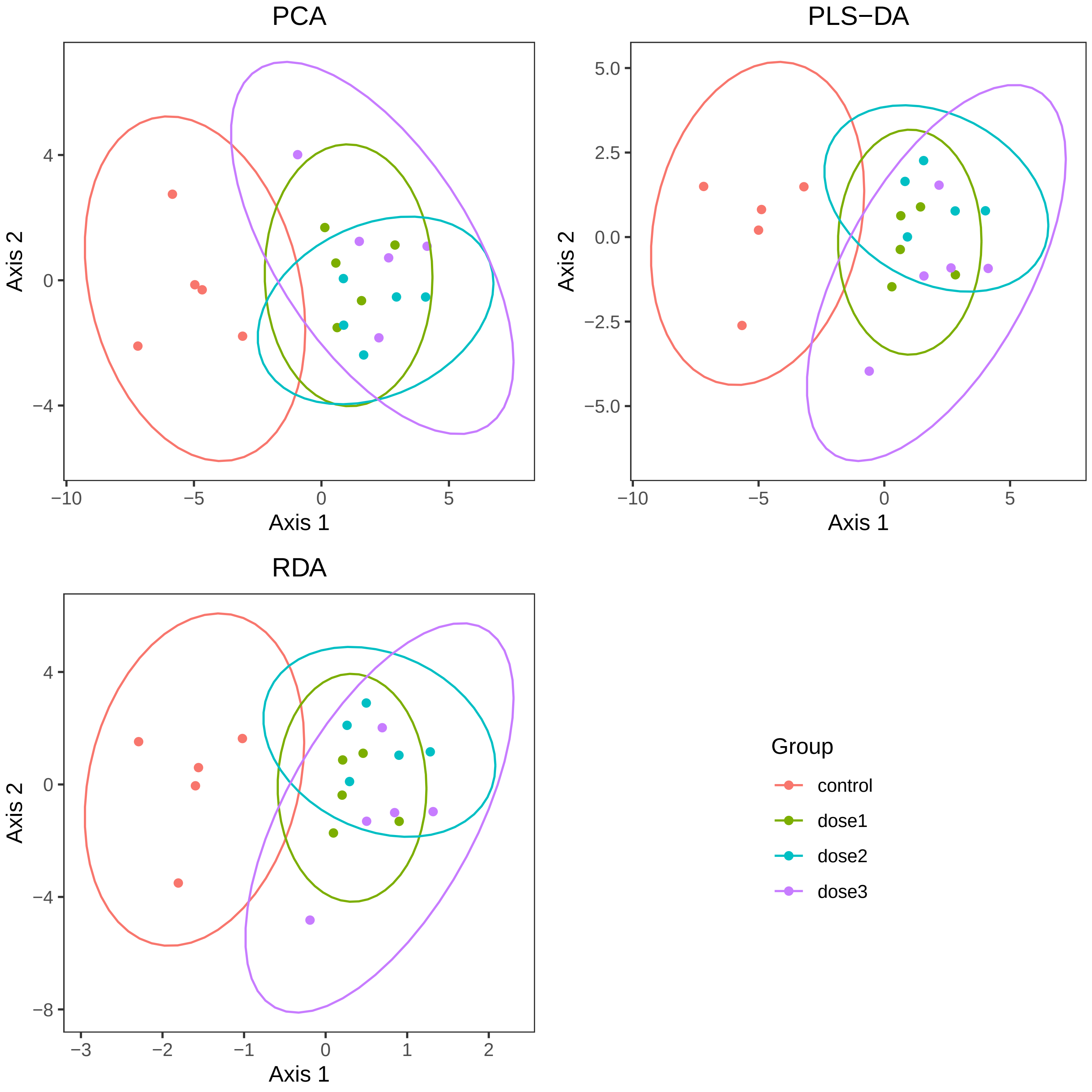

PCA
PLS−DA
RDA
Axis 1
Axis 2
−10
−5
0
5
4
−4
5.0
2.5
0.0
−2.5
−5.0
−3
−2
−1
1
2
−8
Group
control
dose1
dose2
dose3

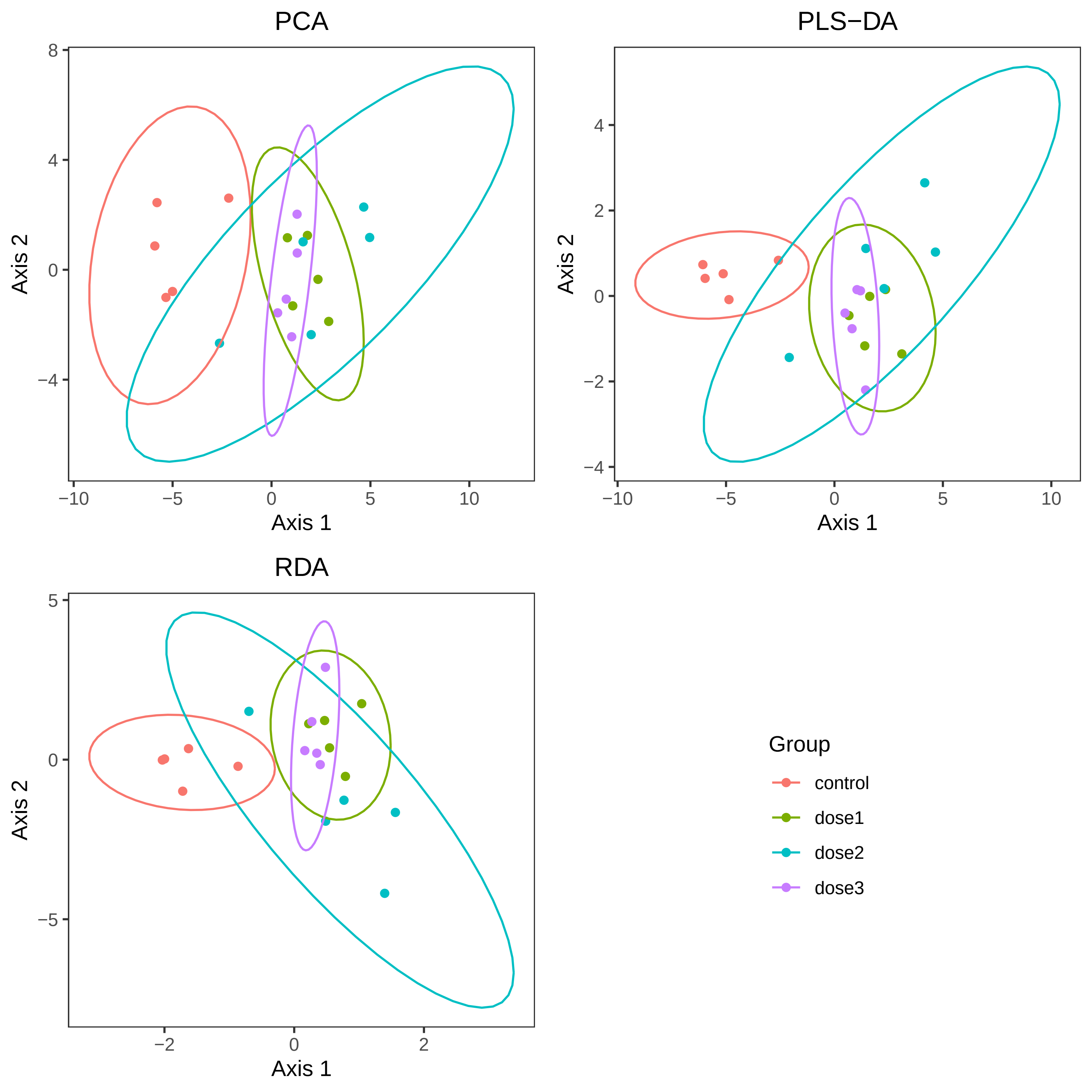

PCA
PLS−DA
RDA
Axis 1
Axis 2
Group
control
dose1
dose2
dose3

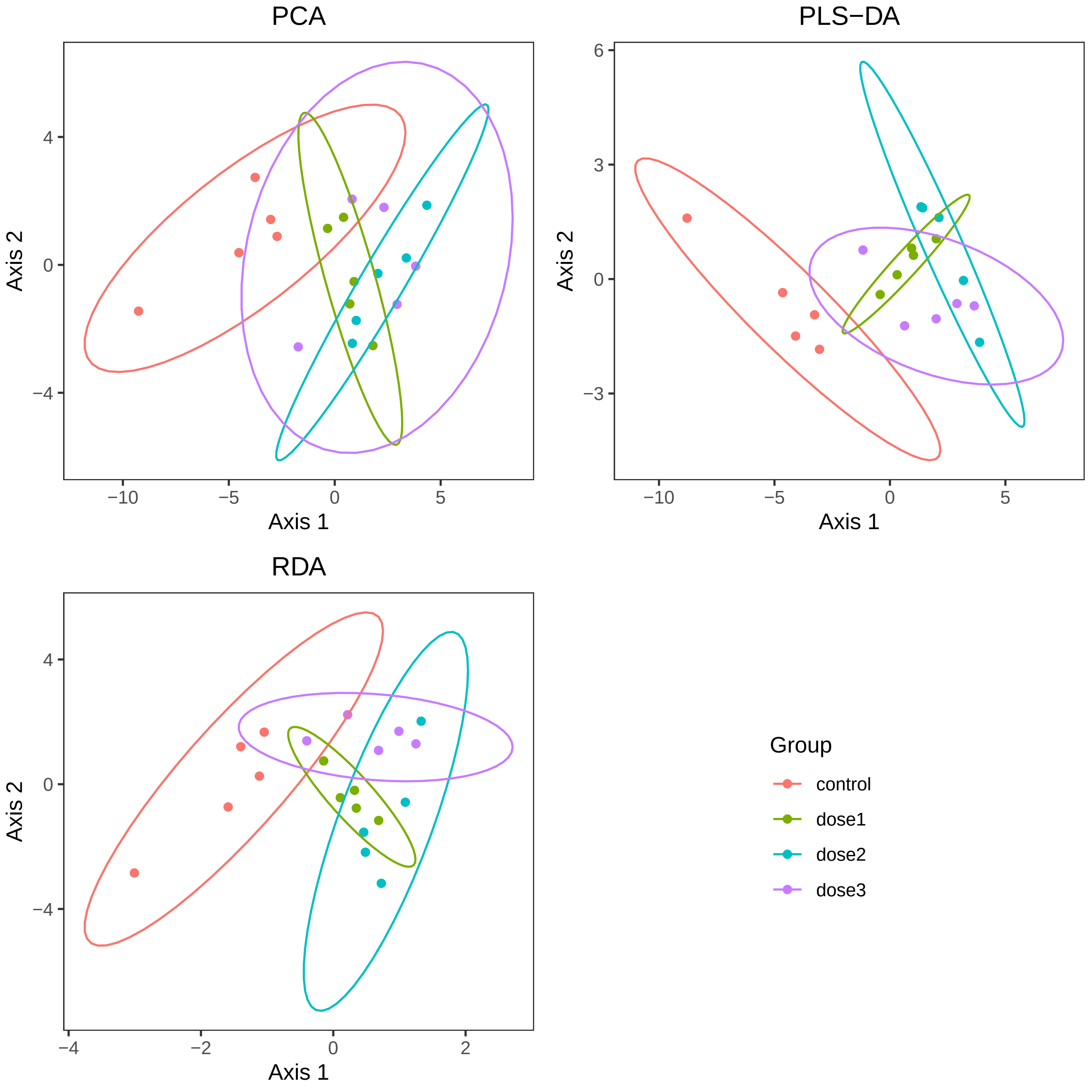

PCA
PLS−DA
RDA
Axis 1
Axis 2
Group
control
dose1
dose2
dose3

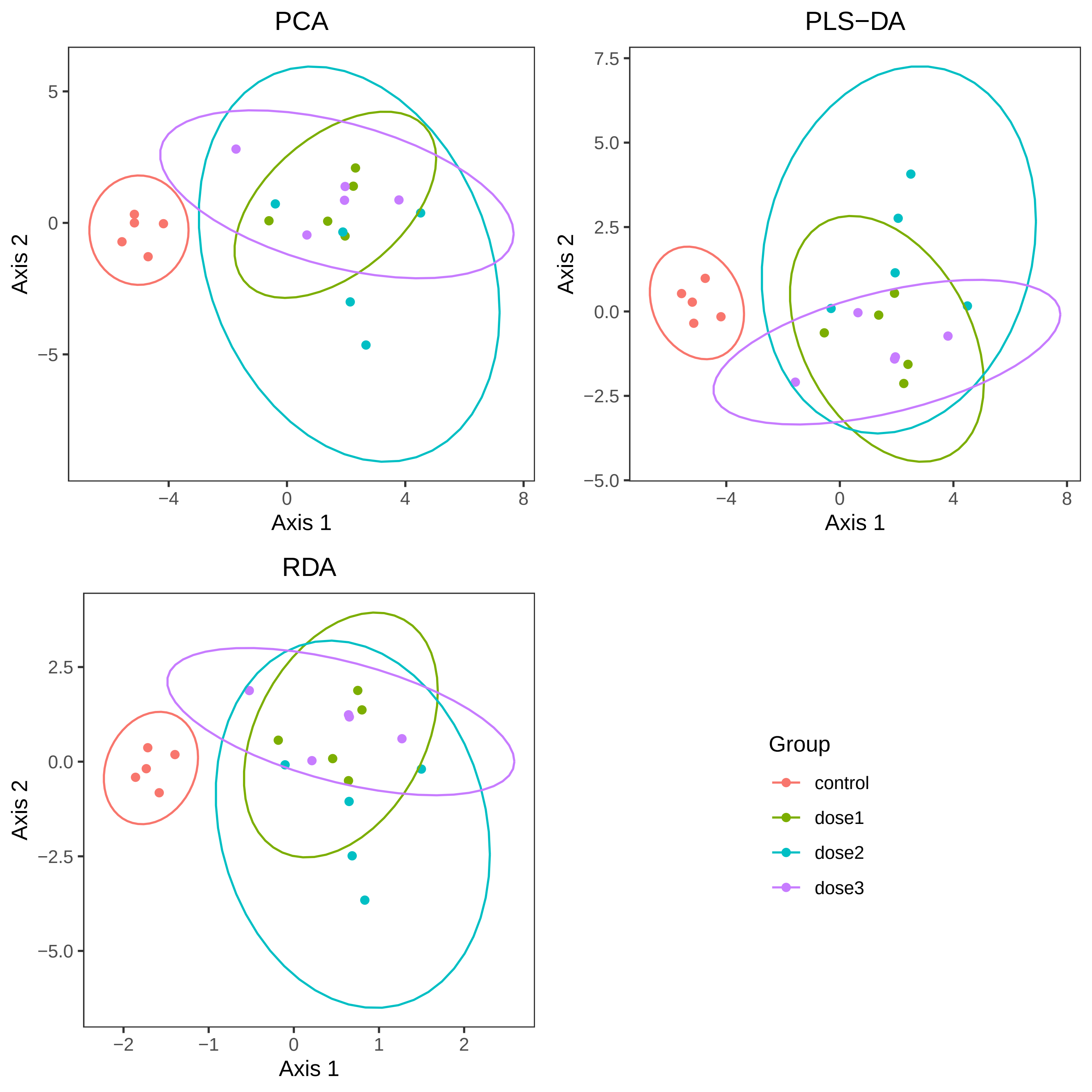
PCA
PLS−DA
RDA
Axis 1
Axis 2
Group
control
dose1
dose2
dose3

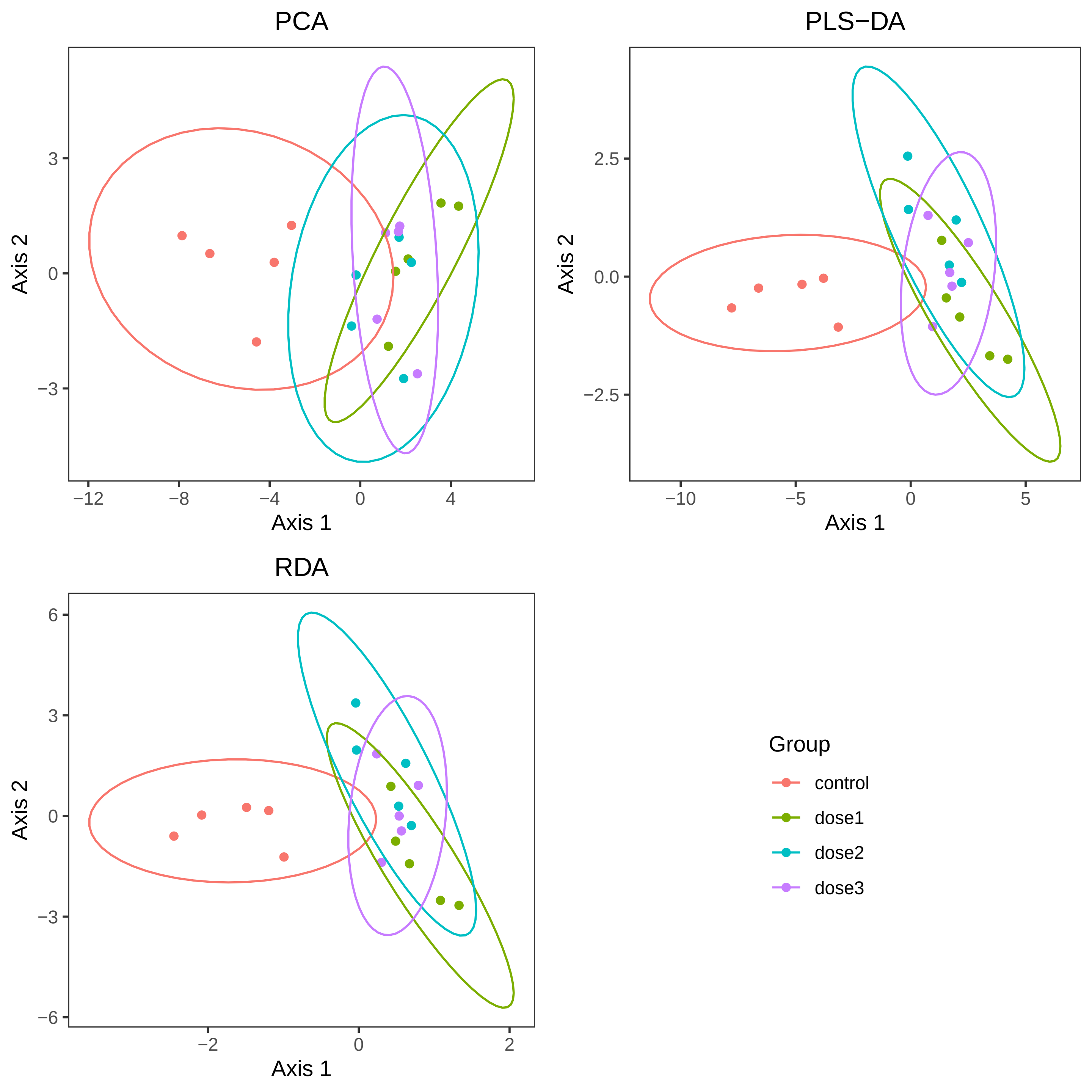
PCA
PLS−DA
RDA
Axis 1
Axis 2
−12
−8
−4
0
4
3
0
−3
−10
−5
0
5
2.5
0.0
−2.5
−2
0
2
6
3
0
−3
−6
Group
control
dose1
dose2
dose3

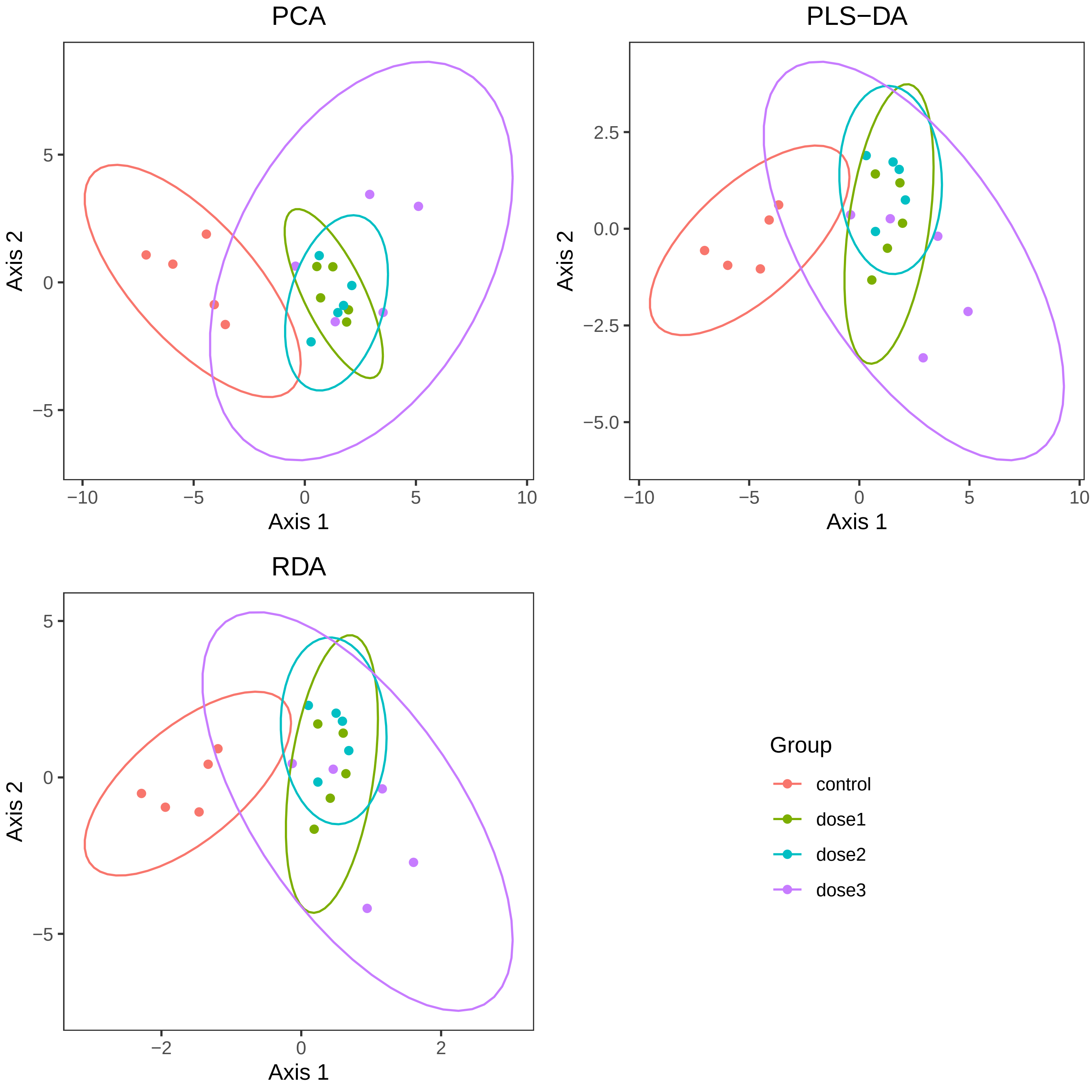

PCA
PLS−DA
RDA
Axis 1
Axis 2
Group
control
dose1
dose2
dose3

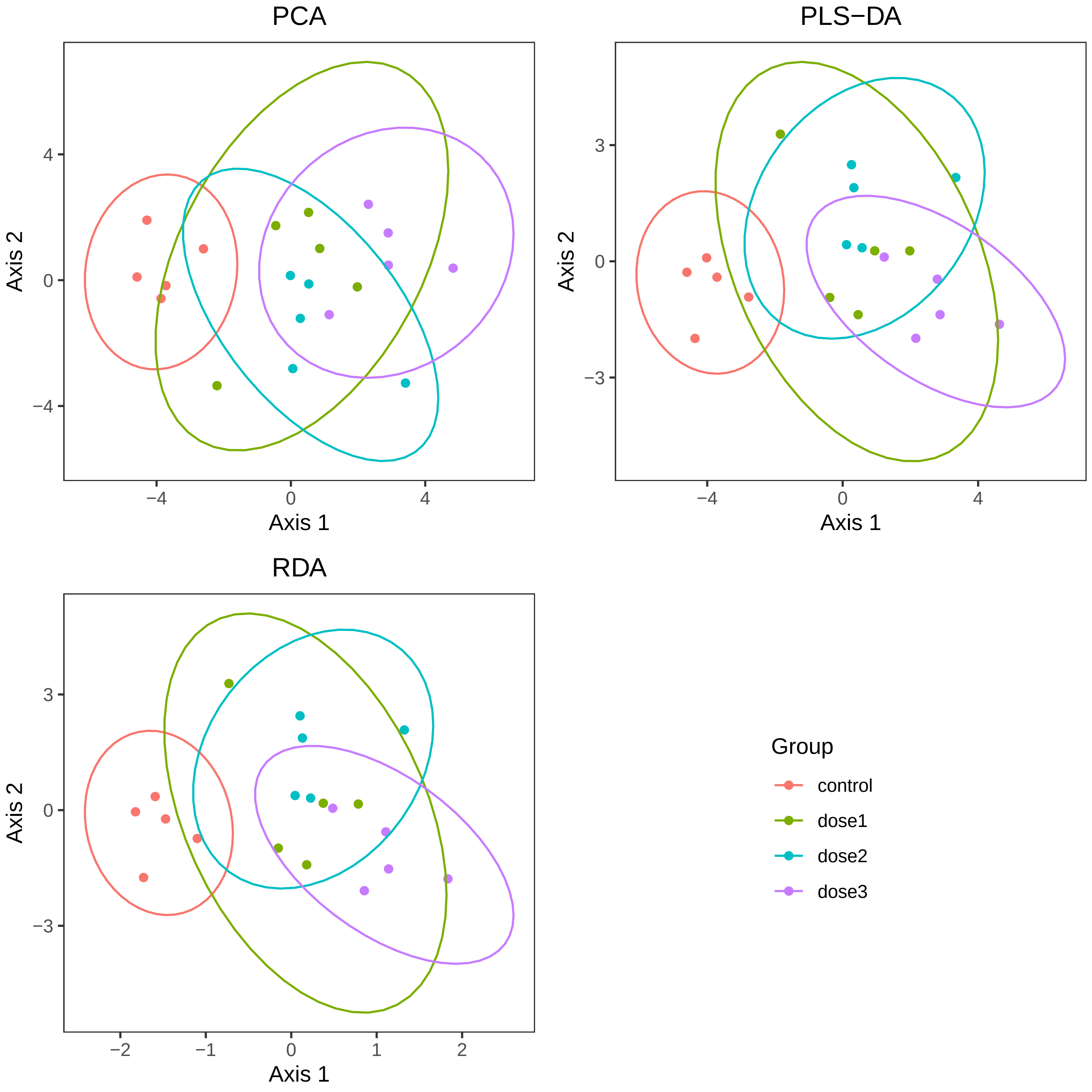

PCA
PLS−DA
RDA
Axis 1
Axis 2
−4
0
4
3
−3
−2
−1
1
2
Group
control
dose1
dose2
dose3

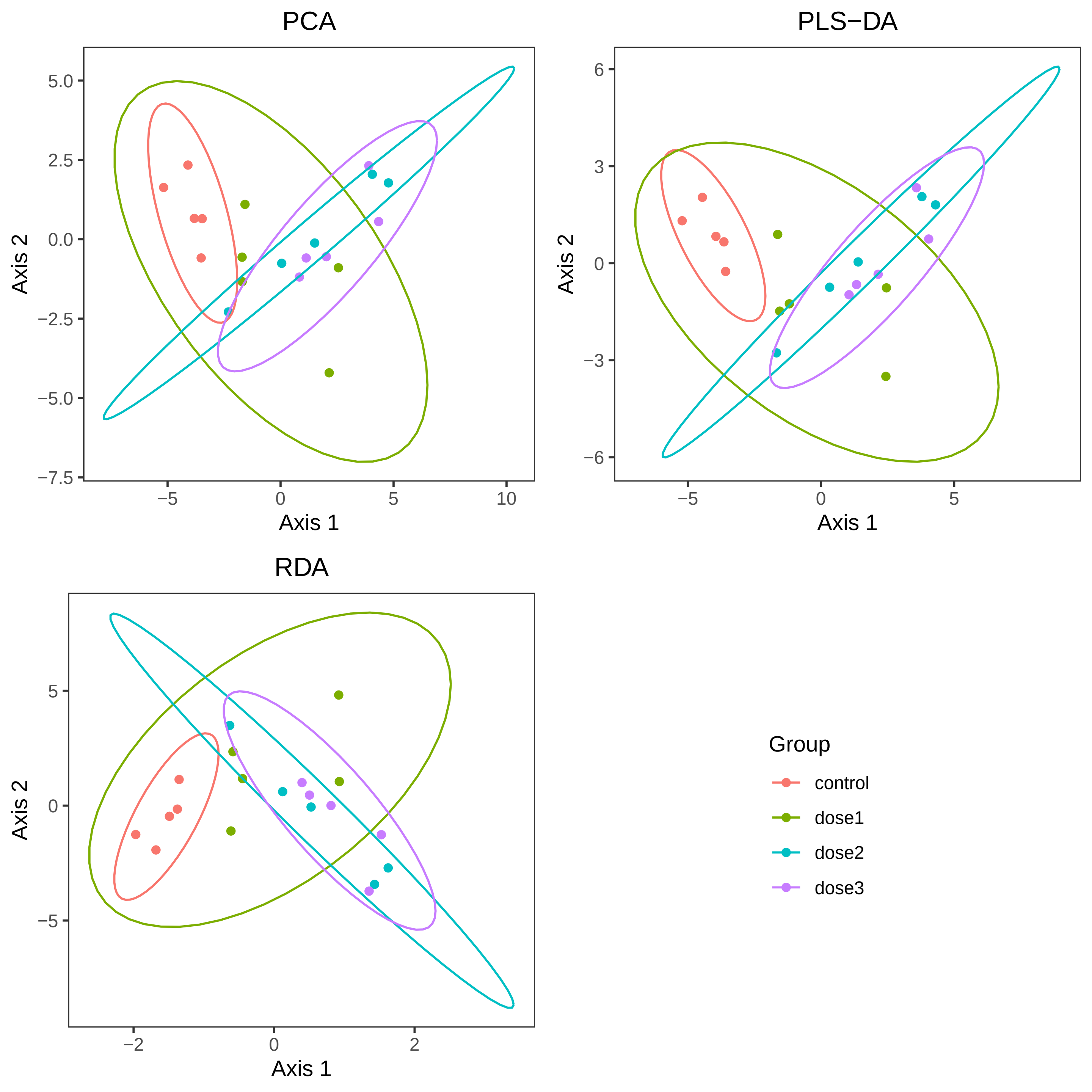

PCA
PLS−DA
RDA
Axis 1
Axis 2
5.0
2.5
0.0
−2.5
−5.0
−7.5
−5
0
5
10
6
3
0
−3
−6
−2
2
Group
control
dose1
dose2
dose3

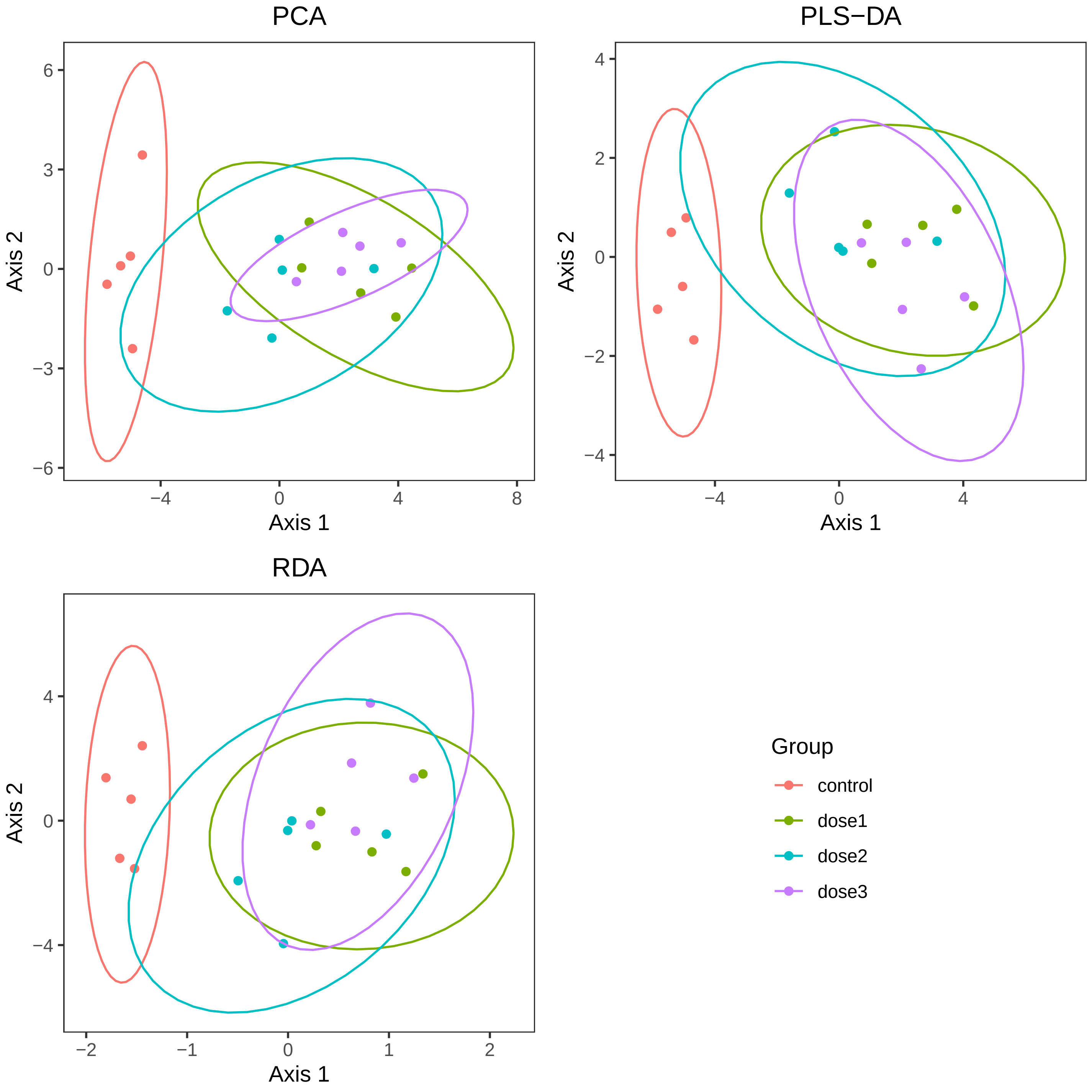
PCA
PLS−DA
RDA
Axis 1
Axis 2
6
3
0
−3
−6
−4
0
4
8
4
2
0
−2
−4
−4
0
4
−2
−1
0
1
2
Group
control
dose1
dose2
dose3

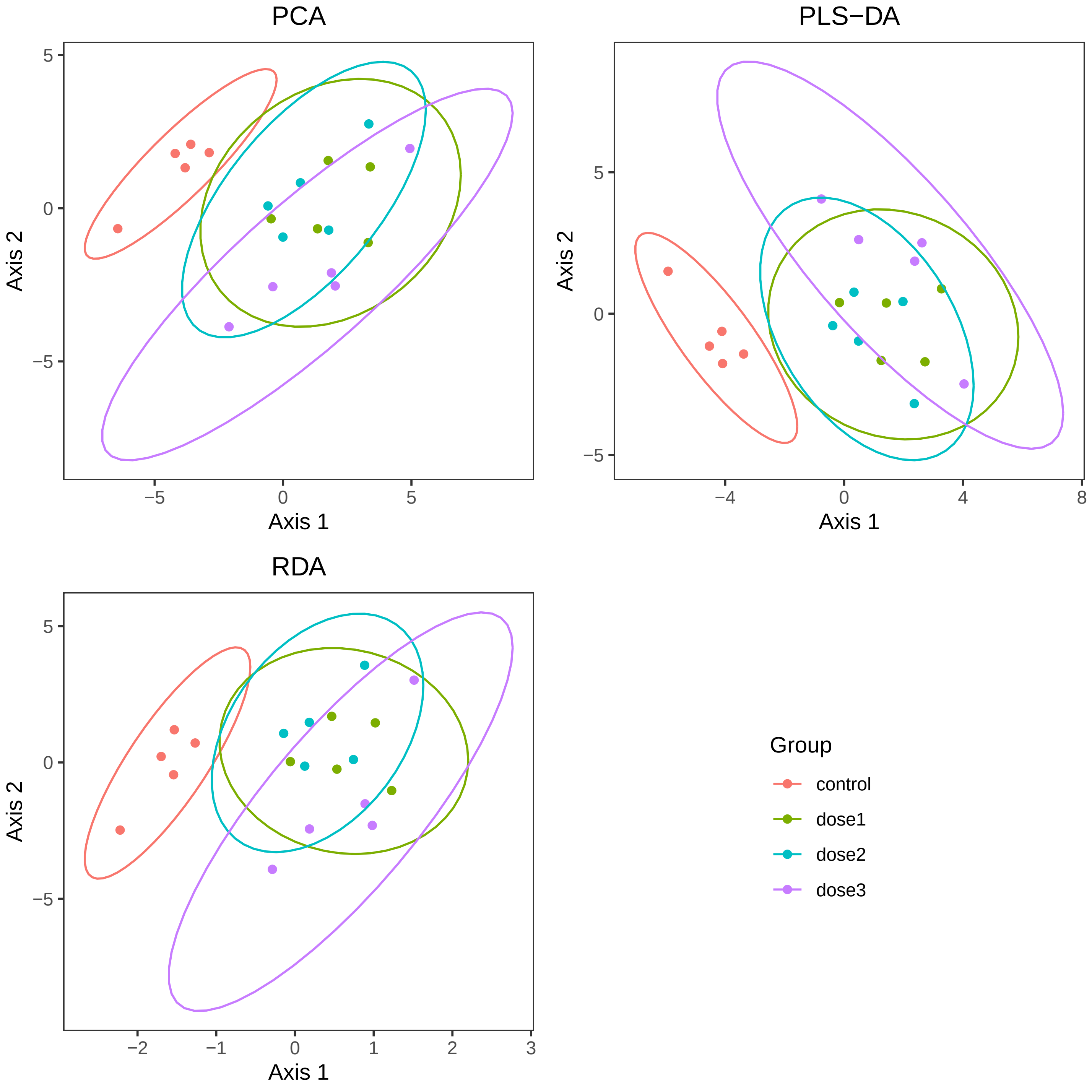

PCA
PLS−DA
RDA
Axis 1
Axis 2
Group
control
dose1
dose2
dose3

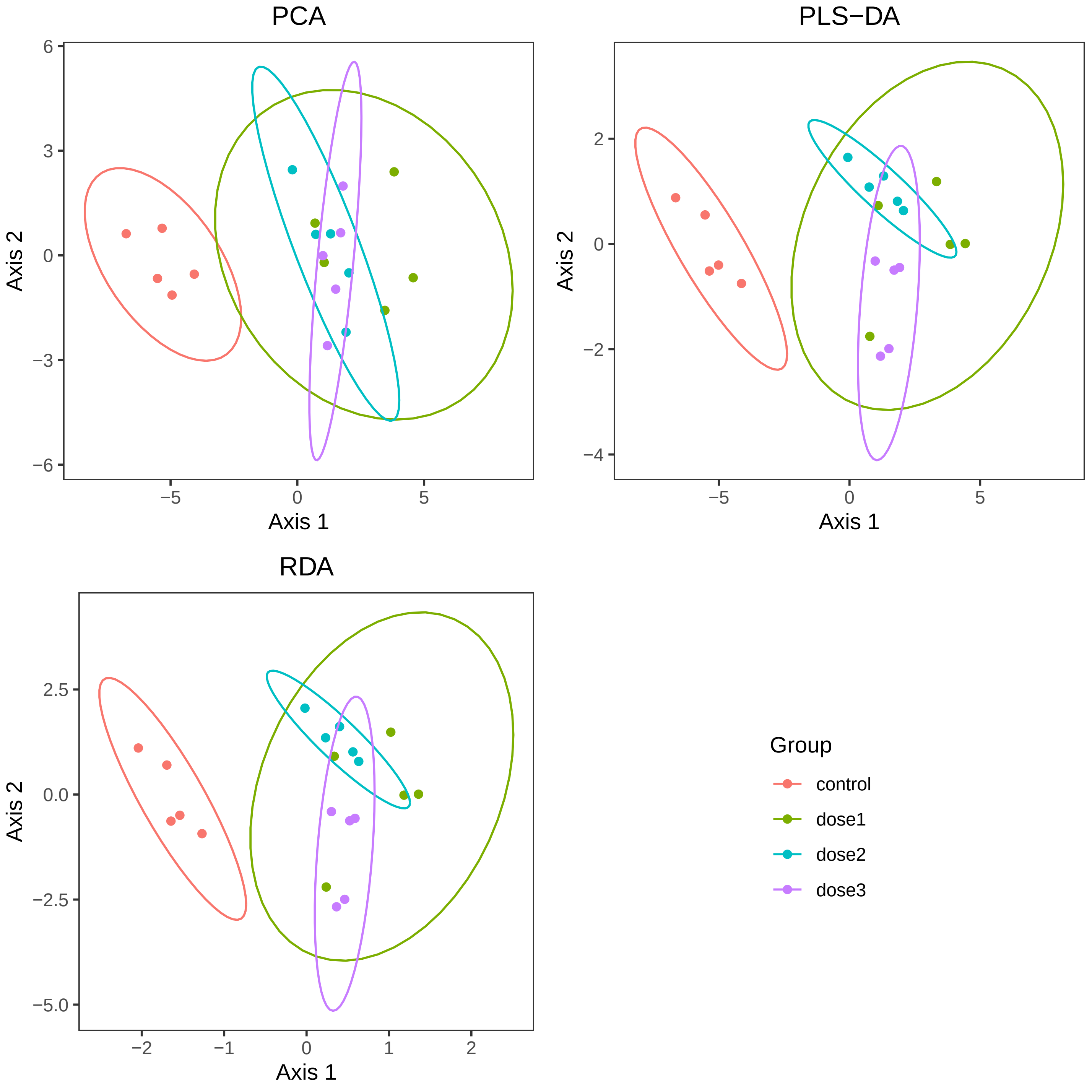

PCA
PLS−DA
RDA
Axis 1
Axis 2
Group
control
dose1
dose2
dose3

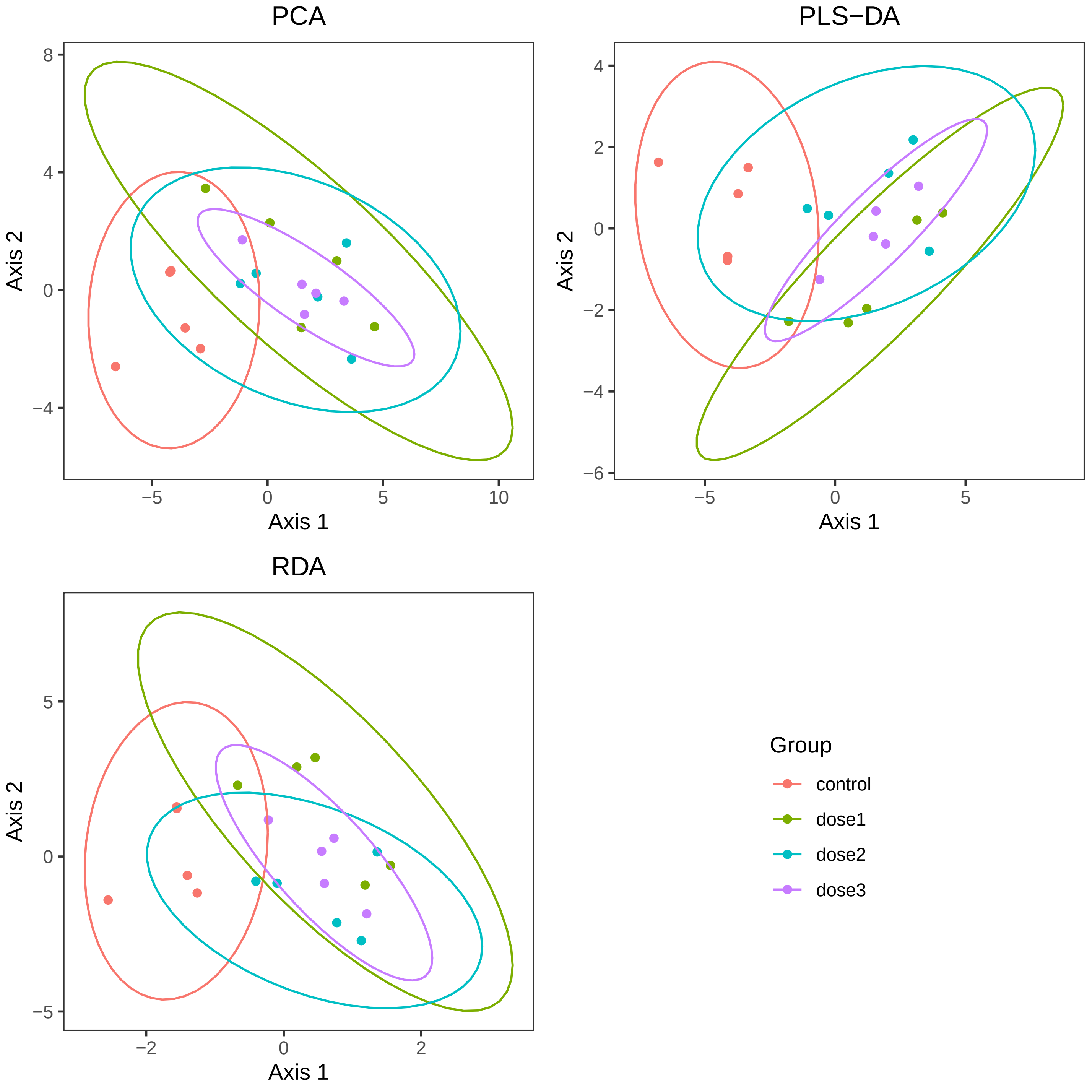

PCA
PLS−DA
RDA
Axis 1
Axis 2
Group
control
dose1
dose2
dose3

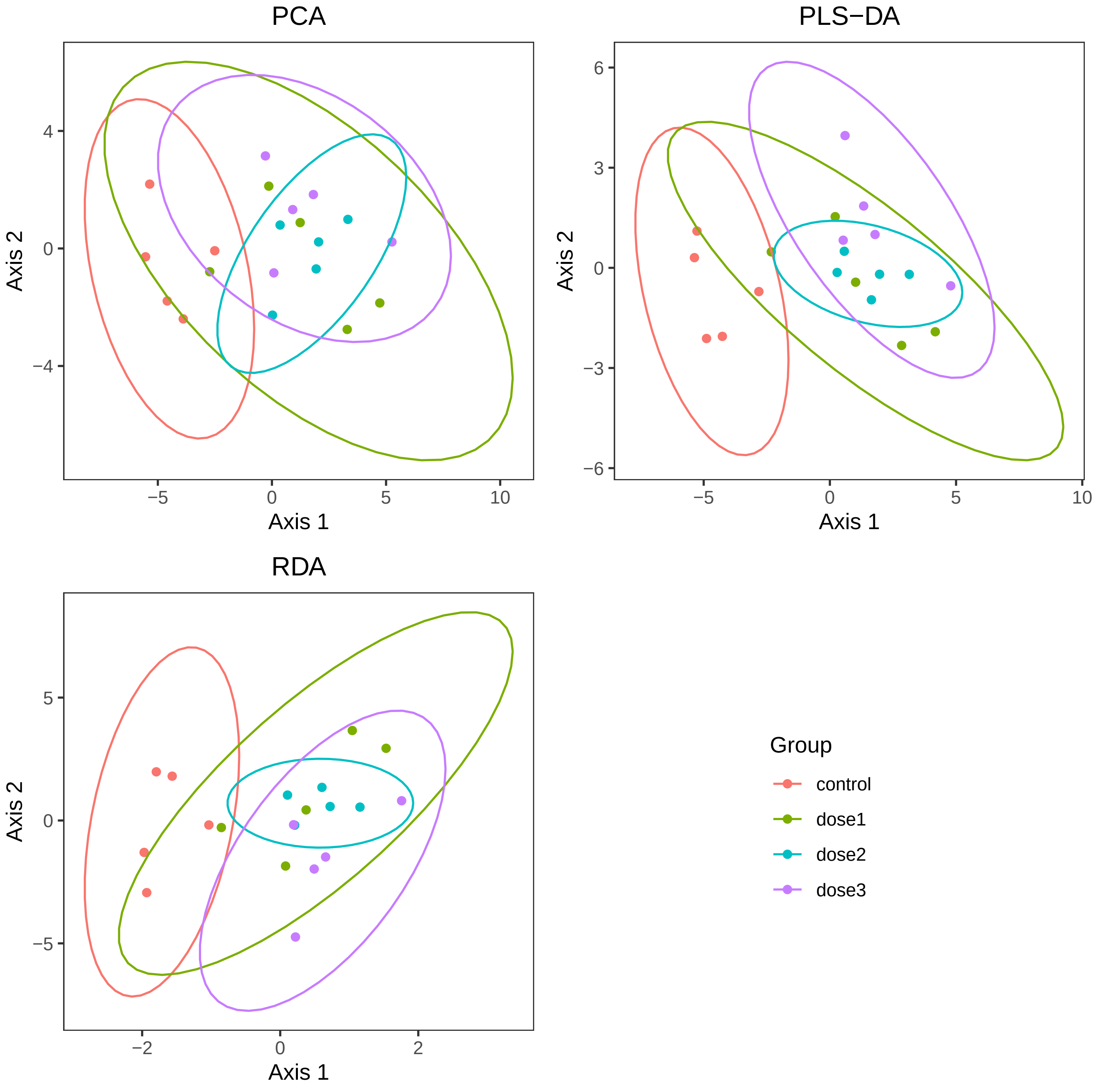
PCA
PLS−DA
RDA
Axis 1
Axis 2
Group
control
dose1
dose2
dose3

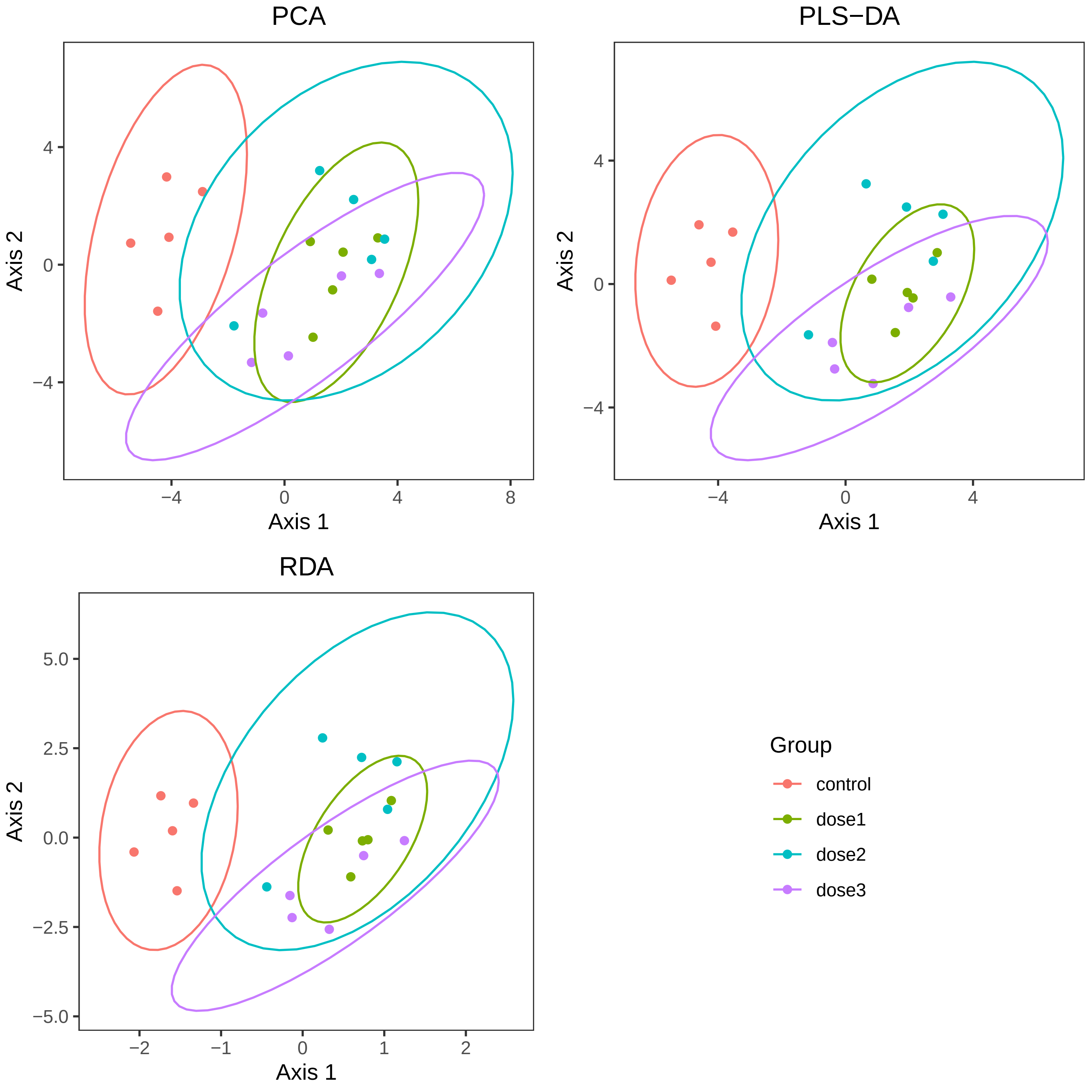
PCA
PLS−DA
RDA
Axis 1
Axis 2
4
0
−4
8
5.0
2.5
0.0
−2.5
−5.0
−2
−1
1
2
Group
control
dose1
dose2
dose3

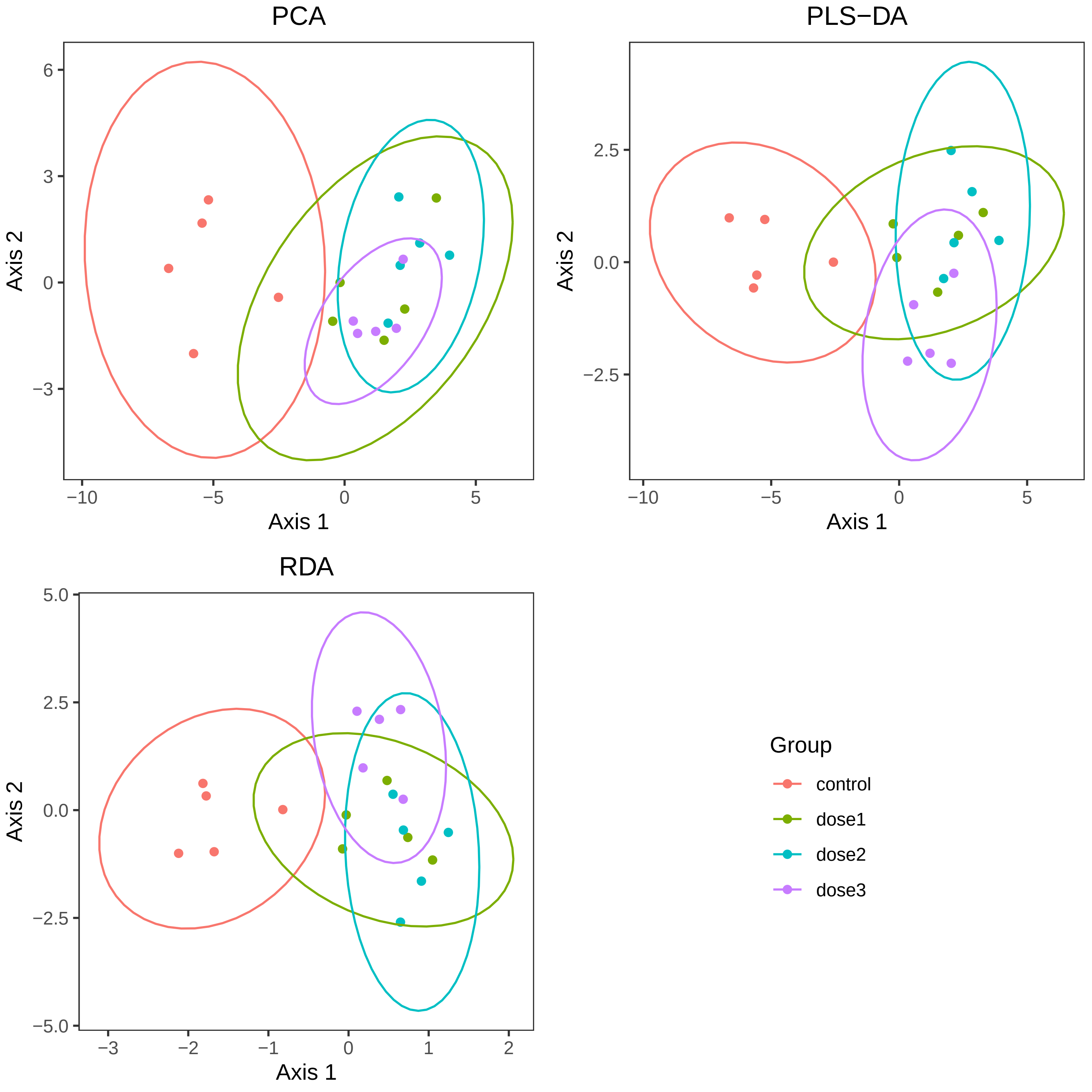

PCA
PLS−DA
RDA
Axis 1
Axis 2
6
3
0
−3
−10
−5
0
5
2.5
0.0
−2.5
5.0
−5.0
−3
−2
−1
1
2
Group
control
dose1
dose2
dose3

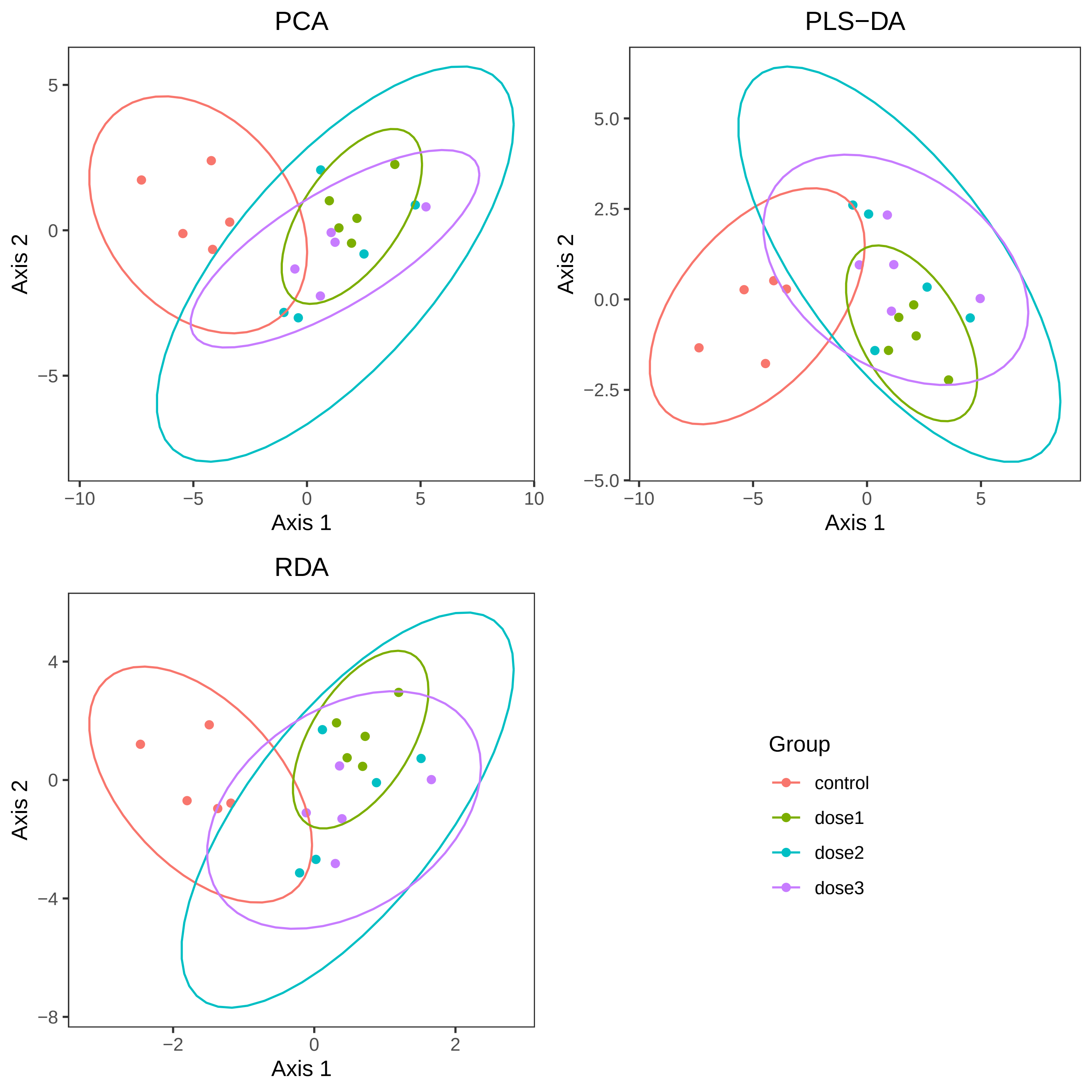

PCA
PLS−DA
RDA
Axis 1
Axis 2
Group
control
dose1
dose2
dose3

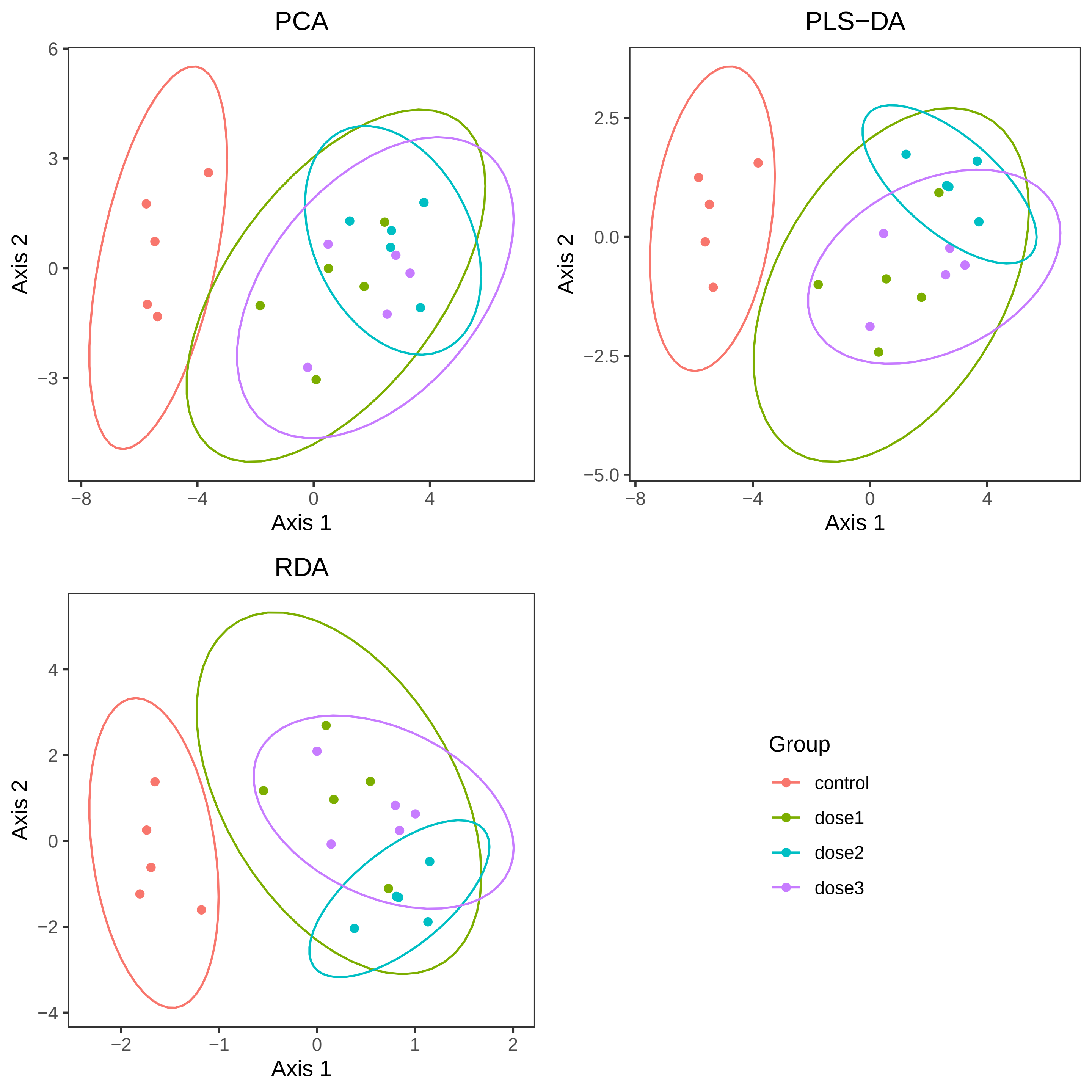
PCA
PLS−DA
RDA
Axis 1
Axis 2
6
3
0
−3
−8
−4
0
4
2.5
0.0
−2.5
−5.0
−2
−1
1
2
4
−4
Group
control
dose1
dose2
dose3

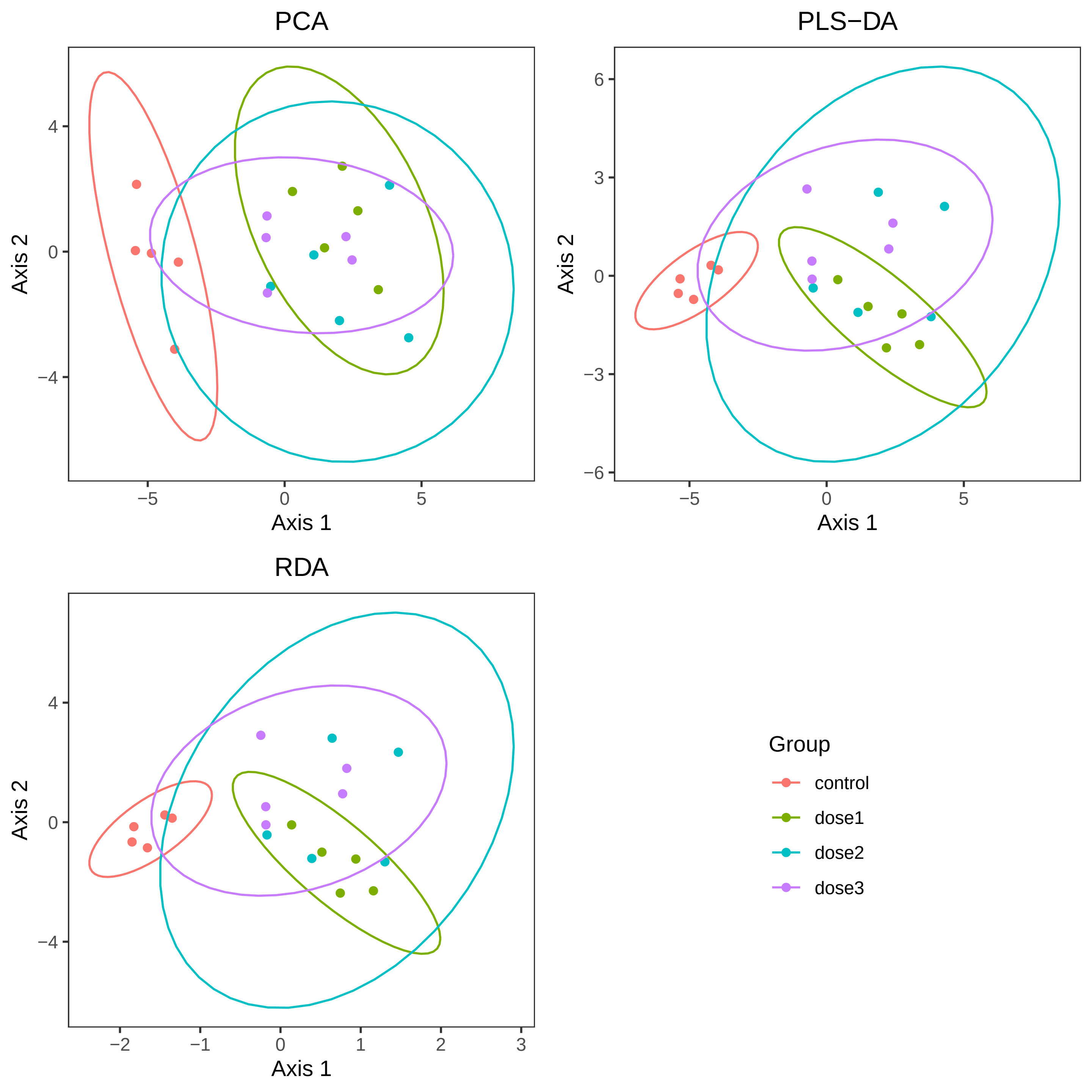
PCA
PLS−DA
RDA
Axis 1
Axis 2
Group
control
dose1
dose2
dose3

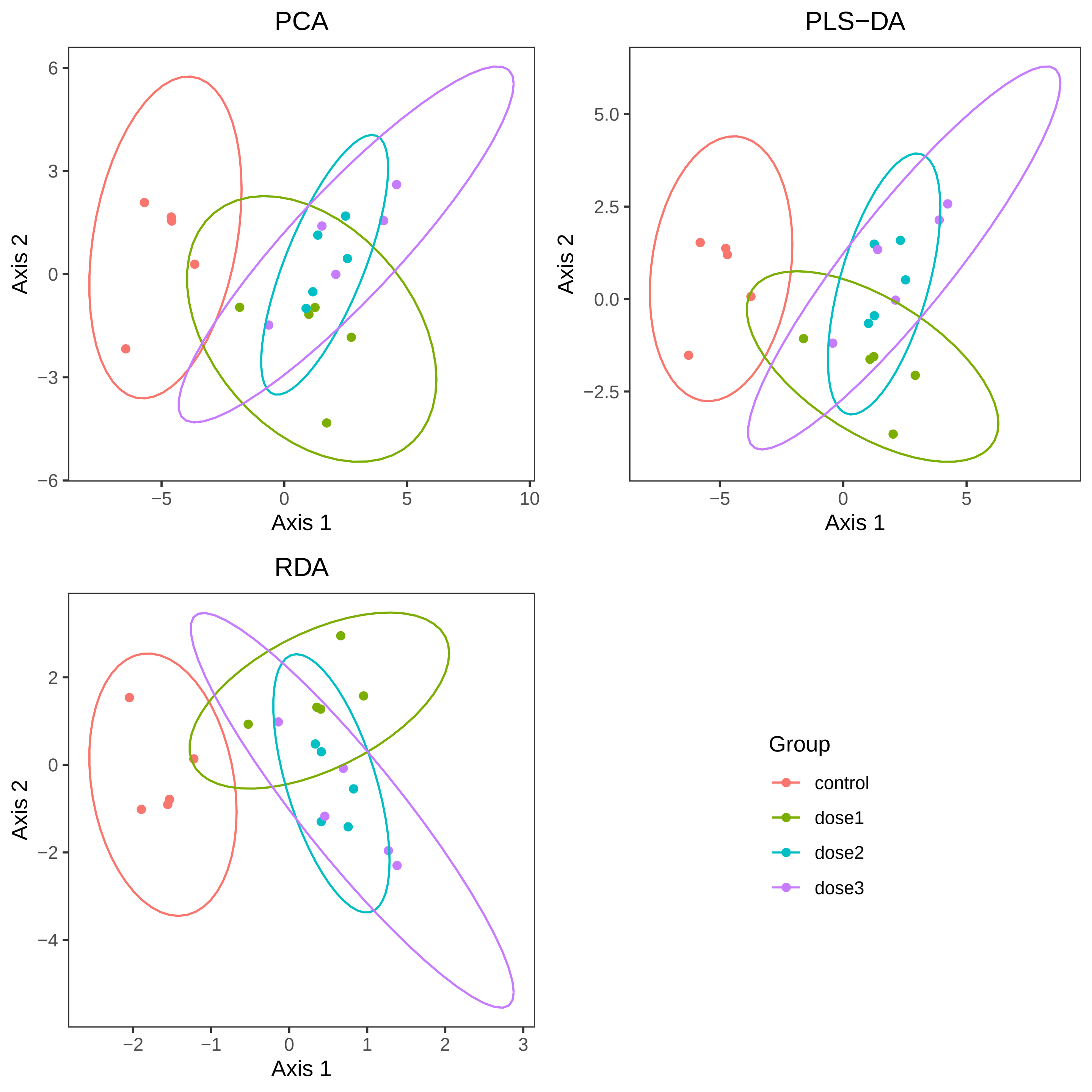

PCA
PLS−DA
RDA
Axis 1
Axis 2
Group
control
dose1
dose2
dose3

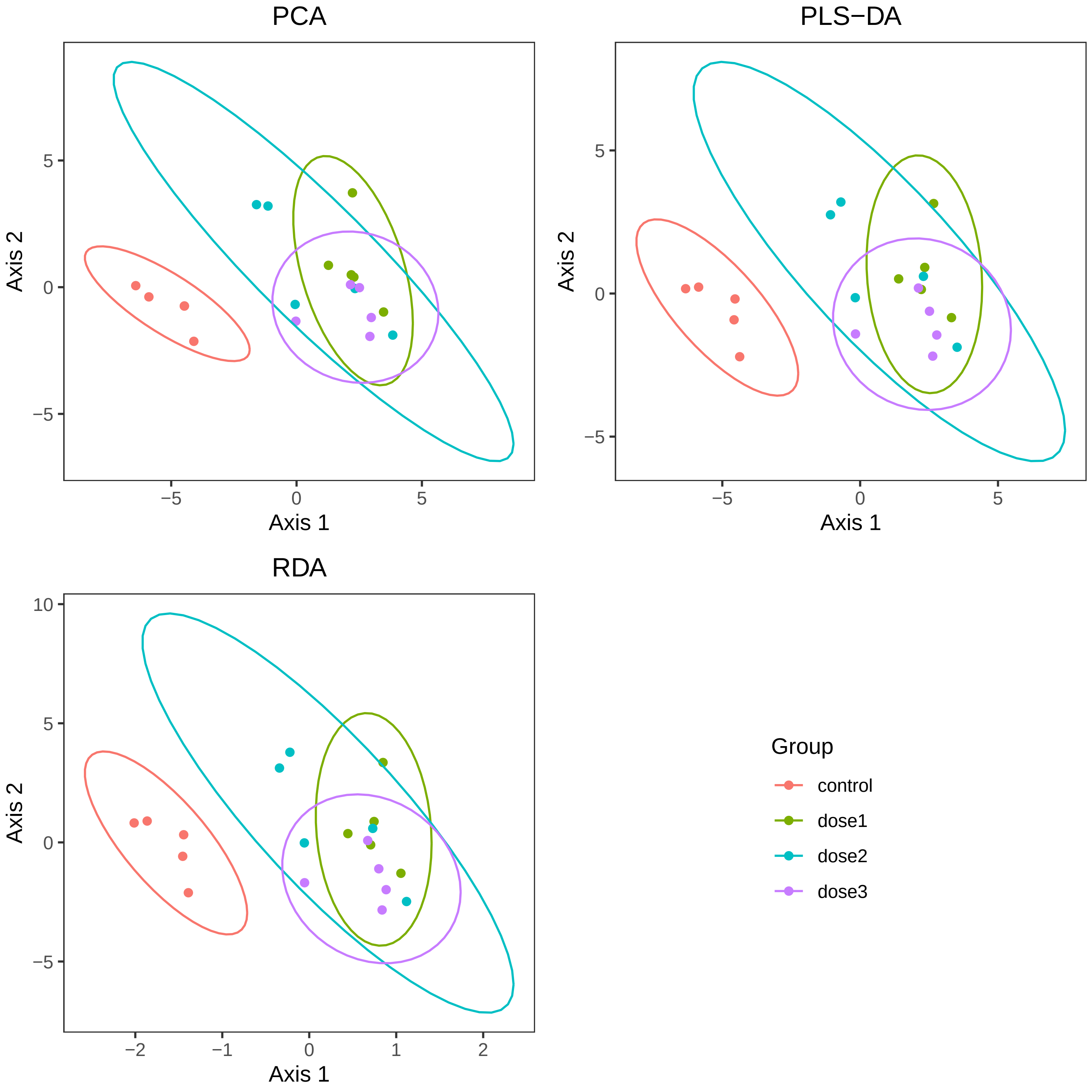

PCA
PLS−DA
RDA
Axis 1
Axis 2
Group
control
dose1
dose2
dose3

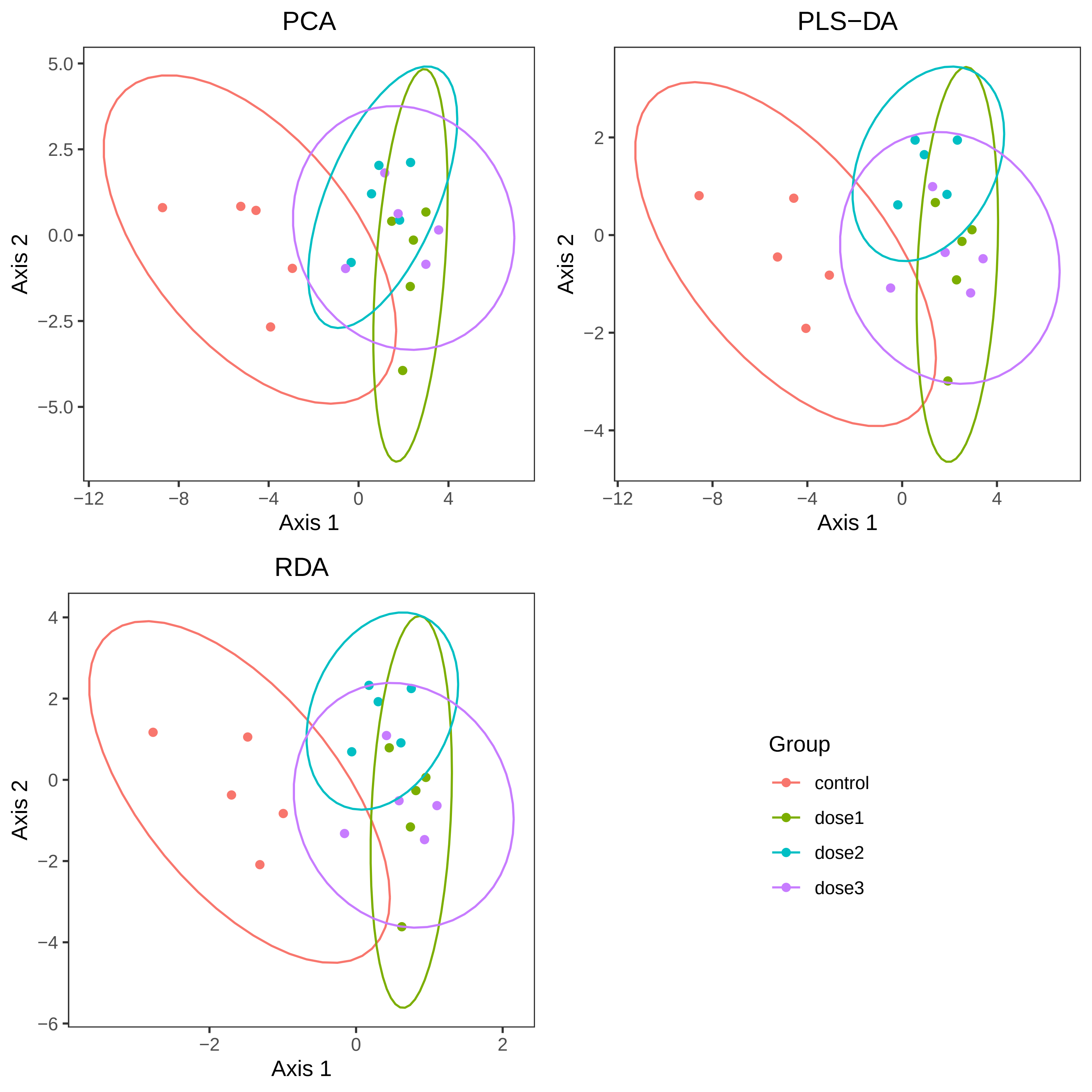
PCA
PLS−DA
RDA
Axis 1
Axis 2
5.0
2.5
0.0
−2.5
−5.0
−12
−8
−4
0
4
2
−2
−4
−6
Group
control
dose1
dose2
dose3

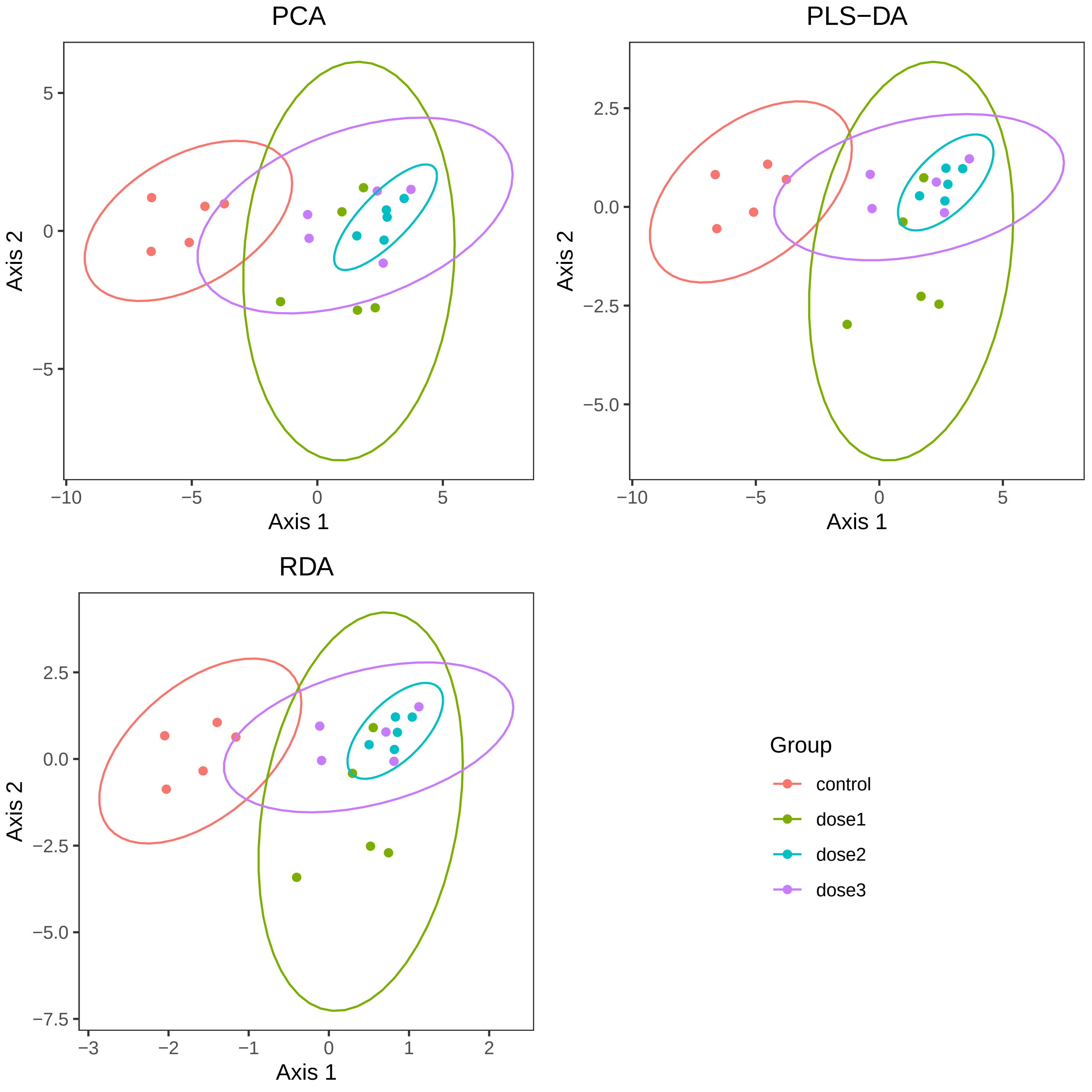
PCA
PLS−DA
RDA
Axis 1
Axis 2
Group
control
dose1
dose2
dose3

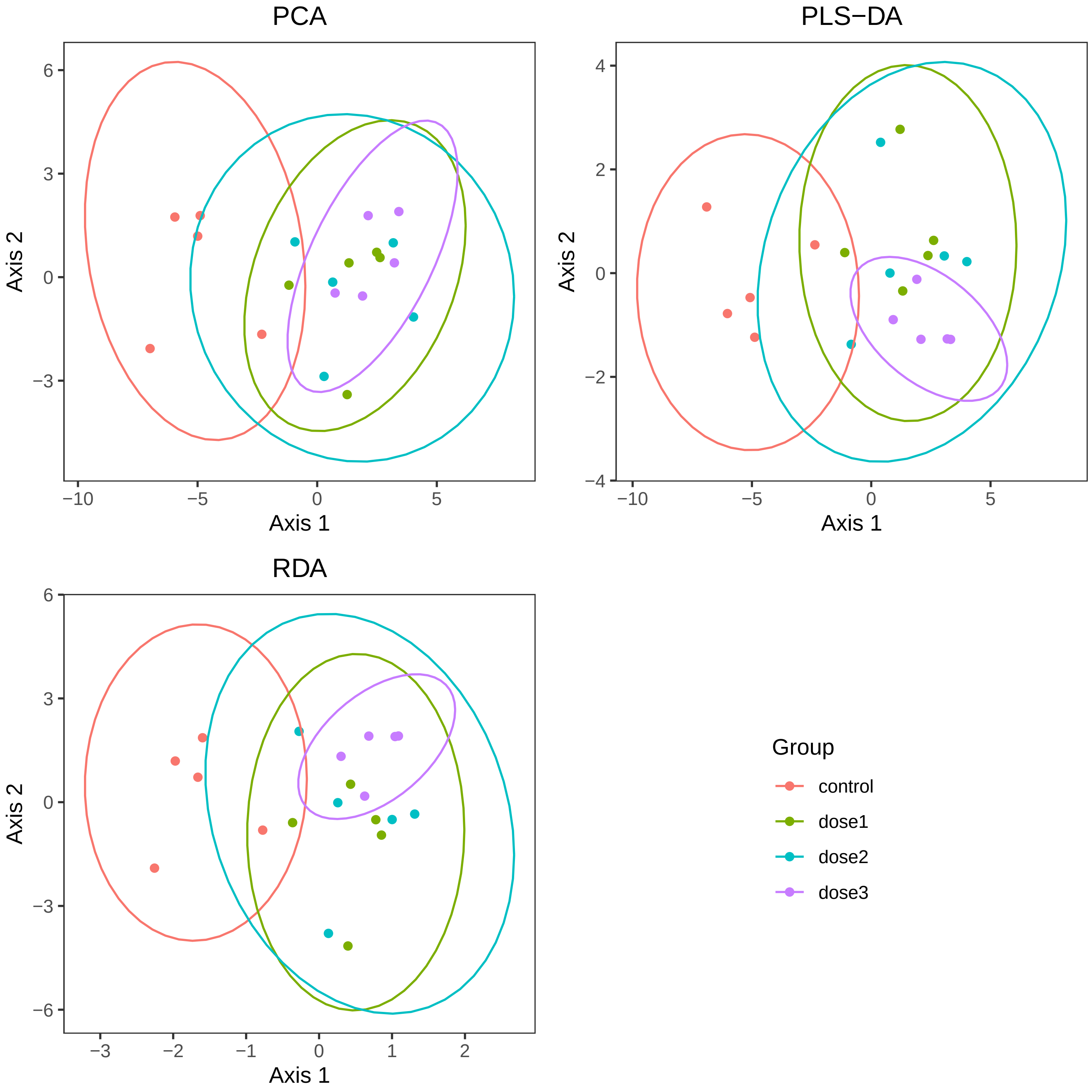
PCA
PLS−DA
RDA
Axis 1
Axis 2
Group
control
dose1
dose2
dose3

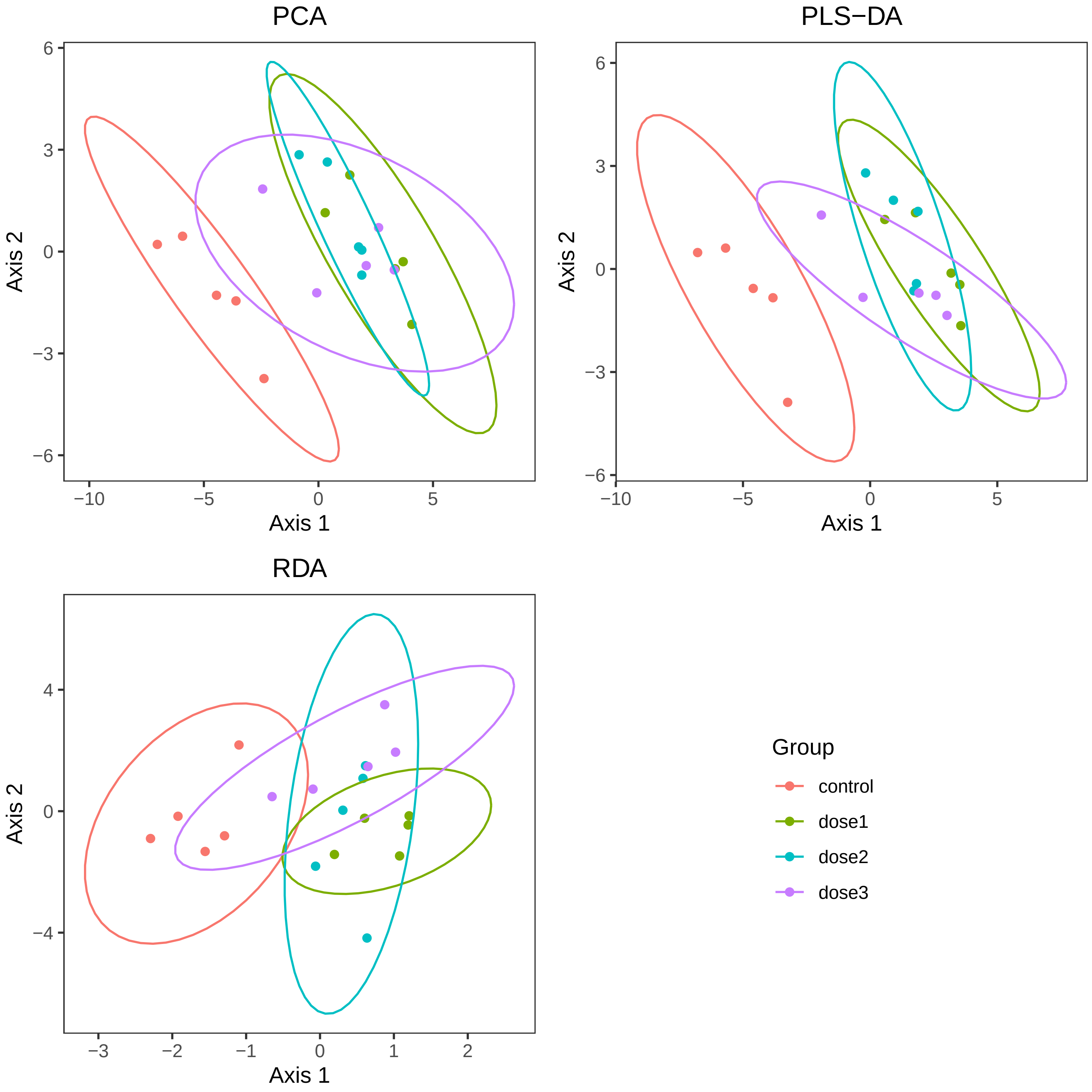
PCA
PLS−DA
RDA
Axis 1
Axis 2
Group
control
dose1
dose2
dose3

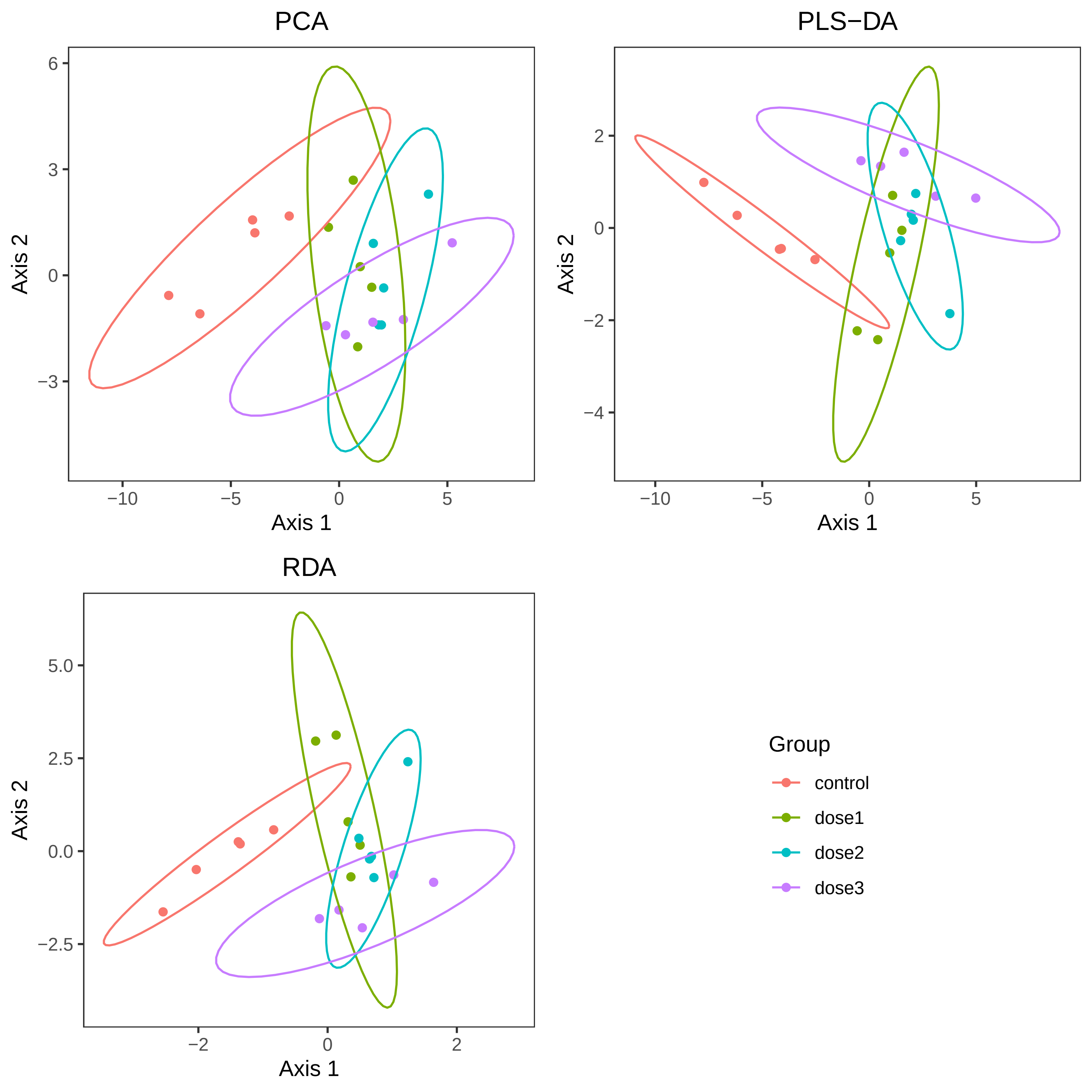
PCA
PLS−DA
RDA
Axis 1
Axis 2
−10
−5
0
5
6
3
0
−3
2
0
−2
−4
5.0
2.5
0.0
−2.5
Group
control
dose1
dose2
dose3

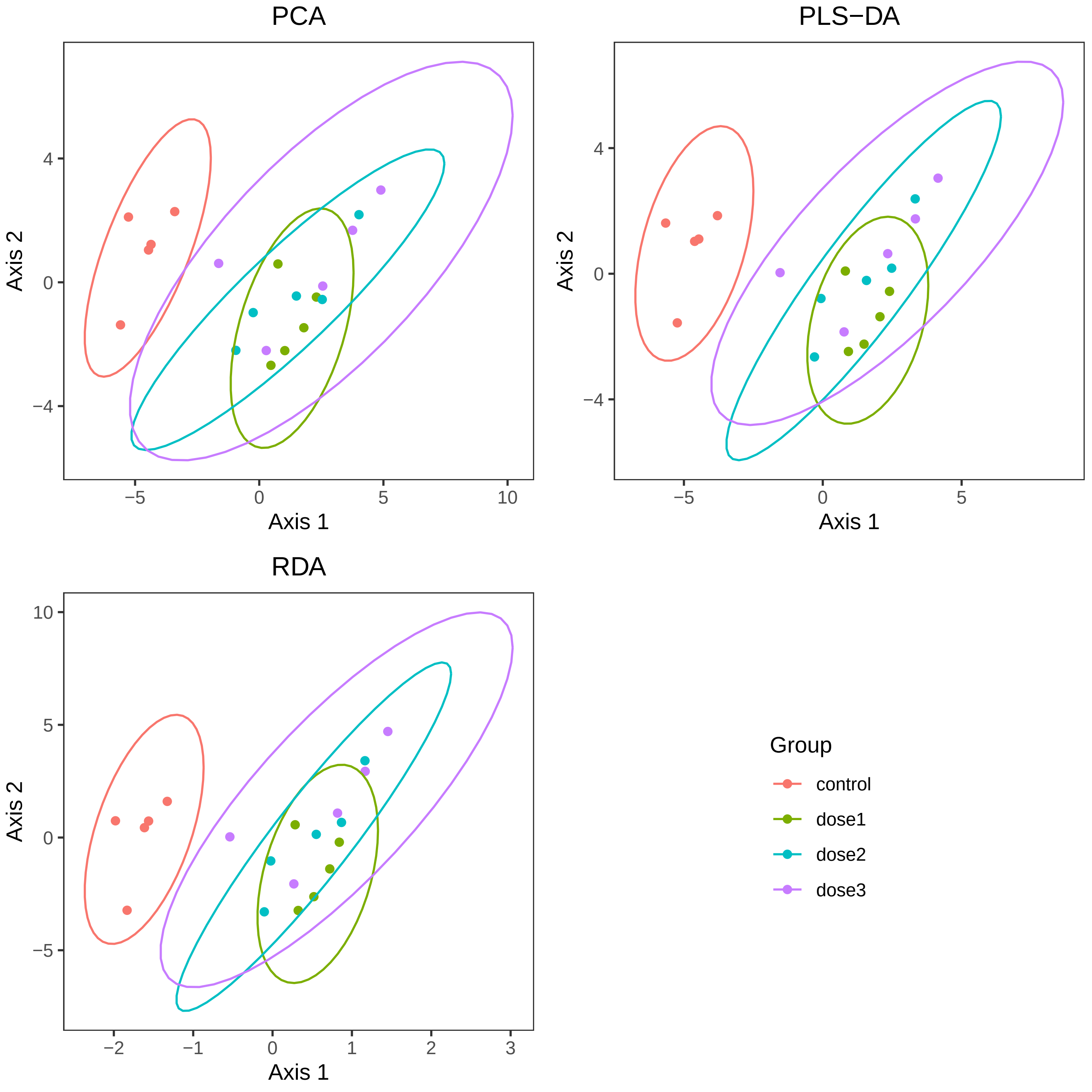

PCA
PLS−DA
RDA
Axis 1
Axis 2
Group
control
dose1
dose2
dose3

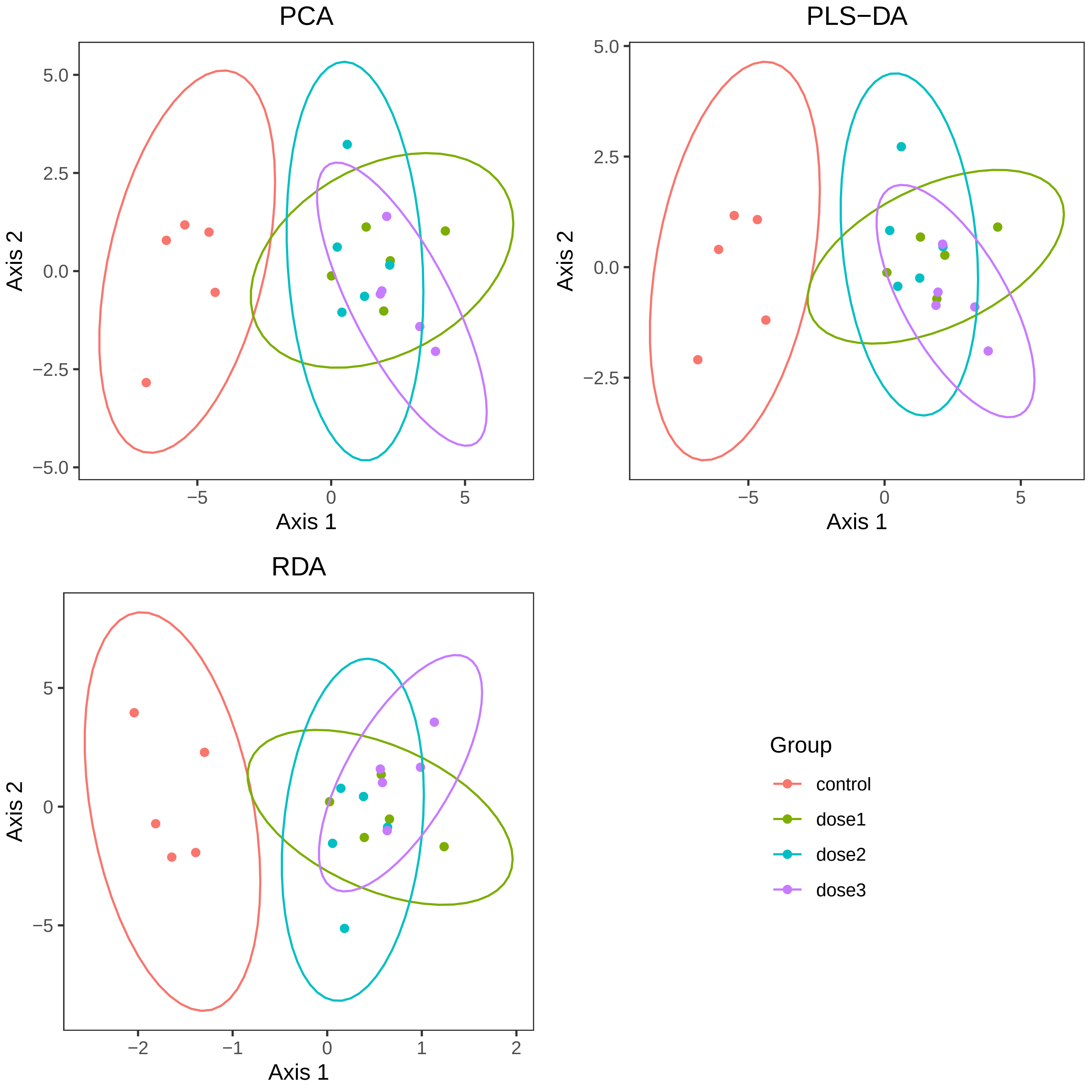

PCA
PLS−DA
RDA
Axis 1
Axis 2
5.0
2.5
0.0
−2.5
−5.0
−5
0
5
−2
−1
1
2
Group
control
dose1
dose2
dose3

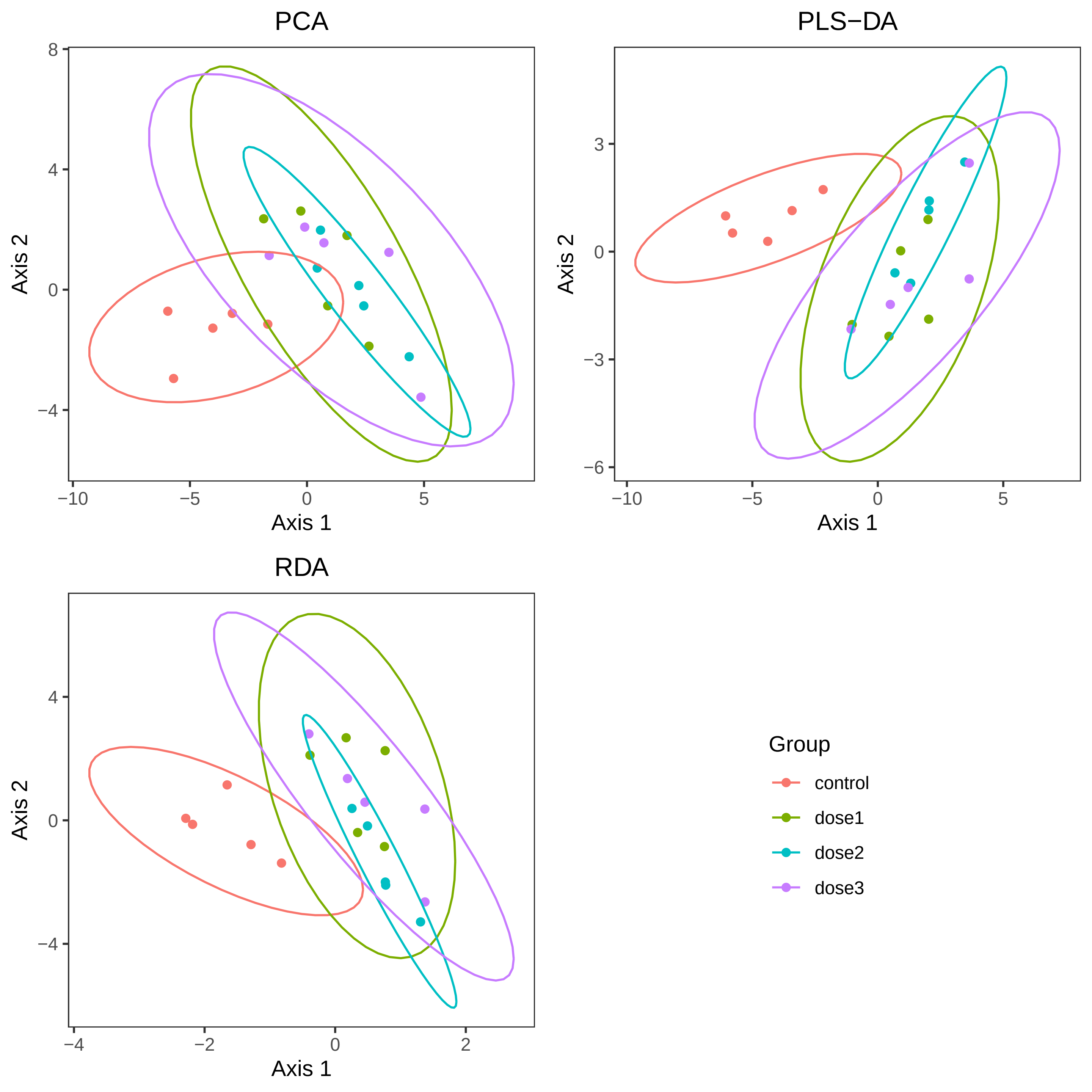

PCA
PLS−DA
RDA
Axis 1
Axis 2
8
4
0
−4
3
−3
−6
−10
−5
5
−2
2
Group
control
dose1
dose2
dose3

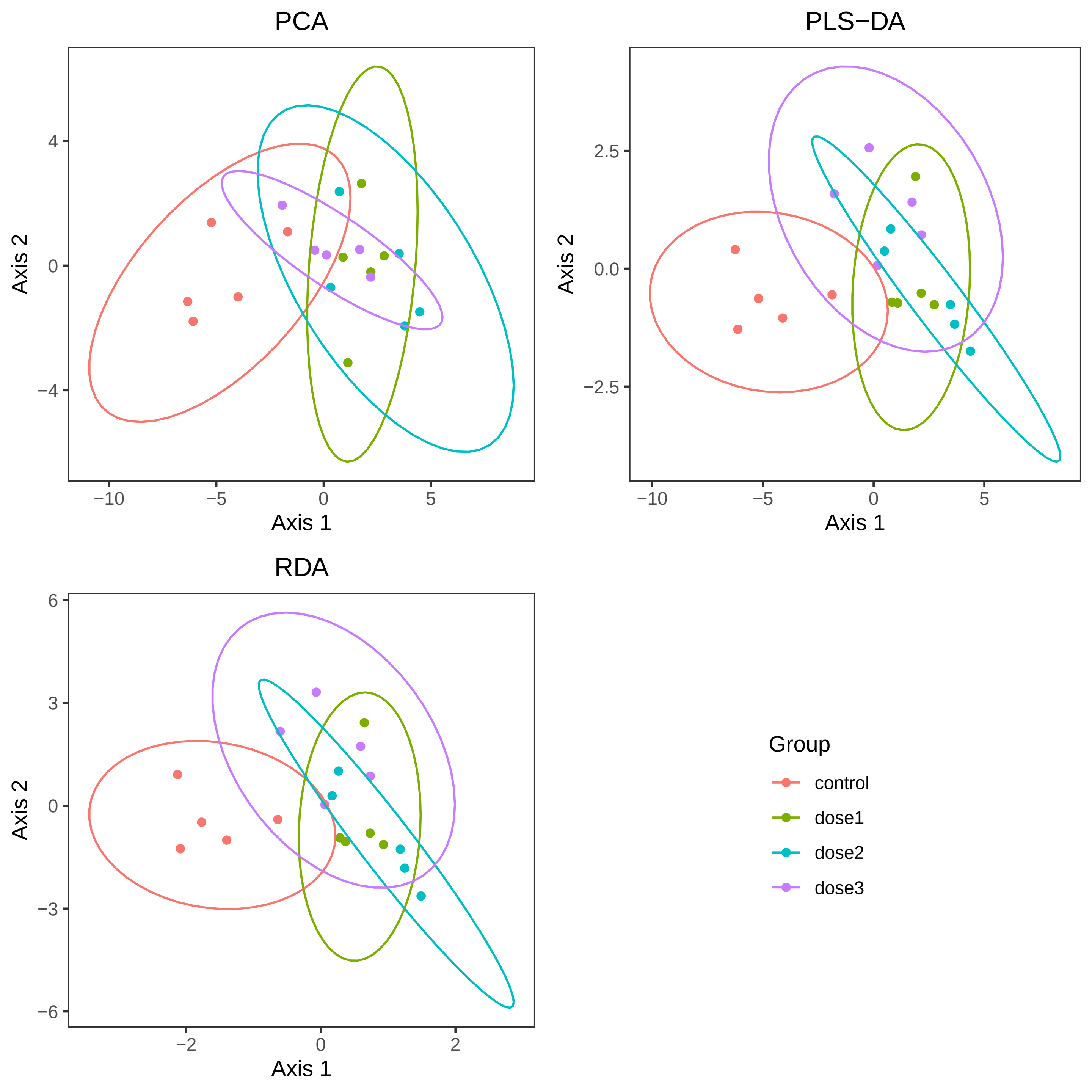
PCA
PLS−DA
RDA
Axis 1
Axis 2
Group
control
dose1
dose2
dose3

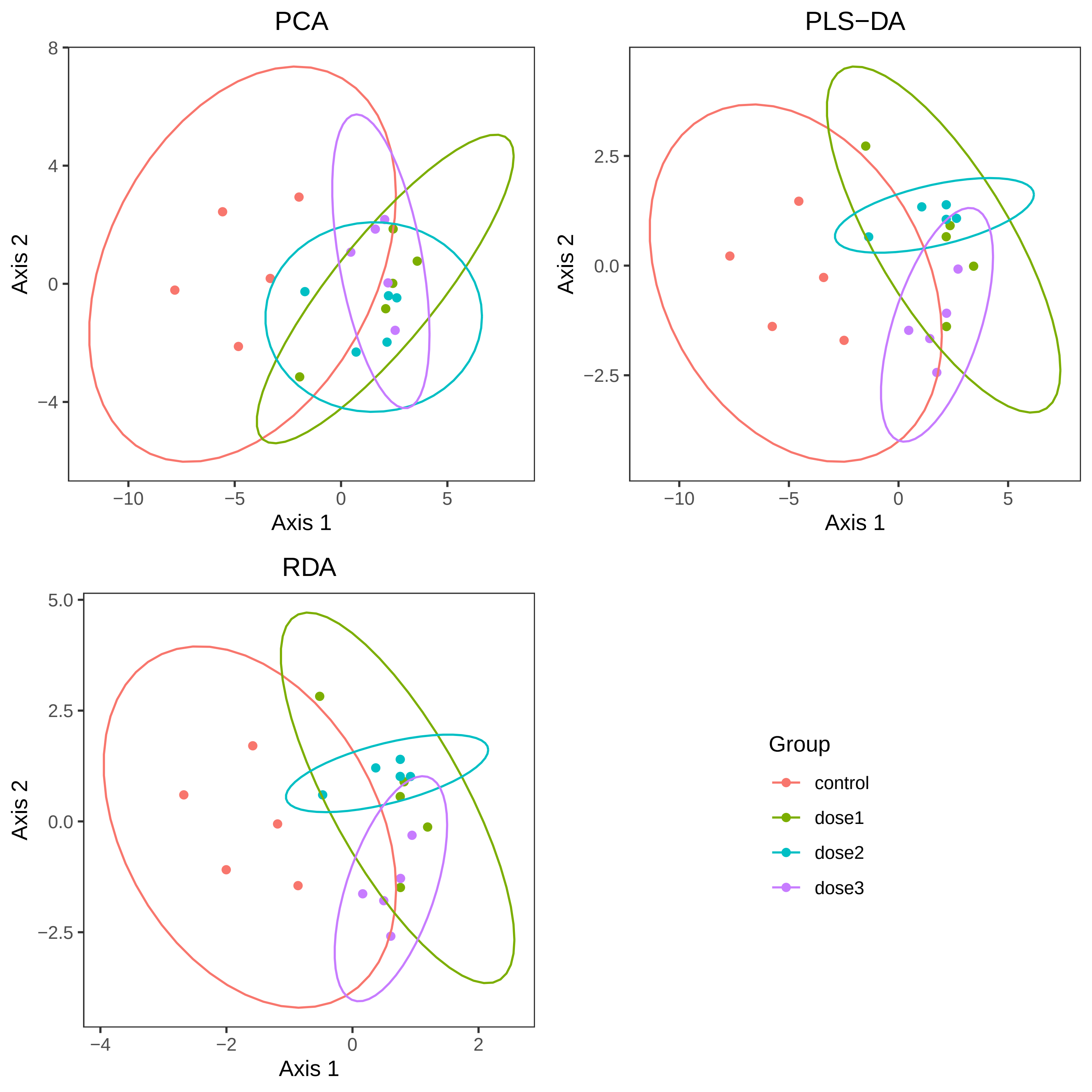

PCA
PLS−DA
RDA
Axis 1
Axis 2
Group
control
dose1
dose2
dose3

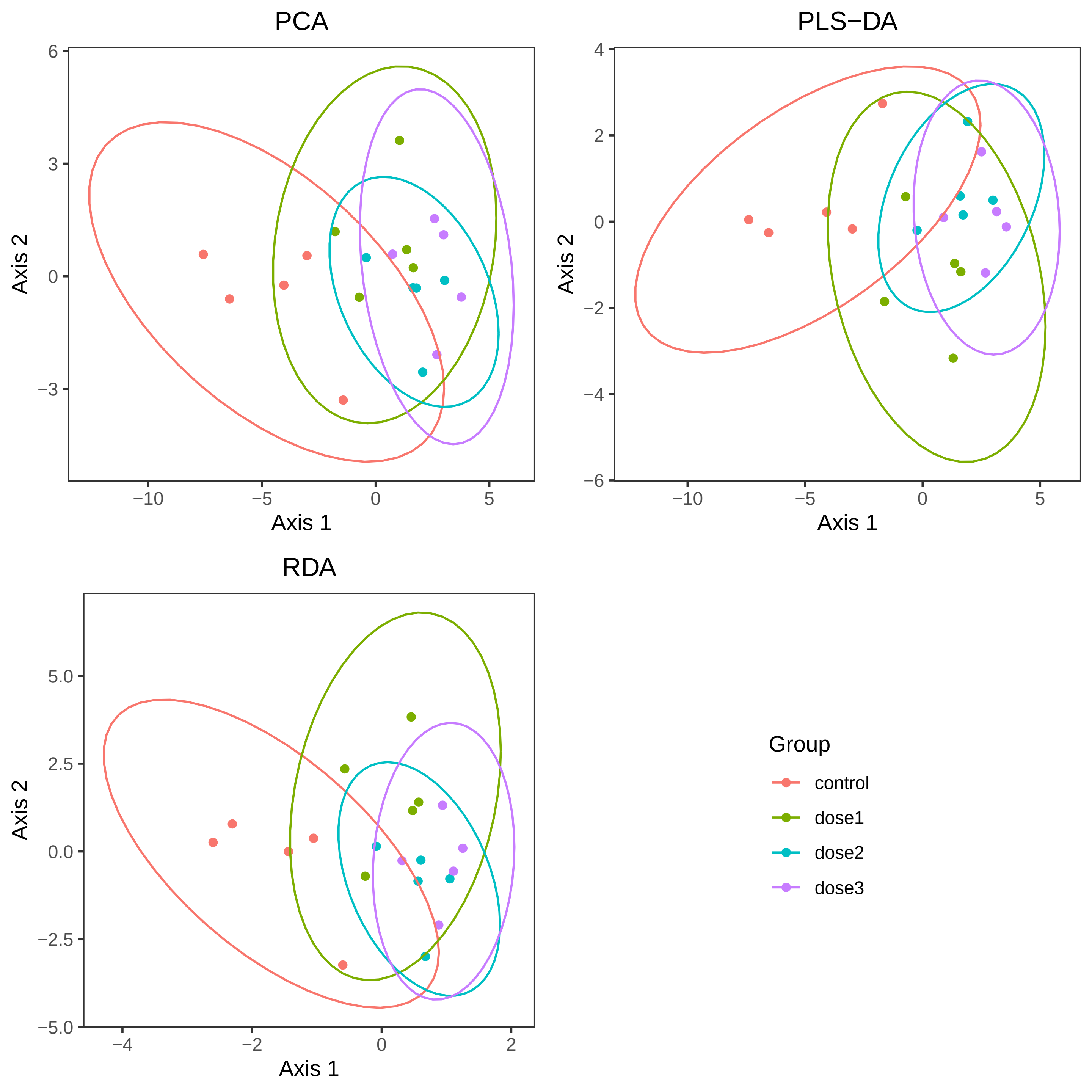
PCA
PLS−DA
RDA
Axis 1
Axis 2
Group
control
dose1
dose2
dose3

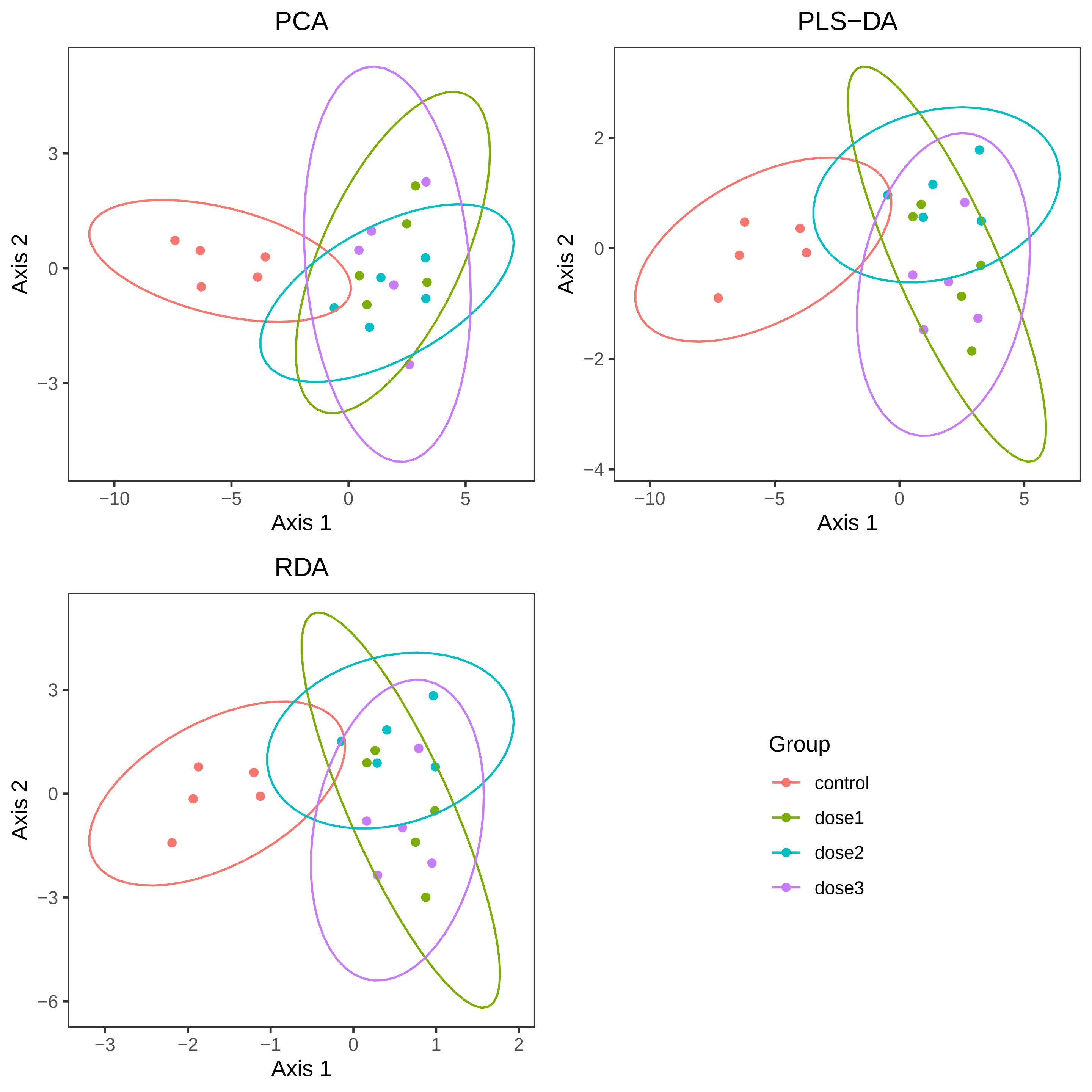
PCA
PLS−DA
RDA
Axis 1
Axis 2
Group
control
dose1
dose2
dose3

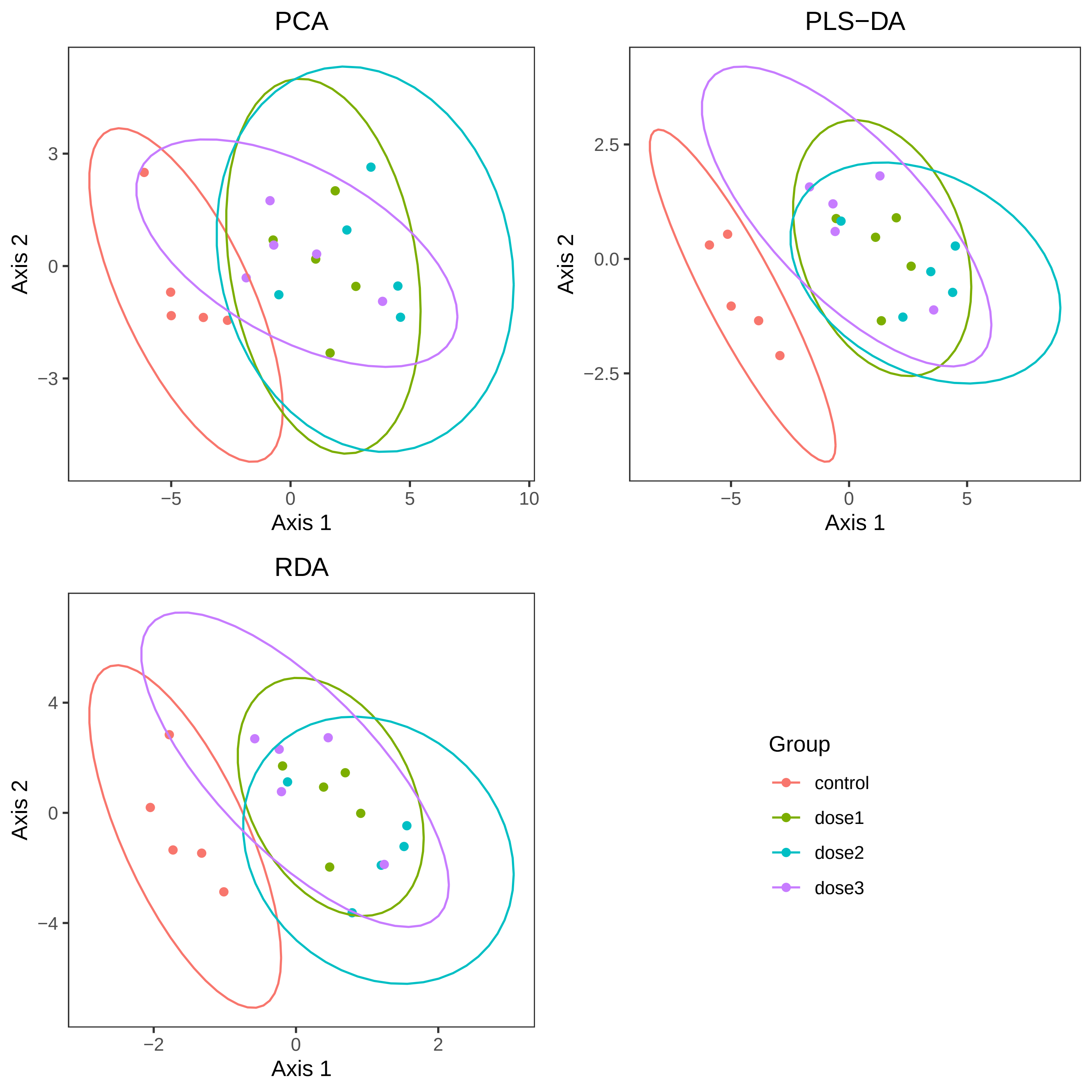
PCA
PLS−DA
RDA
Axis 1
Axis 2
−5
0
5
10
3
0
−3
2.5
0.0
−2.5
−2
2
4
−4
Group
control
dose1
dose2
dose3

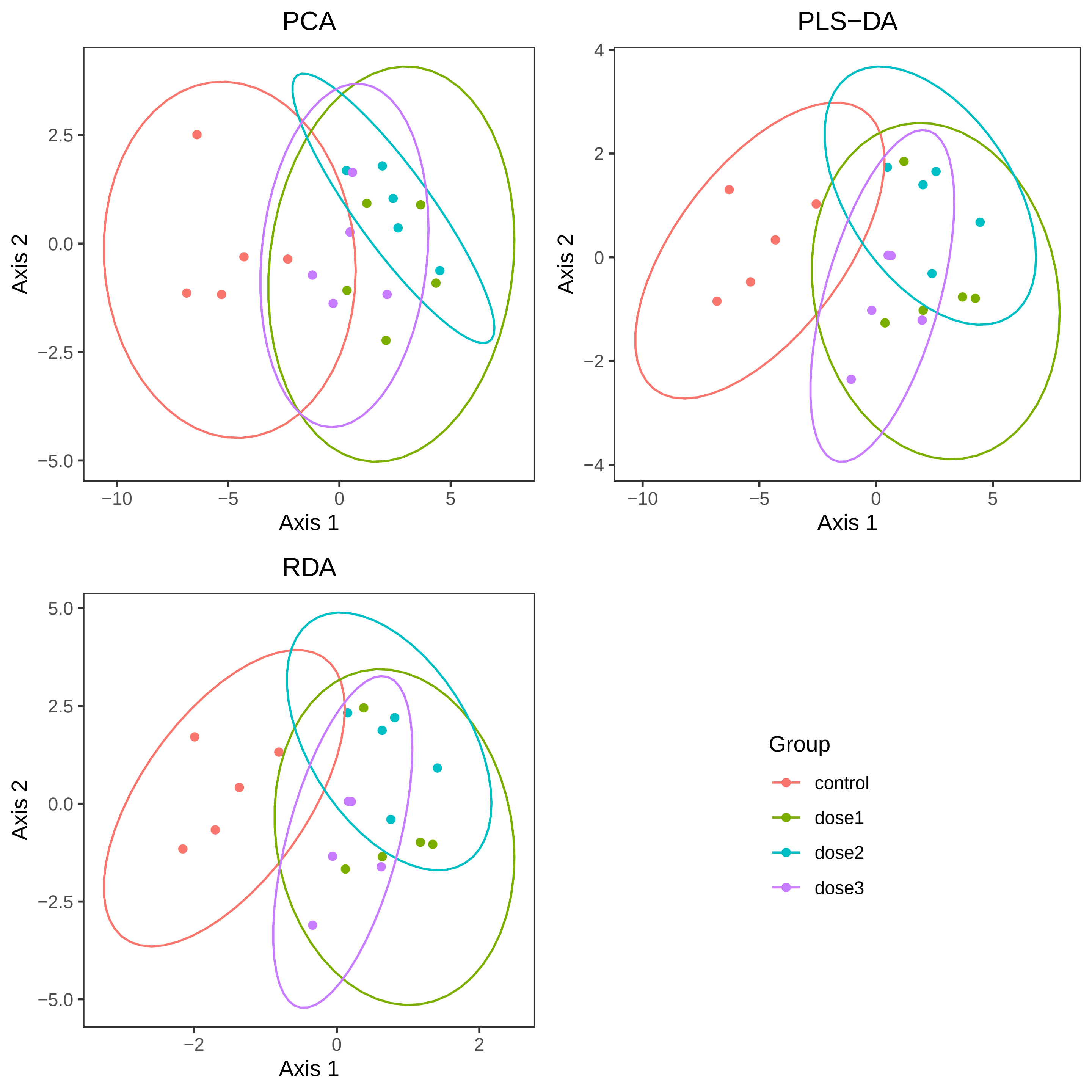

PCA
PLS−DA
RDA
Axis 1
Axis 2
Group
control
dose1
dose2
dose3

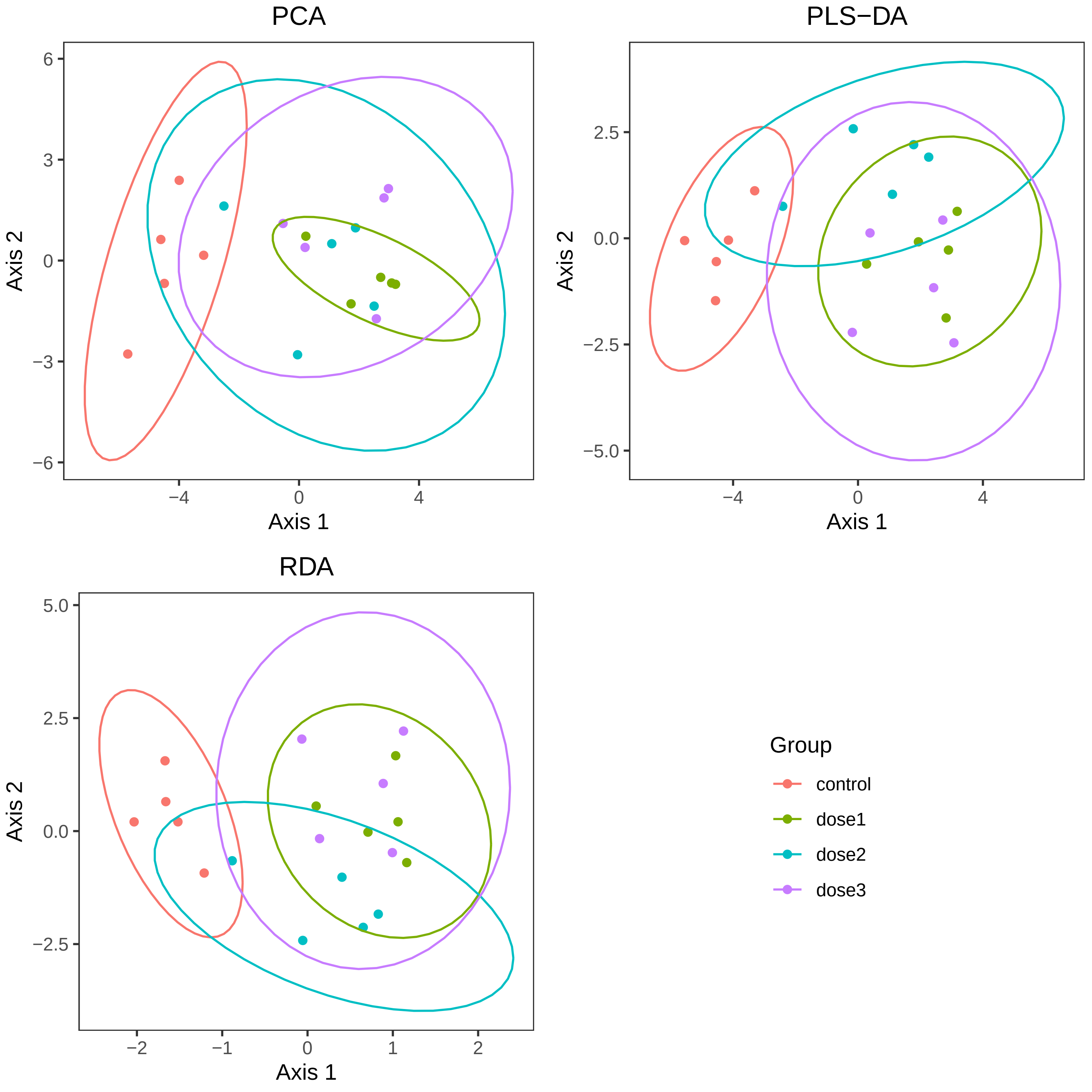

PCA
PLS−DA
RDA
Axis 1
Axis 2
Group
control
dose1
dose2
dose3

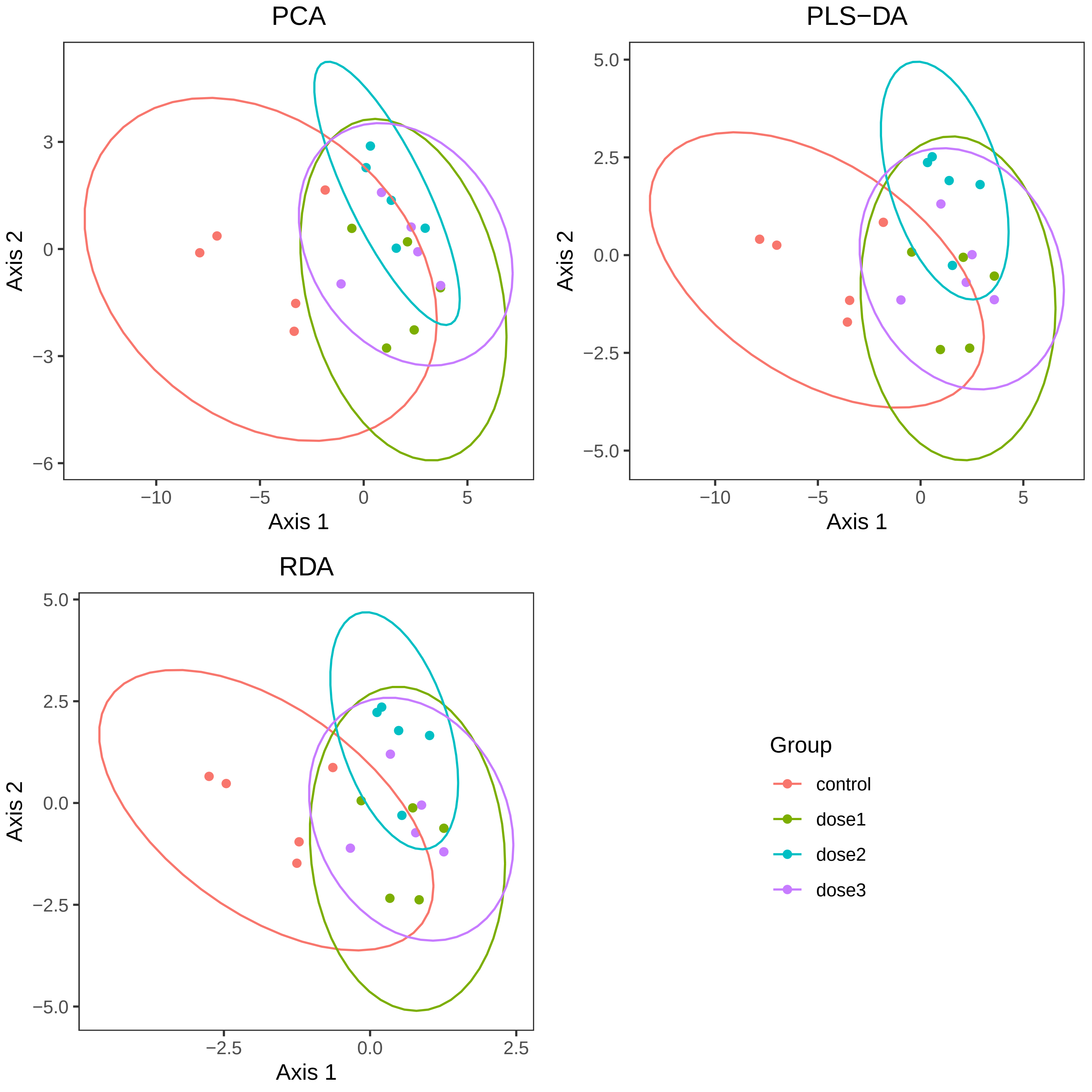
PCA
Axis 1
Axis 2
−10
−5
0
5
3
0
−3
−6
PLS−DA
5.0
2.5
0.0
−2.5
−5.0
RDA
−2.5
0.0
2.5
Group
control
dose1
dose2
dose3

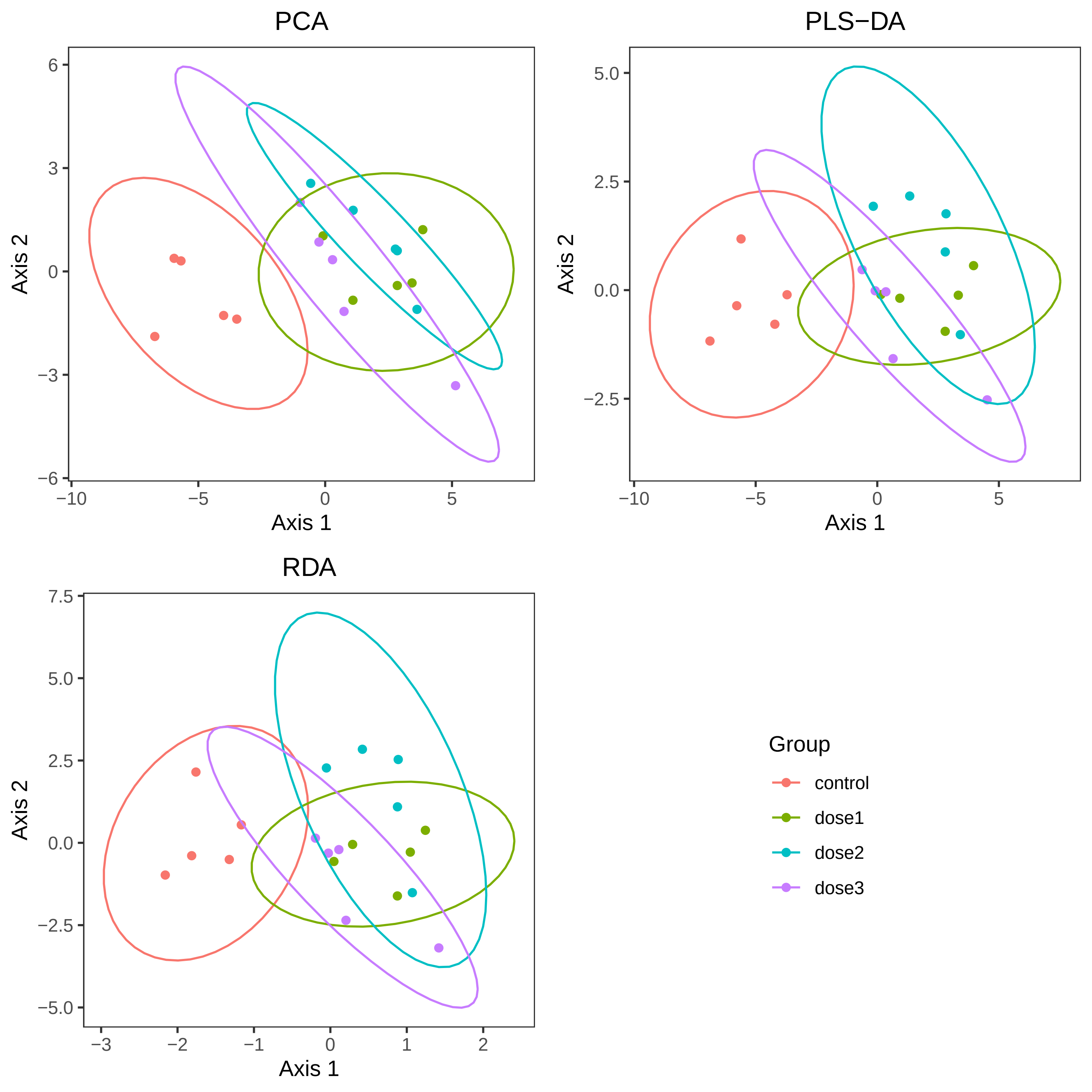
PCA
PLS−DA
RDA
Axis 1
Axis 2
Group
control
dose1
dose2
dose3

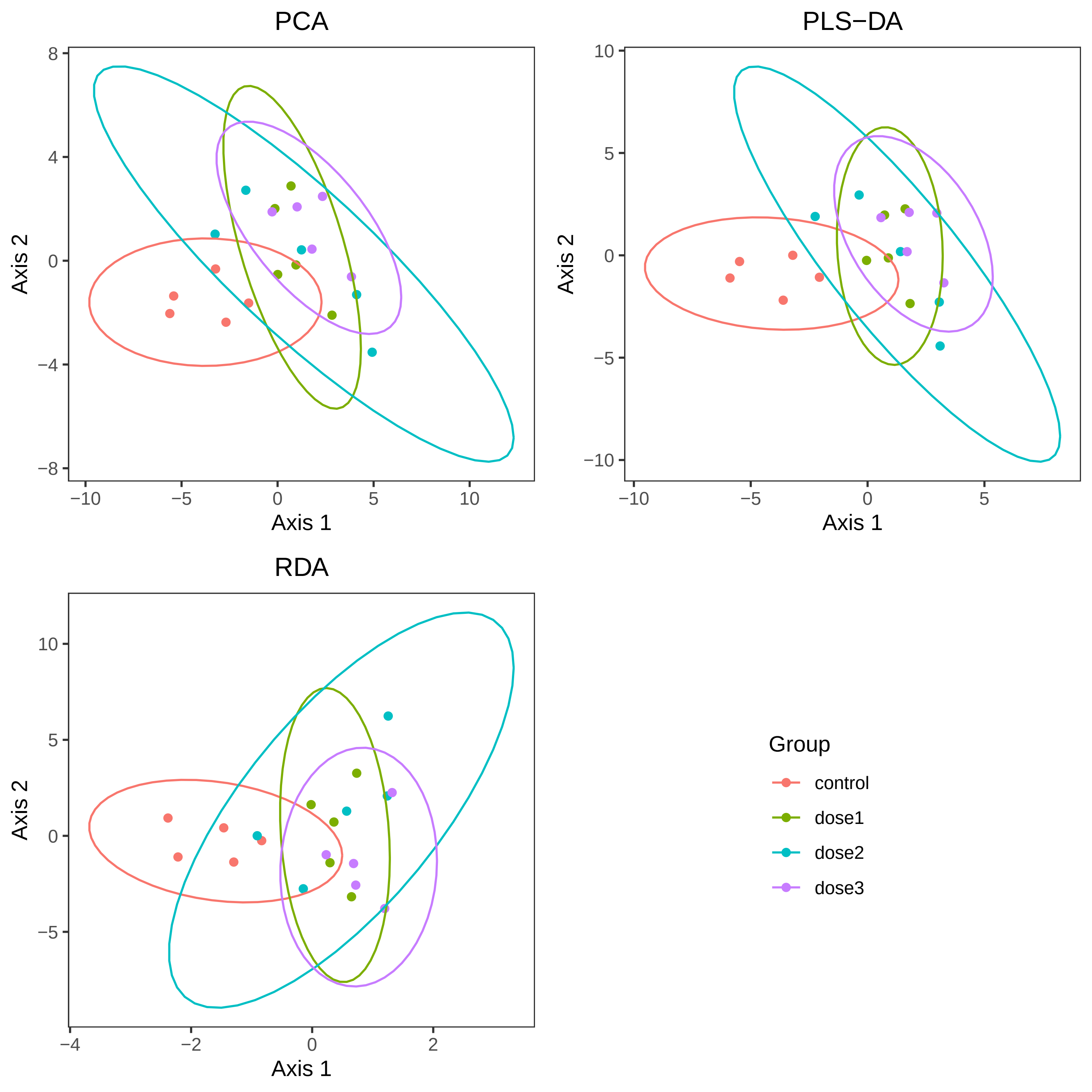

PCA
PLS−DA
RDA
Axis 1
Axis 2
Group
control
dose1
dose2
dose3

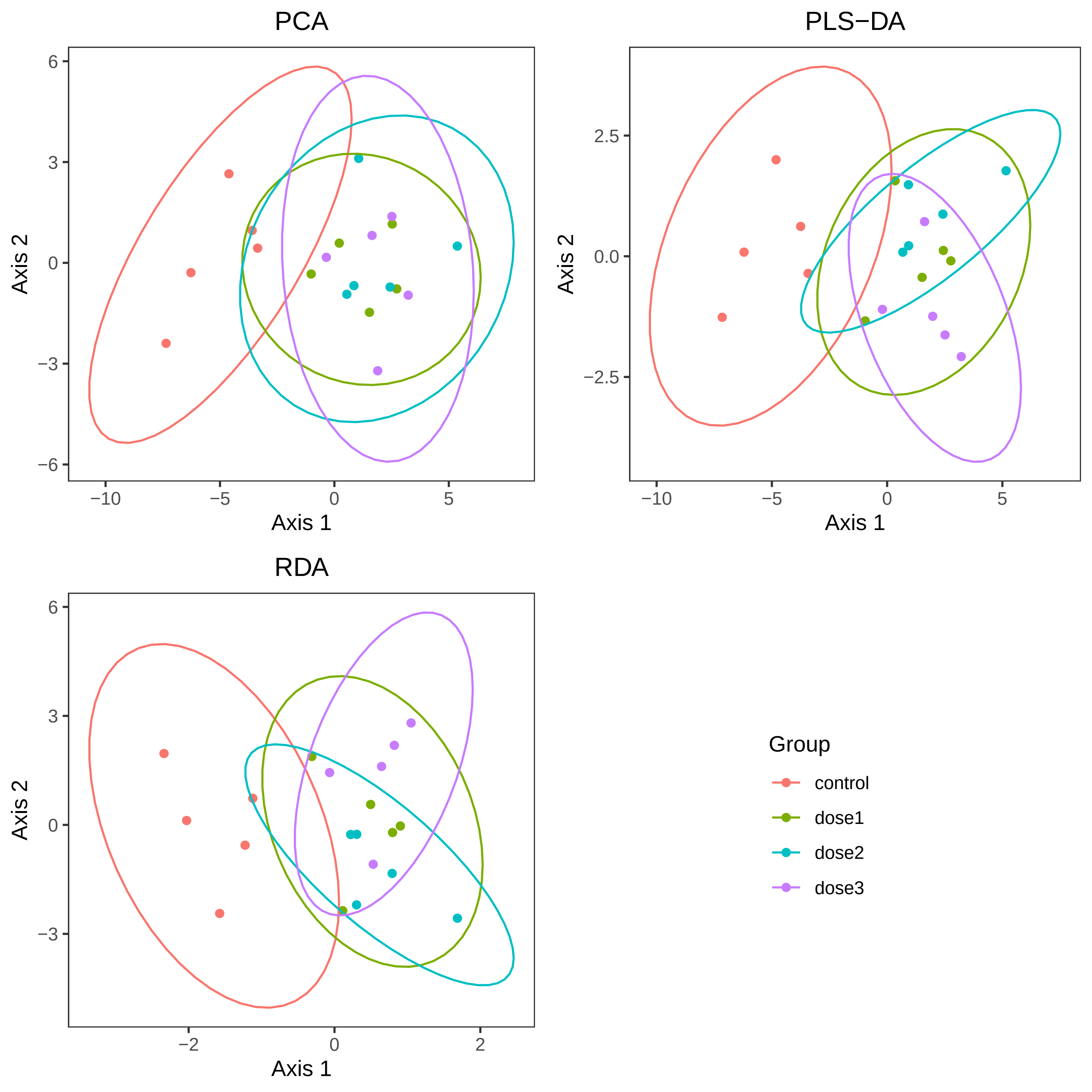

PCA
PLS−DA
RDA
Axis 1
Axis 2
6
3
0
−3
−6
−10
−5
0
5
2.5
0.0
−2.5
−2
2
Group
control
dose1
dose2
dose3

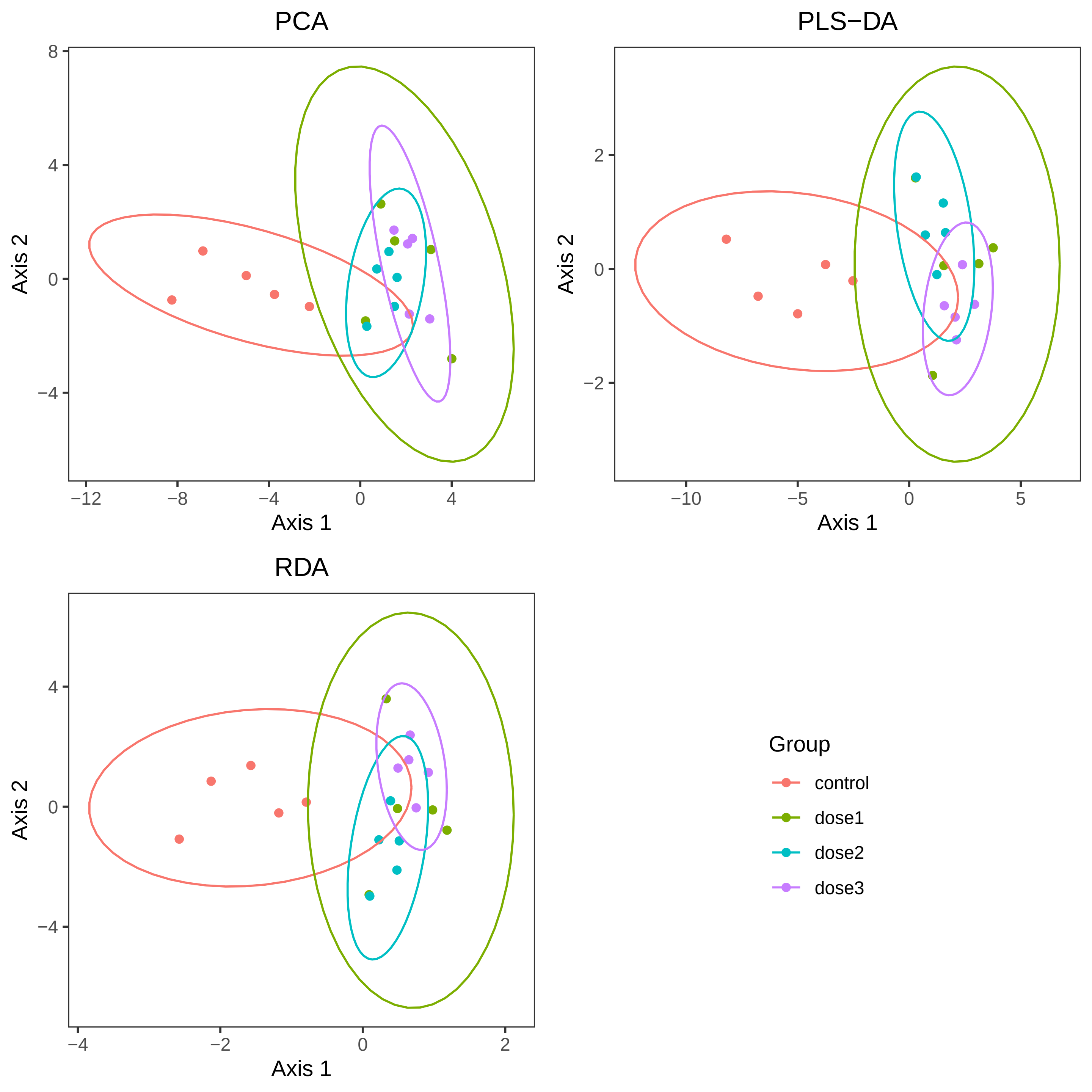

PCA
PLS−DA
RDA
Axis 1
Axis 2
Group
control
dose1
dose2
dose3

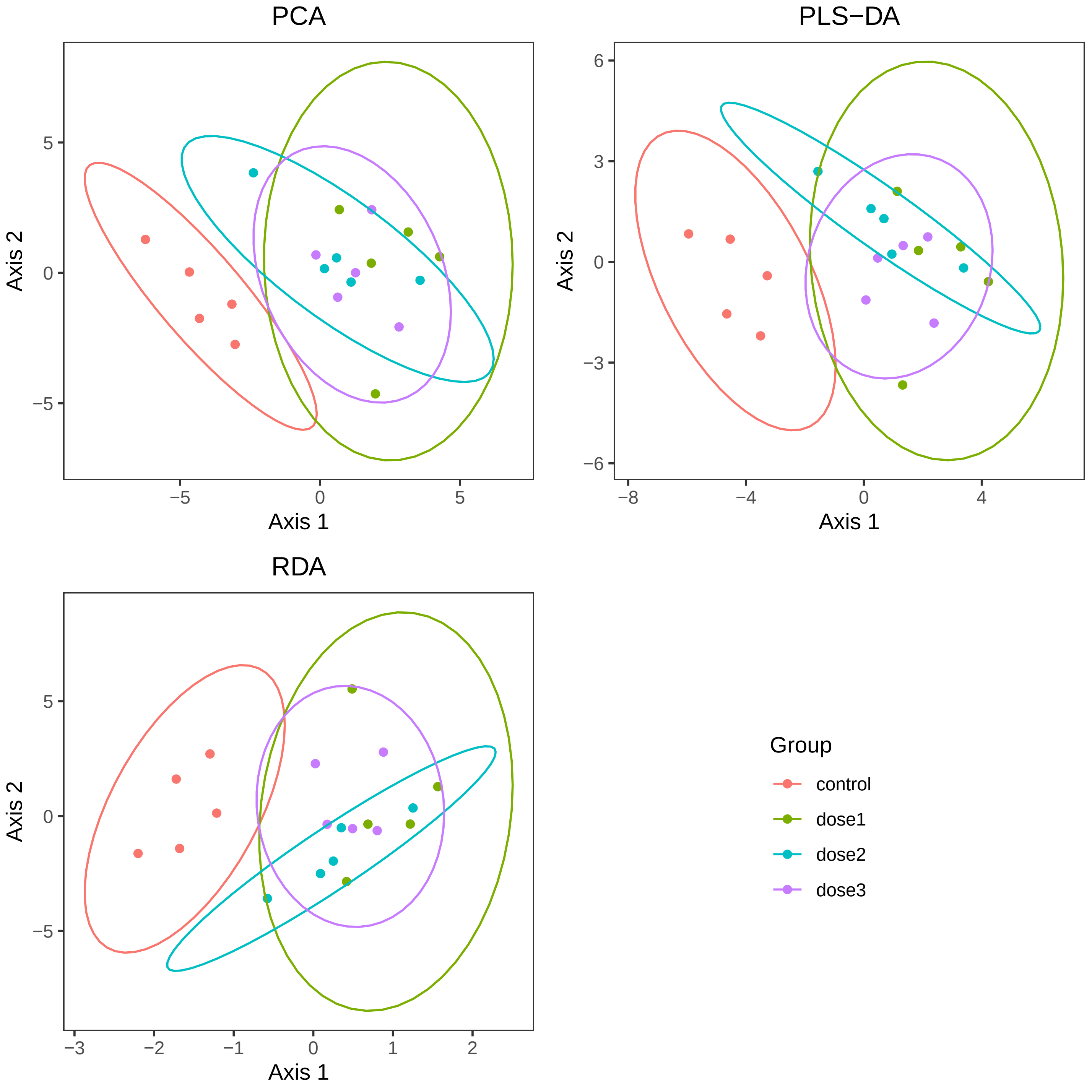
PCA
PLS−DA
RDA
Axis 1
Axis 2
Group
control
dose1
dose2
dose3

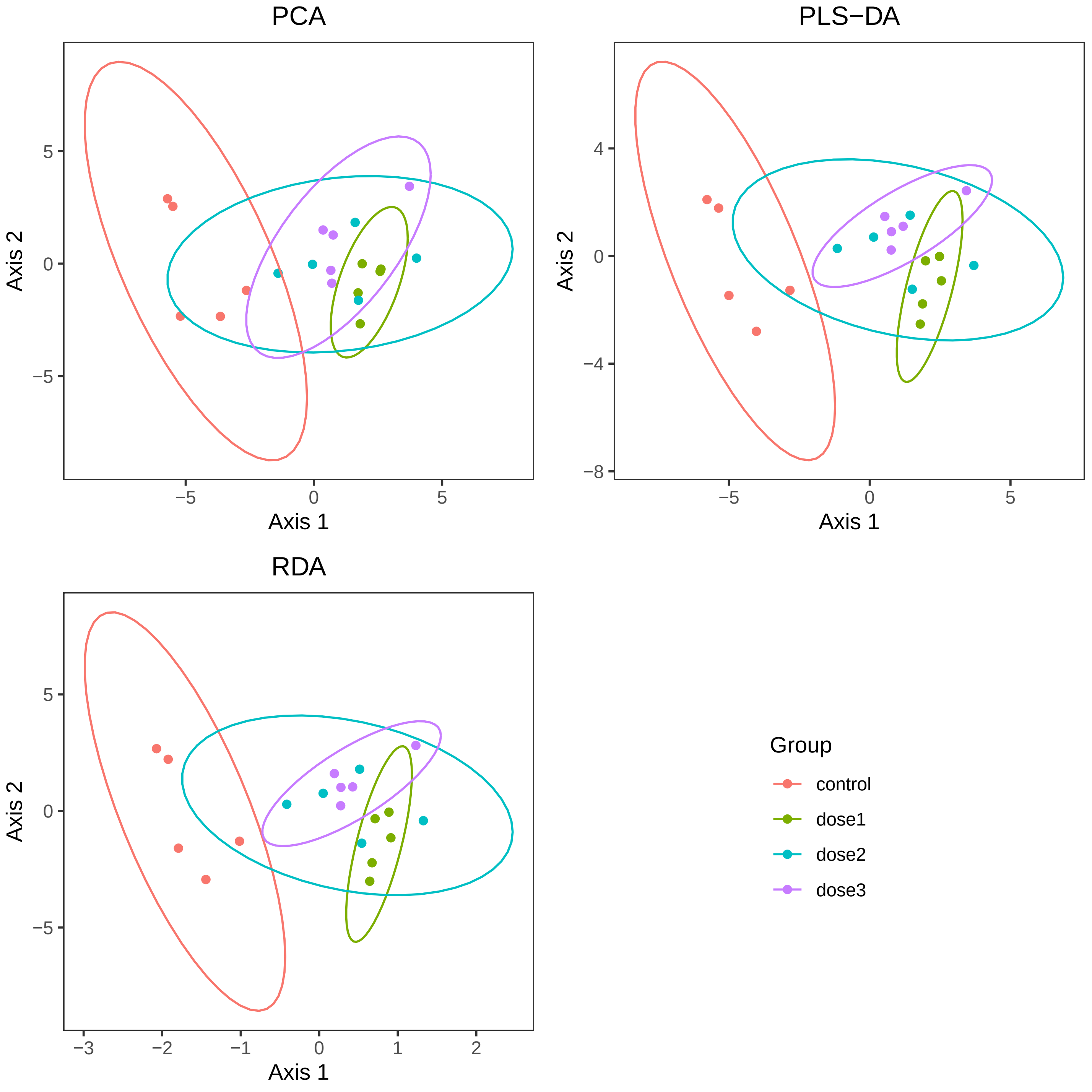

PCA
PLS−DA
RDA
Axis 1
Axis 2
Group
control
dose1
dose2
dose3

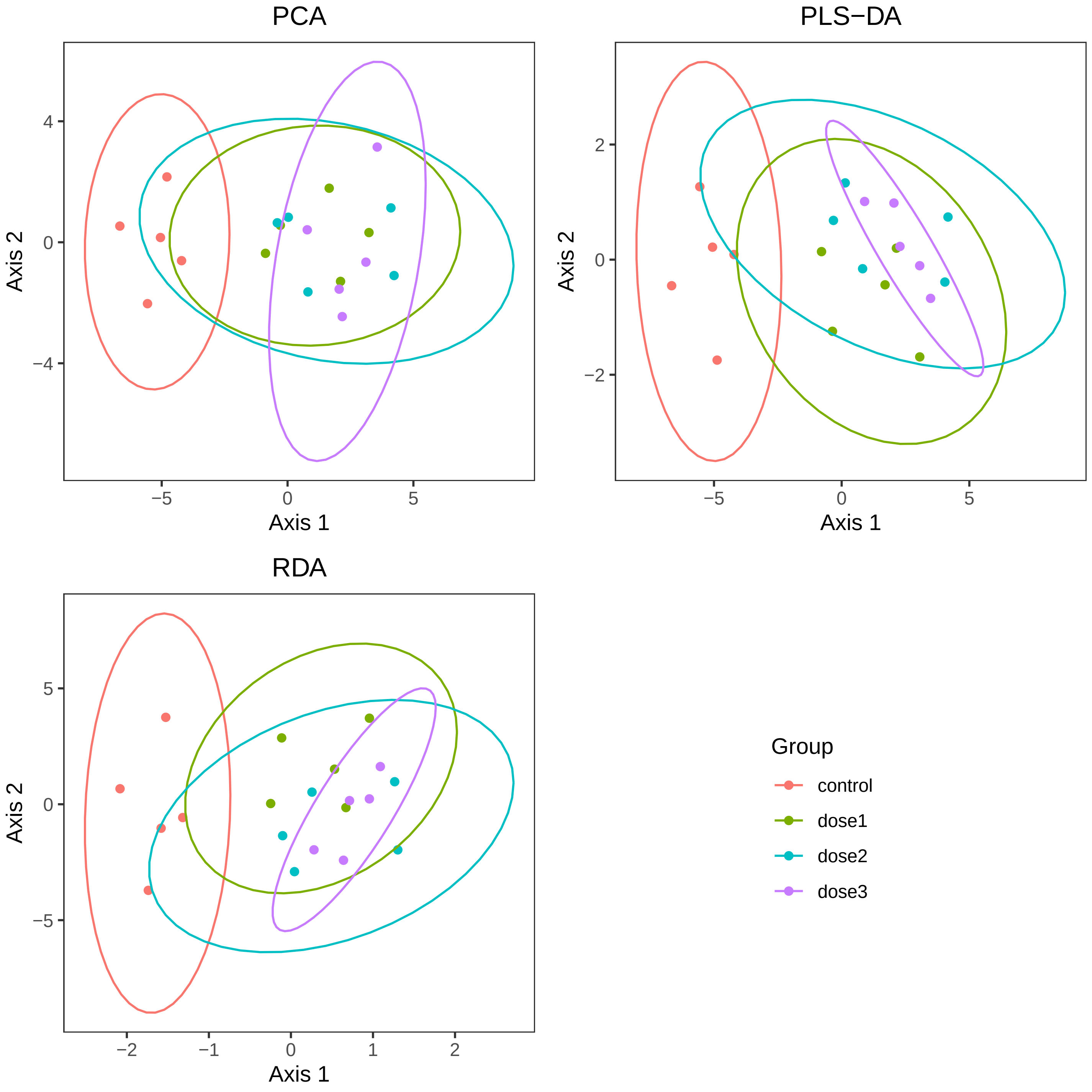

PCA
PLS−DA
RDA
Axis 1
Axis 2
Group
control
dose1
dose2
dose3

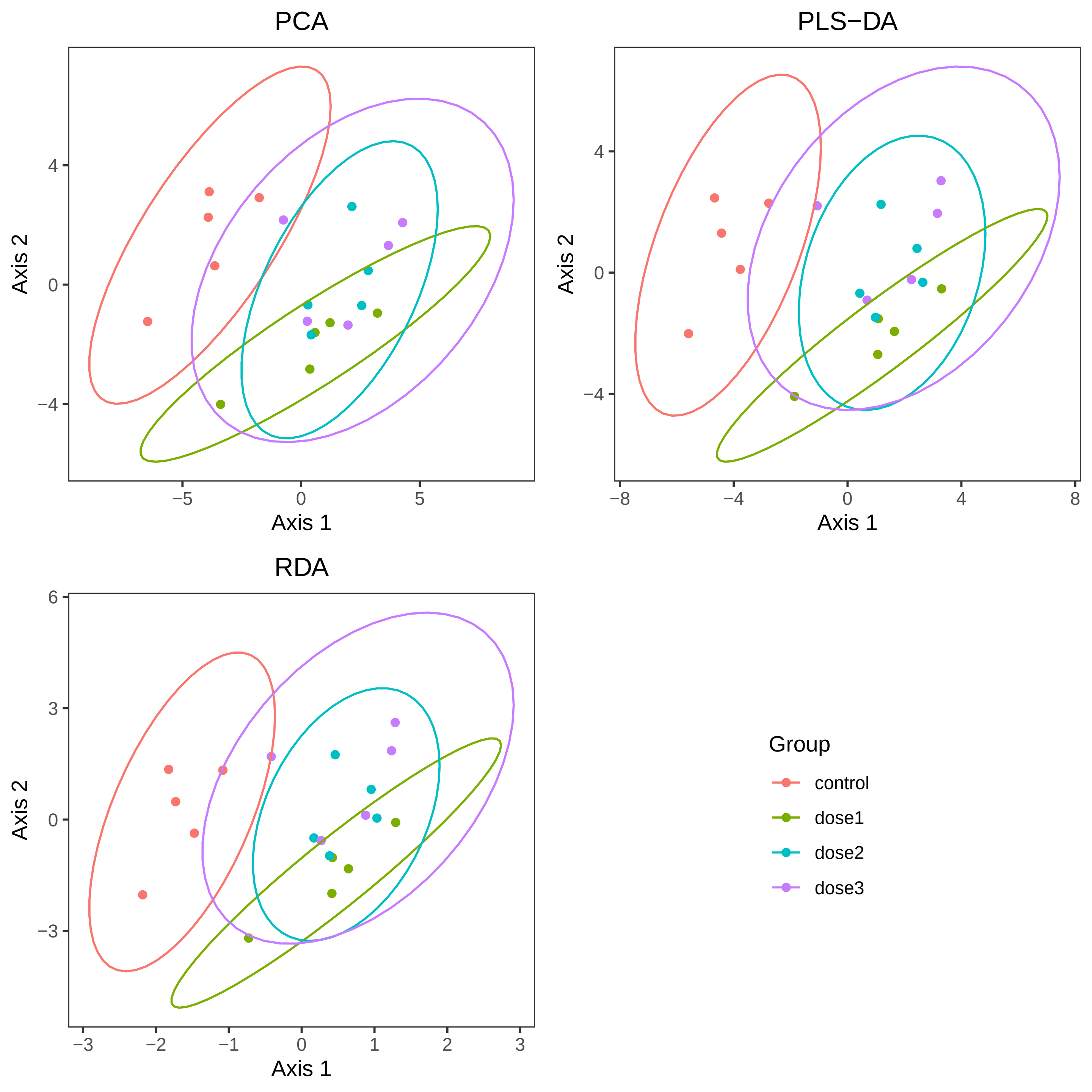

PCA
PLS−DA
RDA
Axis 1
Axis 2
Group
control
dose1
dose2
dose3

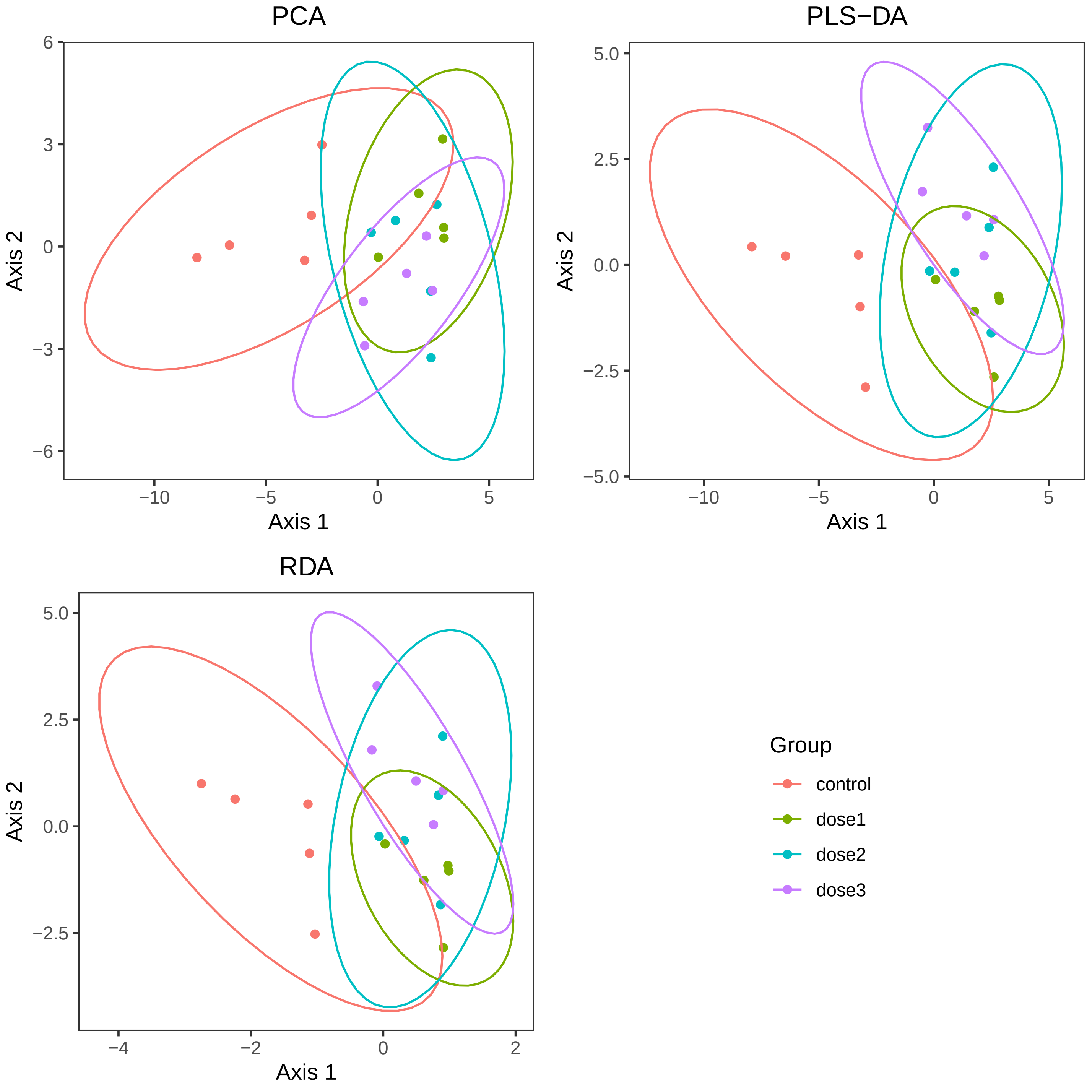
PCA
Axis 1
Axis 2
PLS−DA
Axis 1
Axis 2
RDA
Axis 1
Axis 2
Group
control
dose1
dose2
dose3

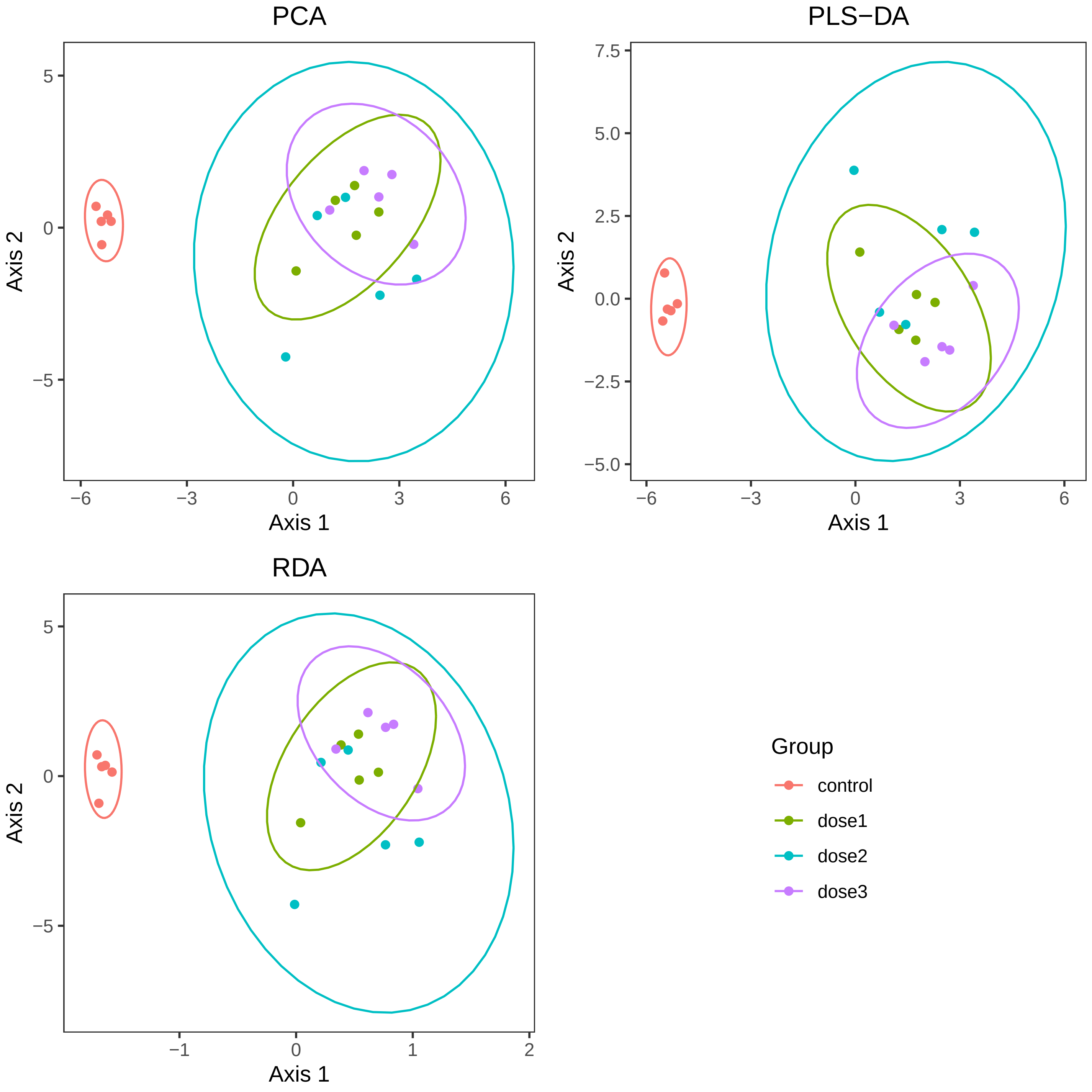

PCA
PLS−DA
RDA
Axis 1
Axis 2
Group
control
dose1
dose2
dose3

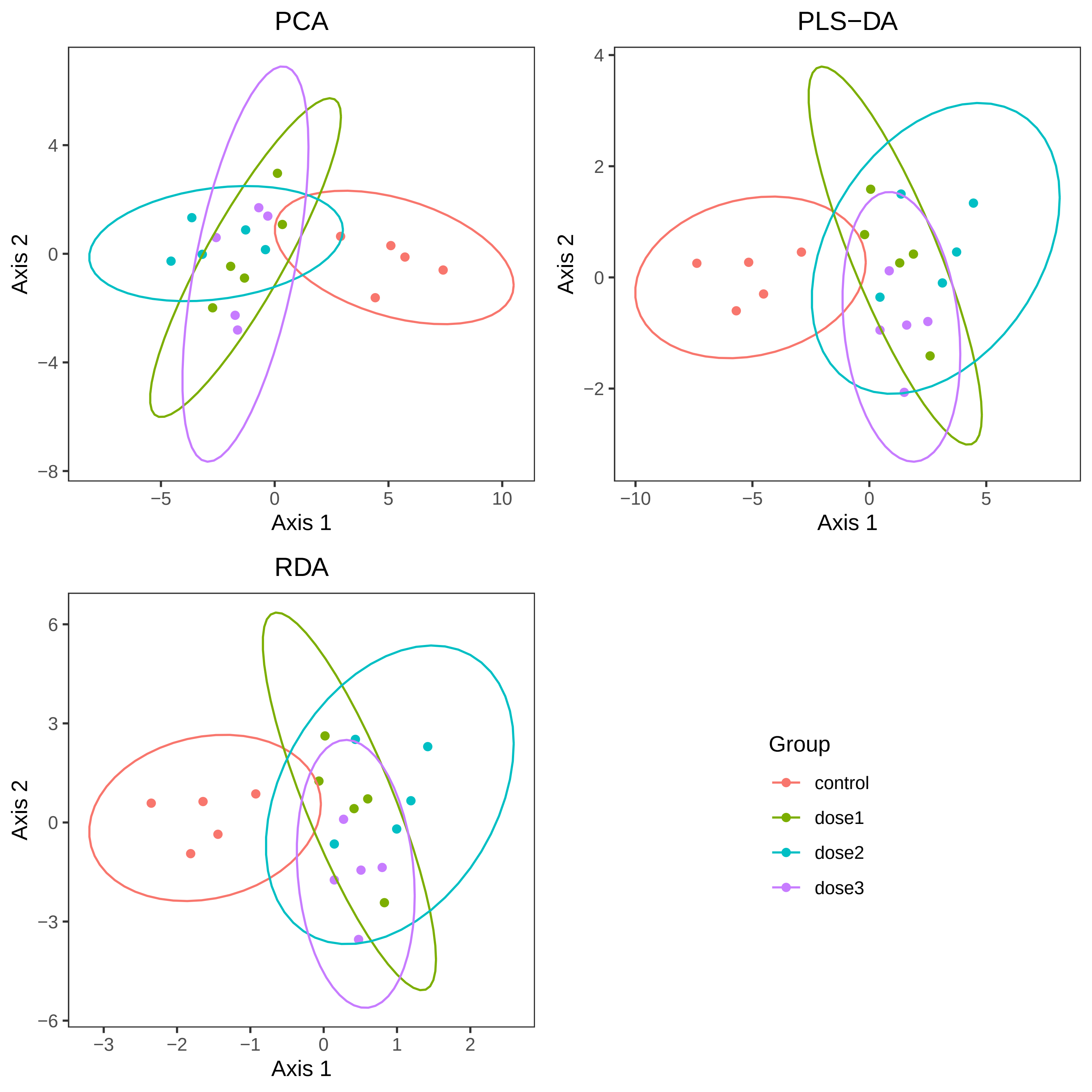

PCA
PLS−DA
RDA
Axis 1
Axis 2
−5
0
5
10
4
0
−4
−8
−10
2
−2
−3
−2
−1
1
3
6
−6
Group
control
dose1
dose2
dose3

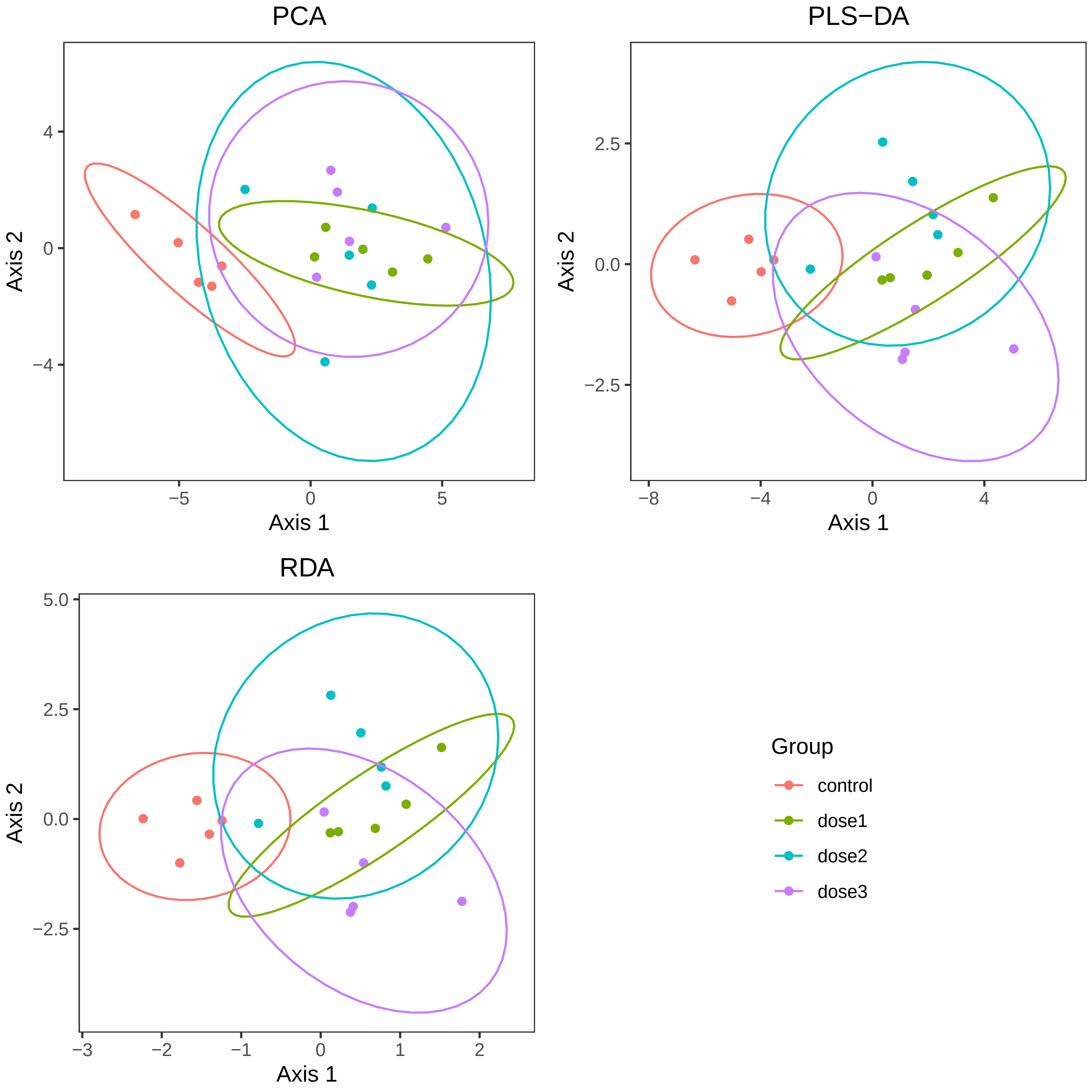
PCA
PLS−DA
RDA
Axis 1
Axis 2
Group
control
dose1
dose2
dose3

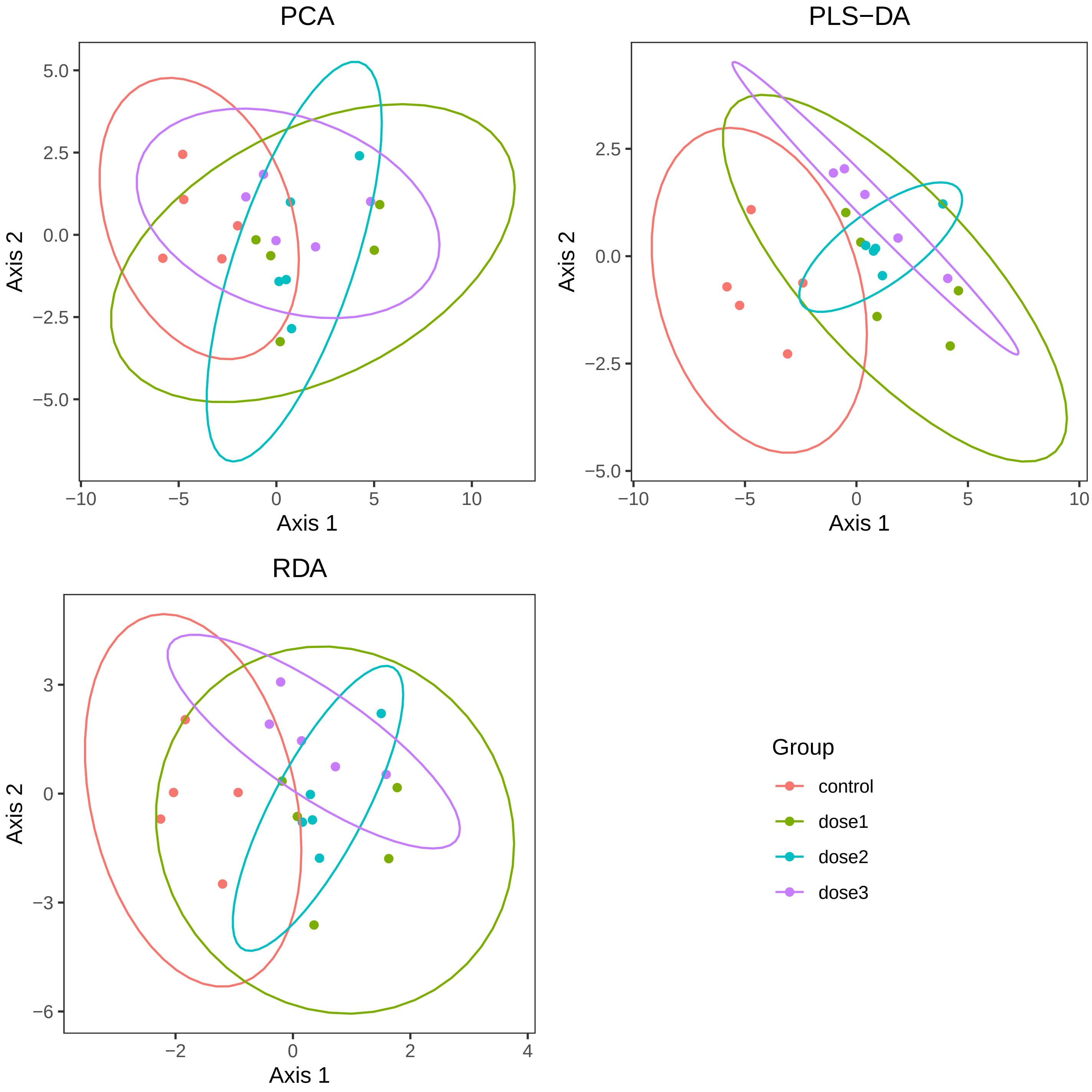
PCA
PLS−DA
RDA
Axis 1
Axis 2
Group
control
dose1
dose2
dose3

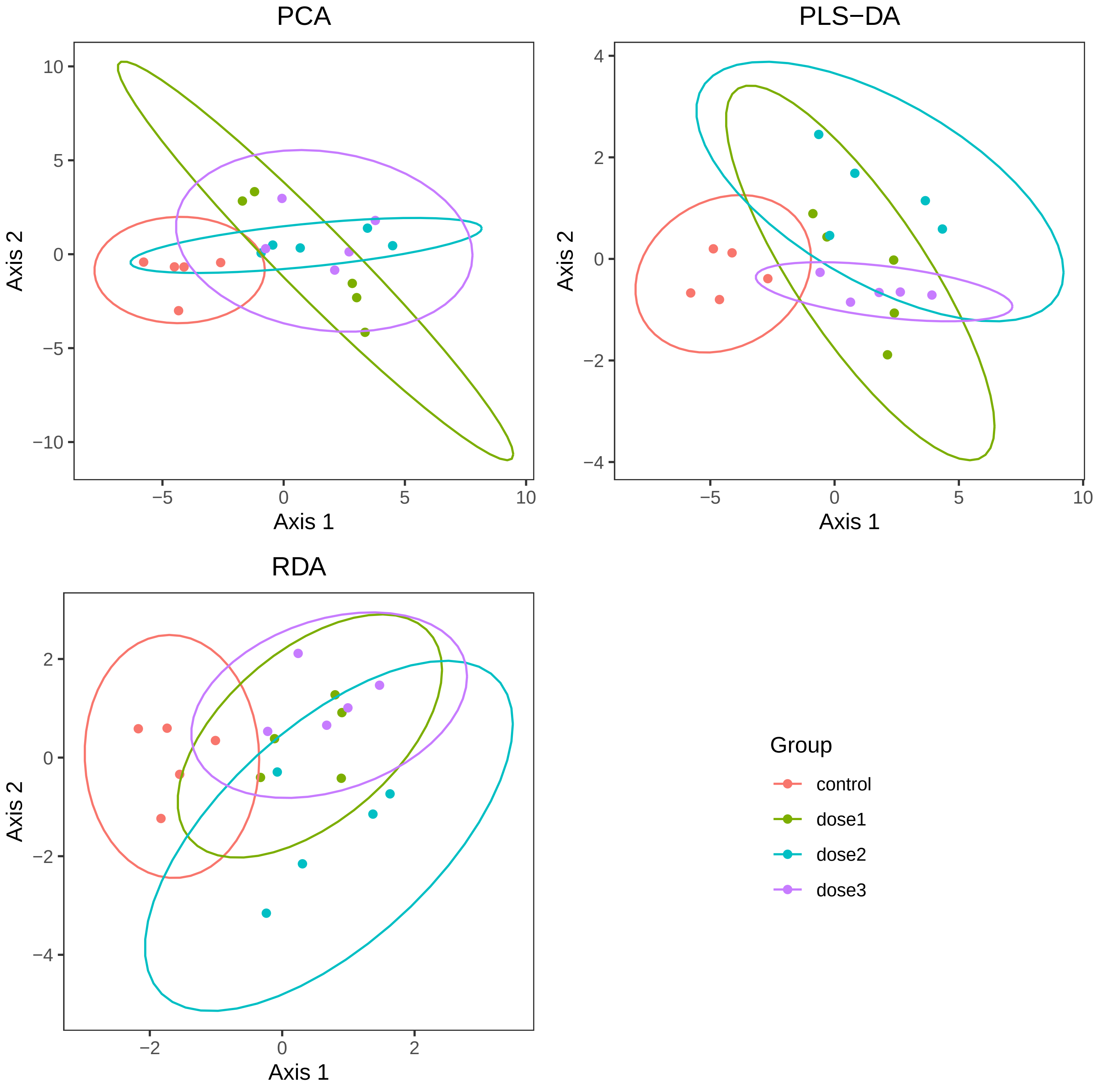
PCA
PLS−DA
RDA
Axis 1
Axis 2
Group
control
dose1
dose2
dose3

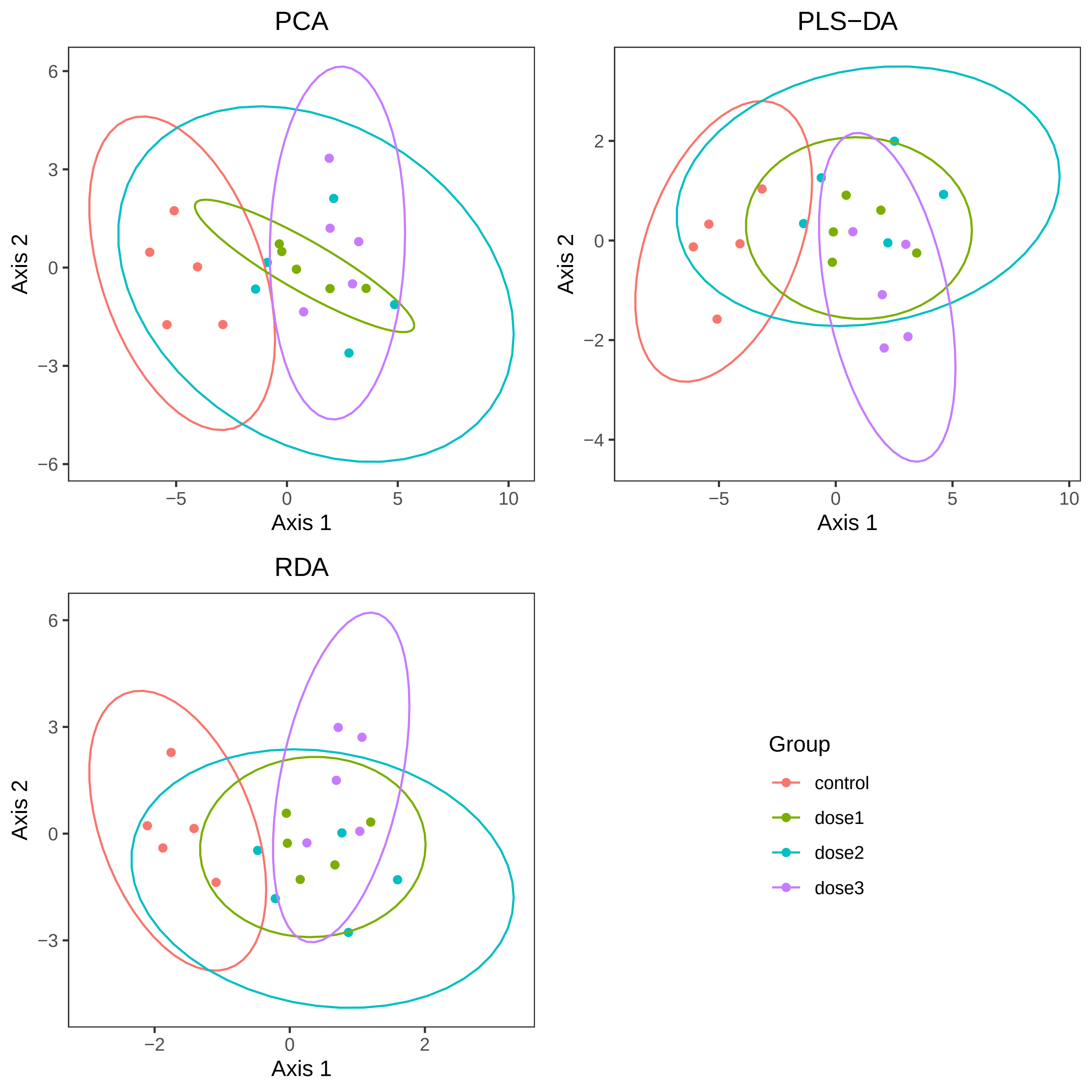

PCA
PLS−DA
RDA
Axis 1
Axis 2
Group
control
dose1
dose2
dose3

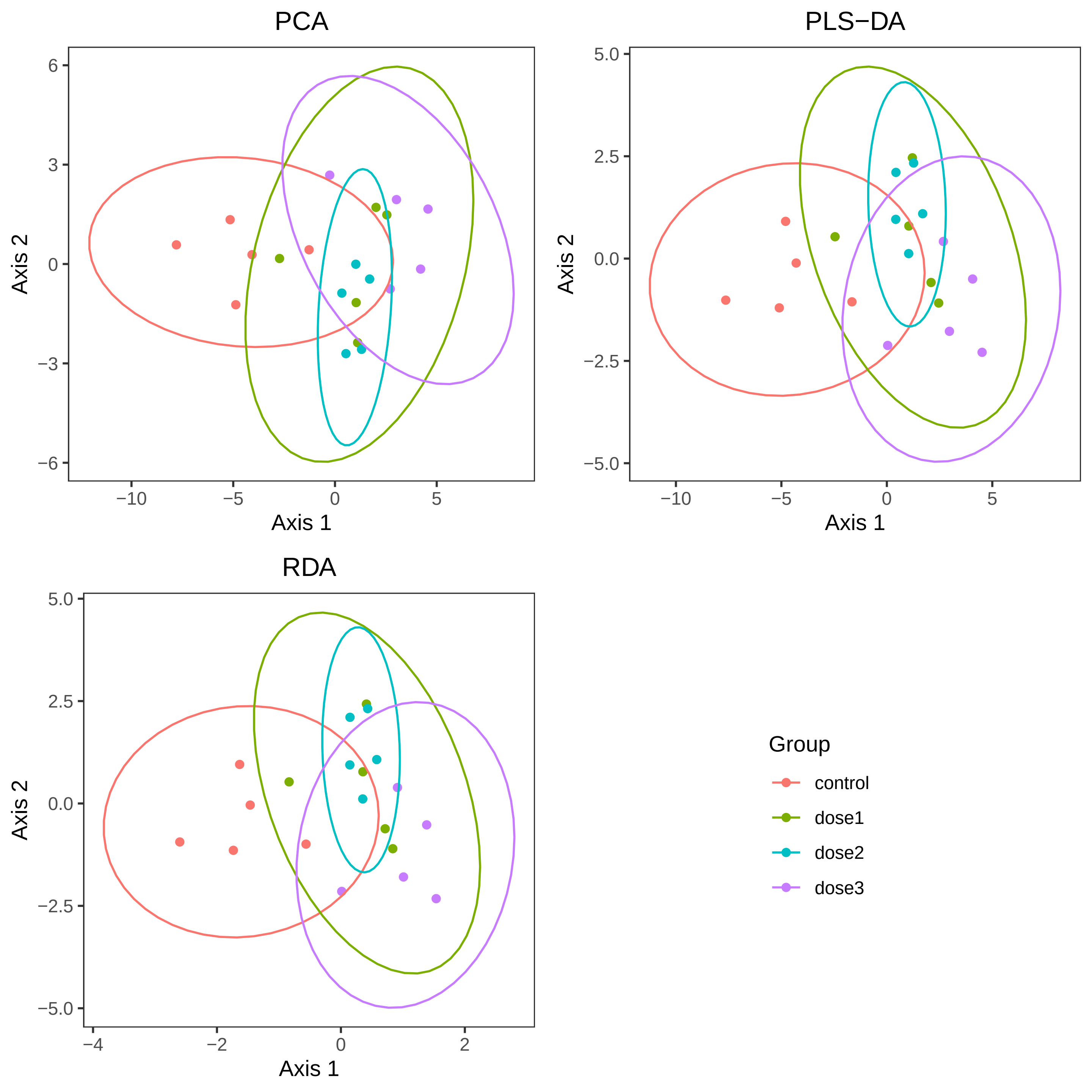

PCA
PLS−DA
RDA
Axis 1
Axis 2
6
3
0
−3
−6
−10
−5
0
5
5.0
2.5
0.0
−2.5
−5.0
−4
−2
2
Group
control
dose1
dose2
dose3

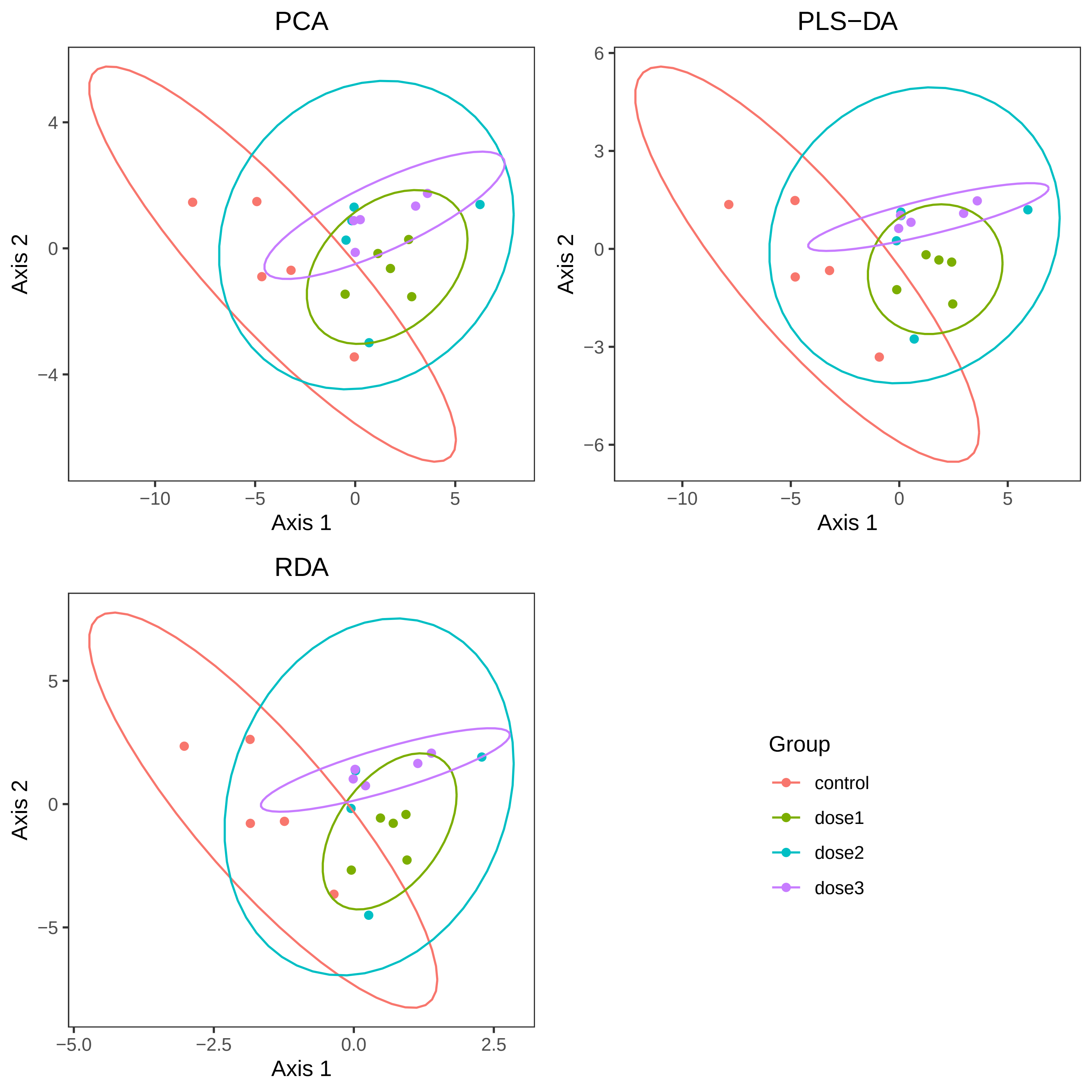
PCA
PLS−DA
RDA
Axis 1
Axis 2
4
0
−4
−10
−5
0
5
6
3
0
−3
−6
−10
−5
0
5
5
0
−5
−5.0
−2.5
0.0
2.5
Group
control
dose1
dose2
dose3

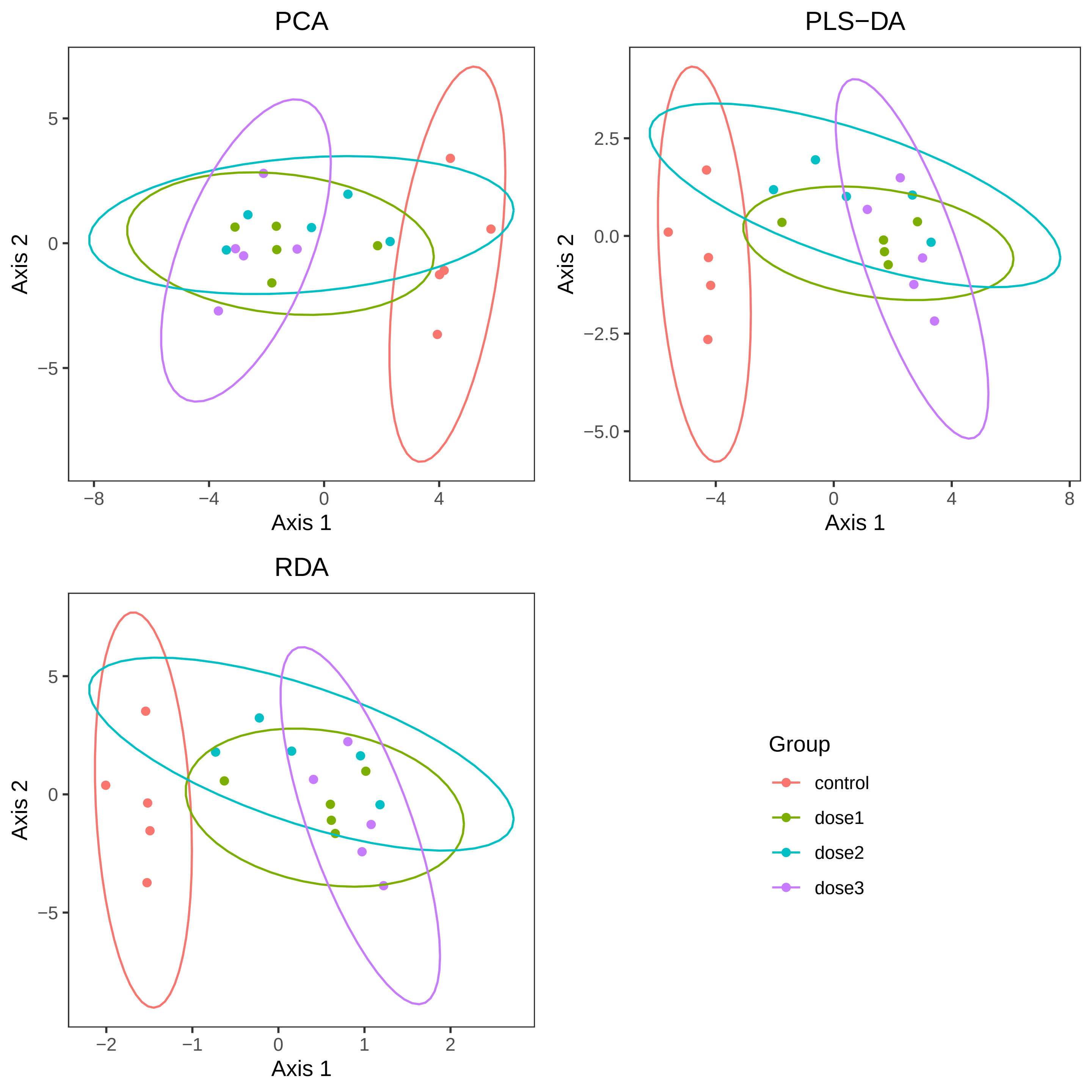

PCA
PLS−DA
RDA
Axis 1
Axis 2
−8
−4
0
4
5
0
−5
8
2.5
0.0
−2.5
−5.0
−2
−1
1
2
Group
control
dose1
dose2
dose3

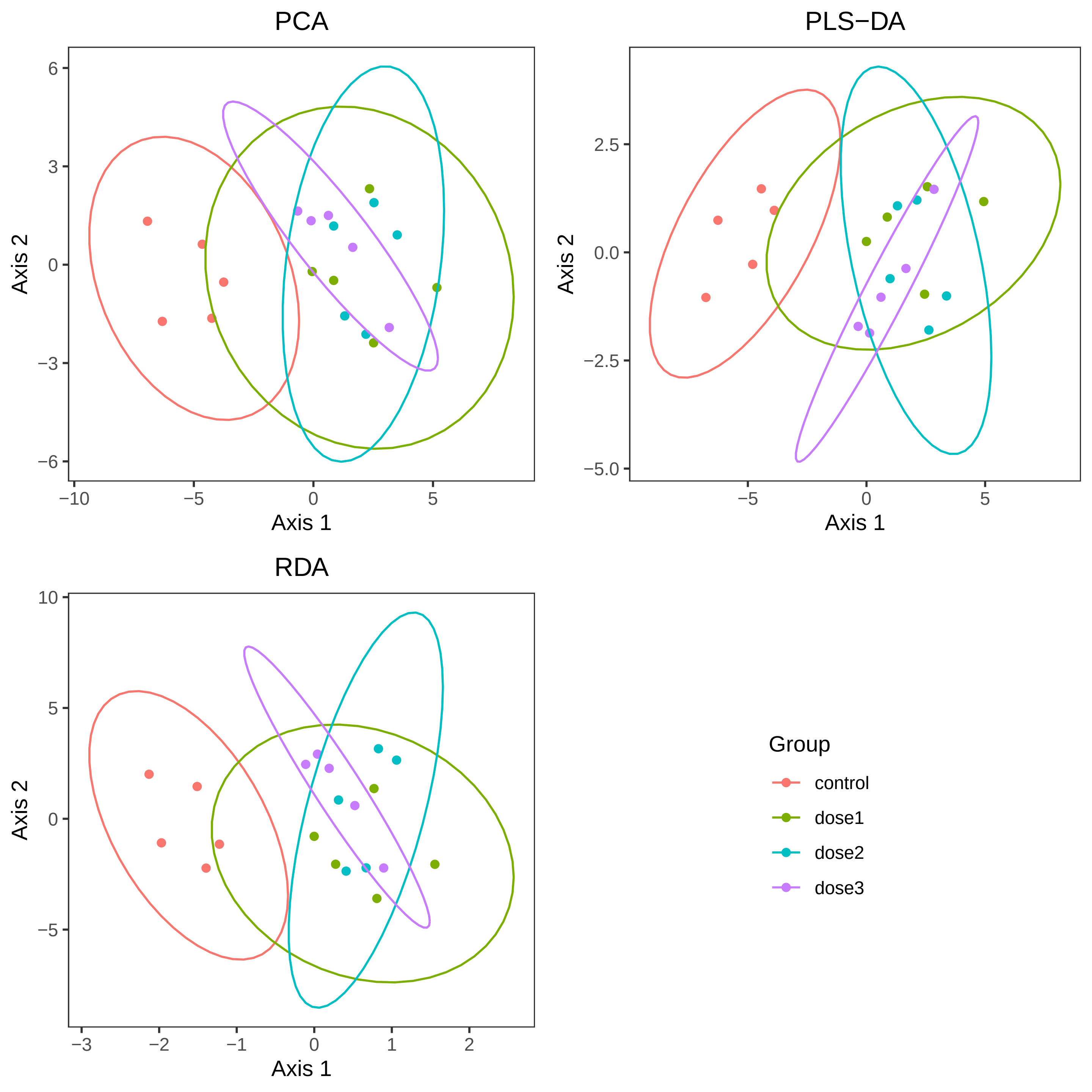
PCA
PLS−DA
RDA
Axis 1
Axis 2
Group
control
dose1
dose2
dose3

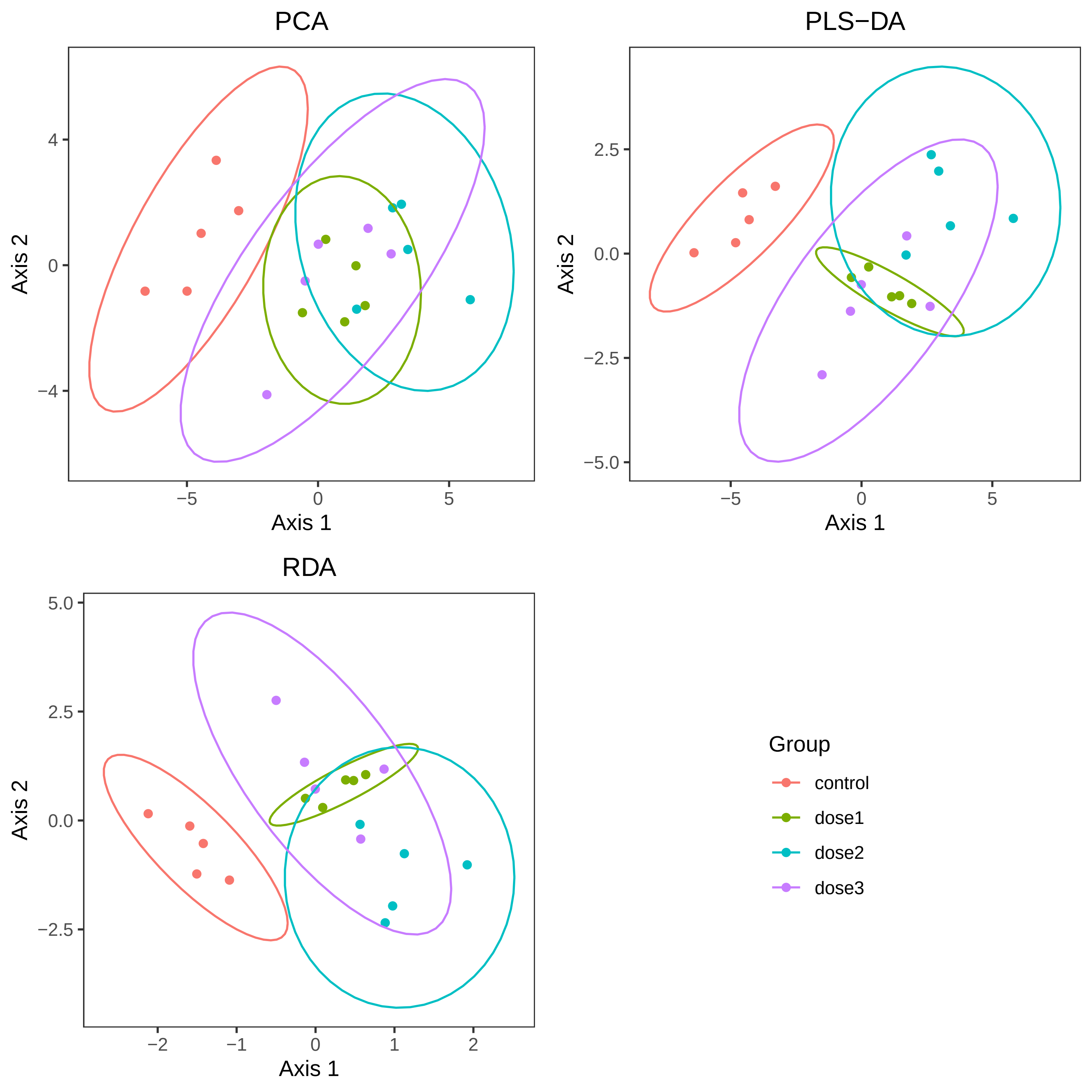

PCA
PLS−DA
RDA
Axis 1
Axis 2
Group
control
dose1
dose2
dose3

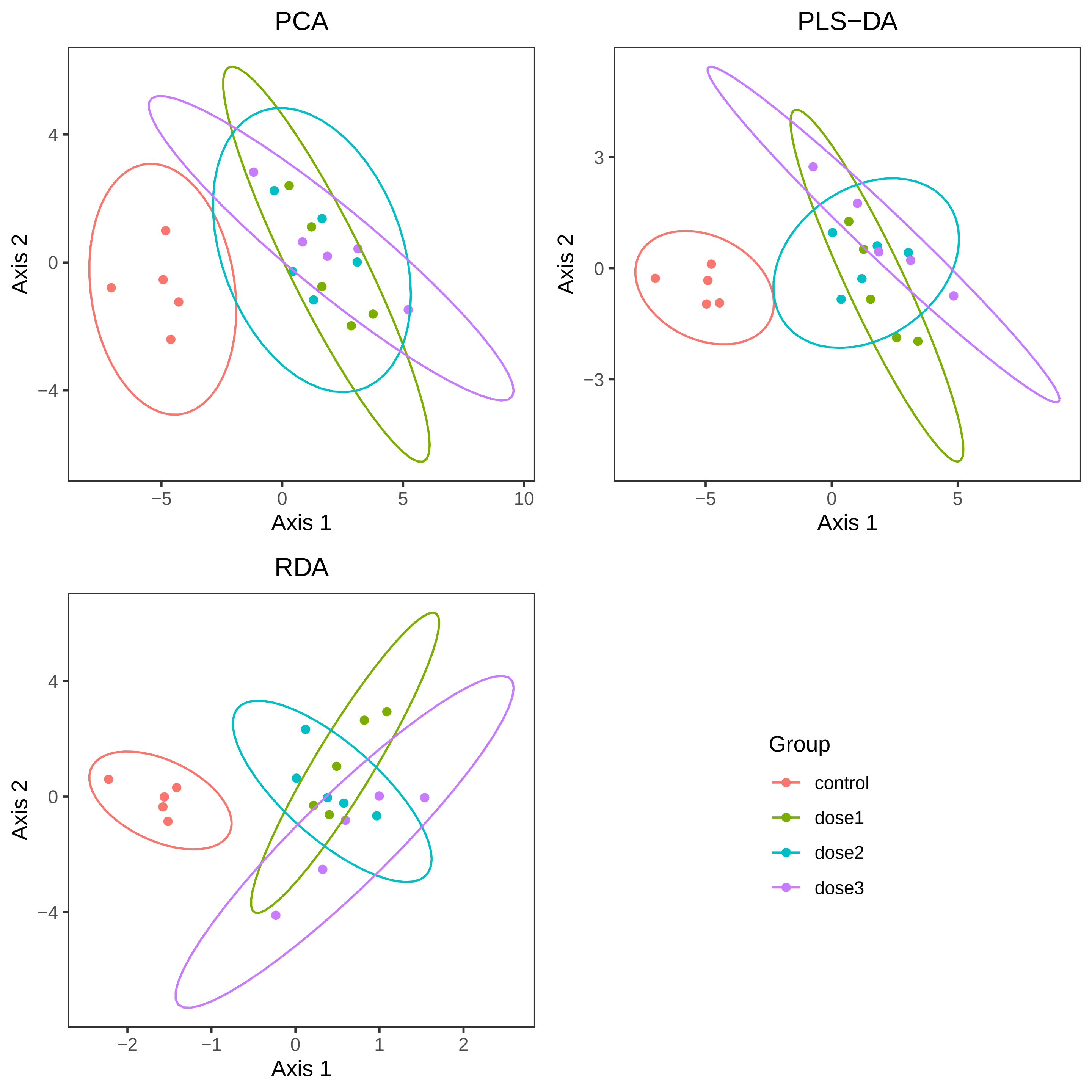
PCA
PLS−DA
RDA
Axis 1
Axis 2
Group
control
dose1
dose2
dose3

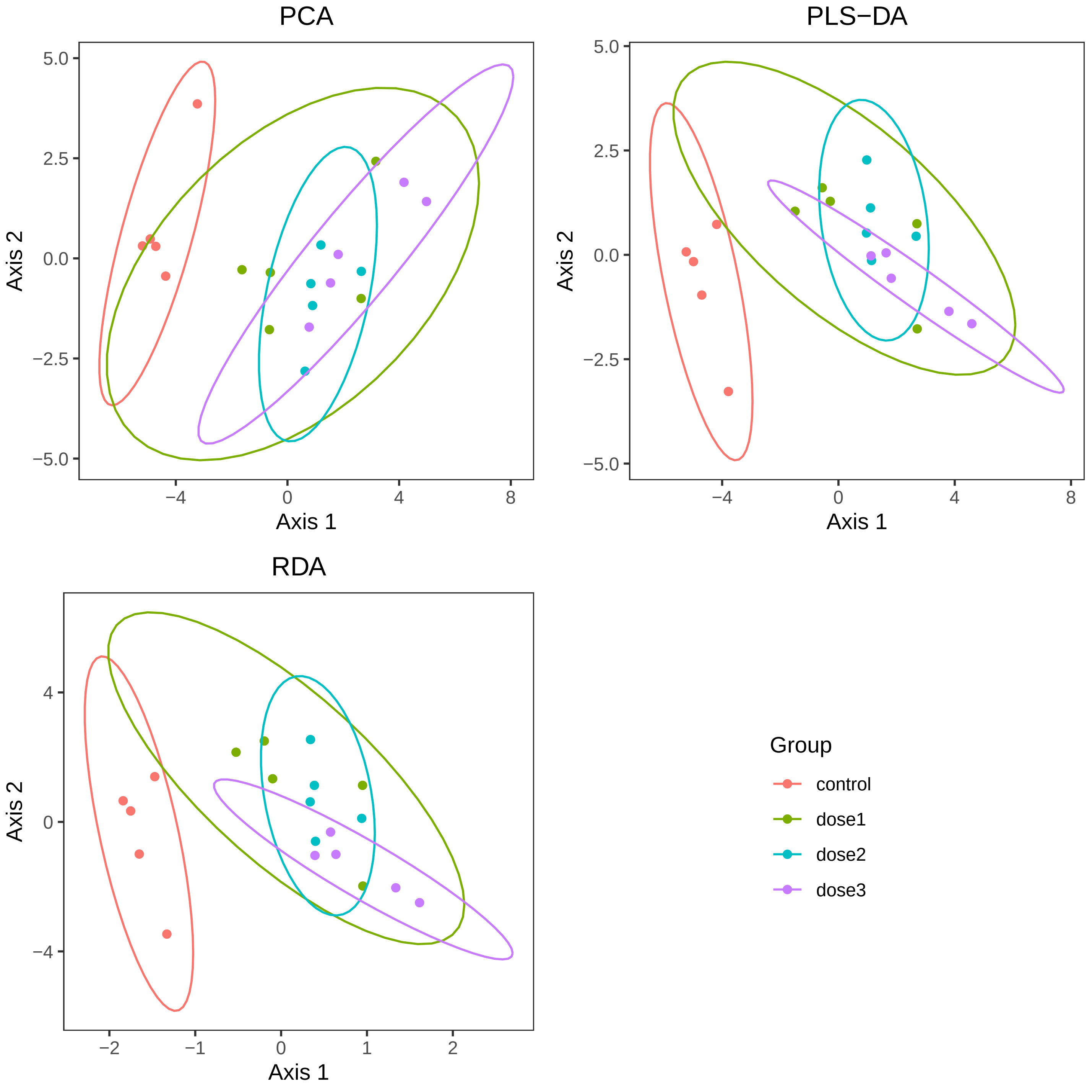

PCA
PLS−DA
RDA
Axis 1
Axis 2
5.0
2.5
0.0
−2.5
−5.0
−4
0
4
8
4
0
−4
−2
−1
1
2
Group
control
dose1
dose2
dose3

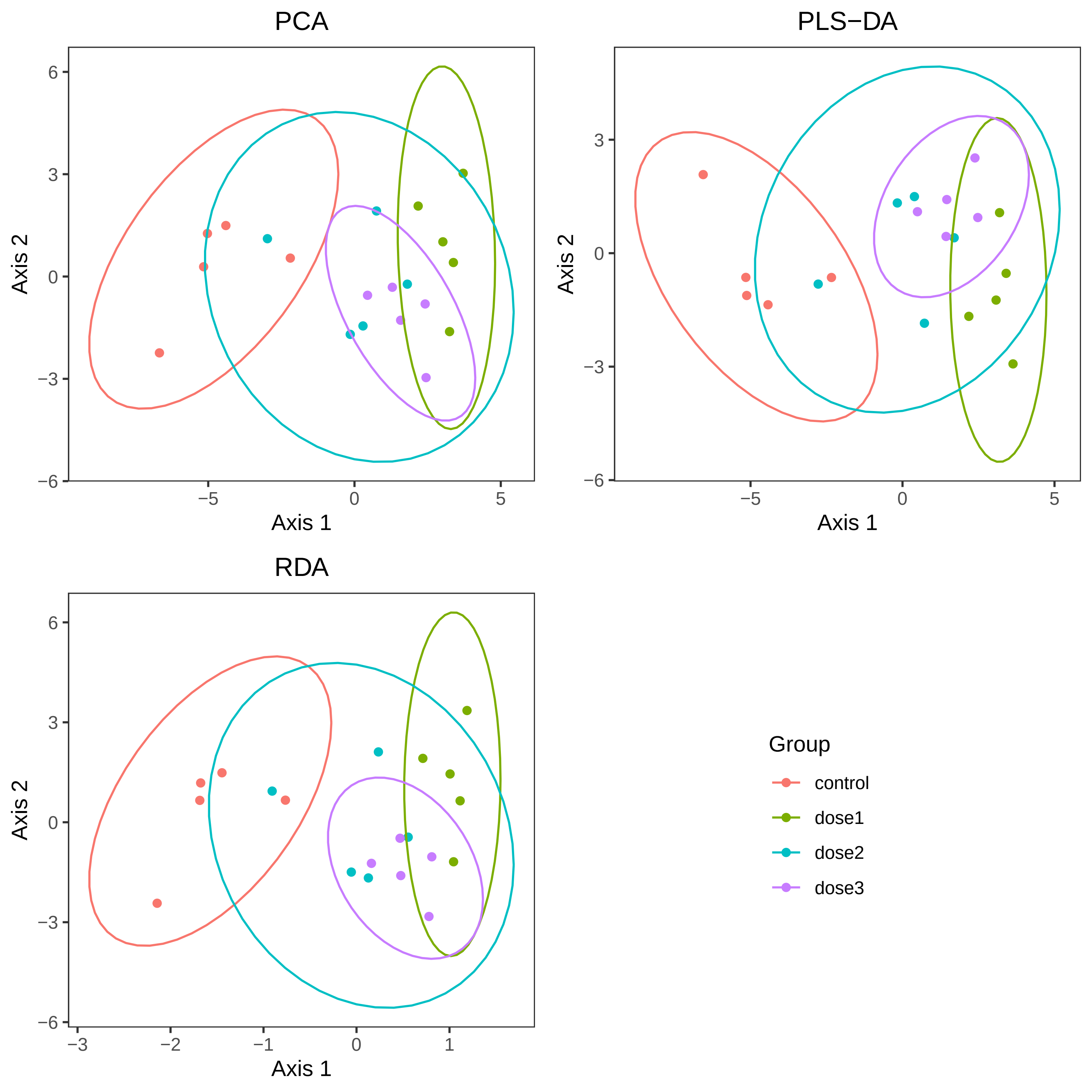
PCA
PLS−DA
RDA
Axis 1
Axis 2
Group
control
dose1
dose2
dose3

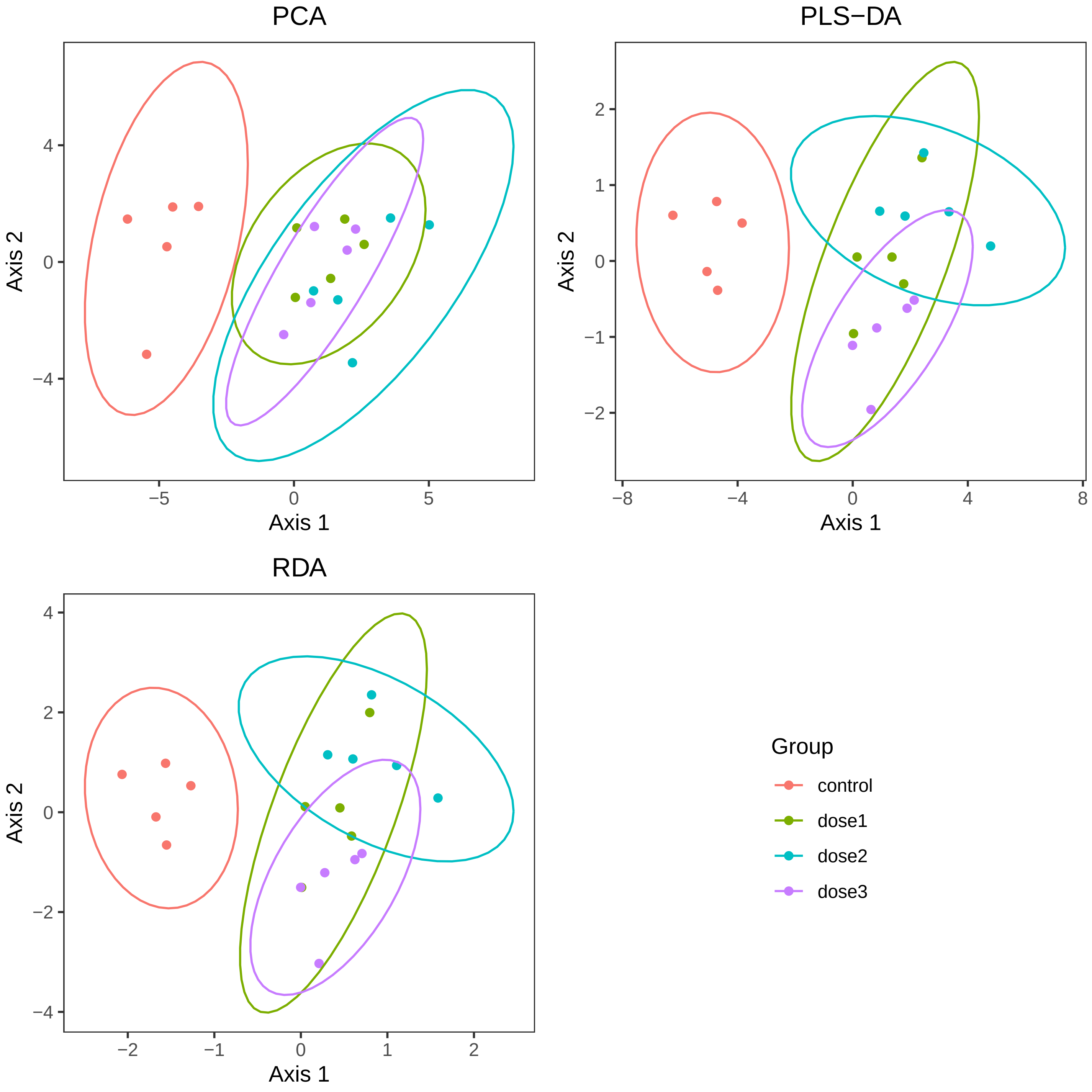

PCA
PLS−DA
RDA
Axis 1
Axis 2
Group
control
dose1
dose2
dose3

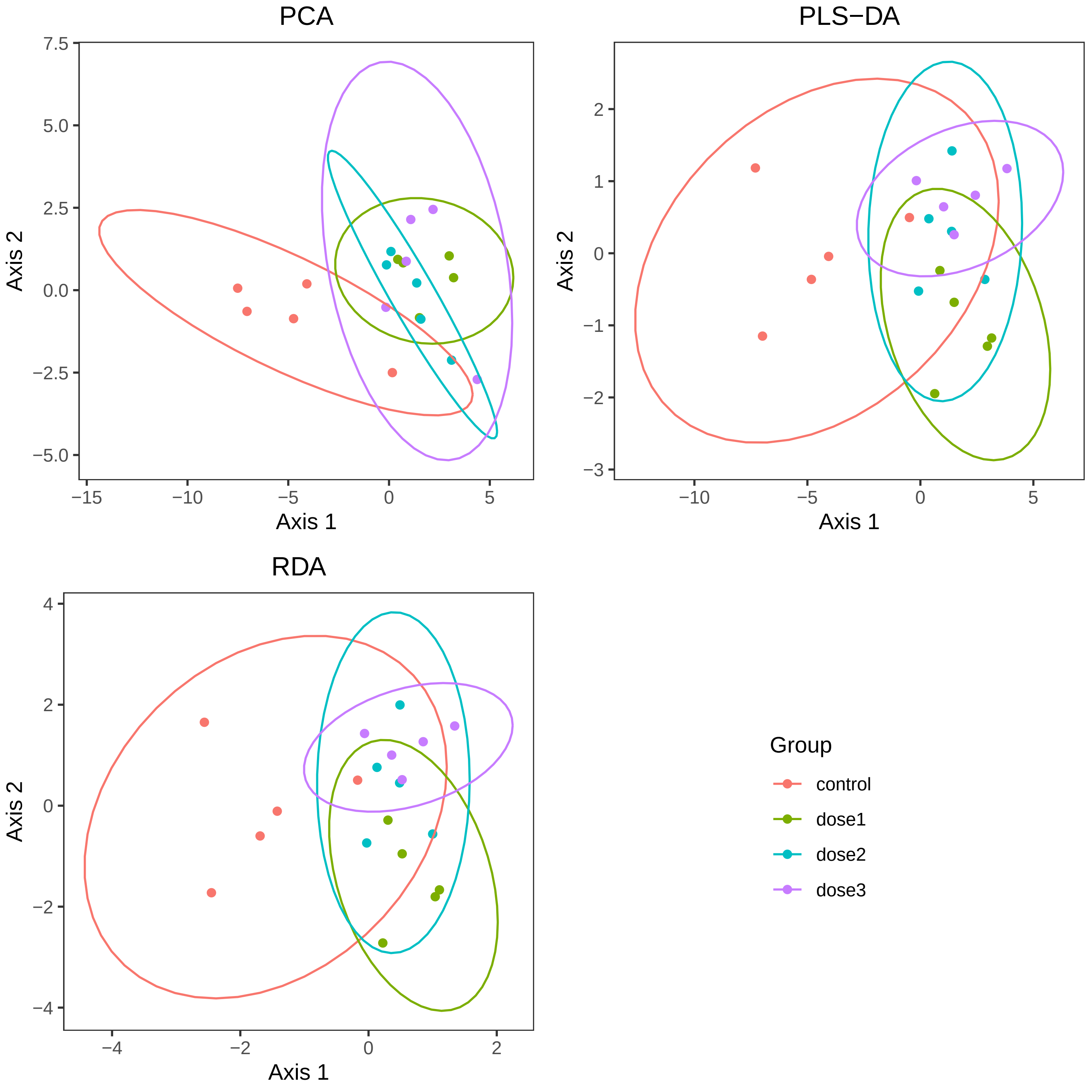
PCA
PLS−DA
RDA
Axis 1
Axis 2
7.5
5.0
2.5
0.0
−2.5
−5.0
−15
−10
−5
0
5
2
1
0
−1
−2
−3
4
−4
Group
control
dose1
dose2
dose3

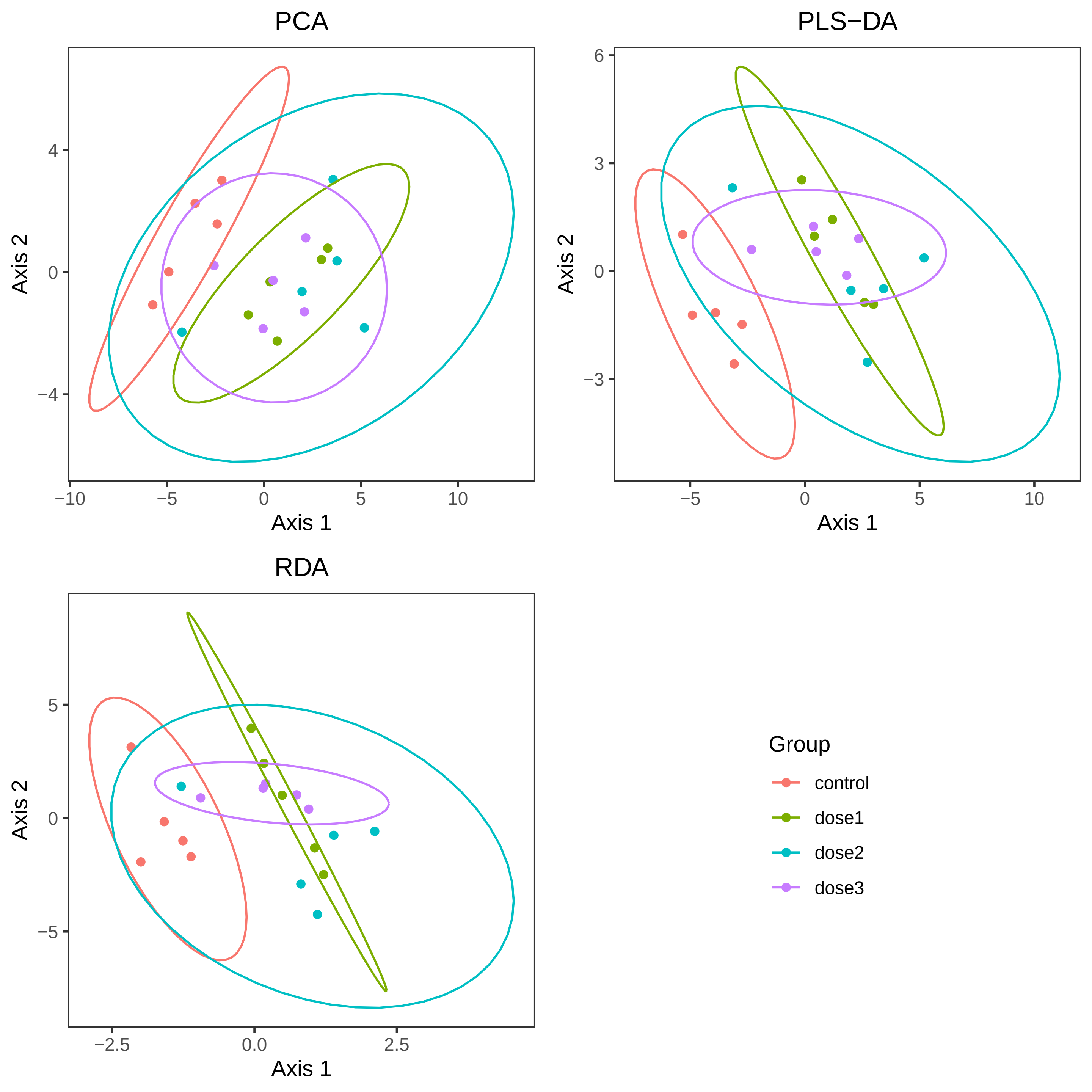
PCA
PLS−DA
RDA
Axis 1
Axis 2
Group
control
dose1
dose2
dose3

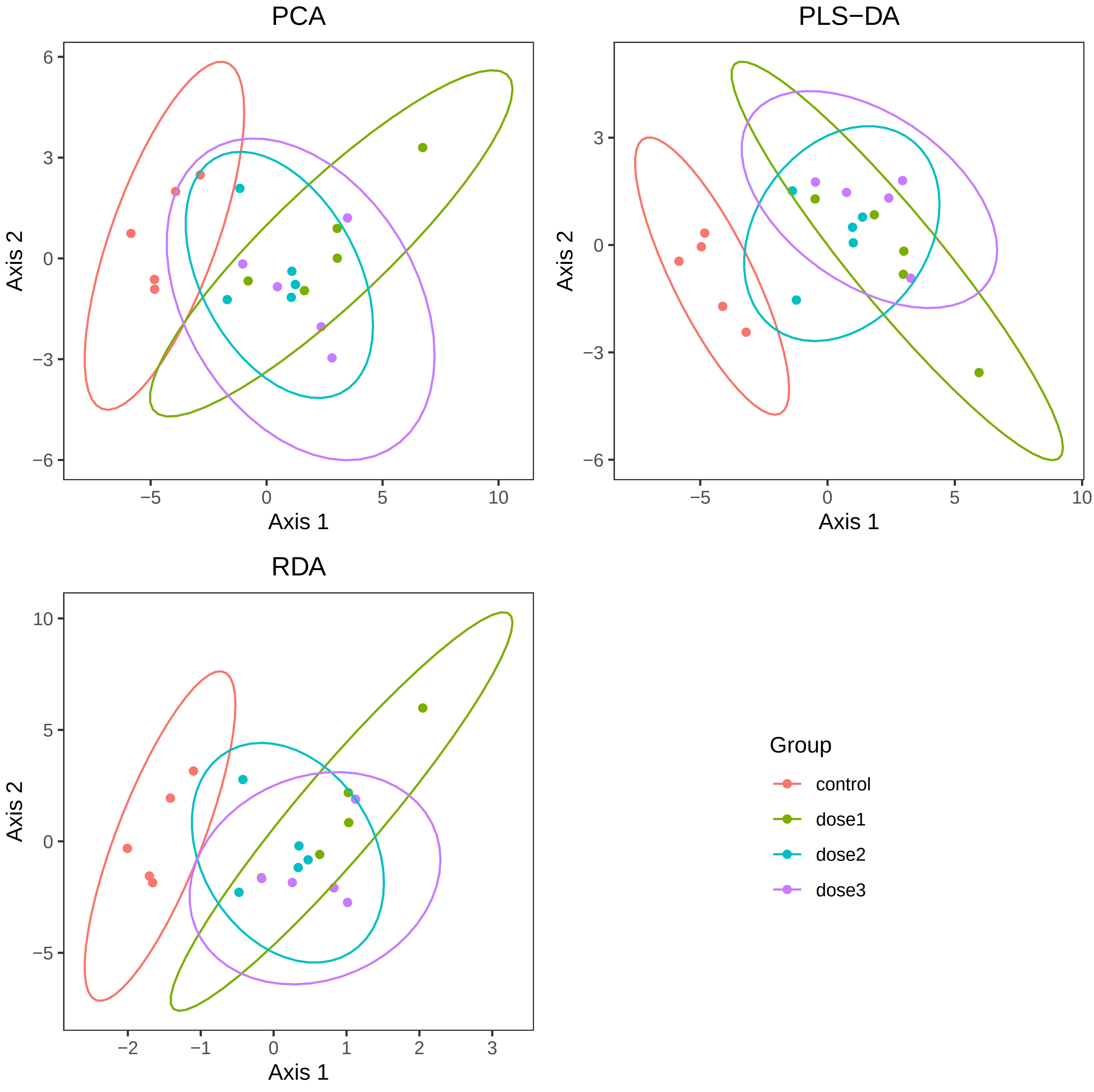
PCA
PLS−DA
RDA
Axis 1
Axis 2
6
3
0
−3
−6
−5
0
5
10
10
5
0
−5
−2
−1
0
1
2
3
Group
control
dose1
dose2
dose3

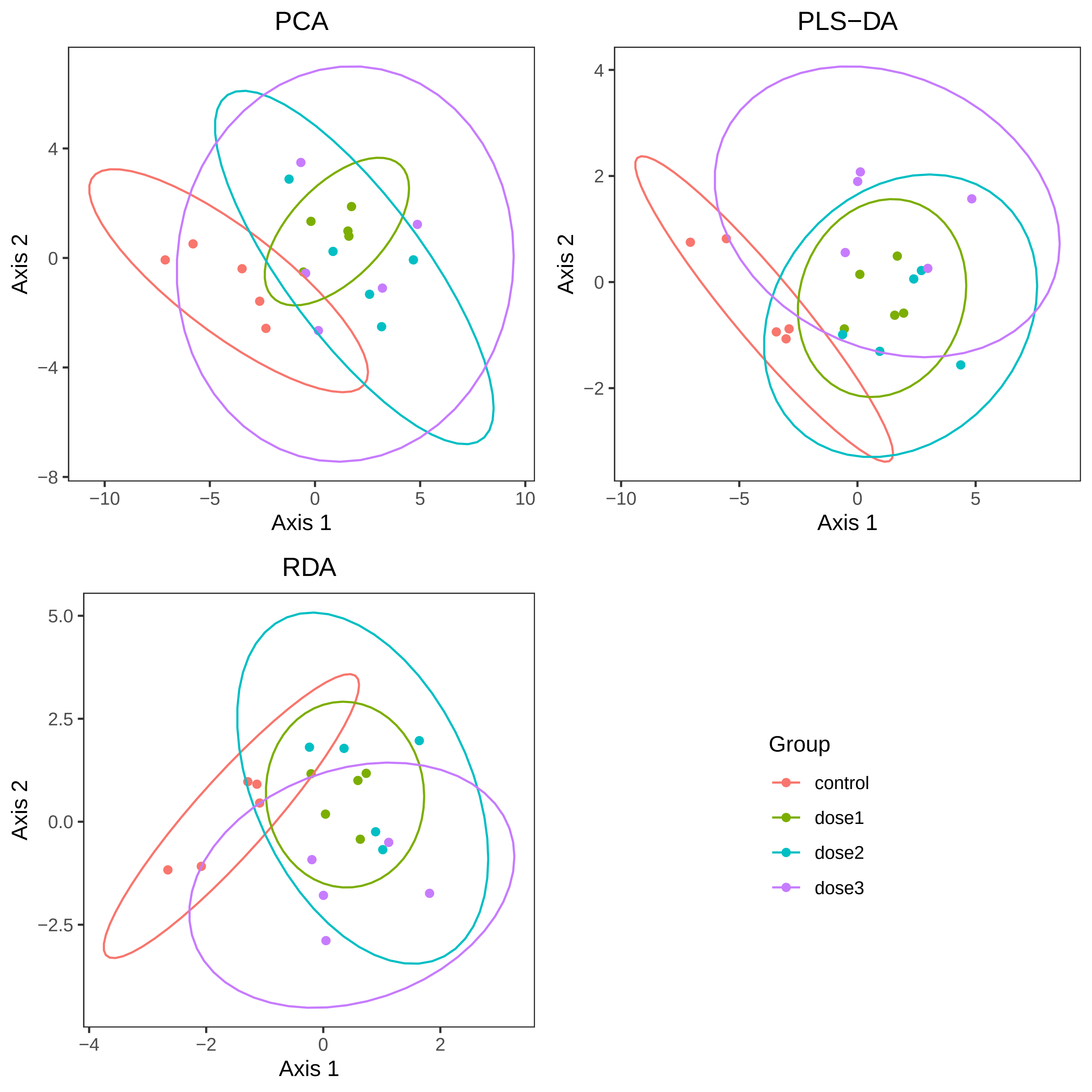

PCA
PLS−DA
RDA
Axis 1
Axis 2
Group
control
dose1
dose2
dose3

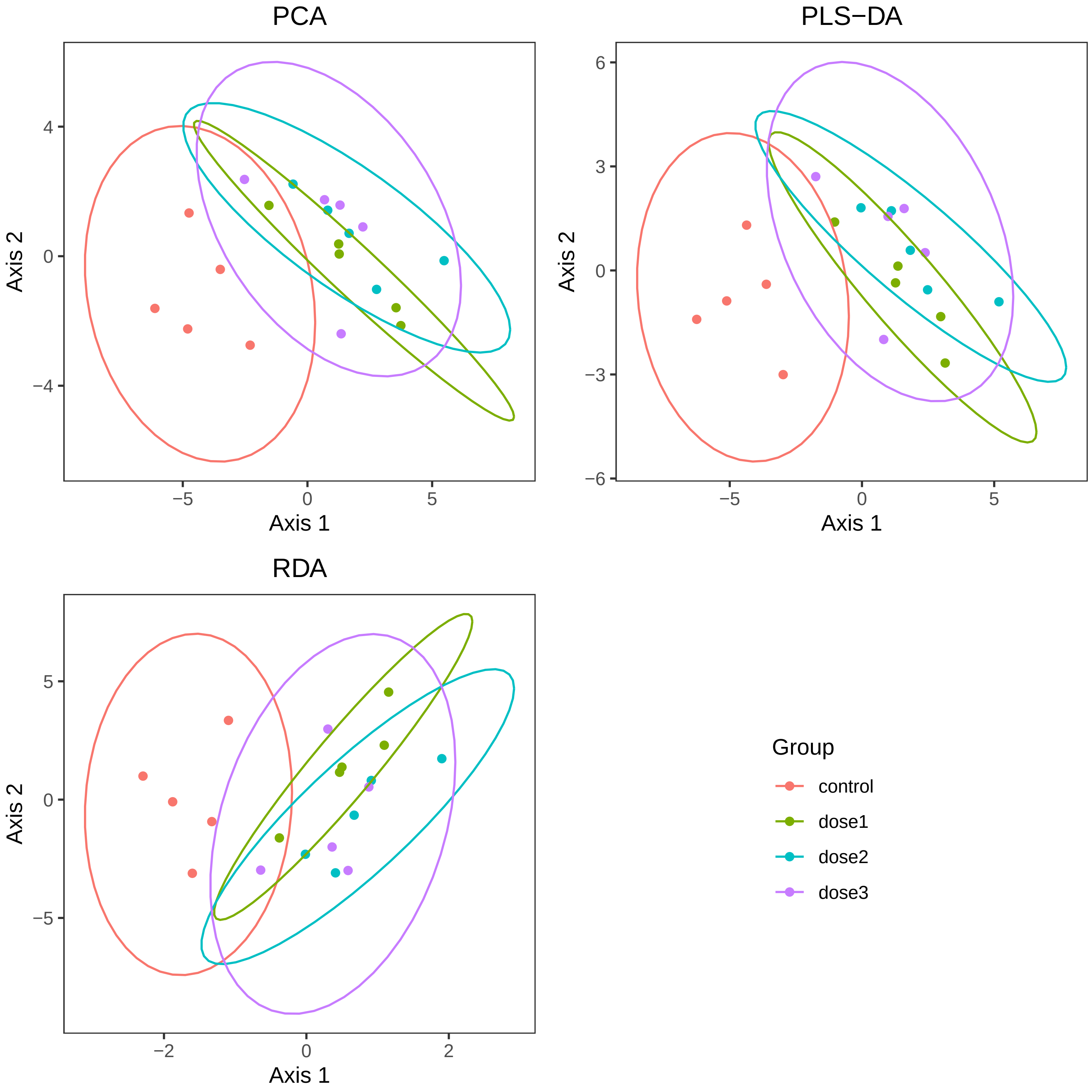
PCA
PLS−DA
RDA
Axis 1
Axis 2
4
0
−4
−5
0
5
6
3
0
−3
−6
5
0
−5
−2
0
2
Group
control
dose1
dose2
dose3

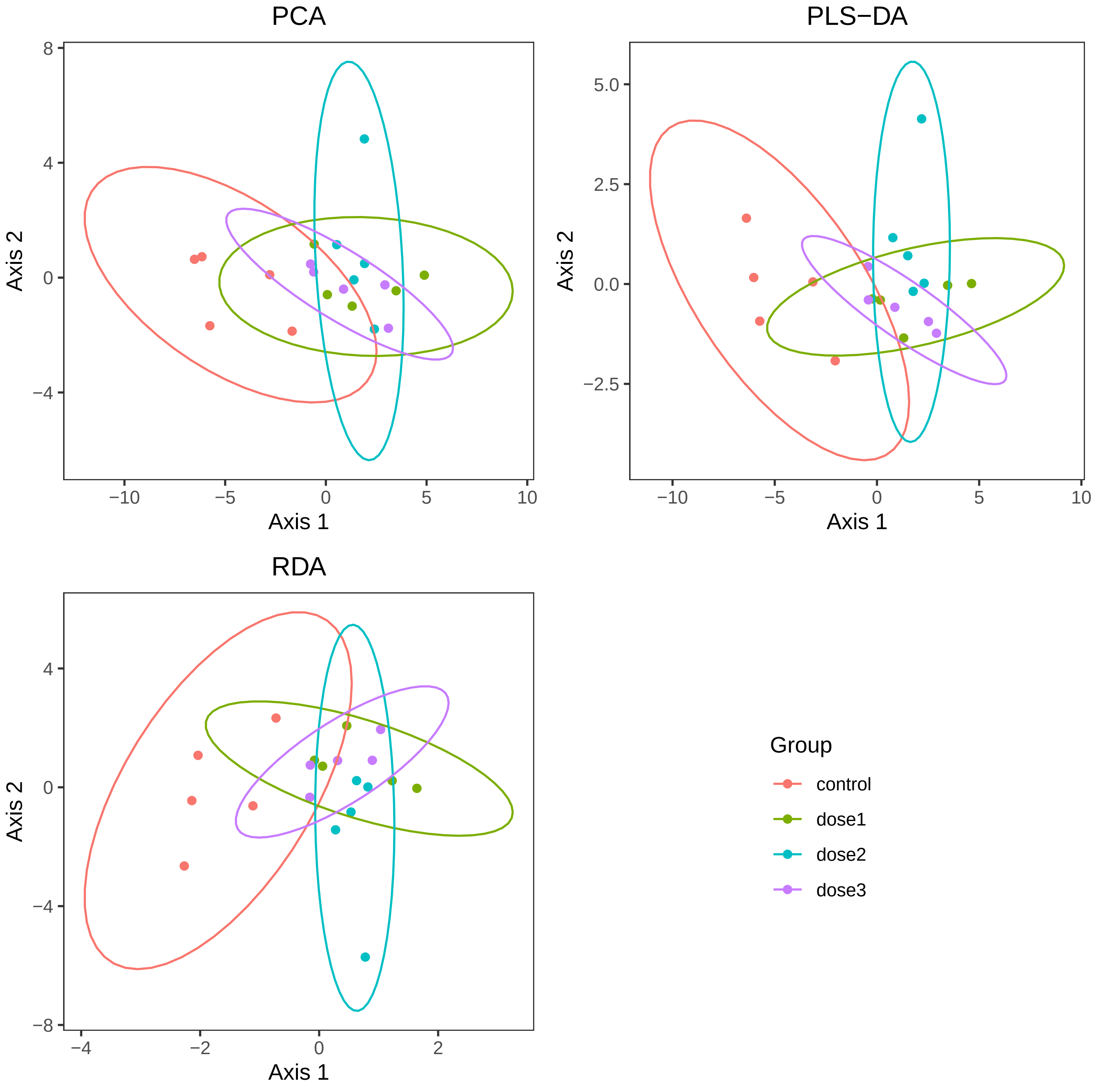

PCA
PLS−DA
RDA
Axis 1
Axis 2
8
4
0
−4
5.0
2.5
0.0
−2.5
−8
−10
−5
0
5
10
−4
−2
2
Group
control
dose1
dose2
dose3

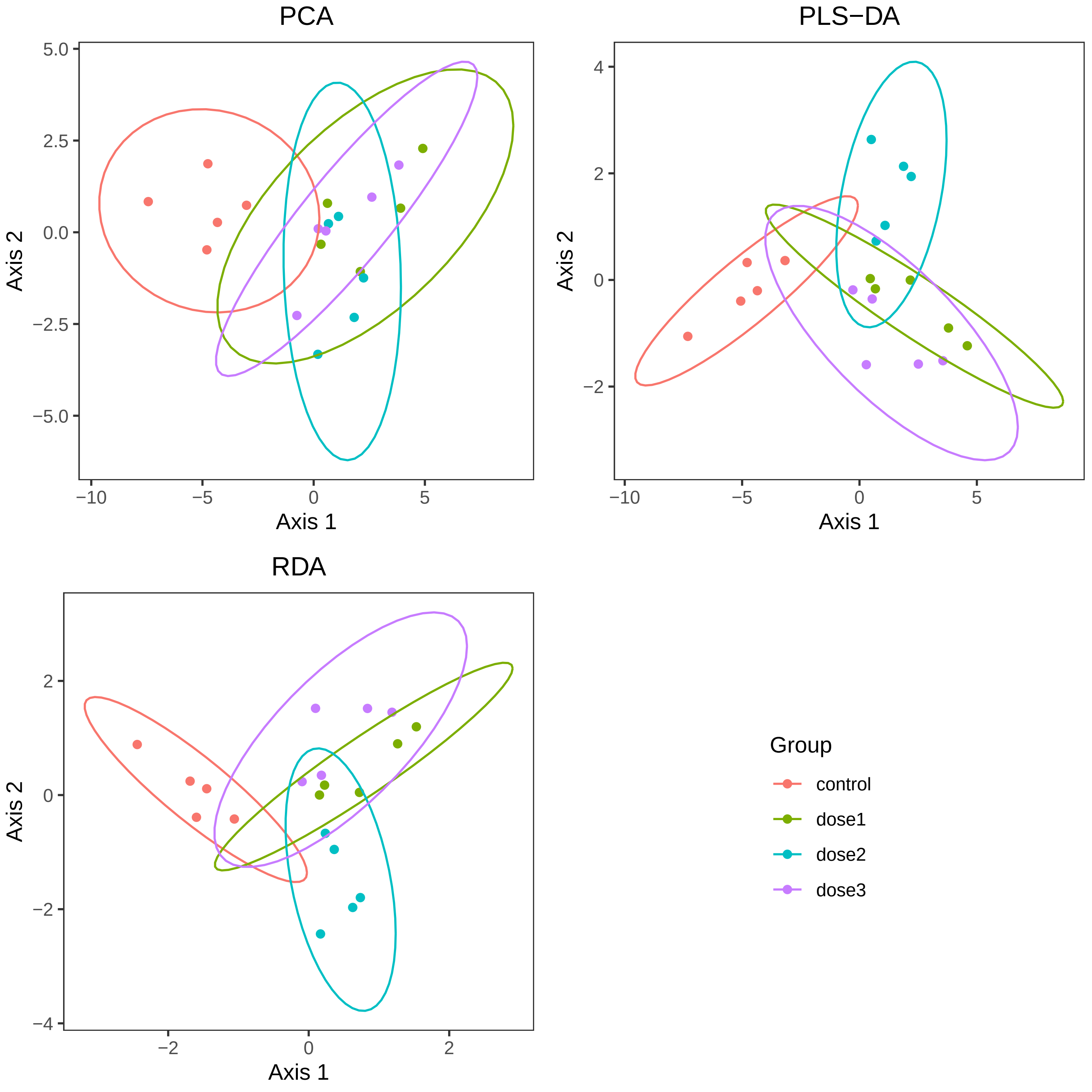

PCA
PLS−DA
RDA
Axis 1
Axis 2
Group
control
dose1
dose2
dose3

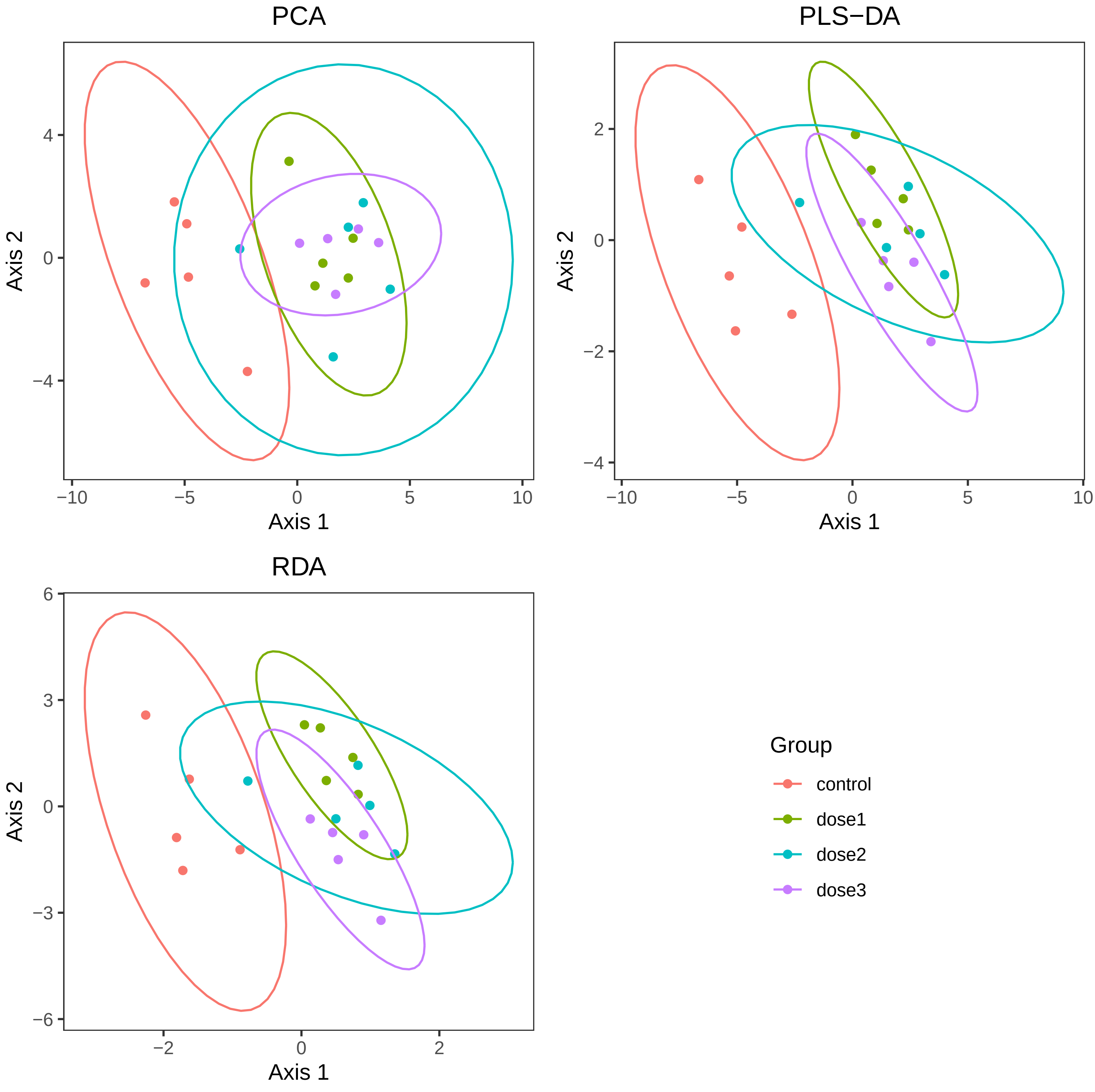
PCA
PLS−DA
RDA
Axis 1
Axis 2
Group
control
dose1
dose2
dose3

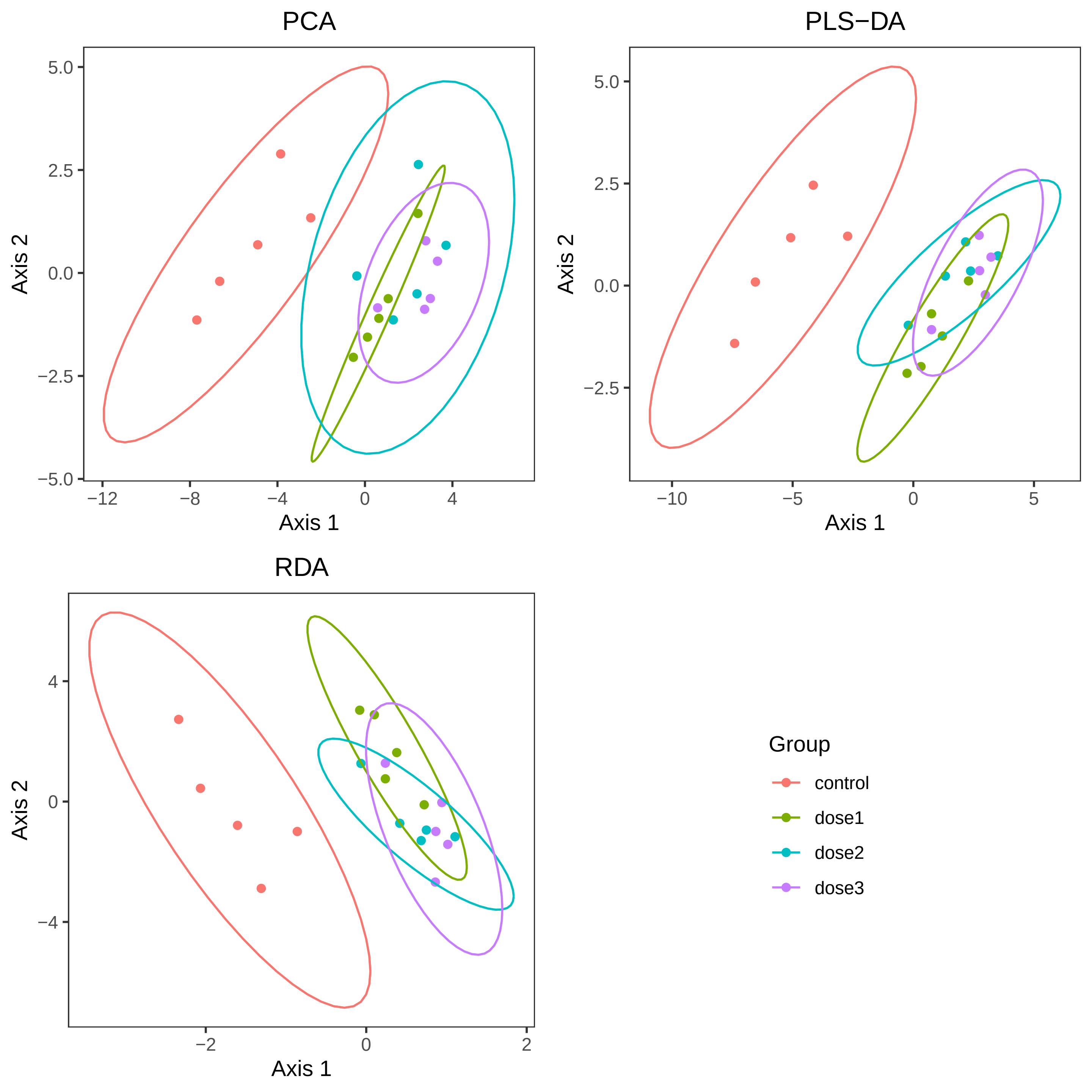

PCA
PLS−DA
RDA
Axis 1
Axis 2
5.0
2.5
0.0
−2.5
−5.0
−12
−8
−4
0
4
−10
−5
5
−2
2
−4
Group
control
dose1
dose2
dose3

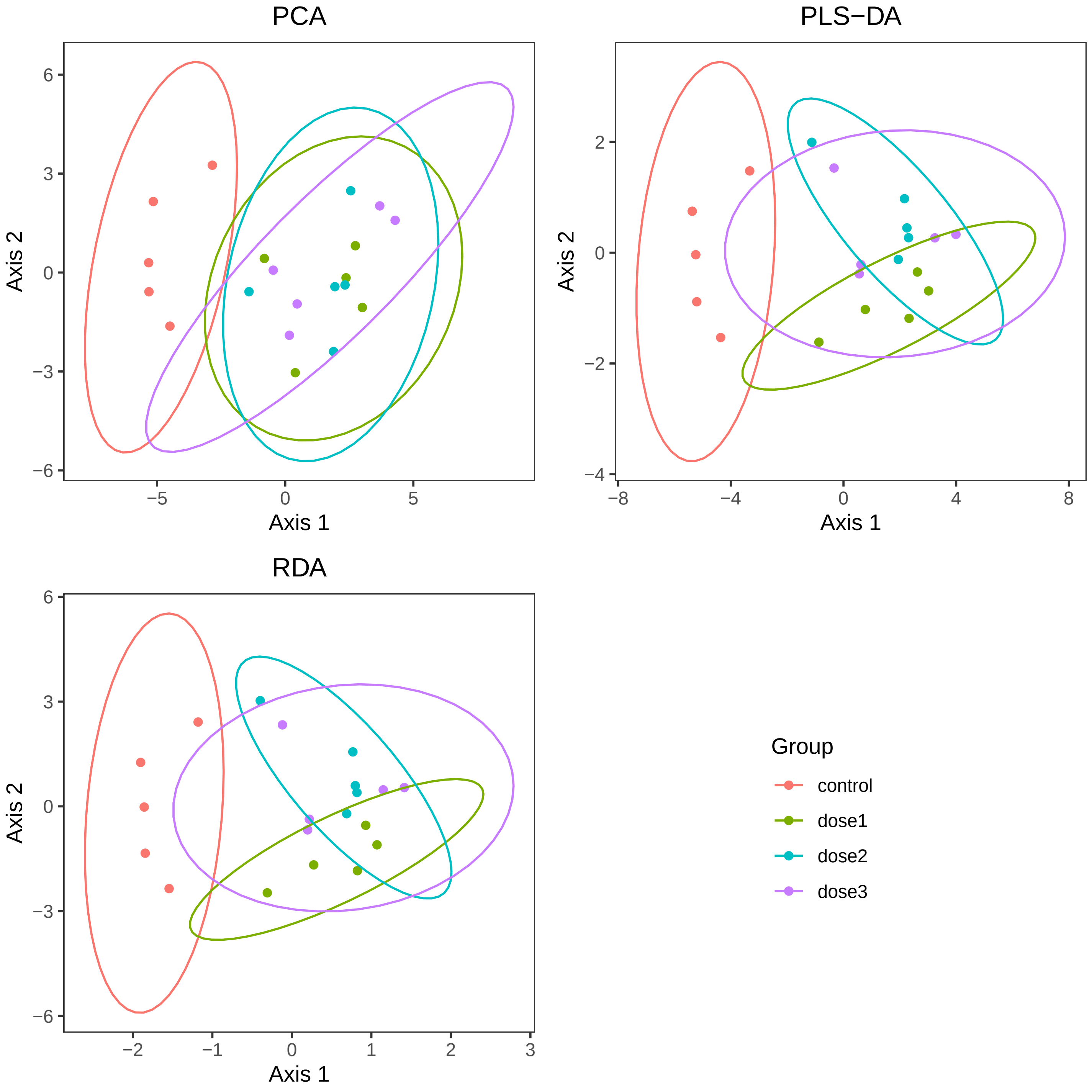

PCA
PLS−DA
RDA
Axis 1
Axis 2
Group
control
dose1
dose2
dose3

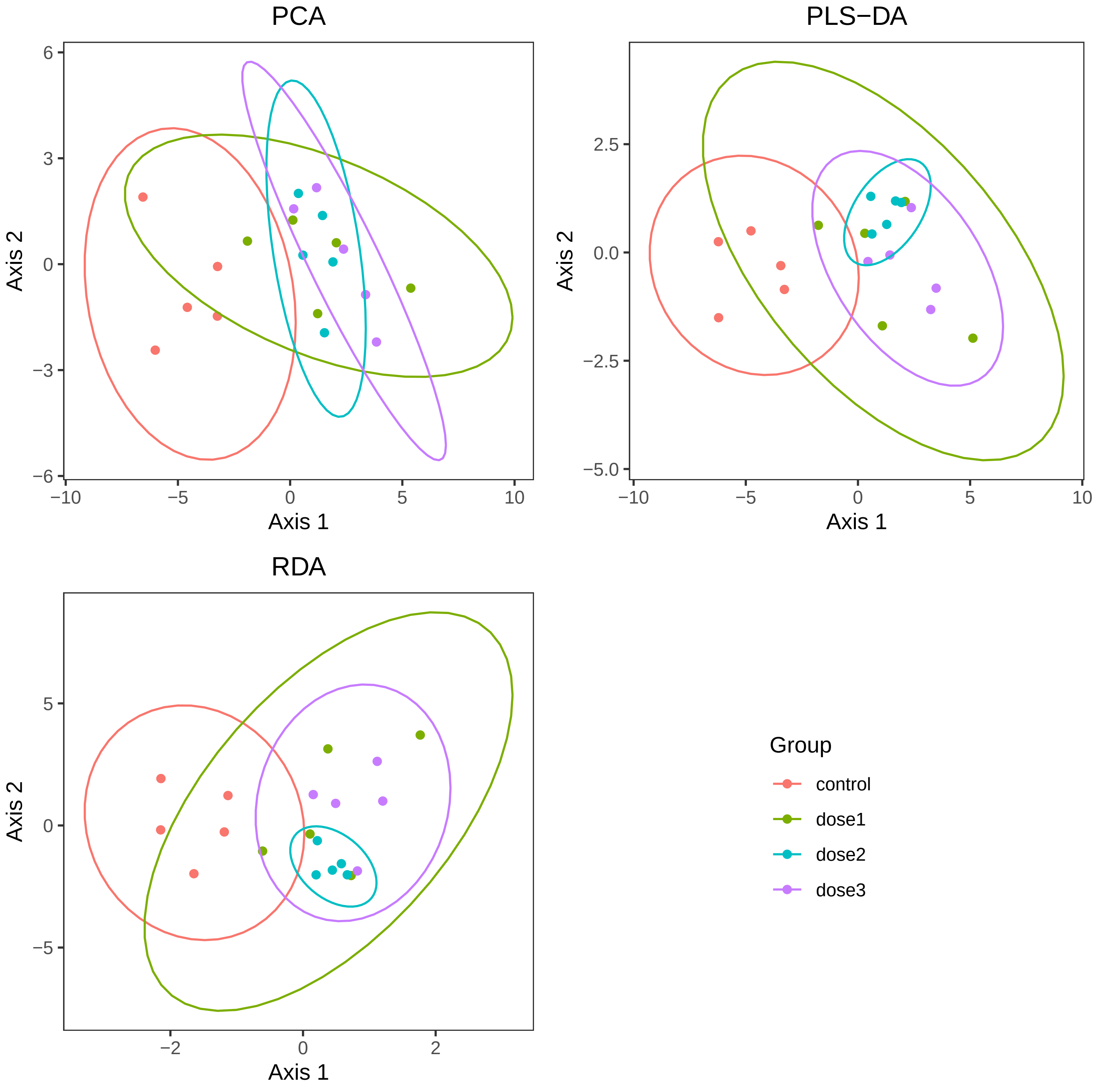

PCA
PLS−DA
RDA
Axis 1
Axis 2
Group
control
dose1
dose2
dose3

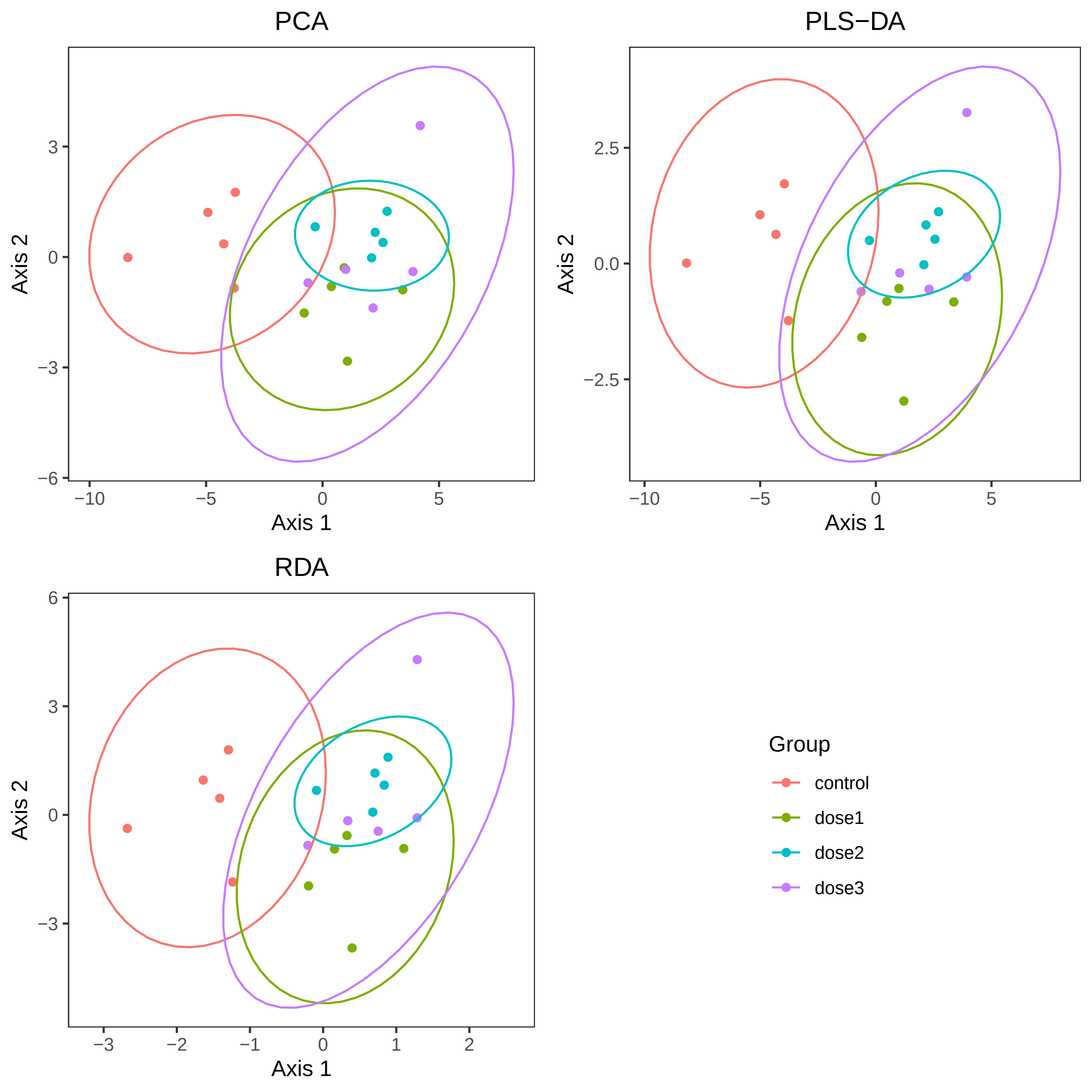
PCA
PLS−DA
RDA
Axis 1
Axis 2
Group
control
dose1
dose2
dose3

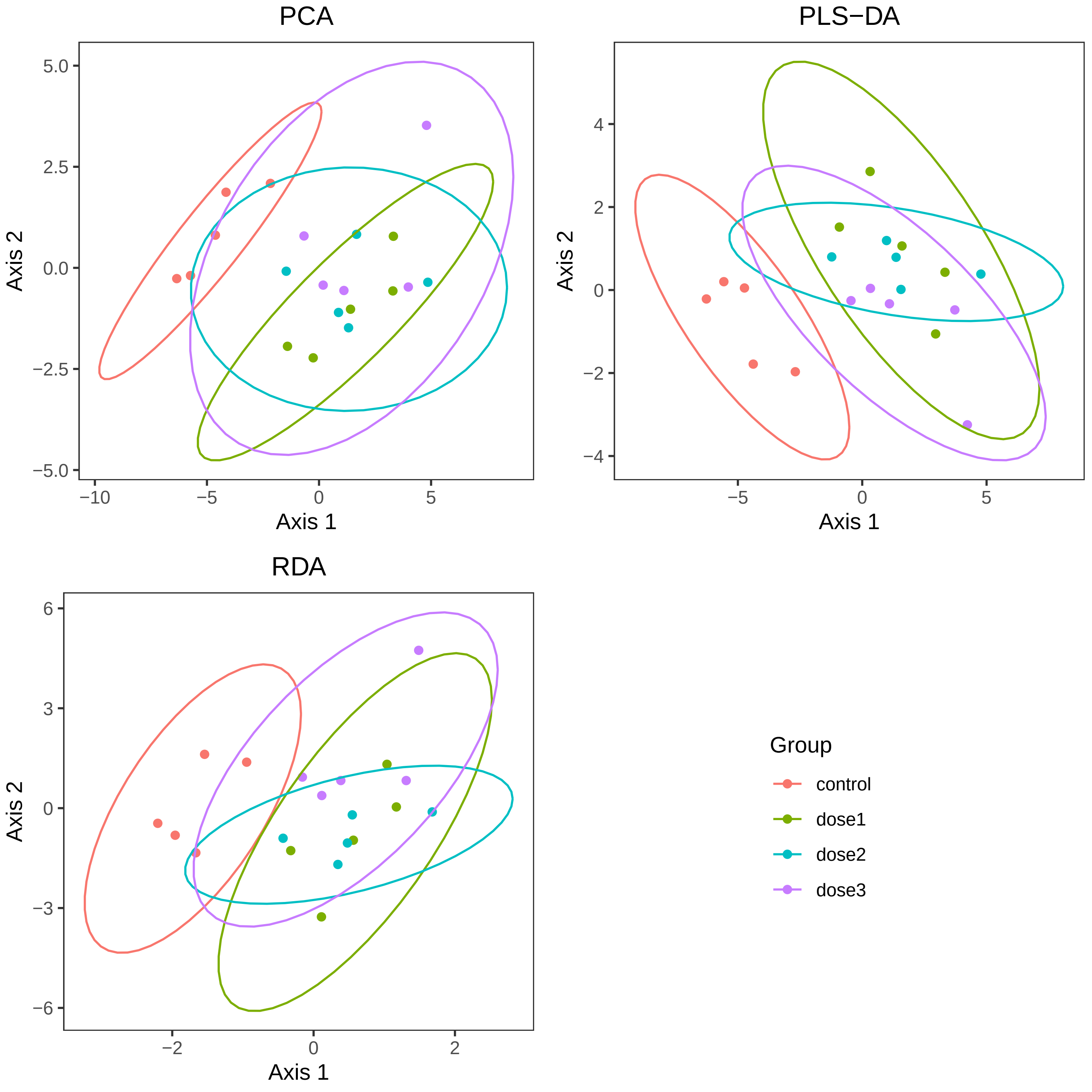
PCA
PLS−DA
RDA
Axis 1
Axis 2
5.0
2.5
0.0
−2.5
−5.0
−10
−5
0
5
4
2
0
−2
−4
6
3
−3
−6
Group
control
dose1
dose2
dose3

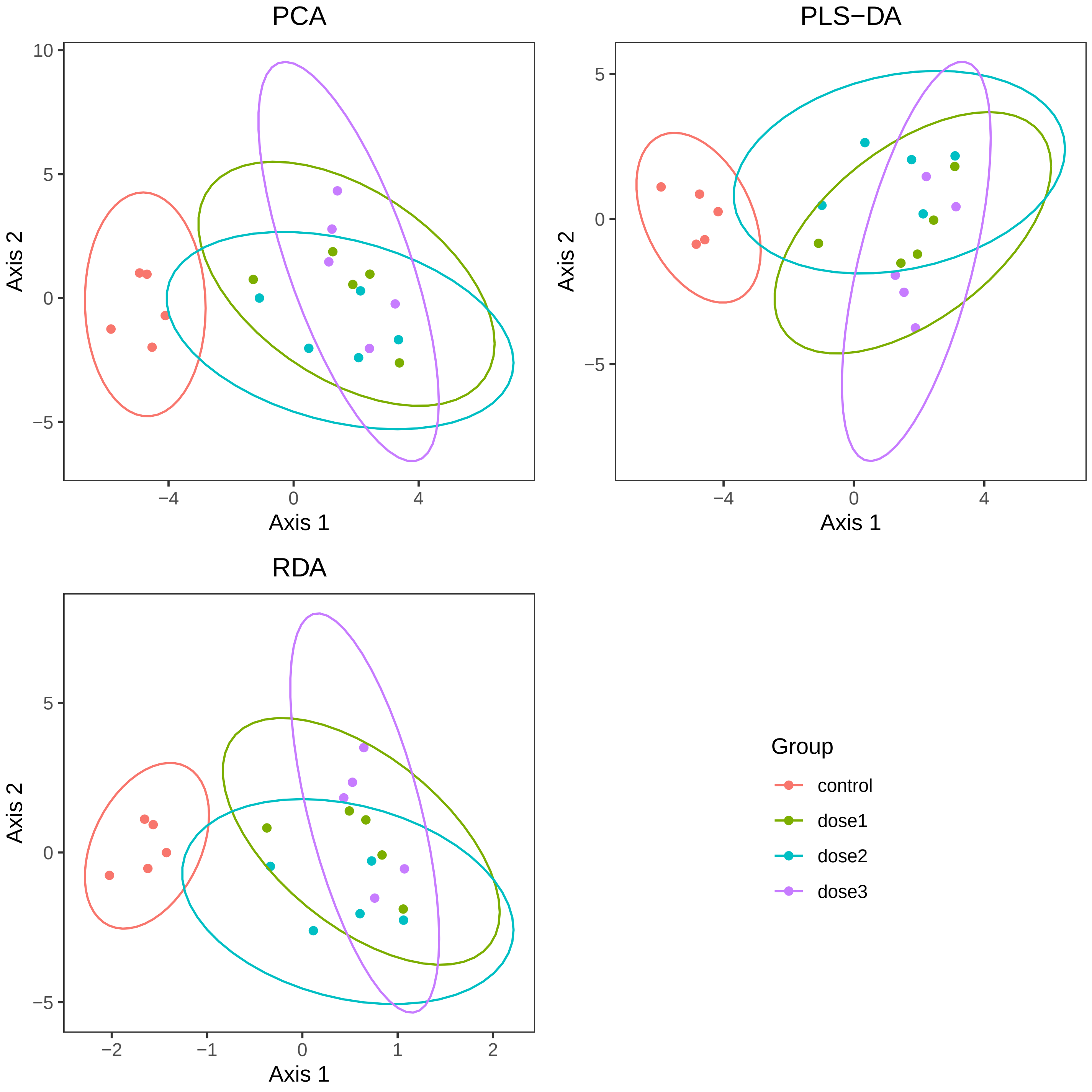
PCA
PLS−DA
RDA
Axis 1
Axis 2
Group
control
dose1
dose2
dose3

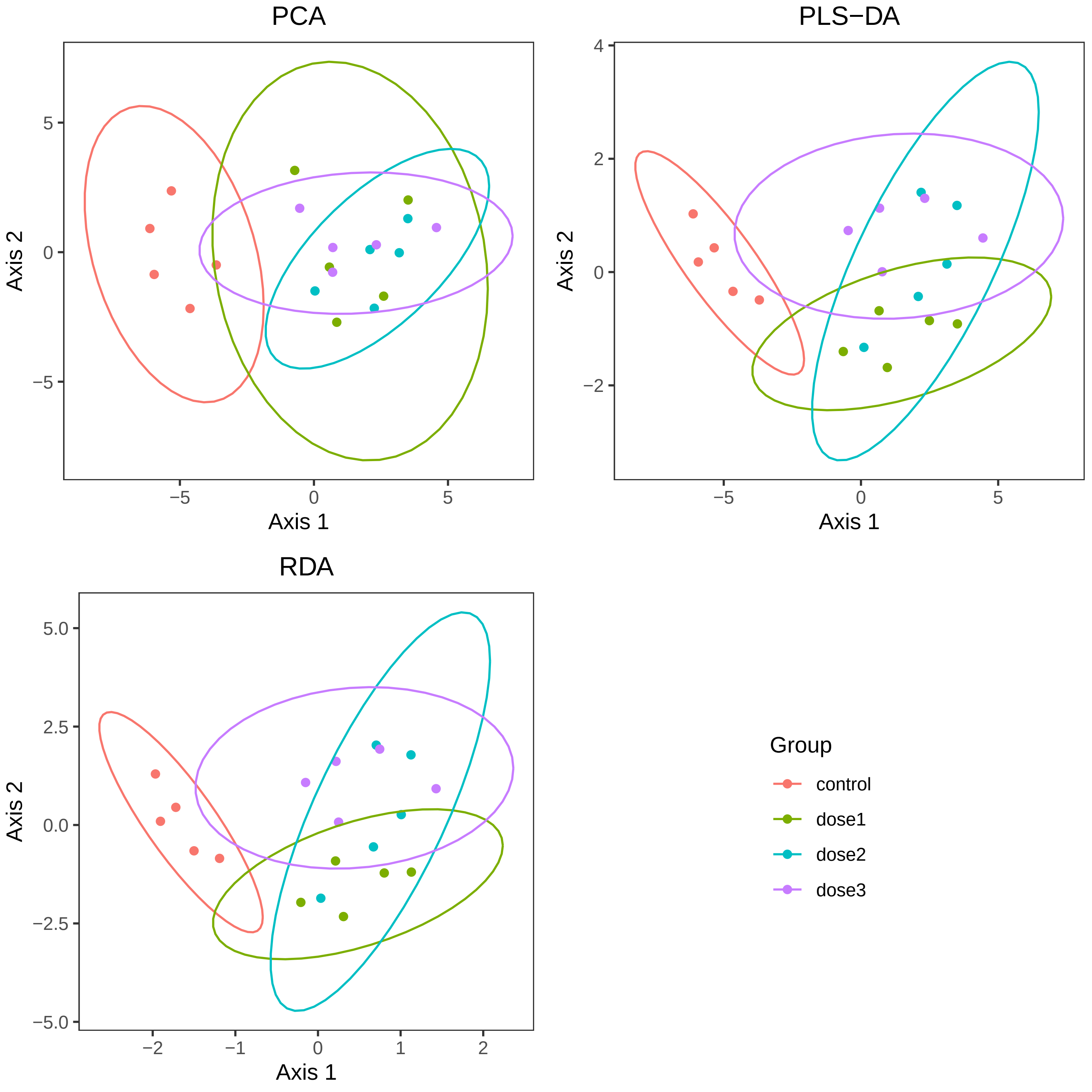
PCA
PLS−DA
RDA
Axis 1
Axis 2
Group
control
dose1
dose2
dose3

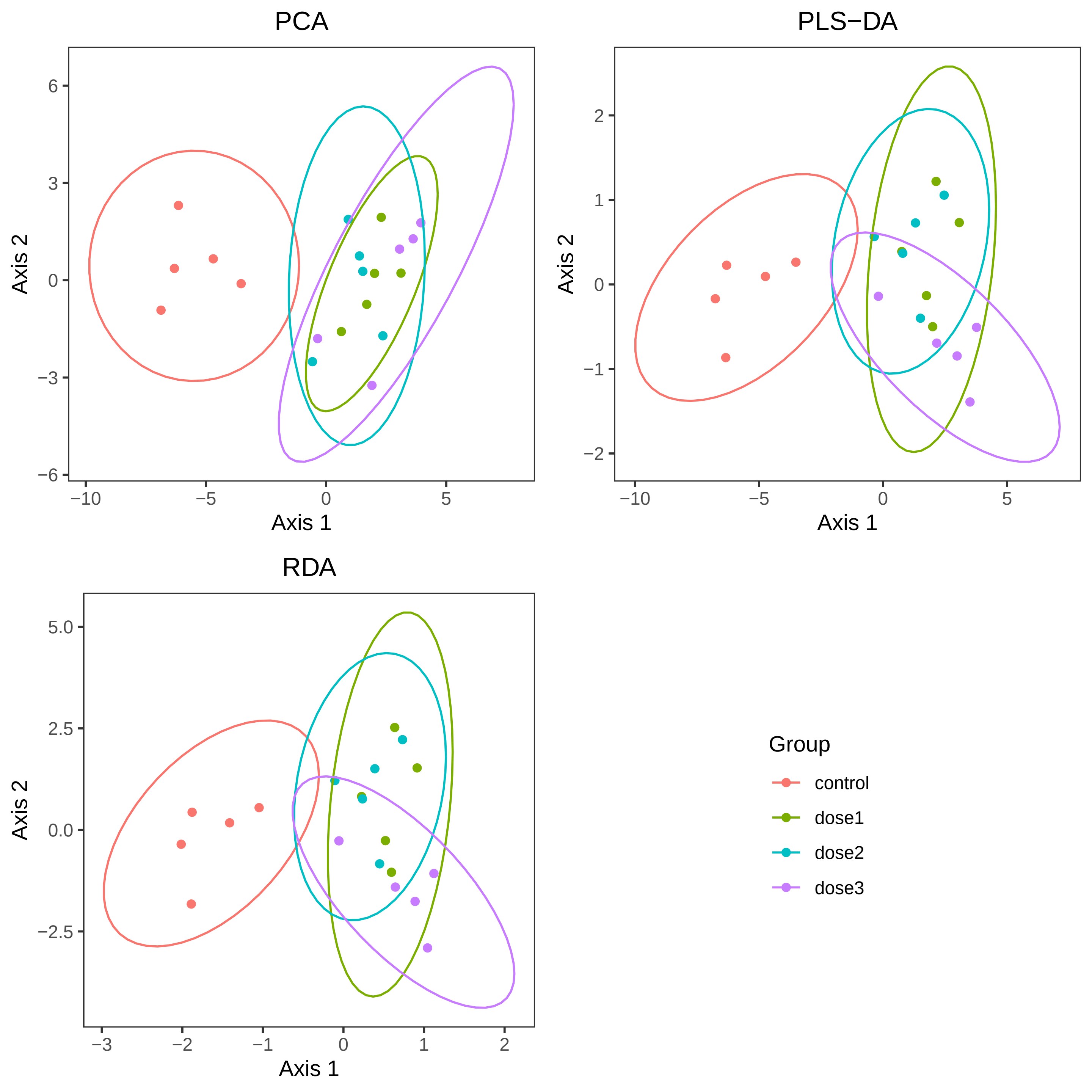

PCA
PLS−DA
RDA
Axis 1
Axis 2
Group
control
dose1
dose2
dose3

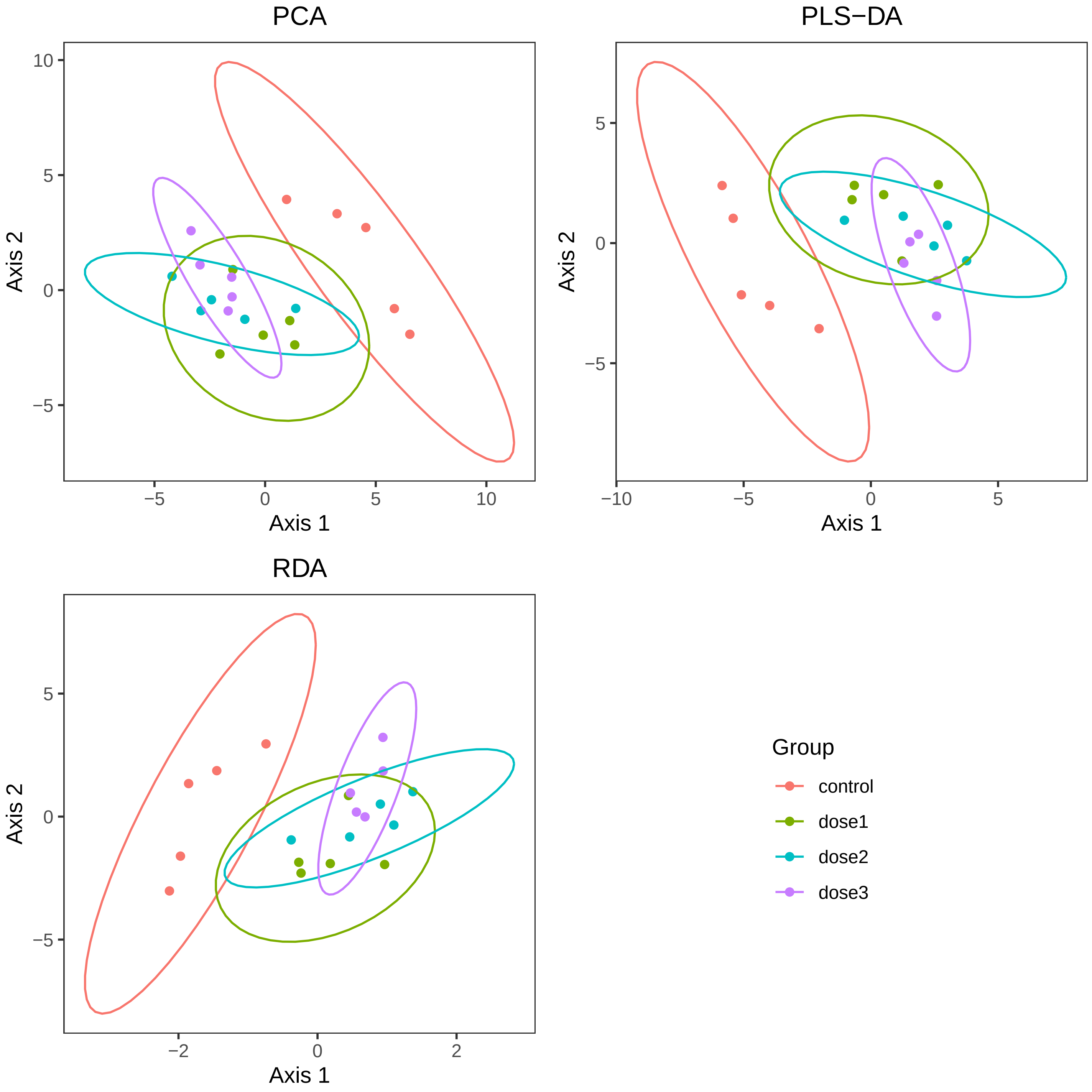

PCA
PLS−DA
RDA
Axis 1
Axis 2
Group
control
dose1
dose2
dose3

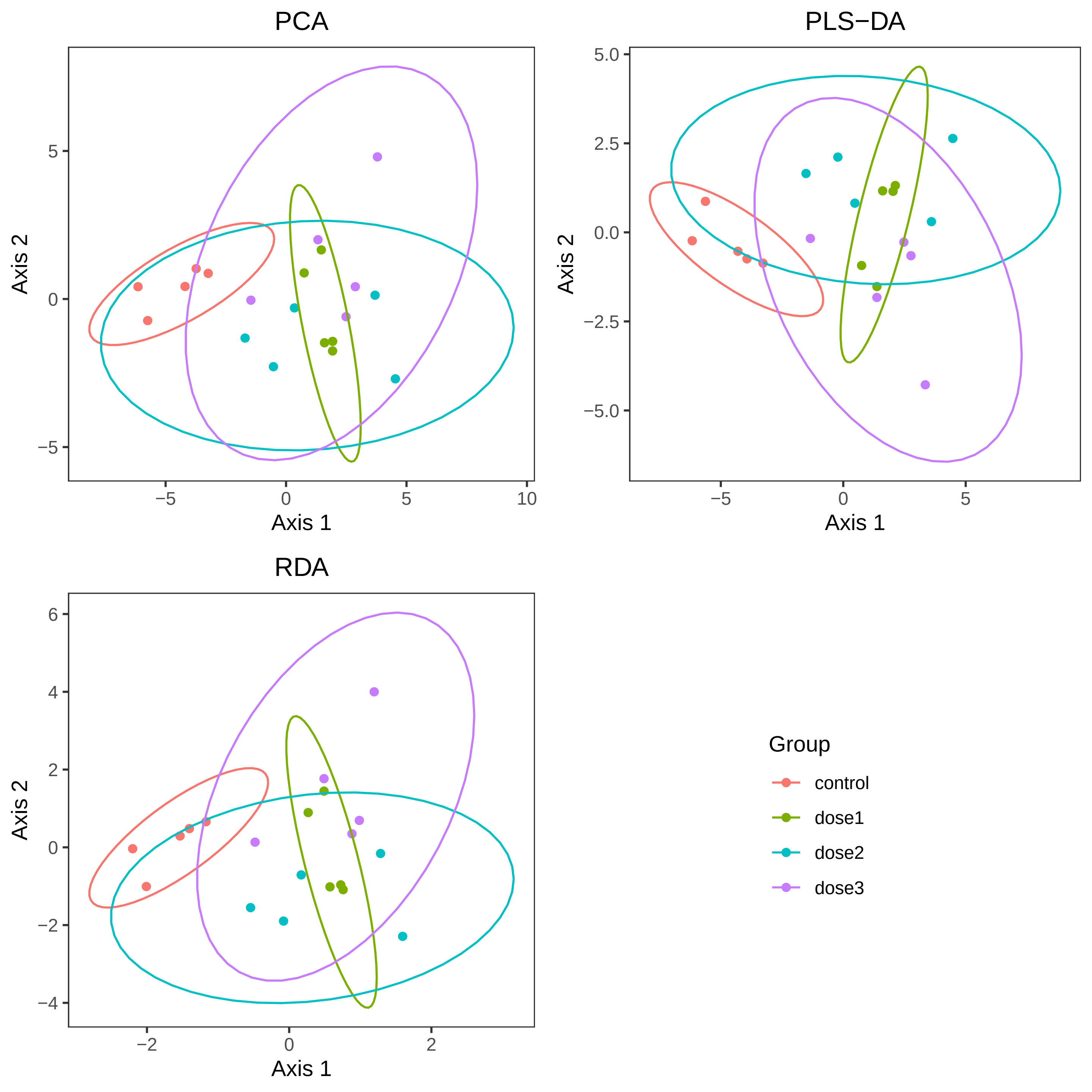
PCA
PLS−DA
RDA
Axis 1
Axis 2
Group
control
dose1
dose2
dose3

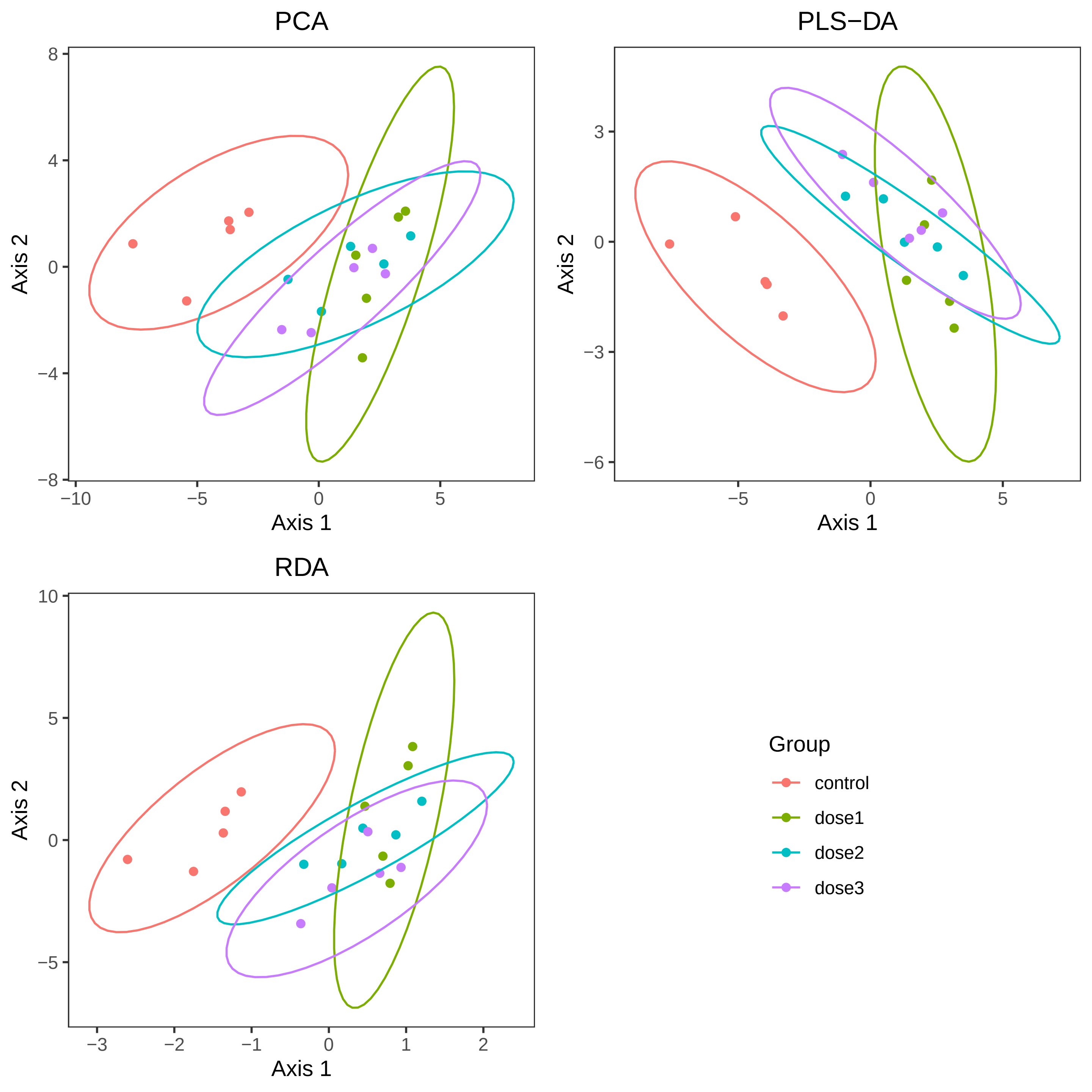

PCA
PLS−DA
RDA
Axis 1
Axis 2
Group
control
dose1
dose2
dose3

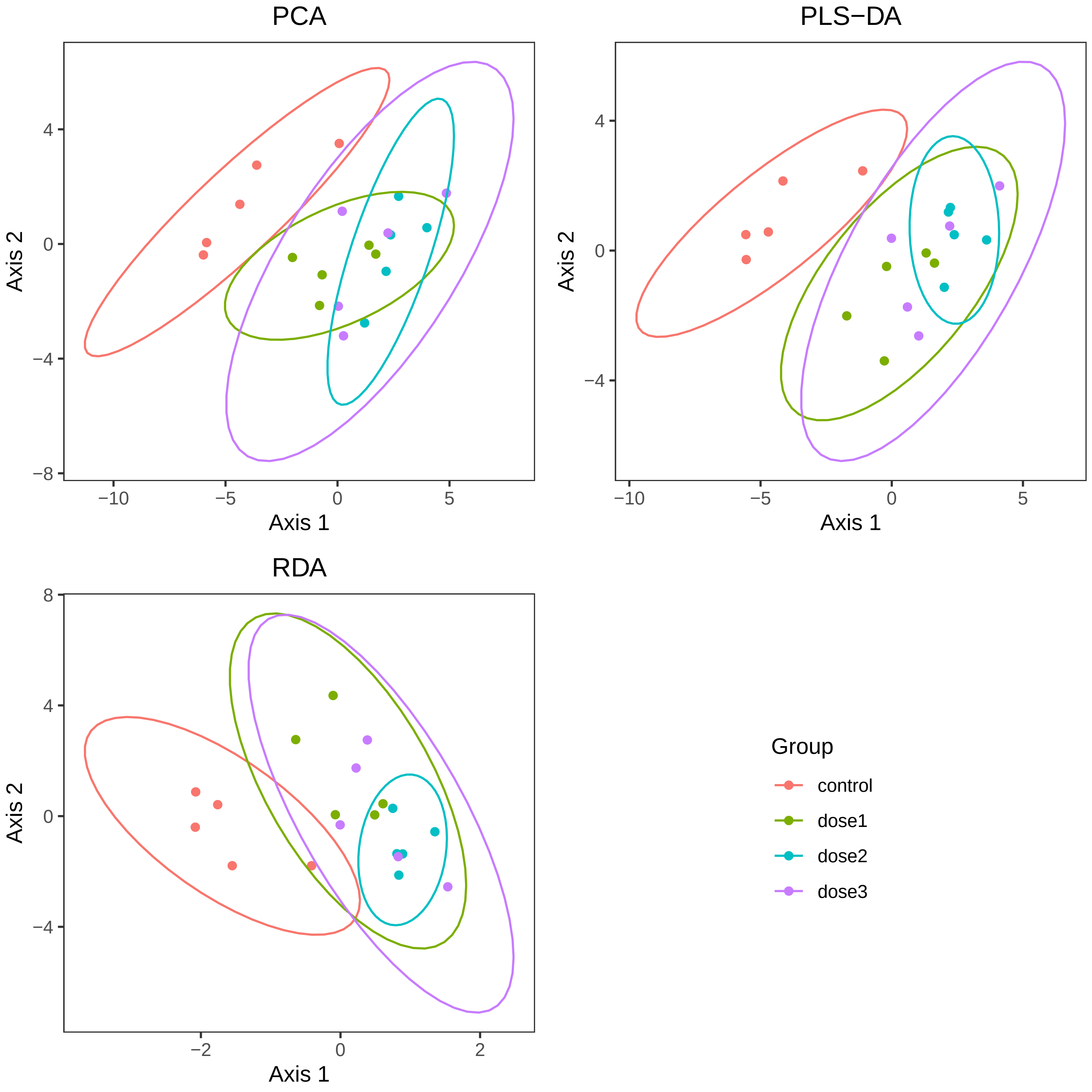

PCA
PLS−DA
RDA
Axis 1
Axis 2
Group
control
dose1
dose2
dose3

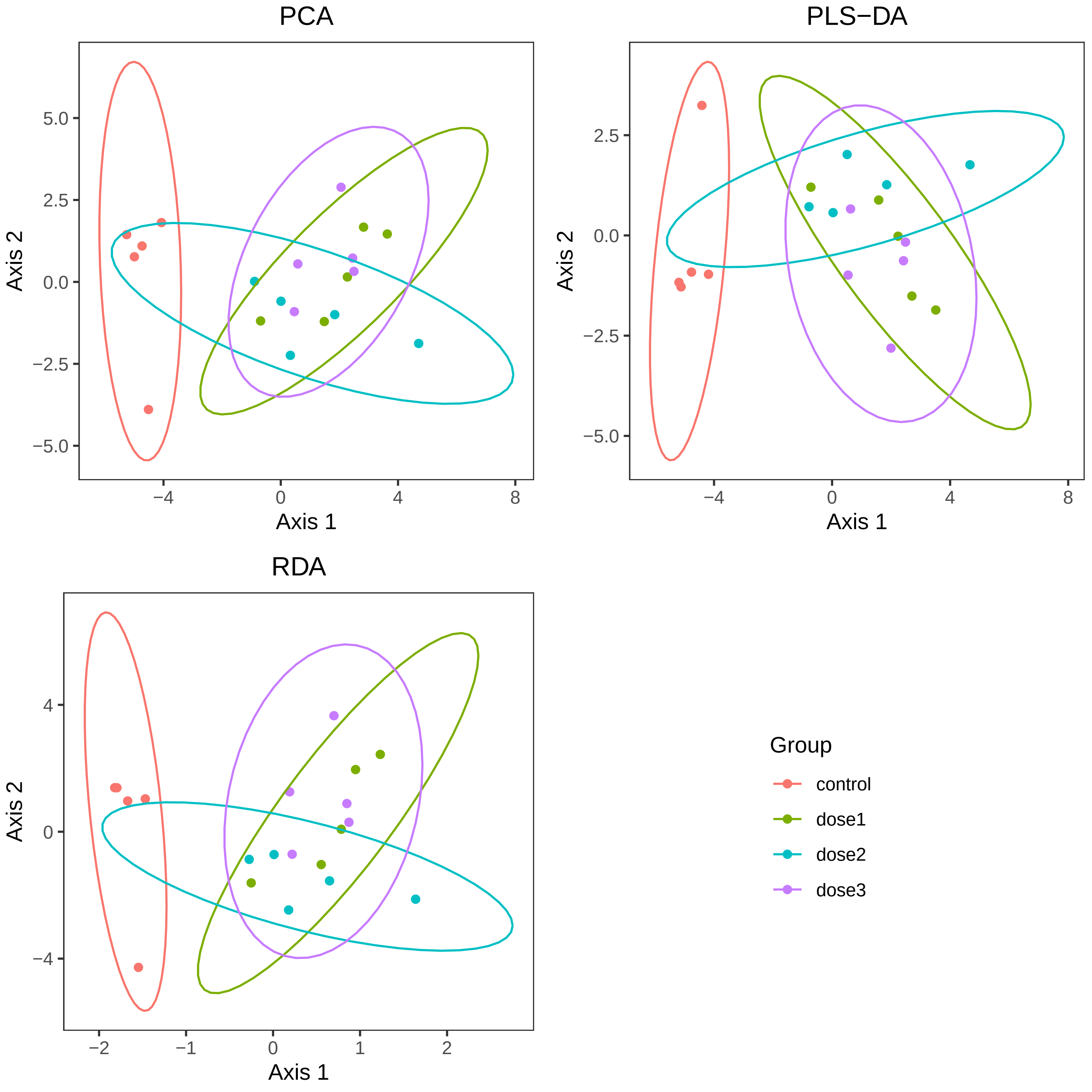

PCA
PLS−DA
RDA
Axis 1
Axis 2
Group
control
dose1
dose2
dose3

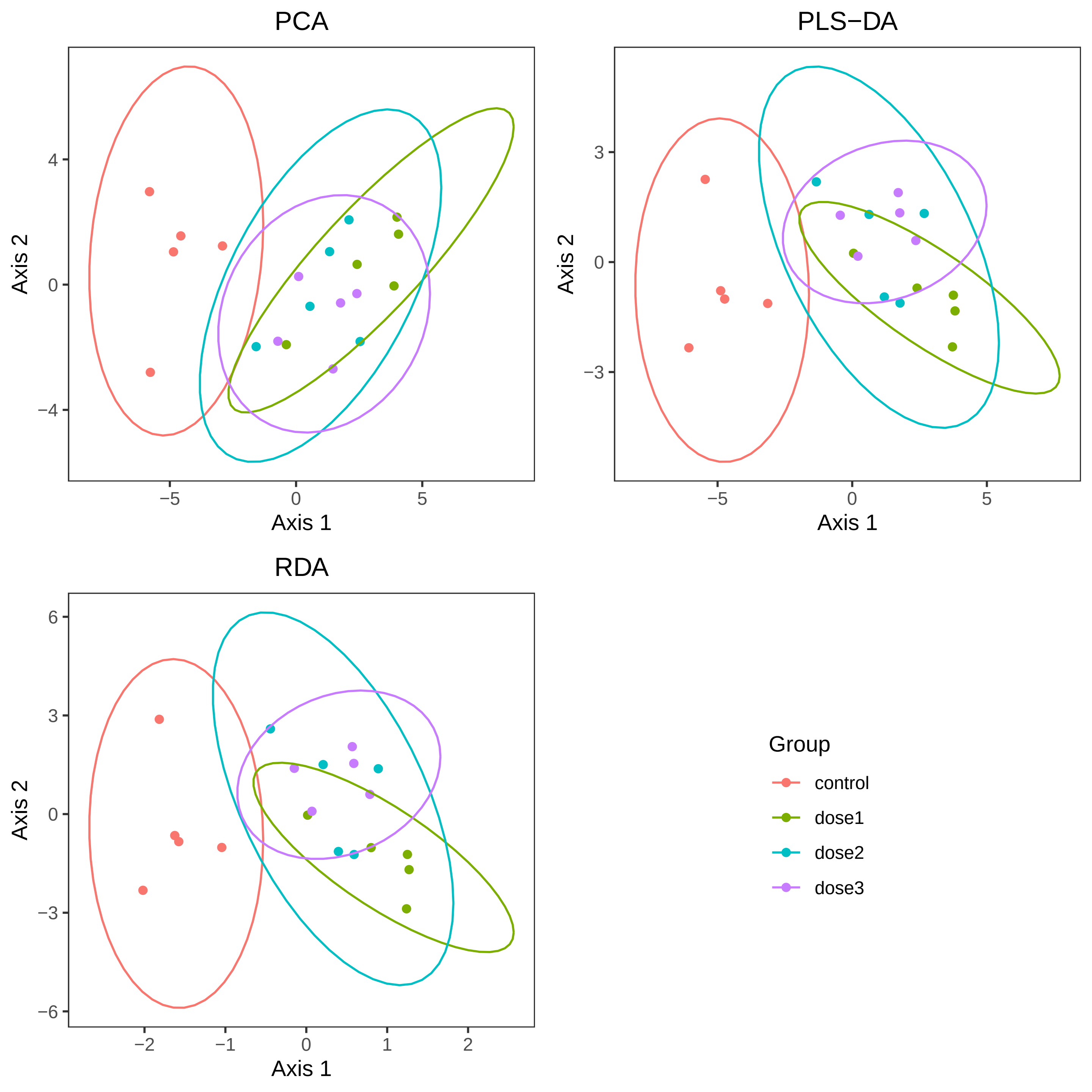
PCA
PLS−DA
RDA
Axis 1
Axis 2
Group
control
dose1
dose2
dose3

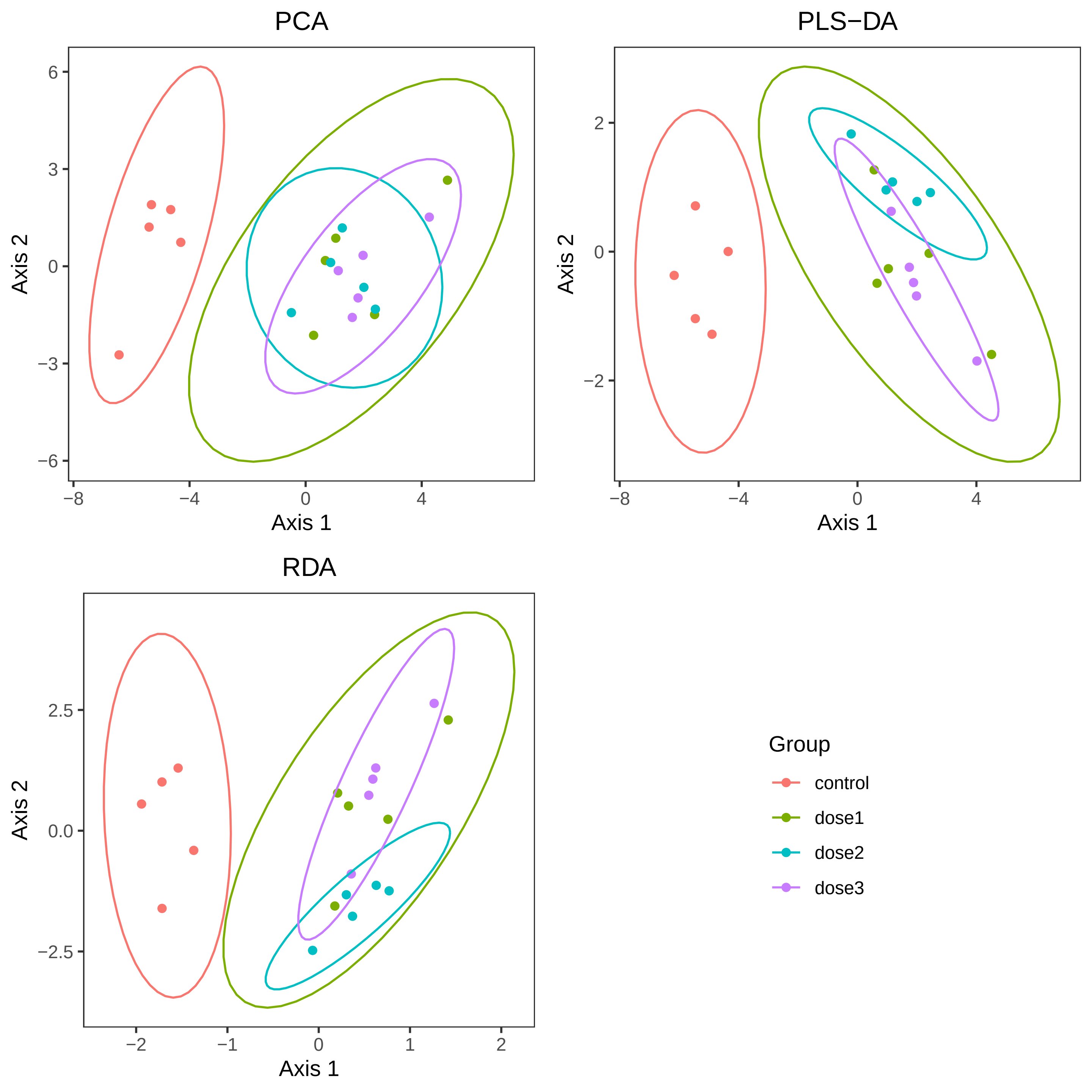
PCA
PLS−DA
RDA
Axis 1
Axis 2
6
3
0
−3
−6
−8
−4
0
4
2
−2
2.5
0.0
−2.5
−1
1
Group
control
dose1
dose2
dose3

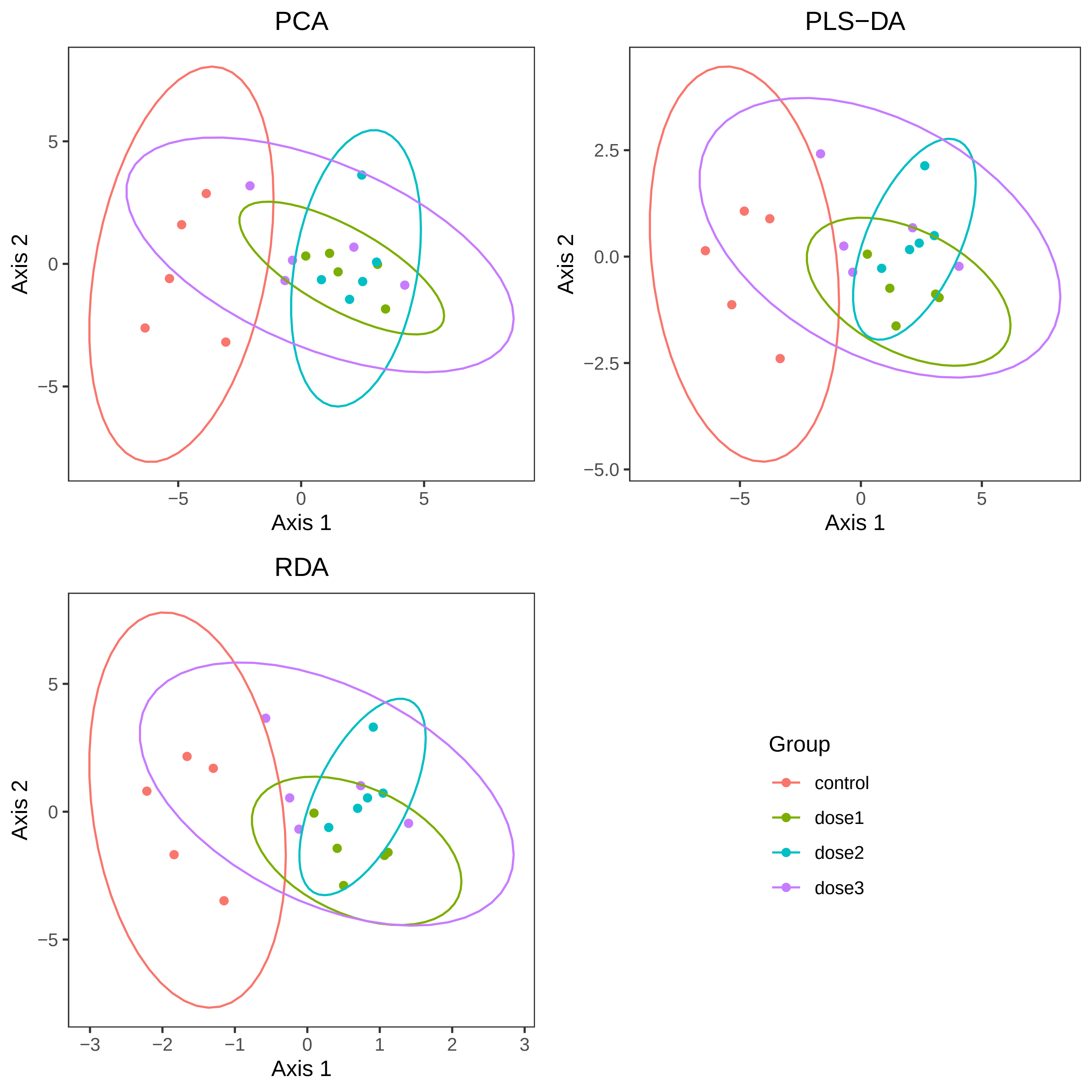
PCA
PLS−DA
RDA
Axis 1
Axis 2
5
0
−5
2.5
0.0
−2.5
−5.0
−3
−2
−1
1
2
3
Group
control
dose1
dose2
dose3

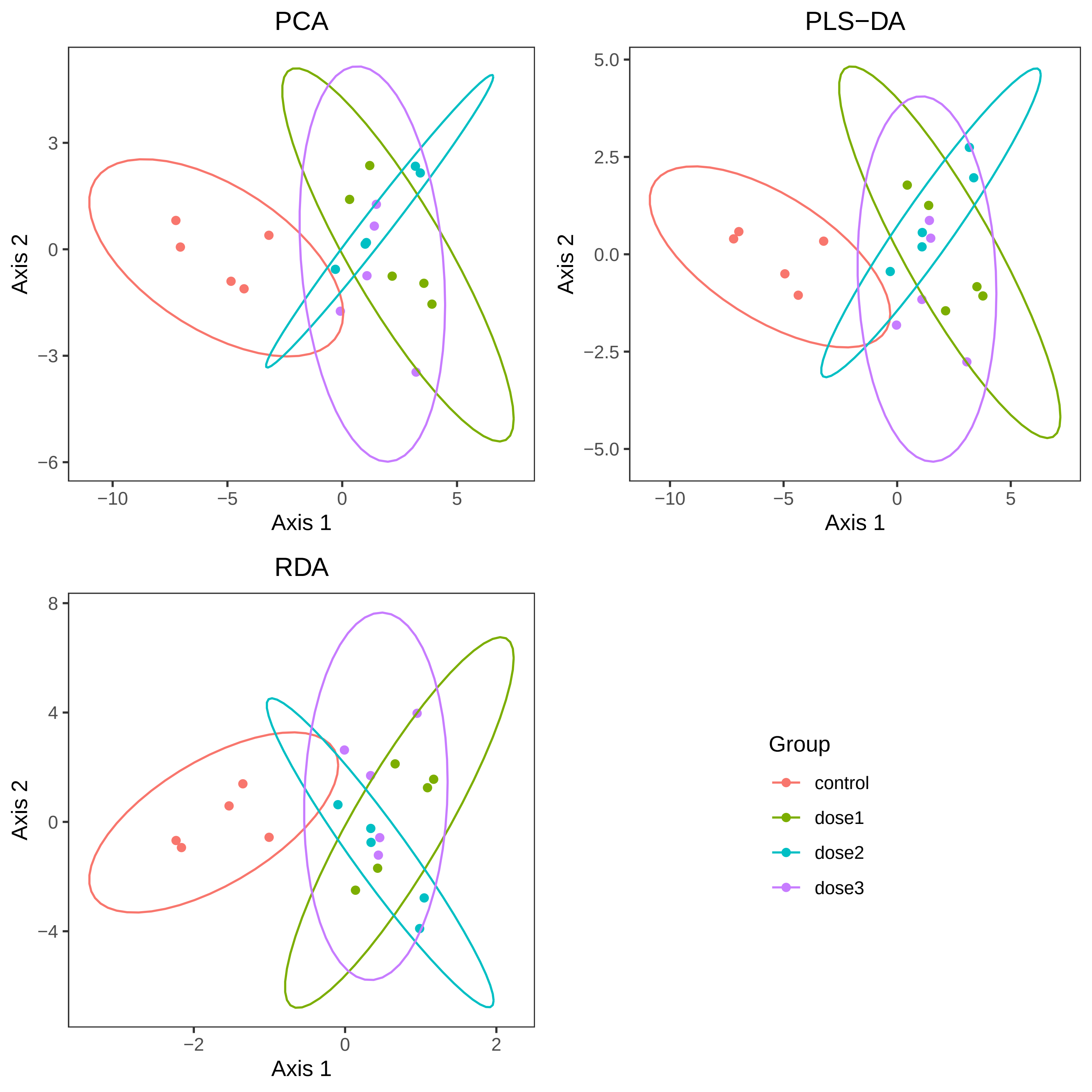

PCA
PLS−DA
RDA
Axis 1
Axis 2
3
0
−3
−6
−10
−5
0
5
5.0
2.5
0.0
−2.5
−5.0
8
4
0
−4
−2
0
2
Group
control
dose1
dose2
dose3

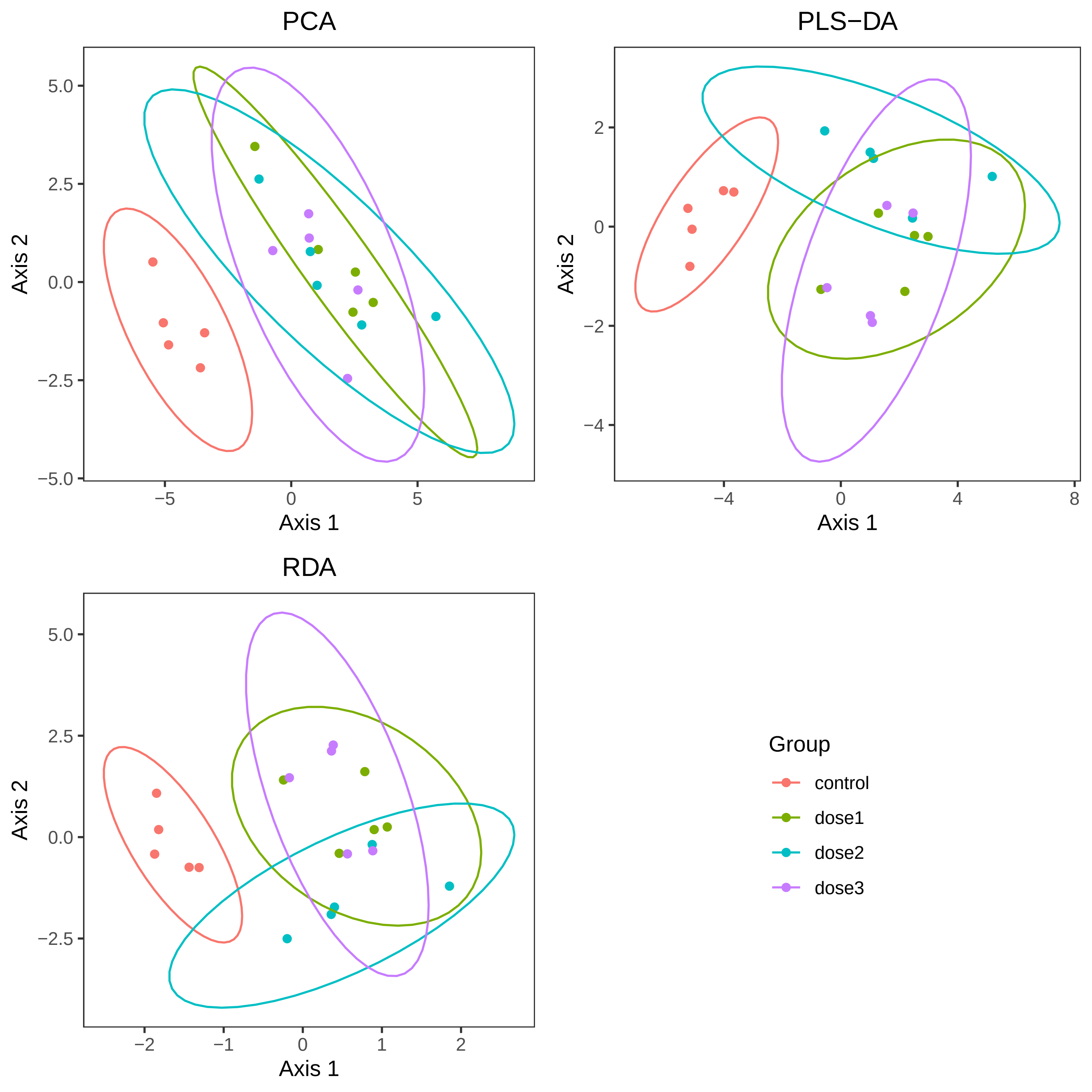

PCA
PLS−DA
RDA
Axis 1
Axis 2
5.0
2.5
0.0
−2.5
−5.0
−5
0
5
2
−2
−4
−4
0
4
8
−2
−1
0
1
2
Group
control
dose1
dose2
dose3

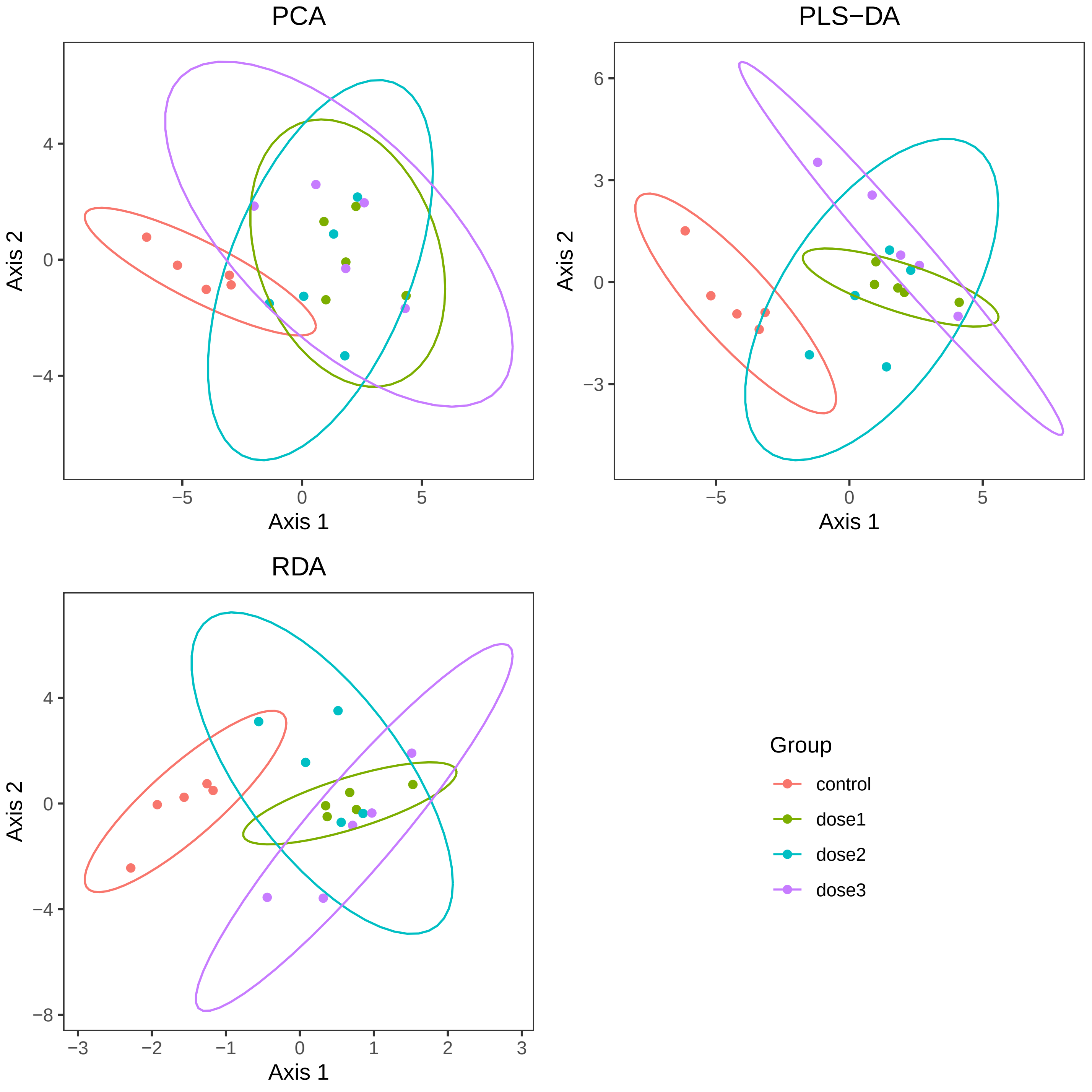
PCA
PLS−DA
RDA
Axis 1
Axis 2
Group
control
dose1
dose2
dose3

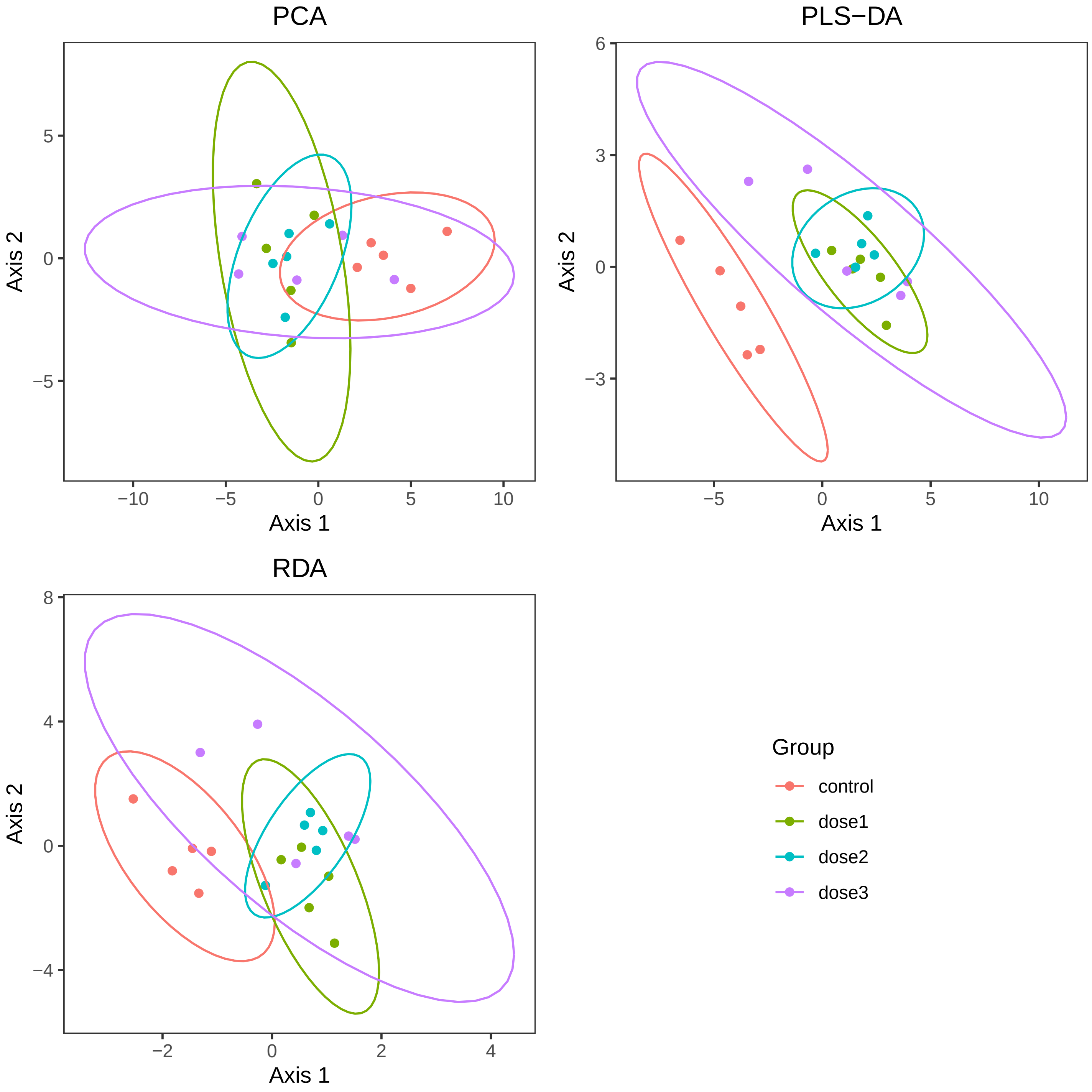
PCA
PLS−DA
RDA
Axis 1
Axis 2
Group
control
dose1
dose2
dose3

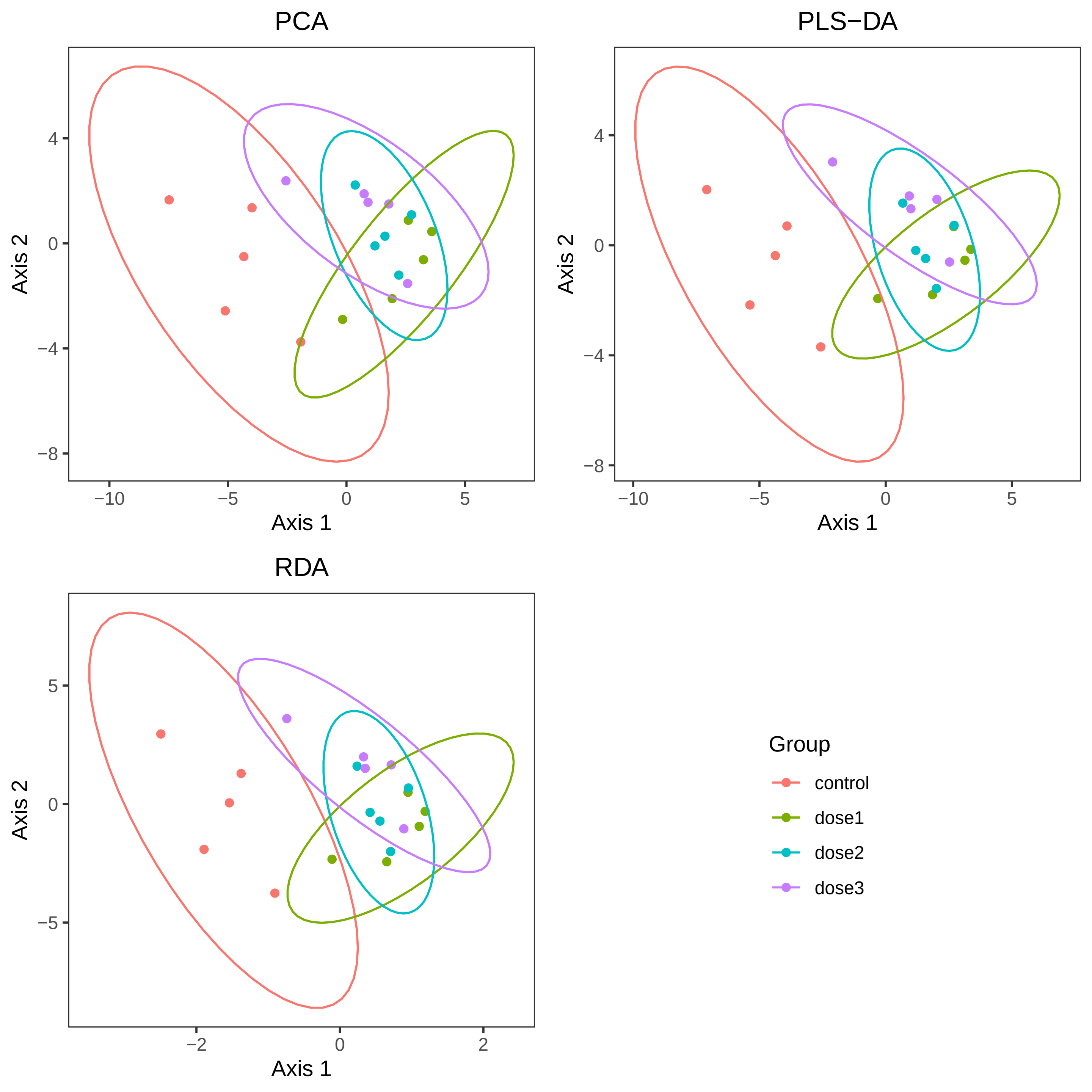
PCA
PLS−DA
RDA
Axis 1
Axis 2
Group
control
dose1
dose2
dose3

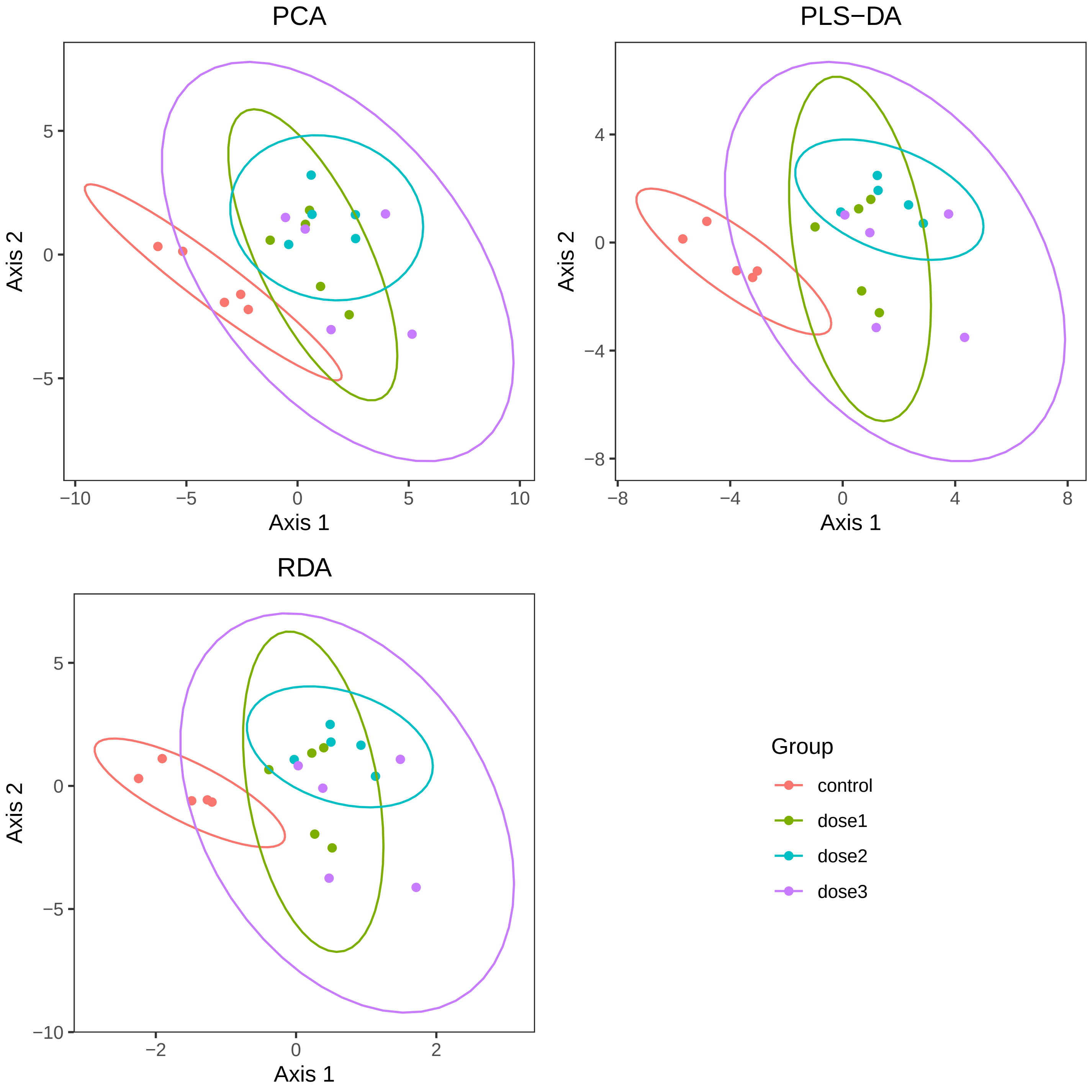
PCA
PLS−DA
RDA
Axis 1
Axis 2
Group
control
dose1
dose2
dose3

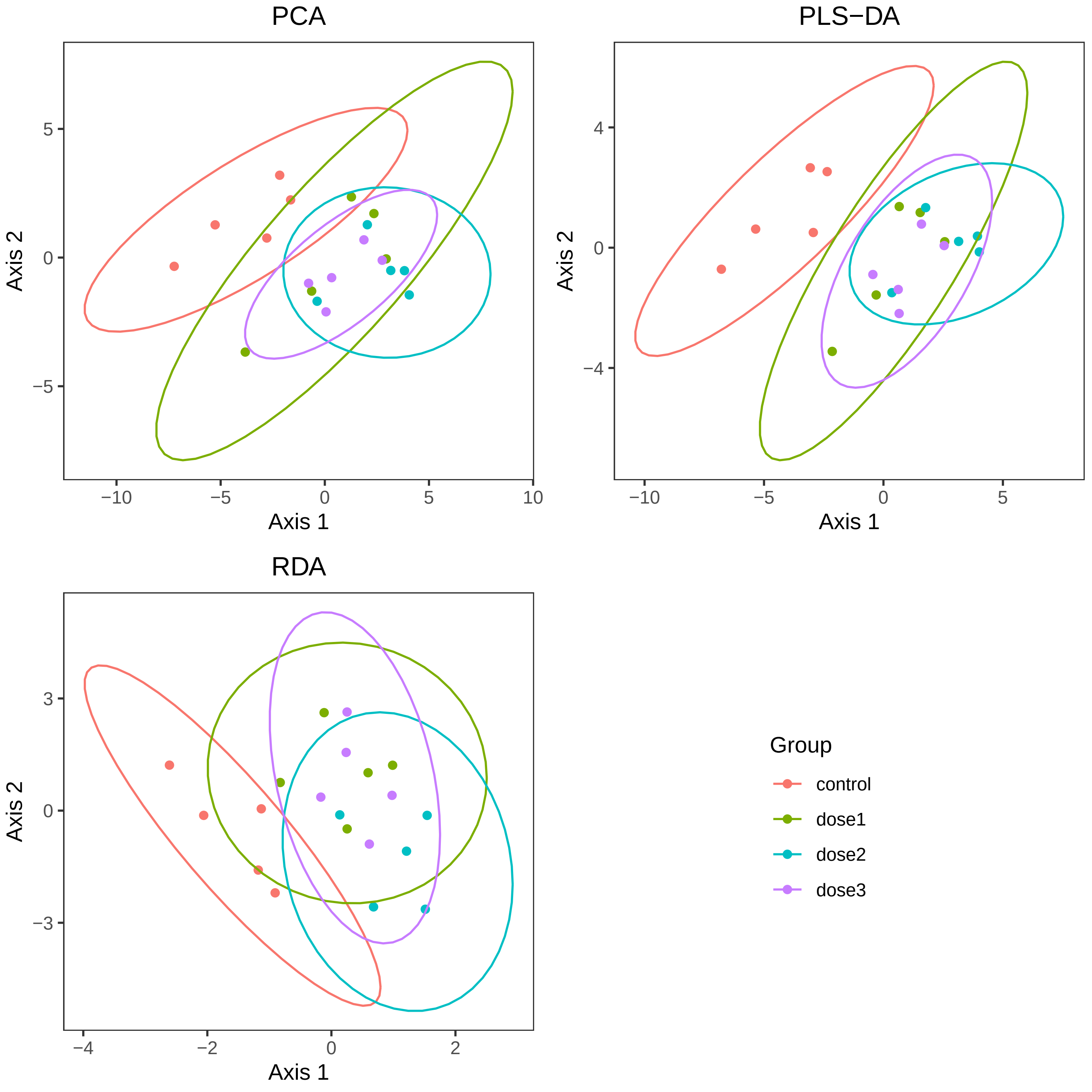
PCA
PLS−DA
RDA
Axis 1
Axis 2
−10
−5
0
5
10
−4
4
−3
3
−2
2
Group
control
dose1
dose2
dose3

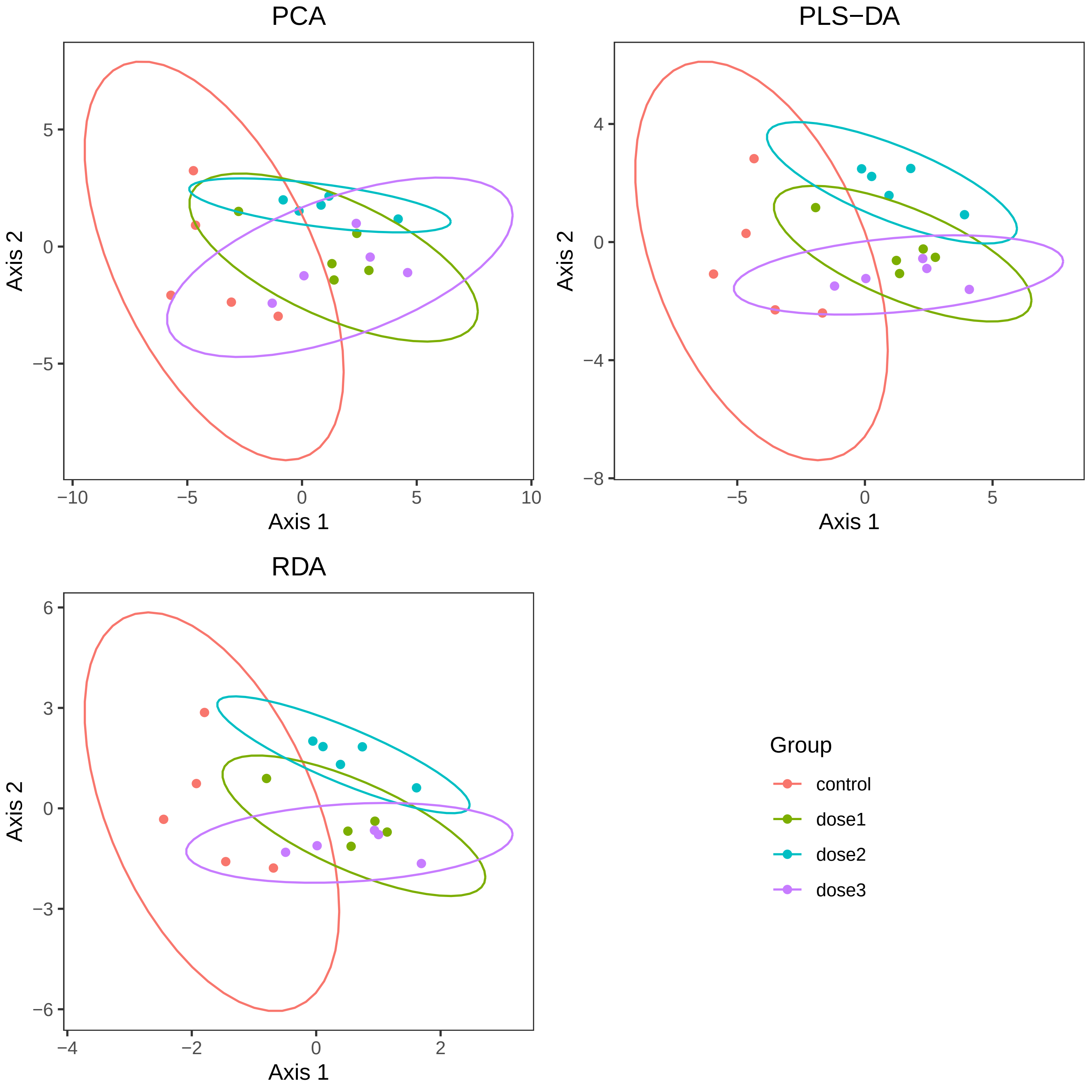

PCA
PLS−DA
RDA
Axis 1
Axis 2
Group
control
dose1
dose2
dose3

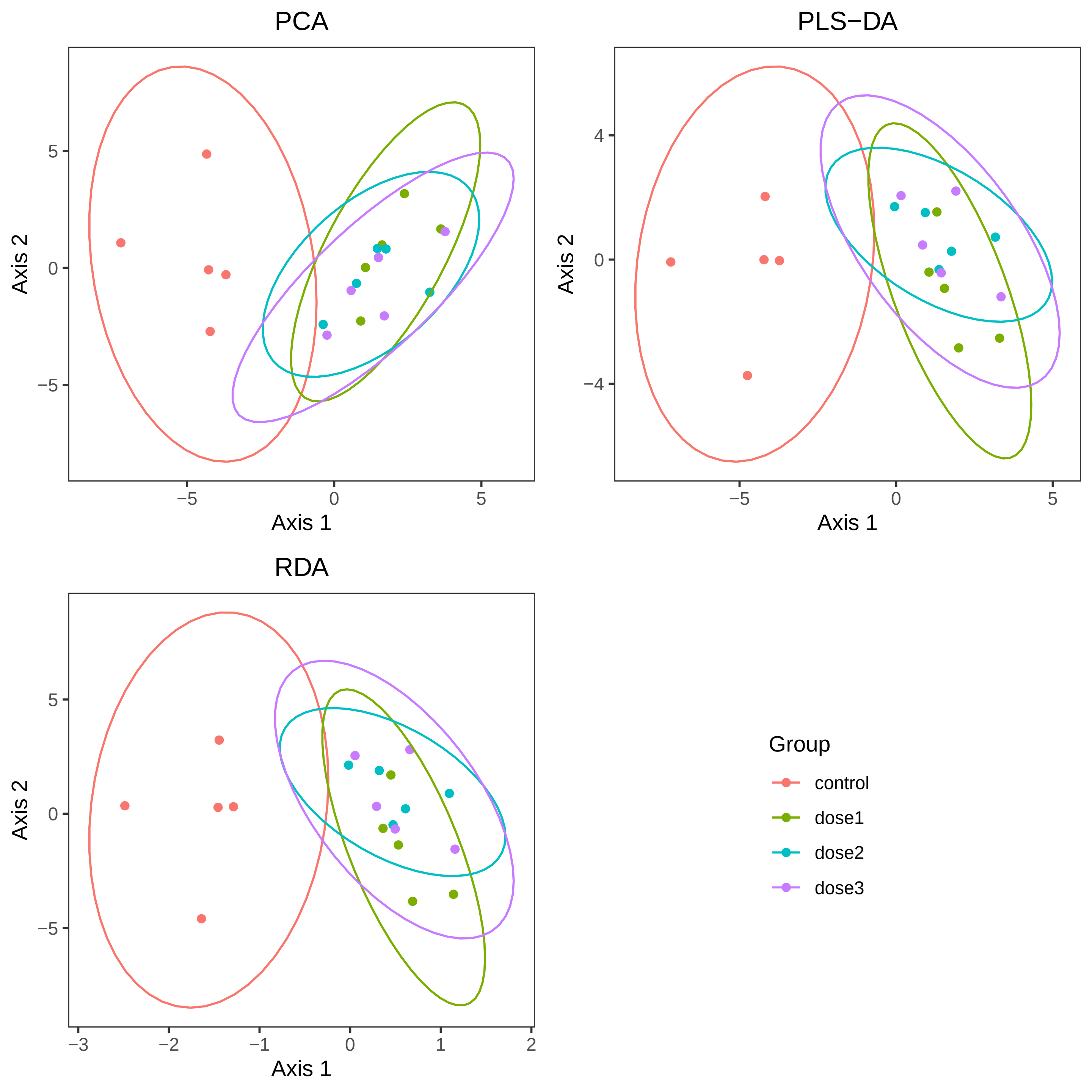
PCA
PLS−DA
RDA
Axis 1
Axis 2
Group
control
dose1
dose2
dose3

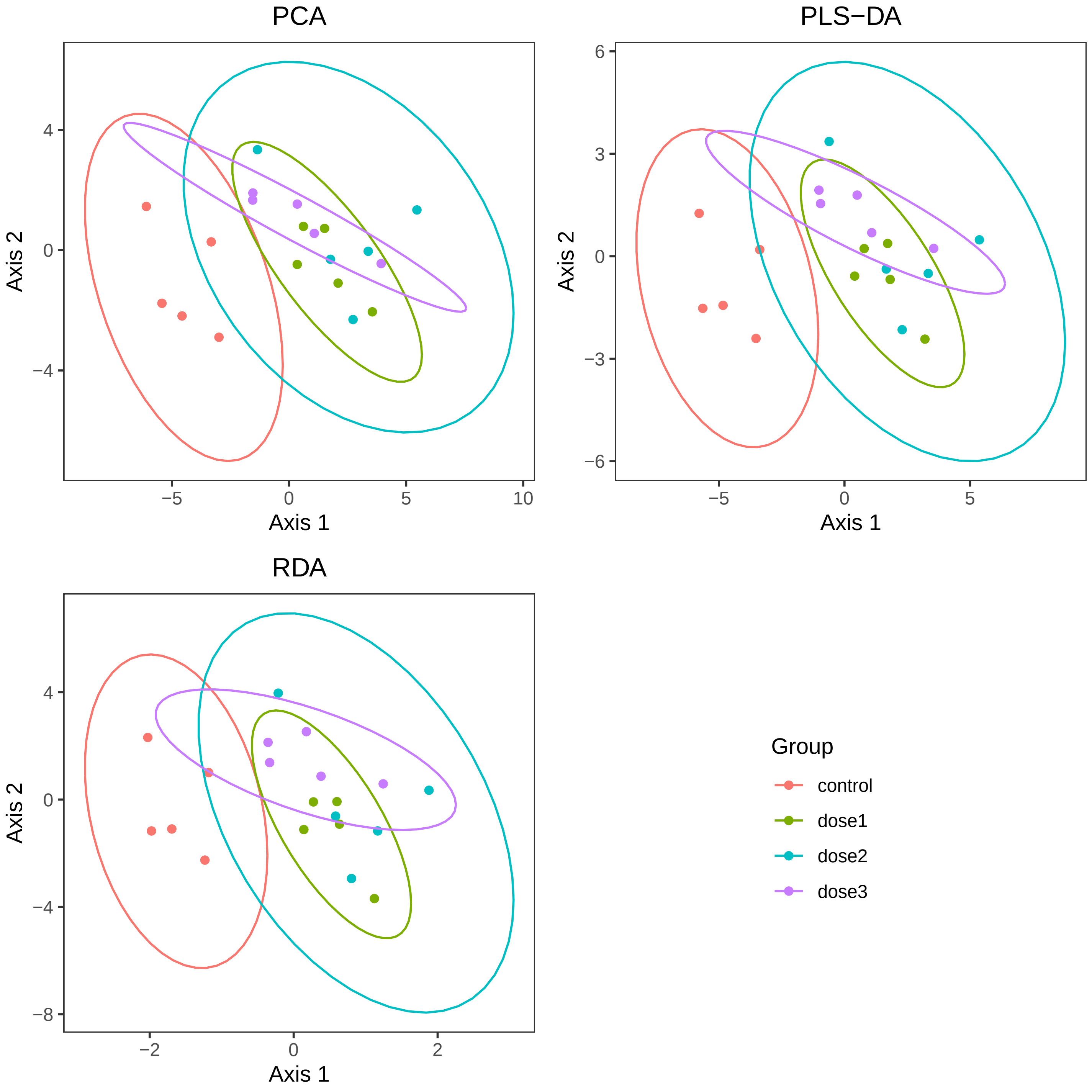
PCA
PLS−DA
RDA
Axis 1
Axis 2
Group
control
dose1
dose2
dose3

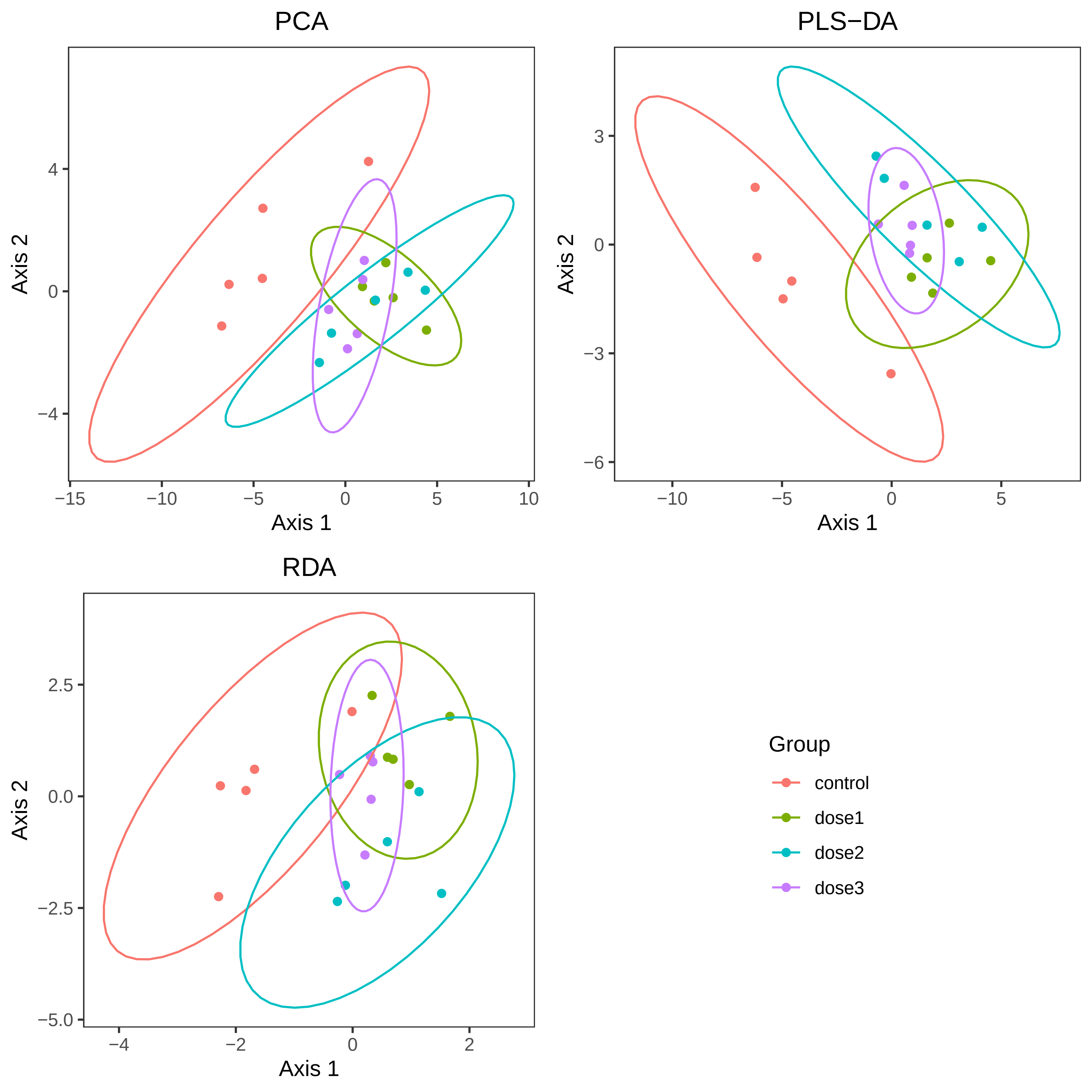

PCA
Axis 2
Axis 1
4
0
−4
−15
−10
−5
0
5
10
PLS−DA
Axis 2
Axis 1
3
0
−3
−6
−10
−5
0
5
RDA
Axis 2
Axis 1
2.5
0.0
−2.5
−5.0
−4
−2
0
2
Group
control
dose1
dose2
dose3

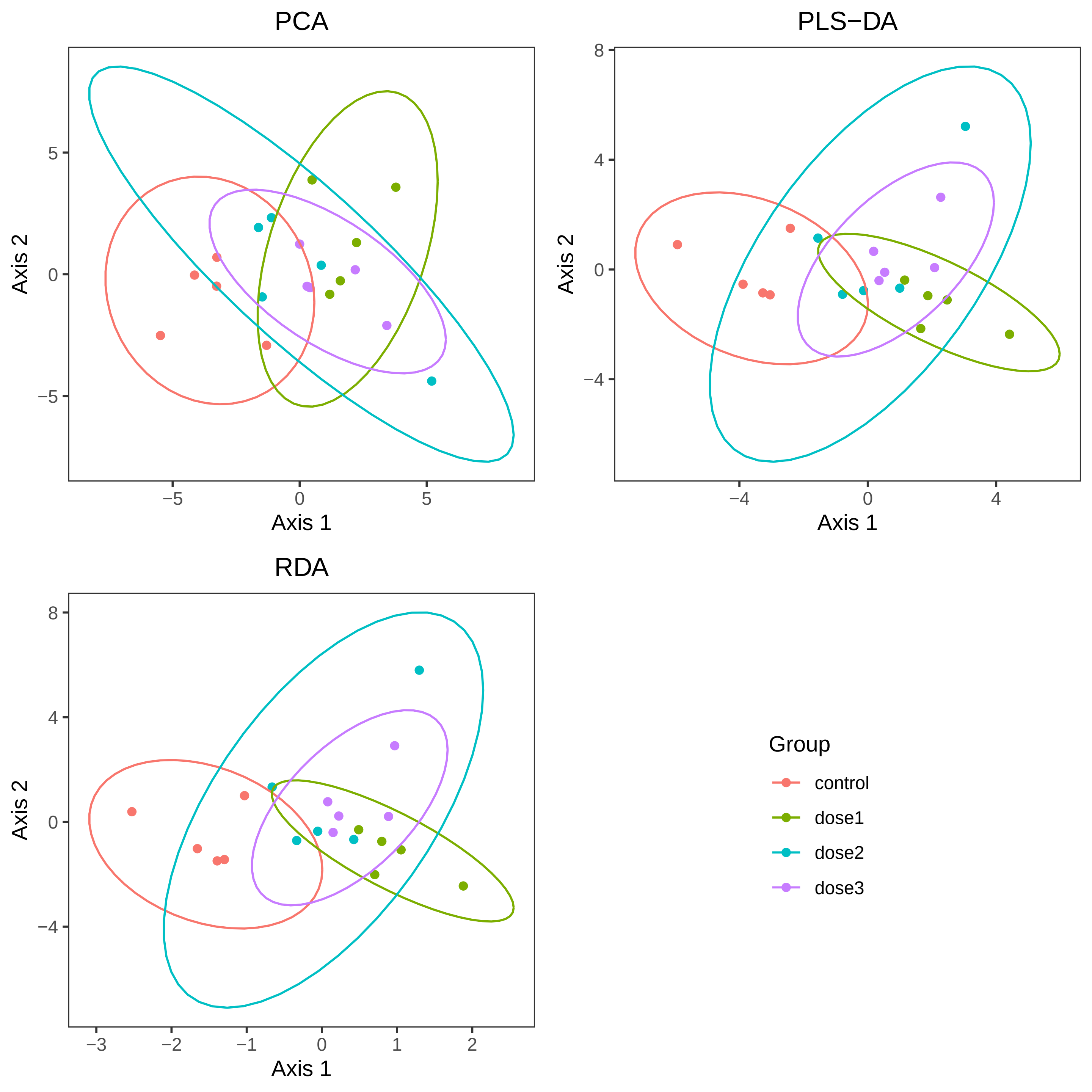

PCA
PLS−DA
RDA
Axis 1
Axis 2
Group
control
dose1
dose2
dose3

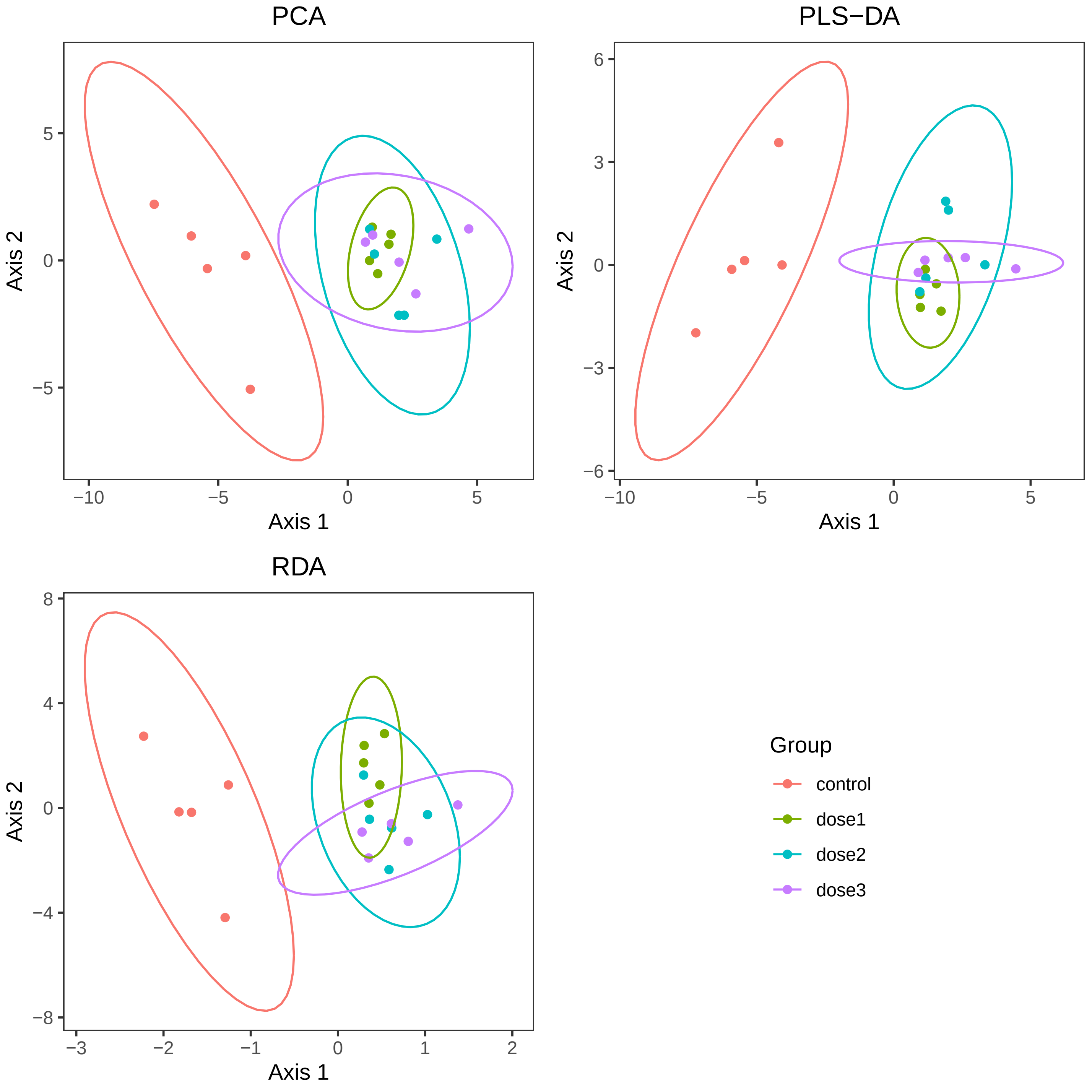

PCA
PLS−DA
RDA
Axis 1
Axis 2
Group
control
dose1
dose2
dose3

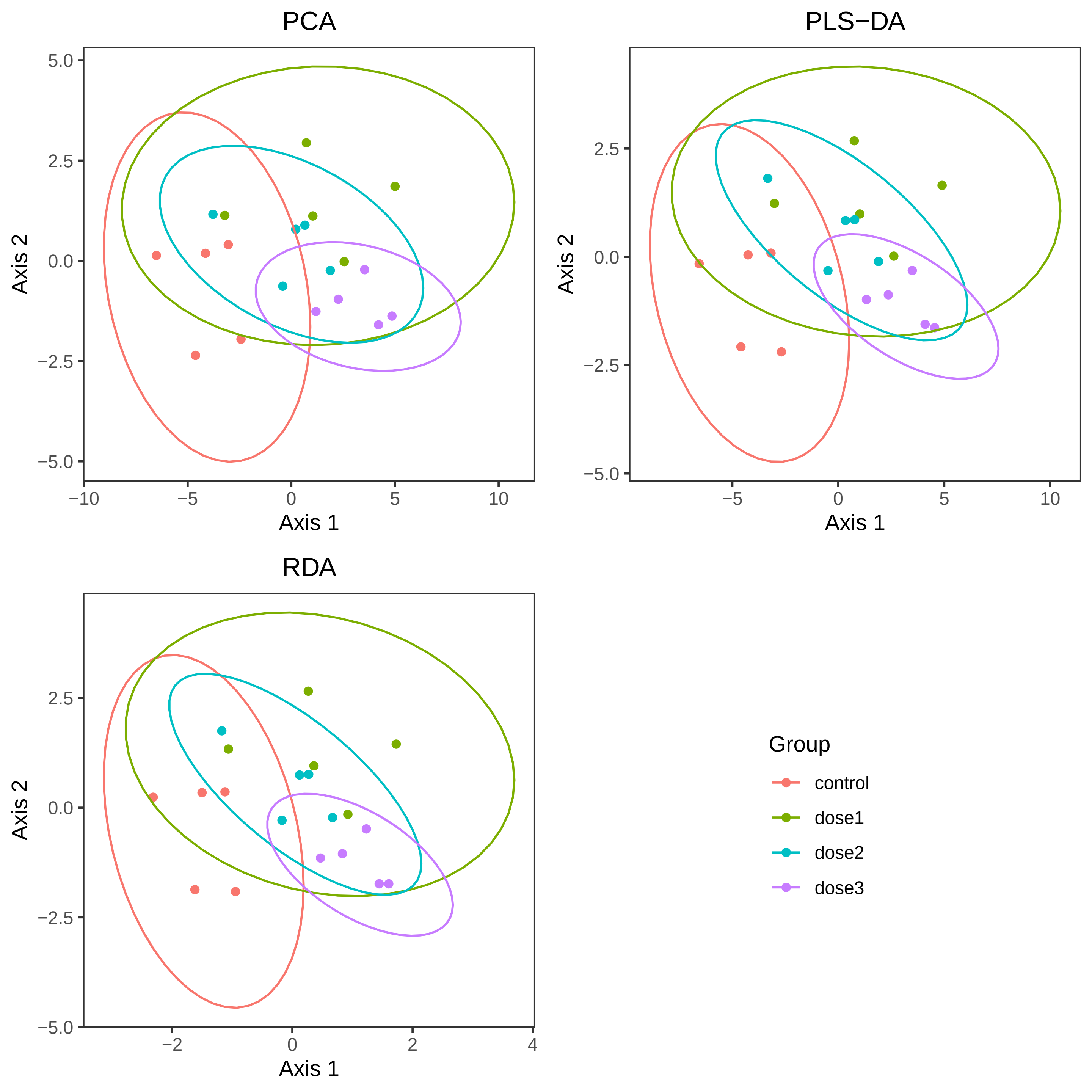

PCA
PLS−DA
RDA
Axis 1
Axis 2
5.0
2.5
0.0
−2.5
−5.0
−10
−5
0
5
10
−2
2
4
Group
control
dose1
dose2
dose3

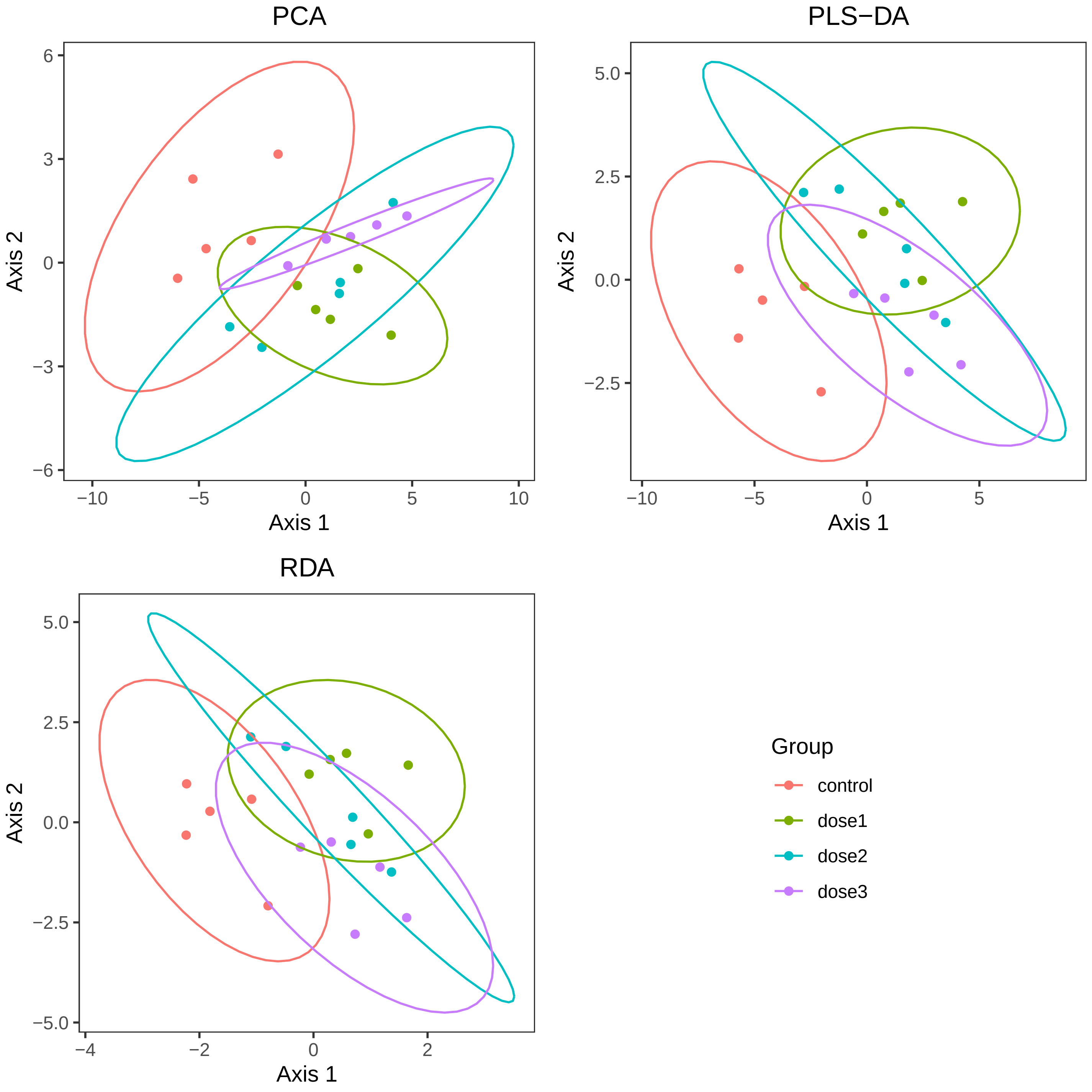
PCA
PLS−DA
RDA
Axis 1
Axis 2
Group
control
dose1
dose2
dose3

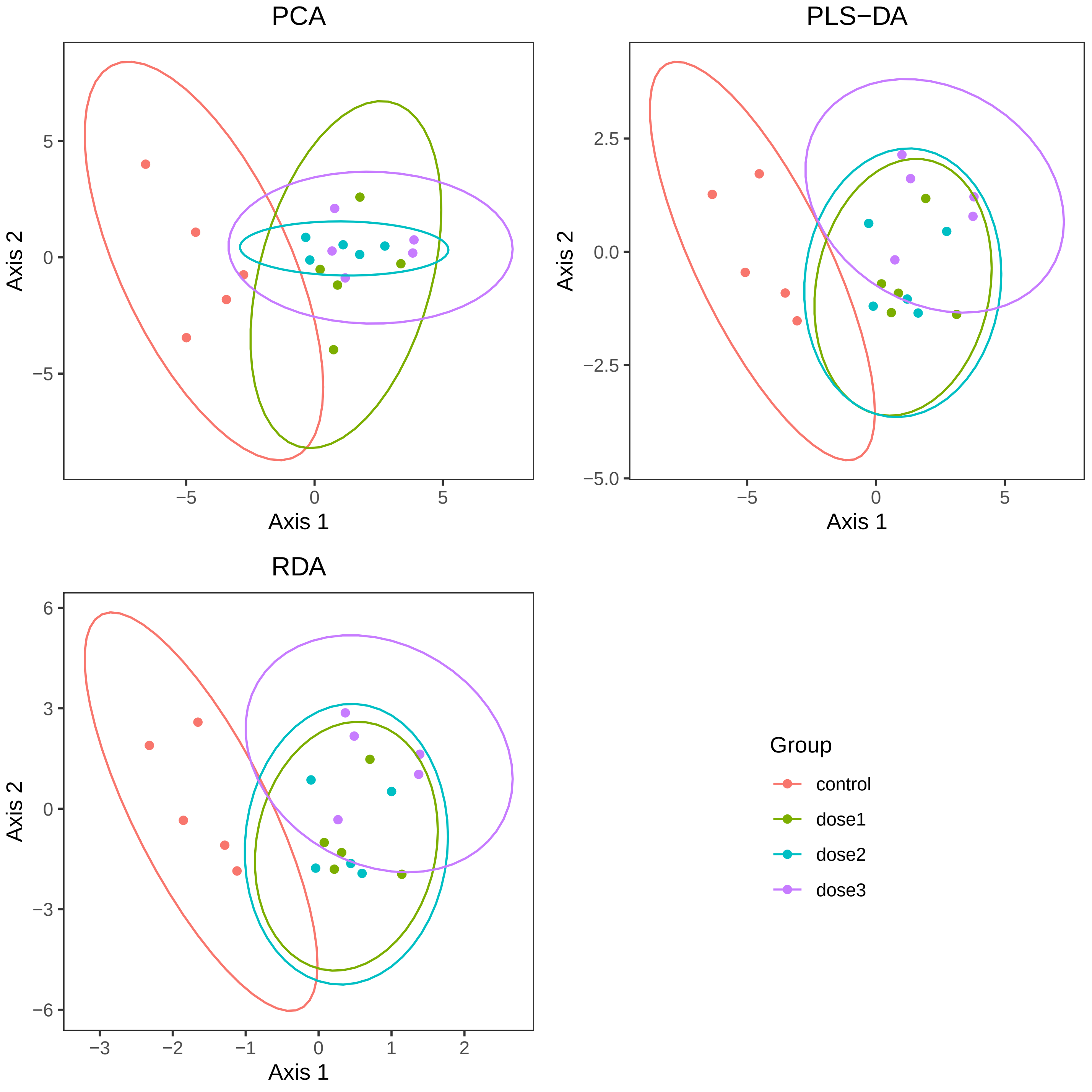
PCA
PLS−DA
RDA
Axis 1
Axis 2
Group
control
dose1
dose2
dose3

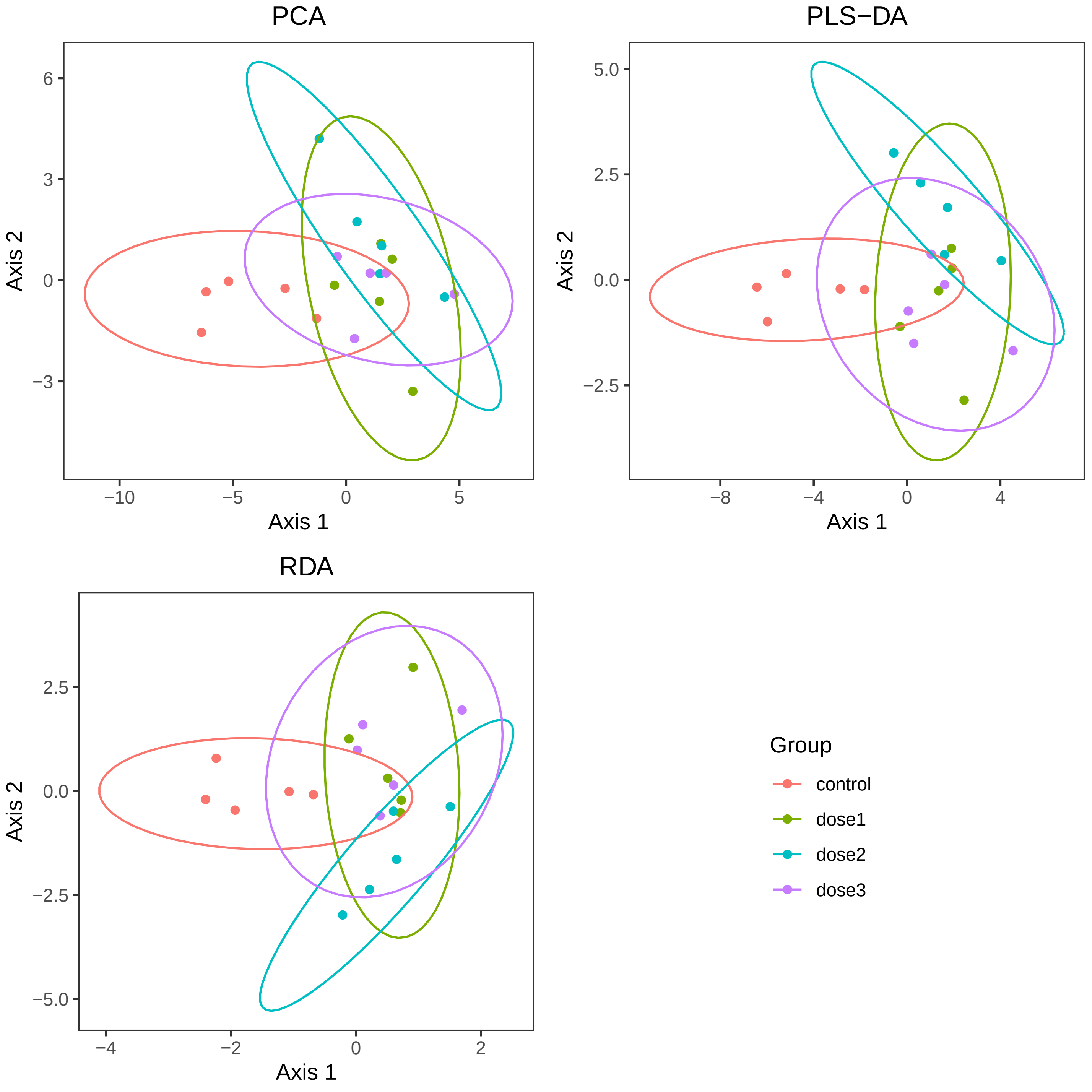

PCA
PLS−DA
RDA
Axis 1
Axis 2
Group
control
dose1
dose2
dose3

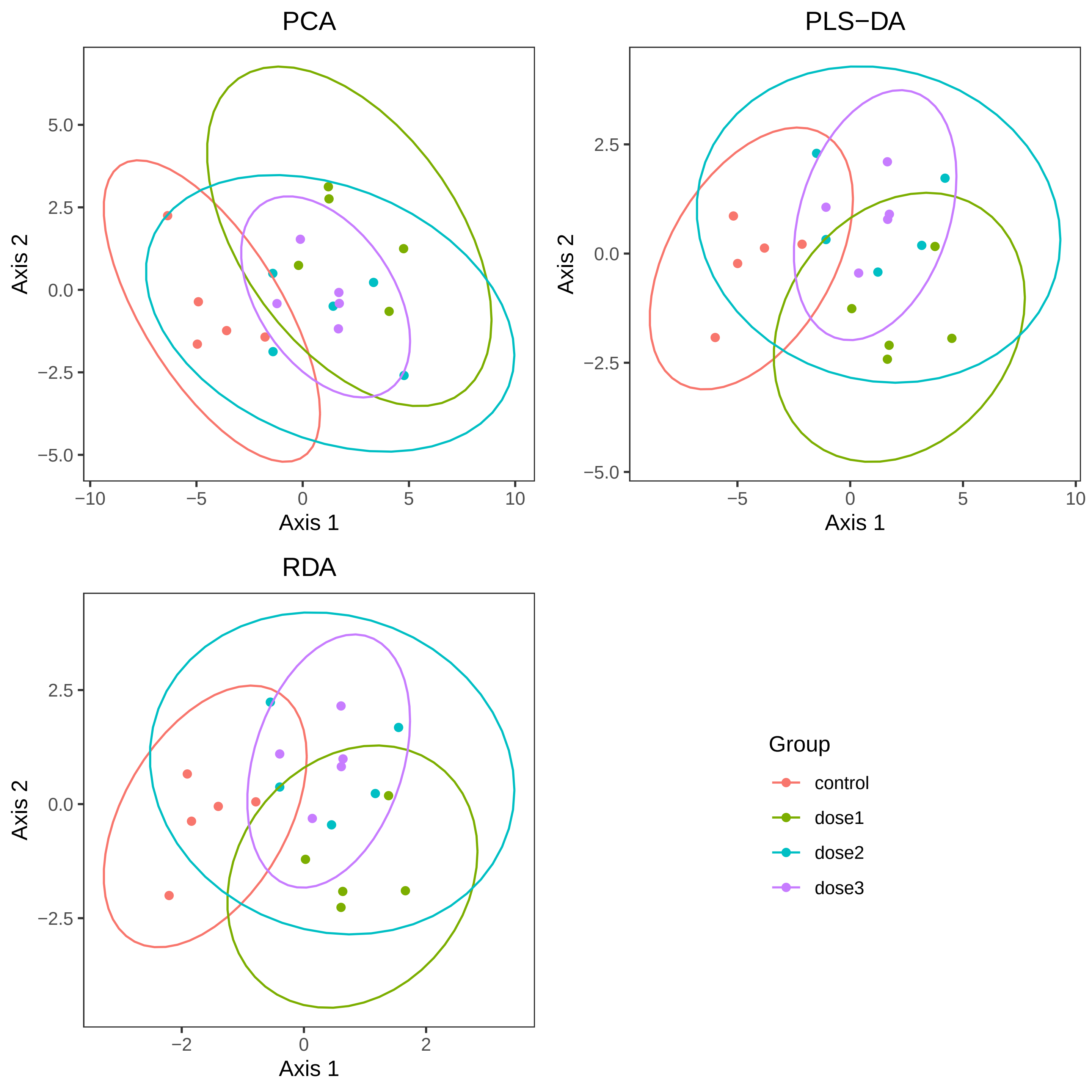

PCA
Axis 2
5.0
2.5
0.0
−2.5
−5.0
−10
−5
0
5
10
Axis 1
PLS−DA
Axis 2
2.5
0.0
−2.5
−5.0
−5
0
5
10
Axis 1
RDA
Axis 2
2.5
0.0
−2.5
−2
0
2
Axis 1
Group
control
dose1
dose2
dose3

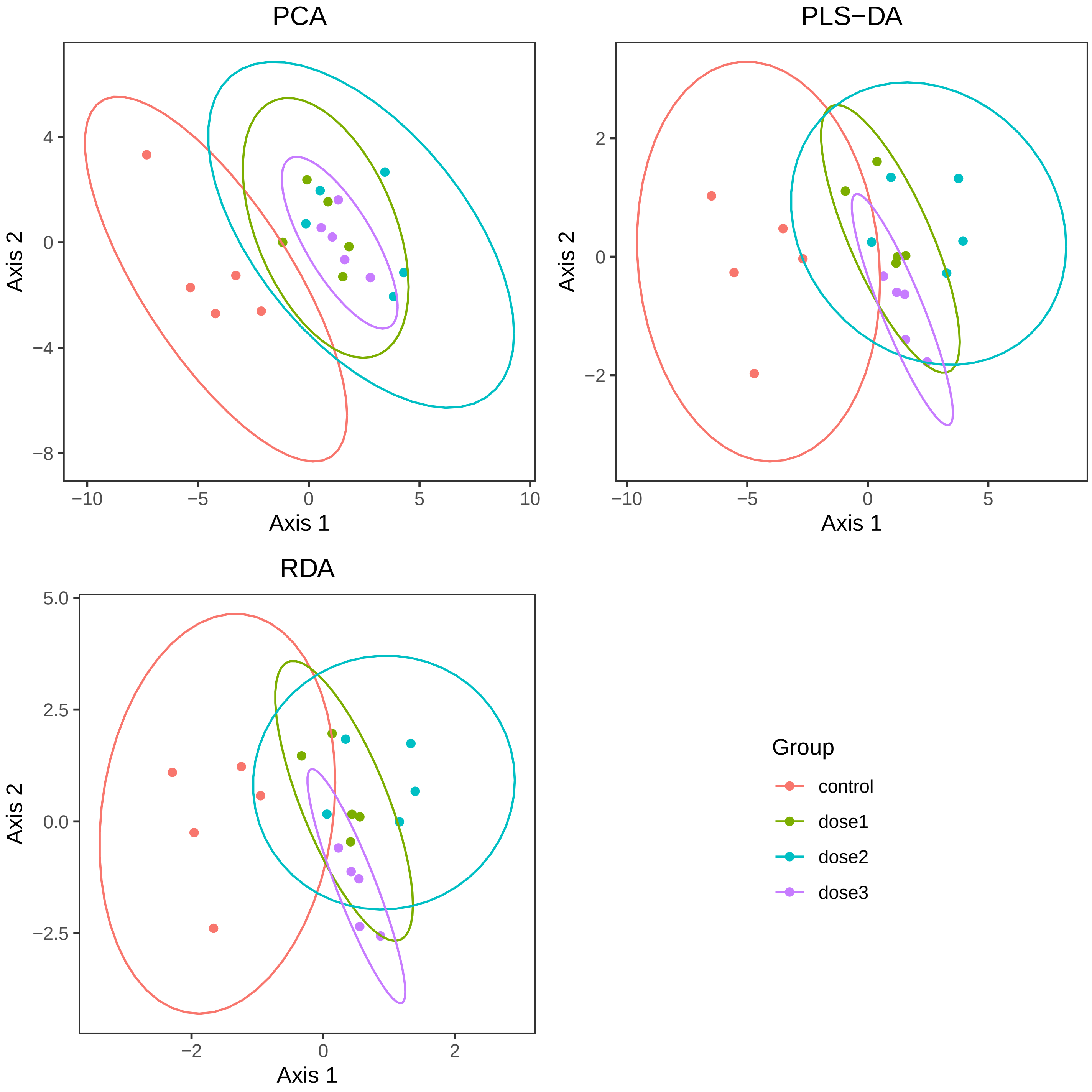
PCA
PLS−DA
RDA
Axis 1
Axis 2
Group
control
dose1
dose2
dose3

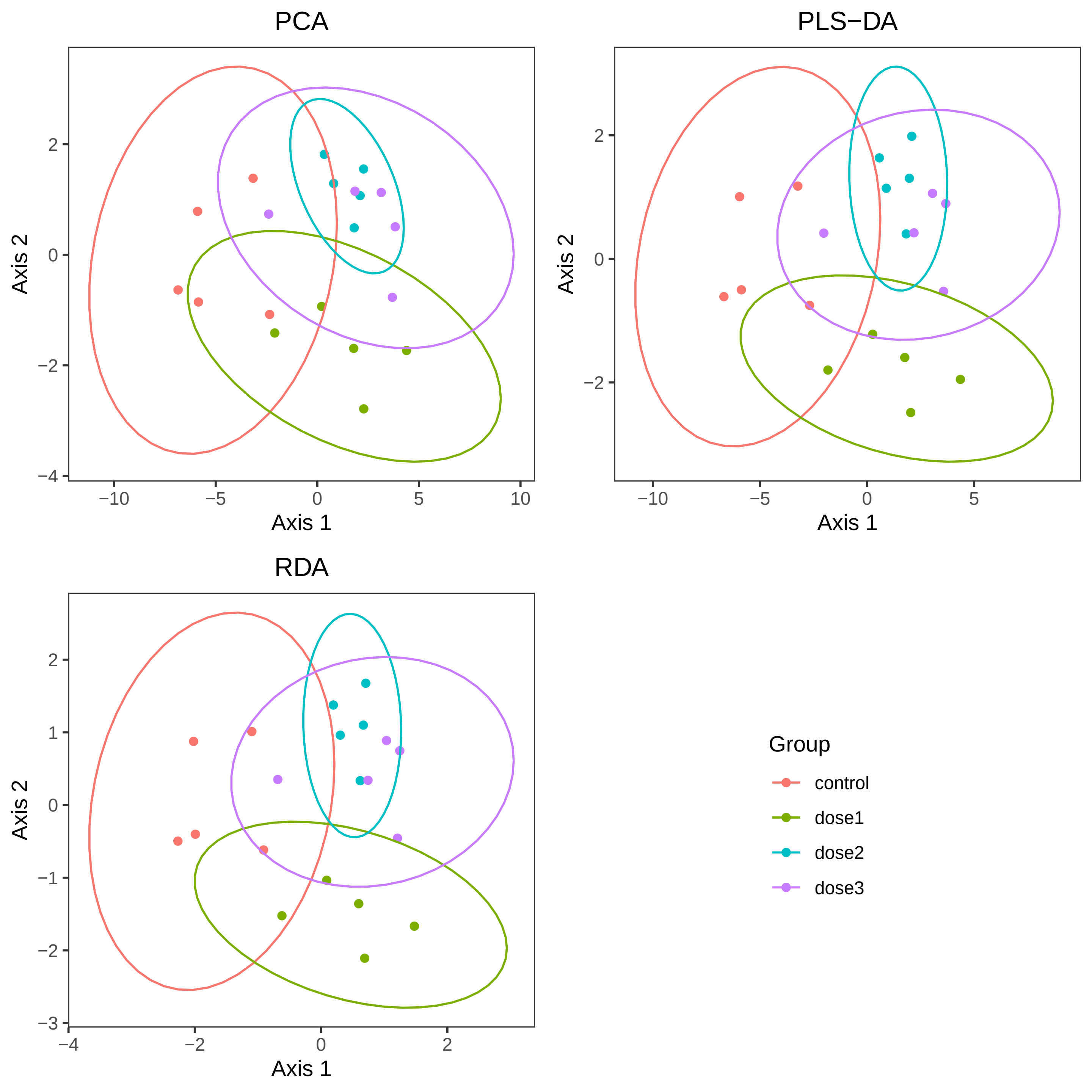

PCA
PLS−DA
RDA
Axis 1
Axis 2
Group
control
dose1
dose2
dose3

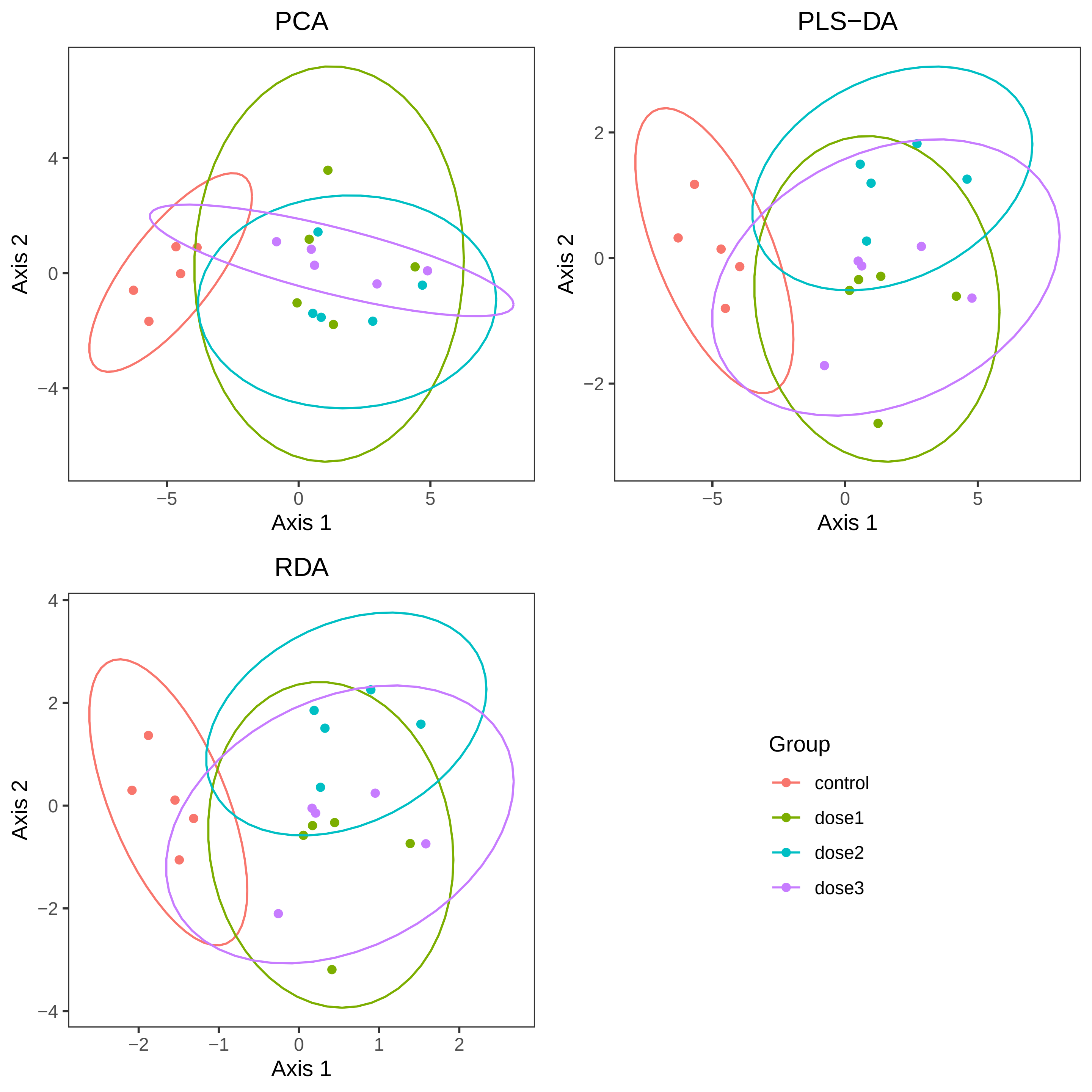

PCA
PLS−DA
RDA
Axis 1
Axis 2
Group
control
dose1
dose2
dose3

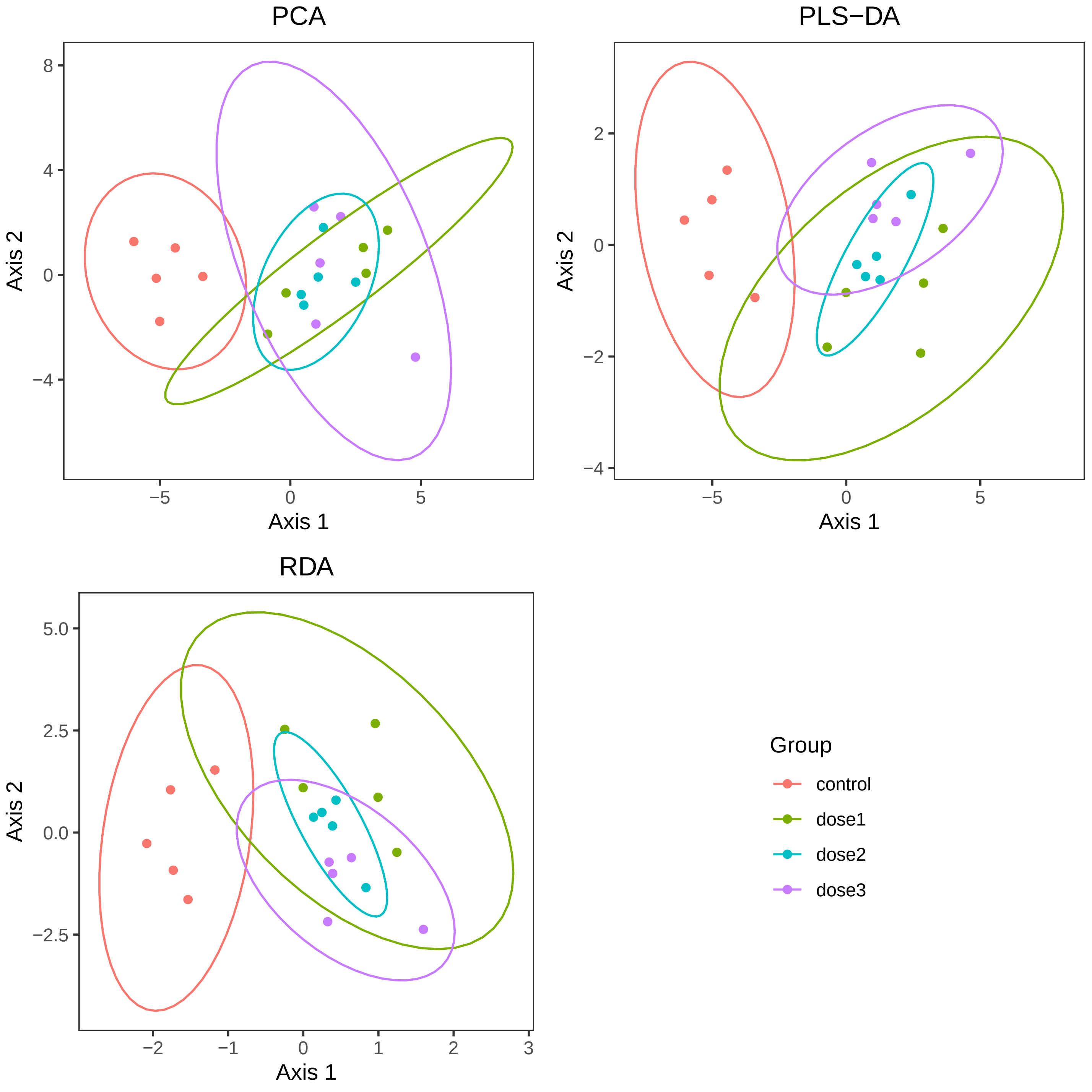
PCA
PLS−DA
RDA
Axis 1
Axis 2
Group
control
dose1
dose2
dose3

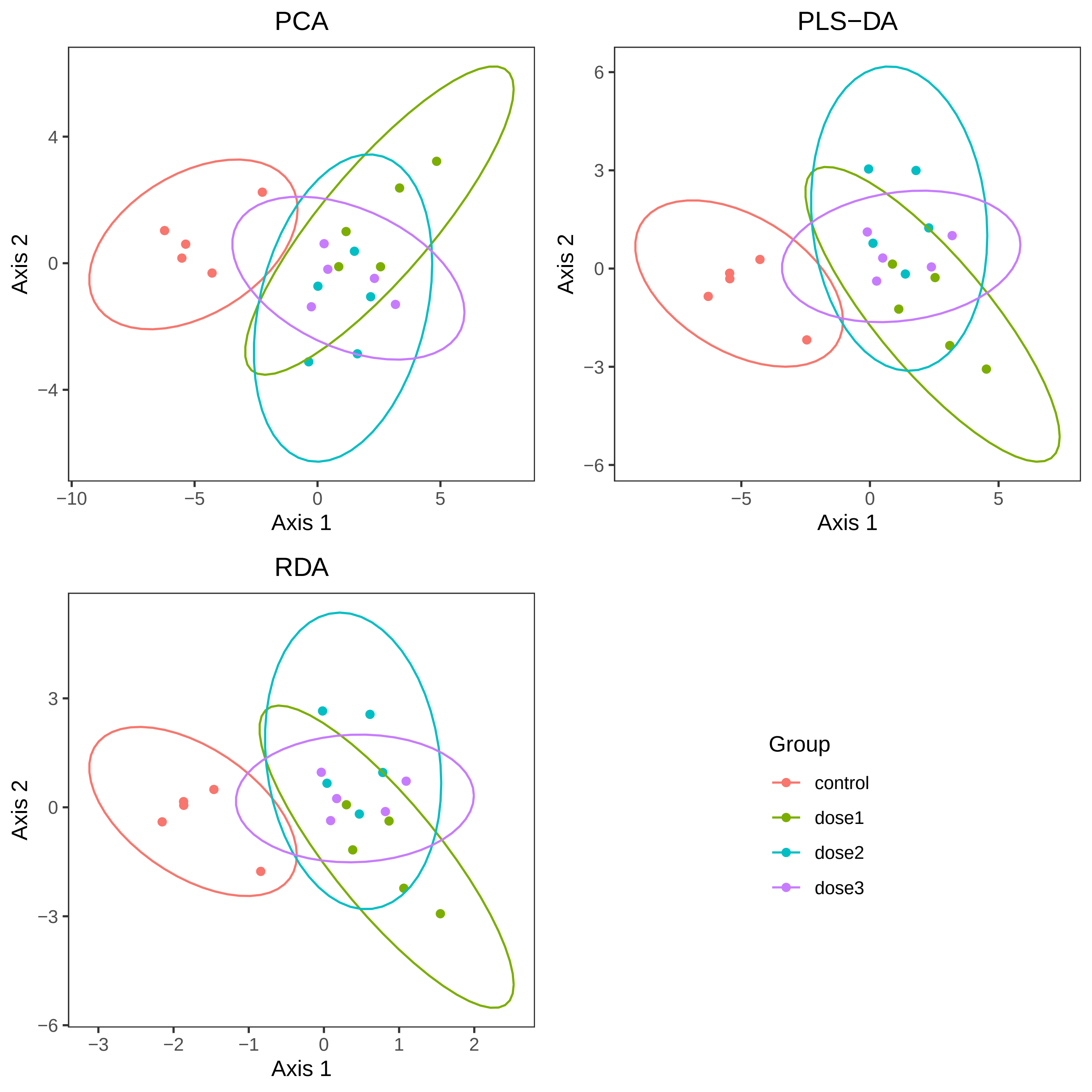
PCA
PLS−DA
RDA
Axis 1
Axis 2
Group
control
dose1
dose2
dose3

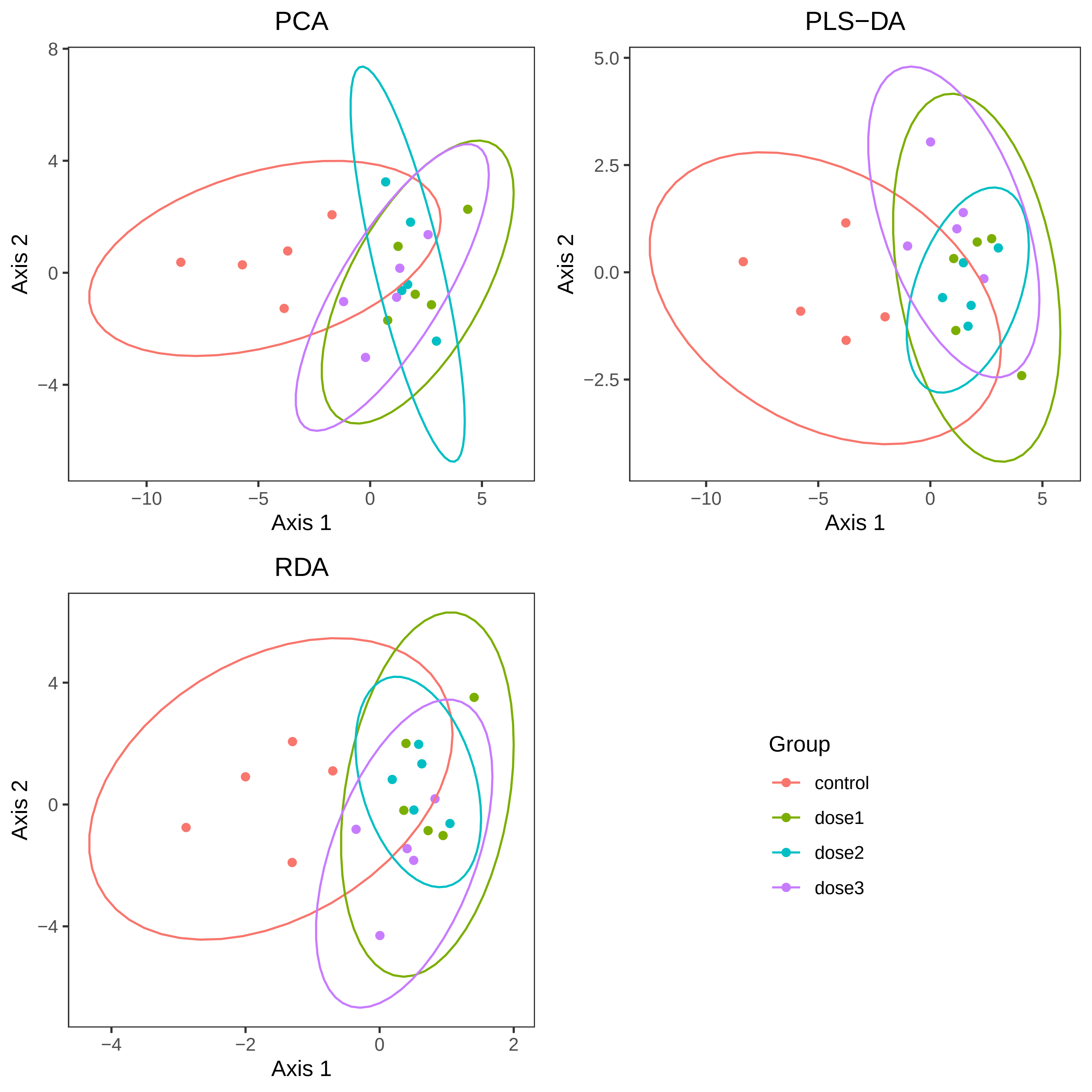

PCA
PLS−DA
RDA
Axis 1
Axis 2
Group
control
dose1
dose2
dose3

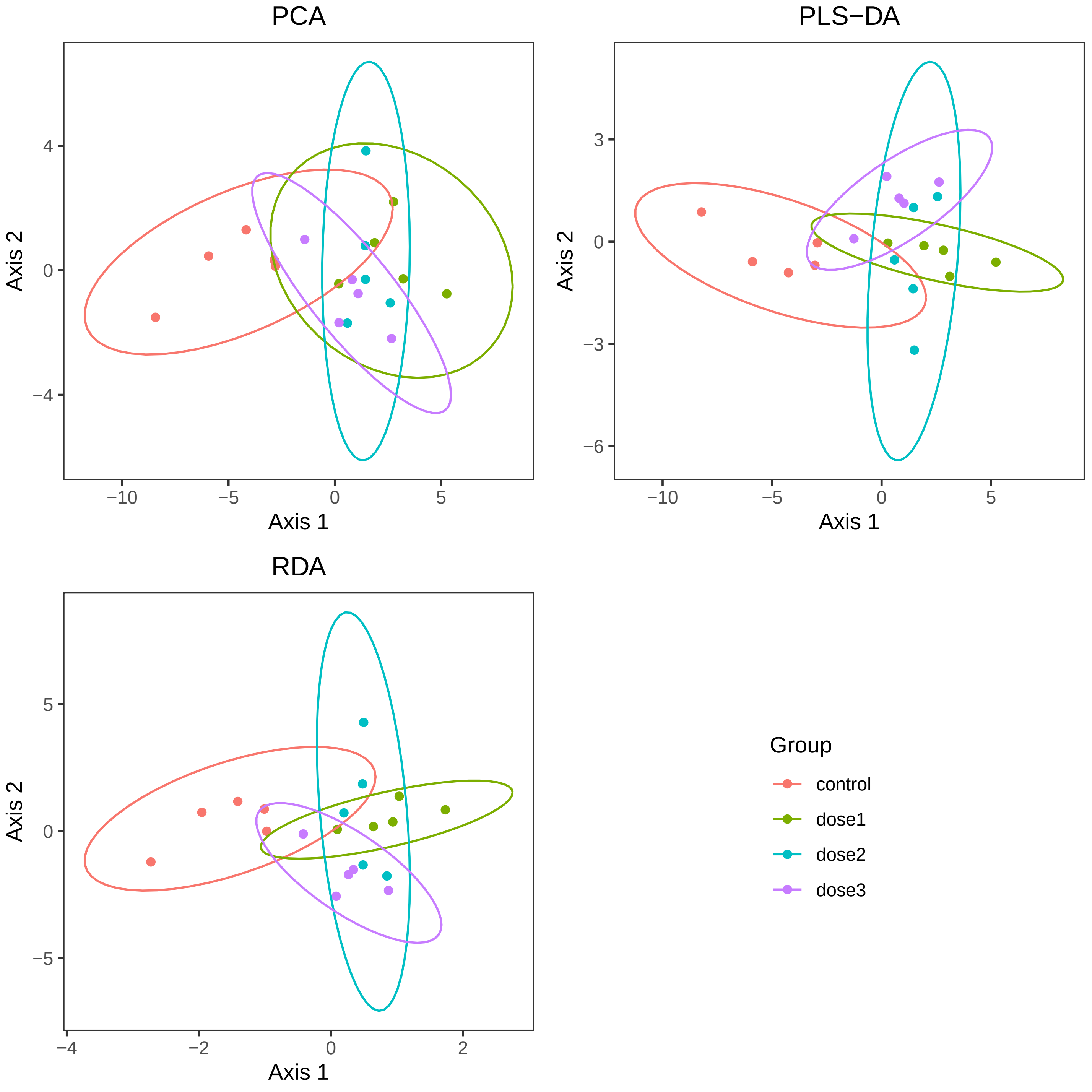
PCA
PLS−DA
RDA
Axis 1
Axis 2
Group
control
dose1
dose2
dose3

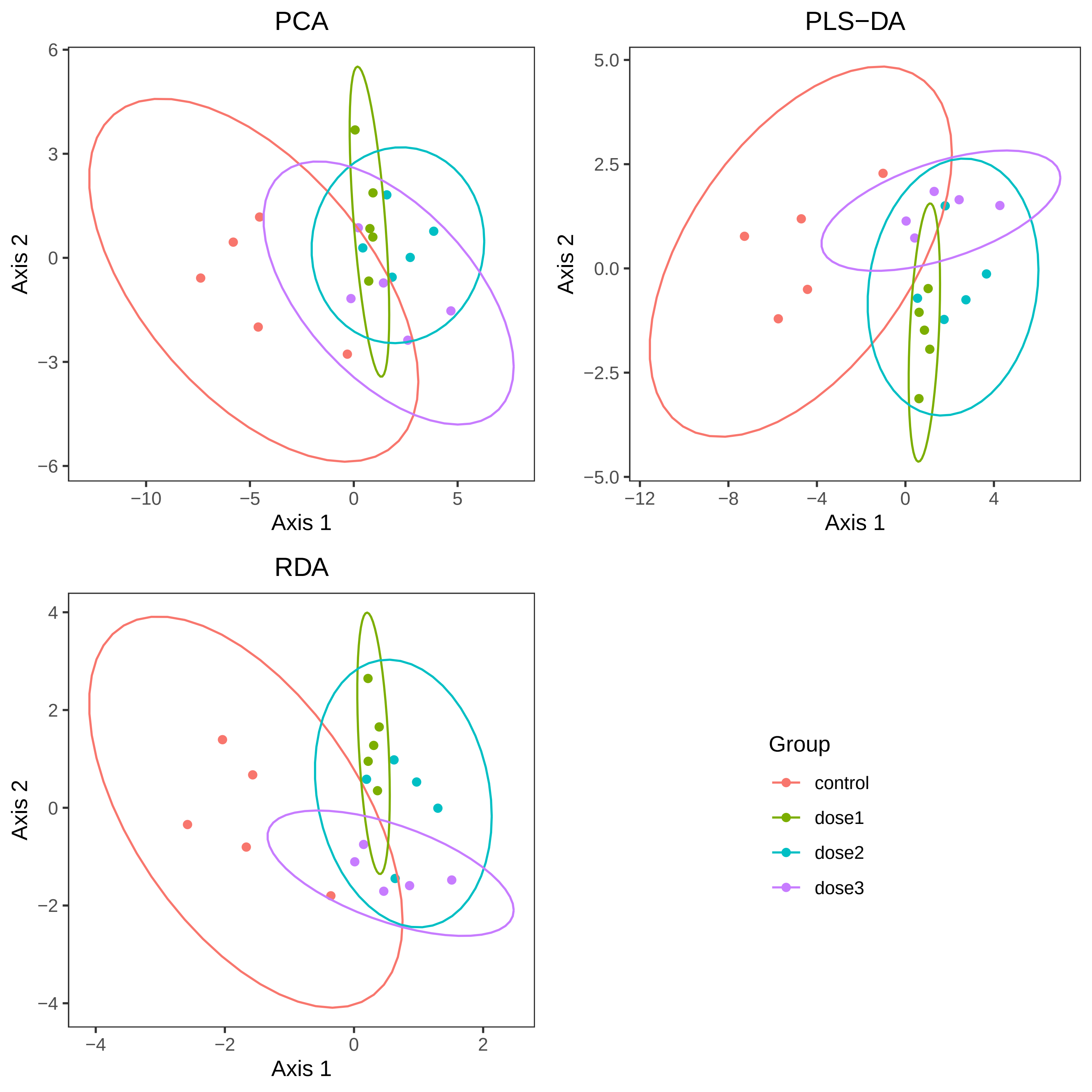

PCA
PLS−DA
RDA
Axis 1
Axis 2
6
3
0
−3
−6
−10
−5
5
5.0
2.5
0.0
−2.5
−5.0
−12
−8
−4
4
2
−2
−4
Group
control
dose1
dose2
dose3

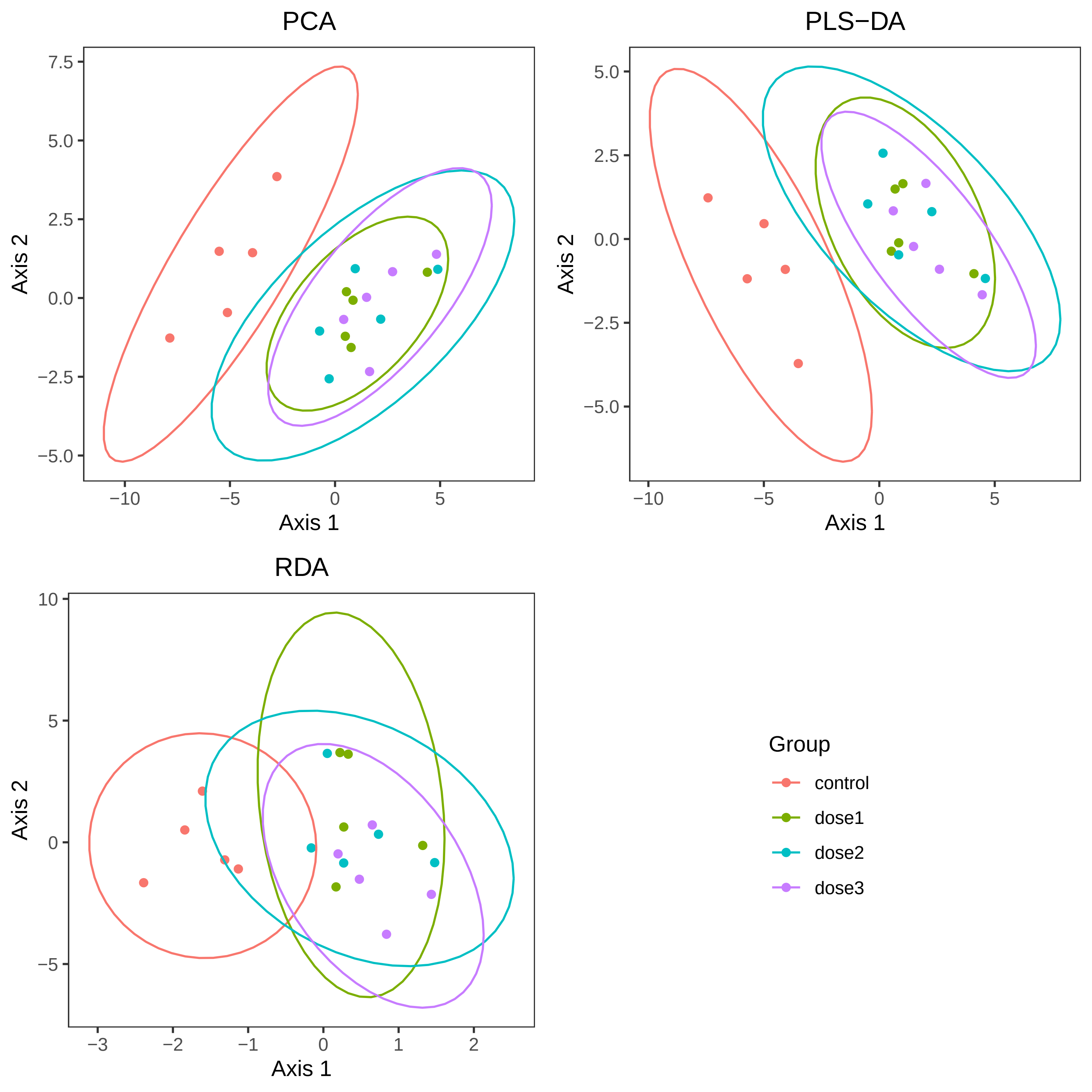

PCA
PLS−DA
RDA
Axis 1
Axis 2
Group
control
dose1
dose2
dose3

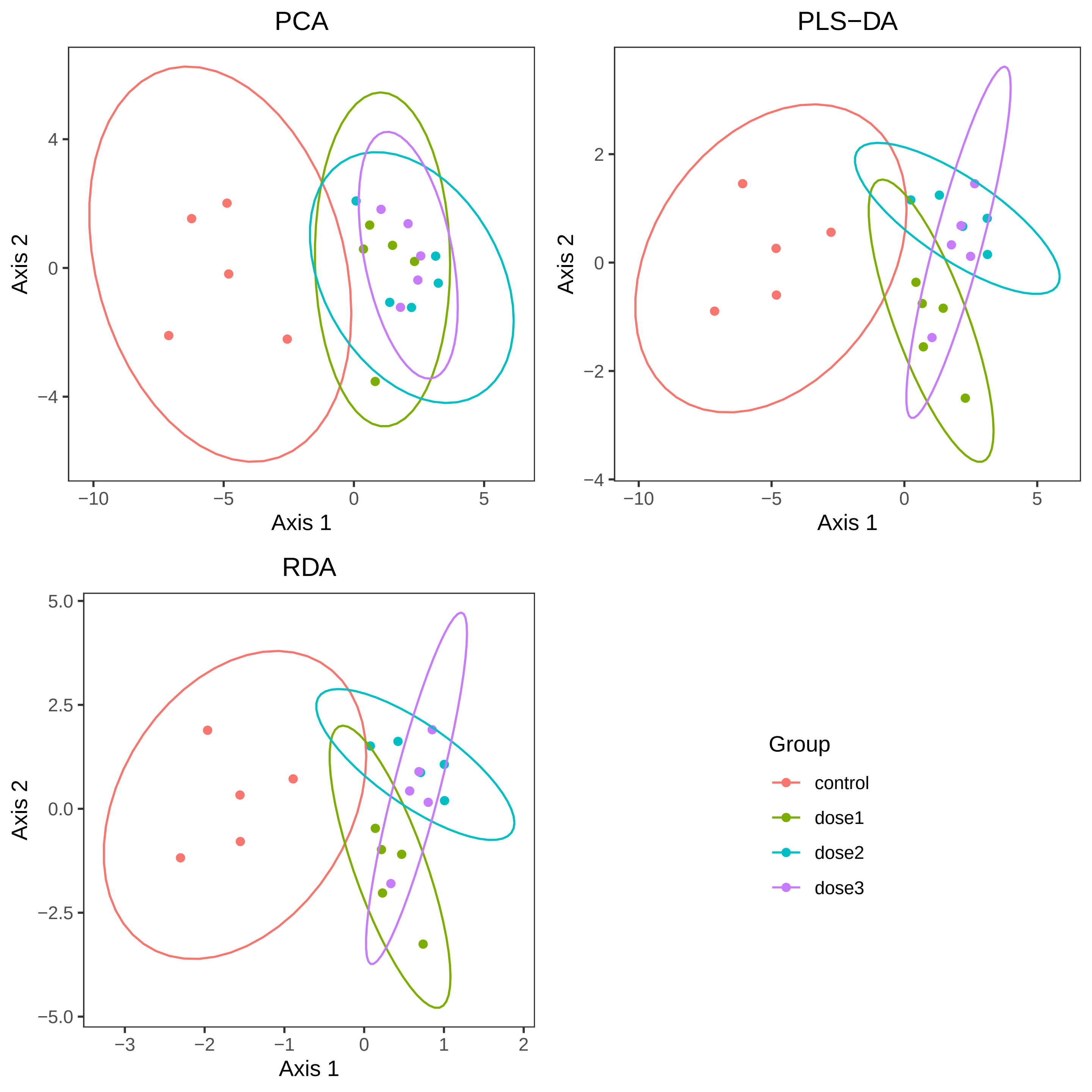

PCA
PLS−DA
RDA
Axis 1
Axis 2
Group
control
dose1
dose2
dose3

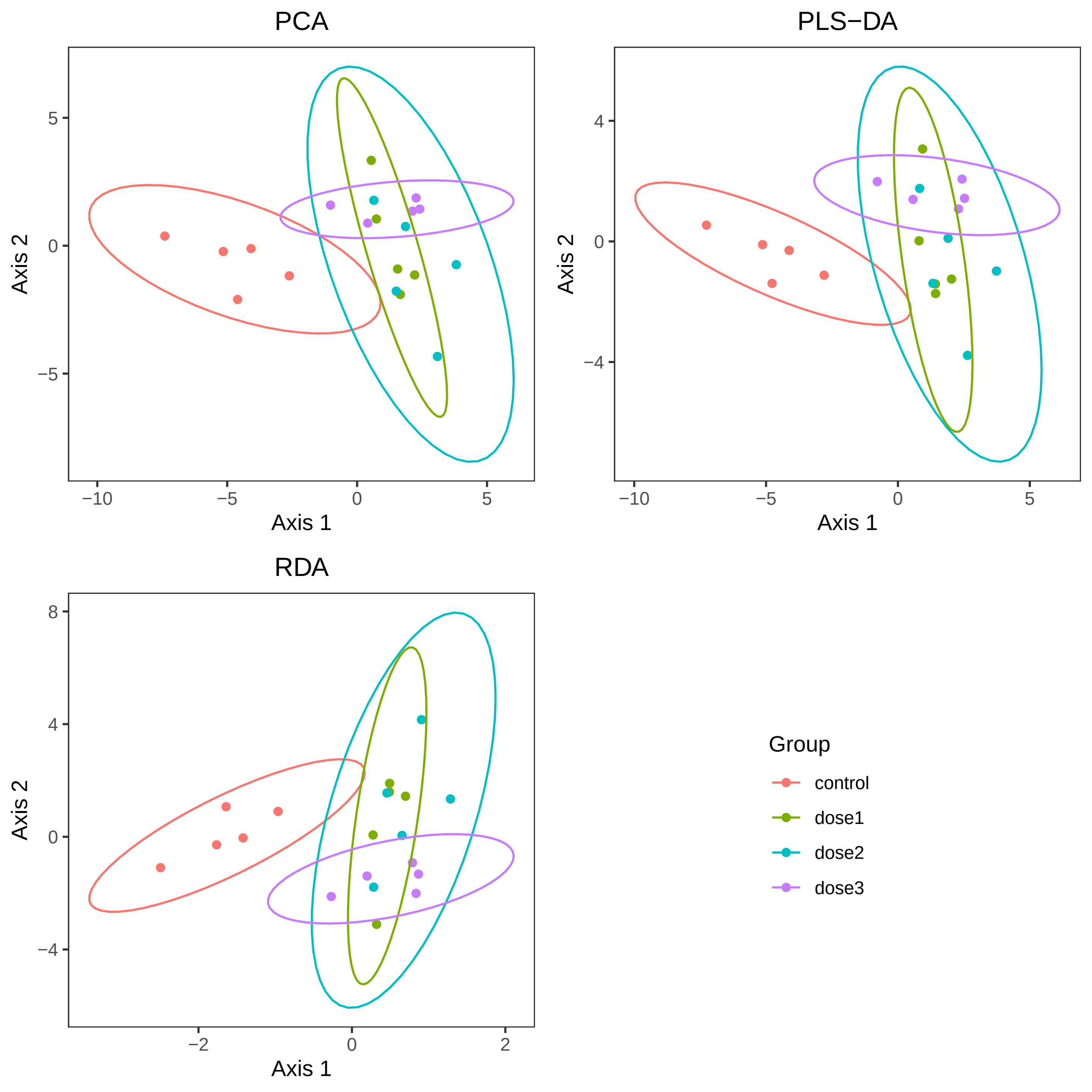

PCA
PLS−DA
RDA
Axis 1
Axis 2
Group
control
dose1
dose2
dose3

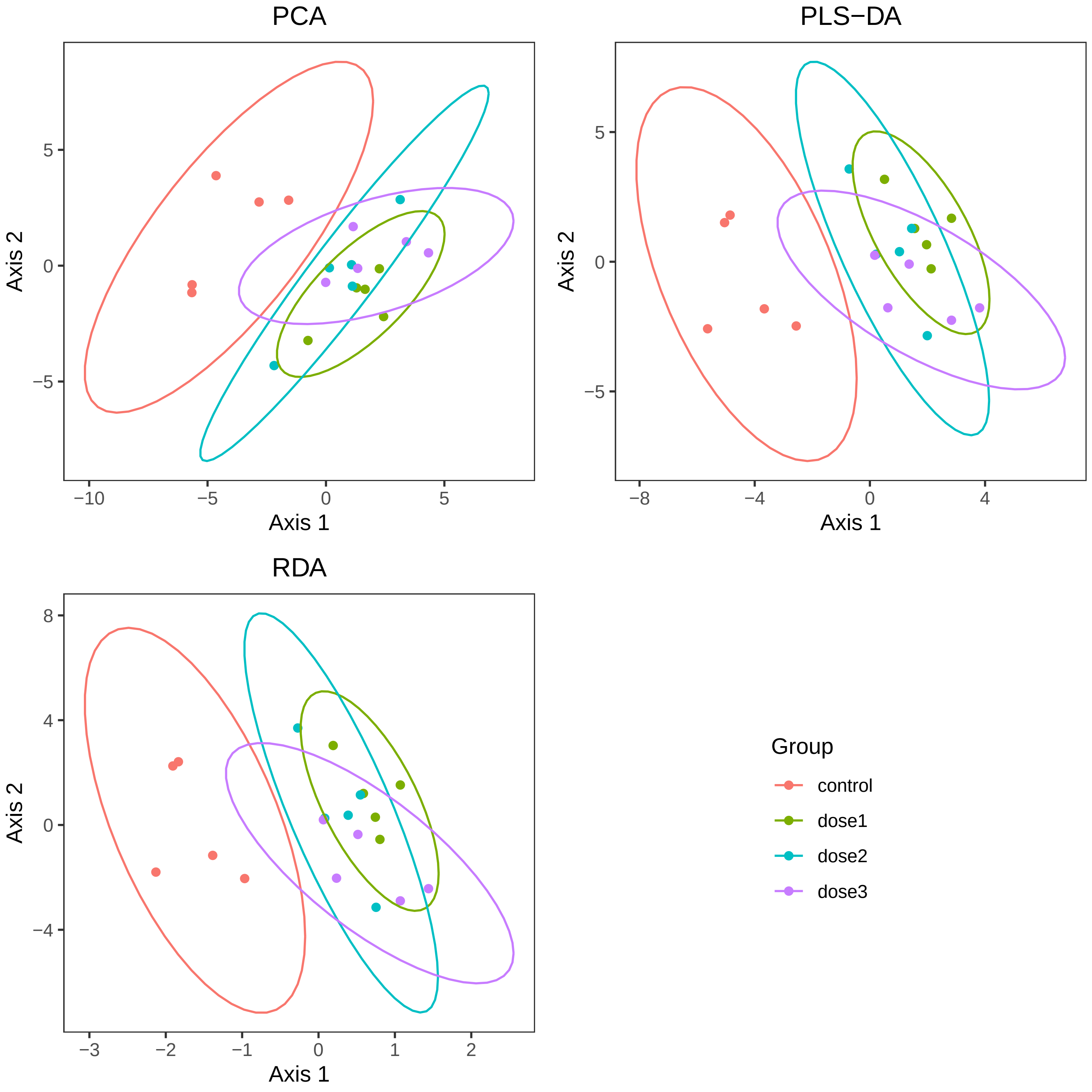
PCA
PLS−DA
RDA
Axis 1
Axis 2
Group
control
dose1
dose2
dose3

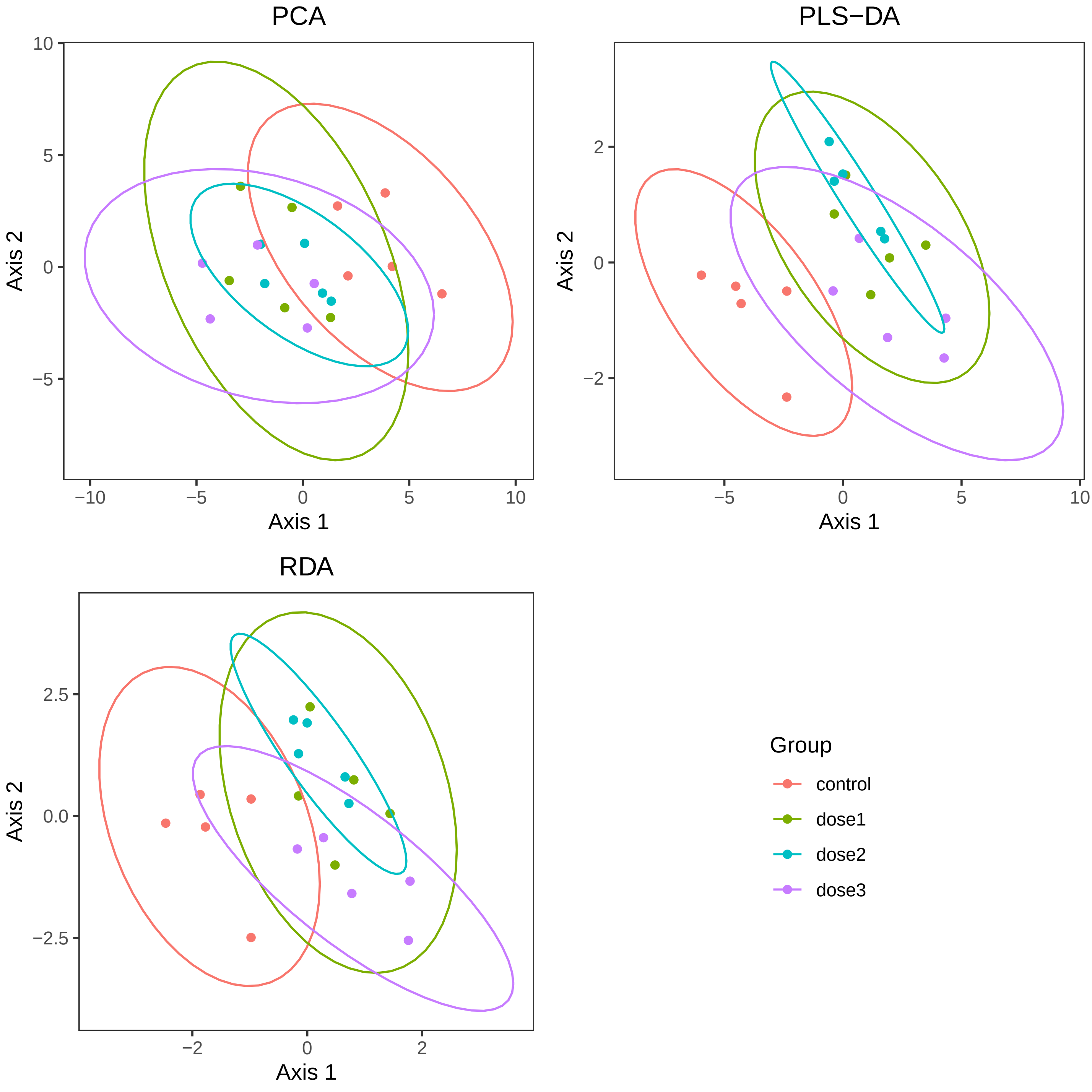
PCA
PLS−DA
RDA
Axis 1
Axis 2
10
5
0
−5
−10
2
−2
2.5
0.0
−2.5
Group
control
dose1
dose2
dose3

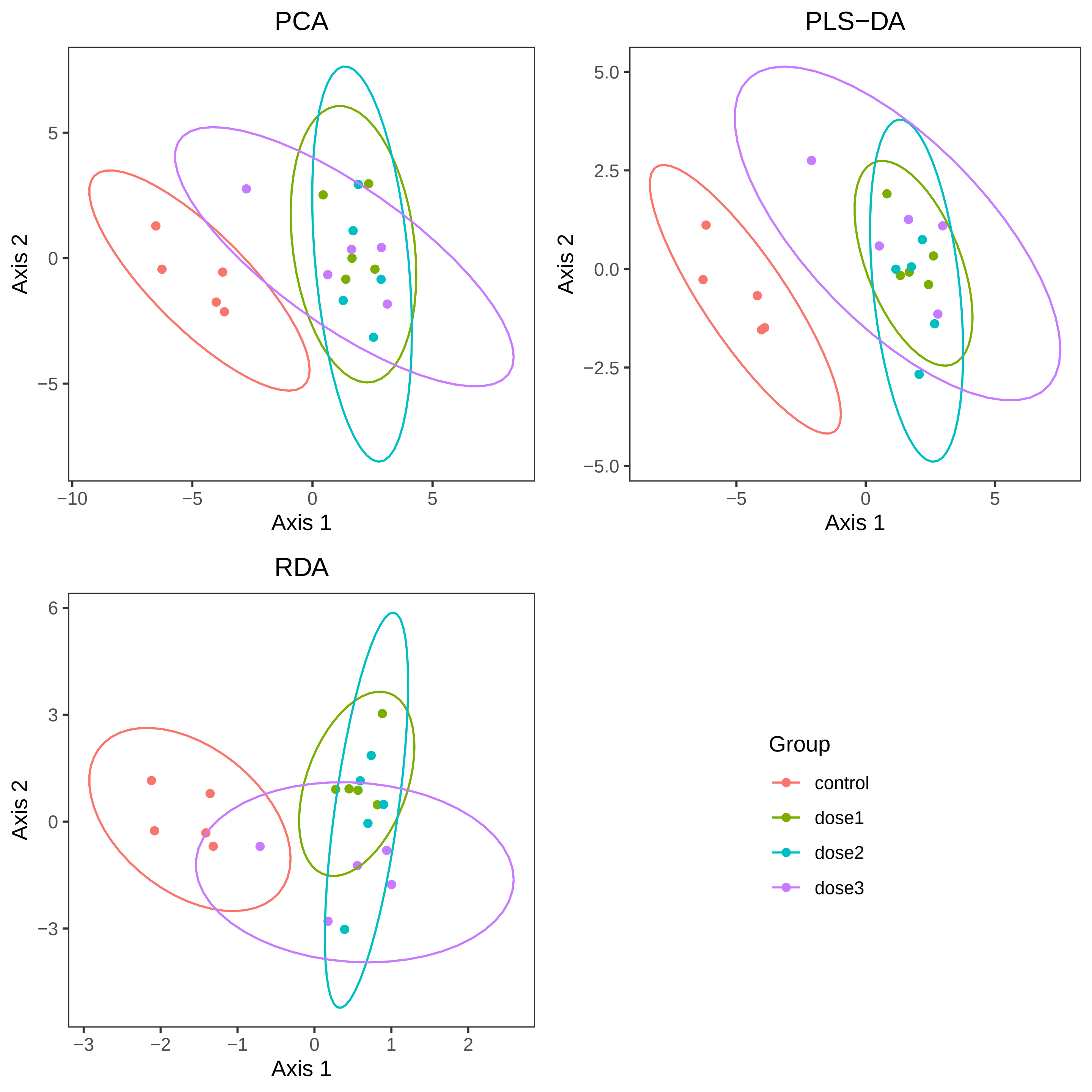

PCA
PLS−DA
RDA
Axis 1
Axis 2
−10
−5
0
5
5.0
2.5
0.0
−2.5
−5.0
−3
−2
−1
1
2
3
6
Group
control
dose1
dose2
dose3

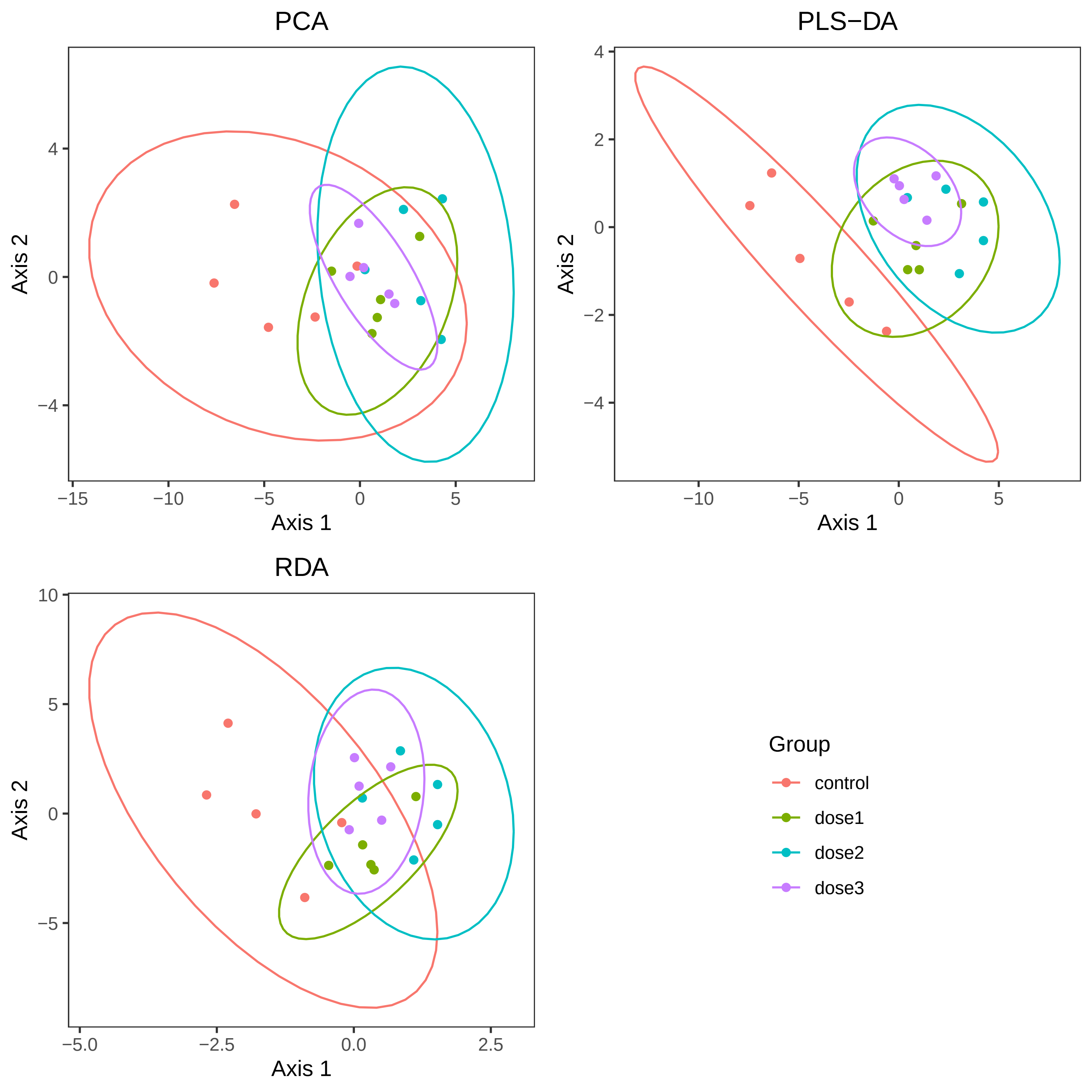
PCA
PLS−DA
RDA
Axis 1
Axis 2
Group
control
dose1
dose2
dose3

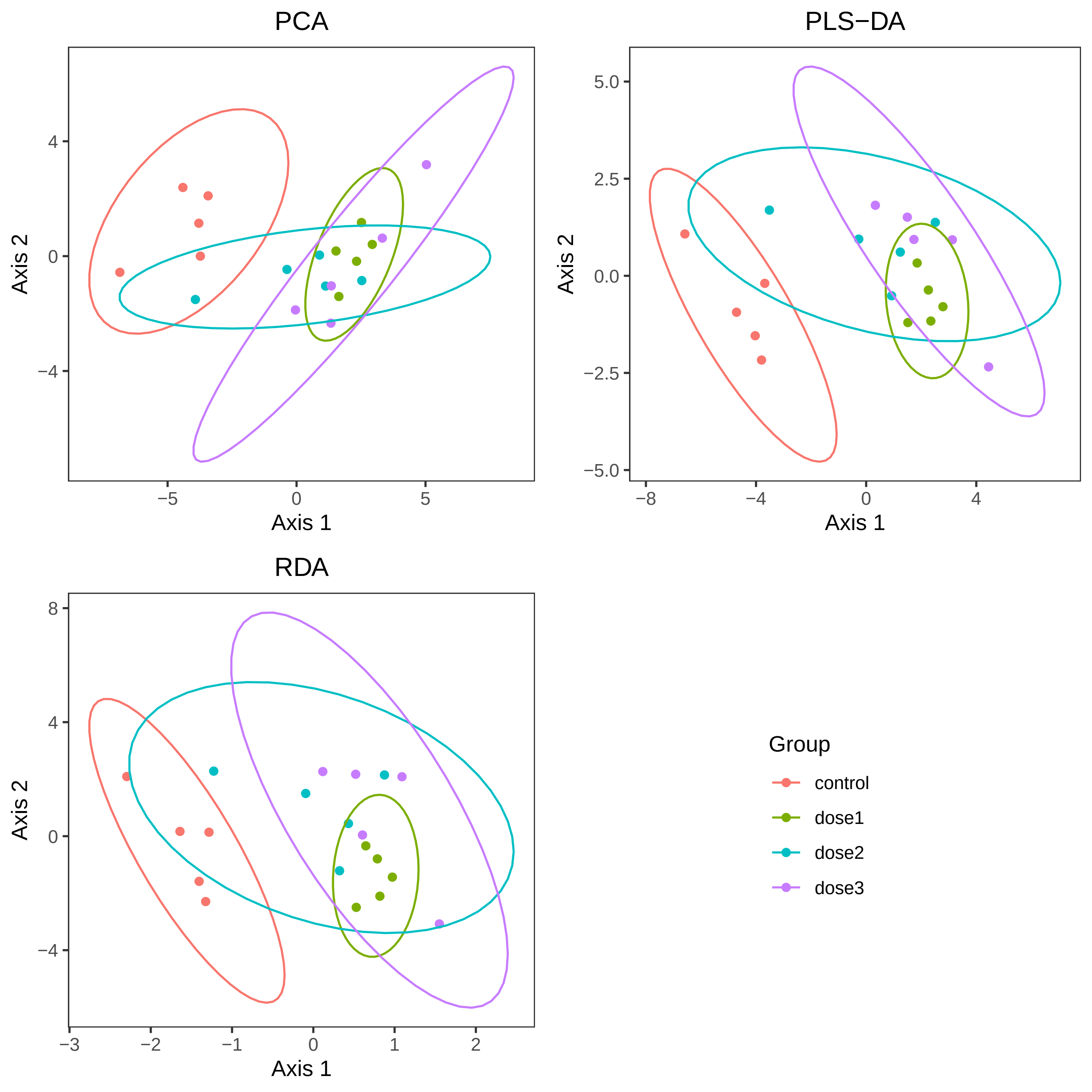
PCA
PLS−DA
RDA
Axis 1
Axis 2
Group
control
dose1
dose2
dose3

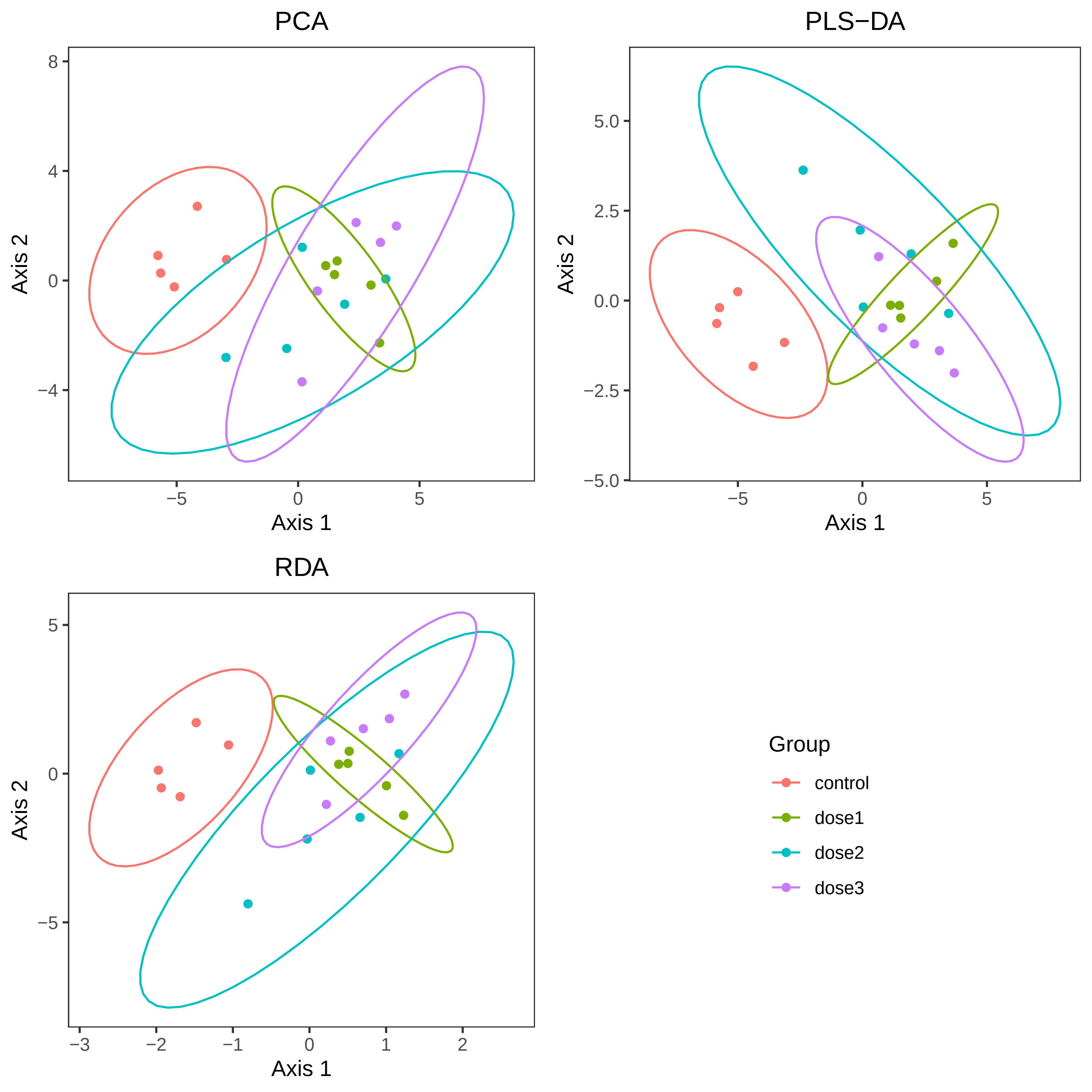
PCA
Axis 1
Axis 2
PLS−DA
Axis 1
Axis 2
RDA
Axis 1
Axis 2
Group
control
dose1
dose2
dose3

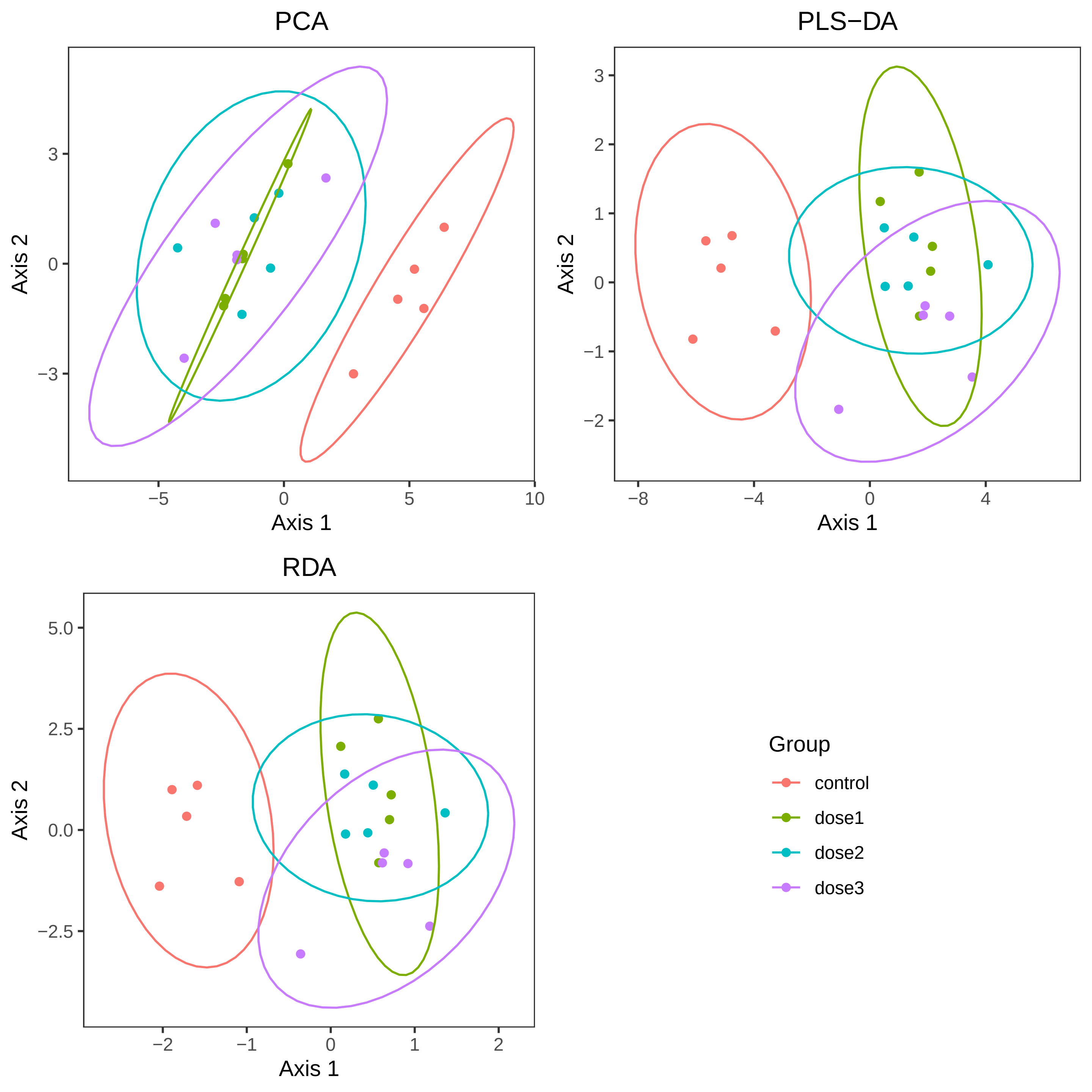
PCA
PLS−DA
RDA
Axis 1
Axis 2
Group
control
dose1
dose2
dose3

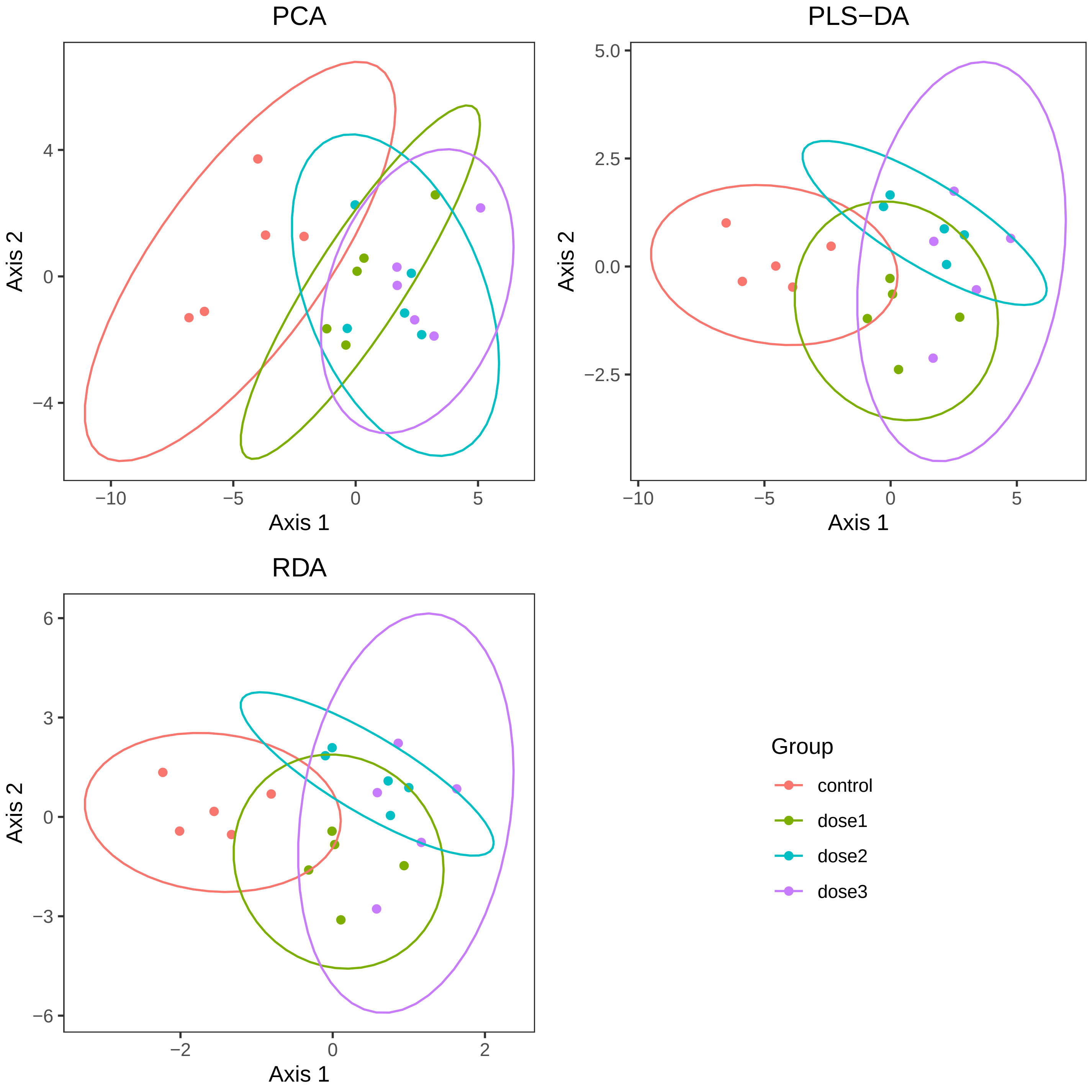

PCA
PLS−DA
RDA
Axis 1
Axis 2
Group
control
dose1
dose2
dose3

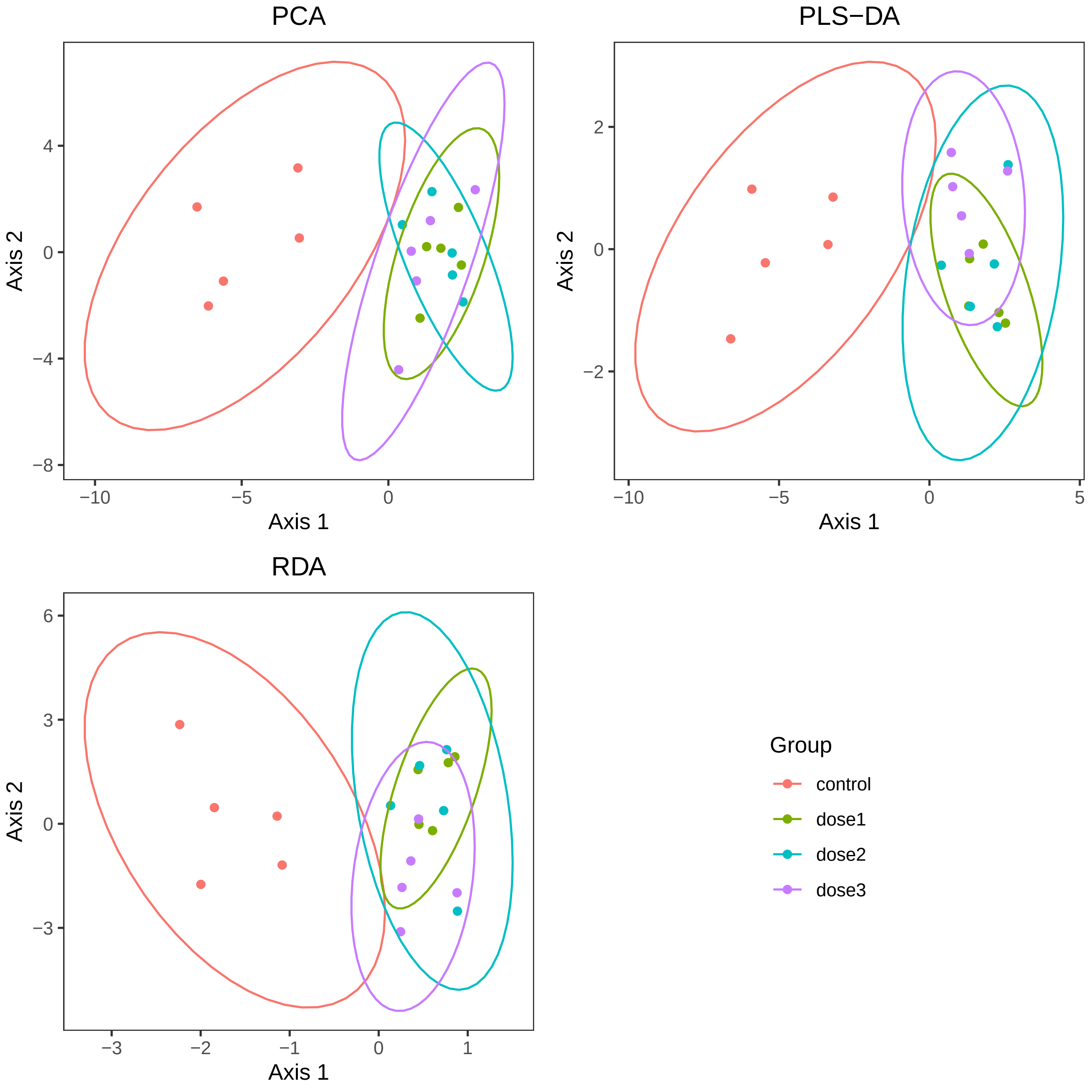

PCA
PLS−DA
RDA
Axis 1
Axis 2
Group
control
dose1
dose2
dose3

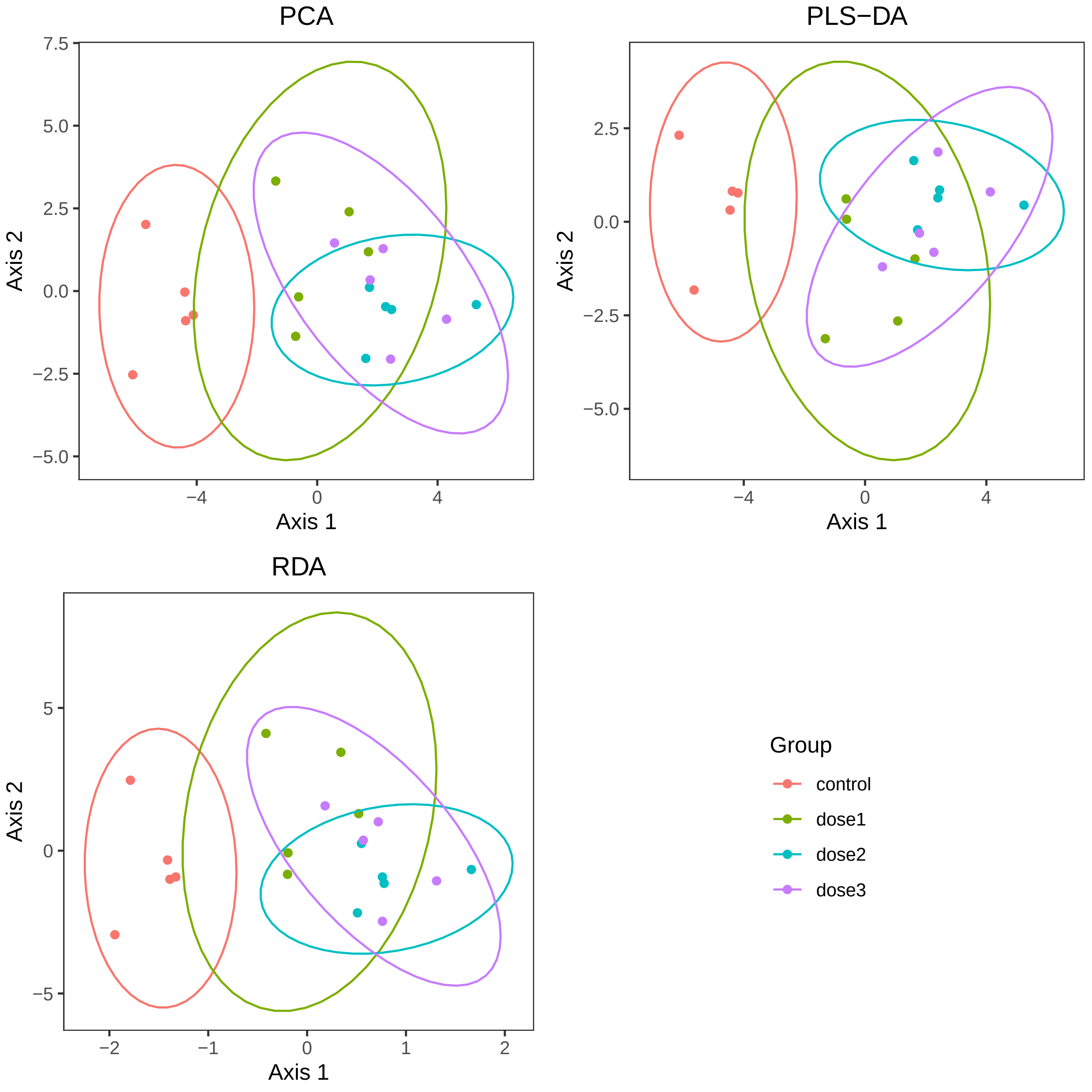
PCA
PLS−DA
RDA
Axis 1
Axis 2
7.5
5.0
2.5
0.0
−2.5
−5.0
−4
0
4
5
0
−5
−2
−1
1
2
Group
control
dose1
dose2
dose3

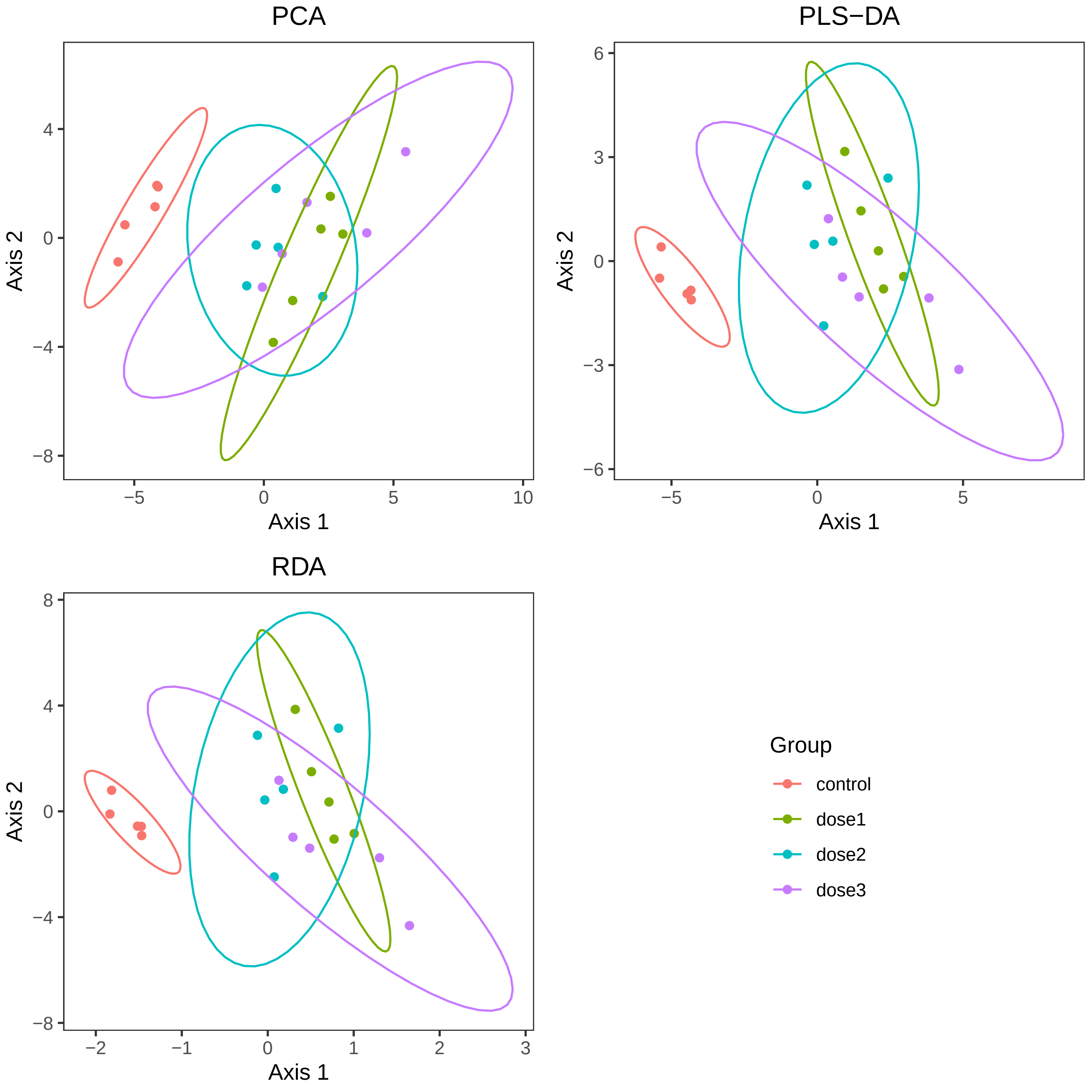

PCA
PLS−DA
RDA
Axis 1
Axis 2
Group
control
dose1
dose2
dose3

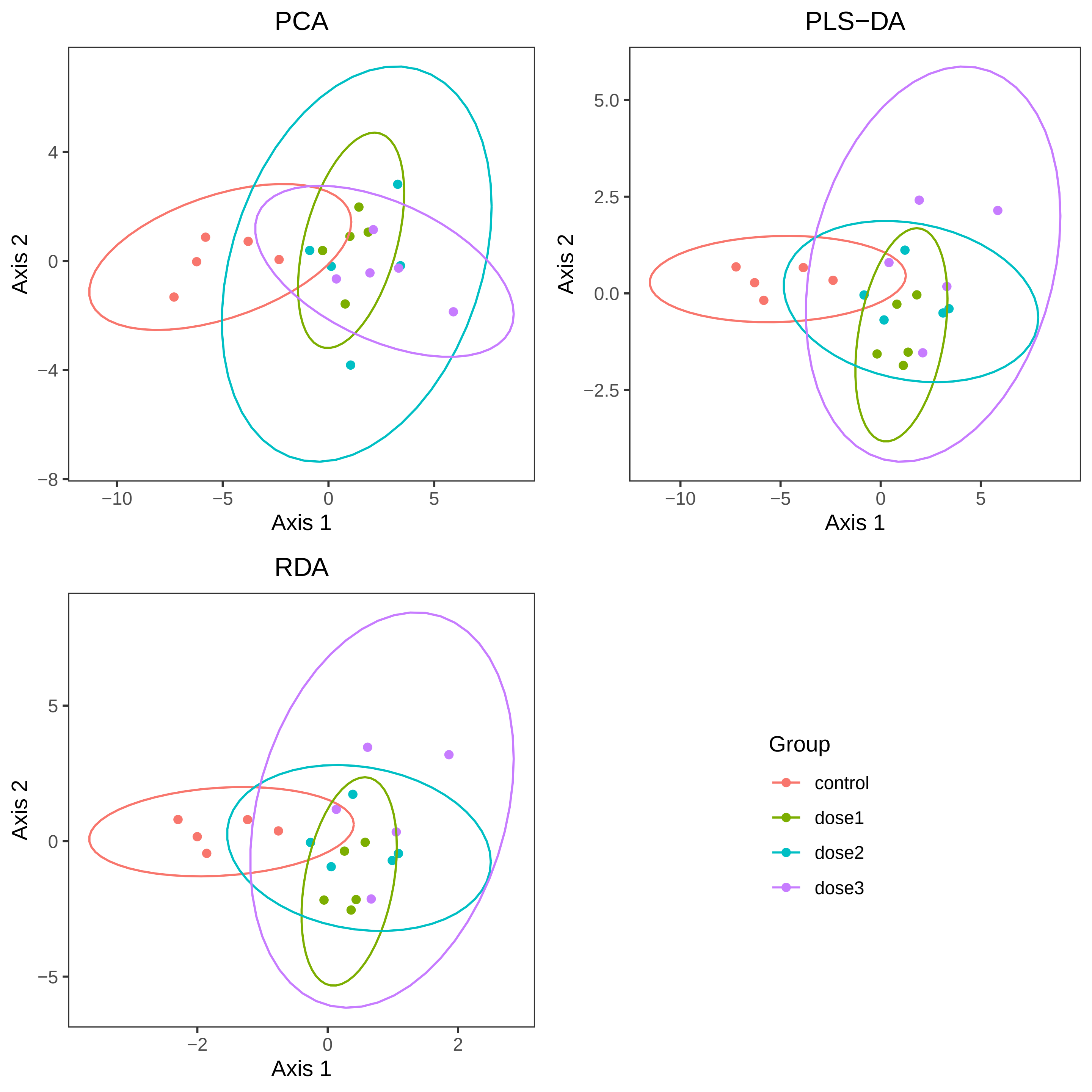

PCA
PLS−DA
RDA
Axis 1
Axis 2
Group
control
dose1
dose2
dose3

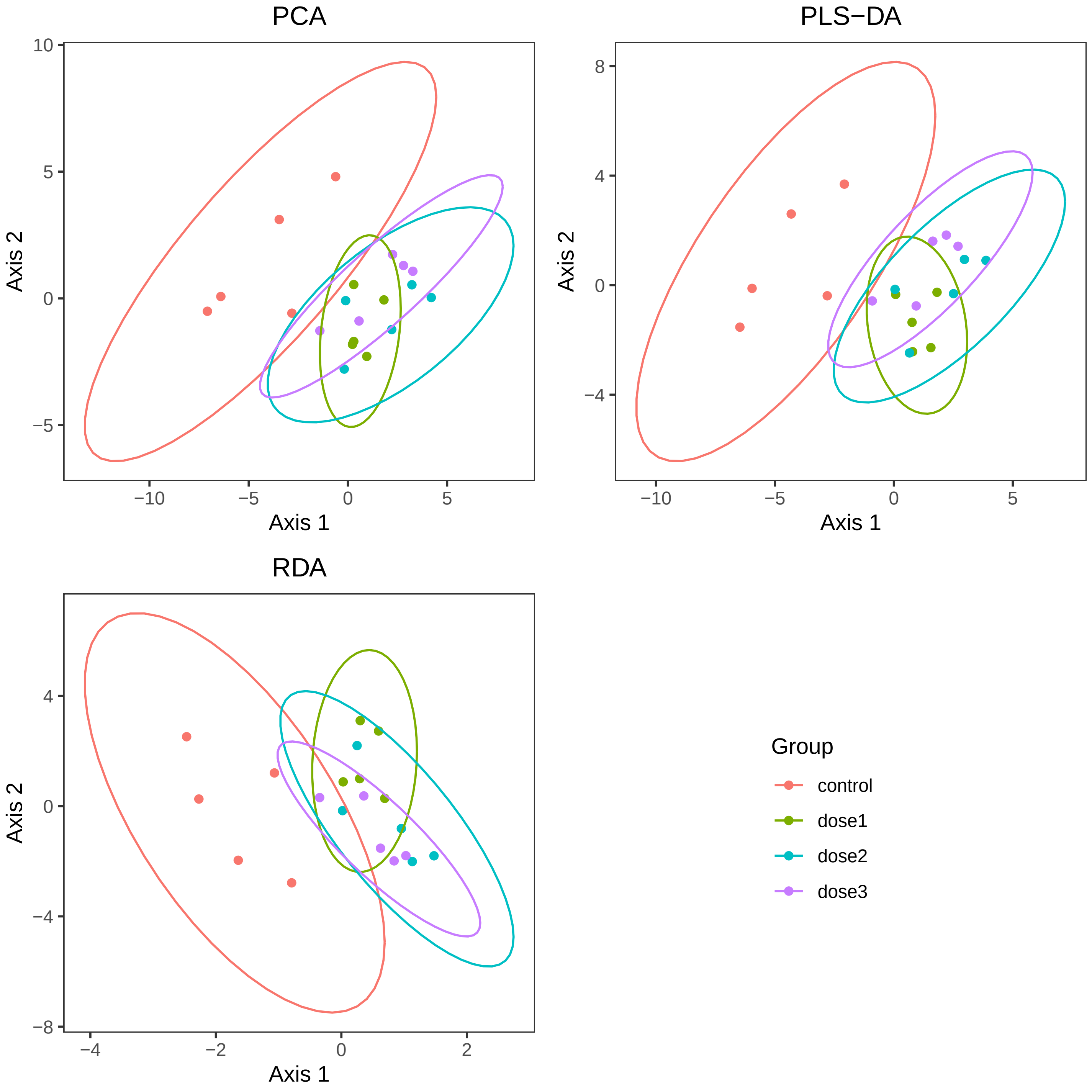
PCA
PLS−DA
RDA
Axis 1
Axis 2
Group
control
dose1
dose2
dose3

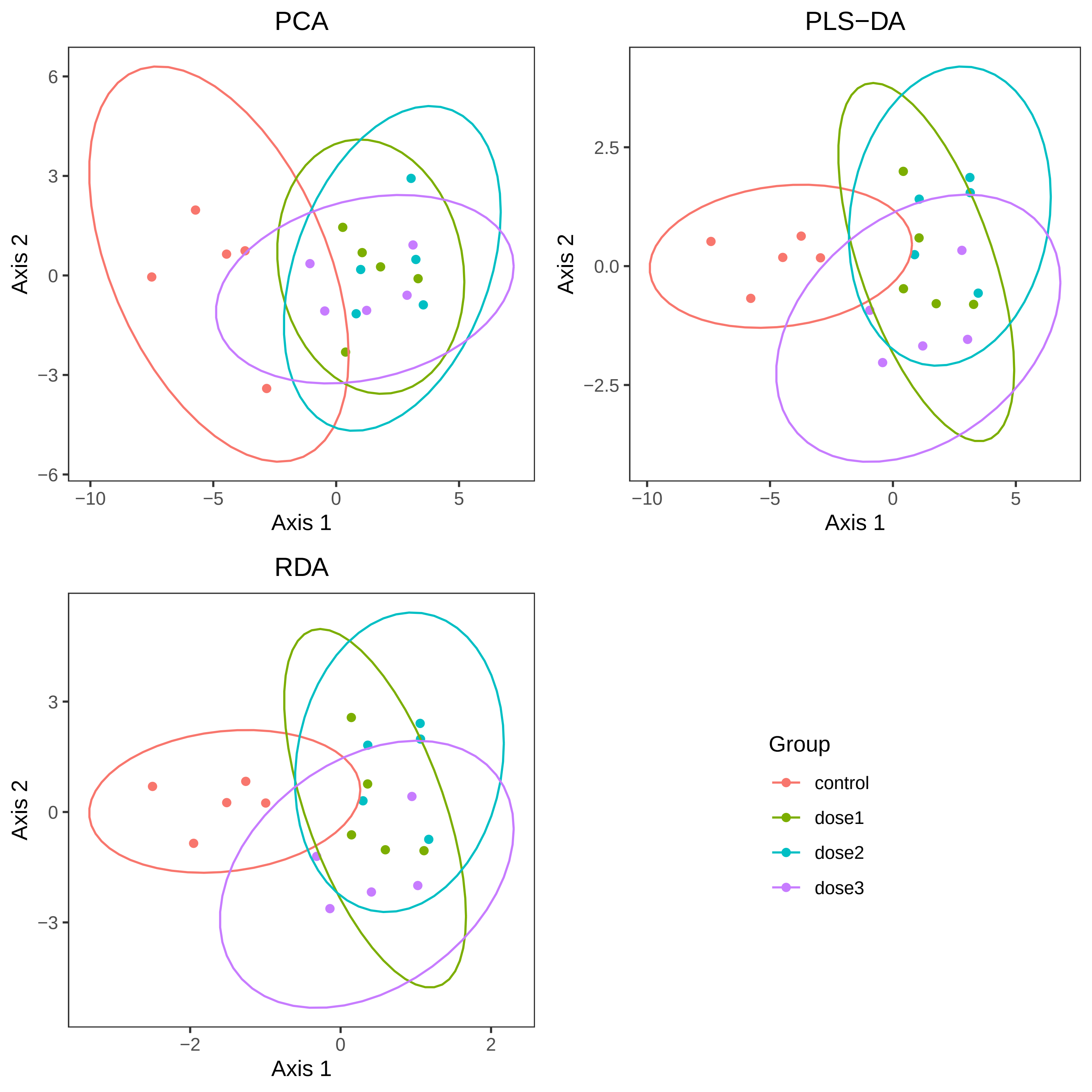

PCA
PLS−DA
RDA
Axis 1
Axis 2
6
3
0
−3
−6
−10
−5
0
5
2.5
0.0
−2.5
−2
2
Group
control
dose1
dose2
dose3

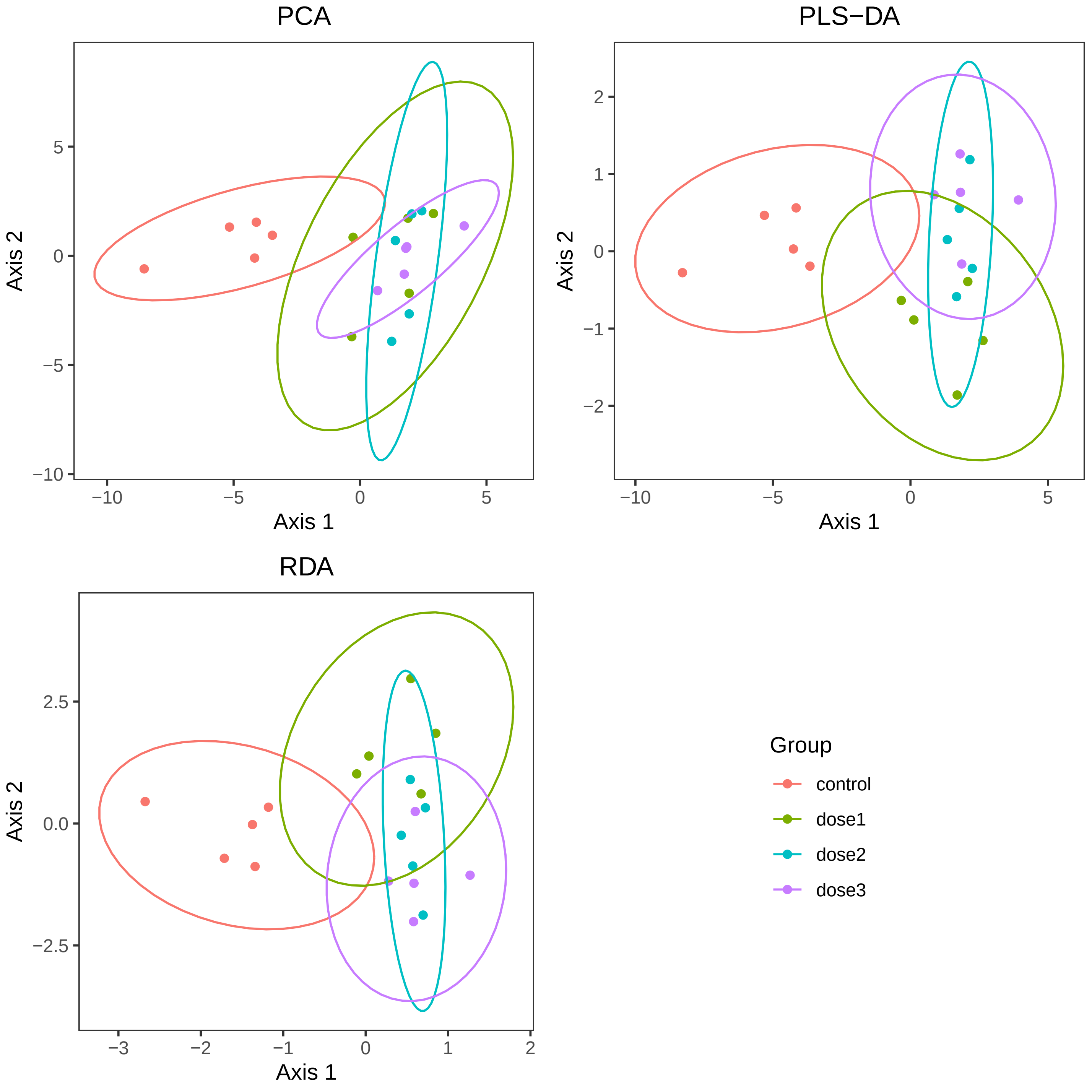

PCA
PLS−DA
RDA
Axis 1
Axis 2
−10
−5
0
5
2.5
0.0
−2.5
−3
−2
−1
1
2
Group
control
dose1
dose2
dose3

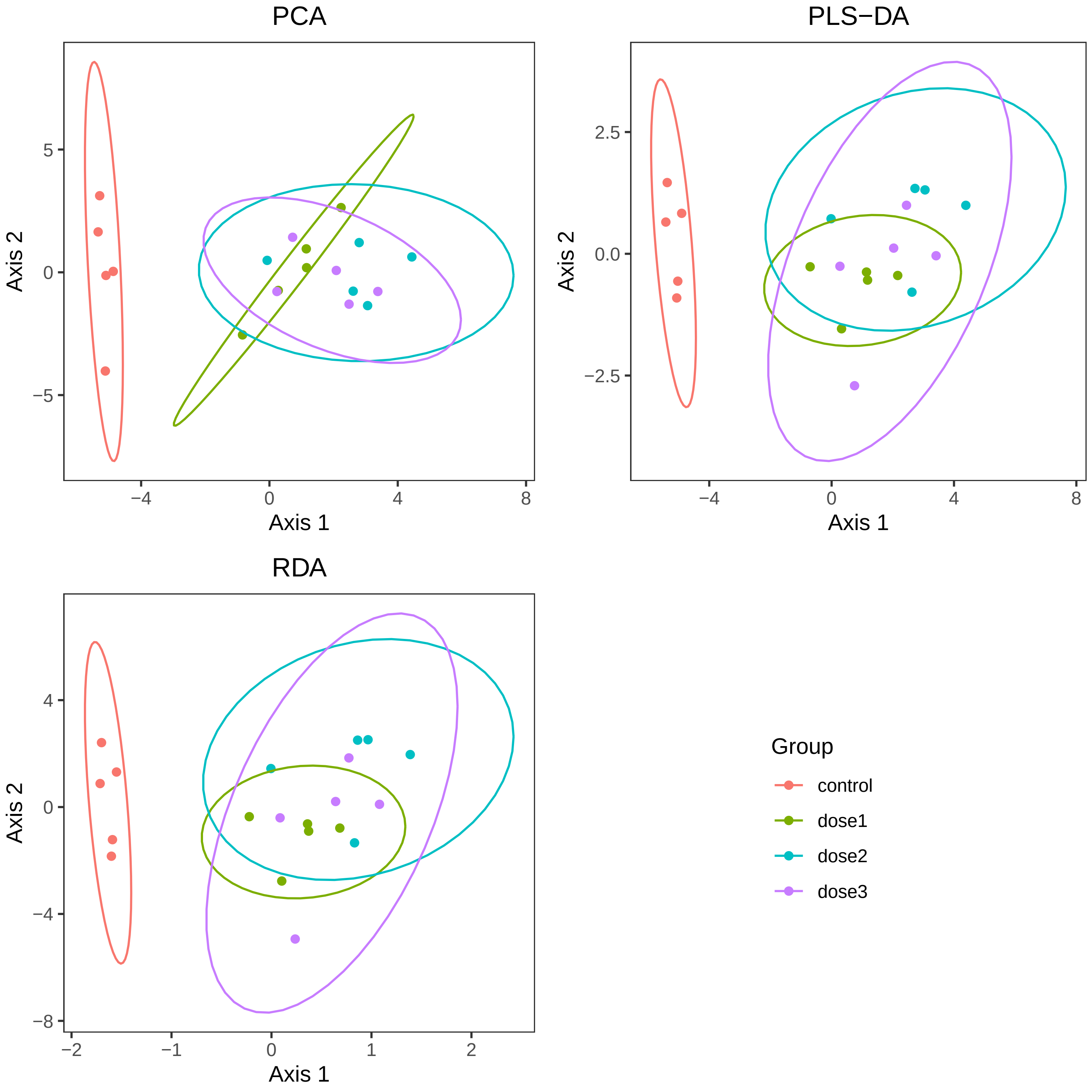

PCA
PLS−DA
RDA
Axis 1
Axis 2
5
0
−5
−4
0
4
8
2.5
0.0
−2.5
4
0
−4
−8
−2
−1
0
1
2
Group
control
dose1
dose2
dose3

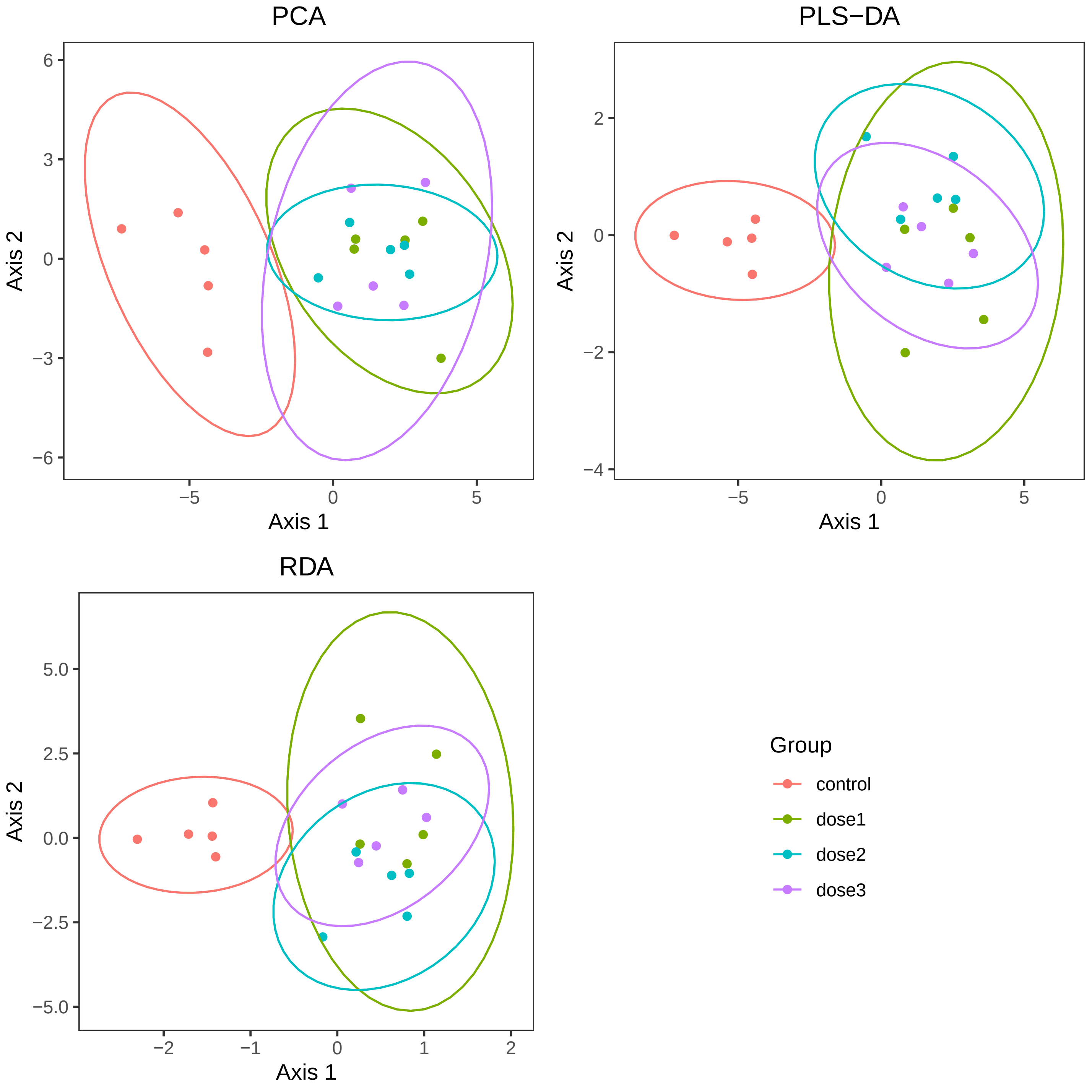

PCA
PLS−DA
RDA
Axis 1
Axis 2
Group
control
dose1
dose2
dose3

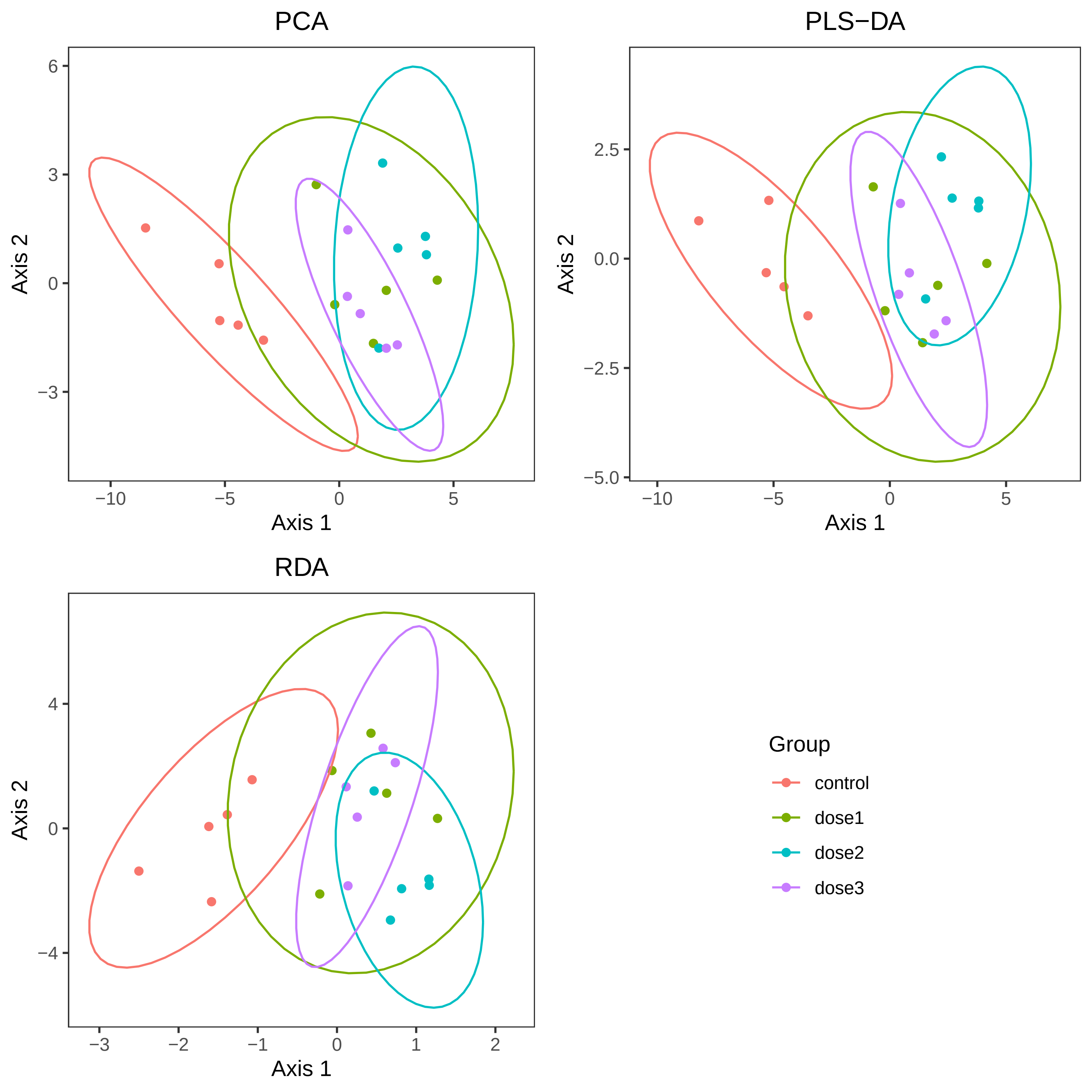

PCA
PLS−DA
RDA
Axis 1
Axis 2
Group
control
dose1
dose2
dose3

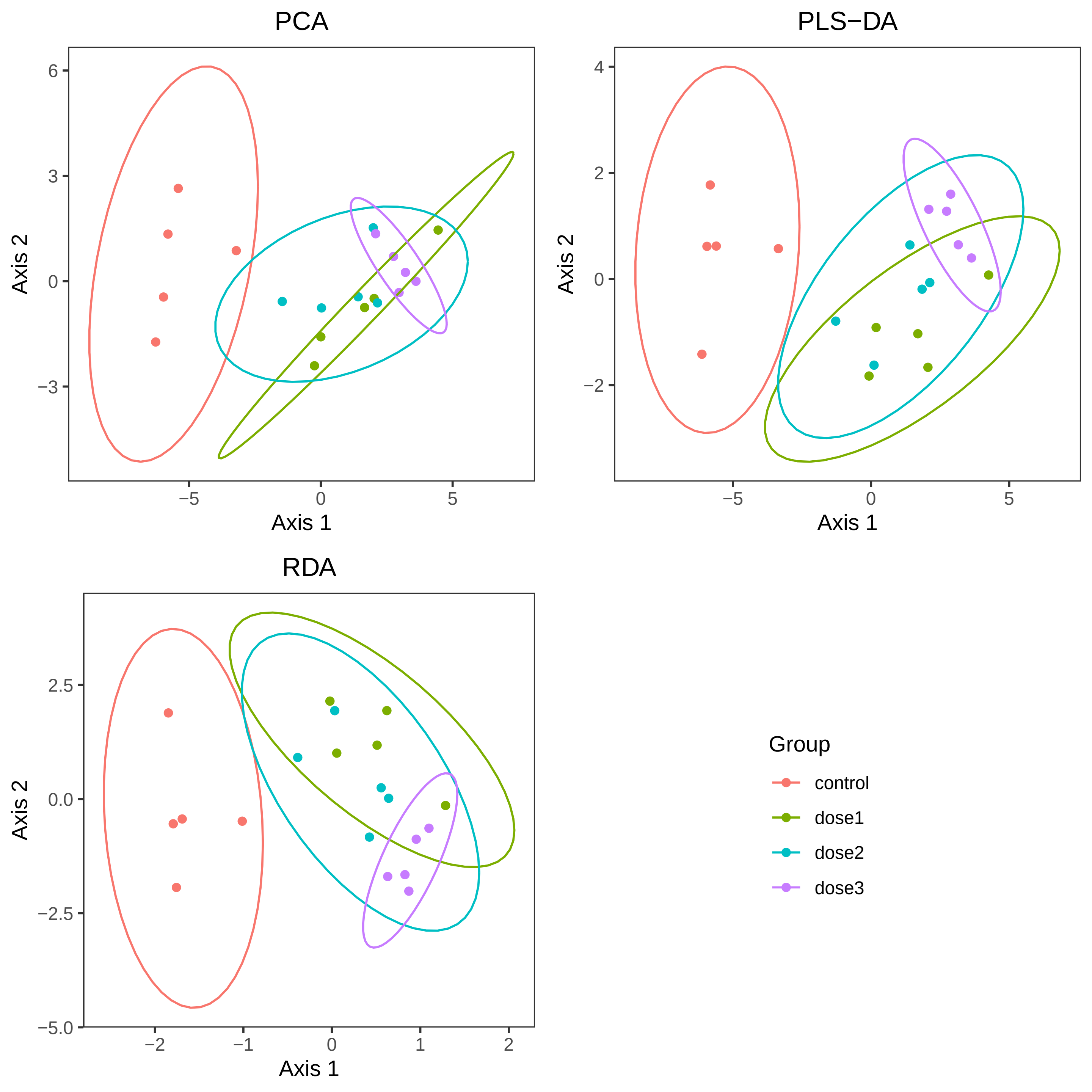

PCA
PLS−DA
RDA
Axis 1
Axis 2
6
3
0
−3
−5
0
5
4
2
0
−2
−5
0
5
2.5
0.0
−2.5
−5.0
−2
−1
0
1
2
Group
control
dose1
dose2
dose3

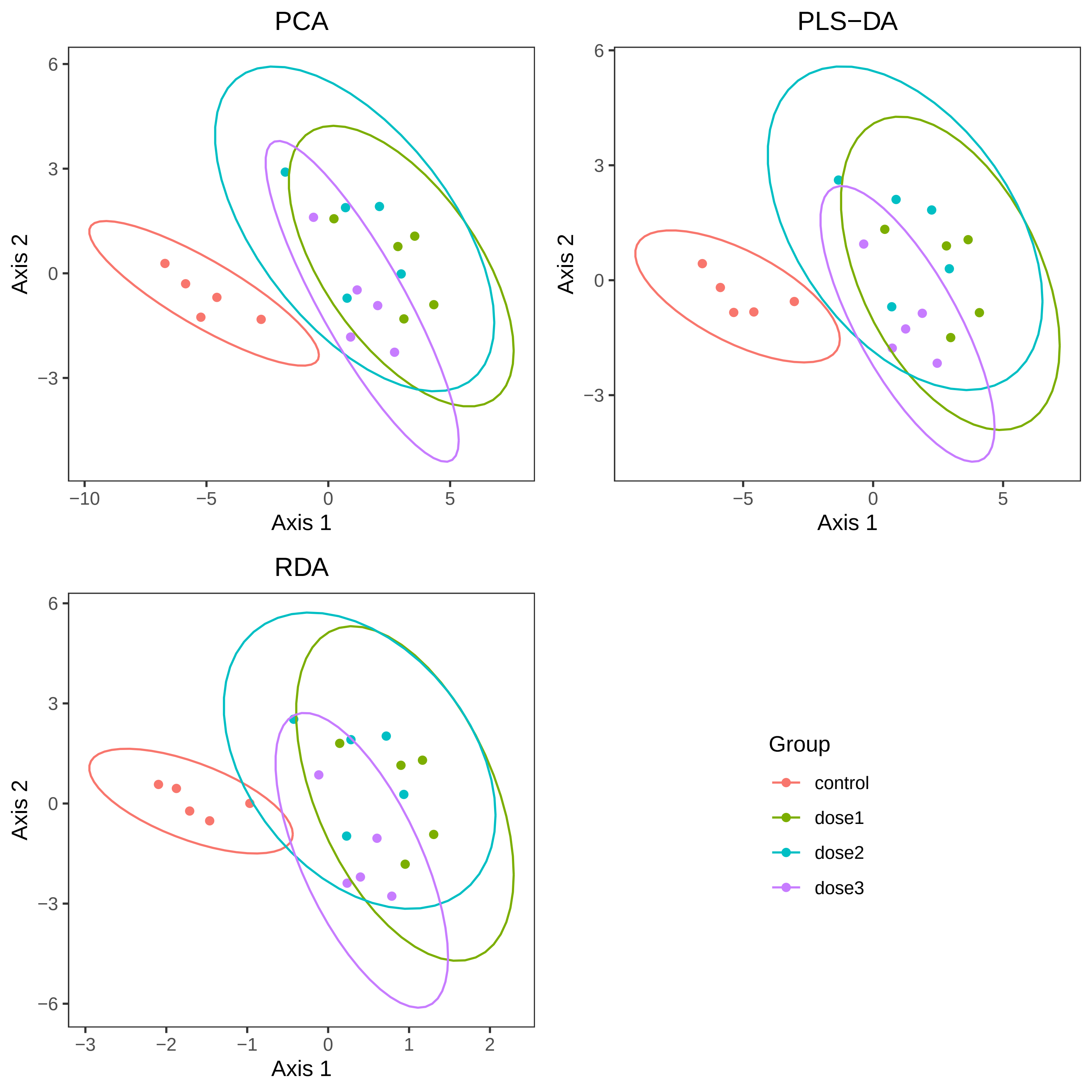

PCA
PLS−DA
RDA
Axis 1
Axis 2
Group
control
dose1
dose2
dose3

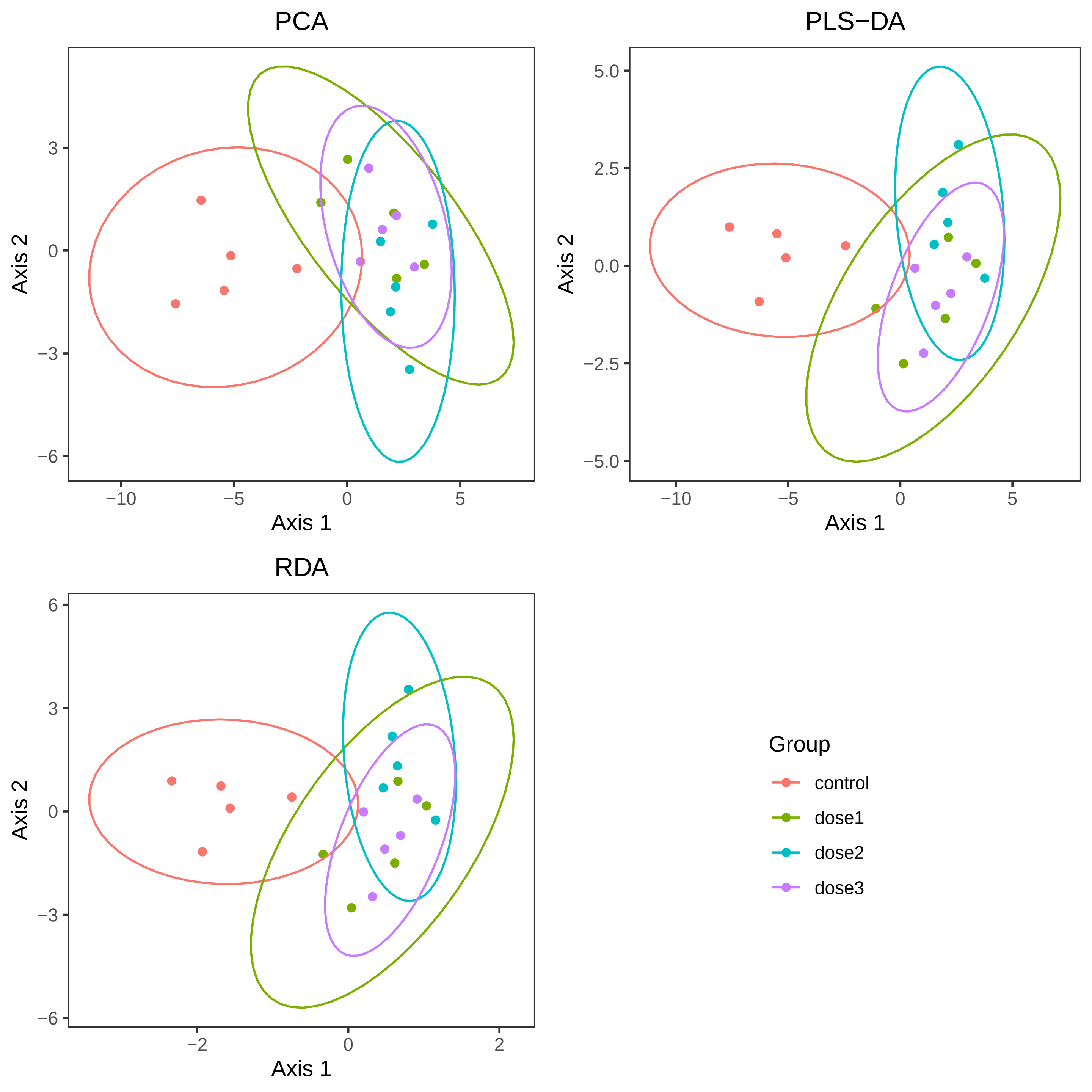
PCA
PLS−DA
RDA
Axis 1
Axis 2
Group
control
dose1
dose2
dose3

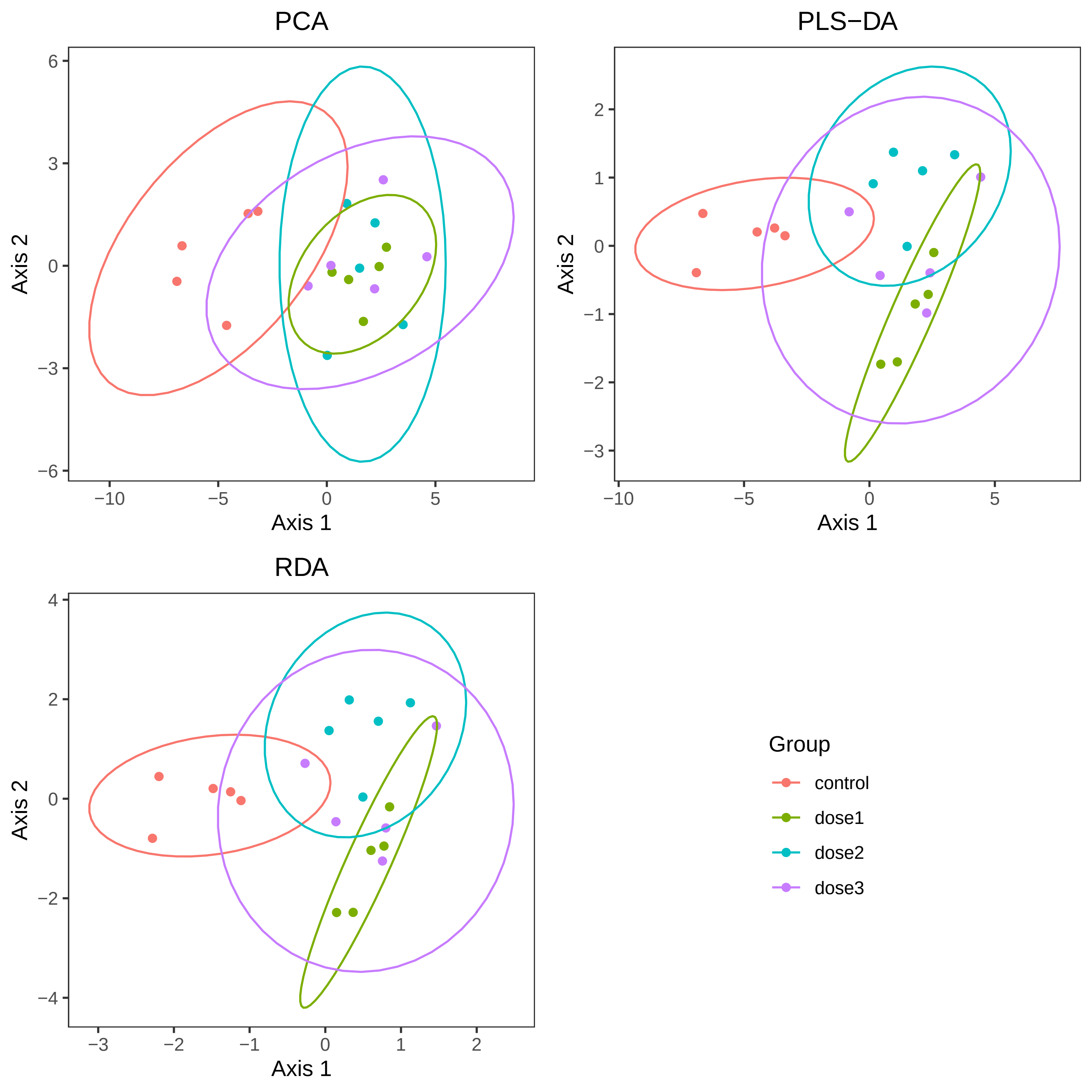
PCA
PLS−DA
RDA
Axis 1
Axis 2
Group
control
dose1
dose2
dose3

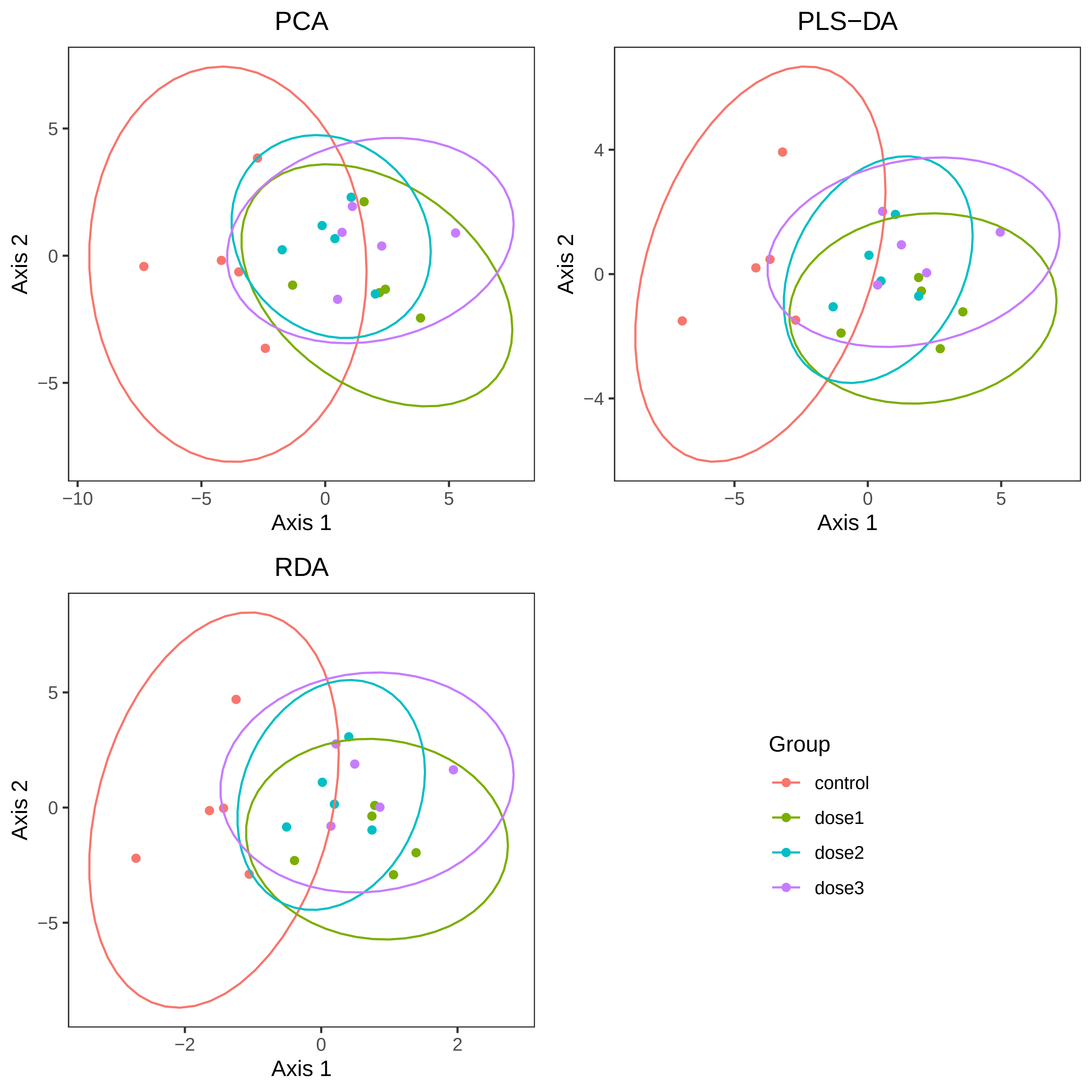
PCA
PLS−DA
RDA
Axis 1
Axis 2
−10
−5
0
5
4
−4
−2
2
Group
control
dose1
dose2
dose3

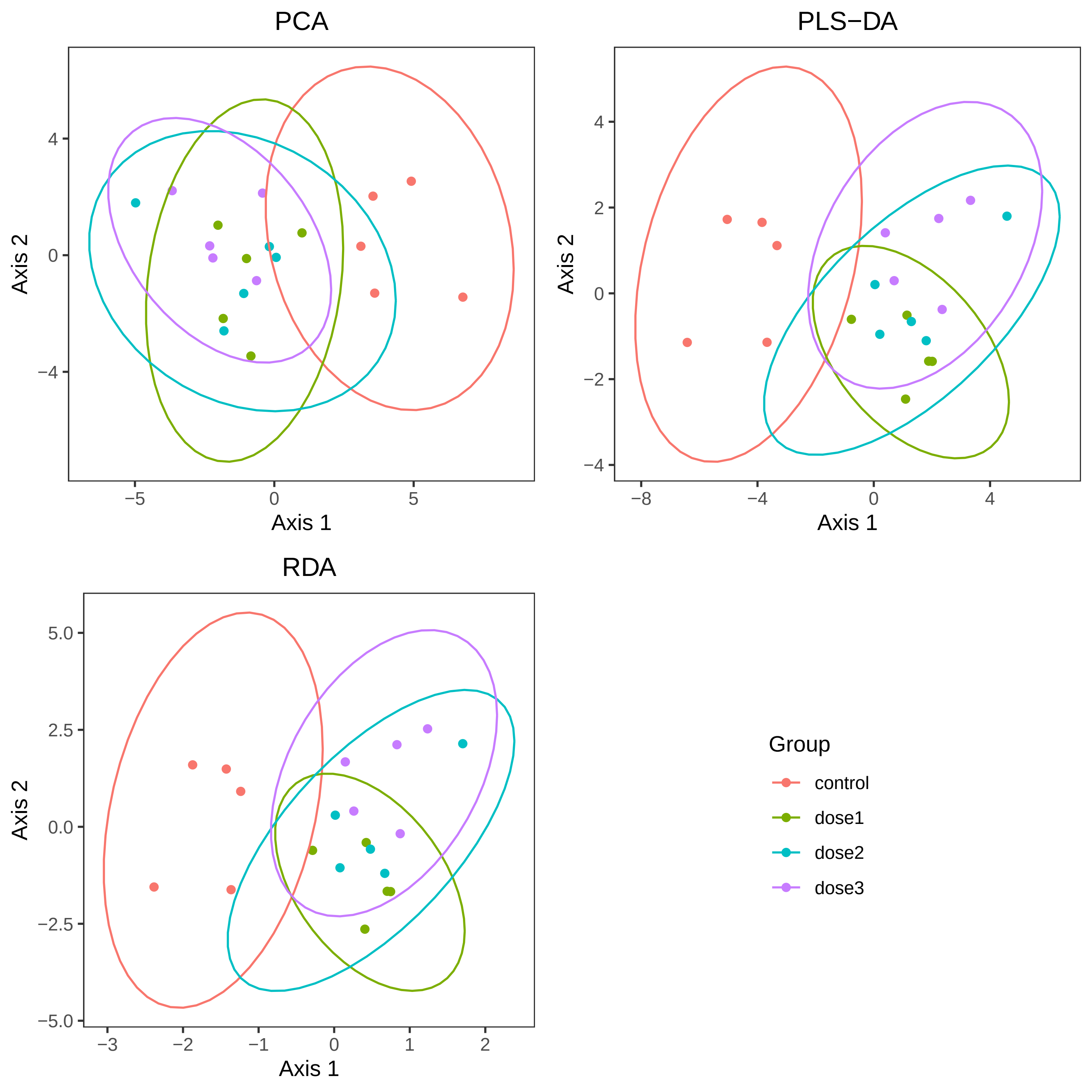

PCA
PLS−DA
RDA
Axis 1
Axis 2
Group
control
dose1
dose2
dose3

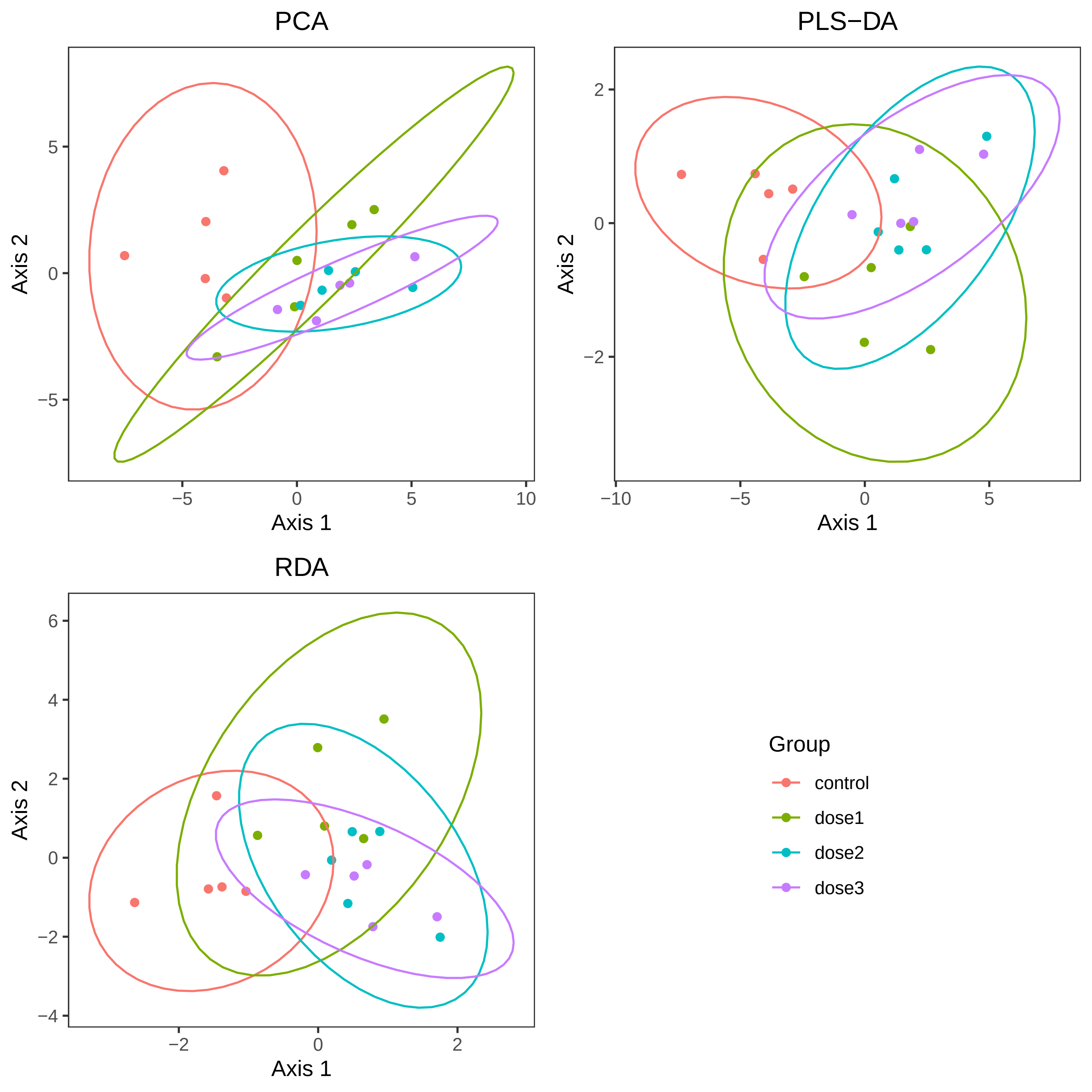

PCA
PLS−DA
RDA
Axis 1
Axis 2
−5
0
5
10
−10
2
−2
4
6
−4
Group
control
dose1
dose2
dose3

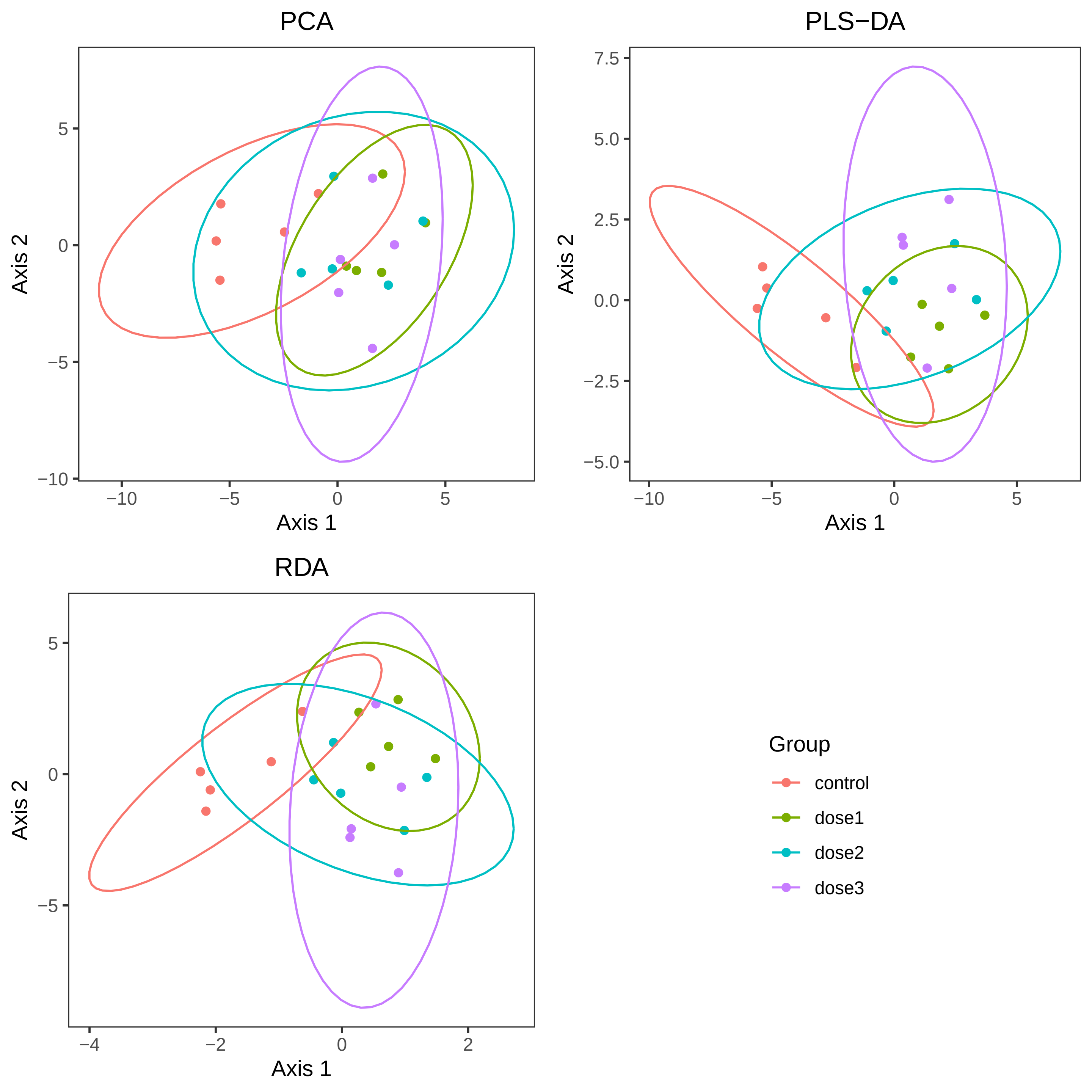
PCA
PLS−DA
RDA
Axis 1
Axis 2
Group
control
dose1
dose2
dose3

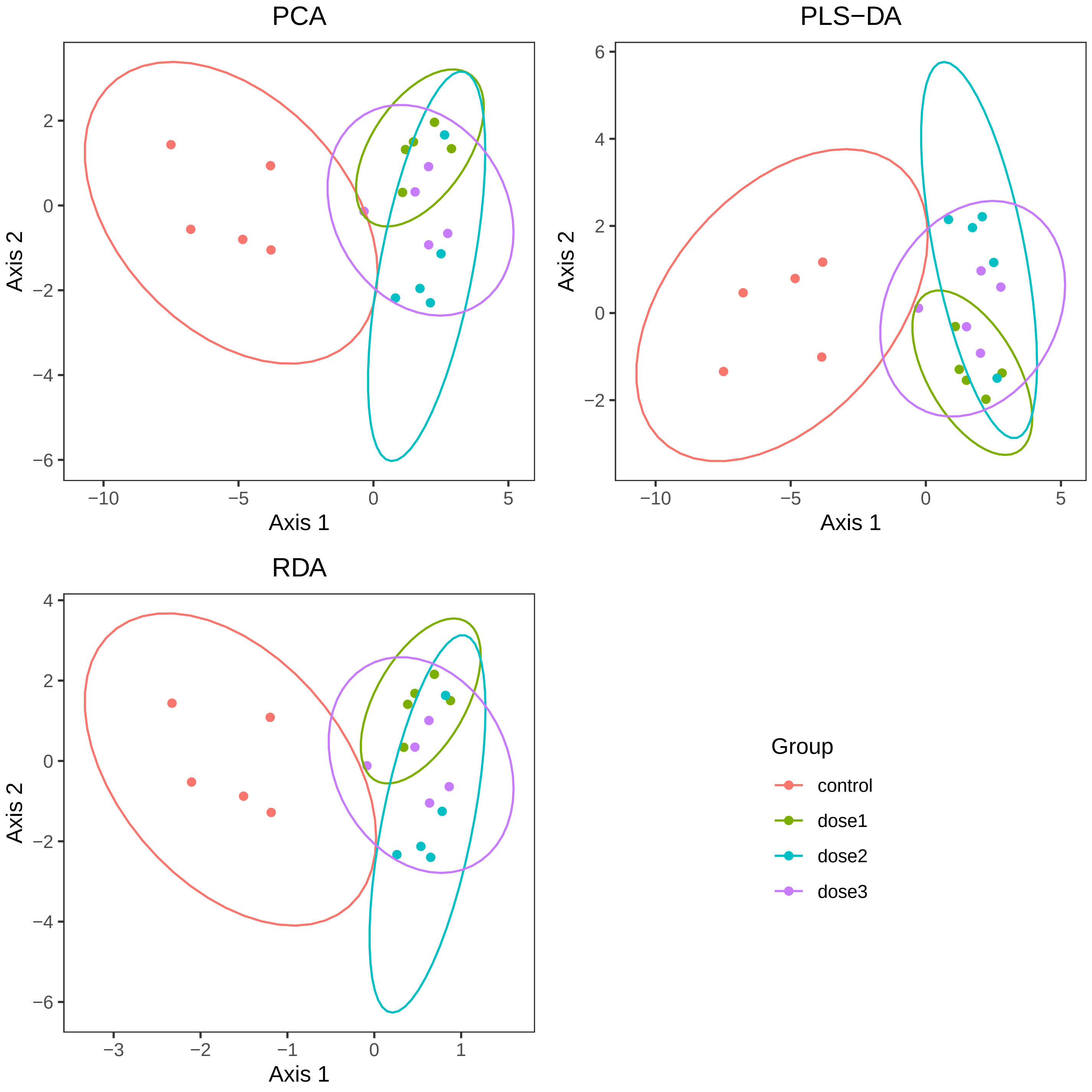

PCA
PLS−DA
RDA
Axis 1
Axis 2
Group
control
dose1
dose2
dose3

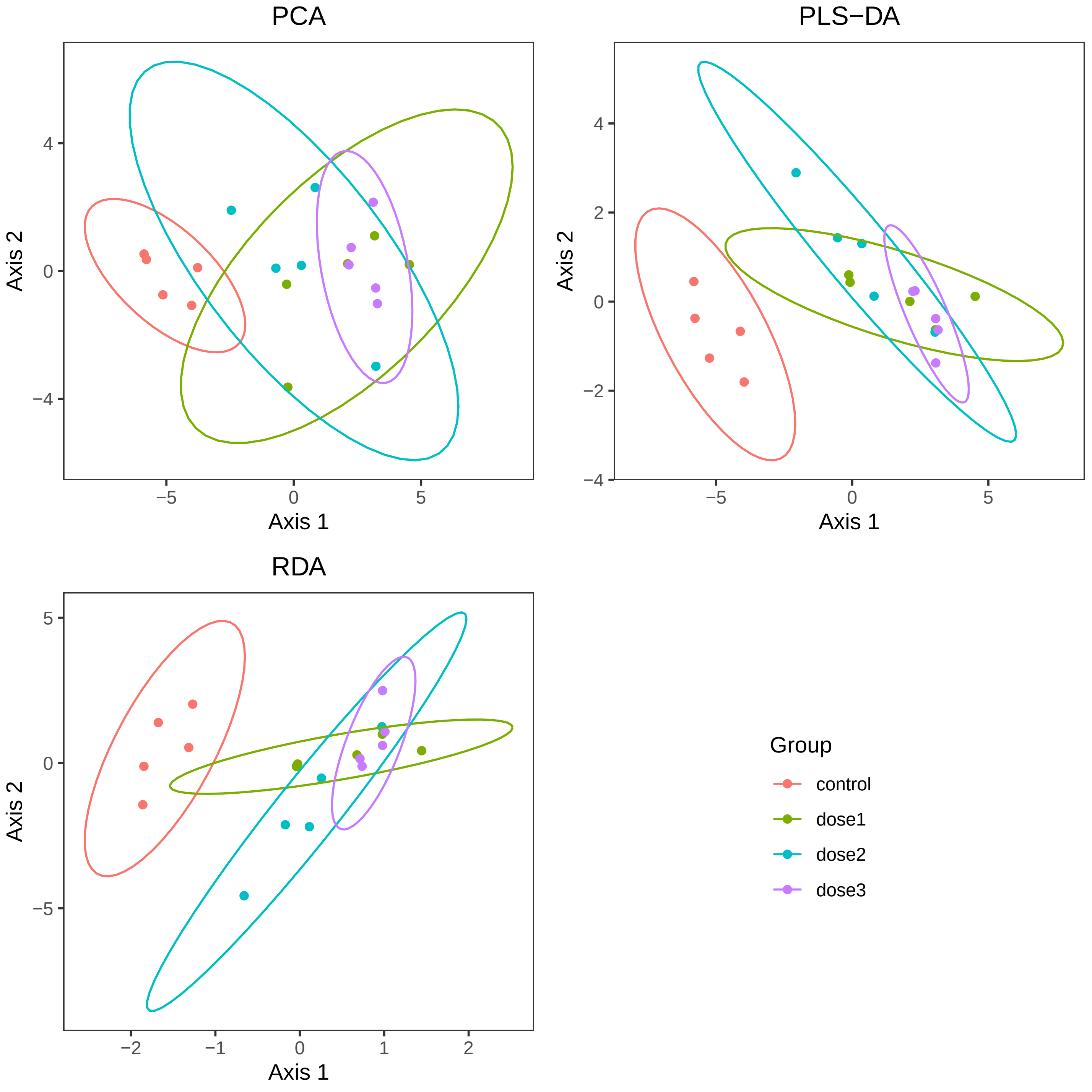
PCA
PLS−DA
RDA
Axis 1
Axis 2
Group
control
dose1
dose2
dose3

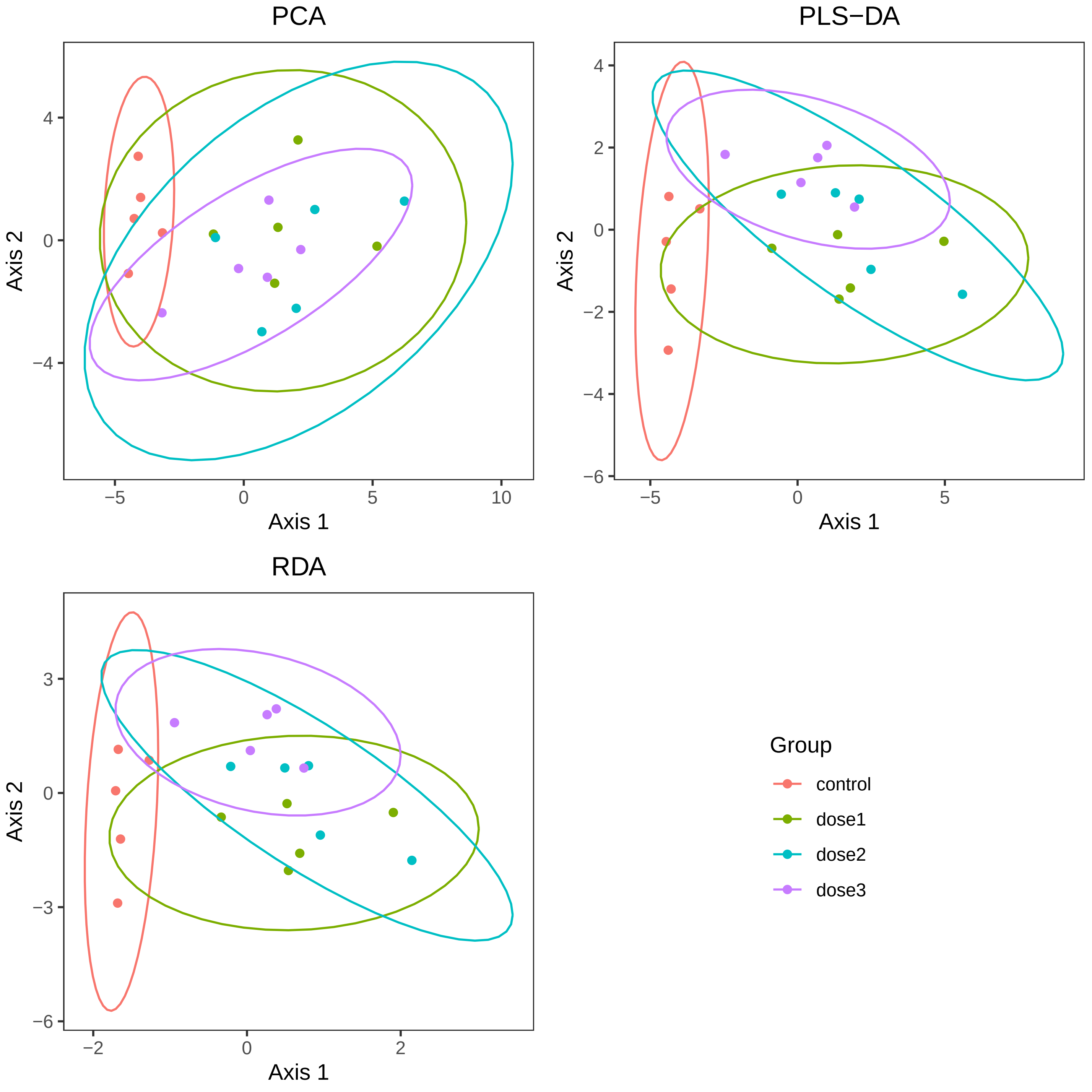

PCA
PLS−DA
RDA
Axis 1
Axis 2
Group
control
dose1
dose2
dose3

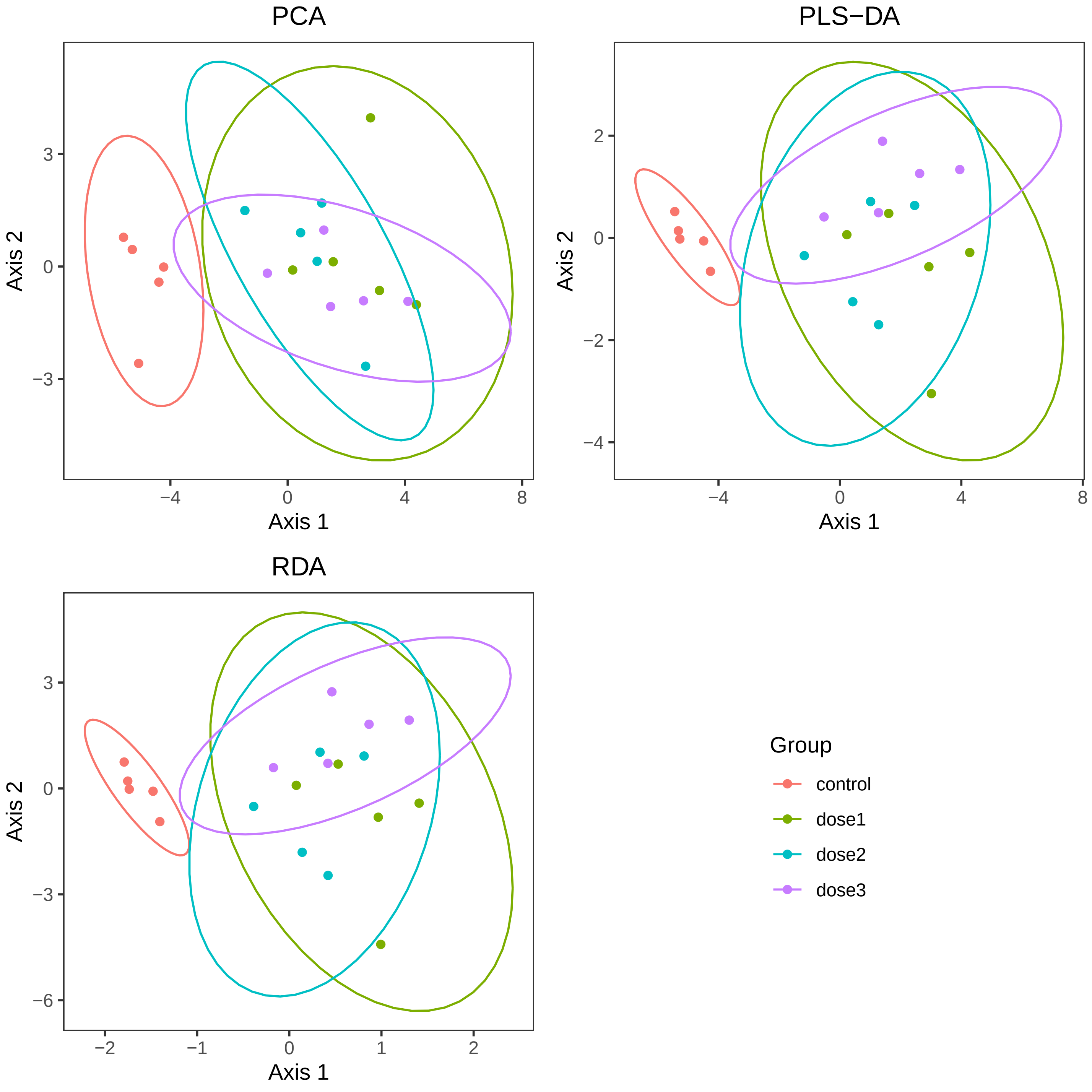
PCA
PLS−DA
RDA
Axis 1
Axis 2
Group
control
dose1
dose2
dose3

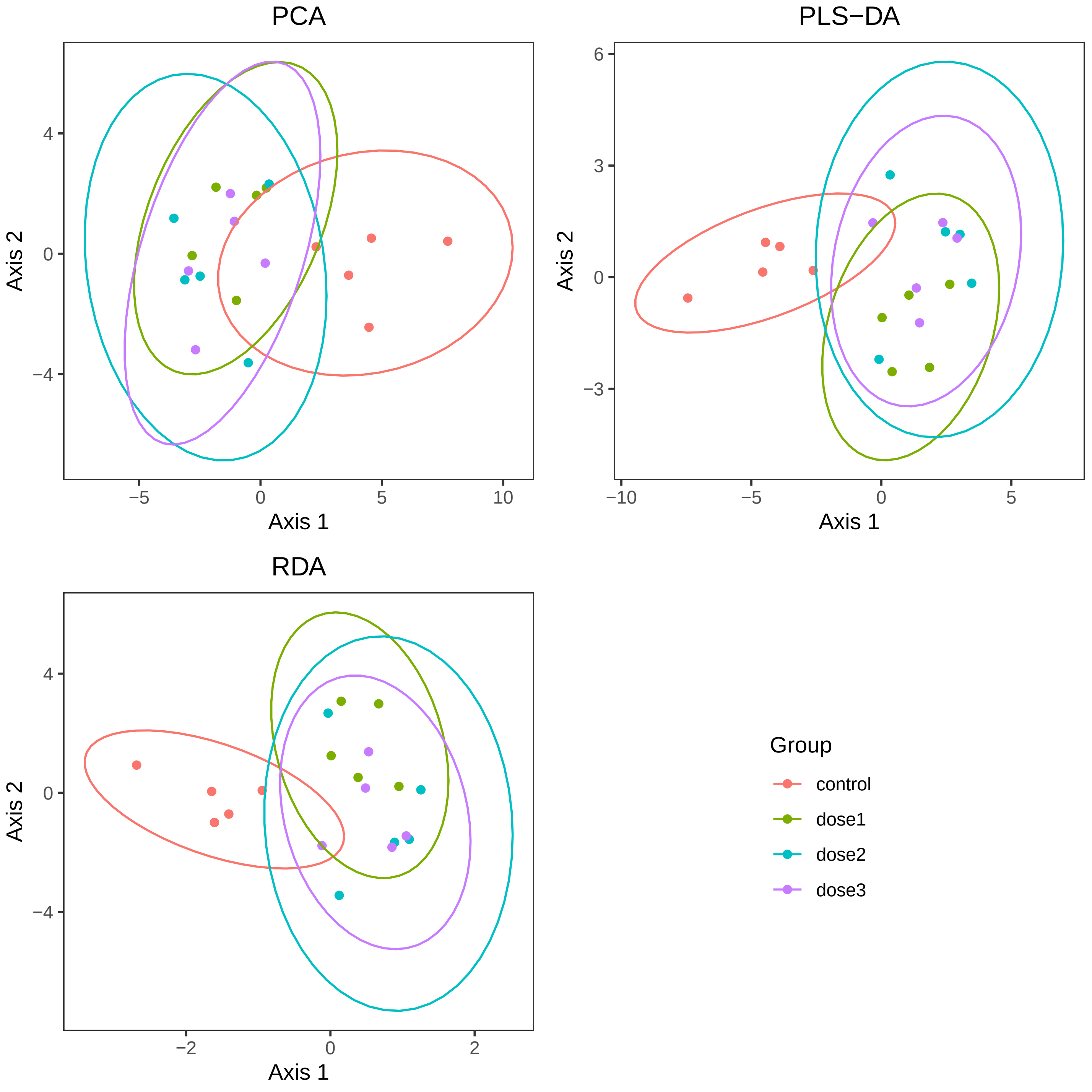
PCA
PLS−DA
RDA
Axis 1
Axis 2
Group
control
dose1
dose2
dose3

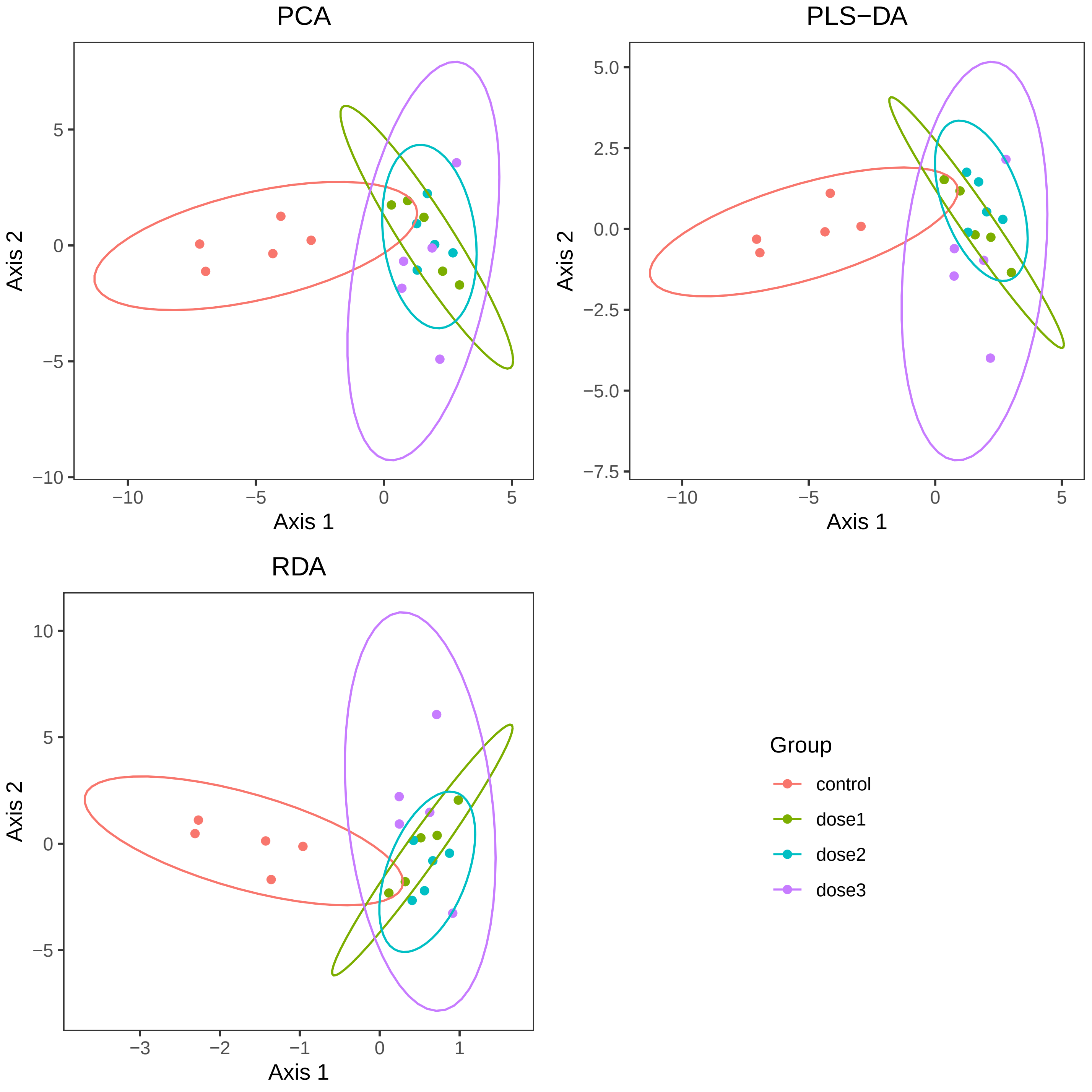
PCA
PLS−DA
RDA
Axis 1
Axis 2
Group
control
dose1
dose2
dose3

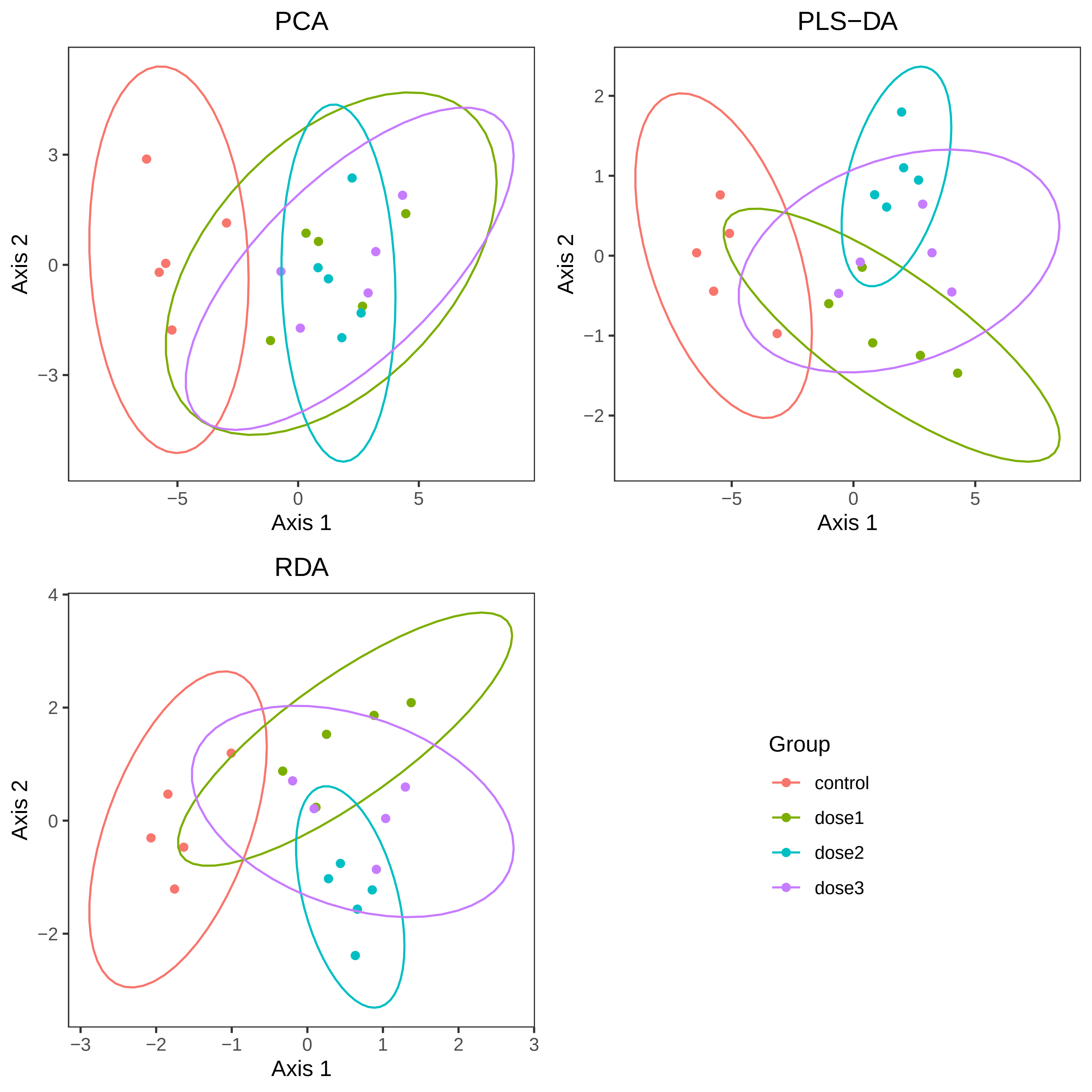
PCA
PLS−DA
RDA
Axis 1
Axis 2
Group
control
dose1
dose2
dose3

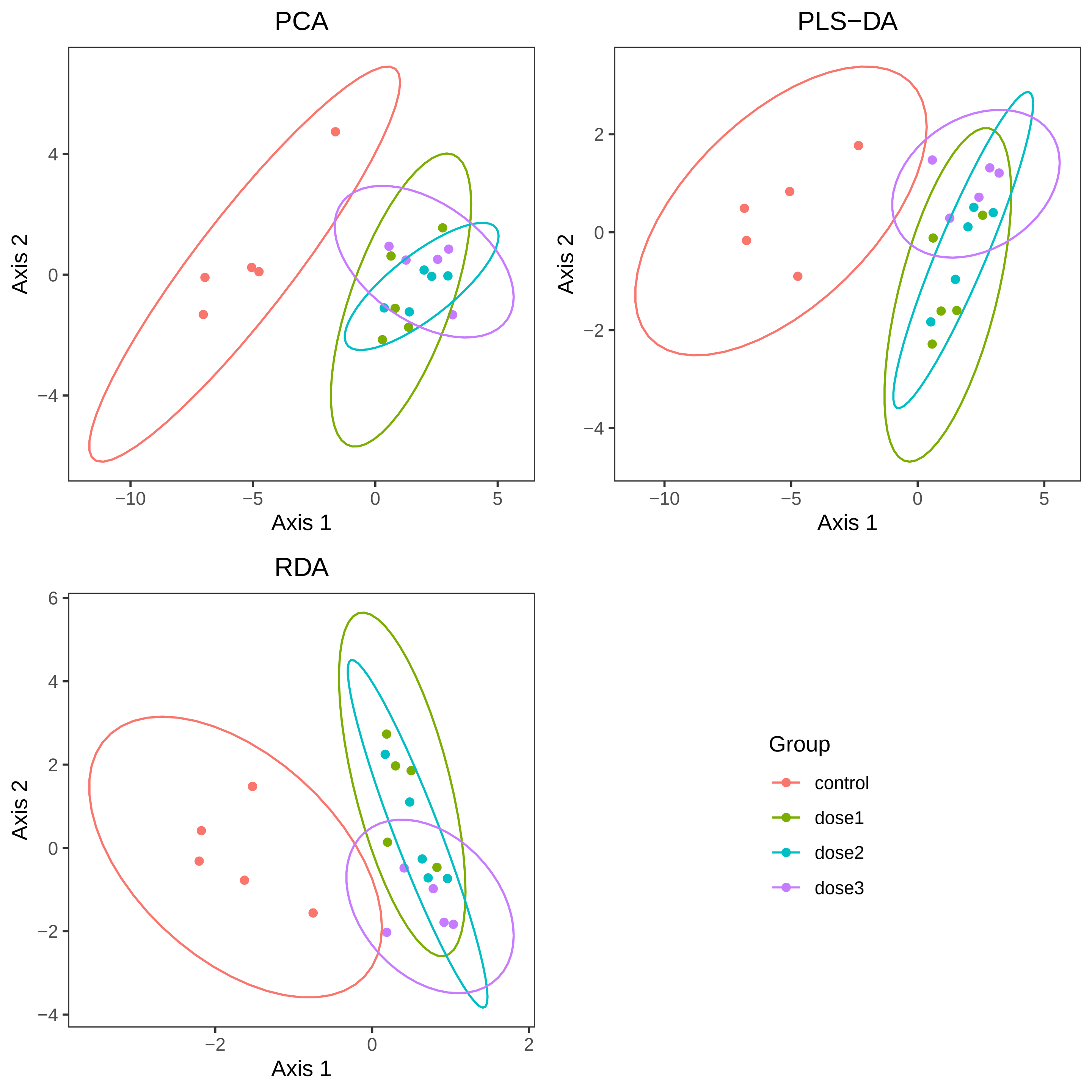

PCA
PLS−DA
RDA
Axis 1
Axis 2
Group
control
dose1
dose2
dose3

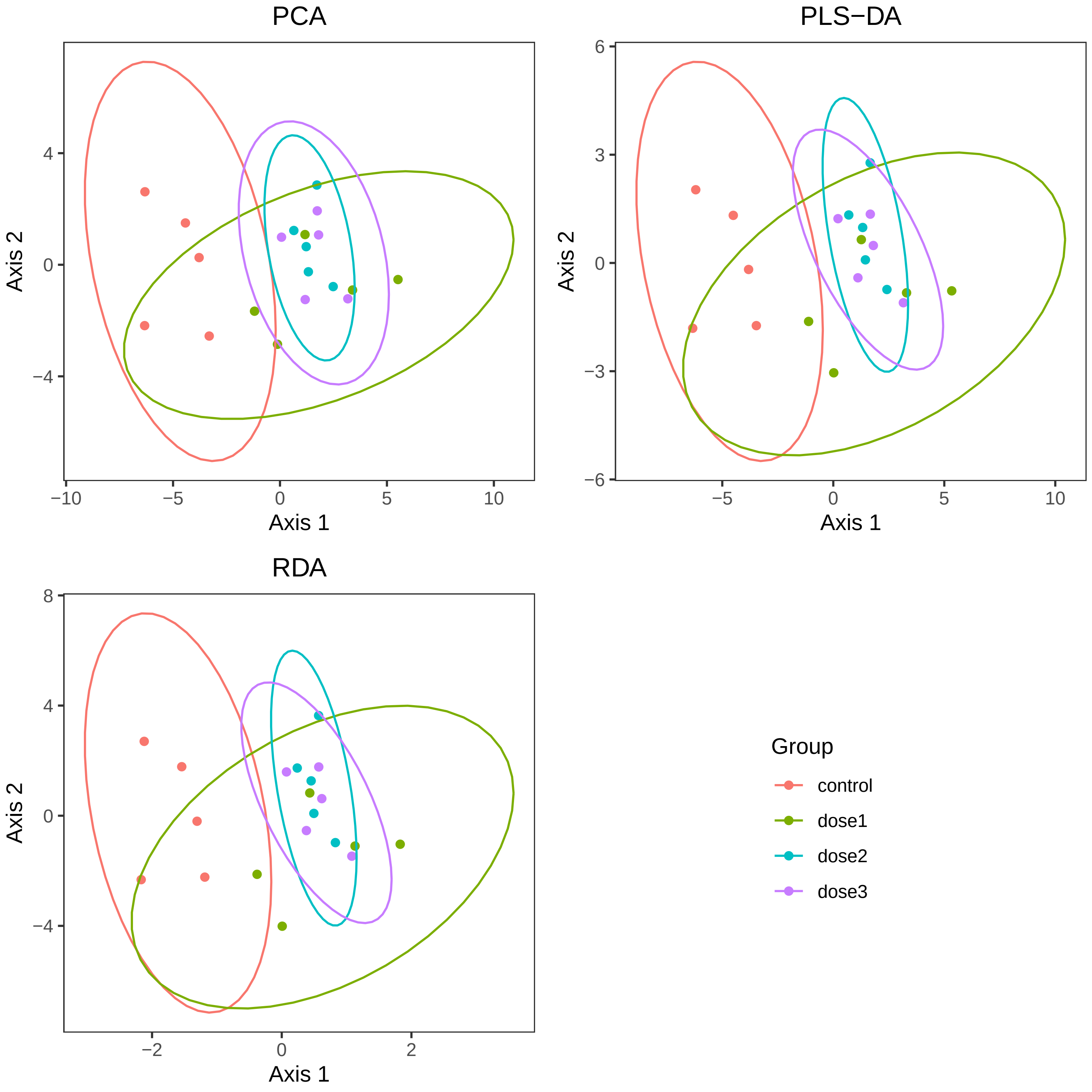

PCA
PLS−DA
RDA
Axis 1
Axis 2
Group
control
dose1
dose2
dose3

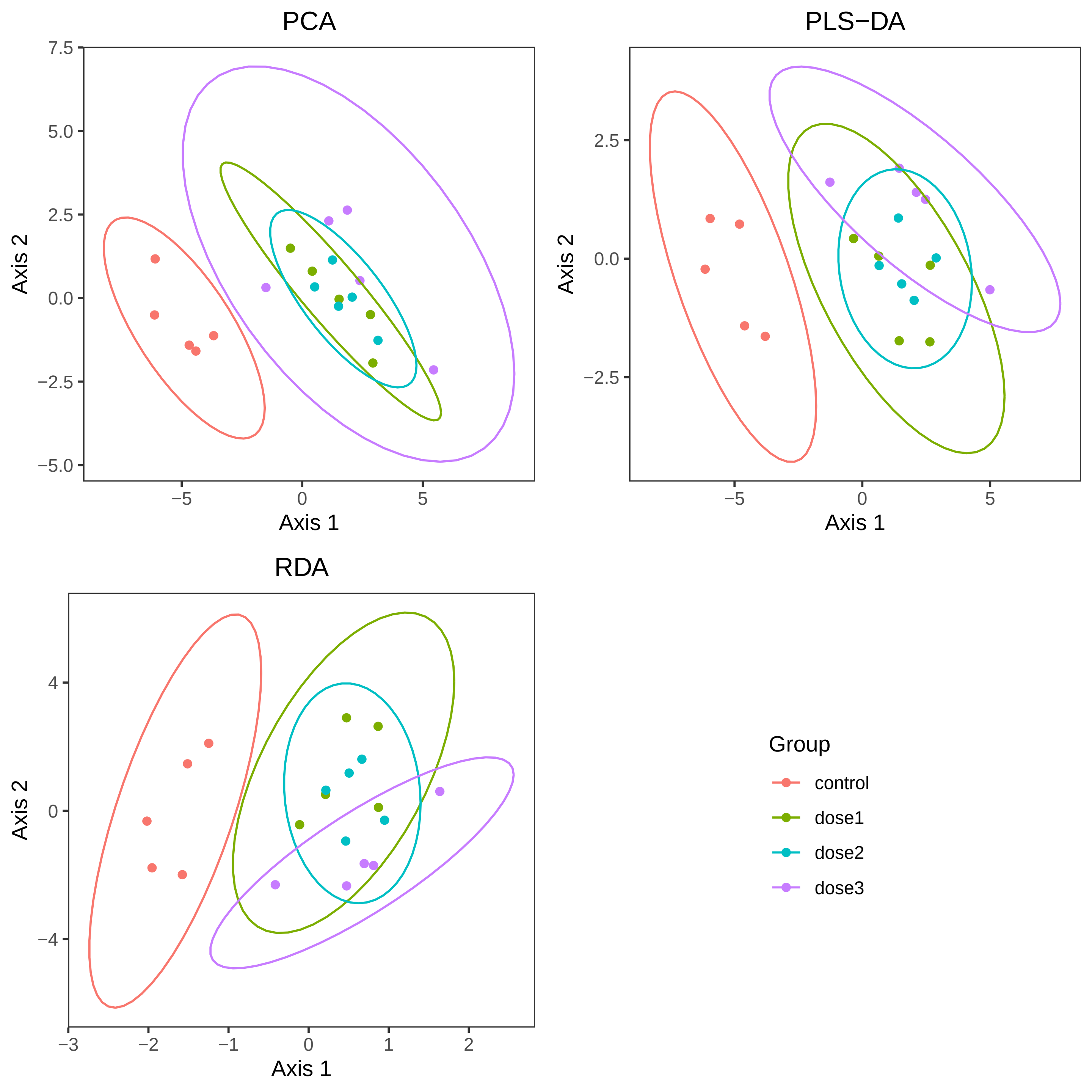
PCA
PLS−DA
RDA
Axis 1
Axis 2
7.5
5.0
2.5
0.0
−2.5
−5.0
−5
0
5
−3
−2
−1
1
2
4
−4
Group
control
dose1
dose2
dose3

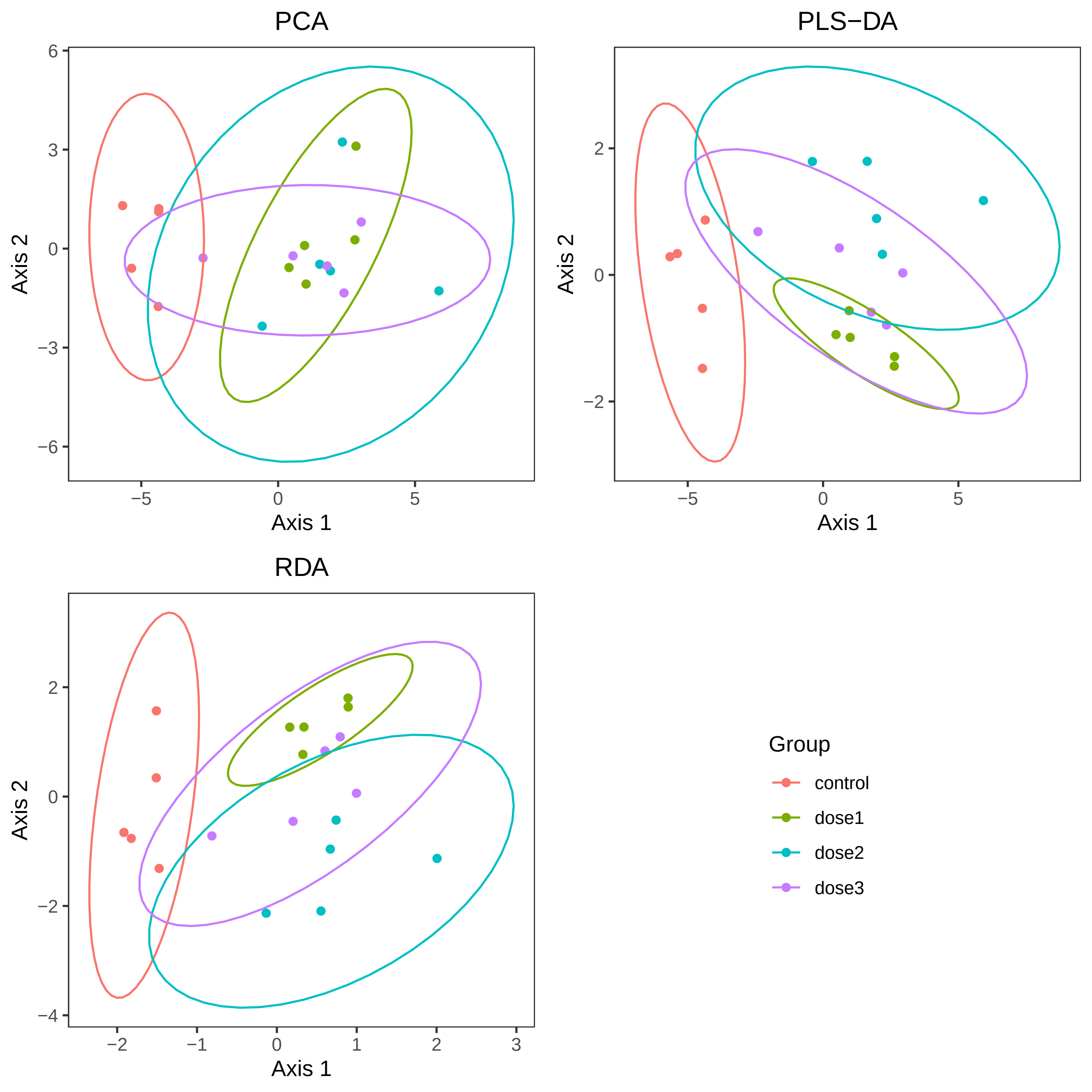

PCA
PLS−DA
RDA
Axis 1
Axis 2
Group
control
dose1
dose2
dose3

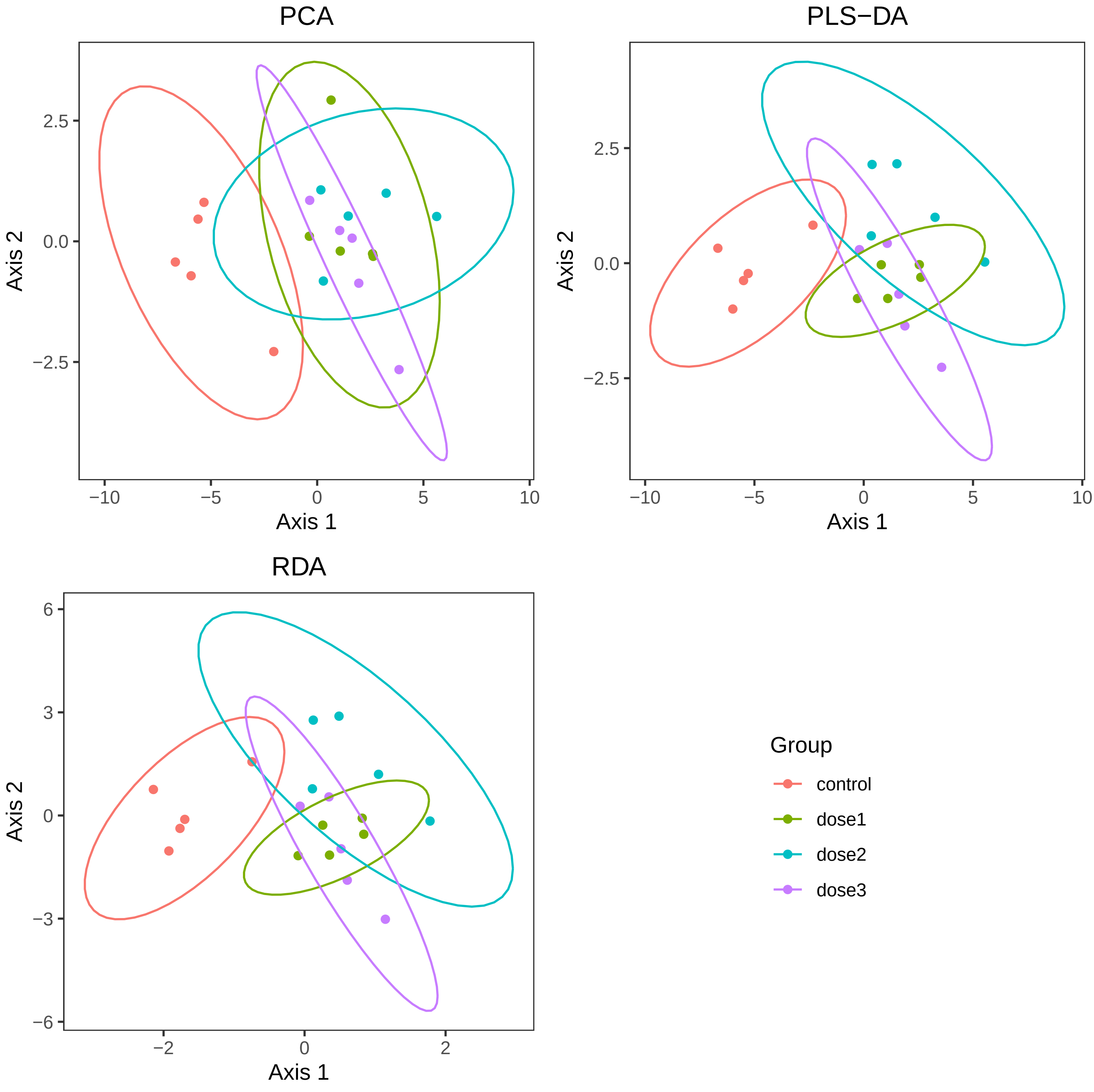
PCA
PLS−DA
RDA
Axis 1
Axis 2
2.5
0.0
−2.5
−10
−5
0
5
10
6
3
−3
−6
−2
2
Group
control
dose1
dose2
dose3

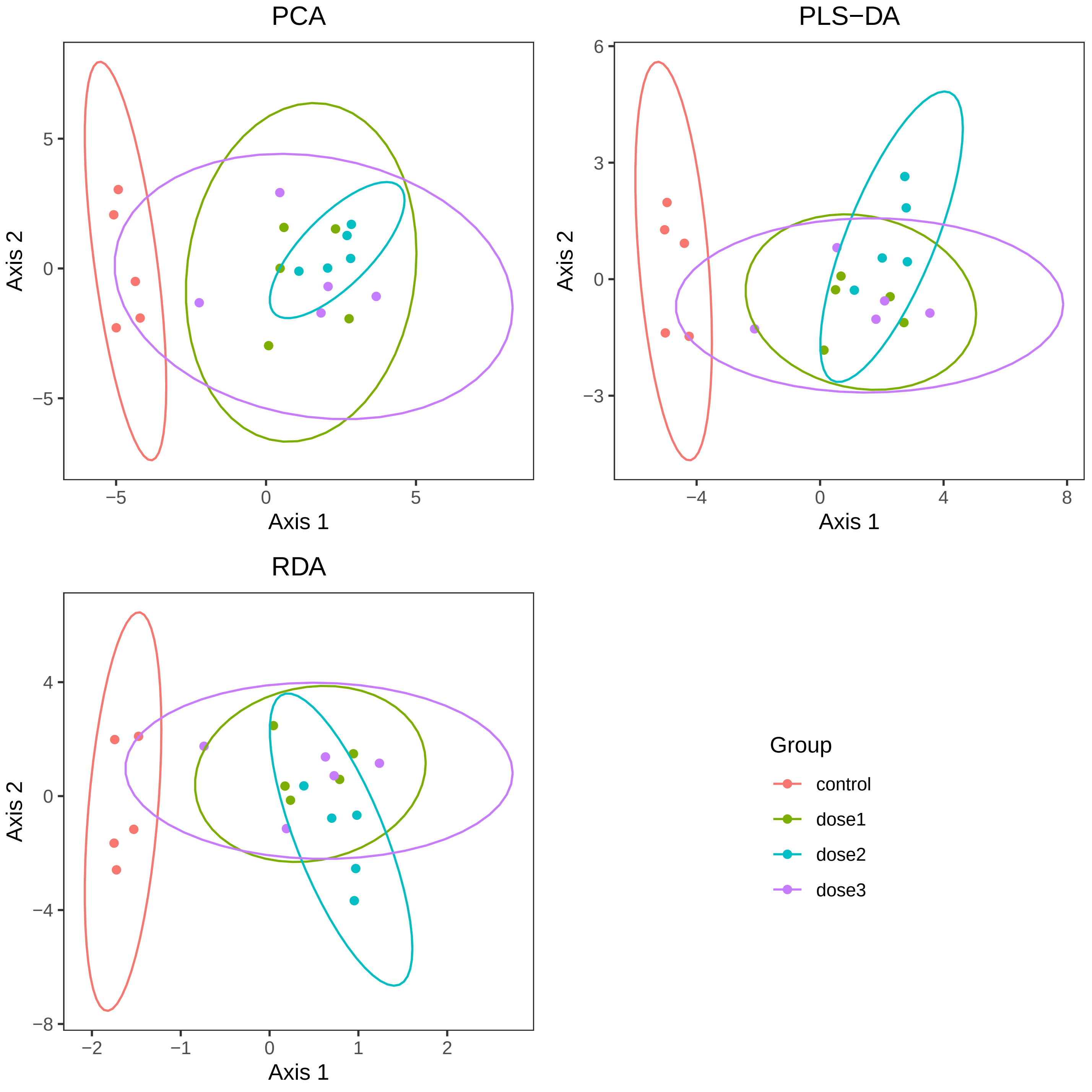

PCA
PLS−DA
RDA
Axis 1
Axis 2
Group
control
dose1
dose2
dose3

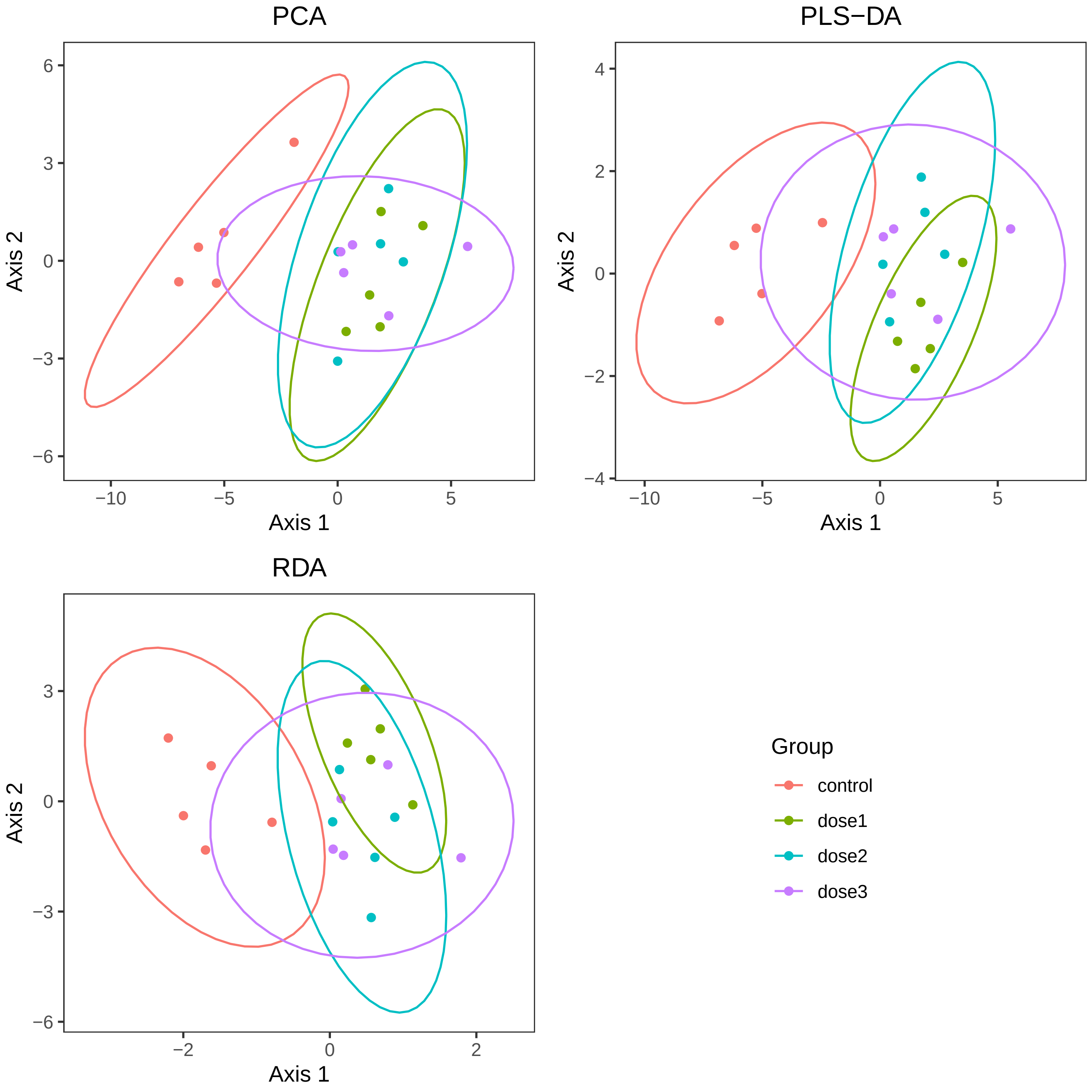
PCA
PLS−DA
RDA
Axis 1
Axis 2
Group
control
dose1
dose2
dose3

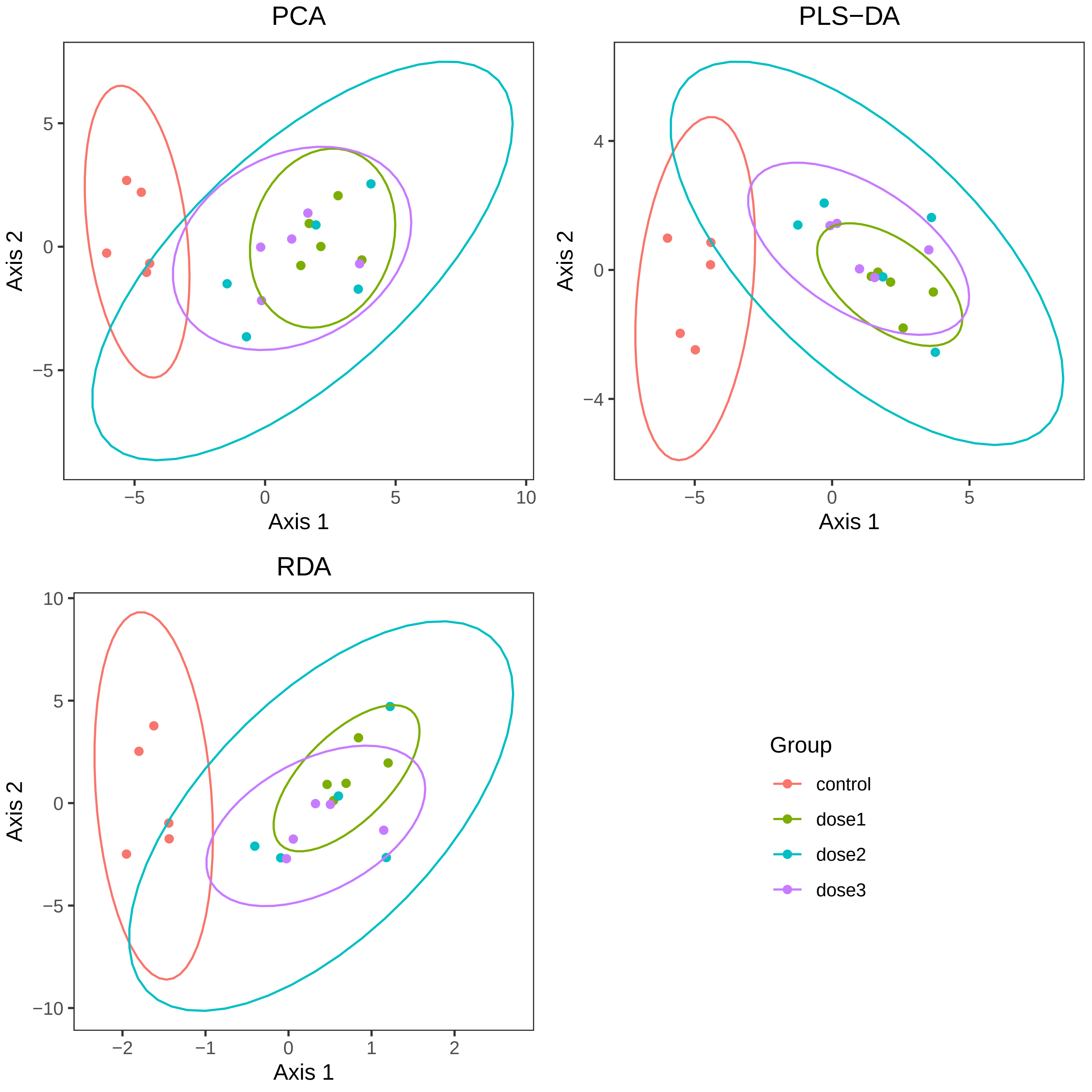

PCA
PLS−DA
RDA
Axis 1
Axis 2
Group
control
dose1
dose2
dose3

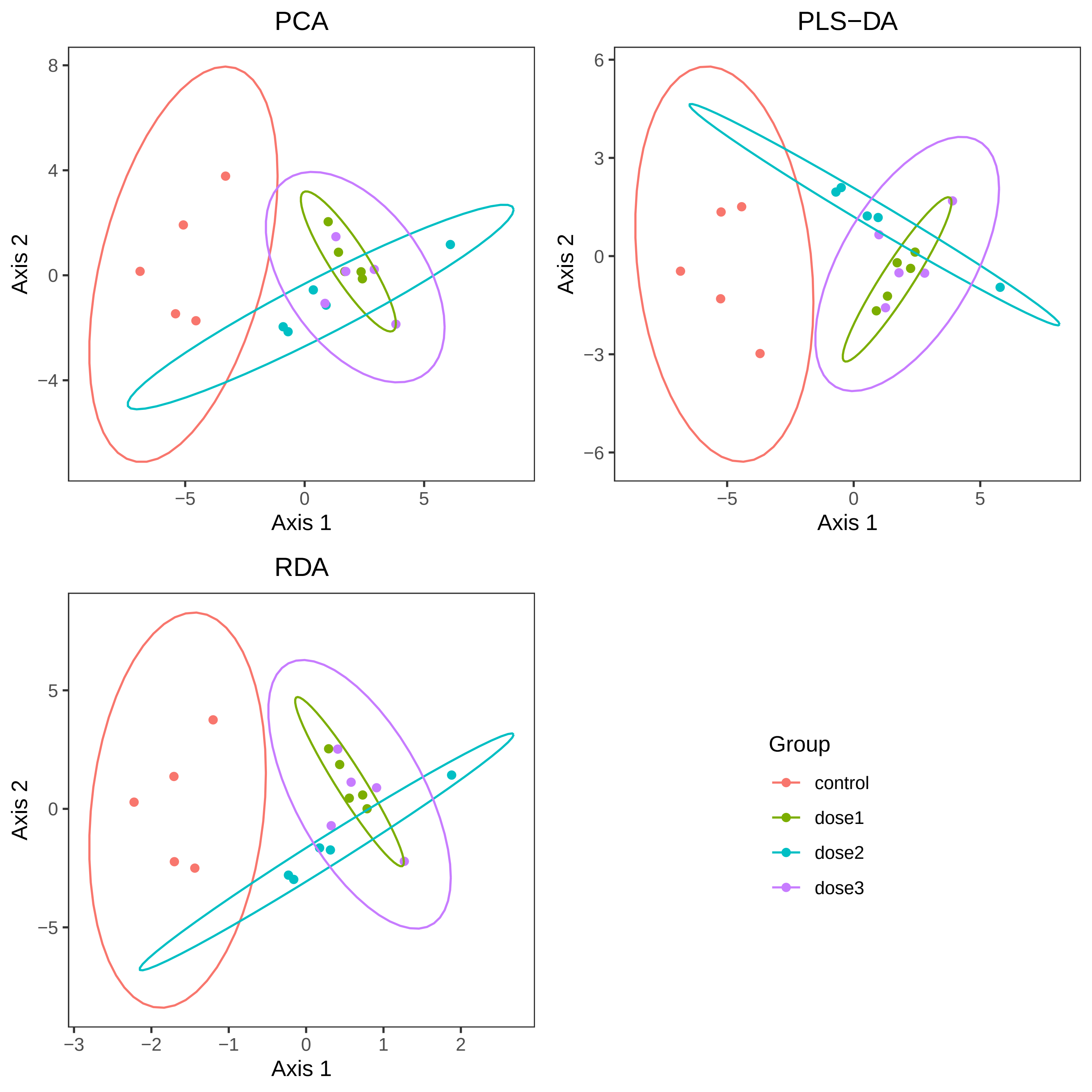
PCA
PLS−DA
RDA
Axis 1
Axis 2
Group
control
dose1
dose2
dose3

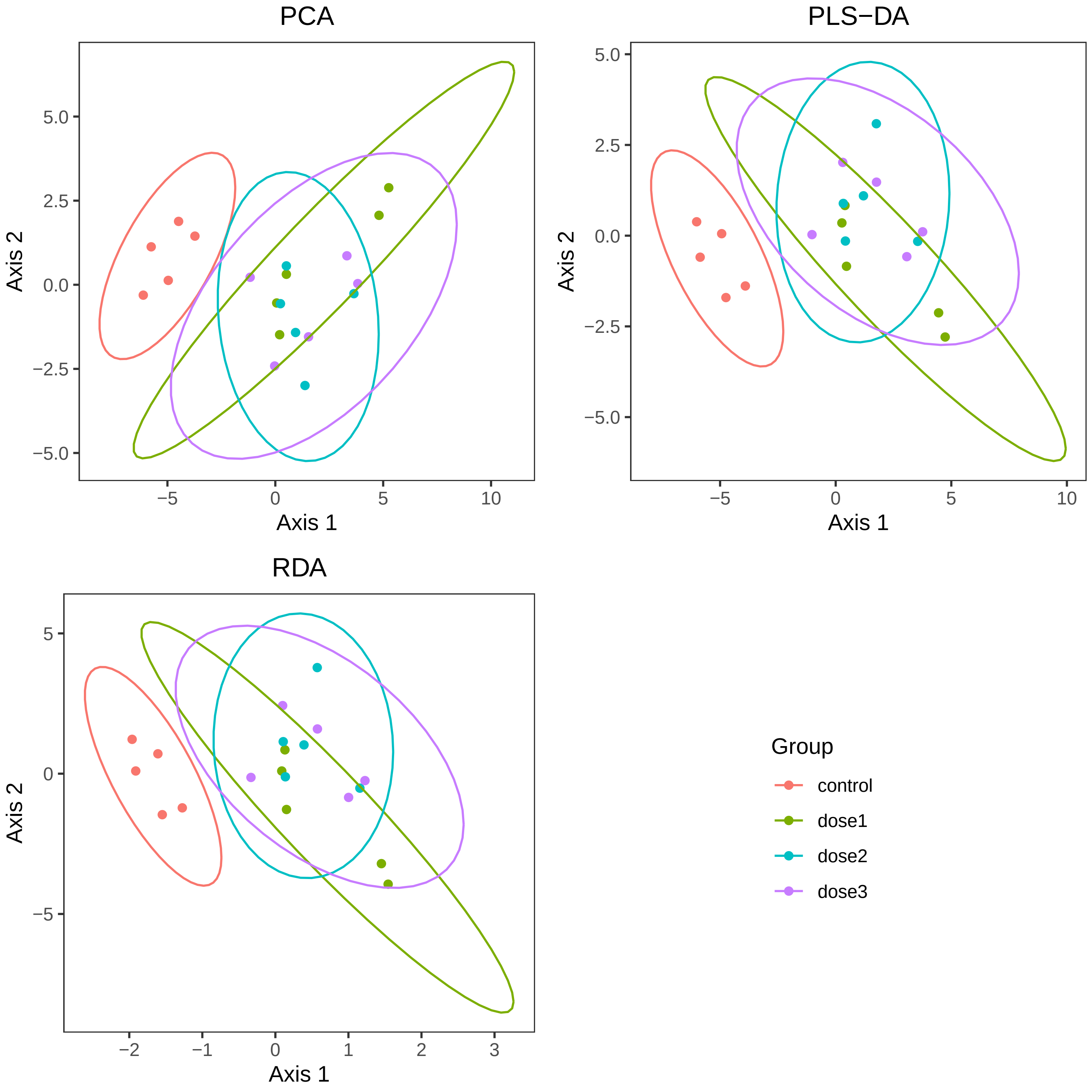
PCA
PLS−DA
RDA
Axis 1
Axis 2
Group
control
dose1
dose2
dose3

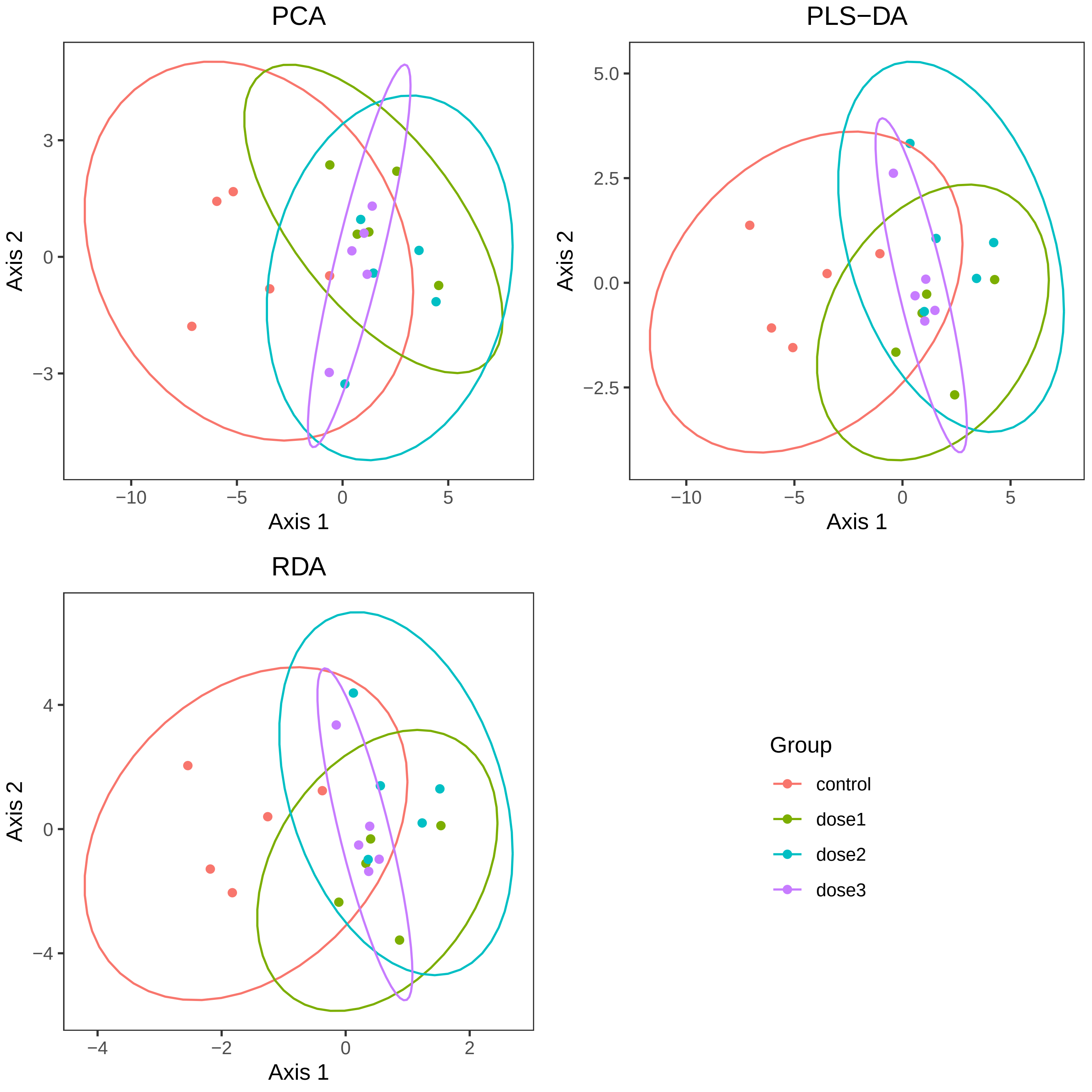
PCA
Axis 2
Axis 1
3
0
−3
−10
−5
0
5
PLS−DA
5.0
2.5
0.0
−2.5
RDA
4
0
−4
−2
2
Group
control
dose1
dose2
dose3

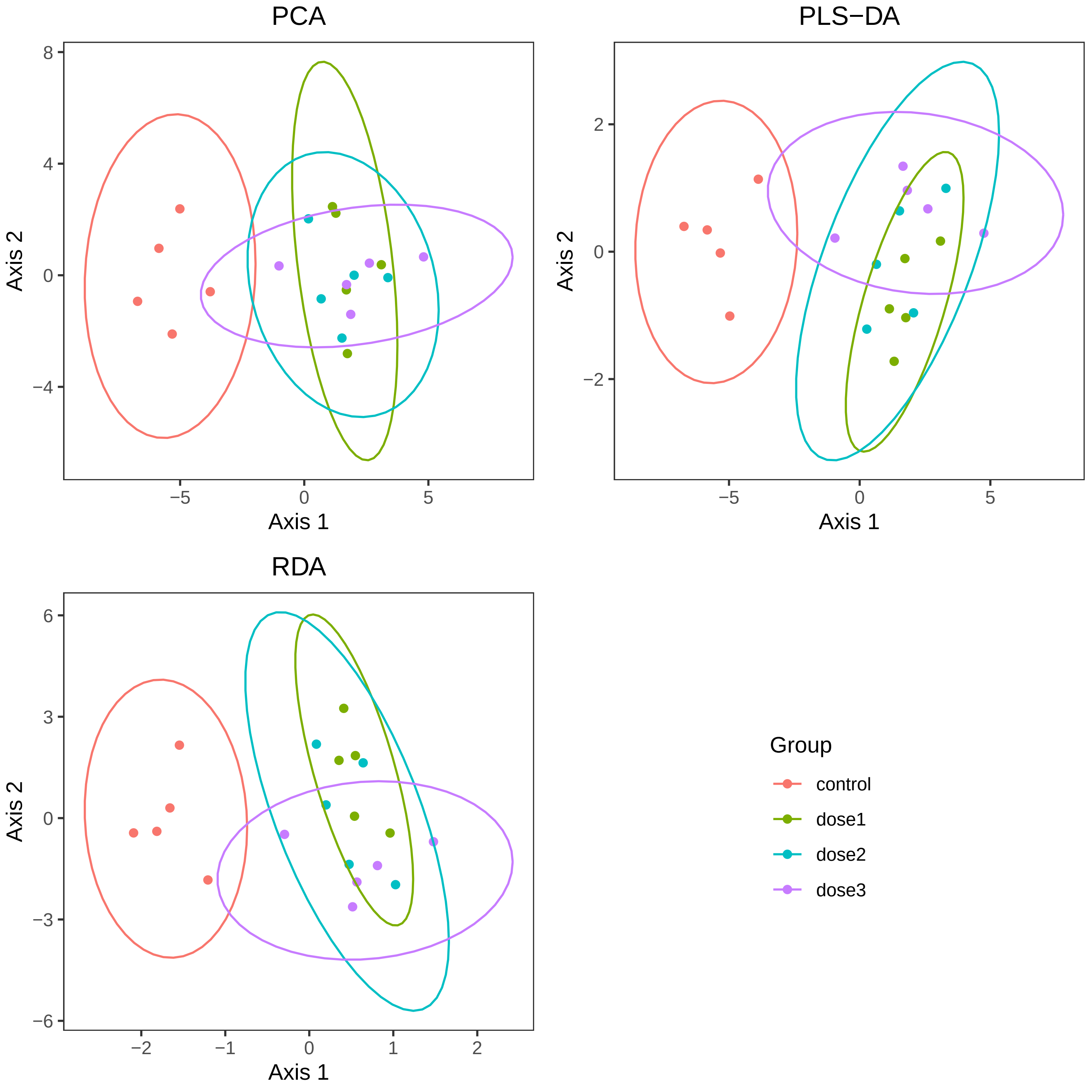
PCA
PLS−DA
RDA
Axis 1
Axis 2
Group
control
dose1
dose2
dose3

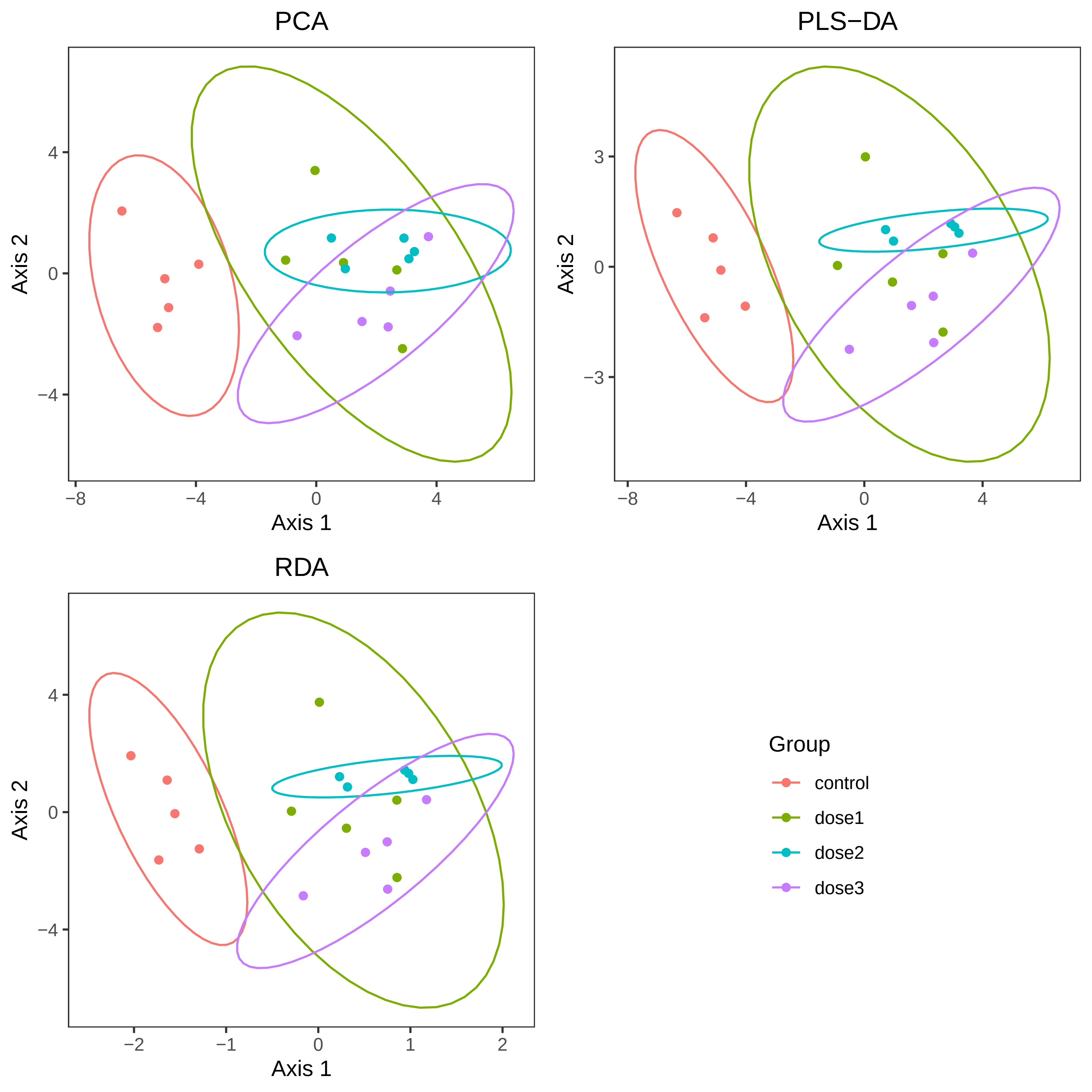

PCA
PLS−DA
RDA
Axis 1
Axis 2
Group
control
dose1
dose2
dose3

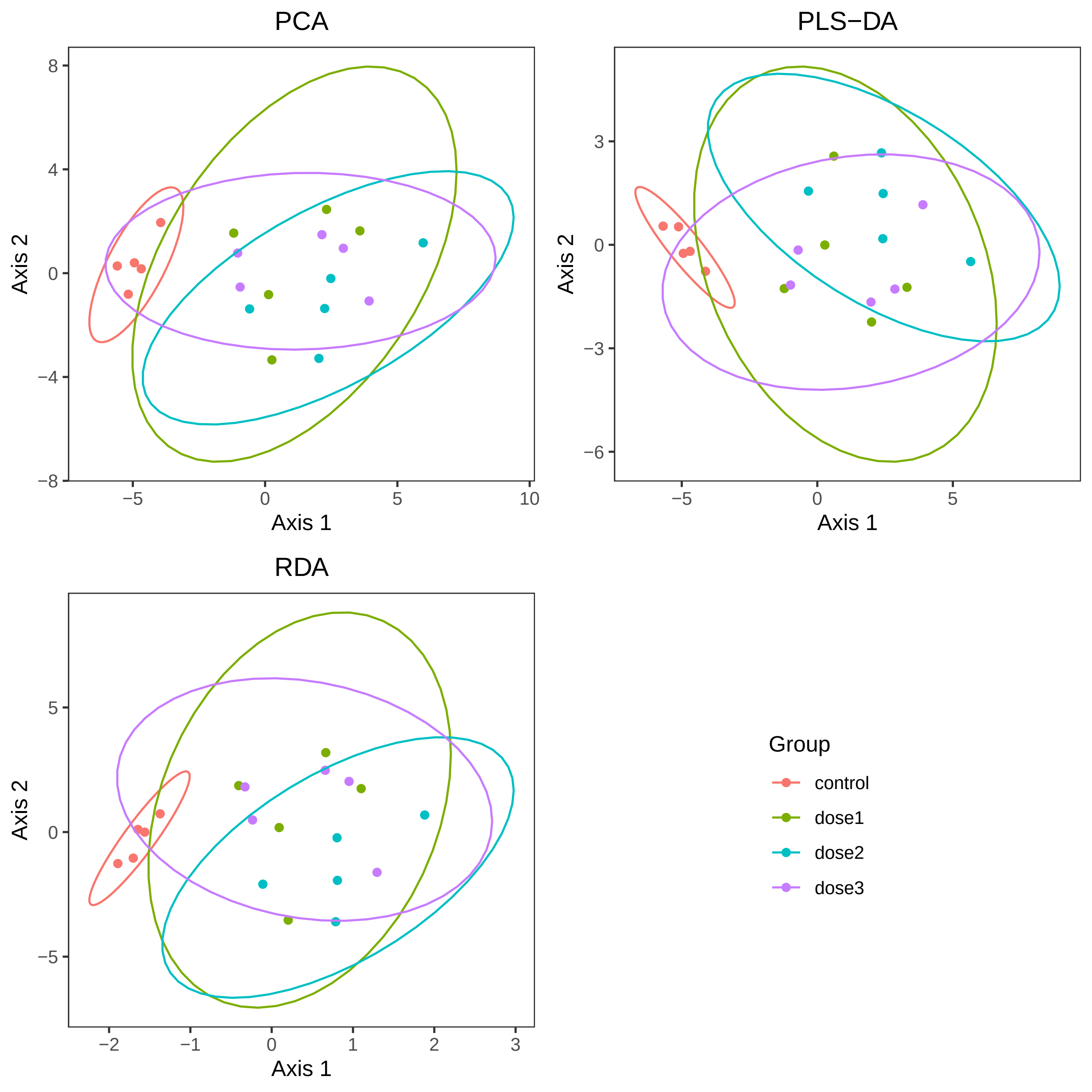
PCA
PLS−DA
RDA
Axis 1
Axis 2
Group
control
dose1
dose2
dose3

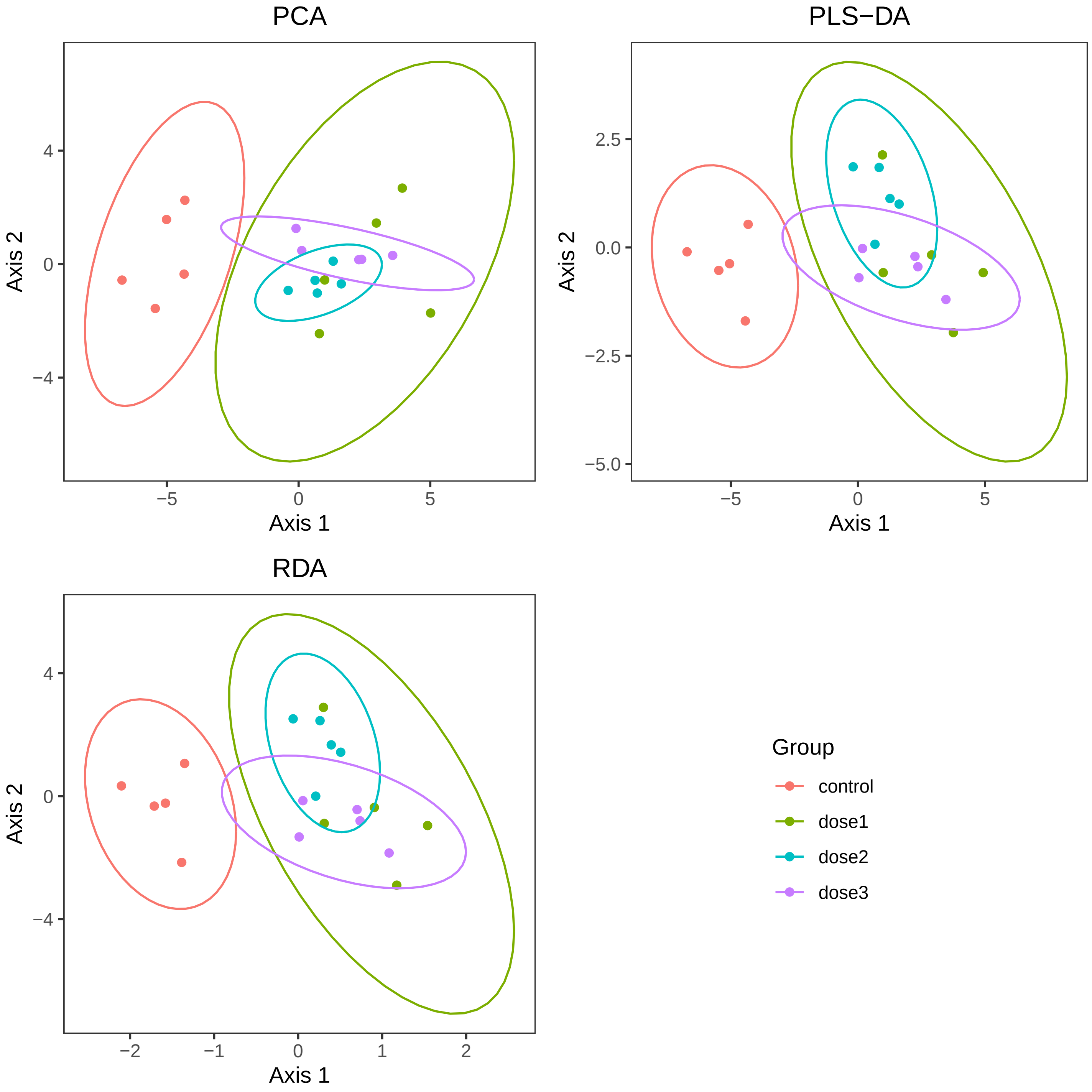
PCA
PLS−DA
RDA
Axis 1
Axis 2
Group
control
dose1
dose2
dose3

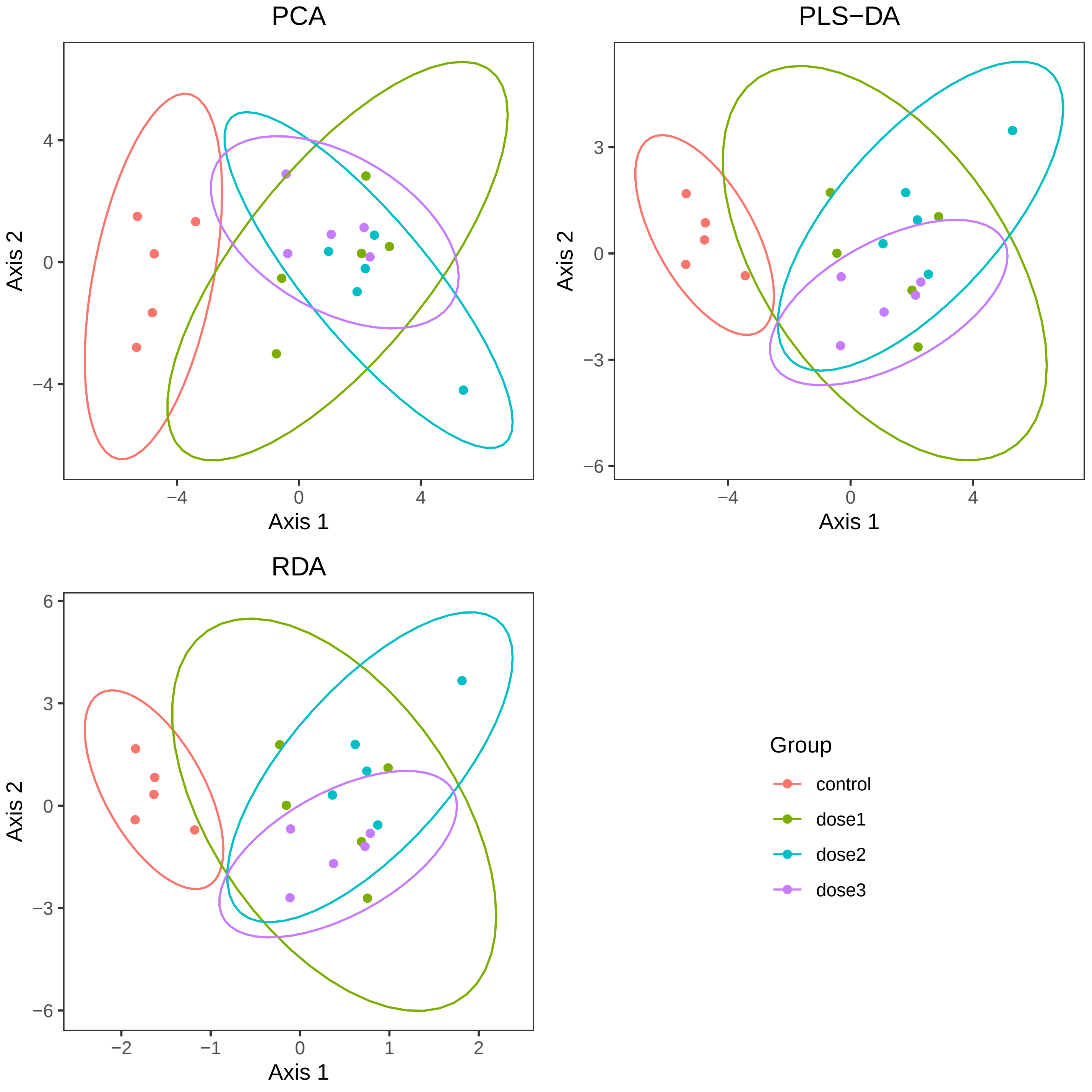

PCA
PLS−DA
RDA
Axis 1
Axis 2
Group
control
dose1
dose2
dose3

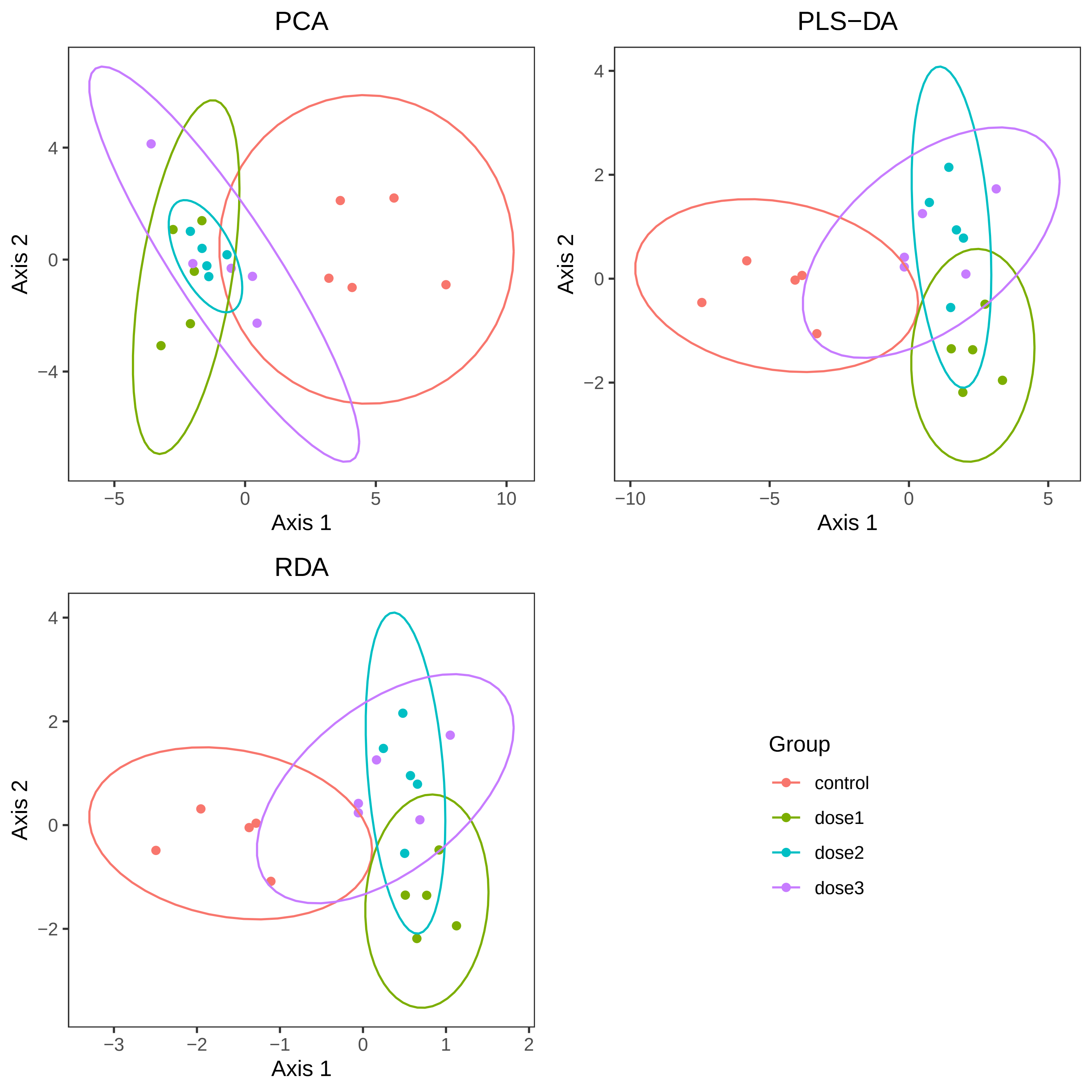
PCA
PLS−DA
RDA
Axis 1
Axis 2
Group
control
dose1
dose2
dose3

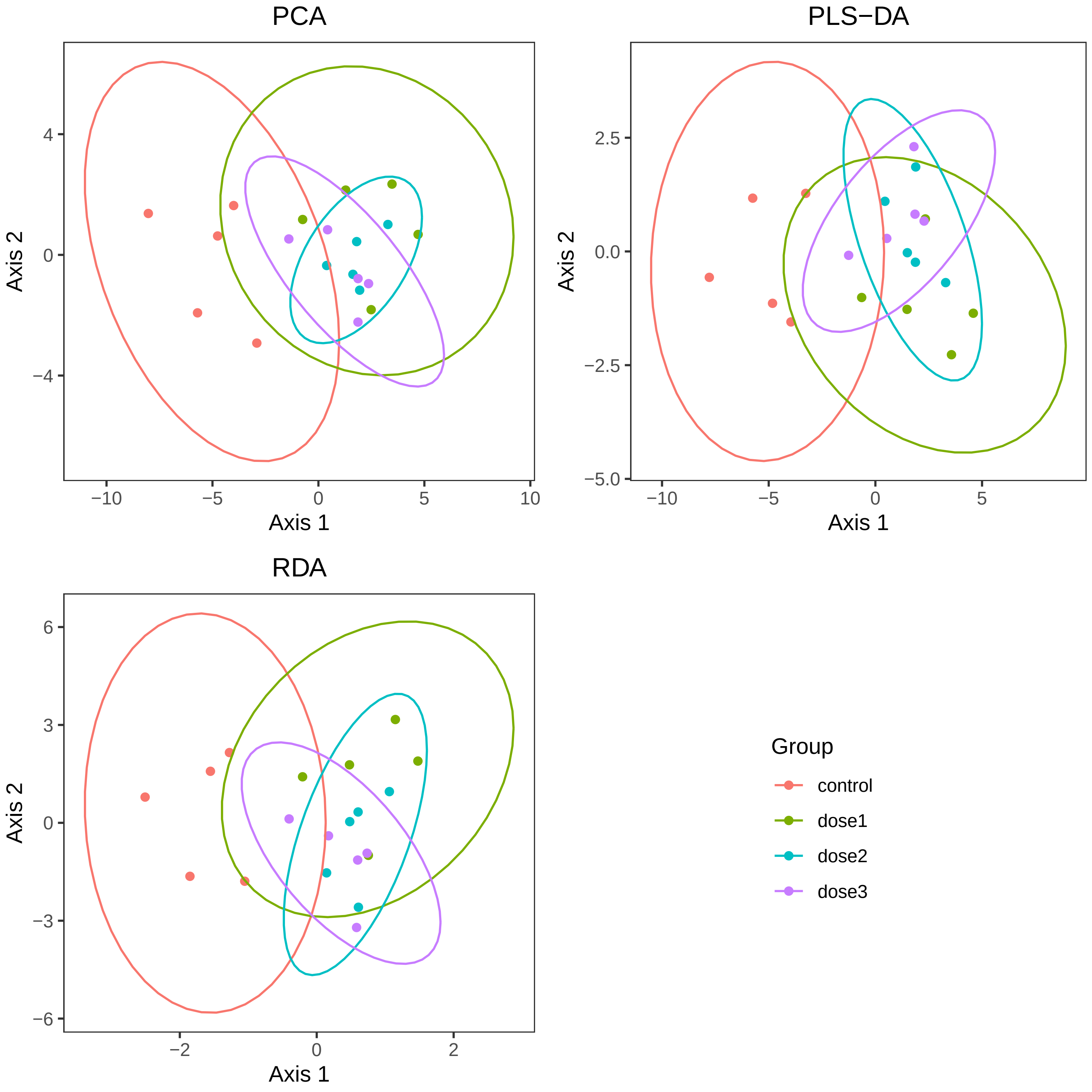

PCA
PLS−DA
RDA
Axis 1
Axis 2
Group
control
dose1
dose2
dose3

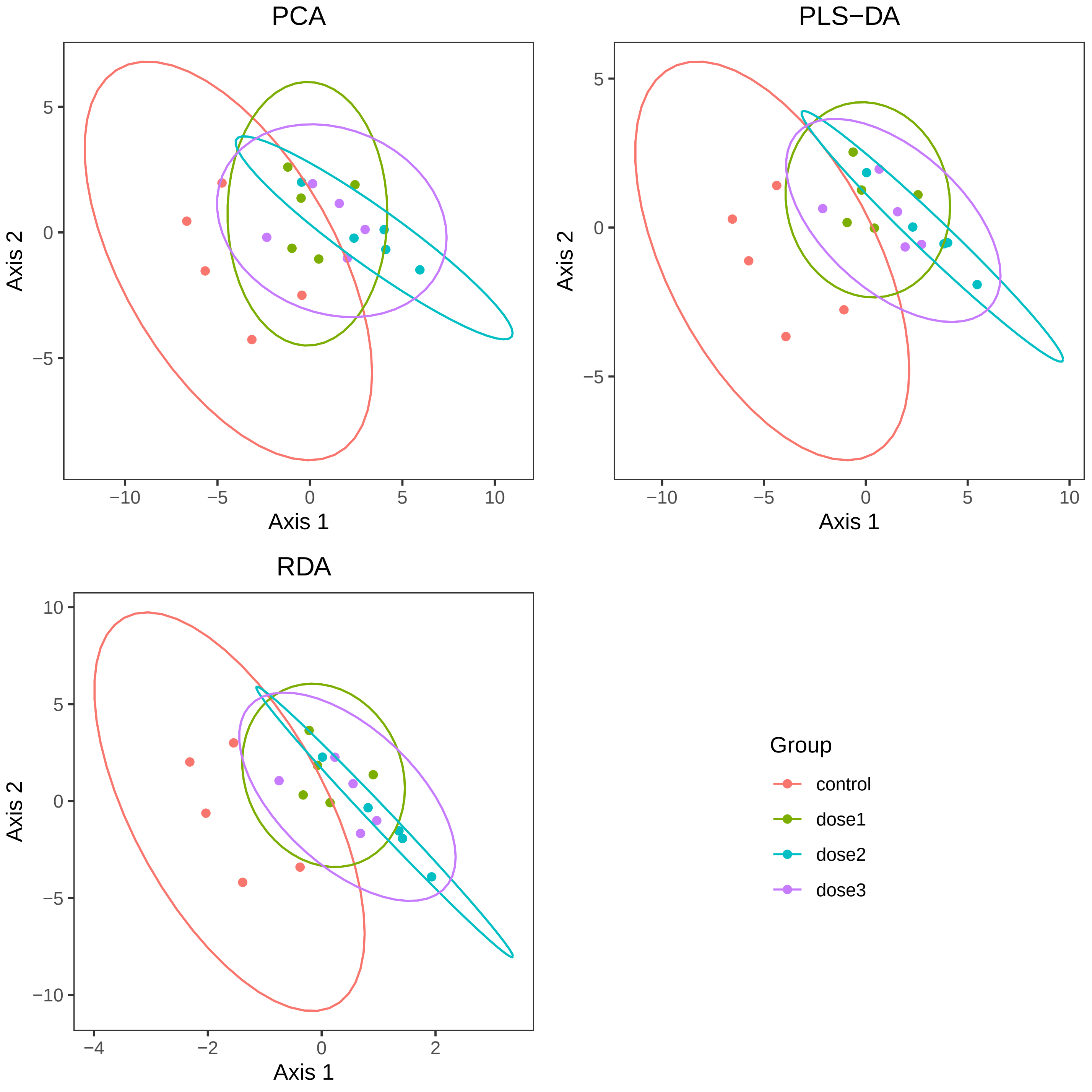
PCA
PLS−DA
RDA
Axis 1
Axis 2
−10
−5
0
5
10
−4
−2
2
Group
control
dose1
dose2
dose3

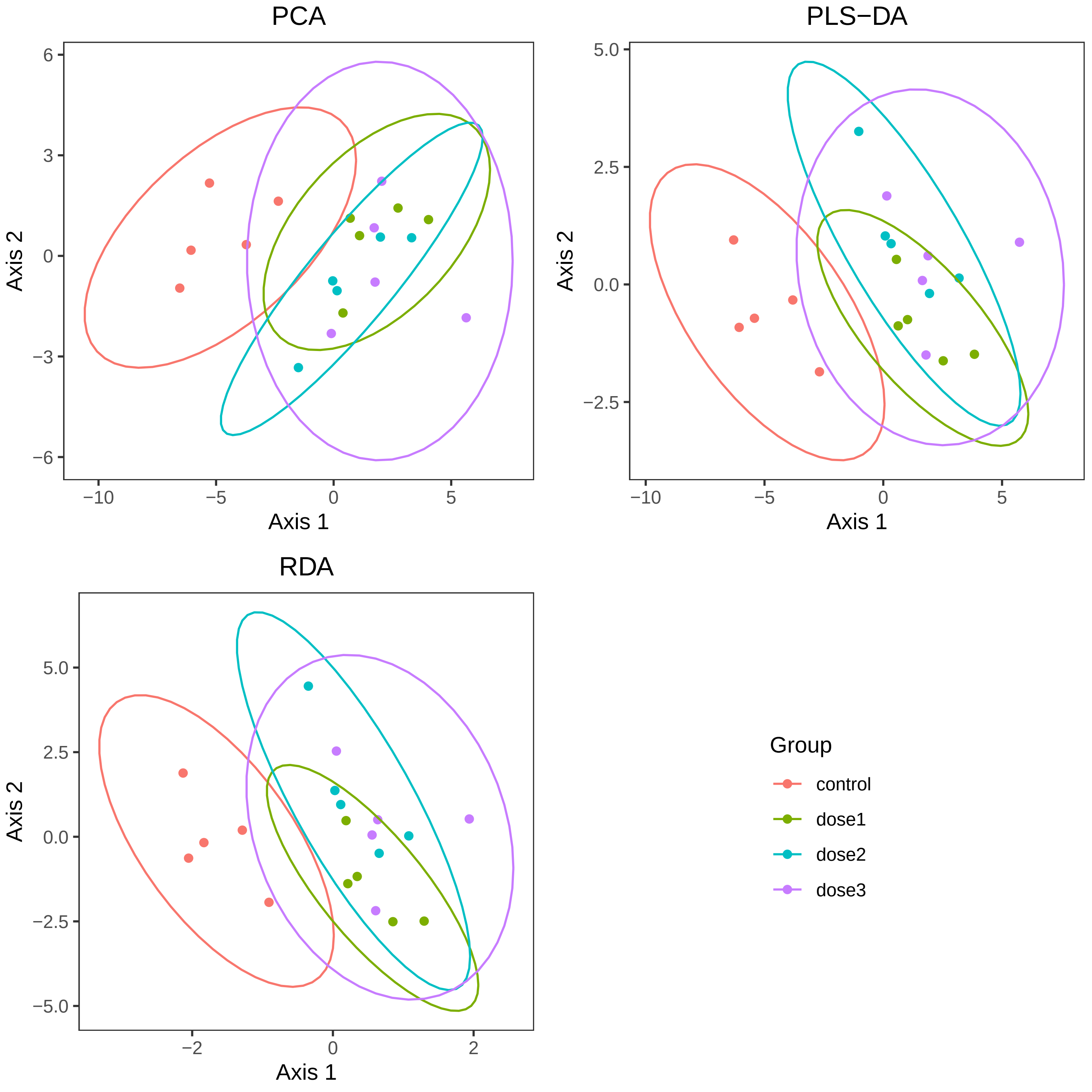

PCA
PLS−DA
RDA
Axis 1
Axis 2
6
3
0
−3
−6
−10
−5
0
5
5.0
2.5
0.0
−2.5
−5.0
−2
2
Group
control
dose1
dose2
dose3

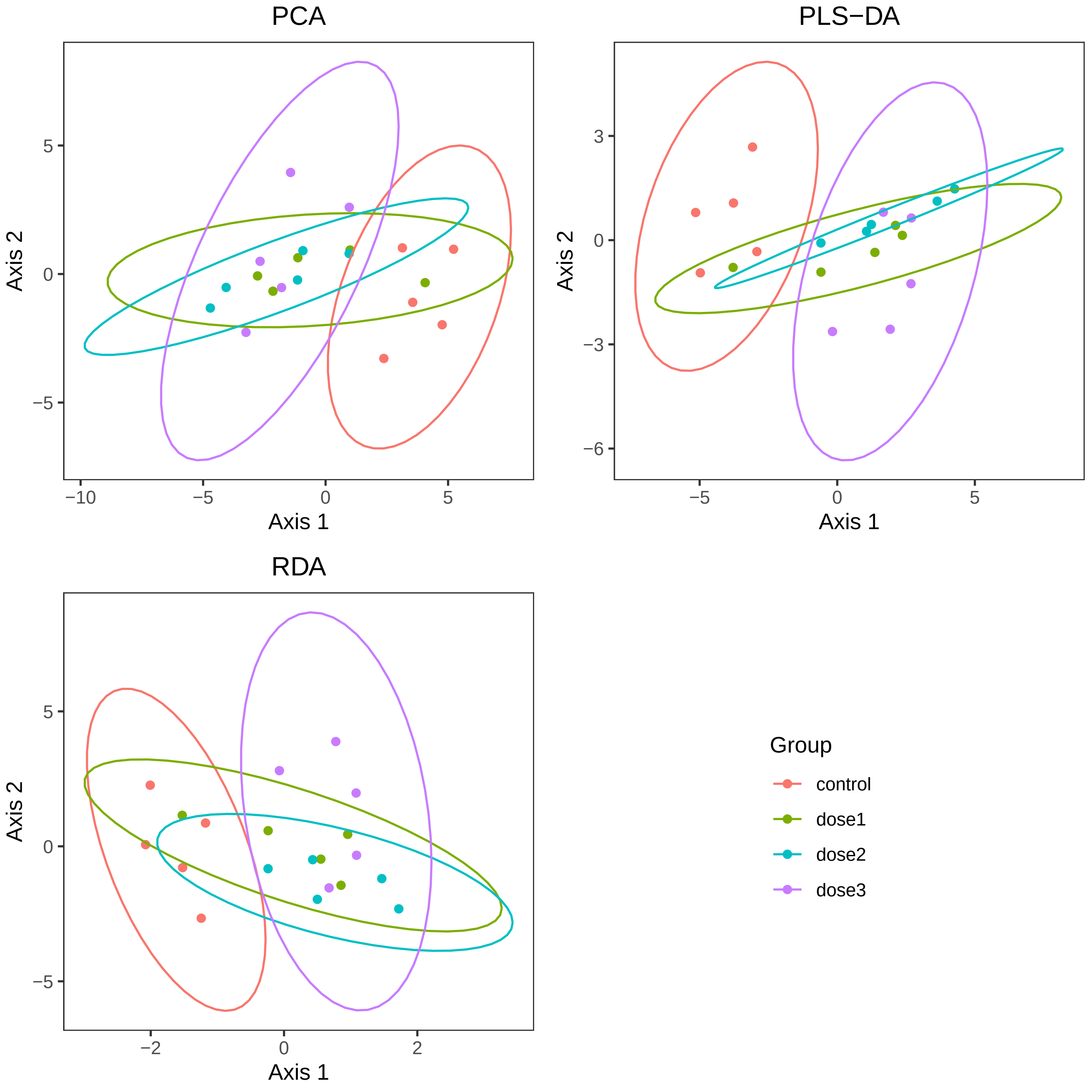

PCA
PLS−DA
RDA
Axis 1
Axis 2
Group
control
dose1
dose2
dose3

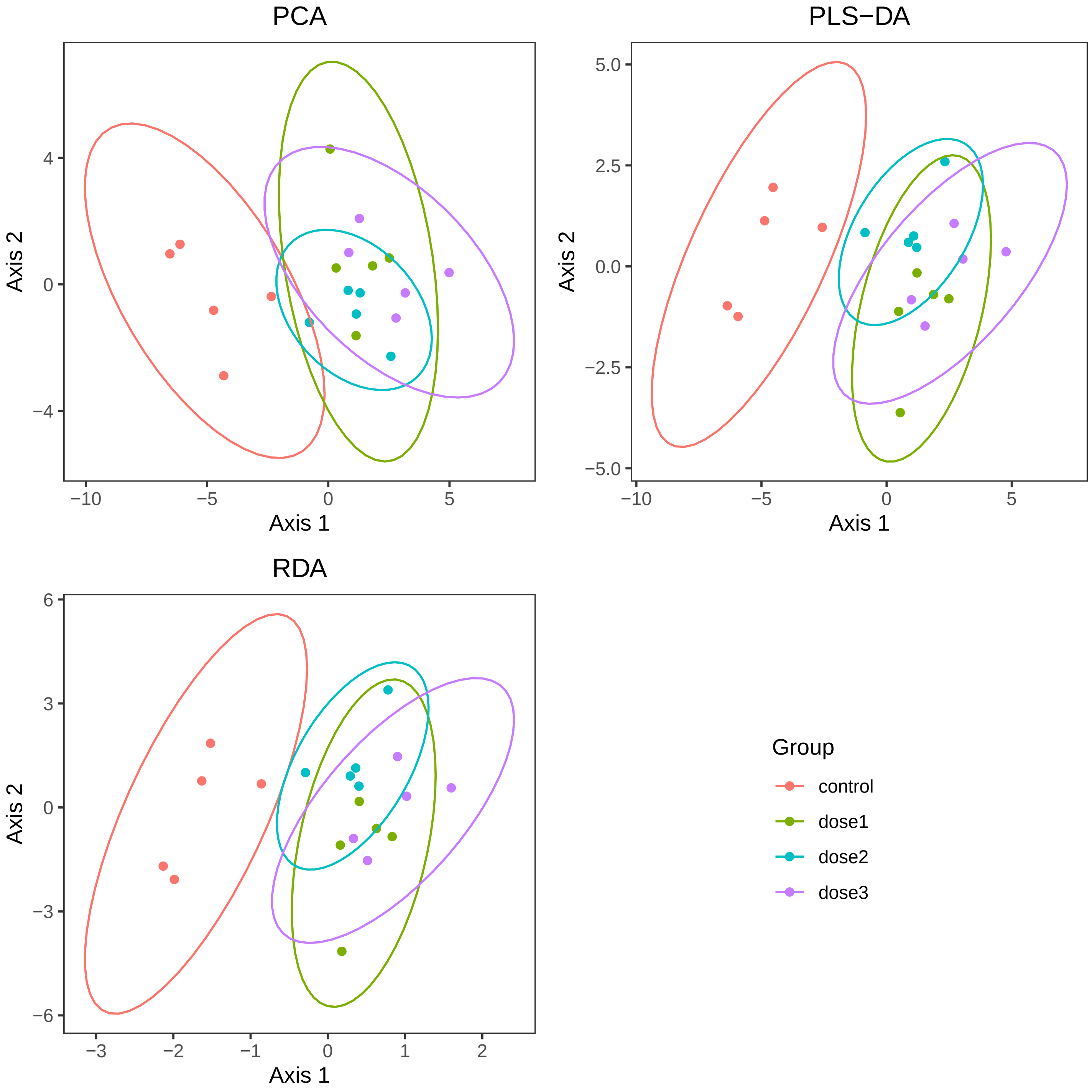
PCA
PLS−DA
RDA
Axis 1
Axis 2
Group
control
dose1
dose2
dose3

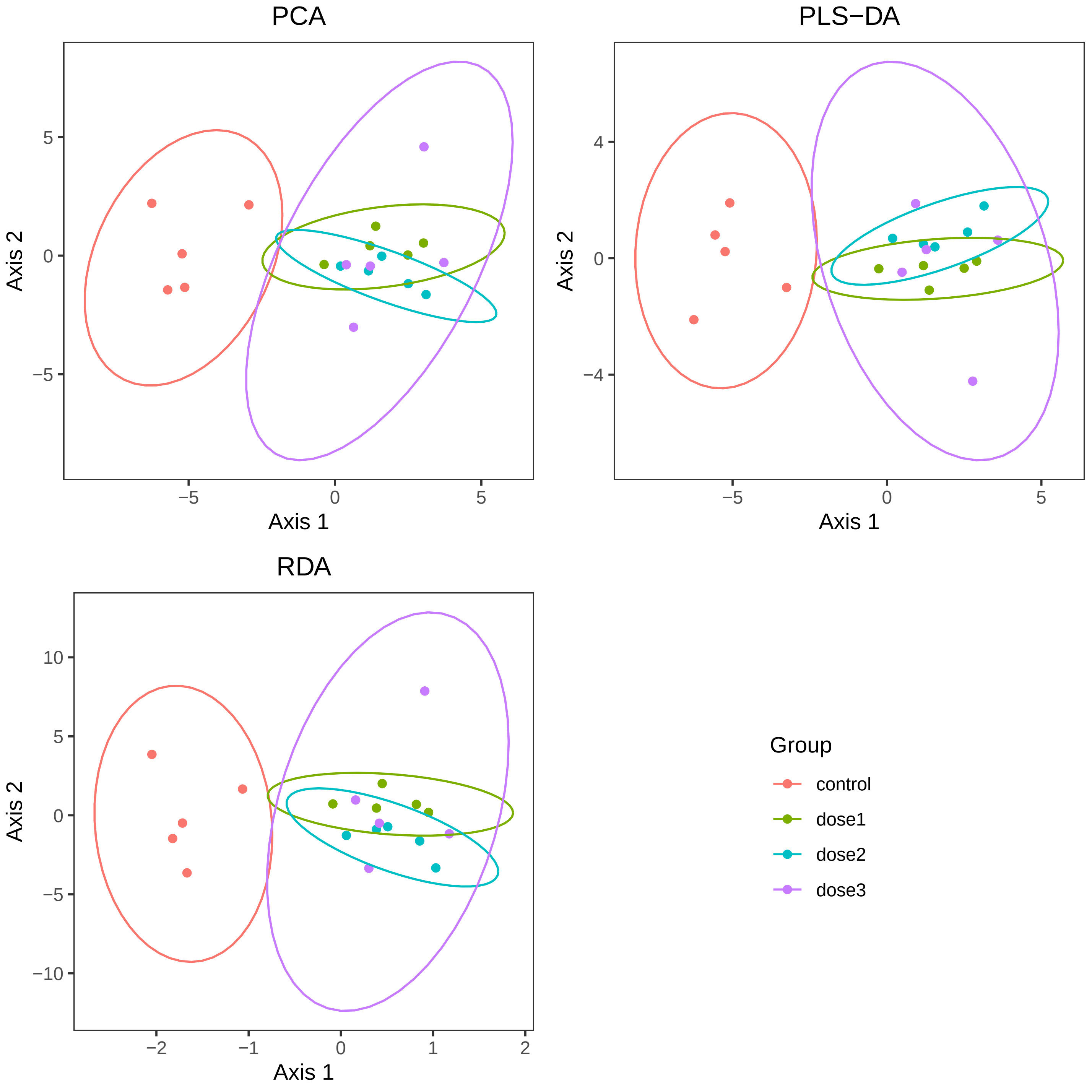
PCA
PLS−DA
RDA
Axis 1
Axis 2
Group
control
dose1
dose2
dose3

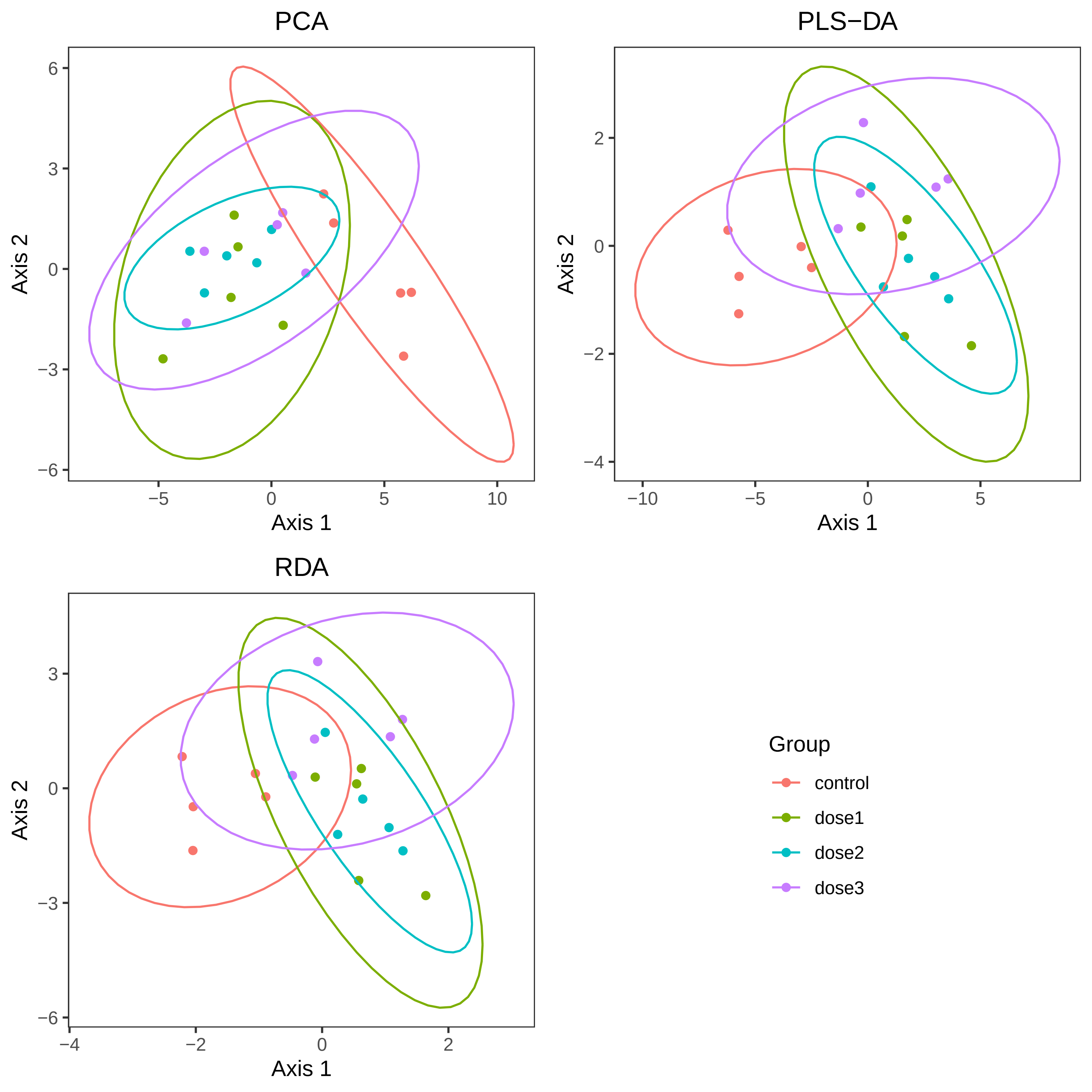

PCA
PLS−DA
RDA
Axis 1
Axis 2
Group
control
dose1
dose2
dose3

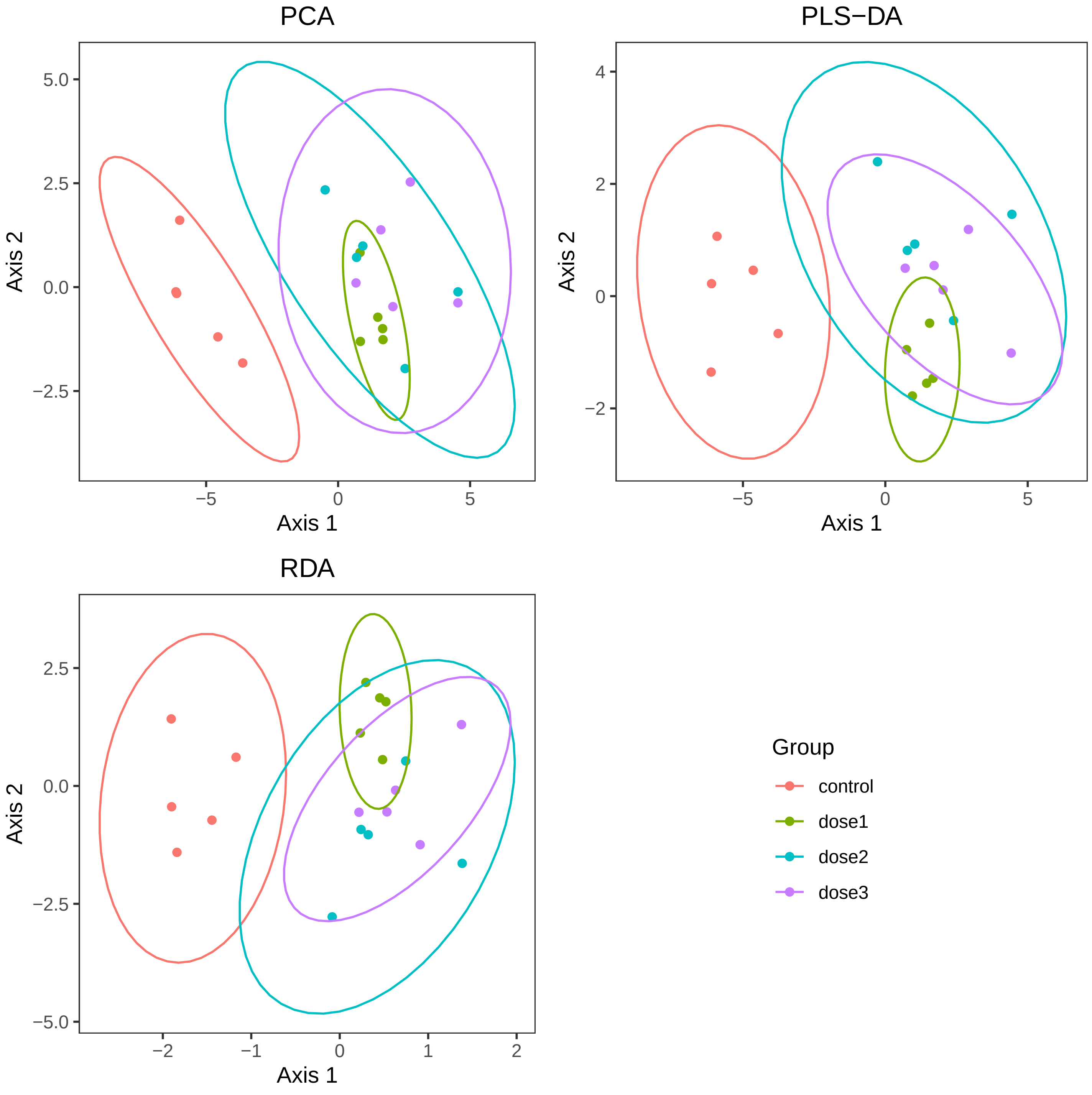
PCA
PLS−DA
RDA
Axis 1
Axis 2
Group
control
dose1
dose2
dose3

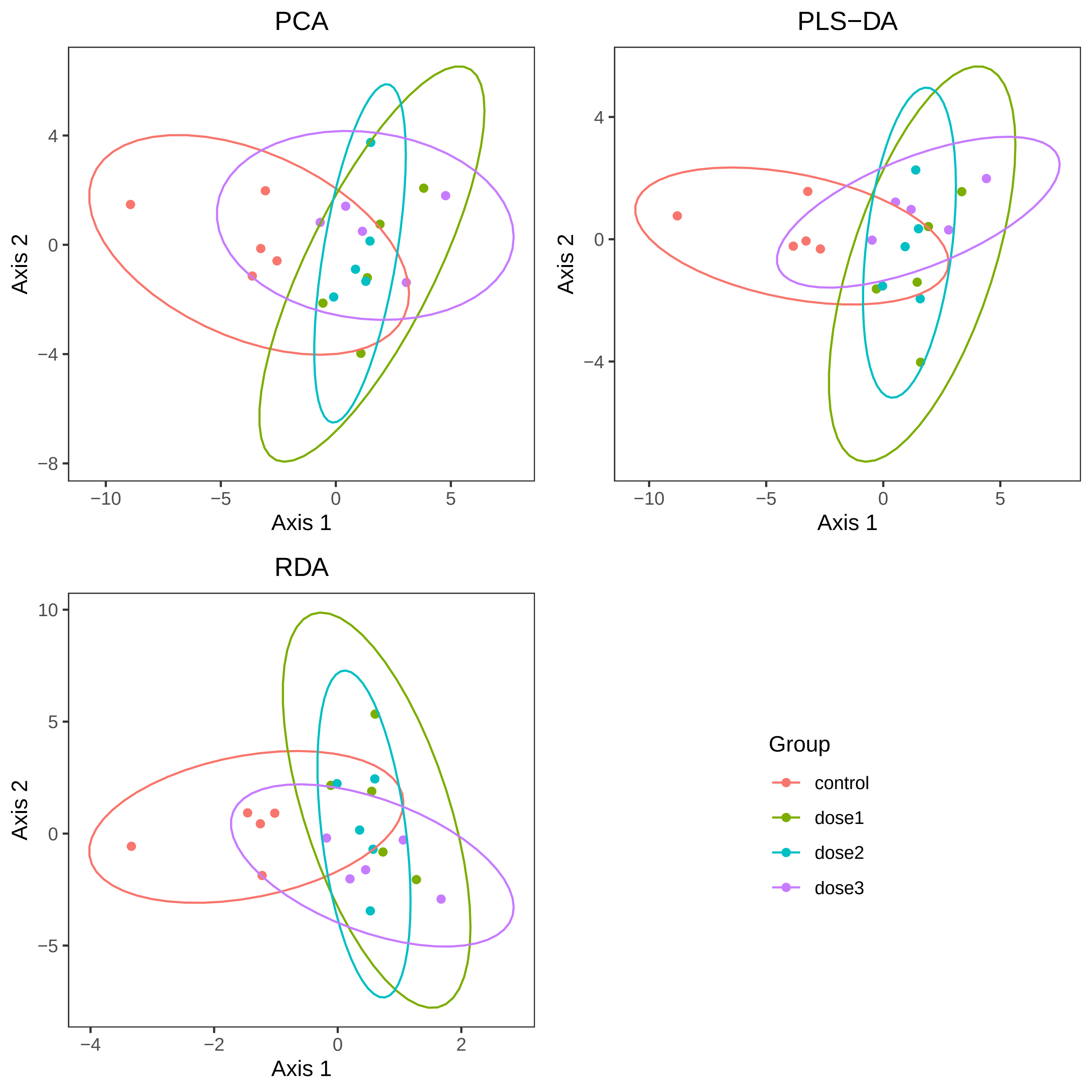
PCA
PLS−DA
RDA
Axis 1
Axis 2
Group
control
dose1
dose2
dose3

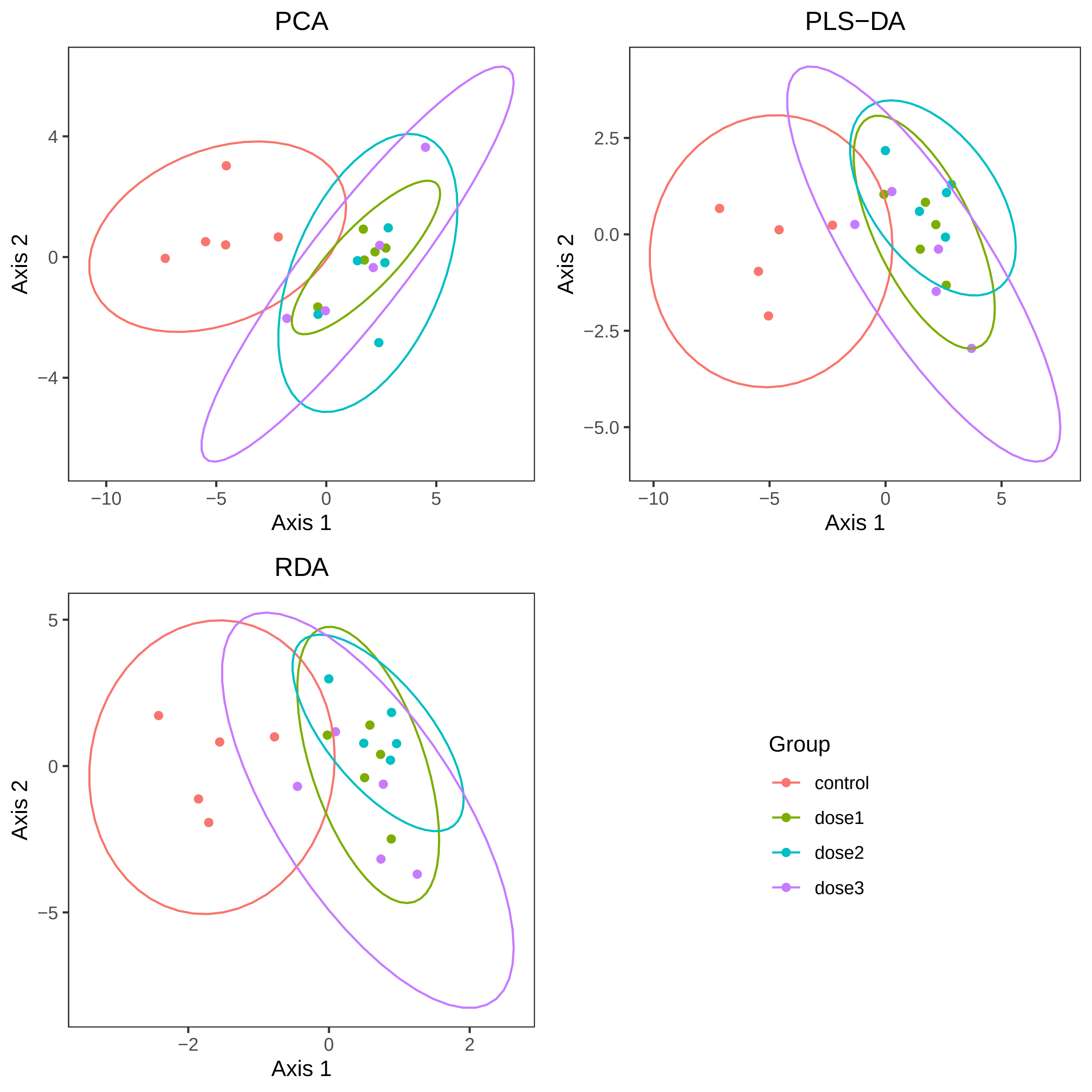
PCA
Axis 2
Axis 1
4
0
−4
−10
−5
0
5
PLS−DA
2.5
0.0
−2.5
−5.0
RDA
5
−5
−2
2
Group
control
dose1
dose2
dose3

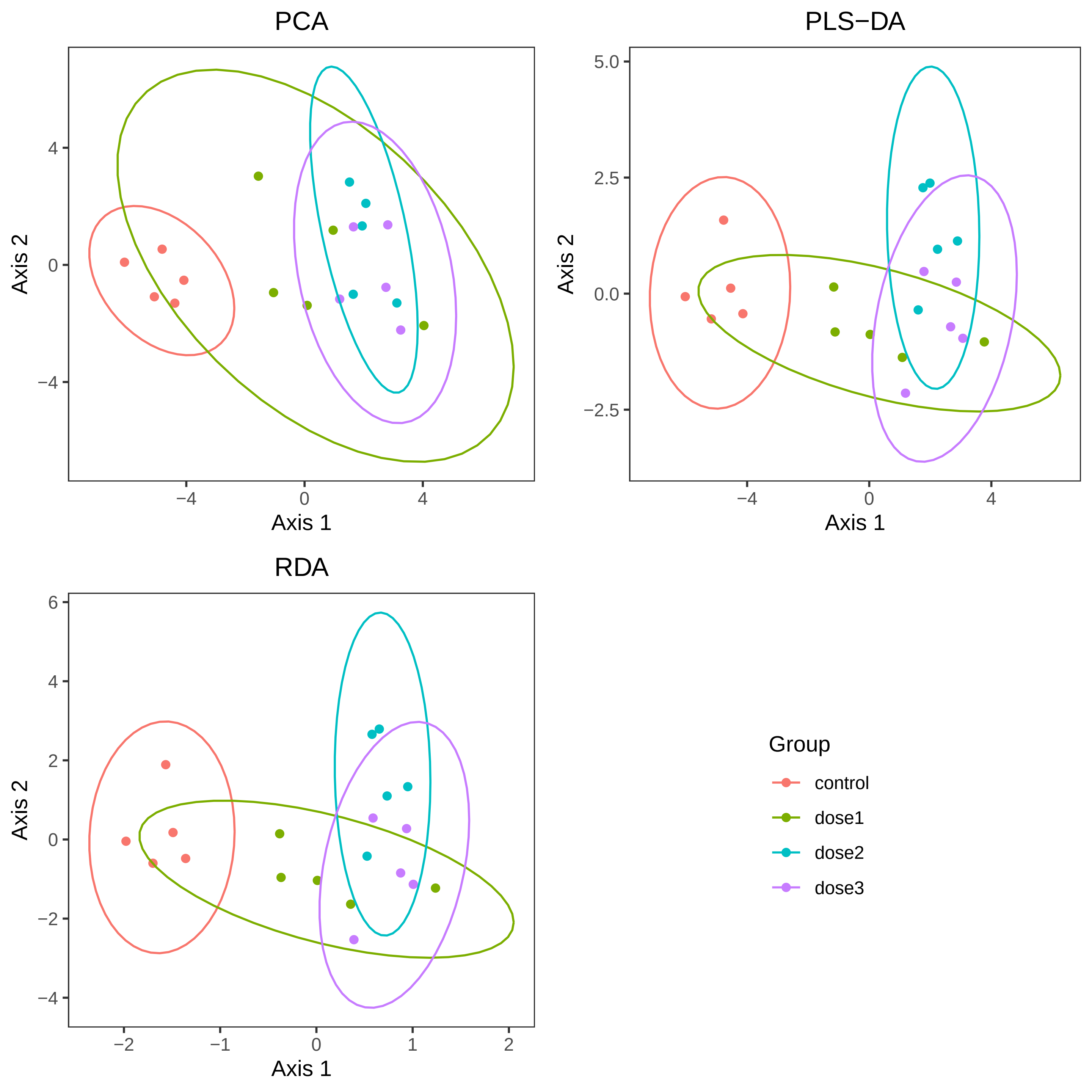
PCA
PLS−DA
RDA
Axis 1
Axis 2
Group
control
dose1
dose2
dose3

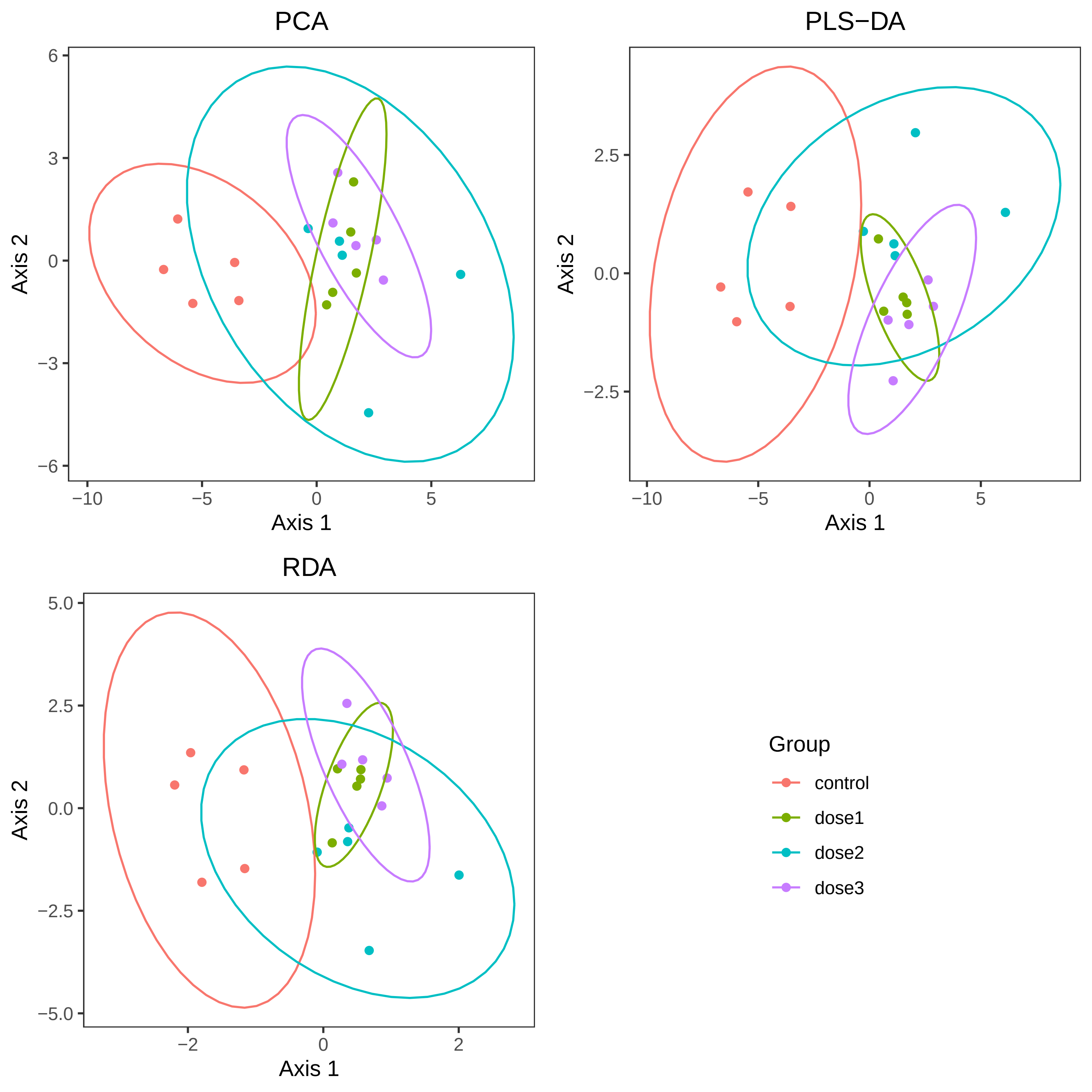

PCA
PLS−DA
RDA
Axis 1
Axis 2
Group
control
dose1
dose2
dose3

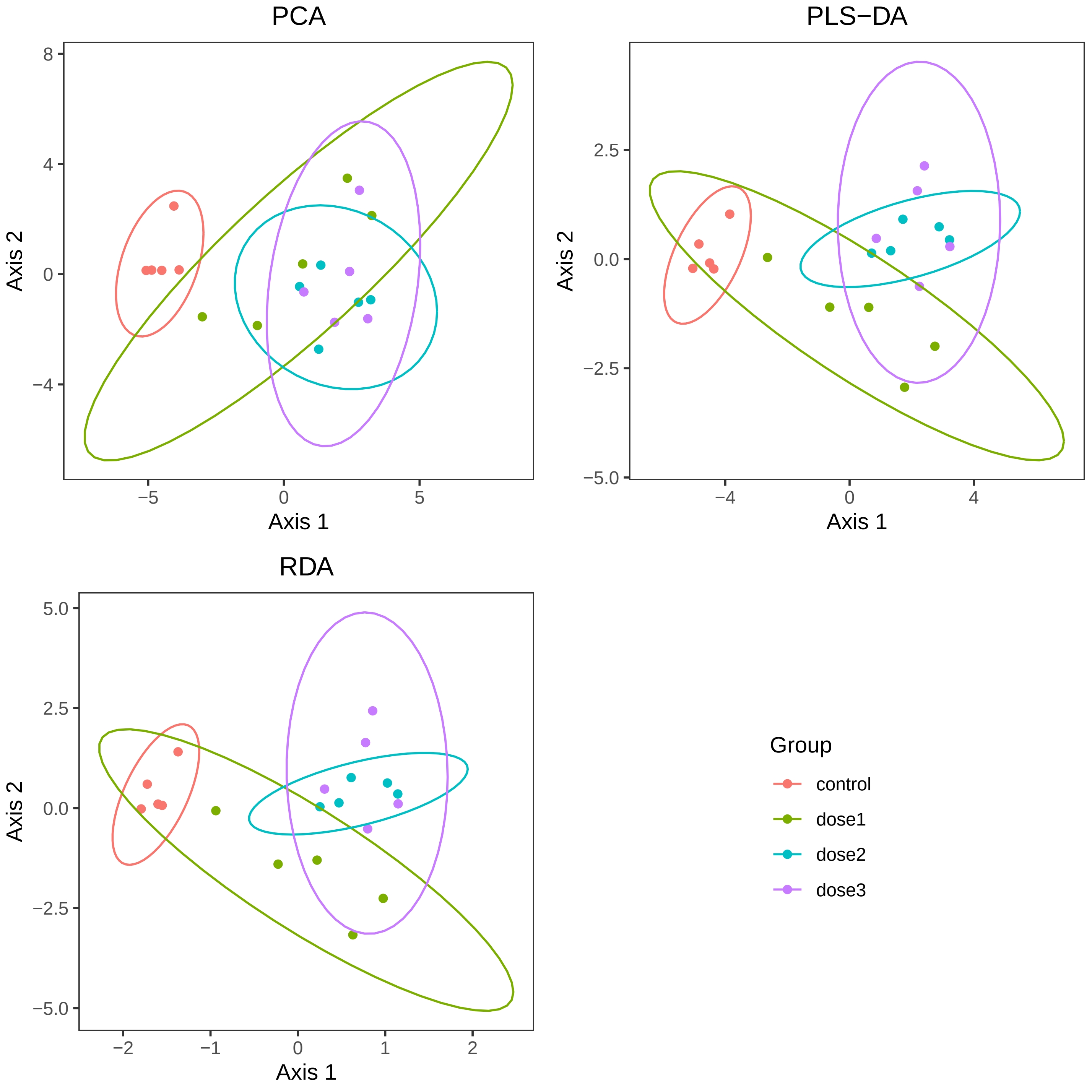

PCA
PLS−DA
RDA
Axis 1
Axis 2
Group
control
dose1
dose2
dose3

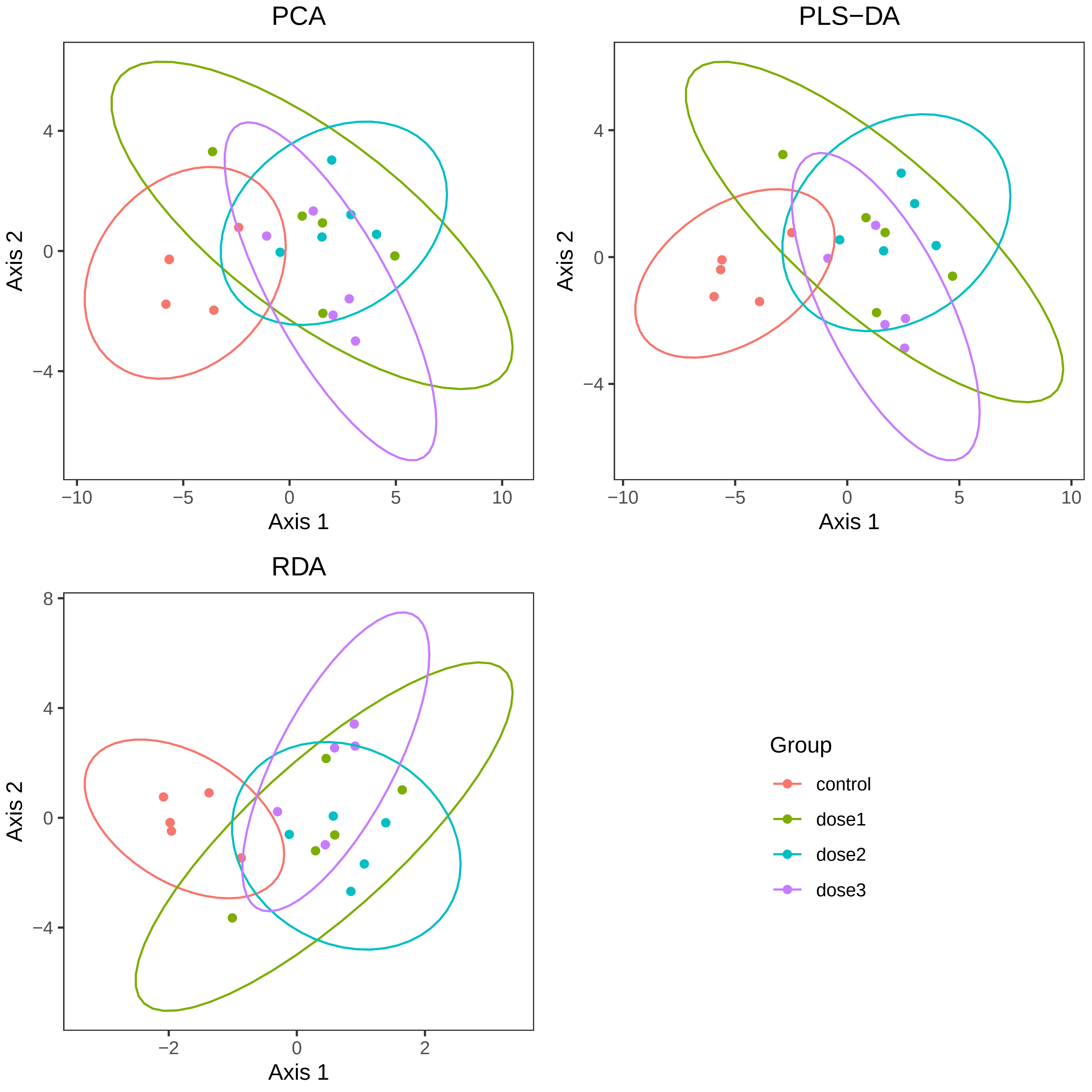

PCA
PLS−DA
RDA
Axis 1
Axis 2
4
0
−4
8
−10
−5
5
10
−2
2
Group
control
dose1
dose2
dose3

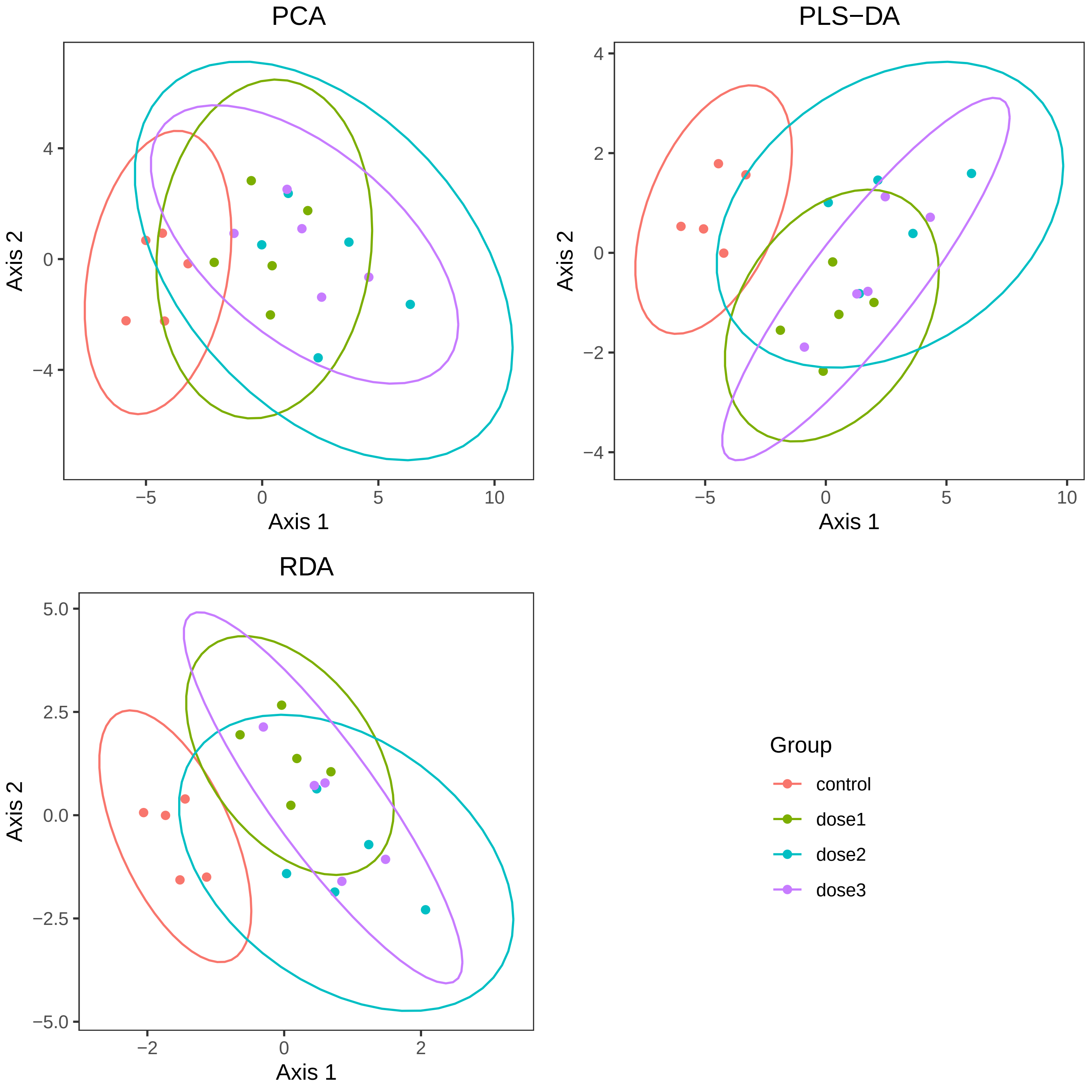
PCA
Axis 2
Axis 1
4
0
−4
−5
0
5
10
PLS−DA
Axis 2
Axis 1
4
2
0
−2
−4
−5
0
5
10
RDA
Axis 2
Axis 1
5.0
2.5
0.0
−2.5
−5.0
−2
0
2
Group
control
dose1
dose2
dose3

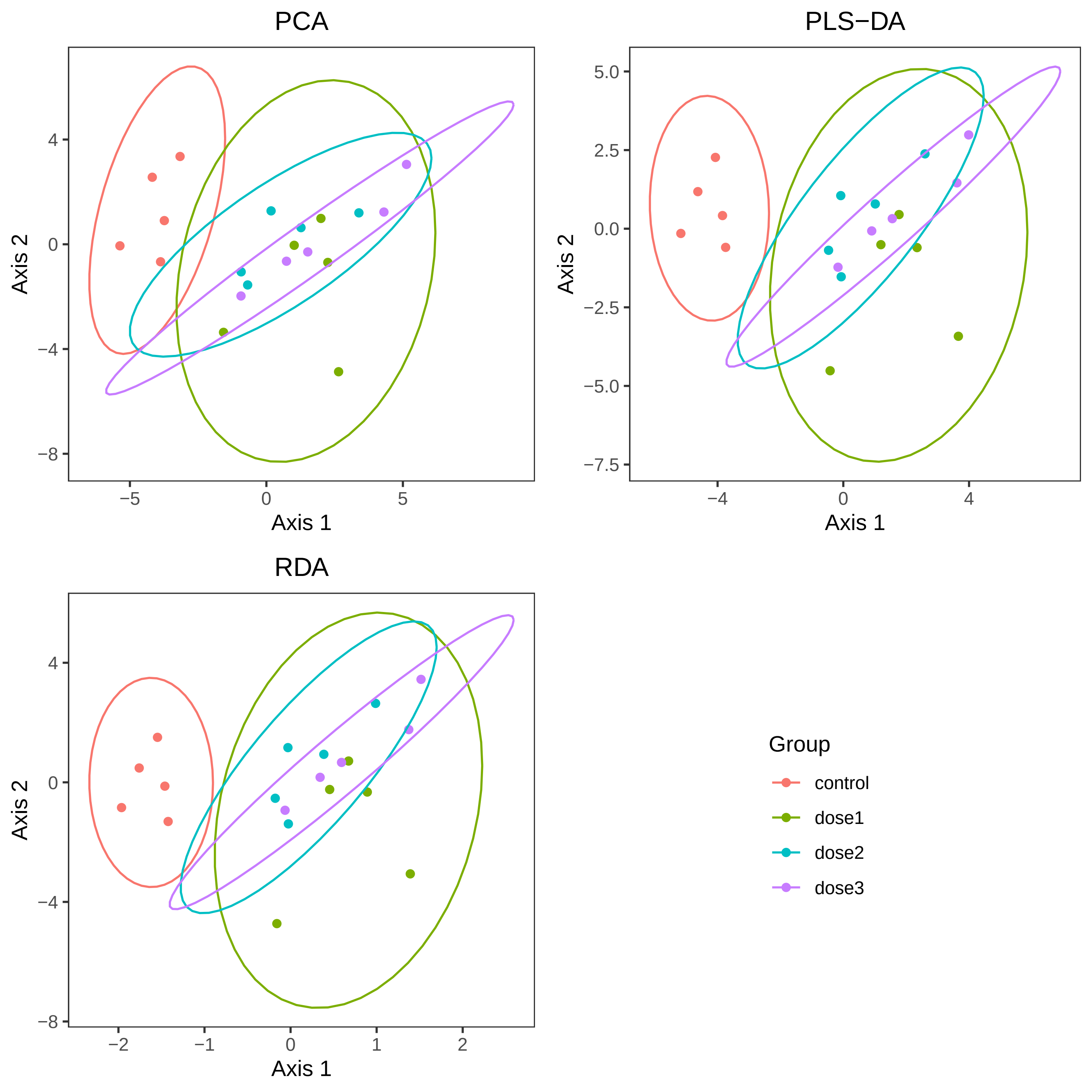

PCA
PLS−DA
RDA
Axis 1
Axis 2
−5
0
5
4
0
−4
−8
5.0
2.5
0.0
−2.5
−5.0
−7.5
−4
0
4
−2
−1
0
1
2
Group
control
dose1
dose2
dose3

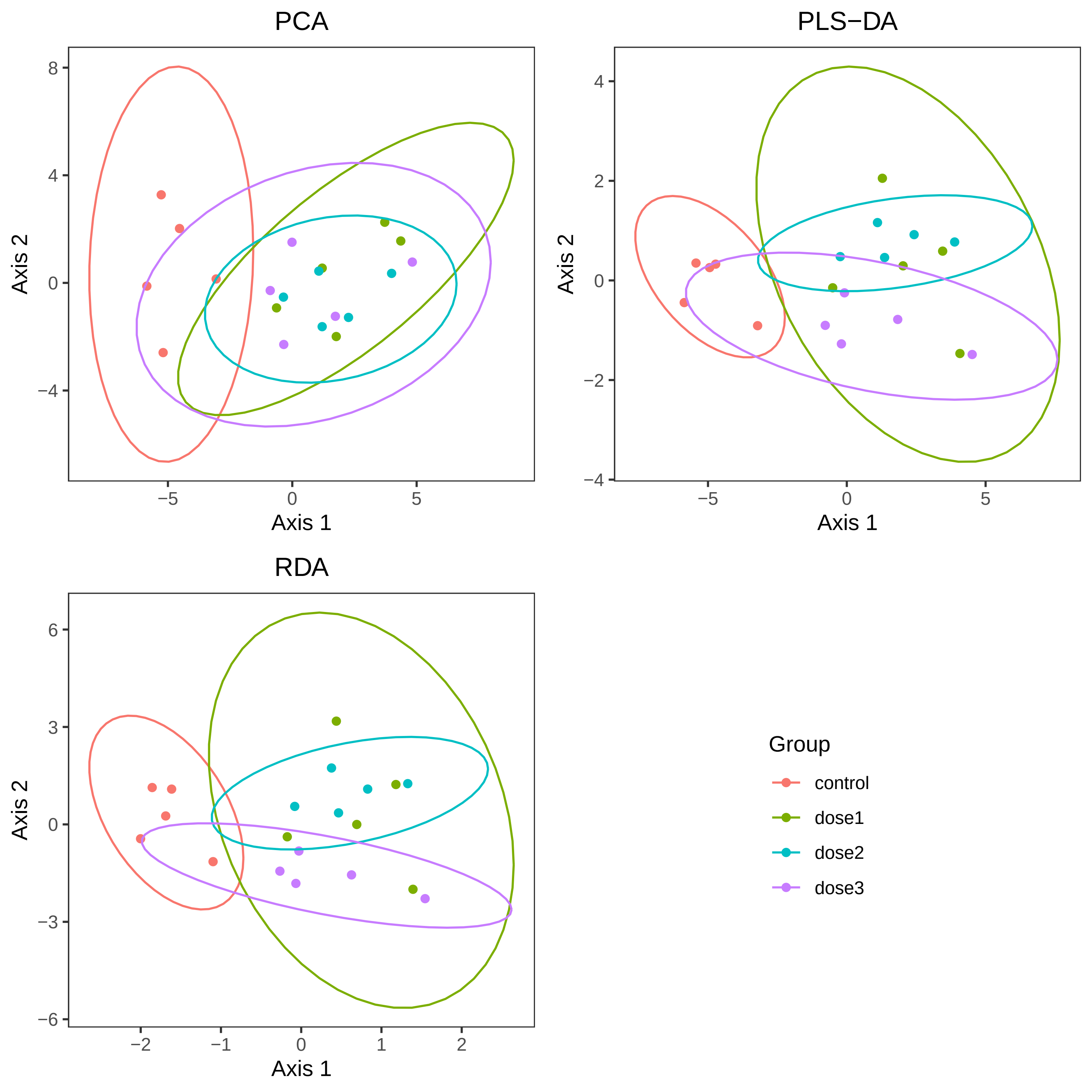

PCA
PLS−DA
RDA
Axis 1
Axis 2
Group
control
dose1
dose2
dose3

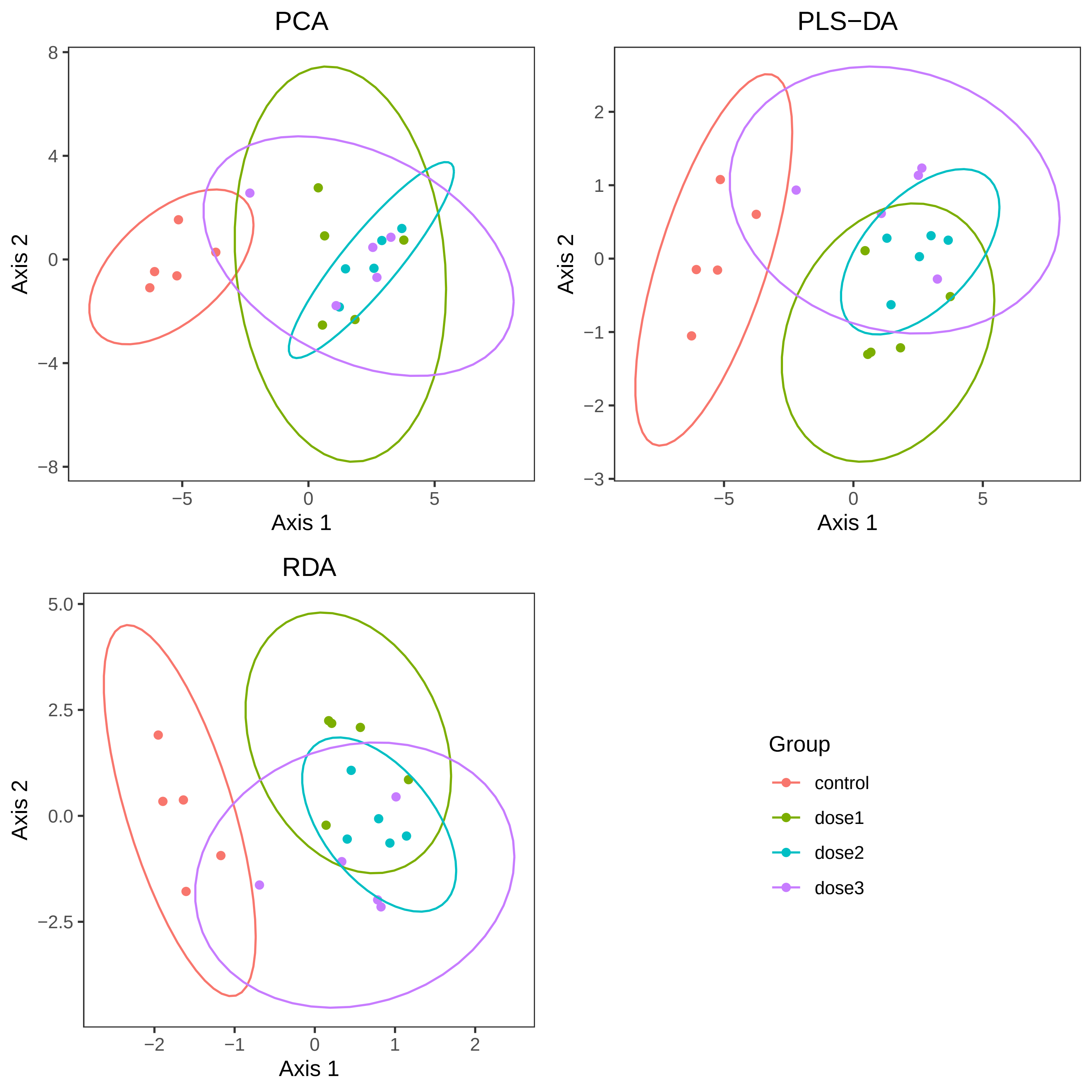

PCA
PLS−DA
RDA
Axis 1
Axis 2
Group
control
dose1
dose2
dose3

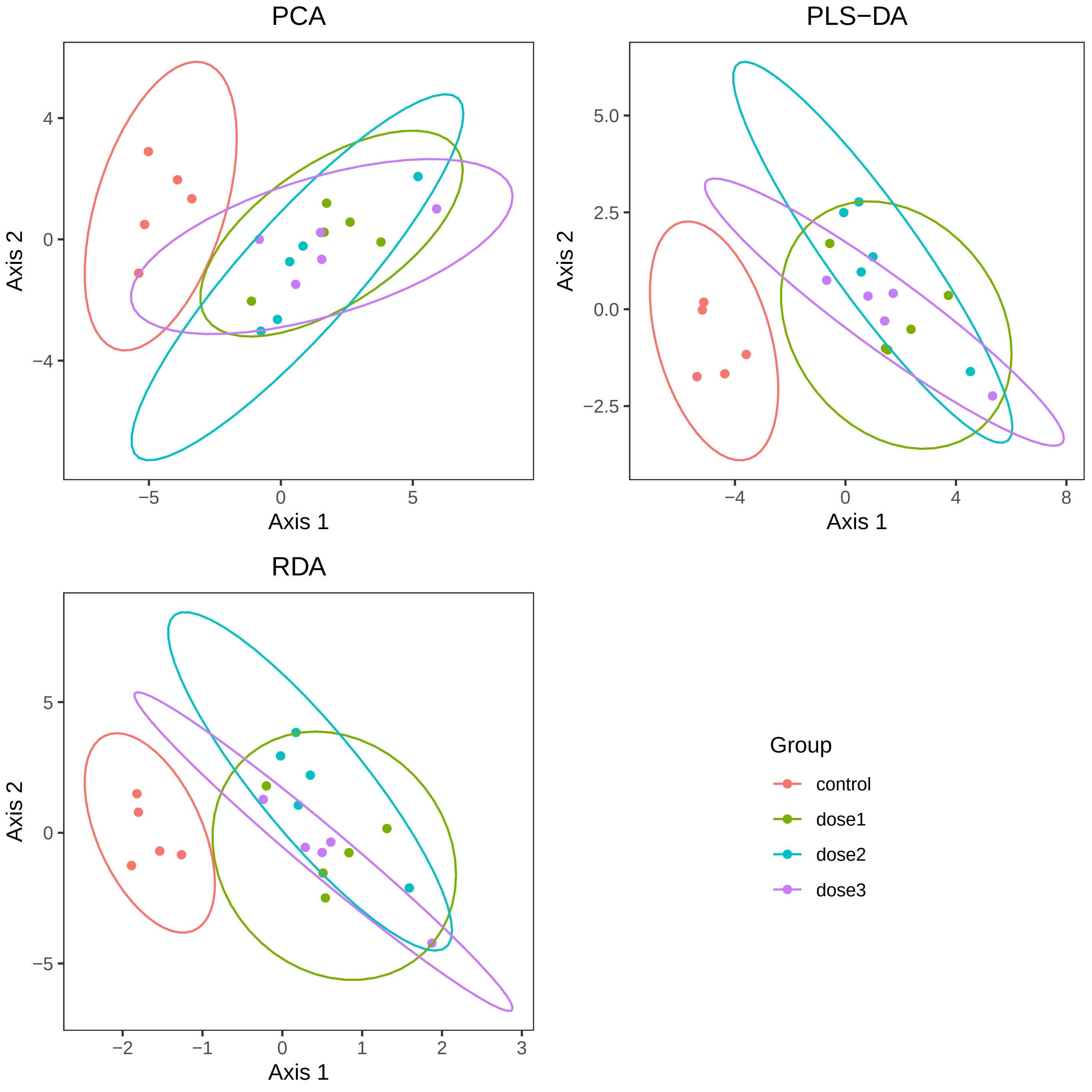

PCA
Axis 2
4
0
−4
−5
0
5
Axis 1
PLS−DA
Axis 2
5.0
2.5
0.0
−2.5
−4
0
4
8
Axis 1
RDA
Axis 2
5
0
−5
−2
−1
0
1
2
3
Axis 1
Group
control
dose1
dose2
dose3

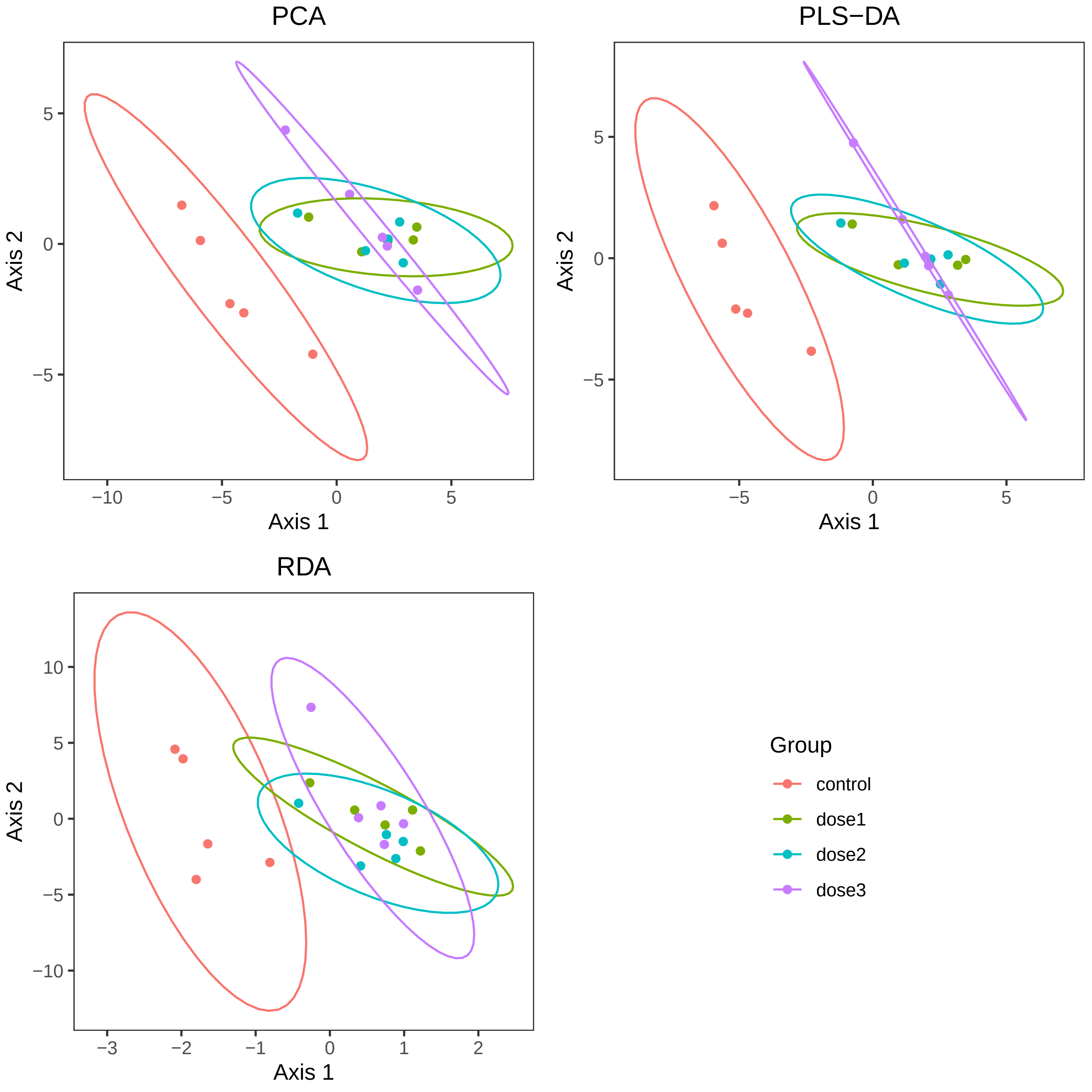

PCA
PLS−DA
RDA
Axis 1
Axis 2
Group
control
dose1
dose2
dose3

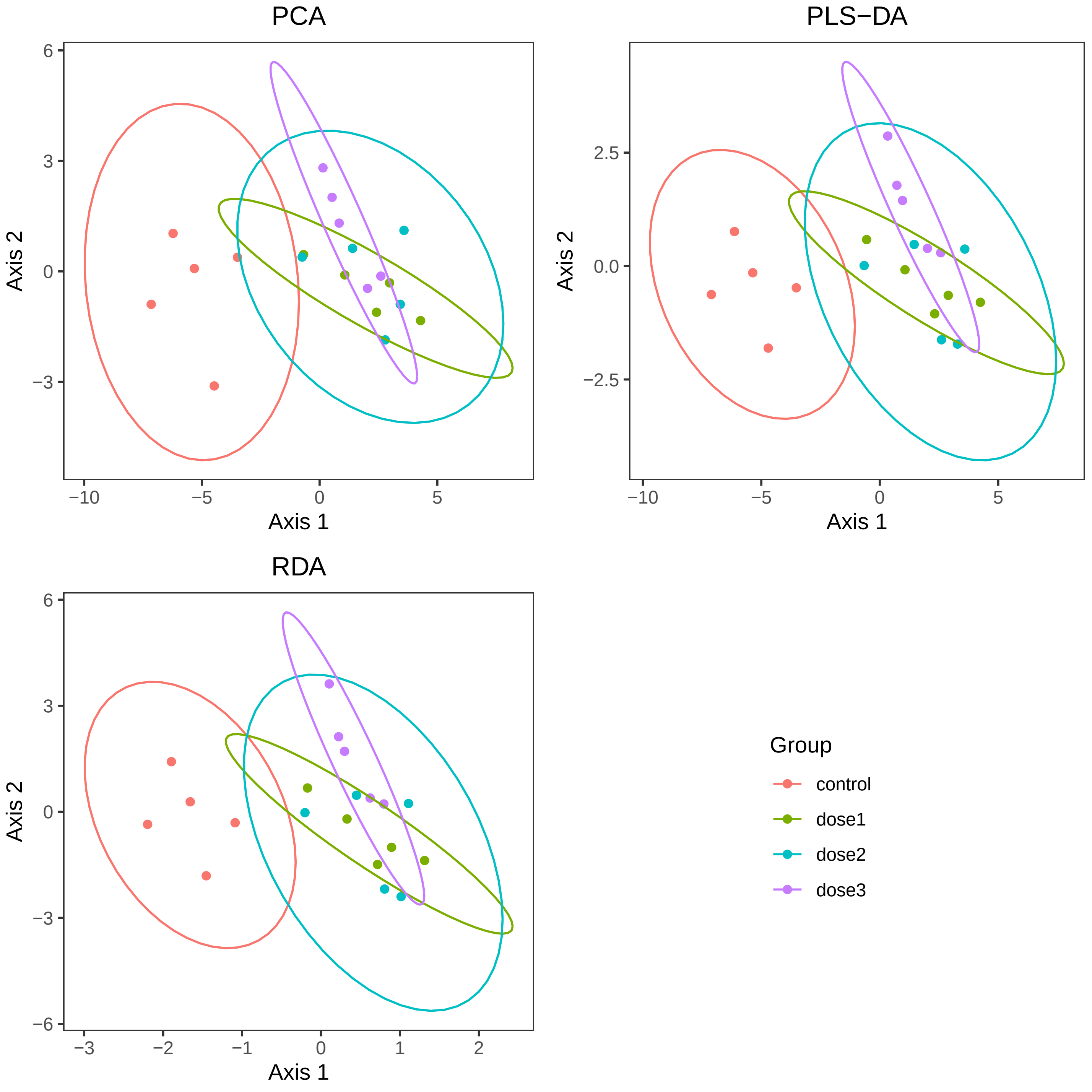

PCA
PLS−DA
RDA
Axis 1
Axis 2
Group
control
dose1
dose2
dose3

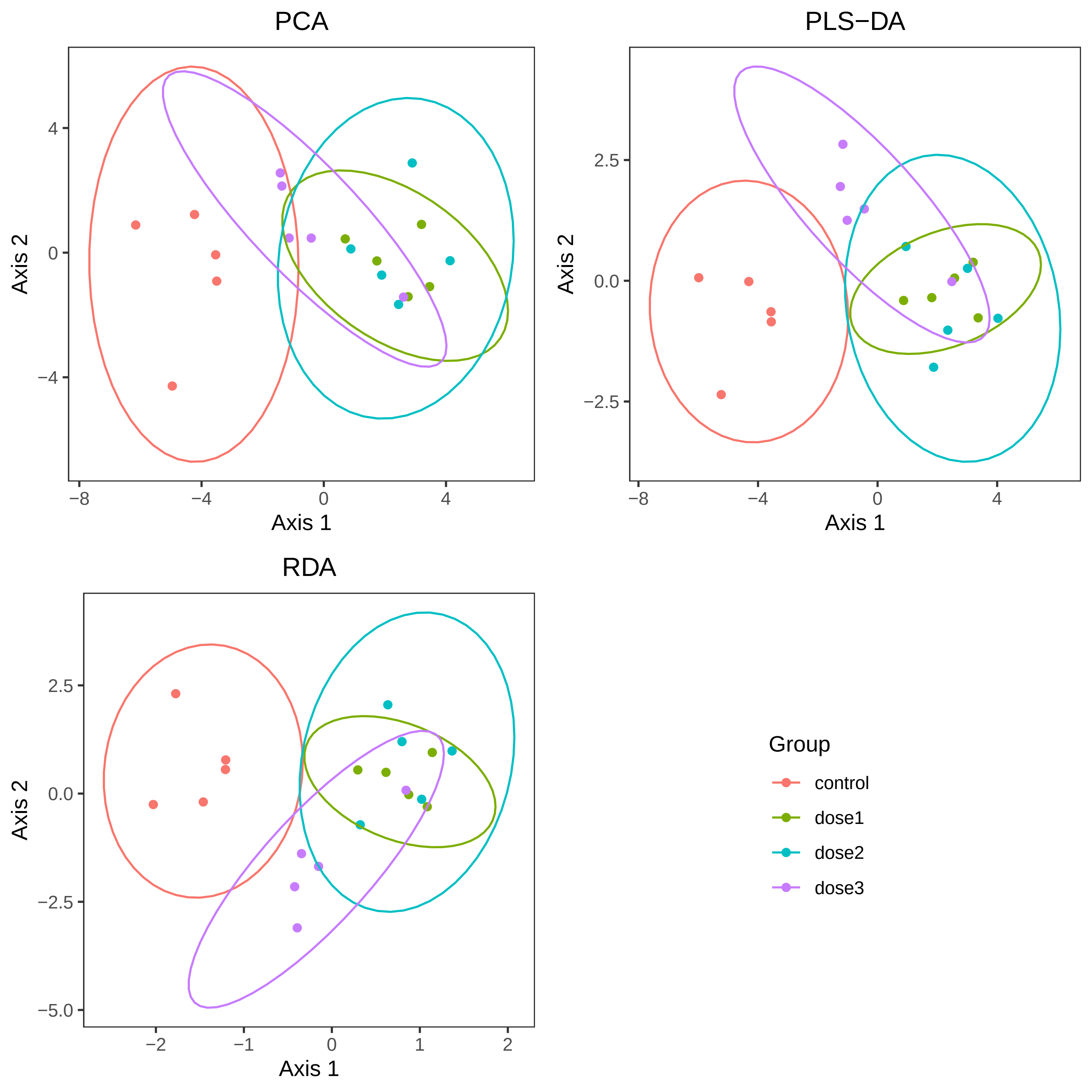

PCA
PLS−DA
RDA
Axis 1
Axis 2
Group
control
dose1
dose2
dose3

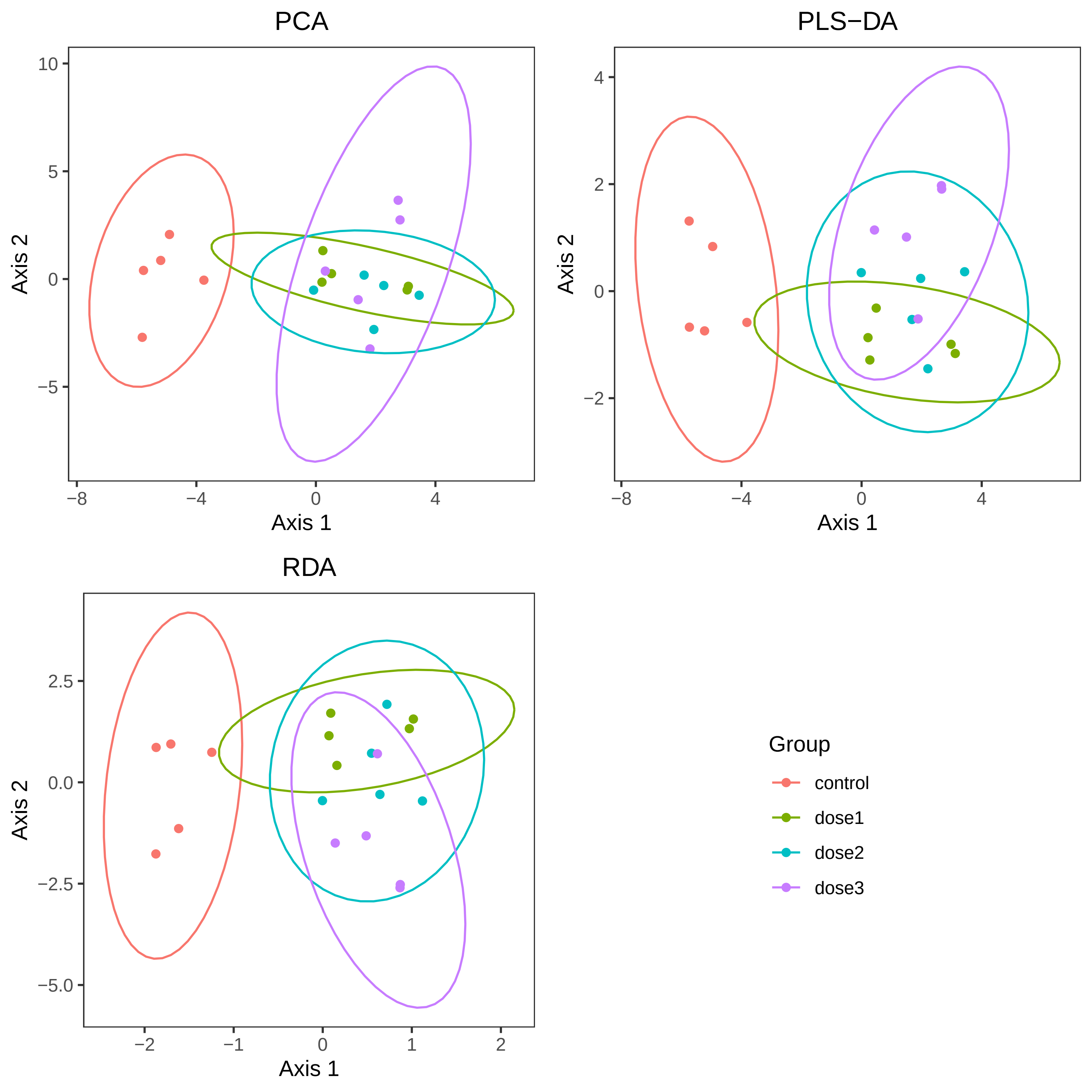
PCA
PLS−DA
RDA
Axis 1
Axis 2
Group
control
dose1
dose2
dose3

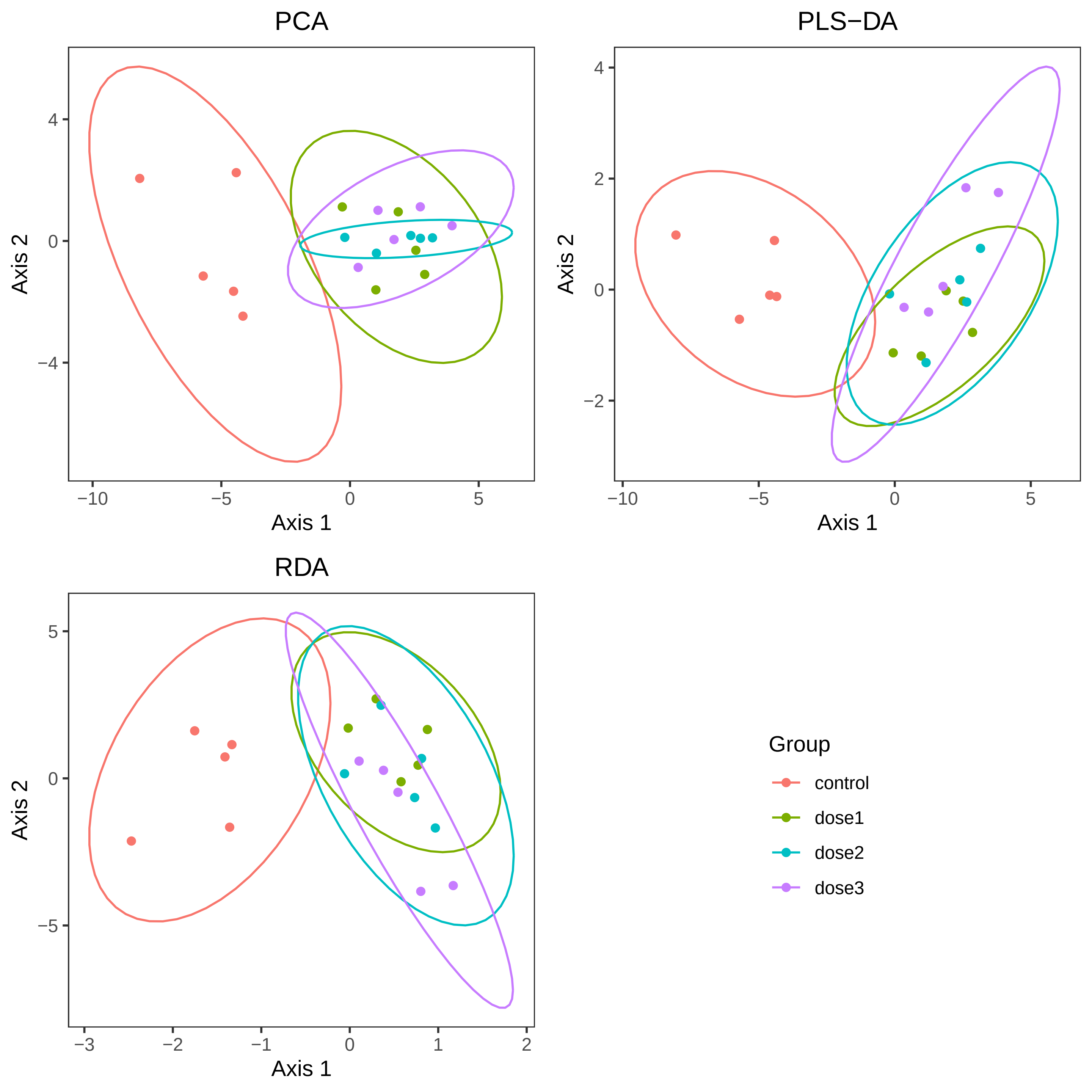
PCA
PLS−DA
RDA
Axis 1
Axis 2
Group
control
dose1
dose2
dose3

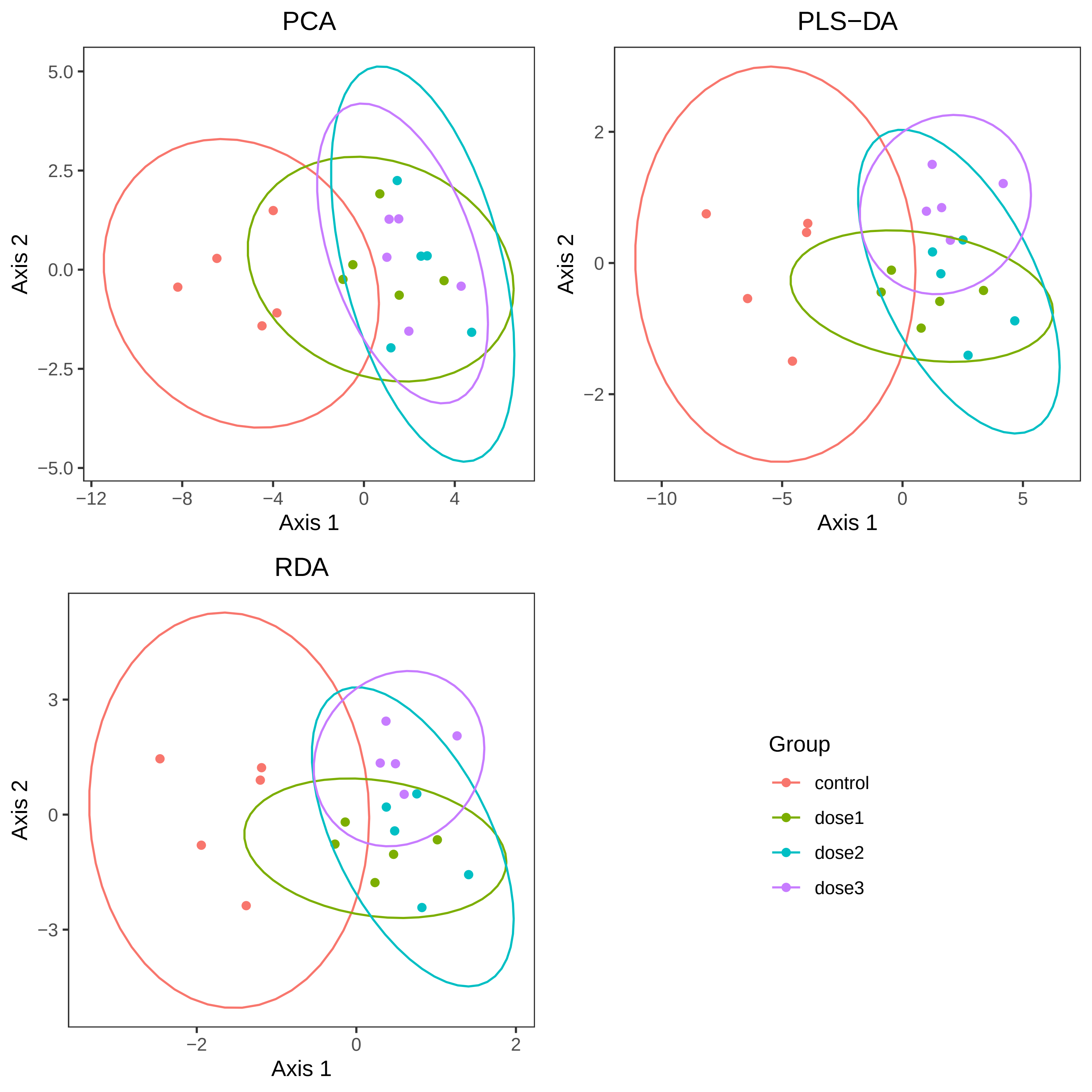
PCA
Axis 1
Axis 2
5.0
2.5
0.0
−2.5
−5.0
−12
−8
−4
0
4
PLS−DA
Axis 1
Axis 2
2
0
−2
−10
−5
0
5
RDA
Axis 1
Axis 2
3
0
−3
−2
0
2
Group
control
dose1
dose2
dose3

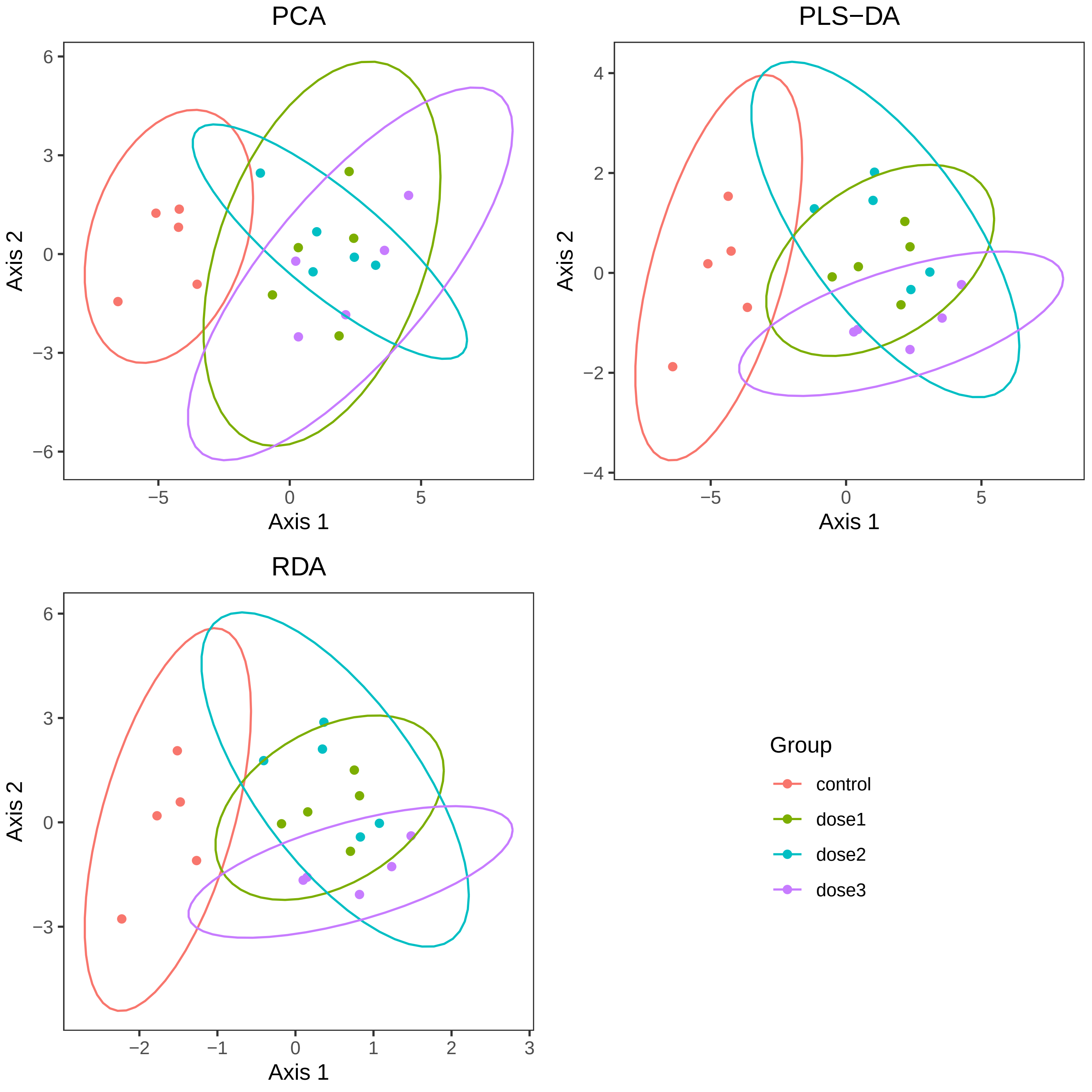
PCA
PLS−DA
RDA
Axis 1
Axis 2
Group
control
dose1
dose2
dose3

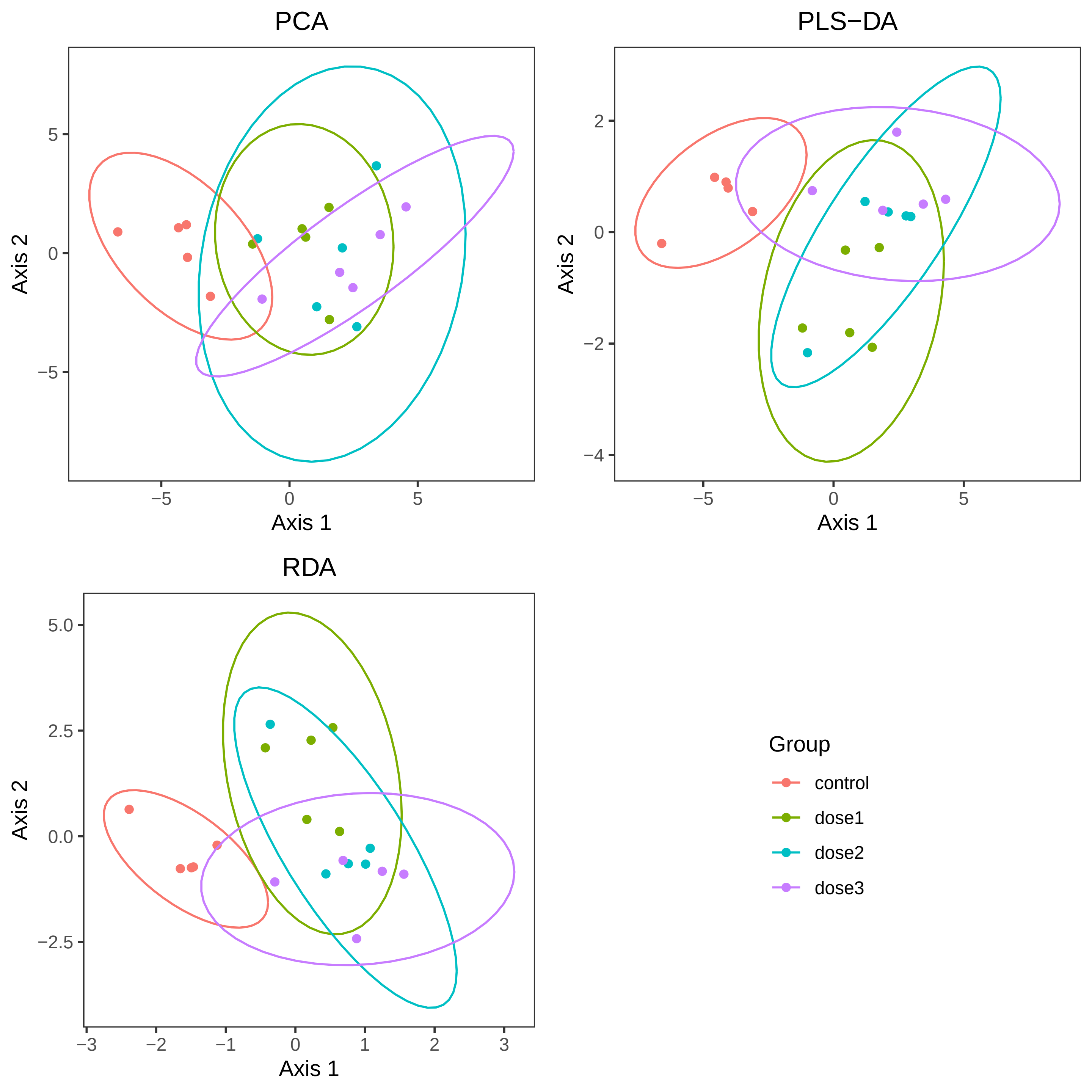

PCA
PLS−DA
RDA
Axis 1
Axis 2
Group
control
dose1
dose2
dose3

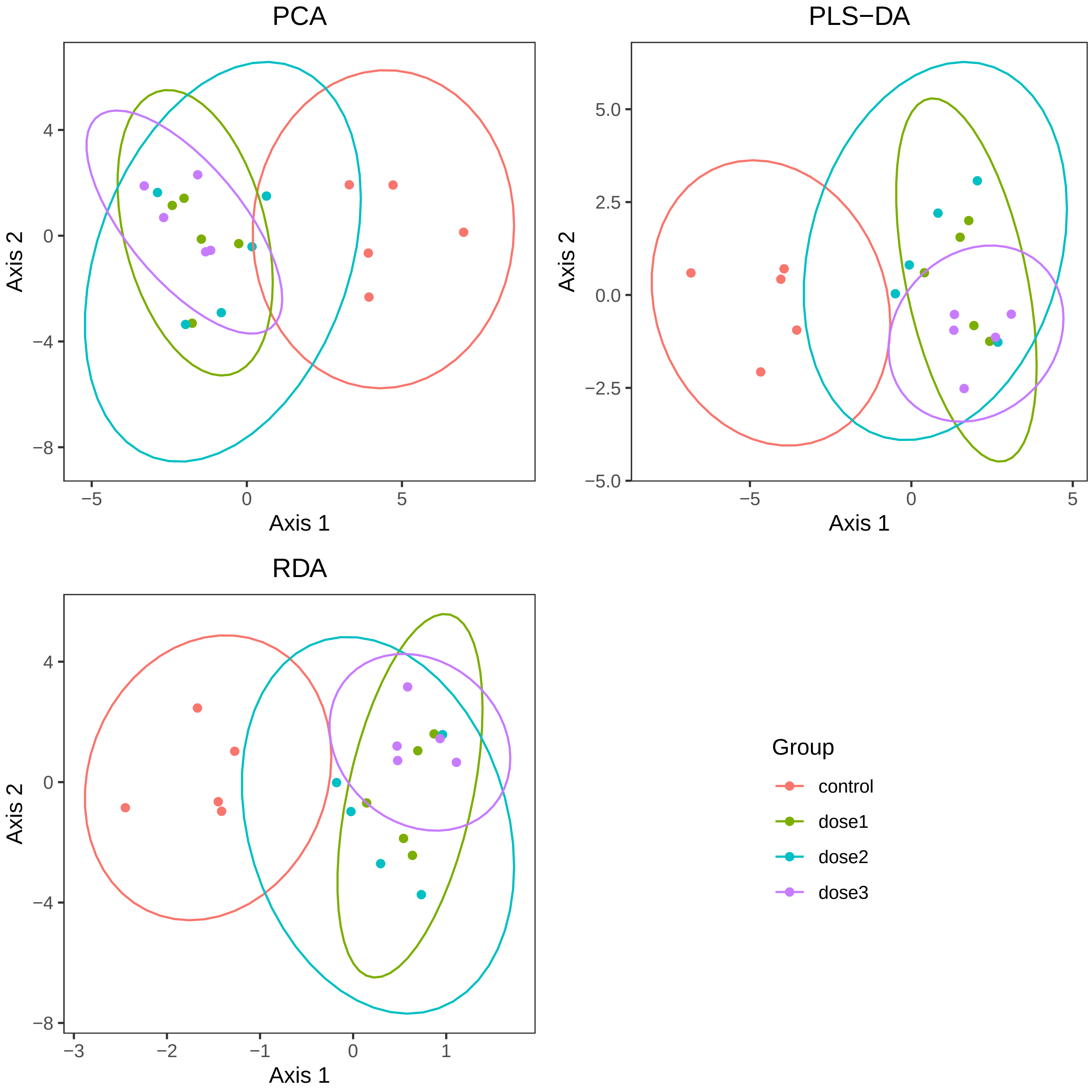

PCA
Axis 2
Axis 1
4
0
−4
−8
−5
0
5
PLS−DA
5.0
2.5
0.0
−2.5
−5.0
RDA
−3
−2
−1
1
Group
control
dose1
dose2
dose3

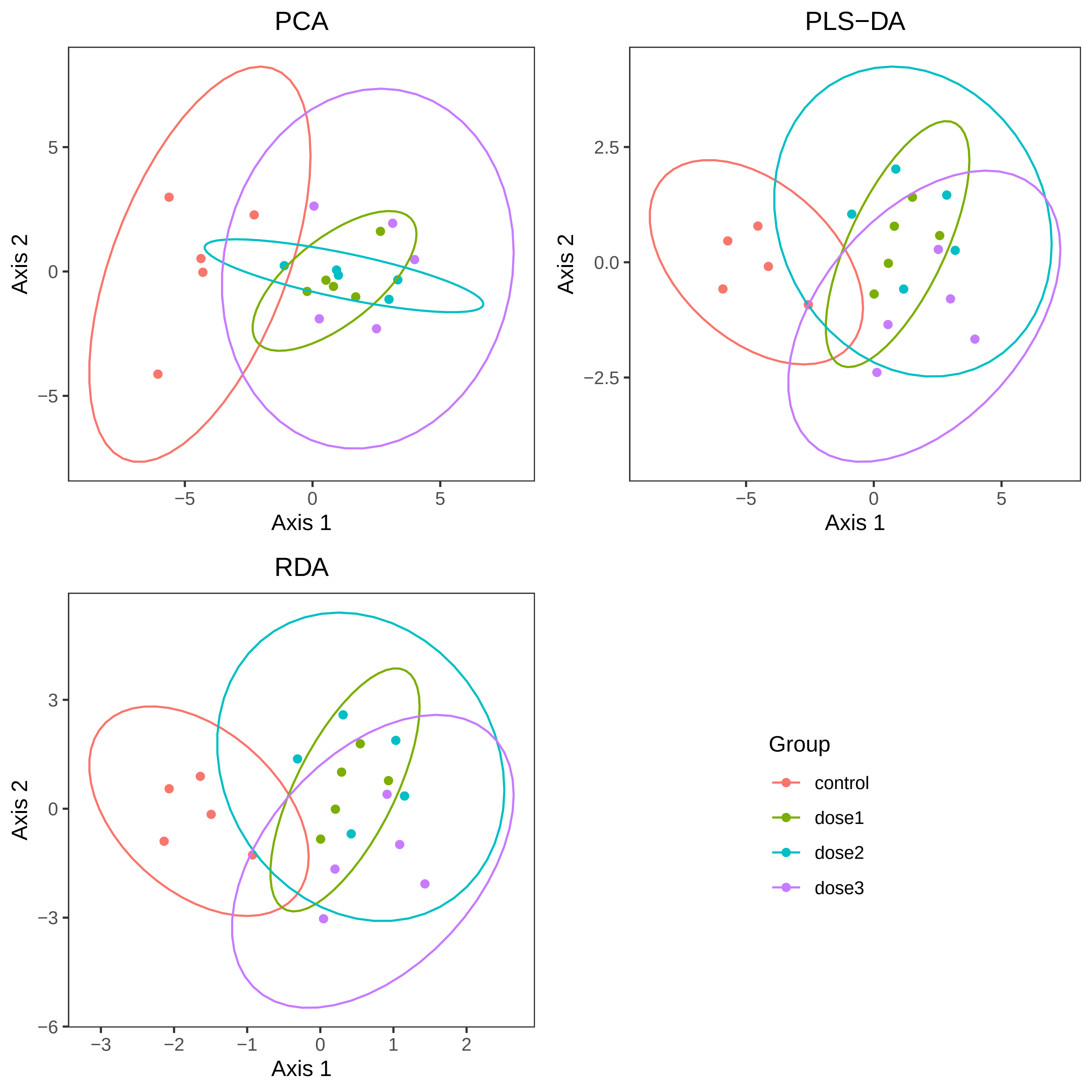
PCA
PLS−DA
RDA
Axis 1
Axis 2
5
0
−5
2.5
0.0
−2.5
3
−3
−6
−3
−2
−1
1
2
Group
control
dose1
dose2
dose3

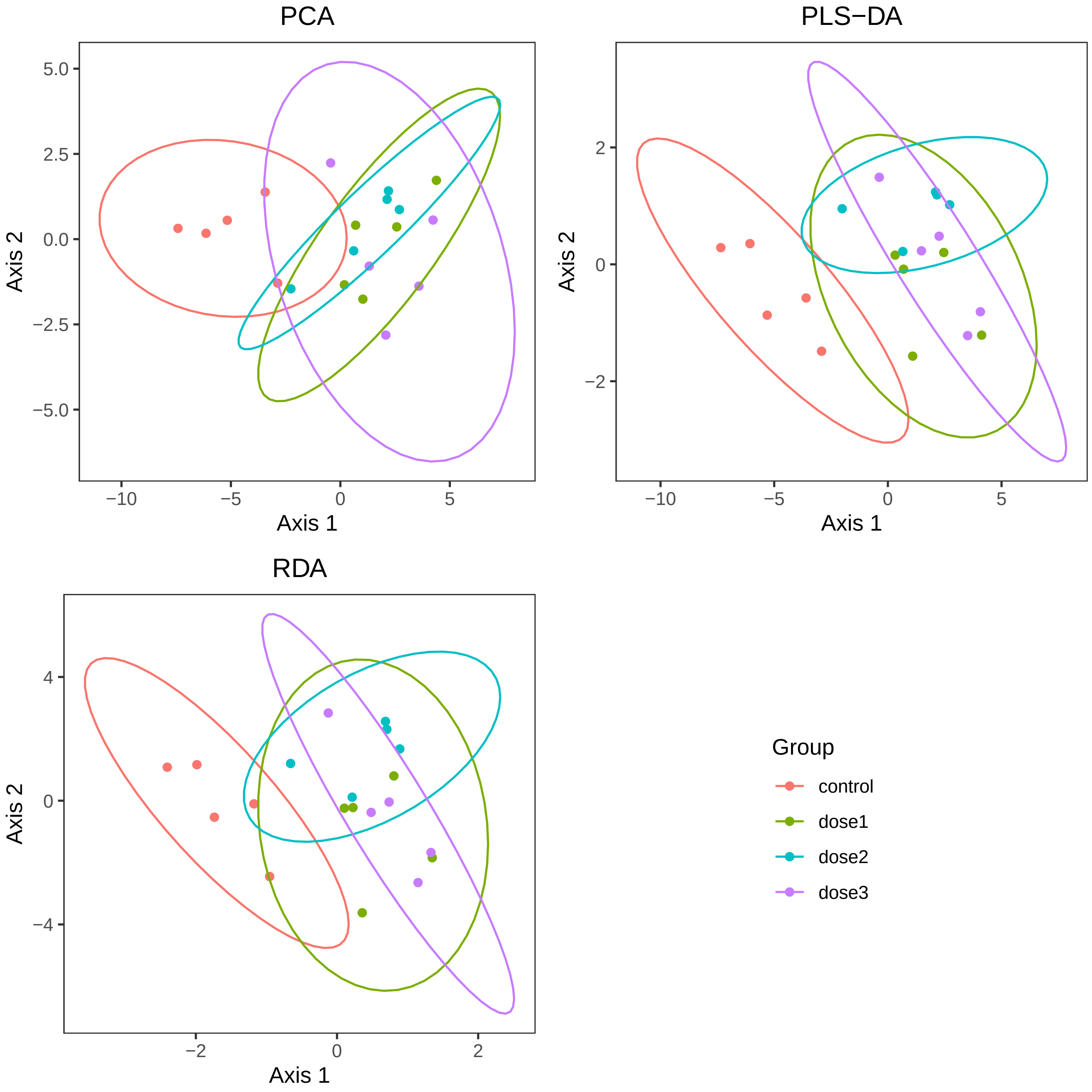

PCA
PLS−DA
RDA
Axis 1
Axis 2
Group
control
dose1
dose2
dose3

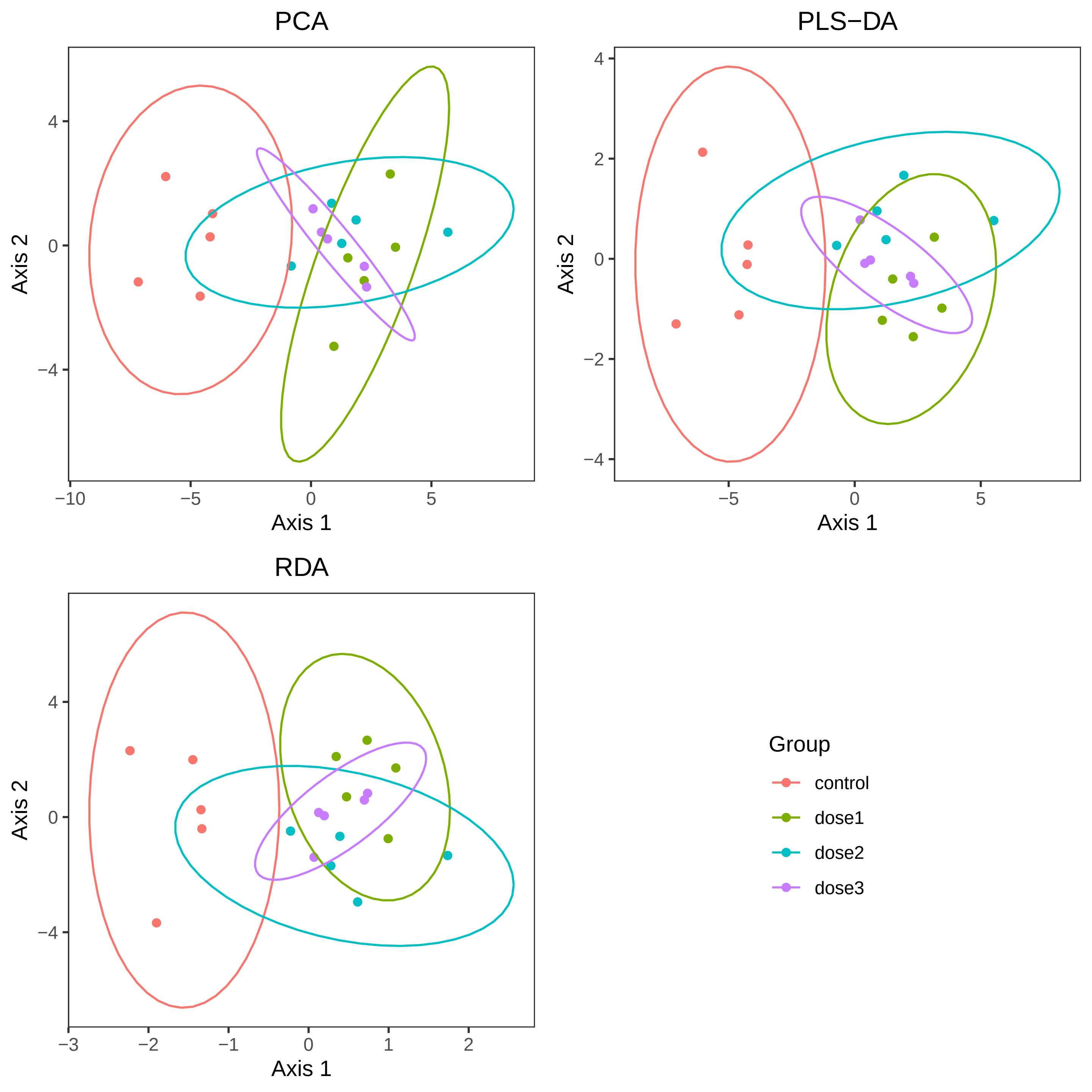
PCA
PLS−DA
RDA
Axis 1
Axis 2
Group
control
dose1
dose2
dose3

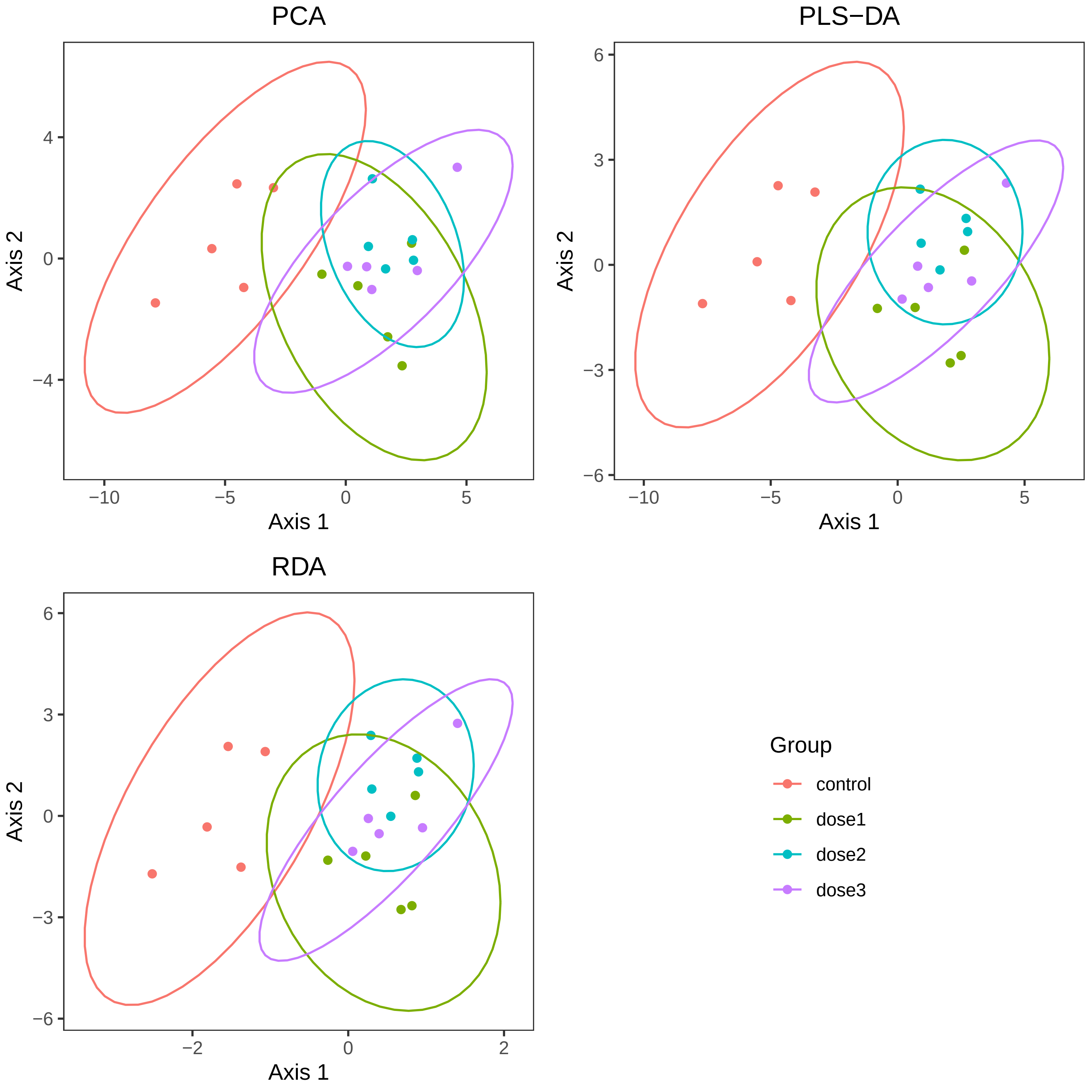
PCA
PLS−DA
RDA
Axis 1
Axis 2
Group
control
dose1
dose2
dose3

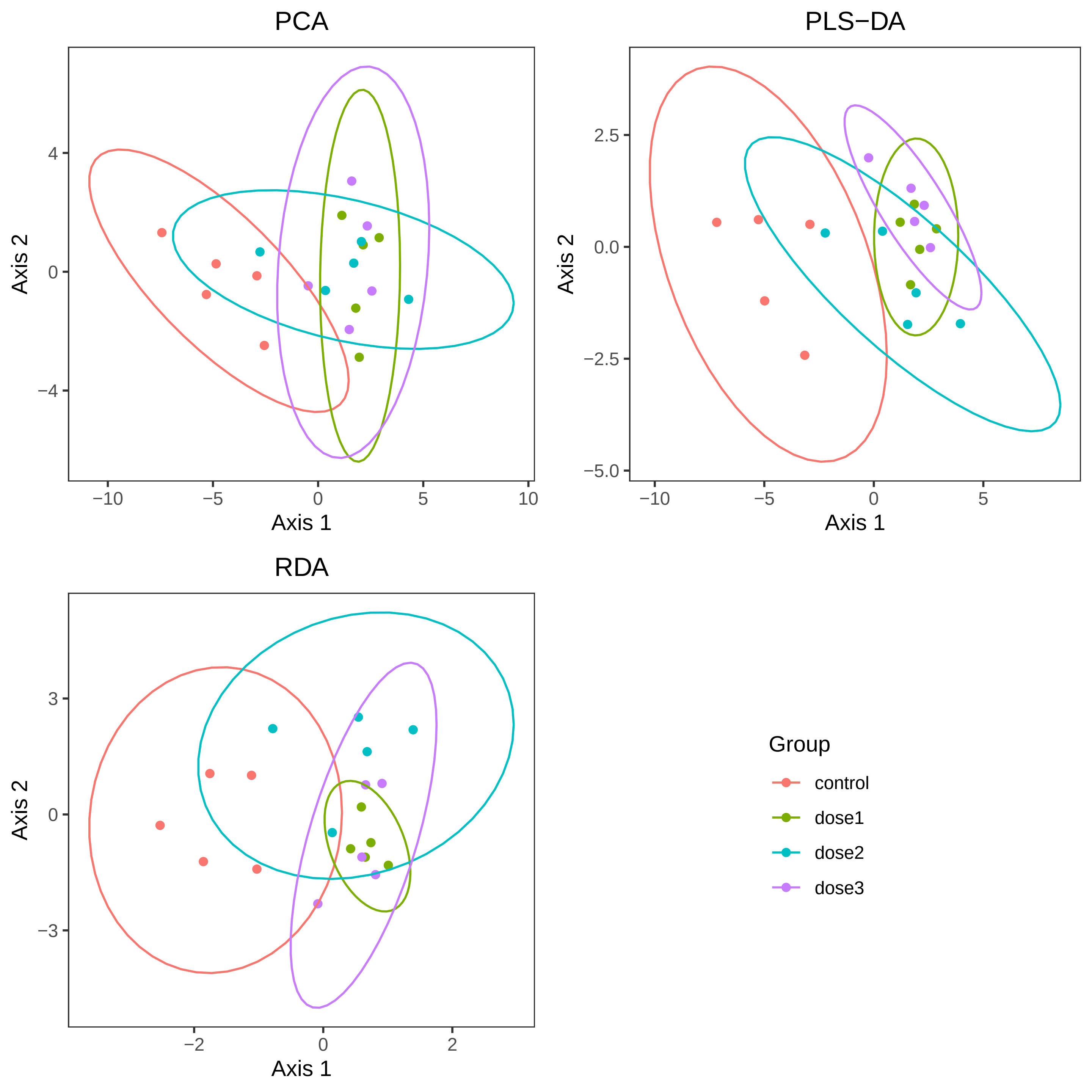
PCA
PLS−DA
RDA
Axis 1
Axis 2
Group
control
dose1
dose2
dose3

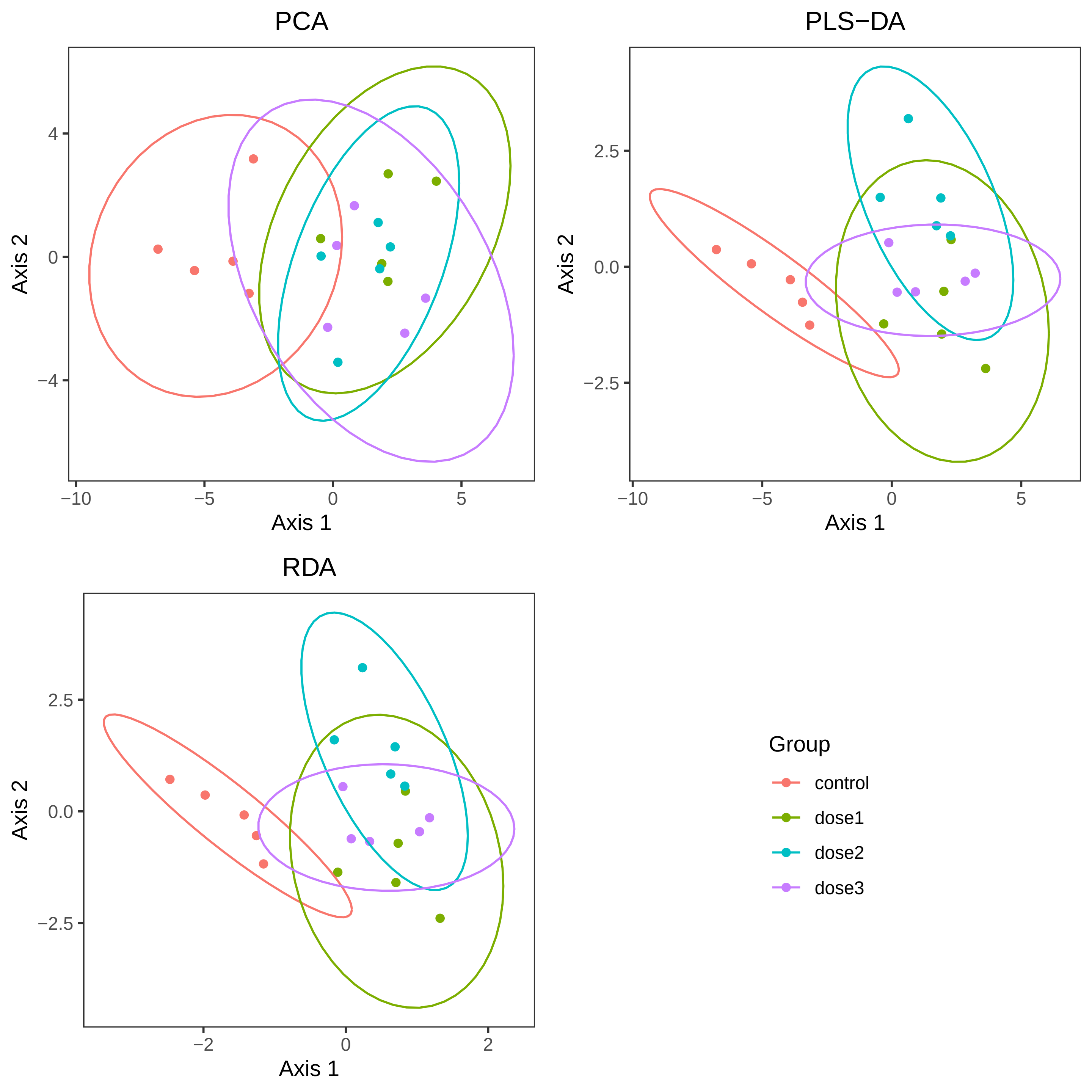

PCA
PLS−DA
RDA
Axis 1
Axis 2
−10
−5
0
5
4
−4
2.5
0.0
−2.5
−2
2
Group
control
dose1
dose2
dose3

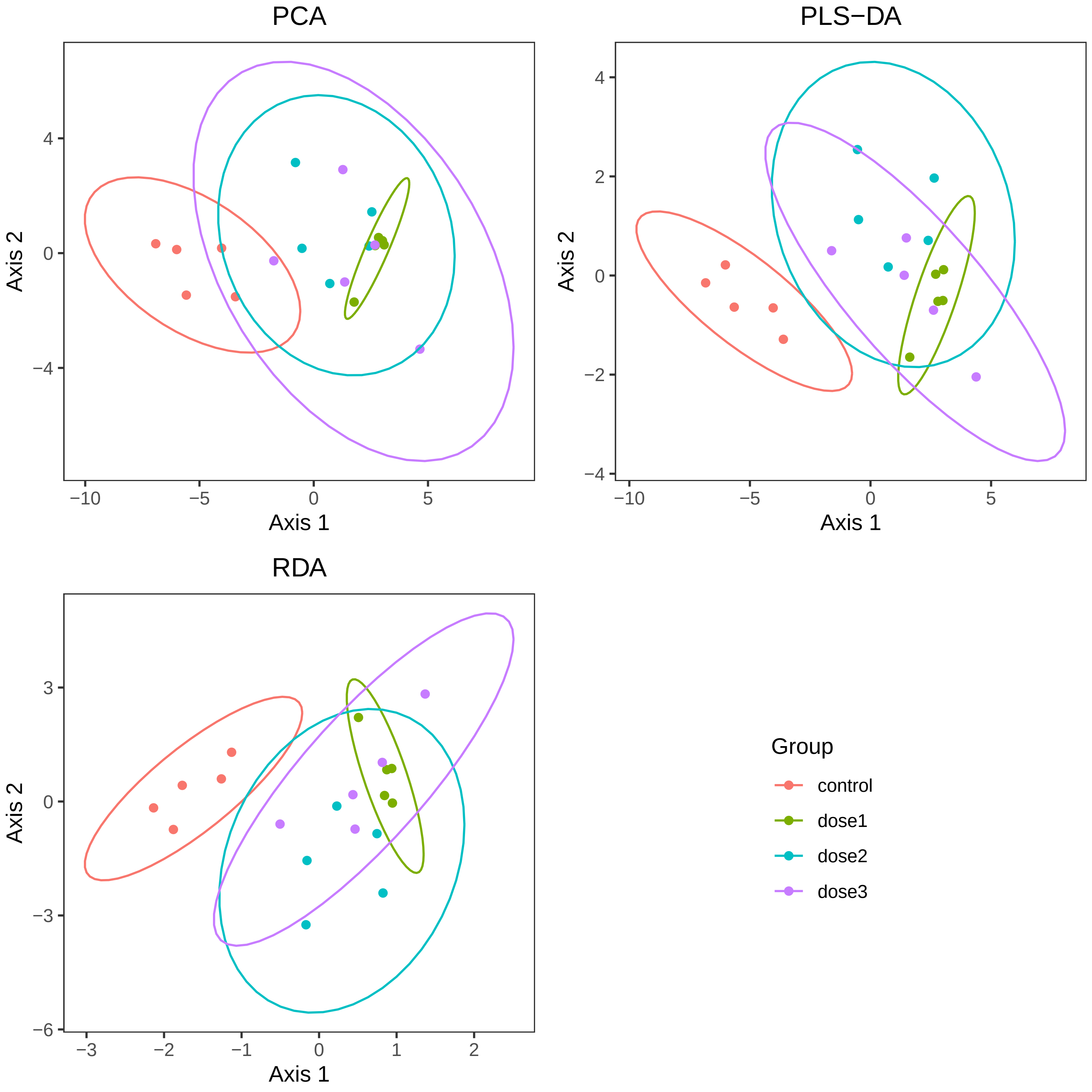

PCA
PLS−DA
RDA
Axis 1
Axis 2
−10
−5
0
5
4
0
−4
2
−2
−3
−6
3
−3
−2
−1
0
1
2
Group
control
dose1
dose2
dose3

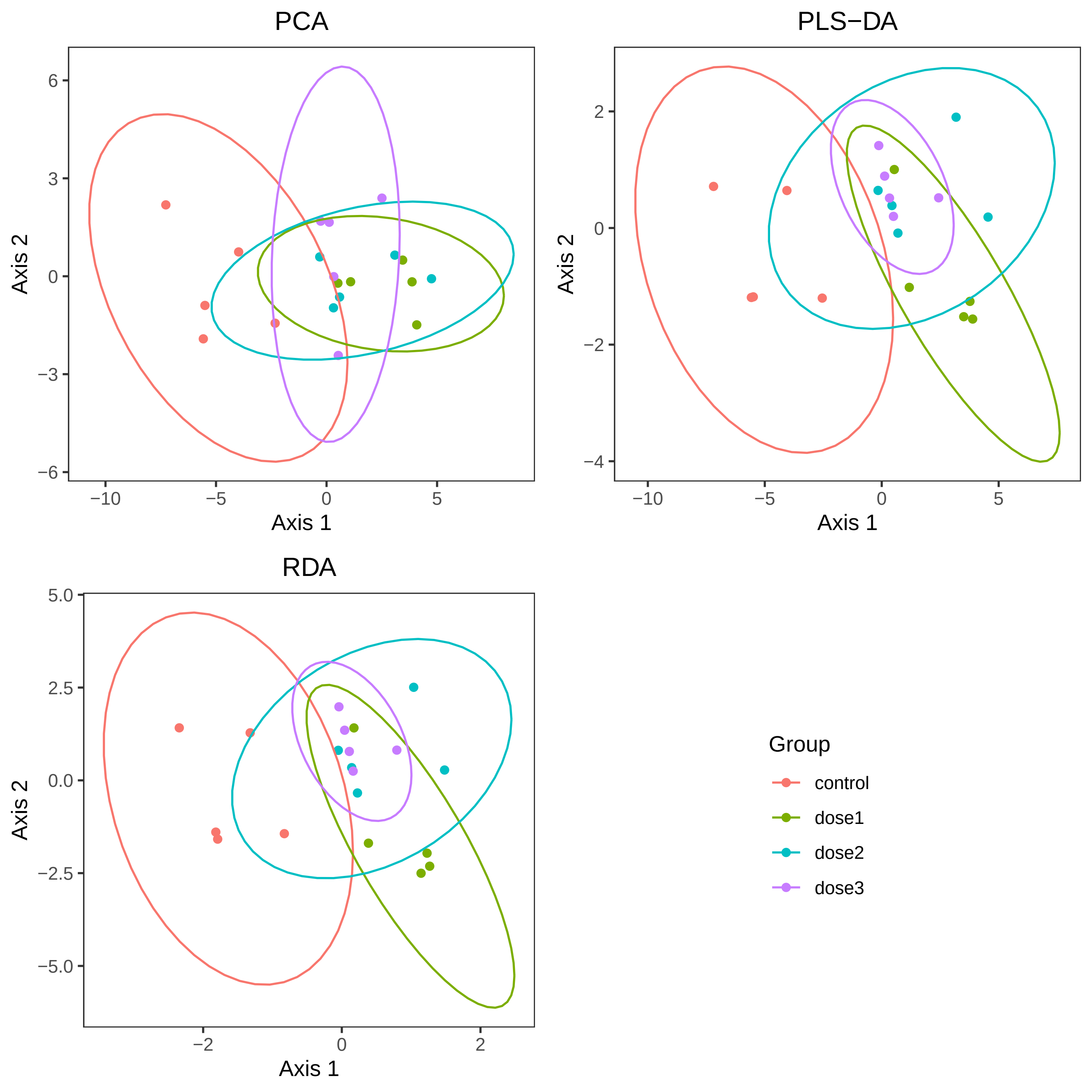

PCA
PLS−DA
RDA
Axis 1
Axis 2
Group
control
dose1
dose2
dose3

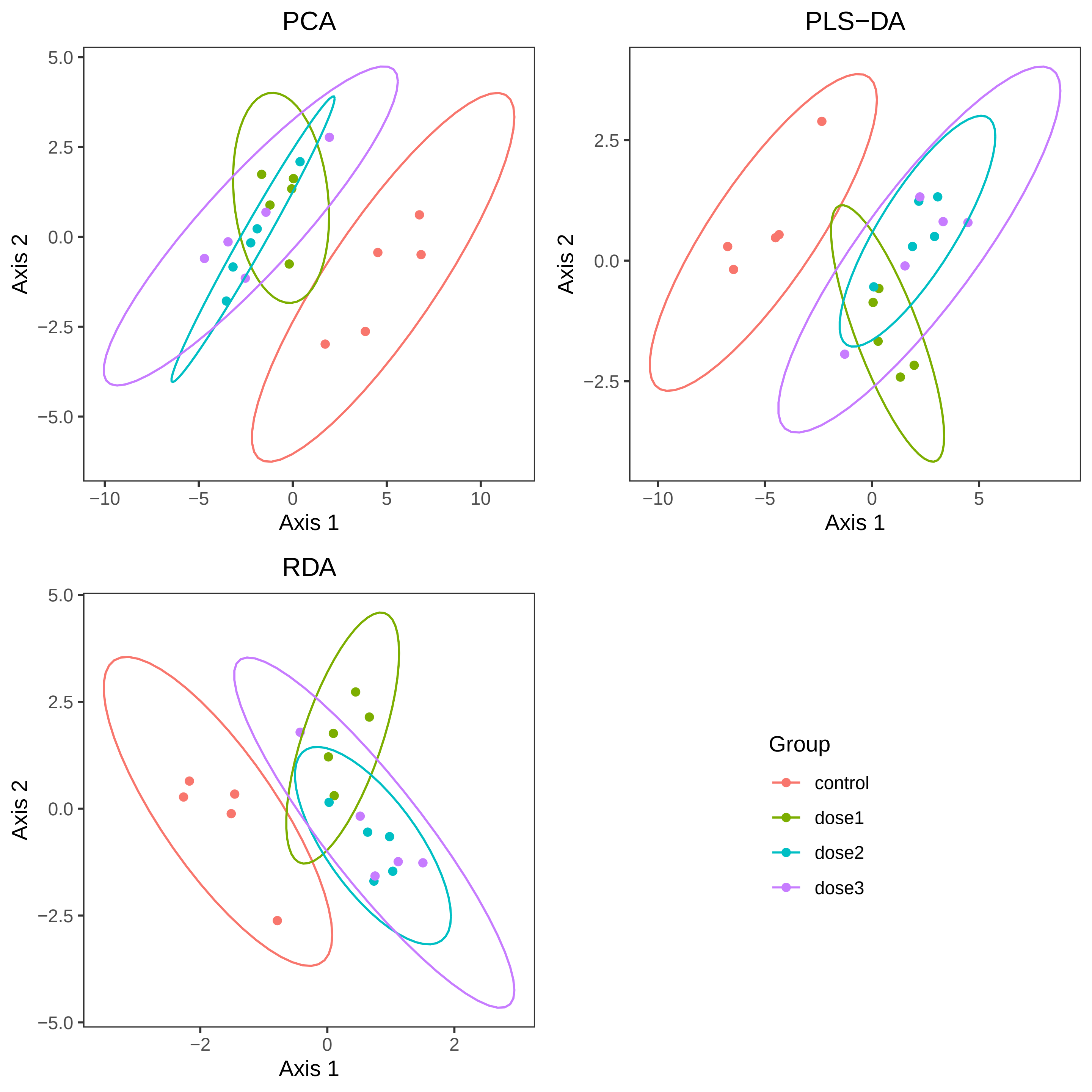

PCA
PLS−DA
RDA
Axis 1
Axis 2
Group
control
dose1
dose2
dose3

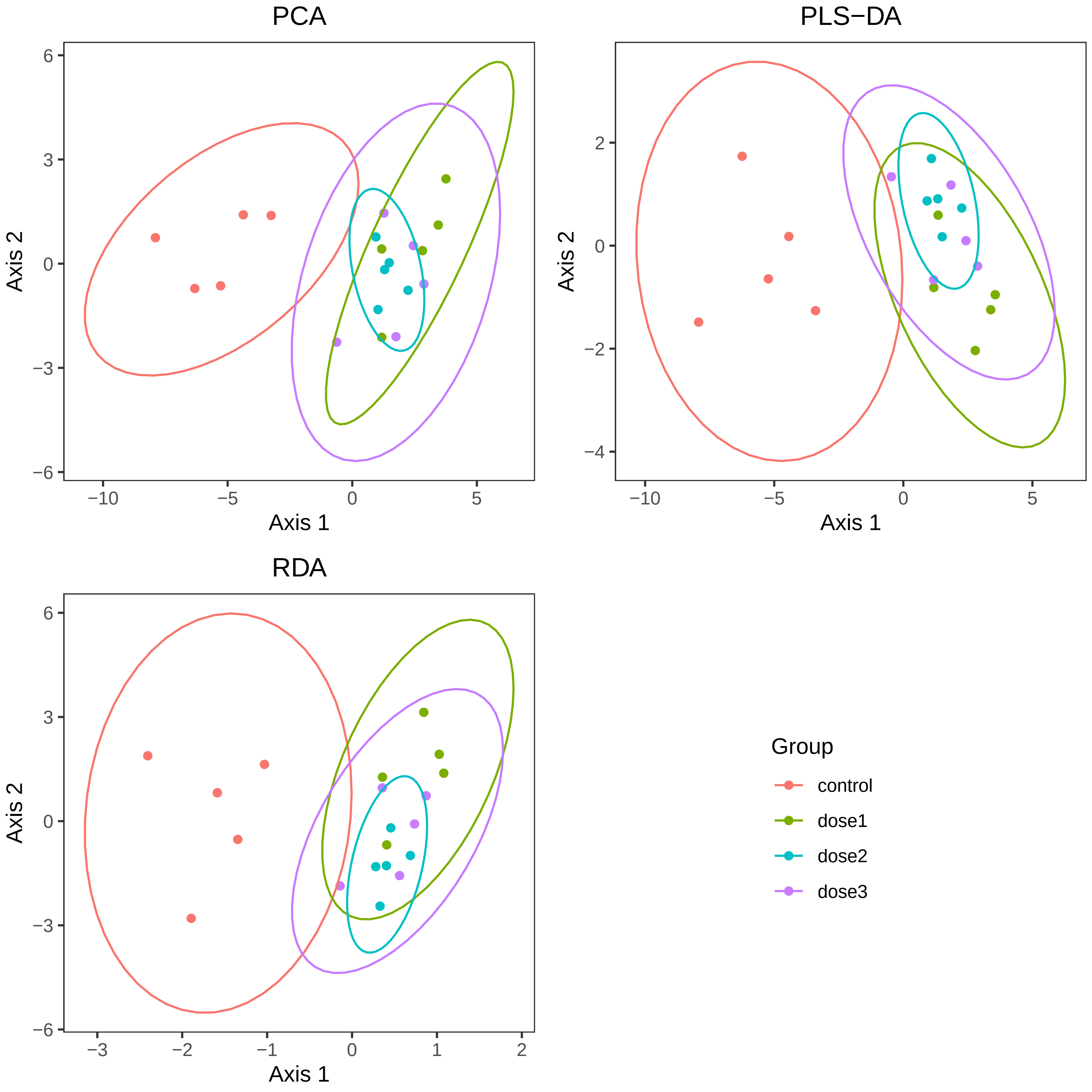

PCA
PLS−DA
RDA
Axis 1
Axis 2
Group
control
dose1
dose2
dose3

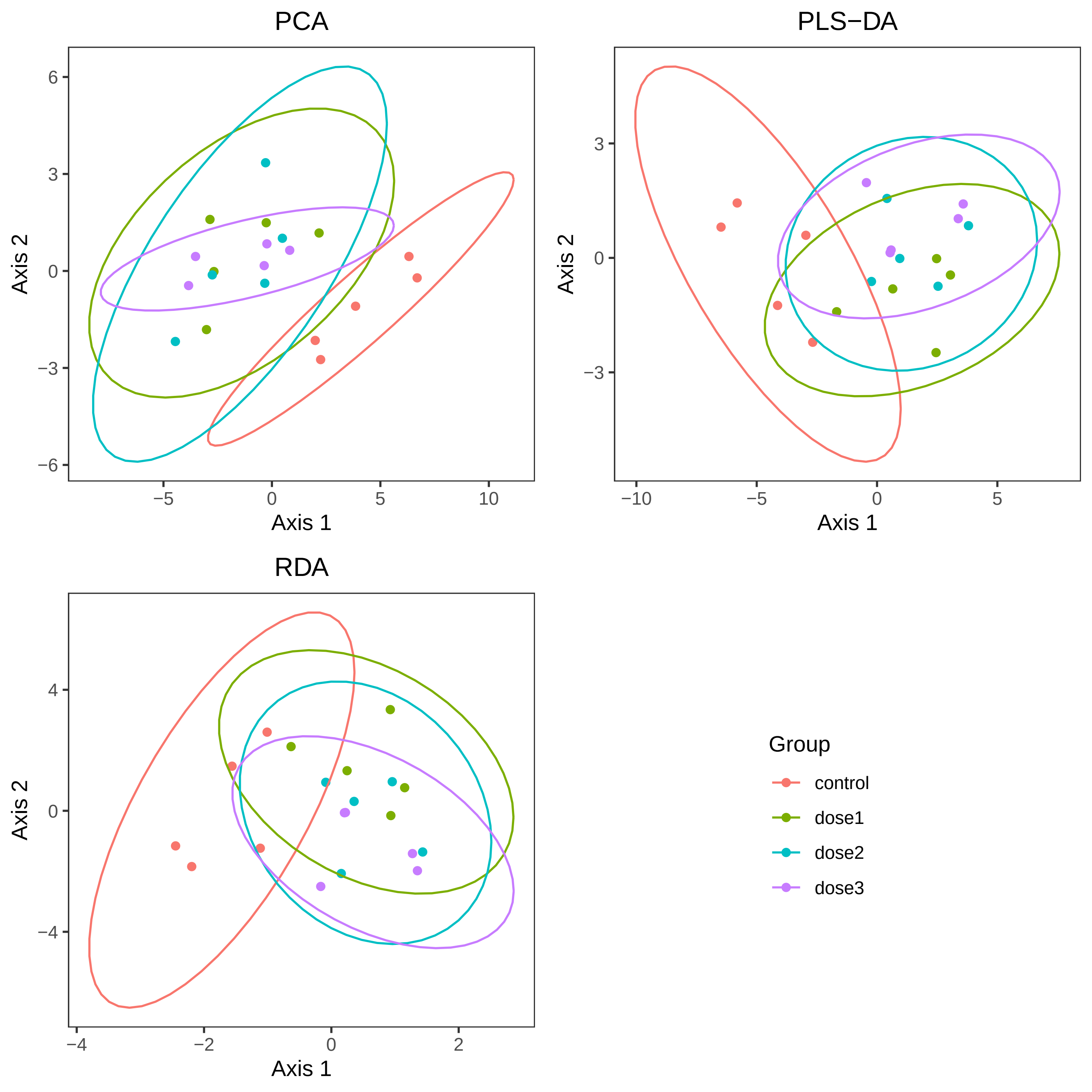
PCA
PLS−DA
RDA
Axis 1
Axis 2
Group
control
dose1
dose2
dose3

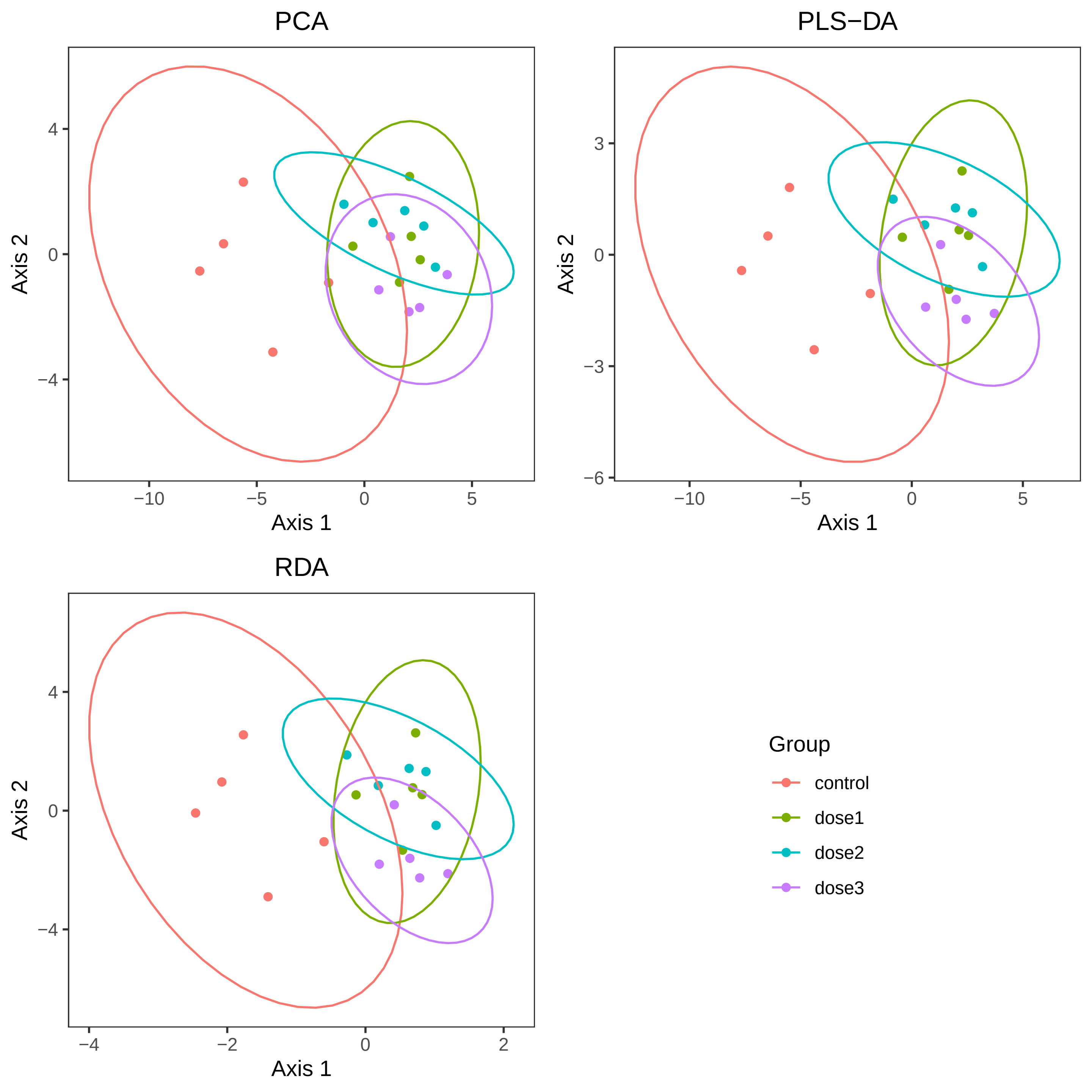
PCA
PLS−DA
RDA
Axis 1
Axis 2
−10
−5
0
5
4
−4
3
−3
−6
−2
2
Group
control
dose1
dose2
dose3

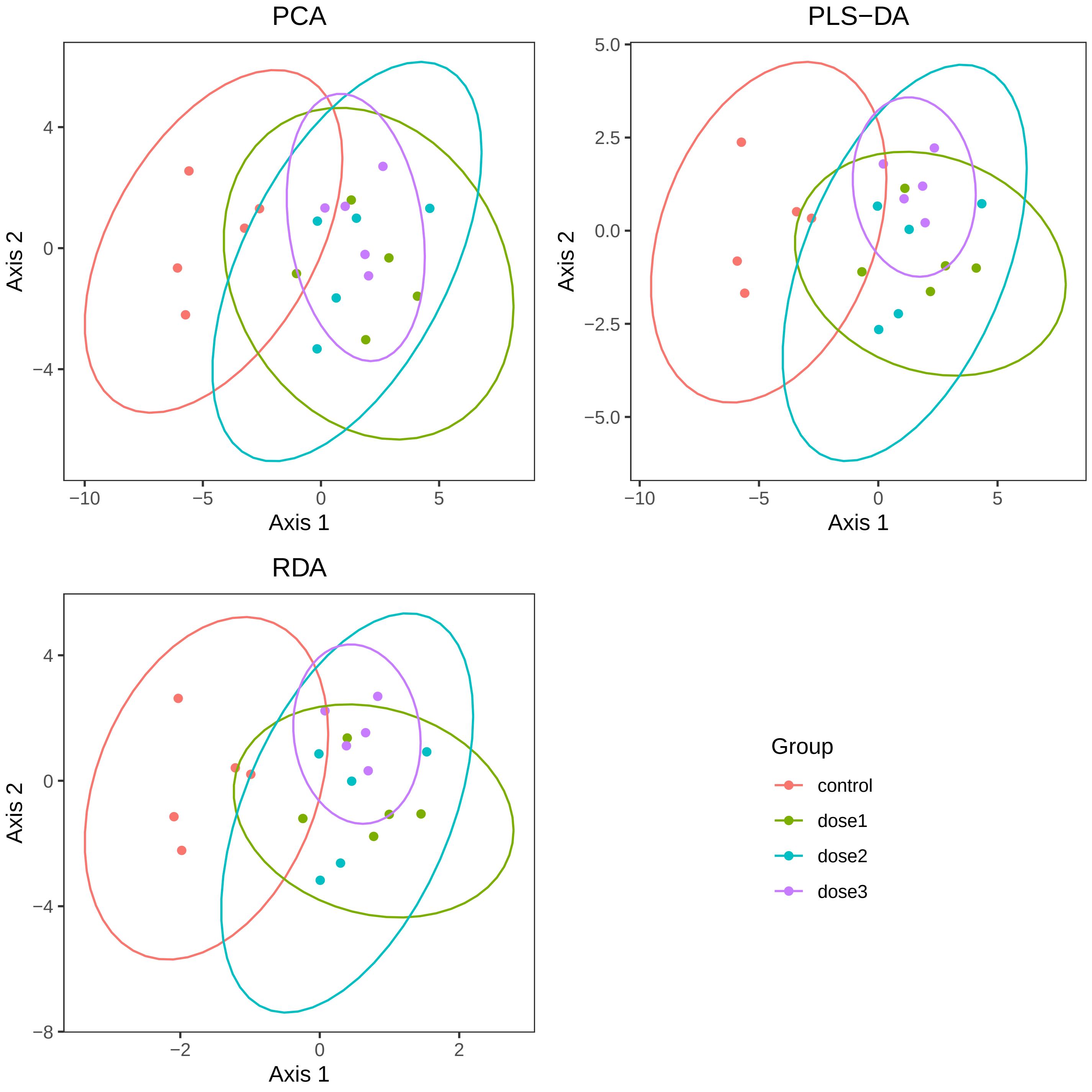

PCA
PLS−DA
RDA
Axis 1
Axis 2
Group
control
dose1
dose2
dose3

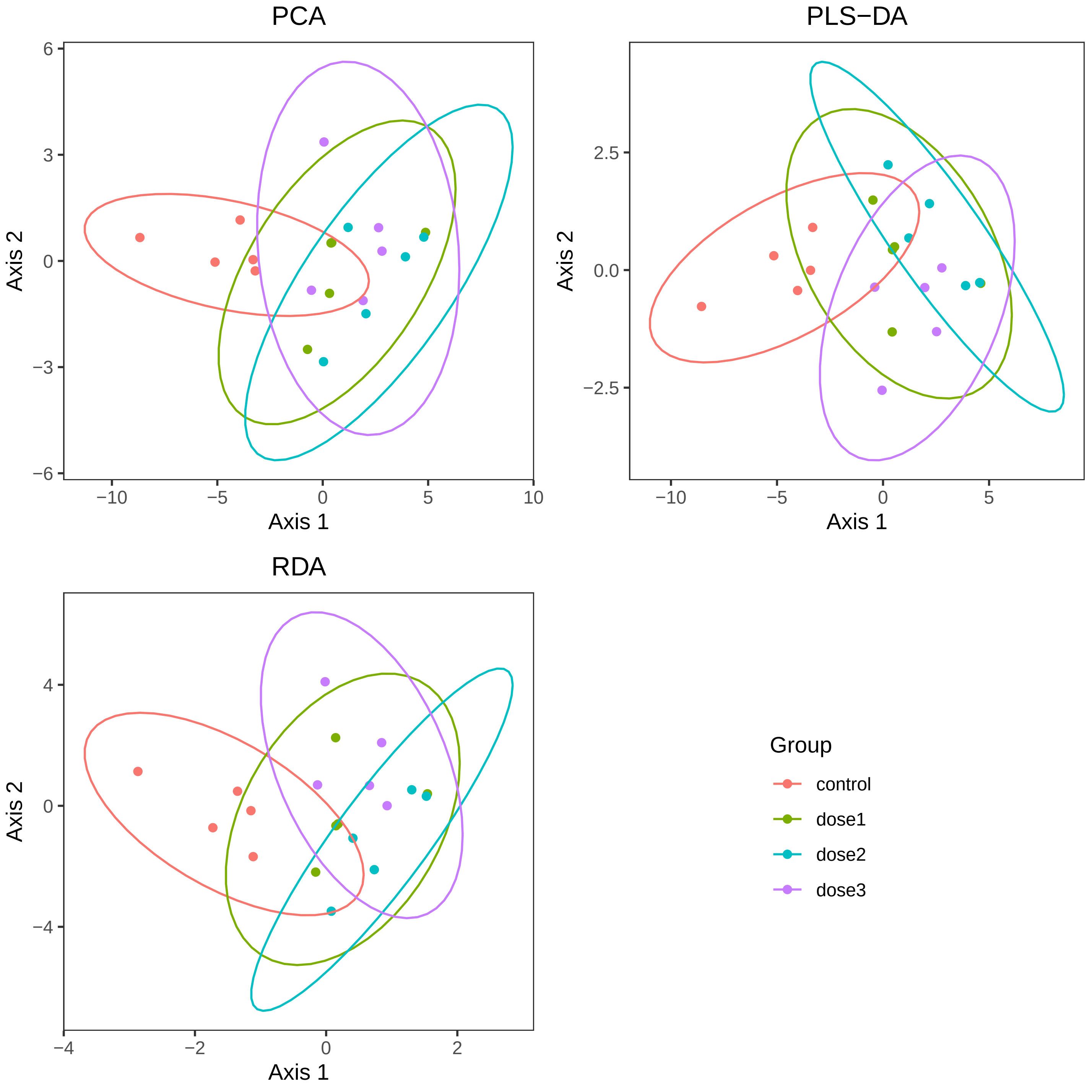
PCA
PLS−DA
RDA
Axis 1
Axis 2
Group
control
dose1
dose2
dose3

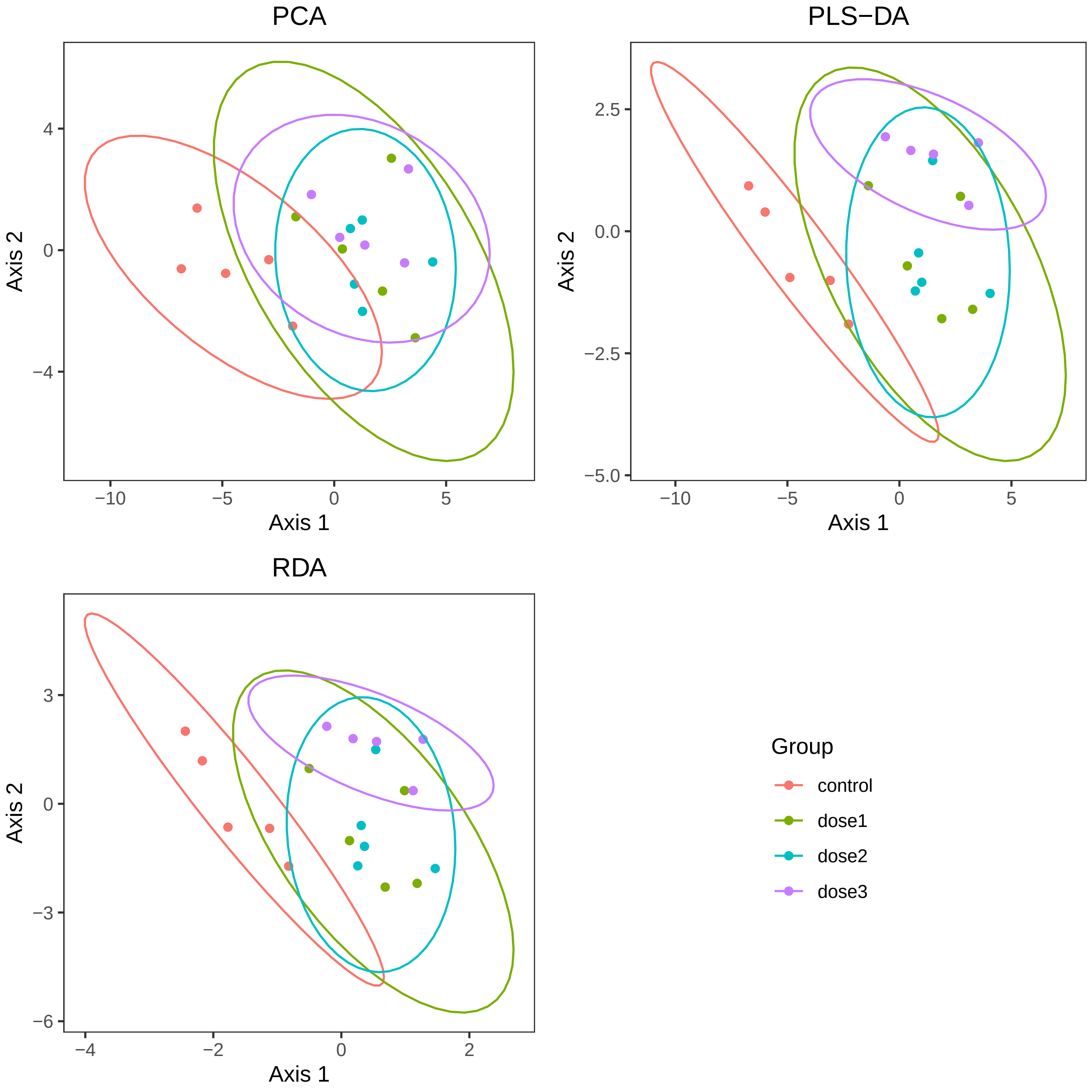
PCA
PLS−DA
RDA
Axis 1
Axis 2
Group
control
dose1
dose2
dose3

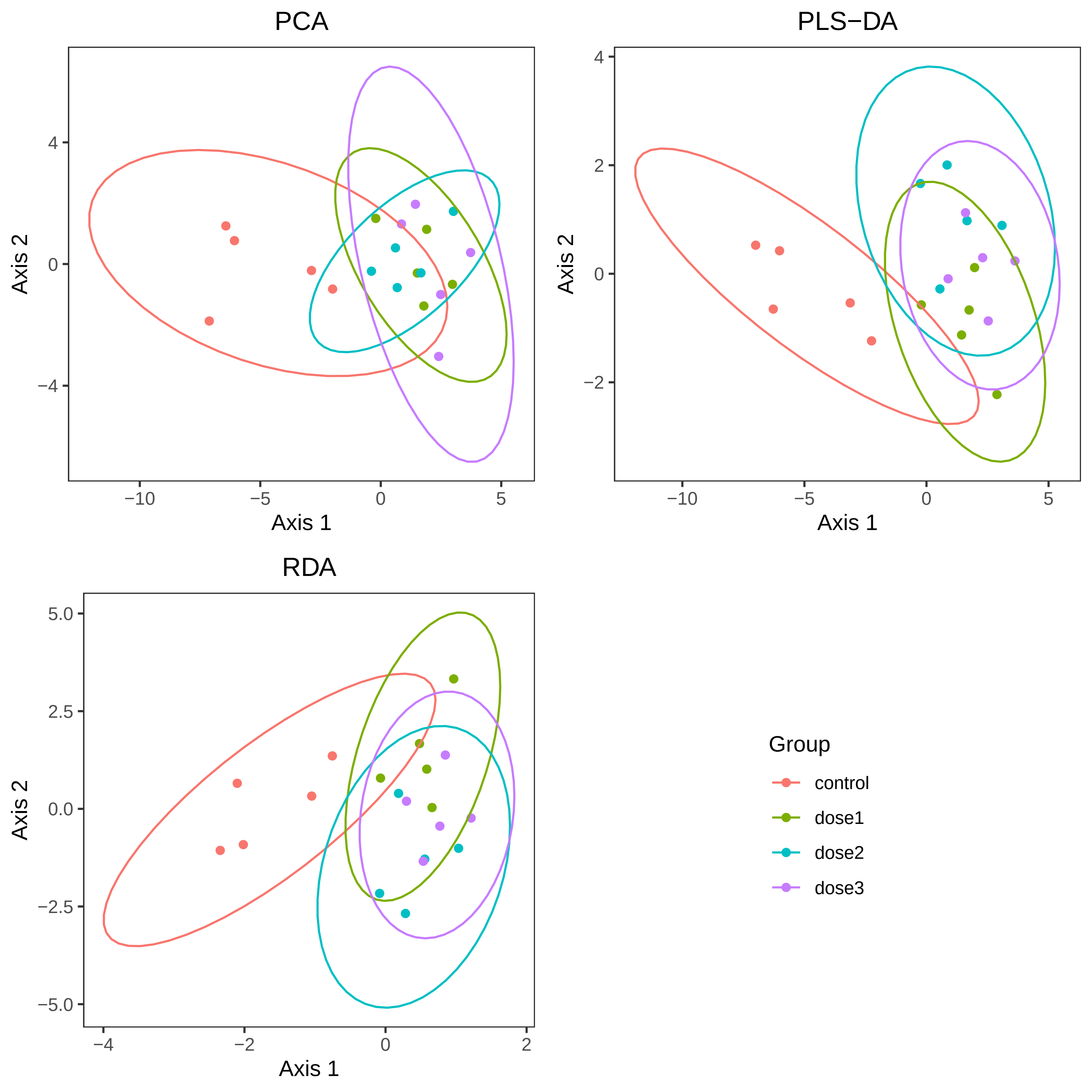
PCA
PLS−DA
RDA
Axis 1
Axis 2
Group
control
dose1
dose2
dose3
−10
−5
0
5
4
2
−2
−4
5.0
2.5
0.0
−2.5
−5.0

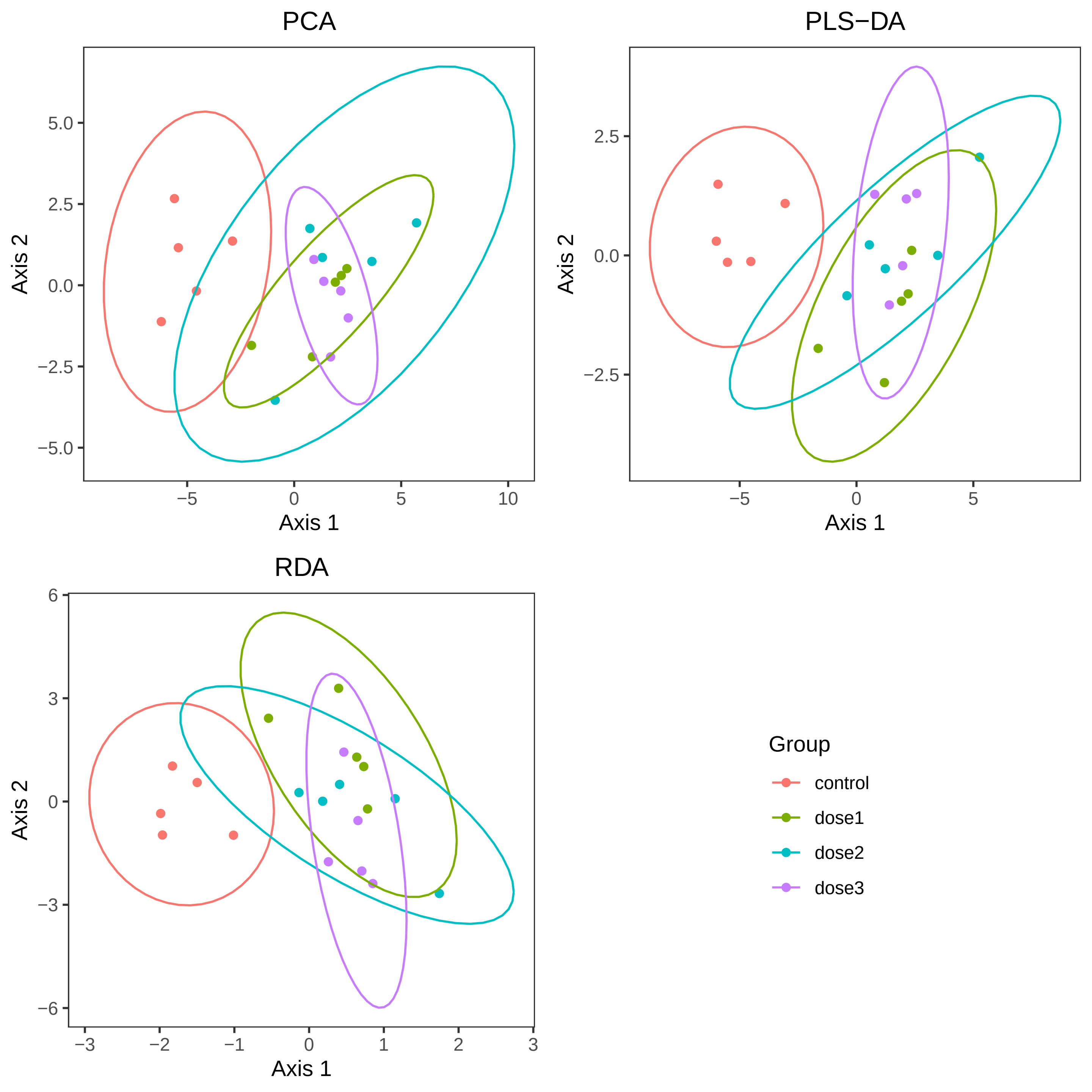
PCA
PLS−DA
RDA
Axis 1
Axis 2
Group
control
dose1
dose2
dose3

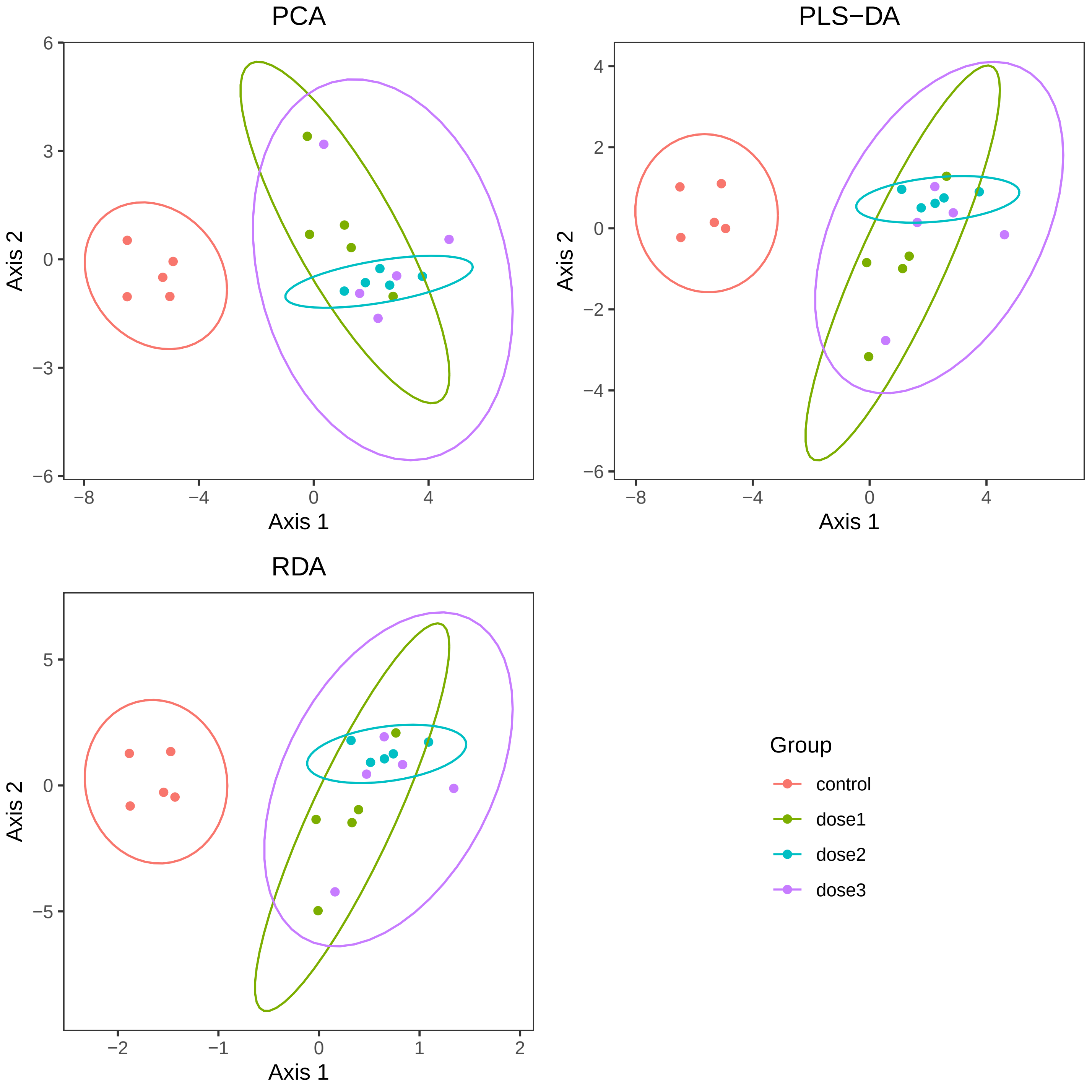

PCA
PLS−DA
RDA
Axis 1
Axis 2
Group
control
dose1
dose2
dose3

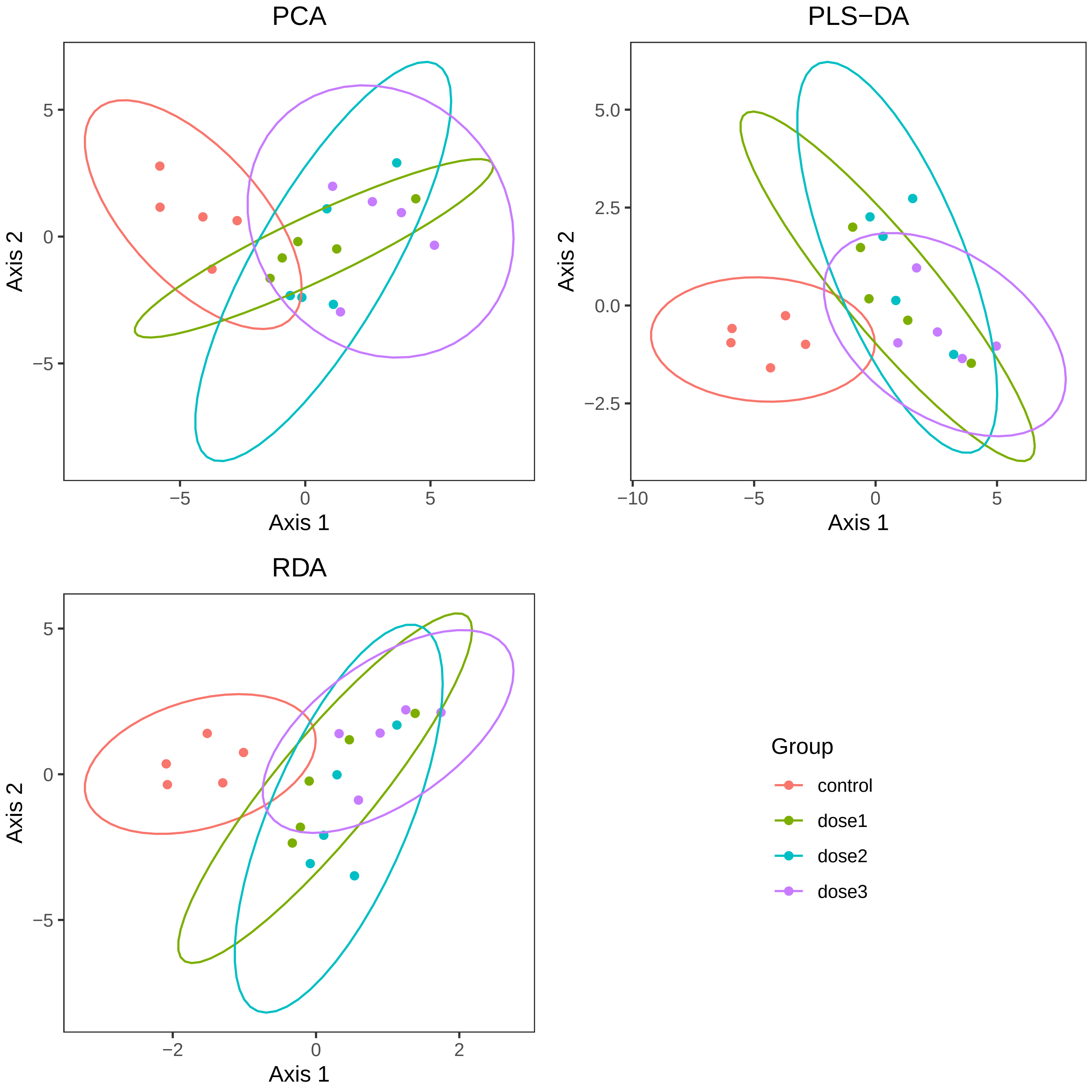

PCA
PLS−DA
RDA
Axis 1
Axis 2
Group
control
dose1
dose2
dose3

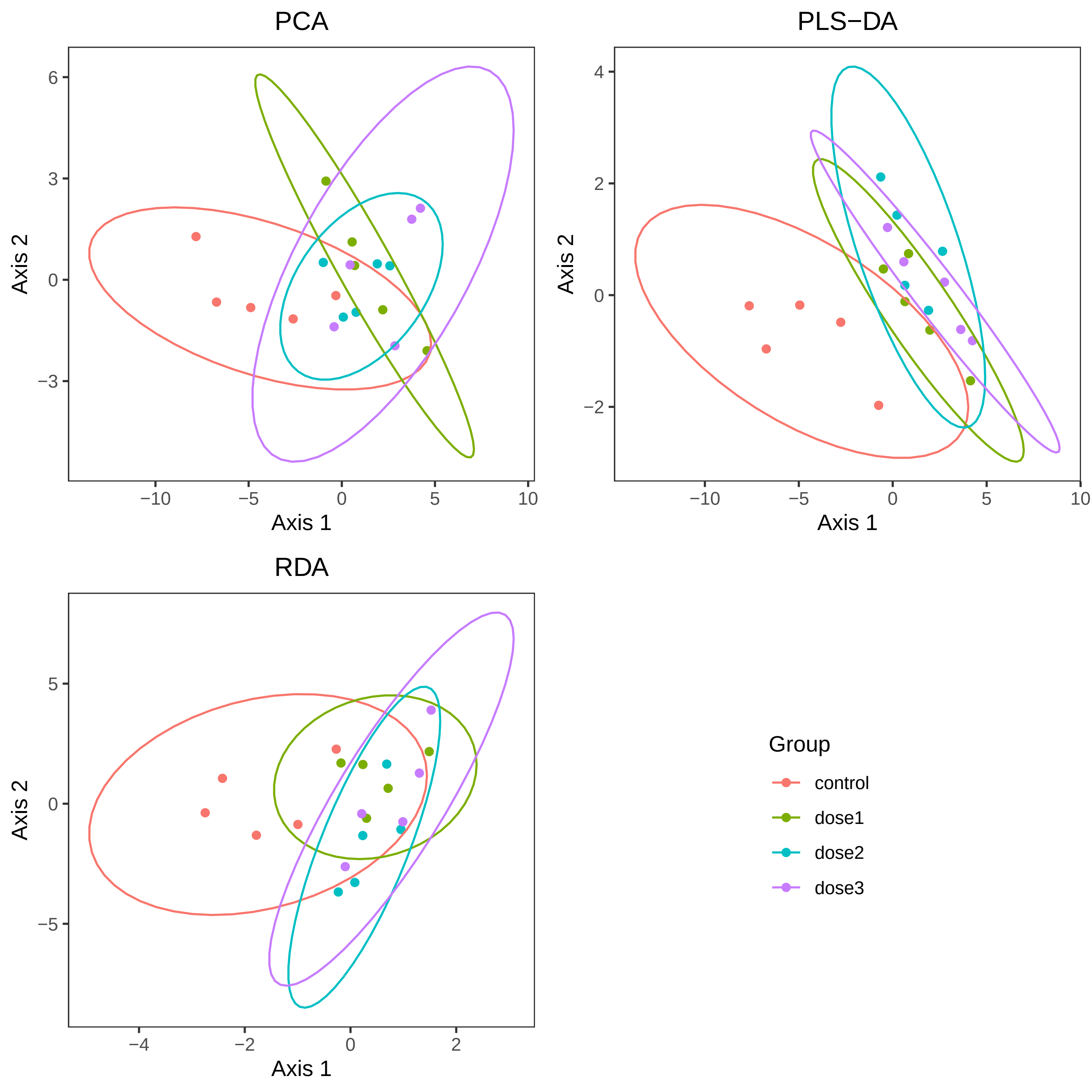

PCA
PLS−DA
RDA
Axis 1
Axis 2
Group
control
dose1
dose2
dose3

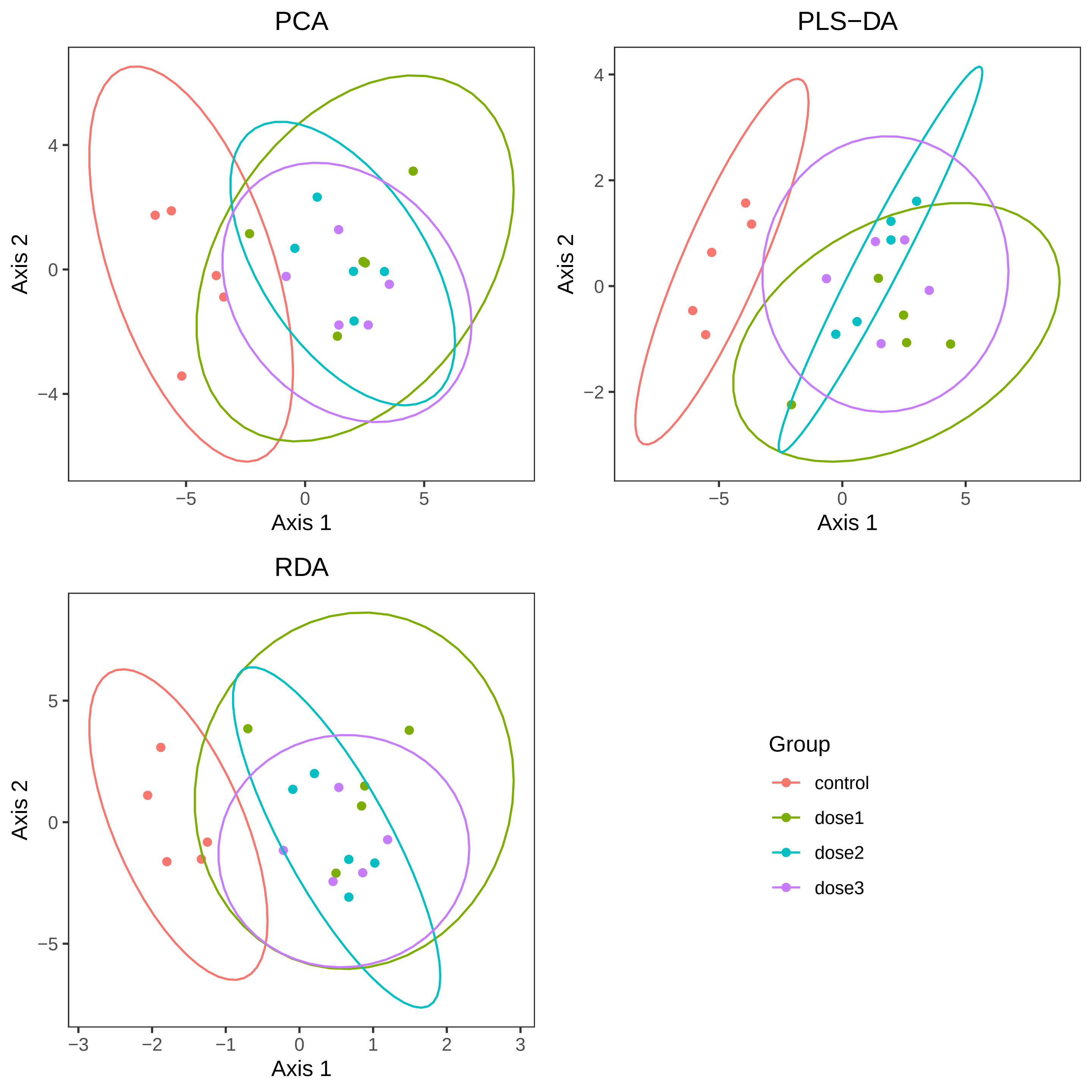
PCA
PLS−DA
RDA
Axis 1
Axis 2
4
0
−4
−5
5
2
−2
−3
−1
1
3
Group
control
dose1
dose2
dose3

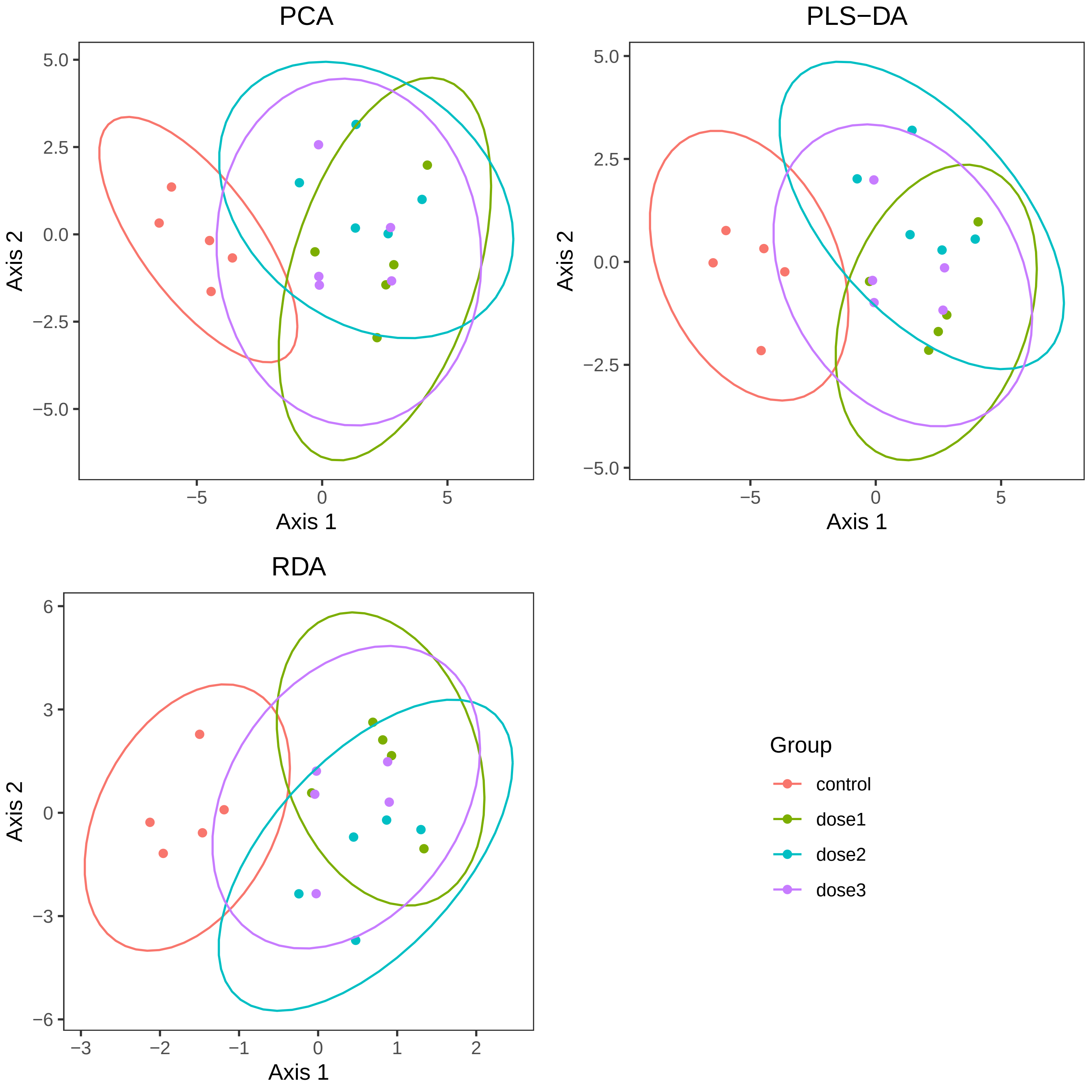

PCA
PLS−DA
RDA
5.0
2.5
0.0
−2.5
−5.0
−5
0
5
6
3
0
−3
−6
−3
−2
−1
0
1
2
Axis 1
Axis 2
Group
control
dose1
dose2
dose3

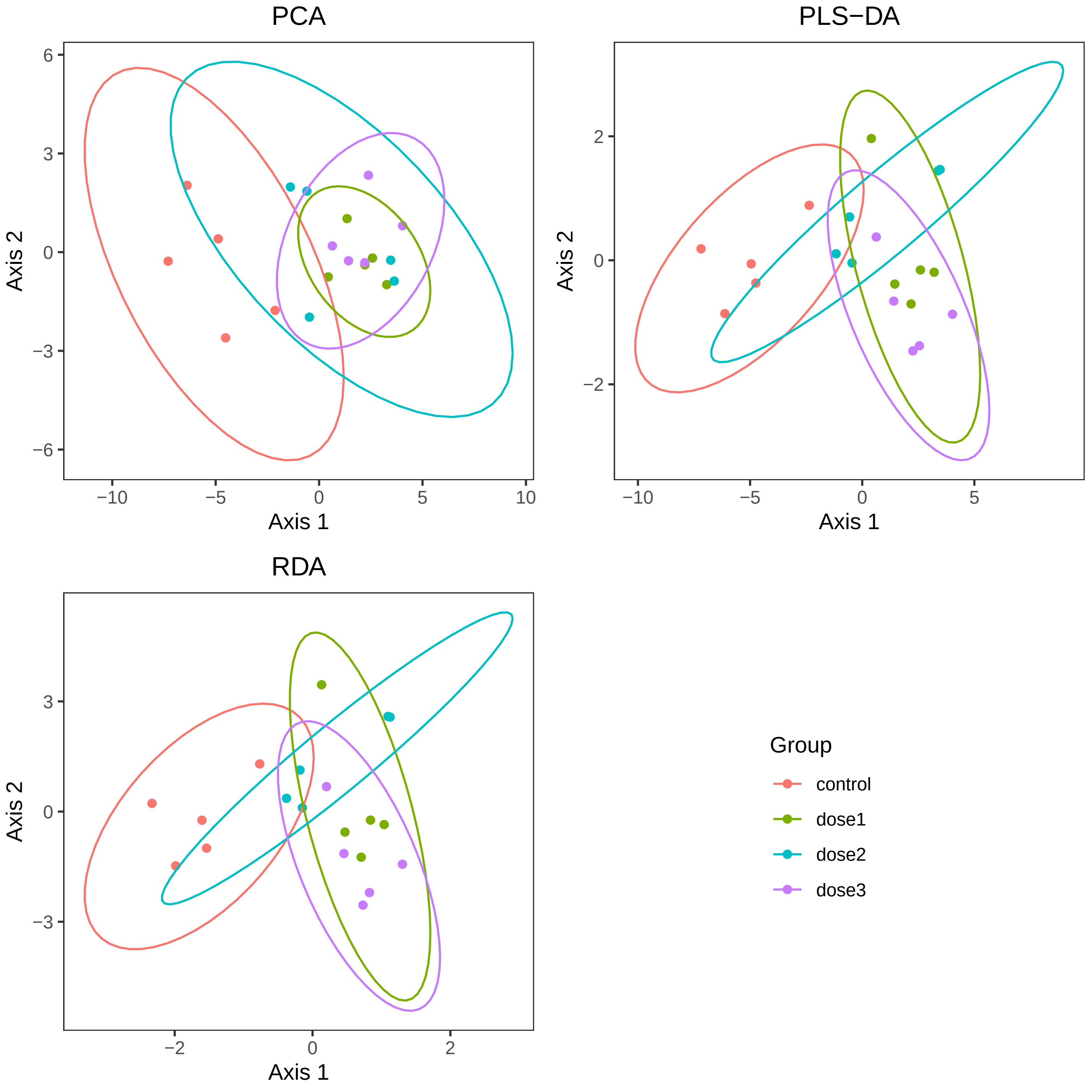

PCA
PLS−DA
RDA
Axis 1
Axis 2
Group
control
dose1
dose2
dose3

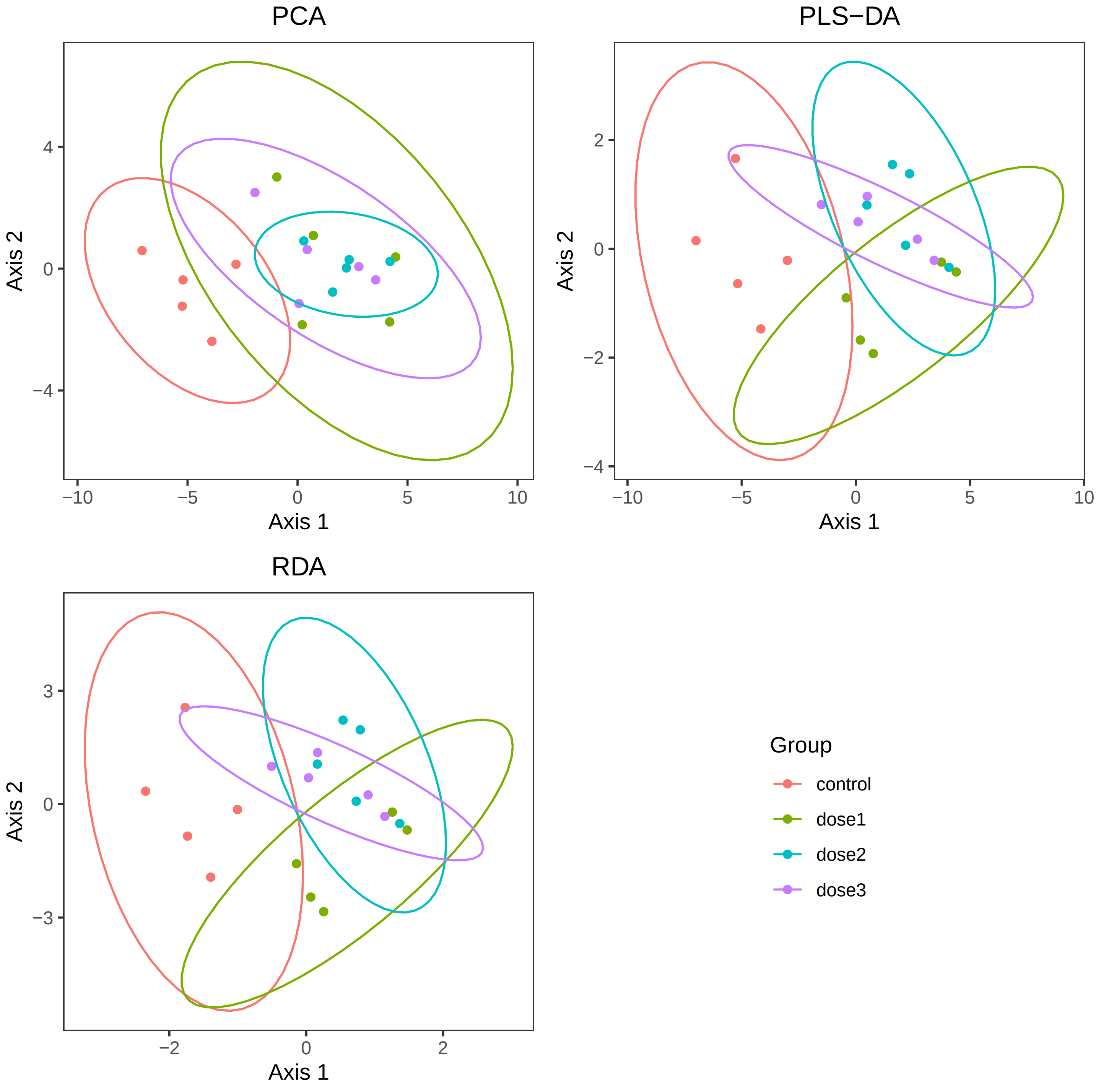

PCA
PLS−DA
RDA
Axis 1
Axis 2
Group
control
dose1
dose2
dose3

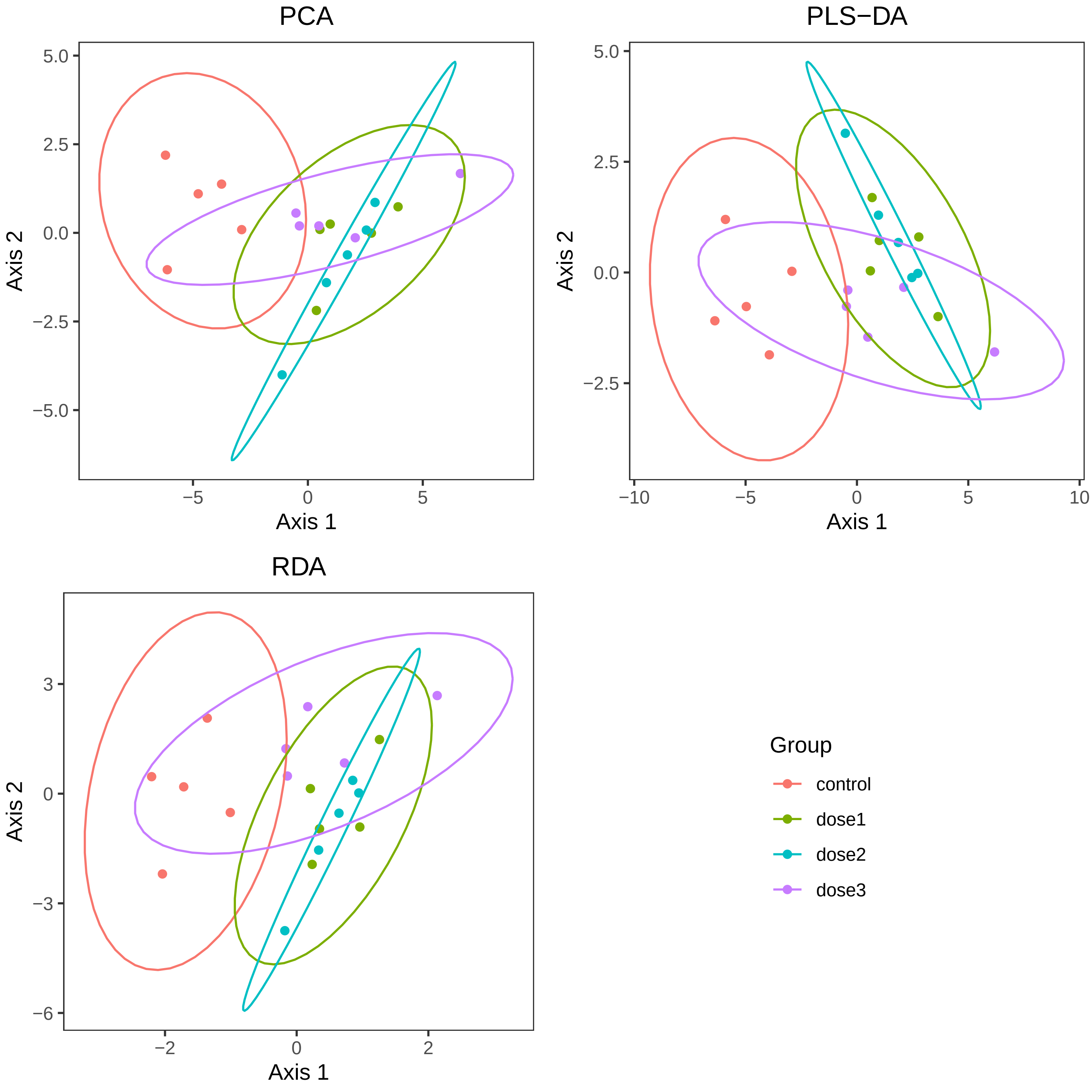
PCA
PLS−DA
RDA
Axis 1
Axis 2
5.0
2.5
0.0
−2.5
−5.0
−5
0
5
−10
10
3
−3
−6
−2
2
Group
control
dose1
dose2
dose3

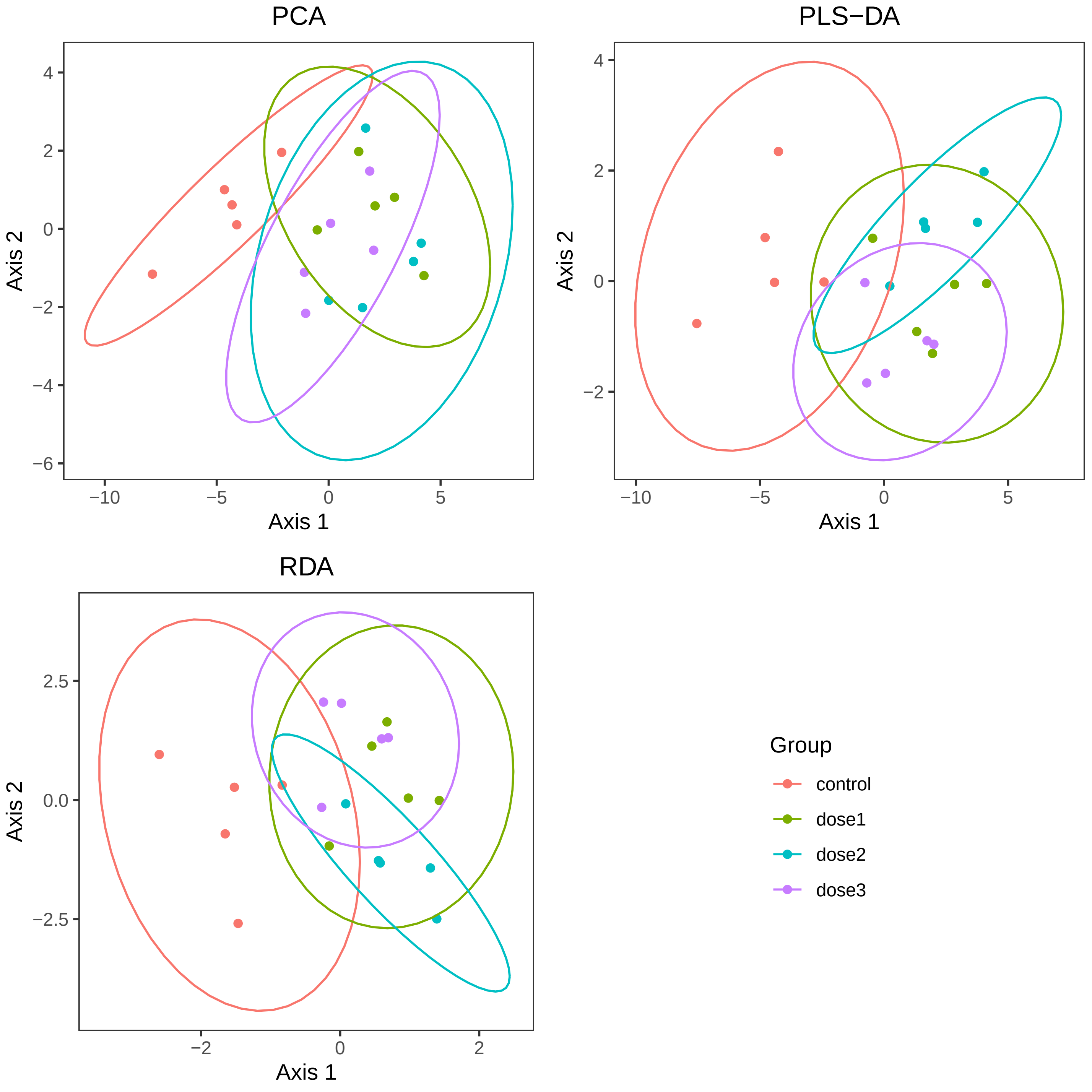
PCA
PLS−DA
RDA
Axis 1
Axis 2
Group
control
dose1
dose2
dose3

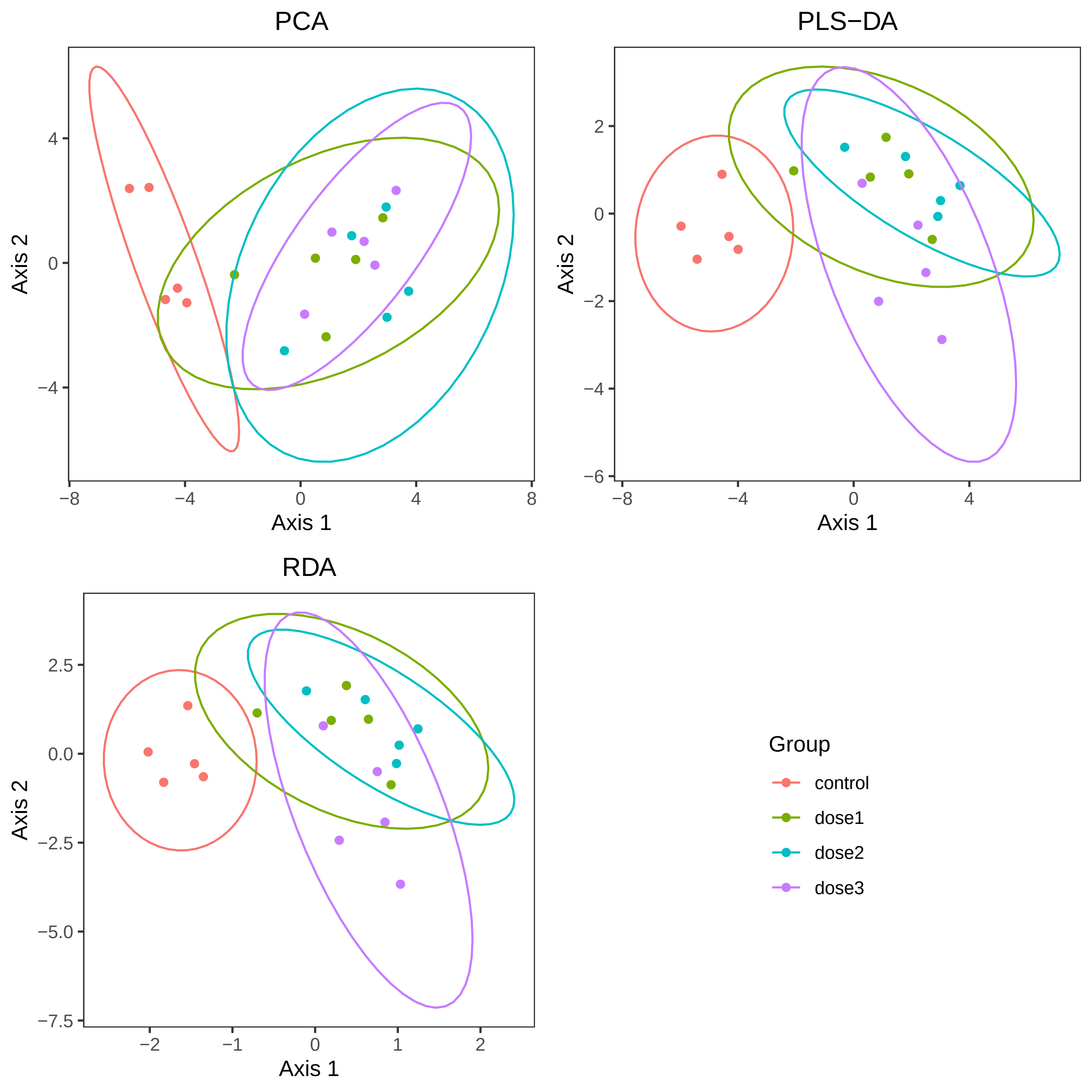

PCA
PLS−DA
RDA
Axis 1
Axis 2
−8
−4
0
4
8
−6
−2
2
2.5
0.0
−2.5
−5.0
−7.5
−1
1
Group
control
dose1
dose2
dose3

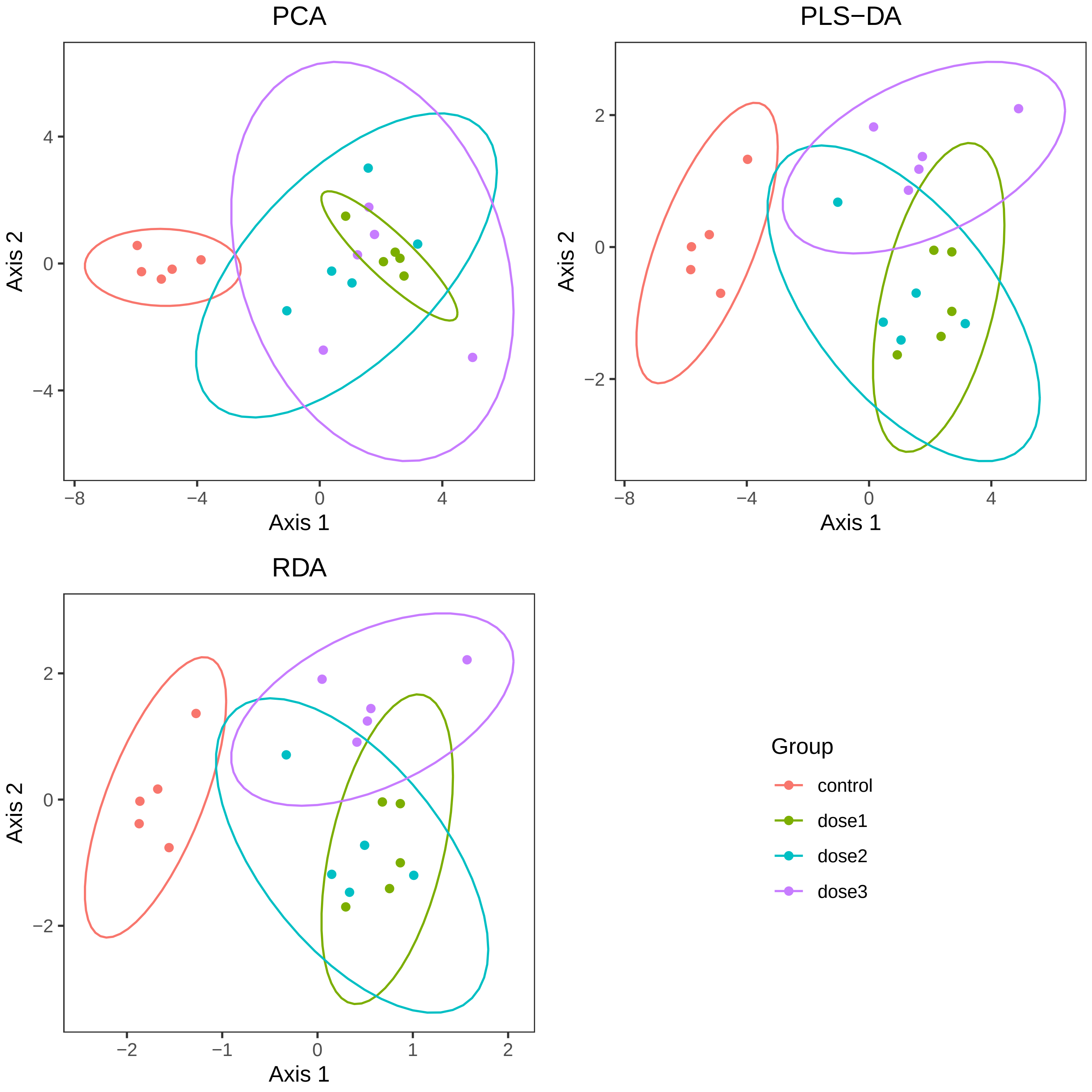

PCA
PLS−DA
RDA
Axis 1
Axis 2
Group
control
dose1
dose2
dose3

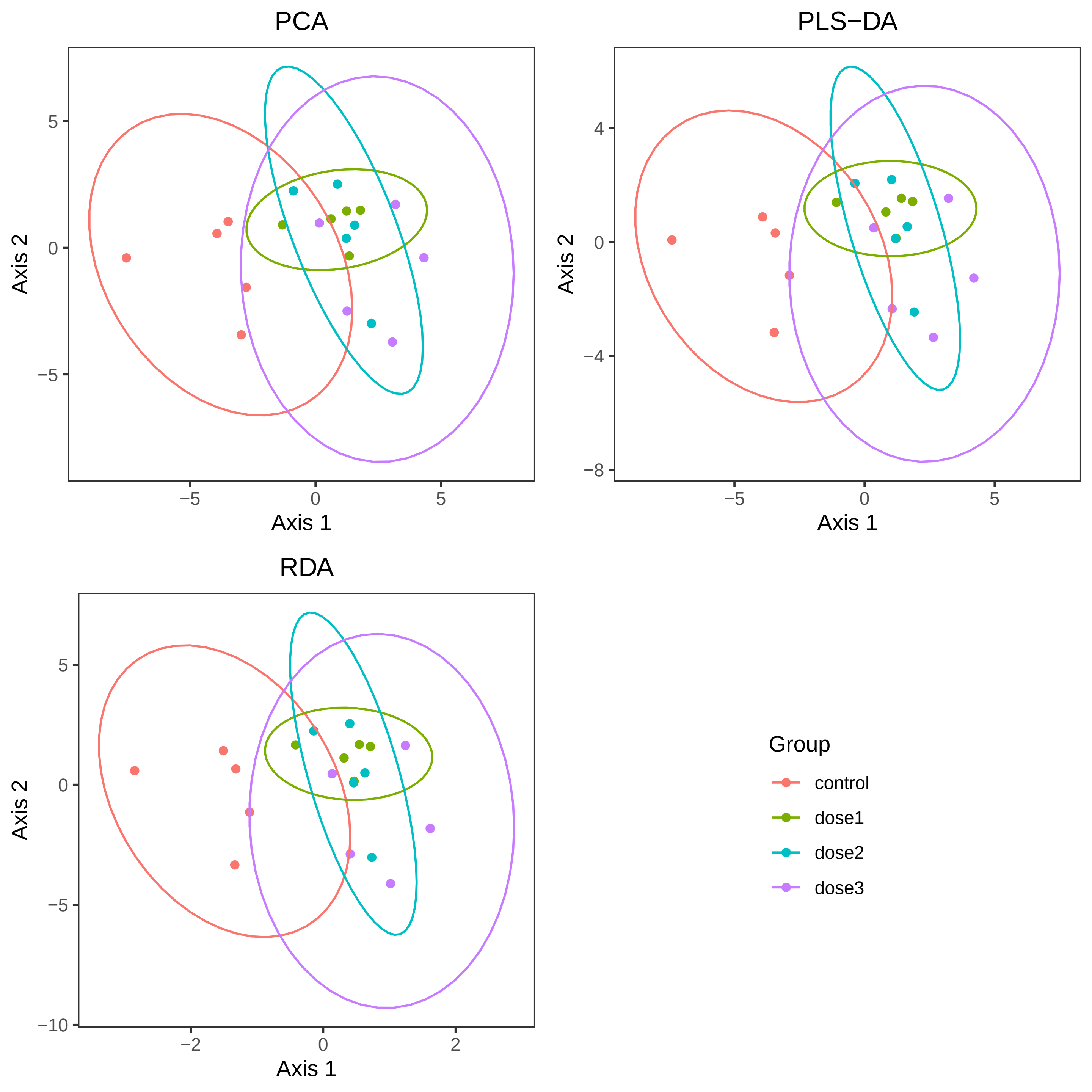
PCA
PLS−DA
RDA
Axis 1
Axis 2
Group
control
dose1
dose2
dose3

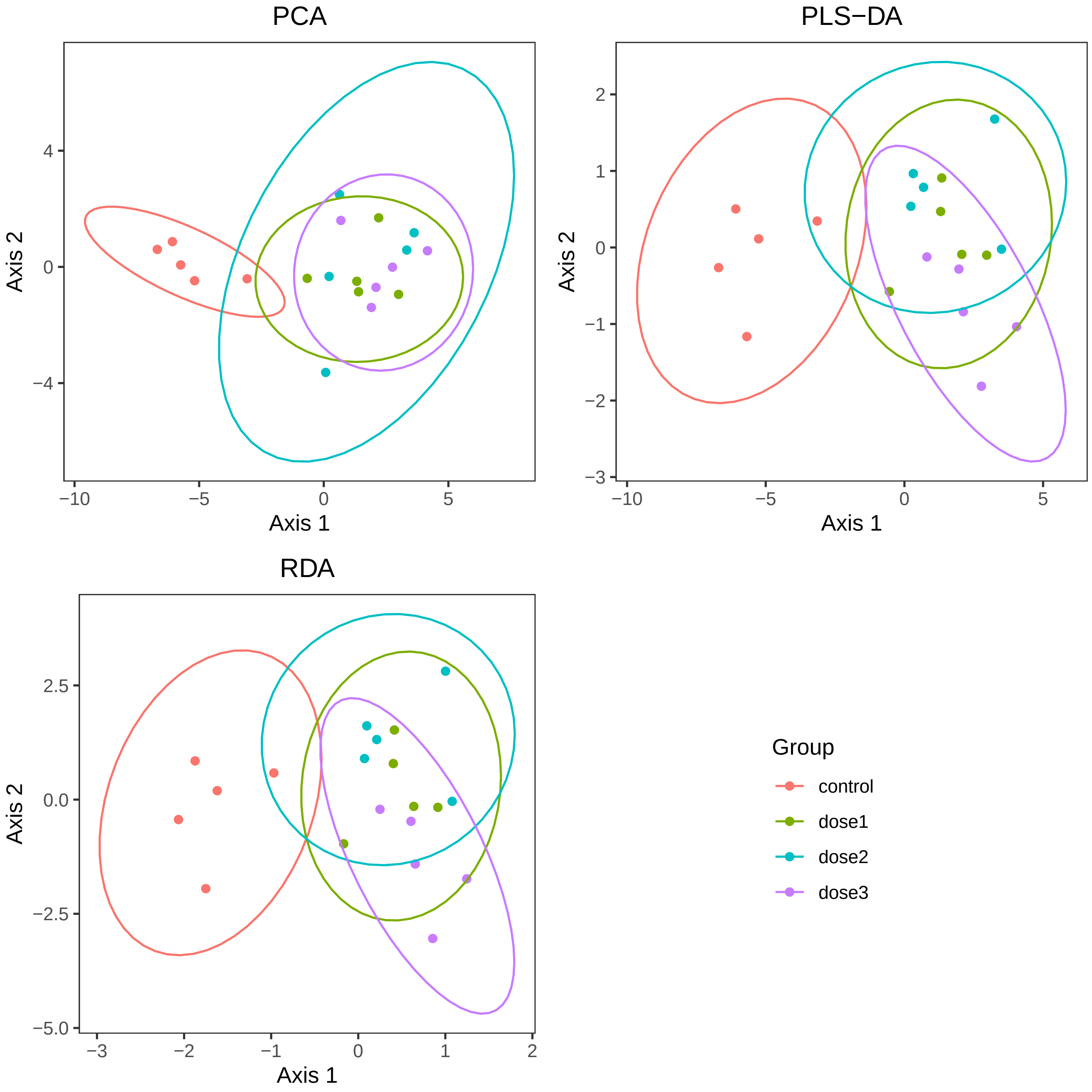

PCA
PLS−DA
RDA
Axis 1
Axis 2
−10
−5
0
5
4
−4
2
1
−1
−2
−3
−3
−2
−1
2.5
0.0
−2.5
−5.0
Group
control
dose1
dose2
dose3

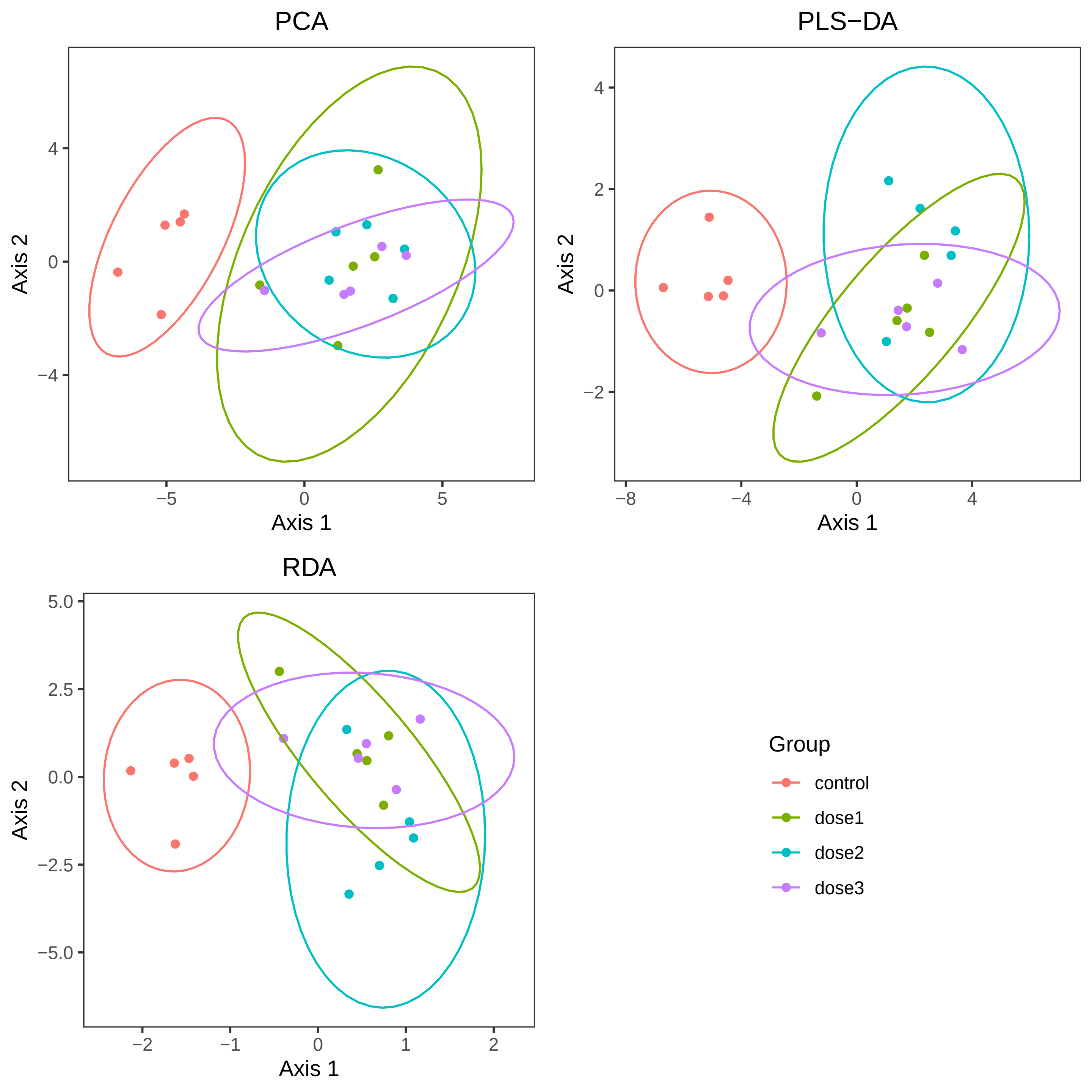
PCA
Axis 2
4
0
−4
−5
0
5
Axis 1
PLS−DA
Axis 2
4
2
0
−2
−8
−4
0
4
Axis 1
RDA
Axis 2
5.0
2.5
0.0
−2.5
−5.0
−2
−1
0
1
2
Axis 1
Group
control
dose1
dose2
dose3

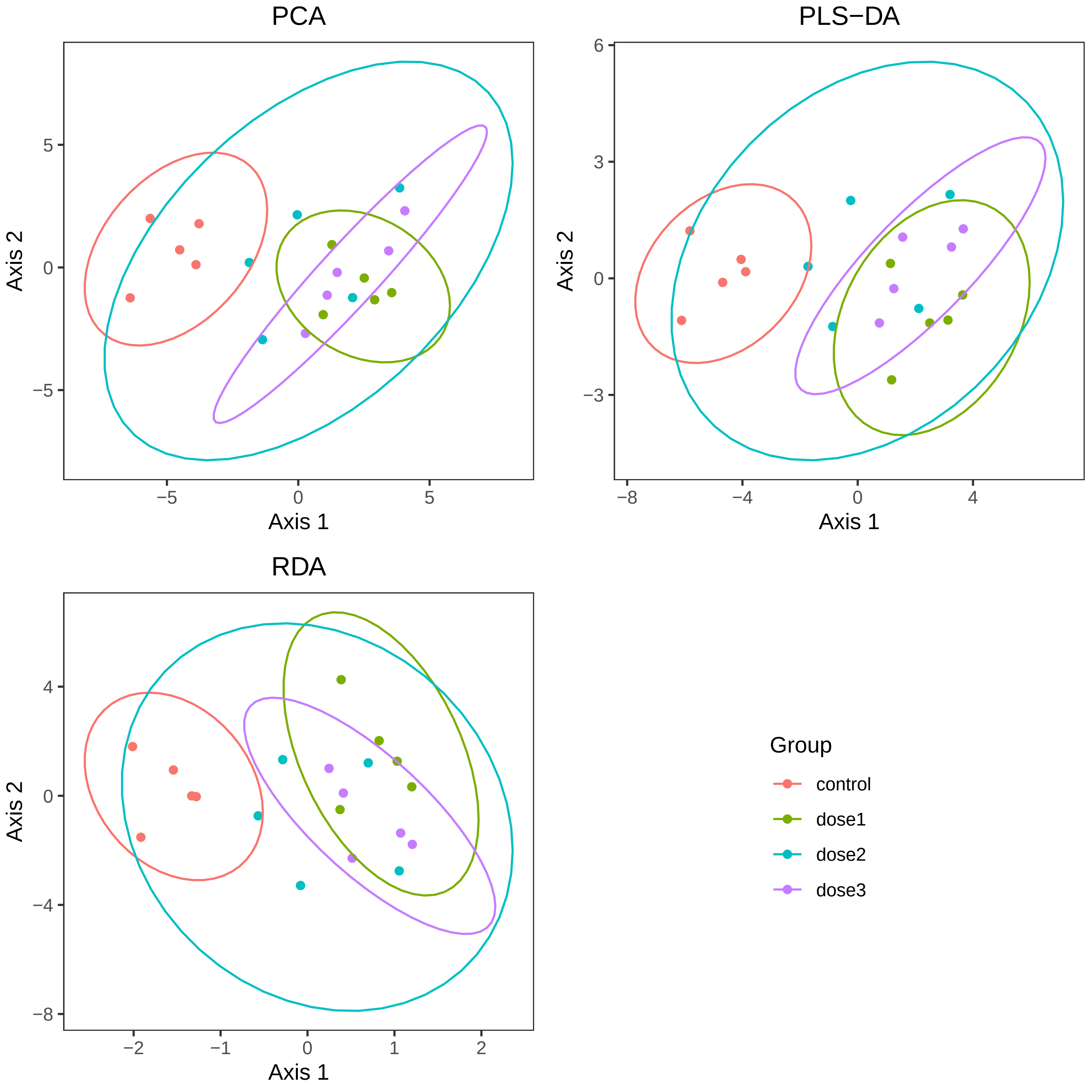

PCA
PLS−DA
RDA
Axis 1
Axis 2
Group
control
dose1
dose2
dose3

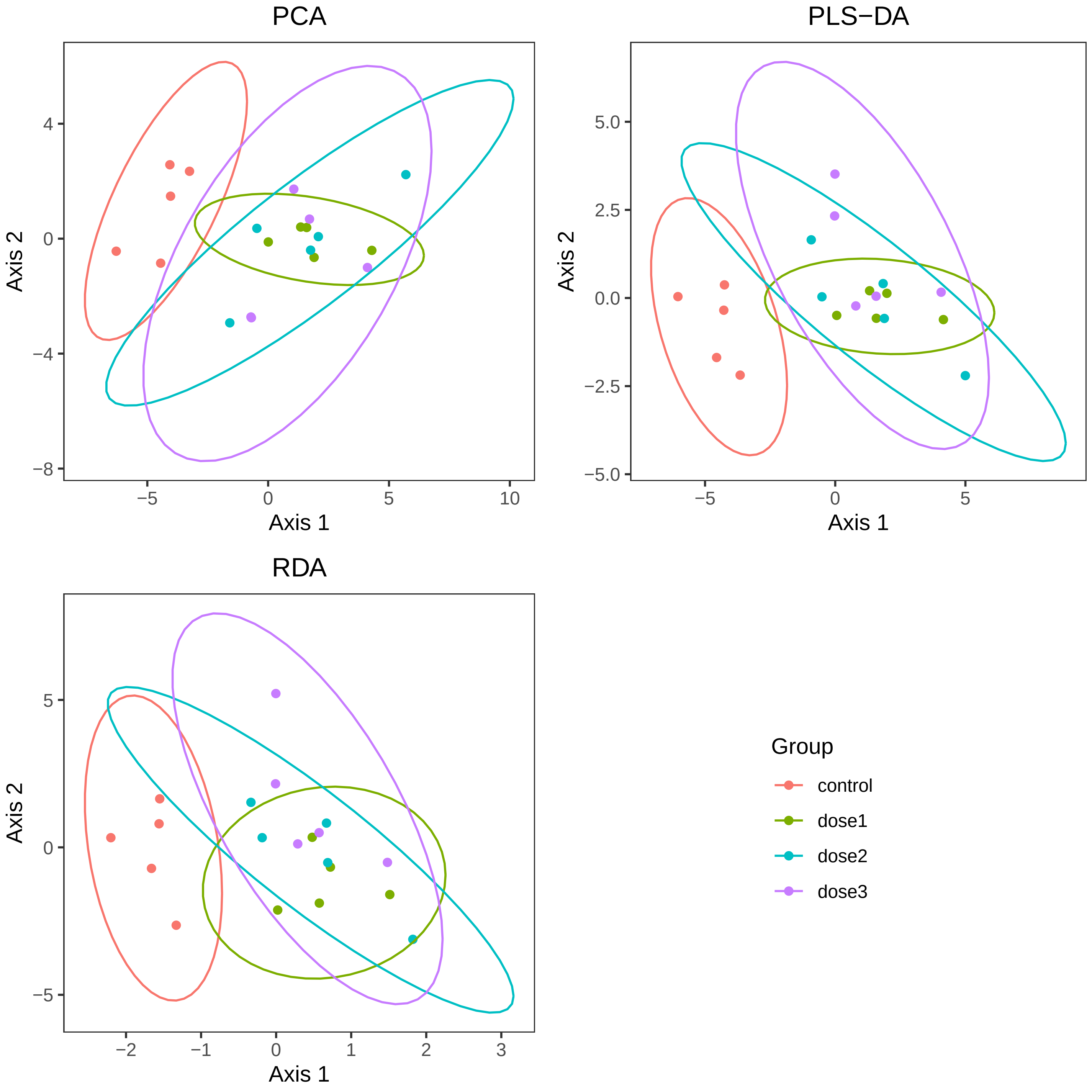
PCA
PLS−DA
RDA
Axis 1
Axis 2
Group
control
dose1
dose2
dose3

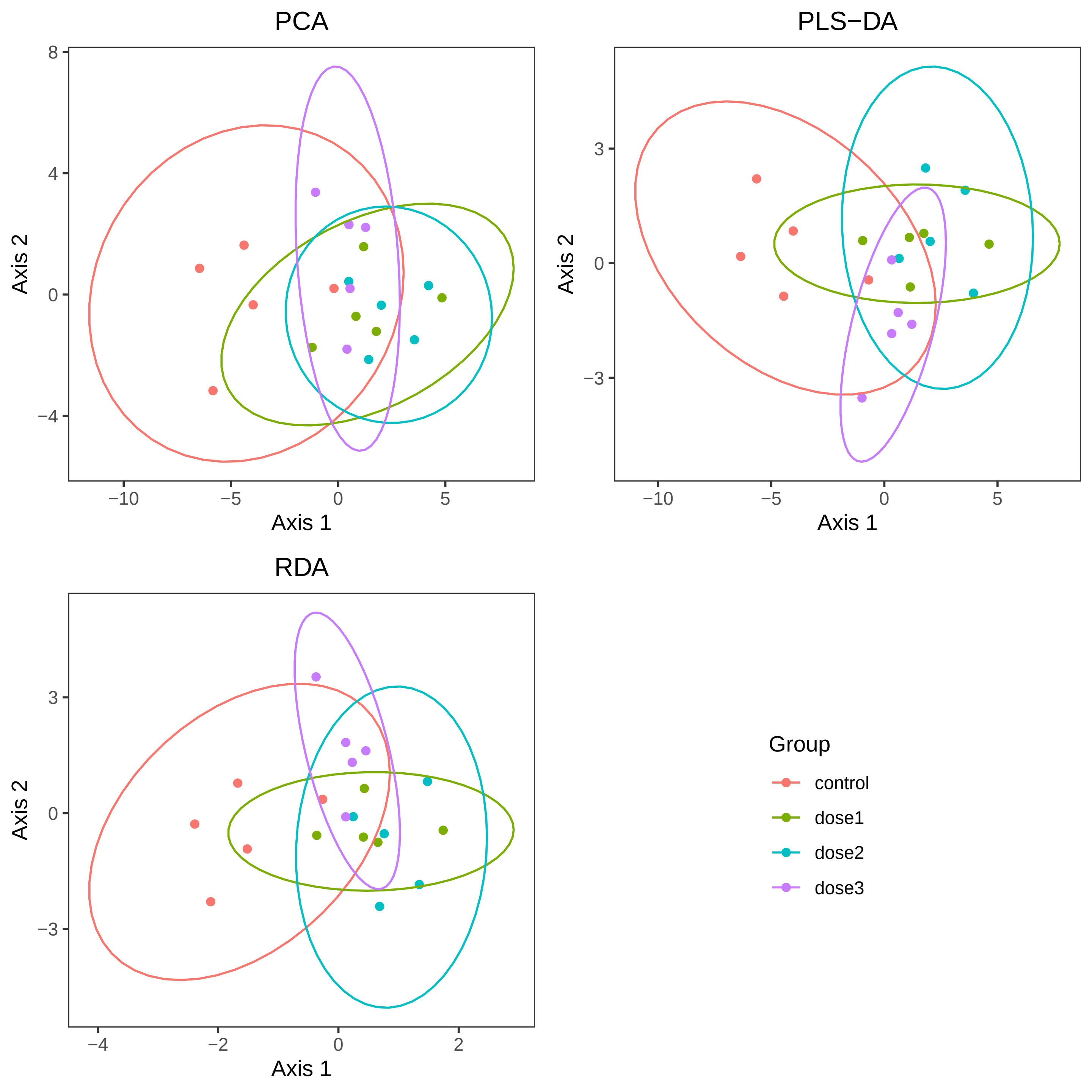

PCA
PLS−DA
RDA
Axis 1
Axis 2
Group
control
dose1
dose2
dose3

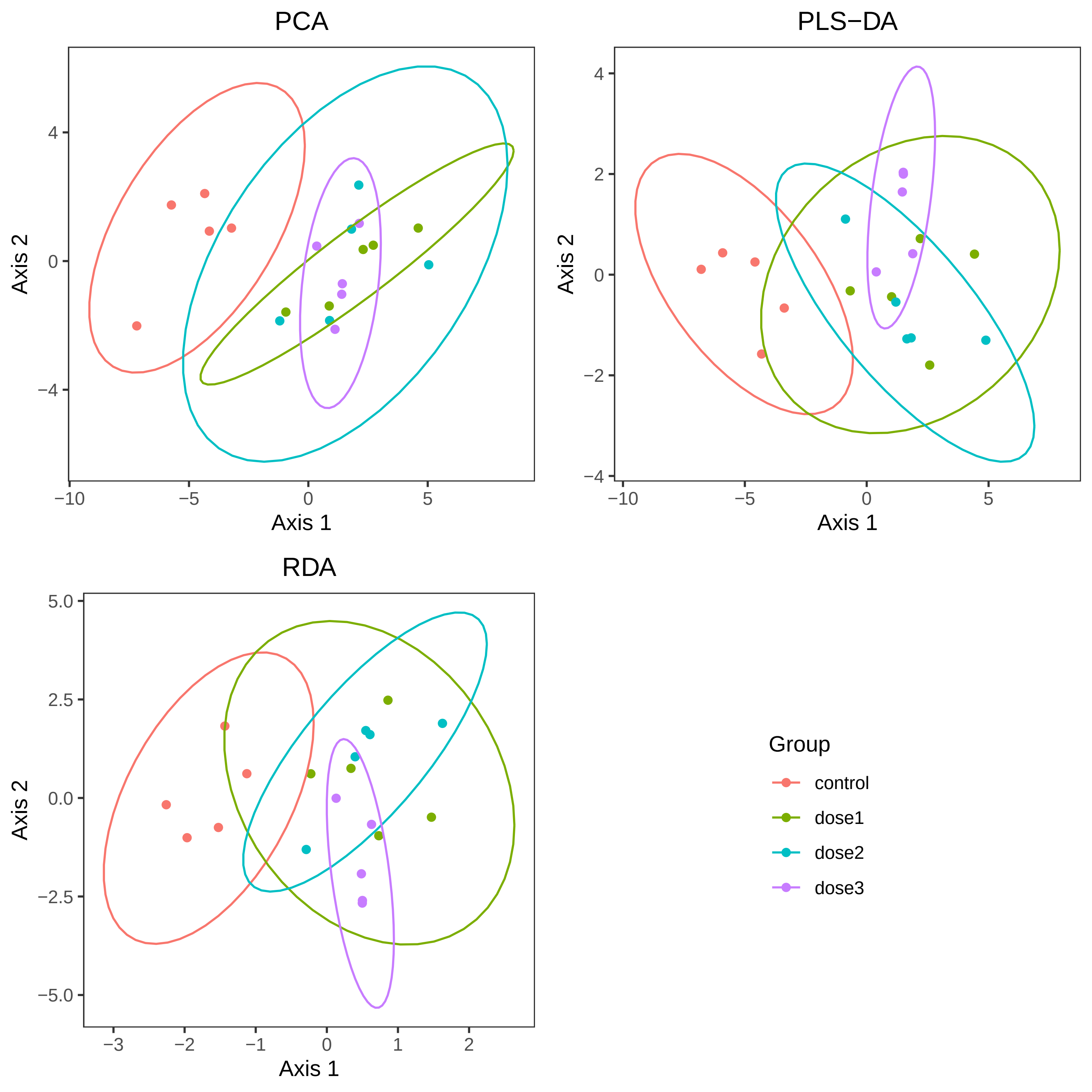

PCA
PLS−DA
RDA
Axis 1
Axis 2
Group
control
dose1
dose2
dose3

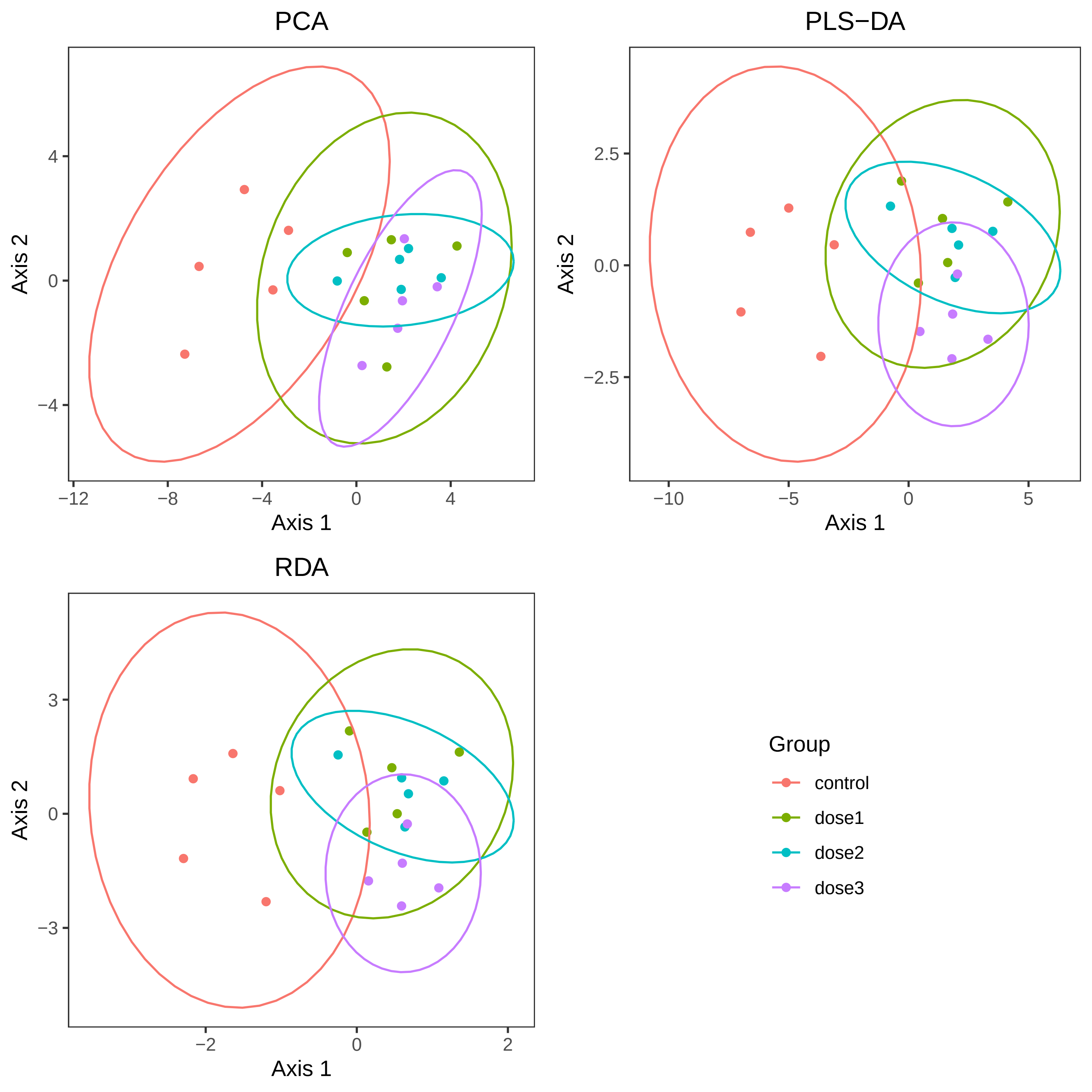

PCA
PLS−DA
RDA
Axis 1
Axis 2
−12
−8
−4
0
4
−10
−5
5
2.5
0.0
−2.5
−2
2
3
−3
Group
control
dose1
dose2
dose3

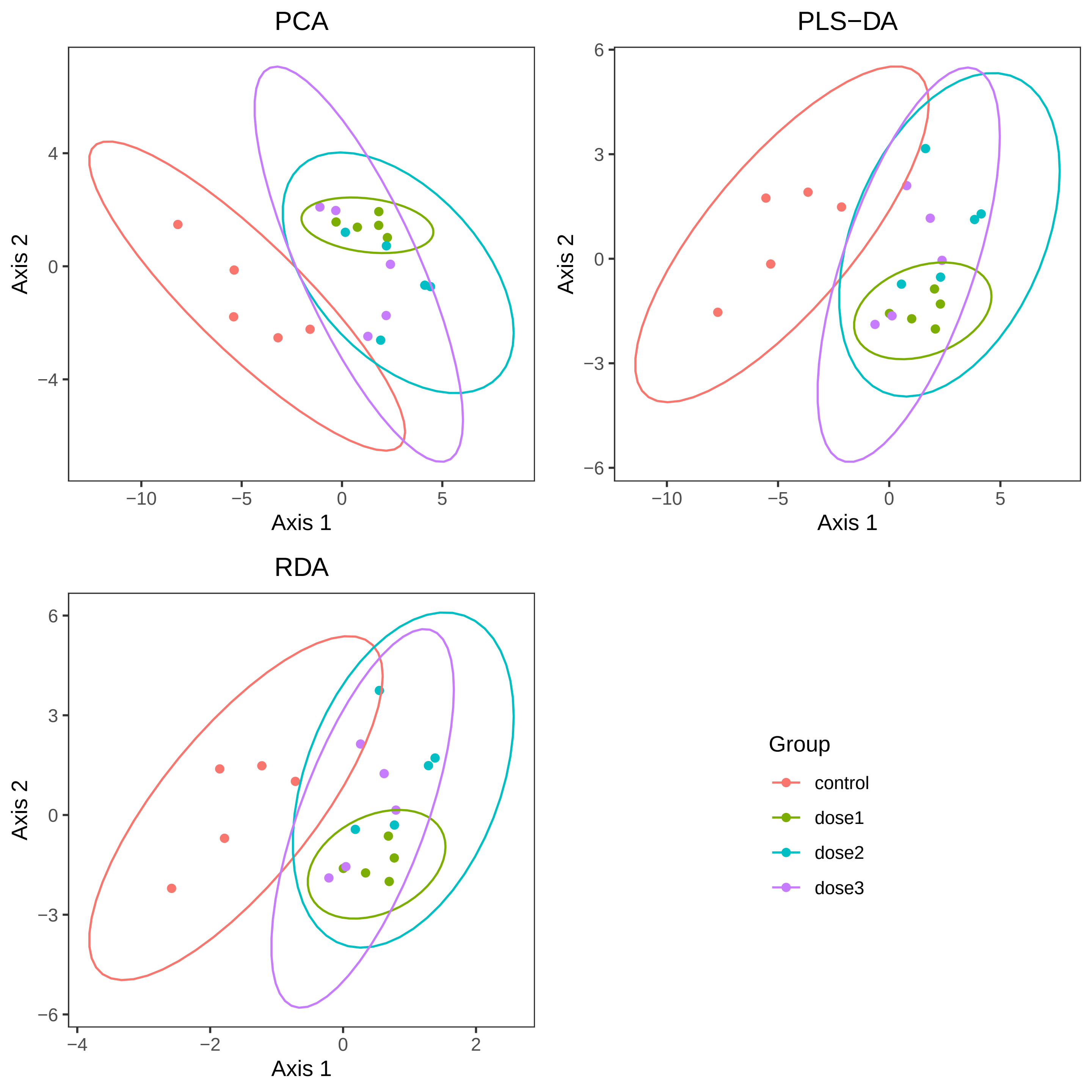
PCA
PLS−DA
RDA
Axis 1
Axis 2
−10
−5
0
5
4
−4
6
3
−3
−6
−4
−2
2
Group
control
dose1
dose2
dose3

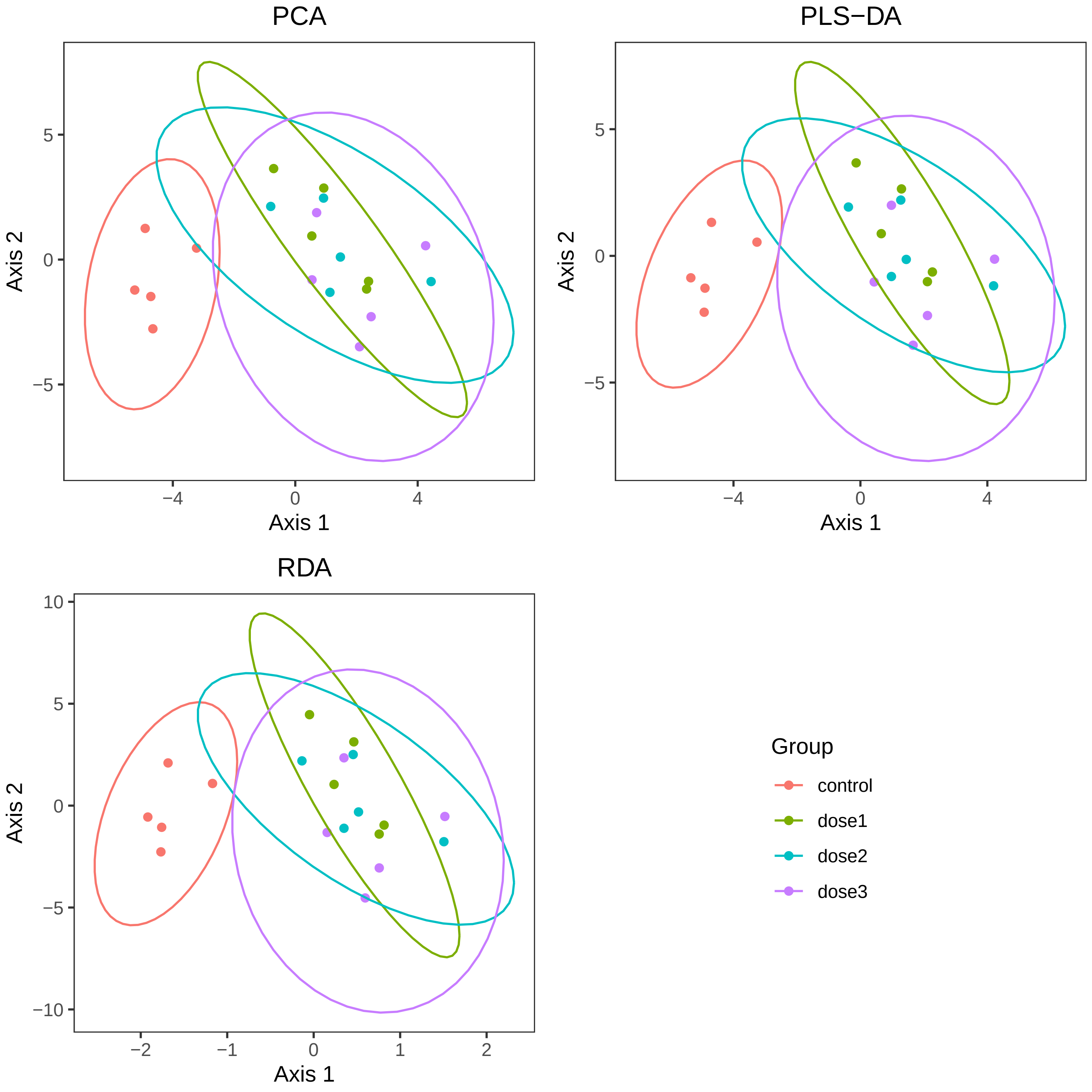
PCA
PLS−DA
RDA
Axis 1
Axis 2
Group
control
dose1
dose2
dose3

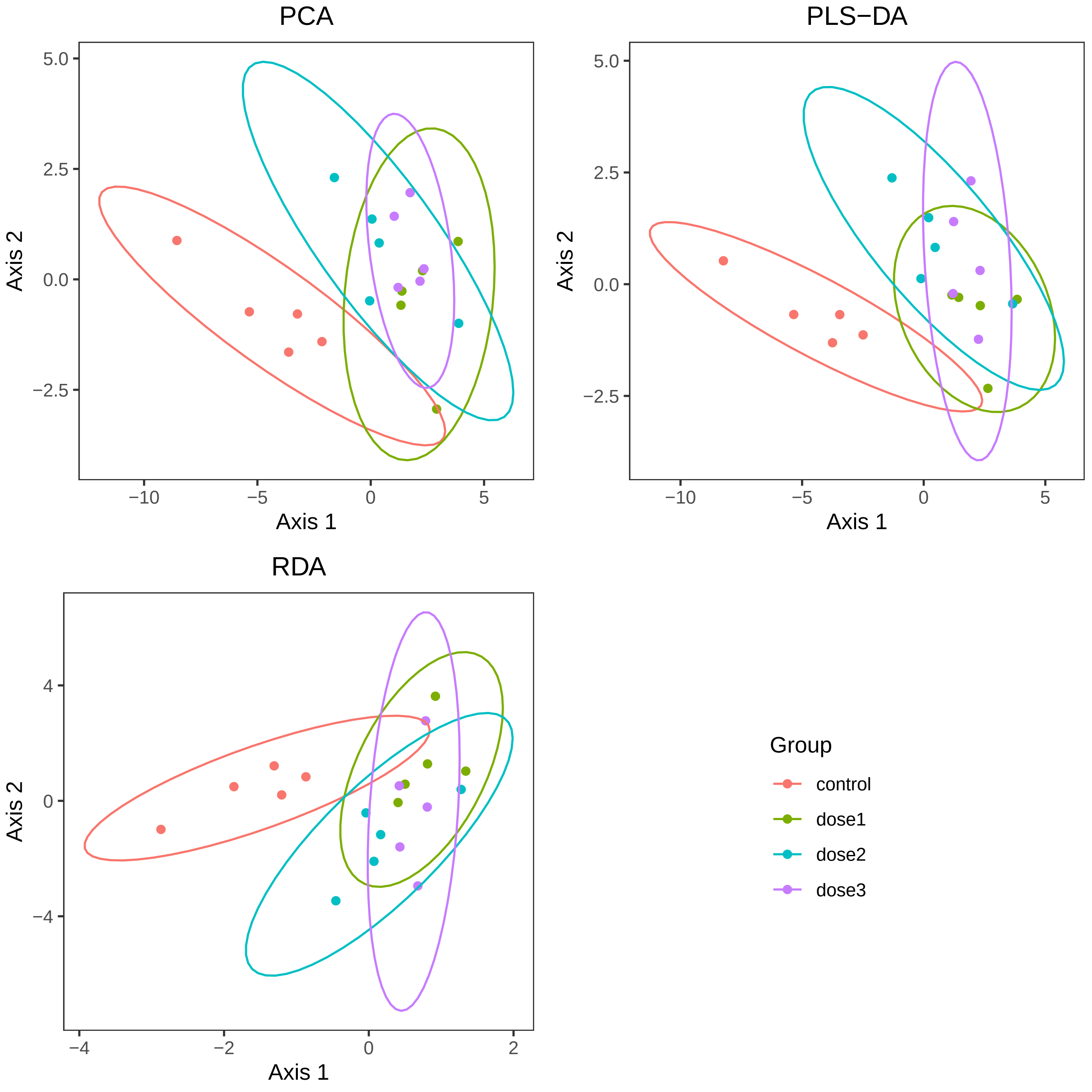
PCA
PLS−DA
RDA
Axis 1
Axis 2
Group
control
dose1
dose2
dose3

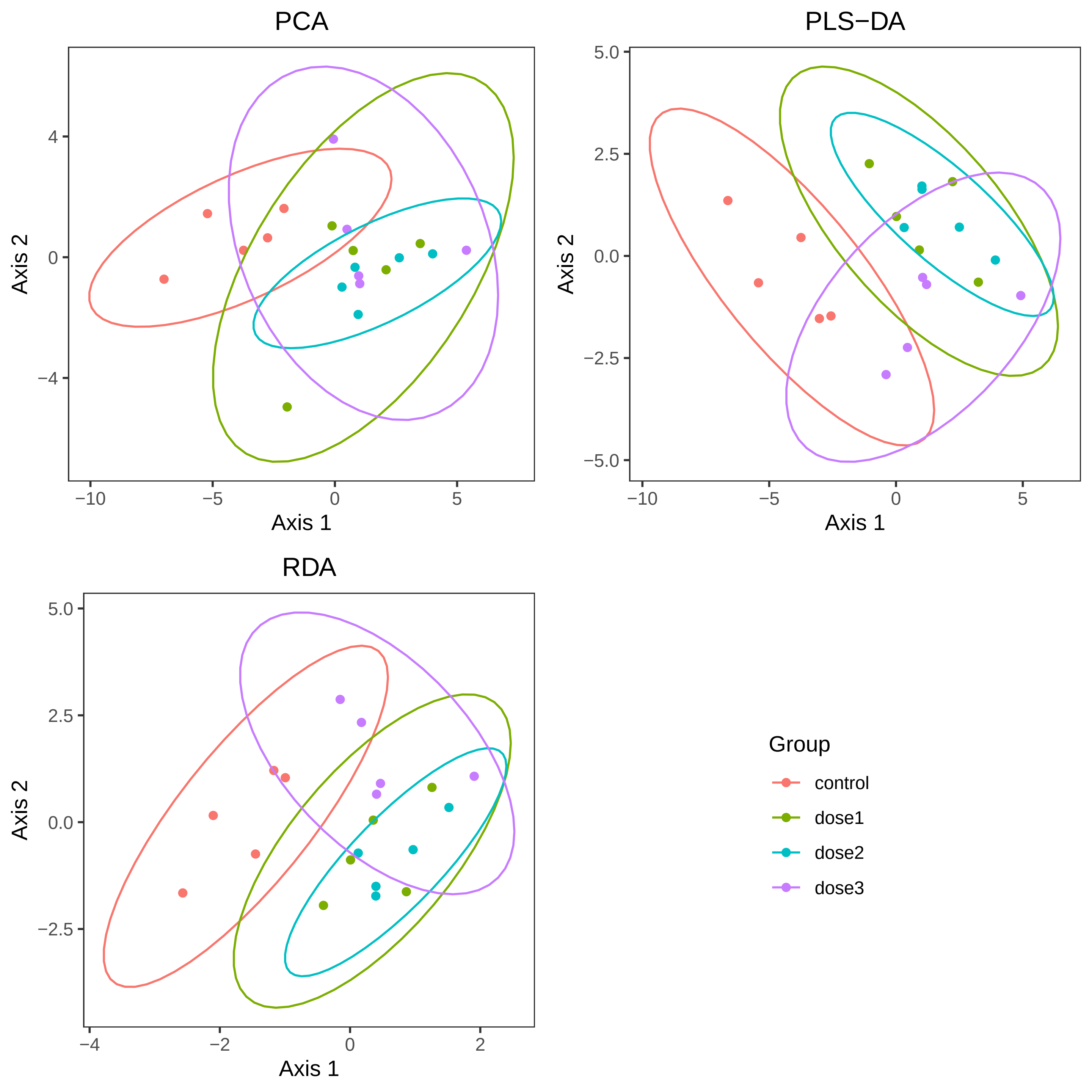
PCA
PLS−DA
RDA
Axis 1
Axis 2
Group
control
dose1
dose2
dose3

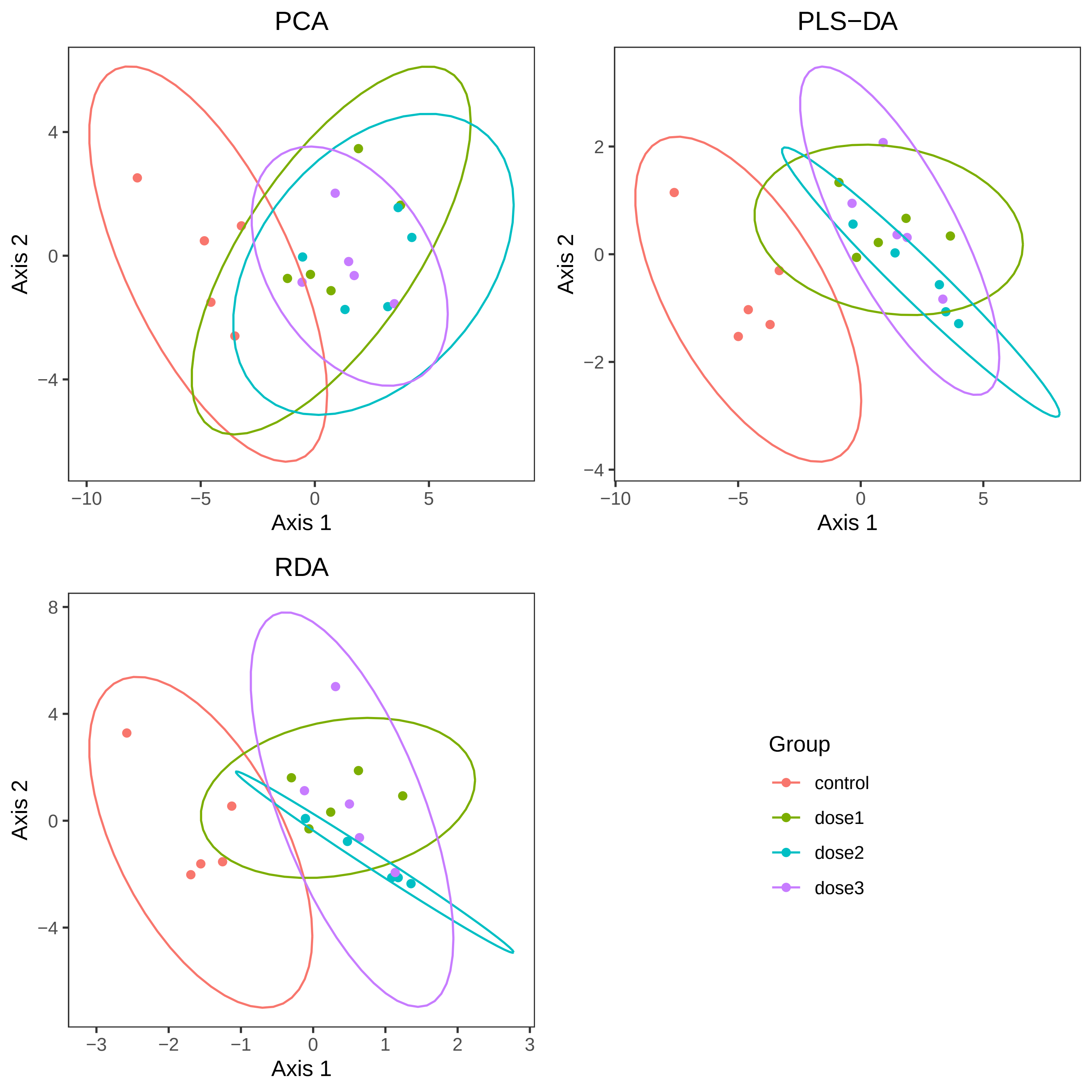
PCA
PLS−DA
RDA
Axis 1
Axis 2
Group
control
dose1
dose2
dose3

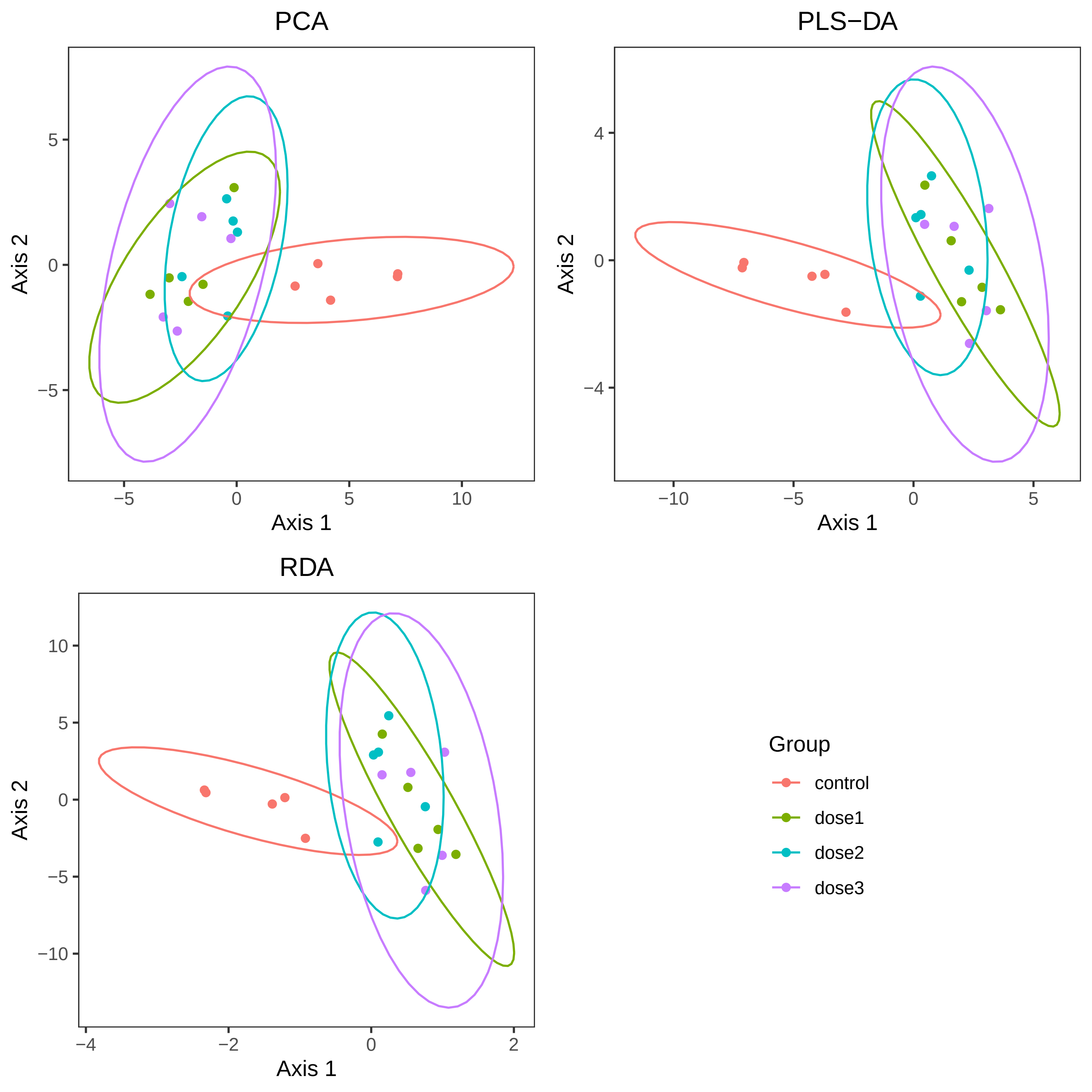

PCA
PLS−DA
RDA
Axis 1
Axis 2
Group
control
dose1
dose2
dose3

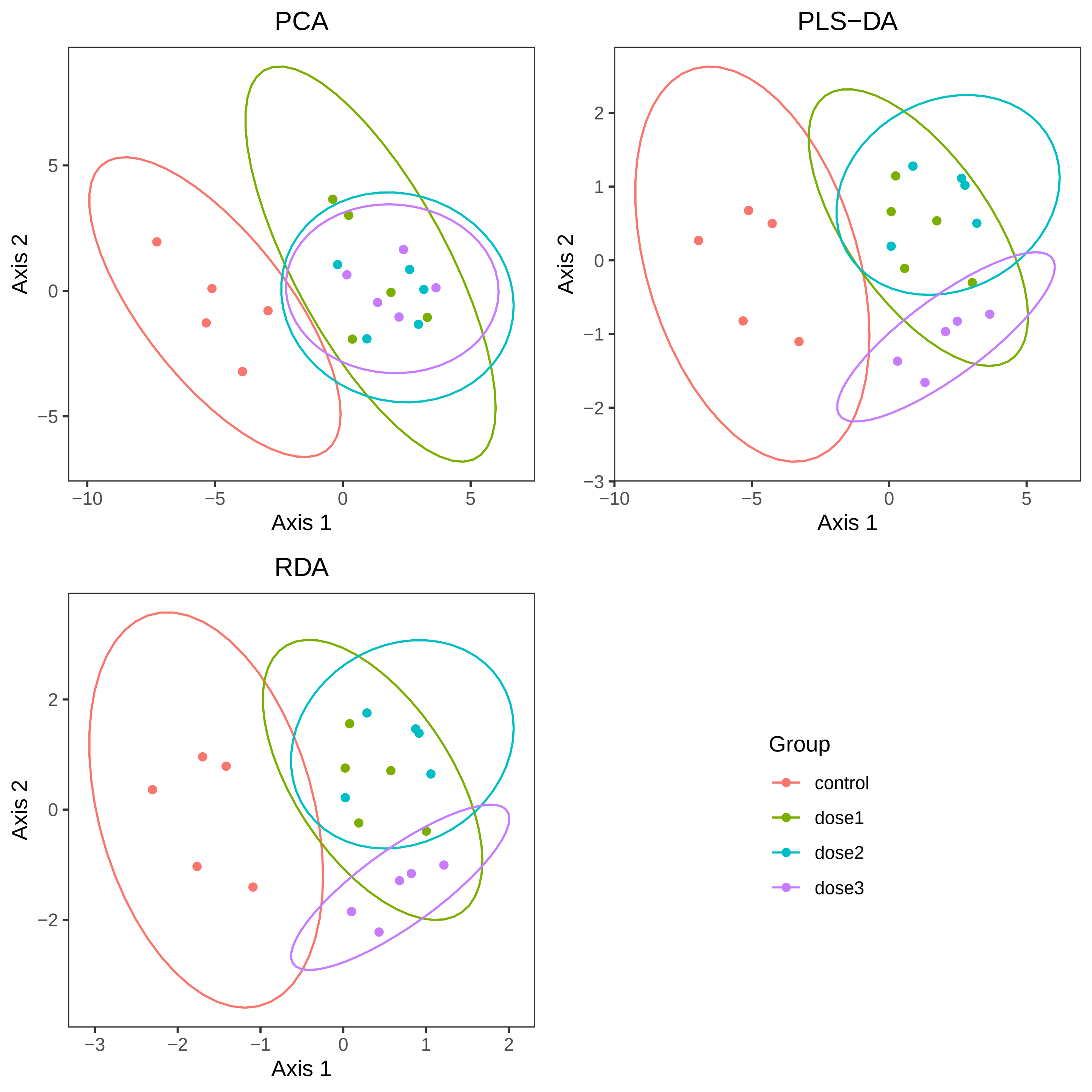
PCA
PLS−DA
RDA
Axis 1
Axis 2
Group
control
dose1
dose2
dose3

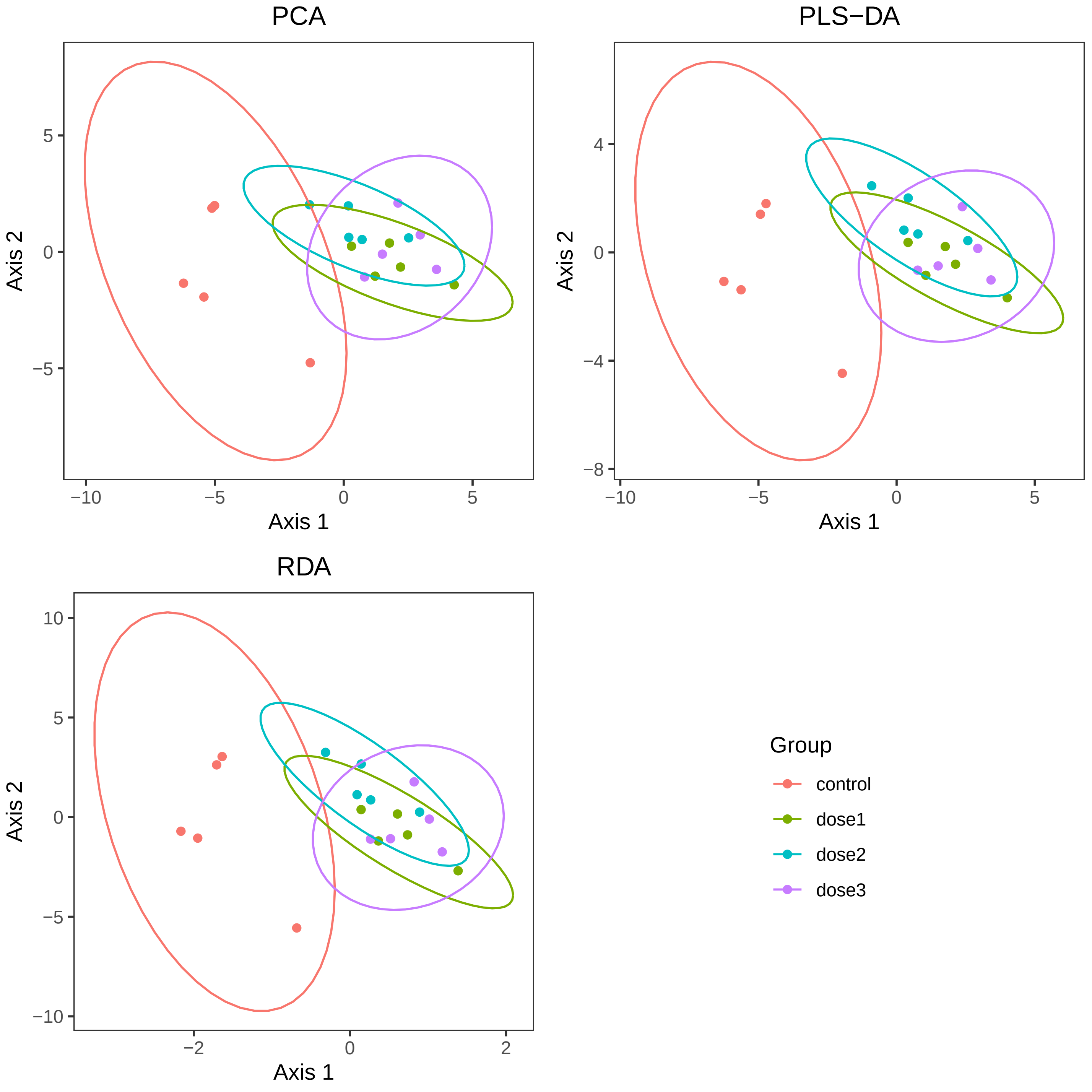
PCA
PLS−DA
RDA
Axis 1
Axis 2
−10
−5
0
5
4
−4
−8
10
−2
2
Group
control
dose1
dose2
dose3

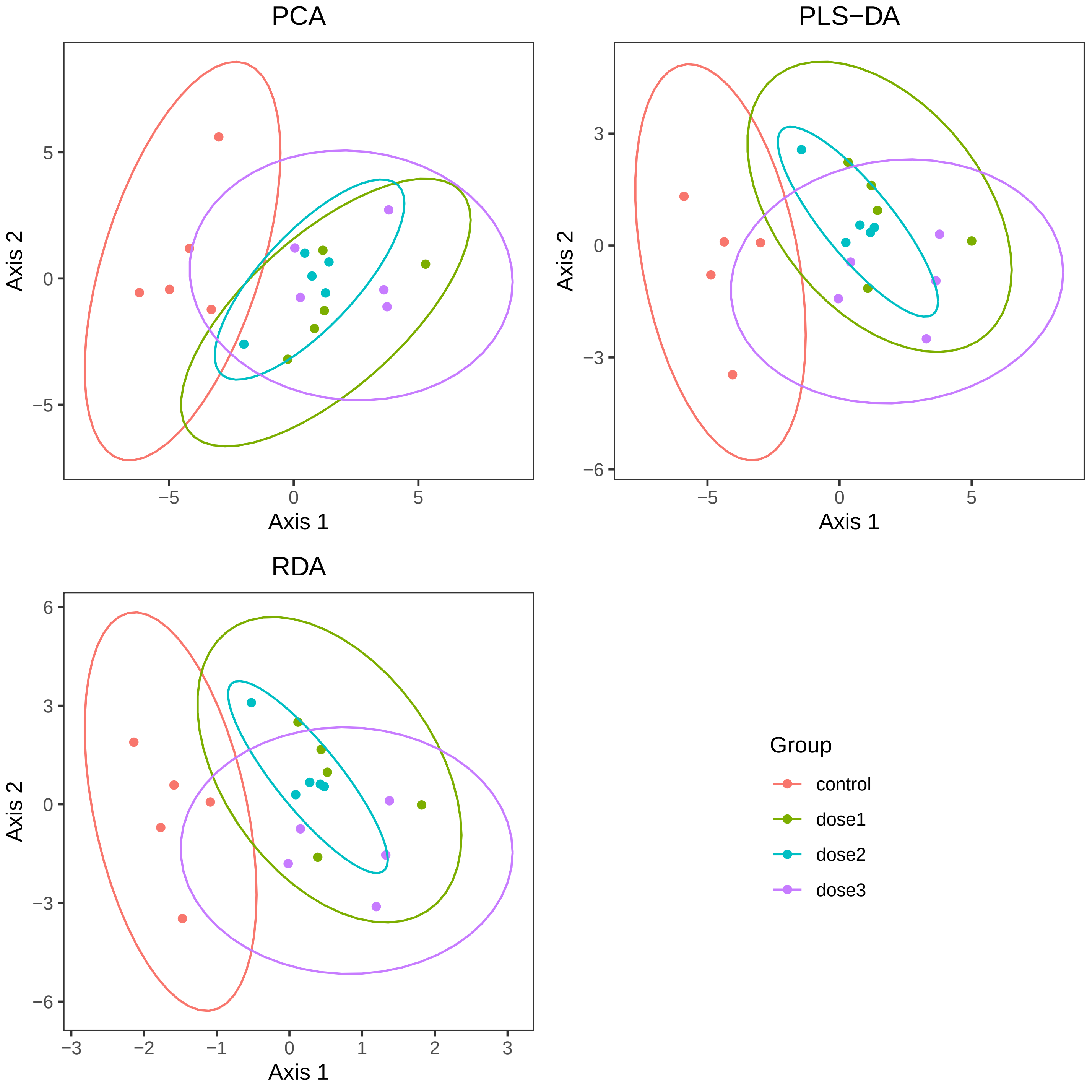
PCA
PLS−DA
RDA
Axis 1
Axis 2
Group
control
dose1
dose2
dose3

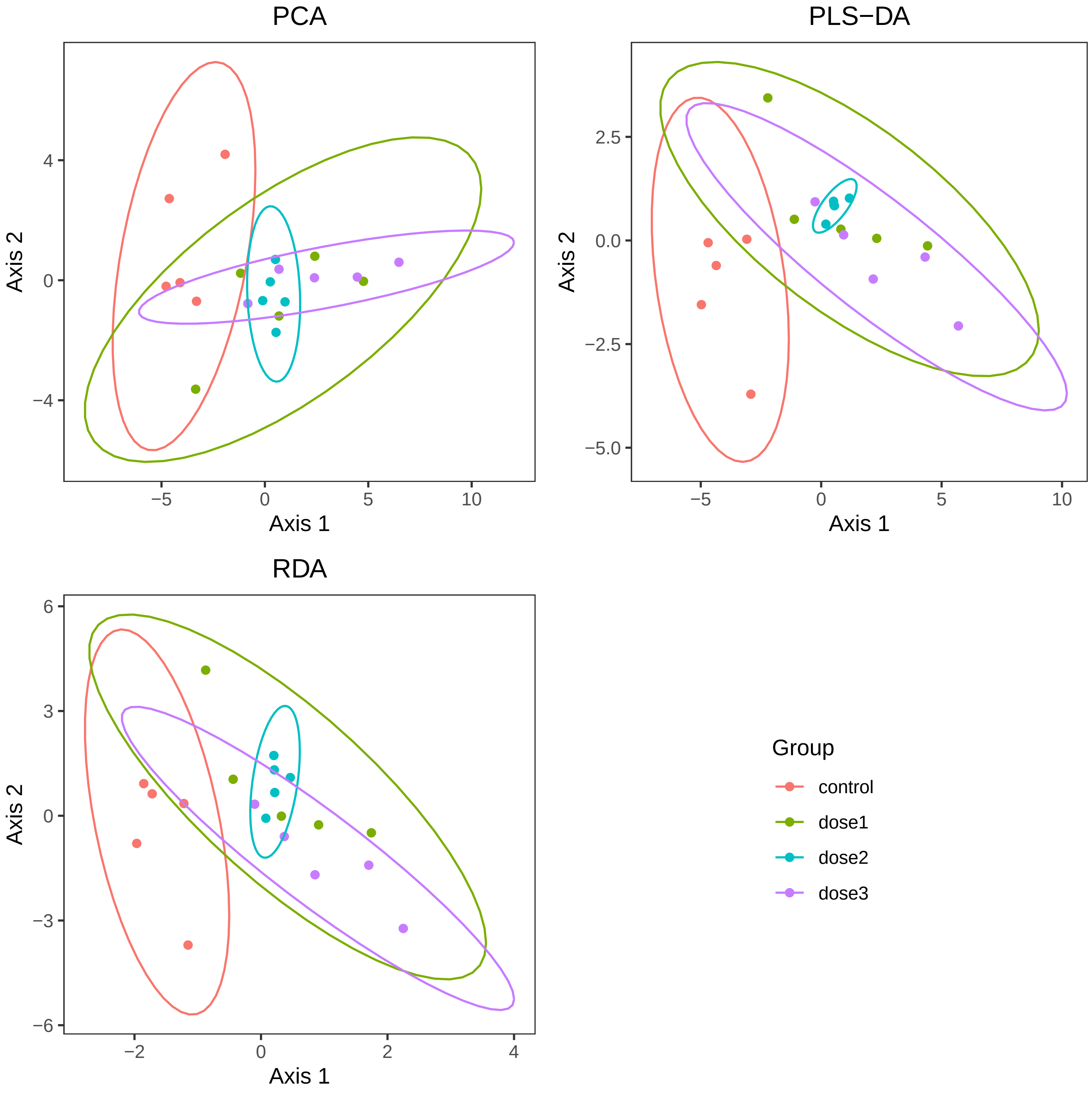
PCA
PLS−DA
RDA
Axis 1
Axis 2
Group
control
dose1
dose2
dose3

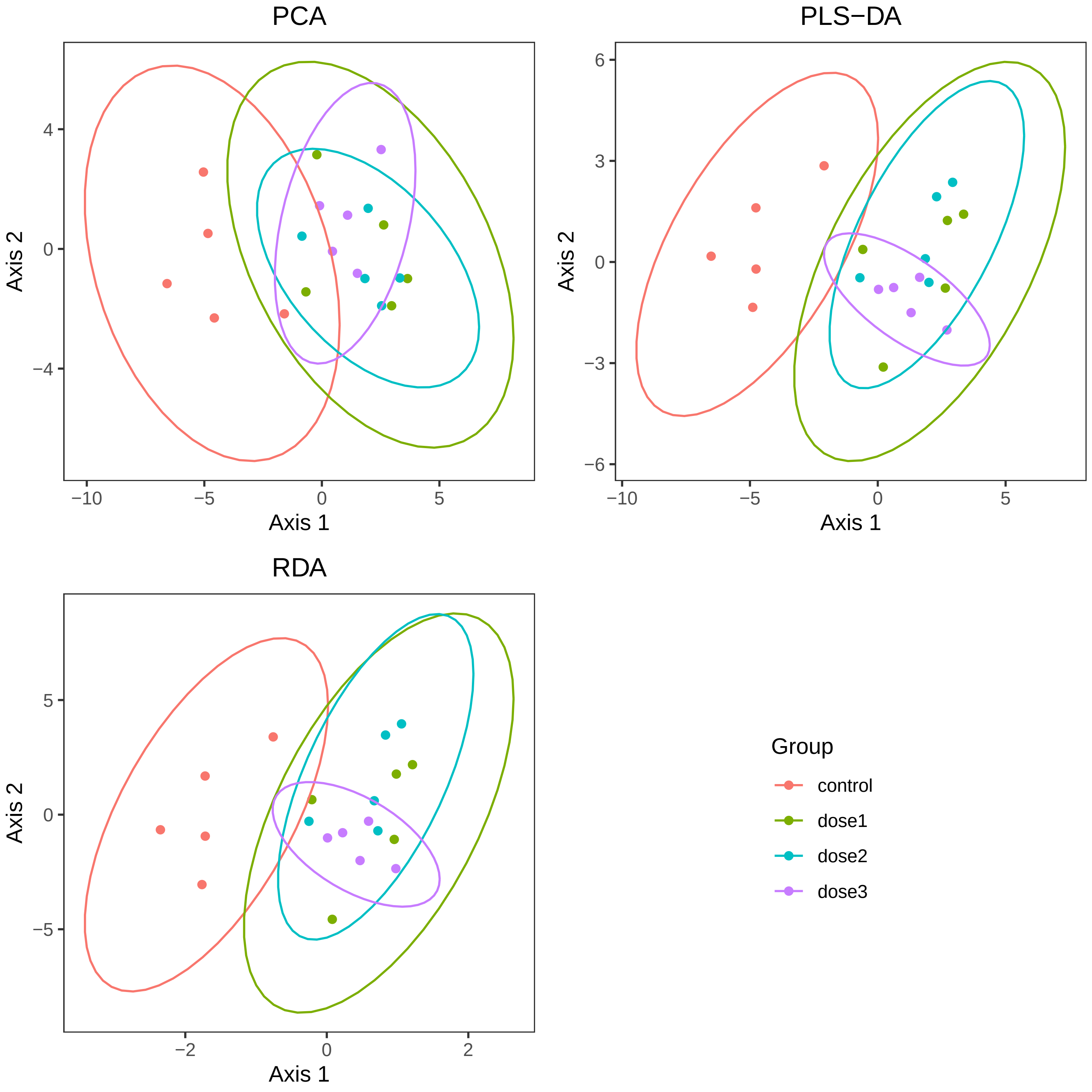

PCA
PLS−DA
RDA
Axis 1
Axis 2
Group
control
dose1
dose2
dose3

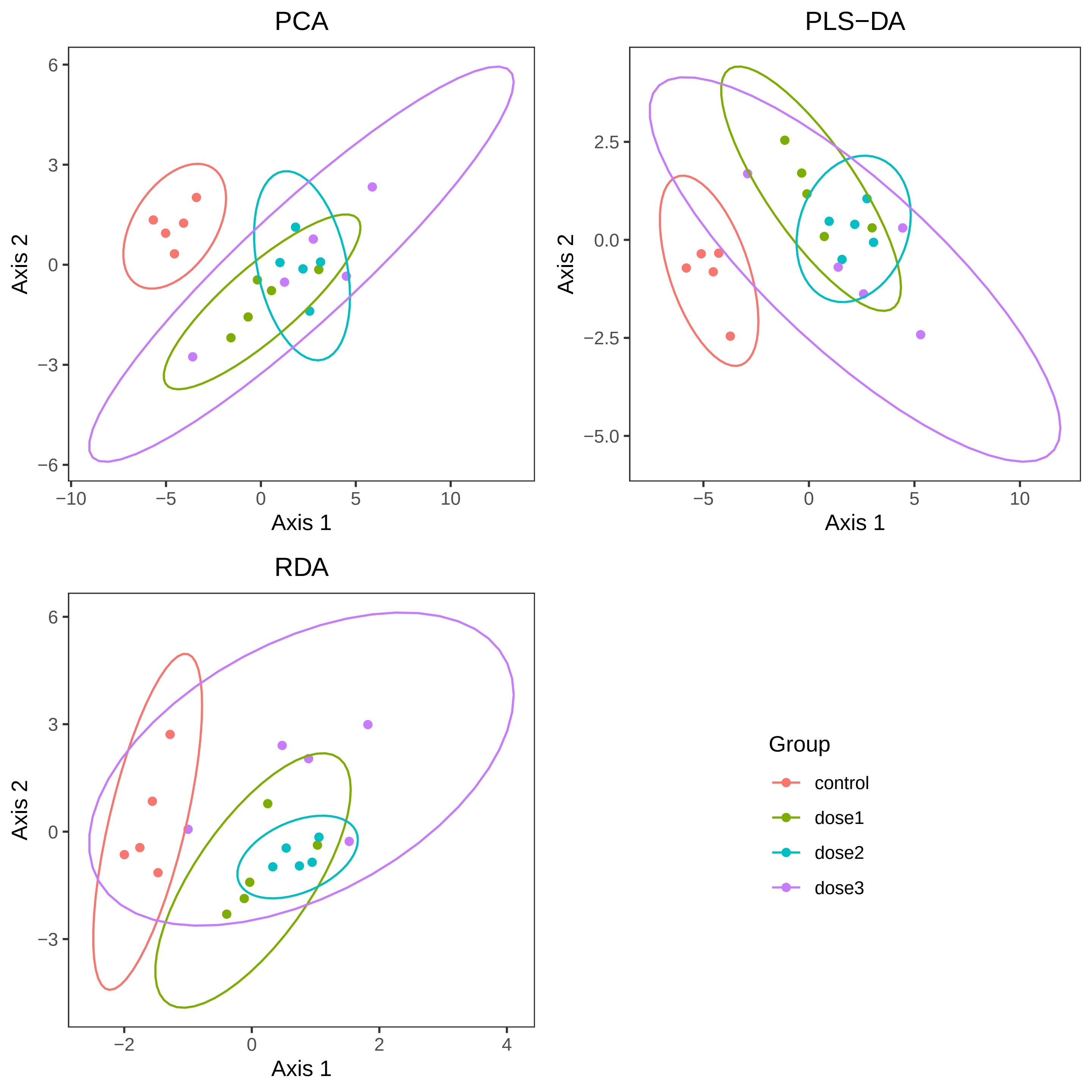

PCA
PLS−DA
RDA
Axis 1
Axis 2
Group
control
dose1
dose2
dose3

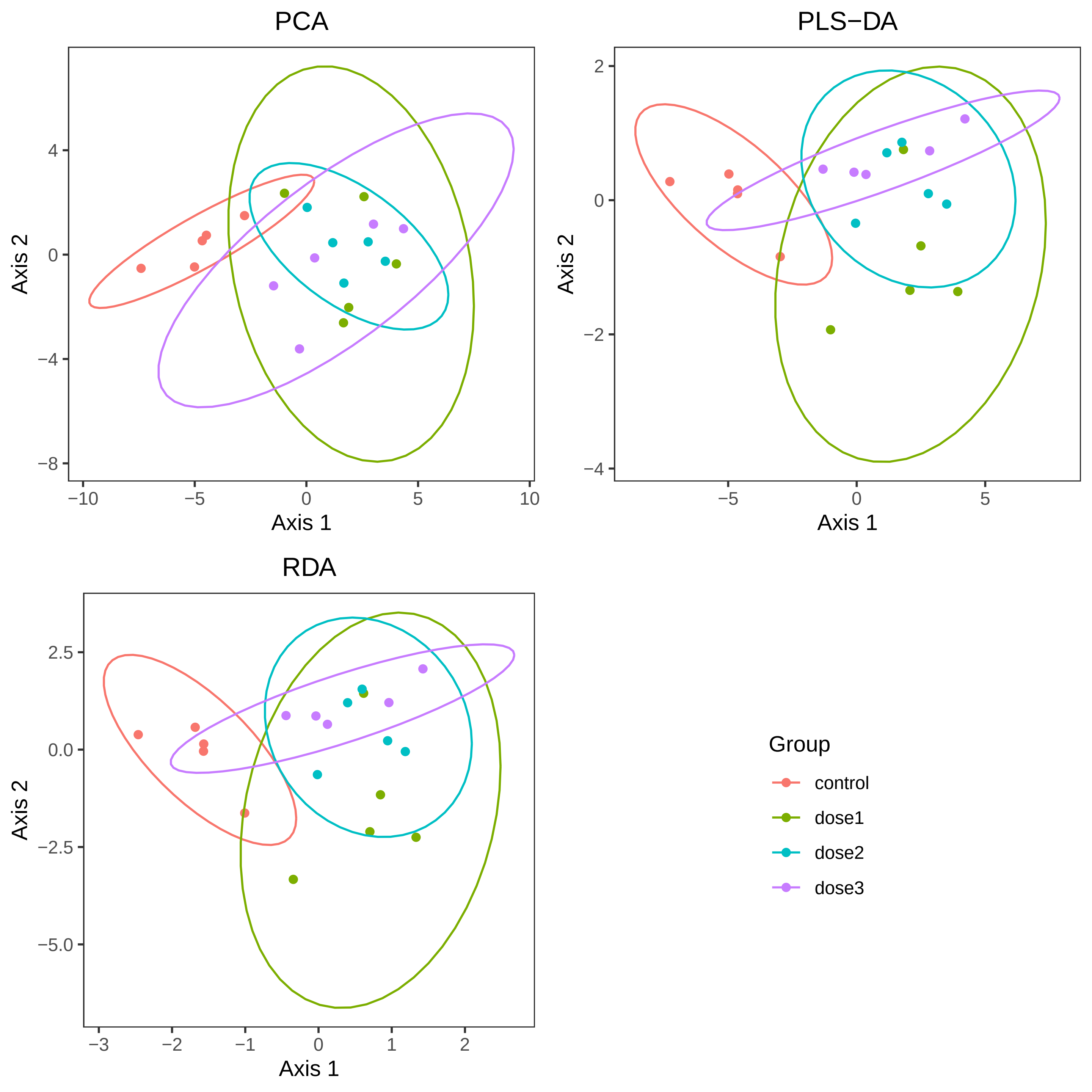
PCA
PLS−DA
RDA
Axis 1
Axis 2
Group
control
dose1
dose2
dose3

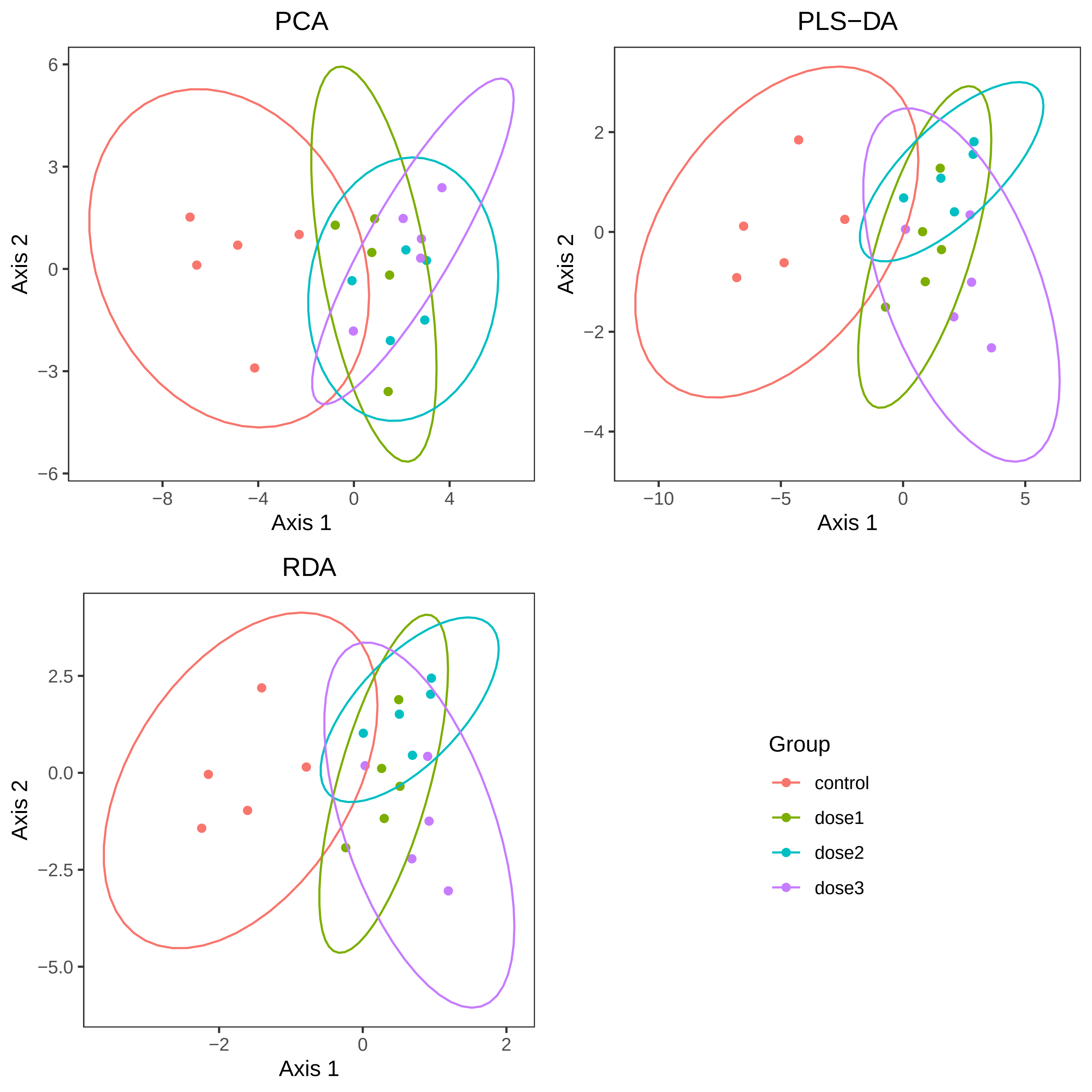
PCA
Axis 2
Axis 1
6
3
0
−3
−6
−8
−4
0
4
PLS−DA
Axis 2
Axis 1
2
0
−2
−4
−10
−5
0
5
RDA
Axis 2
Axis 1
2.5
0.0
−2.5
−5.0
−2
0
2
Group
control
dose1
dose2
dose3

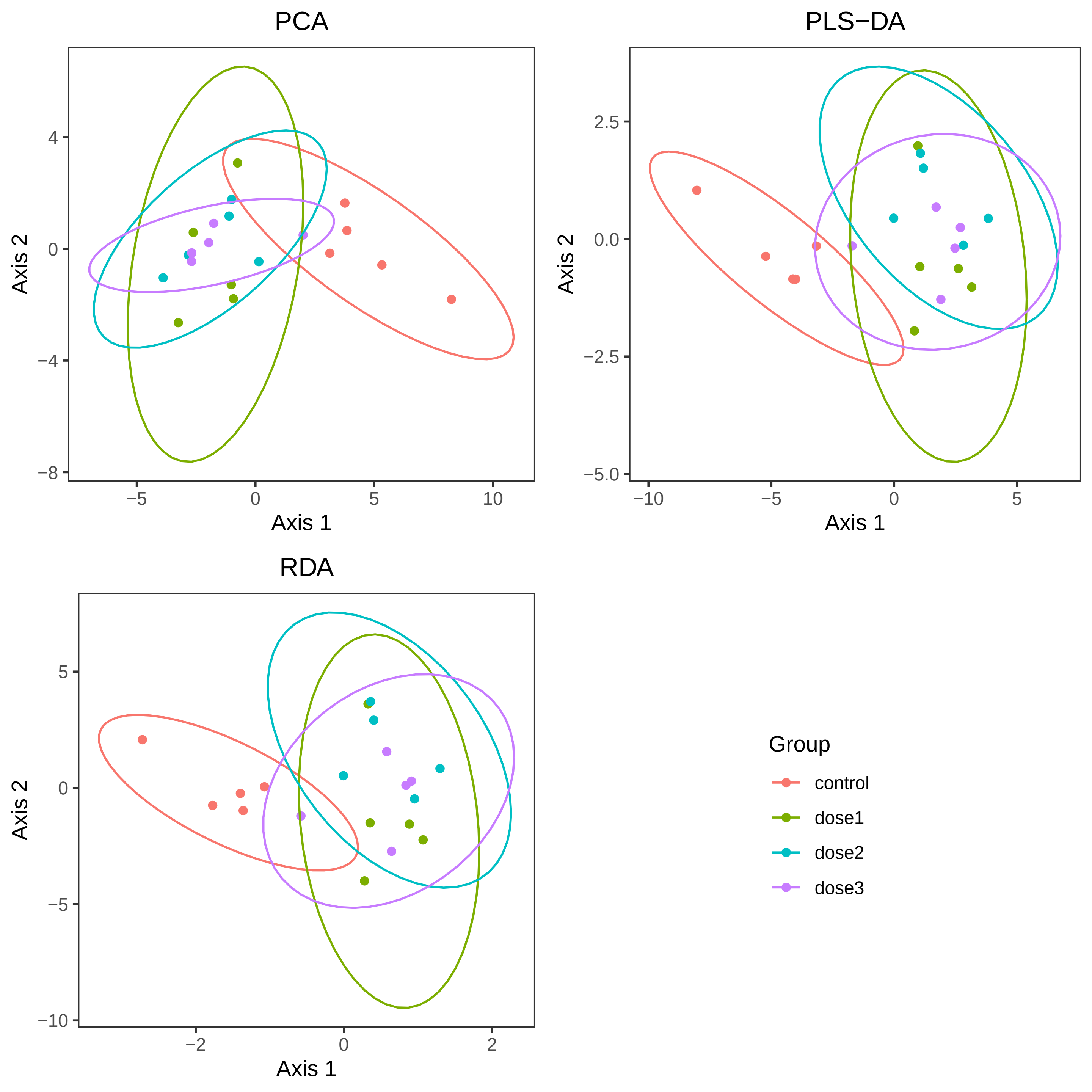
PCA
PLS−DA
RDA
Axis 1
Axis 2
Group
control
dose1
dose2
dose3

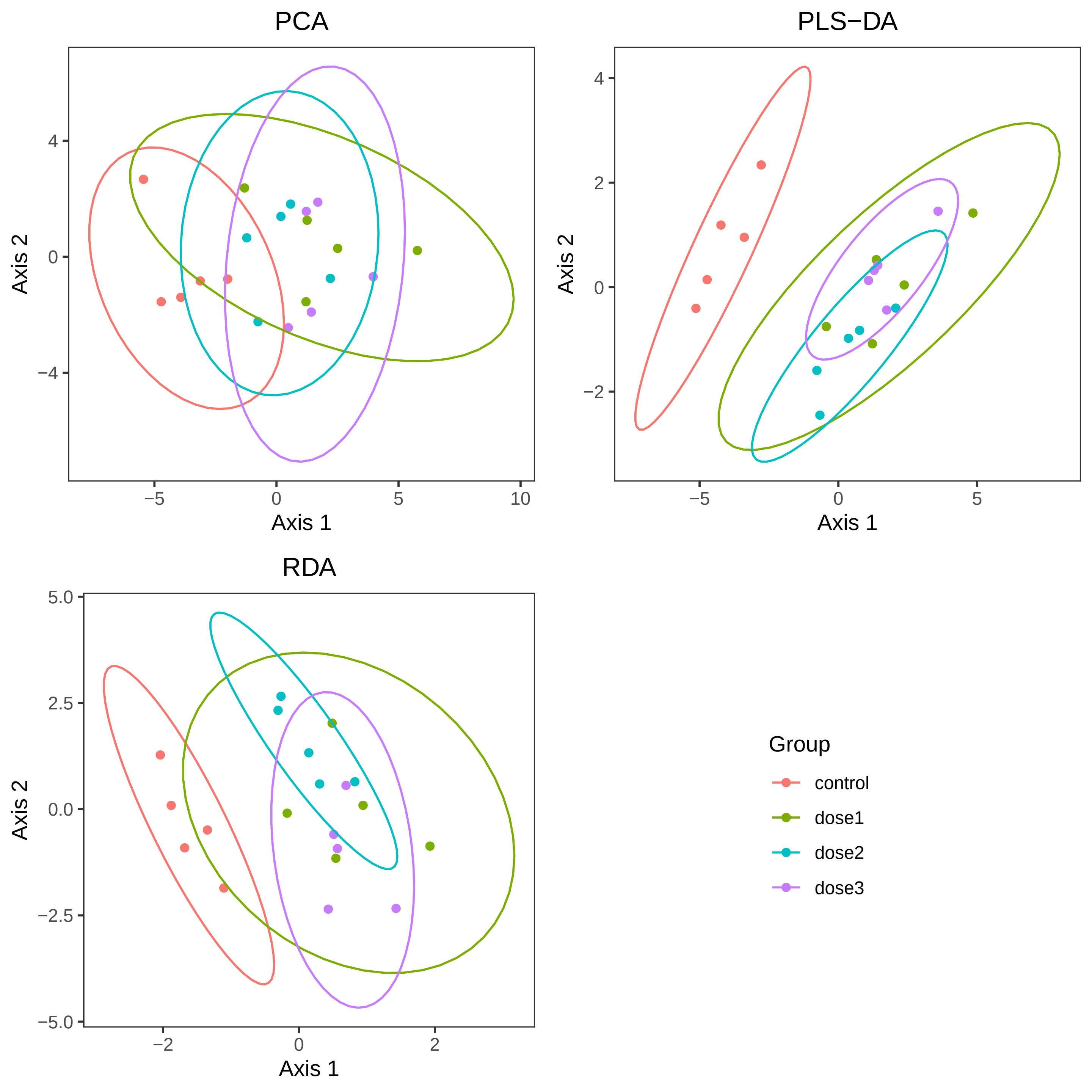

PCA
PLS−DA
RDA
Axis 1
Axis 2
Group
control
dose1
dose2
dose3

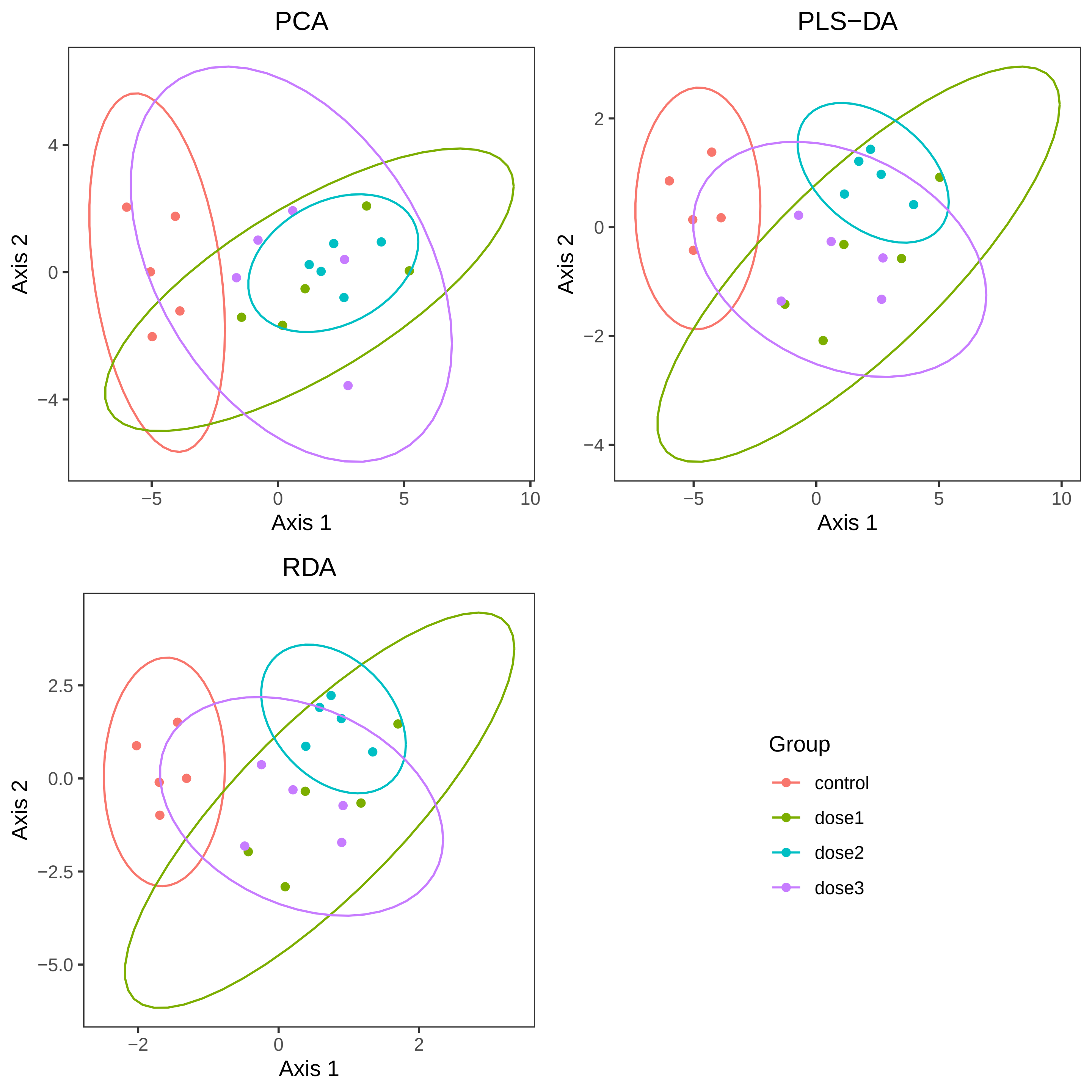

PCA
PLS−DA
RDA
Axis 1
Axis 2
4
0
−4
−5
0
5
10
2
0
−2
−4
2.5
0.0
−2.5
−5.0
−2
0
2
Group
control
dose1
dose2
dose3

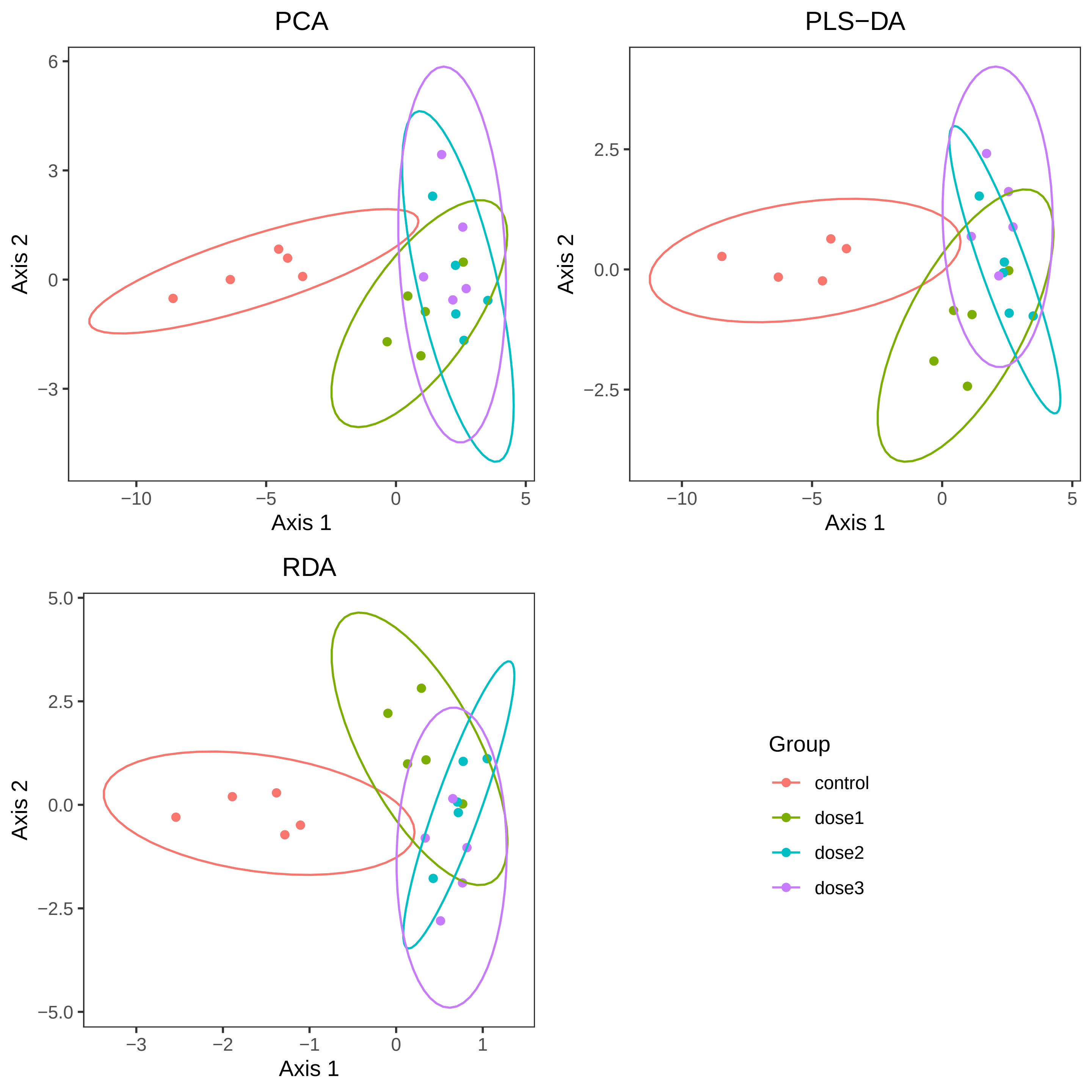

PCA
PLS−DA
RDA
Axis 1
Axis 2
−10
−5
0
5
6
3
0
−3
2.5
0.0
−2.5
5.0
−5.0
−3
−2
−1
1
Group
control
dose1
dose2
dose3

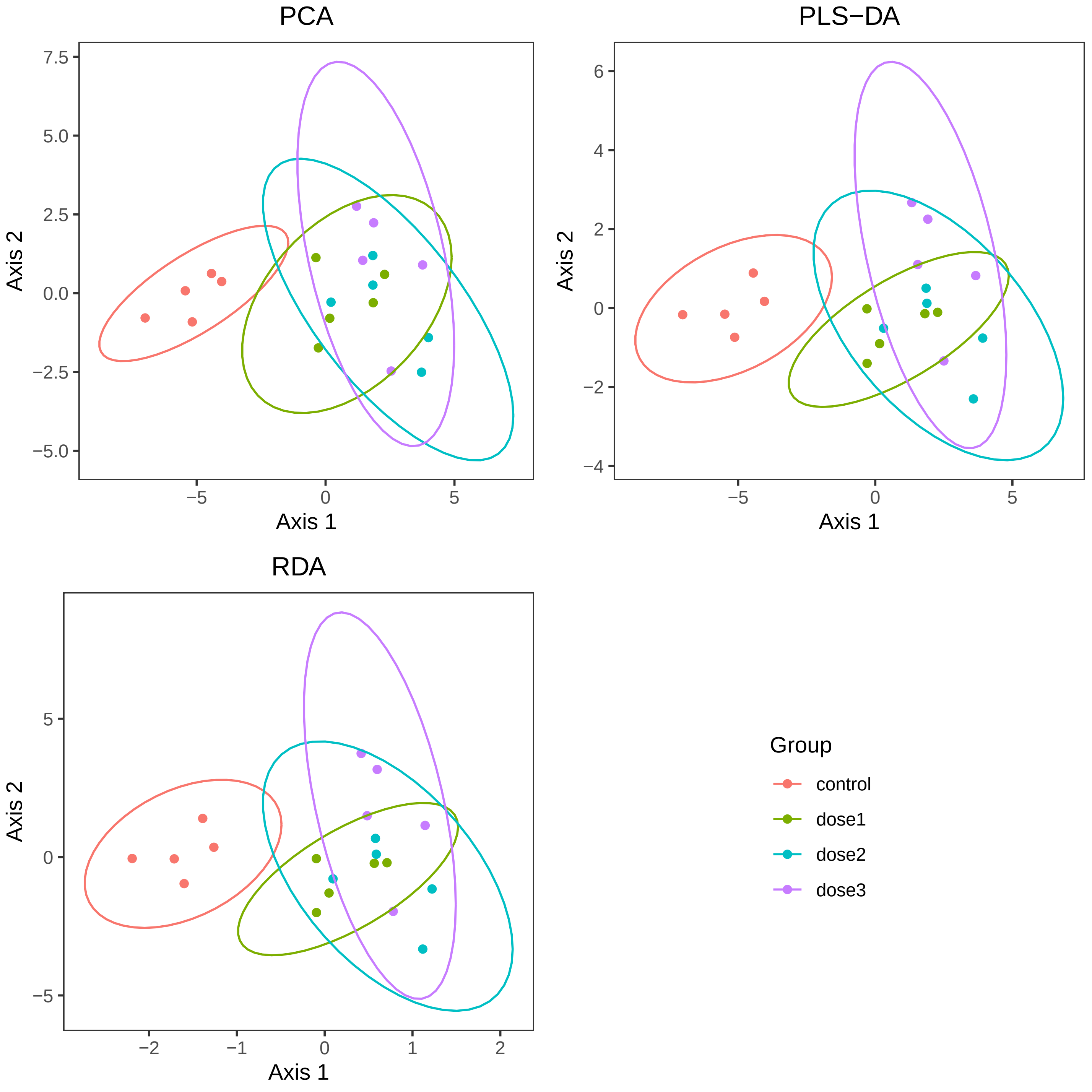

PCA
PLS−DA
RDA
Axis 1
Axis 2
Group
control
dose1
dose2
dose3

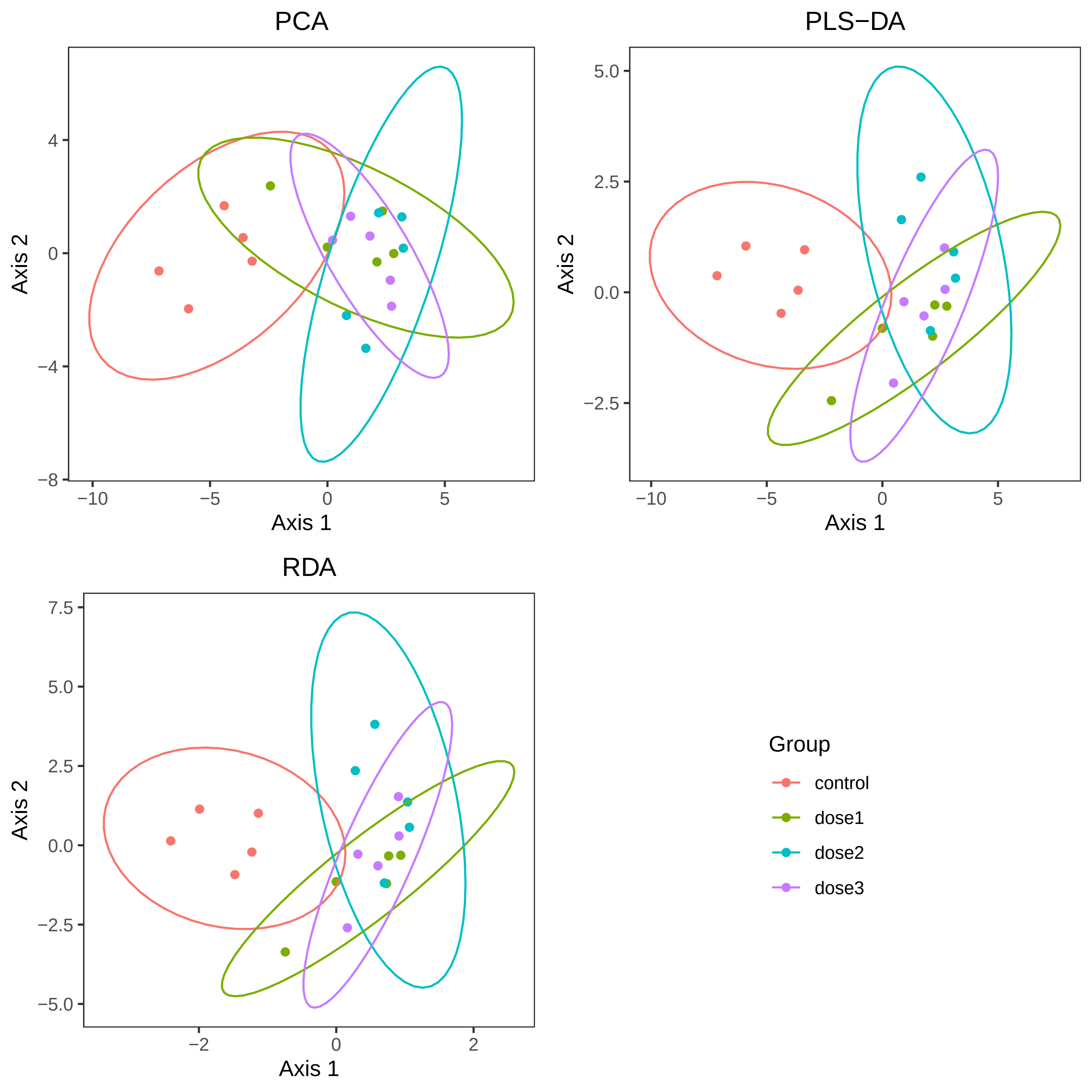
PCA
Axis 1
Axis 2
PLS−DA
Axis 1
Axis 2
RDA
Axis 1
Axis 2
Group
control
dose1
dose2
dose3

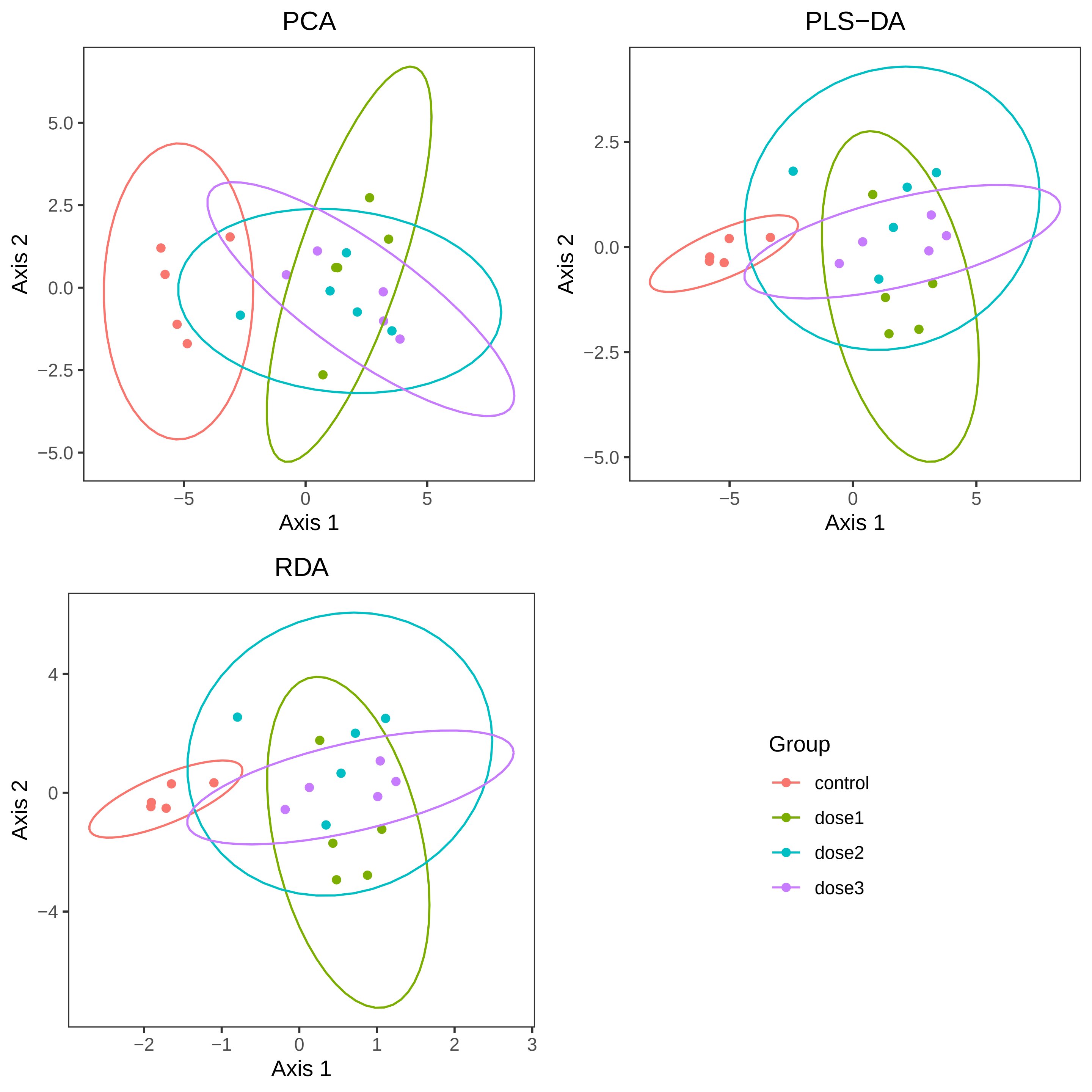

PCA
PLS−DA
RDA
Axis 1
Axis 2
5.0
2.5
0.0
−2.5
−5.0
−5
0
5
4
0
−4
−2
−1
1
2
3
Group
control
dose1
dose2
dose3

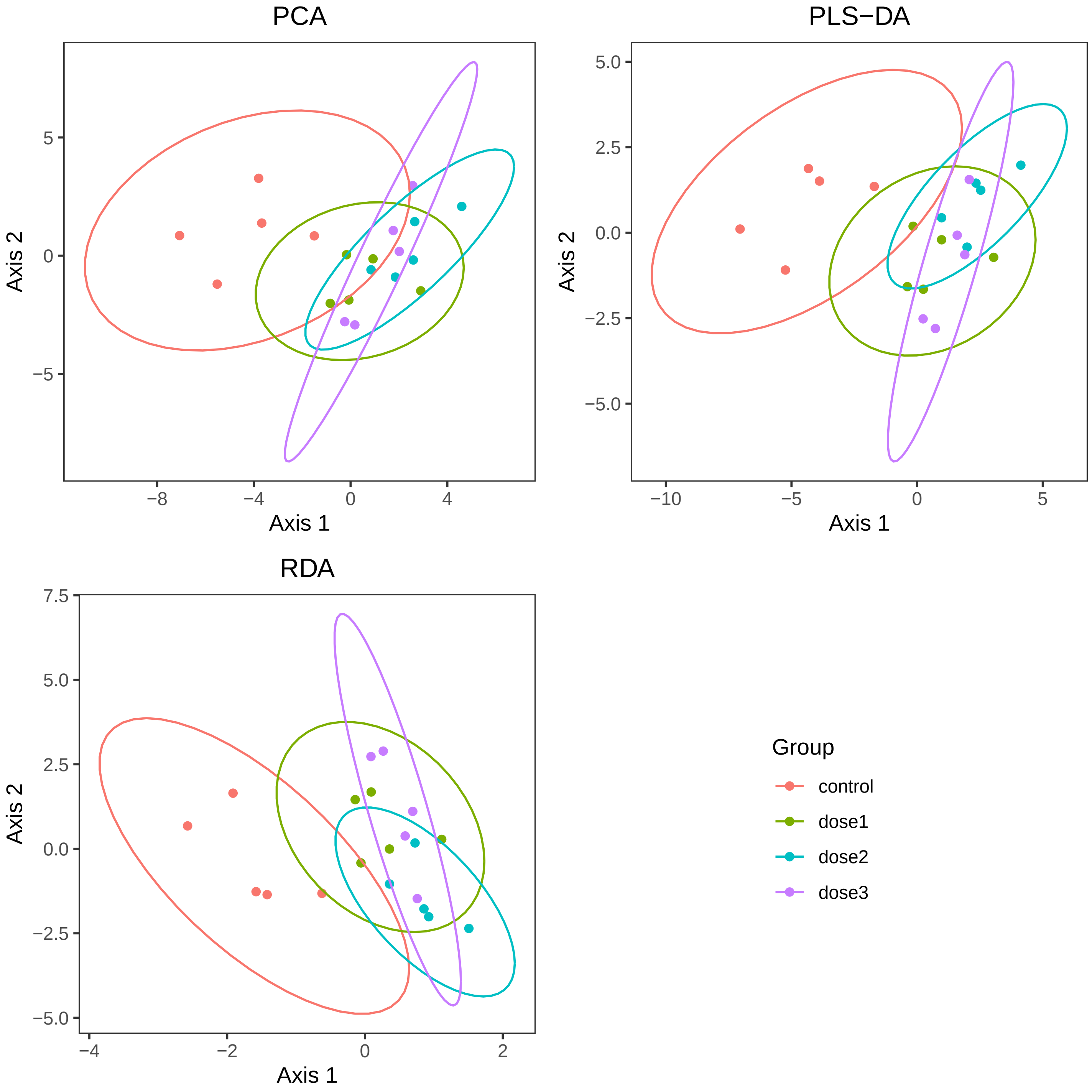
PCA
PLS−DA
RDA
Axis 1
Axis 2
Group
control
dose1
dose2
dose3

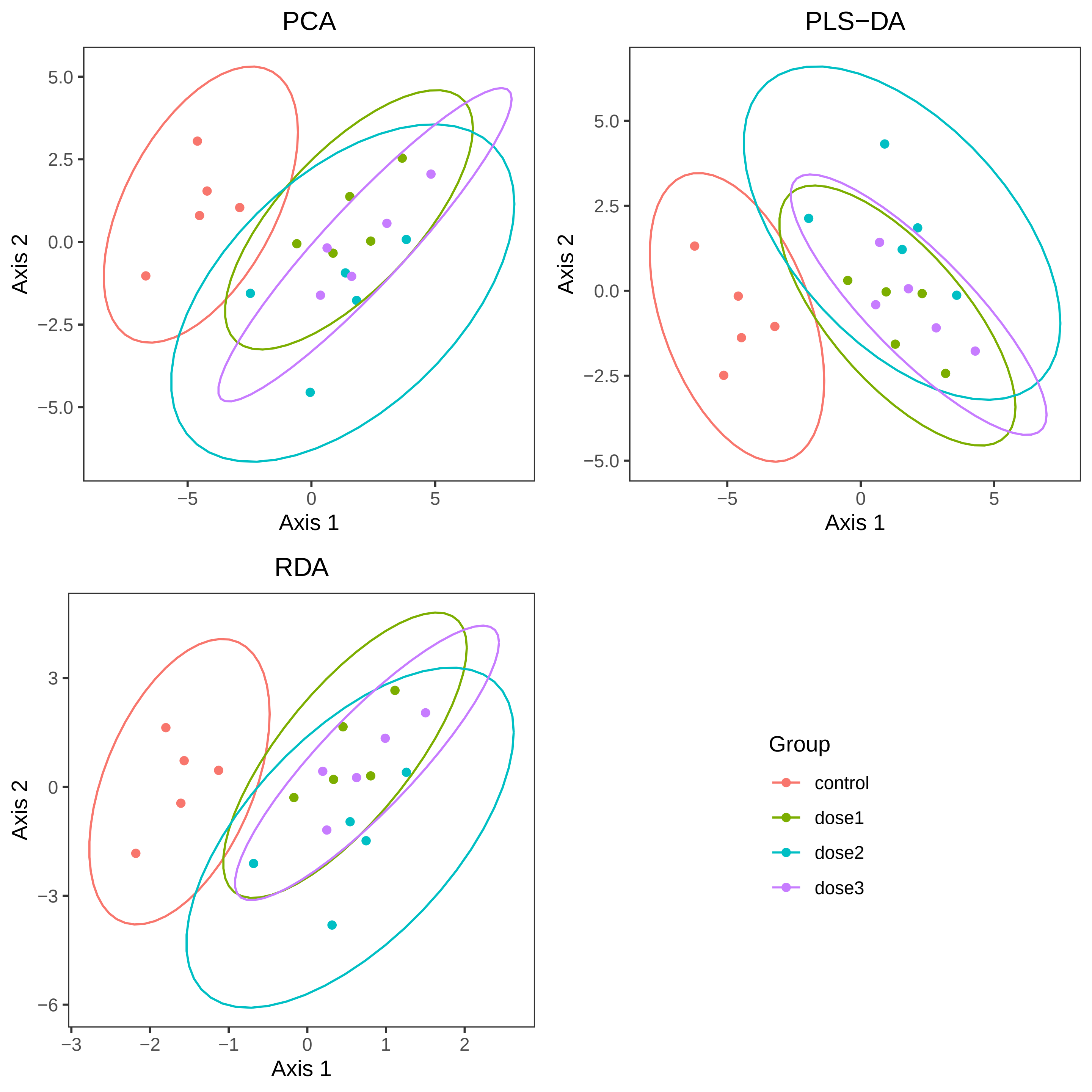

PCA
PLS−DA
RDA
Axis 1
Axis 2
5.0
2.5
0.0
−2.5
−5.0
−5
0
5
3
0
−3
−6
−3
−2
−1
1
2
Group
control
dose1
dose2
dose3

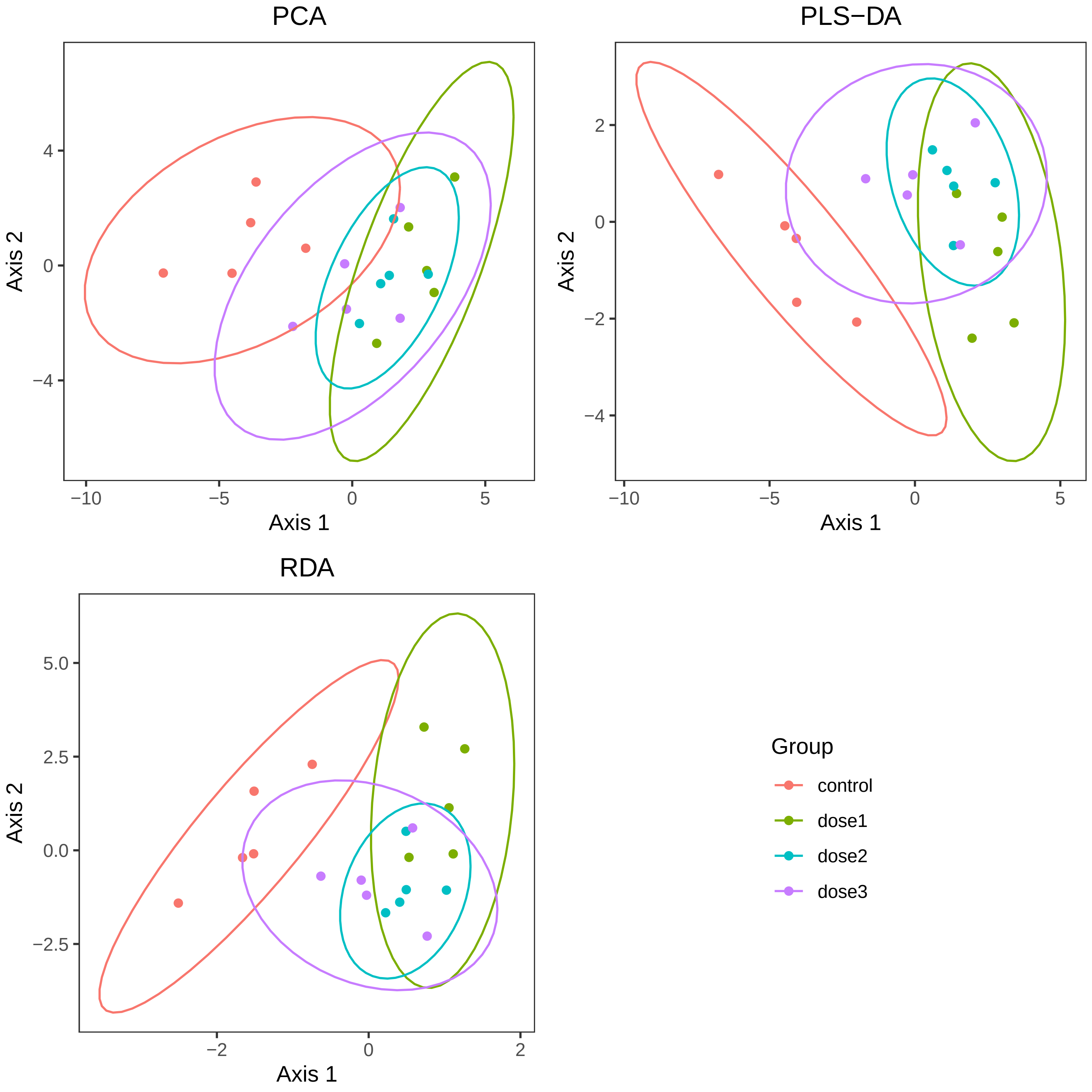

PCA
PLS−DA
RDA
Axis 1
Axis 2
−10
−5
0
5
4
0
−4
2
−2
5.0
2.5
0.0
−2.5
Group
control
dose1
dose2
dose3

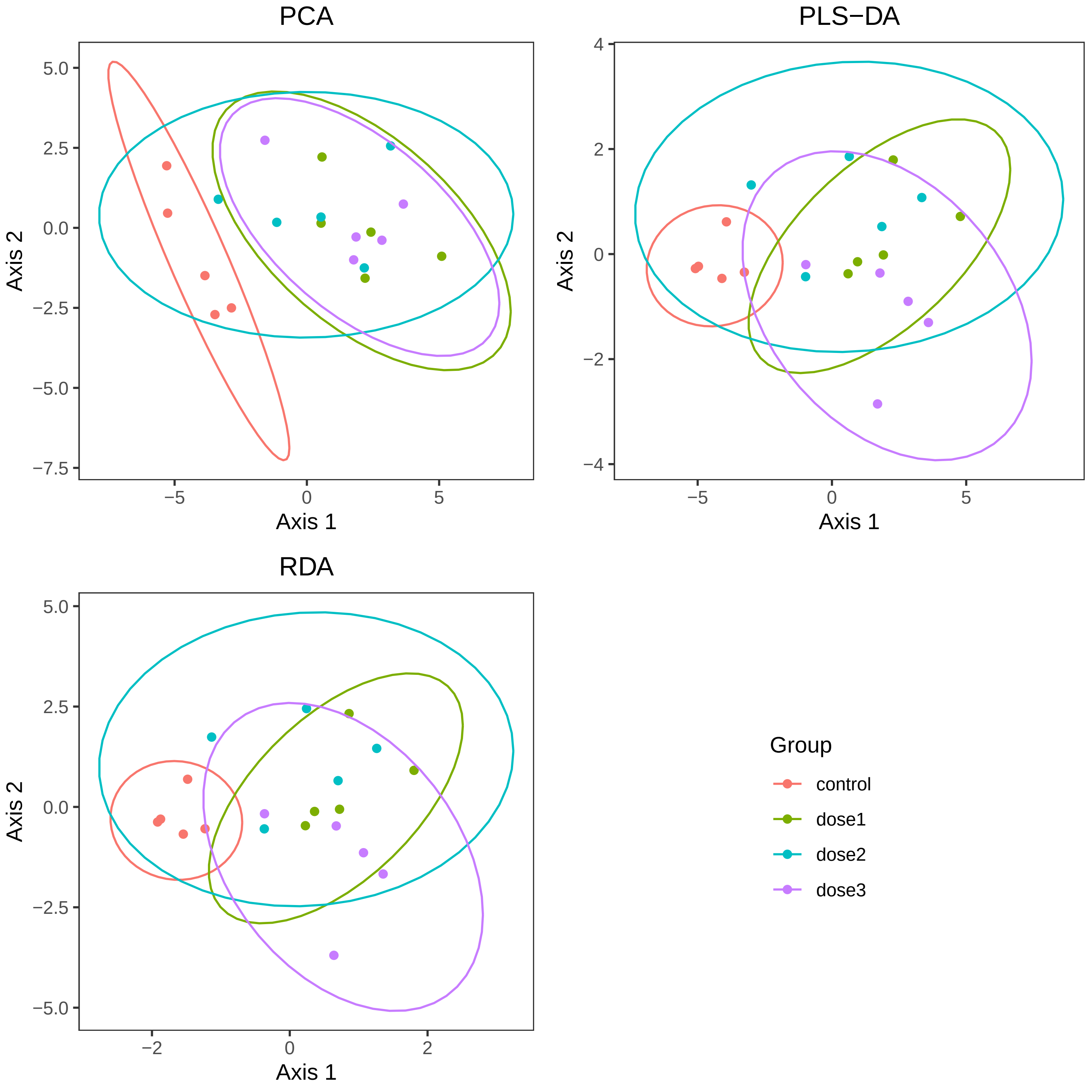
PCA
PLS−DA
RDA
Axis 1
Axis 2
Group
control
dose1
dose2
dose3

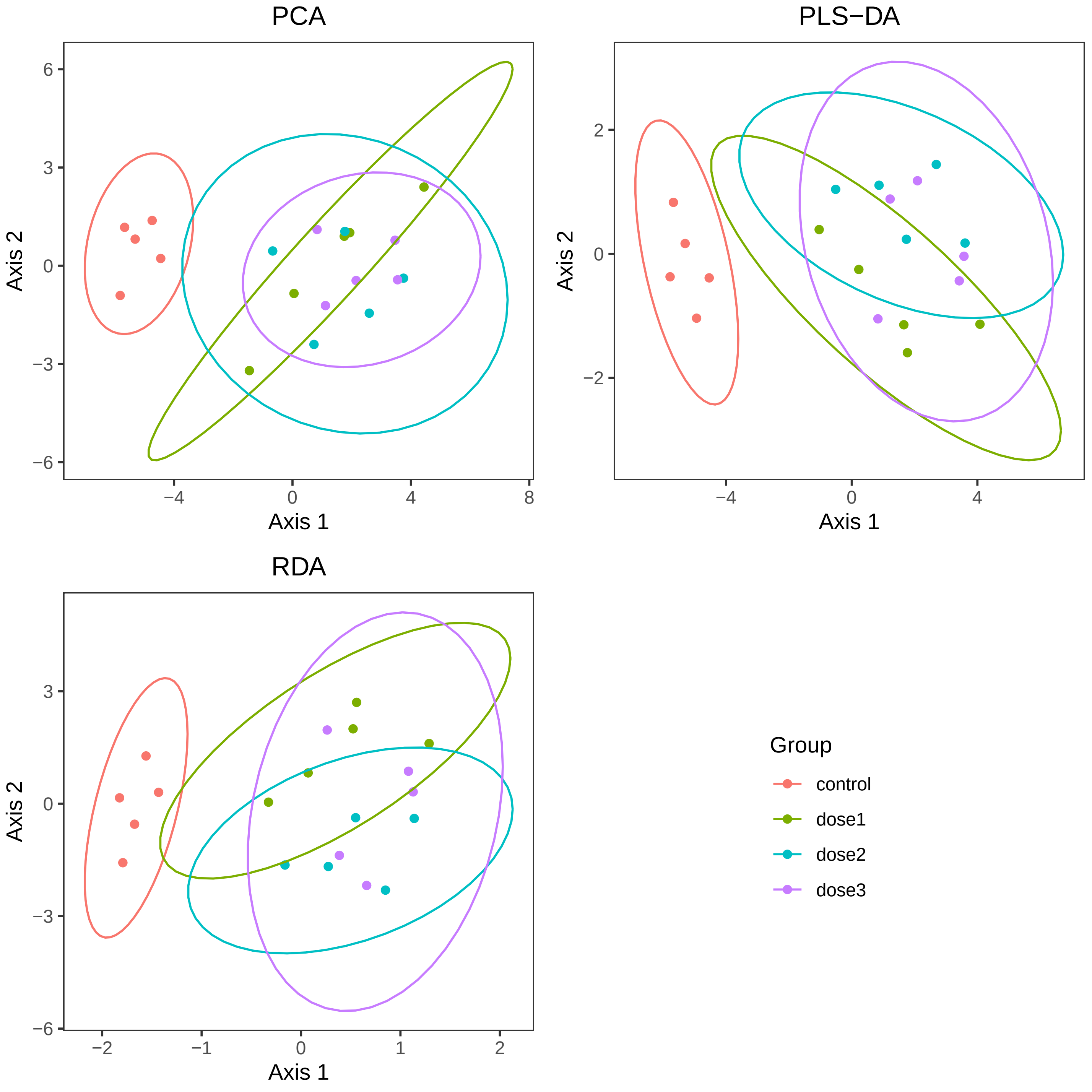

PCA
PLS−DA
RDA
Axis 1
Axis 2
Group
control
dose1
dose2
dose3

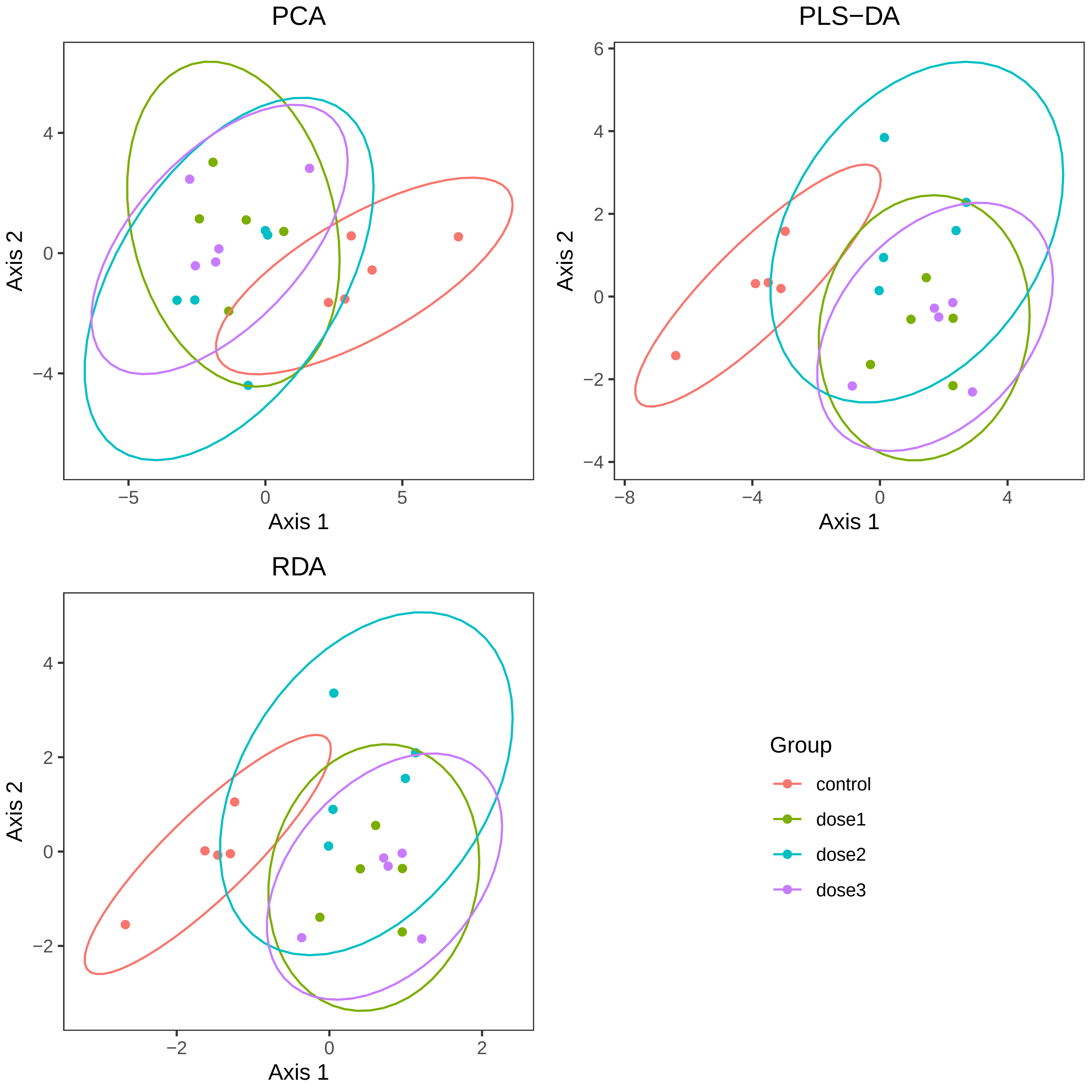

PCA
PLS−DA
RDA
Axis 1
Axis 2
Group
control
dose1
dose2
dose3

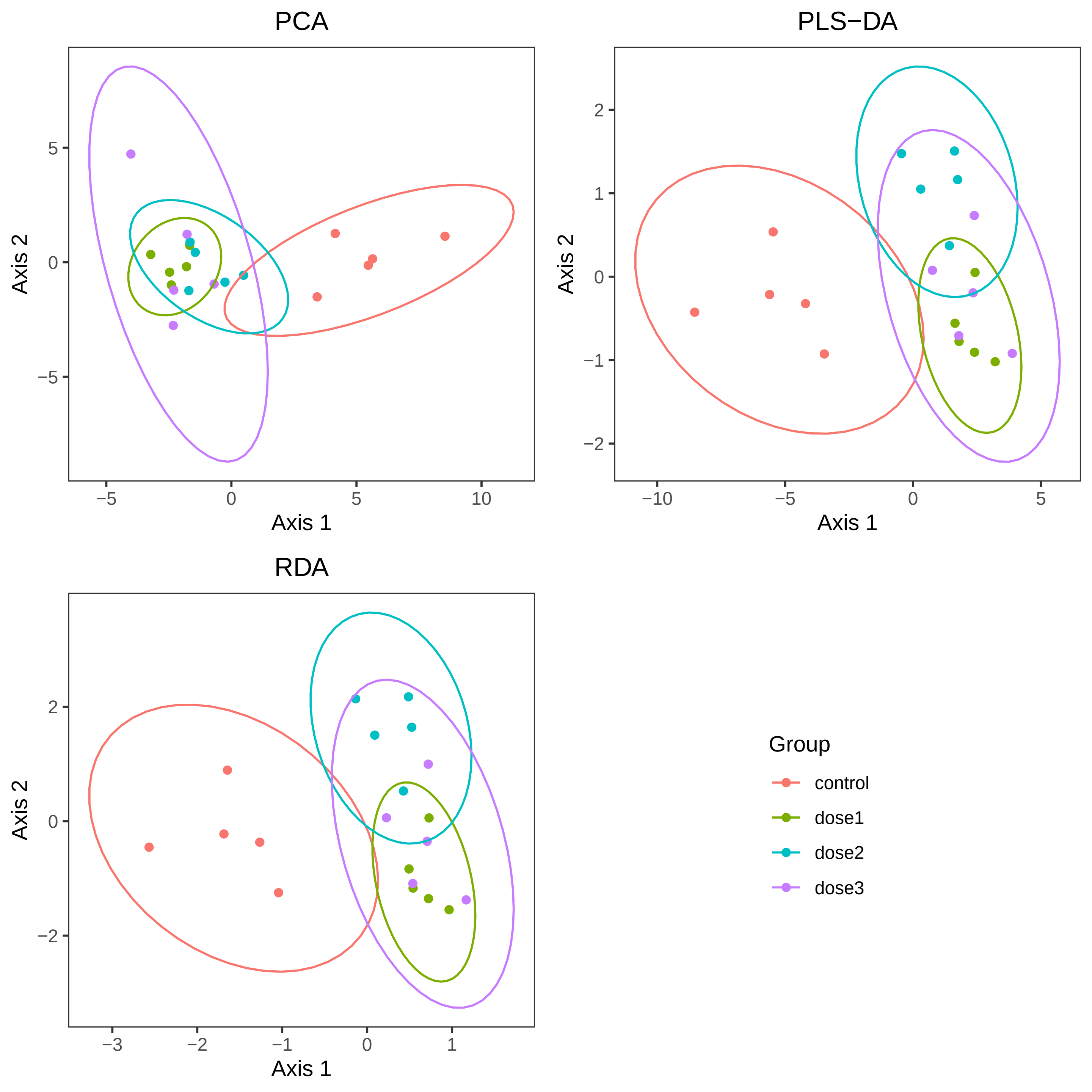

PCA
PLS−DA
RDA
Axis 1
Axis 2
Group
control
dose1
dose2
dose3

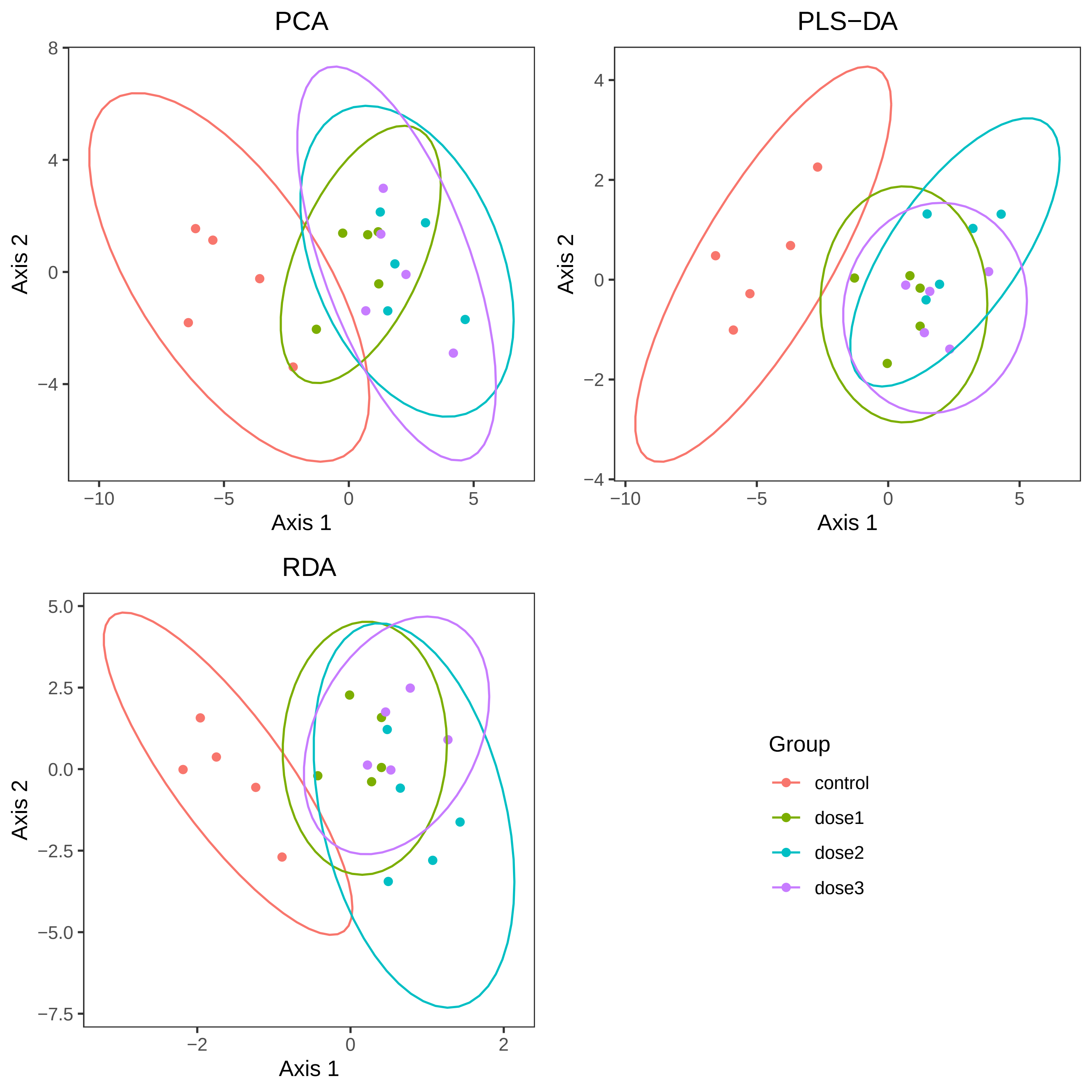

PCA
PLS−DA
RDA
Axis 1
Axis 2
Group
control
dose1
dose2
dose3

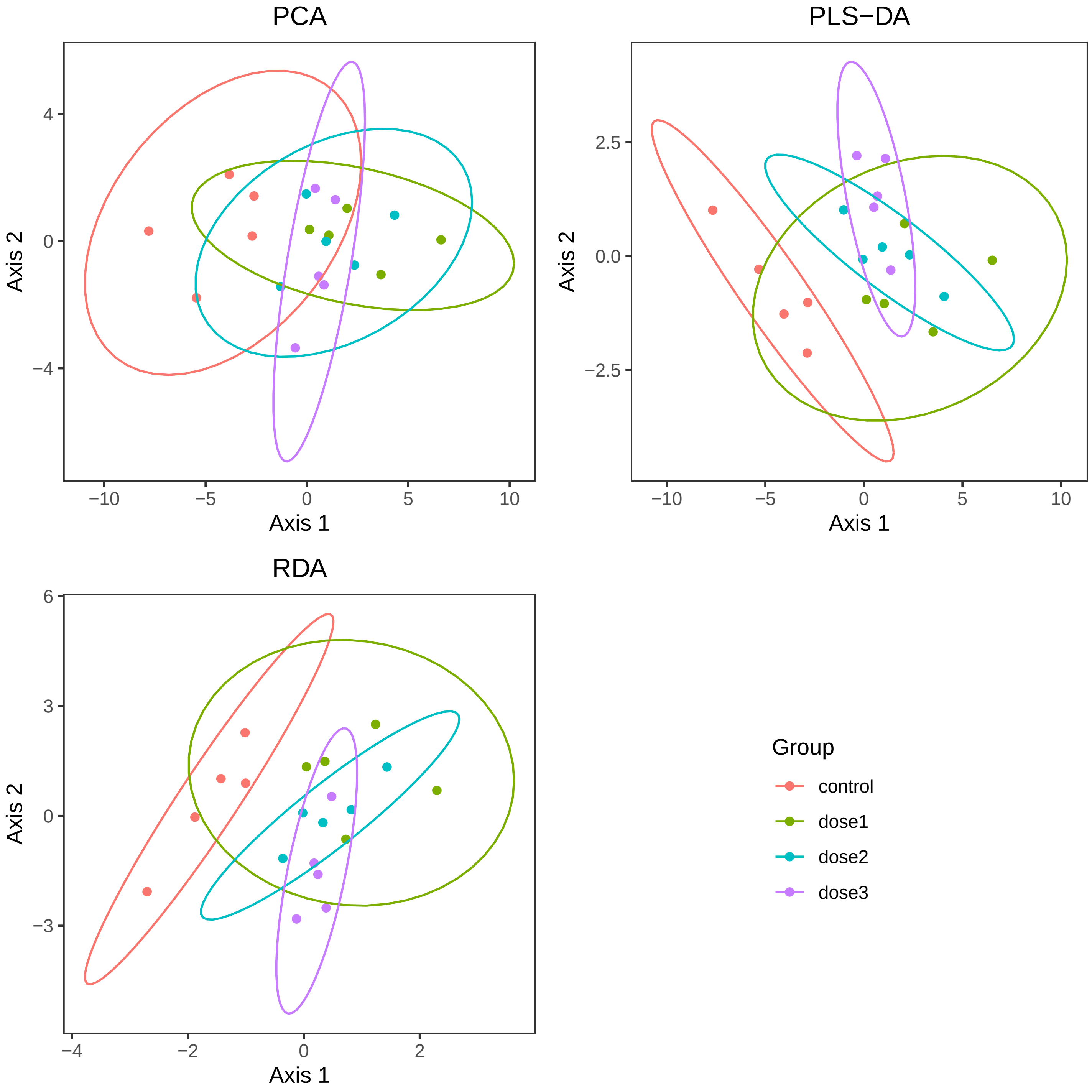
PCA
PLS−DA
RDA
Axis 1
Axis 2
Group
control
dose1
dose2
dose3

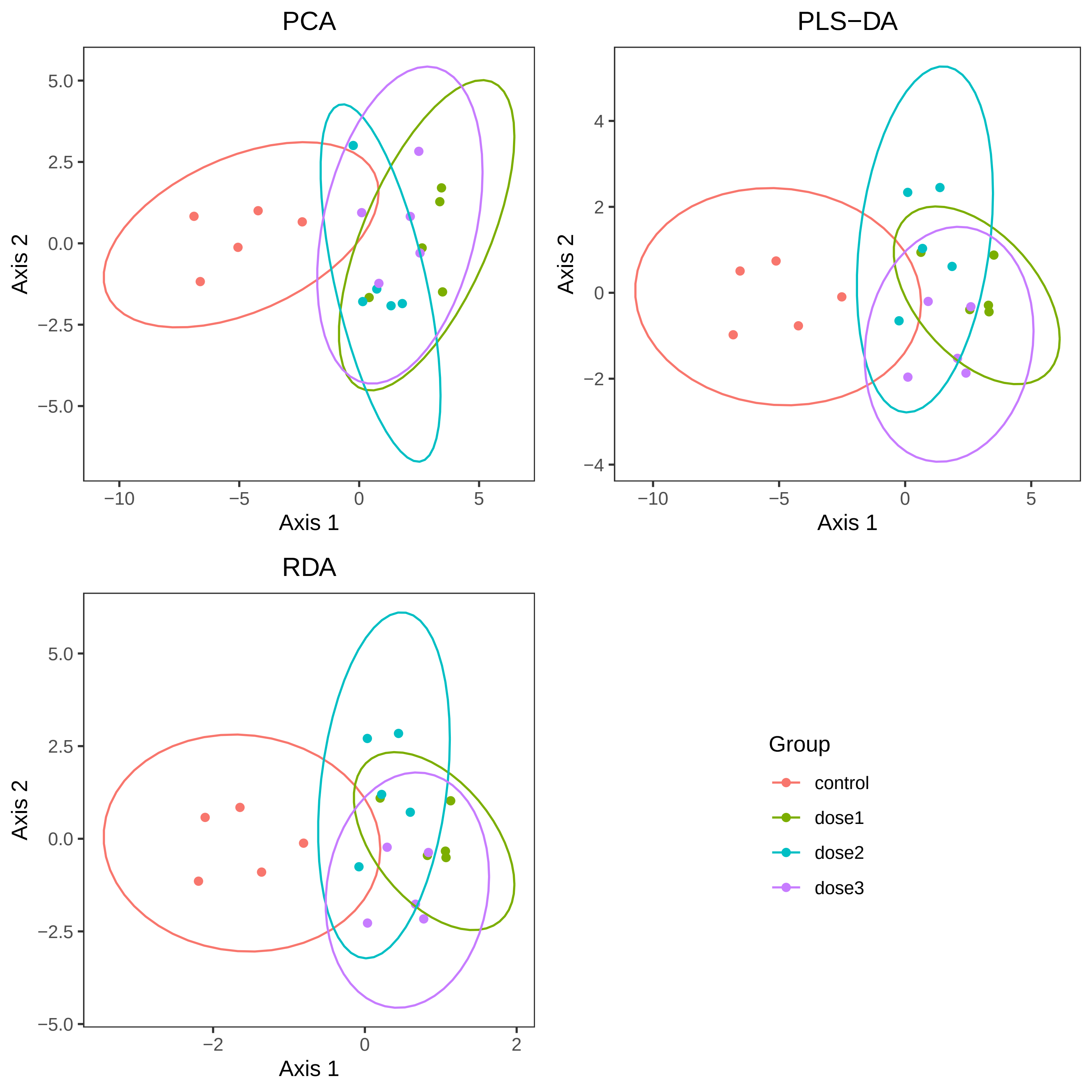

PCA
PLS−DA
RDA
Axis 1
Axis 2
Group
control
dose1
dose2
dose3

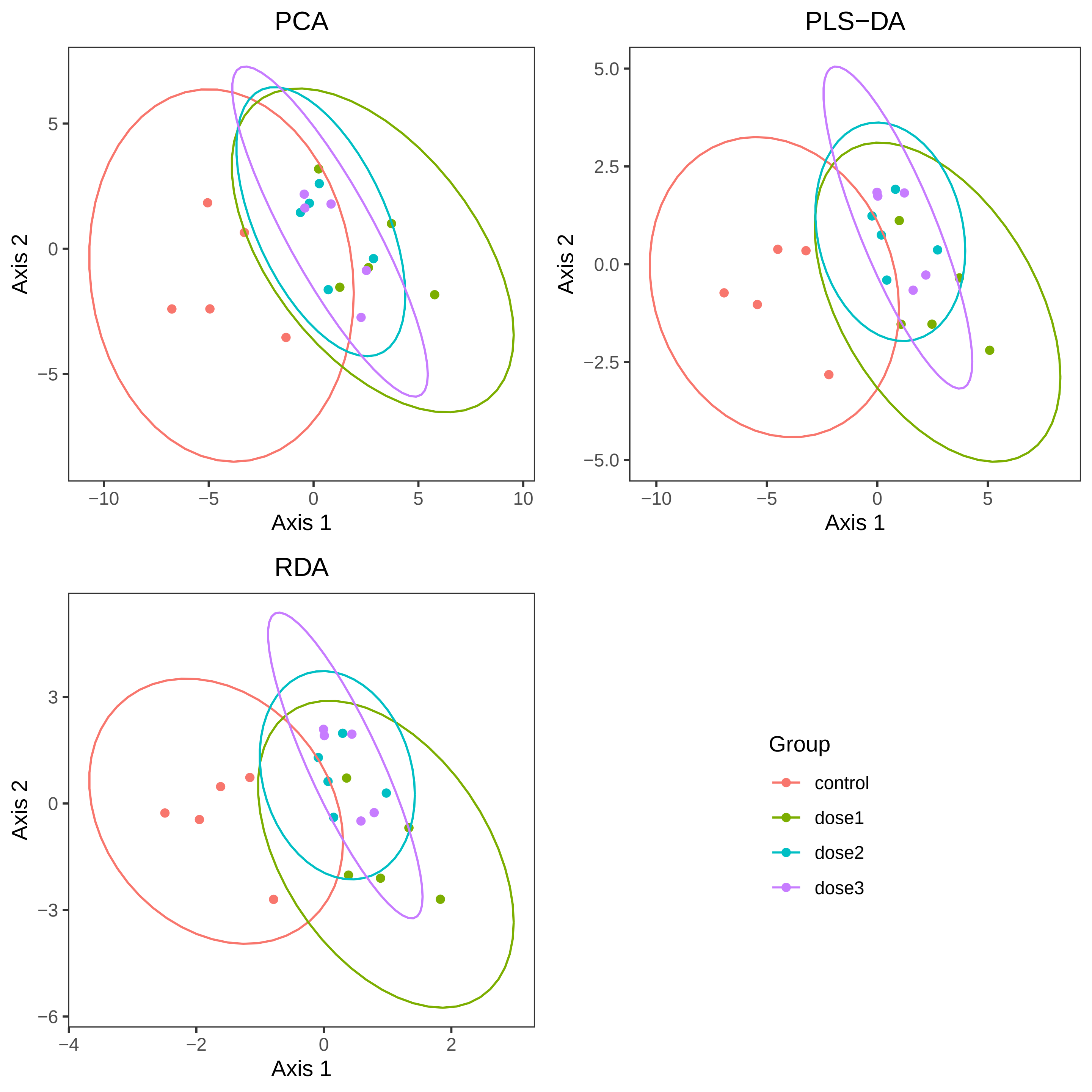
PCA
PLS−DA
RDA
Axis 1
Axis 2
Group
control
dose1
dose2
dose3

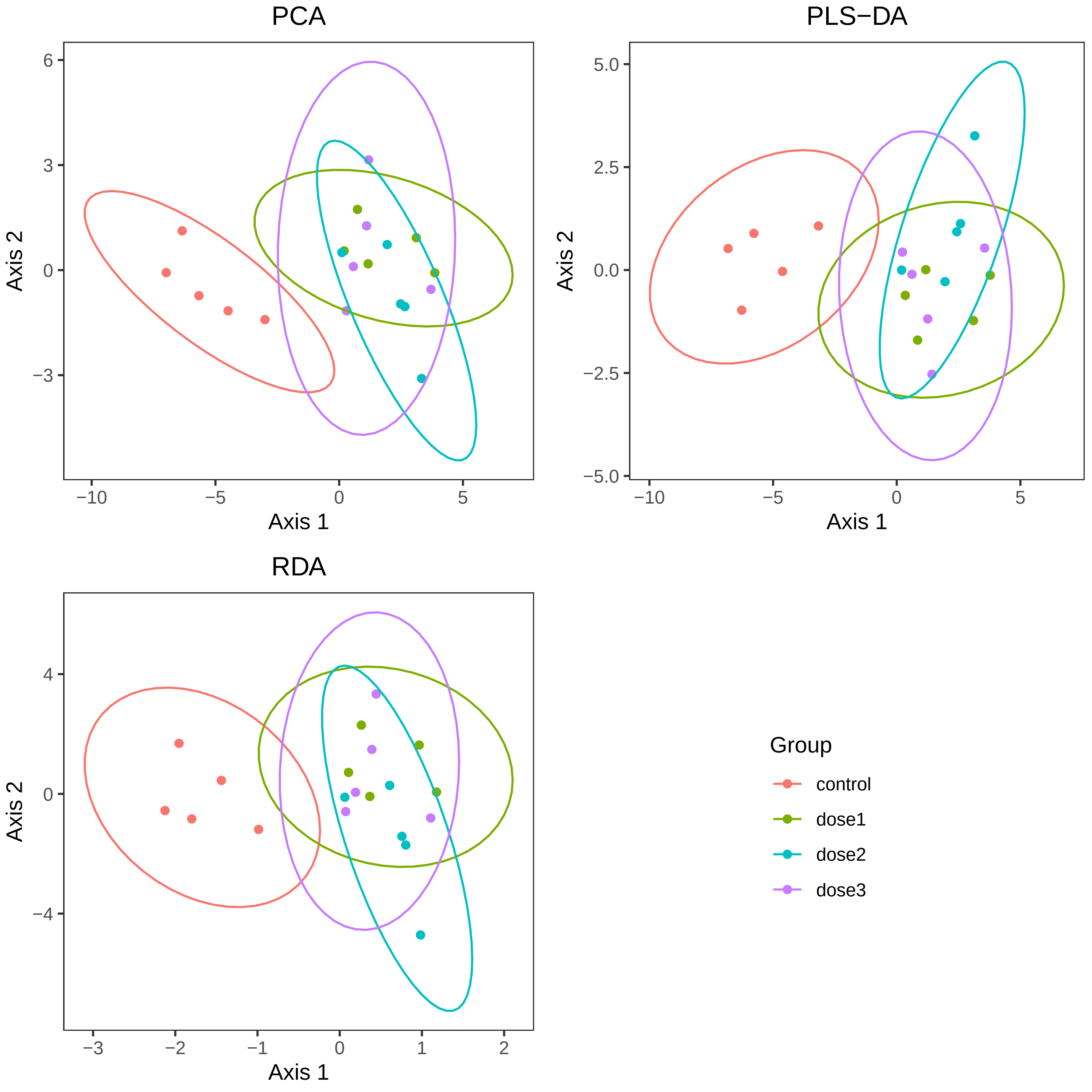
PCA
PLS−DA
RDA
Axis 1
Axis 2
−10
−5
0
5
6
3
0
−3
5.0
2.5
0.0
−2.5
−5.0
−3
−2
−1
1
2
4
−4
Group
control
dose1
dose2
dose3

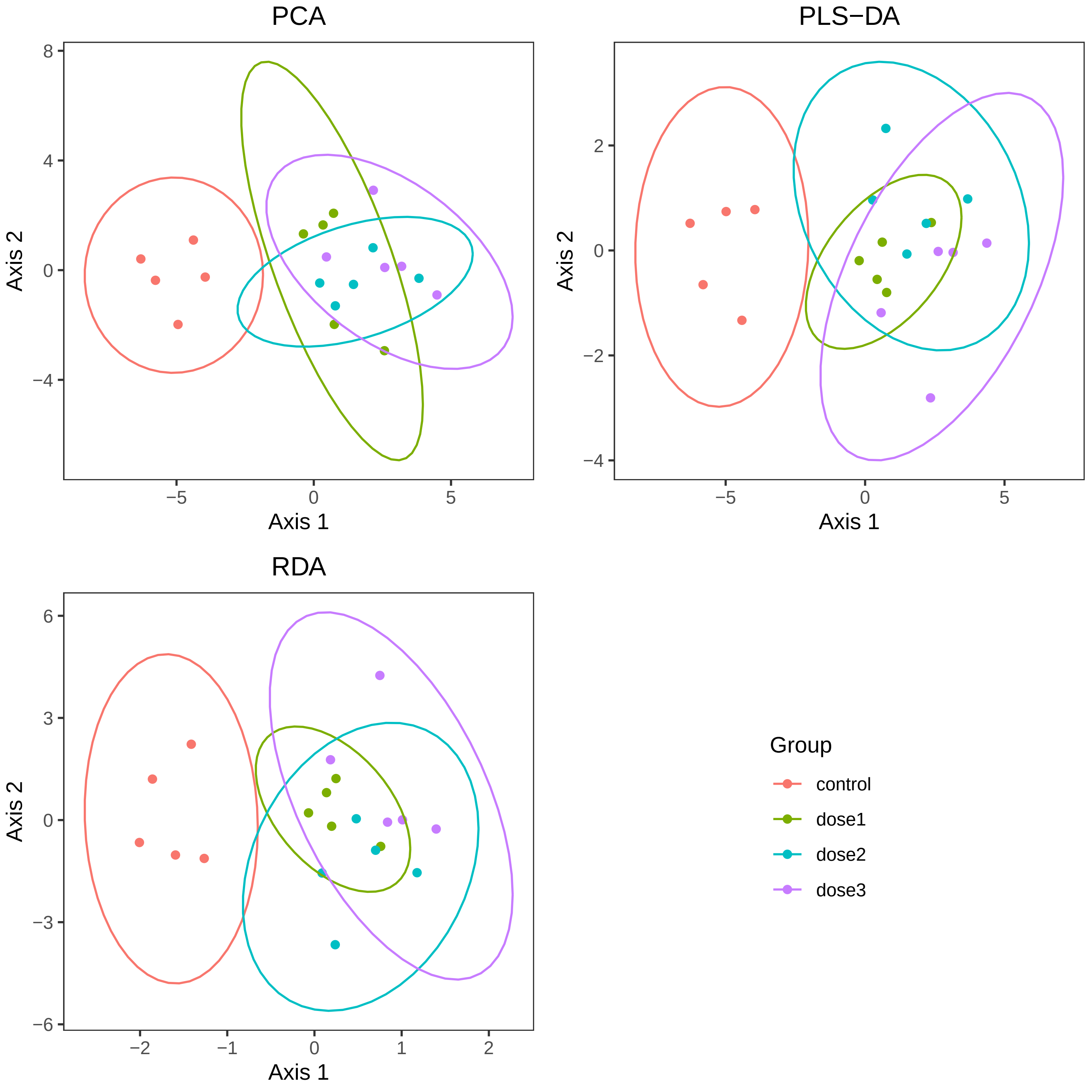
PCA
PLS−DA
RDA
Axis 1
Axis 2
Group
control
dose1
dose2
dose3

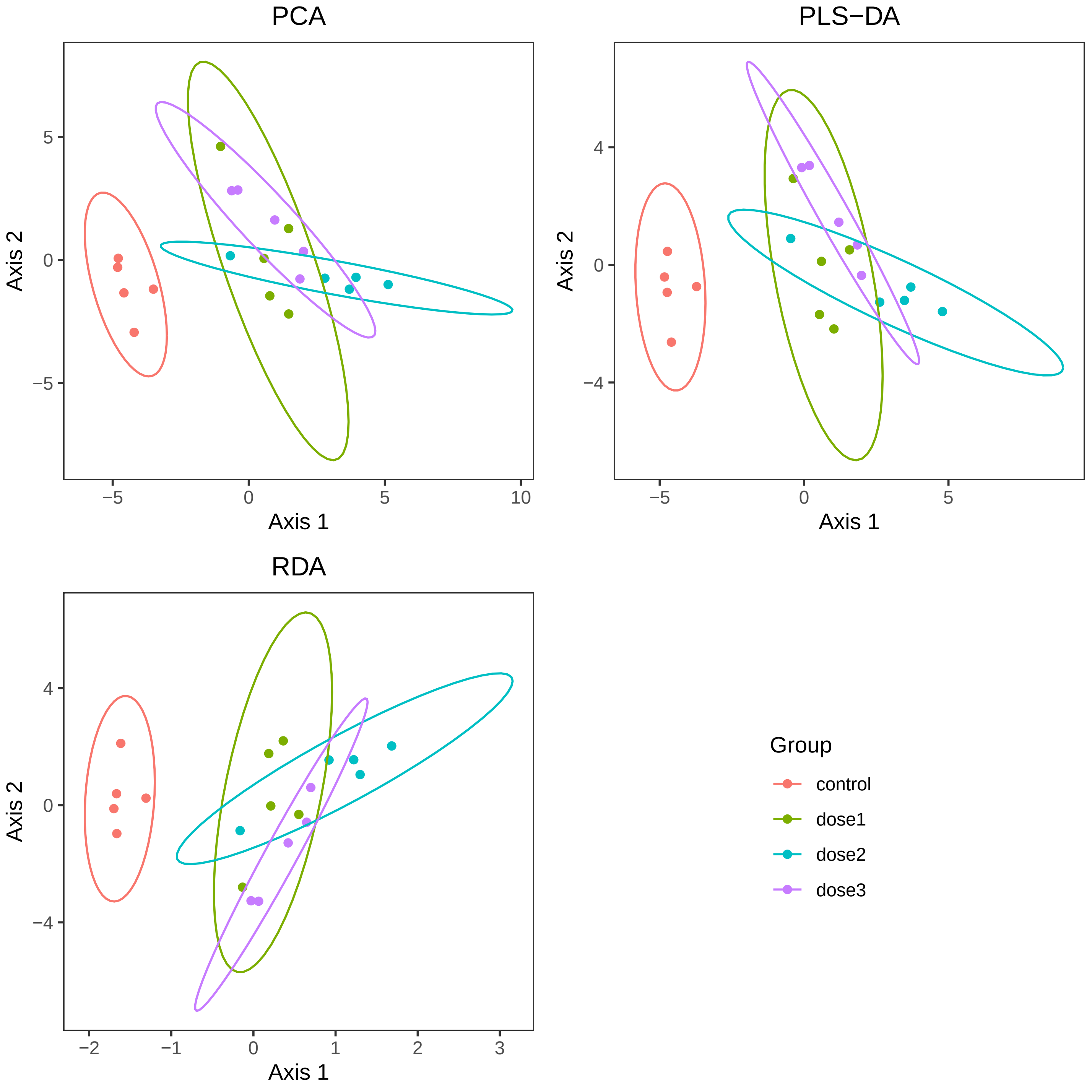

PCA
PLS−DA
RDA
Axis 1
Axis 2
−5
0
5
10
−2
−1
1
2
3
4
−4
Group
control
dose1
dose2
dose3

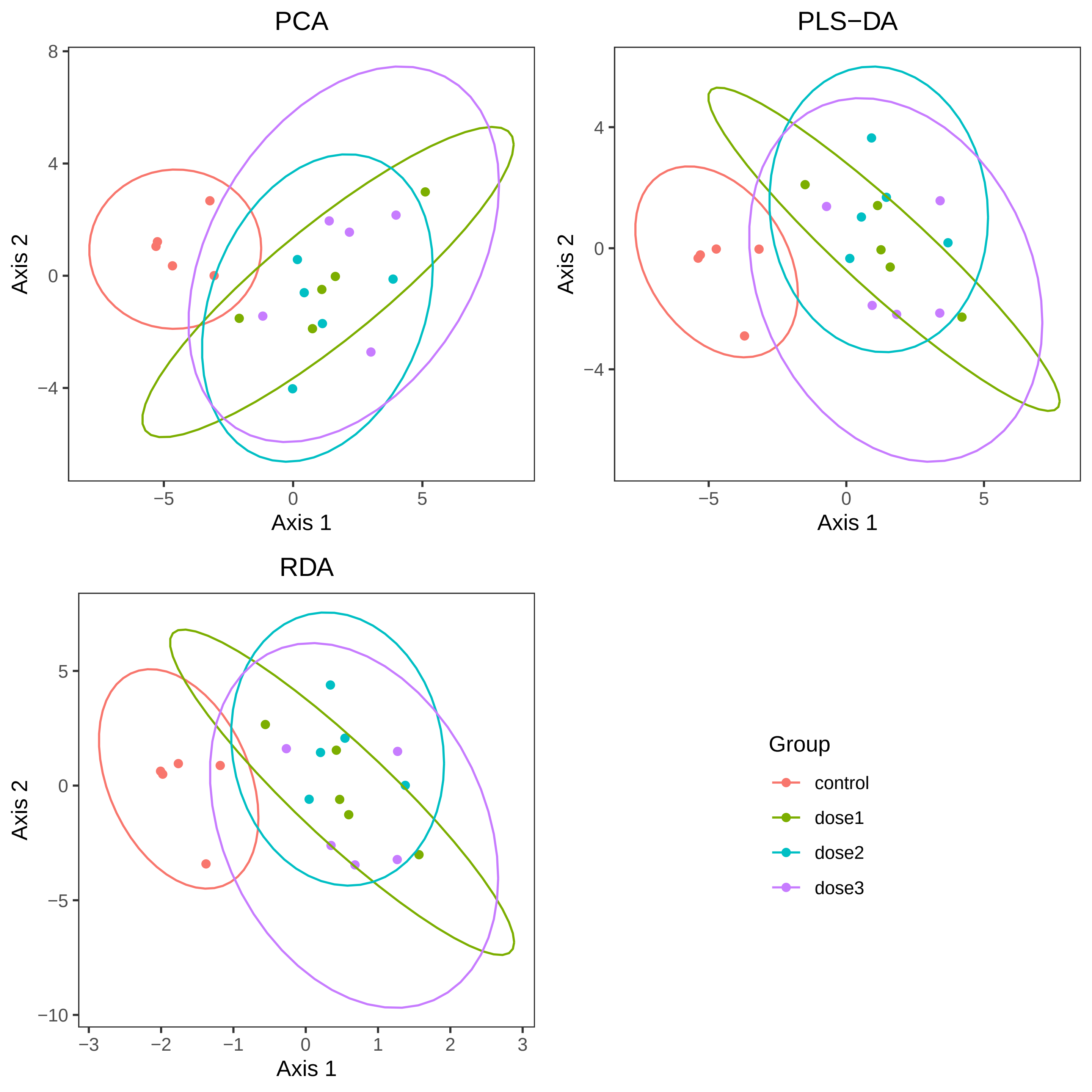
PCA
PLS−DA
RDA
Axis 1
Axis 2
Group
control
dose1
dose2
dose3

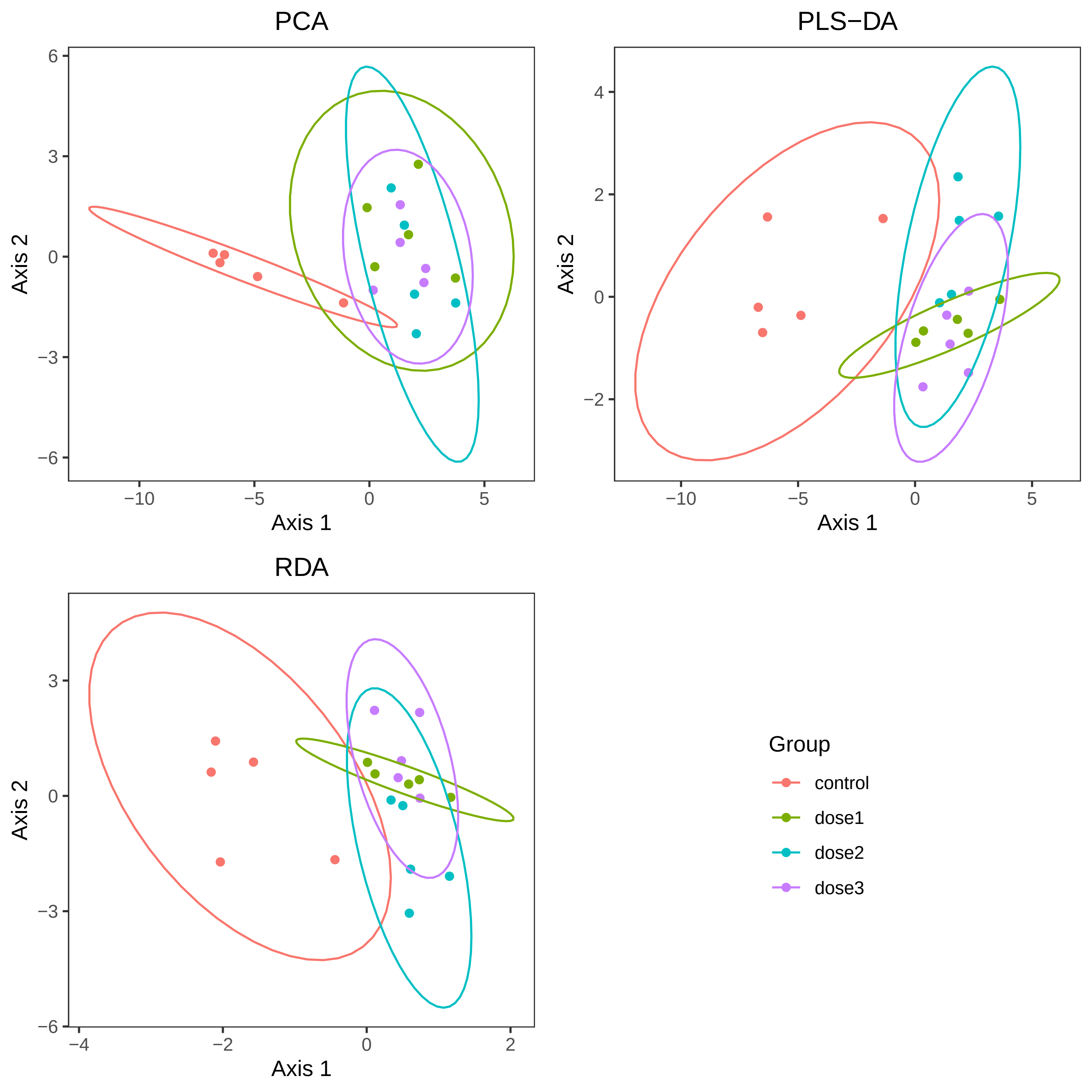

PCA
PLS−DA
RDA
Axis 1
Axis 2
Group
control
dose1
dose2
dose3

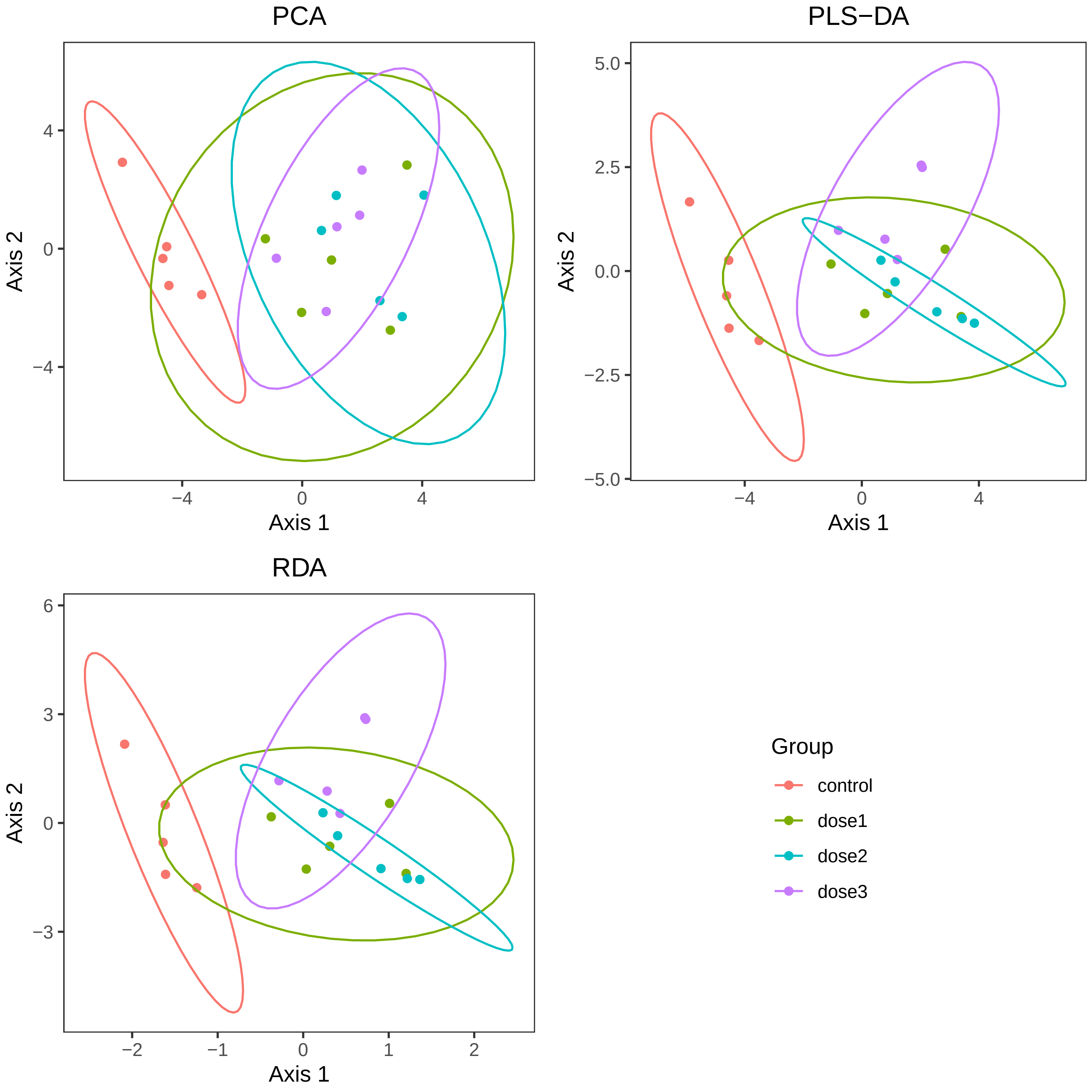
PCA
PLS−DA
RDA
Axis 1
Axis 2
Group
control
dose1
dose2
dose3

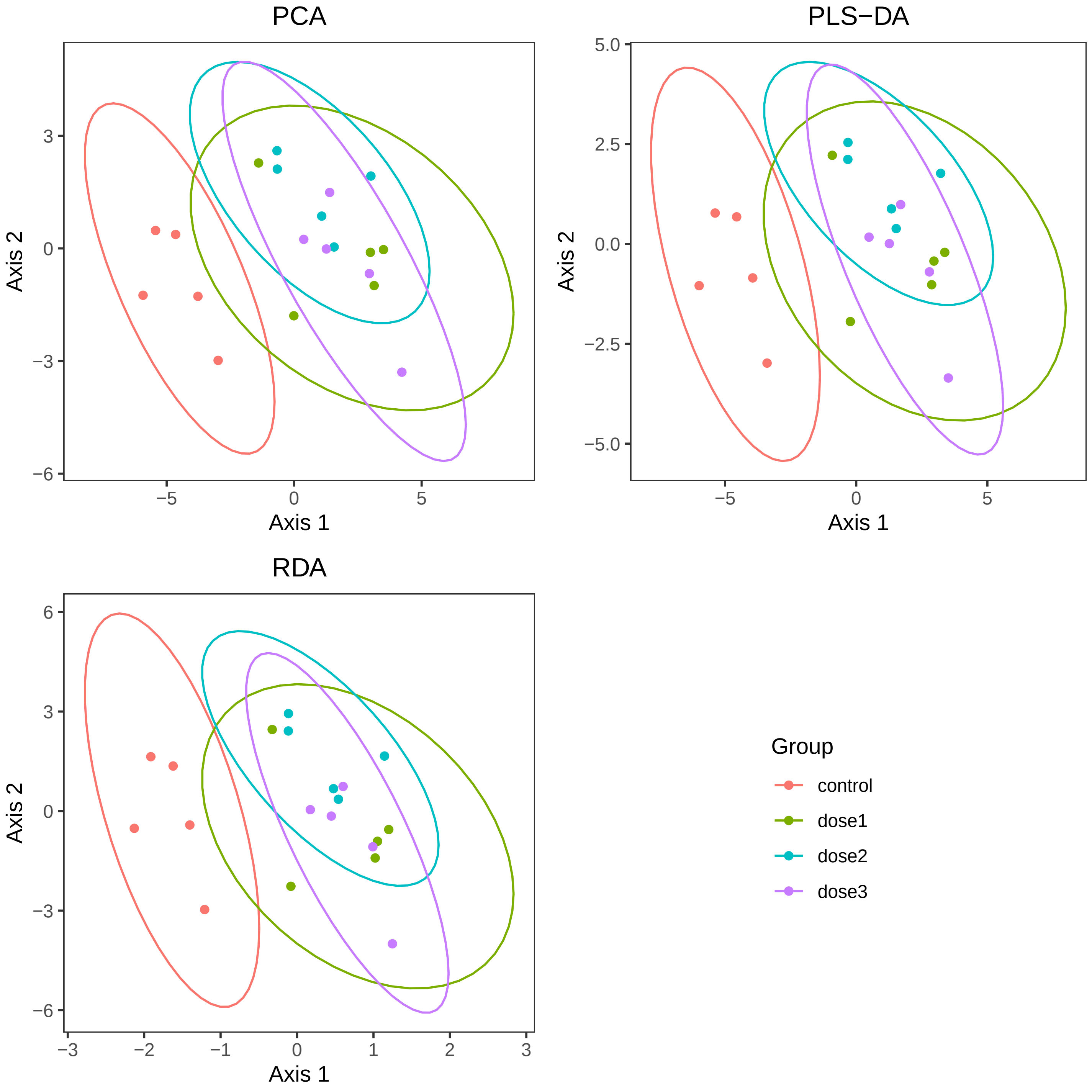

PCA
PLS−DA
RDA
Axis 1
Axis 2
Group
control
dose1
dose2
dose3

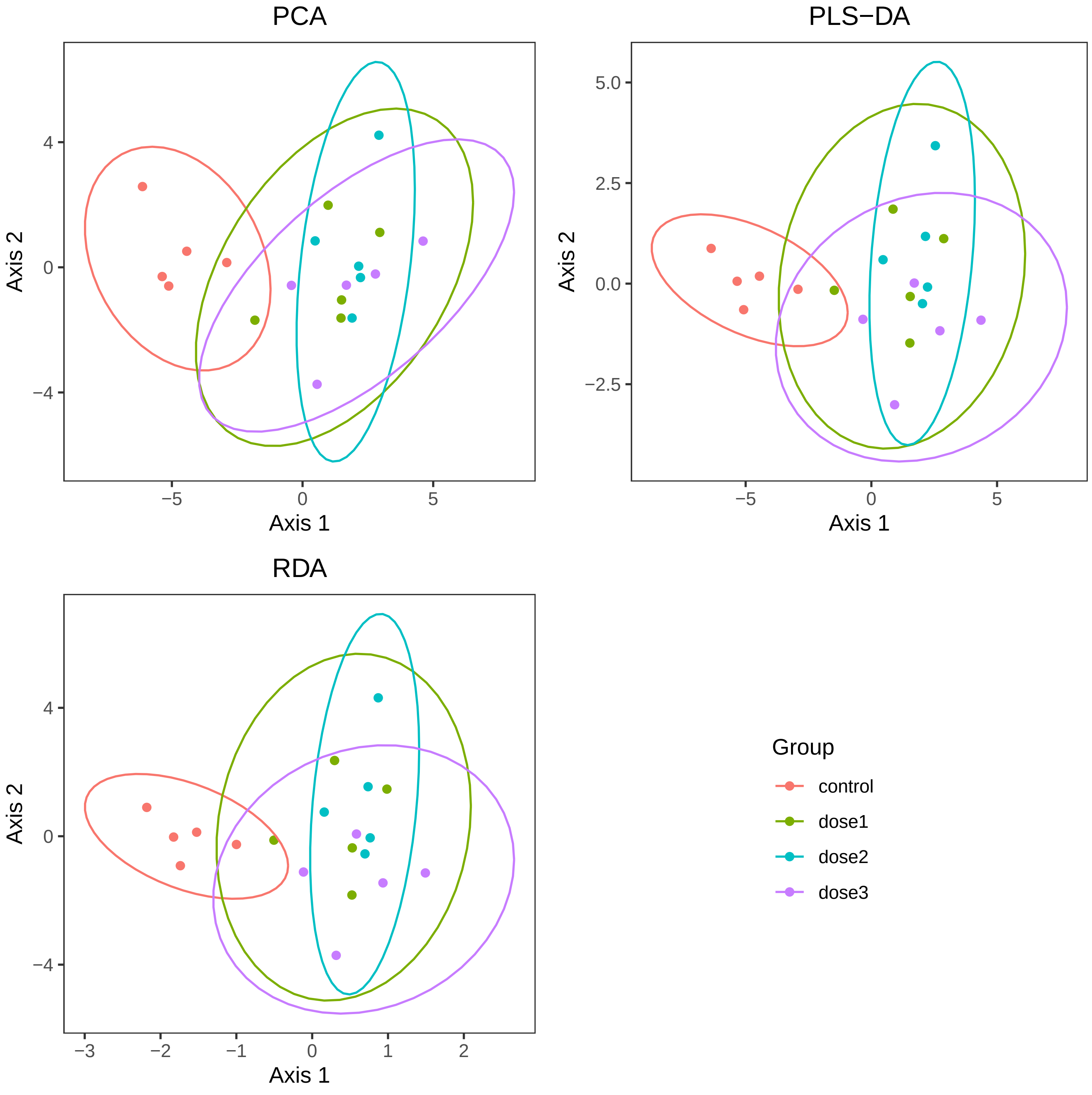
PCA
PLS−DA
RDA
Axis 1
Axis 2
−5
0
5
4
0
−4
5.0
2.5
0.0
−2.5
−3
−2
−1
1
2
Group
control
dose1
dose2
dose3

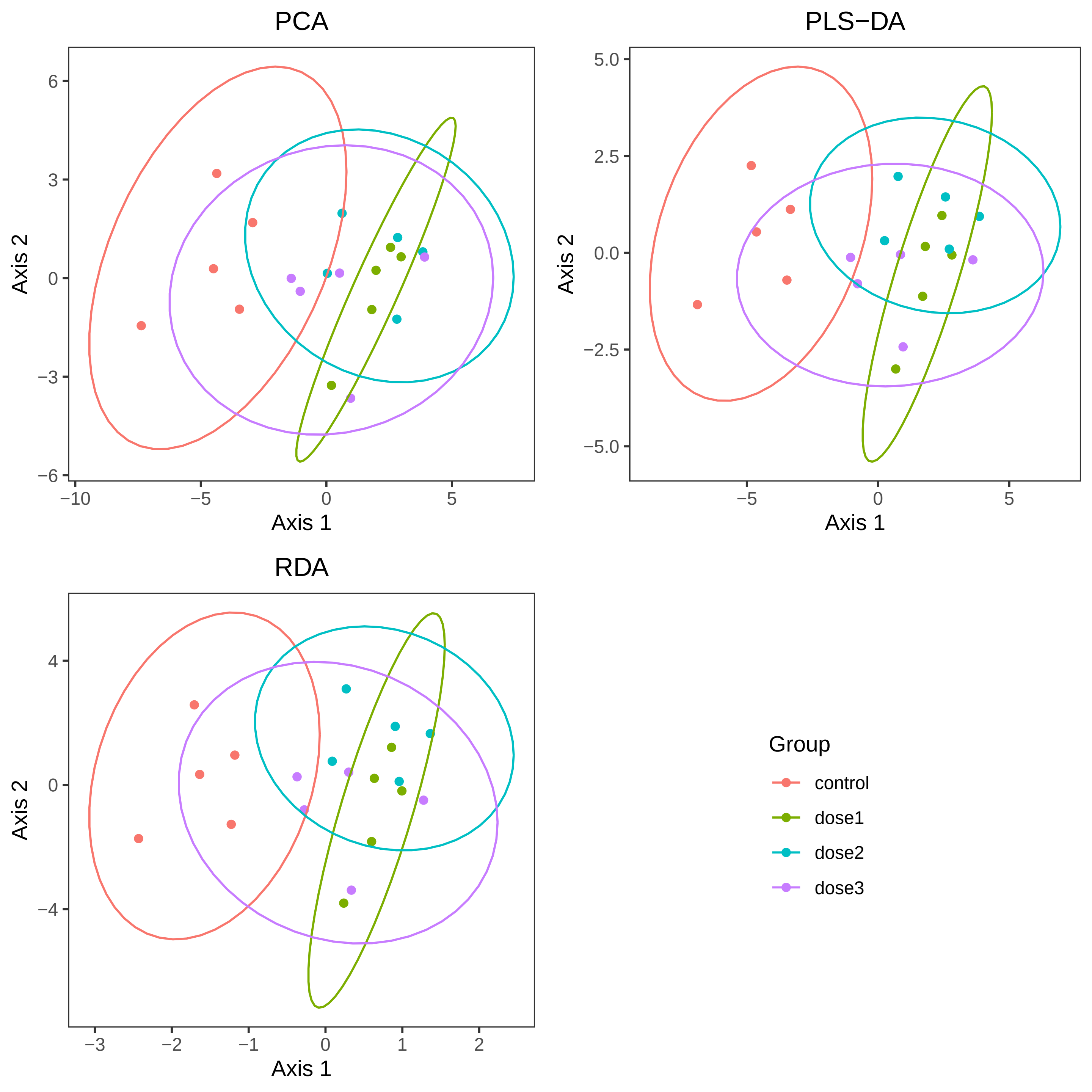

PCA
PLS−DA
RDA
Axis 1
Axis 2
Group
control
dose1
dose2
dose3

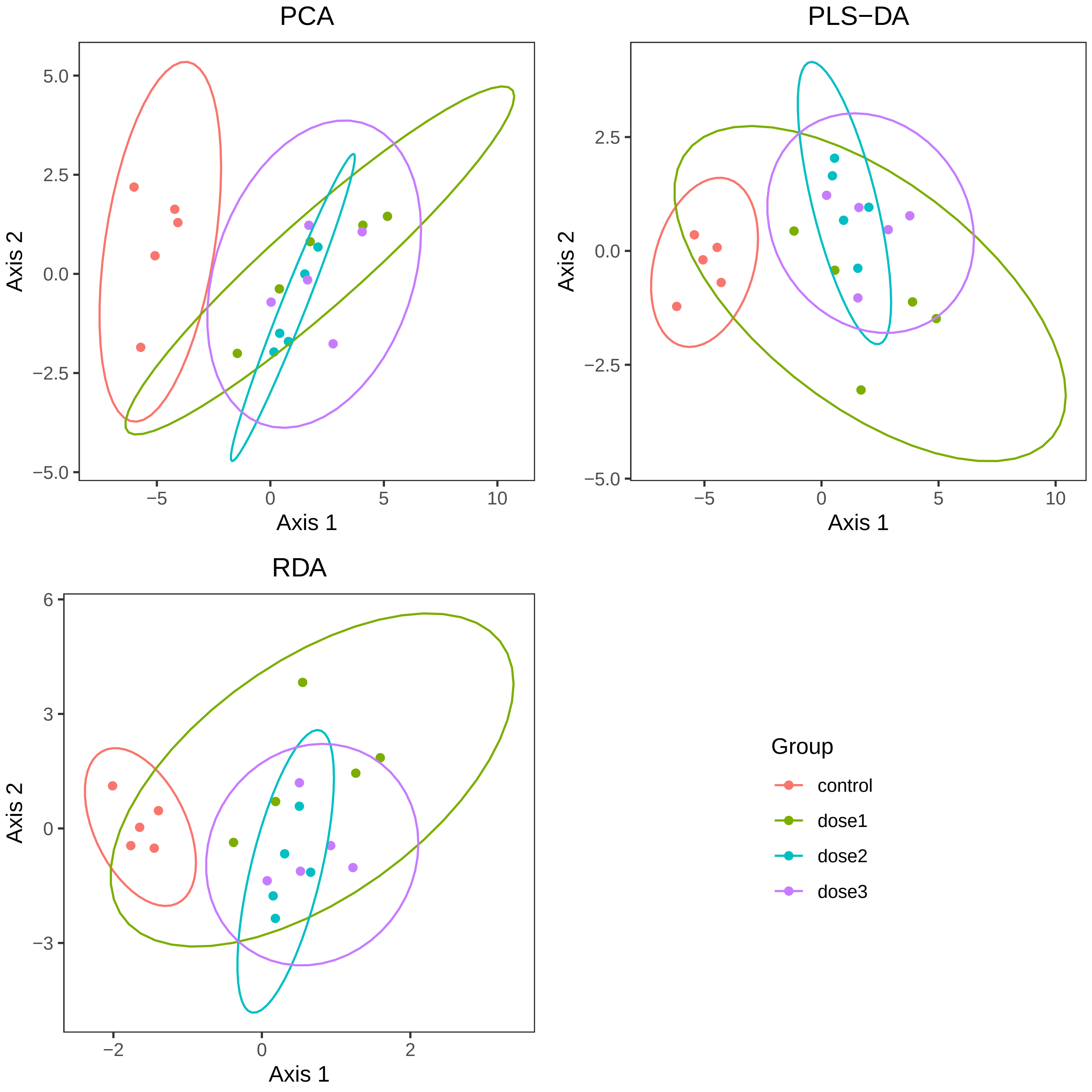

PCA
PLS−DA
RDA
Axis 1
Axis 2
Group
control
dose1
dose2
dose3

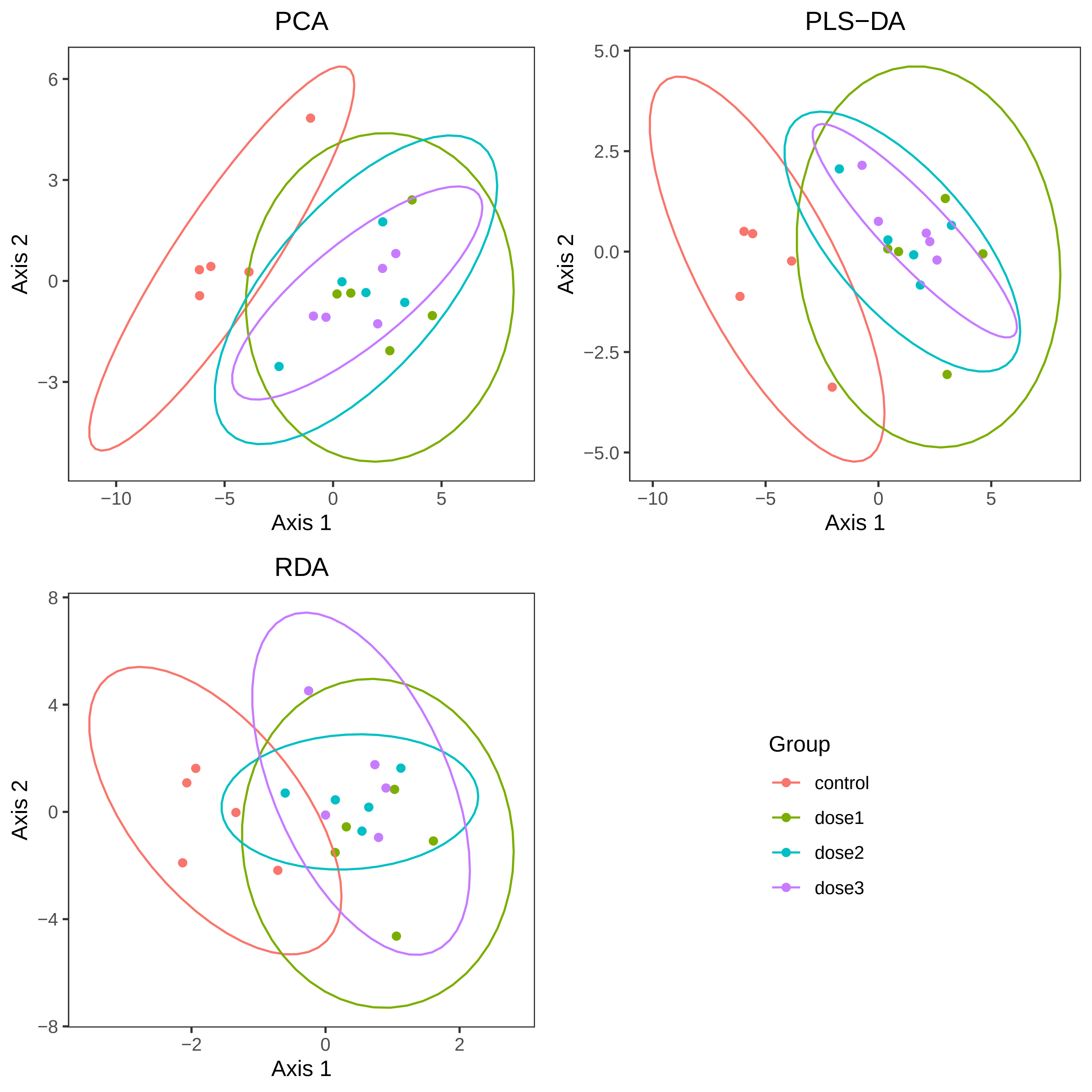
PCA
PLS−DA
RDA
Axis 1
Axis 2
Group
control
dose1
dose2
dose3

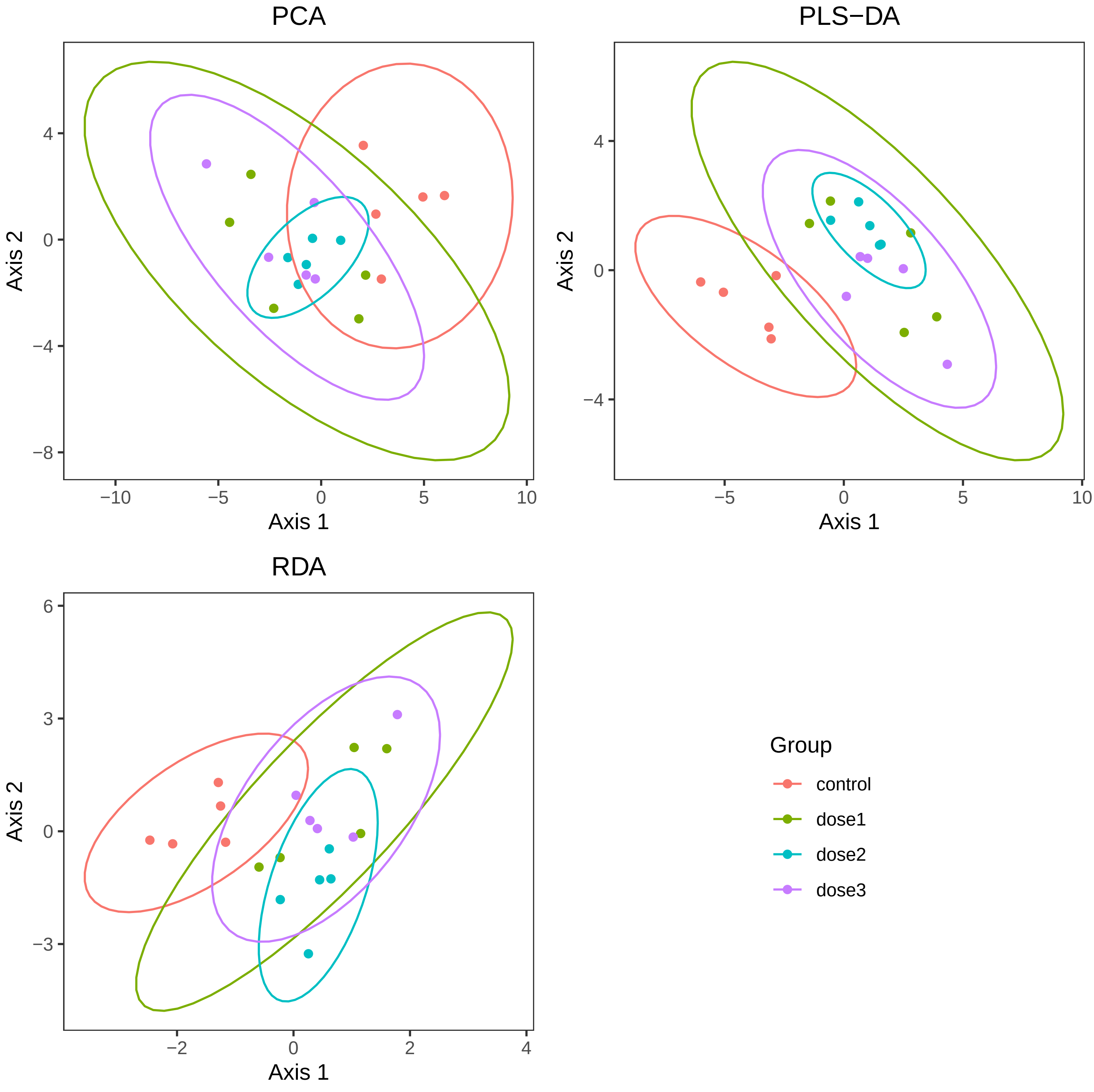
PCA
PLS−DA
RDA
Axis 1
Axis 2
Group
control
dose1
dose2
dose3

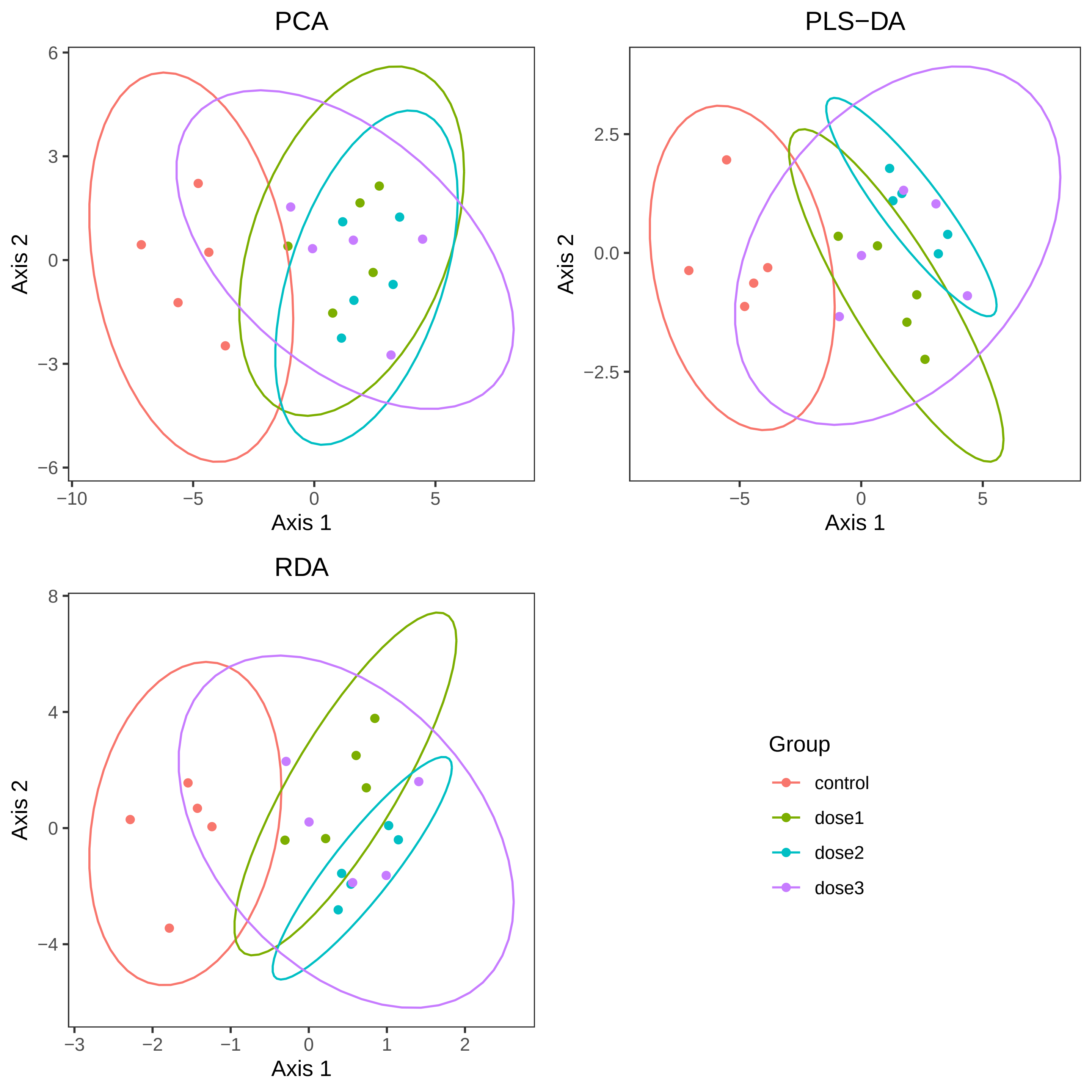
PCA
PLS−DA
RDA
Axis 1
Axis 2
6
3
0
−3
−6
−10
−5
0
5
2.5
0.0
−2.5
8
4
0
−4
−3
−2
−1
1
2
Group
control
dose1
dose2
dose3

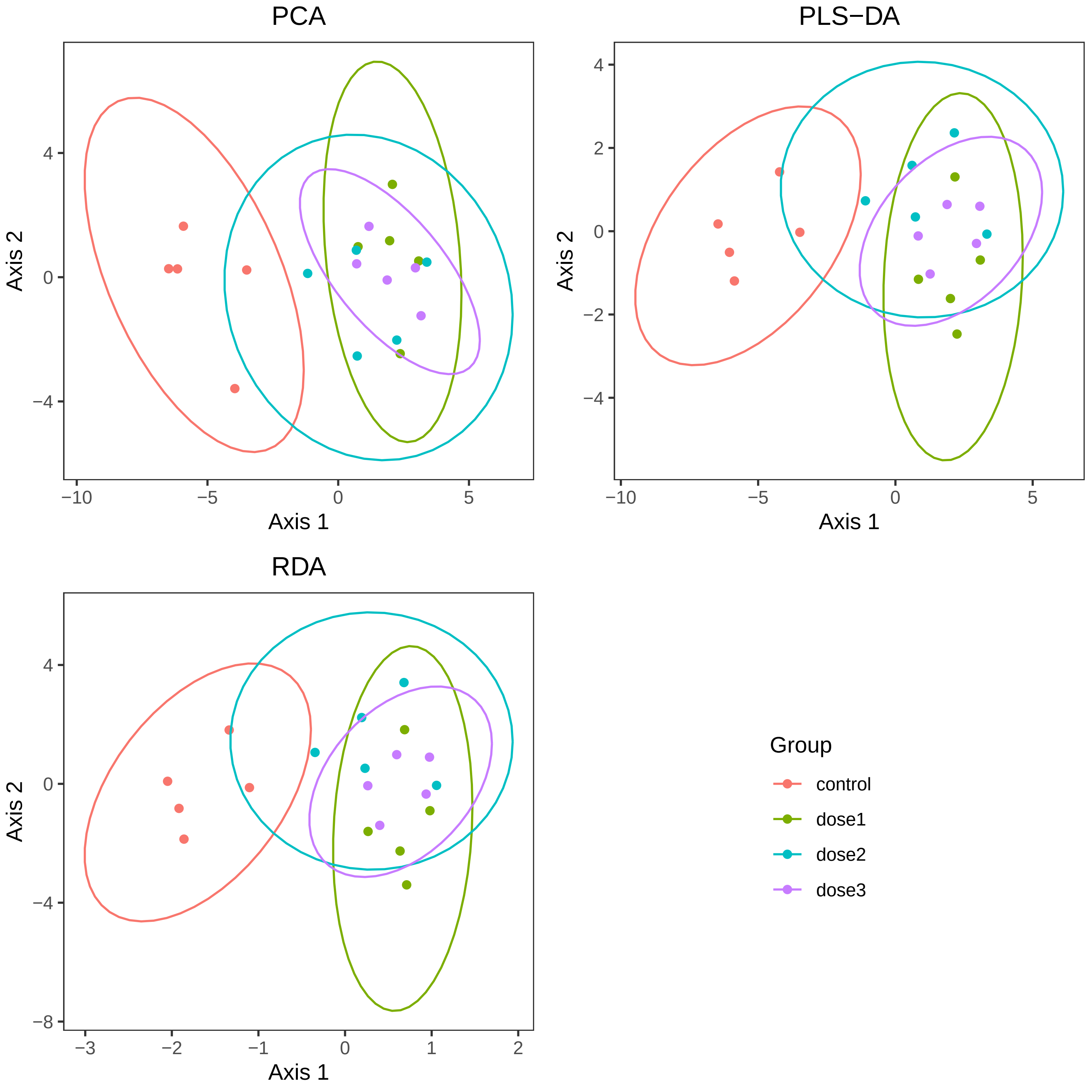

PCA
PLS−DA
RDA
Axis 1
Axis 2
Group
control
dose1
dose2
dose3

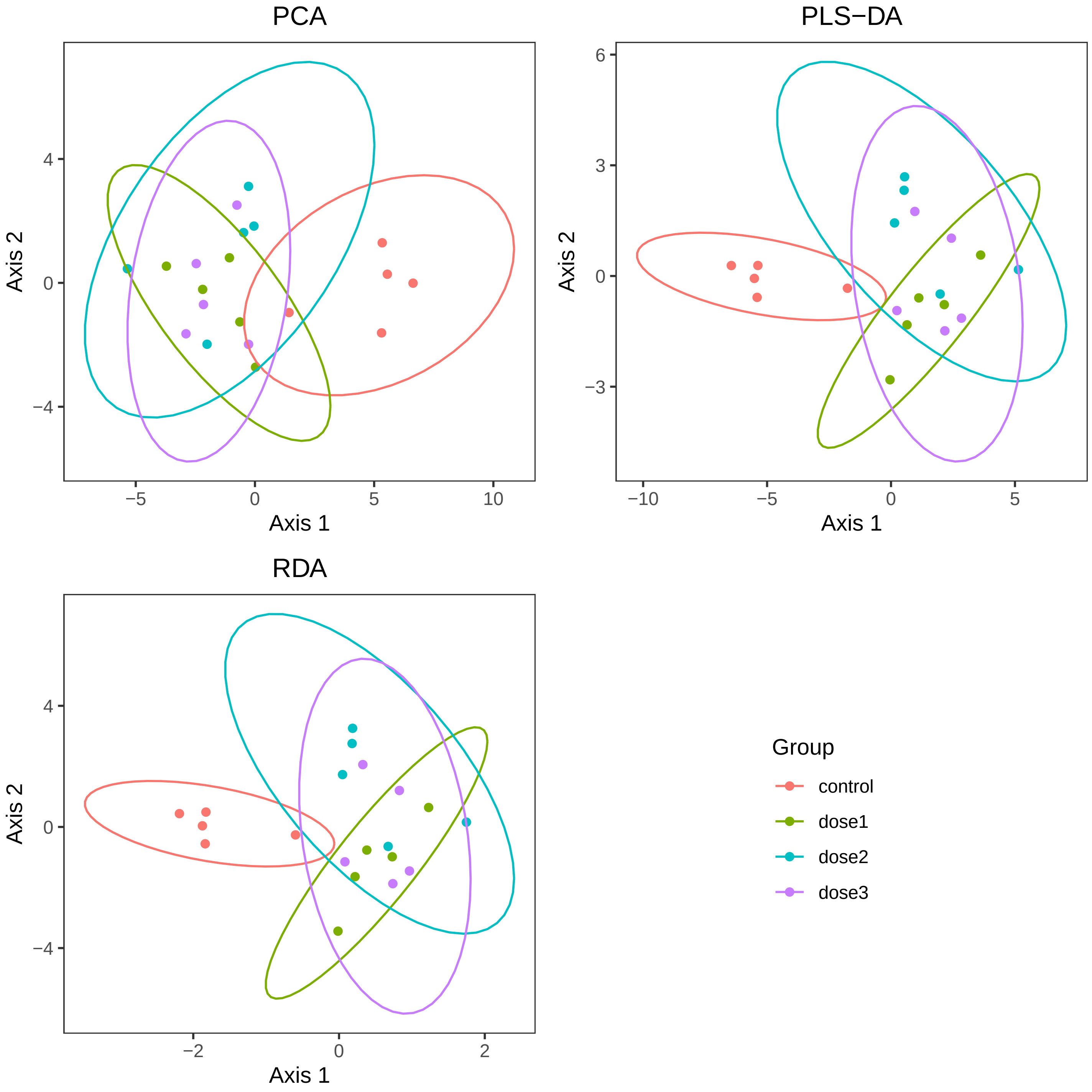
PCA
PLS−DA
RDA
Axis 1
Axis 2
Group
control
dose1
dose2
dose3

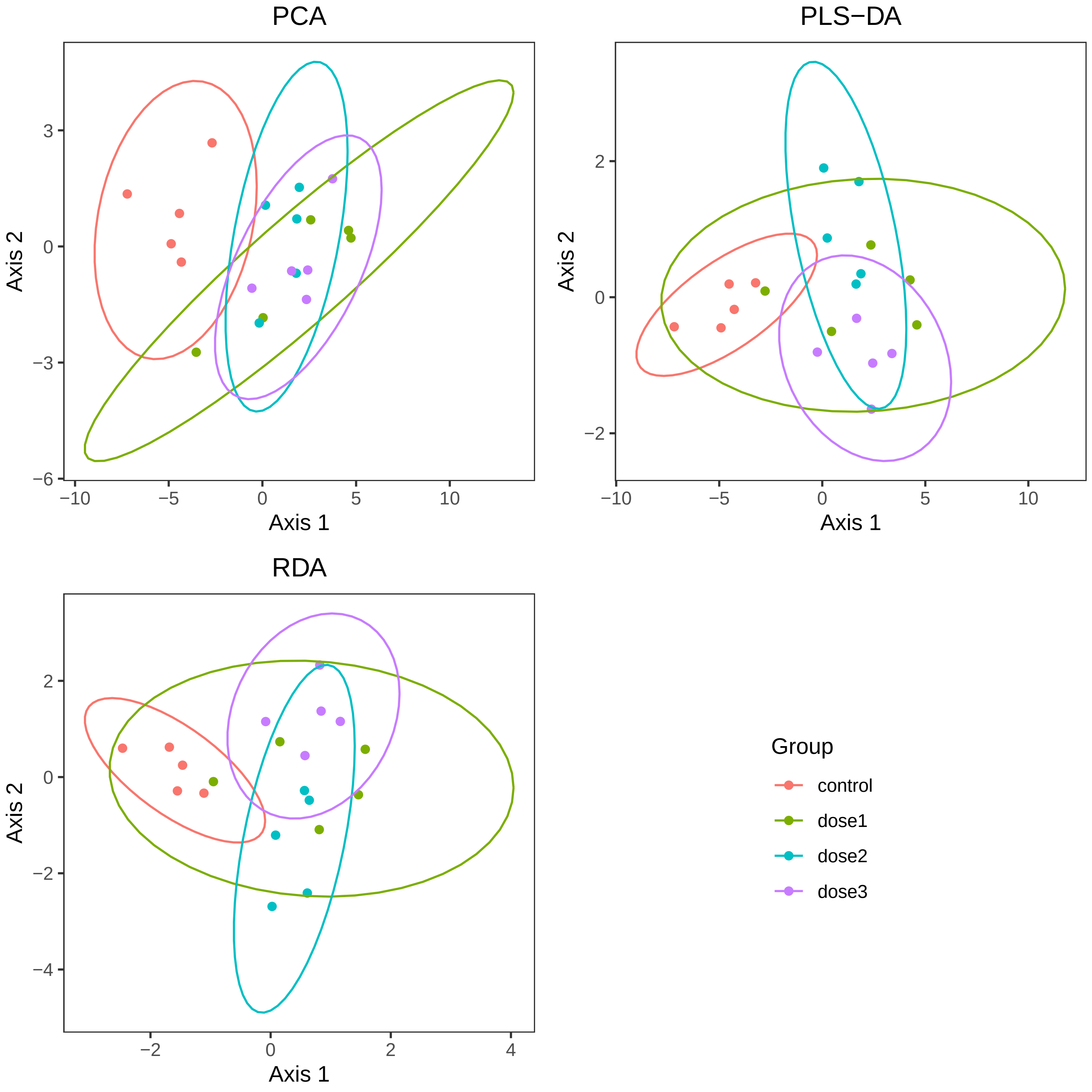

PCA
PLS−DA
RDA
Axis 1
Axis 2
Group
control
dose1
dose2
dose3

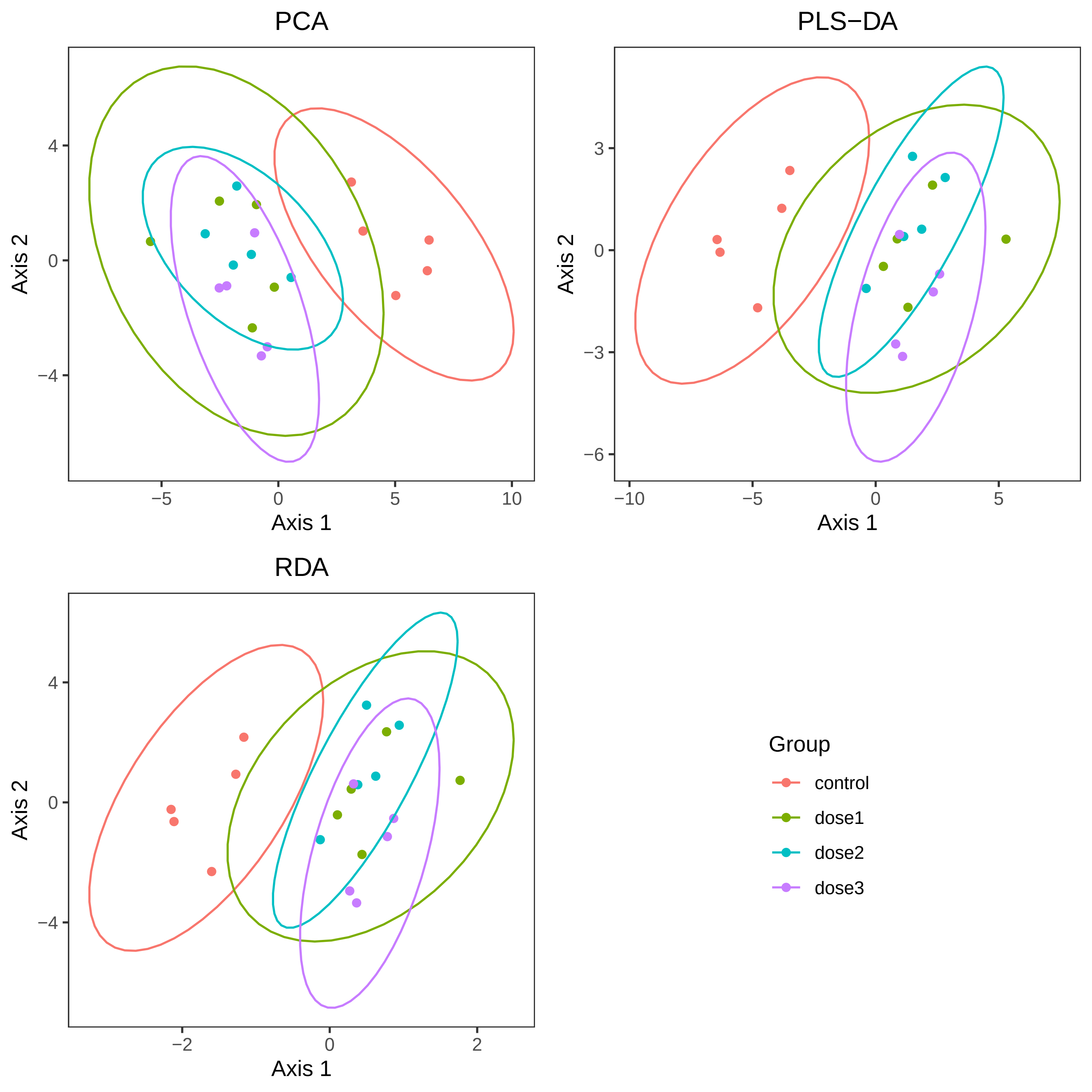
PCA
Axis 1
Axis 2
−5
0
5
10
−4
0
4
PLS−DA
−10
−5
0
5
−6
−3
0
3
RDA
−2
0
2
−4
0
4
Group
control
dose1
dose2
dose3

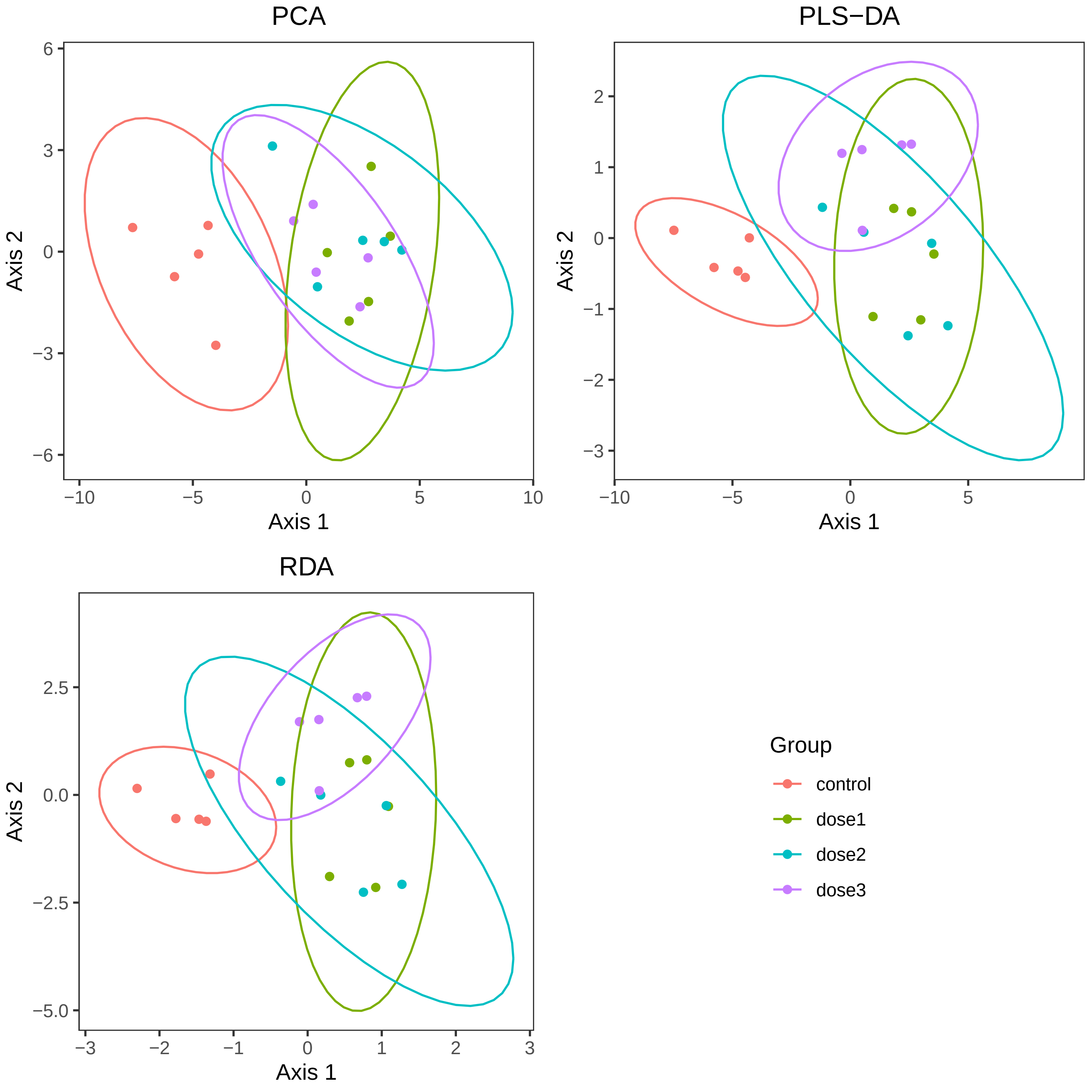

PCA
PLS−DA
RDA
Axis 1
Axis 2
Group
control
dose1
dose2
dose3

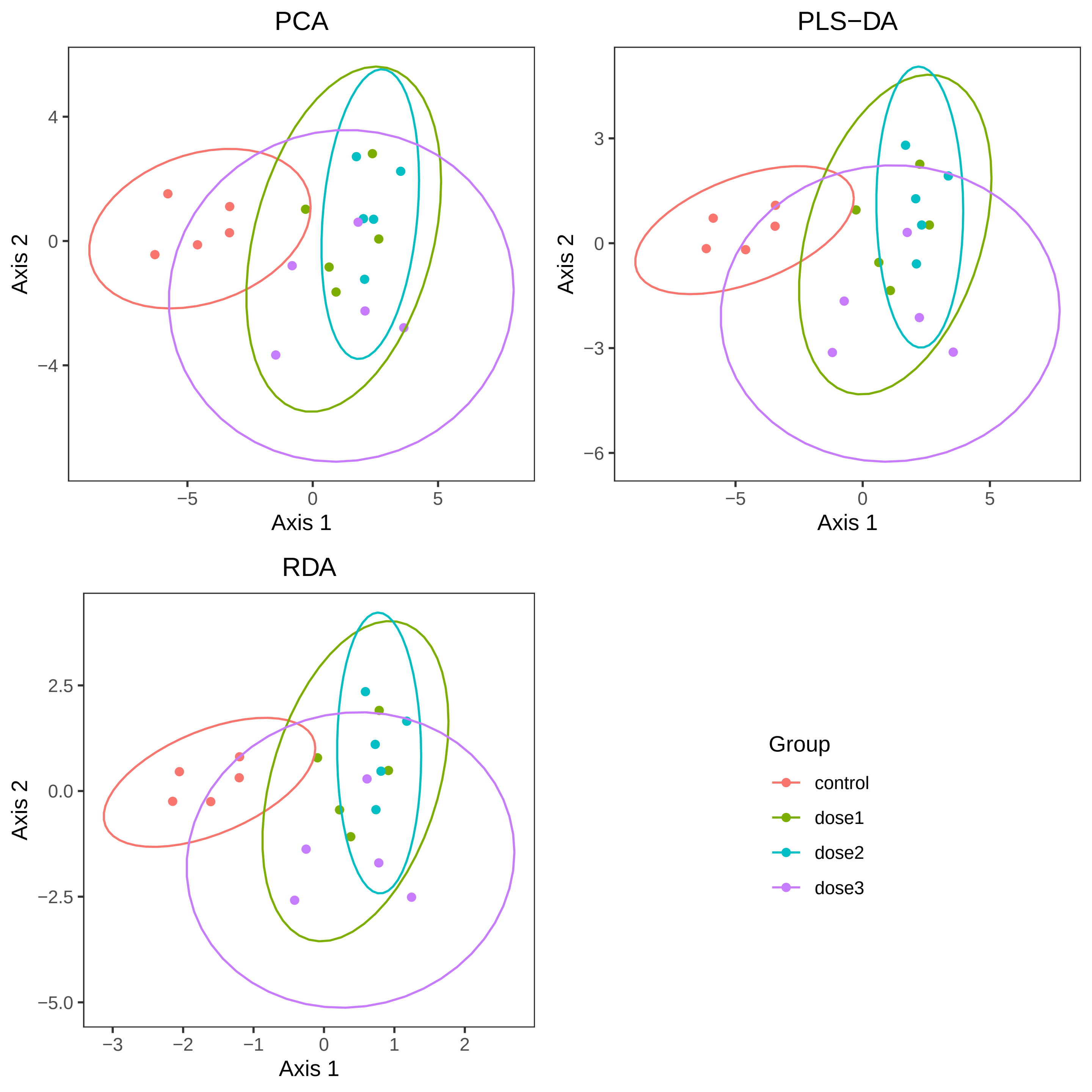
PCA
Axis 1
Axis 2
PLS−DA
Axis 1
Axis 2
RDA
Axis 1
Axis 2
Group
control
dose1
dose2
dose3

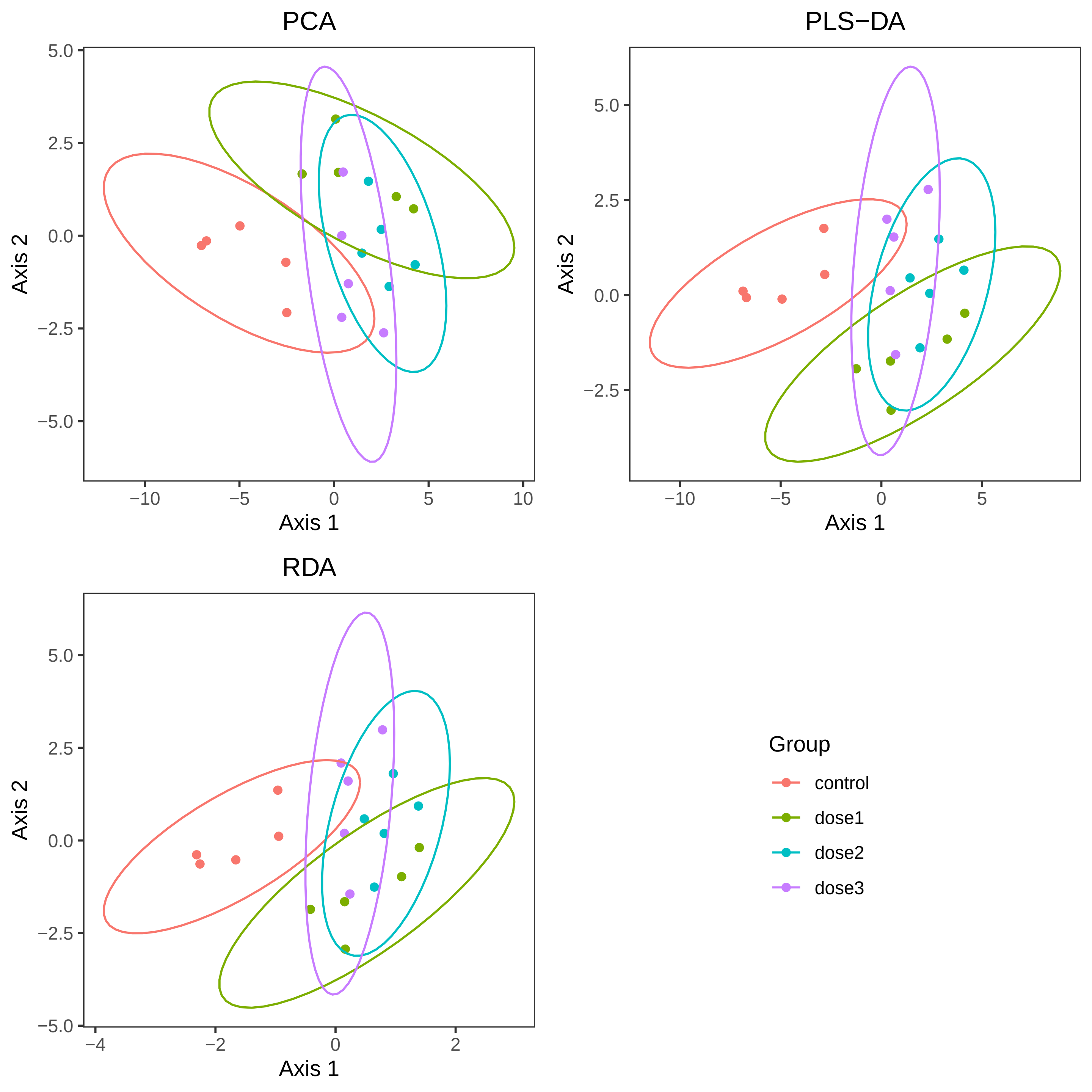

PCA
PLS−DA
RDA
Axis 1
Axis 2
Group
control
dose1
dose2
dose3

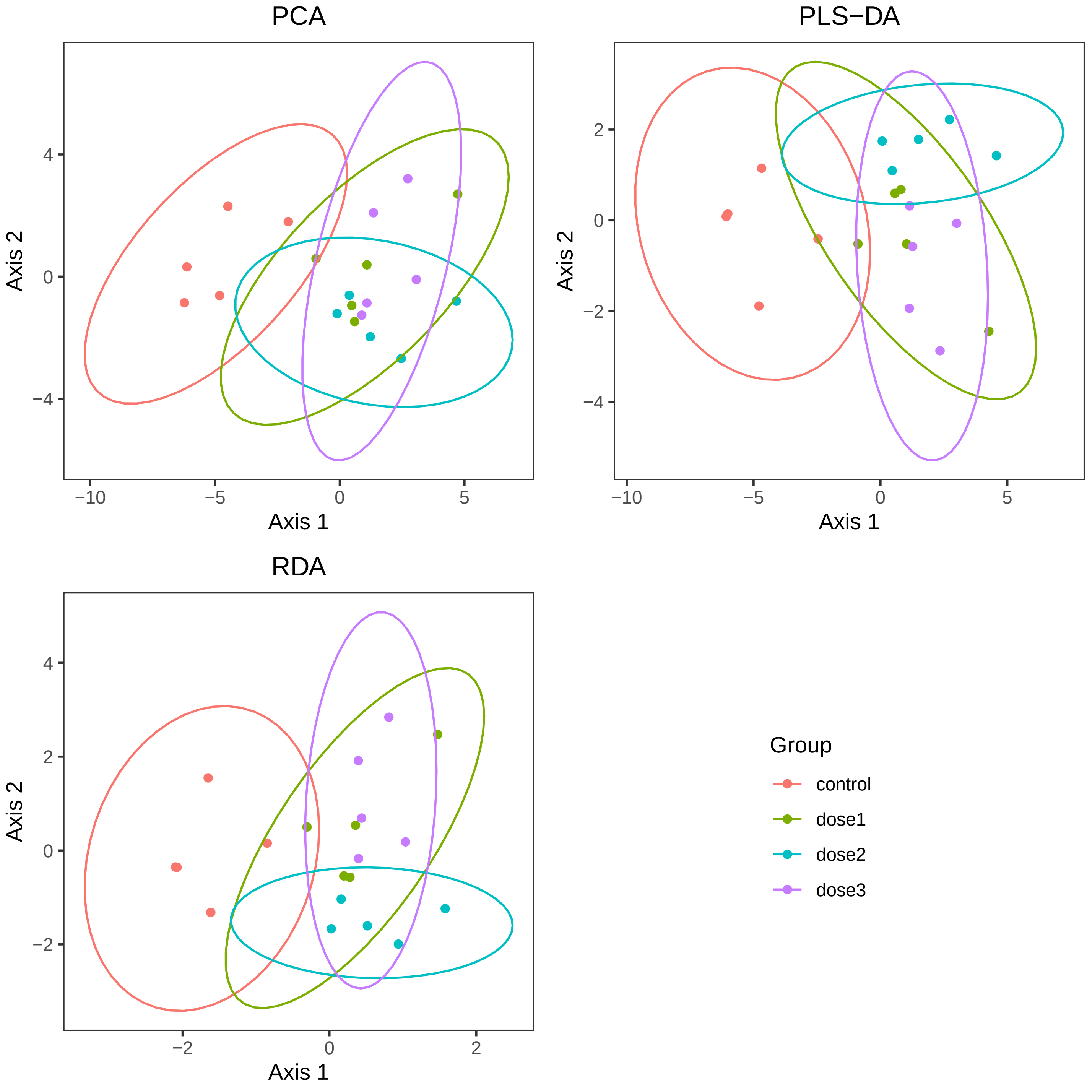

PCA
PLS−DA
RDA
Axis 1
Axis 2
Group
control
dose1
dose2
dose3

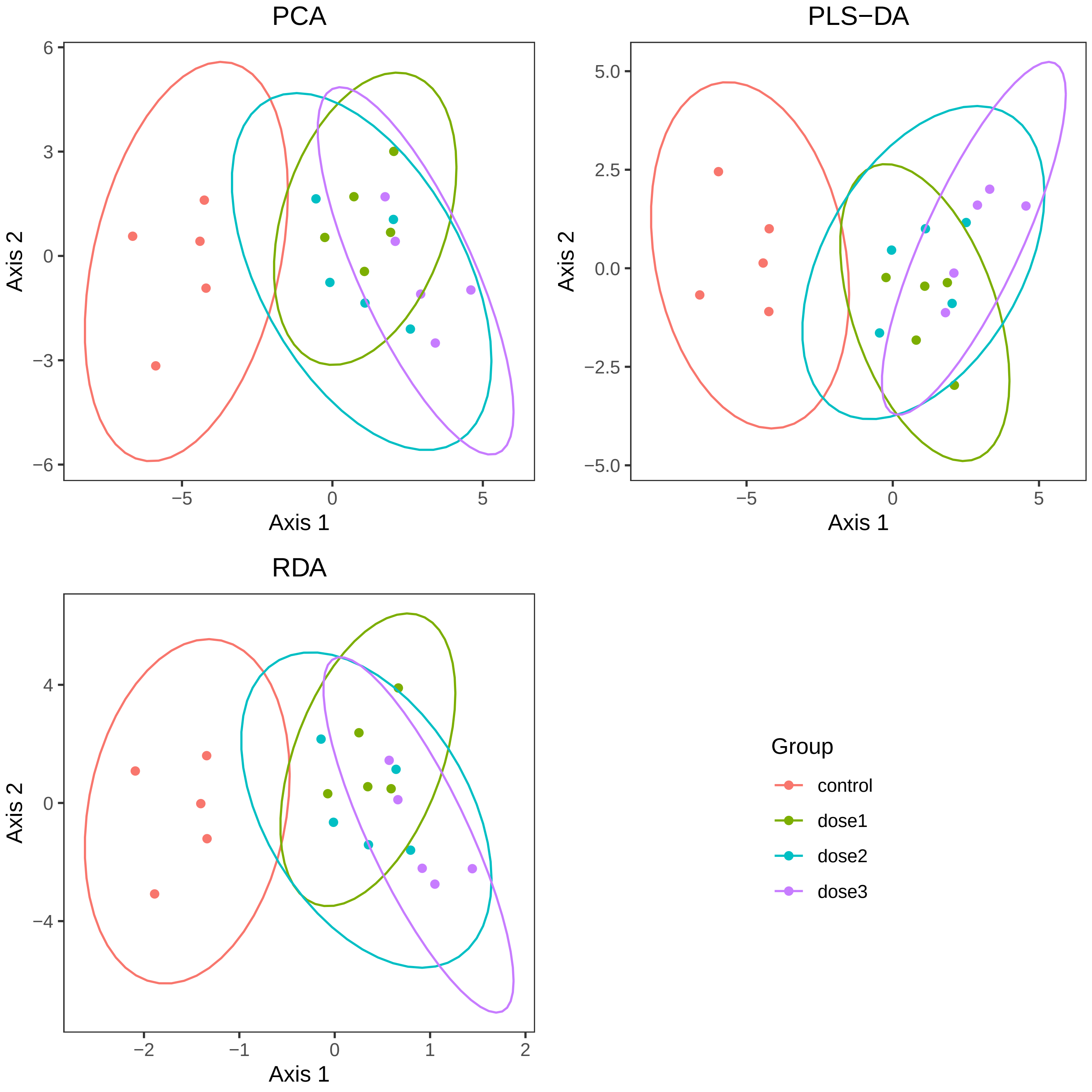
PCA
PLS−DA
RDA
Axis 1
Axis 2
Group
control
dose1
dose2
dose3

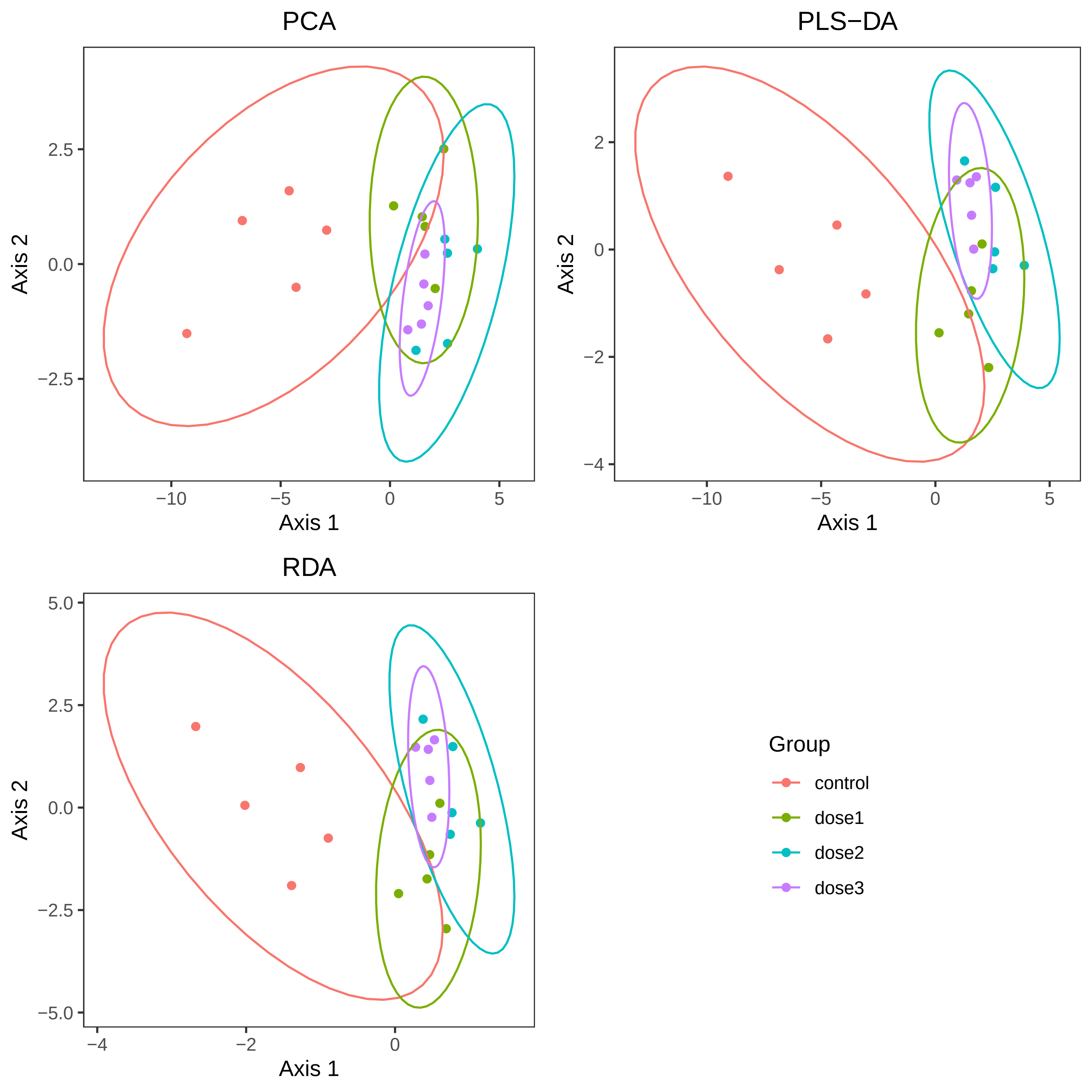
PCA
PLS−DA
RDA
Axis 1
Axis 2
Group
control
dose1
dose2
dose3

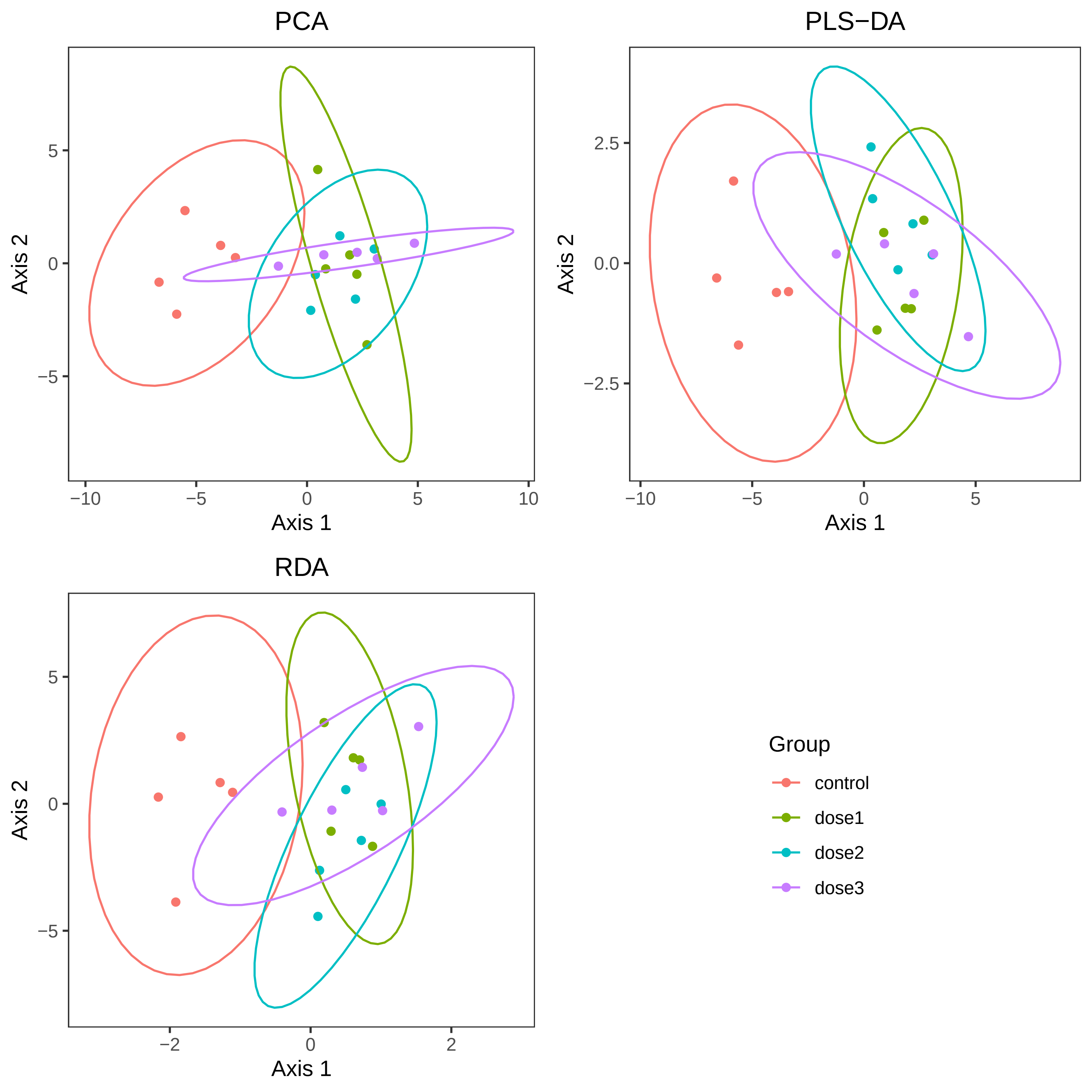

PCA
PLS−DA
RDA
Axis 1
Axis 2
Group
control
dose1
dose2
dose3

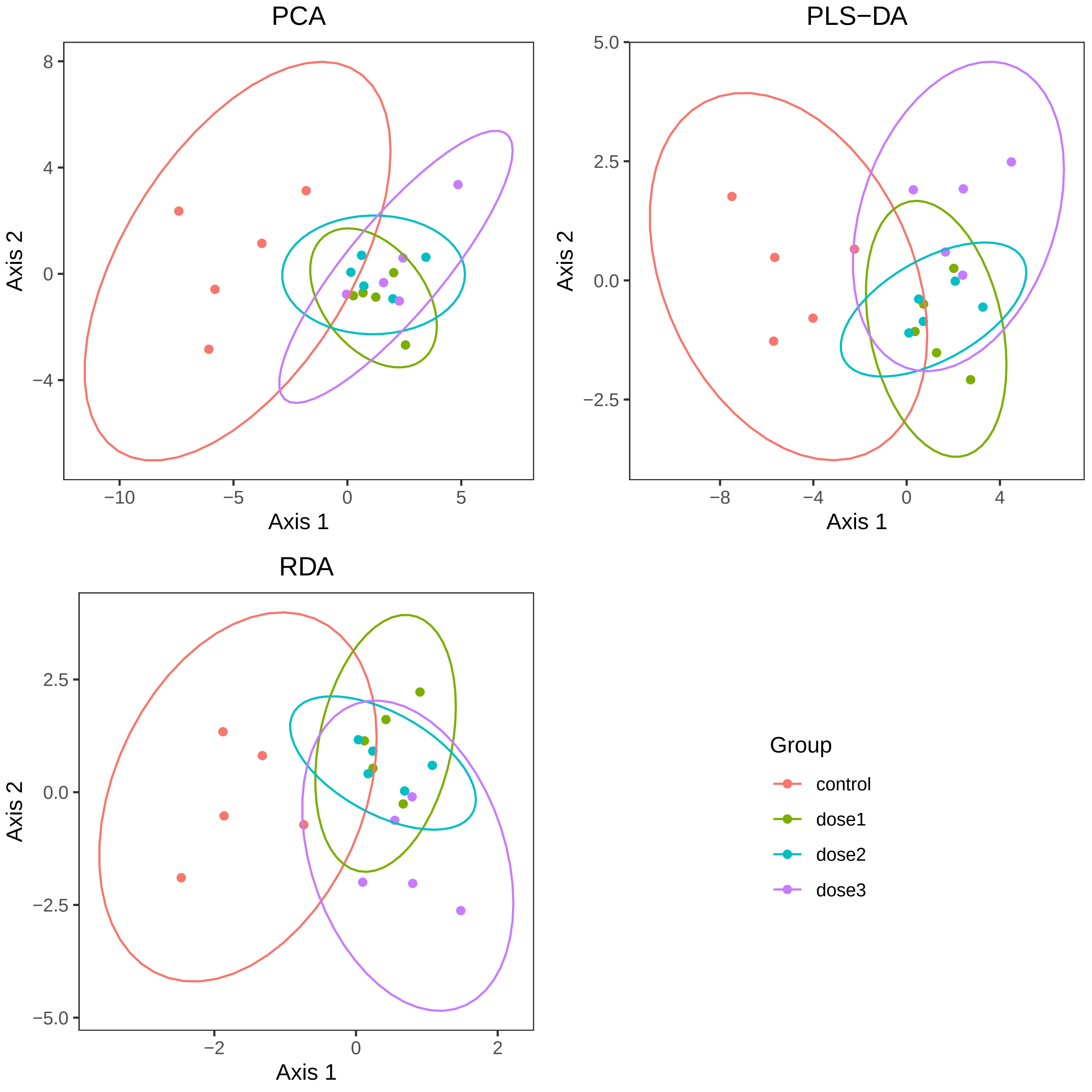
PCA
PLS−DA
RDA
Axis 1
Axis 2
8
4
0
−4
−10
−5
0
5
5.0
2.5
0.0
−2.5
−8
−4
0
4
2.5
0.0
−2.5
−5.0
−2
0
2
Group
control
dose1
dose2
dose3

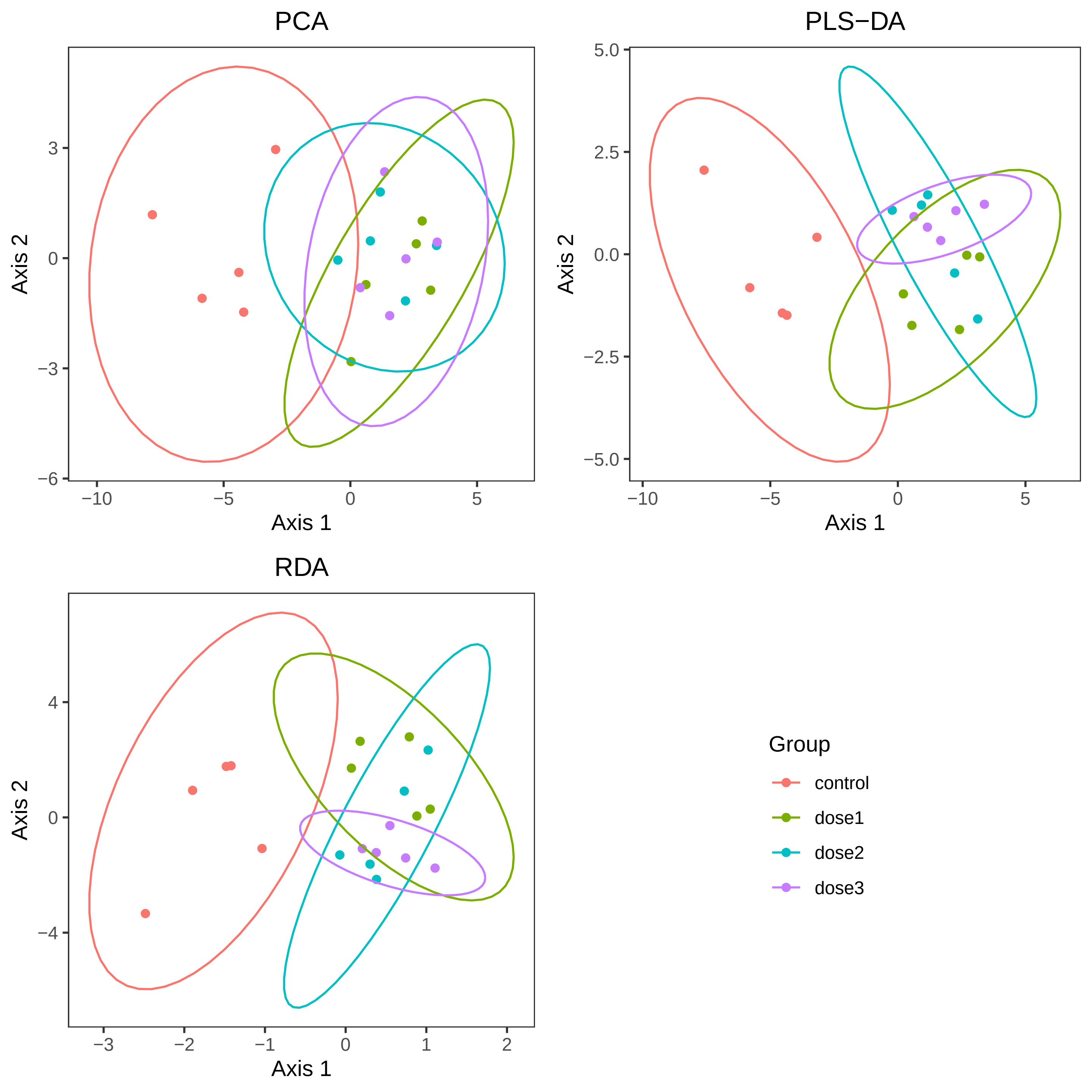
PCA
PLS−DA
RDA
Axis 1
Axis 2
Group
control
dose1
dose2
dose3

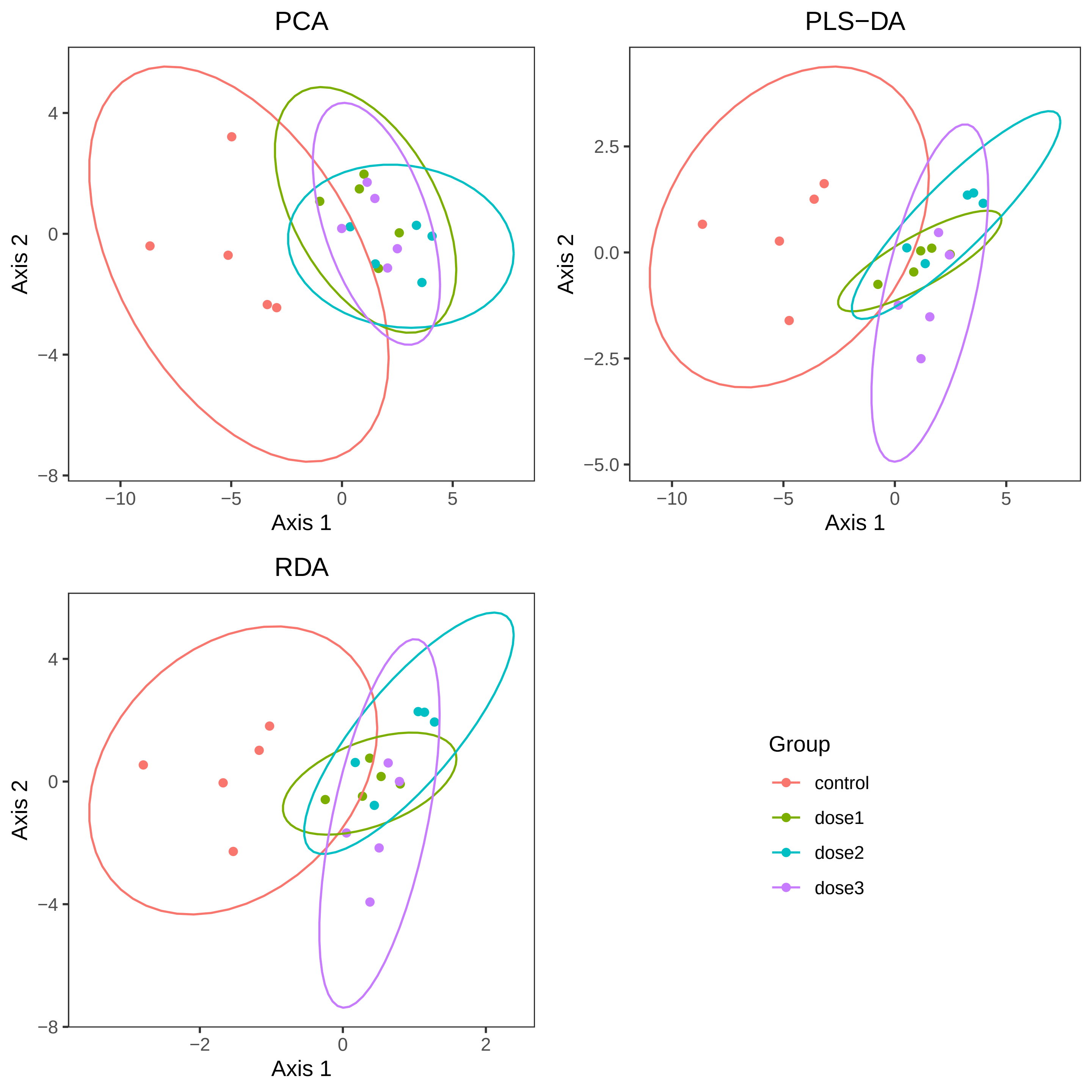

PCA
PLS−DA
RDA
Axis 1
Axis 2
Group
control
dose1
dose2
dose3

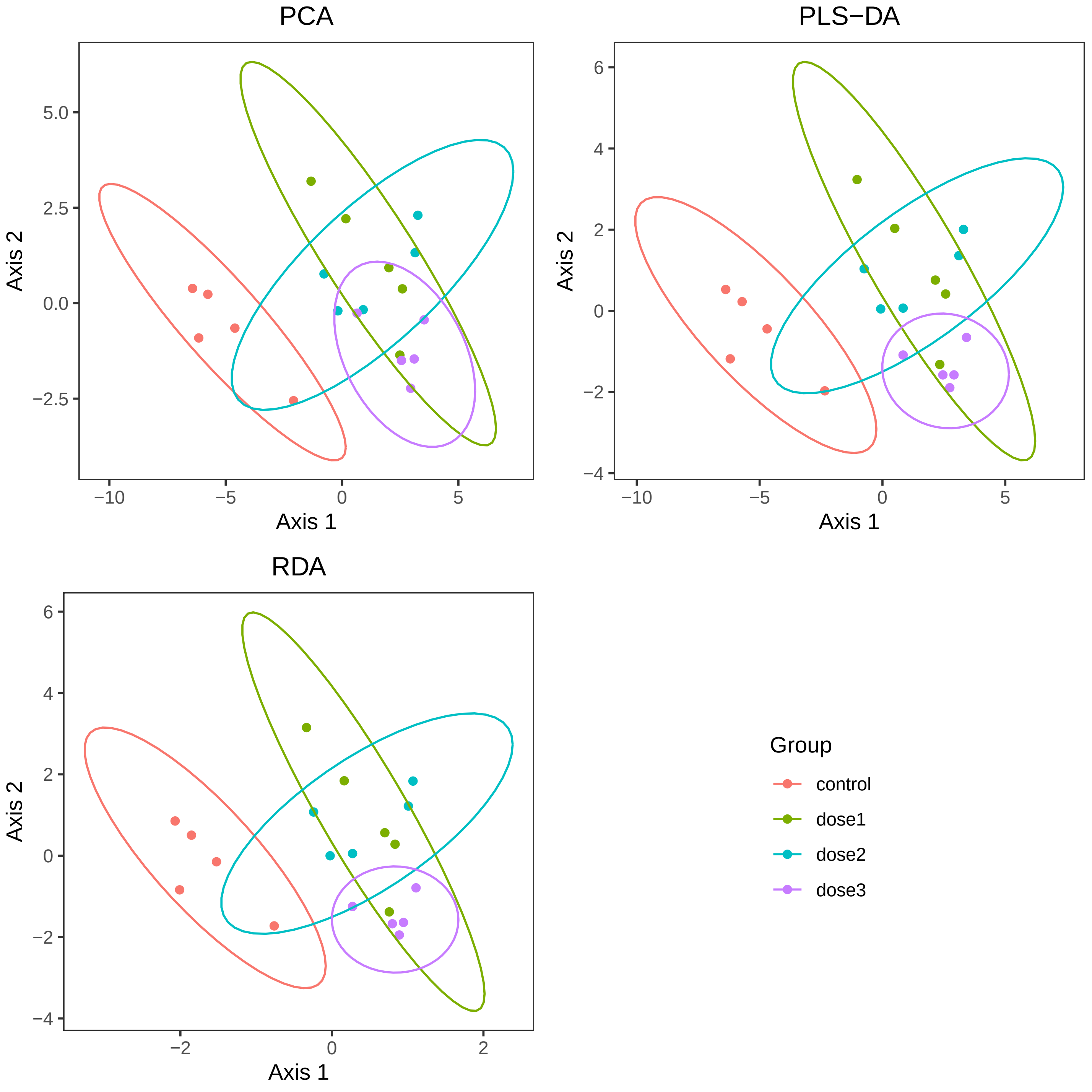

PCA
PLS−DA
RDA
Axis 1
Axis 2
Group
control
dose1
dose2
dose3

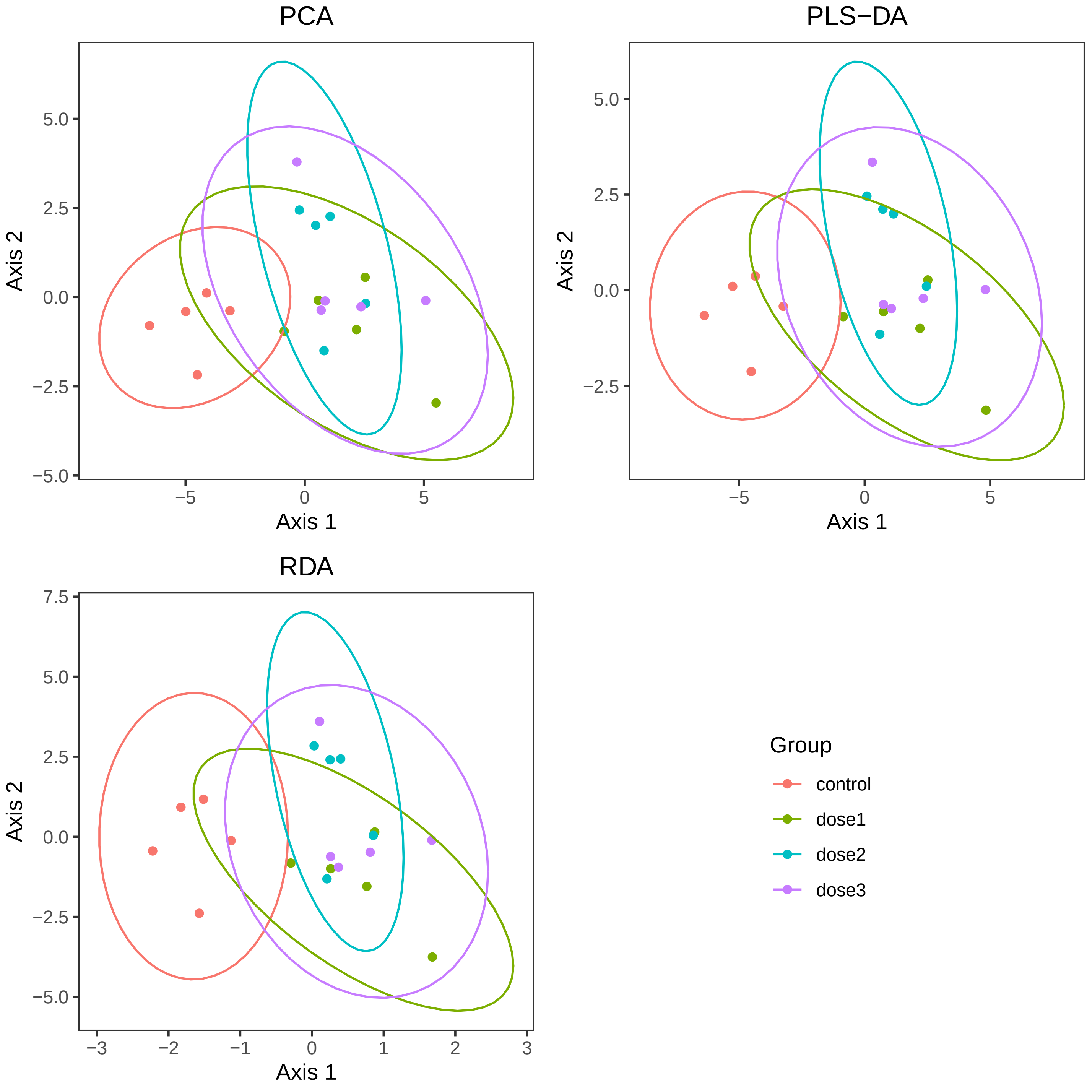
PCA
PLS−DA
RDA
Axis 1
Axis 2
Group
control
dose1
dose2
dose3

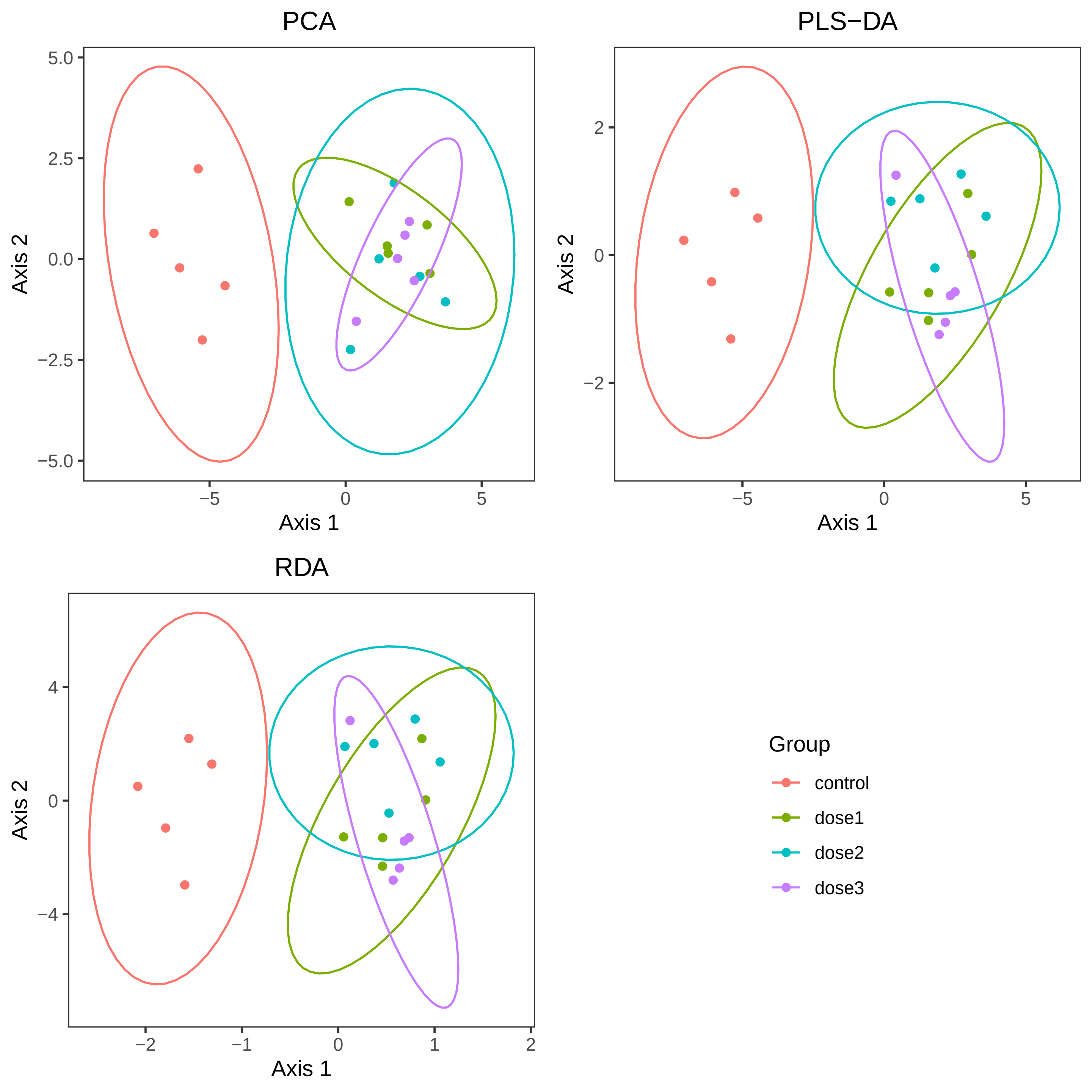

PCA
PLS−DA
RDA
Axis 1
Axis 2
5.0
2.5
0.0
−2.5
−5.0
−5
0
5
2
0
−2
4
0
−4
−2
−1
0
1
2
Group
control
dose1
dose2
dose3

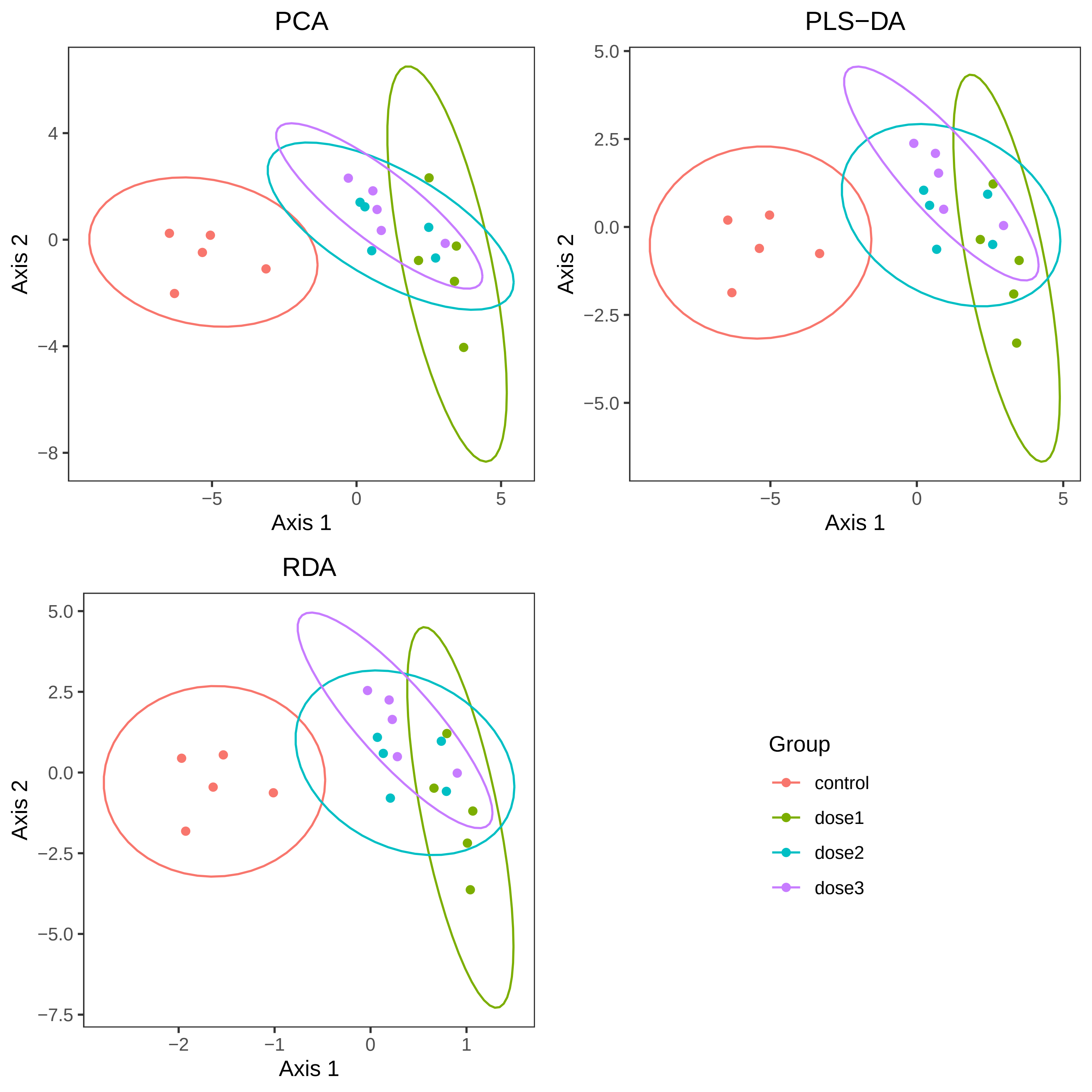

PCA
PLS−DA
RDA
Axis 1
Axis 2
Group
control
dose1
dose2
dose3

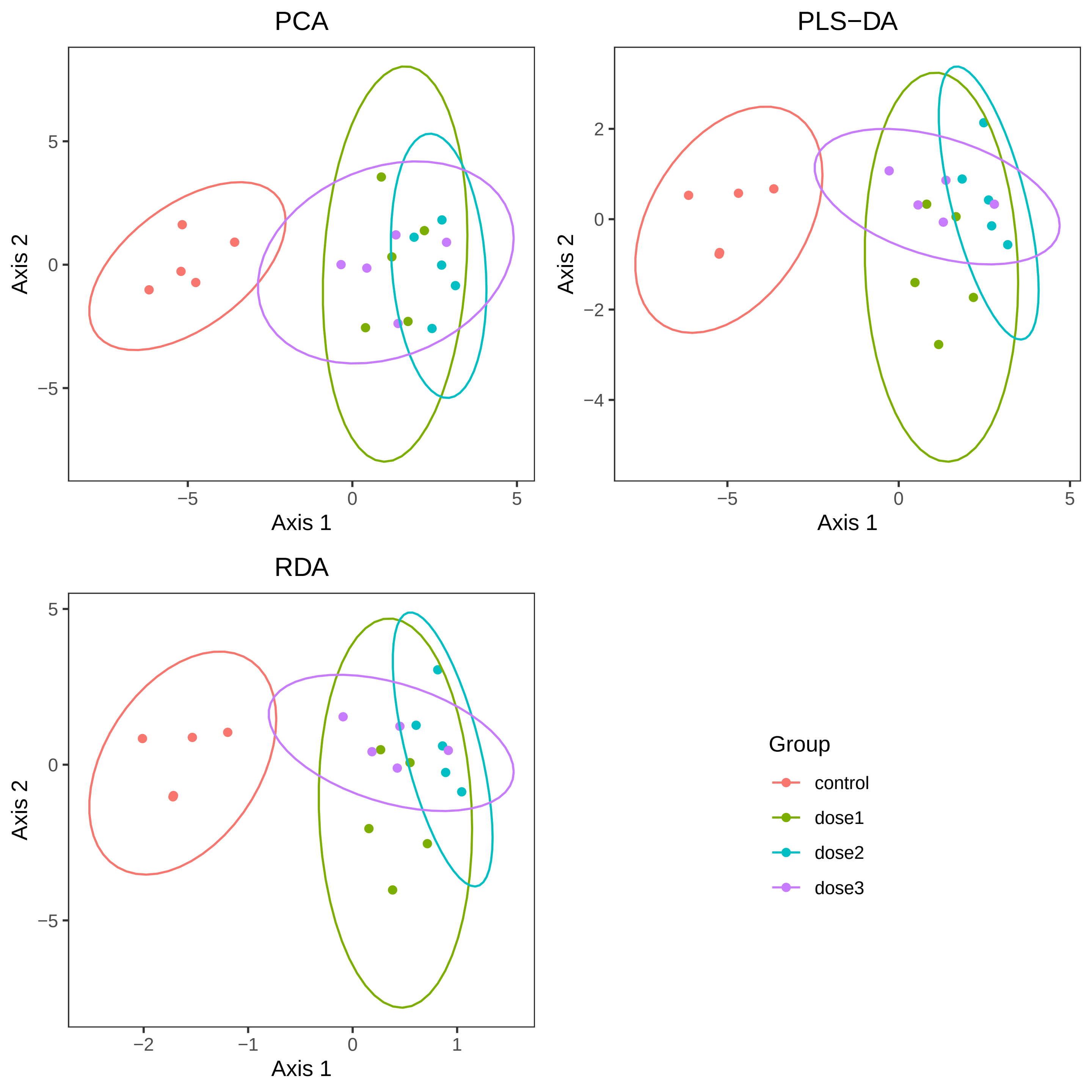

PCA
PLS−DA
RDA
Axis 1
Axis 2
Group
control
dose1
dose2
dose3

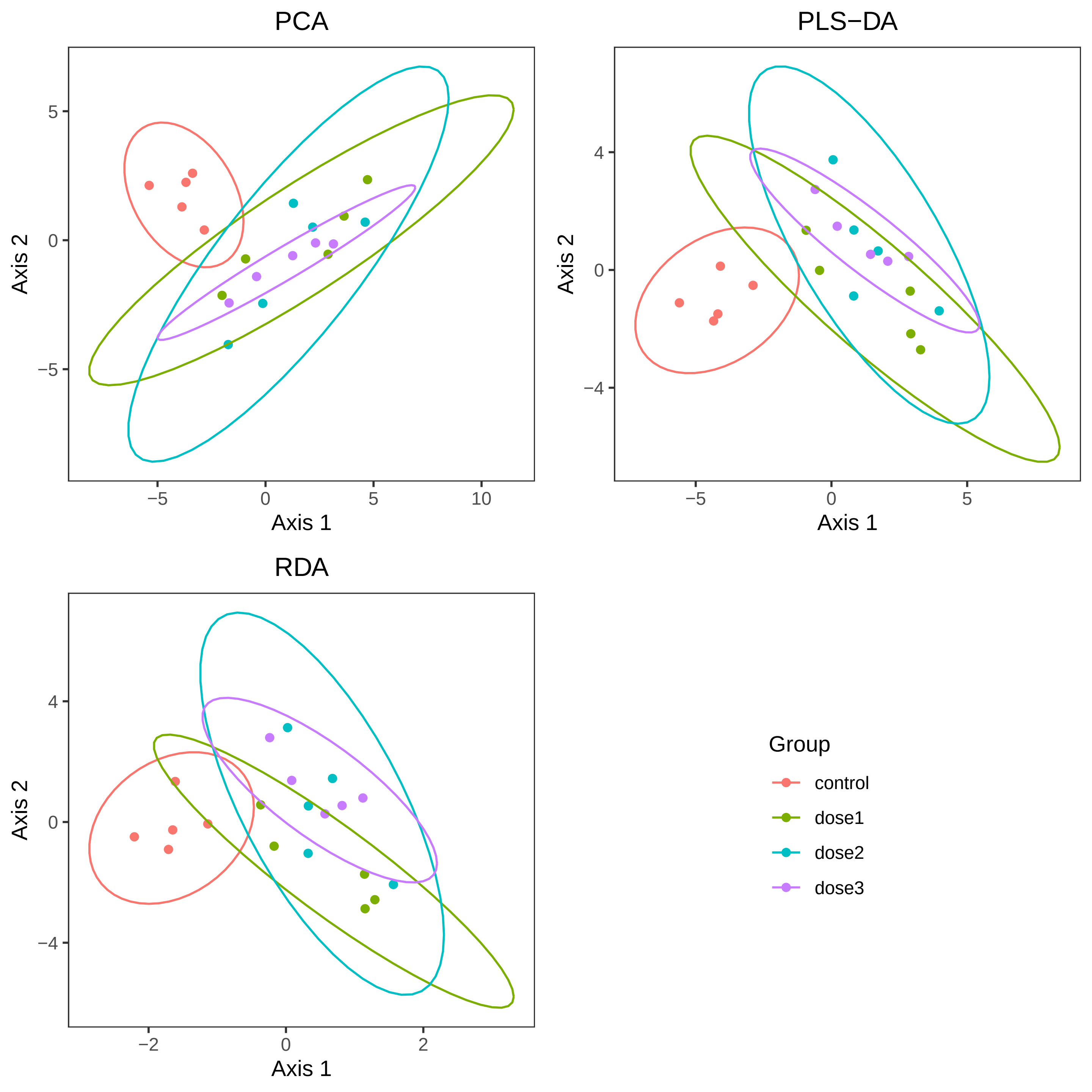

PCA
PLS−DA
RDA
Axis 1
Axis 2
5
0
−5
10
4
−4
−2
2
Group
control
dose1
dose2
dose3

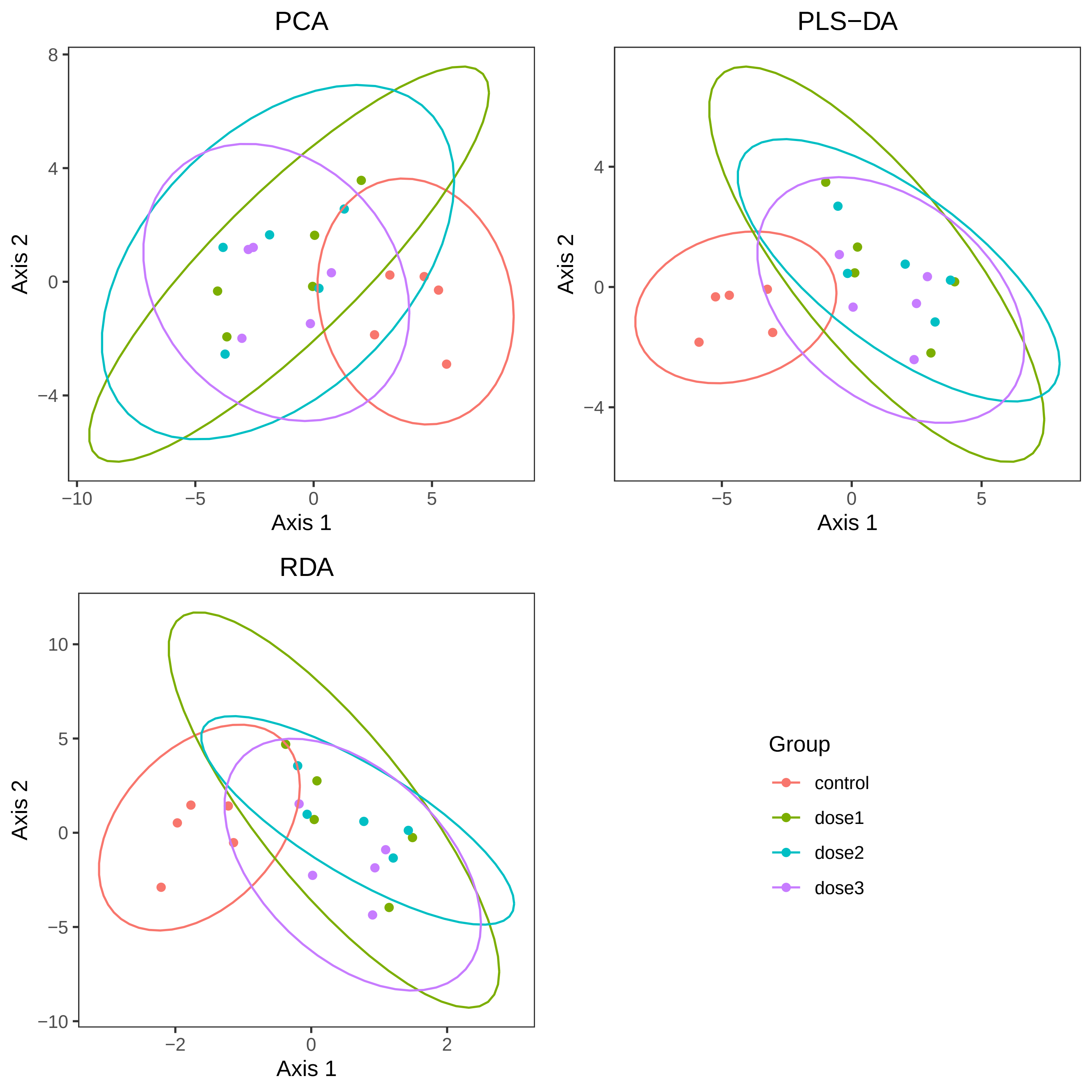
PCA
PLS−DA
RDA
Axis 1
Axis 2
Group
control
dose1
dose2
dose3

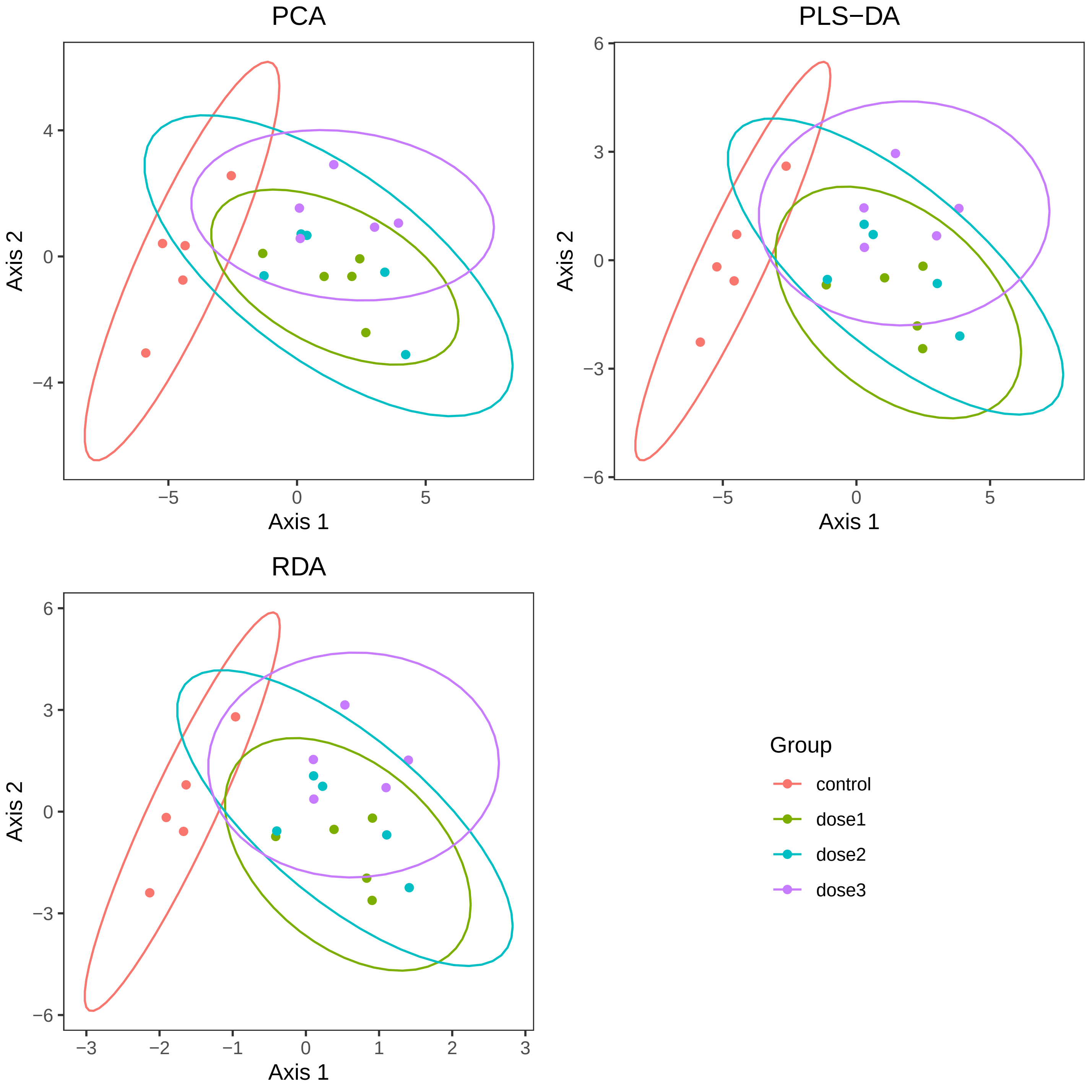
PCA
PLS−DA
RDA
Axis 1
Axis 2
Group
control
dose1
dose2
dose3

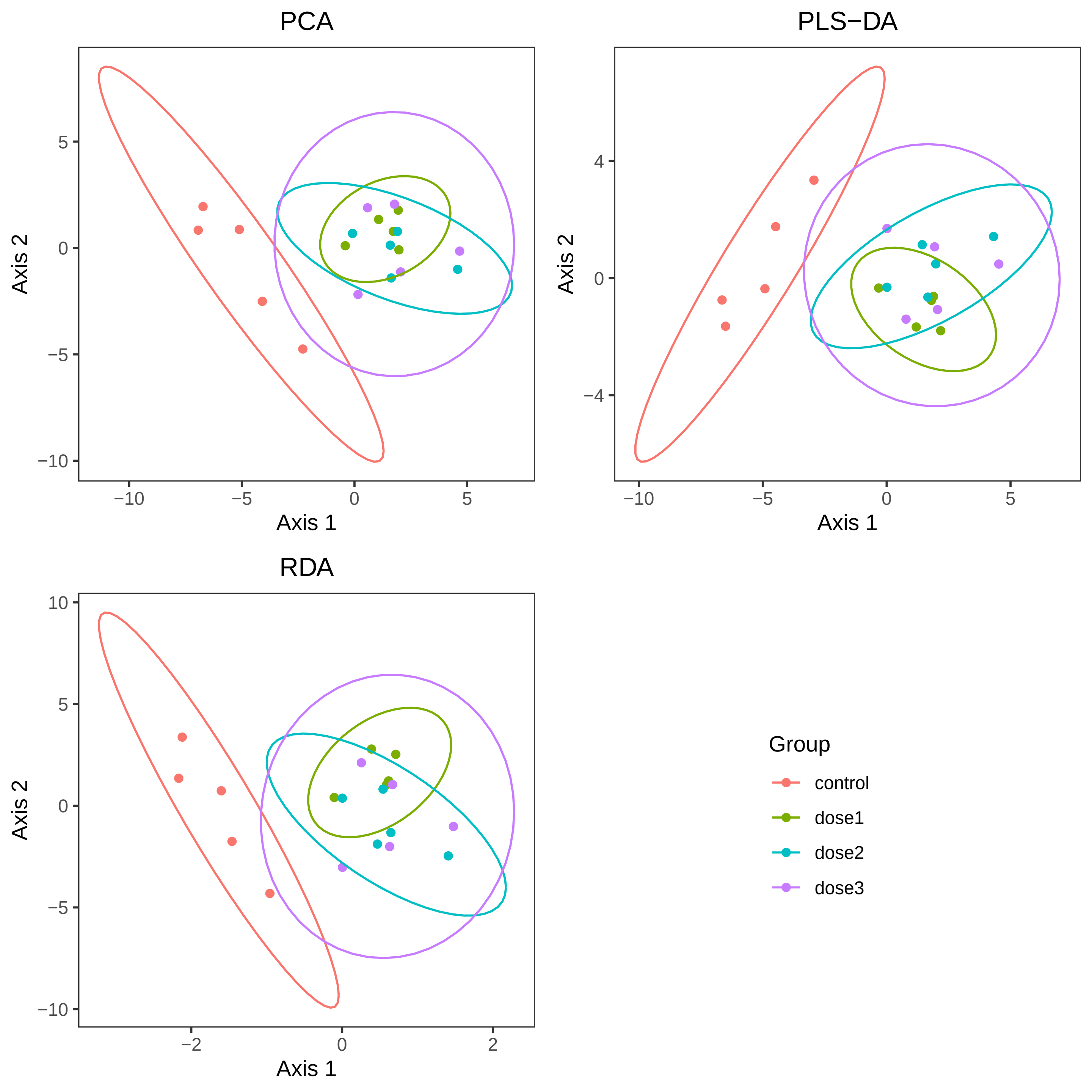
PCA
PLS−DA
RDA
Axis 1
Axis 2
Group
control
dose1
dose2
dose3

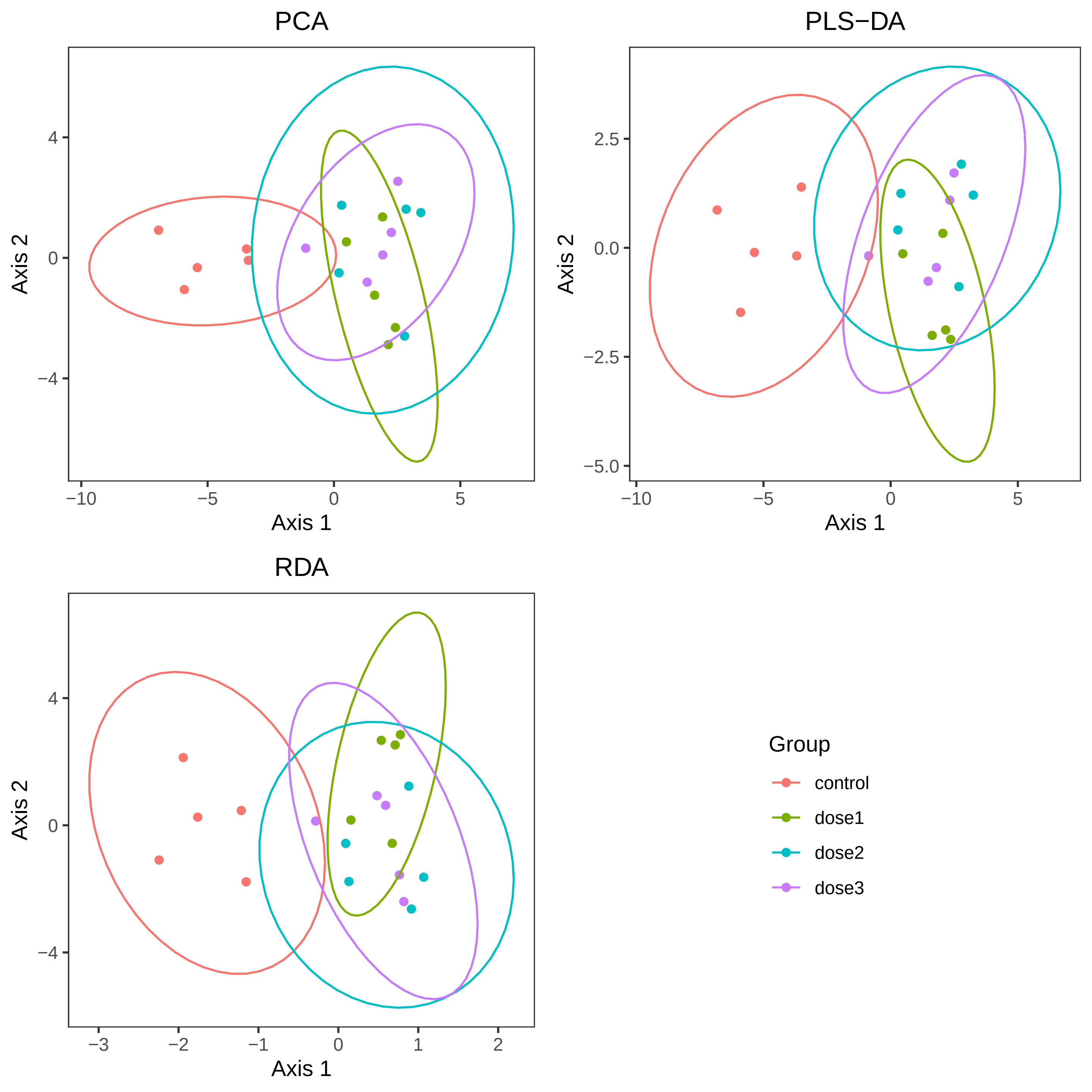

PCA
PLS−DA
RDA
Axis 1
Axis 2
−10
−5
0
5
4
0
−4
2.5
0.0
−2.5
−5.0
−3
−2
−1
1
2
Group
control
dose1
dose2
dose3

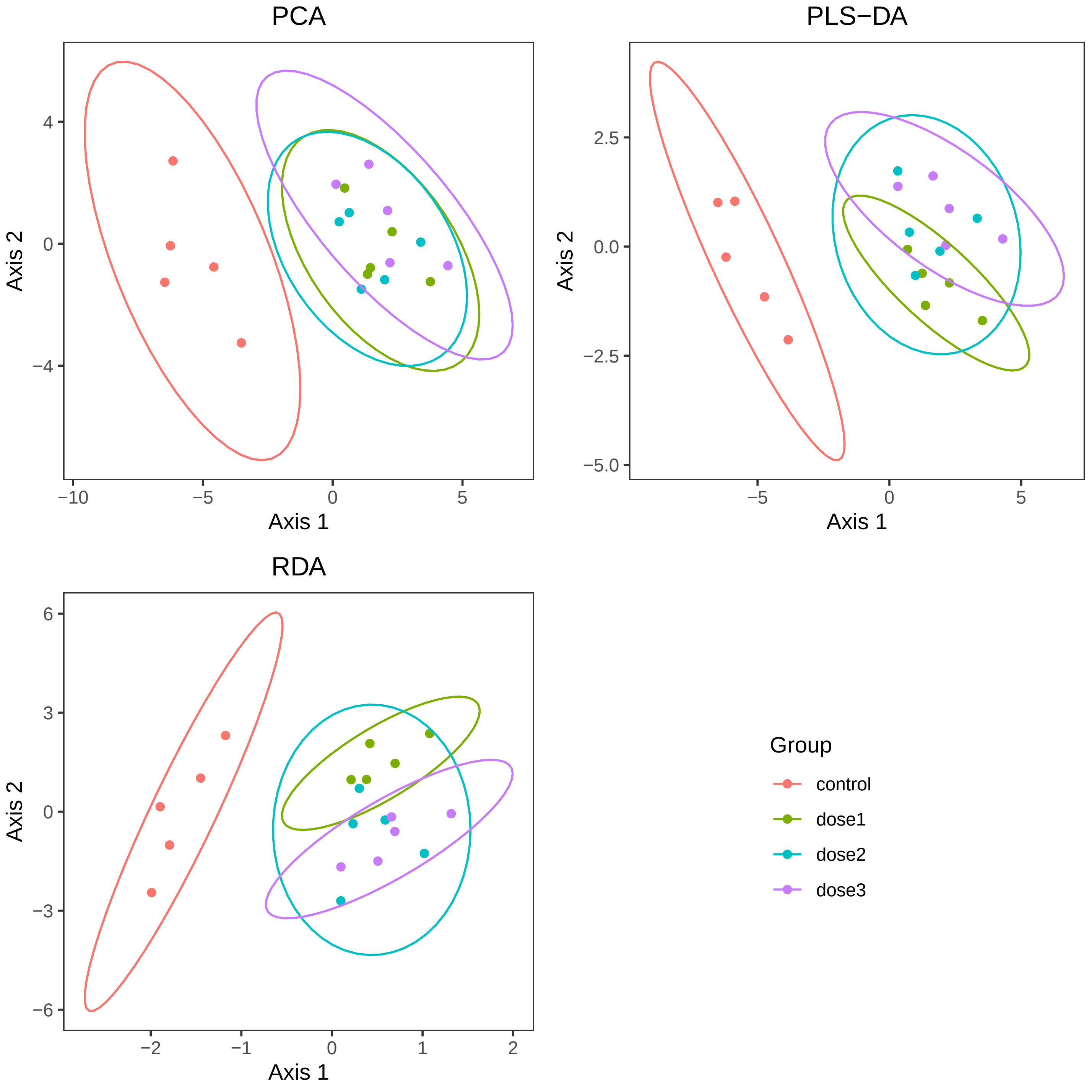

PCA
PLS−DA
RDA
Axis 1
Axis 2
Group
control
dose1
dose2
dose3

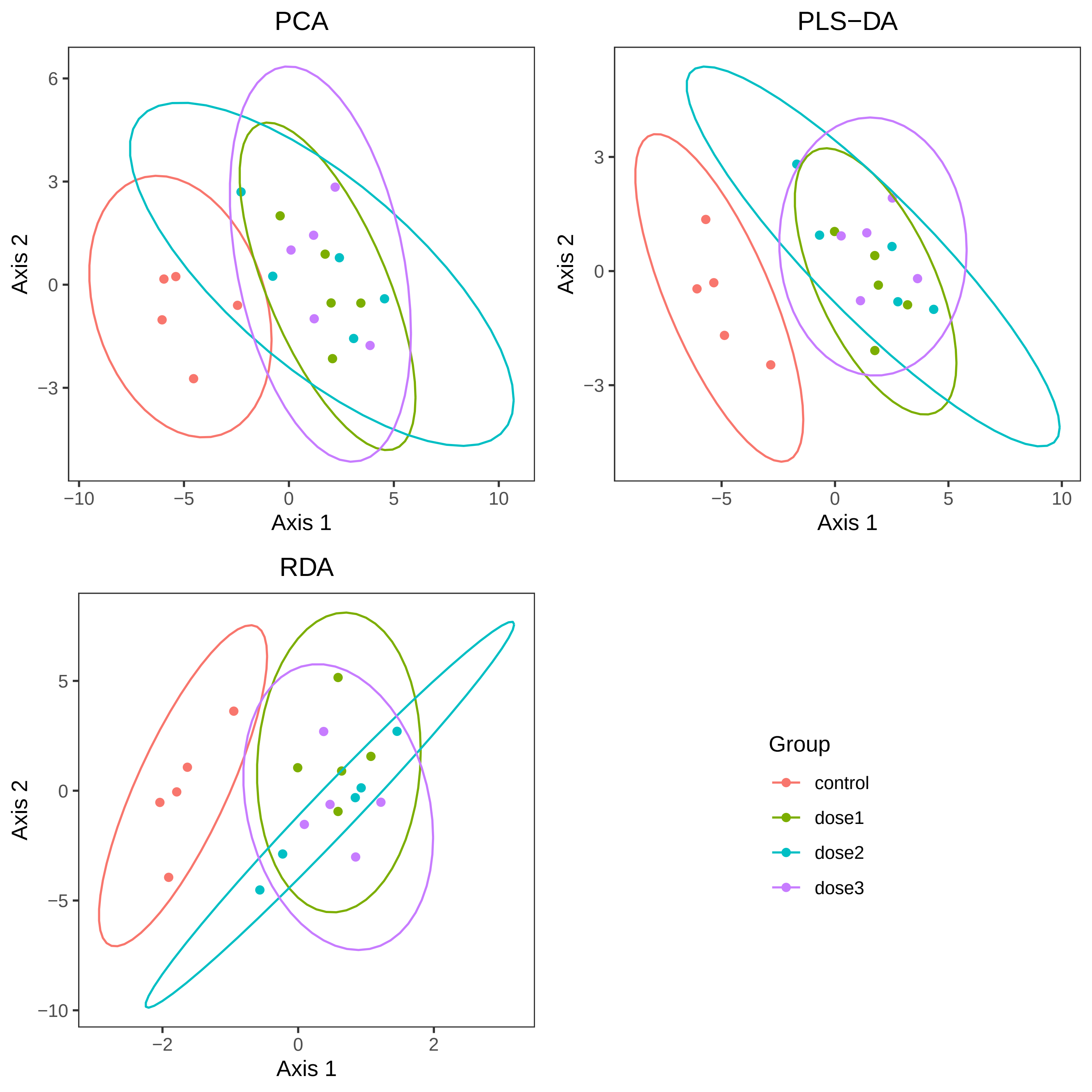
PCA
PLS−DA
RDA
Axis 1
Axis 2
Group
control
dose1
dose2
dose3

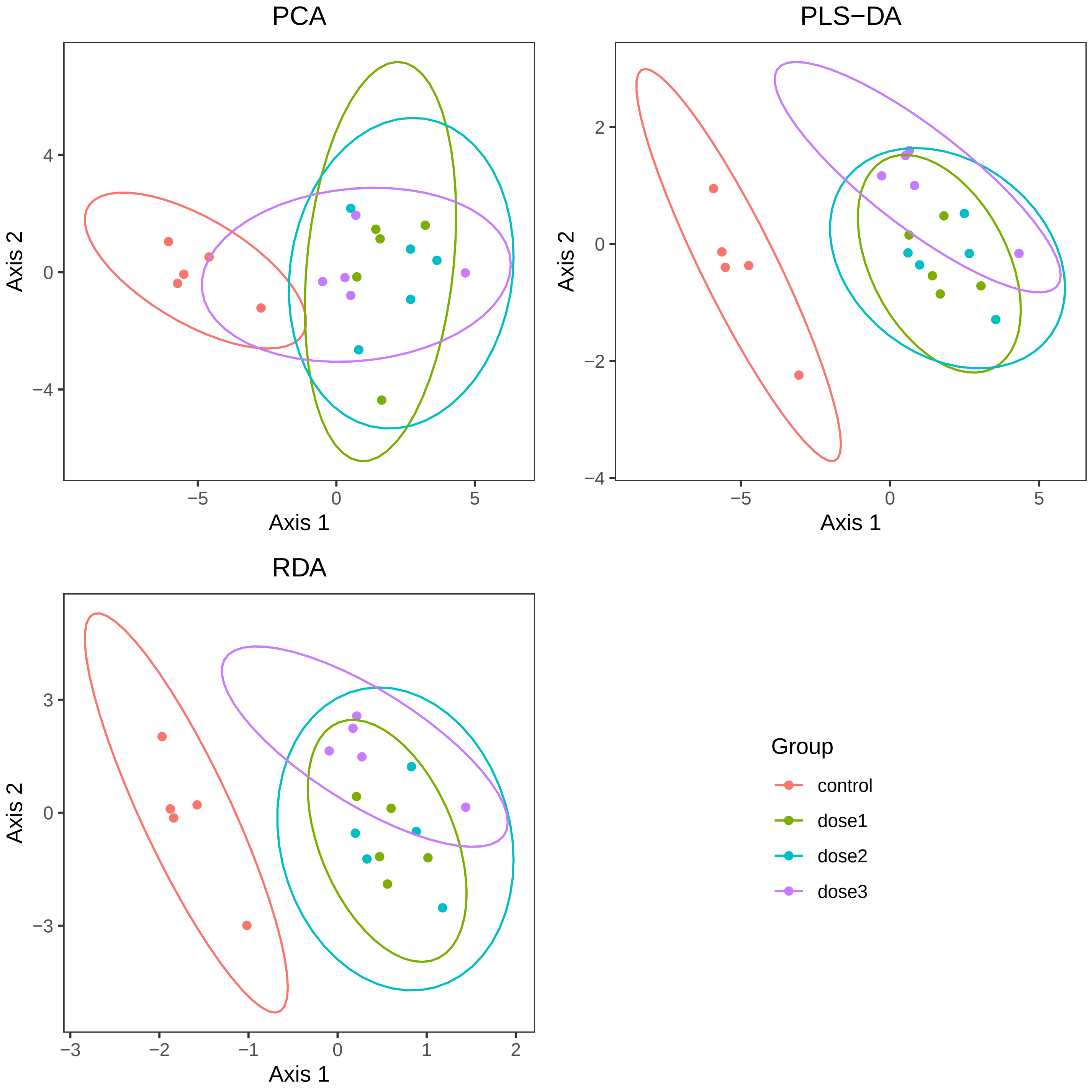

PCA
PLS−DA
RDA
Axis 1
Axis 2
Group
control
dose1
dose2
dose3

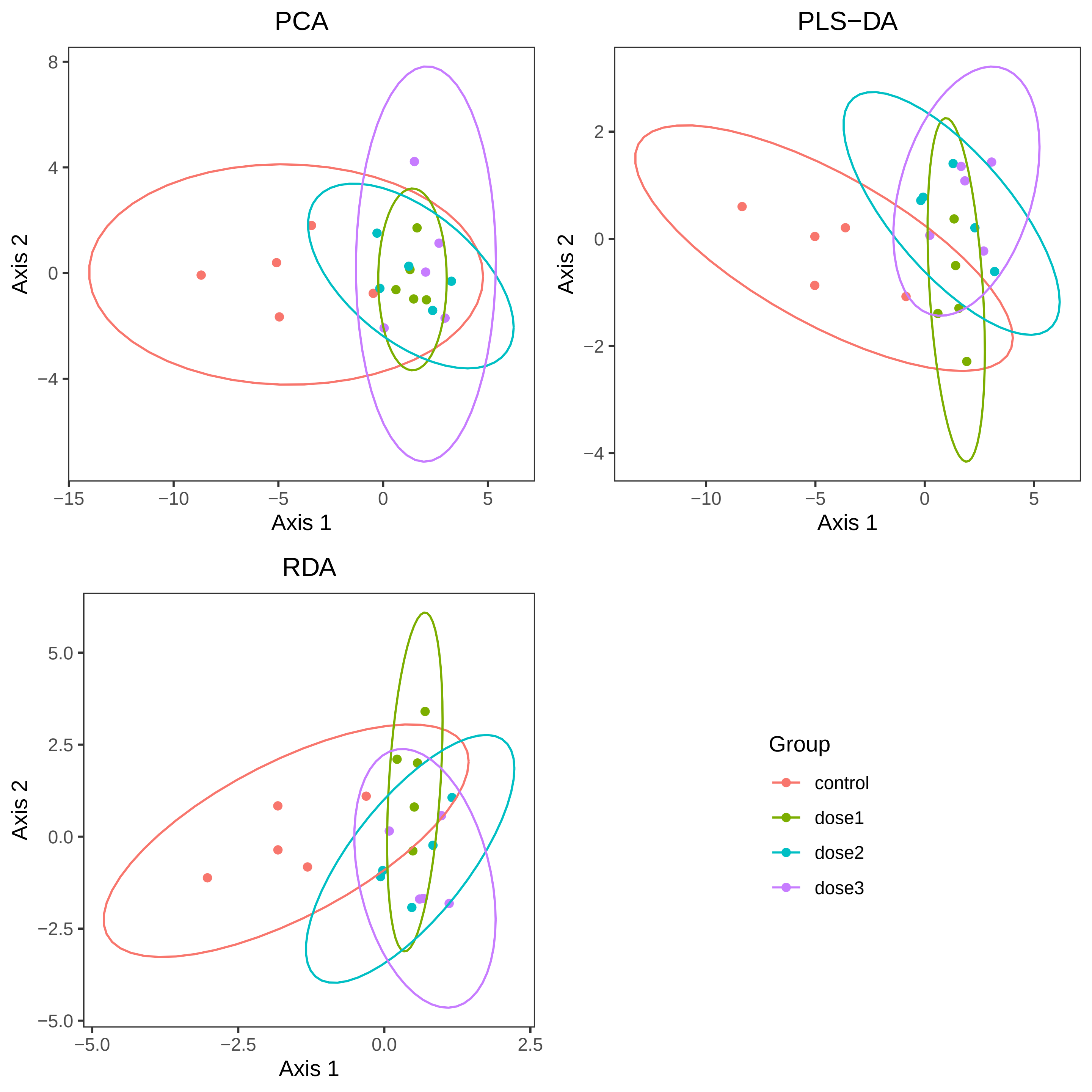
PCA
PLS−DA
RDA
Axis 1
Axis 2
Group
control
dose1
dose2
dose3

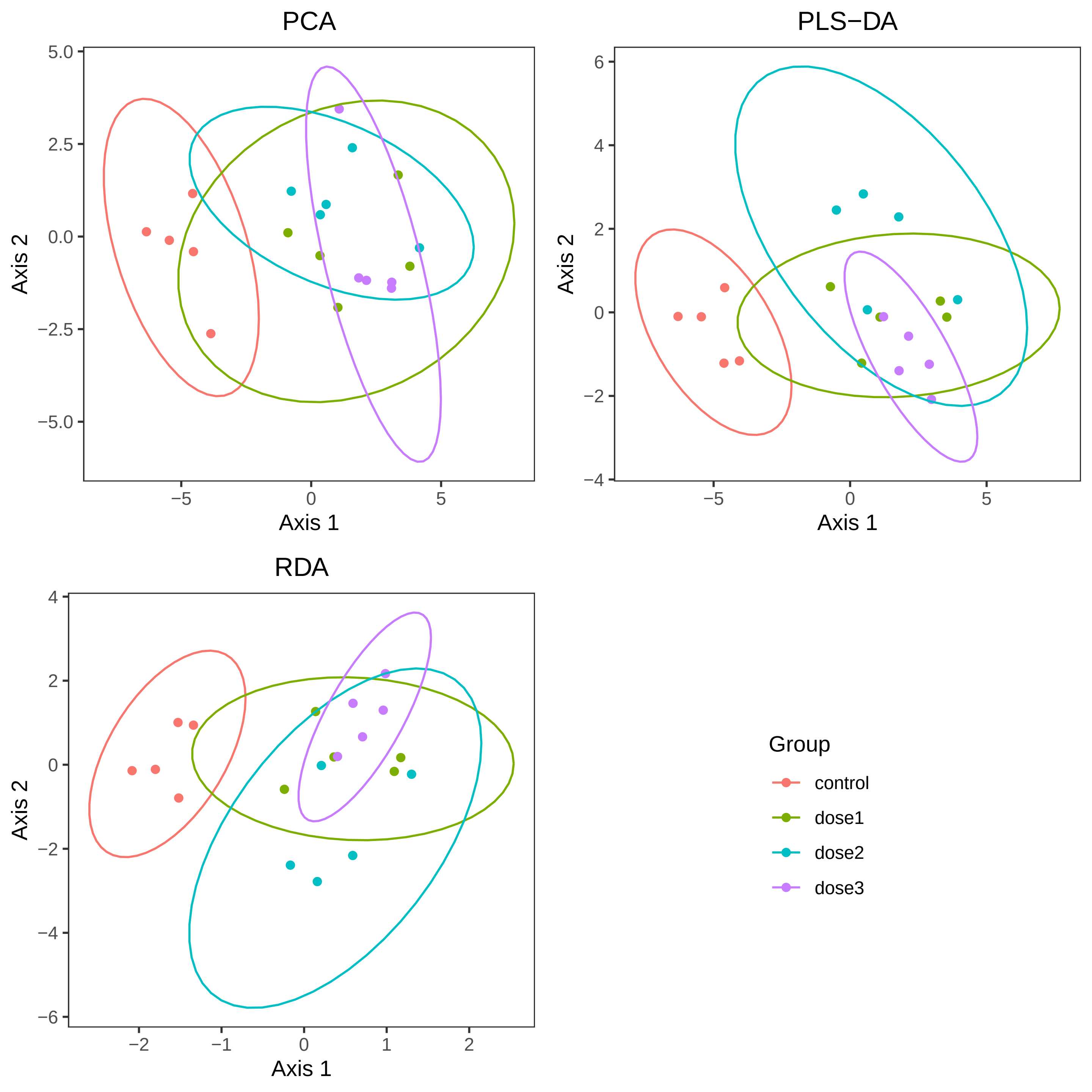
PCA
PLS−DA
RDA
Axis 1
Axis 2
Group
control
dose1
dose2
dose3

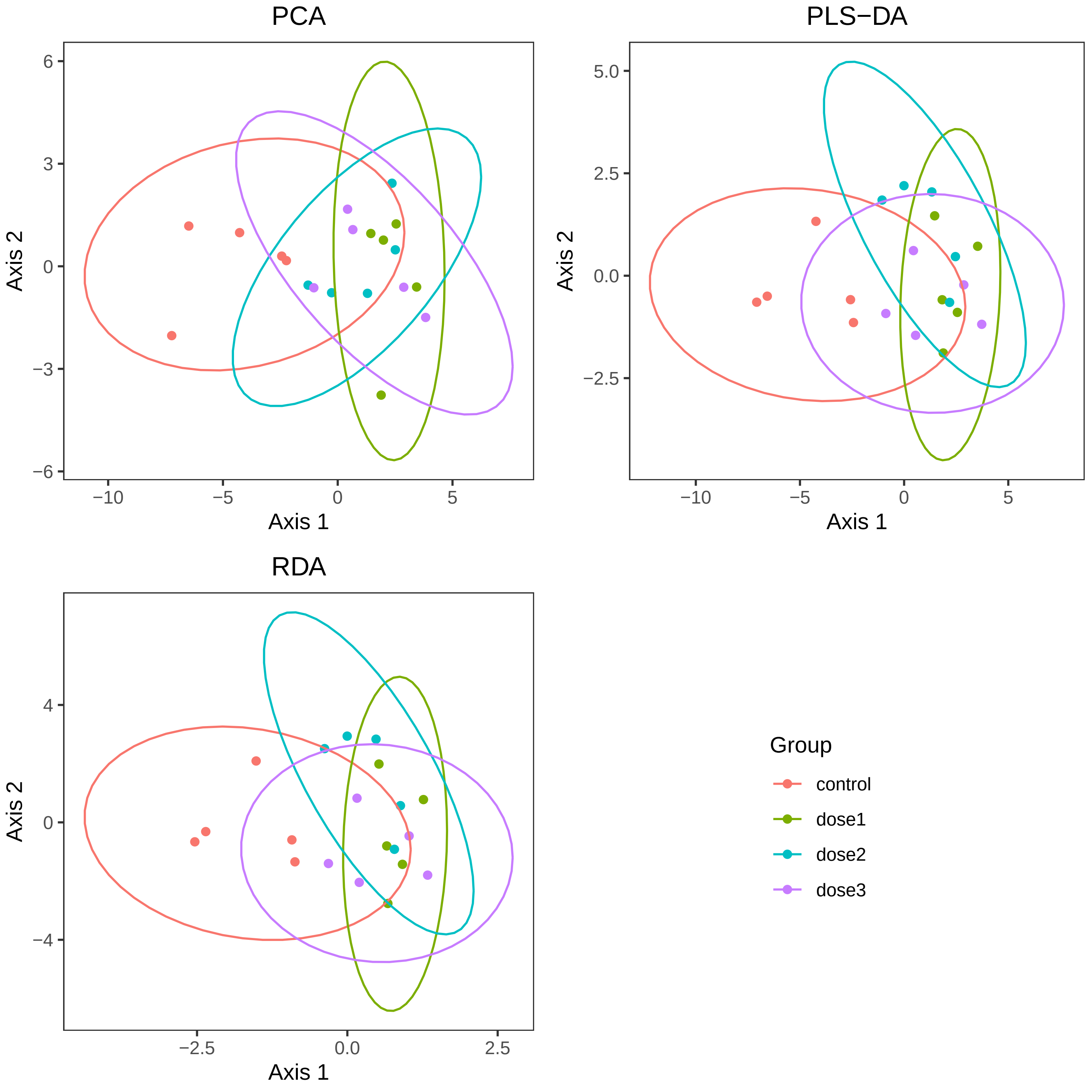
PCA
PLS−DA
RDA
Axis 1
Axis 2
Group
control
dose1
dose2
dose3

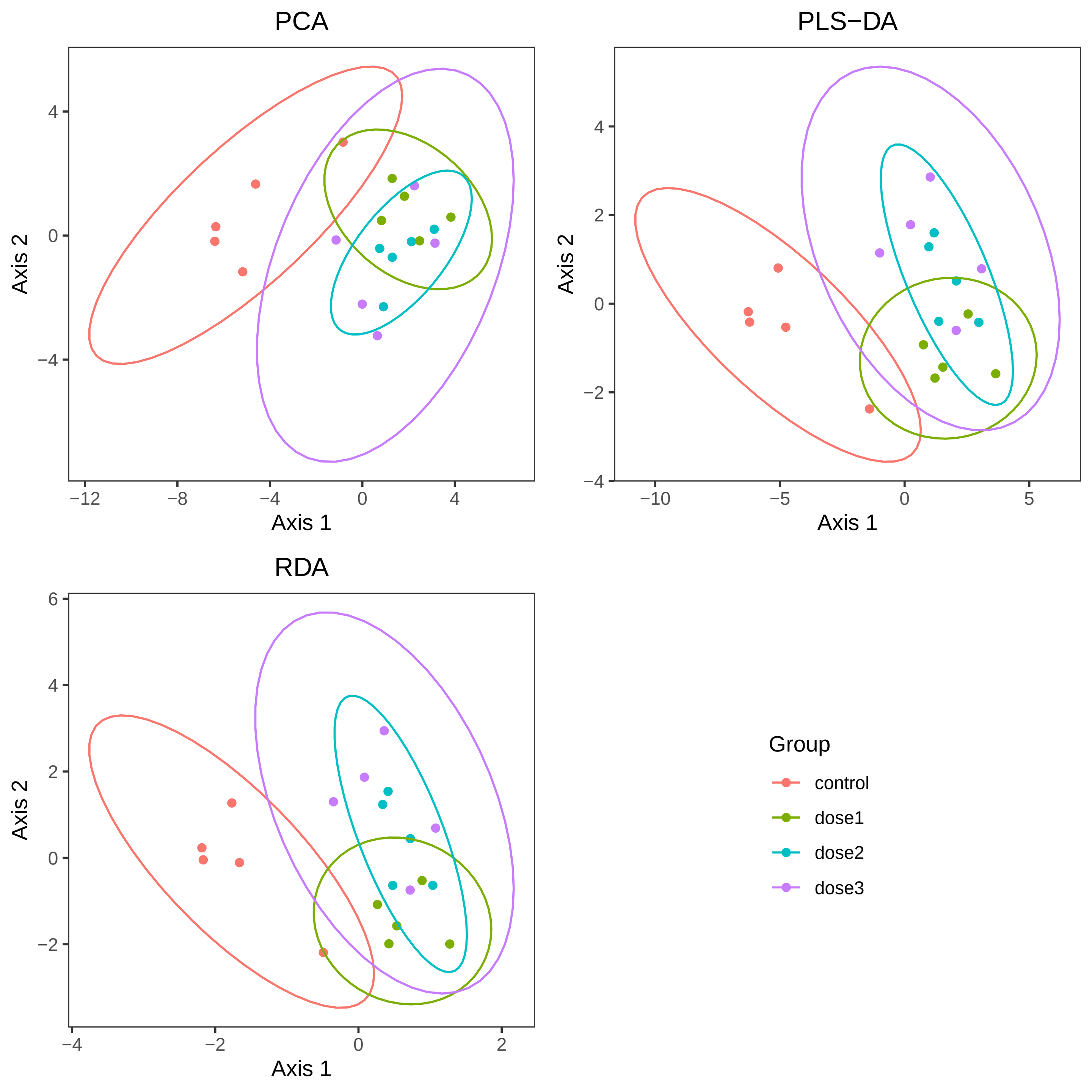

PCA
PLS−DA
RDA
Axis 1
Axis 2
4
0
−4
−12
−8
−4
0
4
−10
−5
0
5
2
−2
6
−4
−2
0
2
Group
control
dose1
dose2
dose3

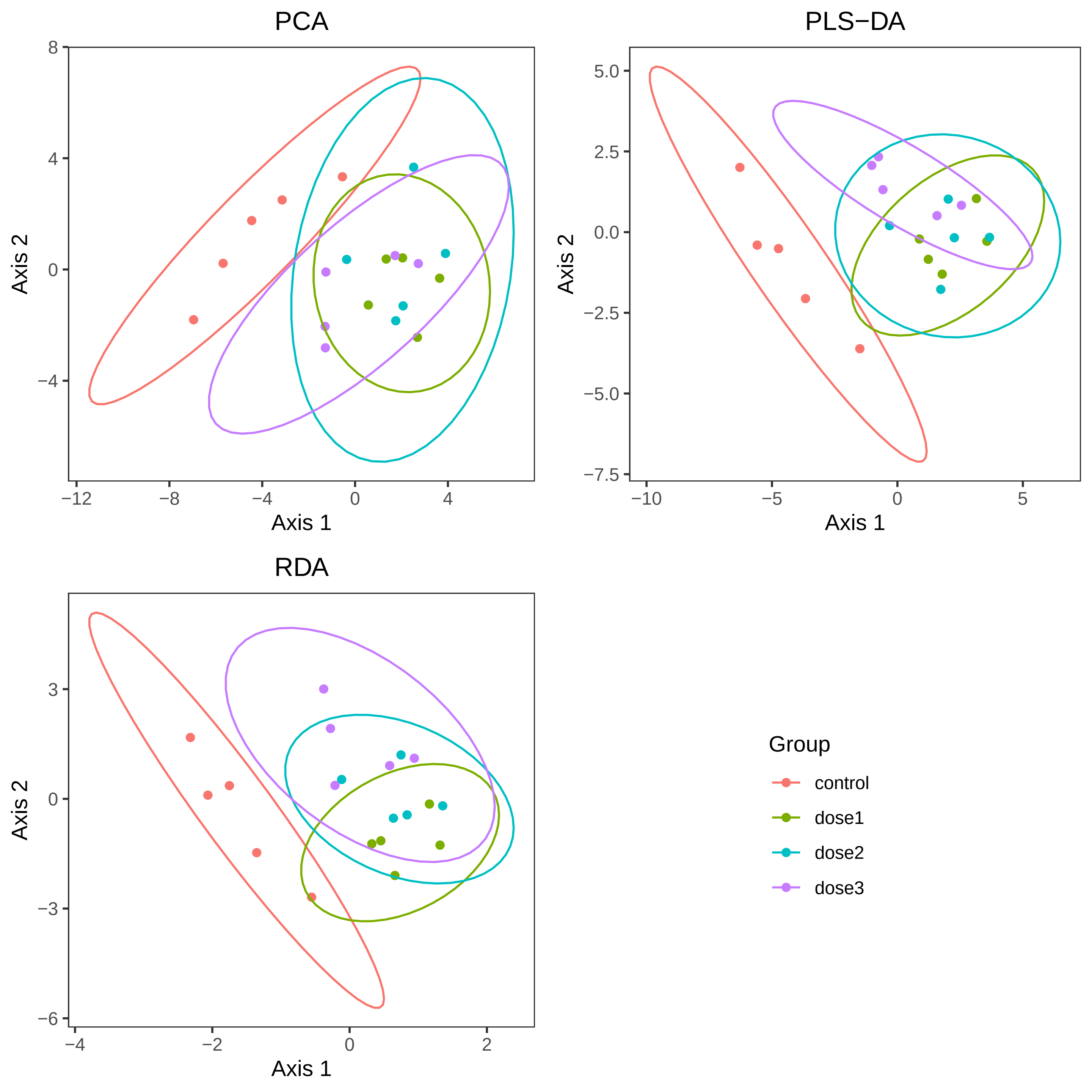

PCA
PLS−DA
RDA
Axis 1
Axis 2
Group
control
dose1
dose2
dose3

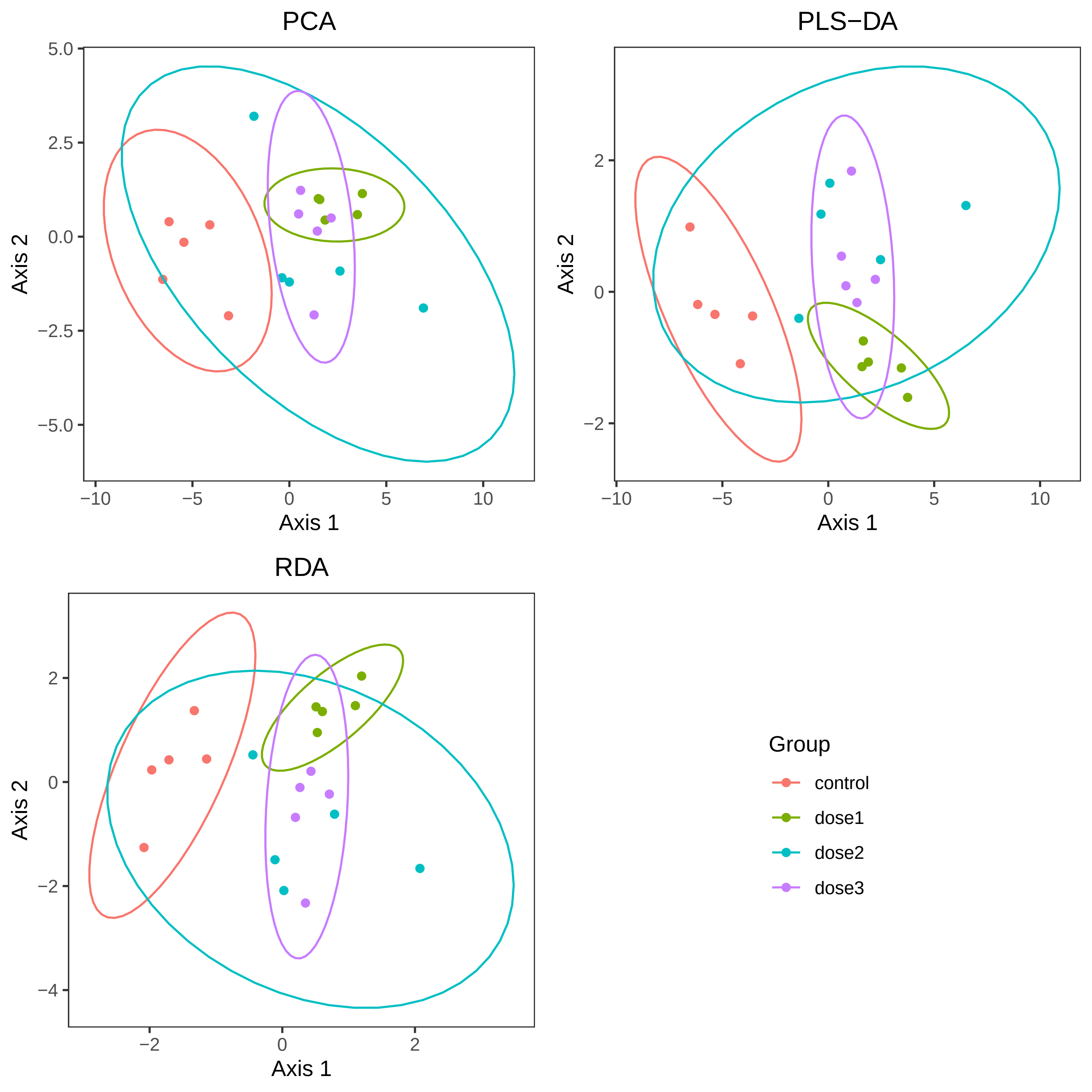
PCA
PLS−DA
RDA
Axis 1
Axis 2
Group
control
dose1
dose2
dose3

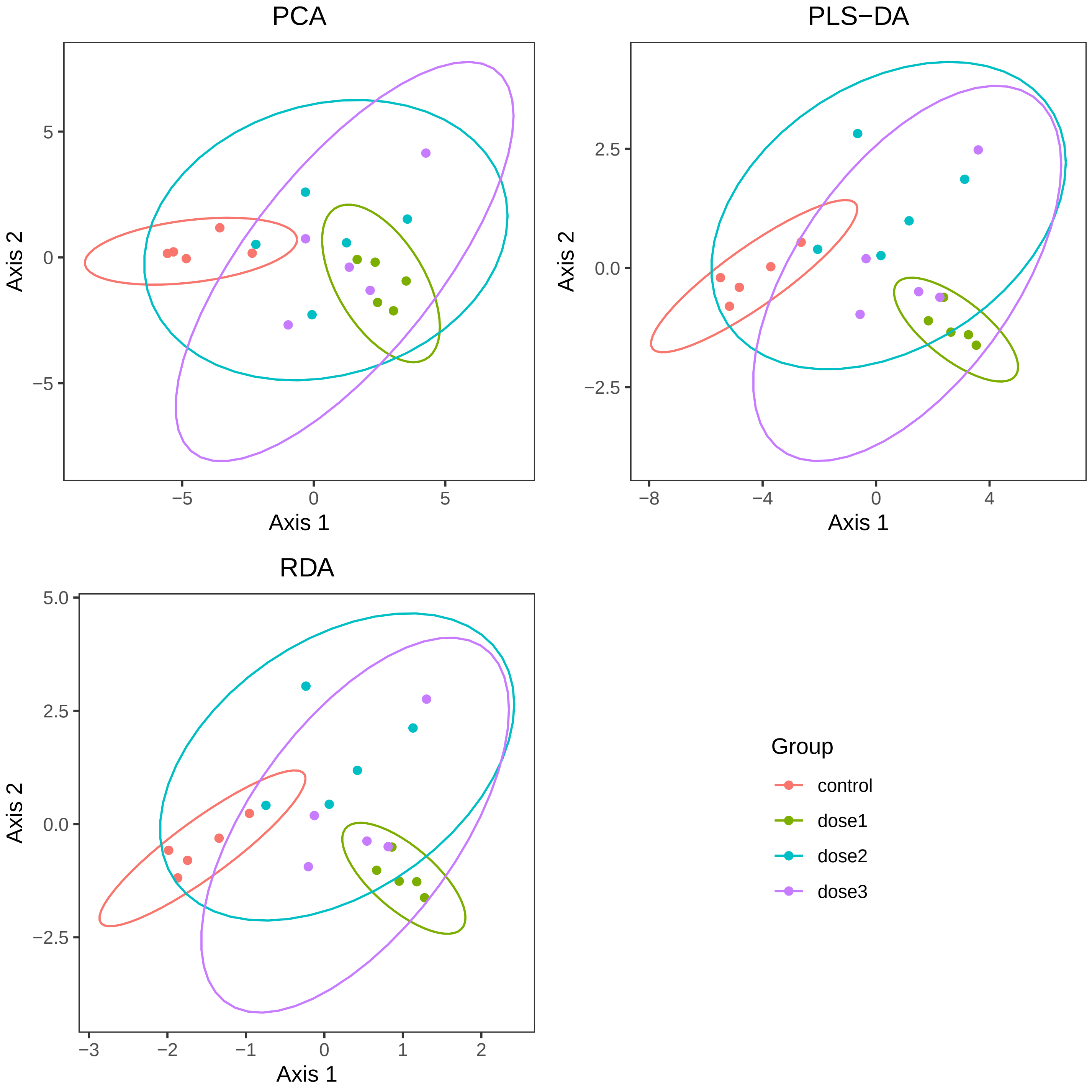
PCA
PLS−DA
RDA
Axis 1
Axis 2
Group
control
dose1
dose2
dose3

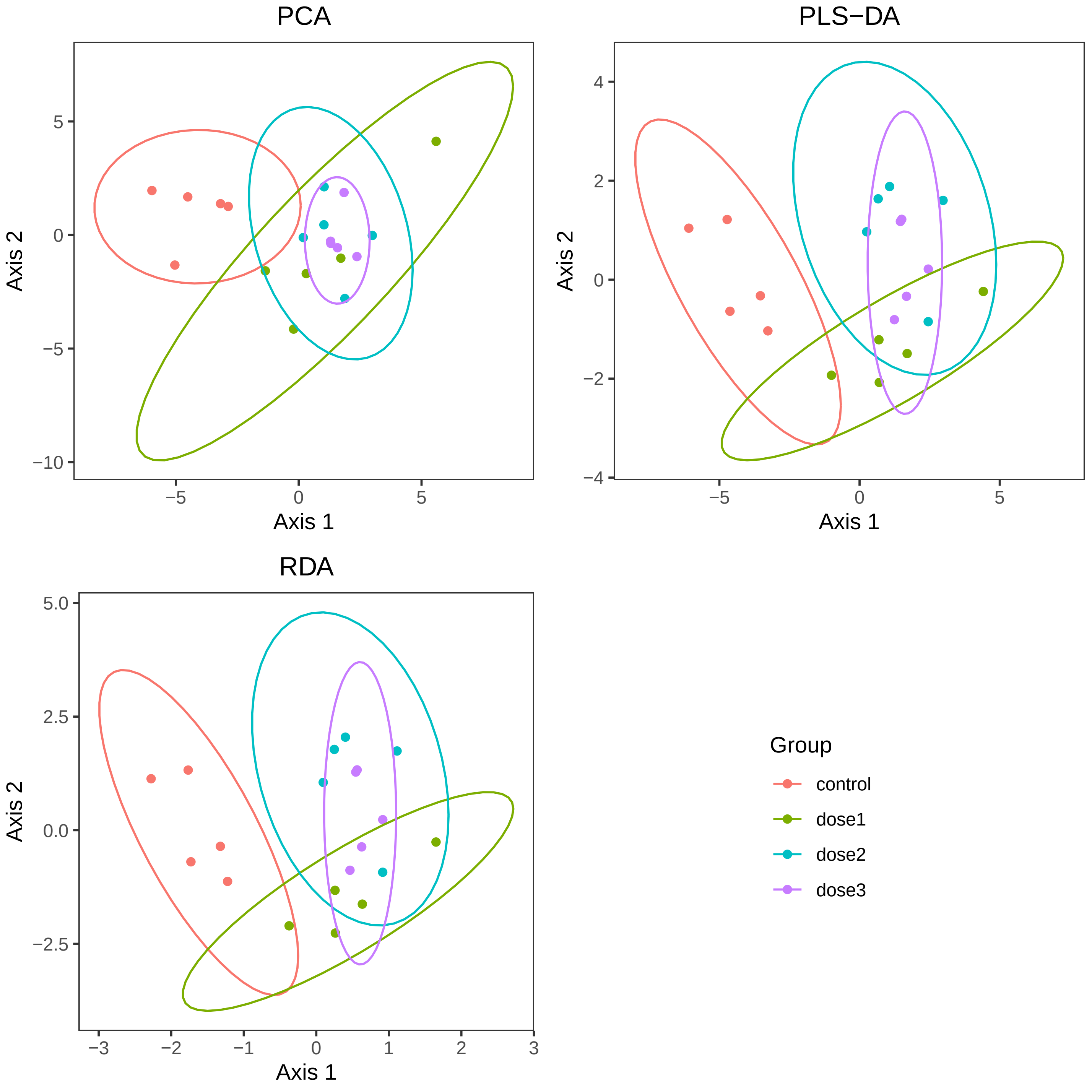

PCA
PLS−DA
RDA
Axis 1
Axis 2
Group
control
dose1
dose2
dose3

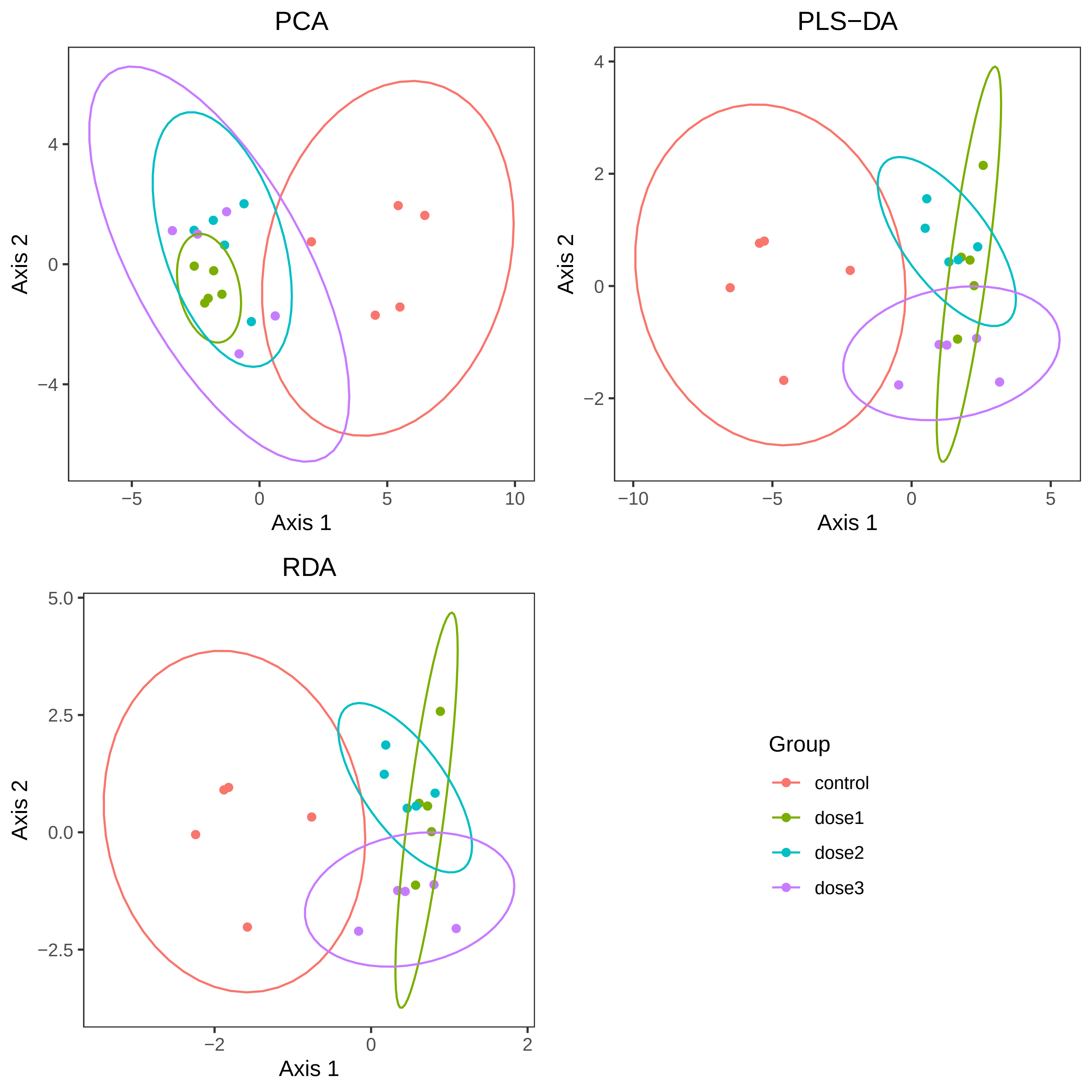

PCA
PLS−DA
RDA
Axis 1
Axis 2
Group
control
dose1
dose2
dose3

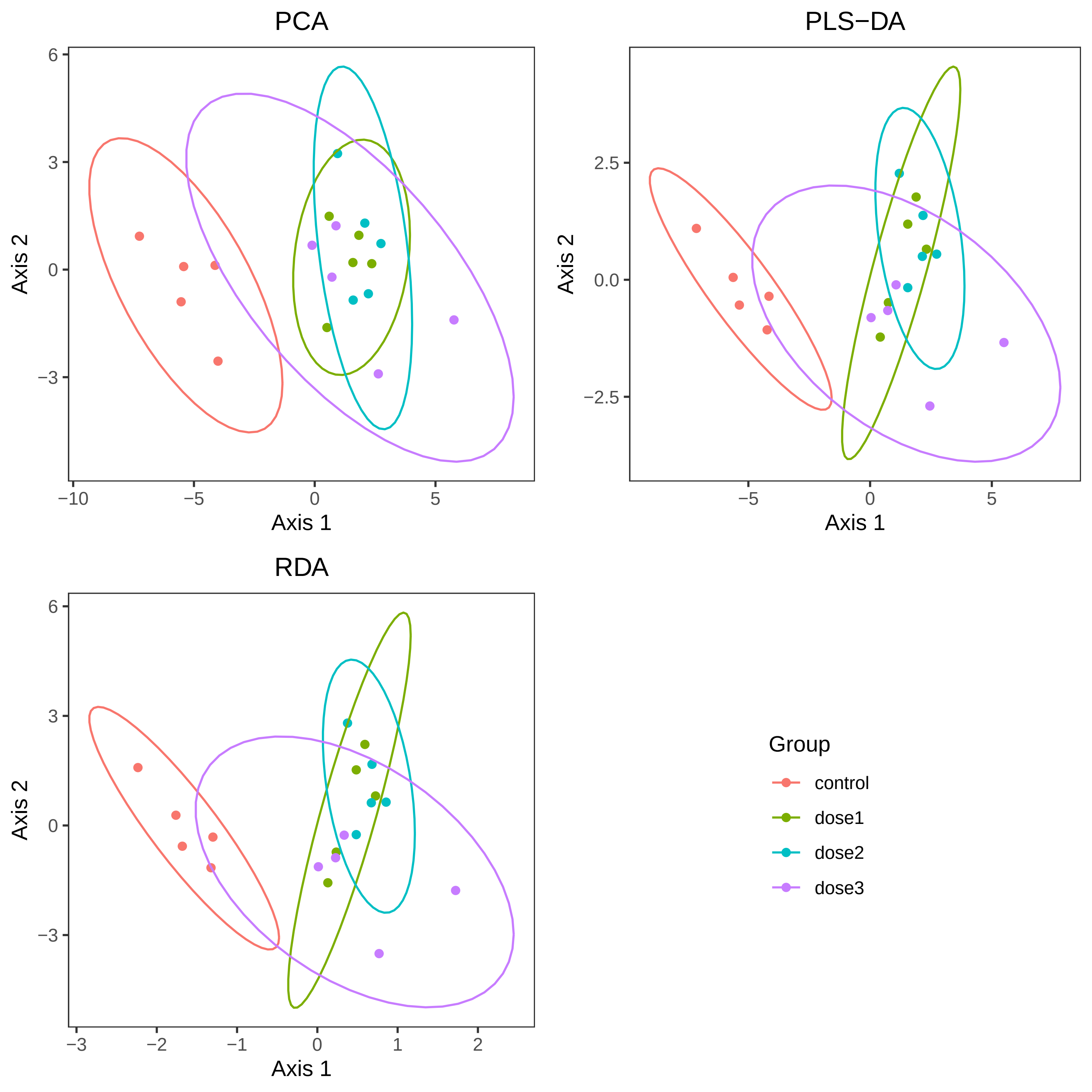
PCA
PLS−DA
RDA
Axis 1
Axis 2
−10
−5
0
5
6
3
0
−3
2.5
0.0
−2.5
−3
−2
−1
1
2
Group
control
dose1
dose2
dose3

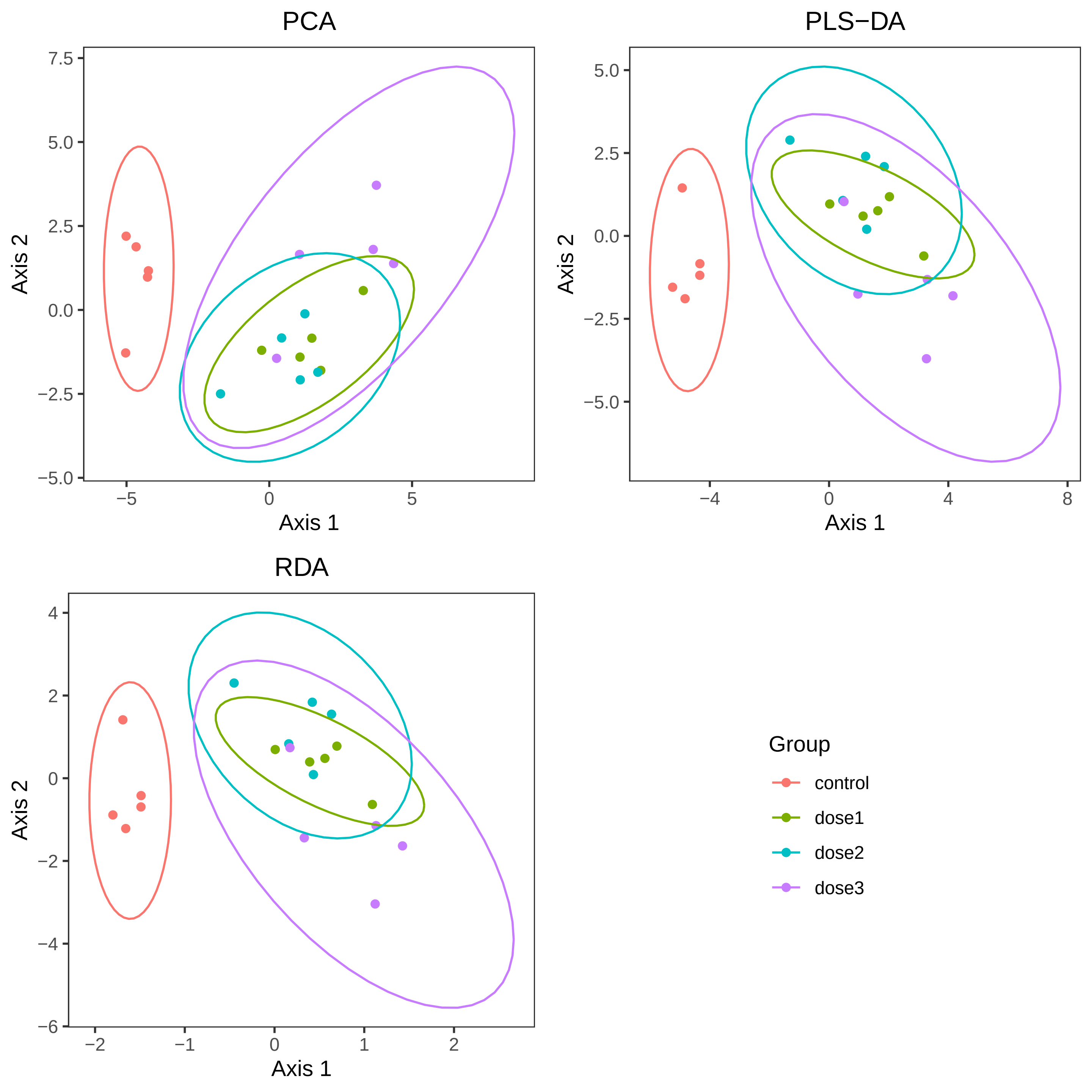

PCA
PLS−DA
RDA
Axis 1
Axis 2
Group
control
dose1
dose2
dose3

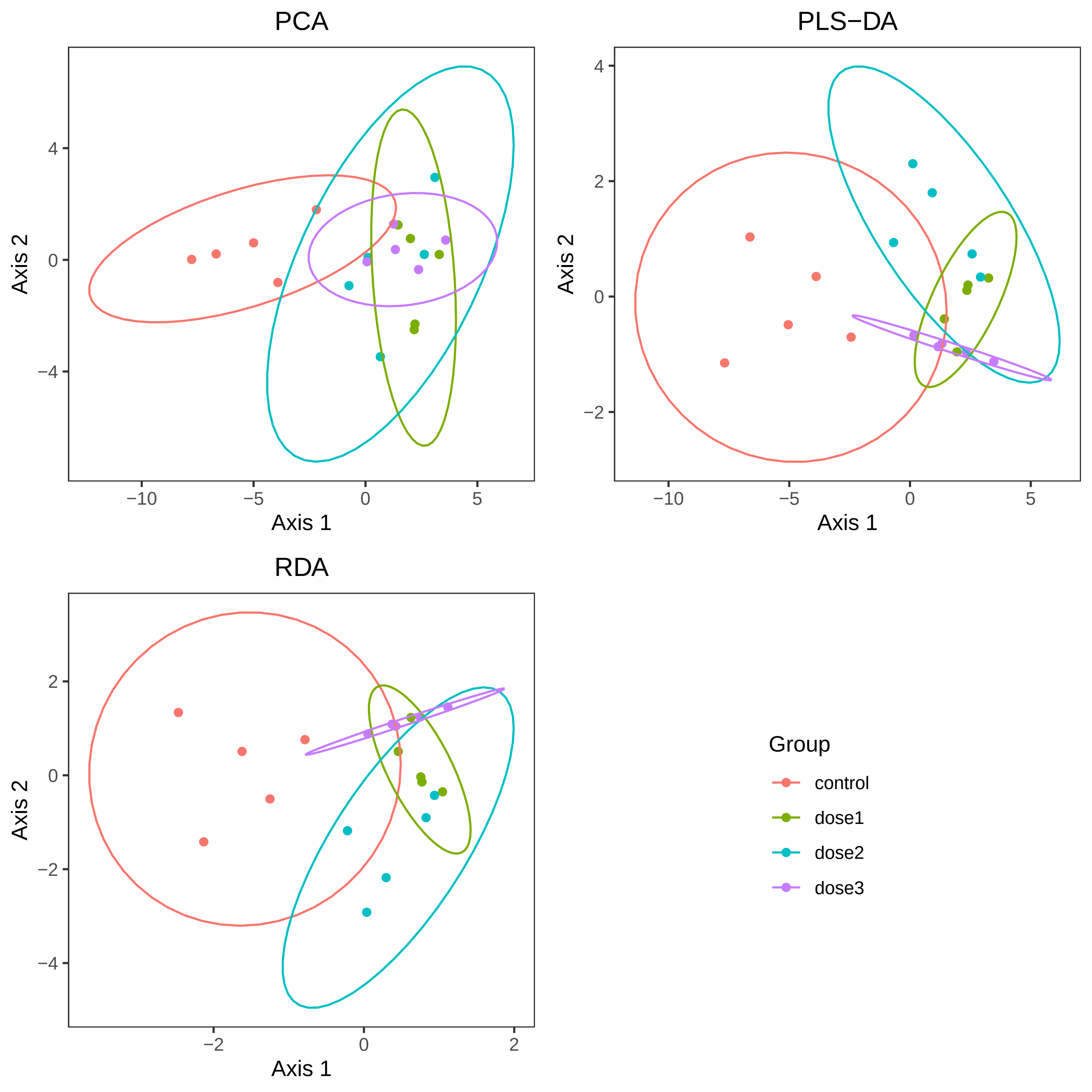
PCA
PLS−DA
RDA
Axis 1
Axis 2
−10
−5
0
5
4
0
−4
2
−2
Group
control
dose1
dose2
dose3

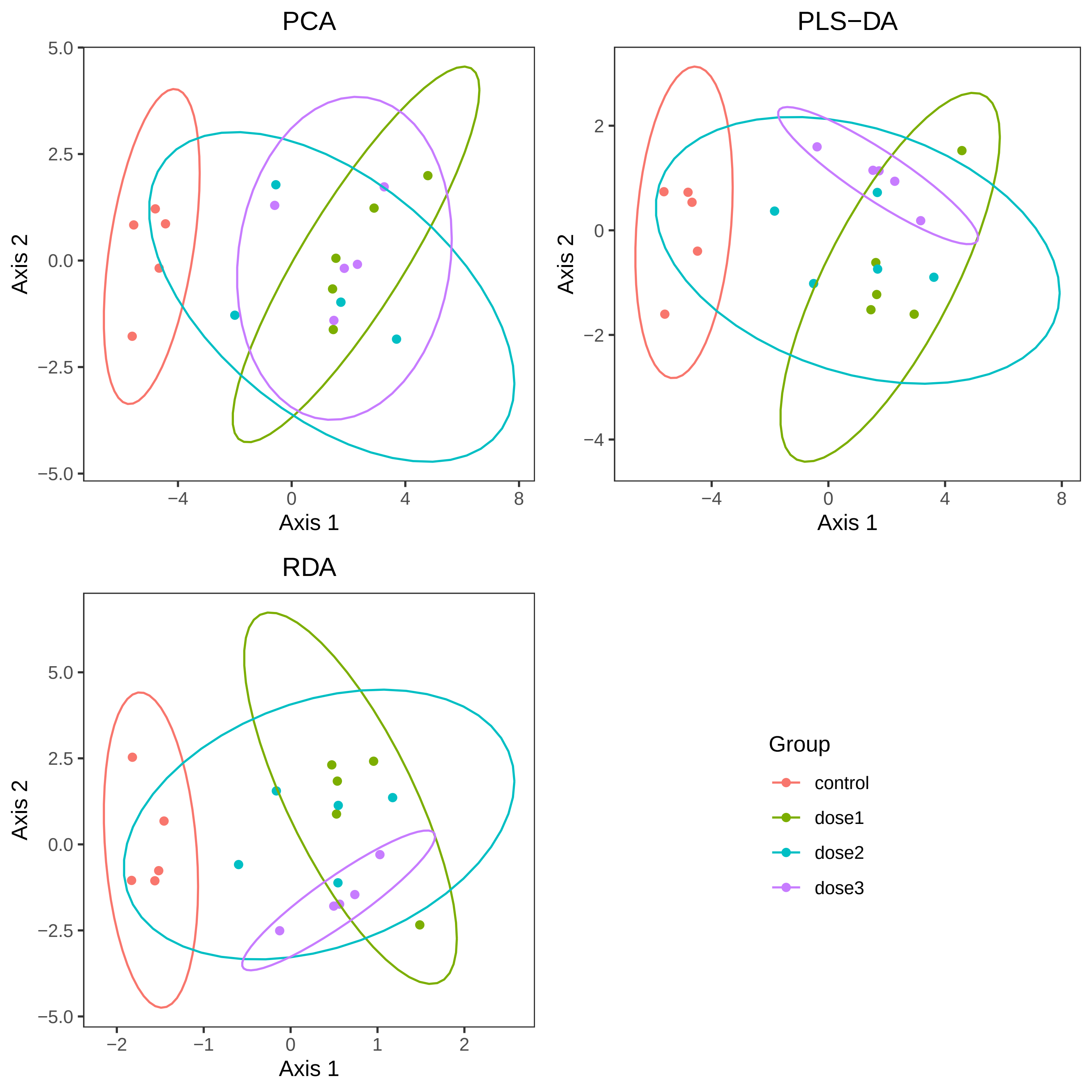

PCA
PLS−DA
RDA
Axis 1
Axis 2
Group
control
dose1
dose2
dose3

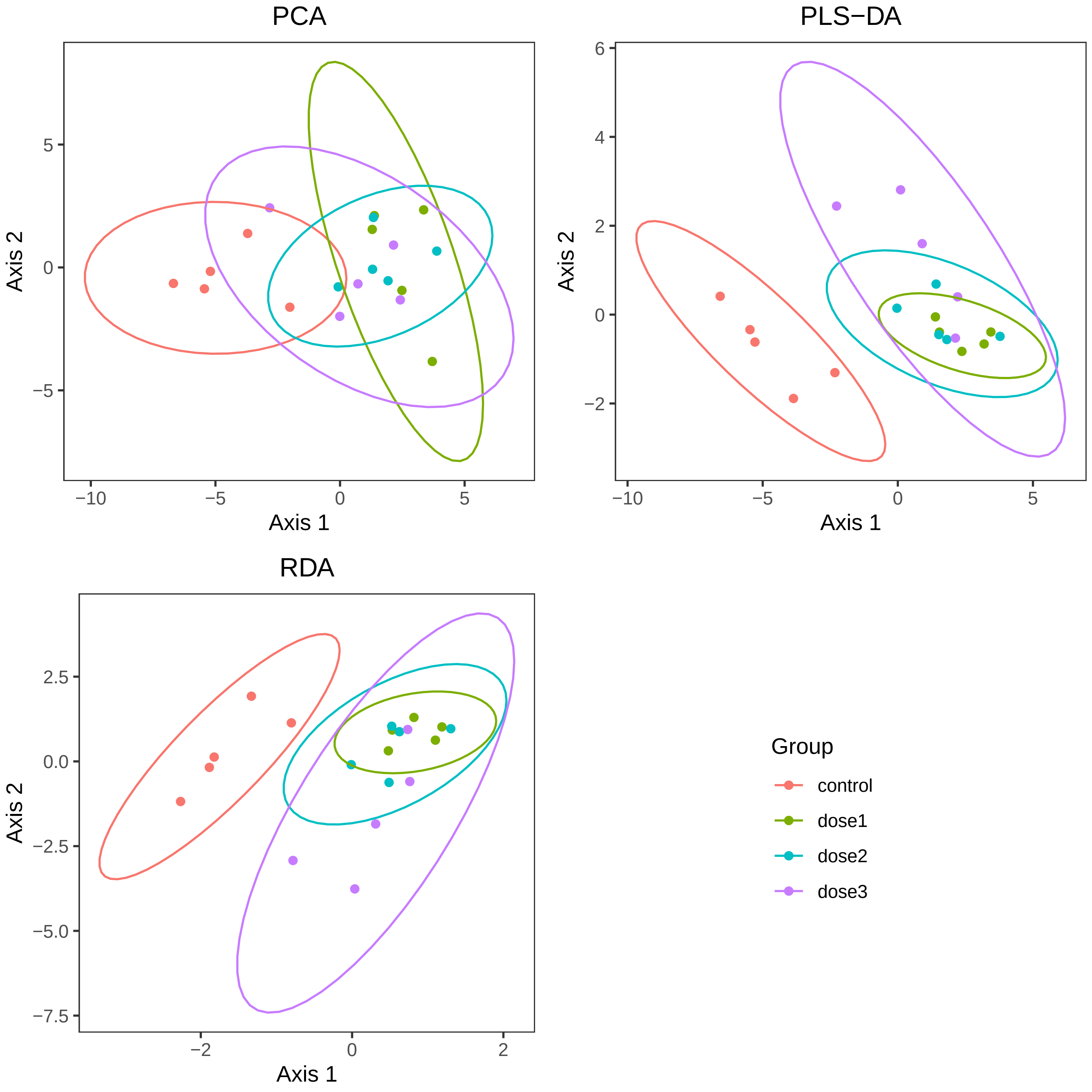

PCA
PLS−DA
RDA
Axis 1
Axis 2
Group
control
dose1
dose2
dose3

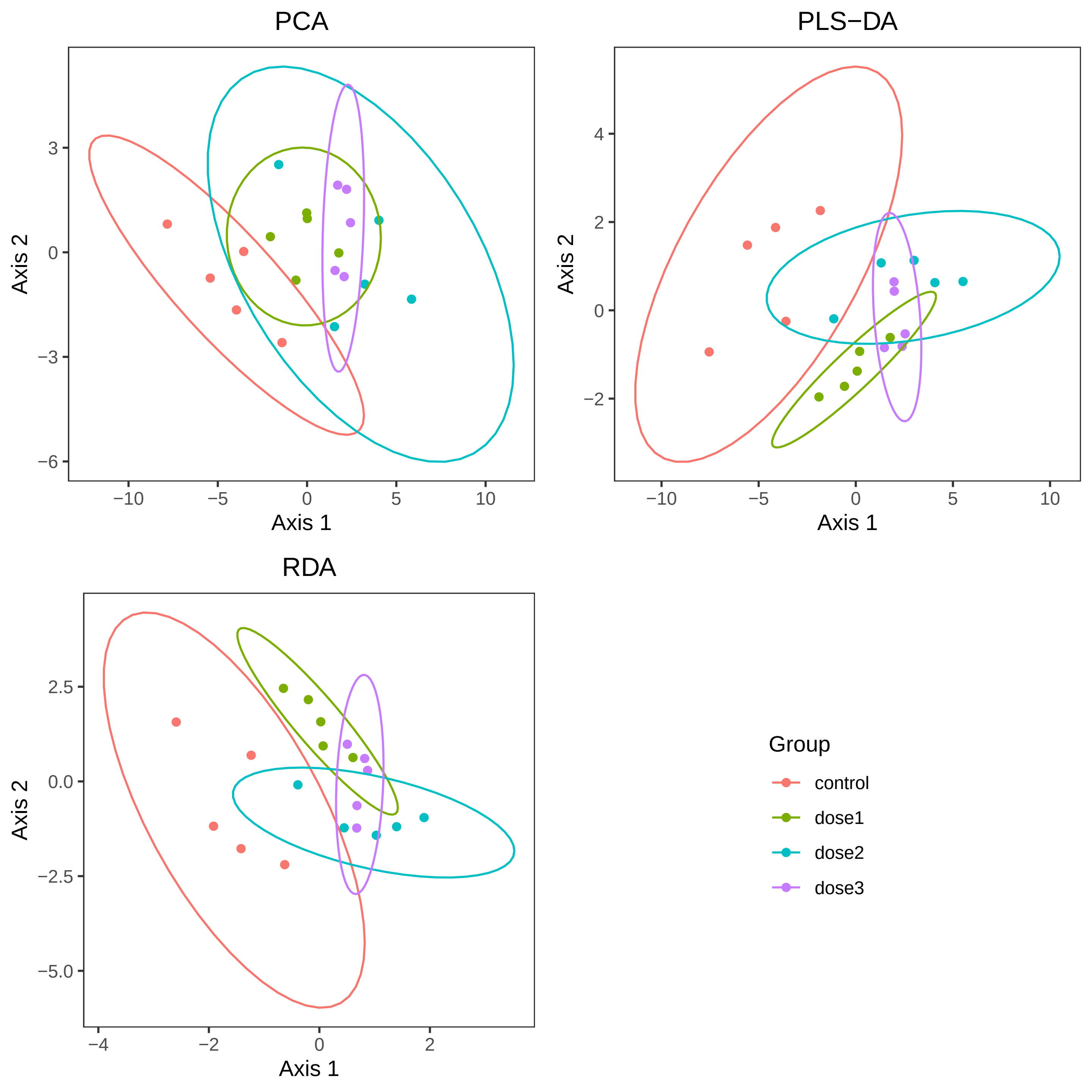
PCA
PLS−DA
RDA
Axis 1
Axis 2
Group
control
dose1
dose2
dose3

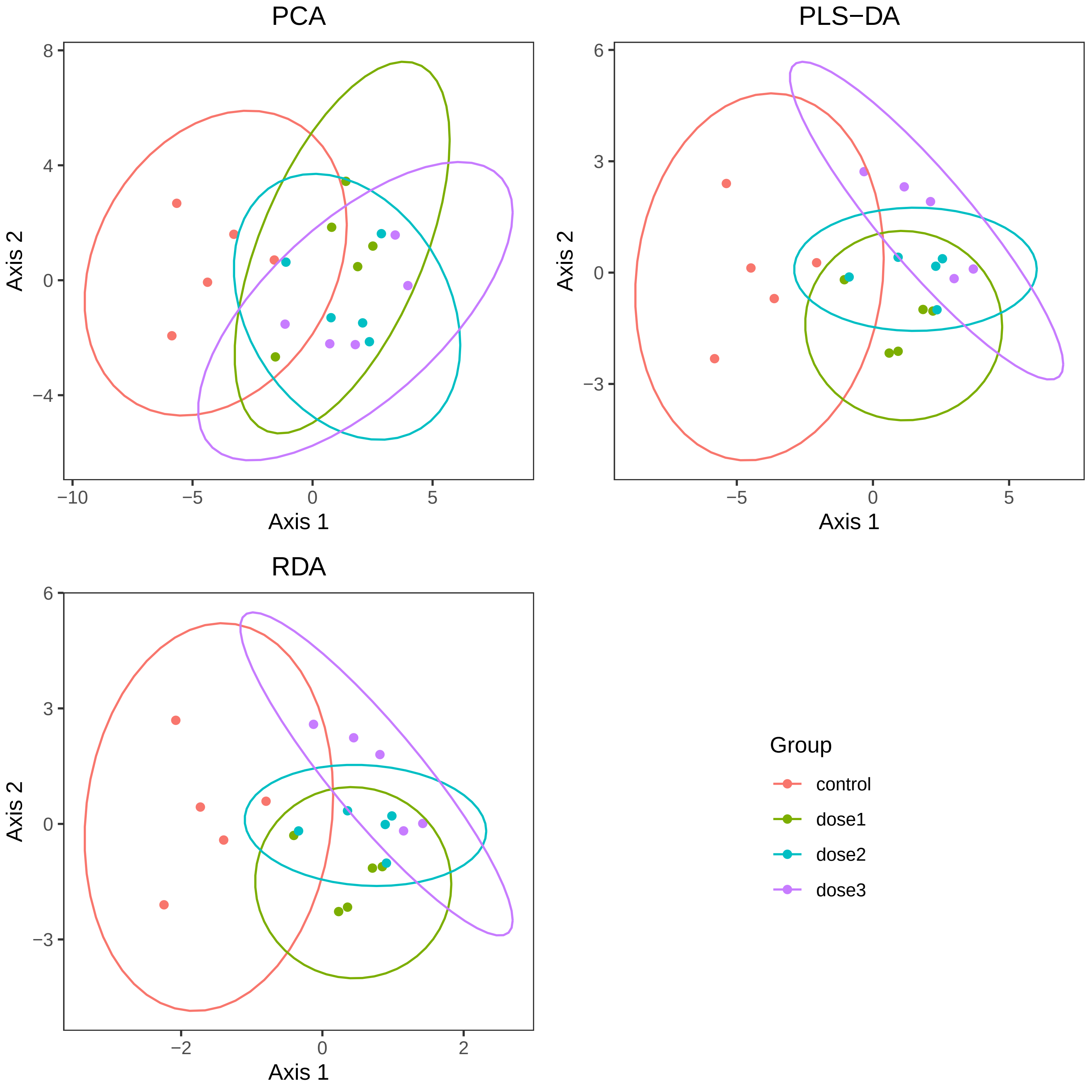
PCA
PLS−DA
RDA
Axis 1
Axis 2
Group
control
dose1
dose2
dose3

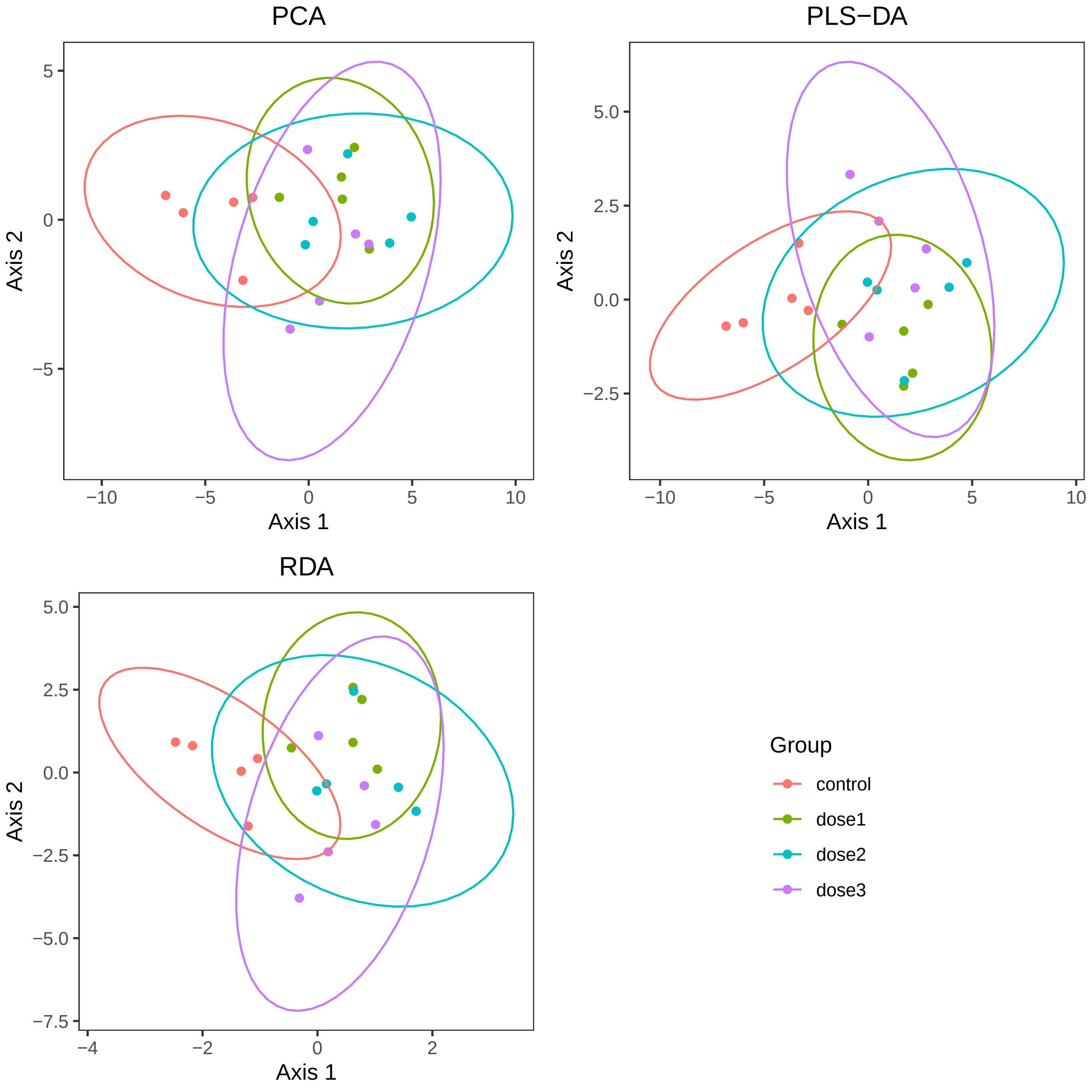

PCA
PLS−DA
RDA
Axis 1
Axis 2
Group
control
dose1
dose2
dose3

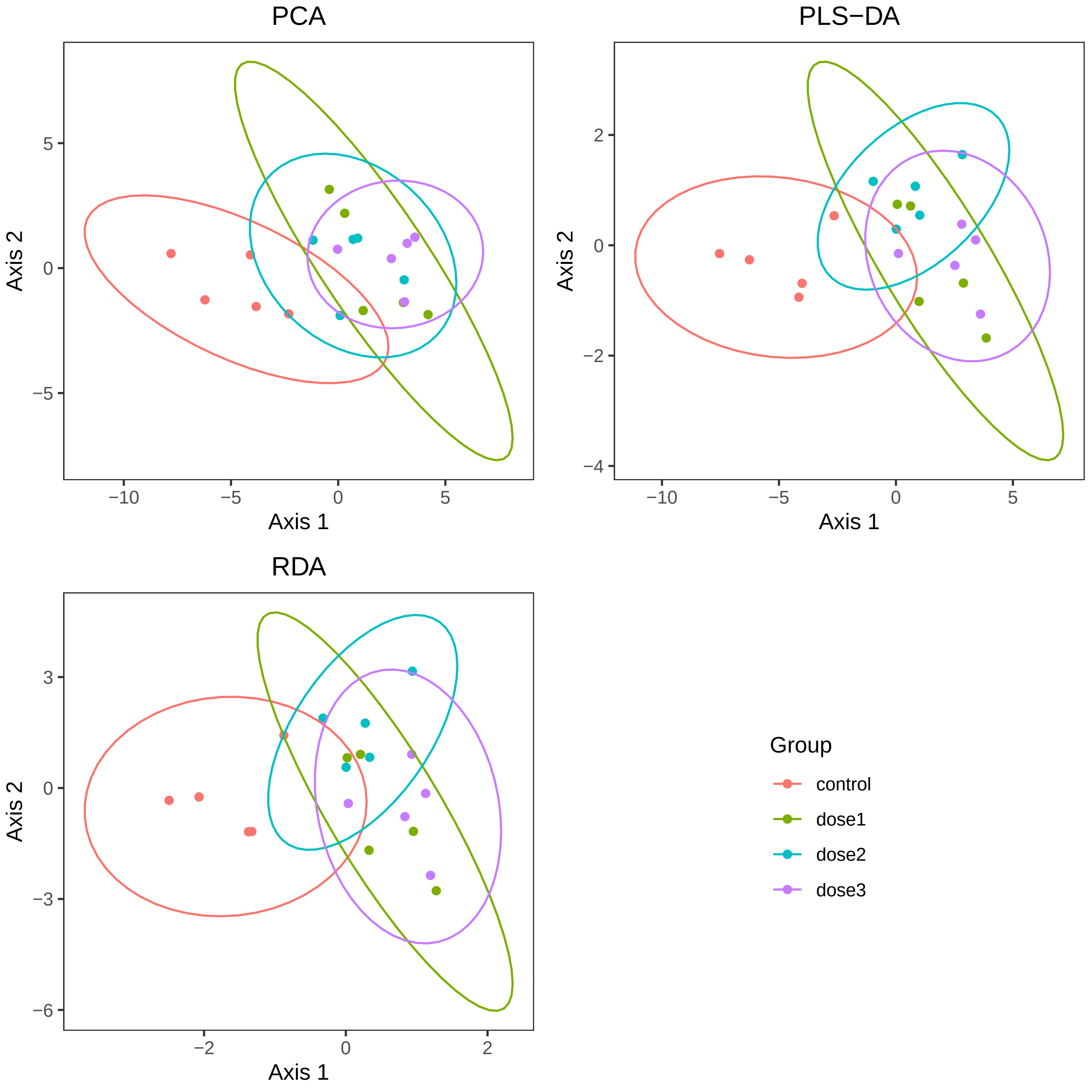

PCA
PLS−DA
RDA
Axis 1
Axis 2
Group
control
dose1
dose2
dose3

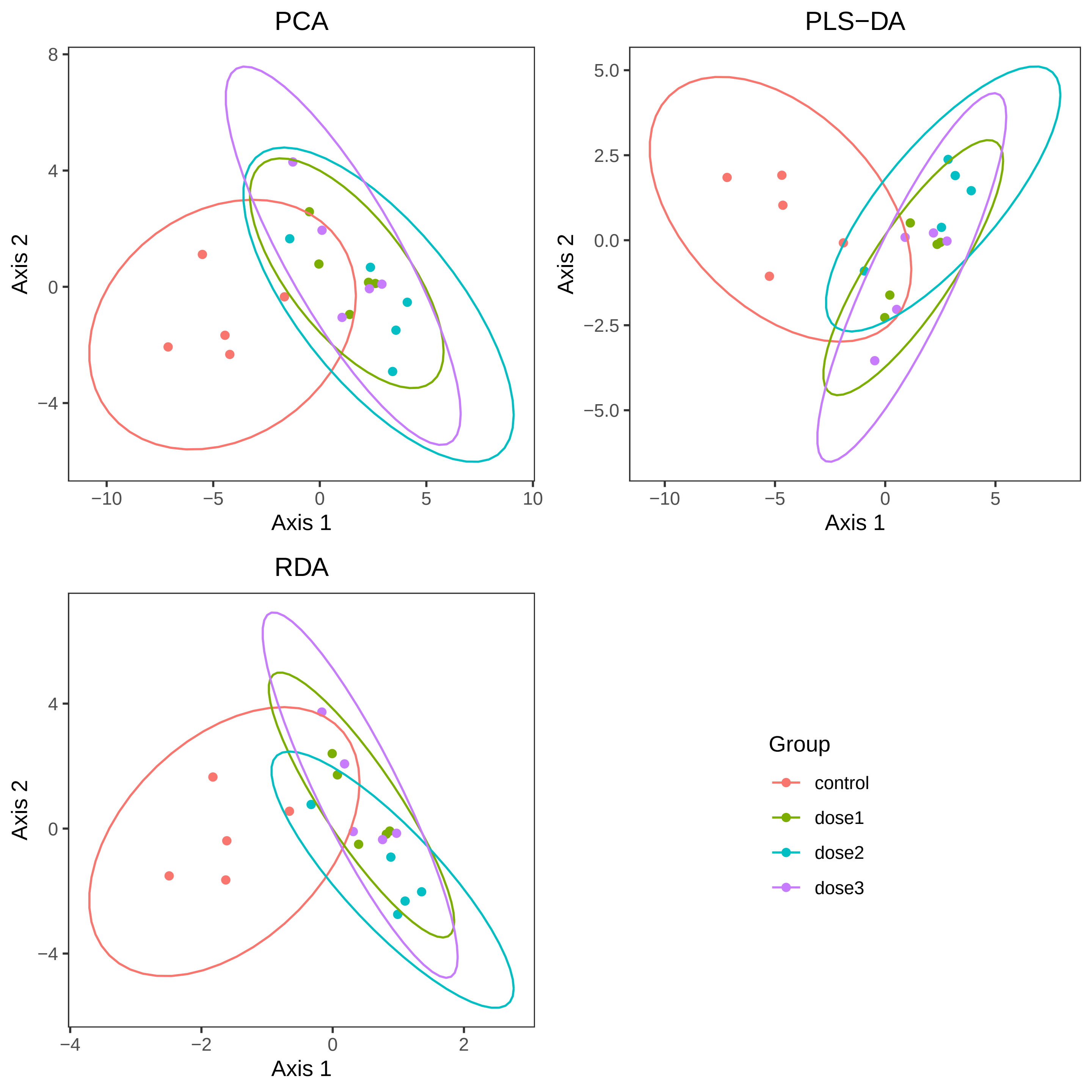
PCA
PLS−DA
RDA
Axis 1
Axis 2
Group
control
dose1
dose2
dose3

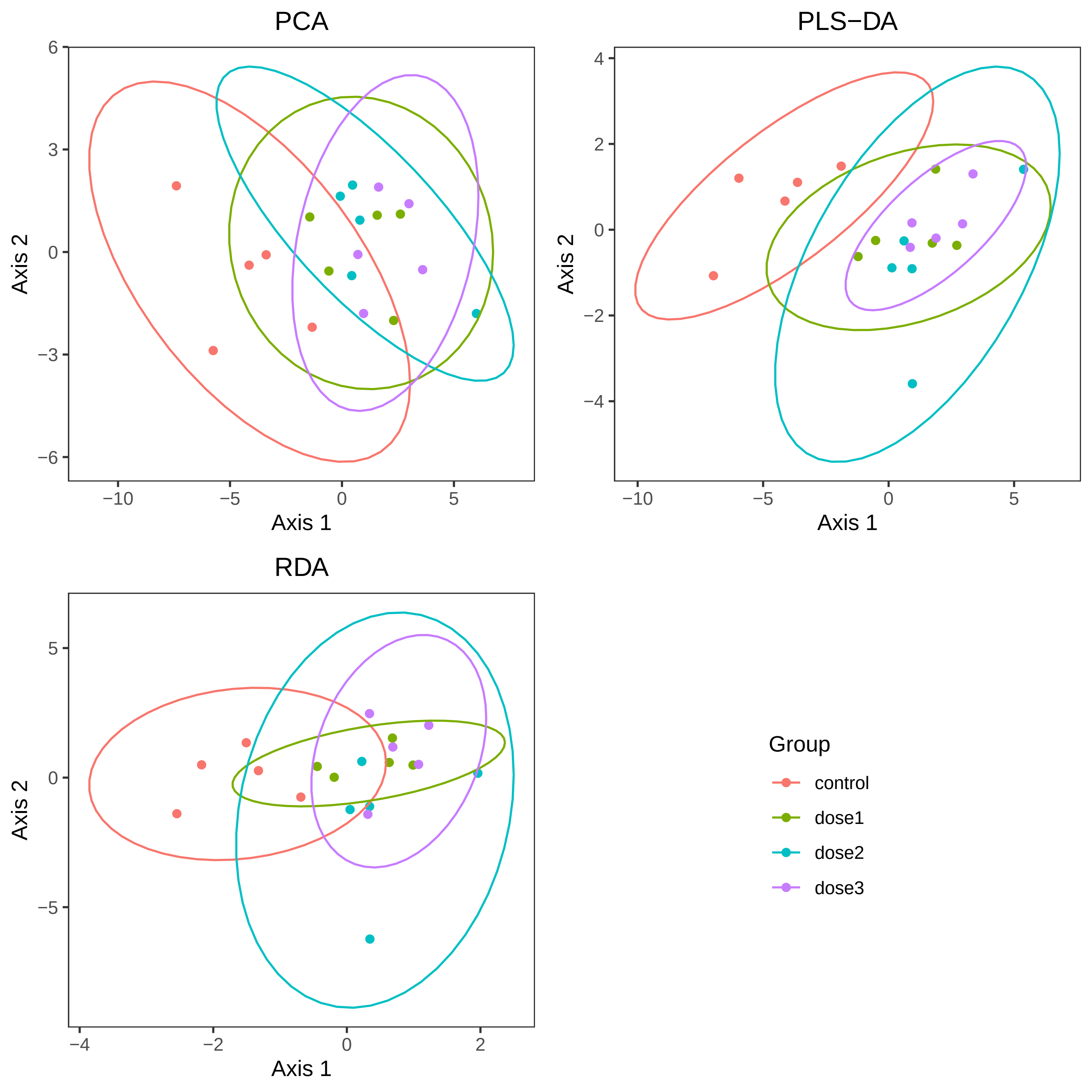
PCA
PLS−DA
RDA
Axis 1
Axis 2
Group
control
dose1
dose2
dose3

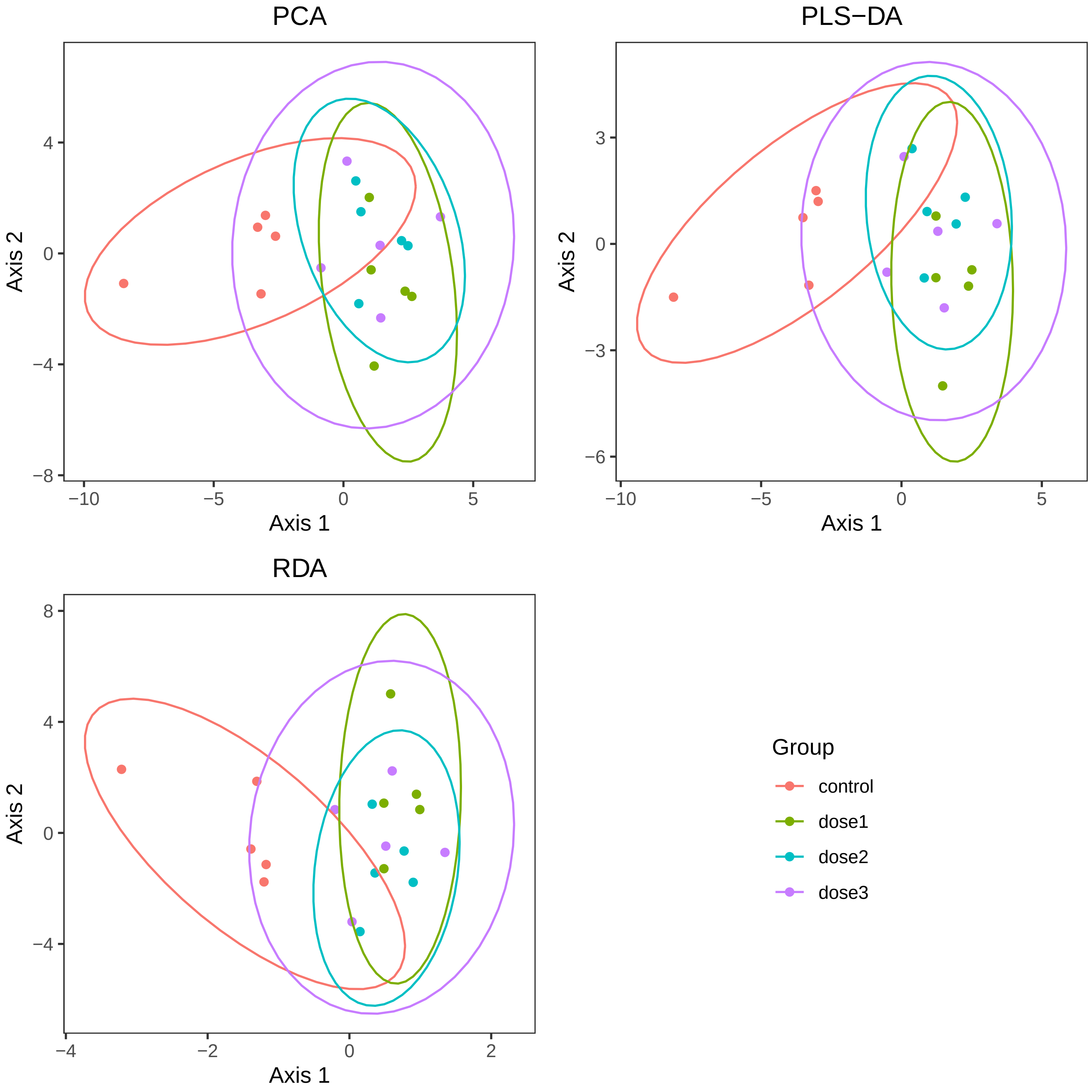
PCA
PLS−DA
RDA
Axis 1
Axis 2
Group
control
dose1
dose2
dose3

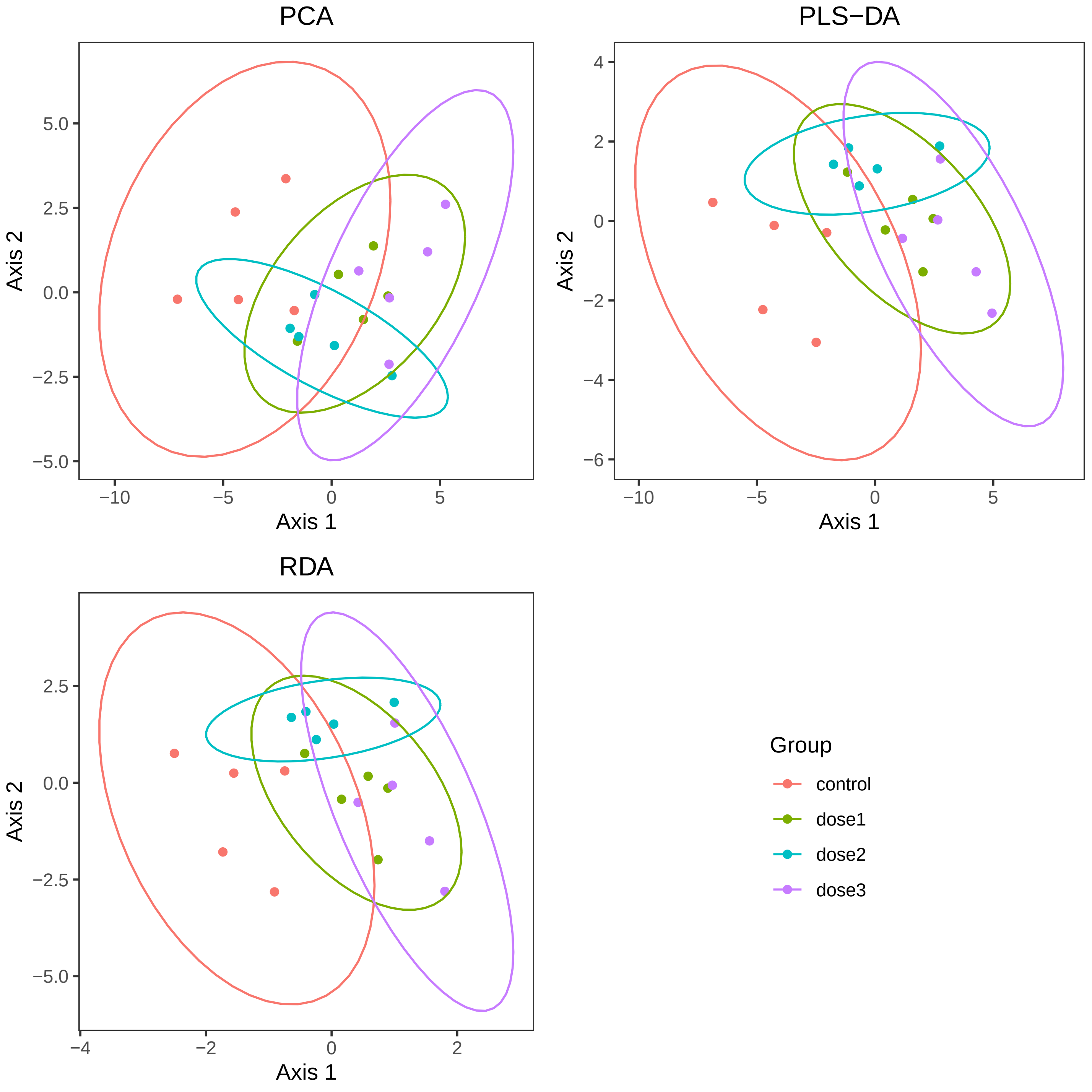
PCA
PLS−DA
RDA
Axis 1
Axis 2
Group
control
dose1
dose2
dose3

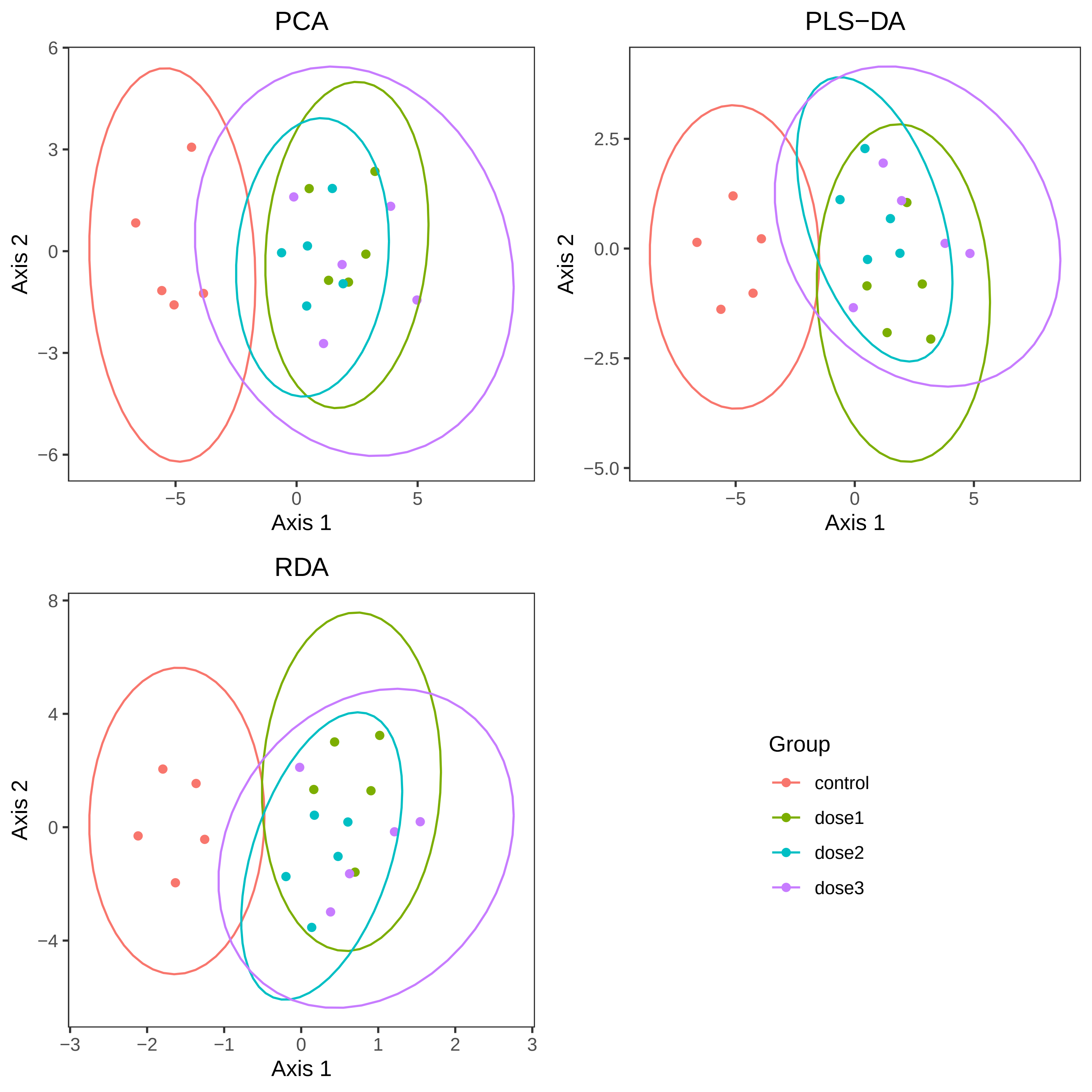

PCA
PLS−DA
RDA
Axis 1
Axis 2
Group
control
dose1
dose2
dose3

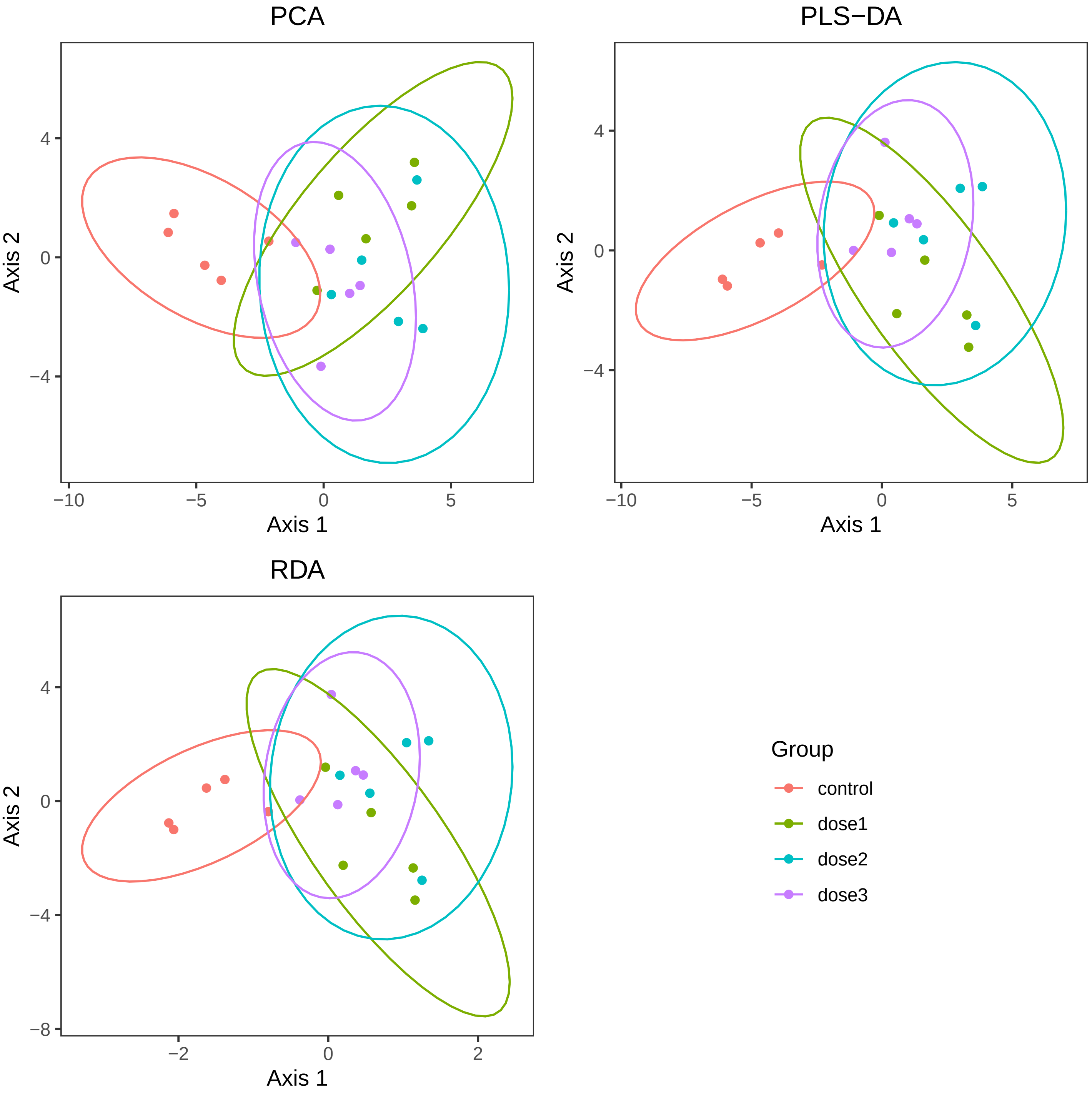
PCA
PLS−DA
RDA
Axis 1
Axis 2
−10
−5
0
5
4
0
−4
−2
2
−8
Group
control
dose1
dose2
dose3

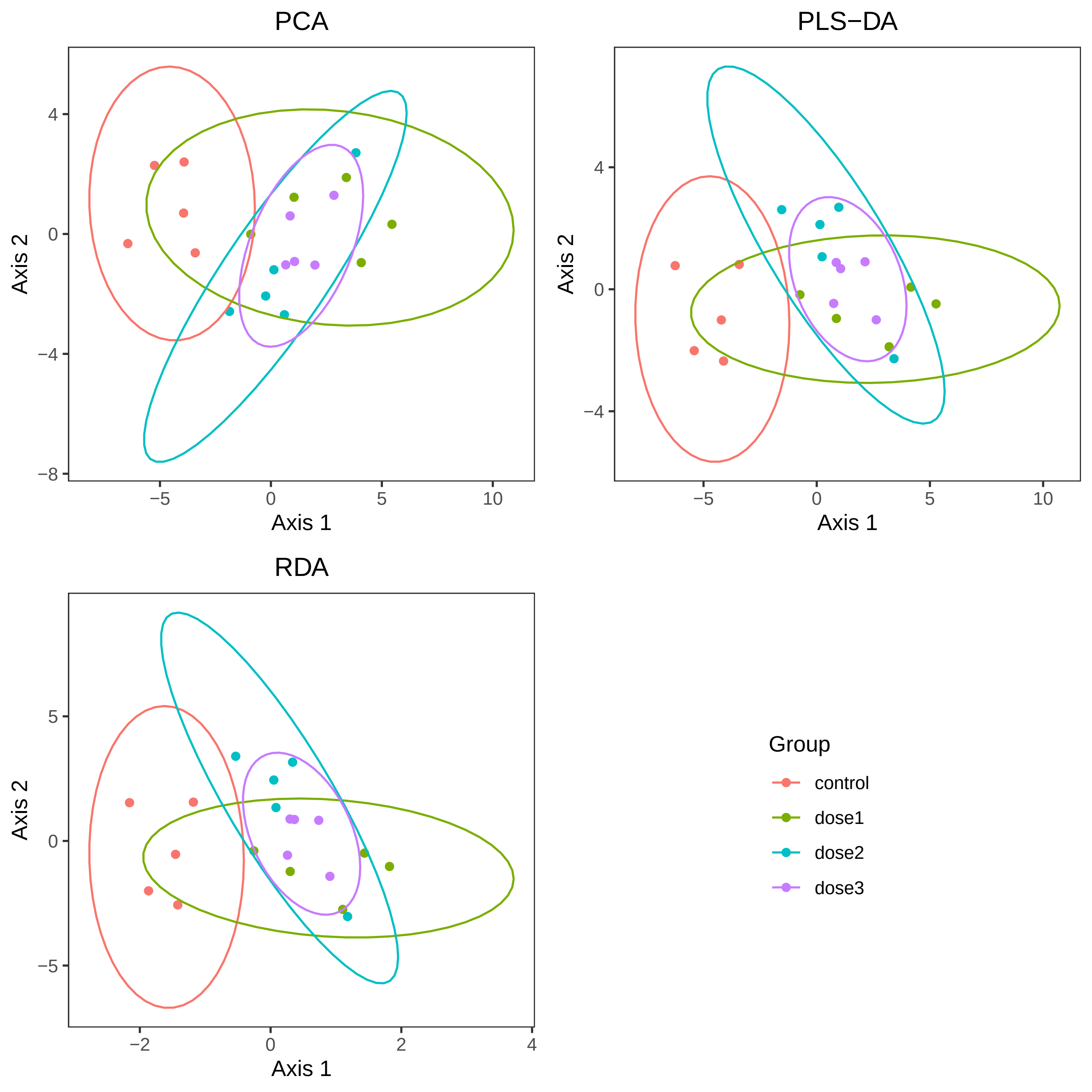

PCA
PLS−DA
RDA
Axis 1
Axis 2
Group
control
dose1
dose2
dose3

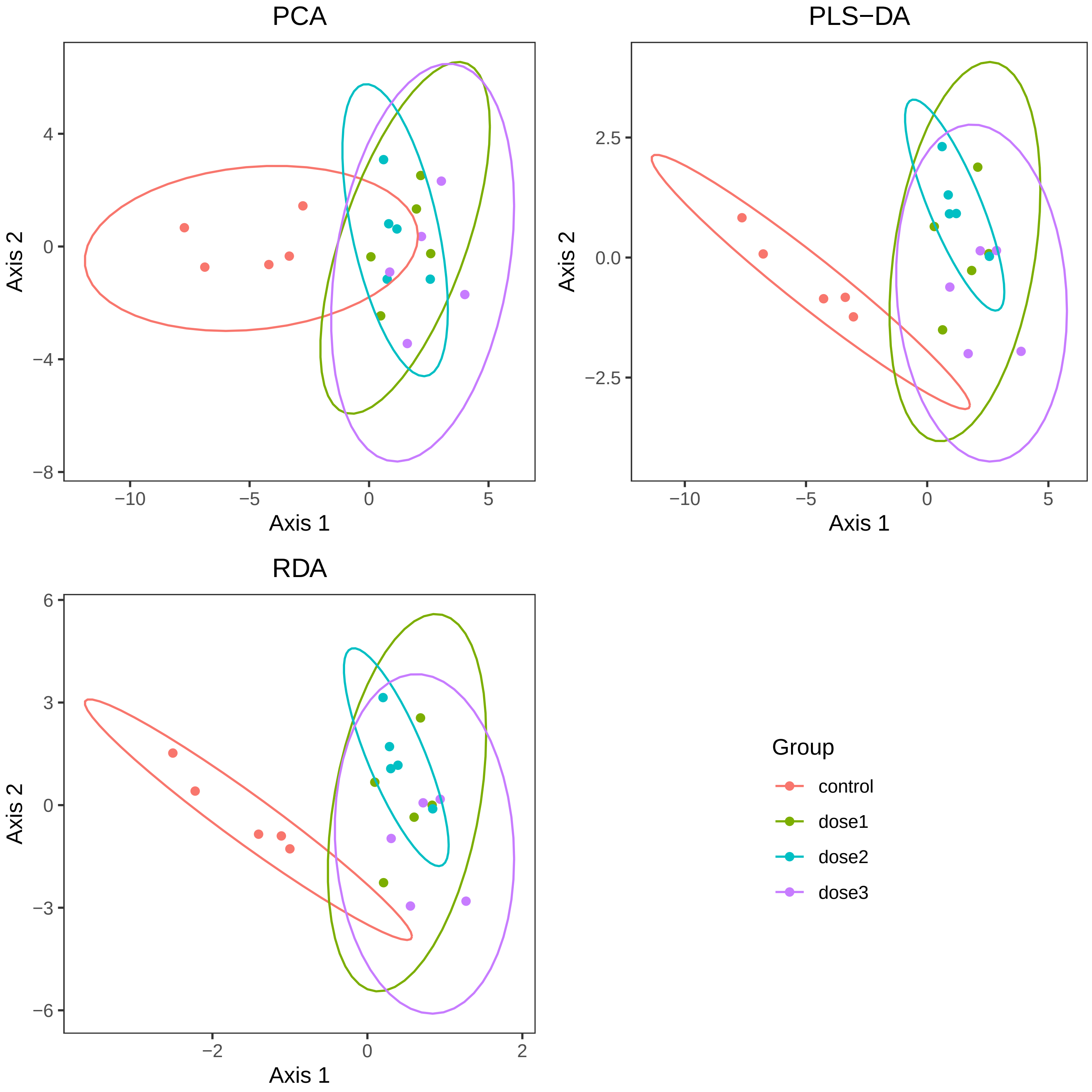
PCA
PLS−DA
RDA
Axis 1
Axis 2
−10
−5
0
5
4
−4
−8
2.5
0.0
−2.5
−2
2
6
3
−3
−6
Group
control
dose1
dose2
dose3
